# Identification of SNPs and INDELS in swine transcribed sequences using short oligonucleotide microarrays

**DOI:** 10.1186/1471-2164-9-252

**Published:** 2008-05-29

**Authors:** Steve R Bischoff, Shengdar Tsai, Nicholas E Hardison, Abby M York, Brad A Freking, Dan Nonneman, Gary Rohrer, Jorge A Piedrahita

**Affiliations:** 1 Department of Molecular Biomedical Sciences, College of Veterinary Medicine, North Carolina State University, Raleigh, NC, 27606, USA; 2 USDA, ARS, U.S. Meat Animal Research Center, Clay Center, NE 68933-0166, USA; 3 Center for Comparative Medicine and Translational Research, North Carolina State University, Raleigh, NC, USA; 4 Program in Statistical Genetics, Department of Statistics, North Carolina State University, Raleigh, NC 27695-7566, USA

## Abstract

**Background:**

Genome-wide detection of single feature polymorphisms (SFP) in swine using transcriptome profiling of day 25 placental RNA by contrasting probe intensities from either Meishan or an occidental composite breed with Affymetrix porcine microarrays is presented. A linear mixed model analysis was used to identify significant breed-by-probe interactions.

**Results:**

Gene specific linear mixed models were fit to each of the log_2 _transformed probe intensities on these arrays, using fixed effects for breed, probe, breed-by-probe interaction, and a random effect for array. After surveying the day 25 placental transcriptome, 857 probes with a q-value ≤ 0.05 and |fold change| ≥ 2 for the breed-by-probe interaction were identified as candidates containing SFP. To address the quality of the bioinformatics approach, universal pyrosequencing assays were designed from Affymetrix exemplar sequences to independently assess polymorphisms within a subset of probes for validation. Additionally probes were randomly selected for sequencing to determine an unbiased confirmation rate. In most cases, the 25-mer probe sequence printed on the microarray diverged from Meishan, not occidental crosses. This analysis was used to define a set of highly reliable predicted SFPs according to their probability scores.

**Conclusion:**

By applying a SFP detection method to two mammalian breeds for the first time, we detected transition and transversion single nucleotide polymorphisms, as well as insertions/deletions which can be used to rapidly develop markers for genetic mapping and association analysis in species where high density genotyping platforms are otherwise unavailable.

SNPs and INDELS discovered by this approach have been publicly deposited in NCBI's SNP repository dbSNP. This method is an attractive bioinformatics tool for uncovering breed-by-probe interactions, for rapidly identifying expressed SNPs, for investigating potential functional correlations between gene expression and breed polymorphisms, and is robust enough to be used on any Affymetrix gene expression platform.

## Background

At the molecular level, naturally occurring DNA sequence variation is comprised of single nucleotide polymorphisms (SNPs) or INDELs (insertions/deletions). Estimates of nucleotide diversity in mammals range from 1:1331 base pairs (bp) in humans [1], 1:400 bp in chimpanzees [2],1:515 bp in mice [3,4], 1:443 bp in cattle [5] and 1:475 bp in pigs [6] from interbreed crosses of the Chinese Meishan and a European white composite. Insights to human diversity after genotyping three major populations (African, European, and Han Chinese) have yielded 4.5 million SNPs that approximately correspond to millions of sequence variation between any two individual human genomes and 100,000 non-synonymous amino acid changes in the proteomes [7]. Discovery of expressed SNPs could arguably represent the most informative and valuable resource to study disease susceptibilities, to determine structural effects on protein sequence, and to design association studies aimed to clarify complex, polygenic phenotypes.

Over the past decade, tremendous progress has been made in developments of high-throughput genome-scale technologies and, collectively, high impact projects like the Human Genome and HapMap have paved the sequencing efforts of livestock genomes. The international Swine Genome Sequencing Consortium (SGSC) has obtained funding to produce a 6X coverage of the domestic swine genome[8]. The pig represents a priority to sequence due to its extensive history in advancing biomedical research to improve human health and agricultural importance [9,10].

**Figure 1:**
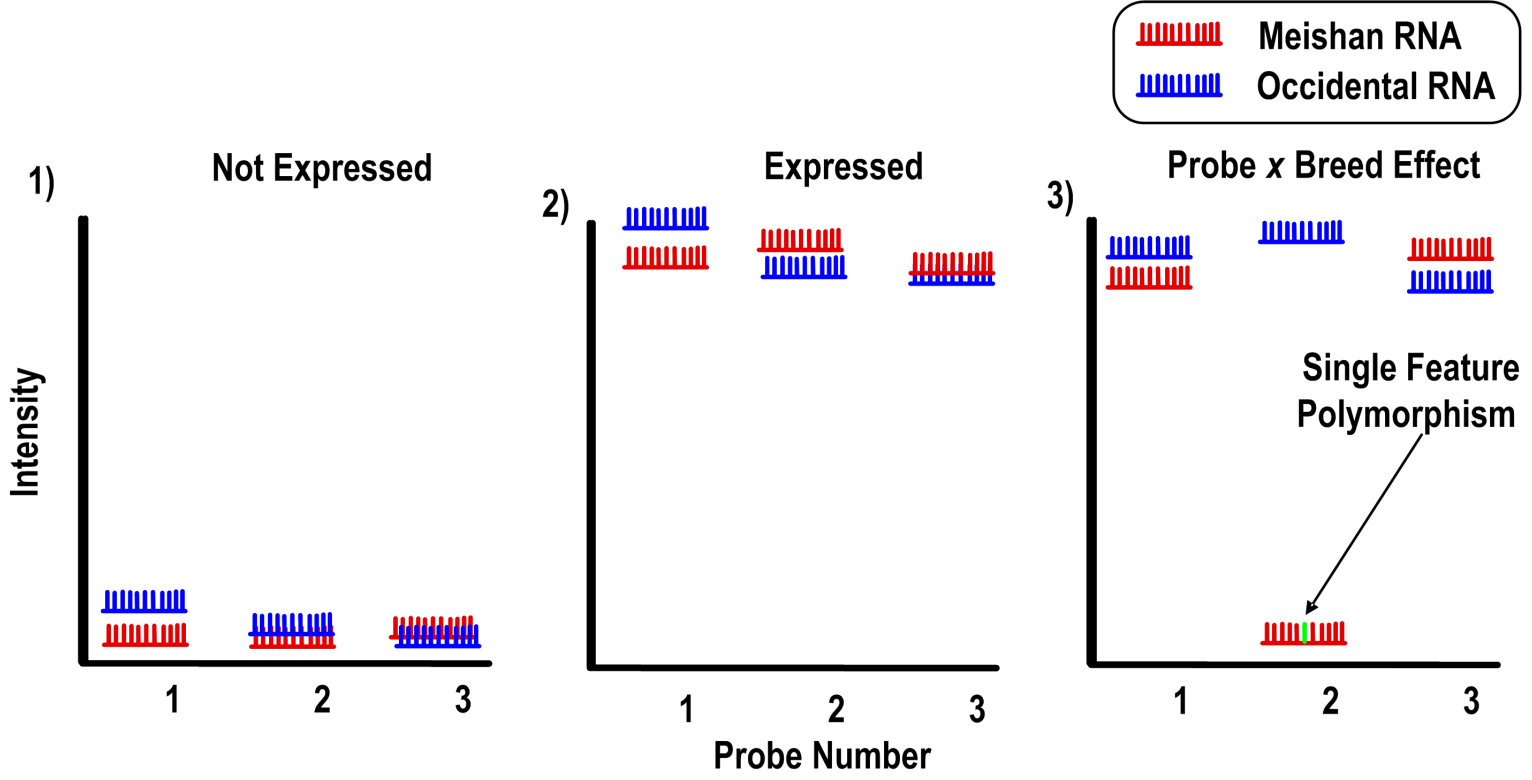
**a, b: Theoretical outcomes of probe hybridization**. A) Affymetrix short oligonucleotide arrays are fabricated with sets of eleven 25-mer probes to interrogate the expression level of a particular mRNA. Because of the short probe length, a SNP falling in the middle of the probe sequence will result in that probe's failure to hybridize. B) Three potential hybridization scenarios within a single probe set are depicted: 1) mRNA to target probes is not expressed in either breed, 2) mRNA to target probes is equivalent in both breeds or, 3) intensity of probes is expressed in one breed and negligible in the other breed. In this latter case, a probe-by-breed effect is seen with Meishan RNA hybridized to probe 2 of the probe set. The green hash represents the SFP located within a central nucleotide position within the probe.

Strategies for SNP discovery roughly dichotomize into experimental or *in silico *approaches. Traditionally, experimental means for uncovering SNPs in resource panels have predominantly relied on polymorphism screening by DNA sequencing [6,11]. Although 'brute force' DNA genotyping by dideoxy sequencing has been the method of choice for *de novo *SNP detection, it suffers from difficulty of DNA template amplification, limitations of PCR multiplexing per reaction, and increasing equipment costs. In light of these limitations, there has been increased demand for affordable, genome-wide strategies for scaleable SNP genotyping to uncover genomic variation [11]. Contemporary, high-throughput strategies for genotyping include bead-based microfluidics, chip-based methods, bioluminescent microfluidics, and multiplex SNP genotyping with MALDI-TOF mass spectrometry to name a few [12-17]. Alternatively, *in silico *SNP discovery pipelines rely on scanning databases for sequence variants via computational tools to examine redundancy and to score for accuracy of SNP detection [18-21]. Recently, chip-based methods [22-32] (and references [26-29]) such as Affymetrix gene expression arrays have been used for SNP discovery Affymetrix short oligonucleotide arrays are fabricated with sets of eleven 25-mer probes to interrogate the expression level of a particular mRNA. Because of the short probe length, a SNP falling in the middle of the probe sequence will result in that probe's failure to hybridize. Figure 1 illustrates three potential hybridization scenarios within a single probe set: 1) mRNA to target probes is not expressed in either breed, 2) mRNA to target probes is equivalent in both breeds or, 3) intensity of a probe(s) within a set is expressed at a high level in one breed and negligible in the other breed. Although several explanations, such as alternative splicing, could account for the latter case, they can also be due to the presence of SNPs within the probe.

Various approaches have been used to detect SFPs. Winzeler et al. [30] provided initial proof-of-principle that single feature polymorphisms in yeast could be detected by hybridizing labeled genomic DNA to short oligonucleotide microarrays. Ronald et al. [32] extended this approach to hybridizing cRNA to identify polymorphisms in yeast with a sensitivity of 45% and a false discovery rate (FDR) of 6%. Borevitz et al. [24] applied the SFP discovery technique to Arabidopsis, an organism with an 120 megabase genome (10-fold larger than yeast) [33], with an average false discovery rate of 35%.

Rostoks et al. [31] reported an average of 35–40% FDR to detect SFPs using cRNA hybridized to Affymetrix barley gene expression short oligonucleotide arrays. However, they observe more noise relative to signal when trying genomic DNA hybridizations to gene expression arrays and offer the suggestion that this may be a consequence of the larger genome size of barley (520 megabases) [31].

Luo et al. [25] compared four different methods of SFP detection using Affymetrix short oligonucleotide arrays in barley. They distinguish between genetic expression markers (GEMs) which affect entire probe sets and SFPs which are localized to individual probes. Four methods are compared: 1) a PM-MM model, 2) a PDNN (position-dependent nearest neighbor model) of Ronald et al. [32], 3) the method of Winzeler et al. [30] based on genomic DNA that does not distinguish between the effects of expression differences across a probe set versus in a specific probe, and 4) a k-means clustering based method. Altogether, their results pointed out the methods uniformly have a high (~64%) false discovery rate (FDR) in analyses of their barley datasets.

Cui et al. [23] report < 20% FDR (13/65) of SFP detection using barley cRNA on Affymetrix short oligonucleotide chips, using a method called robustified projection pursuit. This is a mathematically complex leave-one-out correlation method, where SFPs are predicted on the basis that correlations omitting the feature in a probe set are likely to be higher than correlations calculated including it.

Utilizing the siggene and SAM procedures as described in Rostoks et al [31], Kumar et al [22] reported the identification of SFPs in japonica or saponica rice varieties with a FDR ranging from 10–12%.

In general, successful methods for SFP discovery can reliably distinguish hybridization differences affecting individual probes within the context of expression differences that affect all probes in the probe set. The FDR of predicted SFP prediction appeared to correlate with genome size or complexity.

In this report, we have adapted Rostoks *et al*. [31] method of probe-by-breed analysis to facilitate SNP discovery in two breeds of swine and streamlined and automated the procedure (Click-'N-SNP; supplemental file 1) to interface with SAS/JMP^® ^Genomics.

## Results

### Mining for expressed Single Feature Polymorphisms (SFP) by statistical analysis of expression microarrays

**Figure 2:**
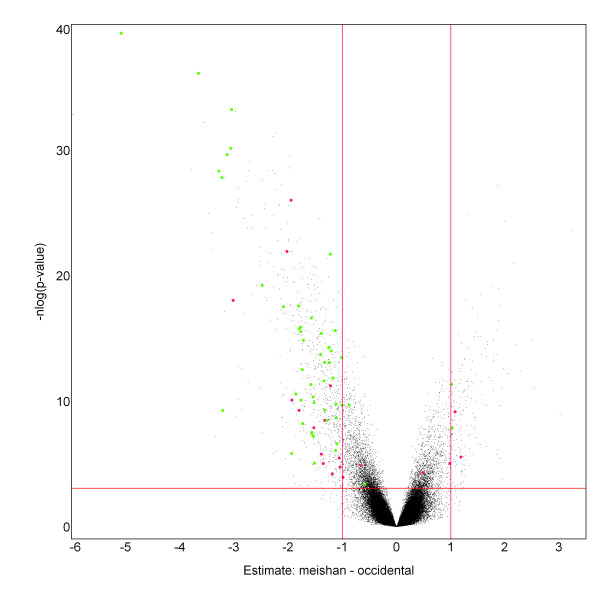
**Volcano plot of corrected probe-by-breed effect**. The volcano plot provides a visualization of significant changes in probe-by-breed expression values between the two swine populations. Using Storey's q-value procedures to correct for multiple testing, 4,635 probes were identified at an estimated false discovery rate of 5%. The points in green are expressed SFPs that have been confirmed by sequencing. The points in red are probes identified as false positives by sequencing. The horizontal red line is a q < 0.05 significance threshold with 4,635 (~1.8%) that are above the threshold. The x-axis shows the log_2 _estimate of Meishan minus occidental probe-by-breed effect. Vertical red lines represent 2-fold expression differences.

Six porcine gene expression chips were hybridized with day 25 placental total RNA of occidental (n = 3) or Chinese Meishan (n = 3) swine origin. A linear-mixed model was fit to the dataset to test for probe-by-breed interaction effects. This model is adapted from the Rostoks *et al*. method with modifications in the correction for the average breed effect. In figure 2, the volcano plot provides a visualization of significant changes in probe-by-breed expression values between the two swine populations. Using Storey's q-value procedures [34] to correct for multiple testing, 4,635 probes were identified at an estimated false discovery rate (FDR) of 5%. Additionally, a threshold of a 2-fold change was imposed to examine the effect on reducing the number of false positives detected. After imposing this filter, which is represented by the vertical red lines in the volcano plot, 857 unique probes at a FDR of q < 0.05 and with greater than 2-fold difference in estimated corrected probe-by-breed interaction effects were identified.

**Figure 3:**
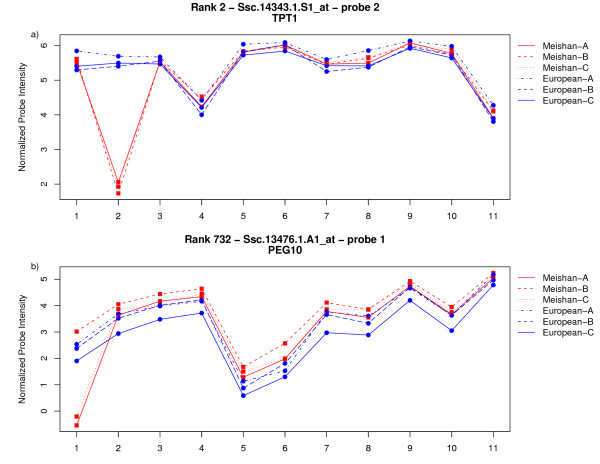
**Probe level plots of expression intensities illustrating a probe-by-breed interaction effect**. These plots demonstrate clear examples of a breed-by-probe interaction. A) Probe 2 of TPT1 gene for all Meishan RNA samples fails to hybridize. B) In the example of PEG10, a non-breed specific polymorphism is detected and confirmed by pyrosequencing. One of the three Meishan samples, '25 MB' is grouped with the occidental placental samples as visualized by the intensity plots.

**Figure 4:**
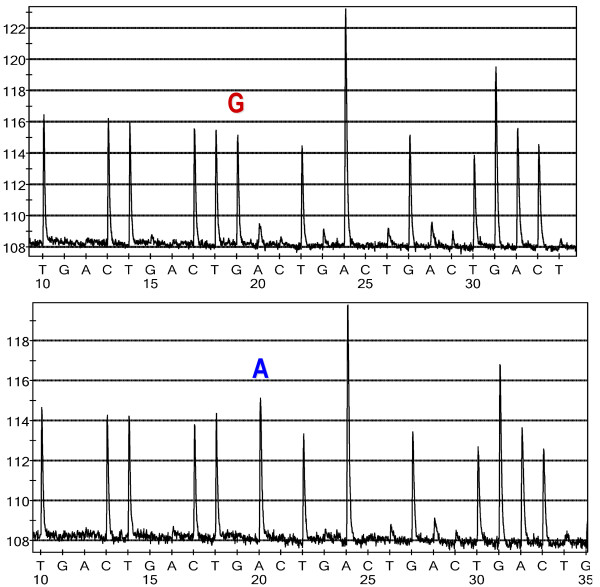
**Pyrograms of PEG10**. The spectral output of a pyrosequencing reaction is a representative pyrogram. PEG10 [rank 732, Ssc.13476.1.A1_at, probe 1] is depicted with an 'R' SFP detected by pyrosequencing of Meishan and occidental placental cDNA. The top panel is Meishan (red 'G' = guanine).

To help interpret the probe-by-breed interaction effect, individual probe plots were constructed for each probe set, represented by 11 unique probes per transcript, using the Affymetrix porcine arrays (see Supplemental Files 2, 3 and 4). Specifically, each of the significant probes was plotted to visually inspect probe behavior within the respective probe set versus its normalized intensity. Representative plots are depicted in Figures 3a and 3b. In Figure 3a Meishan RNA for the TPT1 gene (Affymetrix probe set Ssc.14343.1.S1_at; rank 2) did not hybridize to probe number 2 in all three Meishan arrays (see Table 1); conversely, the occidental breed did hybridize. A rational interpretation of these probe dynamics is that Meishan RNA could be polymorphic with respect to the array probe and is essentially behaving as a mismatch probe instead of a perfect match probe. These efforts were repeated to sample more candidates, including PEG10 (Affymetrix probe set Ssc.13476.1.A1_at, probe 1; figure 3c rank 732). Interestingly, a non-breed specific polymorphism was revealed in one of the Meishan placentas profiled for PEG10 (see Table 1). In other words, because one of the probes with Meishan RNA behaved similar to the three occidental samples, a similar genotype could be inferred for the single Meishan sample. Indeed, the linear-mixed model was sensitive enough to identify a non-breed specific polymorphism as demonstrated by pyrosequencing data in the following section (See Table 1, Figure 4; Table 2).

**Table 1:** Sequence data from pyrosequencing

**Ssc.14343.1.S1_at, probe 2, Rank 2 TPT 1, regulation of apoptosis**	
**25MA**	TAATCACTGGTGT**G**GATATTGTCATGAAC
**25MB**	TAATCACTGGTGT**G**GATATTGTCATGAAC
**25MC**	TAATCACTGGTGT**G**GATATTGTCAACACC
**25WA**	TAATCACTGGTGT**C**GATATTGTCATGAAC
**25WB**	TAATCACTGGTGT**C**GATATTGTCATGAAC
**25WC**	TAATCACTGGTGT**C**GATATTGTCATGAAC
**Probe**	CACTGGTGT**C**GATATTGTCATGAAC
**SNP**	**S**

**Ssc.13476.1.A1_at, probe 1, Rank 732 PEG10, Paternally Expressed 10**	

**25MA**	GATATCTCT**G**TAAGTGGACACGTGT
**25MB**	GATATCTCT**A**TAAGTGGACACGTGT
**25MC**	GATATCTCT**G**TAAGTGGACACGTGT
**25WA**	GATATCTCT**A**TAAGTGGACACGTGT
**25WB**	GATATCTCT**A**TAAGTGGACACGTGT
**25WC**	GATATCTCT**A**TAAGTGGACACGTGT
**probe**	GATATCTCT**A**TAAGTGGACACGTGT
**SNP**	**R**

Pyrosequencing data confirms the intensity plot where a G/C = S polymorphism is identified (top).

Pyrosequencing confirms the non-breed specific nature of the polymorphism, namely A/G = R (bottom).

**Table 2:** Summary of pyrosequencing confirmation, arbitrary probes.

**Probe Set ID**	**Probe No.**	**Gene Name**	**SNP**	**SNP type**	**SNP location**	**Estimate**	**q-value**	**Rank q < 0.05**	**Rank q < 0.05, fold change > 2**
Ssc.14003.2.S1_at	9	RPS4X	R, Y	Transition	15, 19	-5.09342	9.90E-35	1	1
Ssc.14343.1.S1_at	2	TPT1	Y	Transition	10	-3.66704	8.02E-32	2	2
Ssc.1341.1.S1_at	8	TKT	Y	Transition	13	-3.05487	4.13E-29	3	3
Ssc.16710.1.S1_at	4	Q9BTR0	T/-	INDEL	7	-3.07201	1.67E-26	9	9
Ssc.11554.1.S1_at	11	MRPL39	TAAG/----	INDEL	17	-3.13603	4.80E-26	10	10
Ssc.2756.1.A1_at	10	MRPL22	Y	Transition	20	-3.28928	6.28E-25	15	15
Ssc.20986.2.S1_at	4	GRN	R	Transition	13	-3.23164	1.66E-24	20	20
Ssc.24770.1.S1_at	3	PLAGL1	Y	Transition	14	-1.23548	6.85E-19	61	61
Ssc.4324.2.A1_at	4	CAMLG	S	Transversion	6	-1.8121	4.49E-15	124	124
Ssc.55.1.S1_at	9	EGFR	Y	Transition	10	-1.5839	3.27E-14	154	154
Ssc.422.1.S1_at	10	IGF2R	W	Transition	21	-1.41267	1.60E-11	259	253
Ssc.21595.2.S1_at	5	GNAS	R	Transition	19	-1.59188	2.45E-09	401	371
Ssc.2315.1.A1_at	11	Q8N3R3	S	Transversion	8	-1.77102	3.35E-08	521	447
Ssc.26344.1.S1_at	11	PAK3	R	Transition	9	-1.12922	6.41E-08	561	474
Ssc.21857.1.S1_at	11	GRB10	R	Transition	5	-0.88725	7.90E-08	576	no rank
Ssc.6989.1.A1_at	5	SFXN1	Y	Transition	17	-1.33493	1.75E-07	627	509
Ssc.939.1.A1_at	7	RPL12	K	Transversion	20	-3.21892	1.86E-07	637	514
Ssc.26344.1.S1_at	4	PAK3	None	None		1.068089	2.30E-07	655	523
Ssc.12365.1.A1_at	11	ADAMTS1	Y	Transition	5	-1.13449	0.000135	1345	718
Ssc.13476.1.A1_at	1	PEG10	R	Transition	10	-1.94654	0.000221	1444	732
Ssc.2716.1.A1_at	6	NP_055322	None	None		-1.39706	0.000241	1472	736
Ssc.7604.2.A1_a_at	11	C14orf111	M	Transversion	16	-1.52629	0.000994	1871	783
Ssc.1595.1.S1_at	1	PHEMX	None	None		0.973961	0.001016	1884	no rank
Ssc.26460.1.A1_at	1	SNRPN	None	None		-0.66999	0.00147	2003	no rank
Ssc.11508.1.A1_at	7	GABRA5	None	None		0.473879	0.00433	2450	no rank
Ssc.3850.1.S1_at	11	NNAT	None	None		-1.00118	0.008733	2860	822
Ssc.3802.1.S1_at	2	NAP1L4	A/-	INDEL	6	-0.59859	0.02431	3704	no rank

Twenty-seven putative SFPs were selected, and were pyrosequenced to confirm or disprove predicted SNPs at probe locations. The column labeled 'SFP' describes the character of the polymorphism discovered, such as transitions/transversions, INDELs (MRPL39, NAP1L4), or false positives ("None"). When filtering out probes with less than 2-fold expression difference between breeds, 87% of predicted SNPs were true.

### Validation of SFP candidates by pyrosequencing

For the purposes of initial method validation, a subset of 27 probes were arbitrarily selected for reverse transcription-PCR using universally-tagged primers compatible with pyrosequencing technology was performed [35]. We opted to use pyrosequencing on cDNA as this technology has high accuracy in the immediate vicinity of the sequencing primer unlike conventional dye-termination chemistry, and the target sequences were readily available as compared with paucity of swine genomic sequence. Short amplicons (100–200 bp) were designed using the exemplar sequences available from Affymetrix NetAFFX[36] to amplify a region surrounding the 25-mer probe. All primers used for these assays to amplify specific probes were designed using Primer3 [37] or MPrime [38]. Probe behavior as seen in the intensity plots in Figures 3a and 3b was explained by genotyping with pyrosequencing technology (see Table 2). A transversion mutation (G/C = S) in TPT1 was confirmed in position 10 of probe number 1 (Table 1). A forward pyrosequencing assay was generated for PEG10 to confirm the non-breed specific (G/A = R, forward) SNP and is illustrated in Figure 4, respectively. Of the twenty-seven probes examined by pyrosequencing in the absence of the 2-fold cut-off, 22 out of 27 probes were confirmed as containing SNPs. Imposition of the 2-fold expression difference criteria, improved the overall success rate of SNP detection (19 of 22 probes). Validation of SNP discovery efforts is summarized in Table 2.

These results also confirmed that this method tends to identify SFPs that are located near the center portion of the probe sequence rather than at the ends. This is consistent with what one would predict and with the distribution observed in Rostoks et al., 2005 [31].

### Unbiased confirmation rates of SFP detection by random sequencing of genomic DNA

In order to obtain an unbiased confirmation rate of our approach, we randomly selected SFPs for sequencing from the entire pool of 857 candidate SFPs that match a q < 0.05, and |log_2 _fold change| > 1 criteria. As the complete porcine genome assembly is not yet available, these sequences were derived by comparing Affymetrix Porcine target sequences by BLAST to the latest sequence available from the porcine high throughput genomic traces from NCBI's trace archive, and represent 44 predicted SFPs and 222 non-SFPs. Of these, 31 probes were confirmed to be true SFPs, with an unbiased confirmation rate of 70% (false discovery rate (FDR) of 0.30, sensitivity of 0.65, and specificity of 0.94; see Table 3 and Table 4).

**Table 3:** Summary of sequencing confirmation, random probes.

**Probe_Set_ID**	**probe**	**Gene_Name**	**q-value**	**Rank (q < 0.05)**	**Rank (q < 0.05, |fold change| > 1)**	**SNP Code**	**SNP location**
Ssc.19486.1.S1_at	3	CCNB1IP1	7.36E-23	28	28	none	
Ssc.15621.1.A1_at	6	PCYOX1	4.32E-19	59	59	none	
Ssc.24184.1.S1_at	4	GPD1L	1.32E-16	94	94	S	7
Ssc.7266.1.A1_at	2	POSTN	1.71E-15	114	114	none	
Ssc.18458.1.S1_at	6	Q86V57	4.92E-15	126	126	Y	11
Ssc.1447.3.S1_at	4	NP_116255	1.51E-13	174	174	K	14
Ssc.16422.2.A1_at	2	PLAA	2E-13	175	175	S	10
Ssc.21553.1.S1_at	8	HDGF	2.76E-13	179	179	R	10
Ssc.16422.2.A1_at	1	PLAA	3.26E-13	185	185	S	17
Ssc.13948.1.S1_at	3	GET1_HUMAN	4.42E-13	188	188	W	9
Ssc.10435.1.A1_at	1	Q14156	1.47E-12	210	210	M, Y	8,17
Ssc.17315.1.S1_at	3	EIF3S2	4.64E-12	236	233	K	10
Ssc.19651.1.S1_at	10	WDR42A	8.3E-12	249	244	Y	11
Ssc.20060.1.A1_at	8	TRIP12	2.62E-11	269	262	Y	4
Ssc.13817.1.A1_at	6	VDAC3	5.89E-11	286	276	Y	13
Ssc.24025.1.A1_at	9	Q96E16	6.17E-11	287	277	S	19
Ssc.11787.2.A1_at	9	RASSF2	1.96E-10	326	312	M	9
Ssc.21613.1.S1_at	4	TIGD2	8.38E-10	357	339	R	12
Ssc.19447.1.A1_at	2	ARMCX3	1.37E-09	377	353	Y	9
Ssc.30685.1.A1_at	10	C5orf3	2.48E-09	402	372	Y	5
Ssc.16654.1.A2_at	3	HNRPK	3.15E-09	413	379	none	
Ssc.6356.1.S1_at	5	ODC1	1.23E-08	470	419	R	10
Ssc.1442.1.S1_at	8	PHF3	1.98E-08	495	434	Y	7
Ssc.2042.1.S1_at	3	NP_079516	3.39E-08	522	448	none	
Ssc.20974.1.A2_at	7	GNS	5.15E-08	549	467	R	16
Ssc.11173.1.A1_at	1	DEOC_HUMAN	7.88E-08	575	482	Y	17
Ssc.2042.1.S1_at	4	NP_079516	1.83E-07	632	512	none	
Ssc.12013.1.A1_at	1	SYTL4	6.35E-07	726	554	R, Y	15,16
Ssc.10949.1.S1_at	6	GBAS	9.35E-07	757	566	none	
Ssc.5052.1.S1_at	4	KIAA0674	9.74E-07	761	570	R	11
Ssc.25584.1.S1_at	8	CDW92	1.62E-06	797	589	W	6
Ssc.1031.1.S1_at	7	OAS1	3.2E-06	861	612	Y	25
Ssc.28305.1.A1_at	11	TACC1	3.22E-06	862	613	none	
Ssc.17313.2.S1_at	7	CALD1	7.14E-06	938	638	Y	17
Ssc.17423.1.S1_at	4	YWHAZ	1.18E-05	990	661	R	8
Ssc.12029.1.S1_at	6	LAMA2	1.37E-05	1018	670	R	10
Ssc.2466.1.S1_at	5	LRP10	4.59E-05	1177	698	Y	5
Ssc.2466.1.S1_at	5	LRP10	4.59E-05	1177	698	R	5
Ssc.12365.1.A1_at	11	ADAMTS1	0.000135	1345	718	R	5
Ssc.28305.1.A1_at	1	TACC1	0.000376	1585	750	none	
Ssc.18850.1.S1_at	2	GOLPH2	0.000442	1640	758	none	
Ssc.1911.1.A1_at	9	PSMC1	0.001044	1893	788	none	
Ssc.28305.1.A1_at	8	TACC1	0.001842	2108	797	none	
Ssc.1911.1.A1_at	10	PSMC1	0.005343	2571	816	none	

This table summarizes the results of sequencing of randomly selected predicted SFPs meeting the criteria of q ≤ 0.05 and a 2-fold expression difference between interacting probes. We estimate the unbiased confirmation rate from this random sample to be 70% using 6 arrays.

**Table 4:** Confusion matrix for random SFPs sequenced

		**Predicted**	
		
		**Negative**	**Positive**
**Actual**	**Negative**	205	13
	**Positive**	17	31

This is a confusion matrix comparing predicted versus actual SFPs. Sensitivity: 0.65, specificity: 0.94, FDR: 0.30.

### Method comparison to other current approaches

The method of Rostoks et al [31]has been successfully applied to a number of species including arabidopsis, rice, and barley; we were therefore interested to determine whether how our method performed in comparison. In order to directly compare method performance in a species with a larger number of known SNPs, we downloaded barley microarray and SNP data [31] and the respective researcher's website[39]. Both the method described herein and the method described by Rostoks et al. were executed on the six-array GEM subset, and performed within 1–2% of each other as measured by sensitivity and false discovery rate (see Supplemental File 5).

### Mixed model comparison using divergent tissue types

To answer the question of whether we could detect SFPs in the presence of confounding effects such as different tissue types, we tested whether gene profiles obtained from two different tissue types (placenta and fetal fibroblasts) could be used to effectively to detect SFPs. This question is particularly pertinent in the situation where one might download gene expression data online from two different breeds of animals where commonly different treatment or tissue types confound the breed effect. Our test of Spearman's rank correlation coefficient indicates (ρ = 0.1365, p < 0.0001) that there is a significant relationship between the breed-by-probe interaction p-values of a comparison between day 25 Meishan versus occidental placenta and day 25 Meishan versus day 30 occidental fibroblast. 588 SFPs are predicted by both approaches (q < 0.05, |log_2 _fold change| > 1) where 16 are expected by chance indicating that there is a significant overlap (Table 5, chi-squared = 21,205, degrees of freedom (df) = 1, p < 0.0001). Taken together, these statistics suggest that SFPs identified by using divergent tissue types are real.

**Table 5:** Comparison of predicted SFPs using same versus divergent tissues

			**divergent tissues: **day 25 Meishan placenta, day 30 occidental fibroblast	
			
			**SFPs**	**non-SFPs**
			4,873	259, 743
**same tissues: **day 25 Meishan placenta, day 25 occidental placenta	**SFPs**	857	588	269
	**non-SFPs**	263,759	4,285	259,474

Predicted SFPs and non-SFPs from analysis of microarray data from same tissues are in rows; predicted SFPs and non-SFPs from divergent tissues are in columns. 588 are predicted by both analyses, where 16 are expected by random chance.

Chi-squared = 21,205, df = 1, p-value < 0.00001

## Discussion

Traditional methods of SNP discovery via Sanger sequencing are labor intensive. In this study, we tested a bioinformatics method of SNP discovery in a species where presently no SNP-chip genotyping platform exists by using the high-throughput platform of gene expression short oligonucleotide arrays to mine thousands of transcripts simultaneously. The method herein demonstrates the utility of fortuitous SNP discovery exploiting the properties of probe-by-breed interaction effects after transcriptional profiling with Affymetrix GeneChip microarrays.

In this report, we examined Affymetrix probe behavior using a linear-mixed model and reasoned that probes specific to one breed that exhibit outlying intensity profiles may contain expressed single feature polymorphisms. We initially developed 27 pyrosequencing assays to validate the SFP detection method in swine. All sequence initially validated SNPs identified Meishan as the discordant breed on the array due to lack of RNA binding to an individual probe. A more complete description of the porcine genome should allow for localization of those SFPs which fall in coding regions and give rise to non-synonymous changes. These results are also congruent to previous reports [31] in which central nucleotides in the Affymetrix probe were efficiently detected to contain a SFP, however flanking nucleotide positions (1–4; 22–25) were marginally discernible above background (see Tables 2, 3).

After randomly selecting an additional set of predicted SFPs for sequencing, we find that the unbiased confirmation rate (FDR = 0.30), sensitivity, and specificity is comparable to those seen in barley. Our adapted mixed model method highly enriches for SNPs within the SFP region. This mixed model approach is both sensitive and specific. Using this model we do observe a trend correlating lower q-values (higher rank) with the rate of SFP discovery; for example, the lower ranked 50% of samples randomly sequence contained 69% of all false positives detected (see Table 3).

While our approach performs similarly in comparison to other current methods, it is more computationally efficient and scaleable, generating candidate SNPs in approximately 20-fold less time on standard desktop computing workstations. Further, our implementation has been automated for Affymetrix short oligonucleotide microarrays and can generate a list of candidate SFPs when provided only with an experimental design file pointing to raw .CEL files. This method (Click-'N-SNP) is freely available as Supplemental File 1.

However, a limitation of this approach, as evidenced by sequencing of probes such as PAK3 probe 4 Rank 532 (see Supplemental File 3) is that the model is written on the assumption that there is only one polymorphic probe per probe set. The effect of the presence of multiple polymorphic probes within a single probe set is to reduce the accuracy of the correction for the average breed effect, increasing the likely false positive rate.

Another drawback of this approach is that only expressed probe sets are interrogated for SNP discovery. For instance, in the placenta-to-placenta comparison only 13,004 probe sets out of 24,123 were found to be expressed. Thus, only 53.9% of the genes present in the array could be interrogated. When two different profiles were used (placenta versus fibroblast) this number was further decreased to 12,893 (53.4%). An alternative to increase the scope of SFP discovery is to pool different tissues. Noting this potential, executing the model on existing microarray data available through NCBI's expression microarray repository, the Gene Expression Omnibus (GEO) [40], may also lead to new SNPs.

Our comparison of an analysis performed using divergent tissues (placenta versus fibroblast) demonstrate that this method is not limited to gene expression data obtained from the same tissue or treatment and is capable of detecting interacting probes in the context of probe set wide differential expression, whether such differential expression is a result of breed or tissue specific transcriptional differences. There was a high correlation and overlap beyond that expected by chance between the SFP candidates derived from placenta-to-placenta comparison and the placenta-to-fibroblast comparisons. As such this modification broadens the application of the technology and the possibility that existing datasets, even if generated from different tissue types, can be mined for SFPs. Insomuch, existing microarray datasets can quickly be examined for SNPs that are directly associated with the samples used to generate the microarray data, thus, providing SNP information that is directly relevant to the breed, strain, or and sample of interest.

In conclusion, we have adapted a linear mixed model approach to identifying expressed polymorphisms that is portable across any short oligonucleotide platform. The approach was examined by pyrosequencing of cDNA, and 87% of the candidates sampled contained expressed polymorphisms. By this method, expressed transition and transversion SNPs as well as INDELs were detected. The identification of new polymorphisms should: 1) facilitate current swine genetic mapping efforts, 2) enable detailed linkage disequilibrium studies and 3) increase the resolution needed to track quantitative trait loci. As more variation in swine is identified among divergent breeds, the power to identify polygenic traits should also be vastly improved.

## Conclusion

While various methods for the detection of SFPs have been used in other species ranging in genome complexity, we demonstrate in this report the utility to successfully detect SFPs from a mammalian species by using porcine short-oligonucleotide arrays. Indeed, indels, transition and transversion single nucleotide polymorphisms were identified by this approach. Furthermore, the process of SFP detection has been streamlined to simplify detection on almost any short-oligonucleotide array.

## Methods

### Tissue collection and total RNA preparation for SNP discovery panel

For this study, day 25 porcine fetuses were produced from natural matings of purebred white composite gilts [41] or purebred Meishans 6 months of age or older. Pregnant gilts of each breed were sacrificed on the morning of gestational day 25. Immediately following slaughter, the reproductive tracts were excised from the gilts, and fetuses and their placentas were collected from each uterine horn. Physical measurements were taken, including fetal body weight and wet placental weight. Following measurements, whole placental tissue was minced briefly, placed in cryovials and snap-frozen in liquid nitrogen, then stored at -80°C until they could be processed further. Porcine fibroblast lines were established as previously described [42]. Procedures for the handling of animals complied with those specified in the *Guide for the Care and Use of Agricultural Animals in Agricultural Research and Teaching *(1999) [1st rev. ed. Savoy, IL: Federation of Animal Science Societies; 1999]. All animal protocols involving the use of swine for generation of fetal fibroblast lines were approved by the Institutional Animal Care & Use Committee at North Carolina State University.

Total RNA was extracted from each animal's placenta using RNAqueous Kit (Ambion, Austin, TX) following the instructions of the manufacturer with only slight modifications. Briefly, 200–300 mg of placental tissue was pulverized in a mortar and pestle under liquid nitrogen to homogeneity. Powdered tissue was weighed on a microbalance and lysis buffer was added directly to each sample in a weigh-boat (600 μL lysis buffer: 50 mg tissue), vortexed vigorously 60–90 seconds, pre-cleared by centrifugation for 5 min at 3,000 × *g*. In order to remove bulk genomic contamination, 600 μL of the pre-cleared supernatant was applied to an Agilent mini-prefilter column and centrifuged at 10,000 × *g *for 1 min. The filtrate was precipitated with an equal volume of 64% ethanol and the RNAqueous protocol was continued according to the manual.

Integrity of total RNA were crudely assessed by loading 2 μg per well for denaturing agarose gel electrophoresis and quantitated spectrophotometrically by the NanoDrop^® ^ND-1000 spectrophotometer (NanoDrop Technologies, Wilmington, DE). Total RNA (10 μg aliquots in nuclease-free H_2_O) was divided for transcriptome analysis or first-strand cDNA synthesis and then stored at -80°C until they could be processed further. Fetuses were genotyped and selected for all females.

### Experimental Design

Three samples from each breed of swine were hybridized to an Affymetrix Porcine GeneChip microarray.

### Production of cRNA and hybridization to Affymetrix Porcine GeneChip

Gene expression microarrays were hybridized as previously described [42]. The following procedure was performed by Expression Analysis (Durham, NC) in accordance with methods specified by the manufacturer for target preparation and hybridization. Before target production, the quality and quantity of each RNA sample was assessed using a 2100 BioAnalyzer and a RNA 6000 Nano LabChip Kit (Agilent Technologies, Palo Alto, CA). Total RNA (10 μg) was converted into cDNA using SuperScript III Reverse Transcriptase (Invitrogen, Carlsbad, CA) and a modified oligo(dT)_24 _primer that contains T7 promoter sequences (GenSet, San Diego, CA). After first strand synthesis, residual RNA was degraded by the addition of RNase H and a double-stranded cDNA molecule was generated using DNA polymerase I and DNA ligase. The cDNA was then purified and concentrated using a phenol:chloroform:isoamyl alcohol extraction followed by ethanol precipitation. The cDNA products were incubated with T_7 _RNA polymerase and biotinylated ribonucleotides using an In Vitro Transcription kit (Enzo Diagnostics, New York, NY). One-half of the cRNA product was purified using an RNeasy column (Qiagen, Valencia, CA) and quantified with a spectrophotometer. The cRNA target (20 μg) was incubated at 94°C for 35 minutes in fragmentation buffer (tris base, magnesium acetate, potassium acetate). The fragmented cRNA was diluted in hybridization buffer (MES; NaCl, EDTA, tween 20, herring sperm DNA, acetylated BSA) containing biotin-labeled OligoB2 and Eukaryotic Hybridization Controls (Affymetrix, Santa Clara, CA). The hybridization cocktail was denatured at 99°C for 5 minutes, incubated at 45°C for 5 minutes and then injected into a GeneChip cartridge. The GeneChip array was incubated at 42°C for at least 16 hours in a rotating oven at 60 rpm. GeneChips were washed with a series of non-stringent (25°C) and stringent (50°C) solutions containing variable amounts of MES buffer, tween 20 and SSPE buffer. The microarrays were then stained with streptavidin phycoerythrin and the fluorescent signal was amplified using a biotinylated antibody solution. Fluorescent images were detected in GeneChip^® ^Scanner 3000 and expression data was extracted using the MicroArray Suite 5.0 software (Affymetrix, Santa Clara, CA). All GeneChips were scaled to a median intensity setting of 500. The data discussed in this publication have been deposited in NCBIs Gene Expression Omnibus (GEO)[43] and are accessible through GEO Series accession numbers GSE10446, GSE10447.

### Mixed Model Analysis of Variance

Log_2_-transformed perfect-match (PM) intensities for all observations were fit to a linear mixed model that broadly corrected for breed and array effects [44]. A gene-specific mixed model was fit to the normalized intensities (residuals from first model) accounting for fixed breed (B), probe (P), and breed-by-probe interaction effects (BP), and a random array effect.

The model used in PROC MIXED in SAS is given as follows (SAS, Cary, NC):

y_gijk _= B_i _+ P_j _+ (BP)_ij _+ A_k _+ ε_gijk_

g^th ^gene

i^th ^breed

j^th ^probe

k^th ^array

y_ijk _is the normalized perfect match intensity of the j^th ^probe of the *l*^th ^individual of the *i*^th ^breed of the g^th ^gene hybridized on to the *k*^th ^array. We used a perfect-match only model, because it had been suggested that incorporating the mismatch probes increases noisiness of the data when estimating differential expression [45,46].

We then tested the effect of breed (B) for each level of probe (P), resulting in one test for each of approximately 265,000 probes on the array. We corrected for multiple testing by converting the resulting p-values to q-values using Storey's method for controlling FDR [34]. This procedure has been automated and is provided in Supplemental file 1.

### Mixed Model Comparison Using Divergent Tissue Types

The mixed model analysis described above was repeated using our gene expression data from day 25 Meishan placenta and data from day 30 normal occidental fibroblasts previously generated by *Tsai et al *[42]. This represents the situation where gene expression data is downloaded from GEO from two different experiments involving different breeds, using the same short oligonucleotide platform. In this particular model, the fixed breed effect term is confounded by tissue and time. We asked the question of whether we could detect SFPs in the presence of these types of confounding effects by calculating Spearman's correlation coefficient for the p-values of the comparison between day 25 Meishan versus white composite placenta, and day 25 Meishan placenta versus day 30 occidental fibroblasts. This statistic indicates the degree of similarity between the ranks of detected putative SFPs as sorted by p-value.

### First-strand cDNA synthesis and production of universally-tagged amplicons for pyrosequencing (PSQ)

In order to validate a subset of probes identified by the microarray experiments to harbor SNPs, we performed reverse transcription-PCR. The first strand cDNAs were synthesized from 5 μg of day 25 porcine placental total RNA using a 1.5 μM random pentadecamer priming method [47] and SuperScript^® ^III First-Strand Synthesis SuperMix reagents (Invitrogen, Carlsbad, CA USA) in a final volume of 20 μL. Reaction conditions were [50°C, 60 min; 85°C, 5 min]. First-strand product was diluted 1:3 with nuclease-free H_2_O.

Aydin *et al*. 2006 [35] established the feasibility of a universal PCR primer approach compatible with pyrosequencing. The initial PCR was performed with 25 μL reactions containing a final volume/concentration of the following components: 3 μL of reverse transcription product (50 ng cDNA template), 50 nM each gene-specific primers synthesized with the 5'-universal overhangs, 250 nM each of universal primers (with a terminal 5'-biotinylated moiety on one primer based on directionality of PSQ assay design), 3.5 mM MgCl_2_, and 20.5 μL Platinum^® ^Blue PCR SuperMix (Invitrogen, Carlsbad, CA USA). The PCR thermocycling conditions were the following: [95°C, 15 sec, denaturation; 60°C, 15 sec, annealing; 72°C, 30 sec, extension]_n = 8 cycles _followed by [95°C, 15 sec, denaturation; 58°C, 15 sec, annealing; 72°C, 30 sec, extension]_n = 40 cycles _and a final extension at 72°C for 30 sec. Short amplicons (100–200 bp) were designed using the exemplar sequences available from Affymetrix NetAFFX to amplify a region surrounding the 25-mer probe. All primers used for these assays to amplify specific probes were designed using Primer3 [37] or MPrime [38], were chemically synthesized with only desalting purification (IDT, Coralville, IA). A RT-negative control was used to check for DNA contamination and all such controls were negative on PCR amplification. Biotinylated amplicons were visualized on 8% PAGE prior to pyrosequencing.

### Sequencing of individual probes harboring putative single feature polymorphisms

Pyrosequencing was used as a quick and accurate method for SFP discovery in RT-PCR products amplified from cDNA generated from placental tissues of informative subjects (purebred Meishan or white composite porcine embryos).

For template preparation of each sample, 200 μg of streptavidin-coated sepharose beads (GE Biosciences) were washed twice in washing buffer (Biotage, Foxboro, MA) using a vacuum pump manifold and finally re-suspended in 45 μL of binding buffer (Biotage, Foxboro, MA)[35,48]. An equivalent volume (25–45 μL) of biotinylated PCR product was agitated with re-suspended beads for efficient immobilization [15 min, 25°C], then washed briefly in 70% ethanol [10 sec, 25°C]. To remove non-biotinylated DNA strand, immobilized duplexed DNA was melted using a NaOH-containing denaturation buffer [5 sec, 25°C] (Biotage, Foxboro, MA) and subsequently washed once in wash buffer [10 sec, 25°C] (Biotage, Foxboro, MA). Sequencing primer was added in 40 μL annealing buffer at a final concentration of 0.4 μM and hybridized to template by incubating [95°C for 2 min] in a heat block and slowly cooled to ambient temperature.

The pyrosequencing reaction was conducted via an automated PSQ 96MA machine, which uses a disposable cartridge (PSQ 96 Reagent Cartridge, Biotage, Foxboro, MA) to deliver enzymes, substrates, and each of the four nucleotides (PSQ 96 SNP Reagent Kit 5 × 96, Biotage, Foxboro, MA) into the wells of a transparent 96-well micro-titer plate. Successive nucleotides are dispensed in each well at 1 min intervals. Data acquisition of bioluminescence was captured and transcribed into spectra (pyrogram) for analysis.

Post-pyrosequencing, each contig was aligned with MUSCLE [49] and the consensus sequence harboring the polymorphic nucleotide were displayed in the form of IUB codes [50].

Alternatively, fluorescent terminator sequencing was used as previously described [6] to assess SNP true confirmation rate, Genomic DNA from identical animals hybridized to expression arrays was phenol-chloroform extracted.

For the probesets predicted to contain SFPs with a q-value of <= .05 and greater than 2-fold expression difference, the Affymetrix Porcine array target sequence was compared by BLAST to the latest available set of sequences from the porcine high throughput genome sequencing deposited into NCBI's trace repository[51], yielding 584 putative matches. Primer3 [37] was used to generate primers from these targeting an area +/- 200 bp around the probe of interest, with a target amplicon length of 450–500 bp. Primer min, opt, and max Tm were 57°C, 62.5°C, and 65°C respectively; primer_max_poly_x was limited to 3. A list of all primers used in this study is freely available upon request

## Competing interests

The authors declare that they have no competing interests.

## Authors' contributions

SB, ST, NH performed all of the analyses and SB drafted the manuscript. ST, AY, and SB performed pyrosequencing to confirm candidate SFPs. NH automated the SFP detection procedure. JP and BF were involved in the designing and running of the microarray experiments. JP, BF, GR and DN participated in design and coordination of the study. All authors read and approved the final manuscript.

## Supplementary Material

Additional File 1 Click-'N-SNP. Instructions and procedure to execute SAS code for SFP discovery. Click here for file

Additional File 2 Porcine_Candidate_SFPs_NoFC. Intensity plots of SFPs with q < 0.5, no fold change threshold. Click here for file

Additional File 3 Porcine_Candidate_SFPs_2FC. Intensity plots of SFPs with q < 0.5 with |fold change| > 2. Click here for file

Additional File 4 Porcine_Candidate_SFPs. List of all candidate with q < 0.5. Click here for file
