# Supplementary material for: Identification of SNPs and INDELS in swine transcribed sequences using short oligonucleotide microarrays
Source: BMC Genomics. 2008 May 29;9:252. doi: 10.1186/1471-2164-9-252 (PMC2442091; doi:10.1186/1471-2164-9-252)
Supplement: Additional File 1 — Click-'N-SNP. Instructions and procedure to execute SAS code for SFP discovery. [file 1471-2164-9-252-S1.zip › click-'n-snp/README - Click'N'SNP.pdf]

Click-'n-SNP Single Feature Polymorphism Instructions

This implementation takes as input an experimental design file and raw Affymetrix microarray data (.CEL files). A channel definition file specific to the Affymetrix microarray used (available for download from Affymetrix web site: http://www.affymetrix.com/support/technical/libraryfilesmain.affx) is also required.

The script (btmethod.sas) and supporting file (AffymetrixInputEngine.sas) are included in this file.

Requirements

The SAS software is required to run this method.

Method Execution

For the simplest operation, place the program code (.sas scripts), raw Affmetrix microarray data (.CEL files), annotation and experimental design files (SAS datasets) in the same directory. Then, change the following variables at the top of the btmethod.sas script:

1) datadir – this should be the directory chosen above. Trailing slash required.
2) ExpInFile – Change 'porcine.sas7bdat' to be your experimental design file (described below). Please, keep the filename to less than 20 characters.
3) CDFFile – Change 'Porcine.CDF' to be the appropriate array descriptor file (Available from Affymetrix).
4) Annot – name of the annotation dataset (described below)

Then, submit the job to SAS (On windows, right-click btmethod.sas and select 'Batch Submit with SAS'; at a Linux terminal, change to the datadir directory, and execute 'sas –nodms btmethod.sas').

Dataset Descriptions (required input)

Experimental design file - this should be a SAS dataset, with at least the following columns:

1) Array – Number of each array, starting at 1.
2) Breed – Which breed or line each array represents.
3) File – Filename of each array.
4) ColumnName – A unique name for each sample.

Example experimental design file (example_experimental_design.sas7bdat):

Array	breed	File	ColumnName
1	meishan-placenta	EA03004_22695_Porcine_25MA.CEL	_25MA
2	meishan-placenta	EA03004_22696_Porcine_25MB.CEL	_25MB
3	meishan-placenta	EA03004_22697_Porcine_25MC.CEL	_25MC
4	wc-fibroblast	FC1_22148.CEL	FC1
5	wc-fibroblast	FC2-1_22149.CEL	FC2
6	wc-fibroblast	FC3_22152.CEL	FC3

Array Annotation (required input)

This should be a SAS dataset, keyed by a column named Probe_Set_ID. All columns from this dataset will be merged into the results. Alternatively, it is possible to use a comma-separated variable file as an annotation – an example of using proc import to read the file is commented out.

Output Dataset Description

1) results2 – contains results from the mixed model, with FDR calculations (but not annotation information) included.
2) results3 – contains results merged with annotation data
3) snps – contains annotated results with q<.05 and greater than twofold expression difference. A comma-separated version of this dataset is also generated.
