# Supplementary material for: Identification of SNPs and INDELS in swine transcribed sequences using short oligonucleotide microarrays
Source: BMC Genomics. 2008 May 29;9:252. doi: 10.1186/1471-2164-9-252 (PMC2442091; doi:10.1186/1471-2164-9-252)
Supplement: Additional File 2 — Porcine_Candidate_SFPs_NoFC. Intensity plots of SFPs with q < 0.5, no fold change threshold. [file Porcine_Candidate_SFPs_NoFC.pdf › Porcine_Candidate_SFPs_NoFC.pdf]

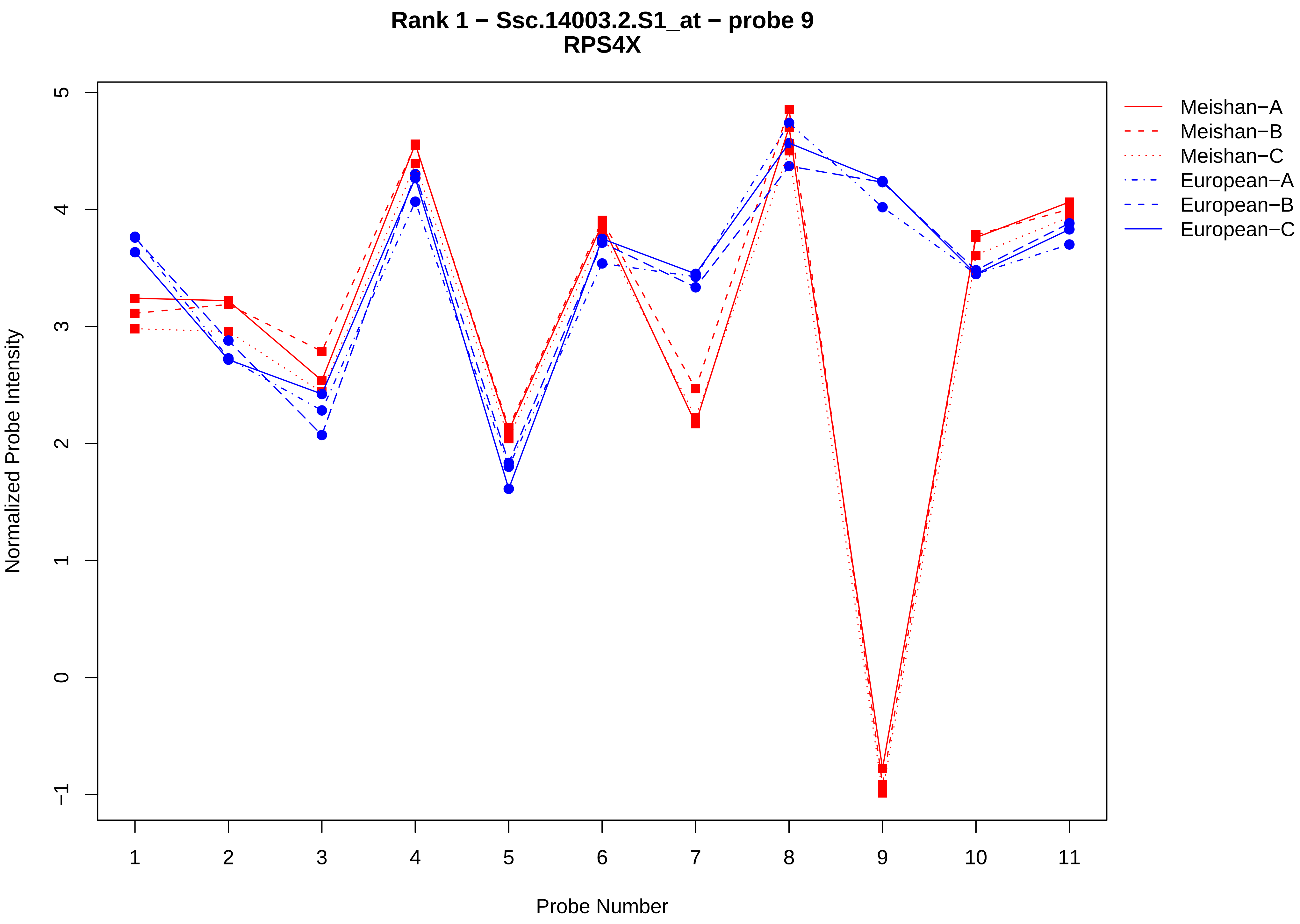
Rank 1 − Ssc.14003.2.S1_at − probe 9
RPS4X
Normalized Probe Intensity
Probe Number
Meishan−A
Meishan−B
Meishan−C
European−A
European−B
European−C
−1
0
1
2
3
4
5
1
2
3
4
5
6
7
8
9
10
11

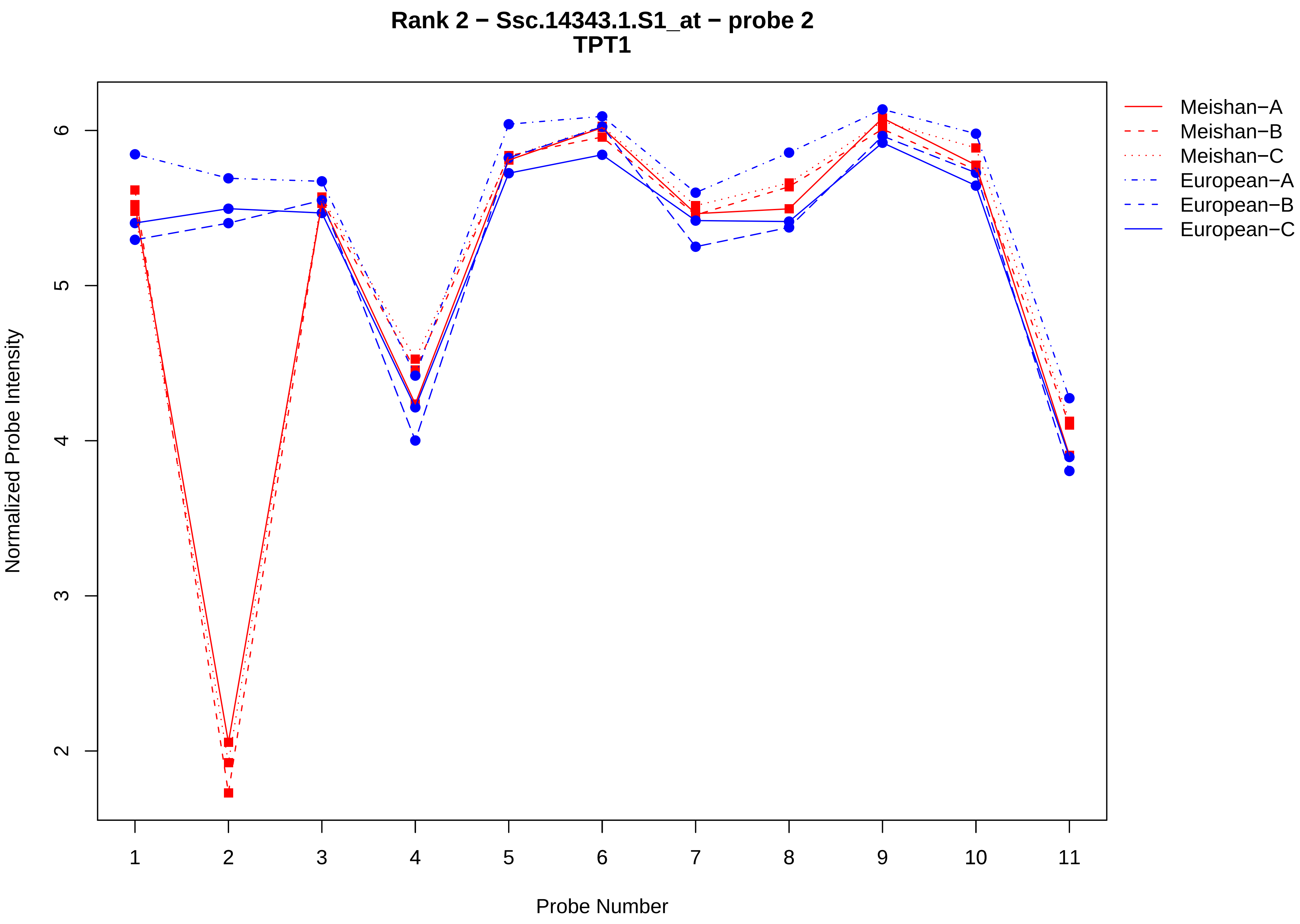
Rank 2 − Ssc.14343.1.S1_at − probe 2
TPT1
Normalized Probe Intensity
Probe Number
2
3
4
5
6
1
2
3
4
5
6
7
8
9
10
11
Meishan−A
Meishan−B
Meishan−C
European−A
European−B
European−C

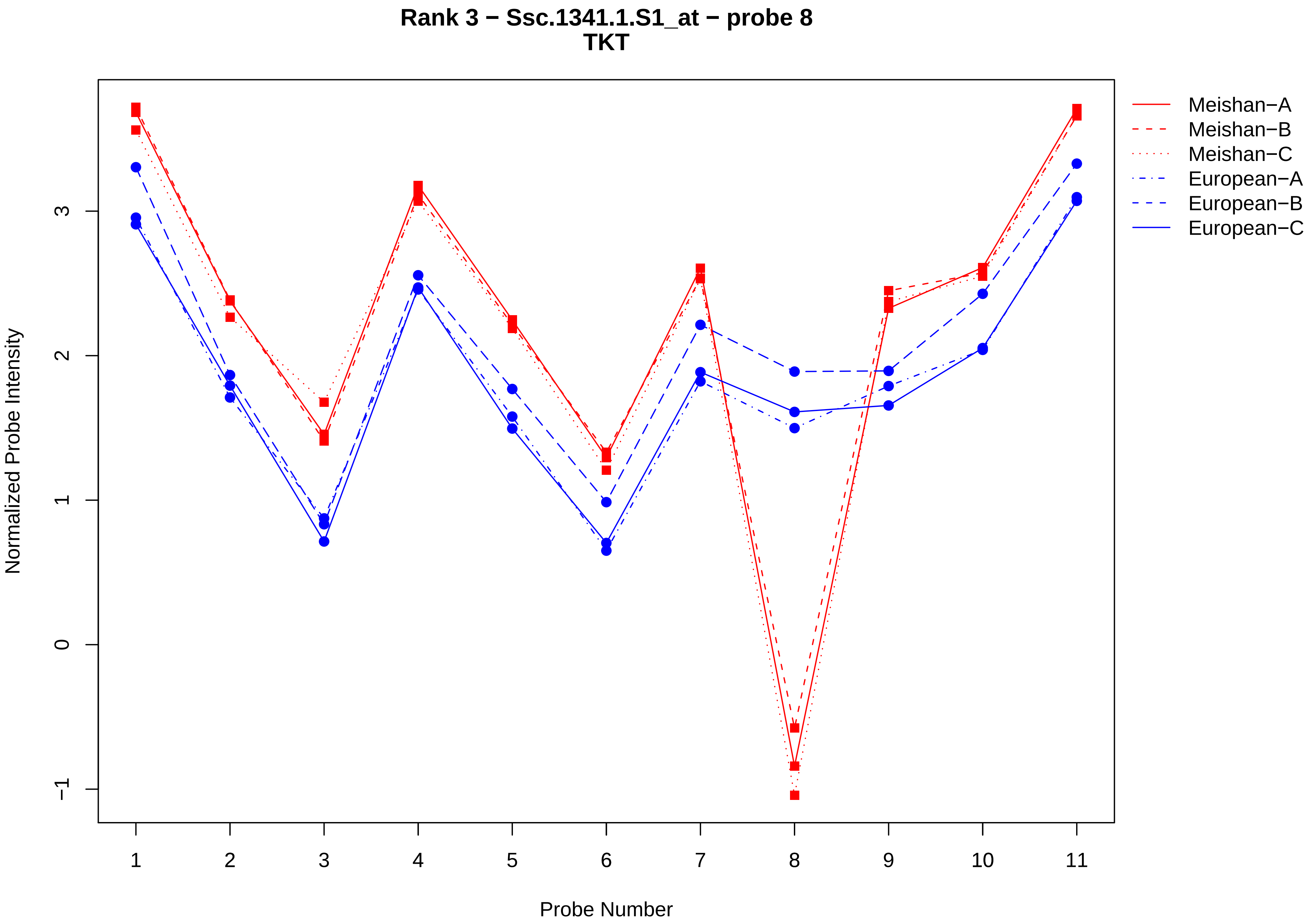

Rank 3 − Ssc.1341.1.S1_at − probe 8
TKT
Normalized Probe Intensity
Probe Number
−1
0
1
2
3
1
2
3
4
5
6
7
8
9
10
11
Meishan−A
Meishan−B
Meishan−C
European−A
European−B
European−C

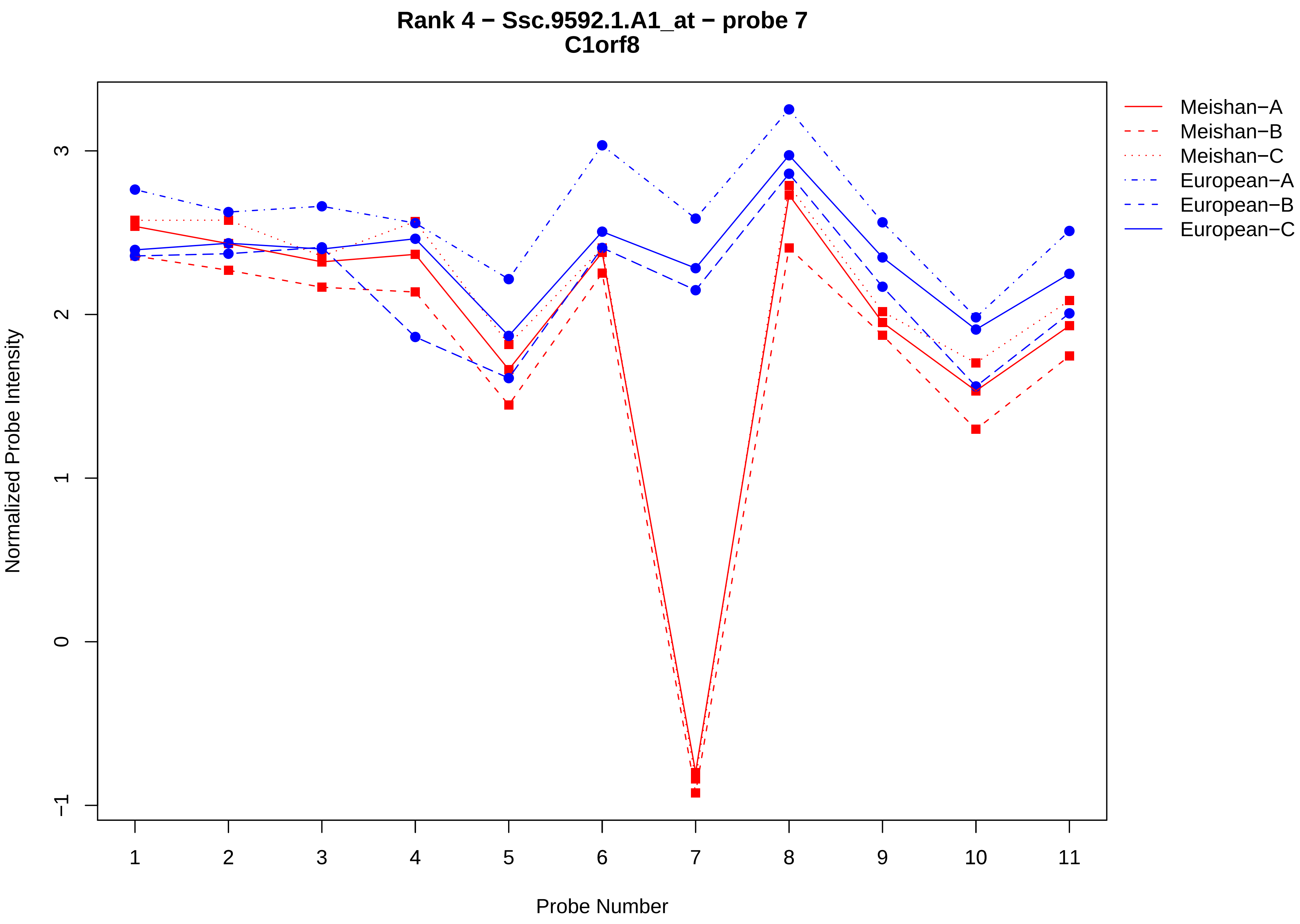
Rank 4 − Ssc.9592.1.A1_at − probe 7
C1orf8
Normalized Probe Intensity
Probe Number
Meishan−A
Meishan−B
Meishan−C
European−A
European−B
European−C
−1
0
1
2
3
1
2
3
4
5
6
7
8
9
10
11

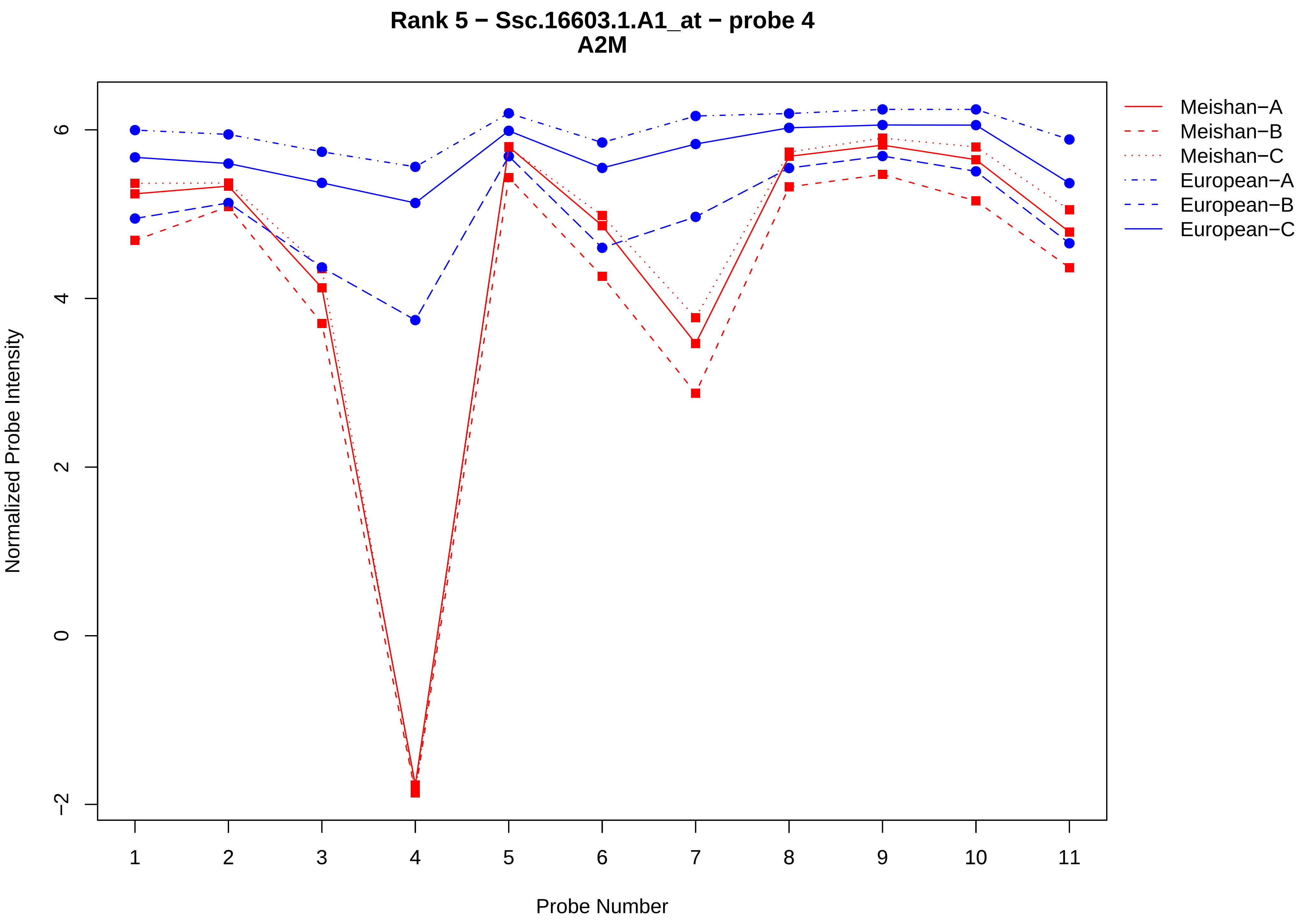
Rank 5 − Ssc.16603.1.A1_at − probe 4
A2M
Normalized Probe Intensity
−2
0
2
4
6
Probe Number
1
2
3
4
5
6
7
8
9
10
11
Meishan−A
Meishan−B
Meishan−C
European−A
European−B
European−C

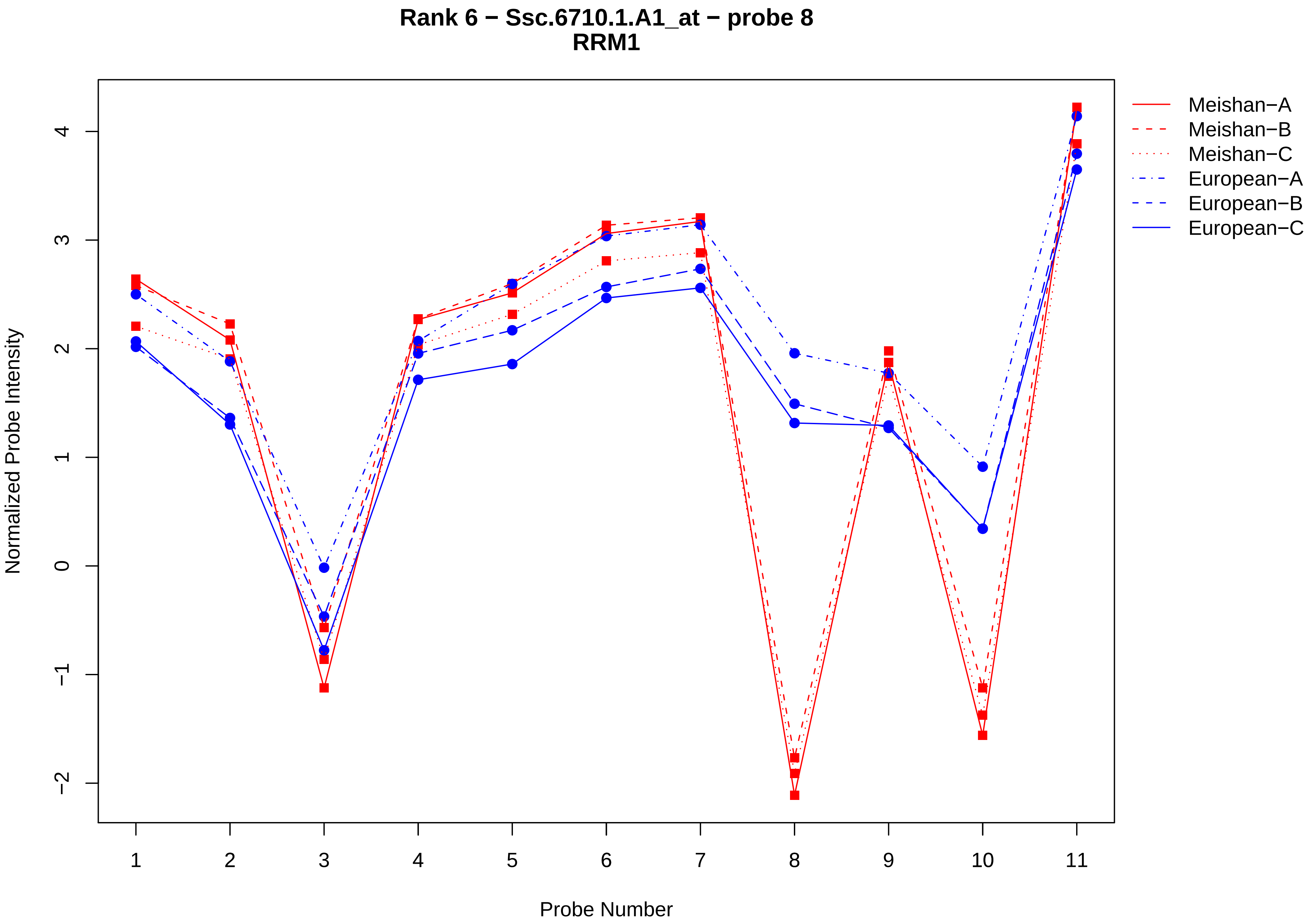
Rank 6 − Ssc.6710.1.A1_at − probe 8
RRM1
Normalized Probe Intensity
Probe Number
−2
−1
0
1
2
3
4
1
2
3
4
5
6
7
8
9
10
11
Meishan−A
Meishan−B
Meishan−C
European−A
European−B
European−C

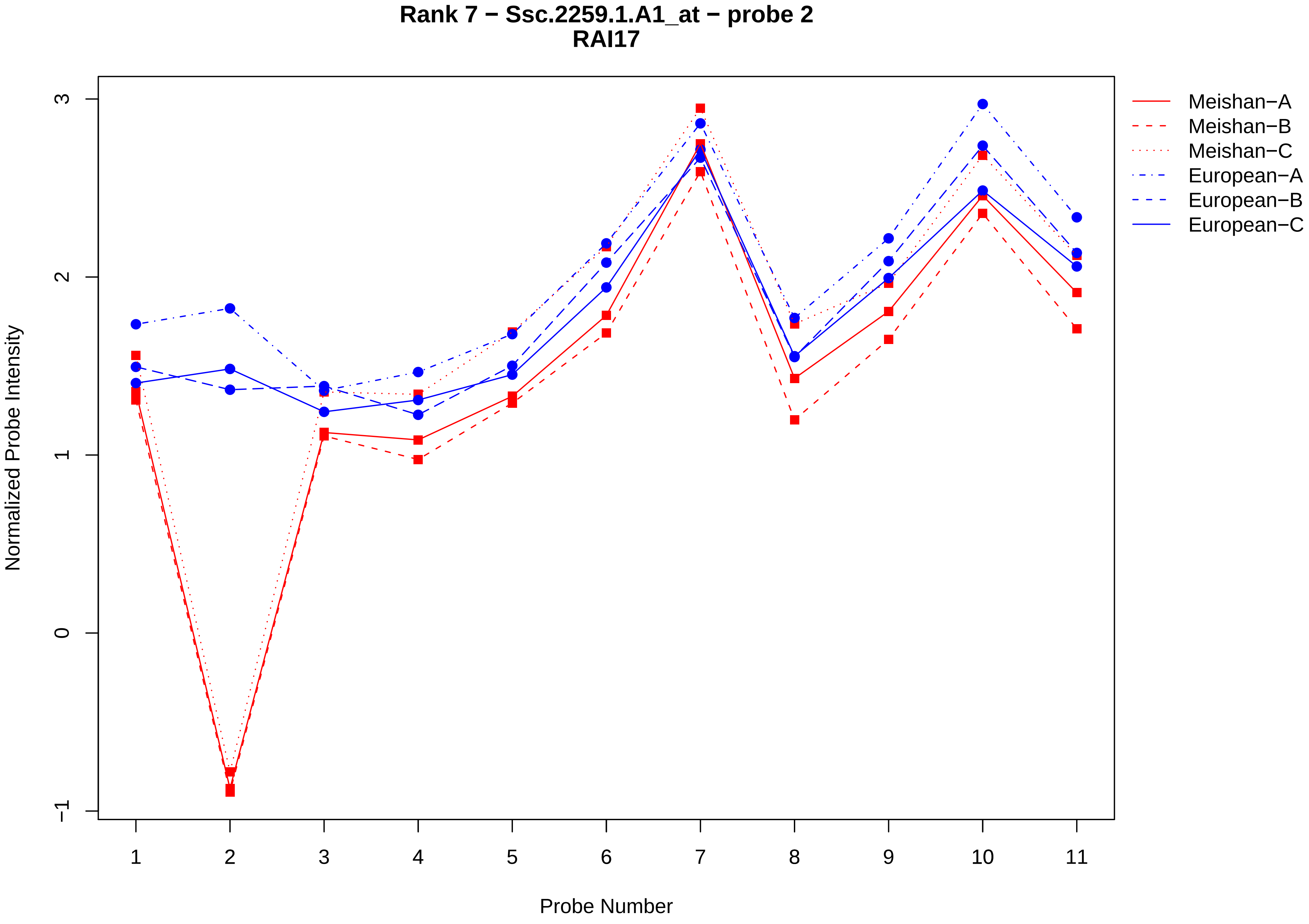

Rank 7 − Ssc.2259.1.A1_at − probe 2
RAI17
Normalized Probe Intensity
Probe Number
−1
0
1
2
3
1
2
3
4
5
6
7
8
9
10
11
Meishan−A
Meishan−B
Meishan−C
European−A
European−B
European−C

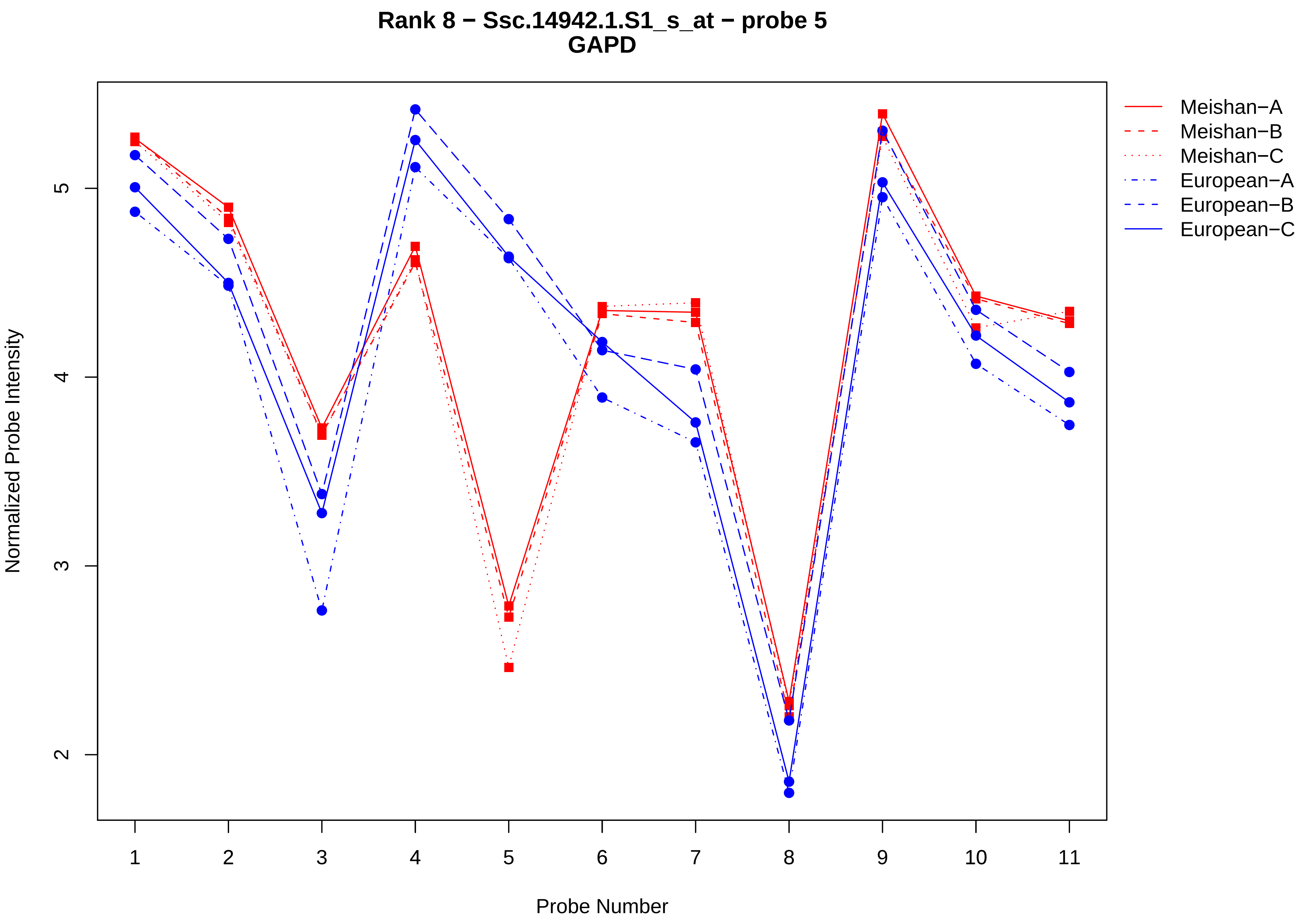

Rank 8 − Ssc.14942.1.S1_s_at − probe 5
GAPD
Normalized Probe Intensity
Probe Number
2
3
4
5
1
2
3
4
5
6
7
8
9
10
11
Meishan−A
Meishan−B
Meishan−C
European−A
European−B
European−C

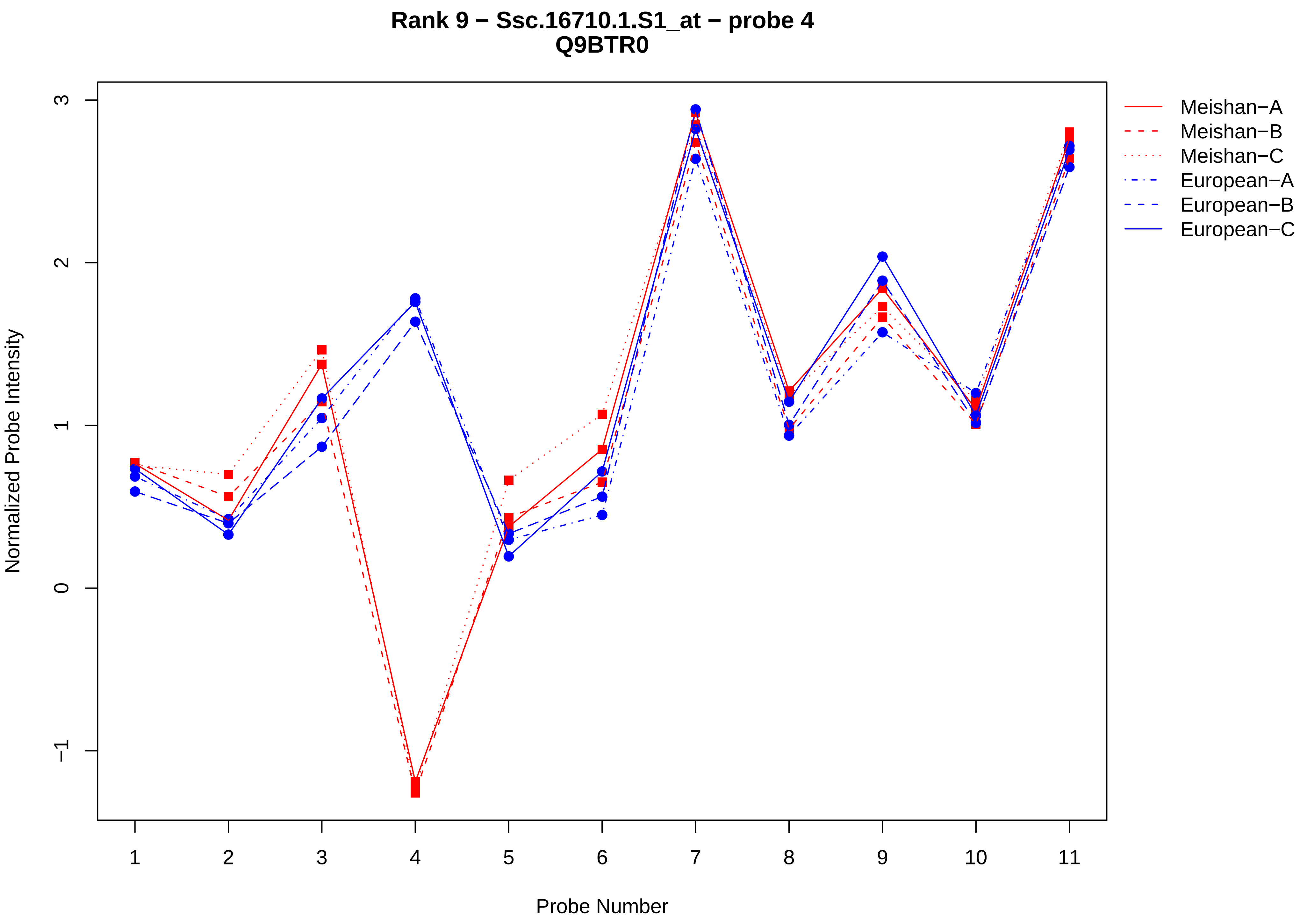
Rank 9 − Ssc.16710.1.S1_at − probe 4
Q9BTR0
Normalized Probe Intensity
Probe Number
Meishan−A
Meishan−B
Meishan−C
European−A
European−B
European−C
−1
0
1
2
3
1
2
3
4
5
6
7
8
9
10
11

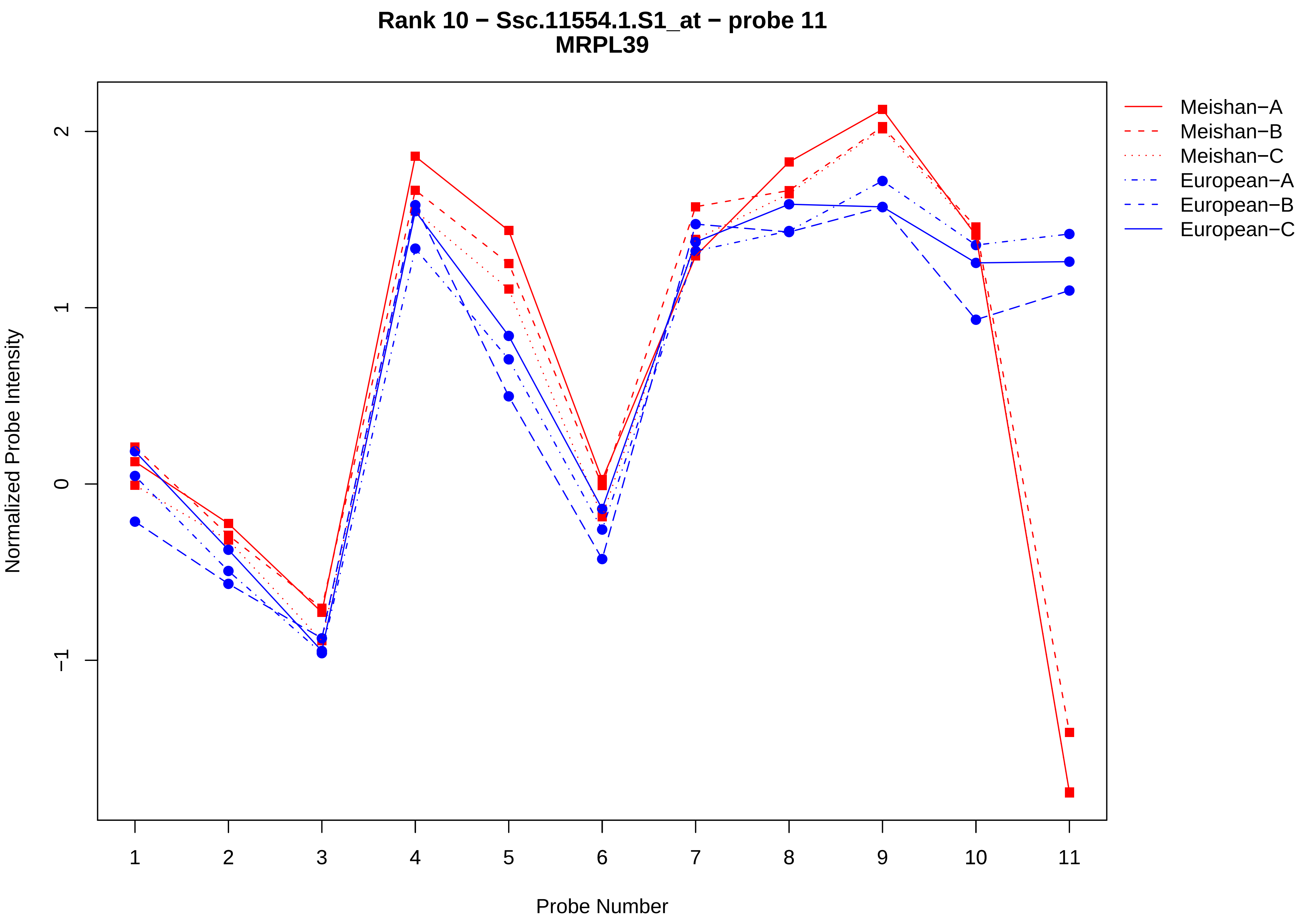

Rank 10 − Ssc.11554.1.S1_at − probe 11
MRPL39
Normalized Probe Intensity
Probe Number
−1
0
1
2
1
2
3
4
5
6
7
8
9
10
11
Meishan−A
Meishan−B
Meishan−C
European−A
European−B
European−C

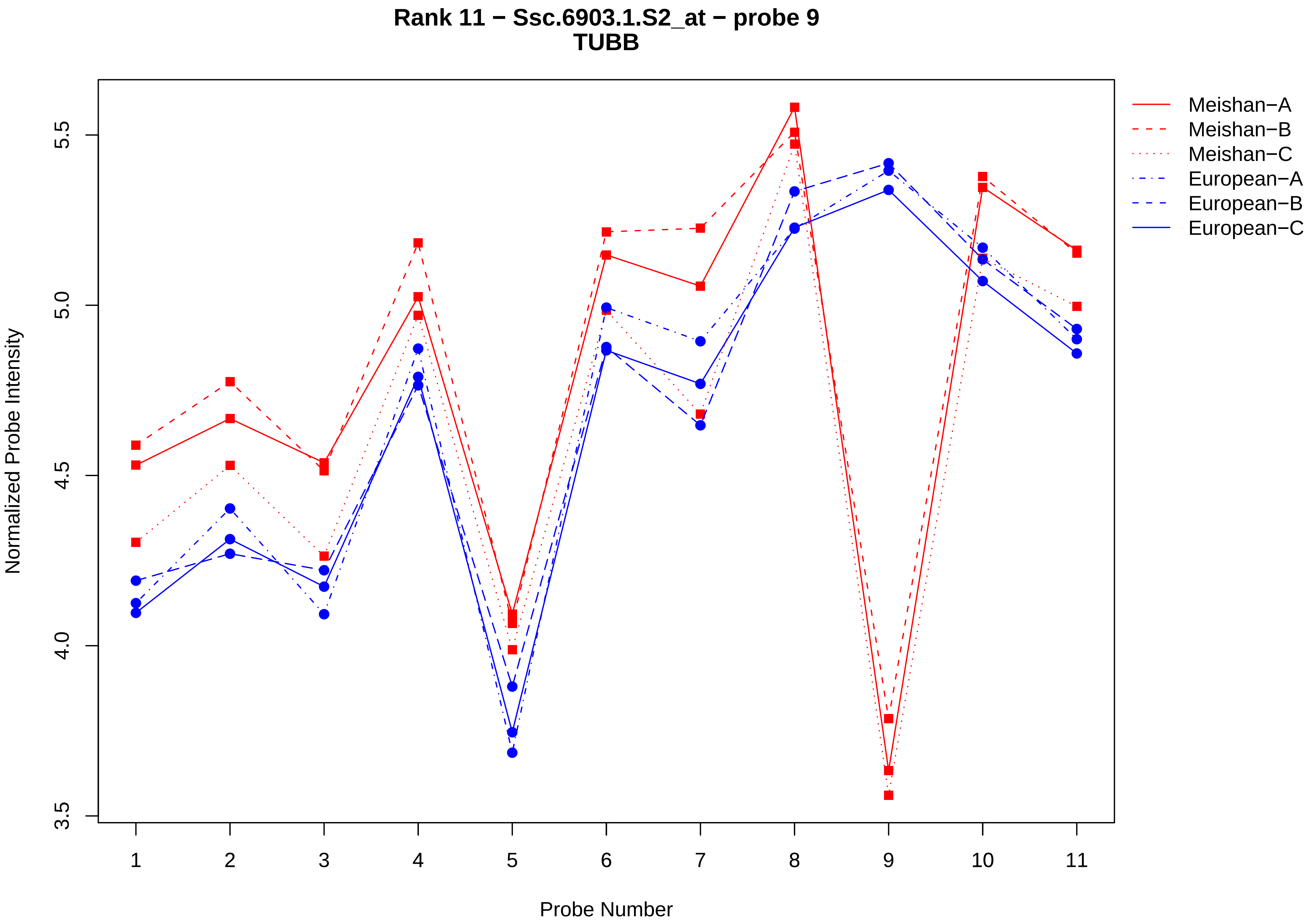

Rank 11 − Ssc.6903.1.S2_at − probe 9
TUBB
Normalized Probe Intensity
3.5
4.0
4.5
5.0
5.5
Probe Number
1
2
3
4
5
6
7
8
9
10
11
Meishan−A
Meishan−B
Meishan−C
European−A
European−B
European−C

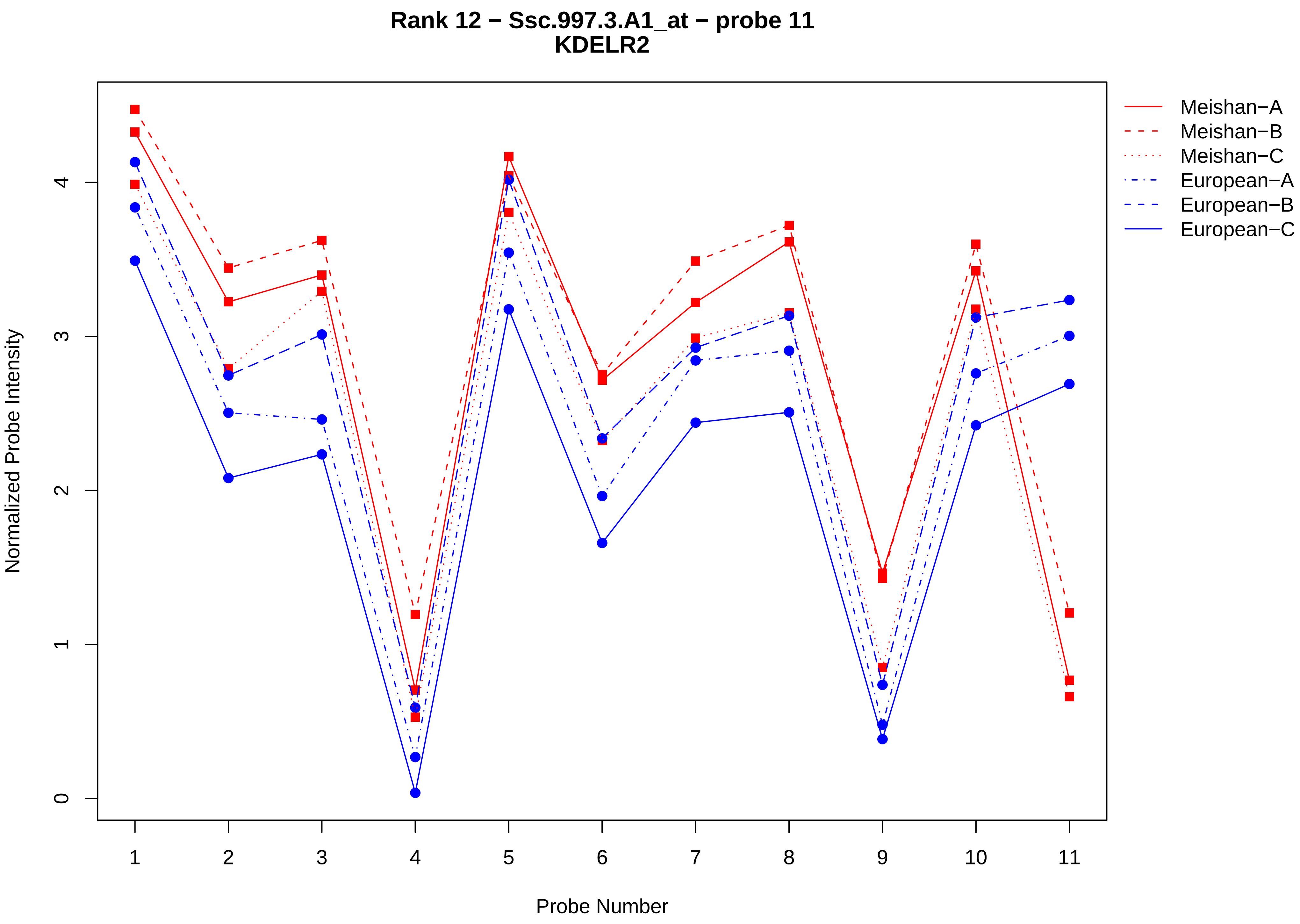
Rank 12 − Ssc.997.3.A1_at − probe 11
KDELR2
Normalized Probe Intensity
0
1
2
3
4
1
2
3
4
5
6
7
8
9
10
11
Probe Number
Meishan−A
Meishan−B
Meishan−C
European−A
European−B
European−C

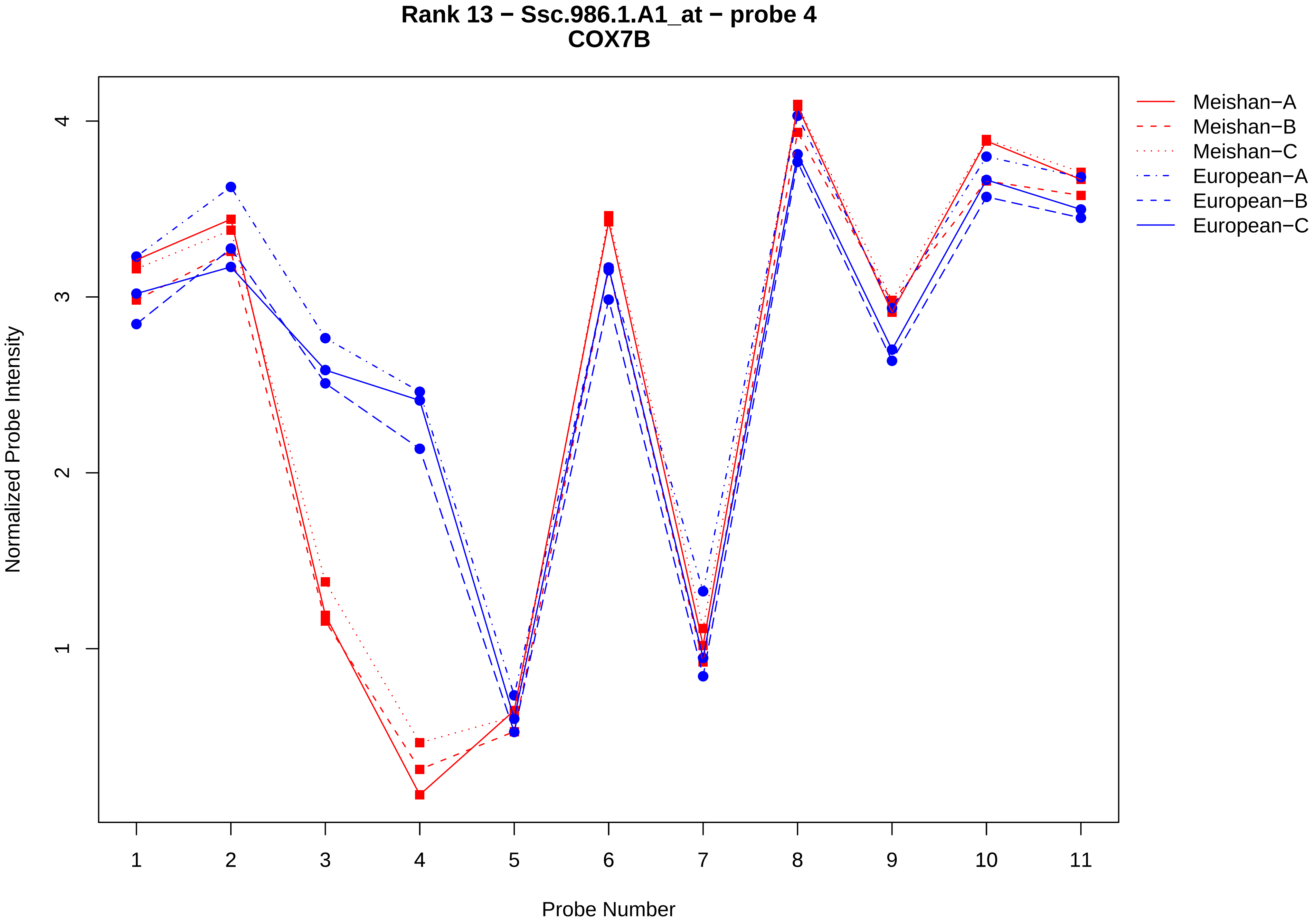
Rank 13 − Ssc.986.1.A1_at − probe 4
COX7B
Normalized Probe Intensity
Probe Number
1
2
3
4
1
2
3
4
5
6
7
8
9
10
11
Meishan−A
Meishan−B
Meishan−C
European−A
European−B
European−C

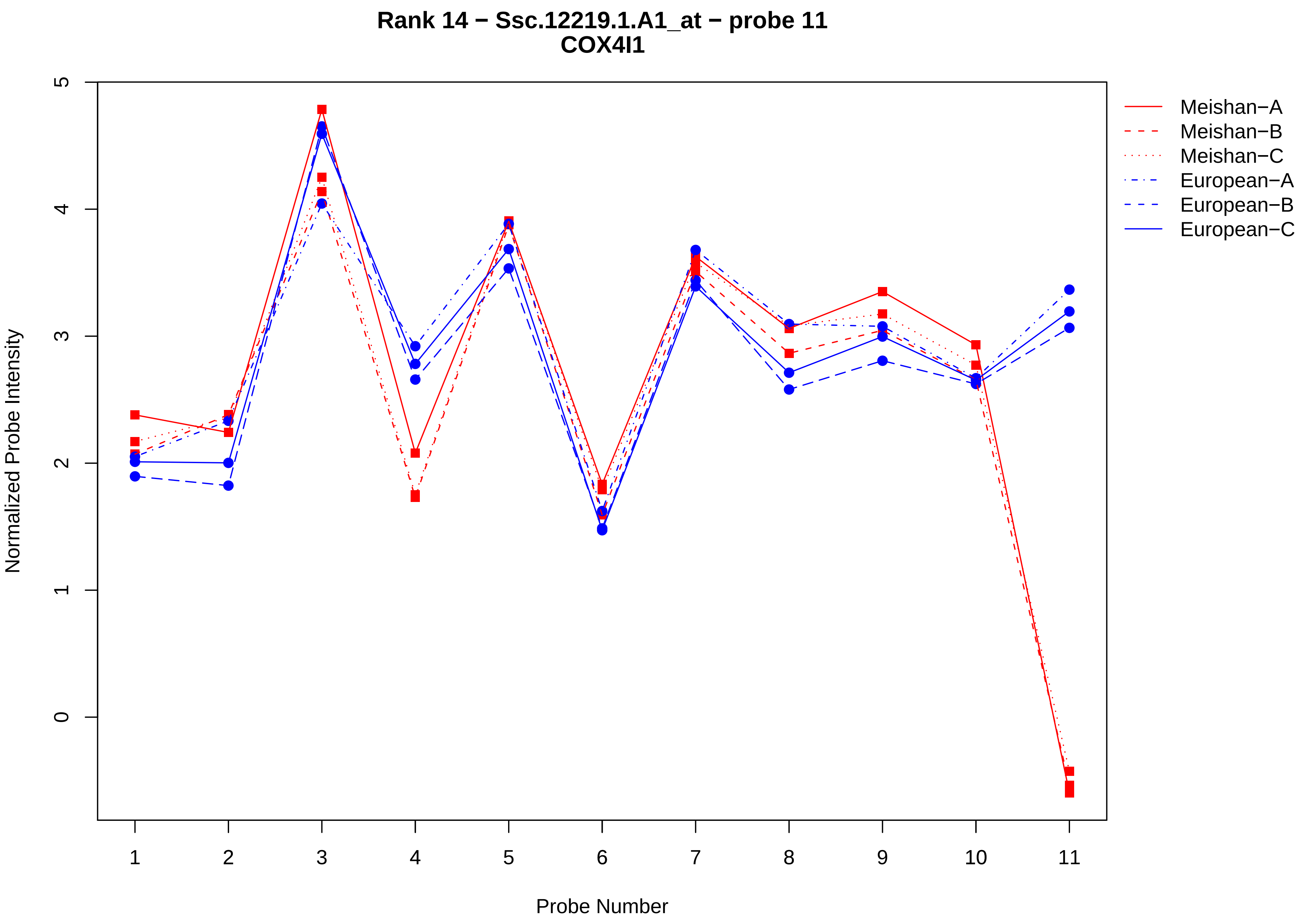
Rank 14 − Ssc.12219.1.A1_at − probe 11
COX4I1
Normalized Probe Intensity
Probe Number
0
1
2
3
4
5
1
2
3
4
5
6
7
8
9
10
11
Meishan−A
Meishan−B
Meishan−C
European−A
European−B
European−C

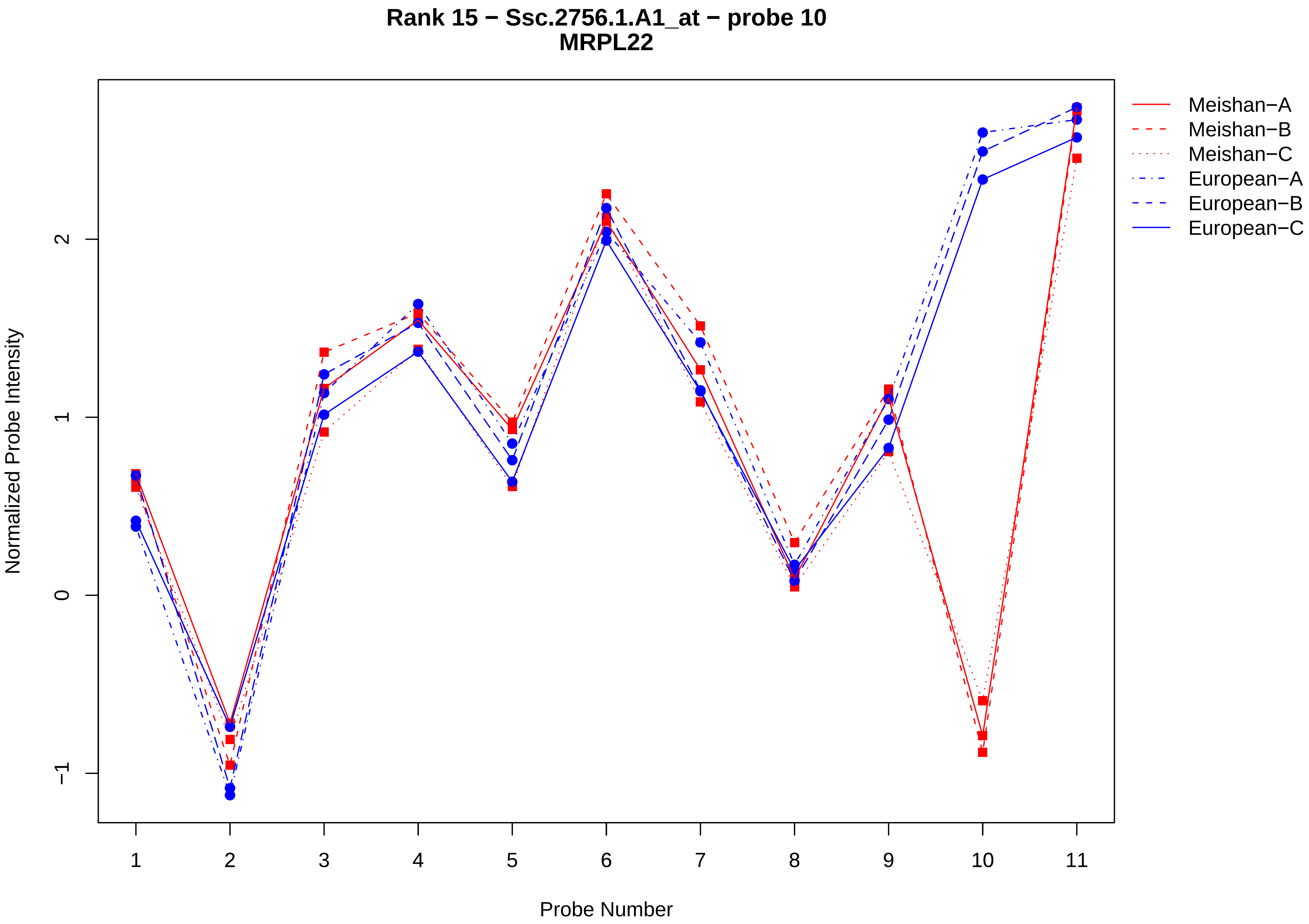

Rank 15 − Ssc.2756.1.A1_at − probe 10
MRPL22
Normalized Probe Intensity
−1
0
1
2
1
2
3
4
5
6
7
8
9
10
11
Probe Number
Meishan−A
Meishan−B
Meishan−C
European−A
European−B
European−C

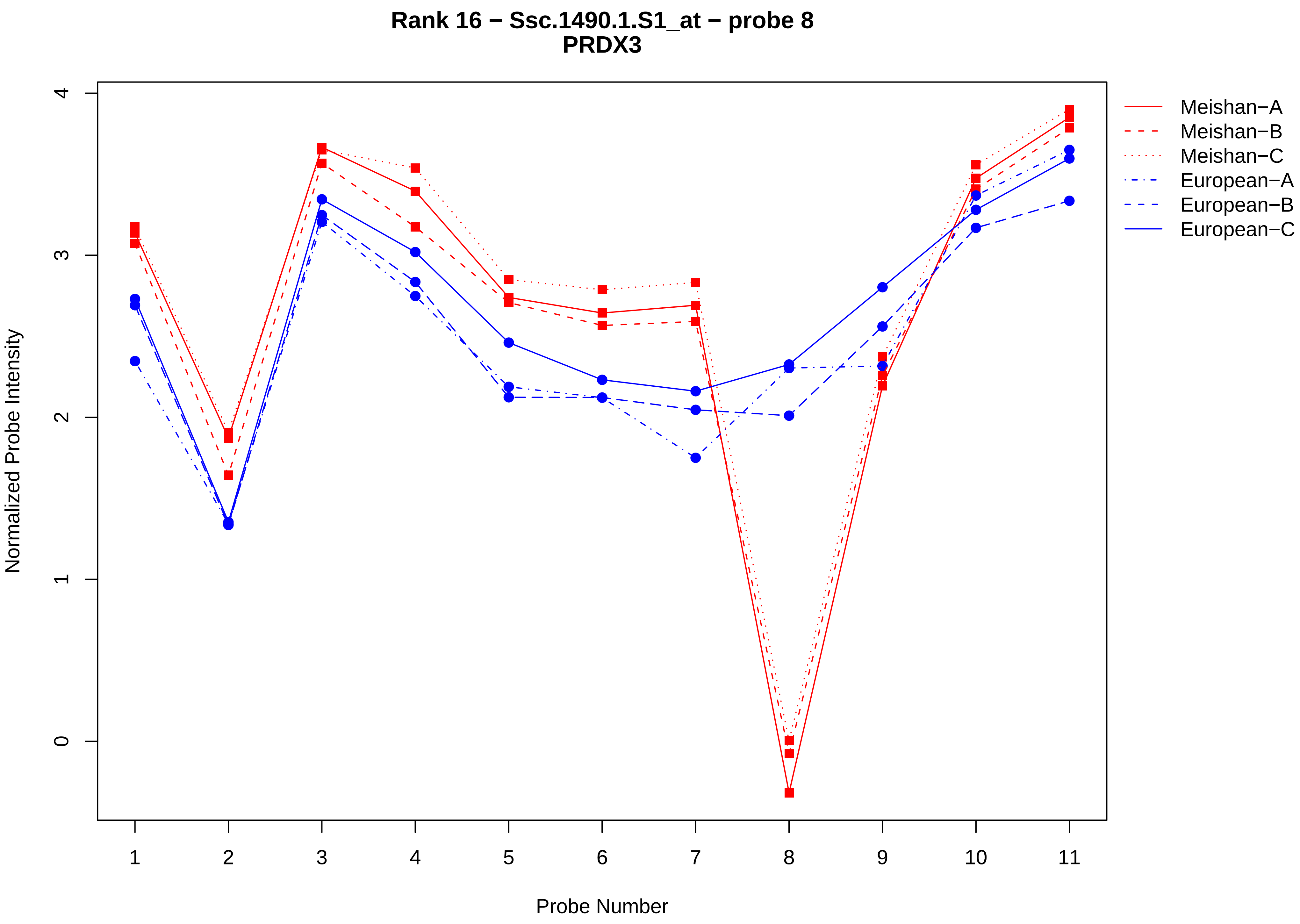
Rank 16 − Ssc.1490.1.S1_at − probe 8
PRDX3
Normalized Probe Intensity
Probe Number
0
1
2
3
4
1
2
3
4
5
6
7
8
9
10
11
Meishan−A
Meishan−B
Meishan−C
European−A
European−B
European−C

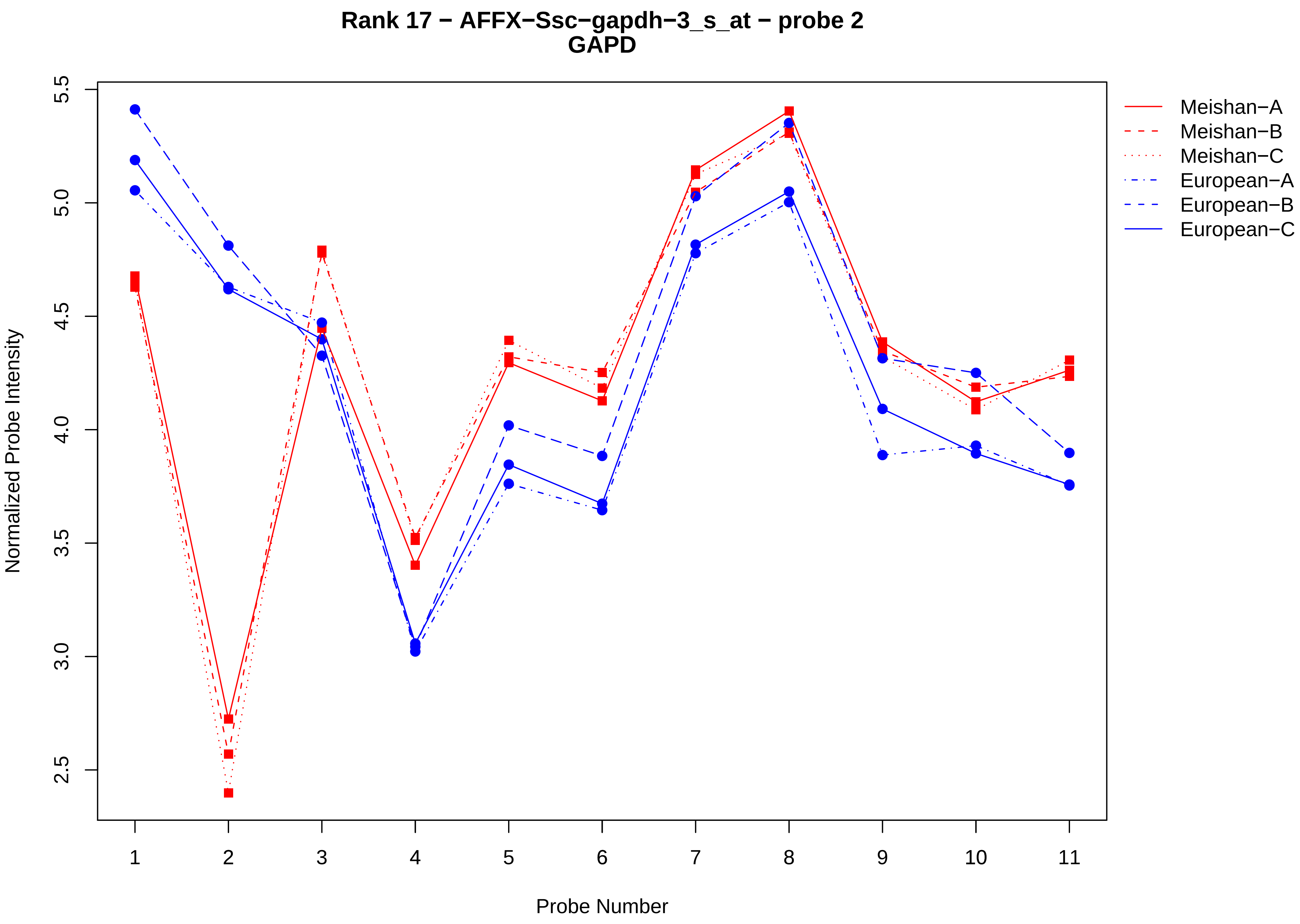

Rank 17 − AFFX−Ssc−gapdh−3_s_at − probe 2
GAPD
Normalized Probe Intensity
Probe Number
2.5
3.0
3.5
4.0
4.5
5.0
5.5
1
2
3
4
5
6
7
8
9
10
11
Meishan−A
Meishan−B
Meishan−C
European−A
European−B
European−C

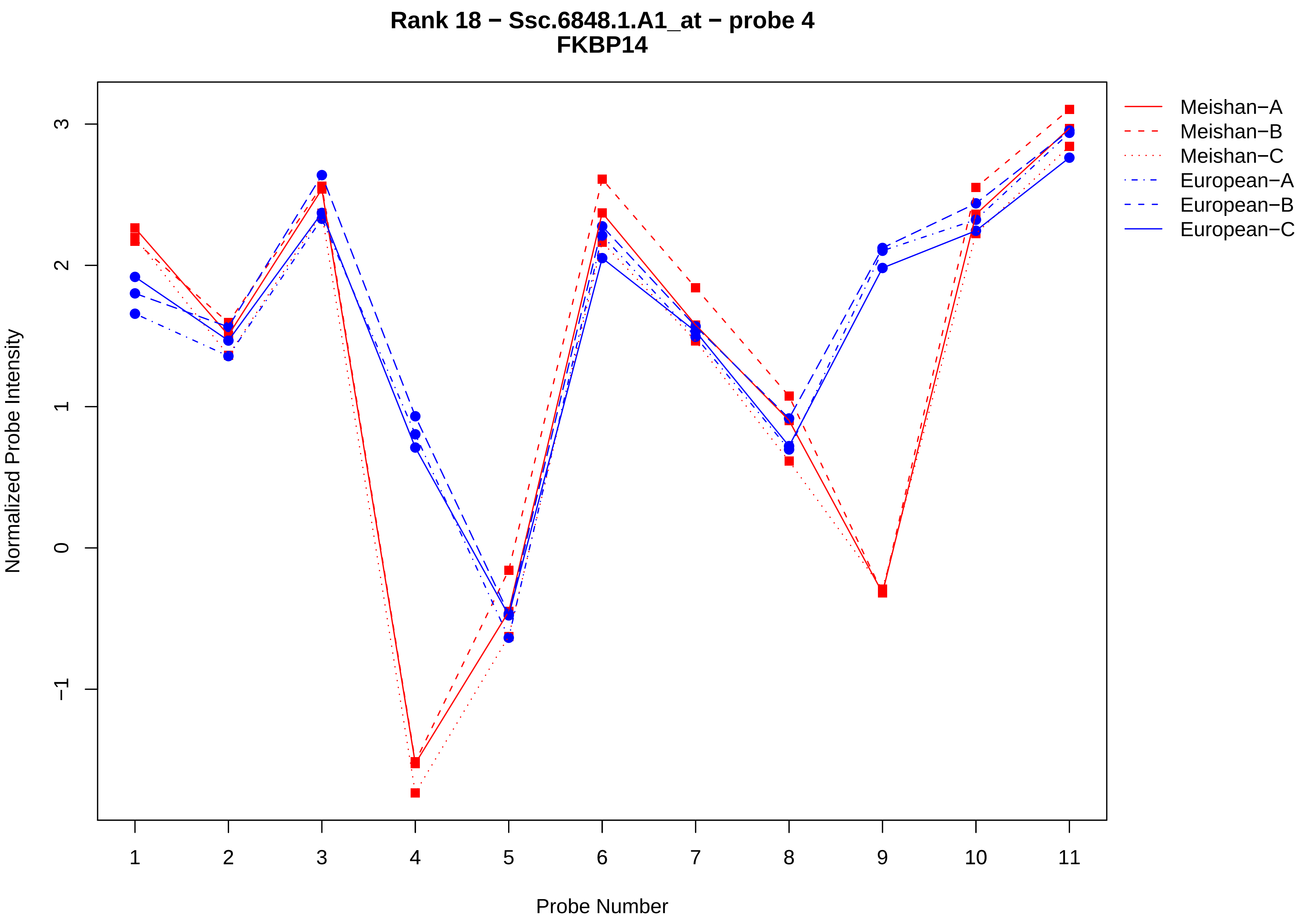

Rank 18 − Ssc.6848.1.A1_at − probe 4
FKBP14
Normalized Probe Intensity
Probe Number
Meishan−A
Meishan−B
Meishan−C
European−A
European−B
European−C
−1
0
1
2
3
1
2
3
4
5
6
7
8
9
10
11

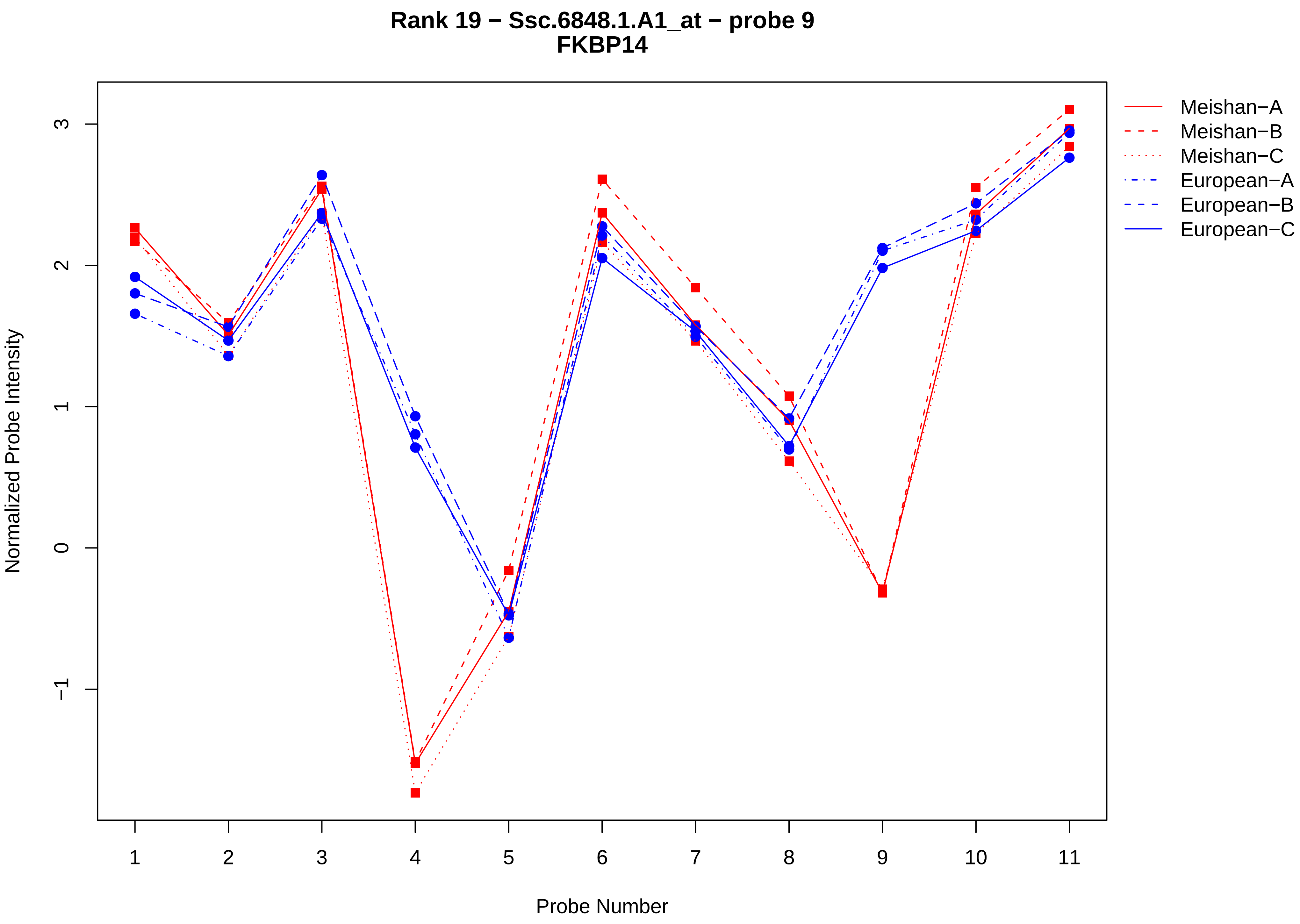
Rank 19 − Ssc.6848.1.A1_at − probe 9
FKBP14
Normalized Probe Intensity
Probe Number
−1
0
1
2
3
1
2
3
4
5
6
7
8
9
10
11
Meishan−A
Meishan−B
Meishan−C
European−A
European−B
European−C

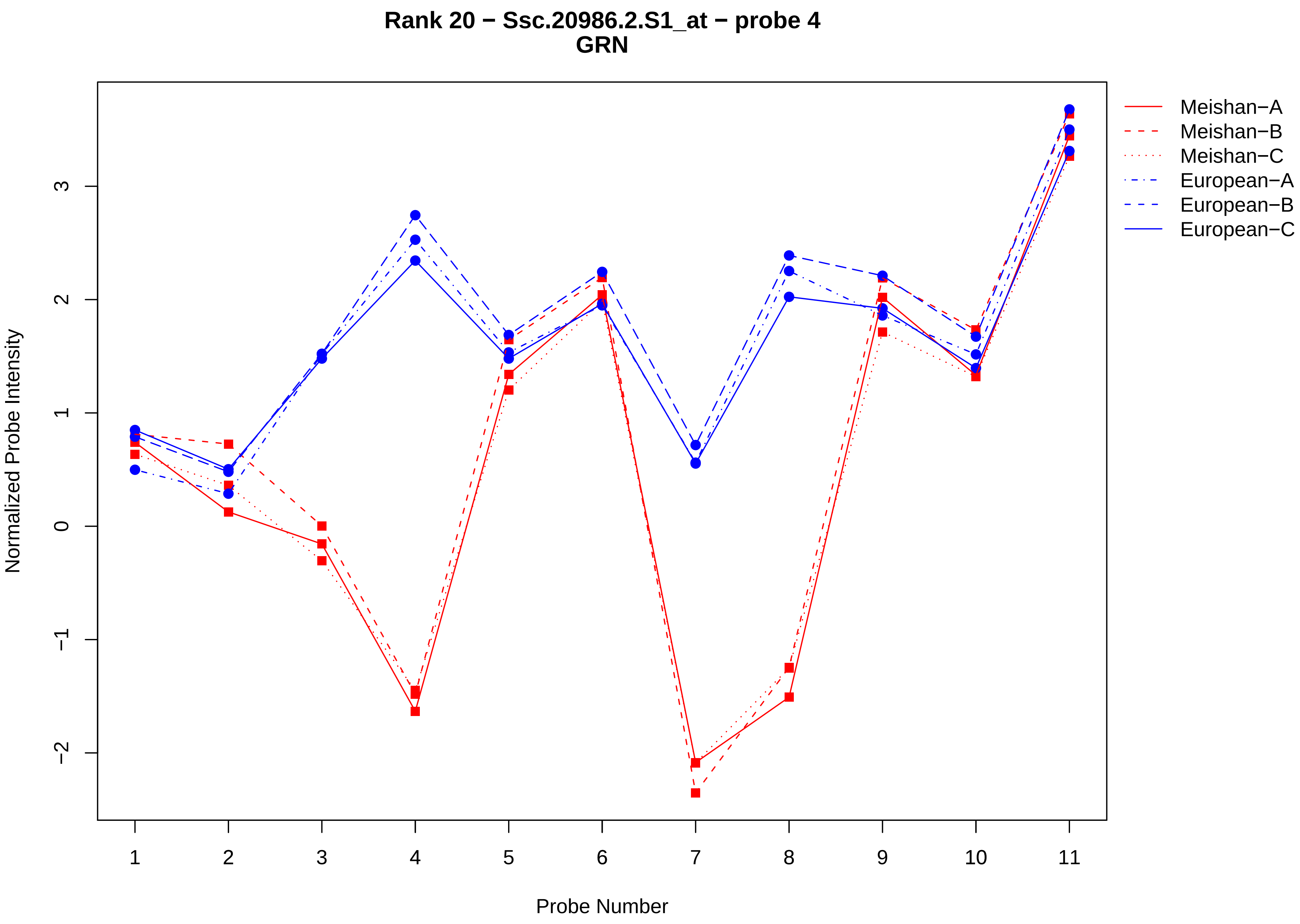
Rank 20 − Ssc.20986.2.S1_at − probe 4
GRN
Normalized Probe Intensity
Probe Number
−2
−1
0
1
2
3
1
2
3
4
5
6
7
8
9
10
11
Meishan−A
Meishan−B
Meishan−C
European−A
European−B
European−C

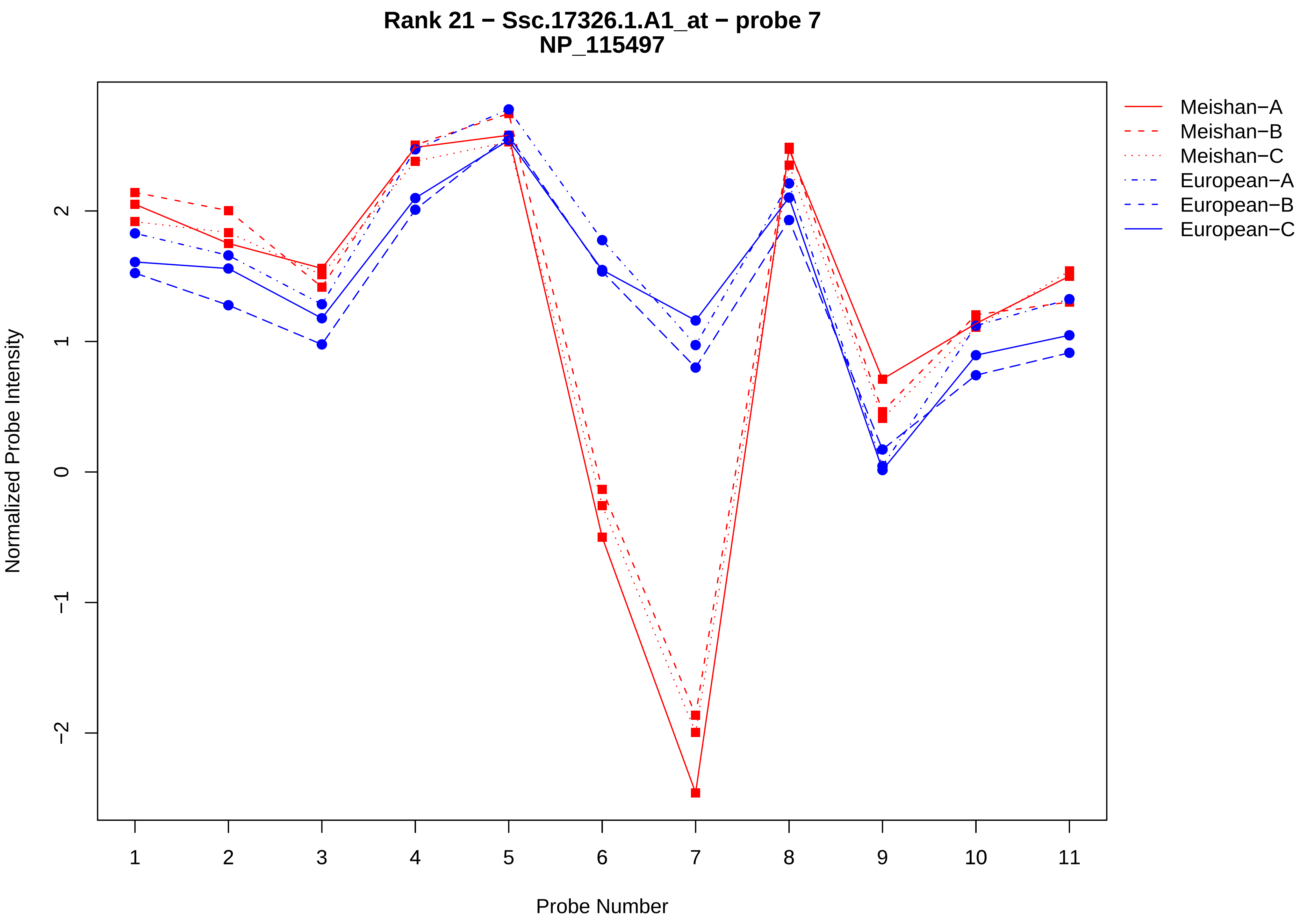
Rank 21 − Ssc.17326.1.A1_at − probe 7
NP_115497
Normalized Probe Intensity
Probe Number
−2
−1
0
1
2
1
2
3
4
5
6
7
8
9
10
11
Meishan−A
Meishan−B
Meishan−C
European−A
European−B
European−C

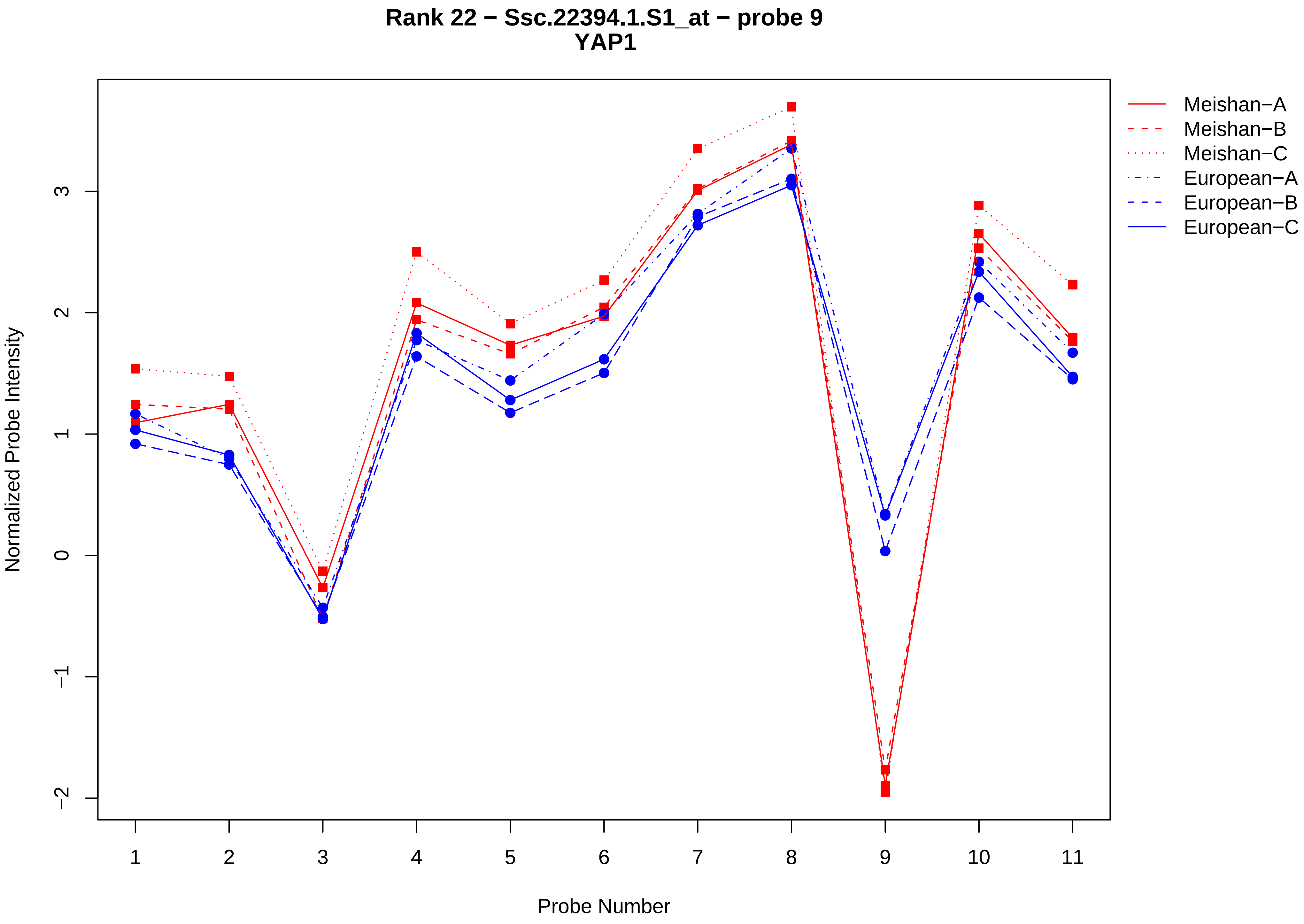

Rank 22 − Ssc.22394.1.S1_at − probe 9
YAP1
Normalized Probe Intensity
Probe Number
−2
−1
0
1
2
3
1
2
3
4
5
6
7
8
9
10
11
Meishan−A
Meishan−B
Meishan−C
European−A
European−B
European−C

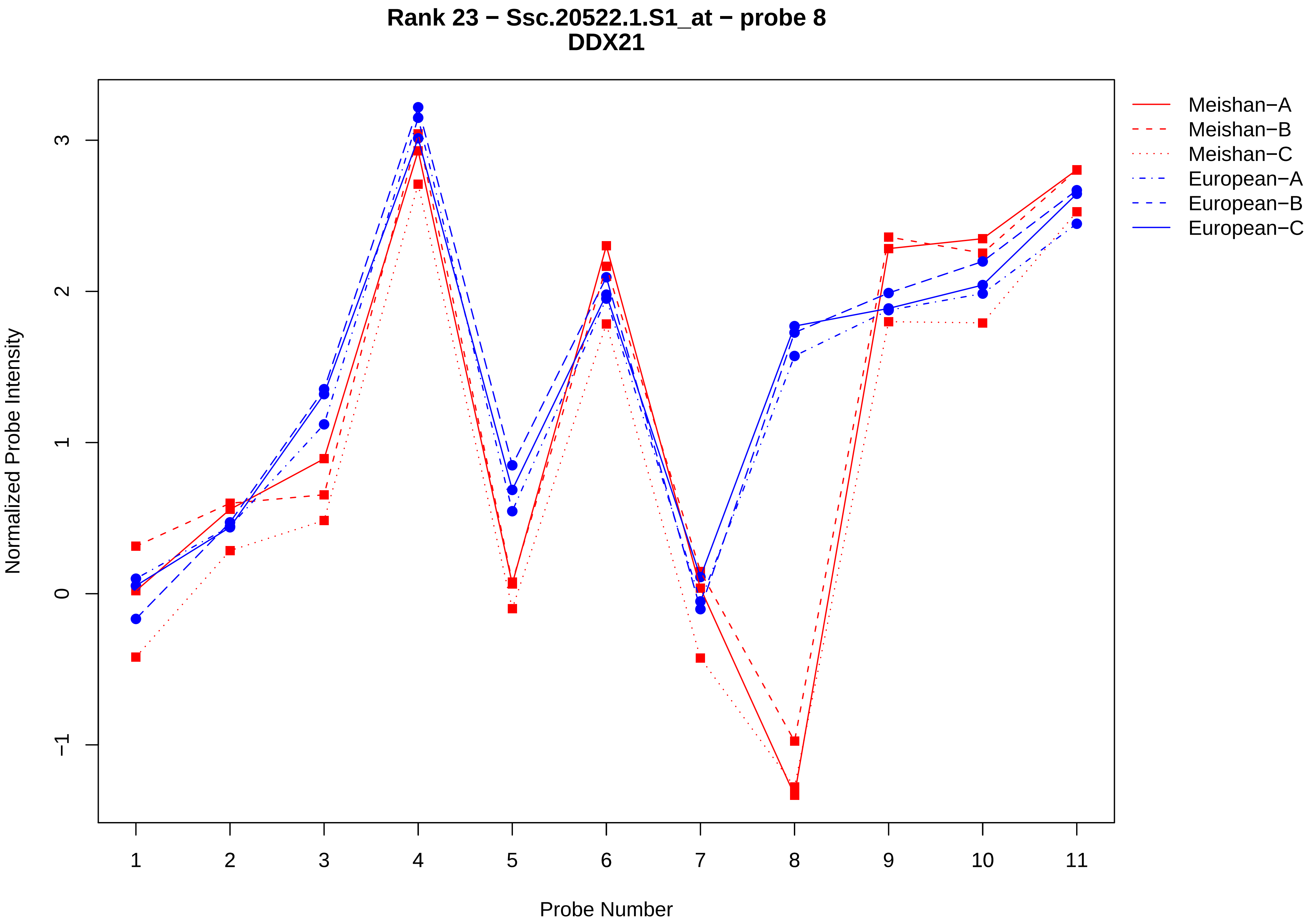
Rank 23 − Ssc.20522.1.S1_at − probe 8
DDX21
Normalized Probe Intensity
Probe Number
−1
0
1
2
3
1
2
3
4
5
6
7
8
9
10
11
Meishan−A
Meishan−B
Meishan−C
European−A
European−B
European−C

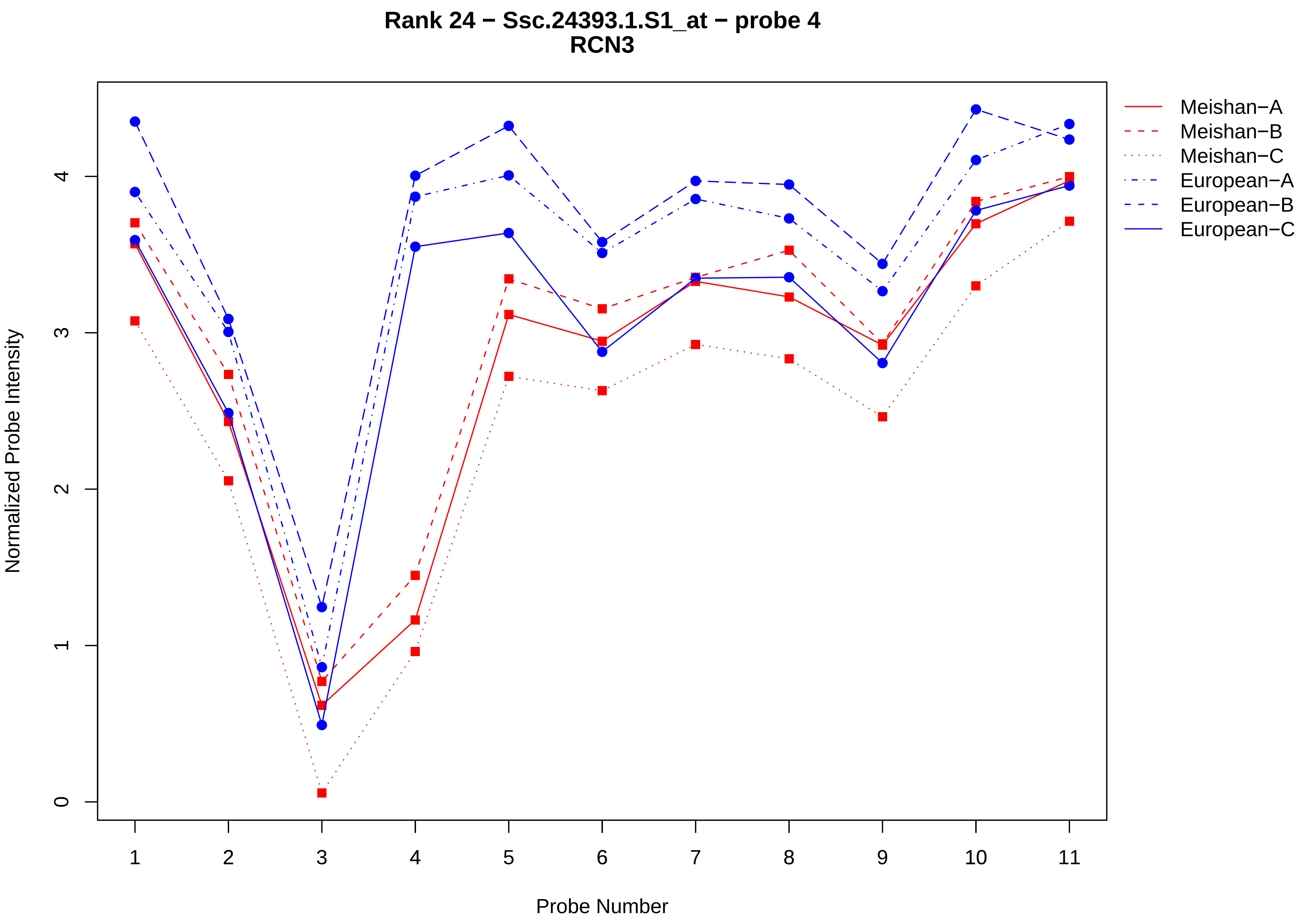
Rank 24 − Ssc.24393.1.S1_at − probe 4
RCN3
Normalized Probe Intensity
0
1
2
3
4
Probe Number
1
2
3
4
5
6
7
8
9
10
11
Meishan−A
Meishan−B
Meishan−C
European−A
European−B
European−C

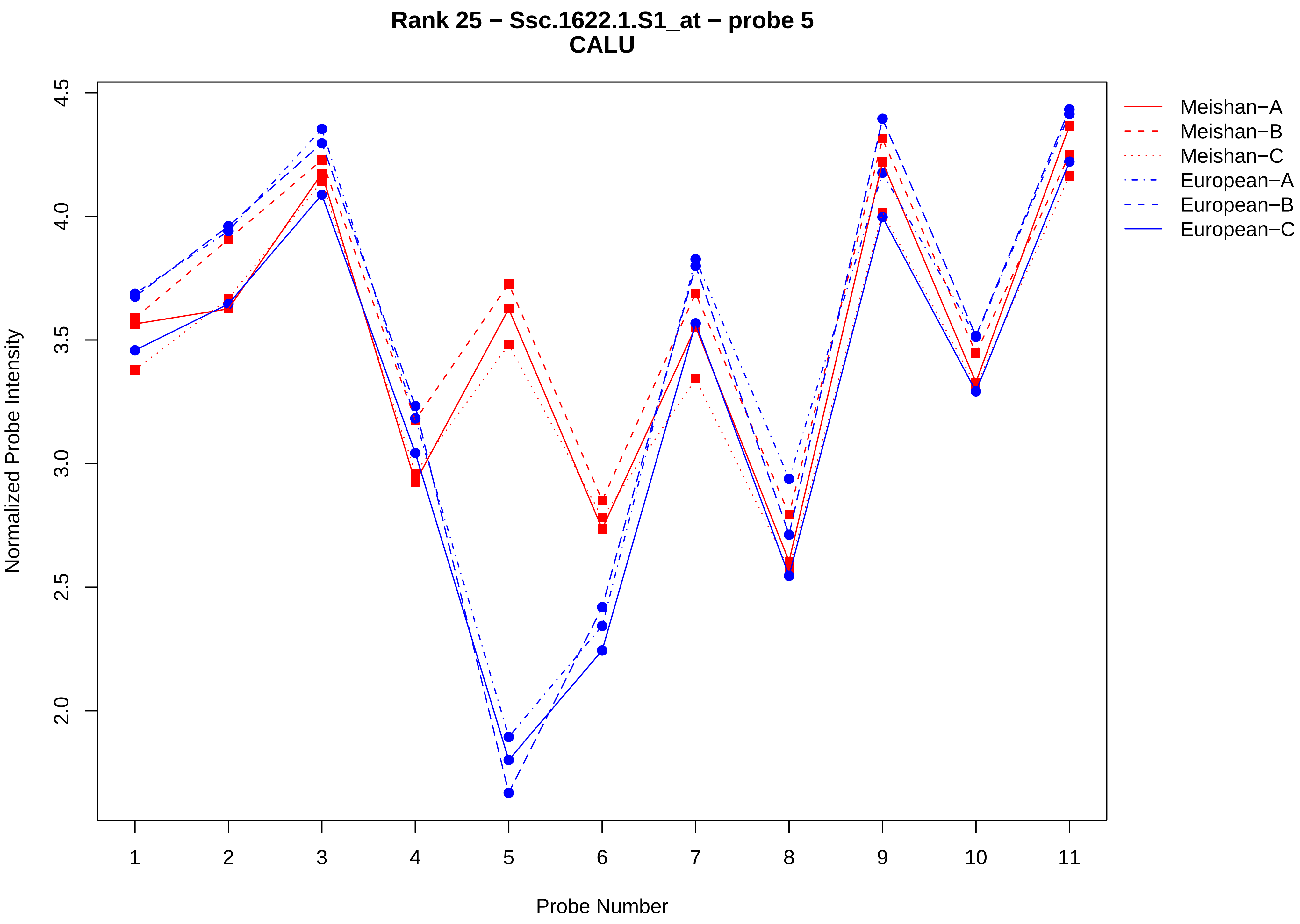
Rank 25 − Ssc.1622.1.S1_at − probe 5
CALU
Normalized Probe Intensity
Probe Number
Meishan−A
Meishan−B
Meishan−C
European−A
European−B
European−C
4.5
4.0
3.5
3.0
2.5
2.0
1
2
3
4
5
6
7
8
9
10
11

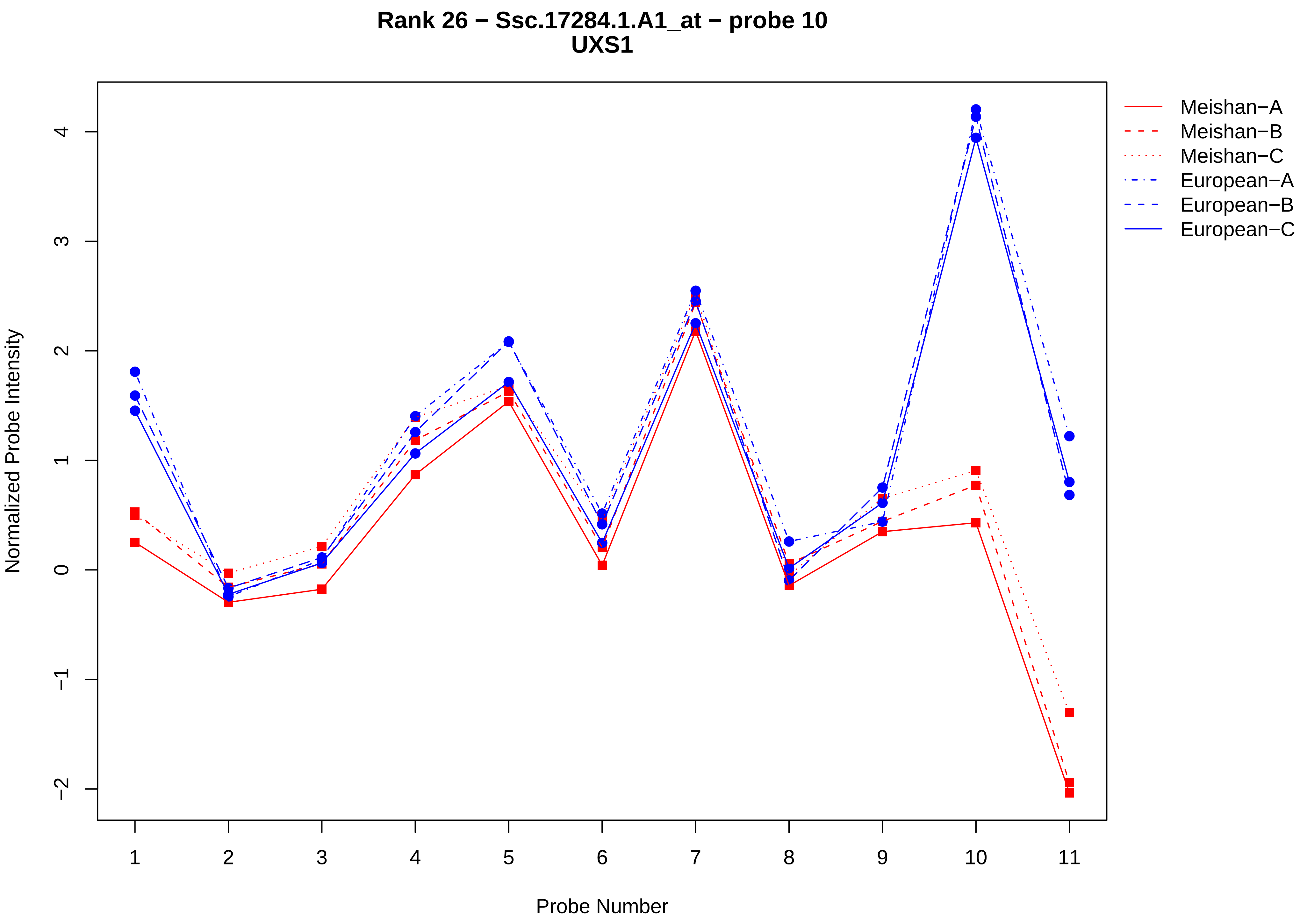
Rank 26 − Ssc.17284.1.A1_at − probe 10
UXS1
Normalized Probe Intensity
Probe Number
−2
−1
0
1
2
3
4
1
2
3
4
5
6
7
8
9
10
11
Meishan−A
Meishan−B
Meishan−C
European−A
European−B
European−C

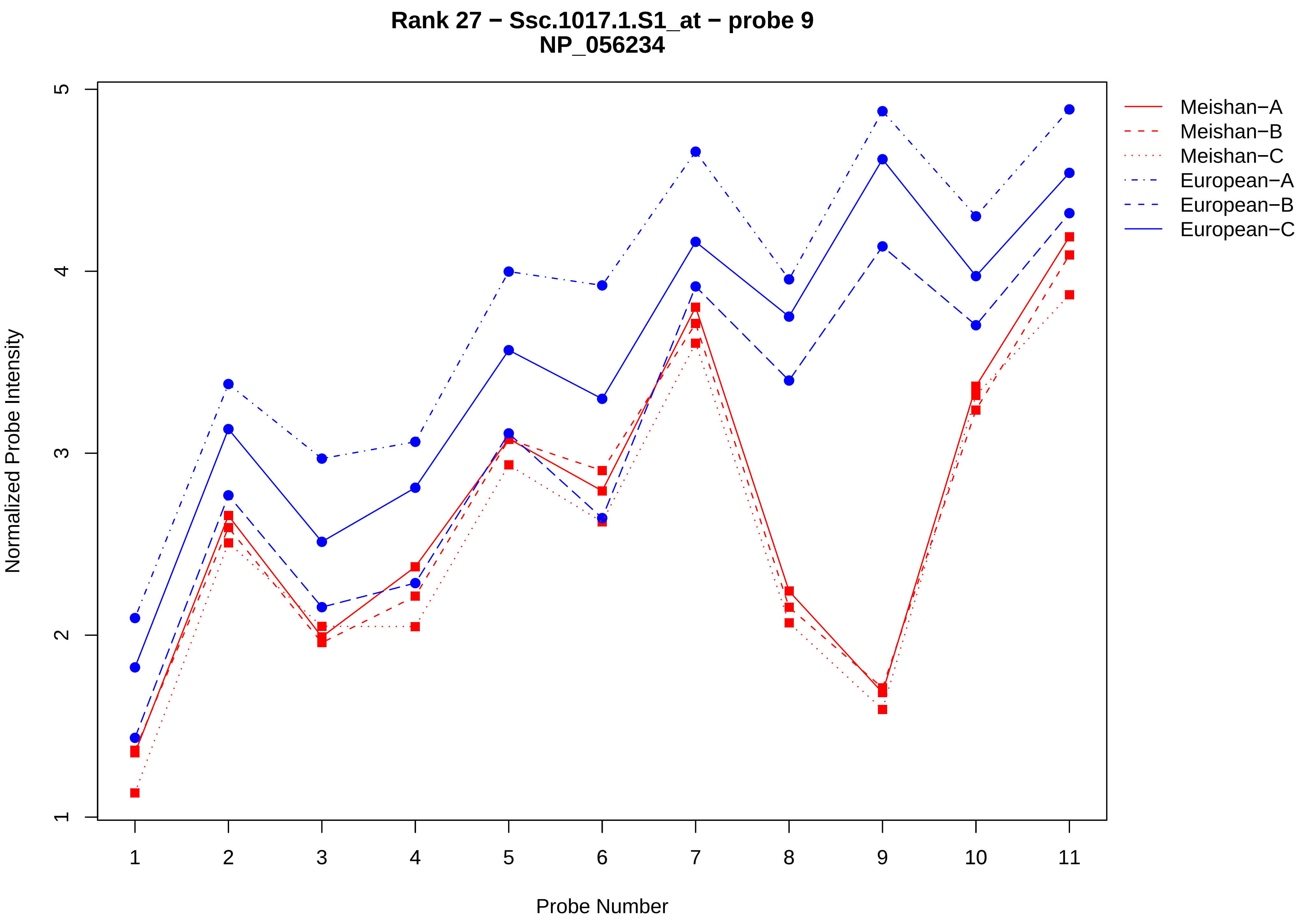

Rank 27 − Ssc.1017.1.S1_at − probe 9
NP_056234
Normalized Probe Intensity
Probe Number
1
2
3
4
5
Meishan−A
Meishan−B
Meishan−C
European−A
European−B
European−C
1
2
3
4
5
6
7
8
9
10
11

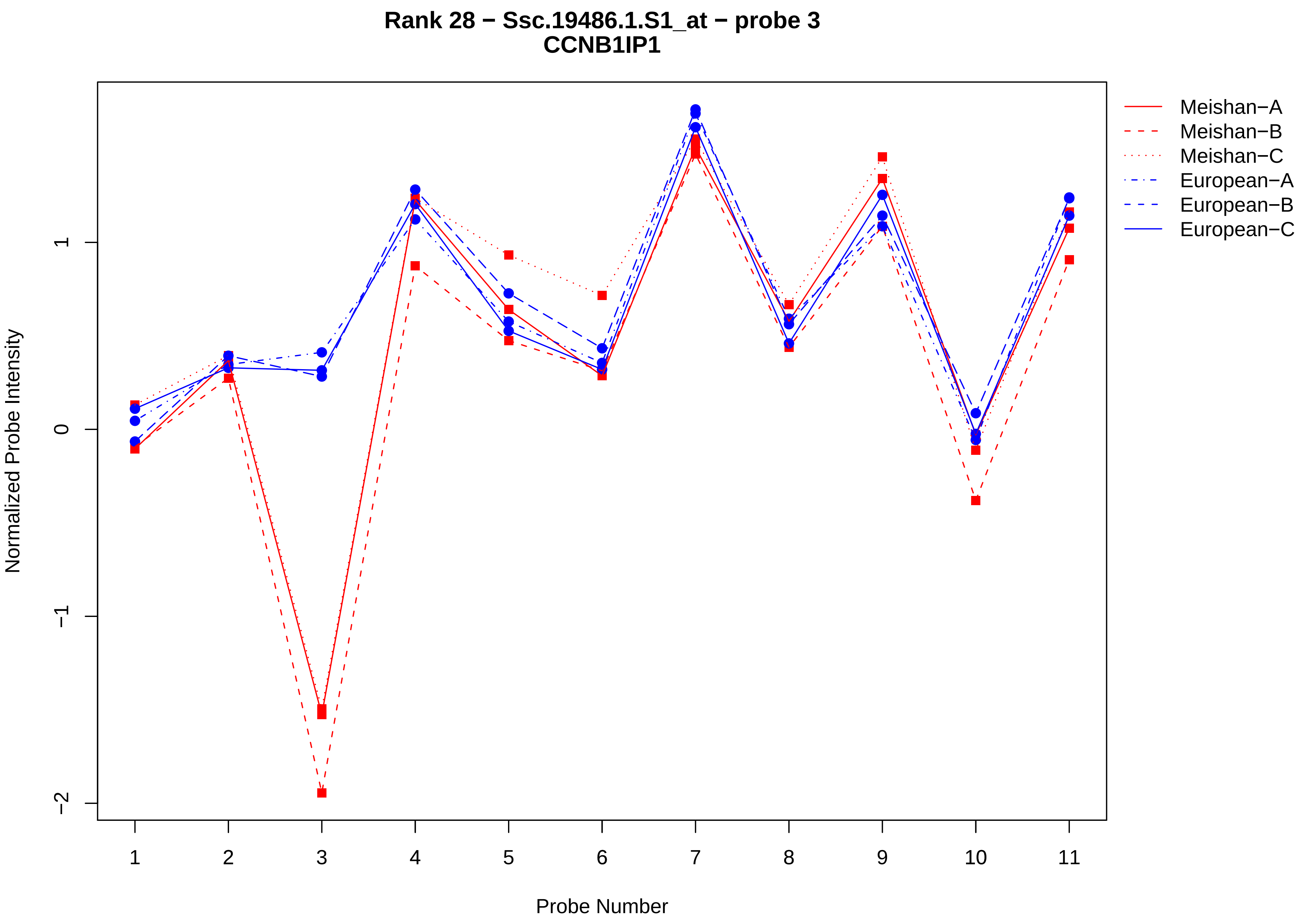

Rank 28 − Ssc.19486.1.S1_at − probe 3
CCNB1IP1
Normalized Probe Intensity
Probe Number
−2
−1
0
1
1
2
3
4
5
6
7
8
9
10
11
Meishan−A
Meishan−B
Meishan−C
European−A
European−B
European−C

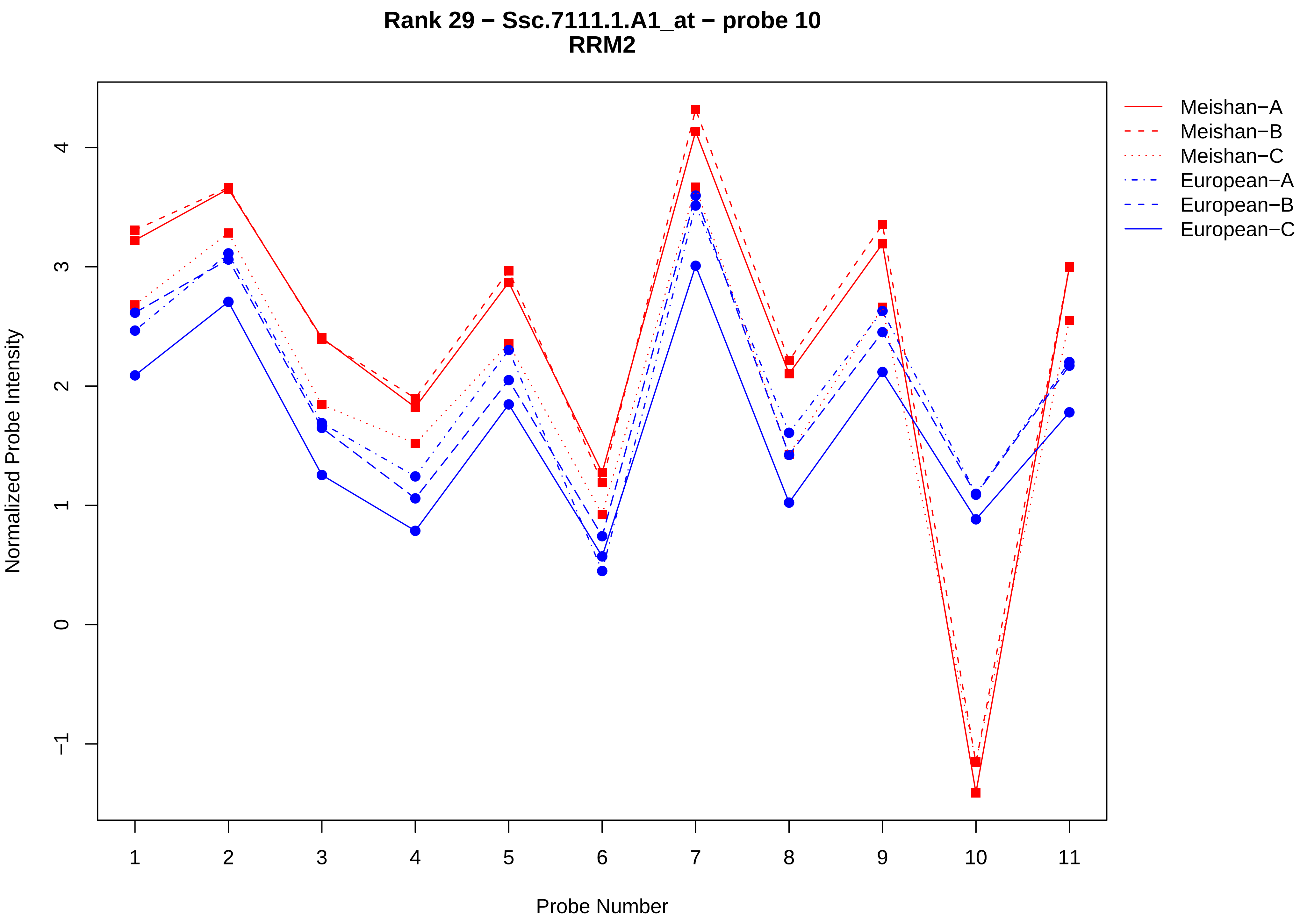
Rank 29 − Ssc.7111.1.A1_at − probe 10
RRM2
Normalized Probe Intensity
Probe Number
−1
0
1
2
3
4
1
2
3
4
5
6
7
8
9
10
11
Meishan−A
Meishan−B
Meishan−C
European−A
European−B
European−C

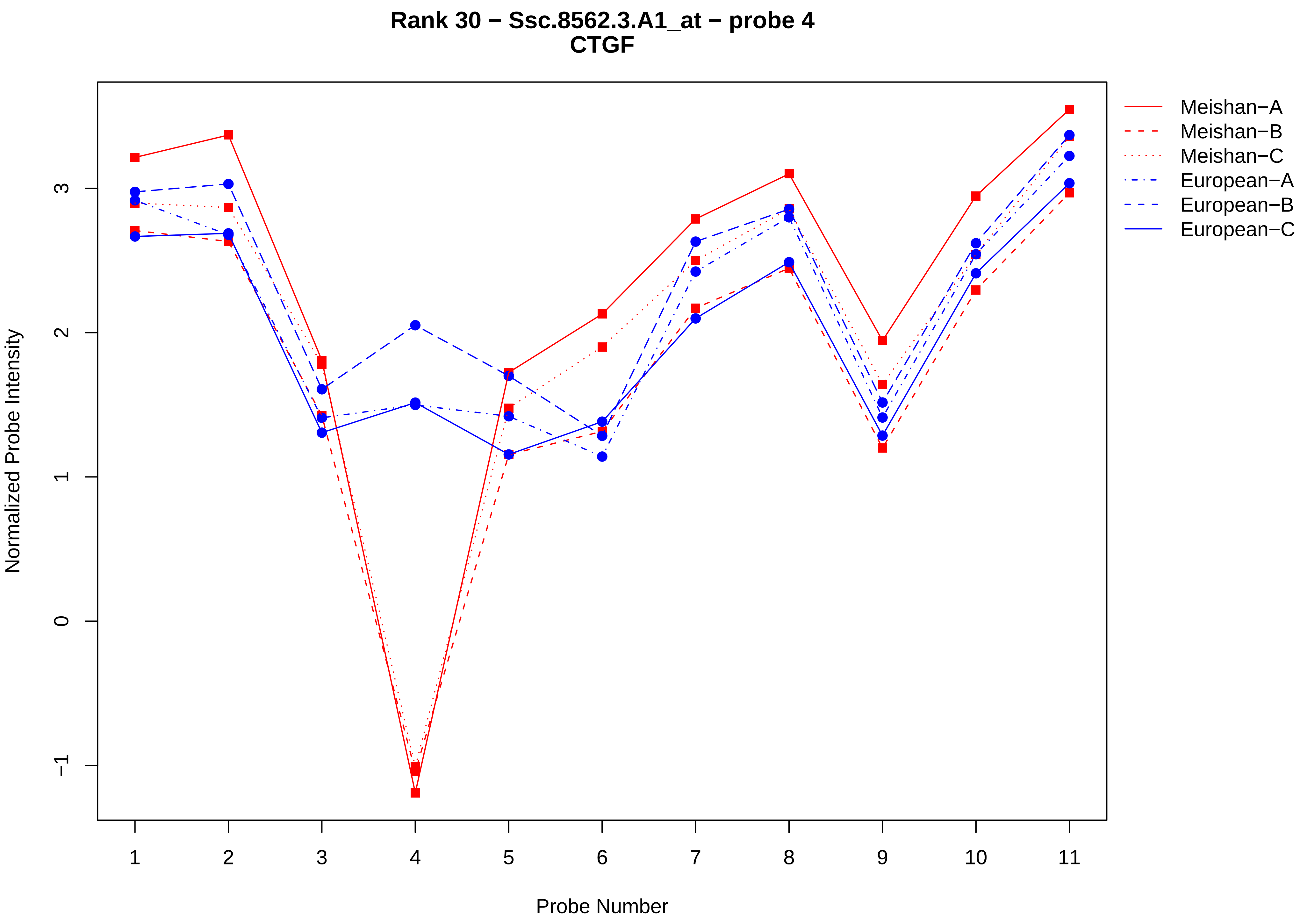

Rank 30 − Ssc.8562.3.A1_at − probe 4
CTGF
Normalized Probe Intensity
Probe Number
Meishan−A
Meishan−B
Meishan−C
European−A
European−B
European−C
−1
0
1
2
3
1
2
3
4
5
6
7
8
9
10
11

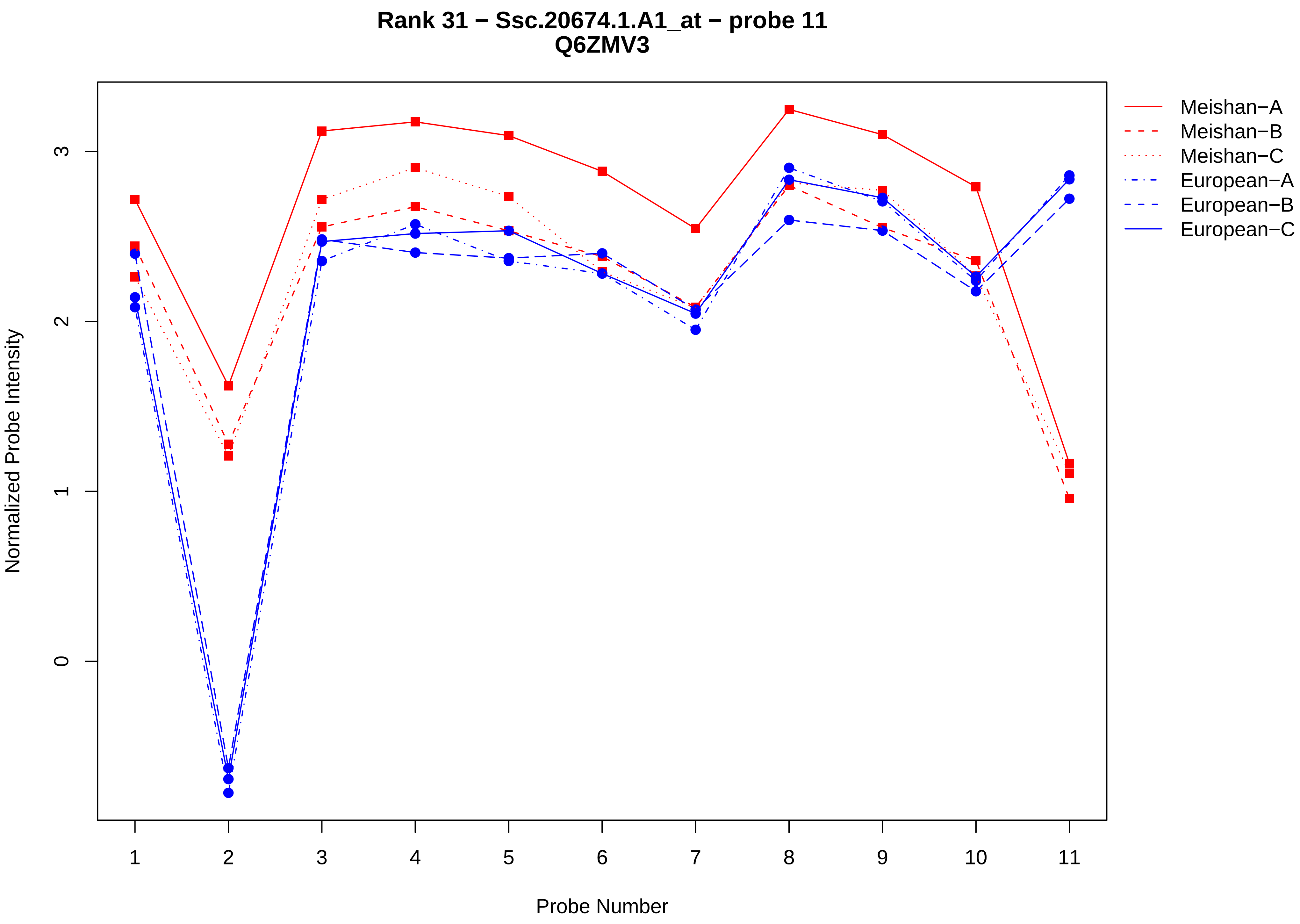
Rank 31 − Ssc.20674.1.A1_at − probe 11
Q6ZMV3
Normalized Probe Intensity
Probe Number
0
1
2
3
1
2
3
4
5
6
7
8
9
10
11
Meishan−A
Meishan−B
Meishan−C
European−A
European−B
European−C

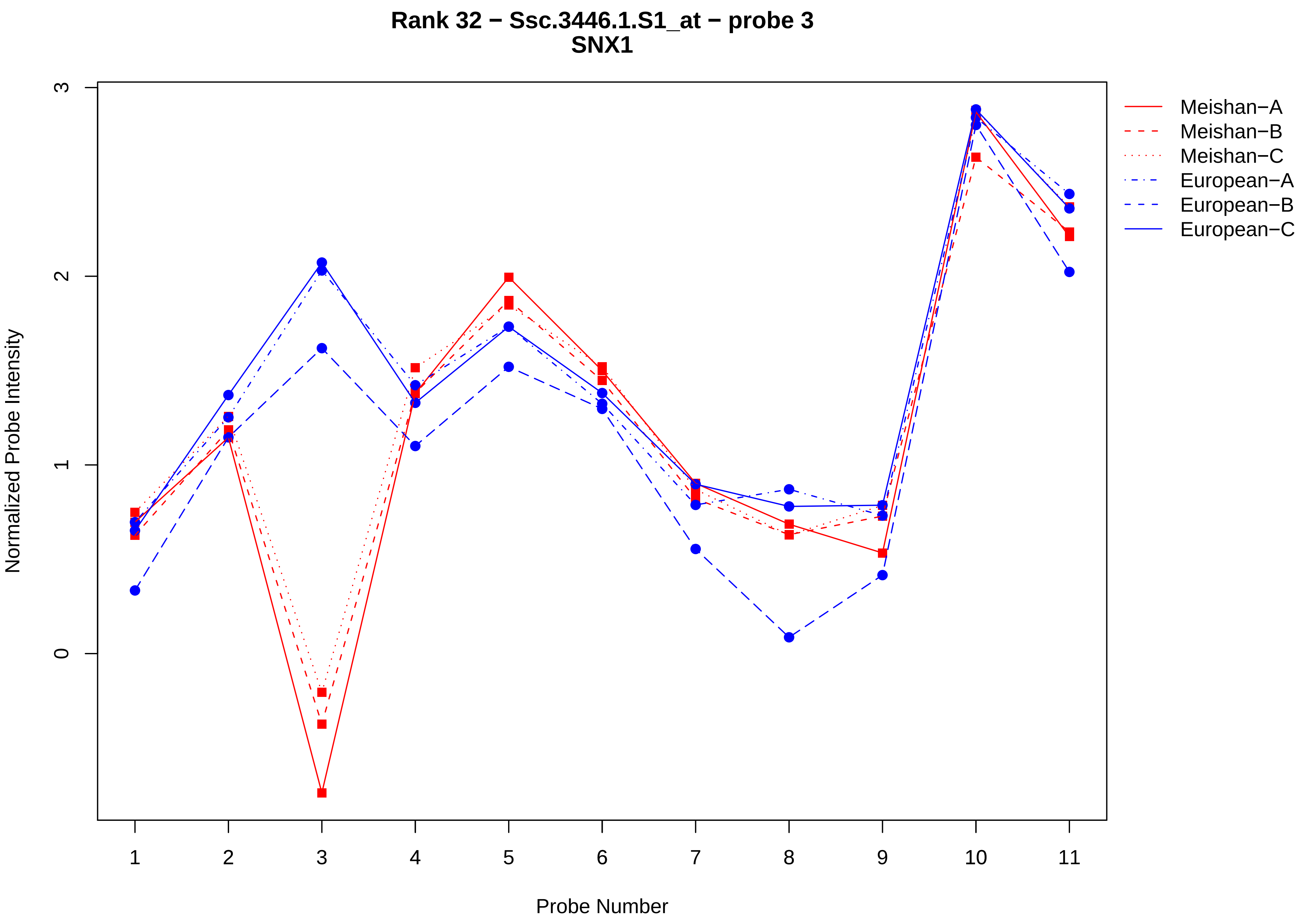
Rank 32 − Ssc.3446.1.S1_at − probe 3
SNX1
Normalized Probe Intensity
Probe Number
0
1
2
3
1
2
3
4
5
6
7
8
9
10
11
Meishan−A
Meishan−B
Meishan−C
European−A
European−B
European−C

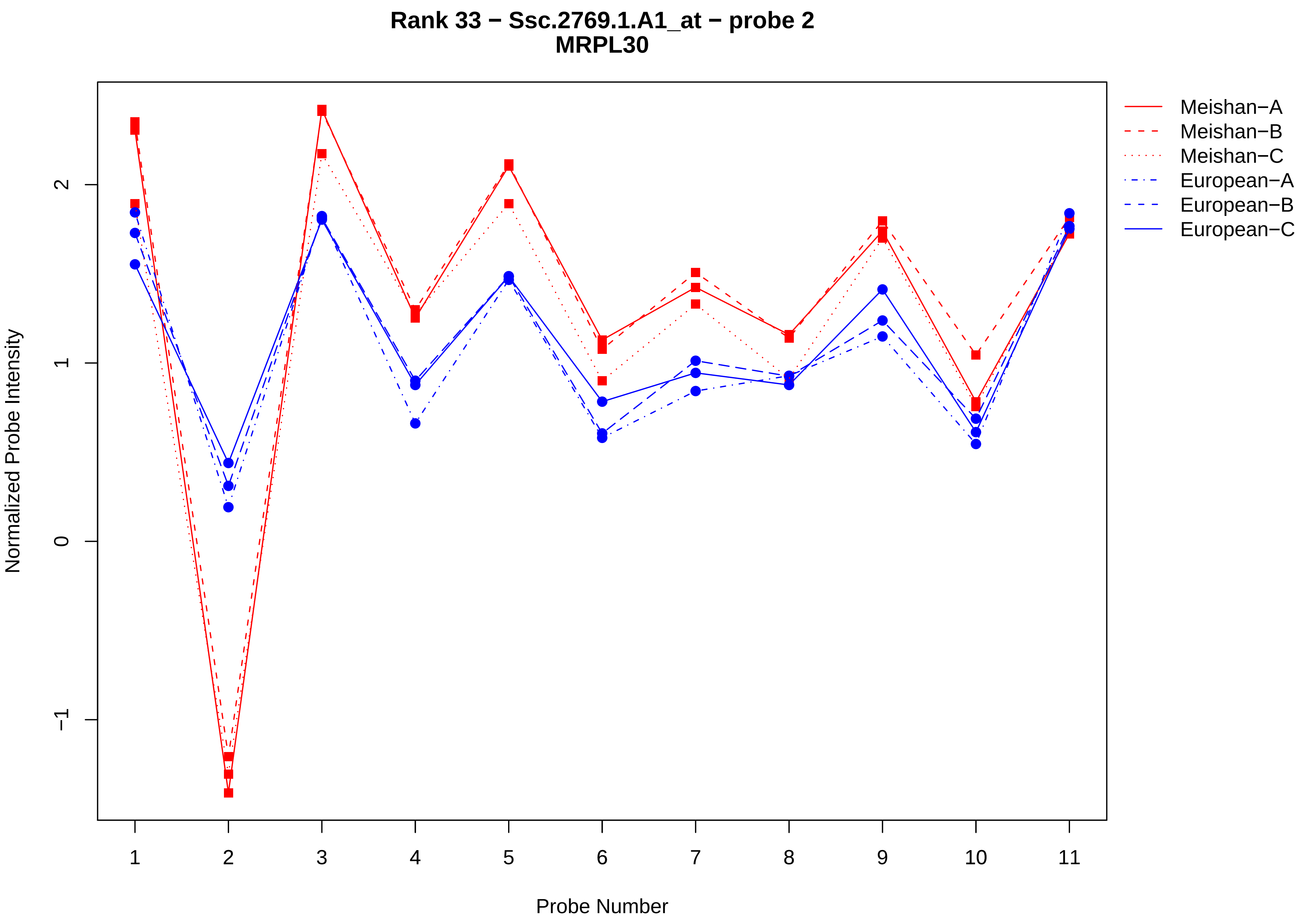
Rank 33 − Ssc.2769.1.A1_at − probe 2
MRPL30
Normalized Probe Intensity
Probe Number
−1
0
1
2
1
2
3
4
5
6
7
8
9
10
11
Meishan−A
Meishan−B
Meishan−C
European−A
European−B
European−C

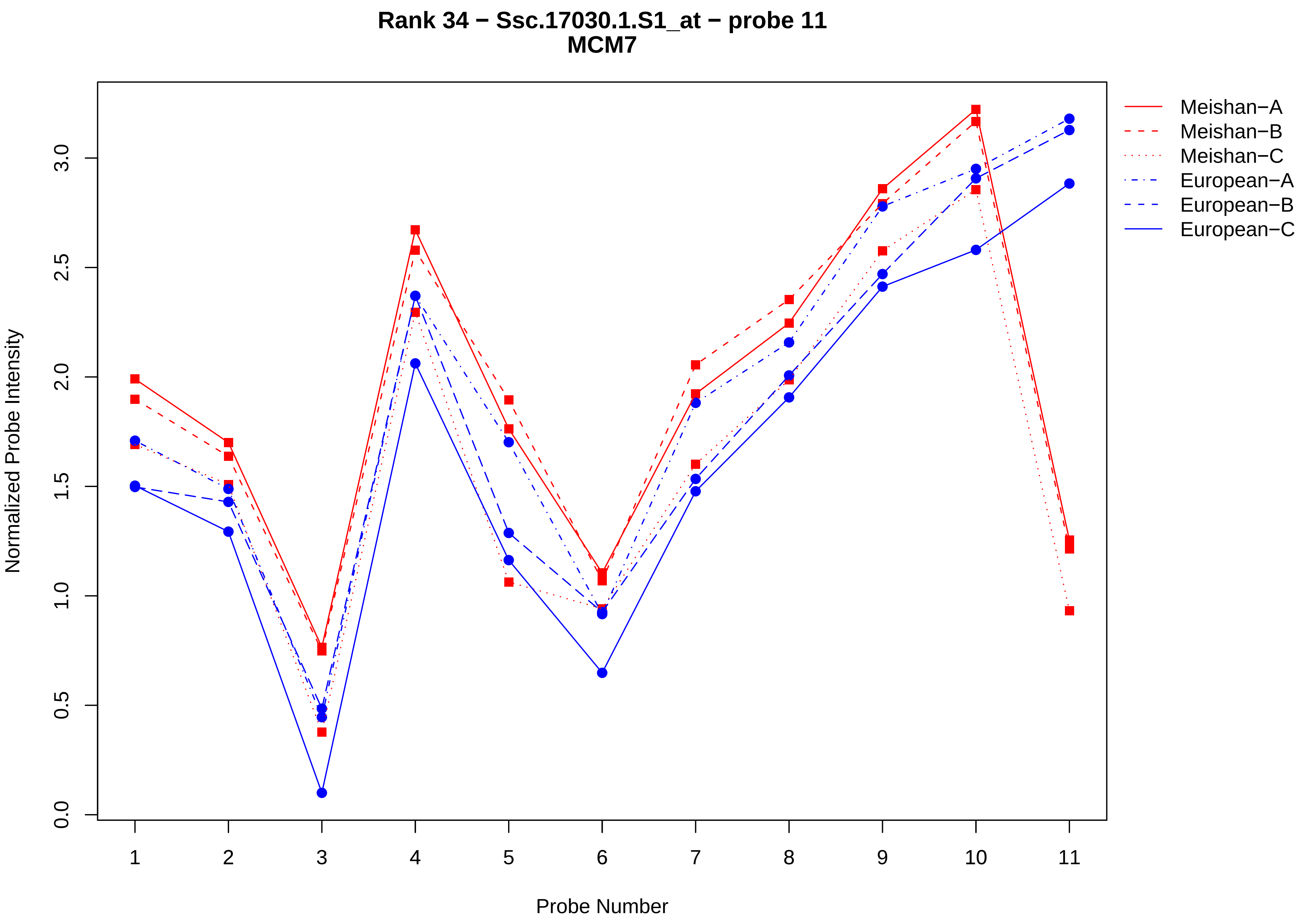

Rank 34 − Ssc.17030.1.S1_at − probe 11
MCM7
Normalized Probe Intensity
0.0
0.5
1.0
1.5
2.0
2.5
3.0
Probe Number
1
2
3
4
5
6
7
8
9
10
11
Meishan−A
Meishan−B
Meishan−C
European−A
European−B
European−C

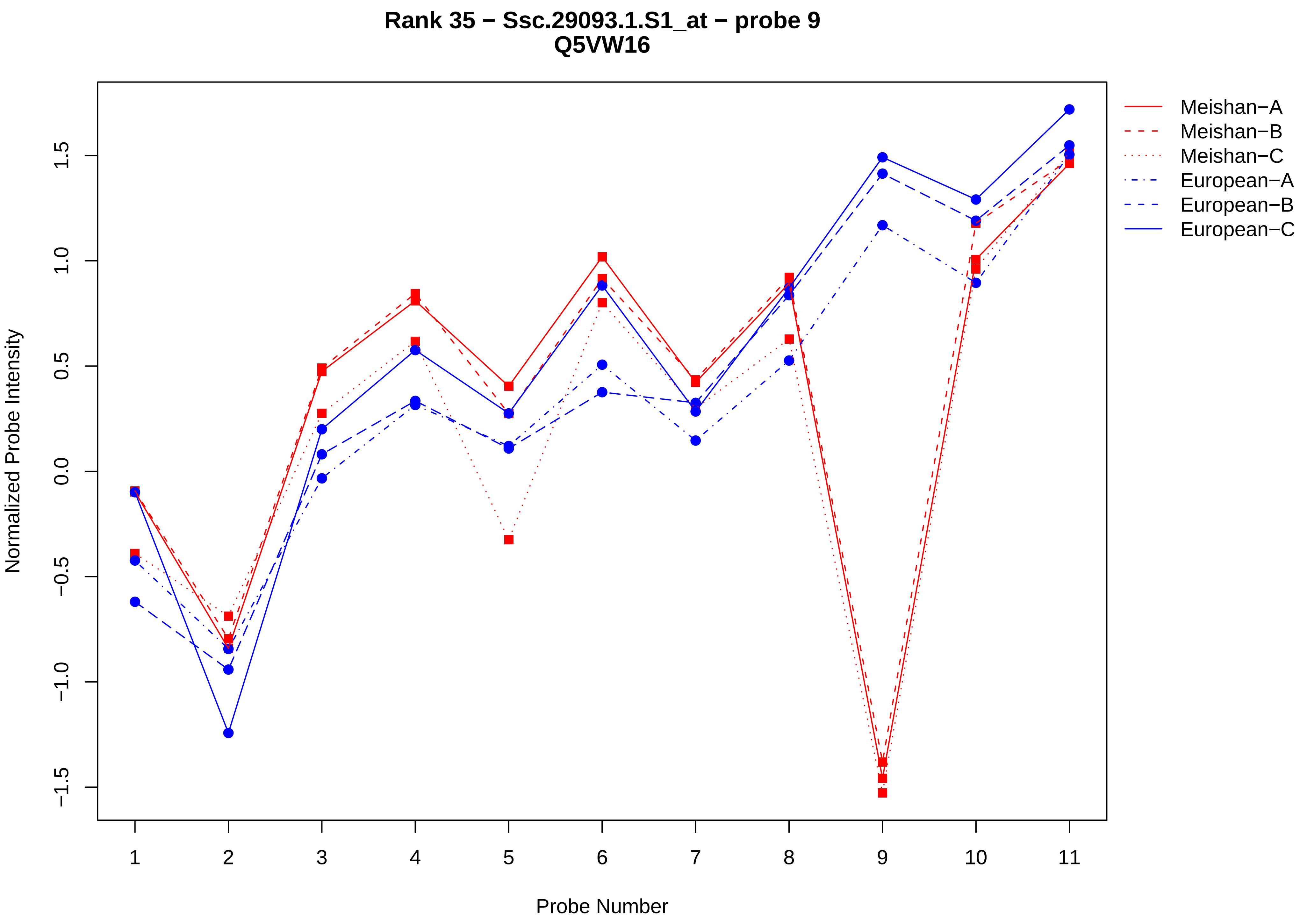
Rank 35 − Ssc.29093.1.S1_at − probe 9
Q5VW16
Normalized Probe Intensity
Probe Number
−1.5
−1.0
−0.5
0.0
0.5
1.0
1.5
1
2
3
4
5
6
7
8
9
10
11
Meishan−A
Meishan−B
Meishan−C
European−A
European−B
European−C

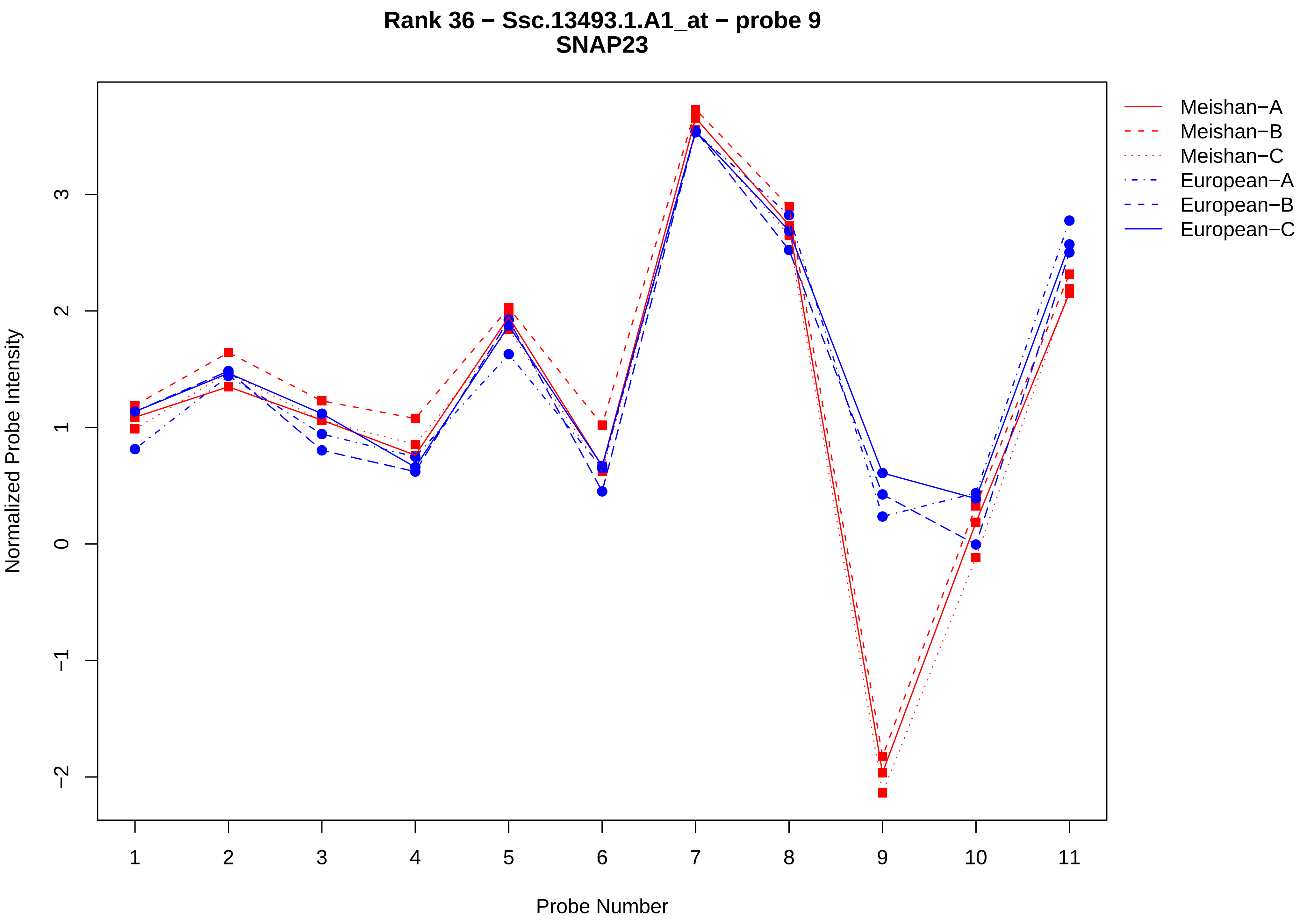

Rank 36 − Ssc.13493.1.A1_at − probe 9
SNAP23
Normalized Probe Intensity
Probe Number
−2
−1
0
1
2
3
1
2
3
4
5
6
7
8
9
10
11
Meishan−A
Meishan−B
Meishan−C
European−A
European−B
European−C

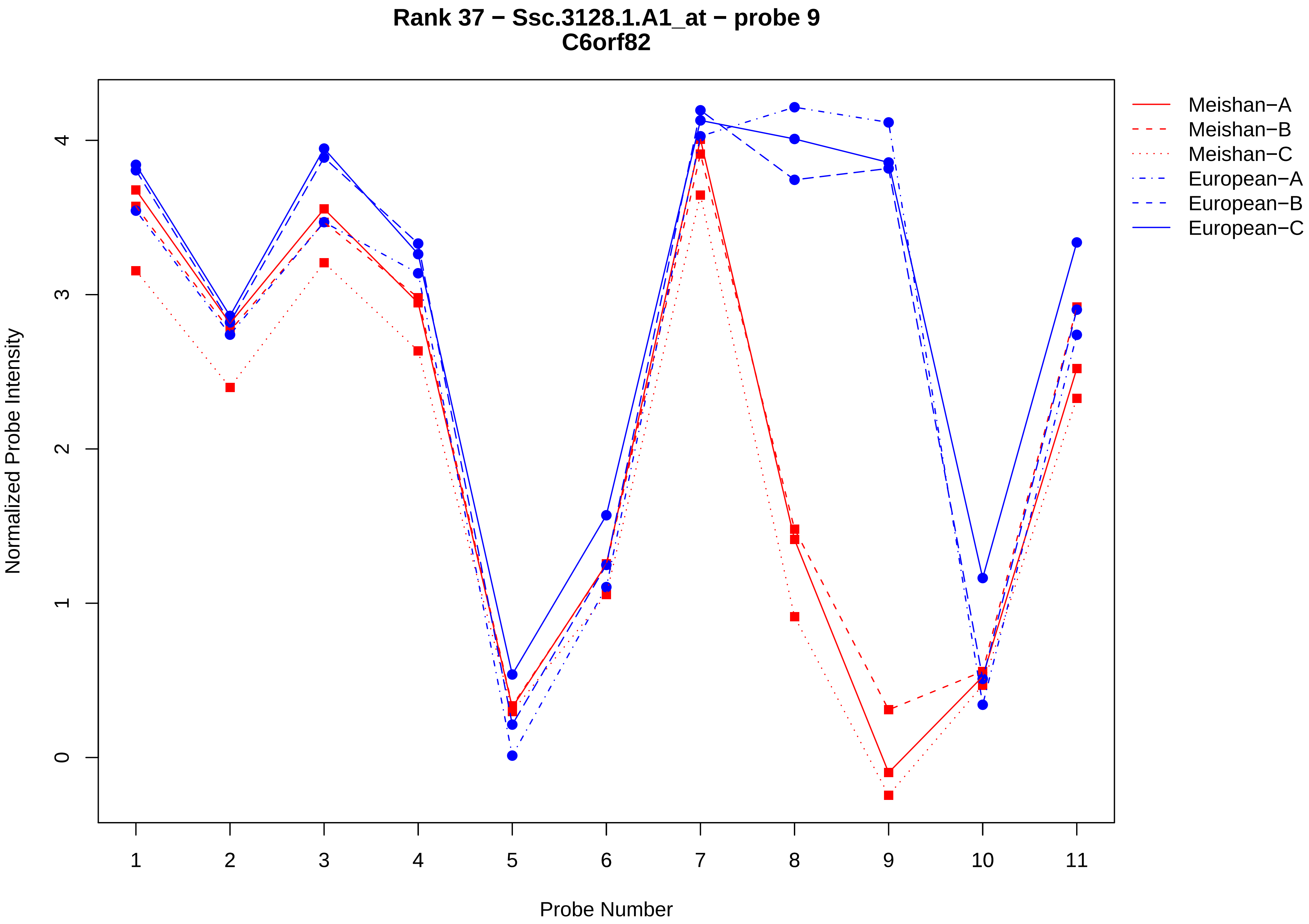

Rank 37 − Ssc.3128.1.A1_at − probe 9
C6orf82
Normalized Probe Intensity
0
1
2
3
4
1
2
3
4
5
6
7
8
9
10
11
Probe Number
Meishan−A
Meishan−B
Meishan−C
European−A
European−B
European−C

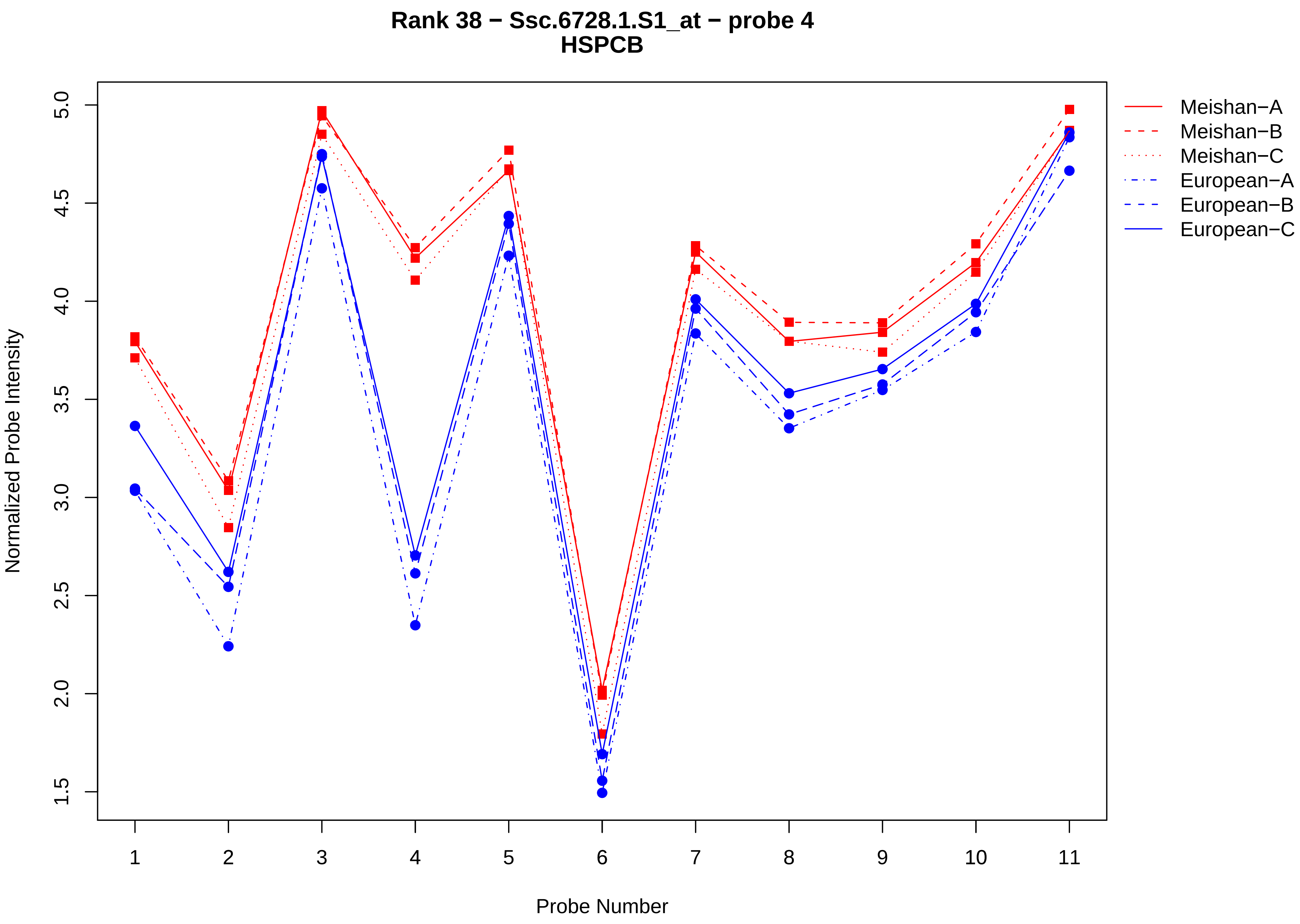

Rank 38 − Ssc.6728.1.S1_at − probe 4
HSPCB
Normalized Probe Intensity
1.5
2.0
2.5
3.0
3.5
4.0
4.5
5.0
Probe Number
1
2
3
4
5
6
7
8
9
10
11
Meishan−A
Meishan−B
Meishan−C
European−A
European−B
European−C

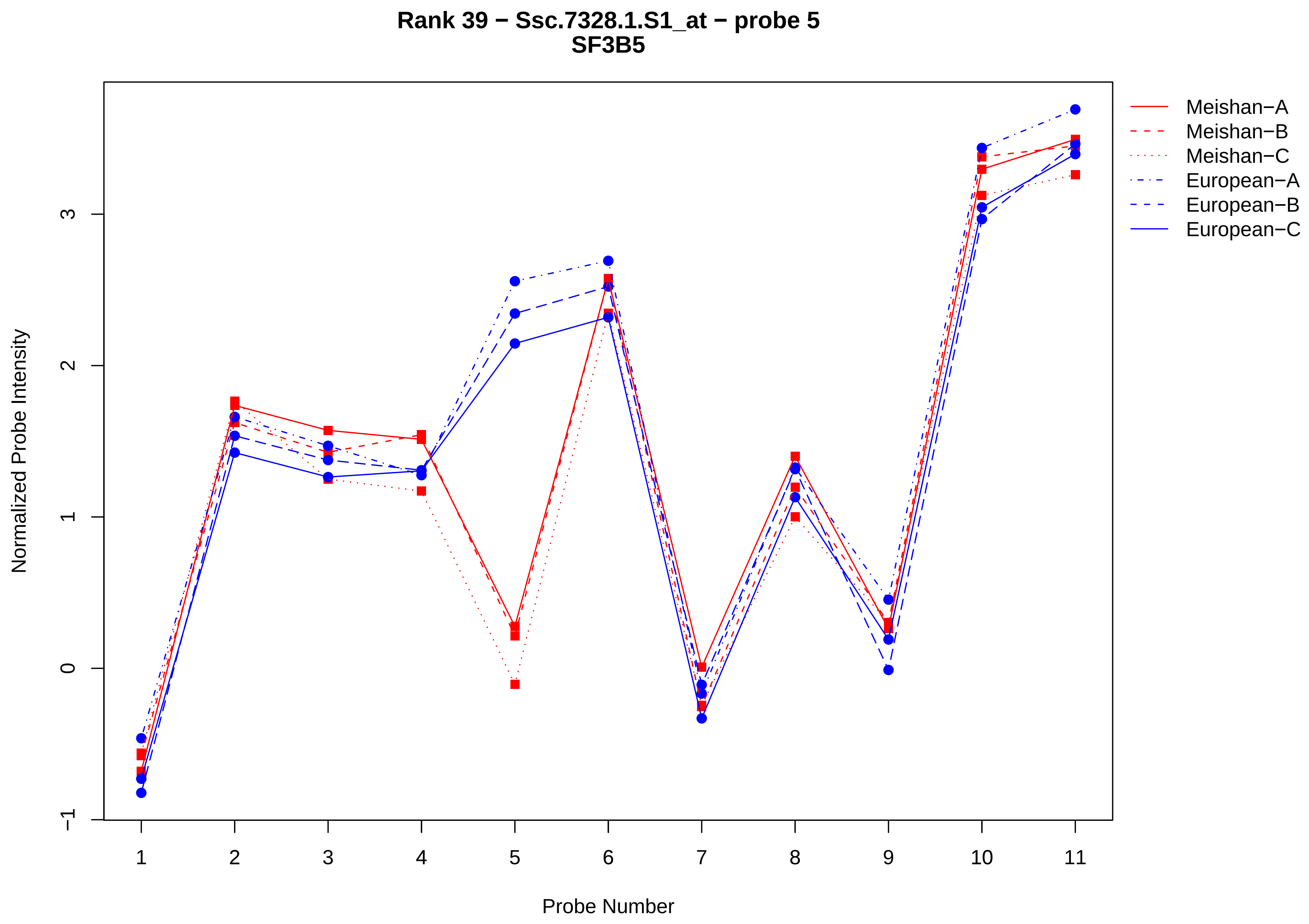

Rank 39 − Ssc.7328.1.S1_at − probe 5
SF3B5
Normalized Probe Intensity
Probe Number
−1
0
1
2
3
1
2
3
4
5
6
7
8
9
10
11
Meishan−A
Meishan−B
Meishan−C
European−A
European−B
European−C

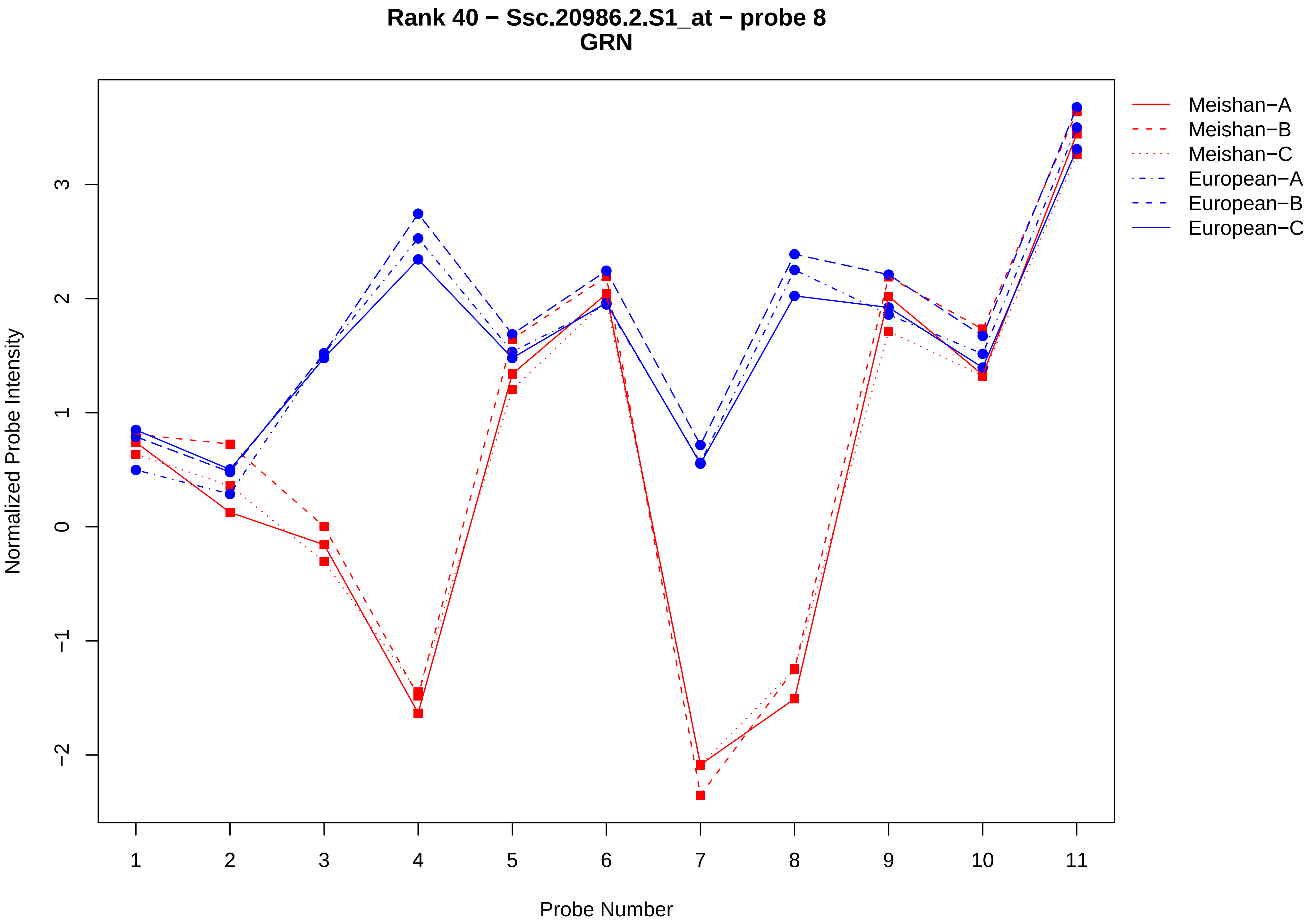

Rank 40 − Ssc.20986.2.S1_at − probe 8
GRN
Normalized Probe Intensity
Probe Number
−2
−1
0
1
2
3
1
2
3
4
5
6
7
8
9
10
11
Meishan−A
Meishan−B
Meishan−C
European−A
European−B
European−C

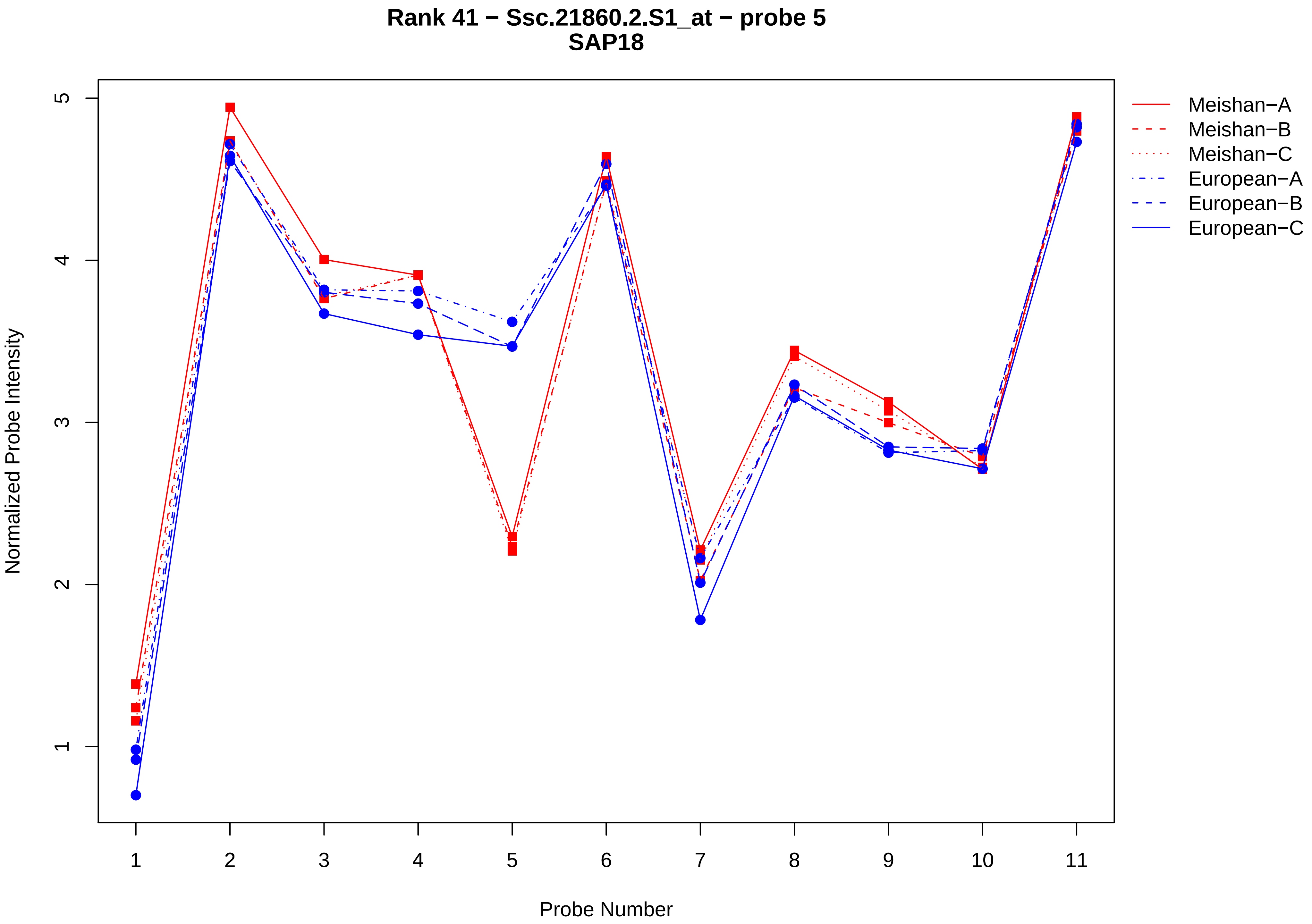
Rank 41 − Ssc.21860.2.S1_at − probe 5
SAP18
Normalized Probe Intensity
Probe Number
1
2
3
4
5
1
2
3
4
5
6
7
8
9
10
11
Meishan−A
Meishan−B
Meishan−C
European−A
European−B
European−C

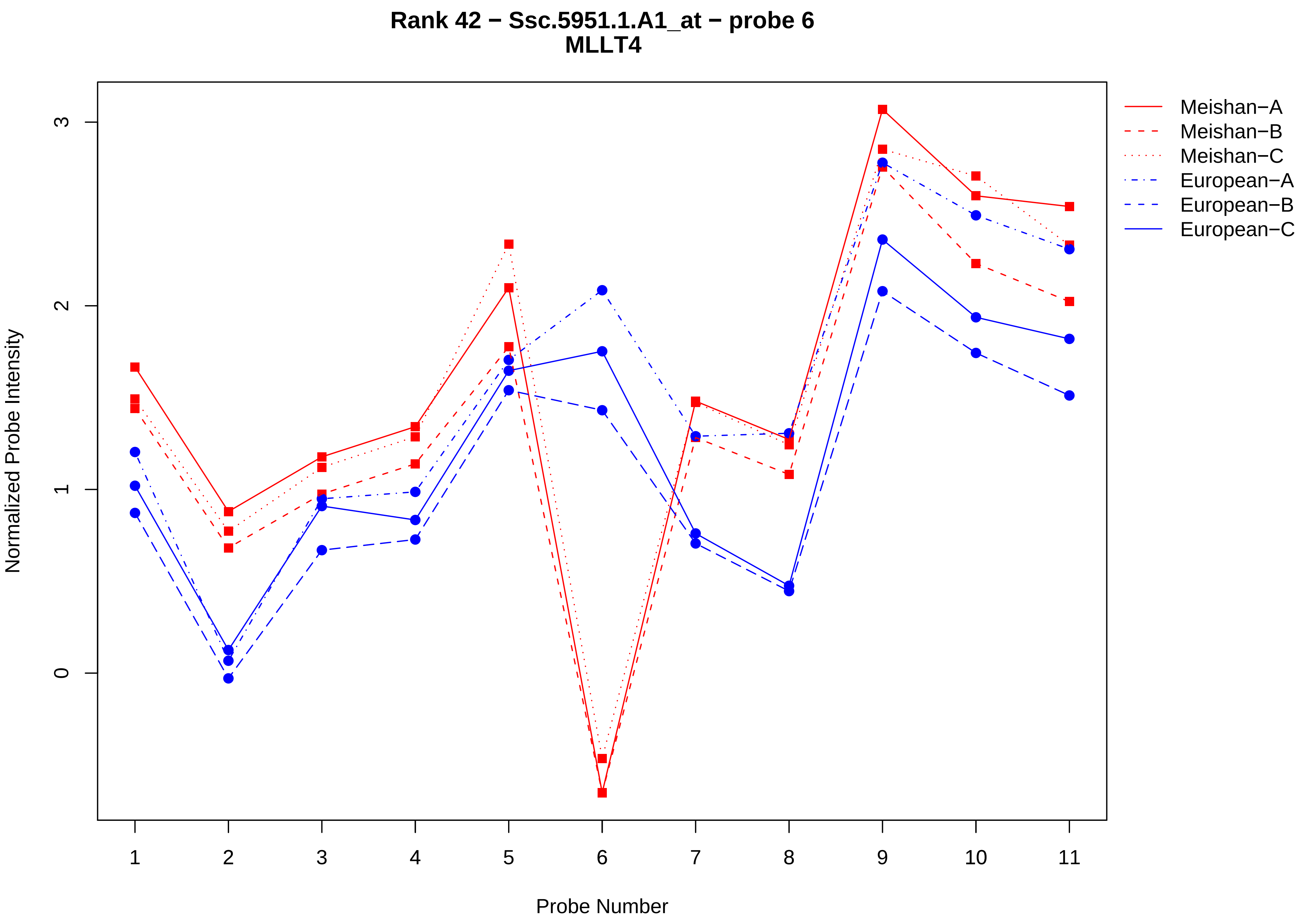

Rank 42 − Ssc.5951.1.A1_at − probe 6
MLLT4
Normalized Probe Intensity
0
1
2
3
1
2
3
4
5
6
7
8
9
10
11
Probe Number
Meishan−A
Meishan−B
Meishan−C
European−A
European−B
European−C

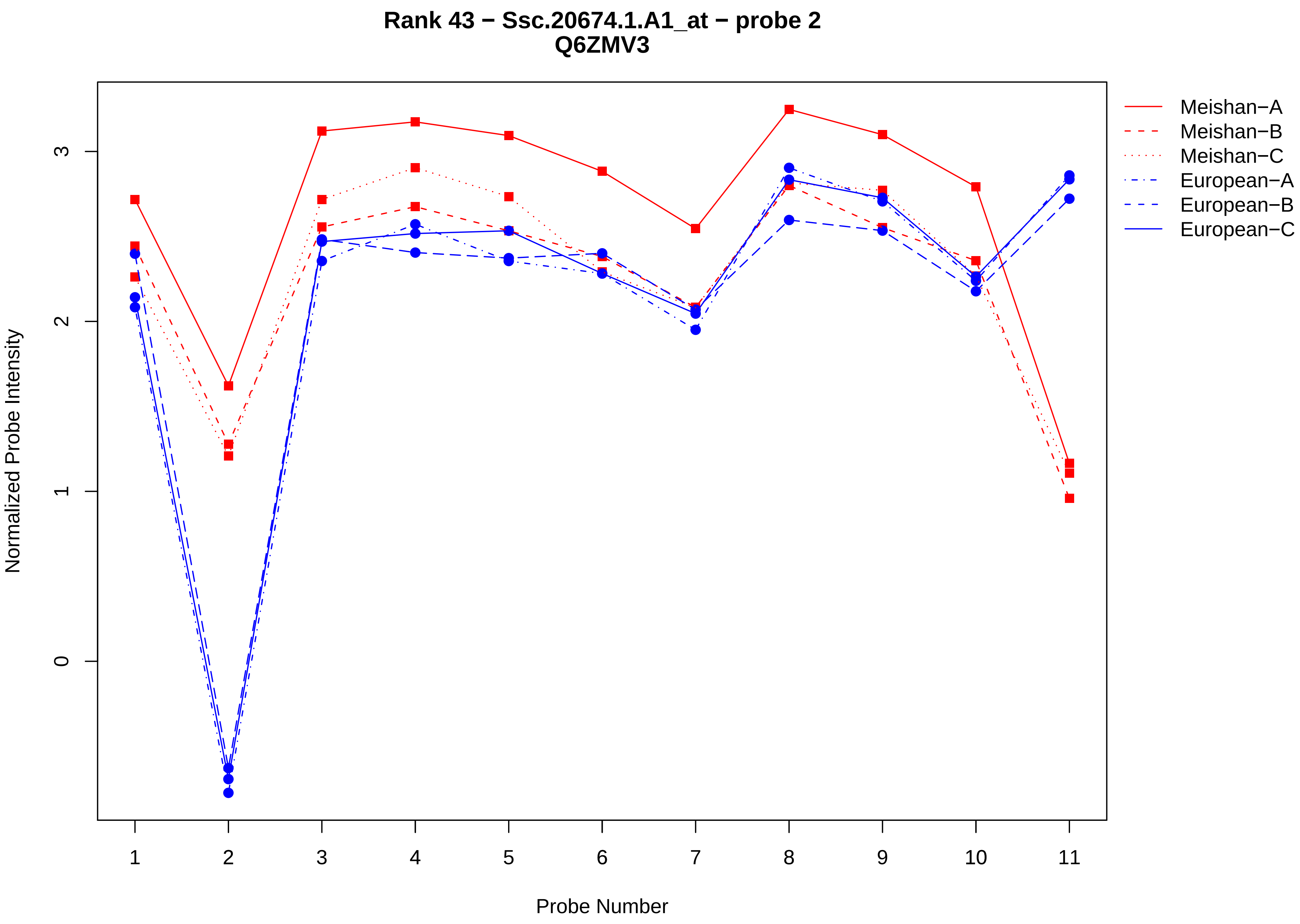

Rank 43 − Ssc.20674.1.A1_at − probe 2
Q6ZMV3
Normalized Probe Intensity
Probe Number
0
1
2
3
1
2
3
4
5
6
7
8
9
10
11
Meishan−A
Meishan−B
Meishan−C
European−A
European−B
European−C

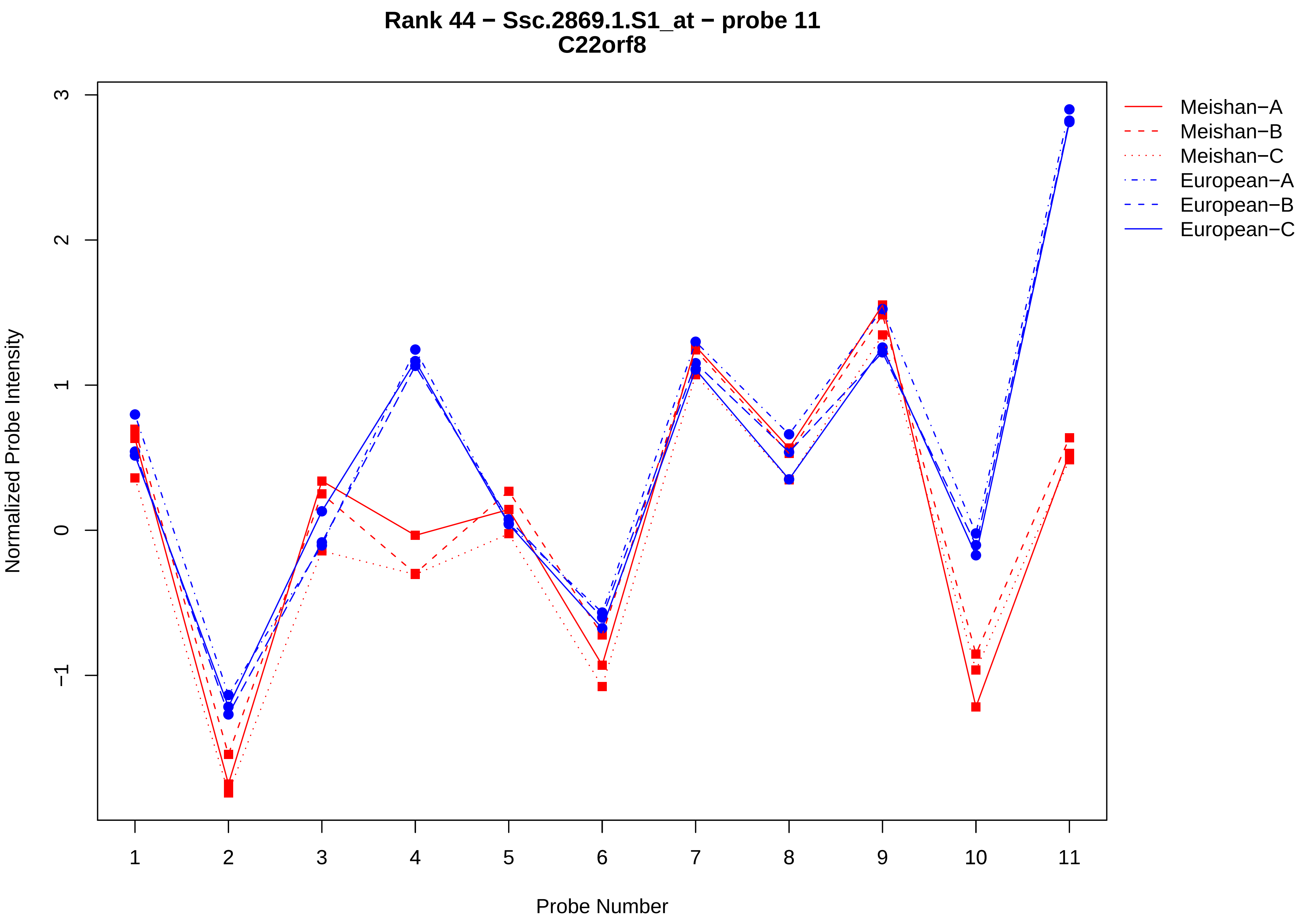
Rank 44 − Ssc.2869.1.S1_at − probe 11
C22orf8
Normalized Probe Intensity
Probe Number
Meishan−A
Meishan−B
Meishan−C
European−A
European−B
European−C
−1
0
1
2
3
1
2
3
4
5
6
7
8
9
10
11

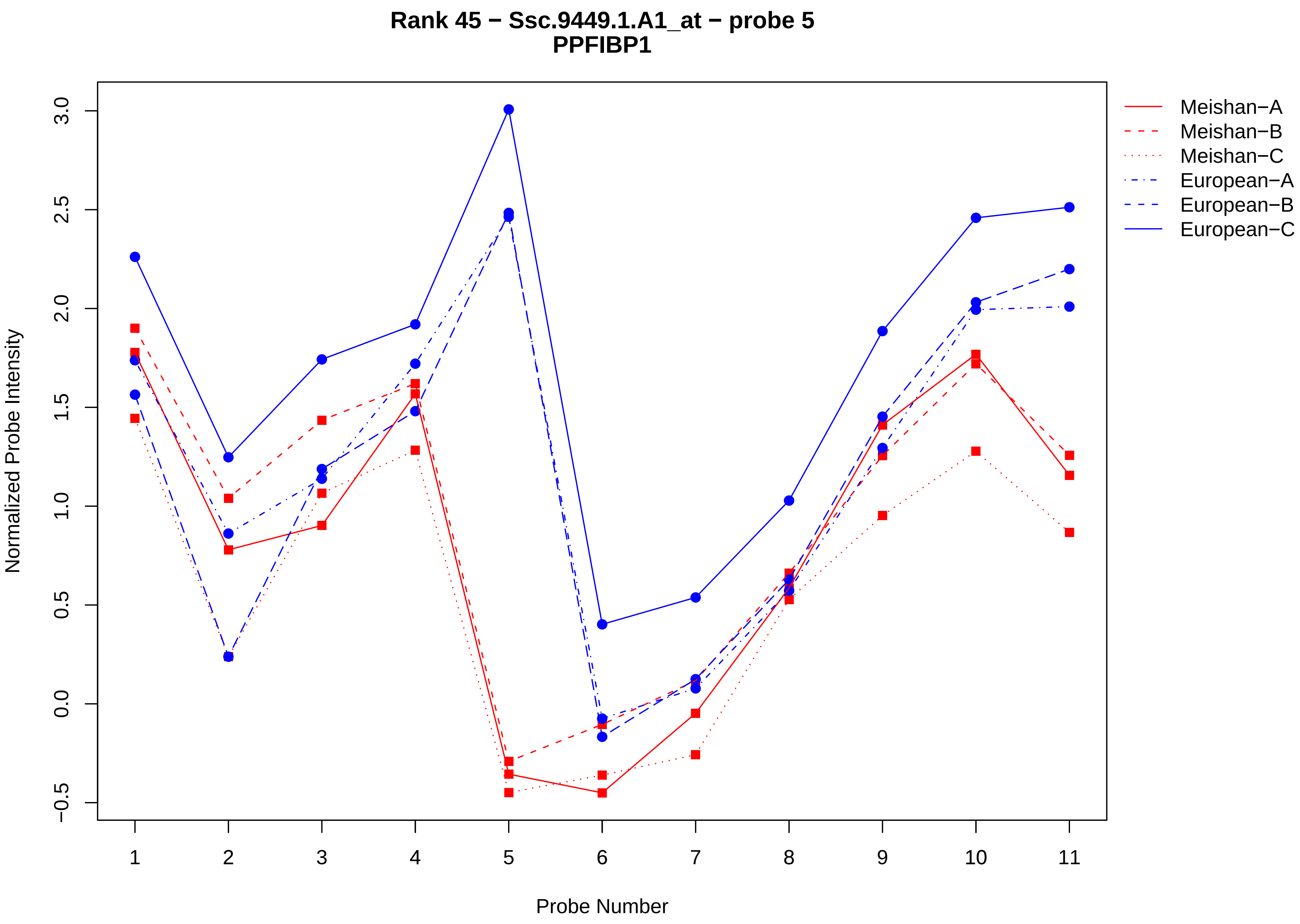

Rank 45 − Ssc.9449.1.A1_at − probe 5
PPFIBP1
Normalized Probe Intensity
Probe Number
Meishan−A
Meishan−B
Meishan−C
European−A
European−B
European−C
−0.5
0.0
0.5
1.0
1.5
2.0
2.5
3.0
1
2
3
4
5
6
7
8
9
10
11

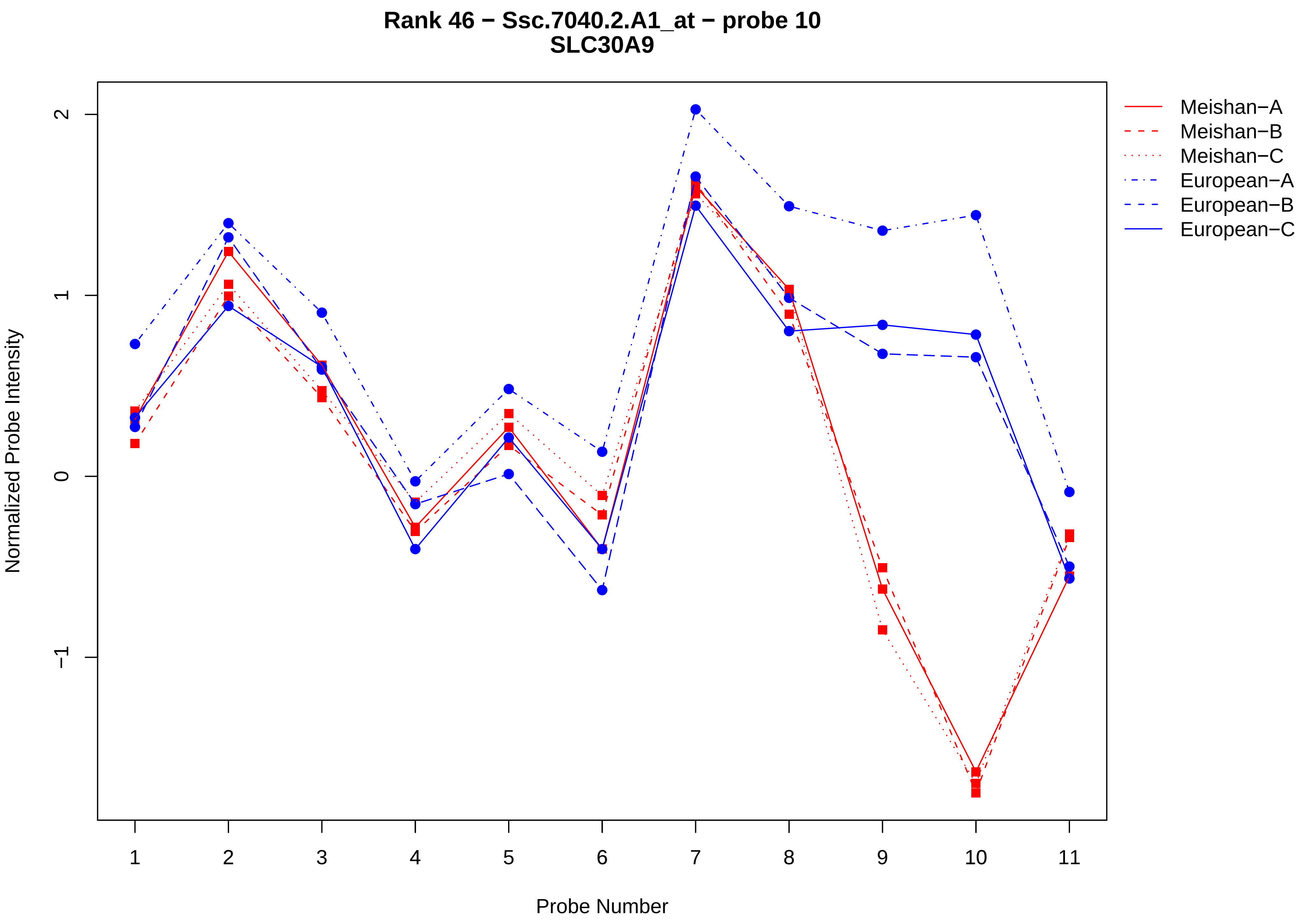
Rank 46 − Ssc.7040.2.A1_at − probe 10
SLC30A9
Normalized Probe Intensity
Probe Number
−1
0
1
2
1
2
3
4
5
6
7
8
9
10
11
Meishan−A
Meishan−B
Meishan−C
European−A
European−B
European−C

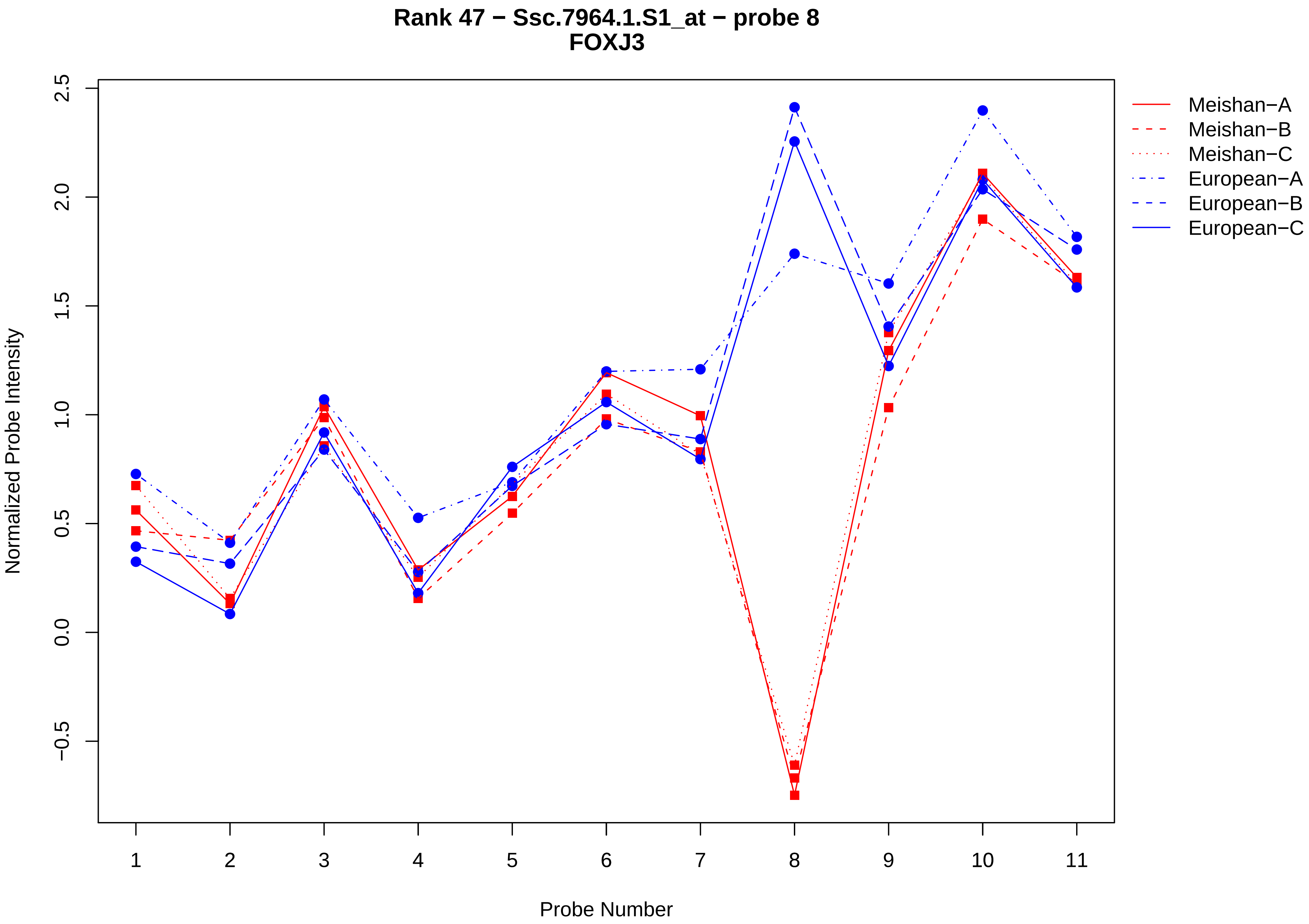

Rank 47 − Ssc.7964.1.S1_at − probe 8
FOXJ3
Normalized Probe Intensity
Probe Number
−0.5
0.0
0.5
1.0
1.5
2.0
2.5
1
2
3
4
5
6
7
8
9
10
11
Meishan−A
Meishan−B
Meishan−C
European−A
European−B
European−C

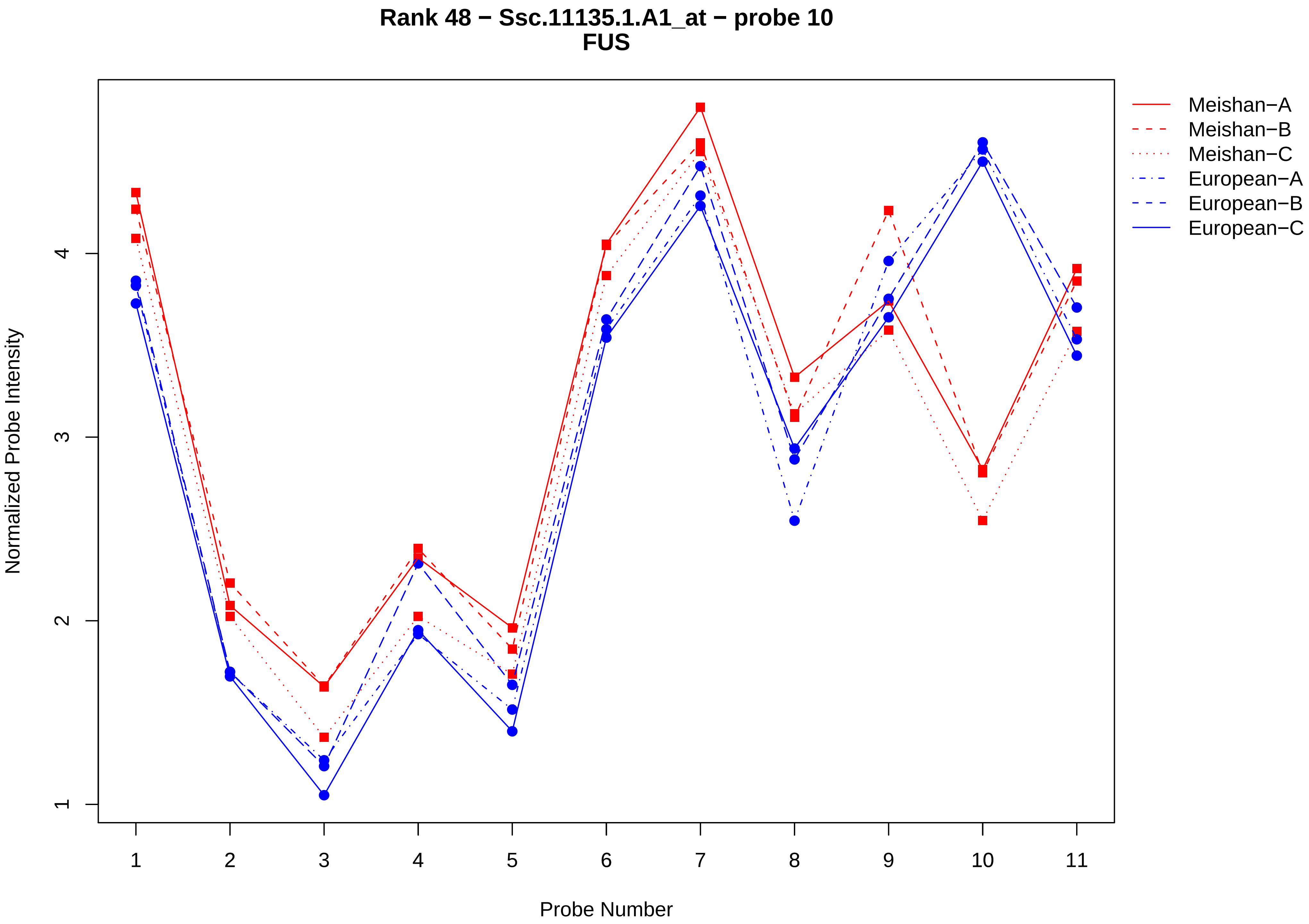
Rank 48 − Ssc.11135.1.A1_at − probe 10
FUS
Normalized Probe Intensity
Probe Number
1
2
3
4
1
2
3
4
5
6
7
8
9
10
11
Meishan−A
Meishan−B
Meishan−C
European−A
European−B
European−C

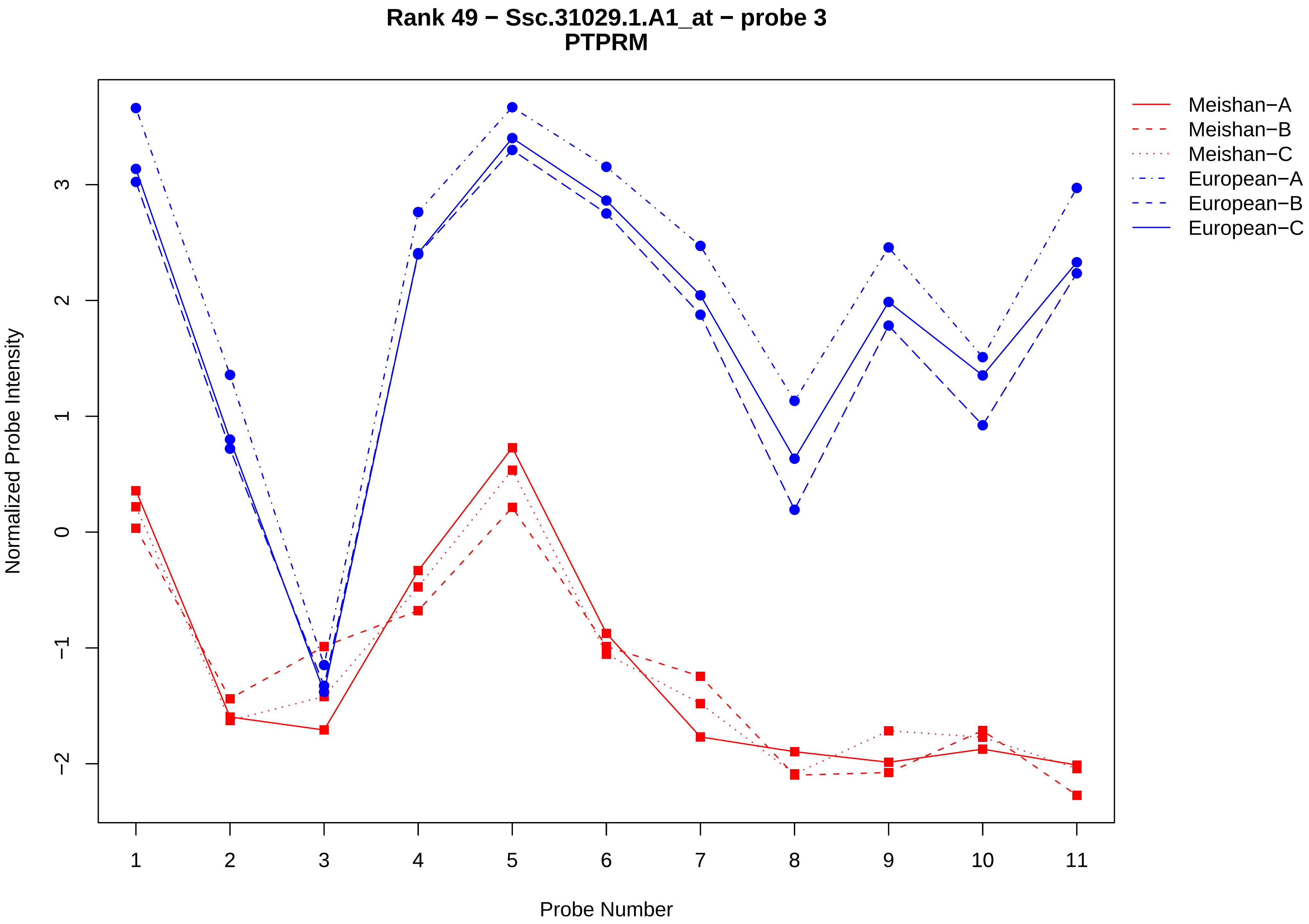

Rank 49 − Ssc.31029.1.A1_at − probe 3
PTPRM
Normalized Probe Intensity
Probe Number
−2
−1
0
1
2
3
1
2
3
4
5
6
7
8
9
10
11
Meishan−A
Meishan−B
Meishan−C
European−A
European−B
European−C

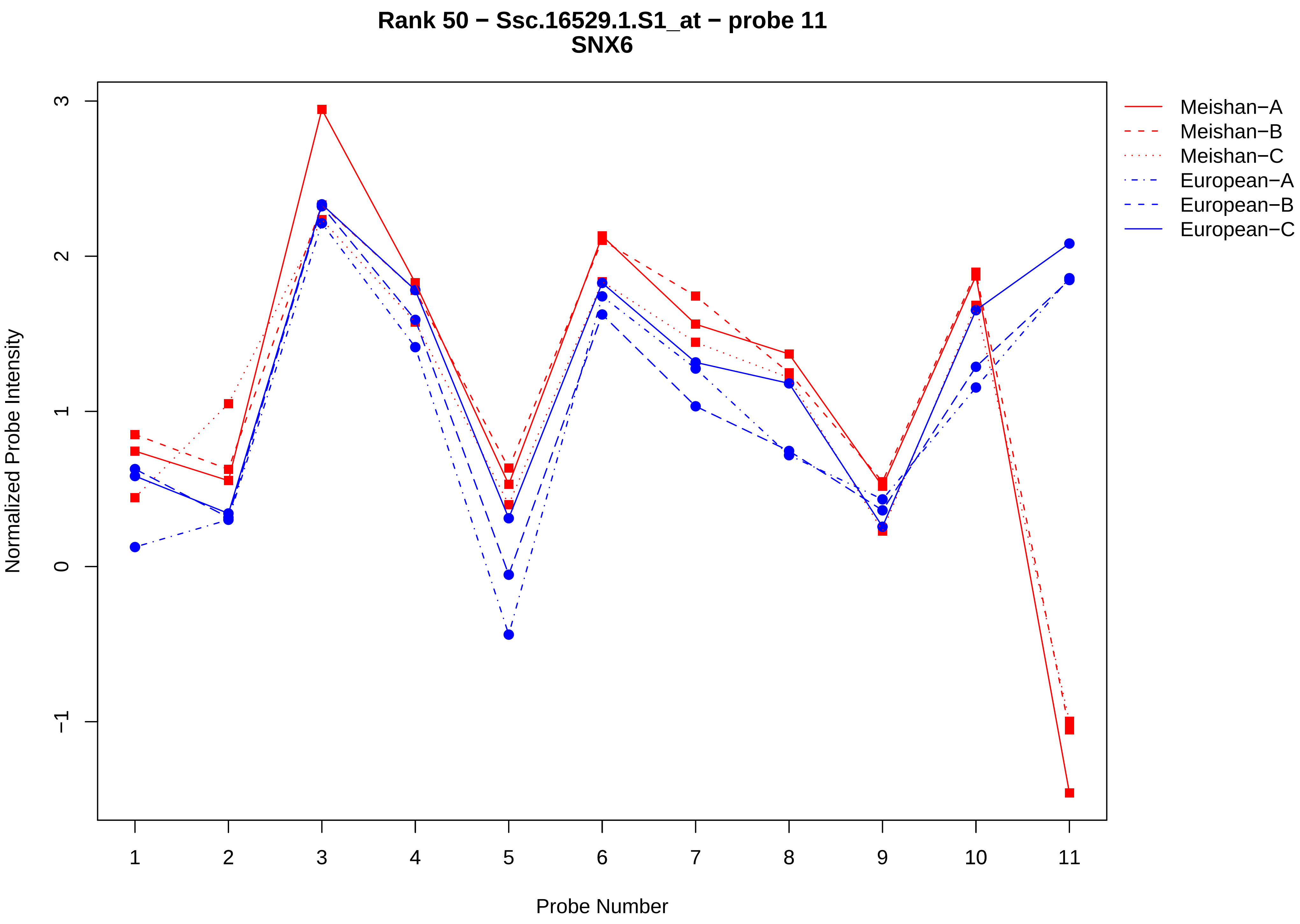

Rank 50 − Ssc.16529.1.S1_at − probe 11
SNX6
Normalized Probe Intensity
Probe Number
−1
0
1
2
3
1
2
3
4
5
6
7
8
9
10
11
Meishan−A
Meishan−B
Meishan−C
European−A
European−B
European−C

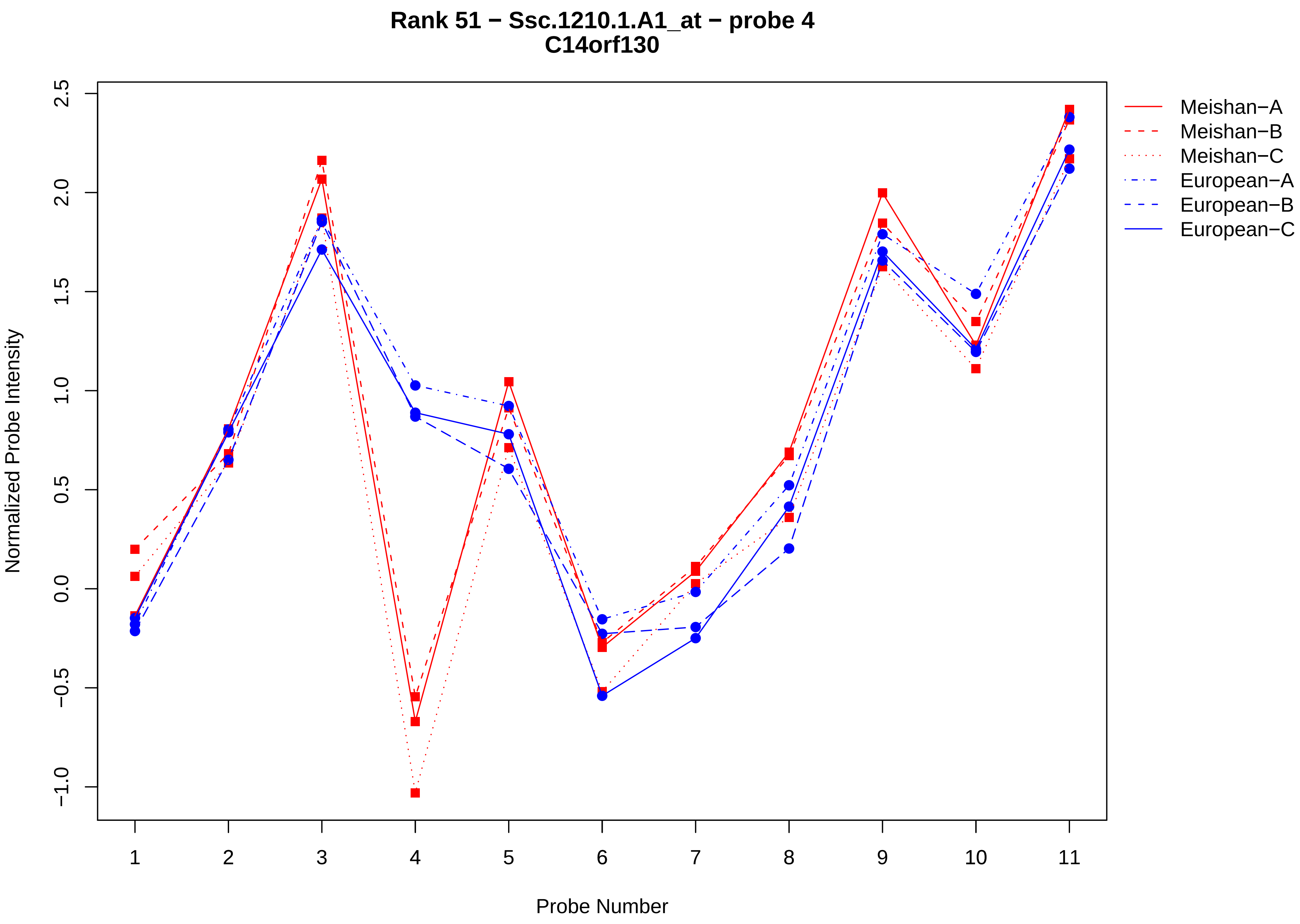
Rank 51 − Ssc.1210.1.A1_at − probe 4
C14orf130
Normalized Probe Intensity
Probe Number
−1.0
−0.5
0.0
0.5
1.0
1.5
2.0
2.5
1
2
3
4
5
6
7
8
9
10
11
Meishan−A
Meishan−B
Meishan−C
European−A
European−B
European−C

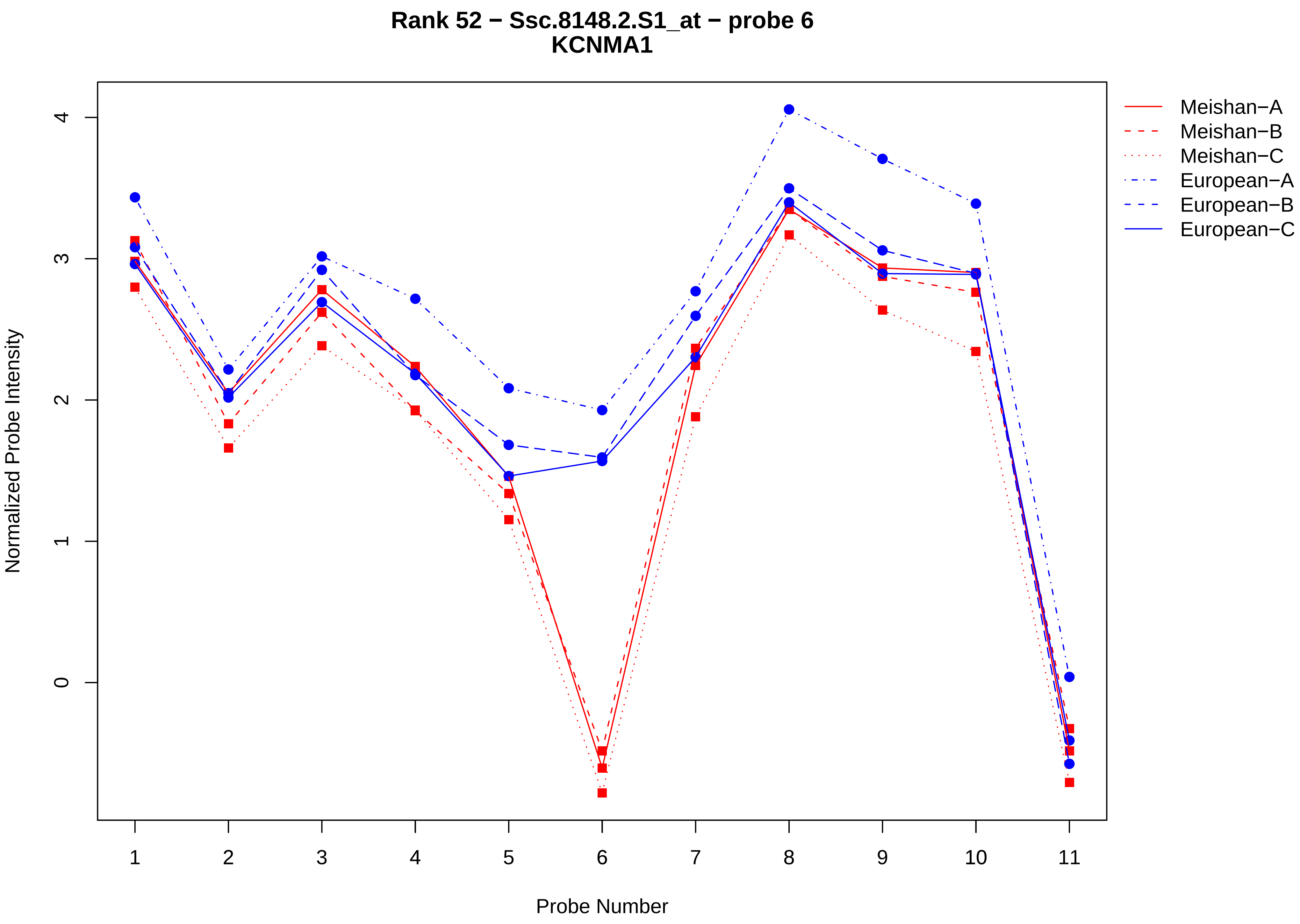
Rank 52 − Ssc.8148.2.S1_at − probe 6
KCNMA1
Normalized Probe Intensity
Probe Number
0
1
2
3
4
1
2
3
4
5
6
7
8
9
10
11
Meishan−A
Meishan−B
Meishan−C
European−A
European−B
European−C

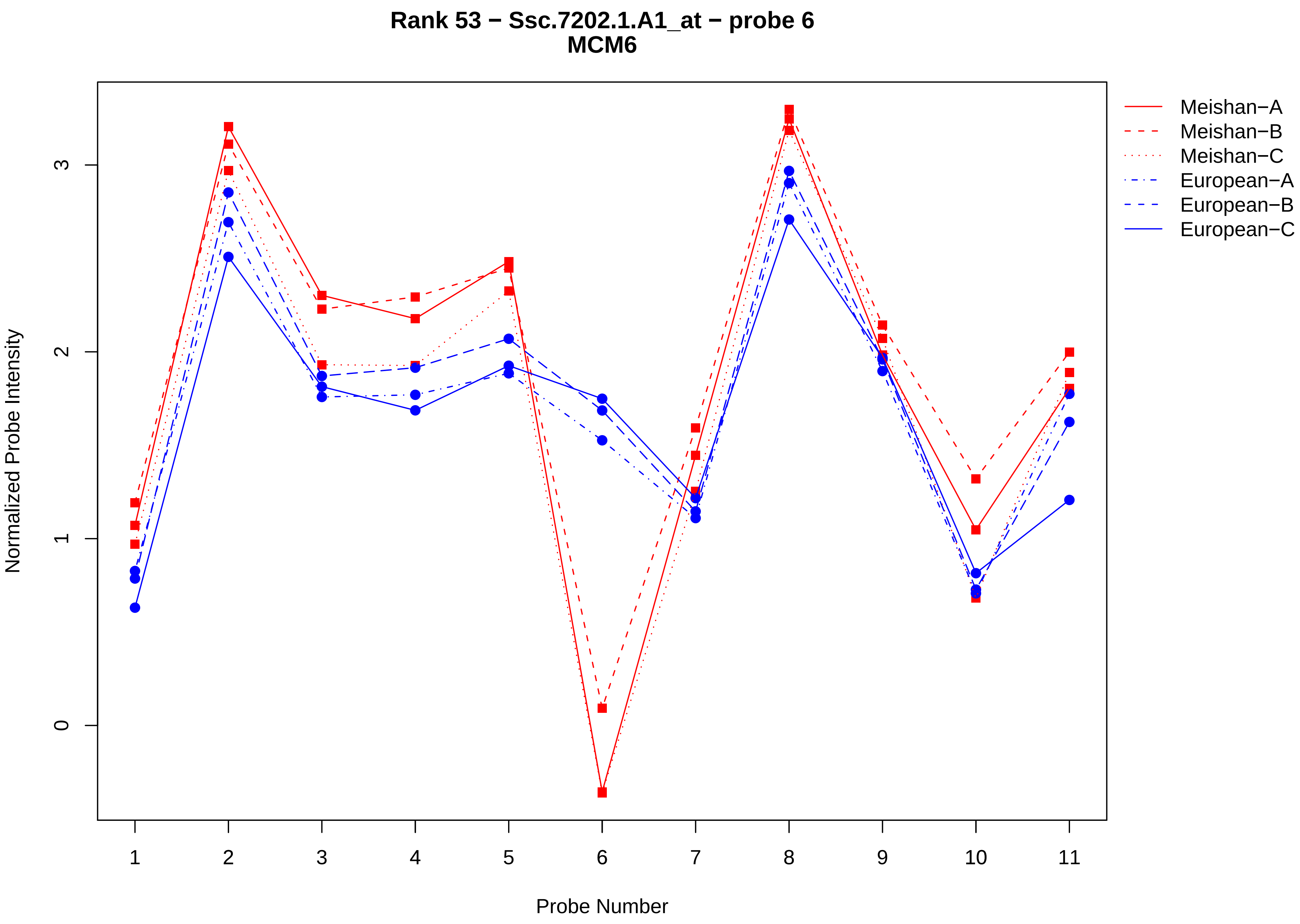

Rank 53 − Ssc.7202.1.A1_at − probe 6
MCM6
Normalized Probe Intensity
0
1
2
3
1
2
3
4
5
6
7
8
9
10
11
Probe Number
Meishan−A
Meishan−B
Meishan−C
European−A
European−B
European−C

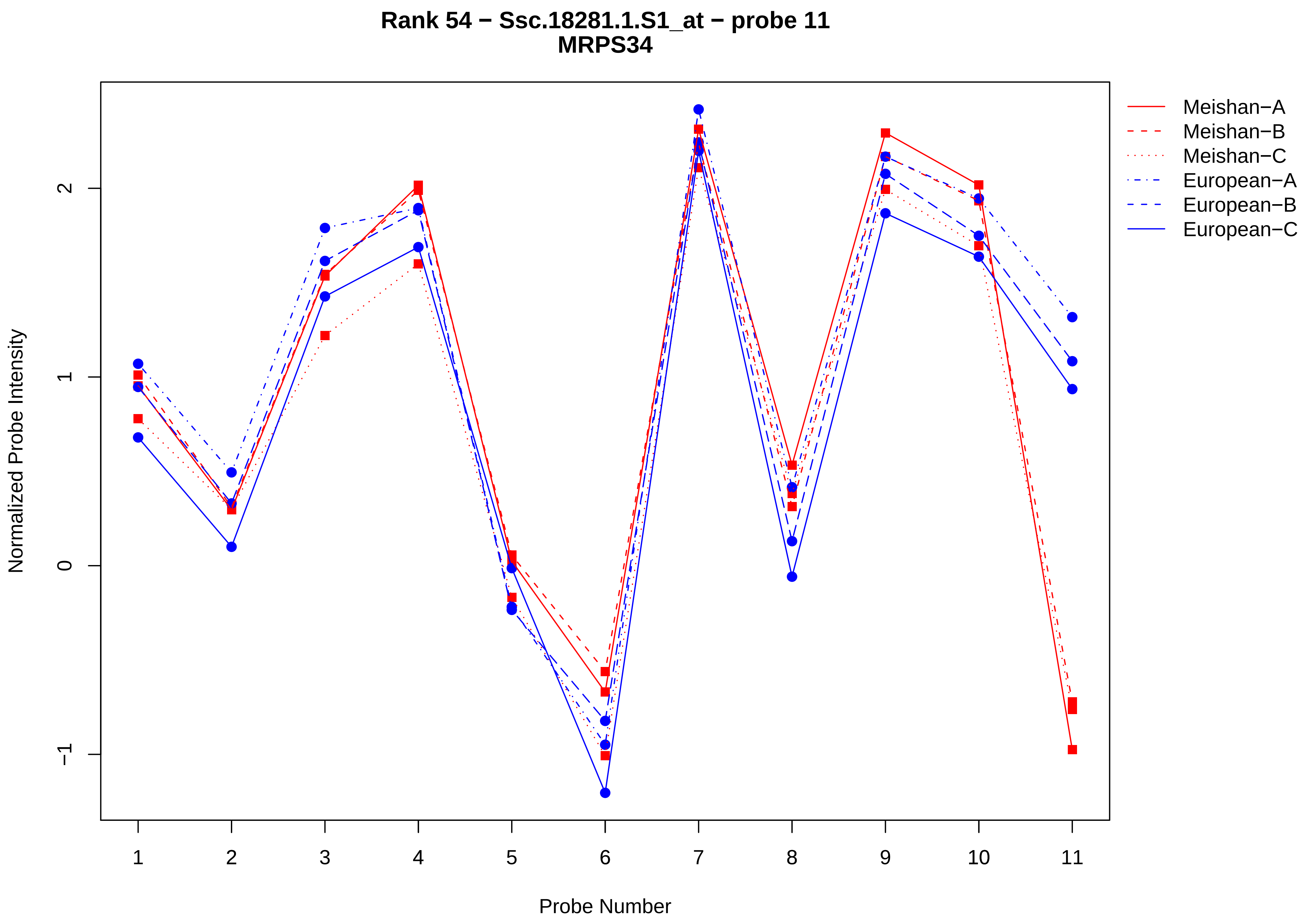
Rank 54 − Ssc.18281.1.S1_at − probe 11
MRPS34
Normalized Probe Intensity
Probe Number
−1
0
1
2
1
2
3
4
5
6
7
8
9
10
11
Meishan−A
Meishan−B
Meishan−C
European−A
European−B
European−C

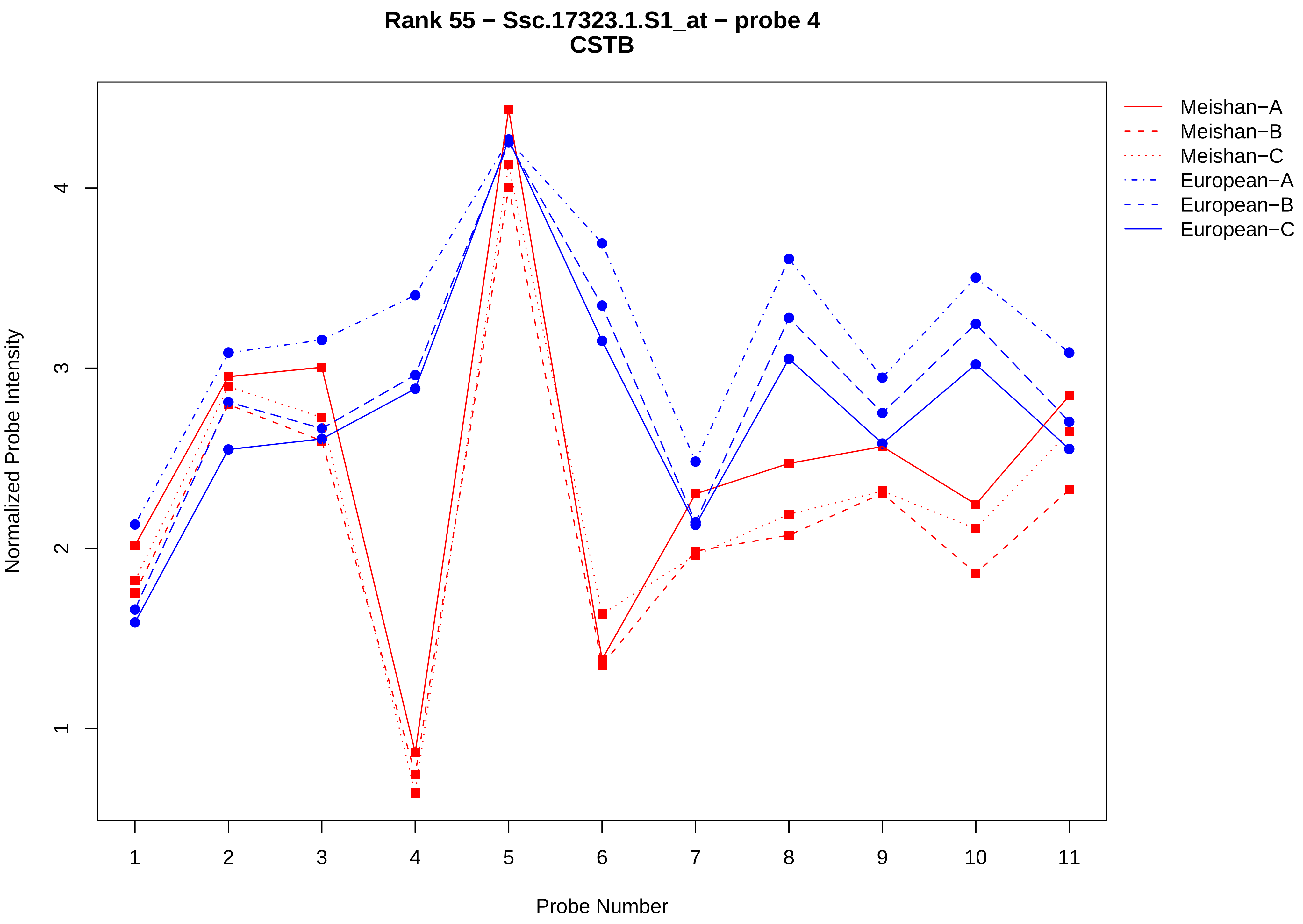
Rank 55 − Ssc.17323.1.S1_at − probe 4
CSTB
Normalized Probe Intensity
Probe Number
1
2
3
4
1
2
3
4
5
6
7
8
9
10
11
Meishan−A
Meishan−B
Meishan−C
European−A
European−B
European−C

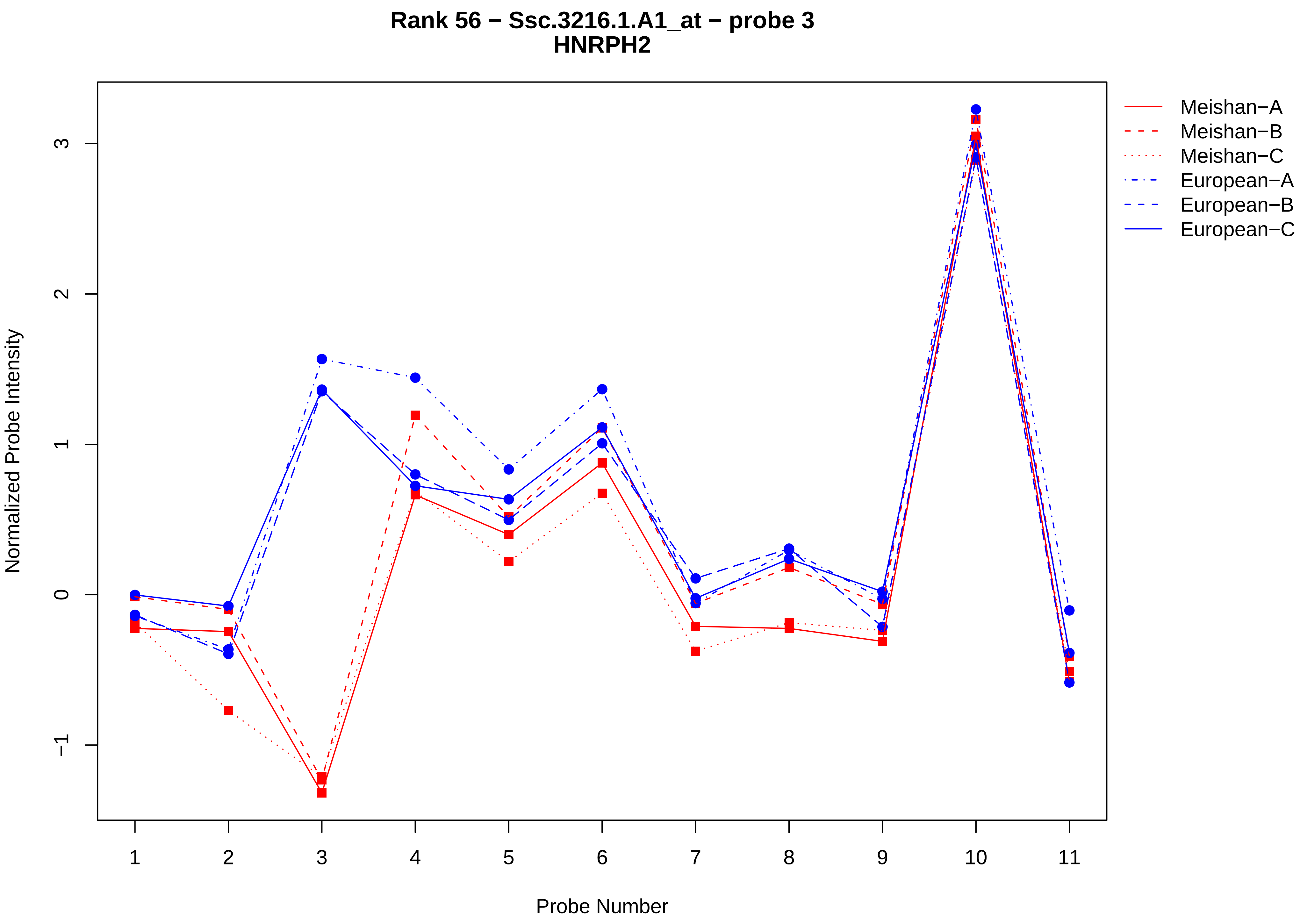
Rank 56 − Ssc.3216.1.A1_at − probe 3
HNRPH2
Normalized Probe Intensity
Probe Number
−1
0
1
2
3
1
2
3
4
5
6
7
8
9
10
11
Meishan−A
Meishan−B
Meishan−C
European−A
European−B
European−C

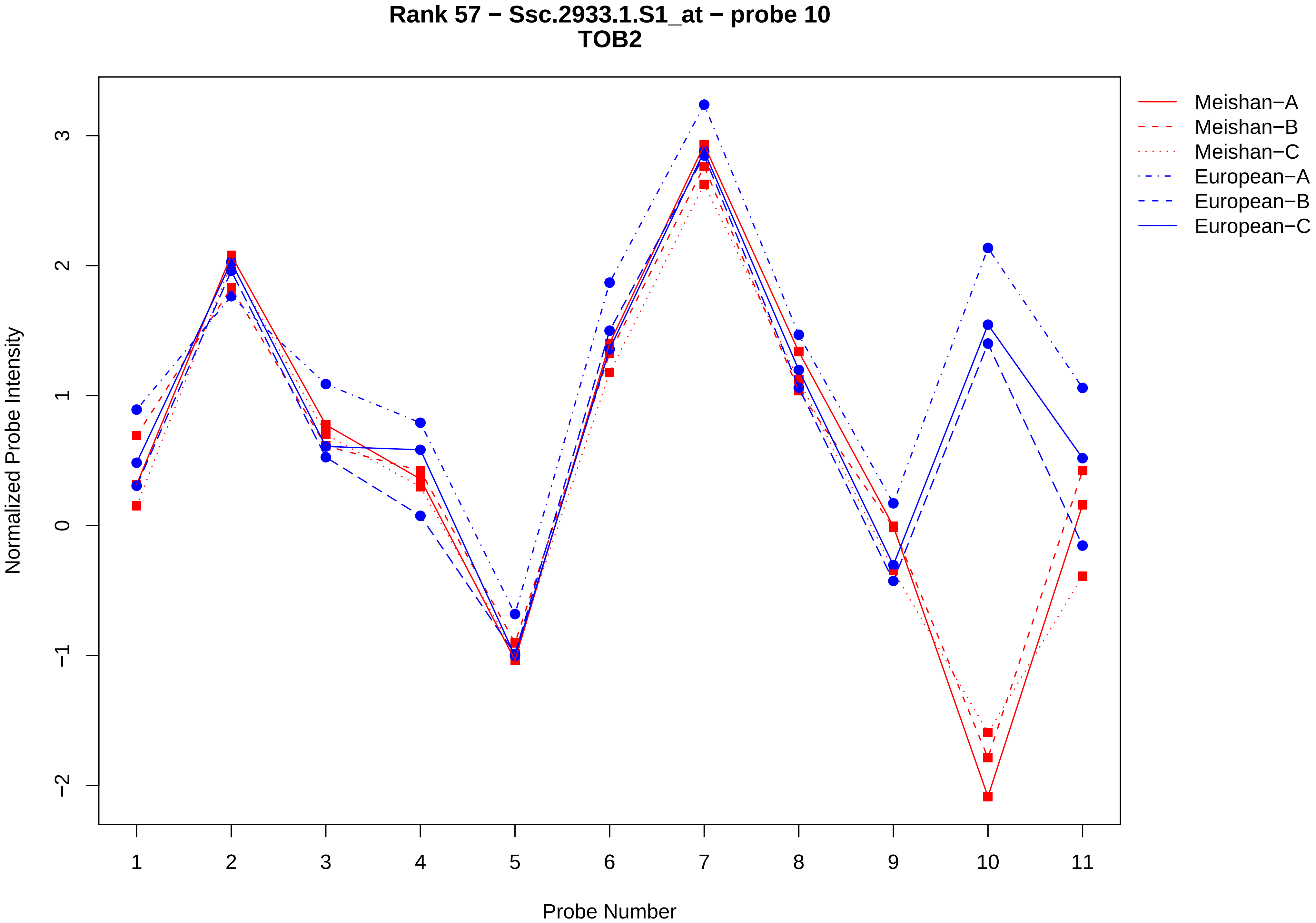

Rank 57 − Ssc.2933.1.S1_at − probe 10
TOB2
Normalized Probe Intensity
Probe Number
3
2
1
0
−1
−2
1
2
3
4
5
6
7
8
9
10
11
Meishan−A
Meishan−B
Meishan−C
European−A
European−B
European−C

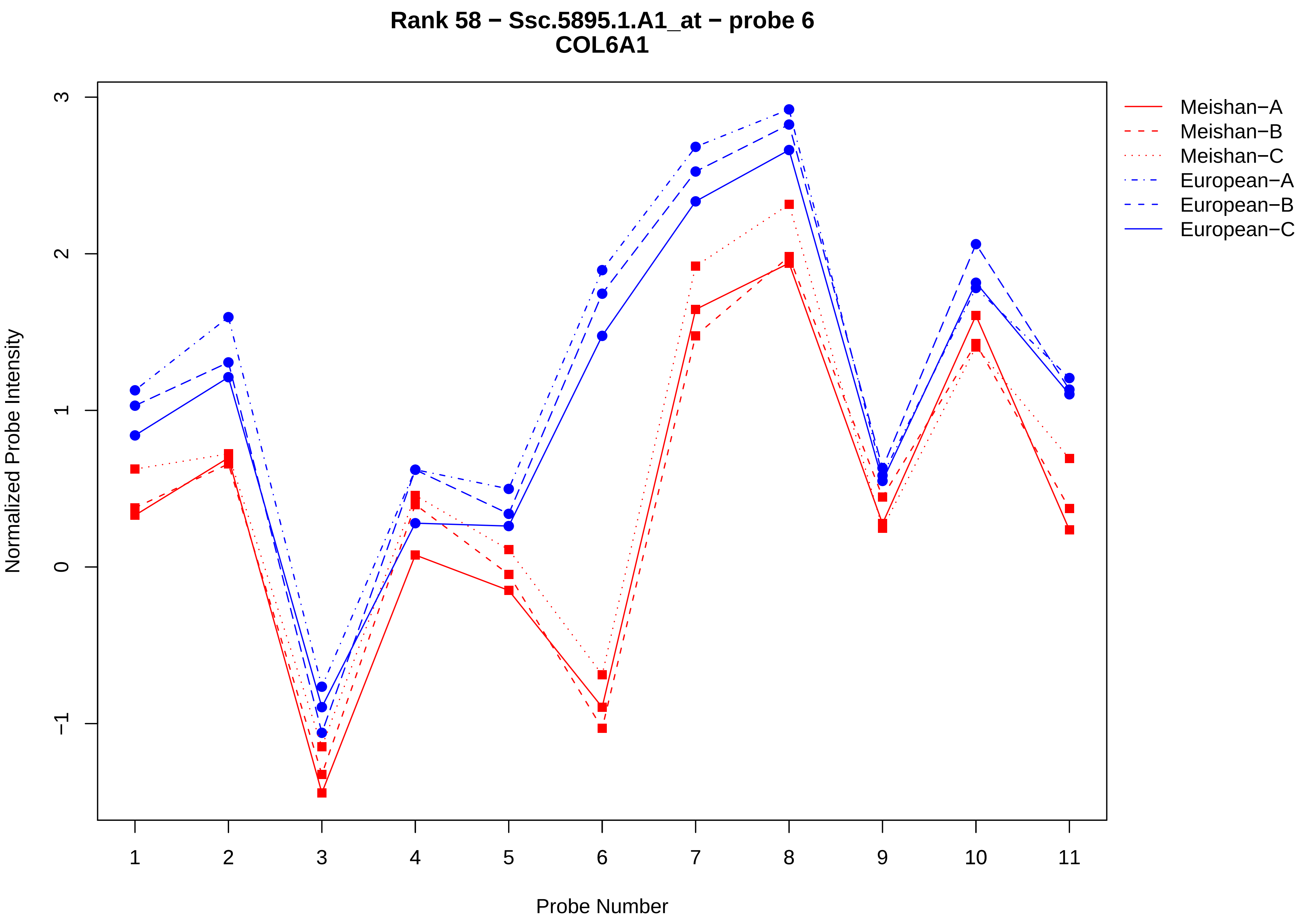
Rank 58 − Ssc.5895.1.A1_at − probe 6
COL6A1
Normalized Probe Intensity
Probe Number
Meishan−A
Meishan−B
Meishan−C
European−A
European−B
European−C
−1
0
1
2
3
1
2
3
4
5
6
7
8
9
10
11

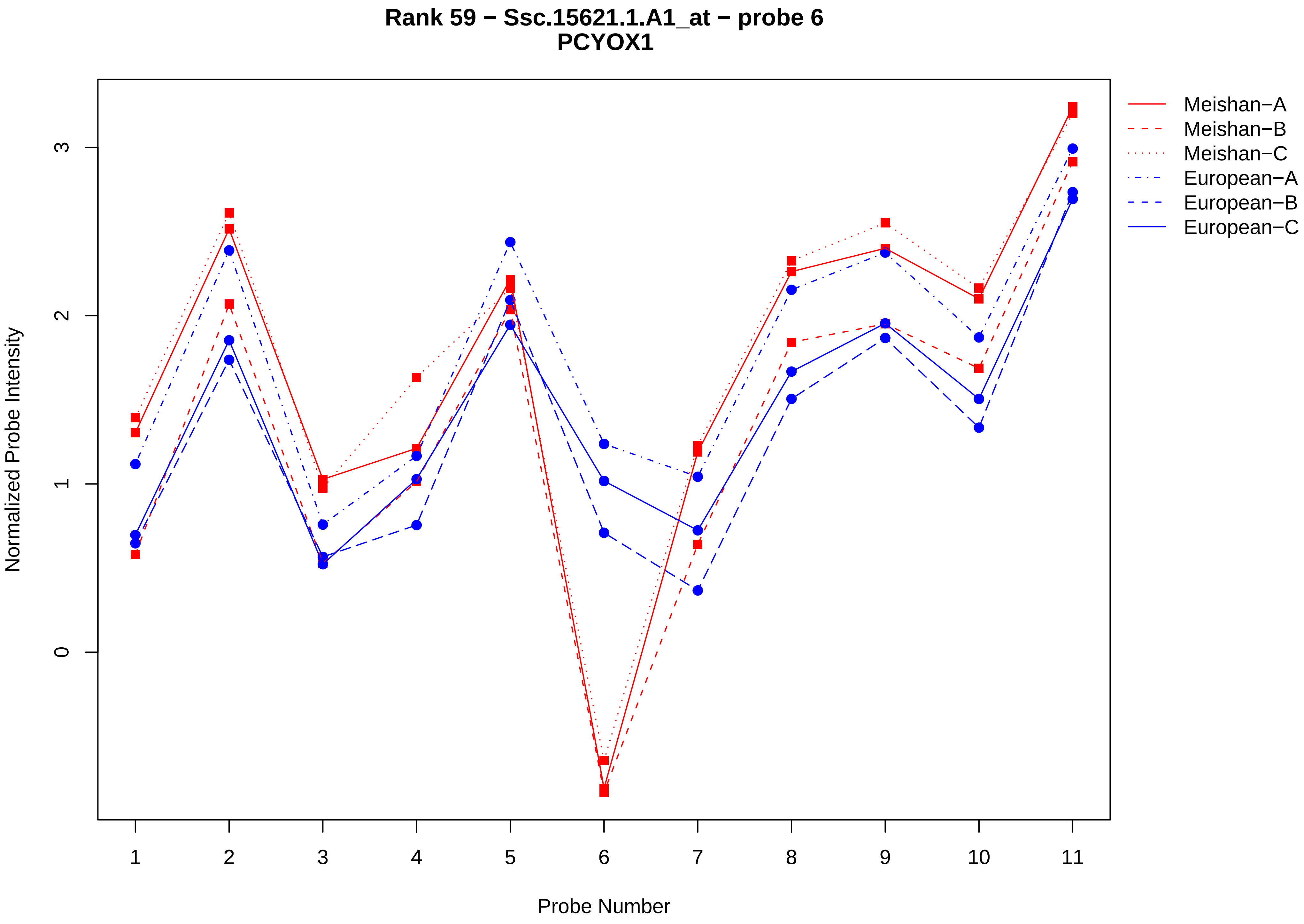
Rank 59 − Ssc.15621.1.A1_at − probe 6
PCYOX1
Normalized Probe Intensity
Probe Number
0
1
2
3
1
2
3
4
5
6
7
8
9
10
11
Meishan−A
Meishan−B
Meishan−C
European−A
European−B
European−C

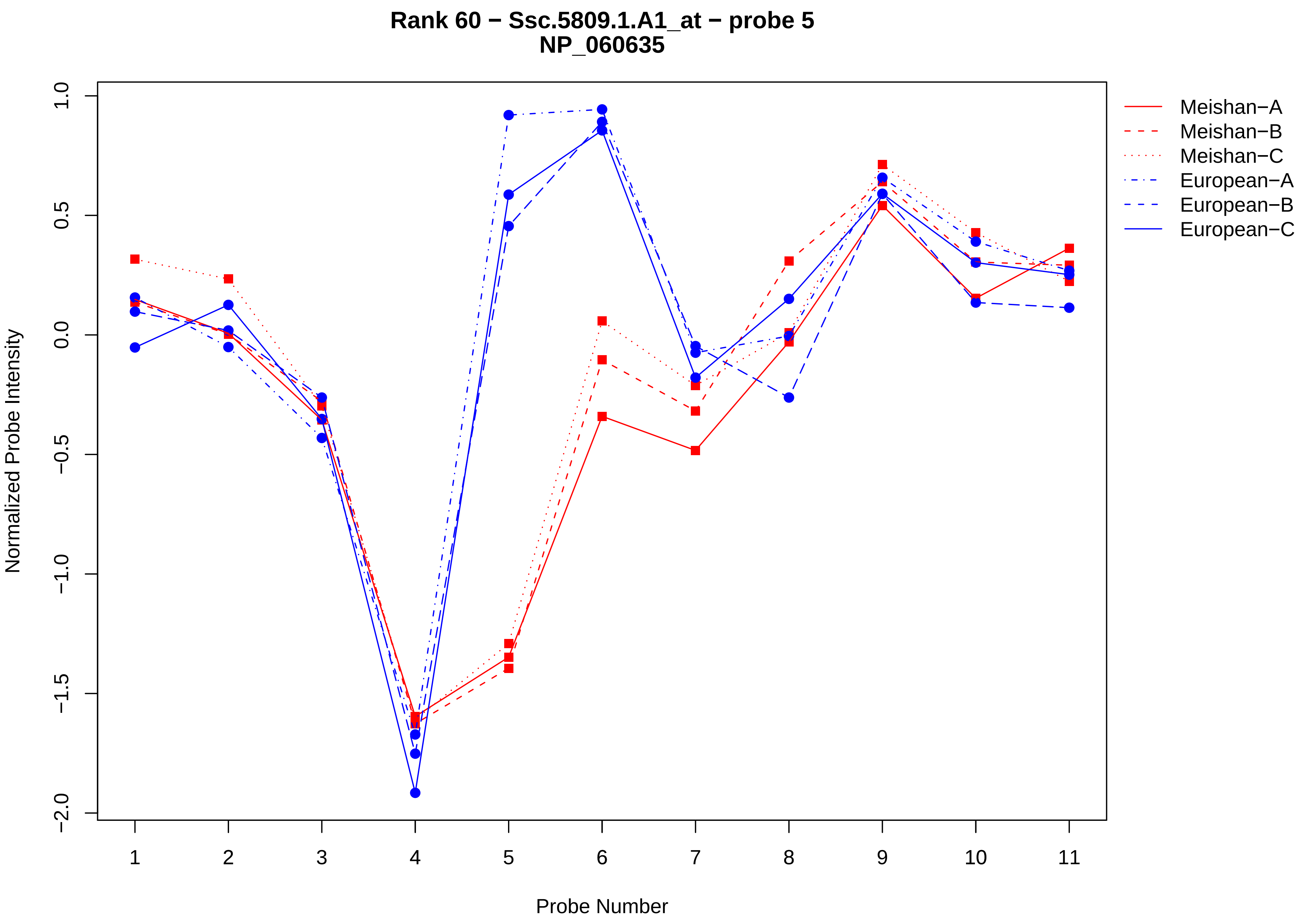
Rank 60 − Ssc.5809.1.A1_at − probe 5
NP_060635
Normalized Probe Intensity
1.0
0.5
0.0
−0.5
−1.0
−1.5
−2.0
1
2
3
4
5
6
7
8
9
10
11
Probe Number
Meishan−A
Meishan−B
Meishan−C
European−A
European−B
European−C

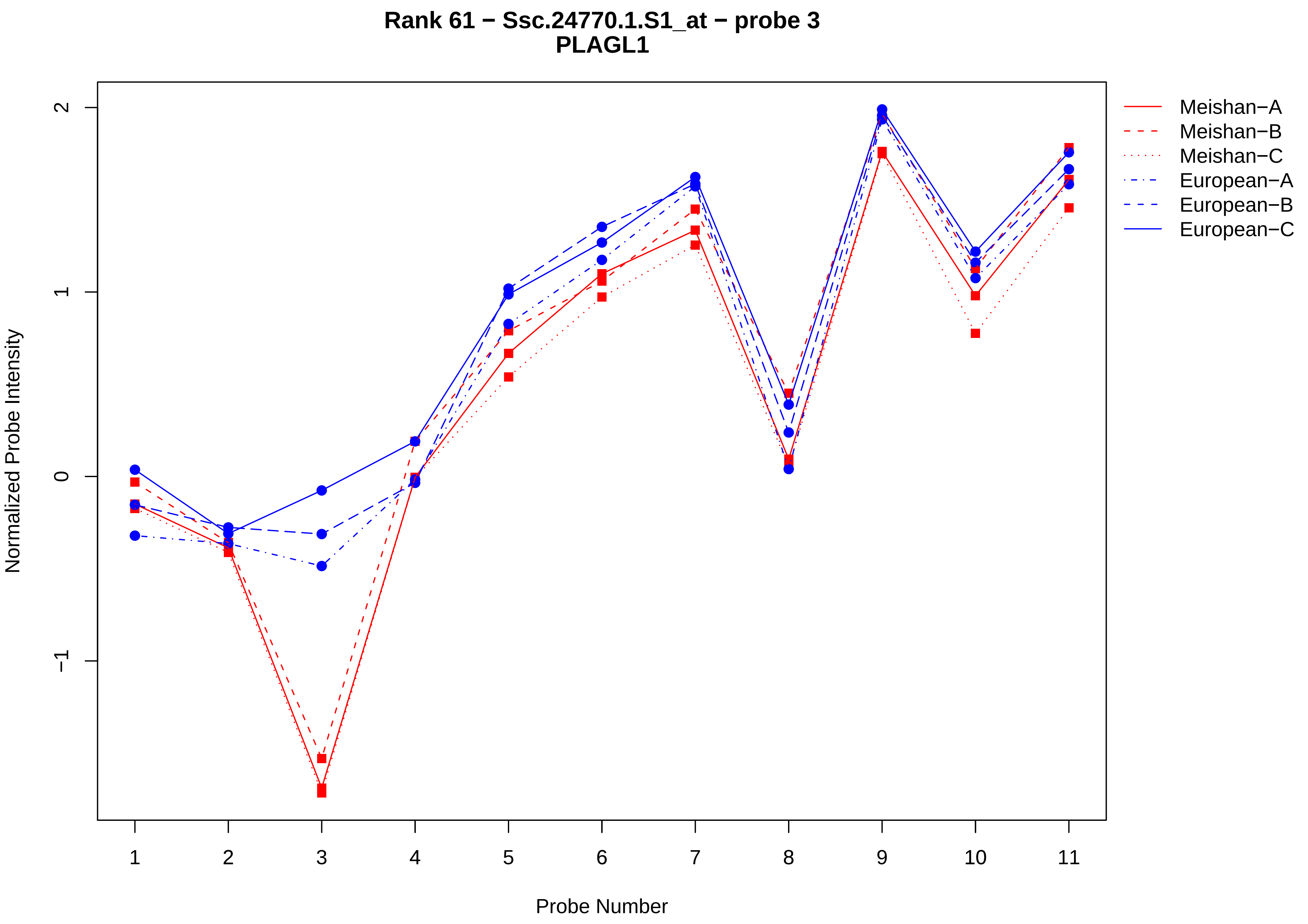
Rank 61 − Ssc.24770.1.S1_at − probe 3
PLAGL1
Normalized Probe Intensity
Probe Number
−1
0
1
2
1
2
3
4
5
6
7
8
9
10
11
Meishan−A
Meishan−B
Meishan−C
European−A
European−B
European−C

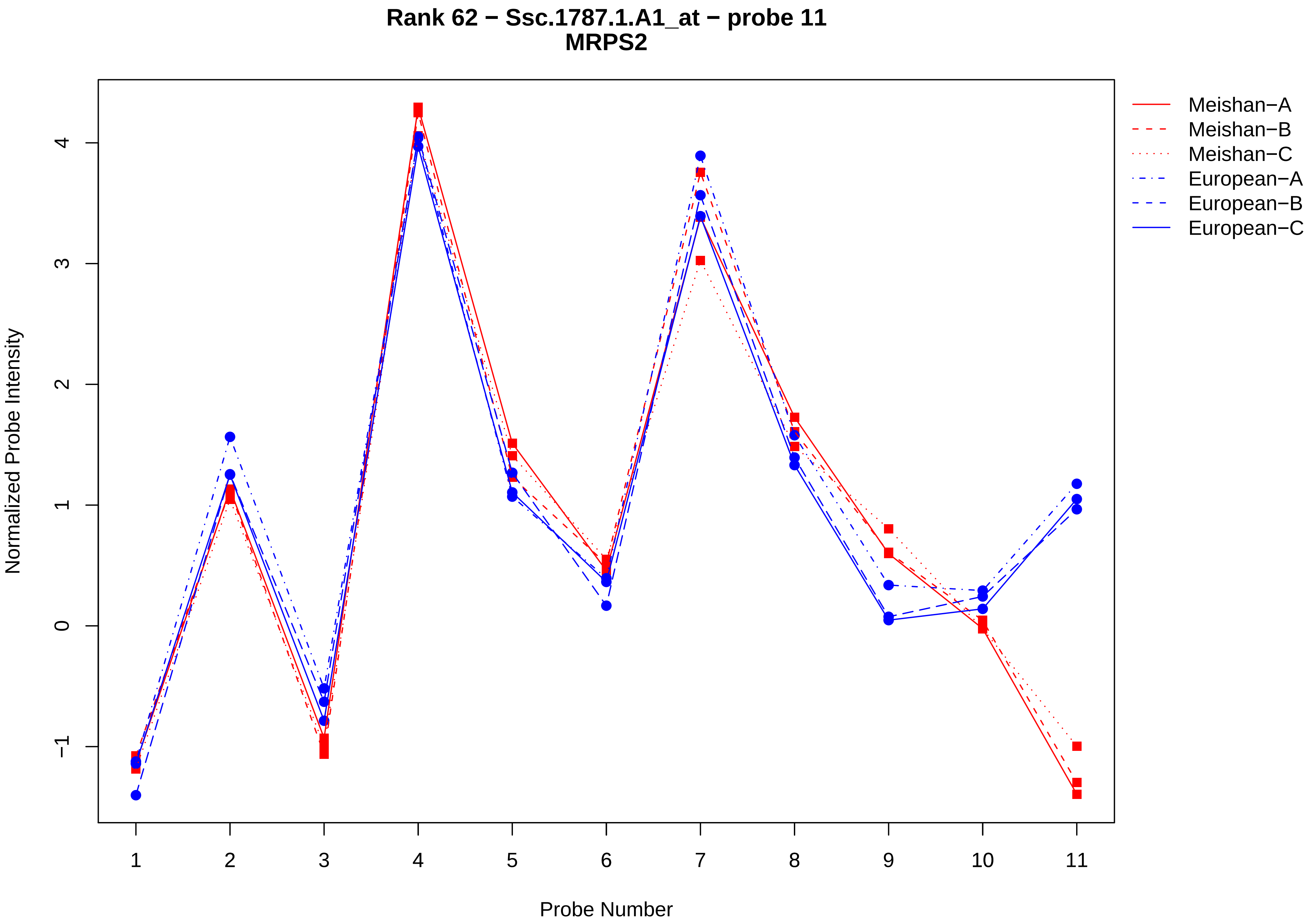
Rank 62 − Ssc.1787.1.A1_at − probe 11
MRPS2
Normalized Probe Intensity
Probe Number
−1
0
1
2
3
4
1
2
3
4
5
6
7
8
9
10
11
Meishan−A
Meishan−B
Meishan−C
European−A
European−B
European−C

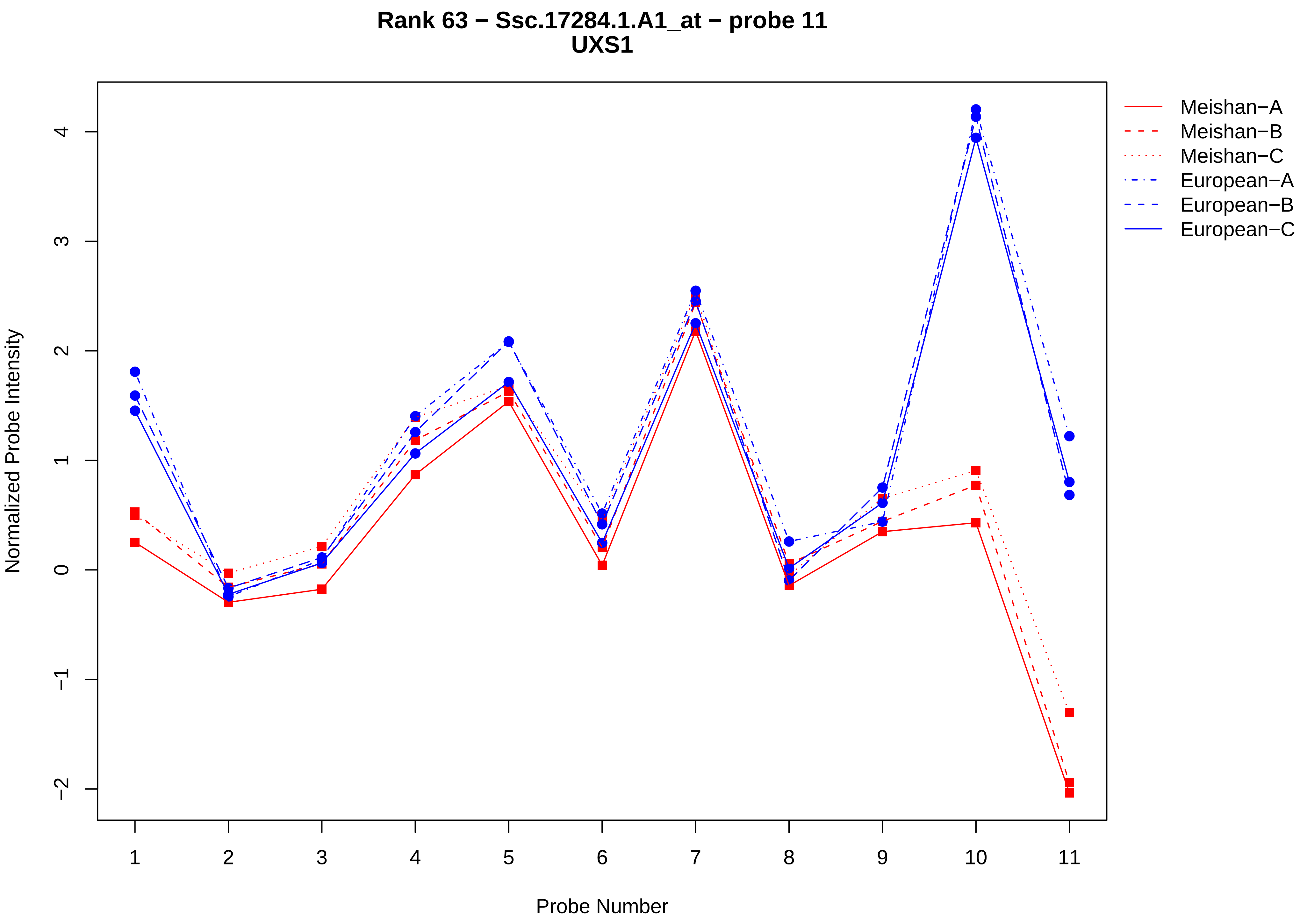

Rank 63 − Ssc.17284.1.A1_at − probe 11
UXS1
Normalized Probe Intensity
Probe Number
−2
−1
0
1
2
3
4
1
2
3
4
5
6
7
8
9
10
11
Meishan−A
Meishan−B
Meishan−C
European−A
European−B
European−C

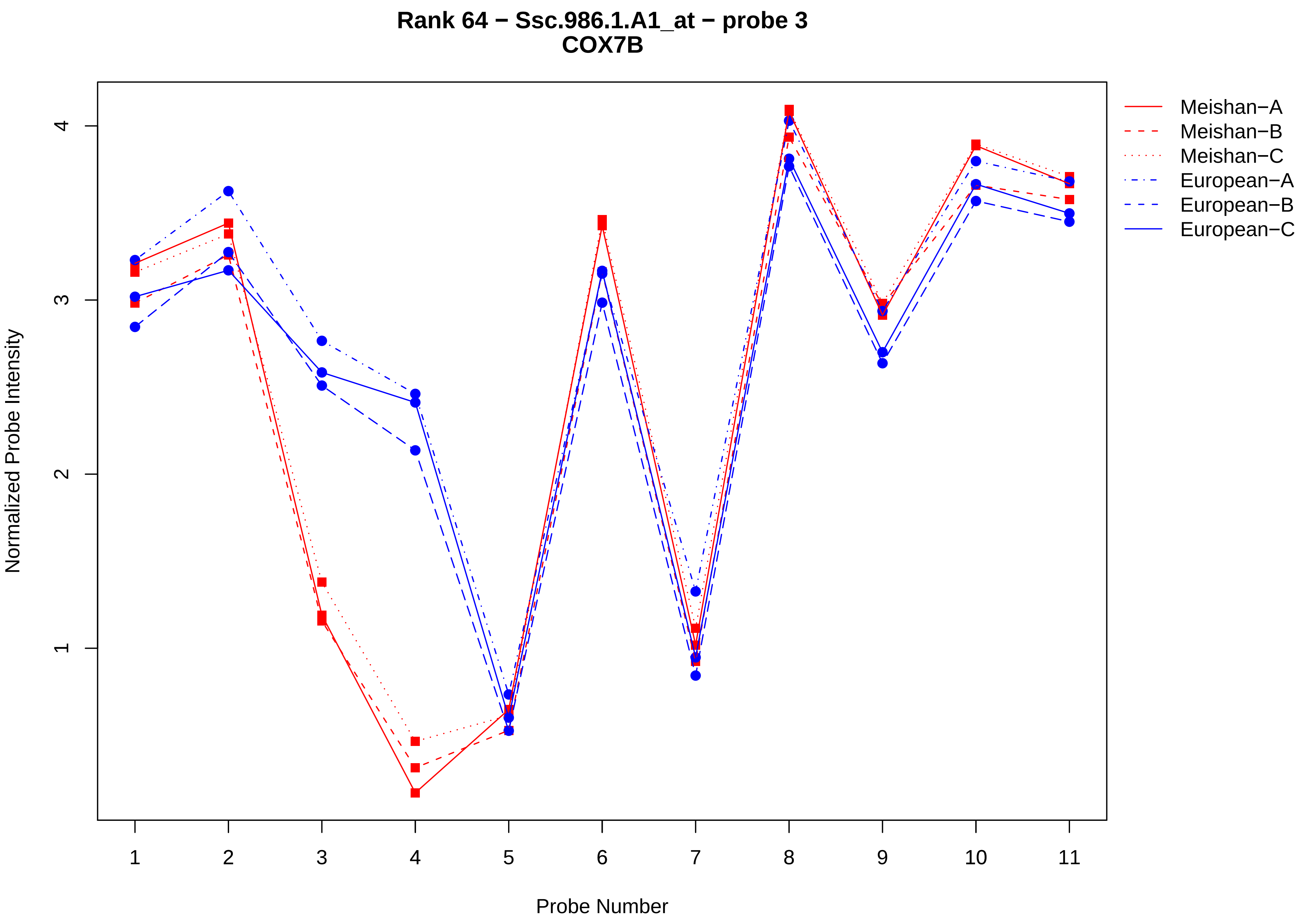
Rank 64 − Ssc.986.1.A1_at − probe 3
COX7B
Normalized Probe Intensity
Probe Number
1
2
3
4
1
2
3
4
5
6
7
8
9
10
11
Meishan−A
Meishan−B
Meishan−C
European−A
European−B
European−C

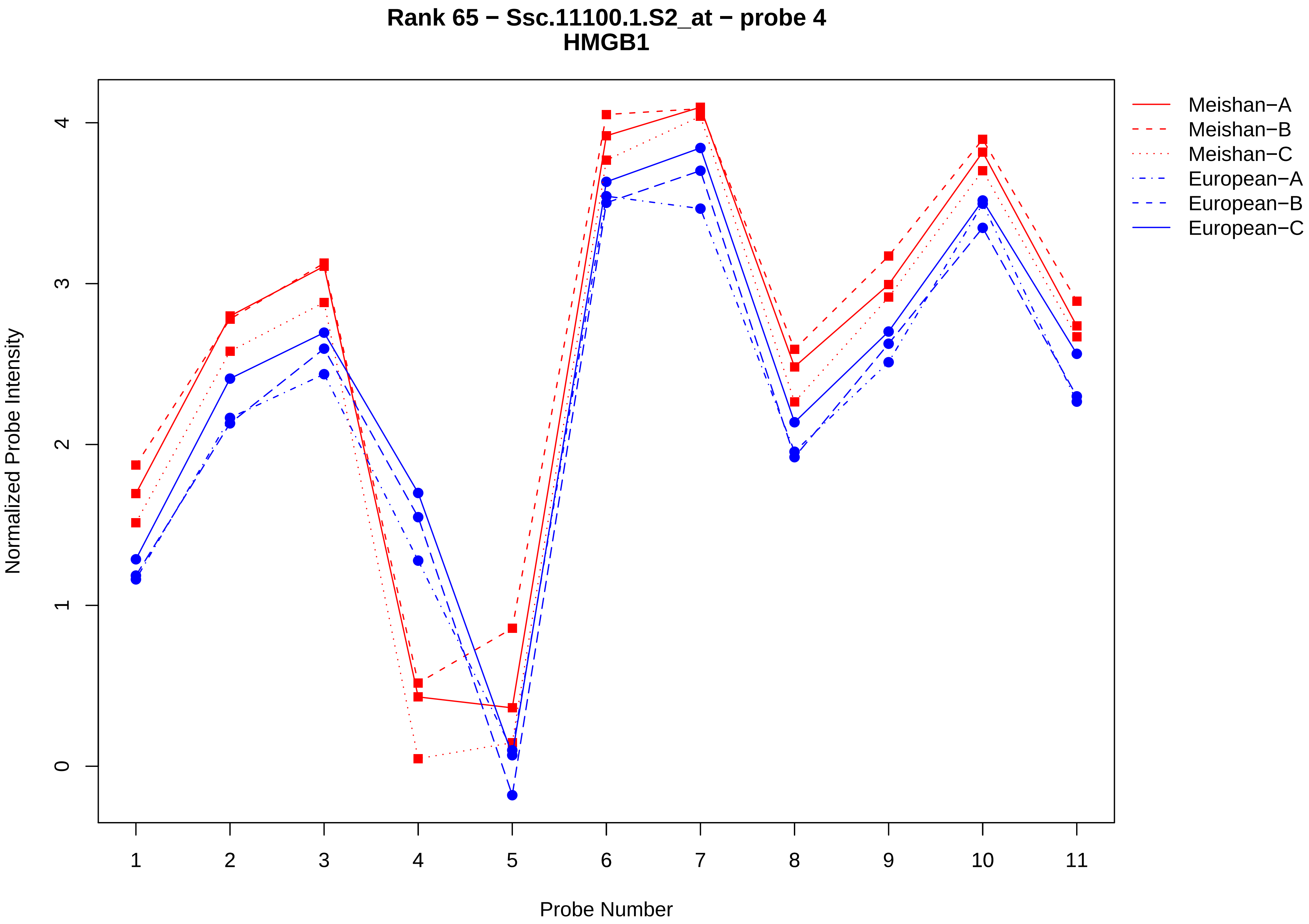
Rank 65 − Ssc.11100.1.S2_at − probe 4
HMGB1
Normalized Probe Intensity
0
1
2
3
4
1
2
3
4
5
6
7
8
9
10
11
Probe Number
Meishan−A
Meishan−B
Meishan−C
European−A
European−B
European−C

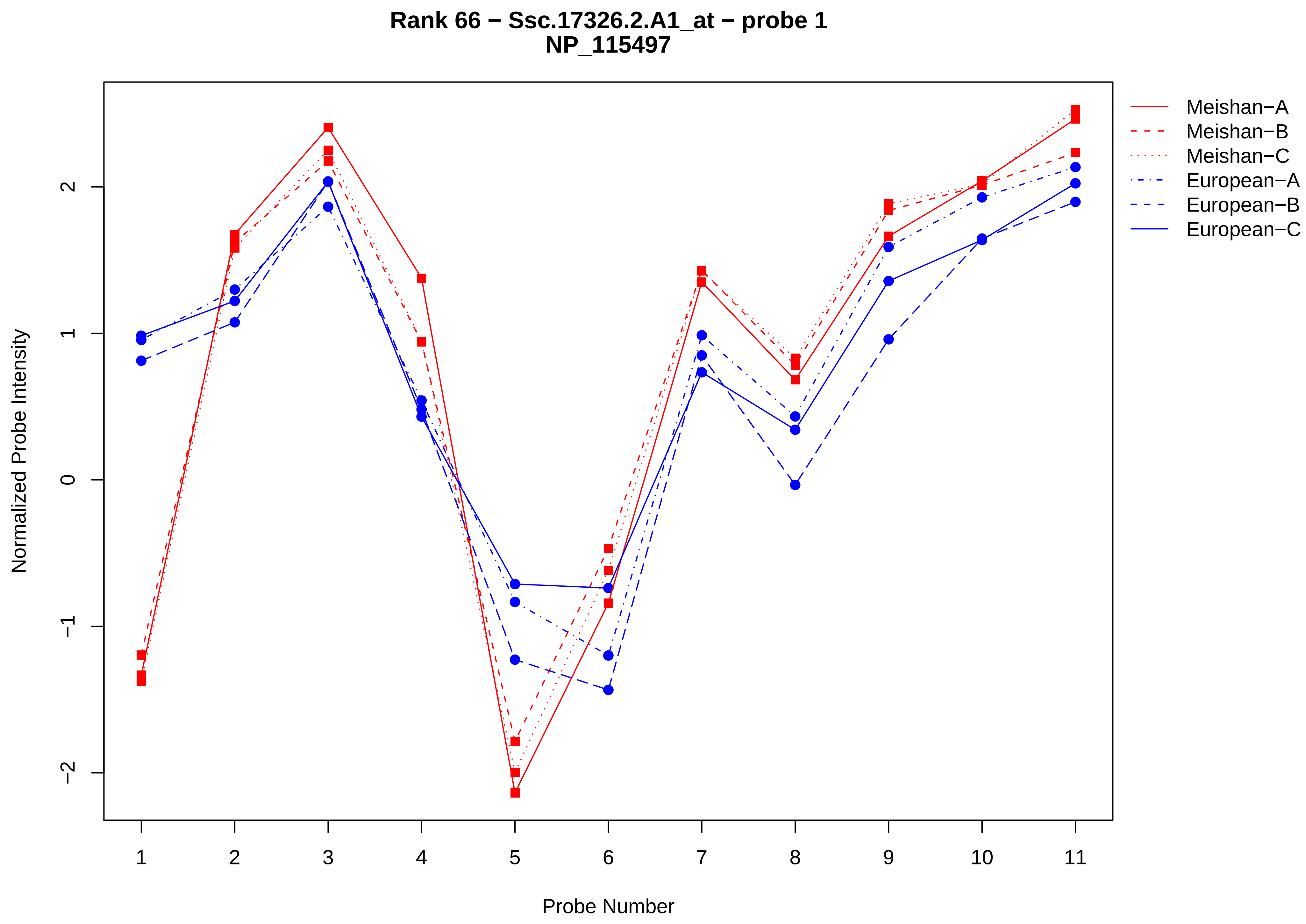

Rank 66 − Ssc.17326.2.A1_at − probe 1
NP_115497
Normalized Probe Intensity
Probe Number
−2
−1
0
1
2
1
2
3
4
5
6
7
8
9
10
11
Meishan−A
Meishan−B
Meishan−C
European−A
European−B
European−C

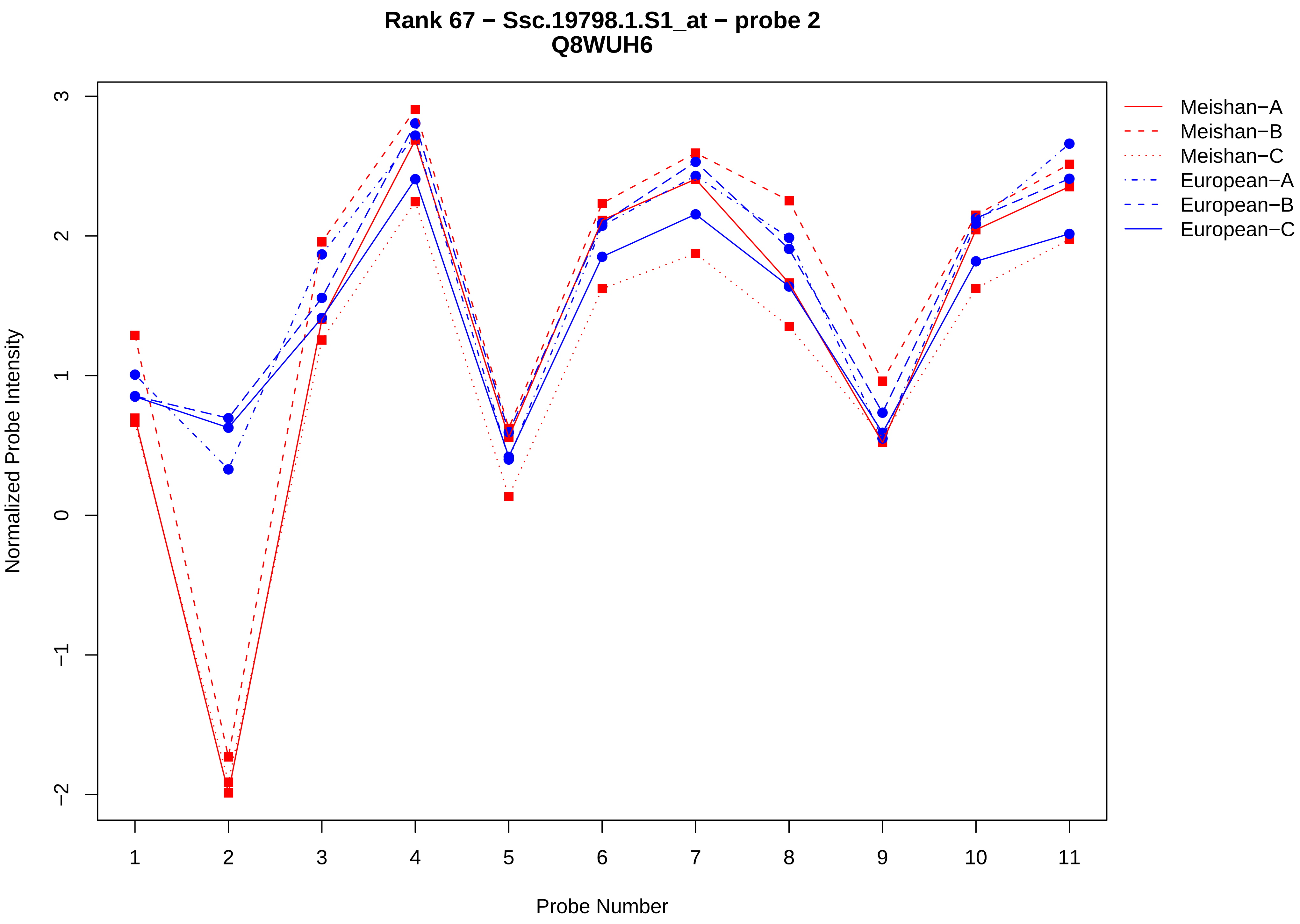
Rank 67 − Ssc.19798.1.S1_at − probe 2
Q8WUH6
Normalized Probe Intensity
Probe Number
−2
−1
0
1
2
3
1
2
3
4
5
6
7
8
9
10
11
Meishan−A
Meishan−B
Meishan−C
European−A
European−B
European−C

Rank 68 − Ssc.734.1.S1_at − probe 11
MMP14

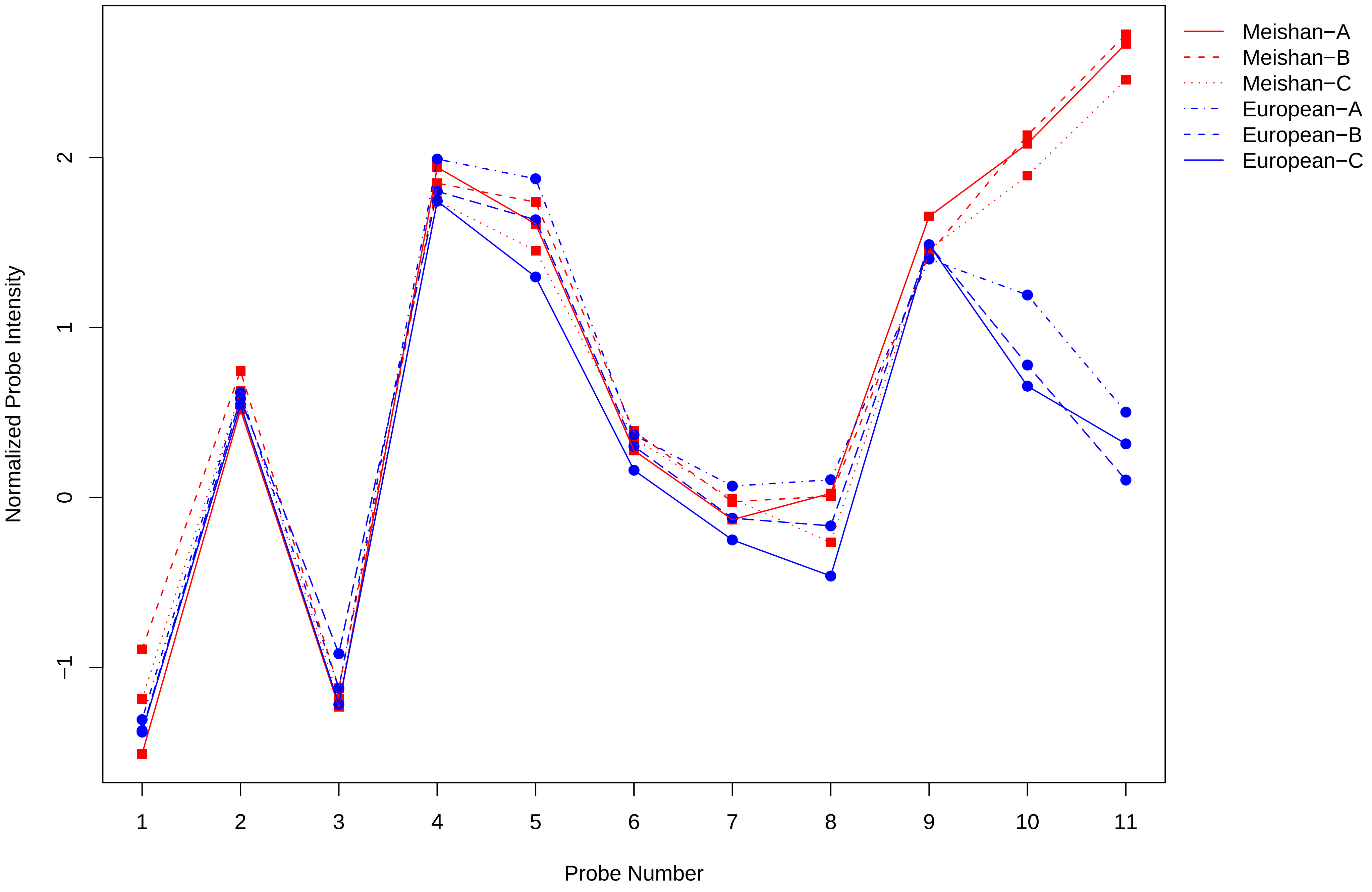

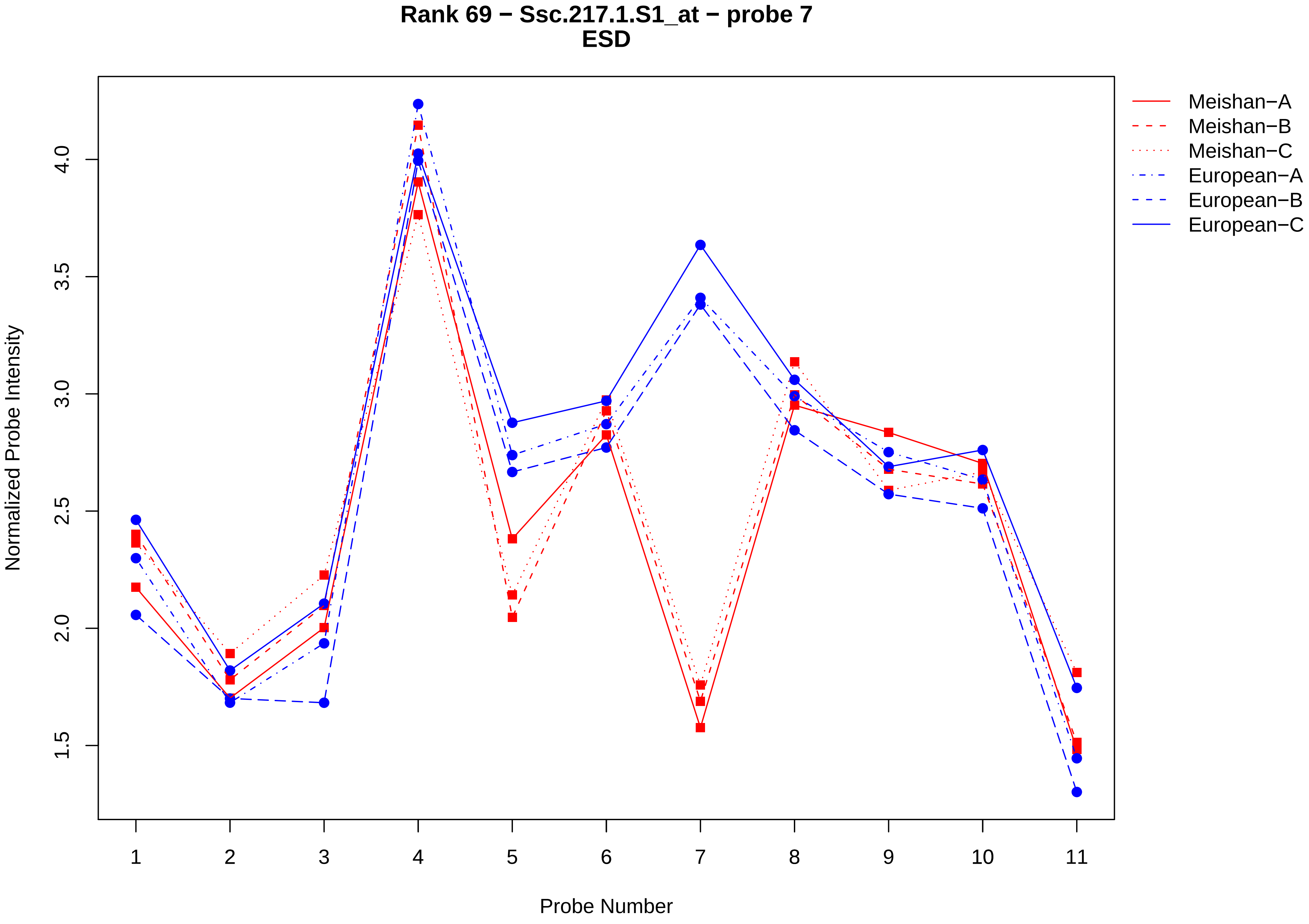
Rank 69 − Ssc.217.1.S1_at − probe 7
ESD
Normalized Probe Intensity
Probe Number
1.5
2.0
2.5
3.0
3.5
4.0
1
2
3
4
5
6
7
8
9
10
11
Meishan−A
Meishan−B
Meishan−C
European−A
European−B
European−C

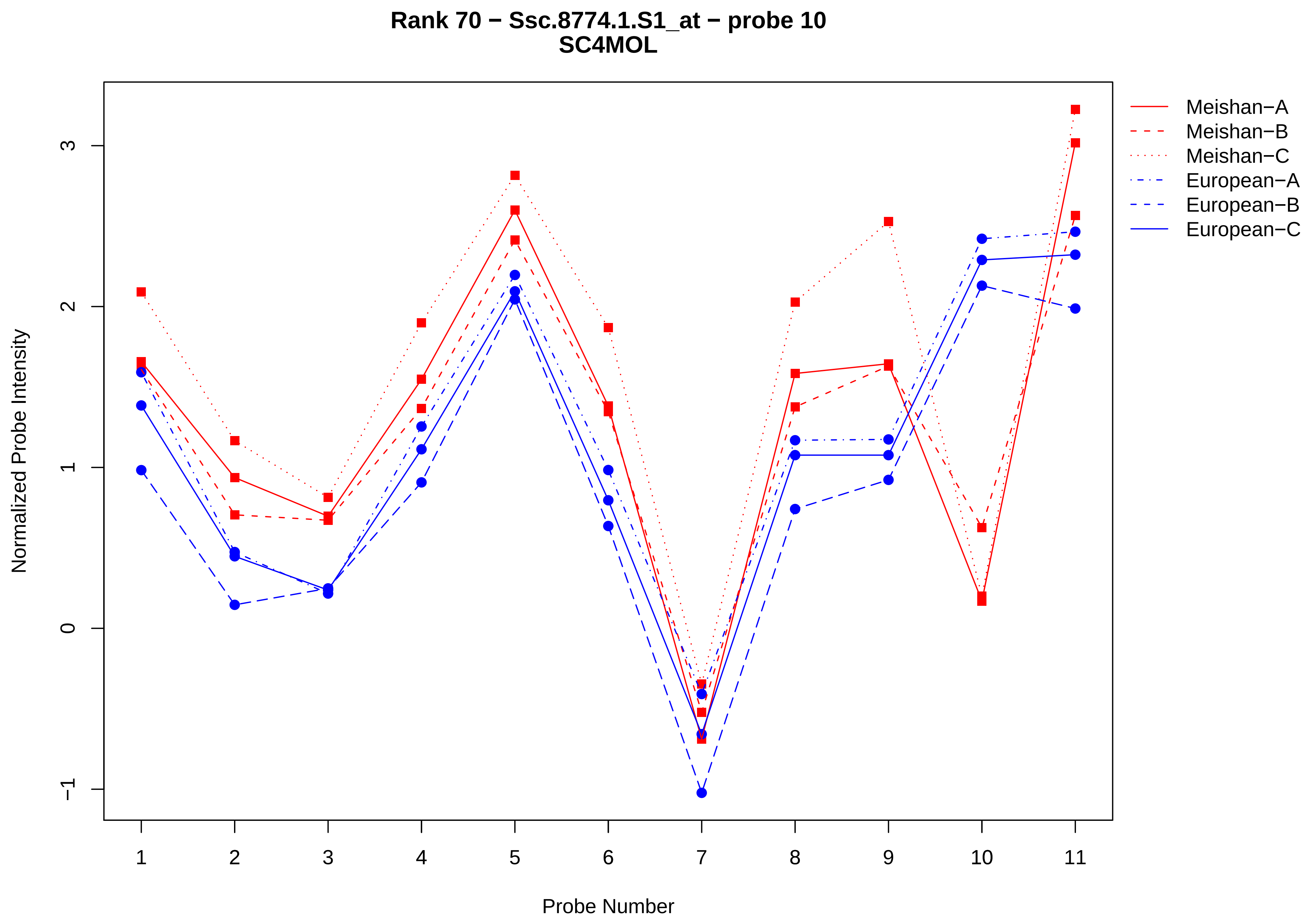
Rank 70 − Ssc.8774.1.S1_at − probe 10
SC4MOL
Normalized Probe Intensity
Probe Number
−1
0
1
2
3
1
2
3
4
5
6
7
8
9
10
11
Meishan−A
Meishan−B
Meishan−C
European−A
European−B
European−C

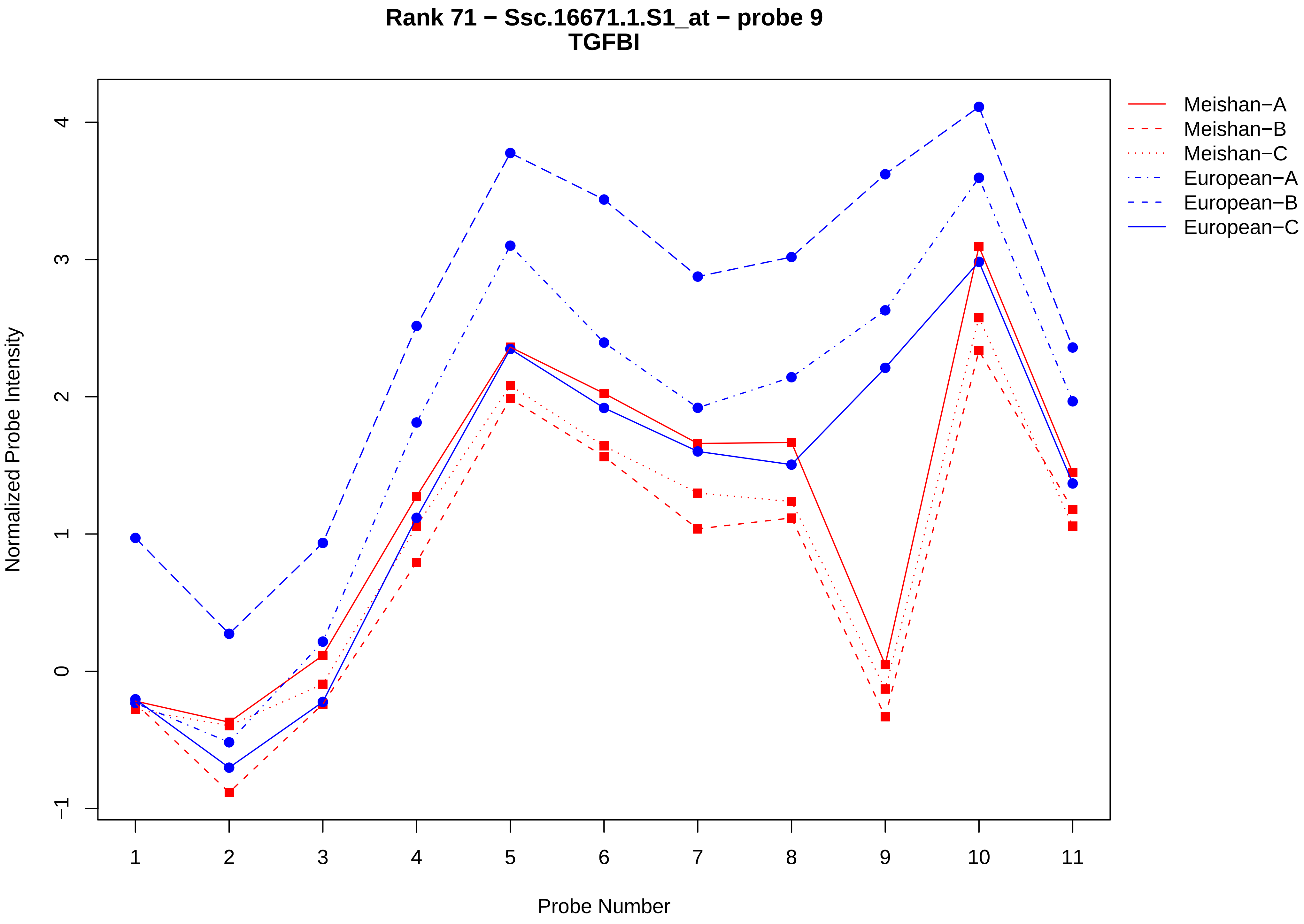

Rank 71 − Ssc.16671.1.S1_at − probe 9
TGFBI
Normalized Probe Intensity
Probe Number
−1
0
1
2
3
4
1
2
3
4
5
6
7
8
9
10
11
Meishan−A
Meishan−B
Meishan−C
European−A
European−B
European−C

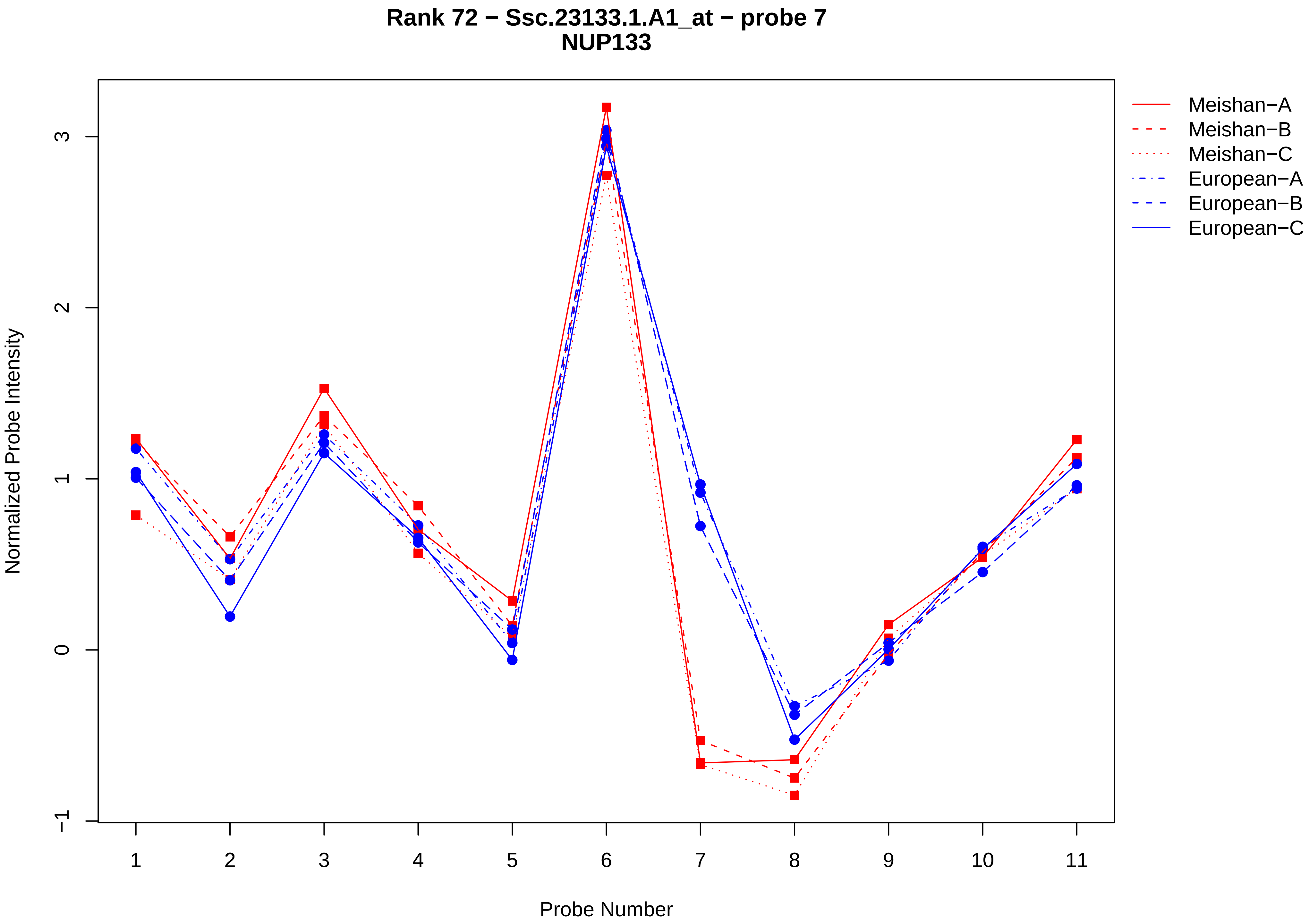

Rank 72 − Ssc.23133.1.A1_at − probe 7
NUP133
Normalized Probe Intensity
Probe Number
−1
0
1
2
3
1
2
3
4
5
6
7
8
9
10
11
Meishan−A
Meishan−B
Meishan−C
European−A
European−B
European−C

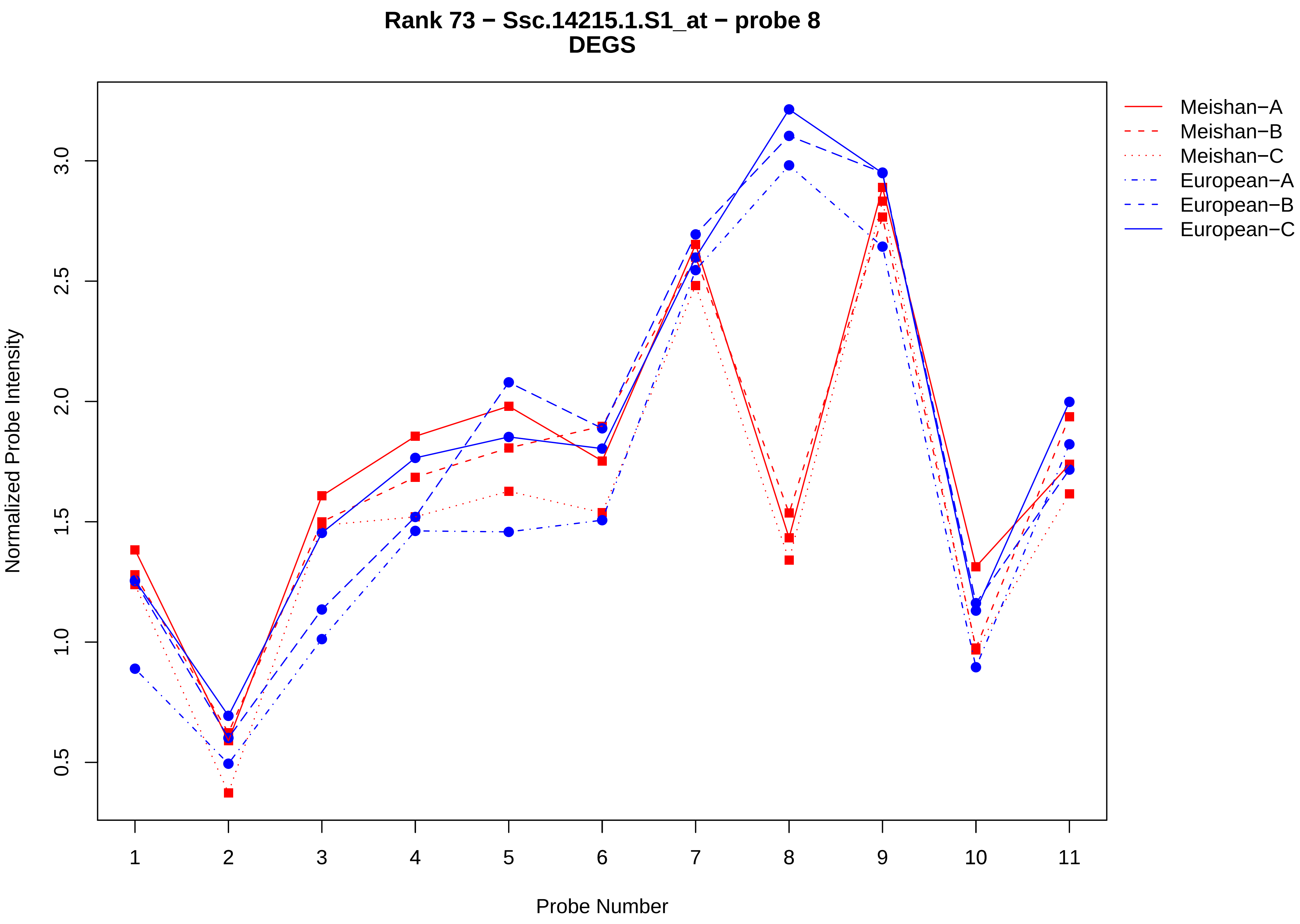
Rank 73 − Ssc.14215.1.S1_at − probe 8
DEGS
Normalized Probe Intensity
0.5
1.0
1.5
2.0
2.5
3.0
Probe Number
1
2
3
4
5
6
7
8
9
10
11
Meishan−A
Meishan−B
Meishan−C
European−A
European−B
European−C

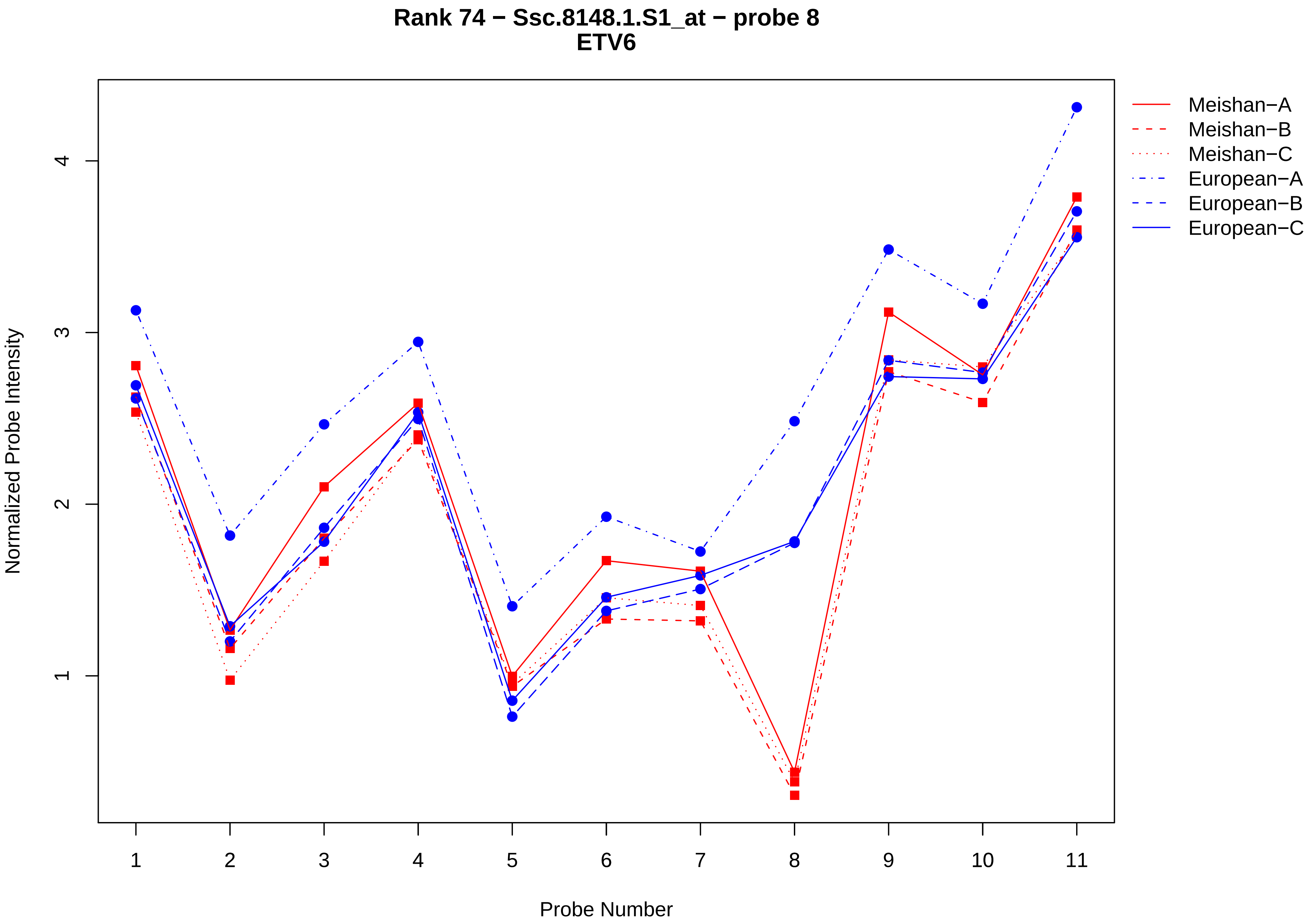
Rank 74 − Ssc.8148.1.S1_at − probe 8
ETV6
Normalized Probe Intensity
Probe Number
1
2
3
4
1
2
3
4
5
6
7
8
9
10
11
Meishan−A
Meishan−B
Meishan−C
European−A
European−B
European−C

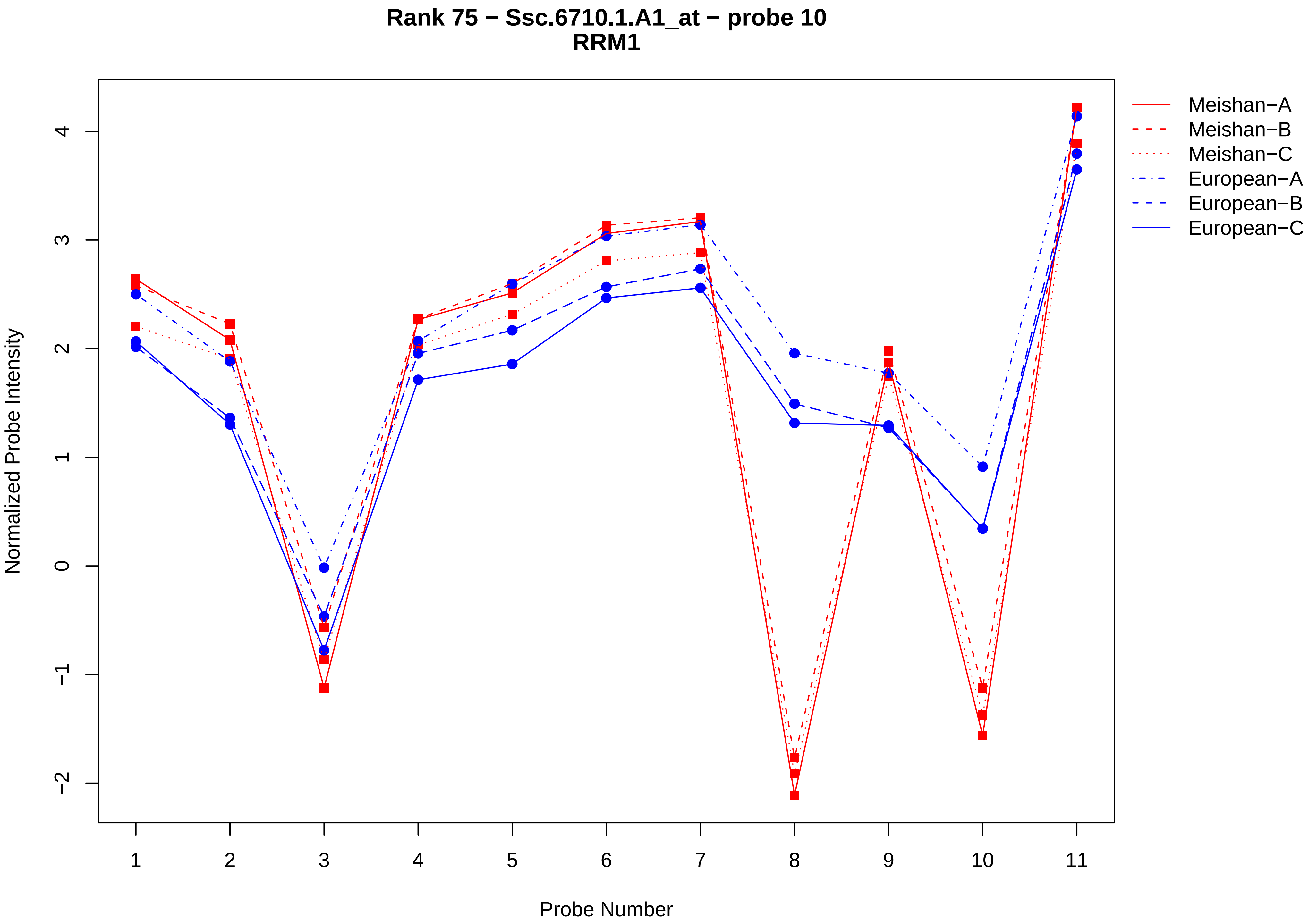
Rank 75 − Ssc.6710.1.A1_at − probe 10
RRM1
Meishan−A
Meishan−B
Meishan−C
European−A
European−B
European−C
Normalized Probe Intensity
−2
−1
0
1
2
3
4
1
2
3
4
5
6
7
8
9
10
11
Probe Number

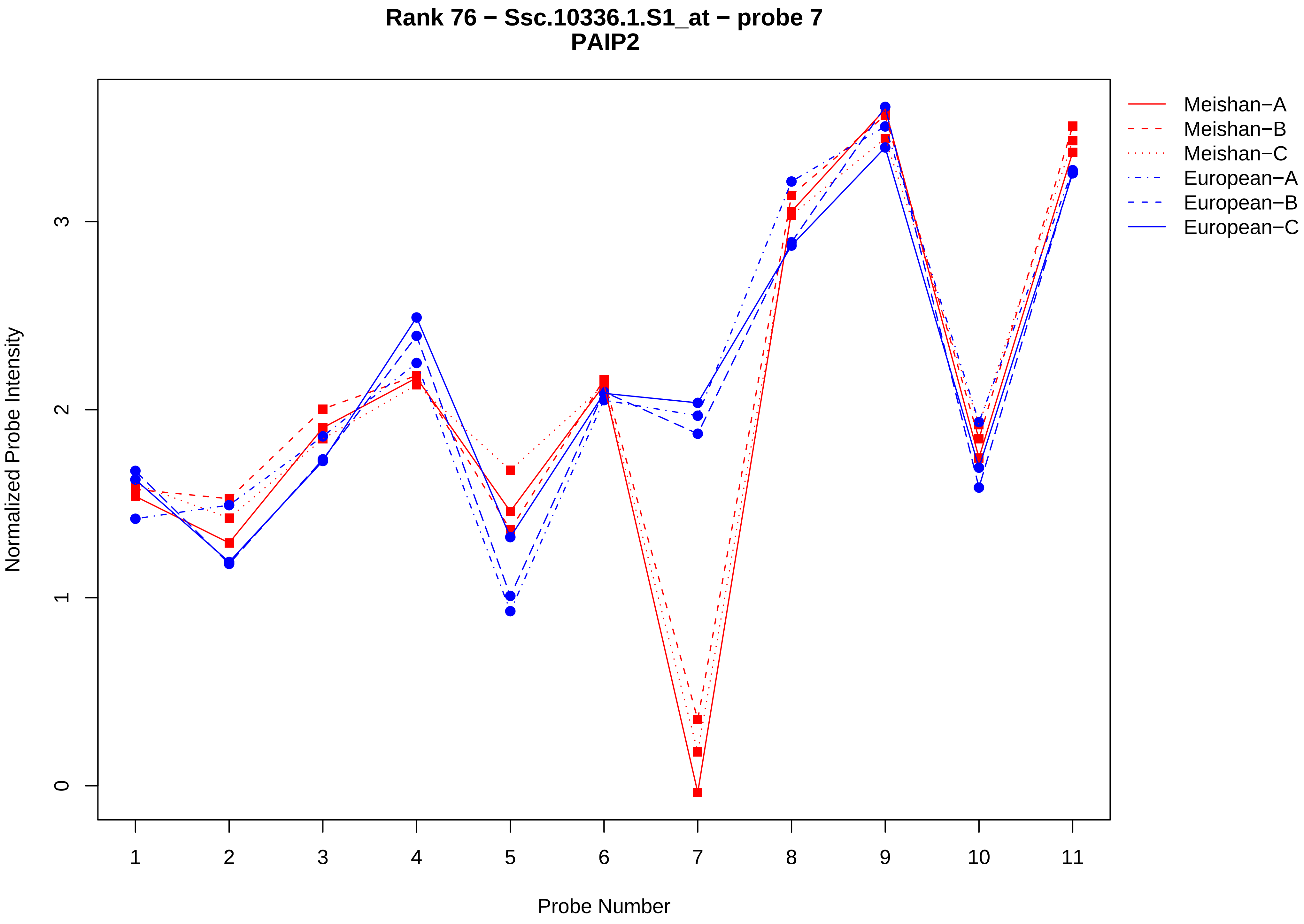

Rank 76 − Ssc.10336.1.S1_at − probe 7
PAIP2
Normalized Probe Intensity
Probe Number
0
1
2
3
1
2
3
4
5
6
7
8
9
10
11
Meishan−A
Meishan−B
Meishan−C
European−A
European−B
European−C

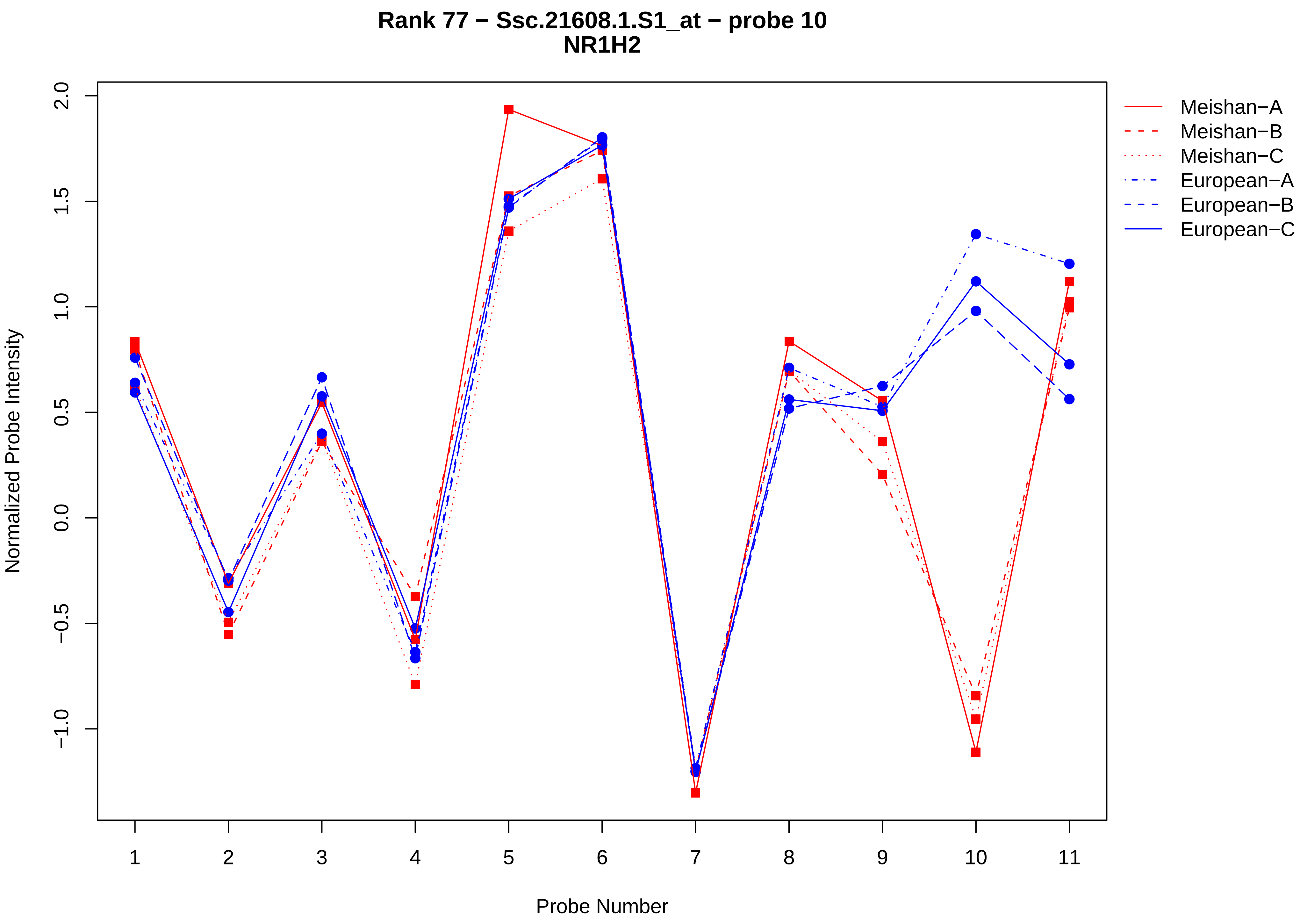

Rank 77 − Ssc.21608.1.S1_at − probe 10
NR1H2
Normalized Probe Intensity
Probe Number
−1.0
−0.5
0.0
0.5
1.0
1.5
2.0
1
2
3
4
5
6
7
8
9
10
11
Meishan−A
Meishan−B
Meishan−C
European−A
European−B
European−C

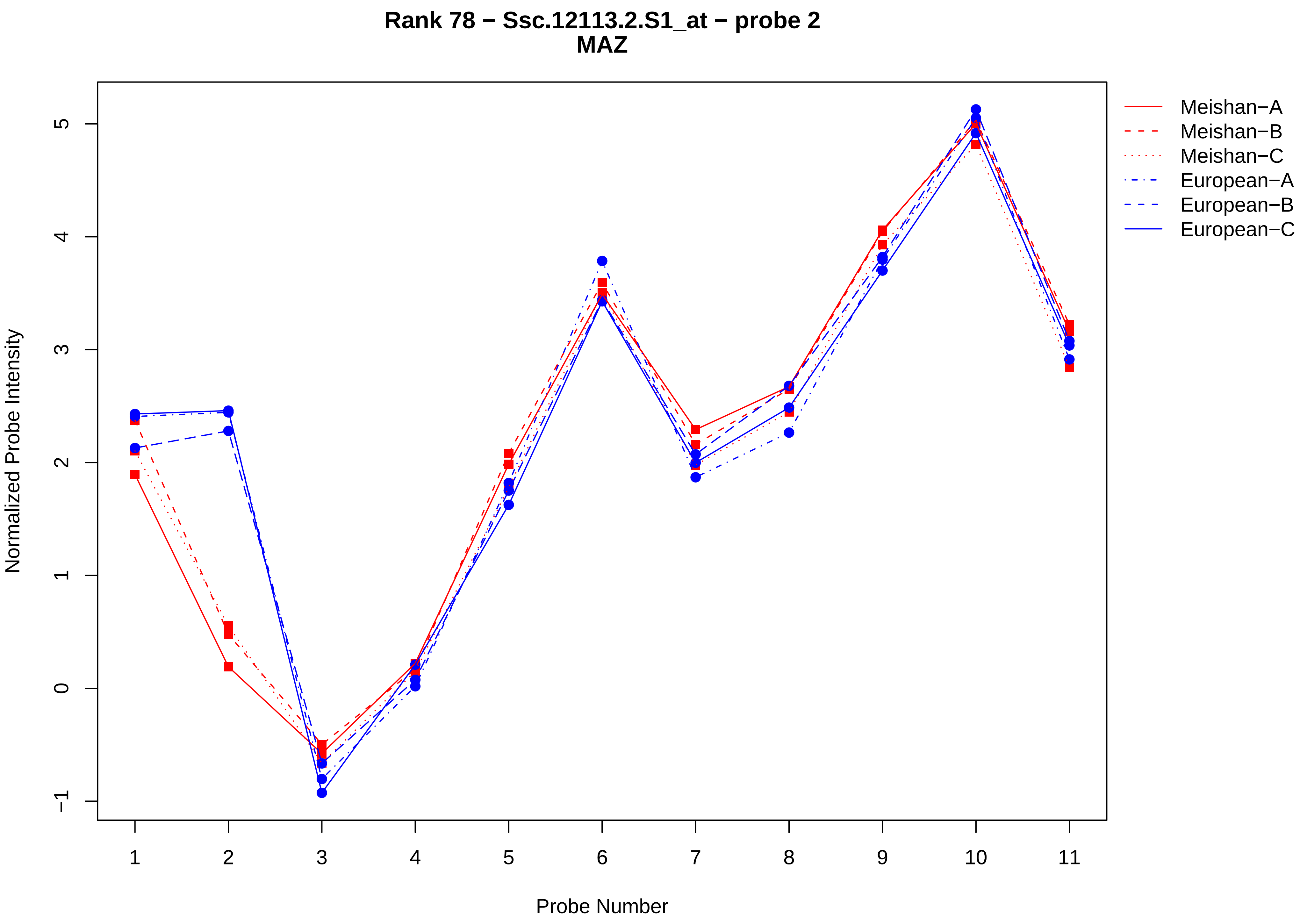

Rank 78 − Ssc.12113.2.S1_at − probe 2
MAZ
Normalized Probe Intensity
Probe Number
−1
0
1
2
3
4
5
1
2
3
4
5
6
7
8
9
10
11
Meishan−A
Meishan−B
Meishan−C
European−A
European−B
European−C

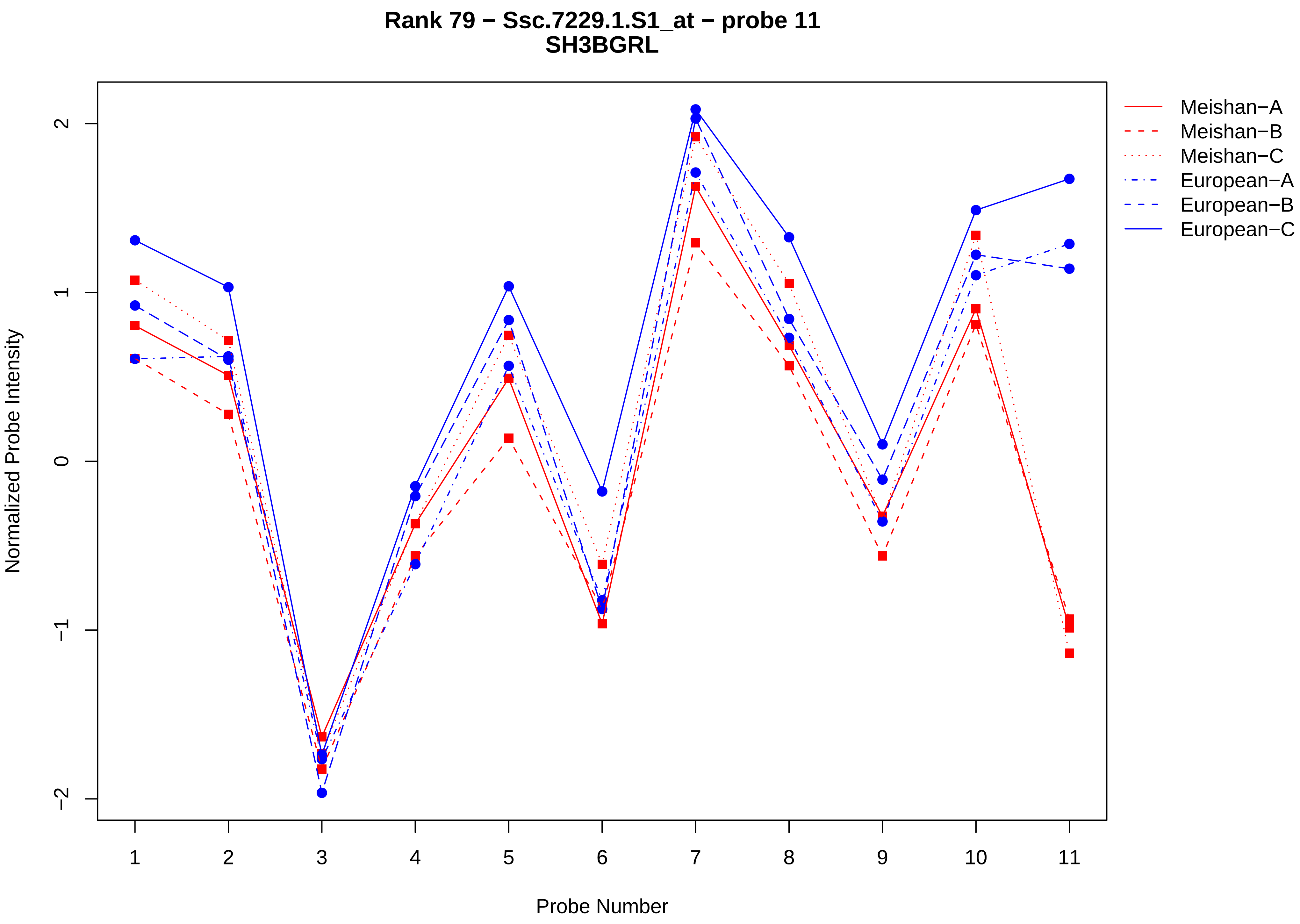

Rank 79 − Ssc.7229.1.S1_at − probe 11
SH3BGRL
Normalized Probe Intensity
Probe Number
Meishan−A
Meishan−B
Meishan−C
European−A
European−B
European−C

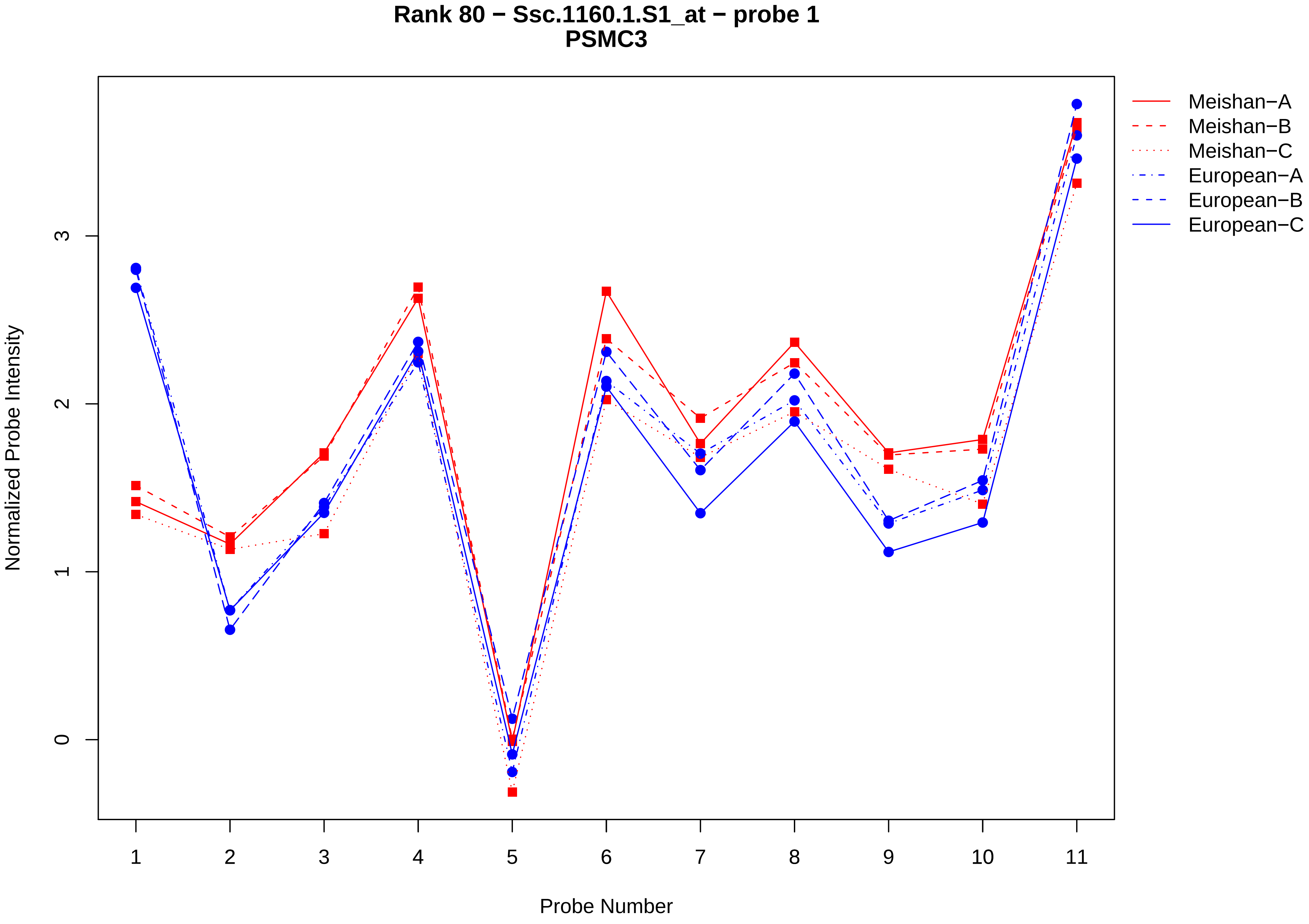

Rank 80 − Ssc.1160.1.S1_at − probe 1
PSMC3
Normalized Probe Intensity
0
1
2
3
1
2
3
4
5
6
7
8
9
10
11
Probe Number
Meishan−A
Meishan−B
Meishan−C
European−A
European−B
European−C

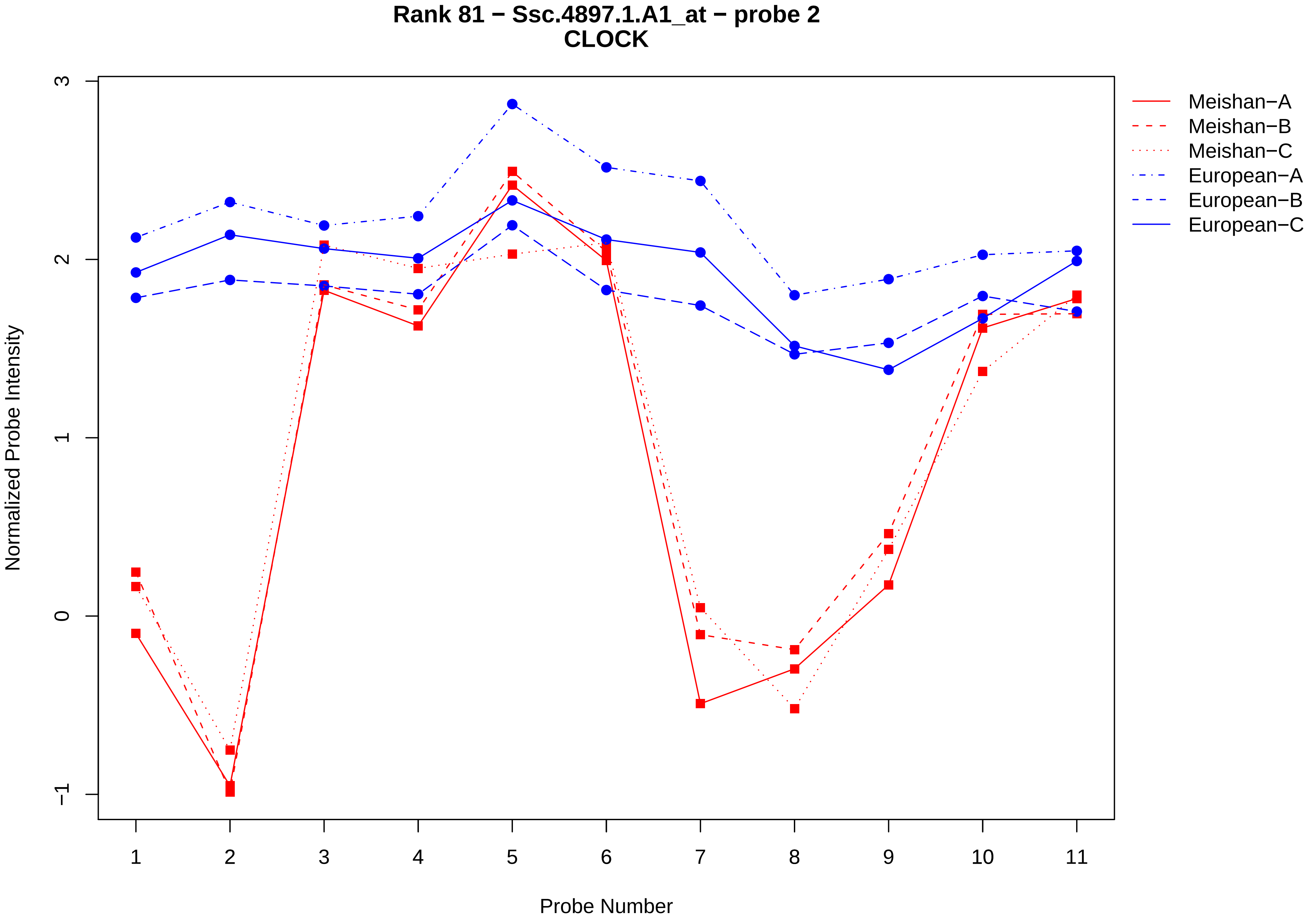

Rank 81 − Ssc.4897.1.A1_at − probe 2
CLOCK
Normalized Probe Intensity
Probe Number
Meishan−A
Meishan−B
Meishan−C
European−A
European−B
European−C
−1
0
1
2
3
1
2
3
4
5
6
7
8
9
10
11

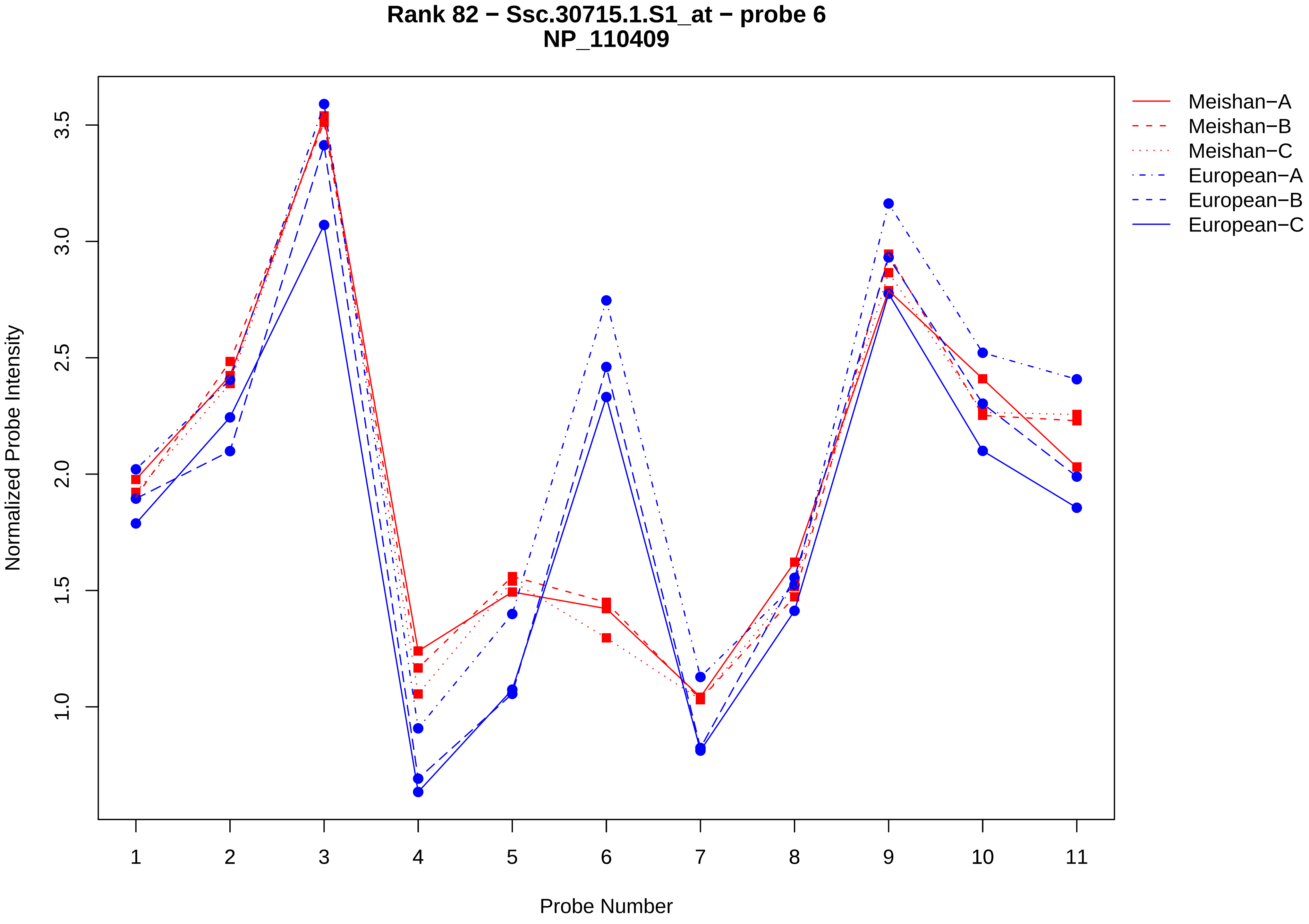

Rank 82 − Ssc.30715.1.S1_at − probe 6
NP_110409
Normalized Probe Intensity
Probe Number
1.0
1.5
2.0
2.5
3.0
3.5
1
2
3
4
5
6
7
8
9
10
11
Meishan−A
Meishan−B
Meishan−C
European−A
European−B
European−C

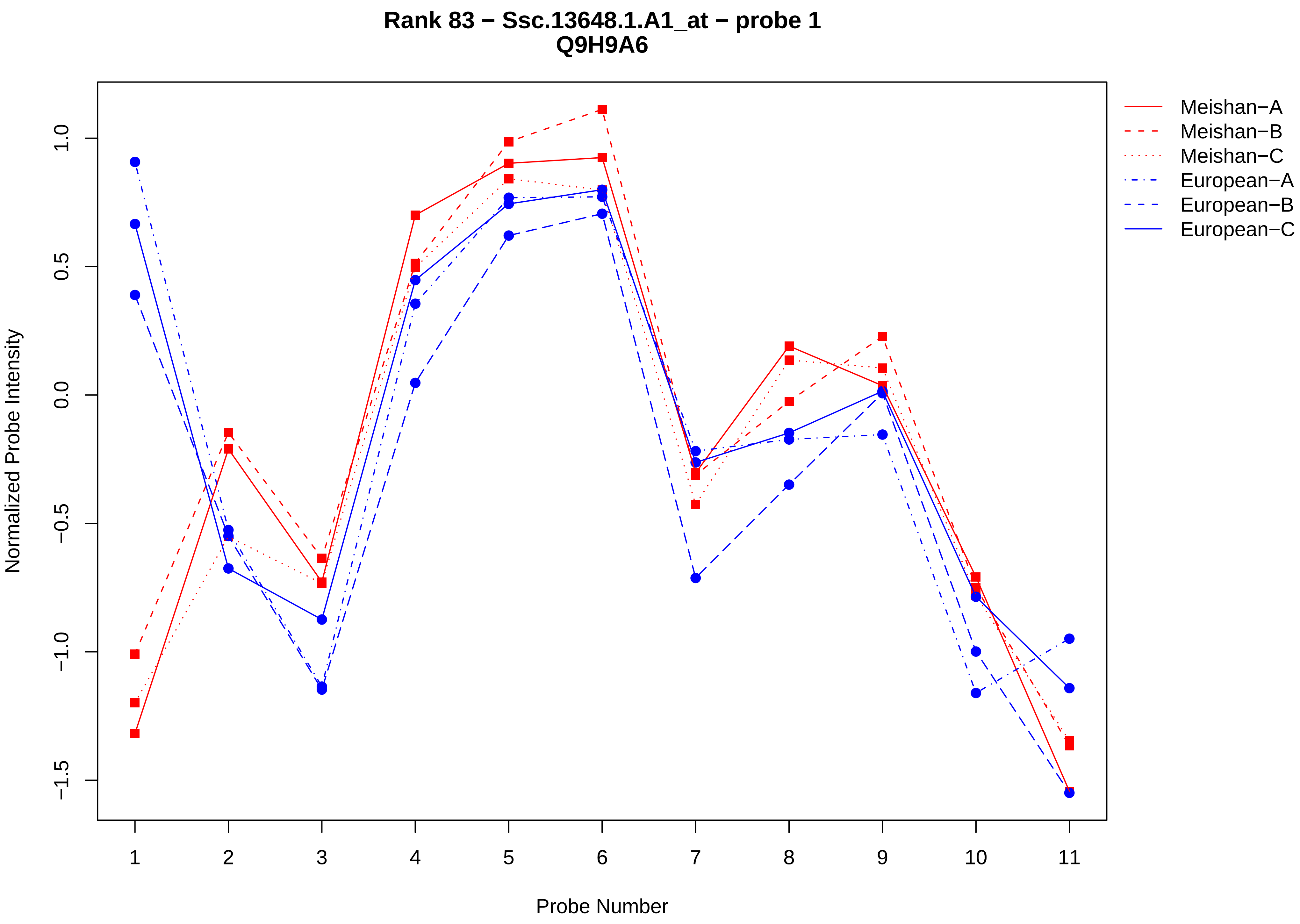

Rank 83 − Ssc.13648.1.A1_at − probe 1
Q9H9A6
Normalized Probe Intensity
1.0
0.5
0.0
−0.5
−1.0
−1.5
1
2
3
4
5
6
7
8
9
10
11
Probe Number
Meishan−A
Meishan−B
Meishan−C
European−A
European−B
European−C

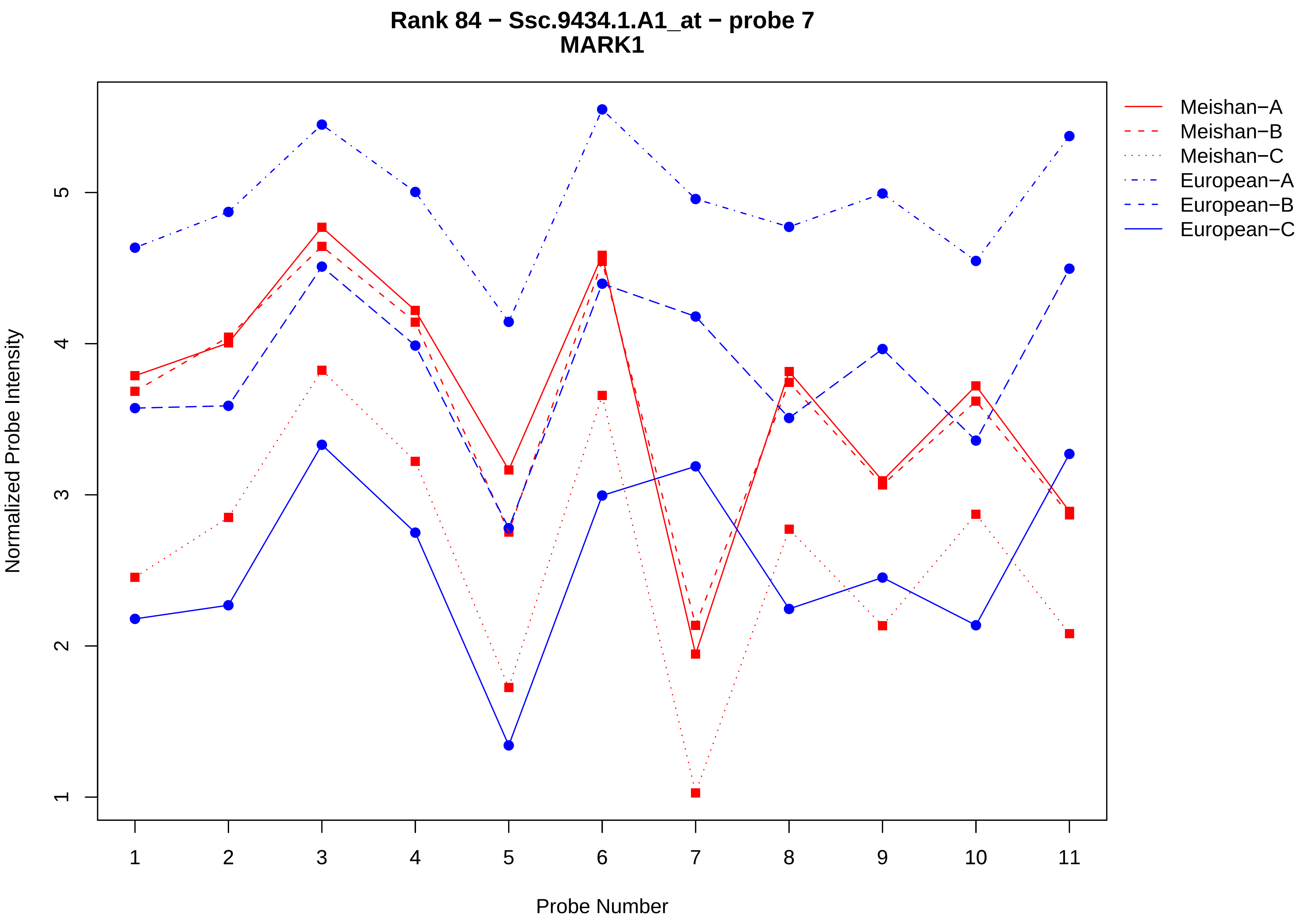

Rank 84 − Ssc.9434.1.A1_at − probe 7
MARK1
Normalized Probe Intensity
Probe Number
1
2
3
4
5
1
2
3
4
5
6
7
8
9
10
11
Meishan−A
Meishan−B
Meishan−C
European−A
European−B
European−C

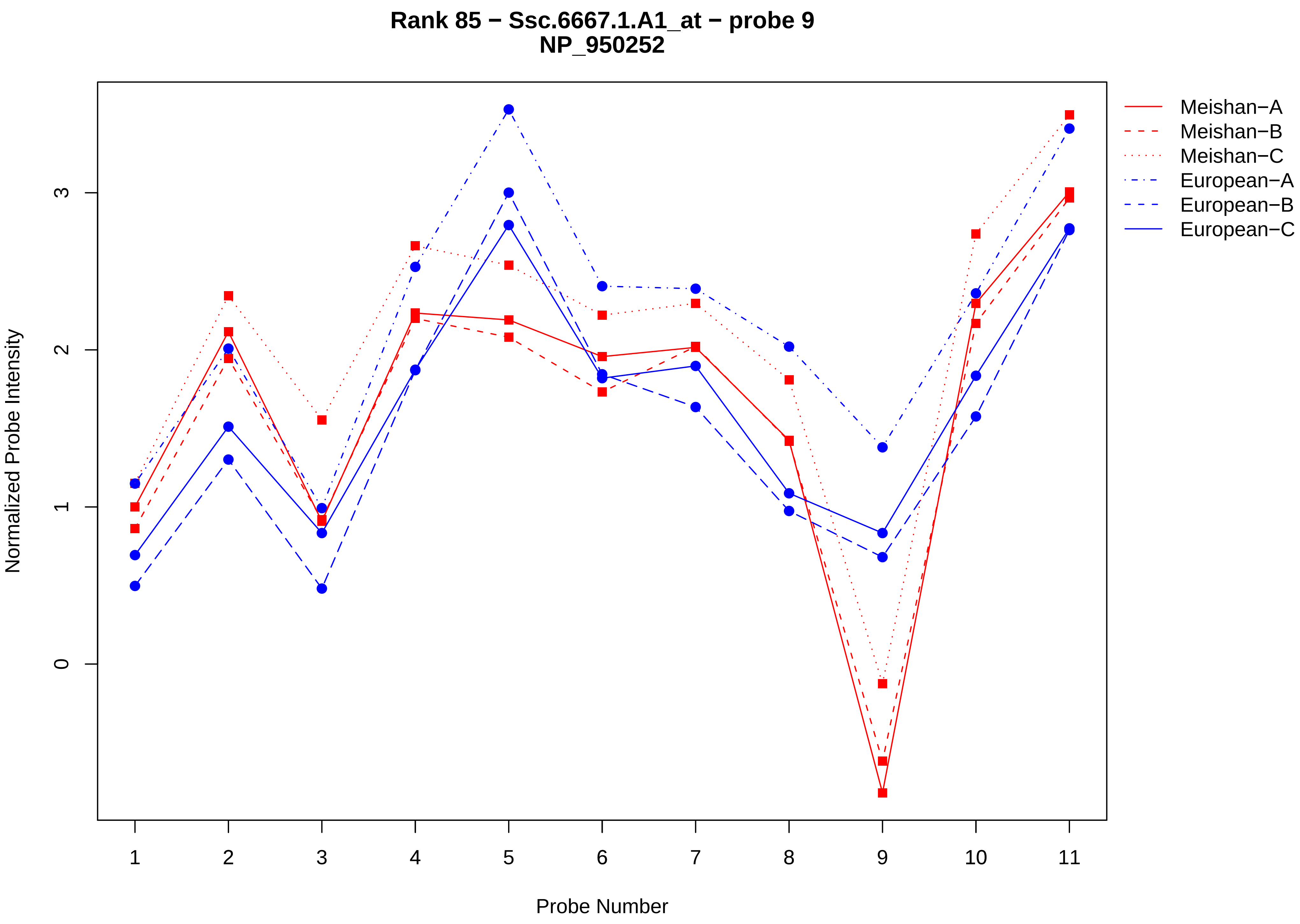

Rank 85 − Ssc.6667.1.A1_at − probe 9
NP_950252
Normalized Probe Intensity
0
1
2
3
1
2
3
4
5
6
7
8
9
10
11
Probe Number
Meishan−A
Meishan−B
Meishan−C
European−A
European−B
European−C

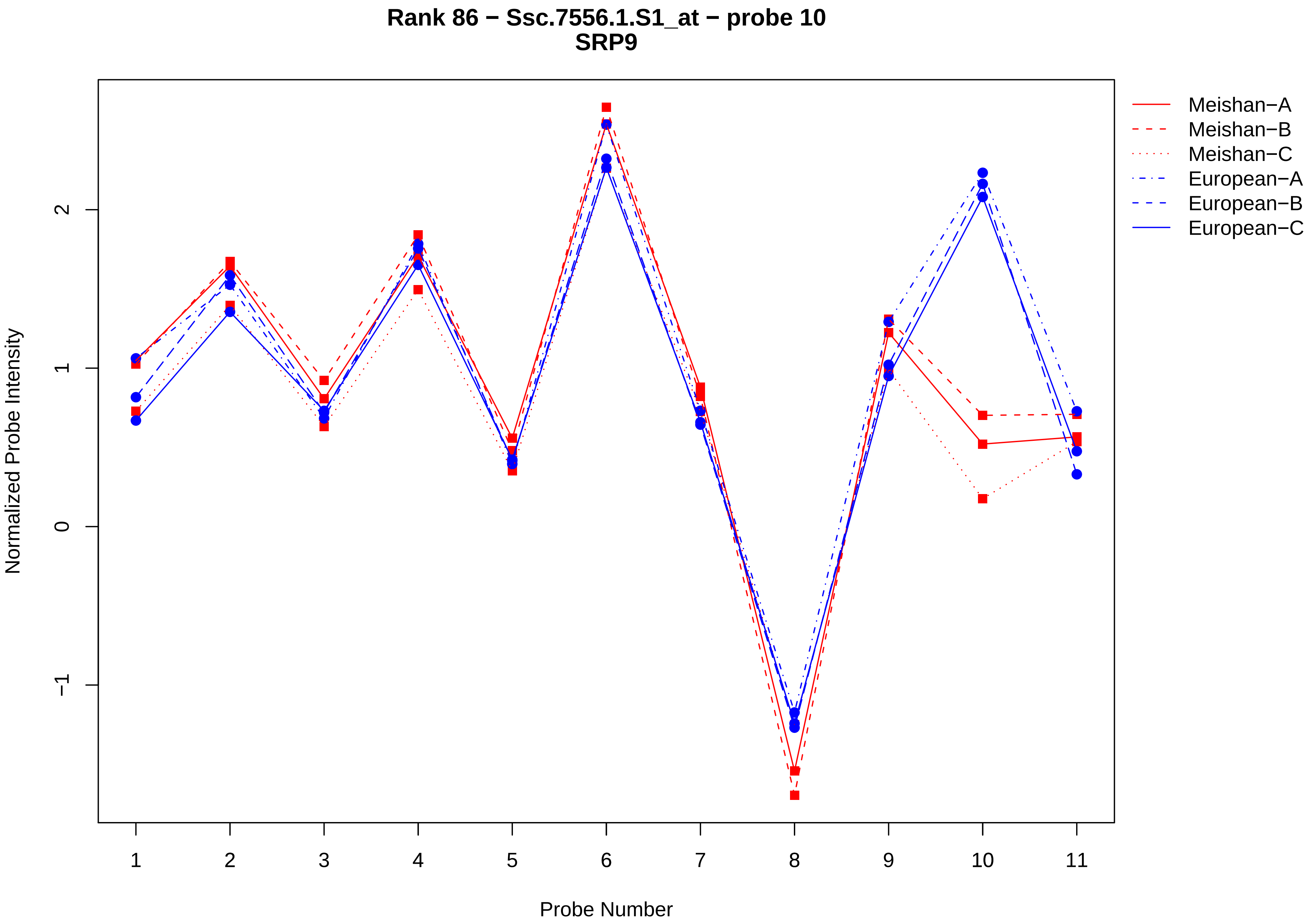
Rank 86 − Ssc.7556.1.S1_at − probe 10
SRP9
Normalized Probe Intensity
Probe Number
−1
0
1
2
1
2
3
4
5
6
7
8
9
10
11
Meishan−A
Meishan−B
Meishan−C
European−A
European−B
European−C

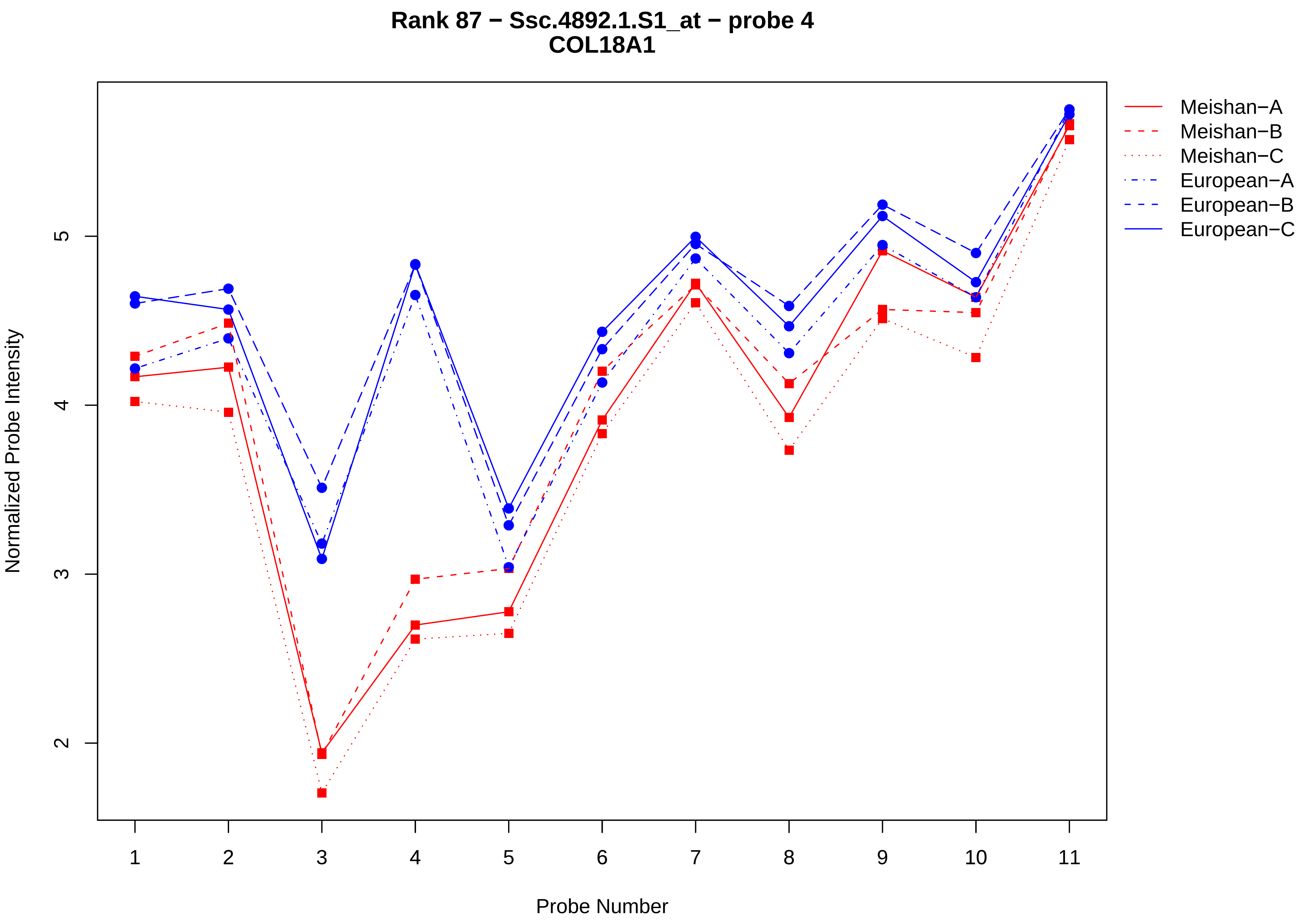

Rank 87 − Ssc.4892.1.S1_at − probe 4
COL18A1
Normalized Probe Intensity
Probe Number
2
3
4
5
1
2
3
4
5
6
7
8
9
10
11
Meishan−A
Meishan−B
Meishan−C
European−A
European−B
European−C

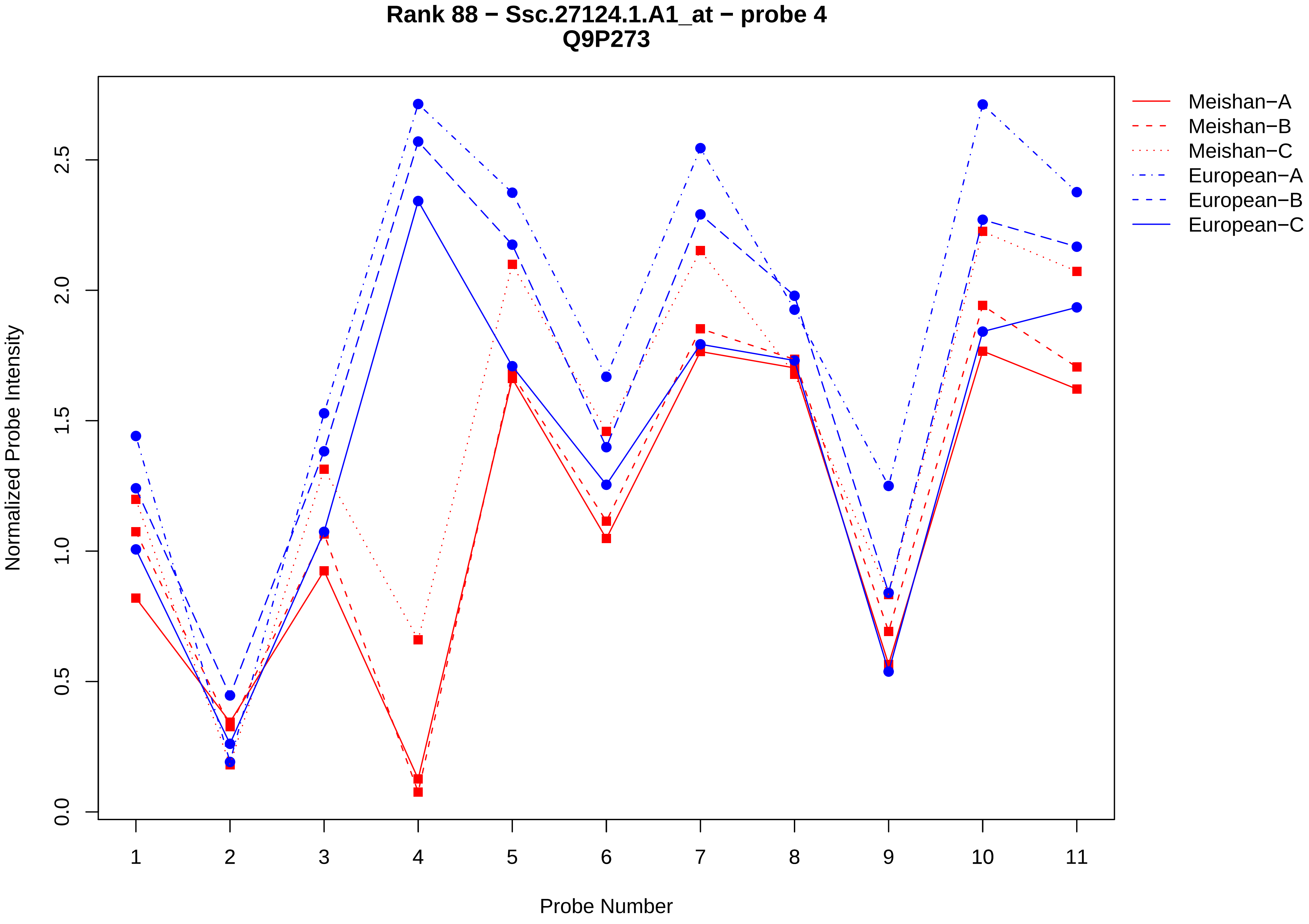

Rank 88 − Ssc.27124.1.A1_at − probe 4
Q9P273
Normalized Probe Intensity
0.0
0.5
1.0
1.5
2.0
2.5
1
2
3
4
5
6
7
8
9
10
11
Probe Number
Meishan−A
Meishan−B
Meishan−C
European−A
European−B
European−C

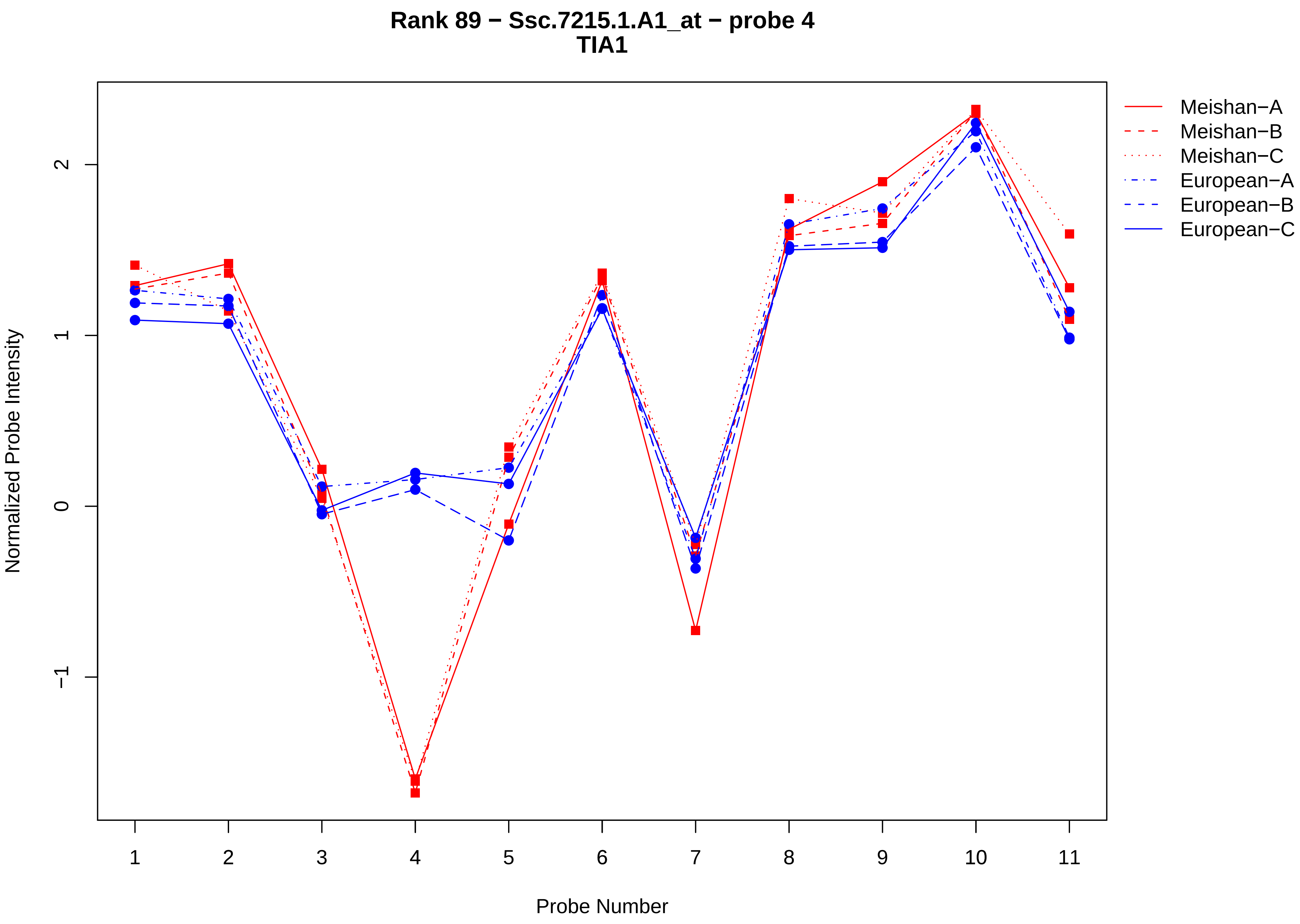
Rank 89 − Ssc.7215.1.A1_at − probe 4
TIA1
Normalized Probe Intensity
Probe Number
Meishan−A
Meishan−B
Meishan−C
European−A
European−B
European−C
−1
0
1
2
1
2
3
4
5
6
7
8
9
10
11

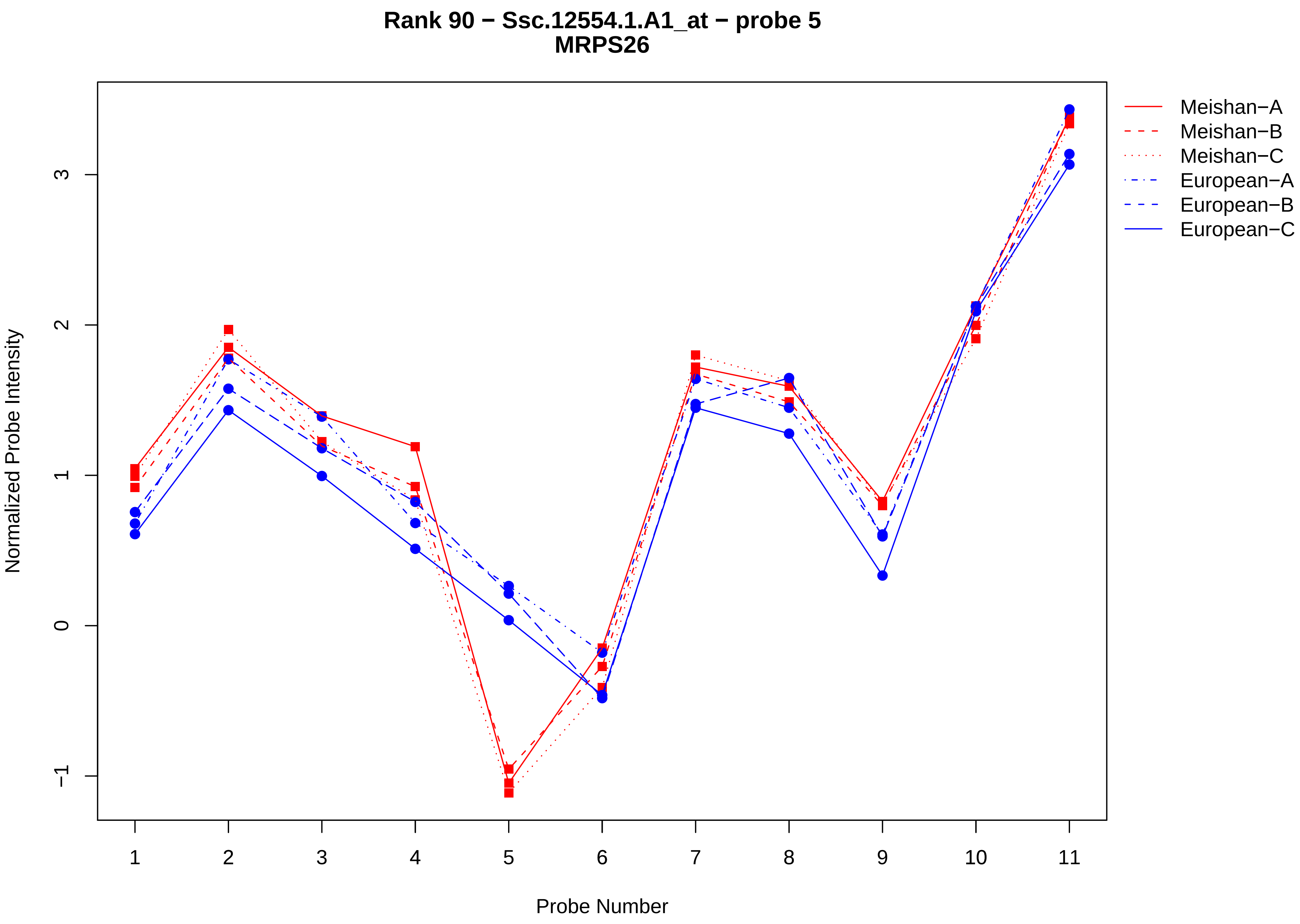

Rank 90 − Ssc.12554.1.A1_at − probe 5
MRPS26
Normalized Probe Intensity
Probe Number
−1
0
1
2
3
1
2
3
4
5
6
7
8
9
10
11
Meishan−A
Meishan−B
Meishan−C
European−A
European−B
European−C

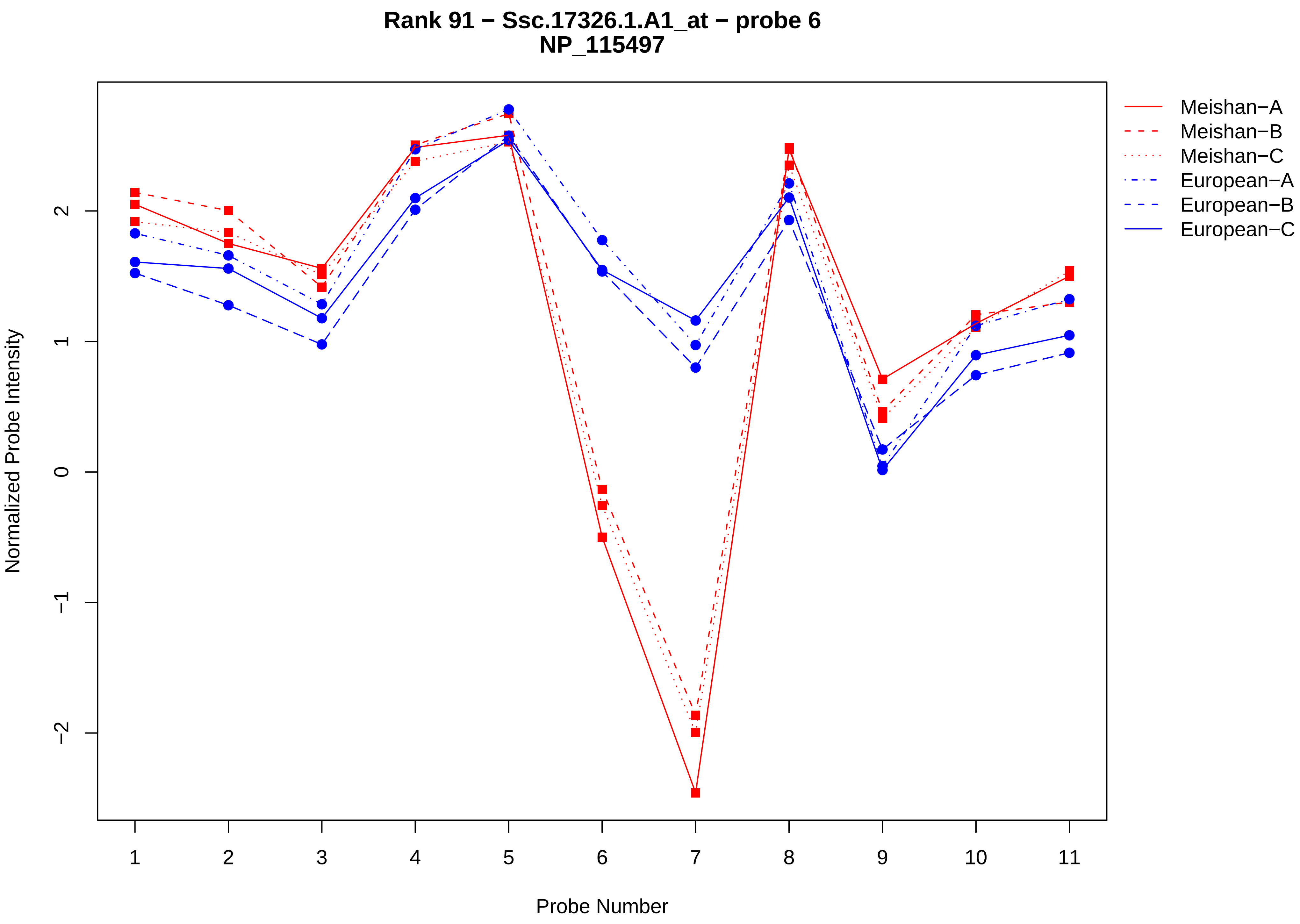
Rank 91 − Ssc.17326.1.A1_at − probe 6
NP_115497
Normalized Probe Intensity
Probe Number
−2
−1
0
1
2
1
2
3
4
5
6
7
8
9
10
11
Meishan−A
Meishan−B
Meishan−C
European−A
European−B
European−C

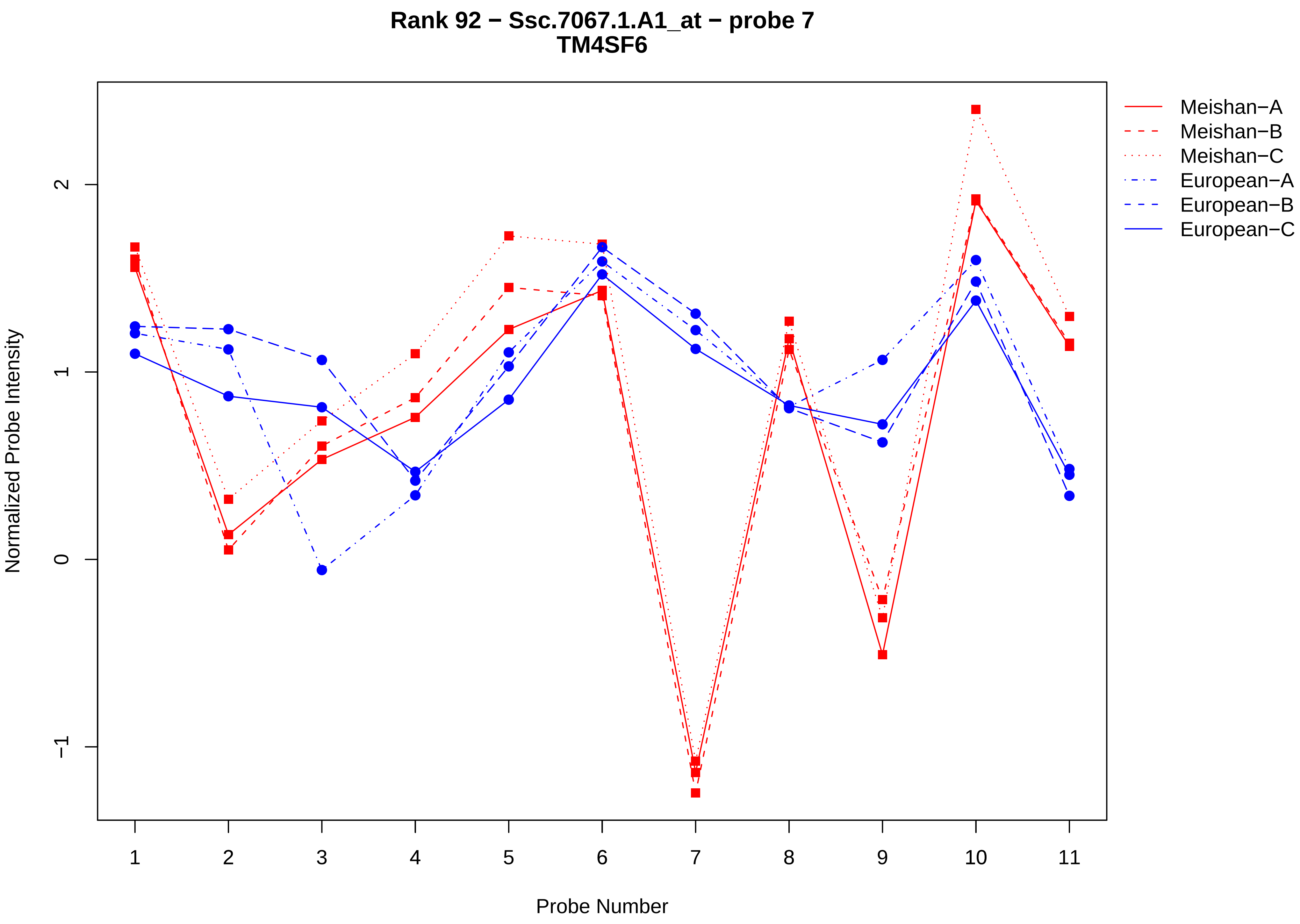

Rank 92 − Ssc.7067.1.A1_at − probe 7
TM4SF6
Normalized Probe Intensity
Probe Number
−1
0
1
2
1
2
3
4
5
6
7
8
9
10
11
Meishan−A
Meishan−B
Meishan−C
European−A
European−B
European−C

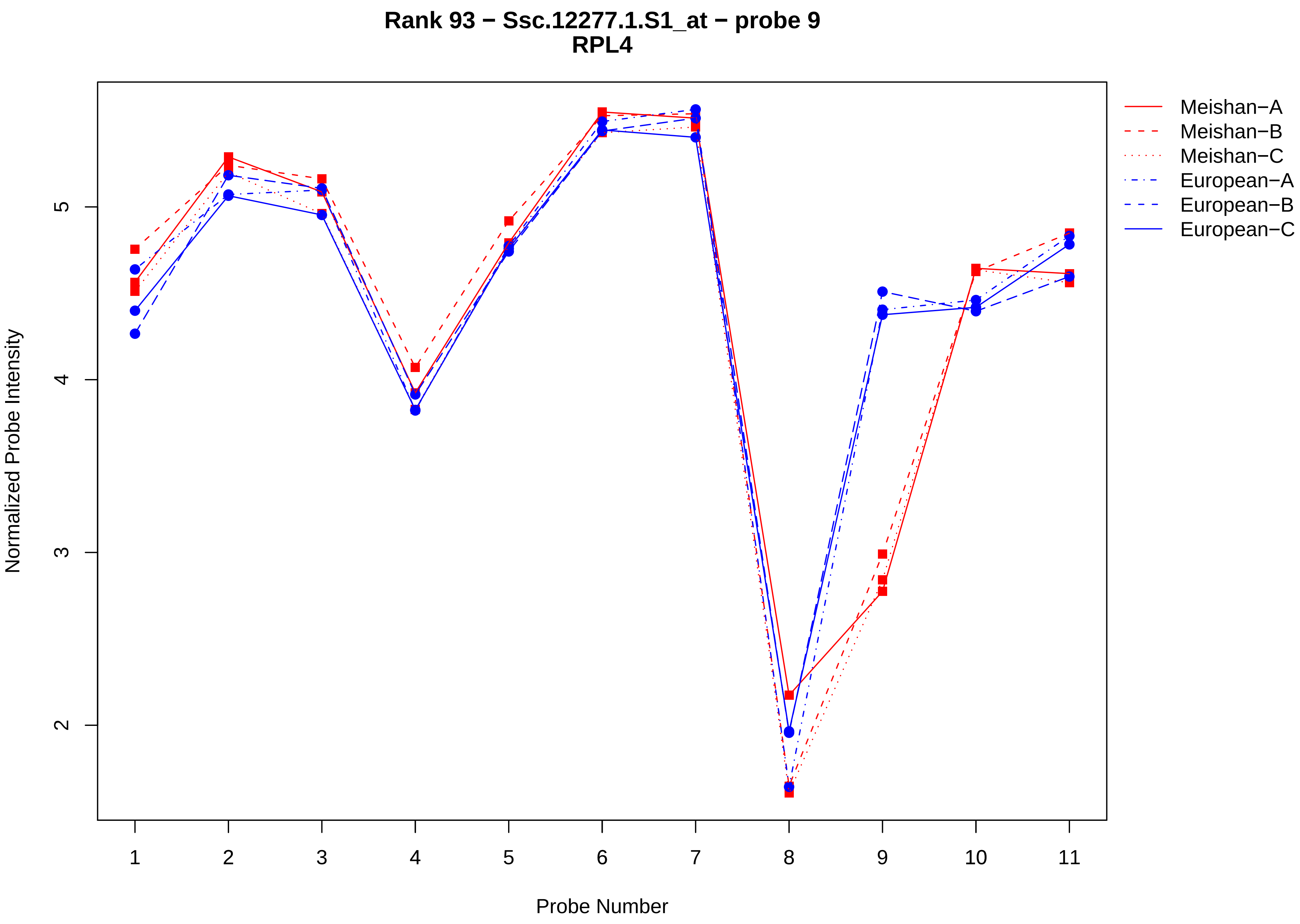

Rank 93 − Ssc.12277.1.S1_at − probe 9
RPL4
Normalized Probe Intensity
Probe Number
2
3
4
5
1
2
3
4
5
6
7
8
9
10
11
Meishan−A
Meishan−B
Meishan−C
European−A
European−B
European−C

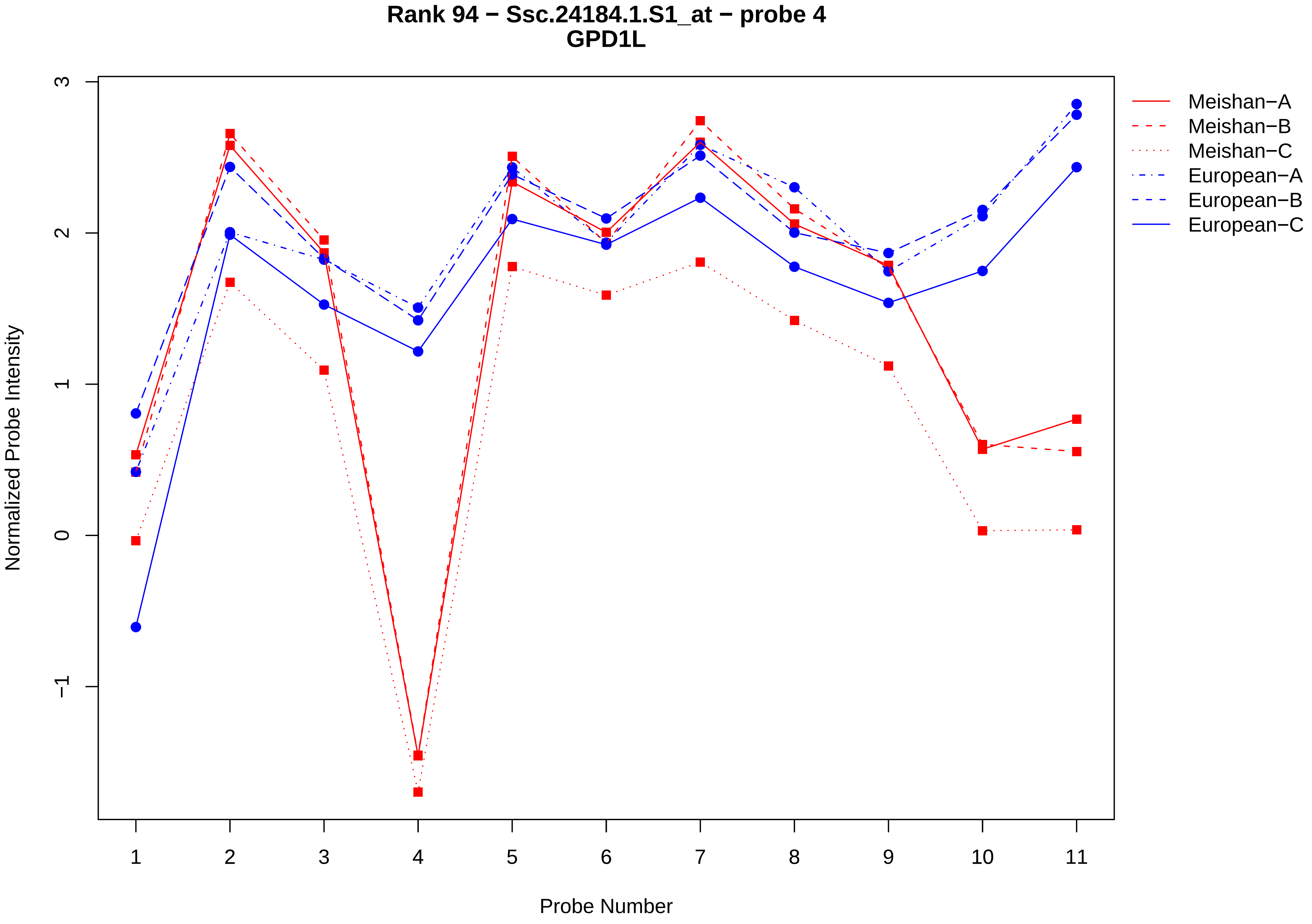

Rank 94 − Ssc.24184.1.S1_at − probe 4
GPD1L
Normalized Probe Intensity
Probe Number
−1
0
1
2
3
1
2
3
4
5
6
7
8
9
10
11
Meishan−A
Meishan−B
Meishan−C
European−A
European−B
European−C

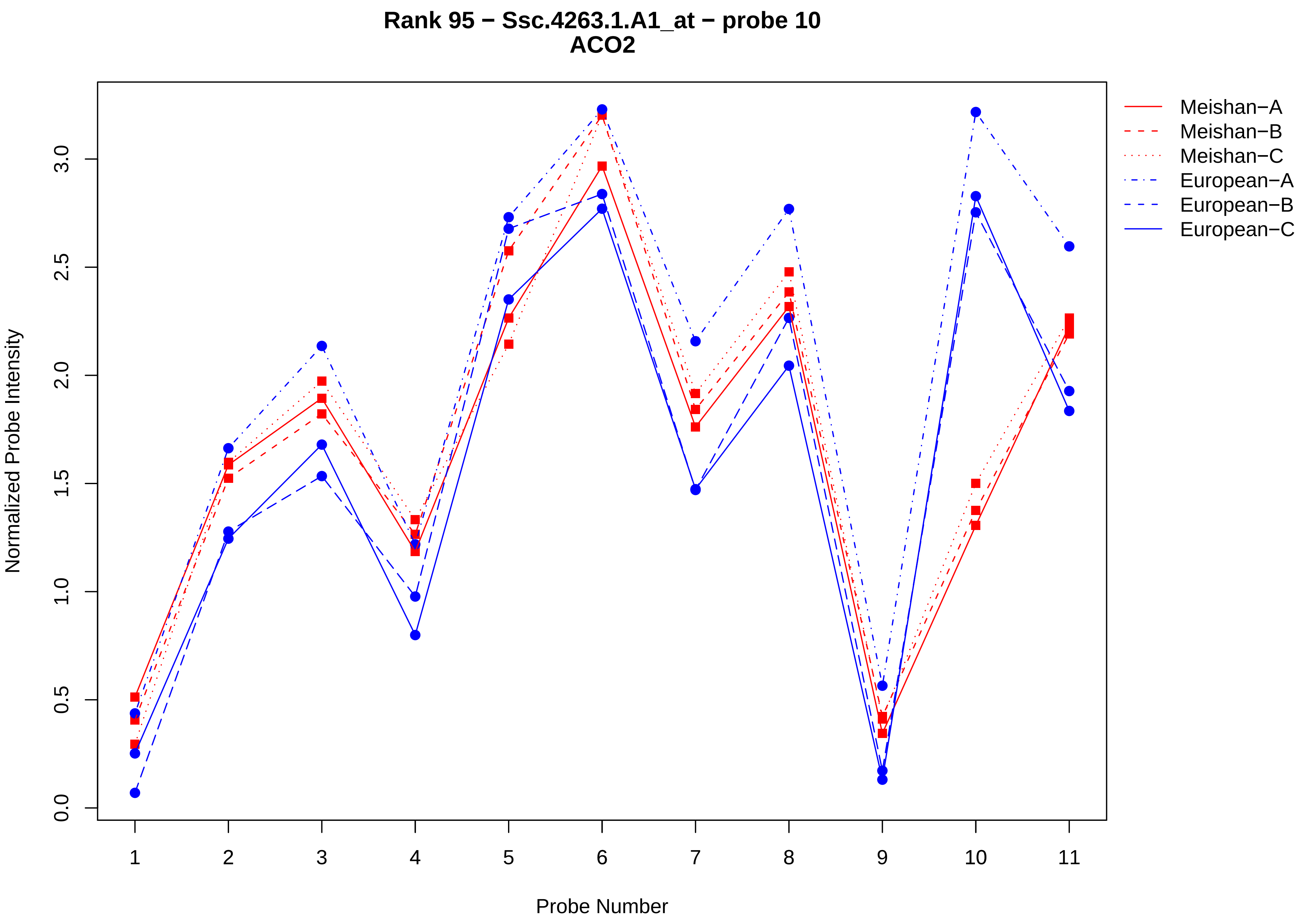
Rank 95 − Ssc.4263.1.A1_at − probe 10
ACO2
Normalized Probe Intensity
Probe Number
0.0
0.5
1.0
1.5
2.0
2.5
3.0
1
2
3
4
5
6
7
8
9
10
11
Meishan−A
Meishan−B
Meishan−C
European−A
European−B
European−C

Rank 96 − Ssc.7039.1.A1_at − probe 4
NGFRAP1

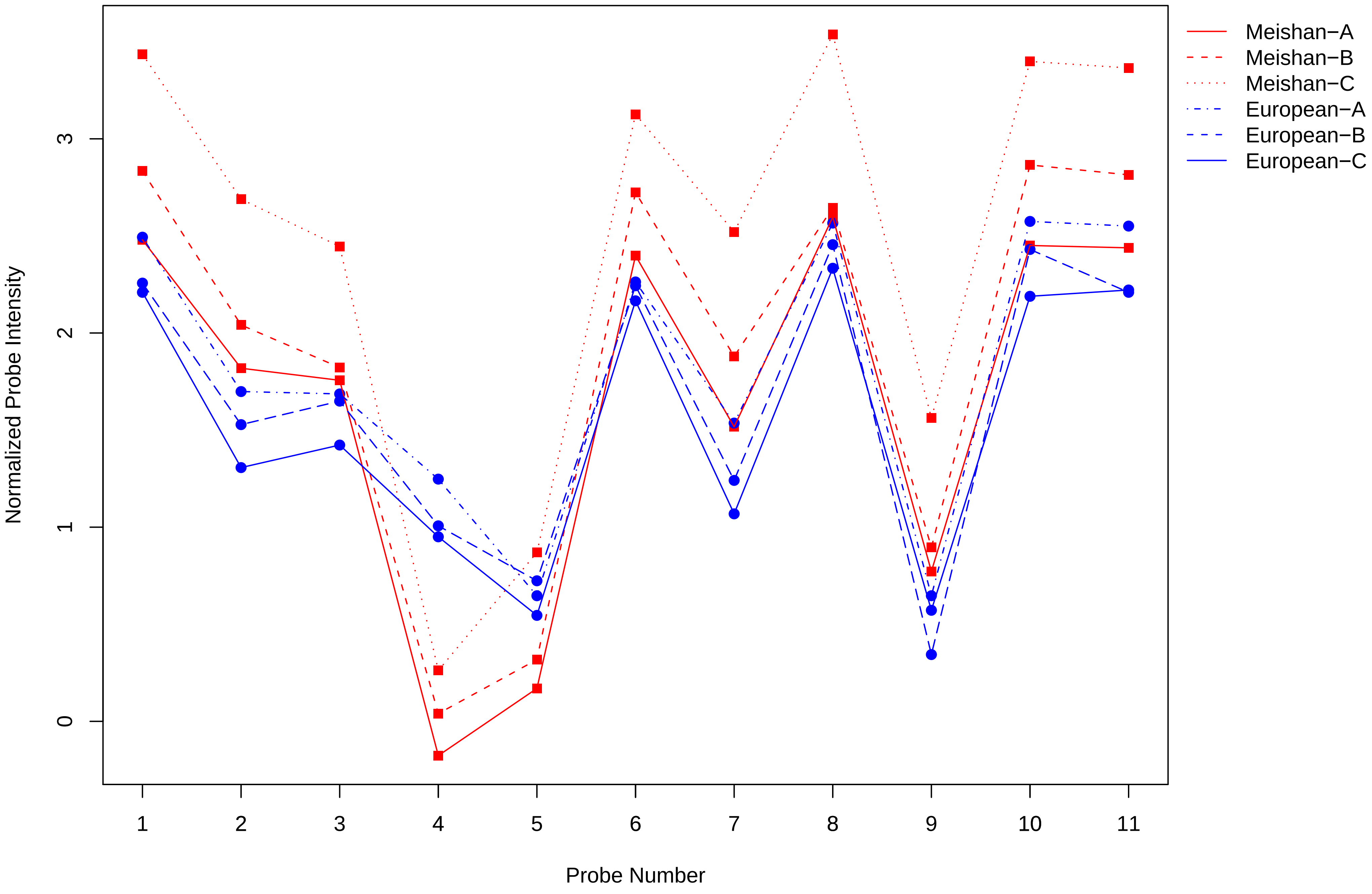

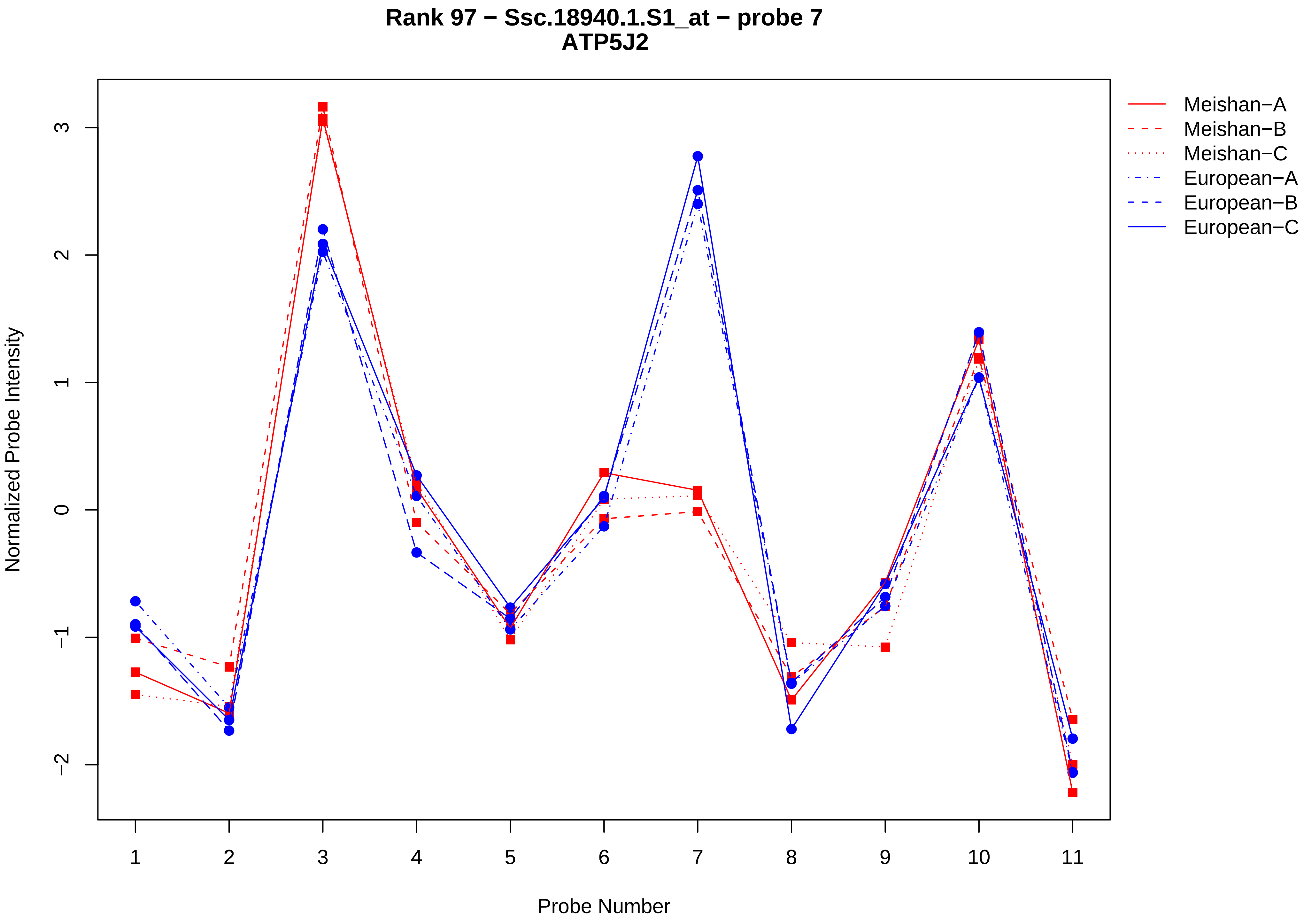

Rank 97 − Ssc.18940.1.S1_at − probe 7
ATP5J2
Normalized Probe Intensity
Probe Number
−2
−1
0
1
2
3
1
2
3
4
5
6
7
8
9
10
11
Meishan−A
Meishan−B
Meishan−C
European−A
European−B
European−C

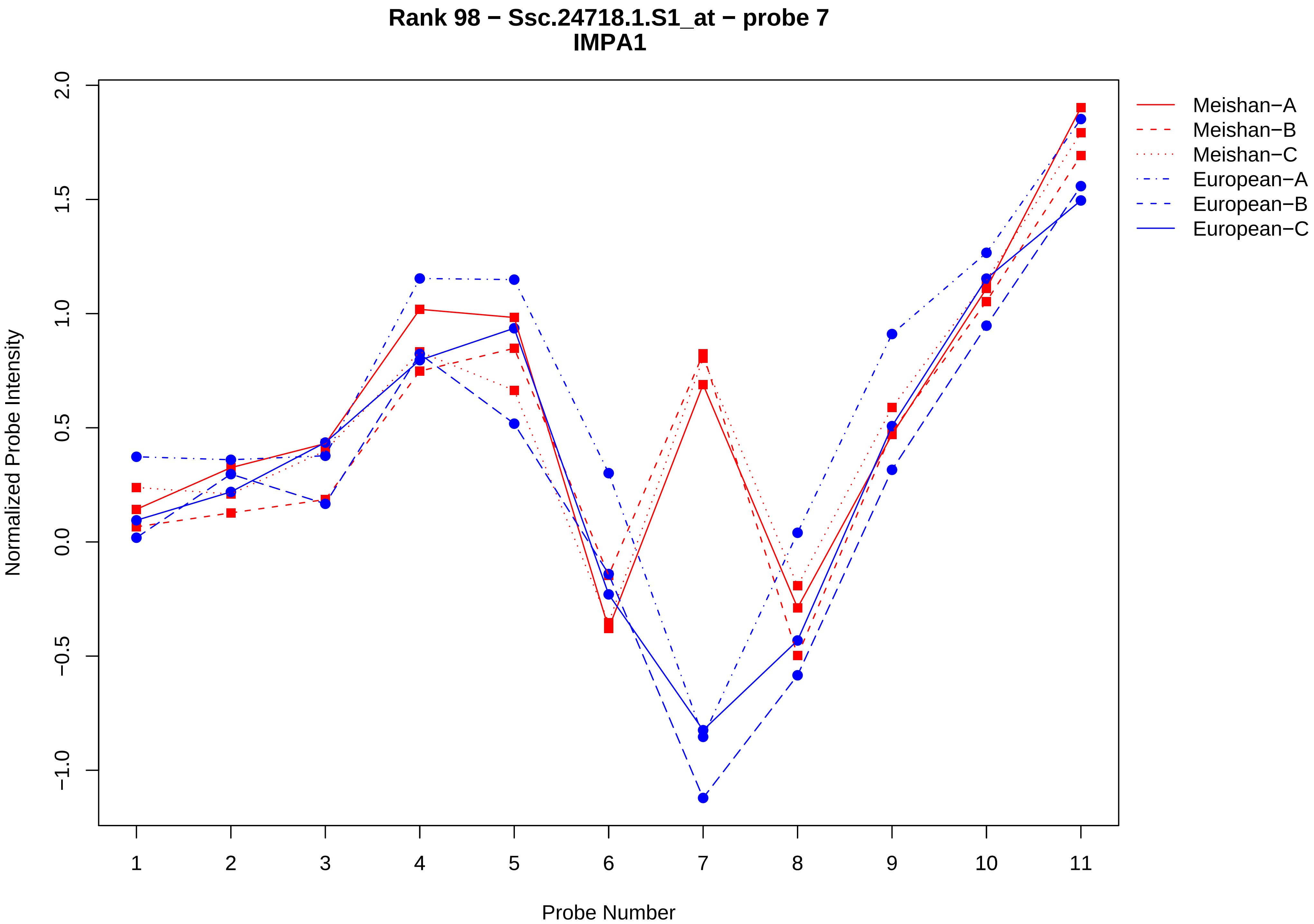

Rank 98 − Ssc.24718.1.S1_at − probe 7
IMPA1
Normalized Probe Intensity
2.0
1.5
1.0
0.5
0.0
−0.5
−1.0
1
2
3
4
5
6
7
8
9
10
11
Probe Number
Meishan−A
Meishan−B
Meishan−C
European−A
European−B
European−C

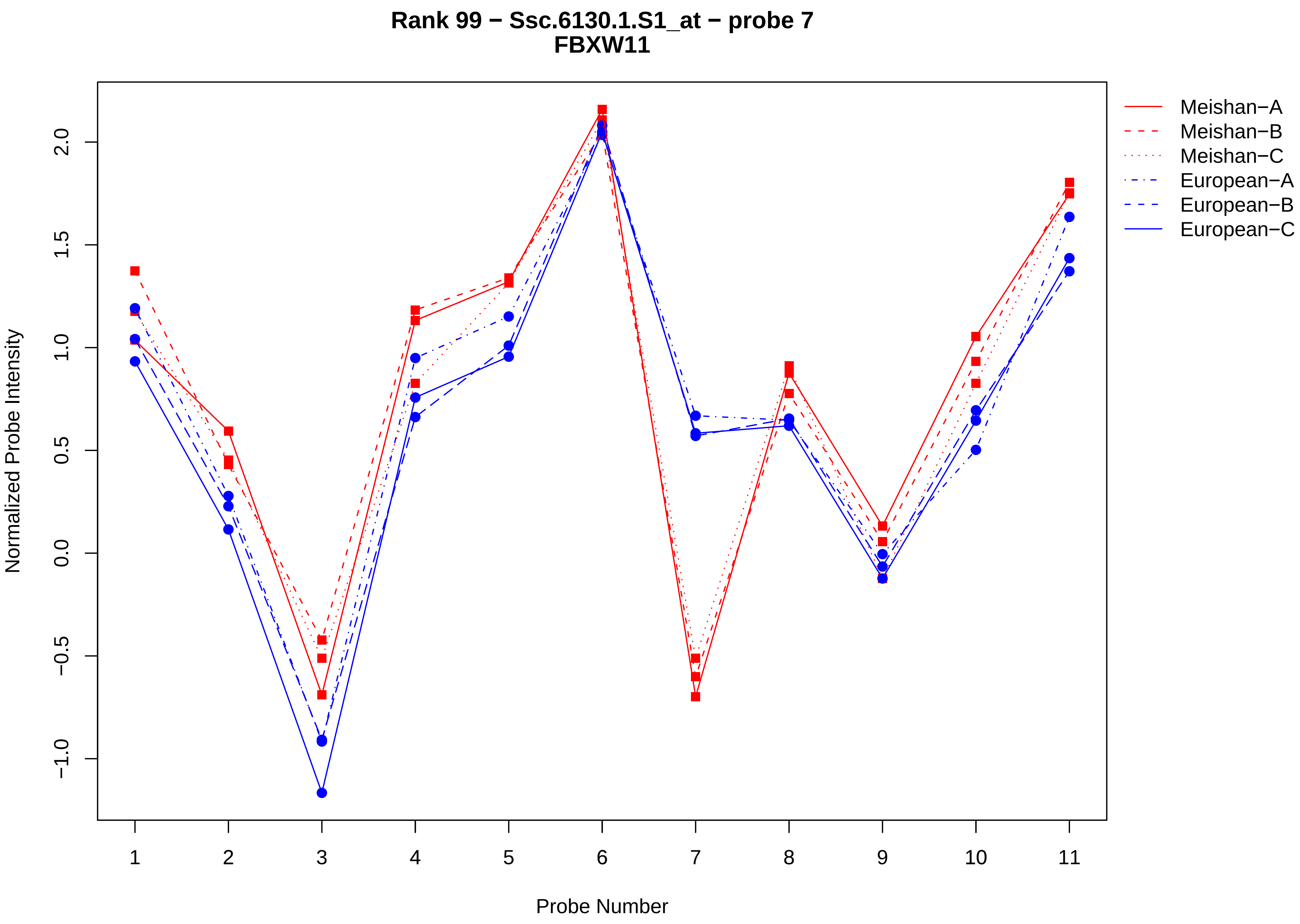
Rank 99 − Ssc.6130.1.S1_at − probe 7
FBXW11
Normalized Probe Intensity
Probe Number
−1.0
−0.5
0.0
0.5
1.0
1.5
2.0
1
2
3
4
5
6
7
8
9
10
11
Meishan−A
Meishan−B
Meishan−C
European−A
European−B
European−C

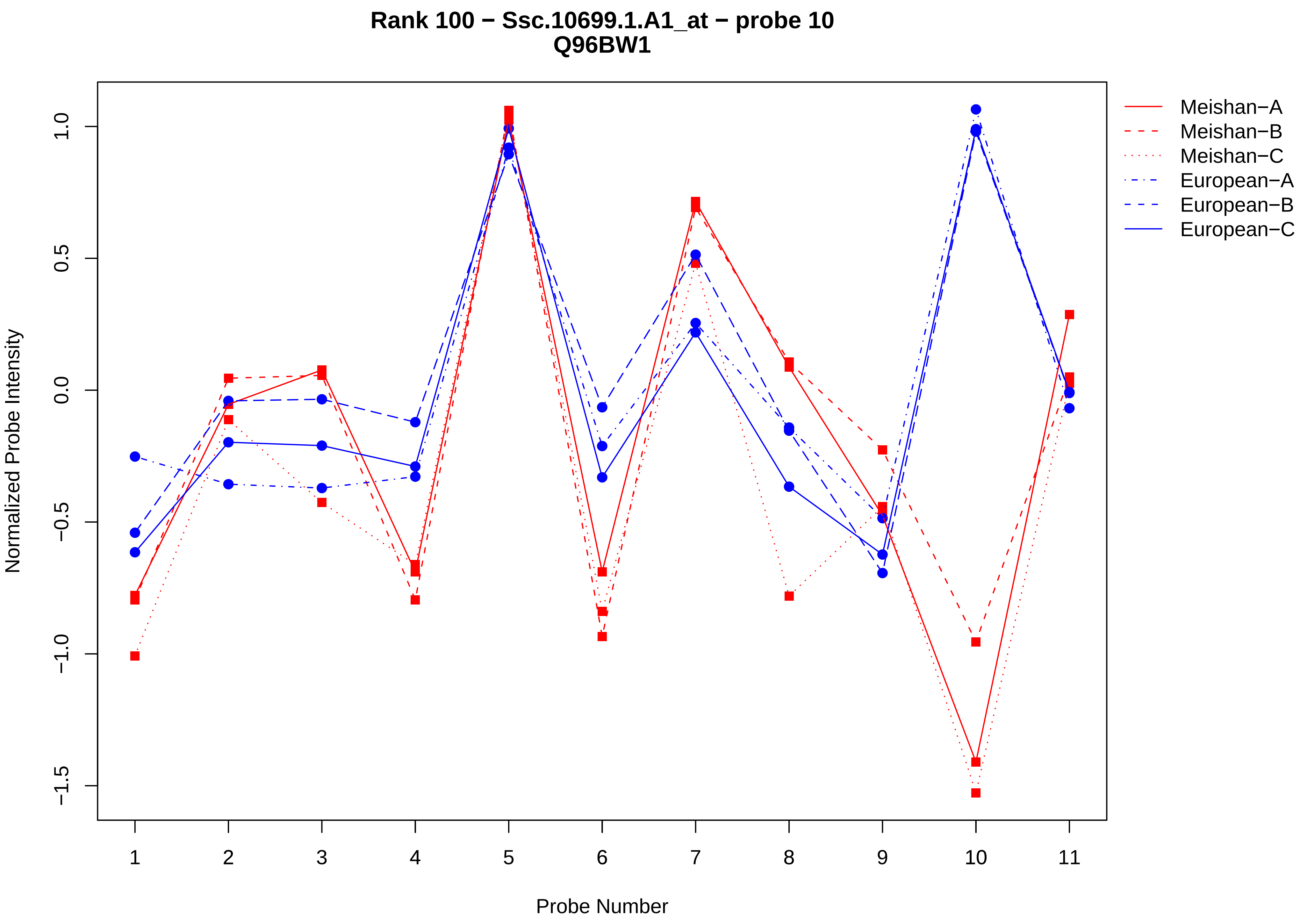
Rank 100 − Ssc.10699.1.A1_at − probe 10
Q96BW1
Normalized Probe Intensity
Probe Number
Meishan−A
Meishan−B
Meishan−C
European−A
European−B
European−C
1.0
0.5
0.0
−0.5
−1.0
−1.5
1
2
3
4
5
6
7
8
9
10
11

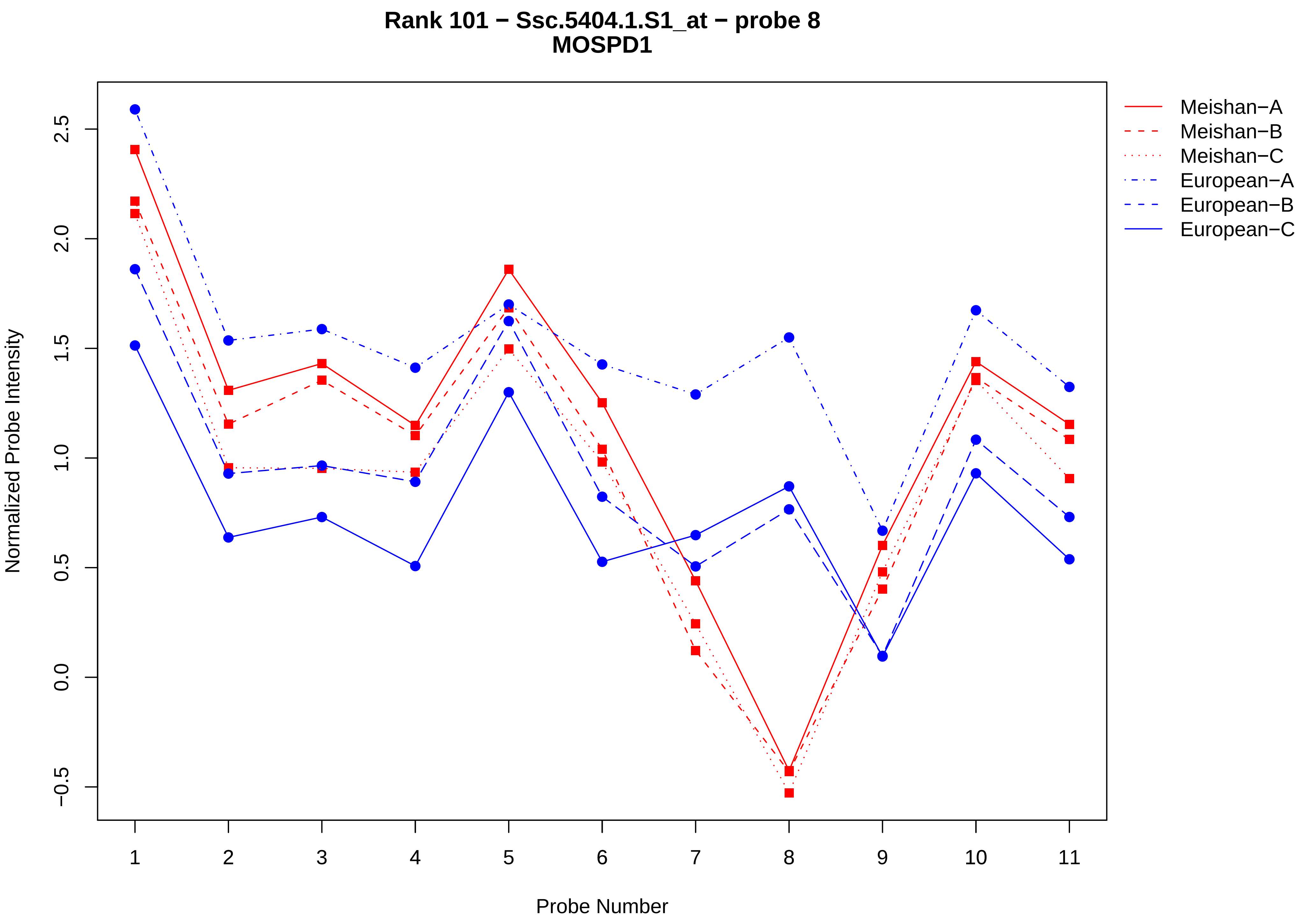

Rank 101 − Ssc.5404.1.S1_at − probe 8
MOSPD1
Normalized Probe Intensity
Probe Number
−0.5
0.0
0.5
1.0
1.5
2.0
2.5
1
2
3
4
5
6
7
8
9
10
11
Meishan−A
Meishan−B
Meishan−C
European−A
European−B
European−C

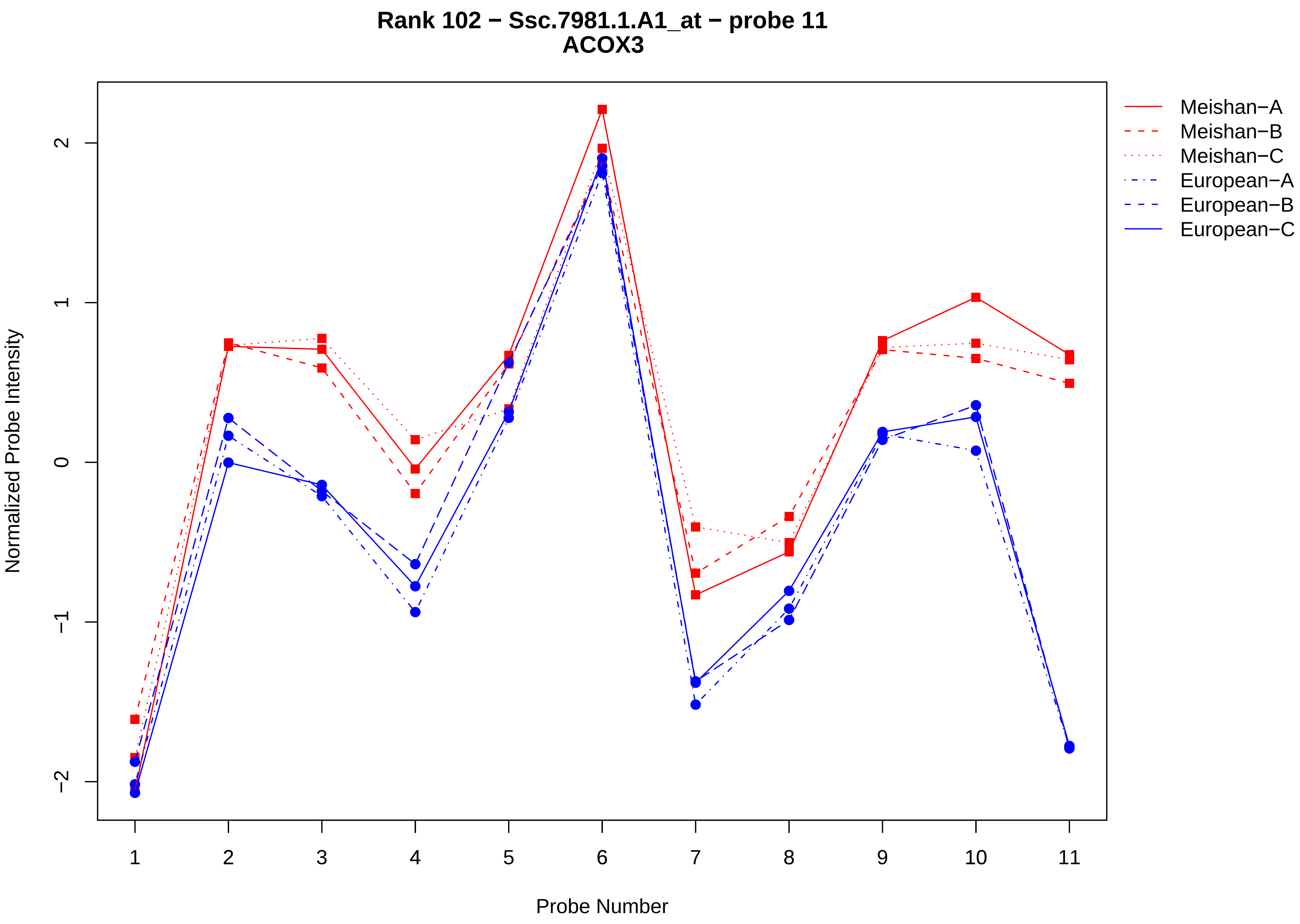

Rank 102 − Ssc.7981.1.A1_at − probe 11
ACOX3
Normalized Probe Intensity
Probe Number
−2
−1
0
1
2
1
2
3
4
5
6
7
8
9
10
11
Meishan−A
Meishan−B
Meishan−C
European−A
European−B
European−C

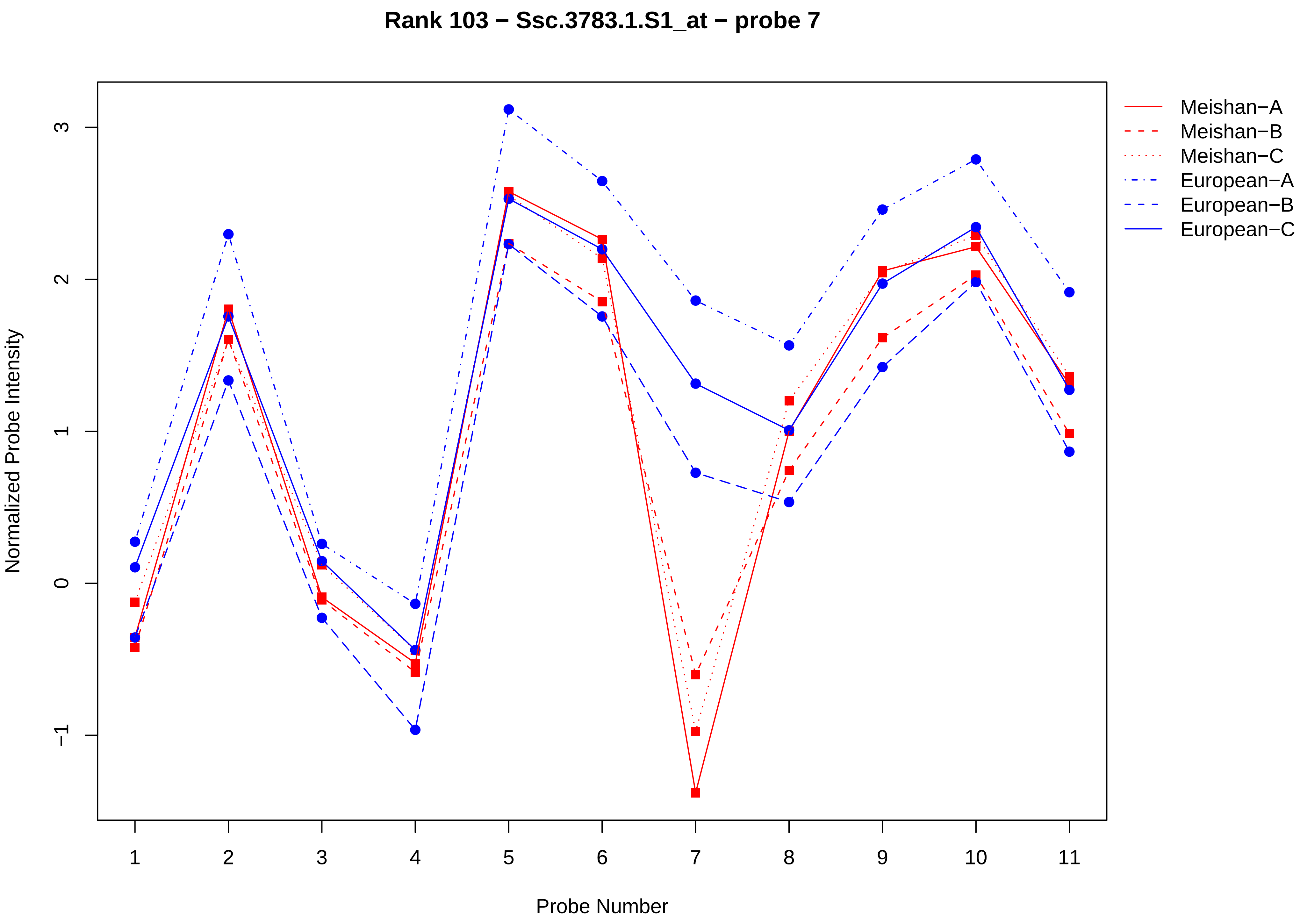

Rank 103 − Ssc.3783.1.S1_at − probe 7
Normalized Probe Intensity
Probe Number
−1
0
1
2
3
1
2
3
4
5
6
7
8
9
10
11
Meishan−A
Meishan−B
Meishan−C
European−A
European−B
European−C

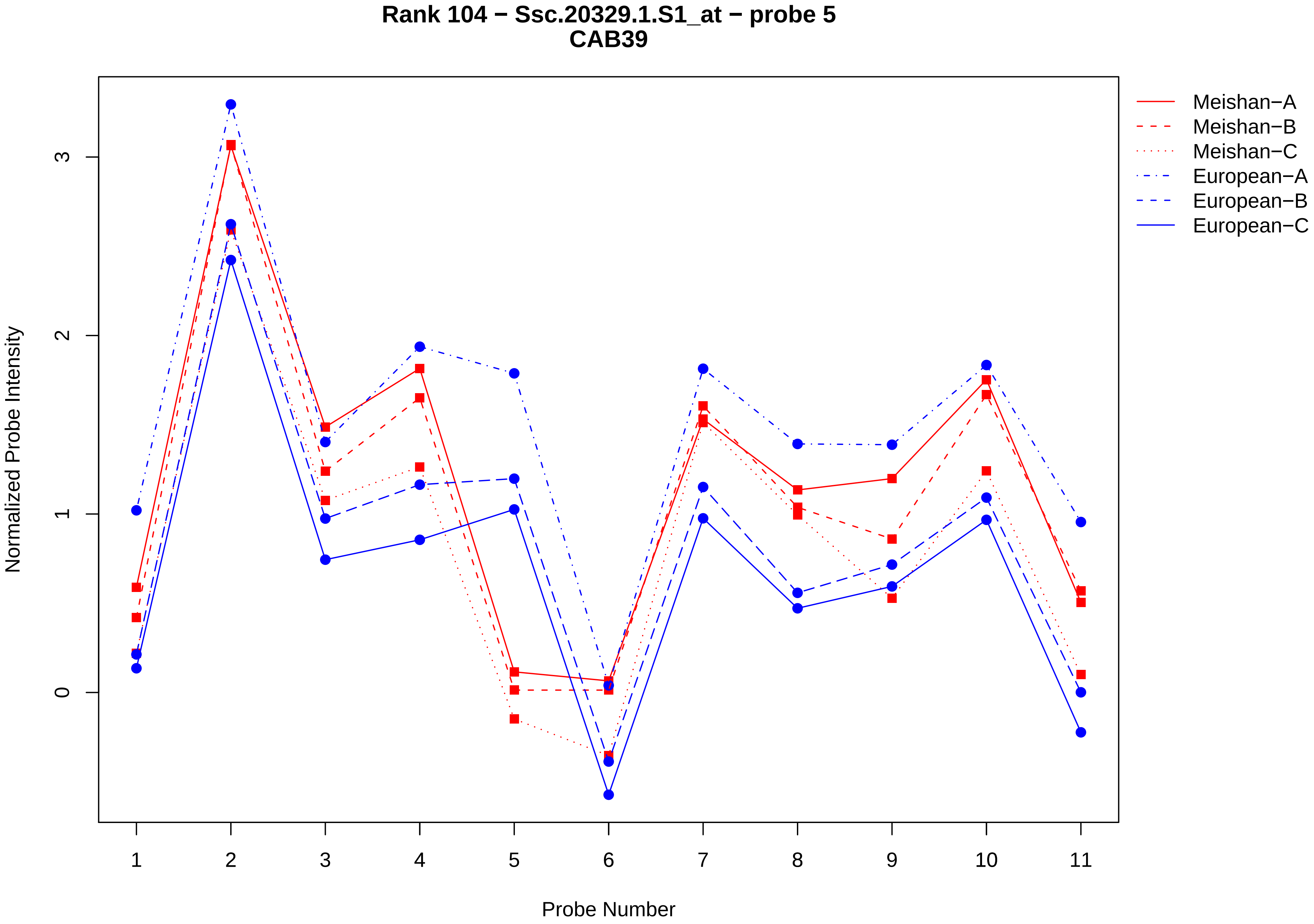

Rank 104 − Ssc.20329.1.S1_at − probe 5
CAB39
Normalized Probe Intensity
Probe Number
0
1
2
3
1
2
3
4
5
6
7
8
9
10
11
Meishan−A
Meishan−B
Meishan−C
European−A
European−B
European−C

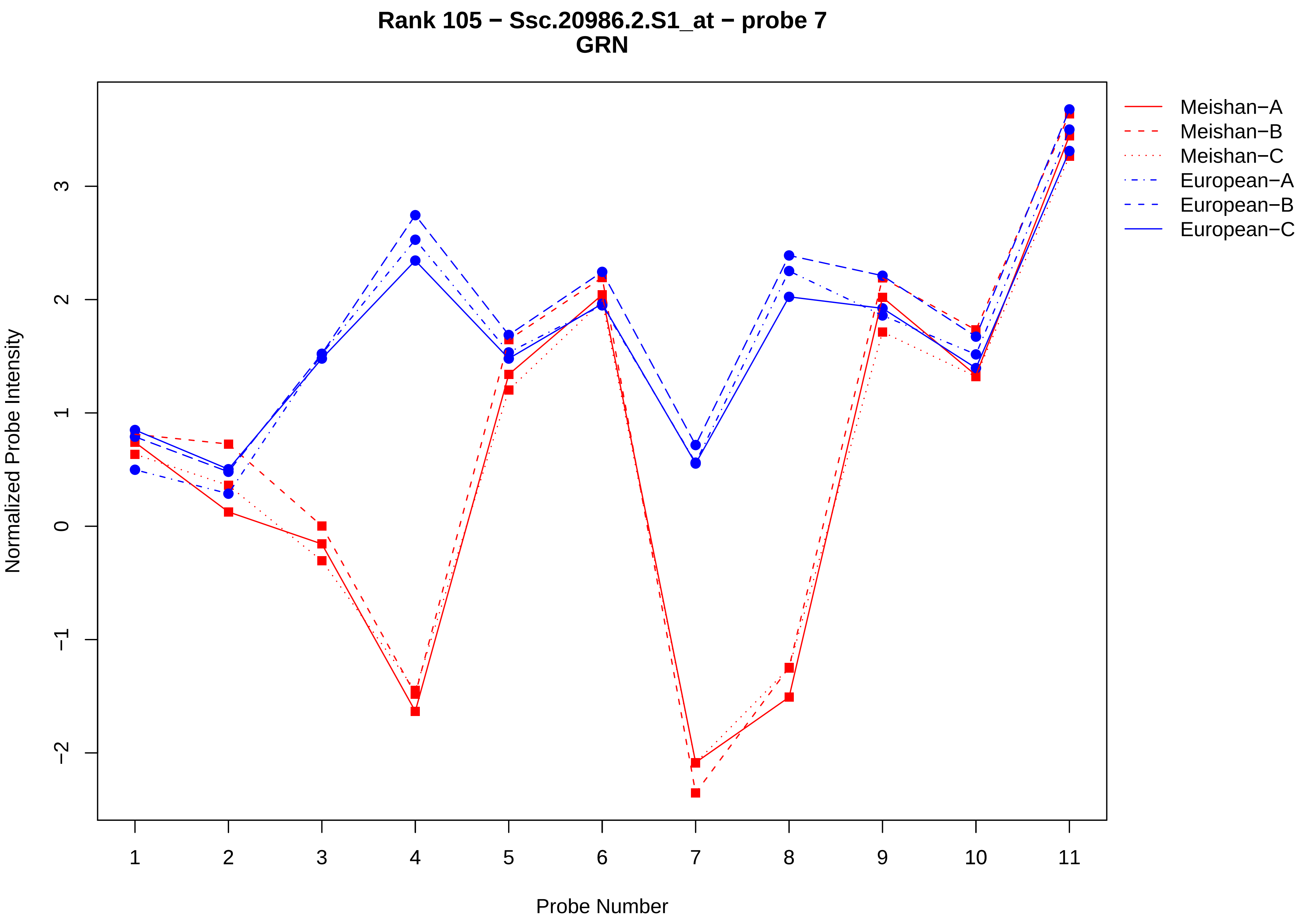

Rank 105 − Ssc.20986.2.S1_at − probe 7
GRN
Normalized Probe Intensity
Probe Number
−2
−1
0
1
2
3
1
2
3
4
5
6
7
8
9
10
11
Meishan−A
Meishan−B
Meishan−C
European−A
European−B
European−C

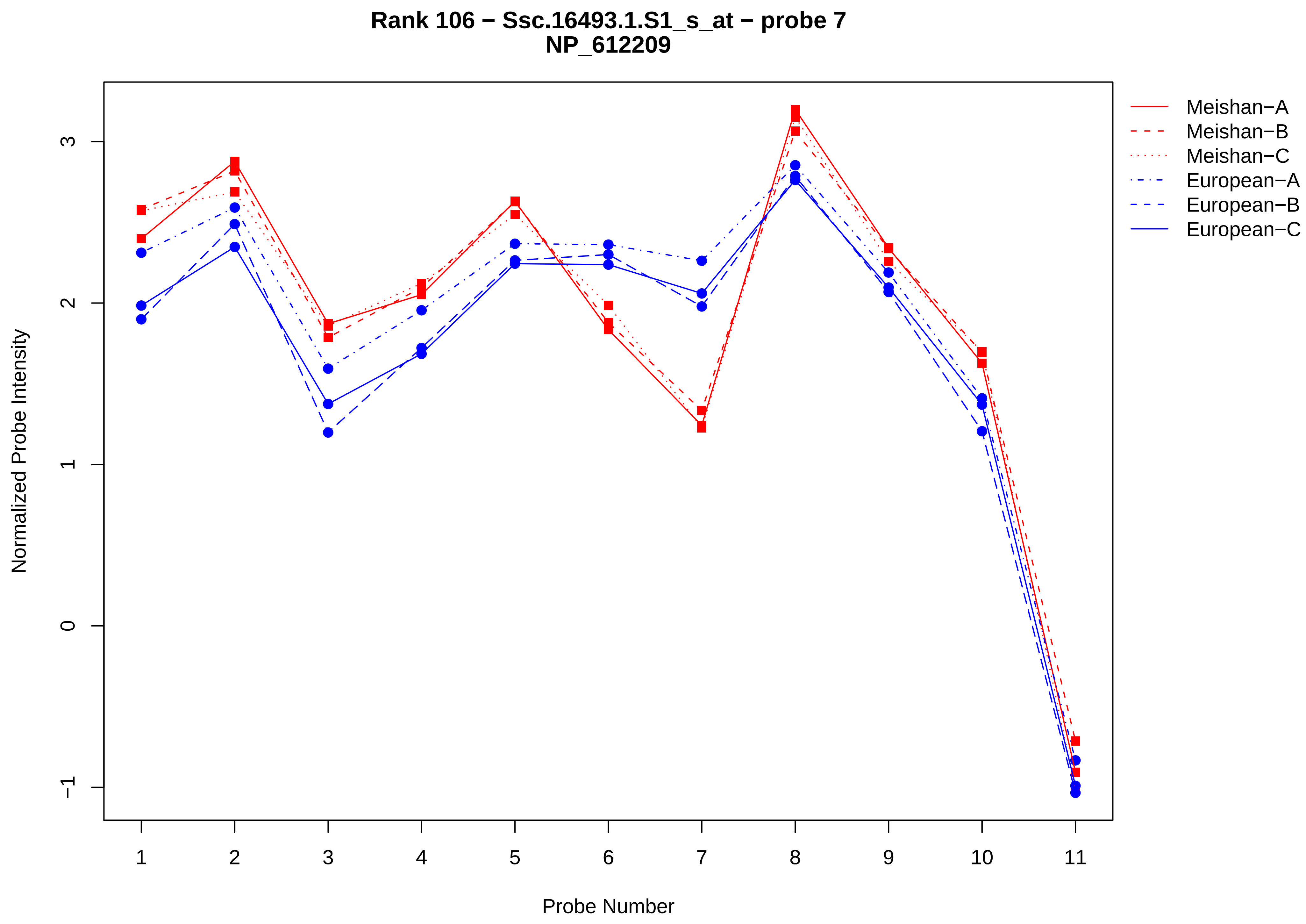
Rank 106 − Ssc.16493.1.S1_s_at − probe 7
NP_612209
Normalized Probe Intensity
Probe Number
−1
0
1
2
3
1
2
3
4
5
6
7
8
9
10
11
Meishan−A
Meishan−B
Meishan−C
European−A
European−B
European−C

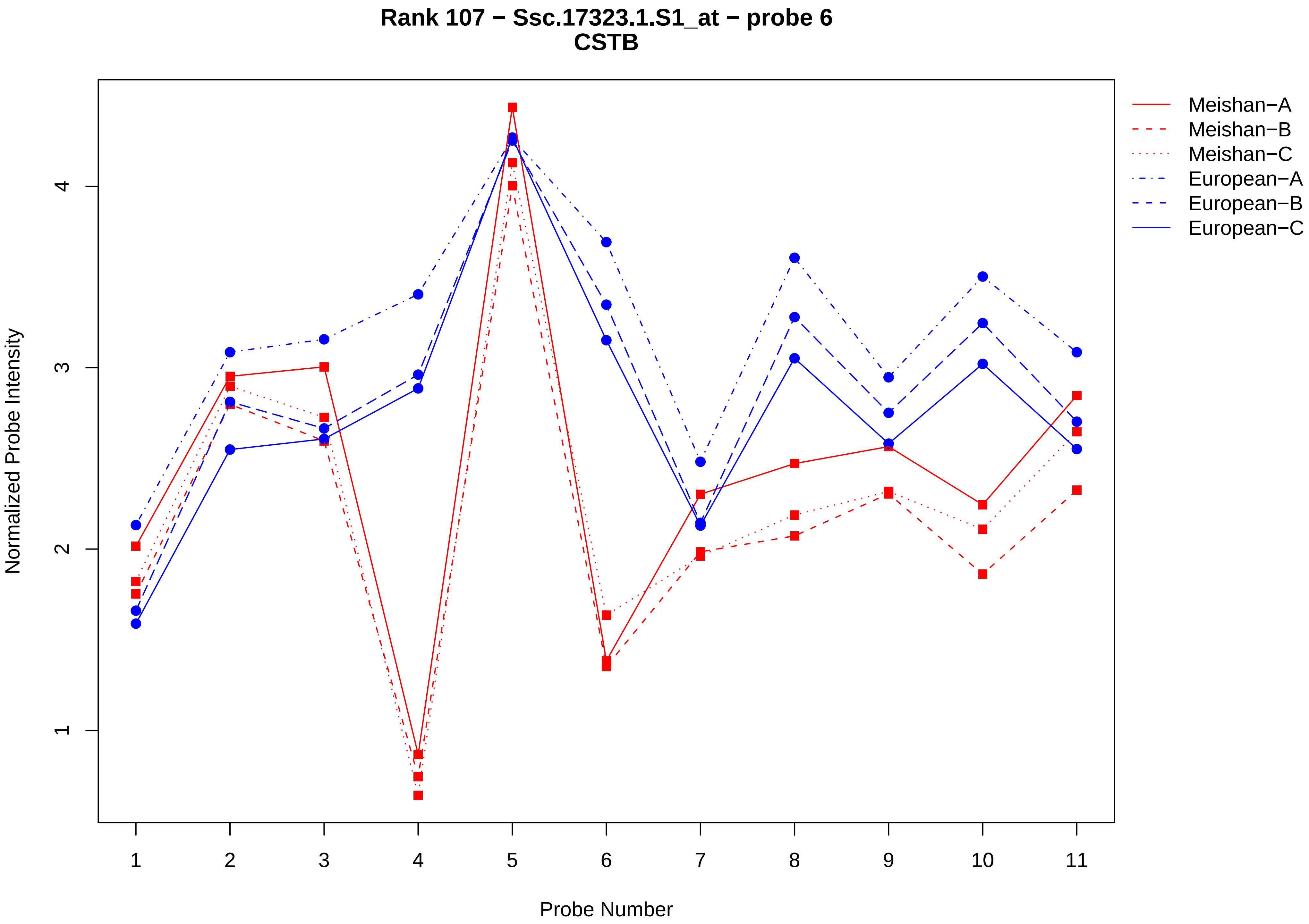
Rank 107 − Ssc.17323.1.S1_at − probe 6
CSTB
Normalized Probe Intensity
Probe Number
1
2
3
4
1
2
3
4
5
6
7
8
9
10
11
Meishan−A
Meishan−B
Meishan−C
European−A
European−B
European−C

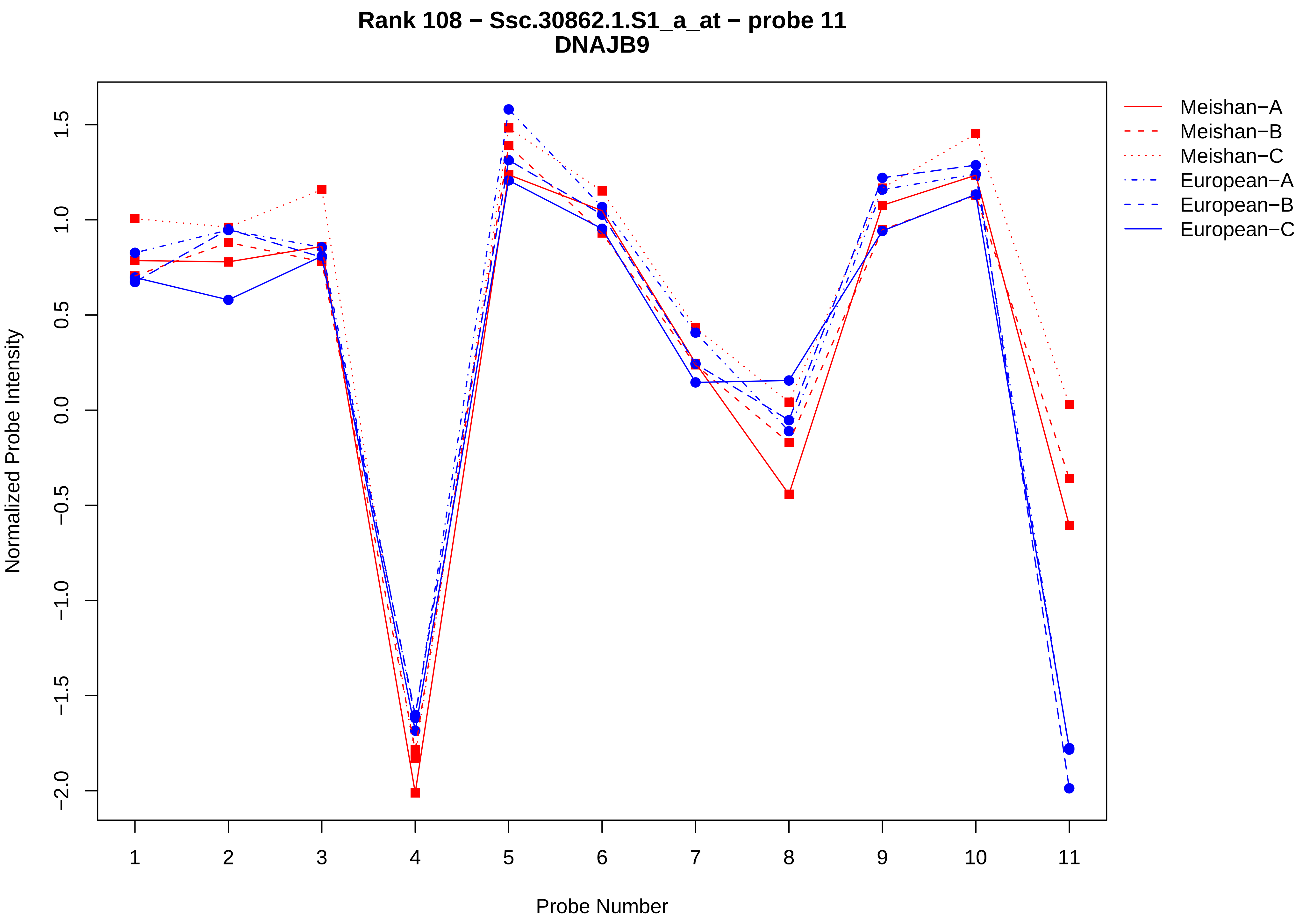

Rank 108 − Ssc.30862.1.S1_a_at − probe 11
DNAJB9
Normalized Probe Intensity
Probe Number
Meishan−A
Meishan−B
Meishan−C
European−A
European−B
European−C
1.5
1.0
0.5
0.0
−0.5
−1.0
−1.5
−2.0
1
2
3
4
5
6
7
8
9
10
11

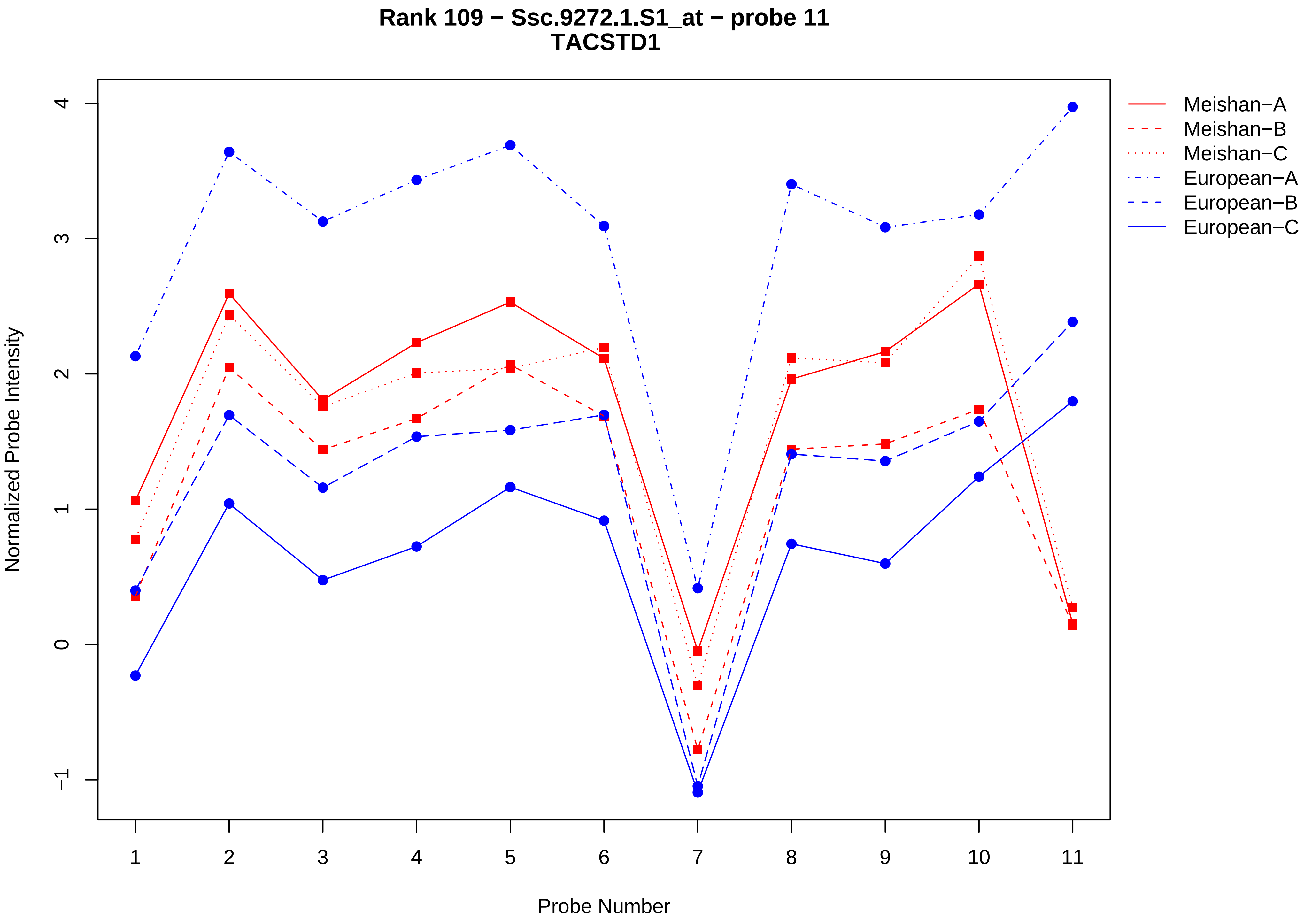

Rank 109 − Ssc.9272.1.S1_at − probe 11
TACSTD1
Normalized Probe Intensity
Probe Number
−1
0
1
2
3
4
1
2
3
4
5
6
7
8
9
10
11
Meishan−A
Meishan−B
Meishan−C
European−A
European−B
European−C

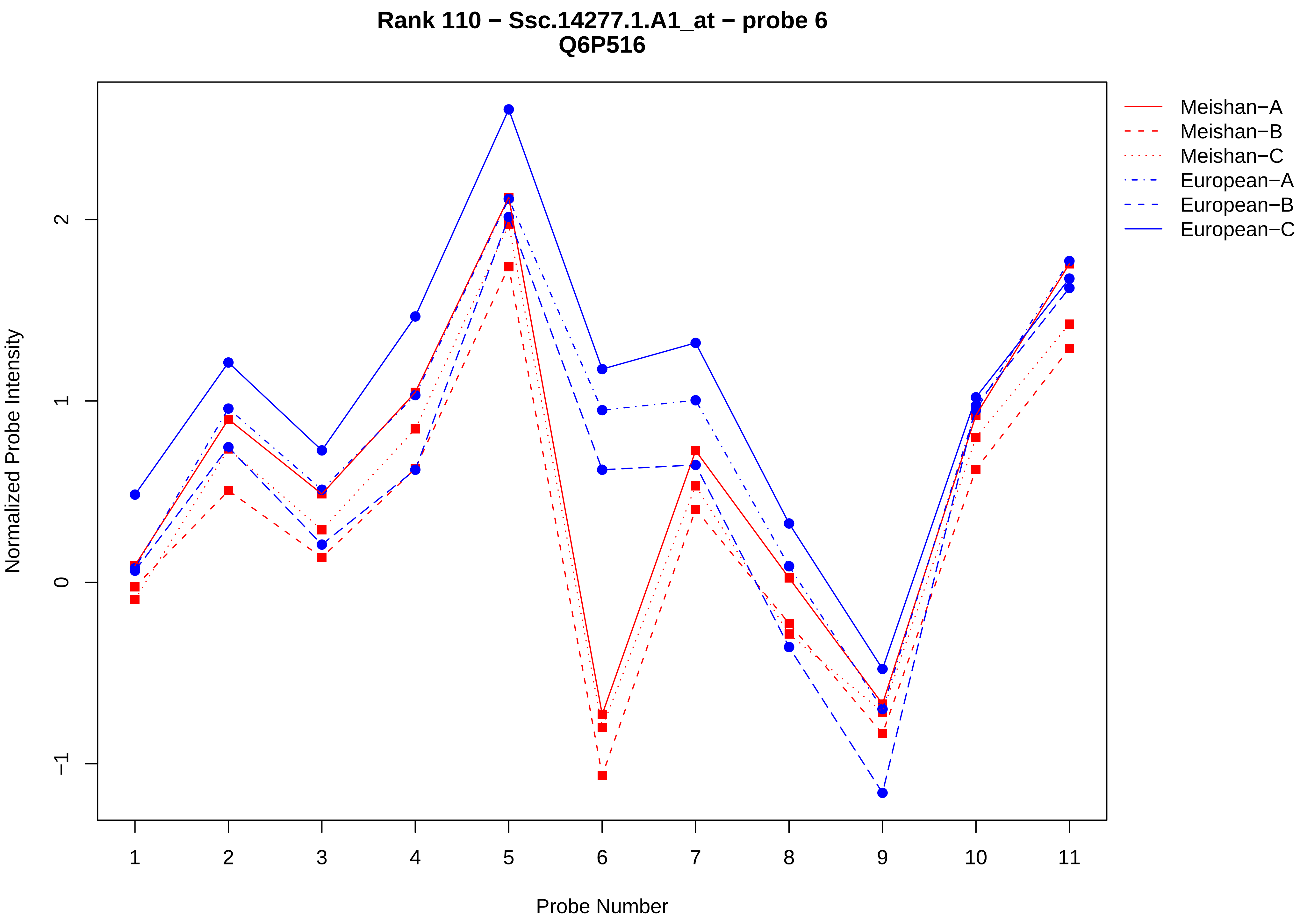

Rank 110 − Ssc.14277.1.A1_at − probe 6
Q6P516
Normalized Probe Intensity
Probe Number
Meishan−A
Meishan−B
Meishan−C
European−A
European−B
European−C
−1
0
1
2
1
2
3
4
5
6
7
8
9
10
11

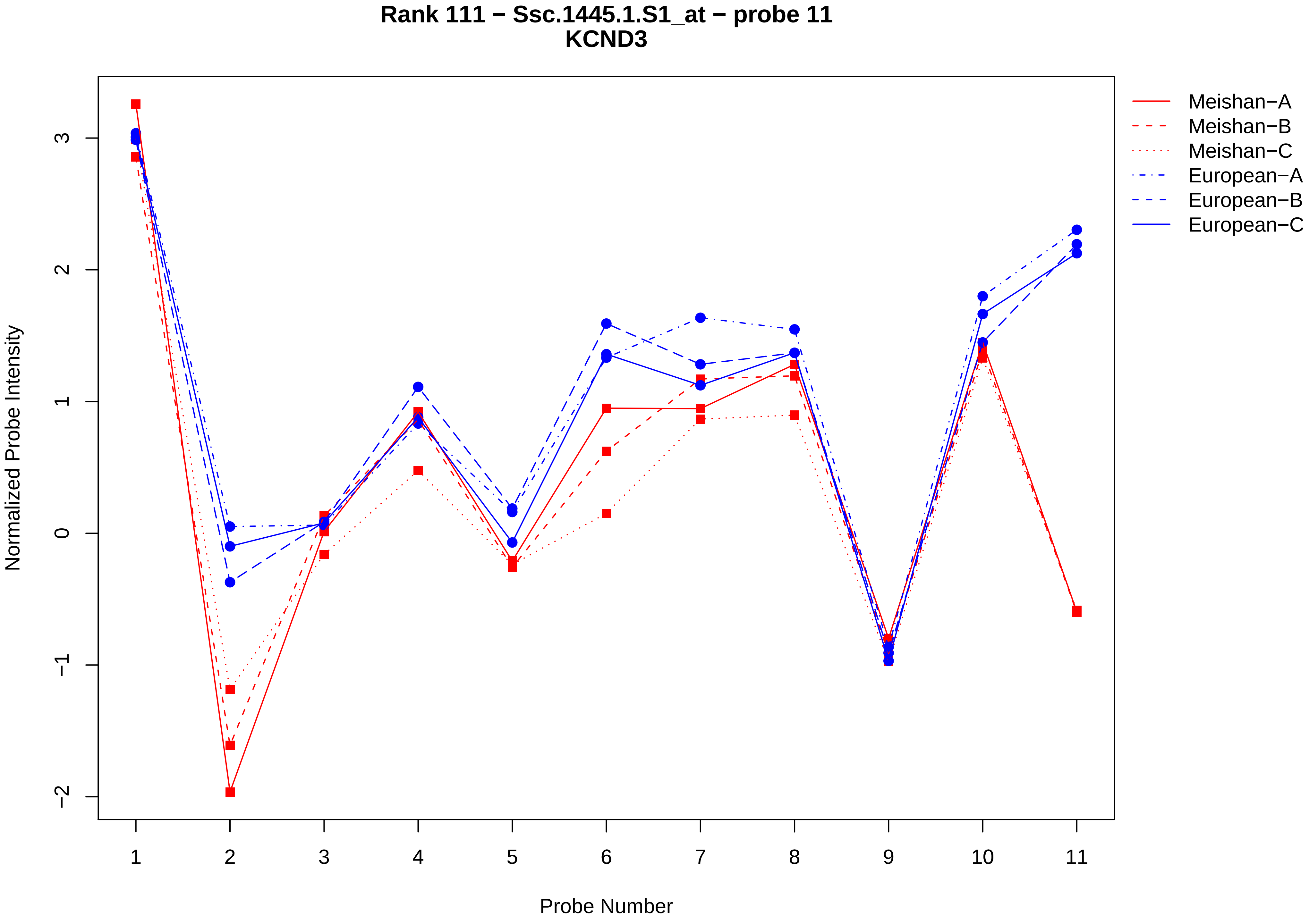

Rank 111 − Ssc.1445.1.S1_at − probe 11
KCND3
Normalized Probe Intensity
Probe Number
−2
−1
0
1
2
3
1
2
3
4
5
6
7
8
9
10
11
Meishan−A
Meishan−B
Meishan−C
European−A
European−B
European−C

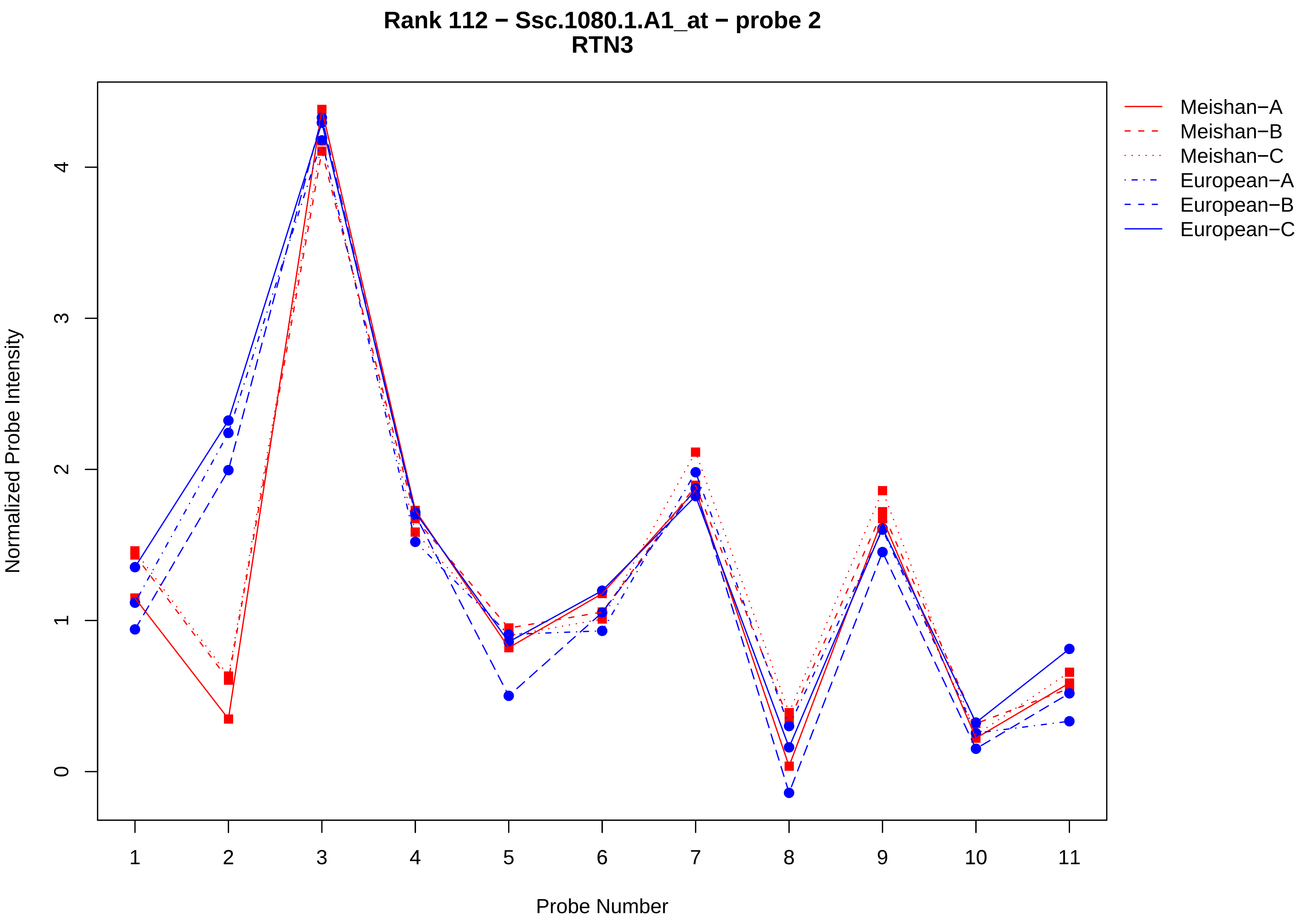

Rank 112 − Ssc.1080.1.A1_at − probe 2
RTN3
Normalized Probe Intensity
Probe Number
0
1
2
3
4
1
2
3
4
5
6
7
8
9
10
11
Meishan−A
Meishan−B
Meishan−C
European−A
European−B
European−C

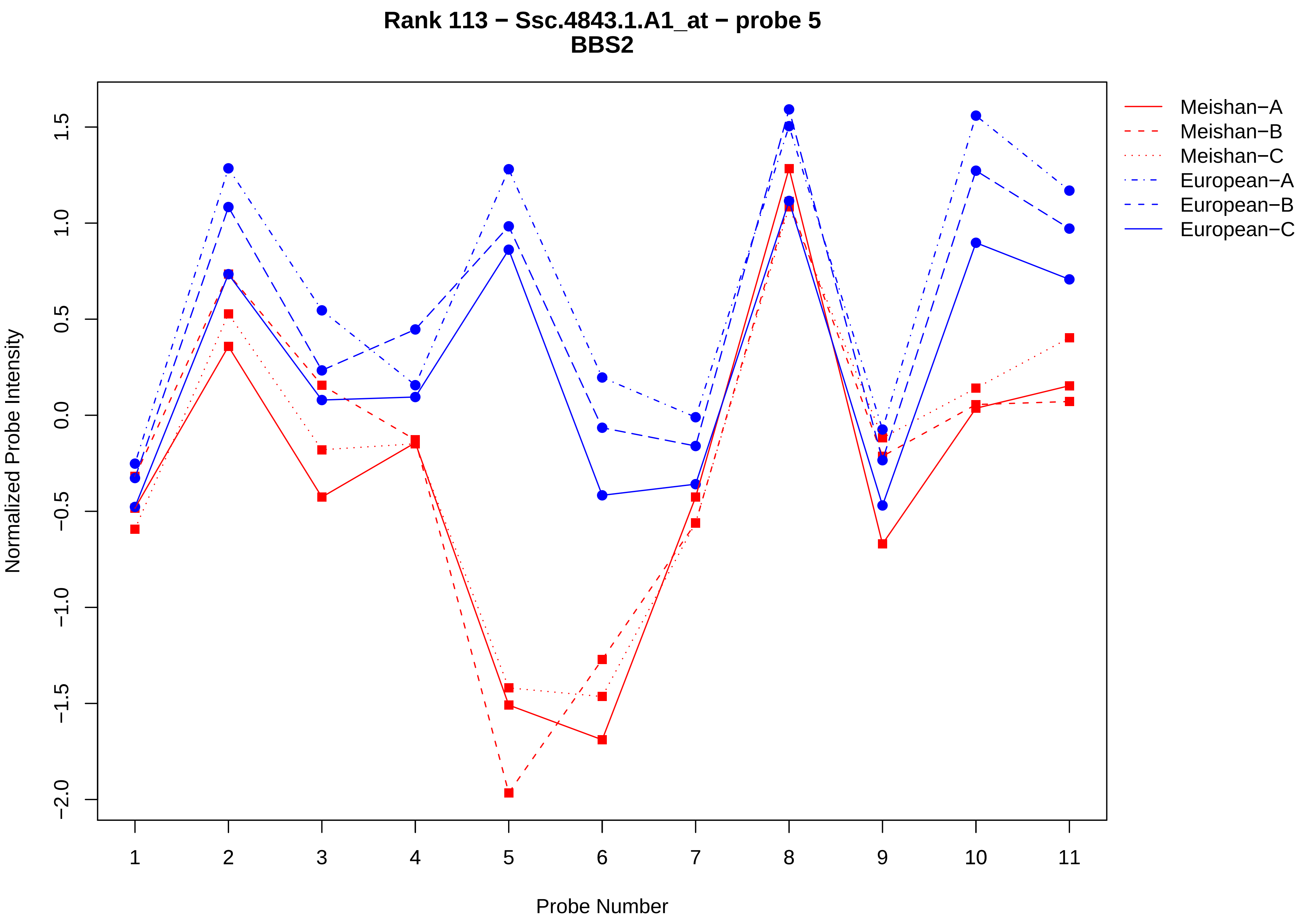
Rank 113 − Ssc.4843.1.A1_at − probe 5
BBS2
Normalized Probe Intensity
Probe Number
Meishan−A
Meishan−B
Meishan−C
European−A
European−B
European−C
1.5
1.0
0.5
0.0
−0.5
−1.0
−1.5
−2.0
1
2
3
4
5
6
7
8
9
10
11

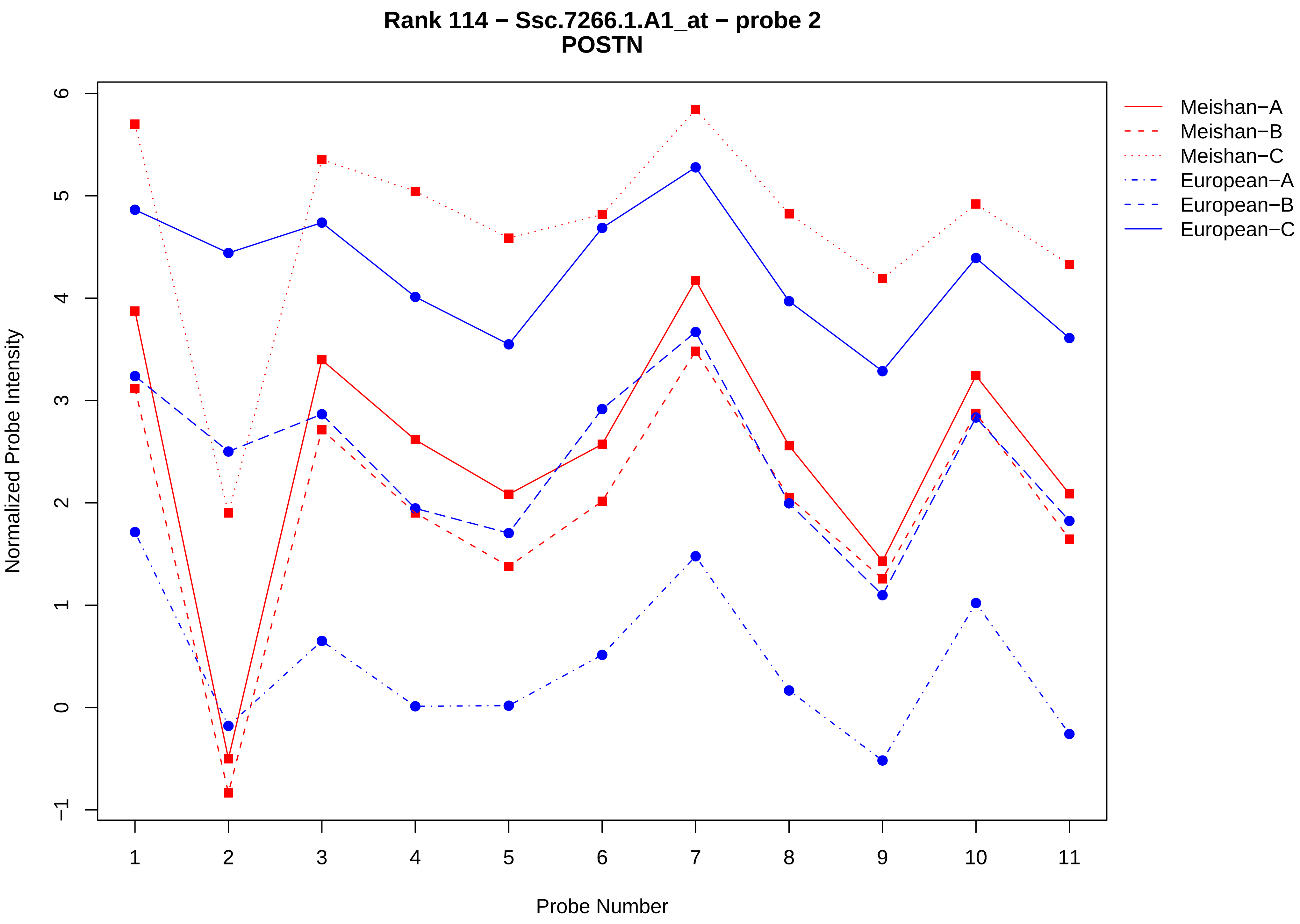
Rank 114 − Ssc.7266.1.A1_at − probe 2
POSTN
Normalized Probe Intensity
Probe Number
Meishan−A
Meishan−B
Meishan−C
European−A
European−B
European−C
−1
0
1
2
3
4
5
6
1
2
3
4
5
6
7
8
9
10
11

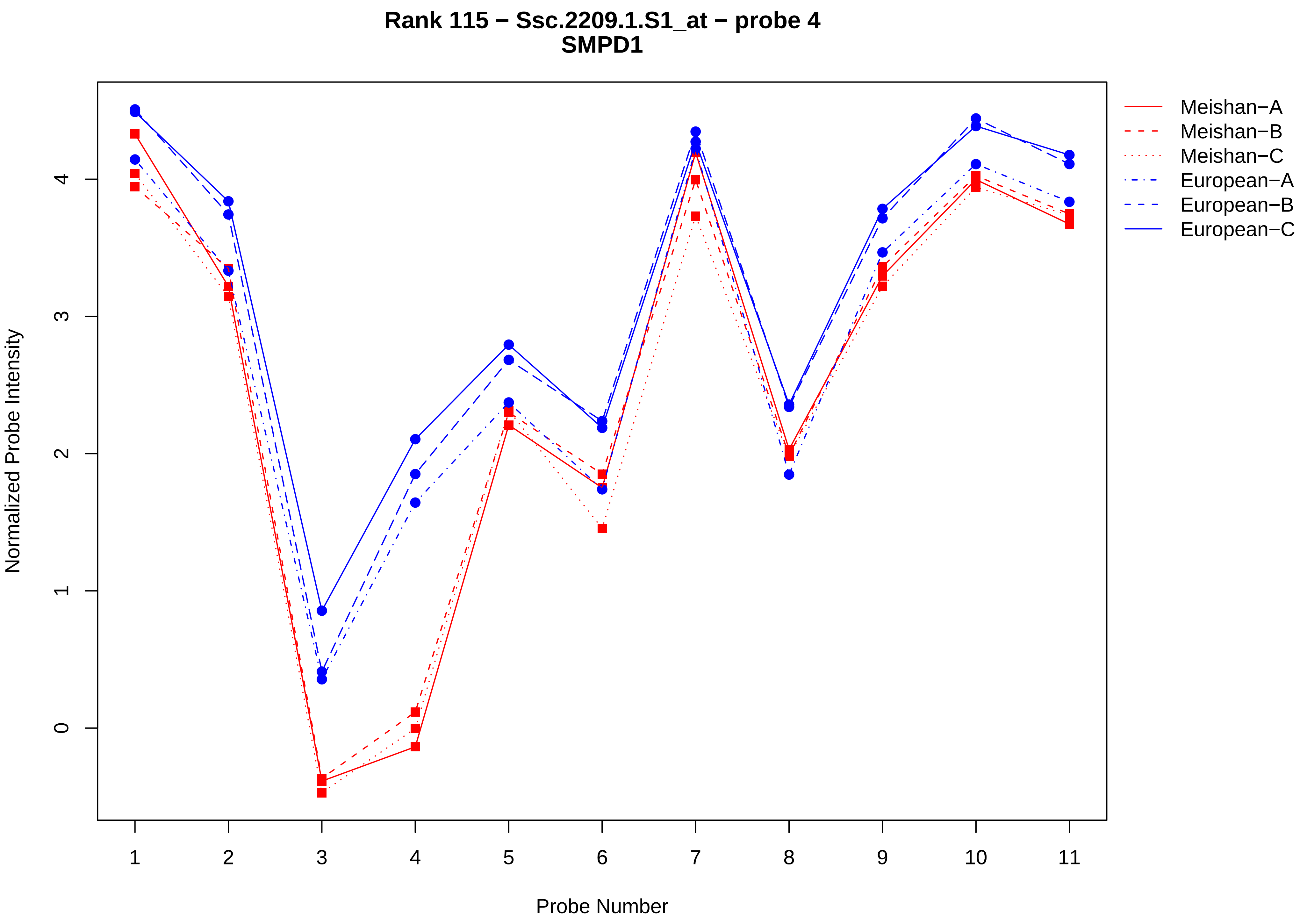
Rank 115 − Ssc.2209.1.S1_at − probe 4
SMPD1
Normalized Probe Intensity
Probe Number
0
1
2
3
4
1
2
3
4
5
6
7
8
9
10
11
Meishan−A
Meishan−B
Meishan−C
European−A
European−B
European−C

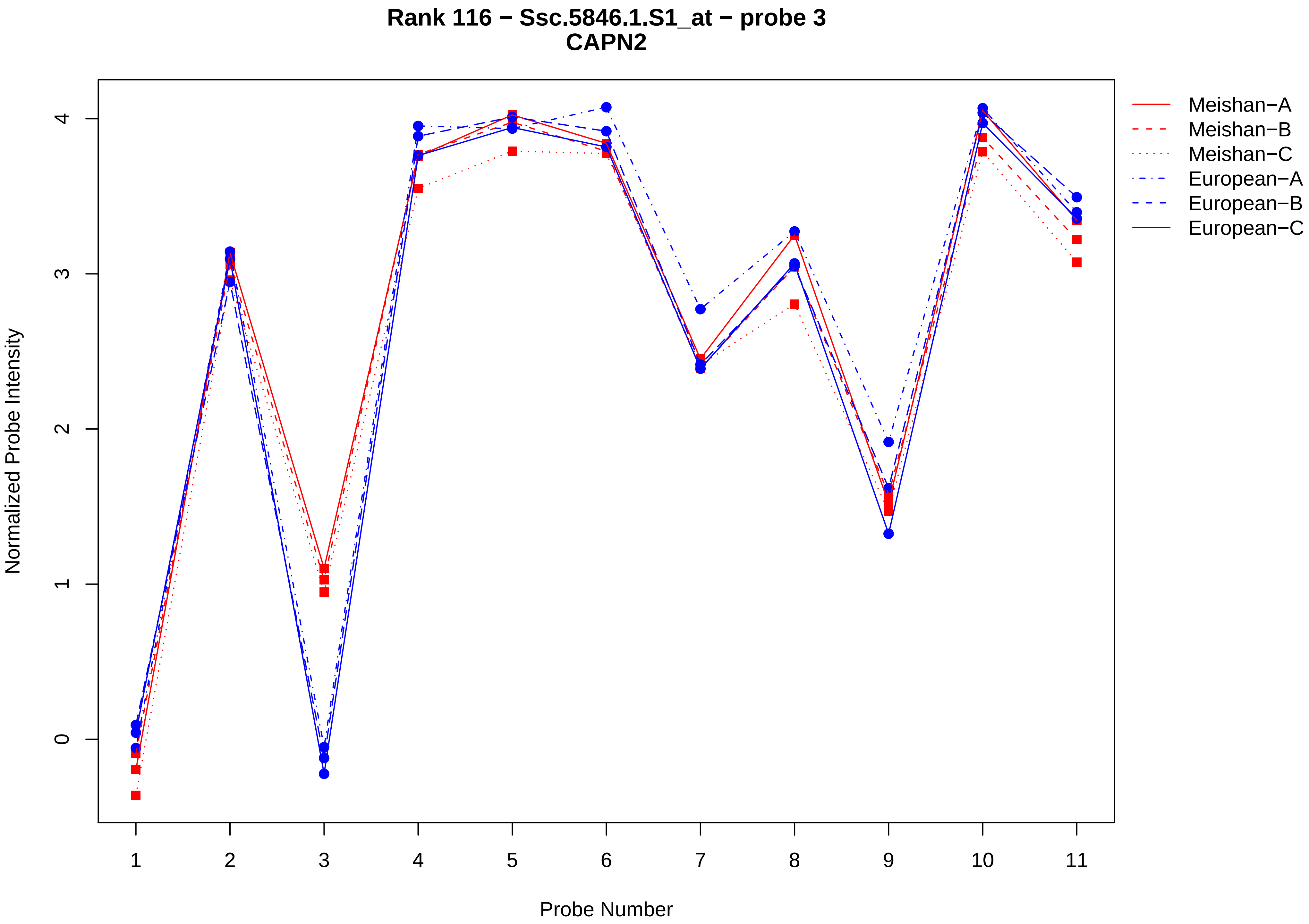
Rank 116 − Ssc.5846.1.S1_at − probe 3
CAPN2
Normalized Probe Intensity
Probe Number
0
1
2
3
4
1
2
3
4
5
6
7
8
9
10
11
Meishan−A
Meishan−B
Meishan−C
European−A
European−B
European−C

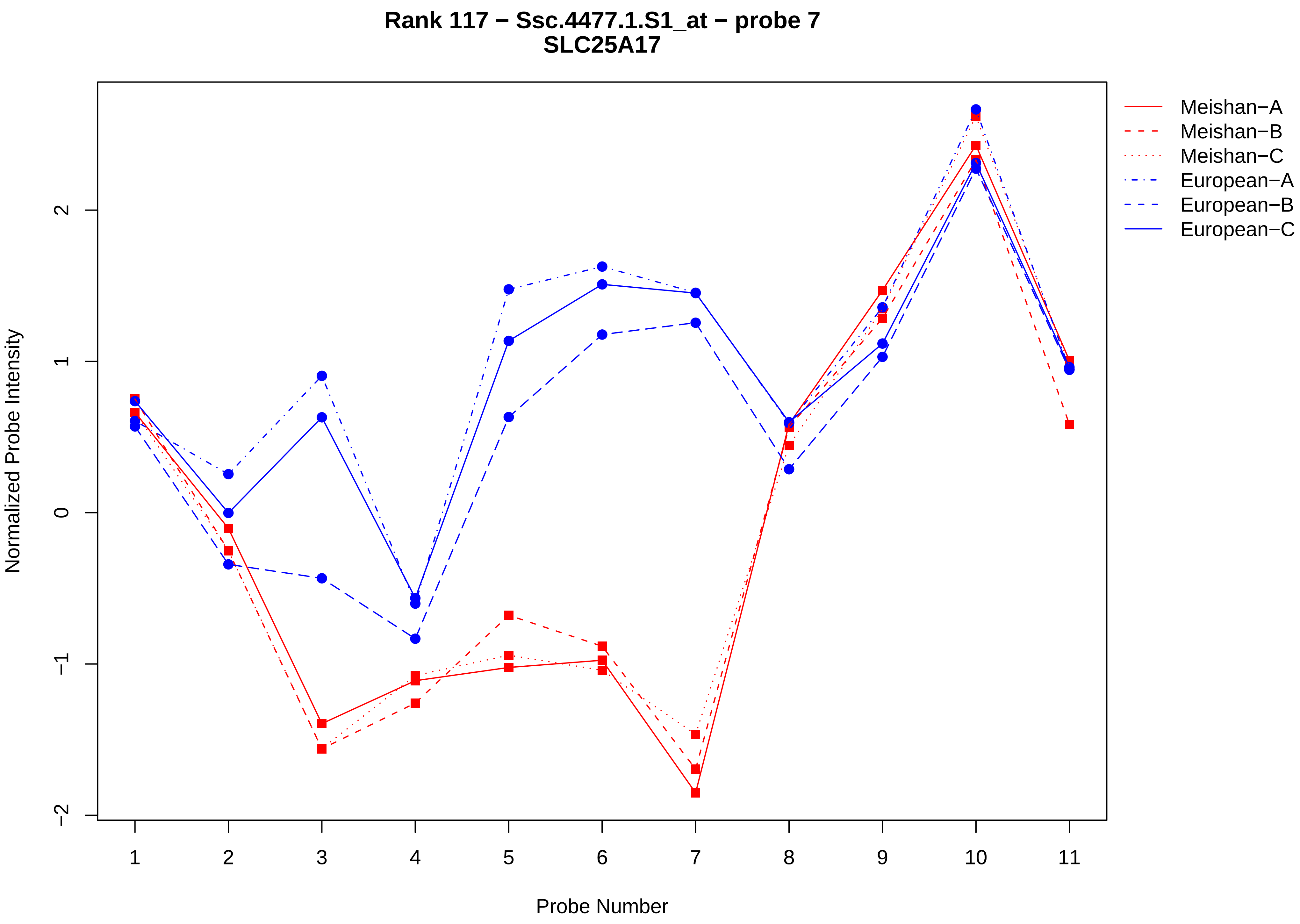
Rank 117 − Ssc.4477.1.S1_at − probe 7
SLC25A17
Normalized Probe Intensity
Probe Number
Meishan−A
Meishan−B
Meishan−C
European−A
European−B
European−C
−2
−1
0
1
2
1
2
3
4
5
6
7
8
9
10
11

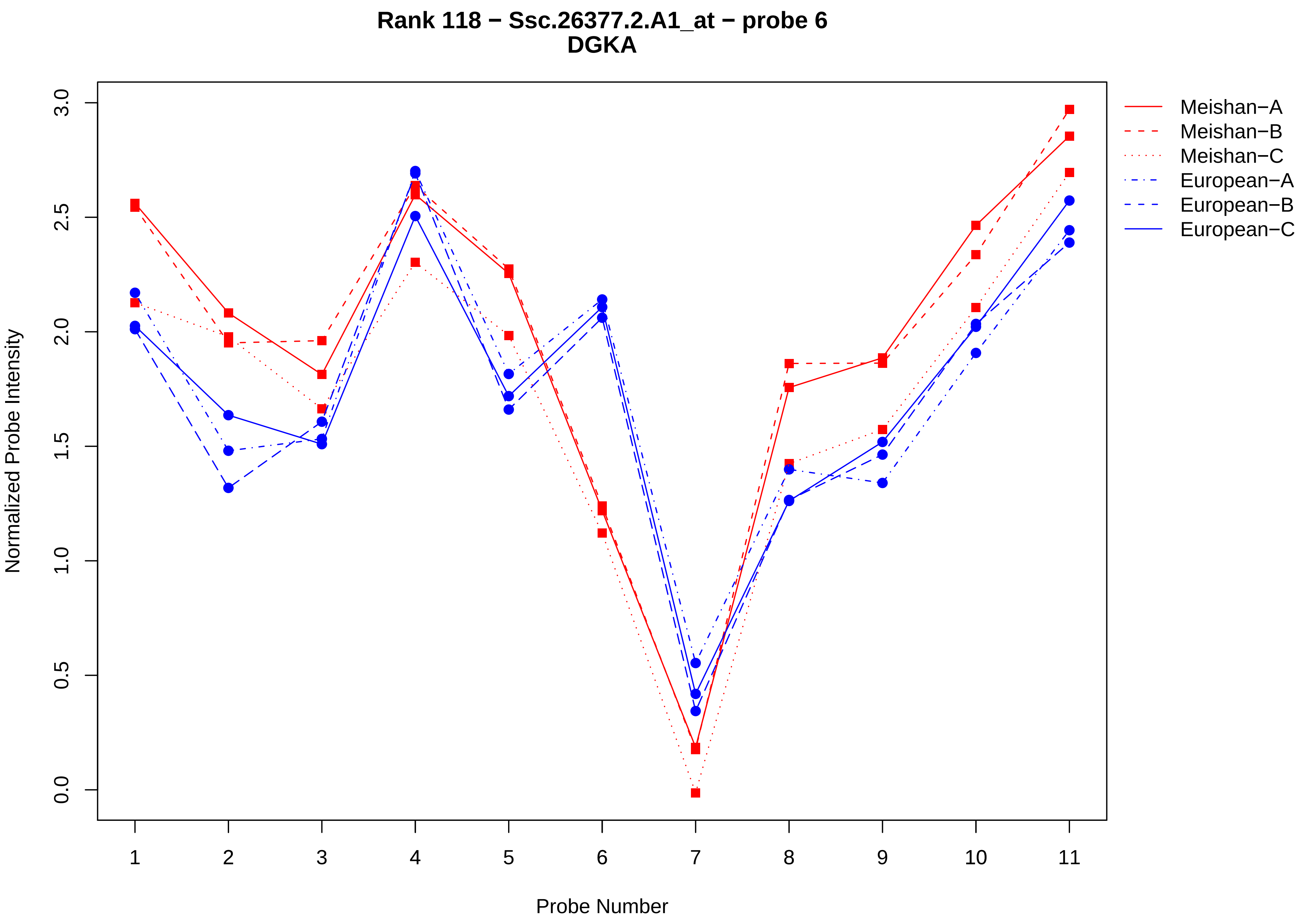
Rank 118 − Ssc.26377.2.A1_at − probe 6
DGKA
Normalized Probe Intensity
Probe Number
0.0
0.5
1.0
1.5
2.0
2.5
3.0
1
2
3
4
5
6
7
8
9
10
11
Meishan−A
Meishan−B
Meishan−C
European−A
European−B
European−C

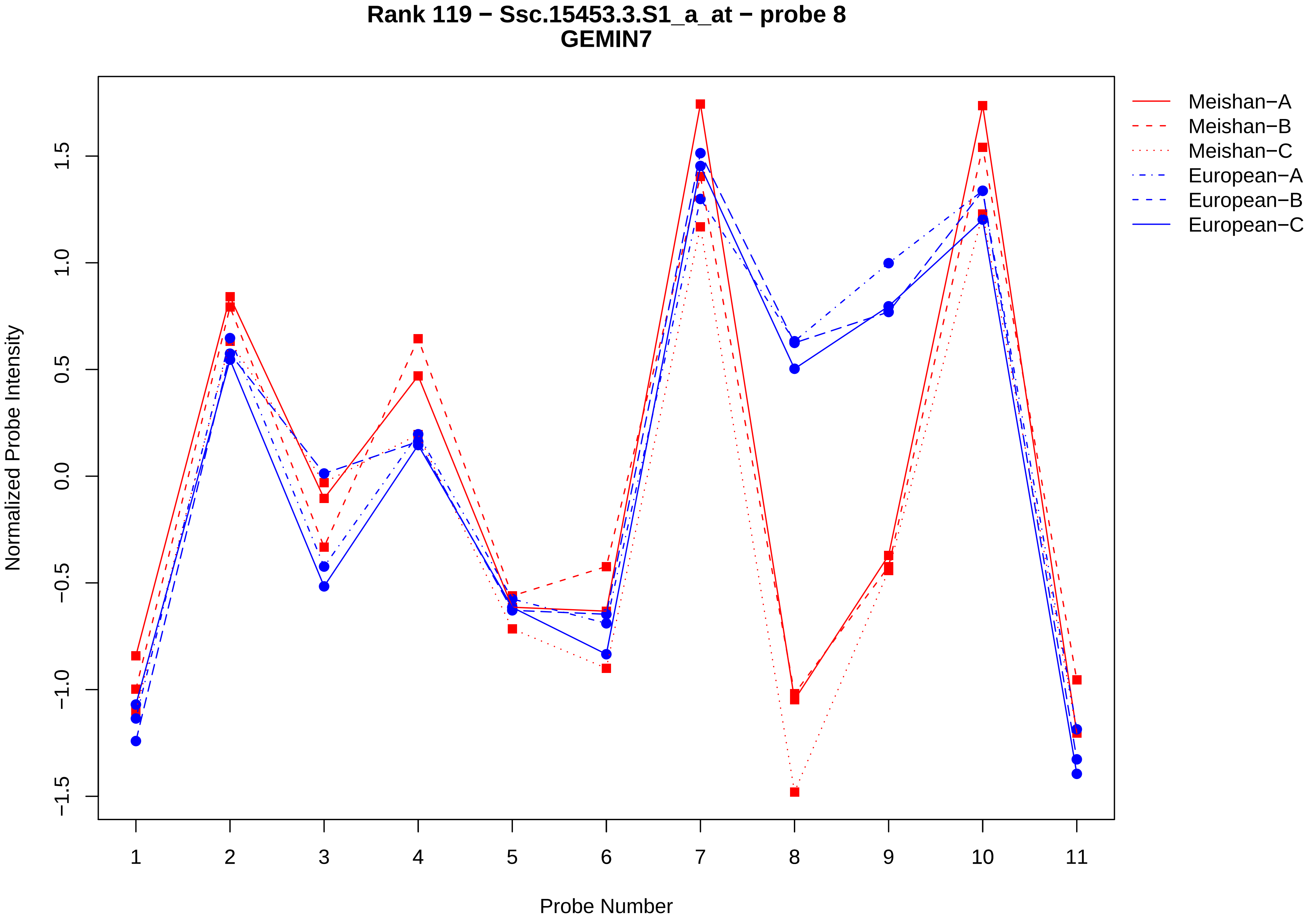
Rank 119 − Ssc.15453.3.S1_a_at − probe 8
GEMIN7
Normalized Probe Intensity
Probe Number
−1.5
−1.0
−0.5
0.0
0.5
1.0
1.5
1
2
3
4
5
6
7
8
9
10
11
Meishan−A
Meishan−B
Meishan−C
European−A
European−B
European−C

Rank 120 − Ssc.4513.2.A1_a_at − probe 3
EMD

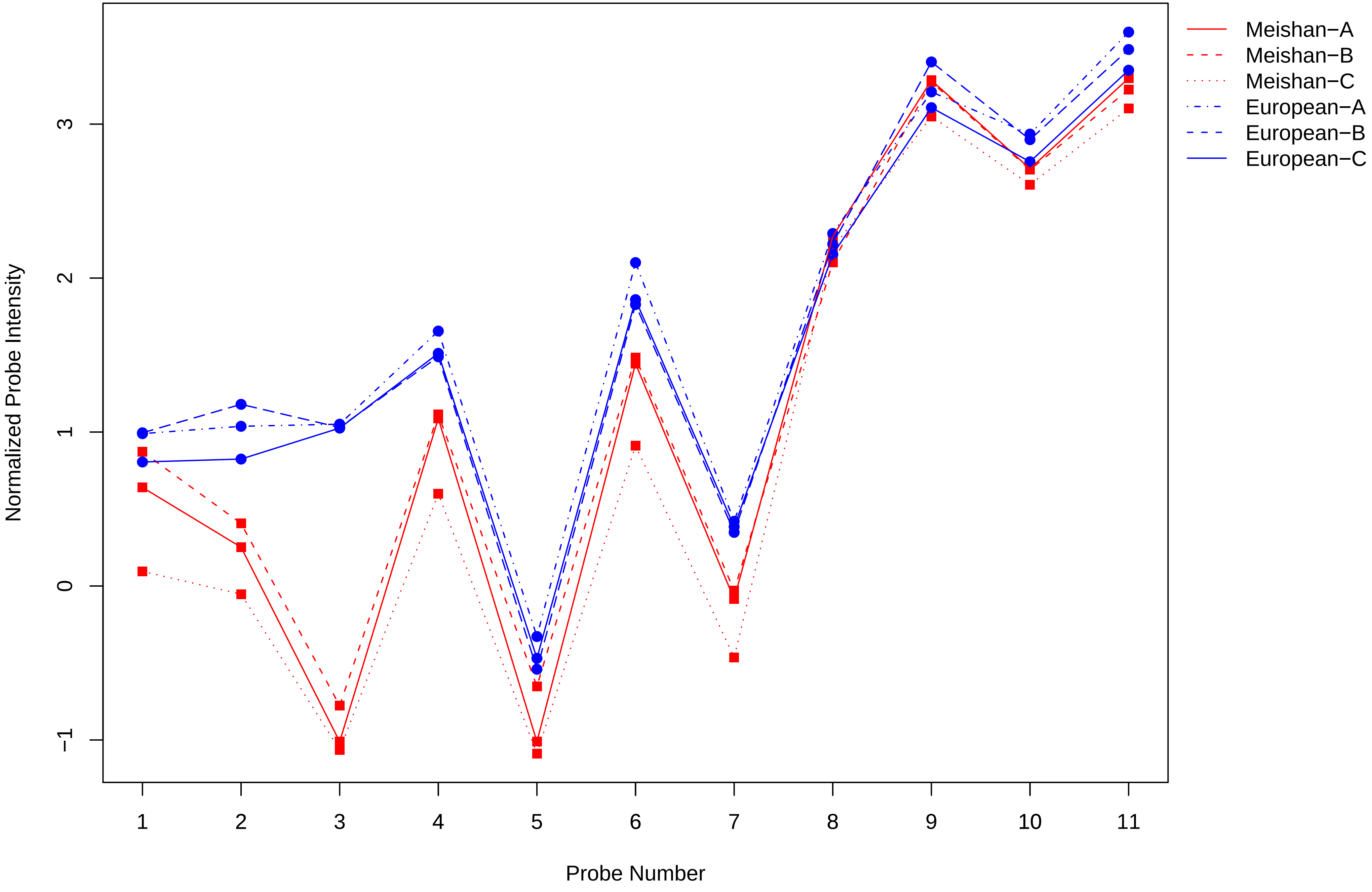

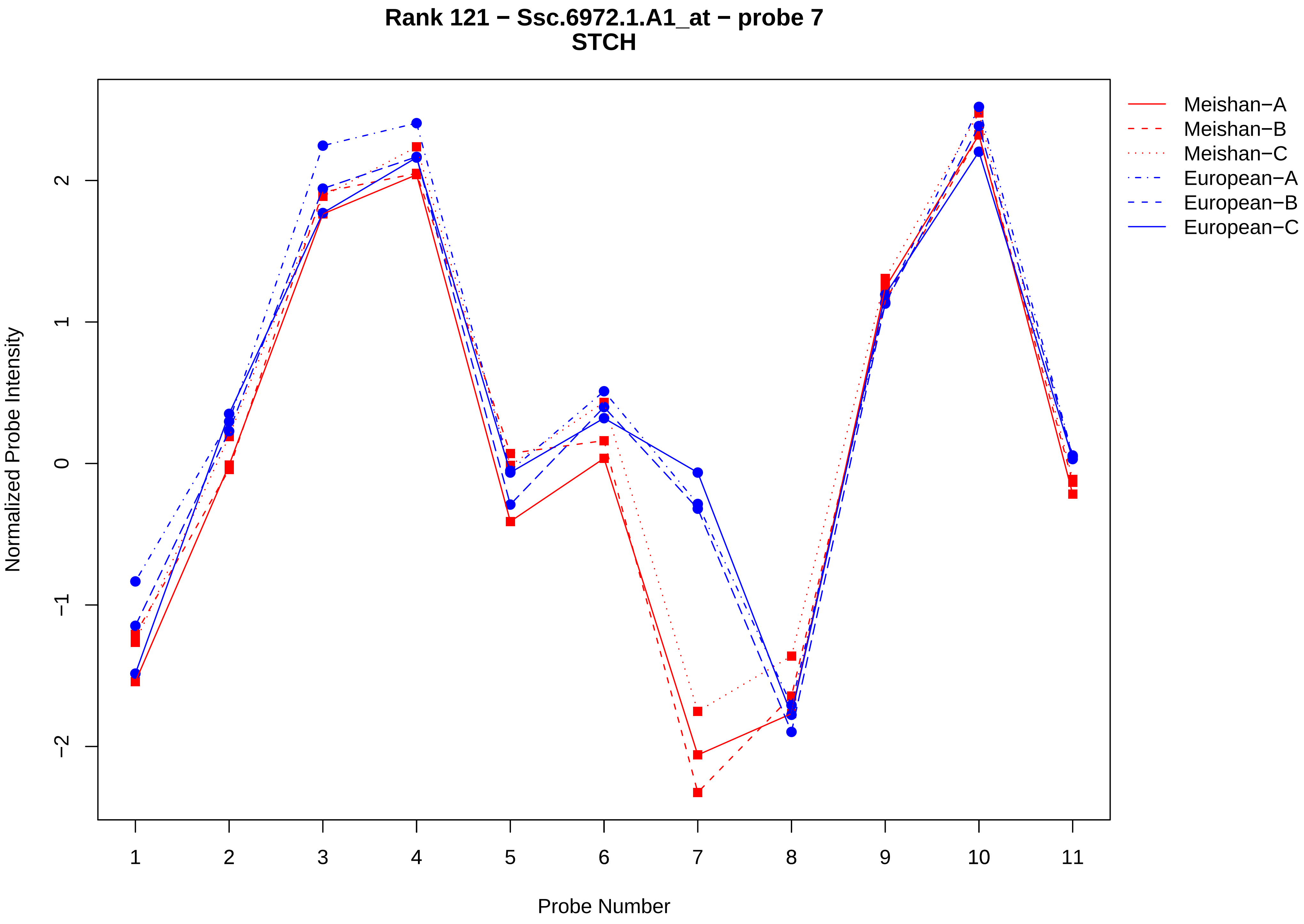

Rank 121 − Ssc.6972.1.A1_at − probe 7
STCH
Normalized Probe Intensity
−2
−1
0
1
2
1
2
3
4
5
6
7
8
9
10
11
Probe Number
Meishan−A
Meishan−B
Meishan−C
European−A
European−B
European−C

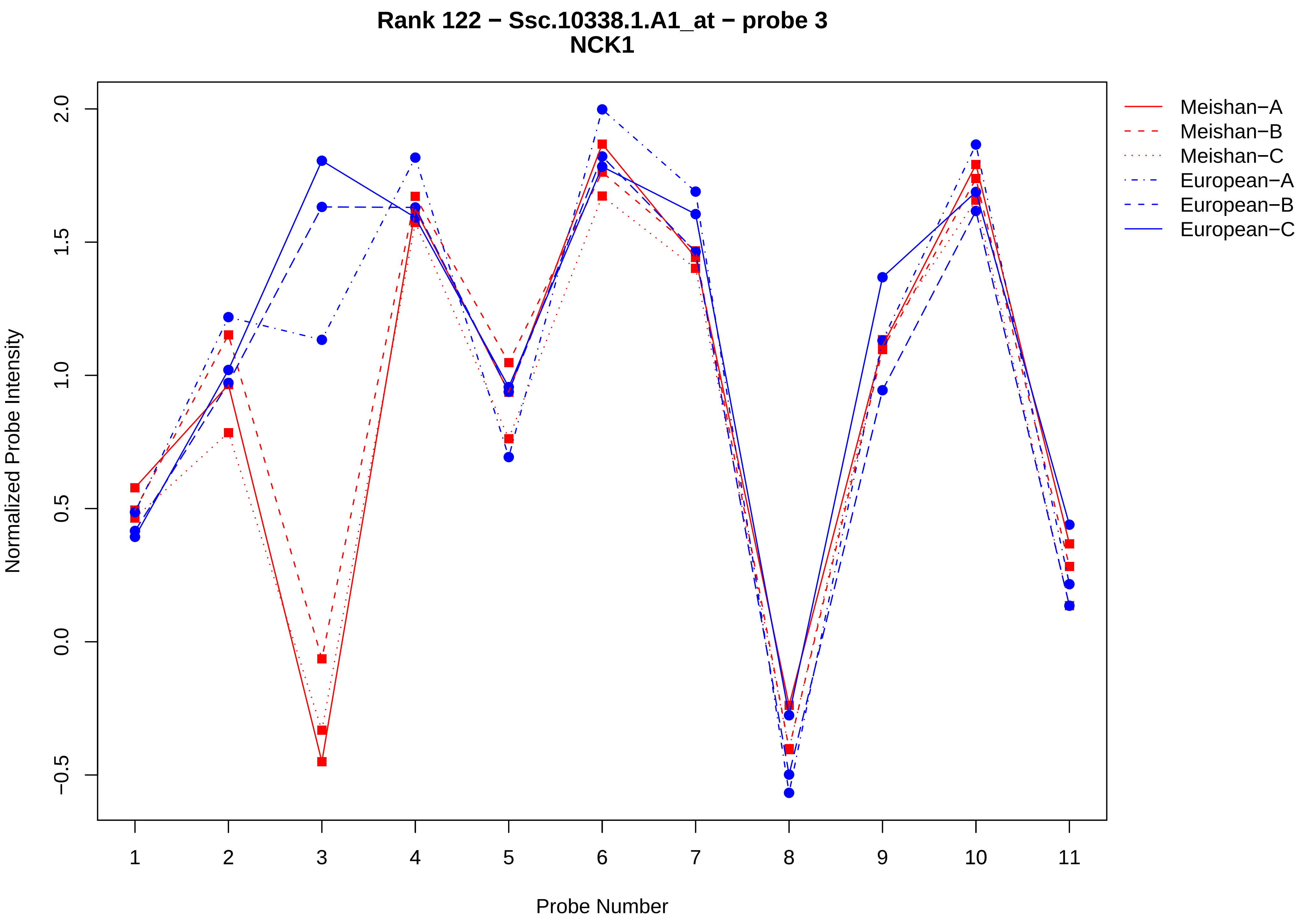

Rank 122 − Ssc.10338.1.A1_at − probe 3
NCK1
Normalized Probe Intensity
Probe Number
Meishan−A
Meishan−B
Meishan−C
European−A
European−B
European−C
−0.5
0.0
0.5
1.0
1.5
2.0
1
2
3
4
5
6
7
8
9
10
11

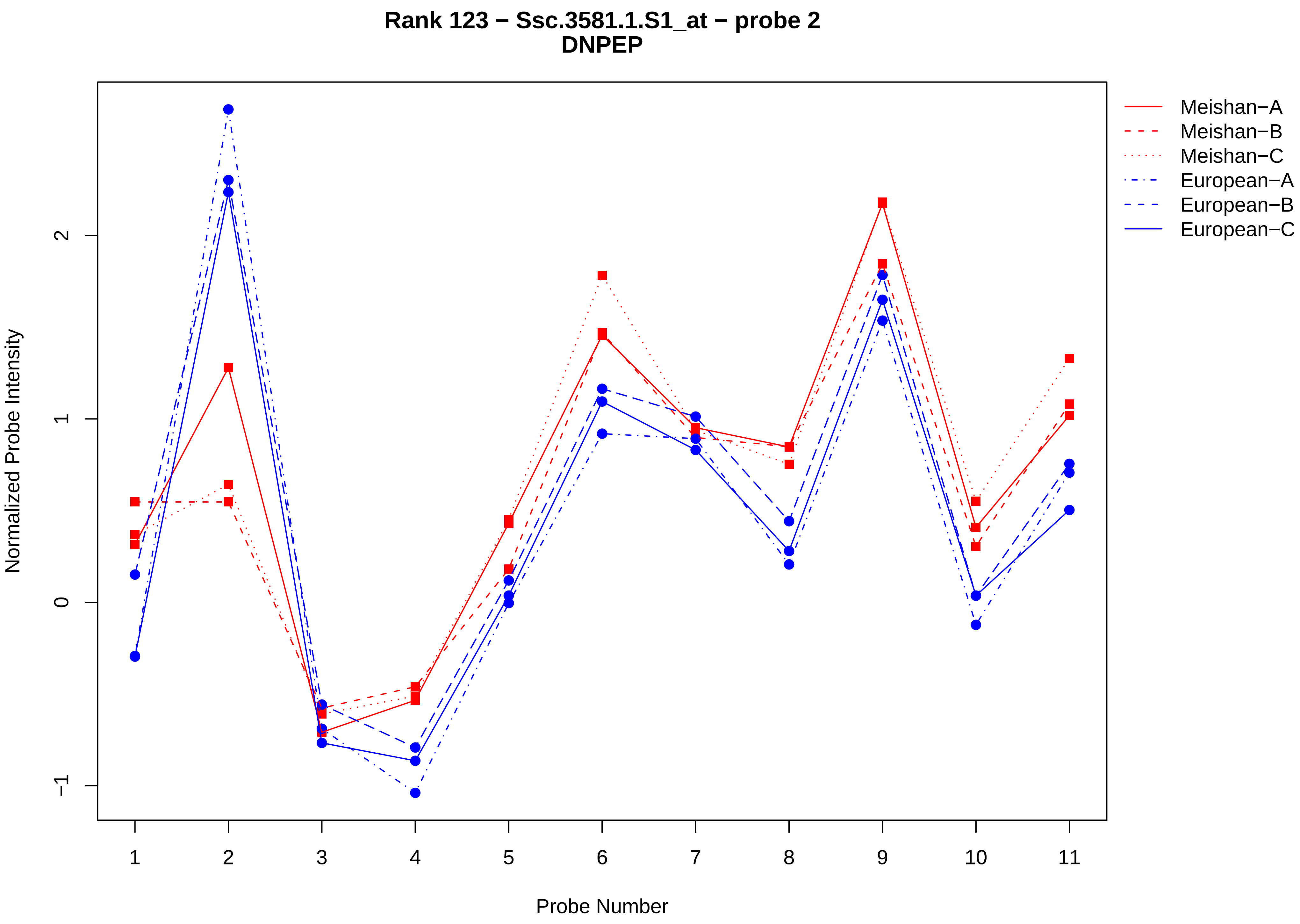
Rank 123 − Ssc.3581.1.S1_at − probe 2
DNPEP
Normalized Probe Intensity
Probe Number
−1
0
1
2
1
2
3
4
5
6
7
8
9
10
11
Meishan−A
Meishan−B
Meishan−C
European−A
European−B
European−C

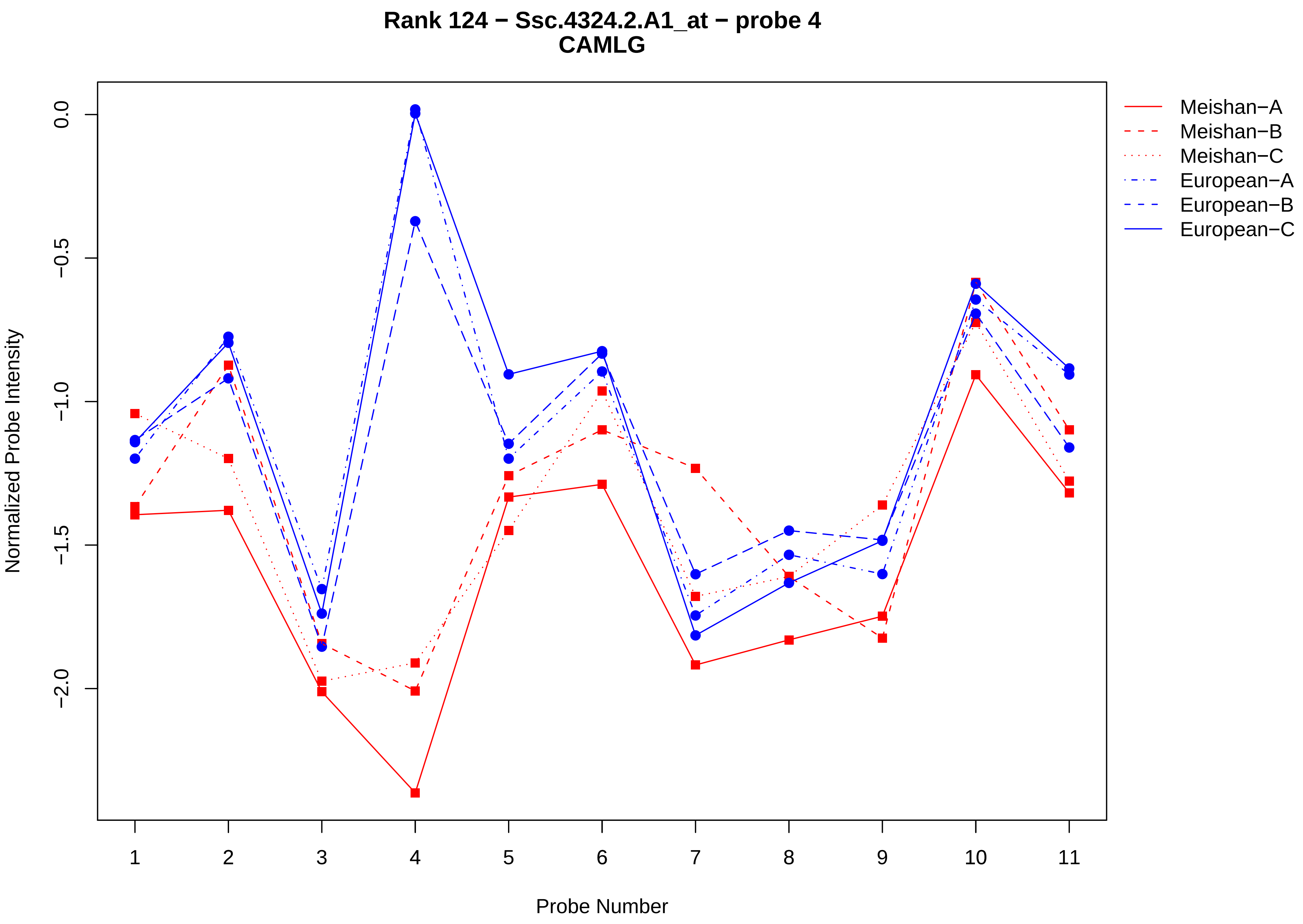

Rank 124 − Ssc.4324.2.A1_at − probe 4
CAMLG
Normalized Probe Intensity
Probe Number
0.0
−0.5
−1.0
−1.5
−2.0
1
2
3
4
5
6
7
8
9
10
11
Meishan−A
Meishan−B
Meishan−C
European−A
European−B
European−C

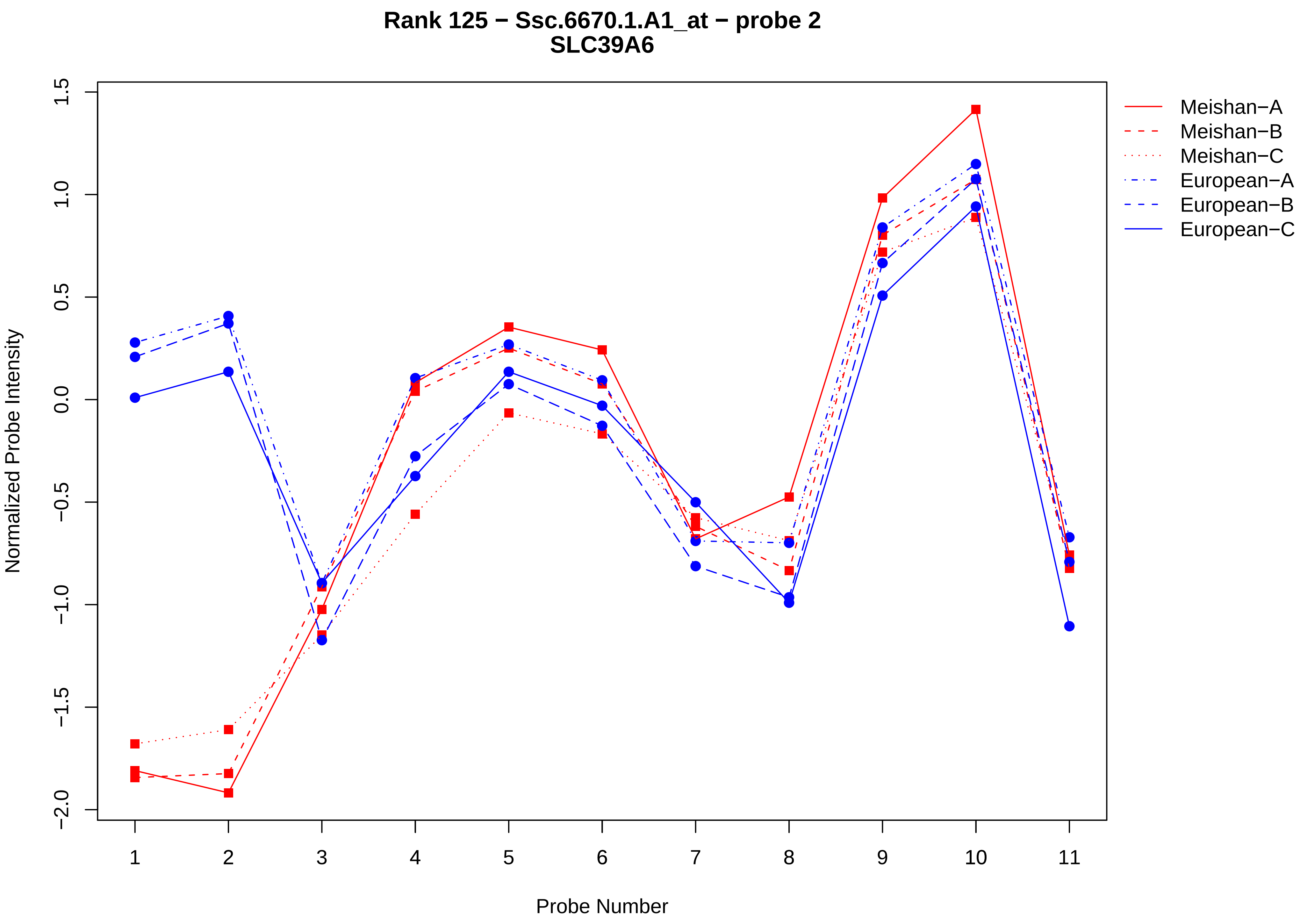
Rank 125 − Ssc.6670.1.A1_at − probe 2
SLC39A6
Normalized Probe Intensity
Probe Number
1.5
1.0
0.5
0.0
−0.5
−1.0
−1.5
−2.0
1
2
3
4
5
6
7
8
9
10
11
Meishan−A
Meishan−B
Meishan−C
European−A
European−B
European−C

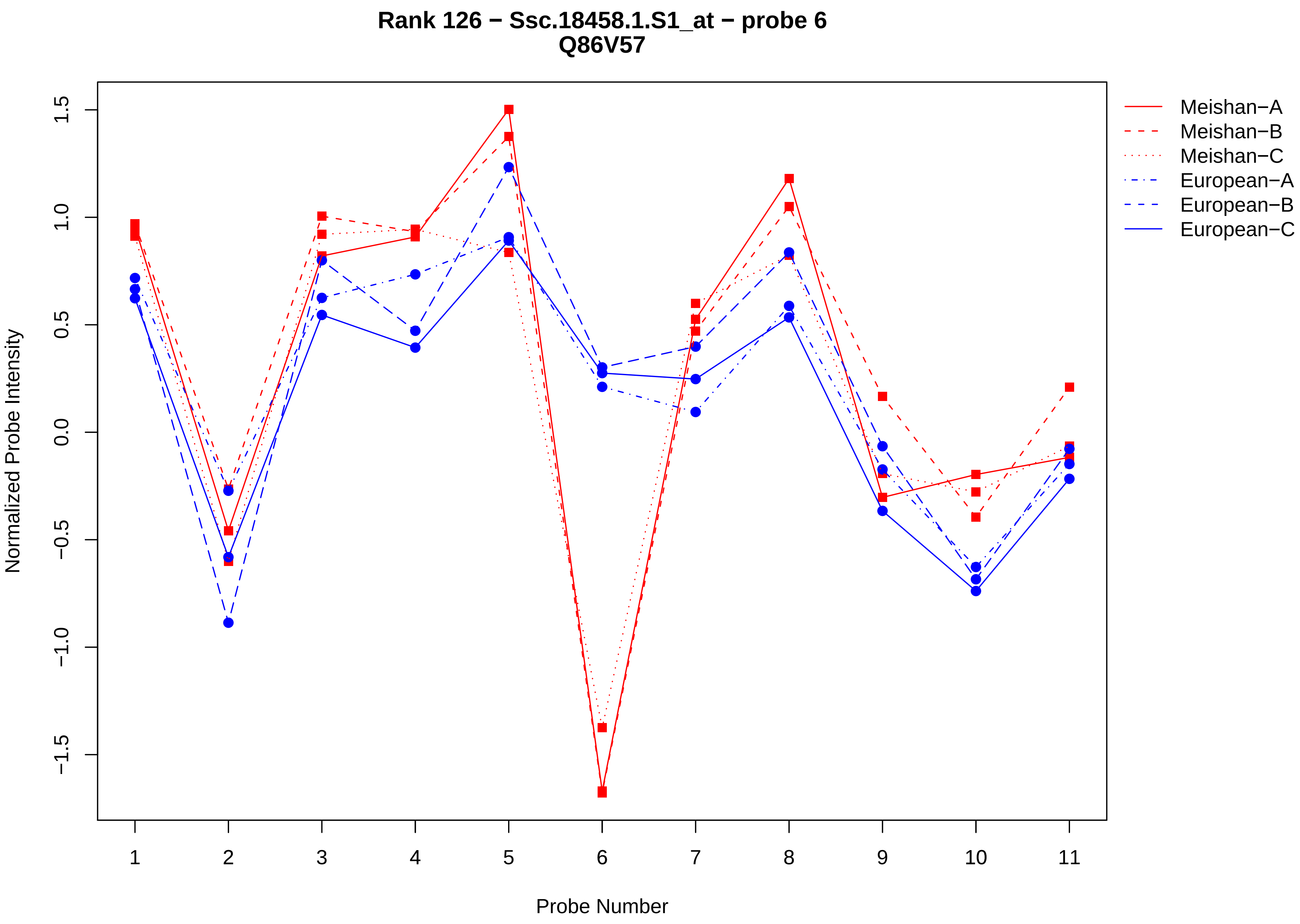

Rank 126 − Ssc.18458.1.S1_at − probe 6
Q86V57
Normalized Probe Intensity
Probe Number
1.5
1.0
0.5
0.0
−0.5
−1.0
−1.5
1
2
3
4
5
6
7
8
9
10
11
Meishan−A
Meishan−B
Meishan−C
European−A
European−B
European−C

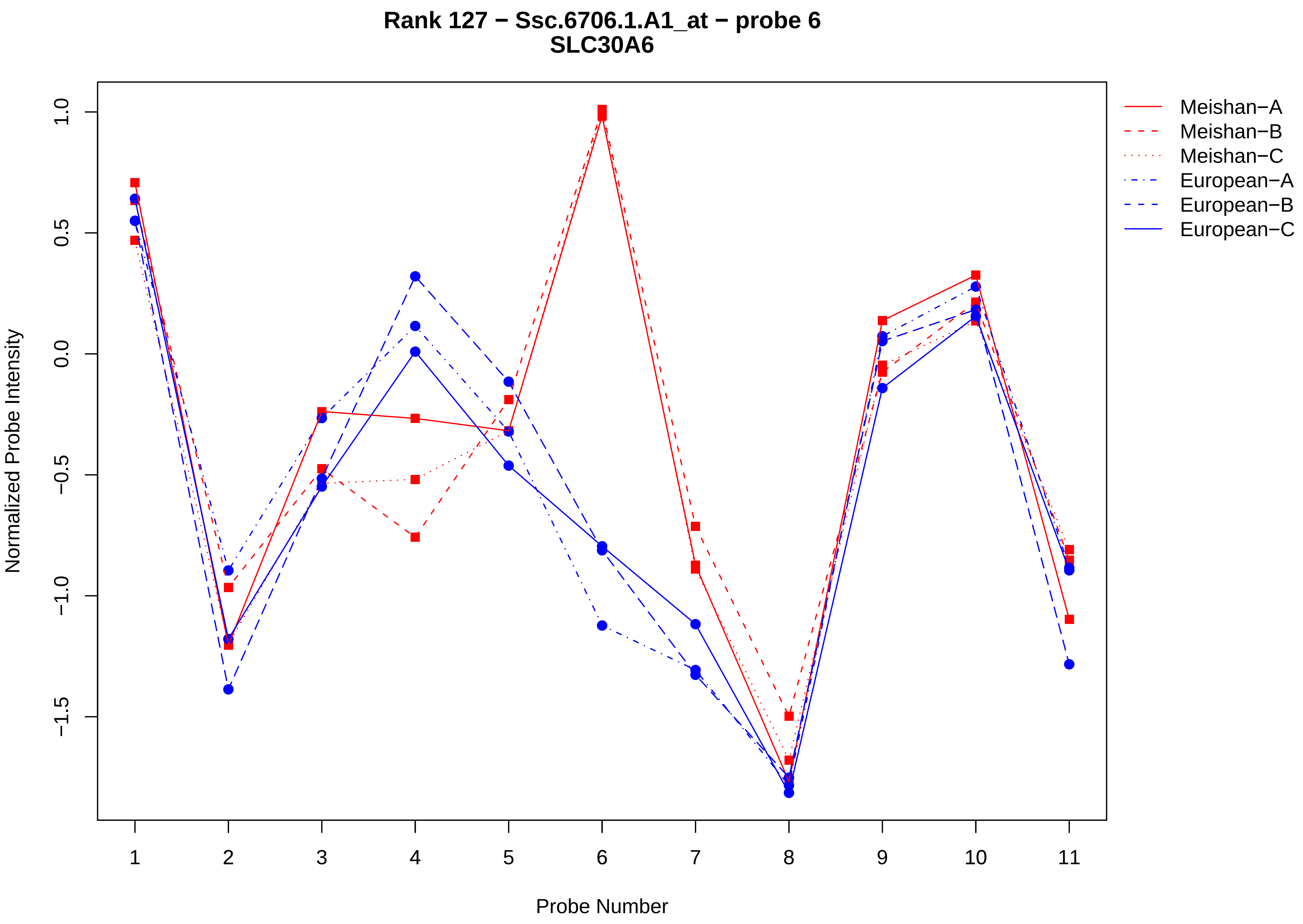

Rank 127 − Ssc.6706.1.A1_at − probe 6
SLC30A6
Normalized Probe Intensity
Probe Number
1.0
0.5
0.0
−0.5
−1.0
−1.5
1
2
3
4
5
6
7
8
9
10
11
Meishan−A
Meishan−B
Meishan−C
European−A
European−B
European−C

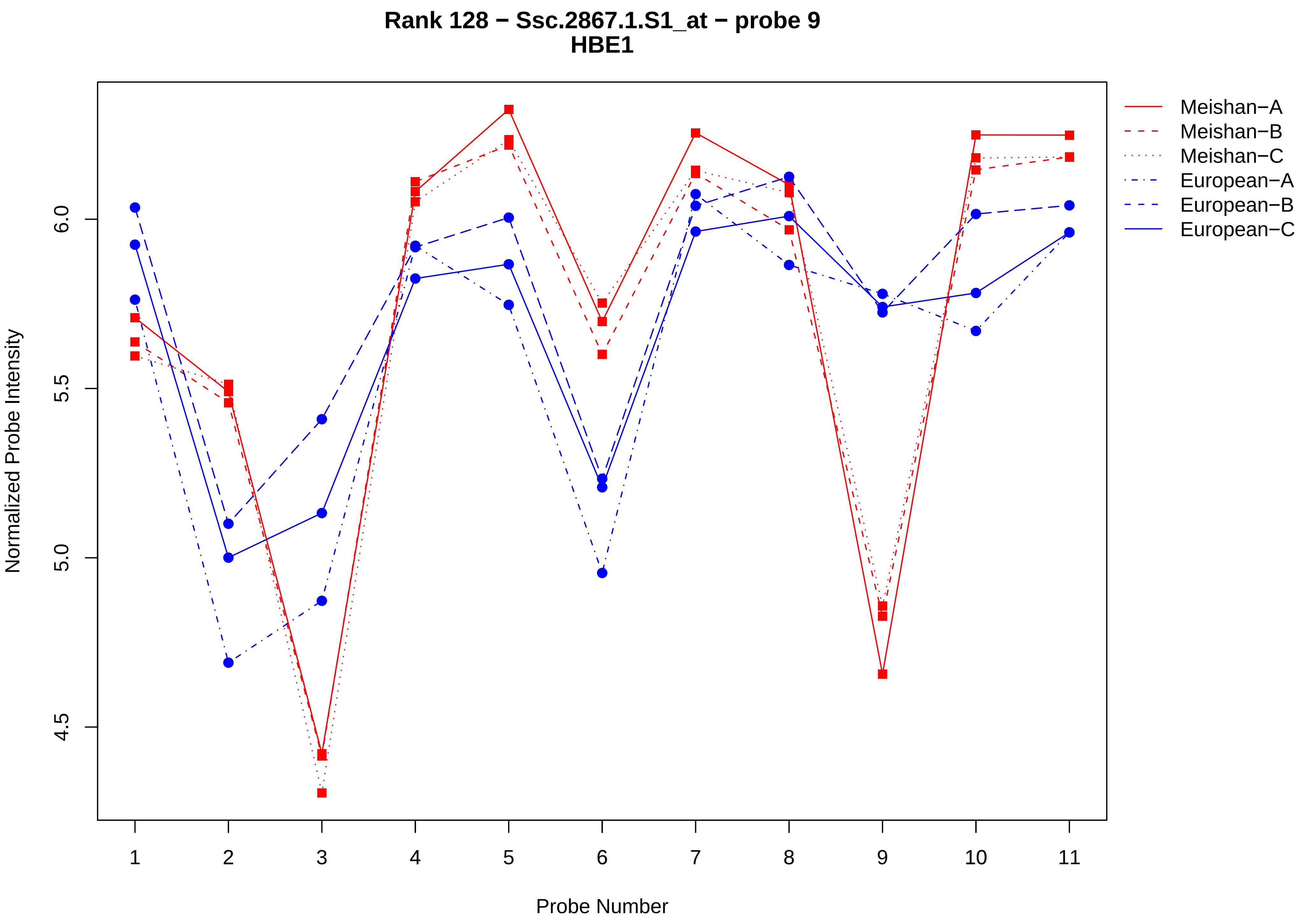

Rank 128 − Ssc.2867.1.S1_at − probe 9
HBE1
Normalized Probe Intensity
4.5
5.0
5.5
6.0
1
2
3
4
5
6
7
8
9
10
11
Probe Number
Meishan−A
Meishan−B
Meishan−C
European−A
European−B
European−C

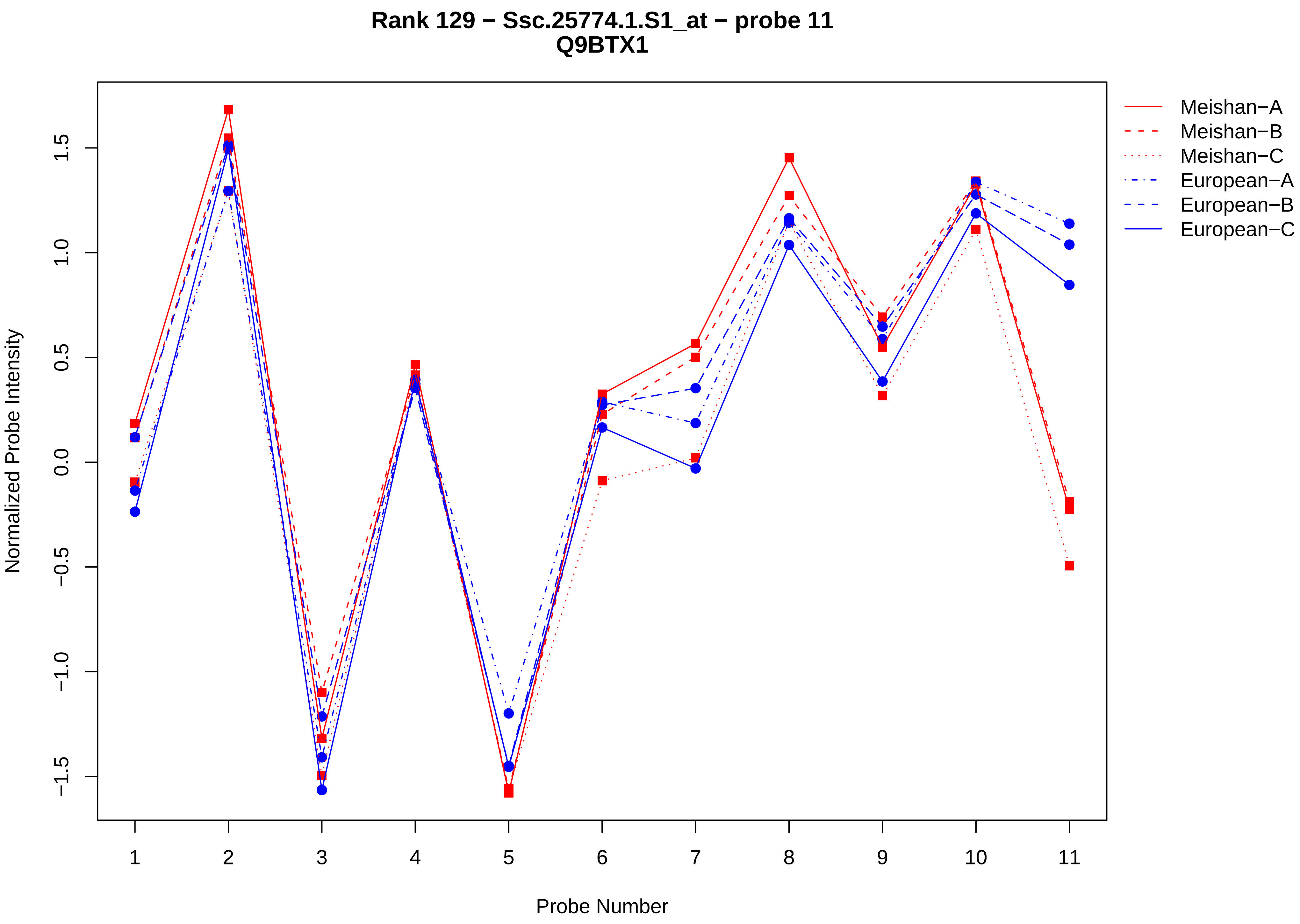

Rank 129 − Ssc.25774.1.S1_at − probe 11
Q9BTX1
Normalized Probe Intensity
Probe Number
Meishan−A
Meishan−B
Meishan−C
European−A
European−B
European−C

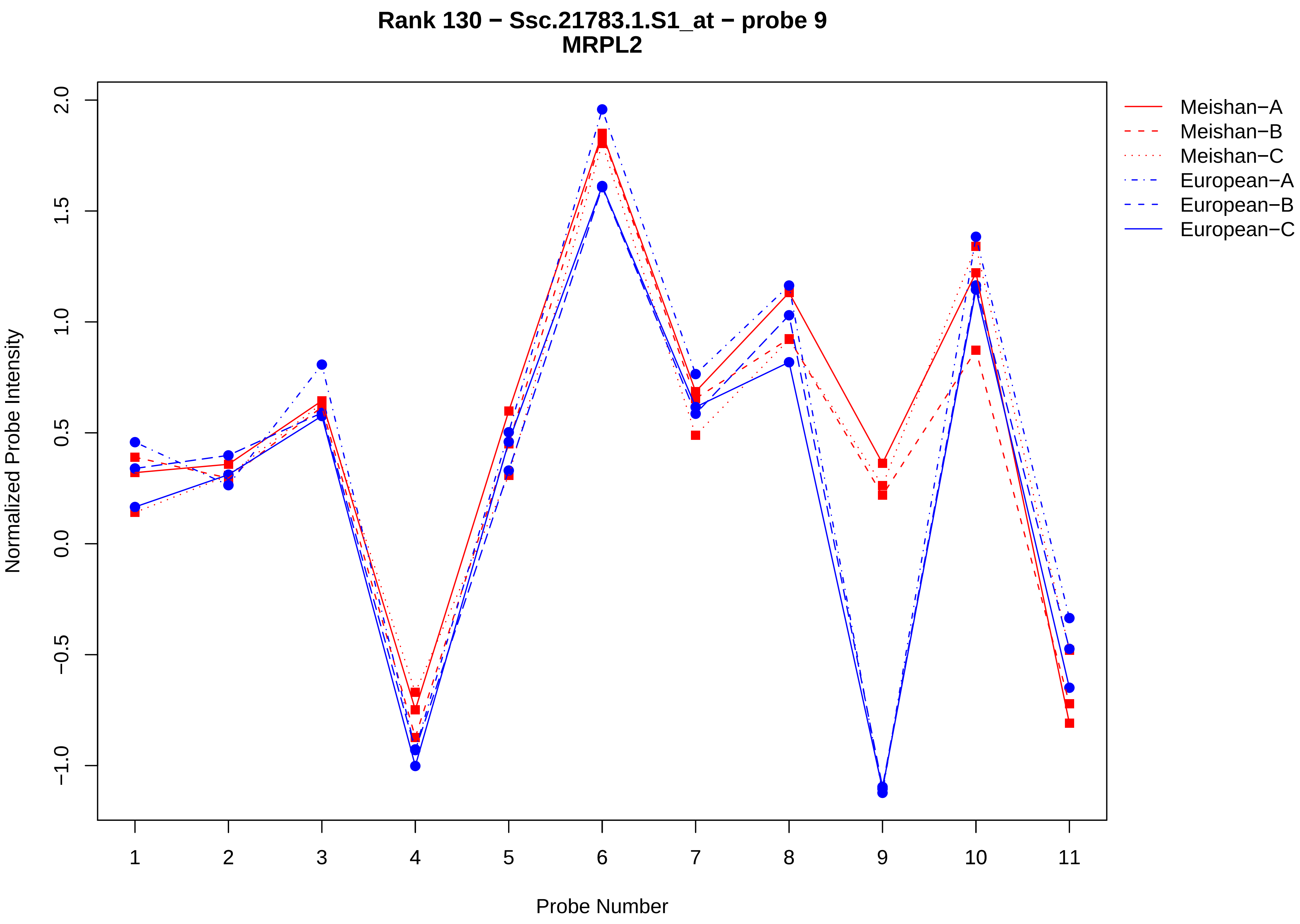

Rank 130 − Ssc.21783.1.S1_at − probe 9
MRPL2
Normalized Probe Intensity
Probe Number
Meishan−A
Meishan−B
Meishan−C
European−A
European−B
European−C
2.0
1.5
1.0
0.5
0.0
−0.5
−1.0
1
2
3
4
5
6
7
8
9
10
11

Rank 131 − Ssc.3908.1.A1_at − probe 2
C21orf4

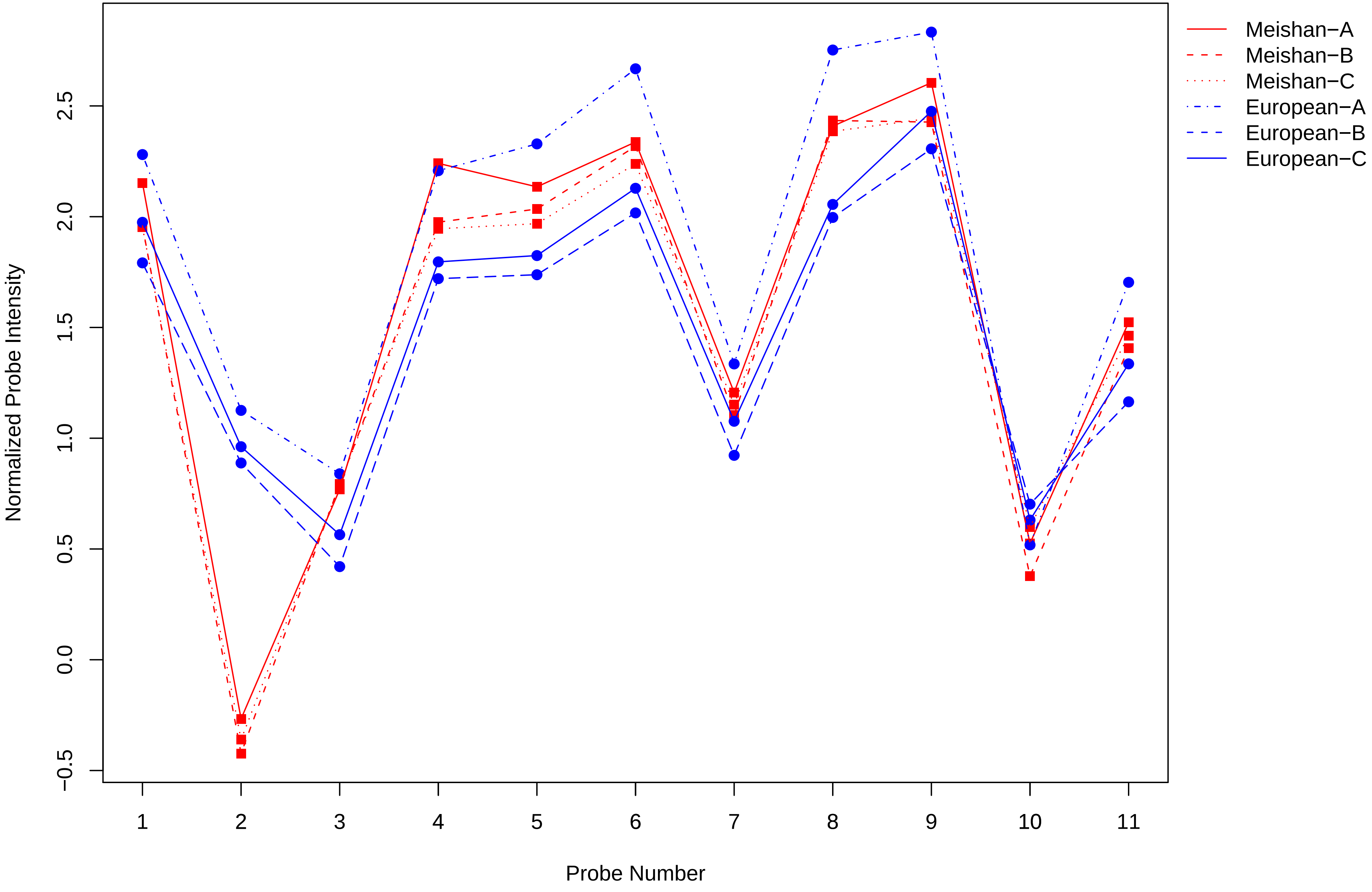

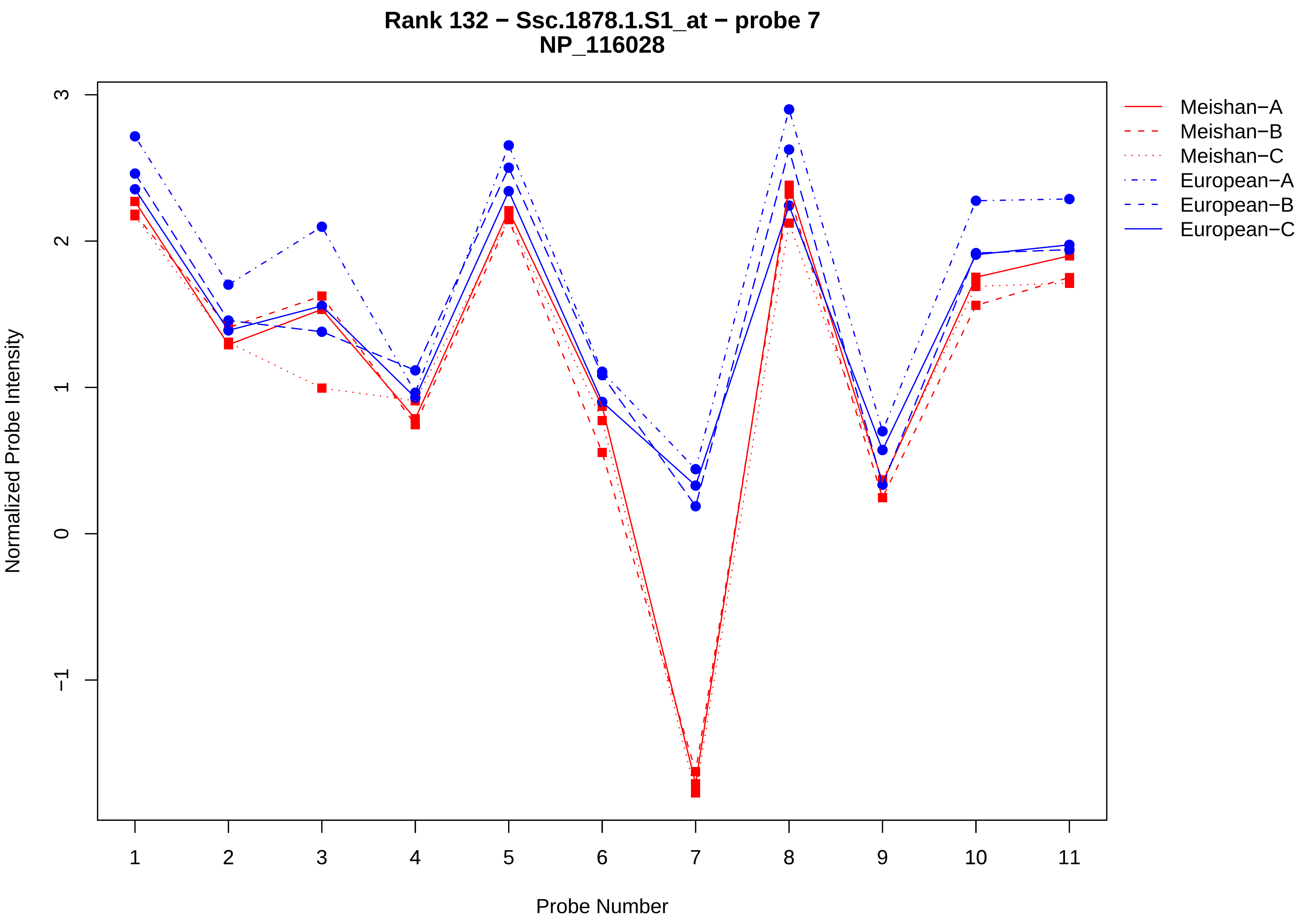

Rank 132 − Ssc.1878.1.S1_at − probe 7
NP_116028
Normalized Probe Intensity
Probe Number
−1
0
1
2
3
1
2
3
4
5
6
7
8
9
10
11
Meishan−A
Meishan−B
Meishan−C
European−A
European−B
European−C

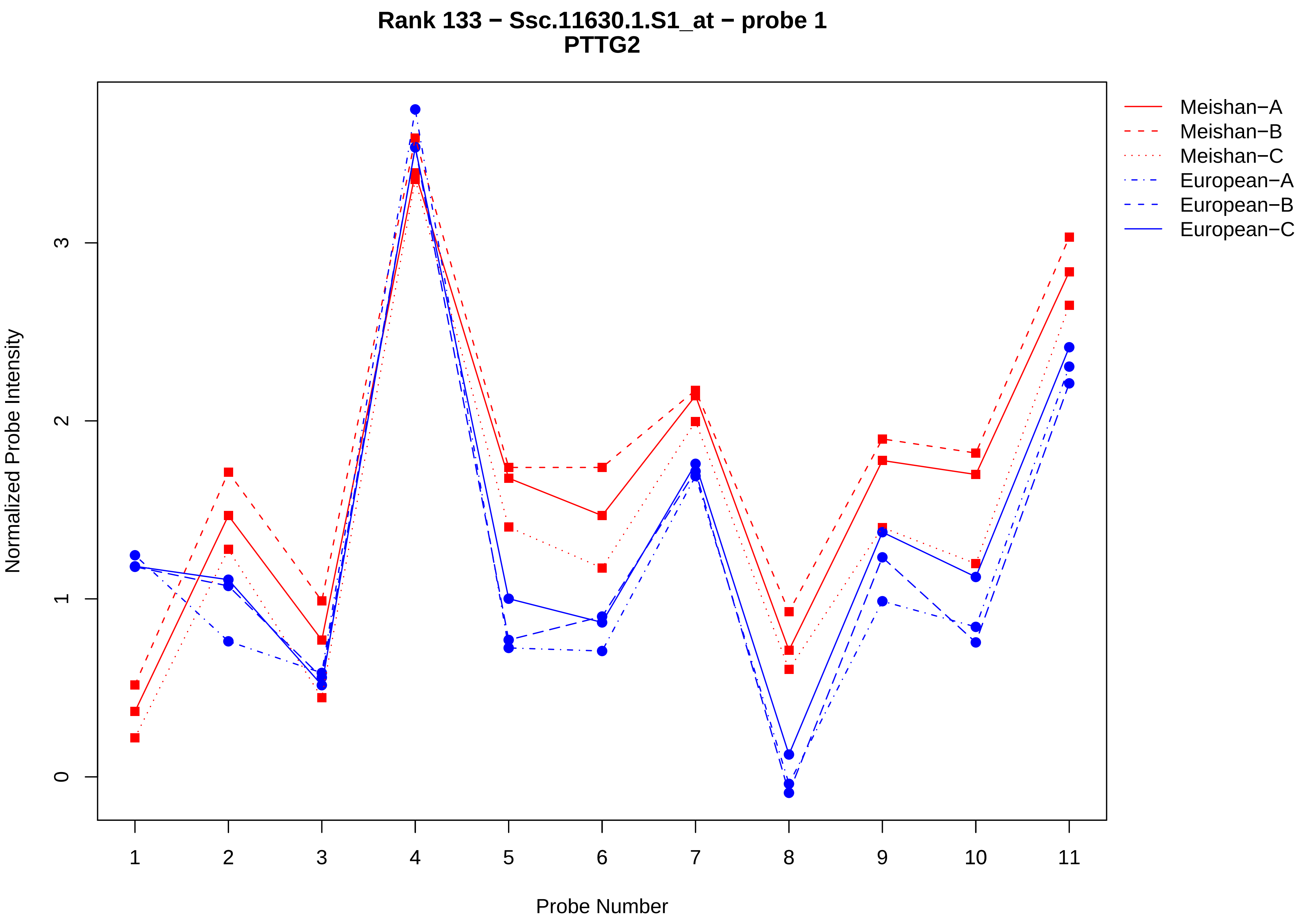

Rank 133 − Ssc.11630.1.S1_at − probe 1
PTTG2
Normalized Probe Intensity
0
1
2
3
1
2
3
4
5
6
7
8
9
10
11
Probe Number
Meishan−A
Meishan−B
Meishan−C
European−A
European−B
European−C

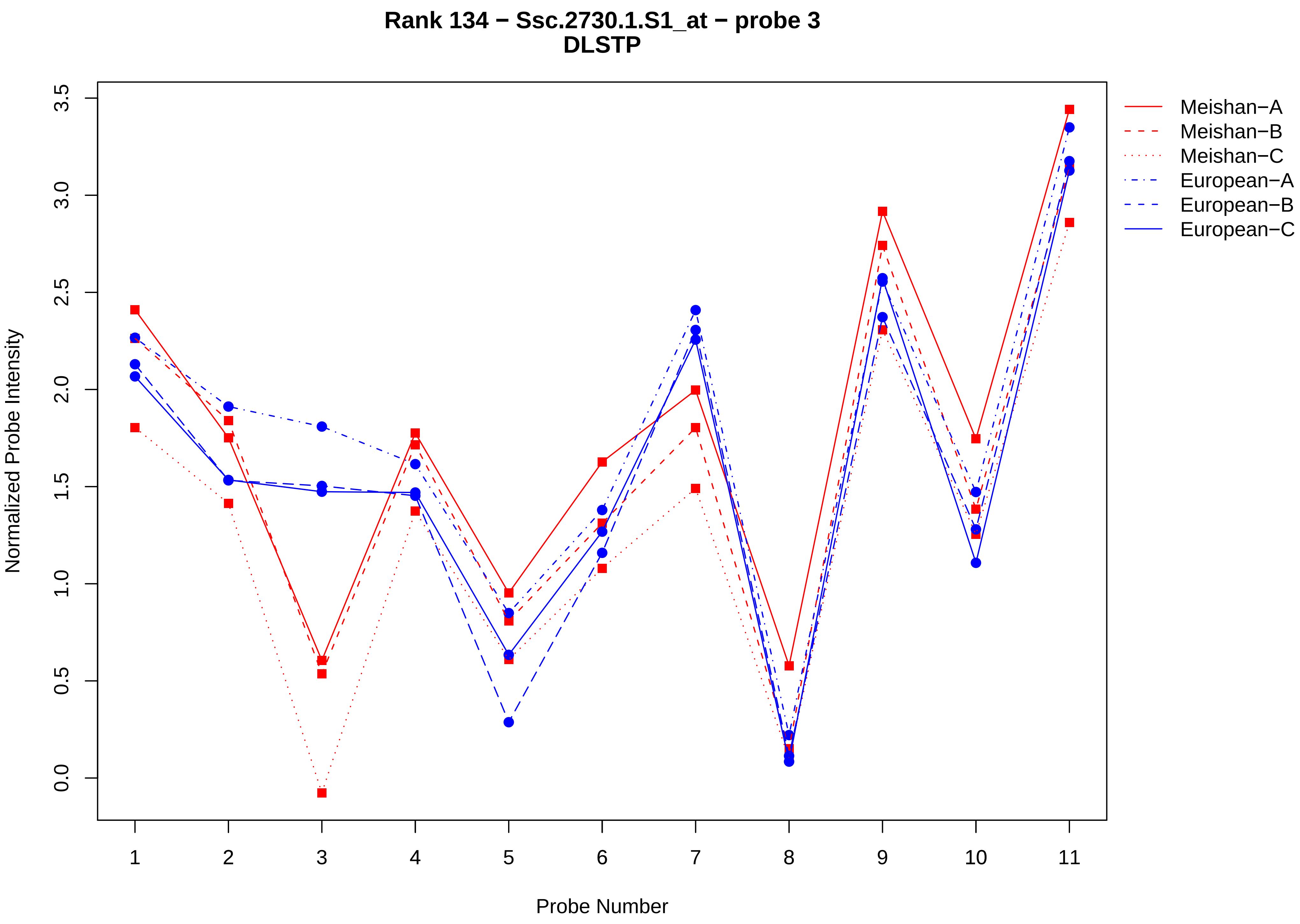
Rank 134 − Ssc.2730.1.S1_at − probe 3
DLSTP
Normalized Probe Intensity
Probe Number
0.0
0.5
1.0
1.5
2.0
2.5
3.0
3.5
1
2
3
4
5
6
7
8
9
10
11
Meishan−A
Meishan−B
Meishan−C
European−A
European−B
European−C

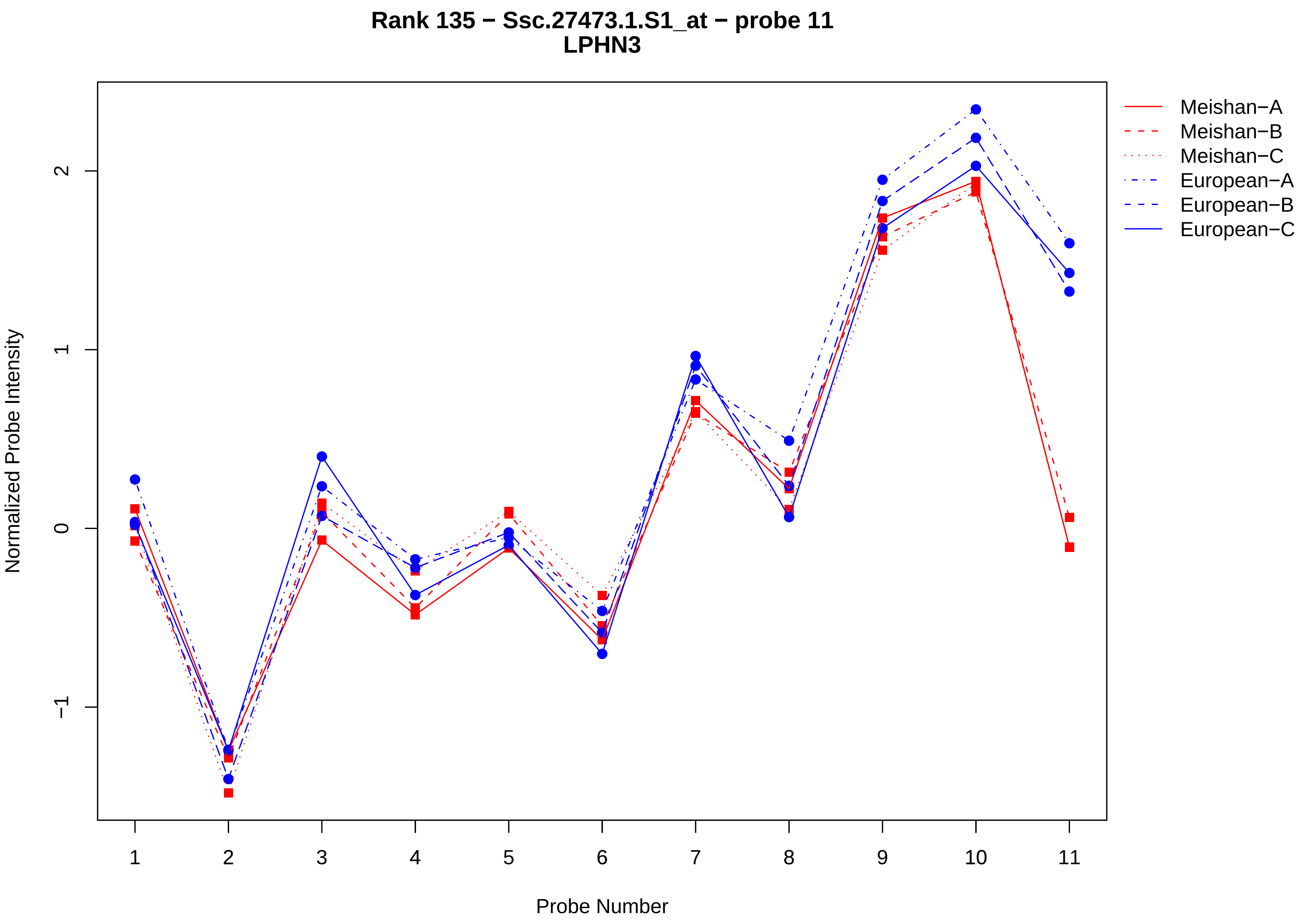

Rank 135 − Ssc.27473.1.S1_at − probe 11
LPHN3
Normalized Probe Intensity
Probe Number
Meishan−A
Meishan−B
Meishan−C
European−A
European−B
European−C

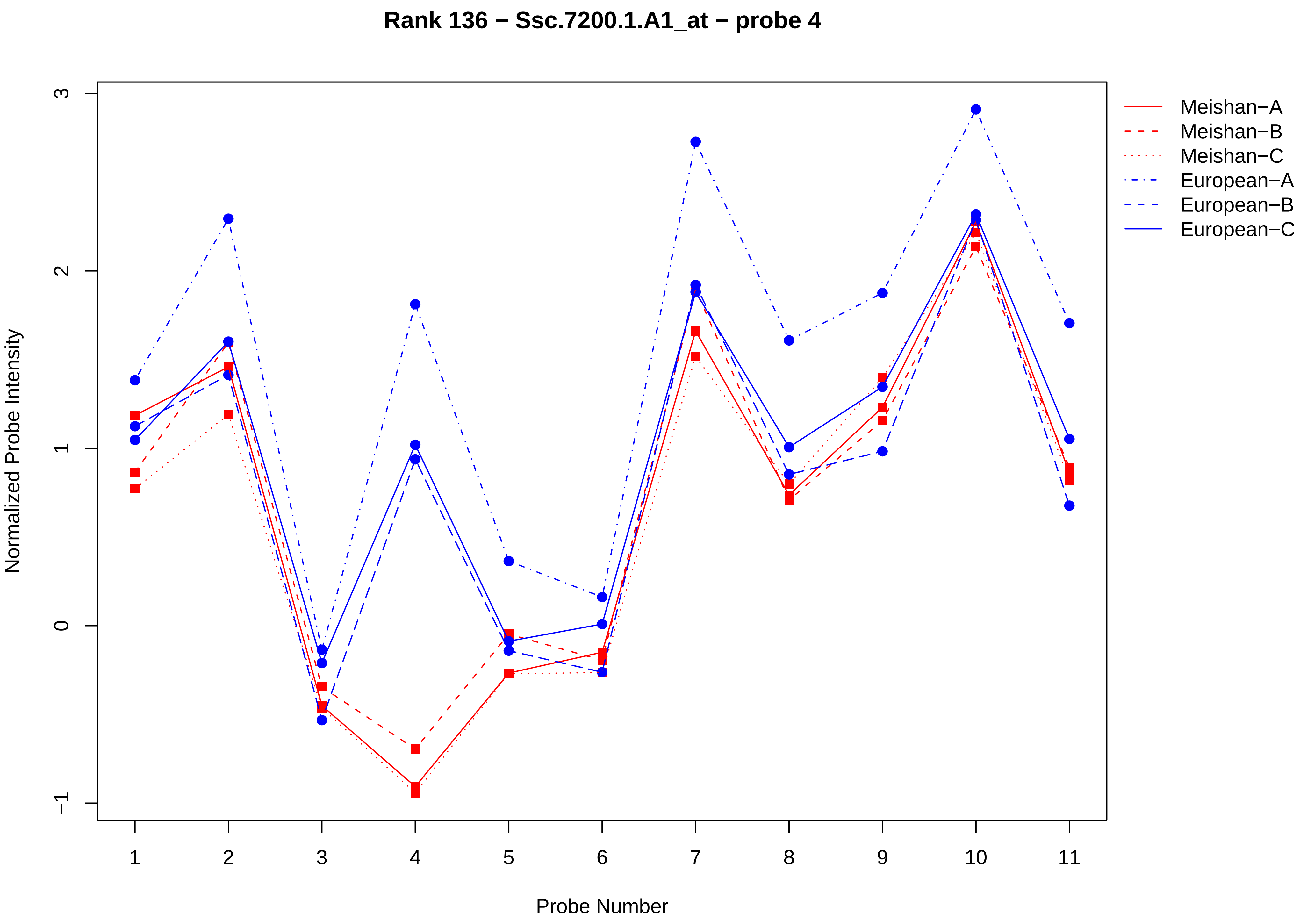
Rank 136 − Ssc.7200.1.A1_at − probe 4
Normalized Probe Intensity
Probe Number
Meishan−A
Meishan−B
Meishan−C
European−A
European−B
European−C
−1
0
1
2
3
1
2
3
4
5
6
7
8
9
10
11

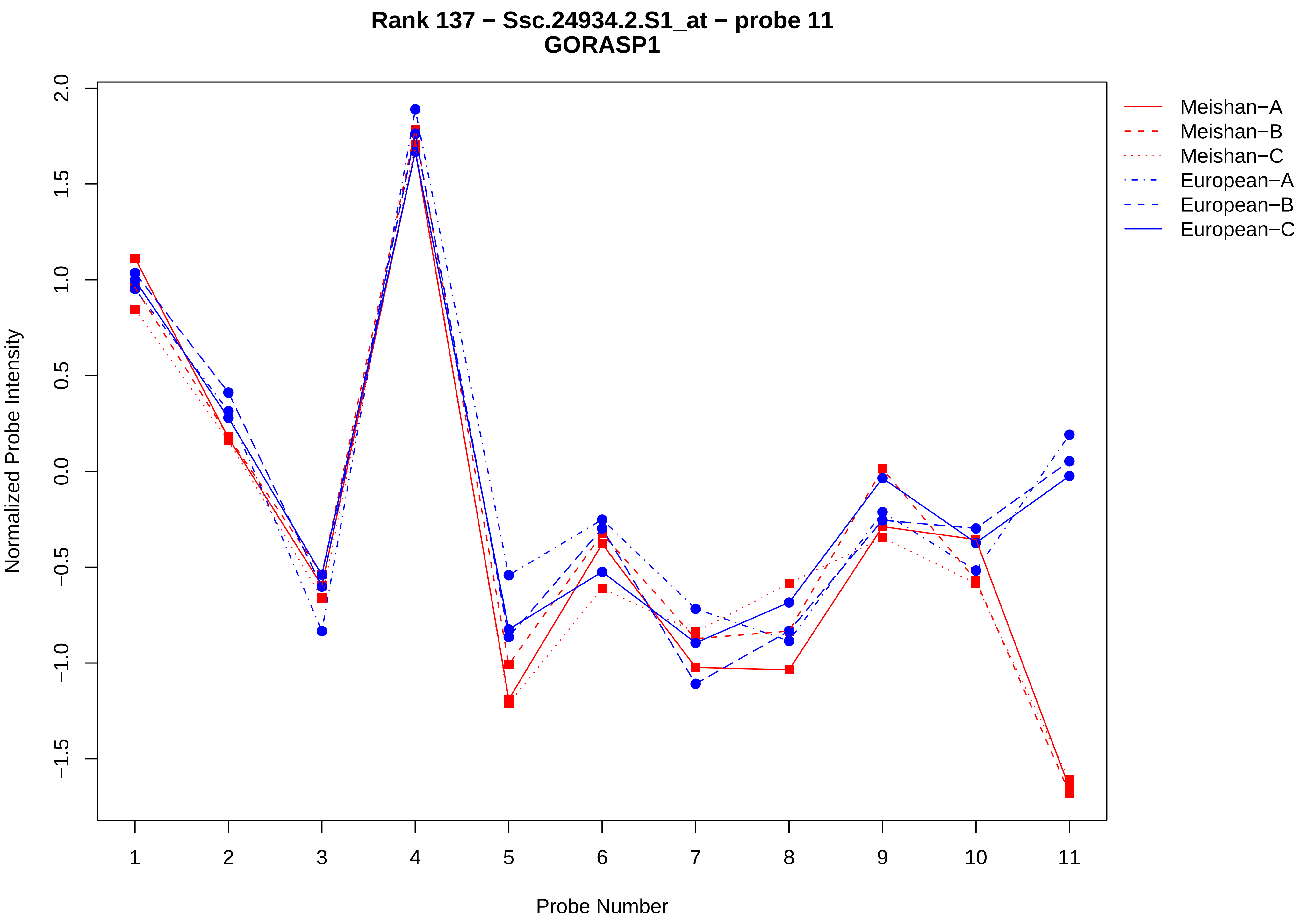

Rank 137 − Ssc.24934.2.S1_at − probe 11
GORASP1
Normalized Probe Intensity
Probe Number
−1.5
−1.0
−0.5
0.0
0.5
1.0
1.5
2.0
1
2
3
4
5
6
7
8
9
10
11
Meishan−A
Meishan−B
Meishan−C
European−A
European−B
European−C

Rank 138 − Ssc.3128.1.A1_at − probe 8
C6orf82

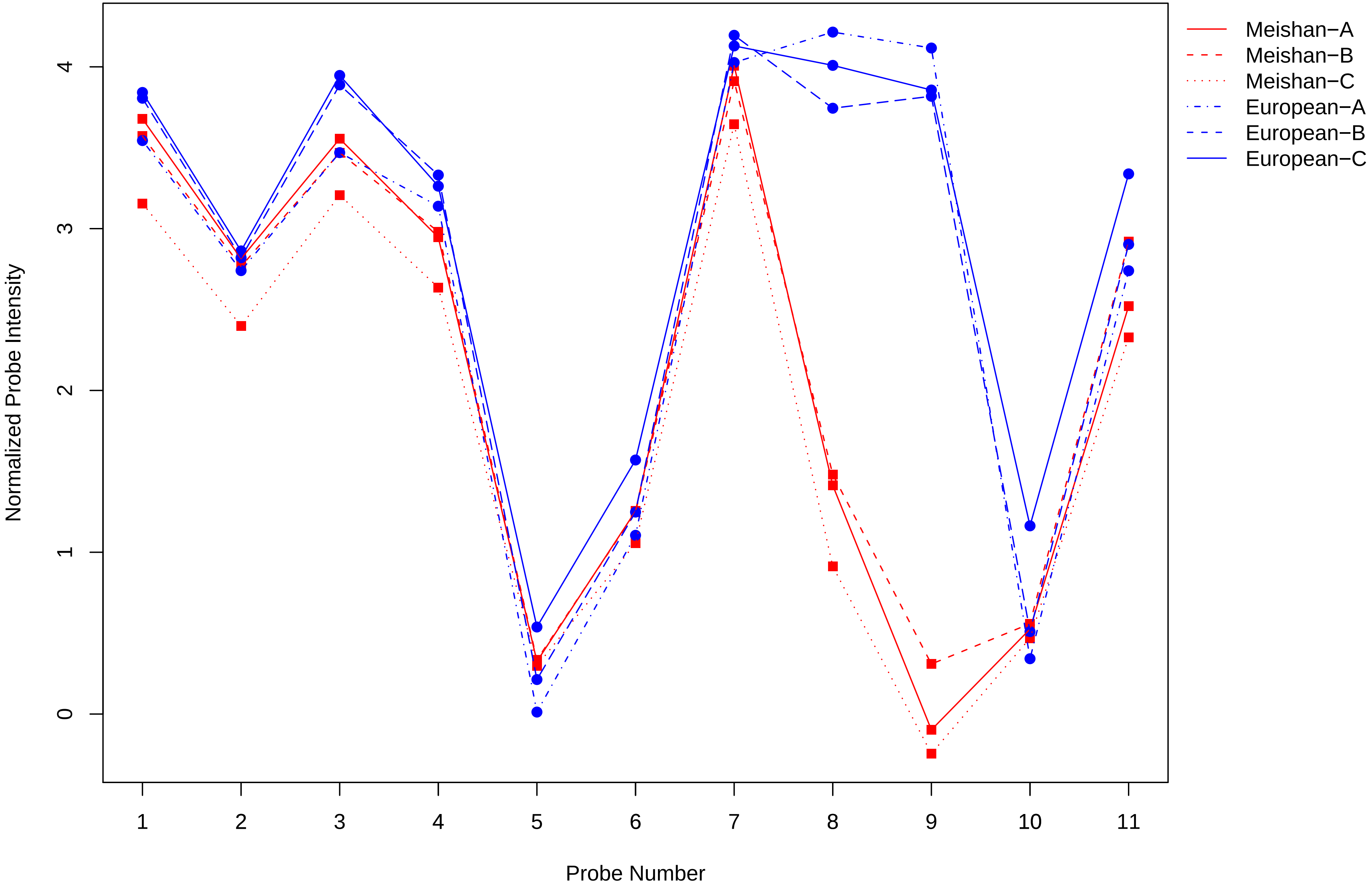

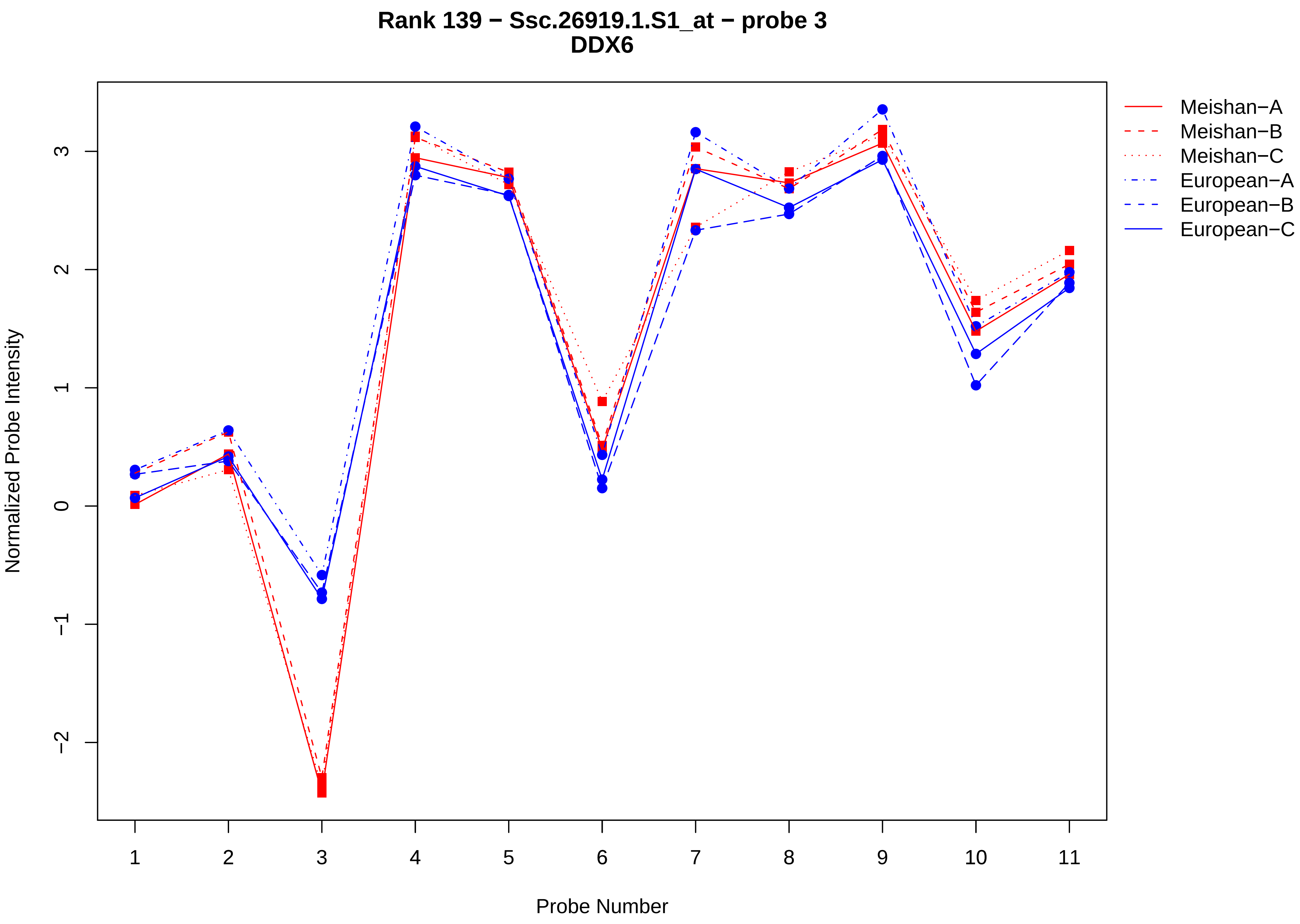

Rank 139 − Ssc.26919.1.S1_at − probe 3
DDX6
Normalized Probe Intensity
Probe Number
Meishan−A
Meishan−B
Meishan−C
European−A
European−B
European−C
−2
−1
0
1
2
3
1
2
3
4
5
6
7
8
9
10
11

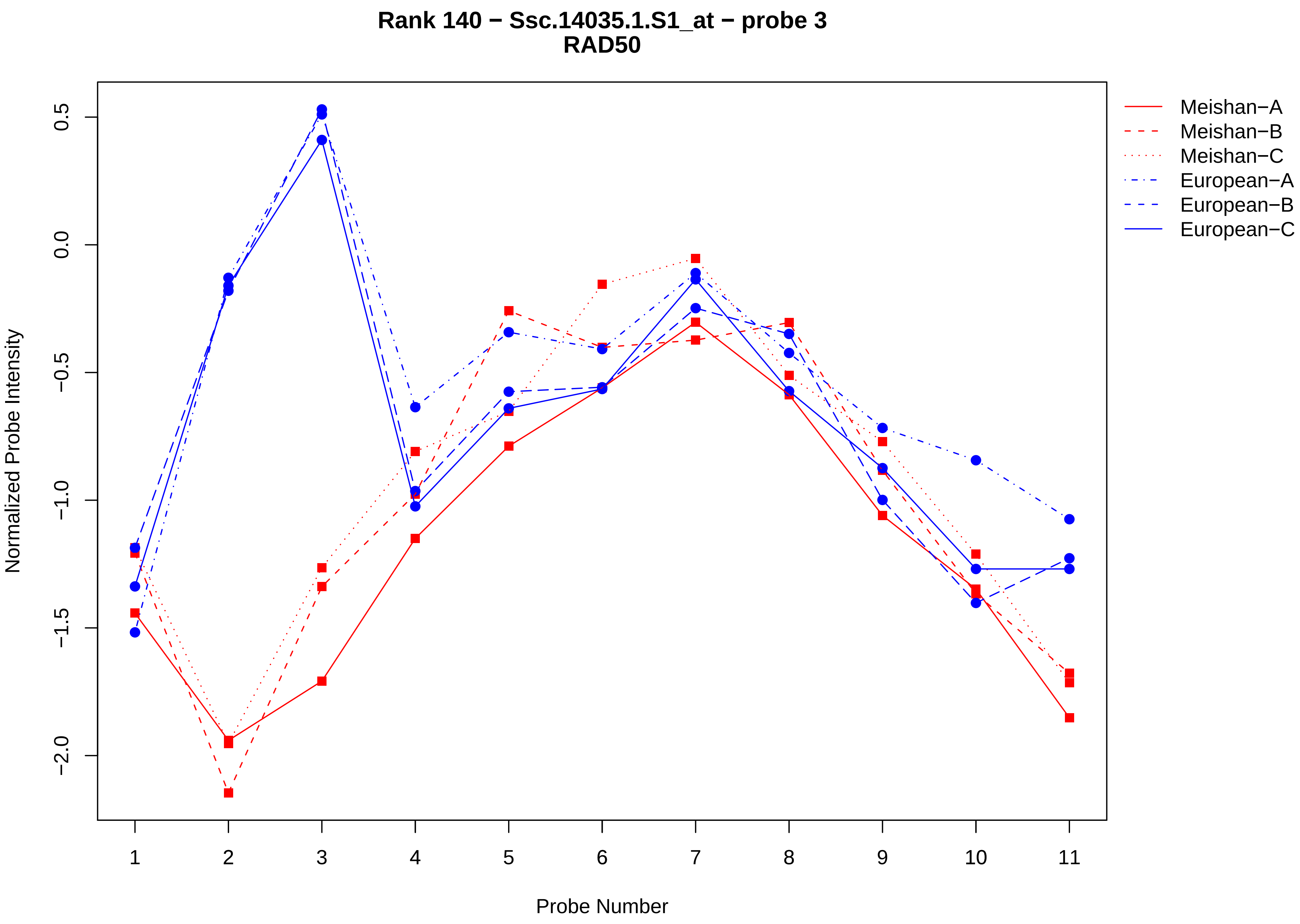

Rank 140 − Ssc.14035.1.S1_at − probe 3
RAD50
Normalized Probe Intensity
Probe Number
0.5
0.0
−0.5
−1.0
−1.5
−2.0
1
2
3
4
5
6
7
8
9
10
11
Meishan−A
Meishan−B
Meishan−C
European−A
European−B
European−C

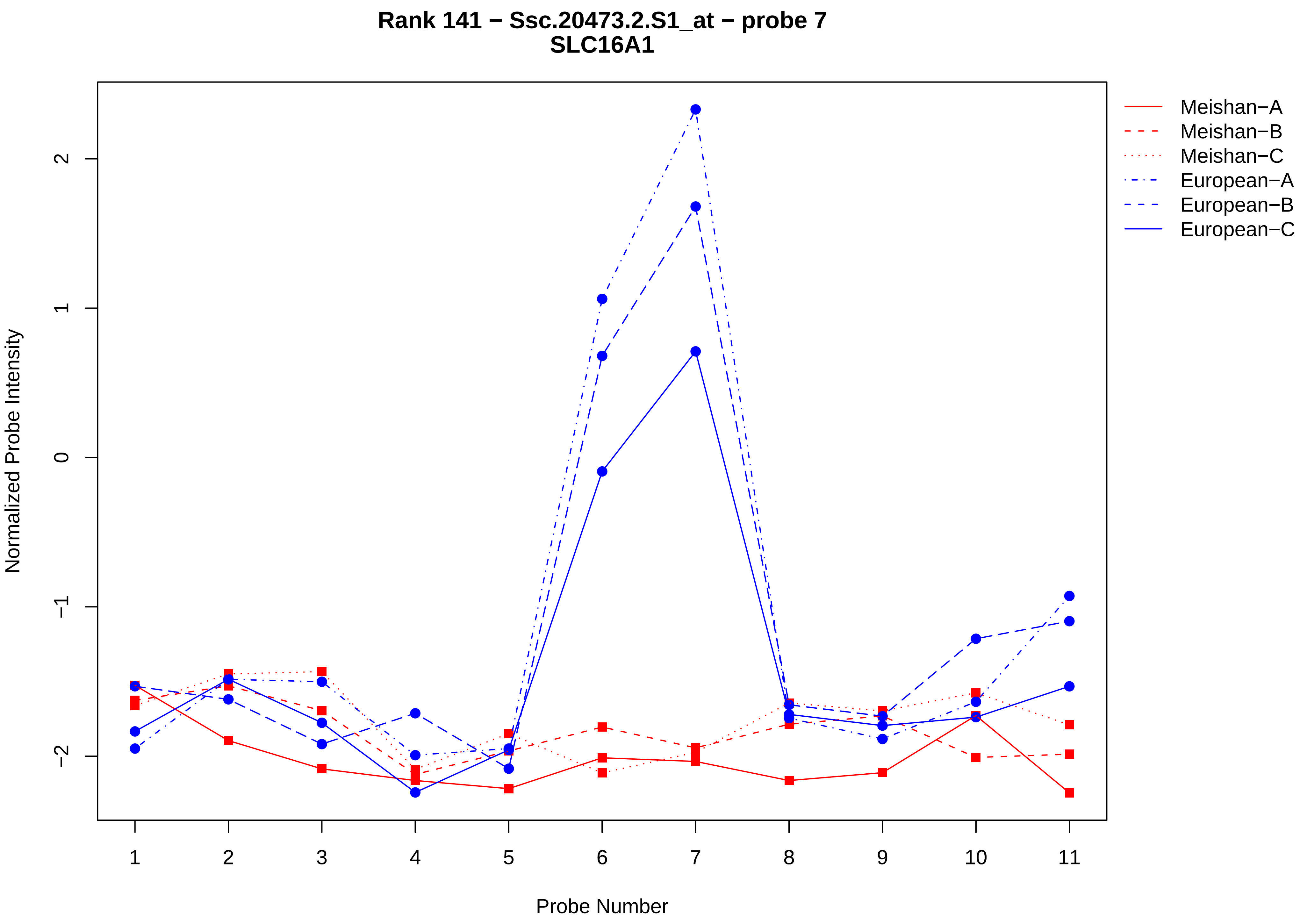
Rank 141 − Ssc.20473.2.S1_at − probe 7
SLC16A1
Normalized Probe Intensity
Probe Number
Meishan−A
Meishan−B
Meishan−C
European−A
European−B
European−C
2
1
0
−1
−2
1
2
3
4
5
6
7
8
9
10
11

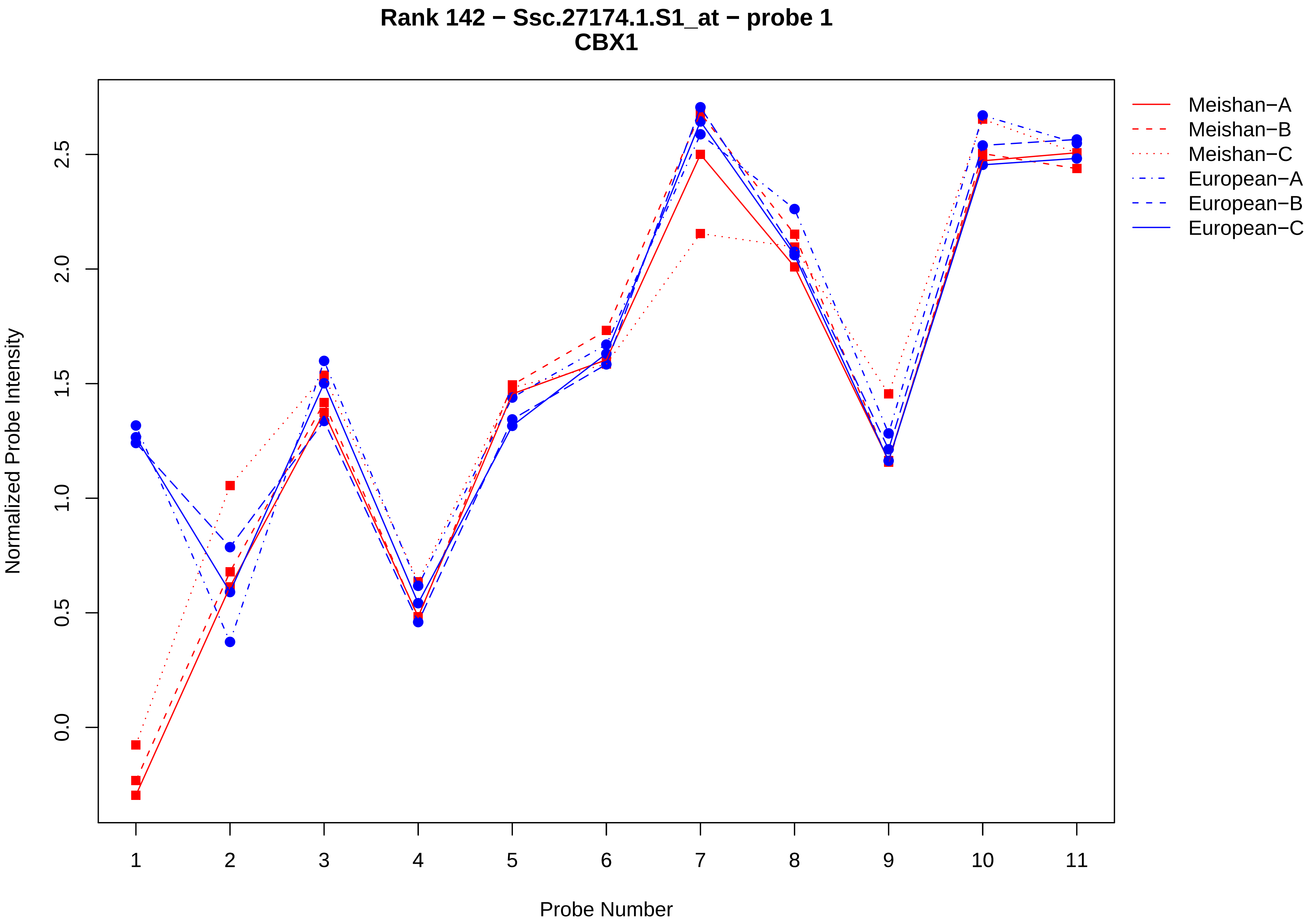

Rank 142 − Ssc.27174.1.S1_at − probe 1
CBX1
Normalized Probe Intensity
Probe Number
0.0
0.5
1.0
1.5
2.0
2.5
1
2
3
4
5
6
7
8
9
10
11
Meishan−A
Meishan−B
Meishan−C
European−A
European−B
European−C

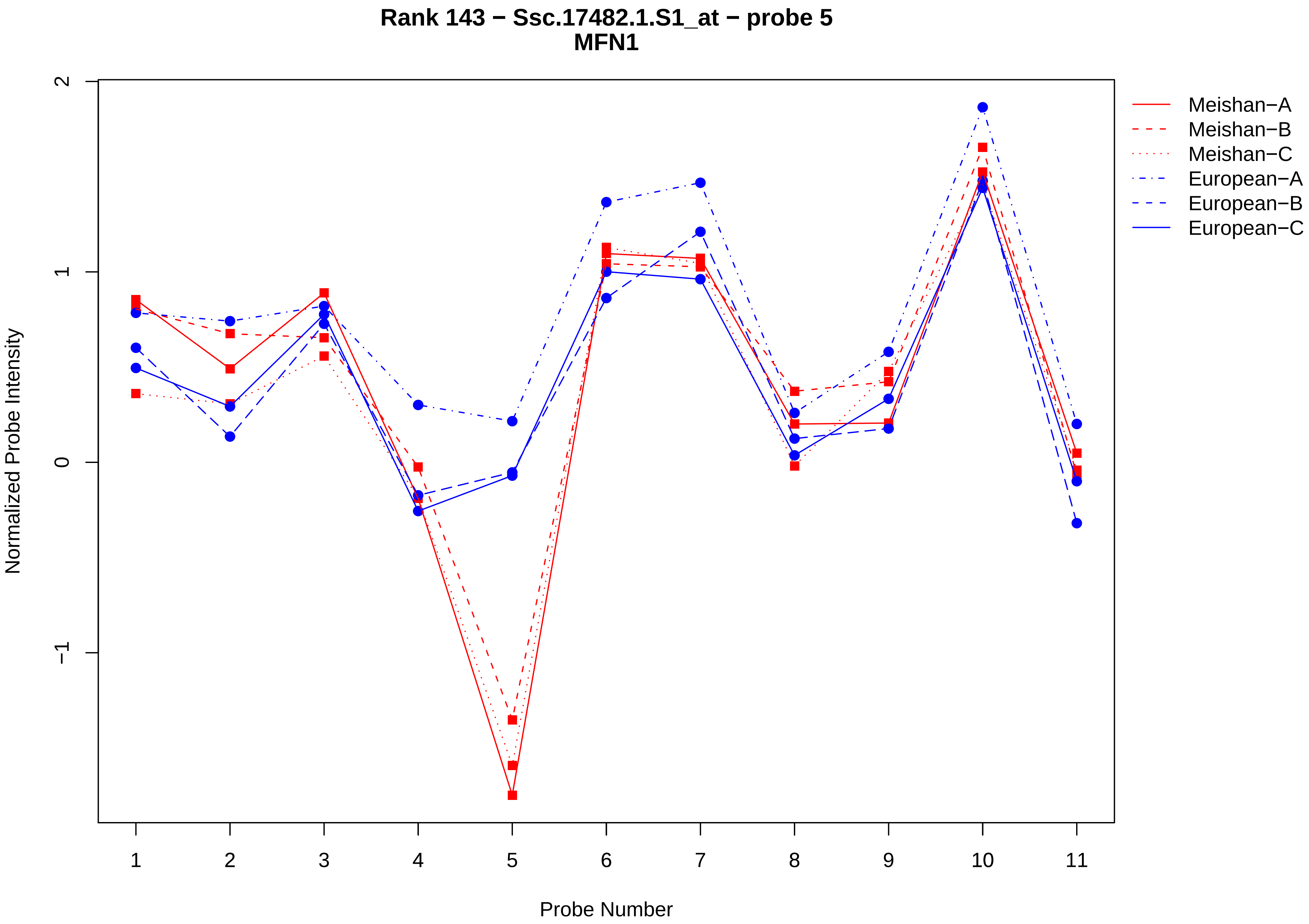

Rank 143 − Ssc.17482.1.S1_at − probe 5
MFN1
Normalized Probe Intensity
Probe Number
2
1
0
−1
1
2
3
4
5
6
7
8
9
10
11
Meishan−A
Meishan−B
Meishan−C
European−A
European−B
European−C

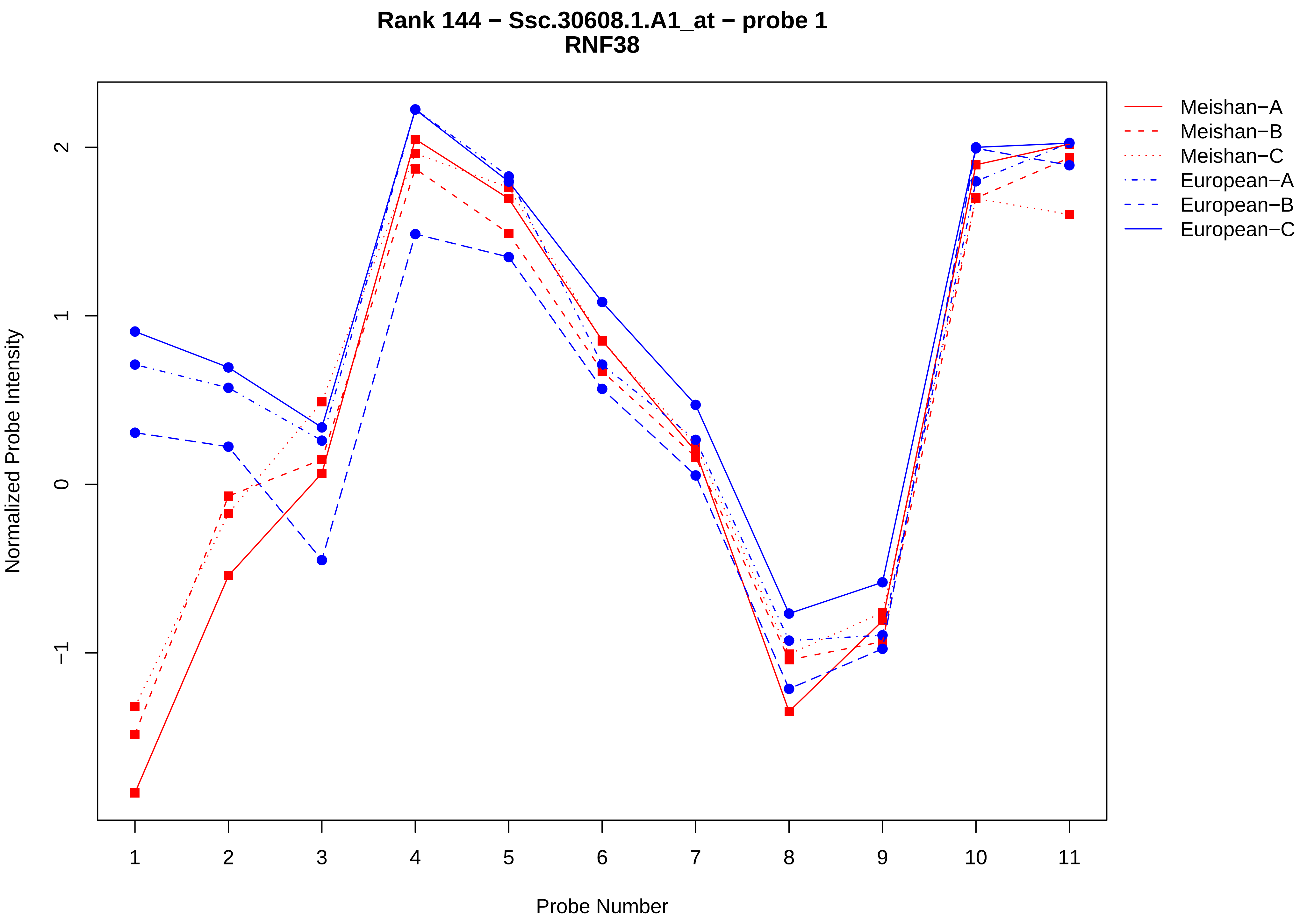

Rank 144 − Ssc.30608.1.A1_at − probe 1
RNF38
Normalized Probe Intensity
Probe Number
−1
0
1
2
1
2
3
4
5
6
7
8
9
10
11
Meishan−A
Meishan−B
Meishan−C
European−A
European−B
European−C

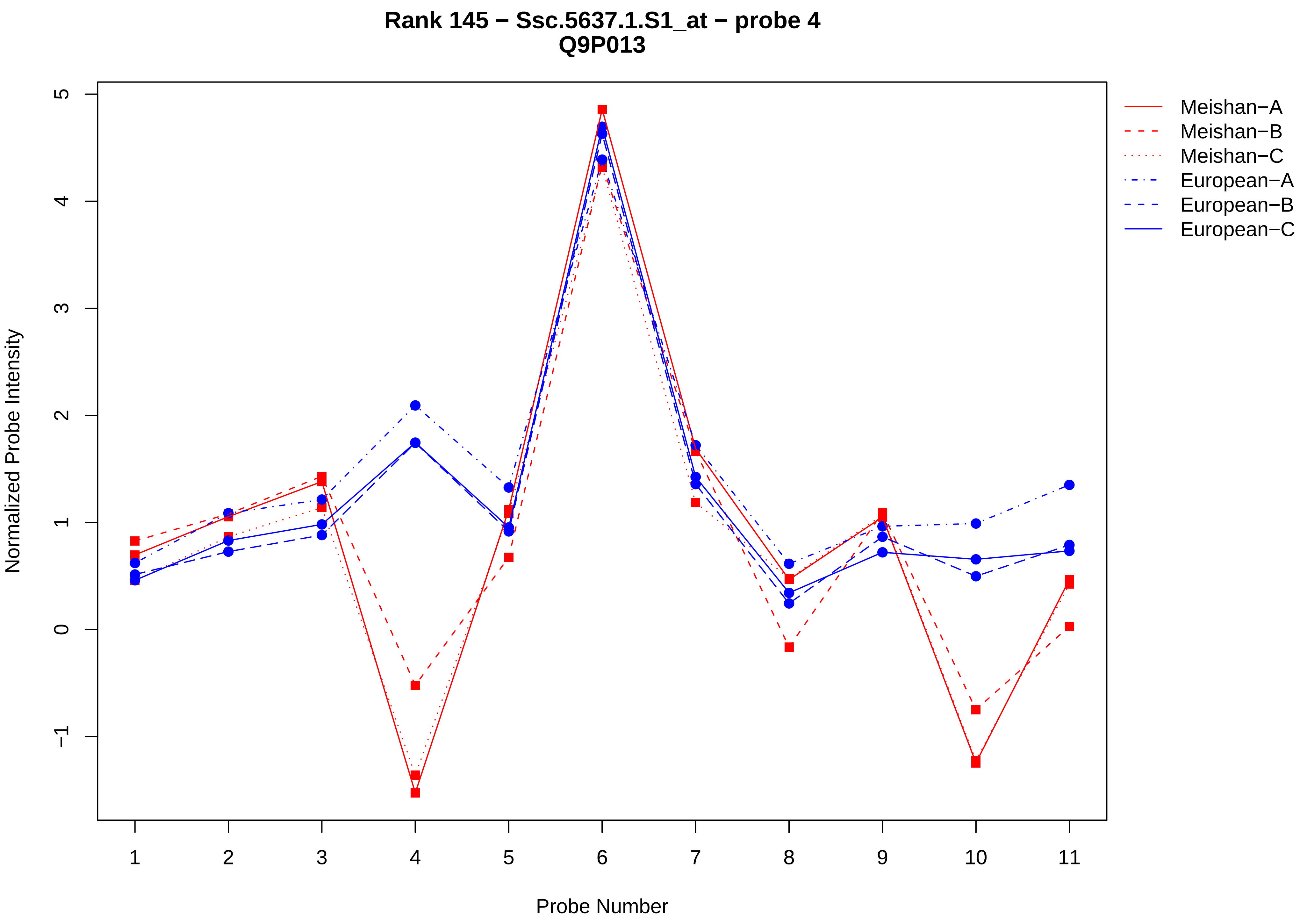
Rank 145 − Ssc.5637.1.S1_at − probe 4
Q9P013
Normalized Probe Intensity
Probe Number
−1
0
1
2
3
4
5
1
2
3
4
5
6
7
8
9
10
11
Meishan−A
Meishan−B
Meishan−C
European−A
European−B
European−C

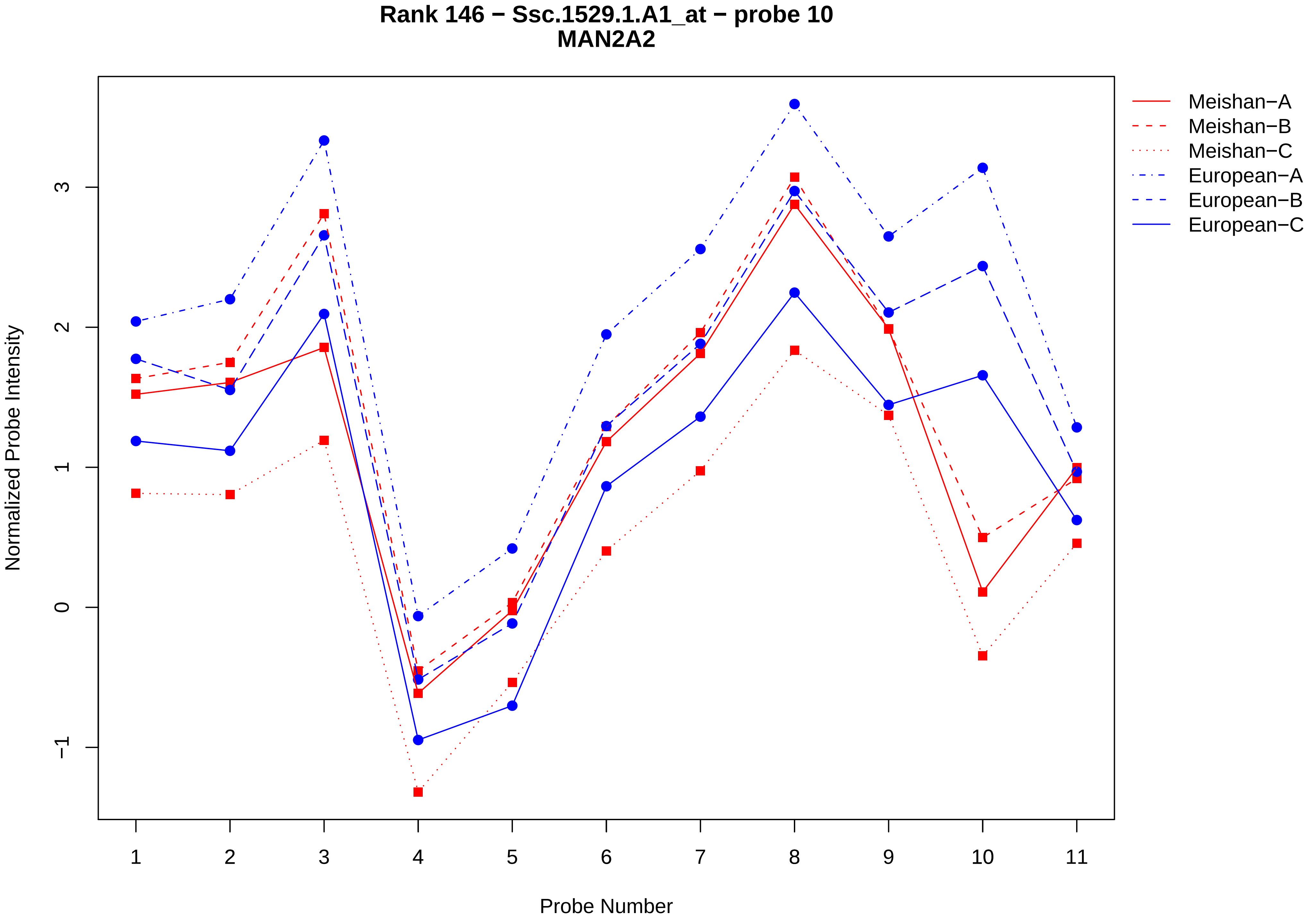
Rank 146 − Ssc.1529.1.A1_at − probe 10
MAN2A2
Normalized Probe Intensity
Probe Number
Meishan−A
Meishan−B
Meishan−C
European−A
European−B
European−C
−1
0
1
2
3
1
2
3
4
5
6
7
8
9
10
11

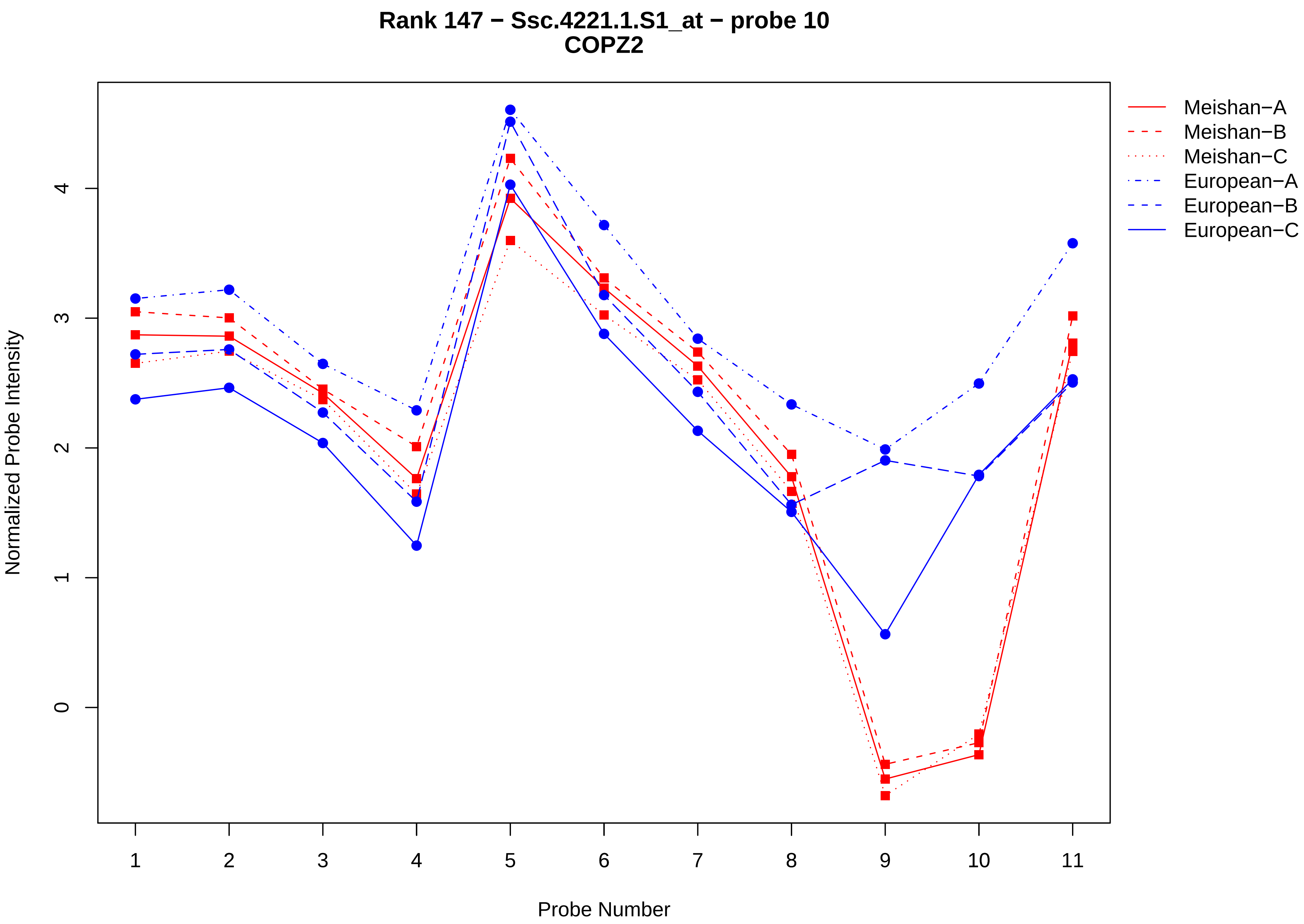

Rank 147 − Ssc.4221.1.S1_at − probe 10
COPZ2
Normalized Probe Intensity
Probe Number
0
1
2
3
4
1
2
3
4
5
6
7
8
9
10
11
Meishan−A
Meishan−B
Meishan−C
European−A
European−B
European−C

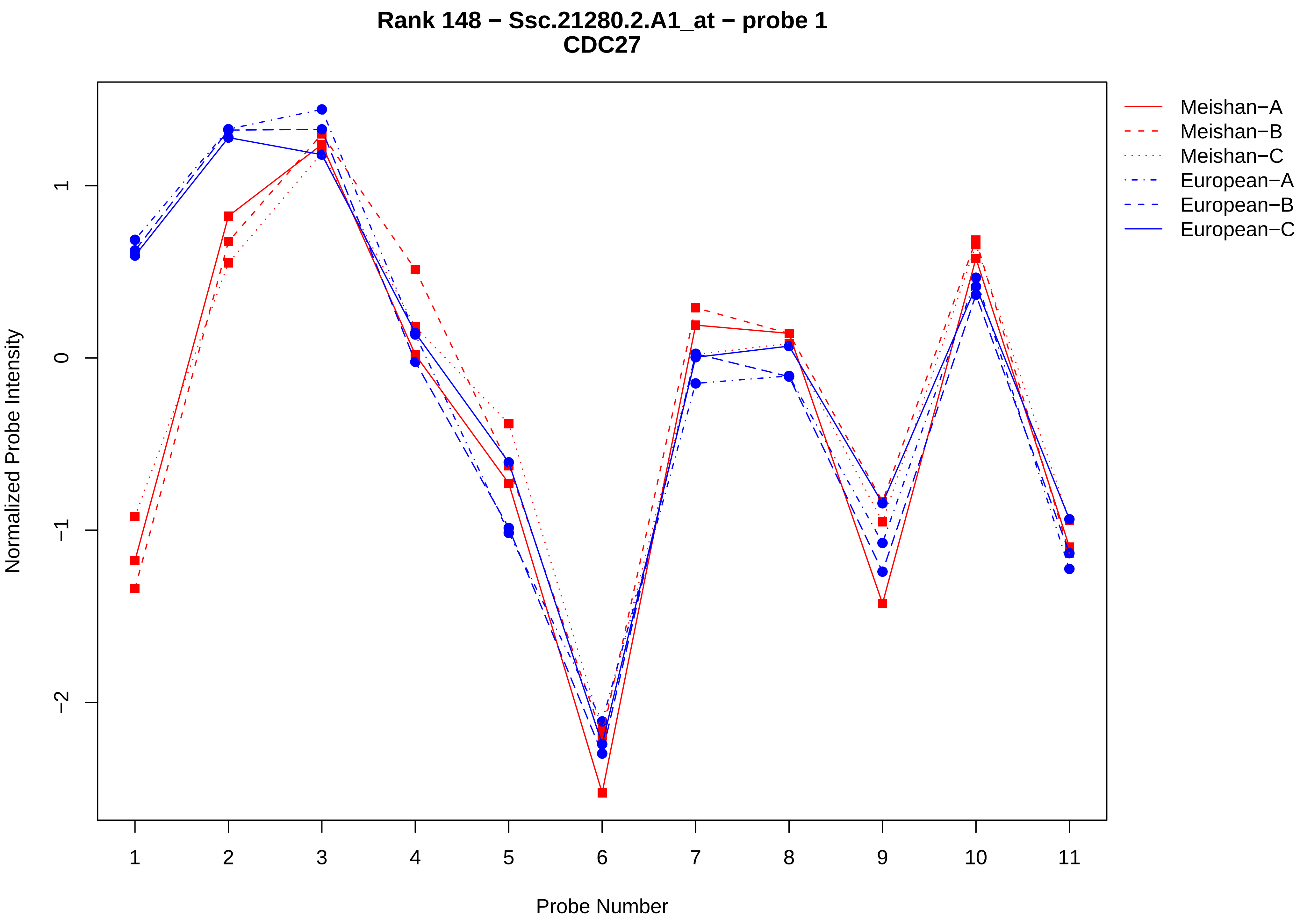
Rank 148 − Ssc.21280.2.A1_at − probe 1
CDC27
Normalized Probe Intensity
Probe Number
1
0
−1
−2
1
2
3
4
5
6
7
8
9
10
11
Meishan−A
Meishan−B
Meishan−C
European−A
European−B
European−C

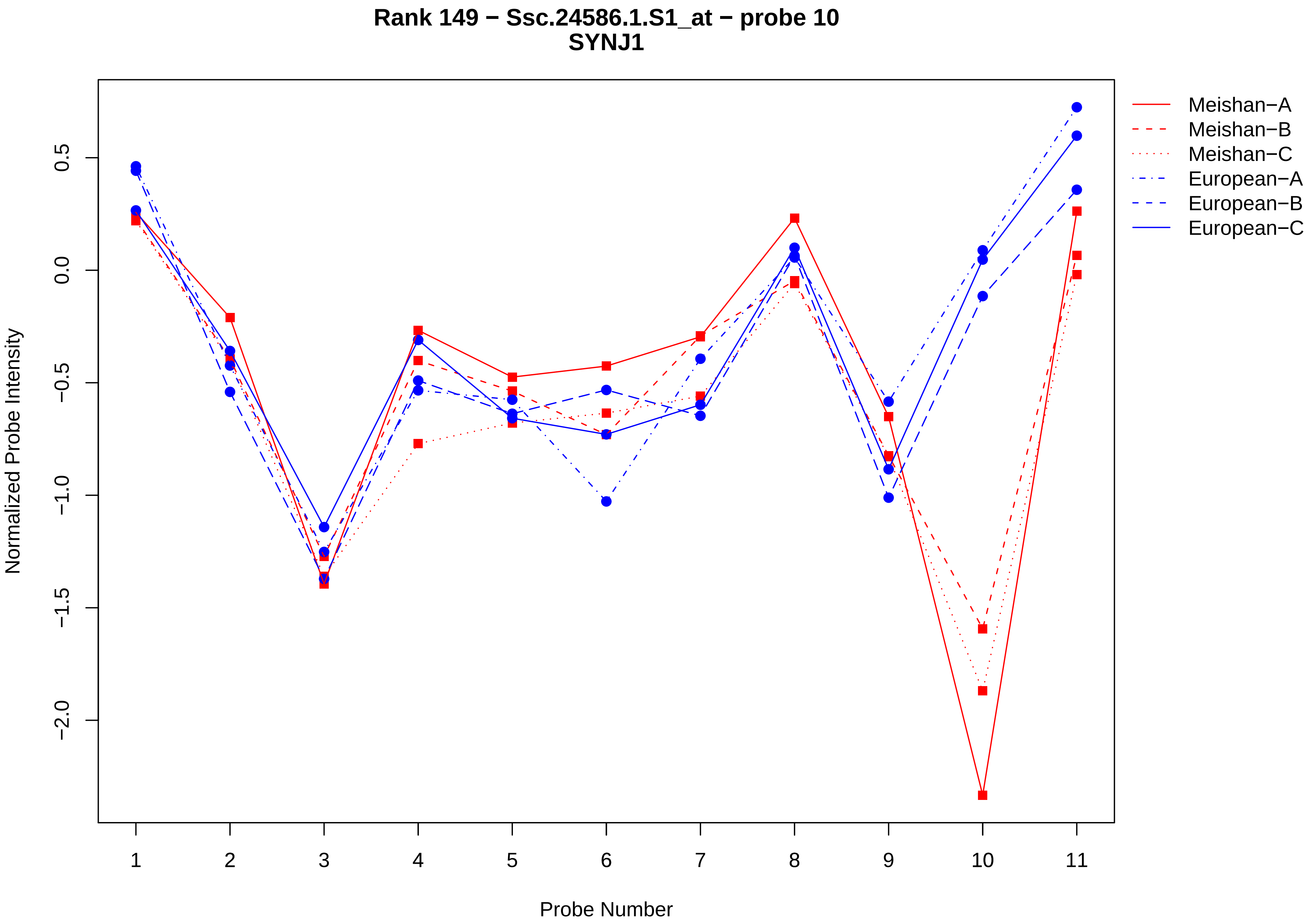

Rank 149 − Ssc.24586.1.S1_at − probe 10
SYNJ1
Normalized Probe Intensity
Probe Number
0.5
0.0
−0.5
−1.0
−1.5
−2.0
1
2
3
4
5
6
7
8
9
10
11
Meishan−A
Meishan−B
Meishan−C
European−A
European−B
European−C

Rank 150 − Ssc.25175.2.S1_at − probe 5
CKLFSF3

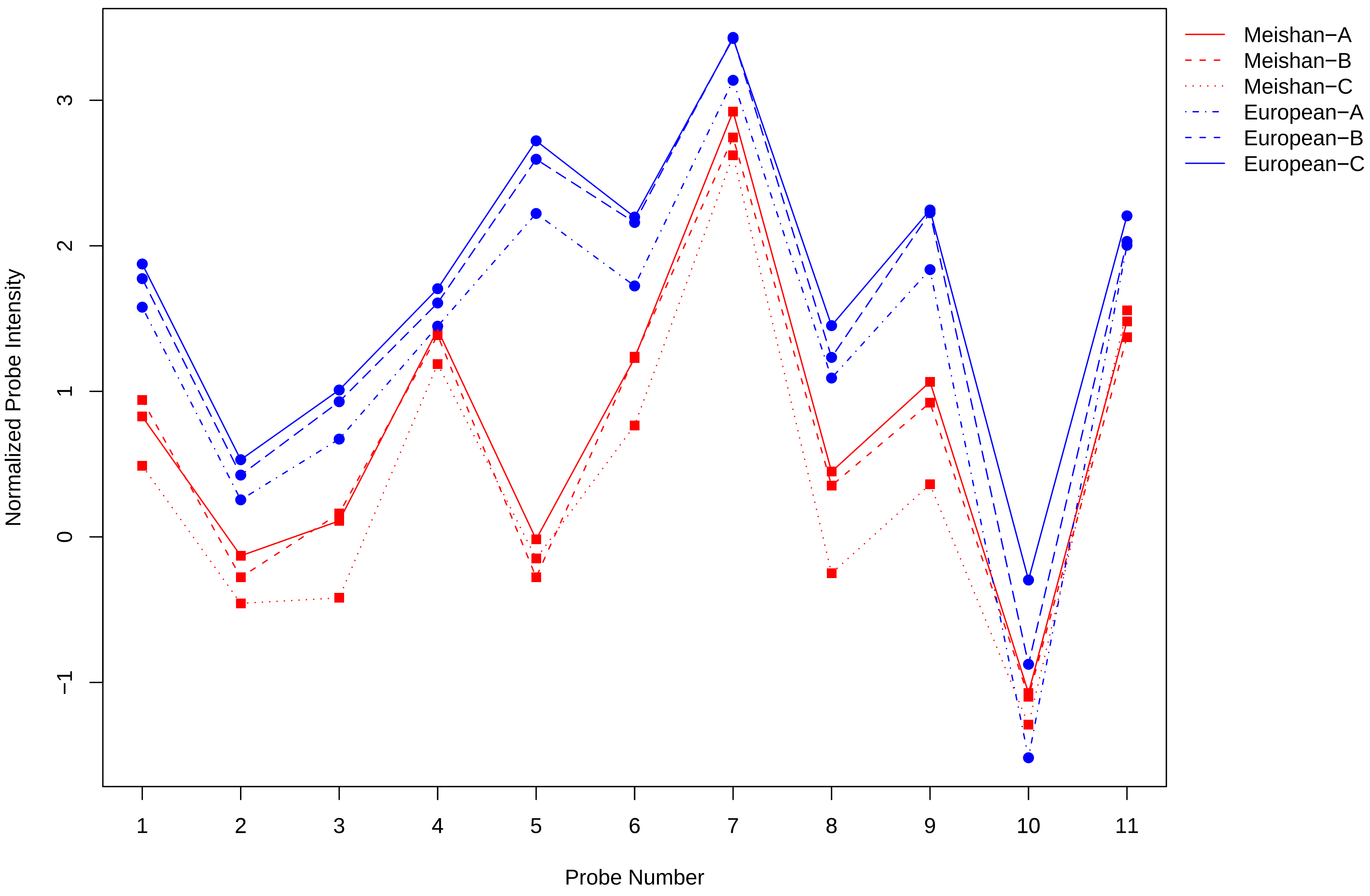

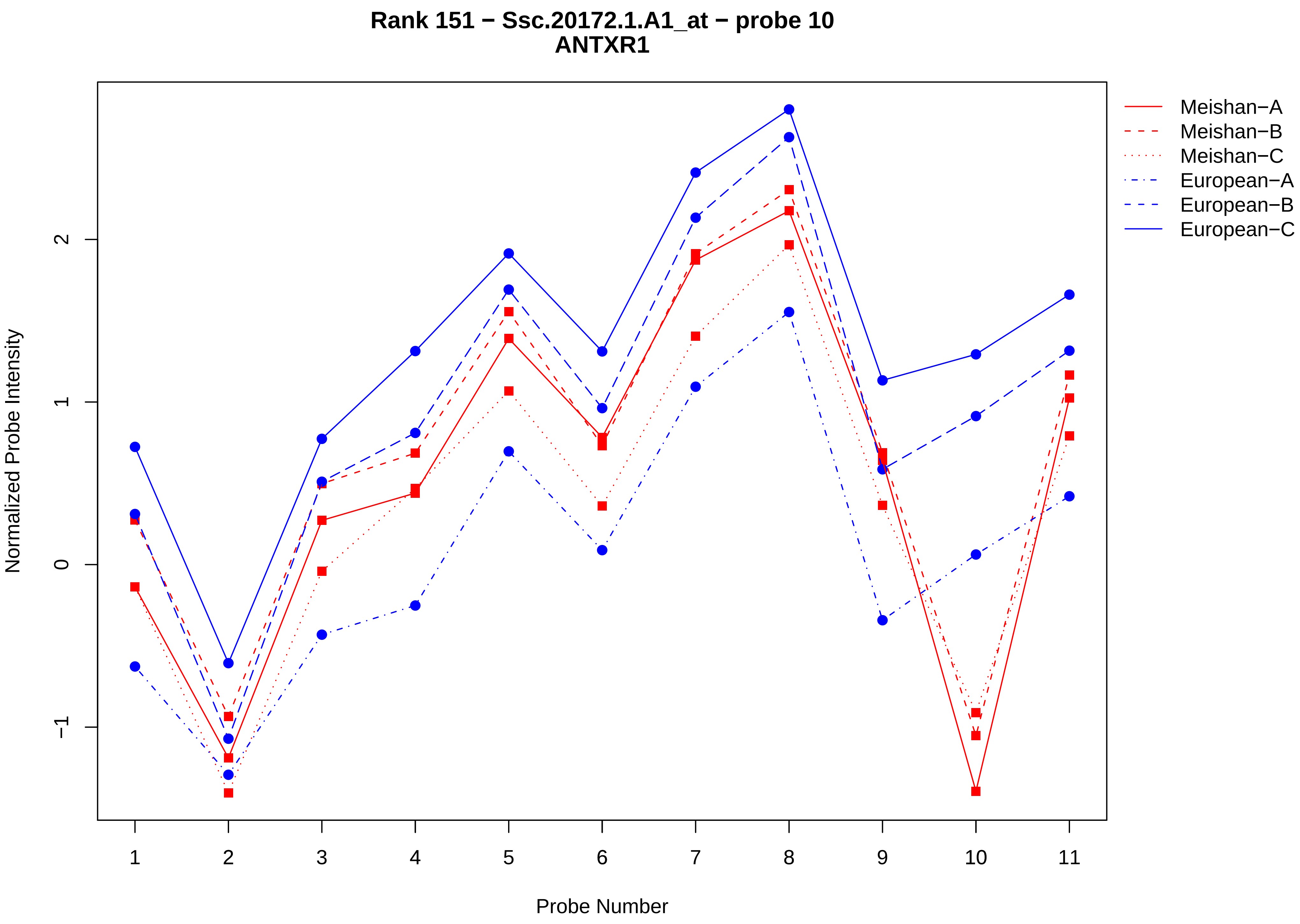
Rank 151 − Ssc.20172.1.A1_at − probe 10
ANTXR1
Normalized Probe Intensity
Probe Number
−1
0
1
2
1
2
3
4
5
6
7
8
9
10
11
Meishan−A
Meishan−B
Meishan−C
European−A
European−B
European−C

Rank 152 − Ssc.974.1.S1_at − probe 10
DCBLD2

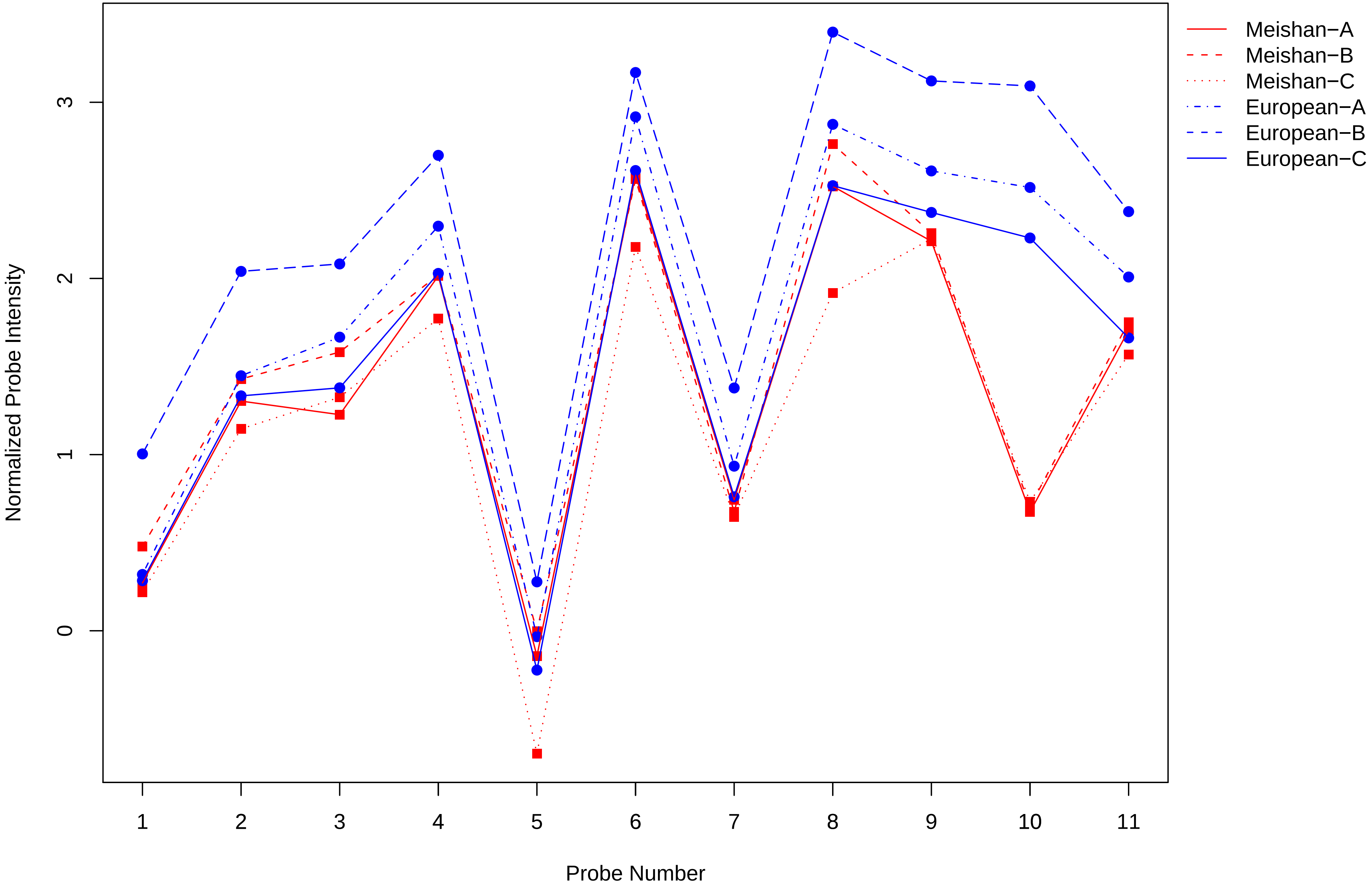

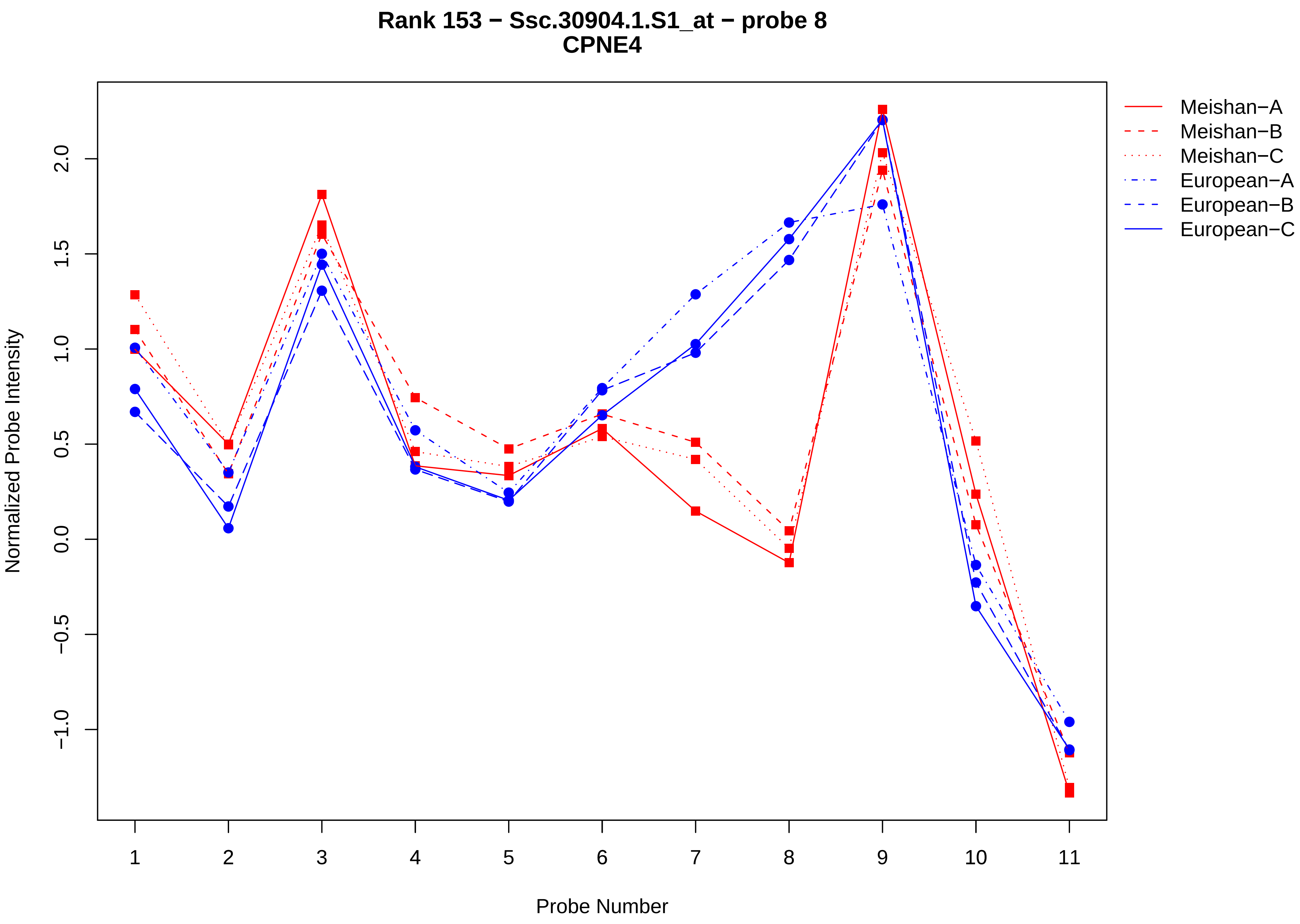

Rank 153 − Ssc.30904.1.S1_at − probe 8
CPNE4
Normalized Probe Intensity
Probe Number
Meishan−A
Meishan−B
Meishan−C
European−A
European−B
European−C
−1.0
−0.5
0.0
0.5
1.0
1.5
2.0
1
2
3
4
5
6
7
8
9
10
11

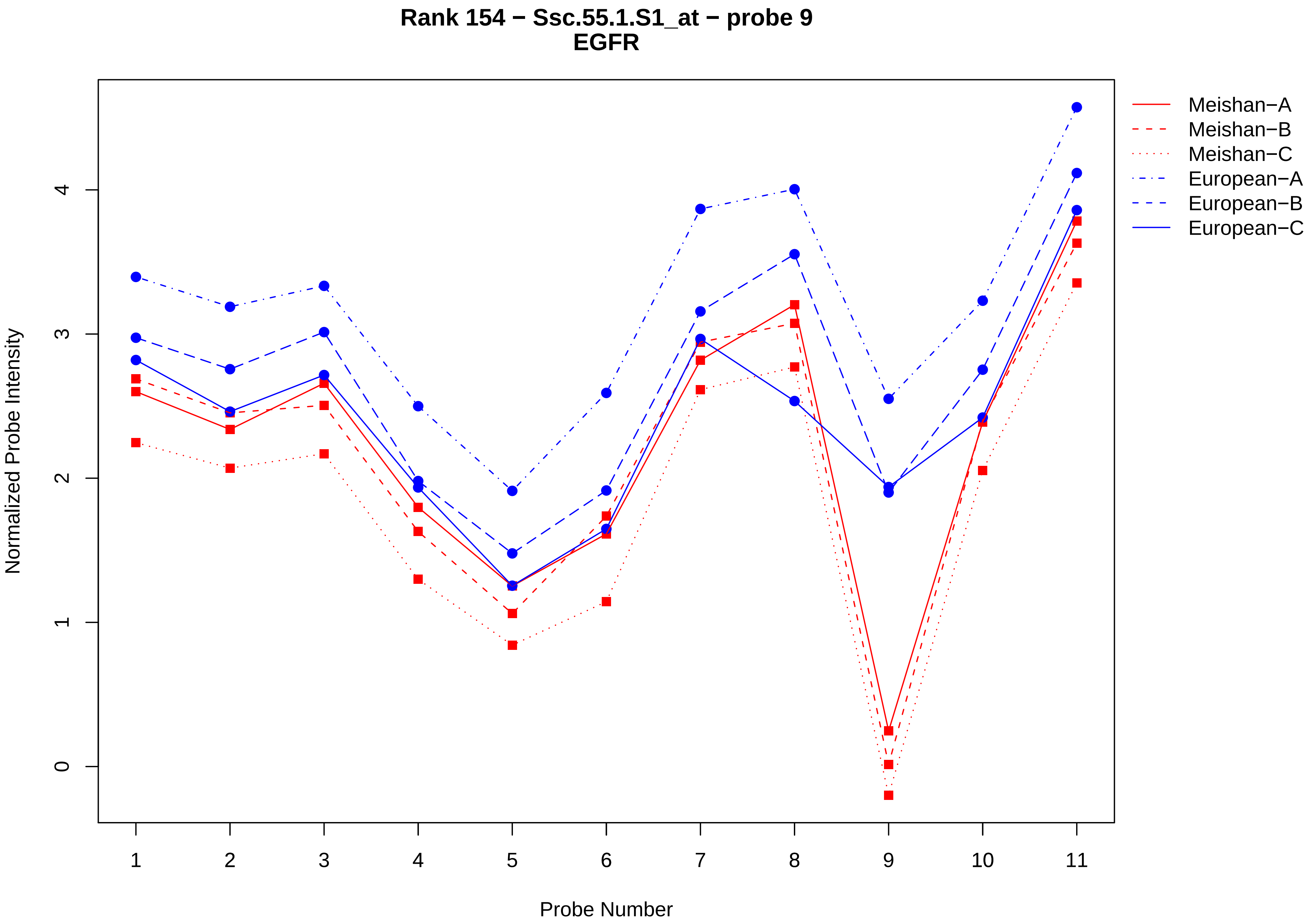

Rank 154 − Ssc.55.1.S1_at − probe 9
EGFR
Normalized Probe Intensity
Probe Number
0
1
2
3
4
1
2
3
4
5
6
7
8
9
10
11
Meishan−A
Meishan−B
Meishan−C
European−A
European−B
European−C

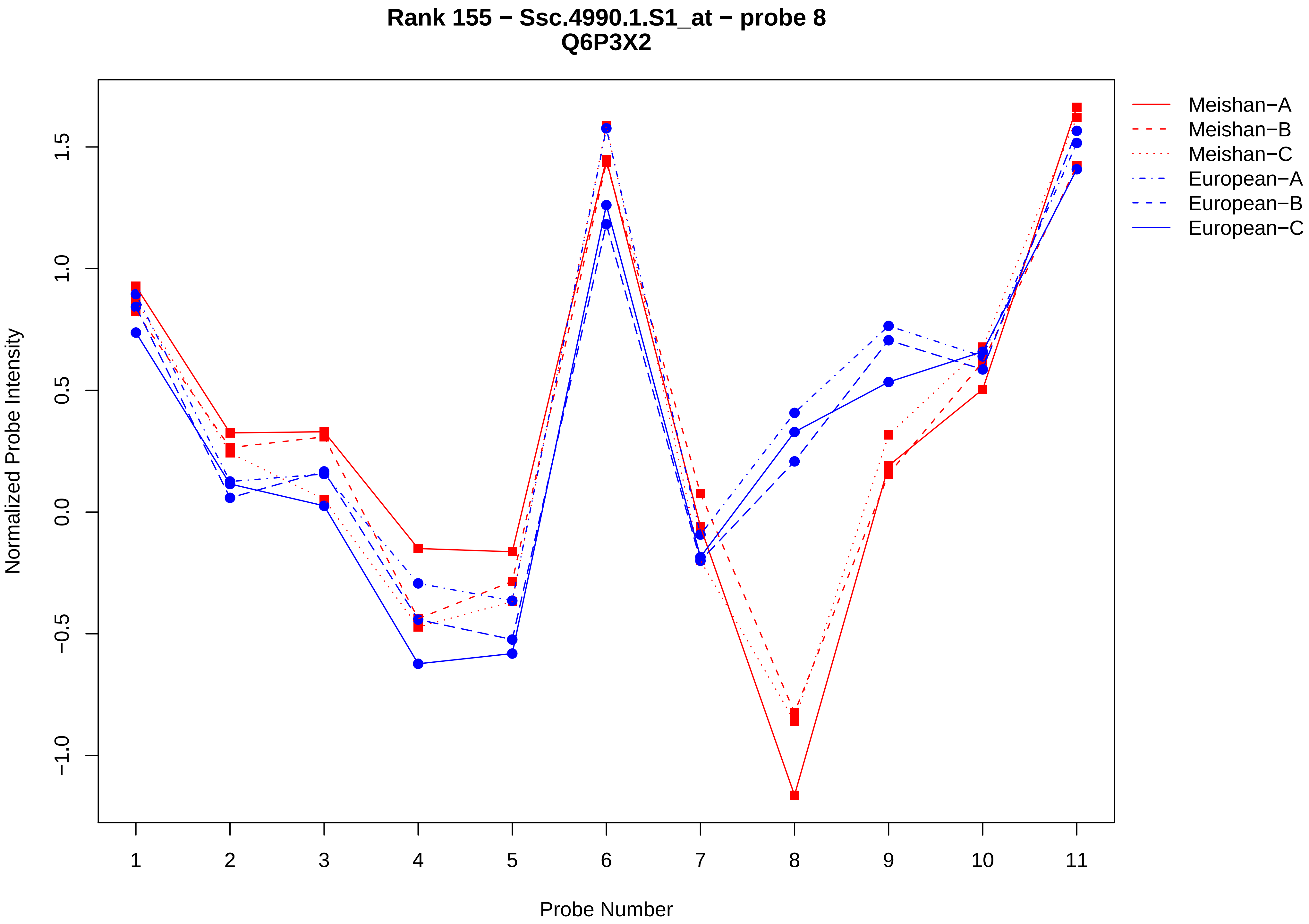
Rank 155 − Ssc.4990.1.S1_at − probe 8
Q6P3X2
Normalized Probe Intensity
Probe Number
Meishan−A
Meishan−B
Meishan−C
European−A
European−B
European−C
−1.0
−0.5
0.0
0.5
1.0
1.5
1
2
3
4
5
6
7
8
9
10
11

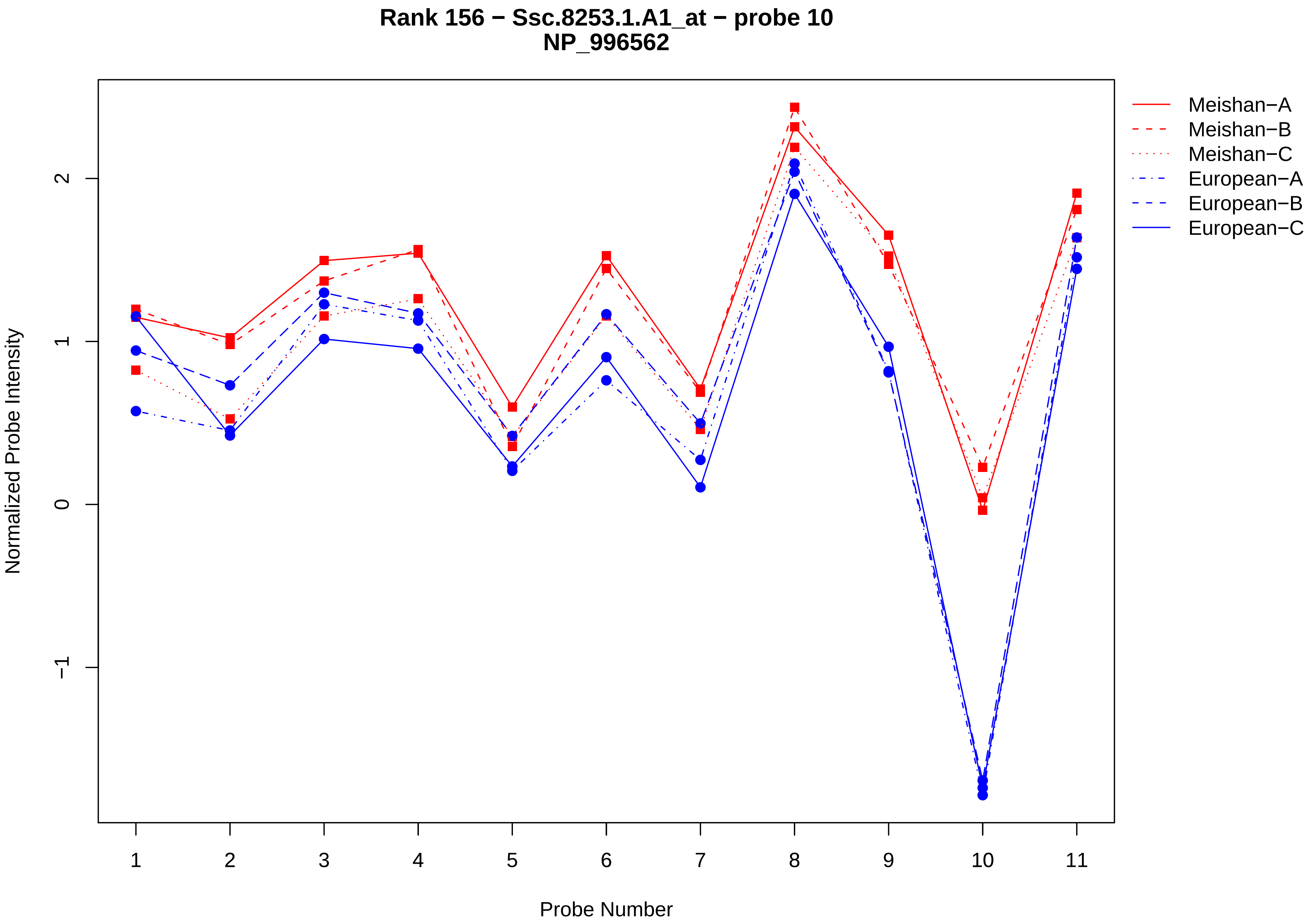

Rank 156 − Ssc.8253.1.A1_at − probe 10
NP_996562
Meishan−A
Meishan−B
Meishan−C
European−A
European−B
European−C
Normalized Probe Intensity
Probe Number
−1
0
1
2
1
2
3
4
5
6
7
8
9
10
11

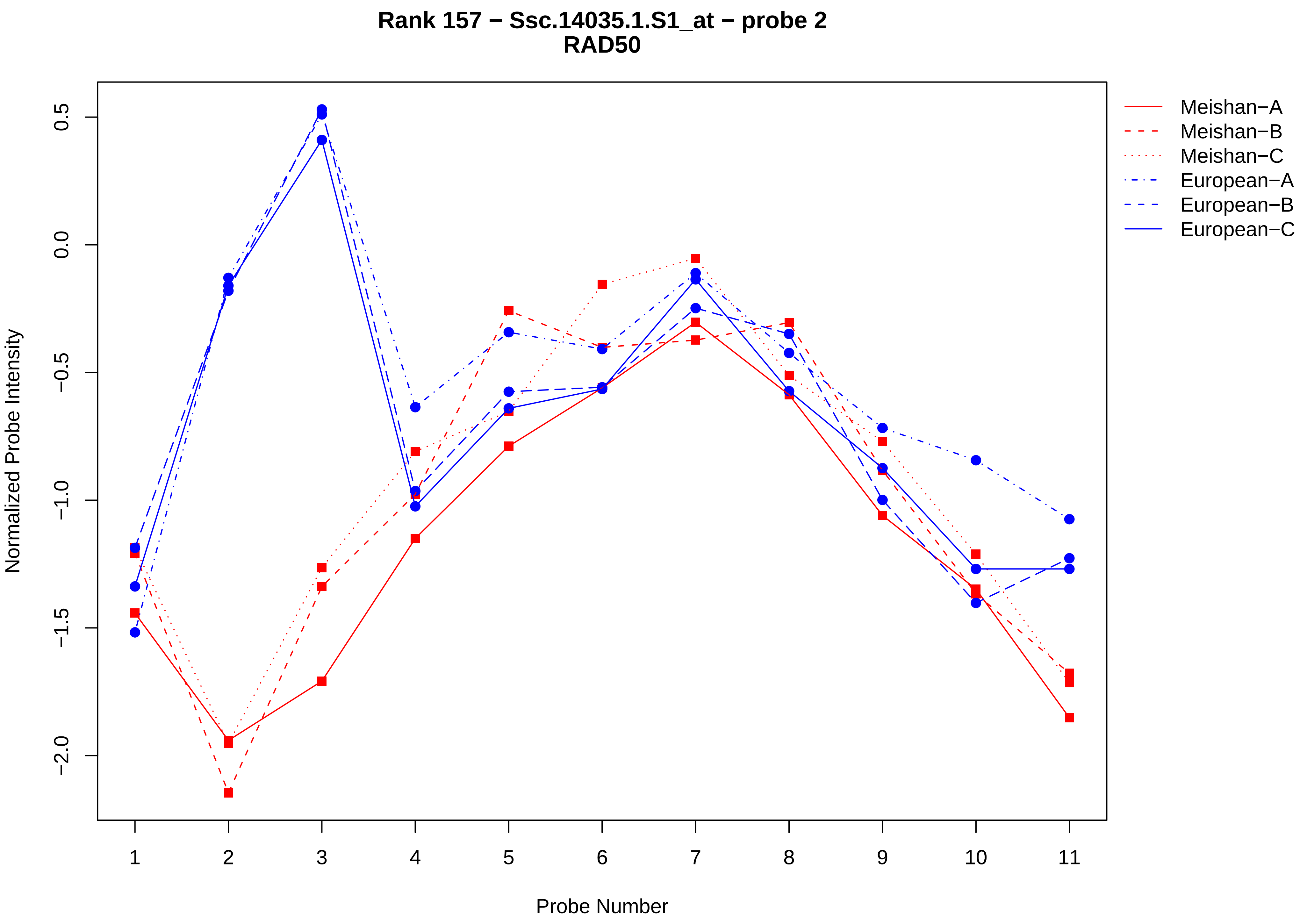

Rank 157 − Ssc.14035.1.S1_at − probe 2
RAD50
Normalized Probe Intensity
Probe Number
0.5
0.0
−0.5
−1.0
−1.5
−2.0
1
2
3
4
5
6
7
8
9
10
11
Meishan−A
Meishan−B
Meishan−C
European−A
European−B
European−C

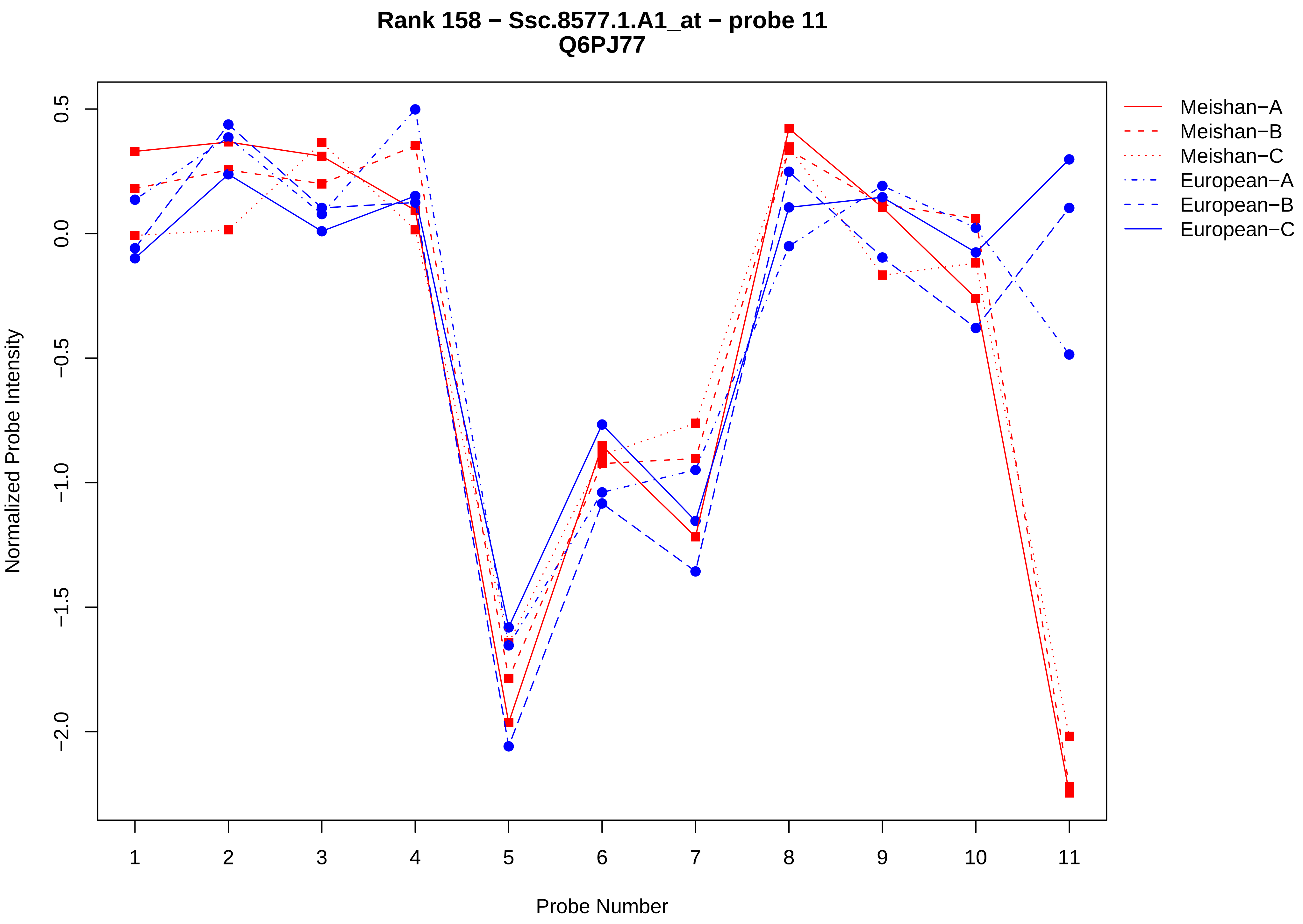
Rank 158 − Ssc.8577.1.A1_at − probe 11
Q6PJ77
Normalized Probe Intensity
Probe Number
0.5
0.0
−0.5
−1.0
−1.5
−2.0
1
2
3
4
5
6
7
8
9
10
11
Meishan−A
Meishan−B
Meishan−C
European−A
European−B
European−C

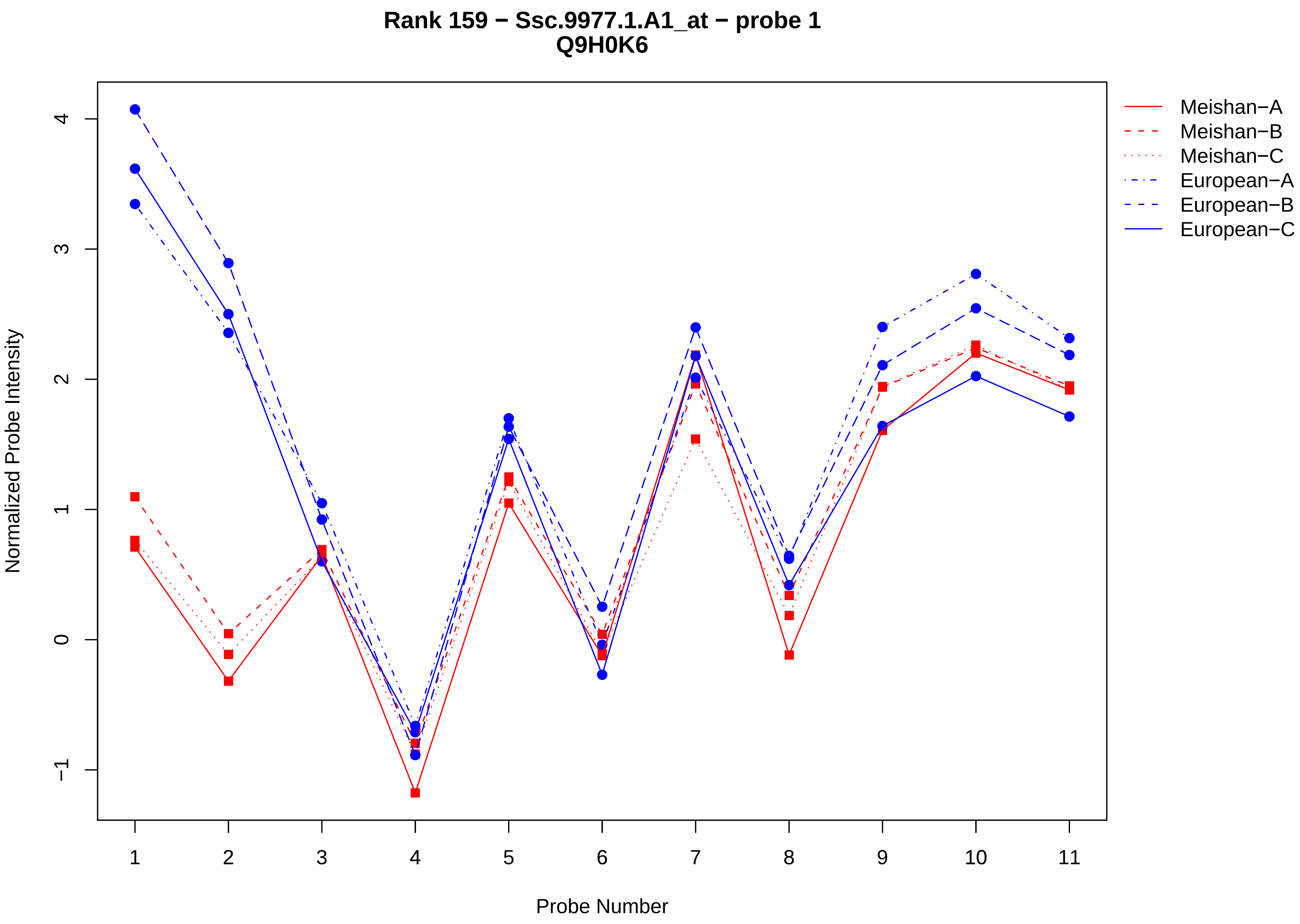

Rank 159 − Ssc.9977.1.A1_at − probe 1
Q9H0K6
Normalized Probe Intensity
Probe Number
−1
0
1
2
3
4
1
2
3
4
5
6
7
8
9
10
11
Meishan−A
Meishan−B
Meishan−C
European−A
European−B
European−C

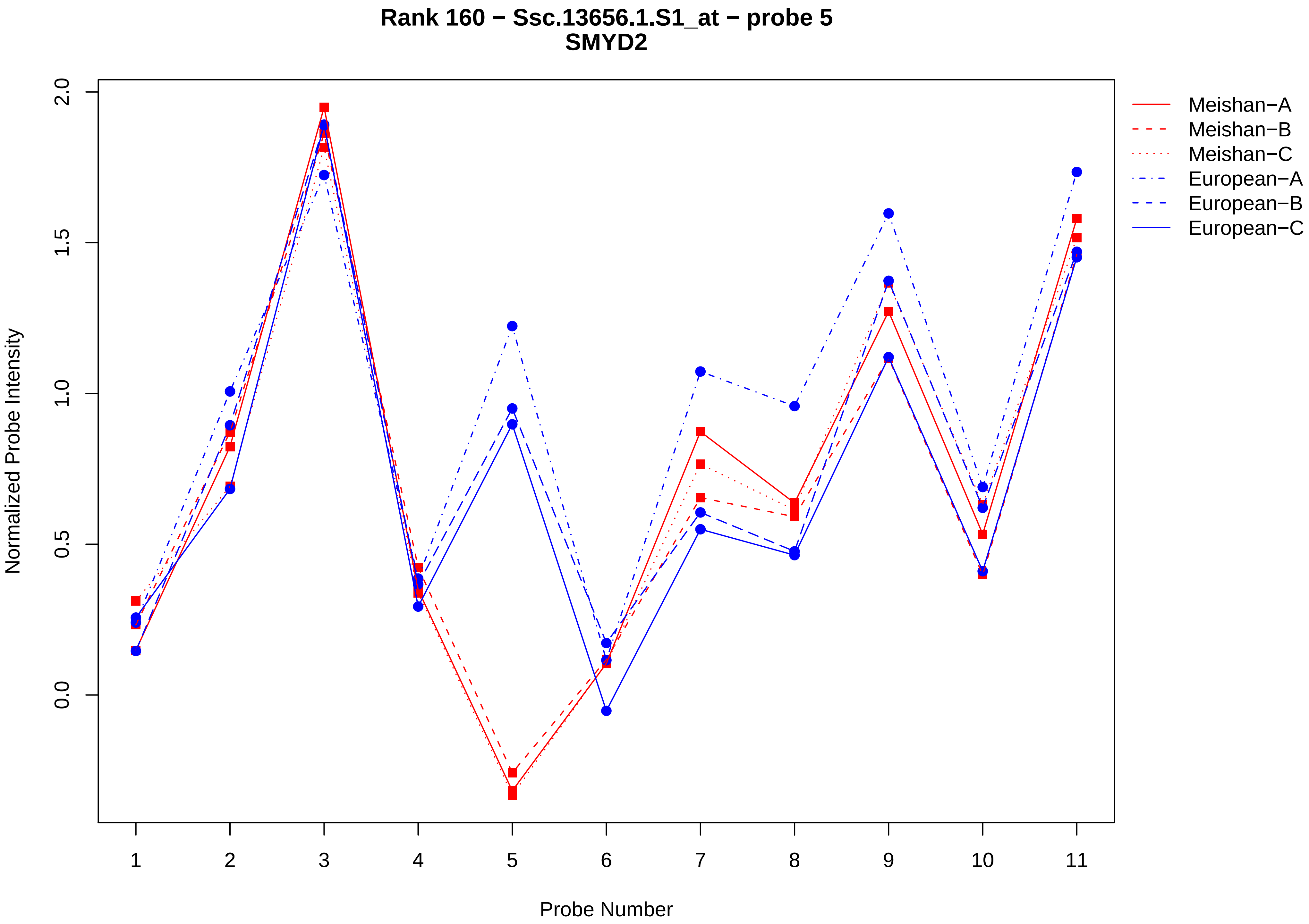

Rank 160 − Ssc.13656.1.S1_at − probe 5
SMYD2
Normalized Probe Intensity
Probe Number
0.0
0.5
1.0
1.5
2.0
1
2
3
4
5
6
7
8
9
10
11
Meishan−A
Meishan−B
Meishan−C
European−A
European−B
European−C

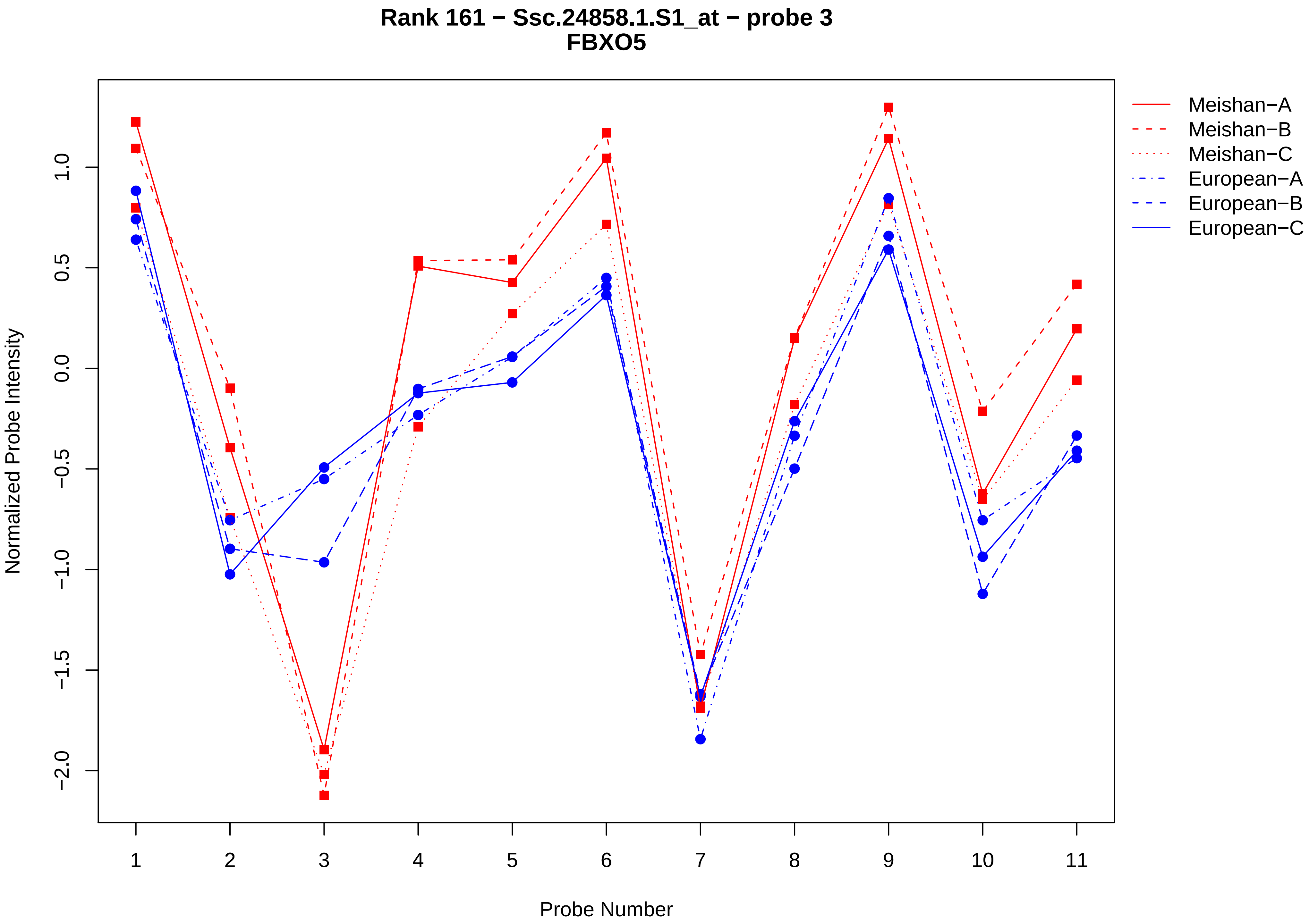

Rank 161 − Ssc.24858.1.S1_at − probe 3
FBXO5
Normalized Probe Intensity
Probe Number
−2.0
−1.5
−1.0
−0.5
0.0
0.5
1.0
1
2
3
4
5
6
7
8
9
10
11
Meishan−A
Meishan−B
Meishan−C
European−A
European−B
European−C

Rank 162 − Ssc.30797.1.S1_at − probe 10
NP_116028

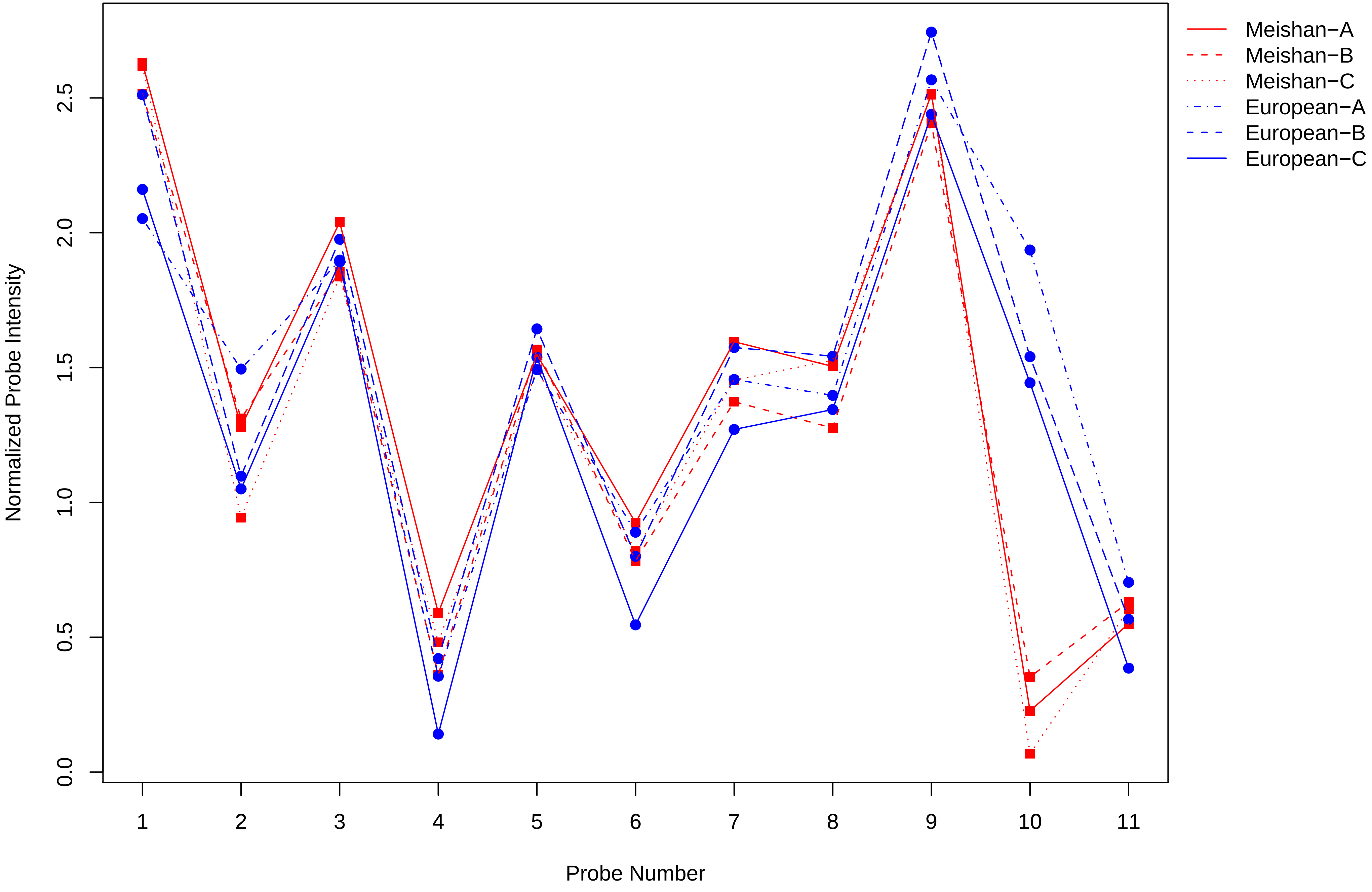

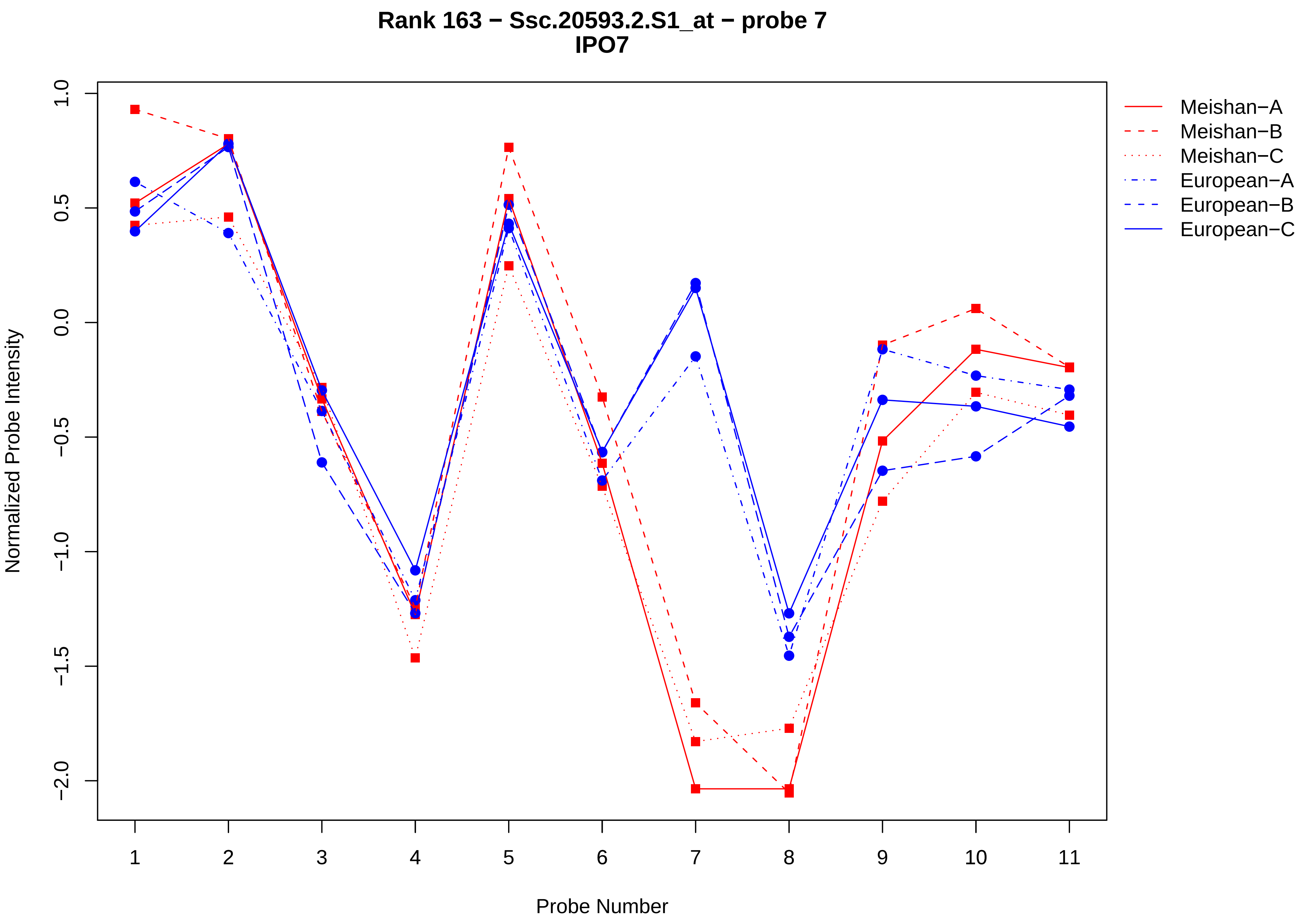

Rank 163 − Ssc.20593.2.S1_at − probe 7
IPO7
Normalized Probe Intensity
Probe Number
1.0
0.5
0.0
−0.5
−1.0
−1.5
−2.0
1
2
3
4
5
6
7
8
9
10
11
Meishan−A
Meishan−B
Meishan−C
European−A
European−B
European−C

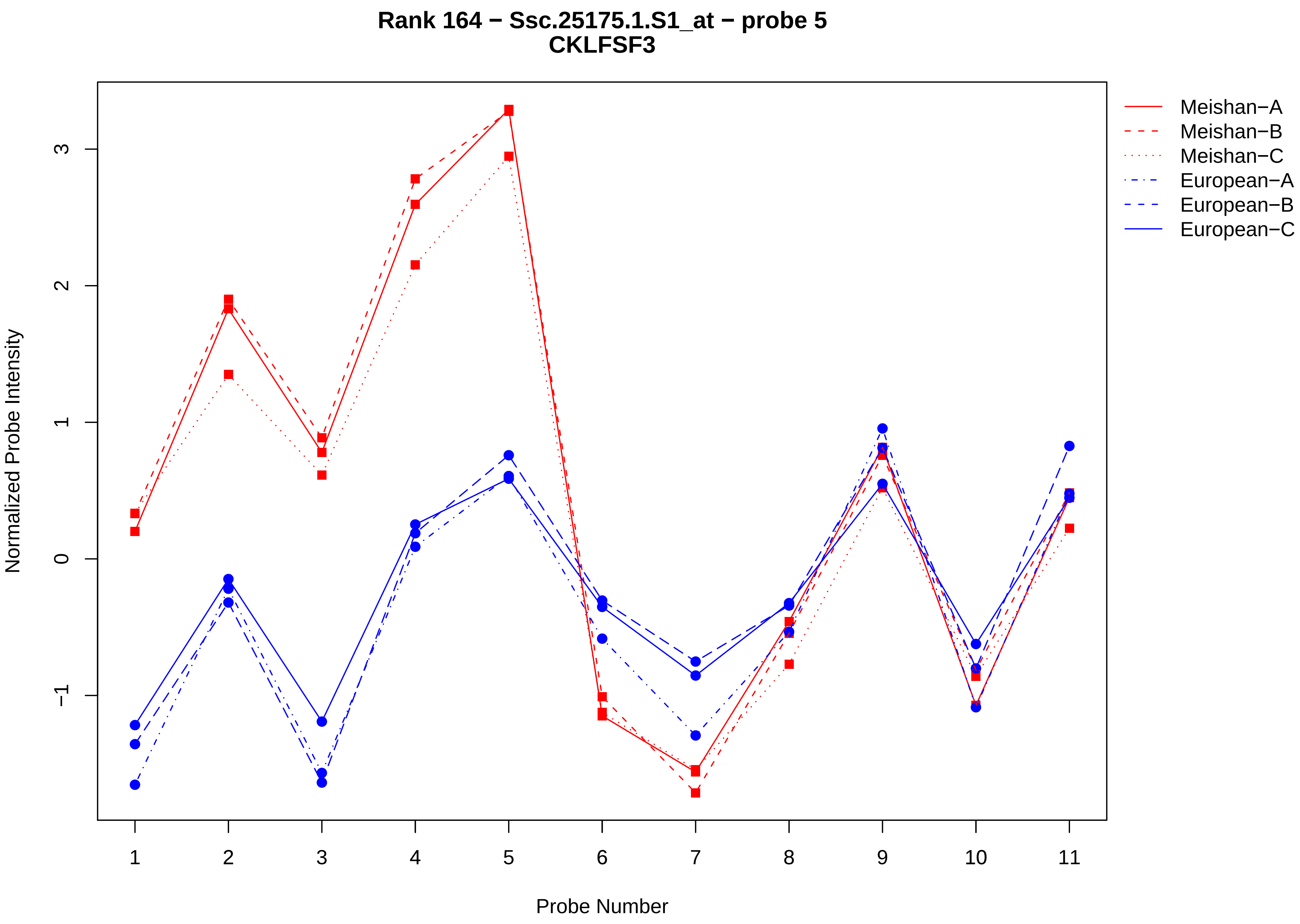
Rank 164 − Ssc.25175.1.S1_at − probe 5
CKLFSF3
Normalized Probe Intensity
Probe Number
−1
0
1
2
3
1
2
3
4
5
6
7
8
9
10
11
Meishan−A
Meishan−B
Meishan−C
European−A
European−B
European−C

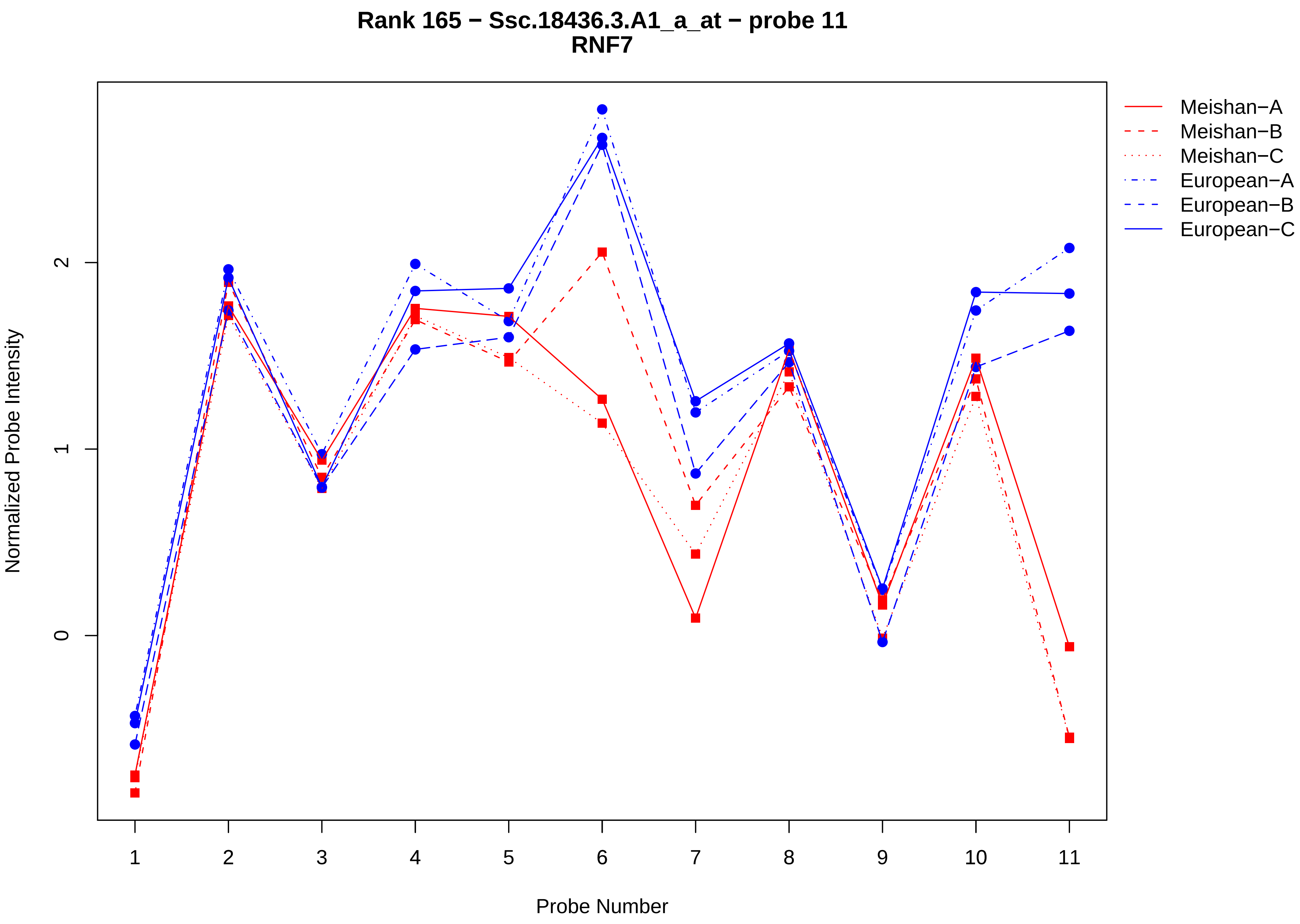

Rank 165 − Ssc.18436.3.A1_a_at − probe 11
RNF7
Normalized Probe Intensity
Probe Number
0
1
2
1
2
3
4
5
6
7
8
9
10
11
Meishan−A
Meishan−B
Meishan−C
European−A
European−B
European−C

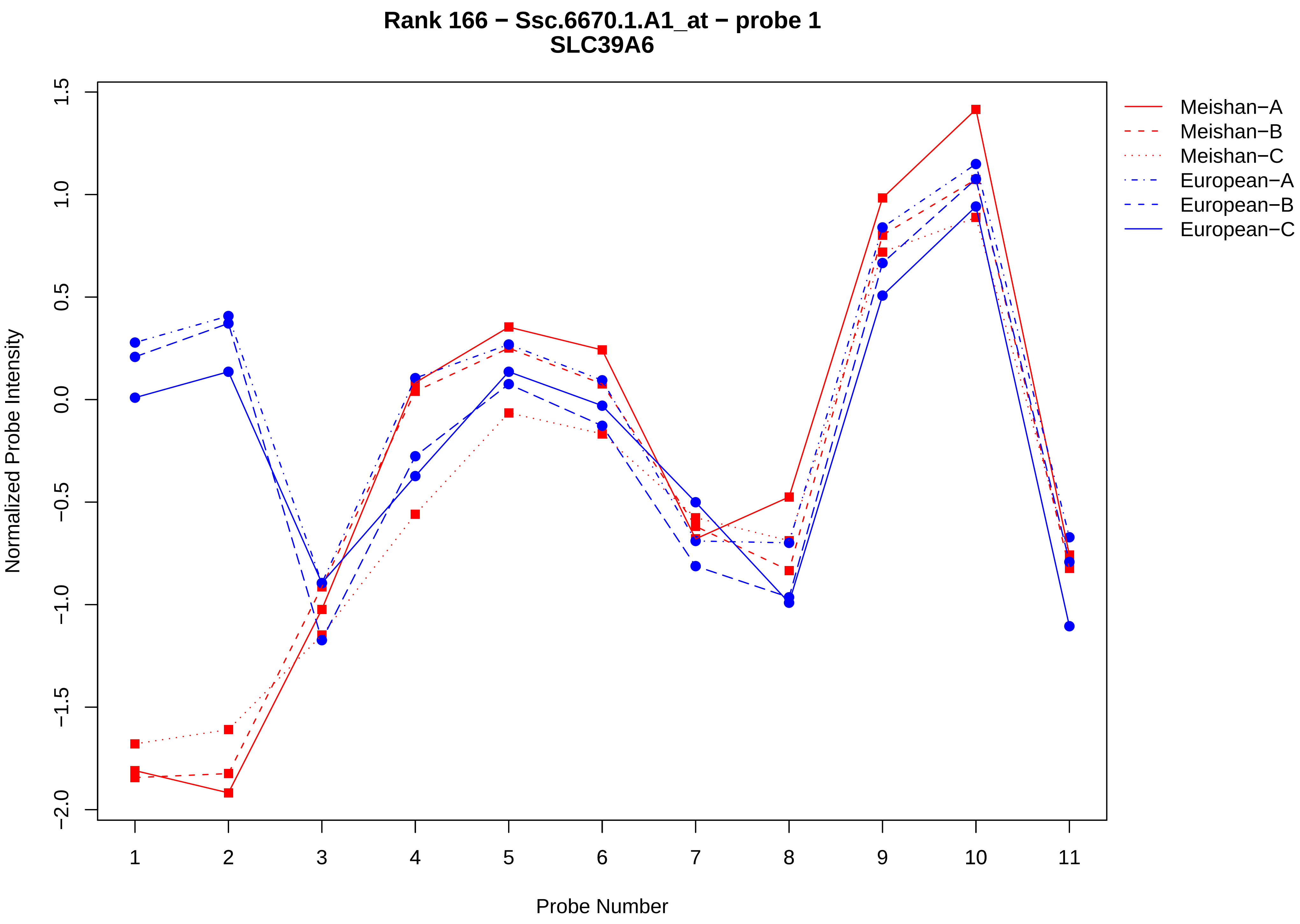

Rank 166 − Ssc.6670.1.A1_at − probe 1
SLC39A6
Normalized Probe Intensity
Probe Number
−2.0
−1.5
−1.0
−0.5
0.0
0.5
1.0
1.5
1
2
3
4
5
6
7
8
9
10
11
Meishan−A
Meishan−B
Meishan−C
European−A
European−B
European−C

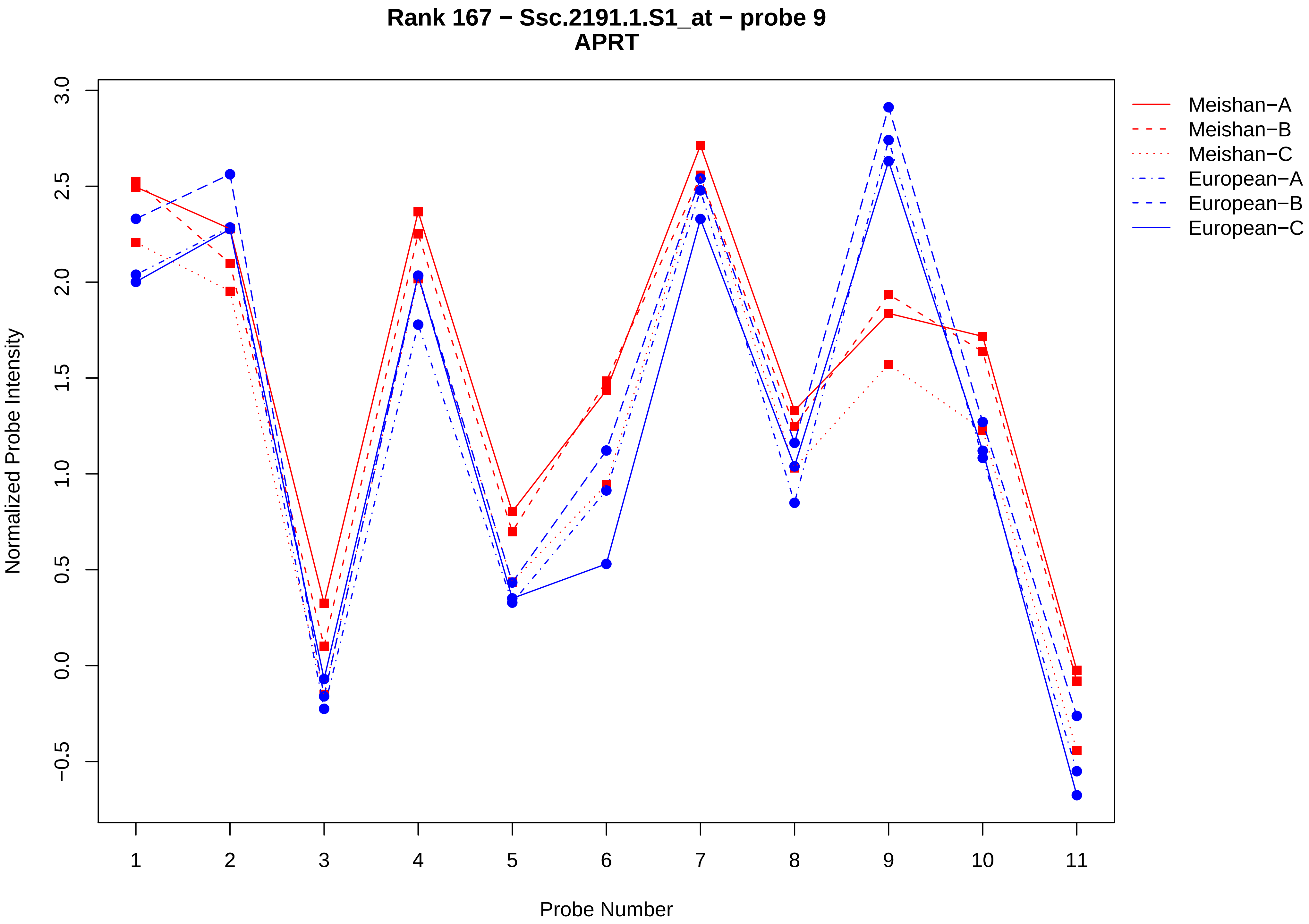

Rank 167 − Ssc.2191.1.S1_at − probe 9
APRT
Normalized Probe Intensity
Probe Number
Meishan−A
Meishan−B
Meishan−C
European−A
European−B
European−C
−0.5
0.0
0.5
1.0
1.5
2.0
2.5
3.0
1
2
3
4
5
6
7
8
9
10
11

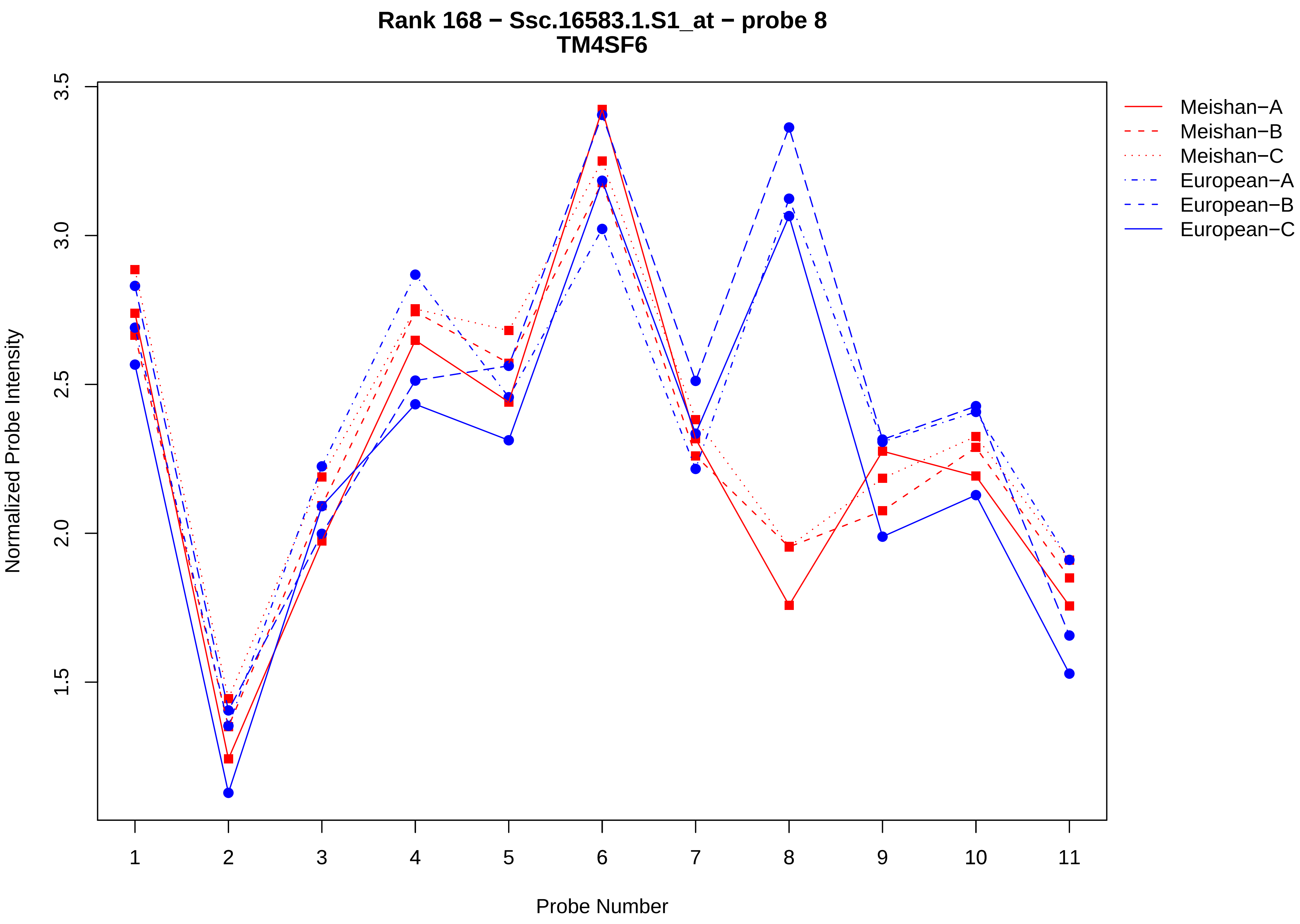
Rank 168 − Ssc.16583.1.S1_at − probe 8
TM4SF6
Normalized Probe Intensity
3.5
3.0
2.5
2.0
1.5
1
2
3
4
5
6
7
8
9
10
11
Probe Number
Meishan−A
Meishan−B
Meishan−C
European−A
European−B
European−C

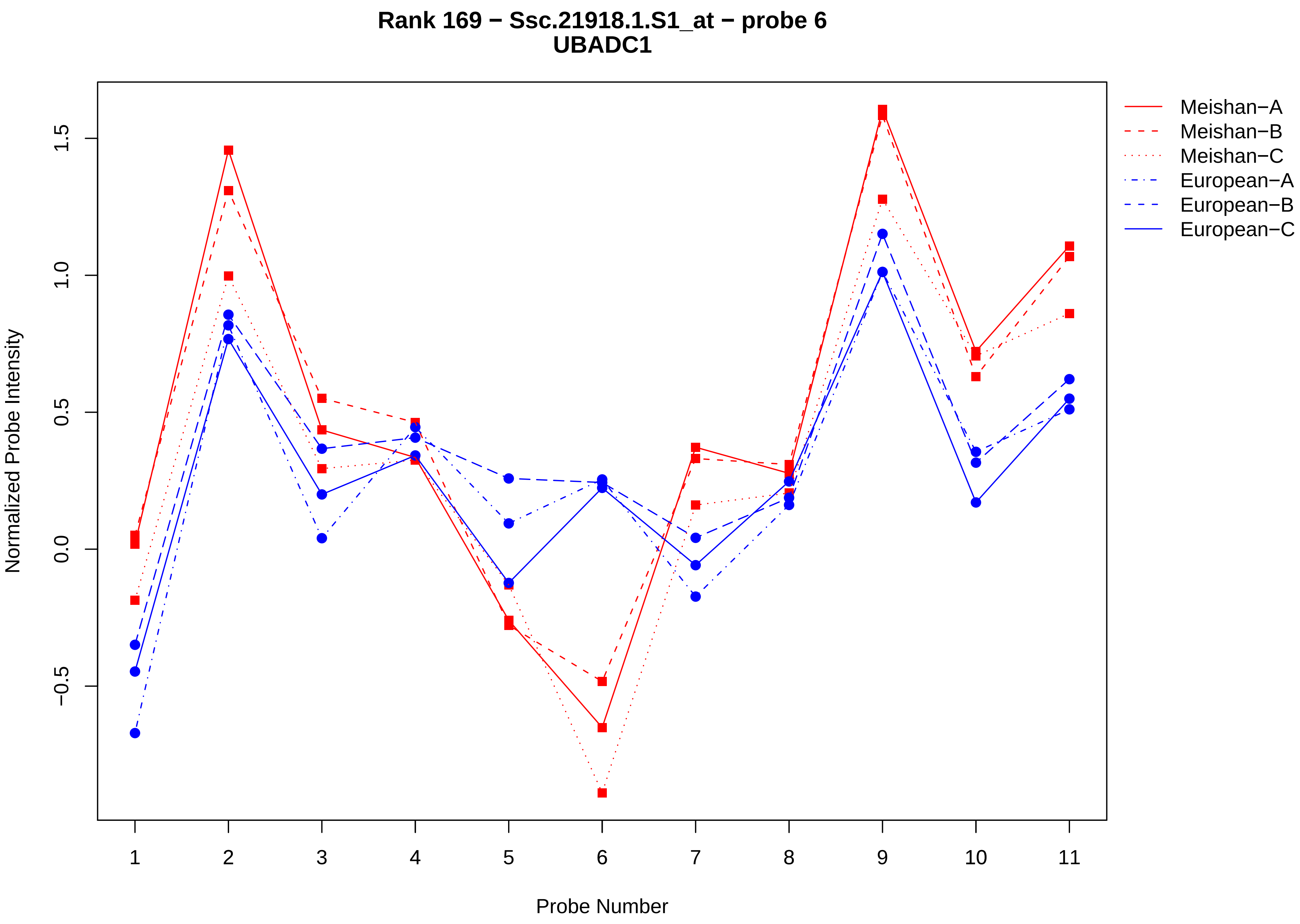
Rank 169 − Ssc.21918.1.S1_at − probe 6
UBADC1
Normalized Probe Intensity
Probe Number
−0.5
0.0
0.5
1.0
1.5
1
2
3
4
5
6
7
8
9
10
11
Meishan−A
Meishan−B
Meishan−C
European−A
European−B
European−C

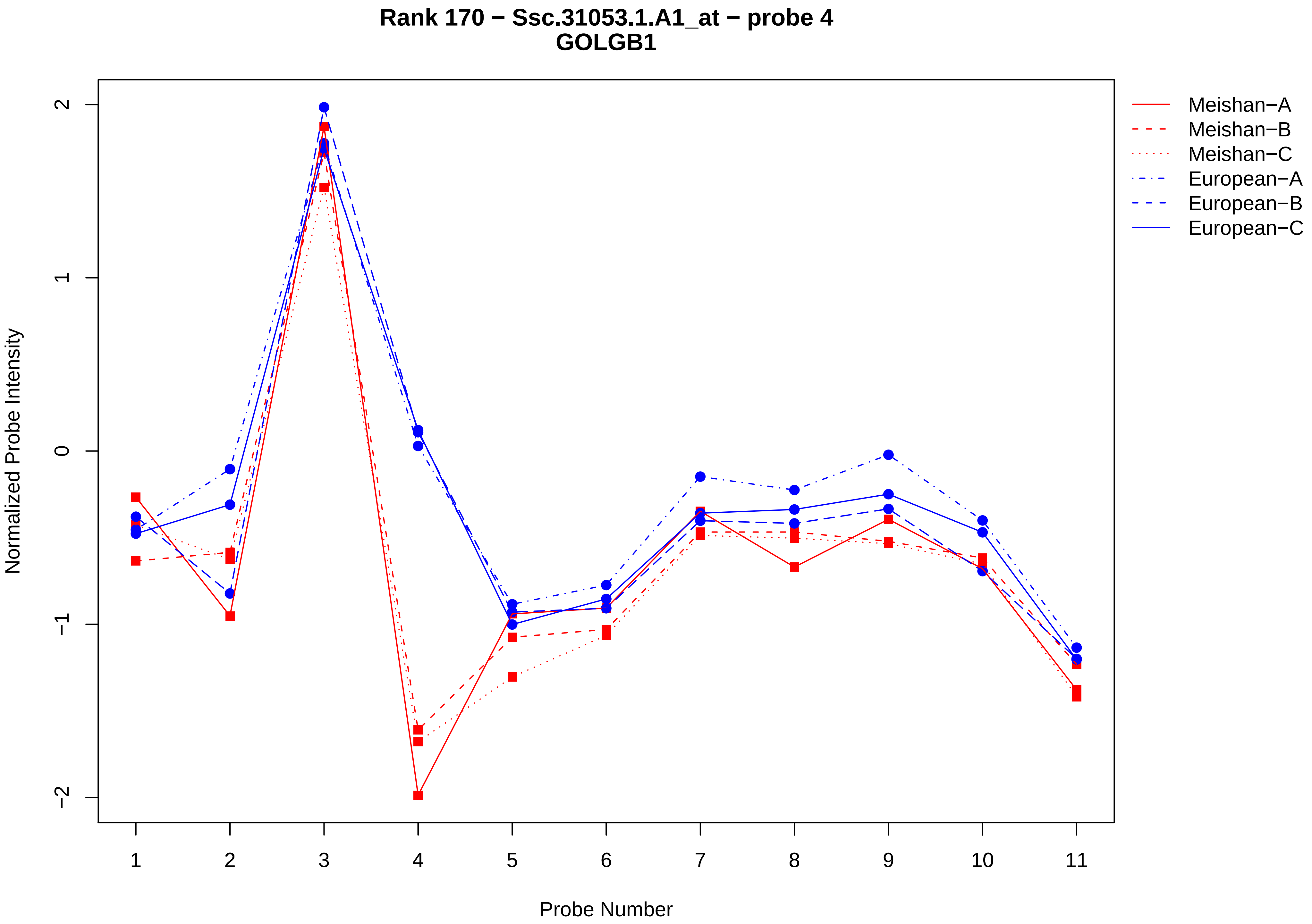
Rank 170 − Ssc.31053.1.A1_at − probe 4
GOLGB1
Normalized Probe Intensity
Probe Number
2
1
0
−1
−2
1
2
3
4
5
6
7
8
9
10
11
Meishan−A
Meishan−B
Meishan−C
European−A
European−B
European−C

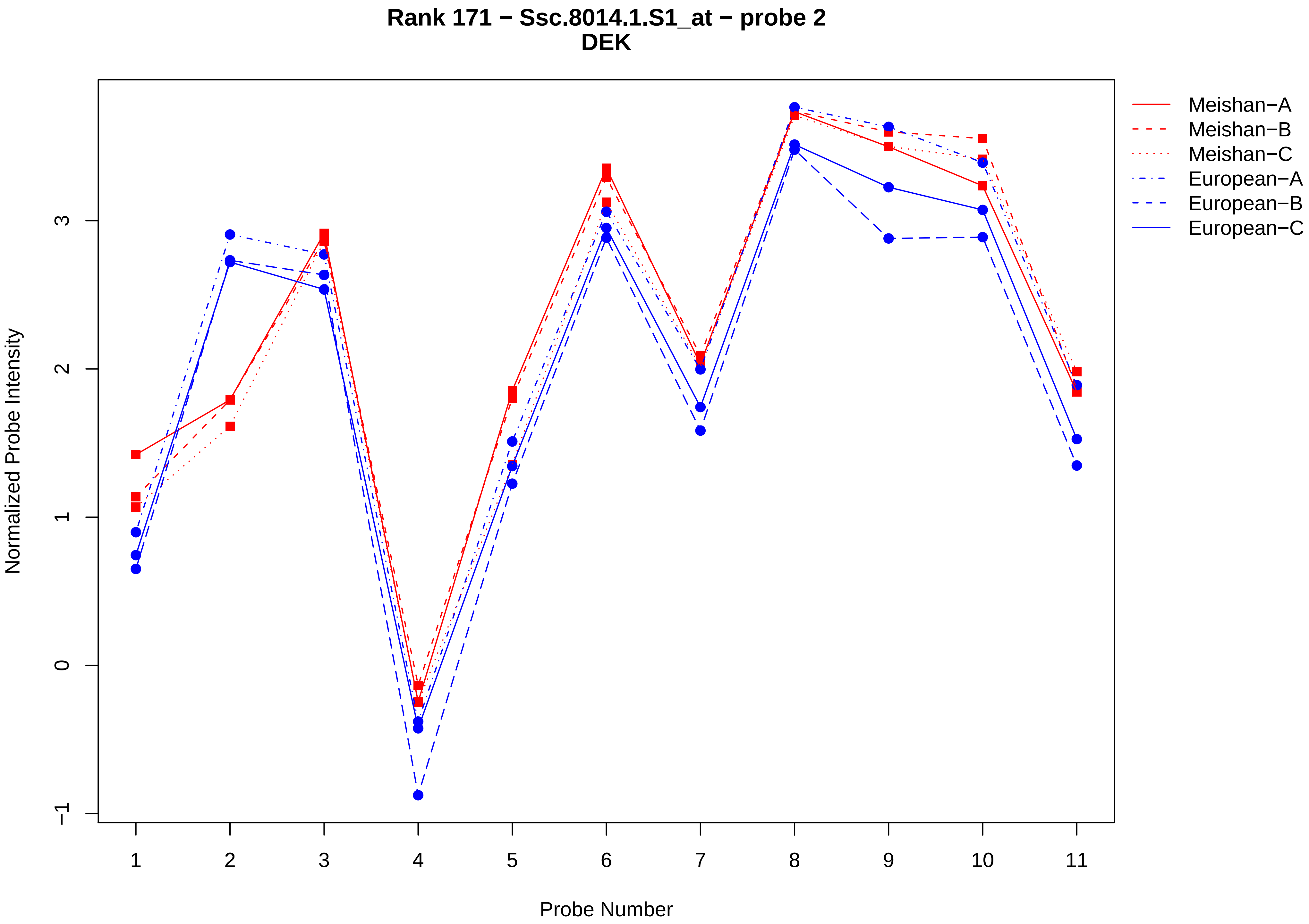

Rank 171 − Ssc.8014.1.S1_at − probe 2
DEK
Normalized Probe Intensity
Probe Number
−1
0
1
2
3
1
2
3
4
5
6
7
8
9
10
11
Meishan−A
Meishan−B
Meishan−C
European−A
European−B
European−C

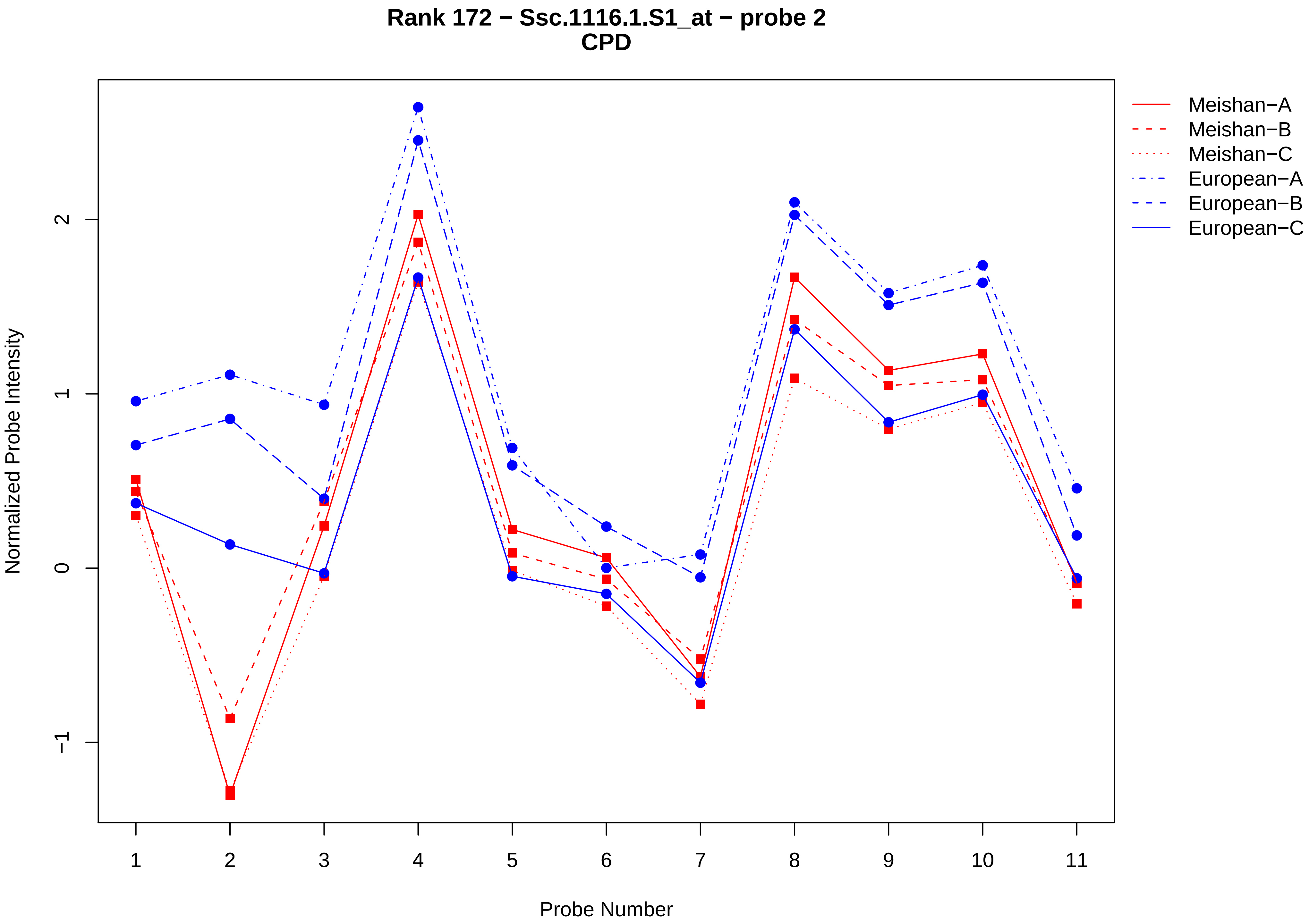

Rank 172 − Ssc.1116.1.S1_at − probe 2
CPD
Normalized Probe Intensity
Probe Number
−1
0
1
2
1
2
3
4
5
6
7
8
9
10
11
Meishan−A
Meishan−B
Meishan−C
European−A
European−B
European−C

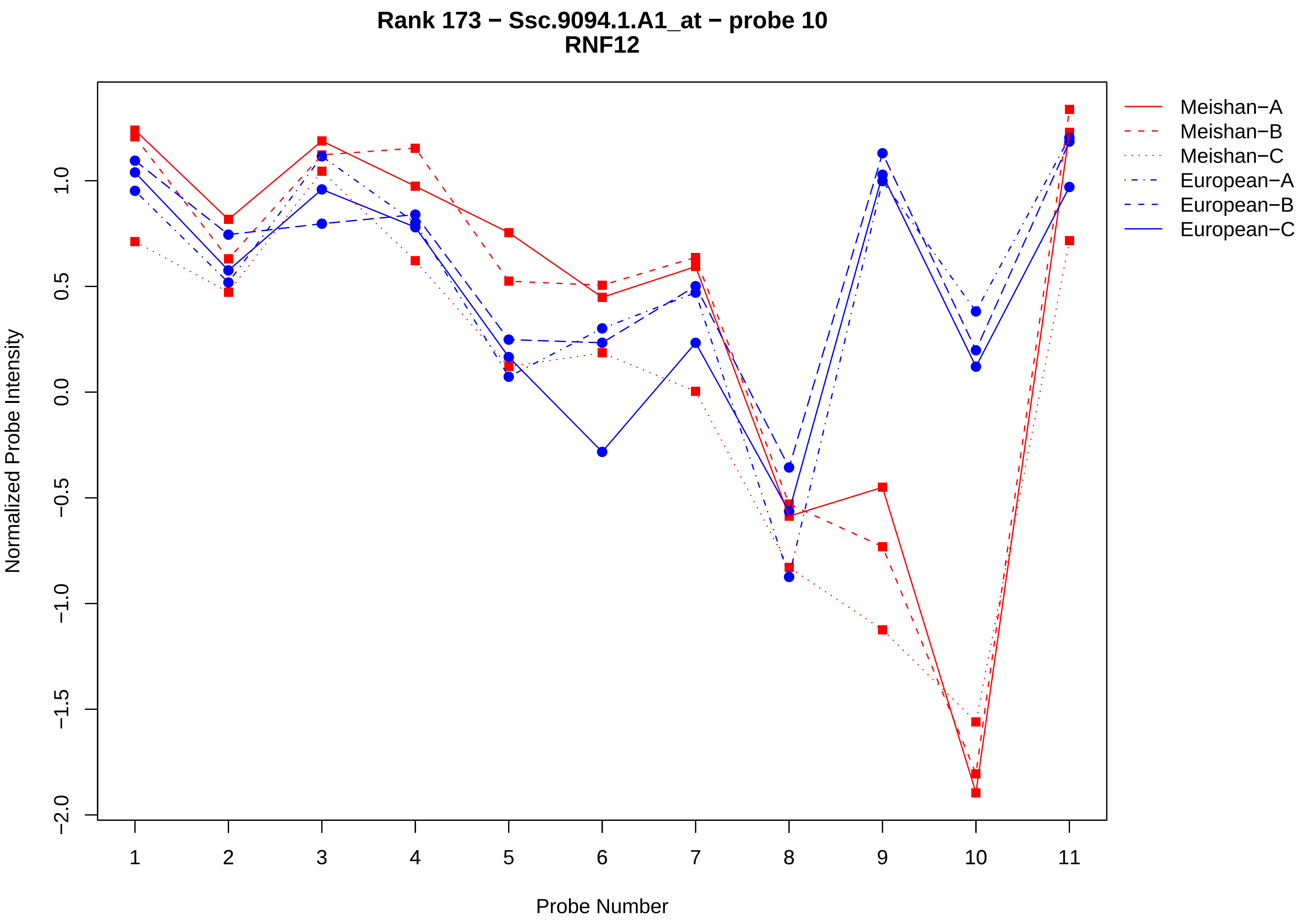
Rank 173 − Ssc.9094.1.A1_at − probe 10
RNF12
Normalized Probe Intensity
Probe Number
Meishan−A
Meishan−B
Meishan−C
European−A
European−B
European−C
−2.0
−1.5
−1.0
−0.5
0.0
0.5
1.0
1
2
3
4
5
6
7
8
9
10
11

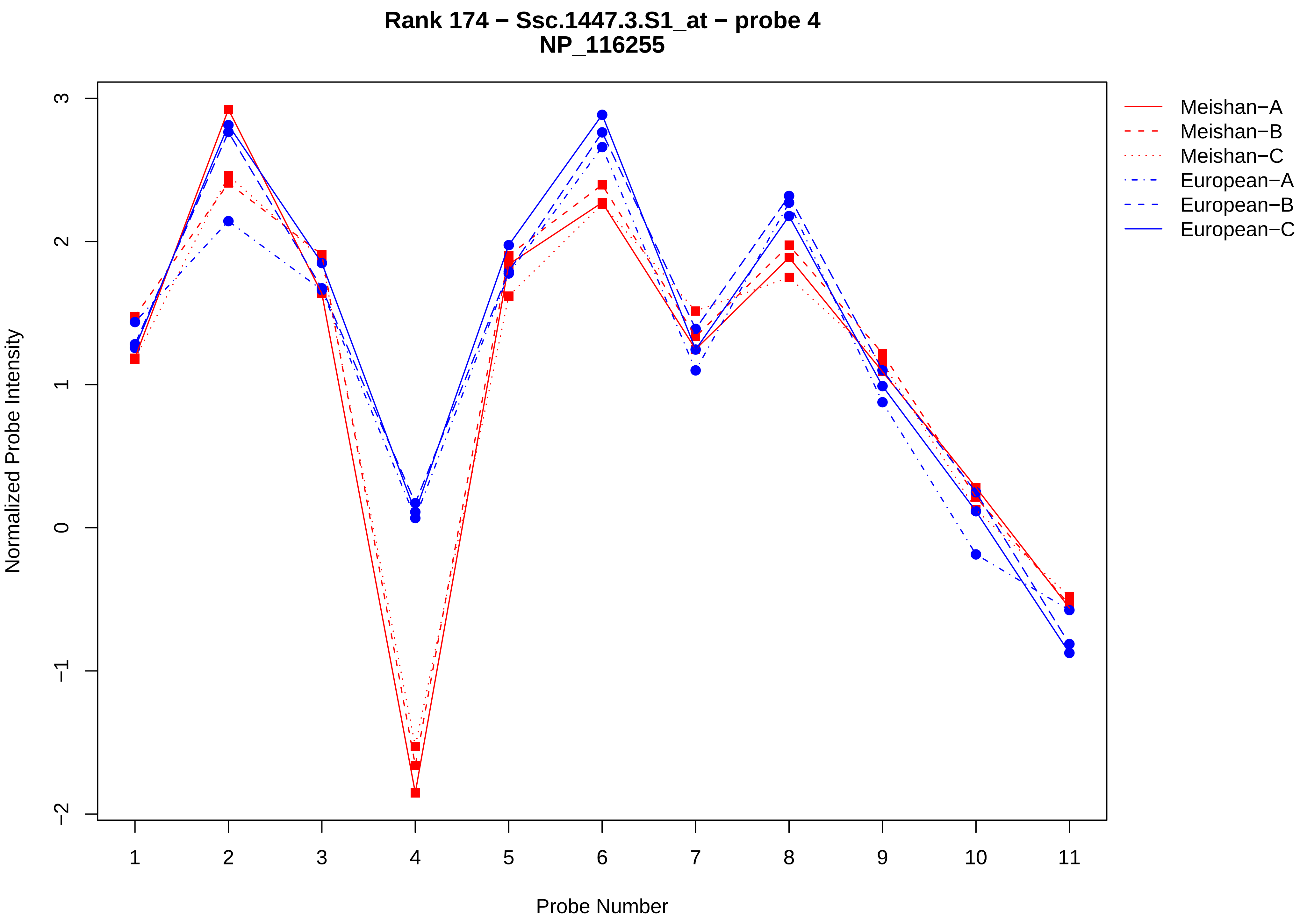

Rank 174 − Ssc.1447.3.S1_at − probe 4
NP_116255
Normalized Probe Intensity
Probe Number
Meishan−A
Meishan−B
Meishan−C
European−A
European−B
European−C

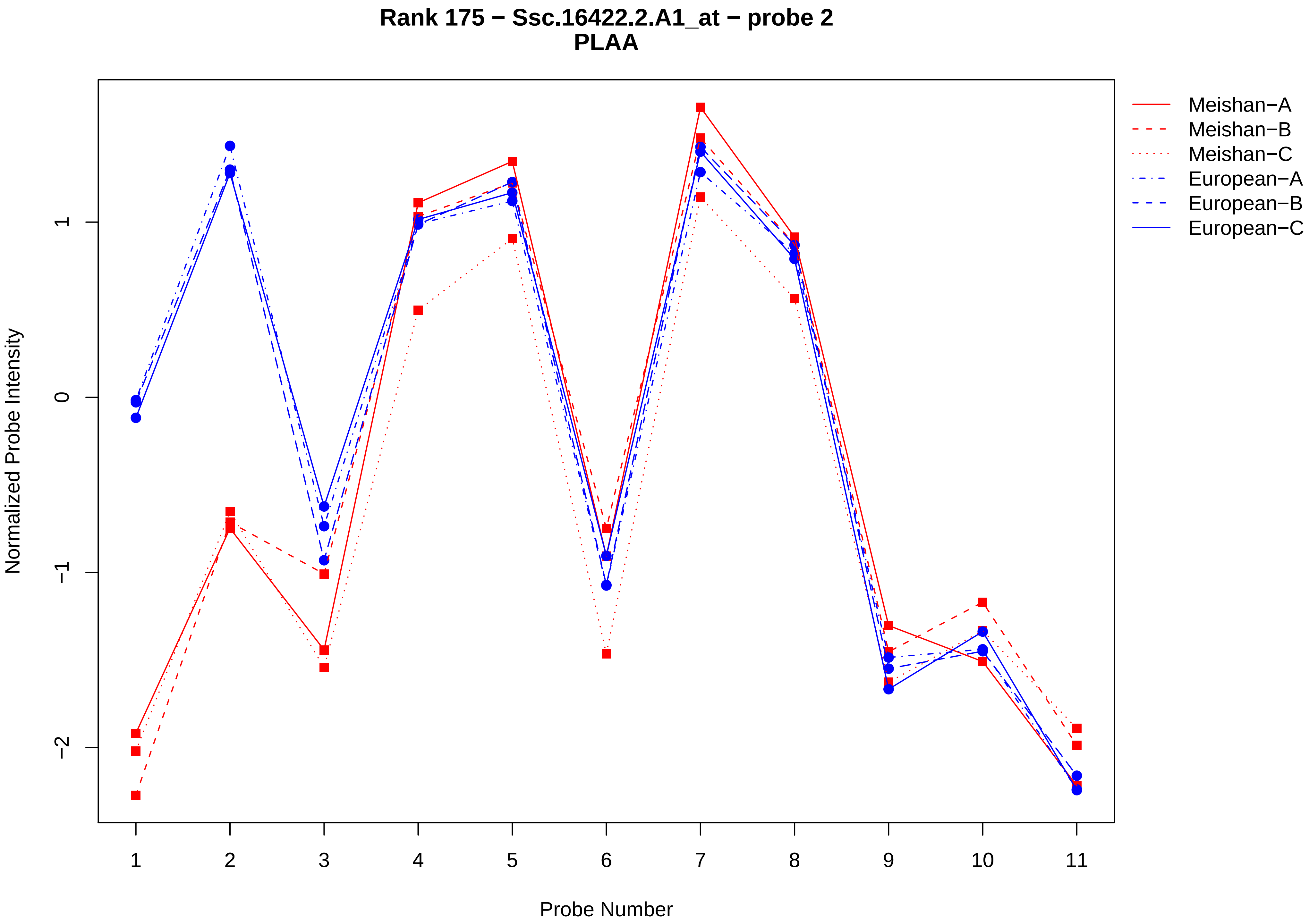

Rank 175 − Ssc.16422.2.A1_at − probe 2
PLAA
Normalized Probe Intensity
Probe Number
−2
−1
0
1
1
2
3
4
5
6
7
8
9
10
11
Meishan−A
Meishan−B
Meishan−C
European−A
European−B
European−C

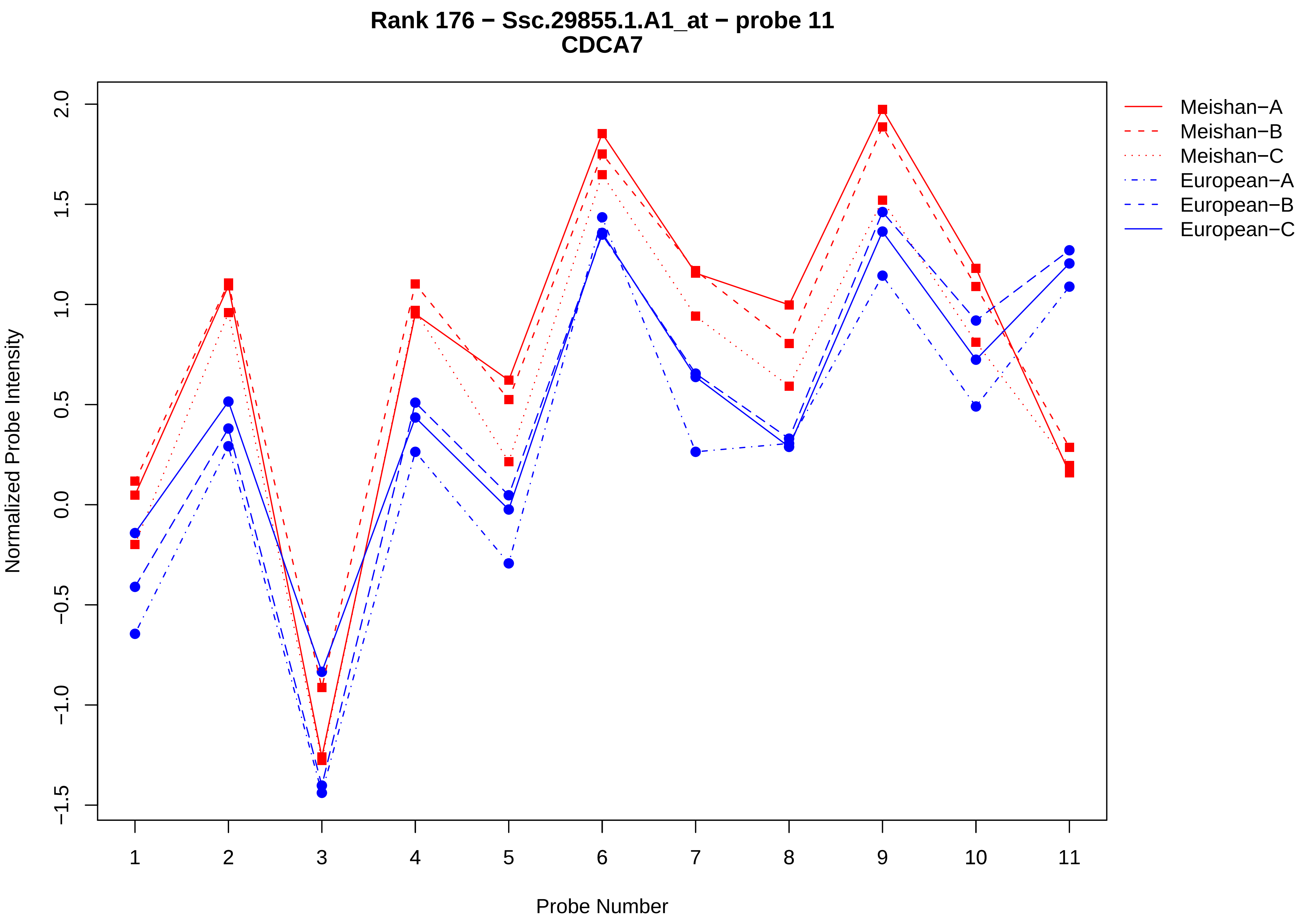

Rank 176 − Ssc.29855.1.A1_at − probe 11
CDCA7
Normalized Probe Intensity
Probe Number
−1.5
−1.0
−0.5
0.0
0.5
1.0
1.5
2.0
1
2
3
4
5
6
7
8
9
10
11
Meishan−A
Meishan−B
Meishan−C
European−A
European−B
European−C

Rank 177 − Ssc.20269.1.A1_at − probe 9
RCBTB1

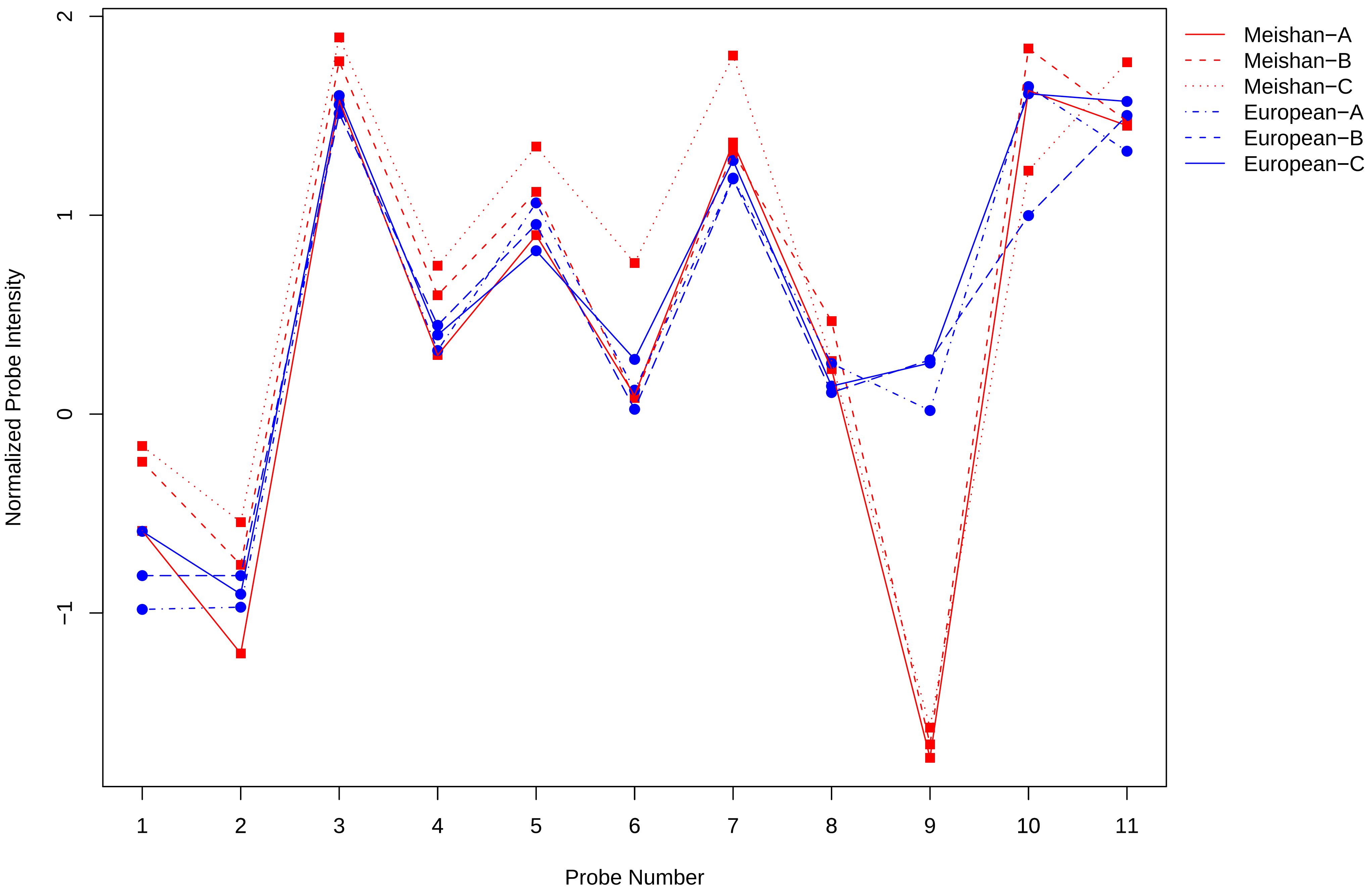

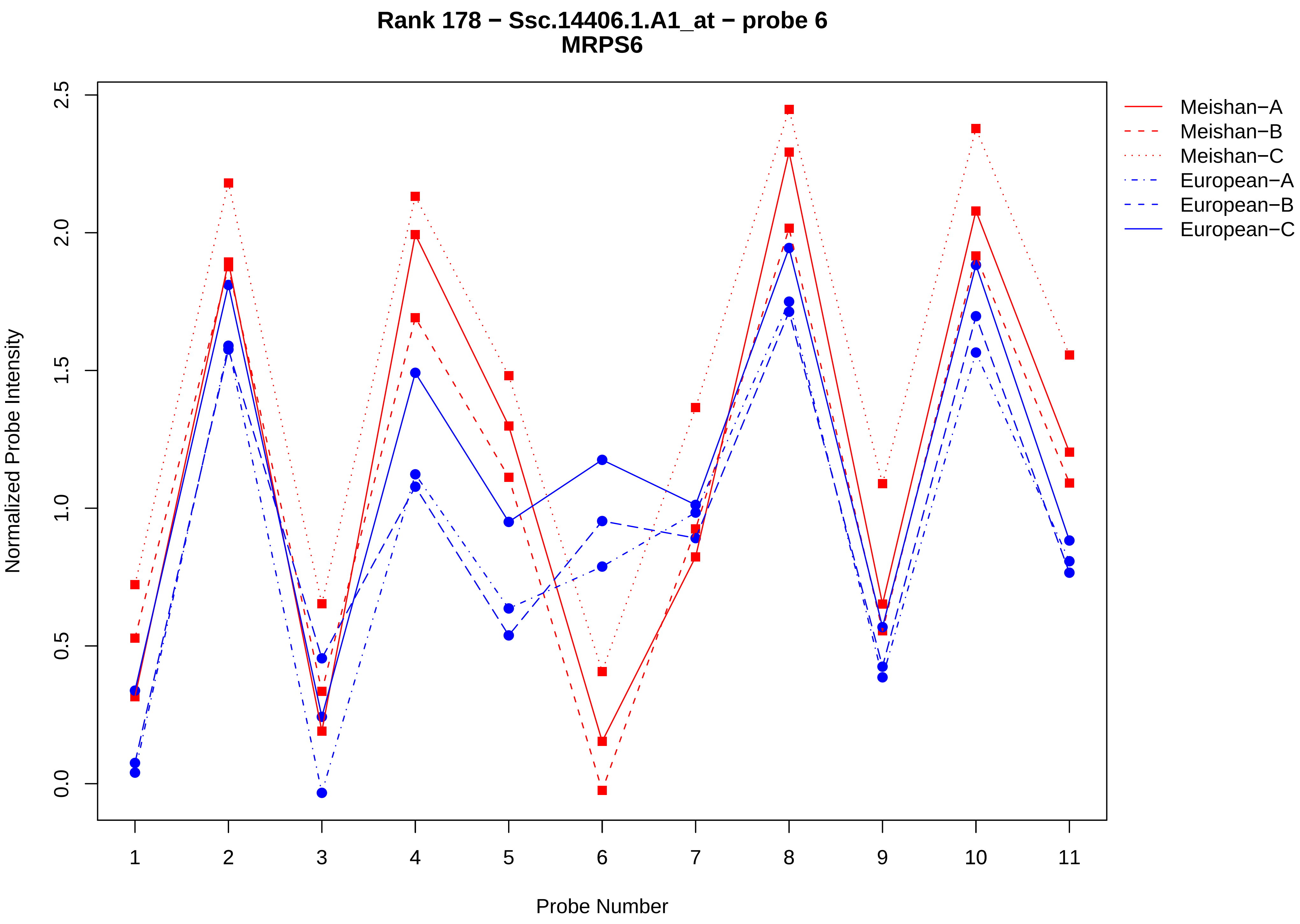
Rank 178 − Ssc.14406.1.A1_at − probe 6
MRPS6
Normalized Probe Intensity
Probe Number
0.0
0.5
1.0
1.5
2.0
2.5
1
2
3
4
5
6
7
8
9
10
11
Meishan−A
Meishan−B
Meishan−C
European−A
European−B
European−C

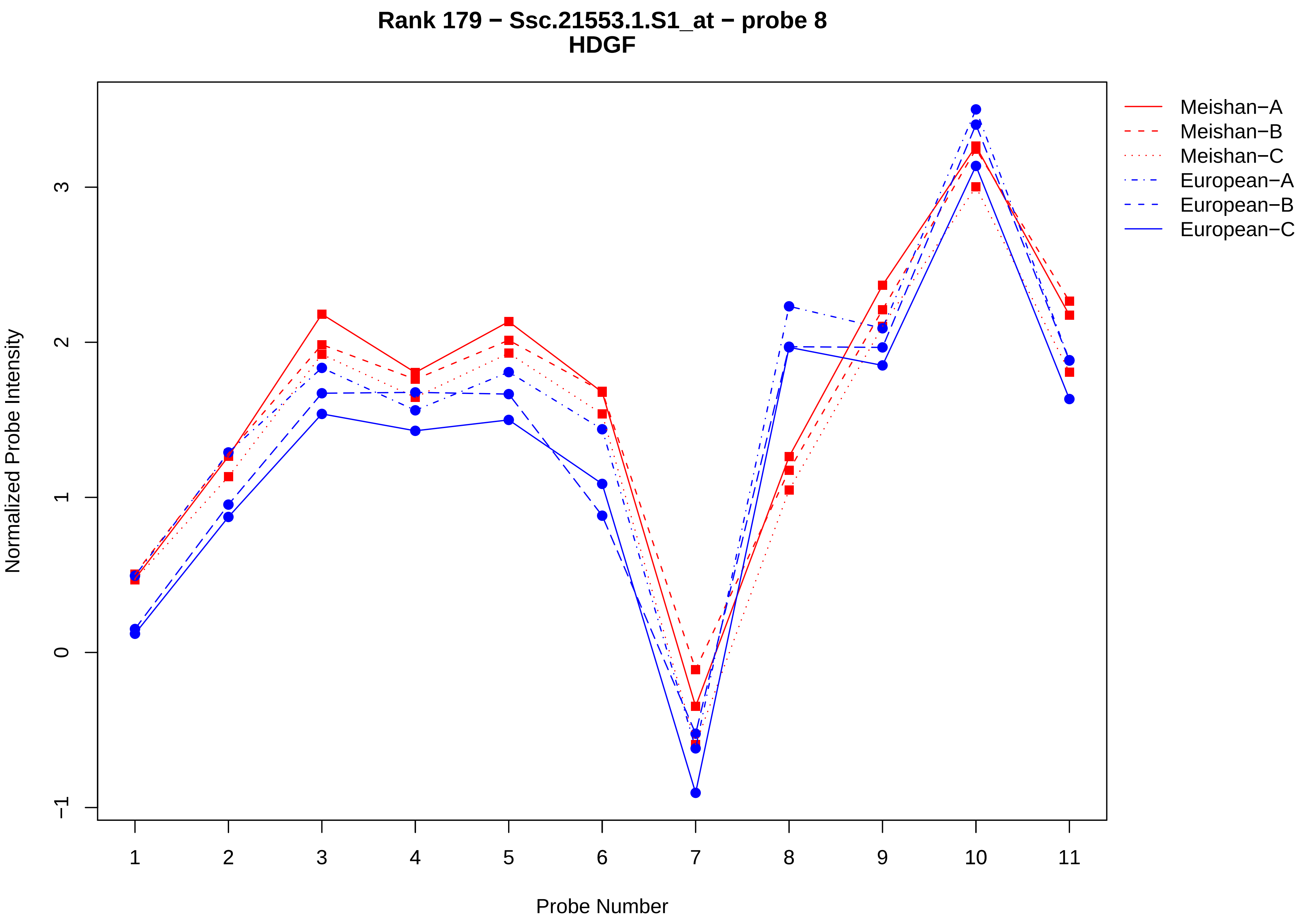

Rank 179 − Ssc.21553.1.S1_at − probe 8
HDGF
Normalized Probe Intensity
−1
0
1
2
3
1
2
3
4
5
6
7
8
9
10
11
Probe Number
Meishan−A
Meishan−B
Meishan−C
European−A
European−B
European−C

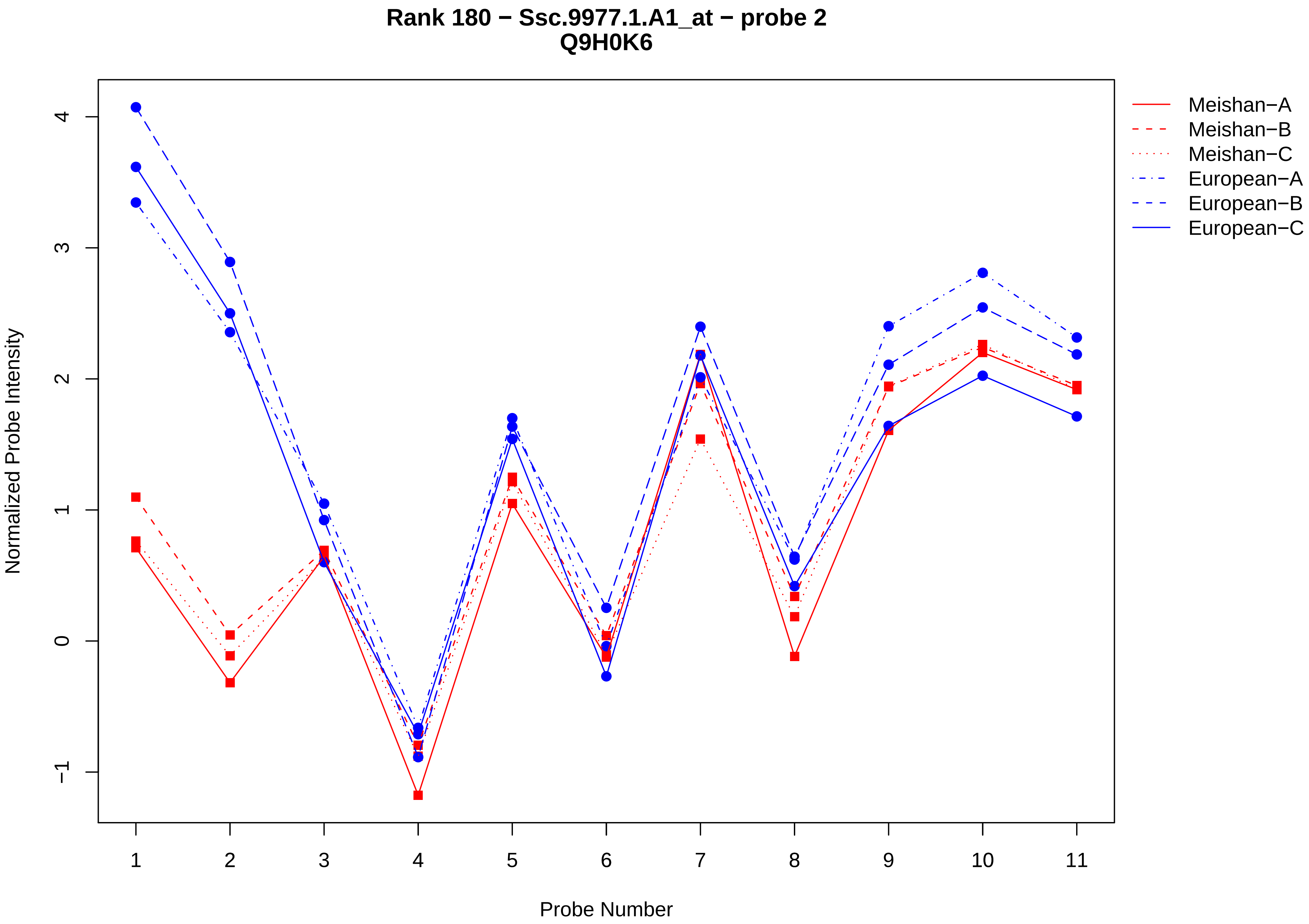
Rank 180 − Ssc.9977.1.A1_at − probe 2
Q9H0K6
Normalized Probe Intensity
Probe Number
Meishan−A
Meishan−B
Meishan−C
European−A
European−B
European−C
−1
0
1
2
3
4
1
2
3
4
5
6
7
8
9
10
11

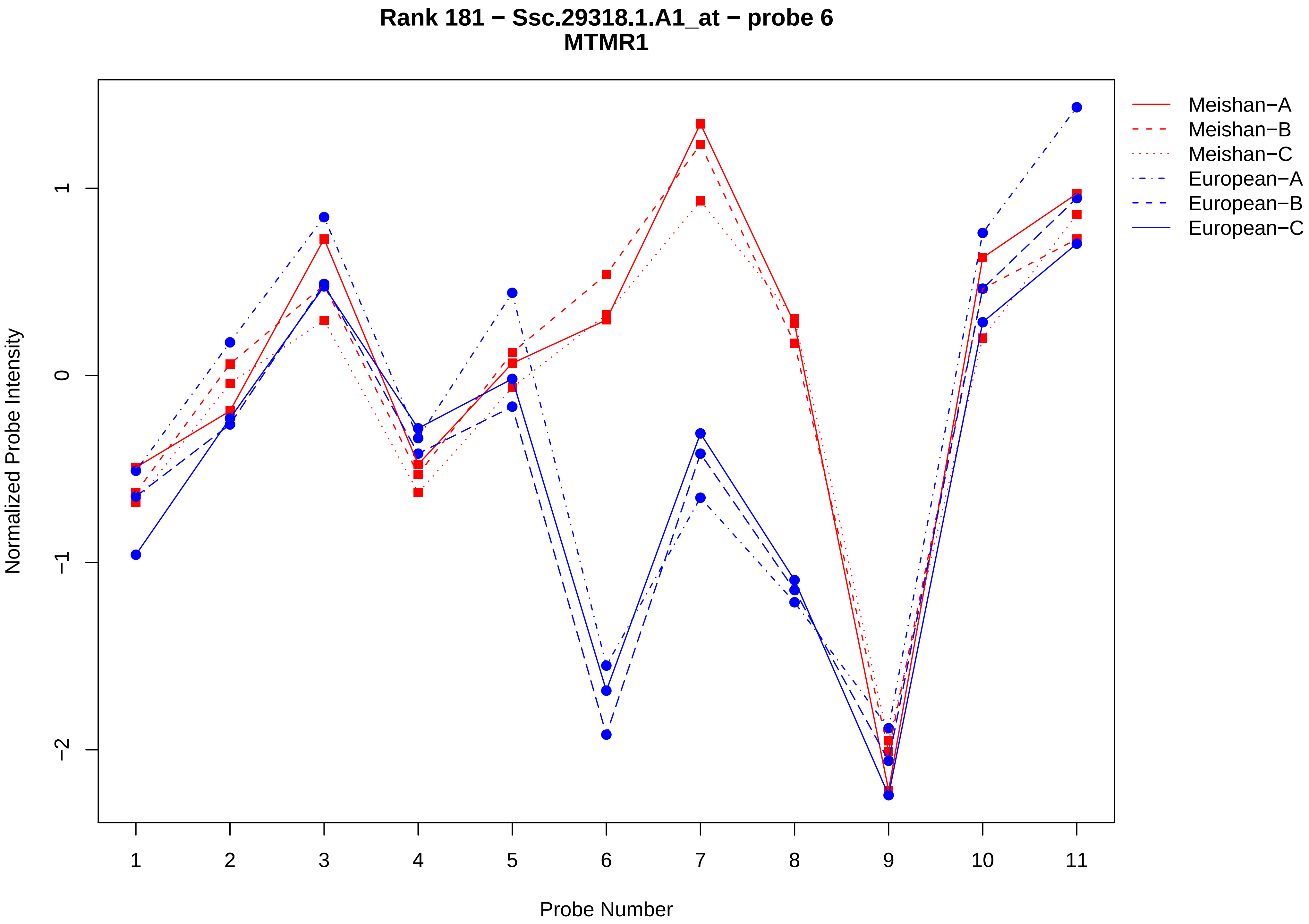

Rank 181 − Ssc.29318.1.A1_at − probe 6
MTMR1
Normalized Probe Intensity
Probe Number
1
0
−1
−2
1
2
3
4
5
6
7
8
9
10
11
Meishan−A
Meishan−B
Meishan−C
European−A
European−B
European−C

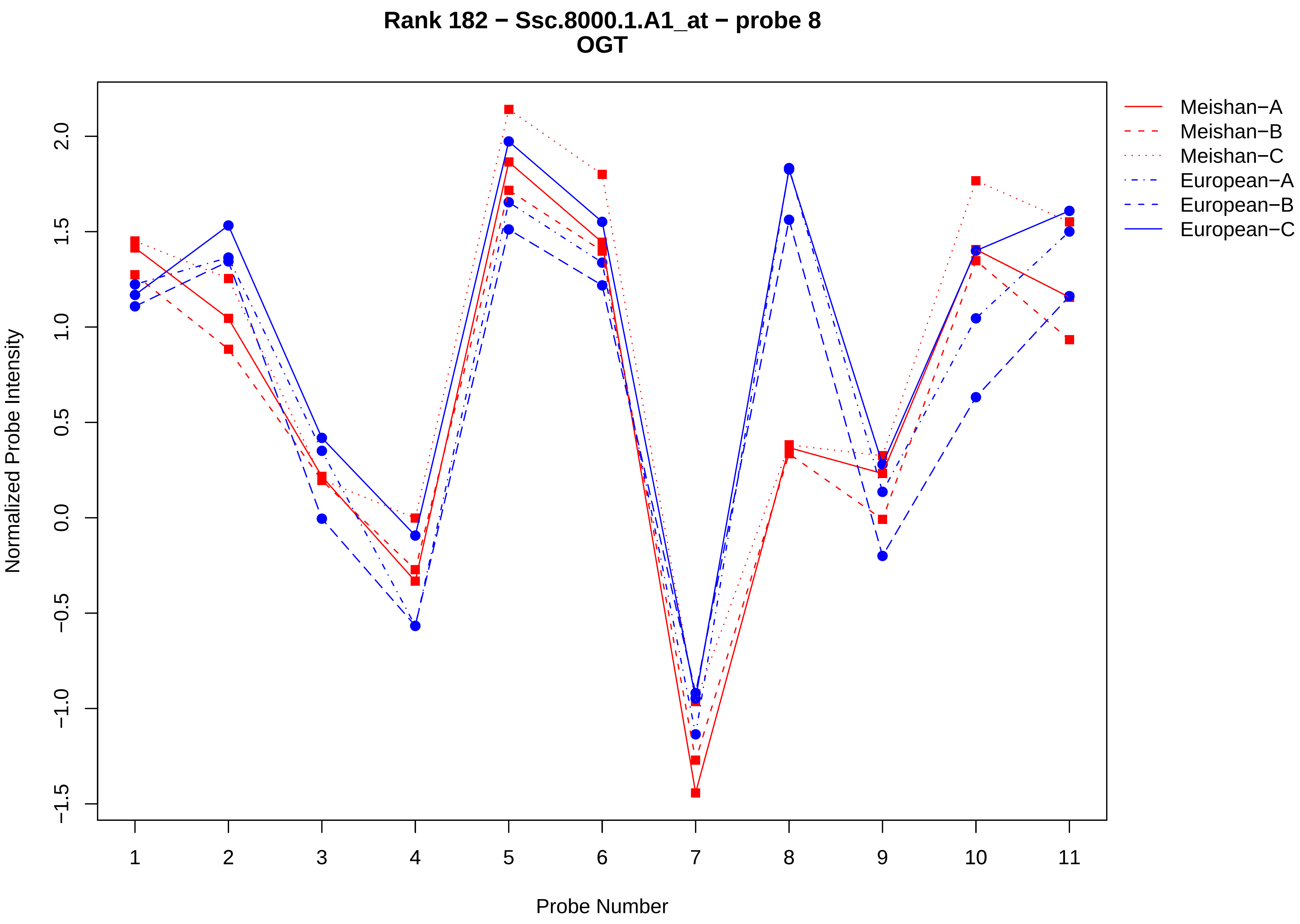

Rank 182 − Ssc.8000.1.A1_at − probe 8
OGT
Normalized Probe Intensity
Probe Number
−1.5
−1.0
−0.5
0.0
0.5
1.0
1.5
2.0
1
2
3
4
5
6
7
8
9
10
11
Meishan−A
Meishan−B
Meishan−C
European−A
European−B
European−C

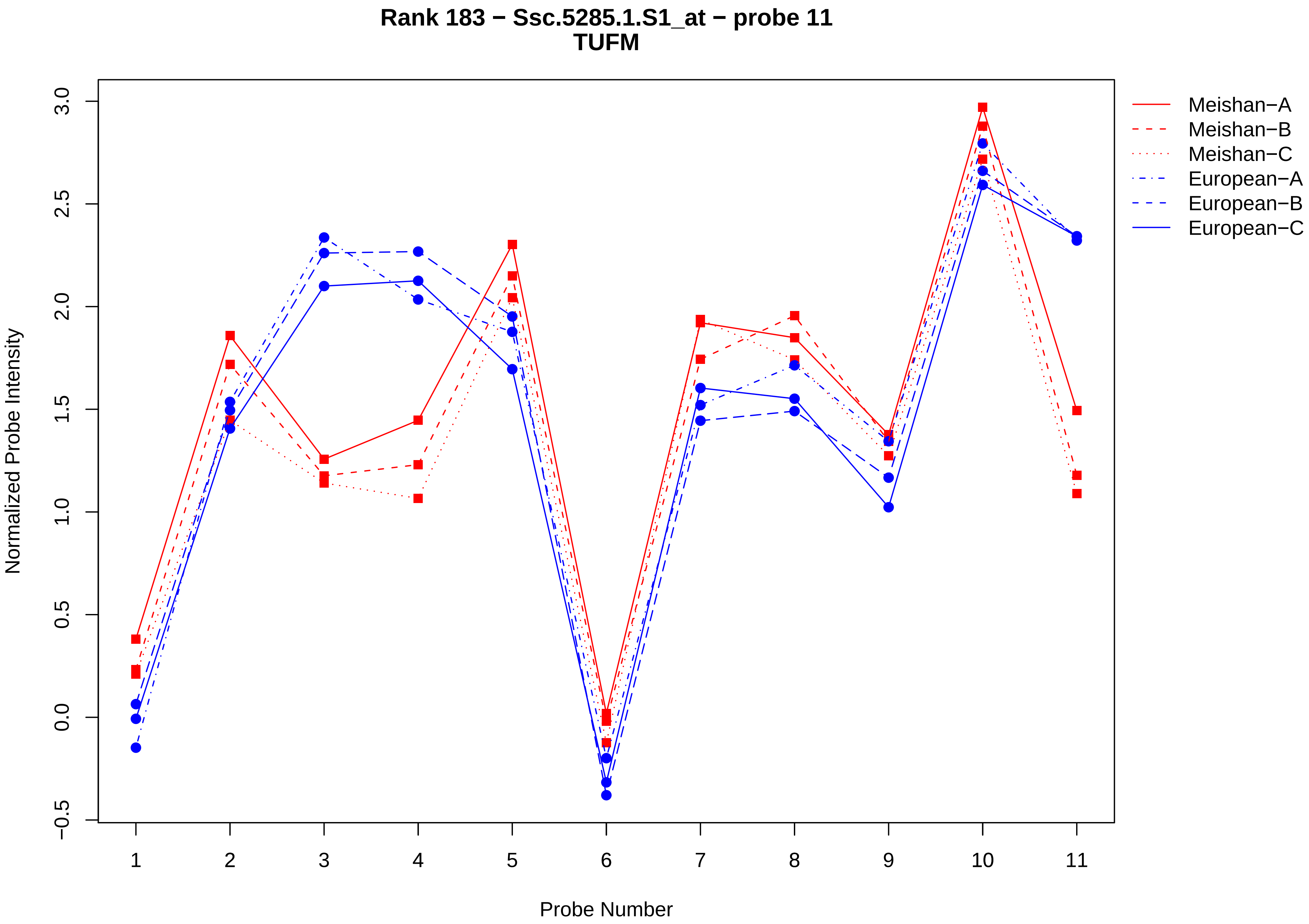

Rank 183 − Ssc.5285.1.S1_at − probe 11
TUFM
Normalized Probe Intensity
Probe Number
−0.5
0.0
0.5
1.0
1.5
2.0
2.5
3.0
1
2
3
4
5
6
7
8
9
10
11
Meishan−A
Meishan−B
Meishan−C
European−A
European−B
European−C

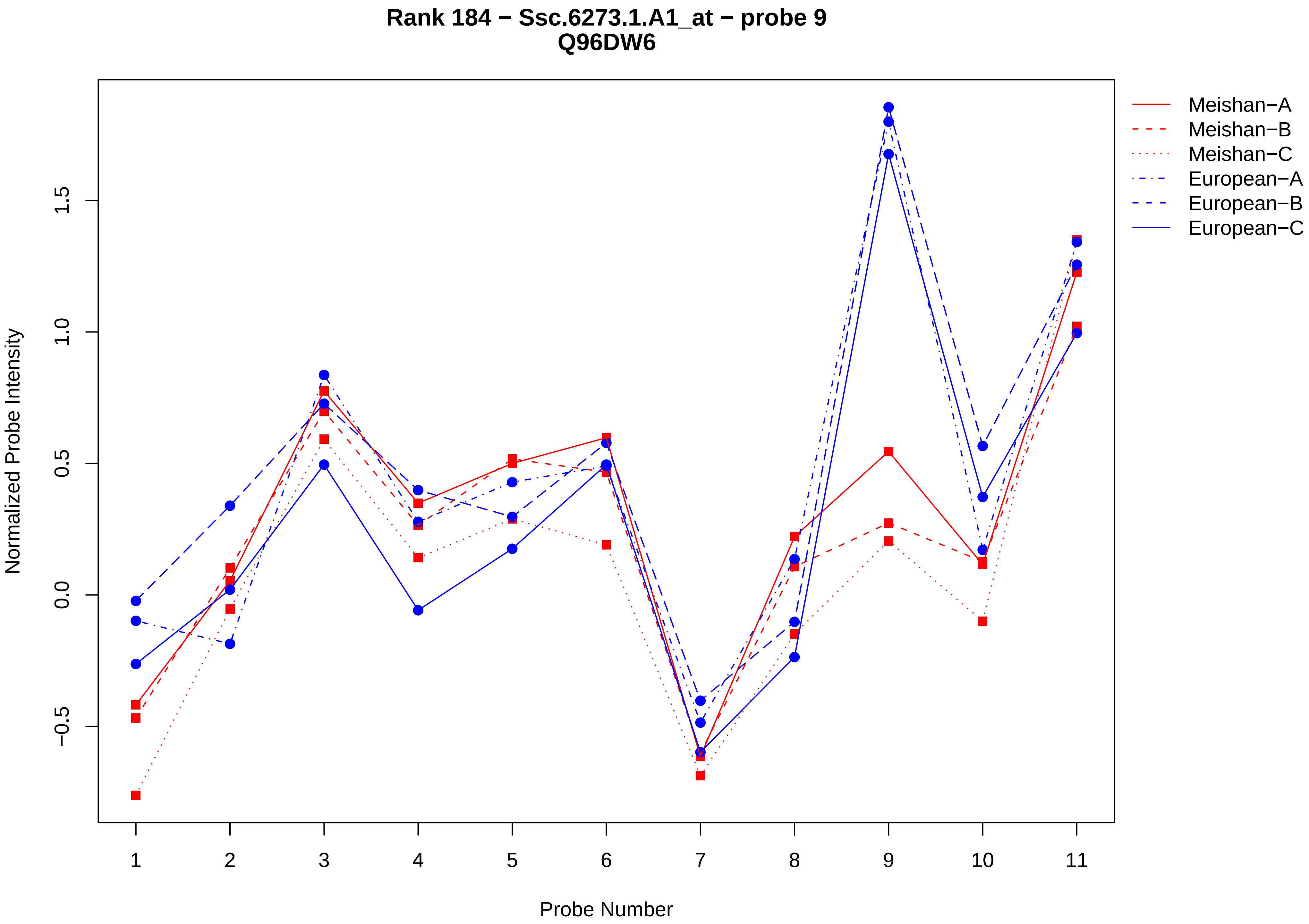

Rank 184 − Ssc.6273.1.A1_at − probe 9
Q96DW6
Normalized Probe Intensity
Probe Number
−0.5
0.0
0.5
1.0
1.5
1
2
3
4
5
6
7
8
9
10
11
Meishan−A
Meishan−B
Meishan−C
European−A
European−B
European−C

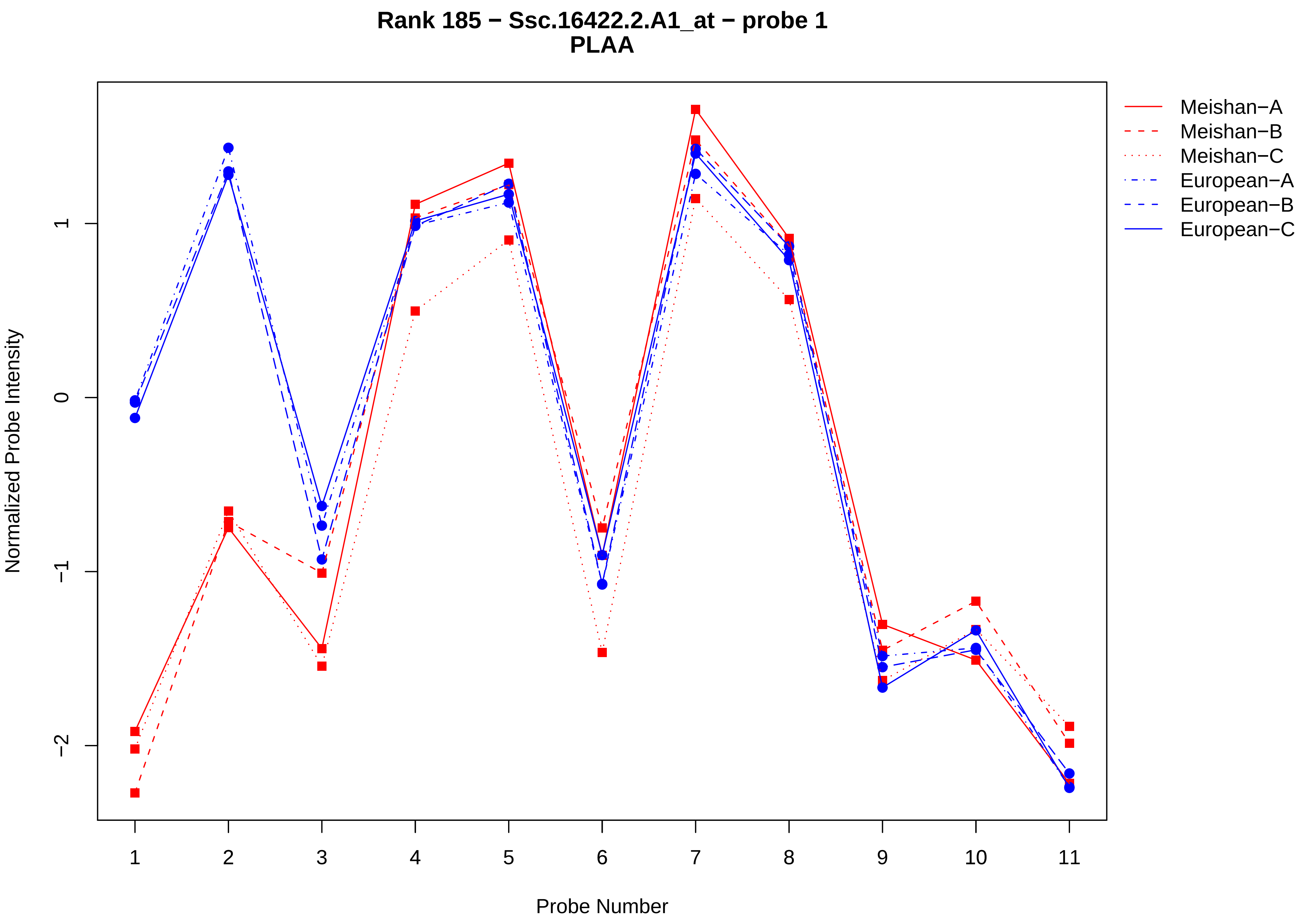
Rank 185 − Ssc.16422.2.A1_at − probe 1
PLAA
Normalized Probe Intensity
Probe Number
−2
−1
0
1
1
2
3
4
5
6
7
8
9
10
11
Meishan−A
Meishan−B
Meishan−C
European−A
European−B
European−C

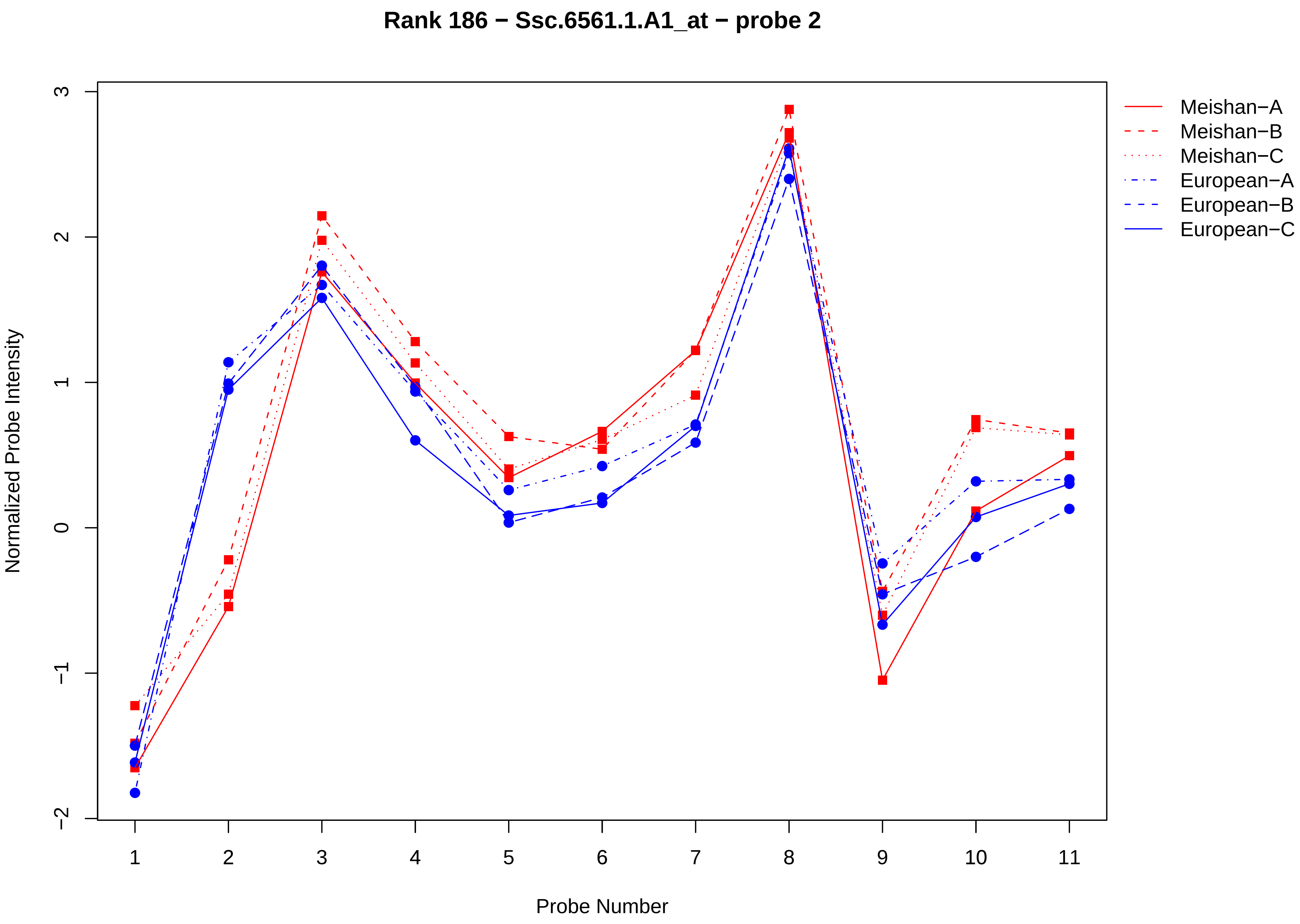
Rank 186 − Ssc.6561.1.A1_at − probe 2
Normalized Probe Intensity
Probe Number
Meishan−A
Meishan−B
Meishan−C
European−A
European−B
European−C
−2
−1
0
1
2
3
1
2
3
4
5
6
7
8
9
10
11

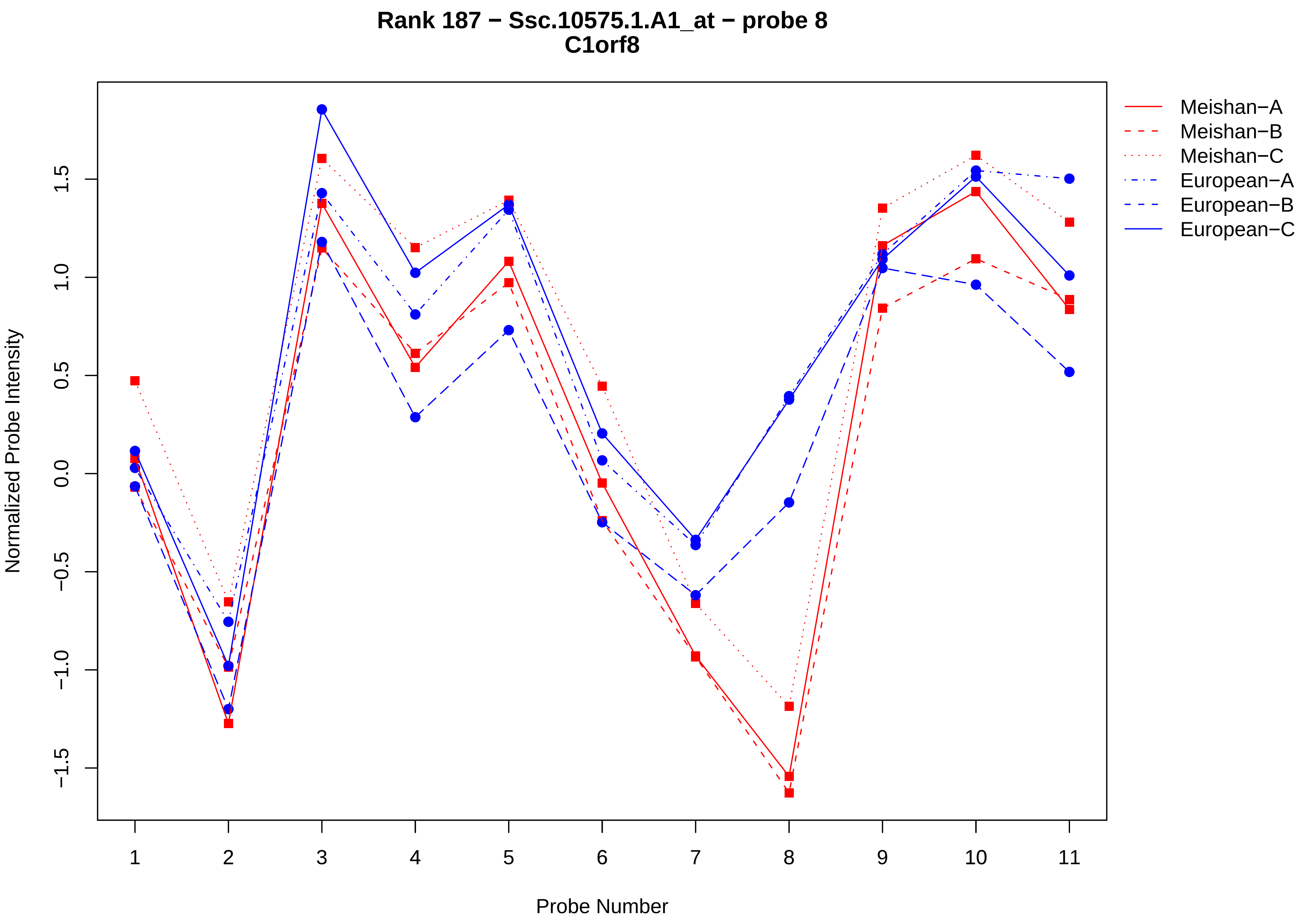
Rank 187 − Ssc.10575.1.A1_at − probe 8
C1orf8
Normalized Probe Intensity
Probe Number
−1.5
−1.0
−0.5
0.0
0.5
1.0
1.5
1
2
3
4
5
6
7
8
9
10
11
Meishan−A
Meishan−B
Meishan−C
European−A
European−B
European−C

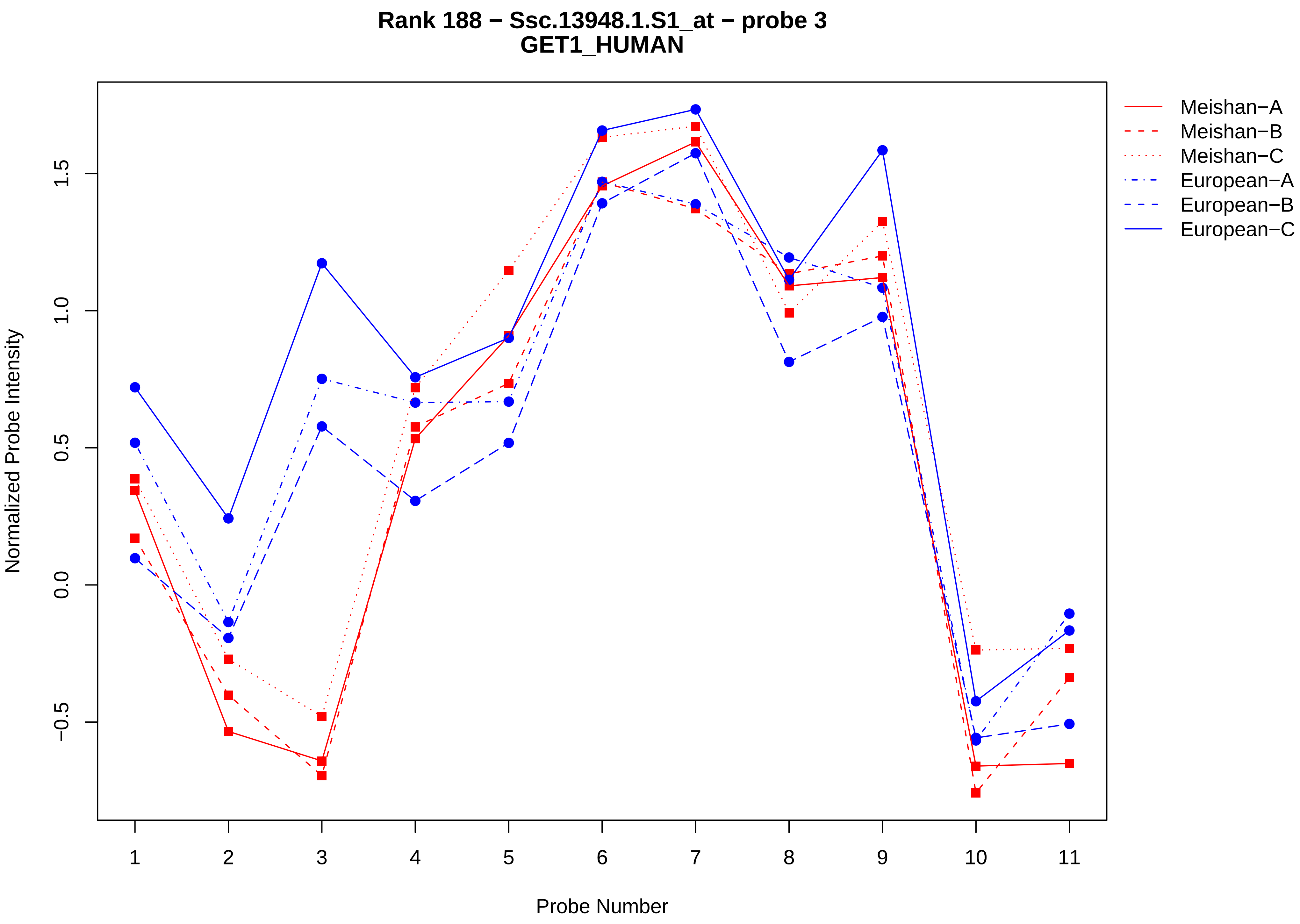
Rank 188 − Ssc.13948.1.S1_at − probe 3
GET1_HUMAN
Normalized Probe Intensity
Probe Number
−0.5
0.0
0.5
1.0
1.5
1
2
3
4
5
6
7
8
9
10
11
Meishan−A
Meishan−B
Meishan−C
European−A
European−B
European−C

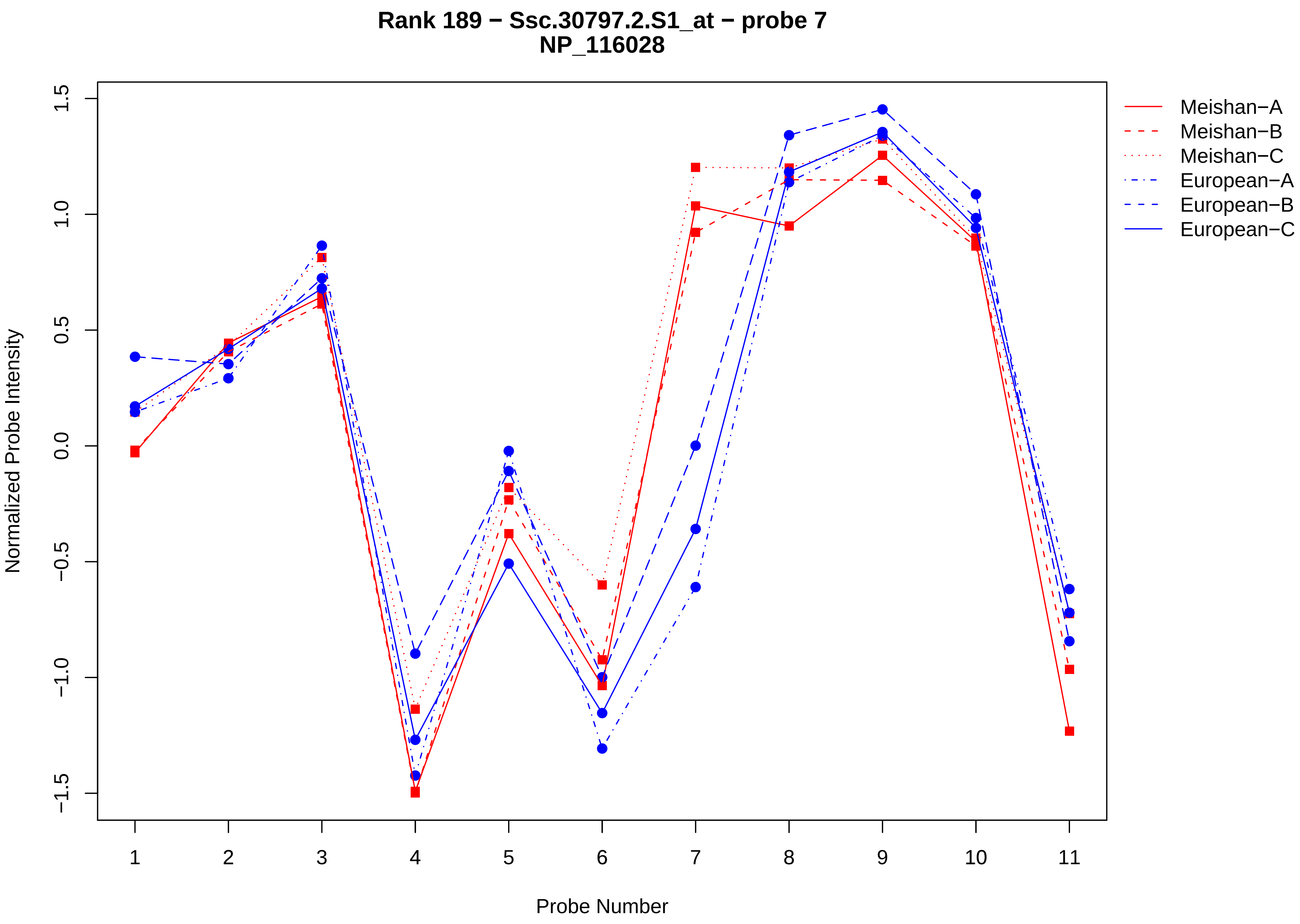
Rank 189 − Ssc.30797.2.S1_at − probe 7
NP_116028
Normalized Probe Intensity
Probe Number
1.5
1.0
0.5
0.0
−0.5
−1.0
−1.5
1
2
3
4
5
6
7
8
9
10
11
Meishan−A
Meishan−B
Meishan−C
European−A
European−B
European−C

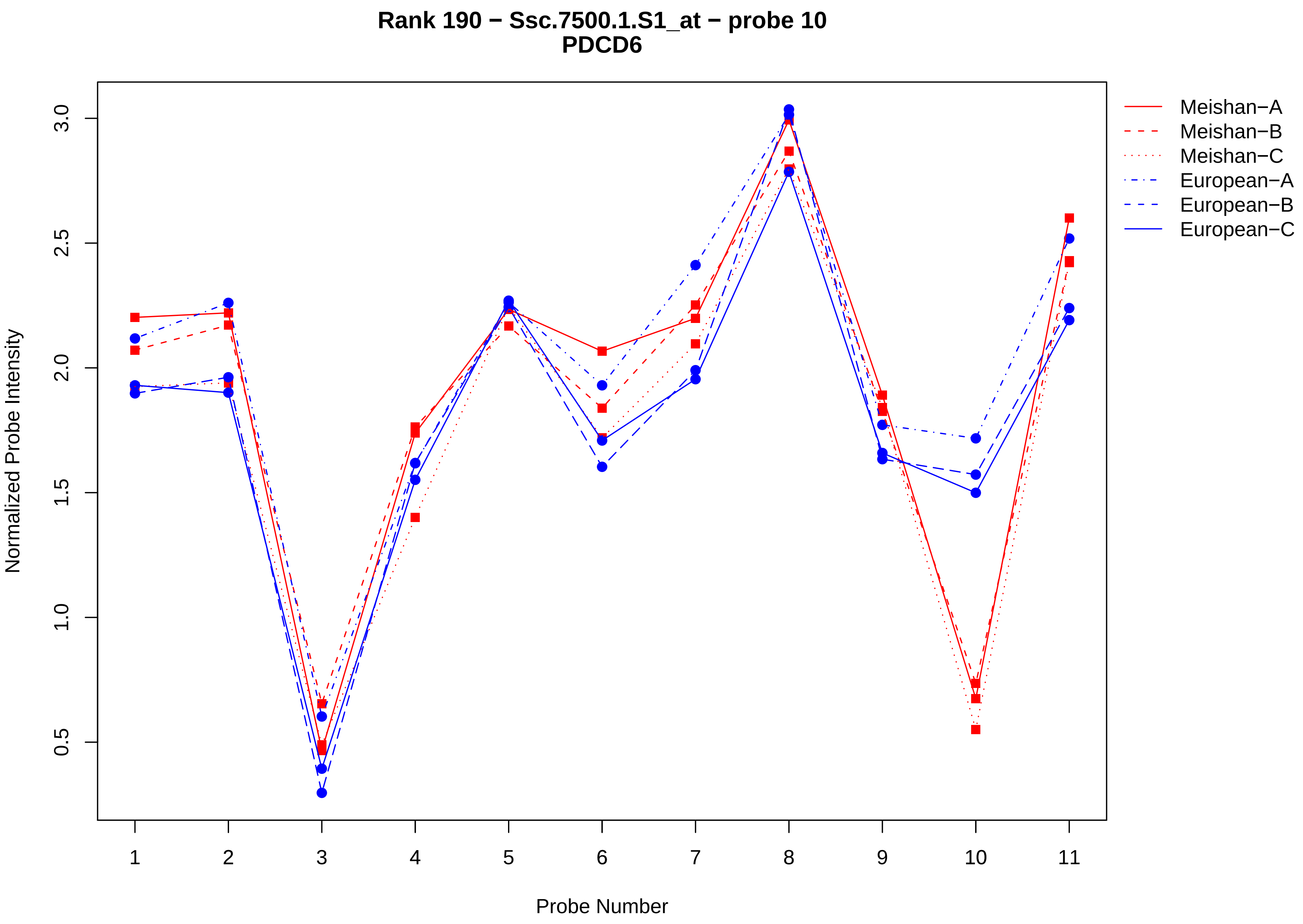

Rank 190 − Ssc.7500.1.S1_at − probe 10
PDCD6
Normalized Probe Intensity
0.5
1.0
1.5
2.0
2.5
3.0
1
2
3
4
5
6
7
8
9
10
11
Probe Number
Meishan−A
Meishan−B
Meishan−C
European−A
European−B
European−C

Rank 191 − Ssc.18069.1.A1_at − probe 6

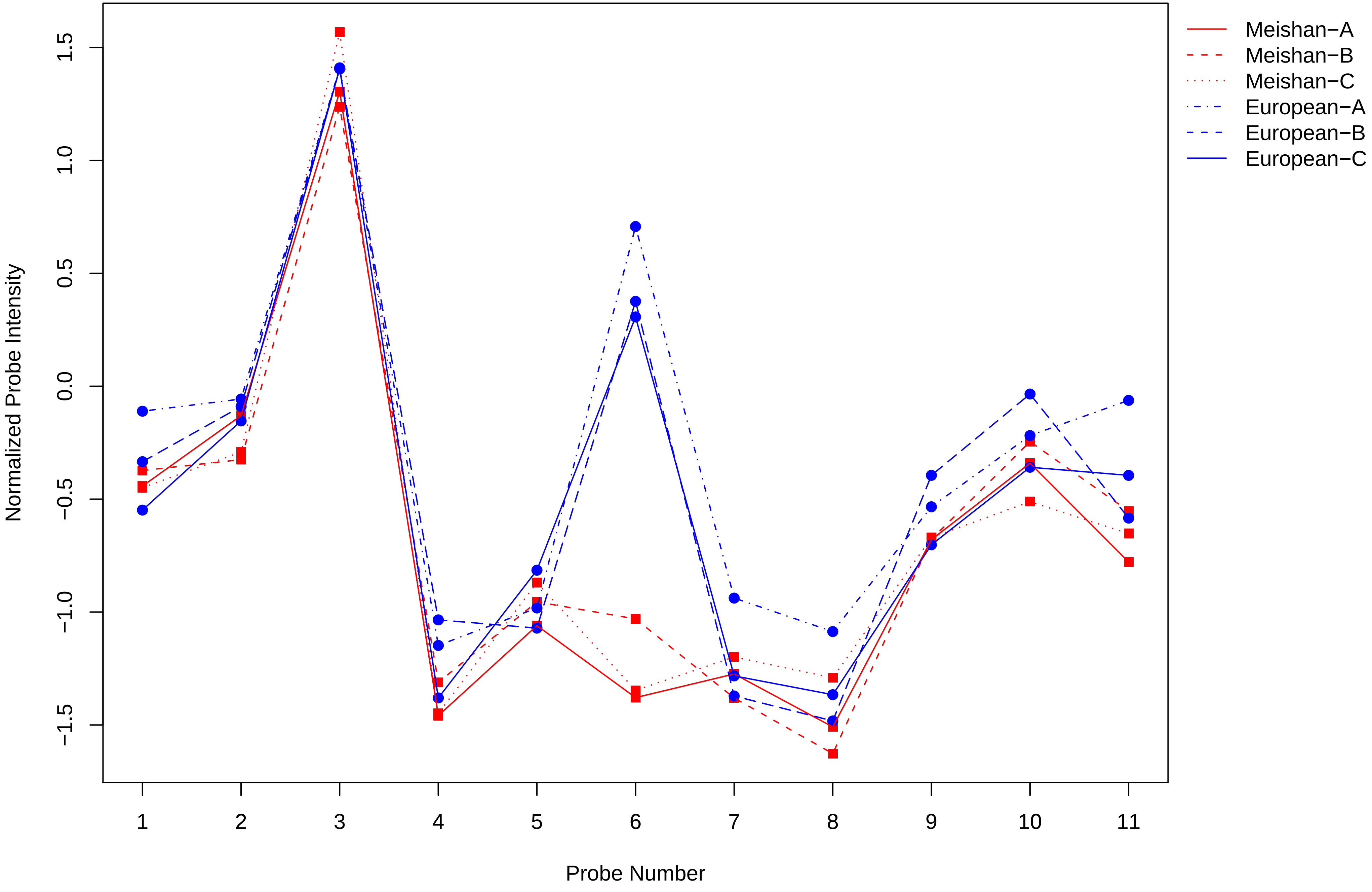

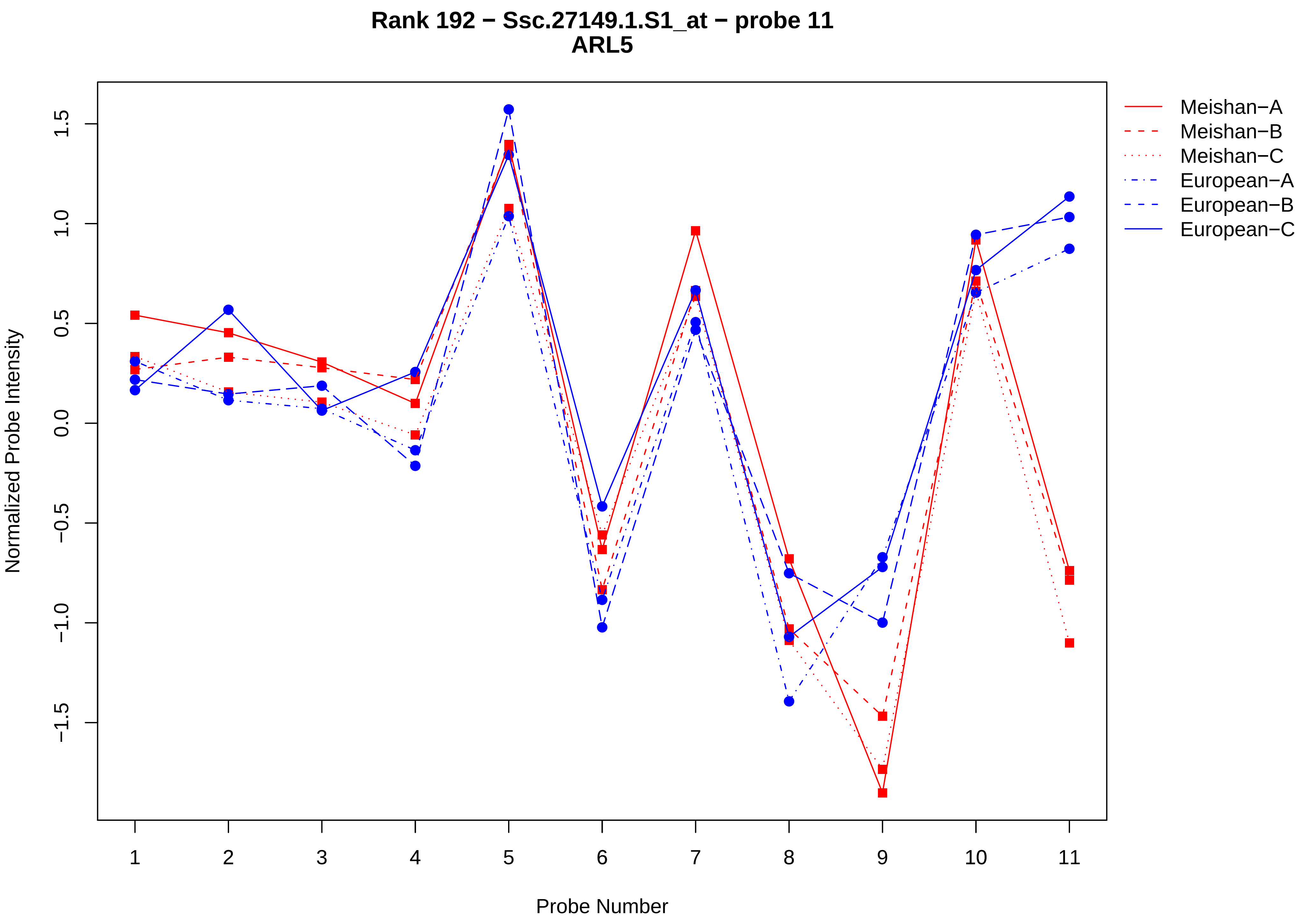

Rank 192 − Ssc.27149.1.S1_at − probe 11
ARL5
Normalized Probe Intensity
Probe Number
1.5
1.0
0.5
0.0
−0.5
−1.0
−1.5
1
2
3
4
5
6
7
8
9
10
11
Meishan−A
Meishan−B
Meishan−C
European−A
European−B
European−C

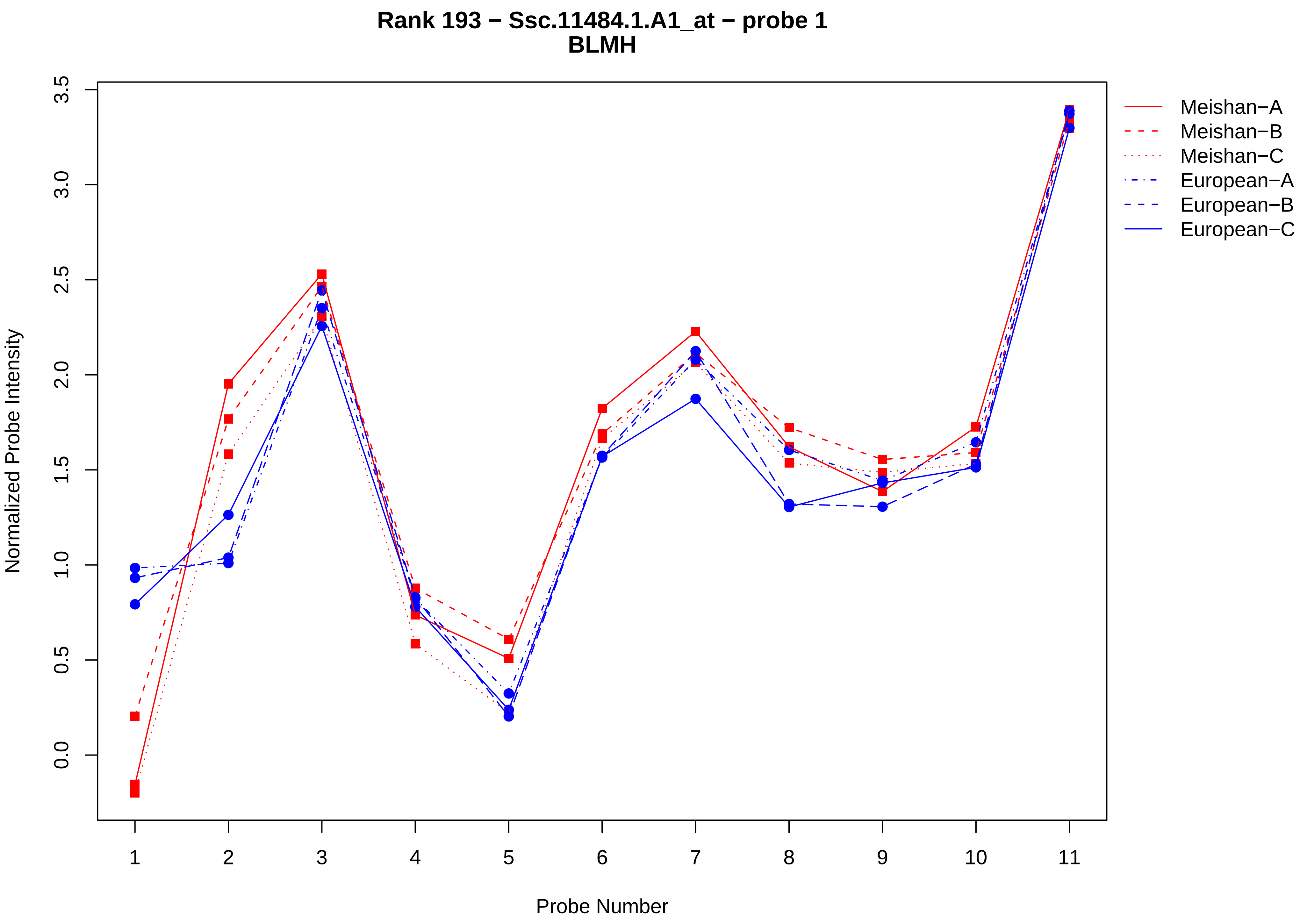

Rank 193 − Ssc.11484.1.A1_at − probe 1
BLMH
Normalized Probe Intensity
Probe Number
0.0
0.5
1.0
1.5
2.0
2.5
3.0
3.5
1
2
3
4
5
6
7
8
9
10
11
Meishan−A
Meishan−B
Meishan−C
European−A
European−B
European−C

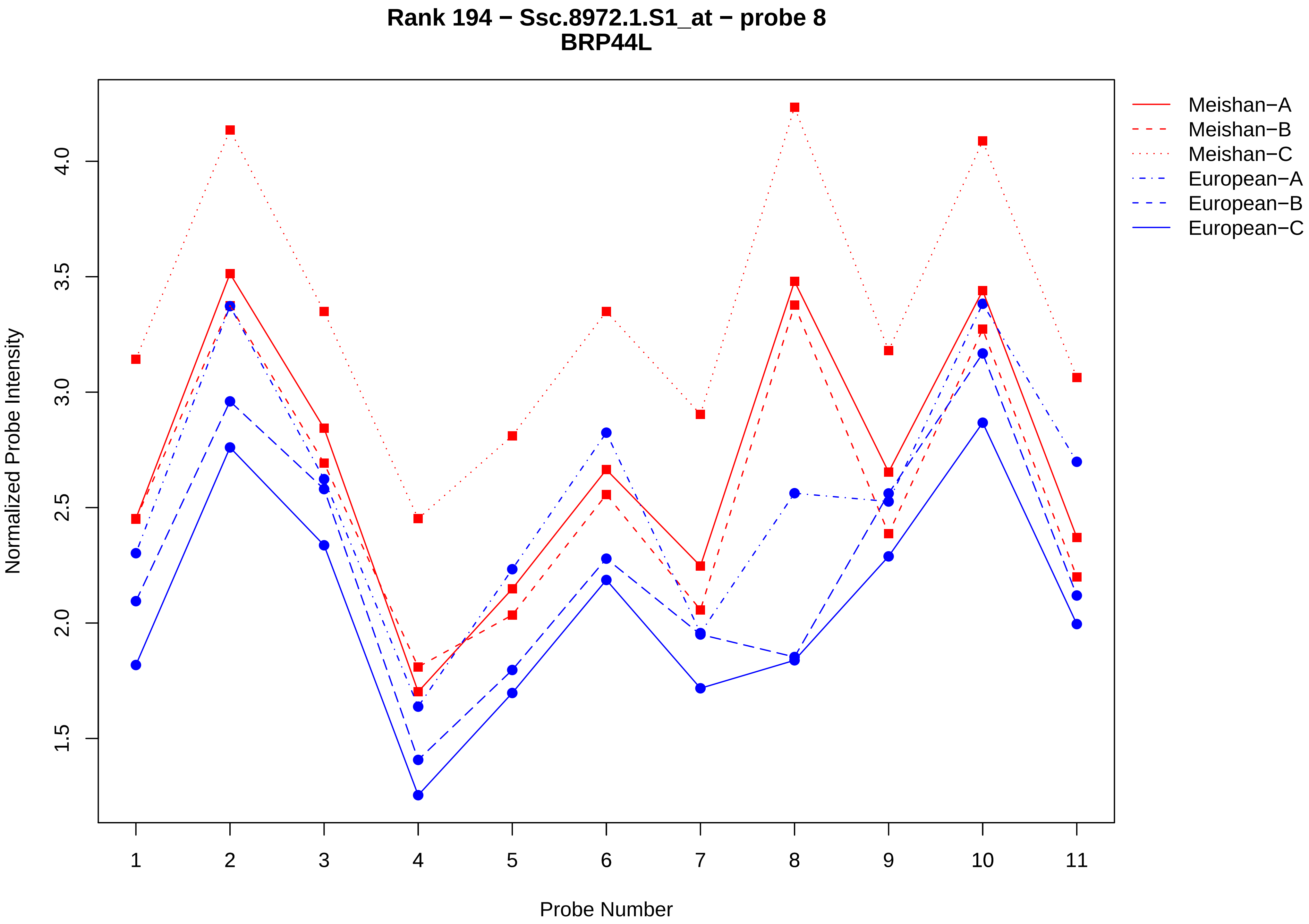
Rank 194 − Ssc.8972.1.S1_at − probe 8
BRP44L
Normalized Probe Intensity
Probe Number
1.5
2.0
2.5
3.0
3.5
4.0
1
2
3
4
5
6
7
8
9
10
11
Meishan−A
Meishan−B
Meishan−C
European−A
European−B
European−C

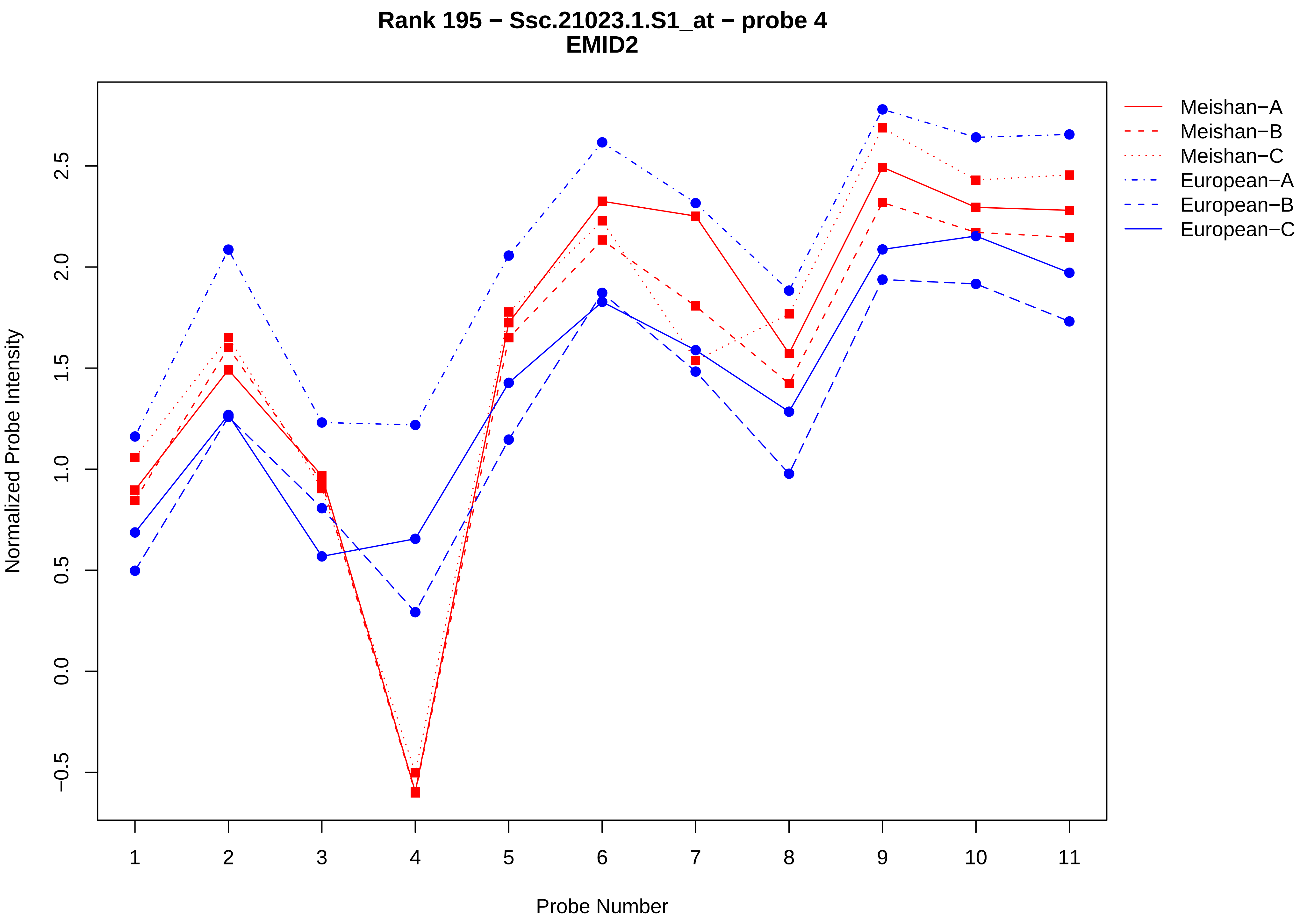

Rank 195 − Ssc.21023.1.S1_at − probe 4
EMID2
Normalized Probe Intensity
Probe Number
Meishan−A
Meishan−B
Meishan−C
European−A
European−B
European−C
−0.5
0.0
0.5
1.0
1.5
2.0
2.5
1
2
3
4
5
6
7
8
9
10
11

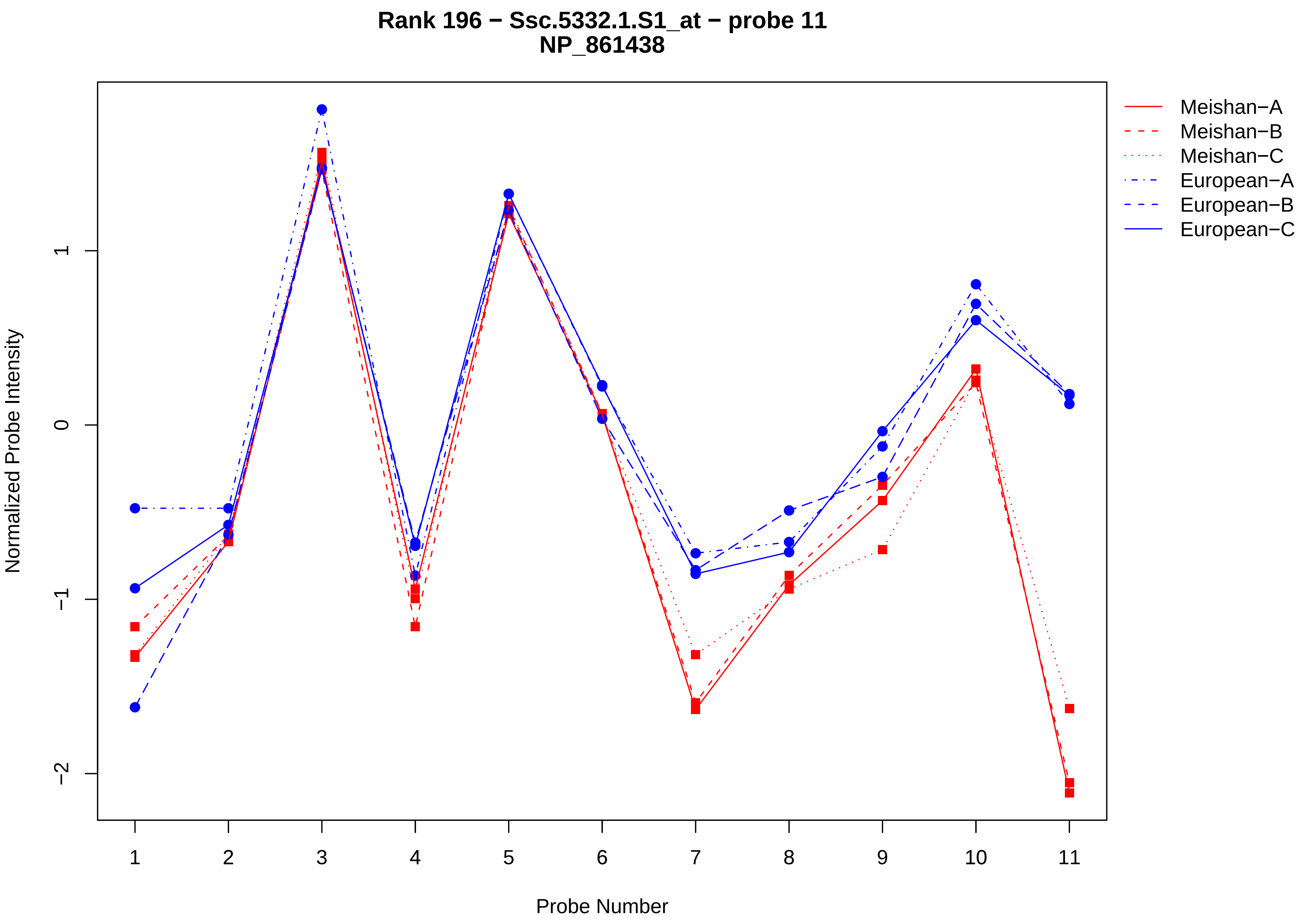
Rank 196 − Ssc.5332.1.S1_at − probe 11
NP_861438
Normalized Probe Intensity
Probe Number
−2
−1
0
1
1
2
3
4
5
6
7
8
9
10
11
Meishan−A
Meishan−B
Meishan−C
European−A
European−B
European−C

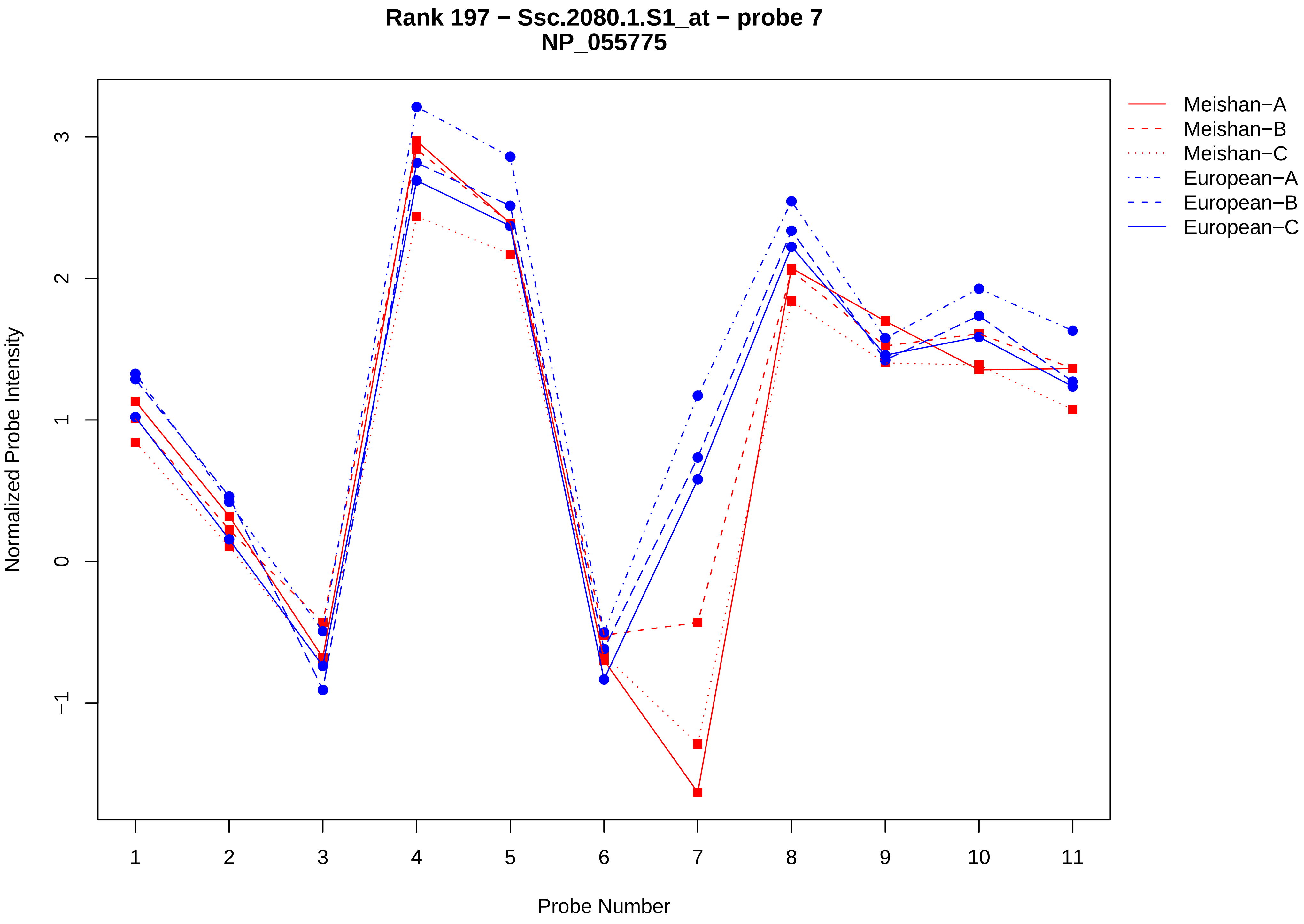
Rank 197 − Ssc.2080.1.S1_at − probe 7
NP_055775
Normalized Probe Intensity
Probe Number
Meishan−A
Meishan−B
Meishan−C
European−A
European−B
European−C

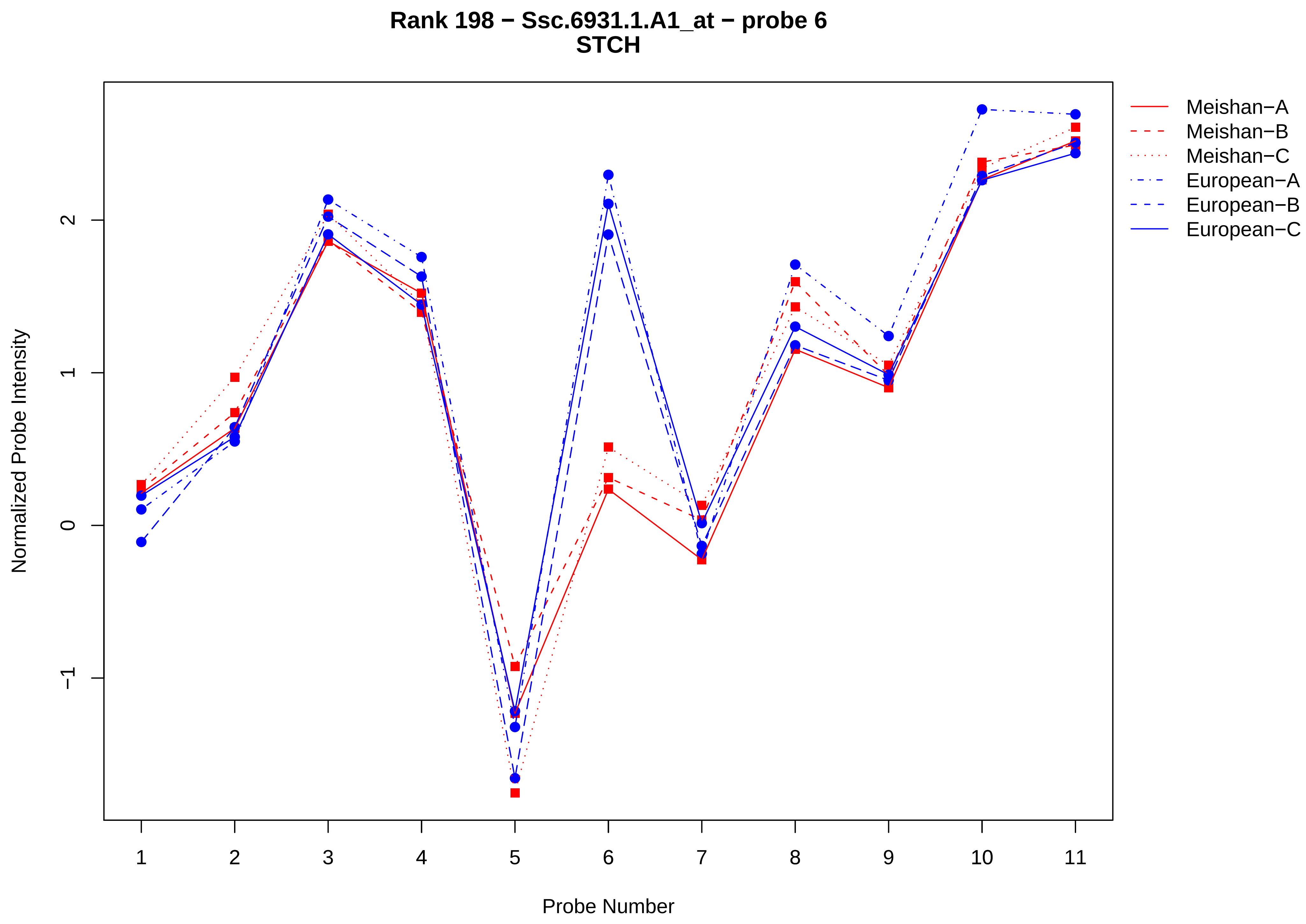

Rank 198 − Ssc.6931.1.A1_at − probe 6
STCH
Normalized Probe Intensity
Probe Number
−1
0
1
2
1
2
3
4
5
6
7
8
9
10
11
Meishan−A
Meishan−B
Meishan−C
European−A
European−B
European−C

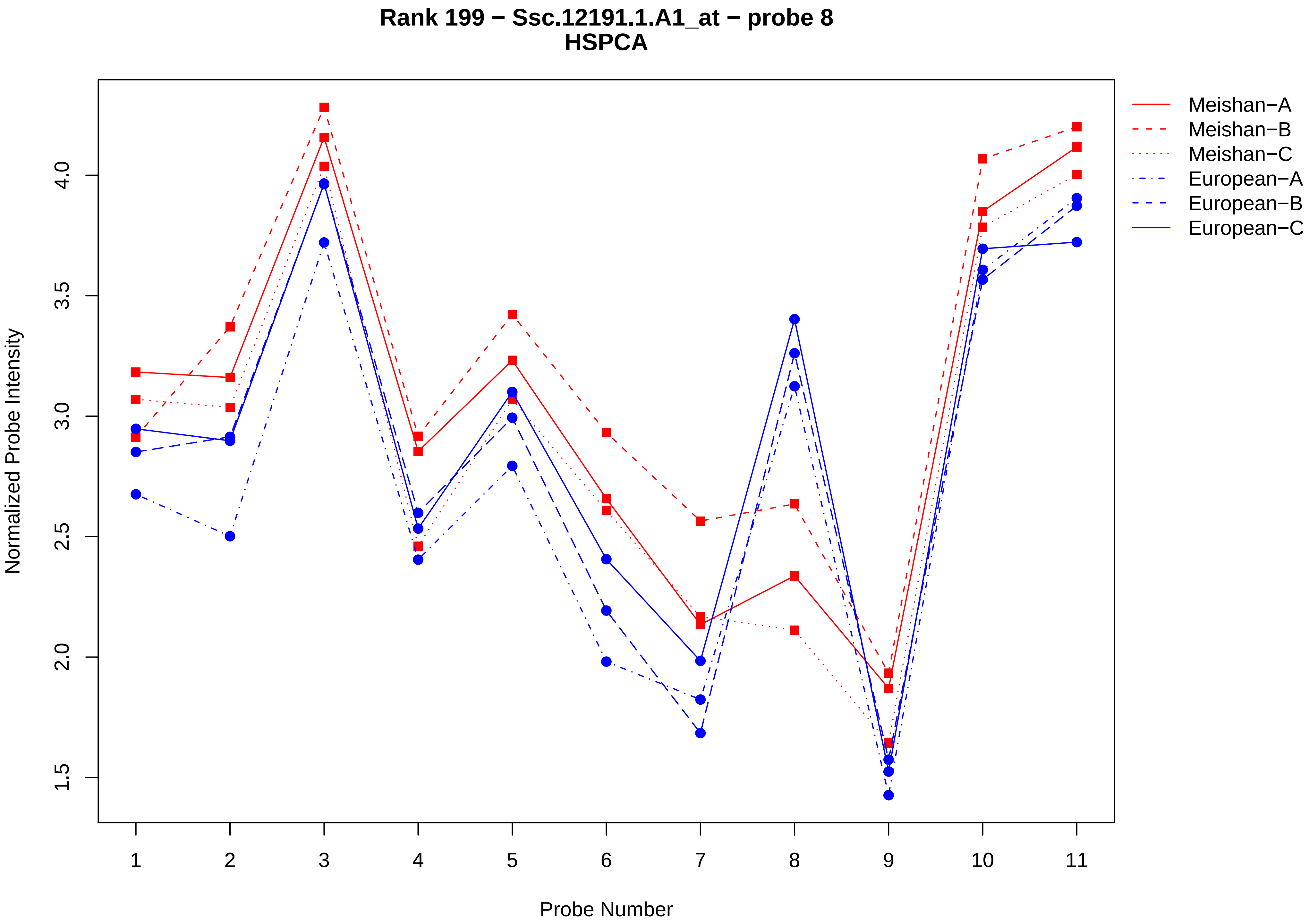

Rank 199 − Ssc.12191.1.A1_at − probe 8
HSPCA
Normalized Probe Intensity
Probe Number
1.5
2.0
2.5
3.0
3.5
4.0
1
2
3
4
5
6
7
8
9
10
11
Meishan−A
Meishan−B
Meishan−C
European−A
European−B
European−C

Rank 200 − Ssc.31190.1.S1_at − probe 11
PDE10A

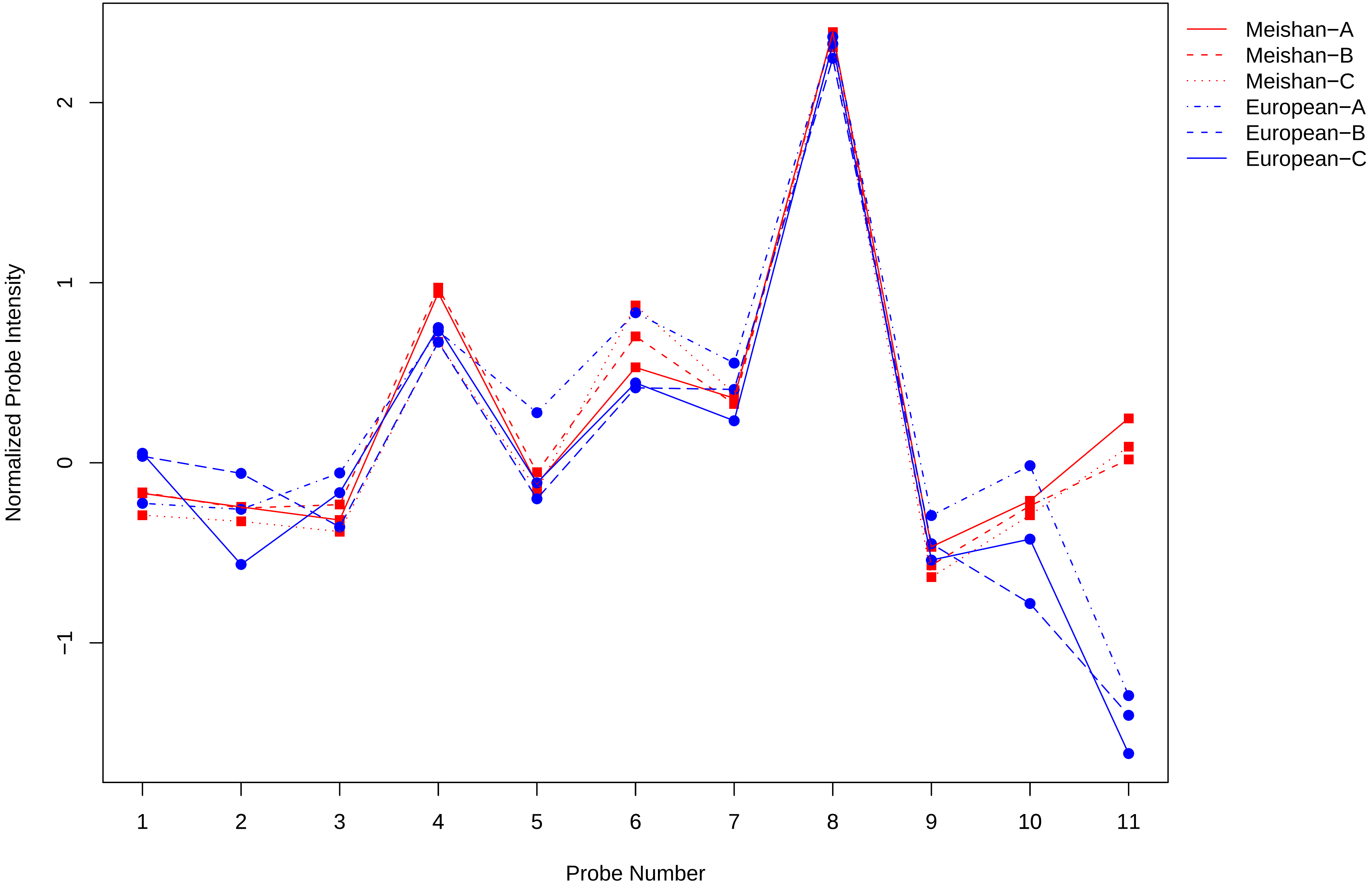

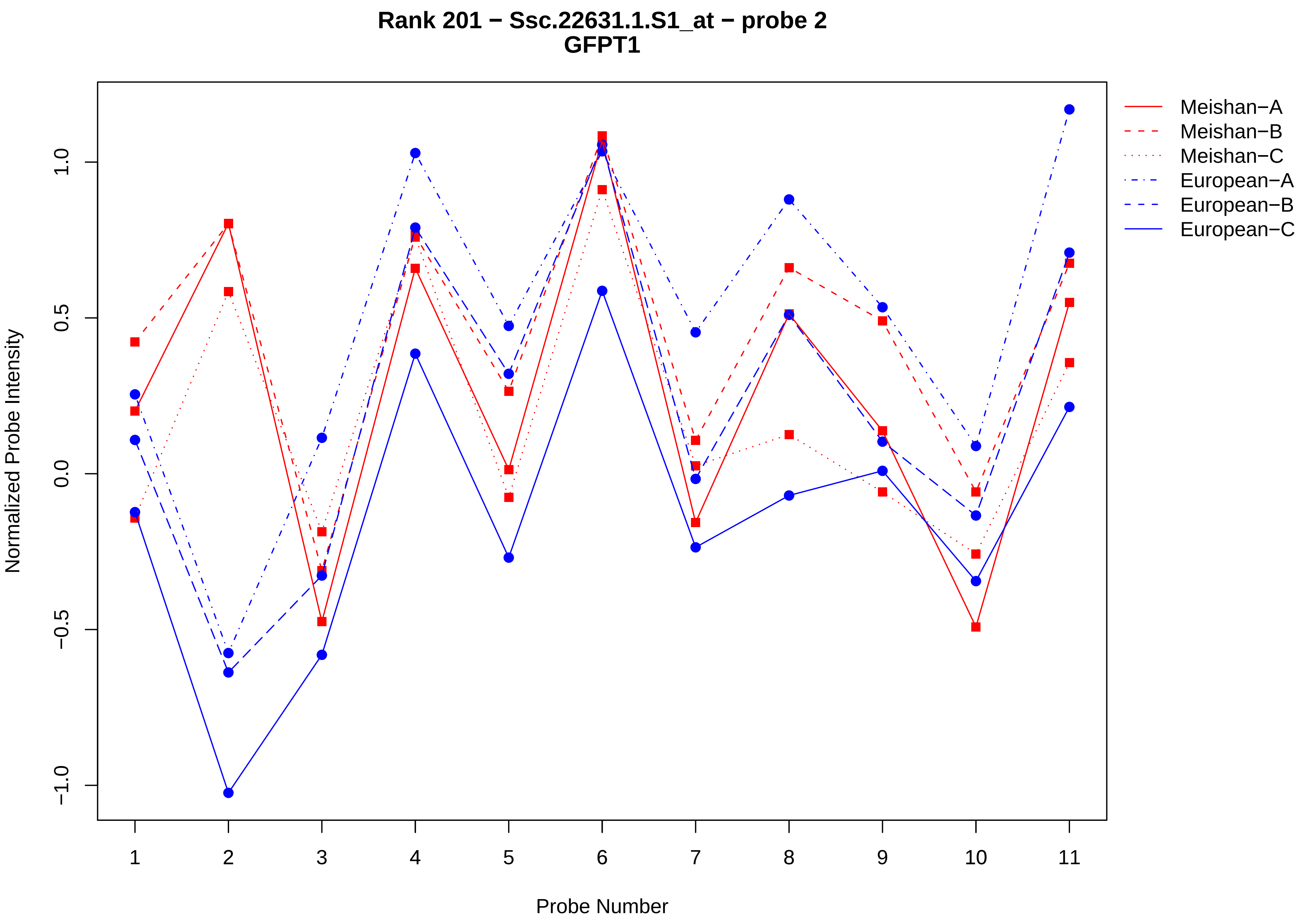
Rank 201 − Ssc.22631.1.S1_at − probe 2
GFPT1
Normalized Probe Intensity
Probe Number
−1.0
−0.5
0.0
0.5
1.0
1
2
3
4
5
6
7
8
9
10
11
Meishan−A
Meishan−B
Meishan−C
European−A
European−B
European−C

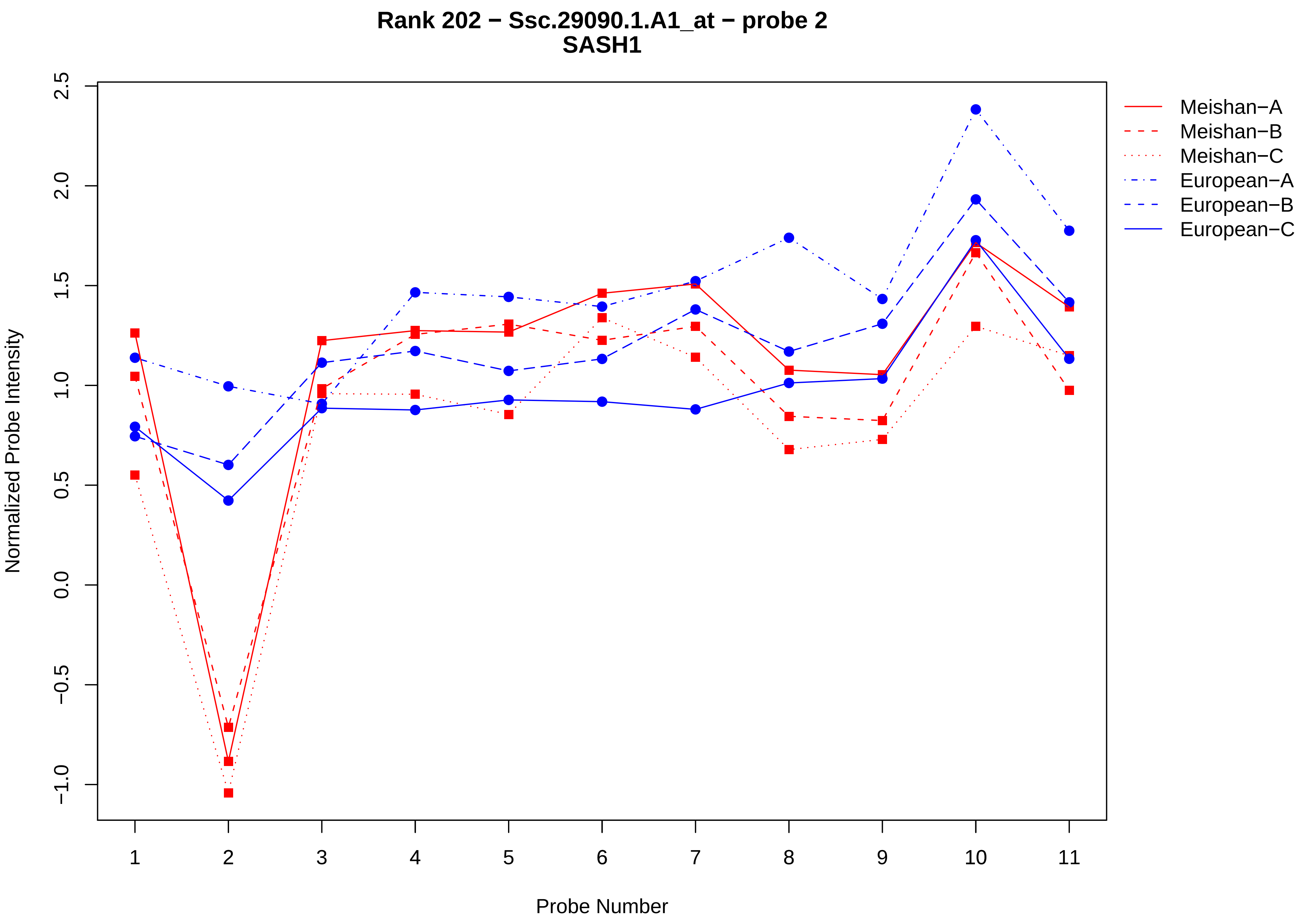

Rank 202 − Ssc.29090.1.A1_at − probe 2
SASH1
Normalized Probe Intensity
Probe Number
−1.0
−0.5
0.0
0.5
1.0
1.5
2.0
2.5
1
2
3
4
5
6
7
8
9
10
11
Meishan−A
Meishan−B
Meishan−C
European−A
European−B
European−C

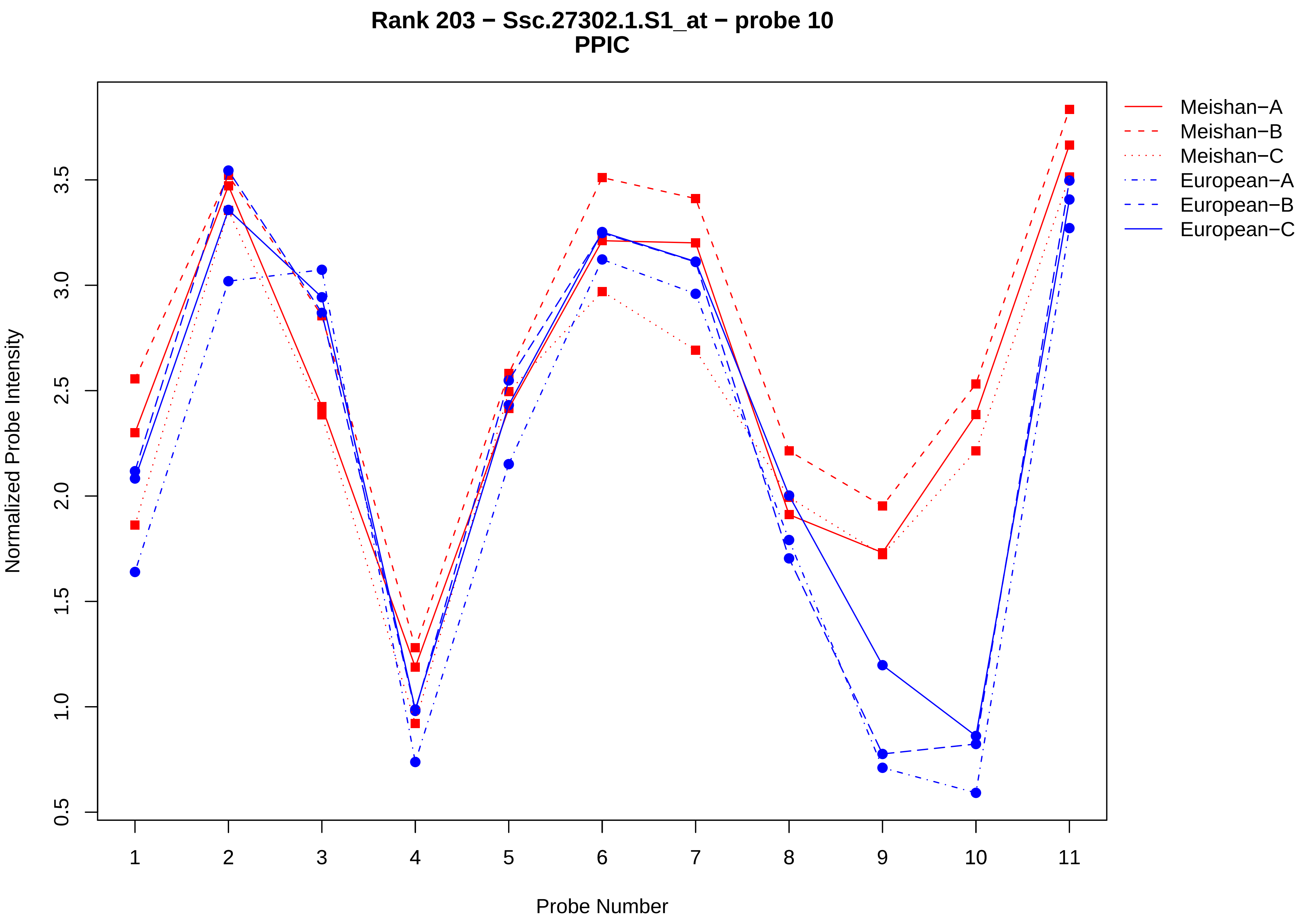

Rank 203 − Ssc.27302.1.S1_at − probe 10
PPIC
Normalized Probe Intensity
Probe Number
0.5
1.0
1.5
2.0
2.5
3.0
3.5
1
2
3
4
5
6
7
8
9
10
11
Meishan−A
Meishan−B
Meishan−C
European−A
European−B
European−C

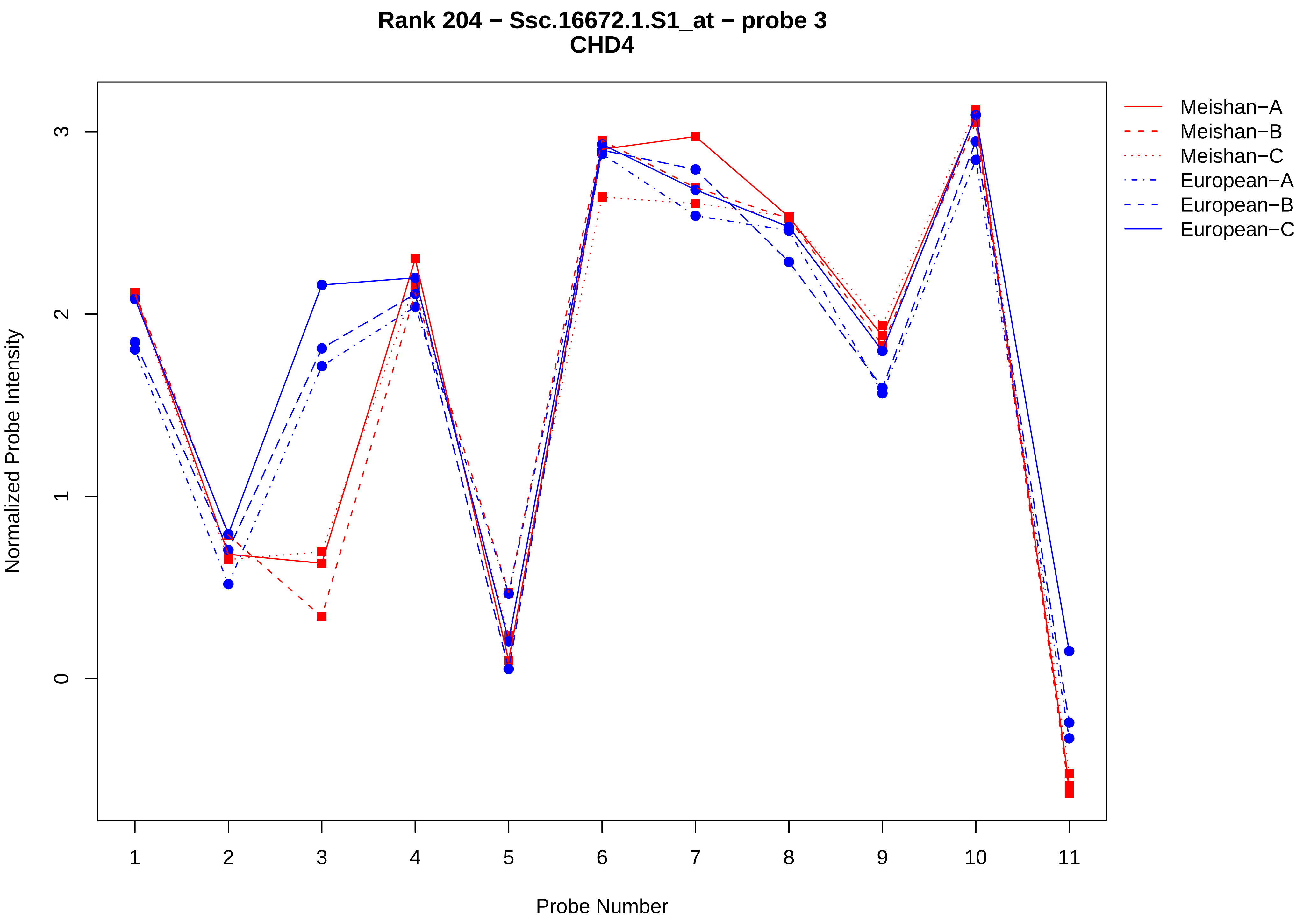

Rank 204 − Ssc.16672.1.S1_at − probe 3
CHD4
Normalized Probe Intensity
Probe Number
0
1
2
3
1
2
3
4
5
6
7
8
9
10
11
Meishan−A
Meishan−B
Meishan−C
European−A
European−B
European−C

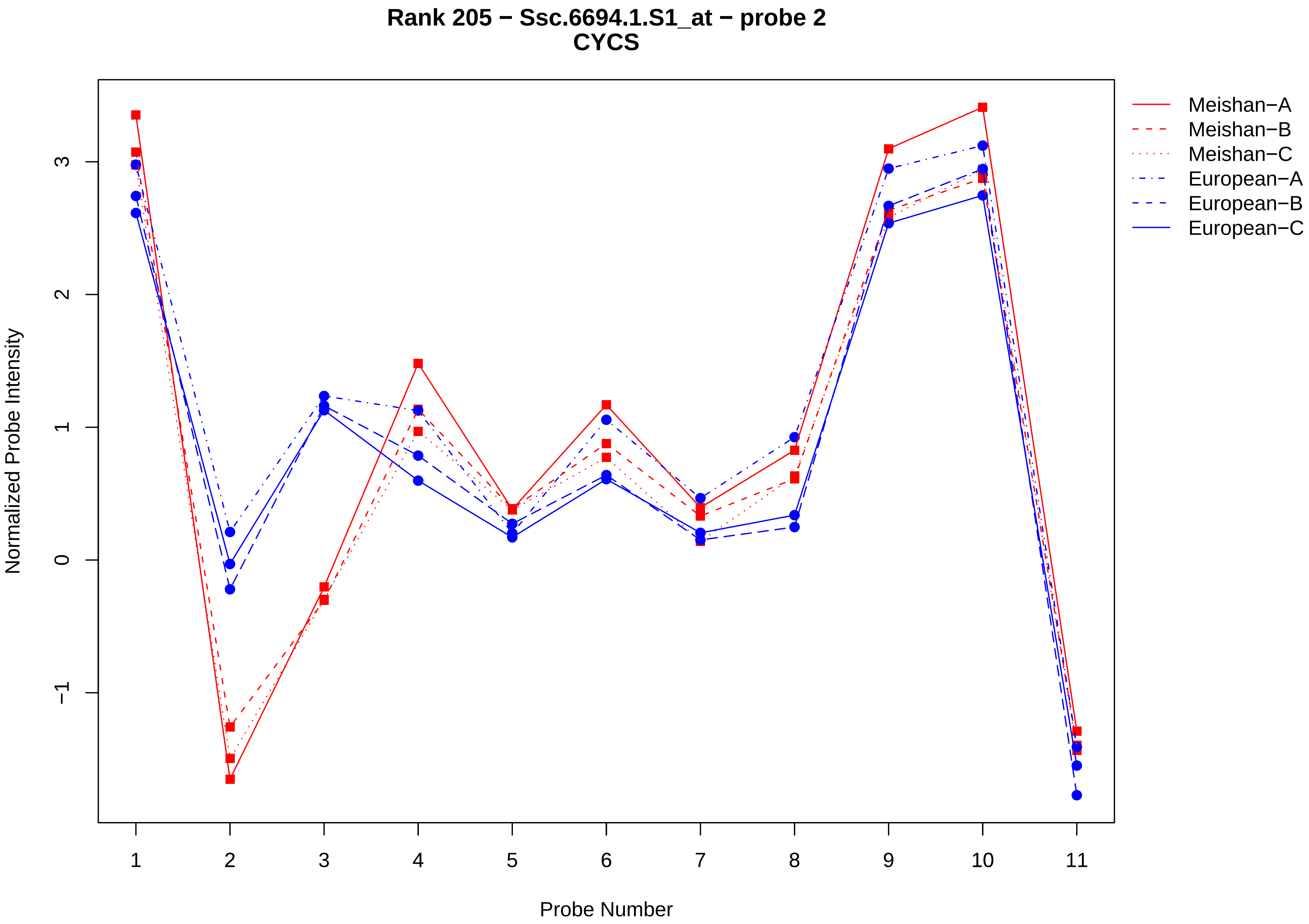

Rank 205 − Ssc.6694.1.S1_at − probe 2
CYCS
Normalized Probe Intensity
Probe Number
−1
0
1
2
3
1
2
3
4
5
6
7
8
9
10
11
Meishan−A
Meishan−B
Meishan−C
European−A
European−B
European−C

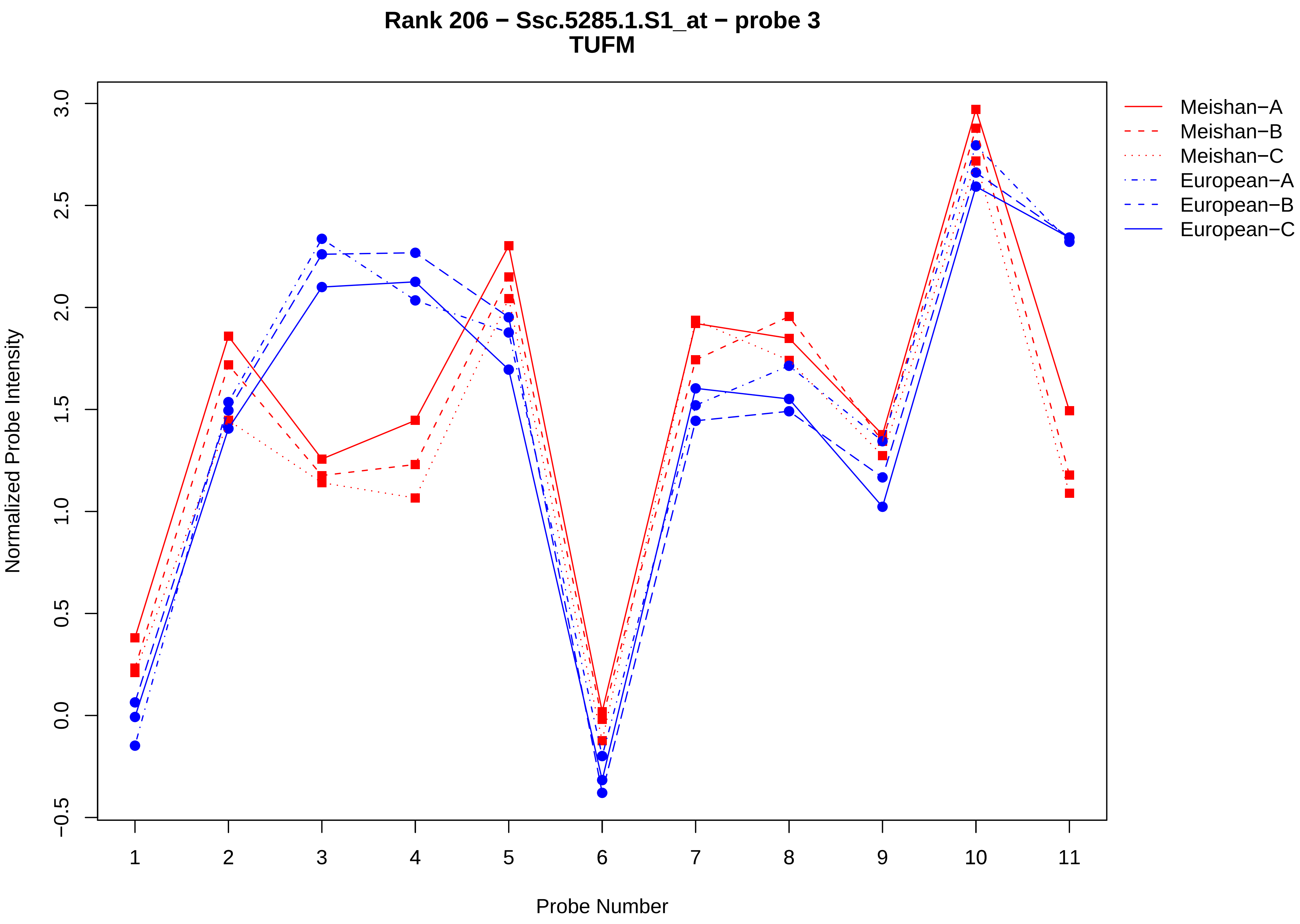

Rank 206 − Ssc.5285.1.S1_at − probe 3
TUFM
Normalized Probe Intensity
Probe Number
−0.5
0.0
0.5
1.0
1.5
2.0
2.5
3.0
1
2
3
4
5
6
7
8
9
10
11
Meishan−A
Meishan−B
Meishan−C
European−A
European−B
European−C

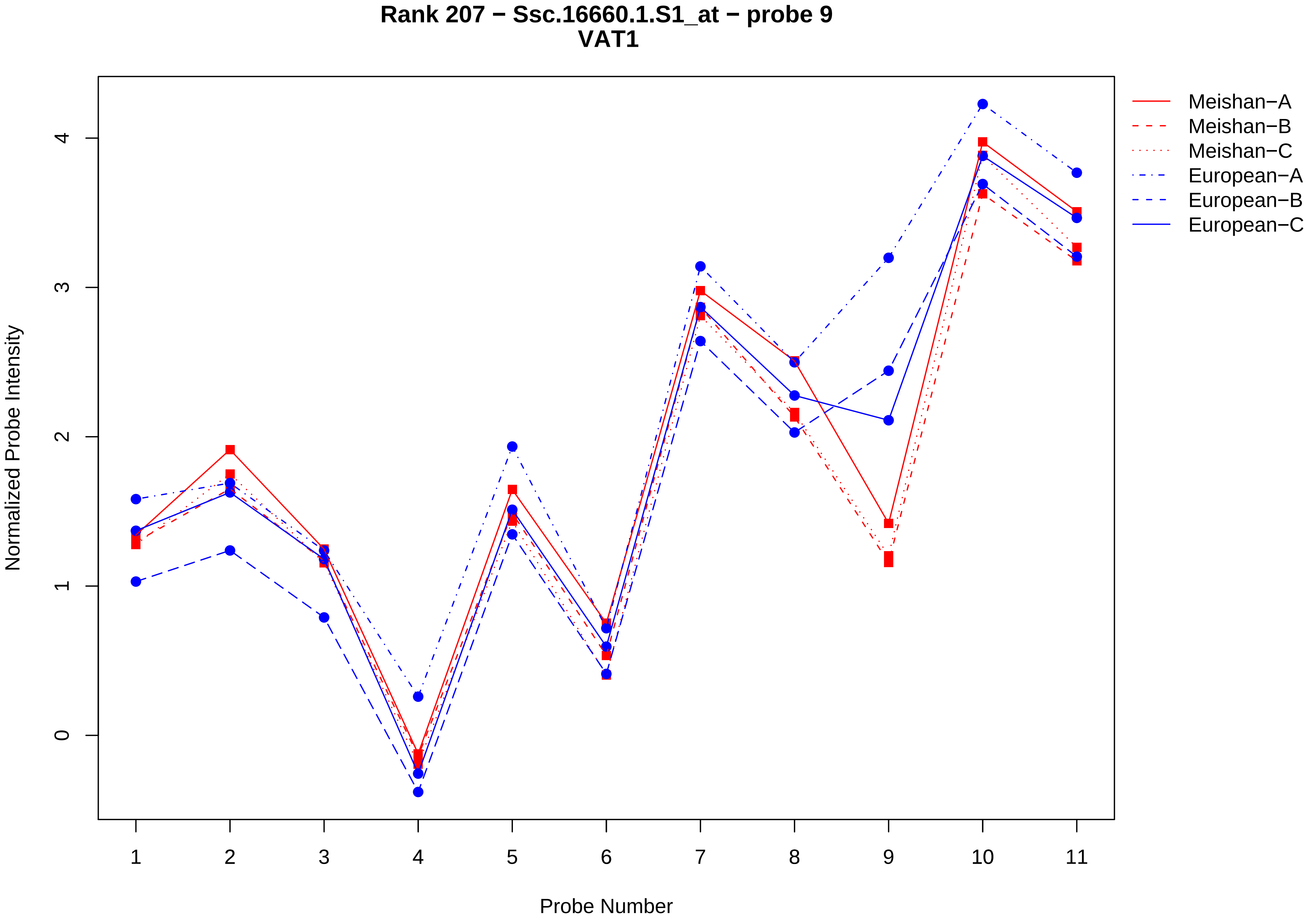
Rank 207 − Ssc.16660.1.S1_at − probe 9
VAT1
Normalized Probe Intensity
Probe Number
0
1
2
3
4
1
2
3
4
5
6
7
8
9
10
11
Meishan−A
Meishan−B
Meishan−C
European−A
European−B
European−C

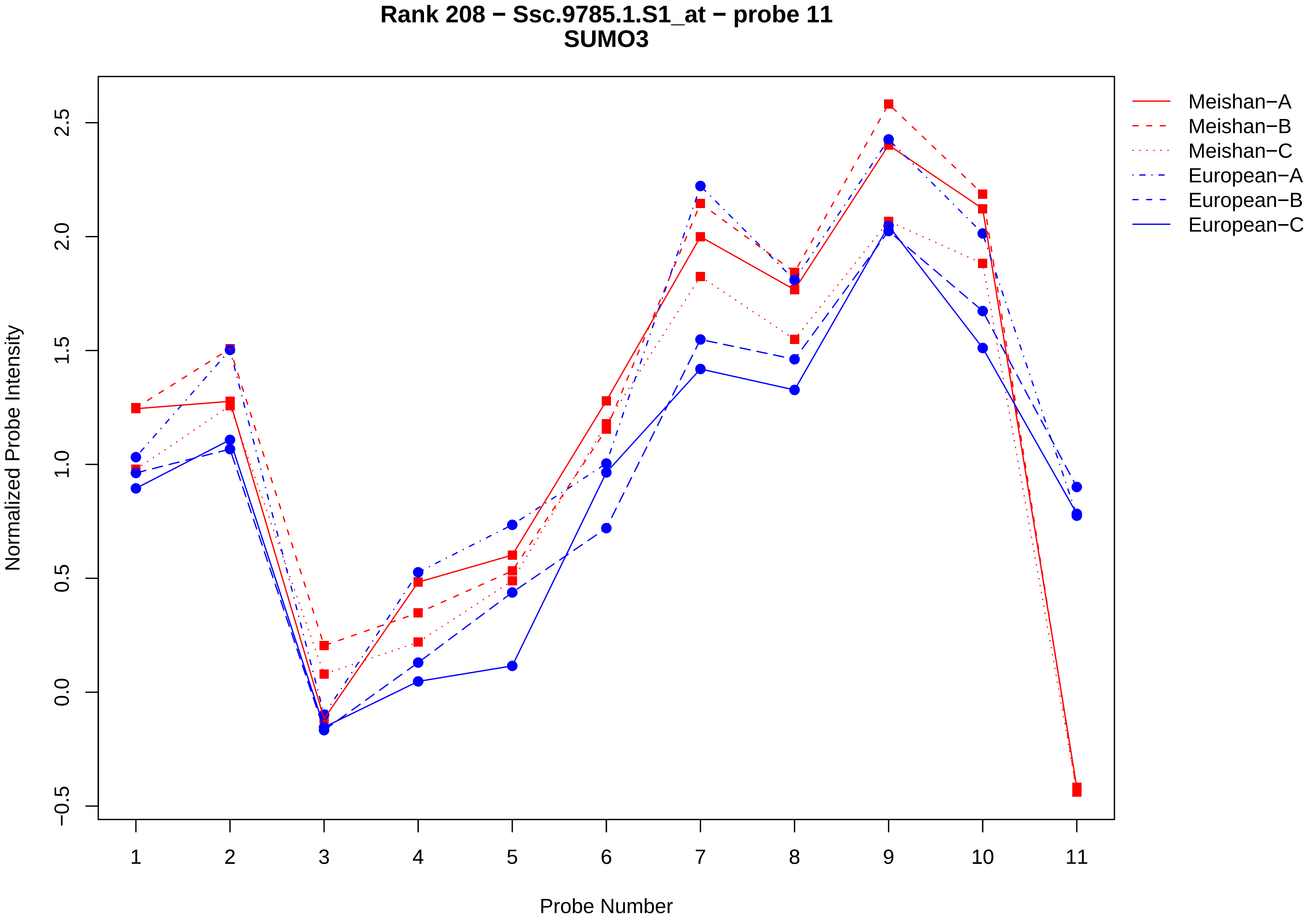
Rank 208 − Ssc.9785.1.S1_at − probe 11
SUMO3
Normalized Probe Intensity
Probe Number
Meishan−A
Meishan−B
Meishan−C
European−A
European−B
European−C
−0.5
0.0
0.5
1.0
1.5
2.0
2.5
1
2
3
4
5
6
7
8
9
10
11

Rank 209 − Ssc.6694.1.S1_at − probe 3
CYCS

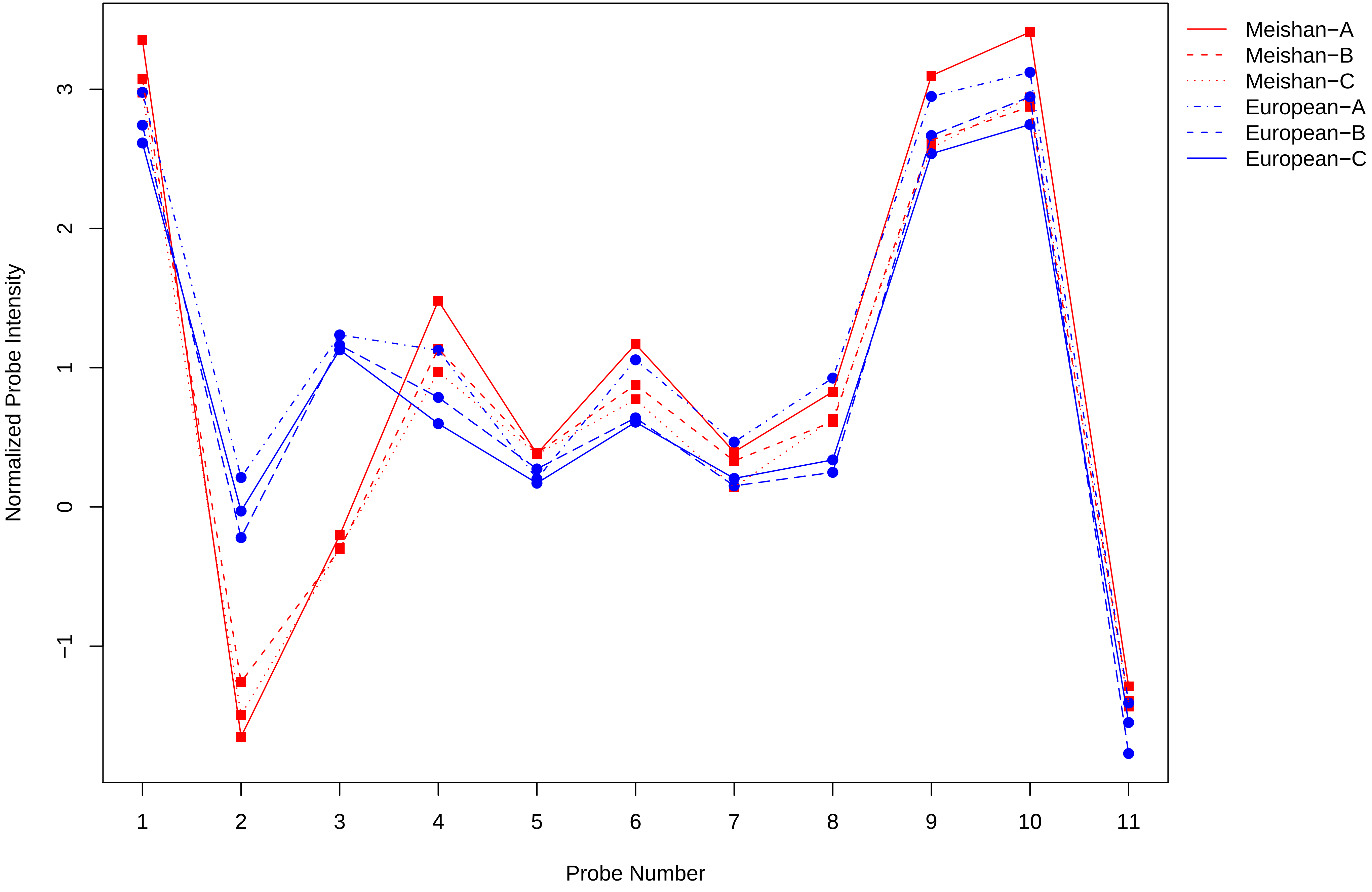

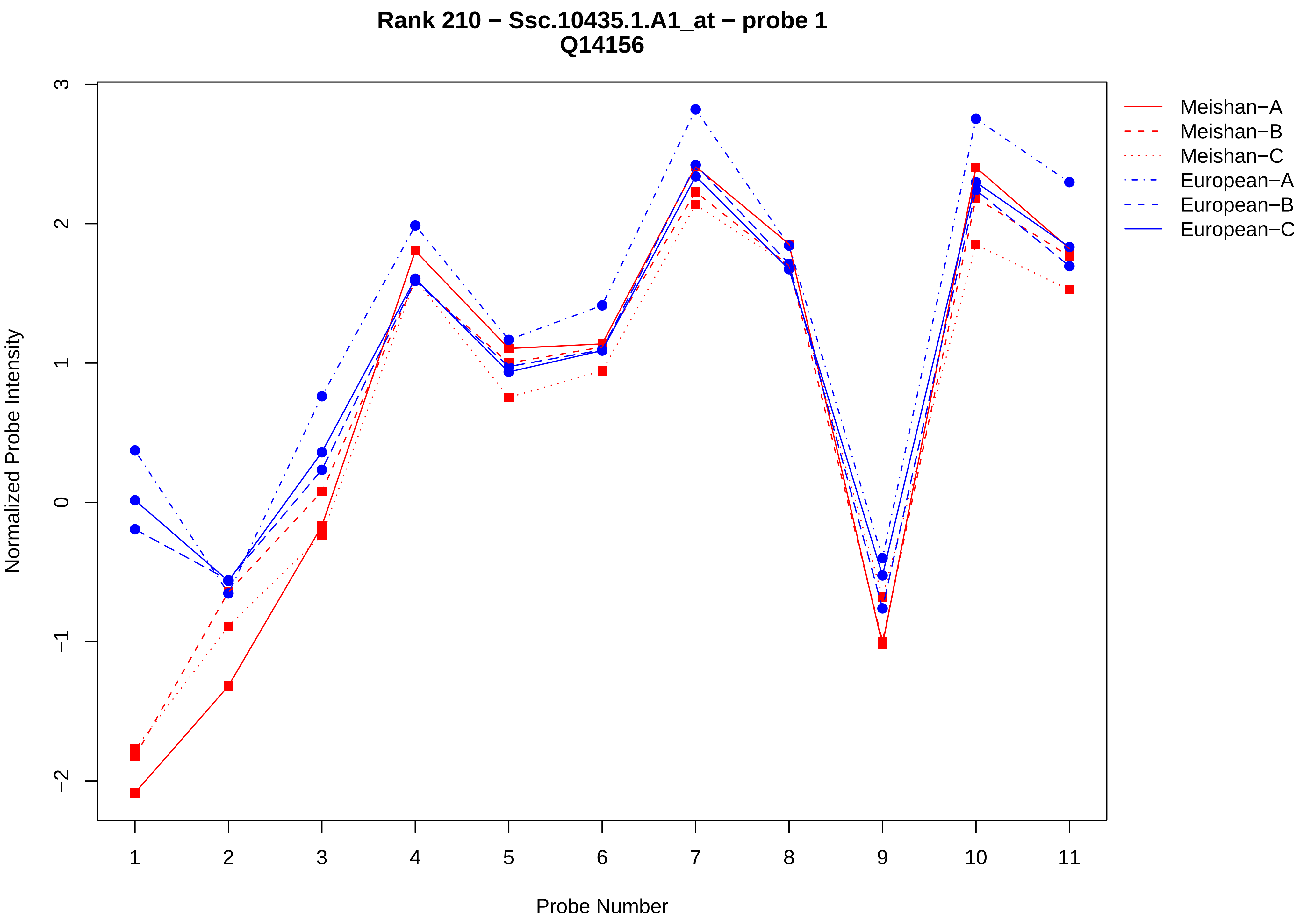

Rank 210 − Ssc.10435.1.A1_at − probe 1
Q14156
Normalized Probe Intensity
Probe Number
Meishan−A
Meishan−B
Meishan−C
European−A
European−B
European−C
3
2
1
0
−1
−2
1
2
3
4
5
6
7
8
9
10
11

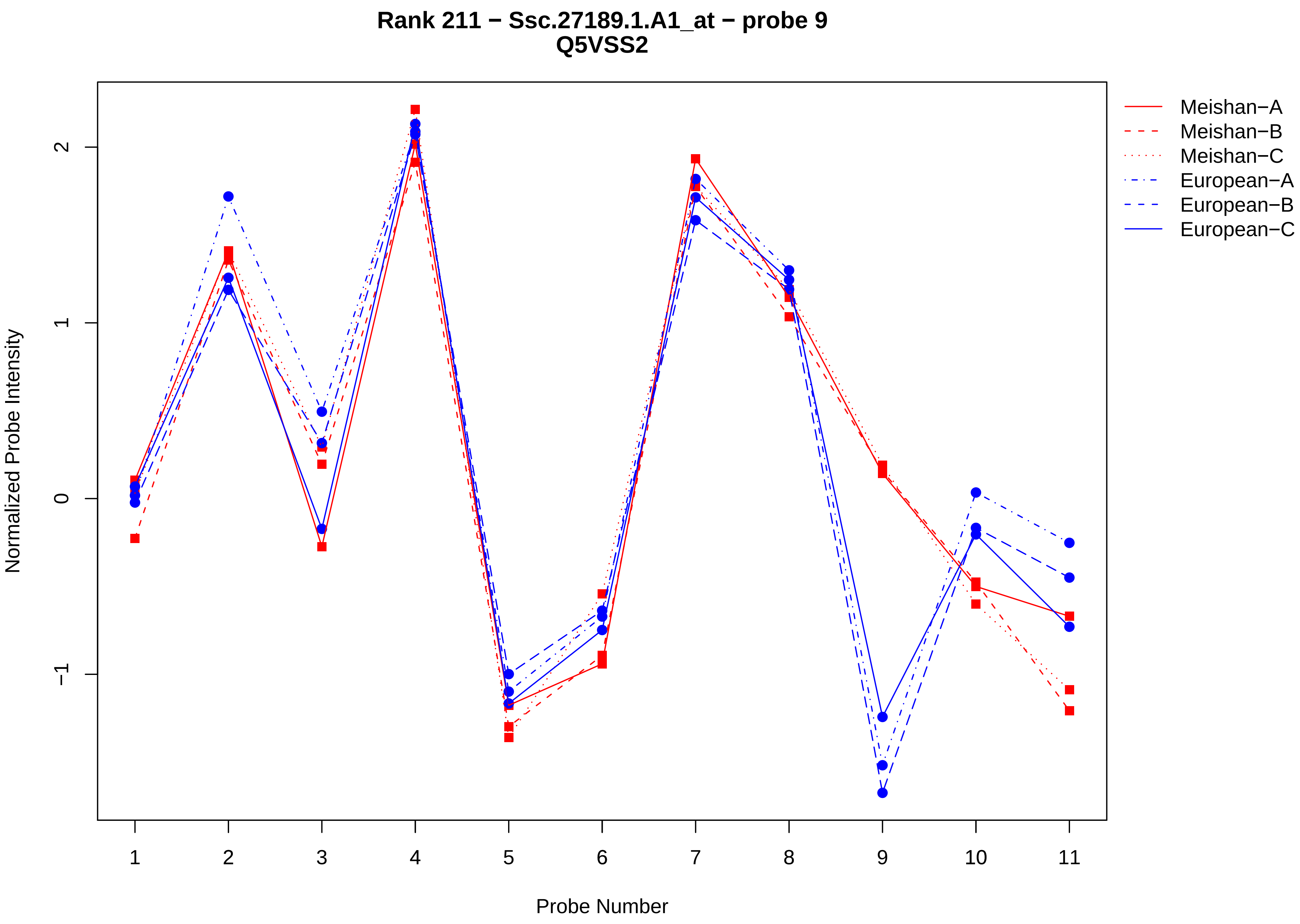

Rank 211 − Ssc.27189.1.A1_at − probe 9
Q5VSS2
Normalized Probe Intensity
Probe Number
Meishan−A
Meishan−B
Meishan−C
European−A
European−B
European−C

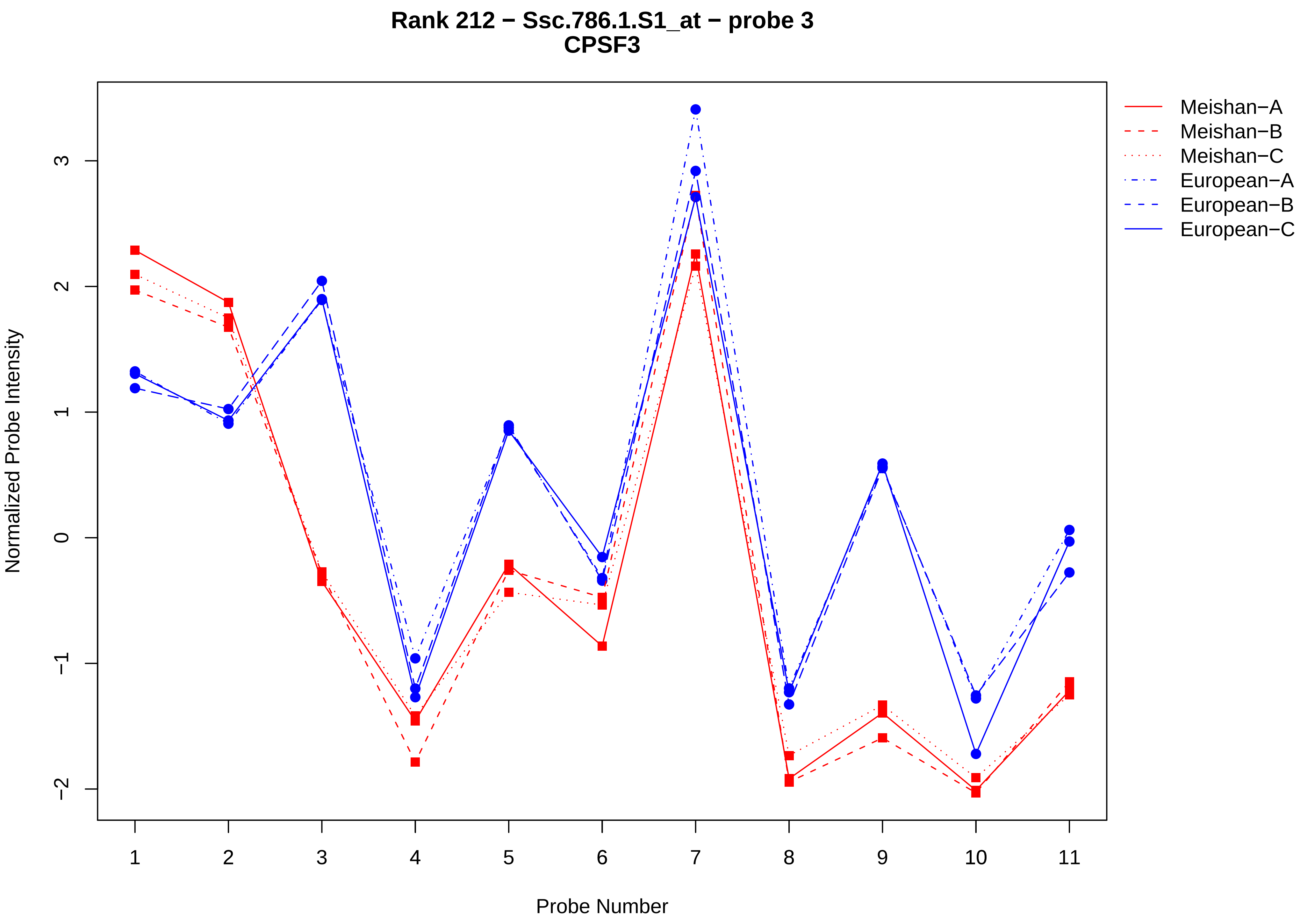
Rank 212 − Ssc.786.1.S1_at − probe 3
CPSF3
Normalized Probe Intensity
Probe Number
Meishan−A
Meishan−B
Meishan−C
European−A
European−B
European−C
−2
−1
0
1
2
3
1
2
3
4
5
6
7
8
9
10
11

Rank 213 − Ssc.21334.1.S1_at − probe 3
SEC31L1

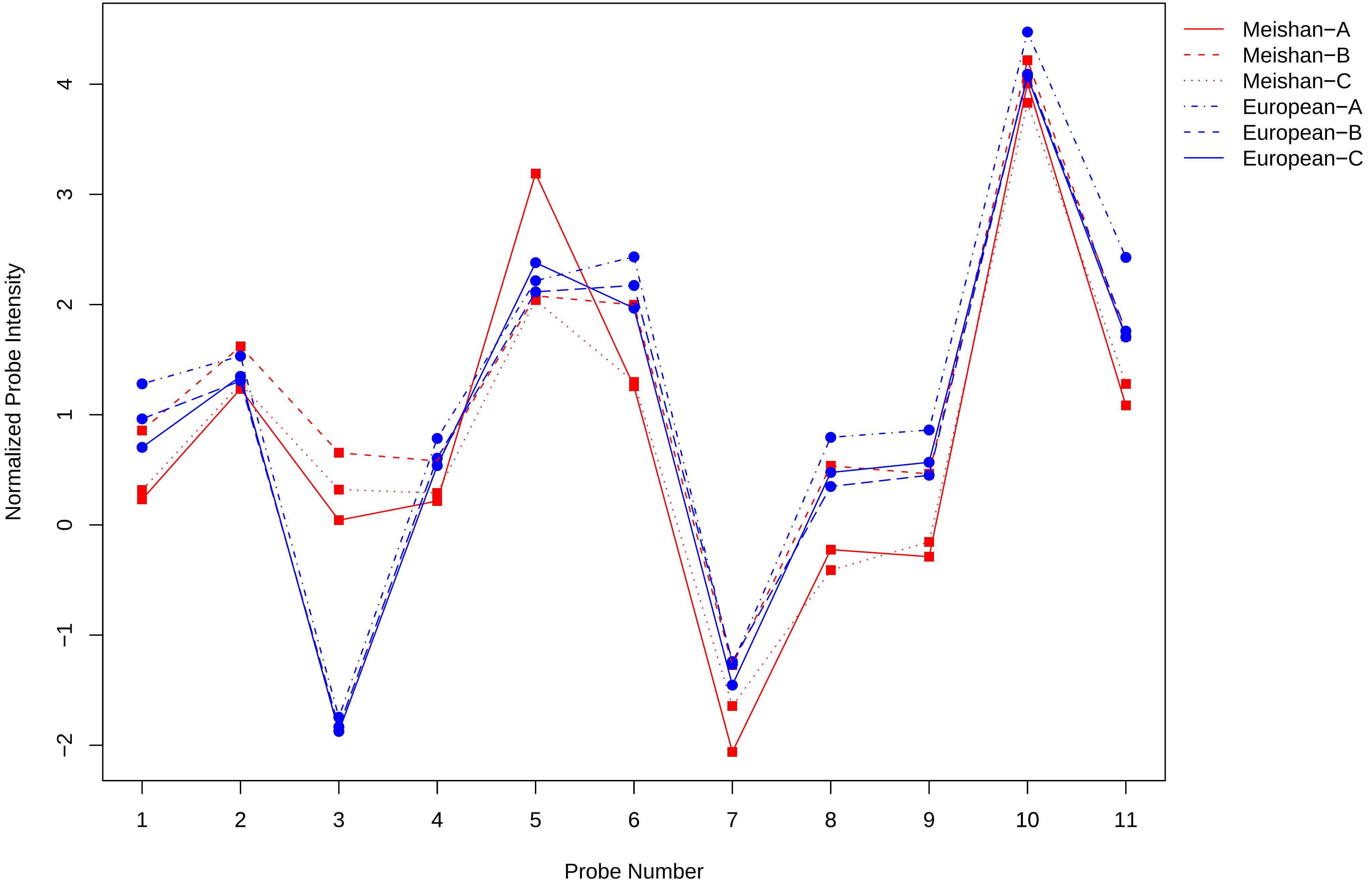

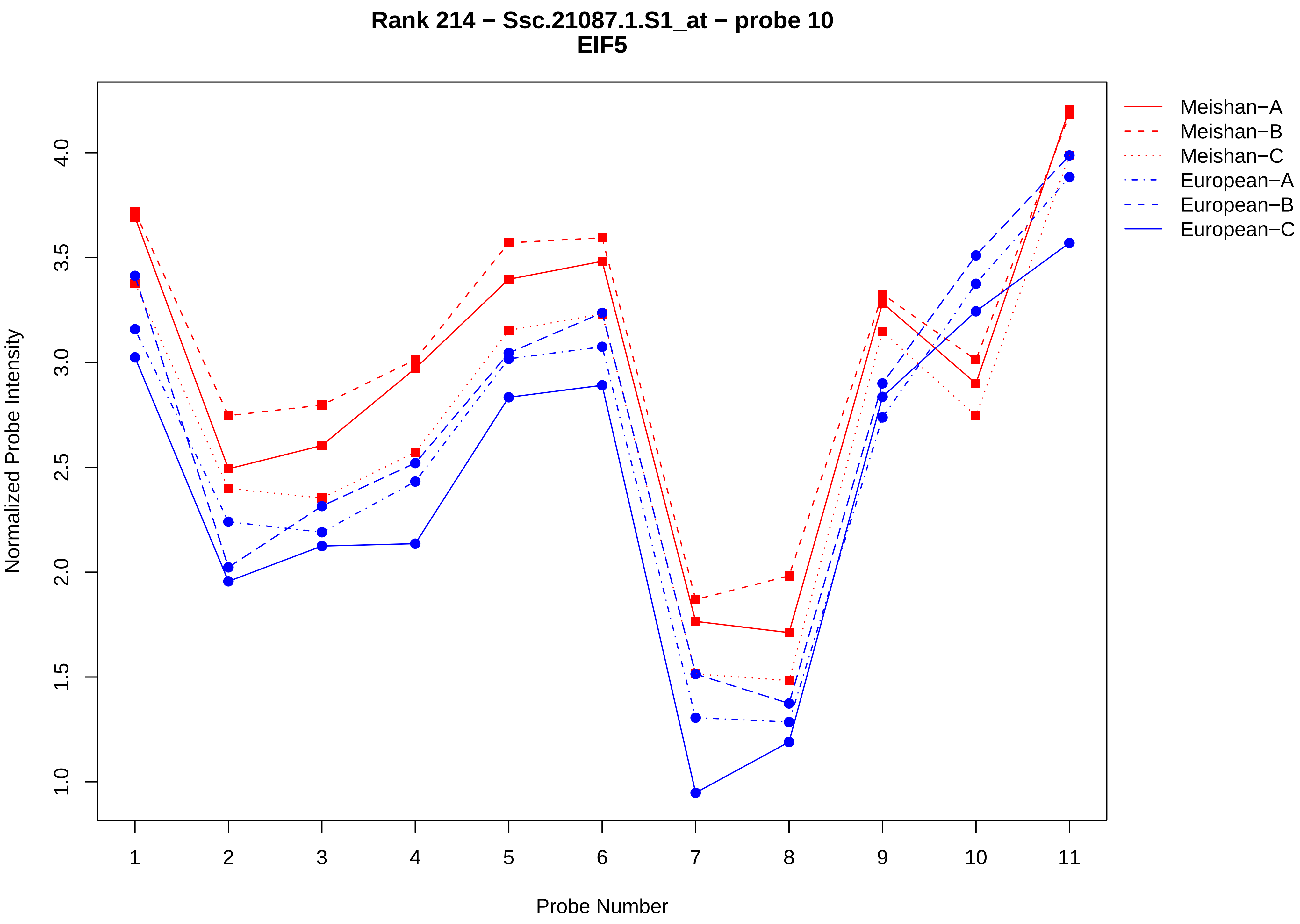
Rank 214 − Ssc.21087.1.S1_at − probe 10
EIF5
Normalized Probe Intensity
Probe Number
1.0
1.5
2.0
2.5
3.0
3.5
4.0
1
2
3
4
5
6
7
8
9
10
11
Meishan−A
Meishan−B
Meishan−C
European−A
European−B
European−C

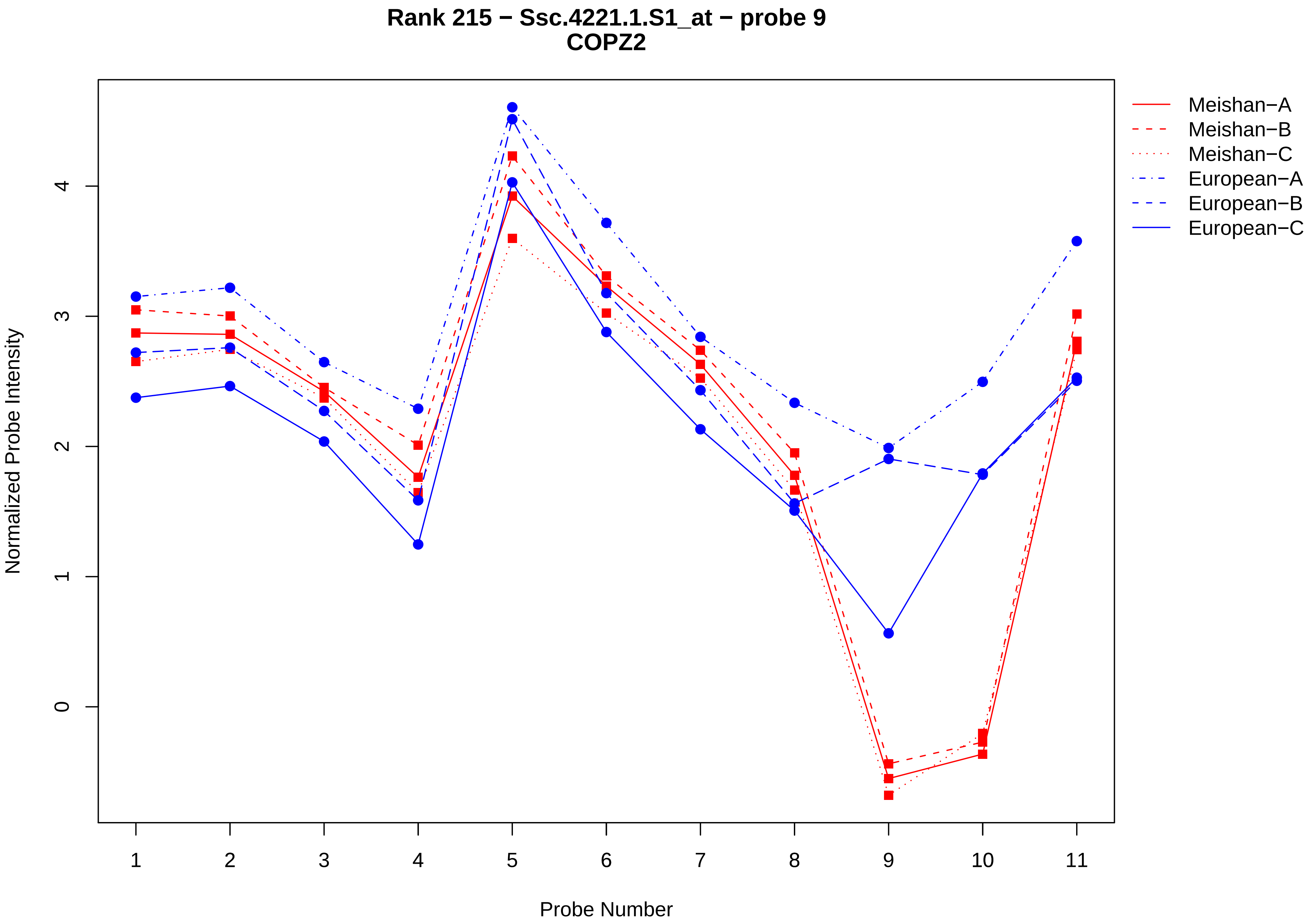

Rank 215 − Ssc.4221.1.S1_at − probe 9
COPZ2
Normalized Probe Intensity
Probe Number
0
1
2
3
4
1
2
3
4
5
6
7
8
9
10
11
Meishan−A
Meishan−B
Meishan−C
European−A
European−B
European−C

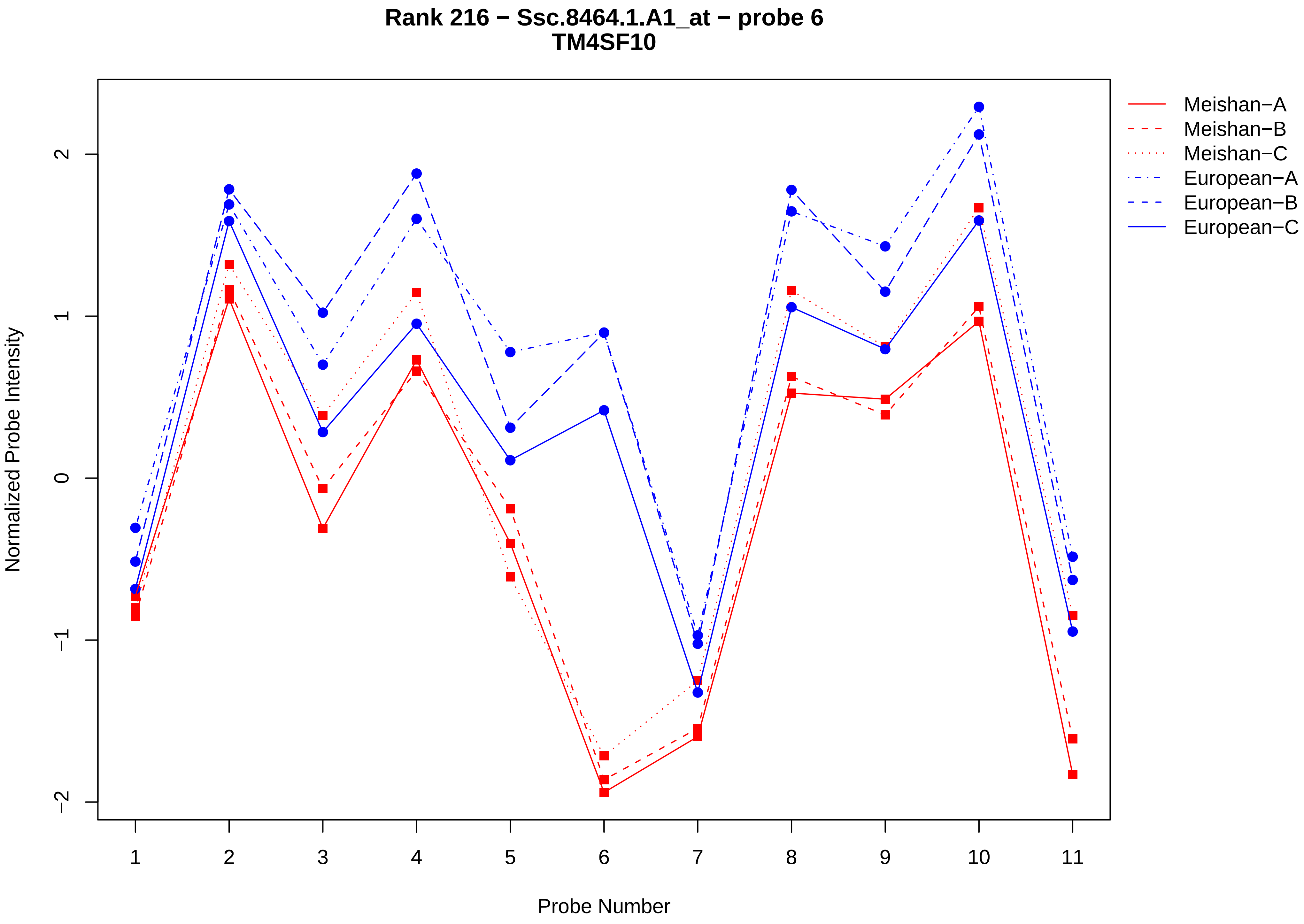
Rank 216 − Ssc.8464.1.A1_at − probe 6
TM4SF10
Normalized Probe Intensity
Probe Number
−2
−1
0
1
2
1
2
3
4
5
6
7
8
9
10
11
Meishan−A
Meishan−B
Meishan−C
European−A
European−B
European−C

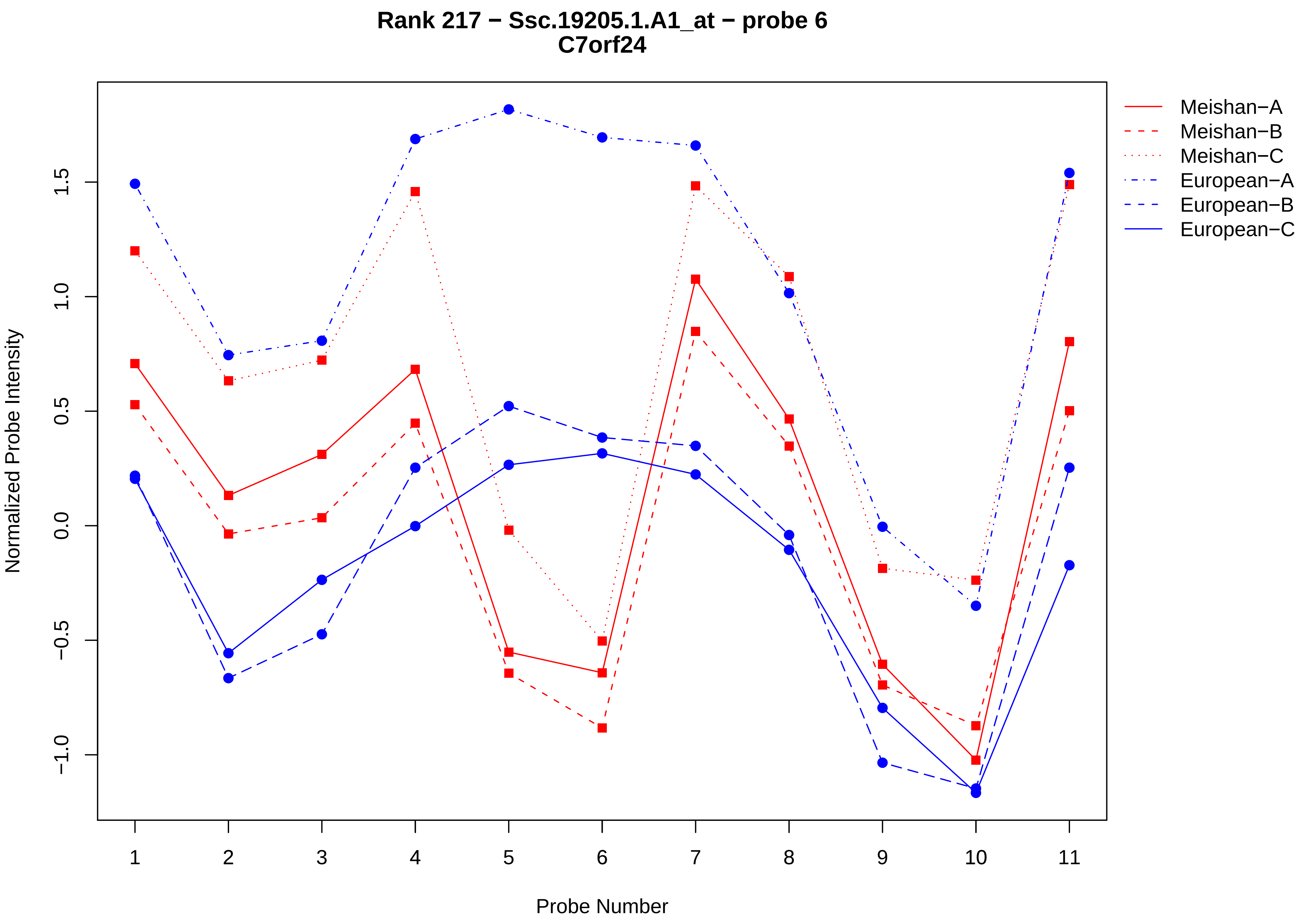

Rank 217 − Ssc.19205.1.A1_at − probe 6
C7orf24
Normalized Probe Intensity
Probe Number
−1.0
−0.5
0.0
0.5
1.0
1.5
1
2
3
4
5
6
7
8
9
10
11
Meishan−A
Meishan−B
Meishan−C
European−A
European−B
European−C

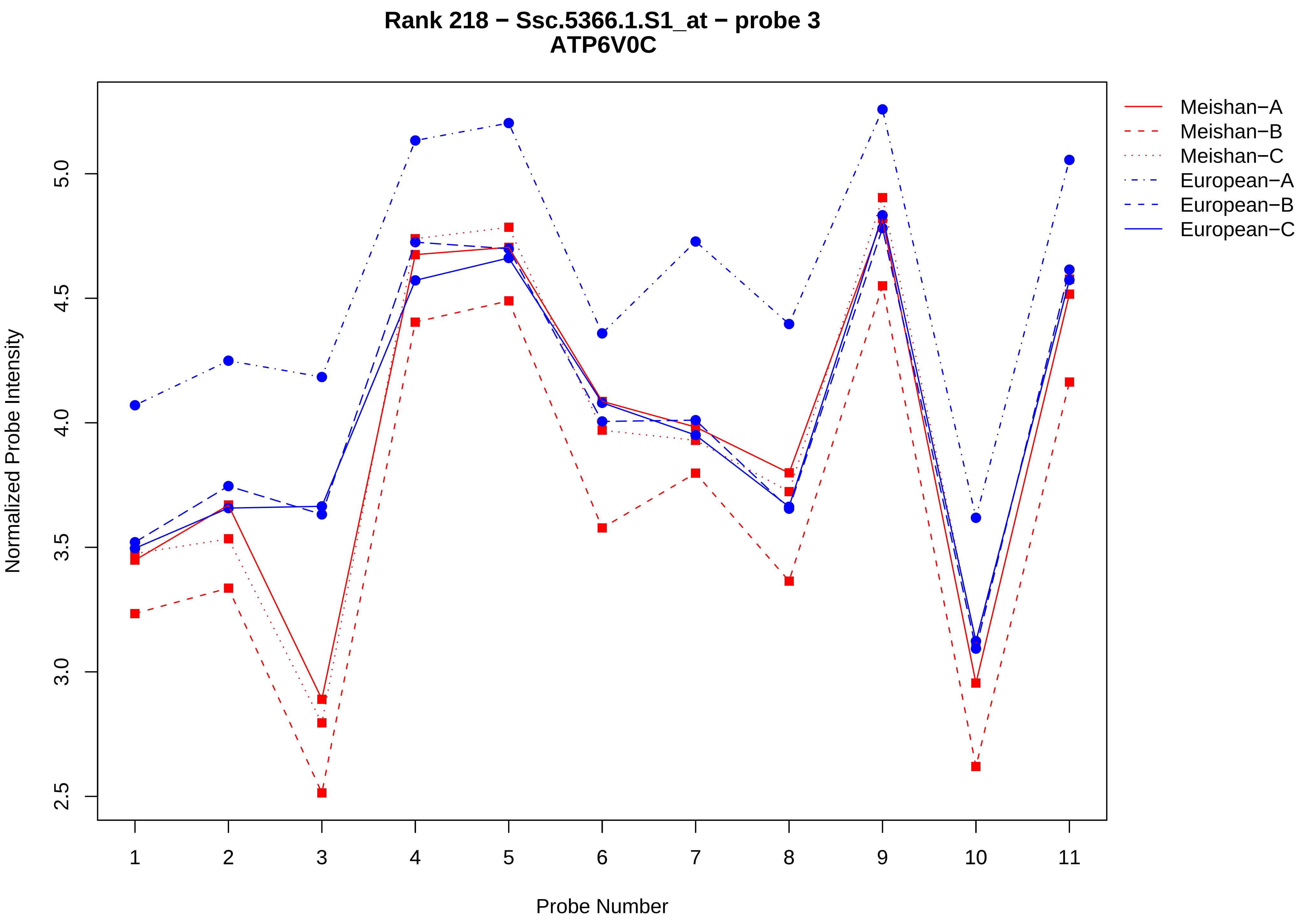

Rank 218 − Ssc.5366.1.S1_at − probe 3
ATP6V0C
Normalized Probe Intensity
Probe Number
2.5
3.0
3.5
4.0
4.5
5.0
1
2
3
4
5
6
7
8
9
10
11
Meishan−A
Meishan−B
Meishan−C
European−A
European−B
European−C

Rank 219 − Ssc.25175.1.S1_at − probe 4
CKLFSF3

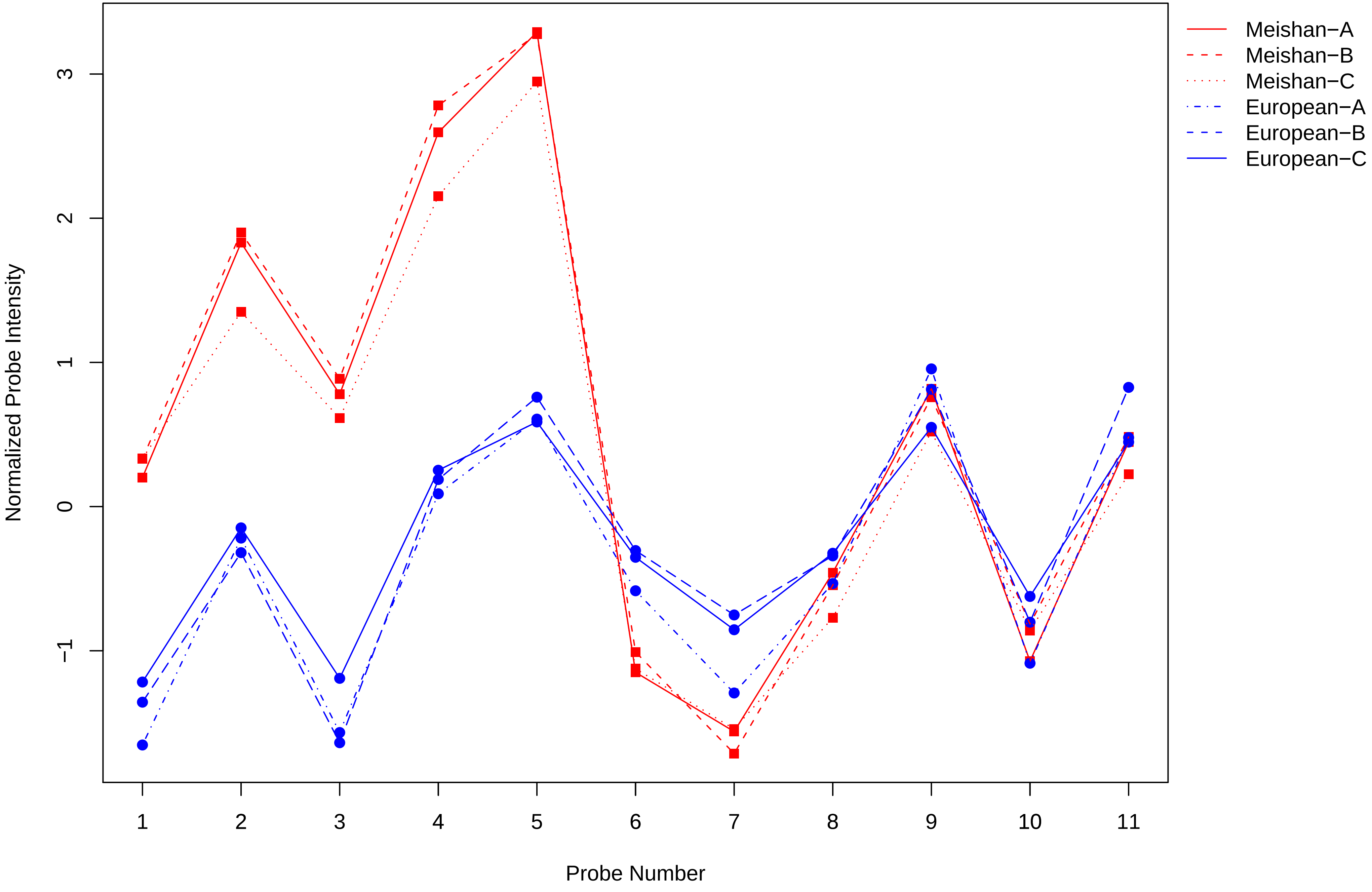

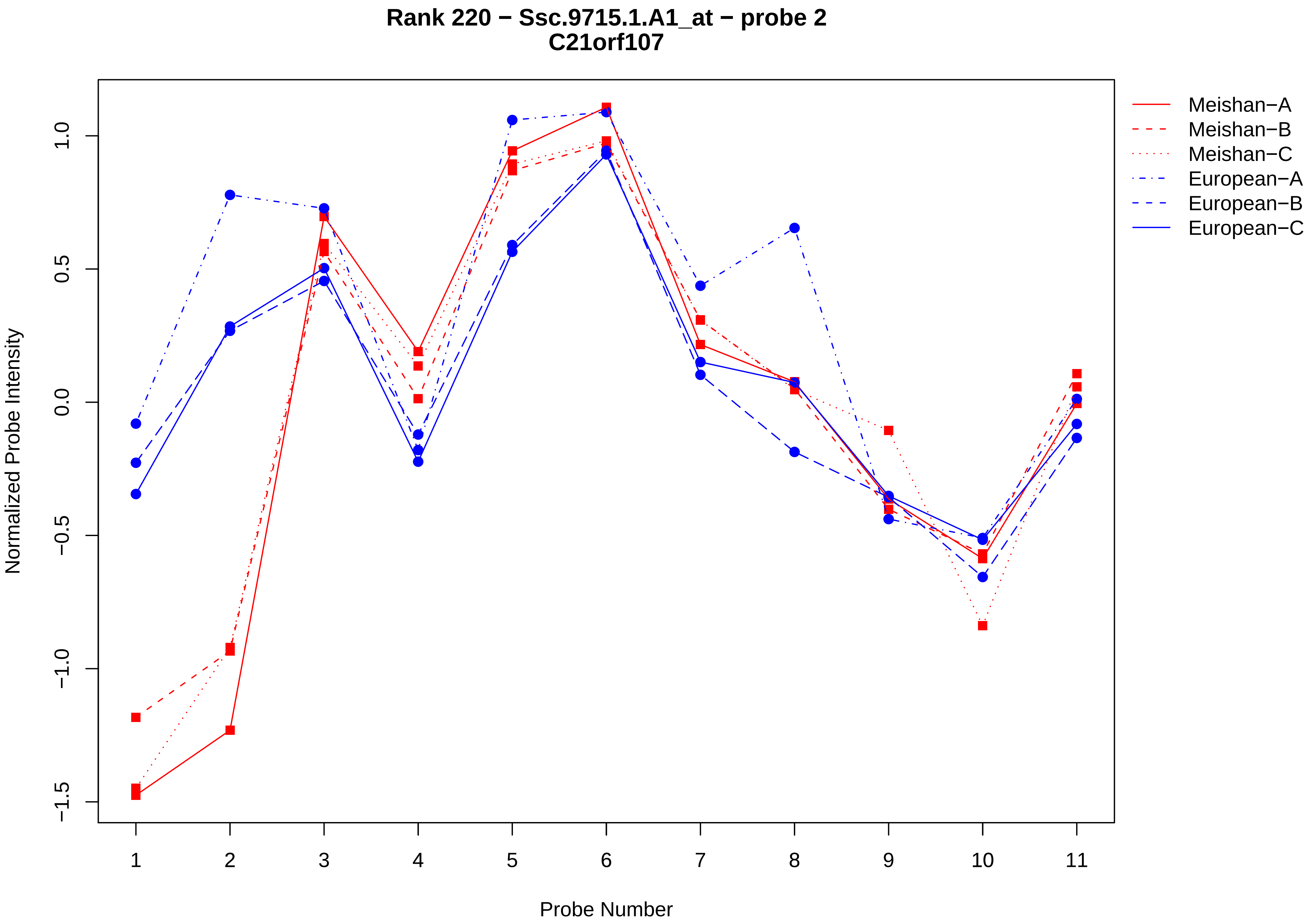
Rank 220 − Ssc.9715.1.A1_at − probe 2
C21orf107
Normalized Probe Intensity
Probe Number
Meishan−A
Meishan−B
Meishan−C
European−A
European−B
European−C

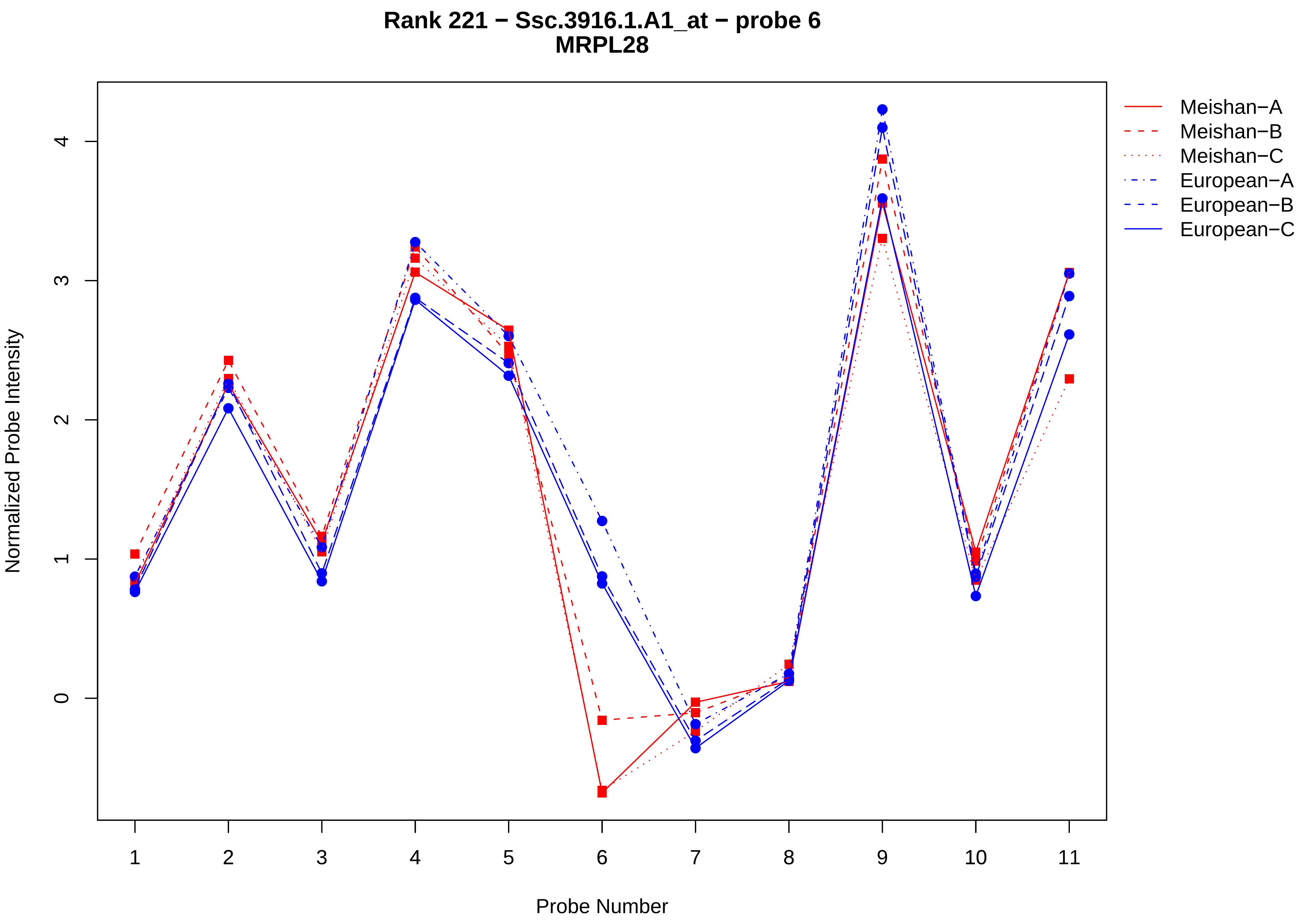

Rank 221 − Ssc.3916.1.A1_at − probe 6
MRPL28
Normalized Probe Intensity
Probe Number
0
1
2
3
4
1
2
3
4
5
6
7
8
9
10
11
Meishan−A
Meishan−B
Meishan−C
European−A
European−B
European−C

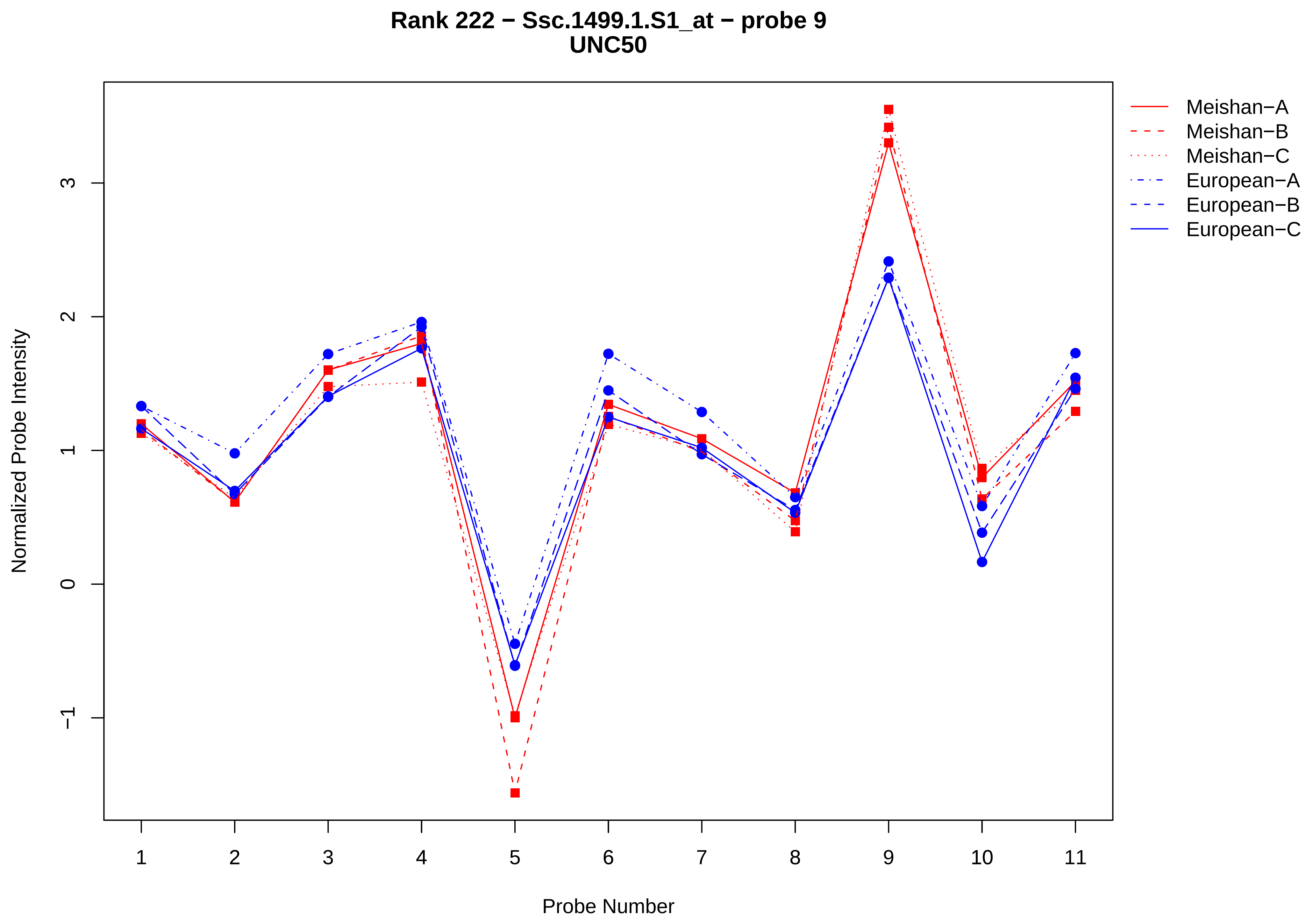

Rank 222 − Ssc.1499.1.S1_at − probe 9
UNC50
Normalized Probe Intensity
Probe Number
−1
0
1
2
3
1
2
3
4
5
6
7
8
9
10
11
Meishan−A
Meishan−B
Meishan−C
European−A
European−B
European−C

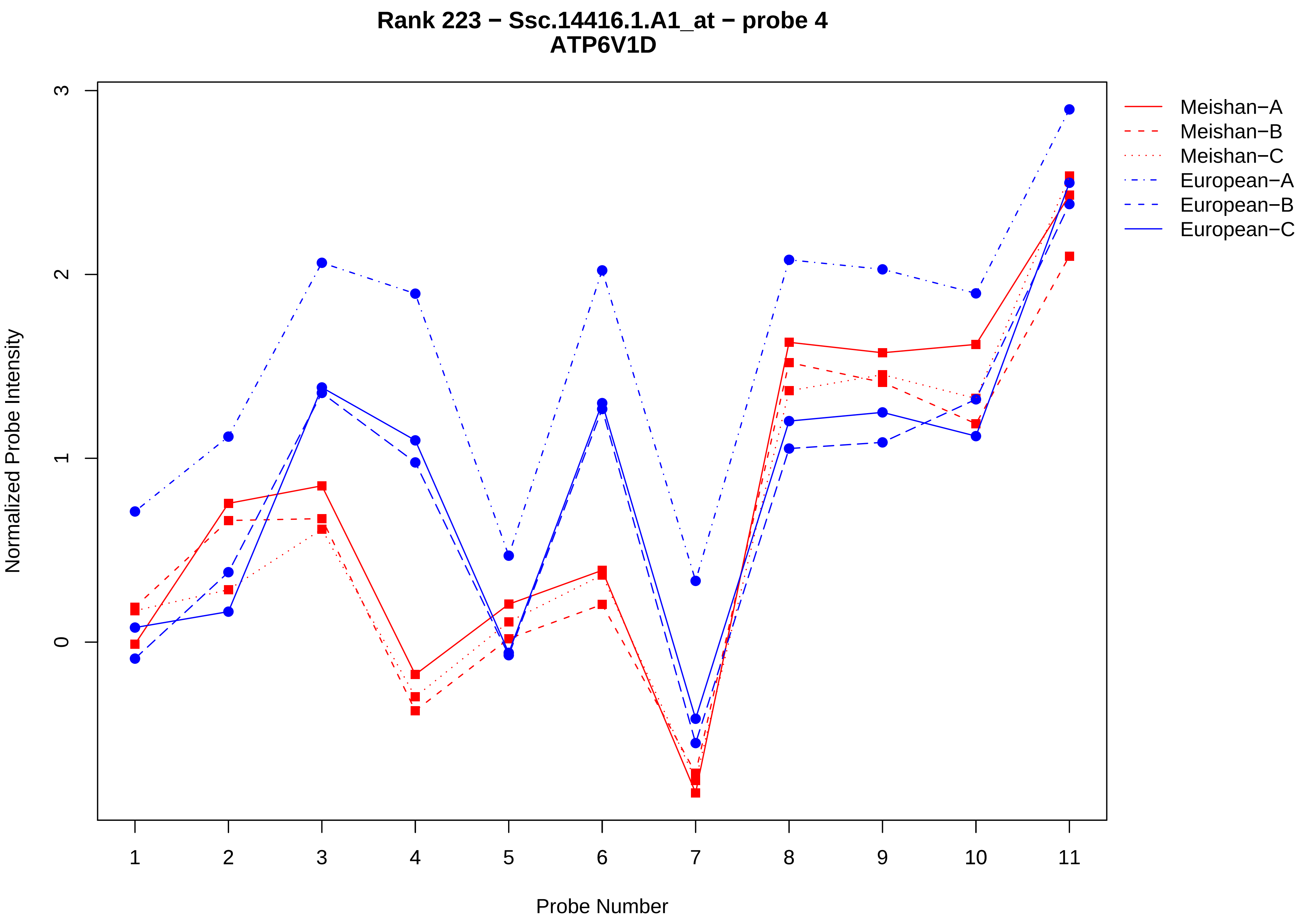

Rank 223 − Ssc.14416.1.A1_at − probe 4
ATP6V1D
Normalized Probe Intensity
Probe Number
0
1
2
3
1
2
3
4
5
6
7
8
9
10
11
Meishan−A
Meishan−B
Meishan−C
European−A
European−B
European−C

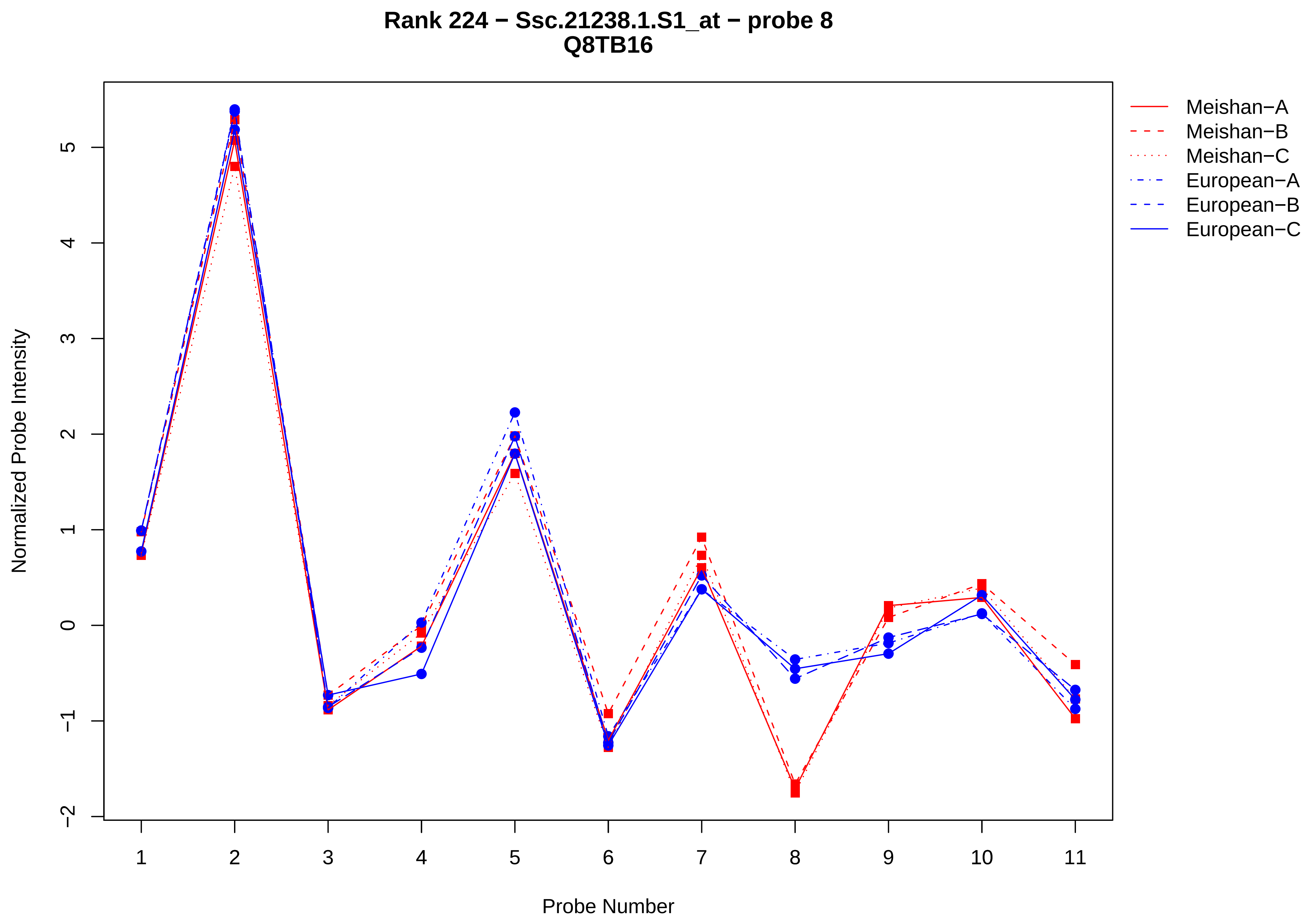
Rank 224 − Ssc.21238.1.S1_at − probe 8
Q8TB16
Normalized Probe Intensity
Probe Number
−2
−1
0
1
2
3
4
5
1
2
3
4
5
6
7
8
9
10
11
Meishan−A
Meishan−B
Meishan−C
European−A
European−B
European−C

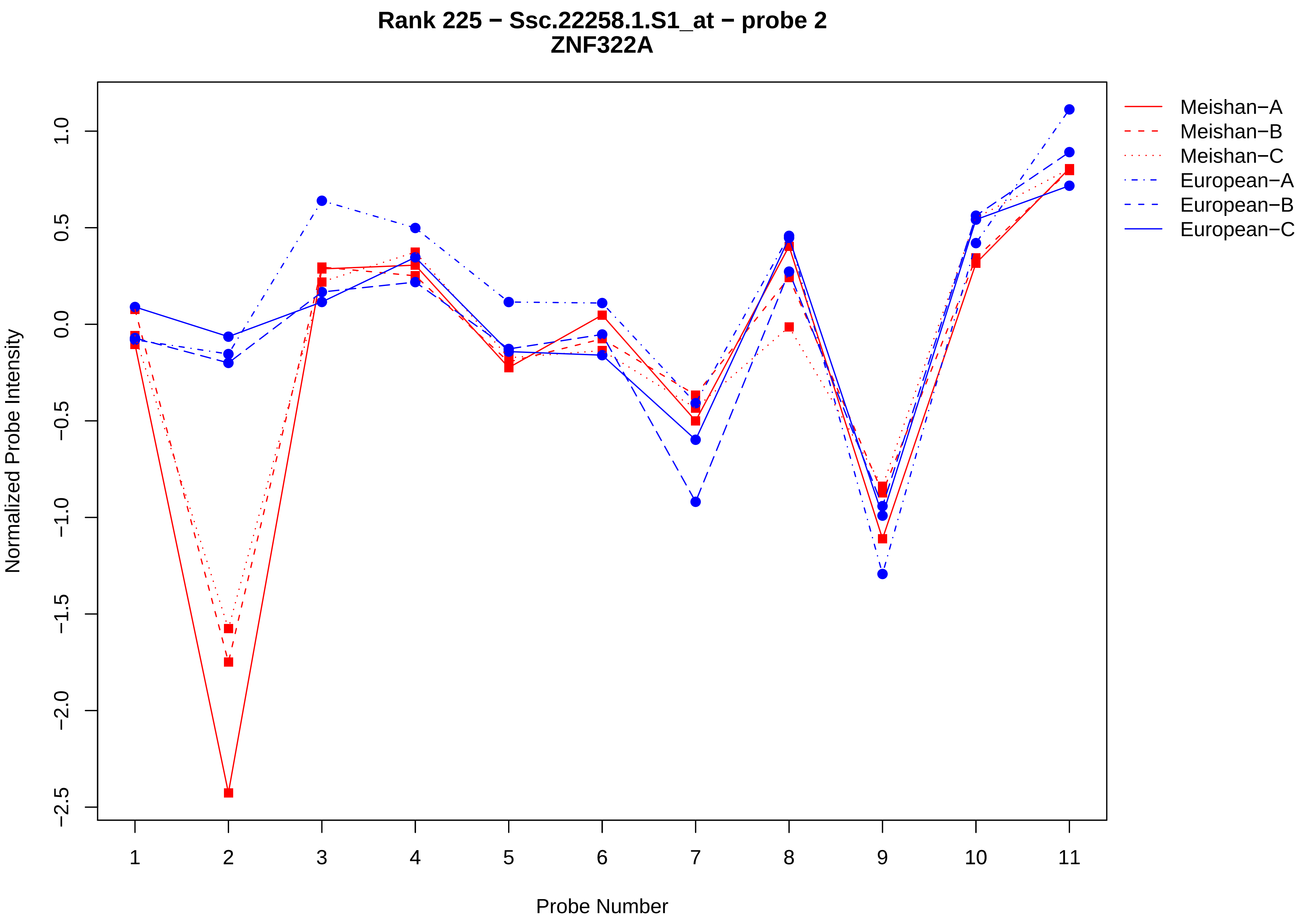

Rank 225 − Ssc.22258.1.S1_at − probe 2
ZNF322A
Normalized Probe Intensity
Probe Number
1.0
0.5
0.0
−0.5
−1.0
−1.5
−2.0
−2.5
1
2
3
4
5
6
7
8
9
10
11
Meishan−A
Meishan−B
Meishan−C
European−A
European−B
European−C

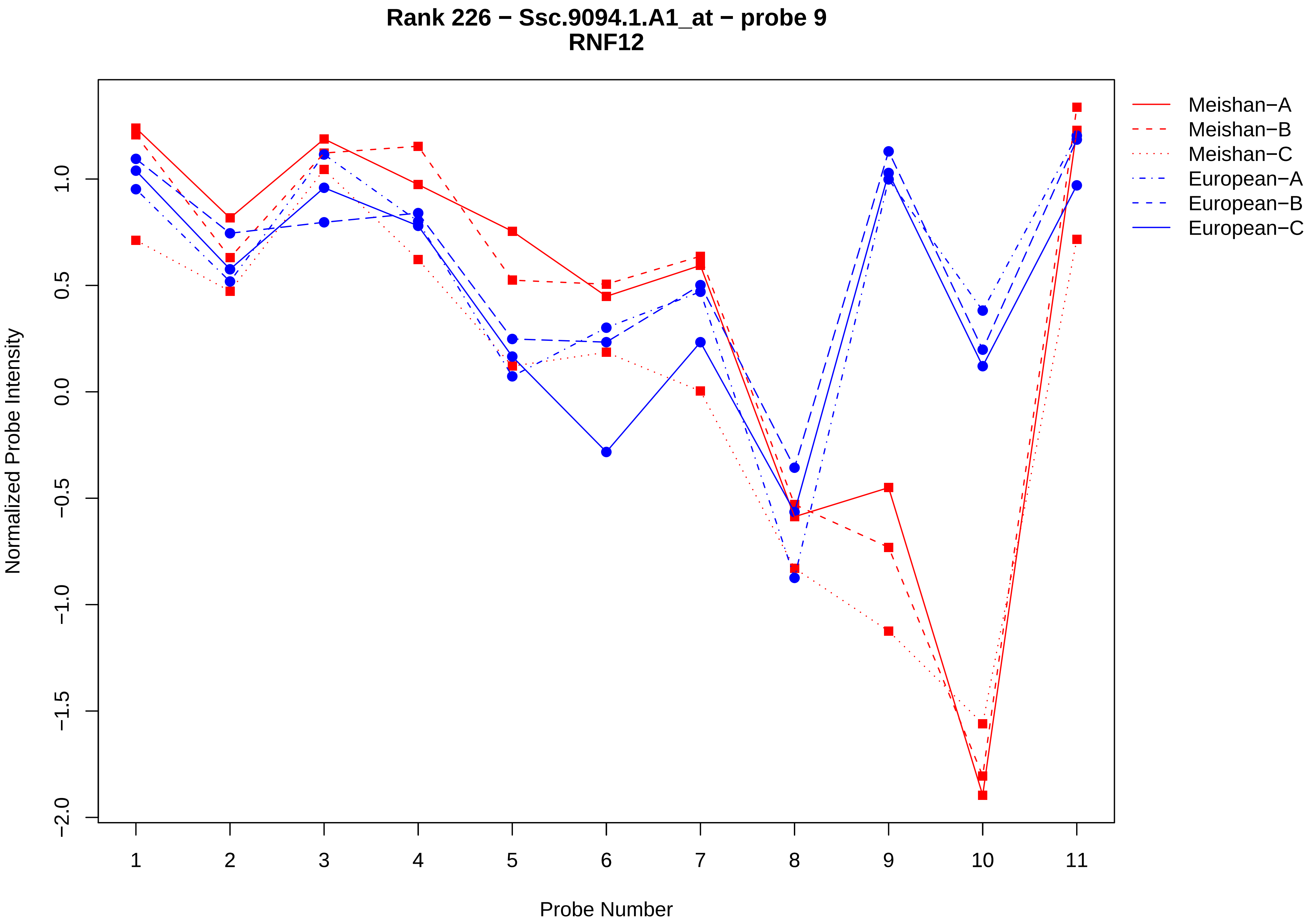

Rank 226 − Ssc.9094.1.A1_at − probe 9
RNF12
Normalized Probe Intensity
Probe Number
−2.0
−1.5
−1.0
−0.5
0.0
0.5
1.0
1
2
3
4
5
6
7
8
9
10
11
Meishan−A
Meishan−B
Meishan−C
European−A
European−B
European−C

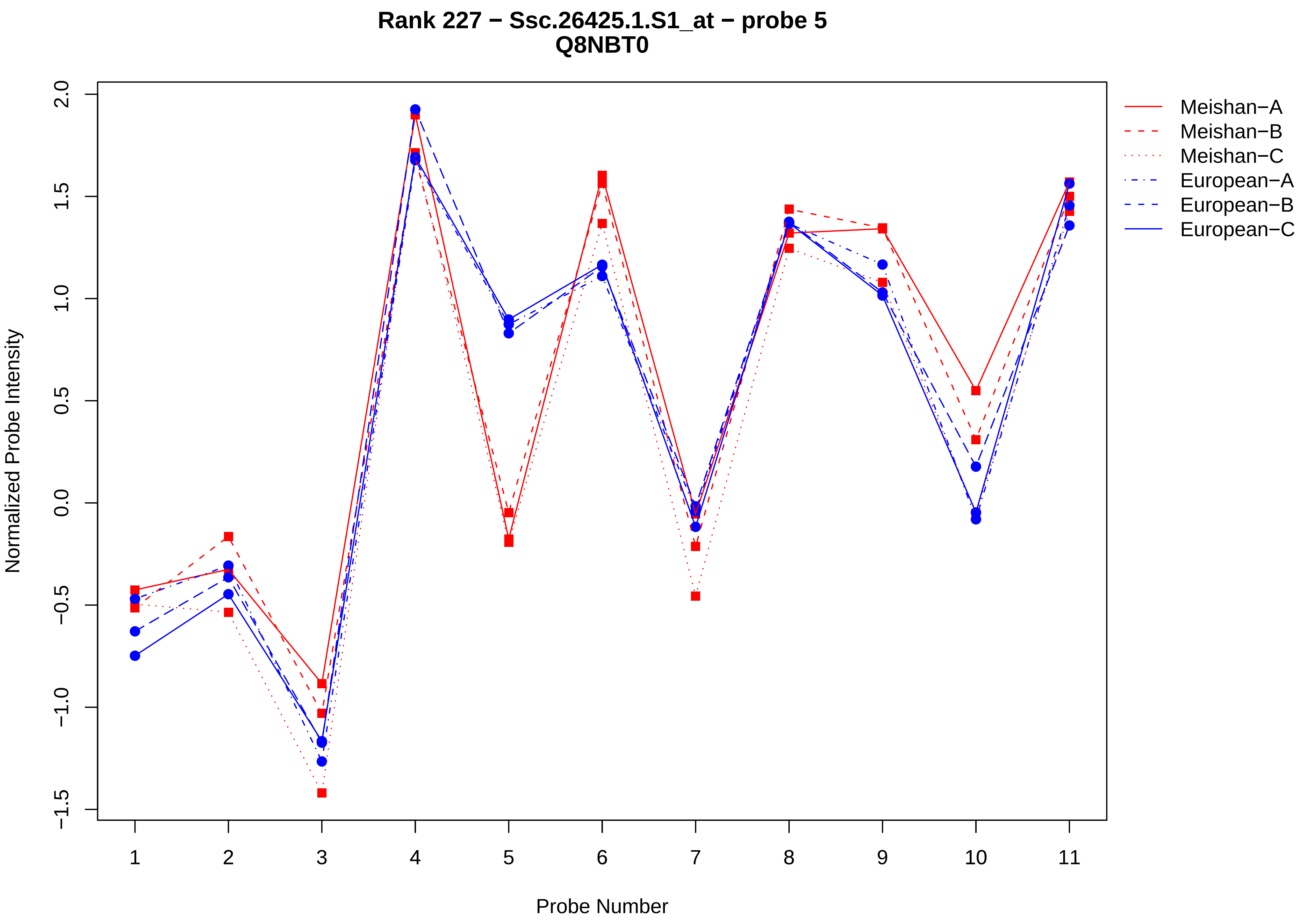

Rank 227 − Ssc.26425.1.S1_at − probe 5
Q8NBT0
Normalized Probe Intensity
Probe Number
Meishan−A
Meishan−B
Meishan−C
European−A
European−B
European−C

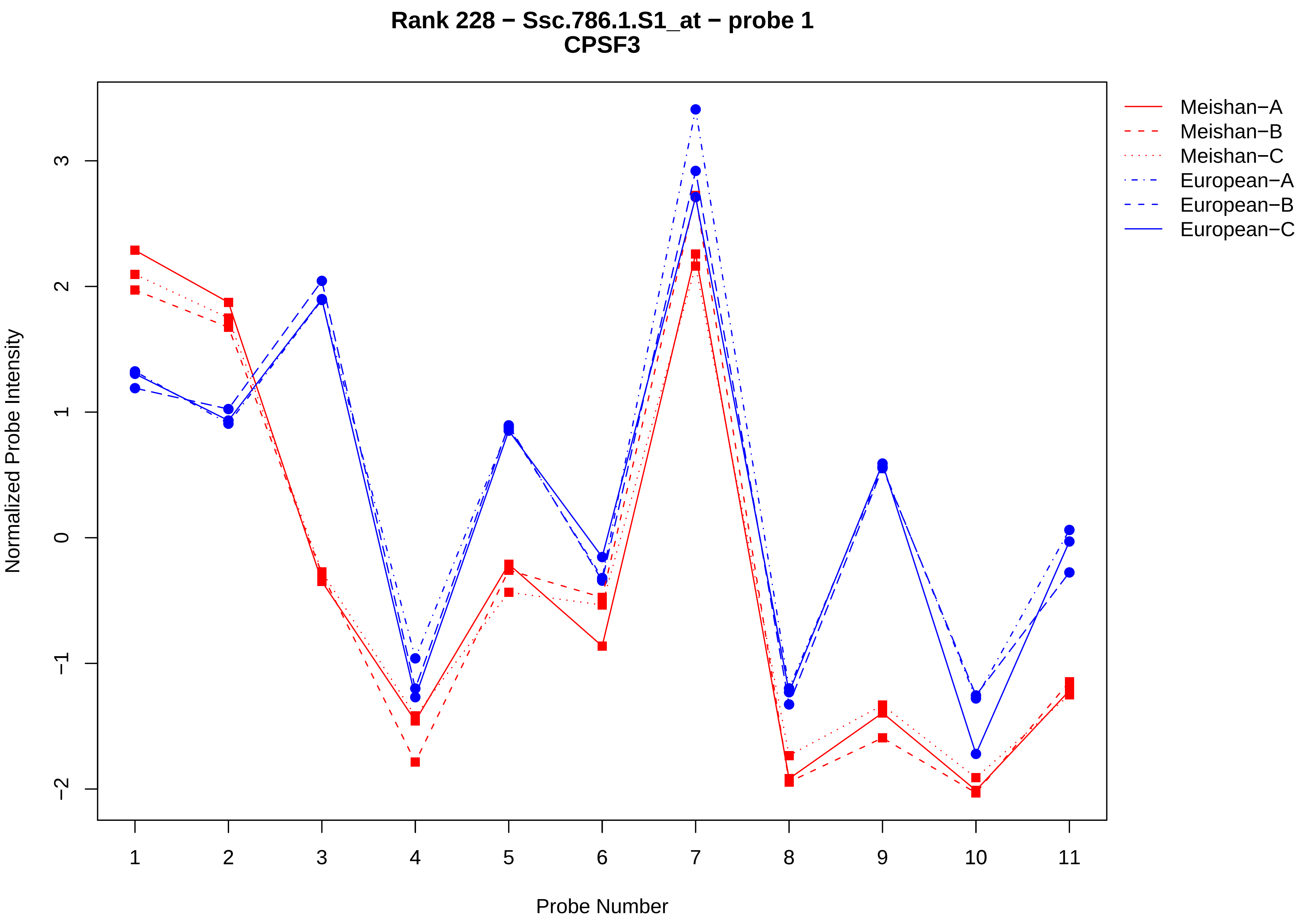

Rank 228 − Ssc.786.1.S1_at − probe 1
CPSF3
Normalized Probe Intensity
Probe Number
Meishan−A
Meishan−B
Meishan−C
European−A
European−B
European−C
−2
−1
0
1
2
3
1
2
3
4
5
6
7
8
9
10
11

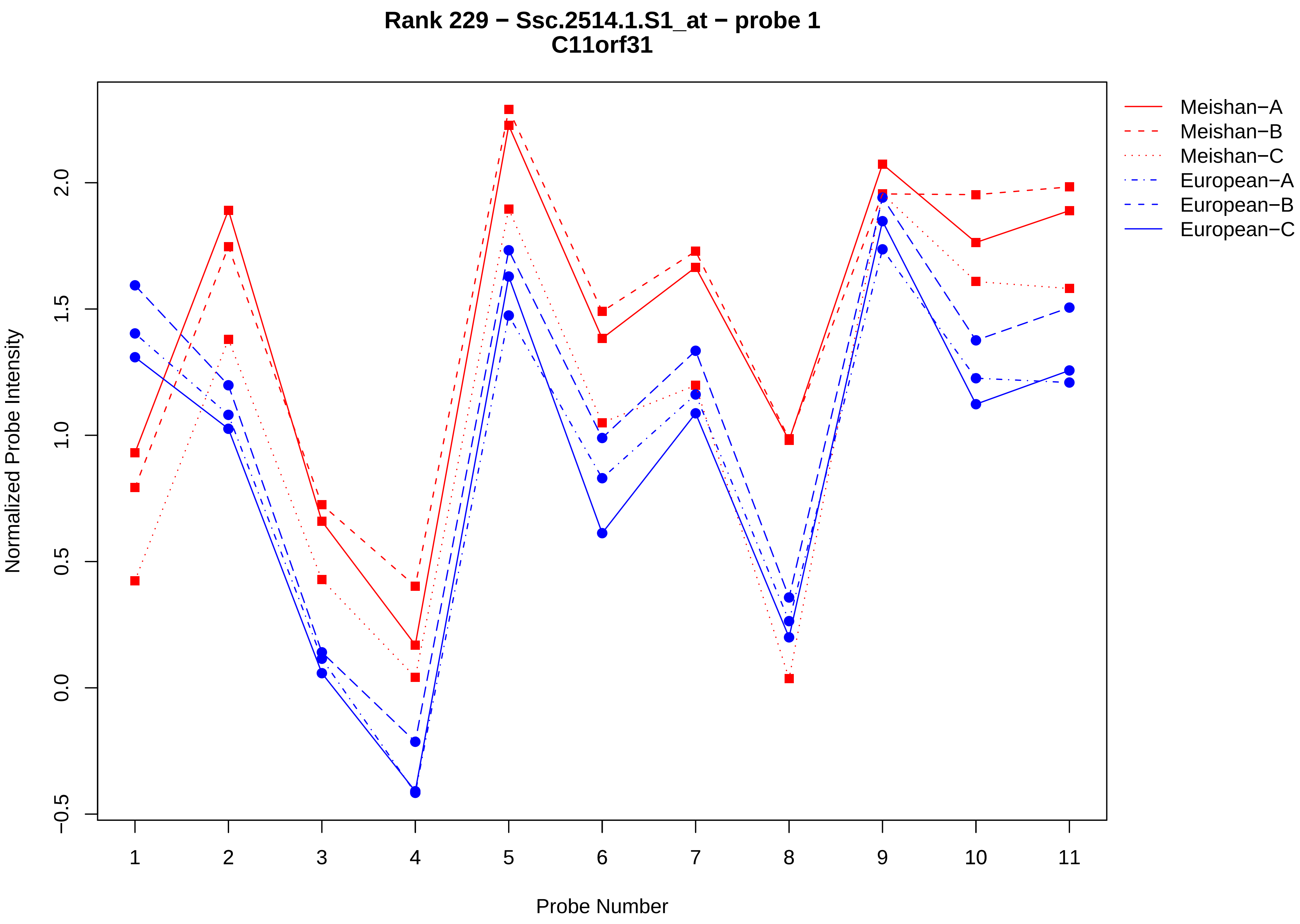

Rank 229 − Ssc.2514.1.S1_at − probe 1
C11orf31
Normalized Probe Intensity
Probe Number
−0.5
0.0
0.5
1.0
1.5
2.0
1
2
3
4
5
6
7
8
9
10
11
Meishan−A
Meishan−B
Meishan−C
European−A
European−B
European−C

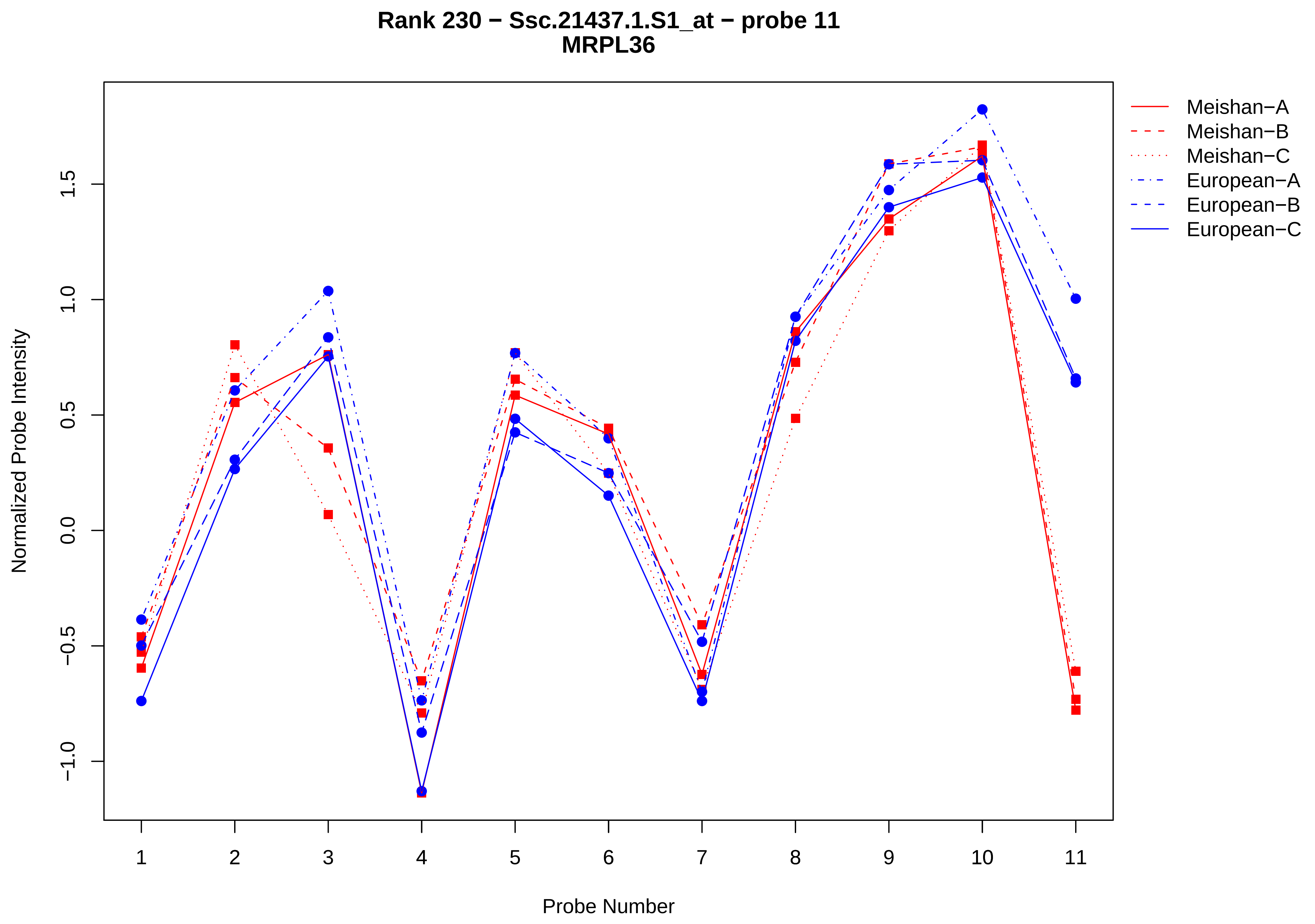

Rank 230 − Ssc.21437.1.S1_at − probe 11
MRPL36
Normalized Probe Intensity
Probe Number
Meishan−A
Meishan−B
Meishan−C
European−A
European−B
European−C
−1.0
−0.5
0.0
0.5
1.0
1.5
1
2
3
4
5
6
7
8
9
10
11

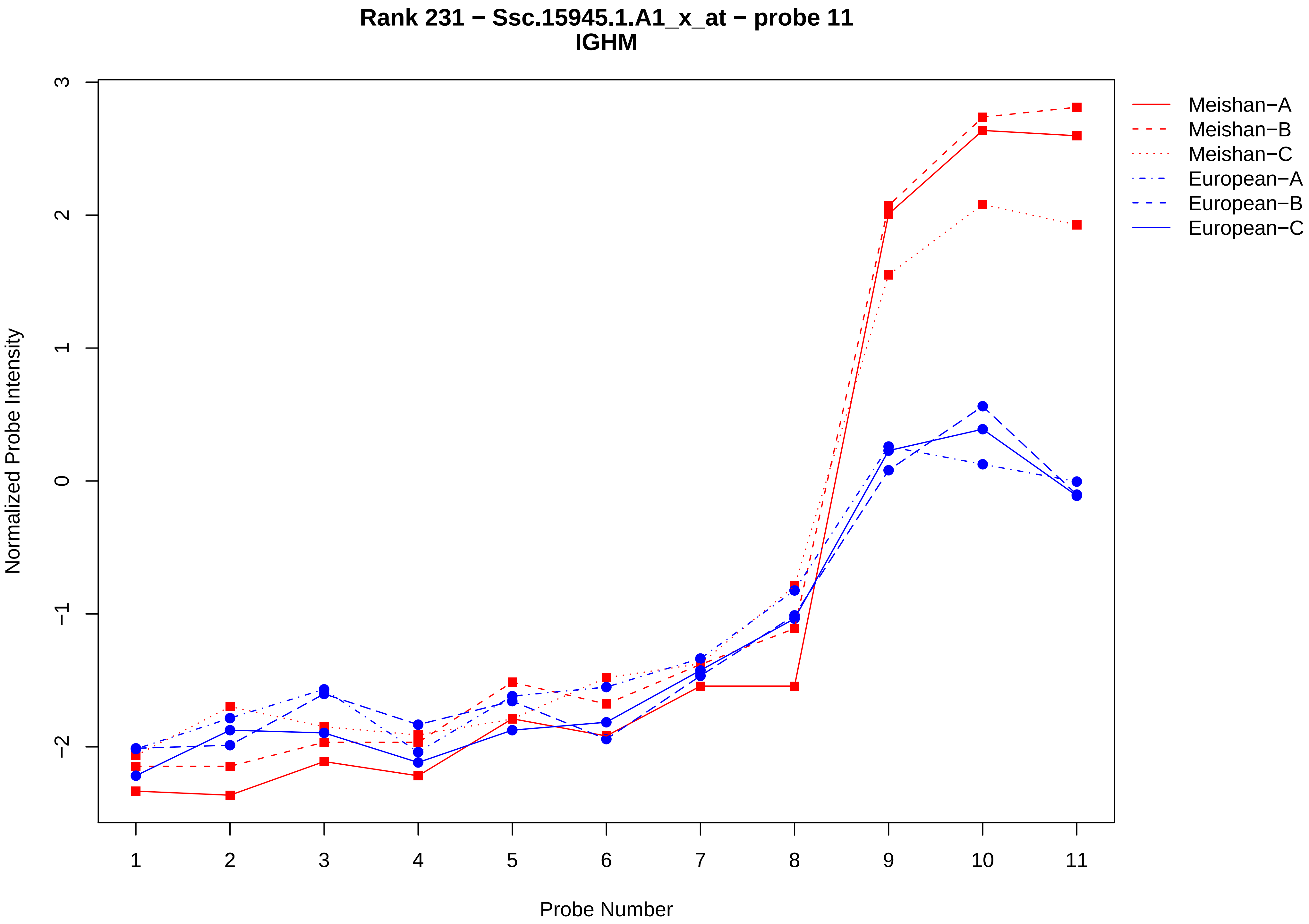
Rank 231 − Ssc.15945.1.A1_x_at − probe 11
IGHM
Normalized Probe Intensity
Probe Number
−2
−1
0
1
2
3
1
2
3
4
5
6
7
8
9
10
11
Meishan−A
Meishan−B
Meishan−C
European−A
European−B
European−C

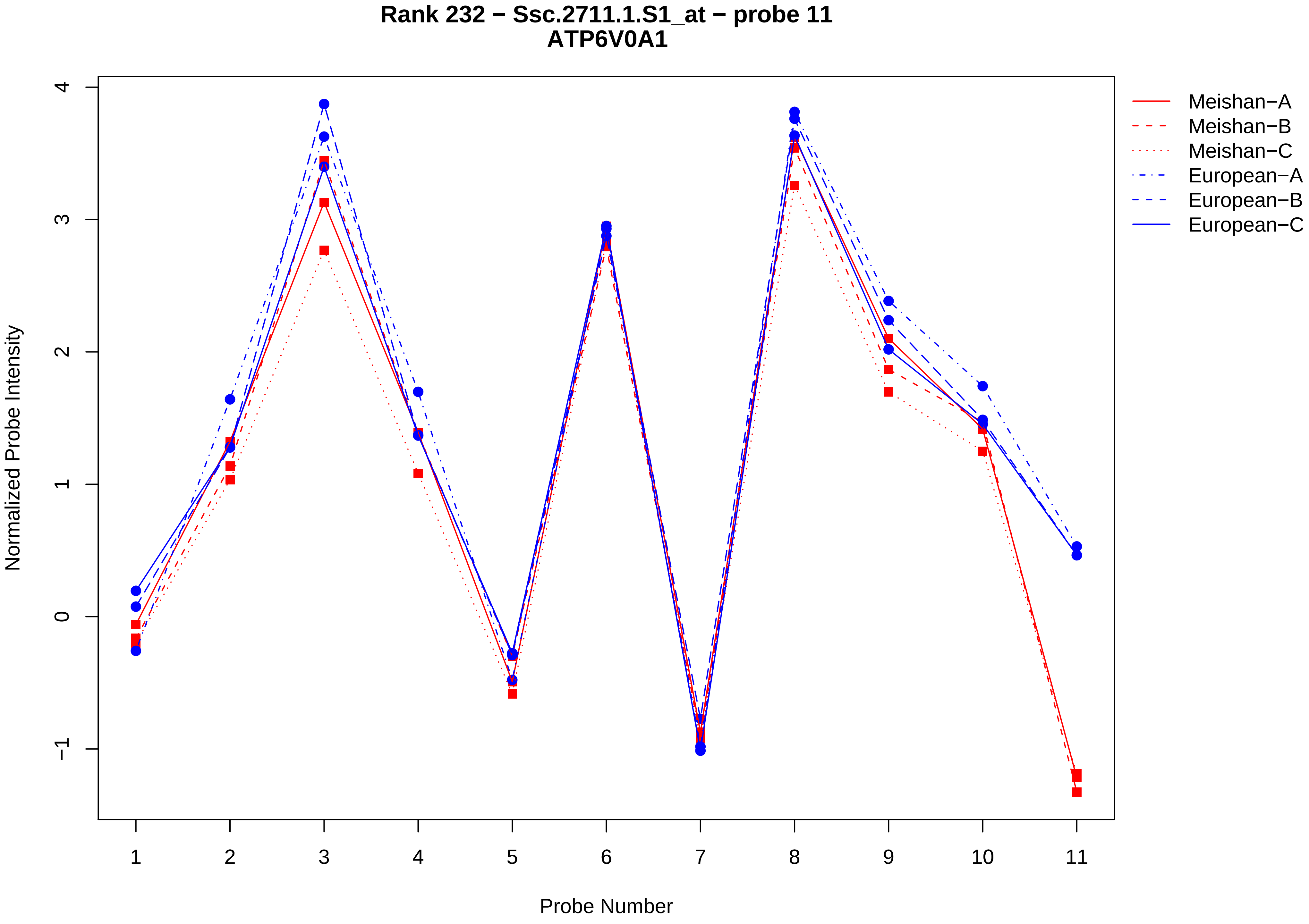

Rank 232 − Ssc.2711.1.S1_at − probe 11
ATP6V0A1
Normalized Probe Intensity
Probe Number
Meishan−A
Meishan−B
Meishan−C
European−A
European−B
European−C

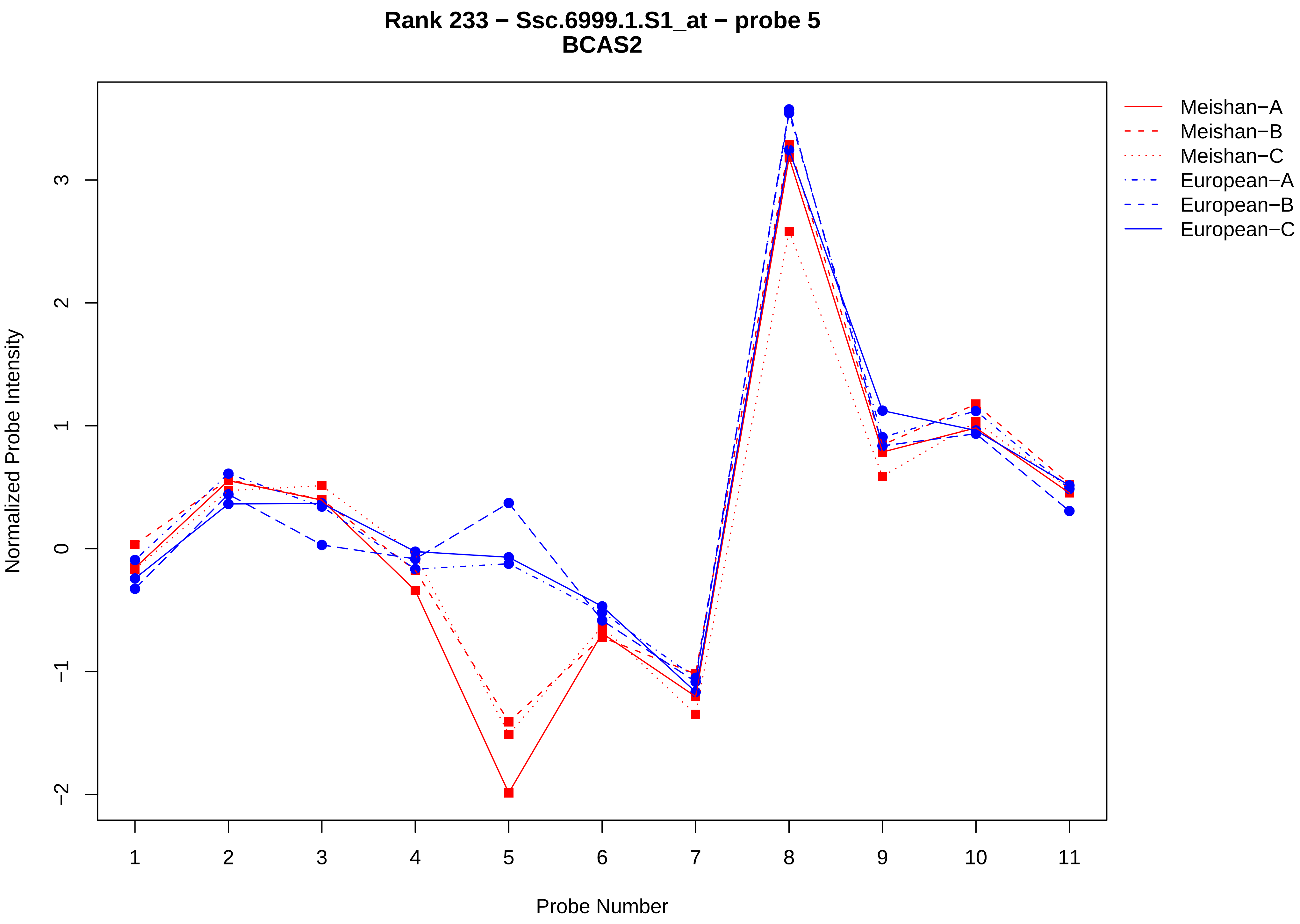
Rank 233 − Ssc.6999.1.S1_at − probe 5
BCAS2
Normalized Probe Intensity
Probe Number
−2
−1
0
1
2
3
1
2
3
4
5
6
7
8
9
10
11
Meishan−A
Meishan−B
Meishan−C
European−A
European−B
European−C

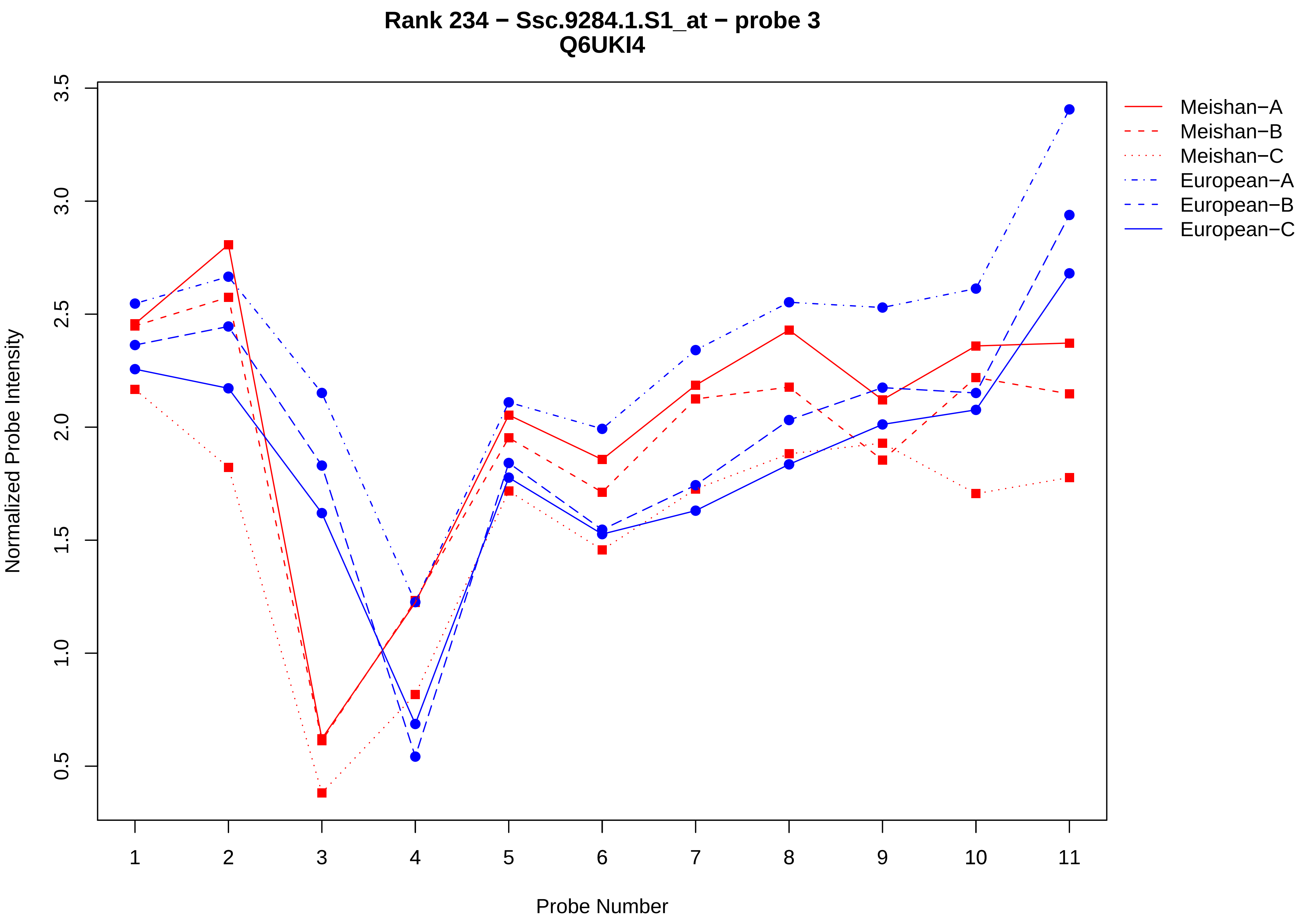

Rank 234 − Ssc.9284.1.S1_at − probe 3
Q6UKI4
Normalized Probe Intensity
Probe Number
0.5
1.0
1.5
2.0
2.5
3.0
3.5
1
2
3
4
5
6
7
8
9
10
11
Meishan−A
Meishan−B
Meishan−C
European−A
European−B
European−C

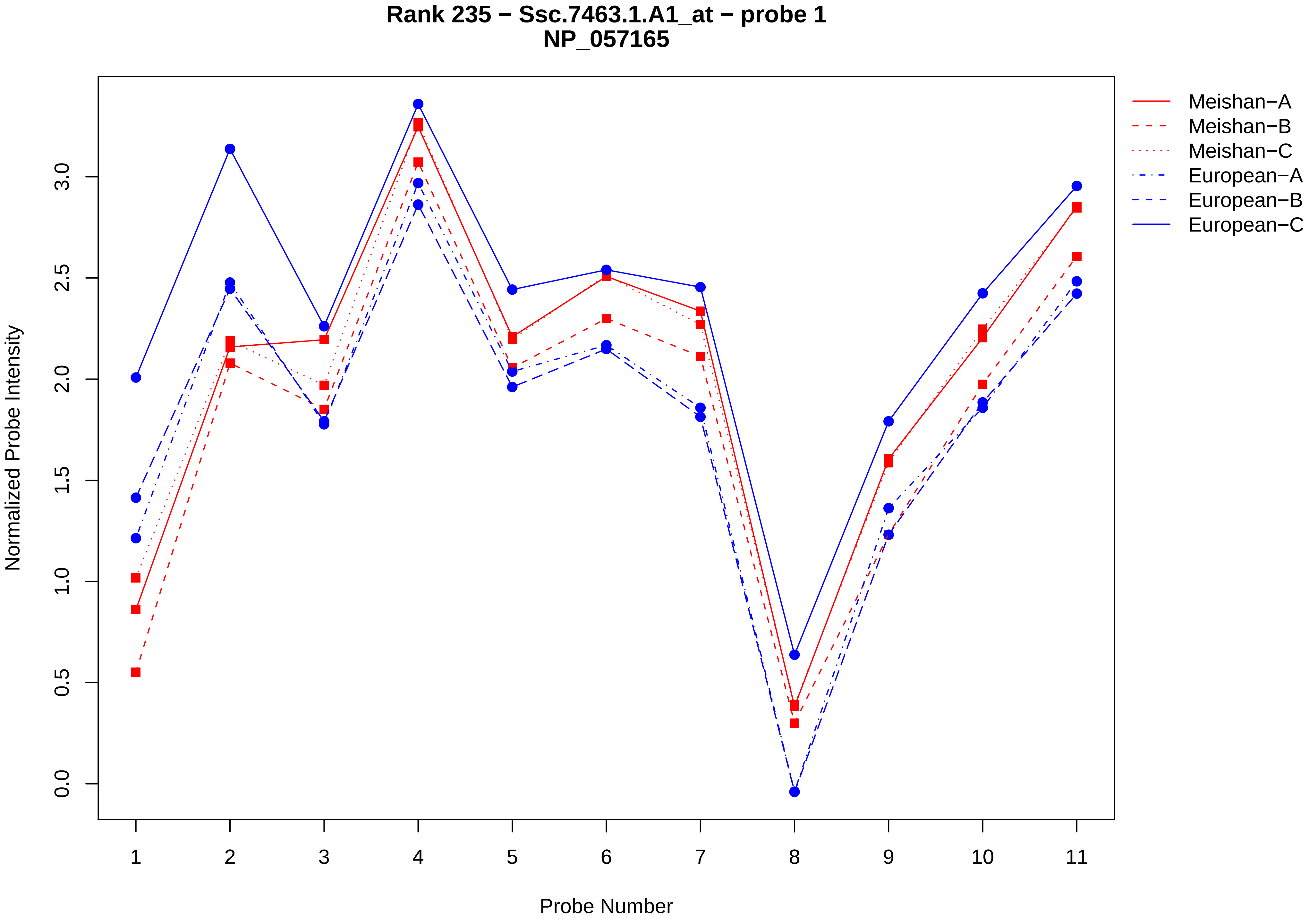

Rank 235 − Ssc.7463.1.A1_at − probe 1
NP_057165
Normalized Probe Intensity
0.0
0.5
1.0
1.5
2.0
2.5
3.0
Probe Number
1
2
3
4
5
6
7
8
9
10
11
Meishan−A
Meishan−B
Meishan−C
European−A
European−B
European−C

Rank 236 − Ssc.17315.1.S1_at − probe 3
EIF3S2

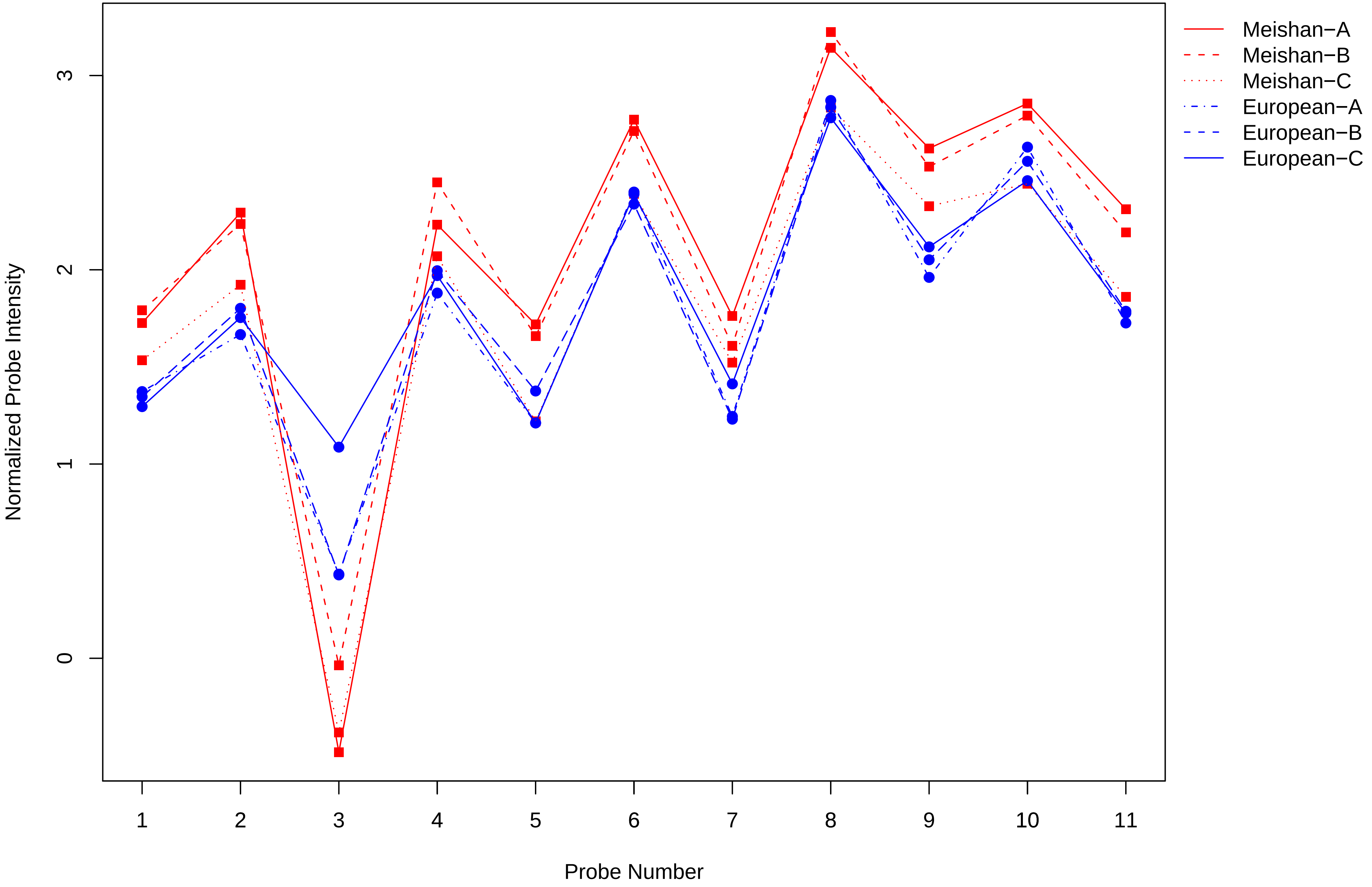

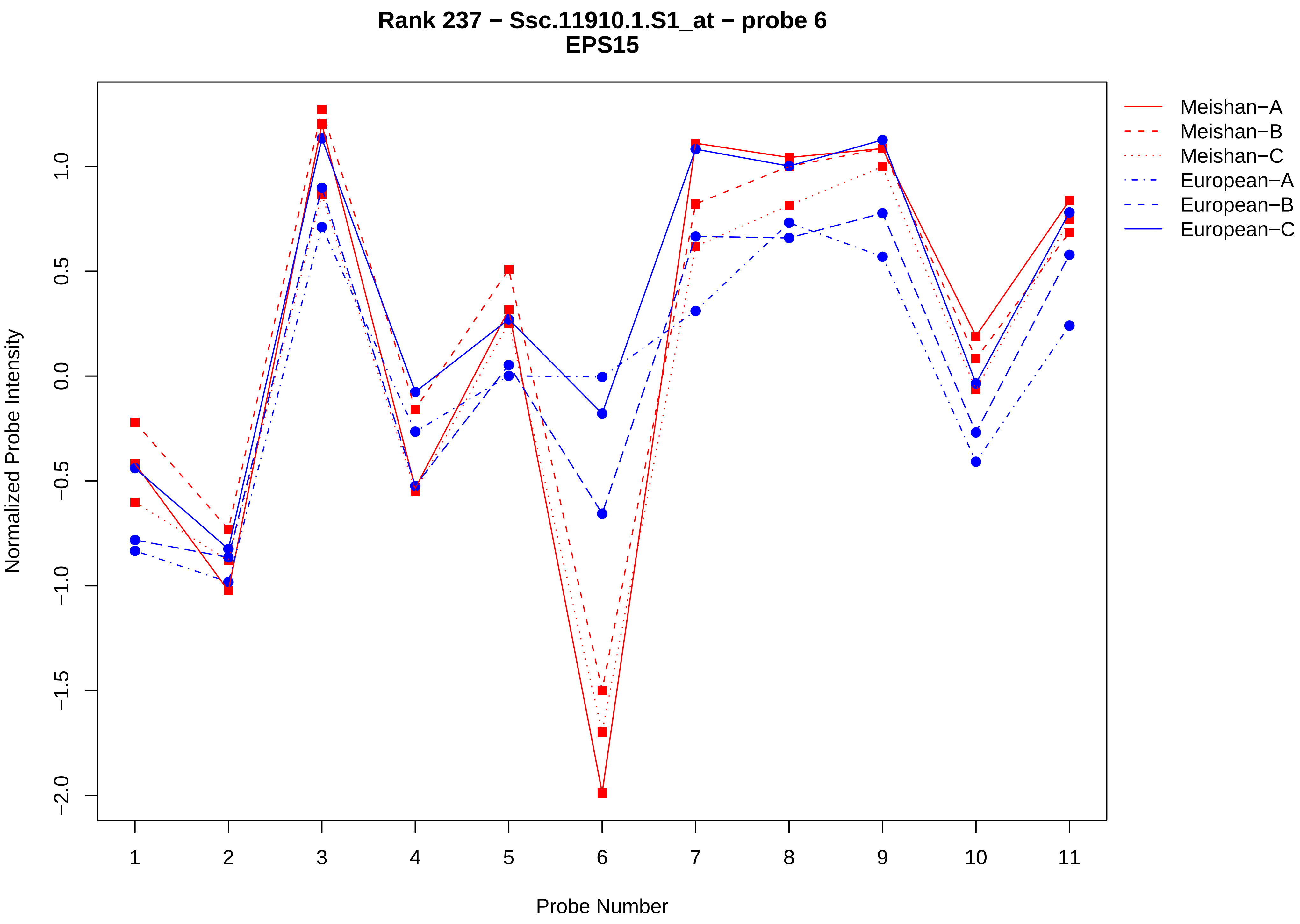

Rank 237 − Ssc.11910.1.S1_at − probe 6
EPS15
Normalized Probe Intensity
Probe Number
1.0
0.5
0.0
−0.5
−1.0
−1.5
−2.0
1
2
3
4
5
6
7
8
9
10
11
Meishan−A
Meishan−B
Meishan−C
European−A
European−B
European−C

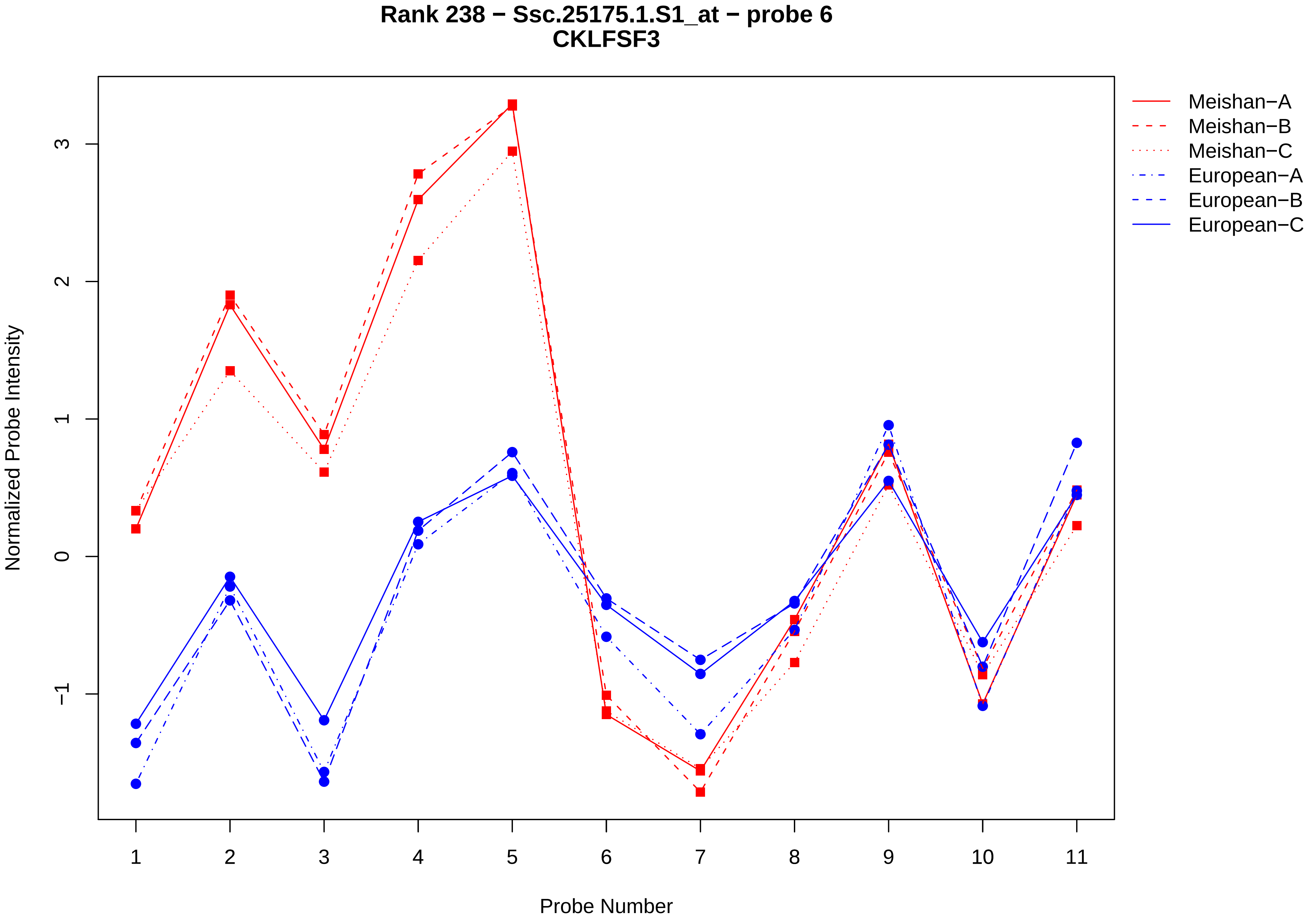

Rank 238 − Ssc.25175.1.S1_at − probe 6
CKLFSF3
Normalized Probe Intensity
Probe Number
Meishan−A
Meishan−B
Meishan−C
European−A
European−B
European−C
−1
0
1
2
3
1
2
3
4
5
6
7
8
9
10
11

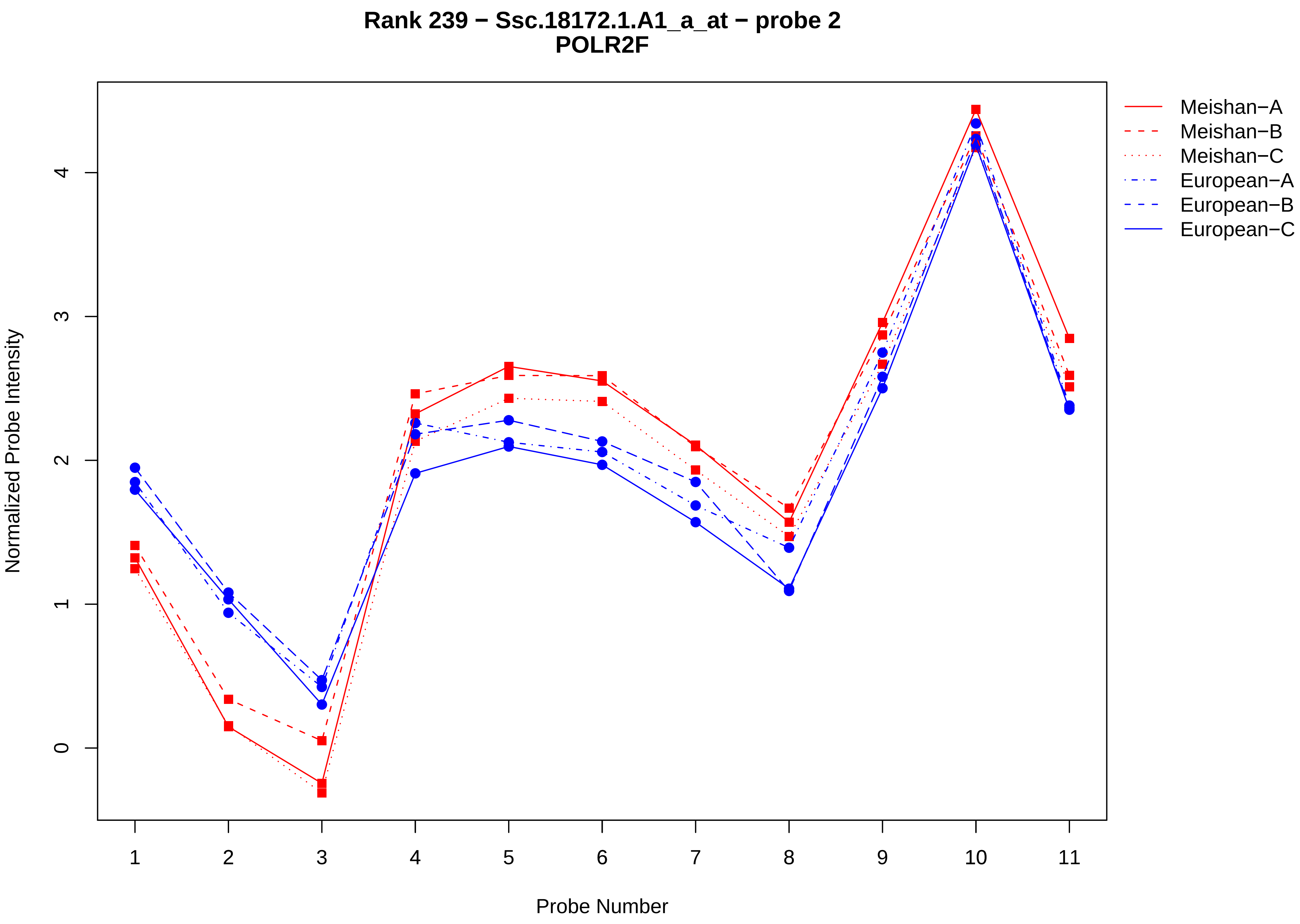

Rank 239 − Ssc.18172.1.A1_a_at − probe 2
POLR2F
Normalized Probe Intensity
0
1
2
3
4
Probe Number
1
2
3
4
5
6
7
8
9
10
11
Meishan−A
Meishan−B
Meishan−C
European−A
European−B
European−C

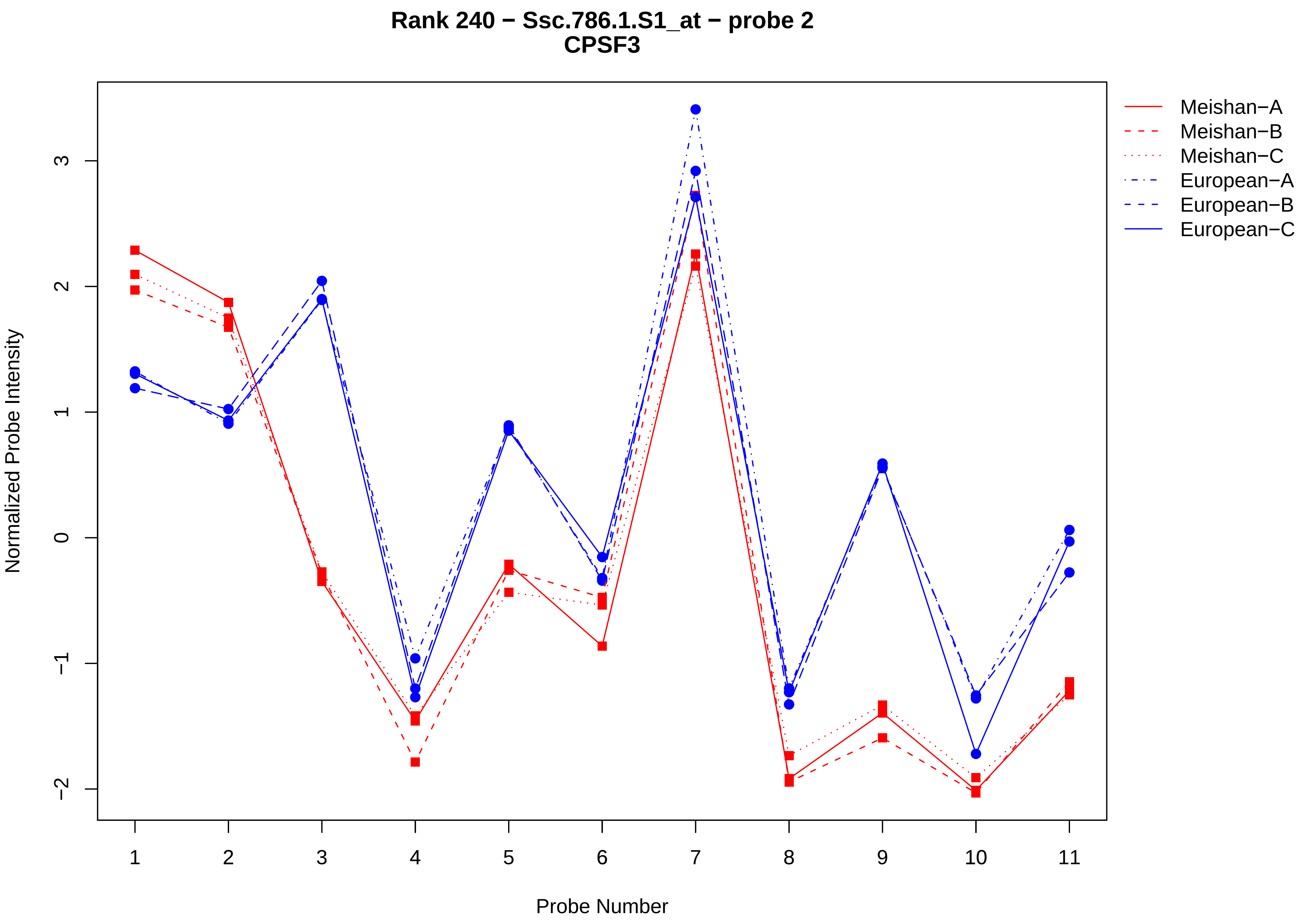

Rank 240 − Ssc.786.1.S1_at − probe 2
CPSF3
Normalized Probe Intensity
Probe Number
Meishan−A
Meishan−B
Meishan−C
European−A
European−B
European−C

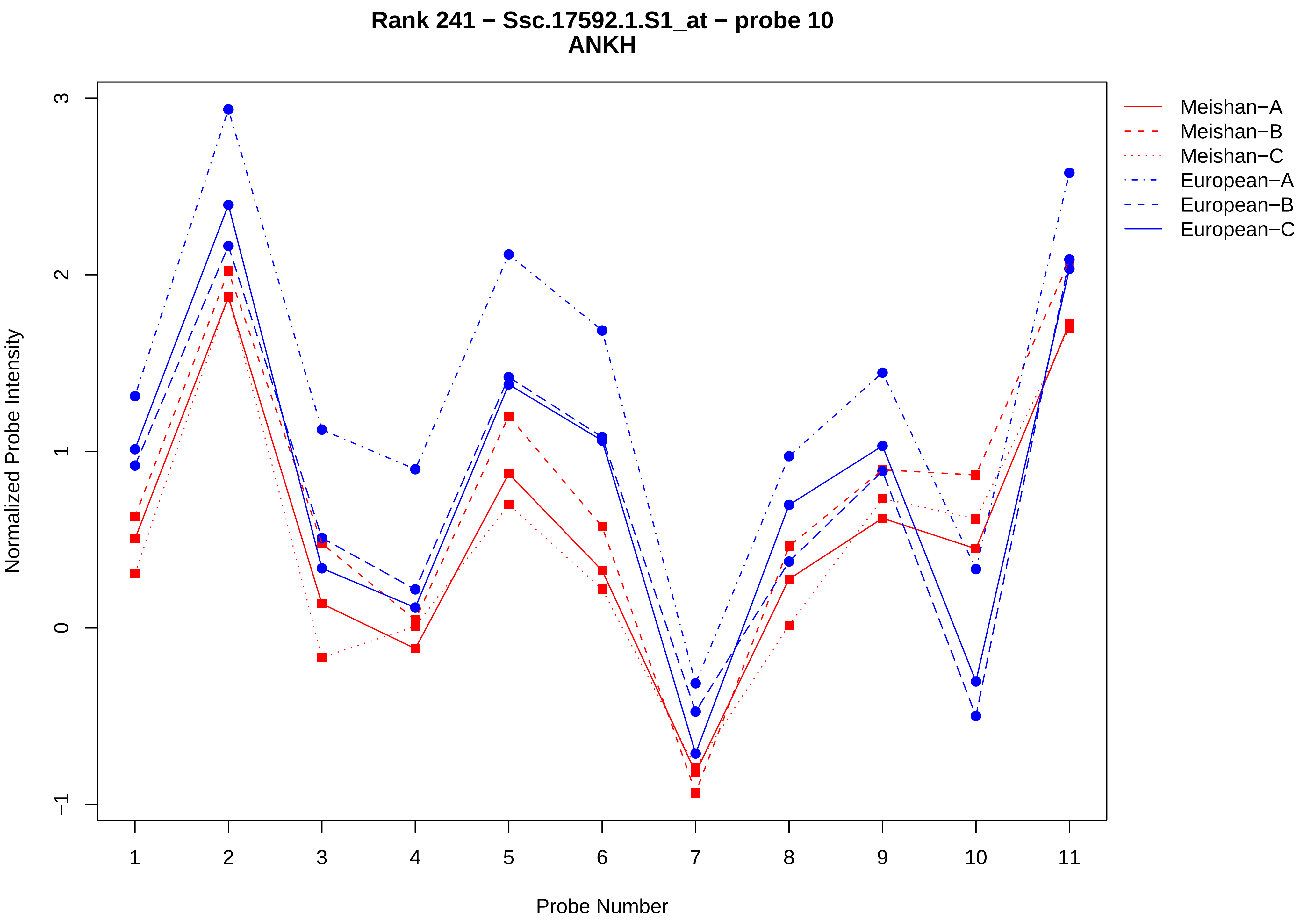

Rank 241 − Ssc.17592.1.S1_at − probe 10
ANKH
Normalized Probe Intensity
Probe Number
−1
0
1
2
3
1
2
3
4
5
6
7
8
9
10
11
Meishan−A
Meishan−B
Meishan−C
European−A
European−B
European−C

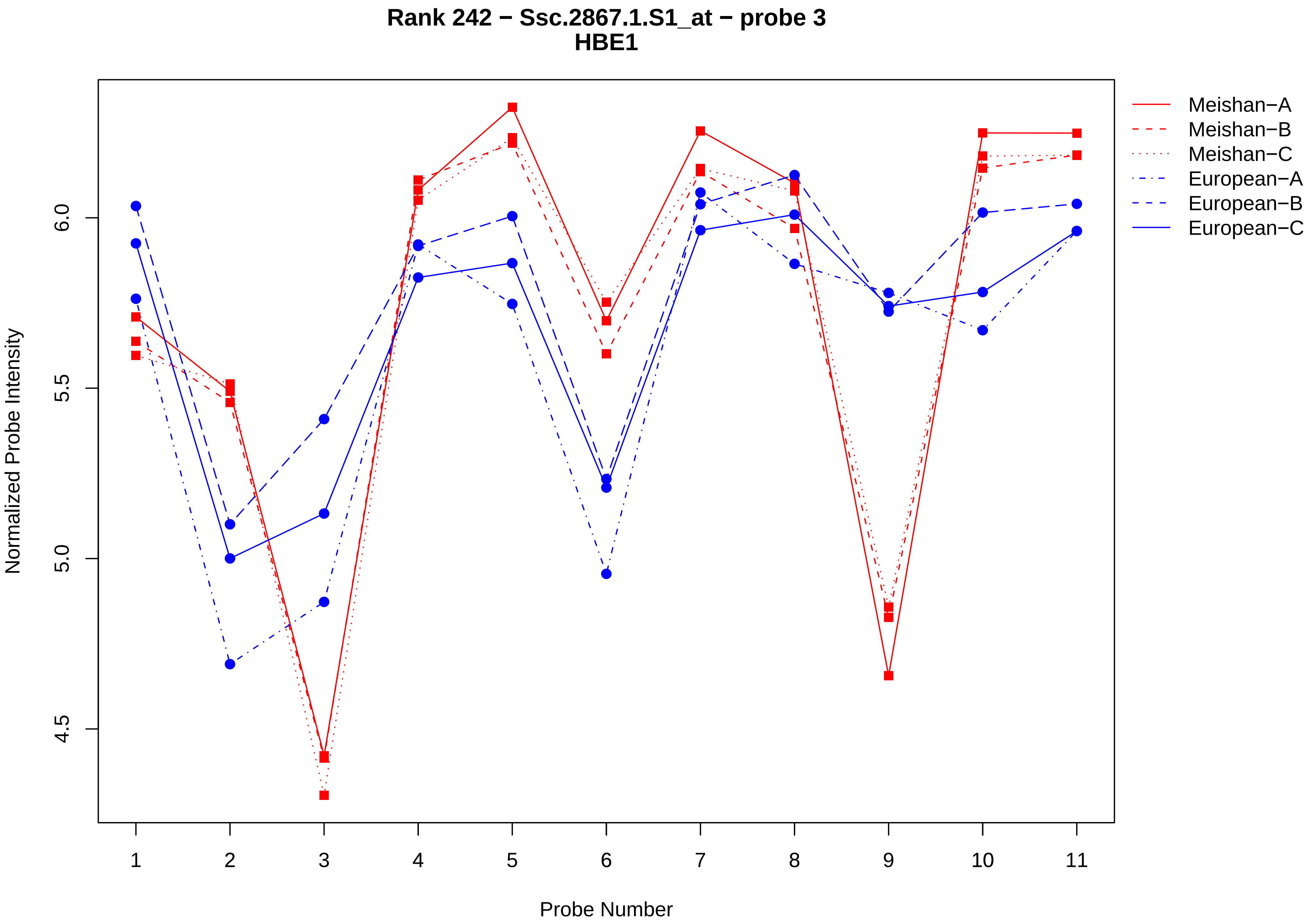

Rank 242 − Ssc.2867.1.S1_at − probe 3
HBE1
Normalized Probe Intensity
Probe Number
4.5
5.0
5.5
6.0
1
2
3
4
5
6
7
8
9
10
11
Meishan−A
Meishan−B
Meishan−C
European−A
European−B
European−C

Rank 243 − Ssc.7040.2.A1_at − probe 9
SLC30A9

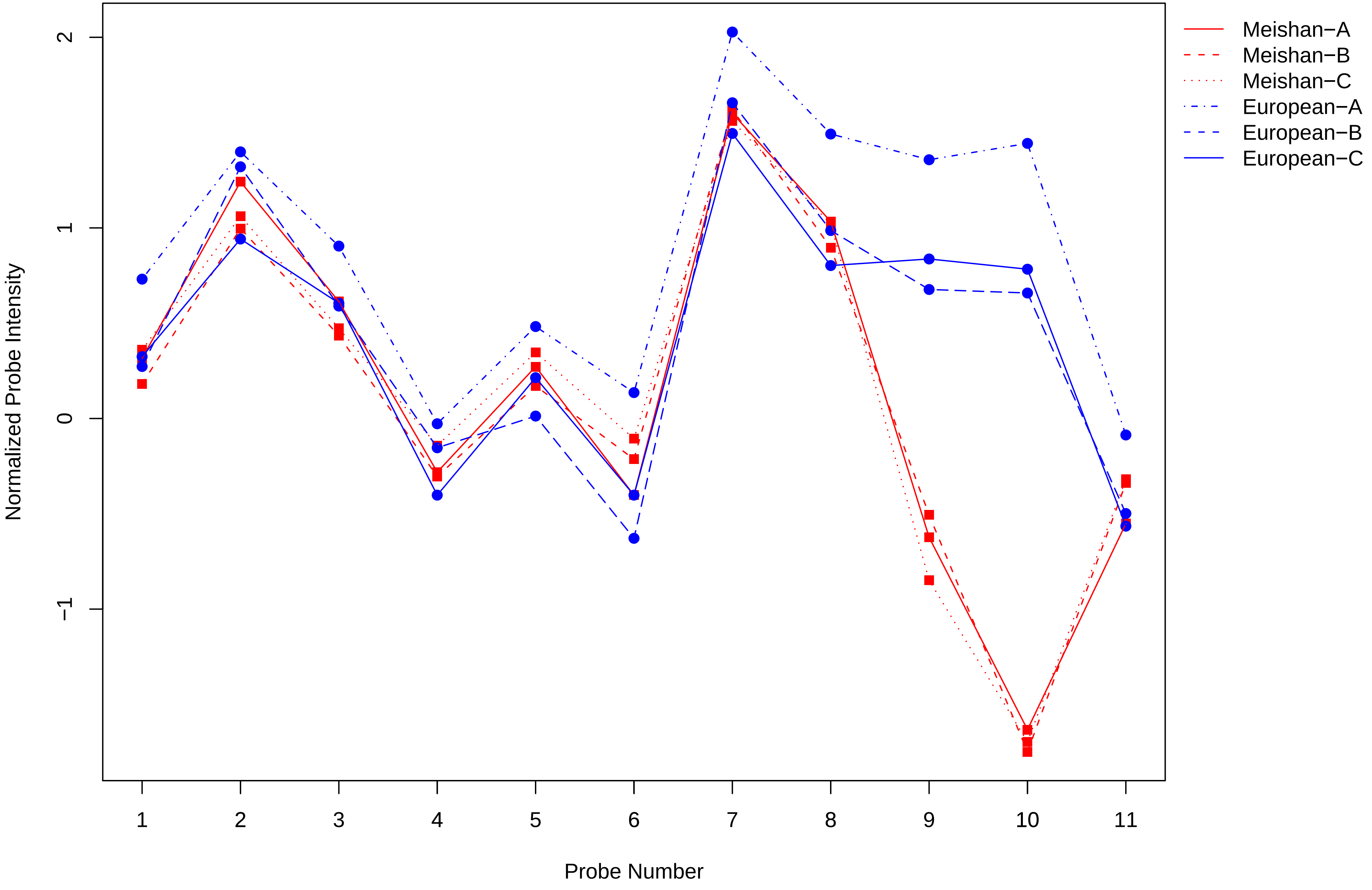

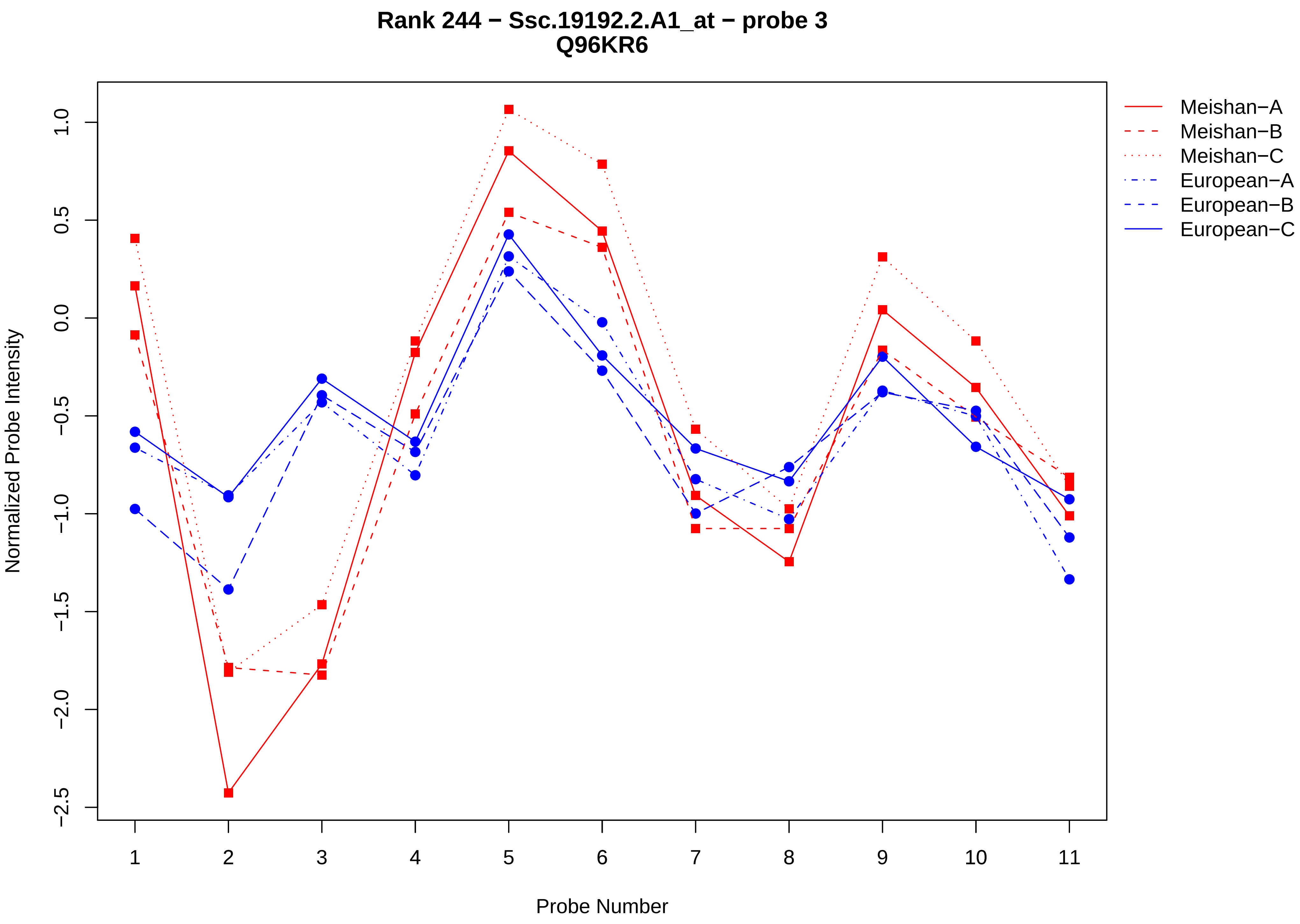
Rank 244 − Ssc.19192.2.A1_at − probe 3
Q96KR6
Normalized Probe Intensity
Probe Number
1.0
0.5
0.0
−0.5
−1.0
−1.5
−2.0
−2.5
1
2
3
4
5
6
7
8
9
10
11
Meishan−A
Meishan−B
Meishan−C
European−A
European−B
European−C

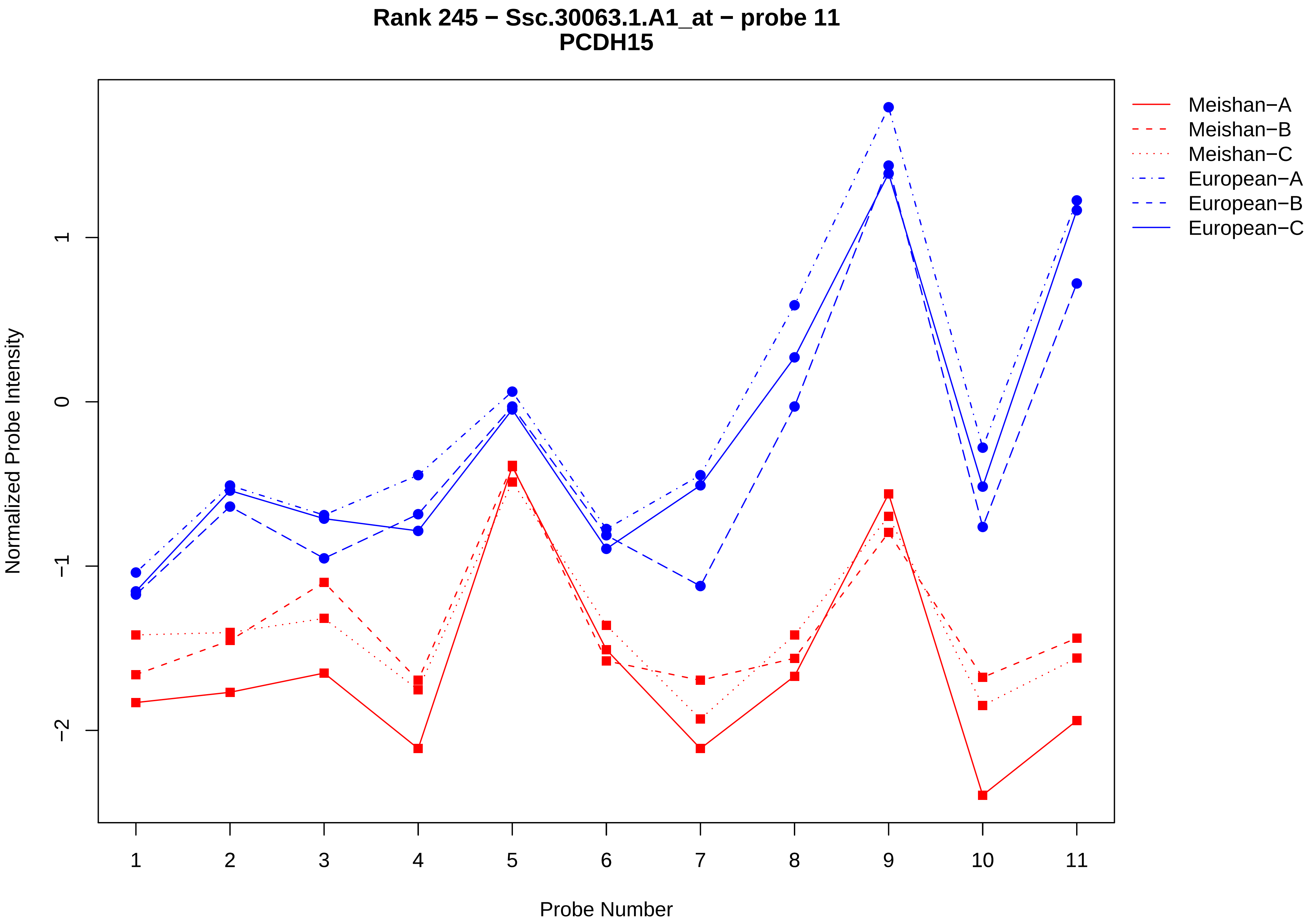

Rank 245 − Ssc.30063.1.A1_at − probe 11
PCDH15
Normalized Probe Intensity
Probe Number
−2
−1
0
1
1
2
3
4
5
6
7
8
9
10
11
Meishan−A
Meishan−B
Meishan−C
European−A
European−B
European−C

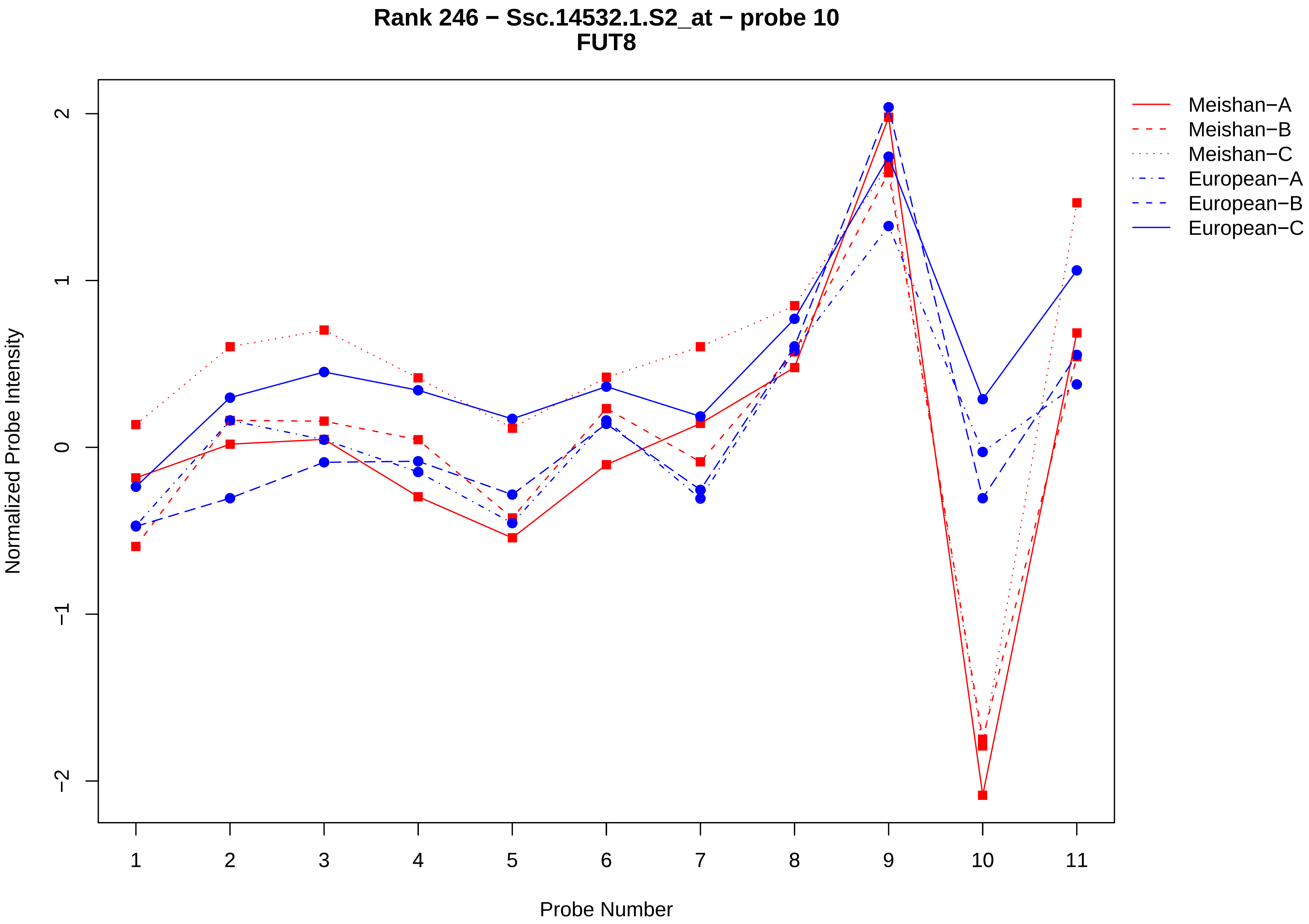

Rank 246 − Ssc.14532.1.S2_at − probe 10
FUT8
Normalized Probe Intensity
Probe Number
−2
−1
0
1
2
1
2
3
4
5
6
7
8
9
10
11
Meishan−A
Meishan−B
Meishan−C
European−A
European−B
European−C

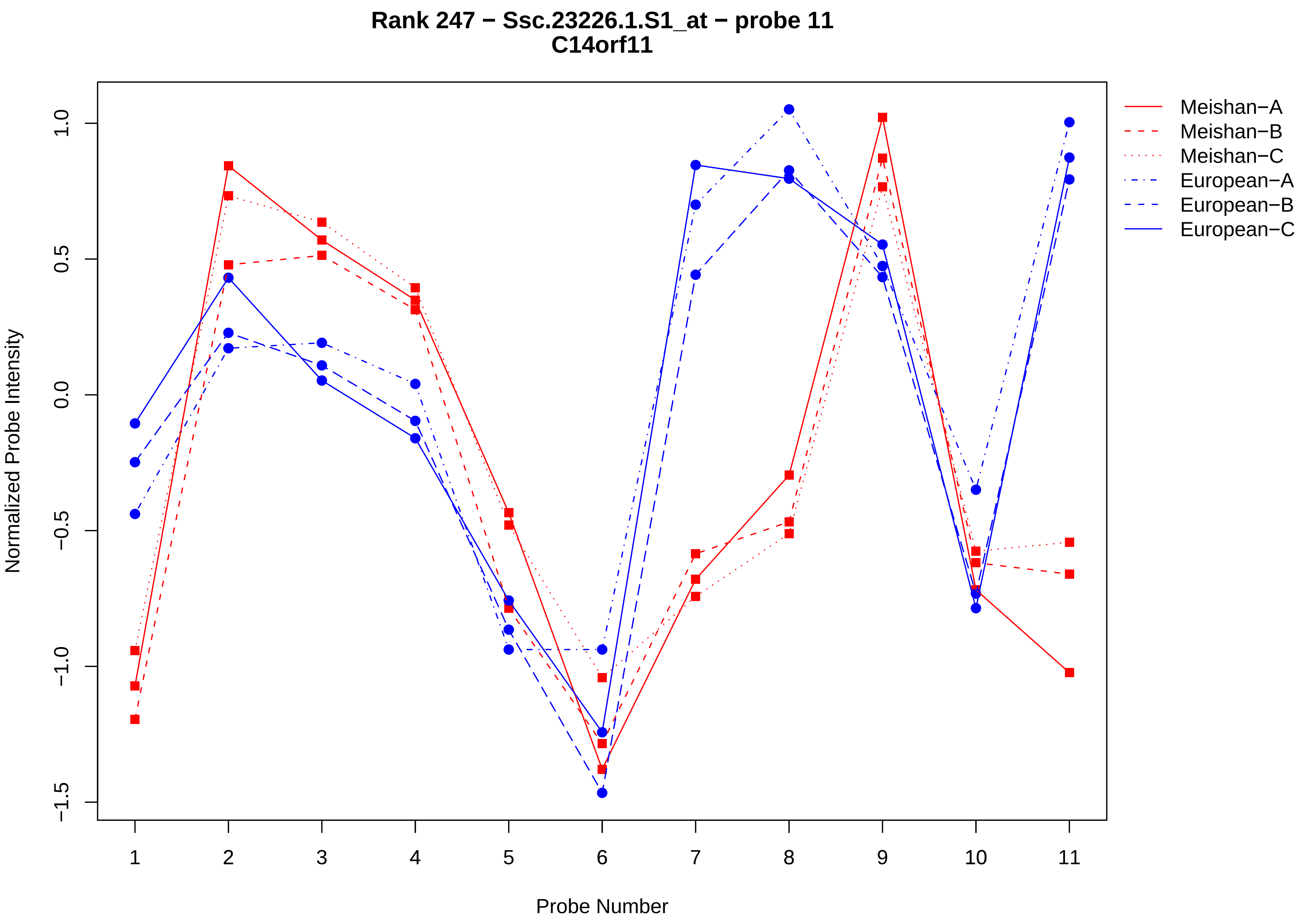
Rank 247 − Ssc.23226.1.S1_at − probe 11
C14orf11
Normalized Probe Intensity
Probe Number
Meishan−A
Meishan−B
Meishan−C
European−A
European−B
European−C
1.0
0.5
0.0
−0.5
−1.0
−1.5
1
2
3
4
5
6
7
8
9
10
11

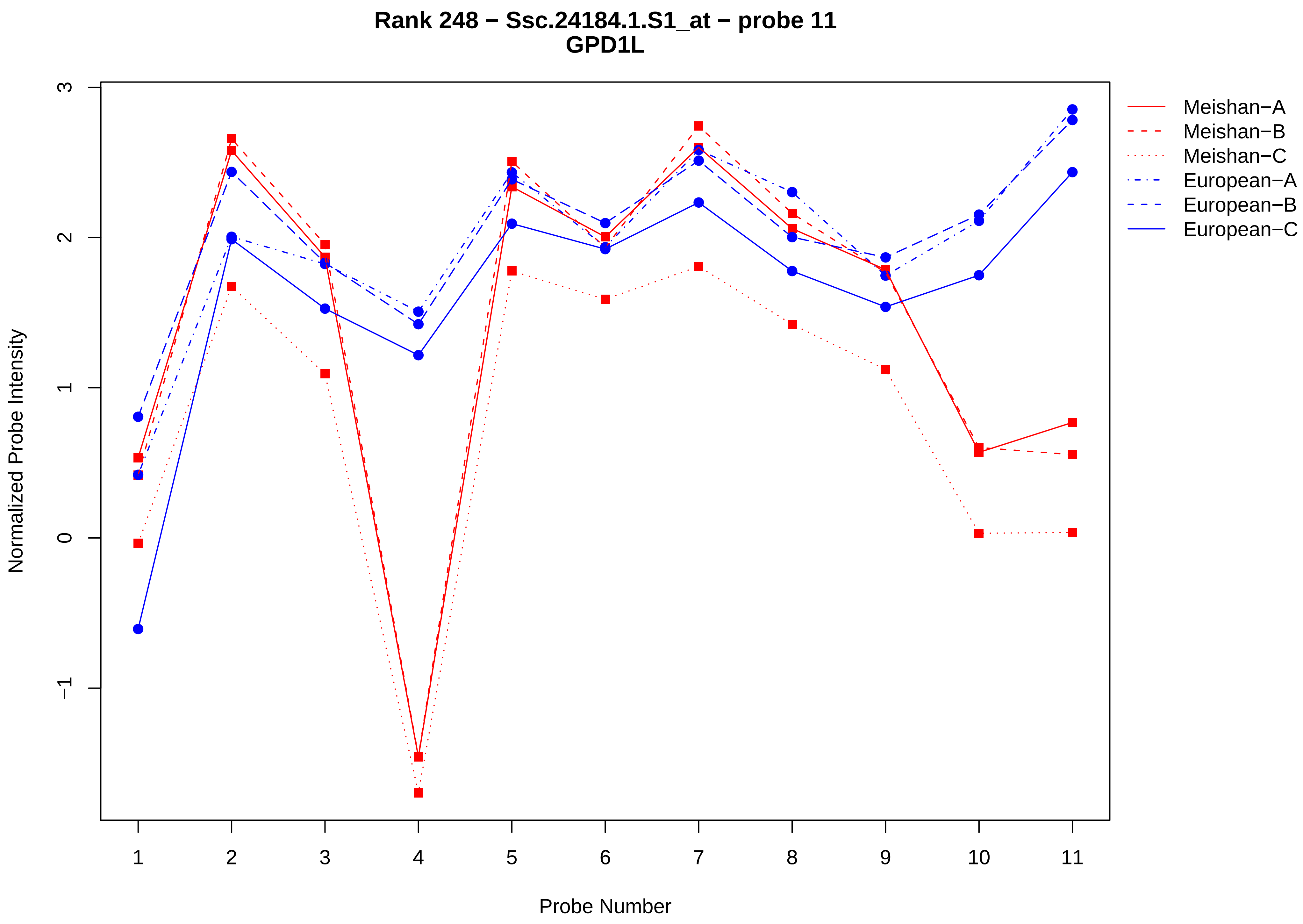
Rank 248 − Ssc.24184.1.S1_at − probe 11
GPD1L
Normalized Probe Intensity
Probe Number
−1
0
1
2
3
1
2
3
4
5
6
7
8
9
10
11
Meishan−A
Meishan−B
Meishan−C
European−A
European−B
European−C

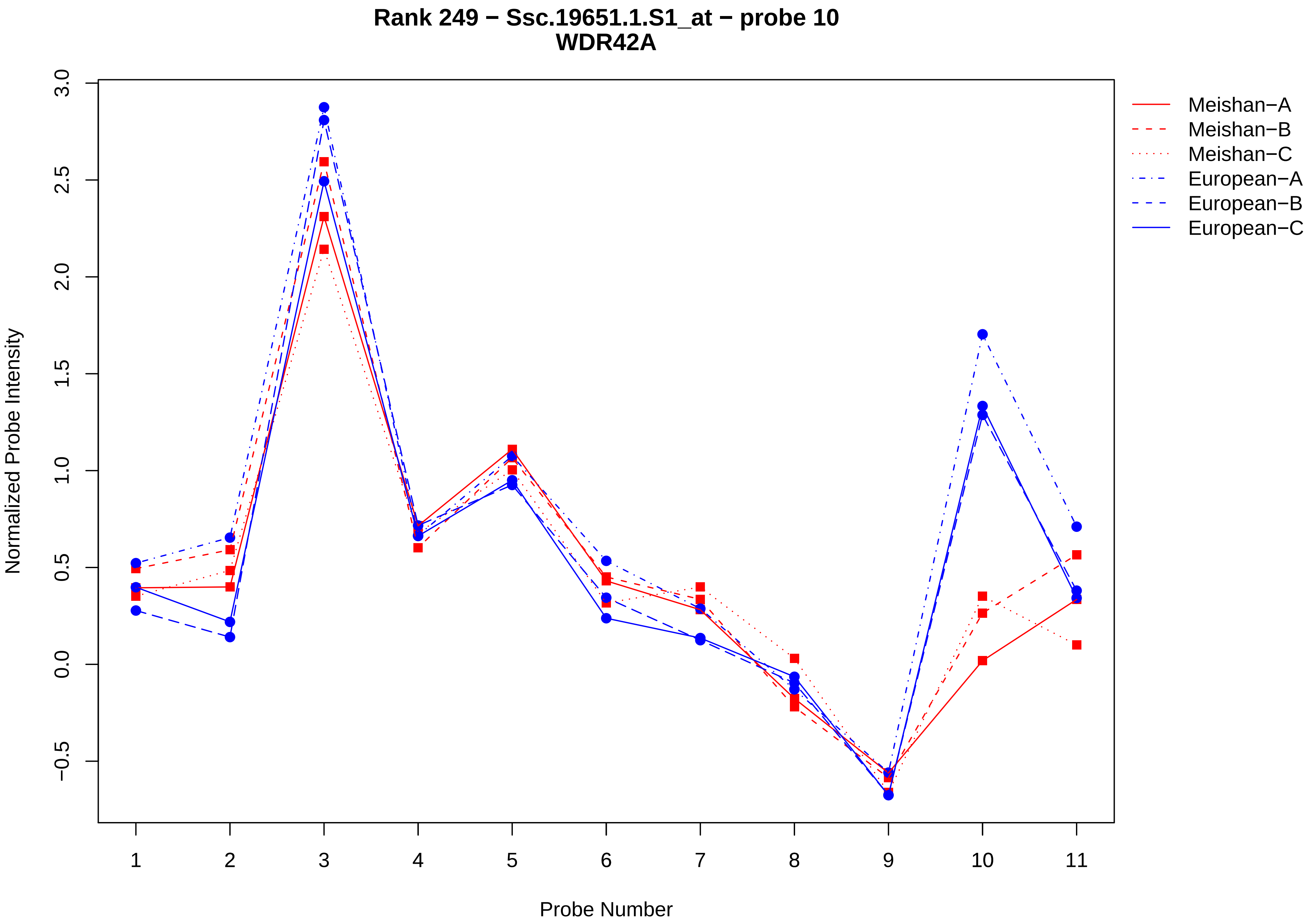
Rank 249 − Ssc.19651.1.S1_at − probe 10
WDR42A
Normalized Probe Intensity
Probe Number
−0.5
0.0
0.5
1.0
1.5
2.0
2.5
3.0
1
2
3
4
5
6
7
8
9
10
11
Meishan−A
Meishan−B
Meishan−C
European−A
European−B
European−C

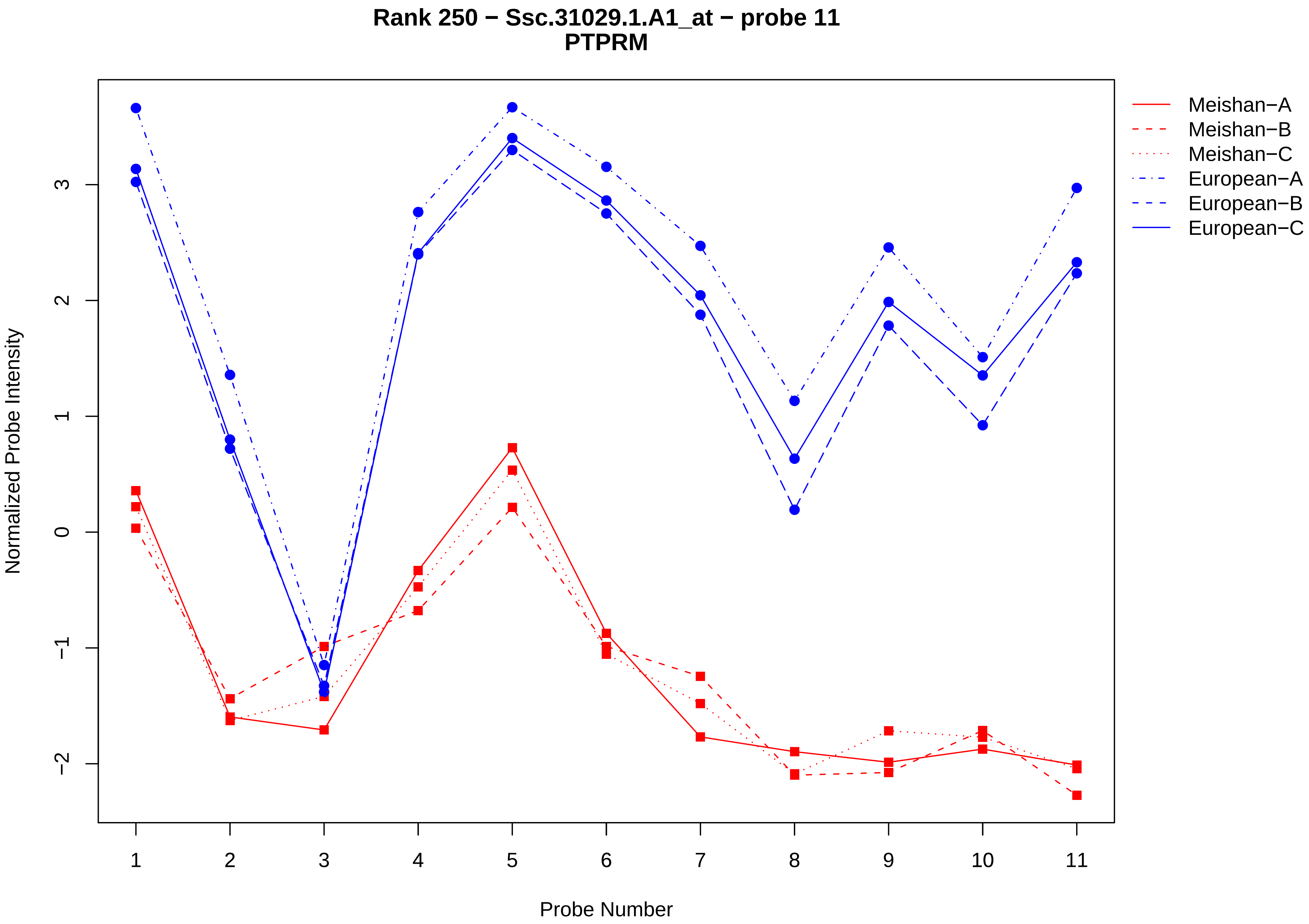
Rank 250 − Ssc.31029.1.A1_at − probe 11
PTPRM
Normalized Probe Intensity
Probe Number
−2
−1
0
1
2
3
1
2
3
4
5
6
7
8
9
10
11
Meishan−A
Meishan−B
Meishan−C
European−A
European−B
European−C

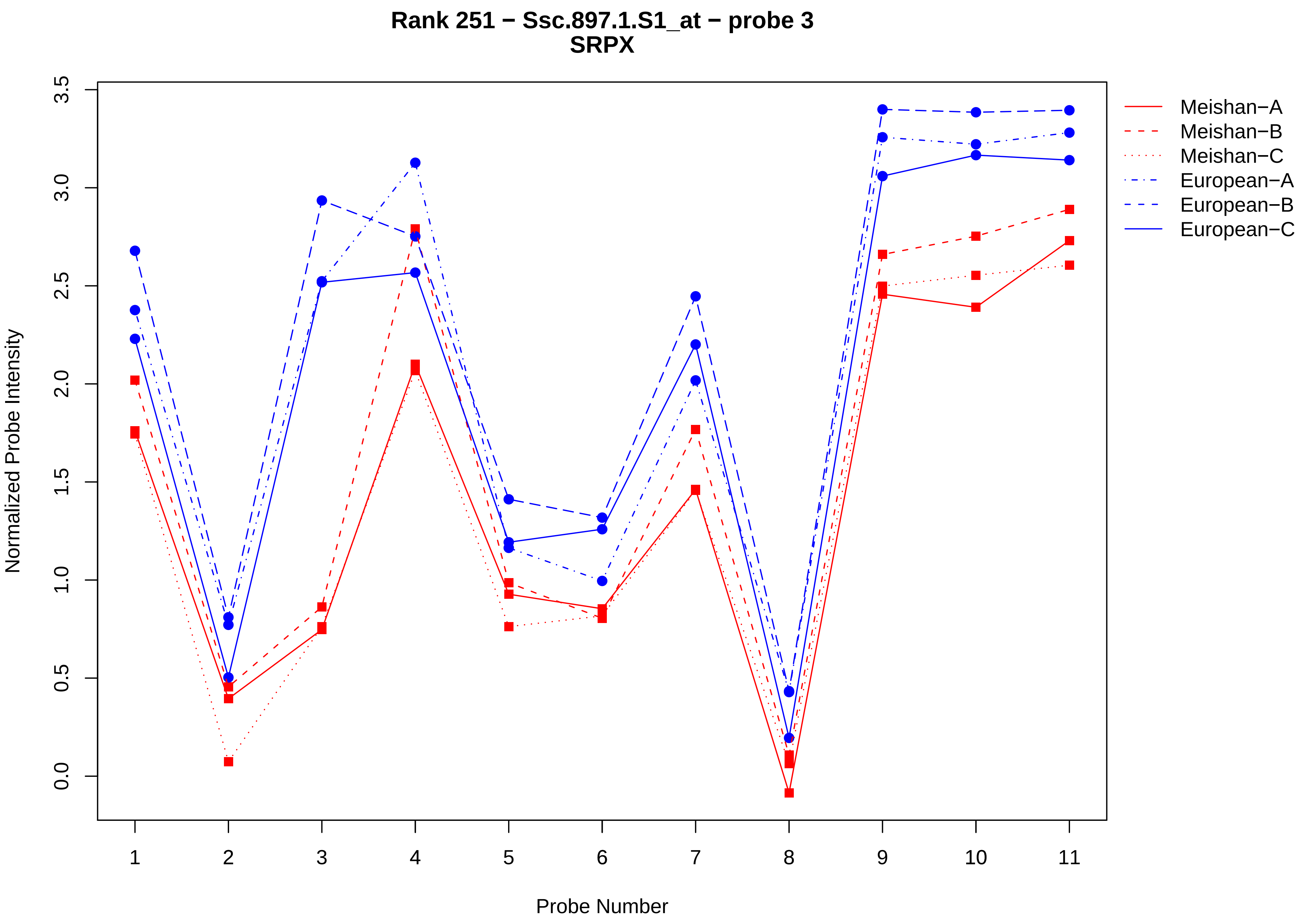

Rank 251 − Ssc.897.1.S1_at − probe 3
SRPX
Normalized Probe Intensity
Probe Number
0.0
0.5
1.0
1.5
2.0
2.5
3.0
3.5
1
2
3
4
5
6
7
8
9
10
11
Meishan−A
Meishan−B
Meishan−C
European−A
European−B
European−C

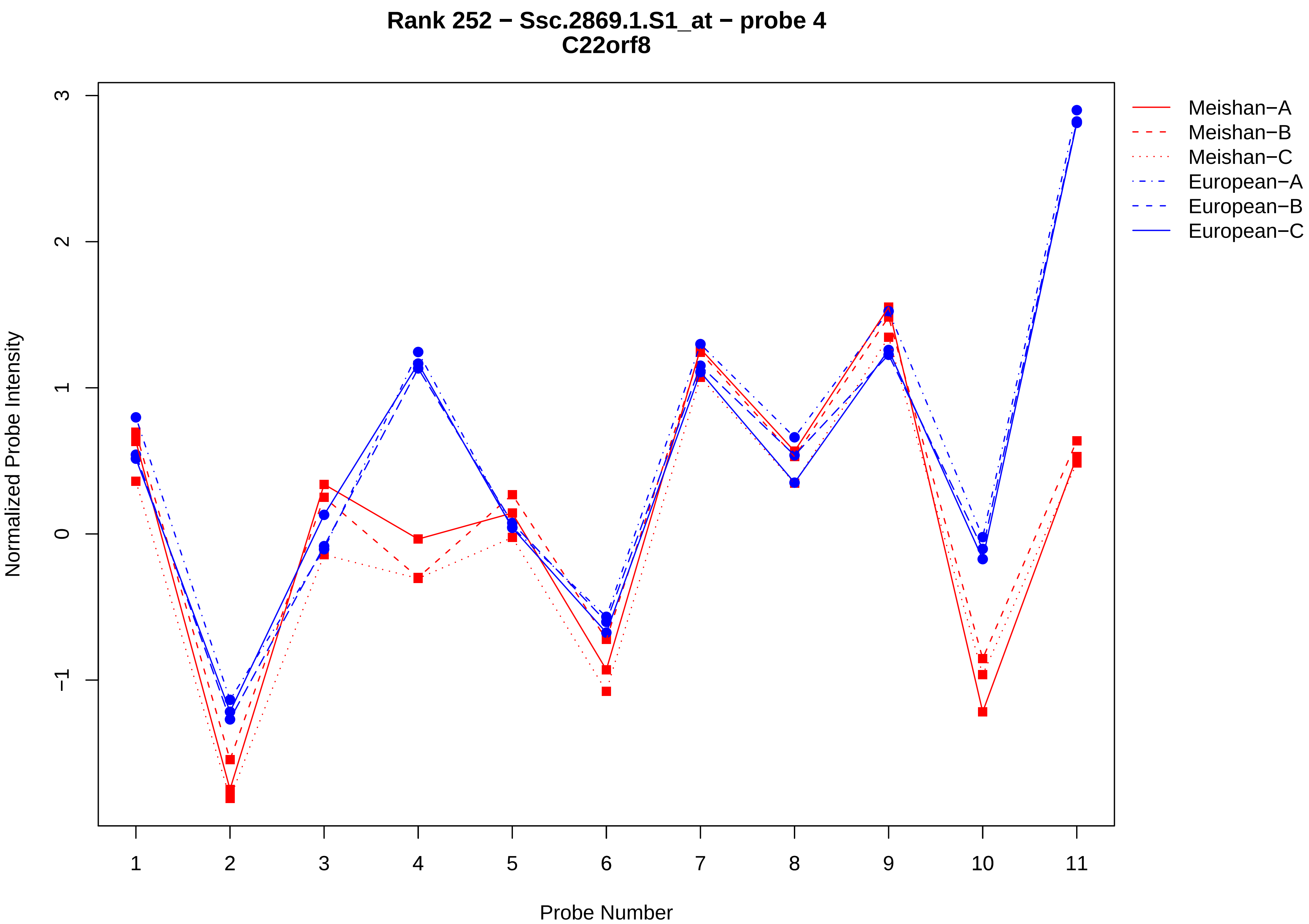

Rank 252 − Ssc.2869.1.S1_at − probe 4
C22orf8
Normalized Probe Intensity
Probe Number
Meishan−A
Meishan−B
Meishan−C
European−A
European−B
European−C
−1
0
1
2
3
1
2
3
4
5
6
7
8
9
10
11

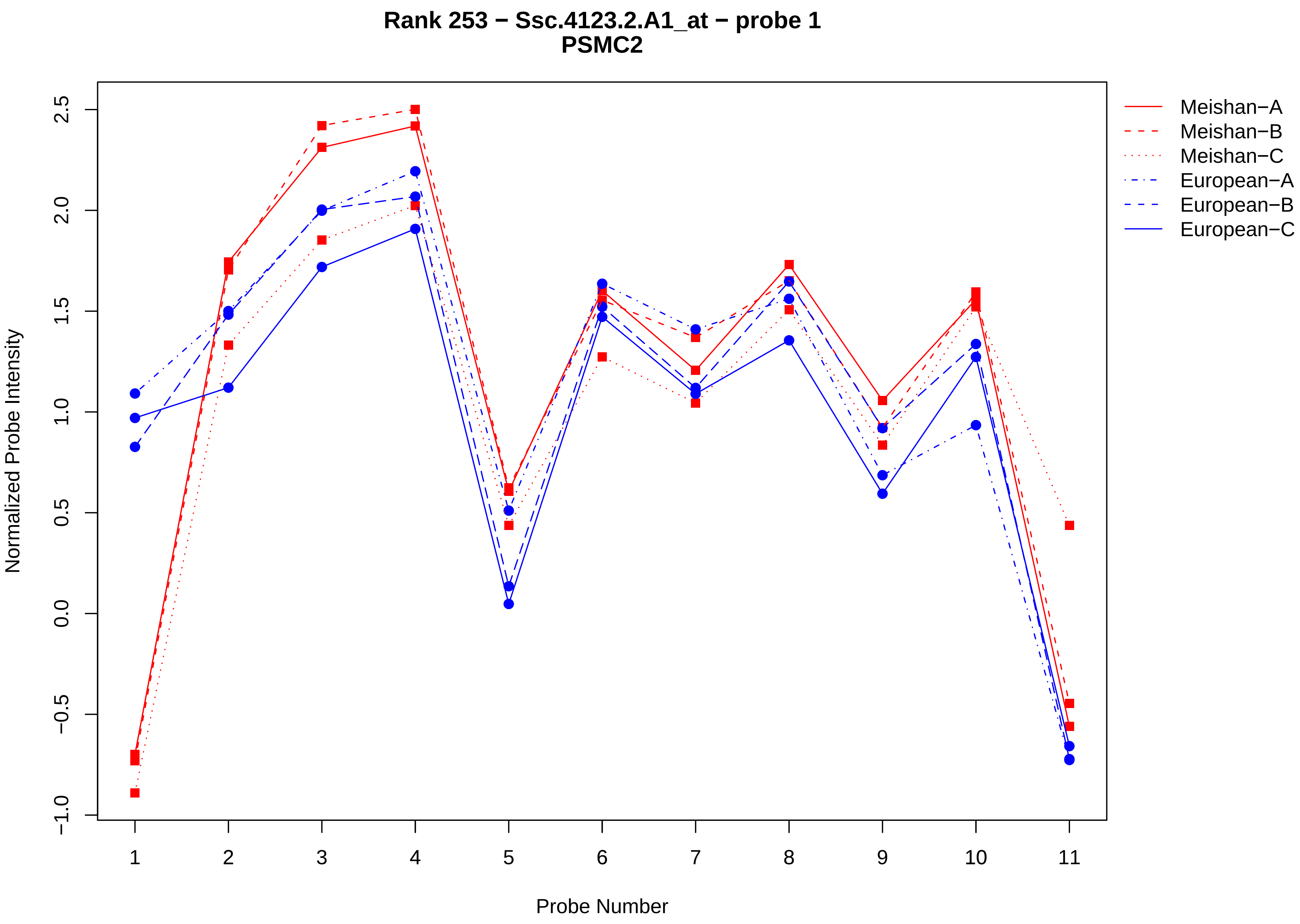

Rank 253 − Ssc.4123.2.A1_at − probe 1
PSMC2
Normalized Probe Intensity
Probe Number
−1.0
−0.5
0.0
0.5
1.0
1.5
2.0
2.5
1
2
3
4
5
6
7
8
9
10
11
Meishan−A
Meishan−B
Meishan−C
European−A
European−B
European−C

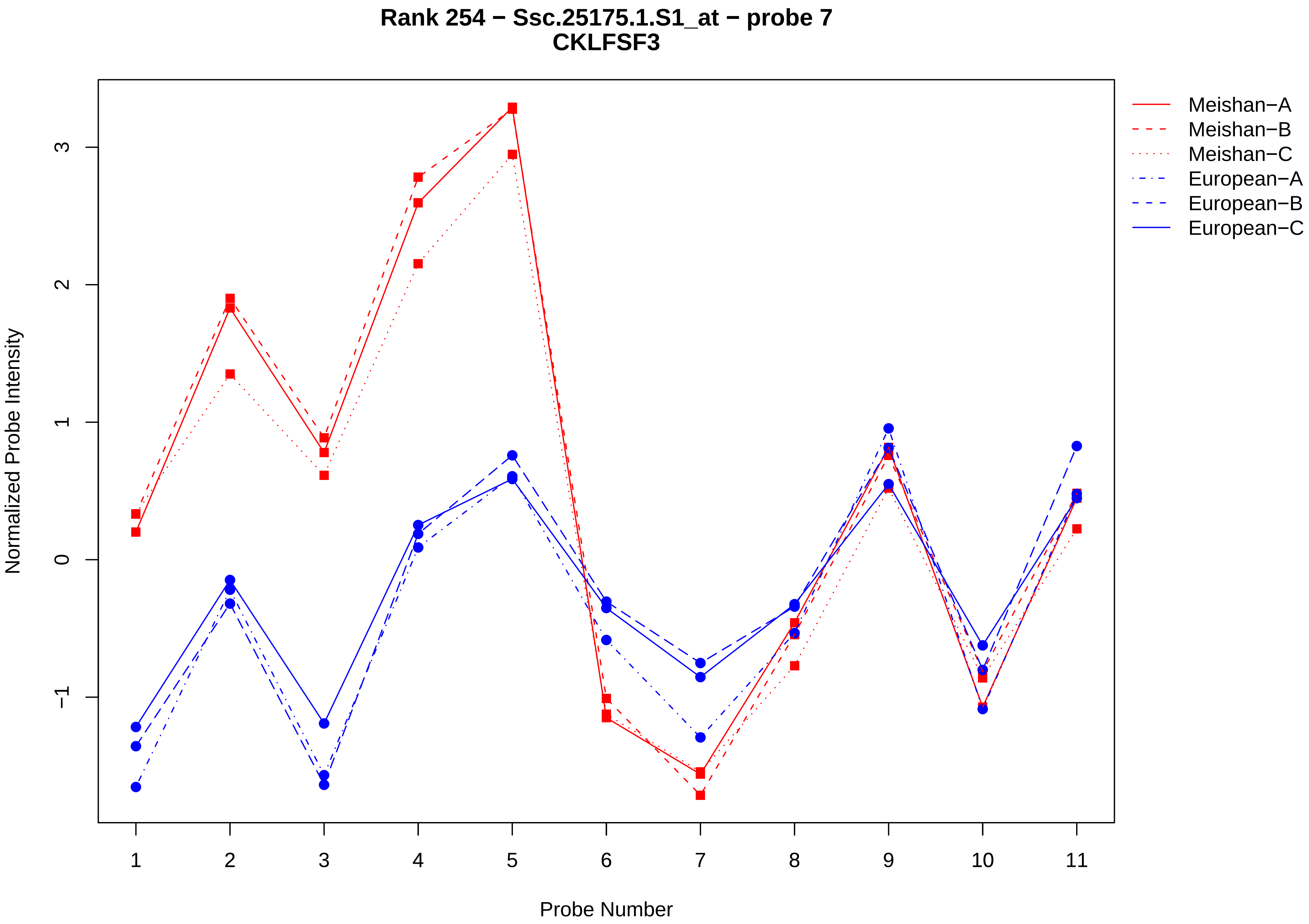

Rank 254 − Ssc.25175.1.S1_at − probe 7
CKLFSF3
Normalized Probe Intensity
Probe Number
−1
0
1
2
3
1
2
3
4
5
6
7
8
9
10
11
Meishan−A
Meishan−B
Meishan−C
European−A
European−B
European−C

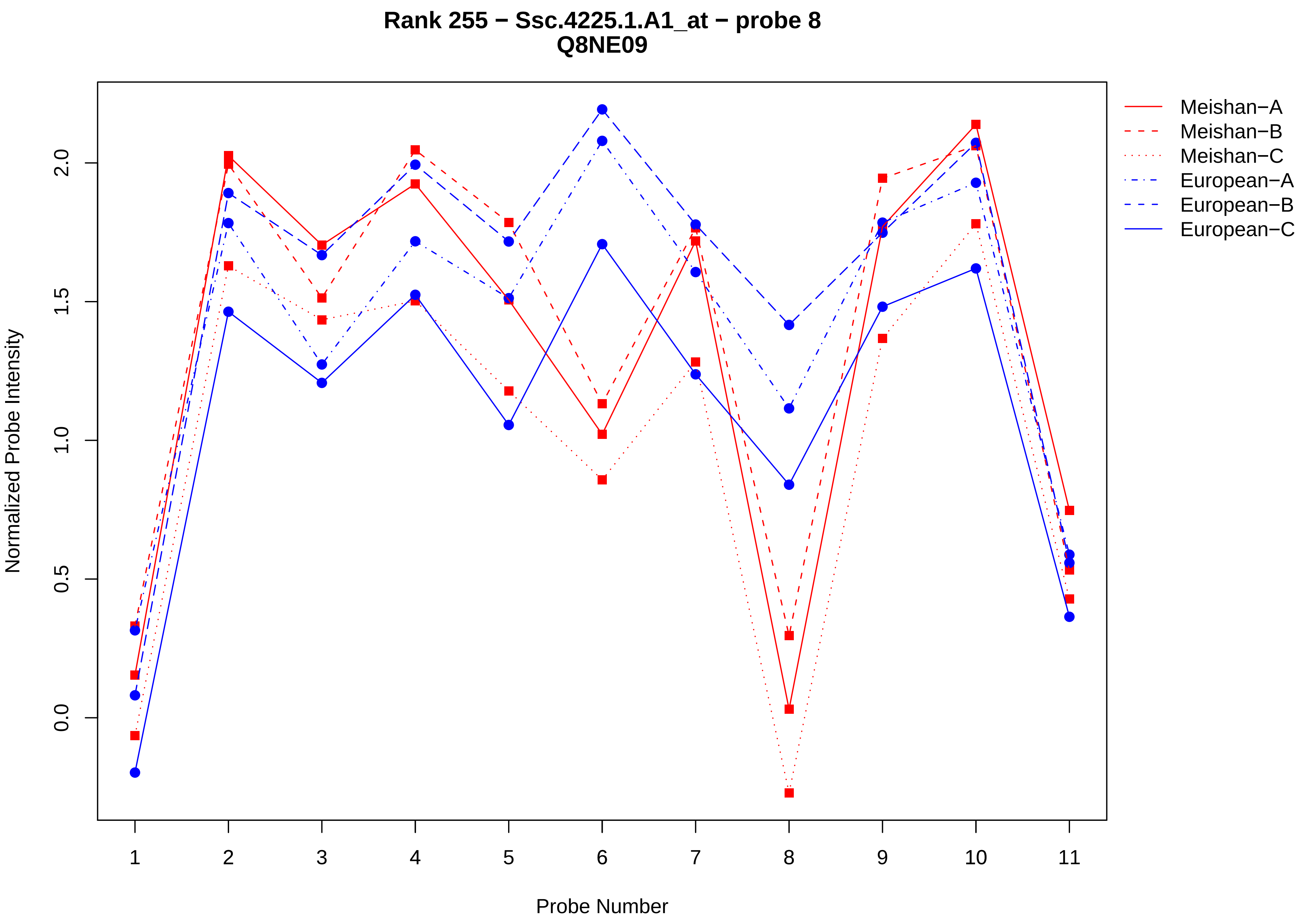
Rank 255 − Ssc.4225.1.A1_at − probe 8
Q8NE09
Normalized Probe Intensity
Probe Number
0.0
0.5
1.0
1.5
2.0
1
2
3
4
5
6
7
8
9
10
11
Meishan−A
Meishan−B
Meishan−C
European−A
European−B
European−C

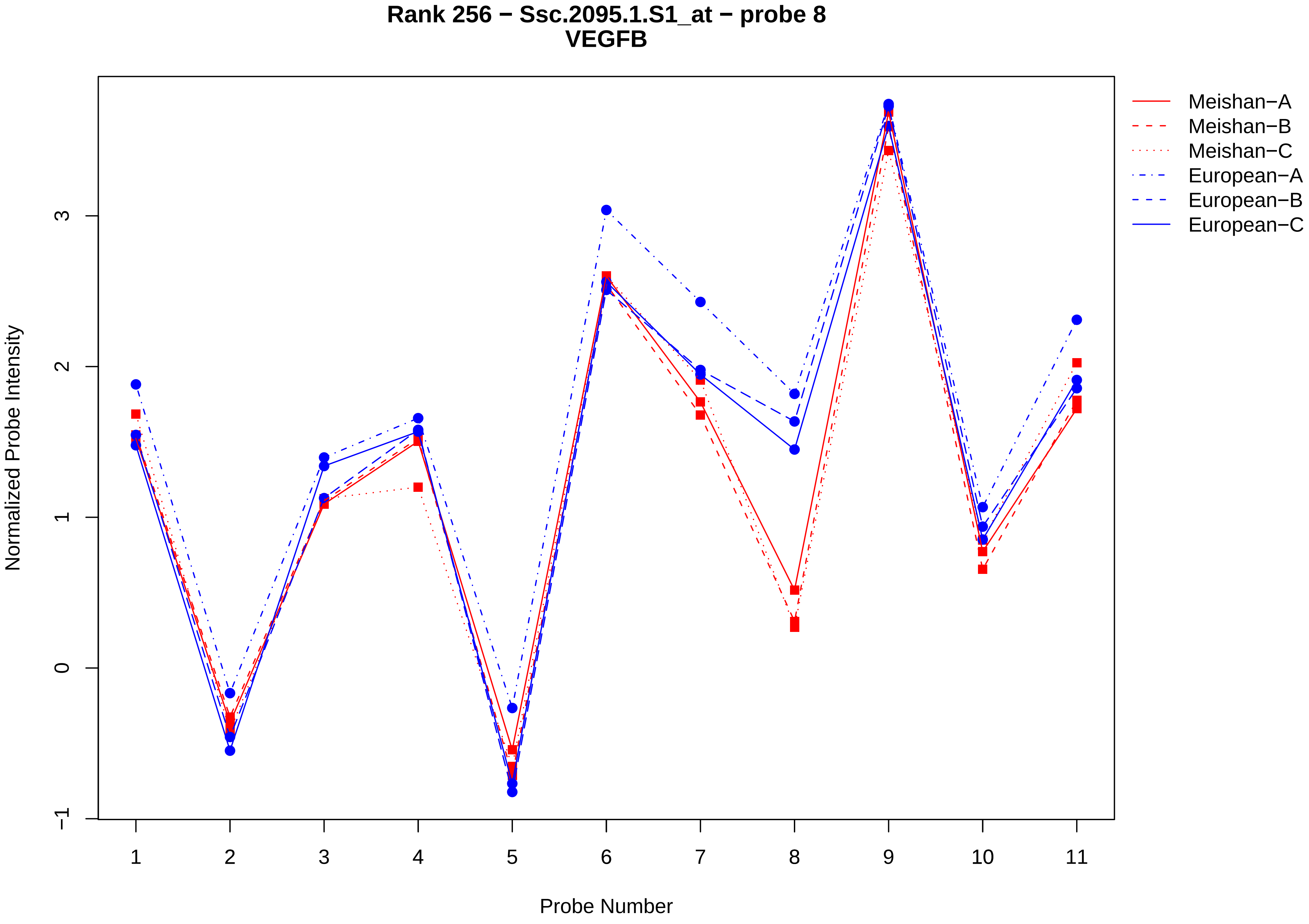

Rank 256 − Ssc.2095.1.S1_at − probe 8
VEGFB
Normalized Probe Intensity
Probe Number
−1
0
1
2
3
1
2
3
4
5
6
7
8
9
10
11
Meishan−A
Meishan−B
Meishan−C
European−A
European−B
European−C

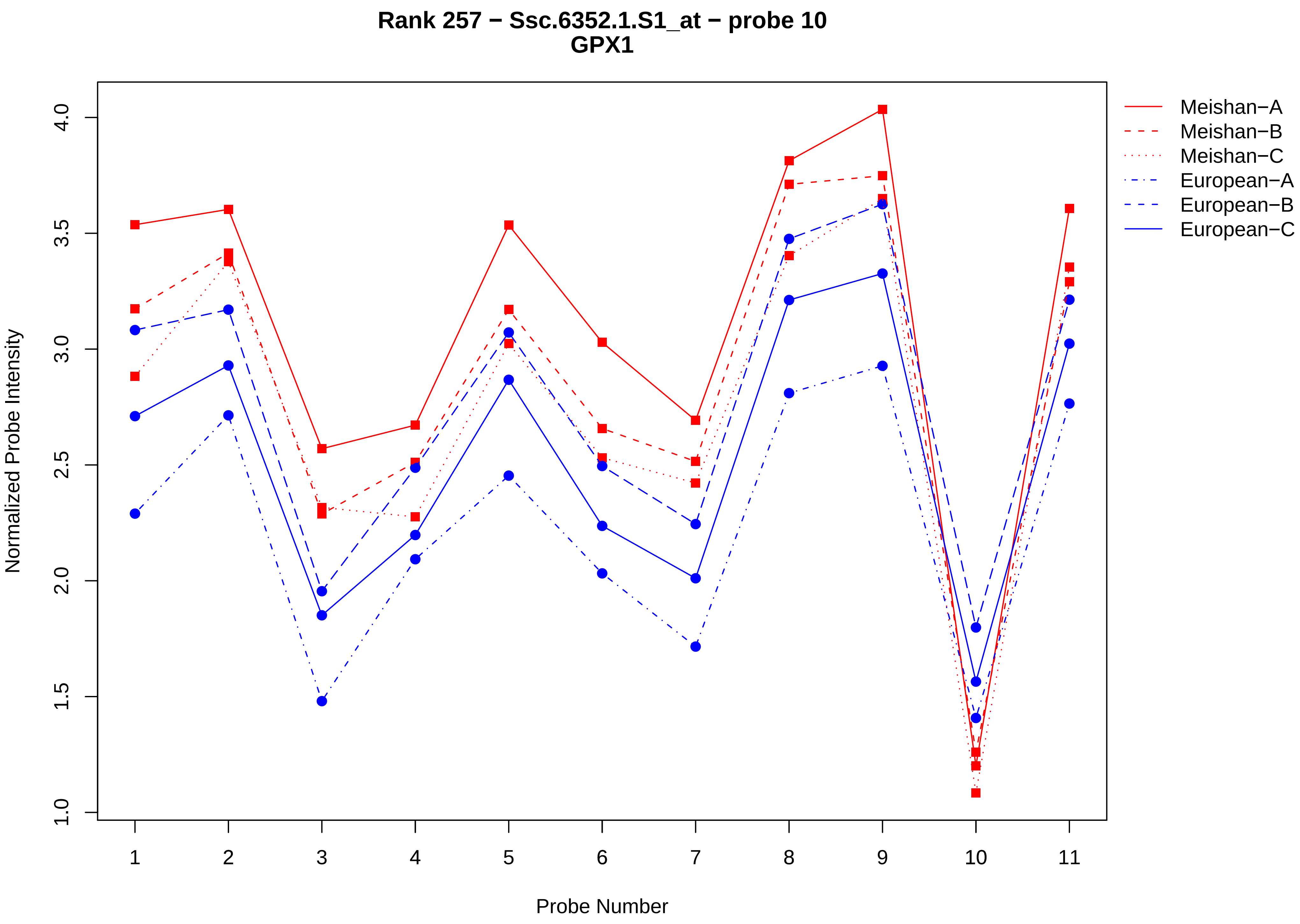

Rank 257 − Ssc.6352.1.S1_at − probe 10
GPX1
Normalized Probe Intensity
Probe Number
1.0
1.5
2.0
2.5
3.0
3.5
4.0
1
2
3
4
5
6
7
8
9
10
11
Meishan−A
Meishan−B
Meishan−C
European−A
European−B
European−C

Rank 258 − Ssc.9434.1.A1_at − probe 11
MARK1

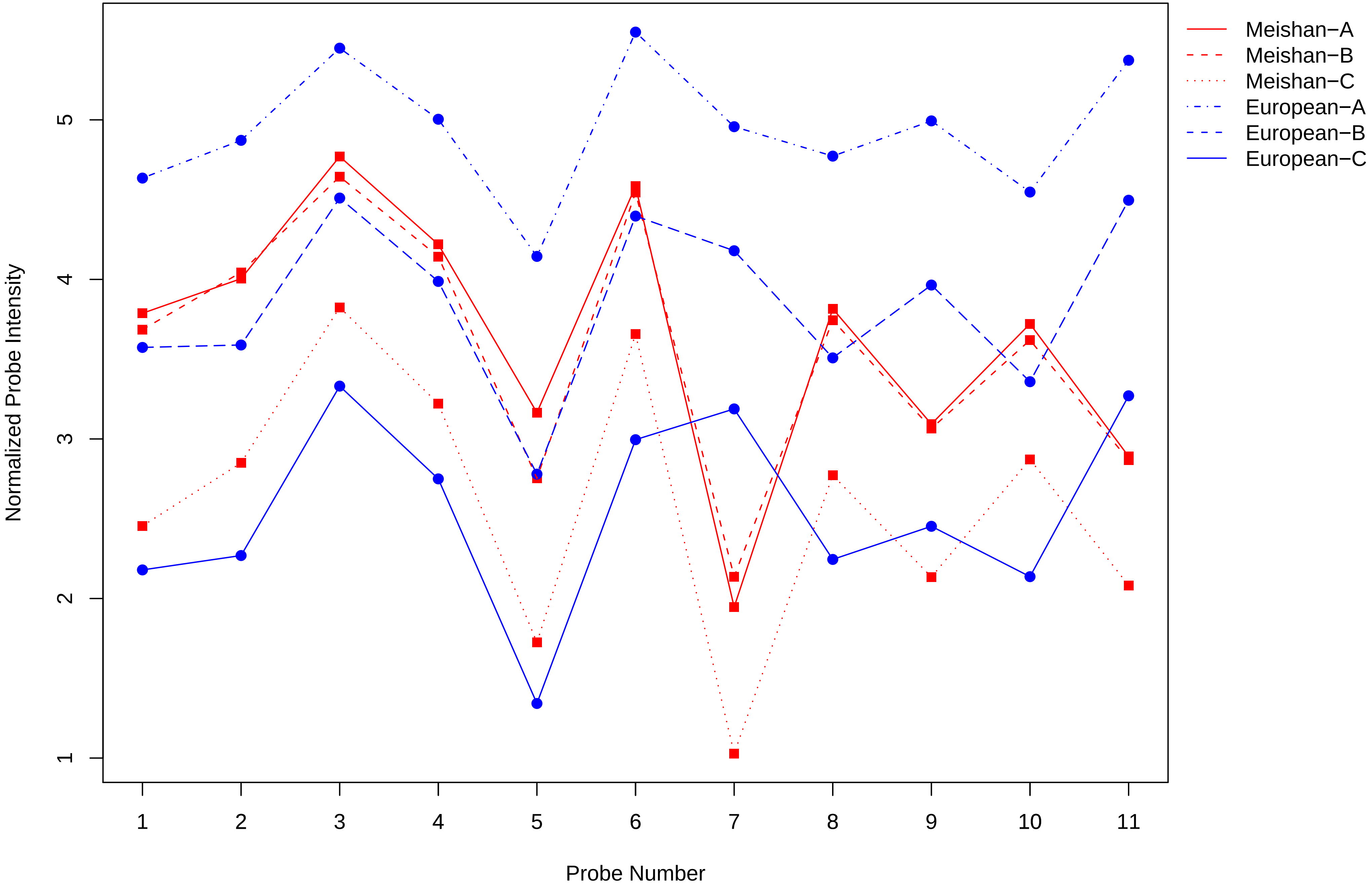

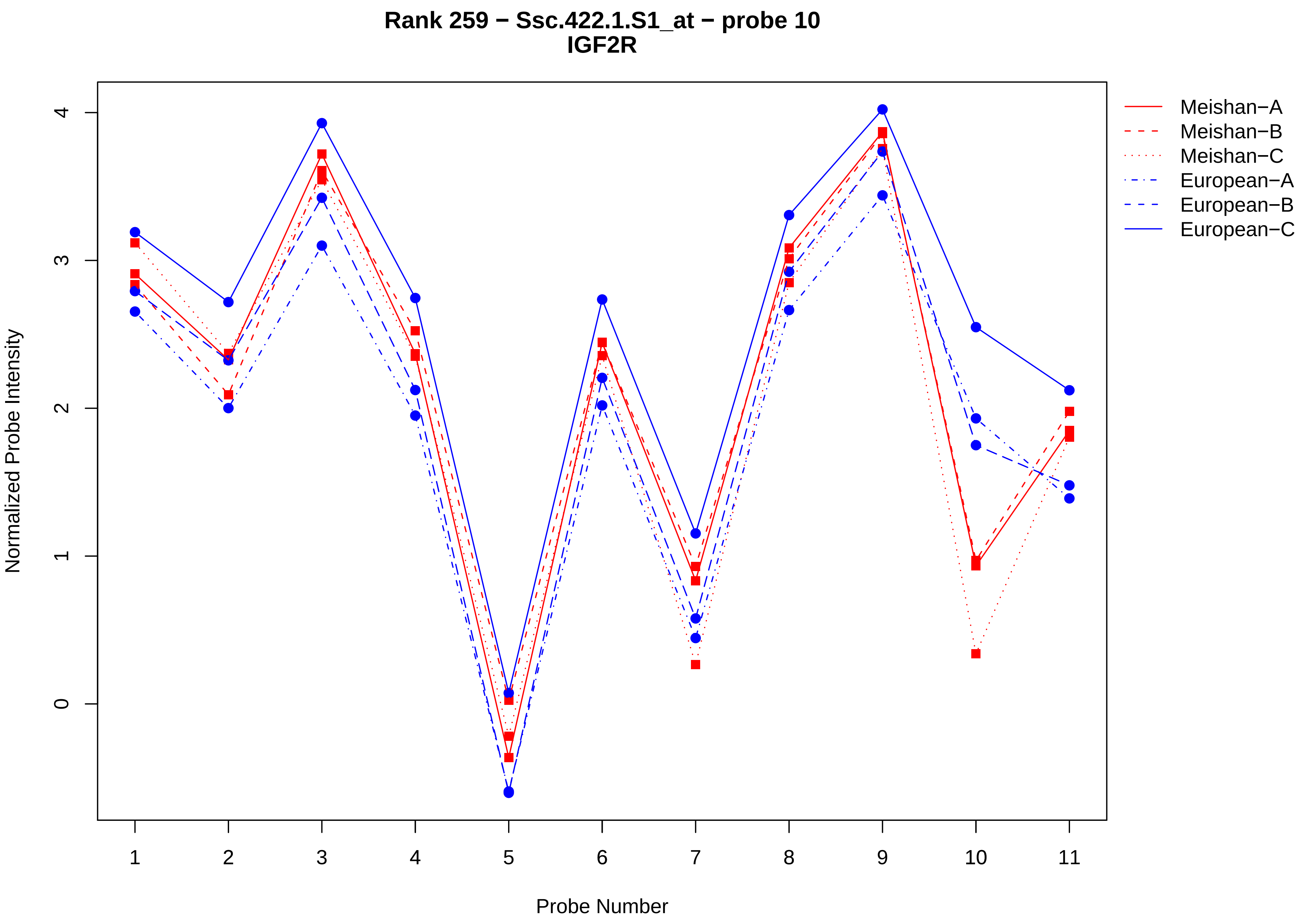

Rank 259 − Ssc.422.1.S1_at − probe 10
IGF2R
Normalized Probe Intensity
Probe Number
0
1
2
3
4
1
2
3
4
5
6
7
8
9
10
11
Meishan−A
Meishan−B
Meishan−C
European−A
European−B
European−C

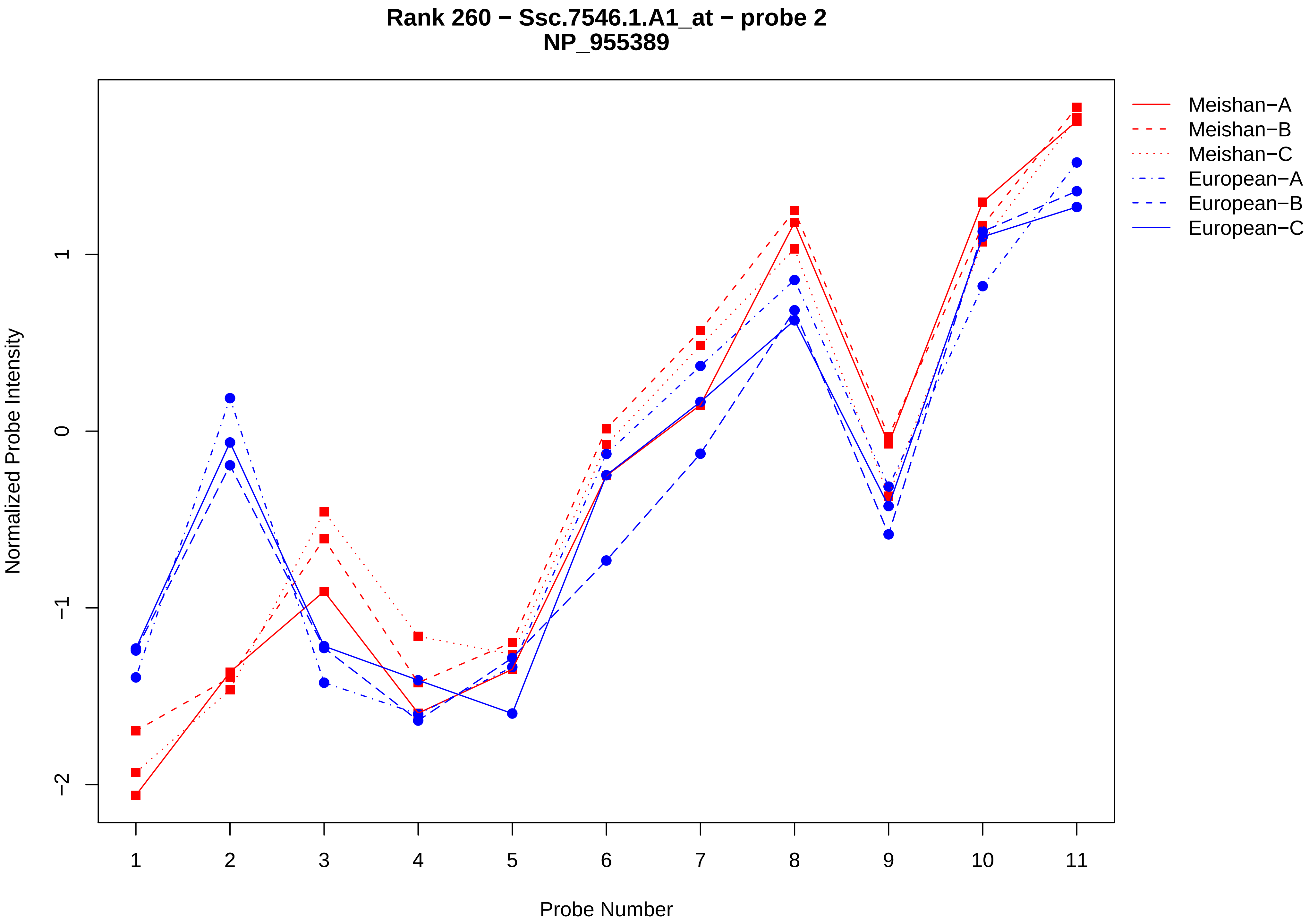
Rank 260 − Ssc.7546.1.A1_at − probe 2
NP_955389
Normalized Probe Intensity
Probe Number
−2
−1
0
1
1
2
3
4
5
6
7
8
9
10
11
Meishan−A
Meishan−B
Meishan−C
European−A
European−B
European−C

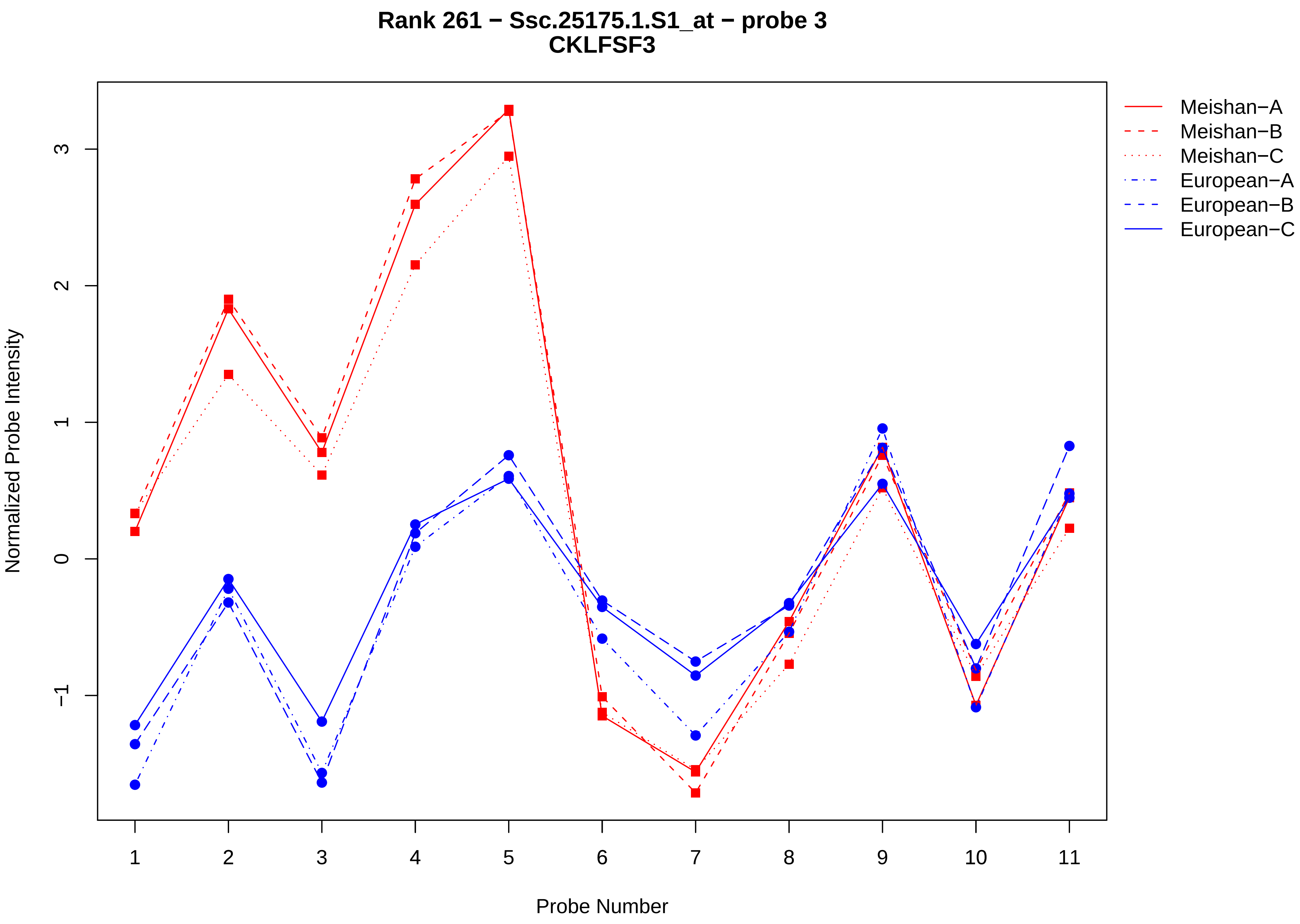
Rank 261 − Ssc.25175.1.S1_at − probe 3
CKLFSF3
Normalized Probe Intensity
Probe Number
Meishan−A
Meishan−B
Meishan−C
European−A
European−B
European−C
−1
0
1
2
3
1
2
3
4
5
6
7
8
9
10
11

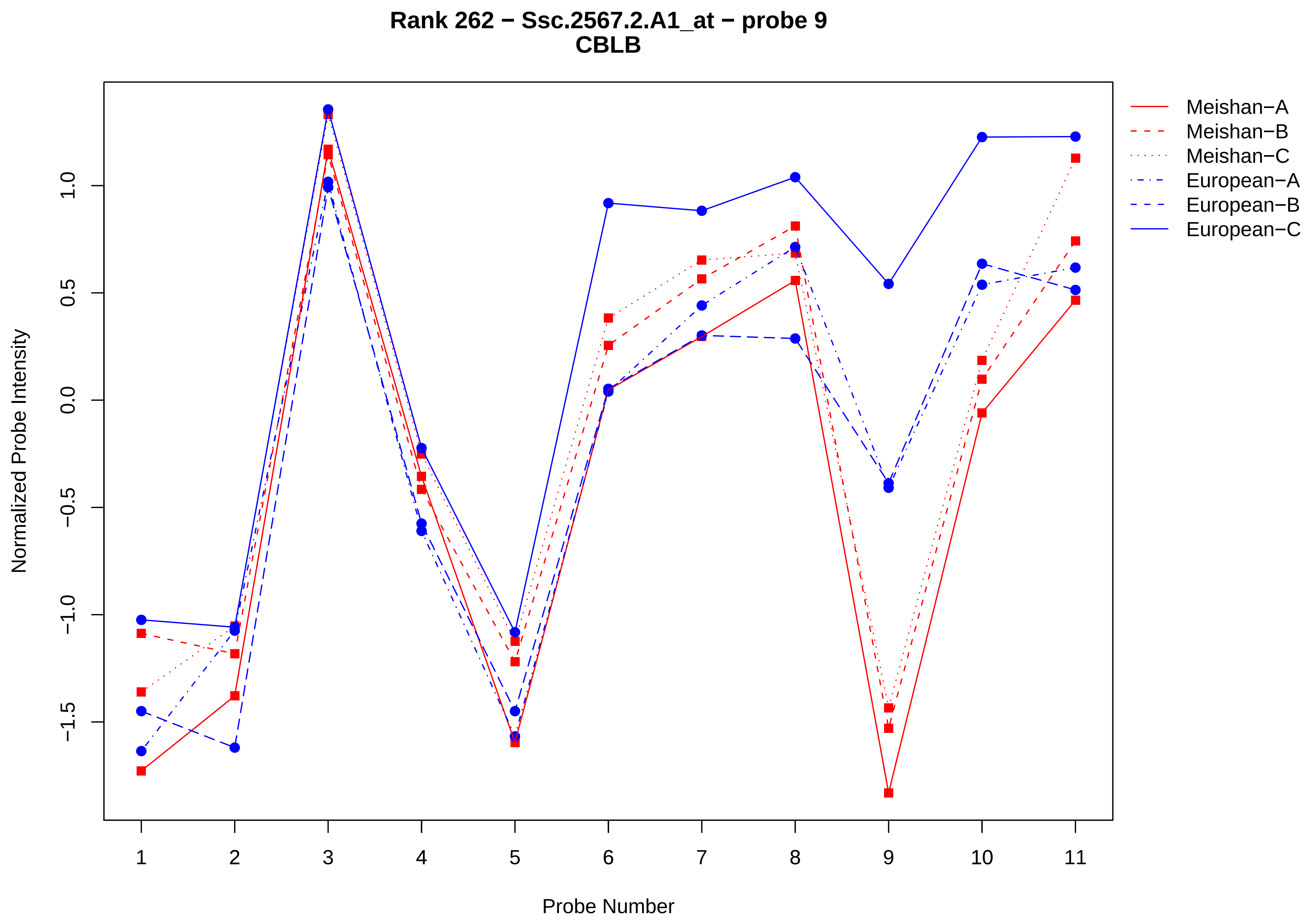

Rank 262 − Ssc.2567.2.A1_at − probe 9
CBLB
Normalized Probe Intensity
Probe Number
1.0
0.5
0.0
−0.5
−1.0
−1.5
1
2
3
4
5
6
7
8
9
10
11
Meishan−A
Meishan−B
Meishan−C
European−A
European−B
European−C

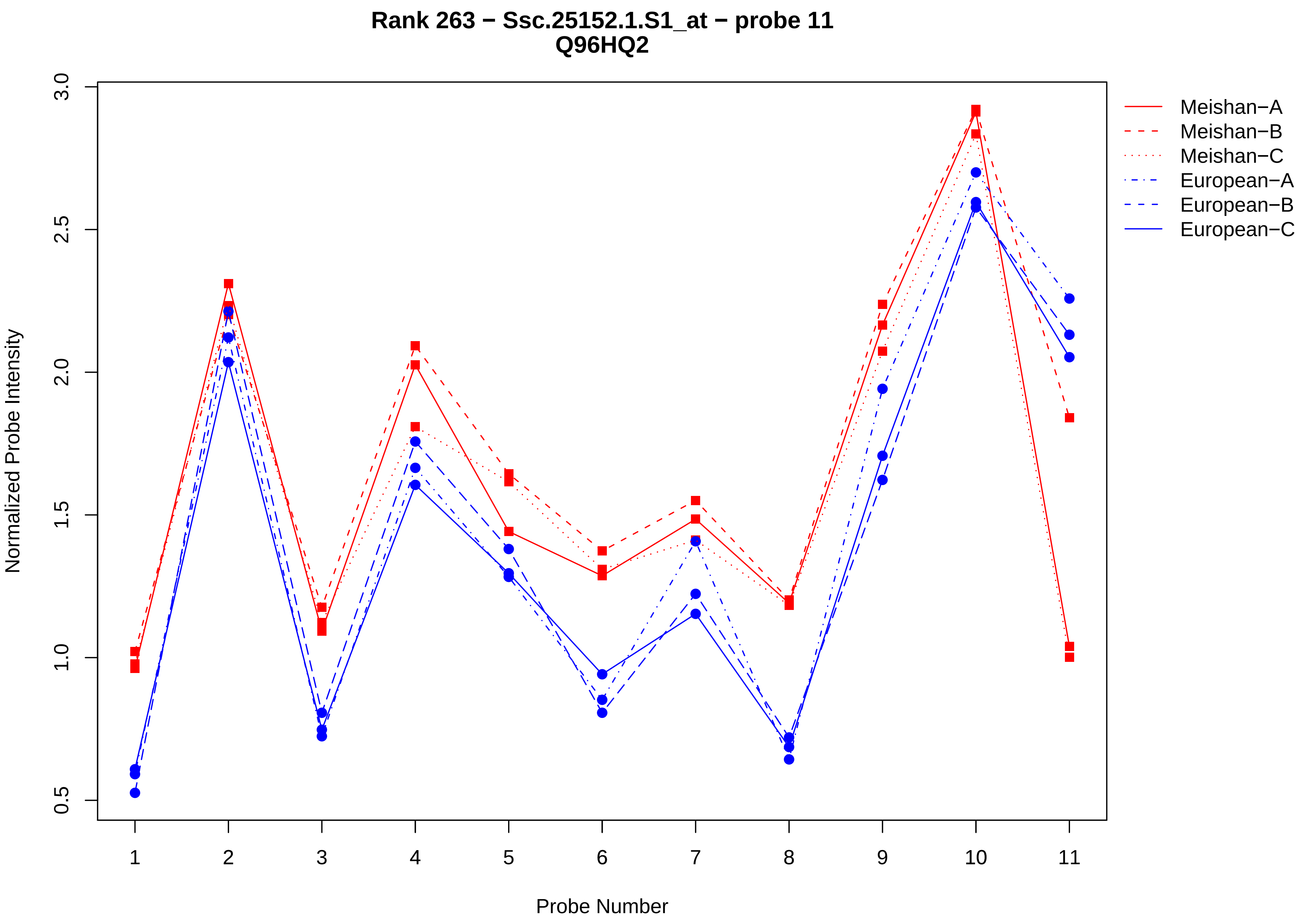
Rank 263 − Ssc.25152.1.S1_at − probe 11
Q96HQ2
Normalized Probe Intensity
0.5
1.0
1.5
2.0
2.5
3.0
Probe Number
1
2
3
4
5
6
7
8
9
10
11
Meishan−A
Meishan−B
Meishan−C
European−A
European−B
European−C

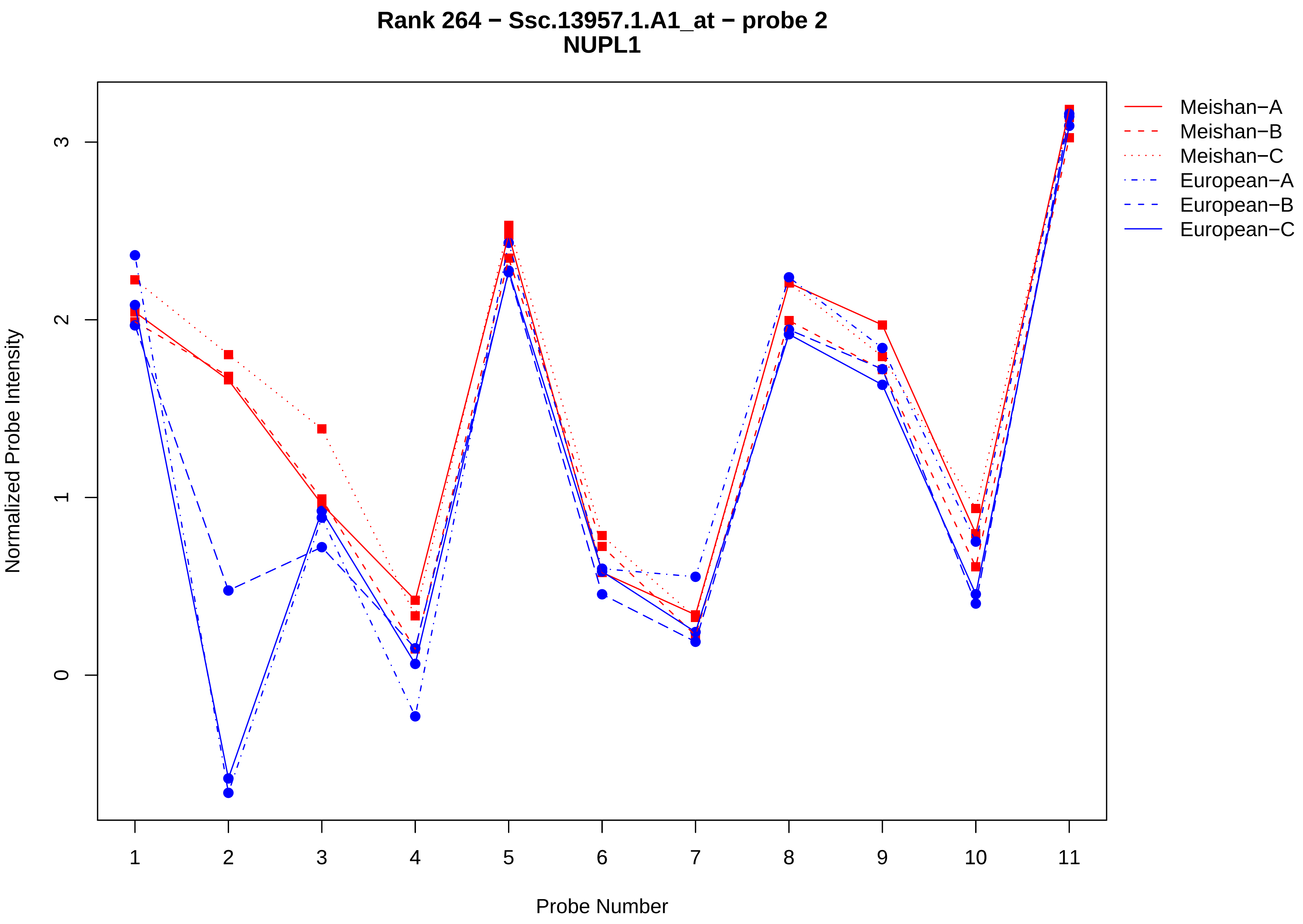

Rank 264 − Ssc.13957.1.A1_at − probe 2
NUPL1
Normalized Probe Intensity
Probe Number
Meishan−A
Meishan−B
Meishan−C
European−A
European−B
European−C
0
1
2
3
1
2
3
4
5
6
7
8
9
10
11

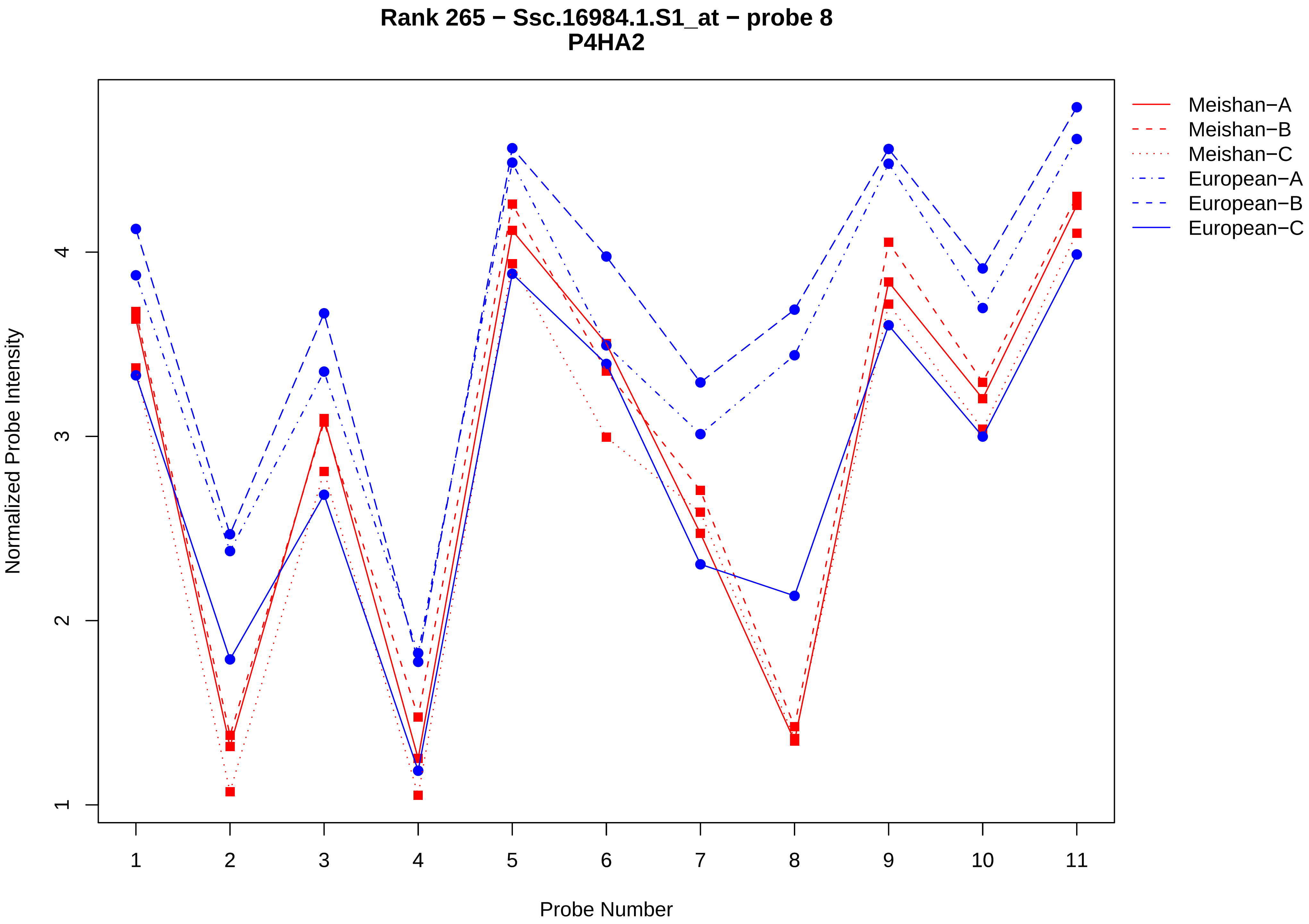

Rank 265 − Ssc.16984.1.S1_at − probe 8
P4HA2
Normalized Probe Intensity
Probe Number
1
2
3
4
1
2
3
4
5
6
7
8
9
10
11
Meishan−A
Meishan−B
Meishan−C
European−A
European−B
European−C

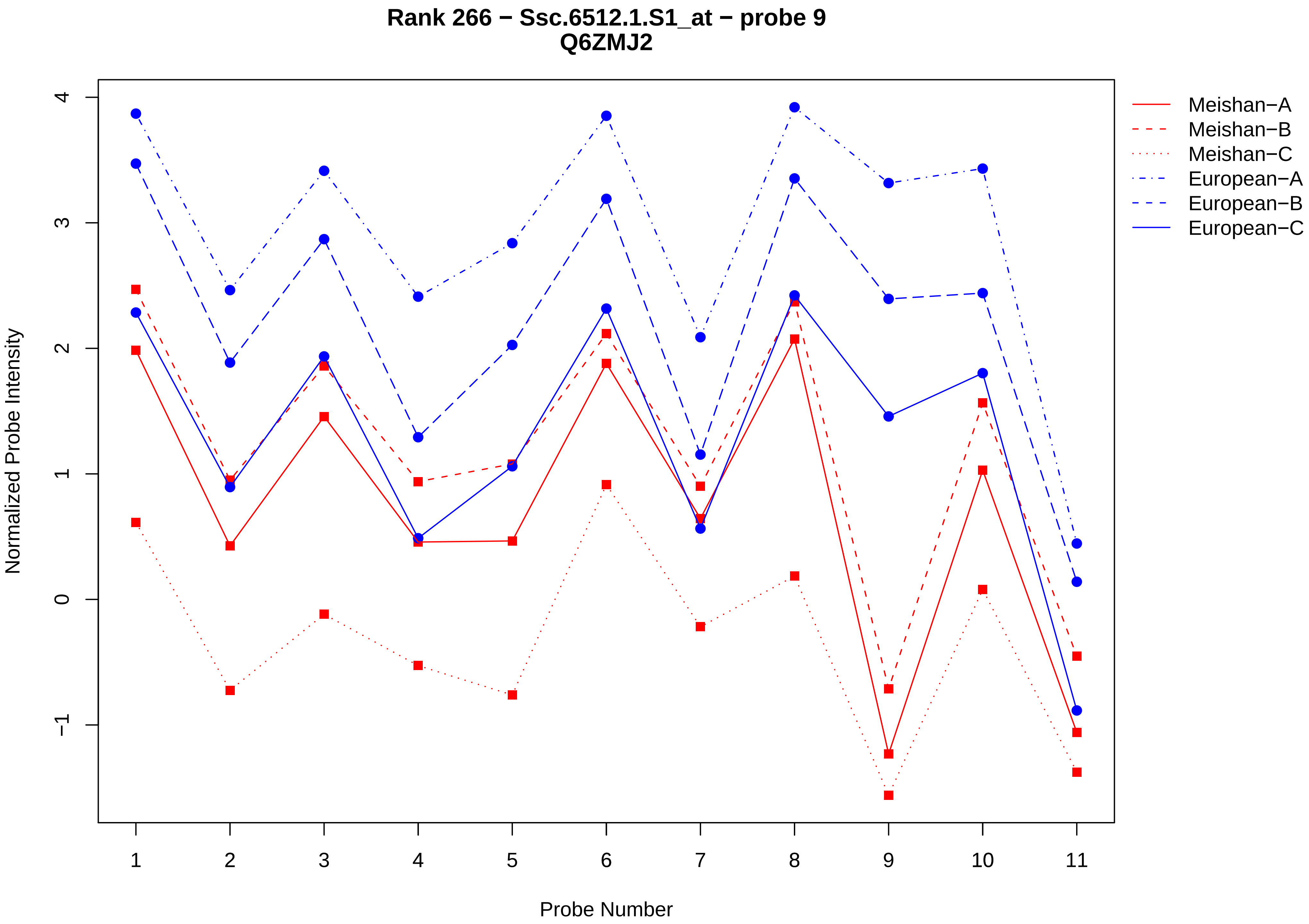

Rank 266 − Ssc.6512.1.S1_at − probe 9
Q6ZMJ2
Normalized Probe Intensity
Probe Number
−1
0
1
2
3
4
1
2
3
4
5
6
7
8
9
10
11
Meishan−A
Meishan−B
Meishan−C
European−A
European−B
European−C

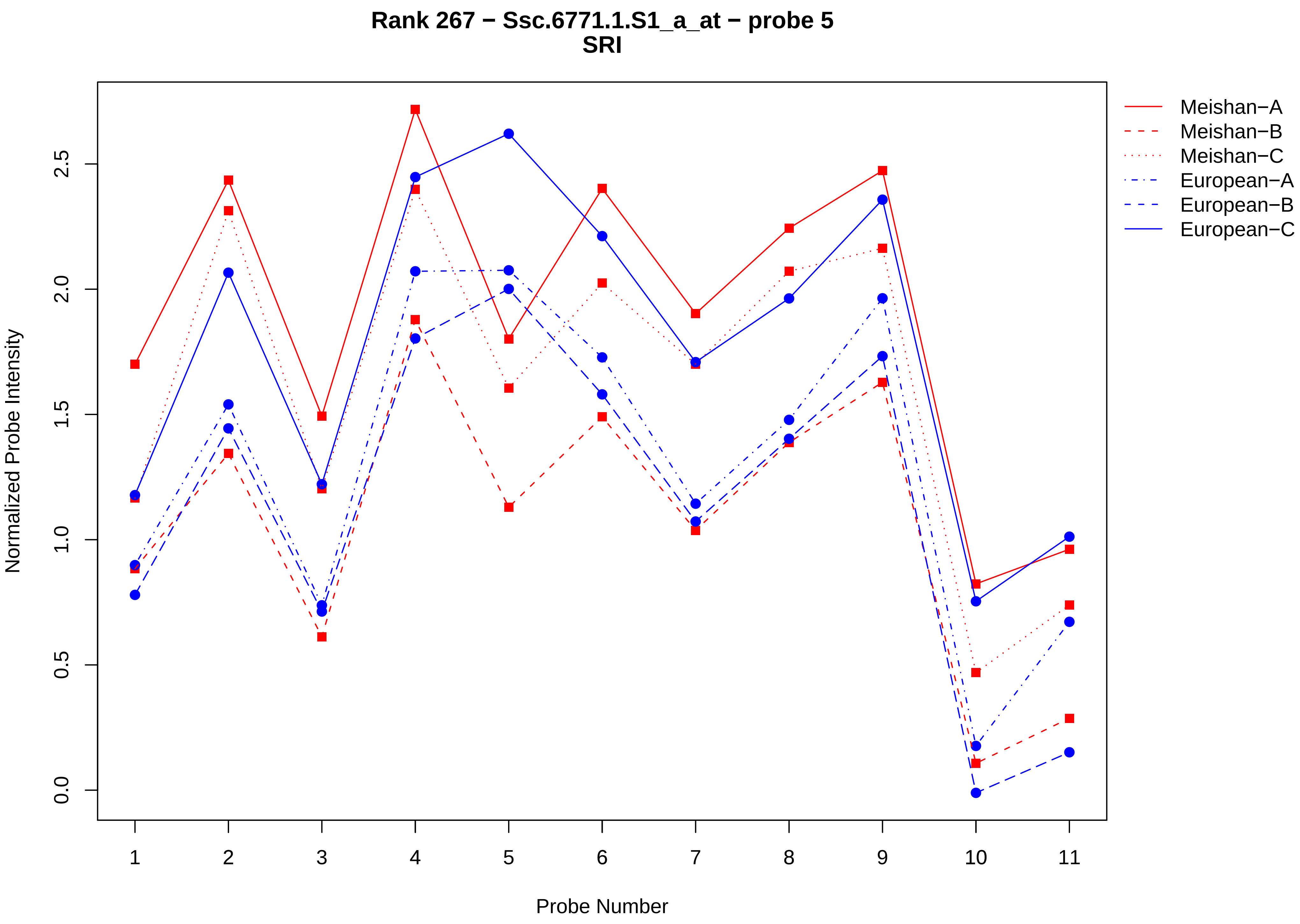
Rank 267 − Ssc.6771.1.S1_a_at − probe 5
SRI
Normalized Probe Intensity
Probe Number
0.0
0.5
1.0
1.5
2.0
2.5
1
2
3
4
5
6
7
8
9
10
11
Meishan−A
Meishan−B
Meishan−C
European−A
European−B
European−C

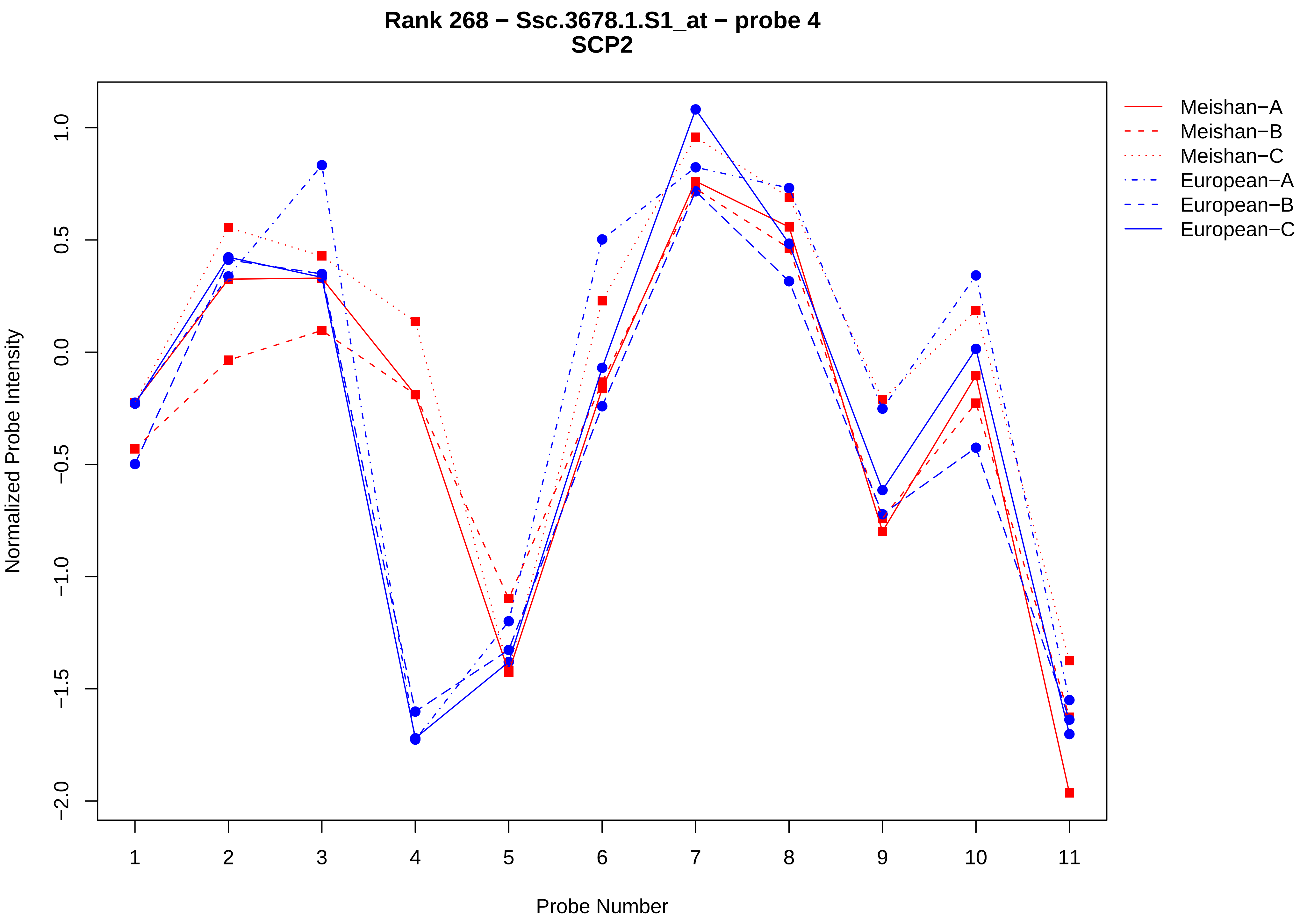

Rank 268 − Ssc.3678.1.S1_at − probe 4
SCP2
Normalized Probe Intensity
Probe Number
1.0
0.5
0.0
−0.5
−1.0
−1.5
−2.0
1
2
3
4
5
6
7
8
9
10
11
Meishan−A
Meishan−B
Meishan−C
European−A
European−B
European−C

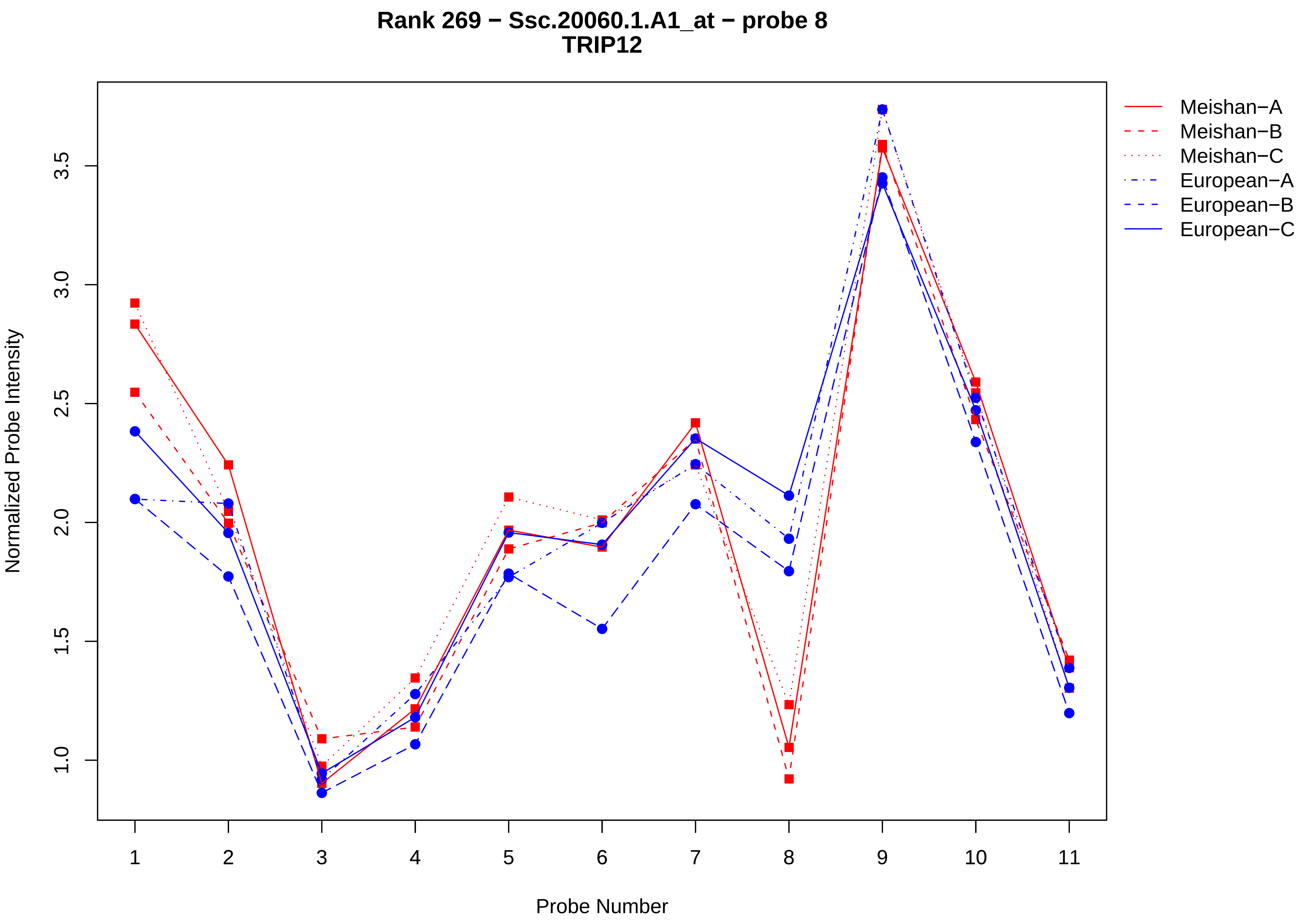

Rank 269 − Ssc.20060.1.A1_at − probe 8
TRIP12
Normalized Probe Intensity
1.0
1.5
2.0
2.5
3.0
3.5
Probe Number
1
2
3
4
5
6
7
8
9
10
11
Meishan−A
Meishan−B
Meishan−C
European−A
European−B
European−C

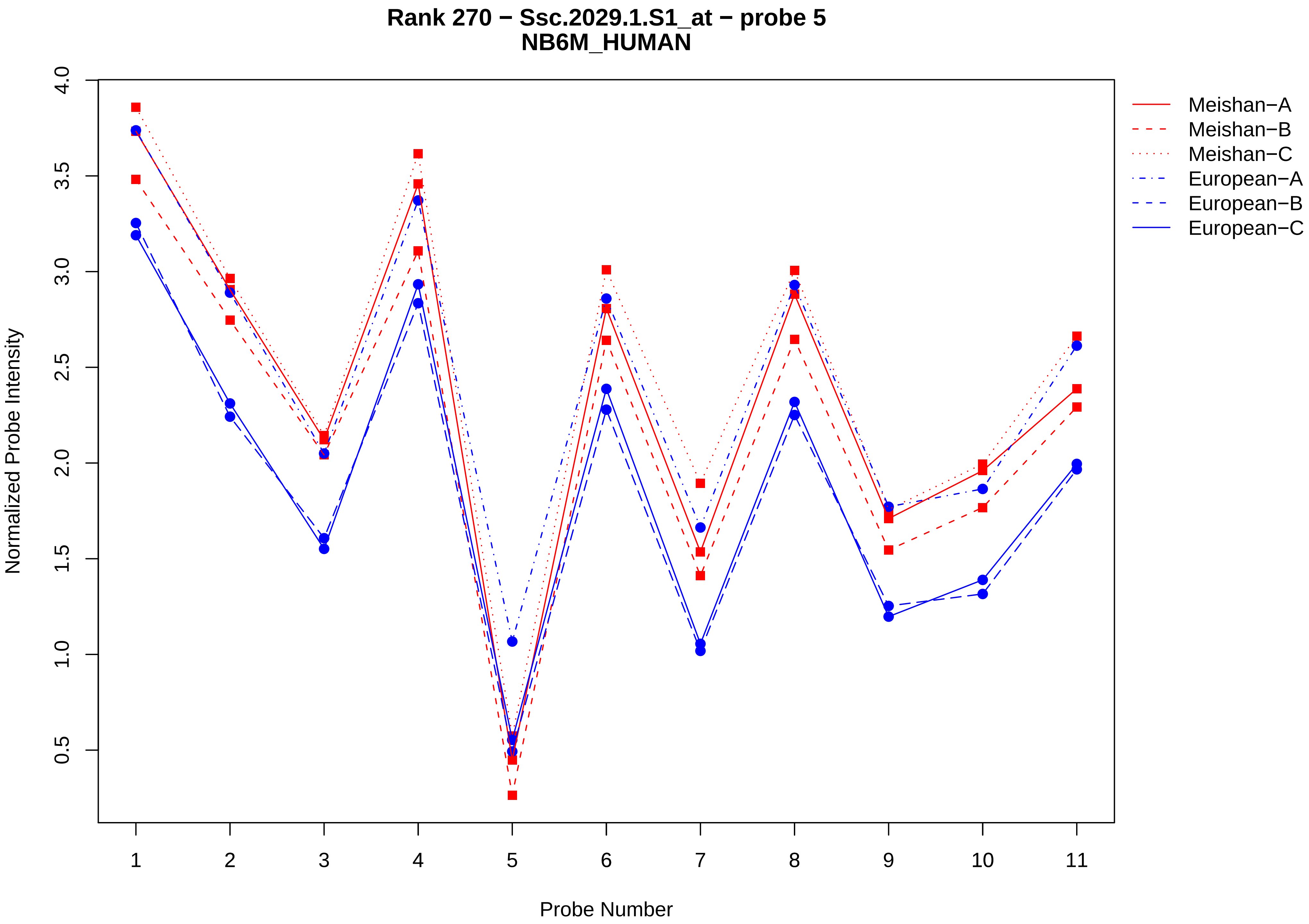
Rank 270 − Ssc.2029.1.S1_at − probe 5
NB6M_HUMAN
Normalized Probe Intensity
Probe Number
Meishan−A
Meishan−B
Meishan−C
European−A
European−B
European−C
0.5
1.0
1.5
2.0
2.5
3.0
3.5
4.0
1
2
3
4
5
6
7
8
9
10
11

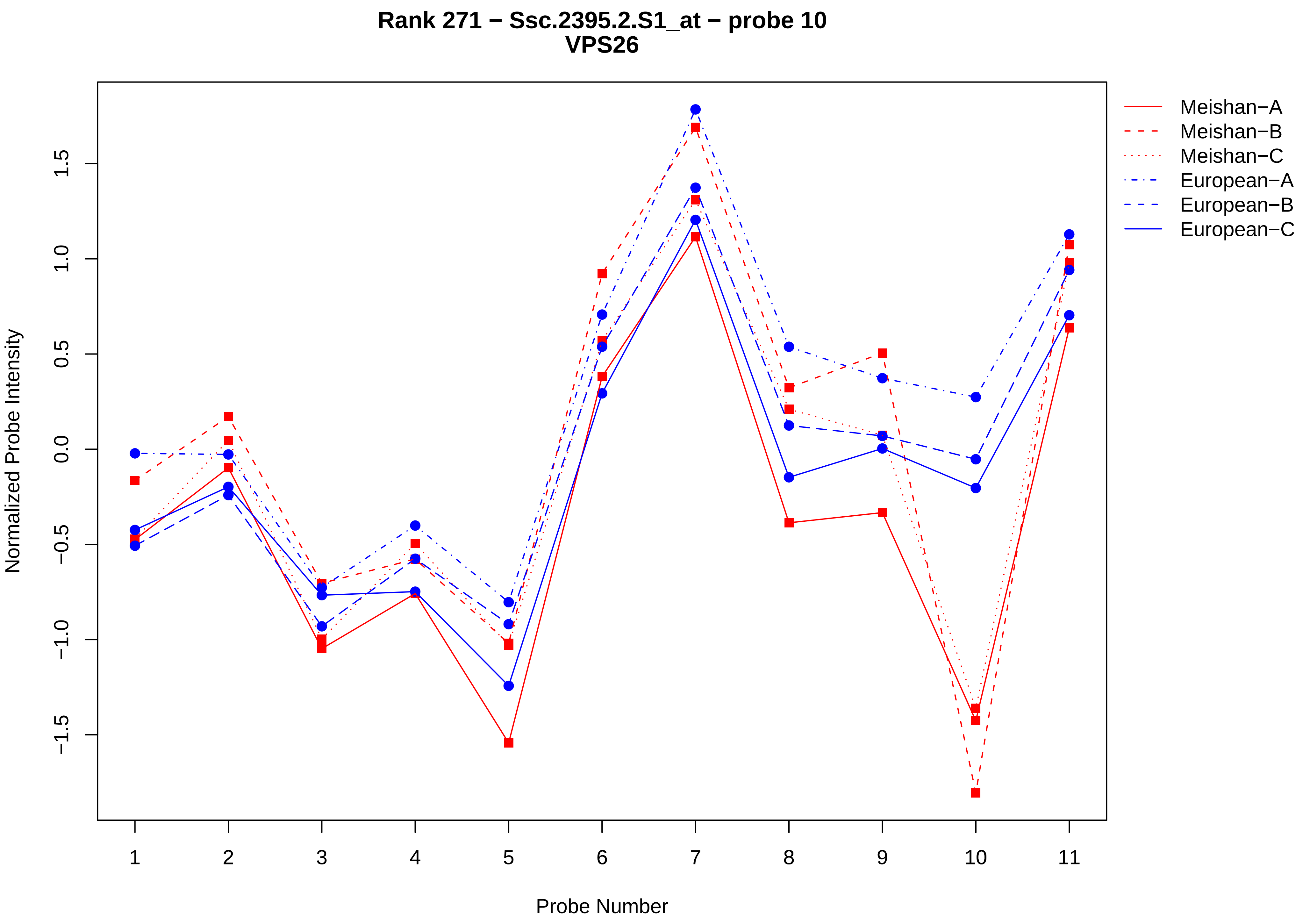

Rank 271 − Ssc.2395.2.S1_at − probe 10
VPS26
Normalized Probe Intensity
Probe Number
1.5
1.0
0.5
0.0
−0.5
−1.0
−1.5
1
2
3
4
5
6
7
8
9
10
11
Meishan−A
Meishan−B
Meishan−C
European−A
European−B
European−C

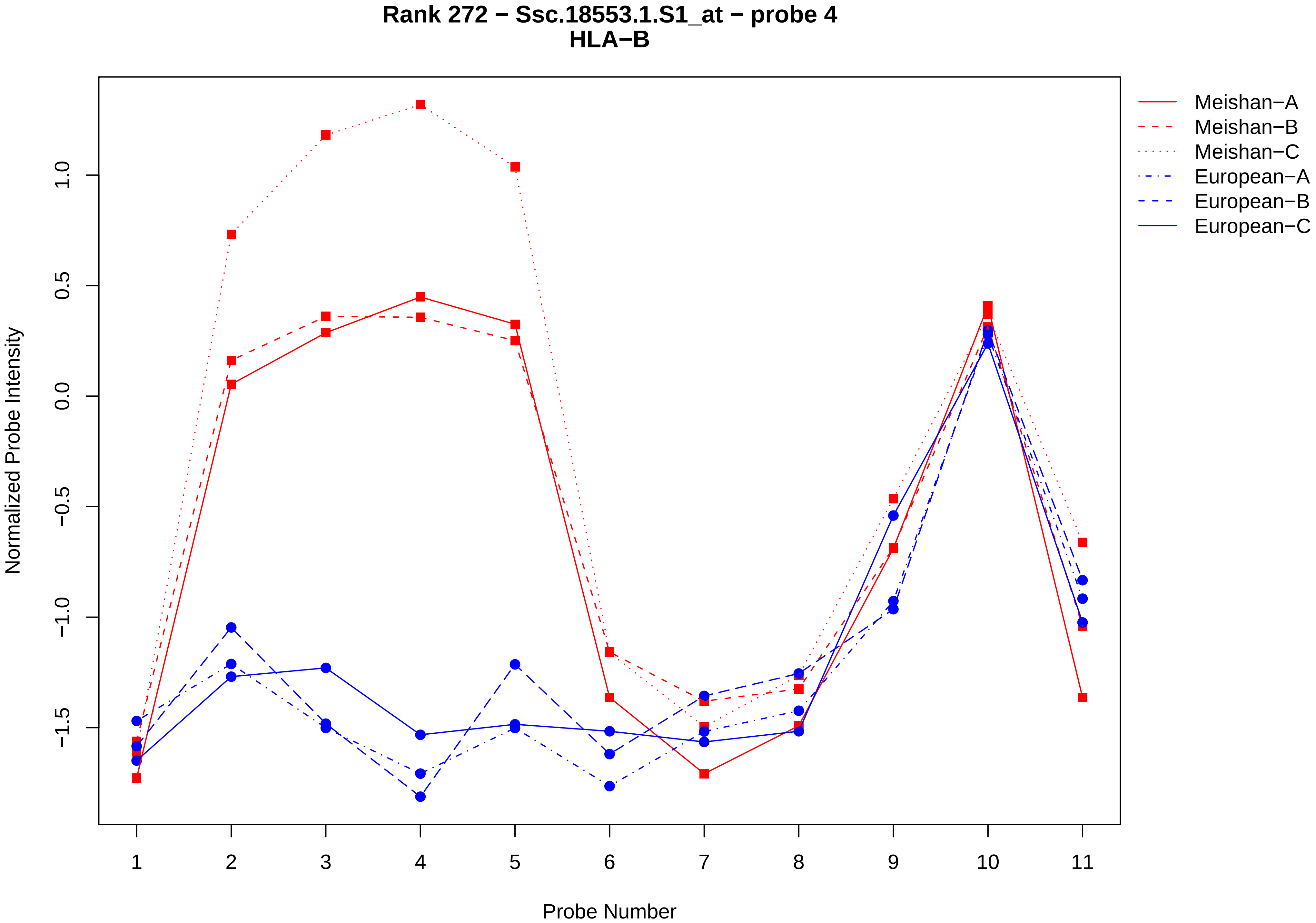

Rank 272 − Ssc.18553.1.S1_at − probe 4
HLA−B
Normalized Probe Intensity
Probe Number
1.0
0.5
0.0
−0.5
−1.0
−1.5
1
2
3
4
5
6
7
8
9
10
11
Meishan−A
Meishan−B
Meishan−C
European−A
European−B
European−C

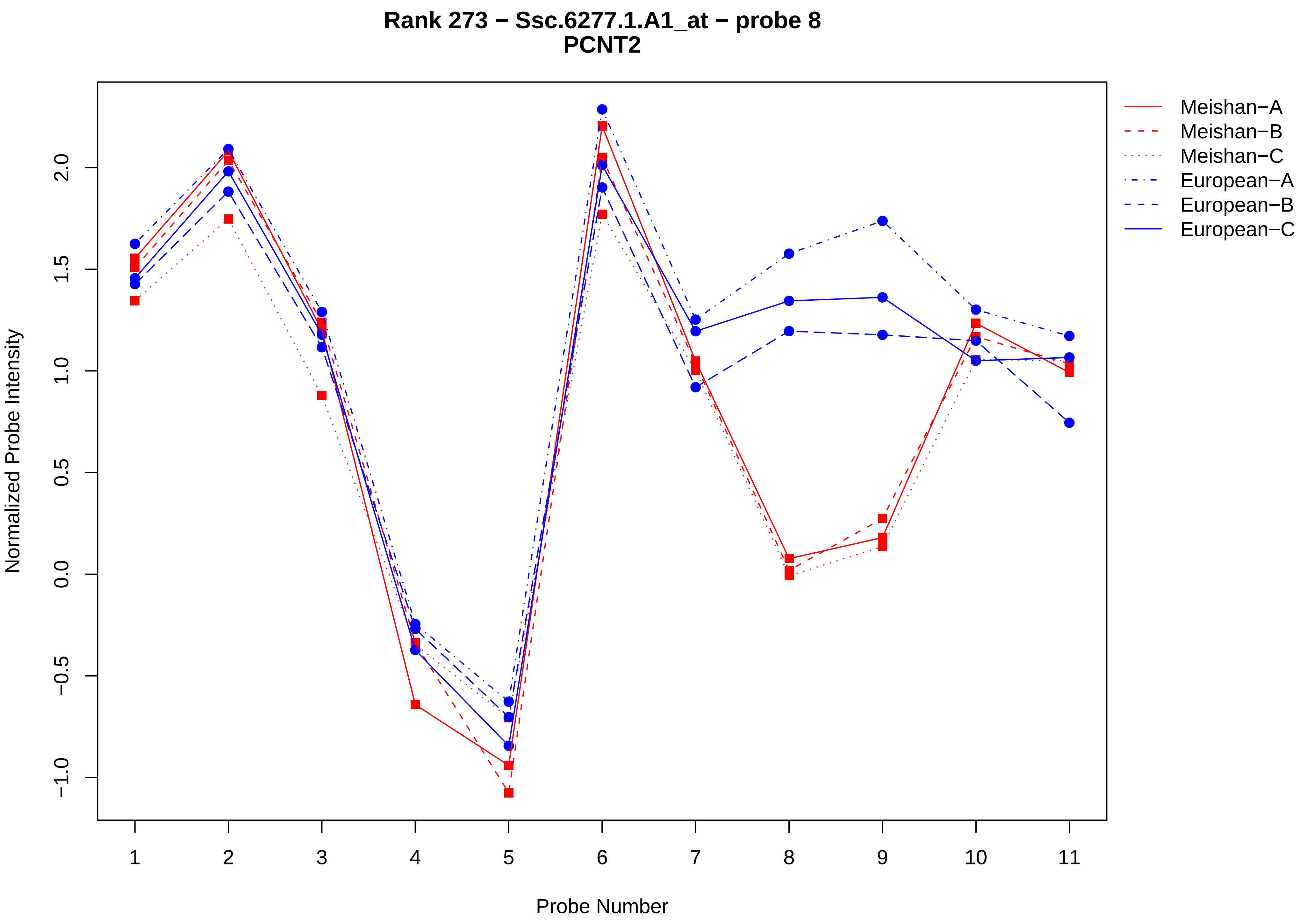
Rank 273 − Ssc.6277.1.A1_at − probe 8
PCNT2
Normalized Probe Intensity
Probe Number
Meishan−A
Meishan−B
Meishan−C
European−A
European−B
European−C
−1.0
−0.5
0.0
0.5
1.0
1.5
2.0
1
2
3
4
5
6
7
8
9
10
11

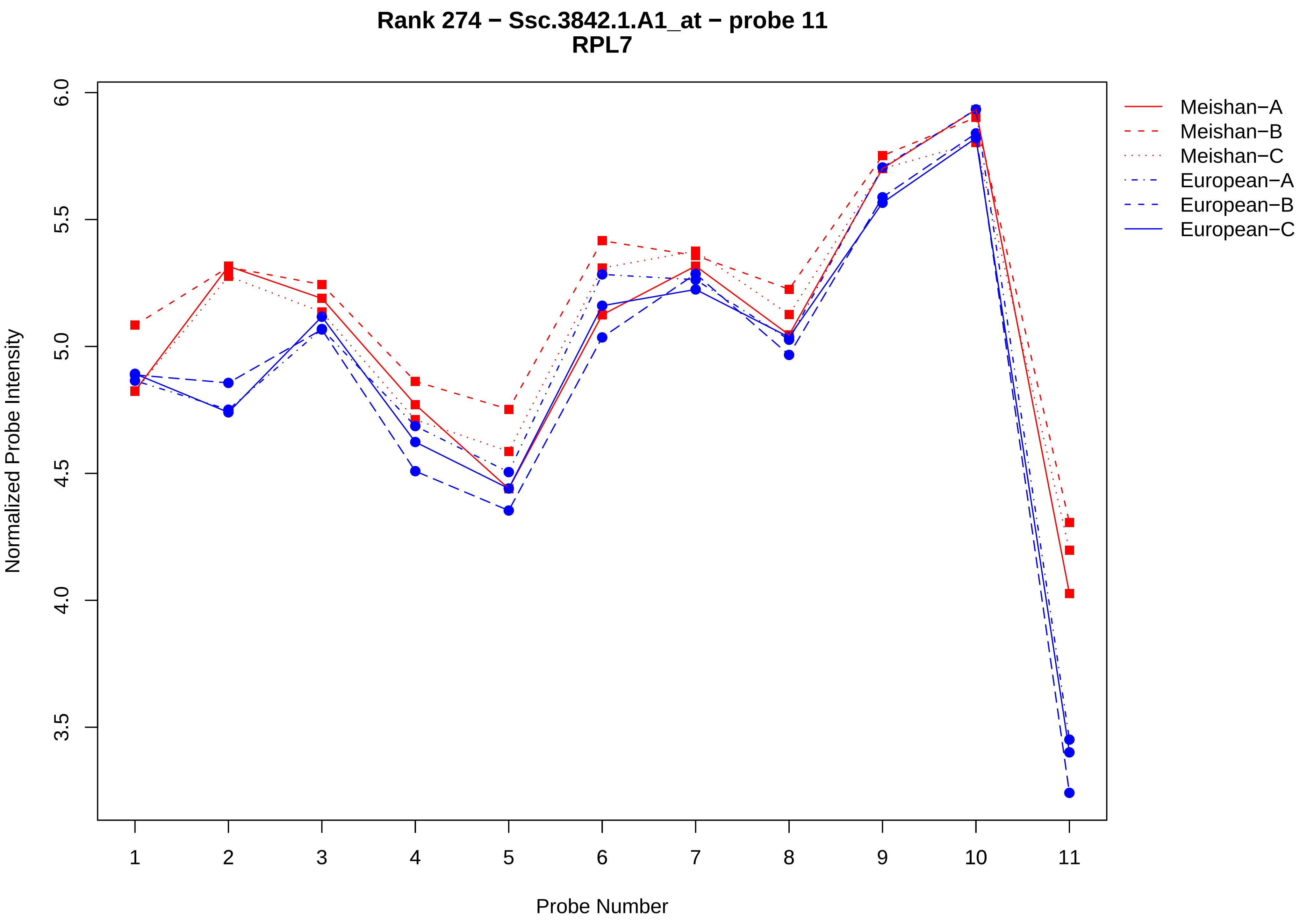
Rank 274 − Ssc.3842.1.A1_at − probe 11
RPL7
Normalized Probe Intensity
6.0
5.5
5.0
4.5
4.0
3.5
1
2
3
4
5
6
7
8
9
10
11
Probe Number
Meishan−A
Meishan−B
Meishan−C
European−A
European−B
European−C

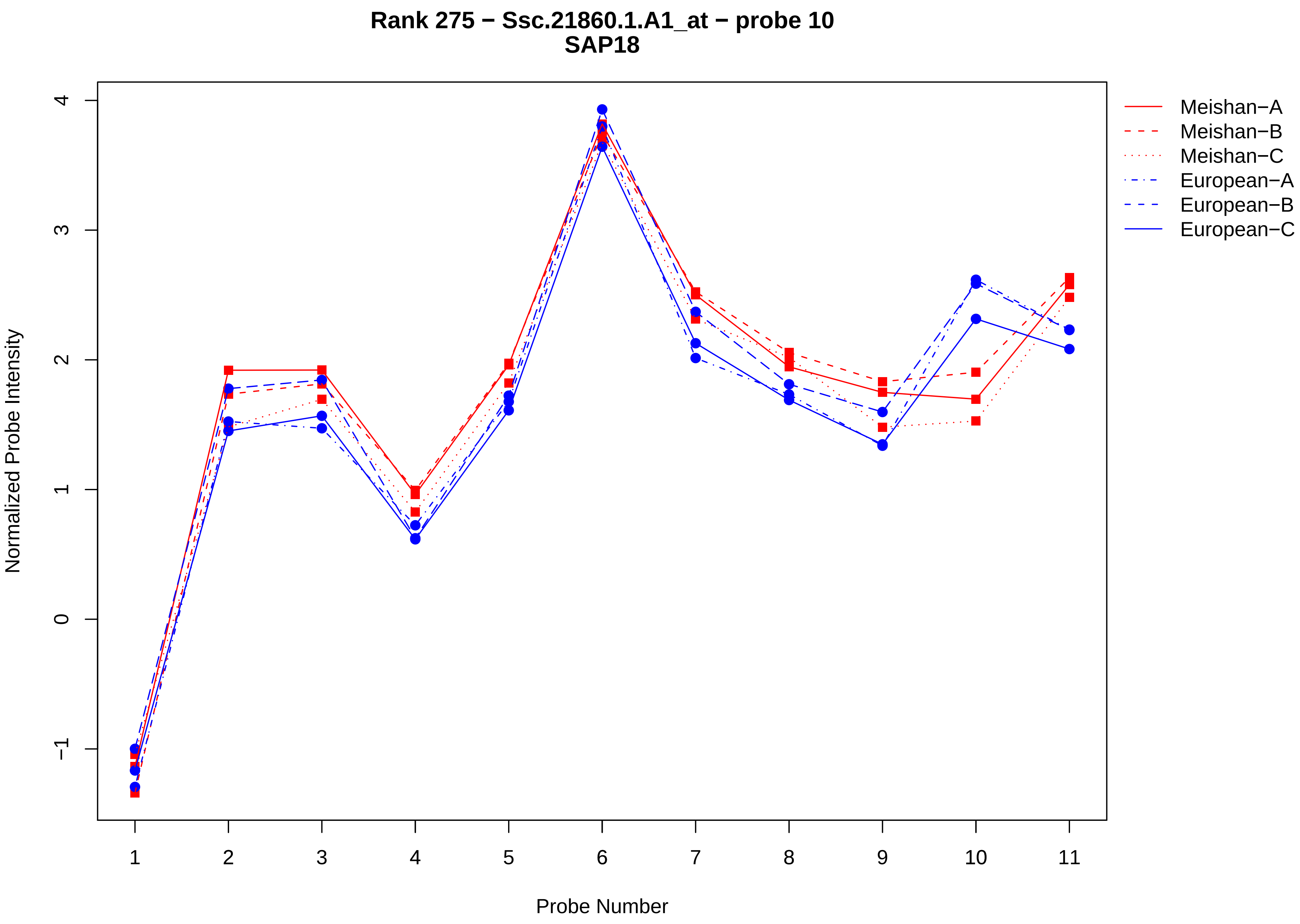
Rank 275 − Ssc.21860.1.A1_at − probe 10
SAP18
Normalized Probe Intensity
Probe Number
−1
0
1
2
3
4
1
2
3
4
5
6
7
8
9
10
11
Meishan−A
Meishan−B
Meishan−C
European−A
European−B
European−C

Rank 276 − Ssc.20986.2.S1_at − probe 6
GRN

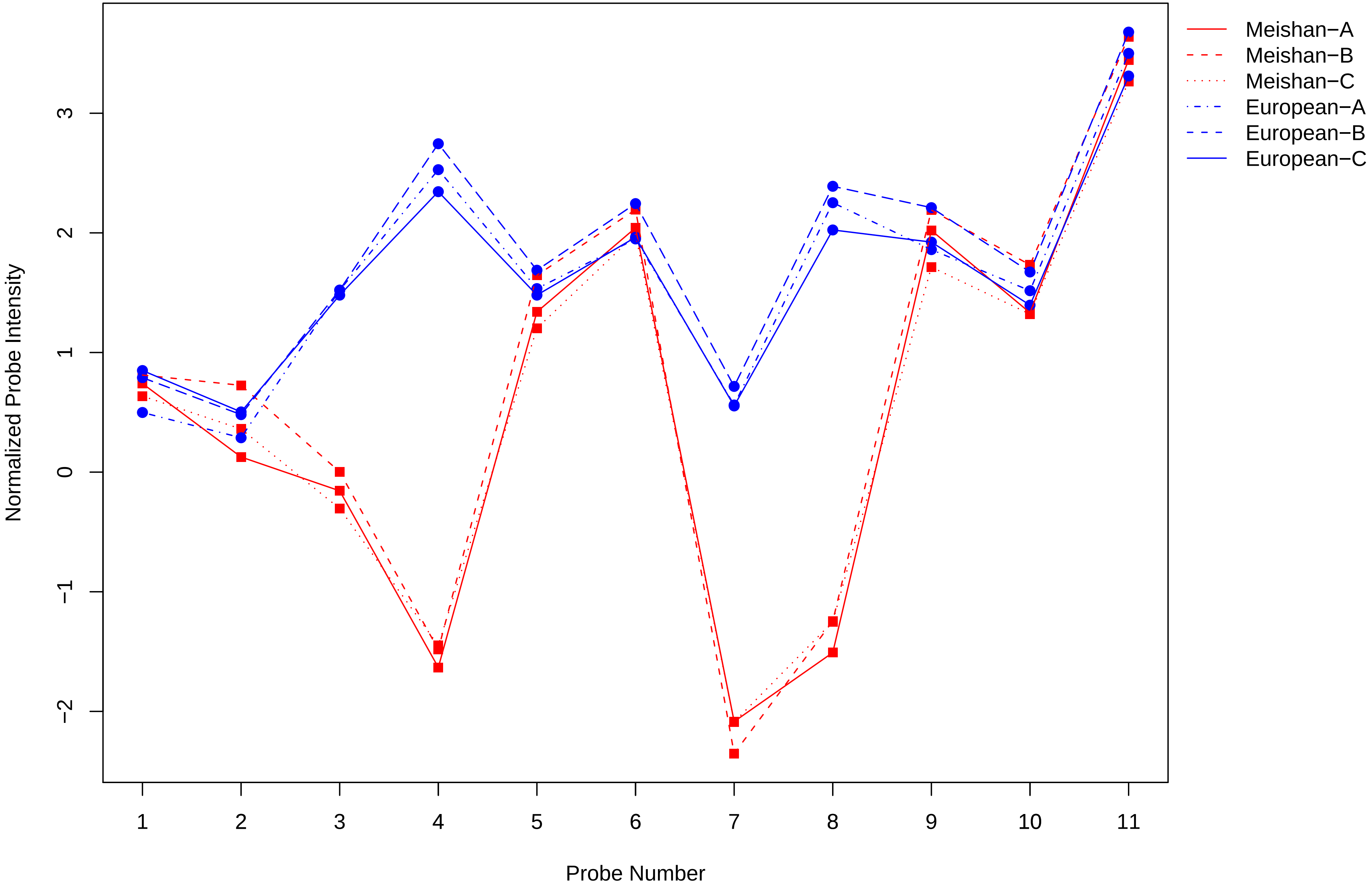

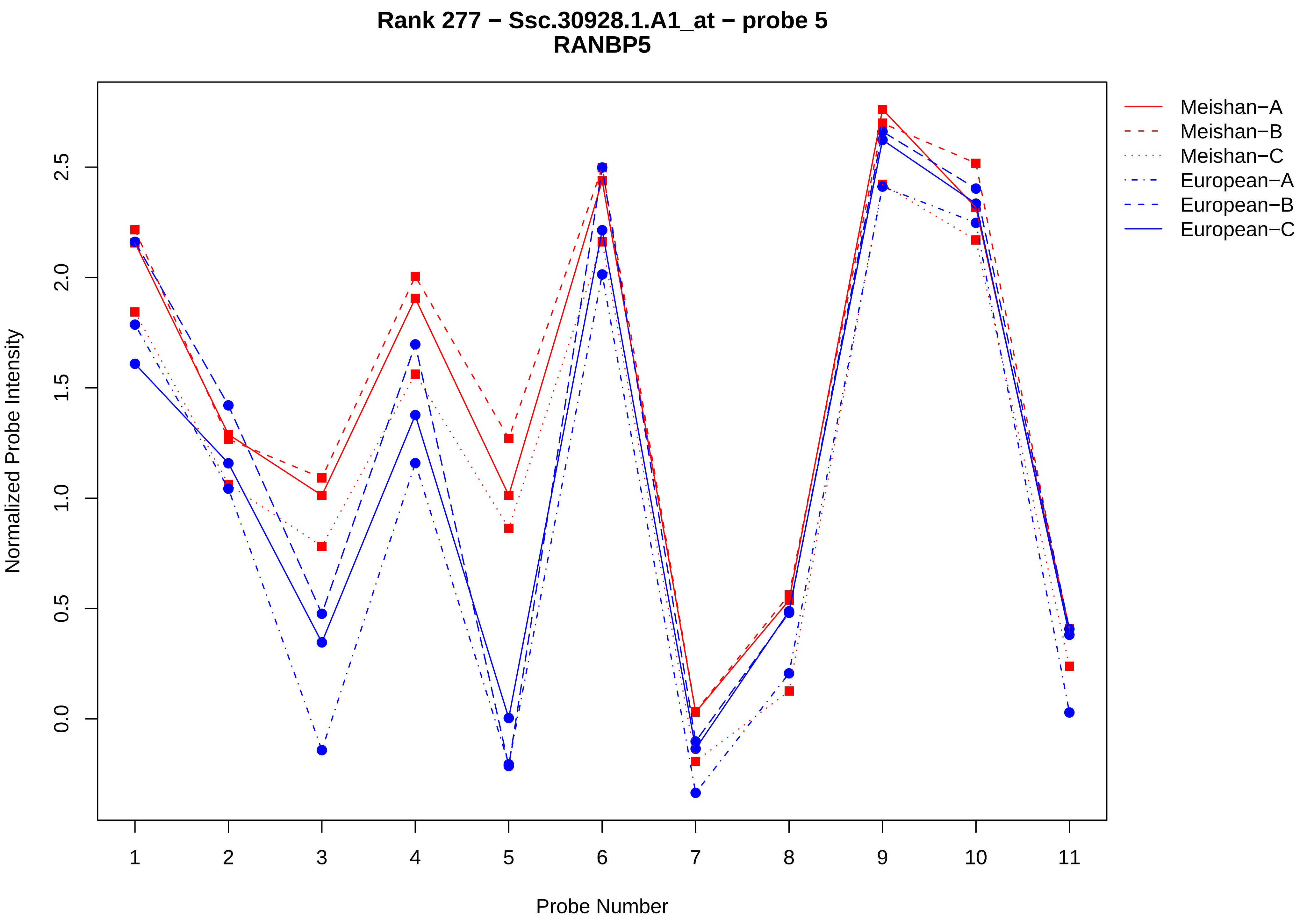

Rank 277 − Ssc.30928.1.A1_at − probe 5
RANBP5
Normalized Probe Intensity
Probe Number
0.0
0.5
1.0
1.5
2.0
2.5
1
2
3
4
5
6
7
8
9
10
11
Meishan−A
Meishan−B
Meishan−C
European−A
European−B
European−C

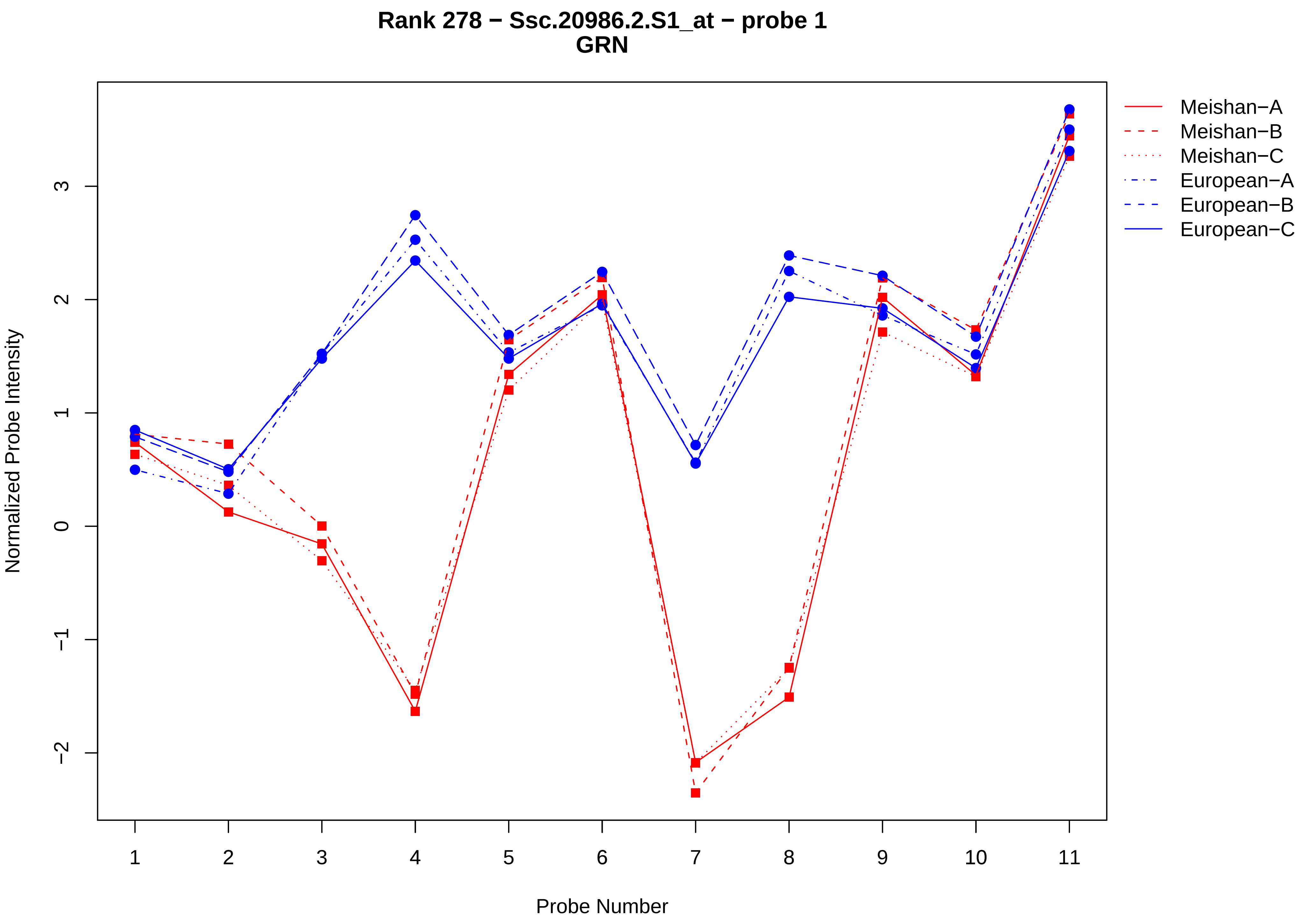

Rank 278 − Ssc.20986.2.S1_at − probe 1
GRN
Normalized Probe Intensity
Probe Number
−2
−1
0
1
2
3
1
2
3
4
5
6
7
8
9
10
11
Meishan−A
Meishan−B
Meishan−C
European−A
European−B
European−C

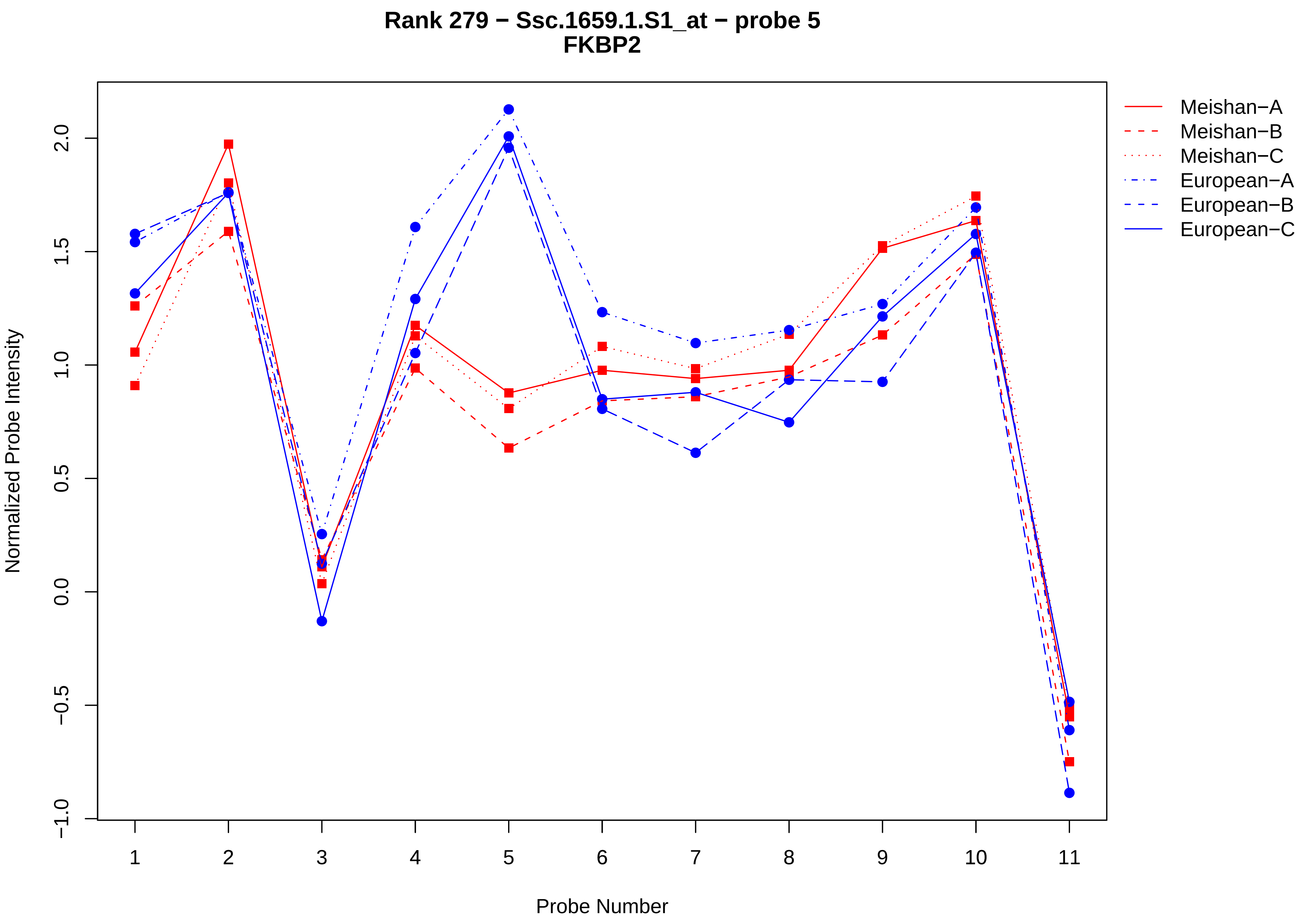
Rank 279 − Ssc.1659.1.S1_at − probe 5
FKBP2
Normalized Probe Intensity
Probe Number
Meishan−A
Meishan−B
Meishan−C
European−A
European−B
European−C
−1.0
−0.5
0.0
0.5
1.0
1.5
2.0
1
2
3
4
5
6
7
8
9
10
11

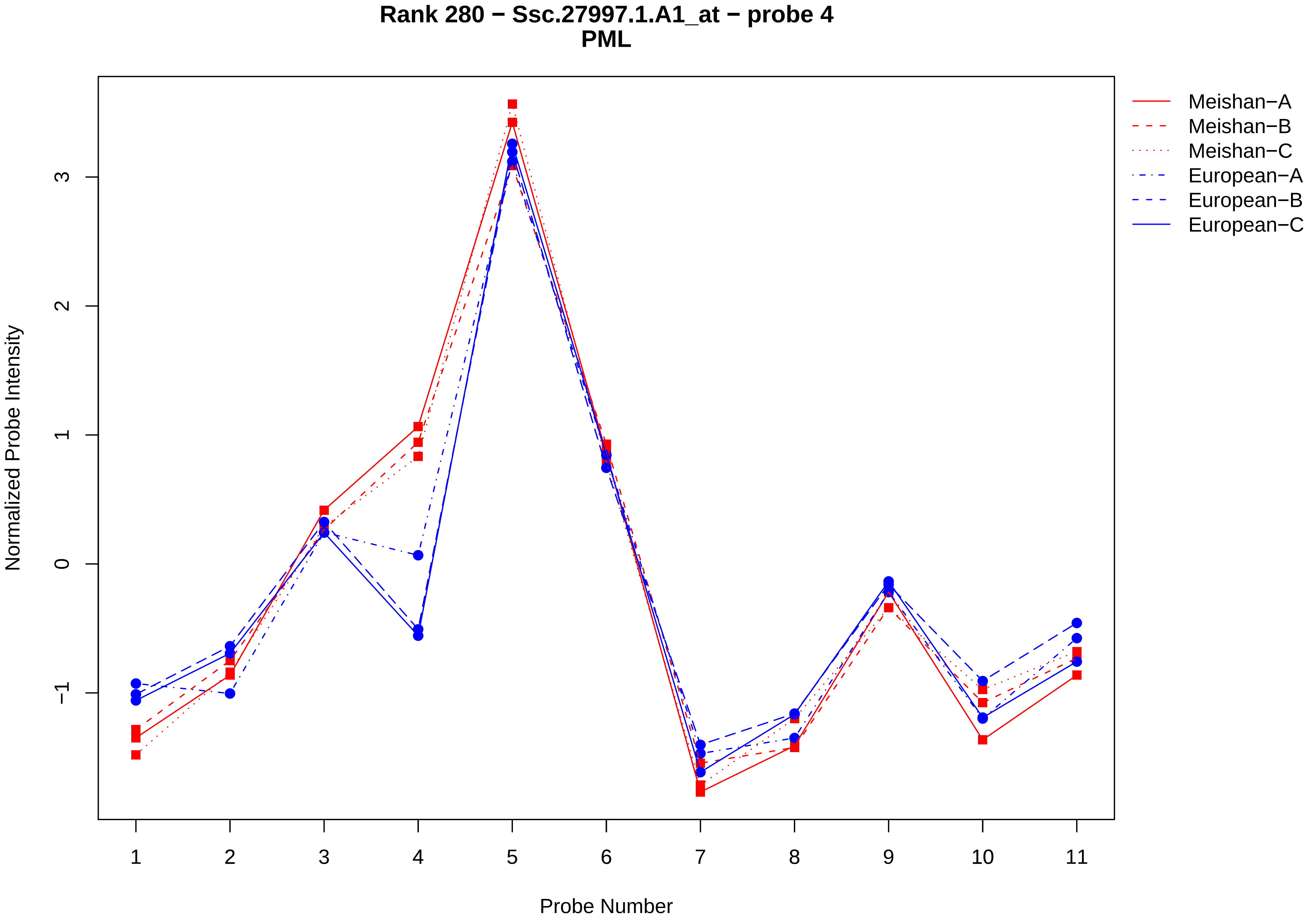

Rank 280 − Ssc.27997.1.A1_at − probe 4
PML
Normalized Probe Intensity
Probe Number
Meishan−A
Meishan−B
Meishan−C
European−A
European−B
European−C
−1
0
1
2
3
1
2
3
4
5
6
7
8
9
10
11

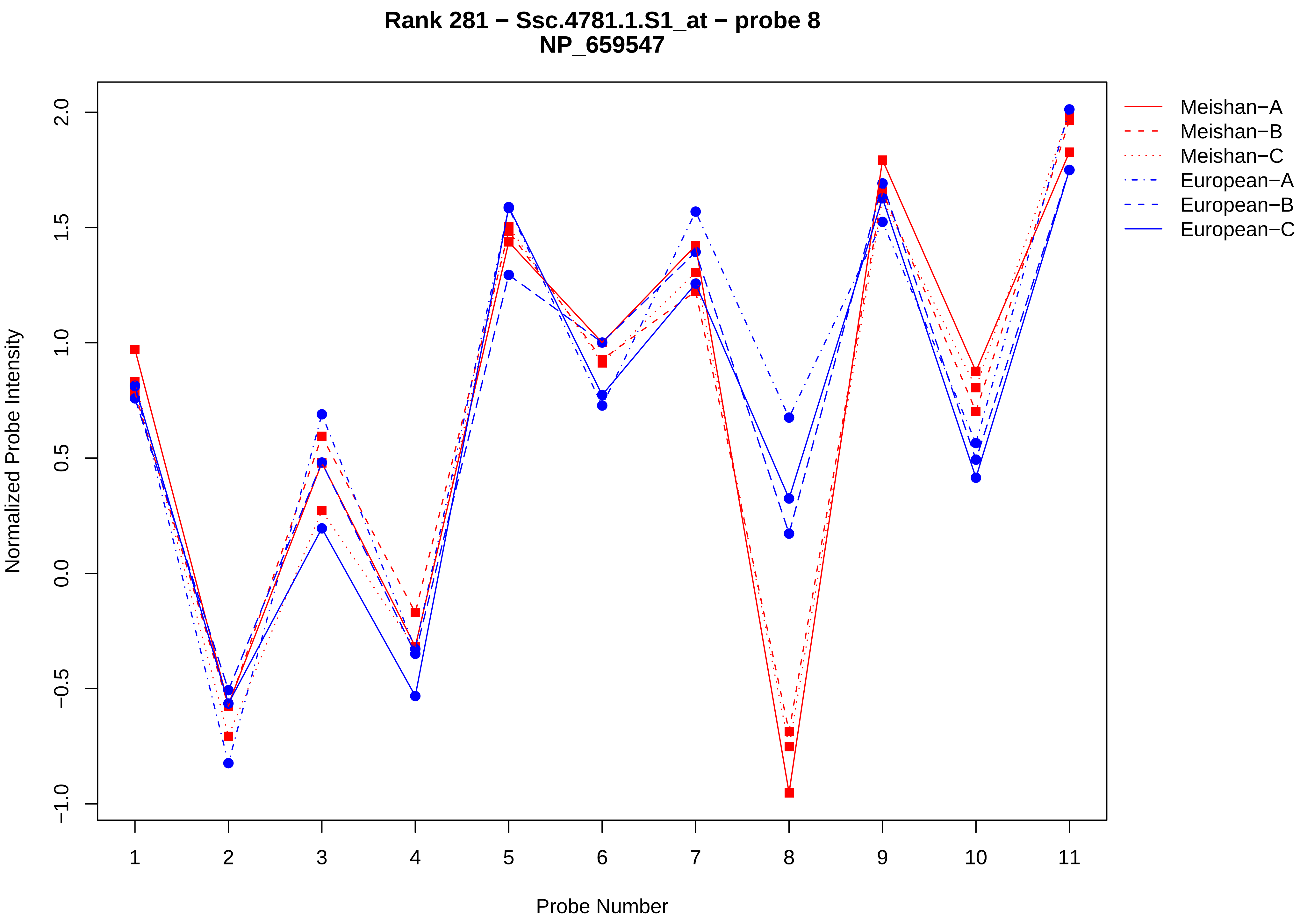
Rank 281 − Ssc.4781.1.S1_at − probe 8
NP_659547
Normalized Probe Intensity
Probe Number
−1.0
−0.5
0.0
0.5
1.0
1.5
2.0
1
2
3
4
5
6
7
8
9
10
11
Meishan−A
Meishan−B
Meishan−C
European−A
European−B
European−C

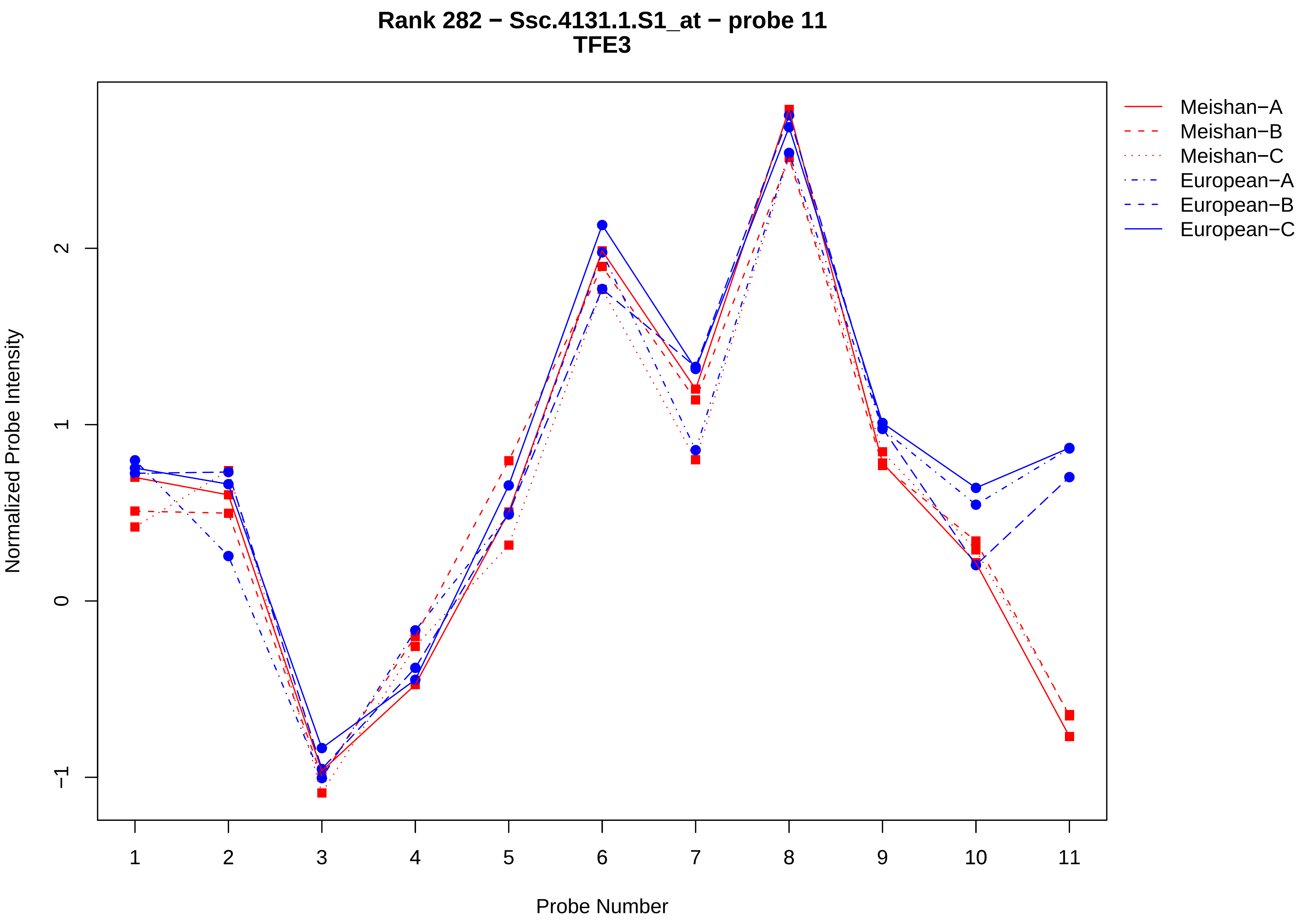

Rank 282 − Ssc.4131.1.S1_at − probe 11
TFE3
Normalized Probe Intensity
Probe Number
−1
0
1
2
1
2
3
4
5
6
7
8
9
10
11
Meishan−A
Meishan−B
Meishan−C
European−A
European−B
European−C

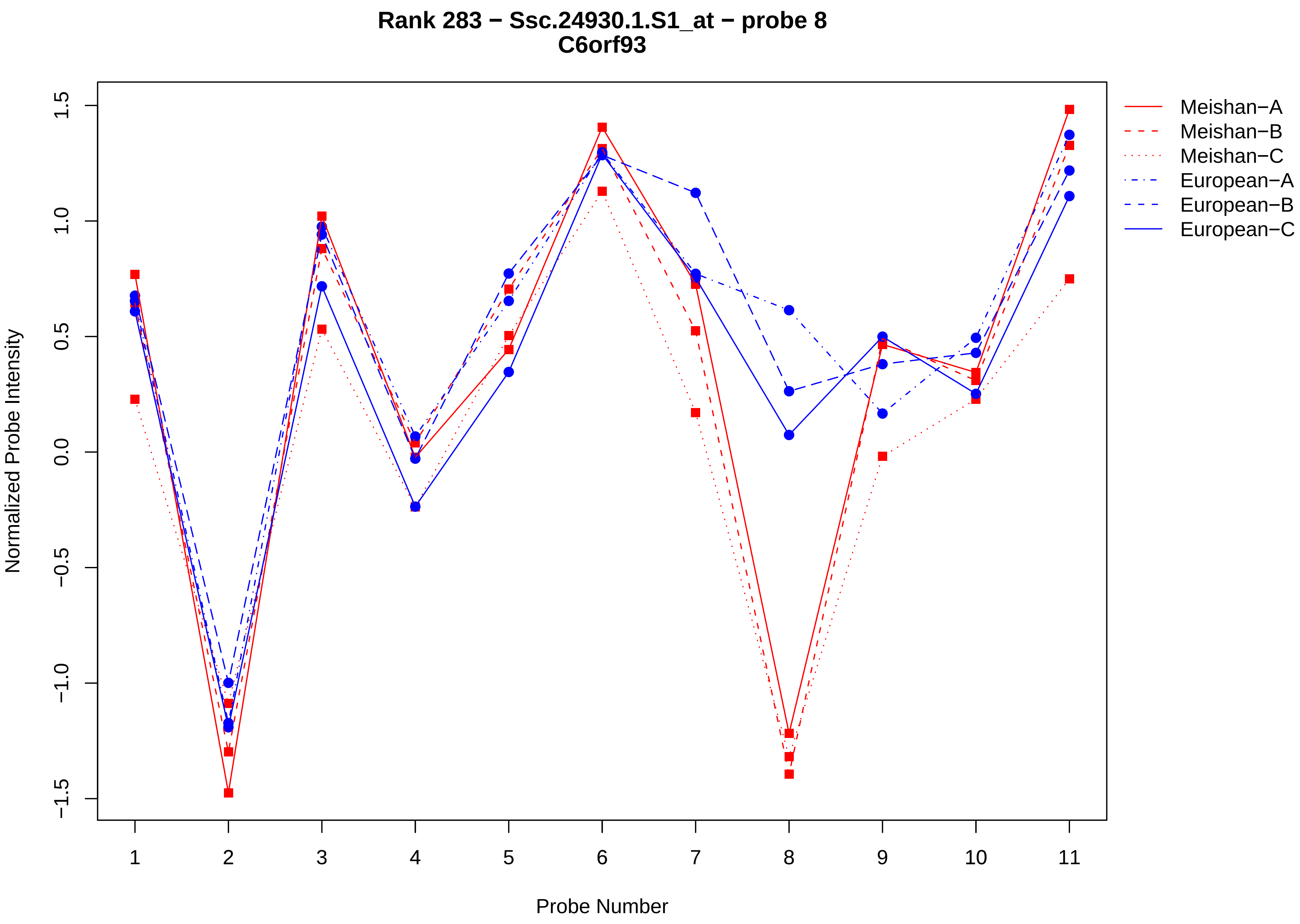

Rank 283 − Ssc.24930.1.S1_at − probe 8
C6orf93
Normalized Probe Intensity
−1.5
−1.0
−0.5
0.0
0.5
1.0
1.5
1
2
3
4
5
6
7
8
9
10
11
Probe Number
Meishan−A
Meishan−B
Meishan−C
European−A
European−B
European−C

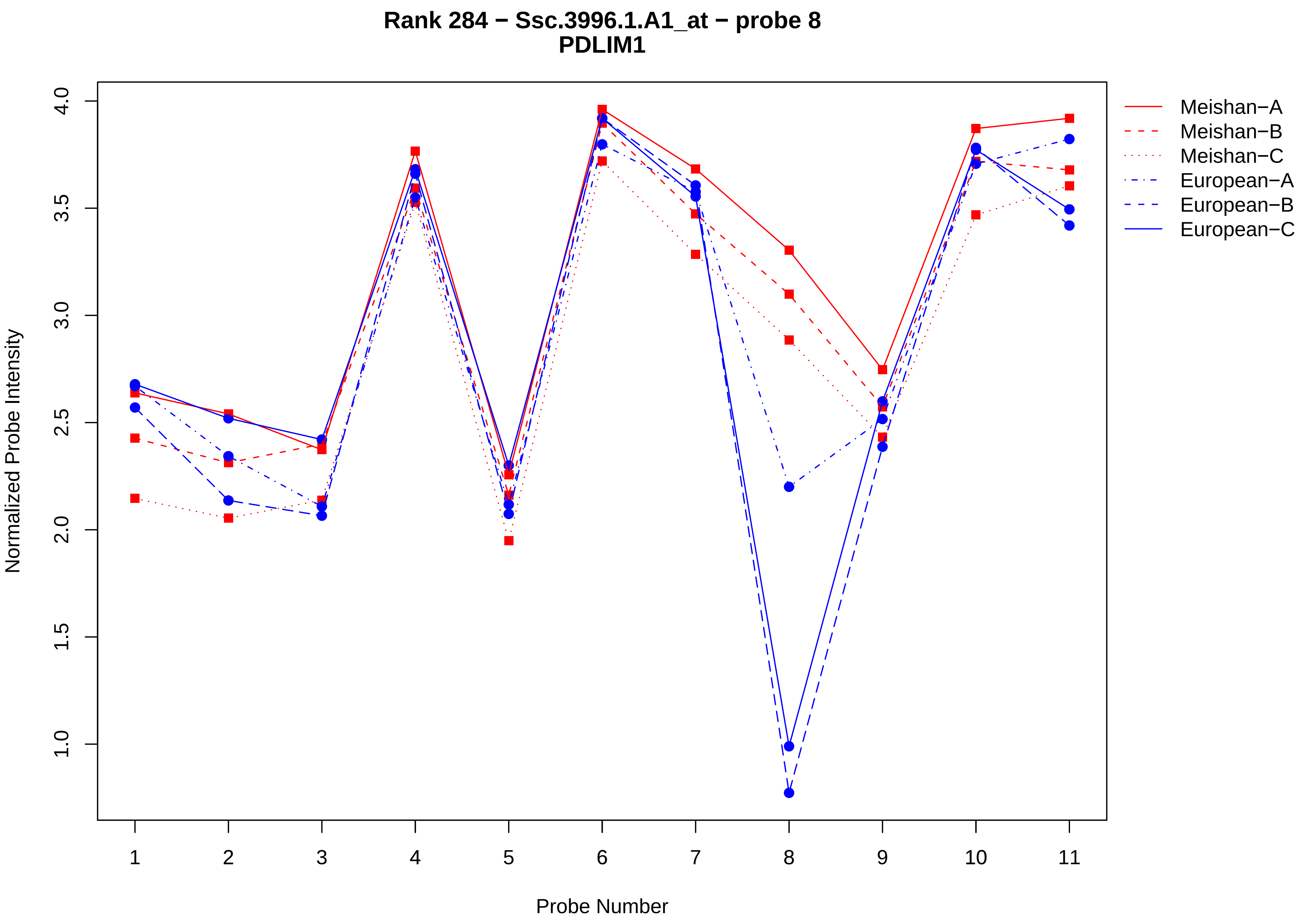

Rank 284 − Ssc.3996.1.A1_at − probe 8
PDLIM1
Normalized Probe Intensity
Probe Number
1.0
1.5
2.0
2.5
3.0
3.5
4.0
1
2
3
4
5
6
7
8
9
10
11
Meishan−A
Meishan−B
Meishan−C
European−A
European−B
European−C

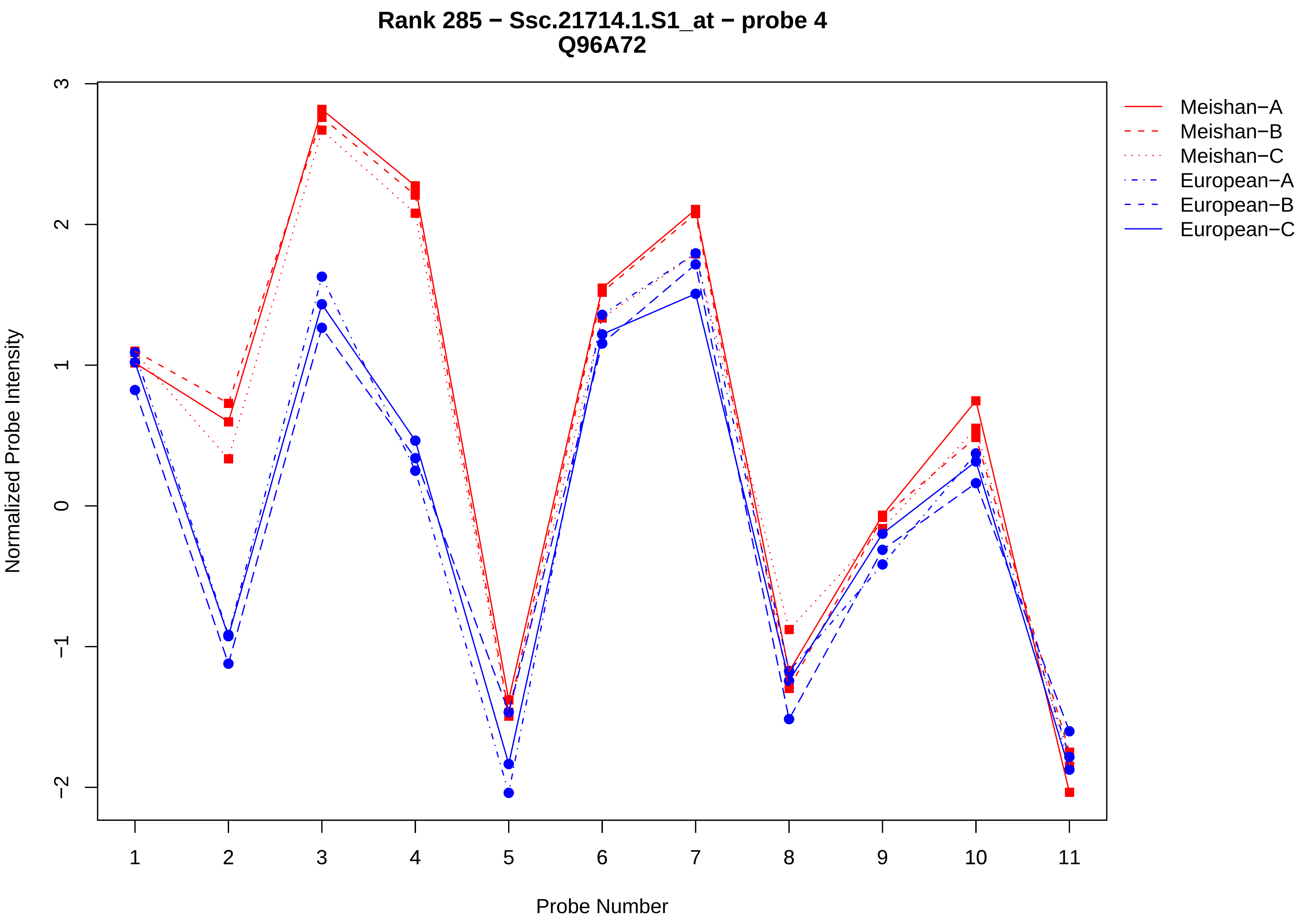

Rank 285 − Ssc.21714.1.S1_at − probe 4
Q96A72
Normalized Probe Intensity
Probe Number
Meishan−A
Meishan−B
Meishan−C
European−A
European−B
European−C

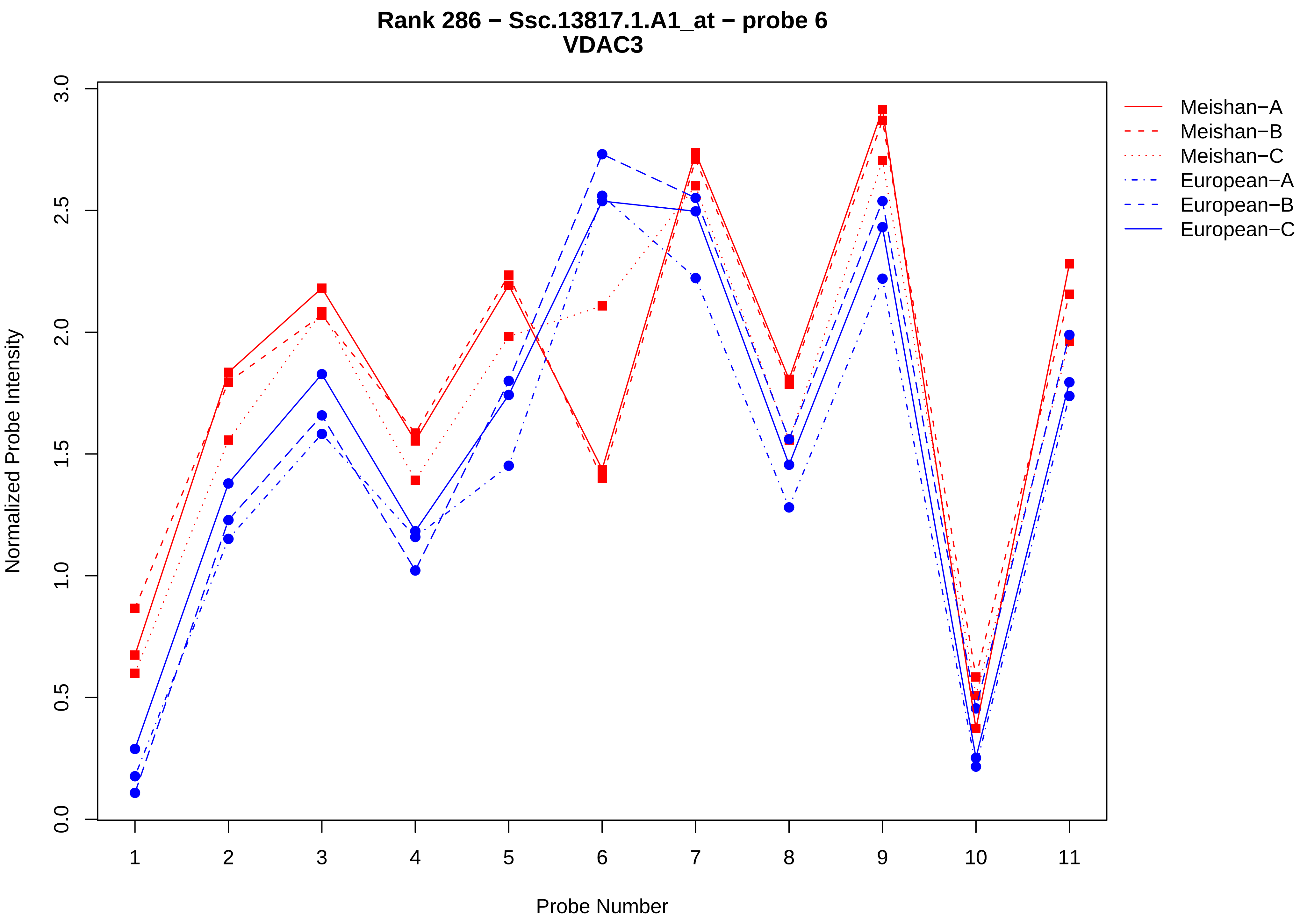

Rank 286 − Ssc.13817.1.A1_at − probe 6
VDAC3
Normalized Probe Intensity
0.0
0.5
1.0
1.5
2.0
2.5
3.0
1
2
3
4
5
6
7
8
9
10
11
Probe Number
Meishan−A
Meishan−B
Meishan−C
European−A
European−B
European−C

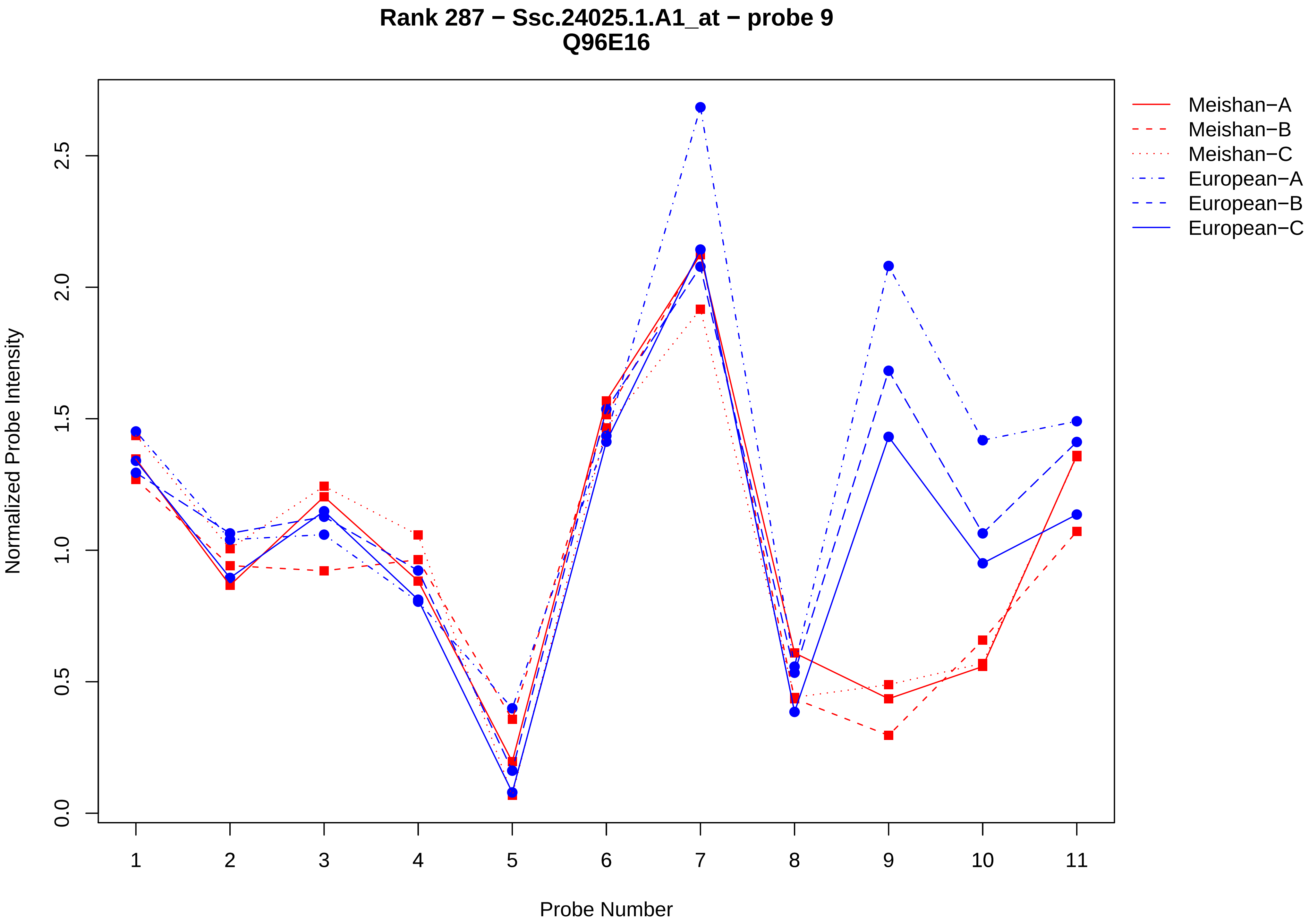

Rank 287 − Ssc.24025.1.A1_at − probe 9
Q96E16
Normalized Probe Intensity
0.0
0.5
1.0
1.5
2.0
2.5
1
2
3
4
5
6
7
8
9
10
11
Probe Number
Meishan−A
Meishan−B
Meishan−C
European−A
European−B
European−C

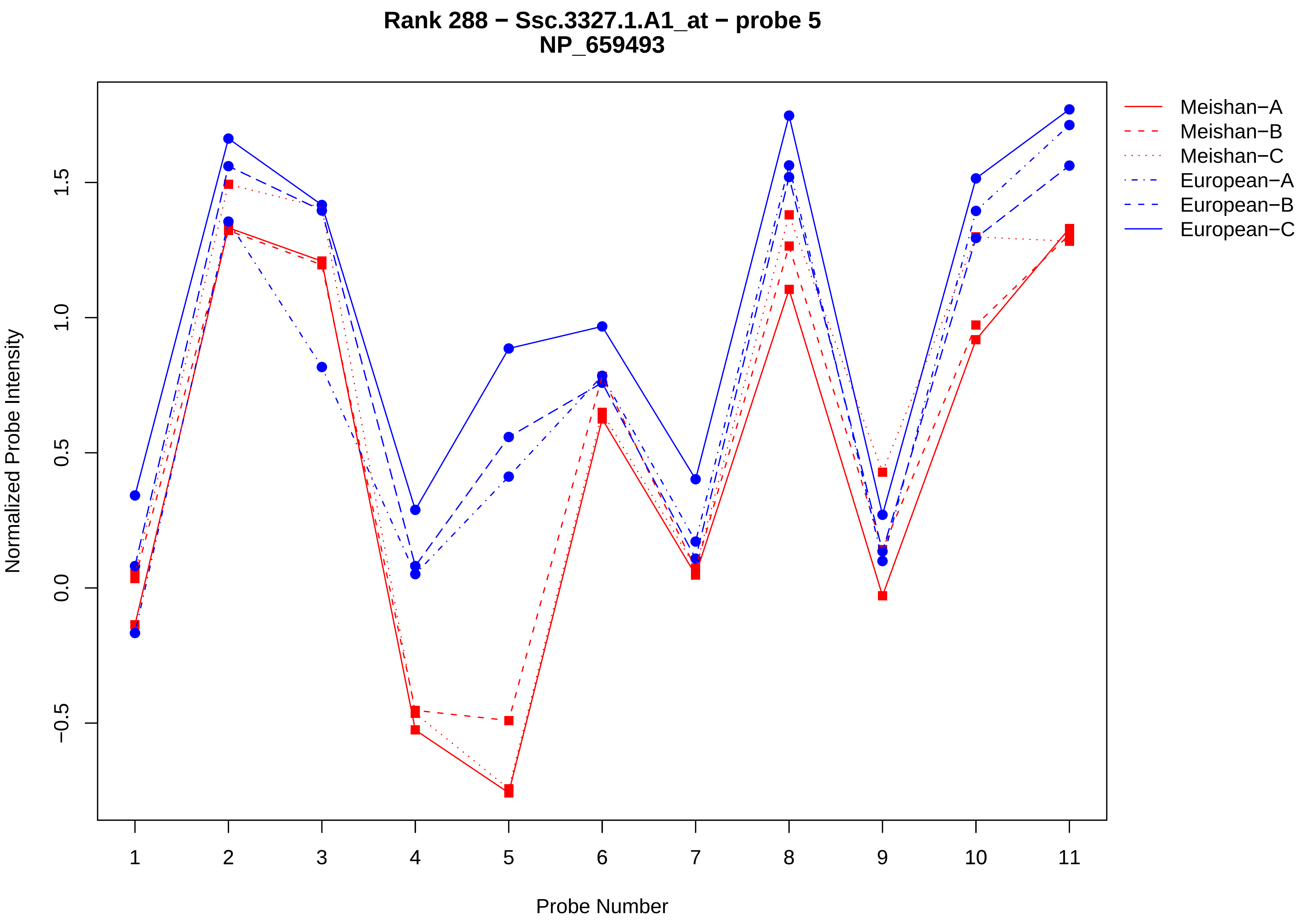
Rank 288 − Ssc.3327.1.A1_at − probe 5
NP_659493
Normalized Probe Intensity
Probe Number
−0.5
0.0
0.5
1.0
1.5
1
2
3
4
5
6
7
8
9
10
11
Meishan−A
Meishan−B
Meishan−C
European−A
European−B
European−C

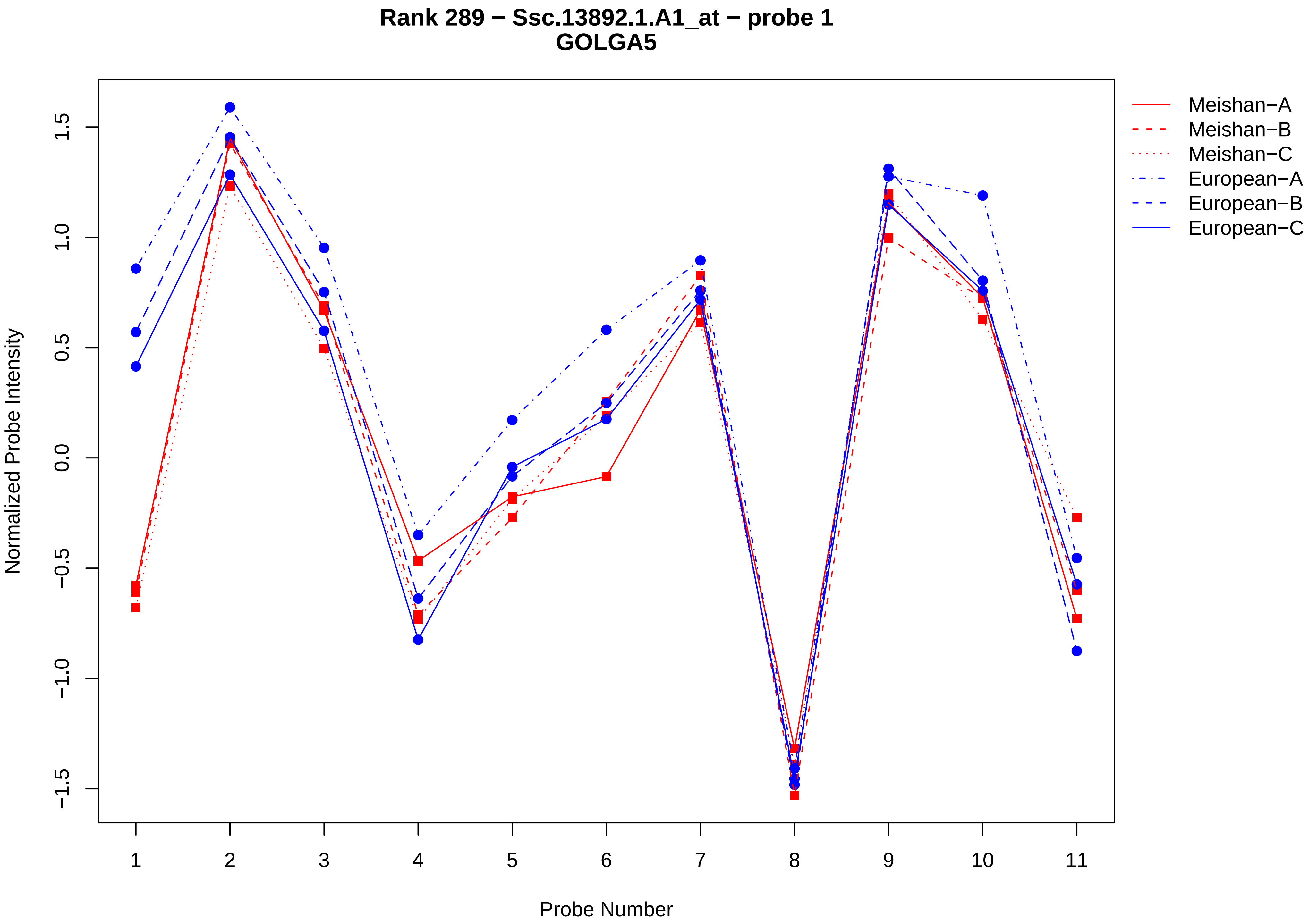

Rank 289 − Ssc.13892.1.A1_at − probe 1
GOLGA5
Normalized Probe Intensity
Probe Number
−1.5
−1.0
−0.5
0.0
0.5
1.0
1.5
1
2
3
4
5
6
7
8
9
10
11
Meishan−A
Meishan−B
Meishan−C
European−A
European−B
European−C

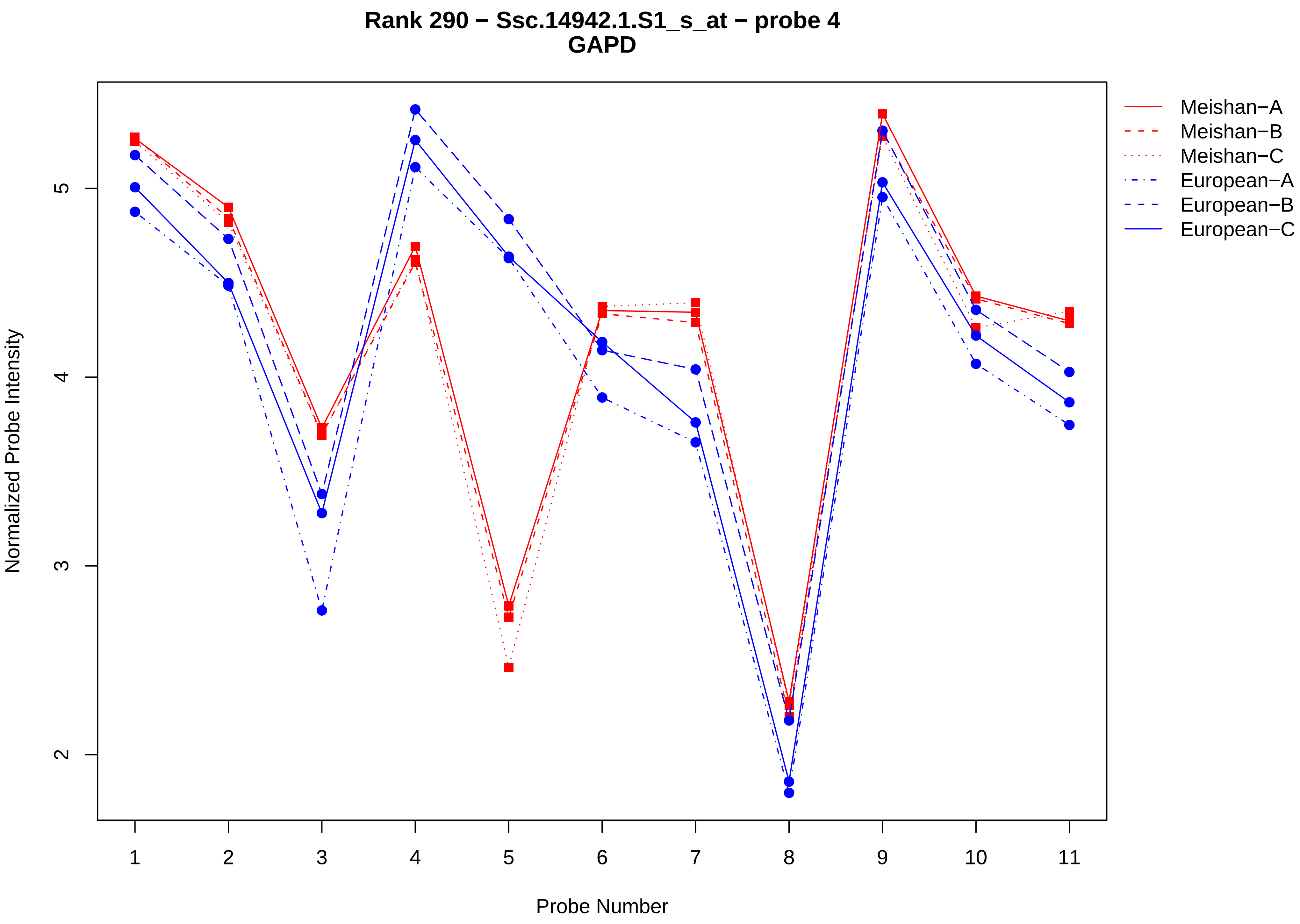
Rank 290 − Ssc.14942.1.S1_s_at − probe 4
GAPD
Normalized Probe Intensity
Probe Number
2
3
4
5
1
2
3
4
5
6
7
8
9
10
11
Meishan−A
Meishan−B
Meishan−C
European−A
European−B
European−C

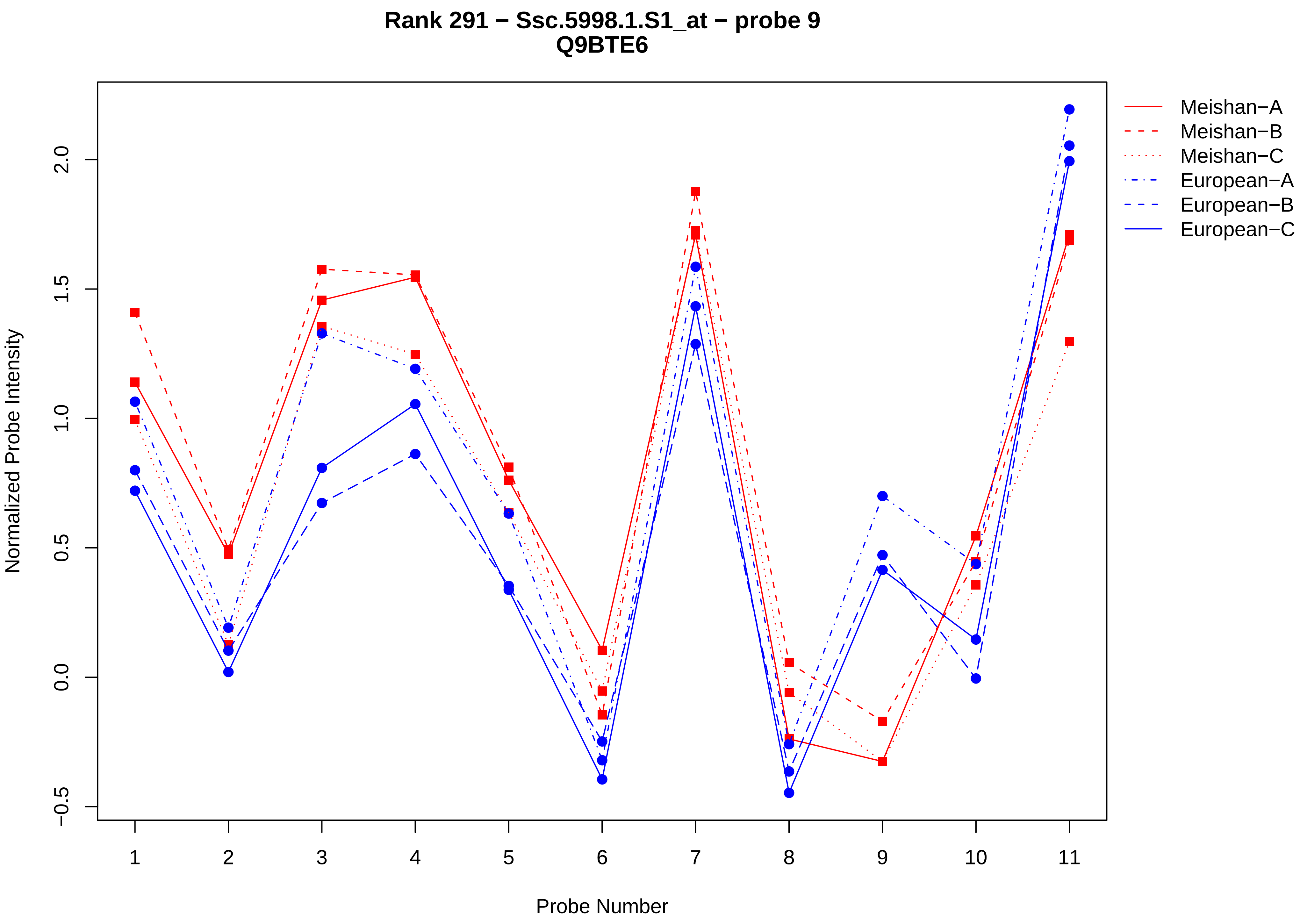
Rank 291 − Ssc.5998.1.S1_at − probe 9
Q9BTE6
Normalized Probe Intensity
Probe Number
Meishan−A
Meishan−B
Meishan−C
European−A
European−B
European−C

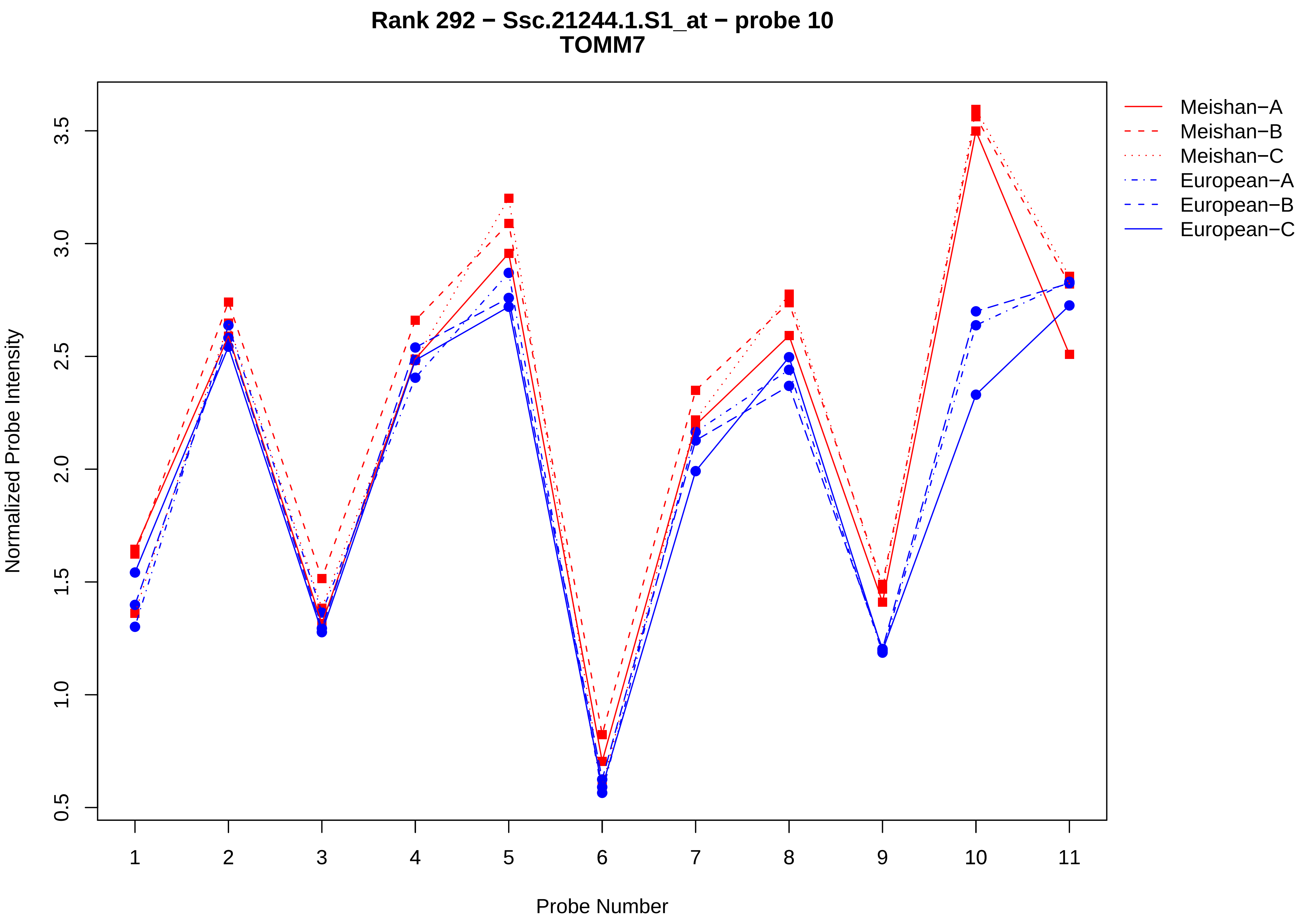
Rank 292 − Ssc.21244.1.S1_at − probe 10
TOMM7
Normalized Probe Intensity
Probe Number
0.5
1.0
1.5
2.0
2.5
3.0
3.5
1
2
3
4
5
6
7
8
9
10
11
Meishan−A
Meishan−B
Meishan−C
European−A
European−B
European−C

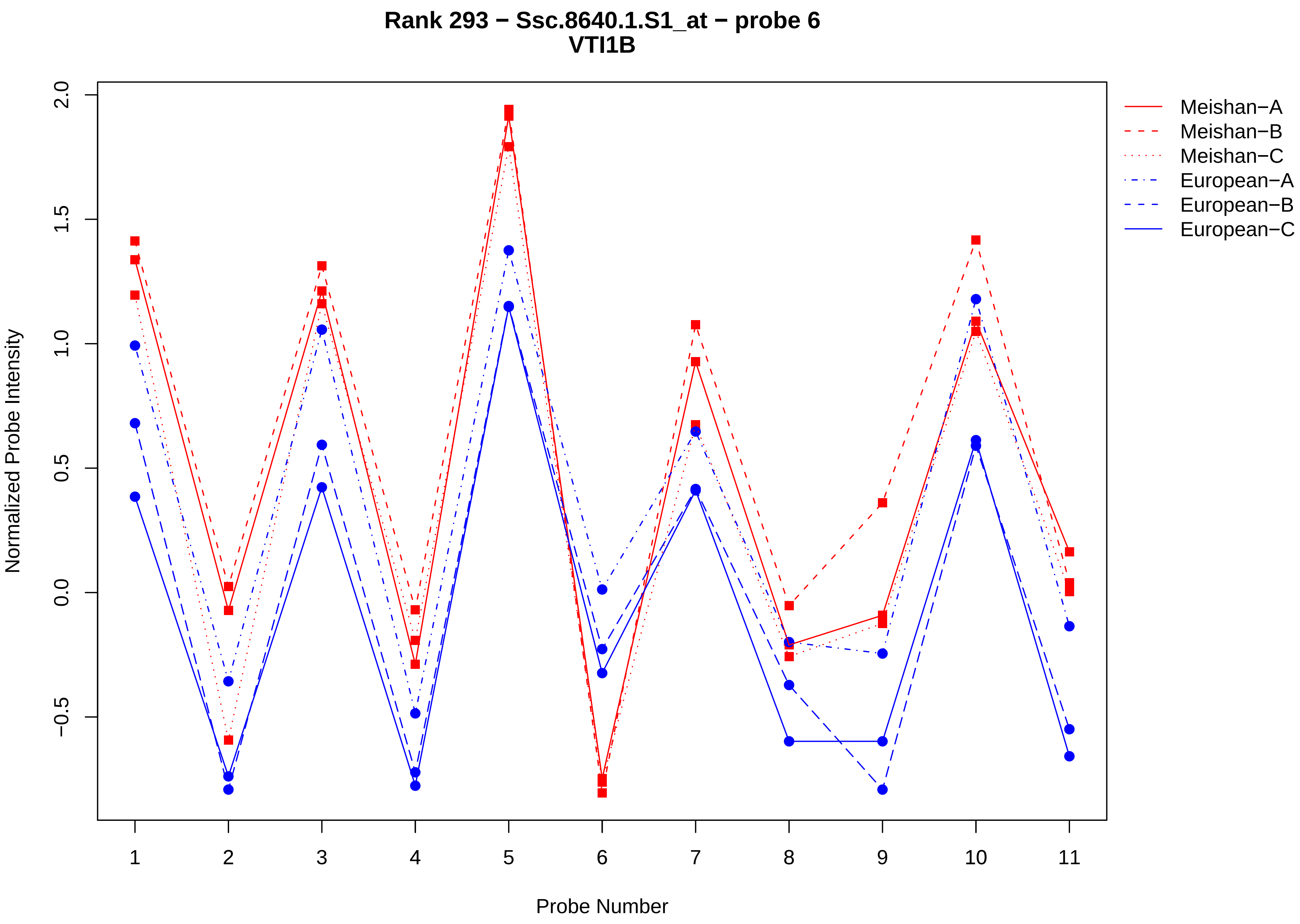

Rank 293 − Ssc.8640.1.S1_at − probe 6
VTI1B
Normalized Probe Intensity
Probe Number
Meishan−A
Meishan−B
Meishan−C
European−A
European−B
European−C
−0.5
0.0
0.5
1.0
1.5
2.0
1
2
3
4
5
6
7
8
9
10
11

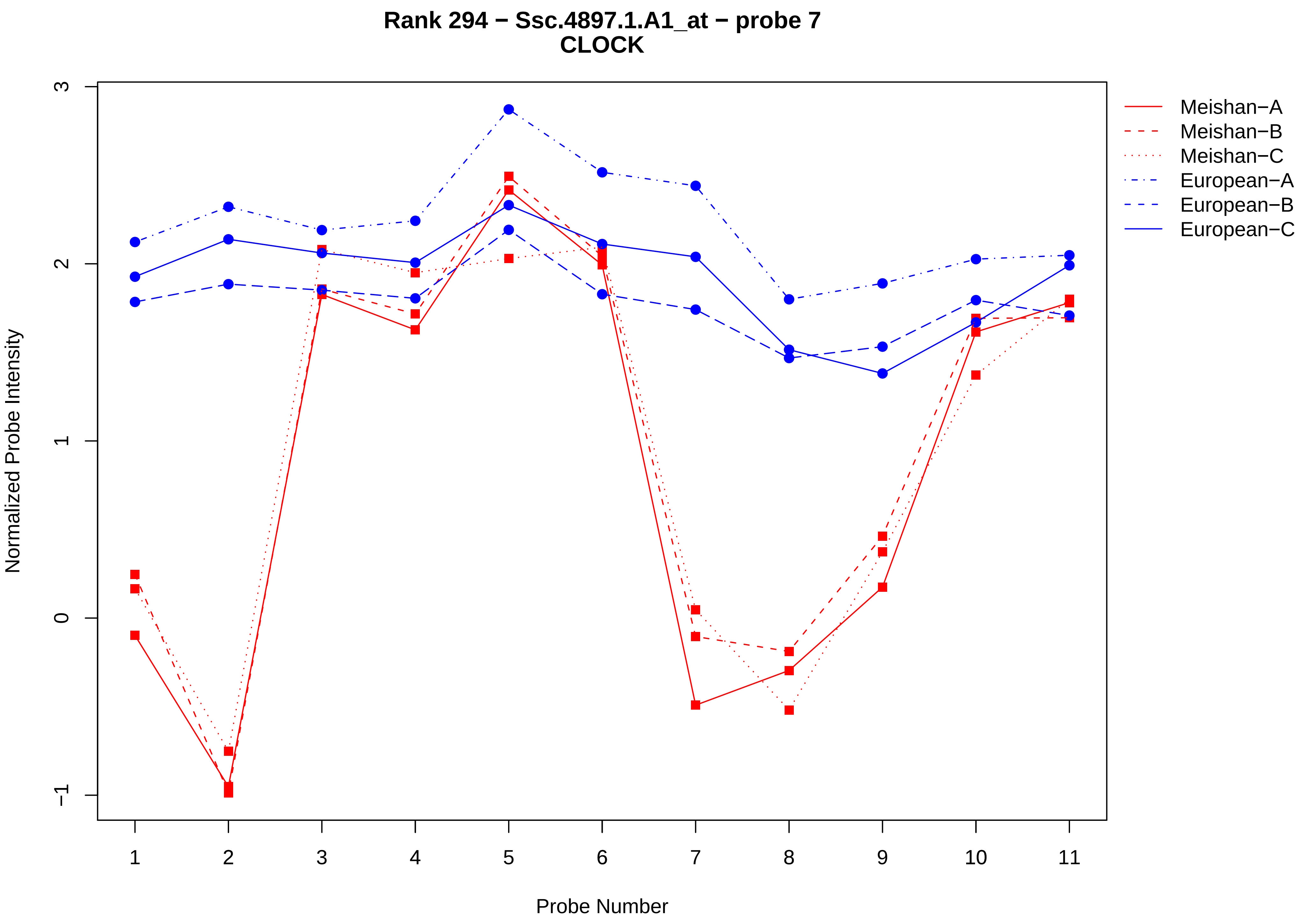

Rank 294 − Ssc.4897.1.A1_at − probe 7
CLOCK
Normalized Probe Intensity
Probe Number
−1
0
1
2
3
1
2
3
4
5
6
7
8
9
10
11
Meishan−A
Meishan−B
Meishan−C
European−A
European−B
European−C

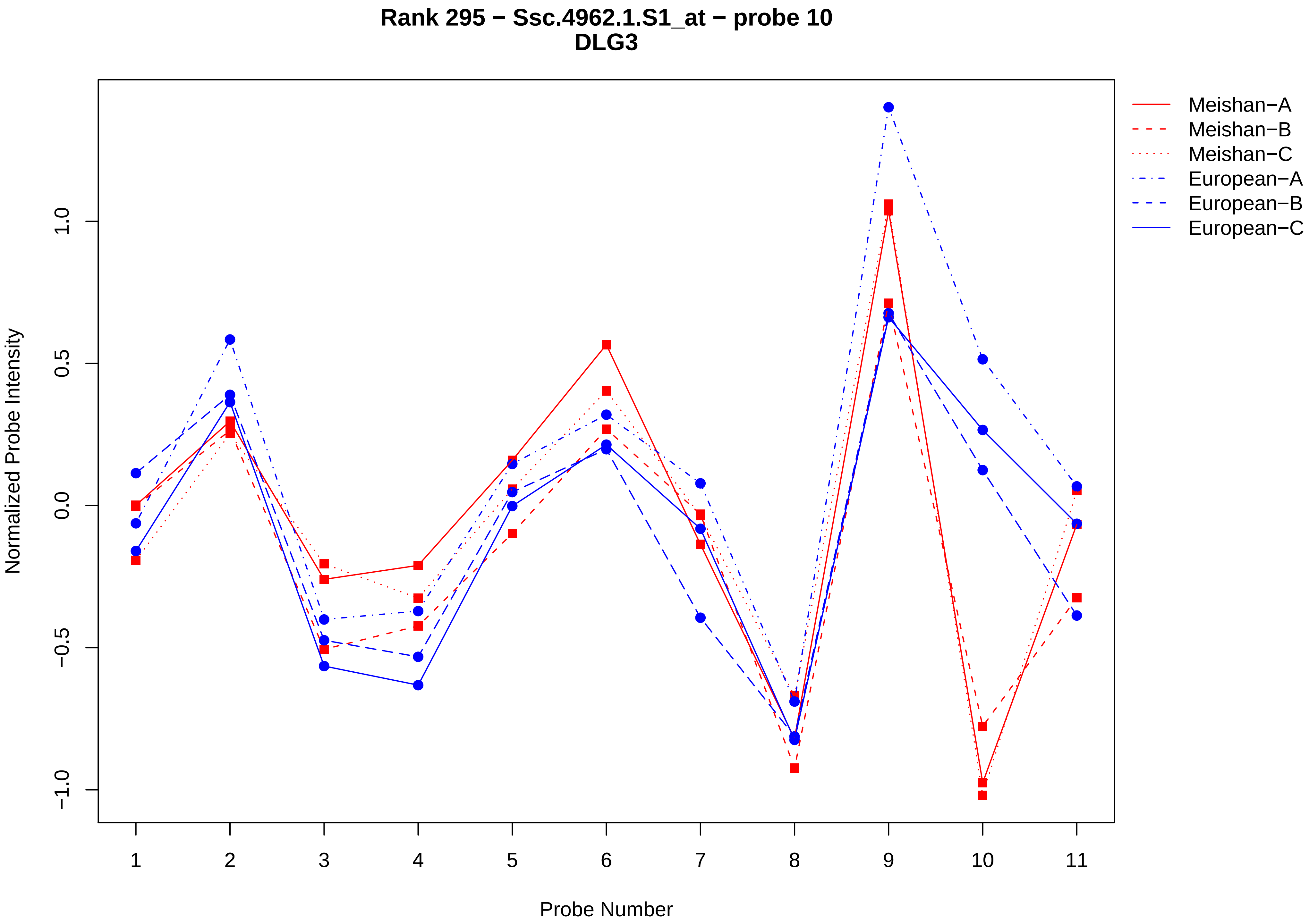

Rank 295 − Ssc.4962.1.S1_at − probe 10
DLG3
Normalized Probe Intensity
Probe Number
1.0
0.5
0.0
−0.5
−1.0
1
2
3
4
5
6
7
8
9
10
11
Meishan−A
Meishan−B
Meishan−C
European−A
European−B
European−C

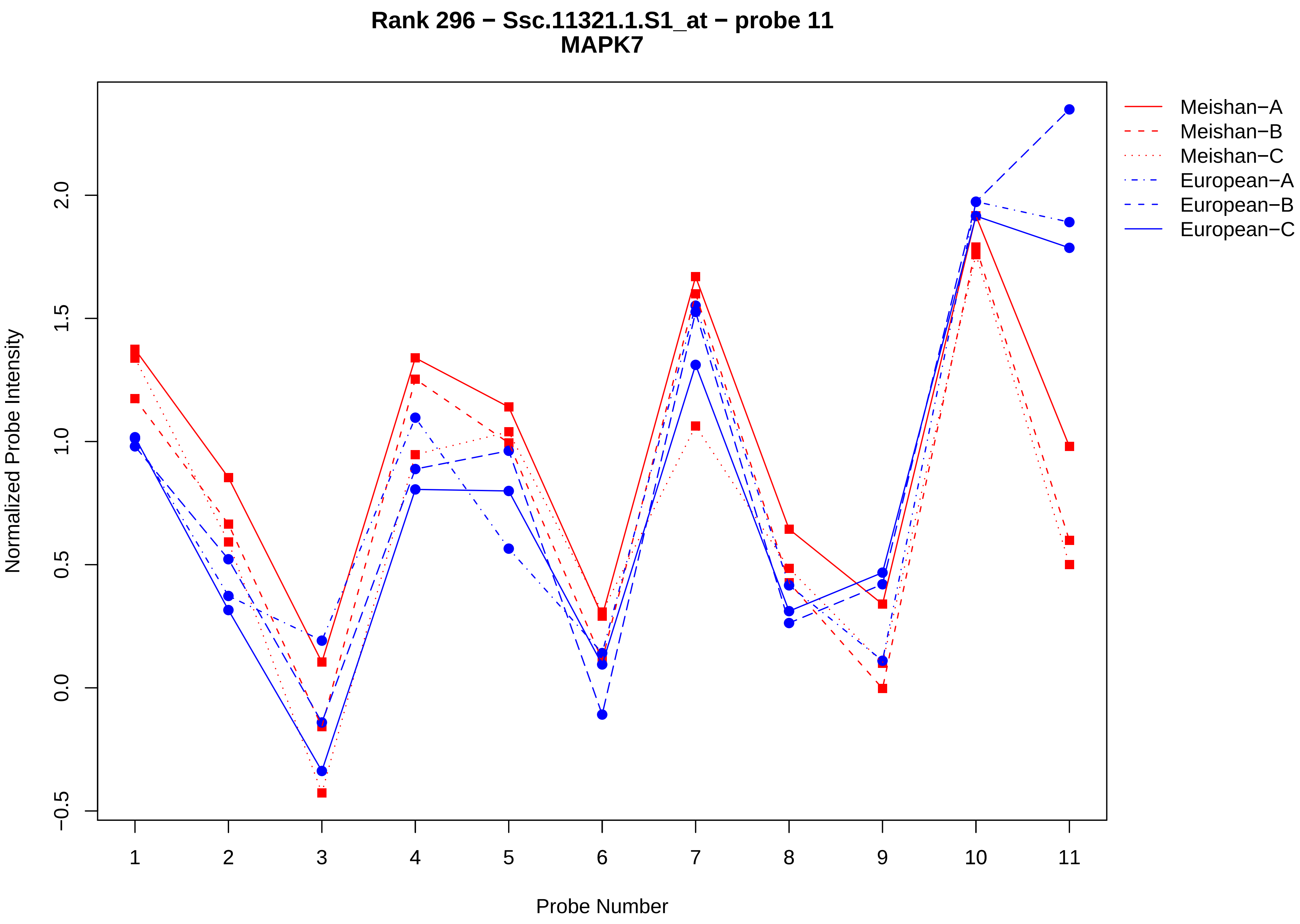
Rank 296 − Ssc.11321.1.S1_at − probe 11
MAPK7
Normalized Probe Intensity
Probe Number
−0.5
0.0
0.5
1.0
1.5
2.0
1
2
3
4
5
6
7
8
9
10
11
Meishan−A
Meishan−B
Meishan−C
European−A
European−B
European−C

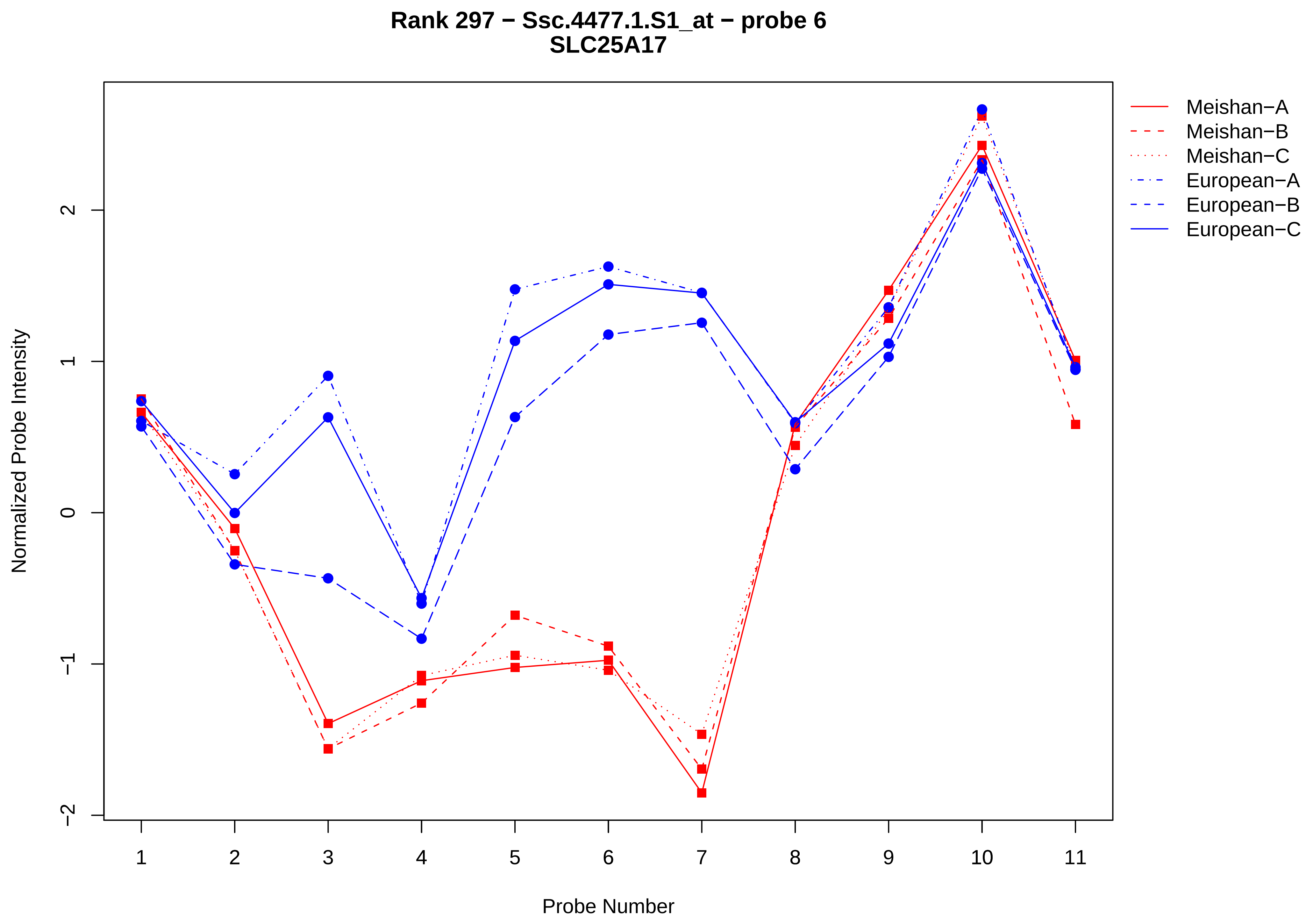

Rank 297 − Ssc.4477.1.S1_at − probe 6
SLC25A17
Normalized Probe Intensity
Probe Number
−2
−1
0
1
2
1
2
3
4
5
6
7
8
9
10
11
Meishan−A
Meishan−B
Meishan−C
European−A
European−B
European−C

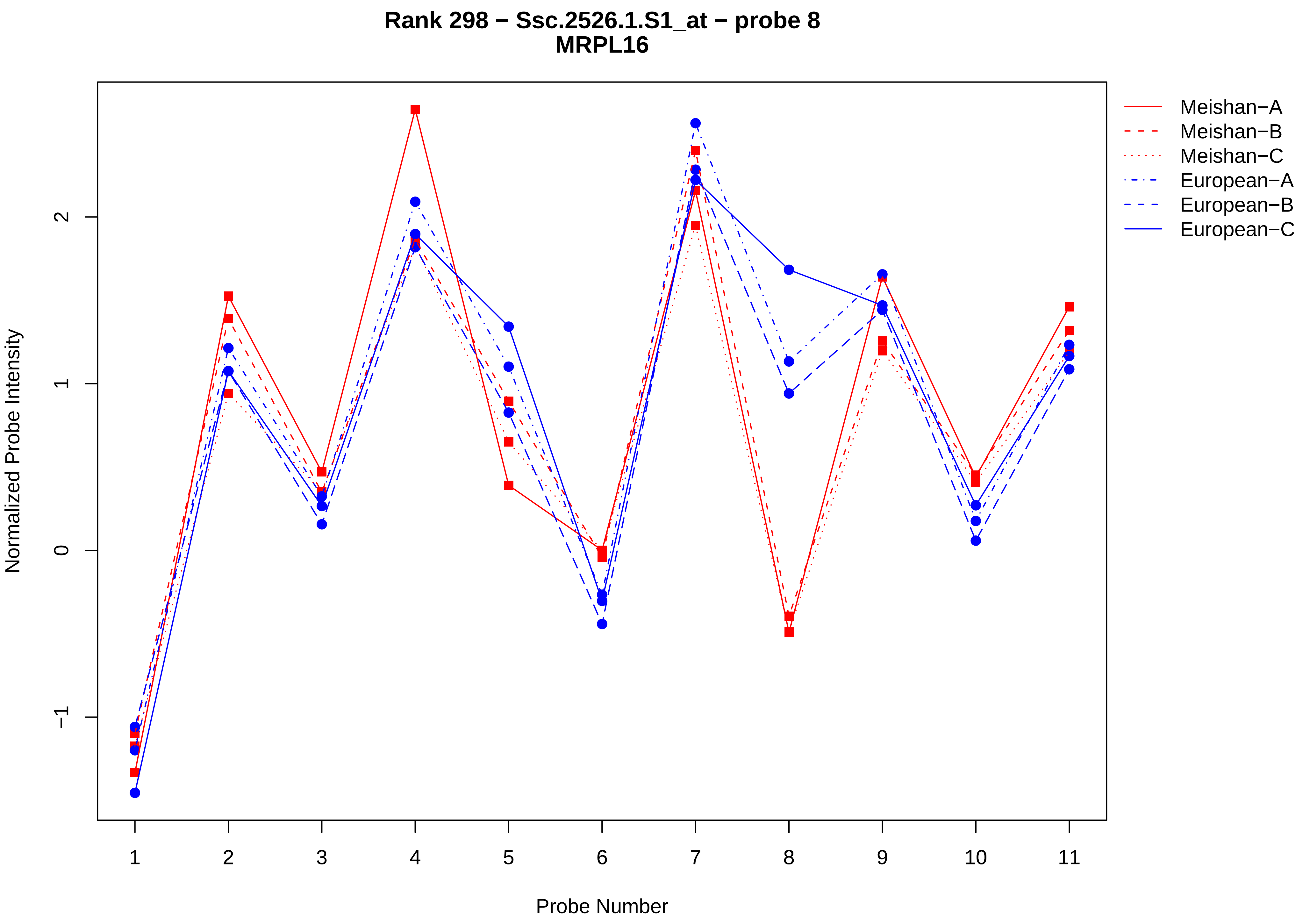

Rank 298 − Ssc.2526.1.S1_at − probe 8
MRPL16
Normalized Probe Intensity
Probe Number
−1
0
1
2
1
2
3
4
5
6
7
8
9
10
11
Meishan−A
Meishan−B
Meishan−C
European−A
European−B
European−C

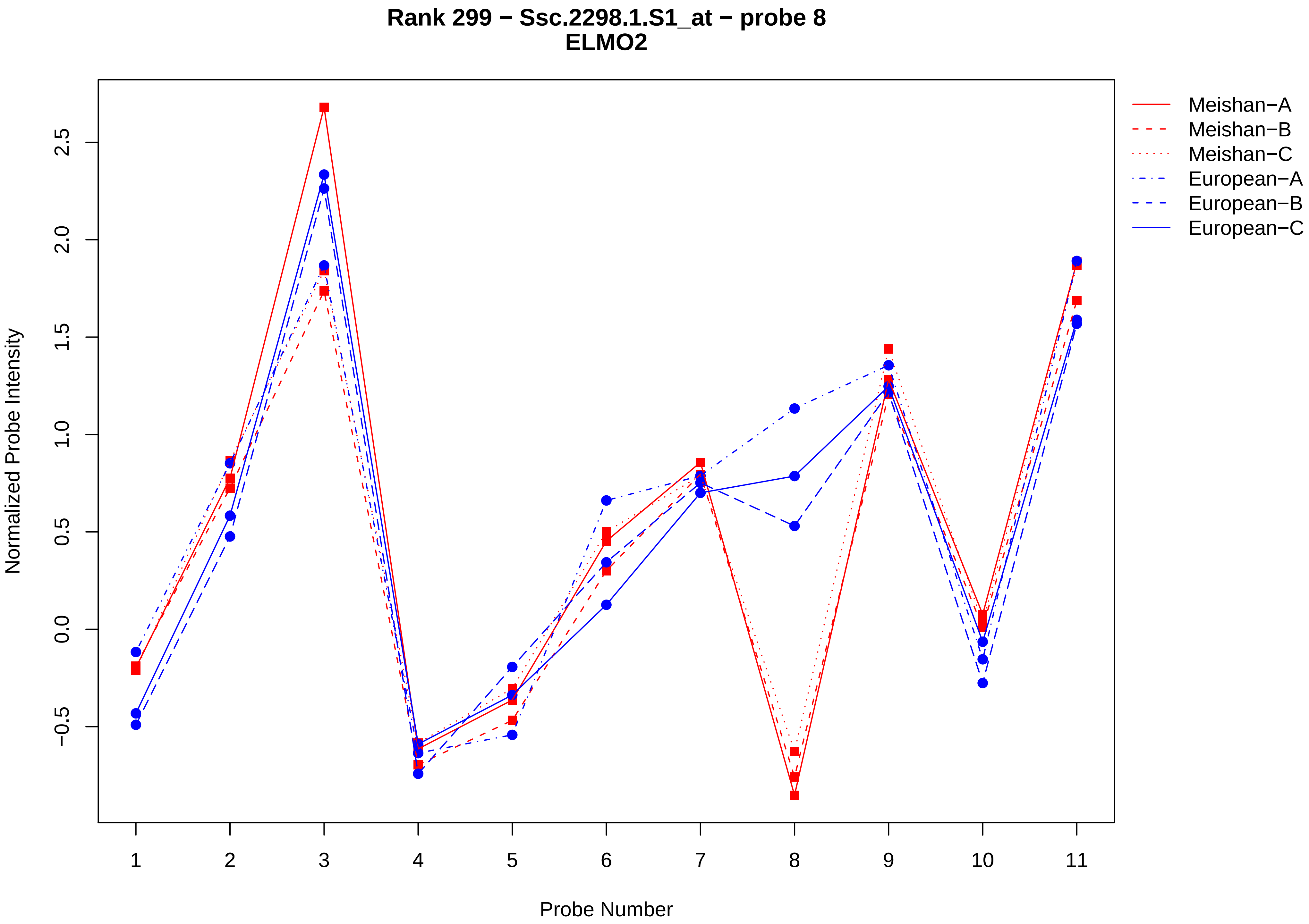

Rank 299 − Ssc.2298.1.S1_at − probe 8
ELMO2
Normalized Probe Intensity
Probe Number
Meishan−A
Meishan−B
Meishan−C
European−A
European−B
European−C
−0.5
0.0
0.5
1.0
1.5
2.0
2.5
1
2
3
4
5
6
7
8
9
10
11

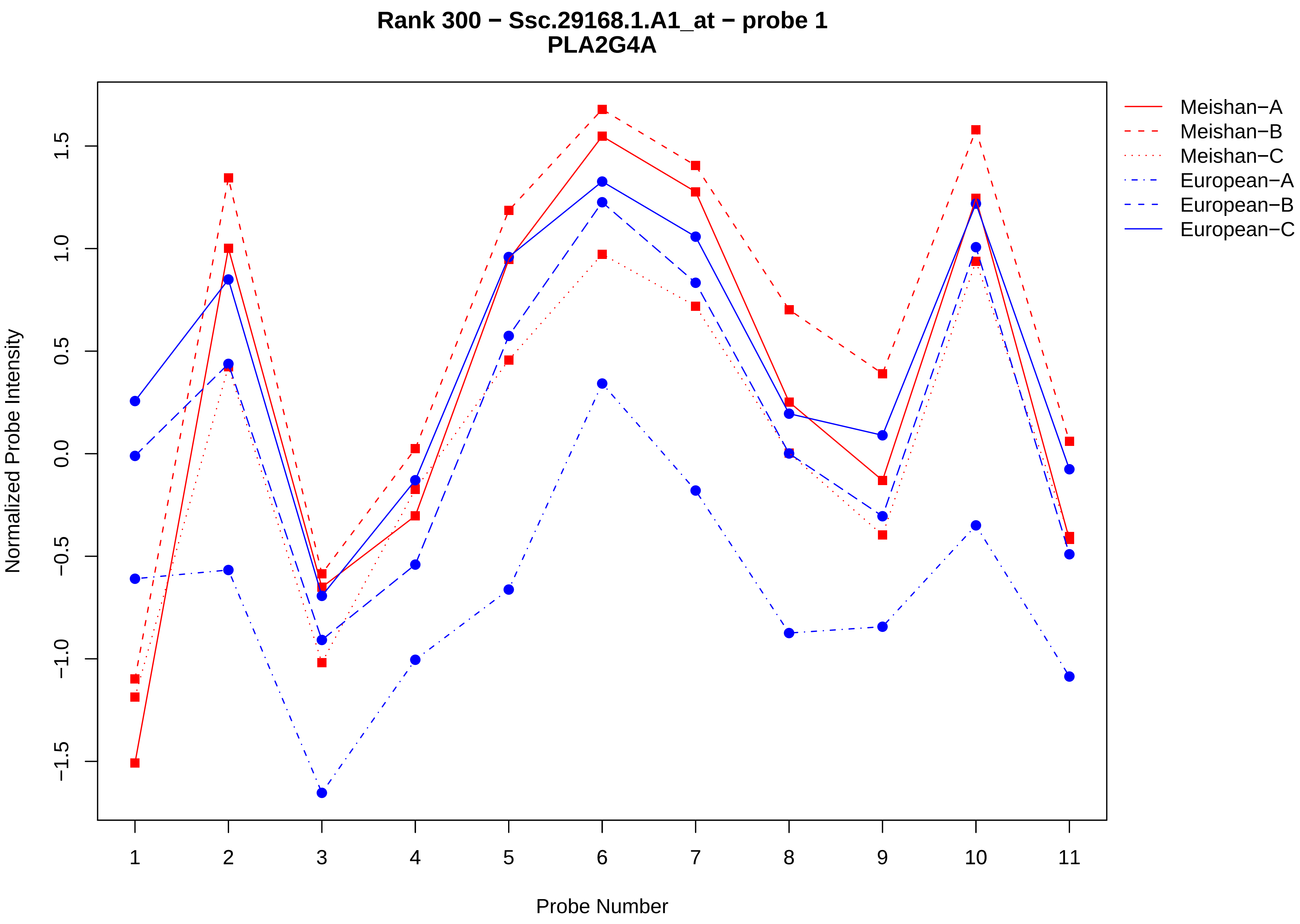

Rank 300 − Ssc.29168.1.A1_at − probe 1
PLA2G4A
Normalized Probe Intensity
Probe Number
Meishan−A
Meishan−B
Meishan−C
European−A
European−B
European−C

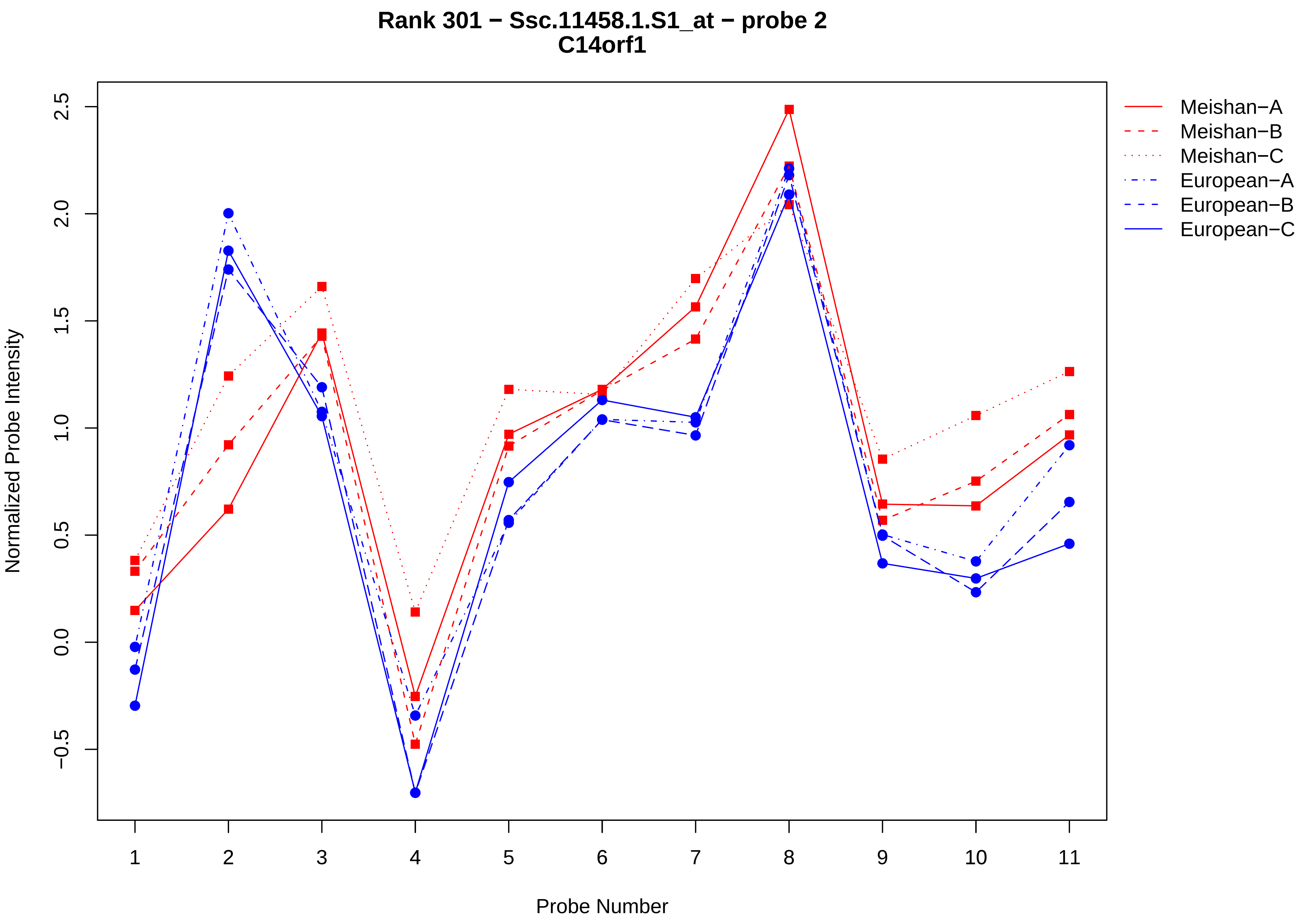

Rank 301 − Ssc.11458.1.S1_at − probe 2
C14orf1
Normalized Probe Intensity
Probe Number
−0.5
0.0
0.5
1.0
1.5
2.0
2.5
1
2
3
4
5
6
7
8
9
10
11
Meishan−A
Meishan−B
Meishan−C
European−A
European−B
European−C

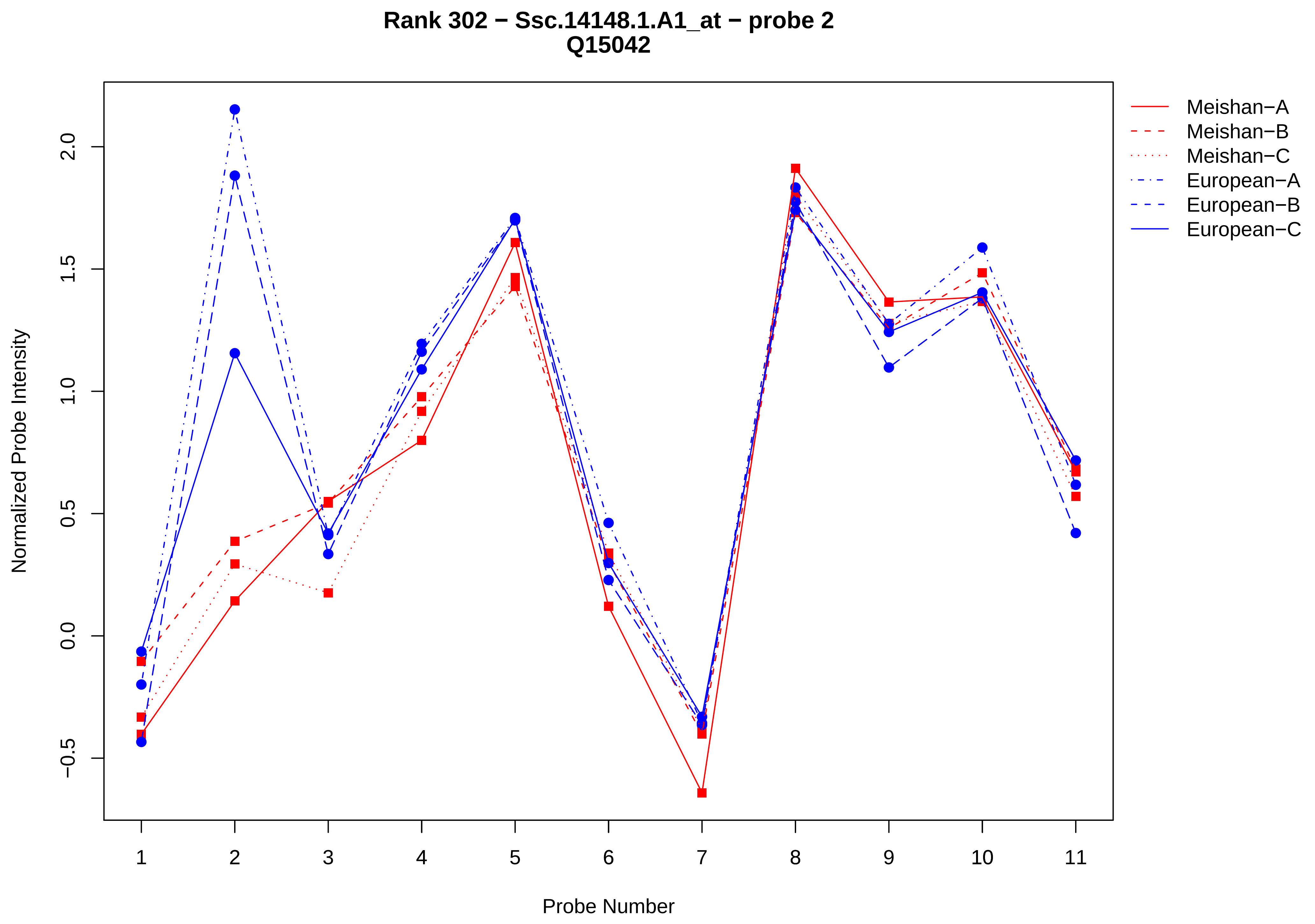
Rank 302 − Ssc.14148.1.A1_at − probe 2
Q15042
Normalized Probe Intensity
−0.5
0.0
0.5
1.0
1.5
2.0
1
2
3
4
5
6
7
8
9
10
11
Probe Number
Meishan−A
Meishan−B
Meishan−C
European−A
European−B
European−C

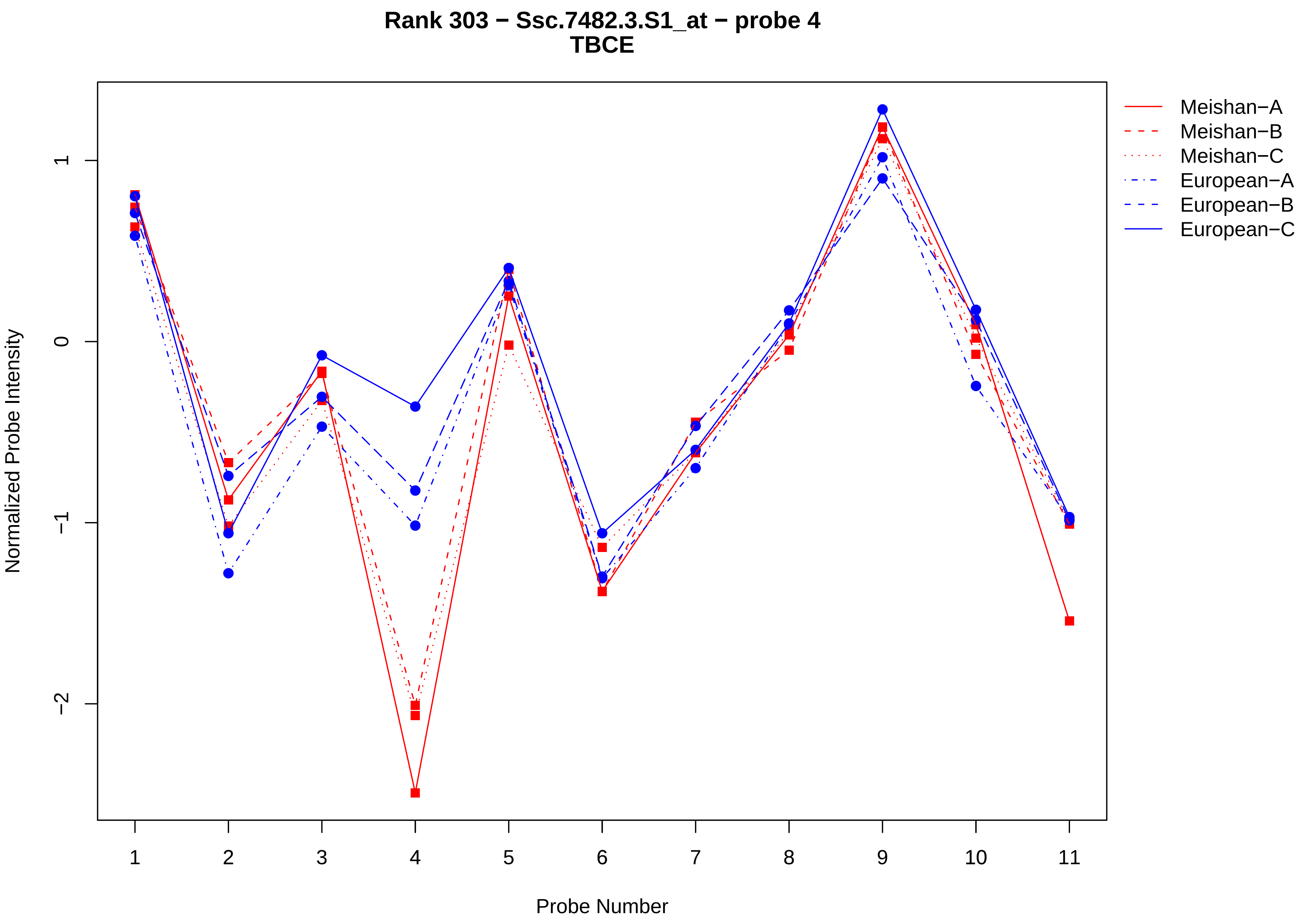

Rank 303 − Ssc.7482.3.S1_at − probe 4
TBCE
Normalized Probe Intensity
Probe Number
1
0
−1
−2
1
2
3
4
5
6
7
8
9
10
11
Meishan−A
Meishan−B
Meishan−C
European−A
European−B
European−C

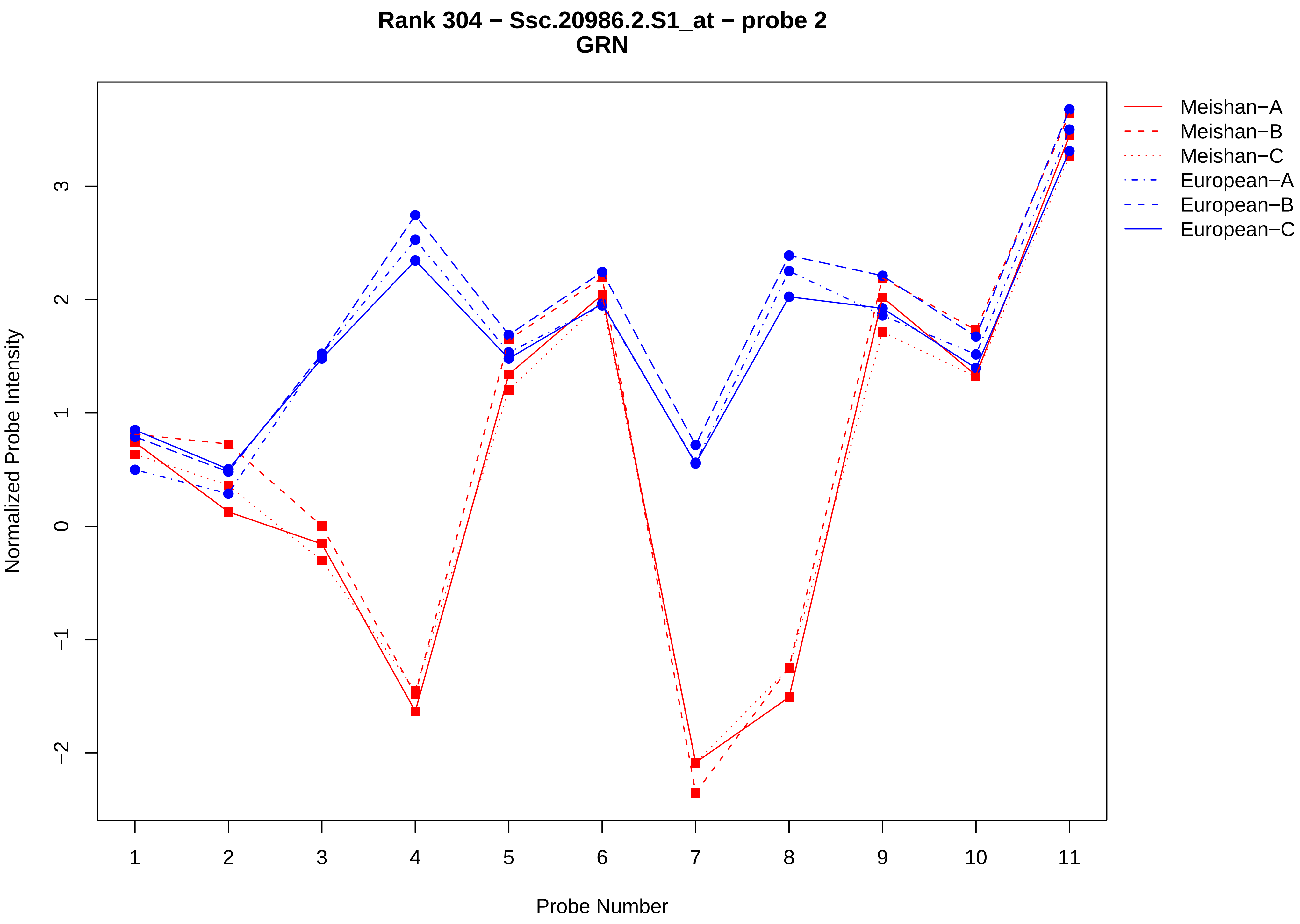

Rank 304 − Ssc.20986.2.S1_at − probe 2
GRN
Normalized Probe Intensity
Probe Number
−2
−1
0
1
2
3
1
2
3
4
5
6
7
8
9
10
11
Meishan−A
Meishan−B
Meishan−C
European−A
European−B
European−C

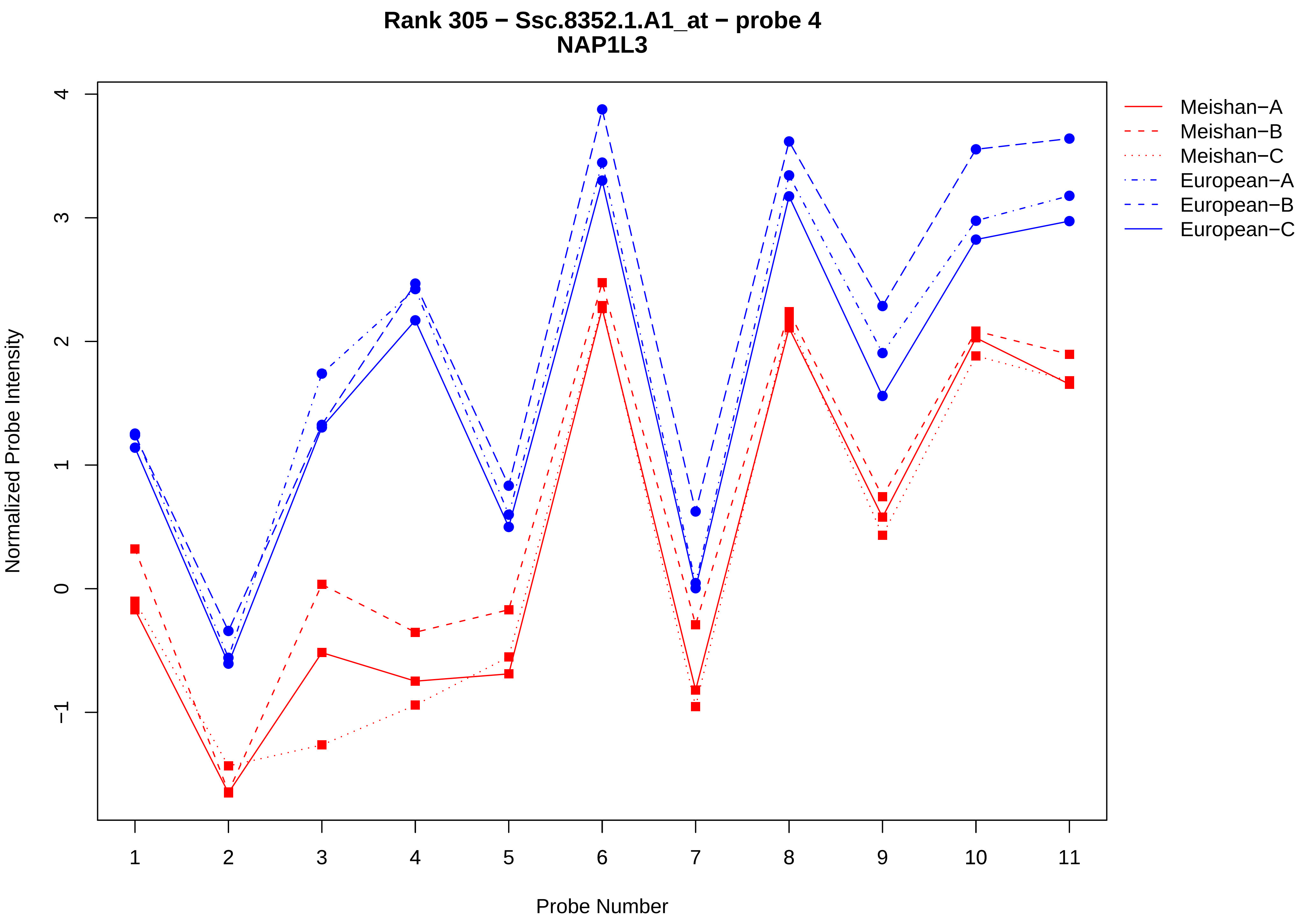

Rank 305 − Ssc.8352.1.A1_at − probe 4
NAP1L3
Normalized Probe Intensity
Probe Number
−1
0
1
2
3
4
1
2
3
4
5
6
7
8
9
10
11
Meishan−A
Meishan−B
Meishan−C
European−A
European−B
European−C

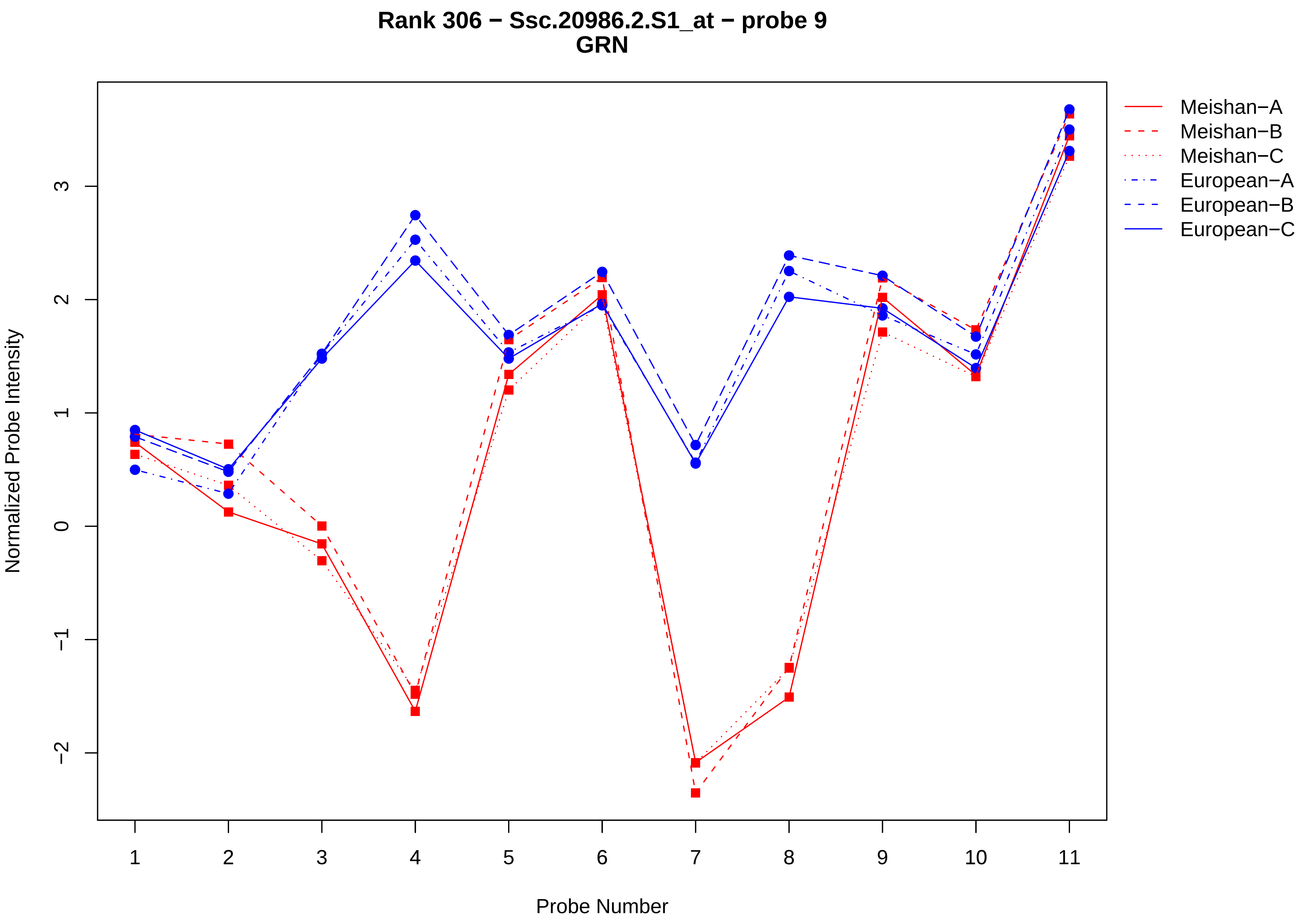

Rank 306 − Ssc.20986.2.S1_at − probe 9
GRN
Normalized Probe Intensity
Probe Number
−2
−1
0
1
2
3
1
2
3
4
5
6
7
8
9
10
11
Meishan−A
Meishan−B
Meishan−C
European−A
European−B
European−C

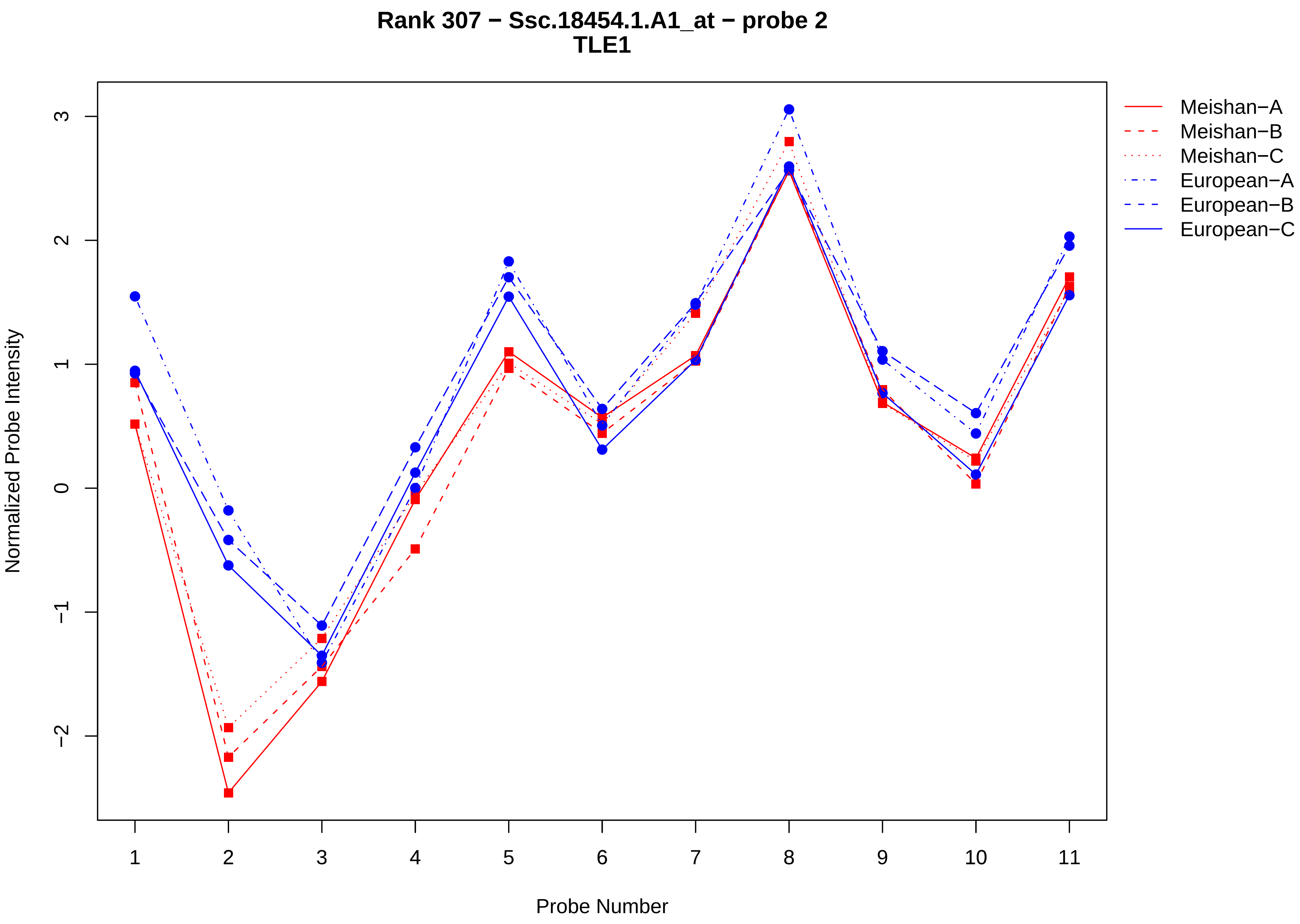

Rank 307 − Ssc.18454.1.A1_at − probe 2
TLE1
Normalized Probe Intensity
Probe Number
Meishan−A
Meishan−B
Meishan−C
European−A
European−B
European−C

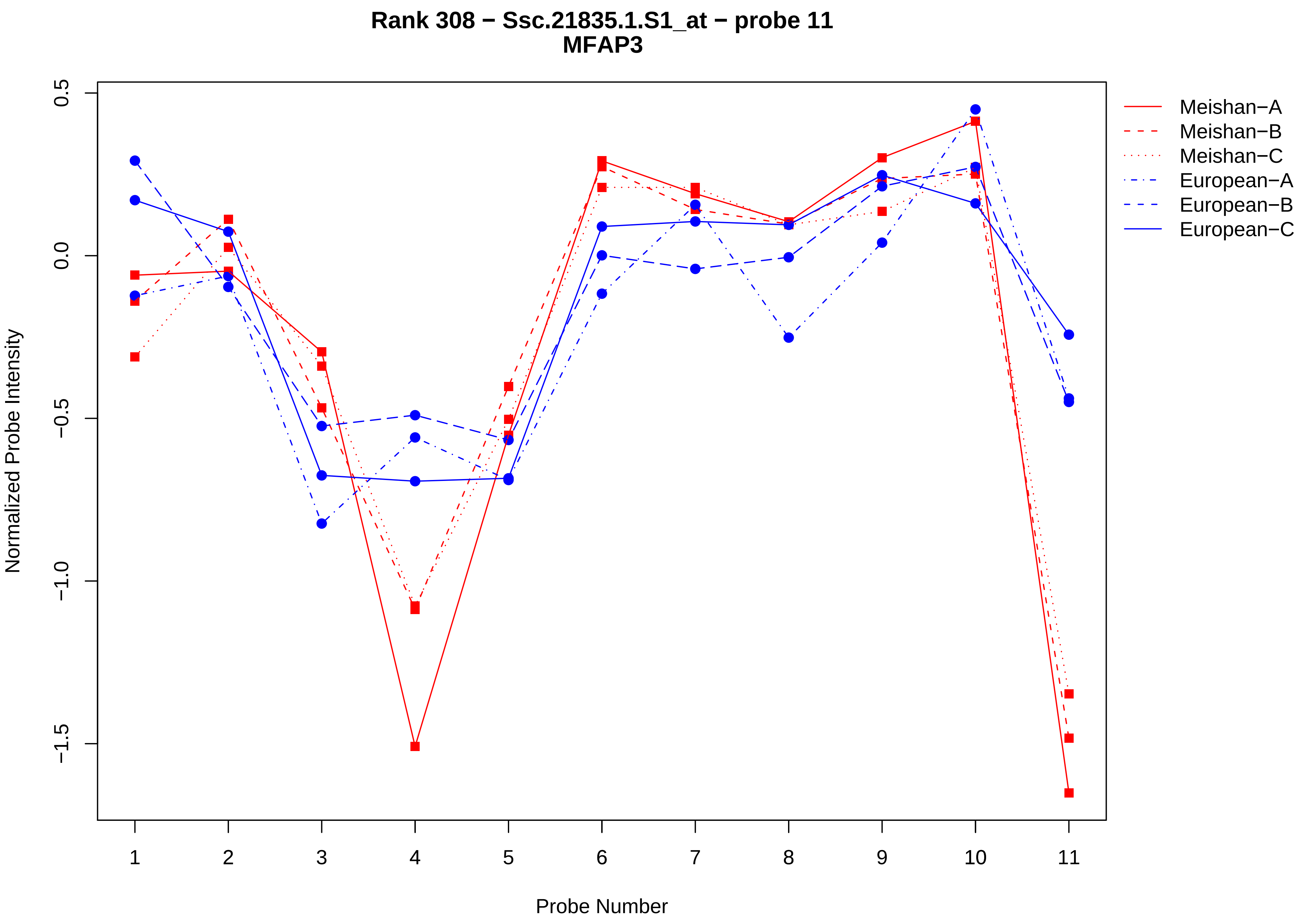
Rank 308 − Ssc.21835.1.S1_at − probe 11
MFAP3
Normalized Probe Intensity
0.5
0.0
−0.5
−1.0
−1.5
Probe Number
1
2
3
4
5
6
7
8
9
10
11
Meishan−A
Meishan−B
Meishan−C
European−A
European−B
European−C

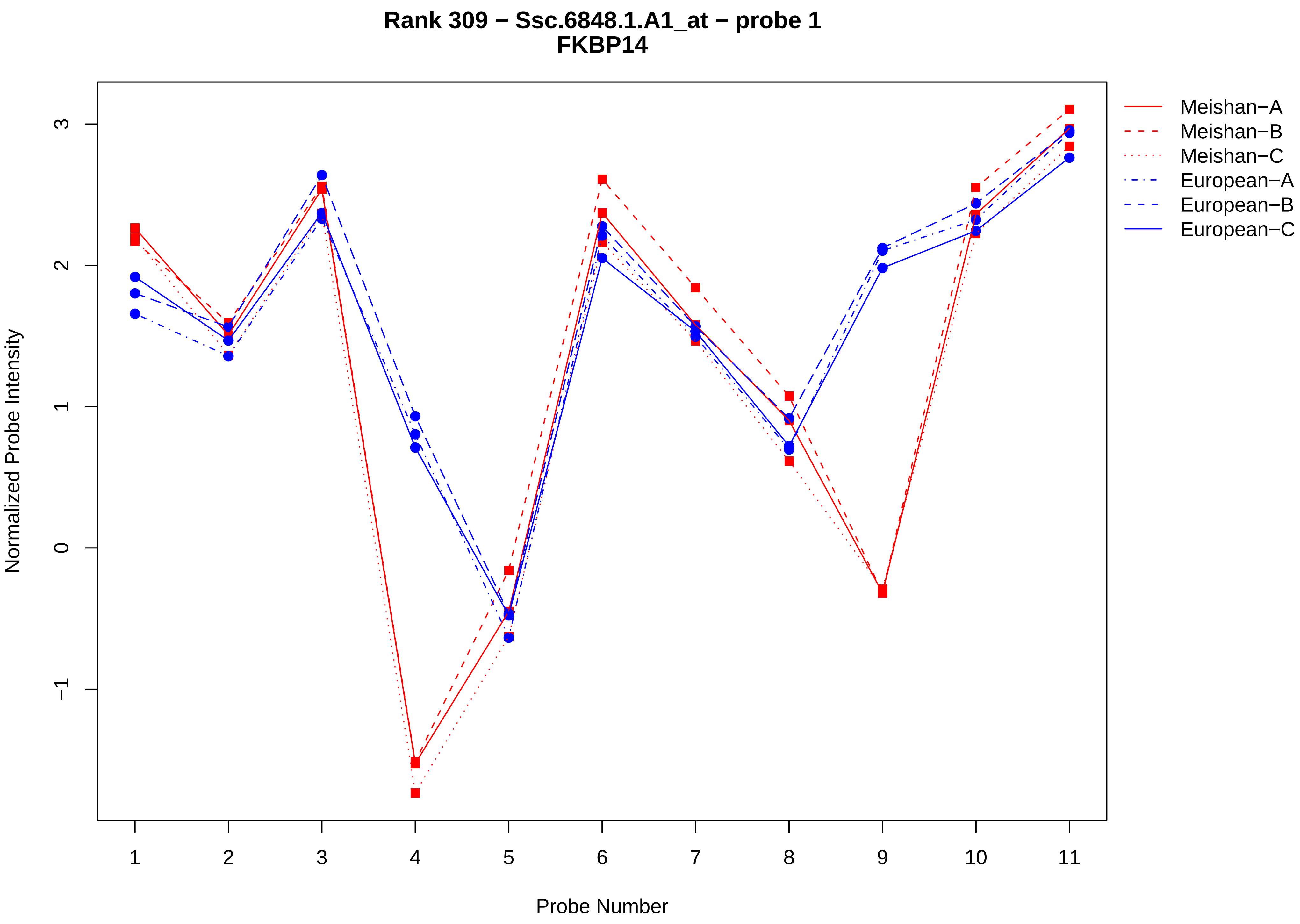

Rank 309 − Ssc.6848.1.A1_at − probe 1
FKBP14
Normalized Probe Intensity
Probe Number
−1
0
1
2
3
1
2
3
4
5
6
7
8
9
10
11
Meishan−A
Meishan−B
Meishan−C
European−A
European−B
European−C

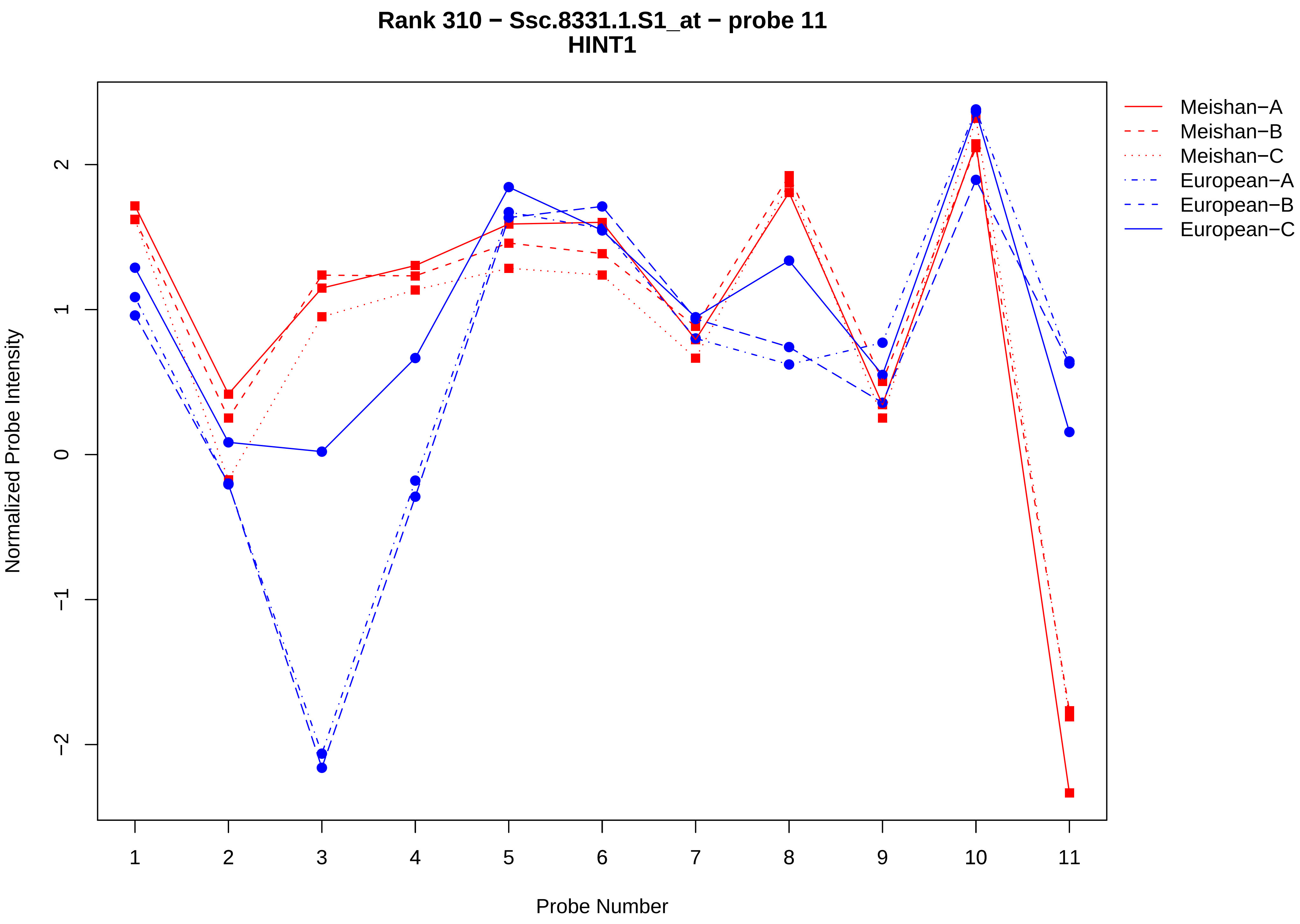

Rank 310 − Ssc.8331.1.S1_at − probe 11
HINT1
Normalized Probe Intensity
Probe Number
−2
−1
0
1
2
1
2
3
4
5
6
7
8
9
10
11
Meishan−A
Meishan−B
Meishan−C
European−A
European−B
European−C

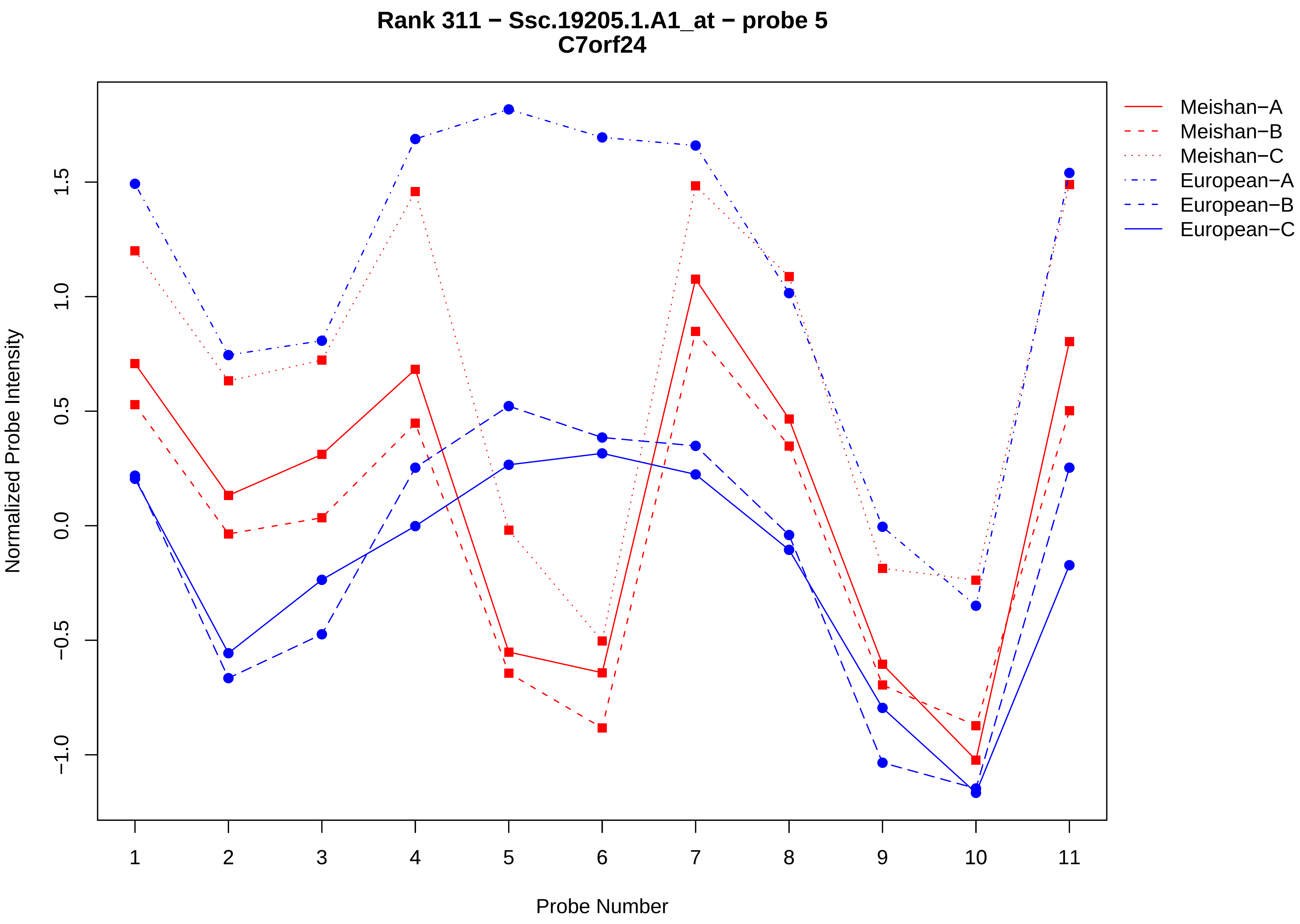
Rank 311 − Ssc.19205.1.A1_at − probe 5
C7orf24
Normalized Probe Intensity
Probe Number
Meishan−A
Meishan−B
Meishan−C
European−A
European−B
European−C
−1.0
−0.5
0.0
0.5
1.0
1.5
1
2
3
4
5
6
7
8
9
10
11

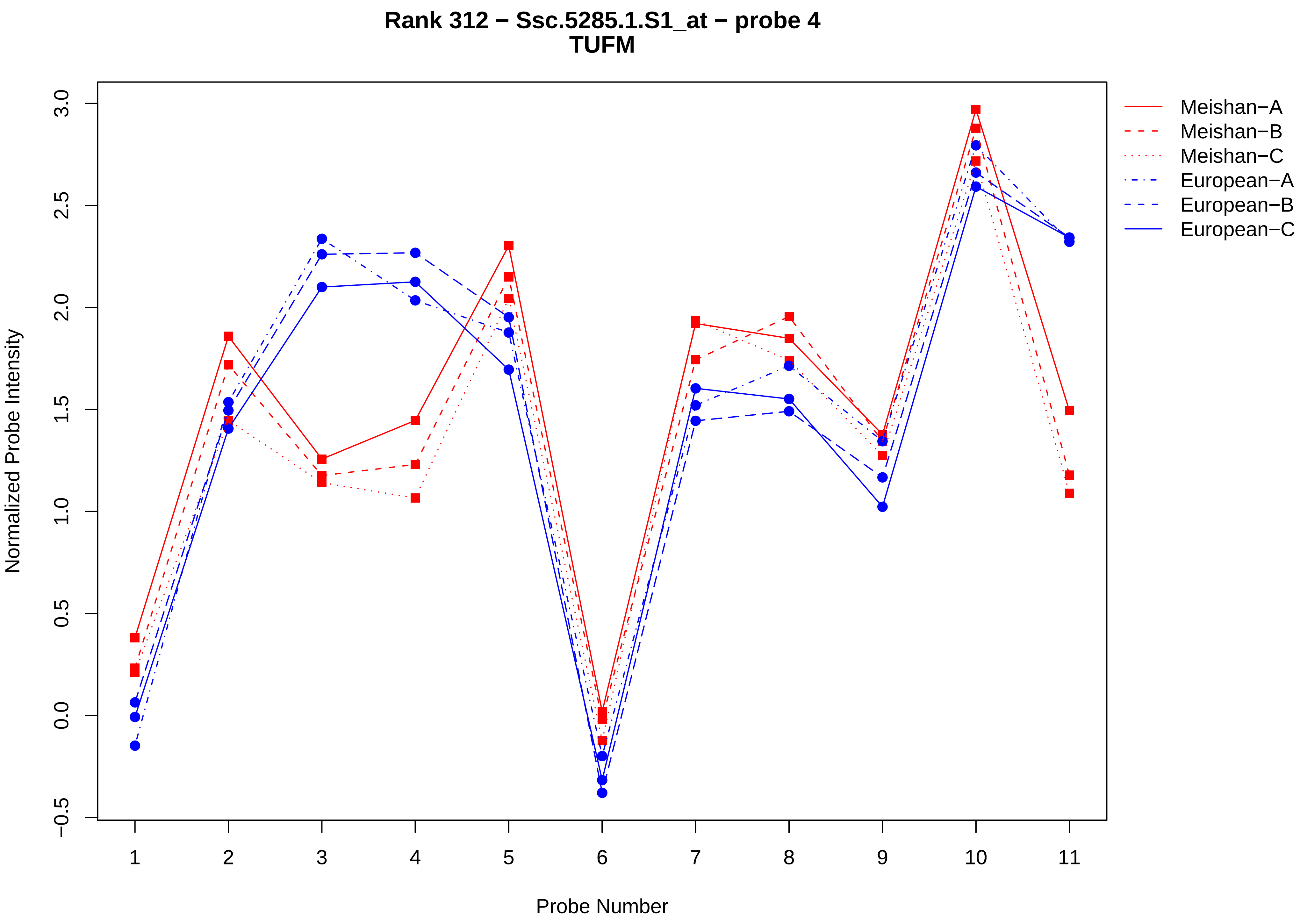

Rank 312 − Ssc.5285.1.S1_at − probe 4
TUFM
Normalized Probe Intensity
Probe Number
−0.5
0.0
0.5
1.0
1.5
2.0
2.5
3.0
1
2
3
4
5
6
7
8
9
10
11
Meishan−A
Meishan−B
Meishan−C
European−A
European−B
European−C

Rank 313 − Ssc.18496.1.S1_at − probe 10
METAP1

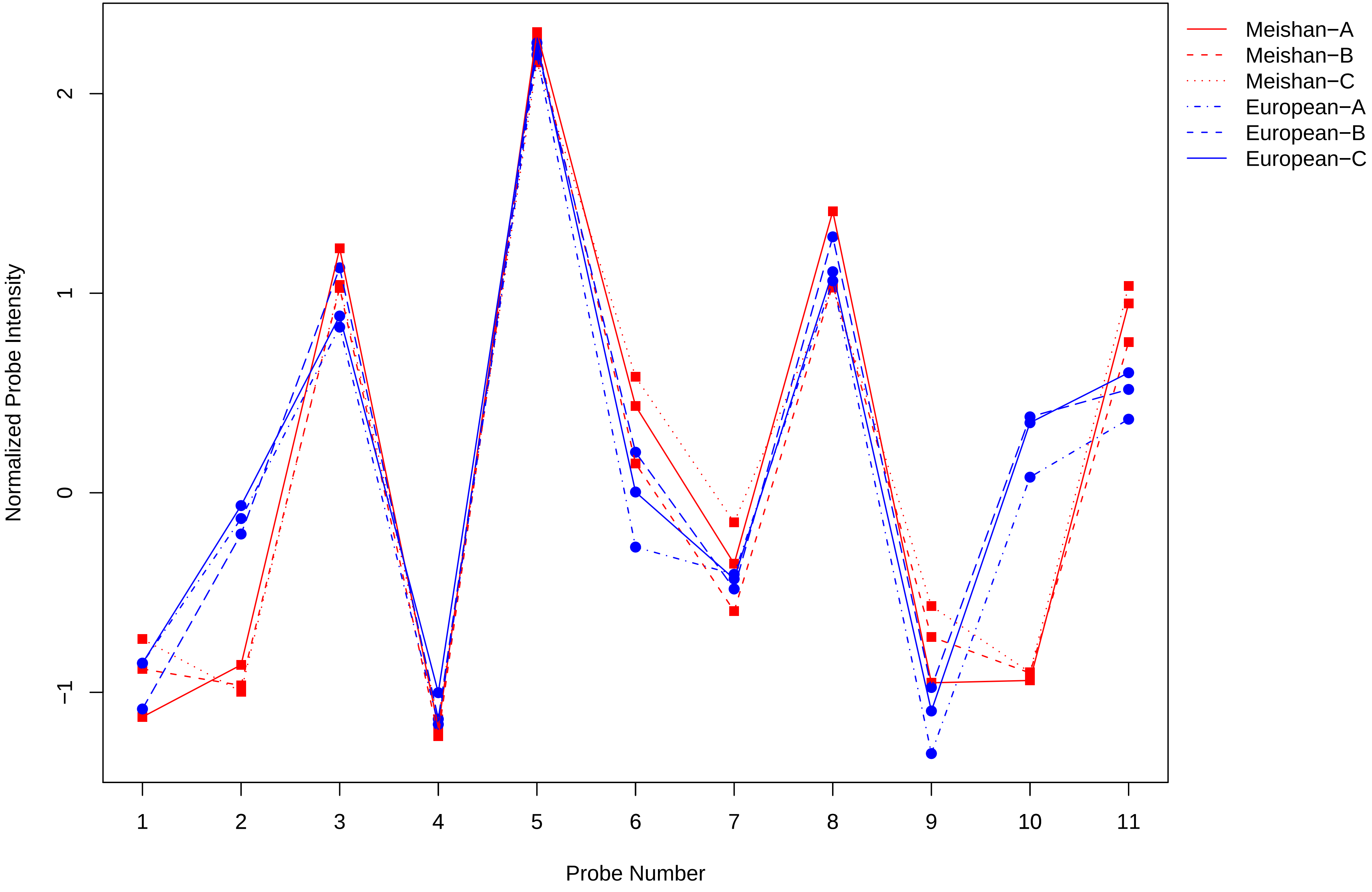

Rank 314 − Ssc.26718.1.S1_at − probe 9
GRB2

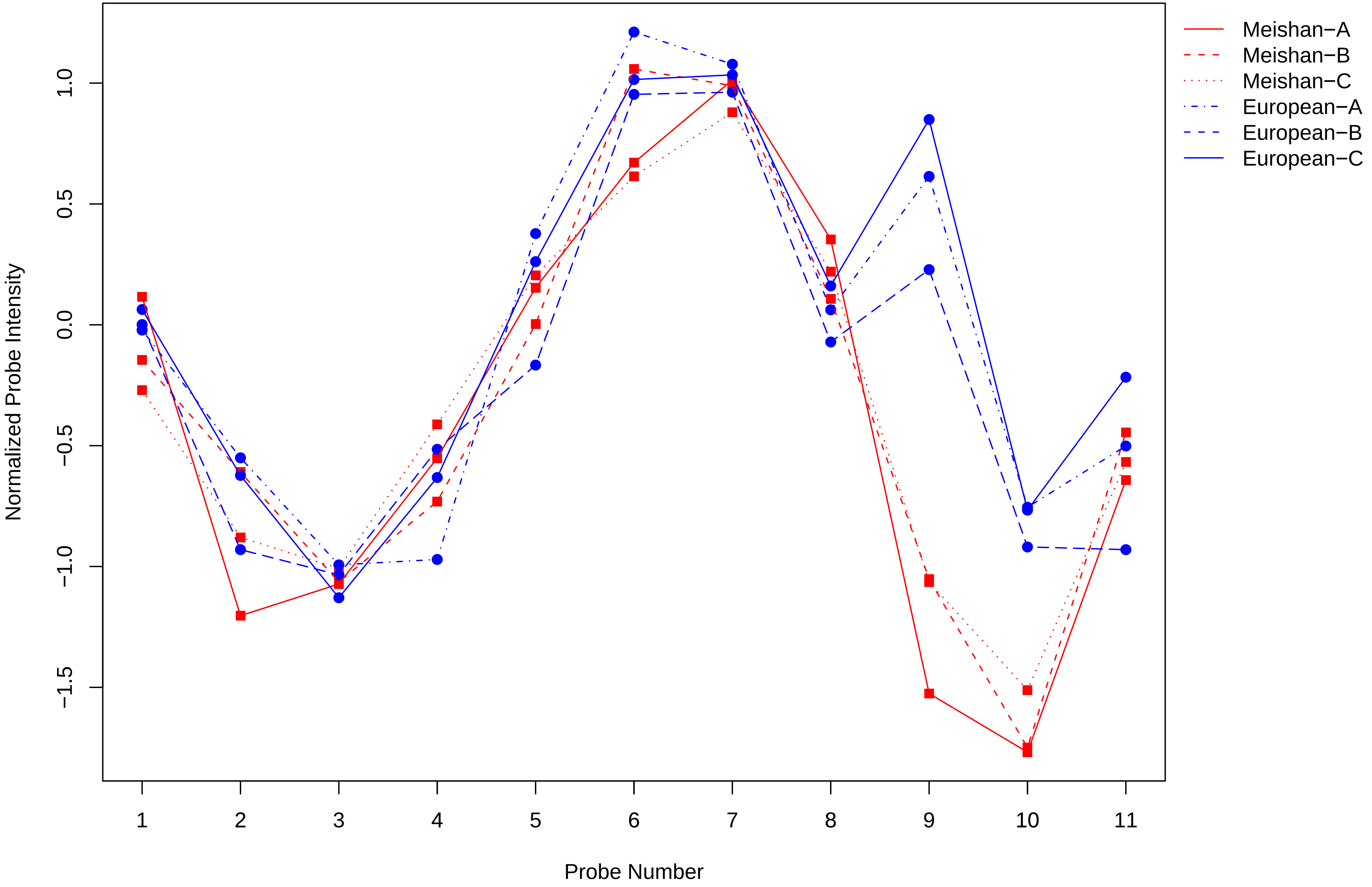

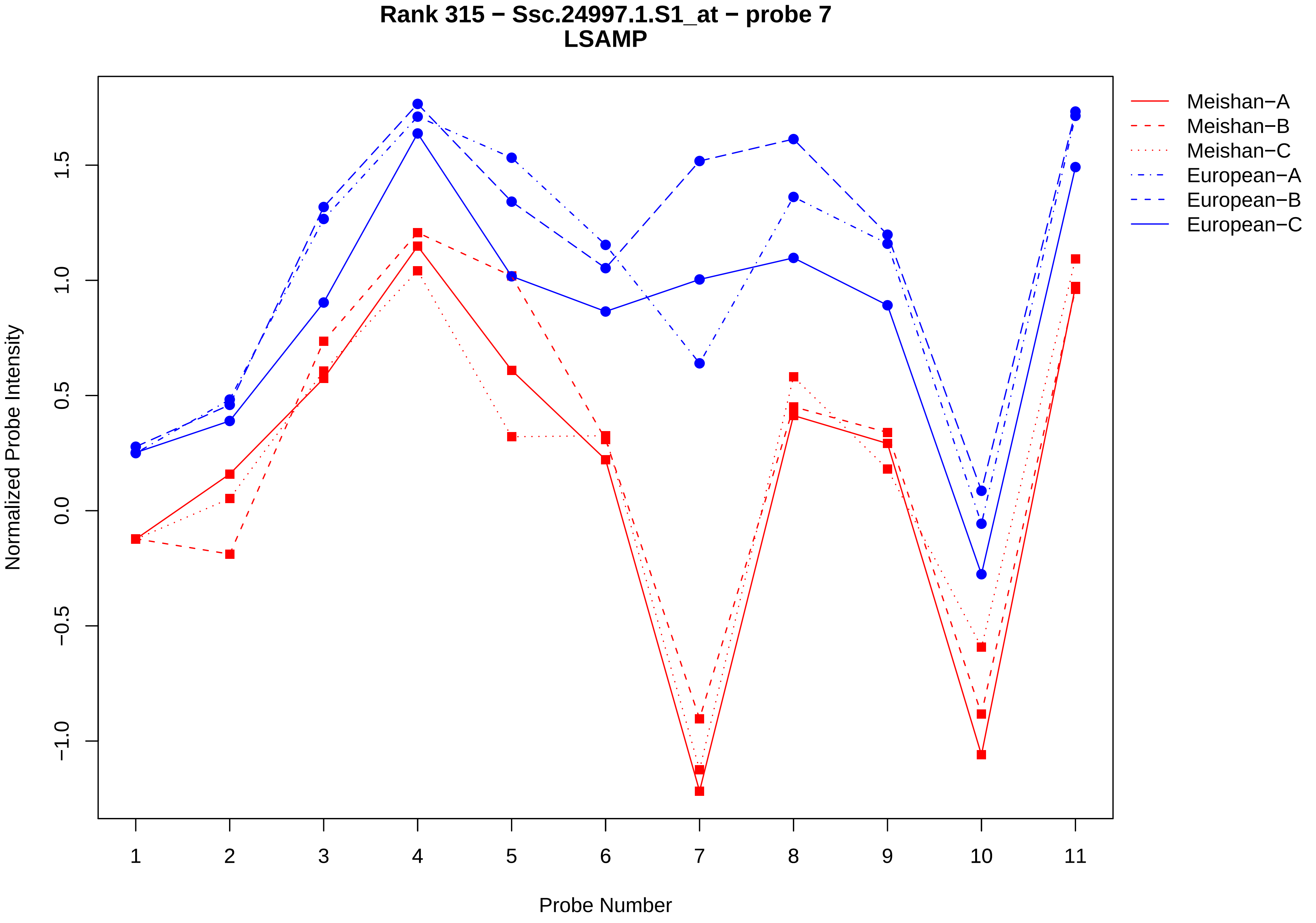

Rank 315 − Ssc.24997.1.S1_at − probe 7
LSAMP
Normalized Probe Intensity
Probe Number
−1.0
−0.5
0.0
0.5
1.0
1.5
1
2
3
4
5
6
7
8
9
10
11
Meishan−A
Meishan−B
Meishan−C
European−A
European−B
European−C

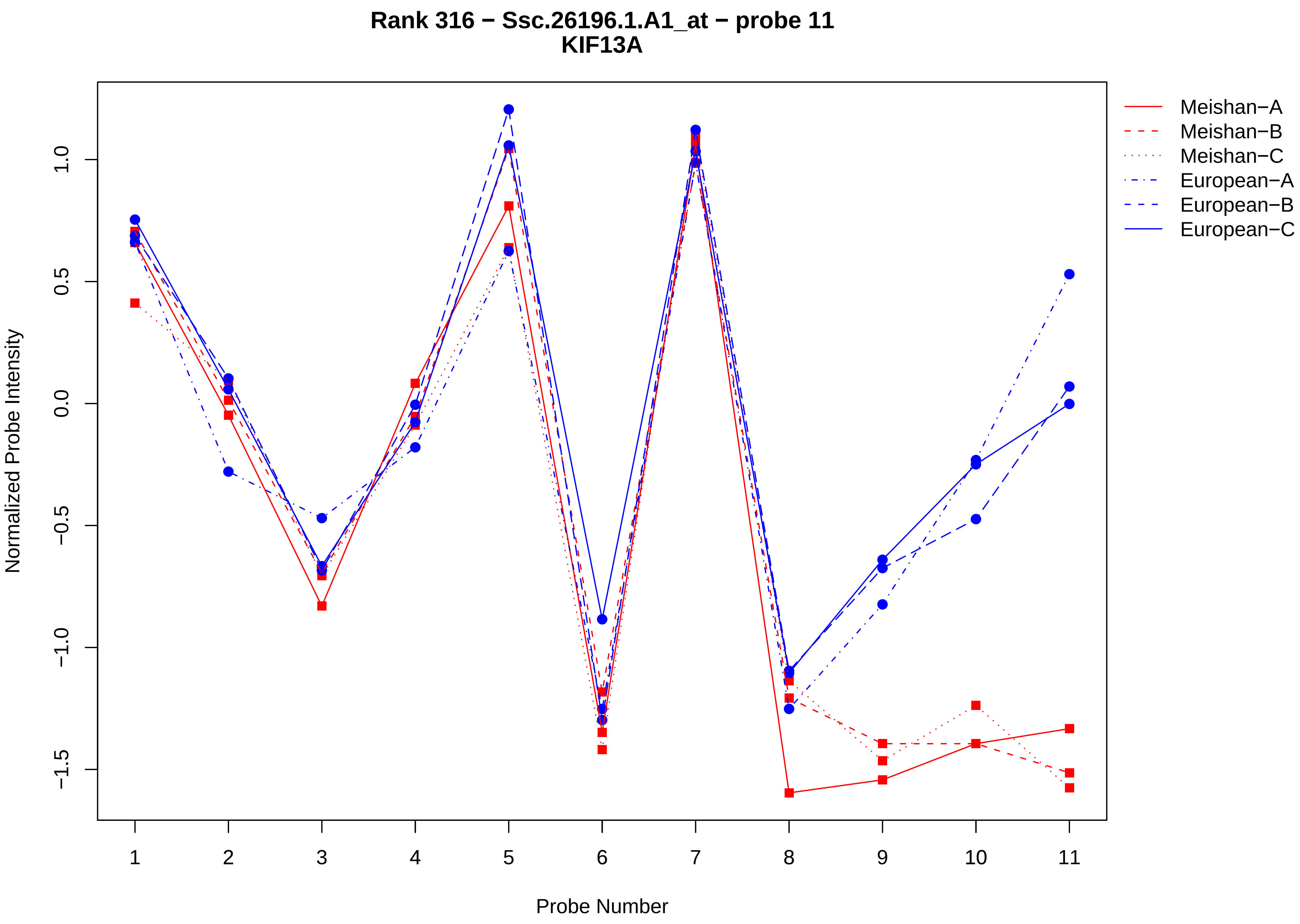

Rank 316 − Ssc.26196.1.A1_at − probe 11
KIF13A
Normalized Probe Intensity
Probe Number
Meishan−A
Meishan−B
Meishan−C
European−A
European−B
European−C
1.0
0.5
0.0
−0.5
−1.0
−1.5
1
2
3
4
5
6
7
8
9
10
11

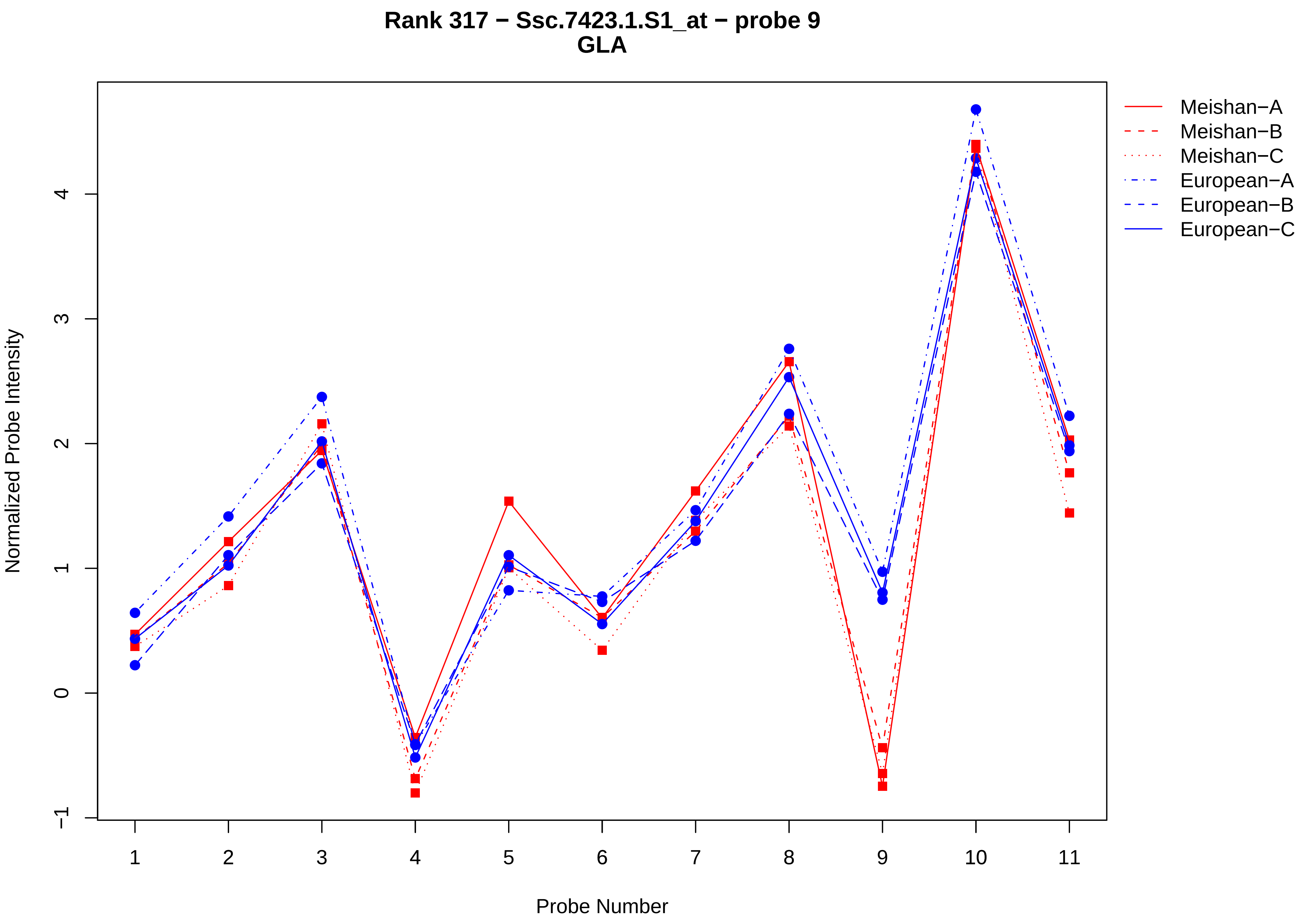

Rank 317 − Ssc.7423.1.S1_at − probe 9
GLA
Normalized Probe Intensity
Probe Number
−1
0
1
2
3
4
1
2
3
4
5
6
7
8
9
10
11
Meishan−A
Meishan−B
Meishan−C
European−A
European−B
European−C

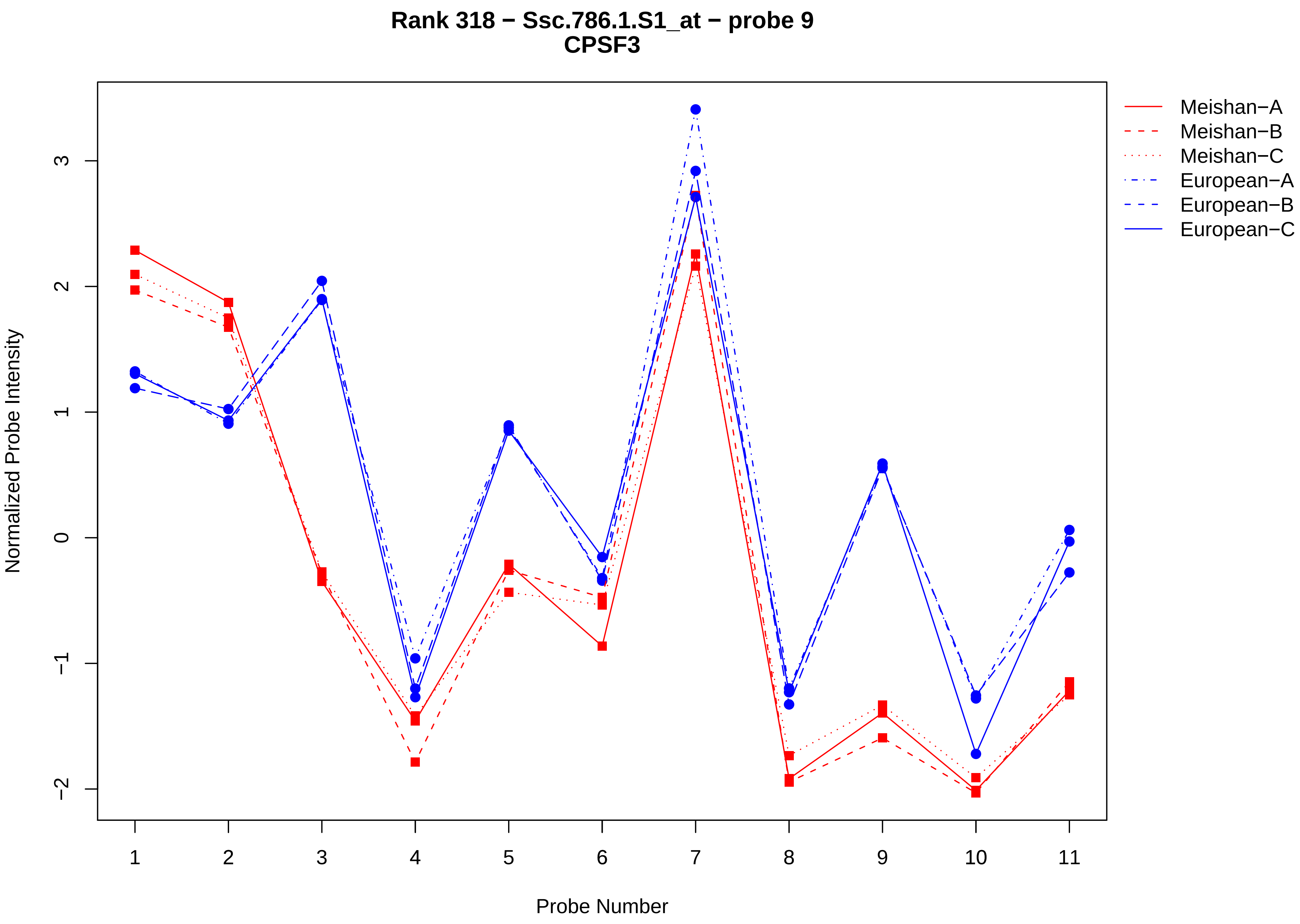

Rank 318 − Ssc.786.1.S1_at − probe 9
CPSF3
Normalized Probe Intensity
Probe Number
−2
−1
0
1
2
3
1
2
3
4
5
6
7
8
9
10
11
Meishan−A
Meishan−B
Meishan−C
European−A
European−B
European−C

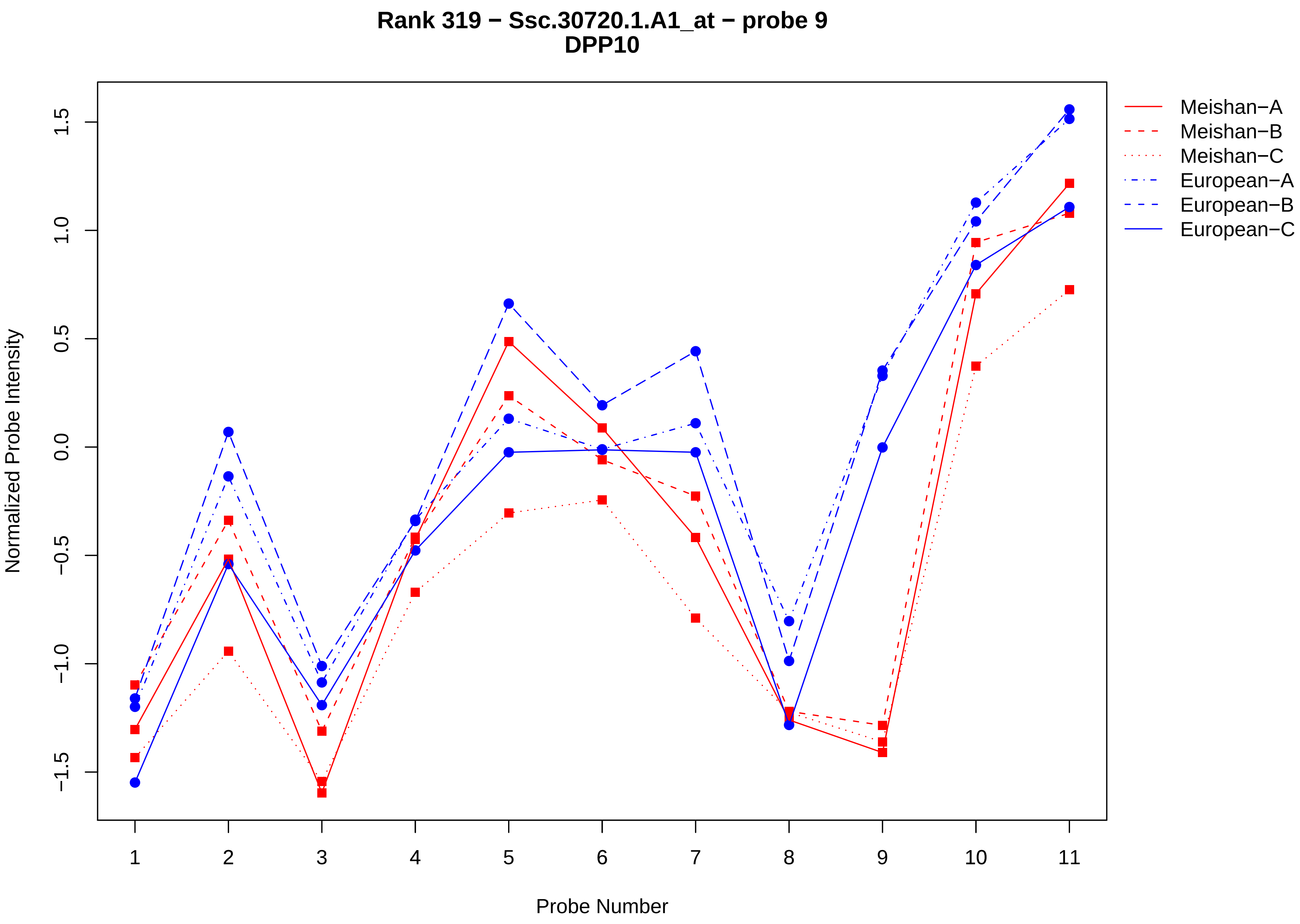

Rank 319 − Ssc.30720.1.A1_at − probe 9
DPP10
Normalized Probe Intensity
Probe Number
−1.5
−1.0
−0.5
0.0
0.5
1.0
1.5
1
2
3
4
5
6
7
8
9
10
11
Meishan−A
Meishan−B
Meishan−C
European−A
European−B
European−C

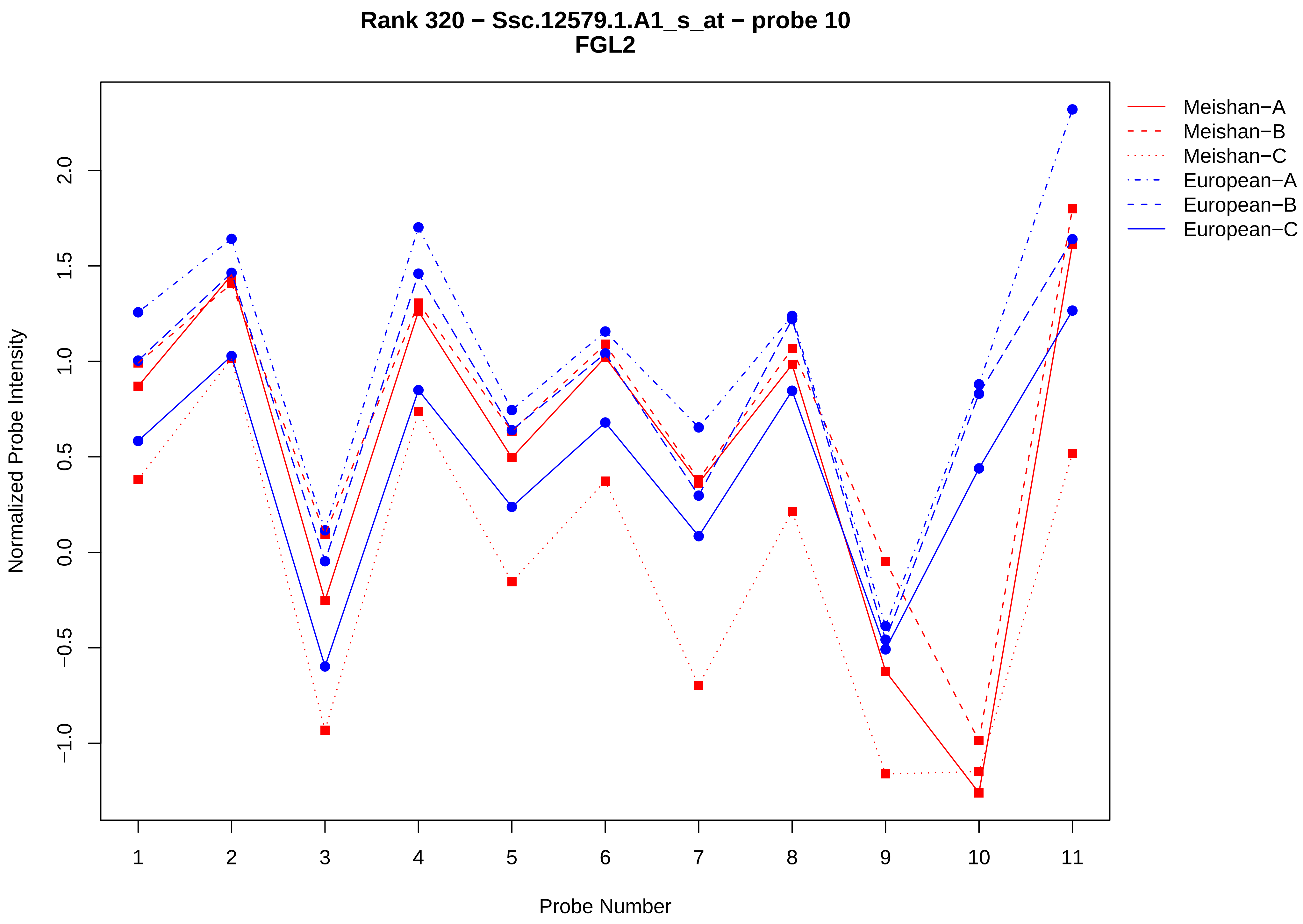

Rank 320 − Ssc.12579.1.A1_s_at − probe 10
FGL2
Normalized Probe Intensity
Probe Number
Meishan−A
Meishan−B
Meishan−C
European−A
European−B
European−C

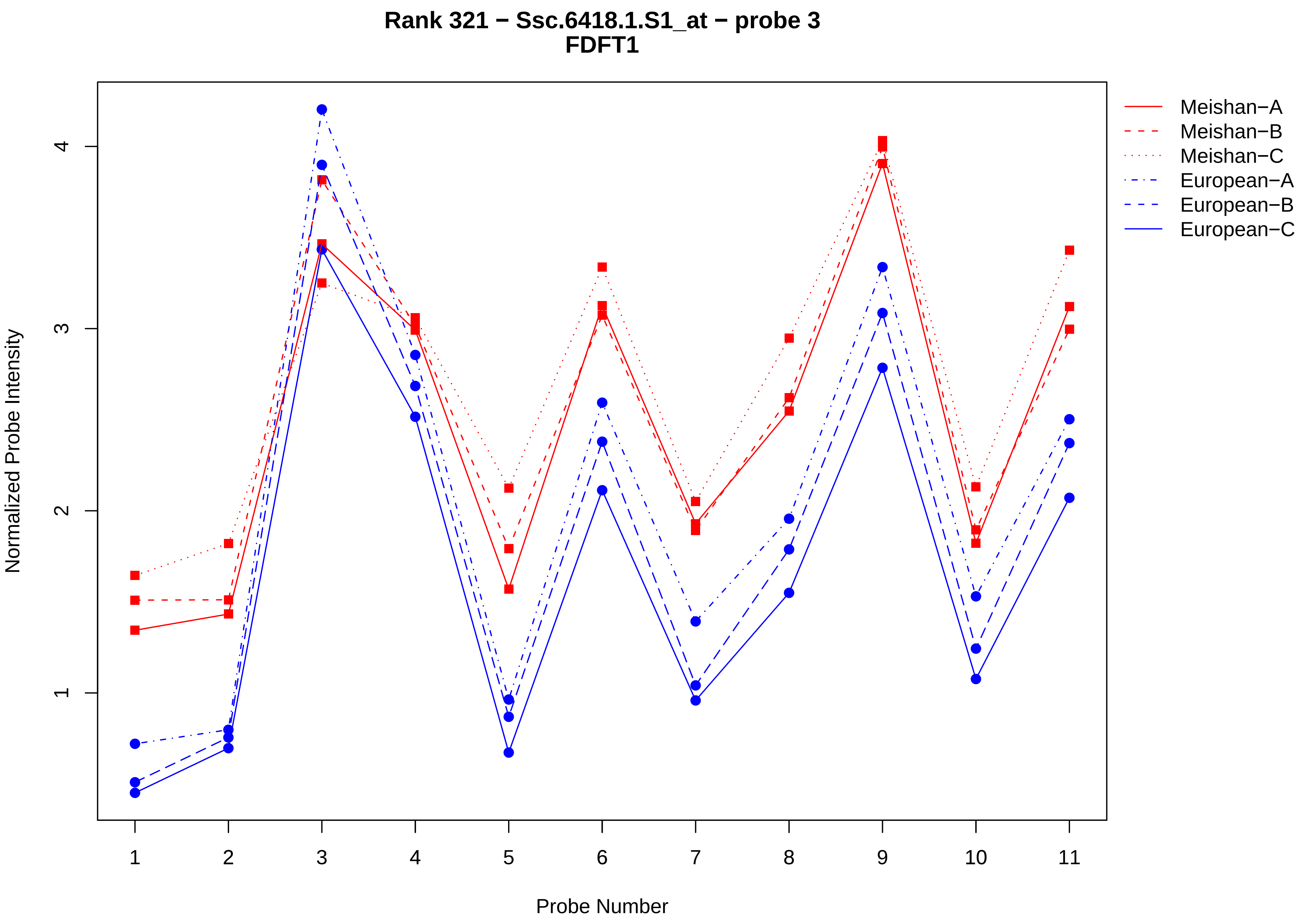

Rank 321 − Ssc.6418.1.S1_at − probe 3
FDFT1
Normalized Probe Intensity
1
2
3
4
Probe Number
1
2
3
4
5
6
7
8
9
10
11
Meishan−A
Meishan−B
Meishan−C
European−A
European−B
European−C

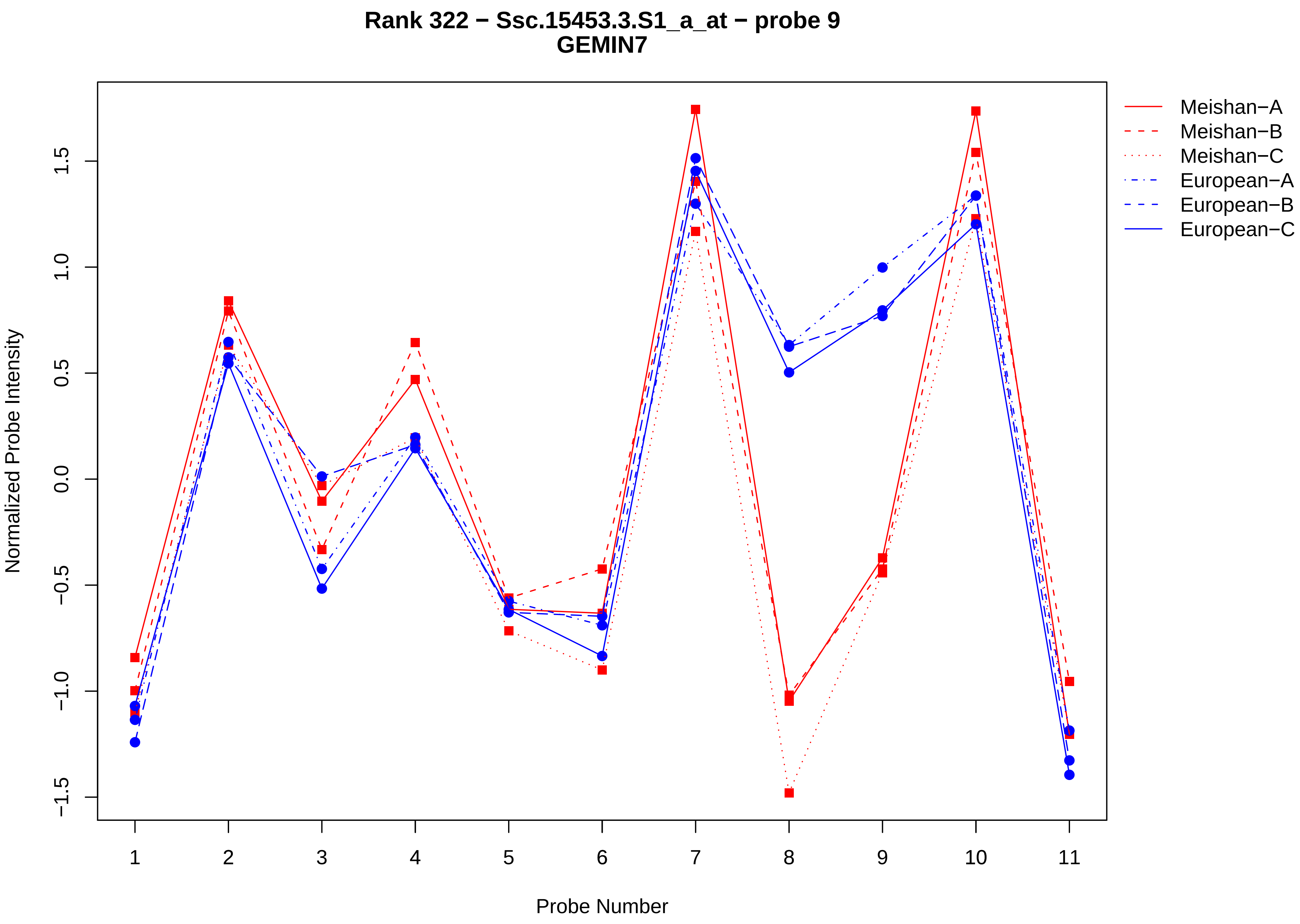

Rank 322 − Ssc.15453.3.S1_a_at − probe 9
GEMIN7
Normalized Probe Intensity
Probe Number
1.5
1.0
0.5
0.0
−0.5
−1.0
−1.5
1 2 3 4 5 6 7 8 9 10 11
Meishan−A
Meishan−B
Meishan−C
European−A
European−B
European−C

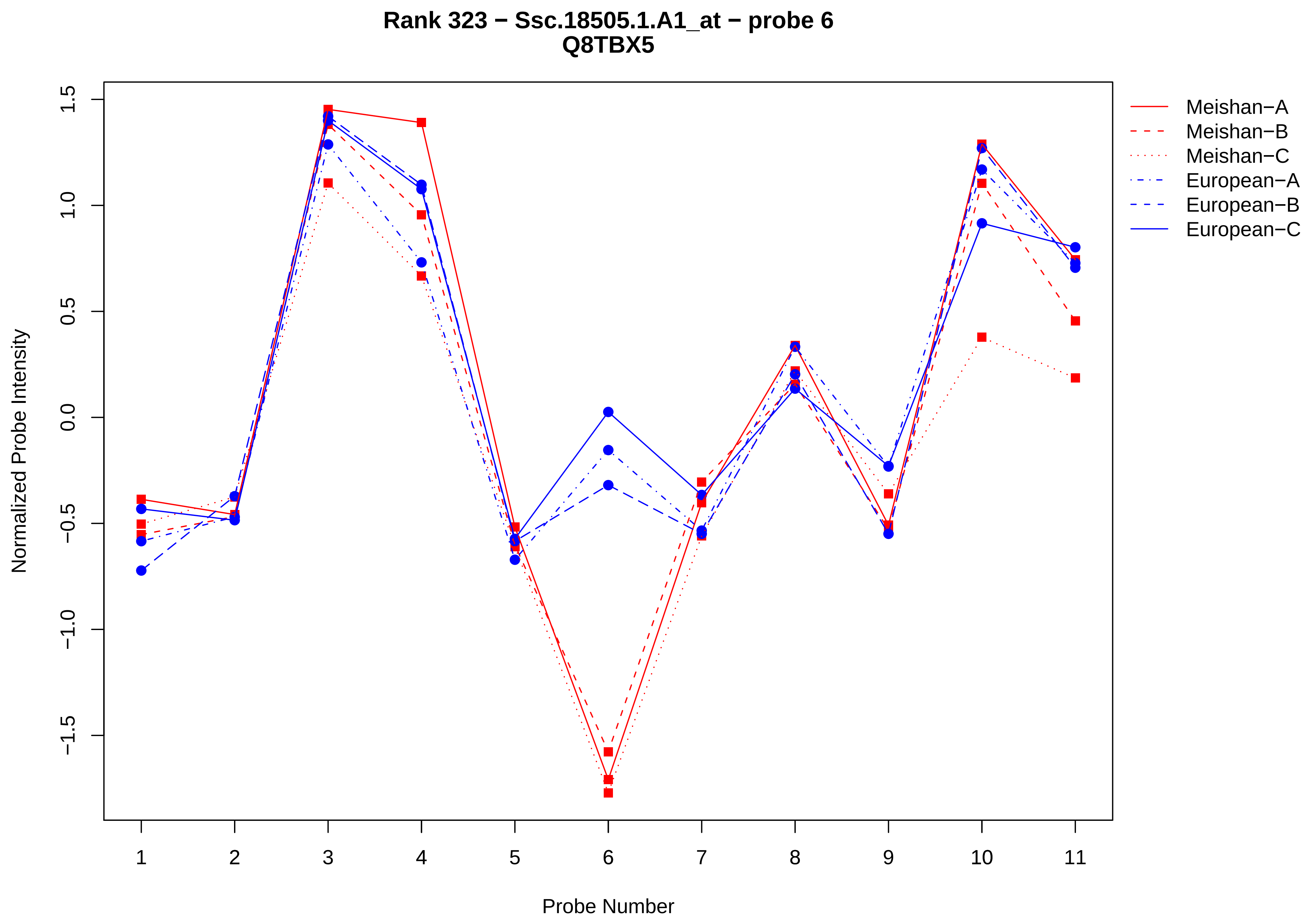

Rank 323 − Ssc.18505.1.A1_at − probe 6
Q8TBX5
Normalized Probe Intensity
Probe Number
1.5
1.0
0.5
0.0
−0.5
−1.0
−1.5
1
2
3
4
5
6
7
8
9
10
11
Meishan−A
Meishan−B
Meishan−C
European−A
European−B
European−C

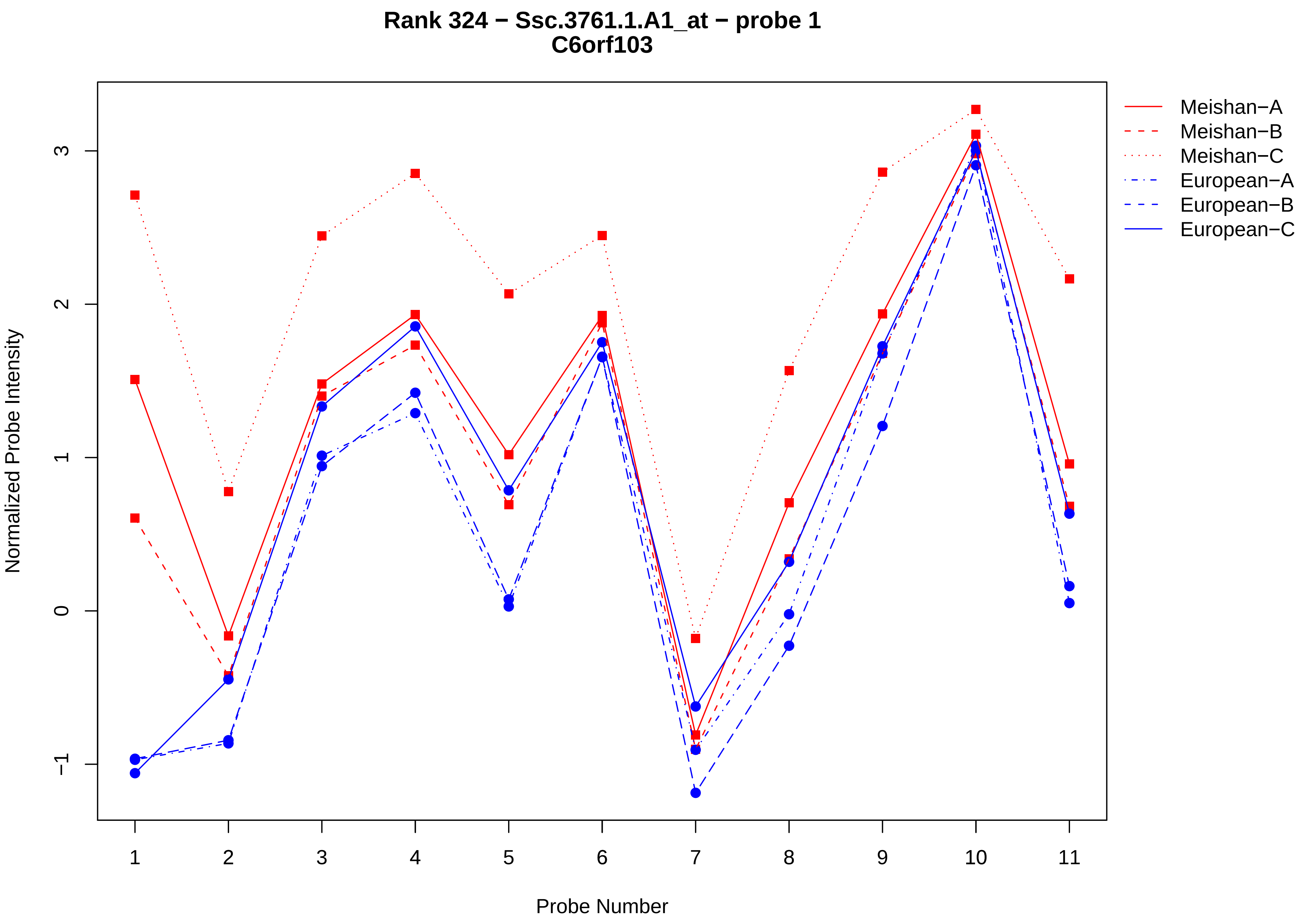
Rank 324 − Ssc.3761.1.A1_at − probe 1
C6orf103
Normalized Probe Intensity
Probe Number
Meishan−A
Meishan−B
Meishan−C
European−A
European−B
European−C
−1
0
1
2
3
1
2
3
4
5
6
7
8
9
10
11

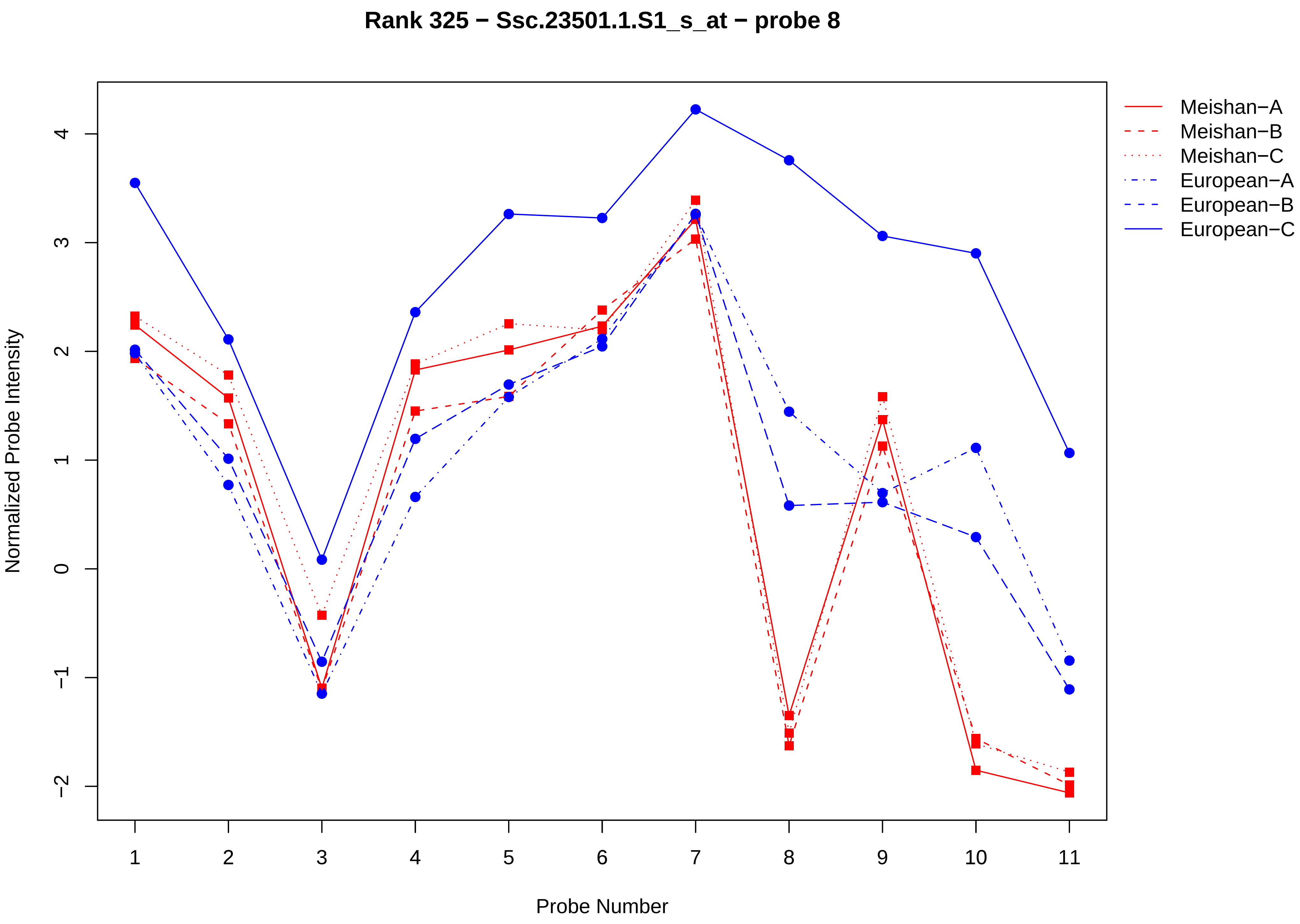
Rank 325 − Ssc.23501.1.S1_s_at − probe 8
Normalized Probe Intensity
Probe Number
−2
−1
0
1
2
3
4
1
2
3
4
5
6
7
8
9
10
11
Meishan−A
Meishan−B
Meishan−C
European−A
European−B
European−C

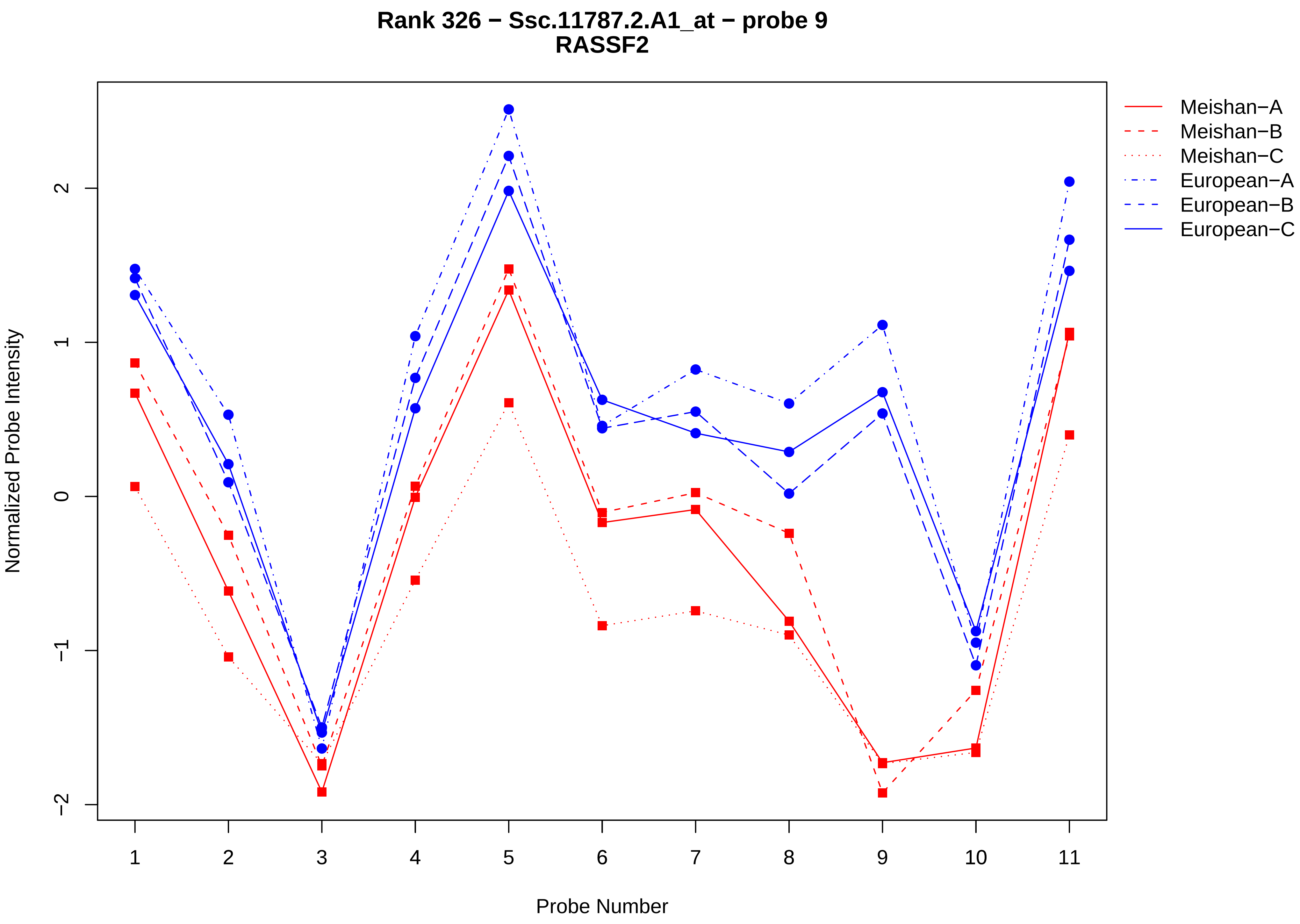

Rank 326 − Ssc.11787.2.A1_at − probe 9
RASSF2
Normalized Probe Intensity
Probe Number
−2
−1
0
1
2
1
2
3
4
5
6
7
8
9
10
11
Meishan−A
Meishan−B
Meishan−C
European−A
European−B
European−C

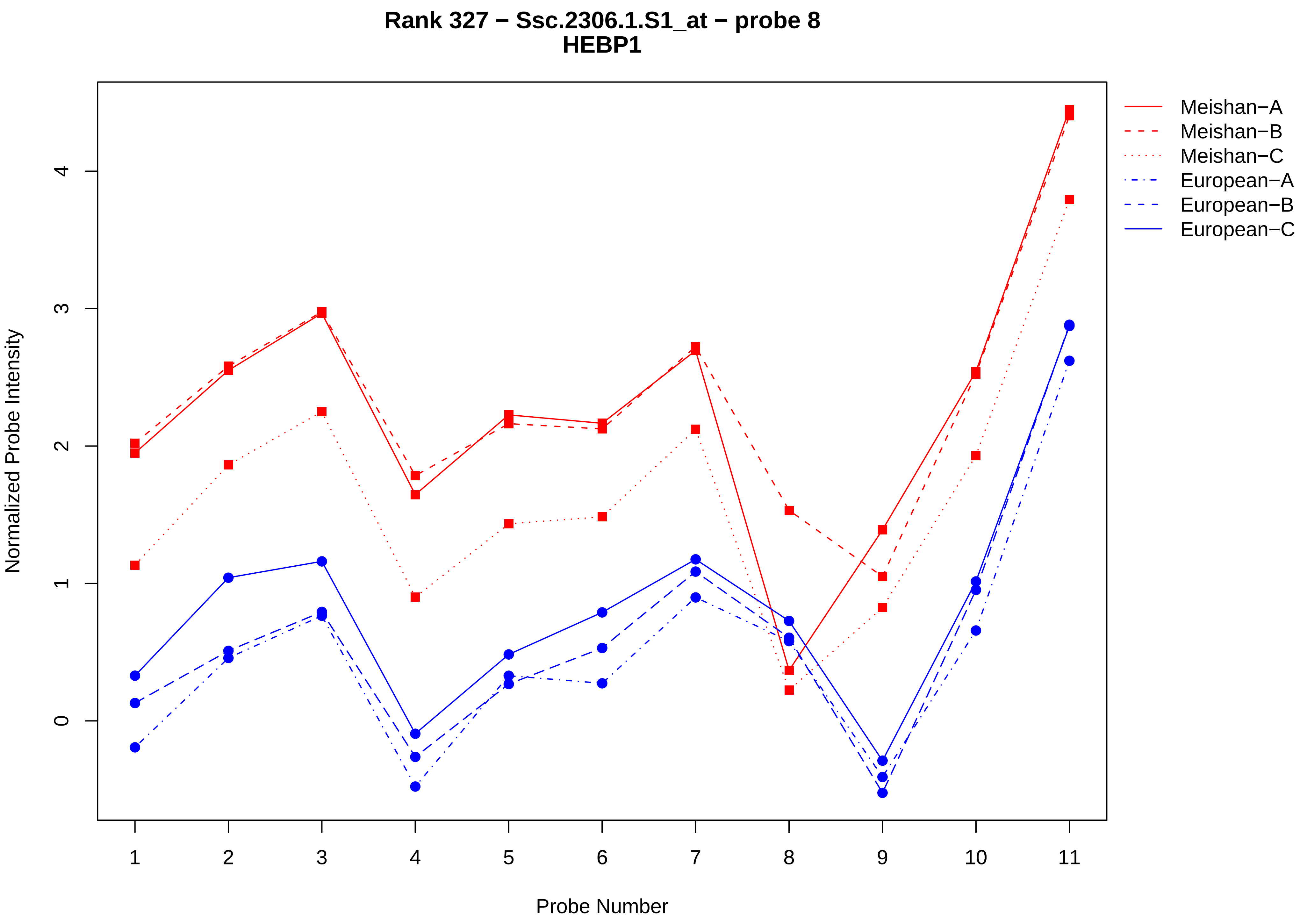
Rank 327 − Ssc.2306.1.S1_at − probe 8
HEBP1
Normalized Probe Intensity
Probe Number
0
1
2
3
4
1
2
3
4
5
6
7
8
9
10
11
Meishan−A
Meishan−B
Meishan−C
European−A
European−B
European−C

Rank 328 − Ssc.20986.2.S1_at − probe 11
GRN

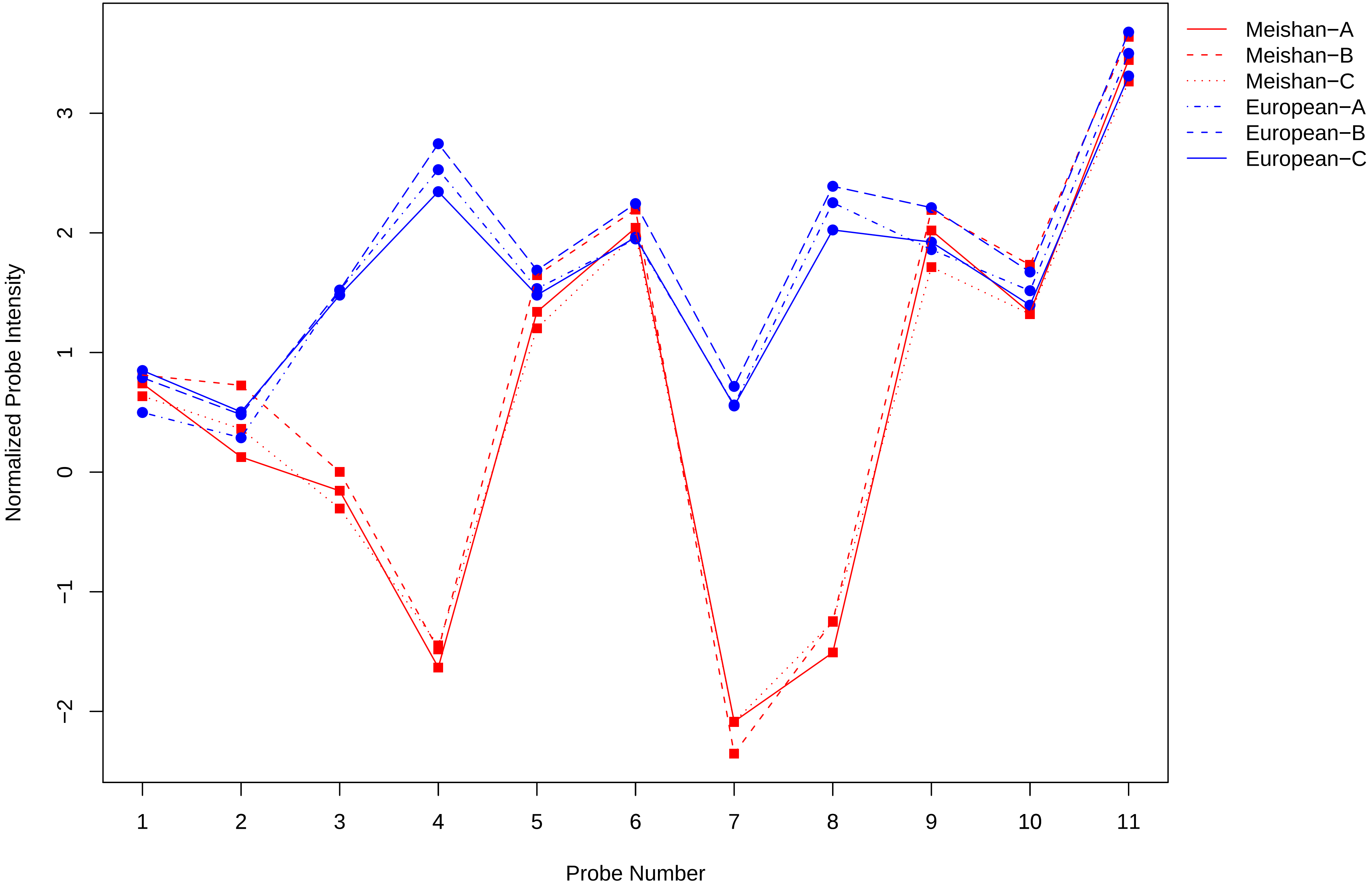

Rank 329 − Ssc.7568.1.A1_at − probe 6
CCL28

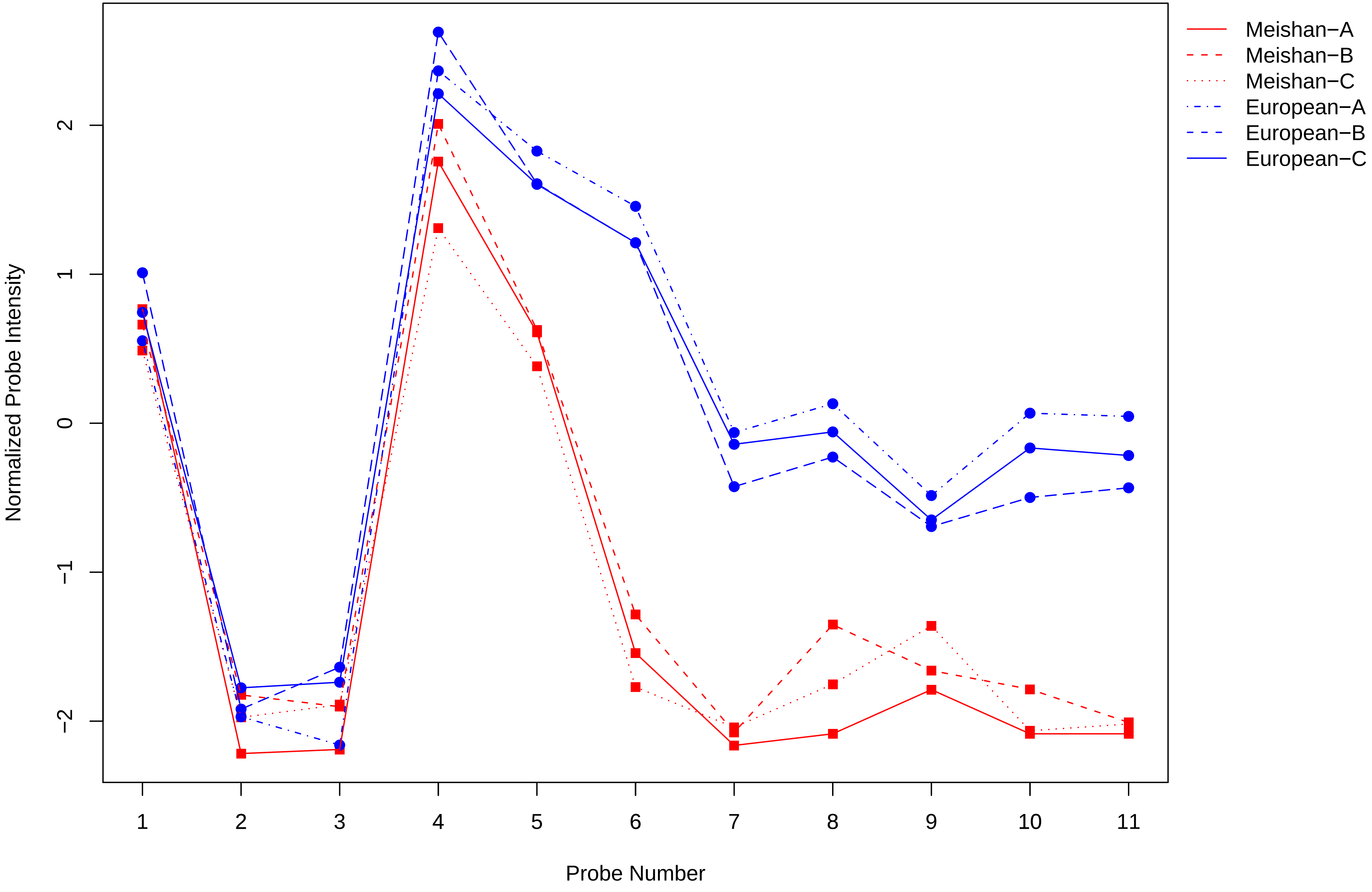

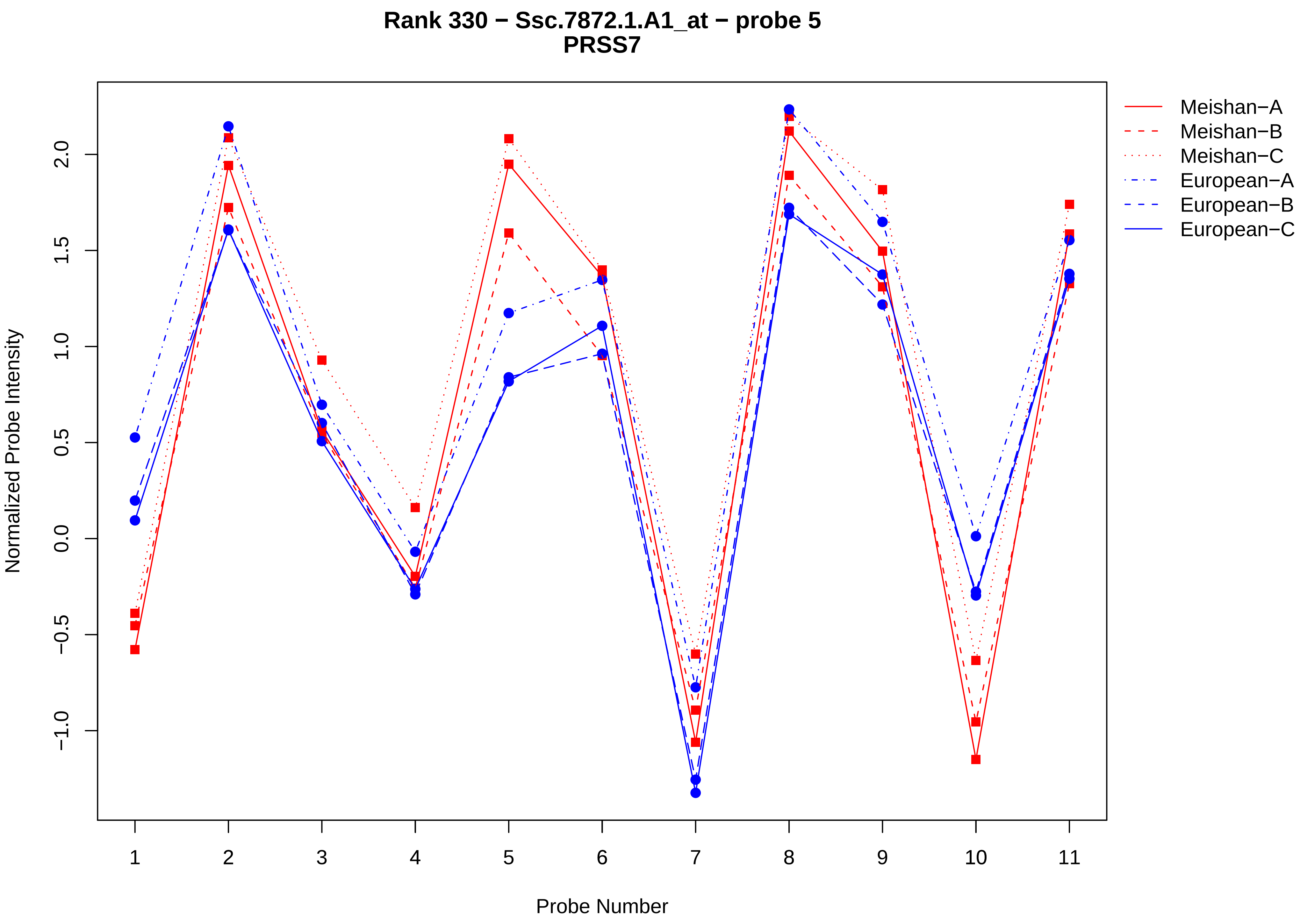

Rank 330 − Ssc.7872.1.A1_at − probe 5
PRSS7
Normalized Probe Intensity
Probe Number
−1.0
−0.5
0.0
0.5
1.0
1.5
2.0
1
2
3
4
5
6
7
8
9
10
11
Meishan−A
Meishan−B
Meishan−C
European−A
European−B
European−C

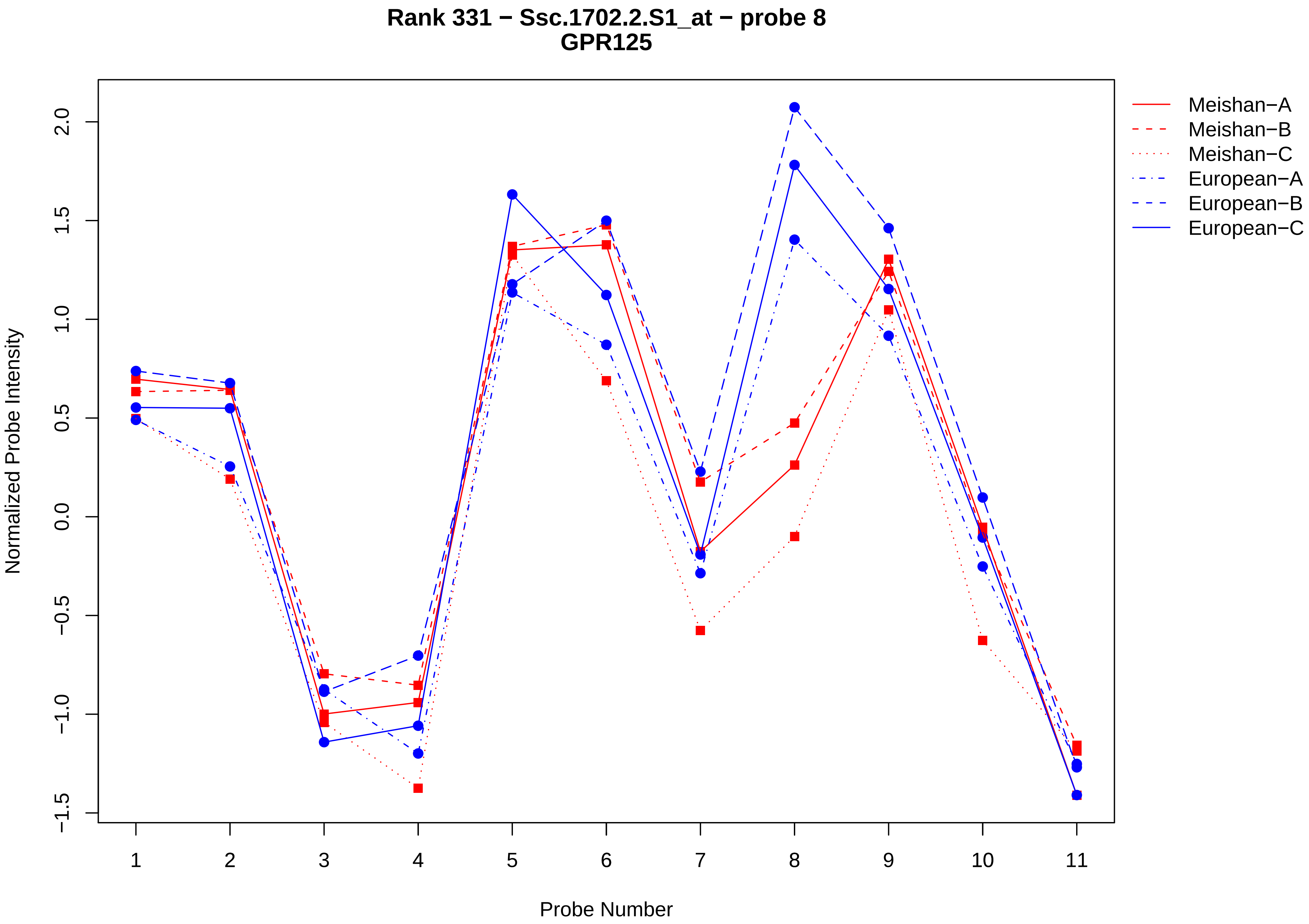

Rank 331 − Ssc.1702.2.S1_at − probe 8
GPR125
Normalized Probe Intensity
Probe Number
Meishan−A
Meishan−B
Meishan−C
European−A
European−B
European−C
−1.5
−1.0
−0.5
0.0
0.5
1.0
1.5
2.0
1
2
3
4
5
6
7
8
9
10
11

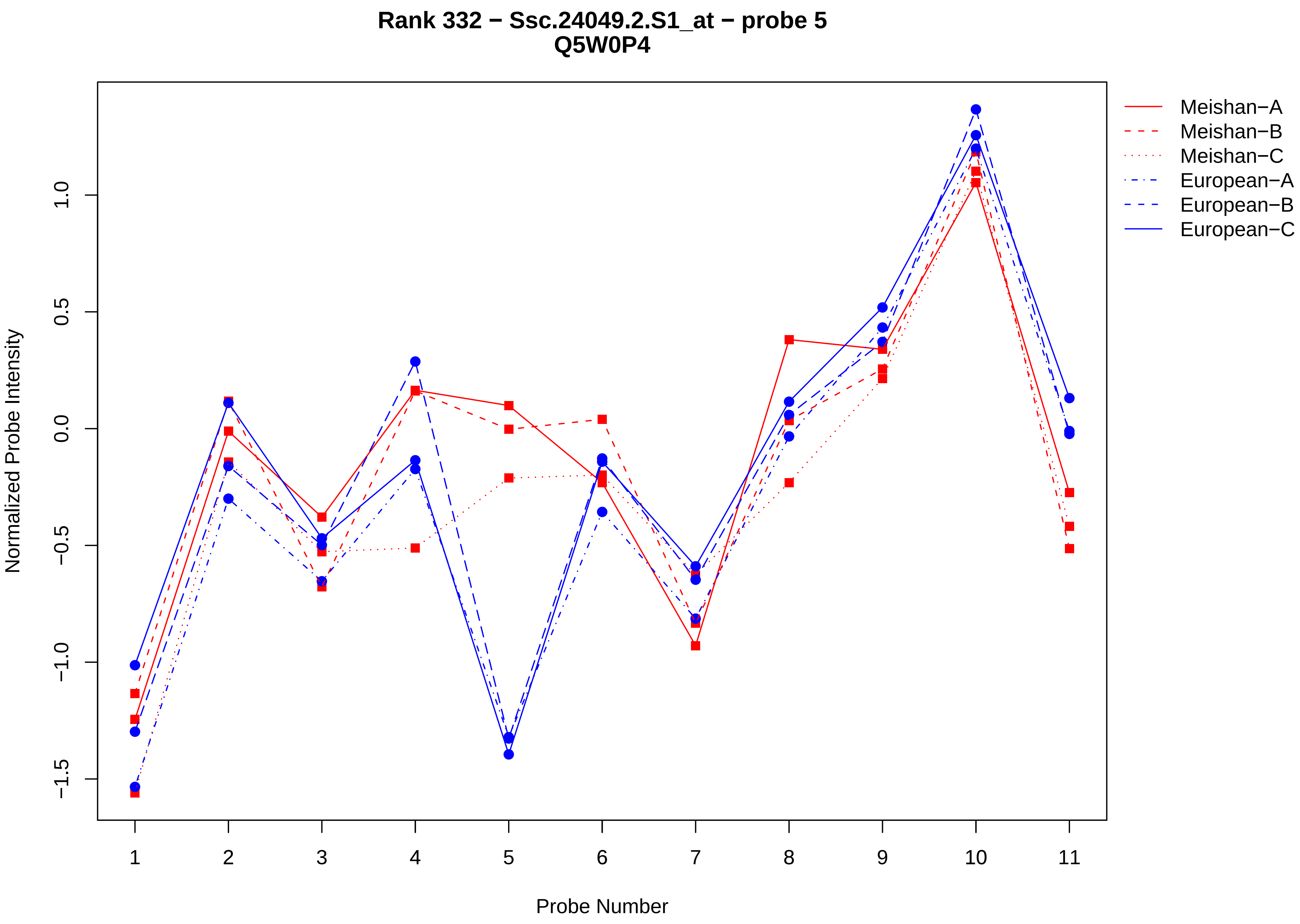
Rank 332 − Ssc.24049.2.S1_at − probe 5
Q5W0P4
Normalized Probe Intensity
Probe Number
Meishan−A
Meishan−B
Meishan−C
European−A
European−B
European−C
−1.5
−1.0
−0.5
0.0
0.5
1.0
1
2
3
4
5
6
7
8
9
10
11

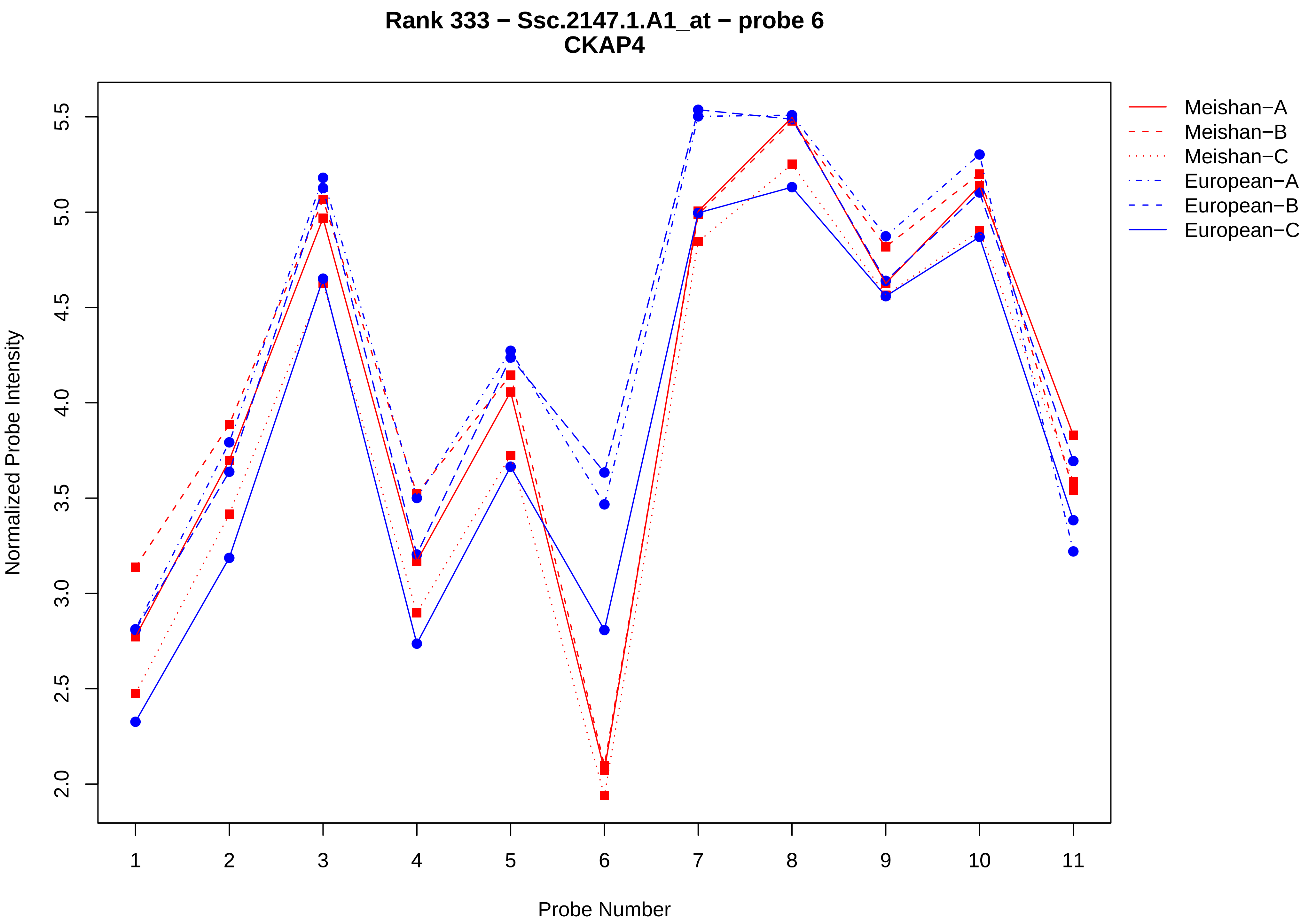

Rank 333 − Ssc.2147.1.A1_at − probe 6
CKAP4
Normalized Probe Intensity
Probe Number
2.0
2.5
3.0
3.5
4.0
4.5
5.0
5.5
1
2
3
4
5
6
7
8
9
10
11
Meishan−A
Meishan−B
Meishan−C
European−A
European−B
European−C

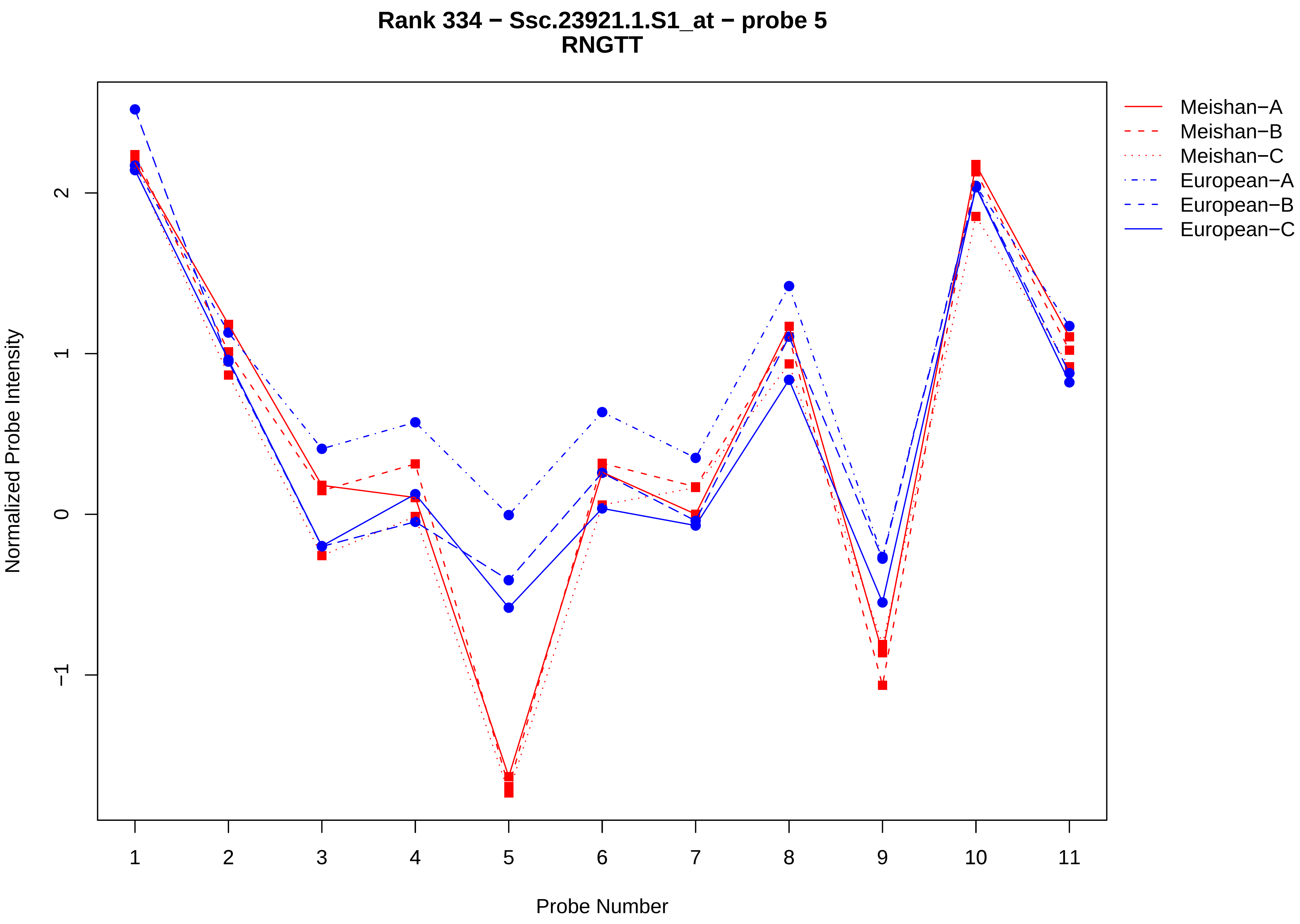

Rank 334 − Ssc.23921.1.S1_at − probe 5
RNGTT
Normalized Probe Intensity
Probe Number
−1
0
1
2
1
2
3
4
5
6
7
8
9
10
11
Meishan−A
Meishan−B
Meishan−C
European−A
European−B
European−C

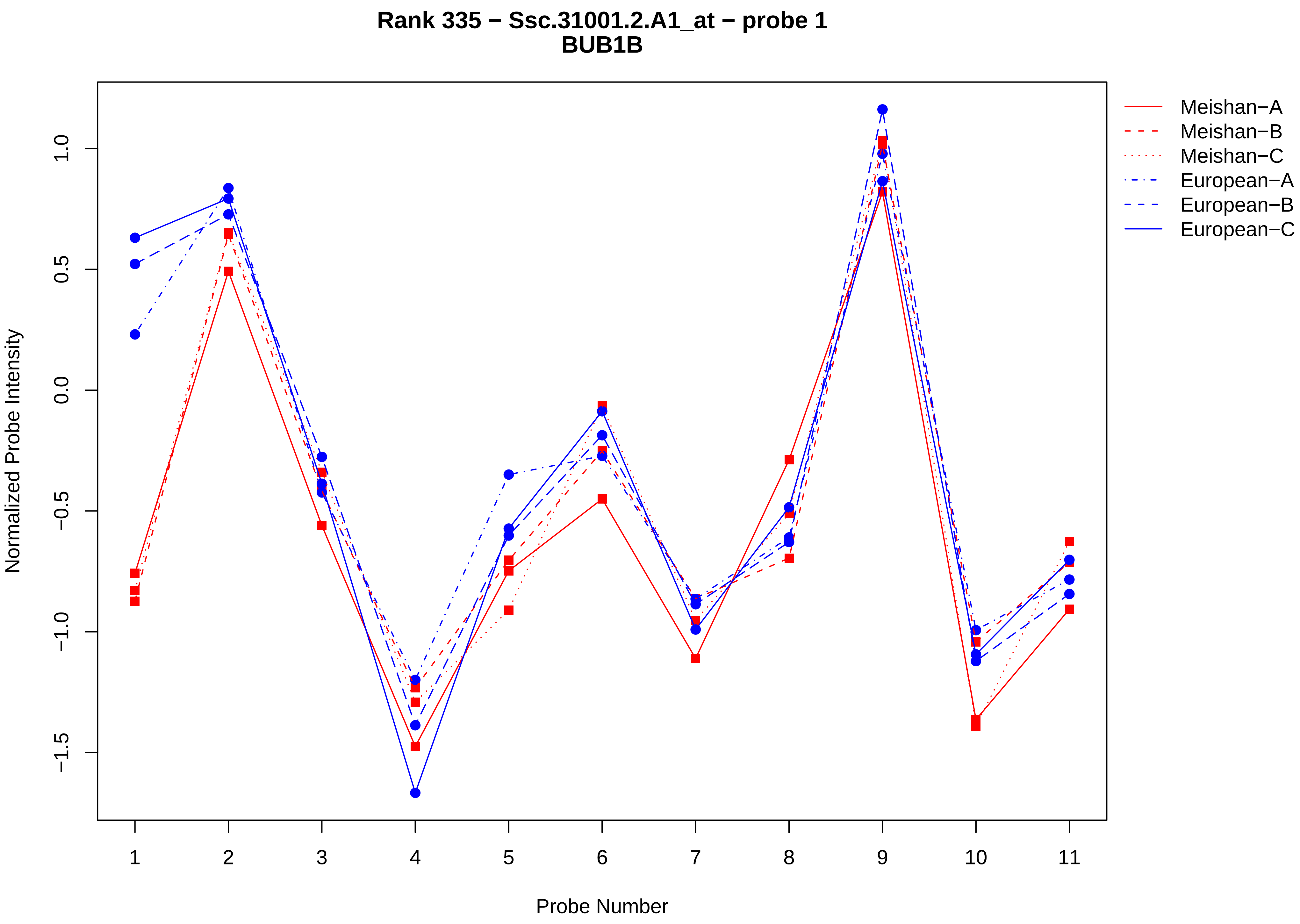
Rank 335 − Ssc.31001.2.A1_at − probe 1
BUB1B
Normalized Probe Intensity
Probe Number
Meishan−A
Meishan−B
Meishan−C
European−A
European−B
European−C
1.0
0.5
0.0
−0.5
−1.0
−1.5
1
2
3
4
5
6
7
8
9
10
11

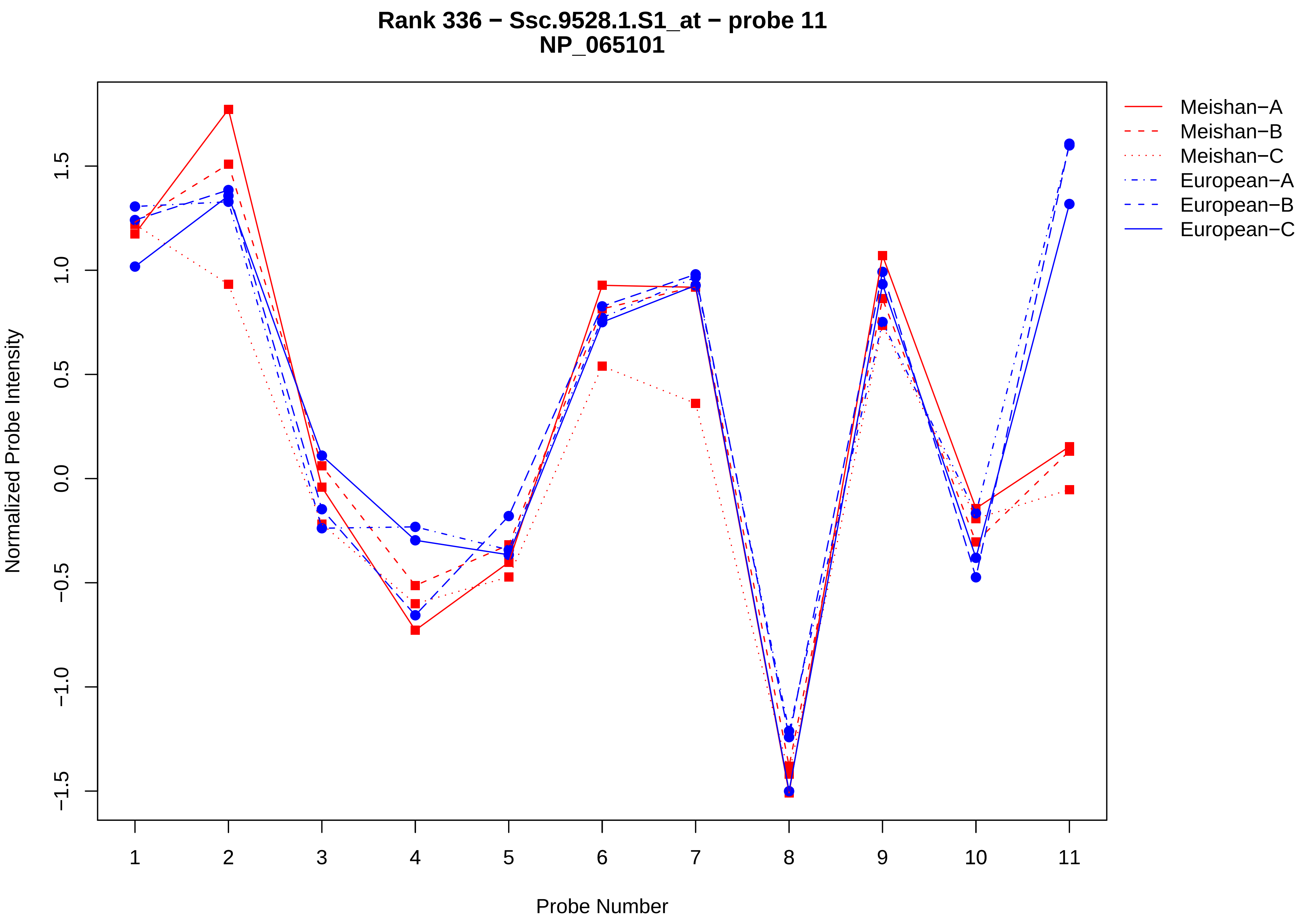

Rank 336 − Ssc.9528.1.S1_at − probe 11
NP_065101
Normalized Probe Intensity
Probe Number
−1.5
−1.0
−0.5
0.0
0.5
1.0
1.5
1
2
3
4
5
6
7
8
9
10
11
Meishan−A
Meishan−B
Meishan−C
European−A
European−B
European−C

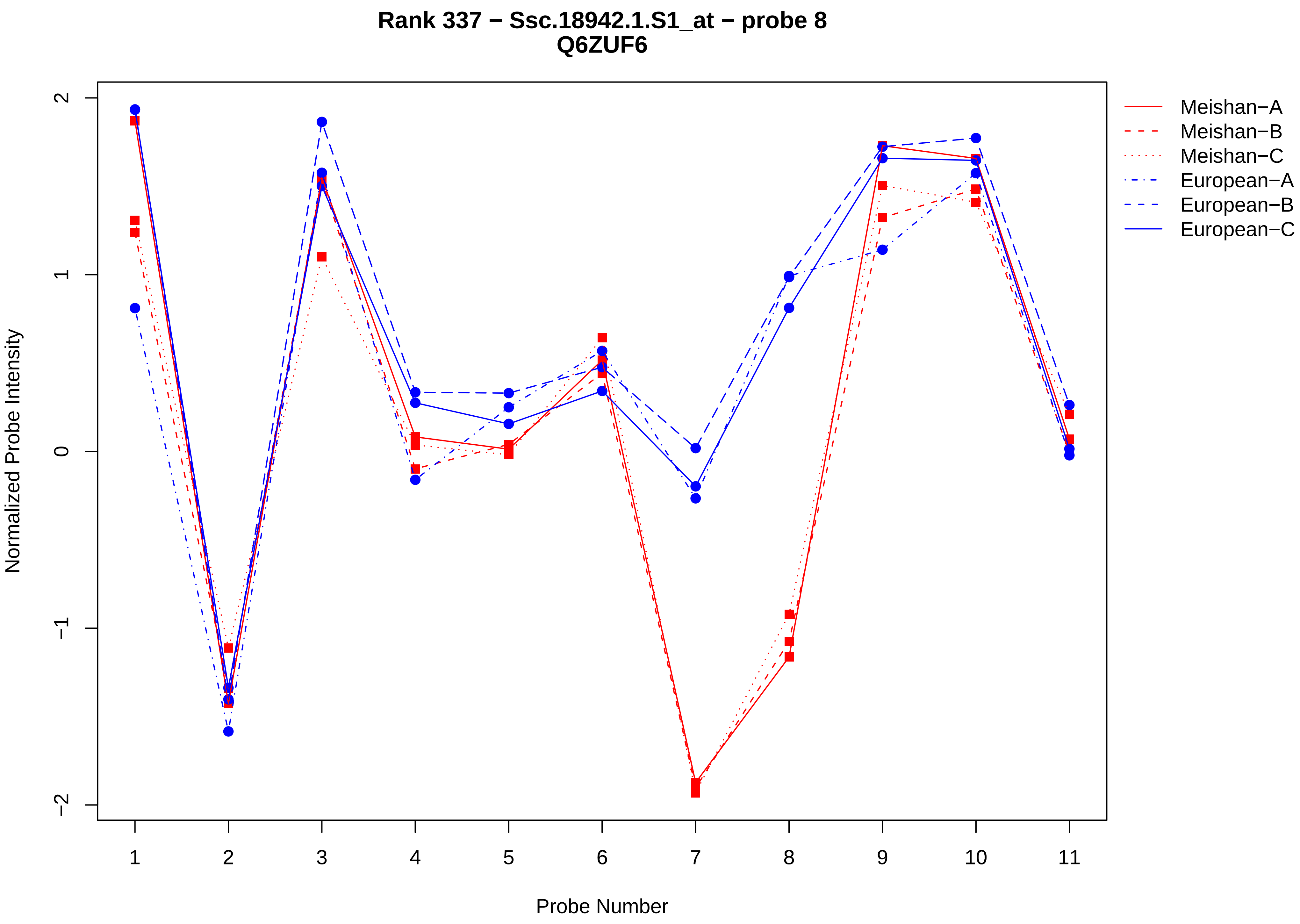

Rank 337 − Ssc.18942.1.S1_at − probe 8
Q6ZUF6
Normalized Probe Intensity
Probe Number
Meishan−A
Meishan−B
Meishan−C
European−A
European−B
European−C
−2
−1
0
1
2
1
2
3
4
5
6
7
8
9
10
11

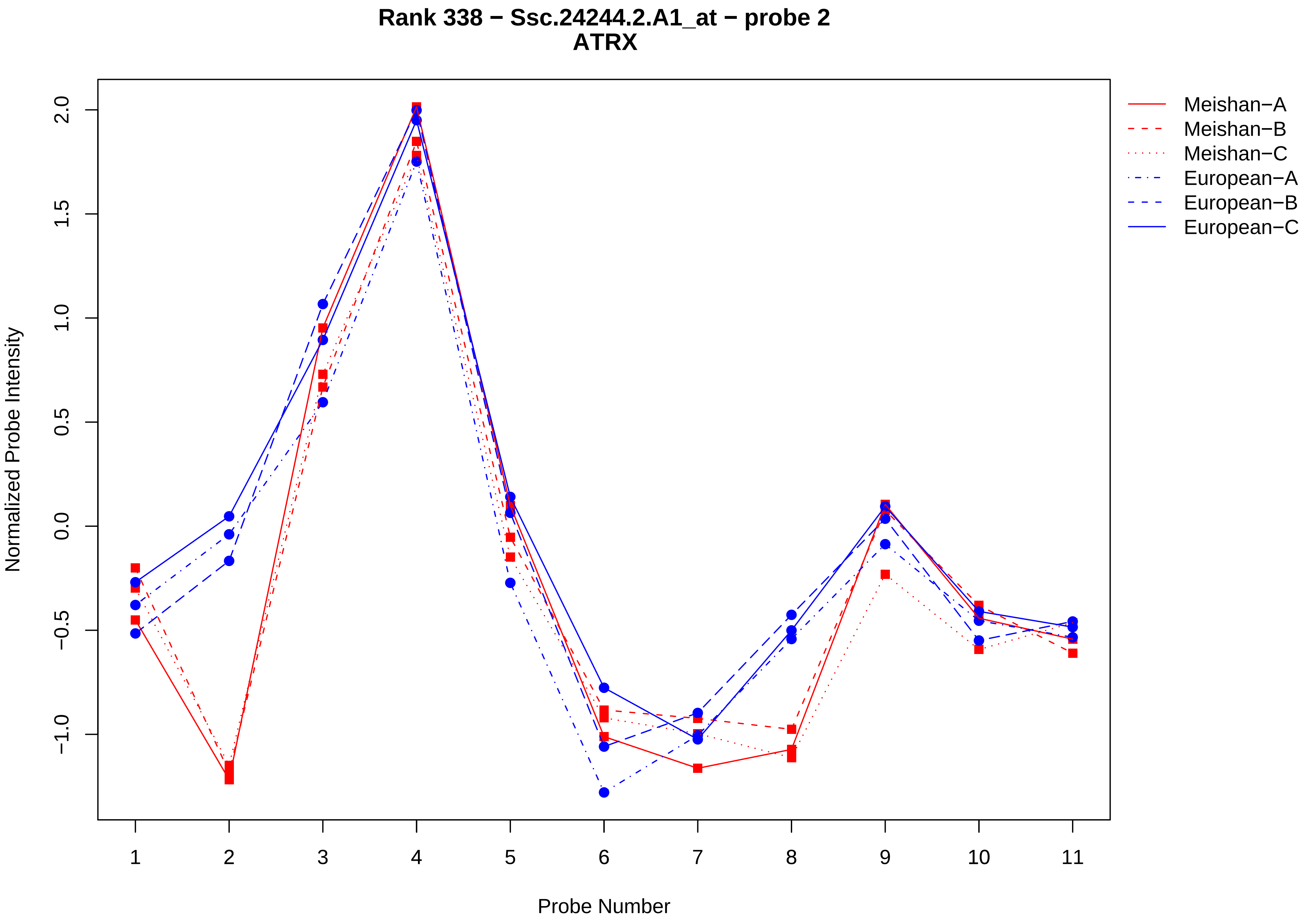

Rank 338 − Ssc.24244.2.A1_at − probe 2
ATRX
Normalized Probe Intensity
Probe Number
2.0
1.5
1.0
0.5
0.0
−0.5
−1.0
1
2
3
4
5
6
7
8
9
10
11
Meishan−A
Meishan−B
Meishan−C
European−A
European−B
European−C

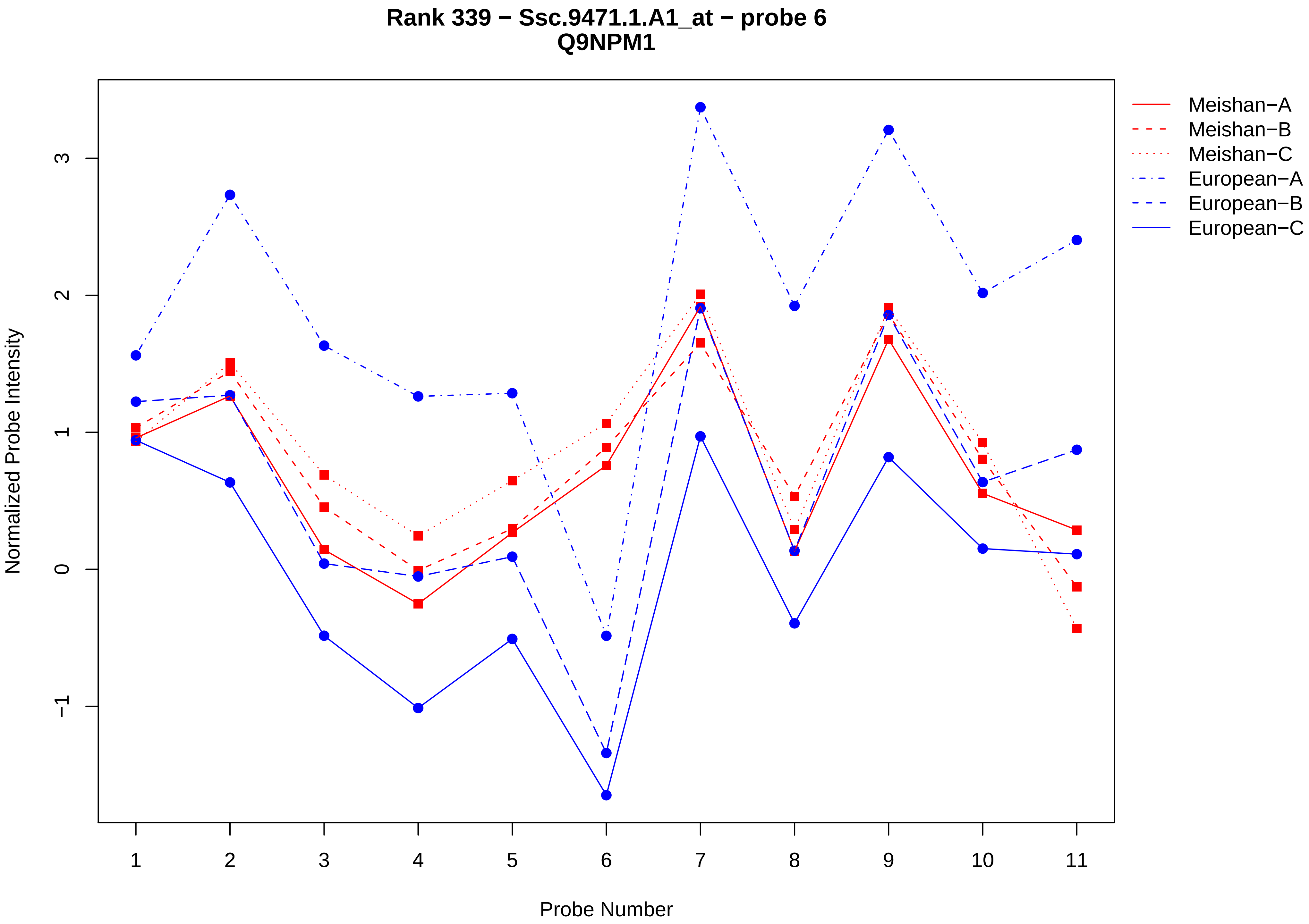

Rank 339 − Ssc.9471.1.A1_at − probe 6
Q9NPM1
Meishan−A
Meishan−B
Meishan−C
European−A
European−B
European−C
Normalized Probe Intensity
Probe Number
−1
0
1
2
3
1
2
3
4
5
6
7
8
9
10
11

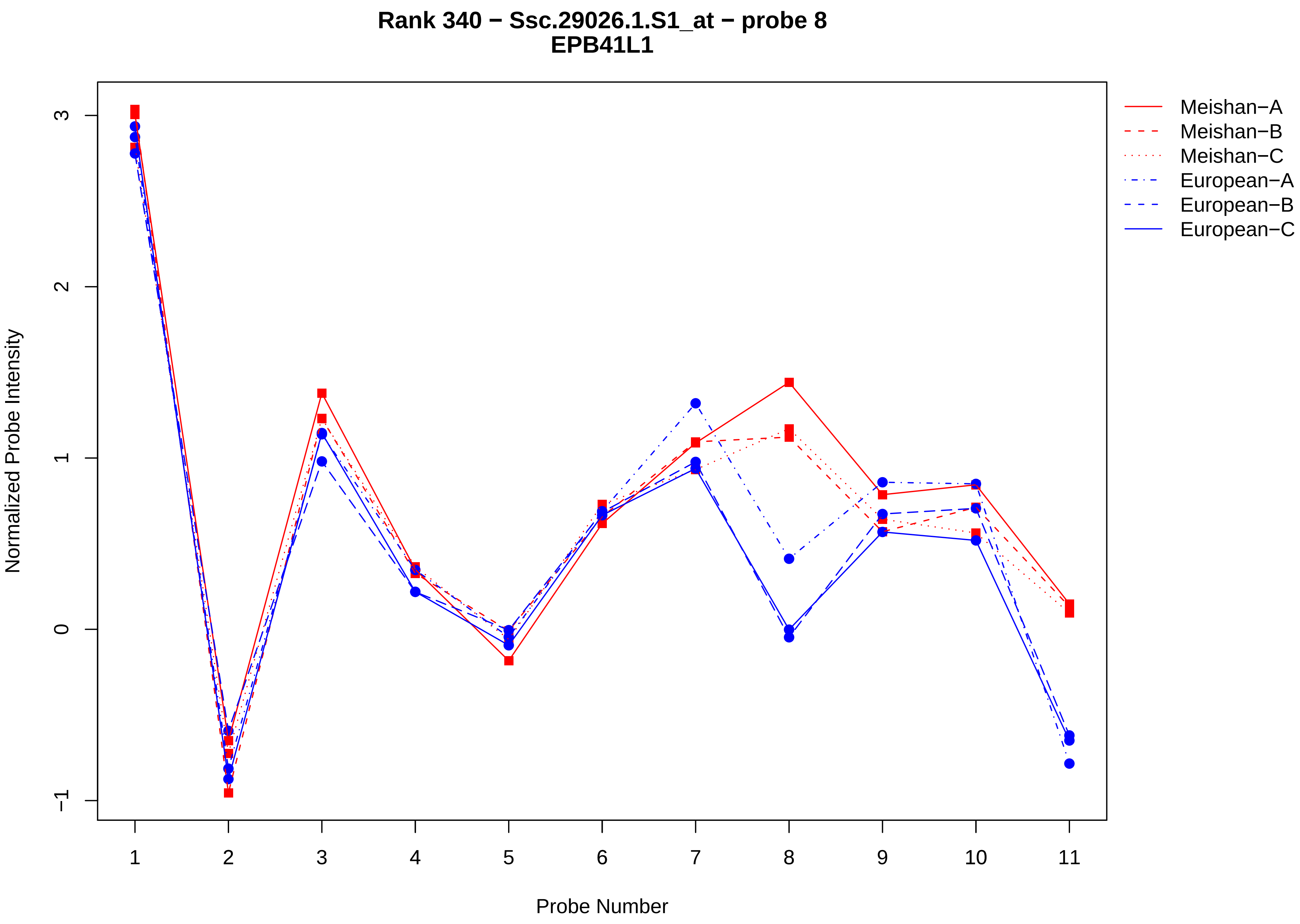
Rank 340 − Ssc.29026.1.S1_at − probe 8
EPB41L1
Normalized Probe Intensity
Probe Number
−1
0
1
2
3
1
2
3
4
5
6
7
8
9
10
11
Meishan−A
Meishan−B
Meishan−C
European−A
European−B
European−C

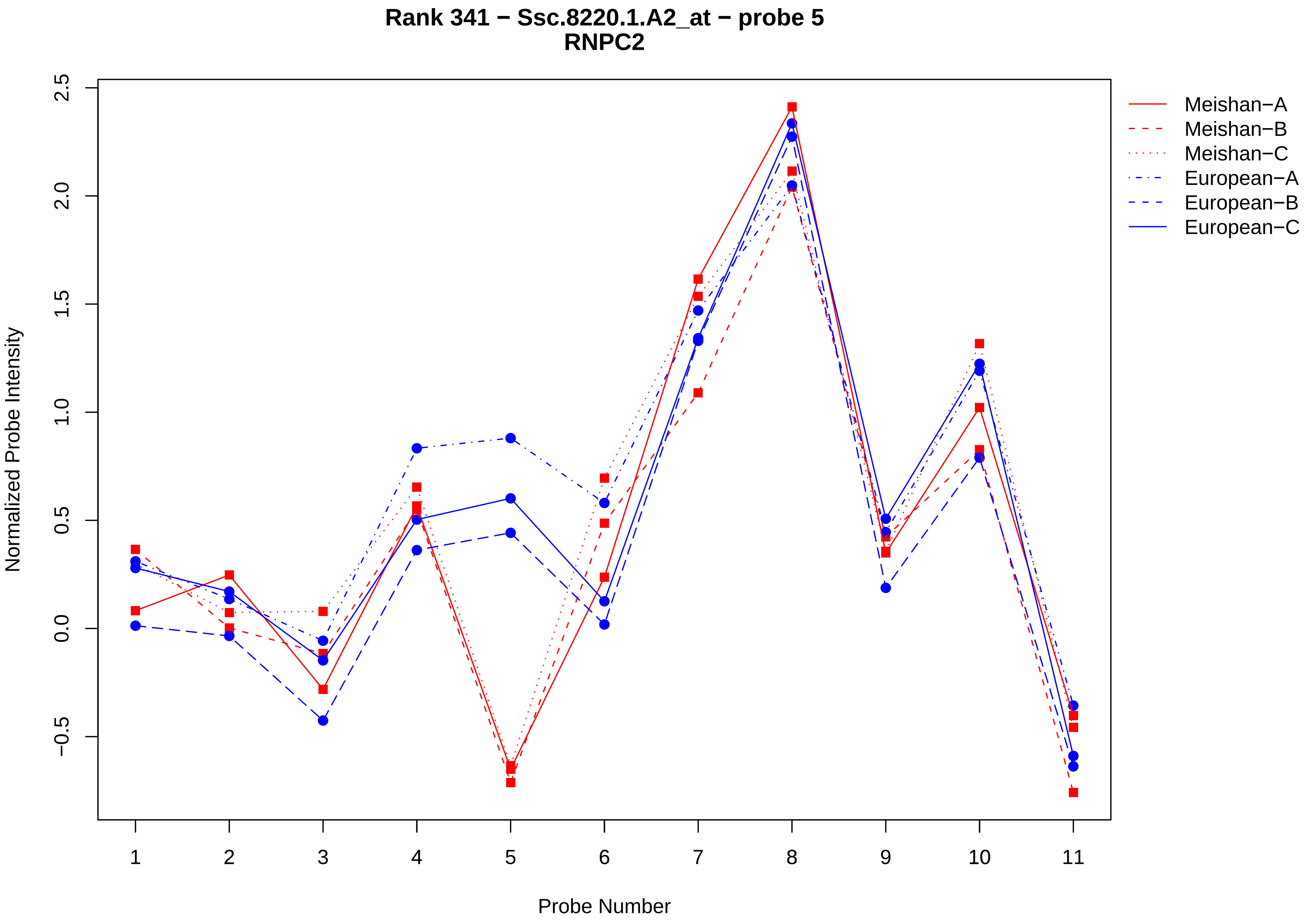

Rank 341 − Ssc.8220.1.A2_at − probe 5
RNPC2
Normalized Probe Intensity
Probe Number
−0.5
0.0
0.5
1.0
1.5
2.0
2.5
1
2
3
4
5
6
7
8
9
10
11
Meishan−A
Meishan−B
Meishan−C
European−A
European−B
European−C

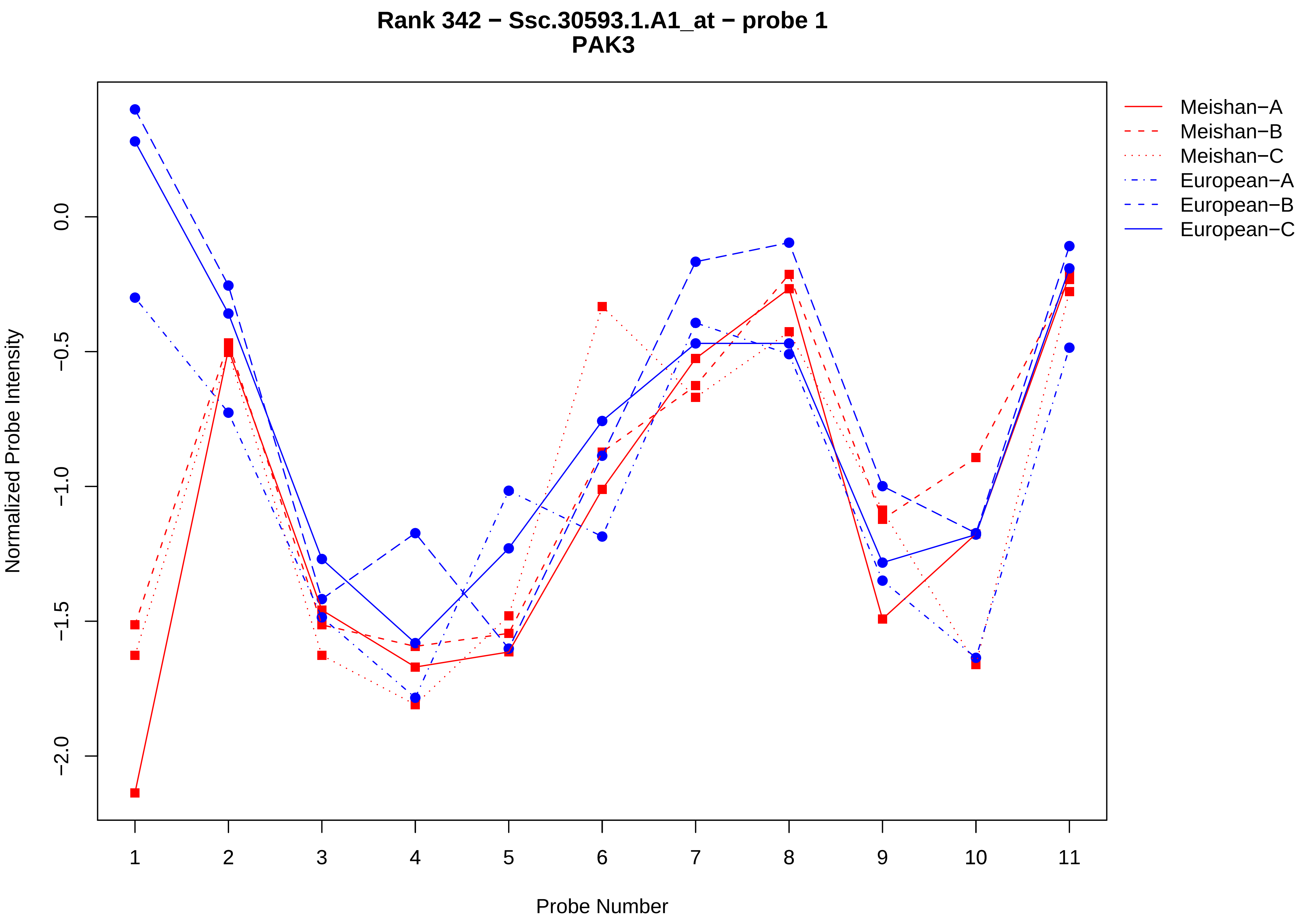
Rank 342 − Ssc.30593.1.A1_at − probe 1
PAK3
Normalized Probe Intensity
Probe Number
0.0
−0.5
−1.0
−1.5
−2.0
1
2
3
4
5
6
7
8
9
10
11
Meishan−A
Meishan−B
Meishan−C
European−A
European−B
European−C

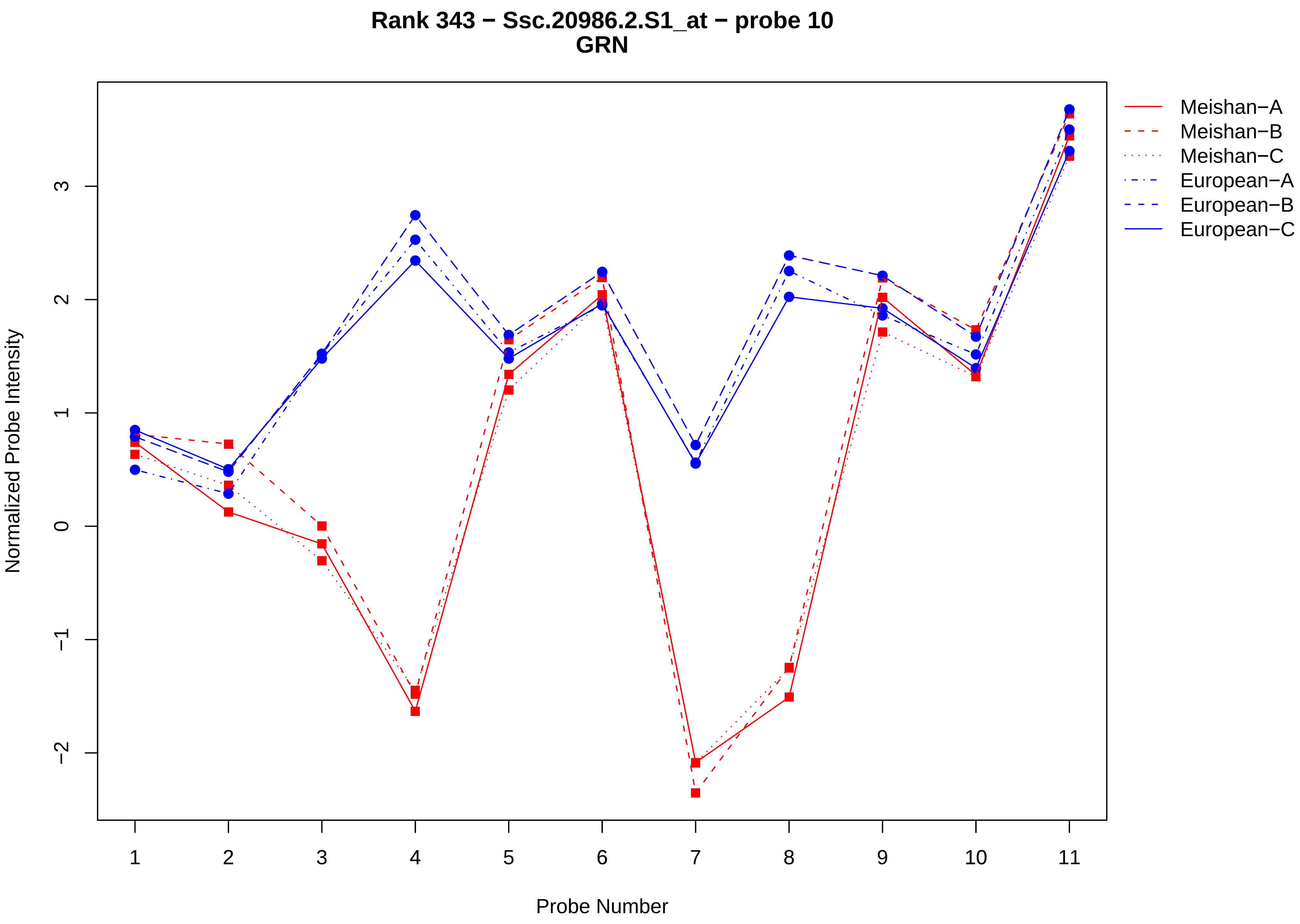
Rank 343 − Ssc.20986.2.S1_at − probe 10
GRN
Normalized Probe Intensity
Probe Number
Meishan−A
Meishan−B
Meishan−C
European−A
European−B
European−C
−2
−1
0
1
2
3
1
2
3
4
5
6
7
8
9
10
11

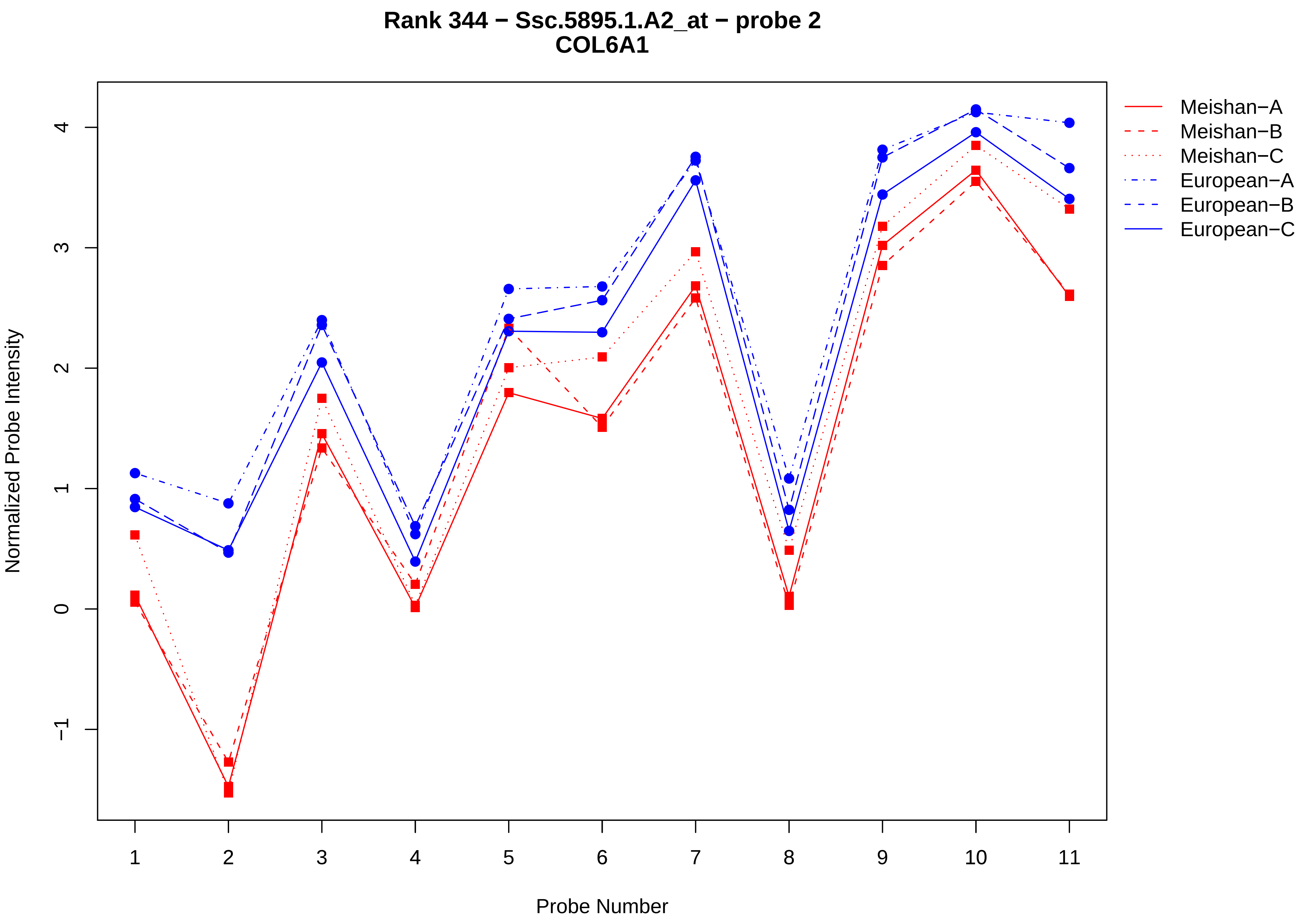
Rank 344 − Ssc.5895.1.A2_at − probe 2
COL6A1
Normalized Probe Intensity
Probe Number
−1
0
1
2
3
4
1
2
3
4
5
6
7
8
9
10
11
Meishan−A
Meishan−B
Meishan−C
European−A
European−B
European−C

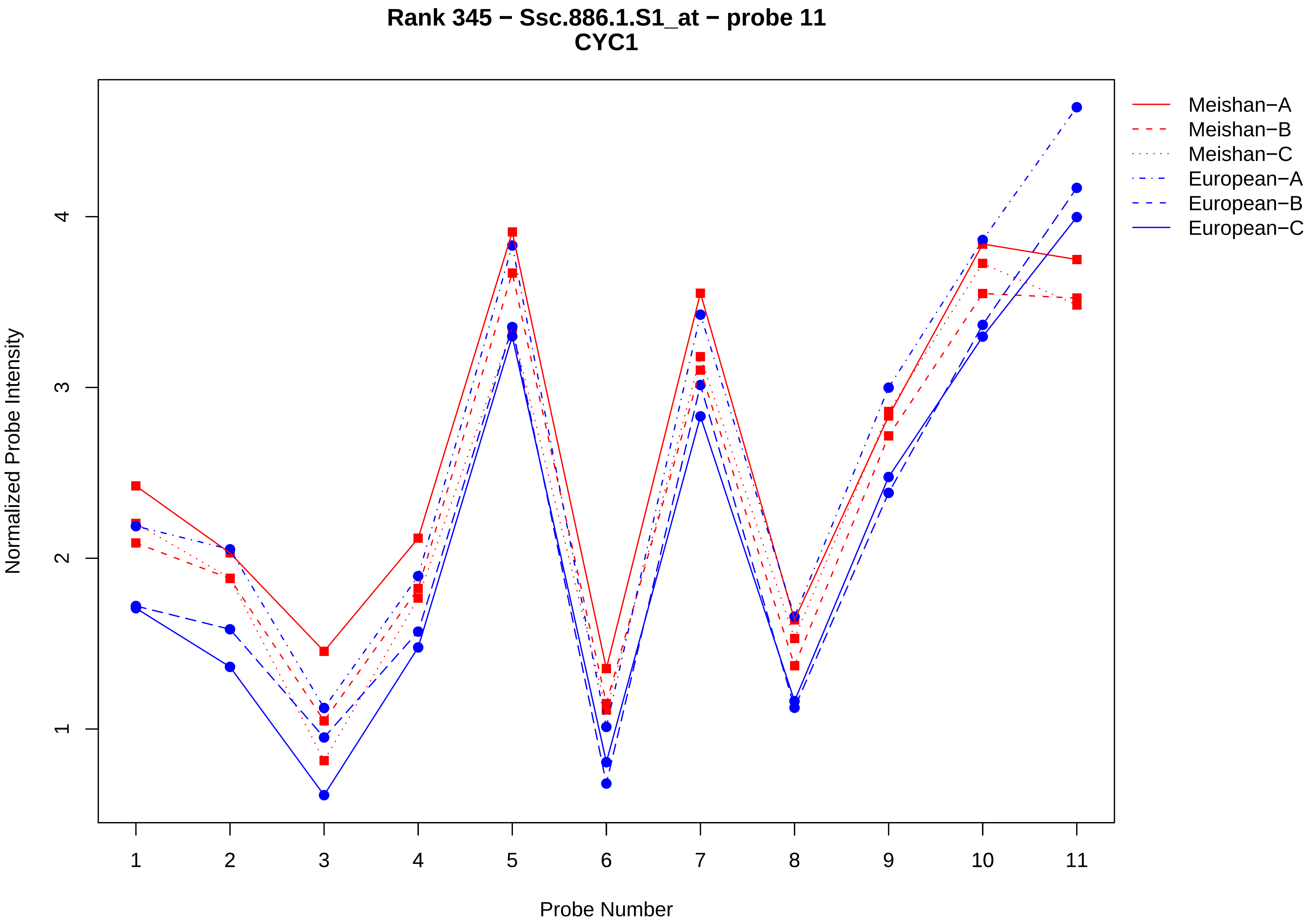

Rank 345 − Ssc.886.1.S1_at − probe 11
CYC1
Normalized Probe Intensity
Probe Number
1
2
3
4
5
6
7
8
9
10
11
Meishan−A
Meishan−B
Meishan−C
European−A
European−B
European−C

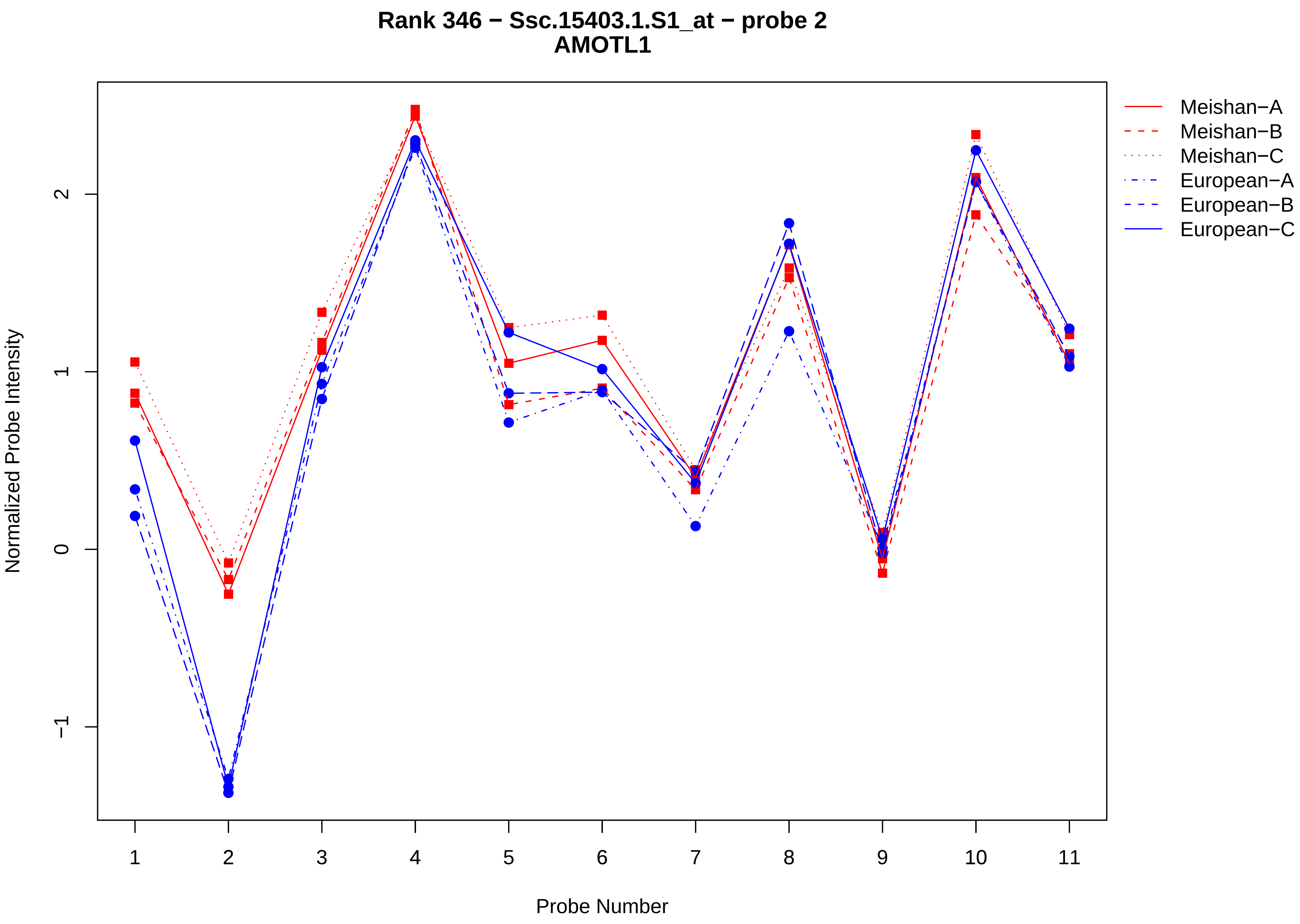

Rank 346 − Ssc.15403.1.S1_at − probe 2
AMOTL1
Normalized Probe Intensity
Probe Number
−1
0
1
2
1
2
3
4
5
6
7
8
9
10
11
Meishan−A
Meishan−B
Meishan−C
European−A
European−B
European−C

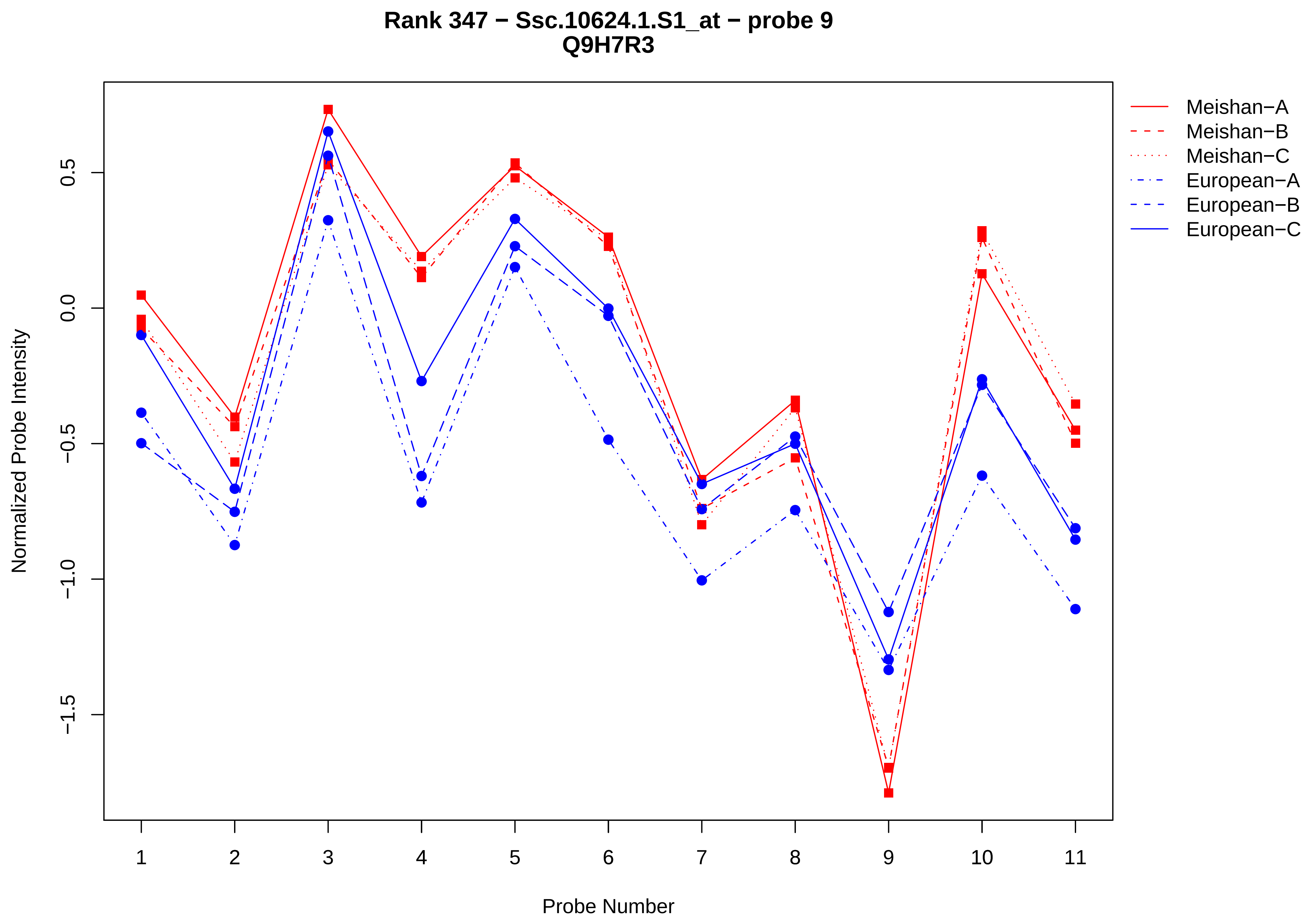

Rank 347 − Ssc.10624.1.S1_at − probe 9
Q9H7R3
Normalized Probe Intensity
Probe Number
0.5
0.0
−0.5
−1.0
−1.5
1
2
3
4
5
6
7
8
9
10
11
Meishan−A
Meishan−B
Meishan−C
European−A
European−B
European−C

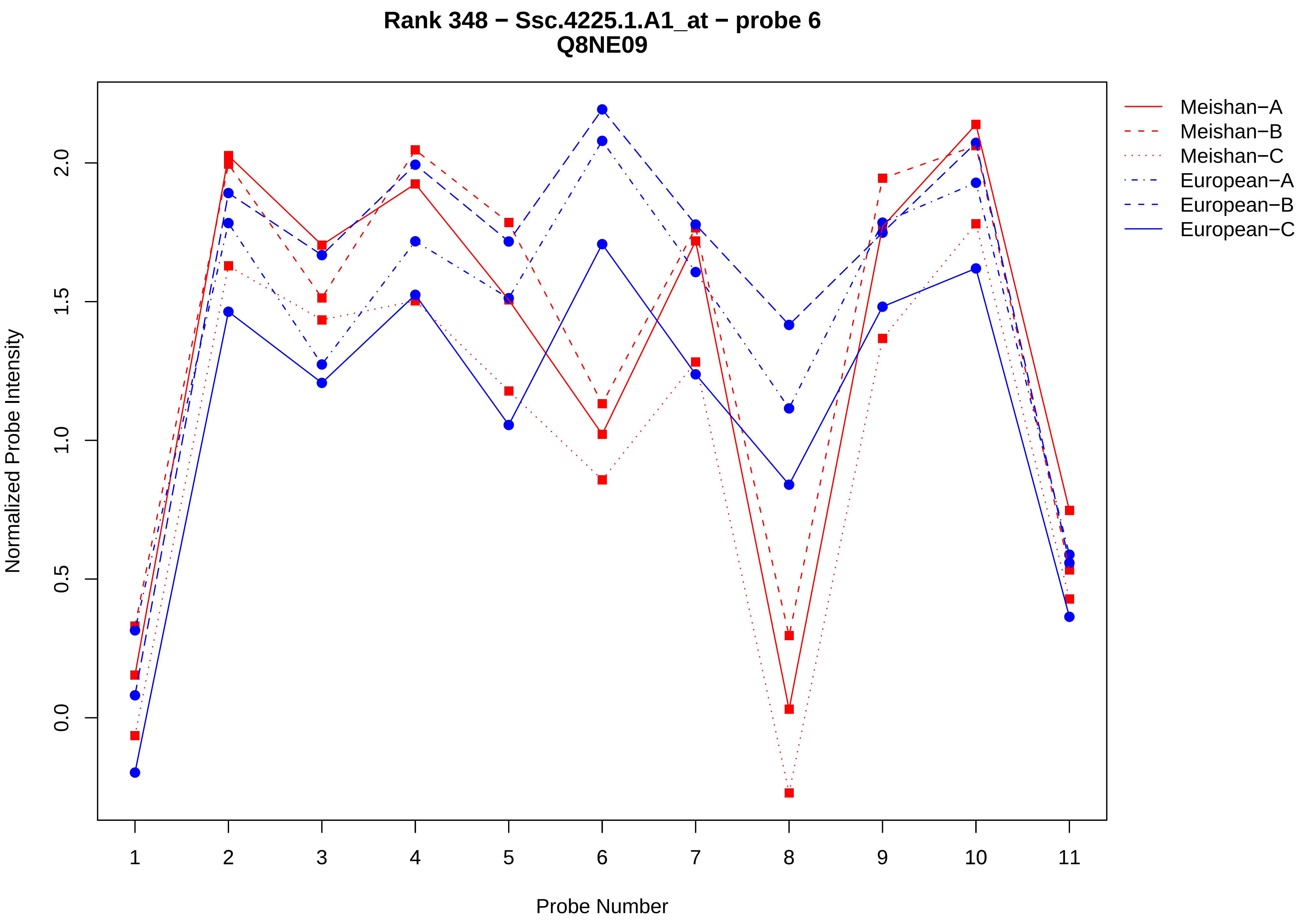
Rank 348 − Ssc.4225.1.A1_at − probe 6
Q8NE09
Normalized Probe Intensity
Probe Number
Meishan−A
Meishan−B
Meishan−C
European−A
European−B
European−C
0.0
0.5
1.0
1.5
2.0
1
2
3
4
5
6
7
8
9
10
11

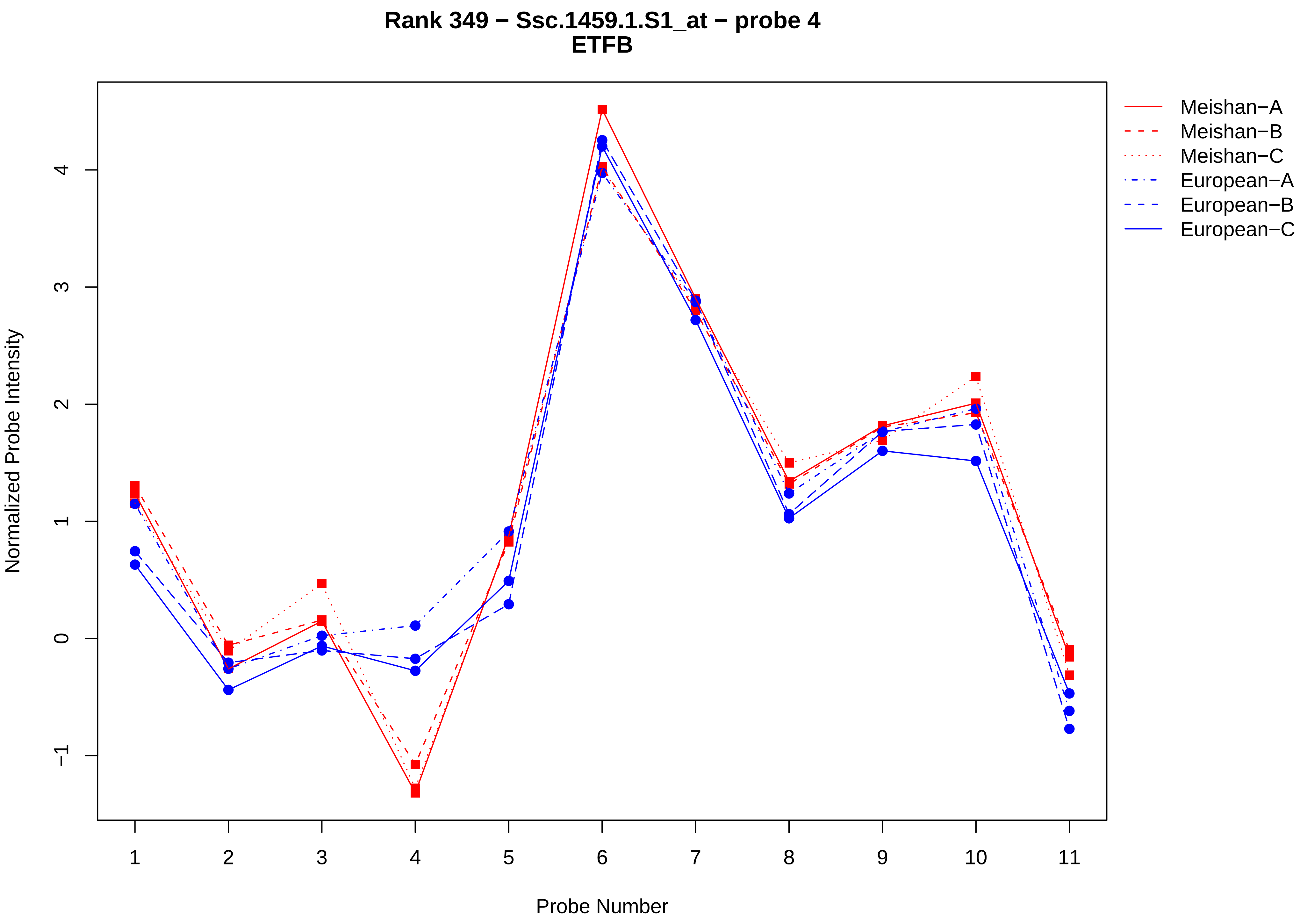

Rank 349 − Ssc.1459.1.S1_at − probe 4
ETFB
Normalized Probe Intensity
Probe Number
Meishan−A
Meishan−B
Meishan−C
European−A
European−B
European−C
−1
0
1
2
3
4
1
2
3
4
5
6
7
8
9
10
11

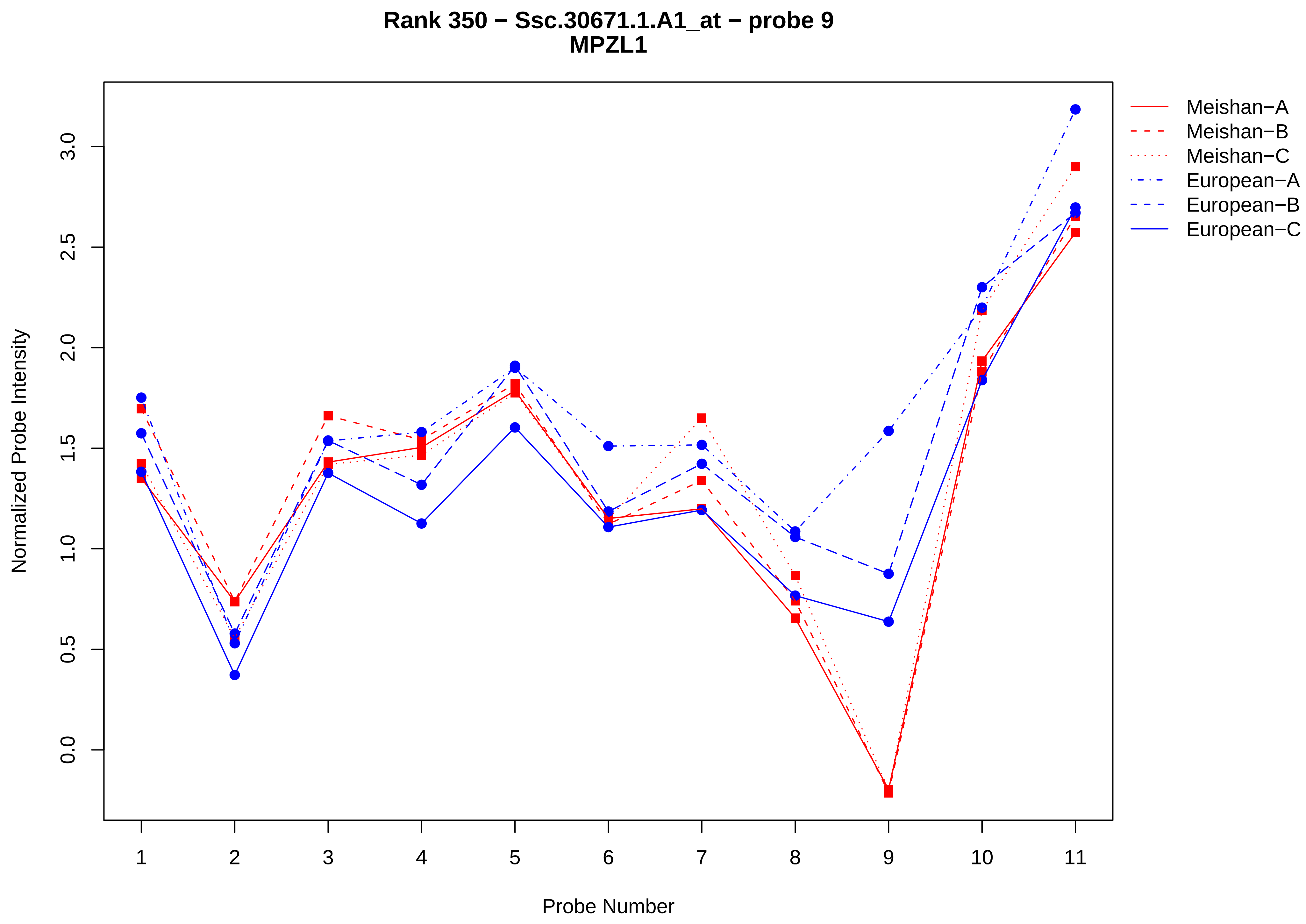

Rank 350 − Ssc.30671.1.A1_at − probe 9
MPZL1
Normalized Probe Intensity
Probe Number
0.0
0.5
1.0
1.5
2.0
2.5
3.0
1
2
3
4
5
6
7
8
9
10
11
Meishan−A
Meishan−B
Meishan−C
European−A
European−B
European−C

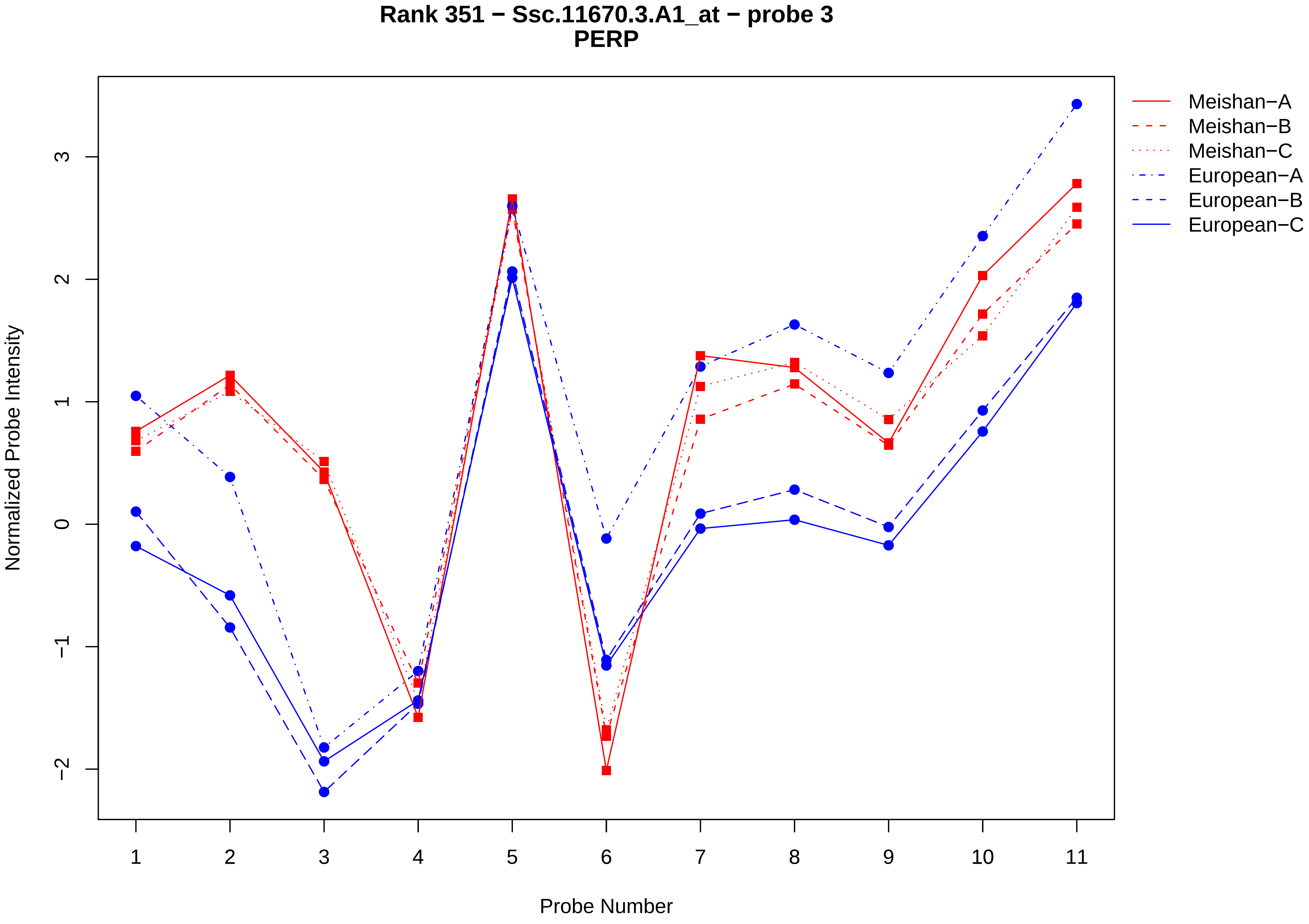
Rank 351 − Ssc.11670.3.A1_at − probe 3
PERP
Normalized Probe Intensity
Probe Number
Meishan−A
Meishan−B
Meishan−C
European−A
European−B
European−C

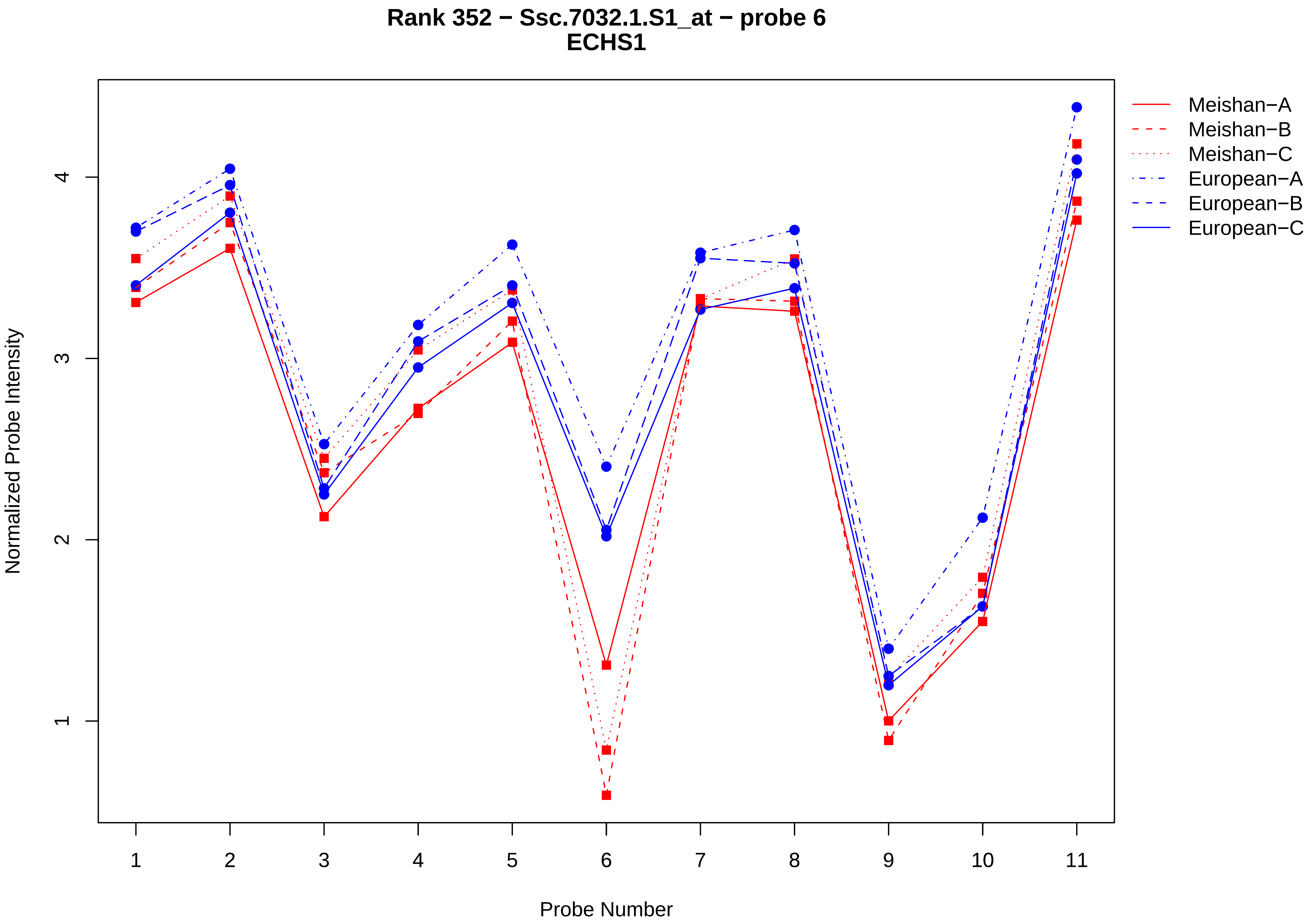

Rank 352 − Ssc.7032.1.S1_at − probe 6
ECHS1
Normalized Probe Intensity
Probe Number
1
2
3
4
1
2
3
4
5
6
7
8
9
10
11
Meishan−A
Meishan−B
Meishan−C
European−A
European−B
European−C

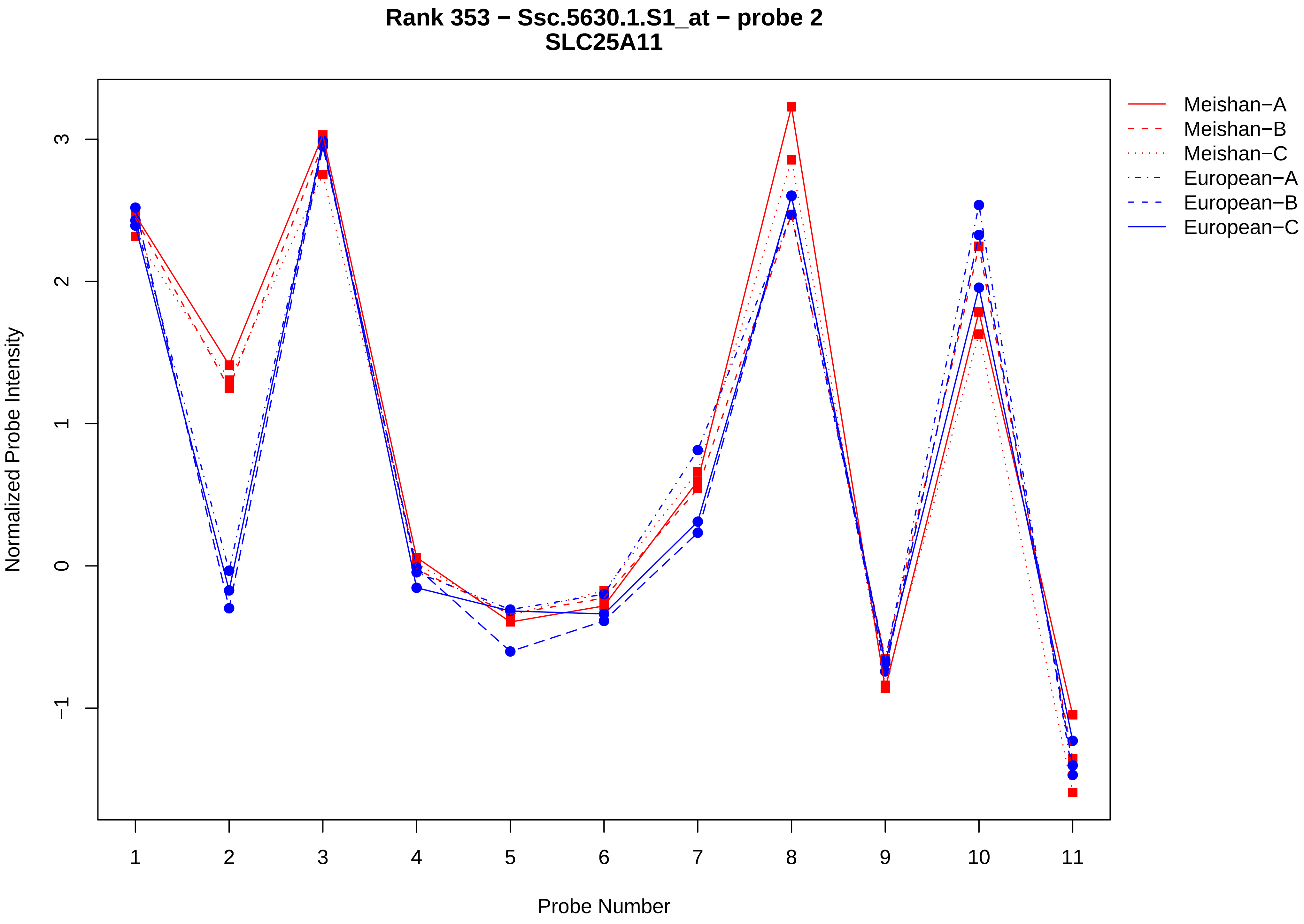

Rank 353 − Ssc.5630.1.S1_at − probe 2
SLC25A11
Normalized Probe Intensity
Probe Number
Meishan−A
Meishan−B
Meishan−C
European−A
European−B
European−C
−1
0
1
2
3
1
2
3
4
5
6
7
8
9
10
11

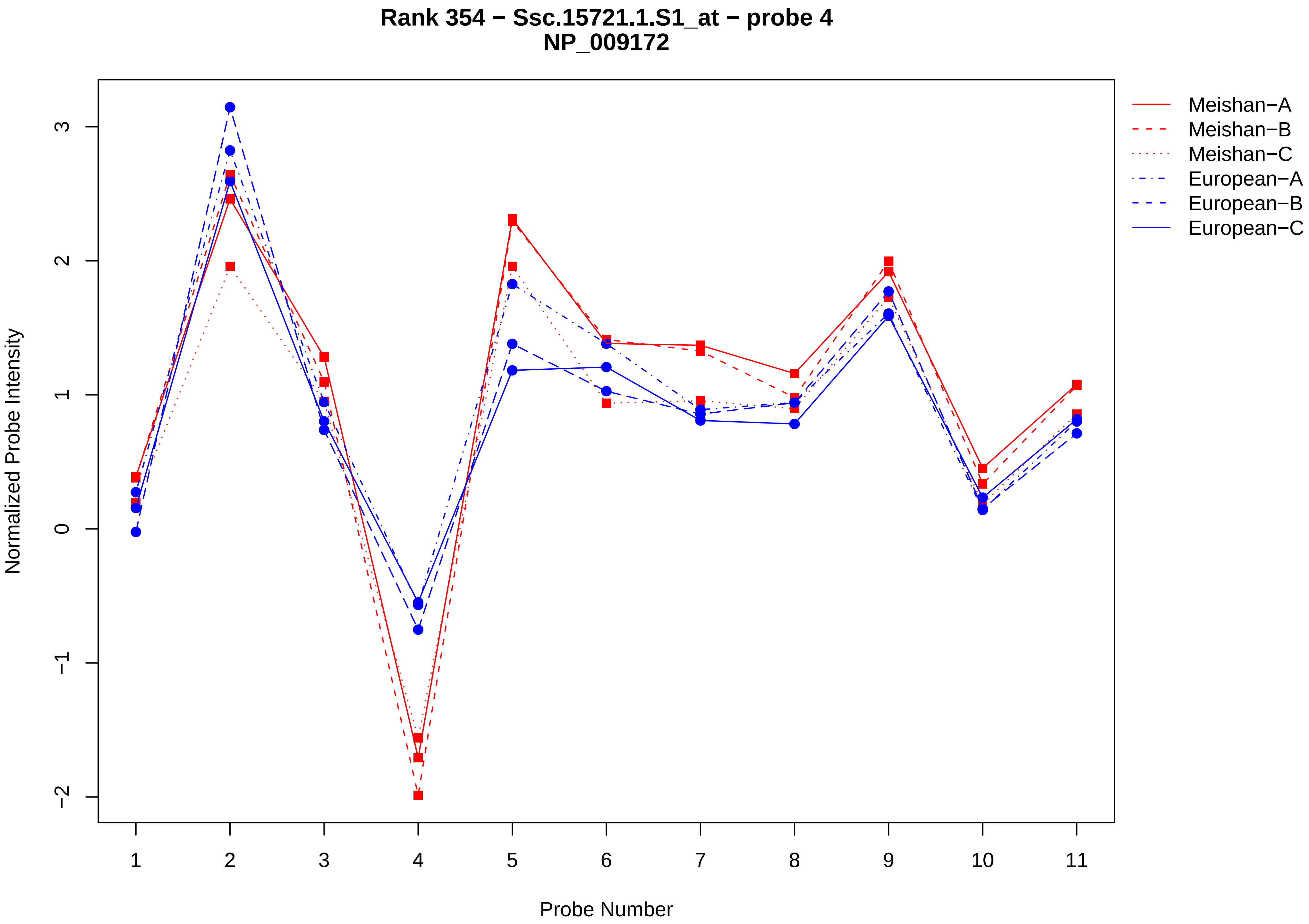

Rank 354 − Ssc.15721.1.S1_at − probe 4
NP_009172
Normalized Probe Intensity
Probe Number
−2
−1
0
1
2
3
1
2
3
4
5
6
7
8
9
10
11
Meishan−A
Meishan−B
Meishan−C
European−A
European−B
European−C

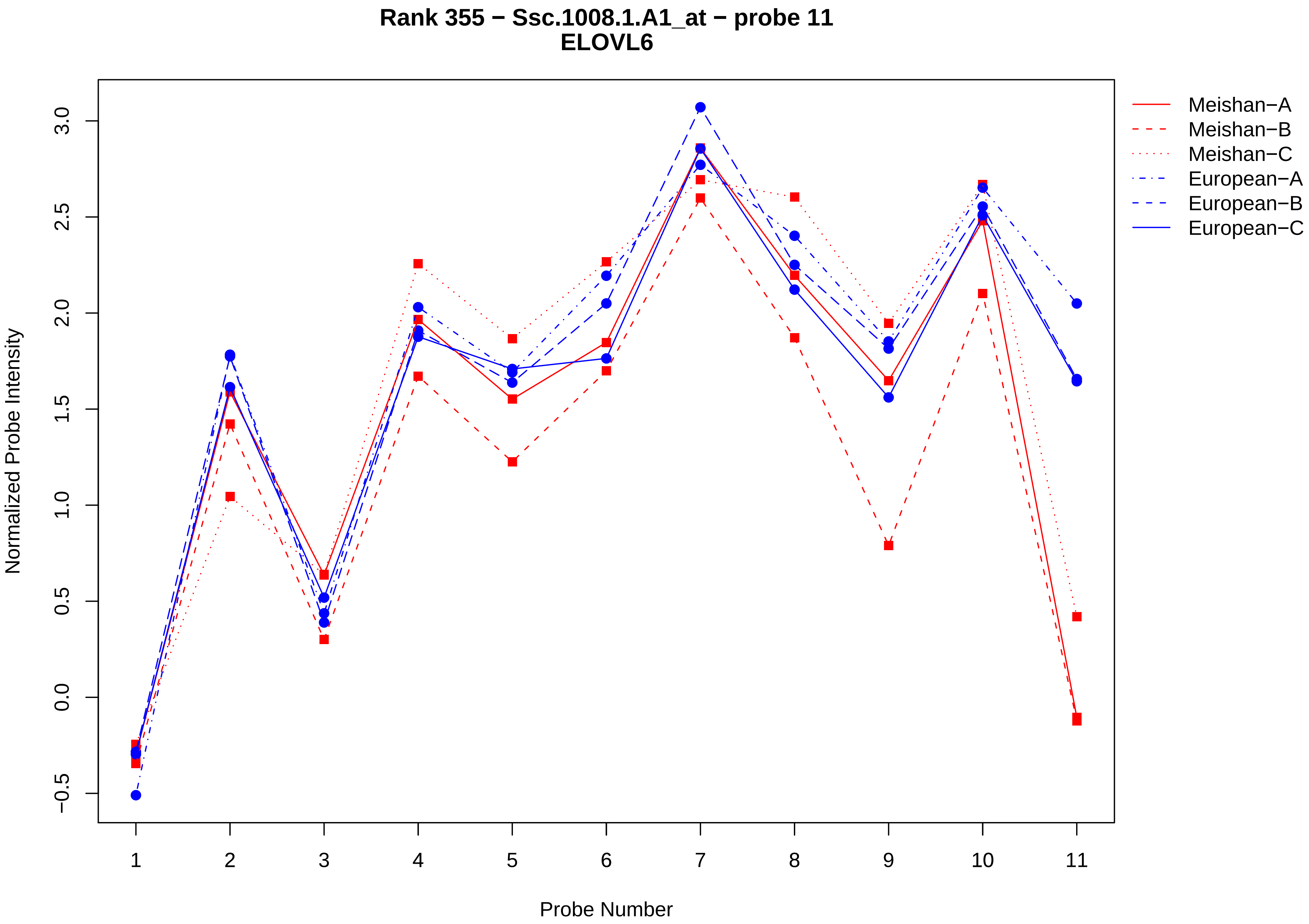

Rank 355 − Ssc.1008.1.A1_at − probe 11
ELOVL6
Normalized Probe Intensity
Probe Number
−0.5
0.0
0.5
1.0
1.5
2.0
2.5
3.0
1
2
3
4
5
6
7
8
9
10
11
Meishan−A
Meishan−B
Meishan−C
European−A
European−B
European−C

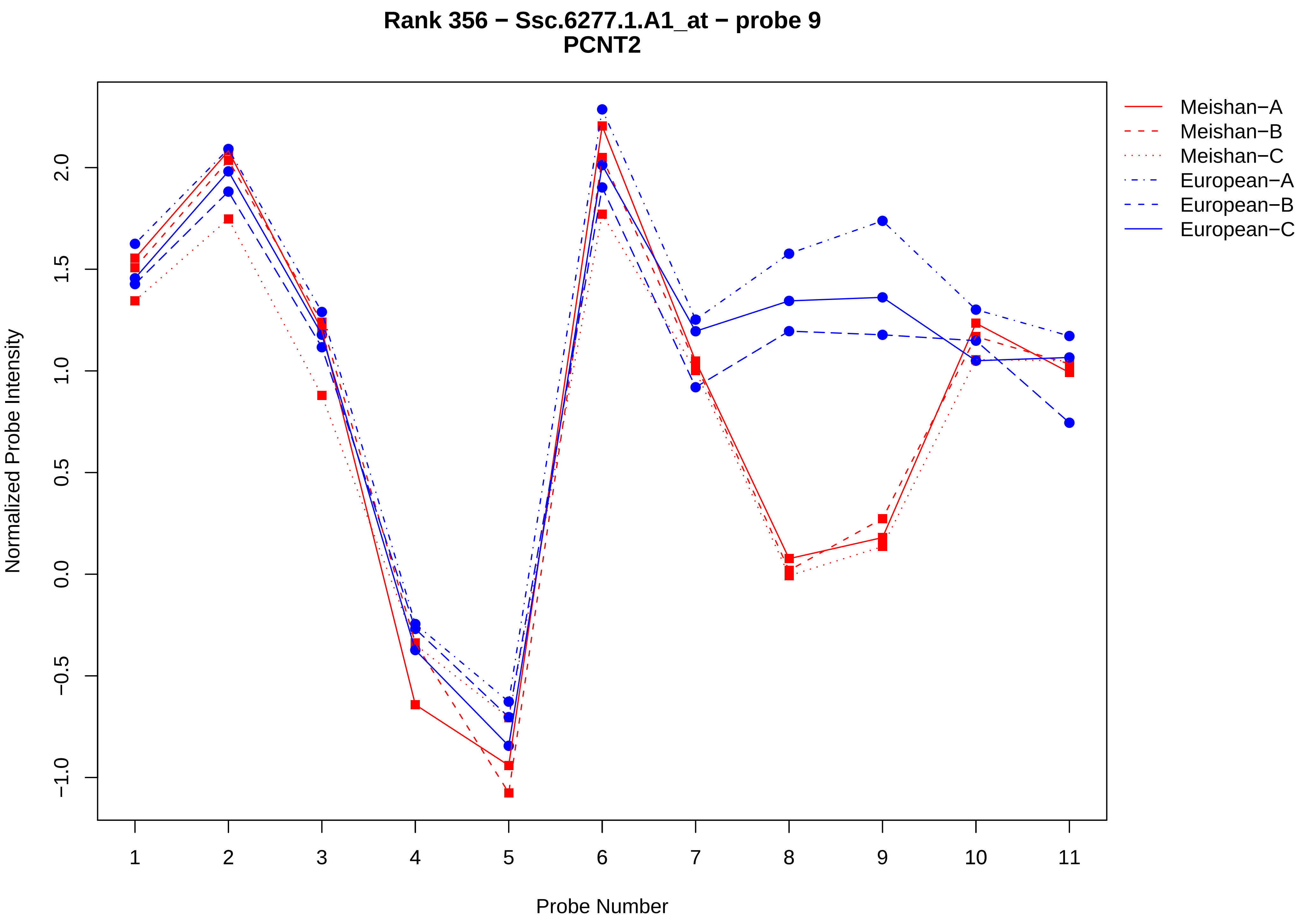

Rank 356 − Ssc.6277.1.A1_at − probe 9
PCNT2
Normalized Probe Intensity
Probe Number
−1.0
−0.5
0.0
0.5
1.0
1.5
2.0
1
2
3
4
5
6
7
8
9
10
11
Meishan−A
Meishan−B
Meishan−C
European−A
European−B
European−C

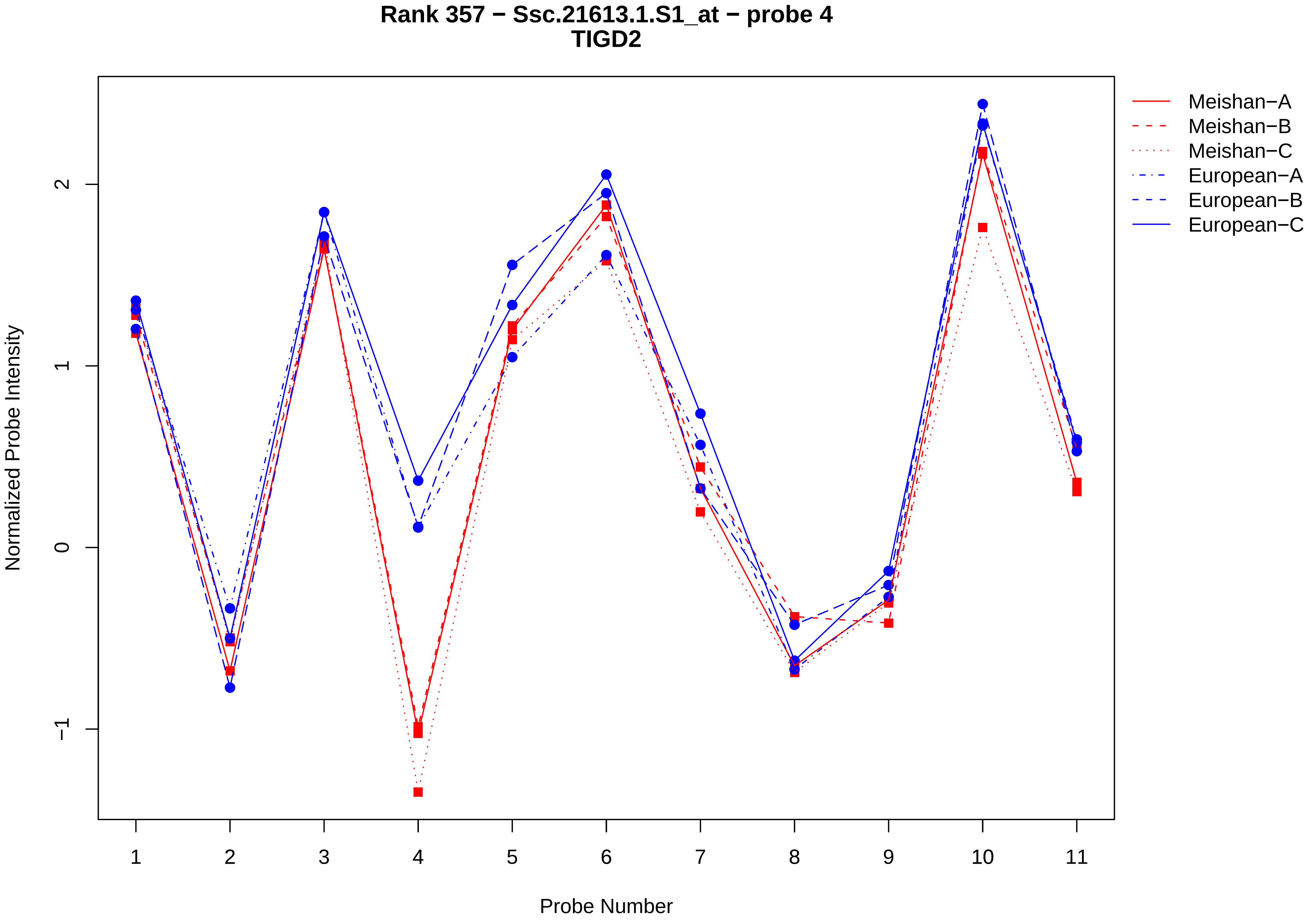

Rank 357 − Ssc.21613.1.S1_at − probe 4
TIGD2
Normalized Probe Intensity
Probe Number
−1
0
1
2
1
2
3
4
5
6
7
8
9
10
11
Meishan−A
Meishan−B
Meishan−C
European−A
European−B
European−C

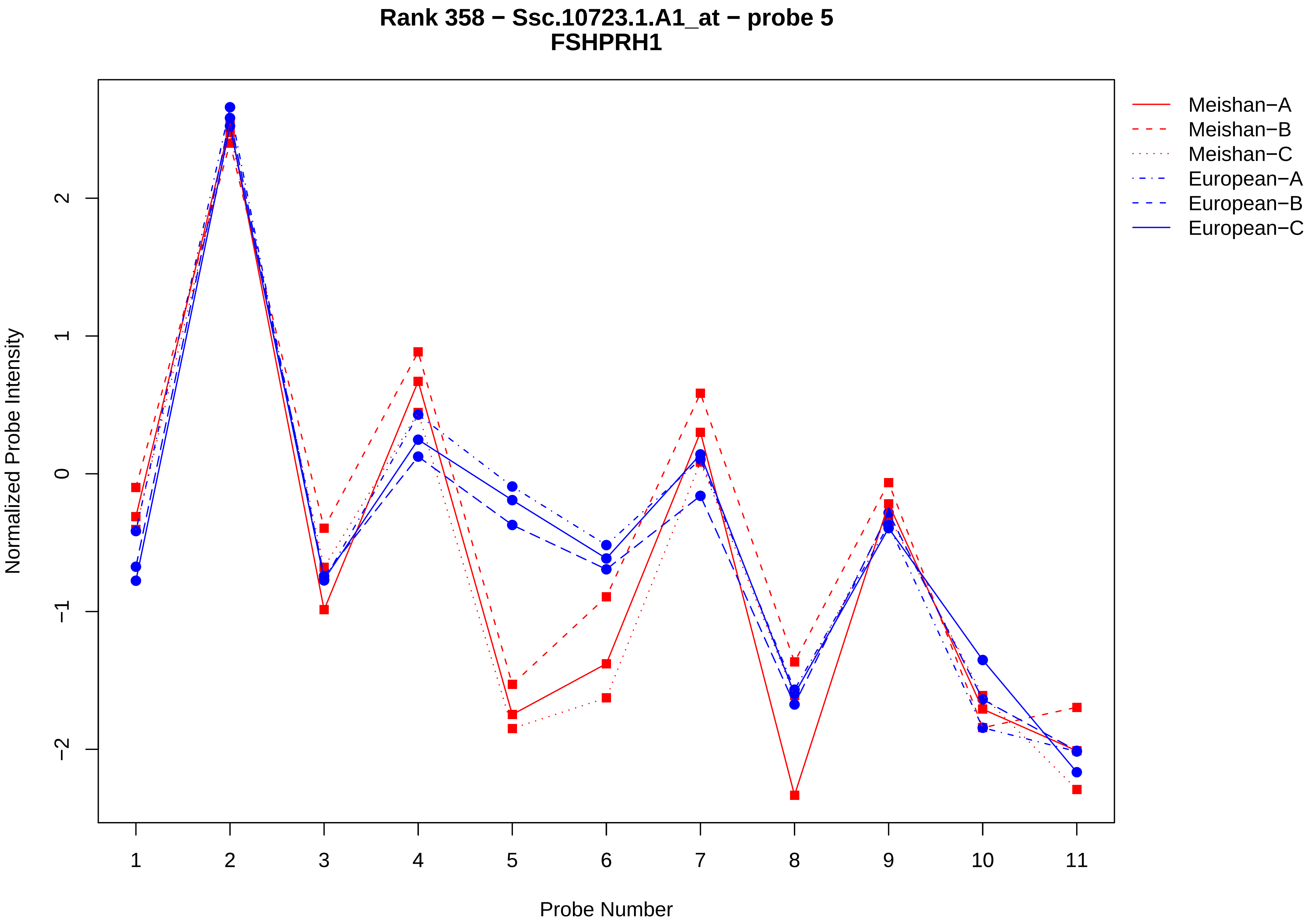

Rank 358 − Ssc.10723.1.A1_at − probe 5
FSHPRH1
Normalized Probe Intensity
Probe Number
−2
−1
0
1
2
1
2
3
4
5
6
7
8
9
10
11
Meishan−A
Meishan−B
Meishan−C
European−A
European−B
European−C

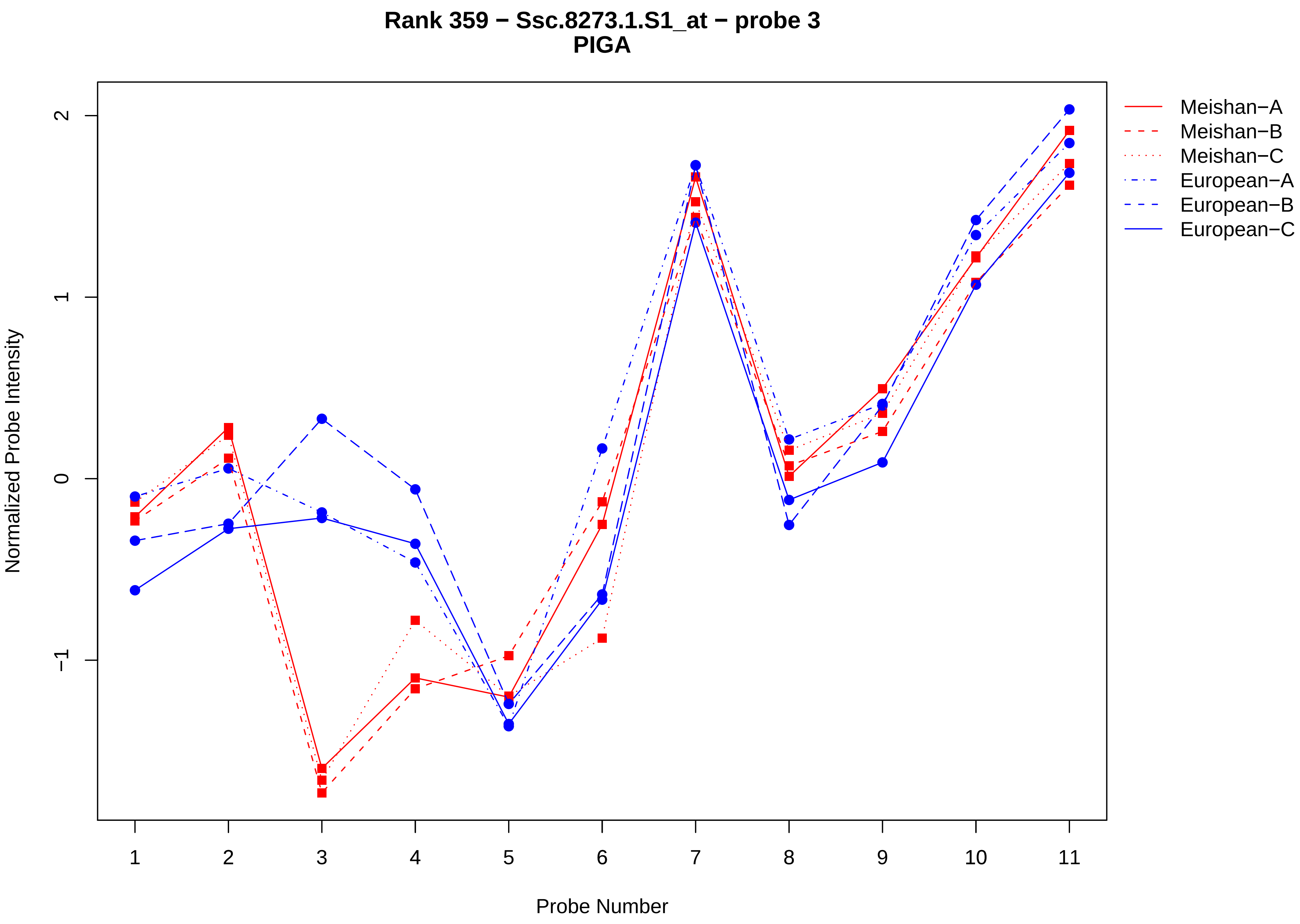

Rank 359 − Ssc.8273.1.S1_at − probe 3
PIGA
Normalized Probe Intensity
Probe Number
−1
0
1
2
1
2
3
4
5
6
7
8
9
10
11
Meishan−A
Meishan−B
Meishan−C
European−A
European−B
European−C

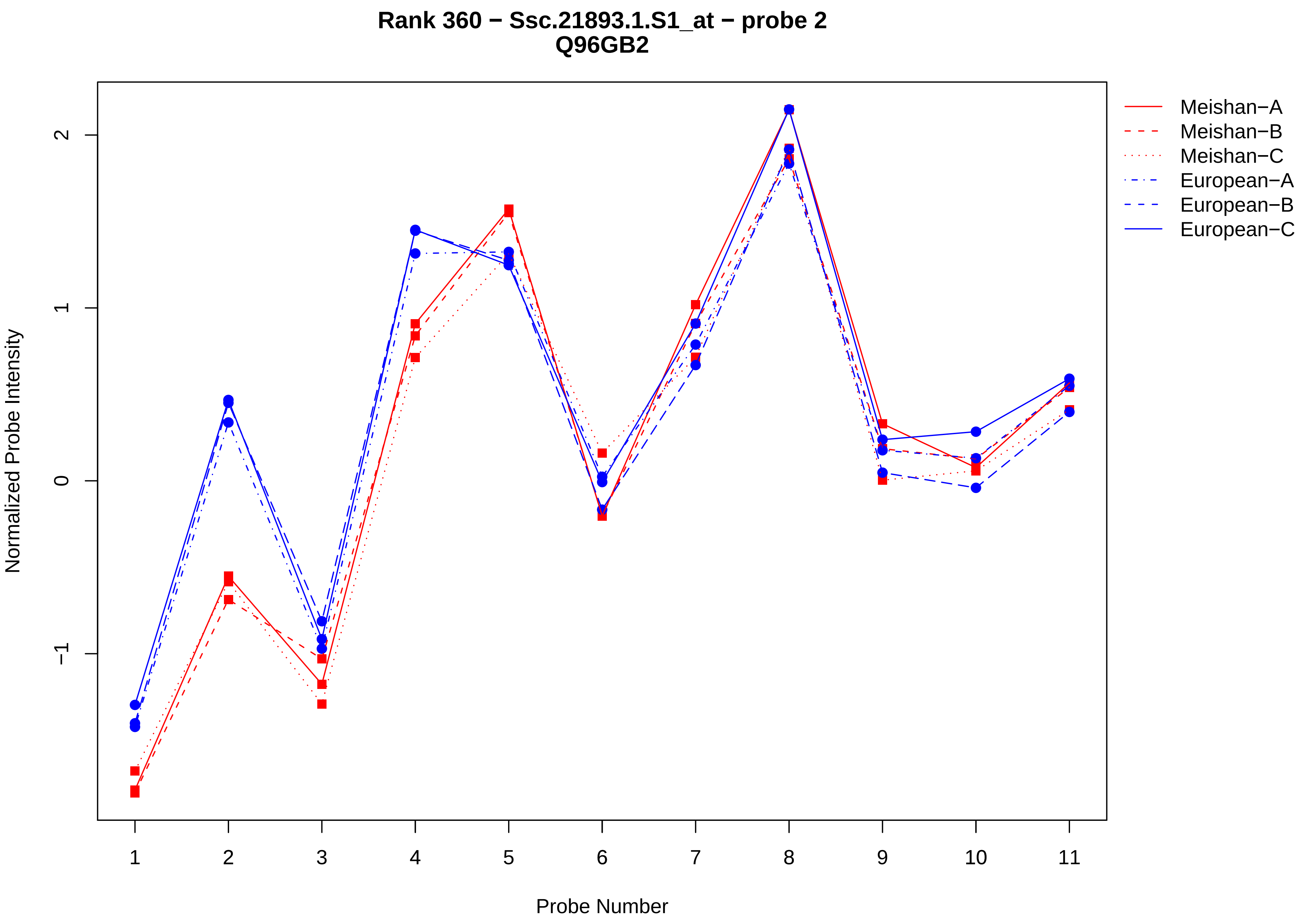

Rank 360 − Ssc.21893.1.S1_at − probe 2
Q96GB2
Normalized Probe Intensity
Probe Number
Meishan−A
Meishan−B
Meishan−C
European−A
European−B
European−C
−1
0
1
2
1
2
3
4
5
6
7
8
9
10
11

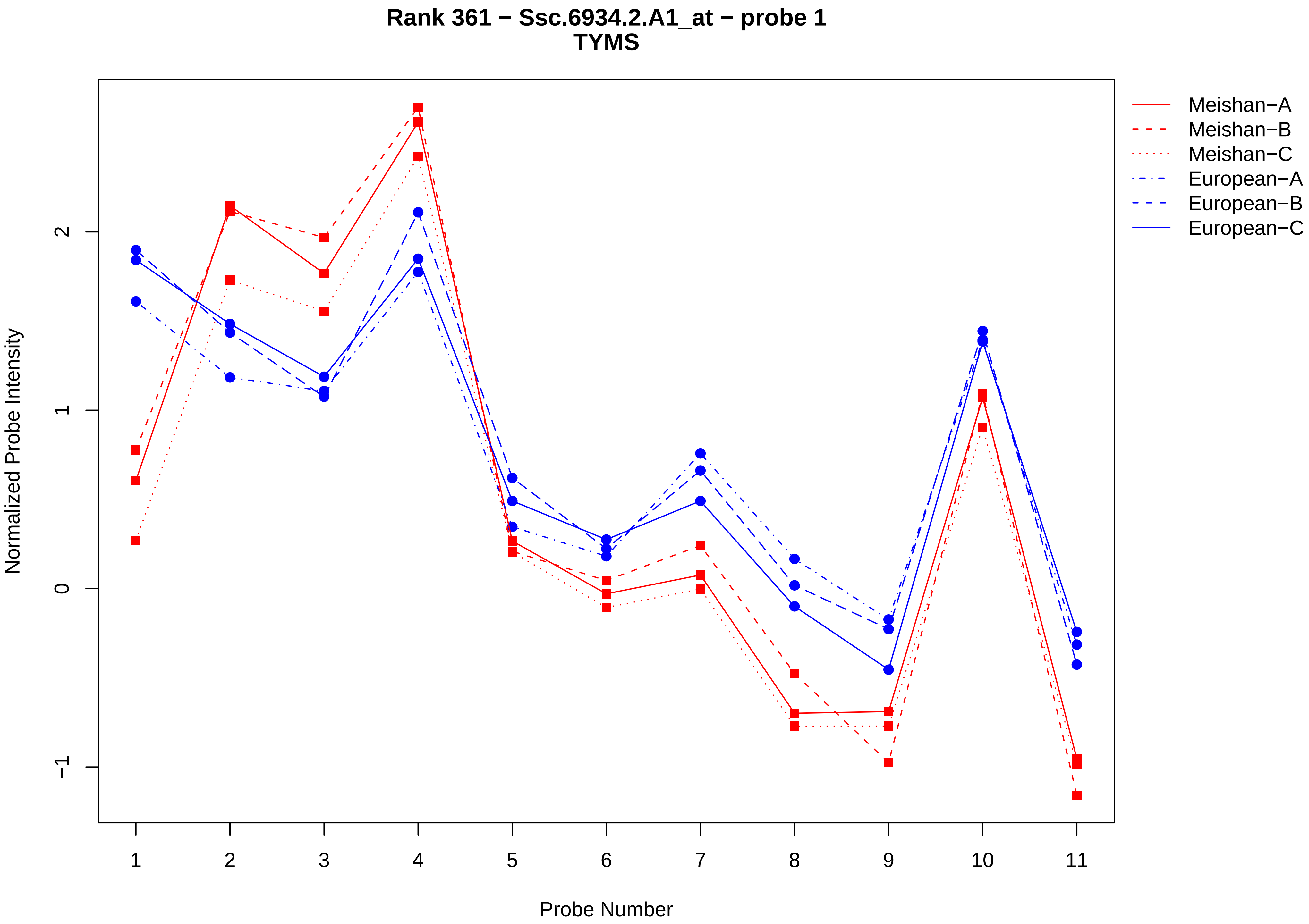

Rank 361 − Ssc.6934.2.A1_at − probe 1
TYMS
Normalized Probe Intensity
Probe Number
−1
0
1
2
1
2
3
4
5
6
7
8
9
10
11
Meishan−A
Meishan−B
Meishan−C
European−A
European−B
European−C

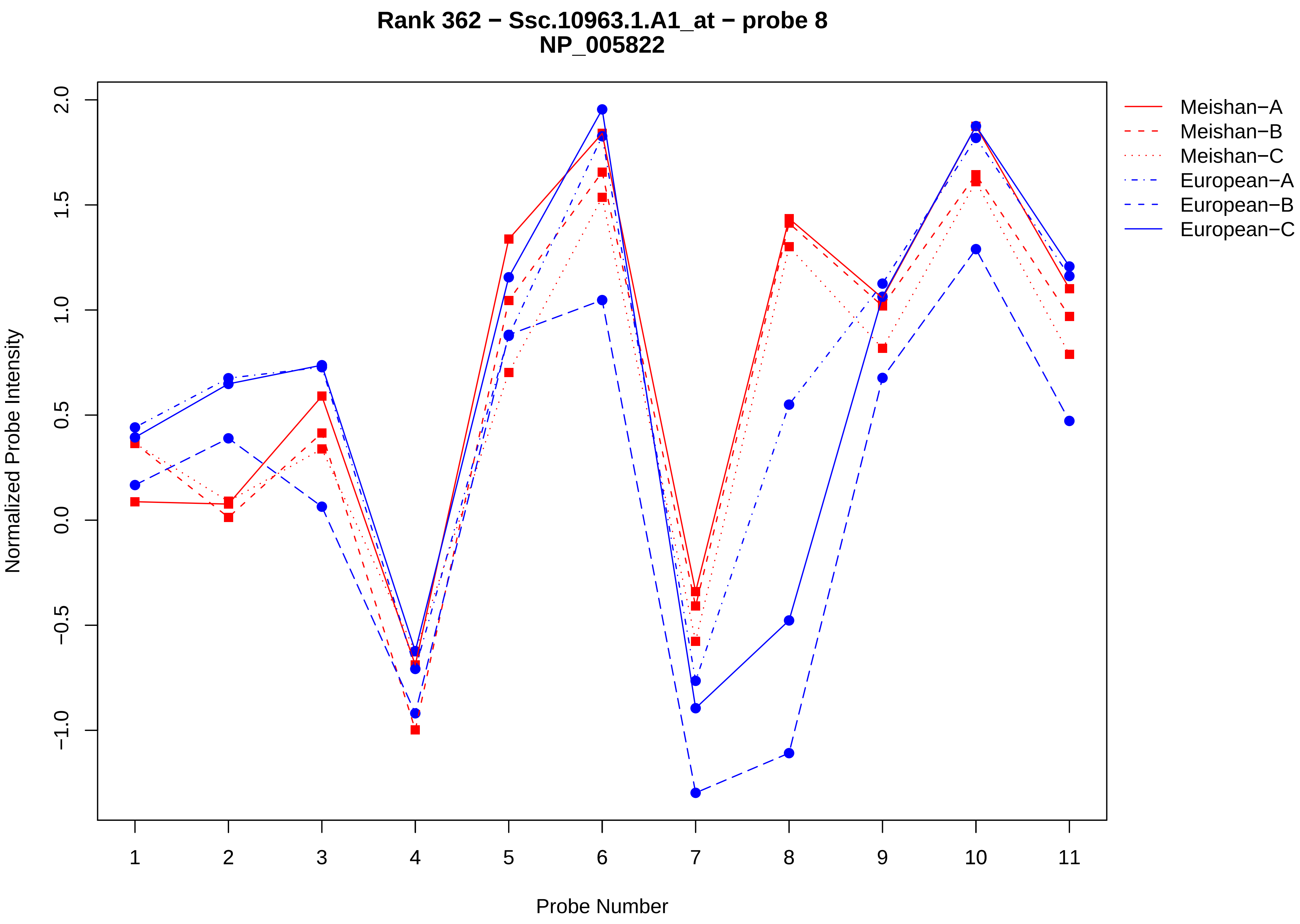

Rank 362 − Ssc.10963.1.A1_at − probe 8
NP_005822
Normalized Probe Intensity
Probe Number
−1.0
−0.5
0.0
0.5
1.0
1.5
2.0
1
2
3
4
5
6
7
8
9
10
11
Meishan−A
Meishan−B
Meishan−C
European−A
European−B
European−C

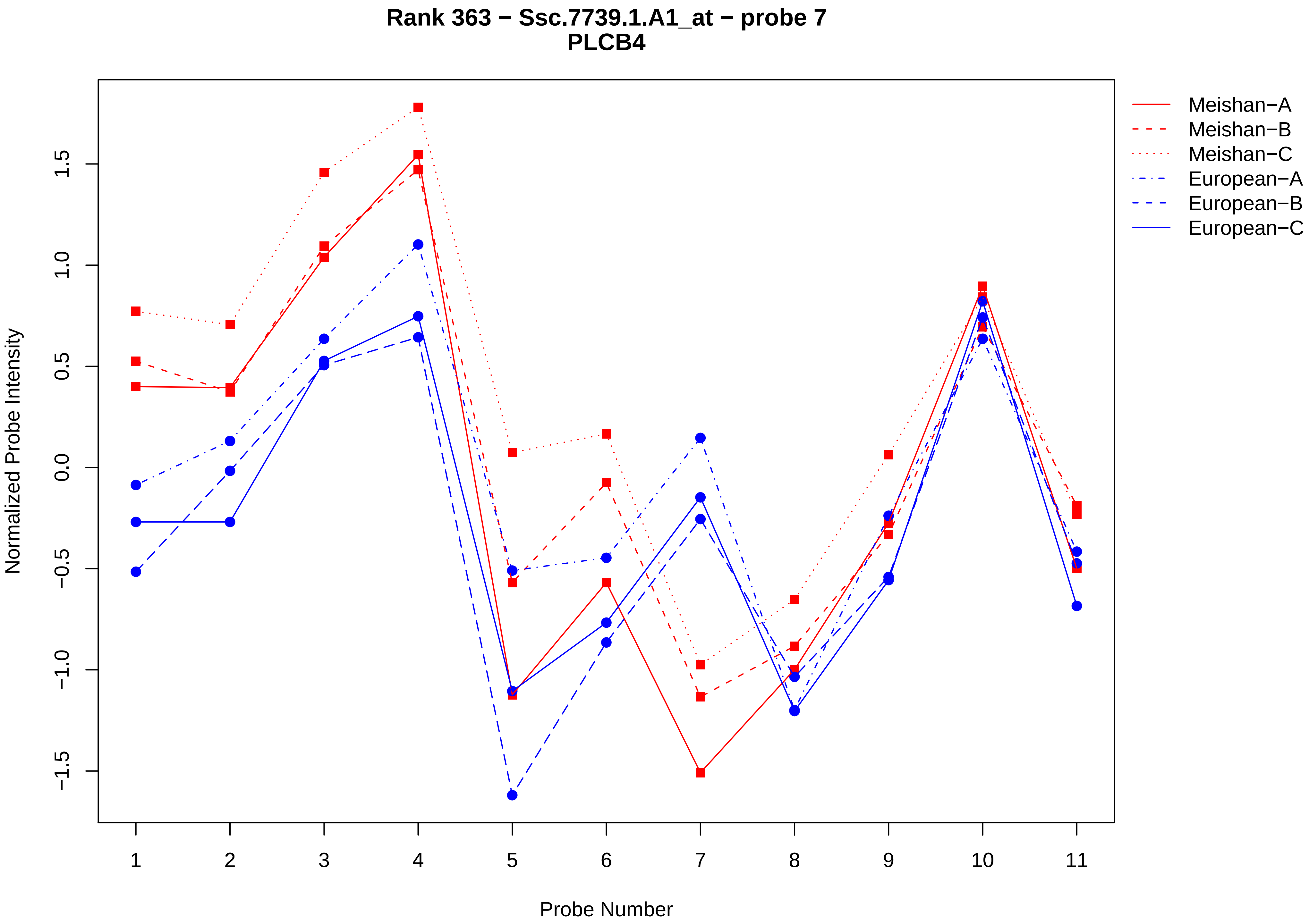

Rank 363 − Ssc.7739.1.A1_at − probe 7
PLCB4
Normalized Probe Intensity
Probe Number
−1.5
−1.0
−0.5
0.0
0.5
1.0
1.5
1
2
3
4
5
6
7
8
9
10
11
Meishan−A
Meishan−B
Meishan−C
European−A
European−B
European−C

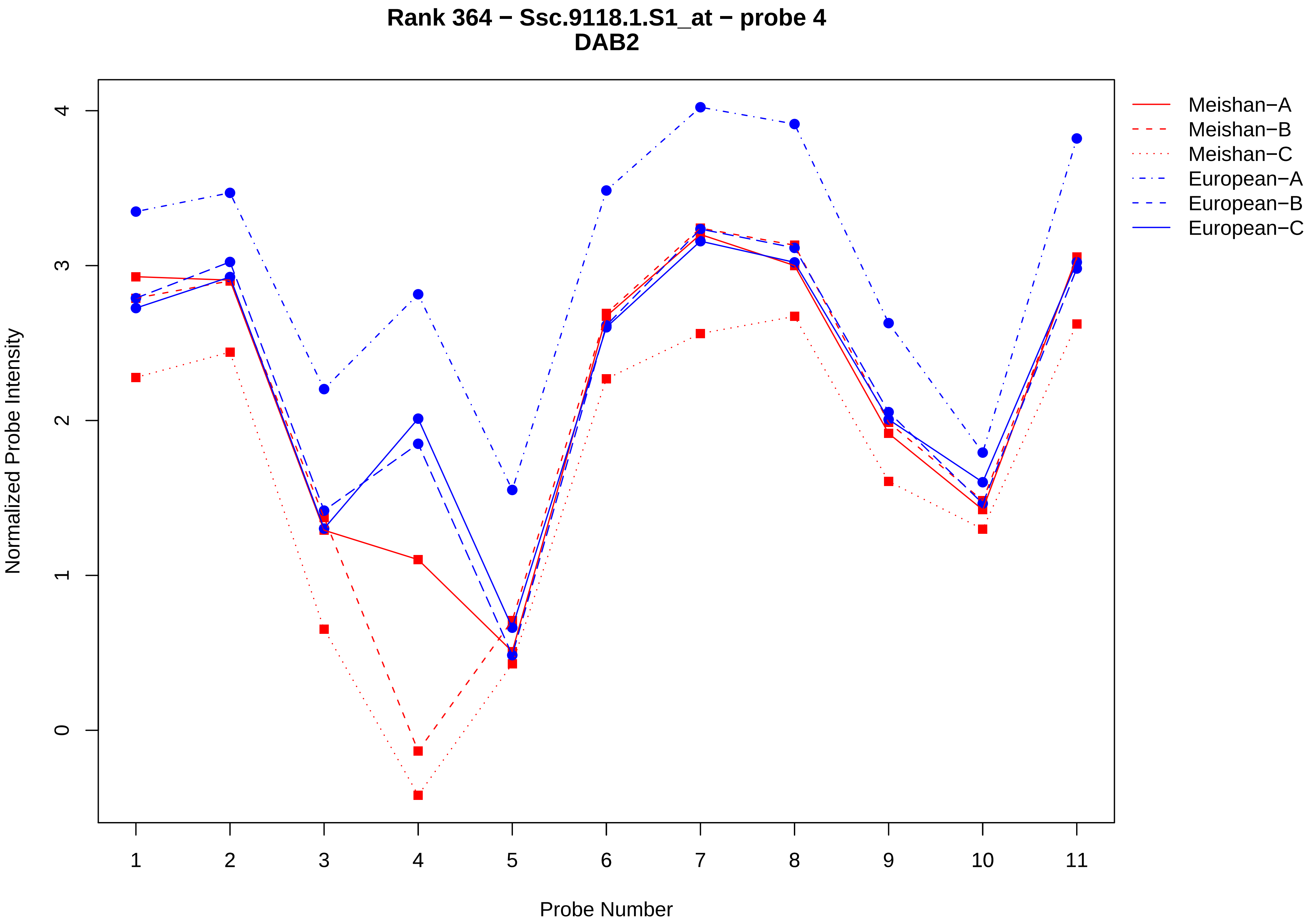
Rank 364 − Ssc.9118.1.S1_at − probe 4
DAB2
Normalized Probe Intensity
Probe Number
Meishan−A
Meishan−B
Meishan−C
European−A
European−B
European−C
0
1
2
3
4
1
2
3
4
5
6
7
8
9
10
11

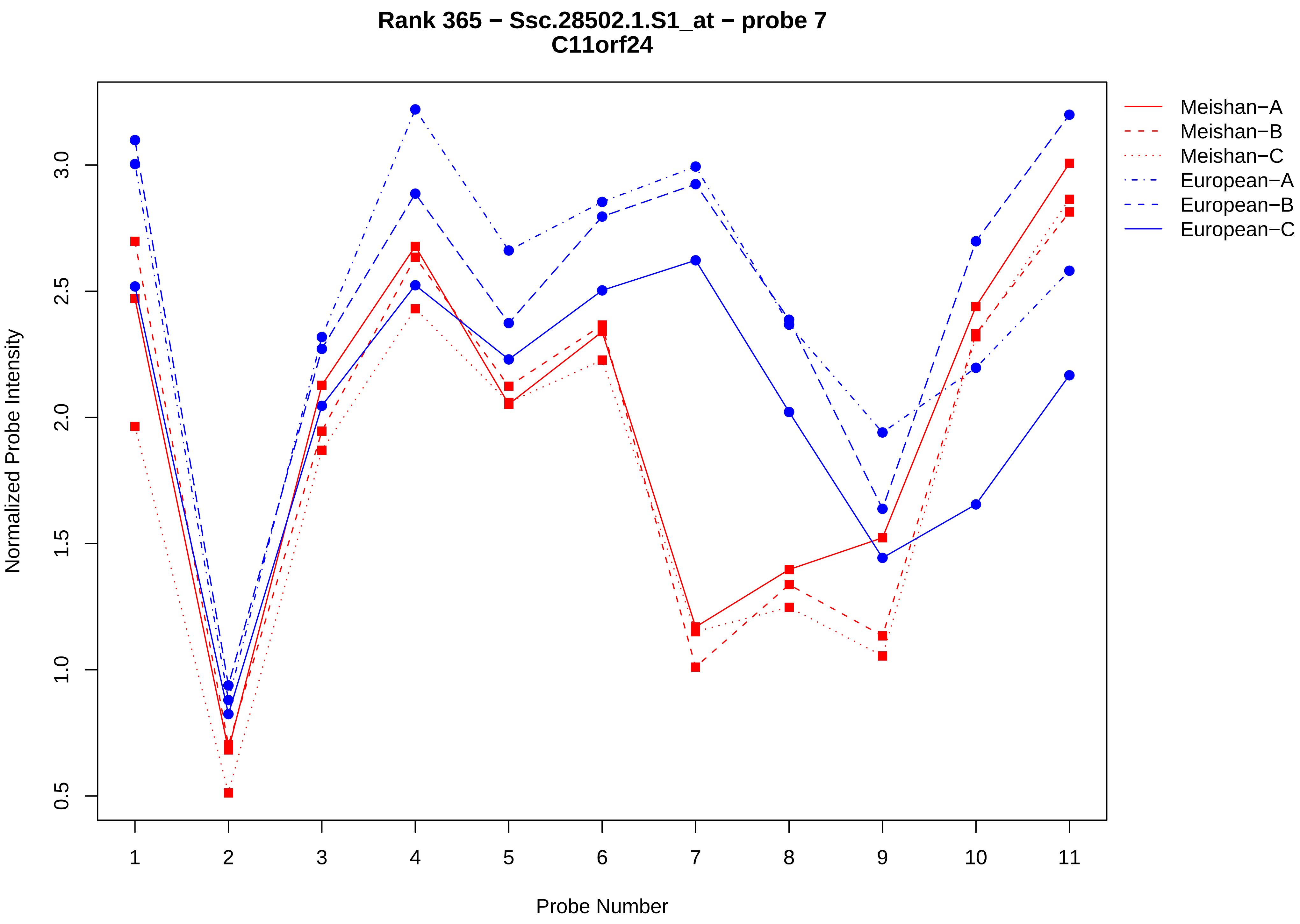
Rank 365 − Ssc.28502.1.S1_at − probe 7
C11orf24
Normalized Probe Intensity
0.5
1.0
1.5
2.0
2.5
3.0
Probe Number
1
2
3
4
5
6
7
8
9
10
11
Meishan−A
Meishan−B
Meishan−C
European−A
European−B
European−C

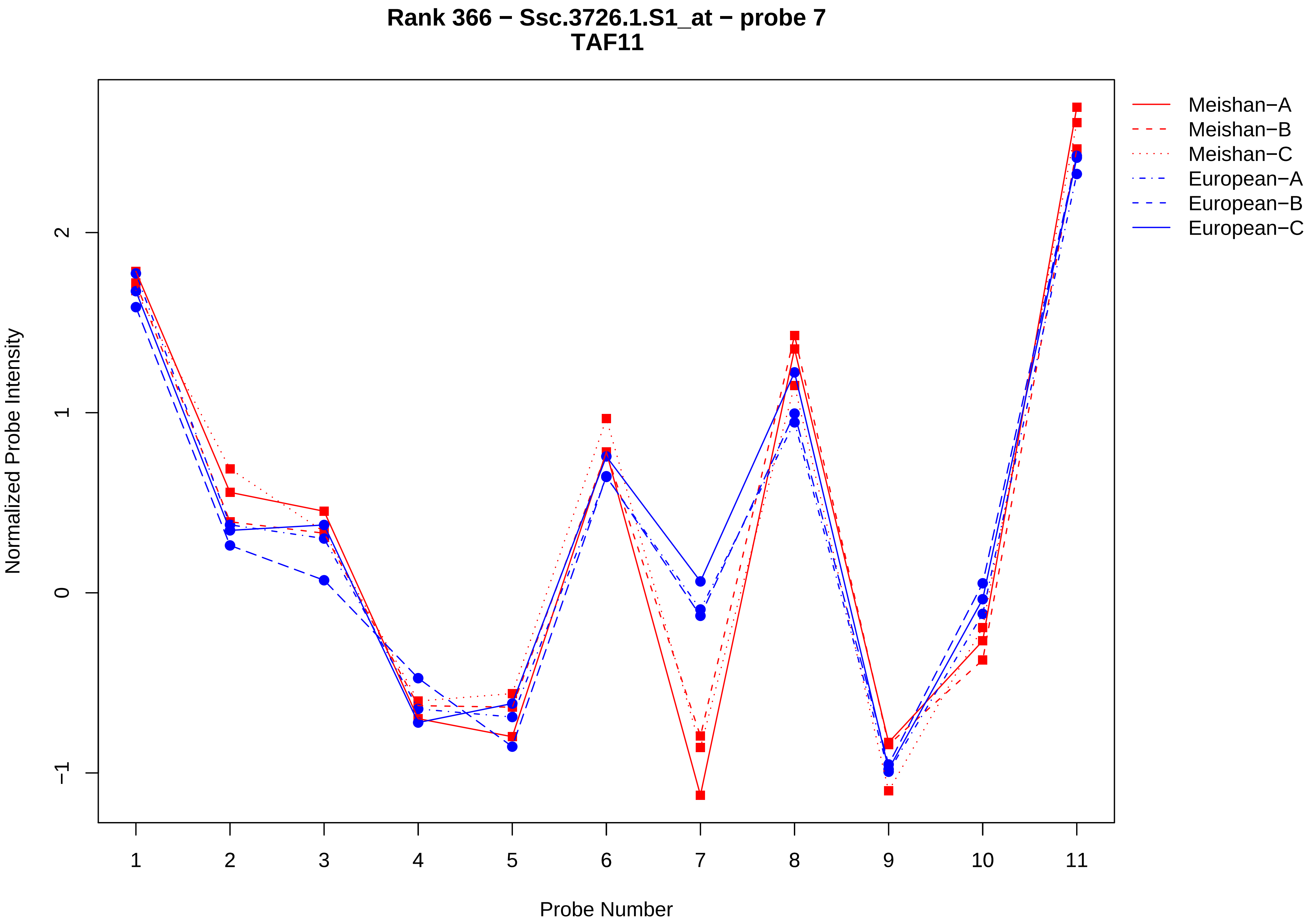

Rank 366 − Ssc.3726.1.S1_at − probe 7
TAF11
Normalized Probe Intensity
Probe Number
−1
0
1
2
1
2
3
4
5
6
7
8
9
10
11
Meishan−A
Meishan−B
Meishan−C
European−A
European−B
European−C

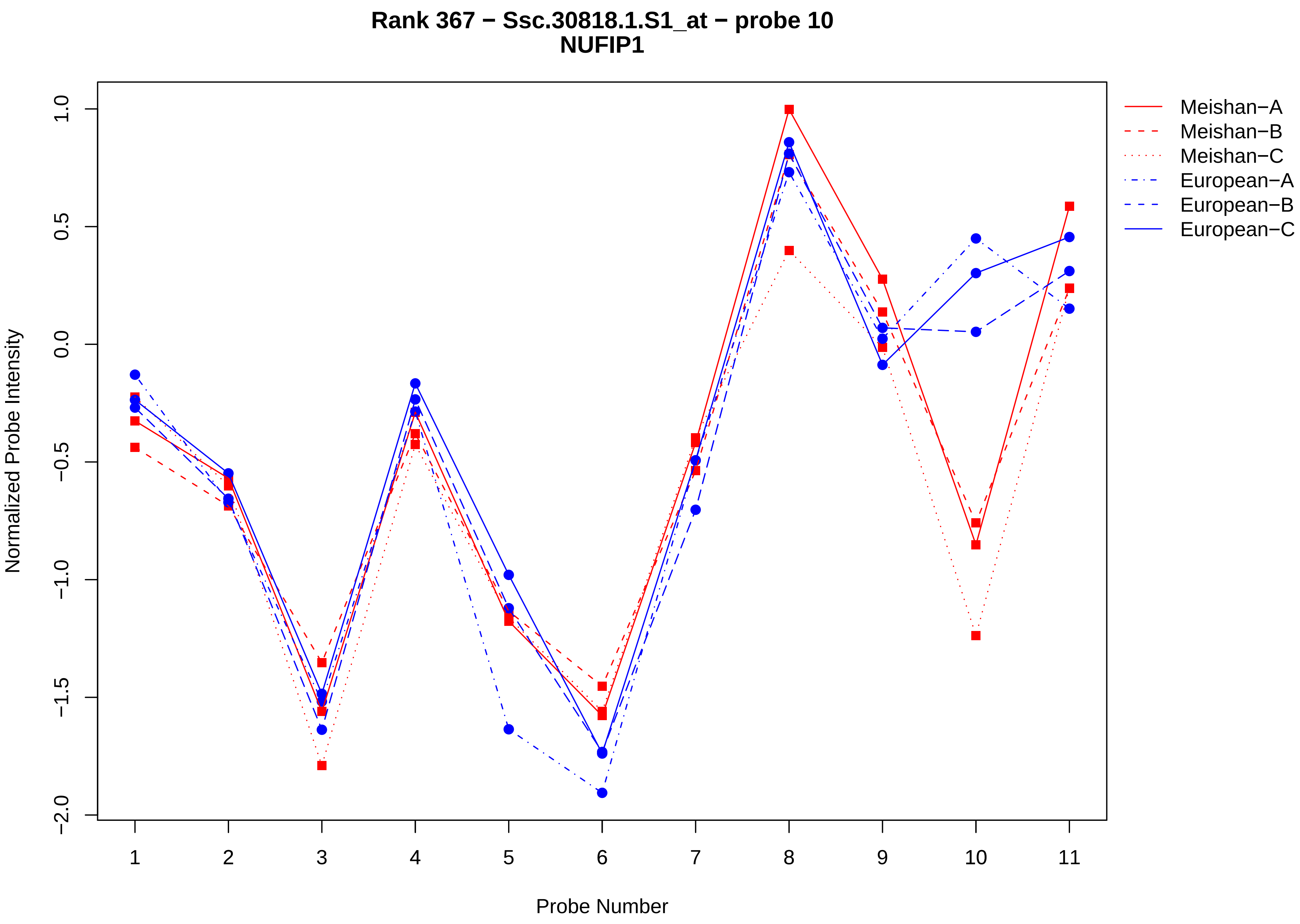

Rank 367 − Ssc.30818.1.S1_at − probe 10
NUFIP1
Normalized Probe Intensity
Probe Number
1.0
0.5
0.0
−0.5
−1.0
−1.5
−2.0
1
2
3
4
5
6
7
8
9
10
11
Meishan−A
Meishan−B
Meishan−C
European−A
European−B
European−C

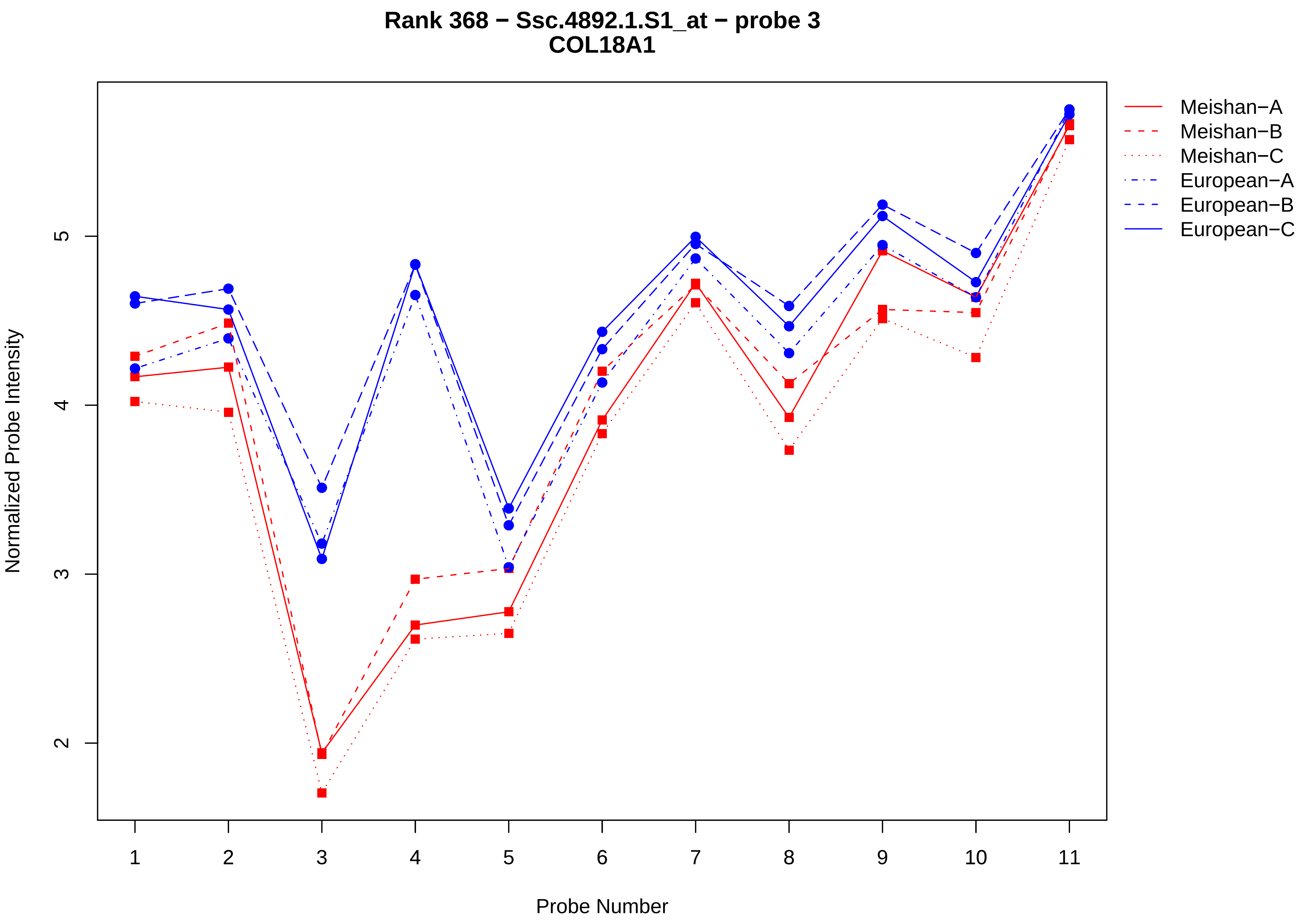
Rank 368 − Ssc.4892.1.S1_at − probe 3
COL18A1
Normalized Probe Intensity
Probe Number
2
3
4
5
1
2
3
4
5
6
7
8
9
10
11
Meishan−A
Meishan−B
Meishan−C
European−A
European−B
European−C

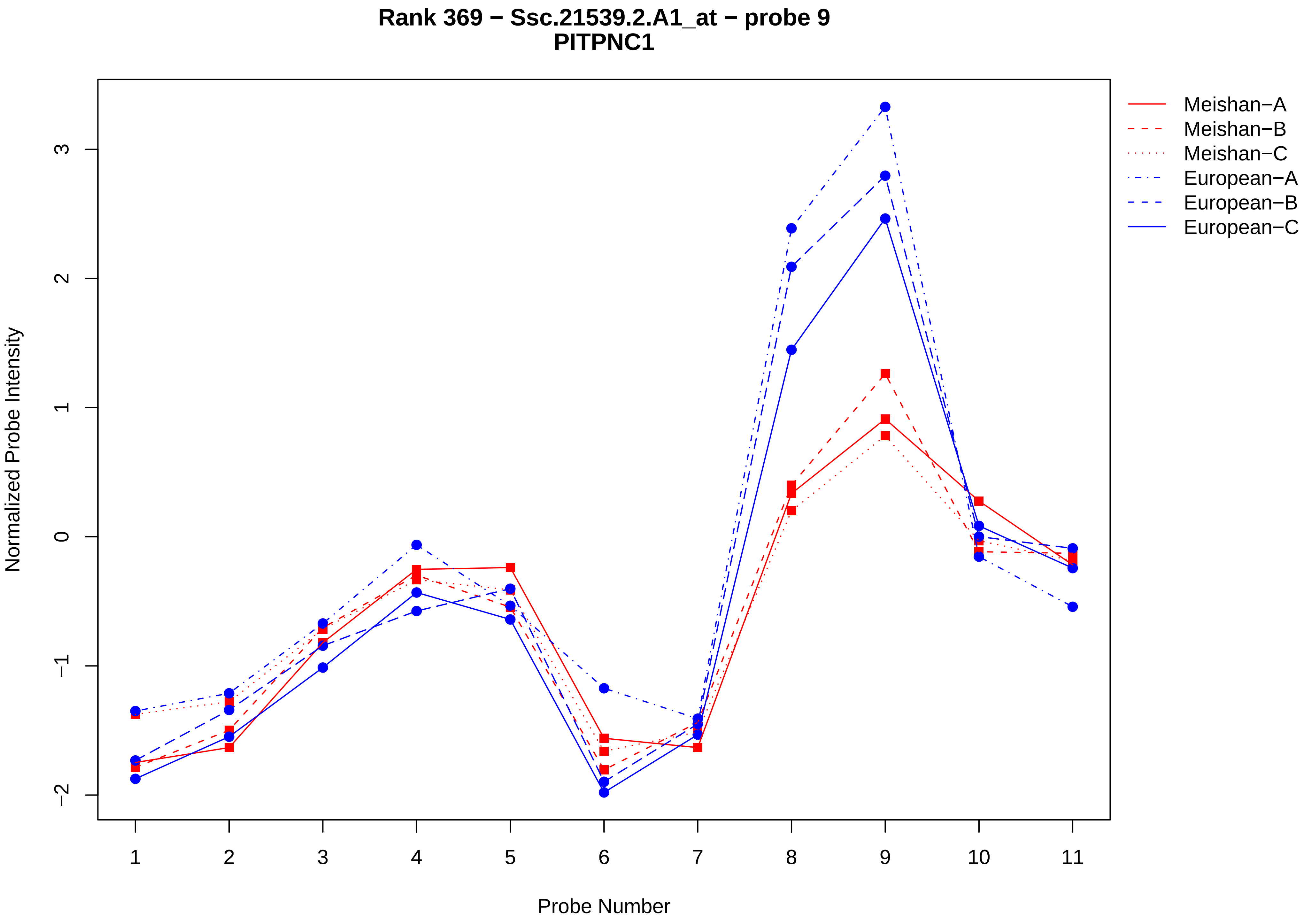
Rank 369 − Ssc.21539.2.A1_at − probe 9
PITPNC1
Normalized Probe Intensity
Probe Number
Meishan−A
Meishan−B
Meishan−C
European−A
European−B
European−C
−2
−1
0
1
2
3
1
2
3
4
5
6
7
8
9
10
11

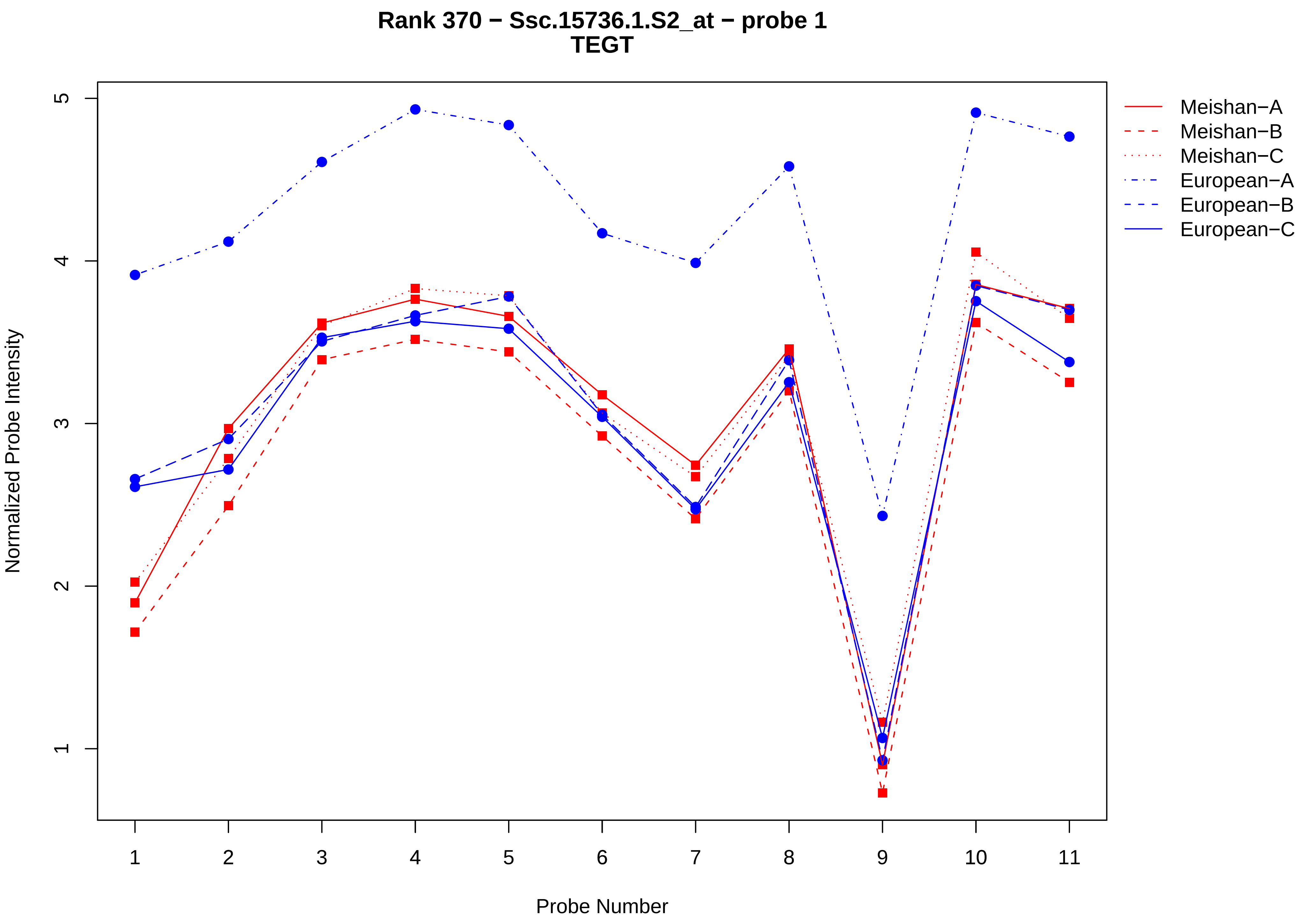
Rank 370 − Ssc.15736.1.S2_at − probe 1
TEGT
Normalized Probe Intensity
Probe Number
1
2
3
4
5
1
2
3
4
5
6
7
8
9
10
11
Meishan−A
Meishan−B
Meishan−C
European−A
European−B
European−C

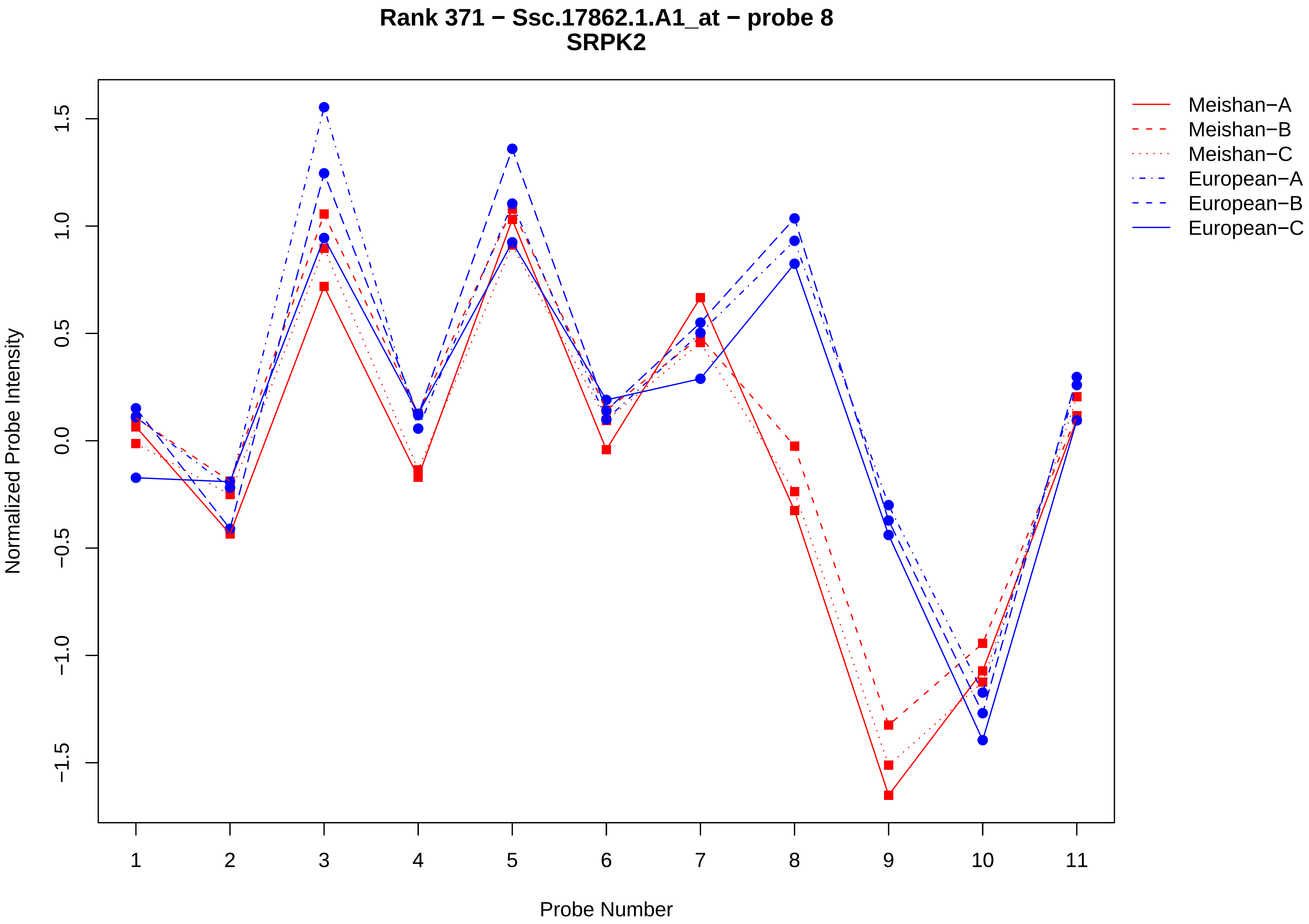
Rank 371 − Ssc.17862.1.A1_at − probe 8
SRPK2
Normalized Probe Intensity
Probe Number
Meishan−A
Meishan−B
Meishan−C
European−A
European−B
European−C

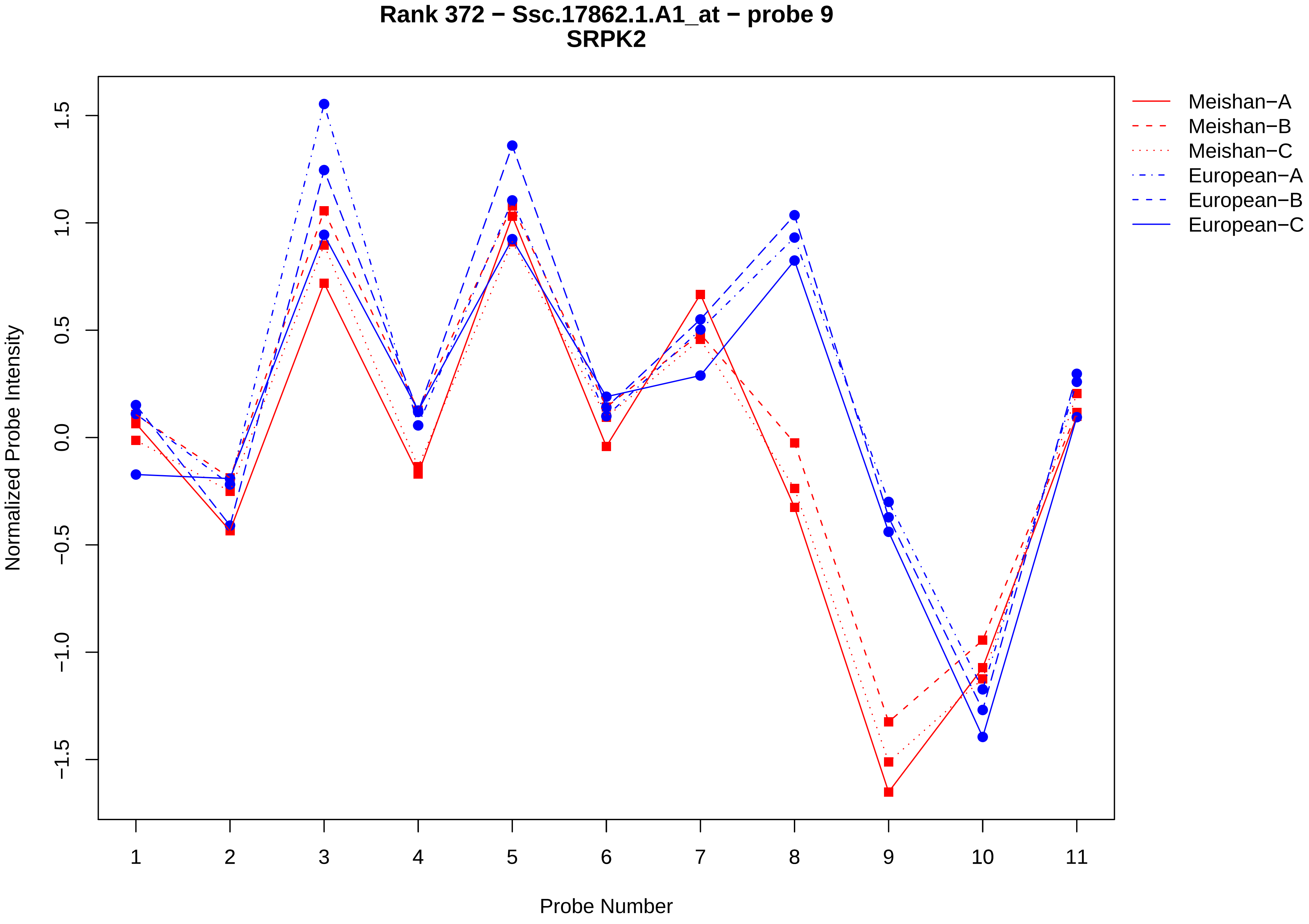

Rank 372 − Ssc.17862.1.A1_at − probe 9
SRPK2
Normalized Probe Intensity
Probe Number
1.5
1.0
0.5
0.0
−0.5
−1.0
−1.5
1
2
3
4
5
6
7
8
9
10
11
Meishan−A
Meishan−B
Meishan−C
European−A
European−B
European−C

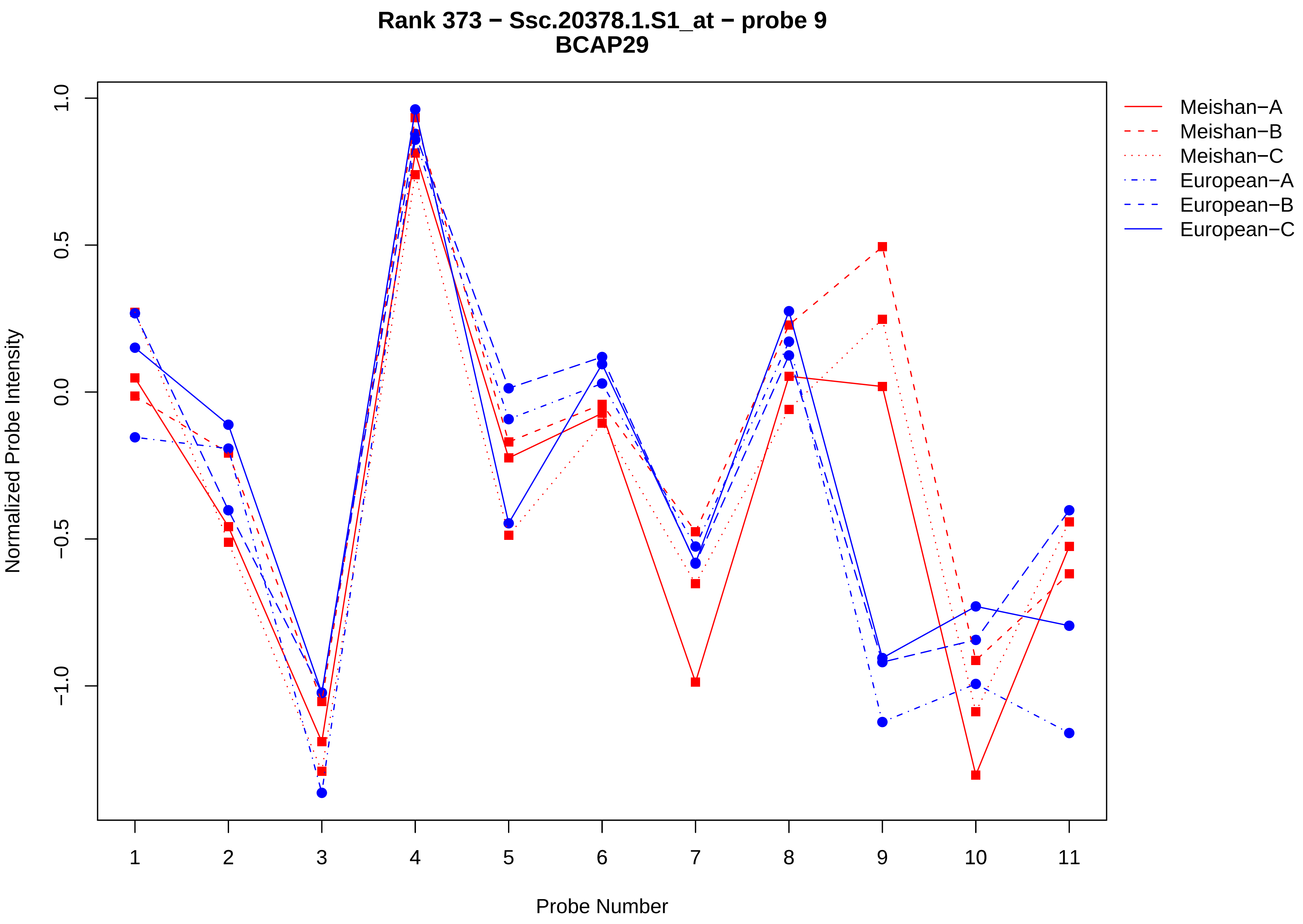

Rank 373 − Ssc.20378.1.S1_at − probe 9
BCAP29
Normalized Probe Intensity
Probe Number
1.0
0.5
0.0
−0.5
−1.0
1
2
3
4
5
6
7
8
9
10
11
Meishan−A
Meishan−B
Meishan−C
European−A
European−B
European−C

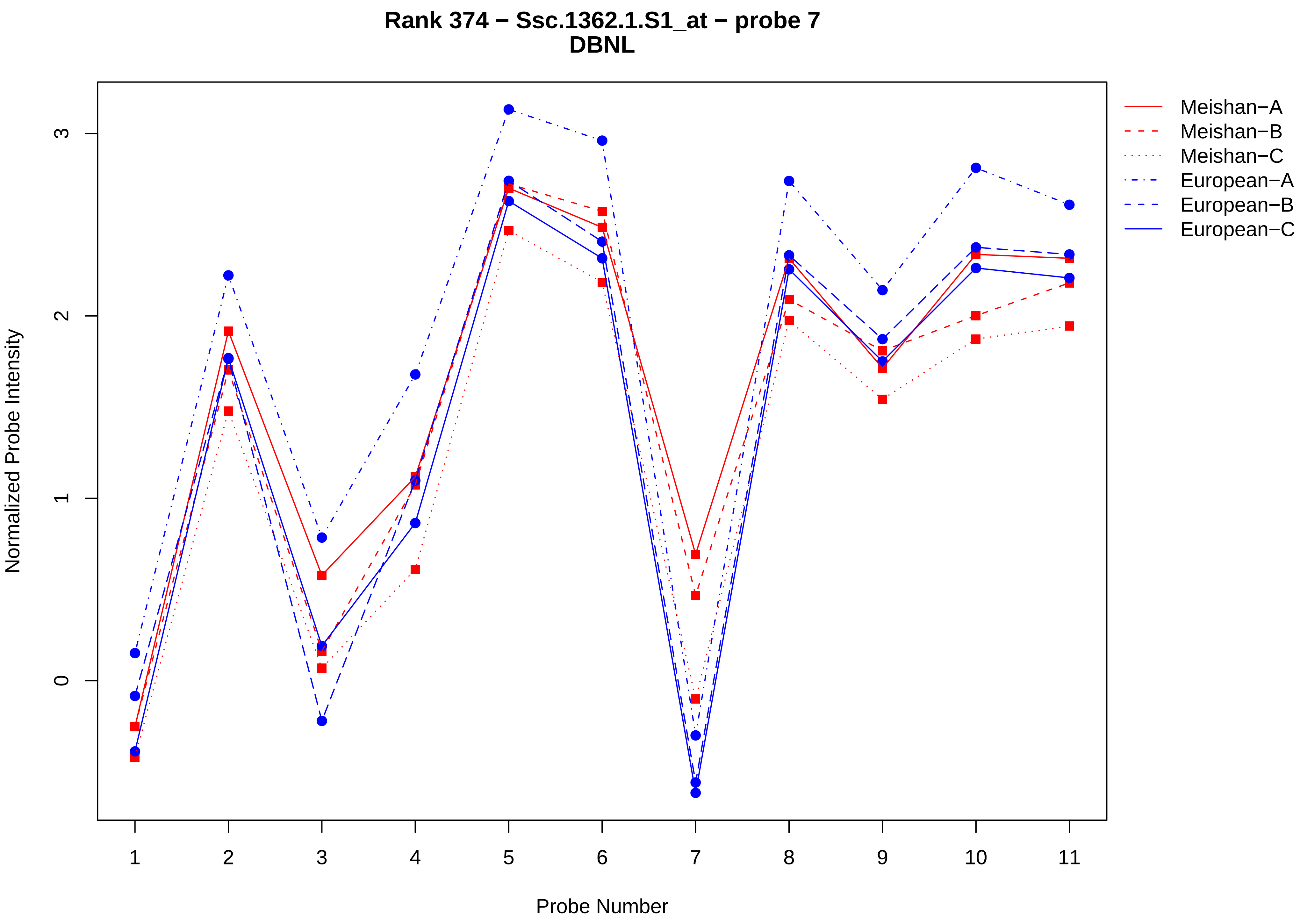
Rank 374 − Ssc.1362.1.S1_at − probe 7
DBNL
Normalized Probe Intensity
Probe Number
0
1
2
3
1
2
3
4
5
6
7
8
9
10
11
Meishan−A
Meishan−B
Meishan−C
European−A
European−B
European−C

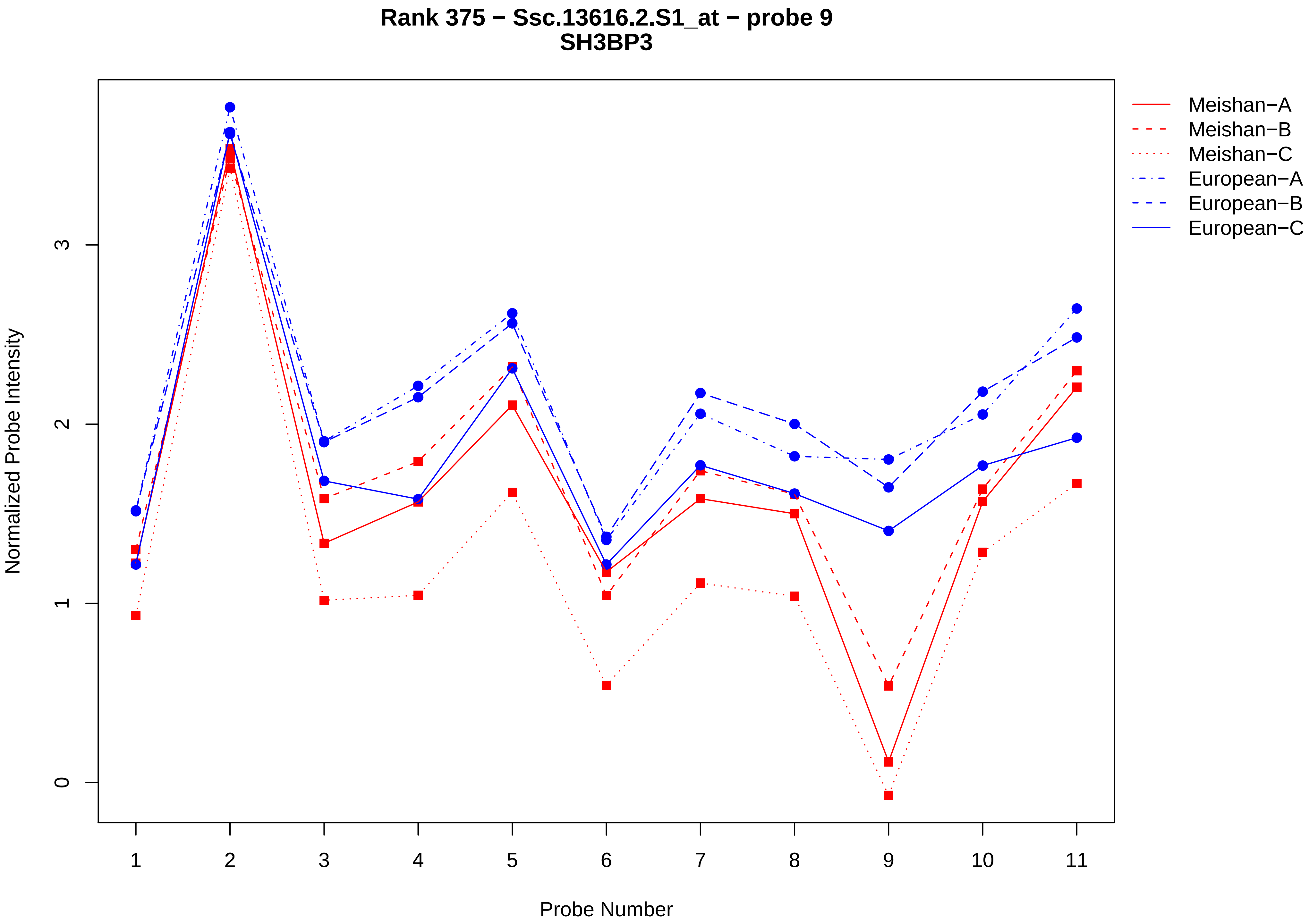

Rank 375 − Ssc.13616.2.S1_at − probe 9
SH3BP3
Normalized Probe Intensity
Probe Number
0
1
2
3
1
2
3
4
5
6
7
8
9
10
11
Meishan−A
Meishan−B
Meishan−C
European−A
European−B
European−C

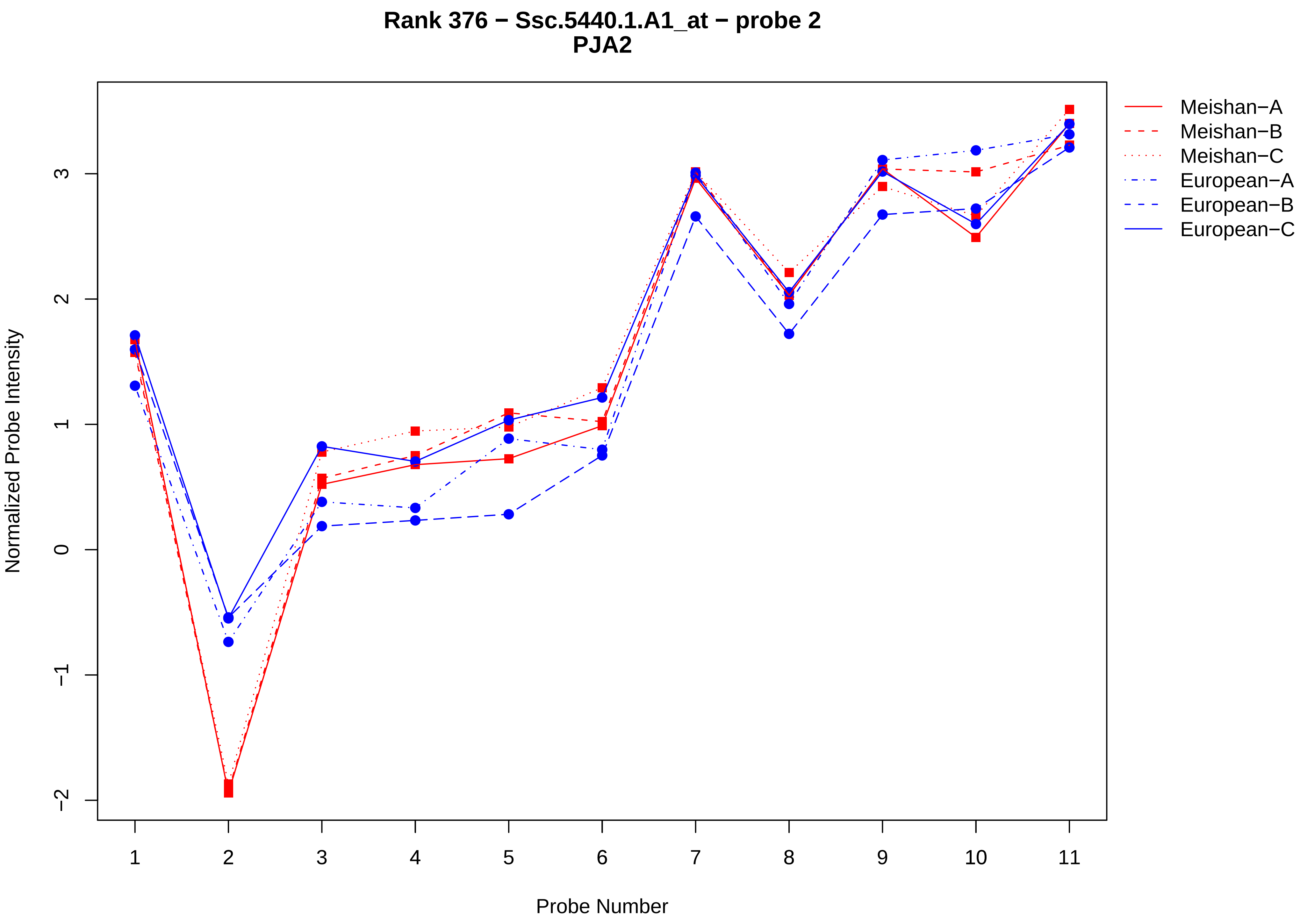
Rank 376 − Ssc.5440.1.A1_at − probe 2
PJA2
Normalized Probe Intensity
Probe Number
−2
−1
0
1
2
3
1
2
3
4
5
6
7
8
9
10
11
Meishan−A
Meishan−B
Meishan−C
European−A
European−B
European−C

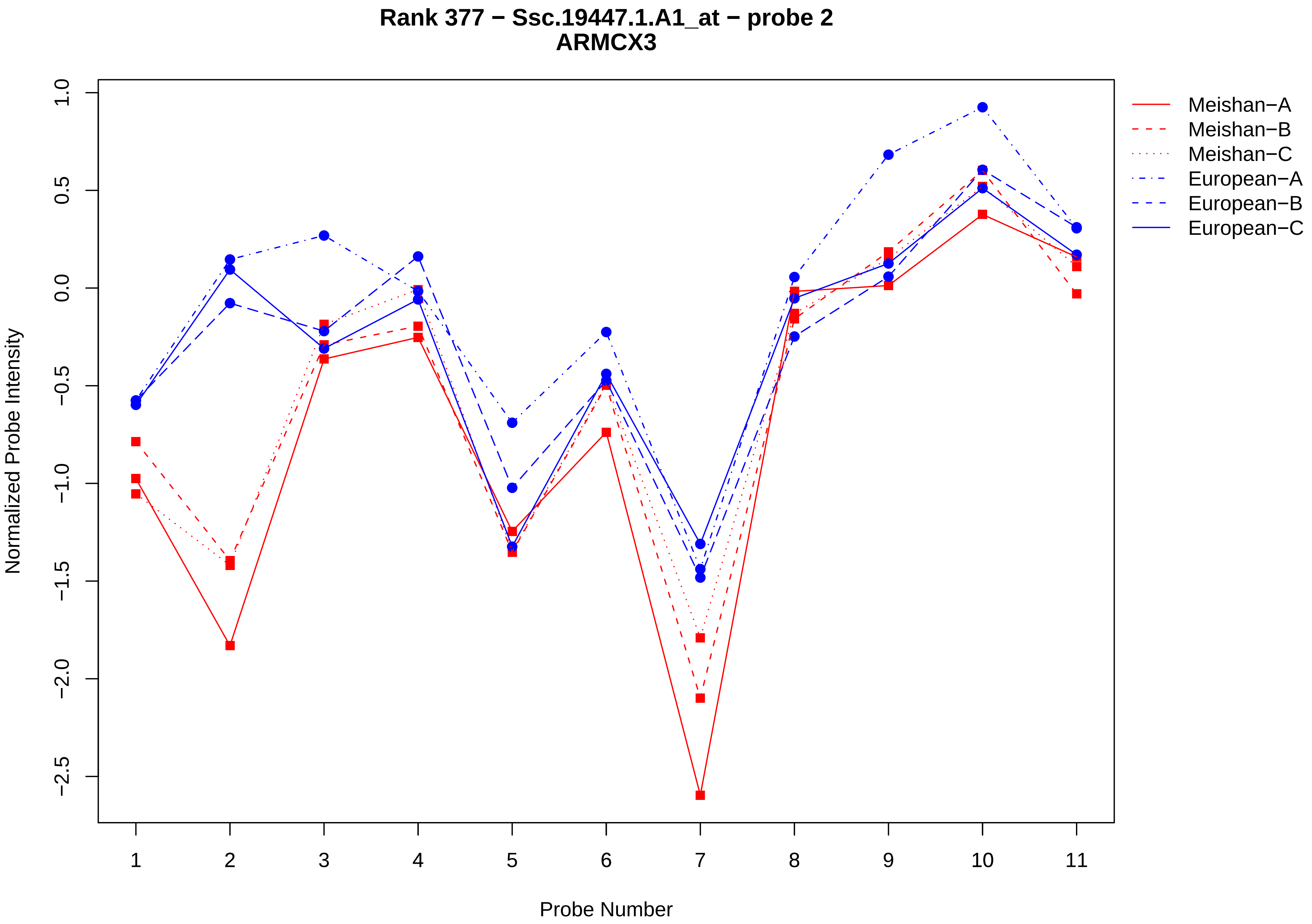

Rank 377 − Ssc.19447.1.A1_at − probe 2
ARMCX3
Normalized Probe Intensity
Probe Number
1.0
0.5
0.0
−0.5
−1.0
−1.5
−2.0
−2.5
1
2
3
4
5
6
7
8
9
10
11
Meishan−A
Meishan−B
Meishan−C
European−A
European−B
European−C

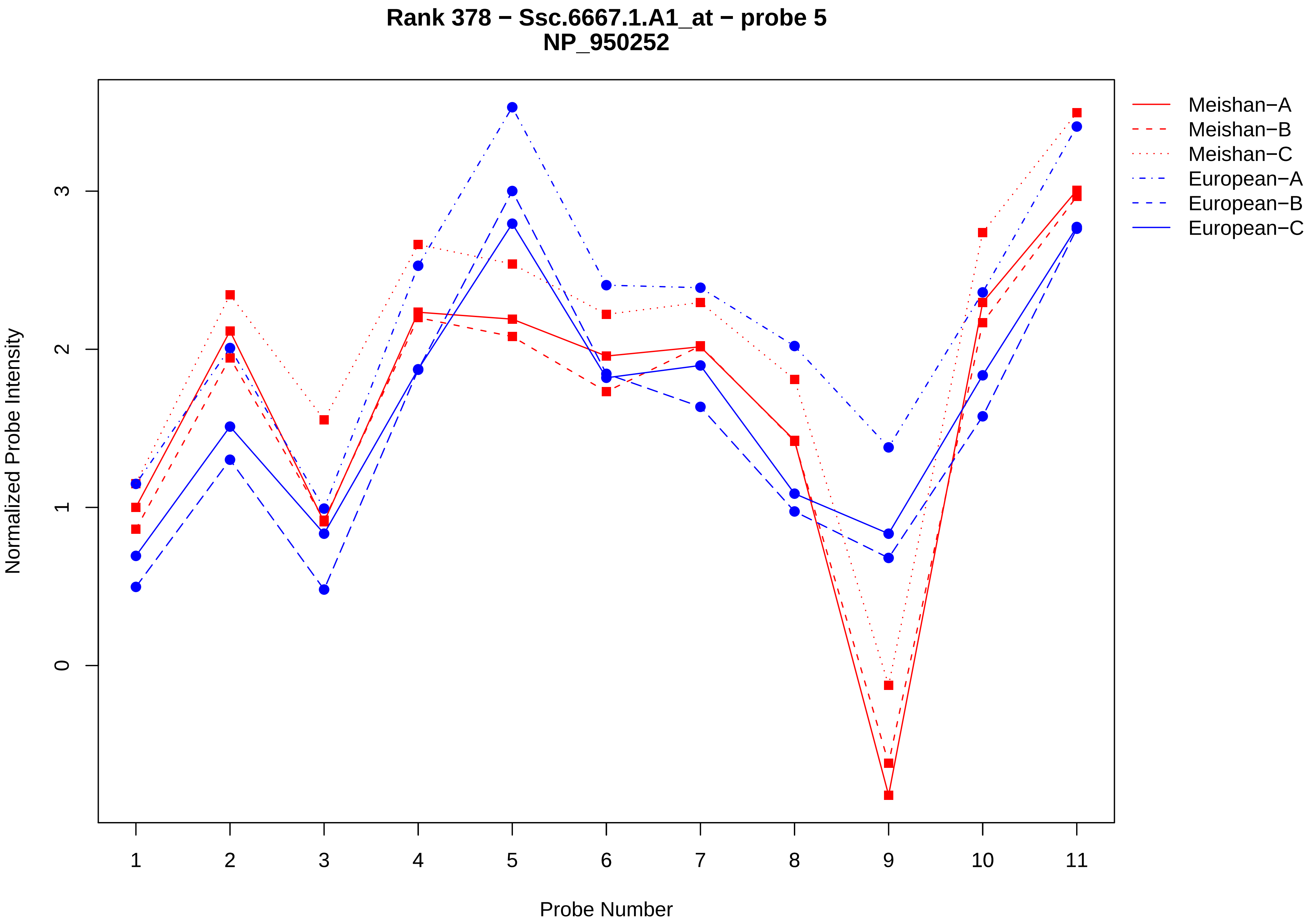
Rank 378 − Ssc.6667.1.A1_at − probe 5
NP_950252
Normalized Probe Intensity
Probe Number
0
1
2
3
1
2
3
4
5
6
7
8
9
10
11
Meishan−A
Meishan−B
Meishan−C
European−A
European−B
European−C

Rank 379 − Ssc.1400.1.A1_at − probe 2
MPDU1

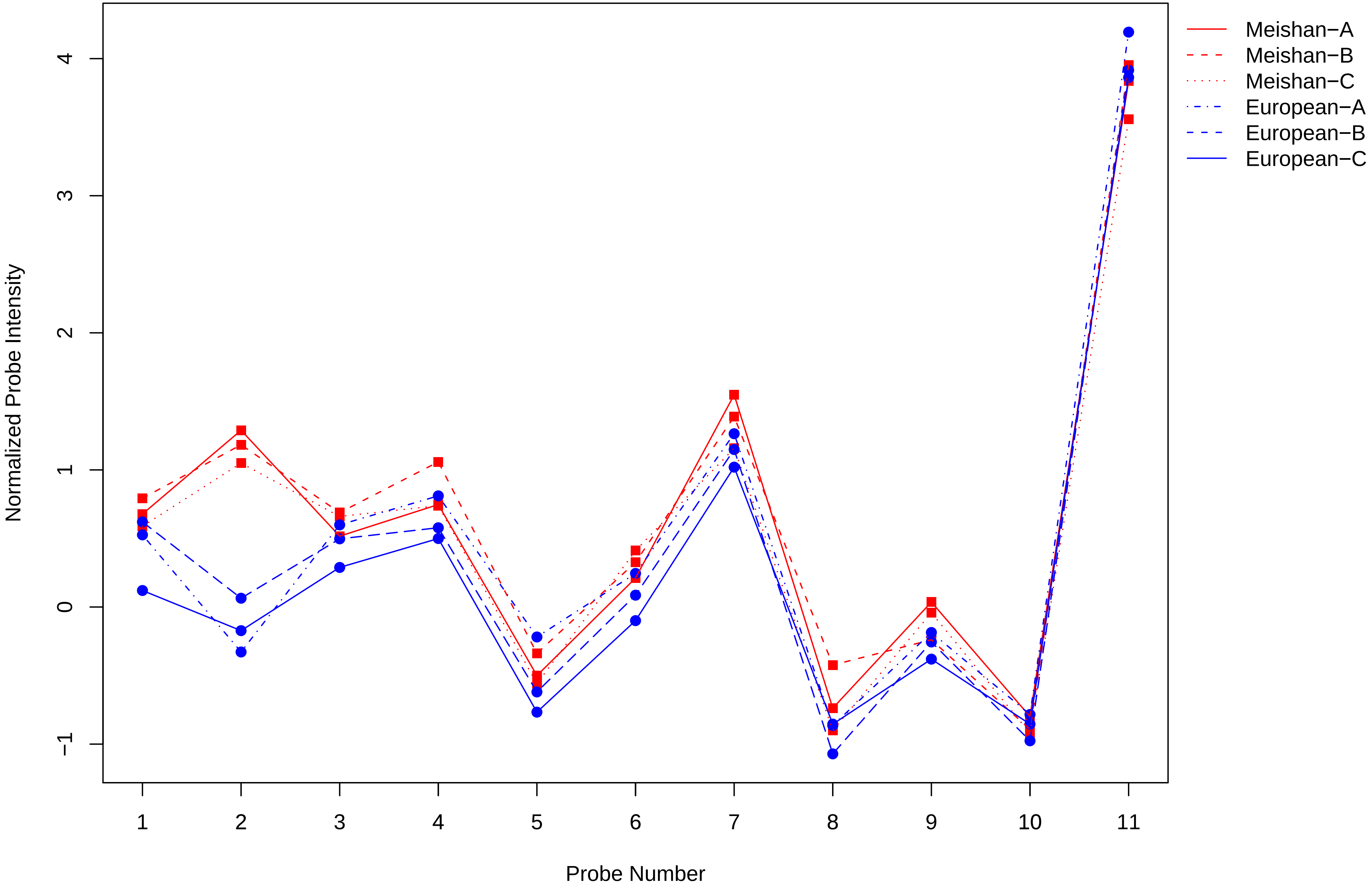

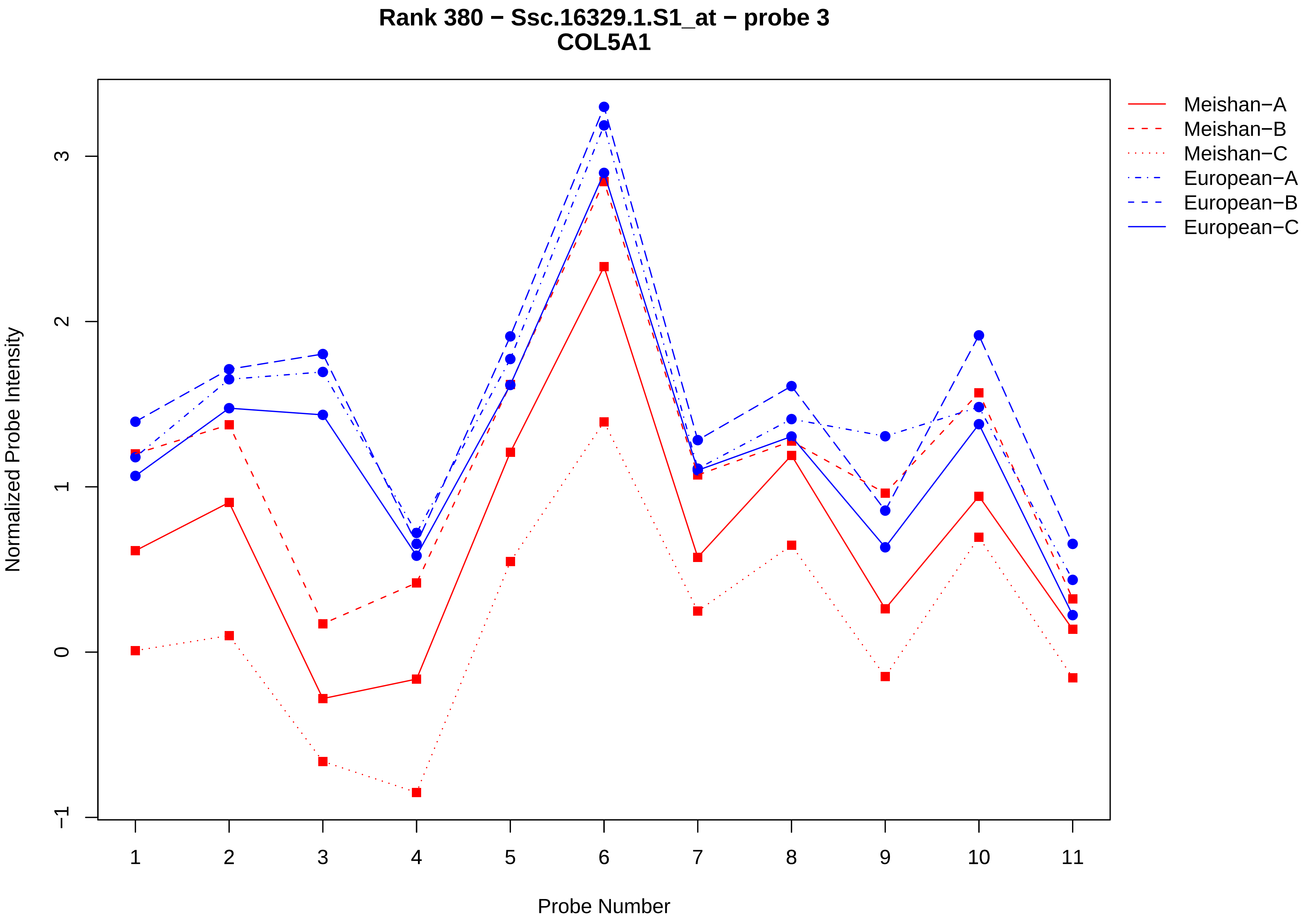

Rank 380 − Ssc.16329.1.S1_at − probe 3
COL5A1
Normalized Probe Intensity
Probe Number
−1
0
1
2
3
1
2
3
4
5
6
7
8
9
10
11
Meishan−A
Meishan−B
Meishan−C
European−A
European−B
European−C

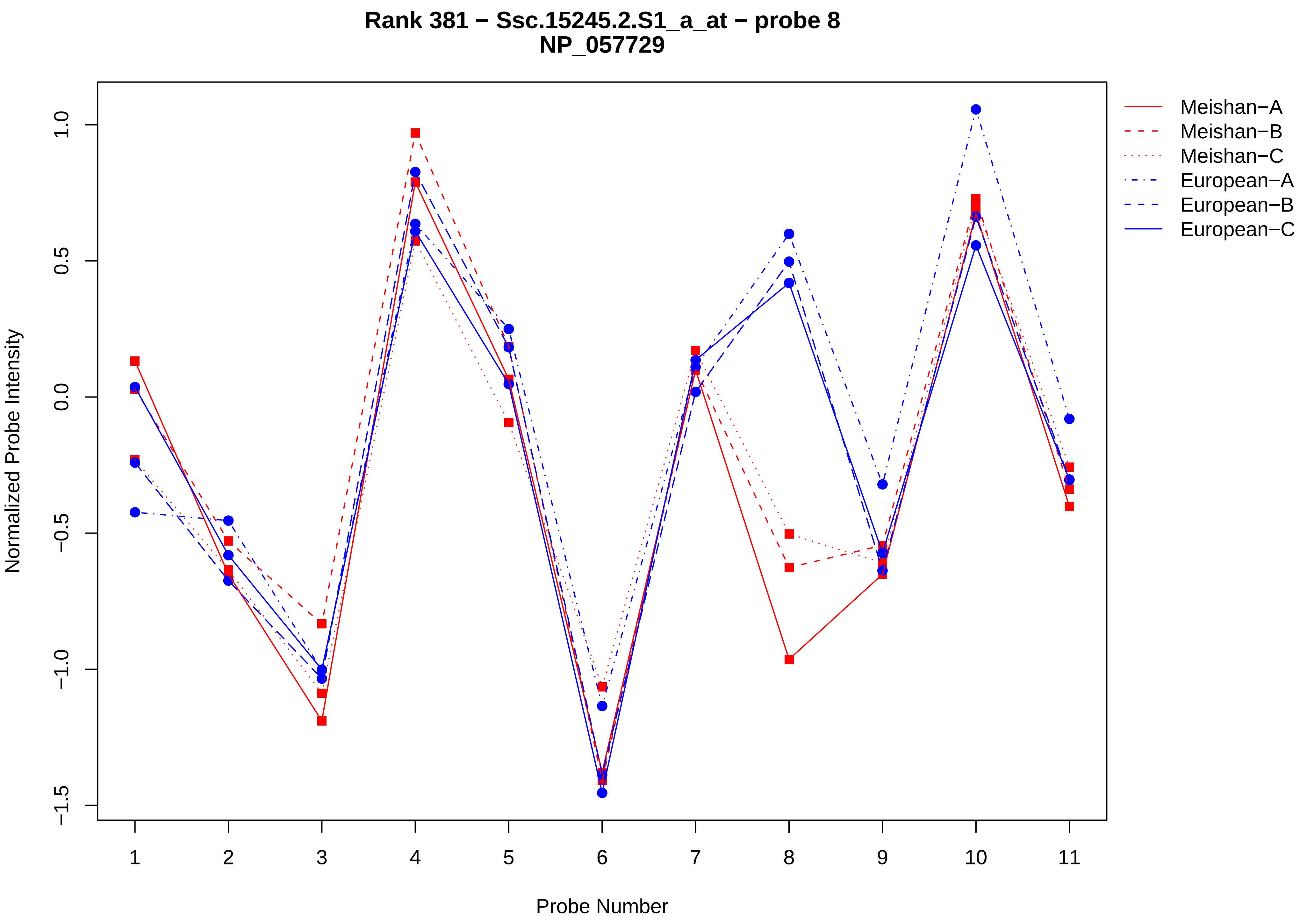

Rank 381 − Ssc.15245.2.S1_a_at − probe 8
NP_057729
Normalized Probe Intensity
Probe Number
Meishan−A
Meishan−B
Meishan−C
European−A
European−B
European−C
1.0
0.5
0.0
−0.5
−1.0
−1.5
1
2
3
4
5
6
7
8
9
10
11

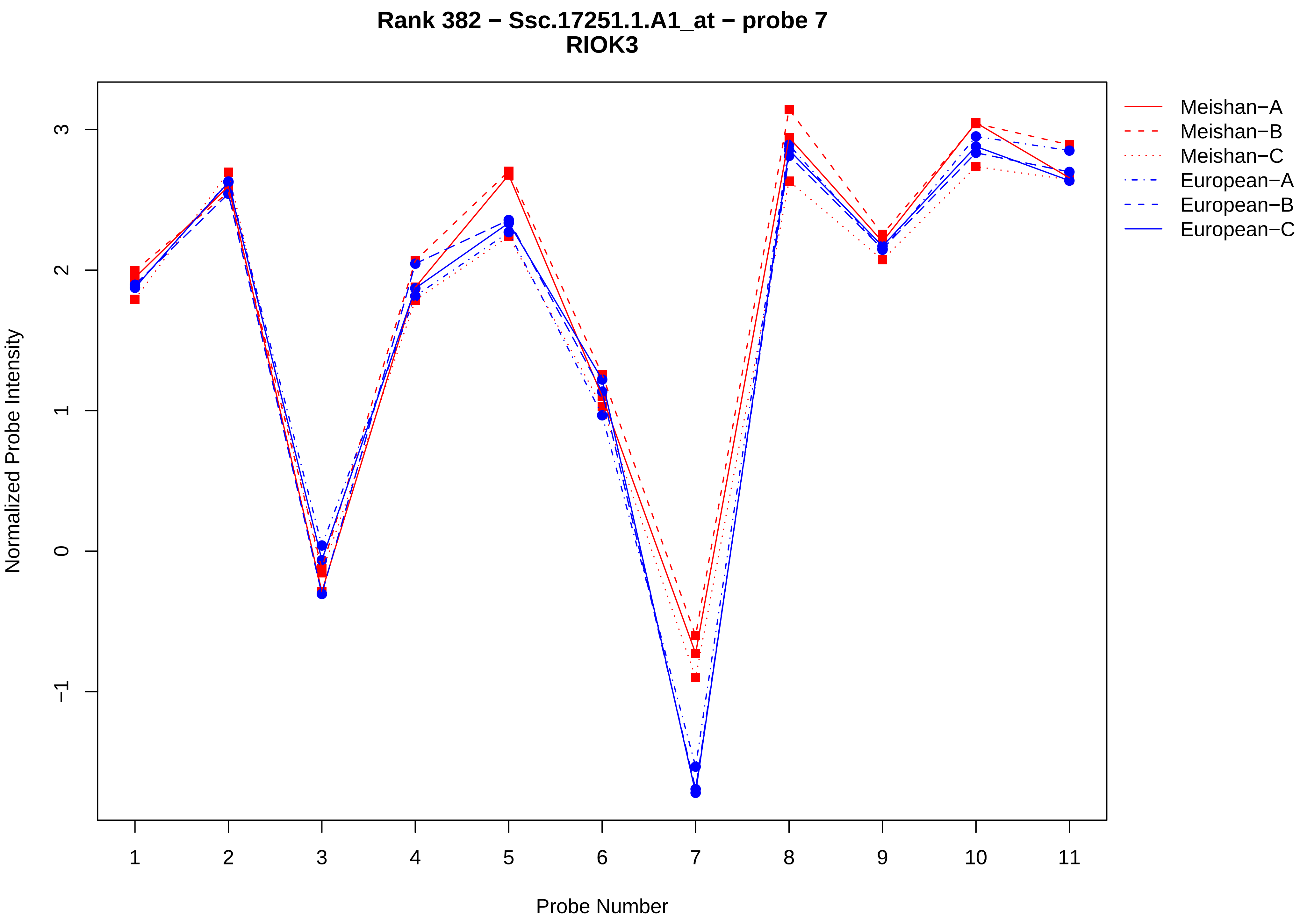

Rank 382 − Ssc.17251.1.A1_at − probe 7
RIOK3
Normalized Probe Intensity
Probe Number
−1
0
1
2
3
1
2
3
4
5
6
7
8
9
10
11
Meishan−A
Meishan−B
Meishan−C
European−A
European−B
European−C

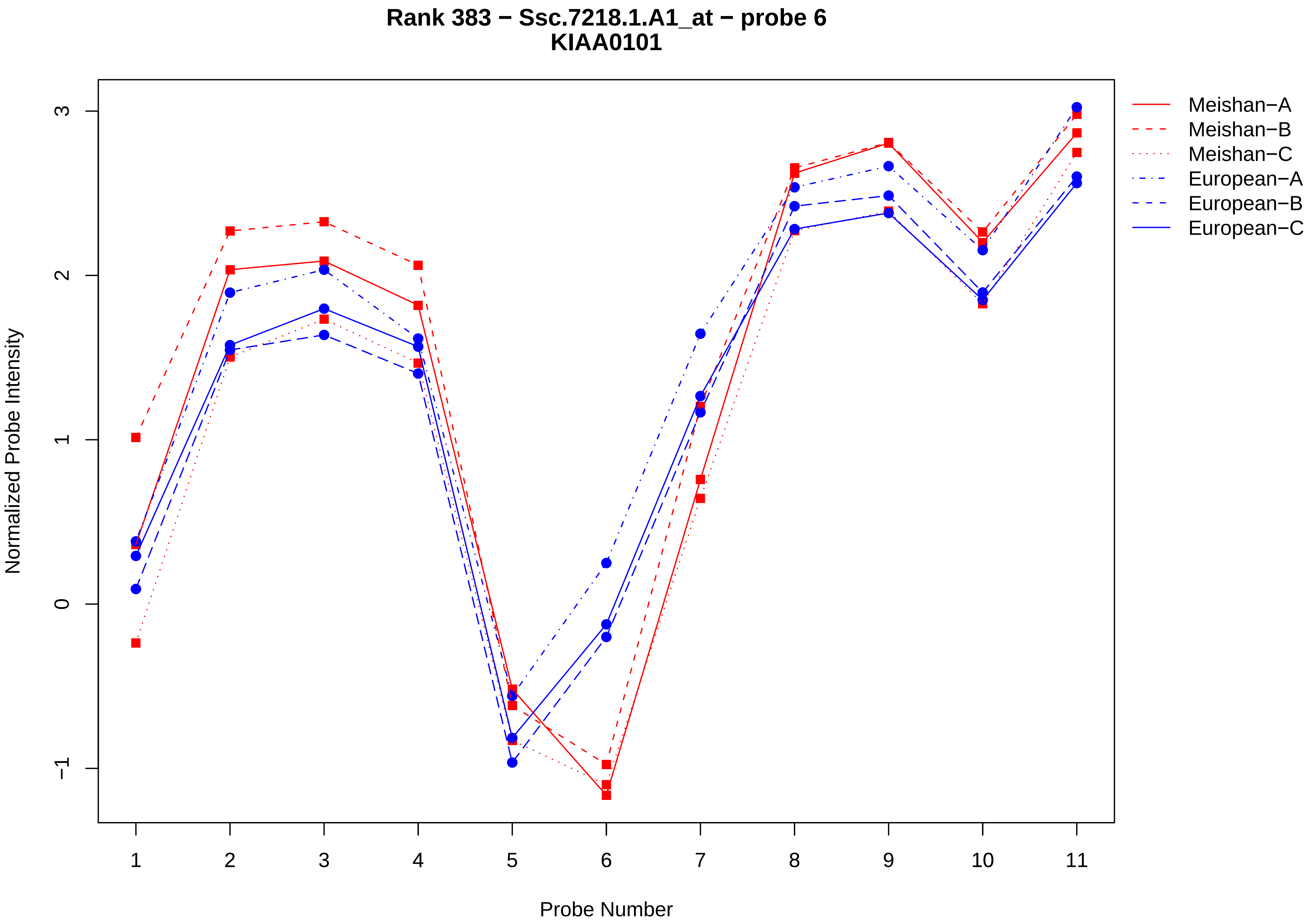
Rank 383 − Ssc.7218.1.A1_at − probe 6
KIAA0101
Normalized Probe Intensity
Probe Number
Meishan−A
Meishan−B
Meishan−C
European−A
European−B
European−C
−1
0
1
2
3
1
2
3
4
5
6
7
8
9
10
11

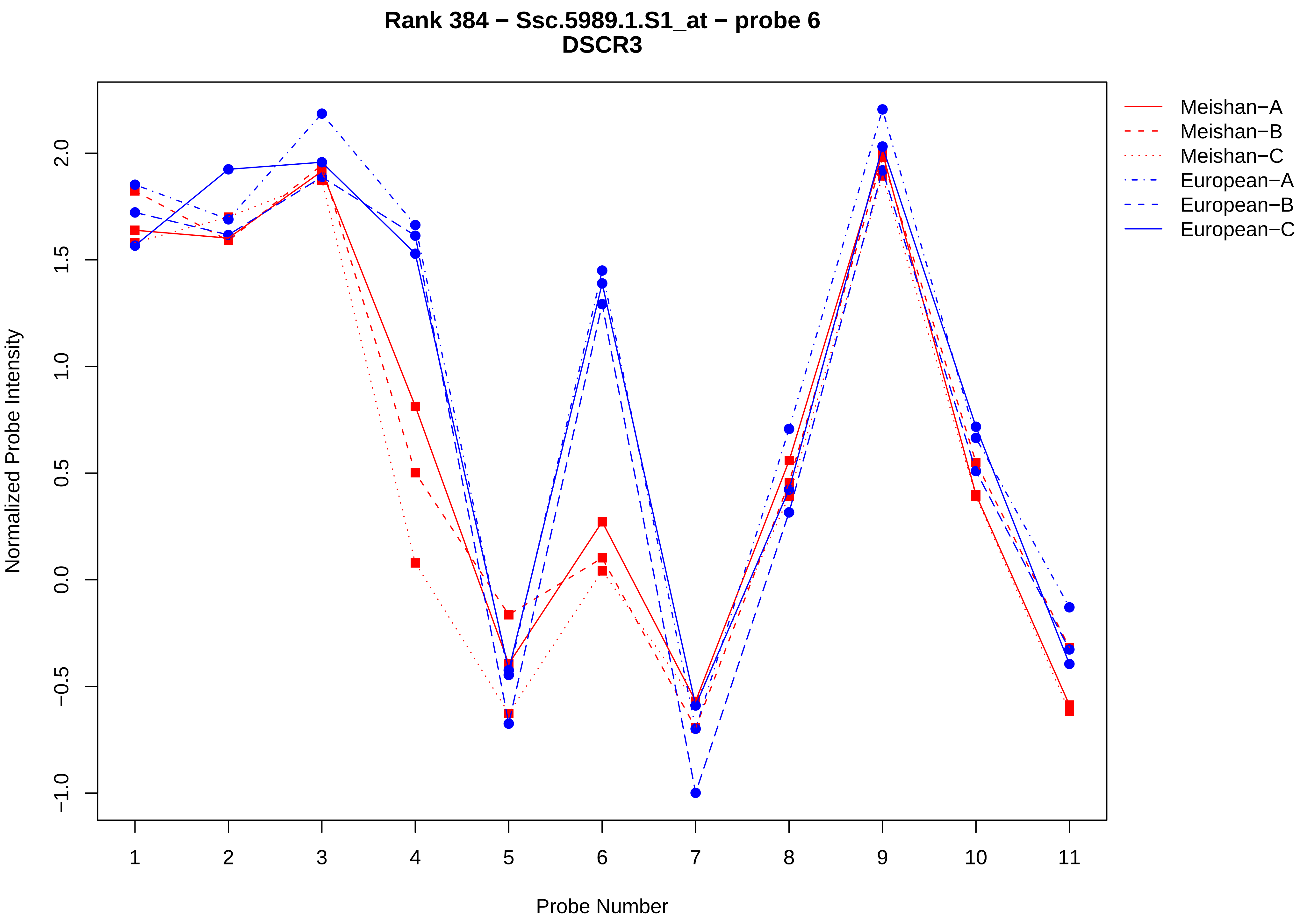

Rank 384 − Ssc.5989.1.S1_at − probe 6
DSCR3
Normalized Probe Intensity
Probe Number
−1.0
−0.5
0.0
0.5
1.0
1.5
2.0
1
2
3
4
5
6
7
8
9
10
11
Meishan−A
Meishan−B
Meishan−C
European−A
European−B
European−C

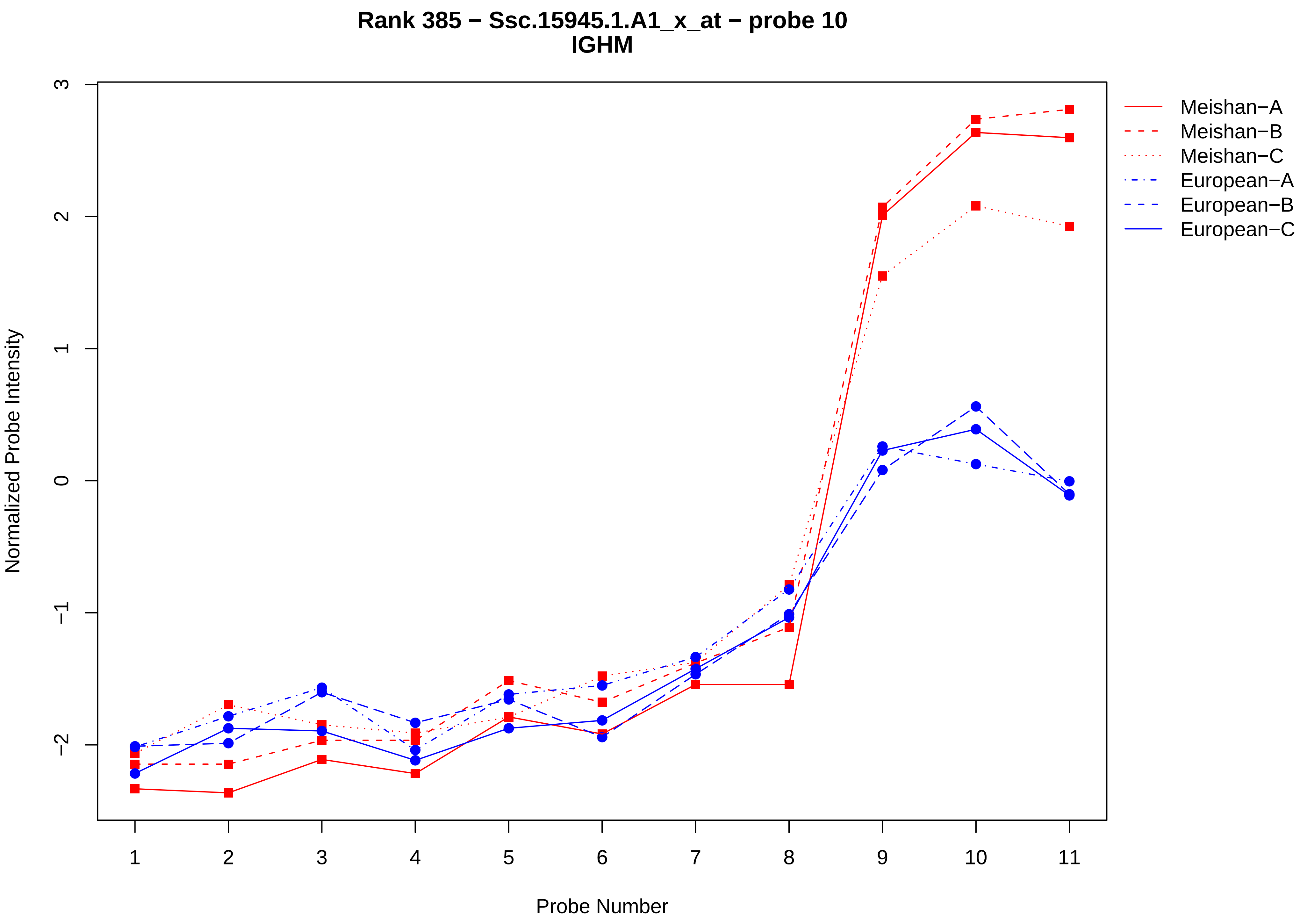
Rank 385 − Ssc.15945.1.A1_x_at − probe 10
IGHM
Normalized Probe Intensity
Probe Number
Meishan−A
Meishan−B
Meishan−C
European−A
European−B
European−C
3
2
1
0
−1
−2
1
2
3
4
5
6
7
8
9
10
11

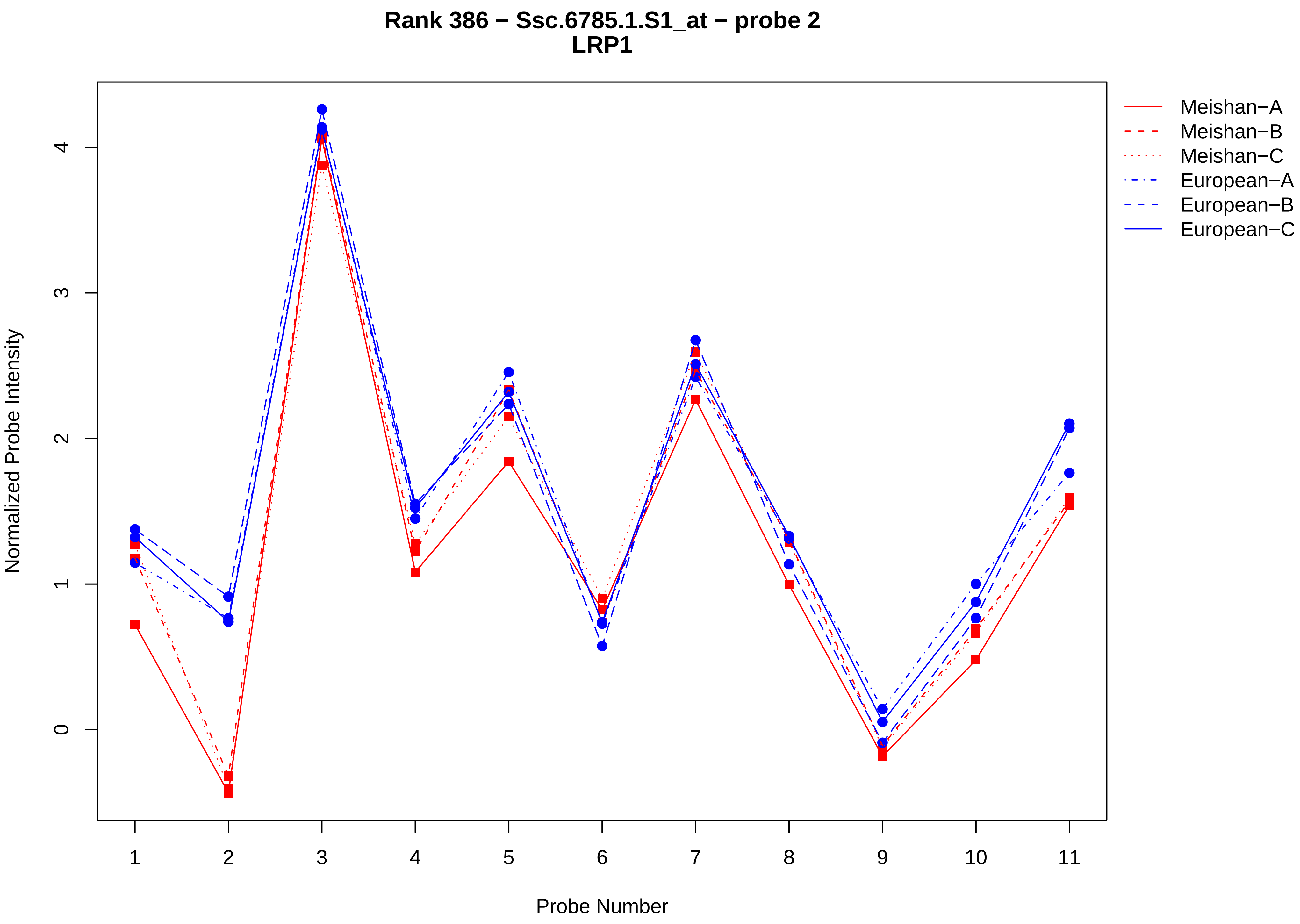

Rank 386 − Ssc.6785.1.S1_at − probe 2
LRP1
Normalized Probe Intensity
Probe Number
Meishan−A
Meishan−B
Meishan−C
European−A
European−B
European−C
0
1
2
3
4
1
2
3
4
5
6
7
8
9
10
11

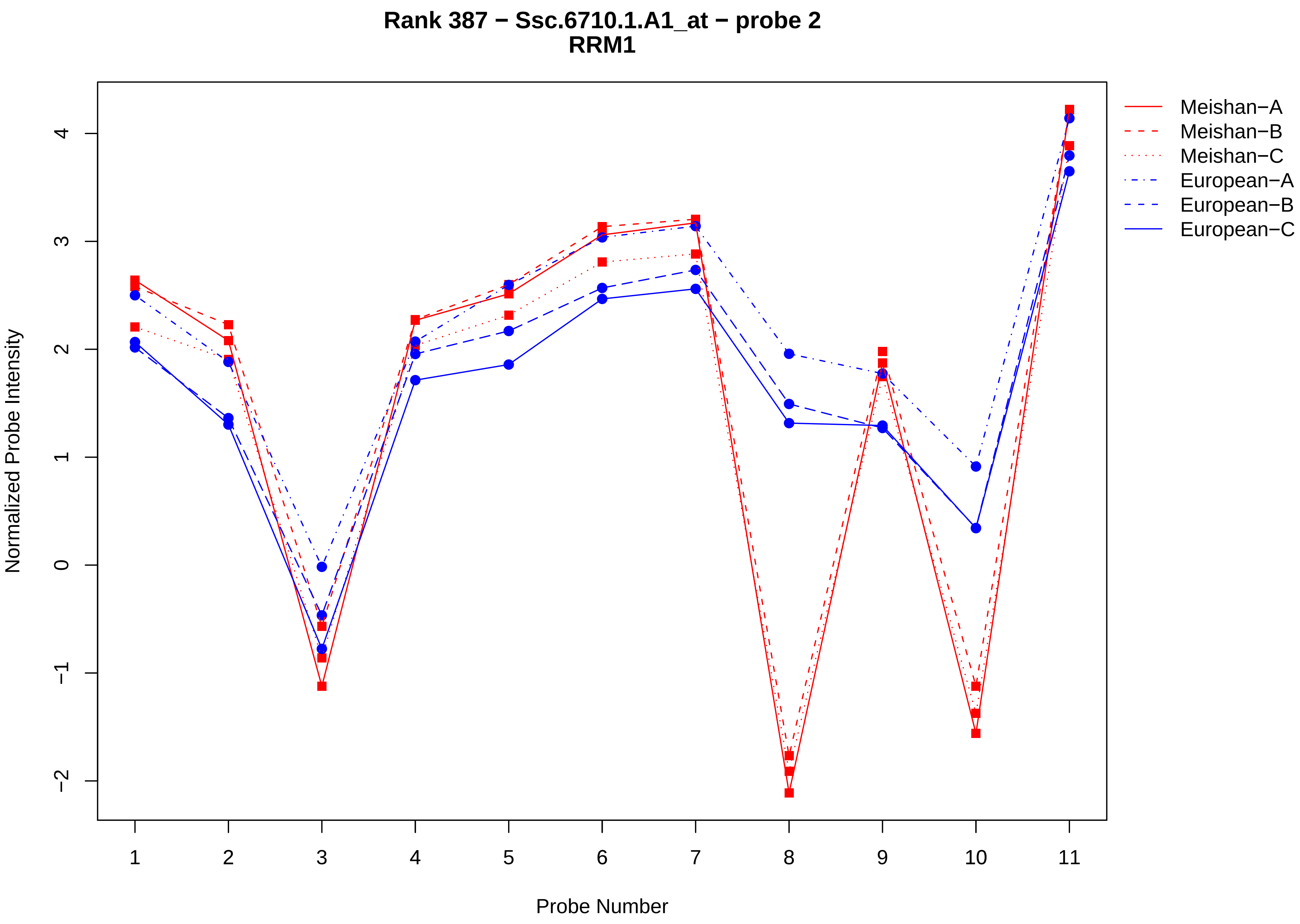

Rank 387 − Ssc.6710.1.A1_at − probe 2
RRM1
Normalized Probe Intensity
Probe Number
Meishan−A
Meishan−B
Meishan−C
European−A
European−B
European−C
−2
−1
0
1
2
3
4
1
2
3
4
5
6
7
8
9
10
11

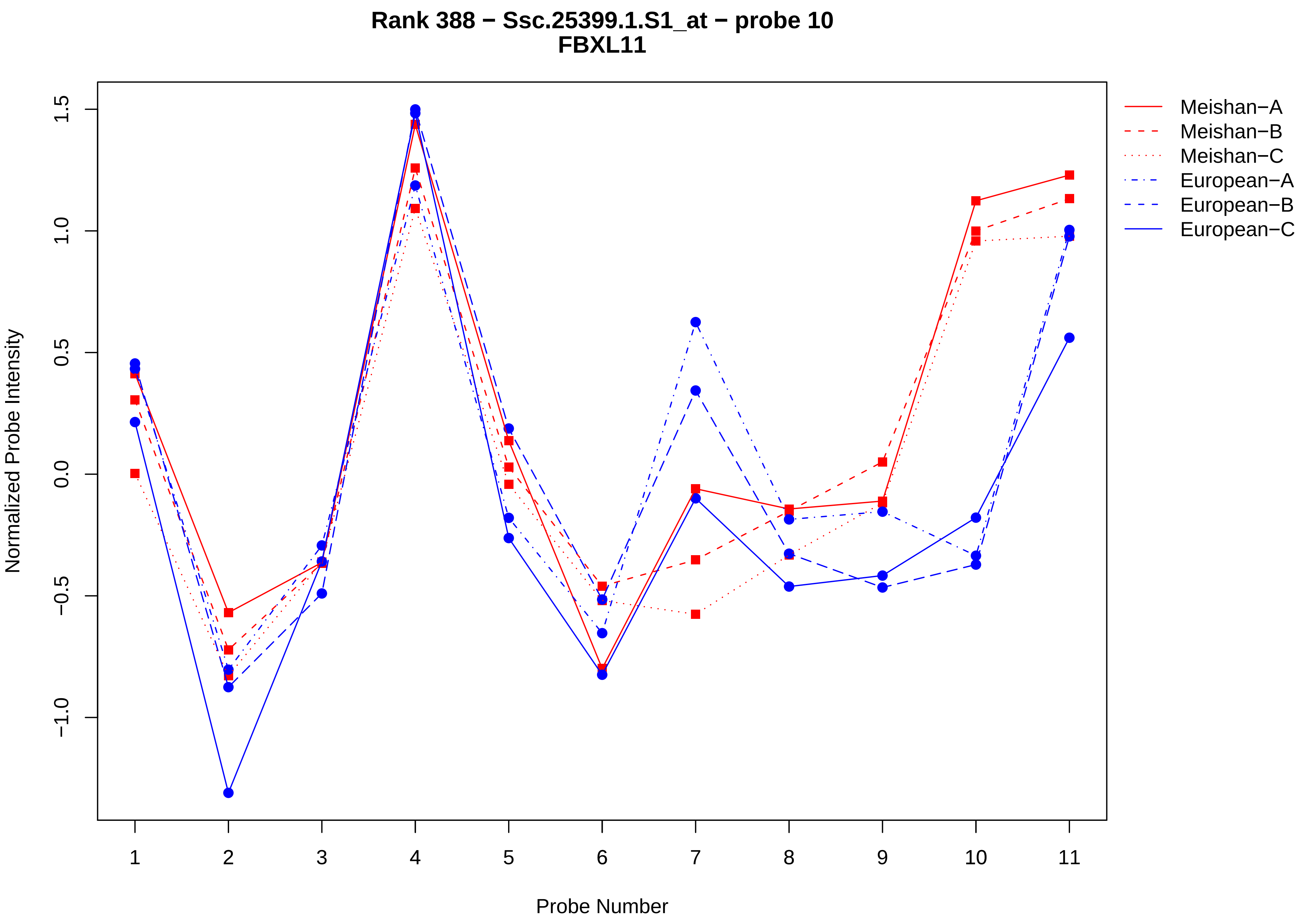

Rank 388 − Ssc.25399.1.S1_at − probe 10
FBXL11
Normalized Probe Intensity
Probe Number
−1.0
−0.5
0.0
0.5
1.0
1.5
1
2
3
4
5
6
7
8
9
10
11
Meishan−A
Meishan−B
Meishan−C
European−A
European−B
European−C

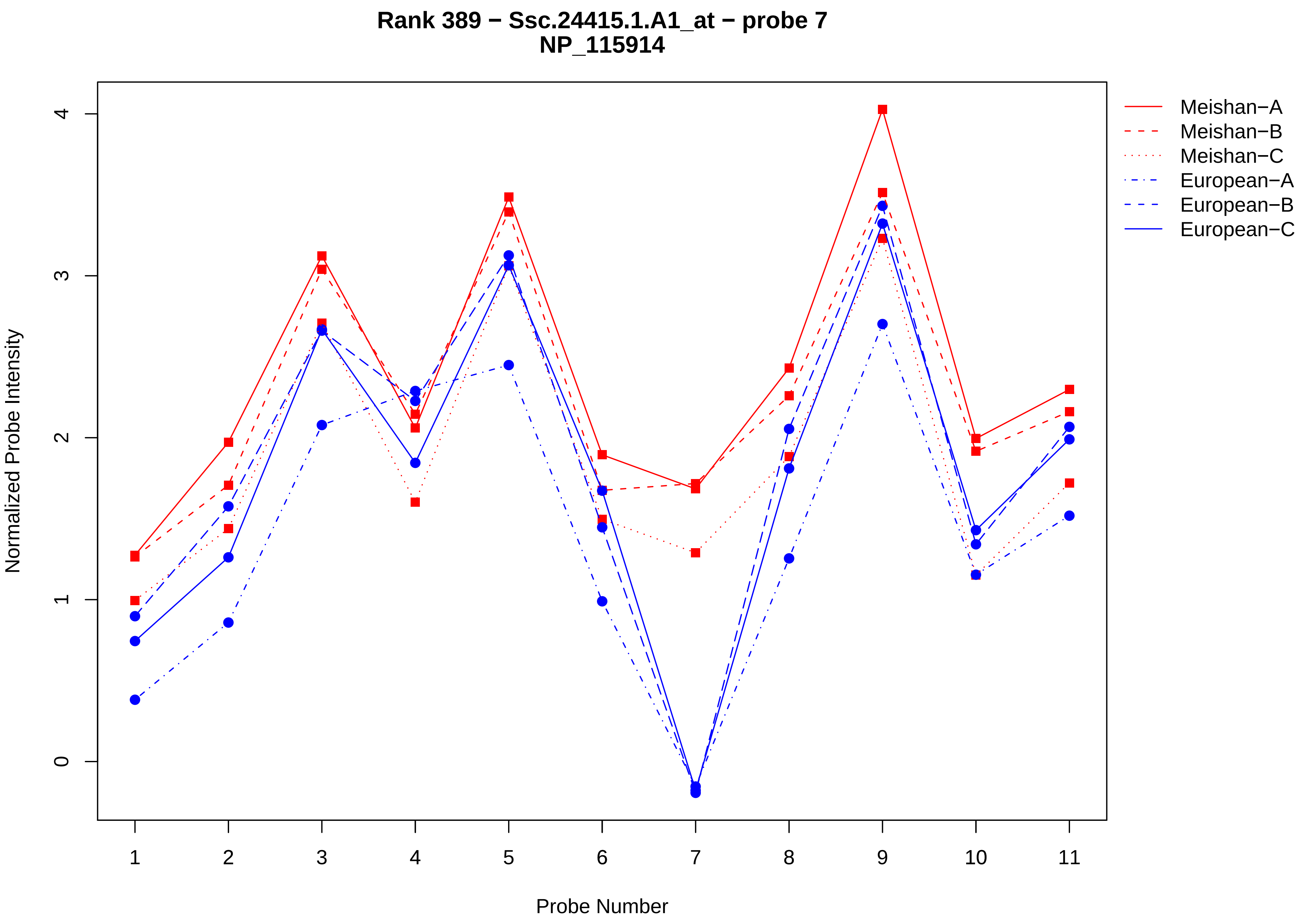

Rank 389 − Ssc.24415.1.A1_at − probe 7
NP_115914
Normalized Probe Intensity
Probe Number
0
1
2
3
4
1
2
3
4
5
6
7
8
9
10
11
Meishan−A
Meishan−B
Meishan−C
European−A
European−B
European−C

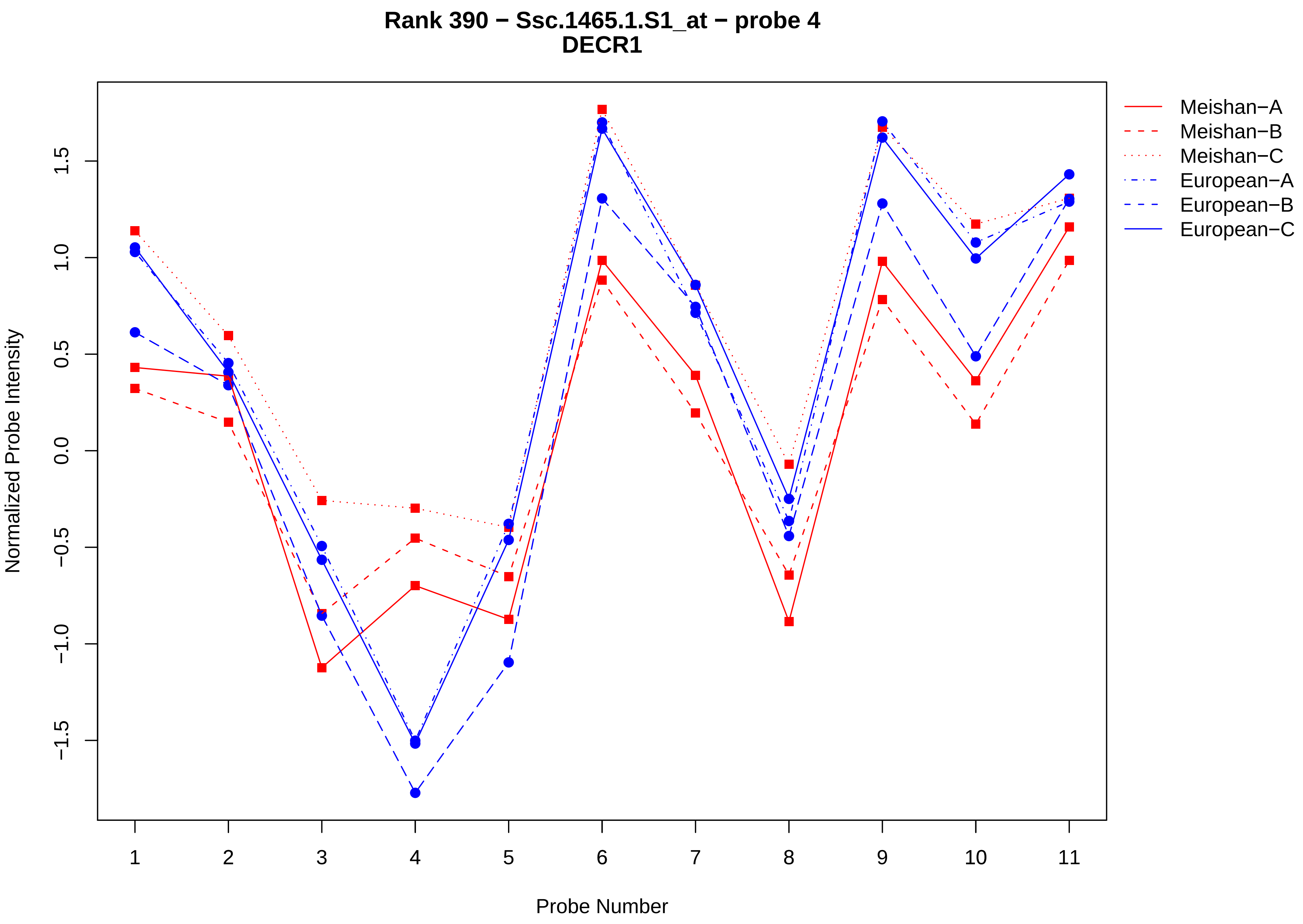

Rank 390 − Ssc.1465.1.S1_at − probe 4
DECR1
Normalized Probe Intensity
−1.5
−1.0
−0.5
0.0
0.5
1.0
1.5
1
2
3
4
5
6
7
8
9
10
11
Probe Number
Meishan−A
Meishan−B
Meishan−C
European−A
European−B
European−C

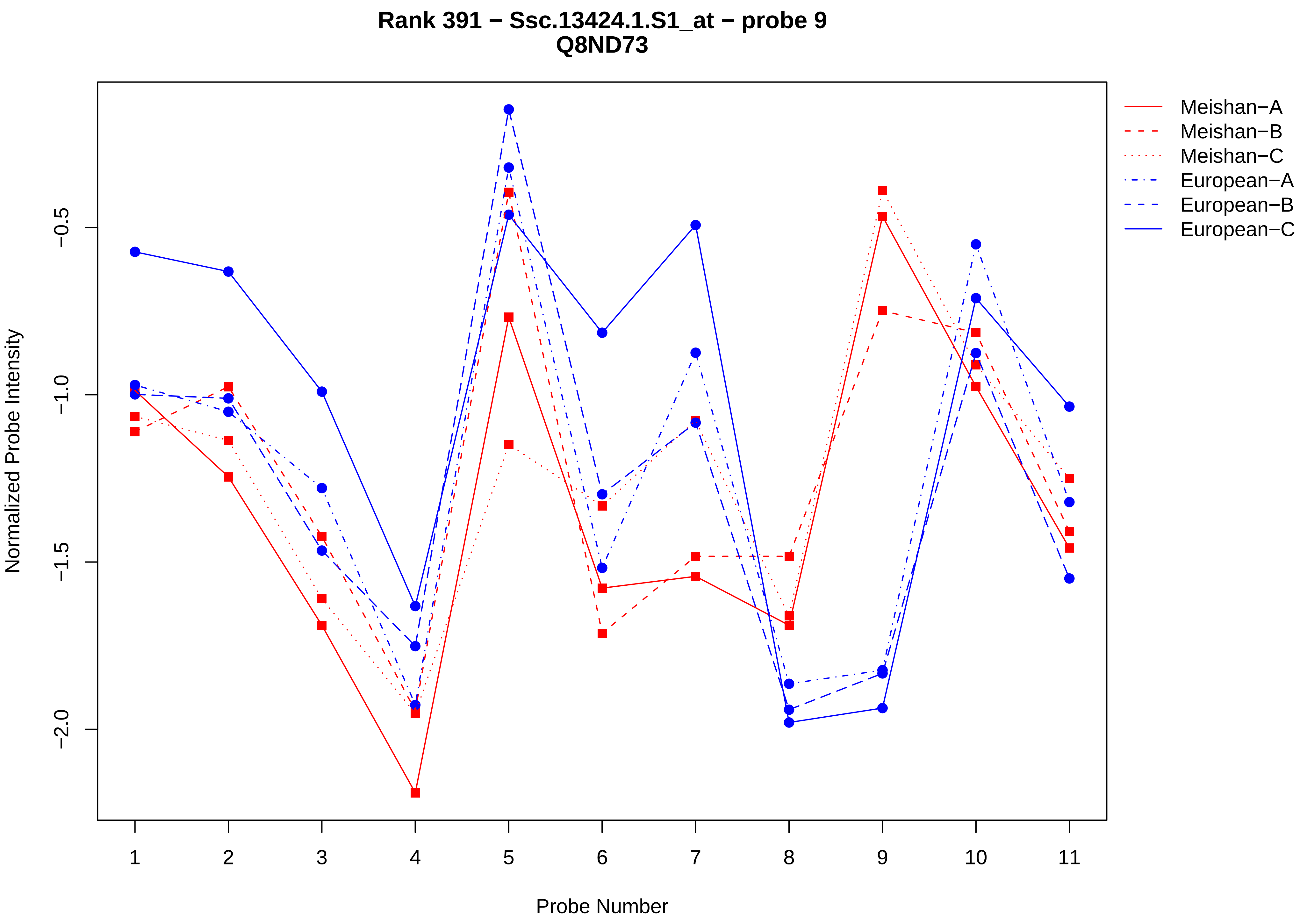
Rank 391 − Ssc.13424.1.S1_at − probe 9
Q8ND73
Normalized Probe Intensity
−0.5
−1.0
−1.5
−2.0
1
2
3
4
5
6
7
8
9
10
11
Probe Number
Meishan−A
Meishan−B
Meishan−C
European−A
European−B
European−C

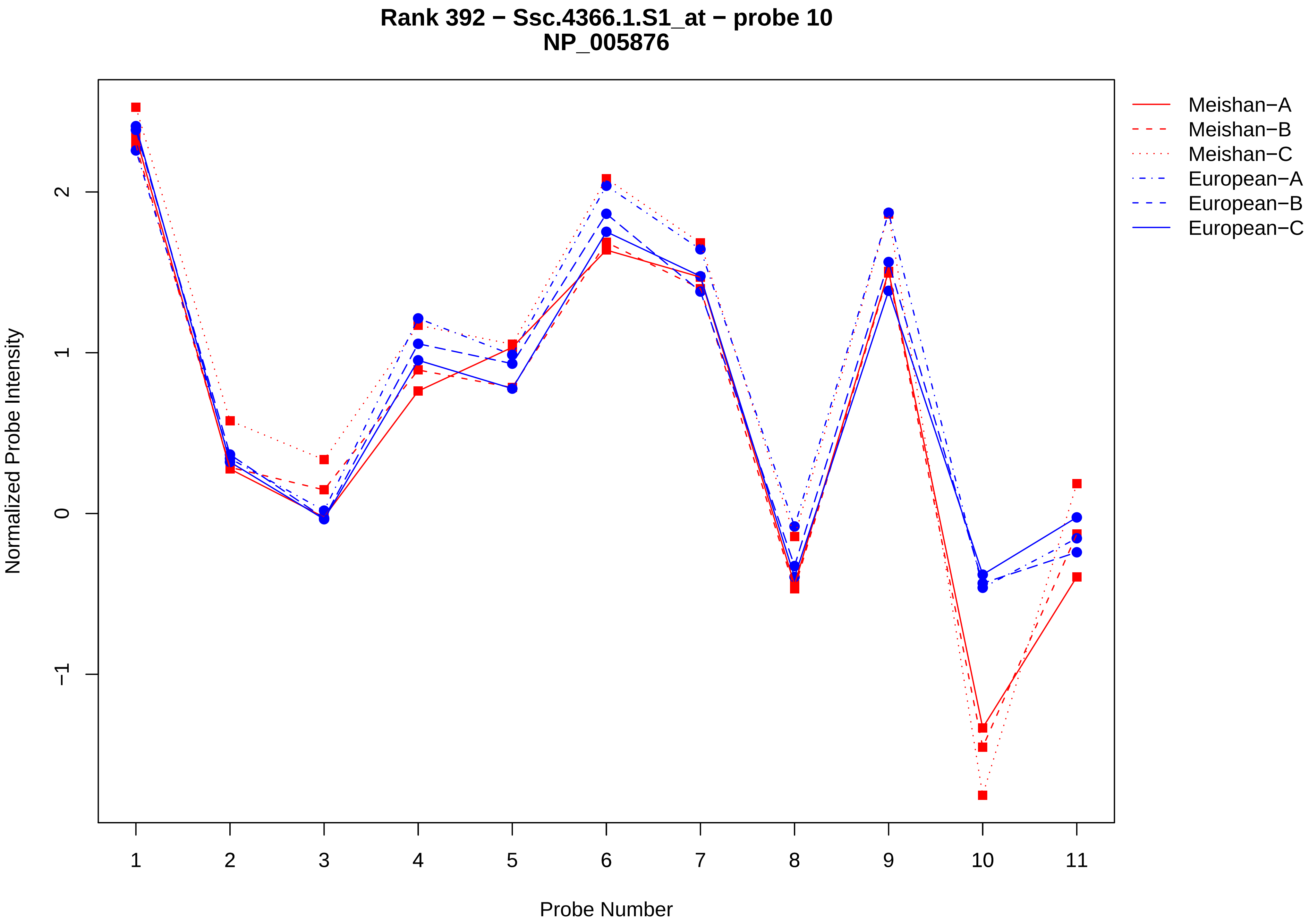
Rank 392 − Ssc.4366.1.S1_at − probe 10
NP_005876
Normalized Probe Intensity
Probe Number
−1
0
1
2
1
2
3
4
5
6
7
8
9
10
11
Meishan−A
Meishan−B
Meishan−C
European−A
European−B
European−C

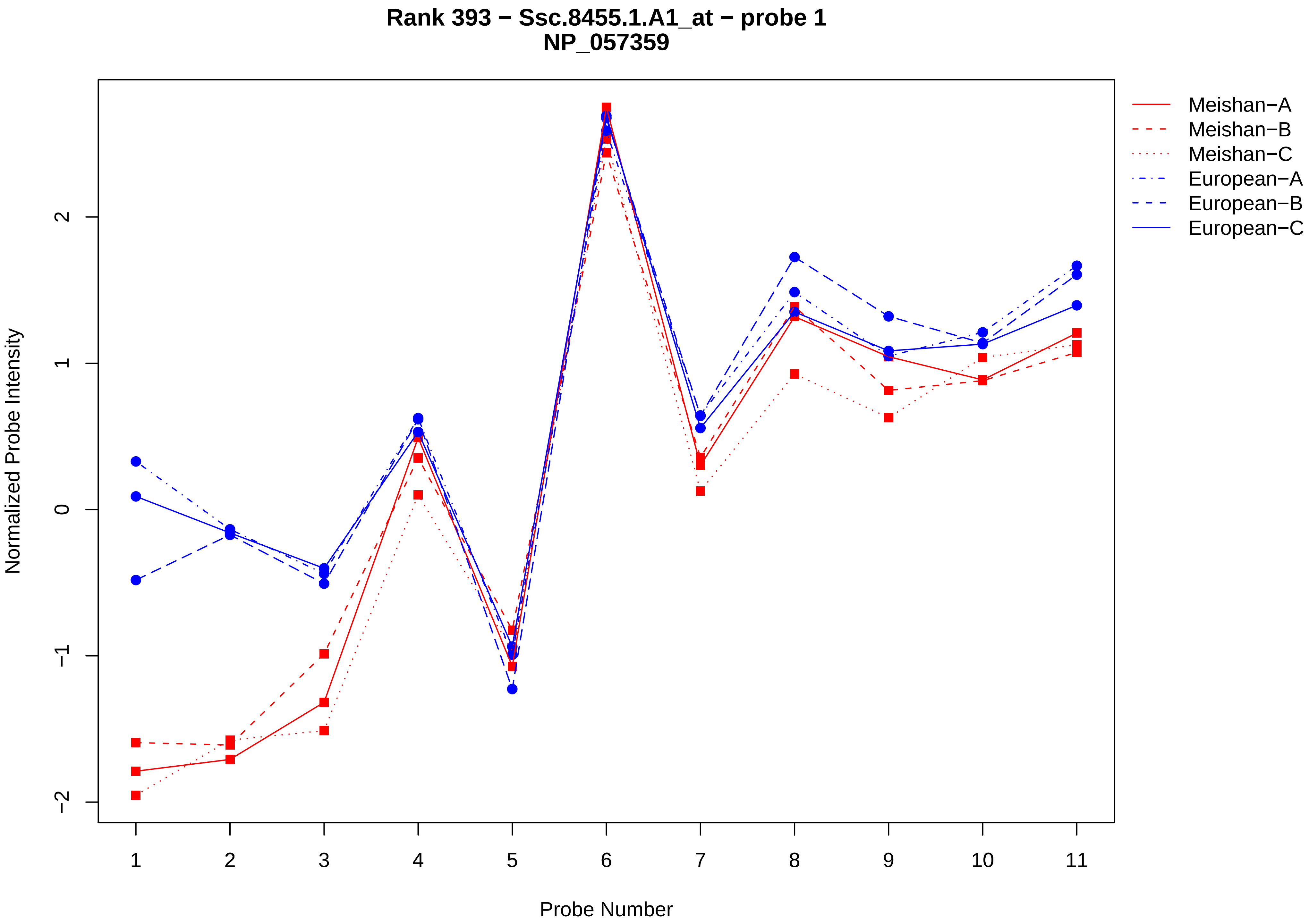

Rank 393 − Ssc.8455.1.A1_at − probe 1
NP_057359
Normalized Probe Intensity
Probe Number
−2
−1
0
1
2
1
2
3
4
5
6
7
8
9
10
11
Meishan−A
Meishan−B
Meishan−C
European−A
European−B
European−C

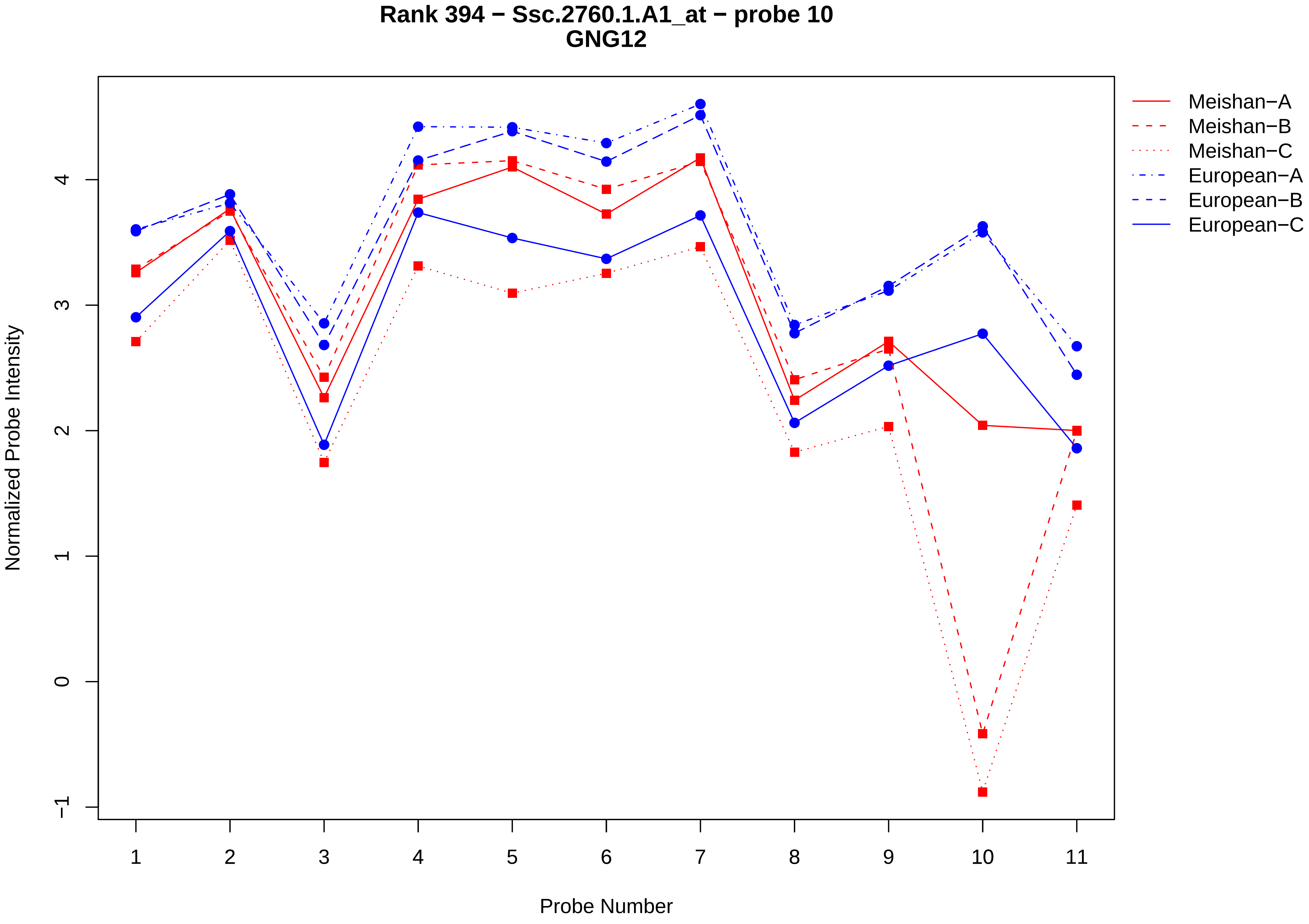

Rank 394 − Ssc.2760.1.A1_at − probe 10
GNG12
Normalized Probe Intensity
Probe Number
−1
0
1
2
3
4
1
2
3
4
5
6
7
8
9
10
11
Meishan−A
Meishan−B
Meishan−C
European−A
European−B
European−C

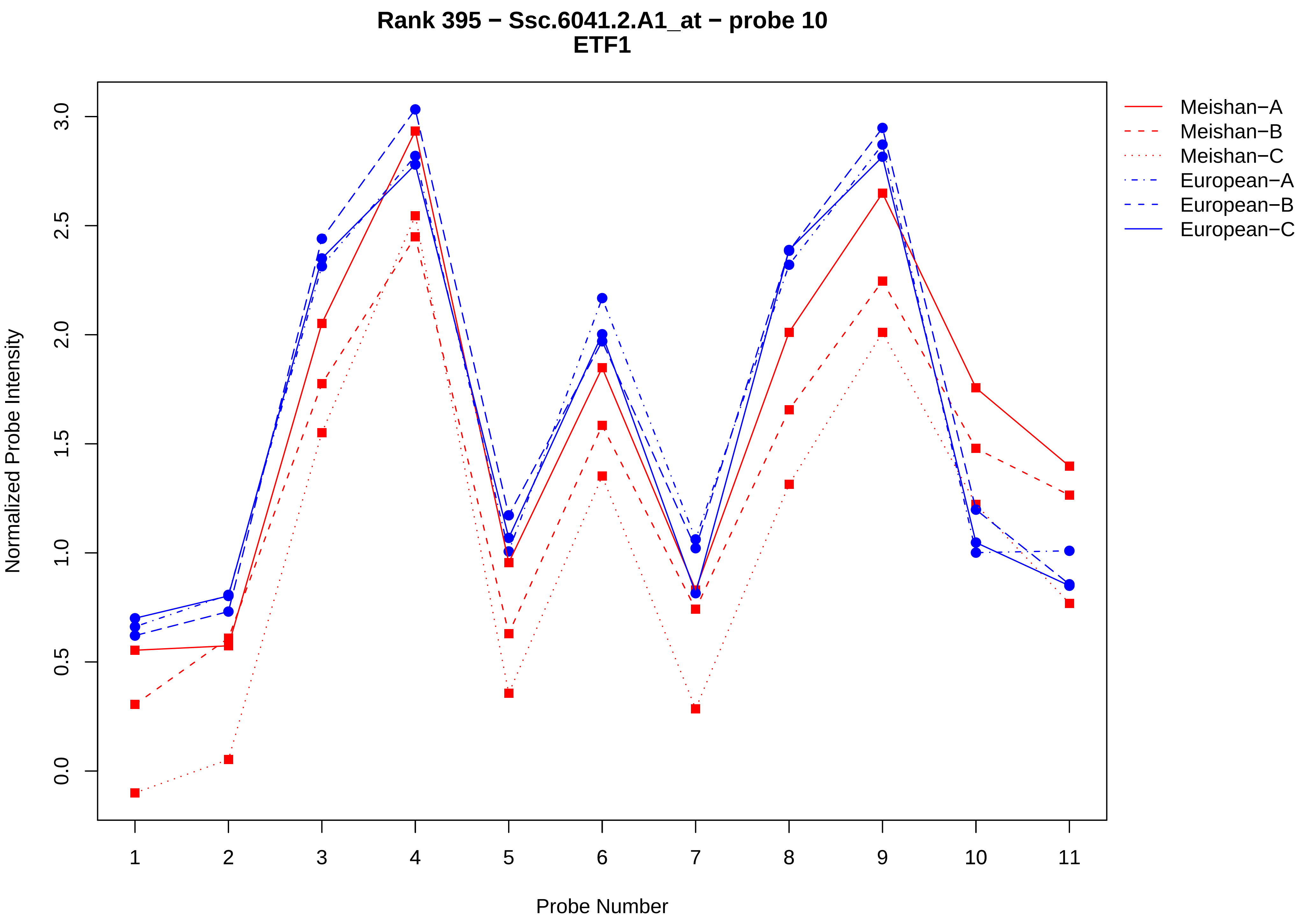
Rank 395 − Ssc.6041.2.A1_at − probe 10
ETF1
Normalized Probe Intensity
0.0
0.5
1.0
1.5
2.0
2.5
3.0
1
2
3
4
5
6
7
8
9
10
11
Probe Number
Meishan−A
Meishan−B
Meishan−C
European−A
European−B
European−C

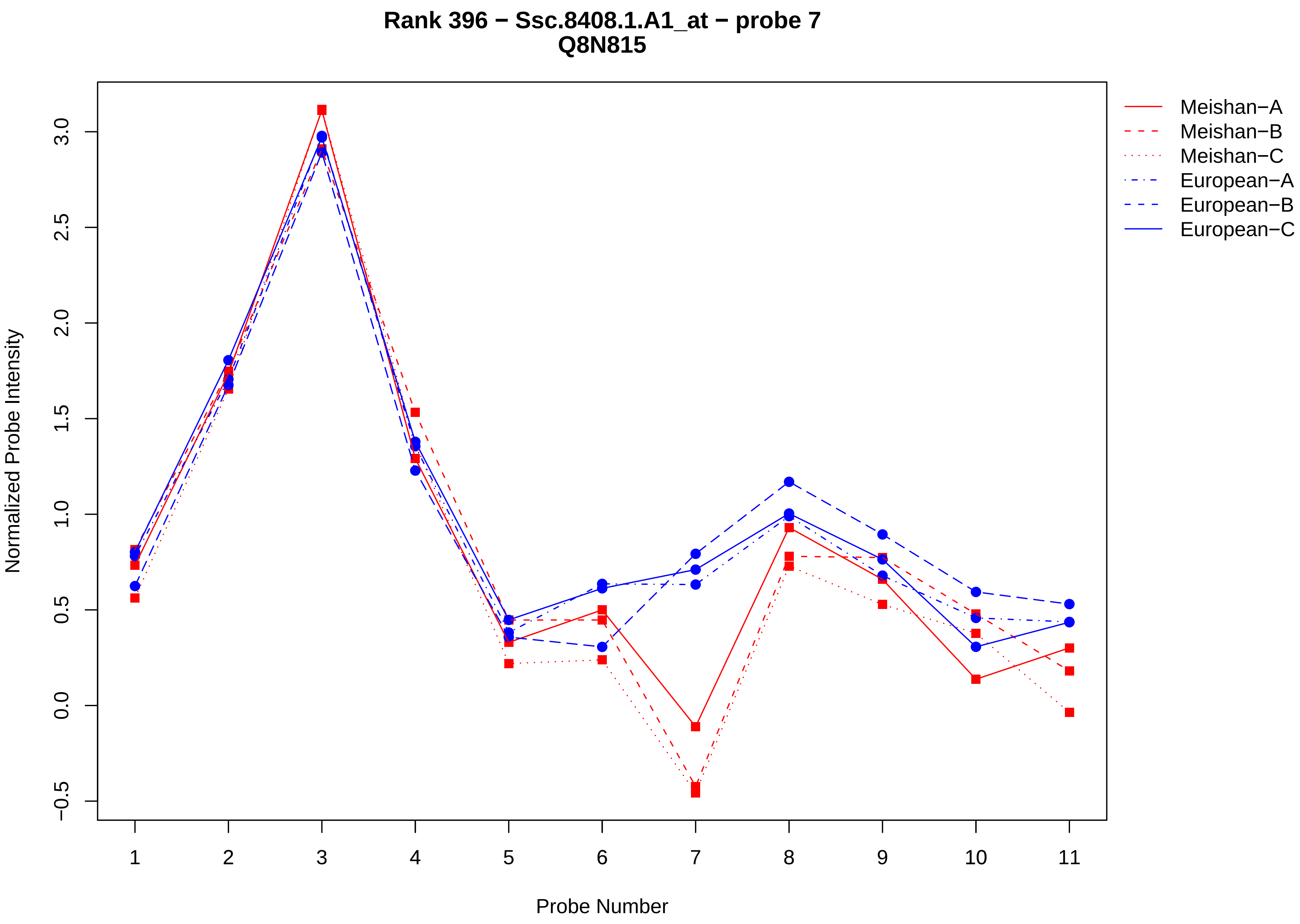
Rank 396 − Ssc.8408.1.A1_at − probe 7
Q8N815
Normalized Probe Intensity
Probe Number
Meishan−A
Meishan−B
Meishan−C
European−A
European−B
European−C
−0.5
0.0
0.5
1.0
1.5
2.0
2.5
3.0
1
2
3
4
5
6
7
8
9
10
11

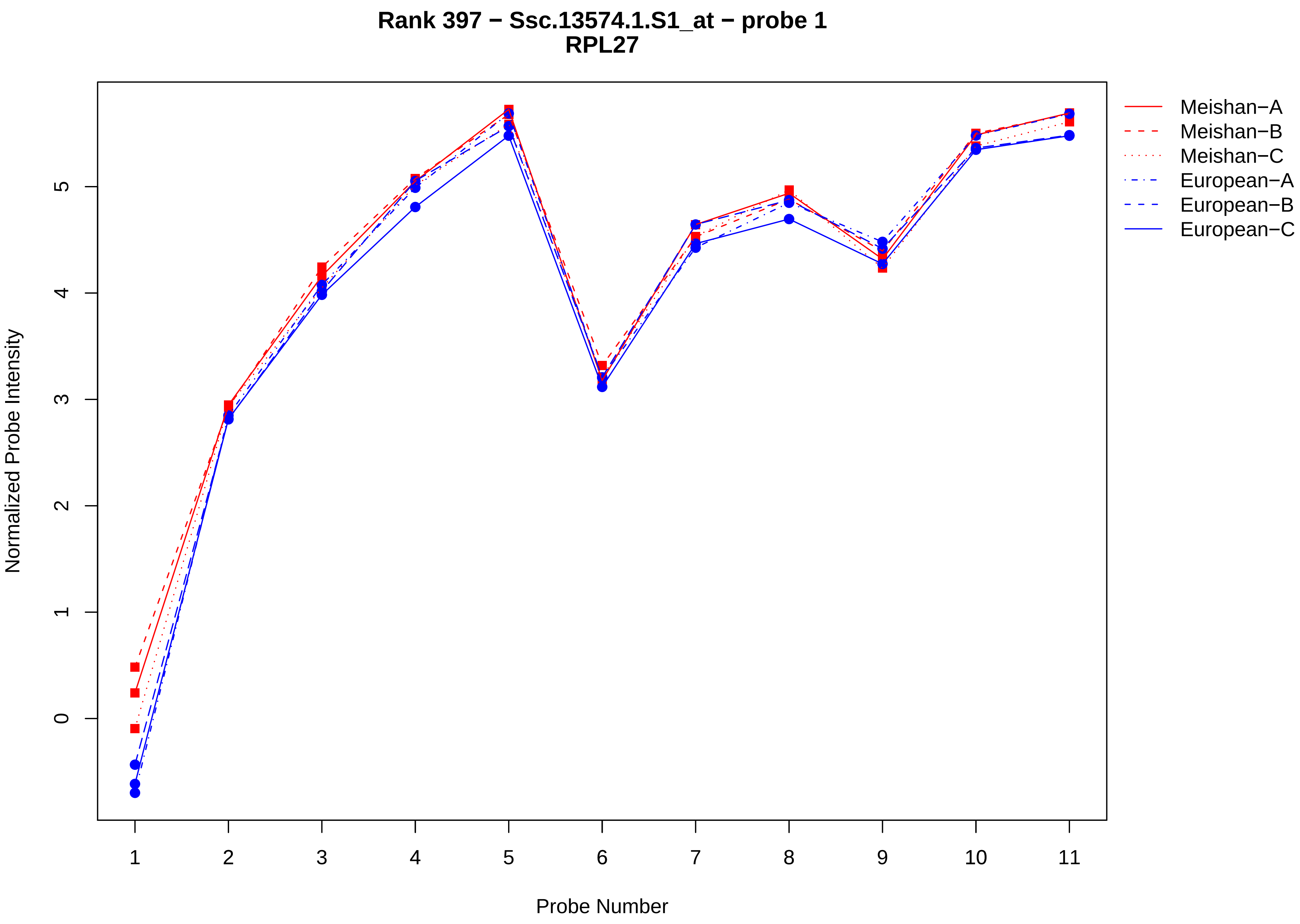

Rank 397 − Ssc.13574.1.S1_at − probe 1
RPL27
Normalized Probe Intensity
Probe Number
0
1
2
3
4
5
1
2
3
4
5
6
7
8
9
10
11
Meishan−A
Meishan−B
Meishan−C
European−A
European−B
European−C

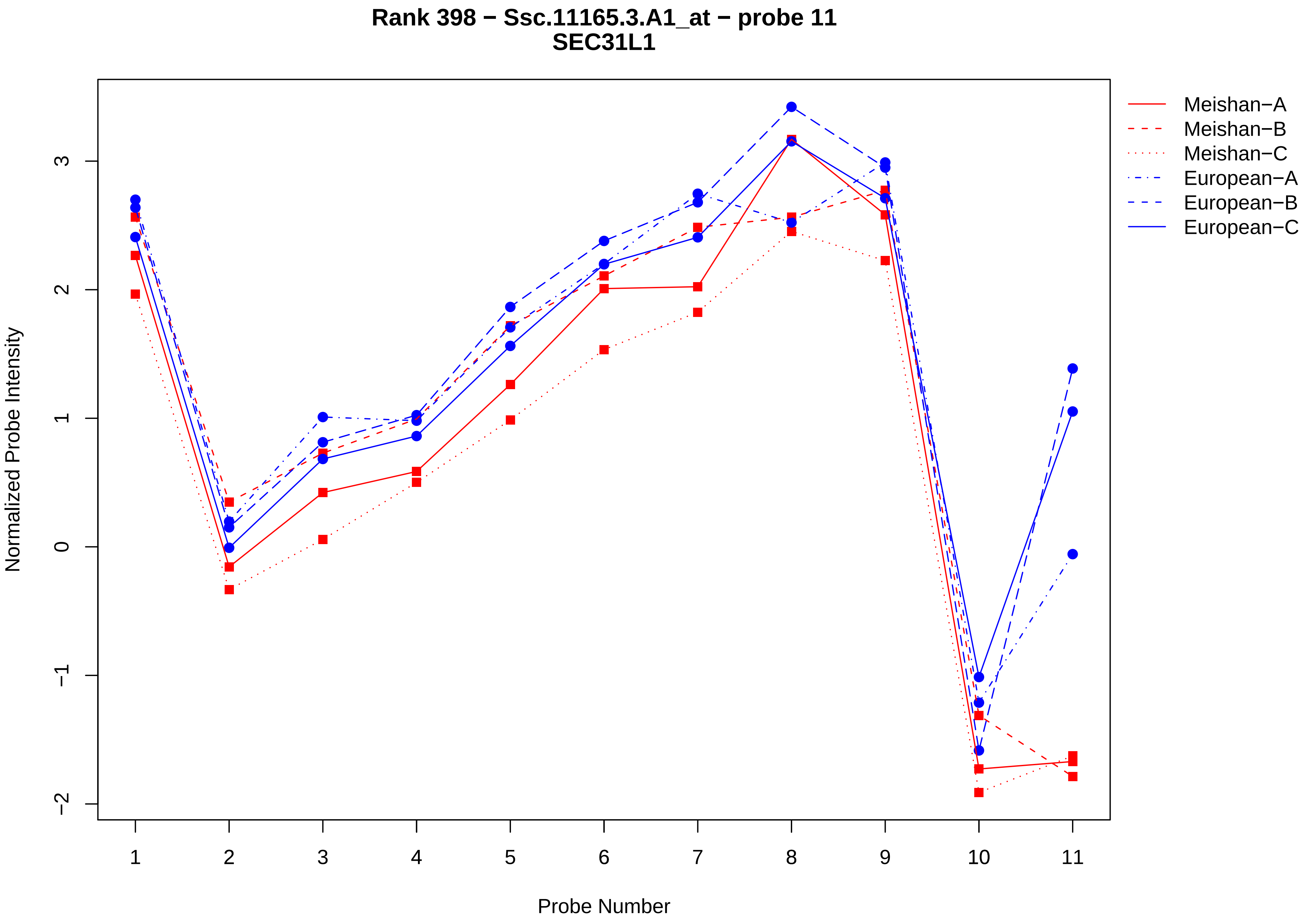

Rank 398 − Ssc.11165.3.A1_at − probe 11
SEC31L1
Normalized Probe Intensity
Probe Number
Meishan−A
Meishan−B
Meishan−C
European−A
European−B
European−C
−2
−1
0
1
2
3
1
2
3
4
5
6
7
8
9
10
11

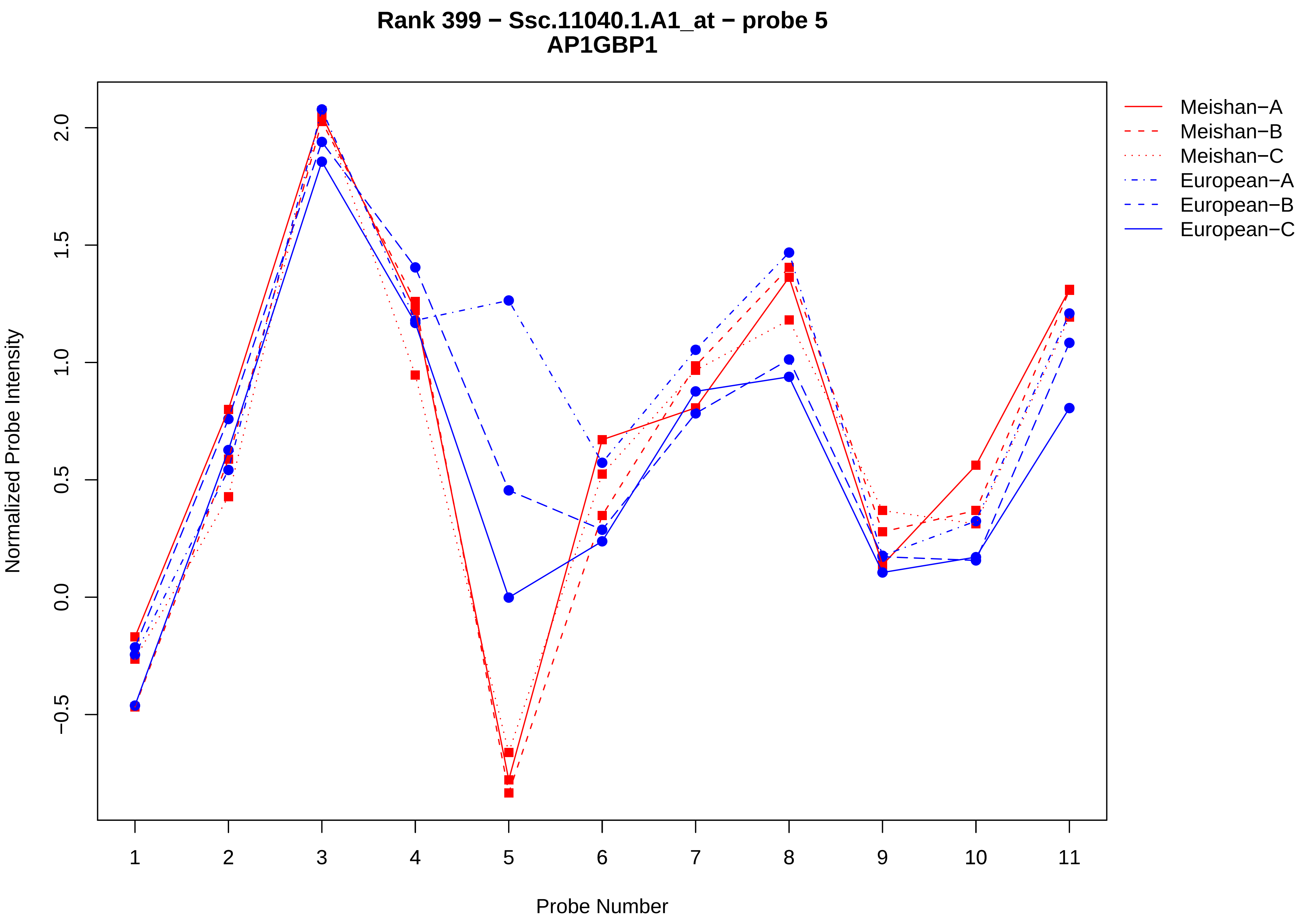

Rank 399 − Ssc.11040.1.A1_at − probe 5
AP1GBP1
Normalized Probe Intensity
Probe Number
−0.5
0.0
0.5
1.0
1.5
2.0
1
2
3
4
5
6
7
8
9
10
11
Meishan−A
Meishan−B
Meishan−C
European−A
European−B
European−C

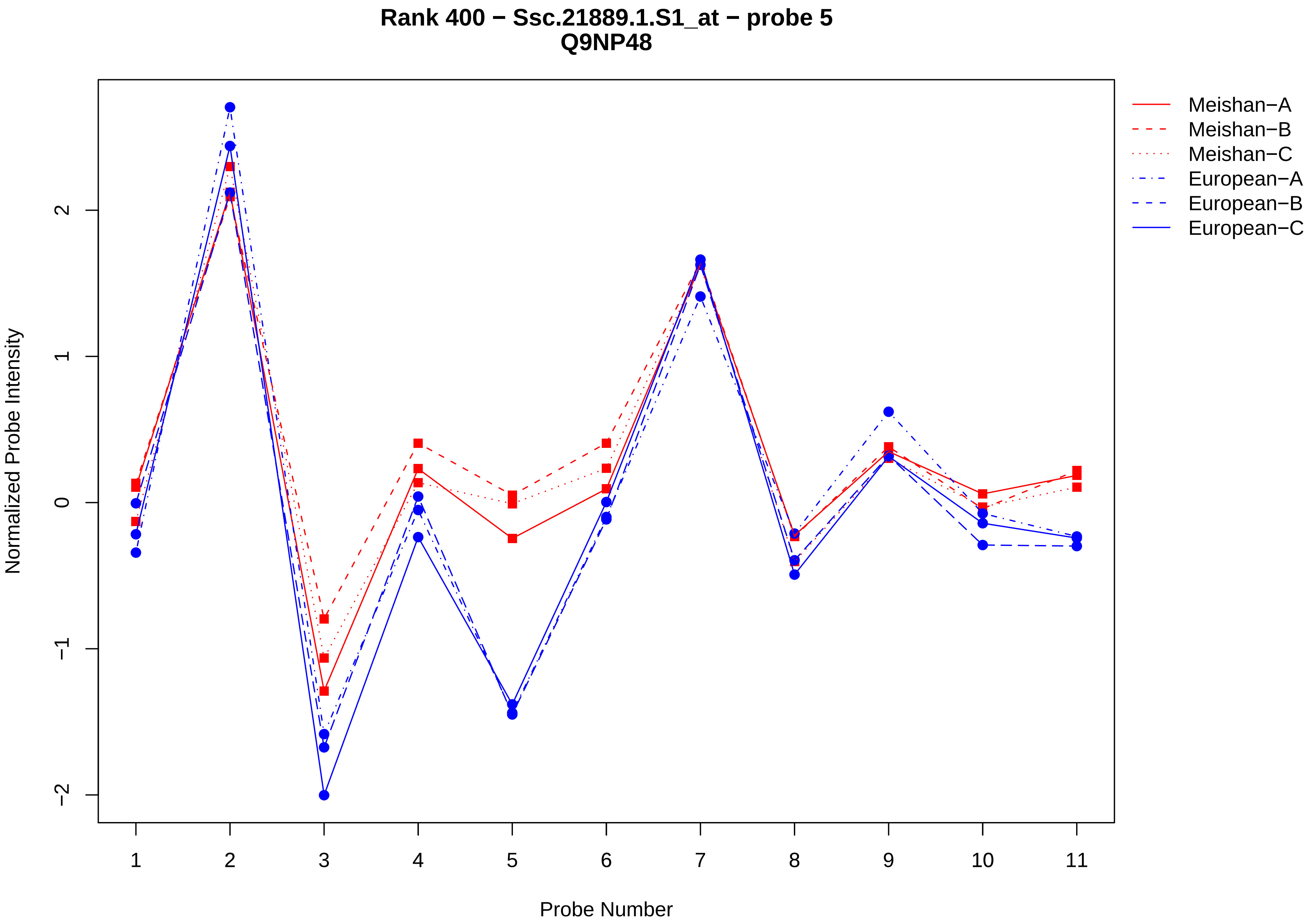
Rank 400 − Ssc.21889.1.S1_at − probe 5
Q9NP48
Normalized Probe Intensity
Probe Number
−2
−1
0
1
2
1
2
3
4
5
6
7
8
9
10
11
Meishan−A
Meishan−B
Meishan−C
European−A
European−B
European−C

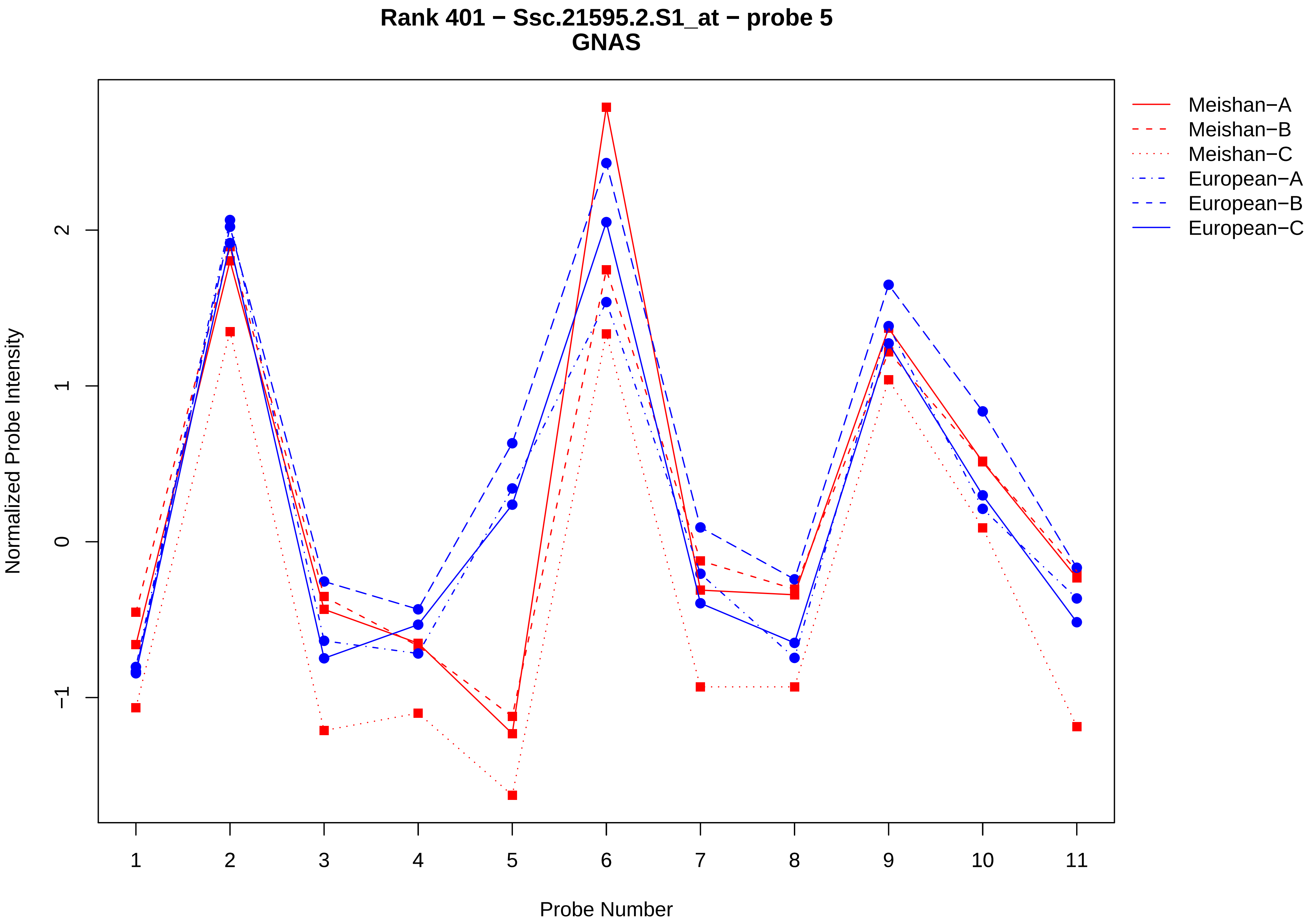

Rank 401 − Ssc.21595.2.S1_at − probe 5
GNAS
Normalized Probe Intensity
−1
0
1
2
1
2
3
4
5
6
7
8
9
10
11
Probe Number
Meishan−A
Meishan−B
Meishan−C
European−A
European−B
European−C

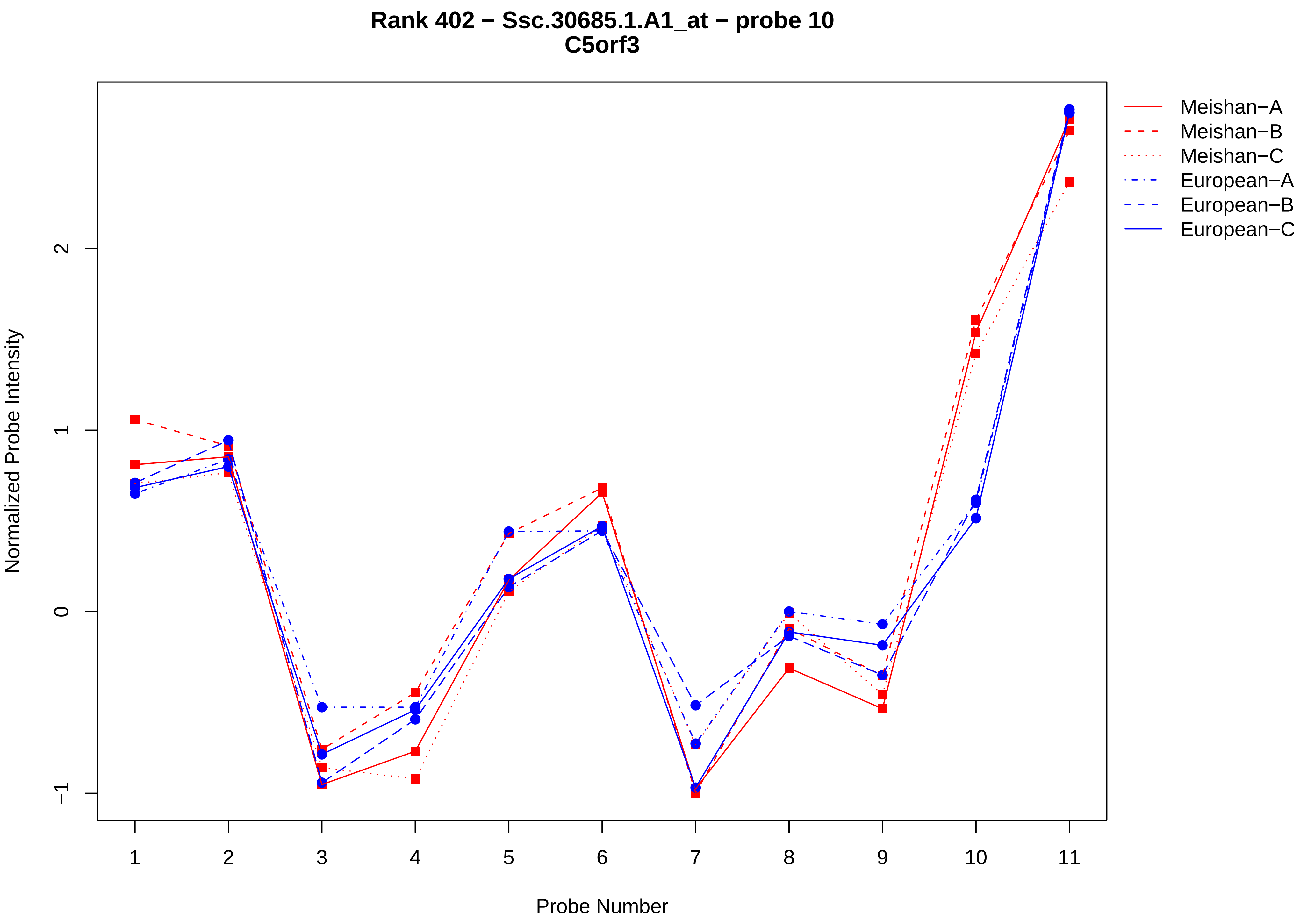

Rank 402 − Ssc.30685.1.A1_at − probe 10
C5orf3
Normalized Probe Intensity
Probe Number
Meishan−A
Meishan−B
Meishan−C
European−A
European−B
European−C
−1
0
1
2
1
2
3
4
5
6
7
8
9
10
11

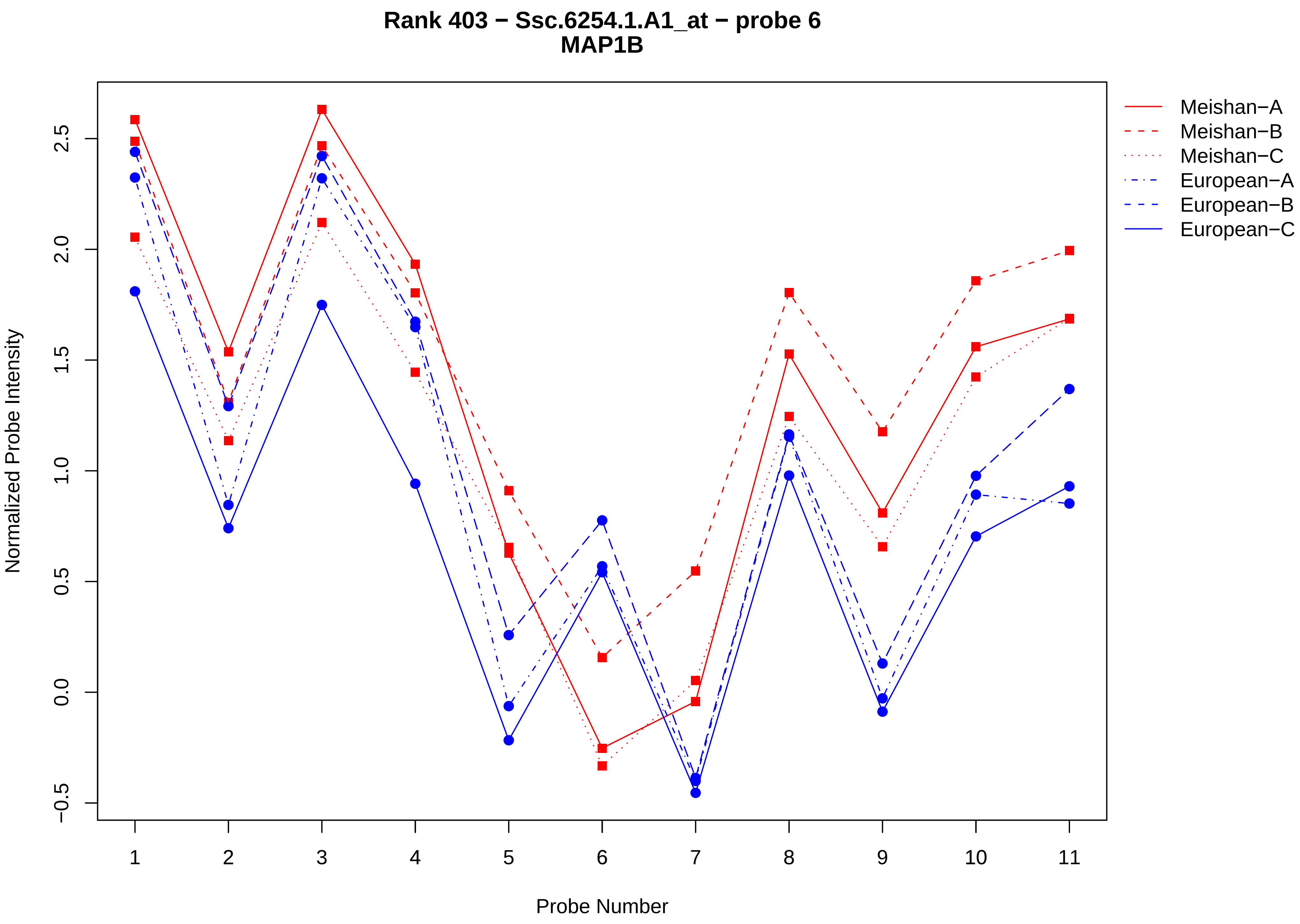
Rank 403 − Ssc.6254.1.A1_at − probe 6
MAP1B
Normalized Probe Intensity
Probe Number
−0.5
0.0
0.5
1.0
1.5
2.0
2.5
1
2
3
4
5
6
7
8
9
10
11
Meishan−A
Meishan−B
Meishan−C
European−A
European−B
European−C

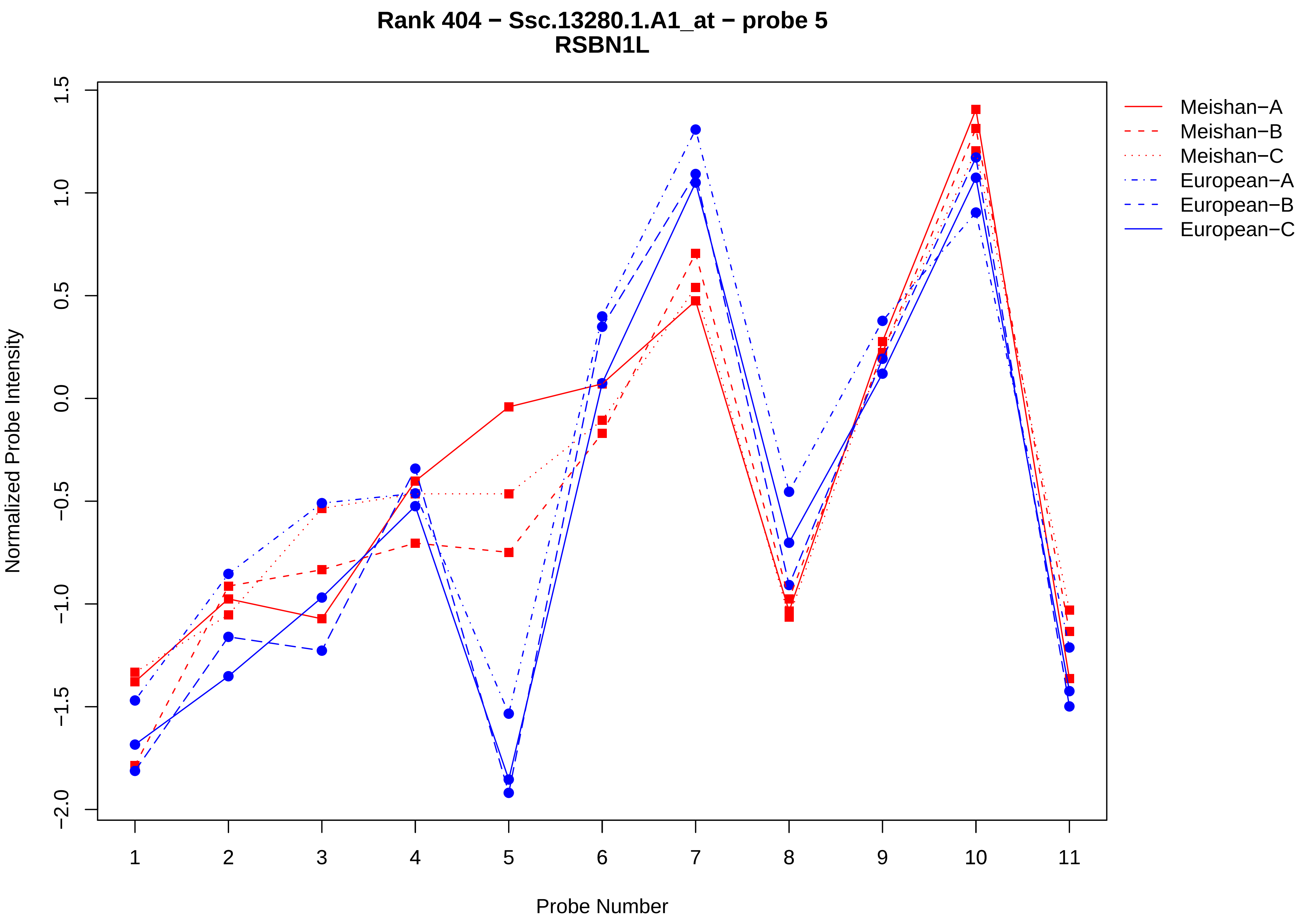

Rank 404 − Ssc.13280.1.A1_at − probe 5
RSBN1L
Normalized Probe Intensity
Probe Number
−2.0
−1.5
−1.0
−0.5
0.0
0.5
1.0
1.5
1
2
3
4
5
6
7
8
9
10
11
Meishan−A
Meishan−B
Meishan−C
European−A
European−B
European−C

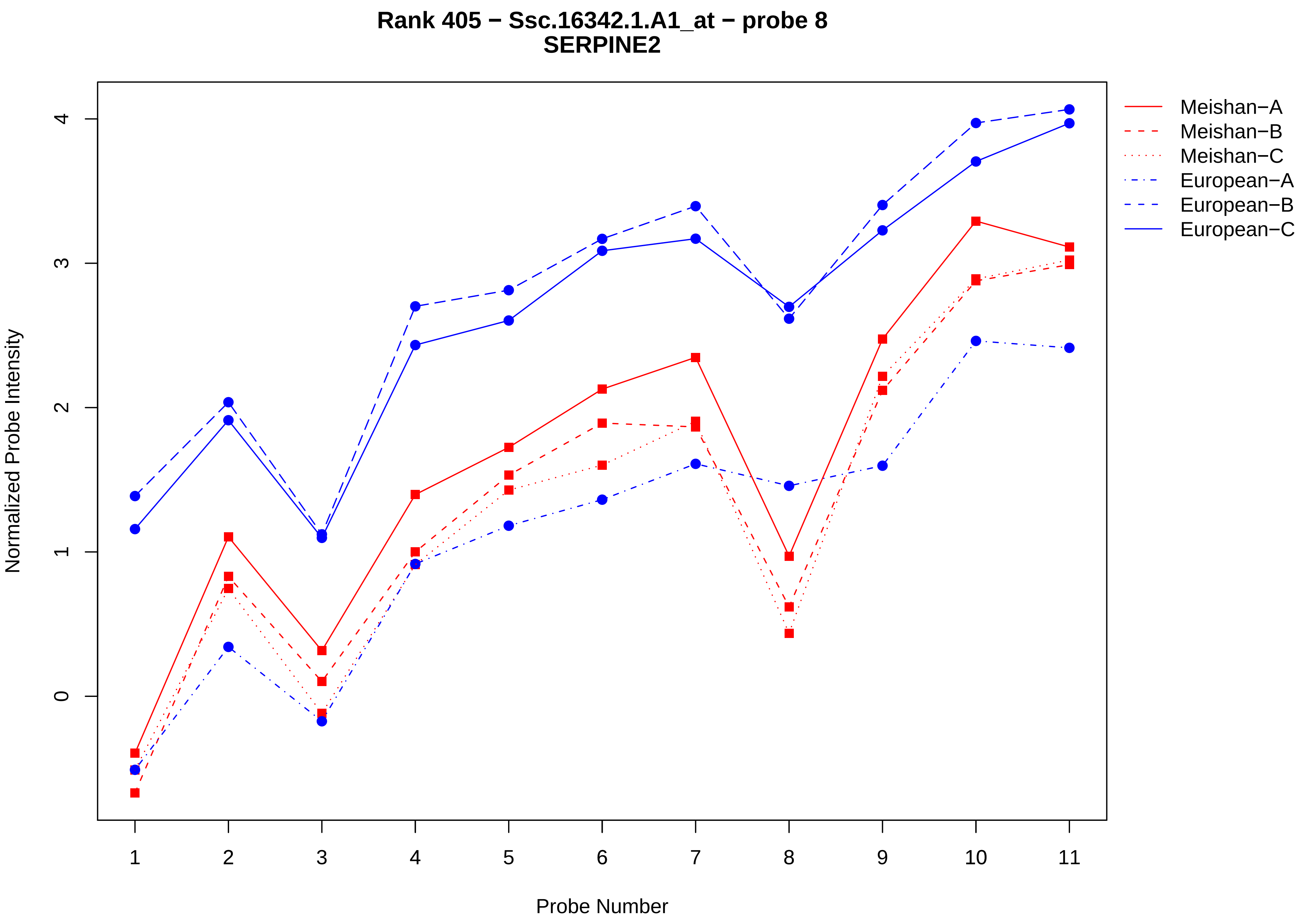

Rank 405 − Ssc.16342.1.A1_at − probe 8
SERPINE2
Normalized Probe Intensity
Probe Number
0
1
2
3
4
1
2
3
4
5
6
7
8
9
10
11
Meishan−A
Meishan−B
Meishan−C
European−A
European−B
European−C

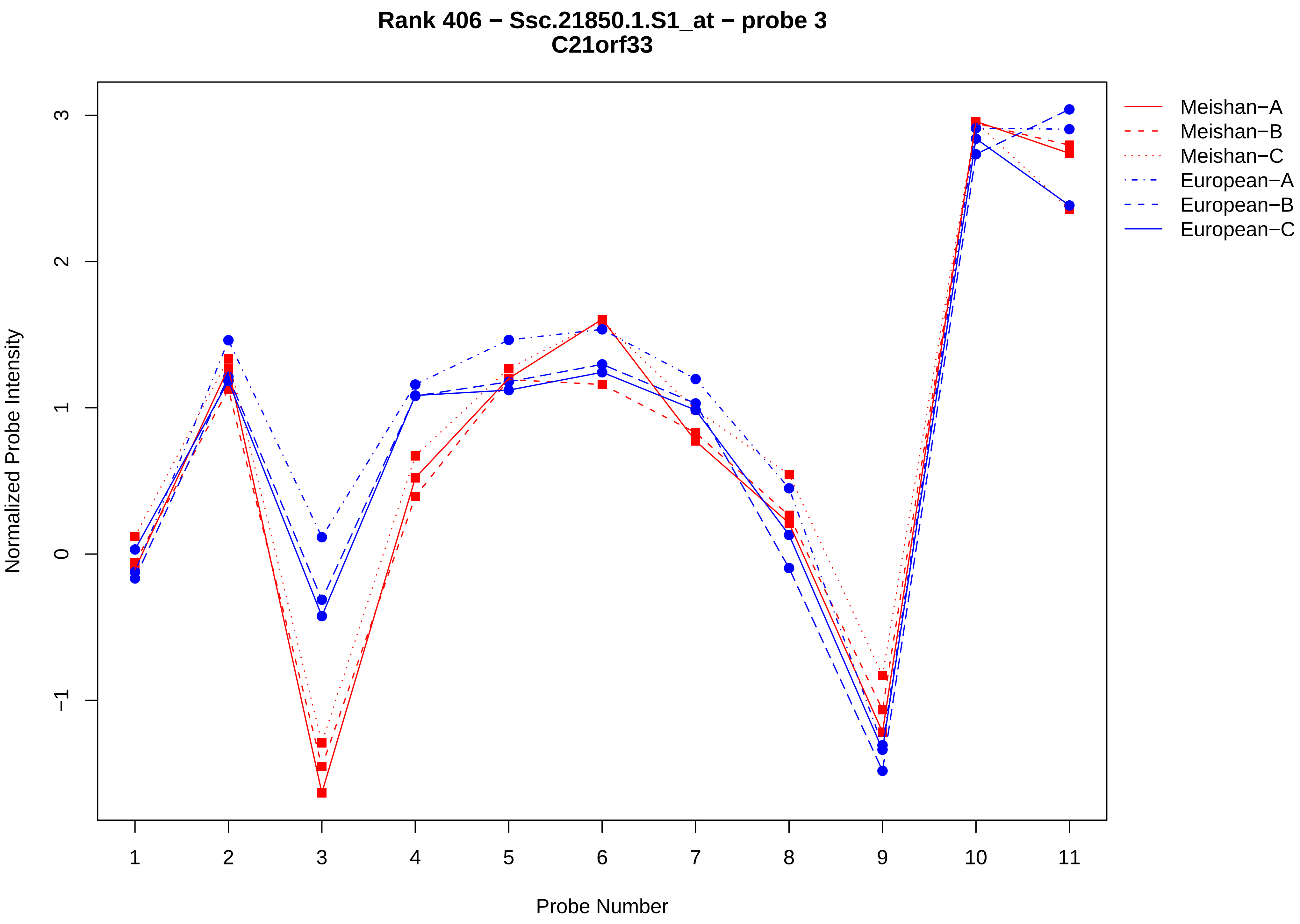

Rank 406 − Ssc.21850.1.S1_at − probe 3
C21orf33
Normalized Probe Intensity
Probe Number
Meishan−A
Meishan−B
Meishan−C
European−A
European−B
European−C

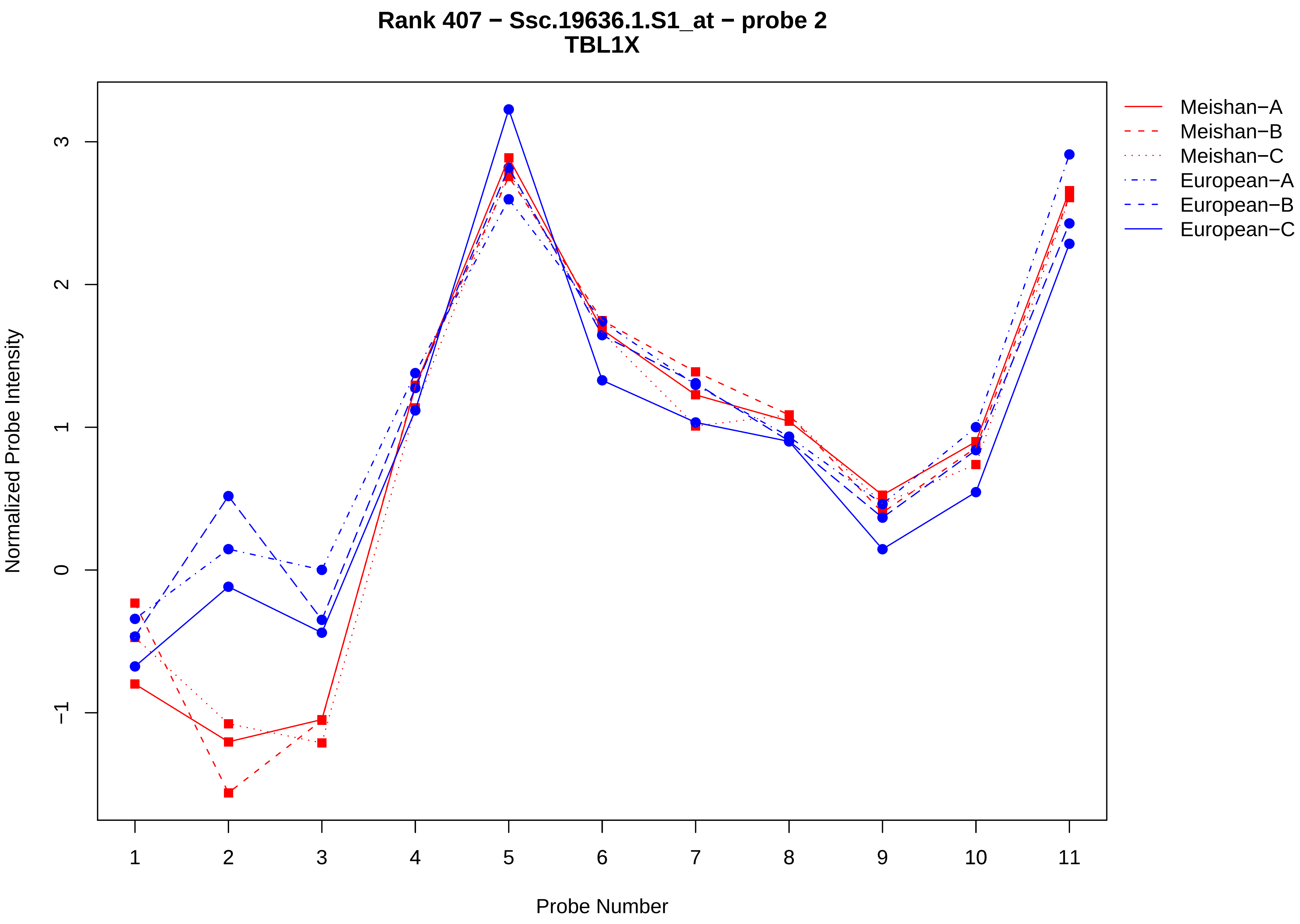

Rank 407 − Ssc.19636.1.S1_at − probe 2
TBL1X
Normalized Probe Intensity
Probe Number
−1
0
1
2
3
1
2
3
4
5
6
7
8
9
10
11
Meishan−A
Meishan−B
Meishan−C
European−A
European−B
European−C

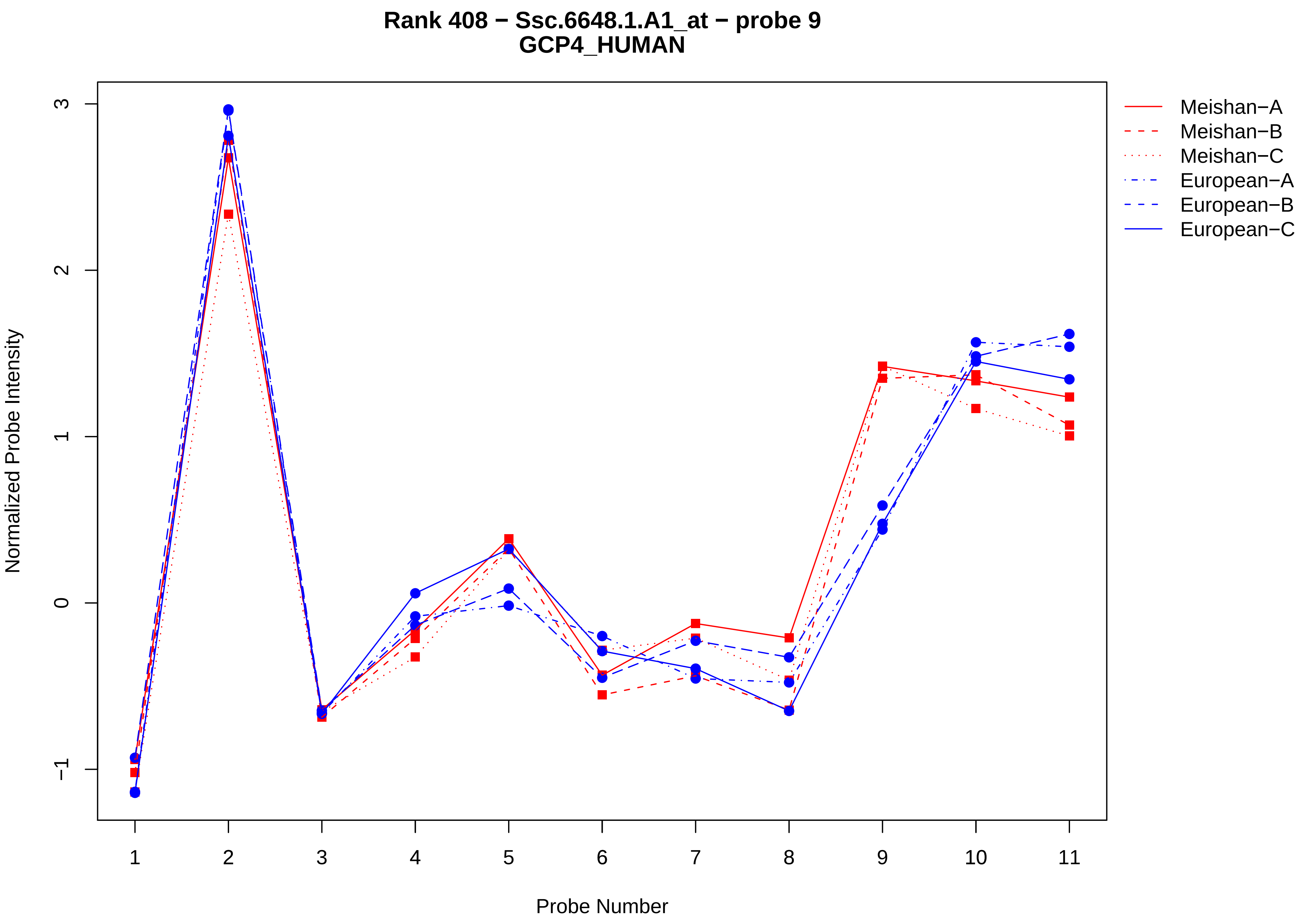

Rank 408 − Ssc.6648.1.A1_at − probe 9
GCP4_HUMAN
Normalized Probe Intensity
Probe Number
Meishan−A
Meishan−B
Meishan−C
European−A
European−B
European−C
−1
0
1
2
3
1
2
3
4
5
6
7
8
9
10
11

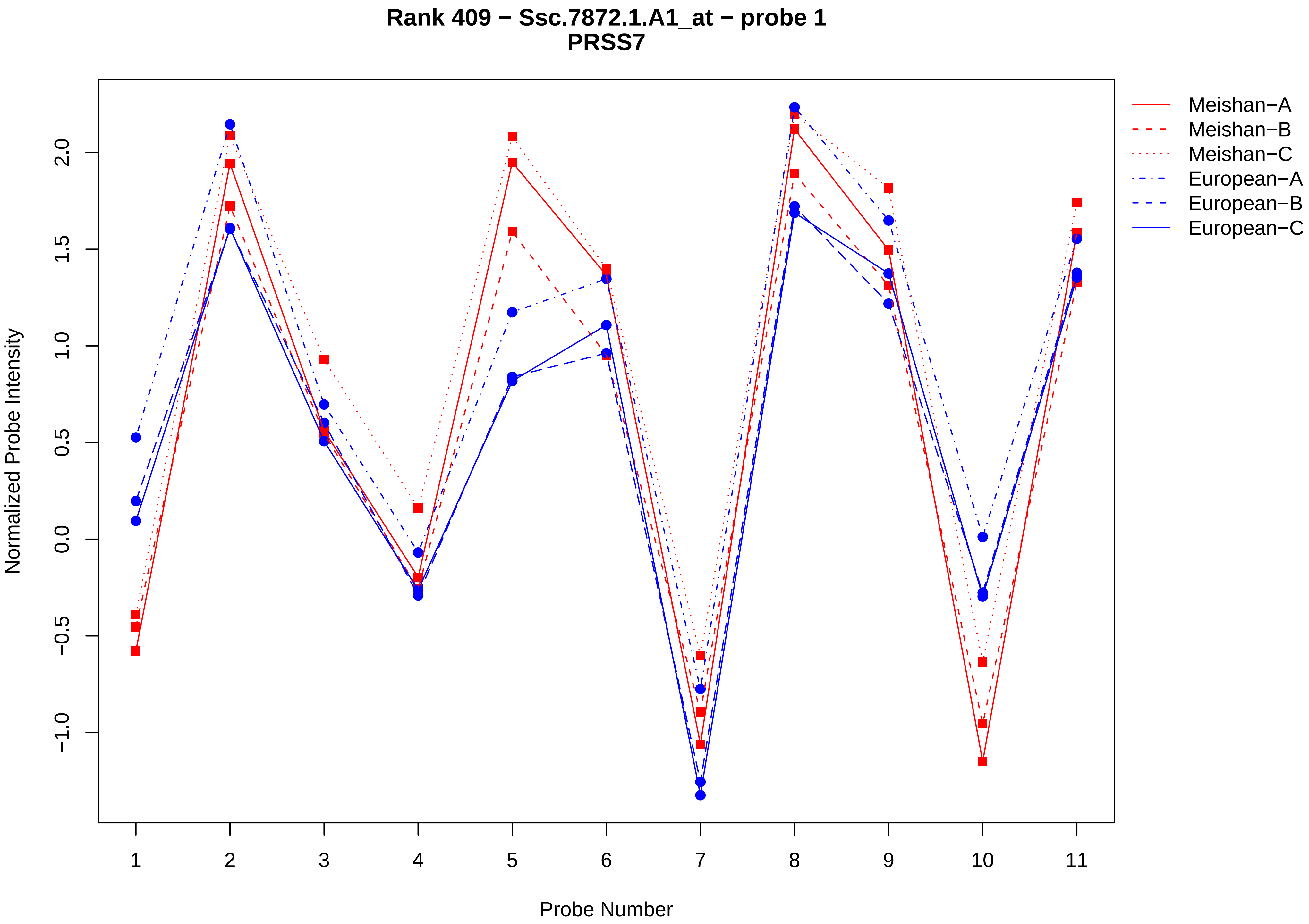

Rank 409 − Ssc.7872.1.A1_at − probe 1
PRSS7
Normalized Probe Intensity
Probe Number
Meishan−A
Meishan−B
Meishan−C
European−A
European−B
European−C
−1.0
−0.5
0.0
0.5
1.0
1.5
2.0
1
2
3
4
5
6
7
8
9
10
11

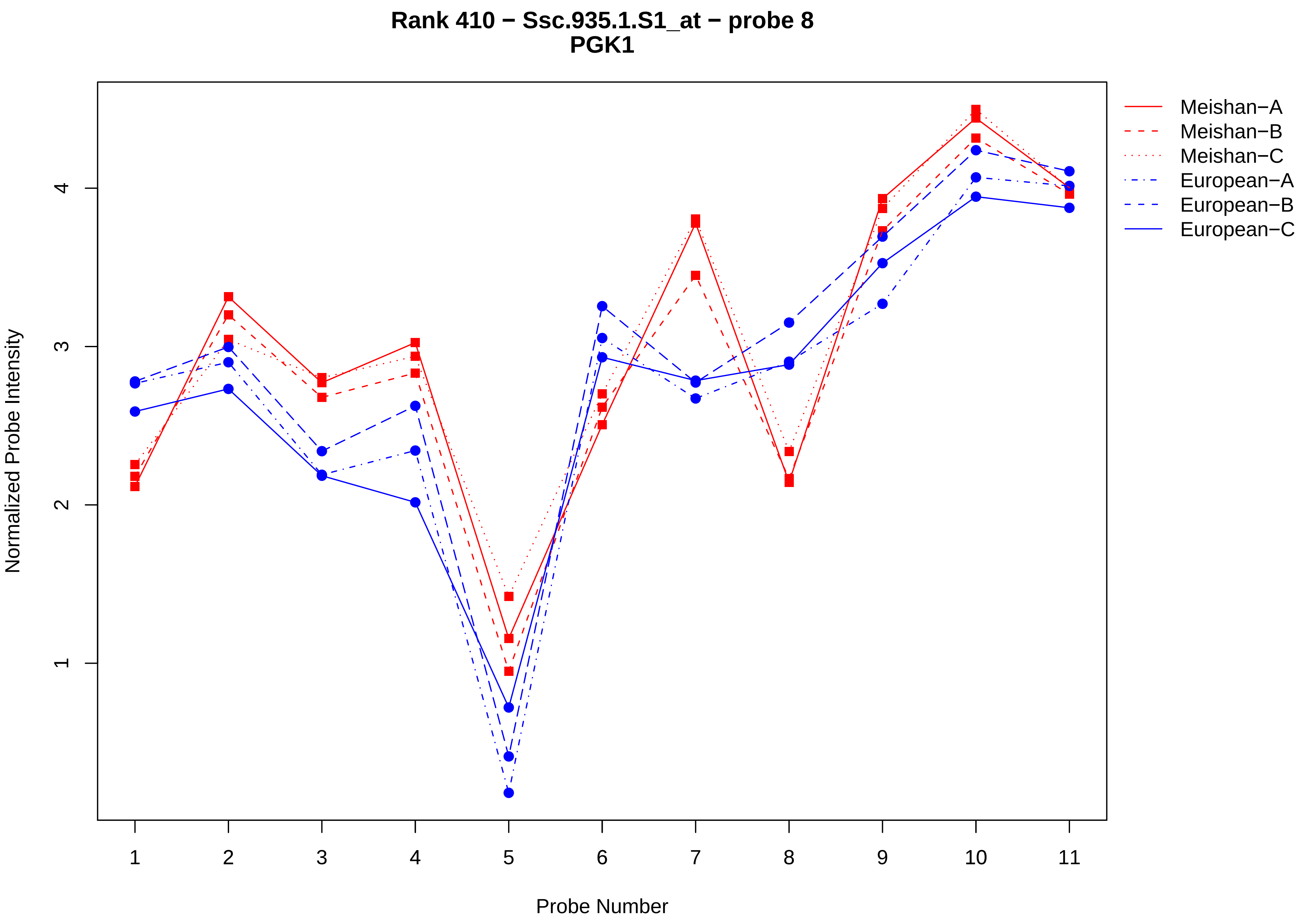
Rank 410 − Ssc.935.1.S1_at − probe 8
PGK1
Normalized Probe Intensity
Probe Number
1
2
3
4
5
6
7
8
9
10
11
Meishan−A
Meishan−B
Meishan−C
European−A
European−B
European−C

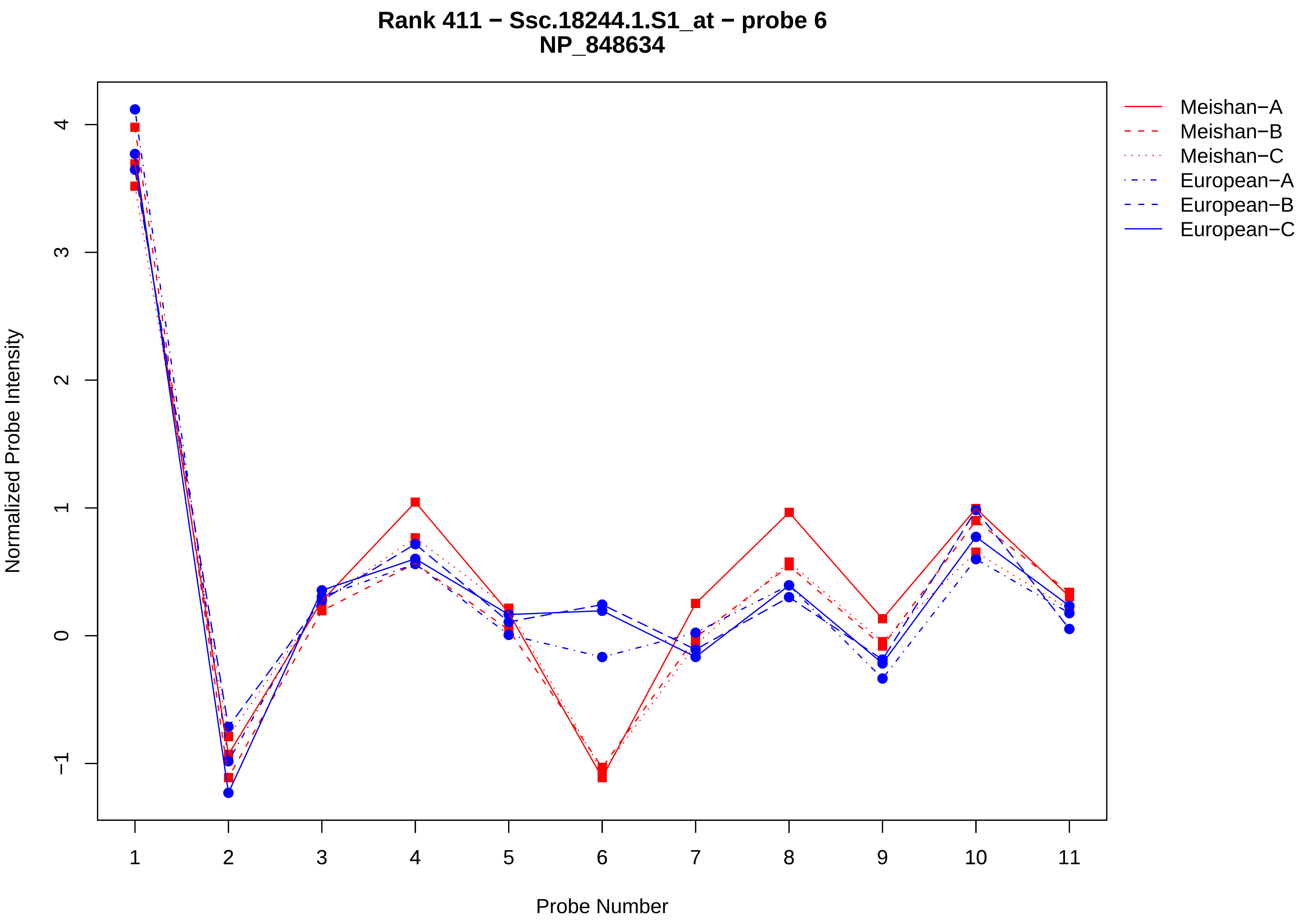
Rank 411 − Ssc.18244.1.S1_at − probe 6
NP_848634
Normalized Probe Intensity
Probe Number
−1
0
1
2
3
4
1
2
3
4
5
6
7
8
9
10
11
Meishan−A
Meishan−B
Meishan−C
European−A
European−B
European−C

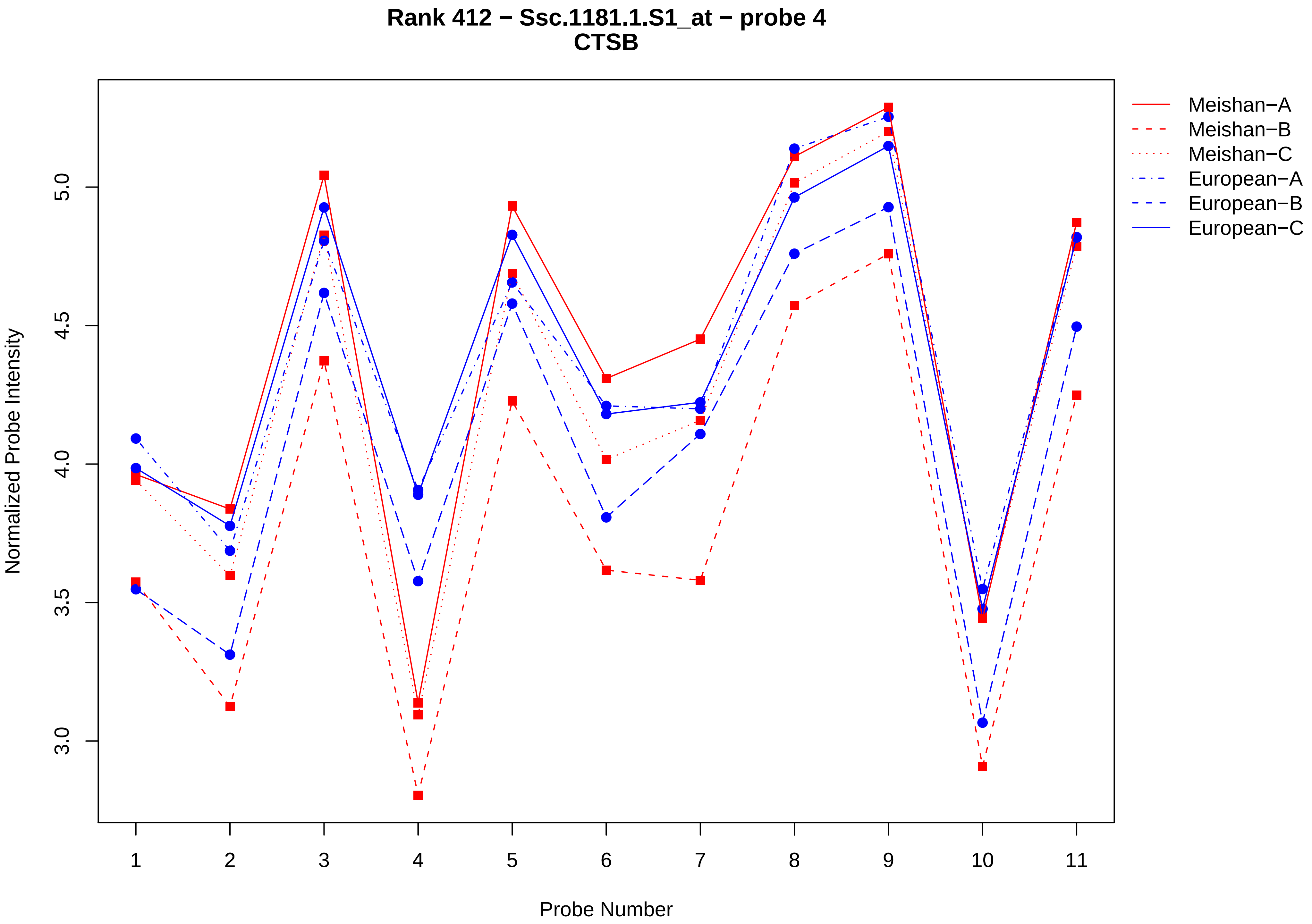

Rank 412 − Ssc.1181.1.S1_at − probe 4
CTSB
Normalized Probe Intensity
Probe Number
3.0
3.5
4.0
4.5
5.0
1
2
3
4
5
6
7
8
9
10
11
Meishan−A
Meishan−B
Meishan−C
European−A
European−B
European−C

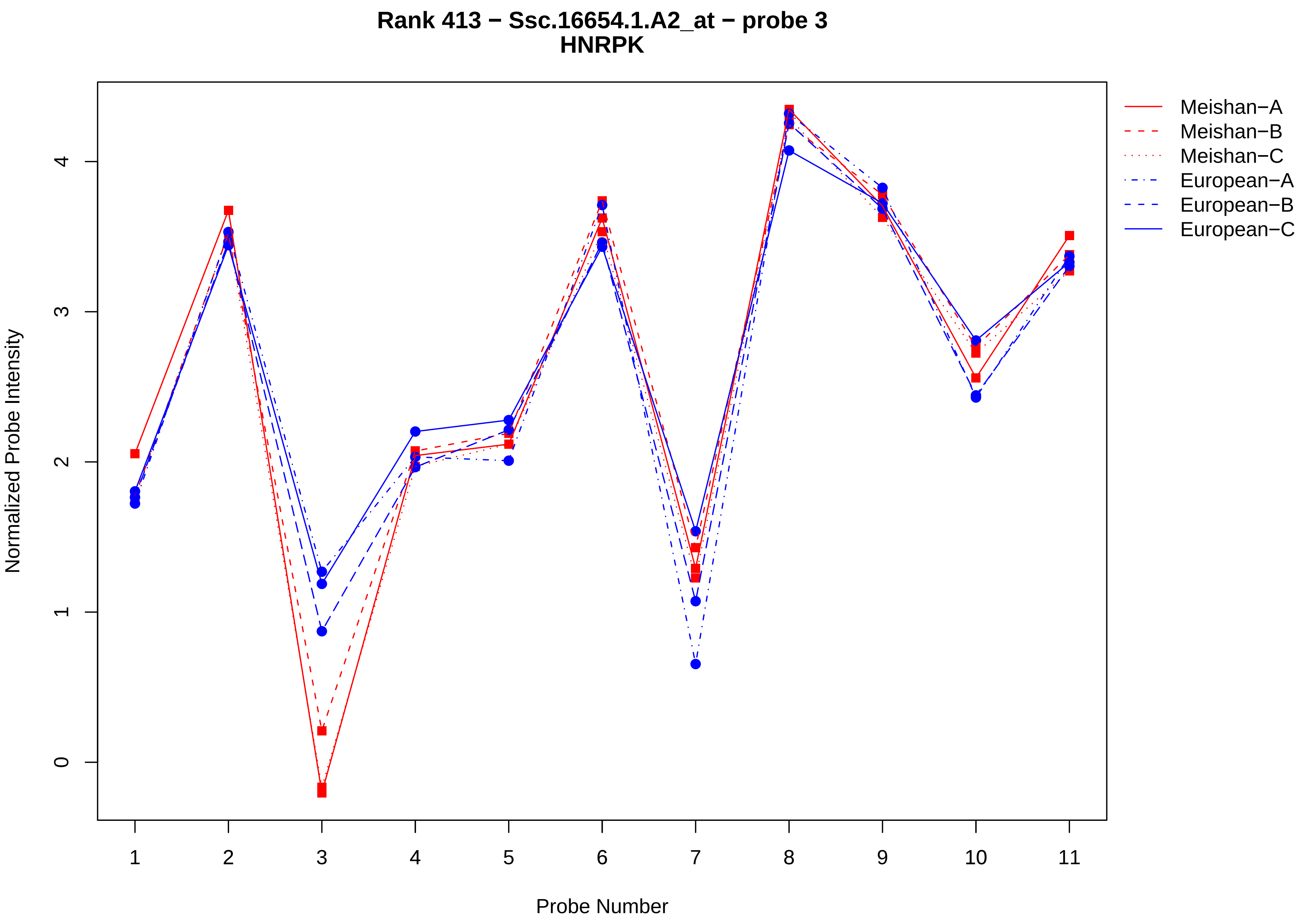

Rank 413 − Ssc.16654.1.A2_at − probe 3
HNRPK
Normalized Probe Intensity
Probe Number
0
1
2
3
4
1
2
3
4
5
6
7
8
9
10
11
Meishan−A
Meishan−B
Meishan−C
European−A
European−B
European−C

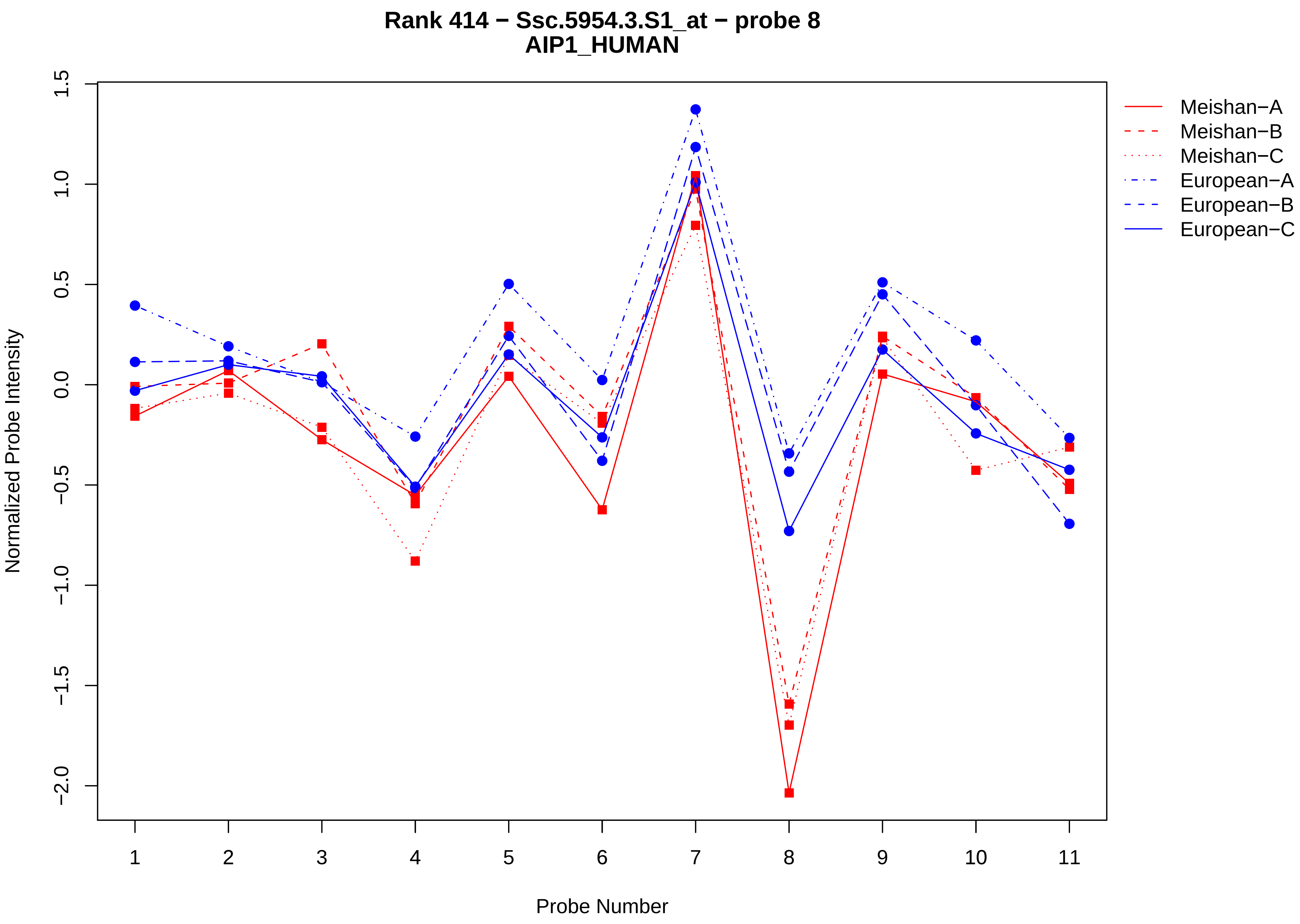
Rank 414 − Ssc.5954.3.S1_at − probe 8
AIP1_HUMAN
Normalized Probe Intensity
Probe Number
Meishan−A
Meishan−B
Meishan−C
European−A
European−B
European−C
1.5
1.0
0.5
0.0
−0.5
−1.0
−1.5
−2.0
1
2
3
4
5
6
7
8
9
10
11

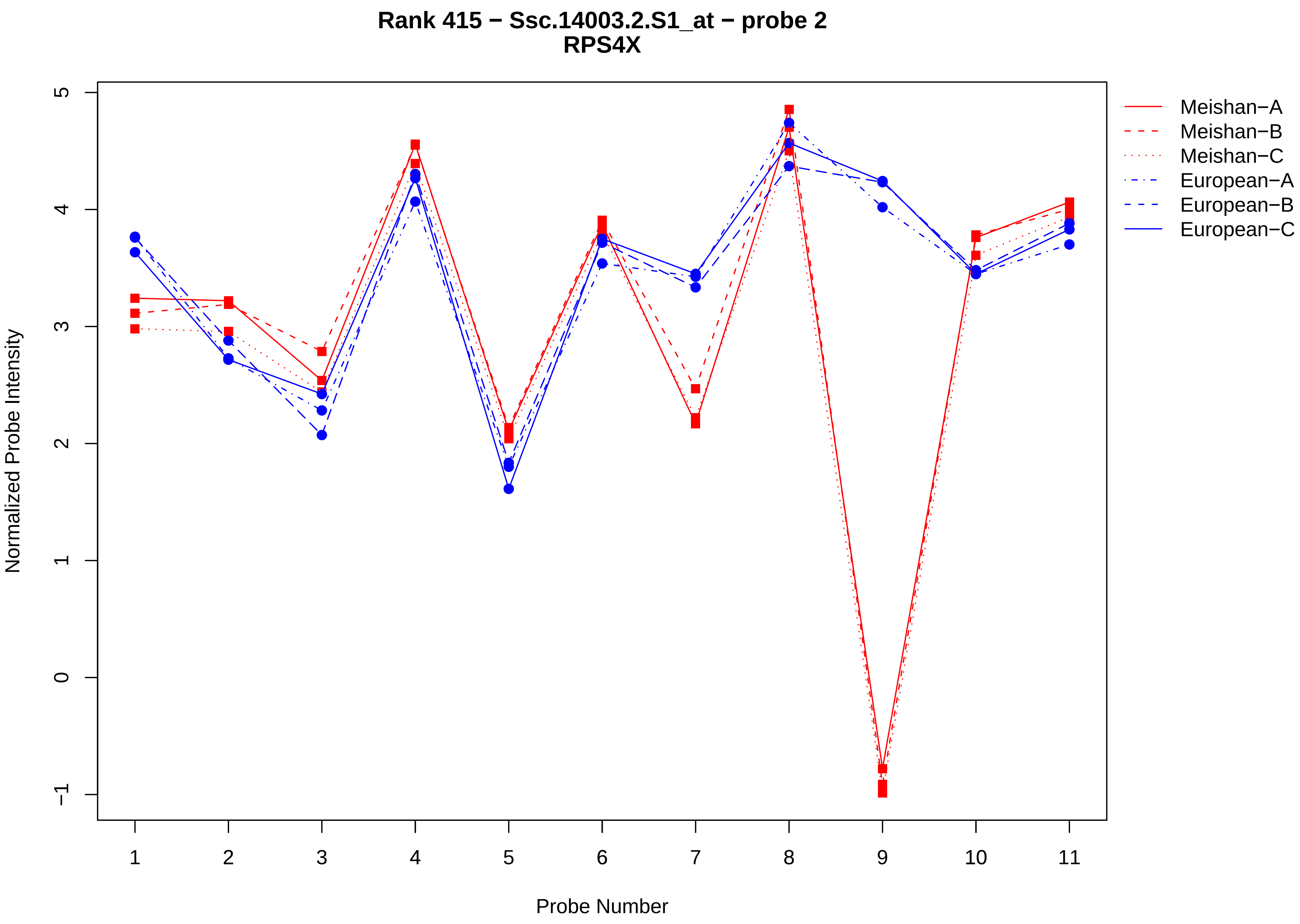
Rank 415 − Ssc.14003.2.S1_at − probe 2
RPS4X
Normalized Probe Intensity
Probe Number
Meishan−A
Meishan−B
Meishan−C
European−A
European−B
European−C
−1
0
1
2
3
4
5
1
2
3
4
5
6
7
8
9
10
11

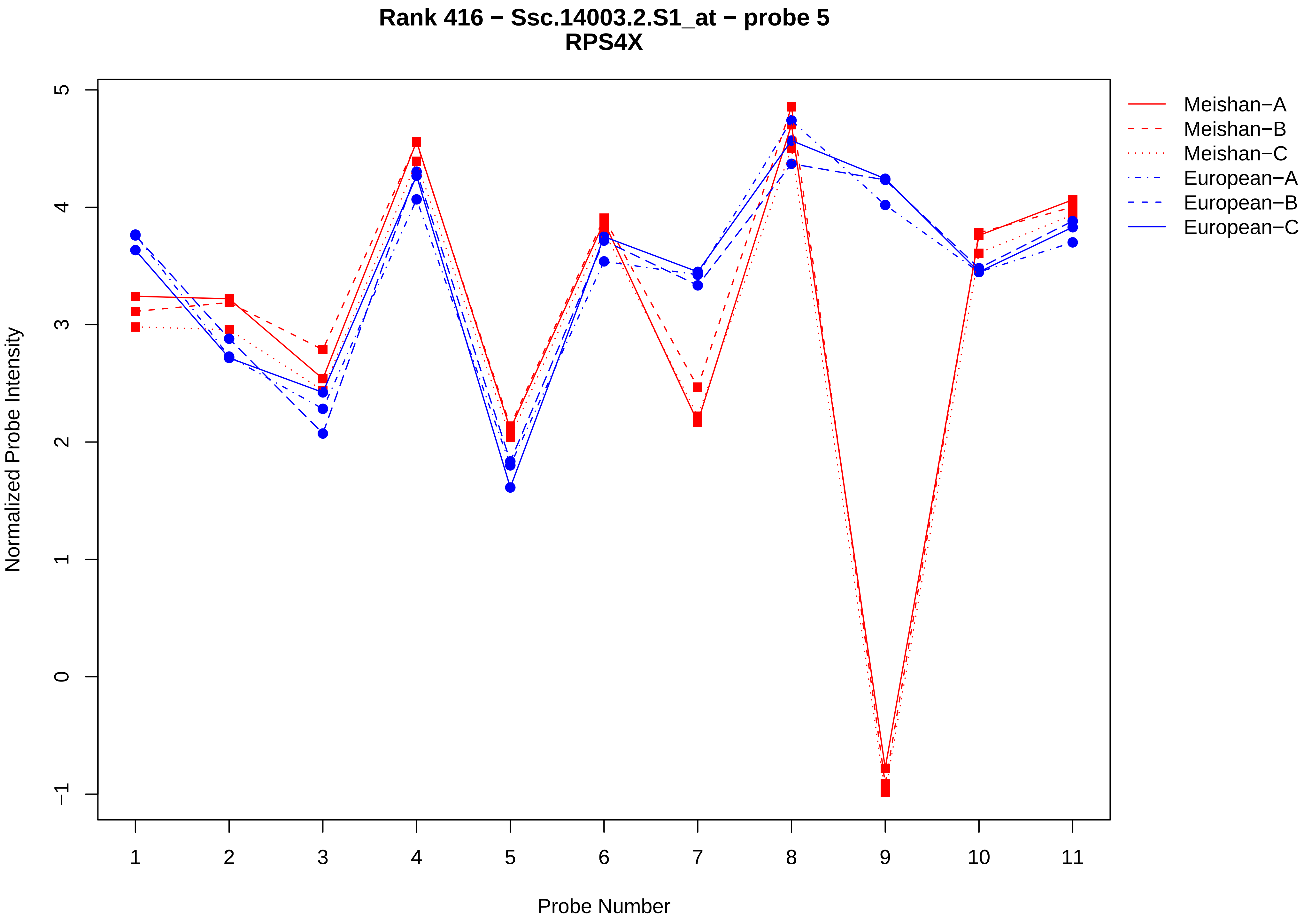

Rank 416 − Ssc.14003.2.S1_at − probe 5
RPS4X
Normalized Probe Intensity
Probe Number
Meishan−A
Meishan−B
Meishan−C
European−A
European−B
European−C
−1
0
1
2
3
4
5
1
2
3
4
5
6
7
8
9
10
11

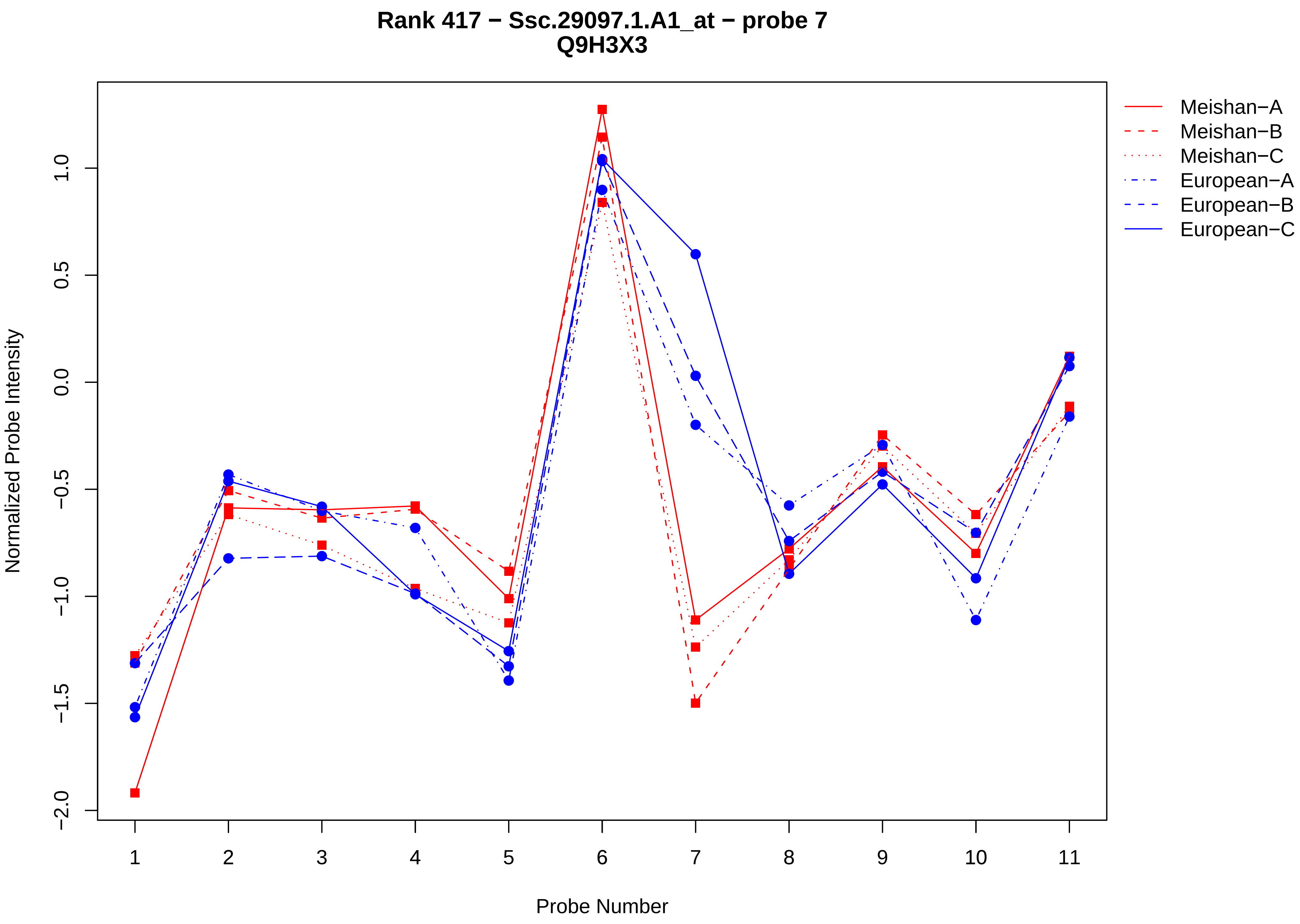

Rank 417 − Ssc.29097.1.A1_at − probe 7
Q9H3X3
Normalized Probe Intensity
Probe Number
−2.0
−1.5
−1.0
−0.5
0.0
0.5
1.0
1
2
3
4
5
6
7
8
9
10
11
Meishan−A
Meishan−B
Meishan−C
European−A
European−B
European−C

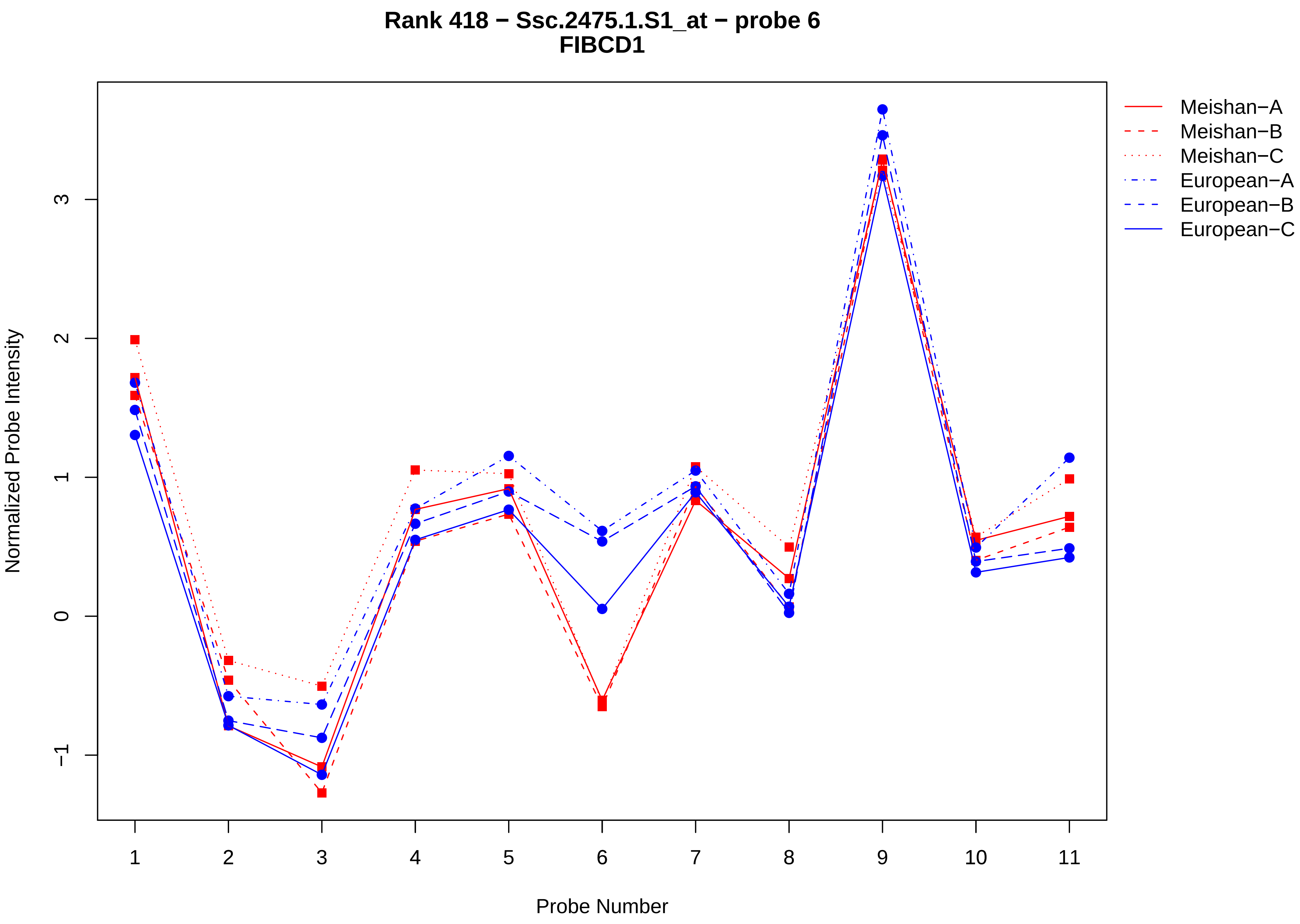
Rank 418 − Ssc.2475.1.S1_at − probe 6
FIBCD1
Normalized Probe Intensity
Probe Number
−1
0
1
2
3
1
2
3
4
5
6
7
8
9
10
11
Meishan−A
Meishan−B
Meishan−C
European−A
European−B
European−C

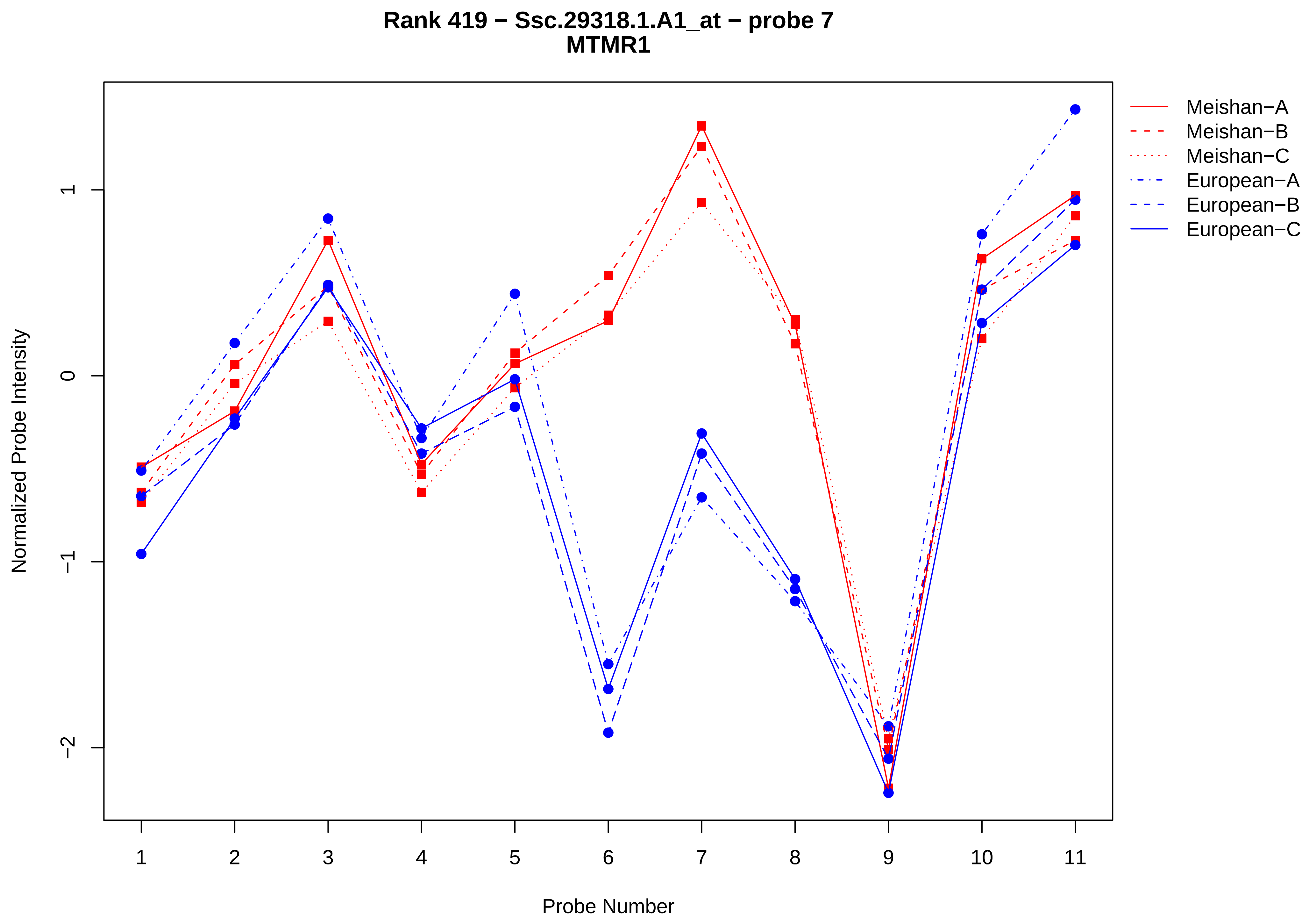
Rank 419 − Ssc.29318.1.A1_at − probe 7
MTMR1
Normalized Probe Intensity
Probe Number
−2
−1
0
1
1
2
3
4
5
6
7
8
9
10
11
Meishan−A
Meishan−B
Meishan−C
European−A
European−B
European−C

Rank 420 − Ssc.10924.1.A1_a_at − probe 11
Q8TAP8

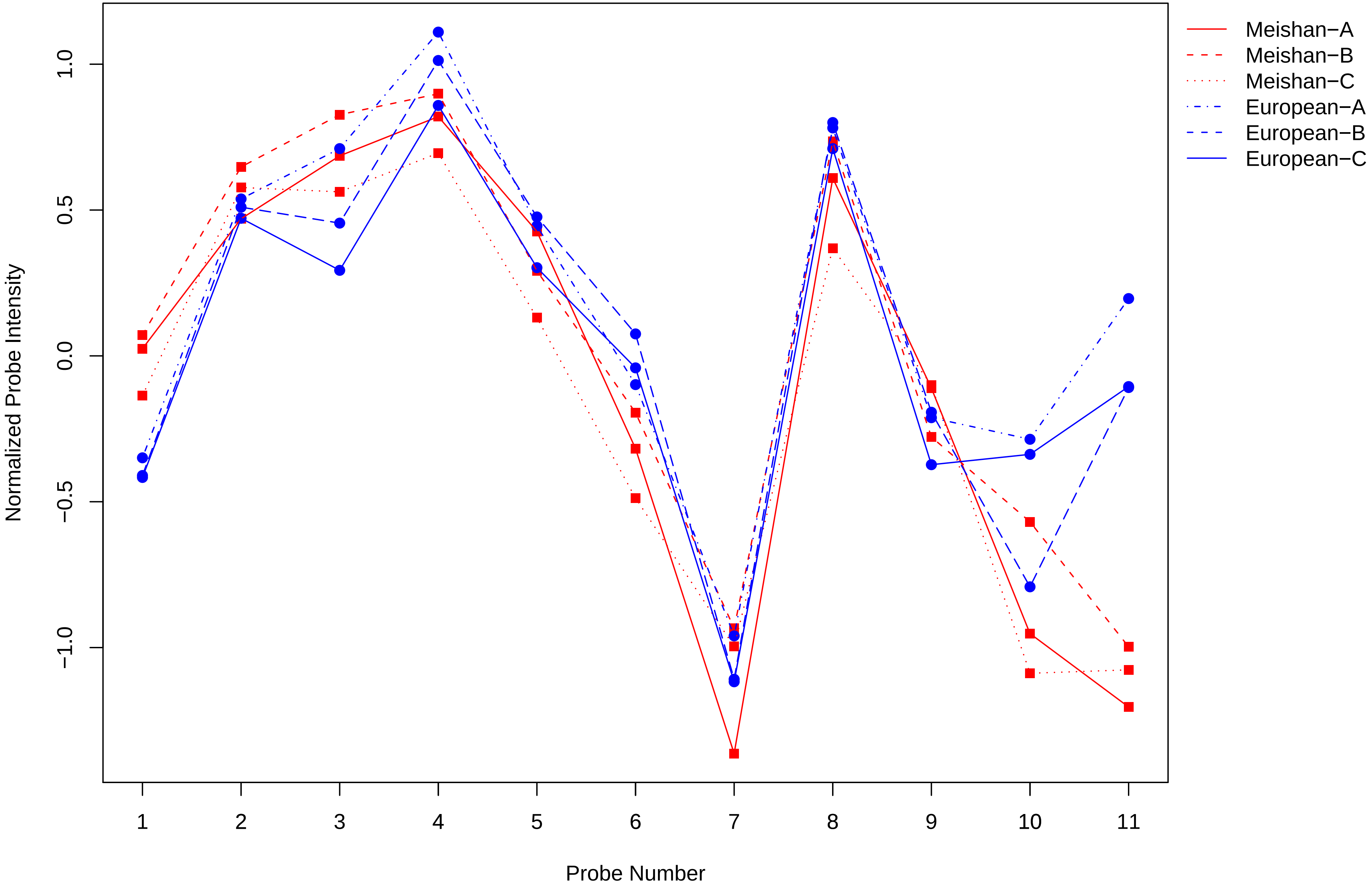

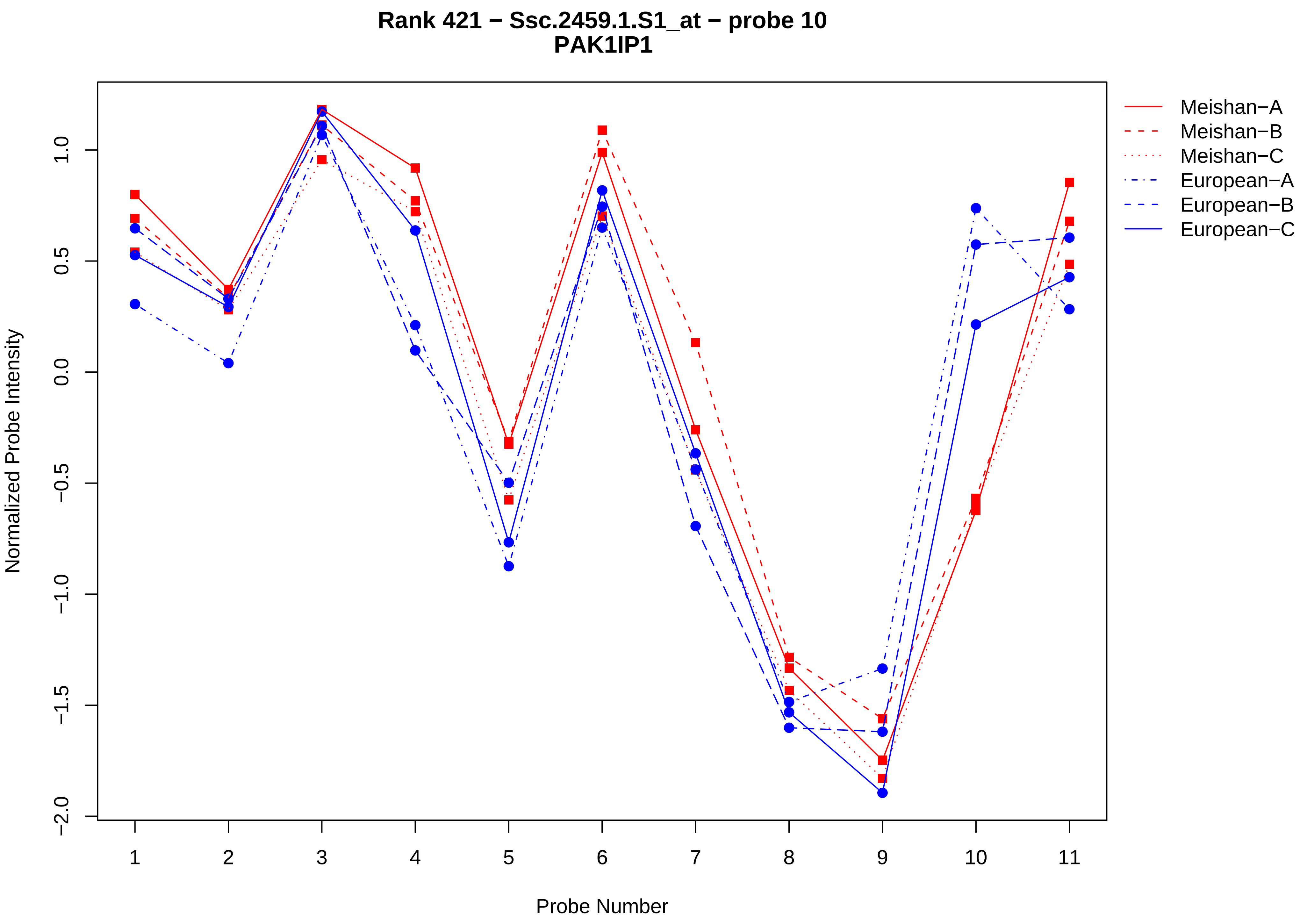

Rank 421 − Ssc.2459.1.S1_at − probe 10
PAK1IP1
Normalized Probe Intensity
Probe Number
Meishan−A
Meishan−B
Meishan−C
European−A
European−B
European−C
1.0
0.5
0.0
−0.5
−1.0
−1.5
−2.0
1
2
3
4
5
6
7
8
9
10
11

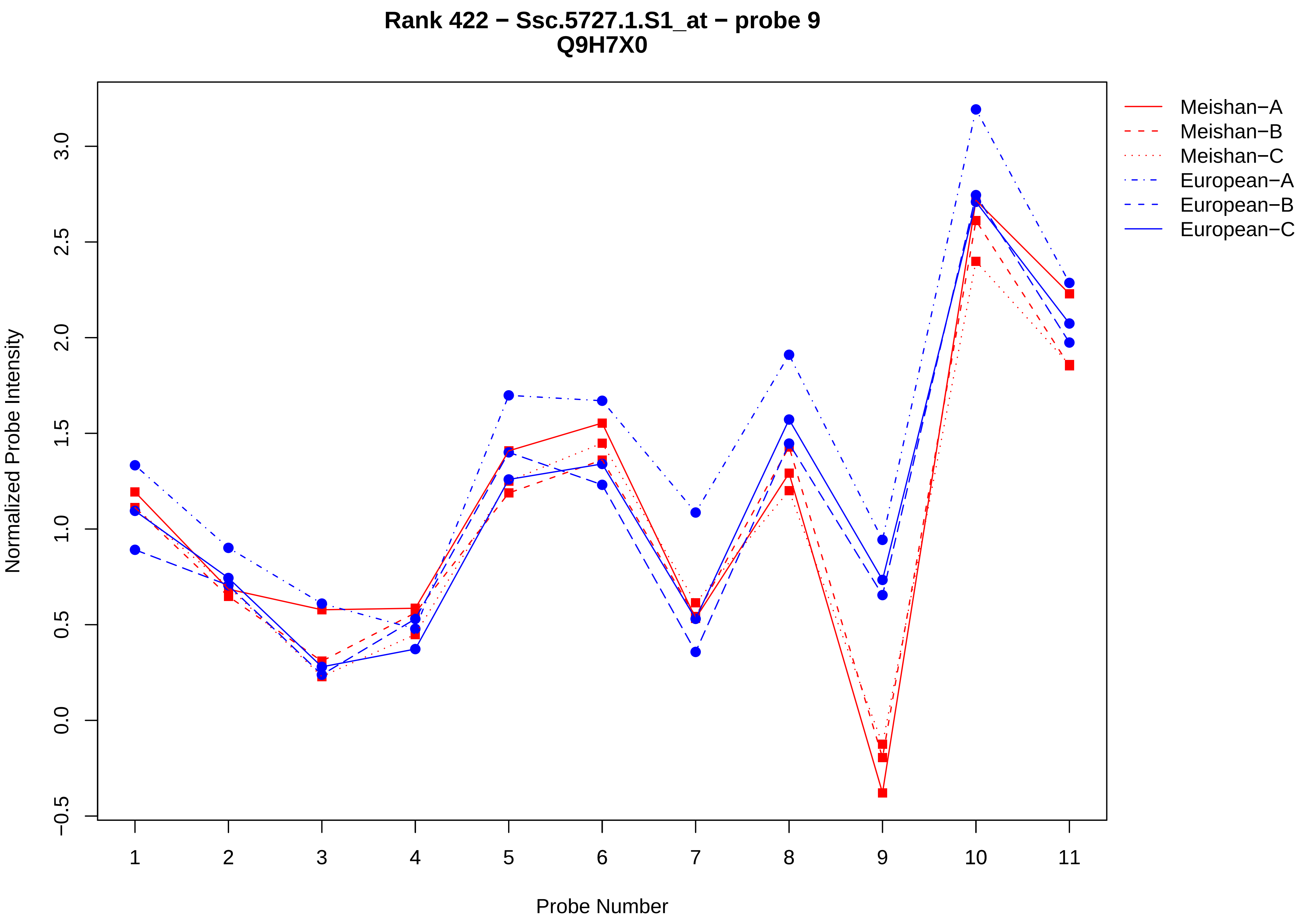
Rank 422 − Ssc.5727.1.S1_at − probe 9
Q9H7X0
Normalized Probe Intensity
Probe Number
−0.5
0.0
0.5
1.0
1.5
2.0
2.5
3.0
1
2
3
4
5
6
7
8
9
10
11
Meishan−A
Meishan−B
Meishan−C
European−A
European−B
European−C

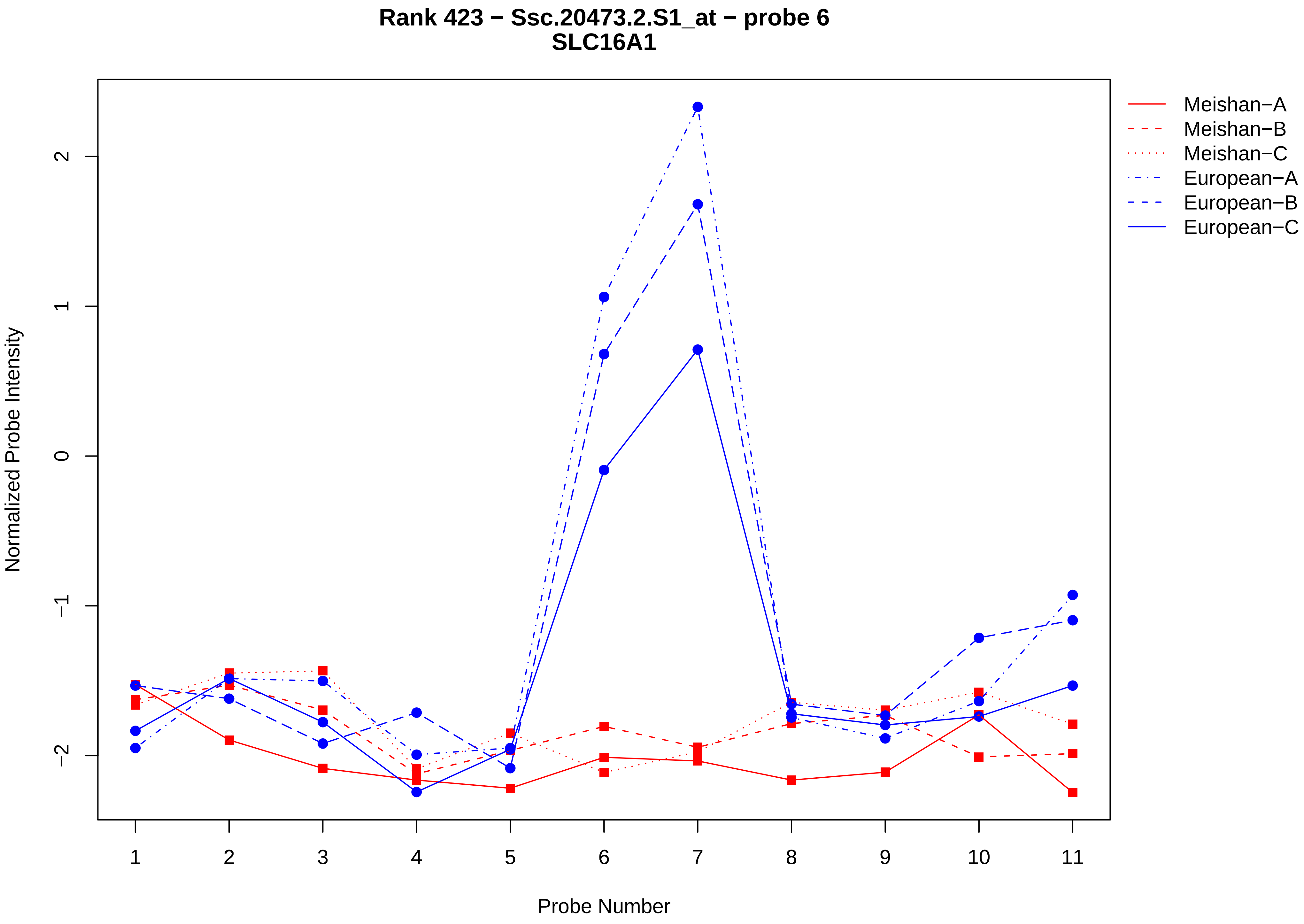

Rank 423 − Ssc.20473.2.S1_at − probe 6
SLC16A1
Normalized Probe Intensity
Probe Number
−2
−1
0
1
2
1
2
3
4
5
6
7
8
9
10
11
Meishan−A
Meishan−B
Meishan−C
European−A
European−B
European−C

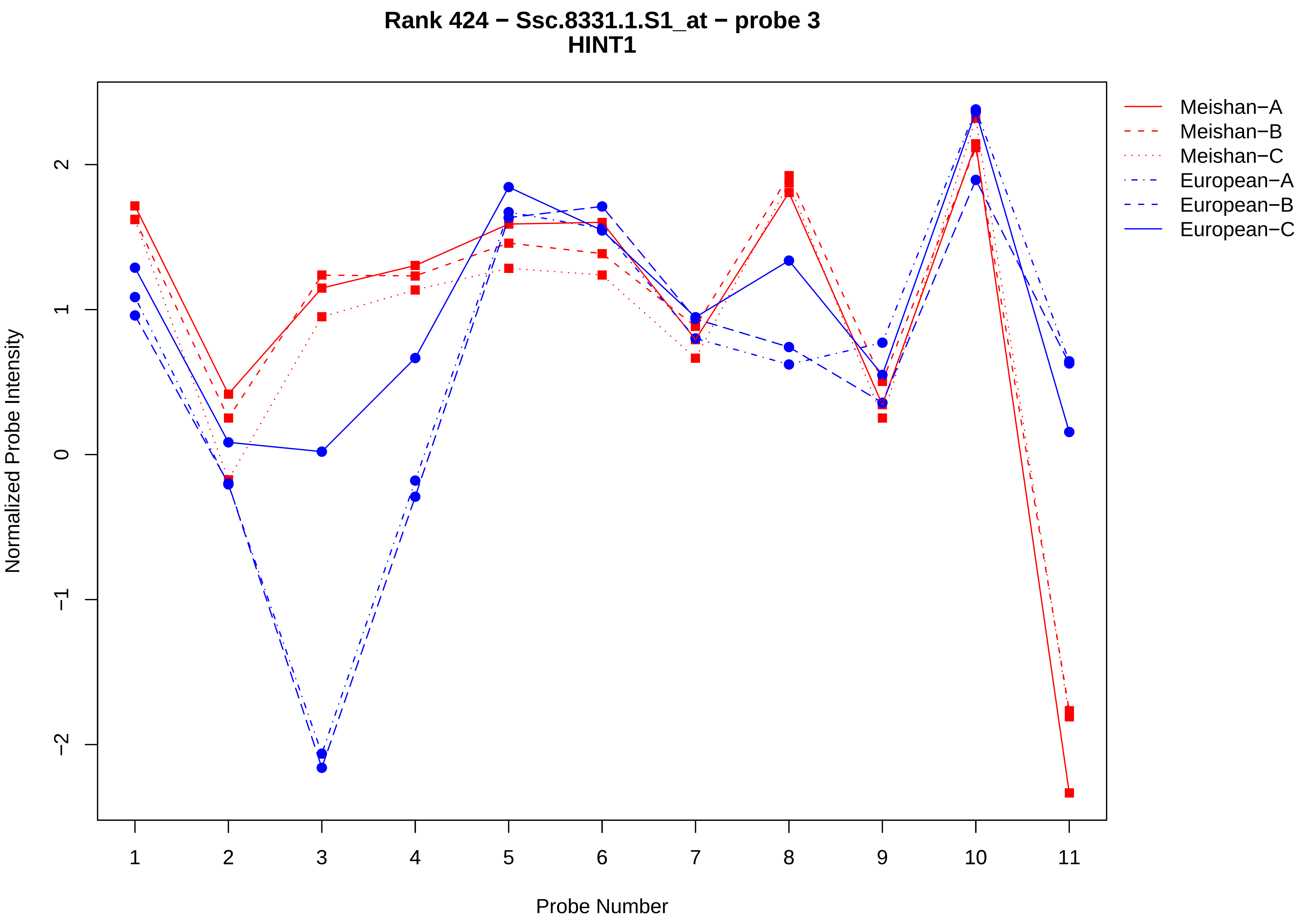
Rank 424 − Ssc.8331.1.S1_at − probe 3
HINT1
Normalized Probe Intensity
Probe Number
Meishan−A
Meishan−B
Meishan−C
European−A
European−B
European−C
−2
−1
0
1
2
1
2
3
4
5
6
7
8
9
10
11

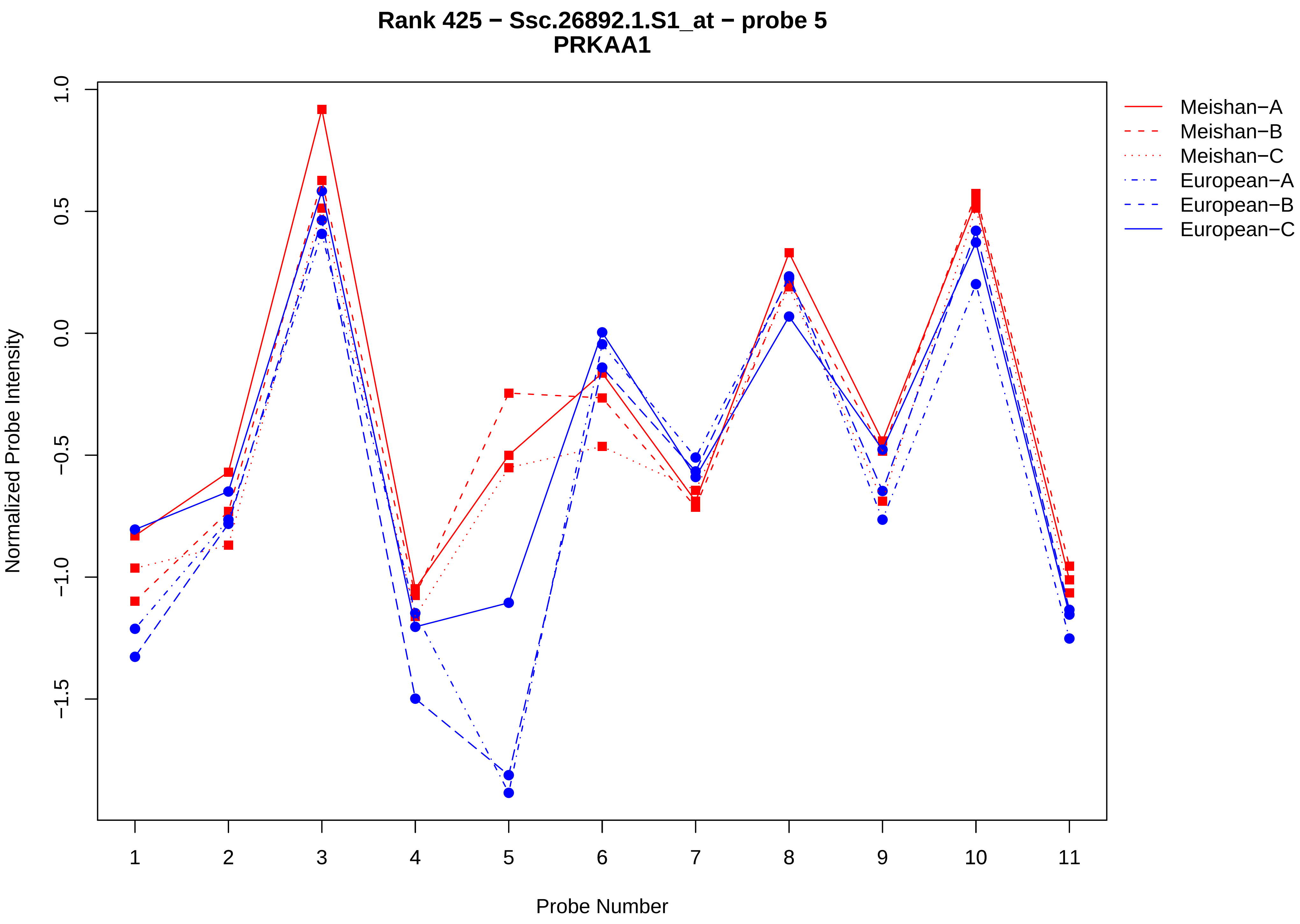
Rank 425 − Ssc.26892.1.S1_at − probe 5
PRKAA1
Normalized Probe Intensity
Probe Number
1.0
0.5
0.0
−0.5
−1.0
−1.5
1
2
3
4
5
6
7
8
9
10
11
Meishan−A
Meishan−B
Meishan−C
European−A
European−B
European−C

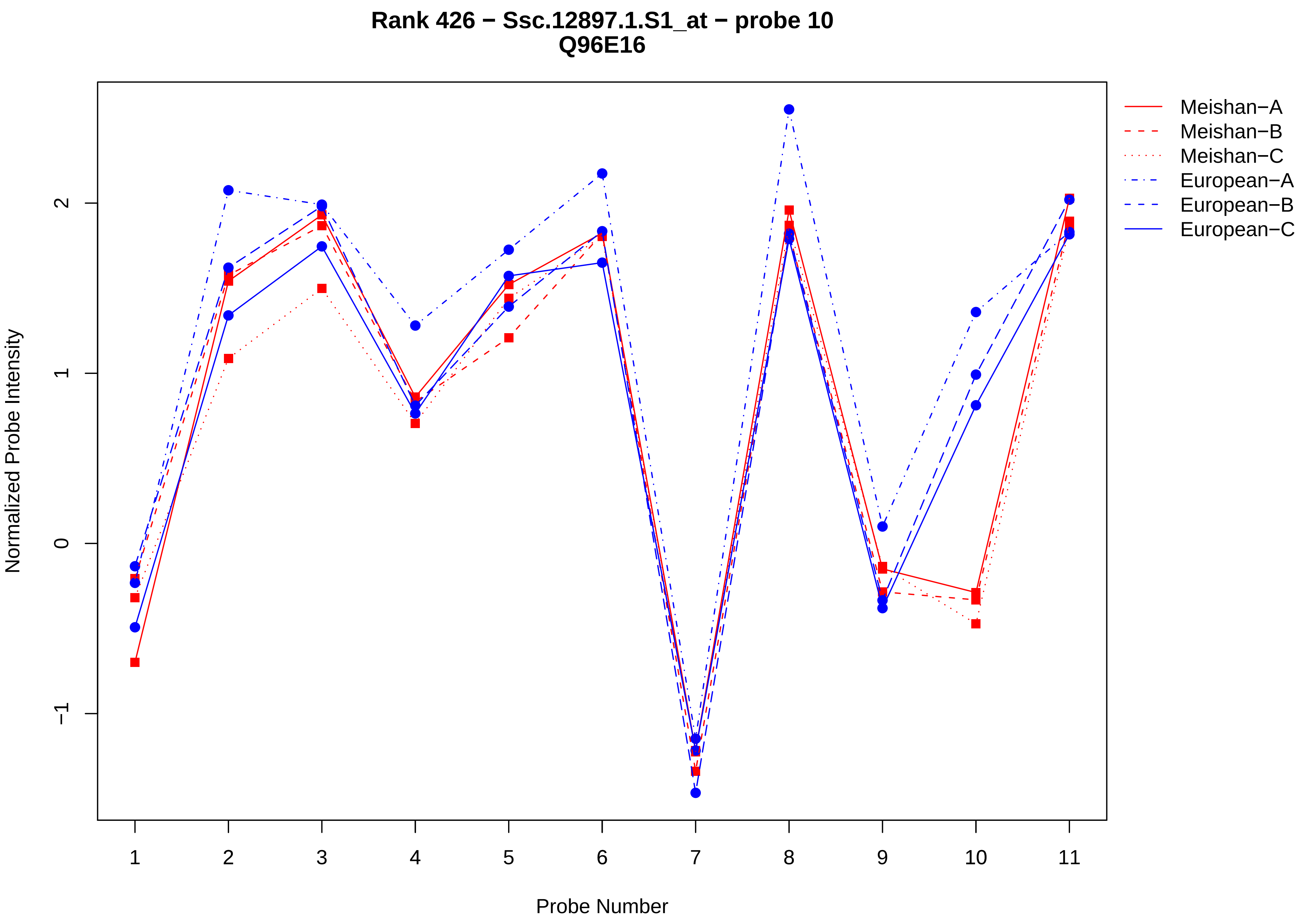

Rank 426 − Ssc.12897.1.S1_at − probe 10
Q96E16
Normalized Probe Intensity
Probe Number
−1
0
1
2
1
2
3
4
5
6
7
8
9
10
11
Meishan−A
Meishan−B
Meishan−C
European−A
European−B
European−C

Rank 427 − Ssc.5681.1.S1_at − probe 9
UBE2V1

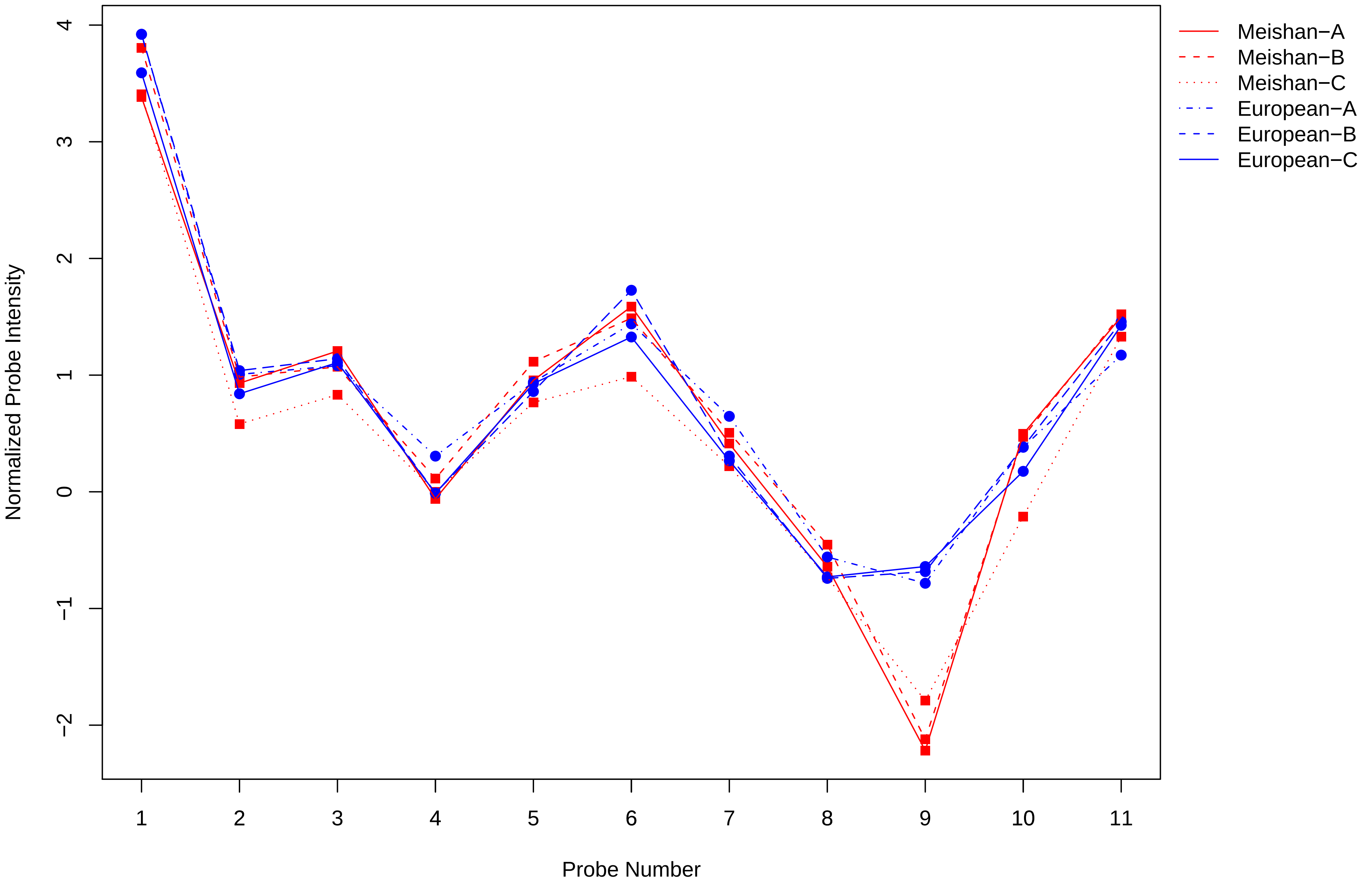

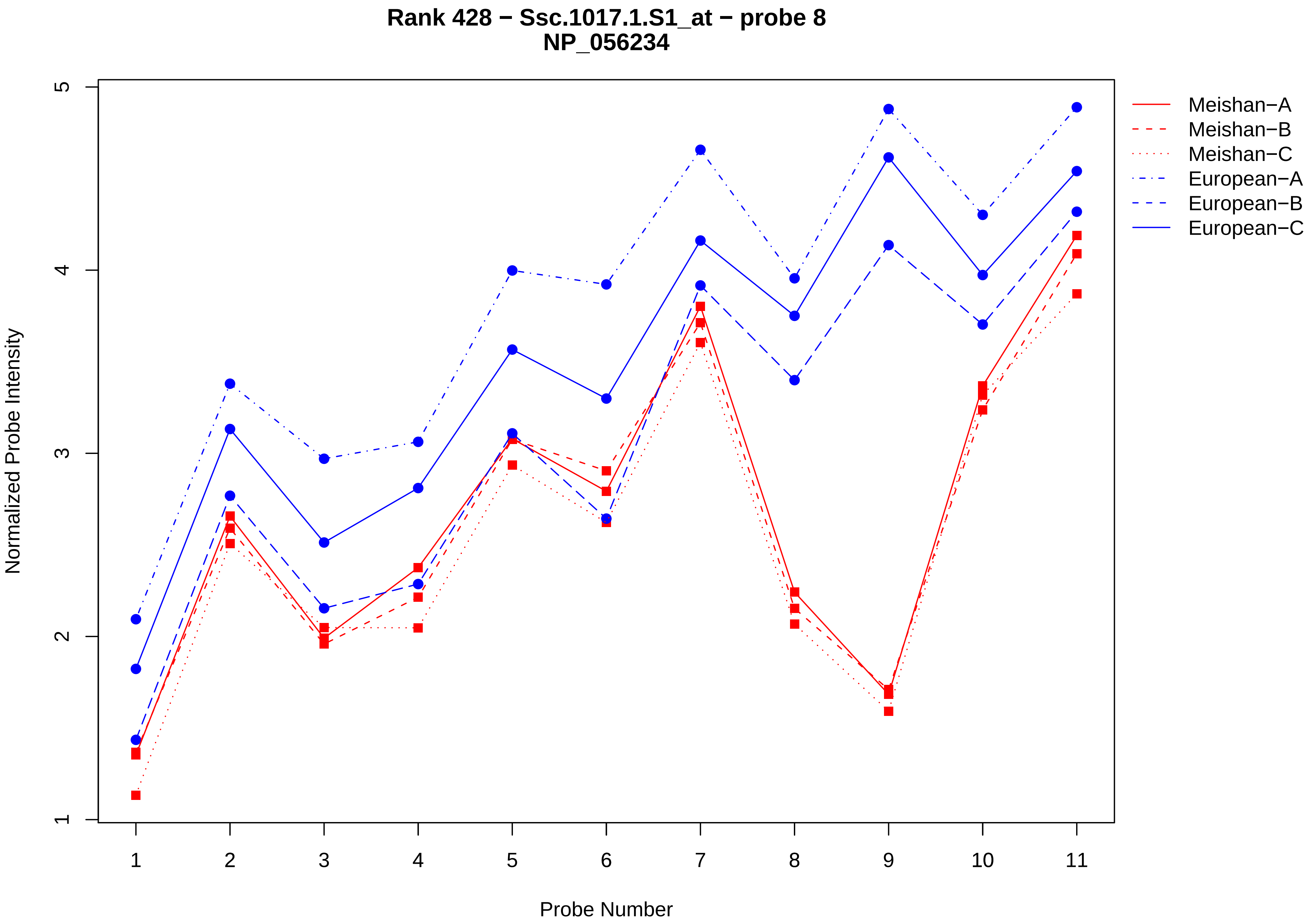

Rank 428 − Ssc.1017.1.S1_at − probe 8
NP_056234
Normalized Probe Intensity
Probe Number
1
2
3
4
5
1
2
3
4
5
6
7
8
9
10
11
Meishan−A
Meishan−B
Meishan−C
European−A
European−B
European−C

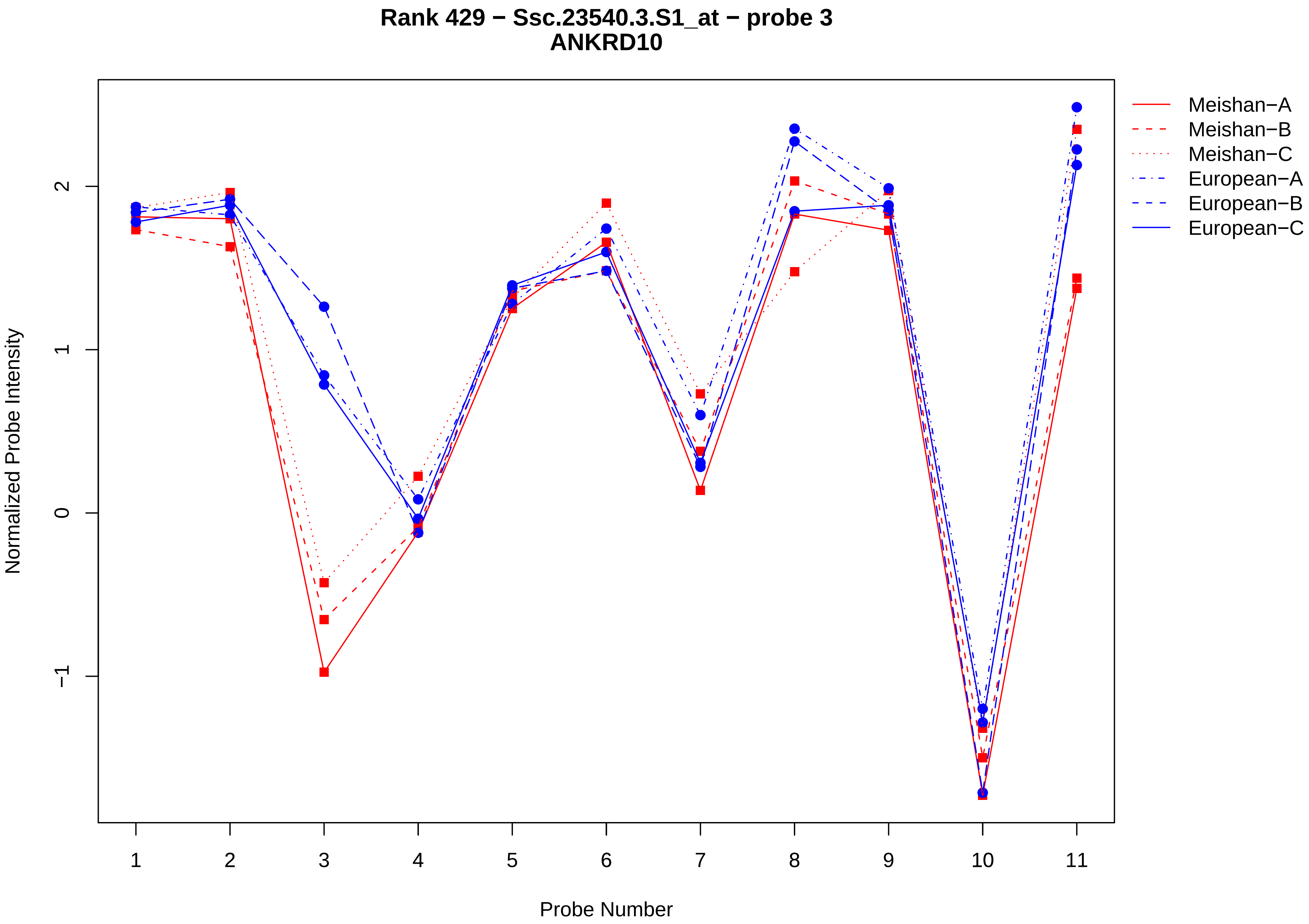

Rank 429 − Ssc.23540.3.S1_at − probe 3
ANKRD10
Normalized Probe Intensity
Probe Number
−1
0
1
2
1
2
3
4
5
6
7
8
9
10
11
Meishan−A
Meishan−B
Meishan−C
European−A
European−B
European−C

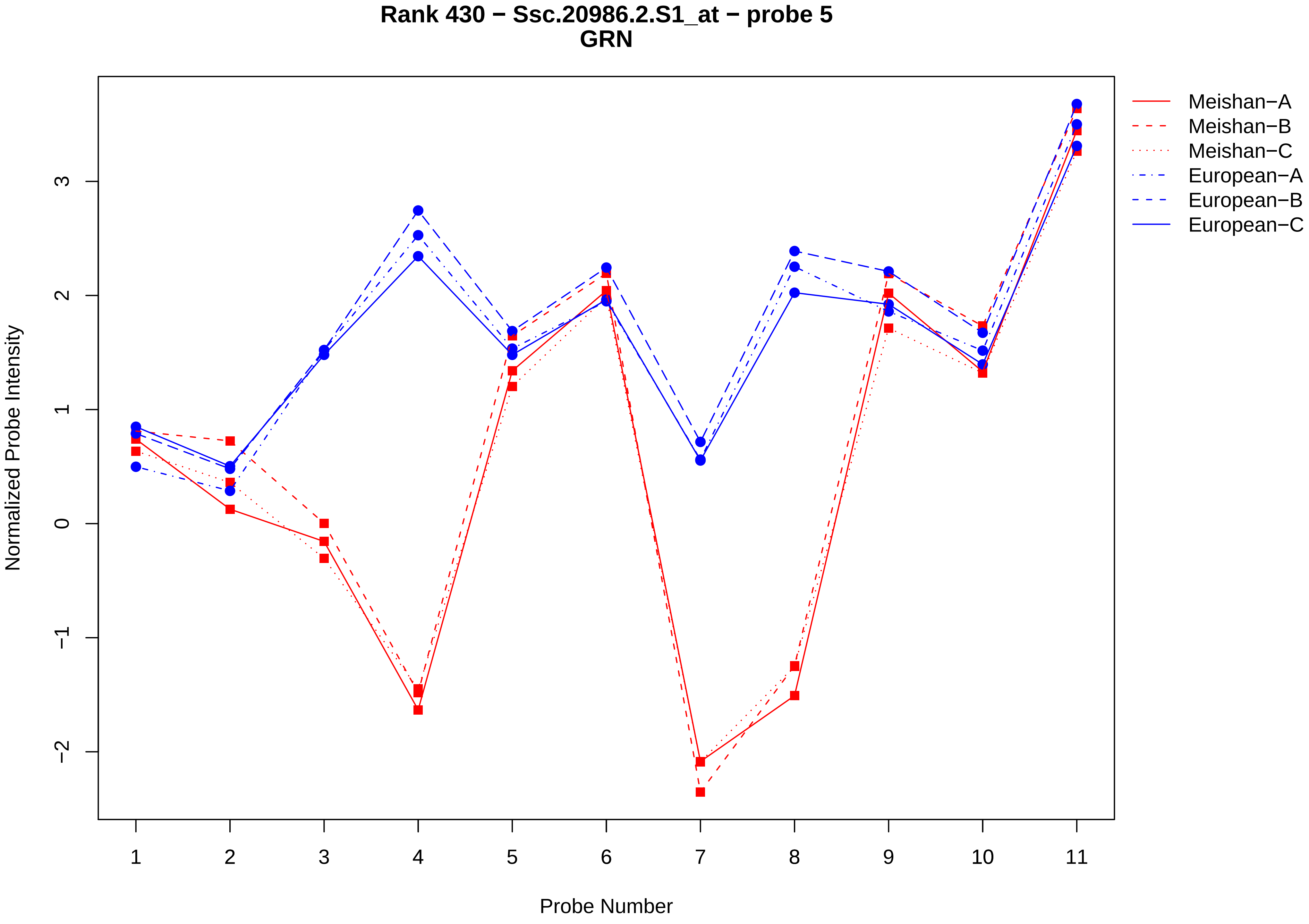

Rank 430 − Ssc.20986.2.S1_at − probe 5
GRN
Normalized Probe Intensity
Probe Number
−2
−1
0
1
2
3
1
2
3
4
5
6
7
8
9
10
11
Meishan−A
Meishan−B
Meishan−C
European−A
European−B
European−C

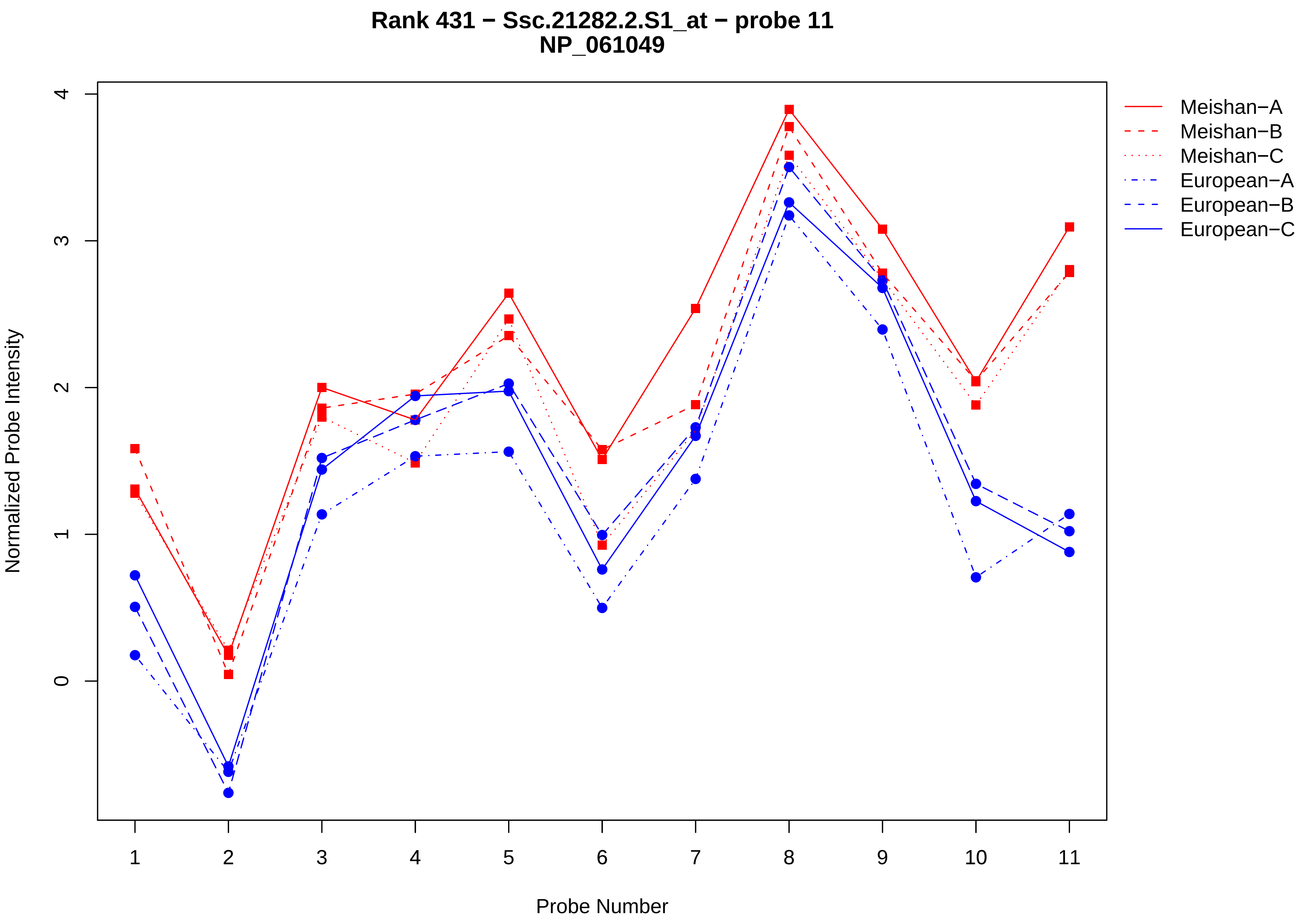

Rank 431 − Ssc.21282.2.S1_at − probe 11
NP_061049
Normalized Probe Intensity
Probe Number
0
1
2
3
4
1
2
3
4
5
6
7
8
9
10
11
Meishan−A
Meishan−B
Meishan−C
European−A
European−B
European−C

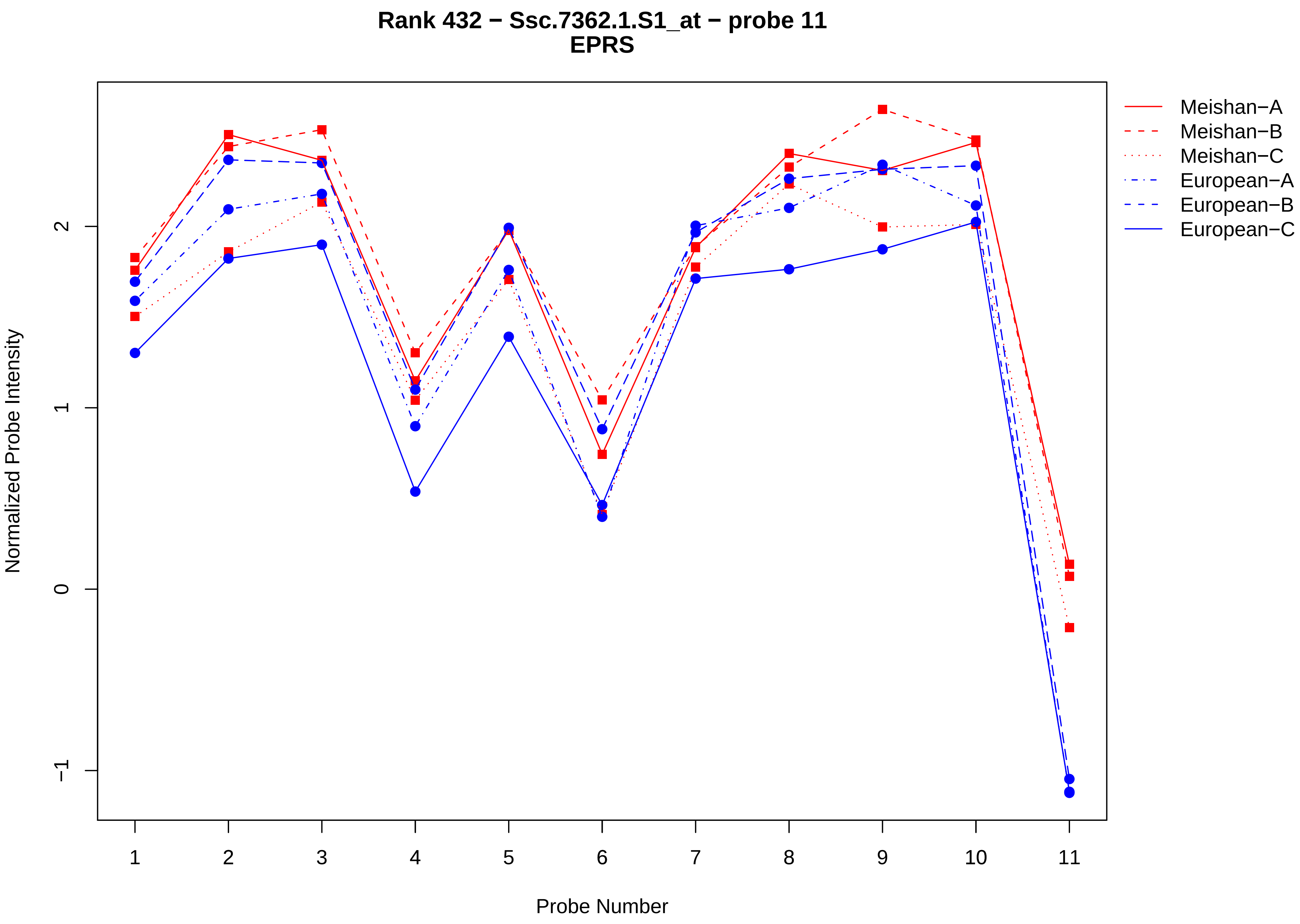
Rank 432 − Ssc.7362.1.S1_at − probe 11
EPRS
Normalized Probe Intensity
Probe Number
−1
0
1
2
1
2
3
4
5
6
7
8
9
10
11
Meishan−A
Meishan−B
Meishan−C
European−A
European−B
European−C

Rank 433 − Ssc.7872.1.A1_at − probe 10
PRSS7

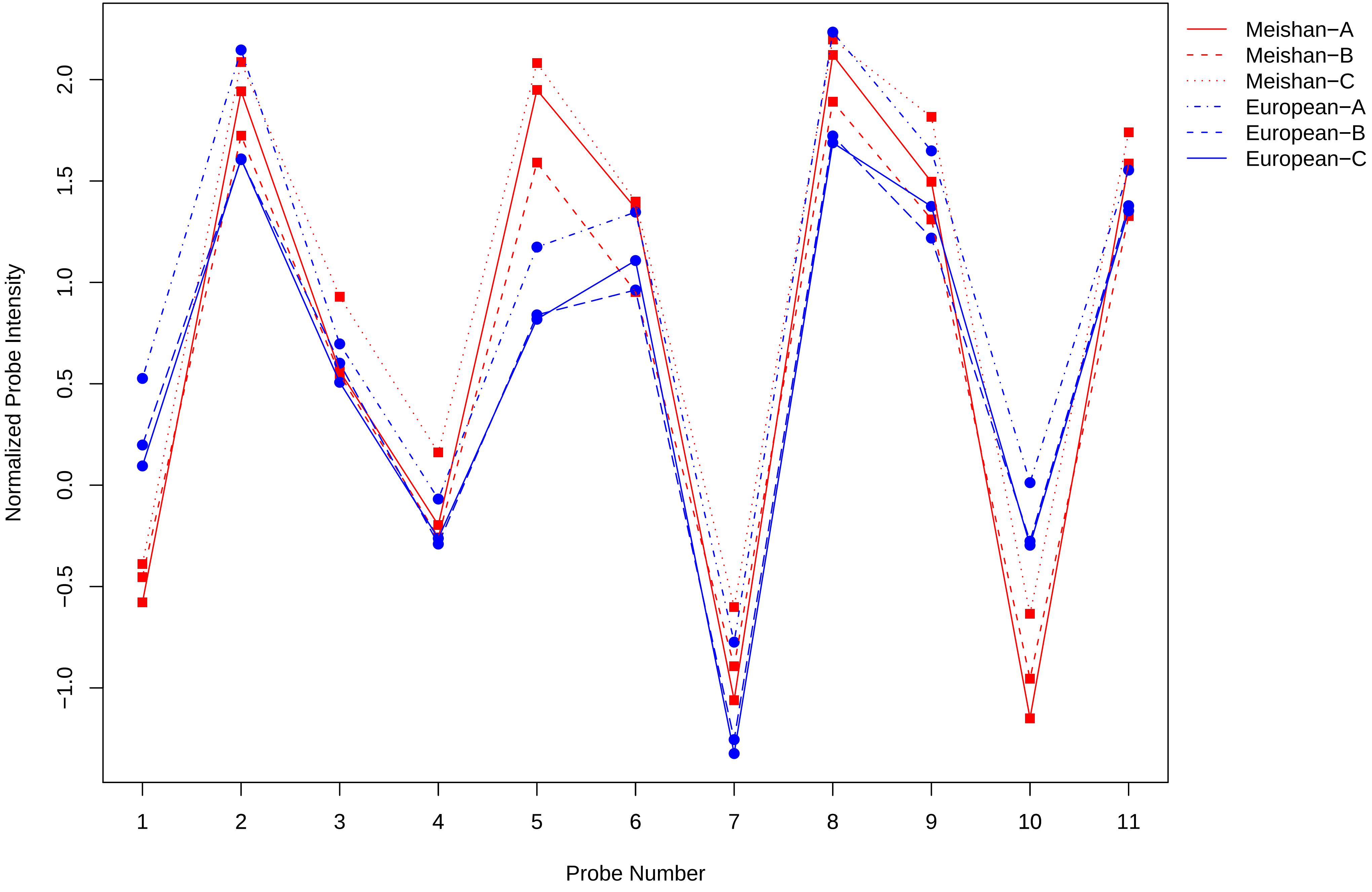

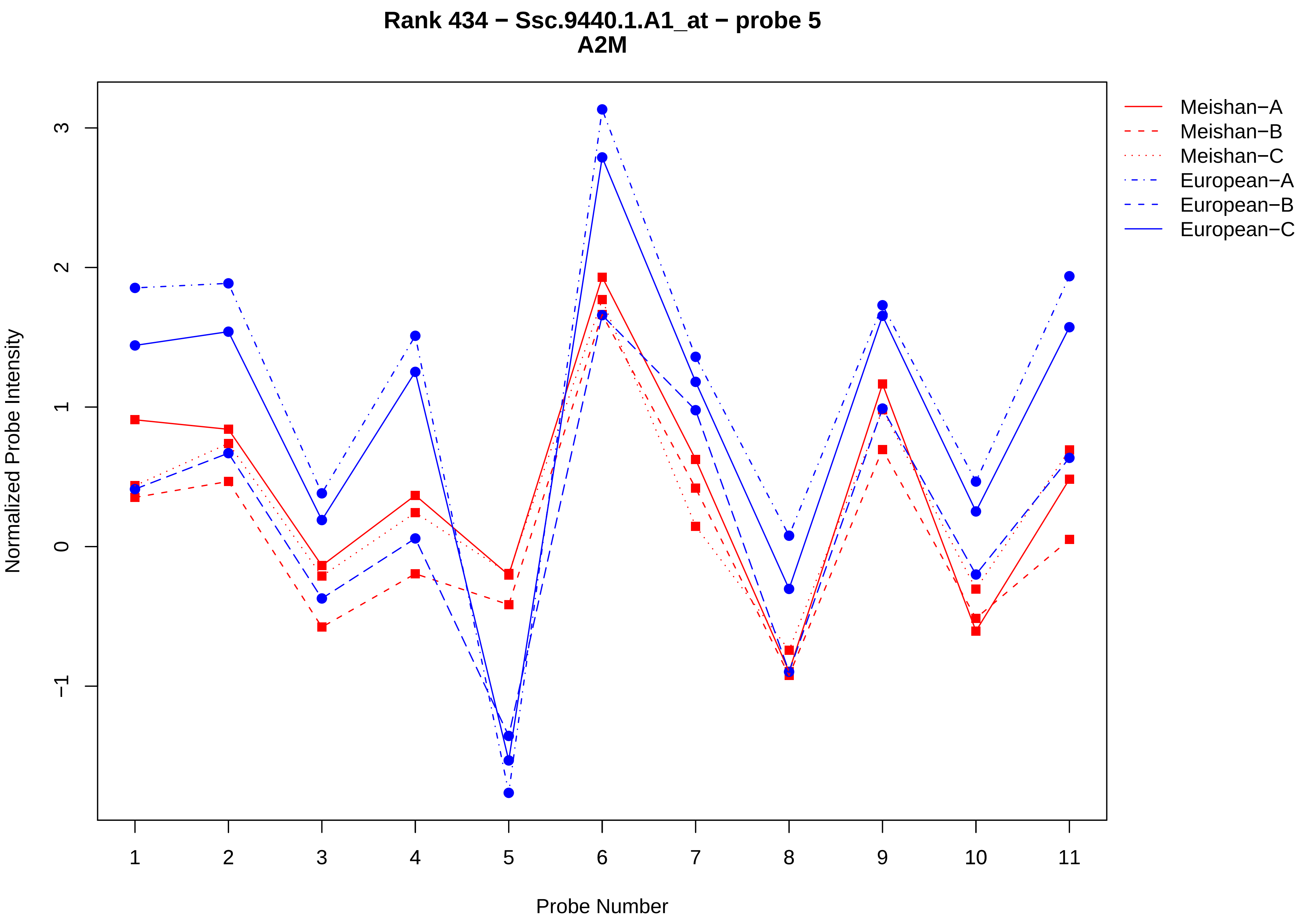
Rank 434 − Ssc.9440.1.A1_at − probe 5
A2M
Normalized Probe Intensity
Probe Number
Meishan−A
Meishan−B
Meishan−C
European−A
European−B
European−C
−1
0
1
2
3
1
2
3
4
5
6
7
8
9
10
11

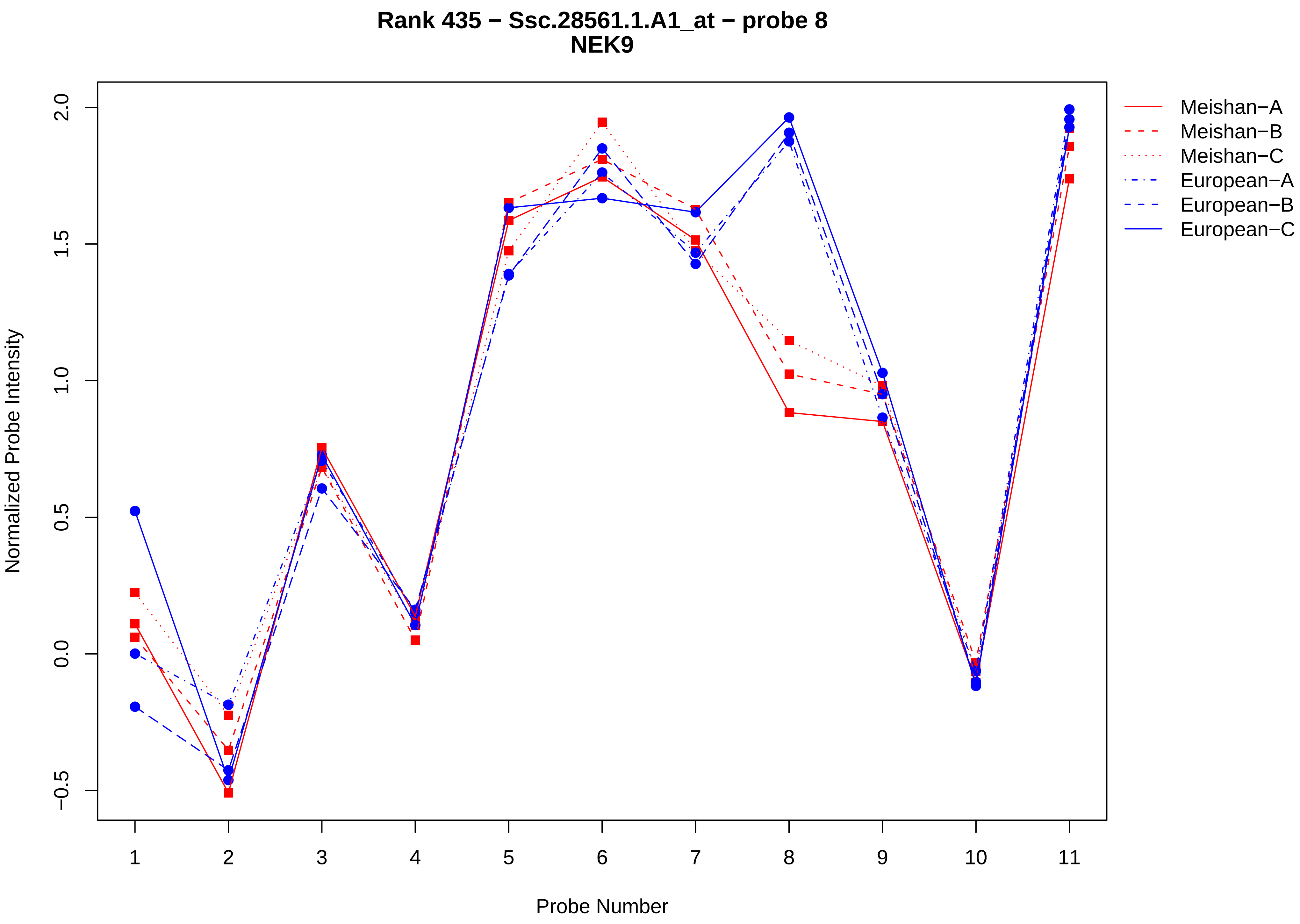

Rank 435 − Ssc.28561.1.A1_at − probe 8
NEK9
Normalized Probe Intensity
Probe Number
−0.5
0.0
0.5
1.0
1.5
2.0
1
2
3
4
5
6
7
8
9
10
11
Meishan−A
Meishan−B
Meishan−C
European−A
European−B
European−C

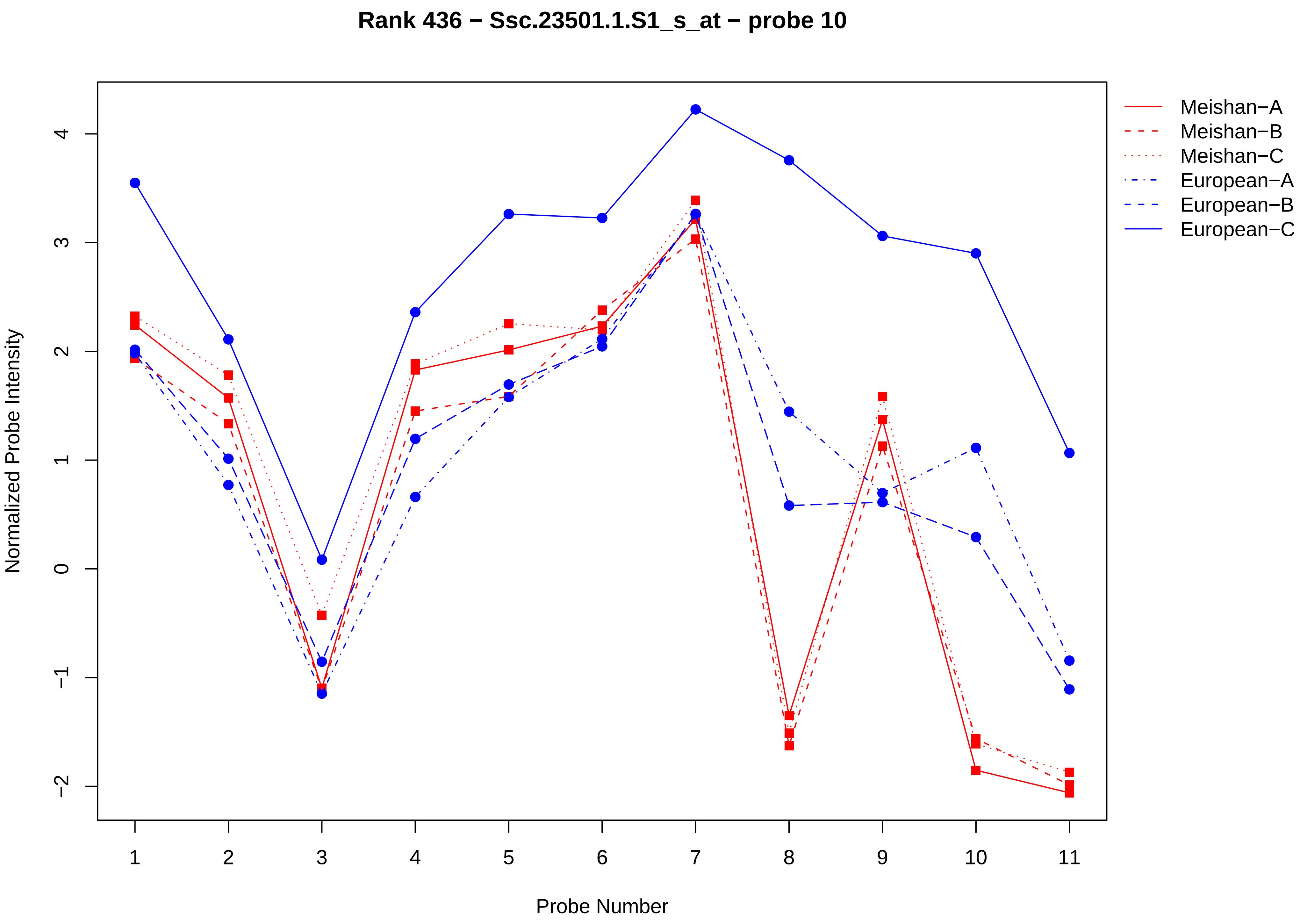

Rank 436 − Ssc.23501.1.S1_s_at − probe 10
Normalized Probe Intensity
Probe Number
−2
−1
0
1
2
3
4
1
2
3
4
5
6
7
8
9
10
11
Meishan−A
Meishan−B
Meishan−C
European−A
European−B
European−C

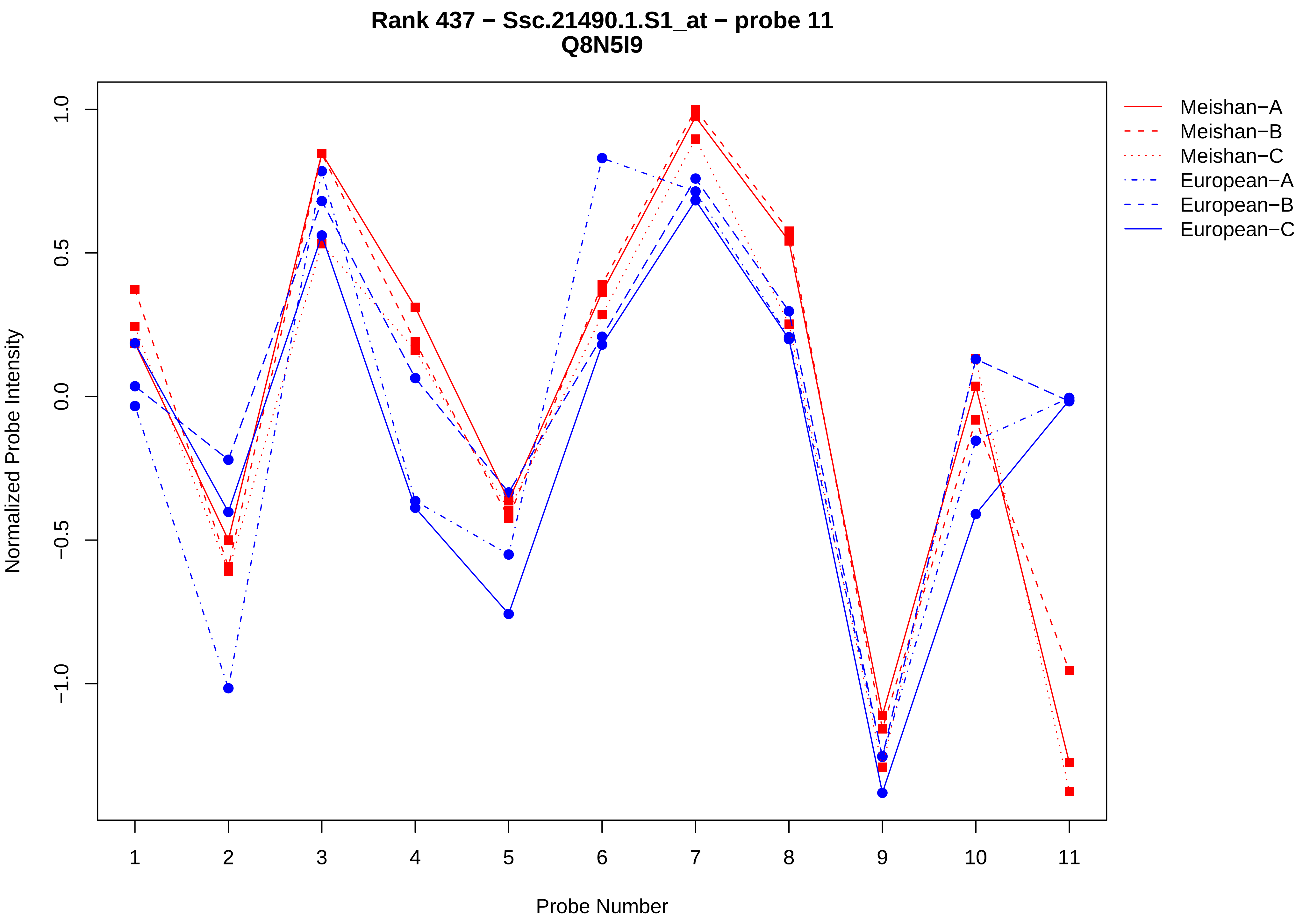

Rank 437 − Ssc.21490.1.S1_at − probe 11
Q8N5I9
Normalized Probe Intensity
Probe Number
1.0
0.5
0.0
−0.5
−1.0
1
2
3
4
5
6
7
8
9
10
11
Meishan−A
Meishan−B
Meishan−C
European−A
European−B
European−C

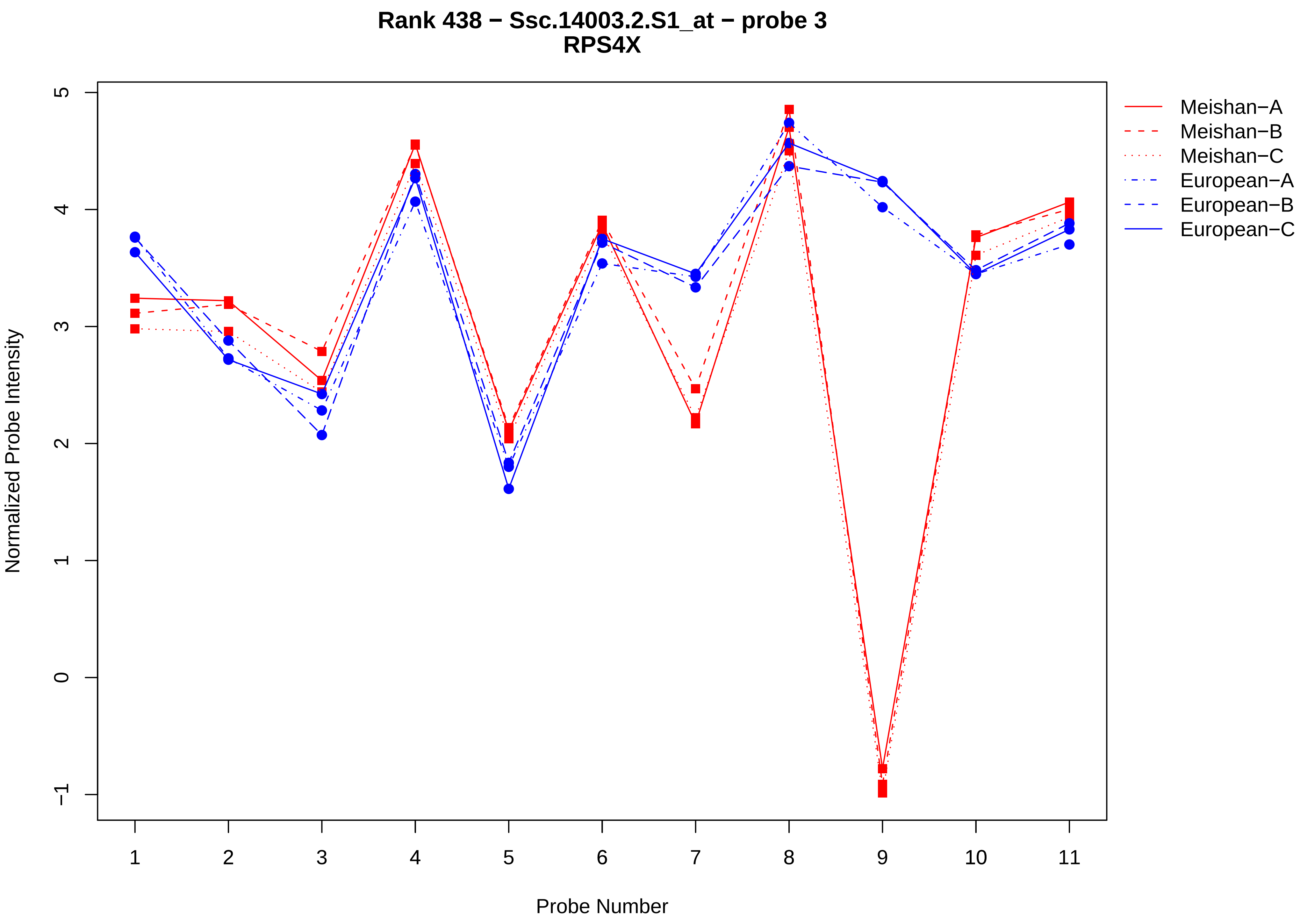
Rank 438 − Ssc.14003.2.S1_at − probe 3
RPS4X
Normalized Probe Intensity
Probe Number
Meishan−A
Meishan−B
Meishan−C
European−A
European−B
European−C
−1
0
1
2
3
4
5
1
2
3
4
5
6
7
8
9
10
11

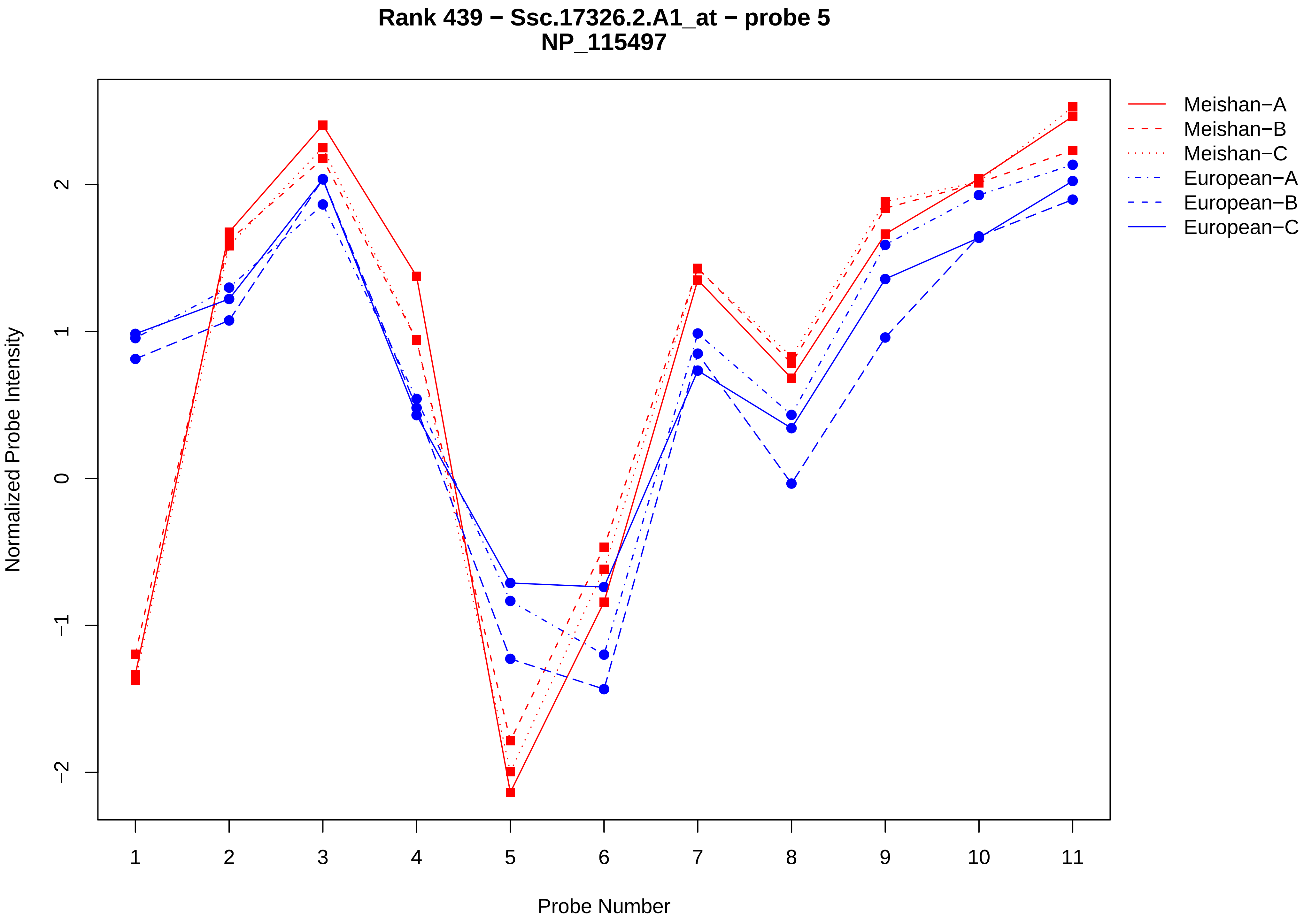
Rank 439 − Ssc.17326.2.A1_at − probe 5
NP_115497
Normalized Probe Intensity
Probe Number
−2
−1
0
1
2
1
2
3
4
5
6
7
8
9
10
11
Meishan−A
Meishan−B
Meishan−C
European−A
European−B
European−C

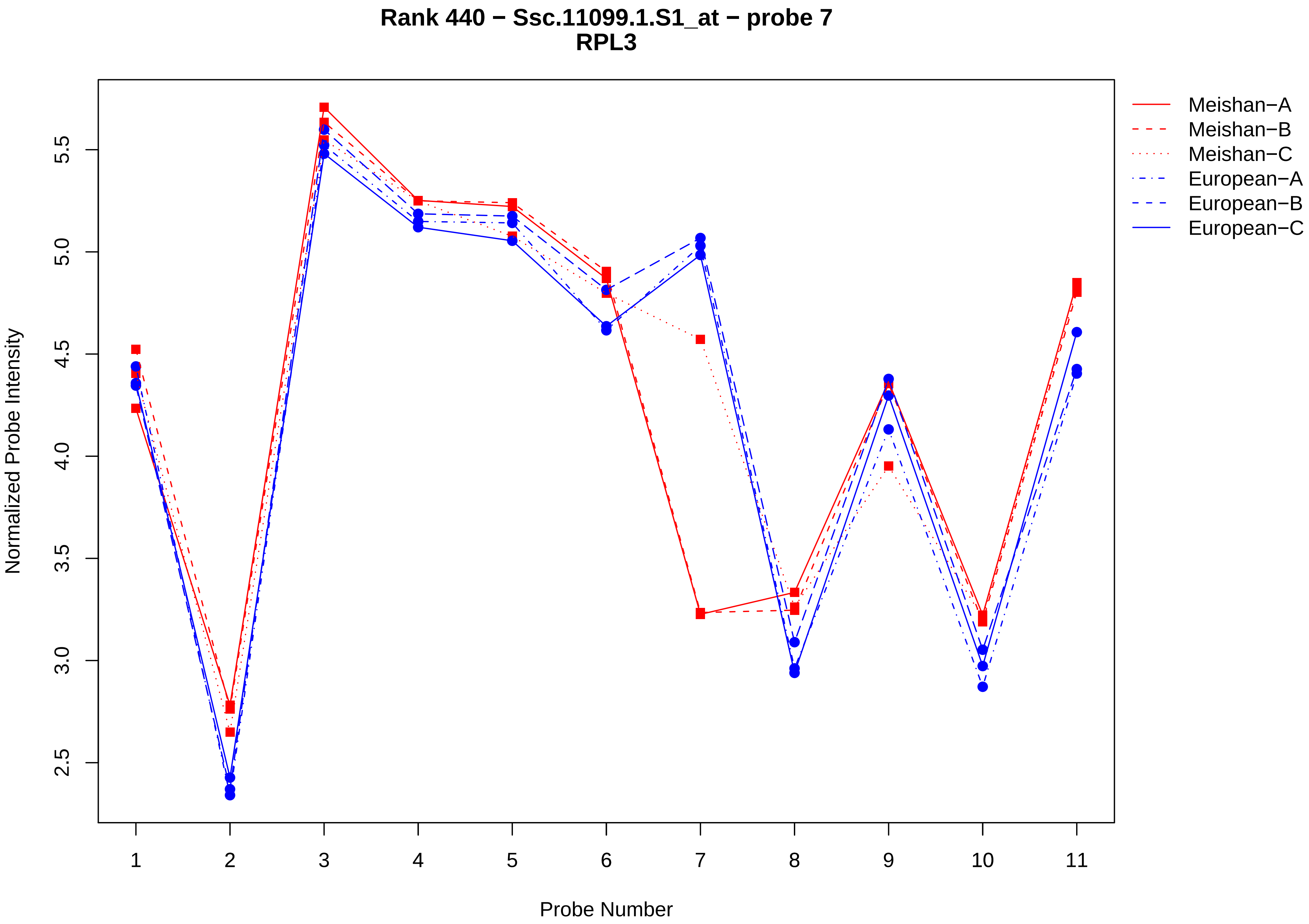
Rank 440 − Ssc.11099.1.S1_at − probe 7
RPL3
Normalized Probe Intensity
Probe Number
2.5
3.0
3.5
4.0
4.5
5.0
5.5
1
2
3
4
5
6
7
8
9
10
11
Meishan−A
Meishan−B
Meishan−C
European−A
European−B
European−C

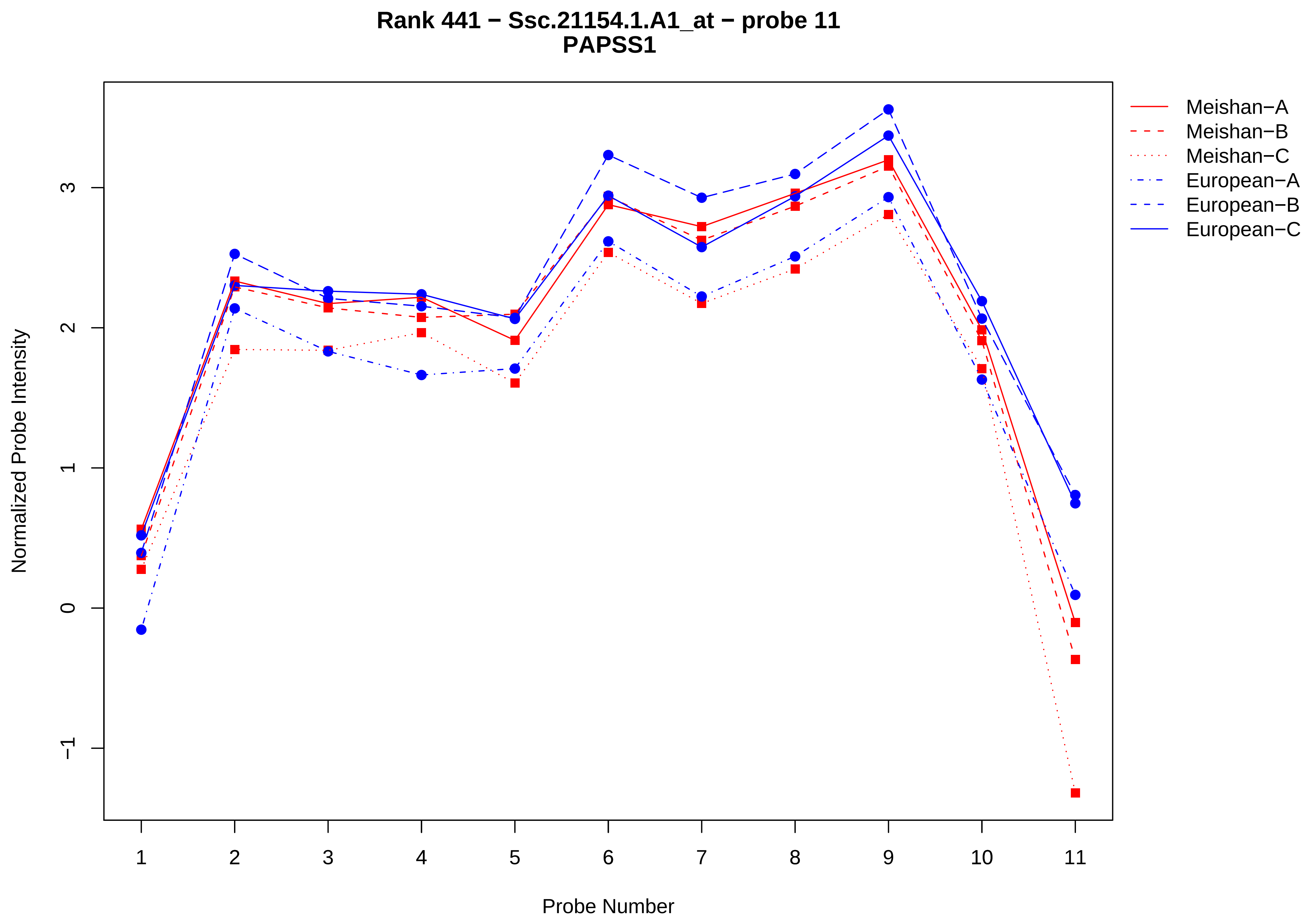
Rank 441 − Ssc.21154.1.A1_at − probe 11
PAPSS1
Normalized Probe Intensity
Probe Number
Meishan−A
Meishan−B
Meishan−C
European−A
European−B
European−C
−1
0
1
2
3
1
2
3
4
5
6
7
8
9
10
11

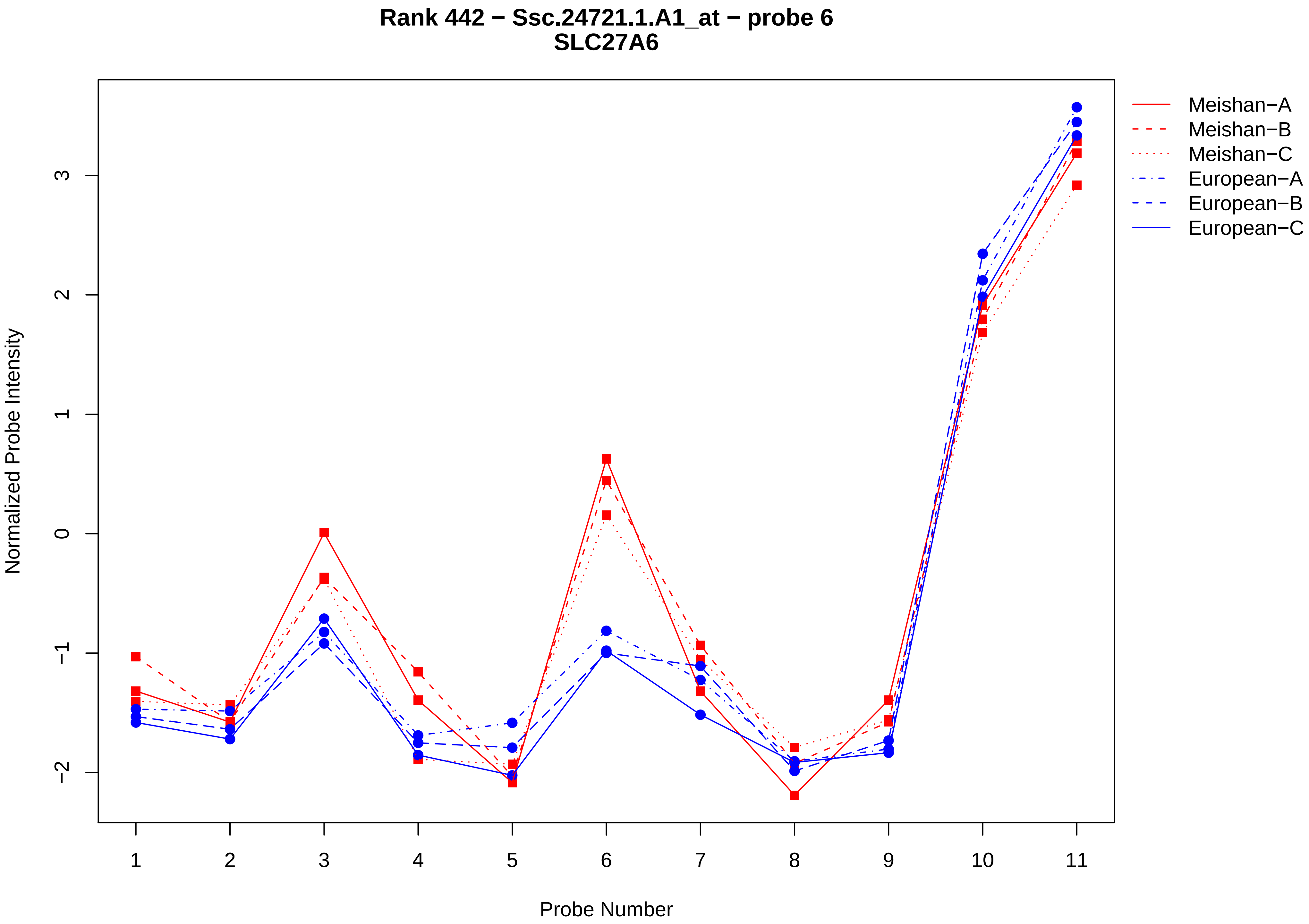

Rank 442 − Ssc.24721.1.A1_at − probe 6
SLC27A6
Normalized Probe Intensity
Probe Number
Meishan−A
Meishan−B
Meishan−C
European−A
European−B
European−C
−2
−1
0
1
2
3
1
2
3
4
5
6
7
8
9
10
11

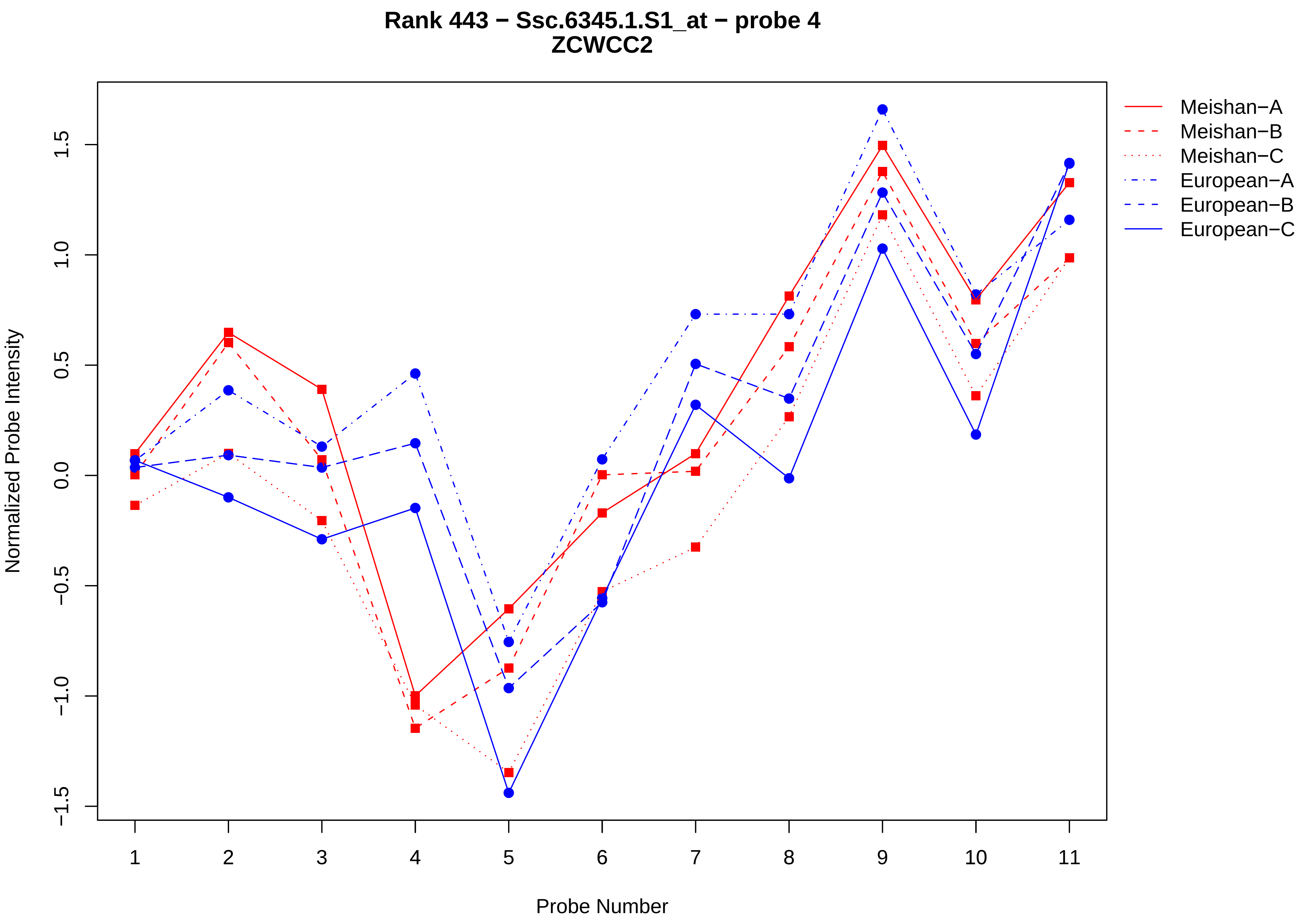

Rank 443 − Ssc.6345.1.S1_at − probe 4
ZCWCC2
Normalized Probe Intensity
Probe Number
−1.5
−1.0
−0.5
0.0
0.5
1.0
1.5
1
2
3
4
5
6
7
8
9
10
11
Meishan−A
Meishan−B
Meishan−C
European−A
European−B
European−C

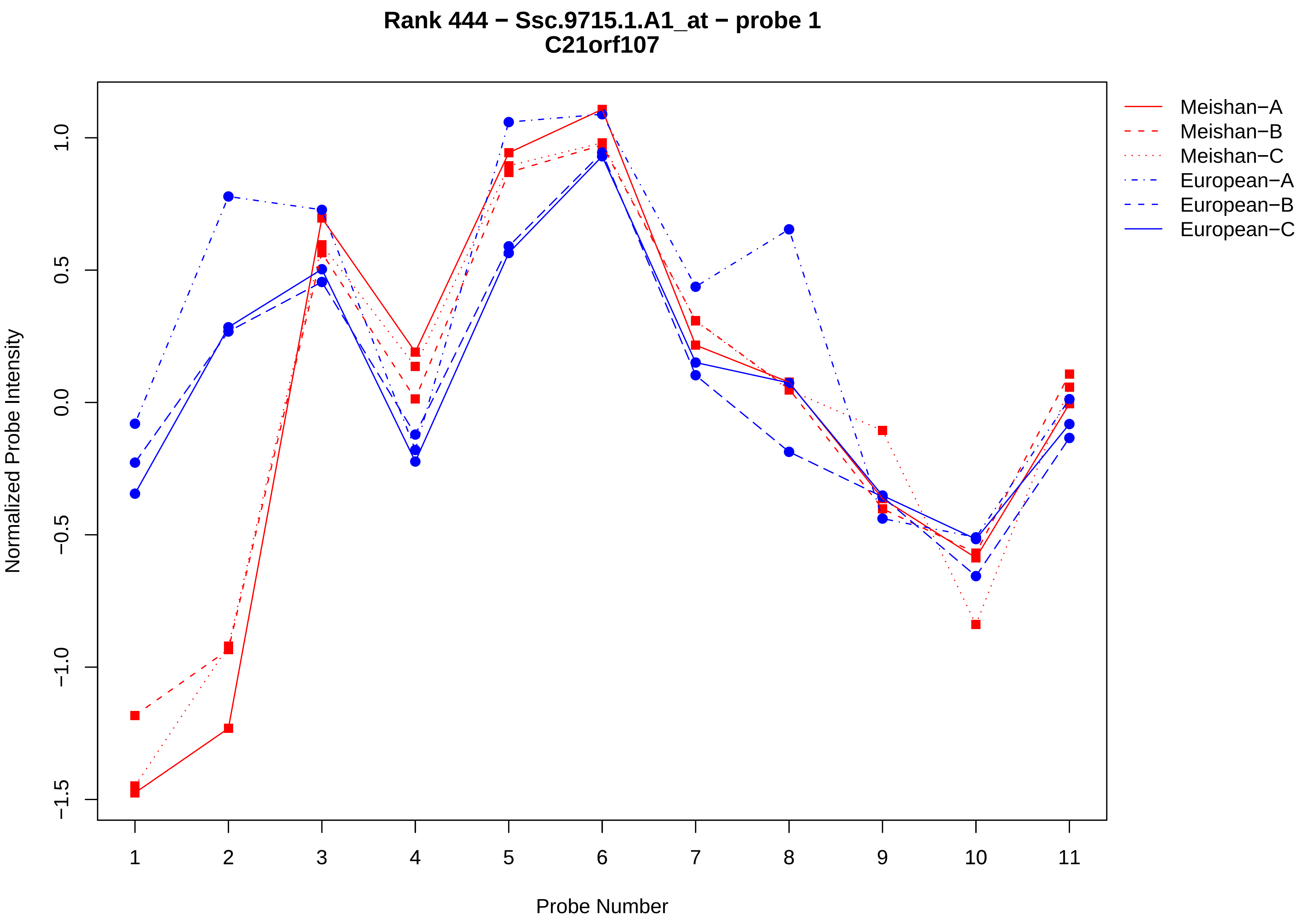

Rank 444 − Ssc.9715.1.A1_at − probe 1
C21orf107
Normalized Probe Intensity
Probe Number
Meishan−A
Meishan−B
Meishan−C
European−A
European−B
European−C
1.0
0.5
0.0
−0.5
−1.0
−1.5
1
2
3
4
5
6
7
8
9
10
11

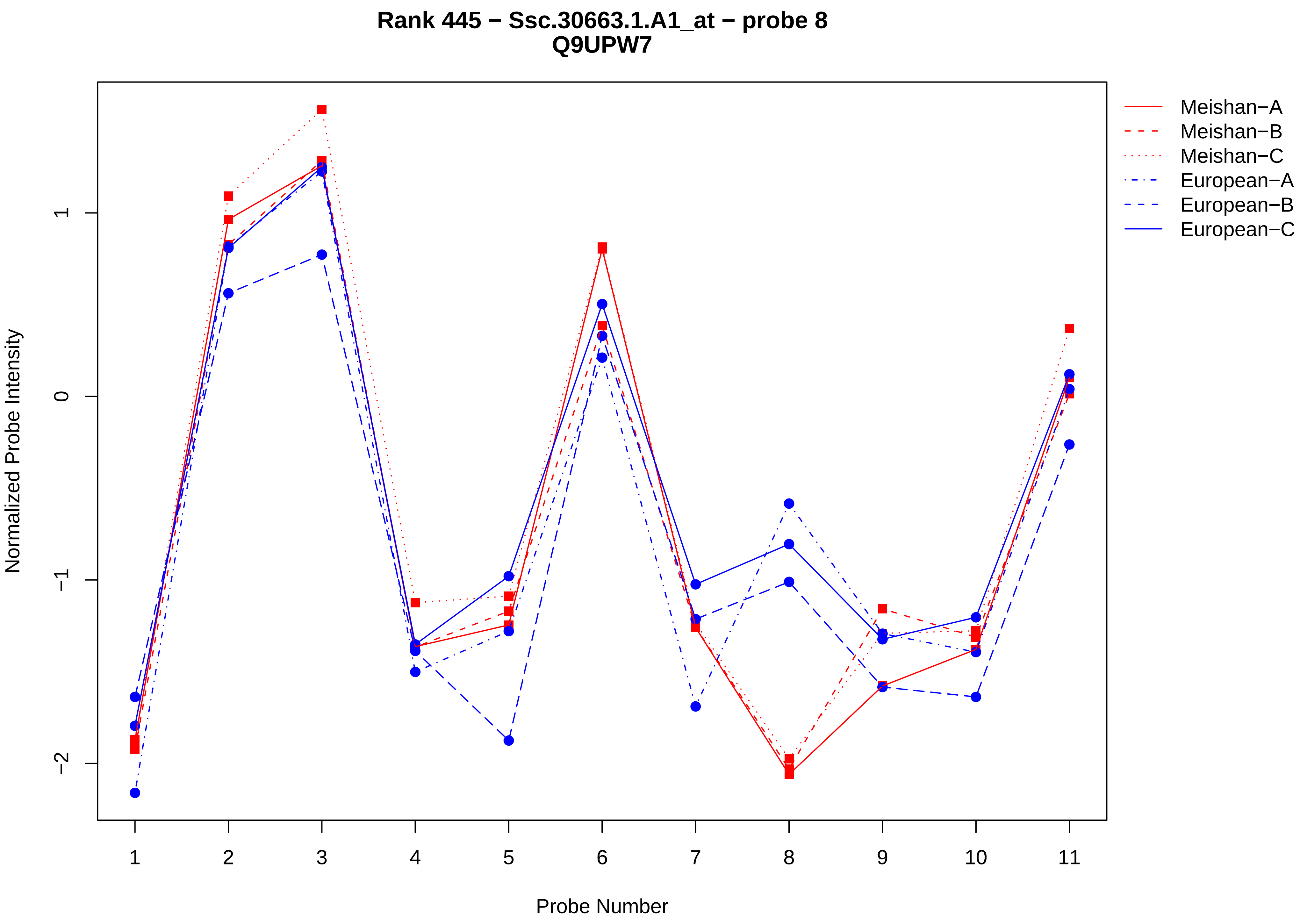
Rank 445 − Ssc.30663.1.A1_at − probe 8
Q9UPW7
Normalized Probe Intensity
Probe Number
1
0
−1
−2
1
2
3
4
5
6
7
8
9
10
11
Meishan−A
Meishan−B
Meishan−C
European−A
European−B
European−C

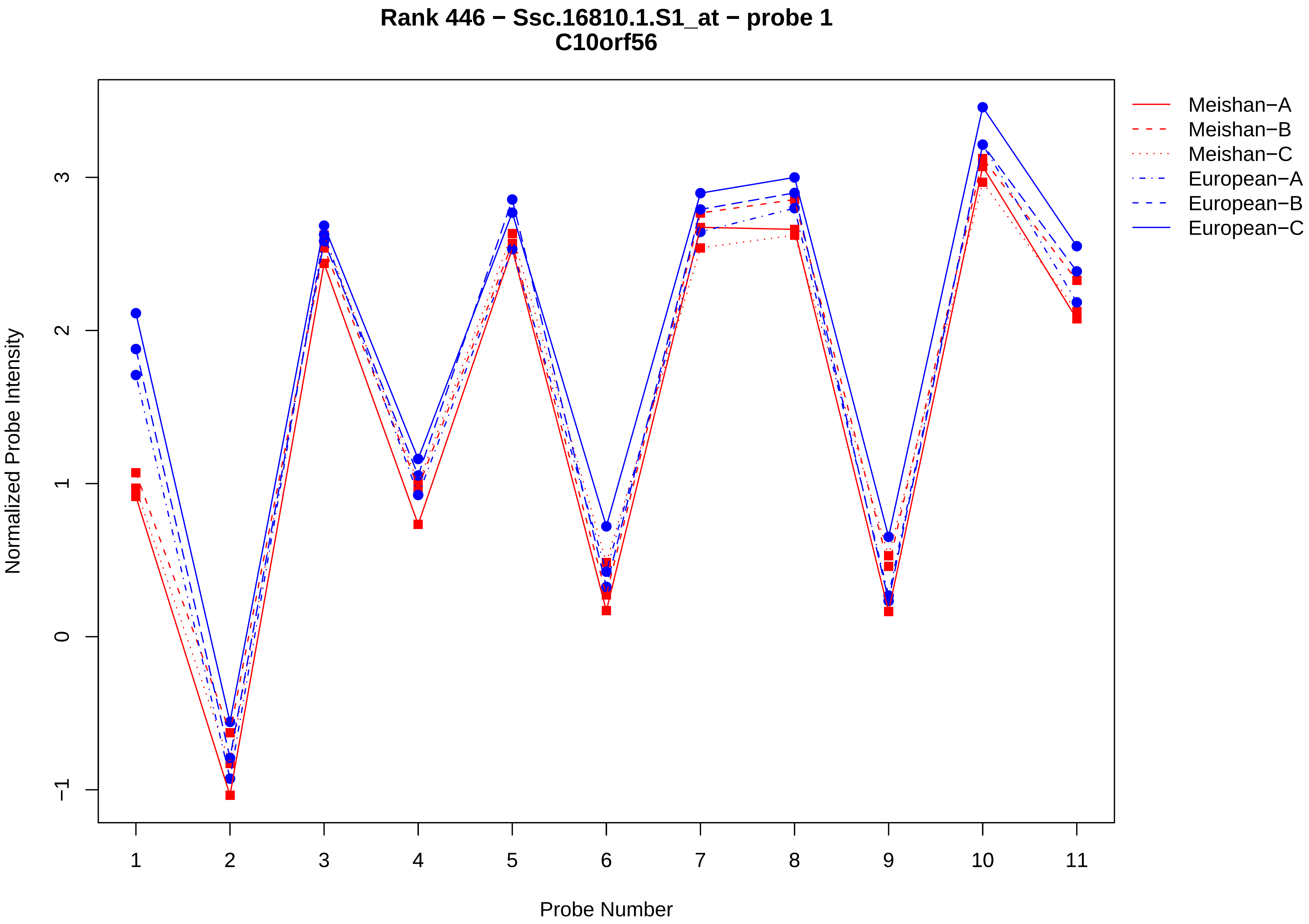

Rank 446 − Ssc.16810.1.S1_at − probe 1
C10orf56
Normalized Probe Intensity
Probe Number
−1
0
1
2
3
1
2
3
4
5
6
7
8
9
10
11
Meishan−A
Meishan−B
Meishan−C
European−A
European−B
European−C

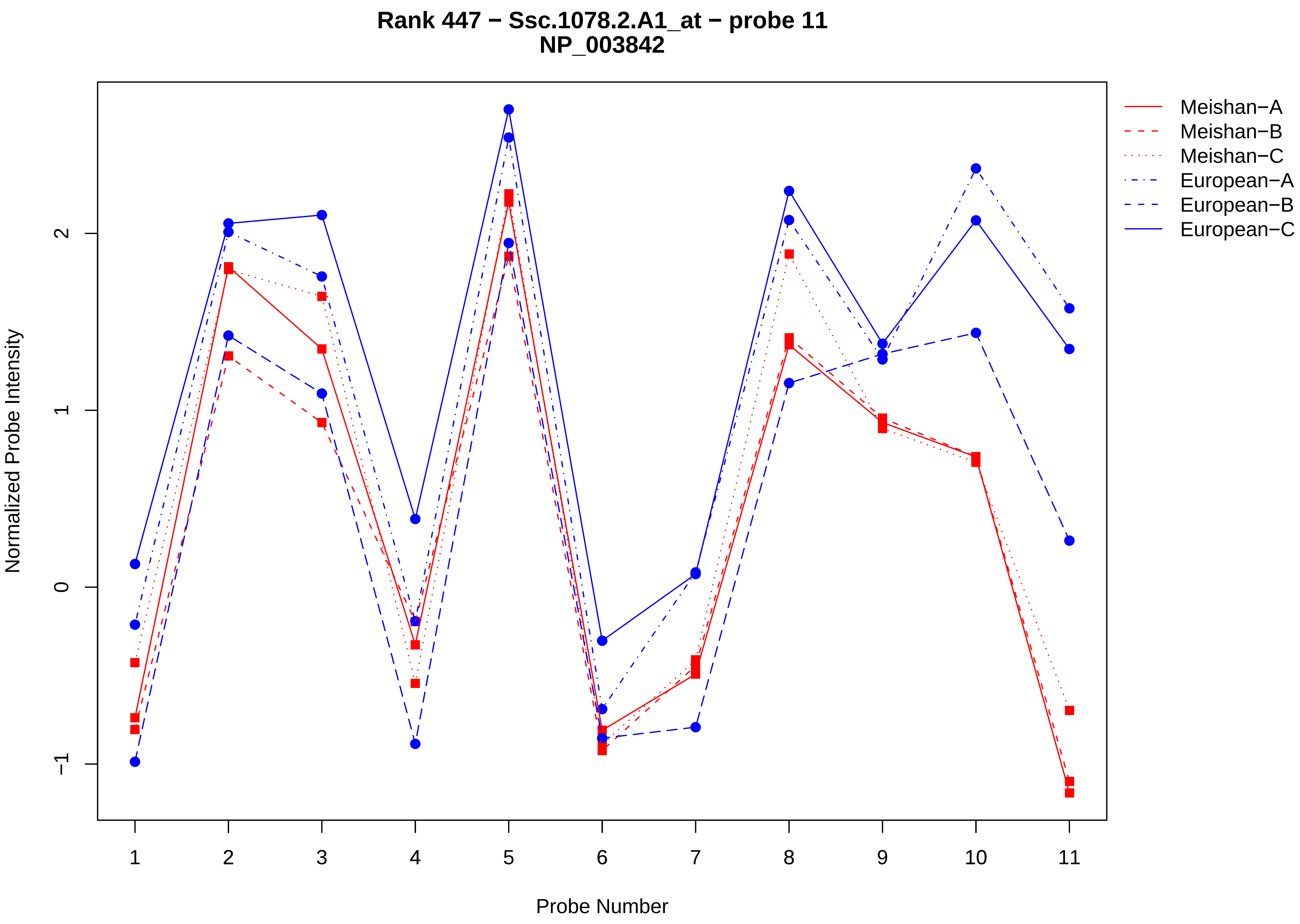

Rank 447 − Ssc.1078.2.A1_at − probe 11
NP_003842
Normalized Probe Intensity
Probe Number
−1
0
1
2
1
2
3
4
5
6
7
8
9
10
11
Meishan−A
Meishan−B
Meishan−C
European−A
European−B
European−C

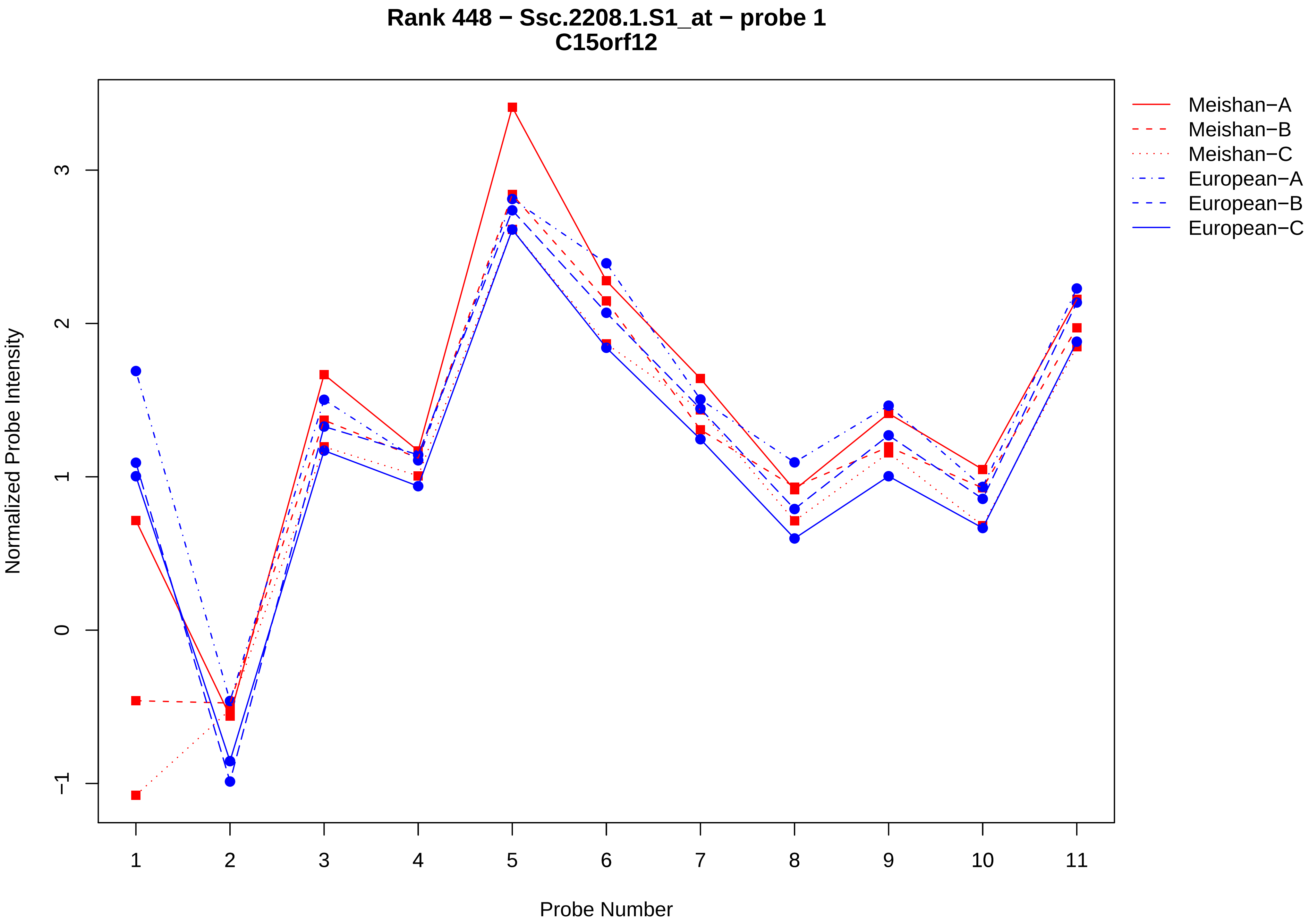

Rank 448 − Ssc.2208.1.S1_at − probe 1
C15orf12
Normalized Probe Intensity
Probe Number
−1
0
1
2
3
1
2
3
4
5
6
7
8
9
10
11
Meishan−A
Meishan−B
Meishan−C
European−A
European−B
European−C

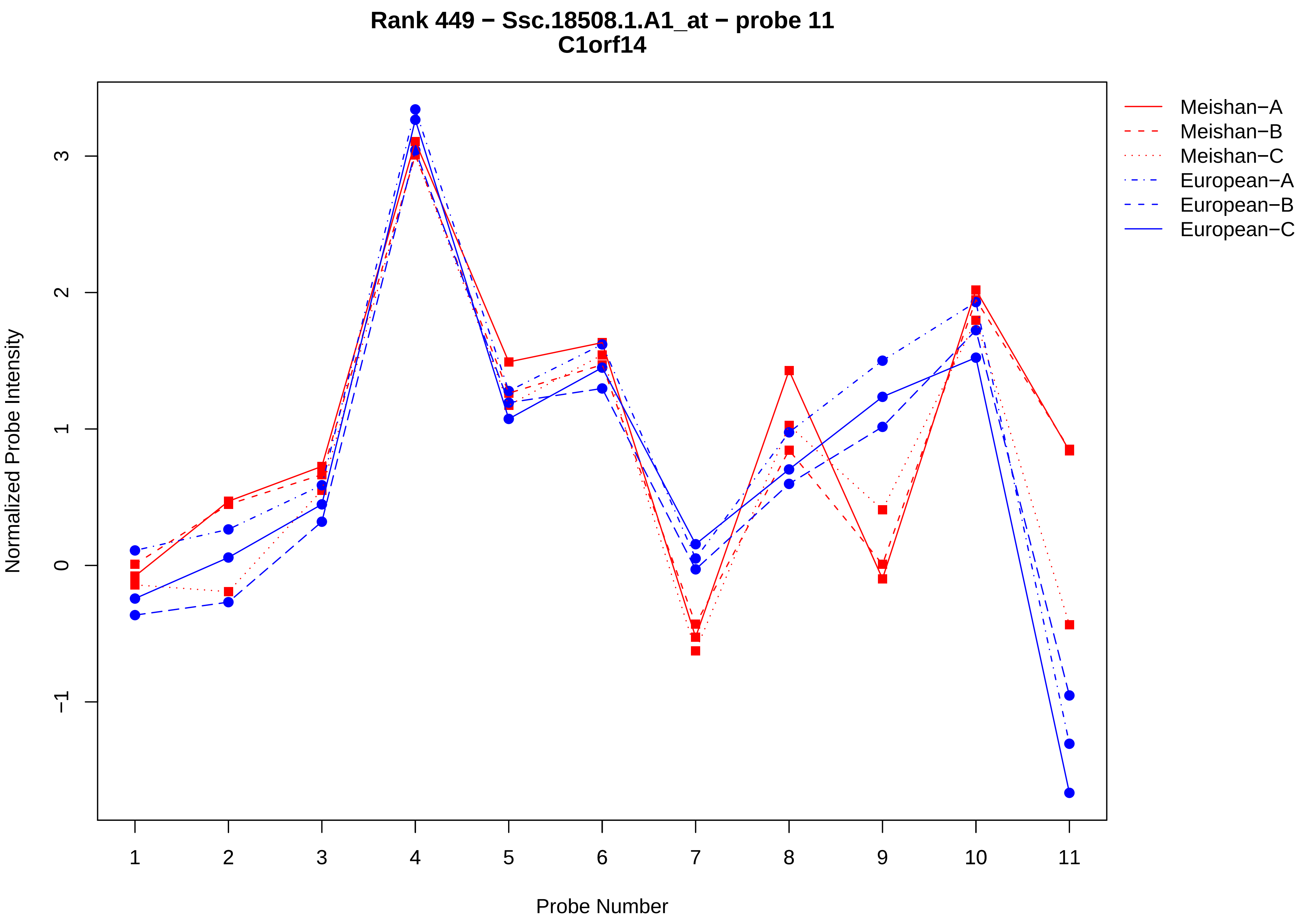
Rank 449 − Ssc.18508.1.A1_at − probe 11
C1orf14
Normalized Probe Intensity
Probe Number
Meishan−A
Meishan−B
Meishan−C
European−A
European−B
European−C
−1
0
1
2
3
1
2
3
4
5
6
7
8
9
10
11

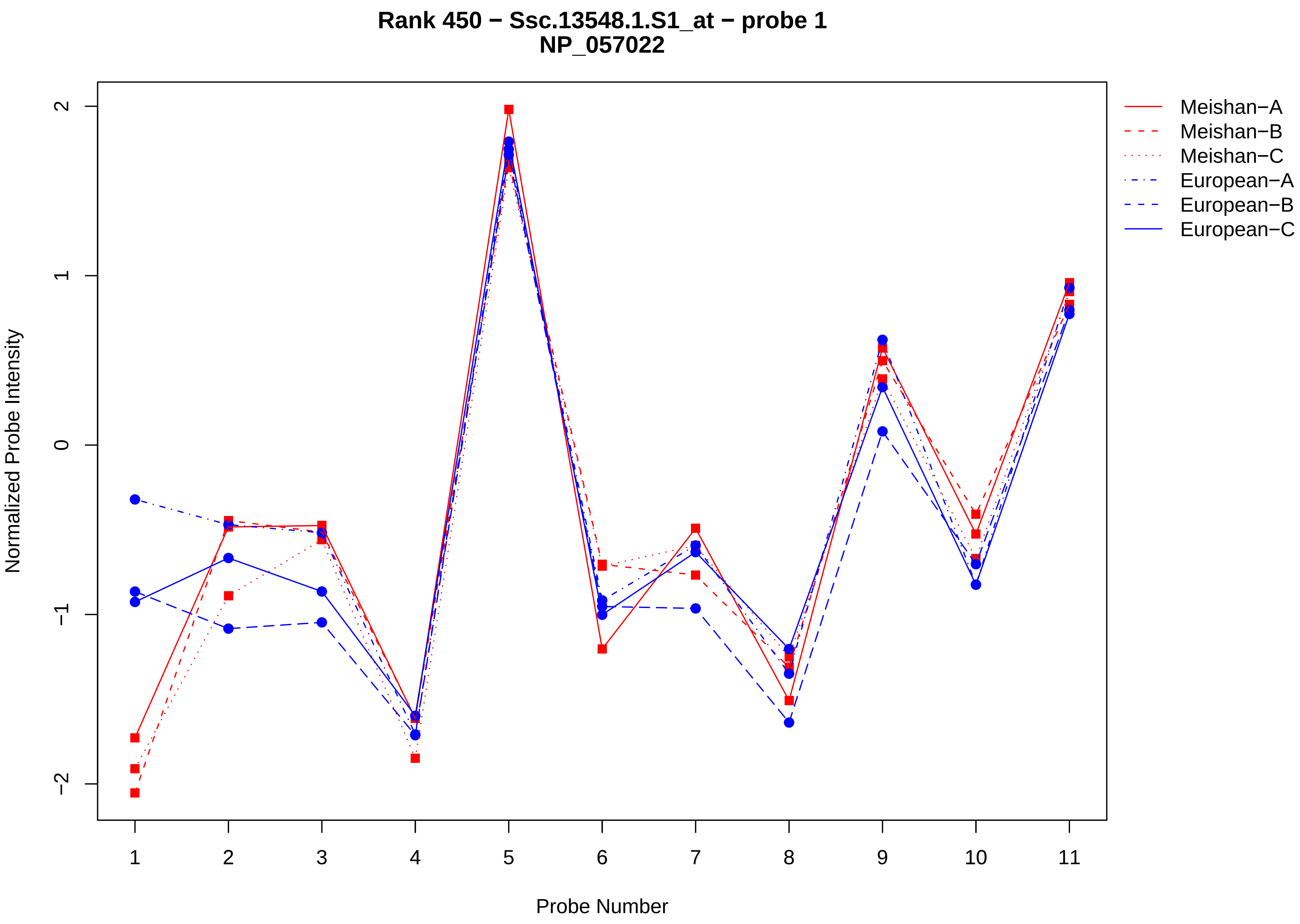
Rank 450 − Ssc.13548.1.S1_at − probe 1
NP_057022
Normalized Probe Intensity
Probe Number
Meishan−A
Meishan−B
Meishan−C
European−A
European−B
European−C
2
1
0
−1
−2
1
2
3
4
5
6
7
8
9
10
11

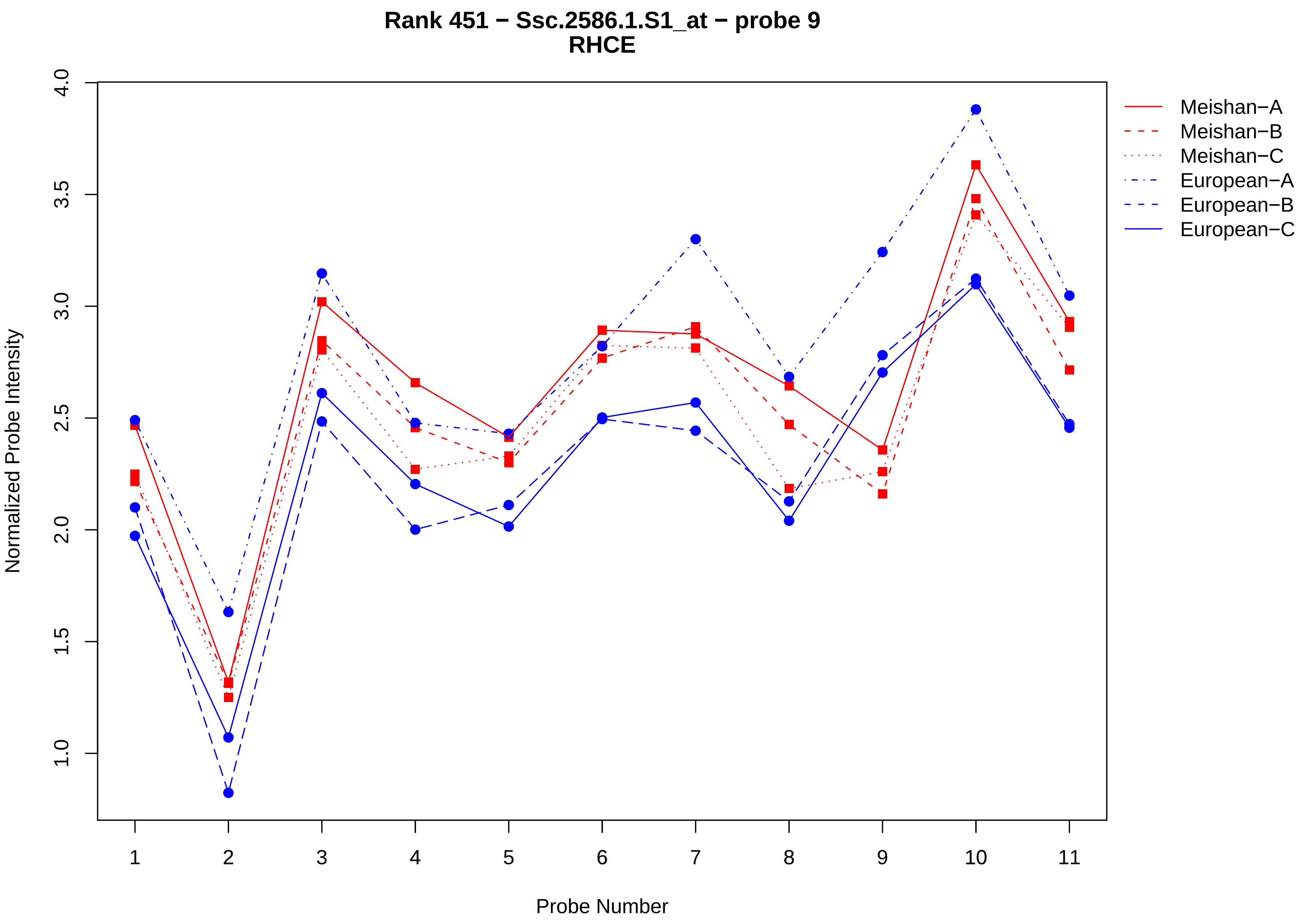

Rank 451 − Ssc.2586.1.S1_at − probe 9
RHCE
Normalized Probe Intensity
Probe Number
1.0
1.5
2.0
2.5
3.0
3.5
4.0
1
2
3
4
5
6
7
8
9
10
11
Meishan−A
Meishan−B
Meishan−C
European−A
European−B
European−C

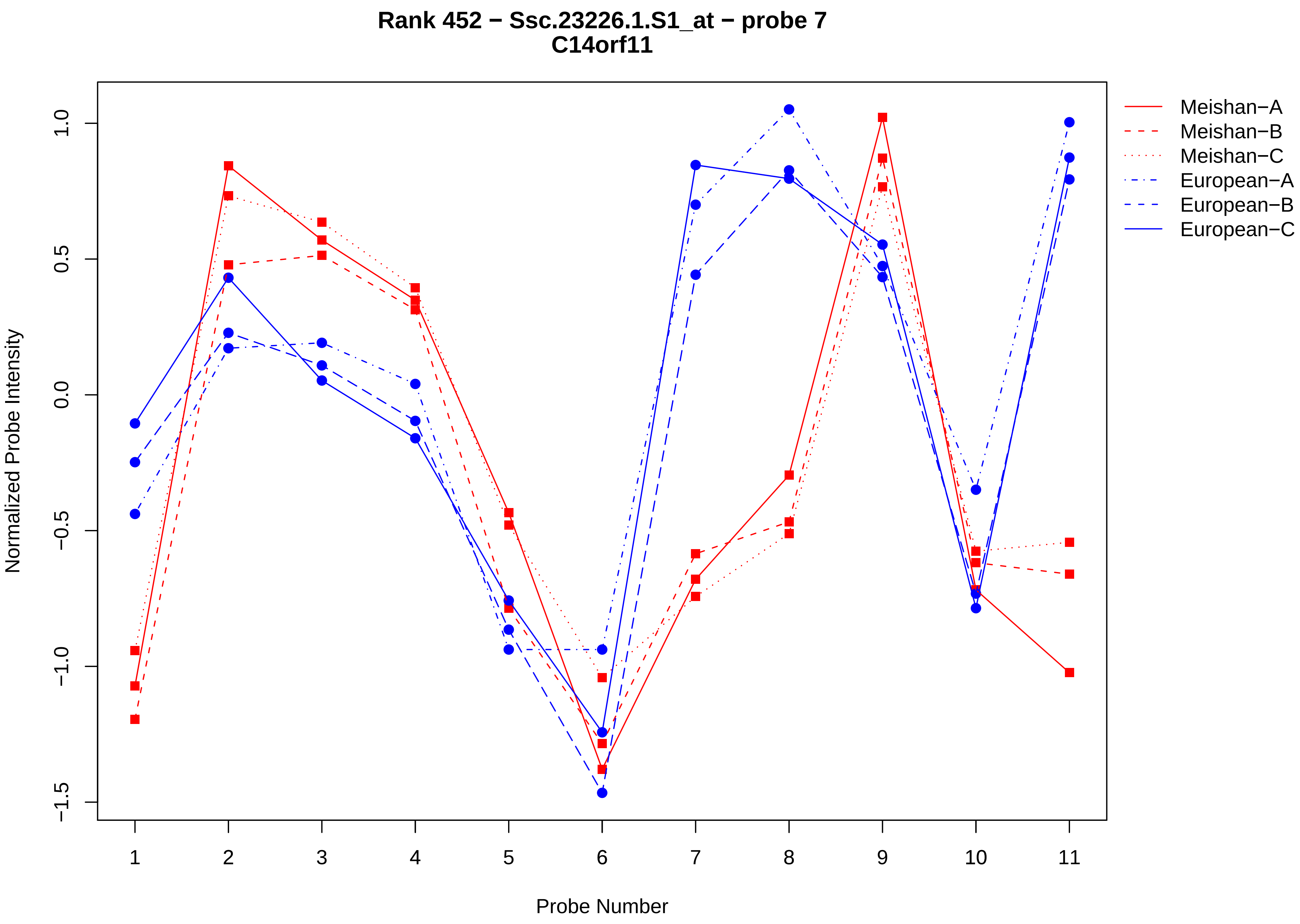

Rank 452 − Ssc.23226.1.S1_at − probe 7
C14orf11
Normalized Probe Intensity
Probe Number
1.0
0.5
0.0
−0.5
−1.0
−1.5
1
2
3
4
5
6
7
8
9
10
11
Meishan−A
Meishan−B
Meishan−C
European−A
European−B
European−C

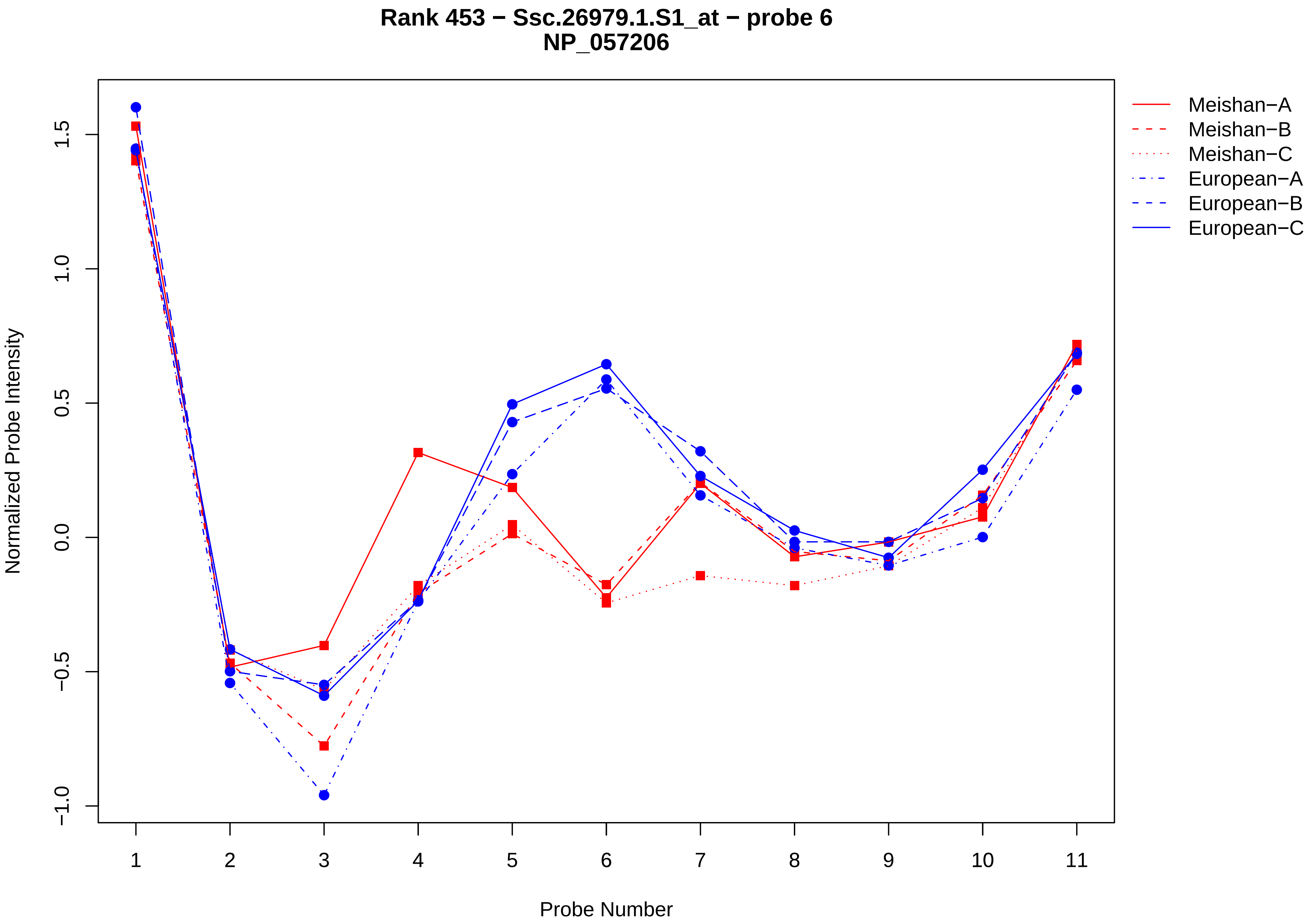

Rank 453 − Ssc.26979.1.S1_at − probe 6
NP_057206
Normalized Probe Intensity
Probe Number
−1.0
−0.5
0.0
0.5
1.0
1.5
1
2
3
4
5
6
7
8
9
10
11
Meishan−A
Meishan−B
Meishan−C
European−A
European−B
European−C

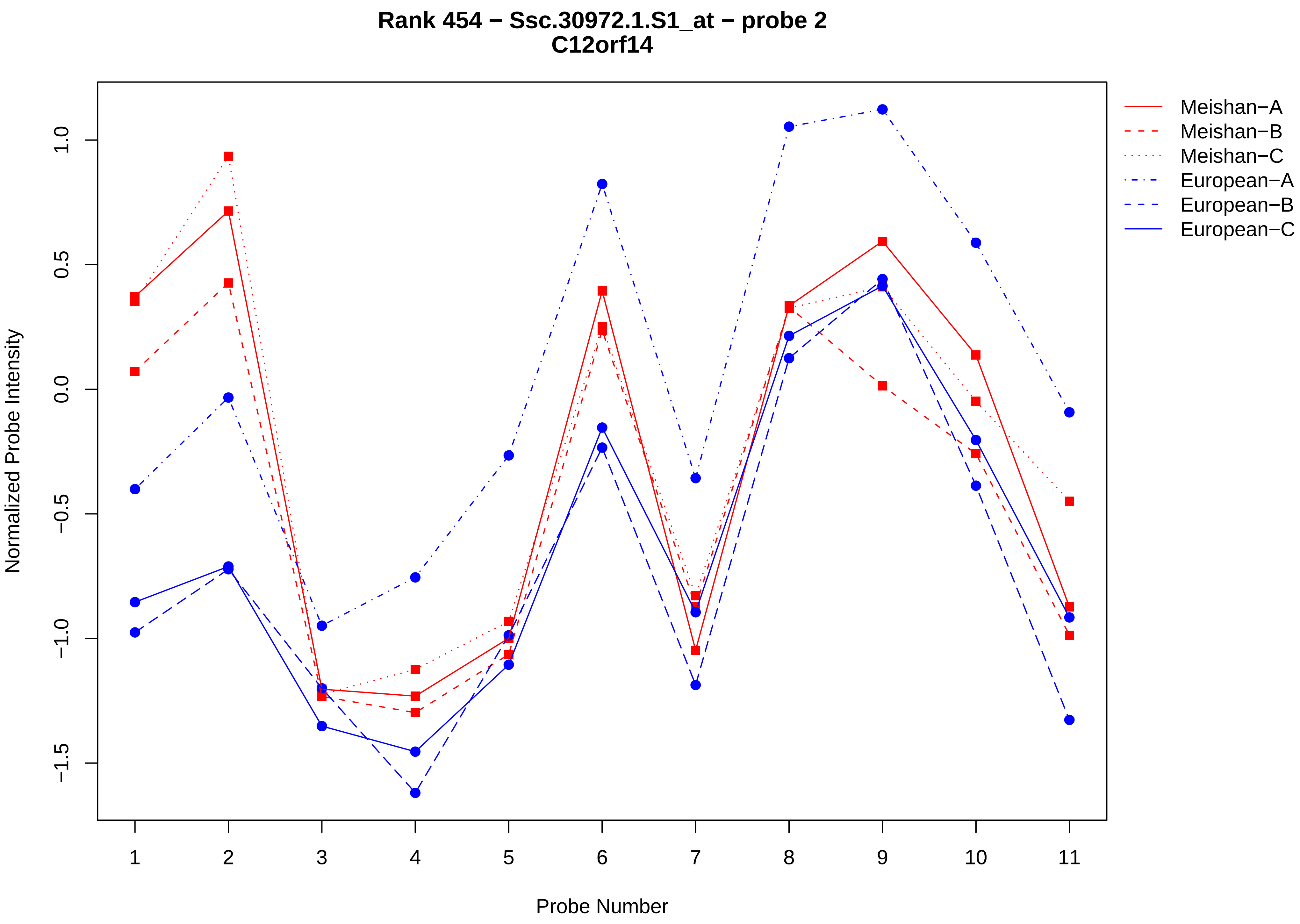
Rank 454 − Ssc.30972.1.S1_at − probe 2
C12orf14
Normalized Probe Intensity
Probe Number
1.0
0.5
0.0
−0.5
−1.0
−1.5
1
2
3
4
5
6
7
8
9
10
11
Meishan−A
Meishan−B
Meishan−C
European−A
European−B
European−C

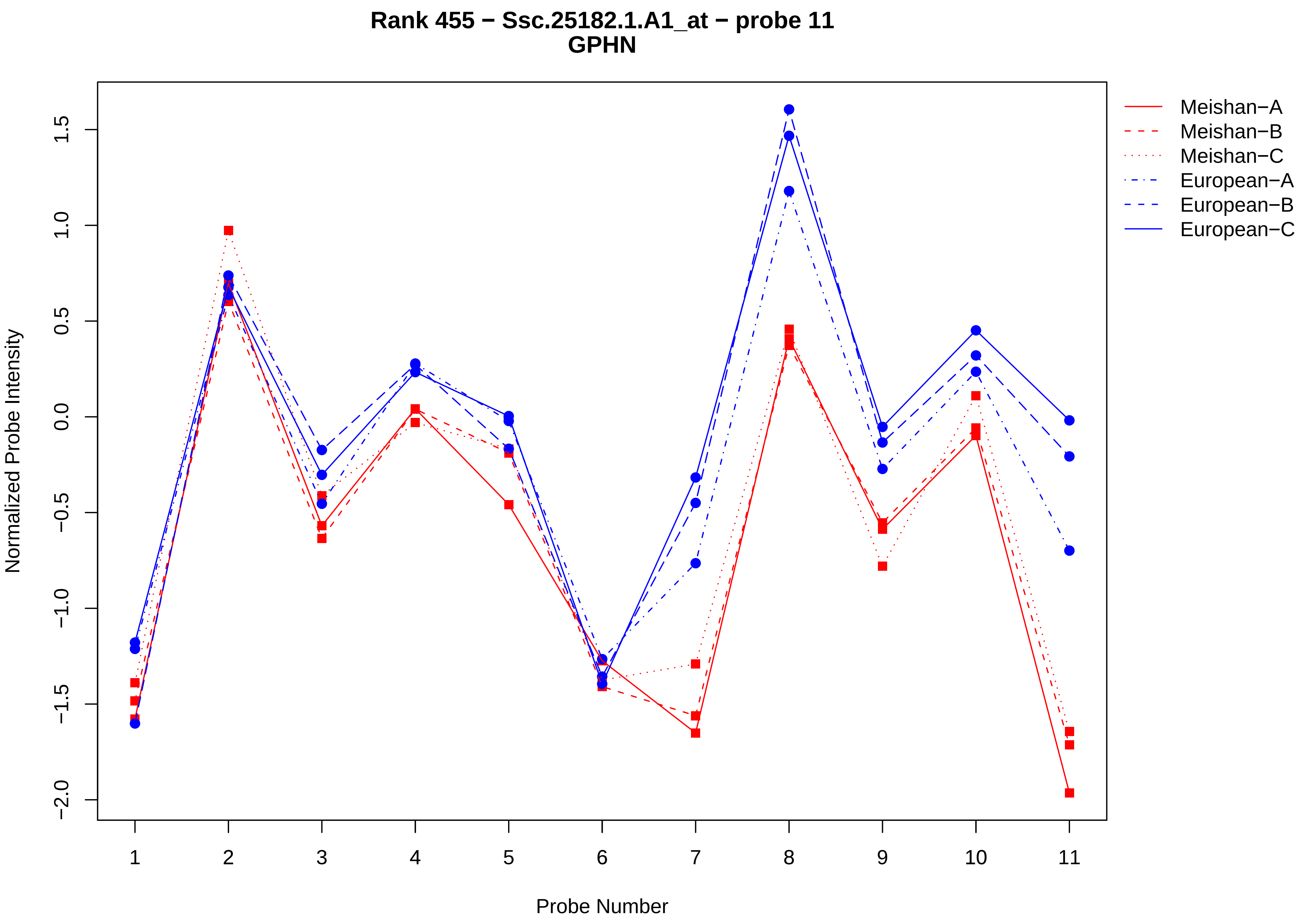

Rank 455 − Ssc.25182.1.A1_at − probe 11
GPHN
Normalized Probe Intensity
Probe Number
Meishan−A
Meishan−B
Meishan−C
European−A
European−B
European−C
1.5
1.0
0.5
0.0
−0.5
−1.0
−1.5
−2.0
1
2
3
4
5
6
7
8
9
10
11

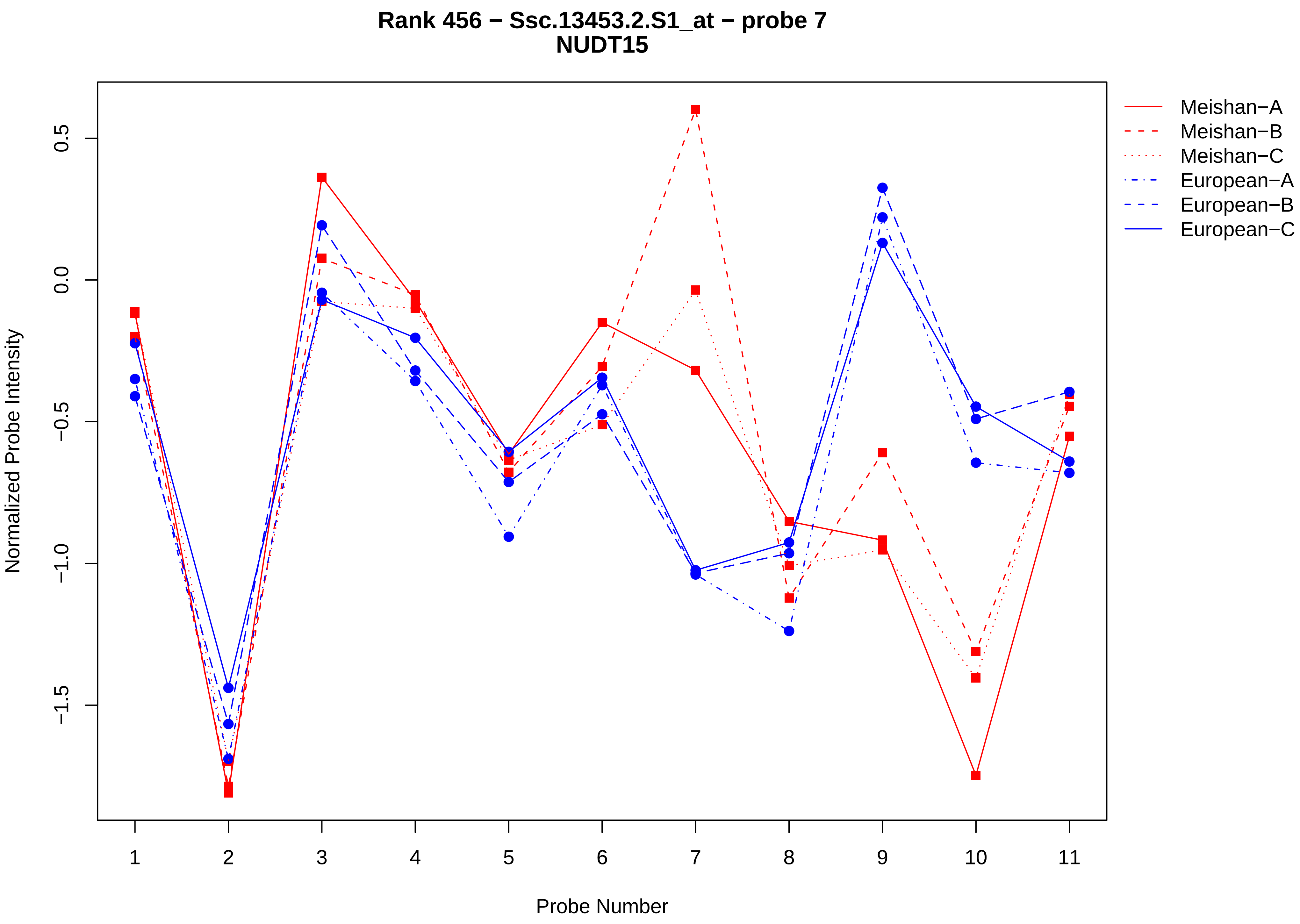

Rank 456 − Ssc.13453.2.S1_at − probe 7
NUDT15
Normalized Probe Intensity
Probe Number
0.5
0.0
−0.5
−1.0
−1.5
1
2
3
4
5
6
7
8
9
10
11
Meishan−A
Meishan−B
Meishan−C
European−A
European−B
European−C

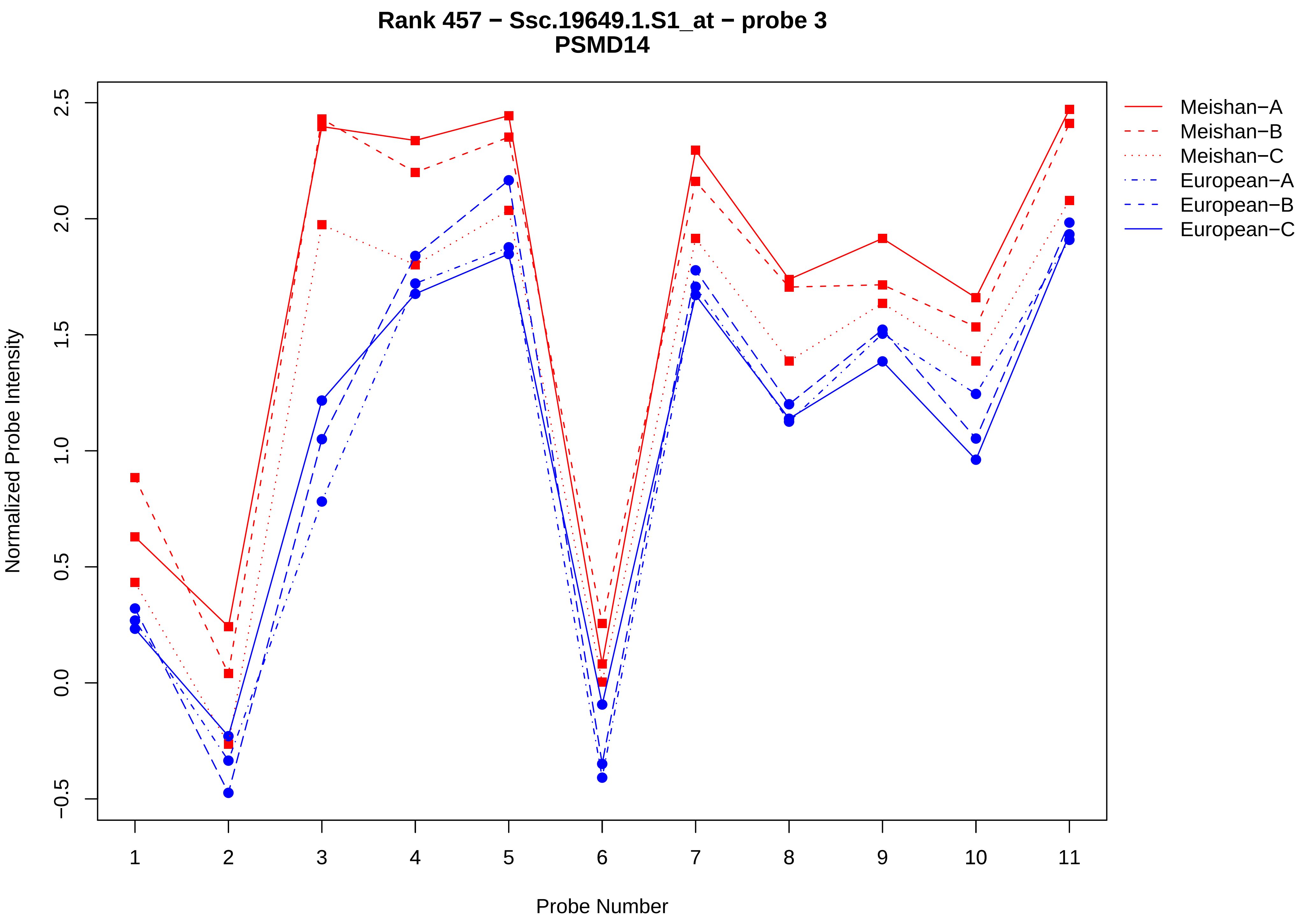

Rank 457 − Ssc.19649.1.S1_at − probe 3
PSMD14
Normalized Probe Intensity
Probe Number
−0.5
0.0
0.5
1.0
1.5
2.0
2.5
1
2
3
4
5
6
7
8
9
10
11
Meishan−A
Meishan−B
Meishan−C
European−A
European−B
European−C

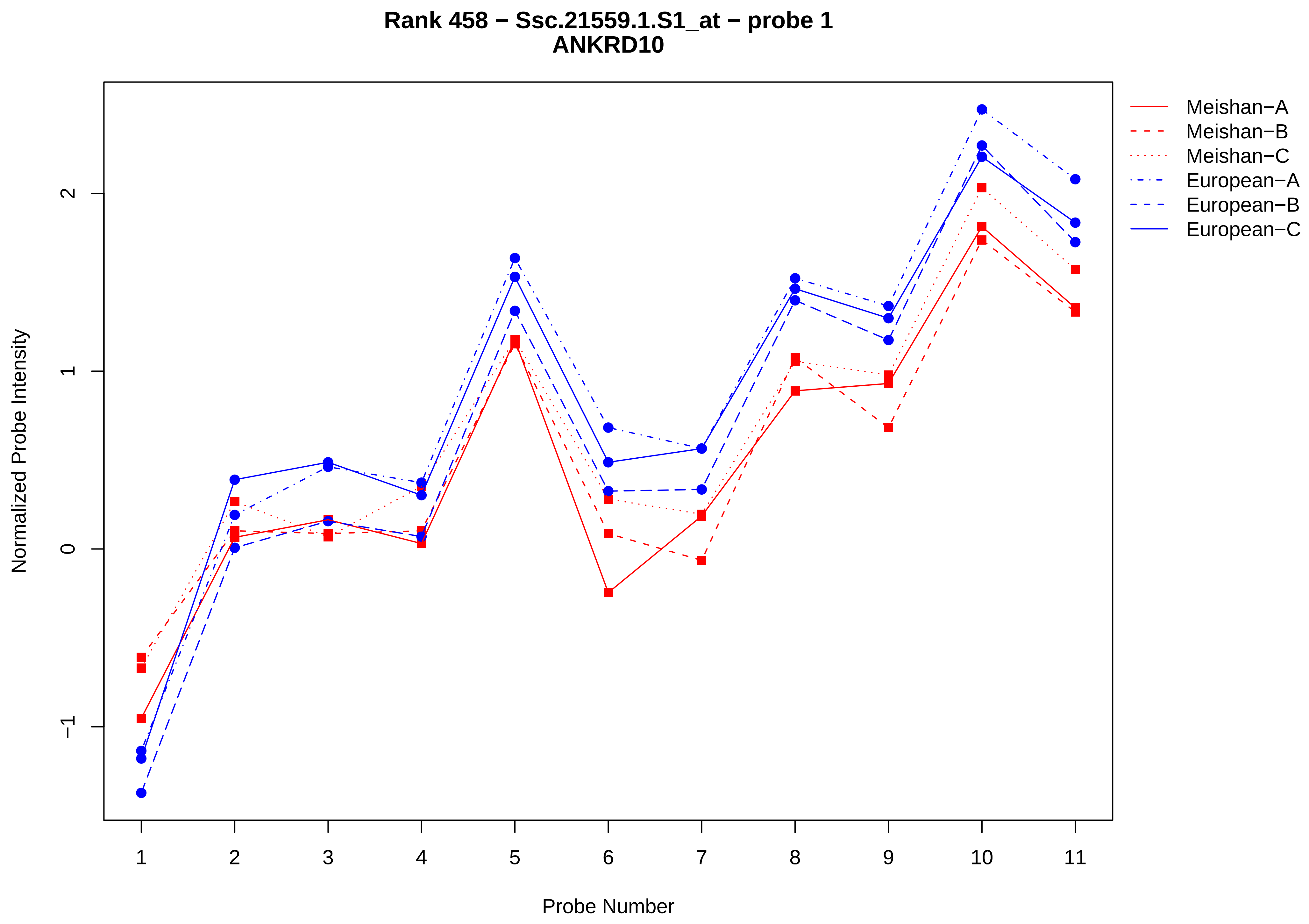

Rank 458 − Ssc.21559.1.S1_at − probe 1
ANKRD10
Normalized Probe Intensity
Probe Number
−1
0
1
2
1
2
3
4
5
6
7
8
9
10
11
Meishan−A
Meishan−B
Meishan−C
European−A
European−B
European−C

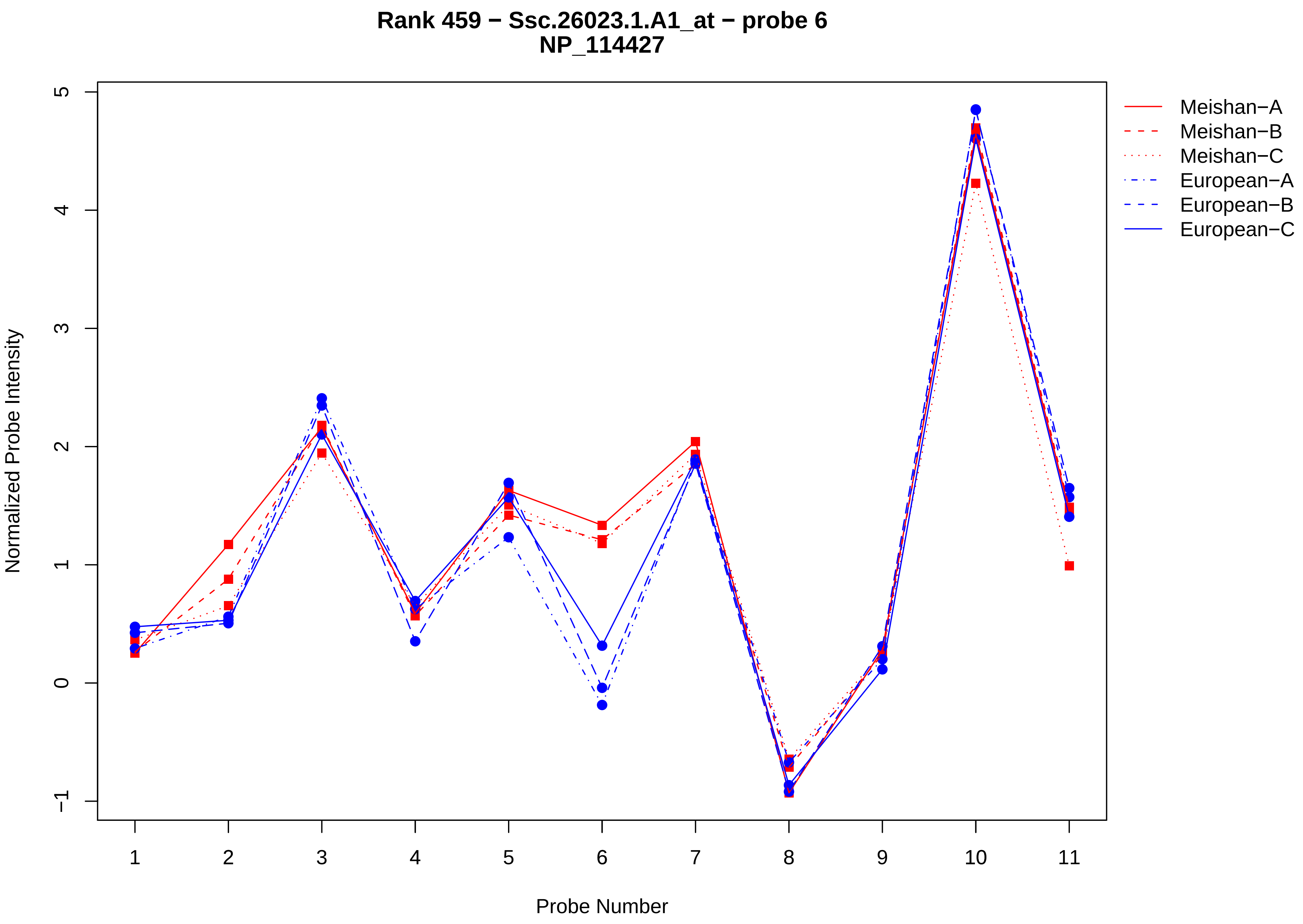

Rank 459 − Ssc.26023.1.A1_at − probe 6
NP_114427
Normalized Probe Intensity
Probe Number
Meishan−A
Meishan−B
Meishan−C
European−A
European−B
European−C

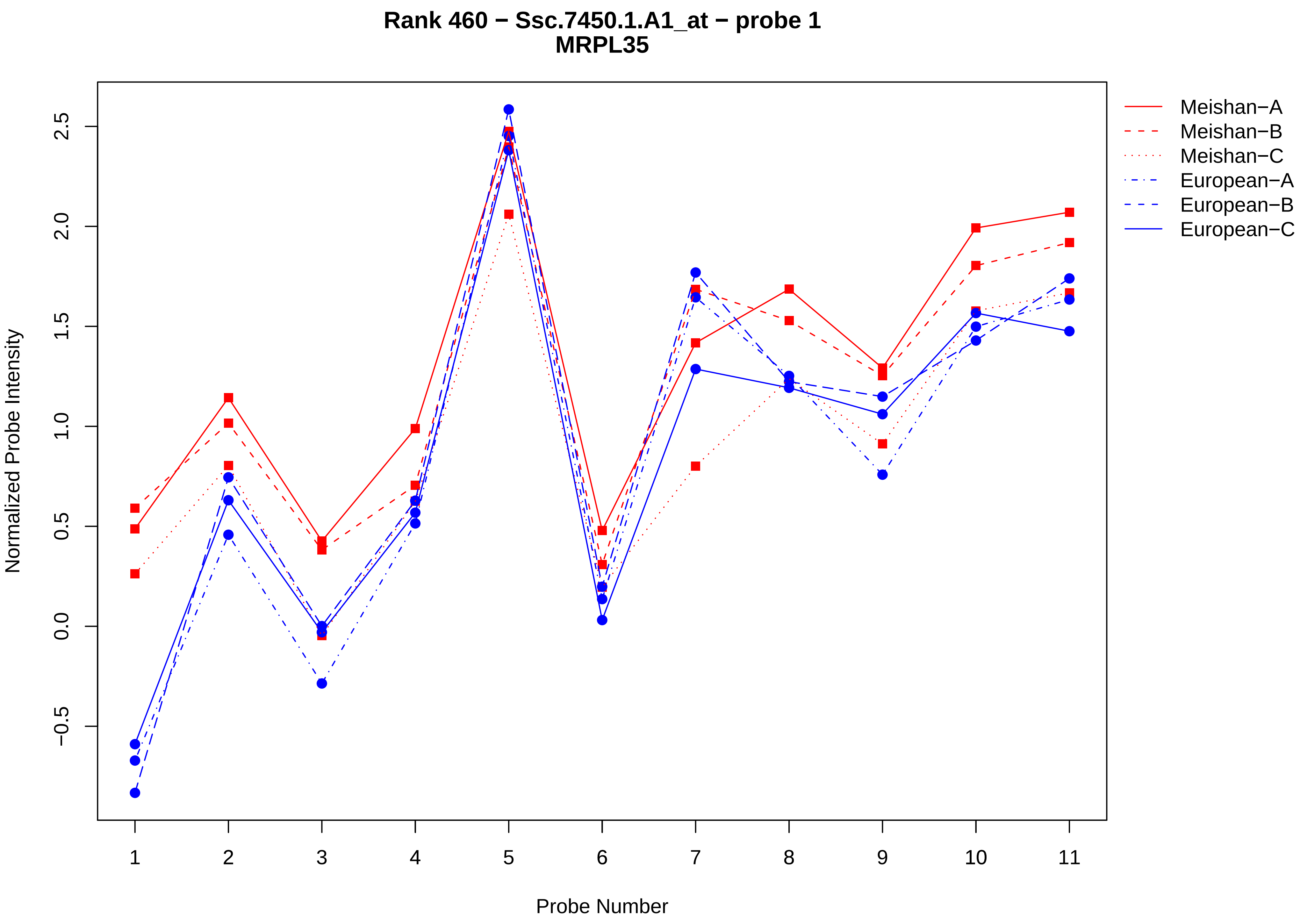

Rank 460 − Ssc.7450.1.A1_at − probe 1
MRPL35
Normalized Probe Intensity
−0.5
0.0
0.5
1.0
1.5
2.0
2.5
1
2
3
4
5
6
7
8
9
10
11
Probe Number
Meishan−A
Meishan−B
Meishan−C
European−A
European−B
European−C

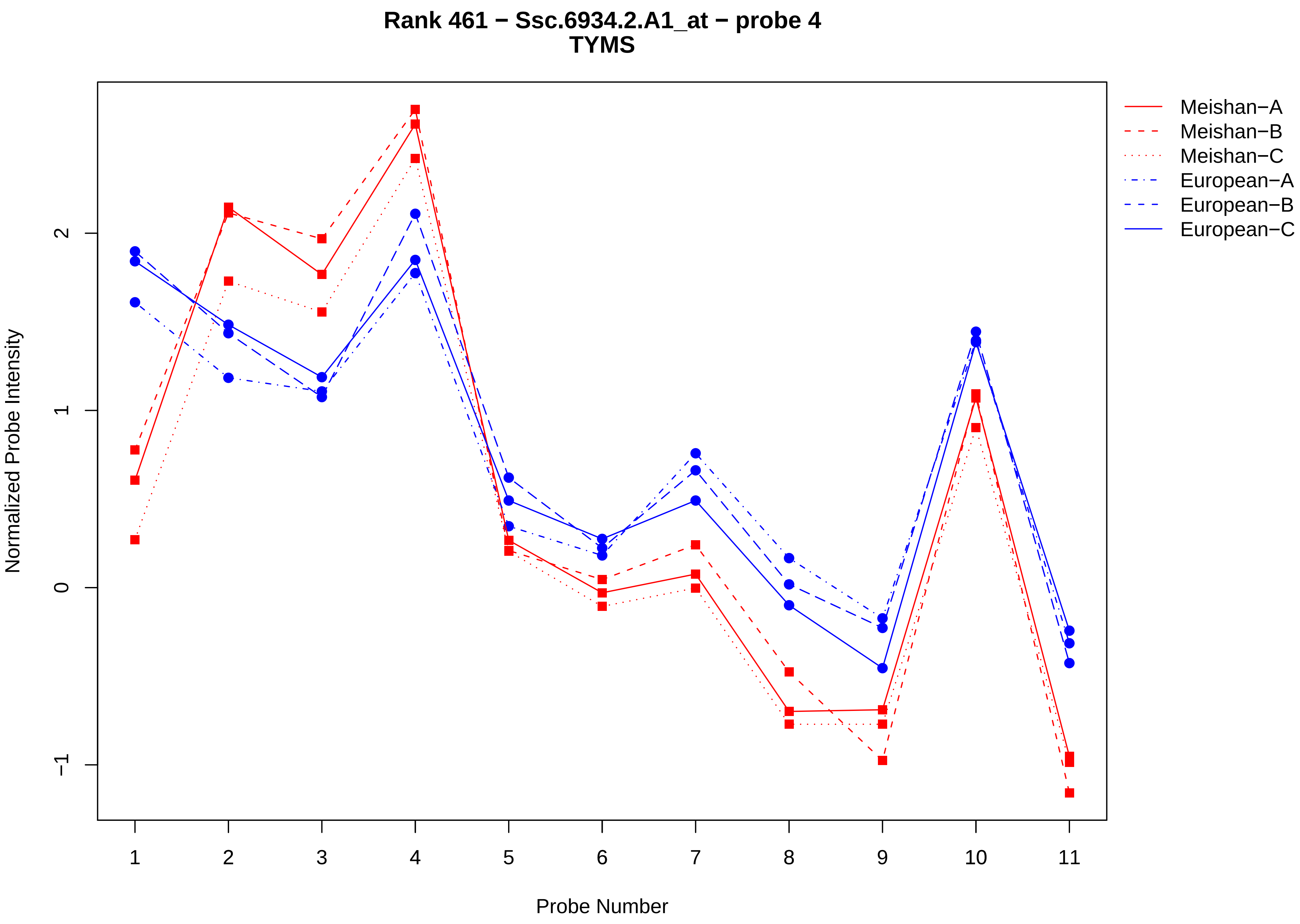

Rank 461 − Ssc.6934.2.A1_at − probe 4
TYMS
Normalized Probe Intensity
Probe Number
−1
0
1
2
1
2
3
4
5
6
7
8
9
10
11
Meishan−A
Meishan−B
Meishan−C
European−A
European−B
European−C

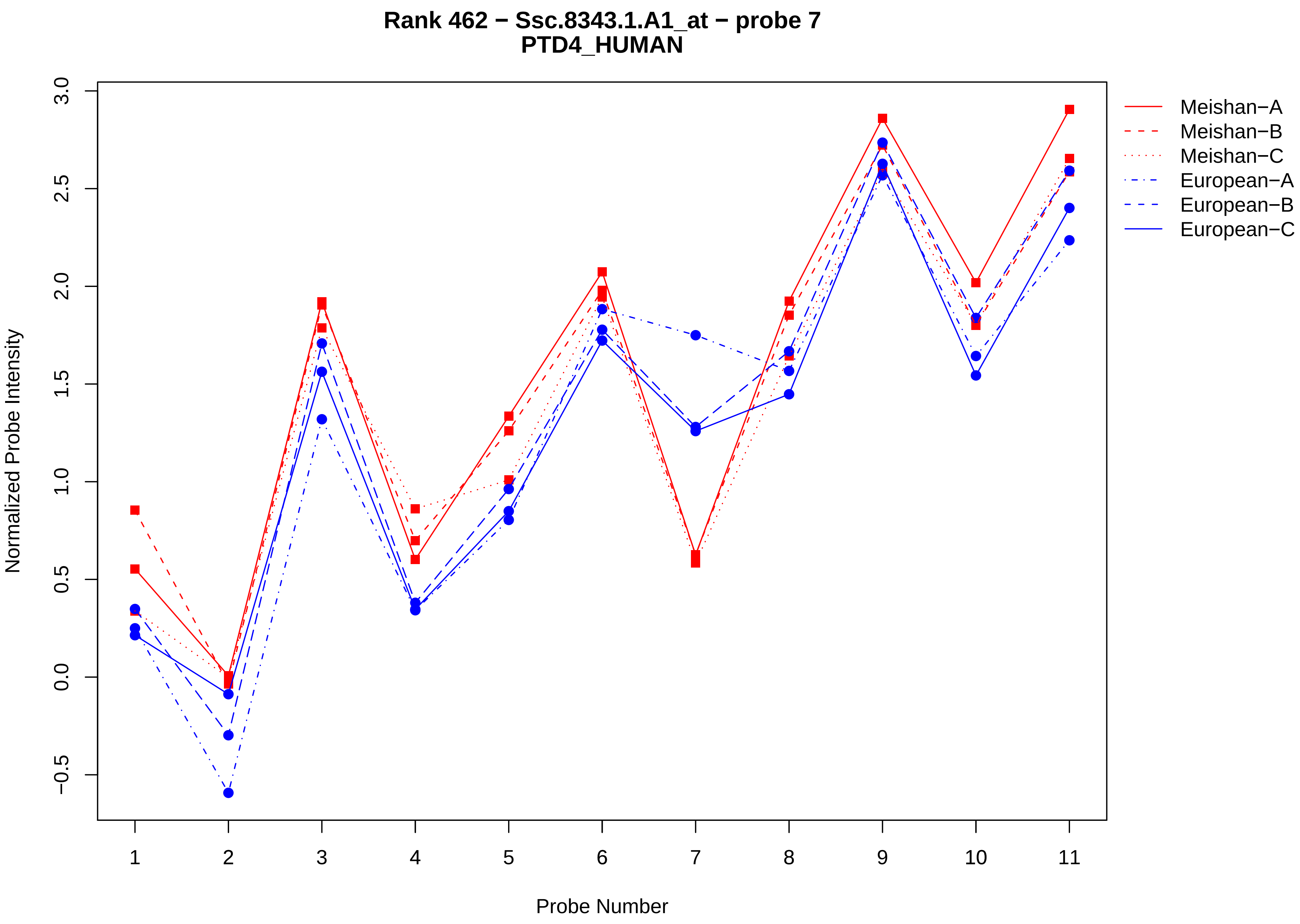

Rank 462 − Ssc.8343.1.A1_at − probe 7
PTD4_HUMAN
Normalized Probe Intensity
Probe Number
Meishan−A
Meishan−B
Meishan−C
European−A
European−B
European−C
−0.5
0.0
0.5
1.0
1.5
2.0
2.5
3.0
1
2
3
4
5
6
7
8
9
10
11

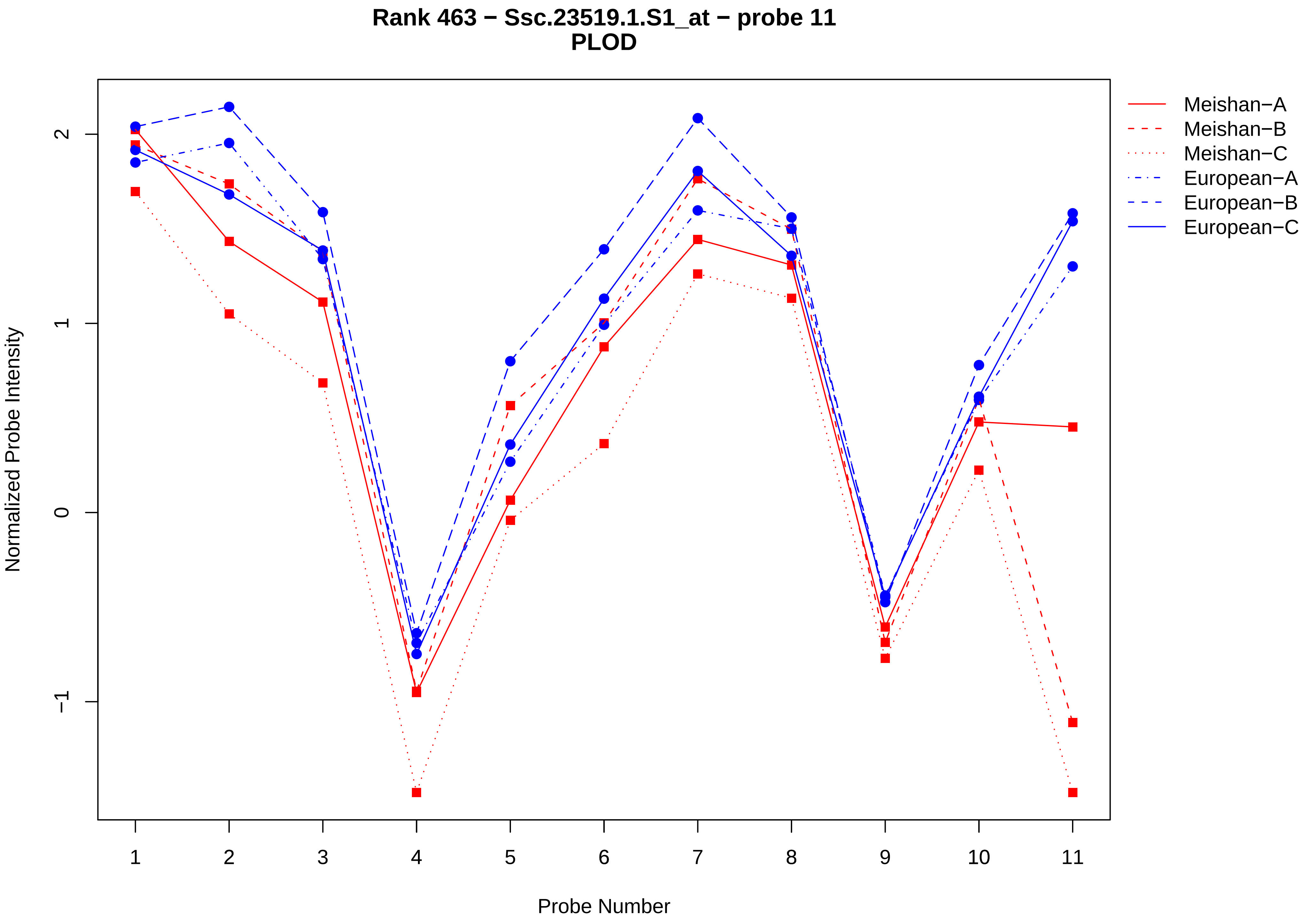

Rank 463 − Ssc.23519.1.S1_at − probe 11
PLOD
Normalized Probe Intensity
Probe Number
−1
0
1
2
1
2
3
4
5
6
7
8
9
10
11
Meishan−A
Meishan−B
Meishan−C
European−A
European−B
European−C

Rank 464 − Ssc.1623.1.S1_at − probe 7
DSP

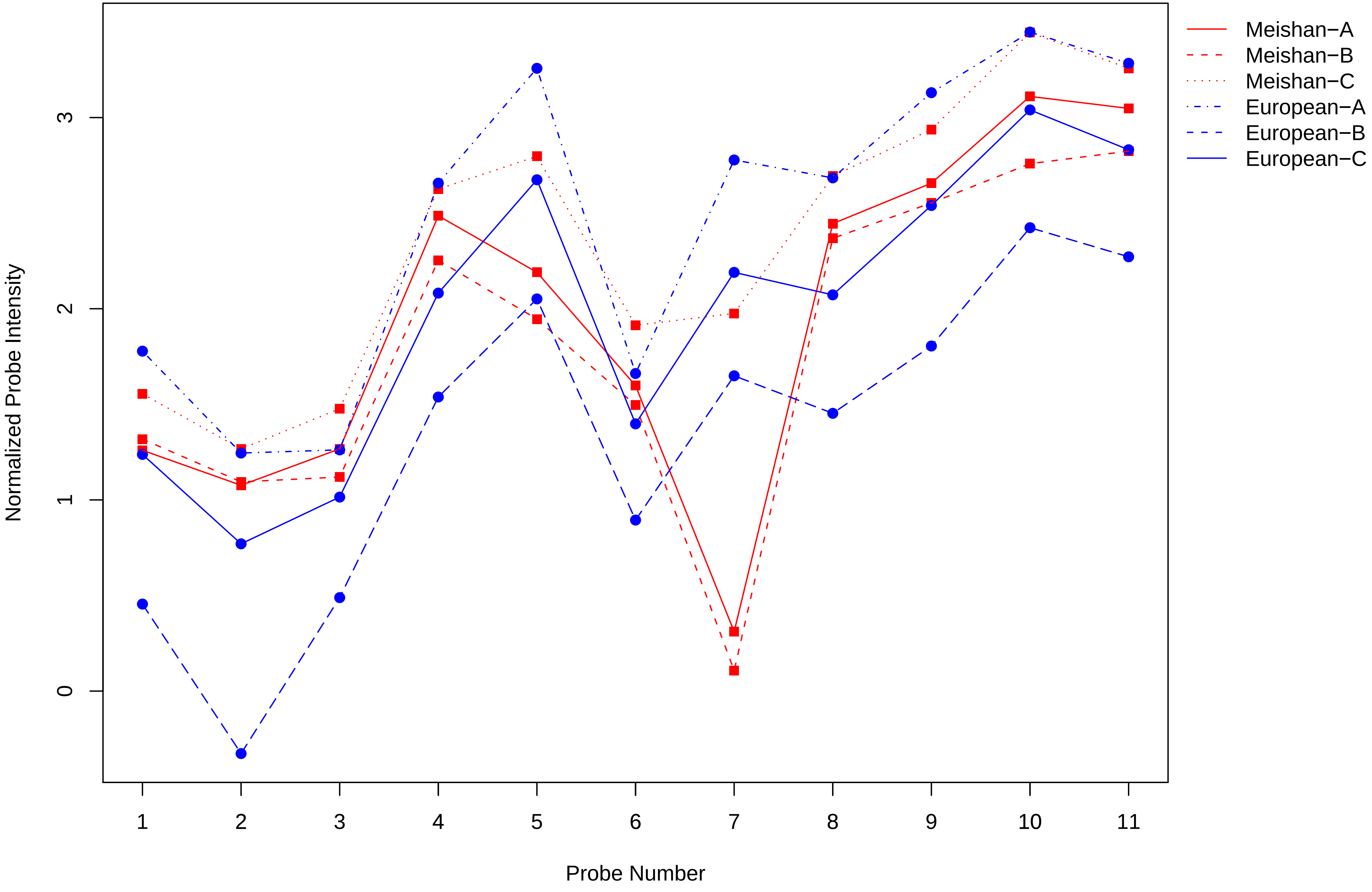

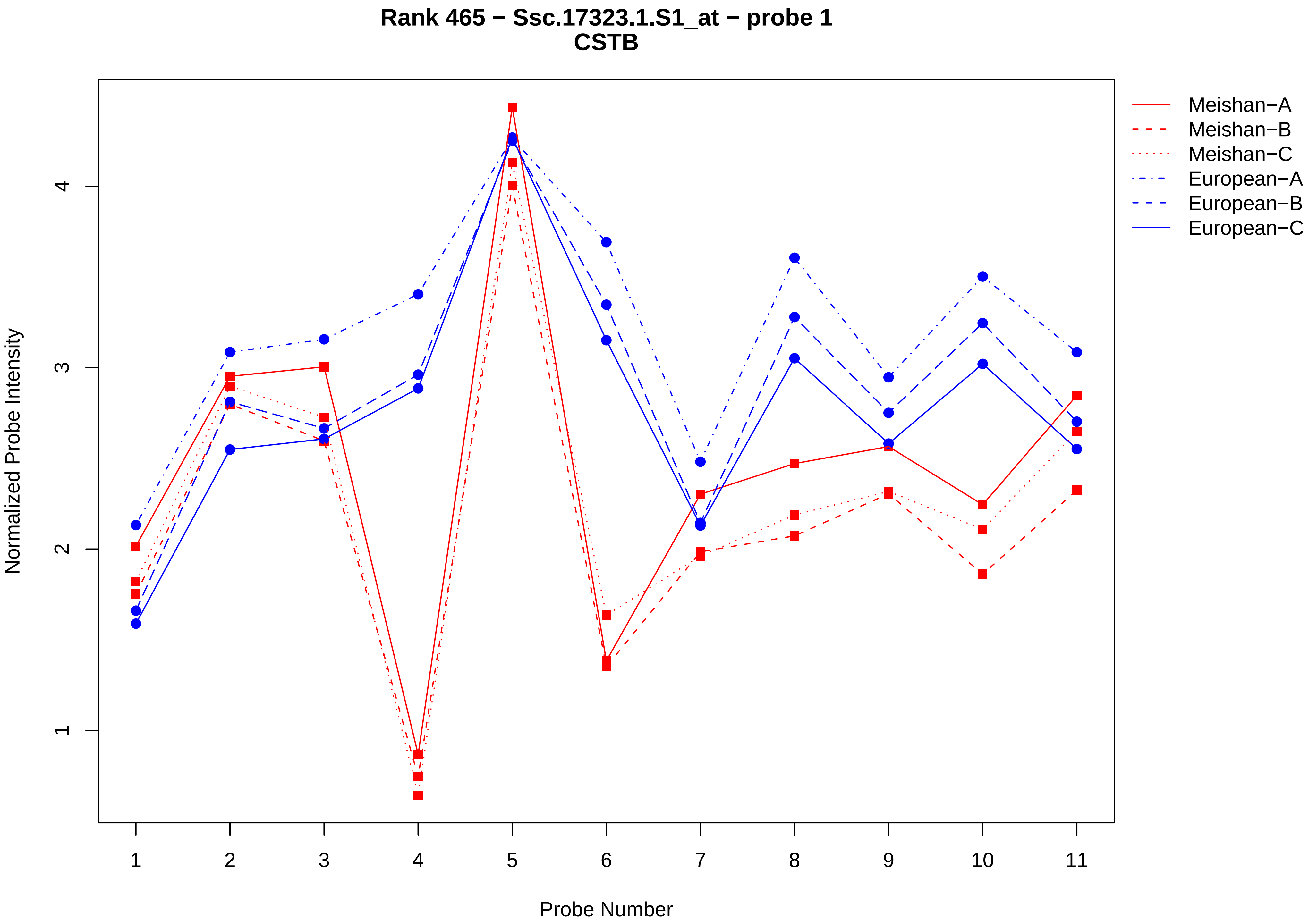

Rank 465 − Ssc.17323.1.S1_at − probe 1
CSTB
Normalized Probe Intensity
Probe Number
1
2
3
4
1
2
3
4
5
6
7
8
9
10
11
Meishan−A
Meishan−B
Meishan−C
European−A
European−B
European−C

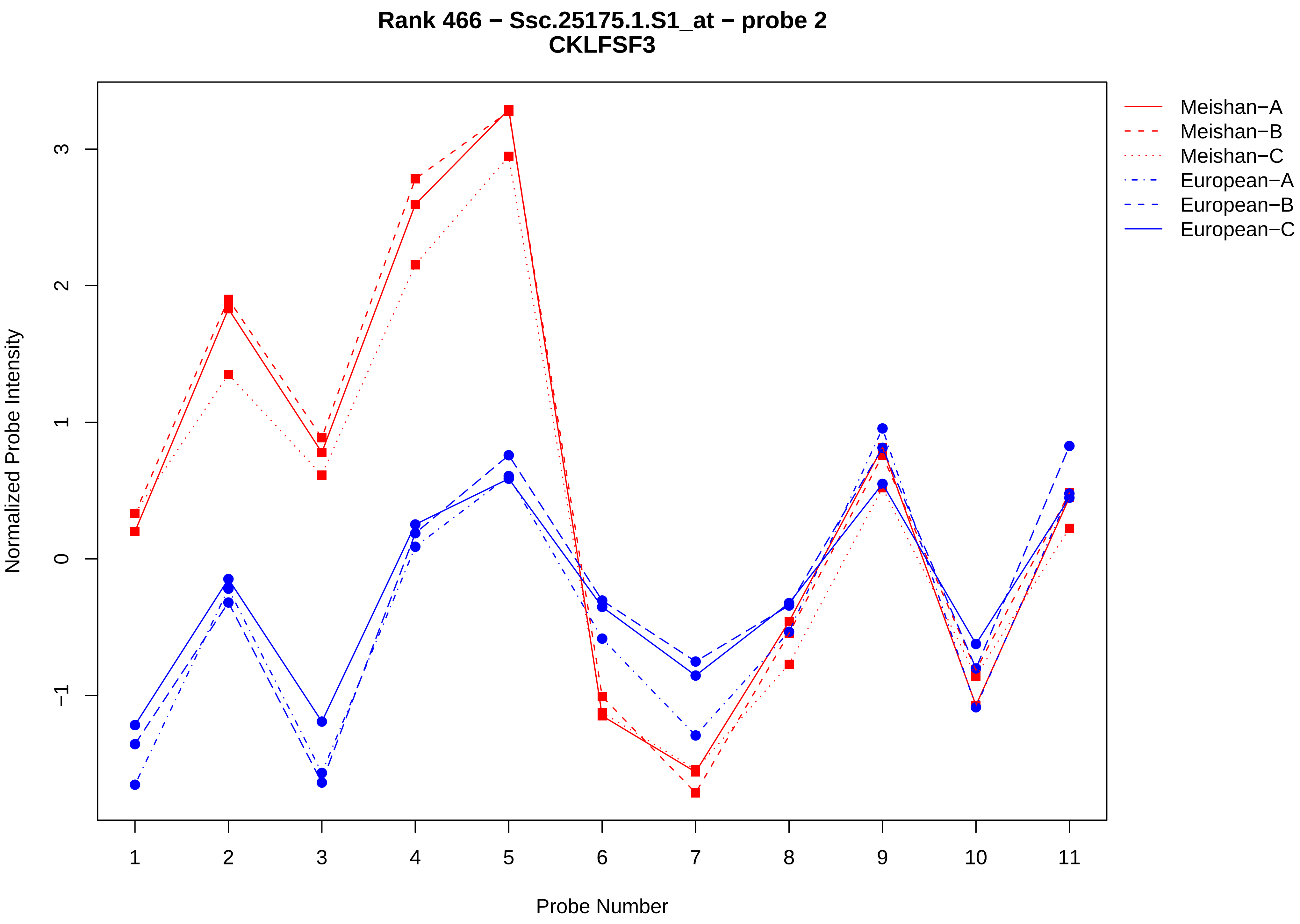

Rank 466 − Ssc.25175.1.S1_at − probe 2
CKLFSF3
Normalized Probe Intensity
Probe Number
−1
0
1
2
3
1
2
3
4
5
6
7
8
9
10
11
Meishan−A
Meishan−B
Meishan−C
European−A
European−B
European−C

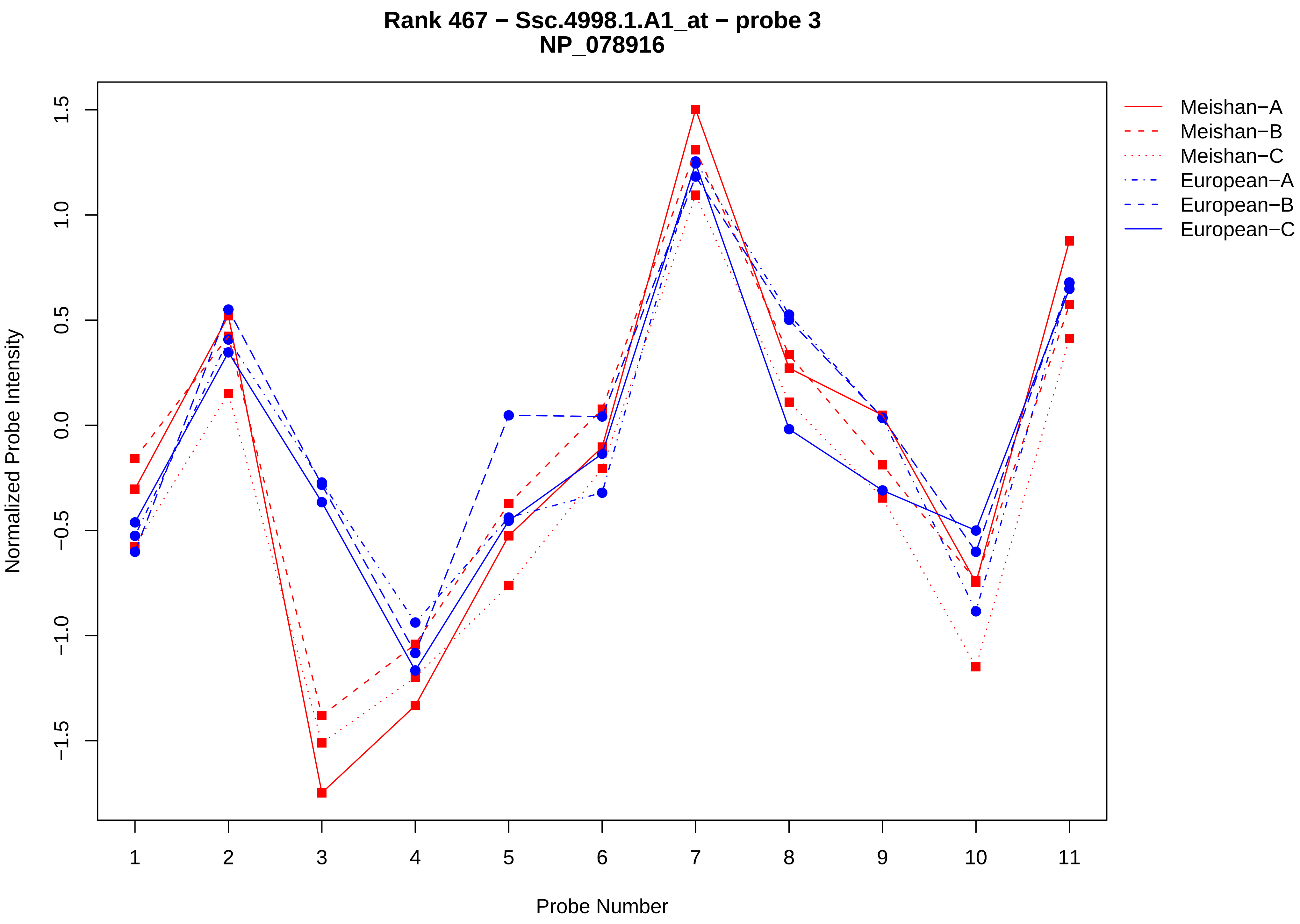
Rank 467 − Ssc.4998.1.A1_at − probe 3
NP_078916
Normalized Probe Intensity
Probe Number
Meishan−A
Meishan−B
Meishan−C
European−A
European−B
European−C
1.5
1.0
0.5
0.0
−0.5
−1.0
−1.5
1
2
3
4
5
6
7
8
9
10
11

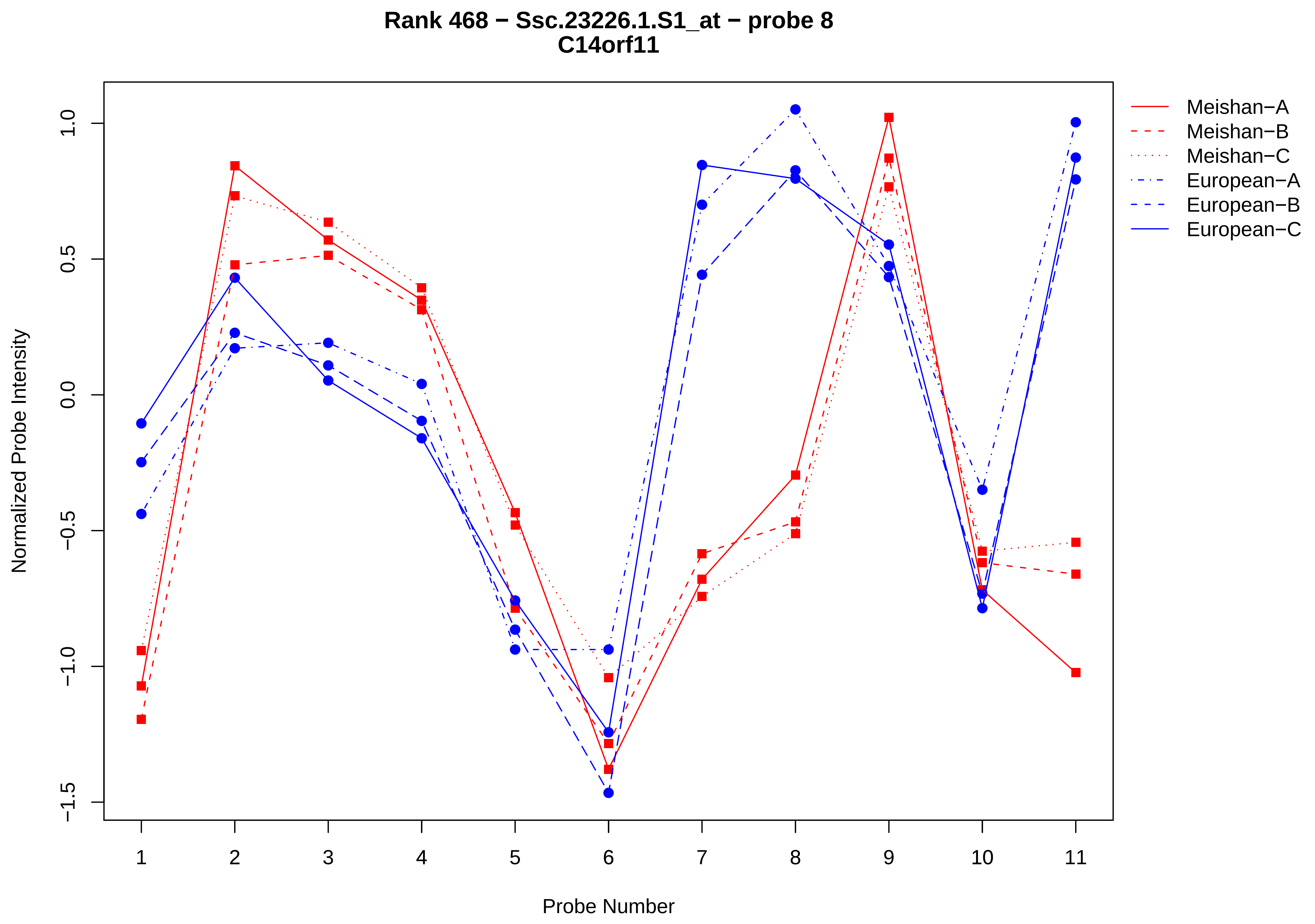

Rank 468 − Ssc.23226.1.S1_at − probe 8
C14orf11
Normalized Probe Intensity
Probe Number
Meishan−A
Meishan−B
Meishan−C
European−A
European−B
European−C
1.0
0.5
0.0
−0.5
−1.0
−1.5
1
2
3
4
5
6
7
8
9
10
11

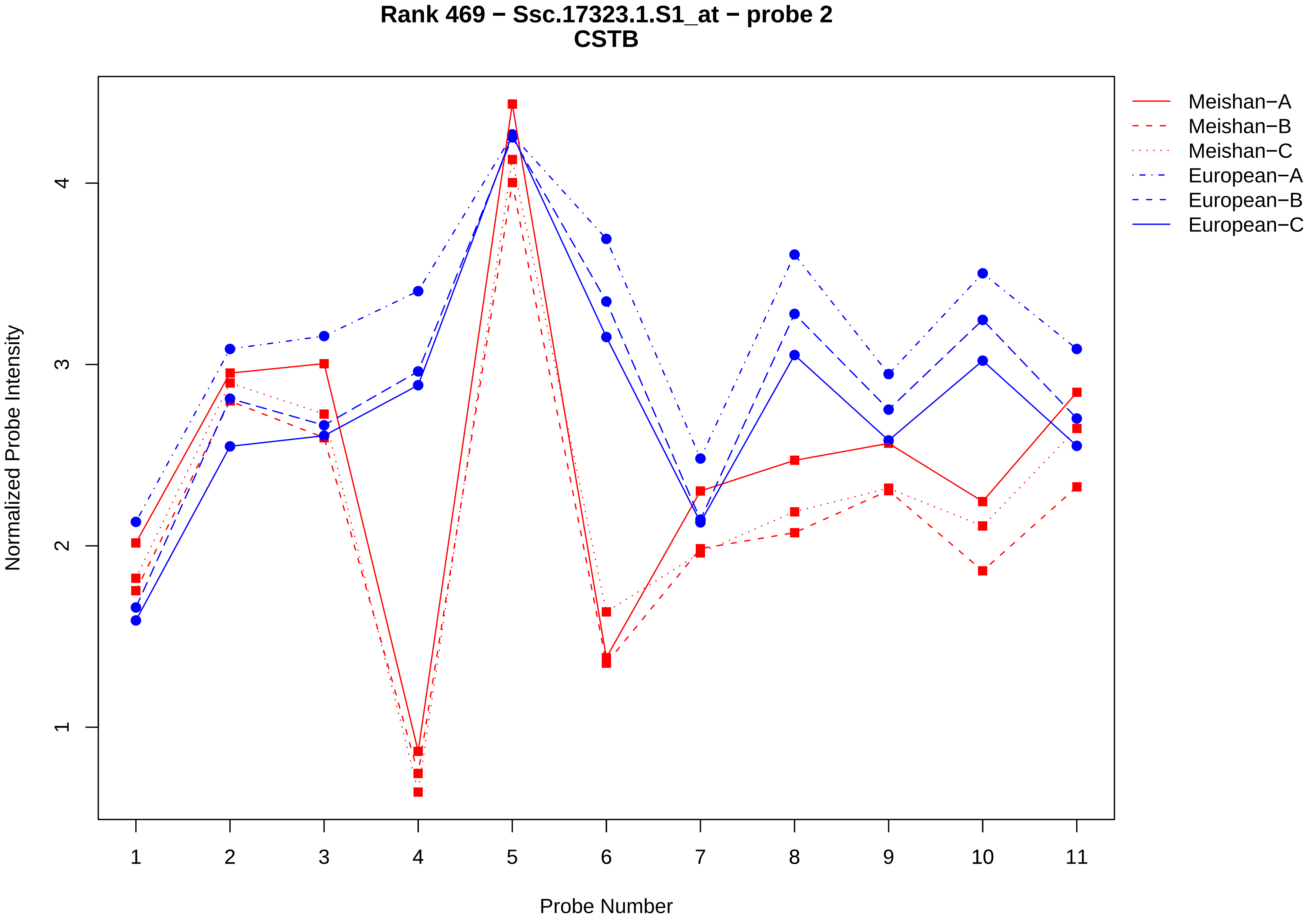
Rank 469 − Ssc.17323.1.S1_at − probe 2
CSTB
Normalized Probe Intensity
Probe Number
1
2
3
4
1
2
3
4
5
6
7
8
9
10
11
Meishan−A
Meishan−B
Meishan−C
European−A
European−B
European−C

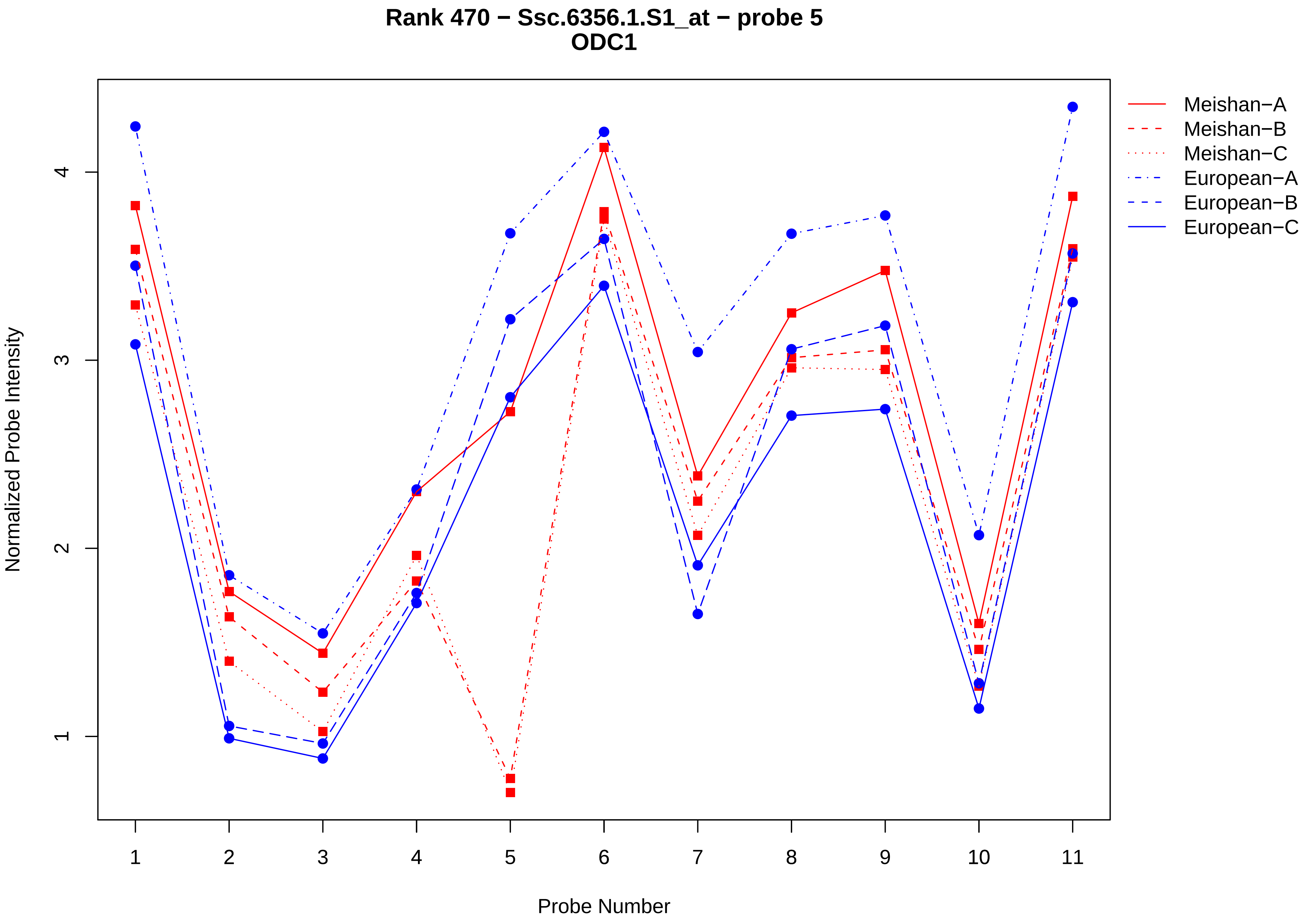

Rank 470 − Ssc.6356.1.S1_at − probe 5
ODC1
Normalized Probe Intensity
Probe Number
1
2
3
4
1
2
3
4
5
6
7
8
9
10
11
Meishan−A
Meishan−B
Meishan−C
European−A
European−B
European−C

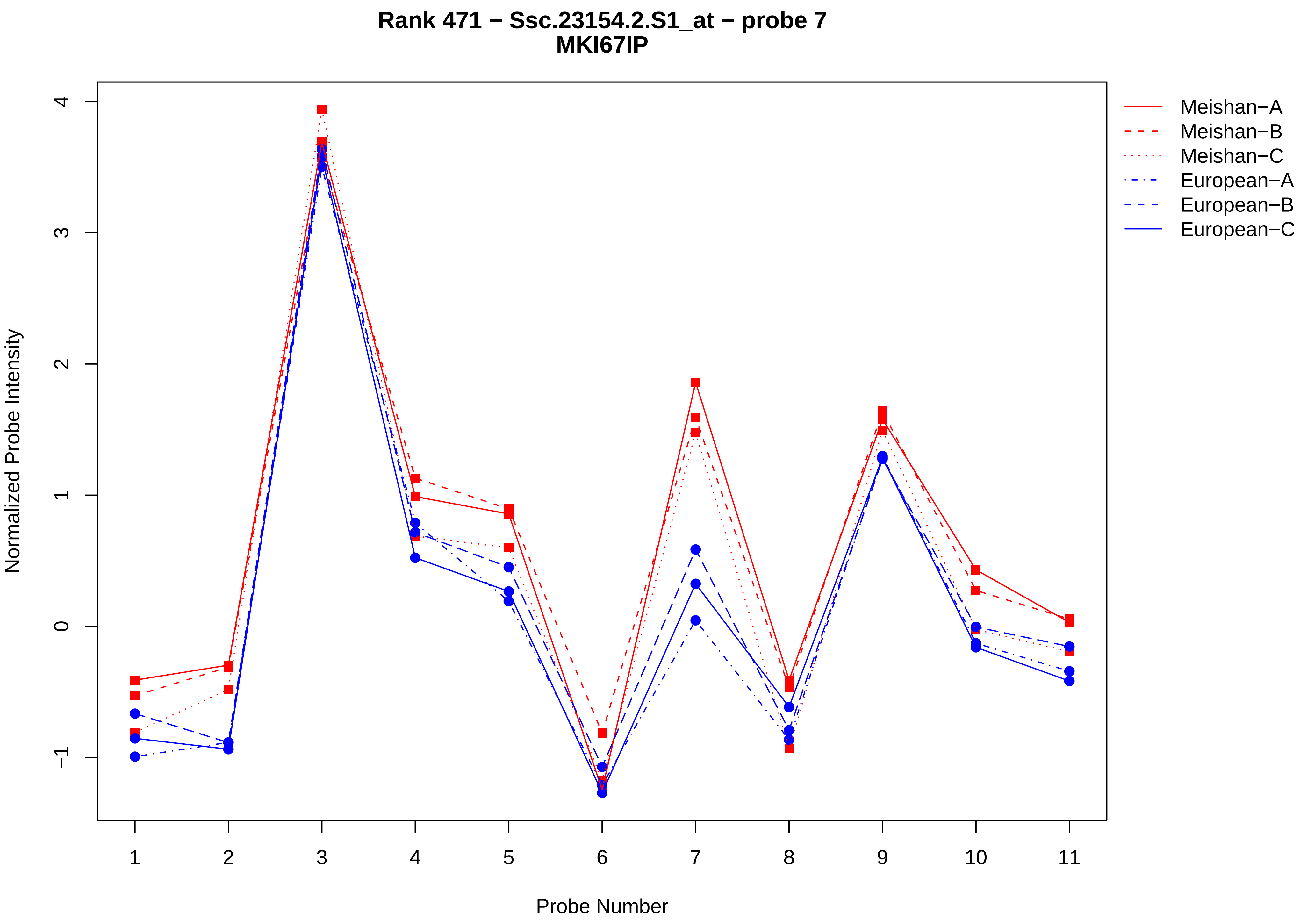

Rank 471 − Ssc.23154.2.S1_at − probe 7
MKI67IP
Normalized Probe Intensity
Probe Number
Meishan−A
Meishan−B
Meishan−C
European−A
European−B
European−C

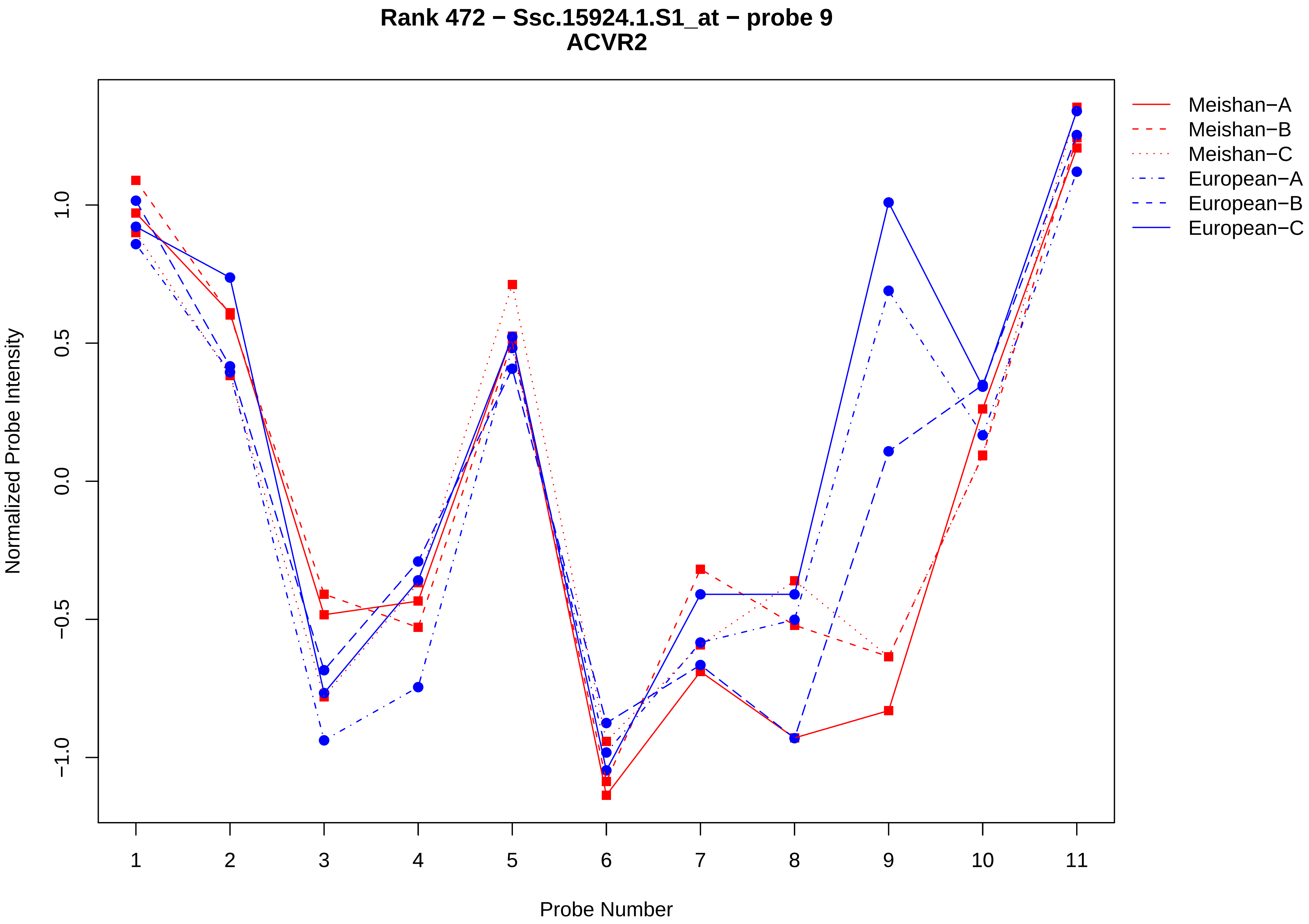

Rank 472 − Ssc.15924.1.S1_at − probe 9
ACVR2
Normalized Probe Intensity
Probe Number
1.0
0.5
0.0
−0.5
−1.0
1
2
3
4
5
6
7
8
9
10
11
Meishan−A
Meishan−B
Meishan−C
European−A
European−B
European−C

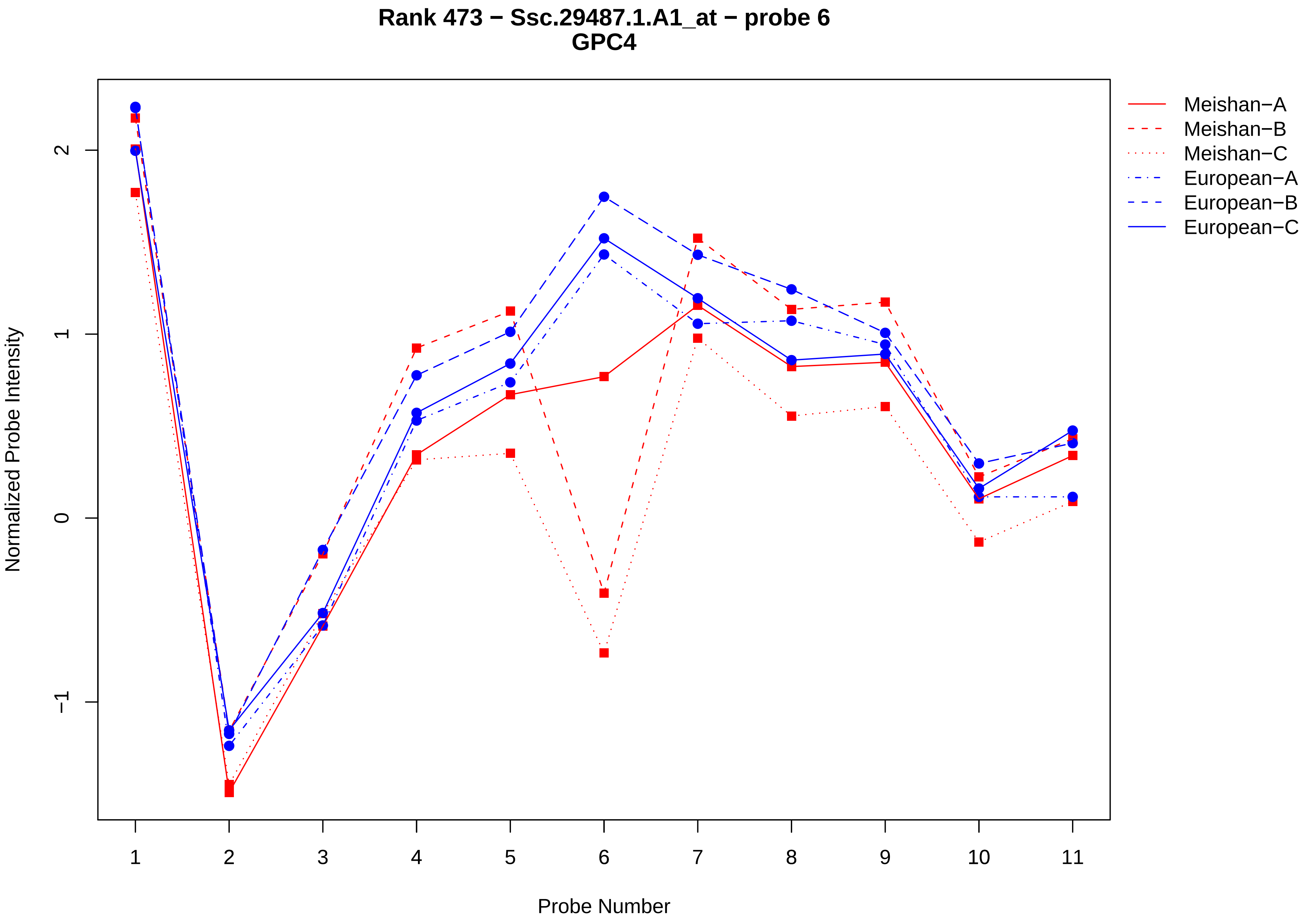

Rank 473 − Ssc.29487.1.A1_at − probe 6
GPC4
Normalized Probe Intensity
Probe Number
−1
0
1
2
1
2
3
4
5
6
7
8
9
10
11
Meishan−A
Meishan−B
Meishan−C
European−A
European−B
European−C

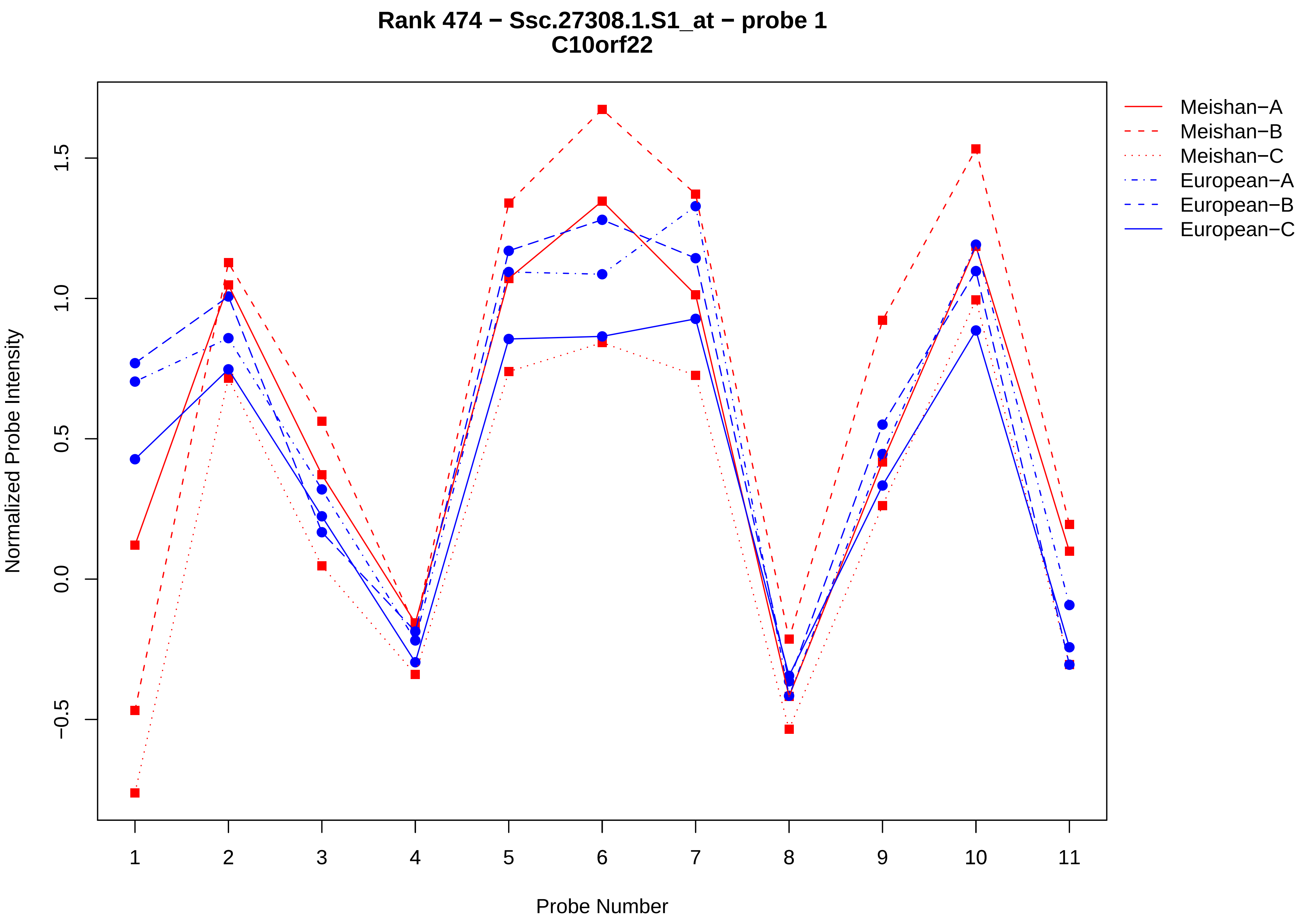
Rank 474 − Ssc.27308.1.S1_at − probe 1
C10orf22
Normalized Probe Intensity
Probe Number
−0.5
0.0
0.5
1.0
1.5
1
2
3
4
5
6
7
8
9
10
11
Meishan−A
Meishan−B
Meishan−C
European−A
European−B
European−C

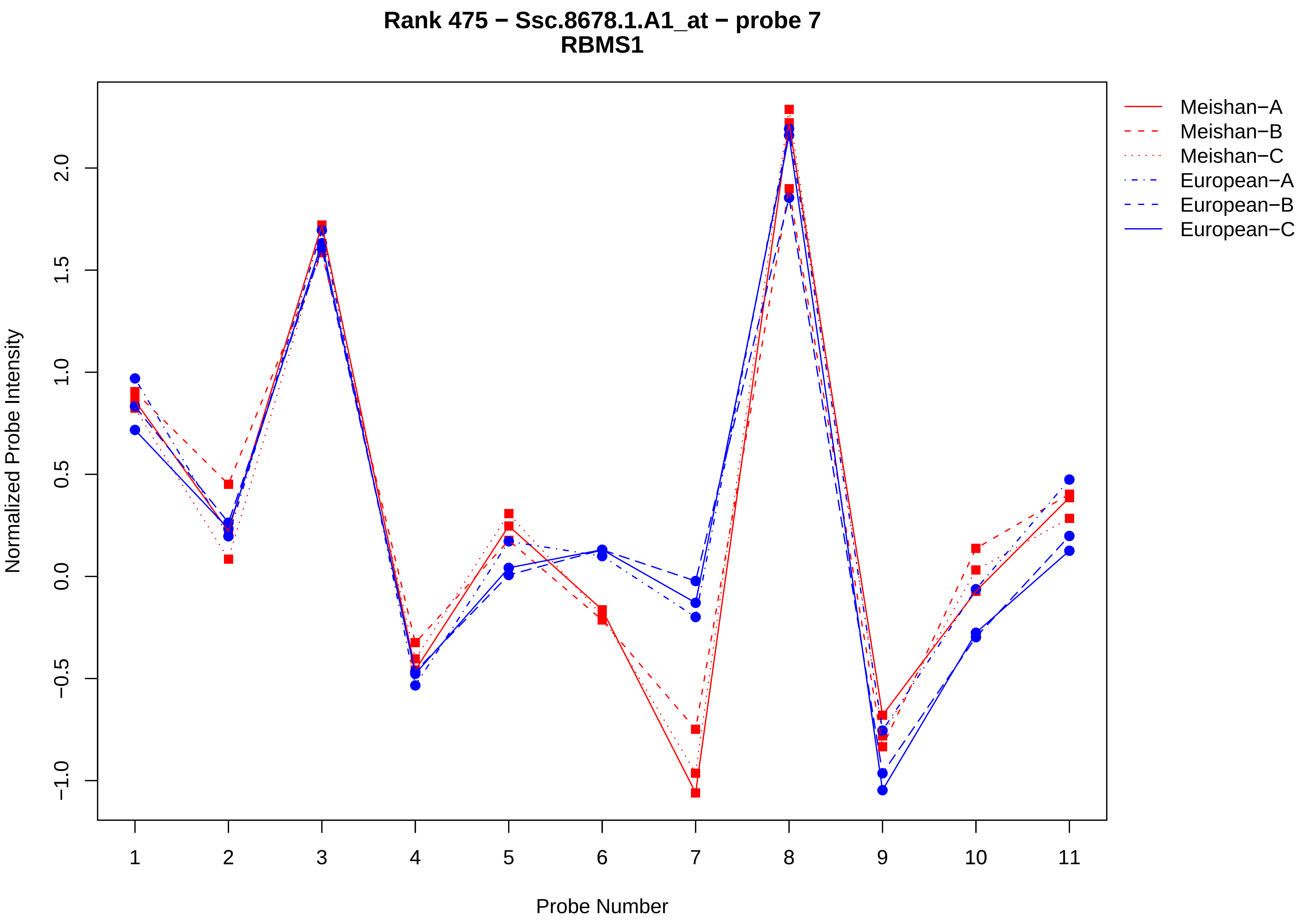

Rank 475 − Ssc.8678.1.A1_at − probe 7
RBMS1
Normalized Probe Intensity
Probe Number
−1.0
−0.5
0.0
0.5
1.0
1.5
2.0
1
2
3
4
5
6
7
8
9
10
11
Meishan−A
Meishan−B
Meishan−C
European−A
European−B
European−C

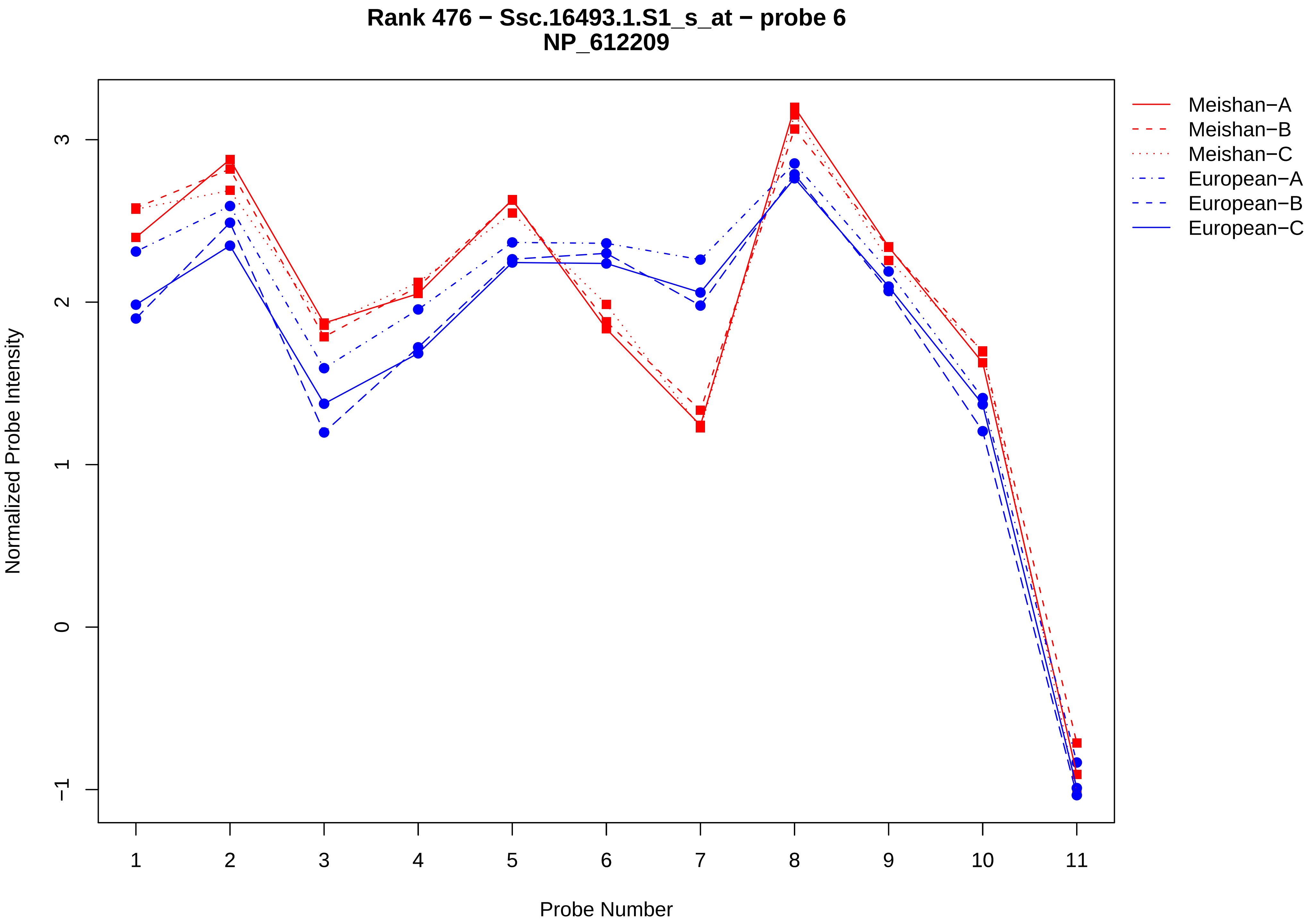
Rank 476 − Ssc.16493.1.S1_s_at − probe 6
NP_612209
Normalized Probe Intensity
Probe Number
Meishan−A
Meishan−B
Meishan−C
European−A
European−B
European−C
−1
0
1
2
3
1
2
3
4
5
6
7
8
9
10
11

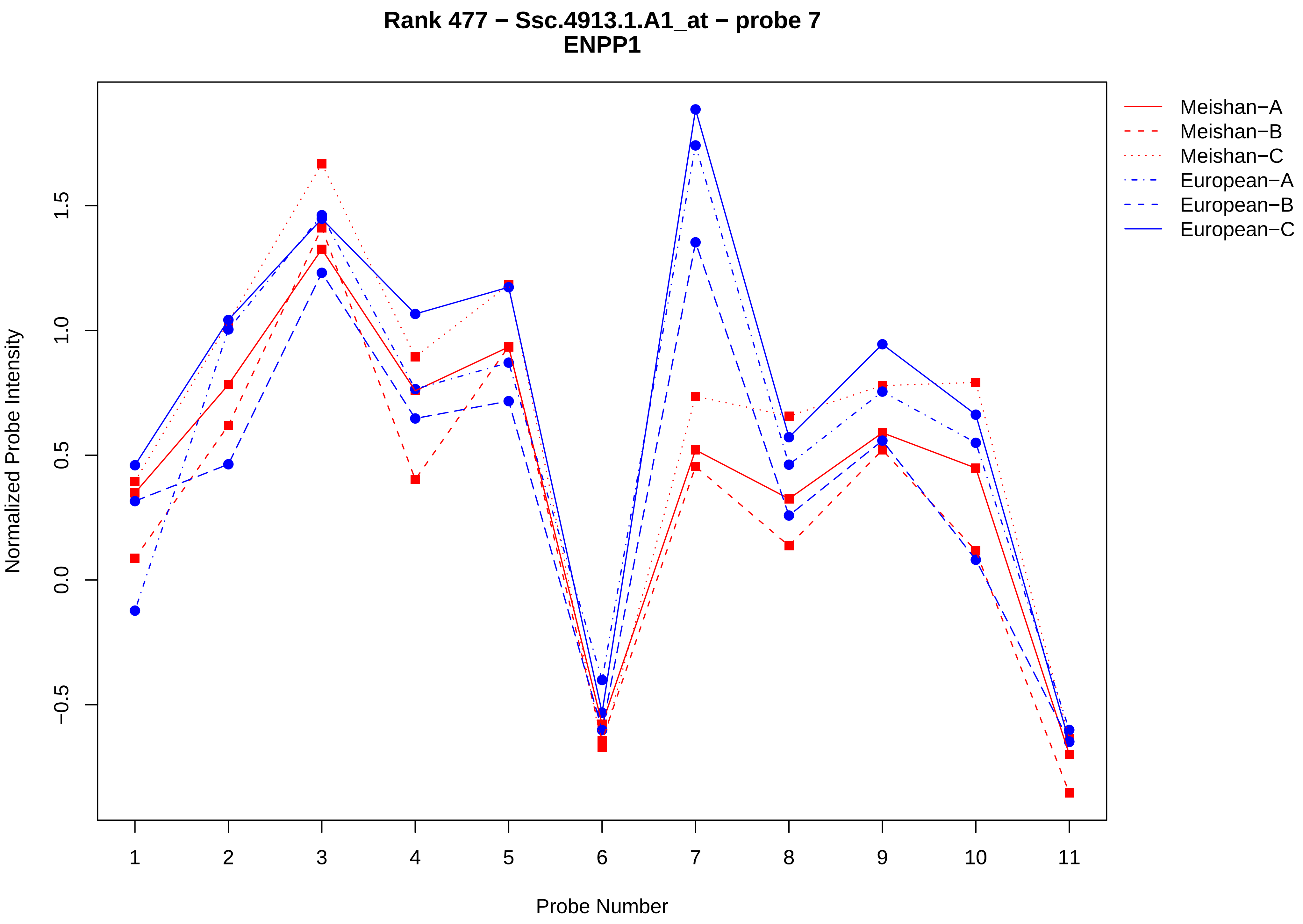

Rank 477 − Ssc.4913.1.A1_at − probe 7
ENPP1
Normalized Probe Intensity
Probe Number
−0.5
0.0
0.5
1.0
1.5
1
2
3
4
5
6
7
8
9
10
11
Meishan−A
Meishan−B
Meishan−C
European−A
European−B
European−C

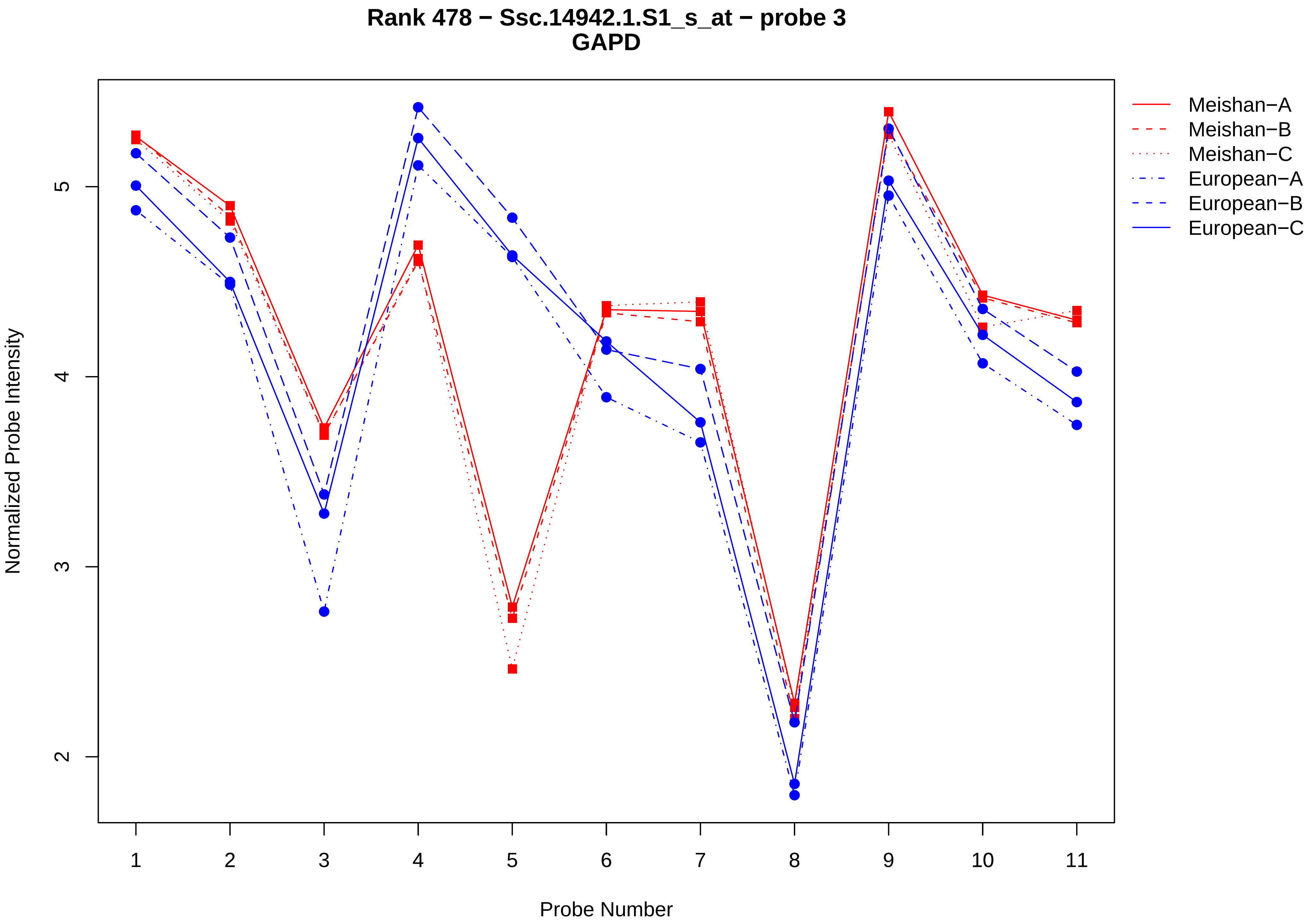

Rank 478 − Ssc.14942.1.S1_s_at − probe 3
GAPD
Normalized Probe Intensity
Probe Number
2
3
4
5
1
2
3
4
5
6
7
8
9
10
11
Meishan−A
Meishan−B
Meishan−C
European−A
European−B
European−C

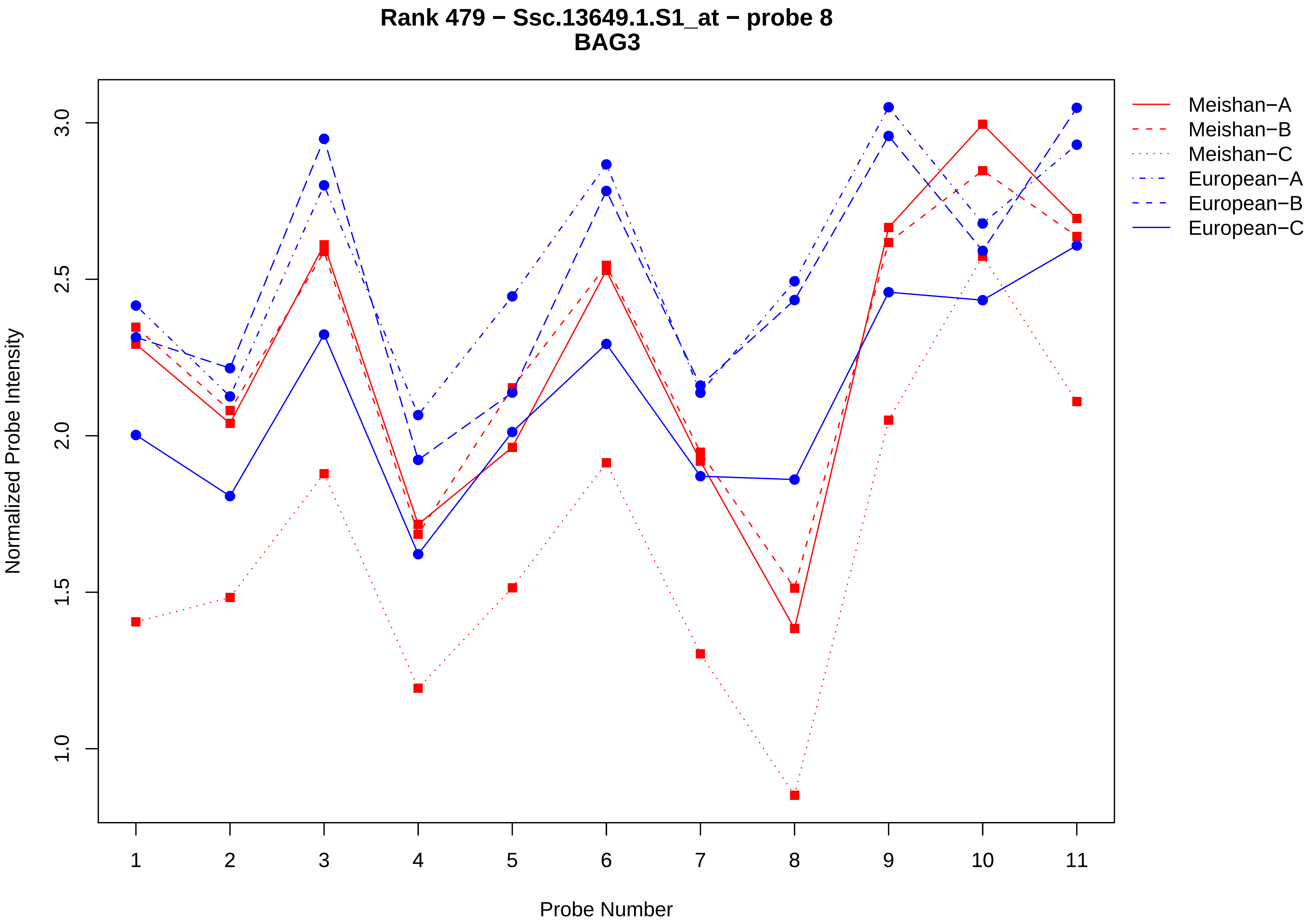
Rank 479 − Ssc.13649.1.S1_at − probe 8
BAG3
Normalized Probe Intensity
Probe Number
1.0
1.5
2.0
2.5
3.0
1
2
3
4
5
6
7
8
9
10
11
Meishan−A
Meishan−B
Meishan−C
European−A
European−B
European−C

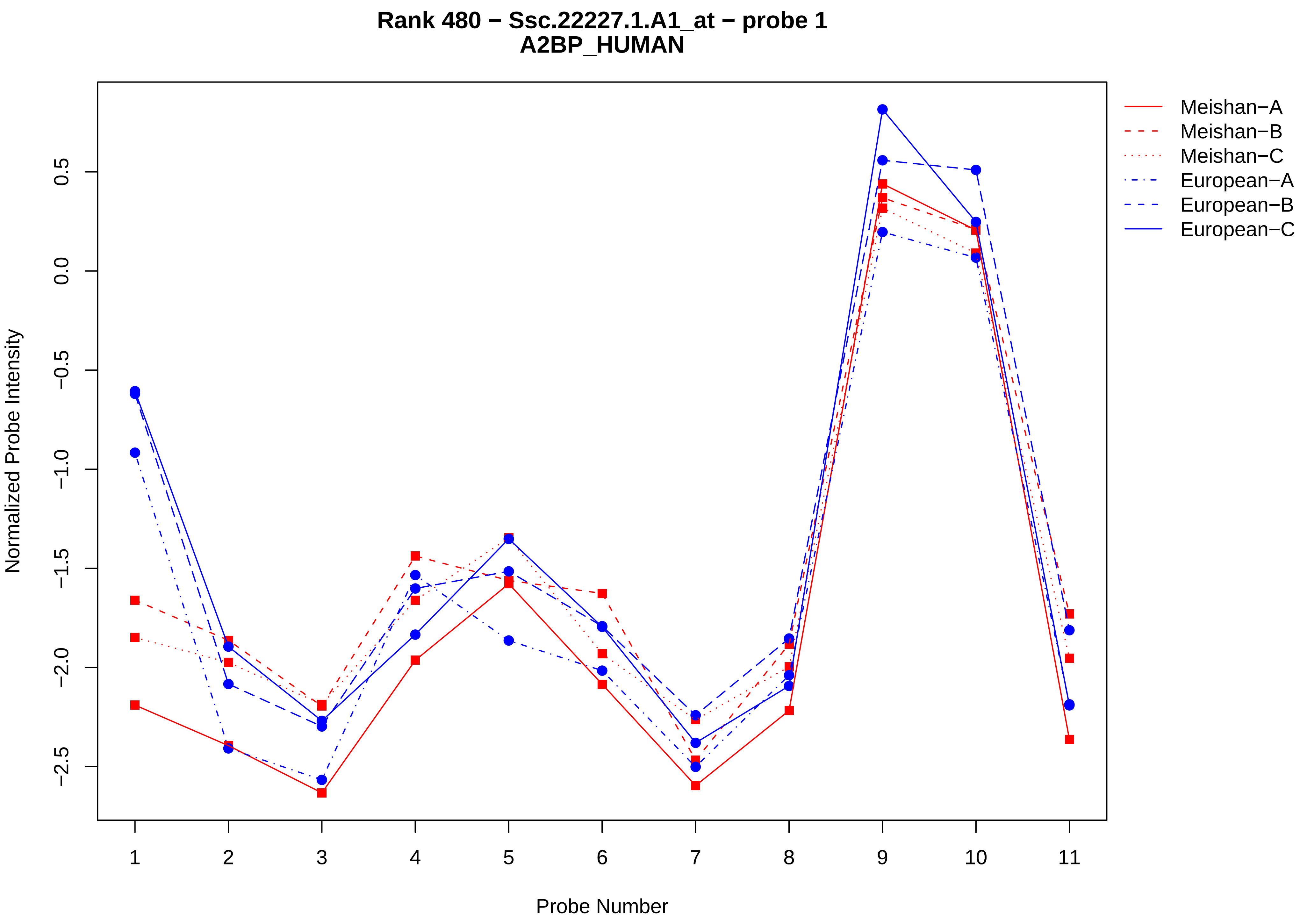
Rank 480 − Ssc.22227.1.A1_at − probe 1
A2BP_HUMAN
Normalized Probe Intensity
Probe Number
Meishan−A
Meishan−B
Meishan−C
European−A
European−B
European−C
0.5
0.0
−0.5
−1.0
−1.5
−2.0
−2.5
1
2
3
4
5
6
7
8
9
10
11

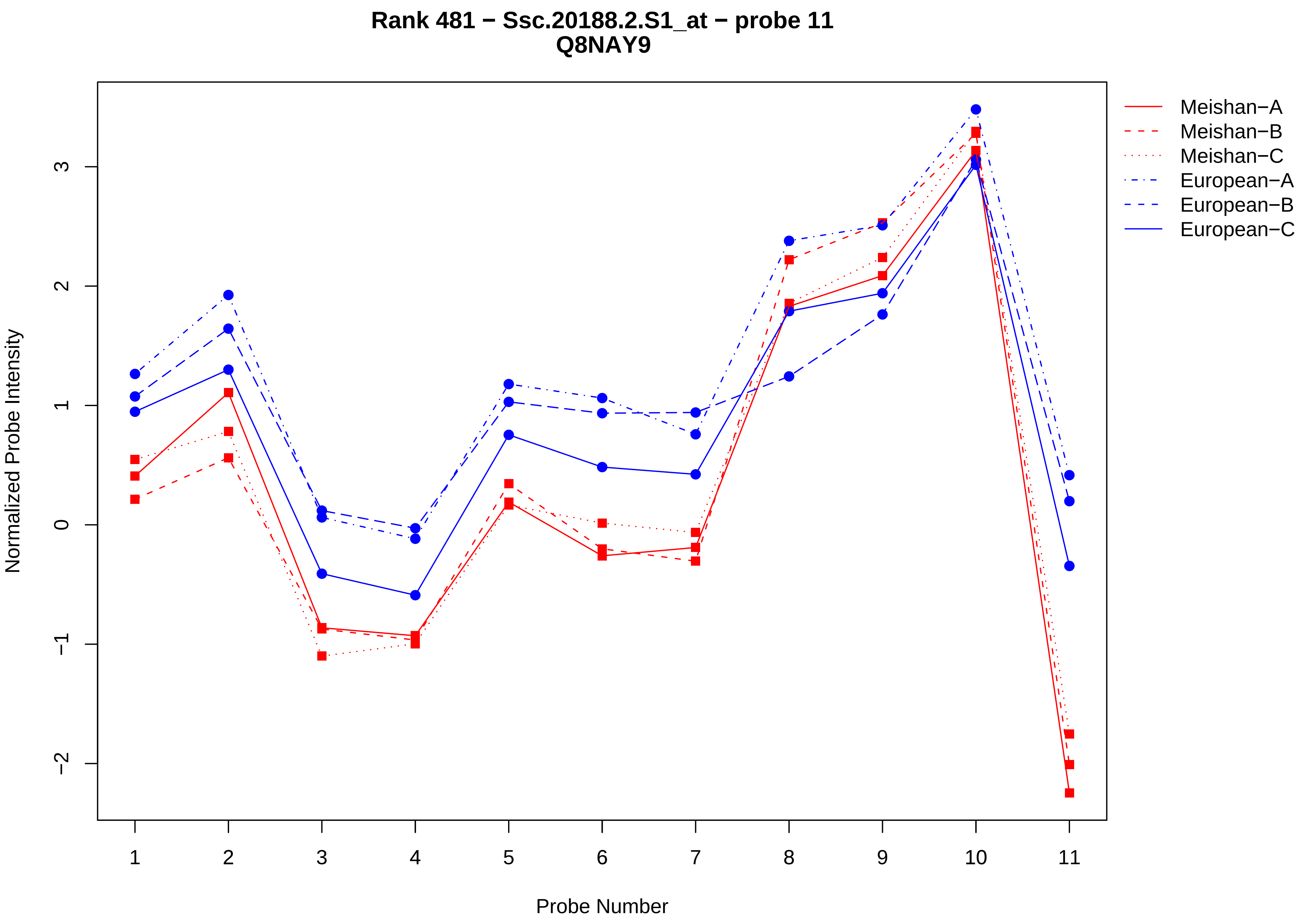

Rank 481 − Ssc.20188.2.S1_at − probe 11
Q8NAY9
Normalized Probe Intensity
Probe Number
−2
−1
0
1
2
3
1
2
3
4
5
6
7
8
9
10
11
Meishan−A
Meishan−B
Meishan−C
European−A
European−B
European−C

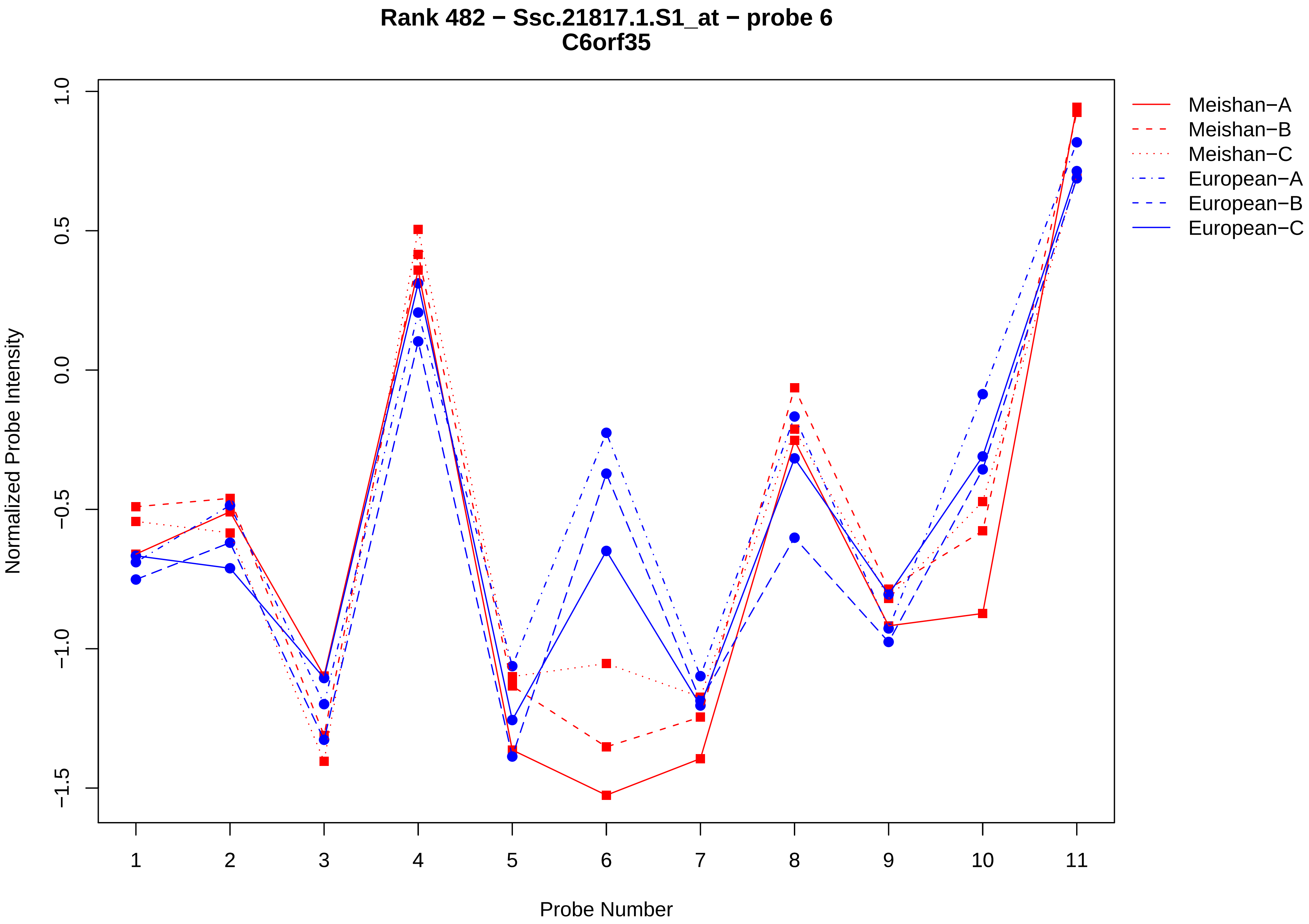

Rank 482 − Ssc.21817.1.S1_at − probe 6
C6orf35
Normalized Probe Intensity
Probe Number
1.0
0.5
0.0
−0.5
−1.0
−1.5
1
2
3
4
5
6
7
8
9
10
11
Meishan−A
Meishan−B
Meishan−C
European−A
European−B
European−C

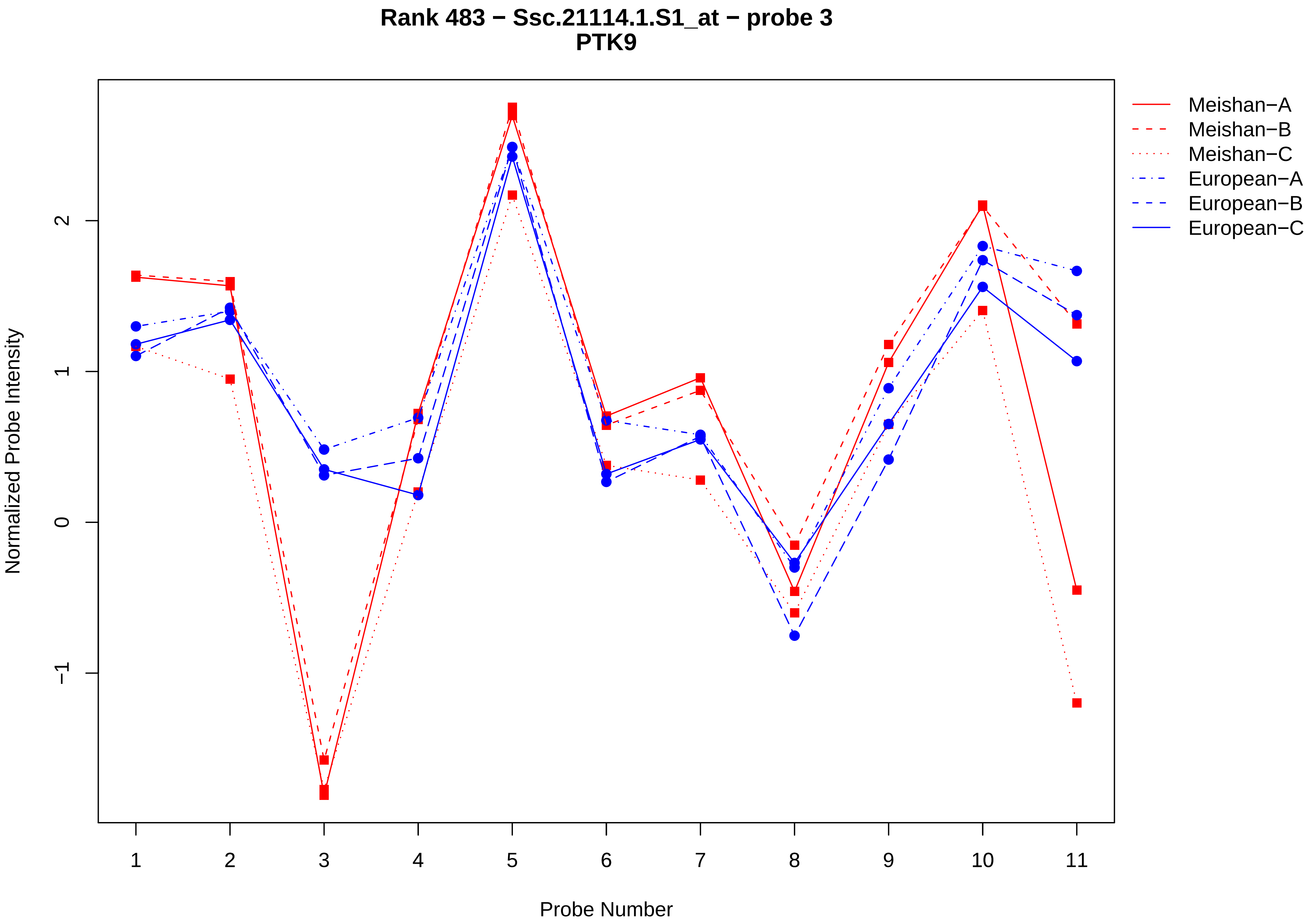
Rank 483 − Ssc.21114.1.S1_at − probe 3
PTK9
Normalized Probe Intensity
Probe Number
−1
0
1
2
1
2
3
4
5
6
7
8
9
10
11
Meishan−A
Meishan−B
Meishan−C
European−A
European−B
European−C

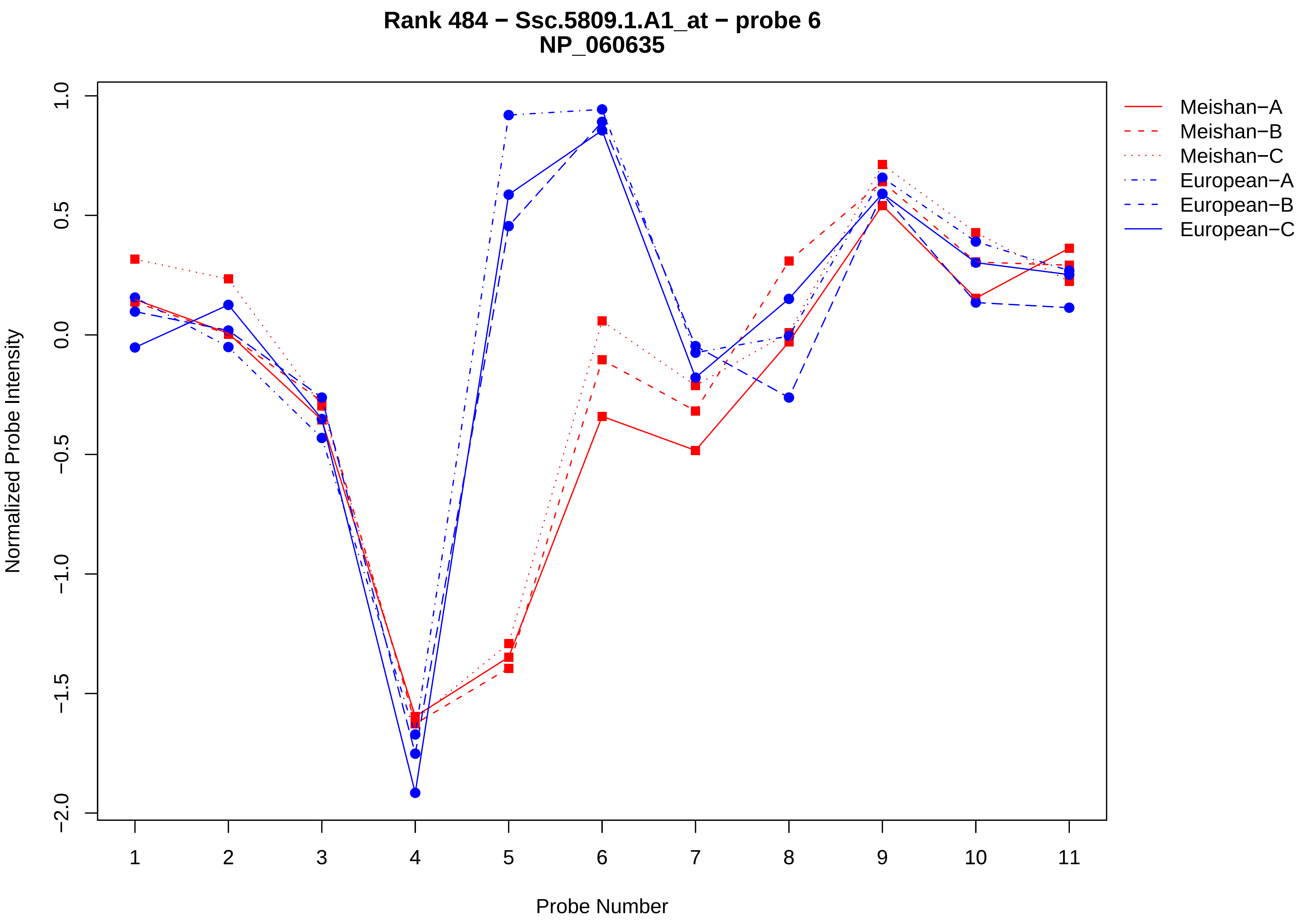
Rank 484 − Ssc.5809.1.A1_at − probe 6
NP_060635
Normalized Probe Intensity
1.0
0.5
0.0
−0.5
−1.0
−1.5
−2.0
1
2
3
4
5
6
7
8
9
10
11
Probe Number
Meishan−A
Meishan−B
Meishan−C
European−A
European−B
European−C

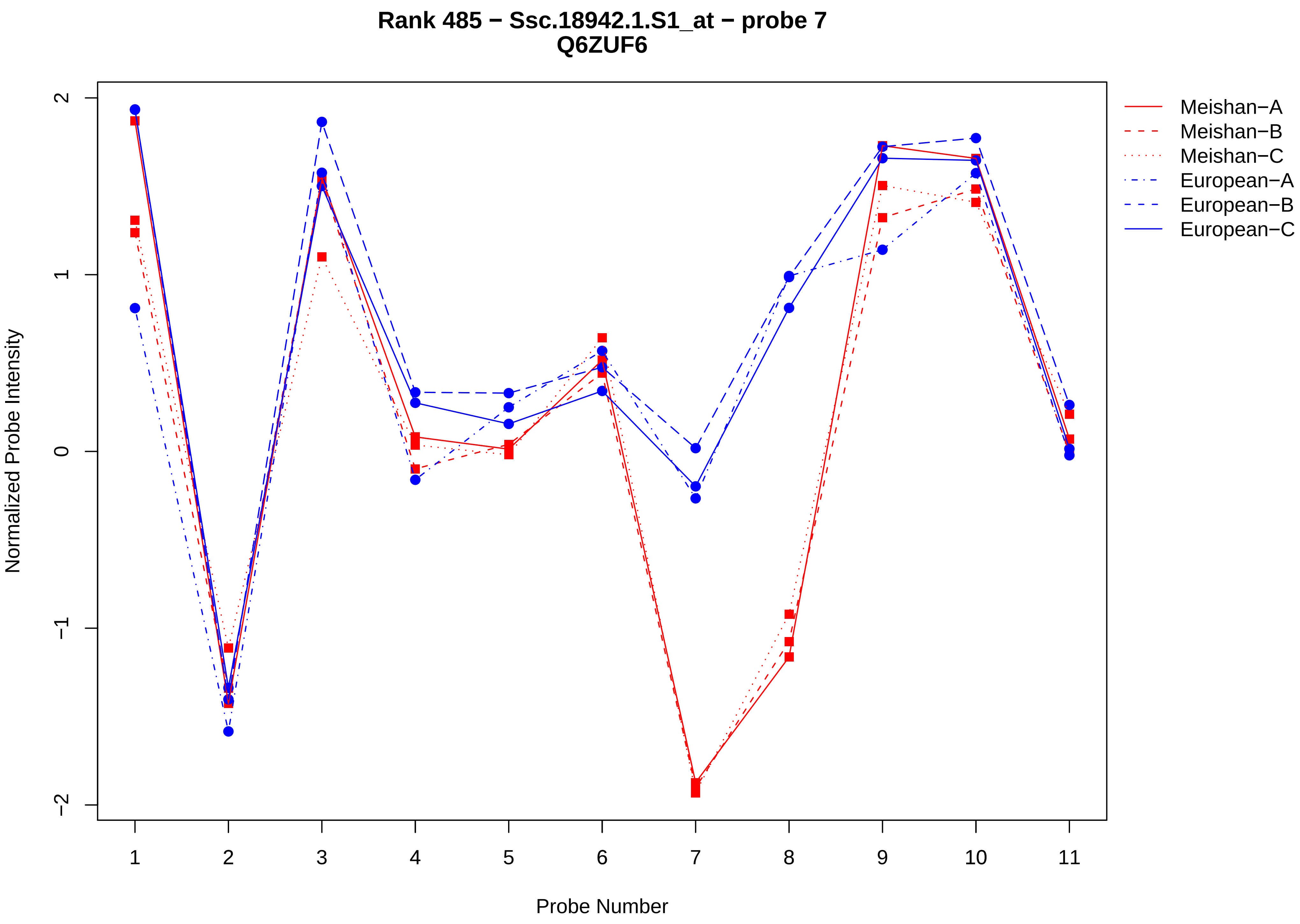
Rank 485 − Ssc.18942.1.S1_at − probe 7
Q6ZUF6
Normalized Probe Intensity
Probe Number
2
1
0
−1
−2
1
2
3
4
5
6
7
8
9
10
11
Meishan−A
Meishan−B
Meishan−C
European−A
European−B
European−C

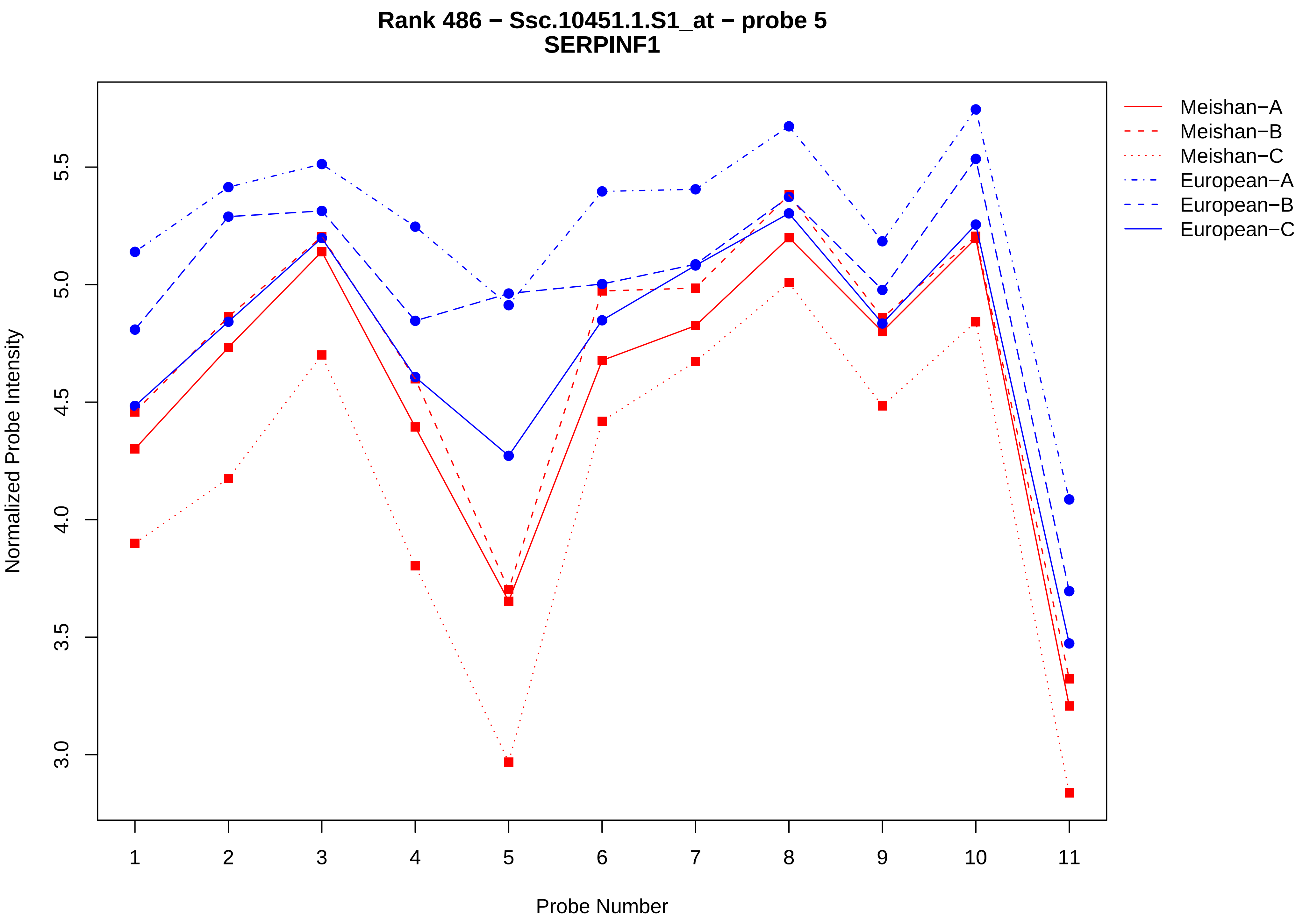
Rank 486 − Ssc.10451.1.S1_at − probe 5
SERPINF1
Normalized Probe Intensity
Probe Number
3.0
3.5
4.0
4.5
5.0
5.5
1
2
3
4
5
6
7
8
9
10
11
Meishan−A
Meishan−B
Meishan−C
European−A
European−B
European−C

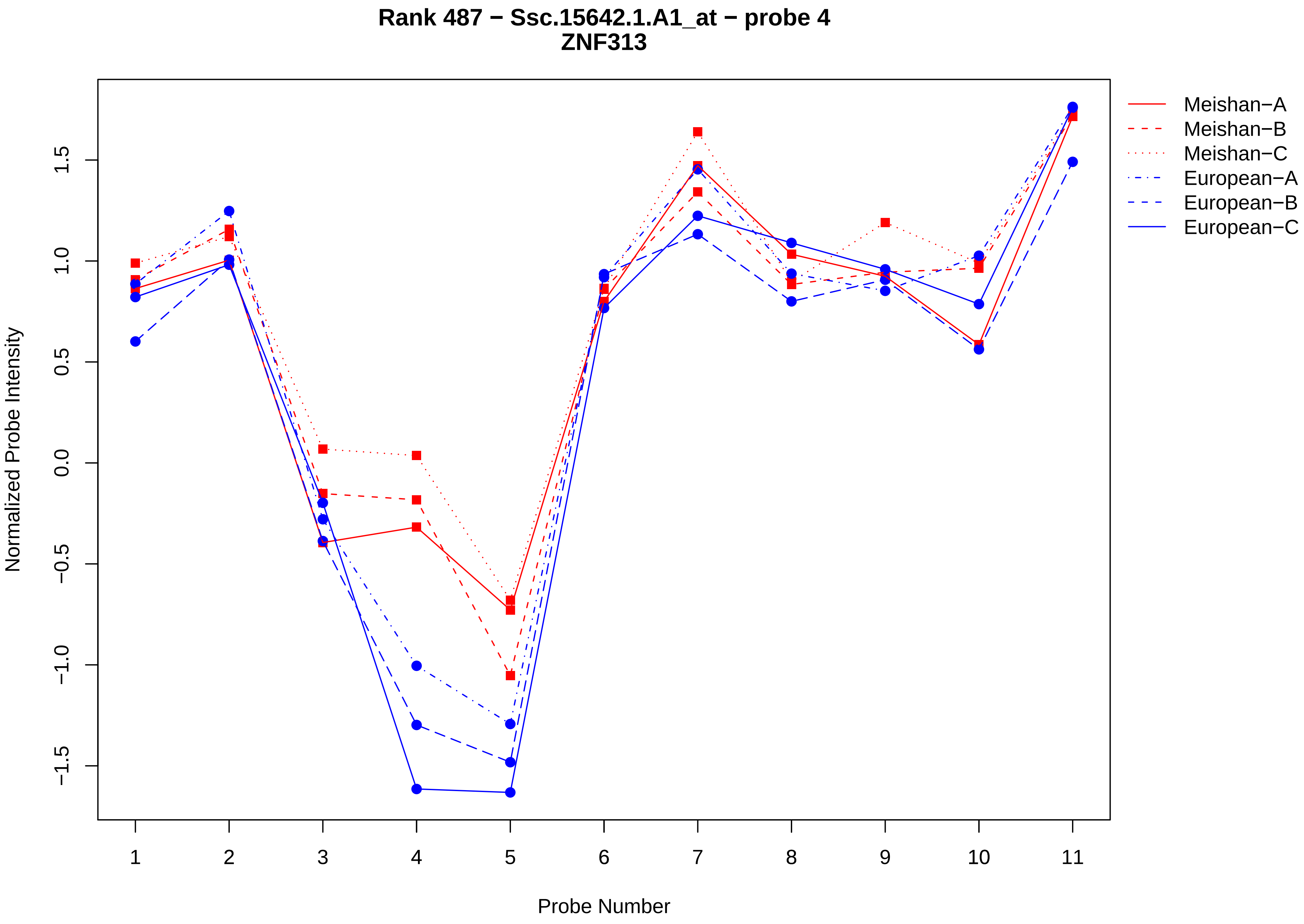

Rank 487 − Ssc.15642.1.A1_at − probe 4
ZNF313
Normalized Probe Intensity
Probe Number
−1.5
−1.0
−0.5
0.0
0.5
1.0
1.5
1
2
3
4
5
6
7
8
9
10
11
Meishan−A
Meishan−B
Meishan−C
European−A
European−B
European−C

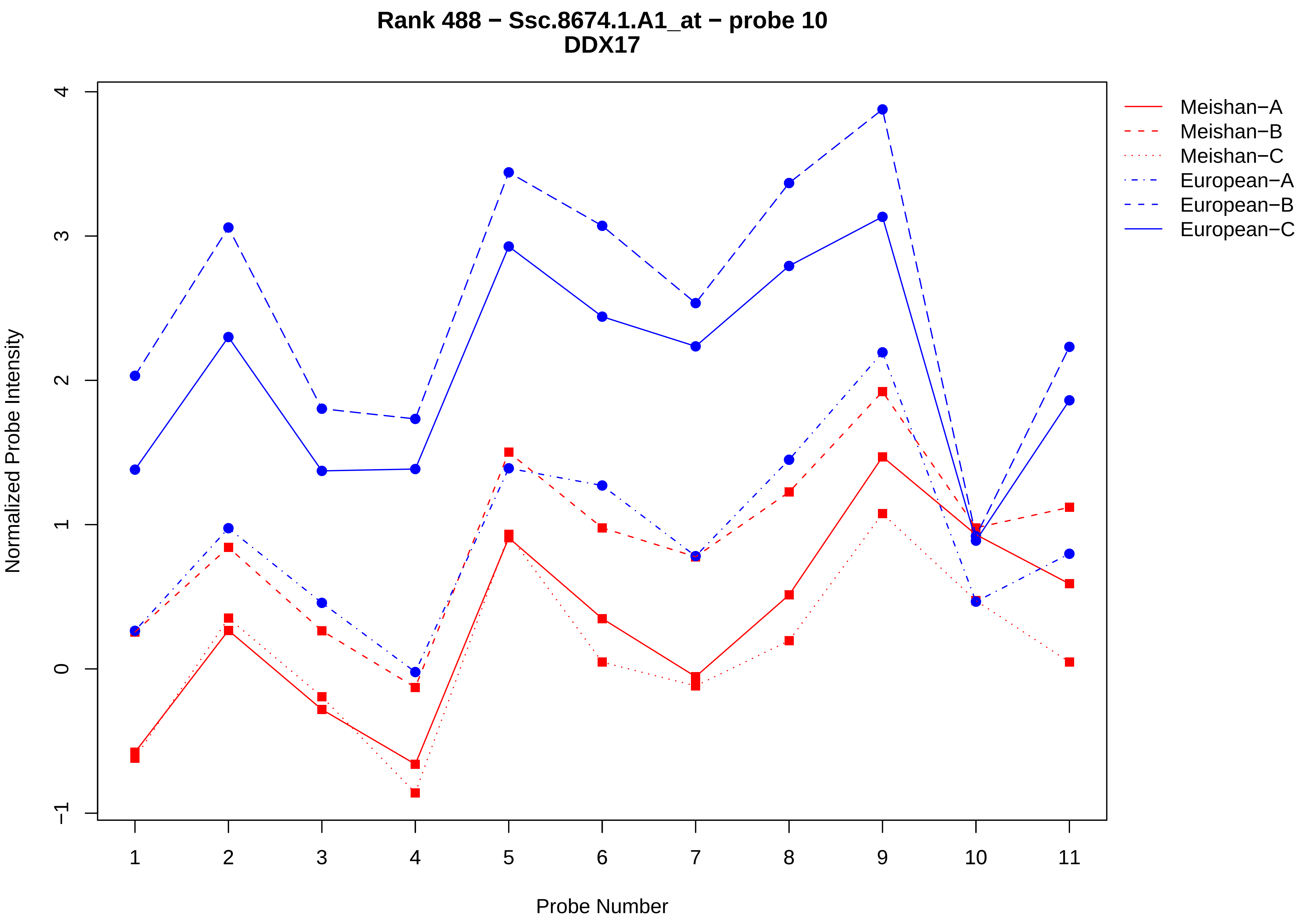
Rank 488 − Ssc.8674.1.A1_at − probe 10
DDX17
Normalized Probe Intensity
Probe Number
Meishan−A
Meishan−B
Meishan−C
European−A
European−B
European−C
−1
0
1
2
3
4
1
2
3
4
5
6
7
8
9
10
11

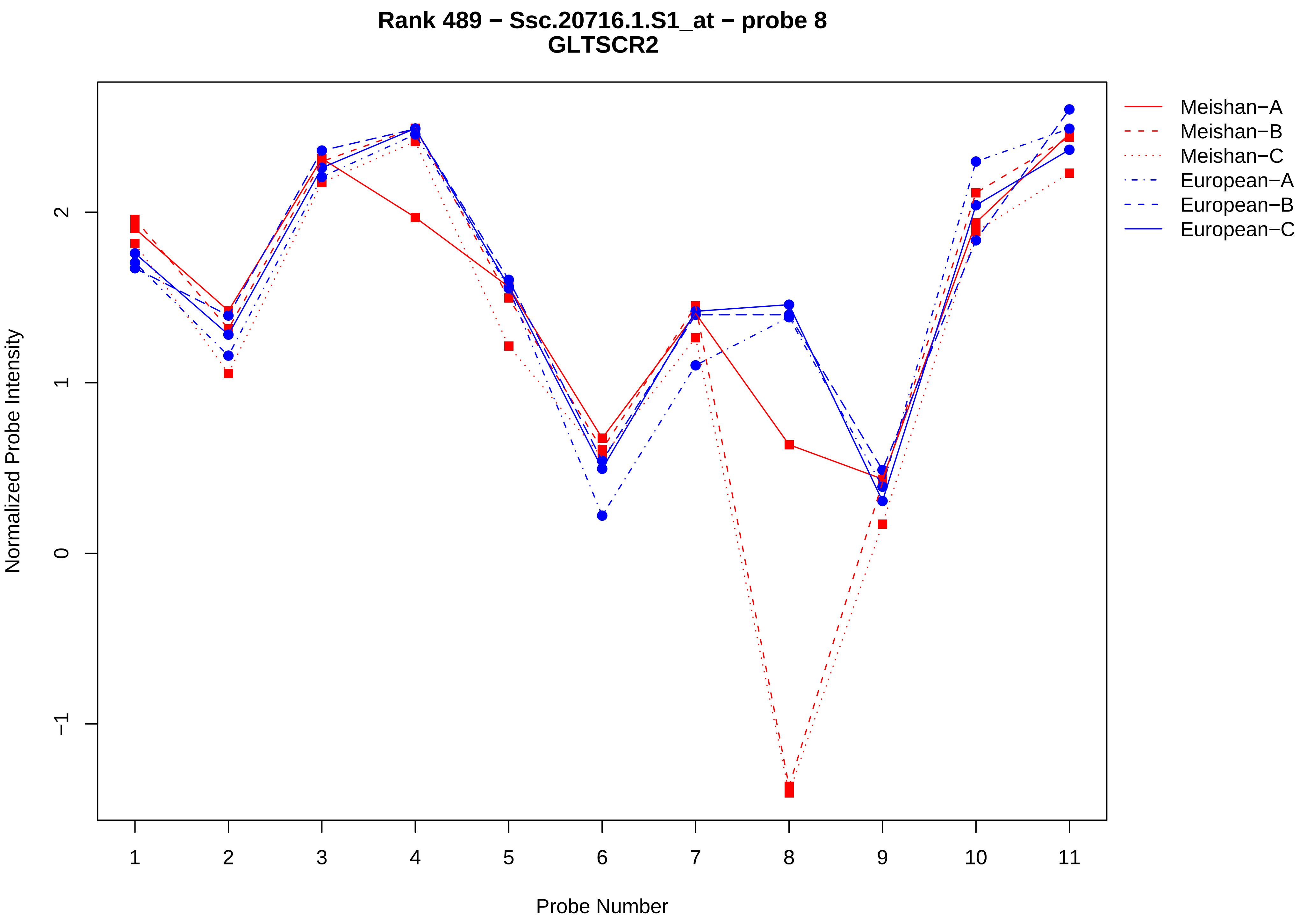

Rank 489 − Ssc.20716.1.S1_at − probe 8
GLTSCR2
Normalized Probe Intensity
Probe Number
−1
0
1
2
1
2
3
4
5
6
7
8
9
10
11
Meishan−A
Meishan−B
Meishan−C
European−A
European−B
European−C

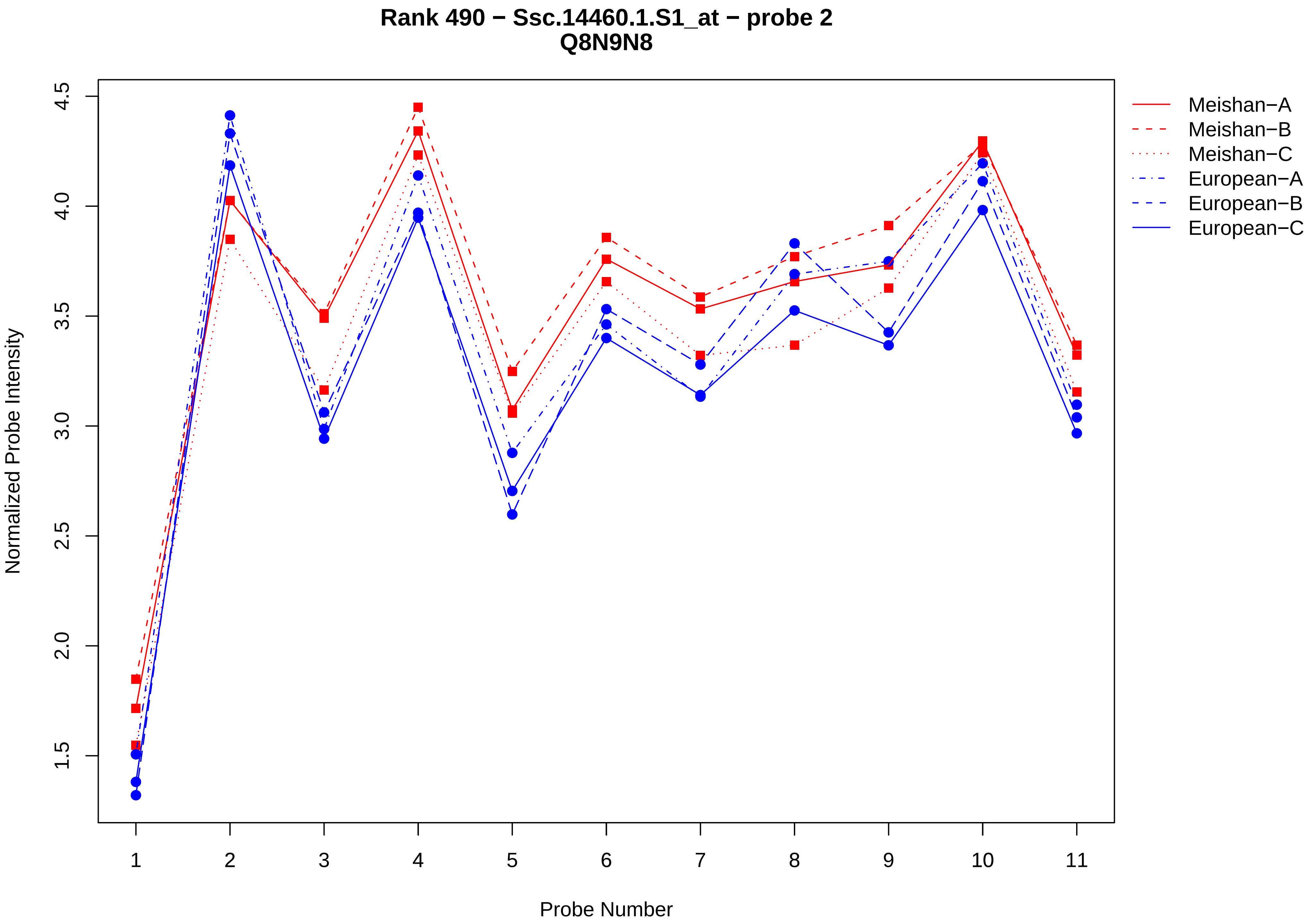
Rank 490 − Ssc.14460.1.S1_at − probe 2
Q8N9N8
Normalized Probe Intensity
Probe Number
1.5
2.0
2.5
3.0
3.5
4.0
4.5
1
2
3
4
5
6
7
8
9
10
11
Meishan−A
Meishan−B
Meishan−C
European−A
European−B
European−C

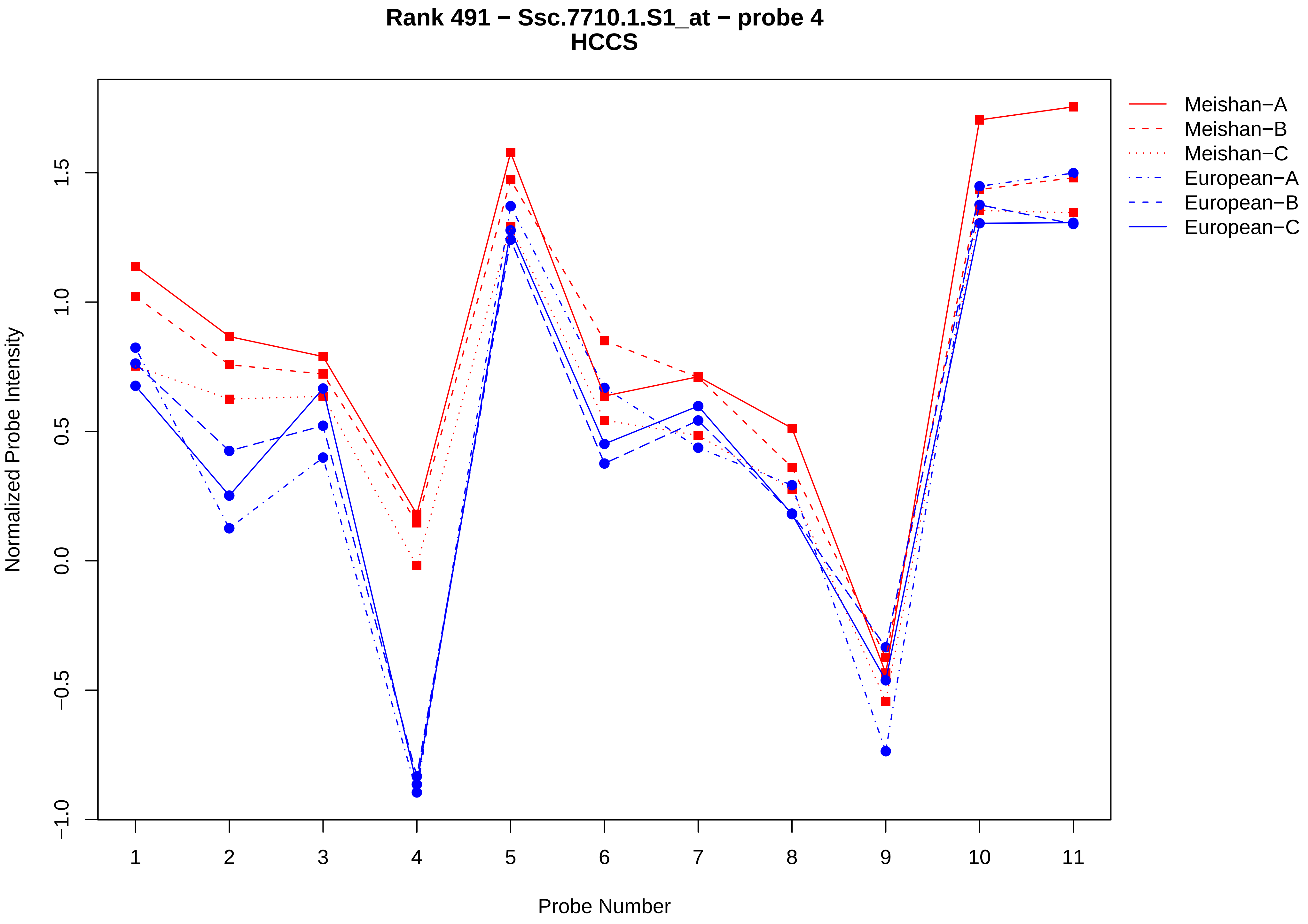
Rank 491 − Ssc.7710.1.S1_at − probe 4
HCCS
Normalized Probe Intensity
Probe Number
−1.0
−0.5
0.0
0.5
1.0
1.5
1
2
3
4
5
6
7
8
9
10
11
Meishan−A
Meishan−B
Meishan−C
European−A
European−B
European−C

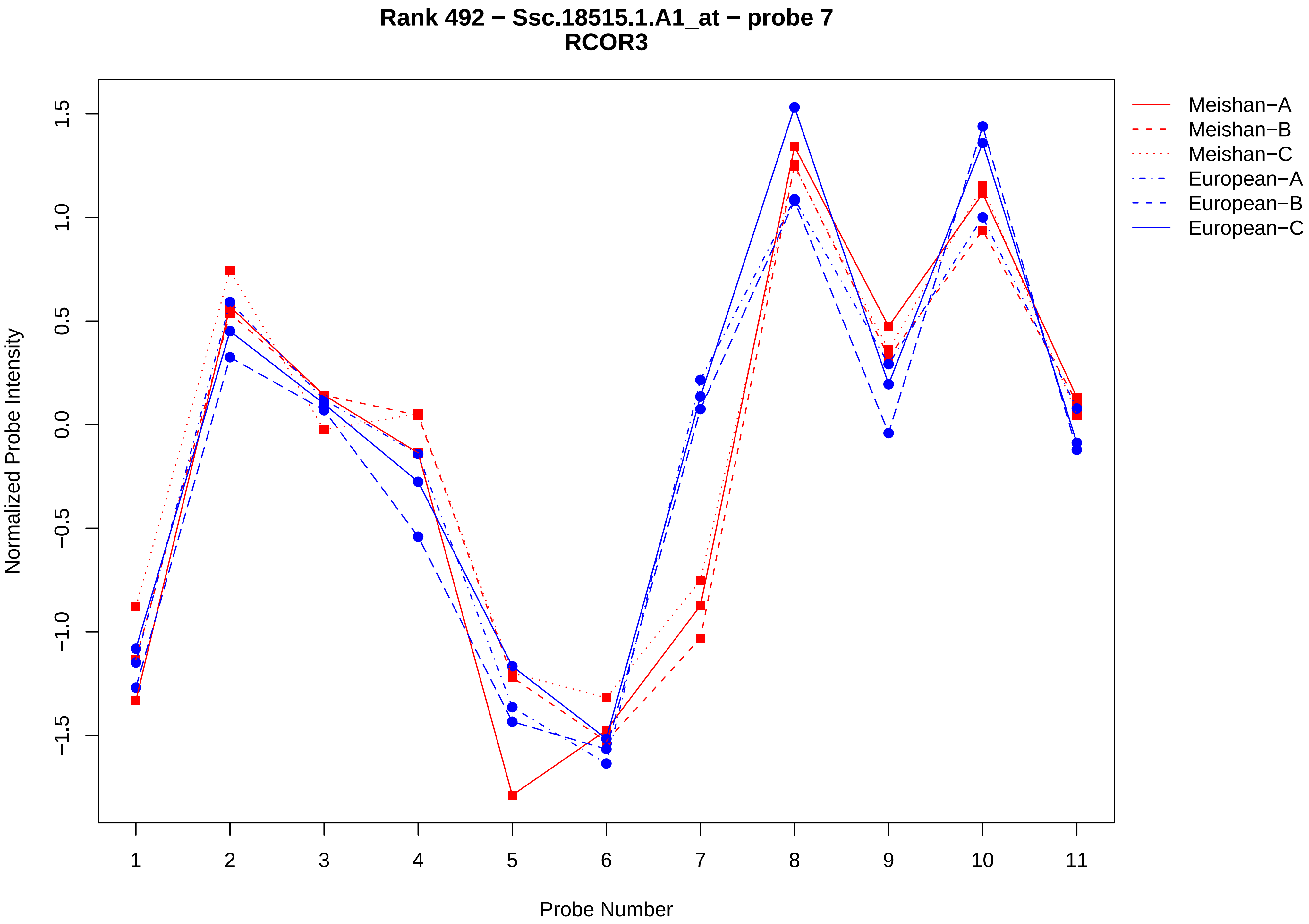
Rank 492 − Ssc.18515.1.A1_at − probe 7
RCOR3
Normalized Probe Intensity
Probe Number
1.5
1.0
0.5
0.0
−0.5
−1.0
−1.5
1
2
3
4
5
6
7
8
9
10
11
Meishan−A
Meishan−B
Meishan−C
European−A
European−B
European−C

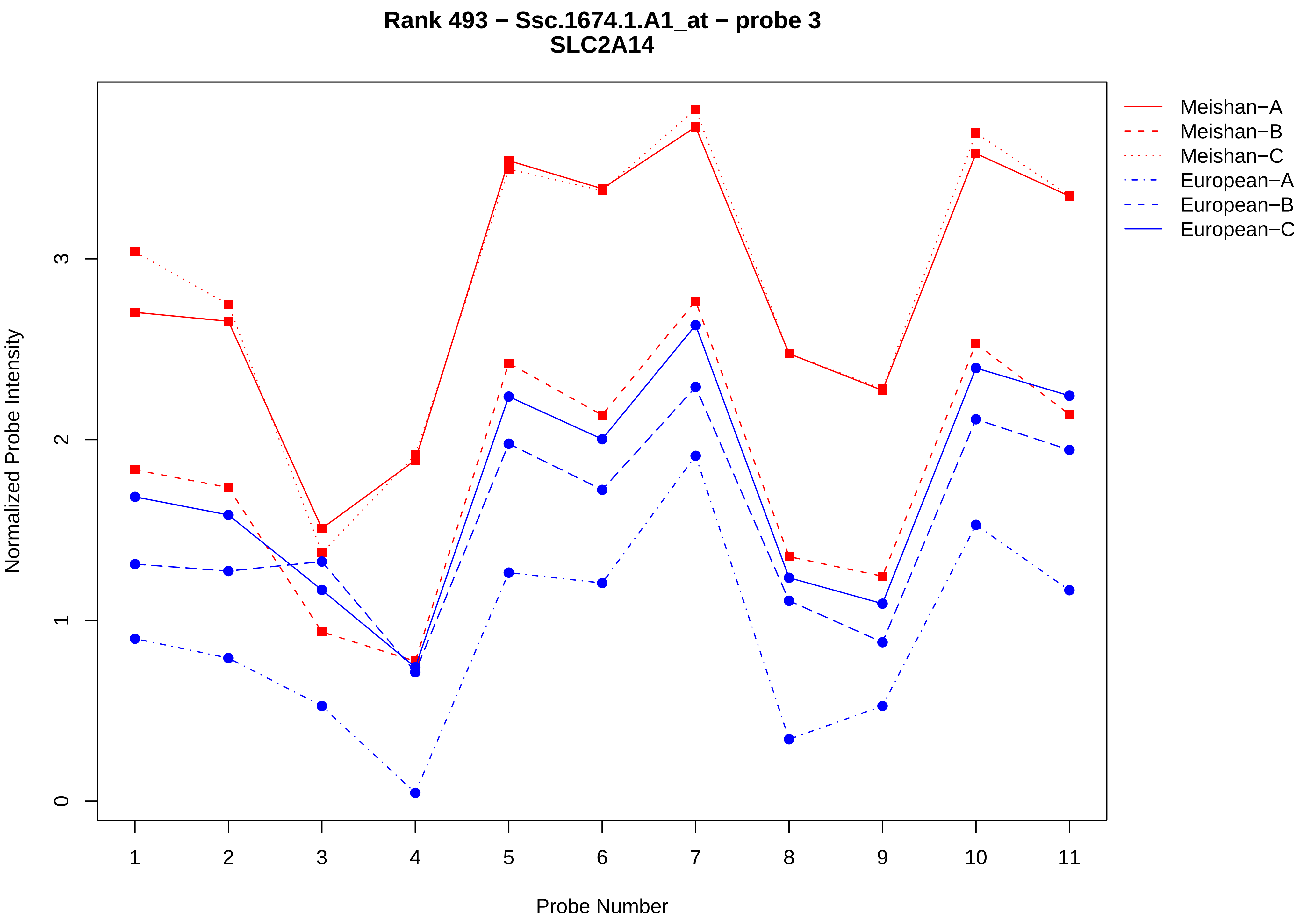

Rank 493 − Ssc.1674.1.A1_at − probe 3
SLC2A14
Normalized Probe Intensity
Probe Number
0
1
2
3
1
2
3
4
5
6
7
8
9
10
11
Meishan−A
Meishan−B
Meishan−C
European−A
European−B
European−C

Rank 494 − Ssc.21290.2.A1_at − probe 11
Q6PI50

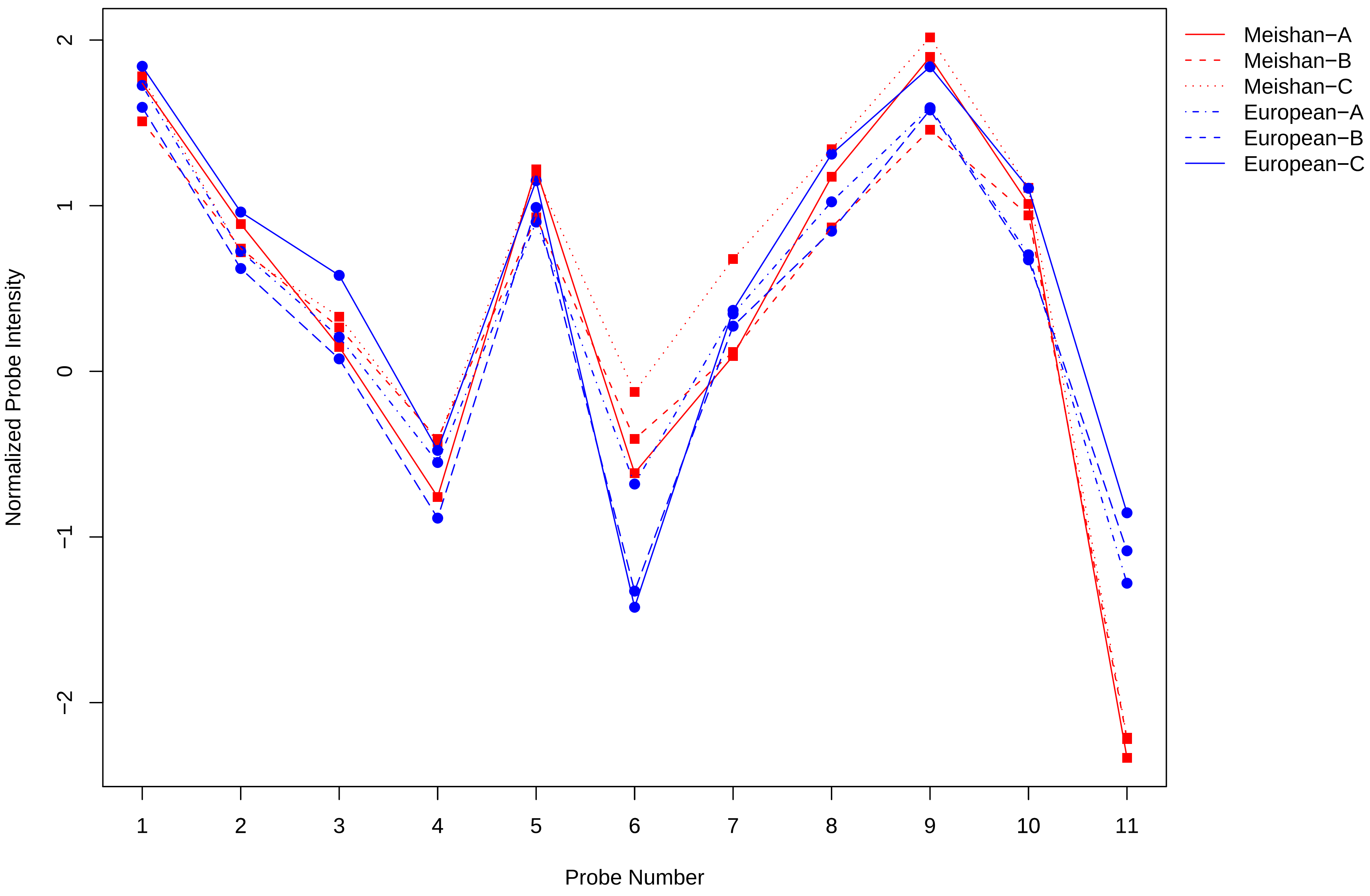

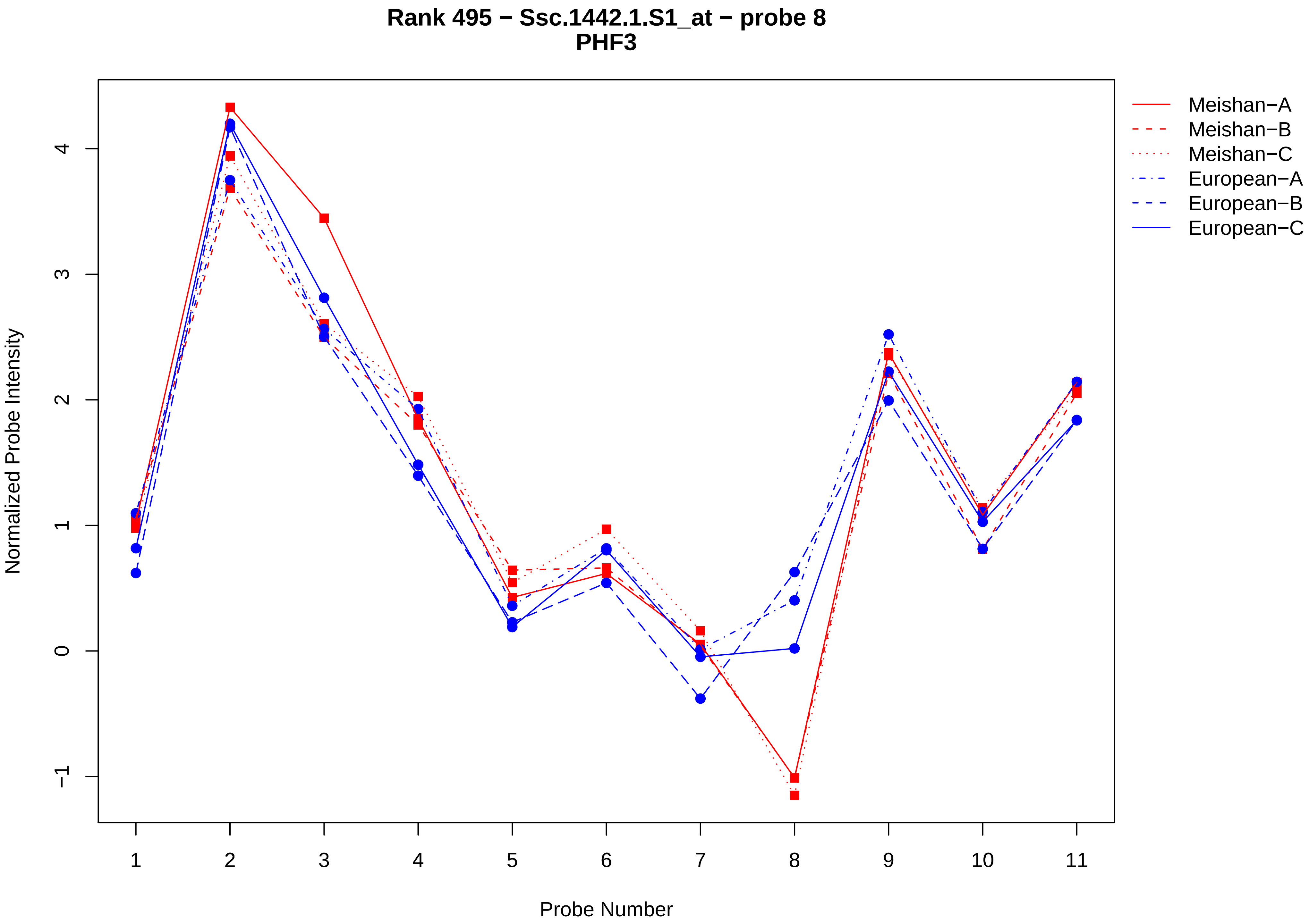

Rank 495 − Ssc.1442.1.S1_at − probe 8
PHF3
Normalized Probe Intensity
Probe Number
Meishan−A
Meishan−B
Meishan−C
European−A
European−B
European−C
−1
0
1
2
3
4
1
2
3
4
5
6
7
8
9
10
11

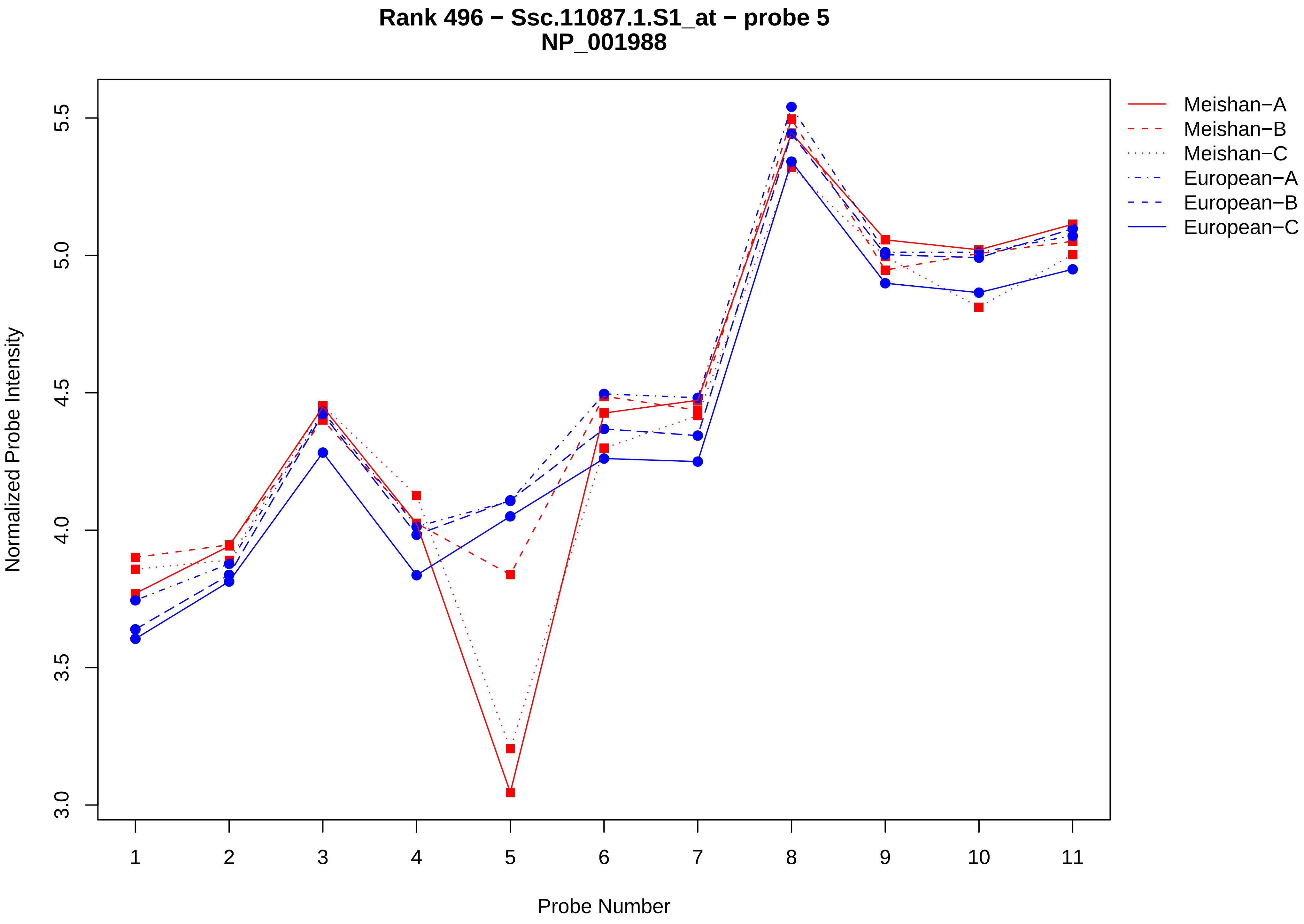

Rank 496 − Ssc.11087.1.S1_at − probe 5
NP_001988
Normalized Probe Intensity
Probe Number
3.0
3.5
4.0
4.5
5.0
5.5
1
2
3
4
5
6
7
8
9
10
11
Meishan−A
Meishan−B
Meishan−C
European−A
European−B
European−C

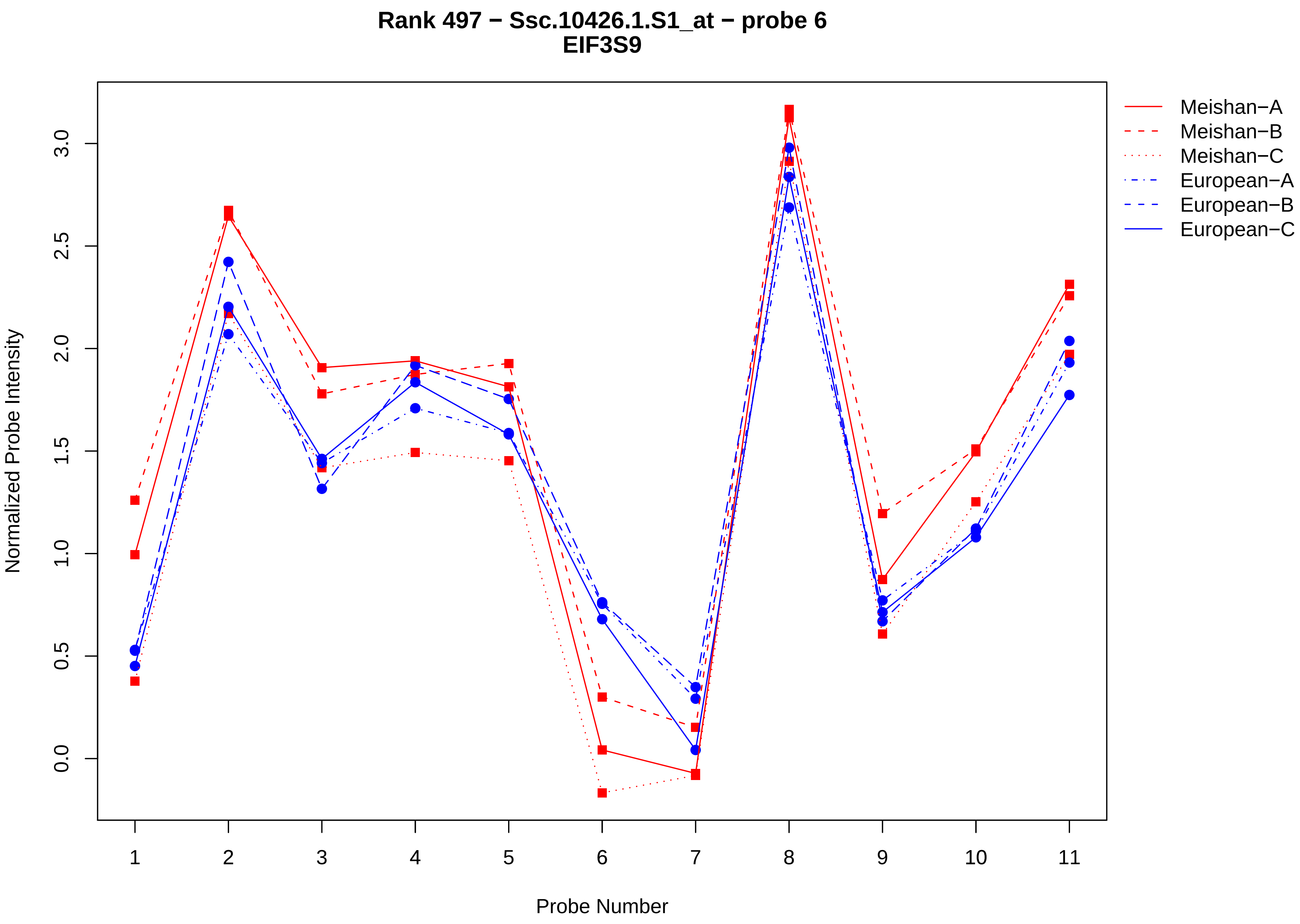
Rank 497 − Ssc.10426.1.S1_at − probe 6
EIF3S9
Normalized Probe Intensity
Probe Number
0.0
0.5
1.0
1.5
2.0
2.5
3.0
1
2
3
4
5
6
7
8
9
10
11
Meishan−A
Meishan−B
Meishan−C
European−A
European−B
European−C

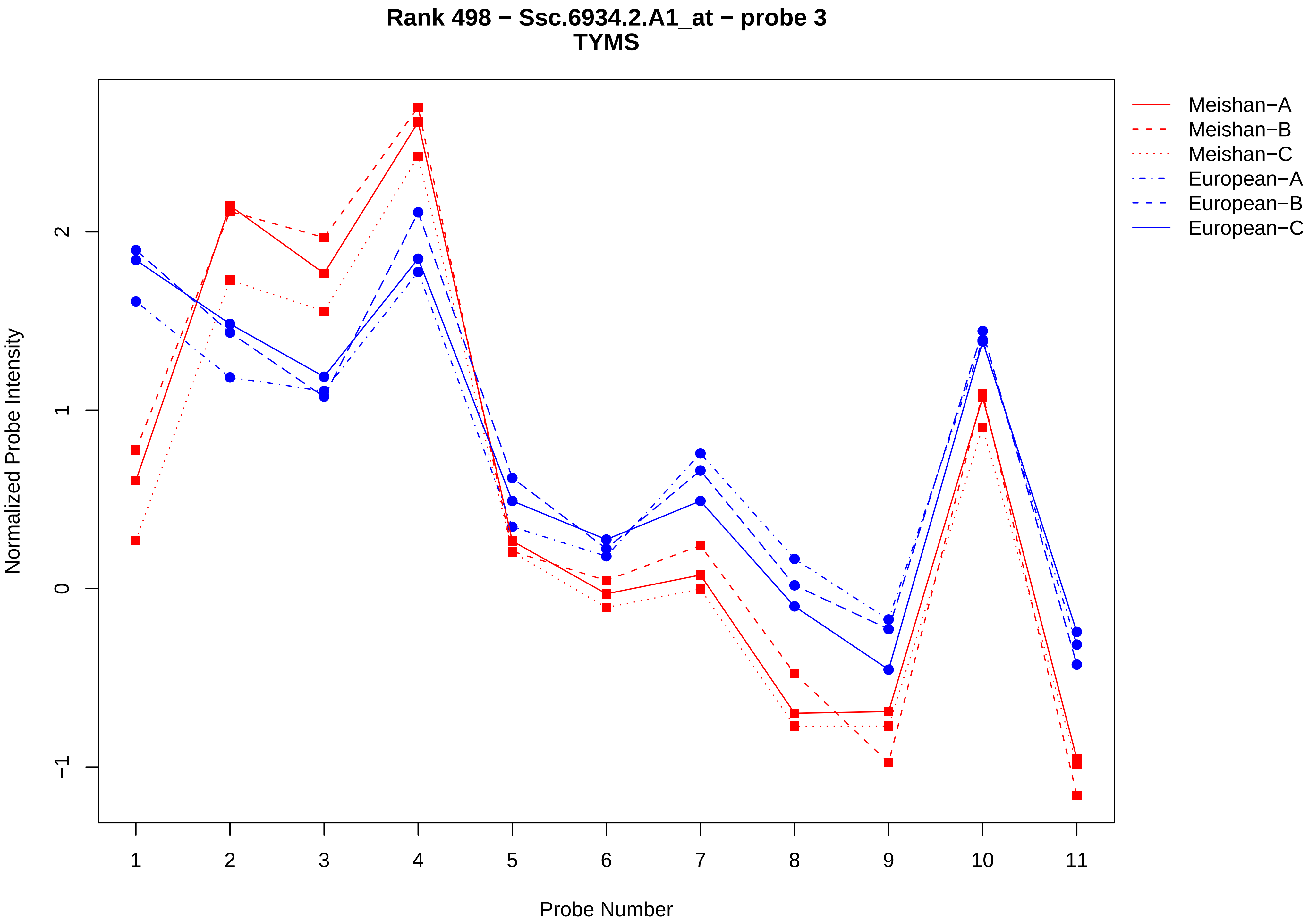

Rank 498 − Ssc.6934.2.A1_at − probe 3
TYMS
Normalized Probe Intensity
Probe Number
−1
0
1
2
1
2
3
4
5
6
7
8
9
10
11
Meishan−A
Meishan−B
Meishan−C
European−A
European−B
European−C

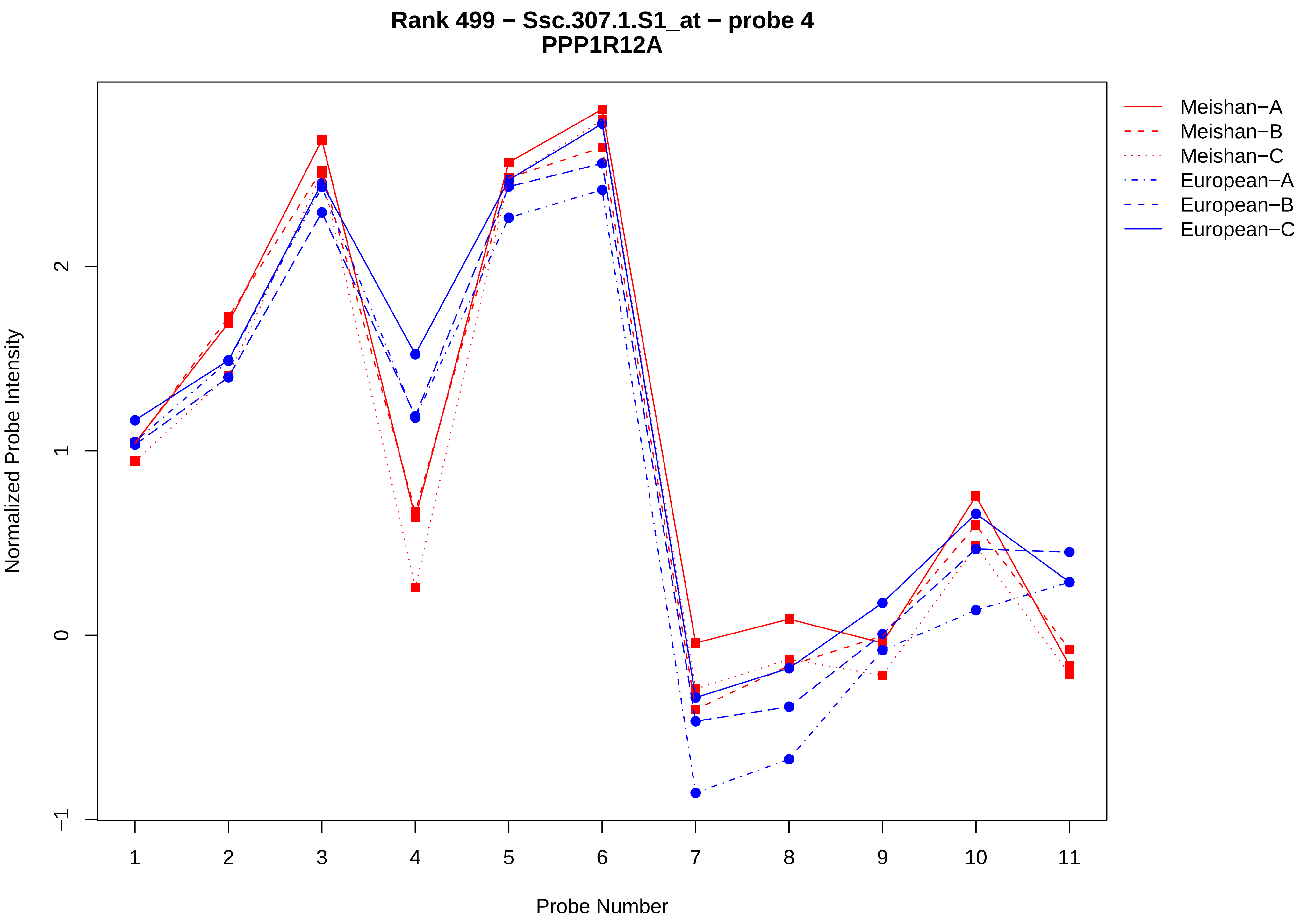

Rank 499 − Ssc.307.1.S1_at − probe 4
PPP1R12A
Normalized Probe Intensity
Probe Number
−1
0
1
2
1
2
3
4
5
6
7
8
9
10
11
Meishan−A
Meishan−B
Meishan−C
European−A
European−B
European−C

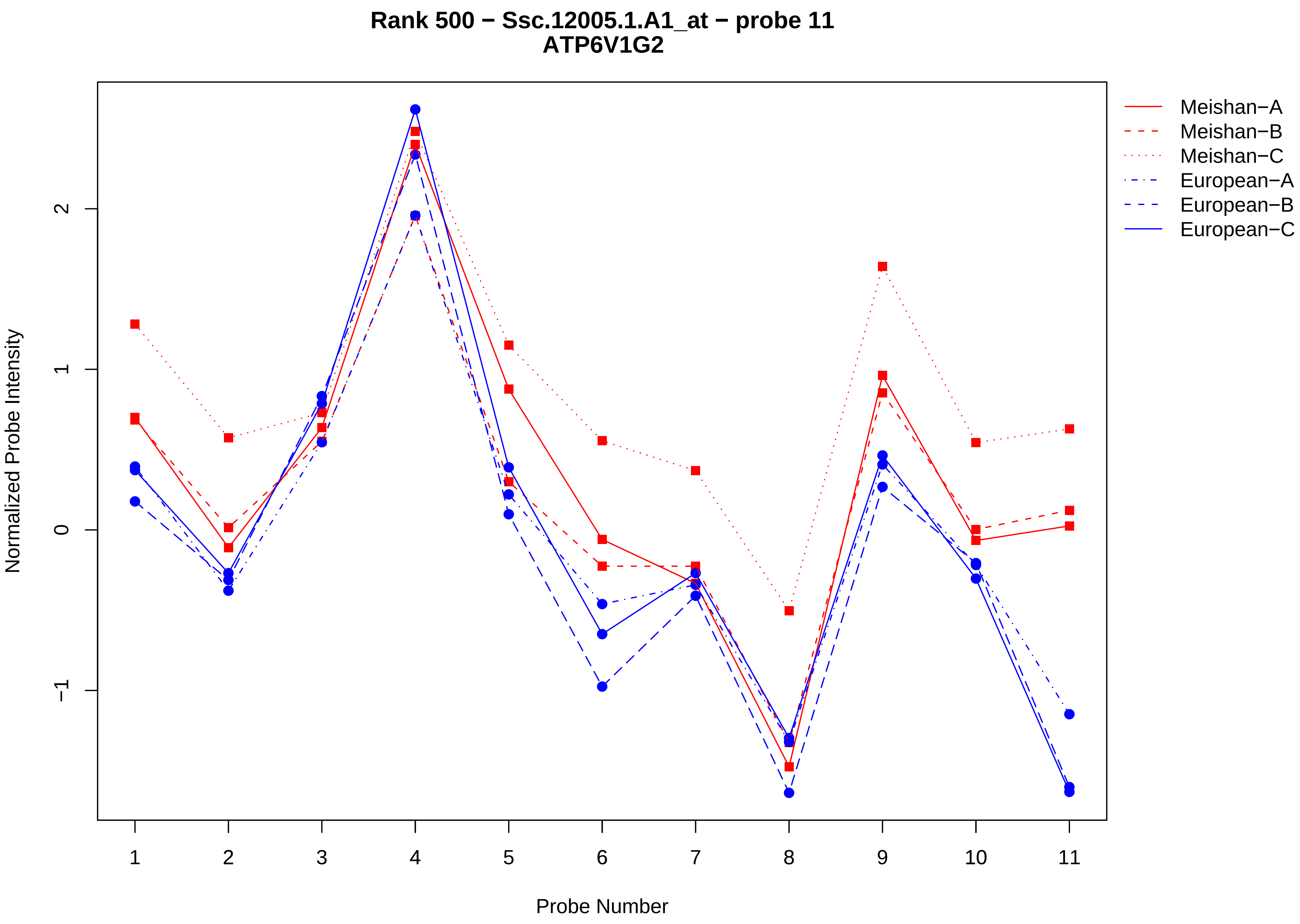

Rank 500 − Ssc.12005.1.A1_at − probe 11
ATP6V1G2
Normalized Probe Intensity
Probe Number
Meishan−A
Meishan−B
Meishan−C
European−A
European−B
European−C
−1
0
1
2
1
2
3
4
5
6
7
8
9
10
11

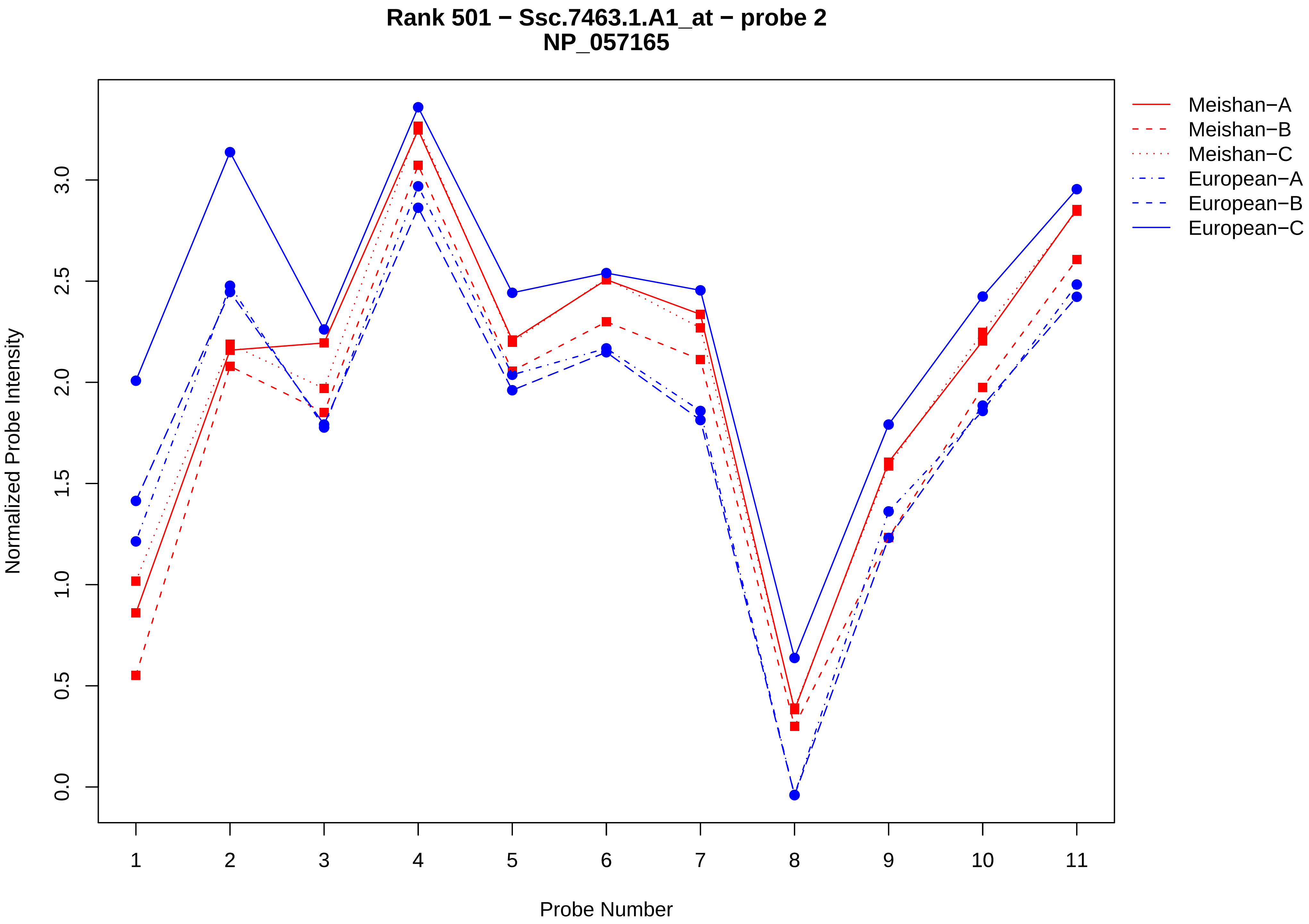

Rank 501 − Ssc.7463.1.A1_at − probe 2
NP_057165
Normalized Probe Intensity
Probe Number
0.0
0.5
1.0
1.5
2.0
2.5
3.0
1
2
3
4
5
6
7
8
9
10
11
Meishan−A
Meishan−B
Meishan−C
European−A
European−B
European−C

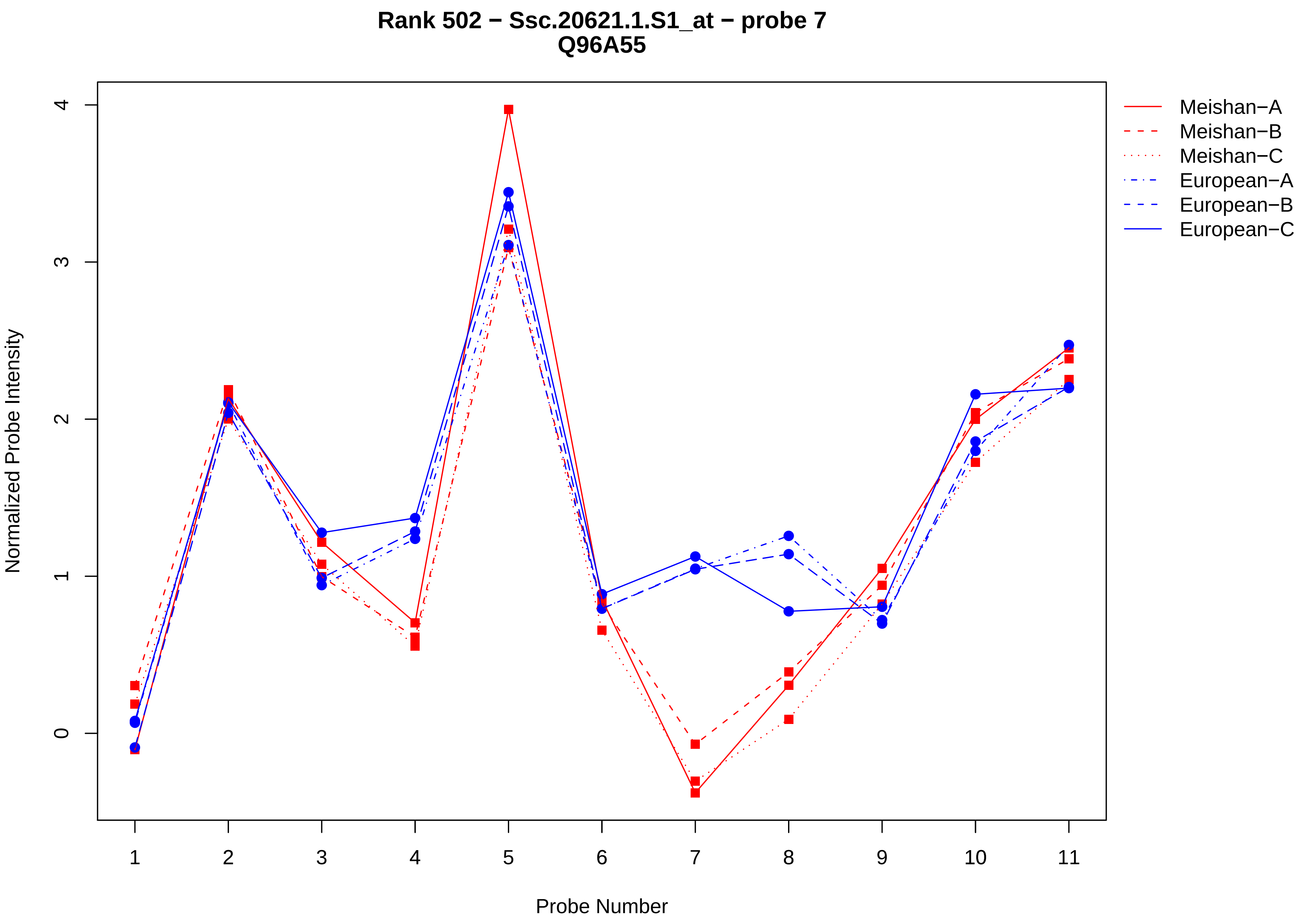

Rank 502 − Ssc.20621.1.S1_at − probe 7
Q96A55
Normalized Probe Intensity
0
1
2
3
4
Probe Number
1
2
3
4
5
6
7
8
9
10
11
Meishan−A
Meishan−B
Meishan−C
European−A
European−B
European−C

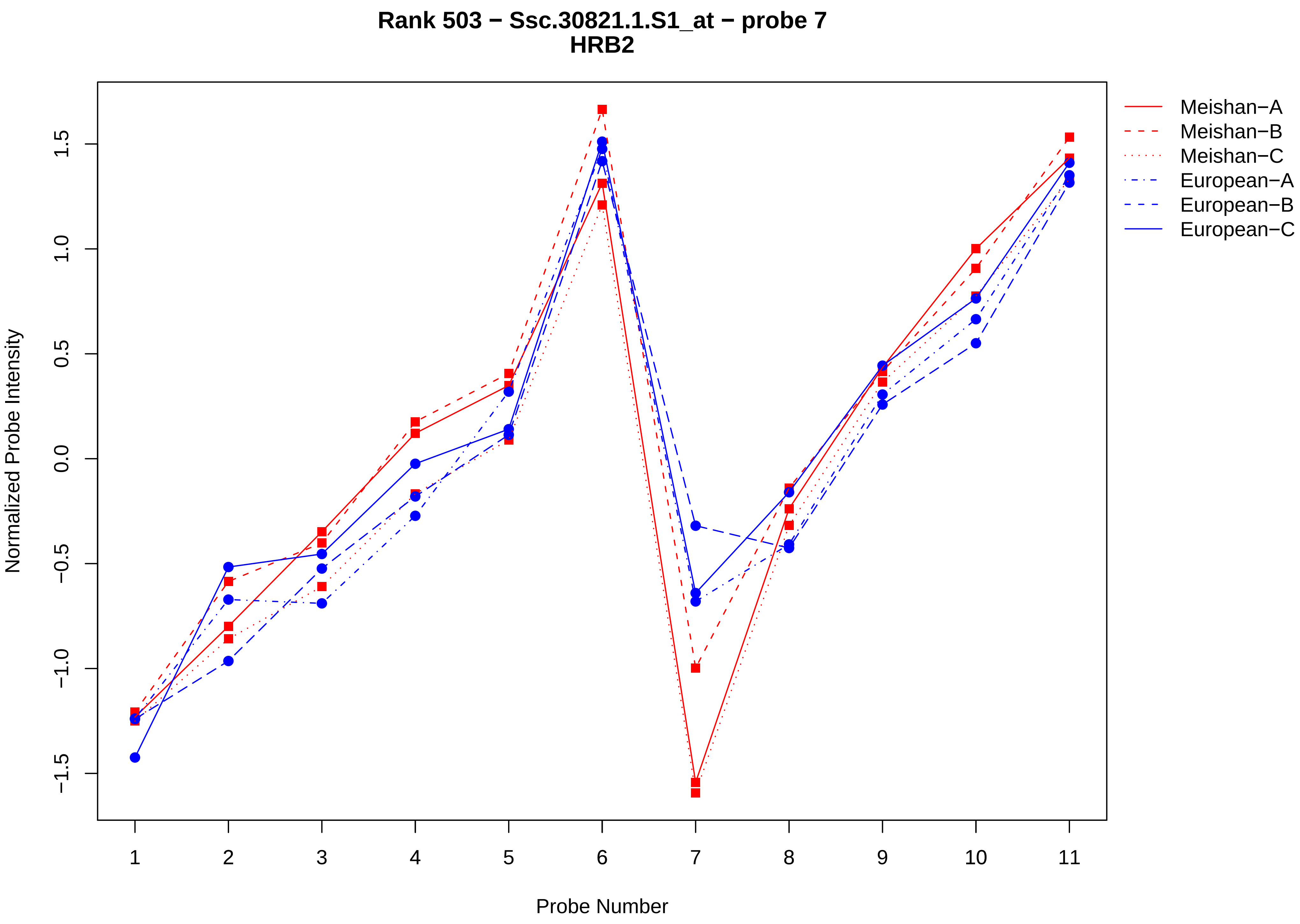

Rank 503 − Ssc.30821.1.S1_at − probe 7
HRB2
Normalized Probe Intensity
Probe Number
−1.5
−1.0
−0.5
0.0
0.5
1.0
1.5
1
2
3
4
5
6
7
8
9
10
11
Meishan−A
Meishan−B
Meishan−C
European−A
European−B
European−C

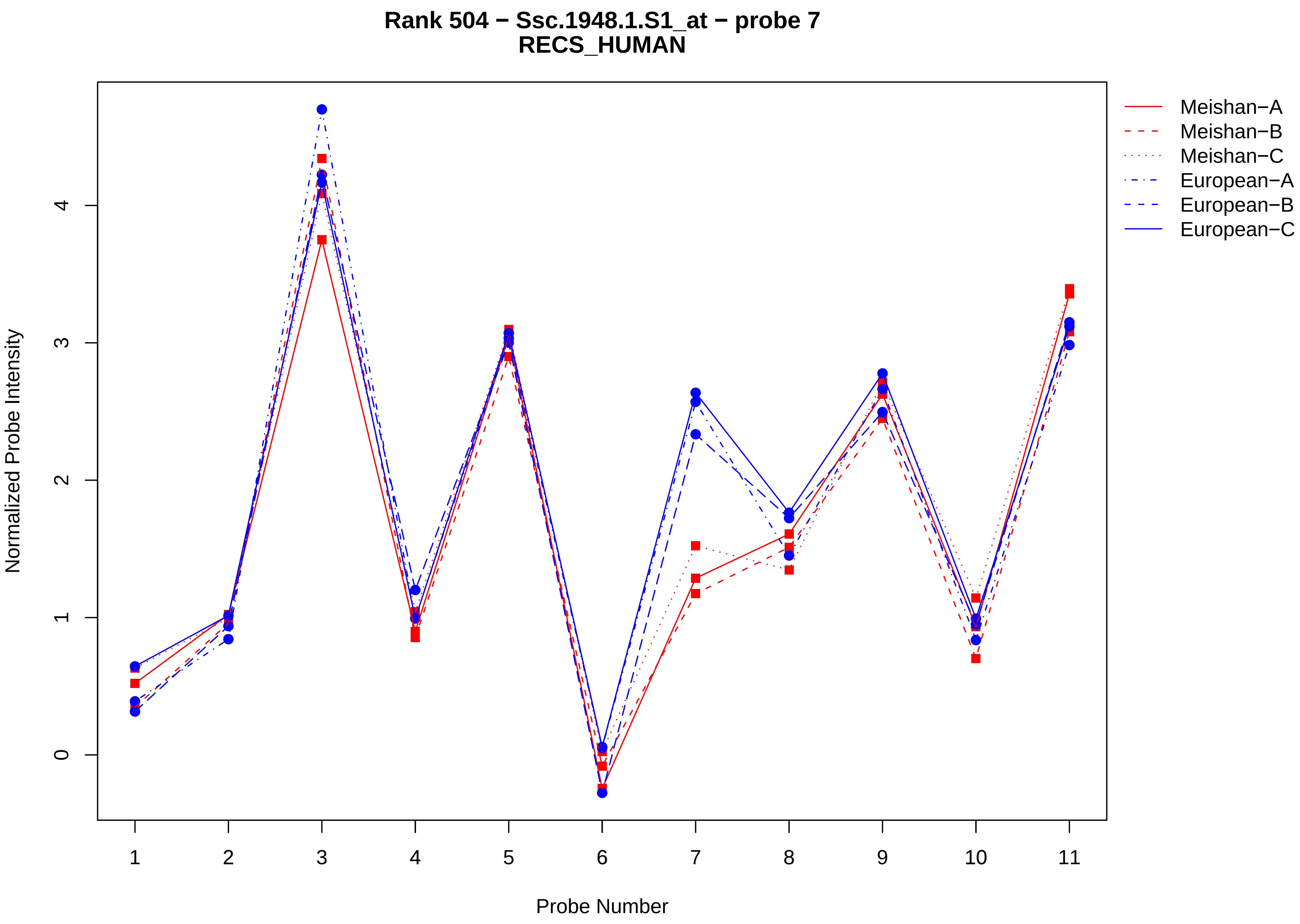

Rank 504 − Ssc.1948.1.S1_at − probe 7
RECS_HUMAN
Normalized Probe Intensity
Probe Number
0
1
2
3
4
1
2
3
4
5
6
7
8
9
10
11
Meishan−A
Meishan−B
Meishan−C
European−A
European−B
European−C

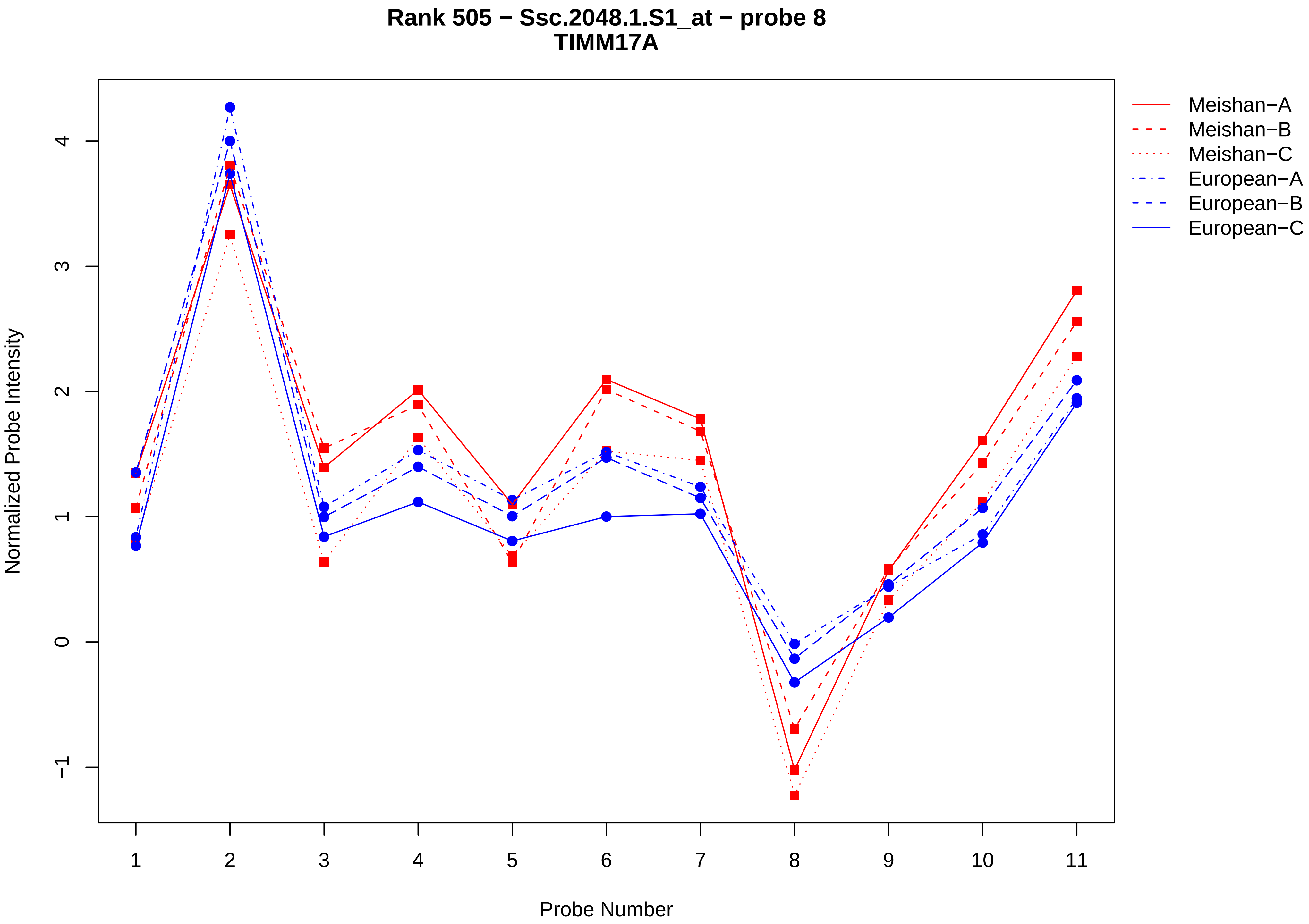

Rank 505 − Ssc.2048.1.S1_at − probe 8
TIMM17A
Normalized Probe Intensity
Probe Number
−1
0
1
2
3
4
1
2
3
4
5
6
7
8
9
10
11
Meishan−A
Meishan−B
Meishan−C
European−A
European−B
European−C

Rank 506 − Ssc.14003.2.S1_at − probe 4
RPS4X

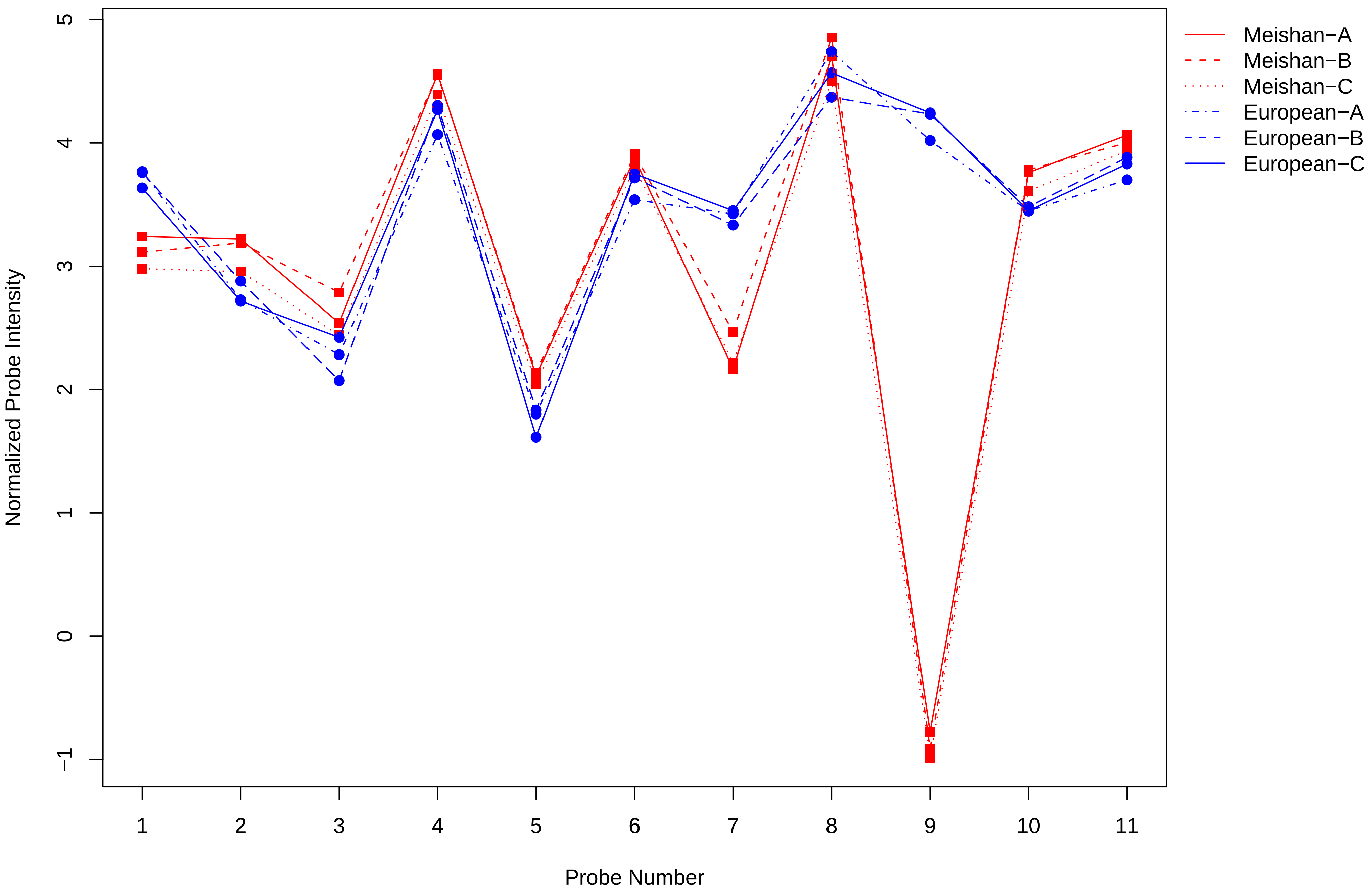

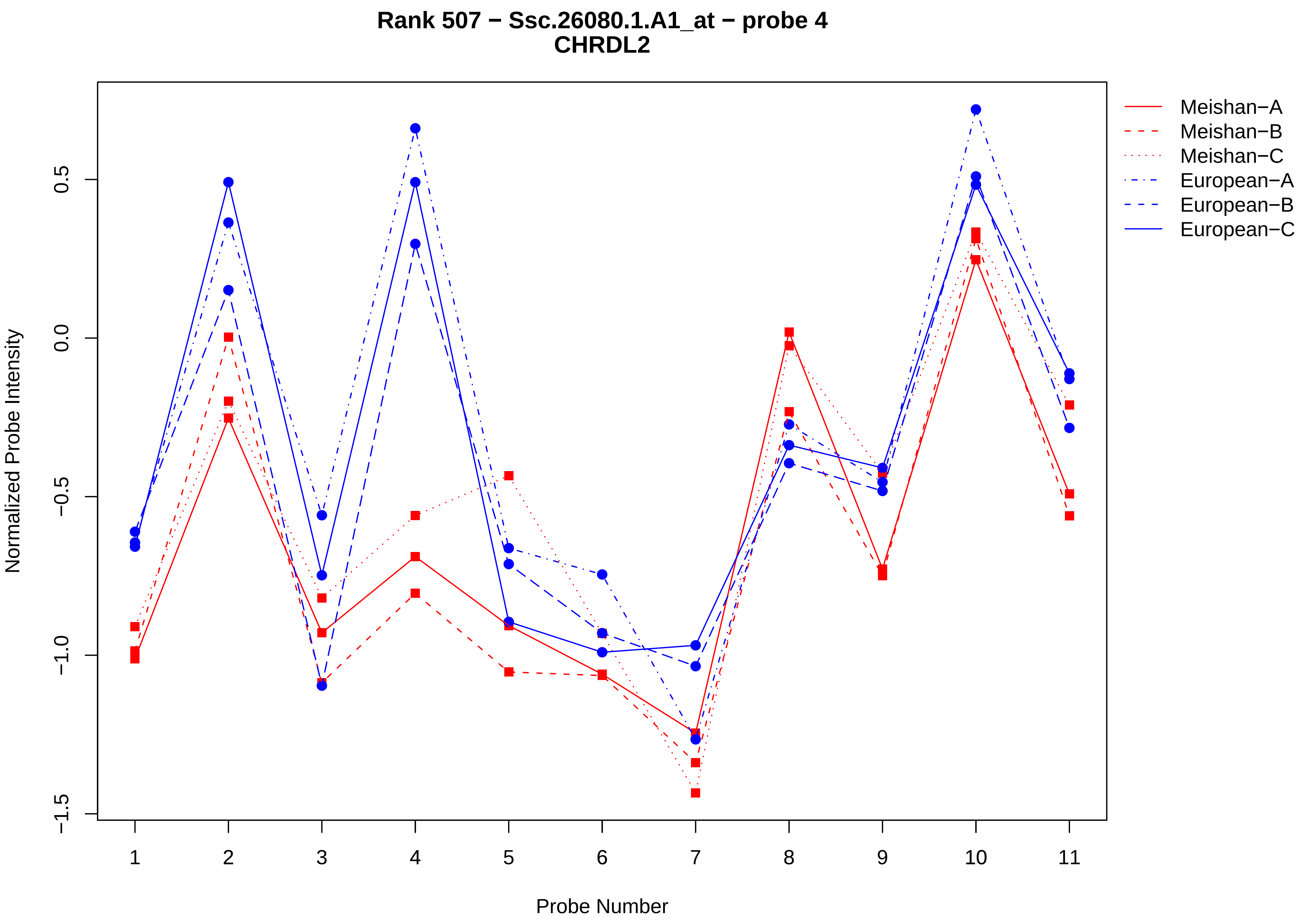
Rank 507 − Ssc.26080.1.A1_at − probe 4
CHRDL2
Normalized Probe Intensity
Probe Number
−1.5
−1.0
−0.5
0.0
0.5
1
2
3
4
5
6
7
8
9
10
11
Meishan−A
Meishan−B
Meishan−C
European−A
European−B
European−C

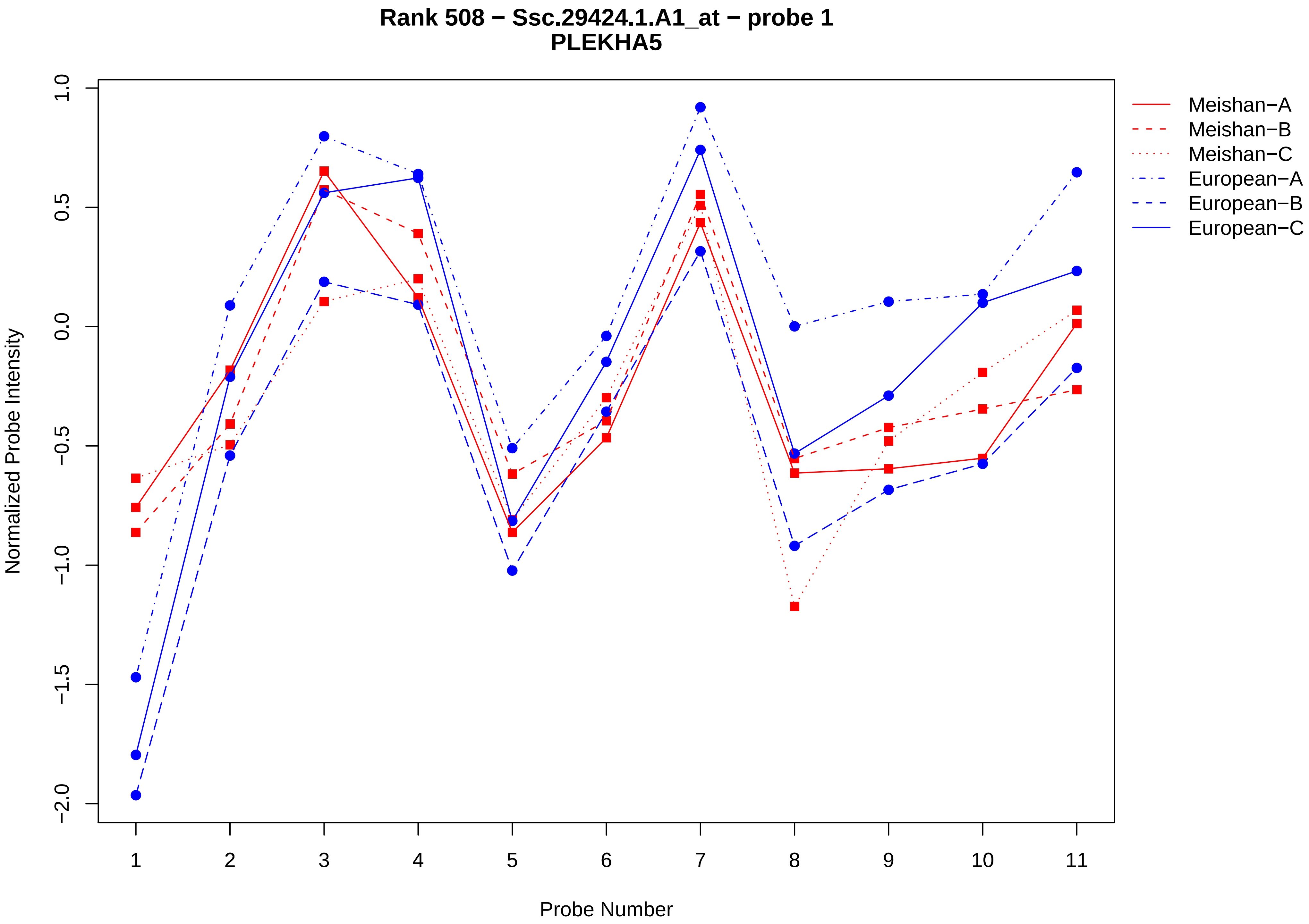

Rank 508 − Ssc.29424.1.A1_at − probe 1
PLEKHA5
Normalized Probe Intensity
Probe Number
1.0
0.5
0.0
−0.5
−1.0
−1.5
−2.0
1
2
3
4
5
6
7
8
9
10
11
Meishan−A
Meishan−B
Meishan−C
European−A
European−B
European−C

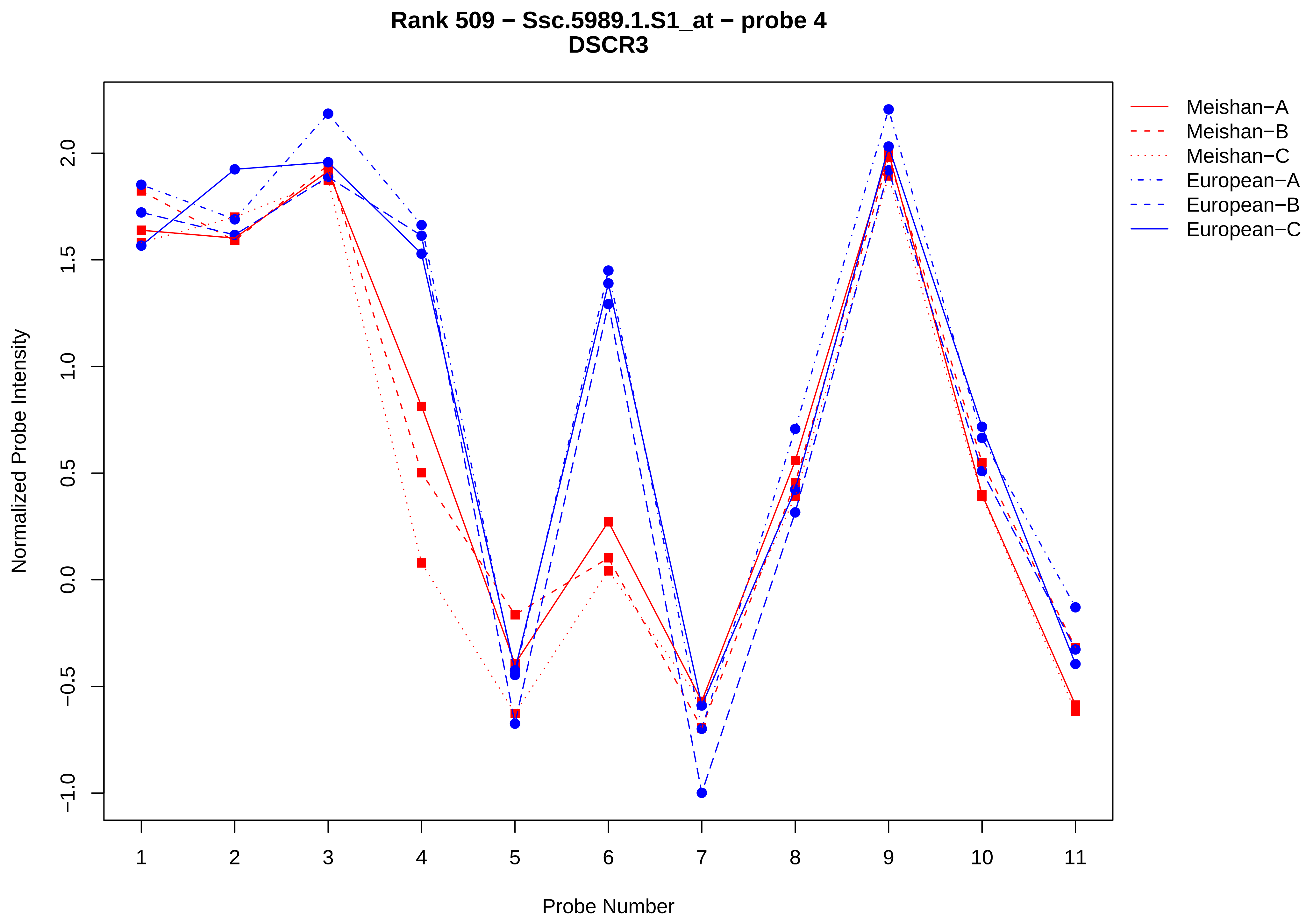

Rank 509 − Ssc.5989.1.S1_at − probe 4
DSCR3
Normalized Probe Intensity
Probe Number
Meishan−A
Meishan−B
Meishan−C
European−A
European−B
European−C
−1.0
−0.5
0.0
0.5
1.0
1.5
2.0
1
2
3
4
5
6
7
8
9
10
11

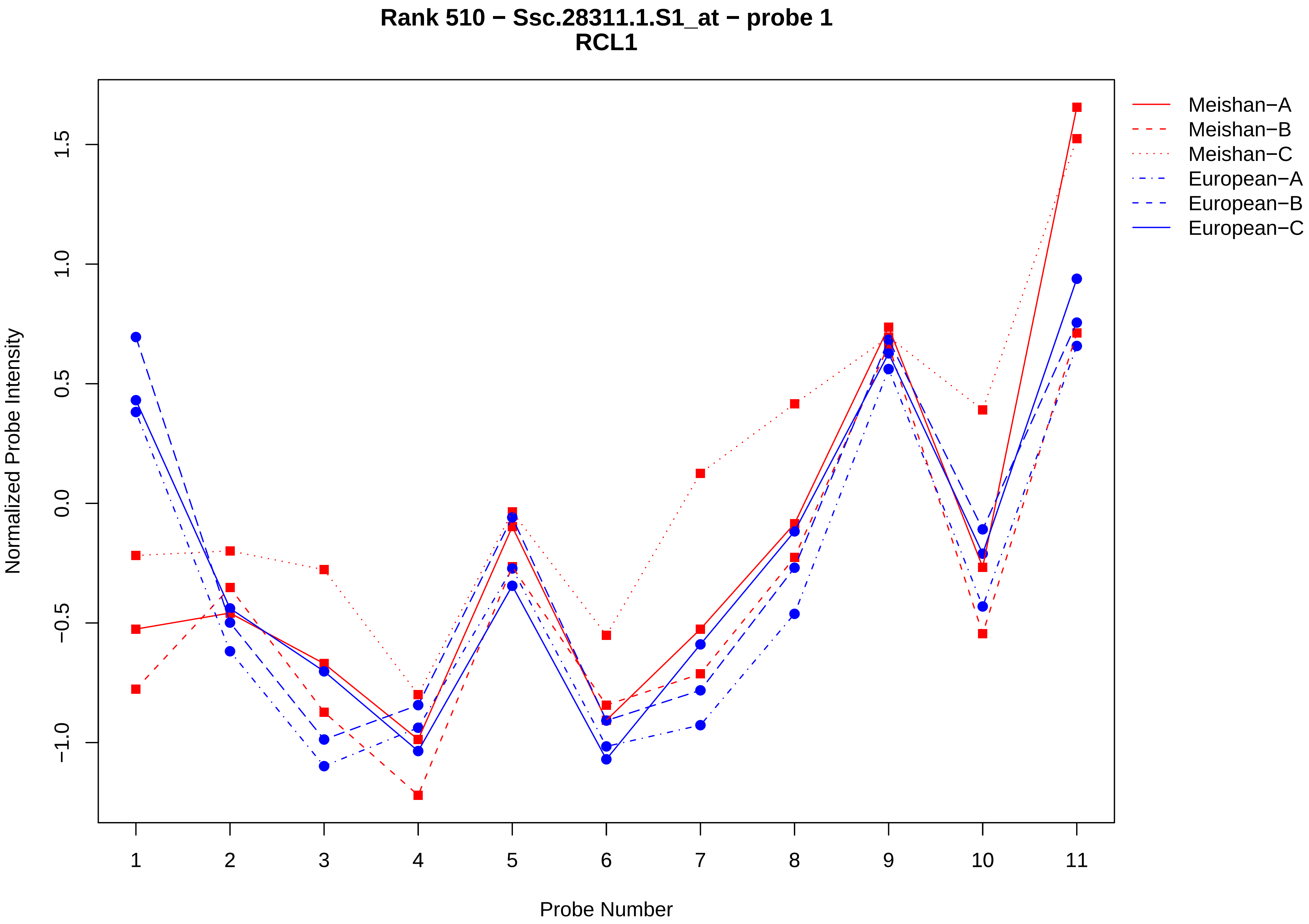

Rank 510 − Ssc.28311.1.S1_at − probe 1
RCL1
Normalized Probe Intensity
Probe Number
Meishan−A
Meishan−B
Meishan−C
European−A
European−B
European−C
1.5
1.0
0.5
0.0
−0.5
−1.0
1
2
3
4
5
6
7
8
9
10
11

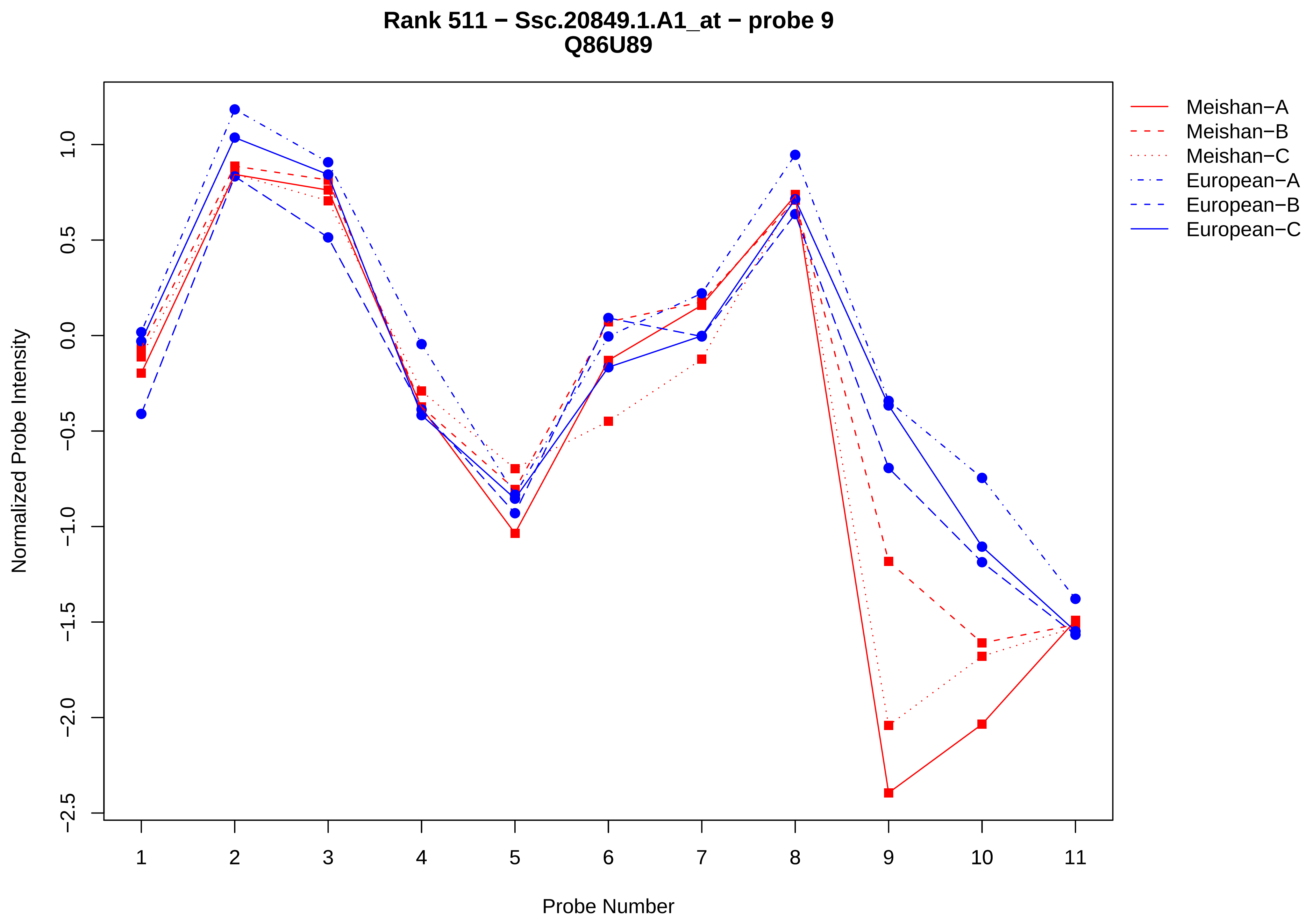
Rank 511 − Ssc.20849.1.A1_at − probe 9
Q86U89
Normalized Probe Intensity
Probe Number
1.0
0.5
0.0
−0.5
−1.0
−1.5
−2.0
−2.5
1
2
3
4
5
6
7
8
9
10
11
Meishan−A
Meishan−B
Meishan−C
European−A
European−B
European−C

Rank 512 − Ssc.2434.1.A1_at − probe 2
HIPK2

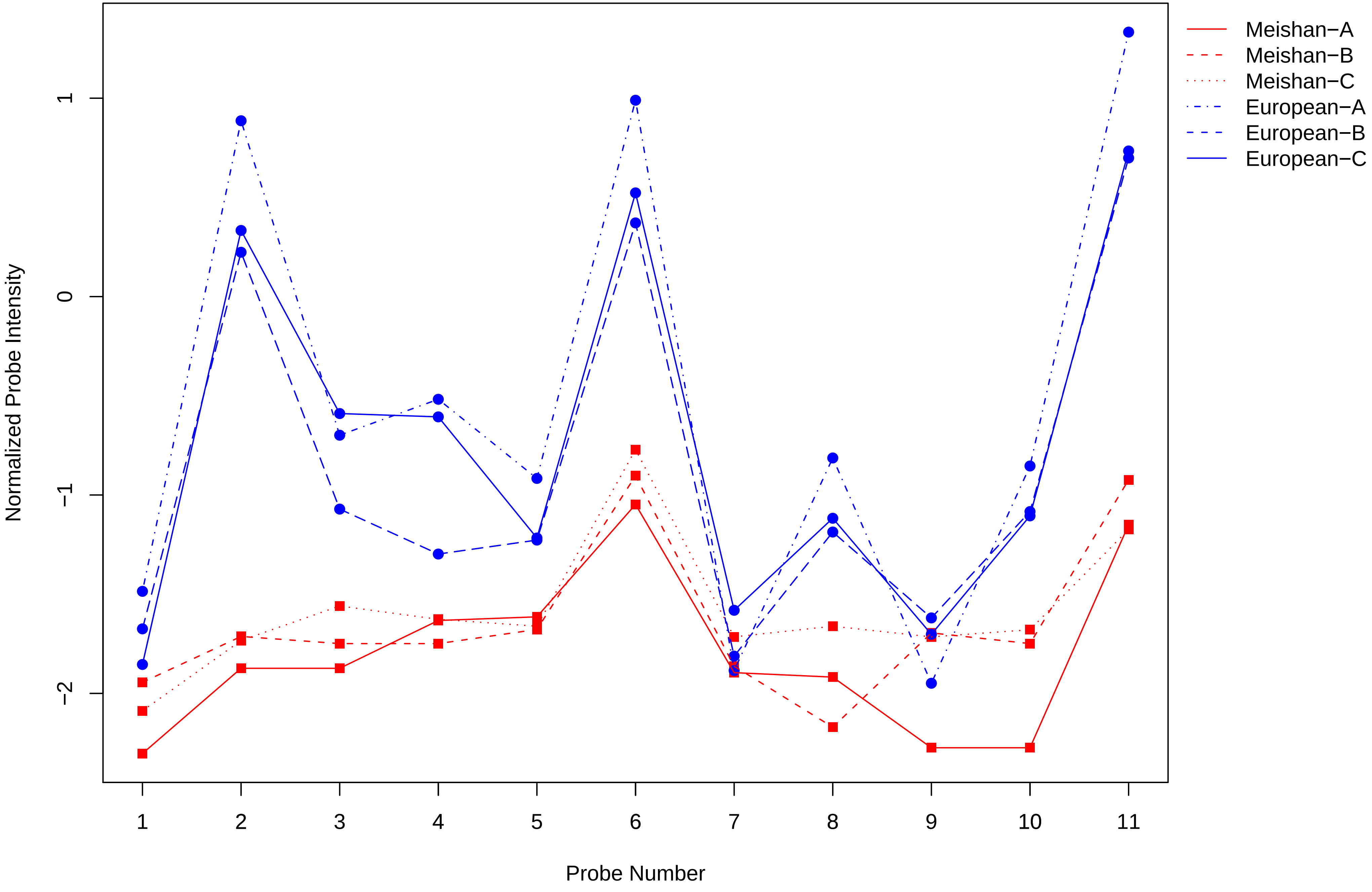

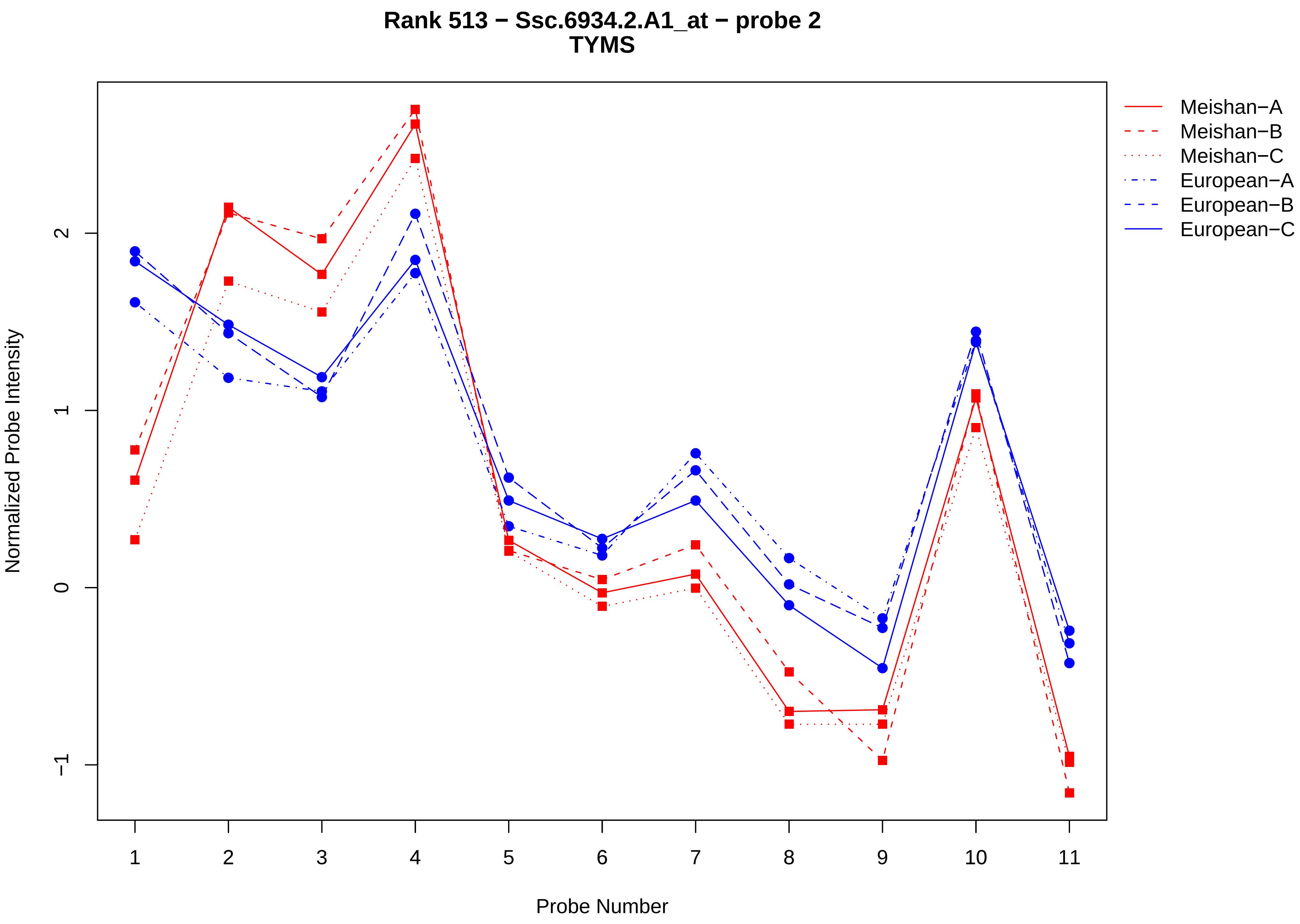
Rank 513 − Ssc.6934.2.A1_at − probe 2
TYMS
Normalized Probe Intensity
Probe Number
−1
0
1
2
1
2
3
4
5
6
7
8
9
10
11
Meishan−A
Meishan−B
Meishan−C
European−A
European−B
European−C

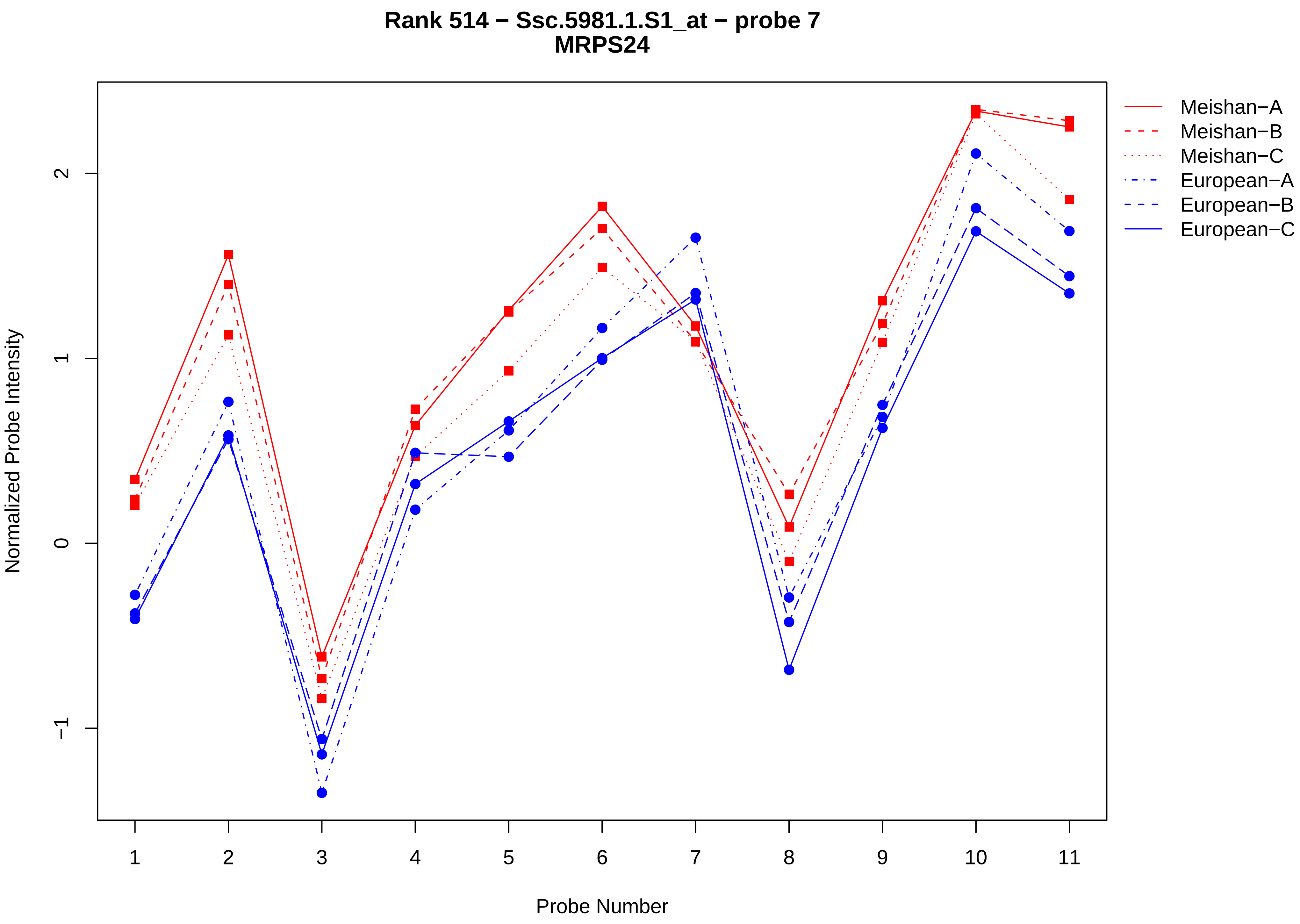

Rank 514 − Ssc.5981.1.S1_at − probe 7
MRPS24
Normalized Probe Intensity
−1
0
1
2
Probe Number
1
2
3
4
5
6
7
8
9
10
11
Meishan−A
Meishan−B
Meishan−C
European−A
European−B
European−C

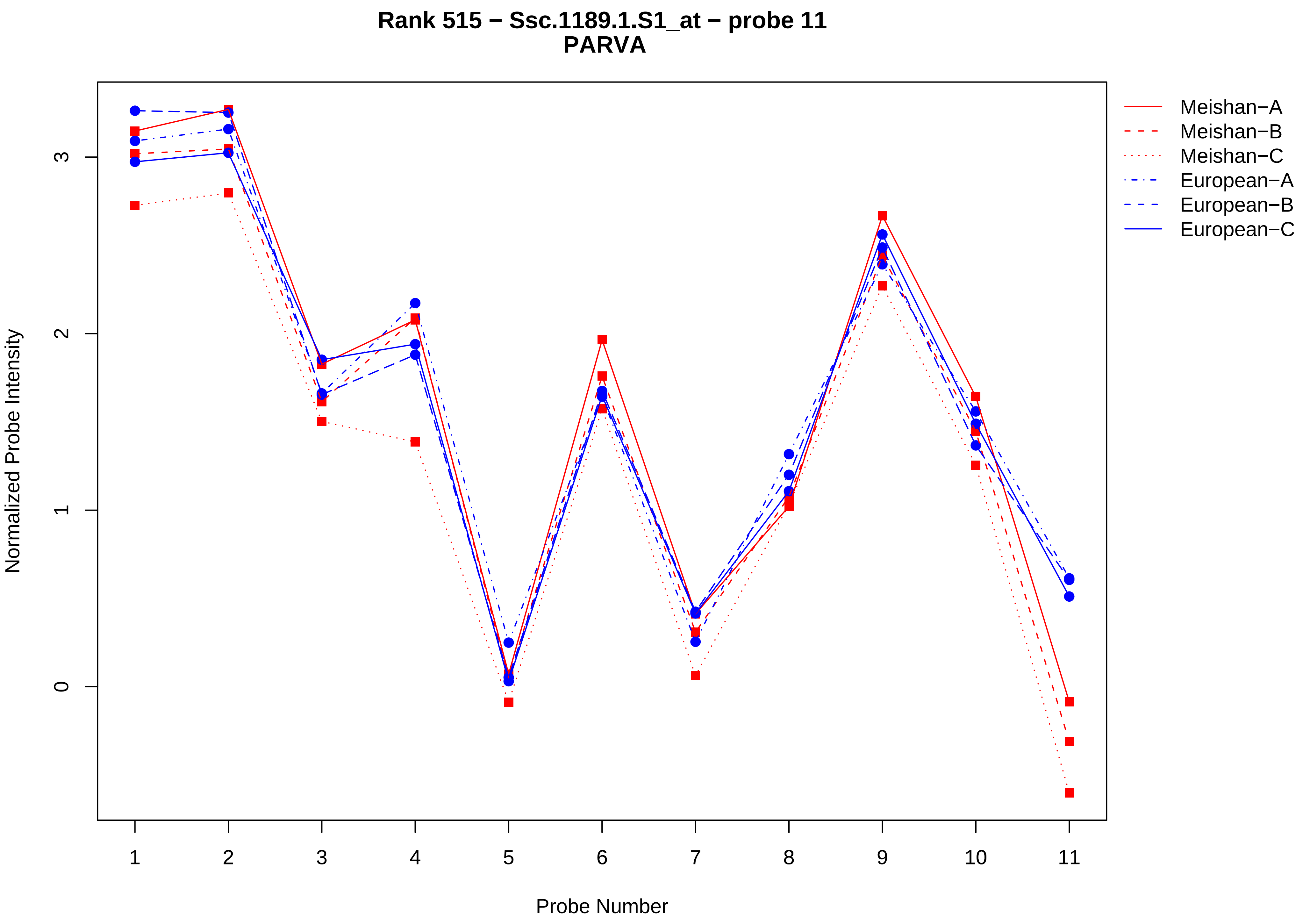
Rank 515 − Ssc.1189.1.S1_at − probe 11
PARVA
Normalized Probe Intensity
Probe Number
0
1
2
3
1
2
3
4
5
6
7
8
9
10
11
Meishan−A
Meishan−B
Meishan−C
European−A
European−B
European−C

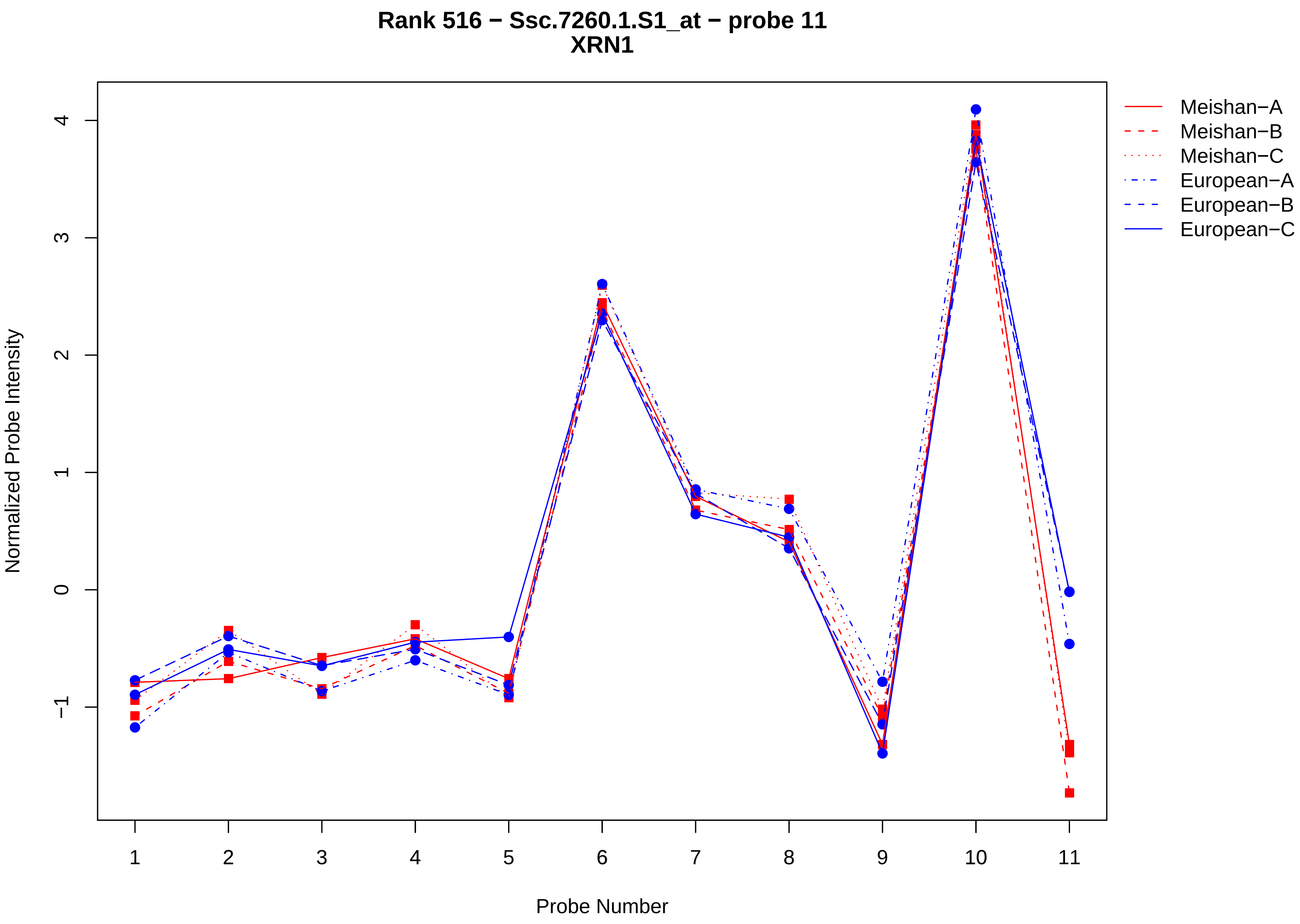

Rank 516 − Ssc.7260.1.S1_at − probe 11
XRN1
Normalized Probe Intensity
Probe Number
Meishan−A
Meishan−B
Meishan−C
European−A
European−B
European−C
−1
0
1
2
3
4
1
2
3
4
5
6
7
8
9
10
11

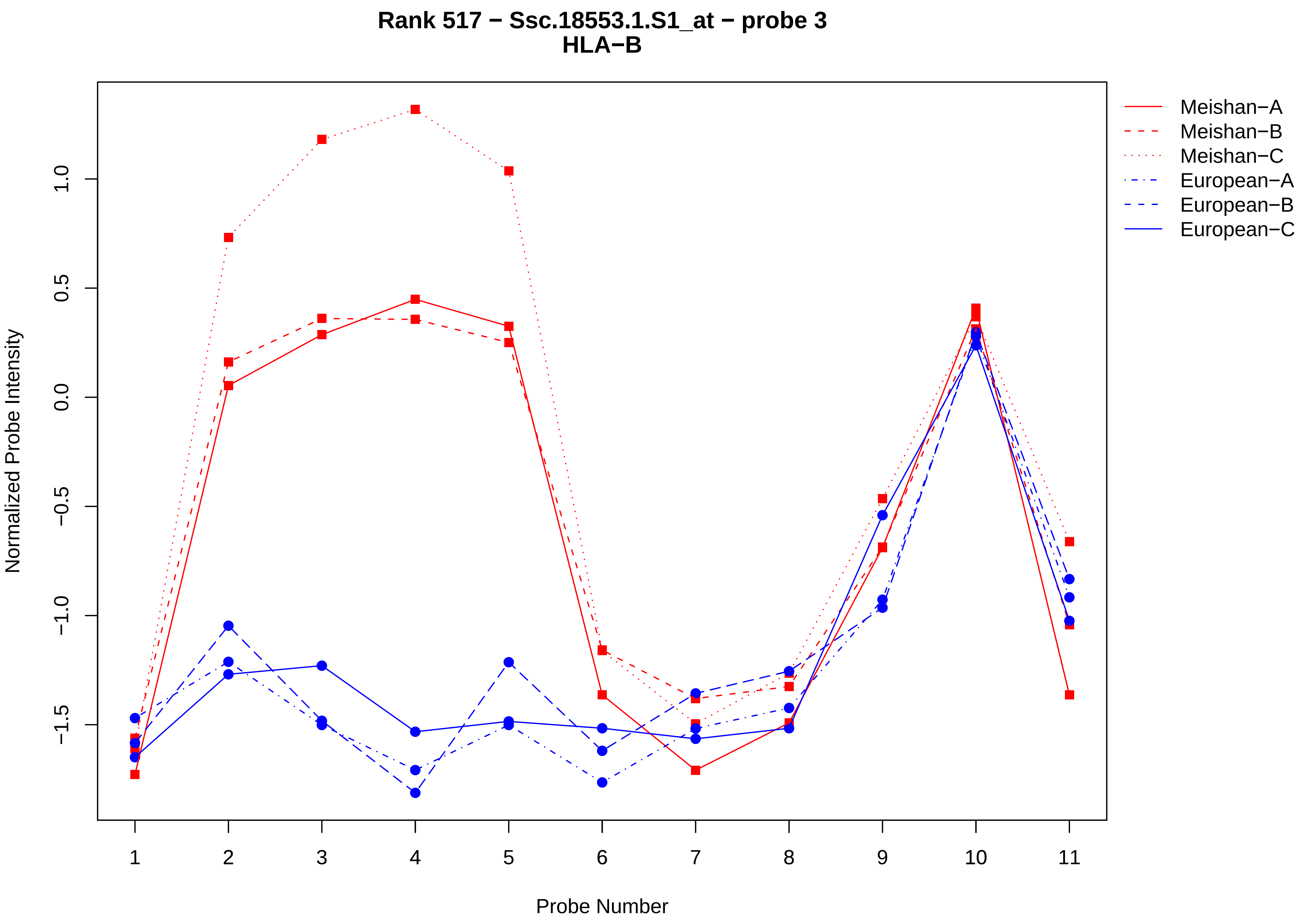

Rank 517 − Ssc.18553.1.S1_at − probe 3
HLA−B
Normalized Probe Intensity
Probe Number
1.0
0.5
0.0
−0.5
−1.0
−1.5
1
2
3
4
5
6
7
8
9
10
11
Meishan−A
Meishan−B
Meishan−C
European−A
European−B
European−C

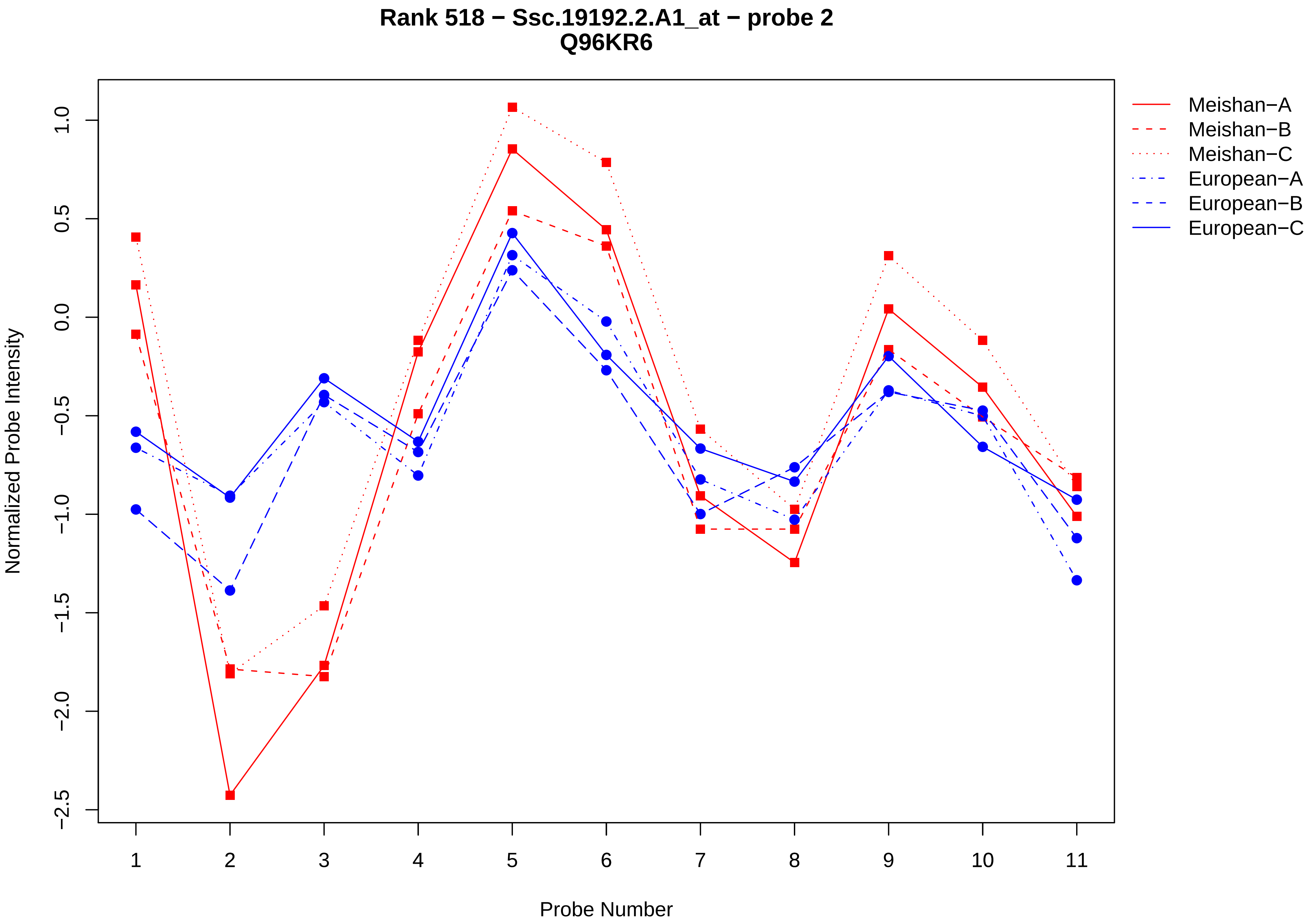
Rank 518 − Ssc.19192.2.A1_at − probe 2
Q96KR6
Normalized Probe Intensity
Probe Number
Meishan−A
Meishan−B
Meishan−C
European−A
European−B
European−C
1.0
0.5
0.0
−0.5
−1.0
−1.5
−2.0
−2.5
1
2
3
4
5
6
7
8
9
10
11

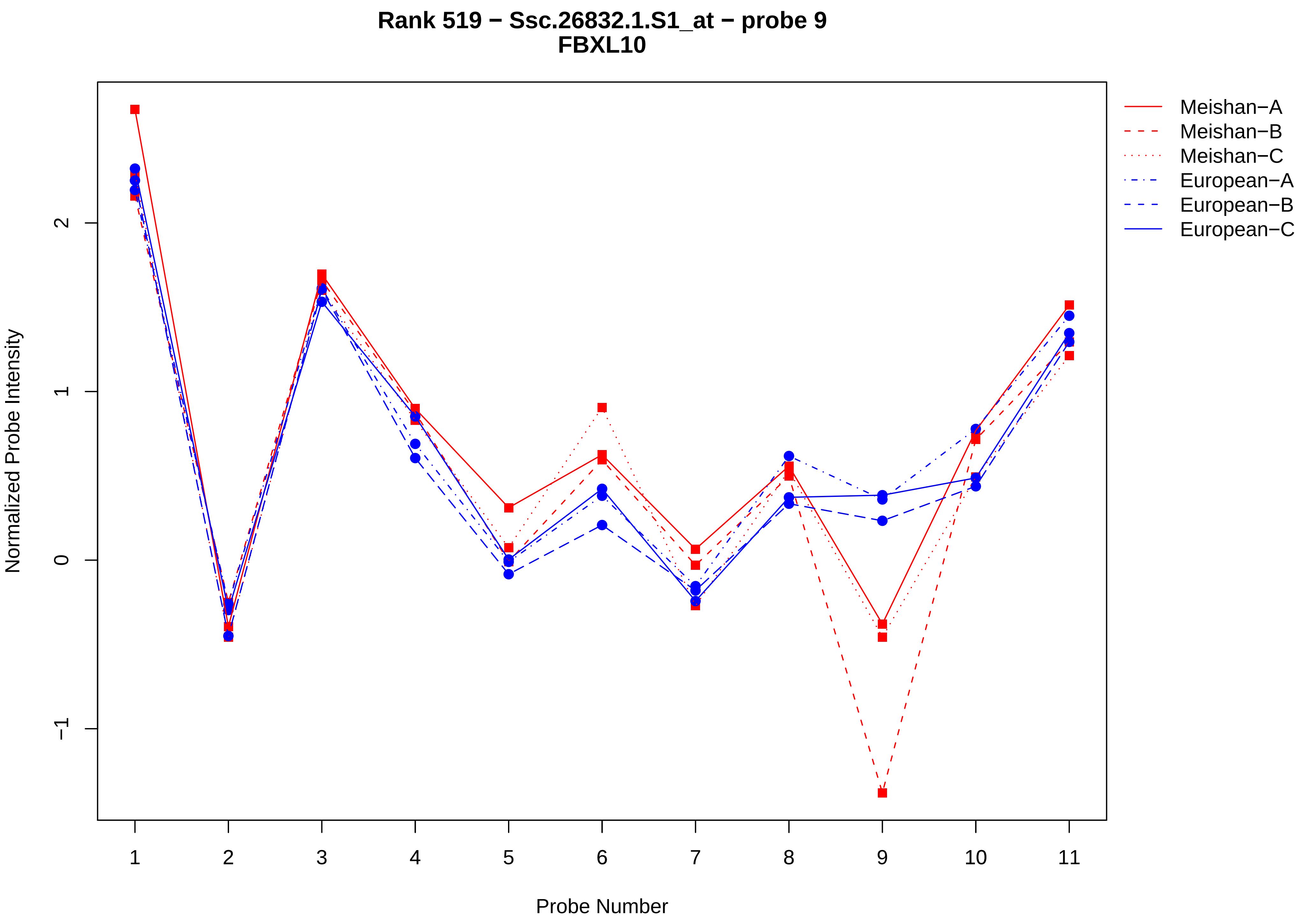

Rank 519 − Ssc.26832.1.S1_at − probe 9
FBXL10
Normalized Probe Intensity
Probe Number
−1
0
1
2
1
2
3
4
5
6
7
8
9
10
11
Meishan−A
Meishan−B
Meishan−C
European−A
European−B
European−C

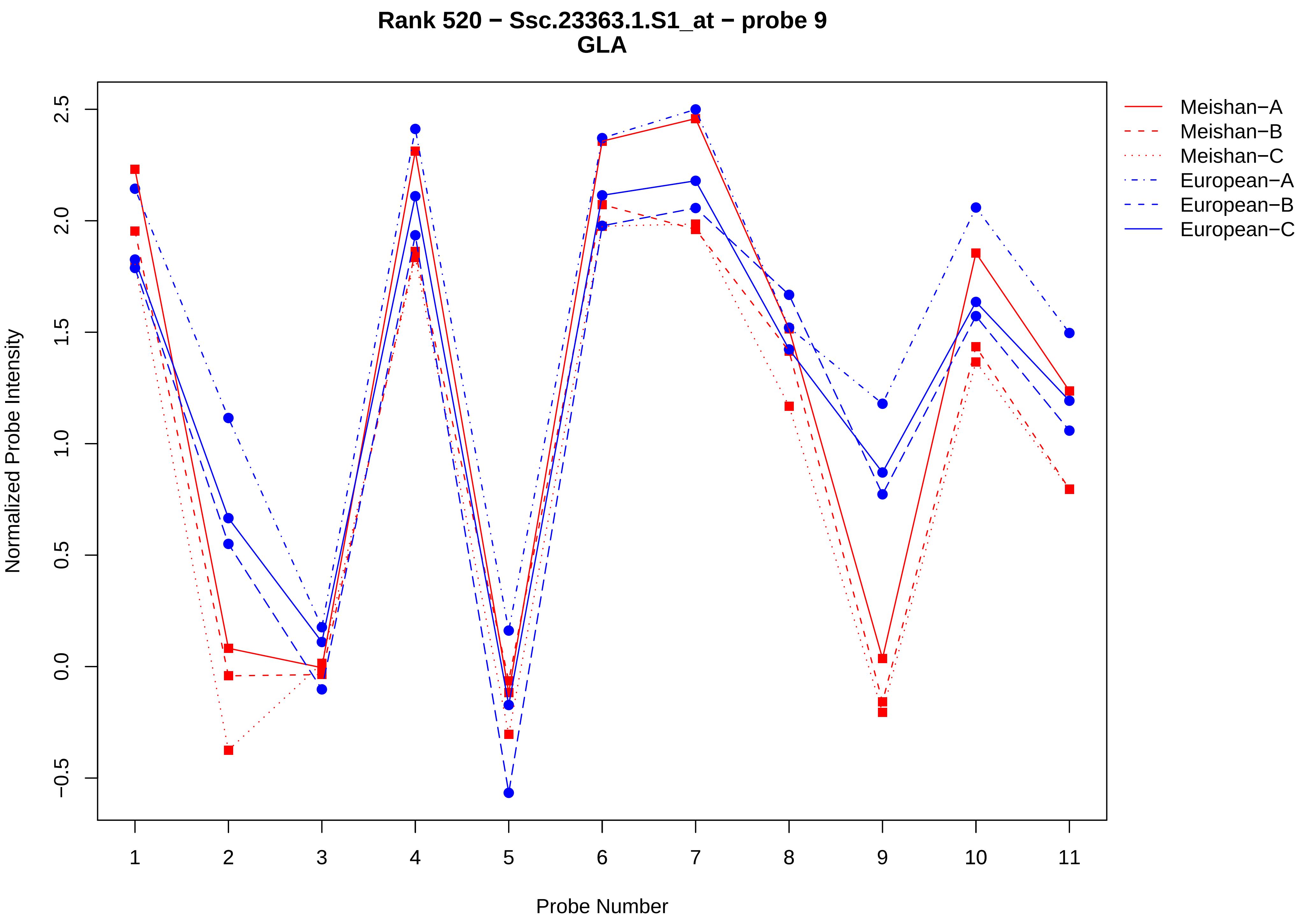
Rank 520 − Ssc.23363.1.S1_at − probe 9
GLA
Normalized Probe Intensity
Probe Number
Meishan−A
Meishan−B
Meishan−C
European−A
European−B
European−C
−0.5
0.0
0.5
1.0
1.5
2.0
2.5
1
2
3
4
5
6
7
8
9
10
11

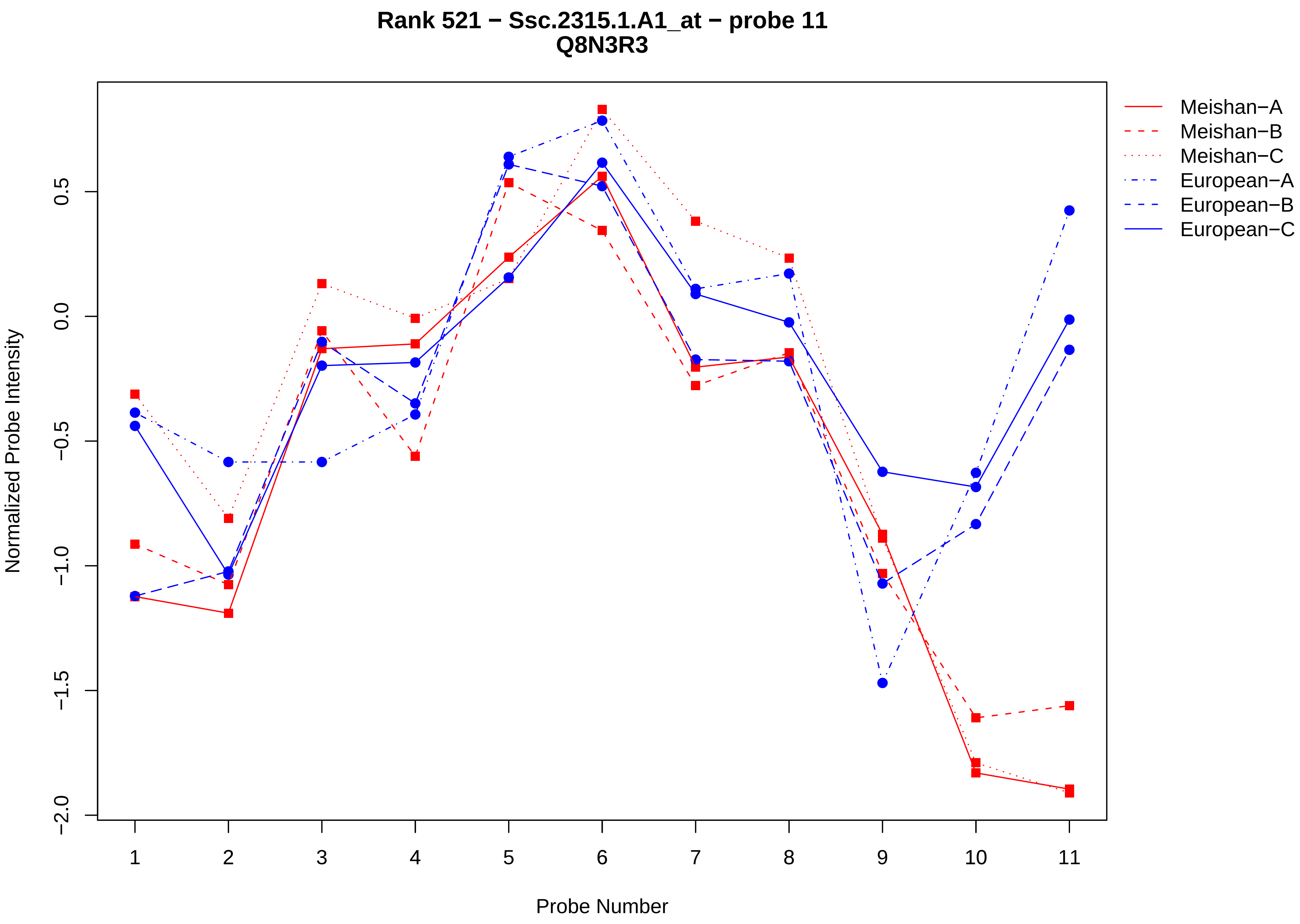
Rank 521 − Ssc.2315.1.A1_at − probe 11
Q8N3R3
Normalized Probe Intensity
Probe Number
0.5
0.0
−0.5
−1.0
−1.5
−2.0
1
2
3
4
5
6
7
8
9
10
11
Meishan−A
Meishan−B
Meishan−C
European−A
European−B
European−C

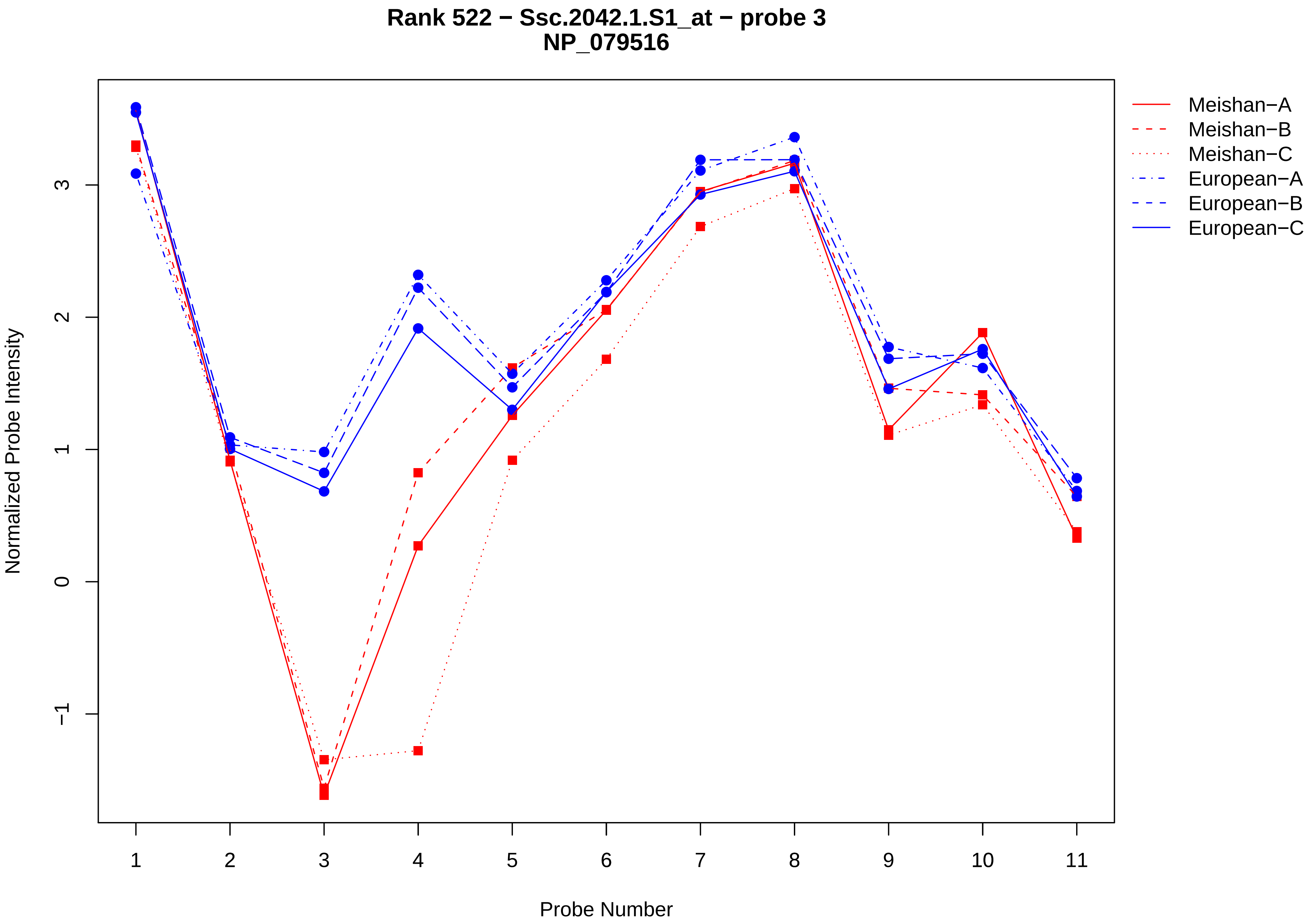

Rank 522 − Ssc.2042.1.S1_at − probe 3
NP_079516
Normalized Probe Intensity
Probe Number
−1
0
1
2
3
1
2
3
4
5
6
7
8
9
10
11
Meishan−A
Meishan−B
Meishan−C
European−A
European−B
European−C

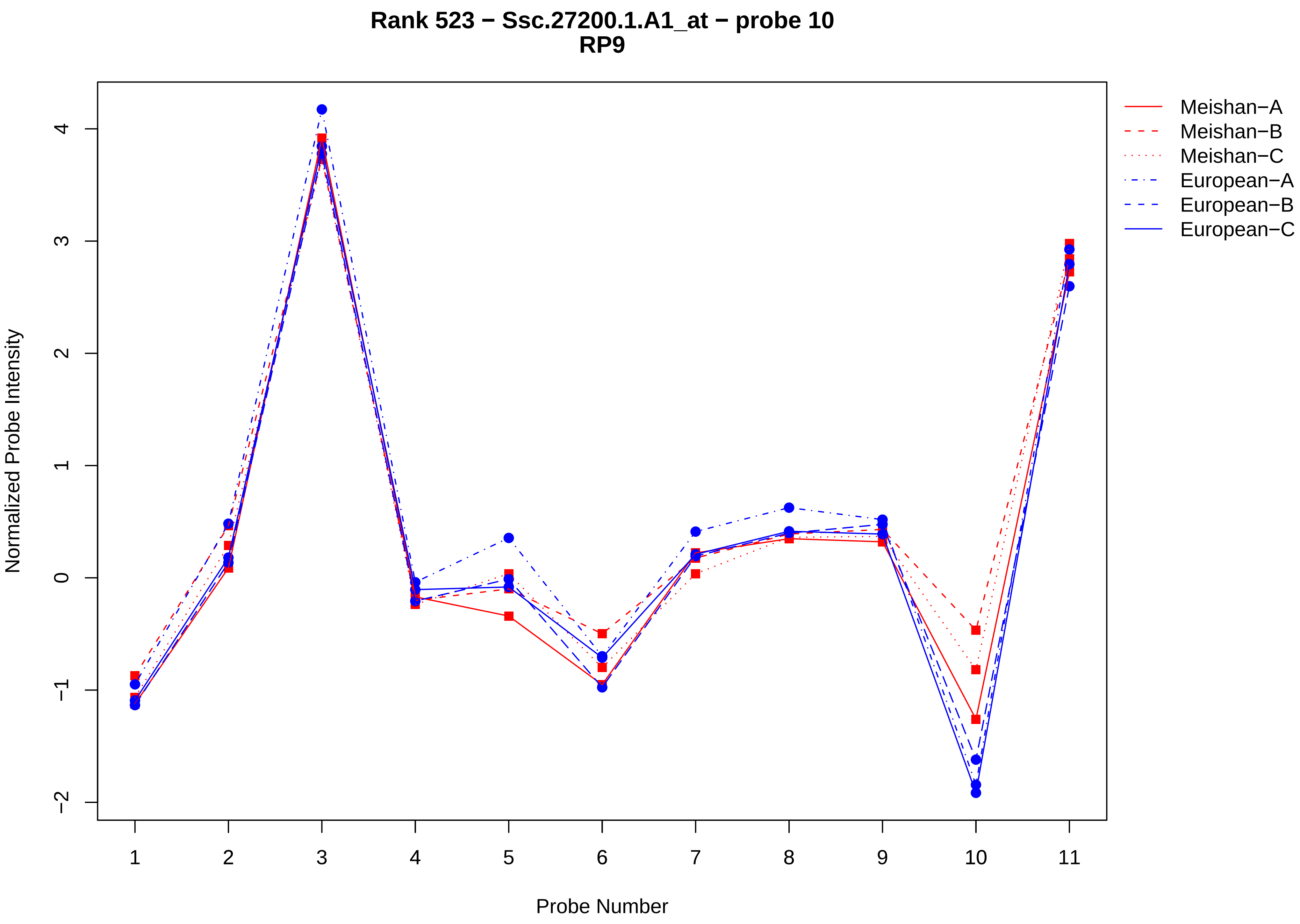

Rank 523 − Ssc.27200.1.A1_at − probe 10
RP9
Normalized Probe Intensity
Probe Number
−2
−1
0
1
2
3
4
1
2
3
4
5
6
7
8
9
10
11
Meishan−A
Meishan−B
Meishan−C
European−A
European−B
European−C

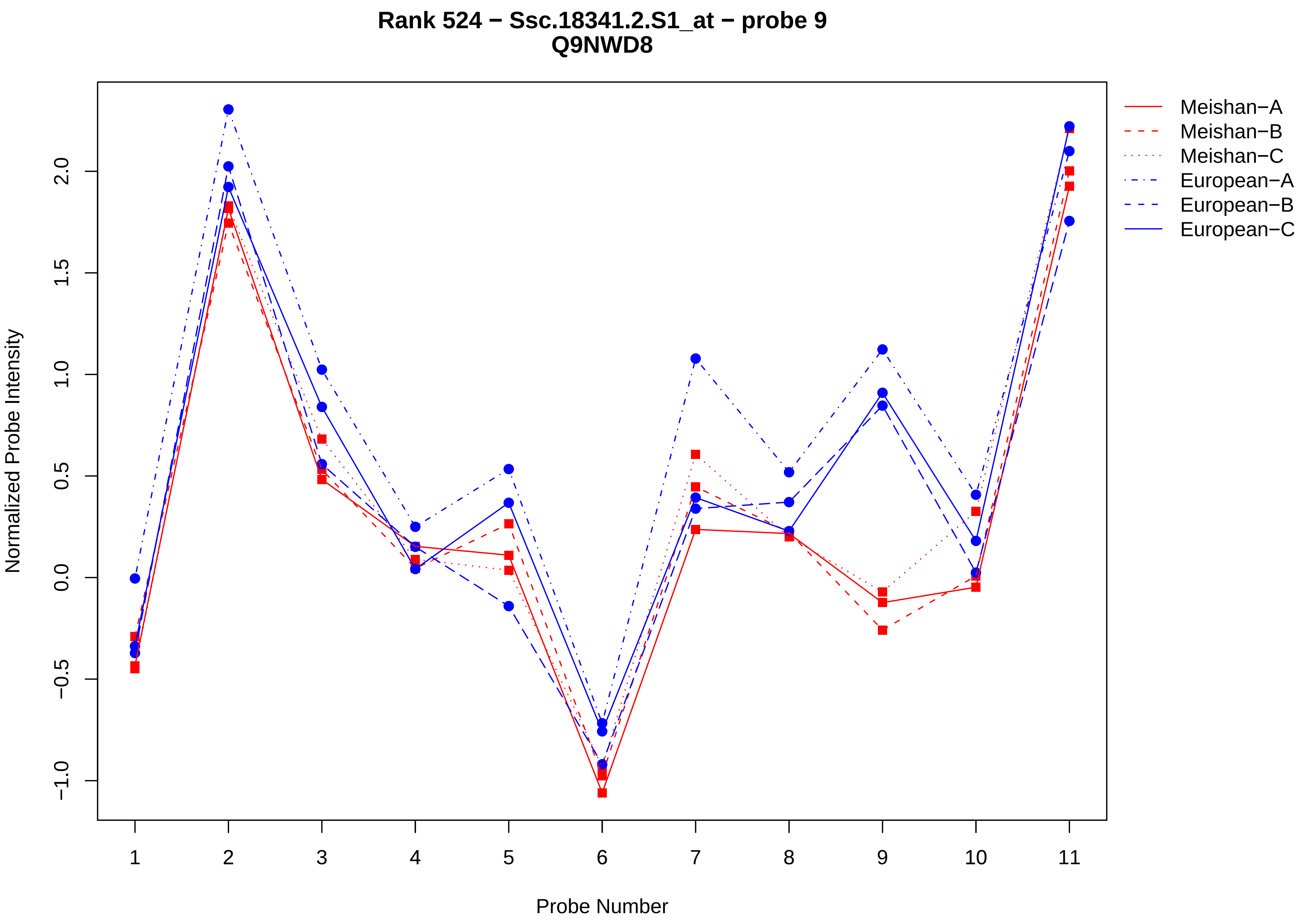

Rank 524 − Ssc.18341.2.S1_at − probe 9
Q9NWD8
Normalized Probe Intensity
Probe Number
−1.0
−0.5
0.0
0.5
1.0
1.5
2.0
1
2
3
4
5
6
7
8
9
10
11
Meishan−A
Meishan−B
Meishan−C
European−A
European−B
European−C

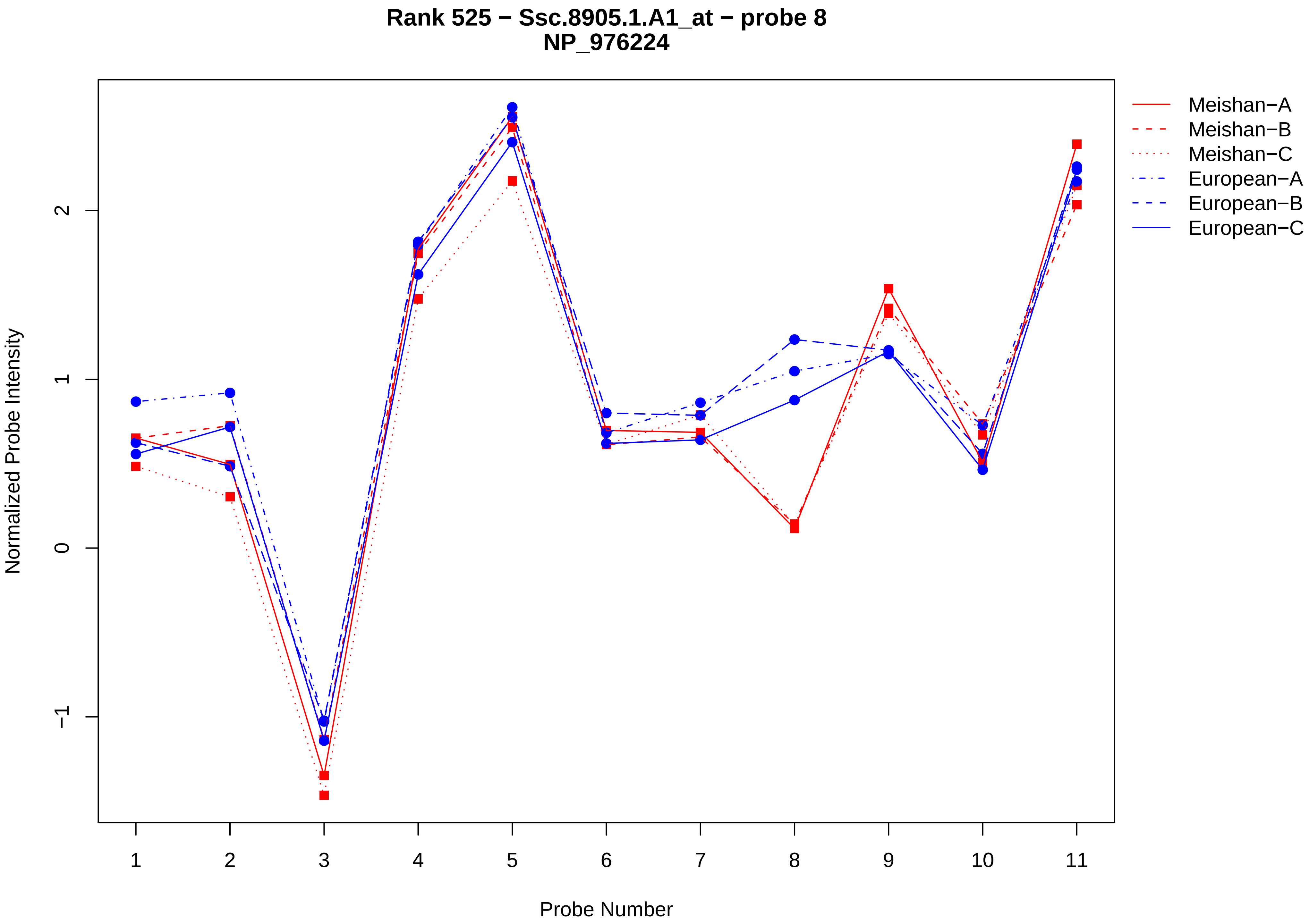
Rank 525 − Ssc.8905.1.A1_at − probe 8
NP_976224
Meishan−A
Meishan−B
Meishan−C
European−A
European−B
European−C
Normalized Probe Intensity
−1
0
1
2
1
2
3
4
5
6
7
8
9
10
11
Probe Number

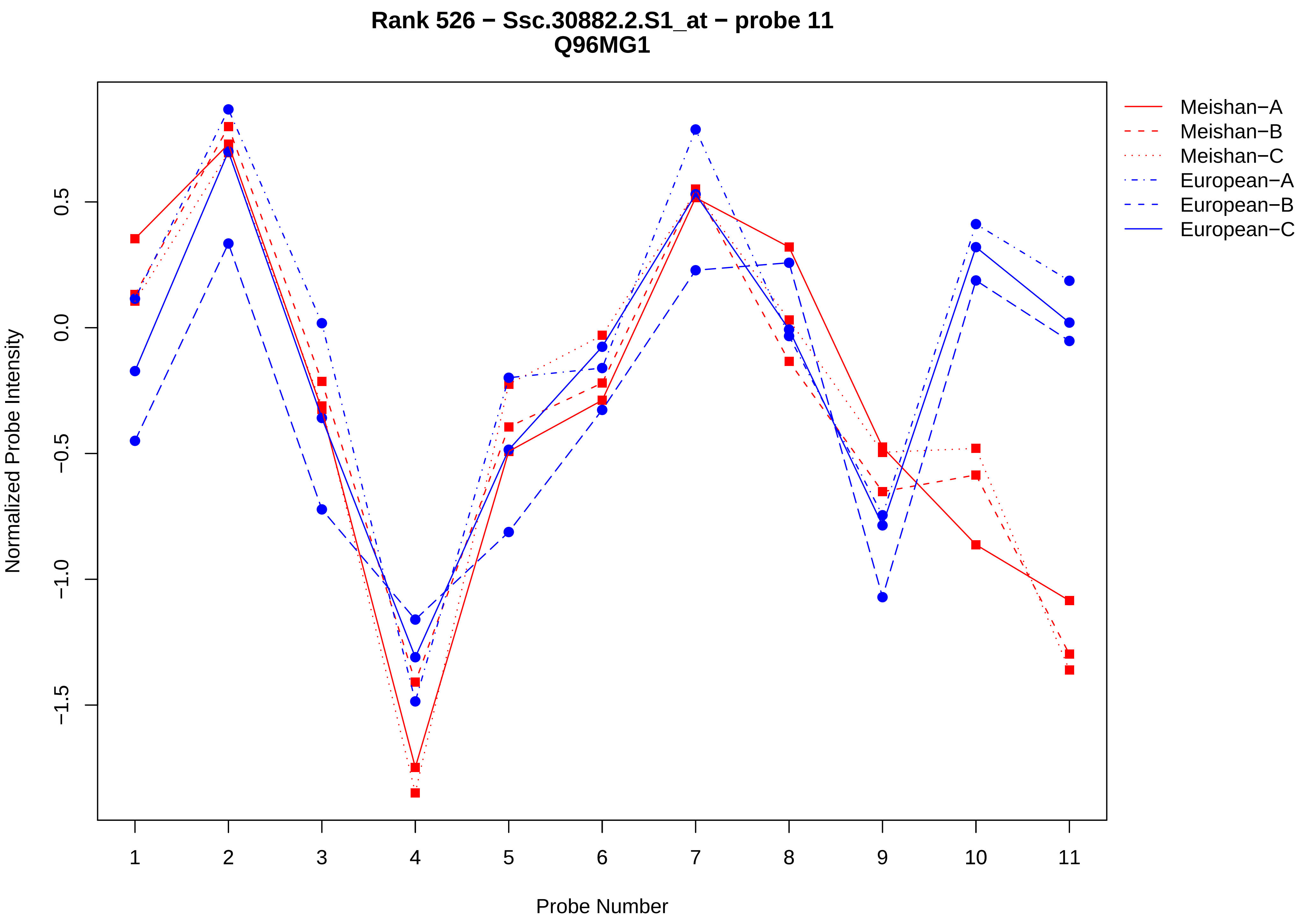
Rank 526 − Ssc.30882.2.S1_at − probe 11
Q96MG1
Normalized Probe Intensity
Probe Number
−1.5
−1.0
−0.5
0.0
0.5
1
2
3
4
5
6
7
8
9
10
11
Meishan−A
Meishan−B
Meishan−C
European−A
European−B
European−C

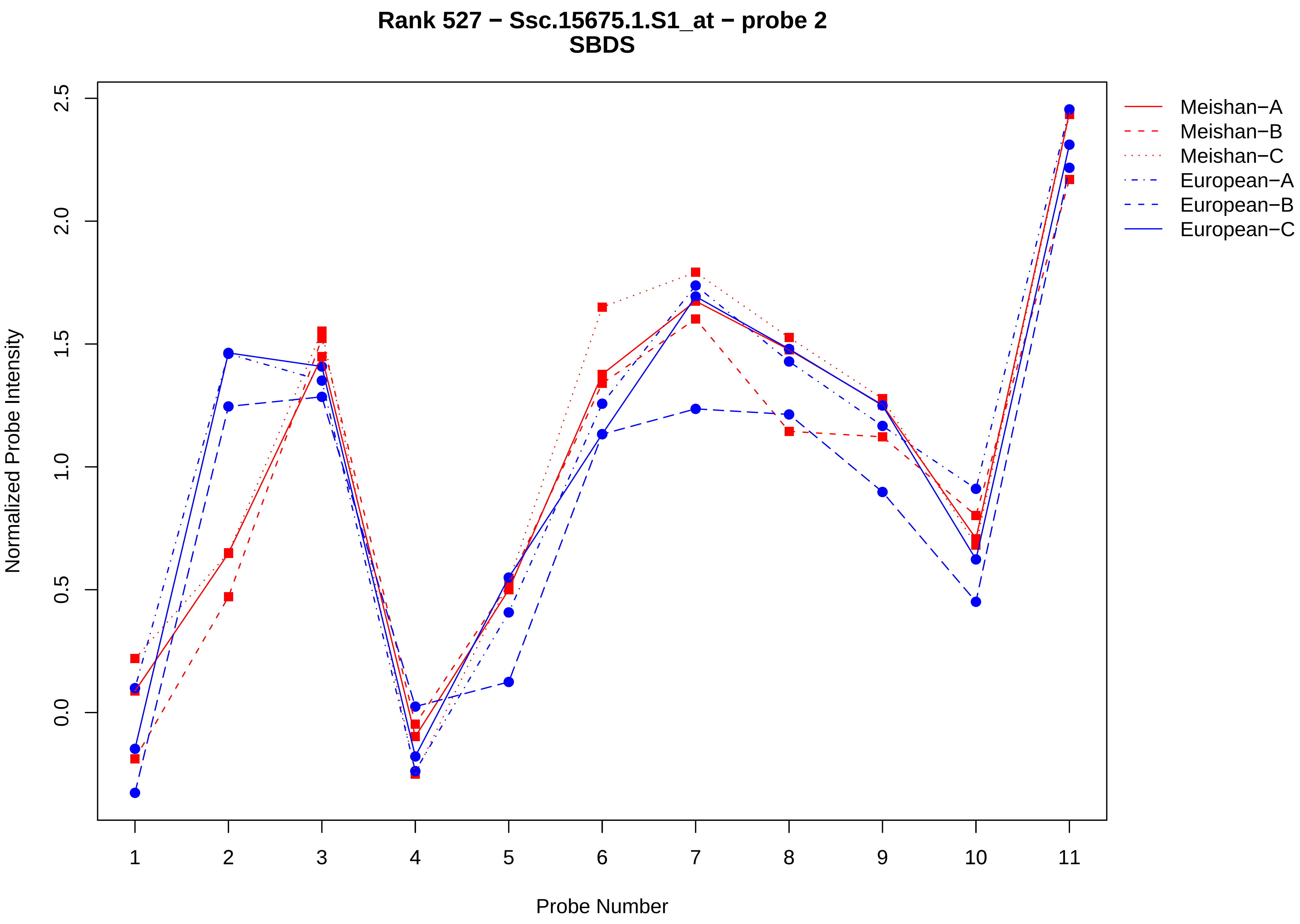
Rank 527 − Ssc.15675.1.S1_at − probe 2
SBDS
Normalized Probe Intensity
Probe Number
0.0
0.5
1.0
1.5
2.0
2.5
1
2
3
4
5
6
7
8
9
10
11
Meishan−A
Meishan−B
Meishan−C
European−A
European−B
European−C

Rank 528 − Ssc.11114.1.A1_at − probe 4
RPA1

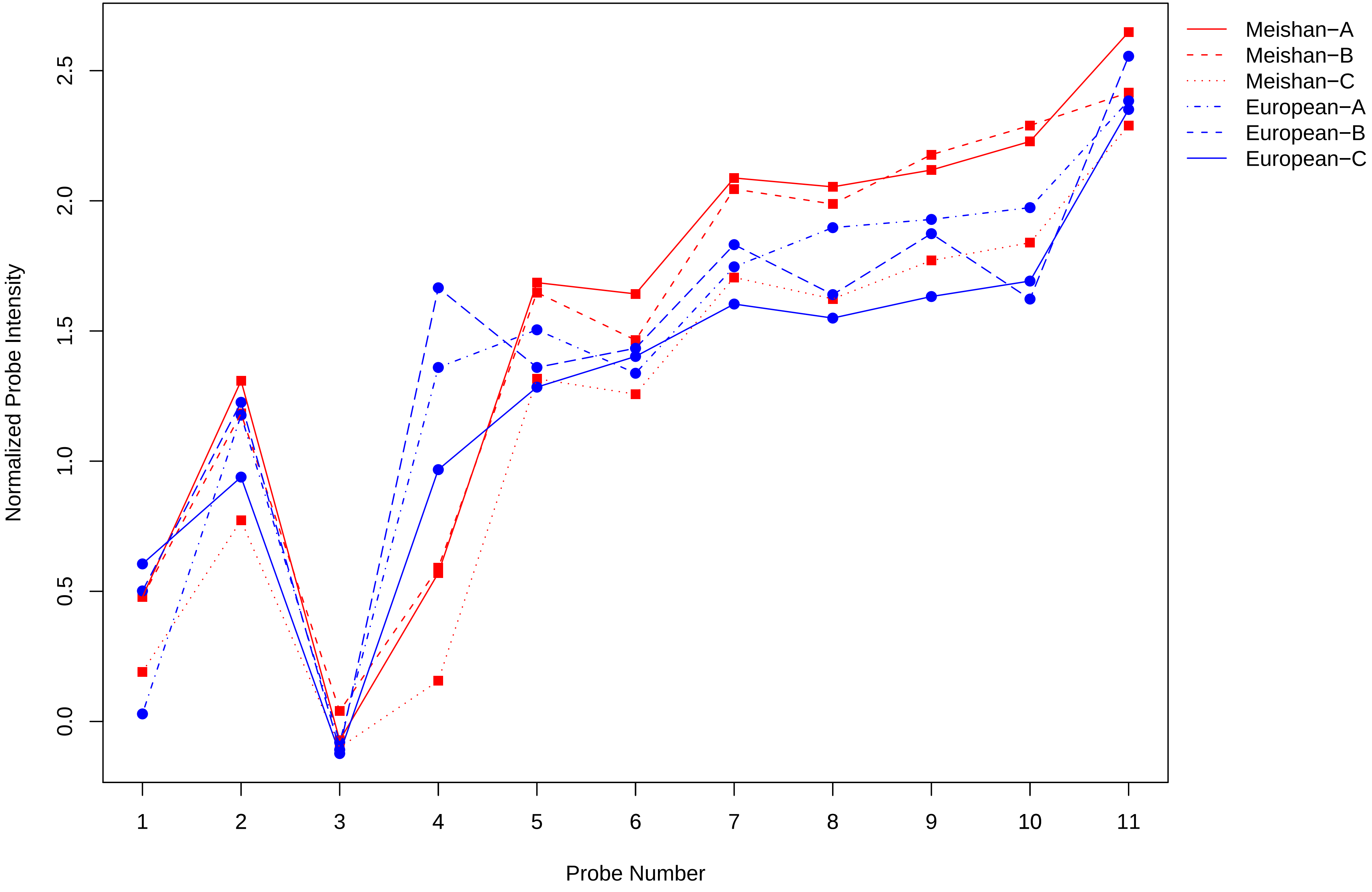

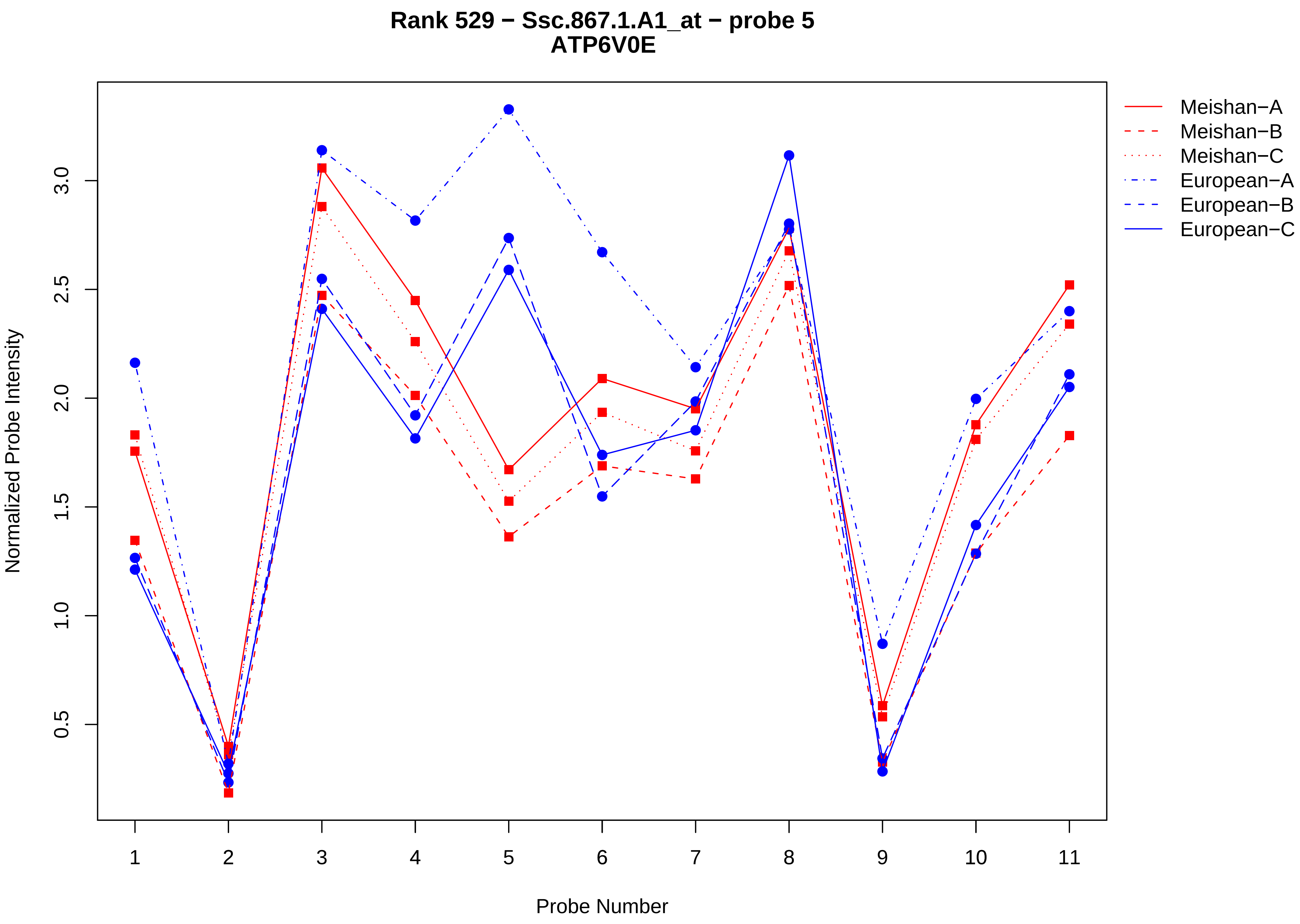

Rank 529 − Ssc.867.1.A1_at − probe 5
ATP6V0E
Normalized Probe Intensity
Probe Number
0.5
1.0
1.5
2.0
2.5
3.0
1
2
3
4
5
6
7
8
9
10
11
Meishan−A
Meishan−B
Meishan−C
European−A
European−B
European−C

Rank 530 − Ssc.3956.1.S1_s_at − probe 1
HBXIP

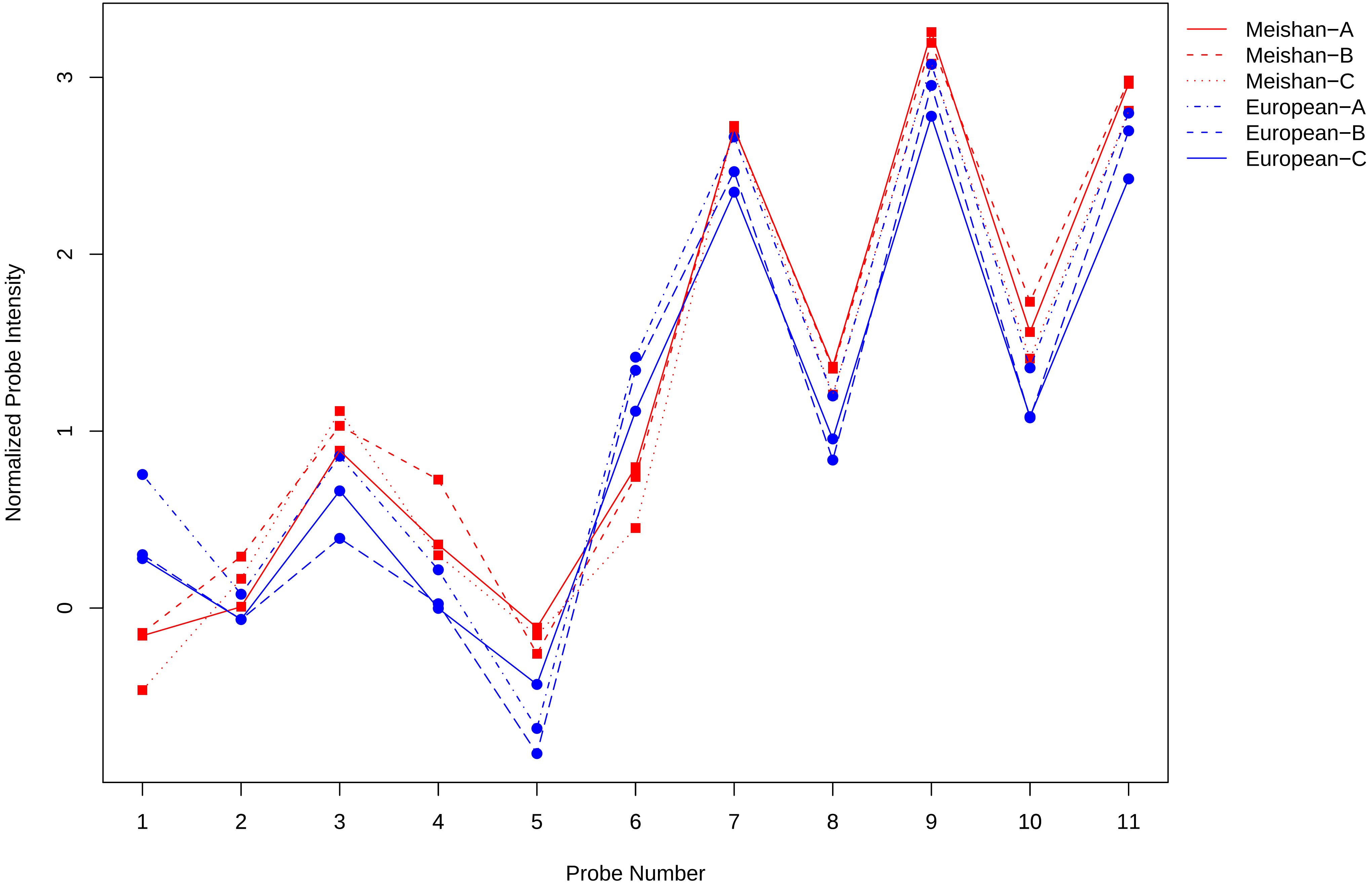

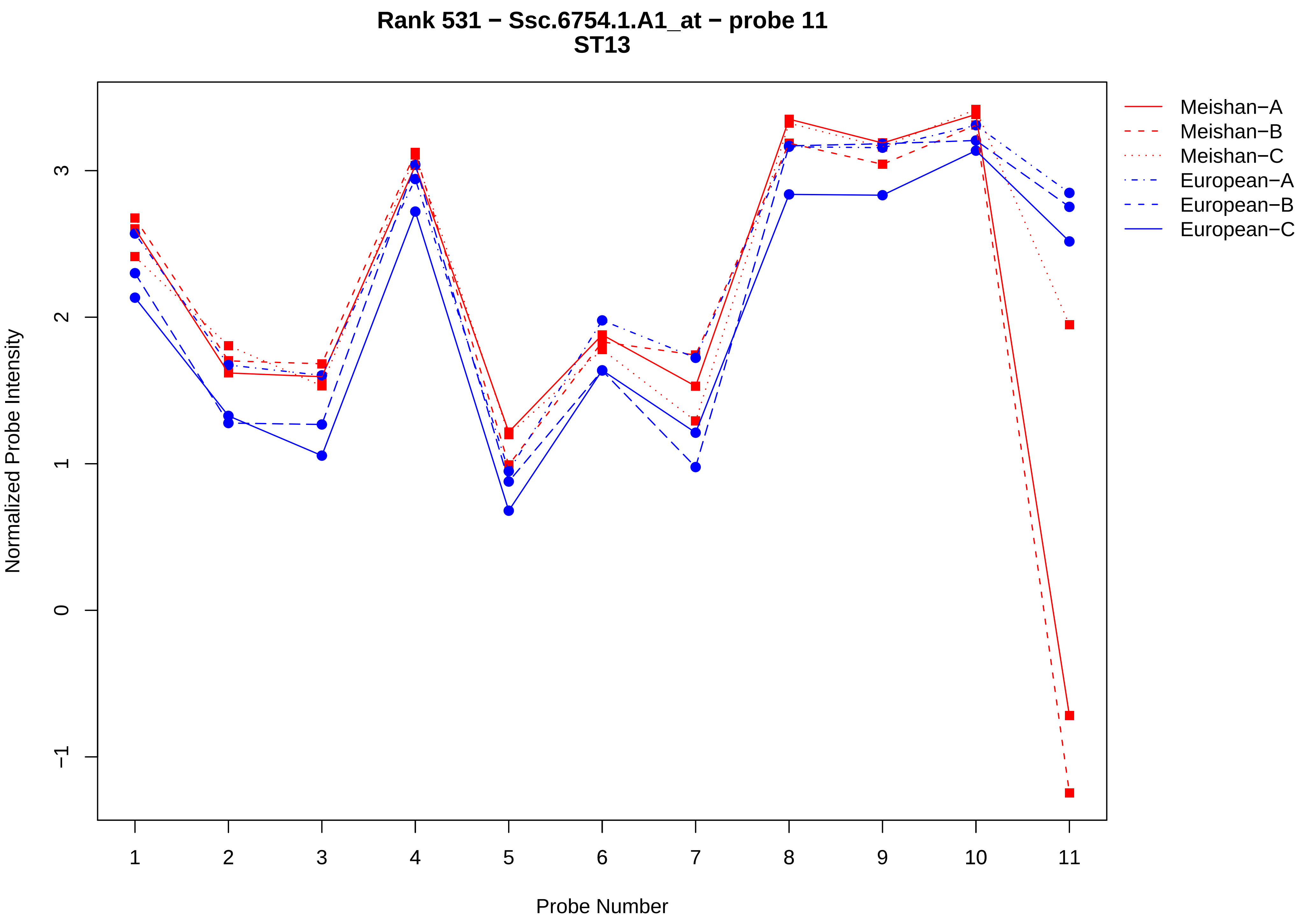
Rank 531 − Ssc.6754.1.A1_at − probe 11
ST13
Normalized Probe Intensity
Probe Number
−1
0
1
2
3
1
2
3
4
5
6
7
8
9
10
11
Meishan−A
Meishan−B
Meishan−C
European−A
European−B
European−C

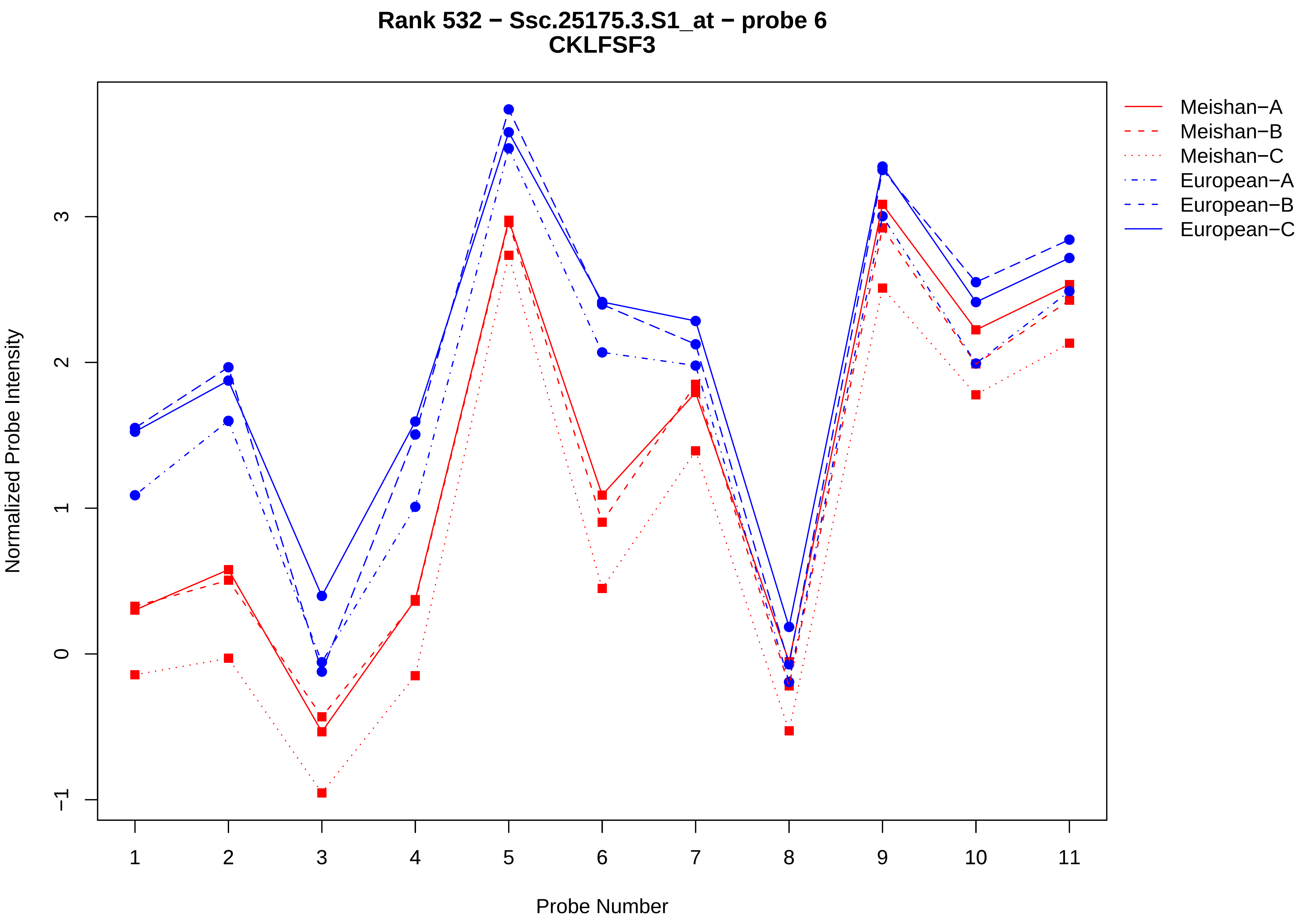

Rank 532 − Ssc.25175.3.S1_at − probe 6
CKLFSF3
Normalized Probe Intensity
Probe Number
−1
0
1
2
3
1
2
3
4
5
6
7
8
9
10
11
Meishan−A
Meishan−B
Meishan−C
European−A
European−B
European−C

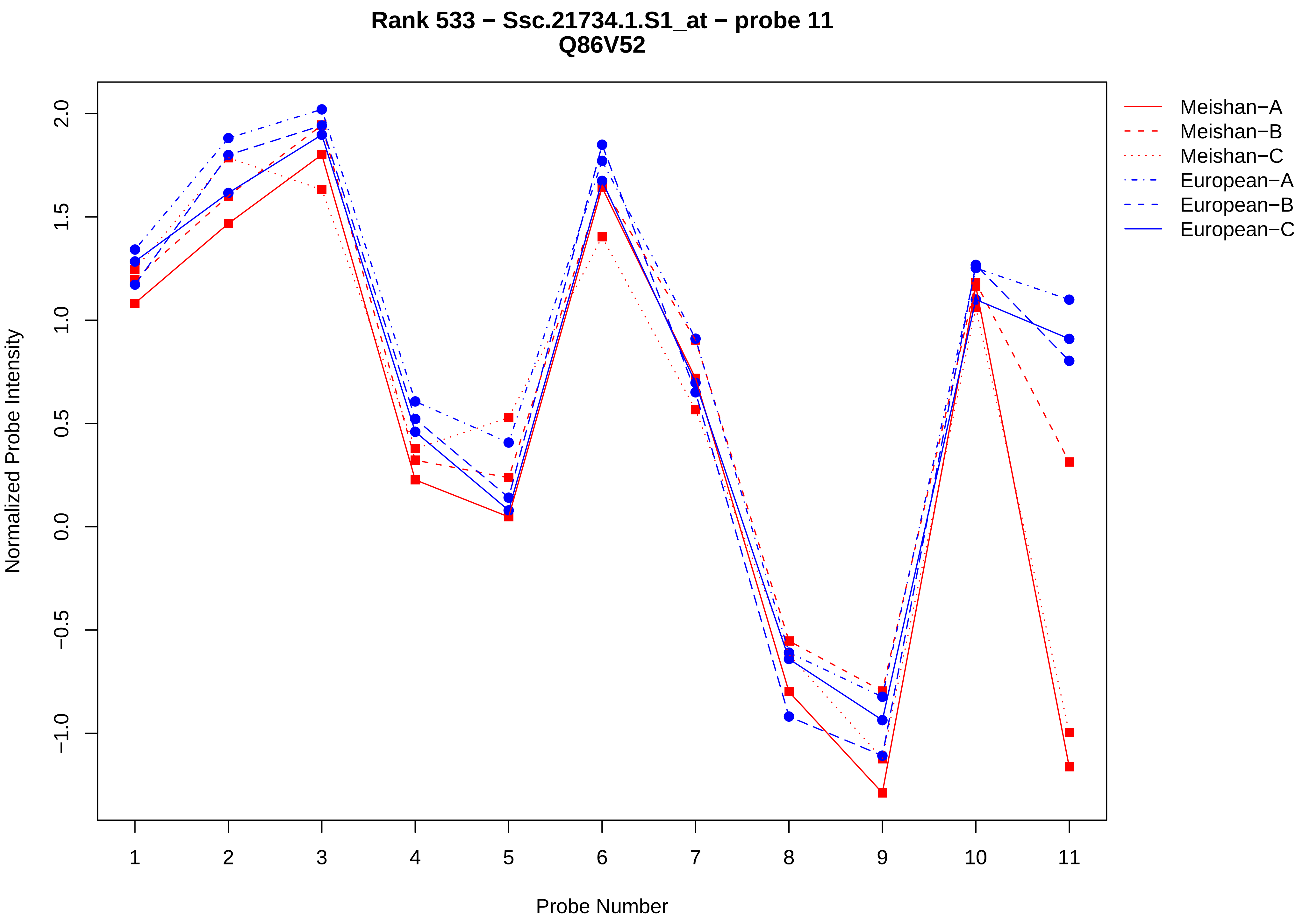

Rank 533 − Ssc.21734.1.S1_at − probe 11
Q86V52
Normalized Probe Intensity
Probe Number
−1.0
−0.5
0.0
0.5
1.0
1.5
2.0
1
2
3
4
5
6
7
8
9
10
11
Meishan−A
Meishan−B
Meishan−C
European−A
European−B
European−C

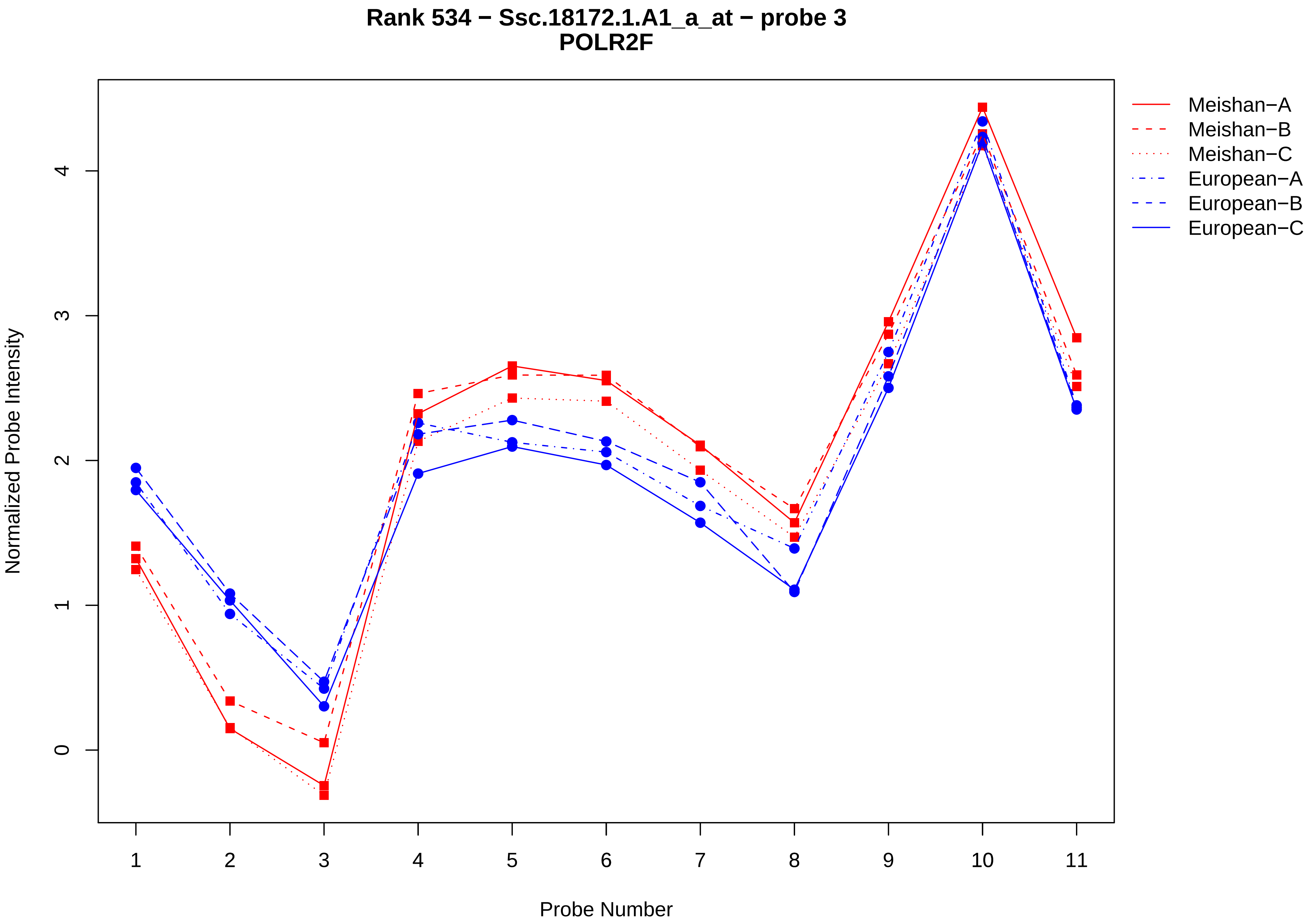

Rank 534 − Ssc.18172.1.A1_a_at − probe 3
POLR2F
Meishan−A
Meishan−B
Meishan−C
European−A
European−B
European−C
Normalized Probe Intensity
Probe Number
0
1
2
3
4
1
2
3
4
5
6
7
8
9
10
11

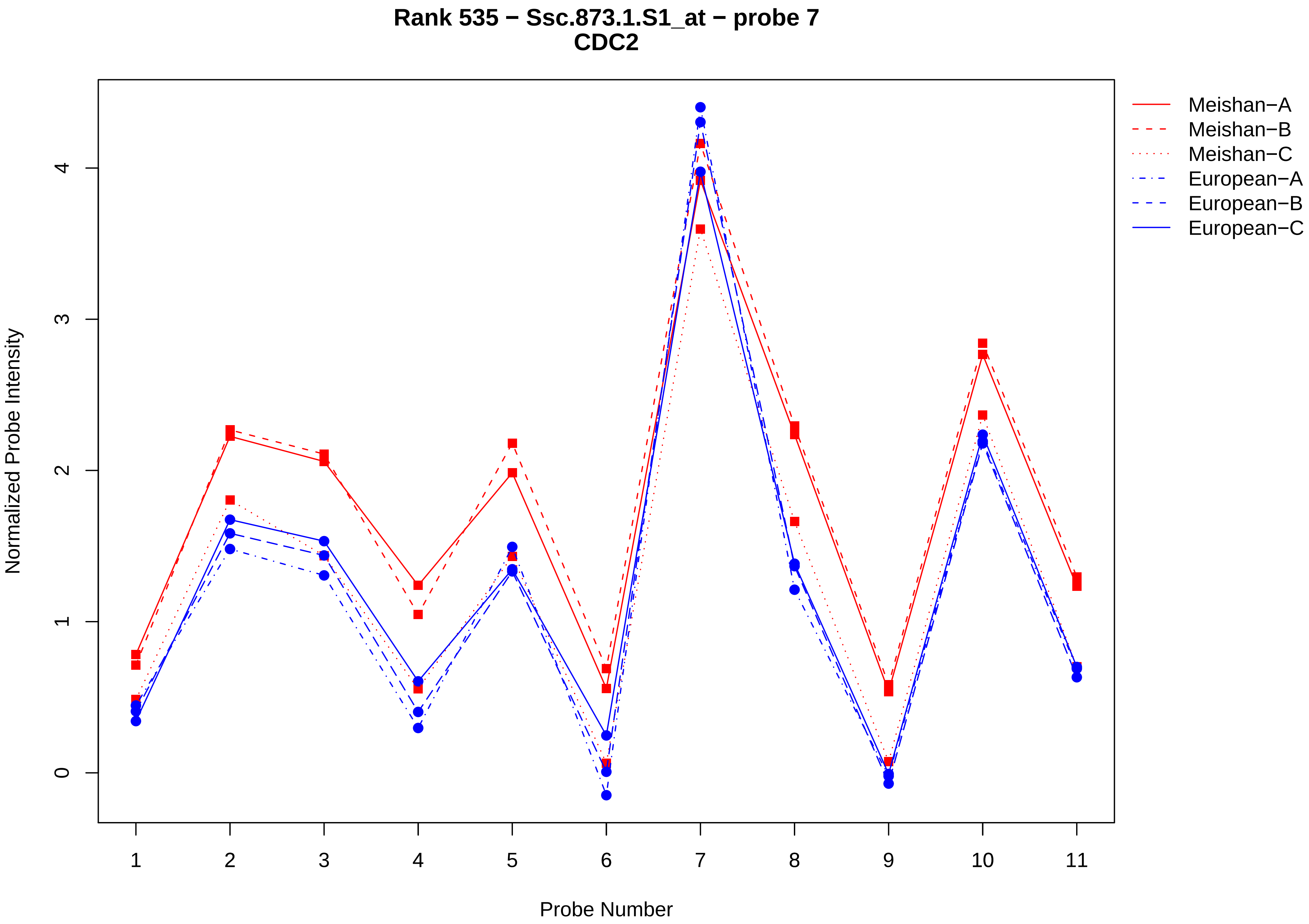

Rank 535 − Ssc.873.1.S1_at − probe 7
CDC2
Normalized Probe Intensity
0
1
2
3
4
1
2
3
4
5
6
7
8
9
10
11
Probe Number
Meishan−A
Meishan−B
Meishan−C
European−A
European−B
European−C

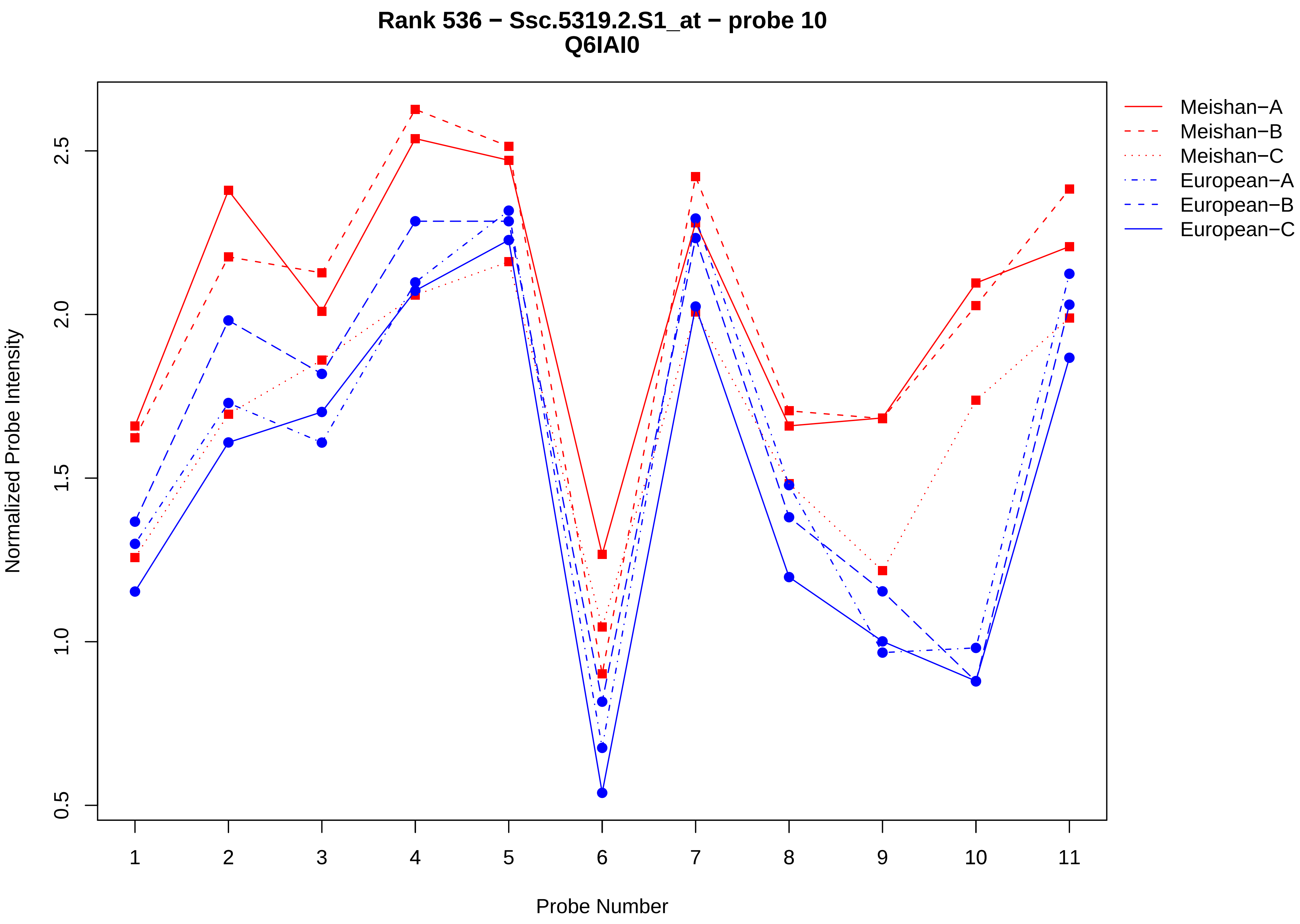

Rank 536 − Ssc.5319.2.S1_at − probe 10
Q6IAI0
Normalized Probe Intensity
0.5
1.0
1.5
2.0
2.5
1
2
3
4
5
6
7
8
9
10
11
Probe Number
Meishan−A
Meishan−B
Meishan−C
European−A
European−B
European−C

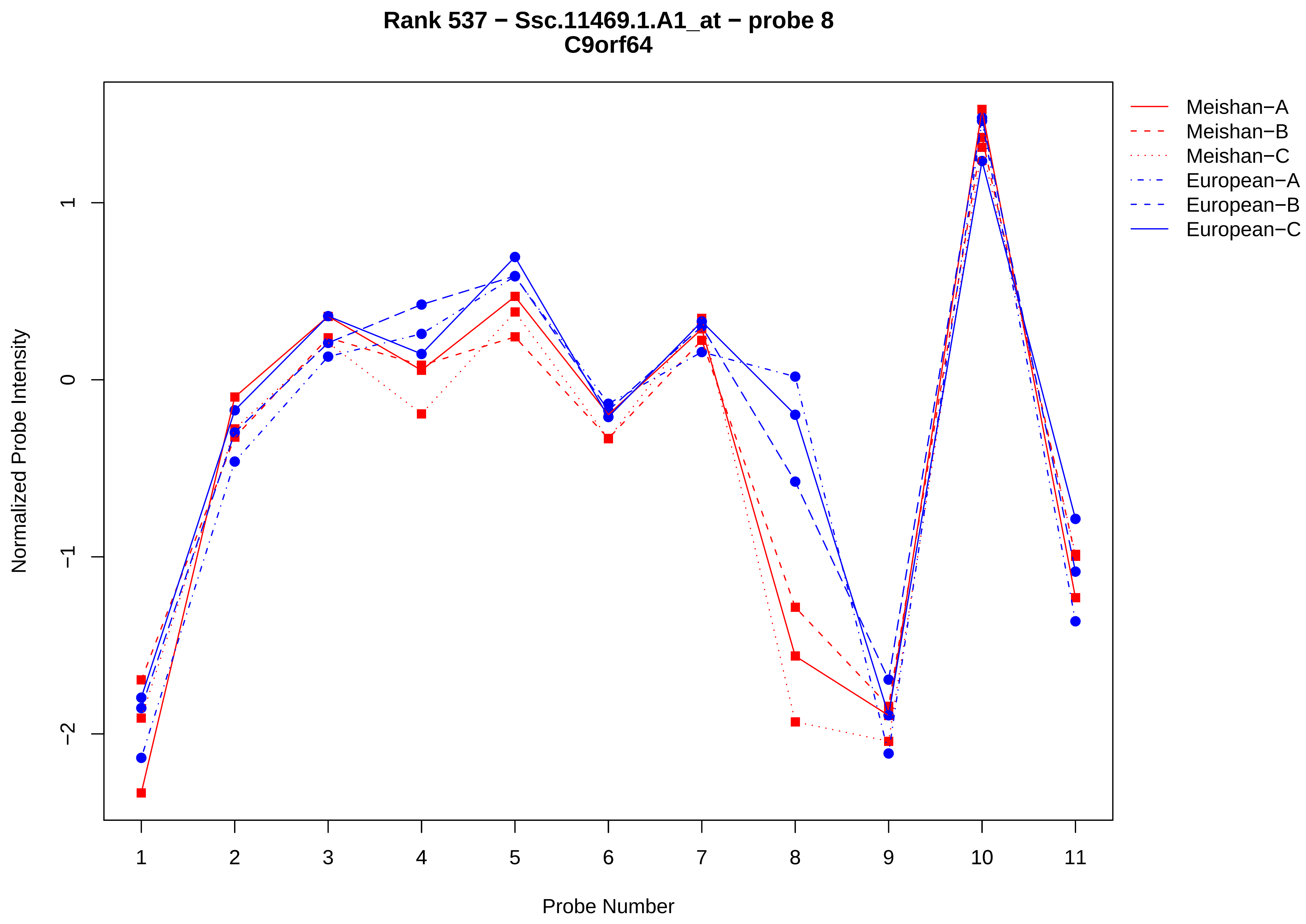

Rank 537 − Ssc.11469.1.A1_at − probe 8
C9orf64
Normalized Probe Intensity
Probe Number
−2
−1
0
1
1
2
3
4
5
6
7
8
9
10
11
Meishan−A
Meishan−B
Meishan−C
European−A
European−B
European−C

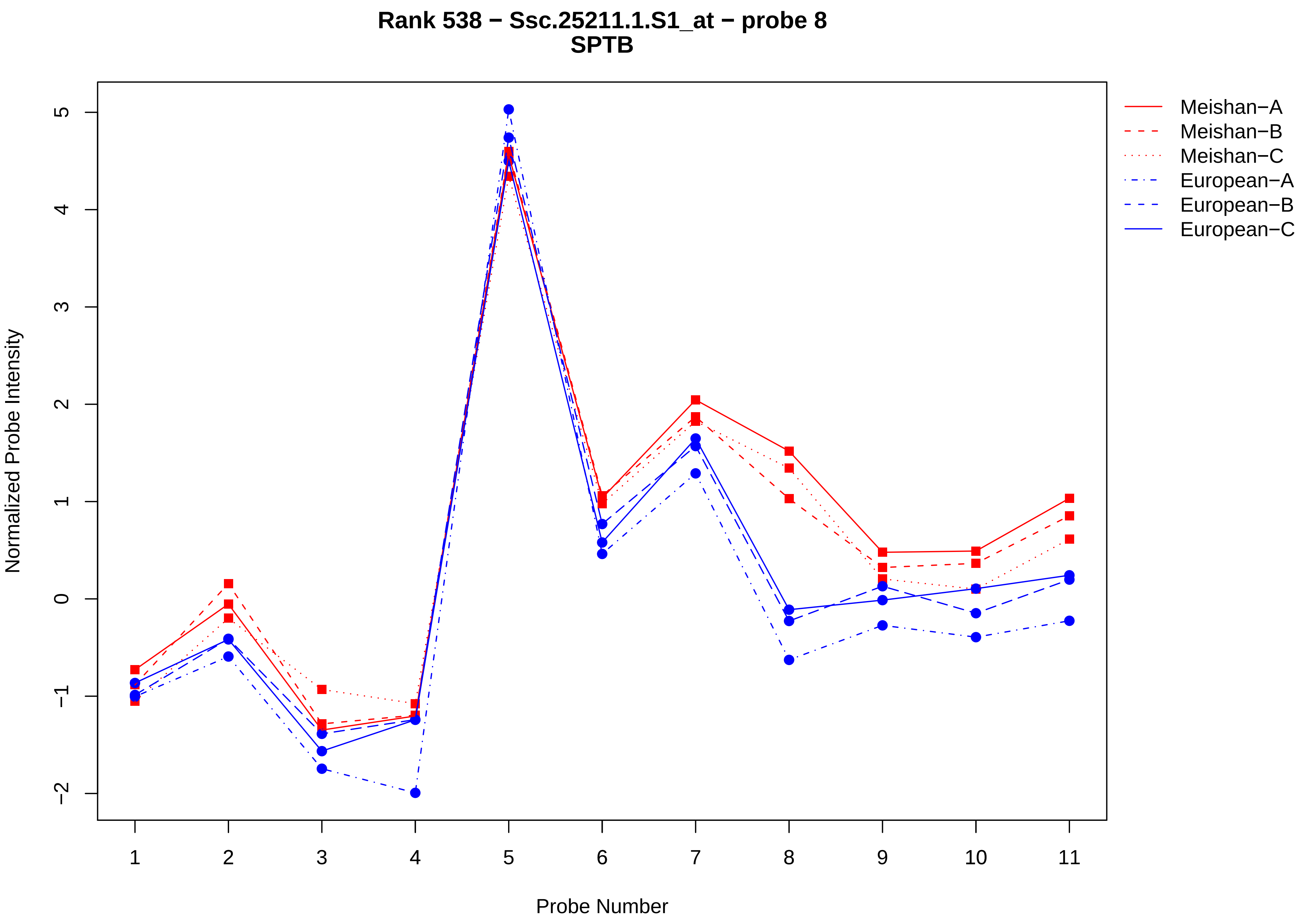

Rank 538 − Ssc.25211.1.S1_at − probe 8
SPTB
Normalized Probe Intensity
Probe Number
Meishan−A
Meishan−B
Meishan−C
European−A
European−B
European−C

Rank 539 − Ssc.19135.1.A1_at − probe 7
O75152

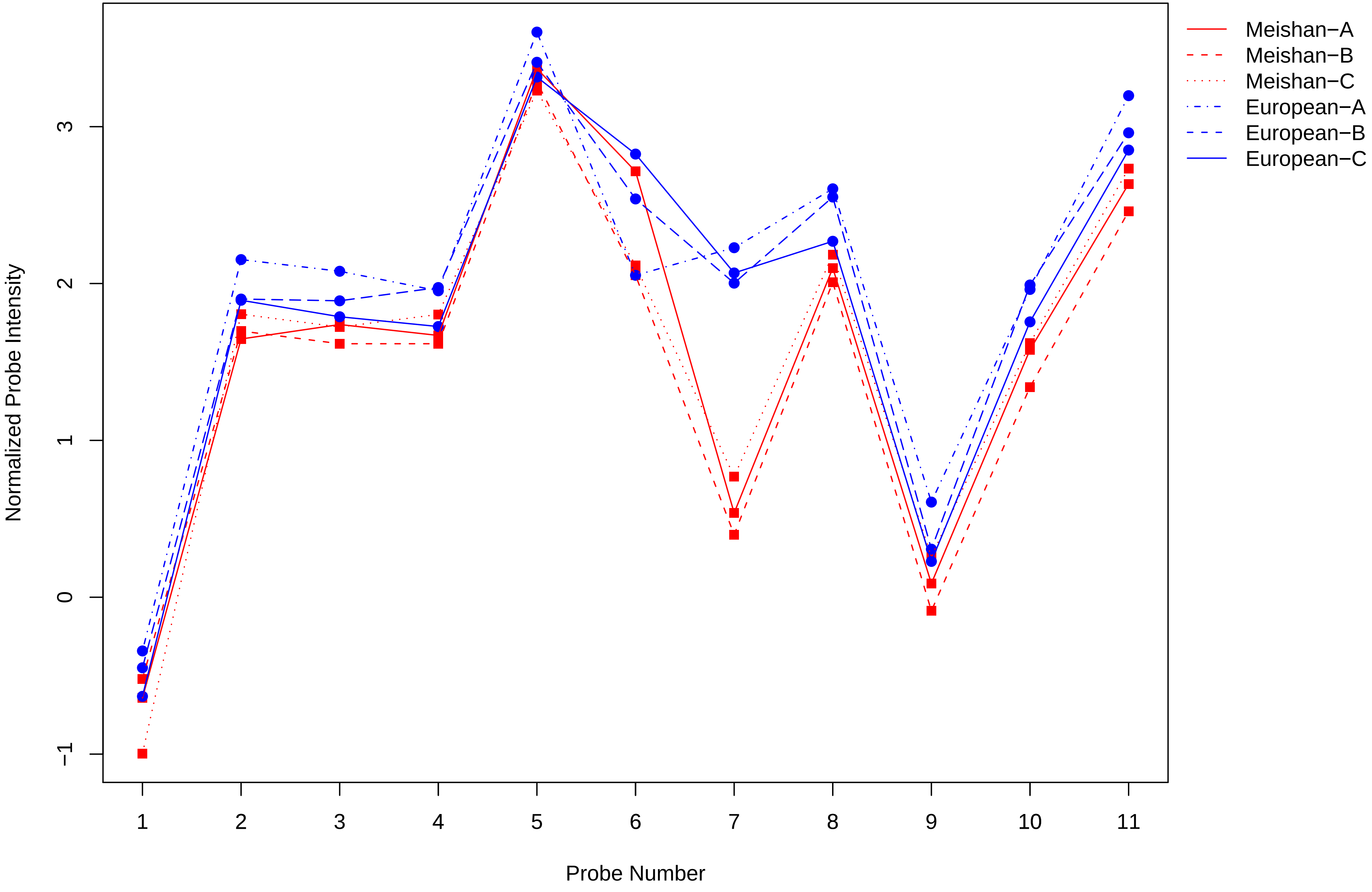

Rank 540 − Ssc.20219.1.S1_at − probe 8
Q9NZJ2

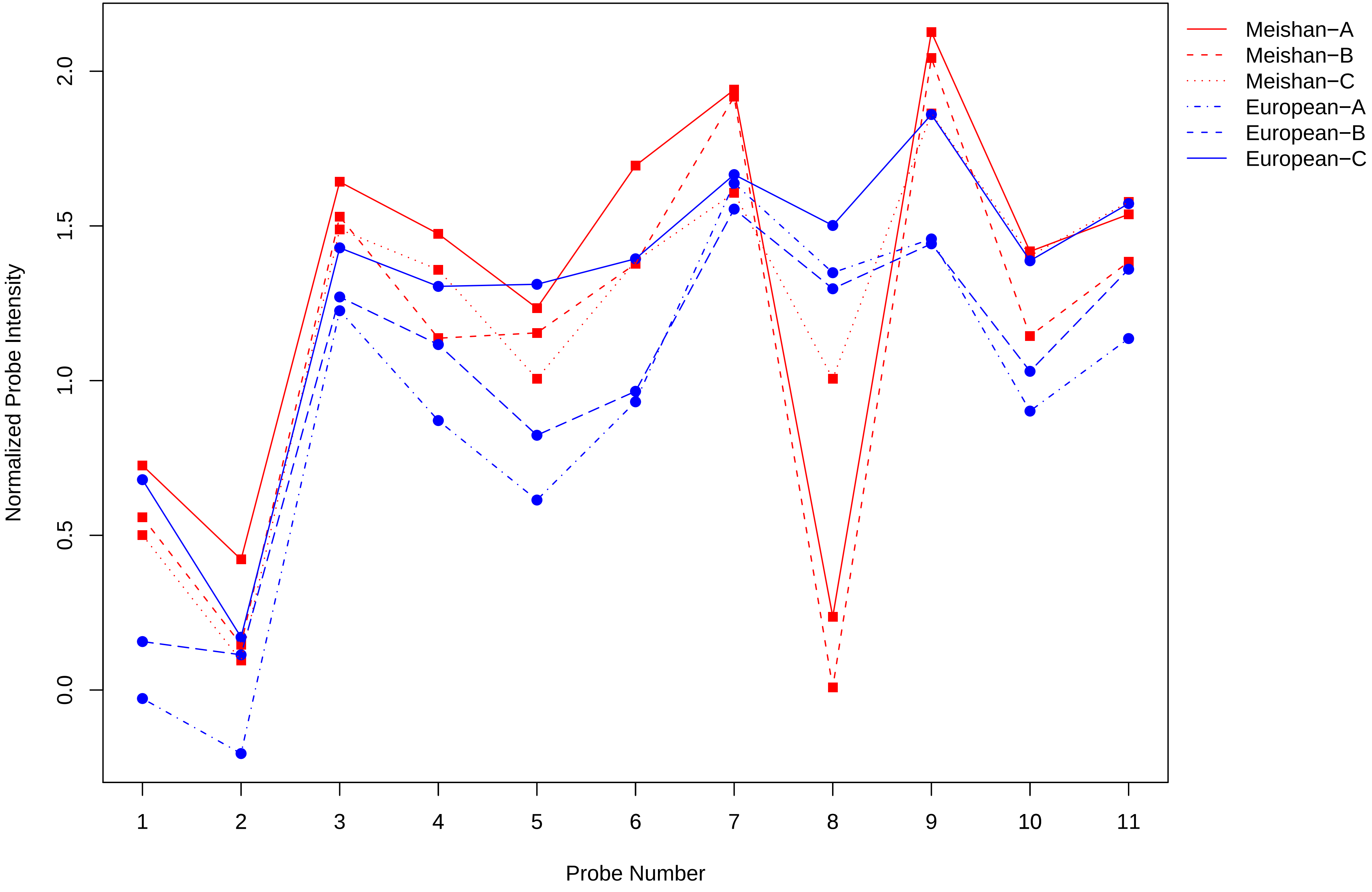

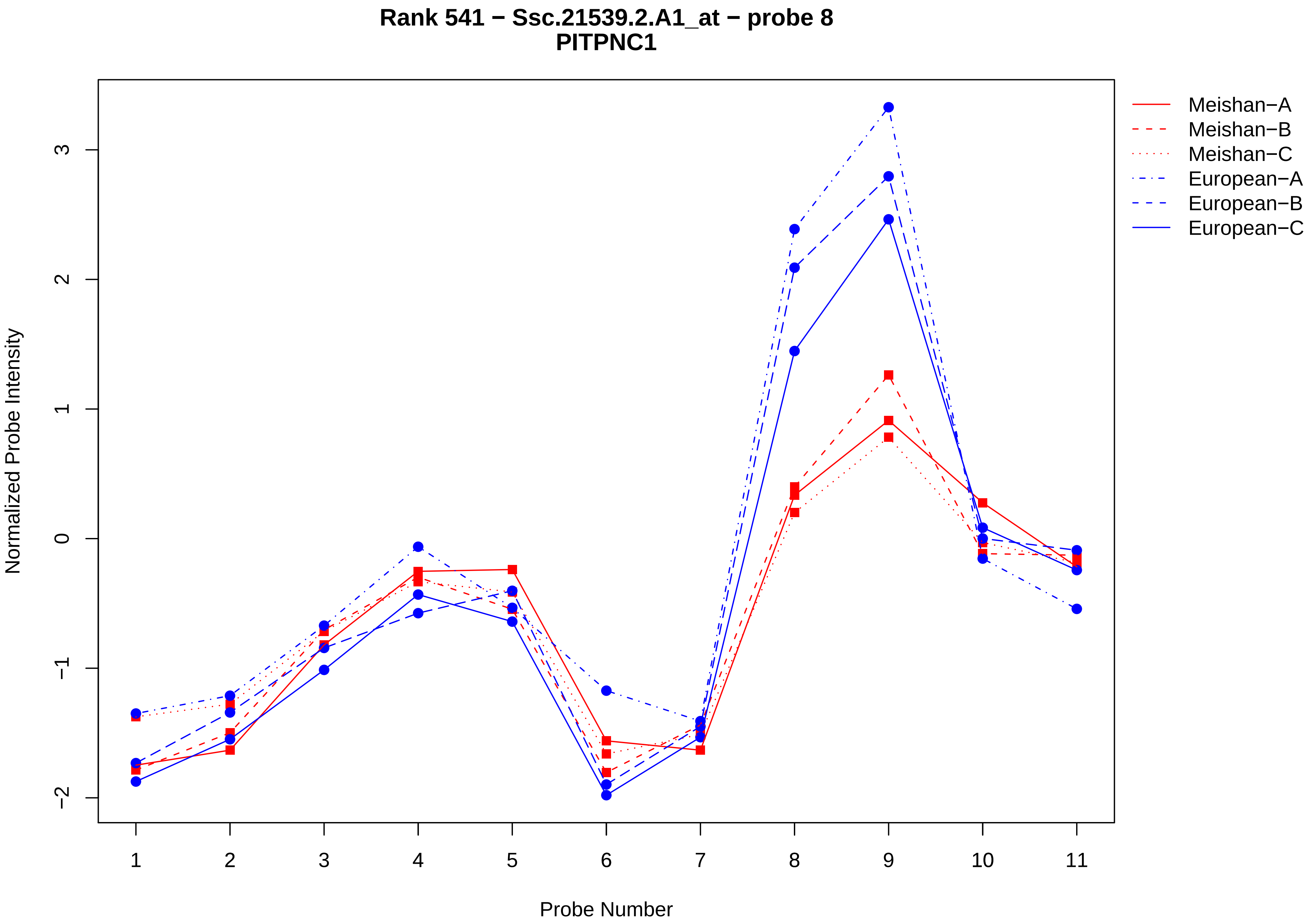

Rank 541 − Ssc.21539.2.A1_at − probe 8
PITPNC1
Normalized Probe Intensity
Probe Number
Meishan−A
Meishan−B
Meishan−C
European−A
European−B
European−C
−2
−1
0
1
2
3
1
2
3
4
5
6
7
8
9
10
11

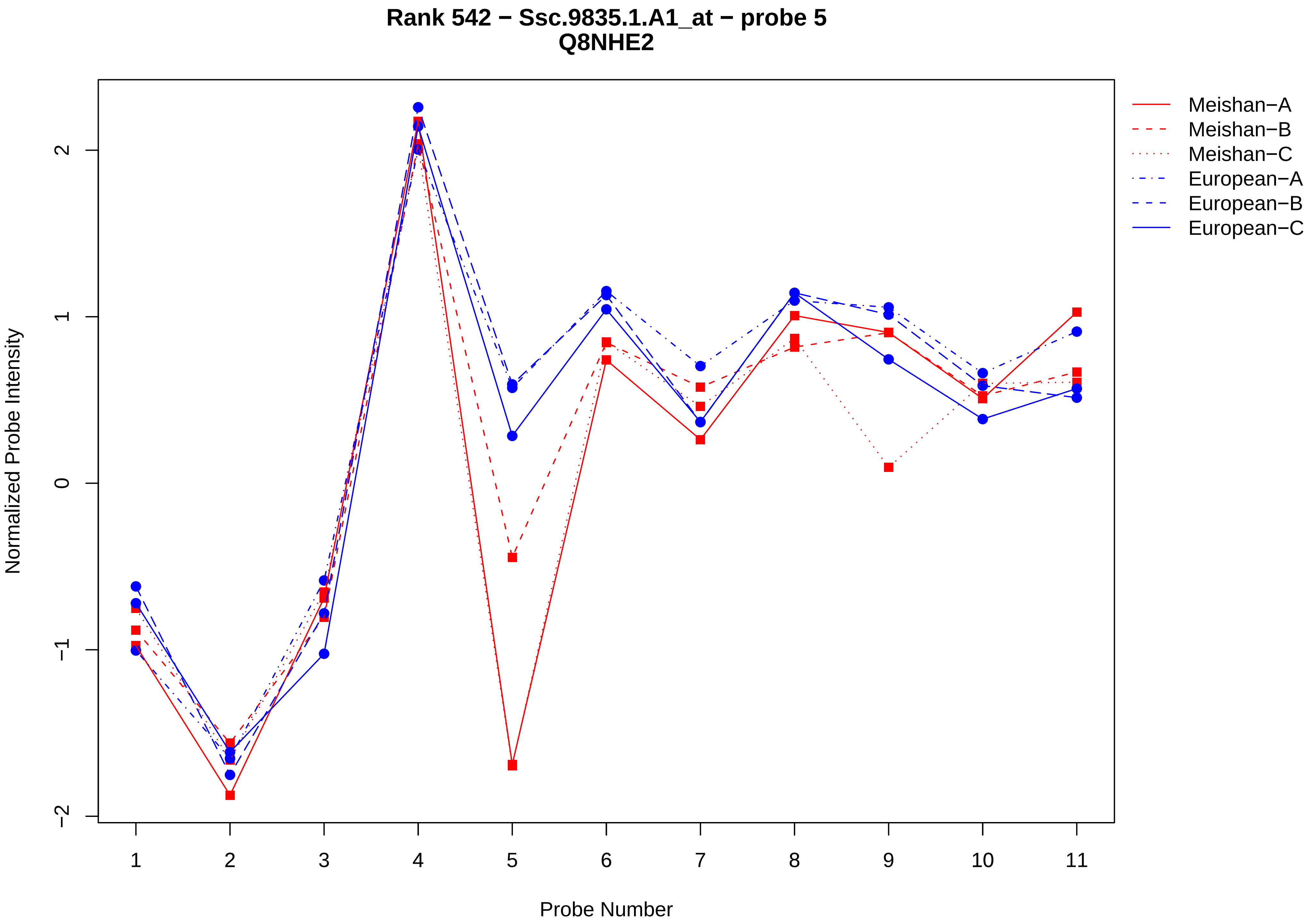

Rank 542 − Ssc.9835.1.A1_at − probe 5
Q8NHE2
Normalized Probe Intensity
Probe Number
−2
−1
0
1
2
1
2
3
4
5
6
7
8
9
10
11
Meishan−A
Meishan−B
Meishan−C
European−A
European−B
European−C

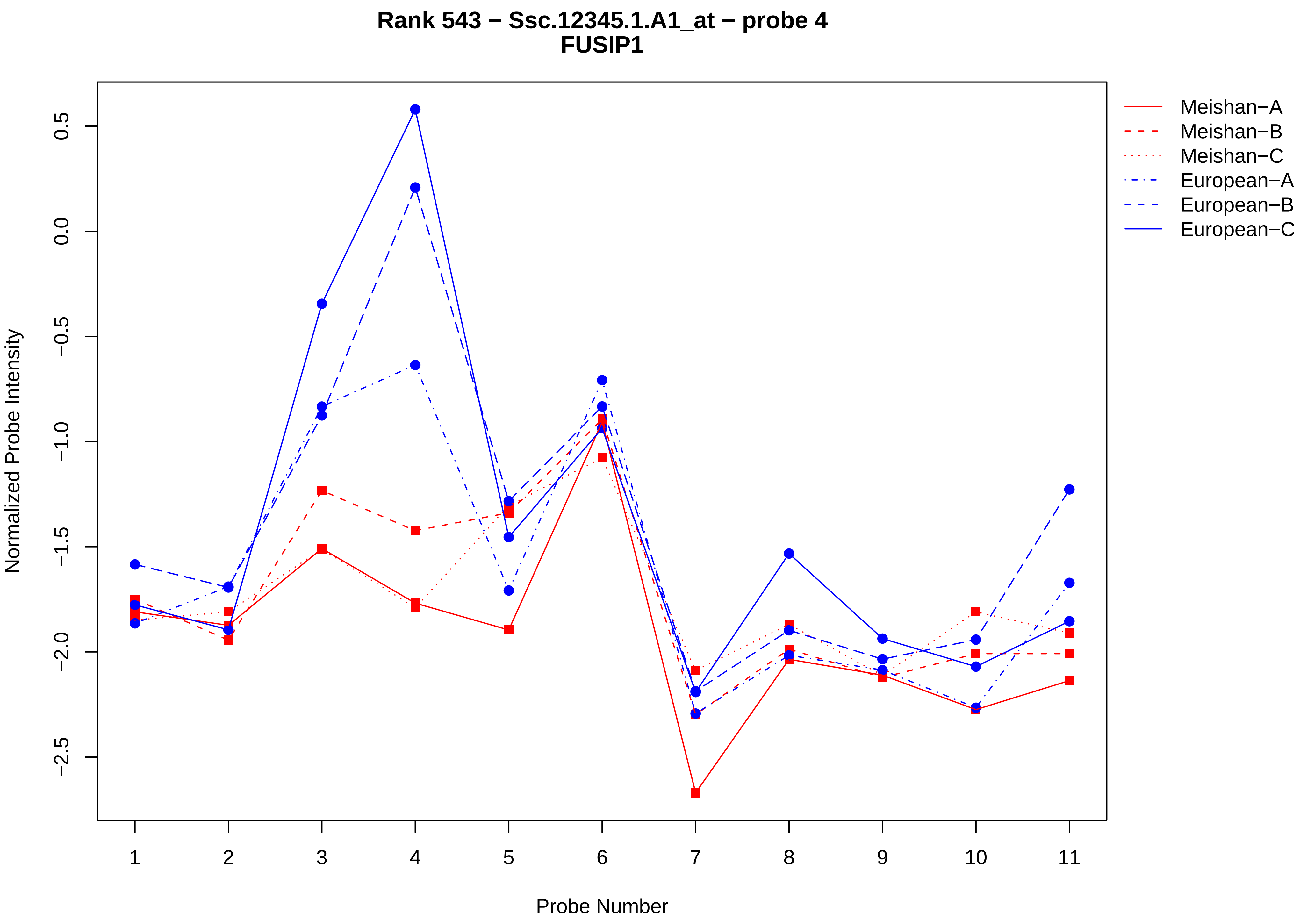

Rank 543 − Ssc.12345.1.A1_at − probe 4
FUSIP1
Normalized Probe Intensity
Probe Number
Meishan−A
Meishan−B
Meishan−C
European−A
European−B
European−C

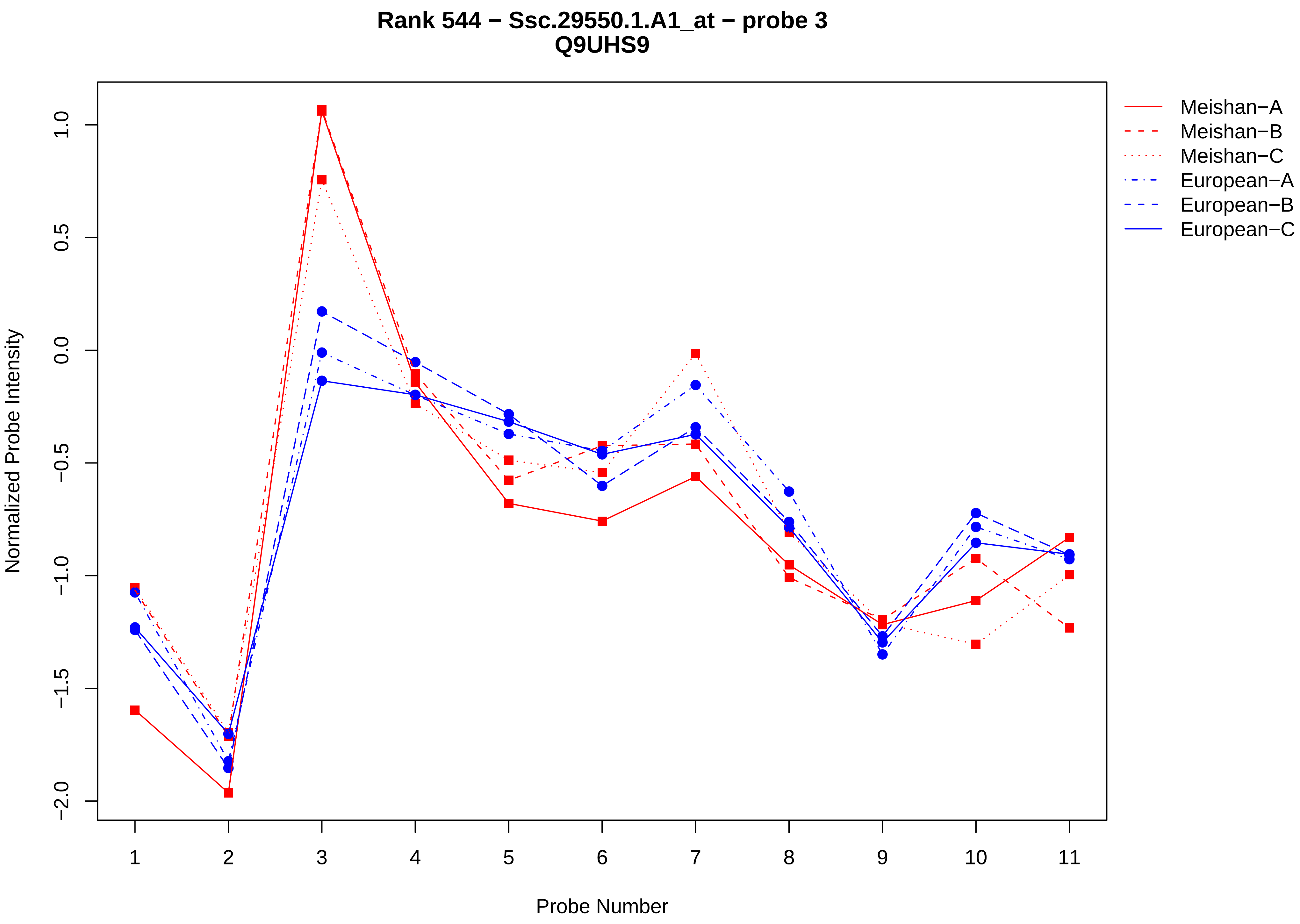

Rank 544 − Ssc.29550.1.A1_at − probe 3
Q9UHS9
Normalized Probe Intensity
Probe Number
1.0
0.5
0.0
−0.5
−1.0
−1.5
−2.0
1
2
3
4
5
6
7
8
9
10
11
Meishan−A
Meishan−B
Meishan−C
European−A
European−B
European−C

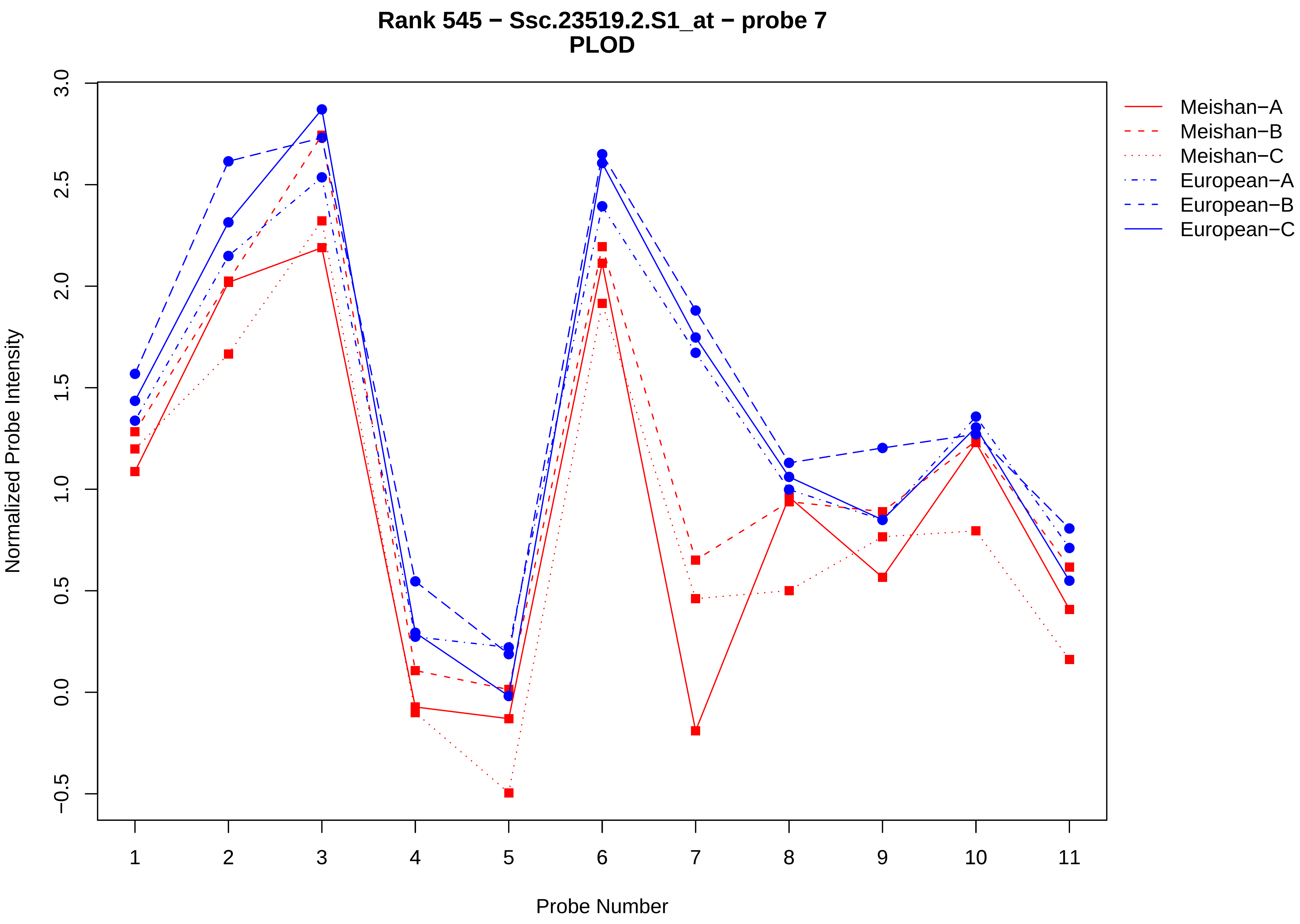

Rank 545 − Ssc.23519.2.S1_at − probe 7
PLOD
Normalized Probe Intensity
−0.5
0.0
0.5
1.0
1.5
2.0
2.5
3.0
1
2
3
4
5
6
7
8
9
10
11
Probe Number
Meishan−A
Meishan−B
Meishan−C
European−A
European−B
European−C

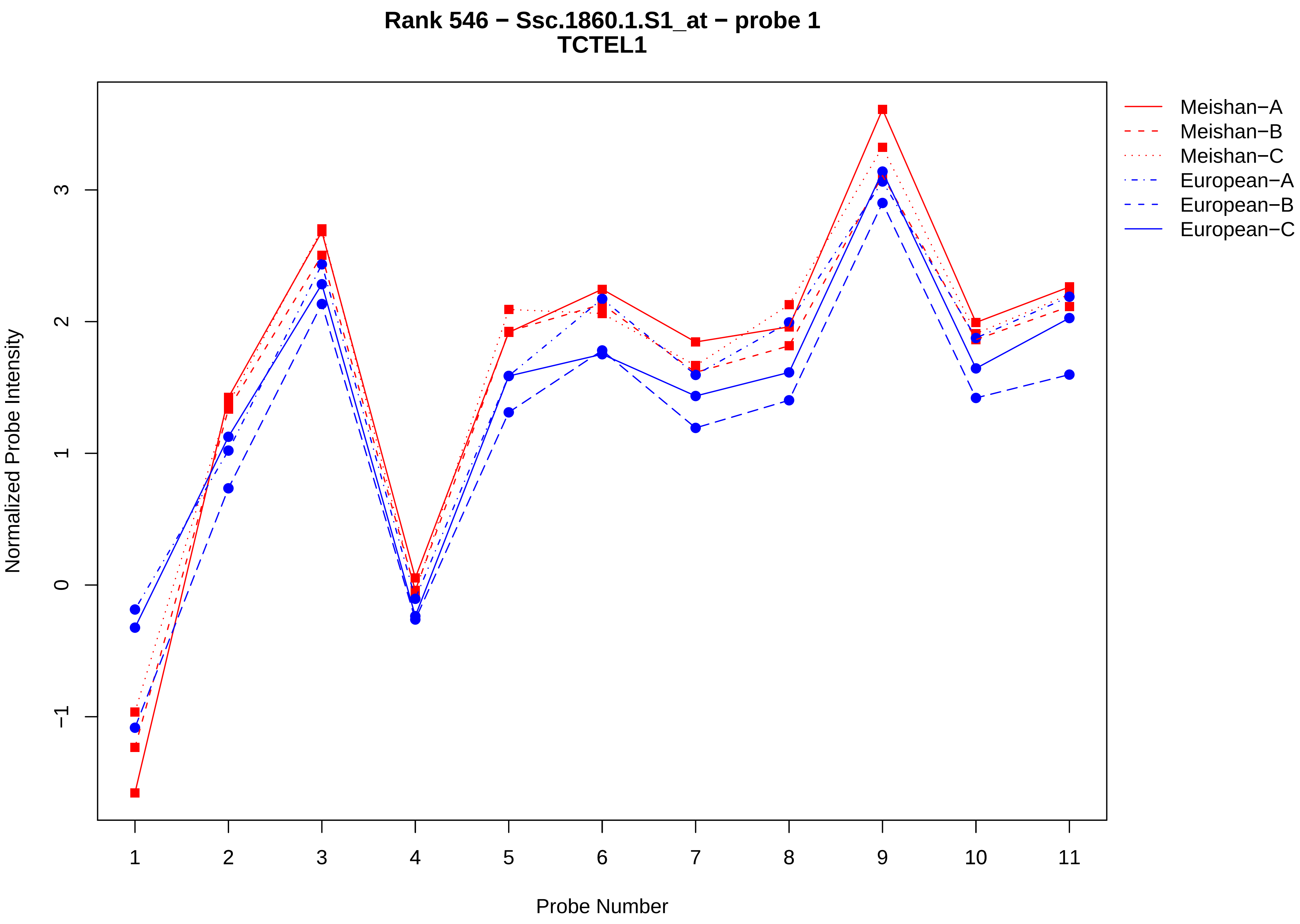

Rank 546 − Ssc.1860.1.S1_at − probe 1
TCTEL1
Normalized Probe Intensity
Probe Number
−1
0
1
2
3
1
2
3
4
5
6
7
8
9
10
11
Meishan−A
Meishan−B
Meishan−C
European−A
European−B
European−C

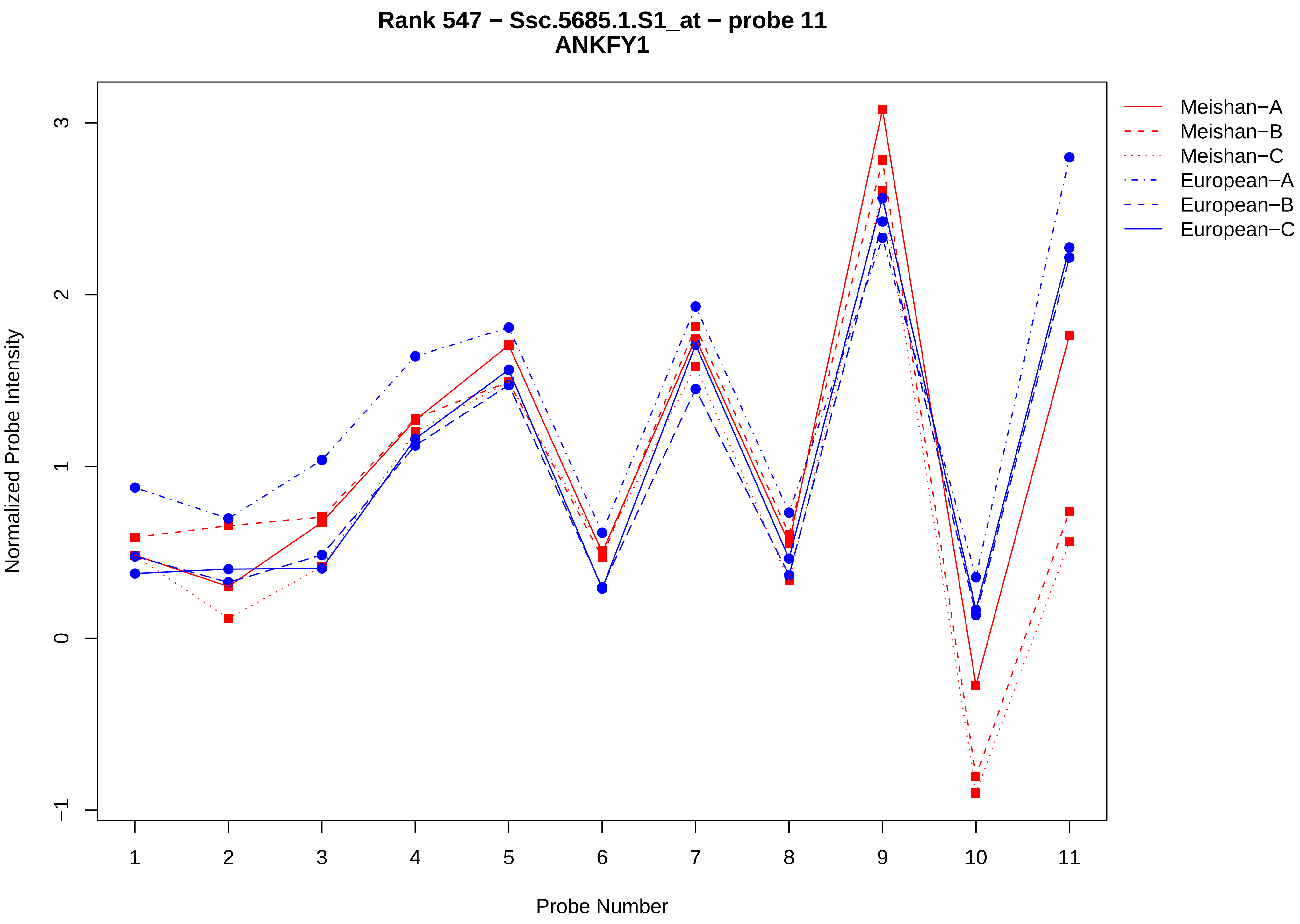

Rank 547 − Ssc.5685.1.S1_at − probe 11
ANKFY1
Normalized Probe Intensity
Probe Number
−1
0
1
2
3
1
2
3
4
5
6
7
8
9
10
11
Meishan−A
Meishan−B
Meishan−C
European−A
European−B
European−C

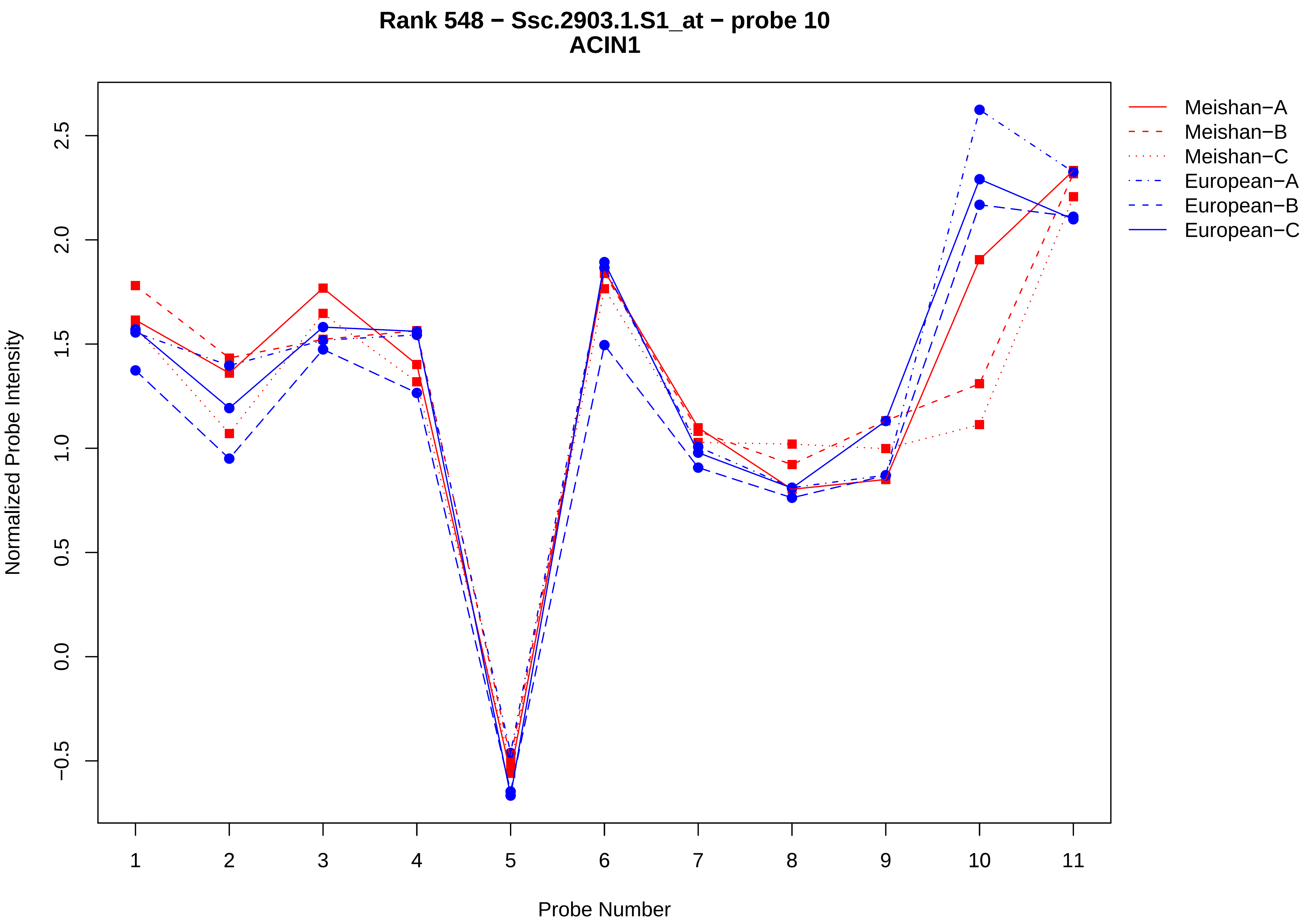

Rank 548 − Ssc.2903.1.S1_at − probe 10
ACIN1
Normalized Probe Intensity
Probe Number
−0.5
0.0
0.5
1.0
1.5
2.0
2.5
1
2
3
4
5
6
7
8
9
10
11
Meishan−A
Meishan−B
Meishan−C
European−A
European−B
European−C

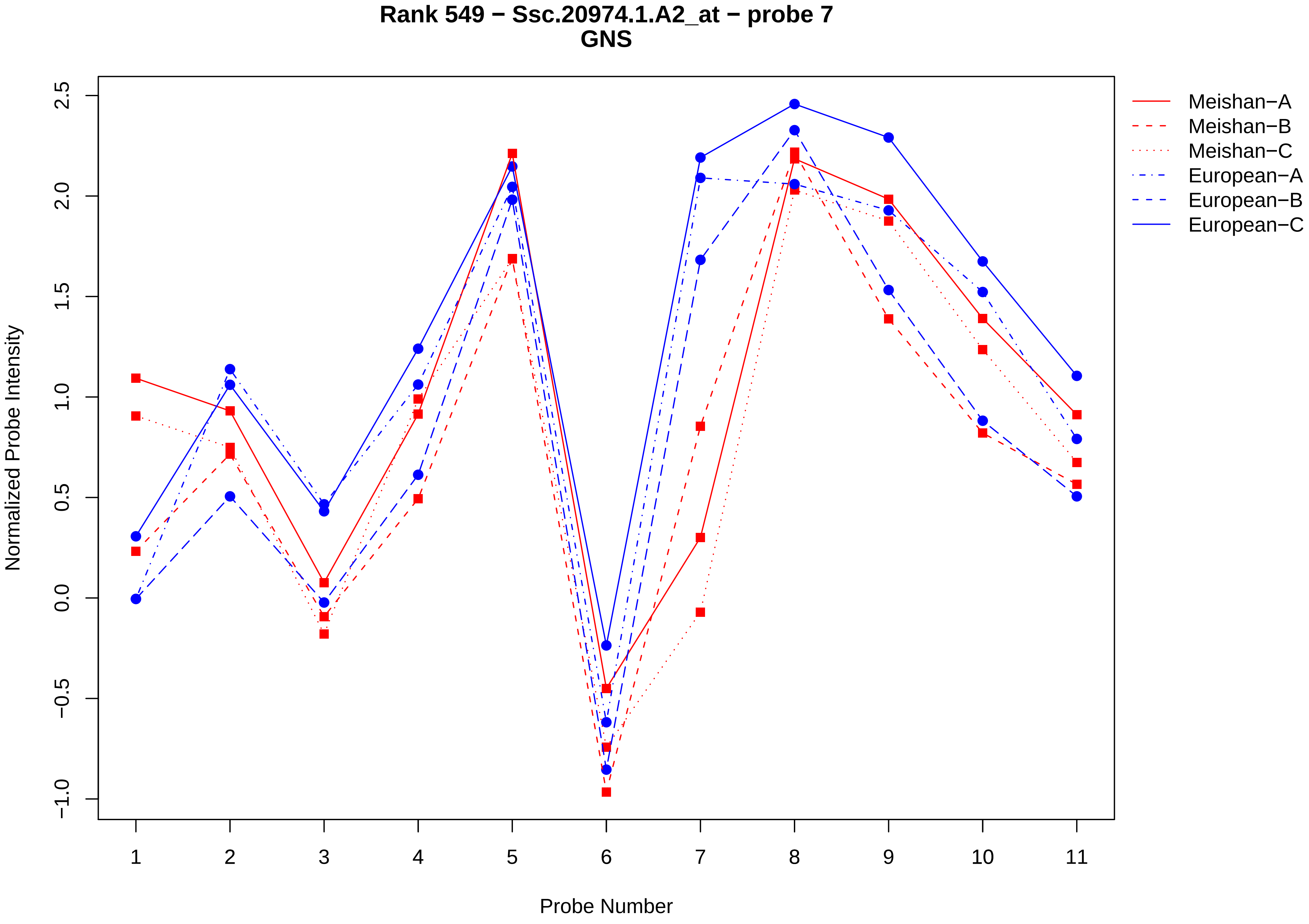
Rank 549 − Ssc.20974.1.A2_at − probe 7
GNS
Normalized Probe Intensity
Probe Number
Meishan−A
Meishan−B
Meishan−C
European−A
European−B
European−C
−1.0
−0.5
0.0
0.5
1.0
1.5
2.0
2.5
1
2
3
4
5
6
7
8
9
10
11

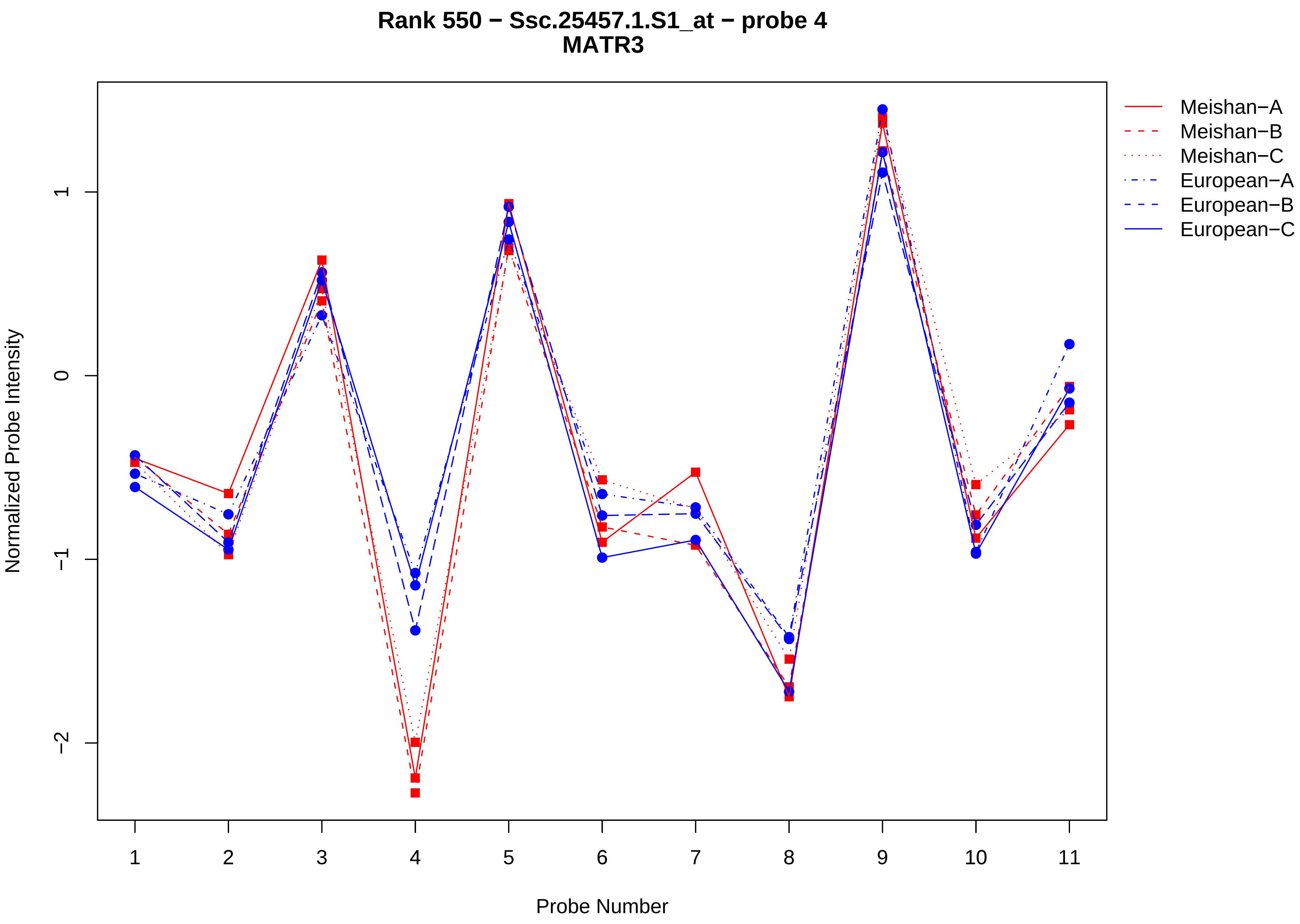

Rank 550 − Ssc.25457.1.S1_at − probe 4
MATR3
Normalized Probe Intensity
Probe Number
−2
−1
0
1
1
2
3
4
5
6
7
8
9
10
11
Meishan−A
Meishan−B
Meishan−C
European−A
European−B
European−C

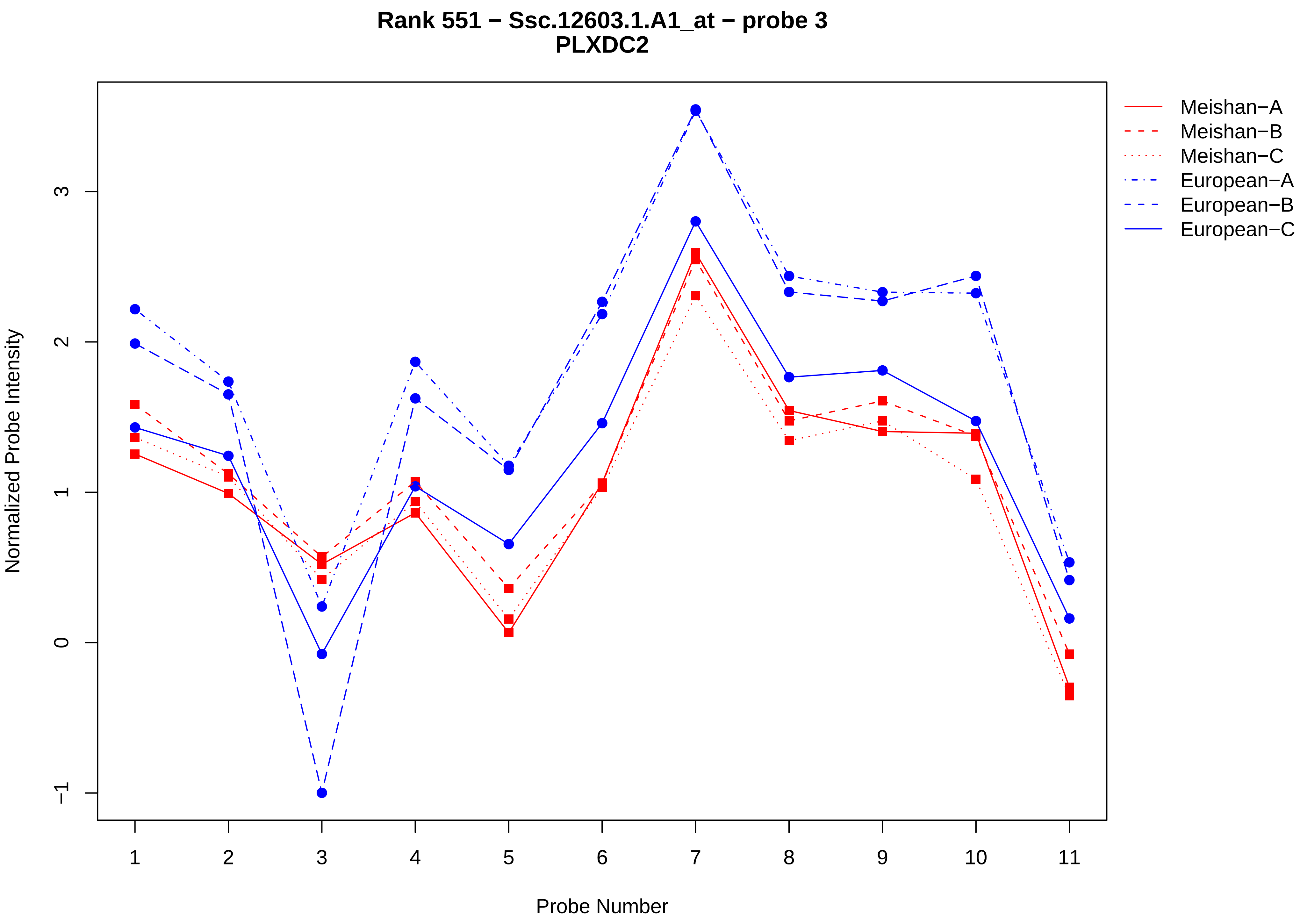
Rank 551 − Ssc.12603.1.A1_at − probe 3
PLXDC2
Normalized Probe Intensity
Probe Number
Meishan−A
Meishan−B
Meishan−C
European−A
European−B
European−C
−1
0
1
2
3
1
2
3
4
5
6
7
8
9
10
11

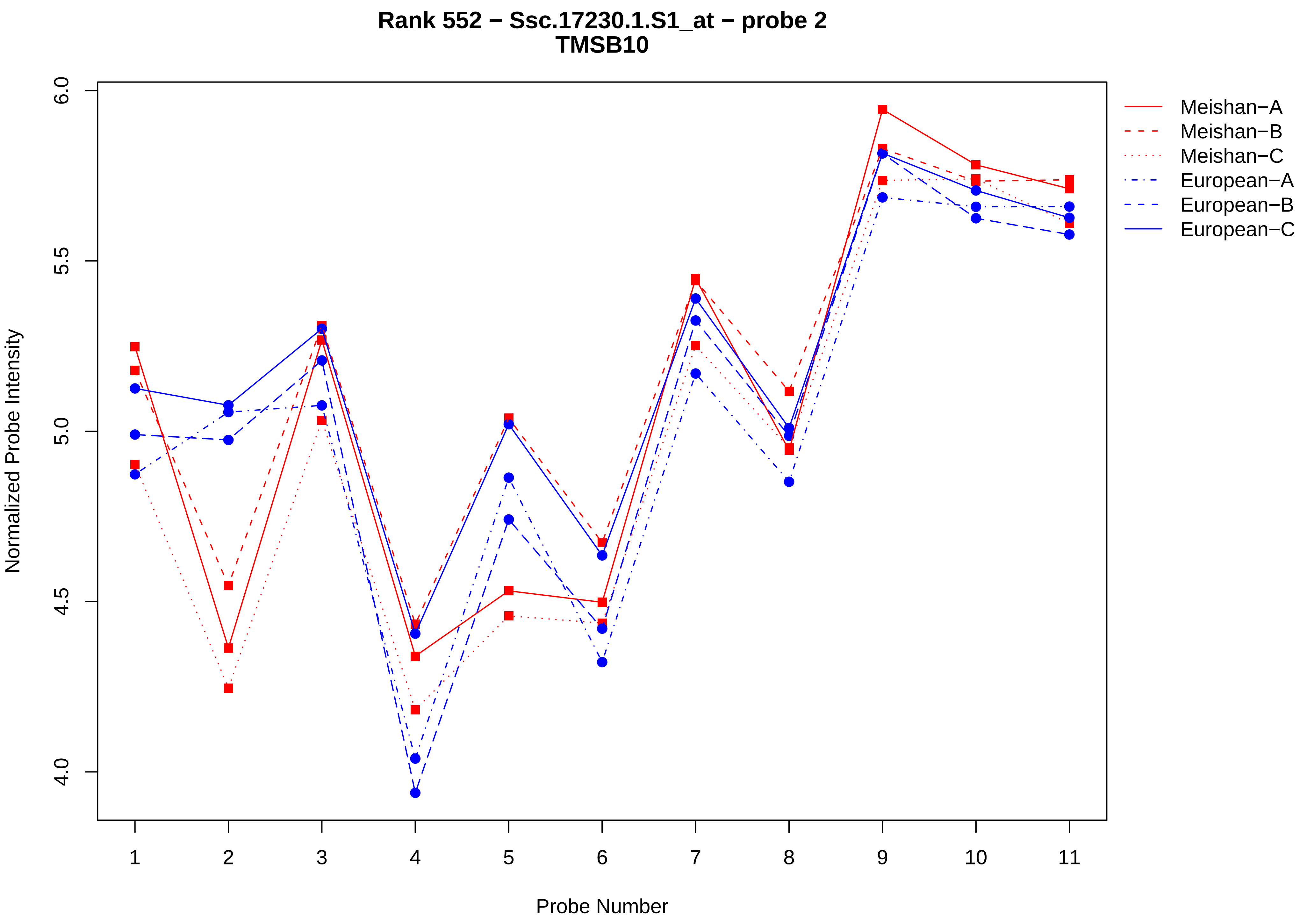
Rank 552 − Ssc.17230.1.S1_at − probe 2
TMSB10
Normalized Probe Intensity
Probe Number
4.0
4.5
5.0
5.5
6.0
1
2
3
4
5
6
7
8
9
10
11
Meishan−A
Meishan−B
Meishan−C
European−A
European−B
European−C

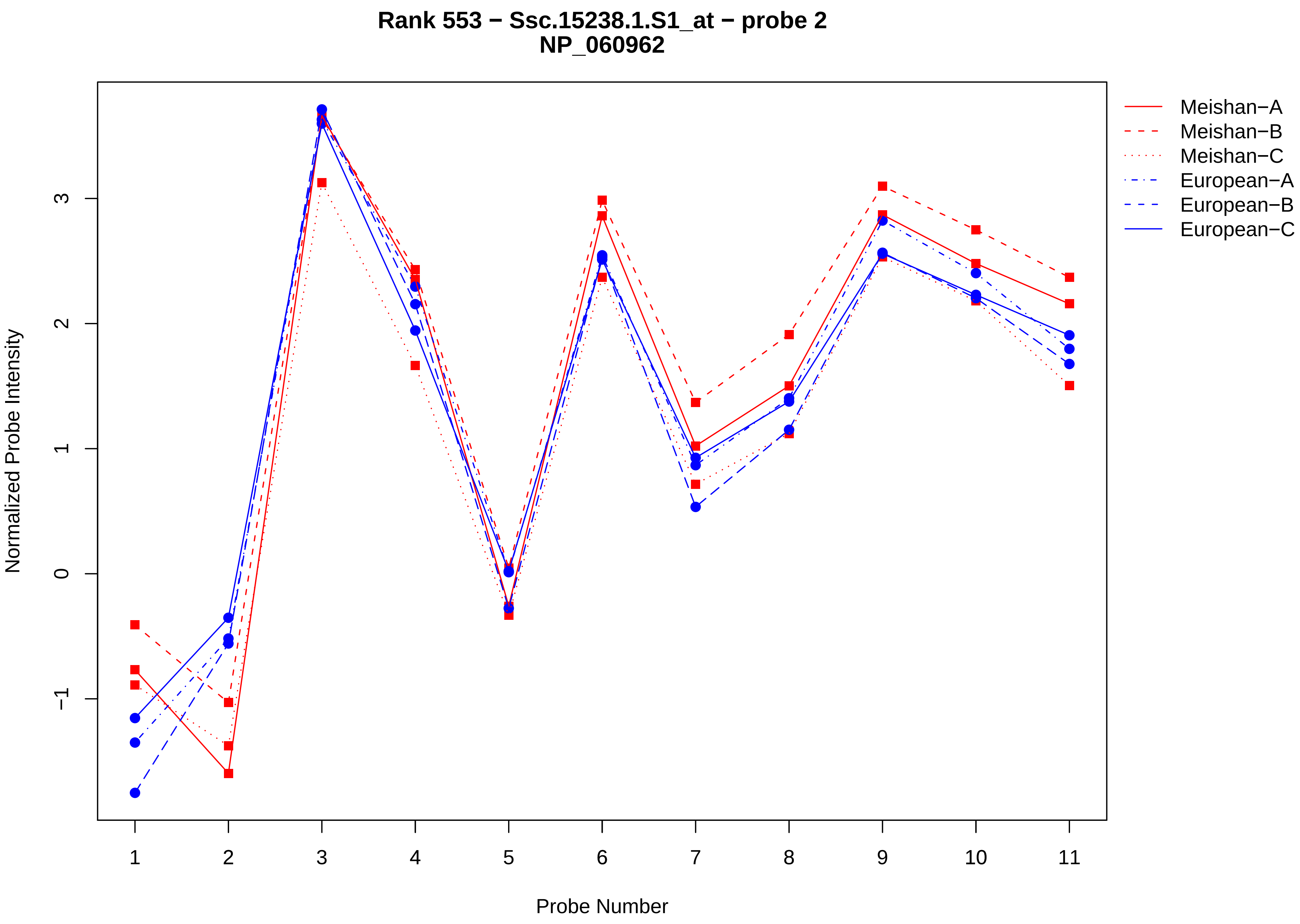
Rank 553 − Ssc.15238.1.S1_at − probe 2
NP_060962
Normalized Probe Intensity
Probe Number
Meishan−A
Meishan−B
Meishan−C
European−A
European−B
European−C
−1
0
1
2
3
1
2
3
4
5
6
7
8
9
10
11

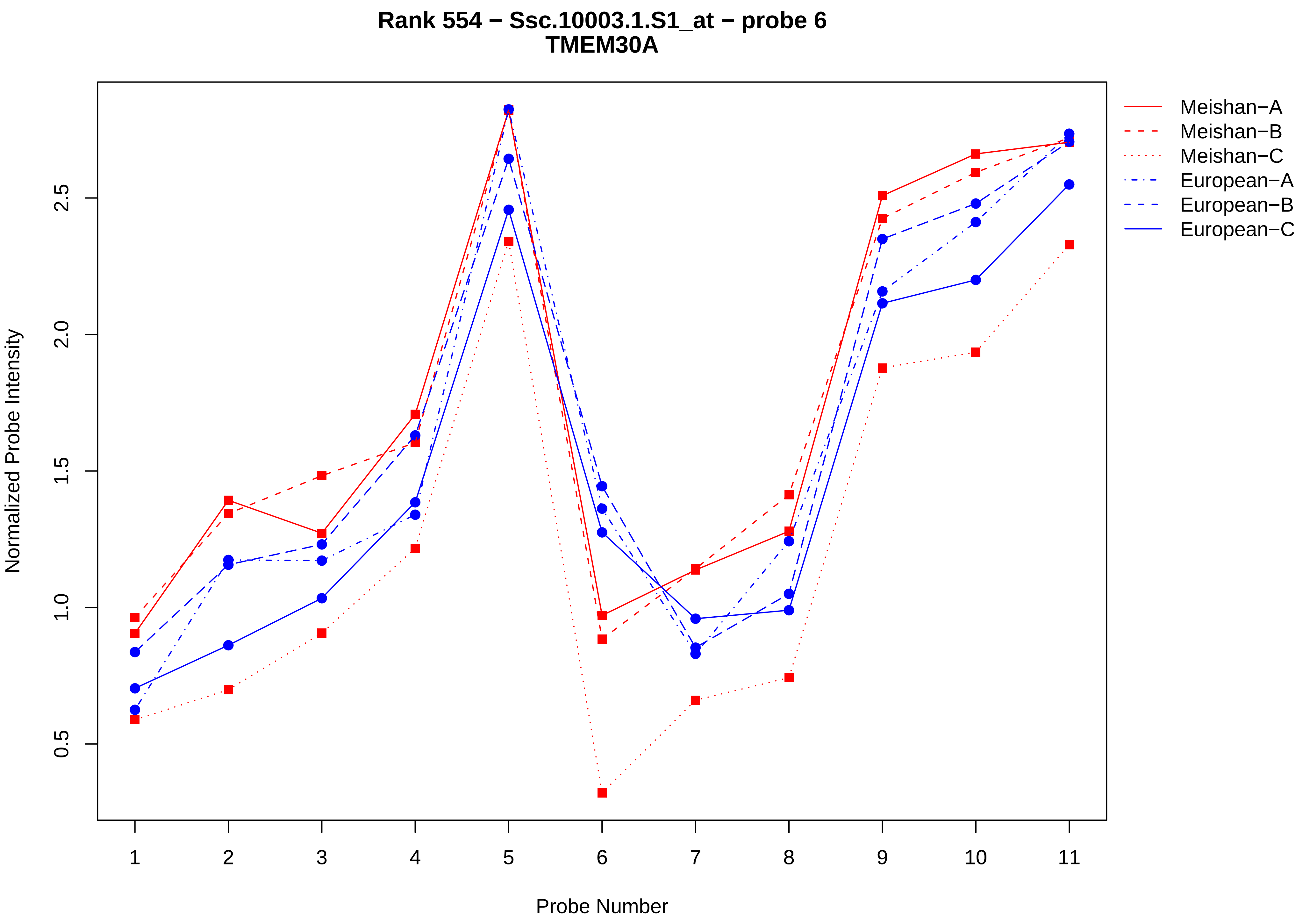

Rank 554 − Ssc.10003.1.S1_at − probe 6
TMEM30A
Normalized Probe Intensity
0.5
1.0
1.5
2.0
2.5
1
2
3
4
5
6
7
8
9
10
11
Probe Number
Meishan−A
Meishan−B
Meishan−C
European−A
European−B
European−C

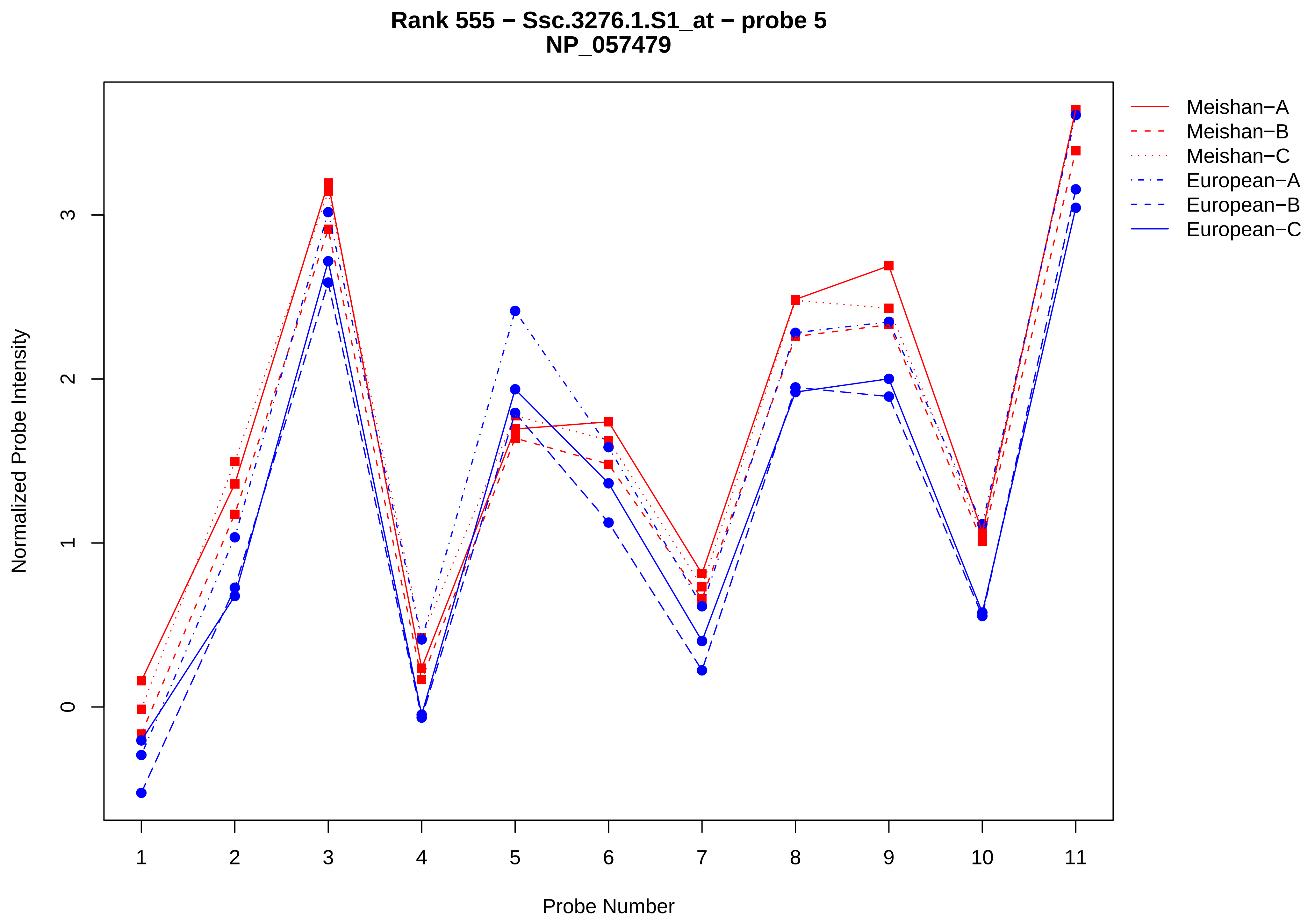

Rank 555 − Ssc.3276.1.S1_at − probe 5
NP_057479
Normalized Probe Intensity
Probe Number
0
1
2
3
1
2
3
4
5
6
7
8
9
10
11
Meishan−A
Meishan−B
Meishan−C
European−A
European−B
European−C

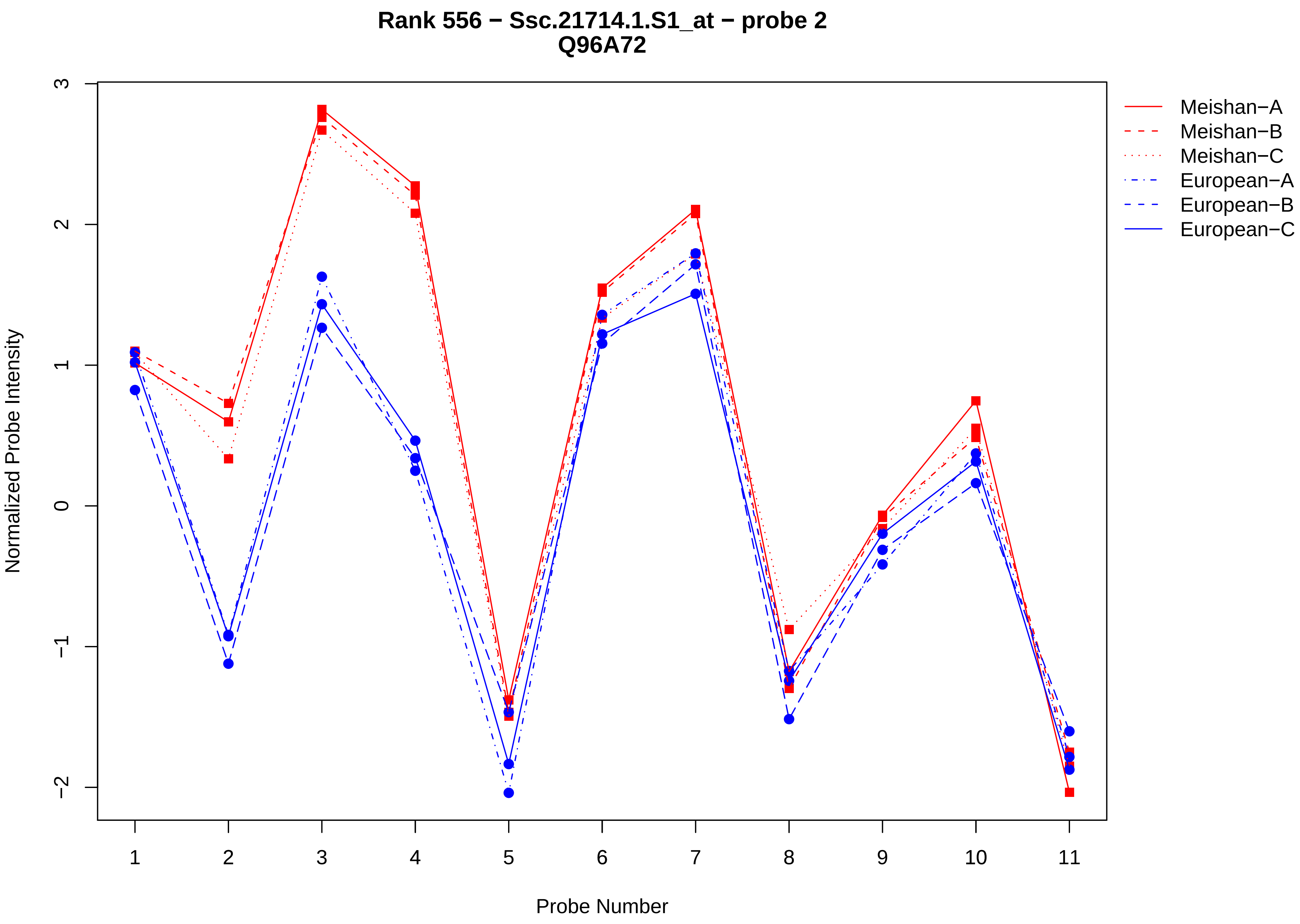
Rank 556 − Ssc.21714.1.S1_at − probe 2
Q96A72
Normalized Probe Intensity
Probe Number
−2
−1
0
1
2
3
1
2
3
4
5
6
7
8
9
10
11
Meishan−A
Meishan−B
Meishan−C
European−A
European−B
European−C

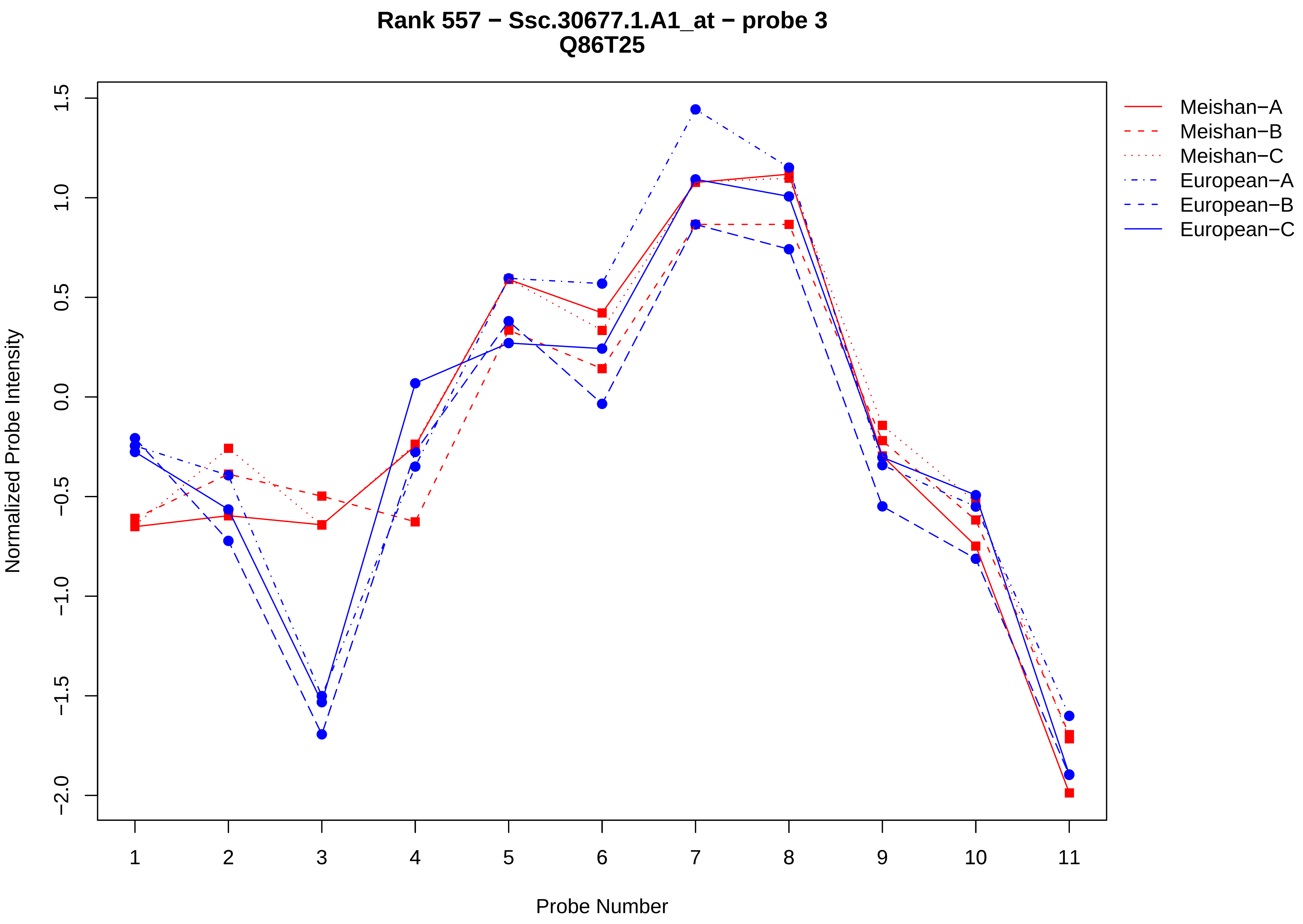
Rank 557 − Ssc.30677.1.A1_at − probe 3
Q86T25
Normalized Probe Intensity
Probe Number
1.5
1.0
0.5
0.0
−0.5
−1.0
−1.5
−2.0
1
2
3
4
5
6
7
8
9
10
11
Meishan−A
Meishan−B
Meishan−C
European−A
European−B
European−C

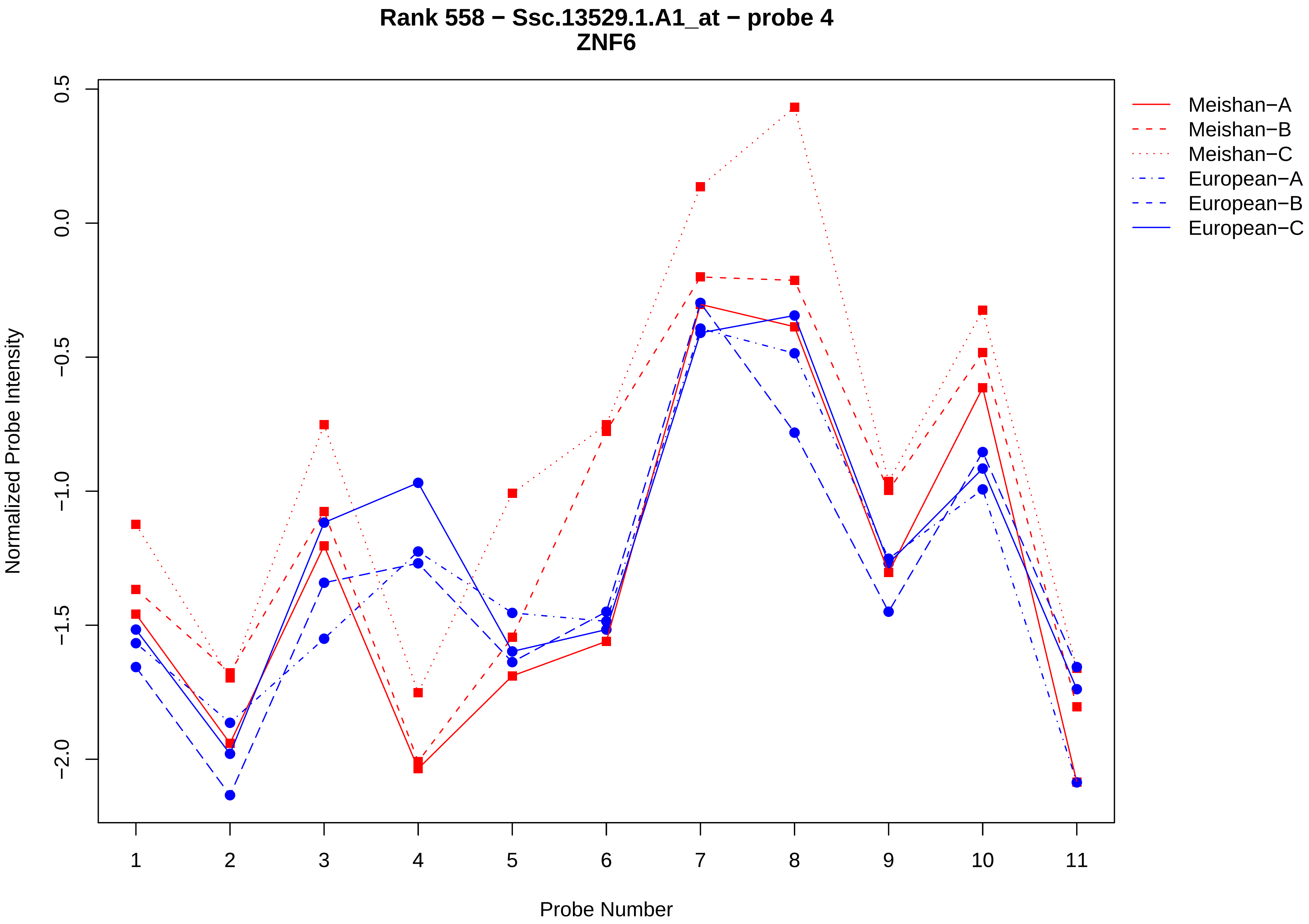

Rank 558 − Ssc.13529.1.A1_at − probe 4
ZNF6
Normalized Probe Intensity
Probe Number
0.5
0.0
−0.5
−1.0
−1.5
−2.0
1
2
3
4
5
6
7
8
9
10
11
Meishan−A
Meishan−B
Meishan−C
European−A
European−B
European−C

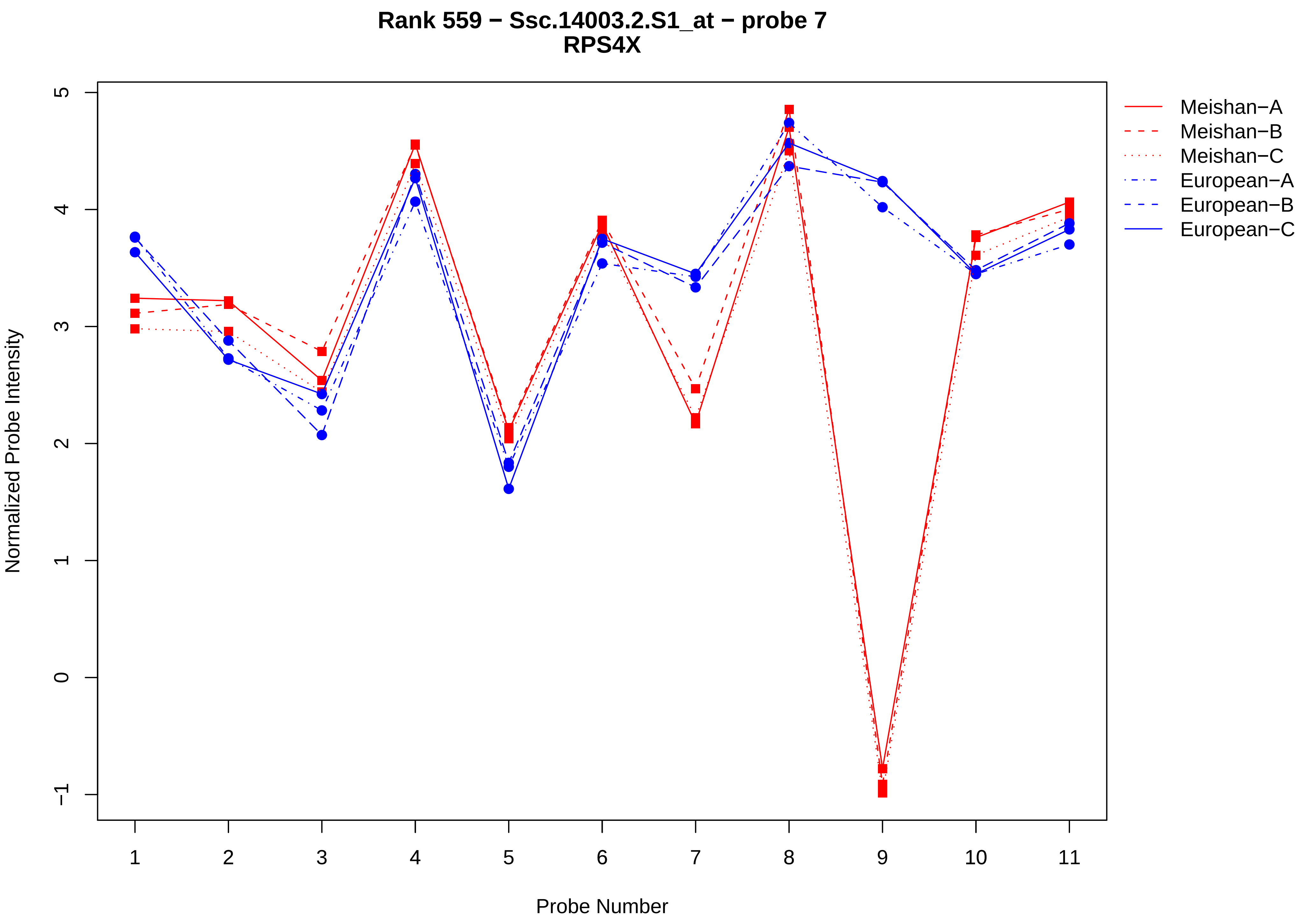

Rank 559 − Ssc.14003.2.S1_at − probe 7
RPS4X
Normalized Probe Intensity
Probe Number
-1
0
1
2
3
4
5
1
2
3
4
5
6
7
8
9
10
11
Meishan−A
Meishan−B
Meishan−C
European−A
European−B
European−C

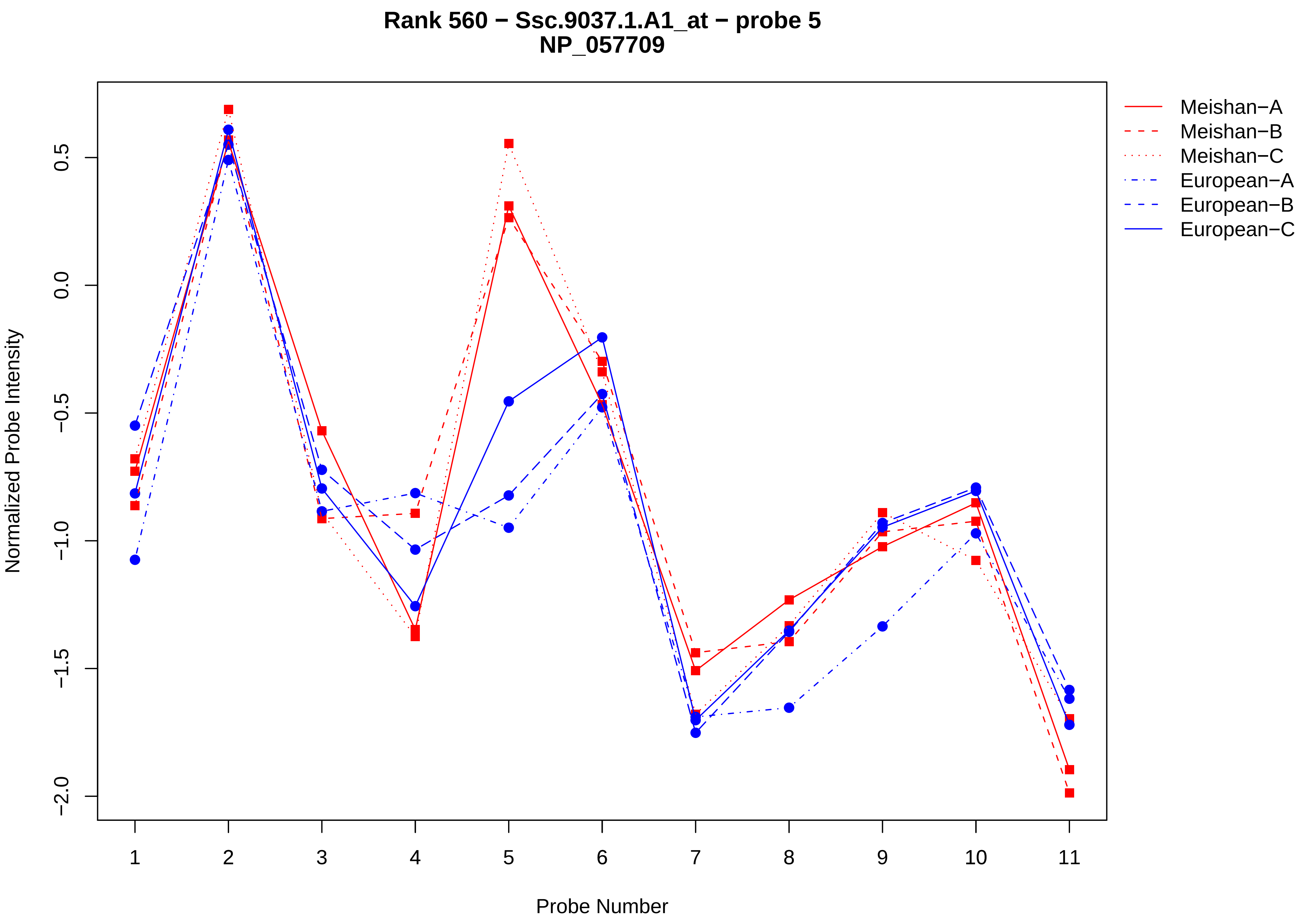

Rank 560 − Ssc.9037.1.A1_at − probe 5
NP_057709
Normalized Probe Intensity
Probe Number
0.5
0.0
−0.5
−1.0
−1.5
−2.0
1
2
3
4
5
6
7
8
9
10
11
Meishan−A
Meishan−B
Meishan−C
European−A
European−B
European−C

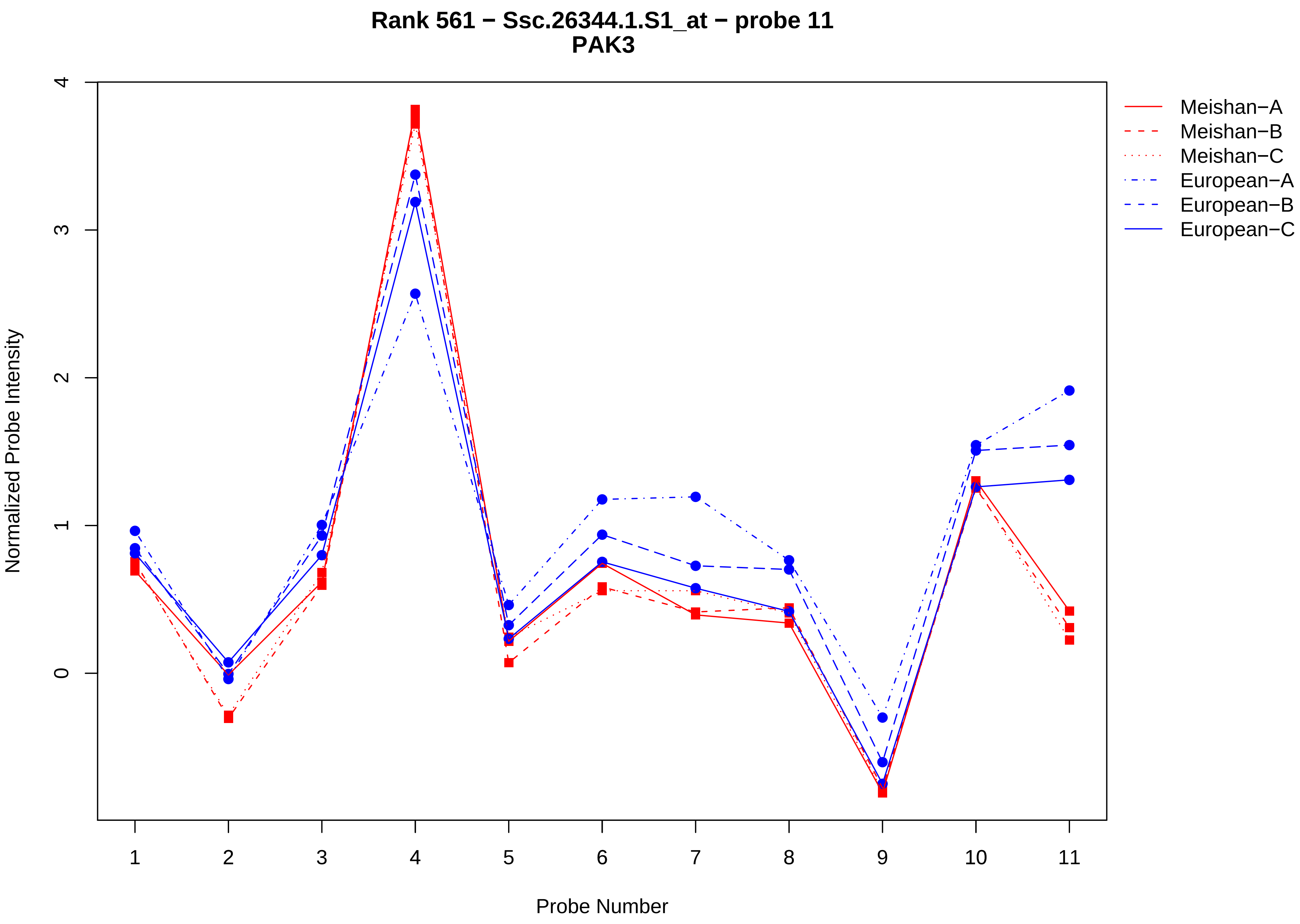

Rank 561 − Ssc.26344.1.S1_at − probe 11
PAK3
Normalized Probe Intensity
Probe Number
0
1
2
3
4
1
2
3
4
5
6
7
8
9
10
11
Meishan−A
Meishan−B
Meishan−C
European−A
European−B
European−C

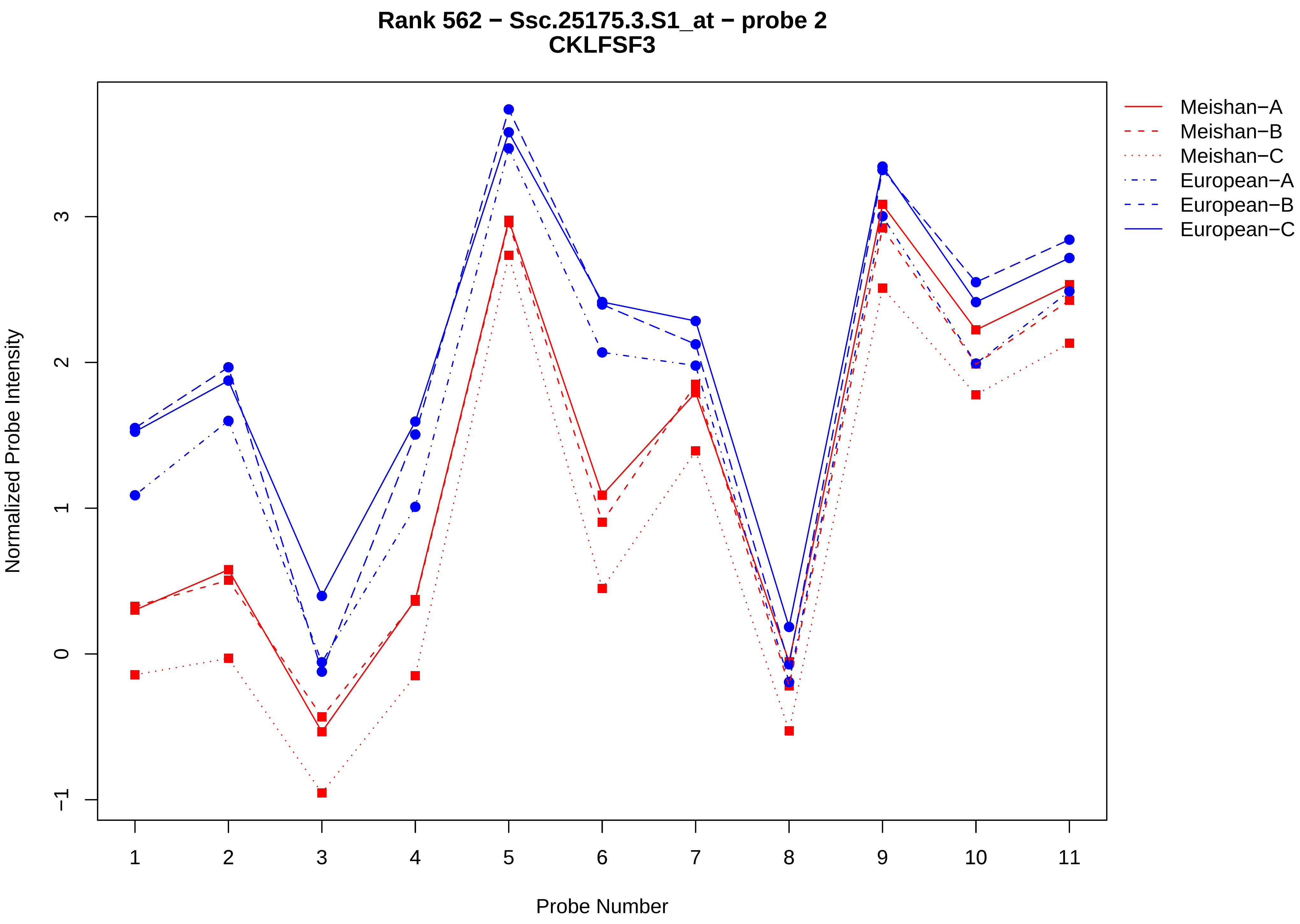

Rank 562 − Ssc.25175.3.S1_at − probe 2
CKLFSF3
Normalized Probe Intensity
Probe Number
−1
0
1
2
3
1
2
3
4
5
6
7
8
9
10
11
Meishan−A
Meishan−B
Meishan−C
European−A
European−B
European−C

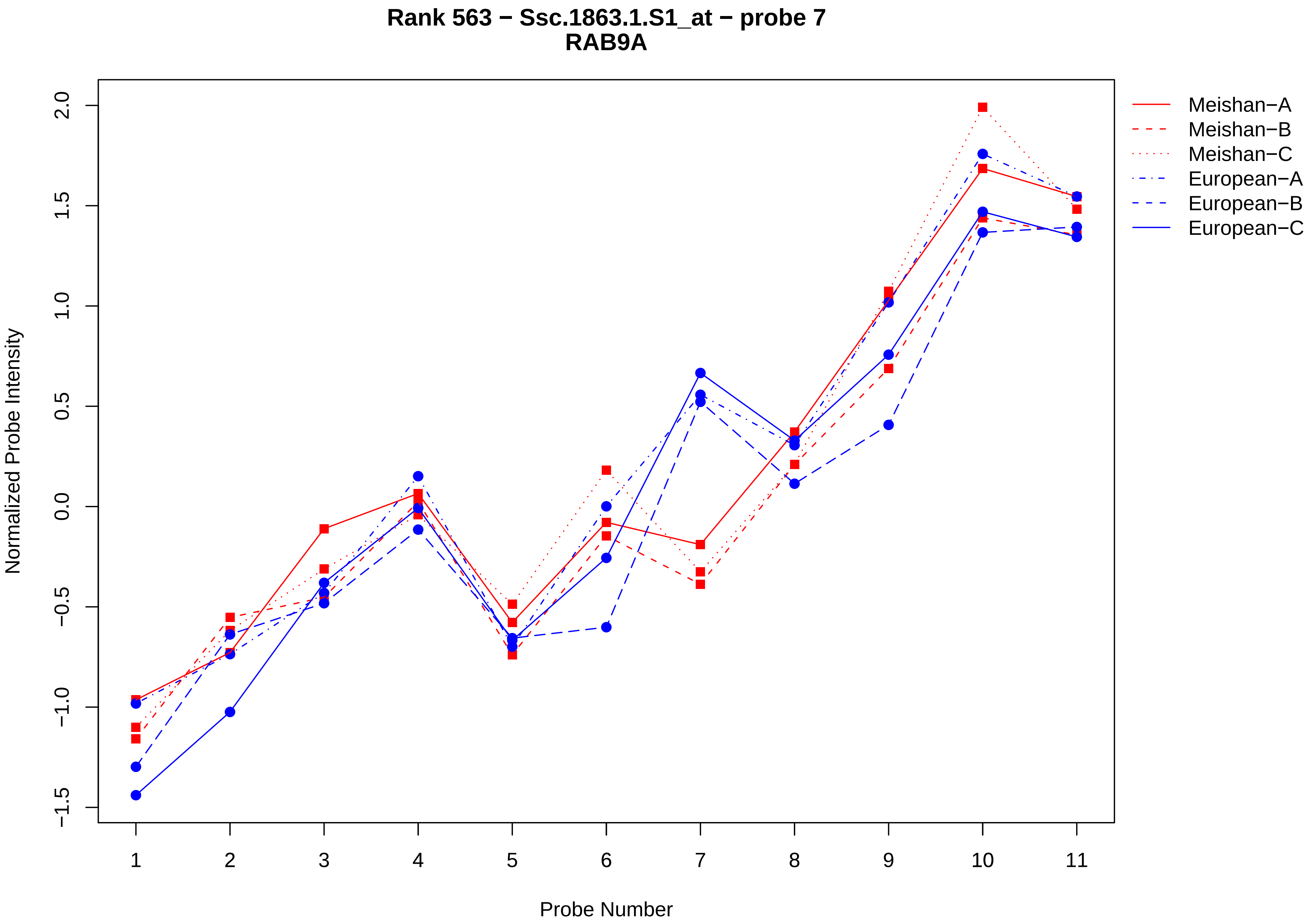

Rank 563 − Ssc.1863.1.S1_at − probe 7
RAB9A
Normalized Probe Intensity
Probe Number
−1.5
−1.0
−0.5
0.0
0.5
1.0
1.5
2.0
1
2
3
4
5
6
7
8
9
10
11
Meishan−A
Meishan−B
Meishan−C
European−A
European−B
European−C

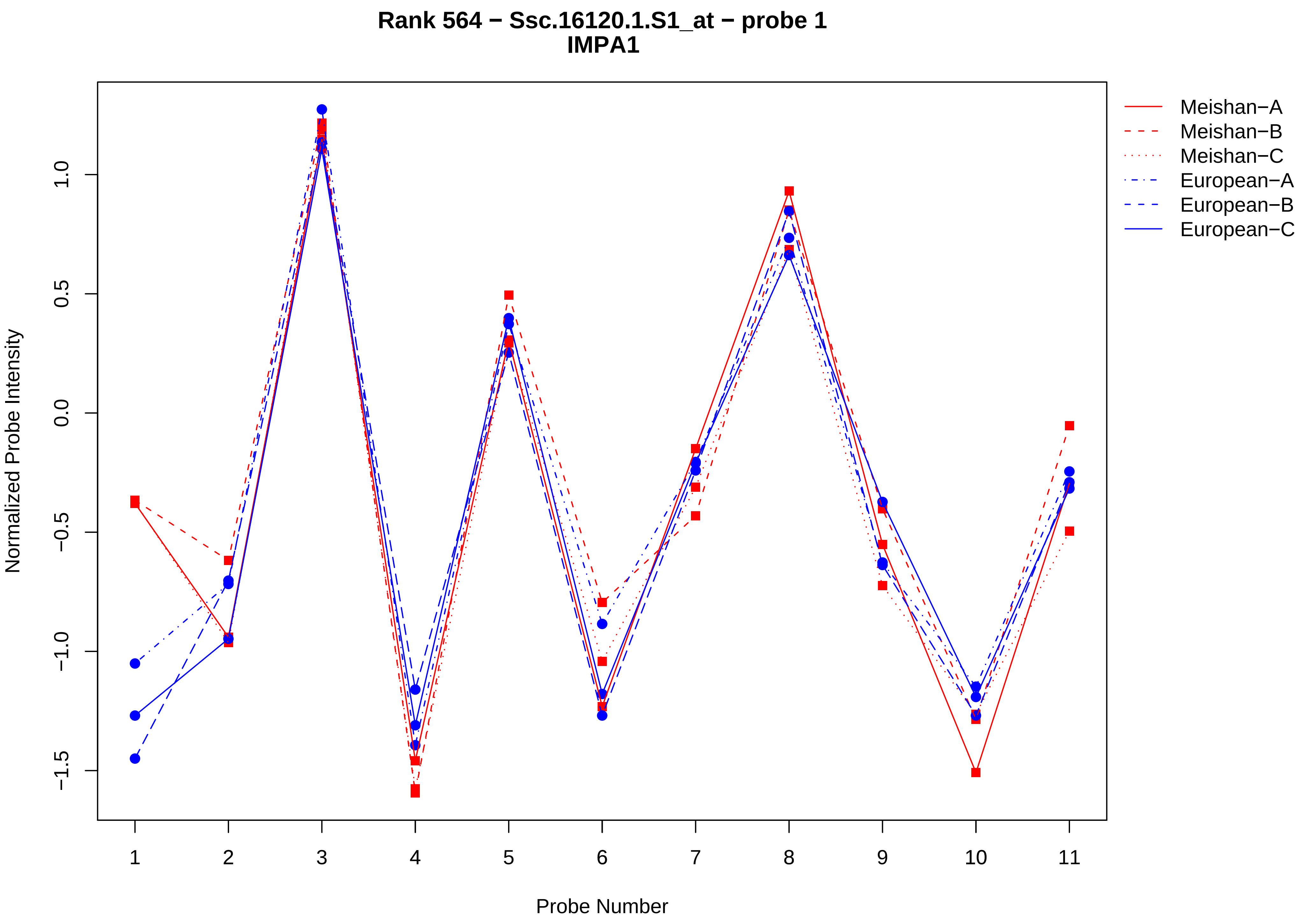

Rank 564 − Ssc.16120.1.S1_at − probe 1
IMPA1
Normalized Probe Intensity
Probe Number
Meishan−A
Meishan−B
Meishan−C
European−A
European−B
European−C
1.0
0.5
0.0
−0.5
−1.0
−1.5
1
2
3
4
5
6
7
8
9
10
11

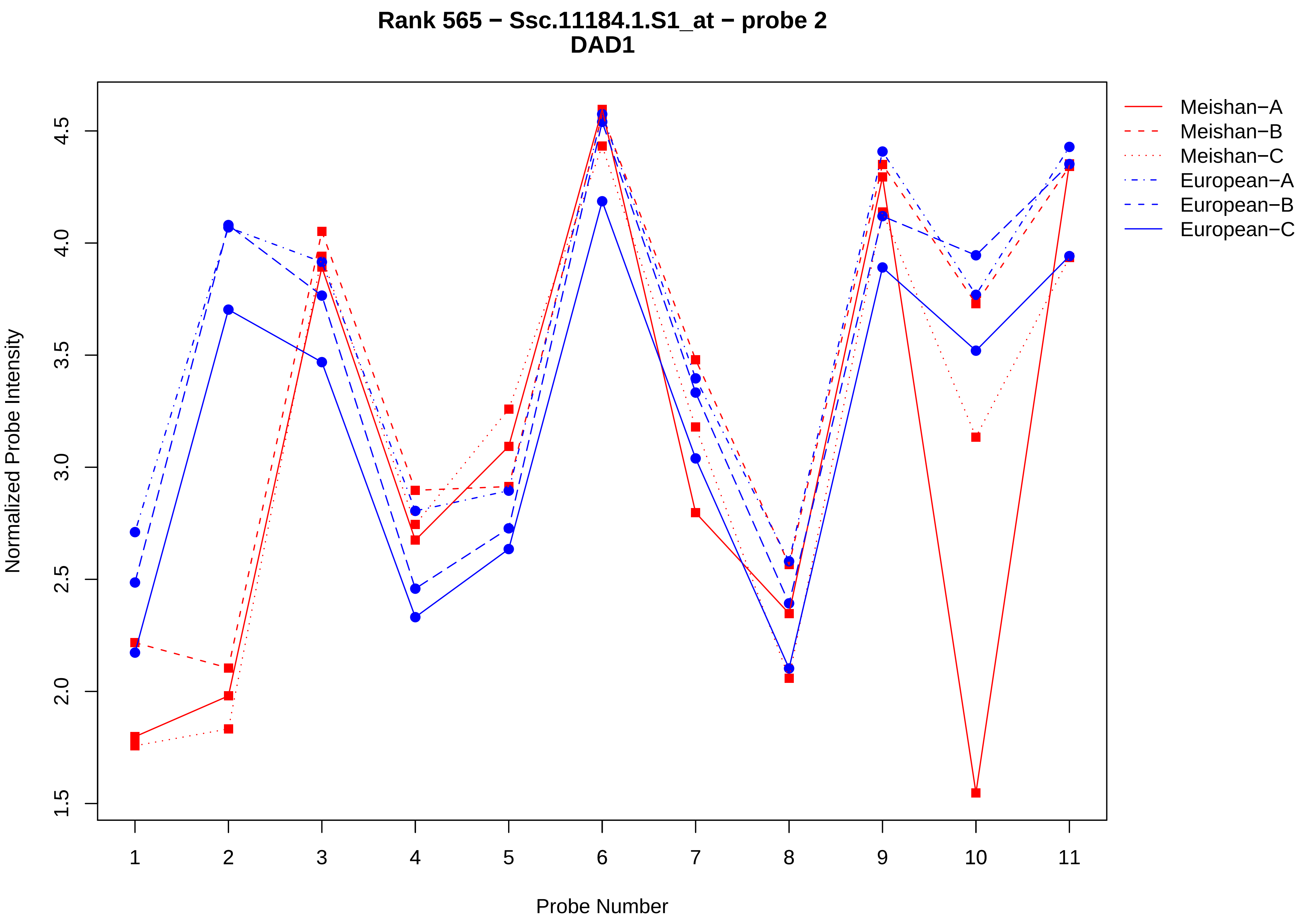

Rank 565 − Ssc.11184.1.S1_at − probe 2
DAD1
Normalized Probe Intensity
Probe Number
1.5
2.0
2.5
3.0
3.5
4.0
4.5
1
2
3
4
5
6
7
8
9
10
11
Meishan−A
Meishan−B
Meishan−C
European−A
European−B
European−C

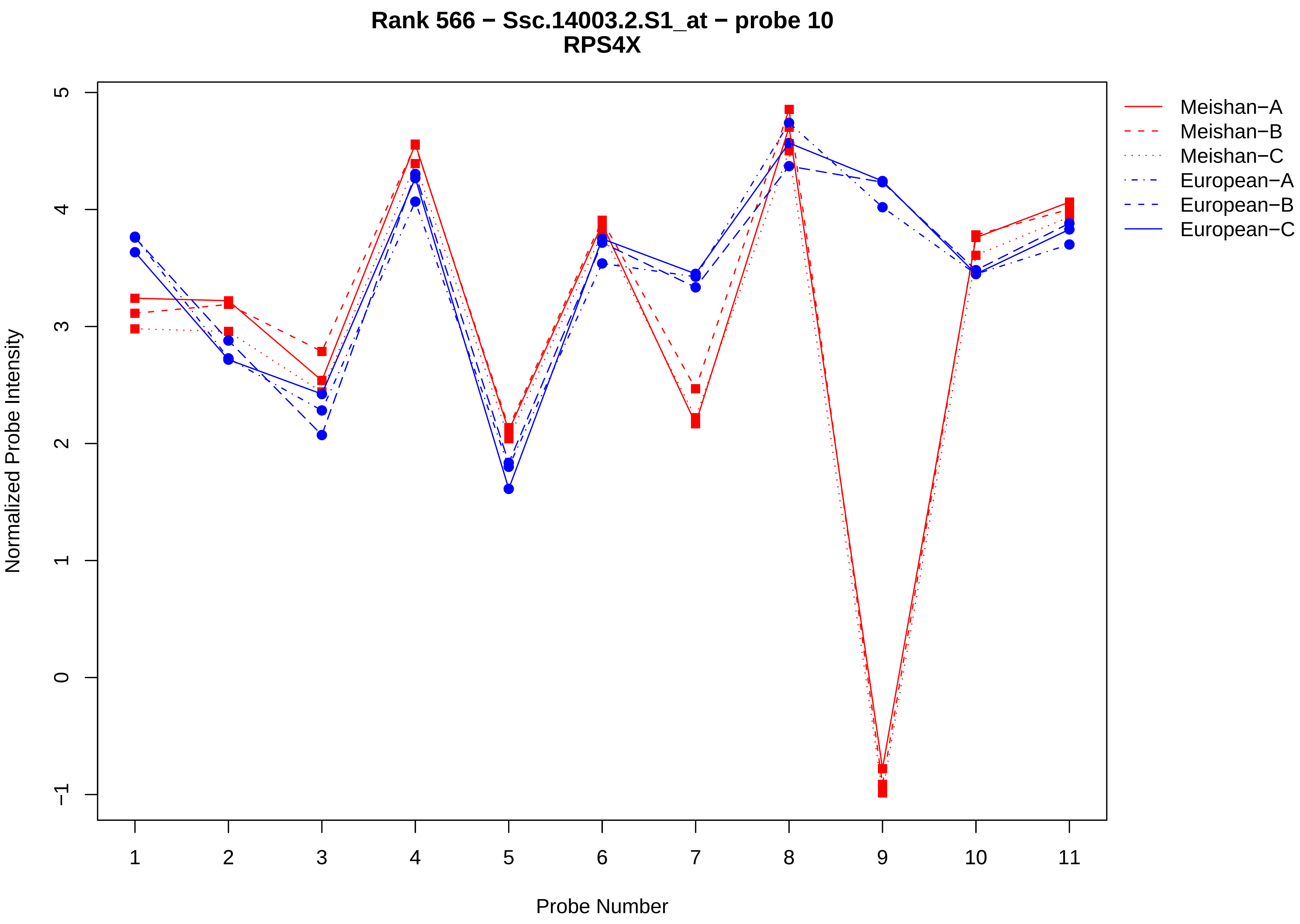

Rank 566 − Ssc.14003.2.S1_at − probe 10
RPS4X
Normalized Probe Intensity
Probe Number
−1
0
1
2
3
4
5
1
2
3
4
5
6
7
8
9
10
11
Meishan−A
Meishan−B
Meishan−C
European−A
European−B
European−C

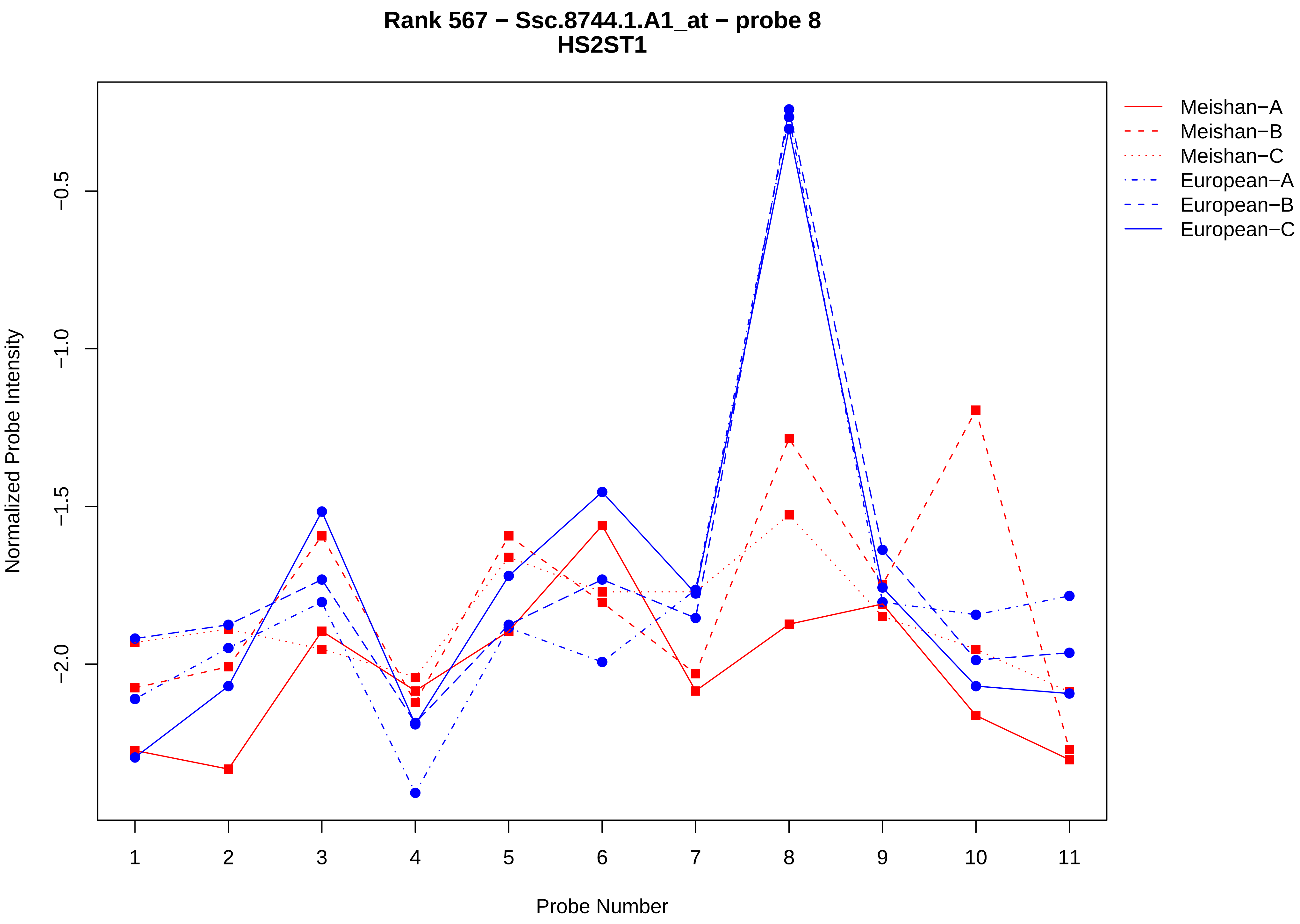
Rank 567 − Ssc.8744.1.A1_at − probe 8
HS2ST1
Normalized Probe Intensity
−0.5
−1.0
−1.5
−2.0
Probe Number
1
2
3
4
5
6
7
8
9
10
11
Meishan−A
Meishan−B
Meishan−C
European−A
European−B
European−C

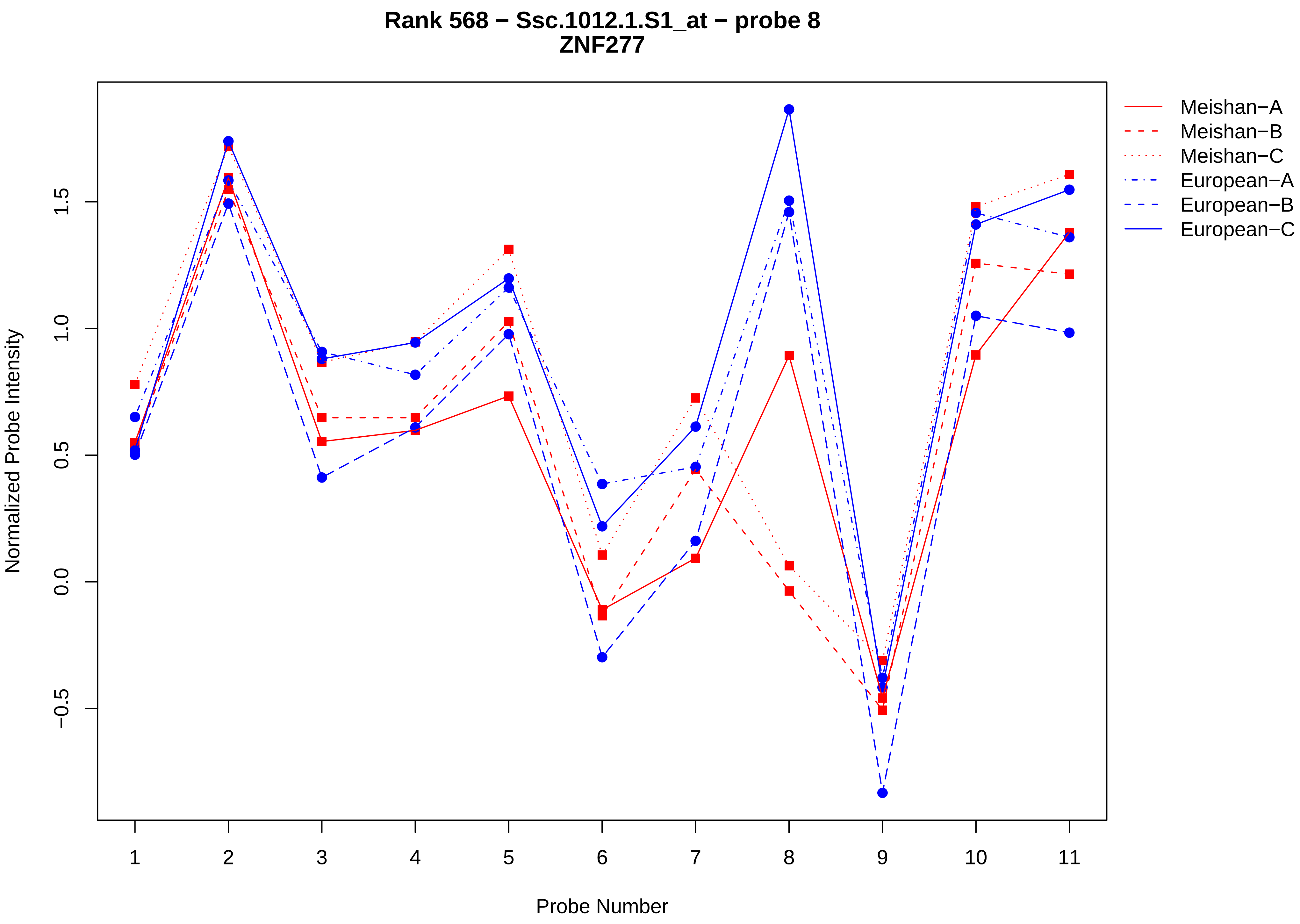
Rank 568 − Ssc.1012.1.S1_at − probe 8
ZNF277
Normalized Probe Intensity
Probe Number
−0.5
0.0
0.5
1.0
1.5
1
2
3
4
5
6
7
8
9
10
11
Meishan−A
Meishan−B
Meishan−C
European−A
European−B
European−C

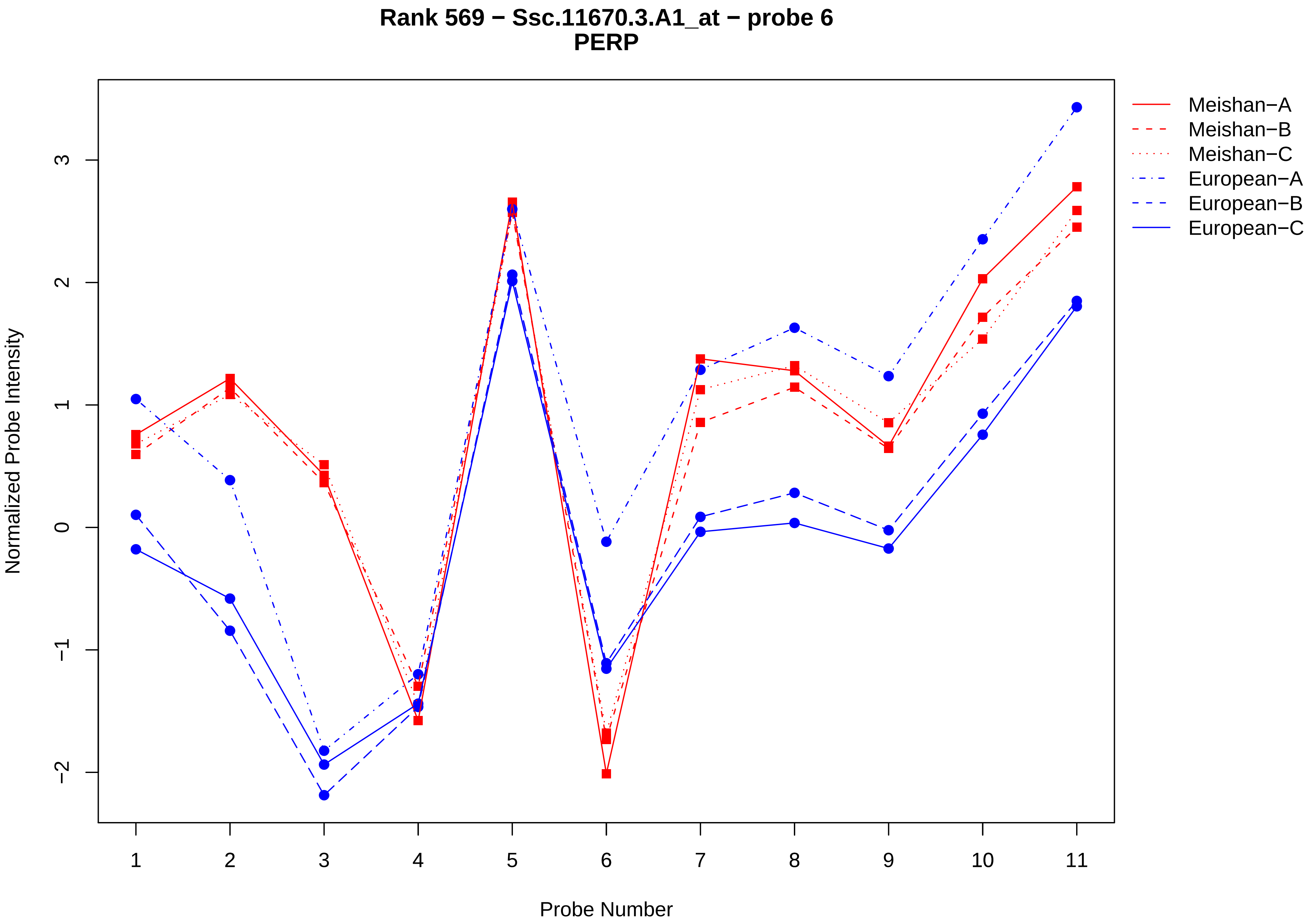

Rank 569 − Ssc.11670.3.A1_at − probe 6
PERP
Normalized Probe Intensity
Probe Number
−2
−1
0
1
2
3
1
2
3
4
5
6
7
8
9
10
11
Meishan−A
Meishan−B
Meishan−C
European−A
European−B
European−C

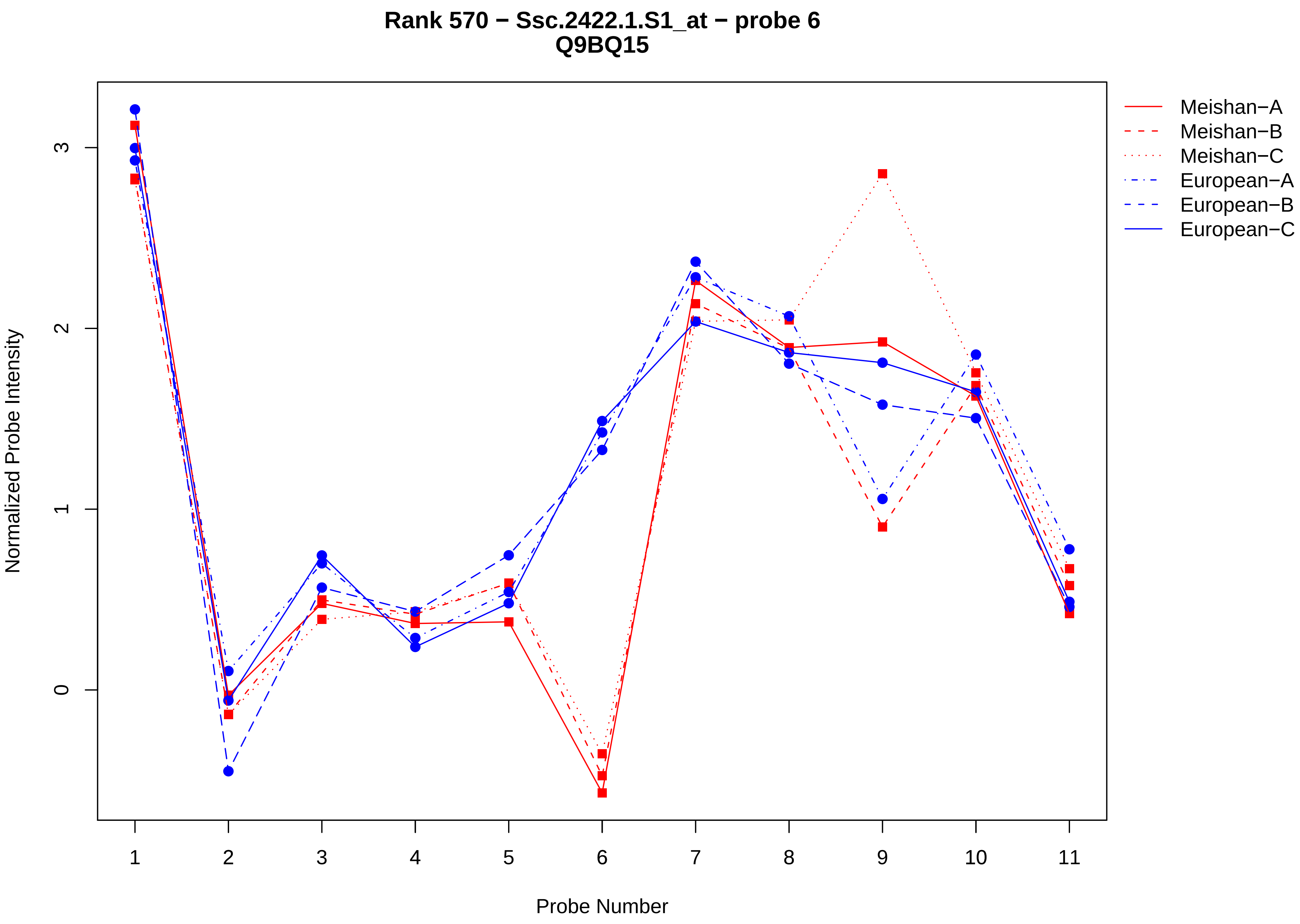
Rank 570 − Ssc.2422.1.S1_at − probe 6
Q9BQ15
Normalized Probe Intensity
0
1
2
3
Probe Number
1
2
3
4
5
6
7
8
9
10
11
Meishan−A
Meishan−B
Meishan−C
European−A
European−B
European−C

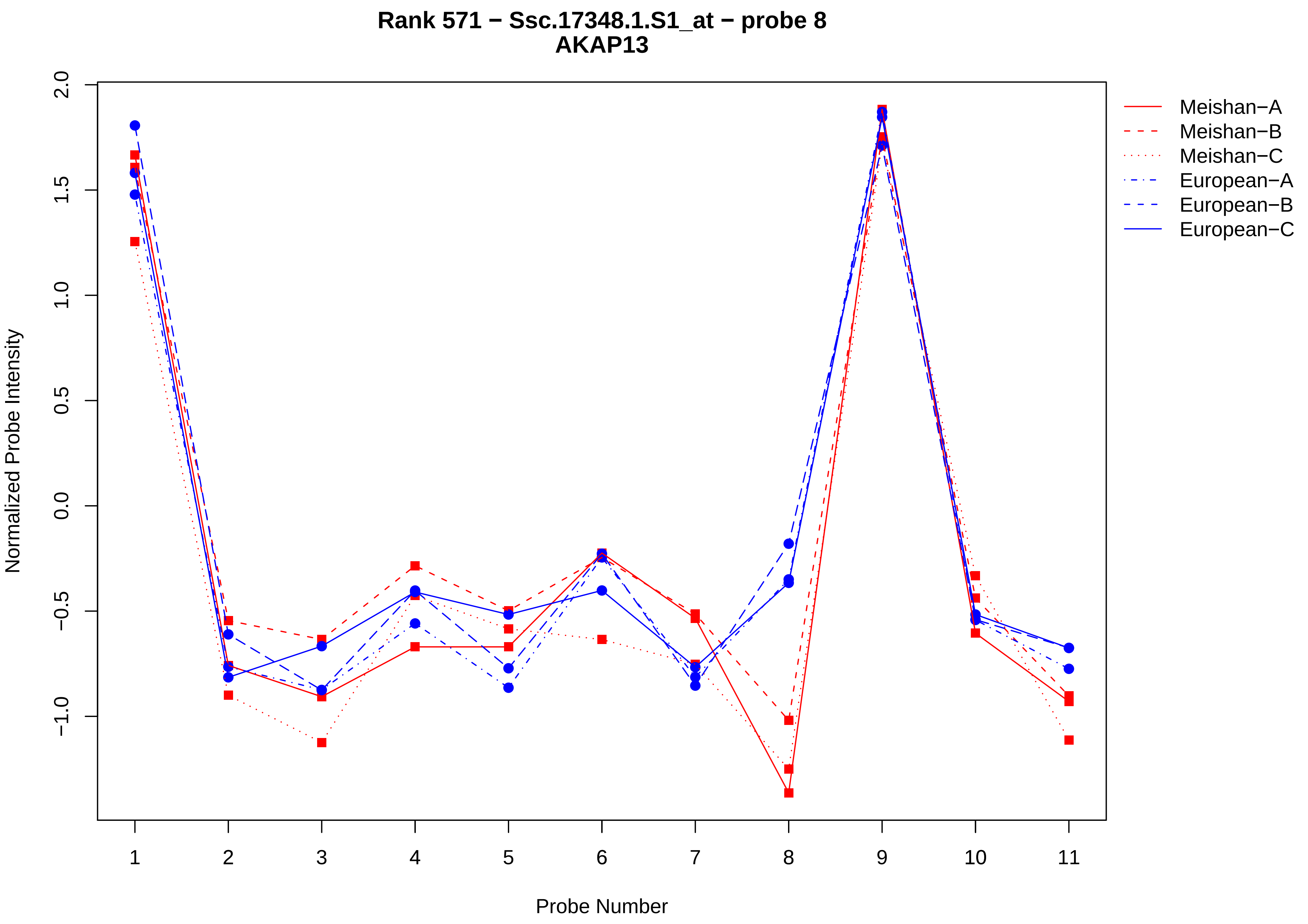

Rank 571 − Ssc.17348.1.S1_at − probe 8
AKAP13
Normalized Probe Intensity
Probe Number
2.0
1.5
1.0
0.5
0.0
−0.5
−1.0
1
2
3
4
5
6
7
8
9
10
11
Meishan−A
Meishan−B
Meishan−C
European−A
European−B
European−C

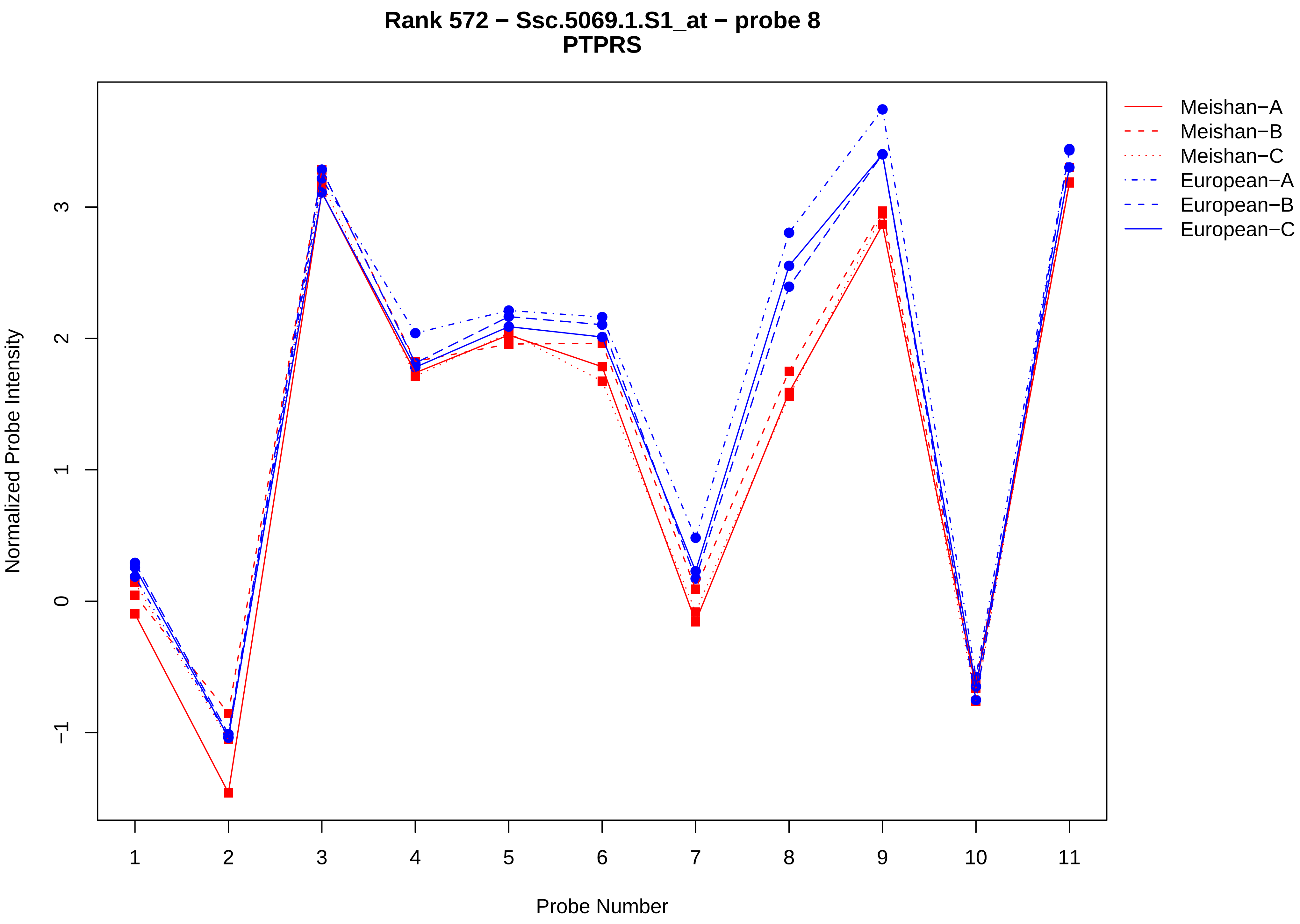

Rank 572 − Ssc.5069.1.S1_at − probe 8
PTPRS
Normalized Probe Intensity
Probe Number
−1
0
1
2
3
1
2
3
4
5
6
7
8
9
10
11
Meishan−A
Meishan−B
Meishan−C
European−A
European−B
European−C

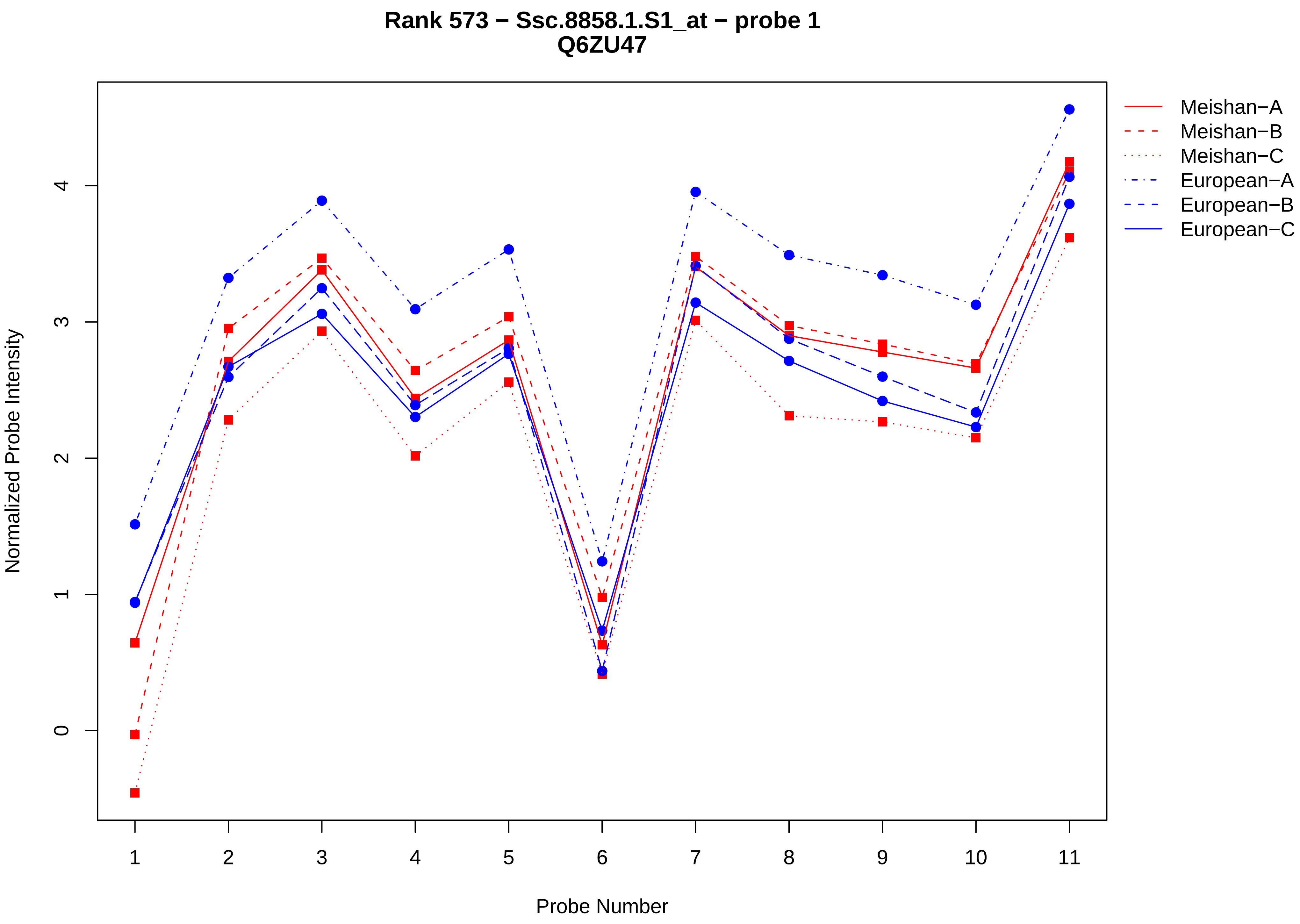
Rank 573 − Ssc.8858.1.S1_at − probe 1
Q6ZU47
Normalized Probe Intensity
Probe Number
0
1
2
3
4
1
2
3
4
5
6
7
8
9
10
11
Meishan−A
Meishan−B
Meishan−C
European−A
European−B
European−C

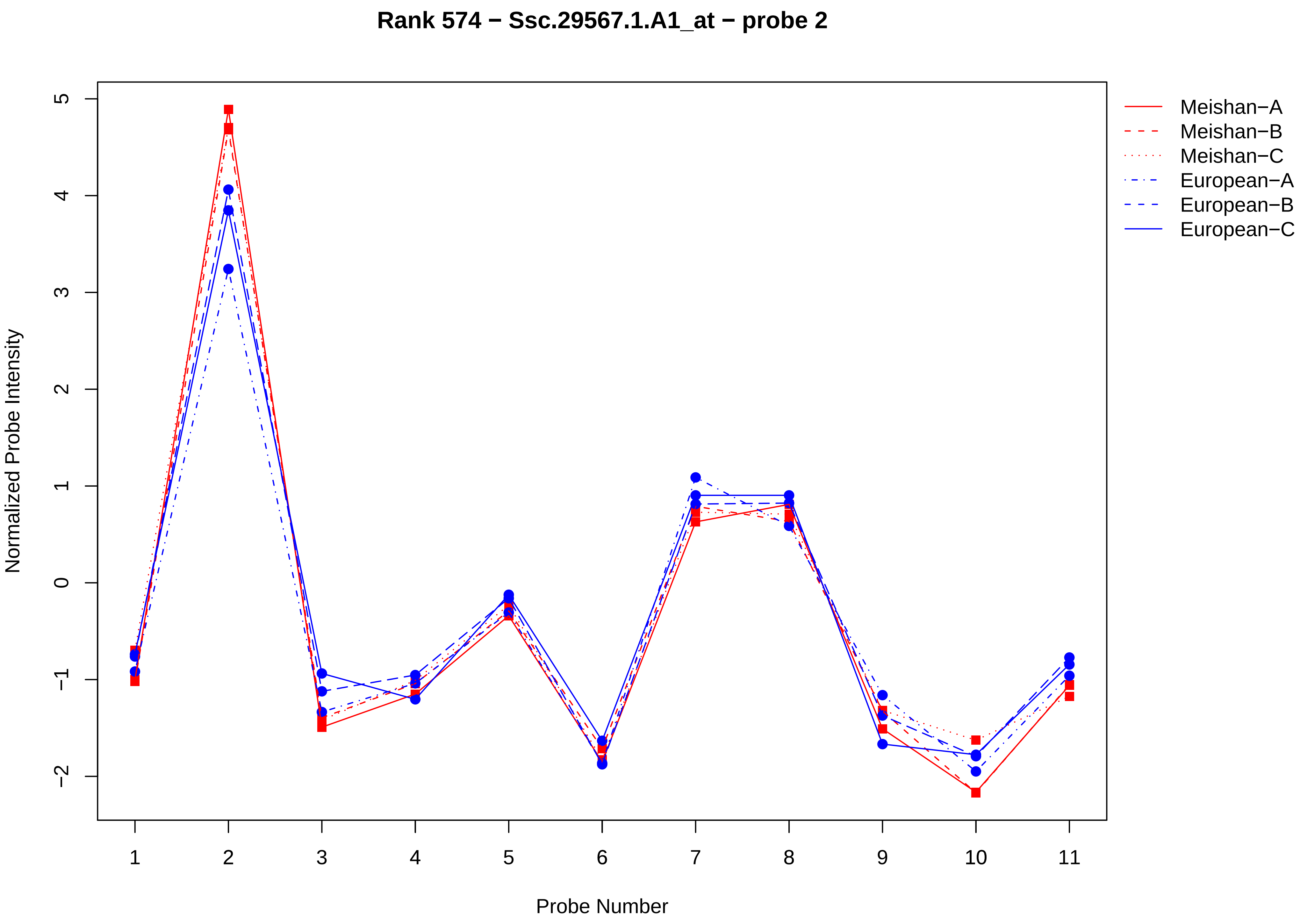
Rank 574 − Ssc.29567.1.A1_at − probe 2
Normalized Probe Intensity
Probe Number
Meishan−A
Meishan−B
Meishan−C
European−A
European−B
European−C
5
4
3
2
1
0
−1
−2
1
2
3
4
5
6
7
8
9
10
11

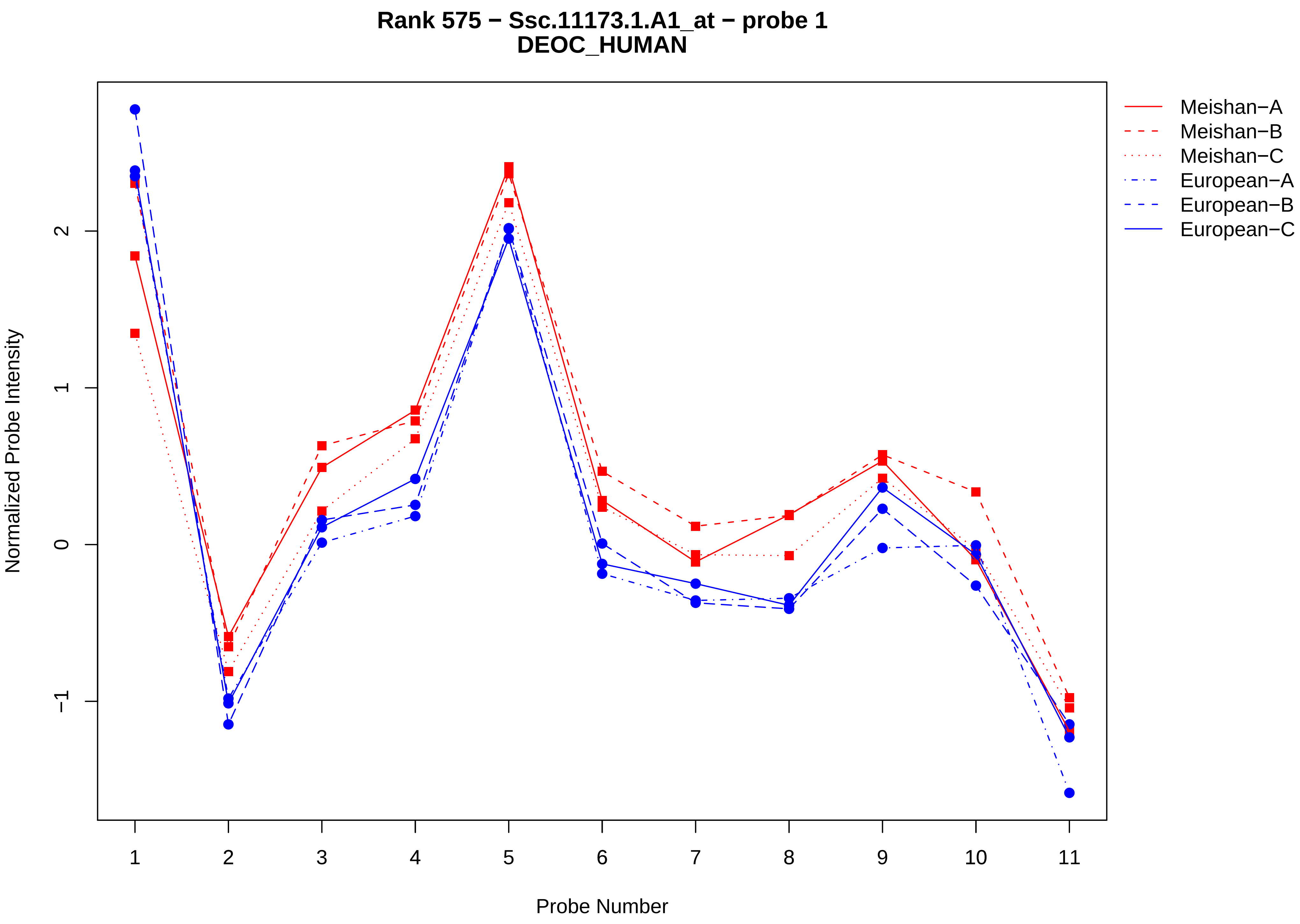

Rank 575 − Ssc.11173.1.A1_at − probe 1
DEOC_HUMAN
Normalized Probe Intensity
Probe Number
−1
0
1
2
1
2
3
4
5
6
7
8
9
10
11
Meishan−A
Meishan−B
Meishan−C
European−A
European−B
European−C

Rank 576 − Ssc.21857.1.S1_at − probe 11
GRB10

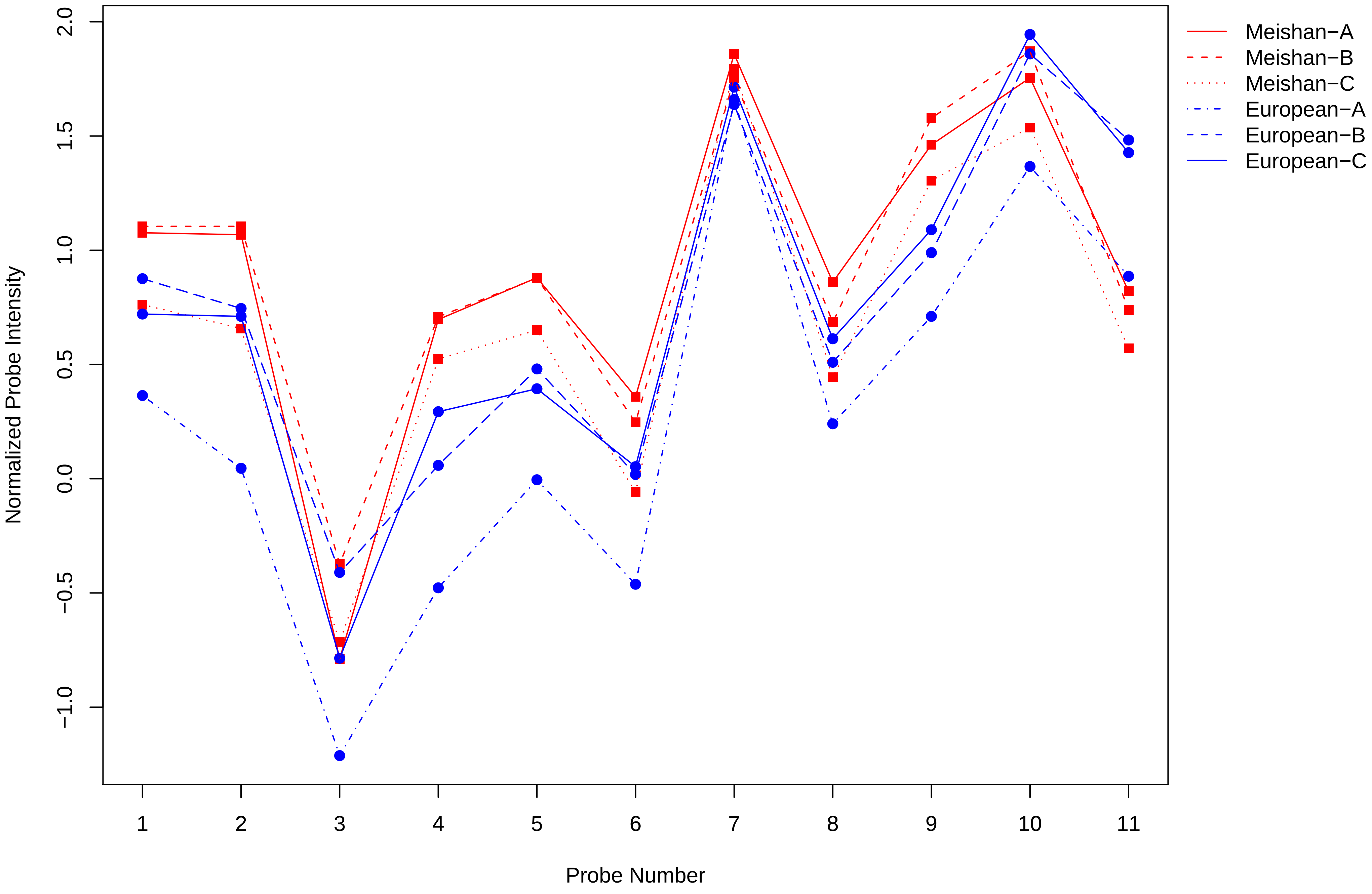

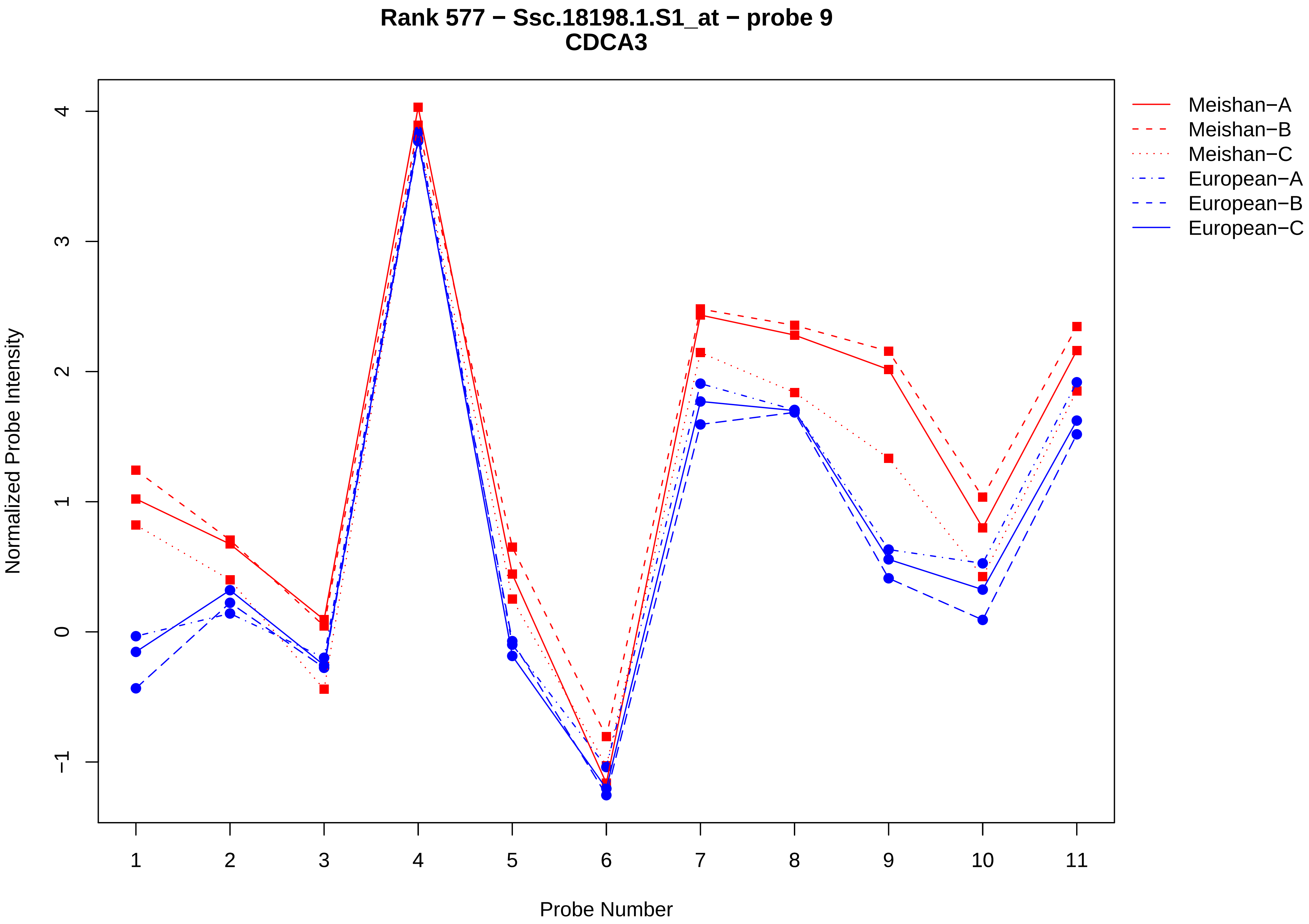

Rank 577 − Ssc.18198.1.S1_at − probe 9
CDCA3
Normalized Probe Intensity
Probe Number
−1
0
1
2
3
4
1
2
3
4
5
6
7
8
9
10
11
Meishan−A
Meishan−B
Meishan−C
European−A
European−B
European−C

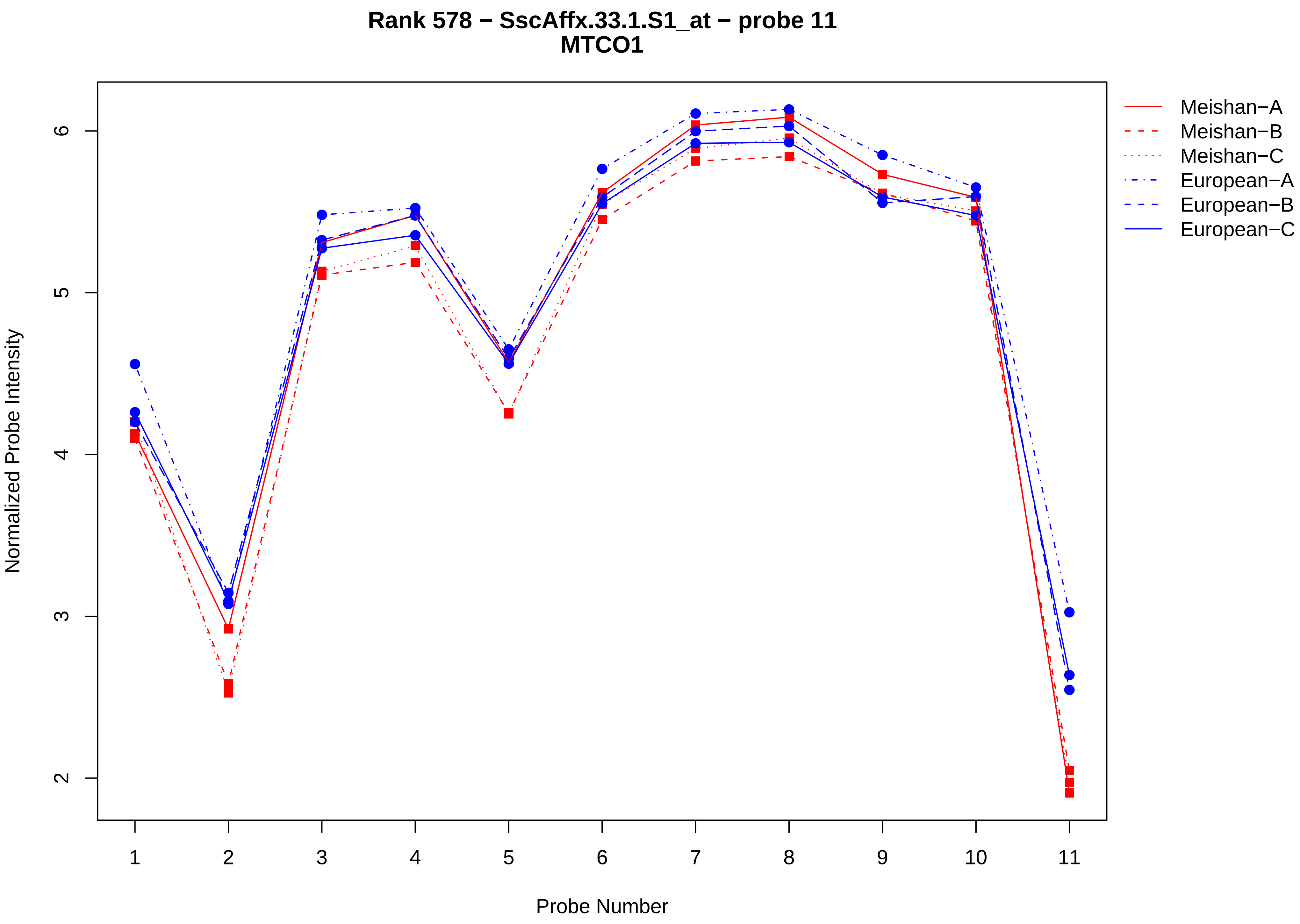

Rank 578 − SscAffx.33.1.S1_at − probe 11
MTCO1
Normalized Probe Intensity
Probe Number
2
3
4
5
6
1
2
3
4
5
6
7
8
9
10
11
Meishan−A
Meishan−B
Meishan−C
European−A
European−B
European−C

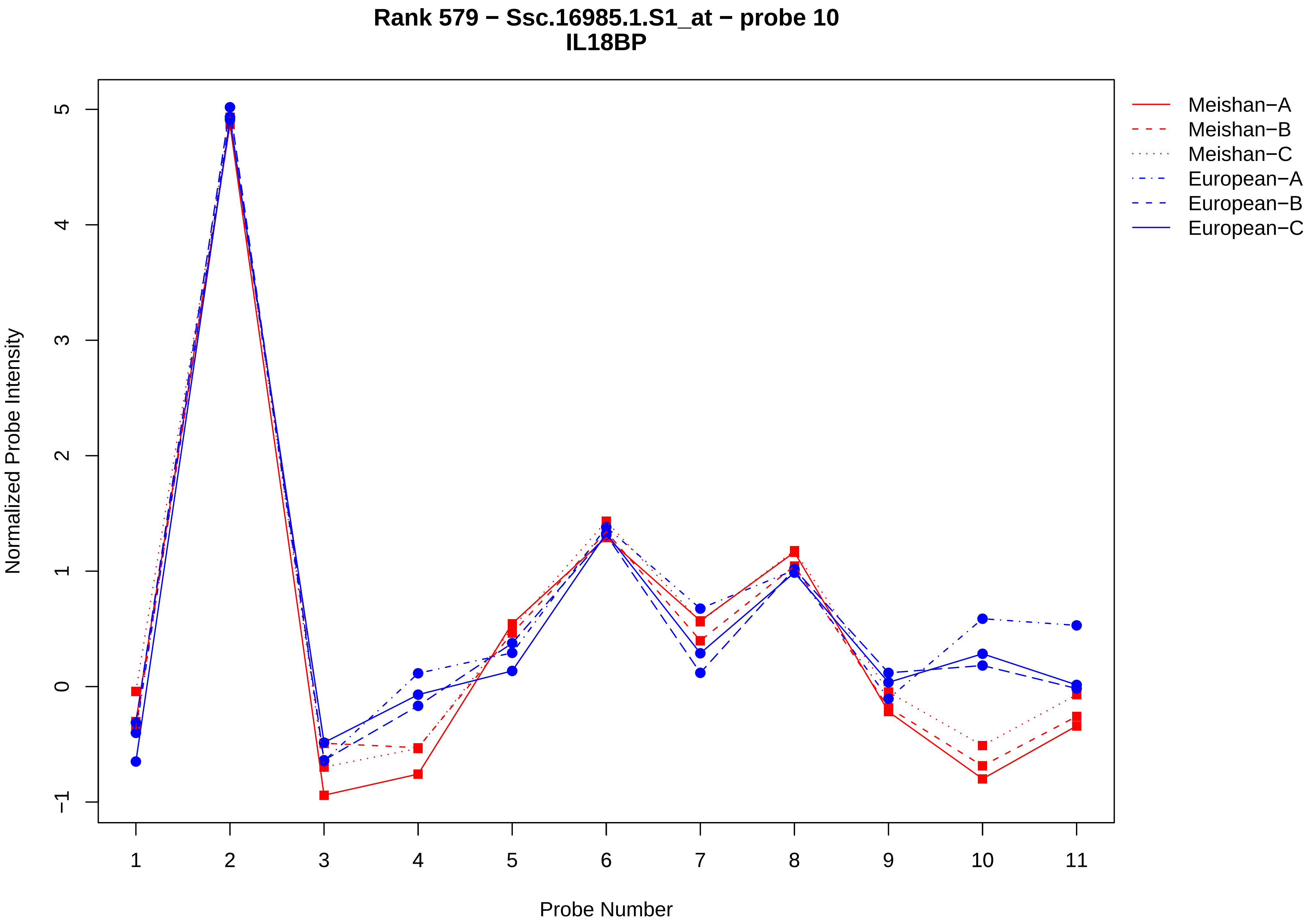

Rank 579 − Ssc.16985.1.S1_at − probe 10
IL18BP
Normalized Probe Intensity
Probe Number
−1
0
1
2
3
4
5
1
2
3
4
5
6
7
8
9
10
11
Meishan−A
Meishan−B
Meishan−C
European−A
European−B
European−C

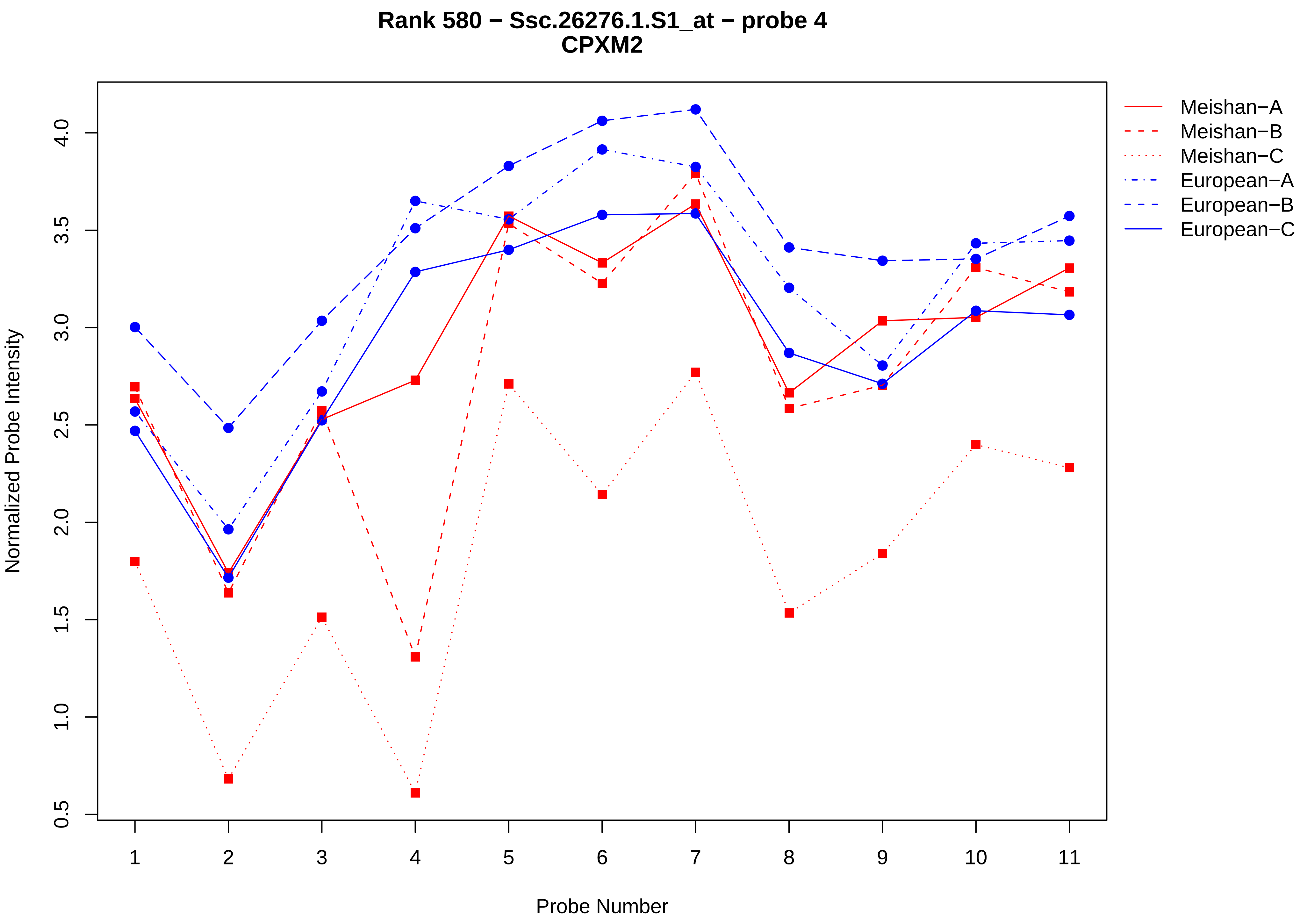
Rank 580 − Ssc.26276.1.S1_at − probe 4
CPXM2
Normalized Probe Intensity
Probe Number
Meishan−A
Meishan−B
Meishan−C
European−A
European−B
European−C

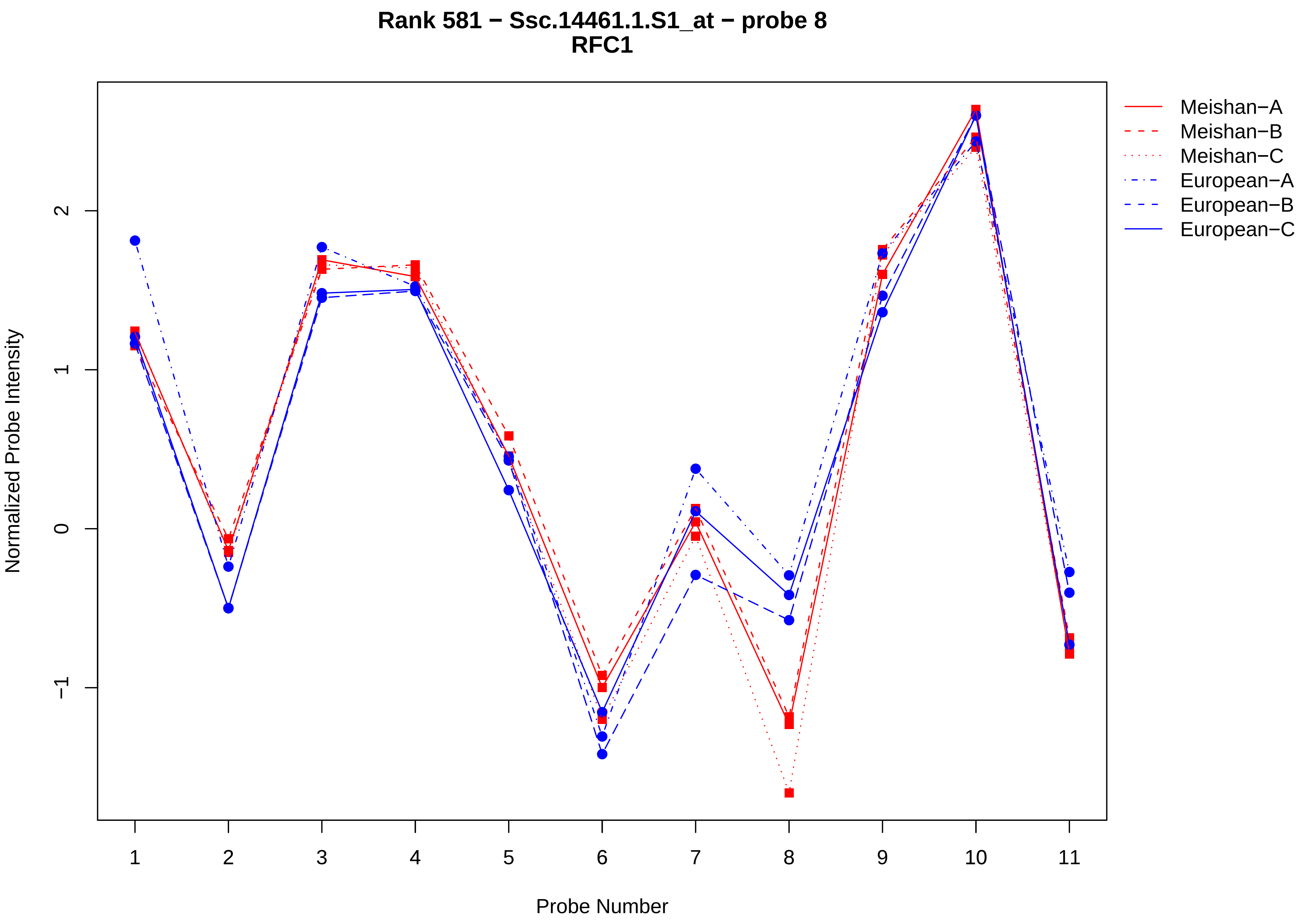

Rank 581 − Ssc.14461.1.S1_at − probe 8
RFC1
Normalized Probe Intensity
Probe Number
−1
0
1
2
1
2
3
4
5
6
7
8
9
10
11
Meishan−A
Meishan−B
Meishan−C
European−A
European−B
European−C

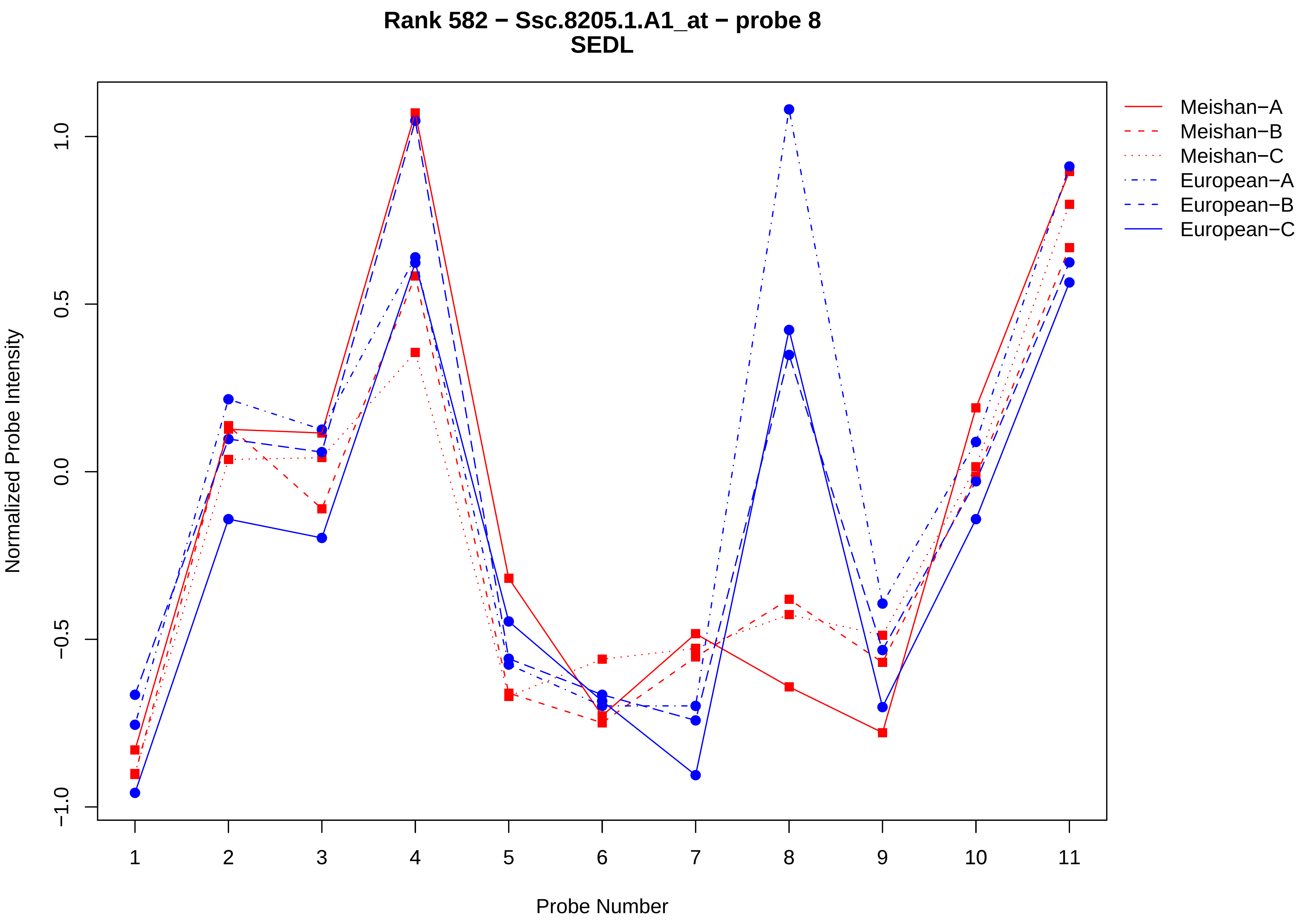

Rank 582 − Ssc.8205.1.A1_at − probe 8
SEDL
Normalized Probe Intensity
Probe Number
Meishan−A
Meishan−B
Meishan−C
European−A
European−B
European−C

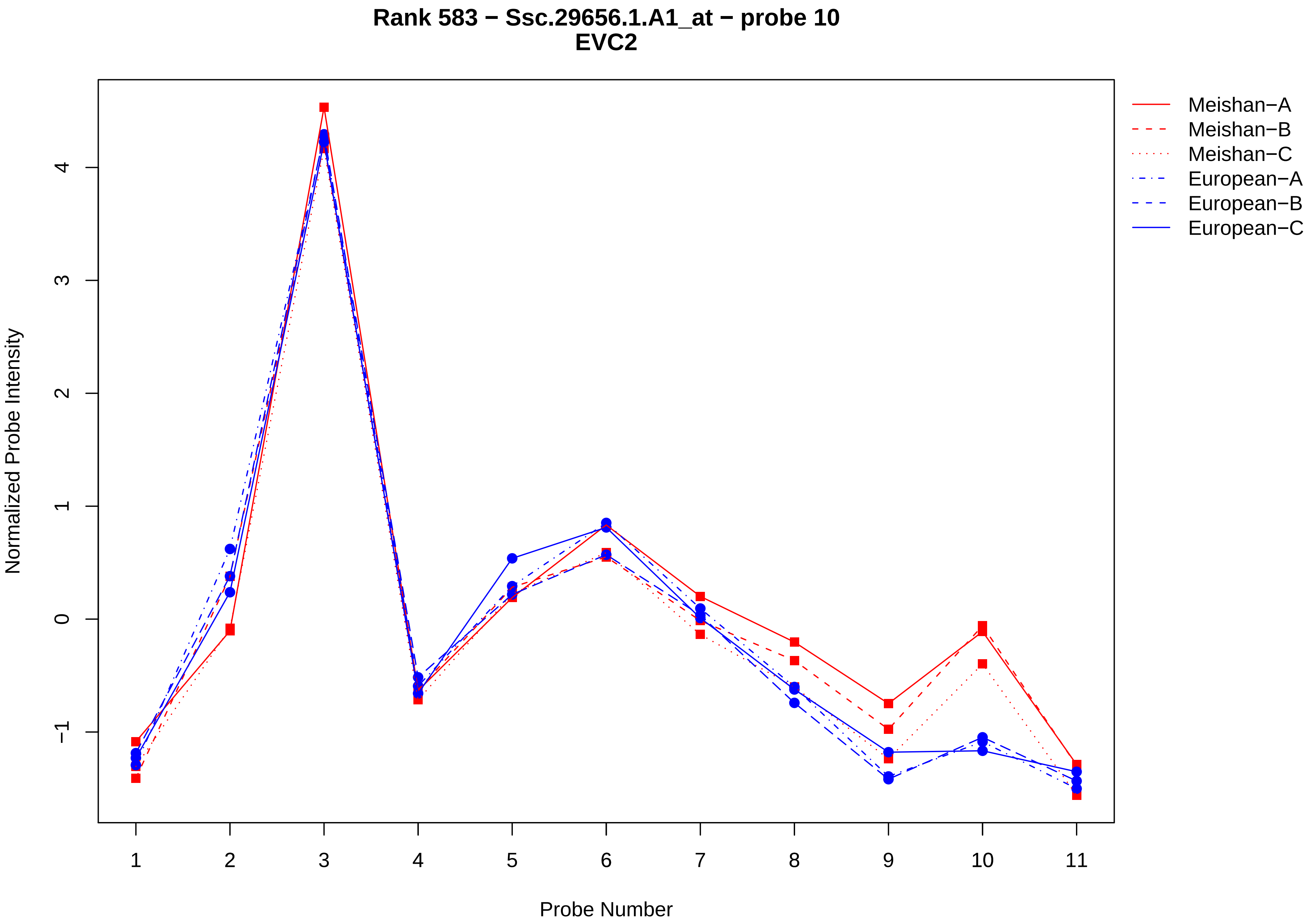

Rank 583 − Ssc.29656.1.A1_at − probe 10
EVC2
Normalized Probe Intensity
Probe Number
−1
0
1
2
3
4
1
2
3
4
5
6
7
8
9
10
11
Meishan−A
Meishan−B
Meishan−C
European−A
European−B
European−C

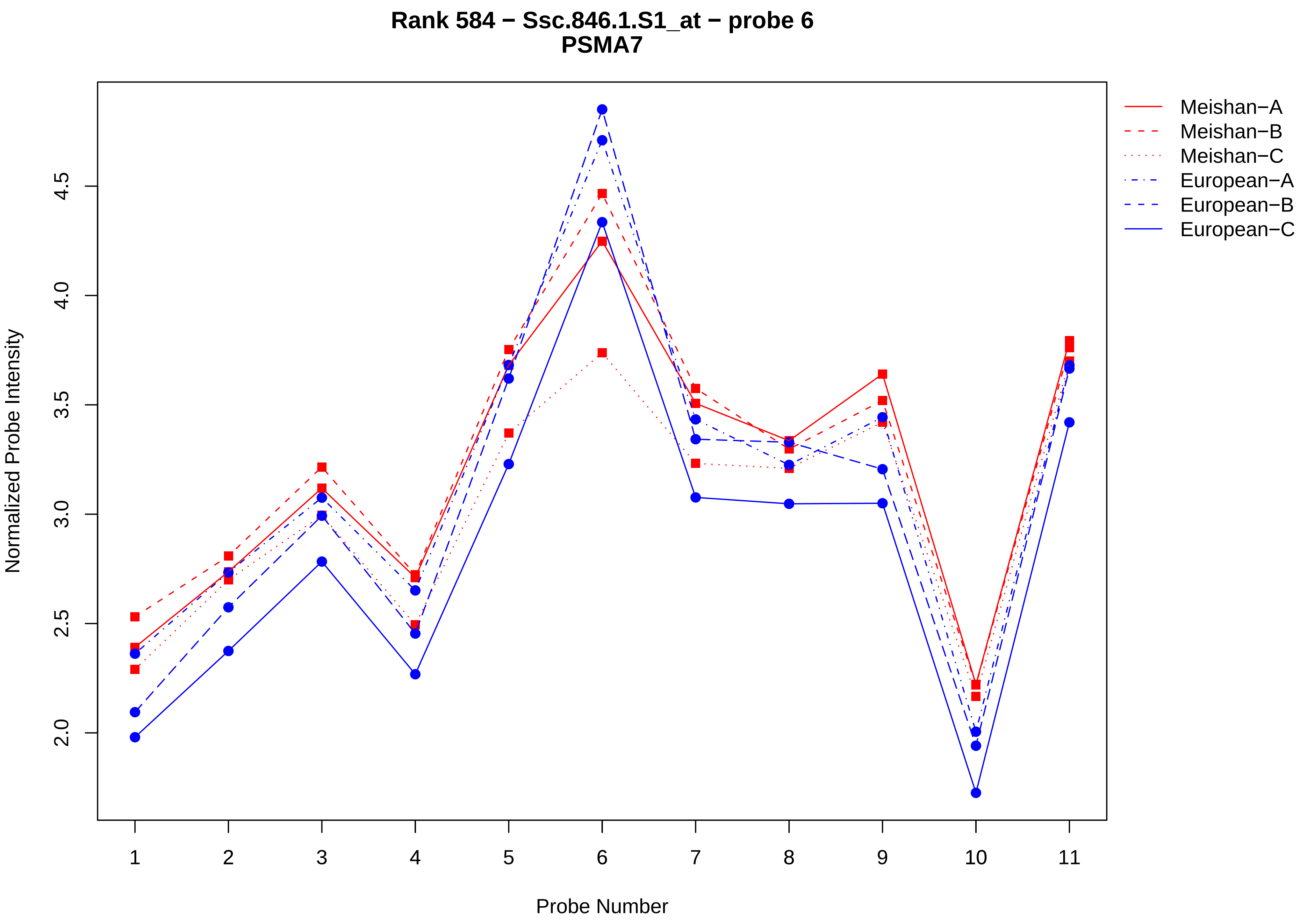

Rank 584 − Ssc.846.1.S1_at − probe 6
PSMA7
Normalized Probe Intensity
Probe Number
2.0
2.5
3.0
3.5
4.0
4.5
1
2
3
4
5
6
7
8
9
10
11
Meishan−A
Meishan−B
Meishan−C
European−A
European−B
European−C

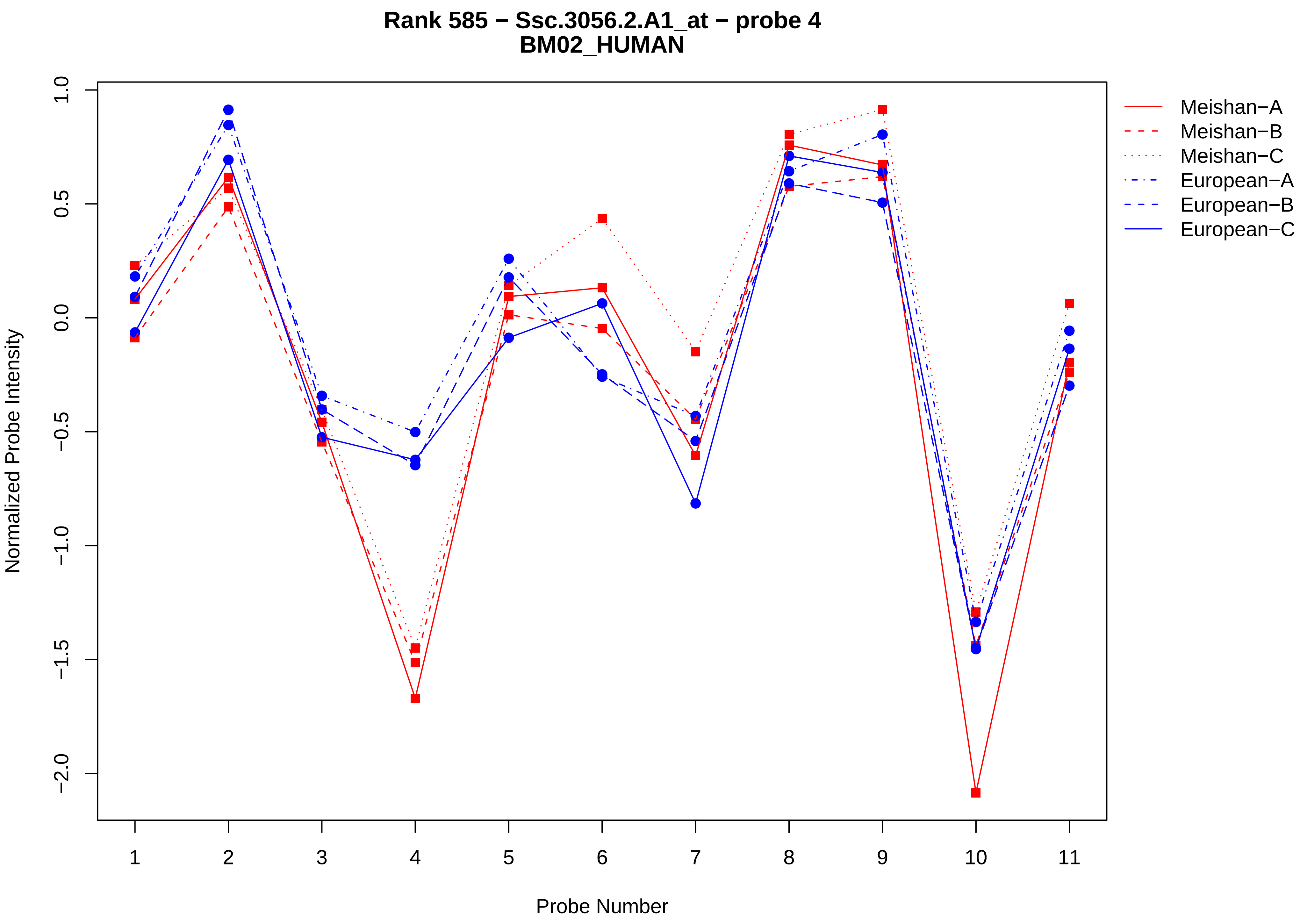

Rank 585 − Ssc.3056.2.A1_at − probe 4
BM02_HUMAN
Normalized Probe Intensity
Probe Number
Meishan−A
Meishan−B
Meishan−C
European−A
European−B
European−C
1.0
0.5
0.0
−0.5
−1.0
−1.5
−2.0
1
2
3
4
5
6
7
8
9
10
11

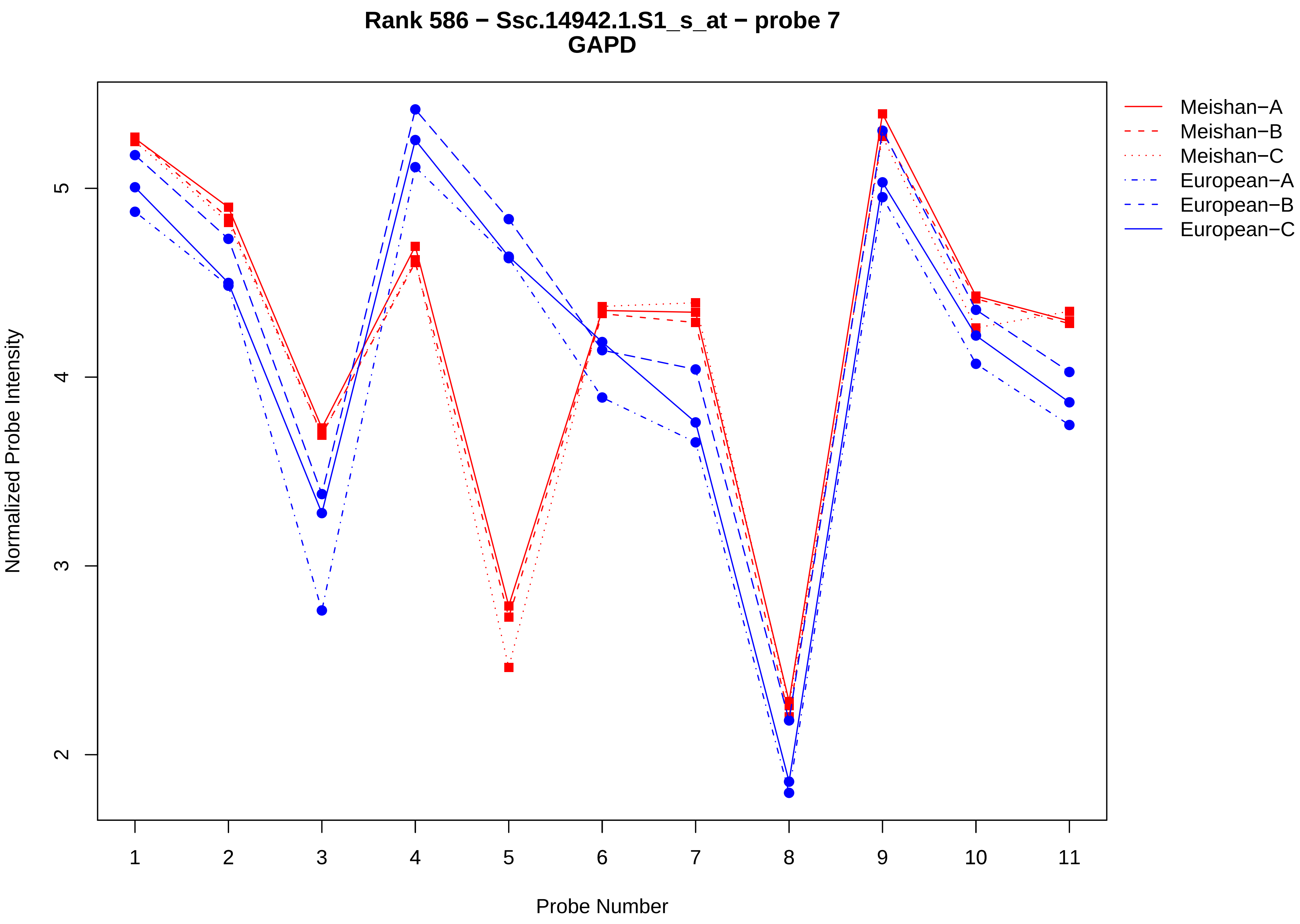
Rank 586 − Ssc.14942.1.S1_s_at − probe 7
GAPD
Normalized Probe Intensity
Probe Number
2
3
4
5
1
2
3
4
5
6
7
8
9
10
11
Meishan−A
Meishan−B
Meishan−C
European−A
European−B
European−C

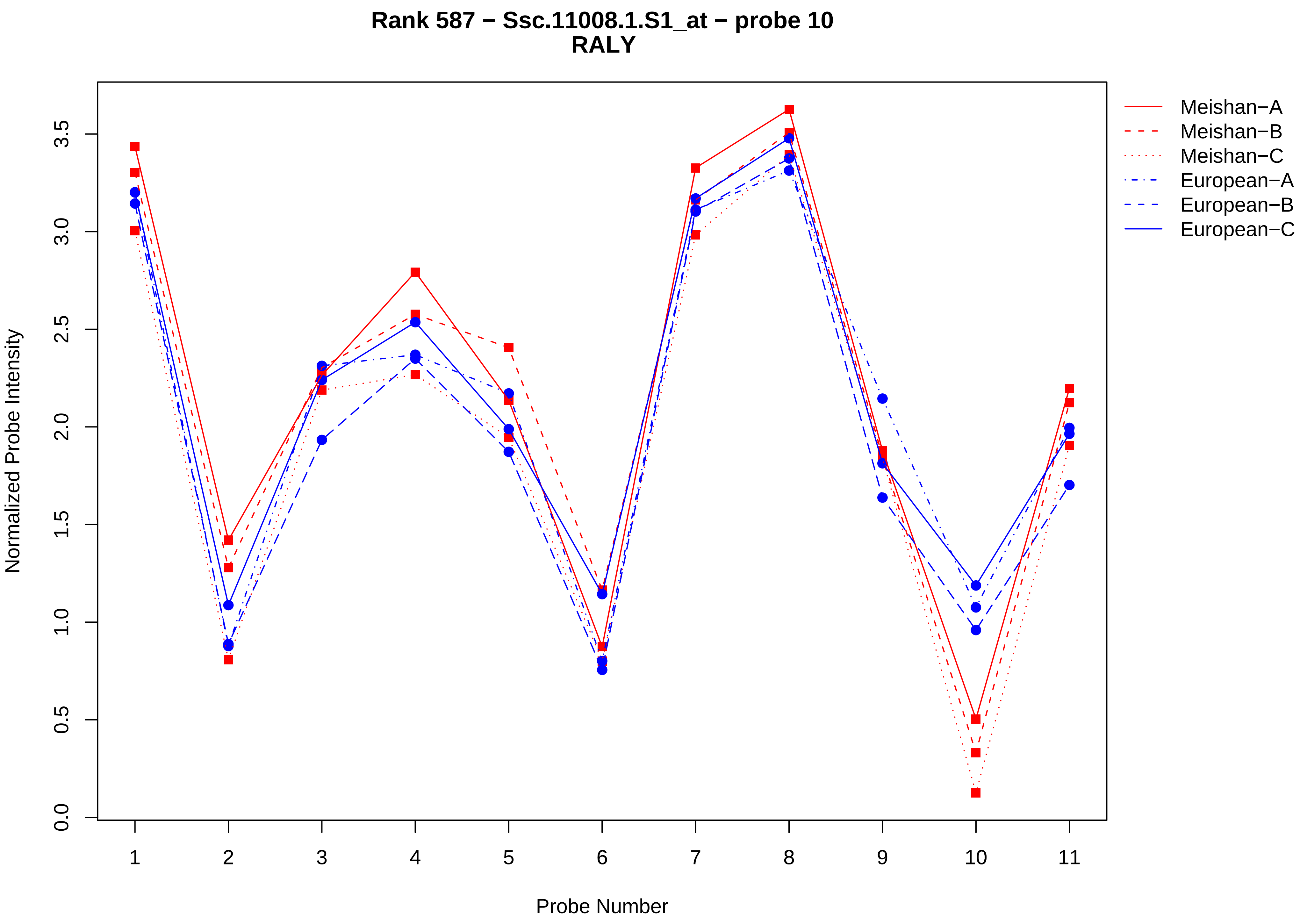
Rank 587 − Ssc.11008.1.S1_at − probe 10
RALY
Normalized Probe Intensity
0.0
0.5
1.0
1.5
2.0
2.5
3.0
3.5
1
2
3
4
5
6
7
8
9
10
11
Probe Number
Meishan−A
Meishan−B
Meishan−C
European−A
European−B
European−C

Rank 588 − Ssc.24741.1.A1_at − probe 11
UTRN

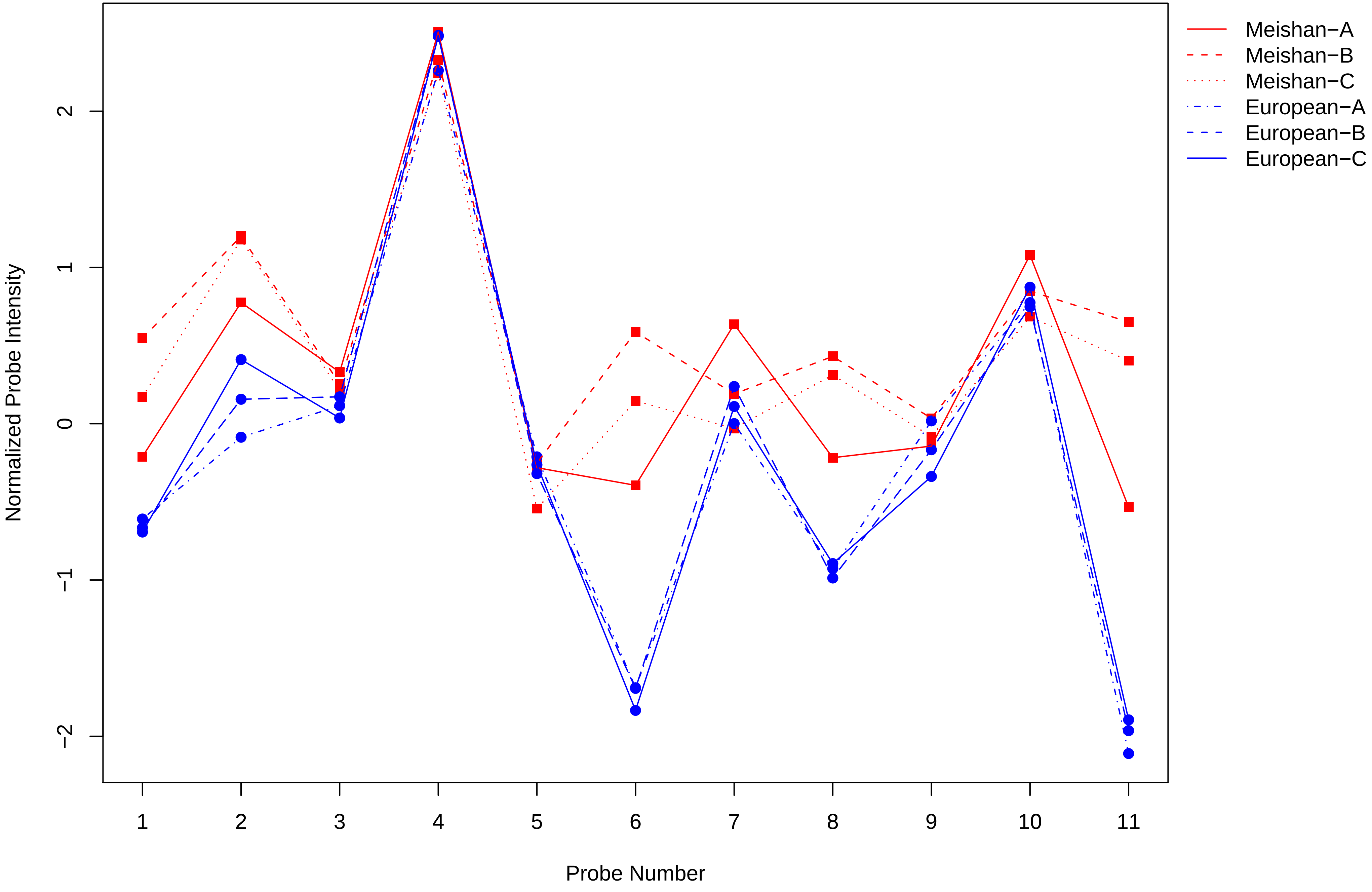

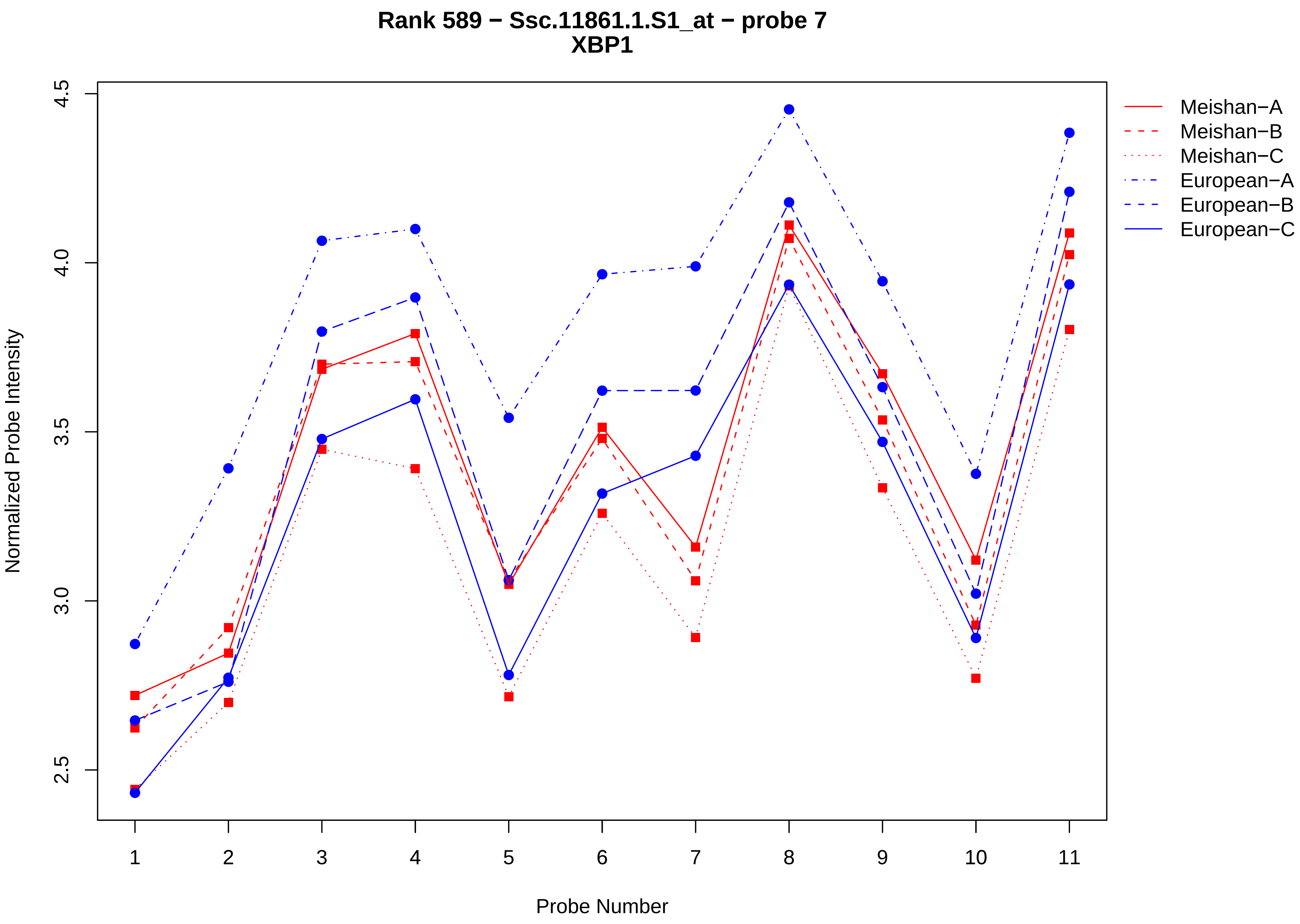
Rank 589 − Ssc.11861.1.S1_at − probe 7
XBP1
Normalized Probe Intensity
Probe Number
2.5
3.0
3.5
4.0
4.5
1
2
3
4
5
6
7
8
9
10
11
Meishan−A
Meishan−B
Meishan−C
European−A
European−B
European−C

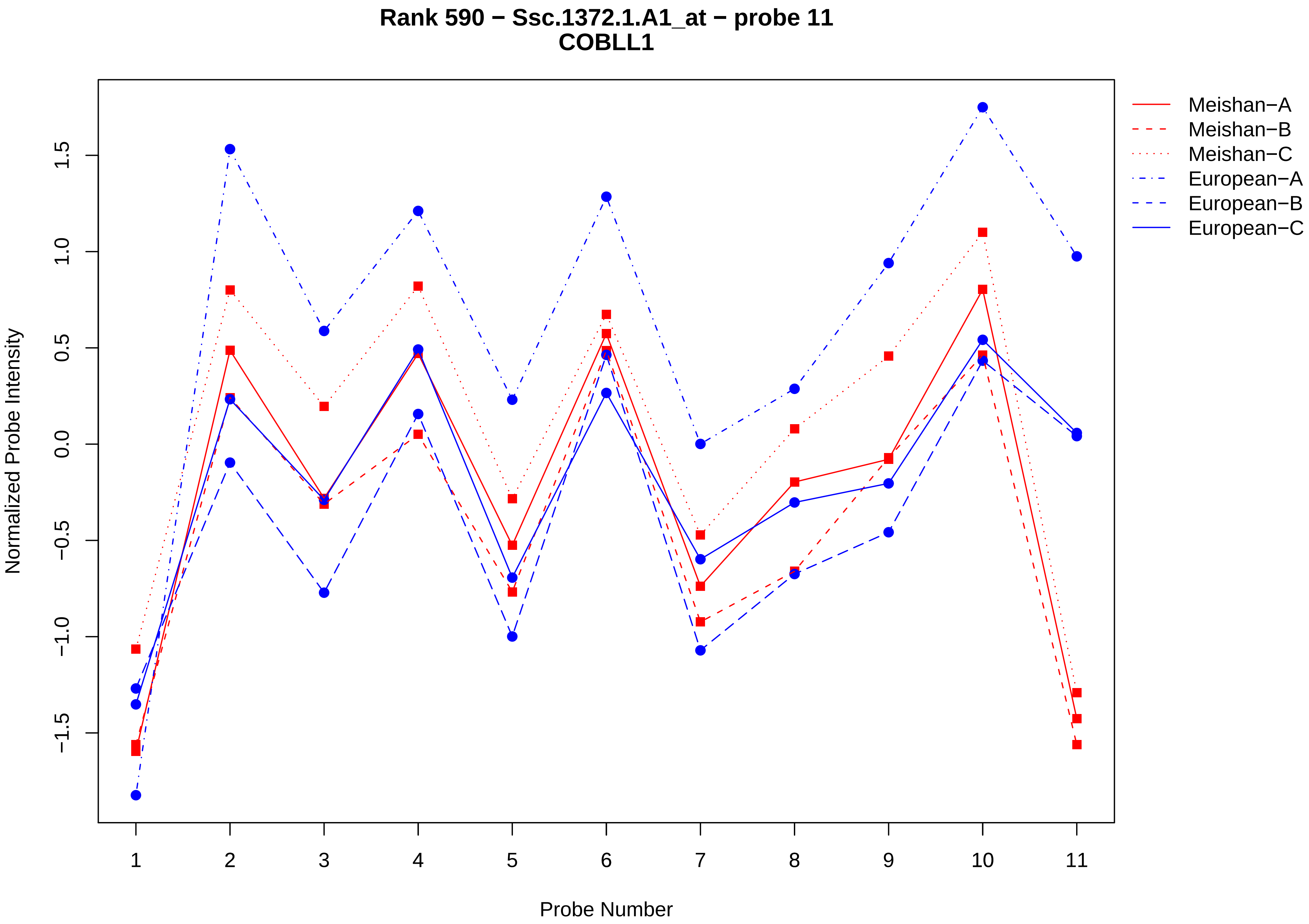

Rank 590 − Ssc.1372.1.A1_at − probe 11
COBLL1
Normalized Probe Intensity
Probe Number
−1.5
−1.0
−0.5
0.0
0.5
1.0
1.5
1
2
3
4
5
6
7
8
9
10
11
Meishan−A
Meishan−B
Meishan−C
European−A
European−B
European−C

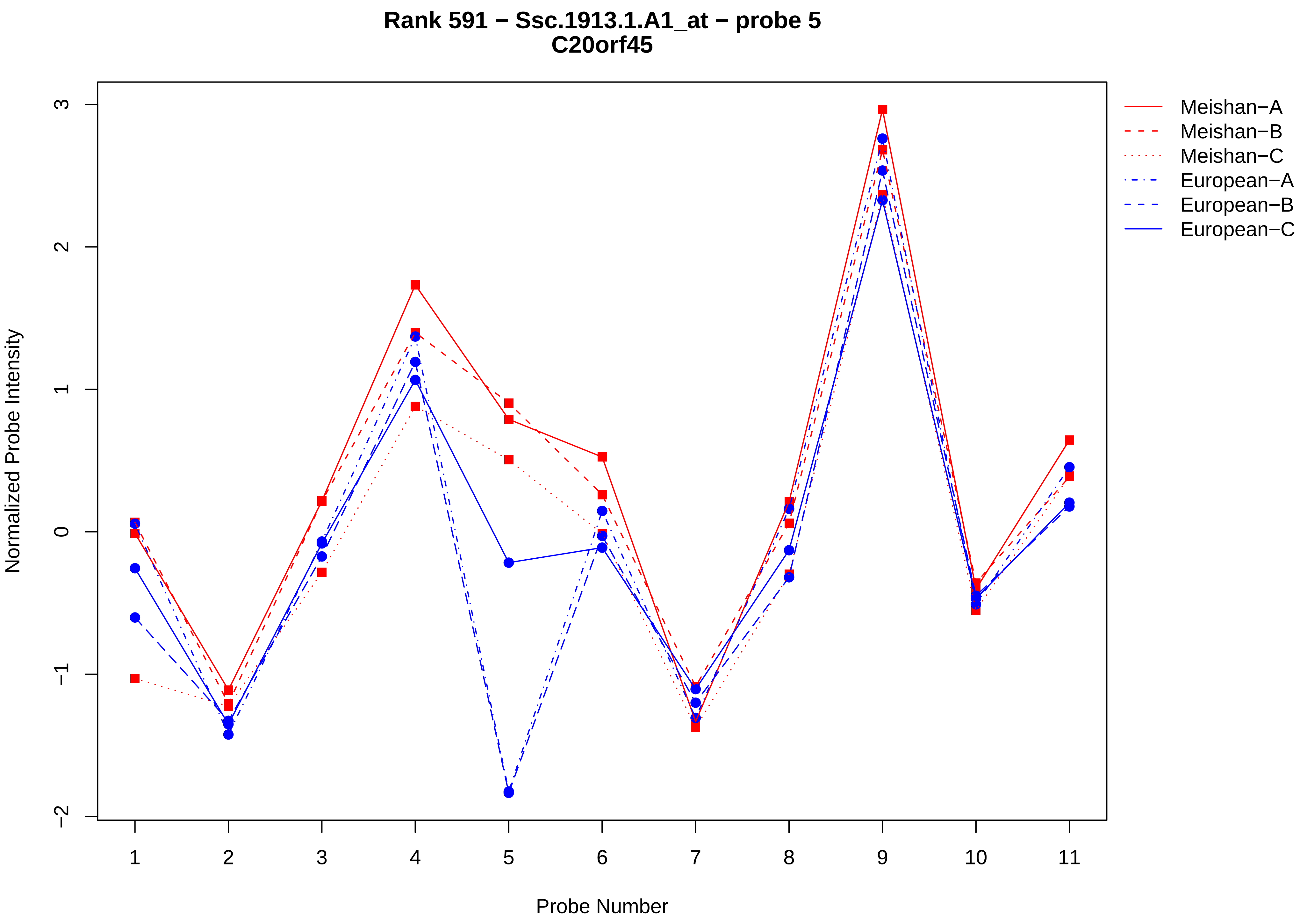
Rank 591 − Ssc.1913.1.A1_at − probe 5
C20orf45
Normalized Probe Intensity
Probe Number
−2
−1
0
1
2
3
1
2
3
4
5
6
7
8
9
10
11
Meishan−A
Meishan−B
Meishan−C
European−A
European−B
European−C

Rank 592 − Ssc.20017.2.S1_at − probe 11
MYH10

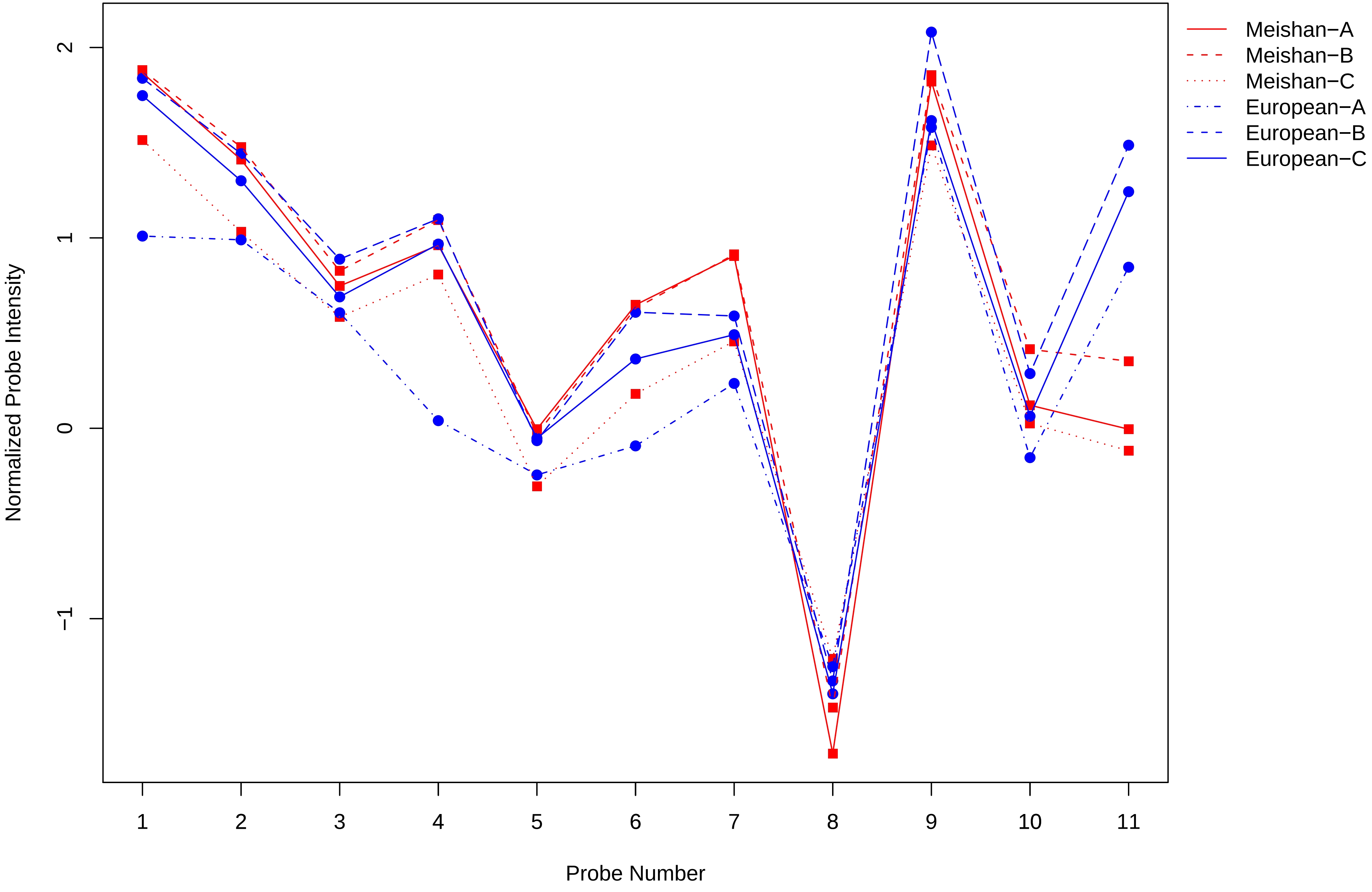

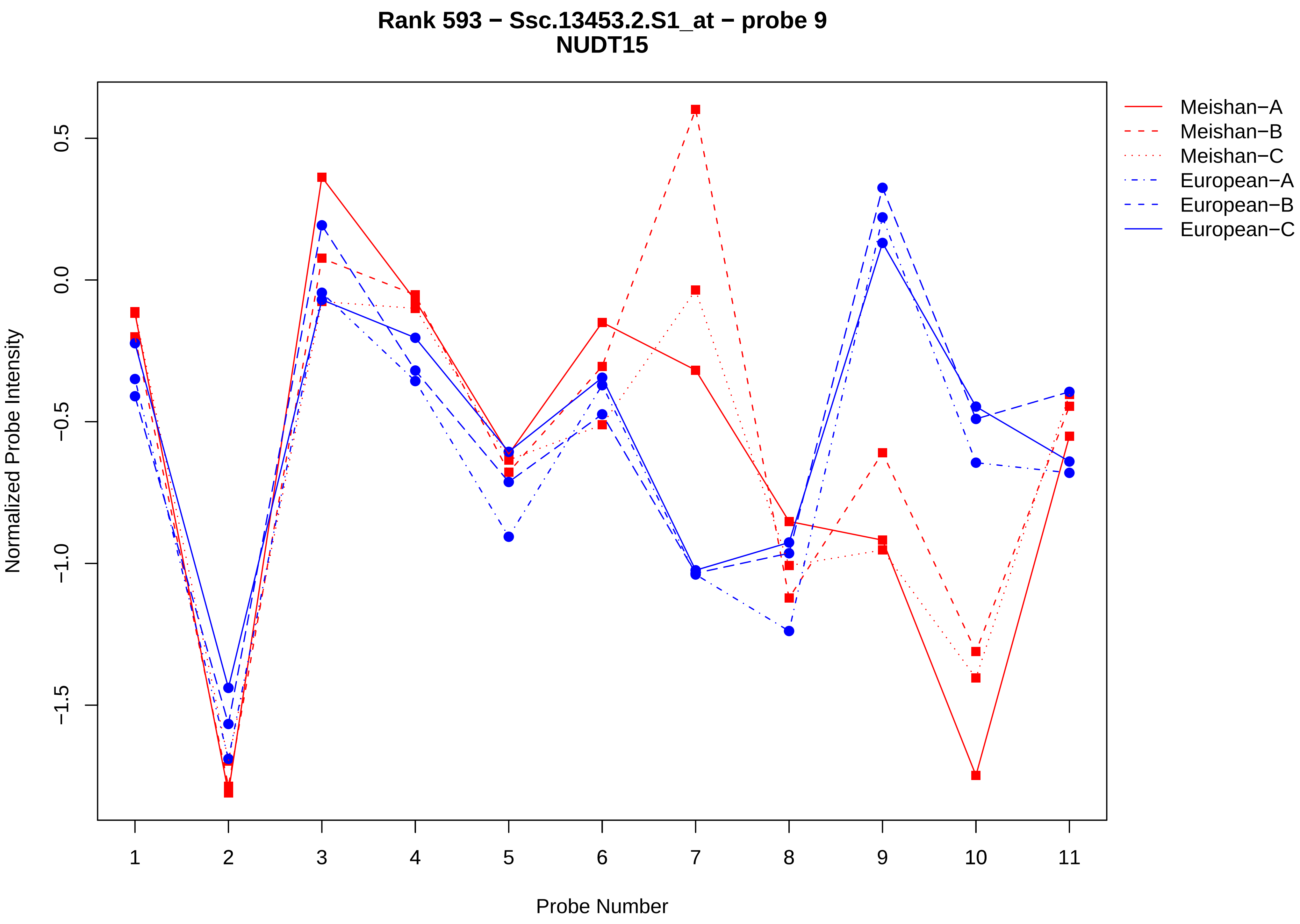

Rank 593 − Ssc.13453.2.S1_at − probe 9
NUDT15
Normalized Probe Intensity
Probe Number
0.5
0.0
−0.5
−1.0
−1.5
1
2
3
4
5
6
7
8
9
10
11
Meishan−A
Meishan−B
Meishan−C
European−A
European−B
European−C

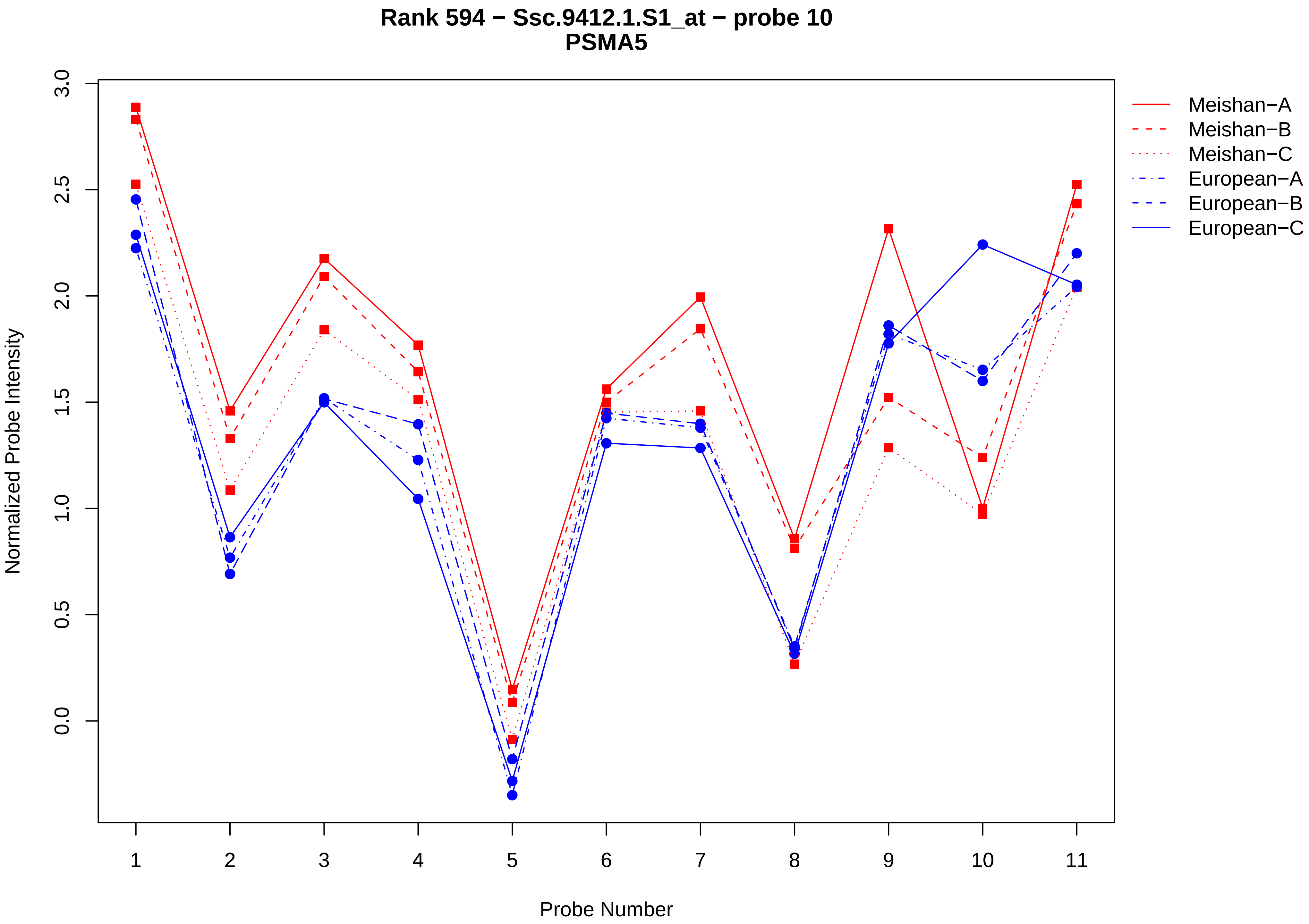

Rank 594 − Ssc.9412.1.S1_at − probe 10
PSMA5
Normalized Probe Intensity
Probe Number
0.0
0.5
1.0
1.5
2.0
2.5
3.0
1
2
3
4
5
6
7
8
9
10
11
Meishan−A
Meishan−B
Meishan−C
European−A
European−B
European−C

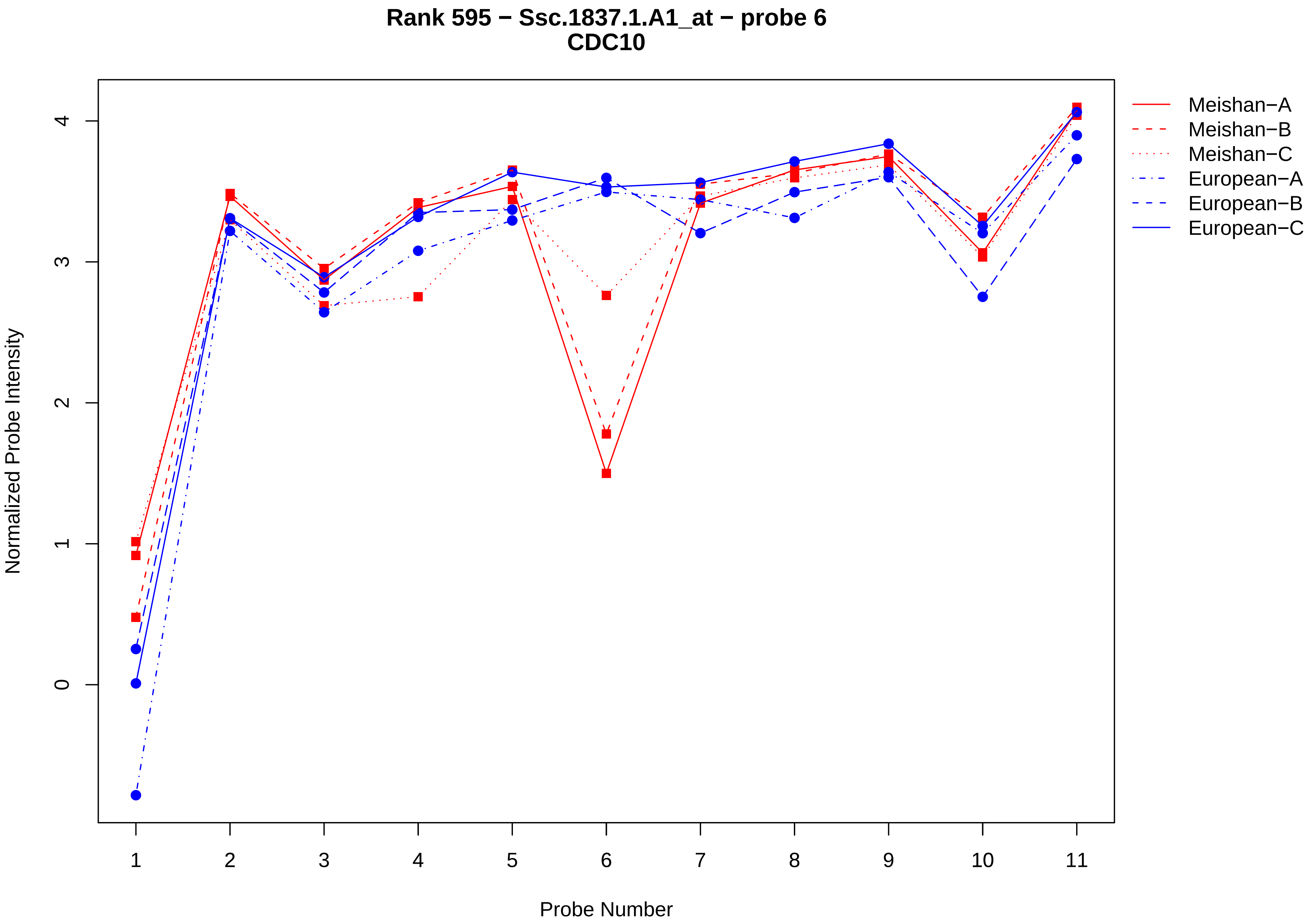

Rank 595 − Ssc.1837.1.A1_at − probe 6
CDC10
Normalized Probe Intensity
Probe Number
0
1
2
3
4
1
2
3
4
5
6
7
8
9
10
11
Meishan−A
Meishan−B
Meishan−C
European−A
European−B
European−C

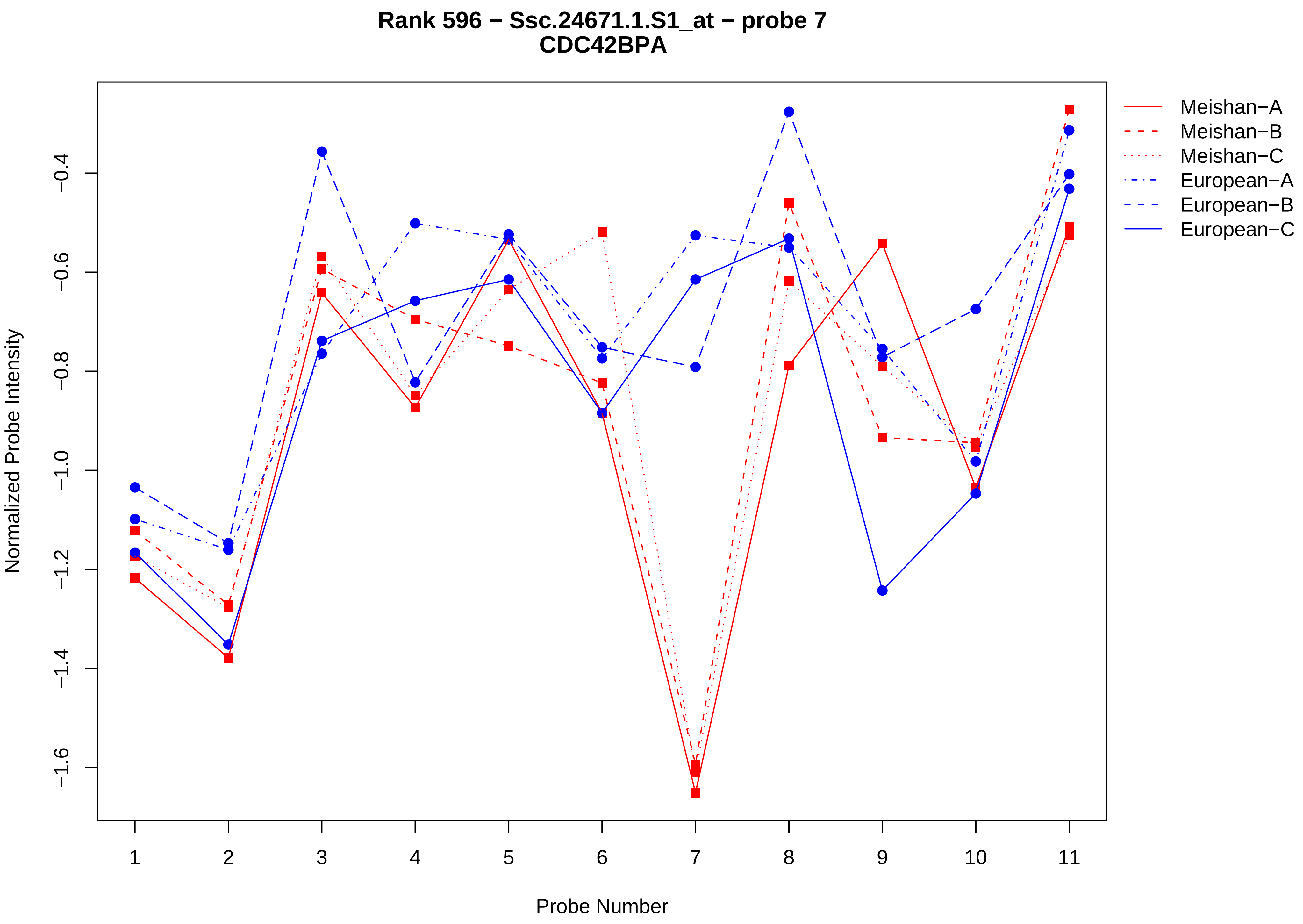
Rank 596 − Ssc.24671.1.S1_at − probe 7
CDC42BPA
Normalized Probe Intensity
Probe Number
−0.4
−0.6
−0.8
−1.0
−1.2
−1.4
−1.6
1
2
3
4
5
6
7
8
9
10
11
Meishan−A
Meishan−B
Meishan−C
European−A
European−B
European−C

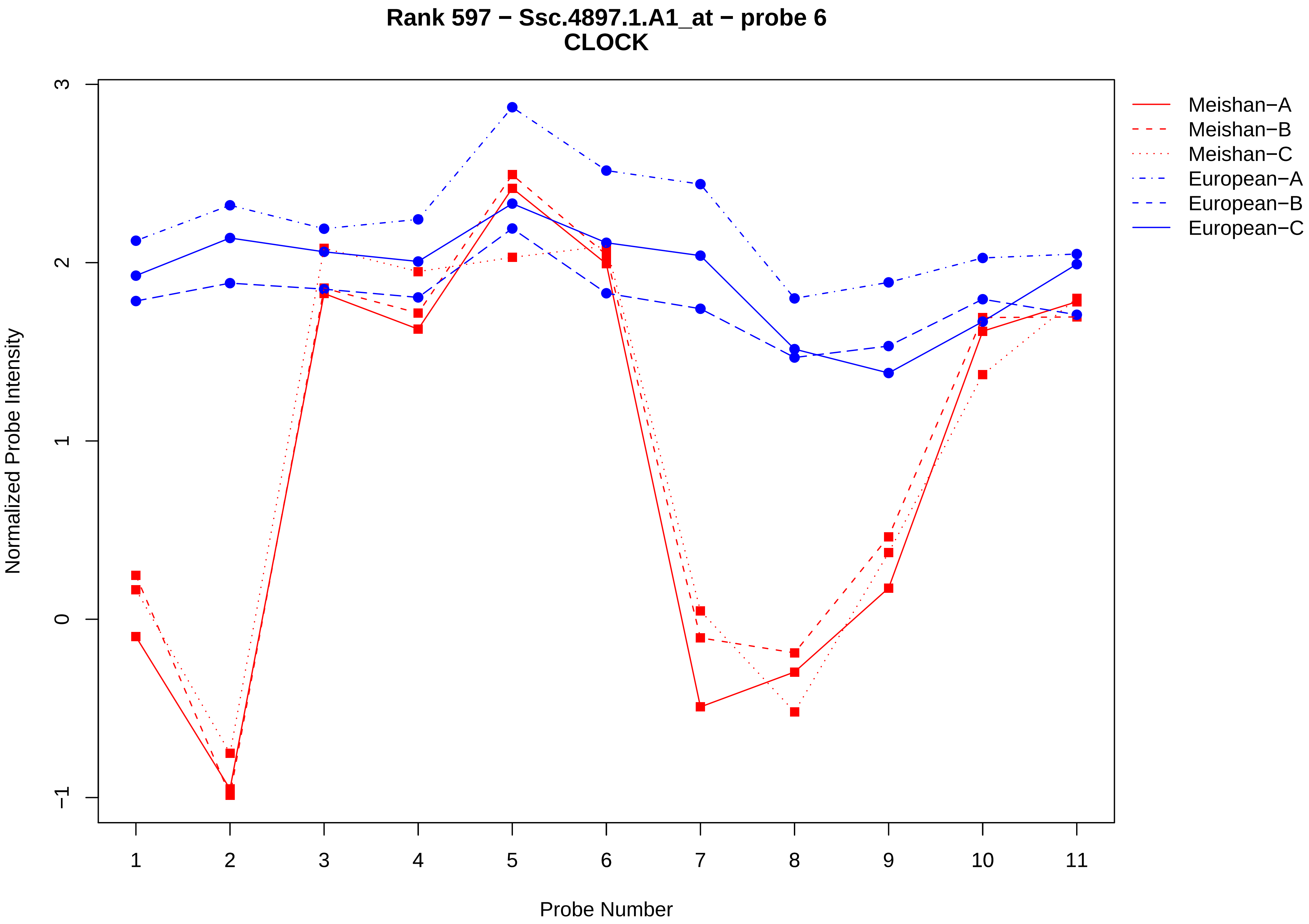
Rank 597 − Ssc.4897.1.A1_at − probe 6
CLOCK
Normalized Probe Intensity
Probe Number
−1
0
1
2
3
1
2
3
4
5
6
7
8
9
10
11
Meishan−A
Meishan−B
Meishan−C
European−A
European−B
European−C

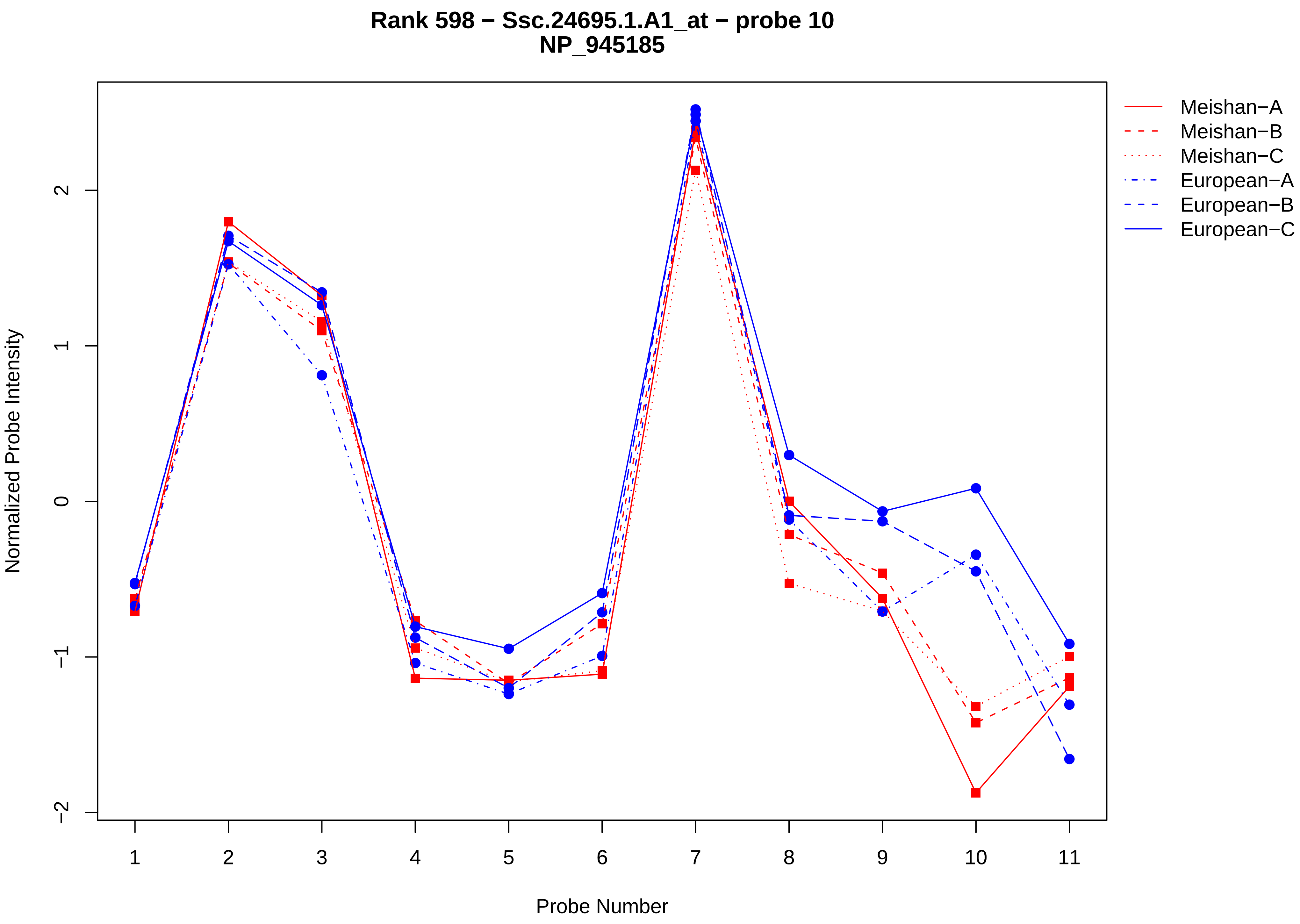

Rank 598 − Ssc.24695.1.A1_at − probe 10
NP_945185
Normalized Probe Intensity
Probe Number
−2
−1
0
1
2
1
2
3
4
5
6
7
8
9
10
11
Meishan−A
Meishan−B
Meishan−C
European−A
European−B
European−C

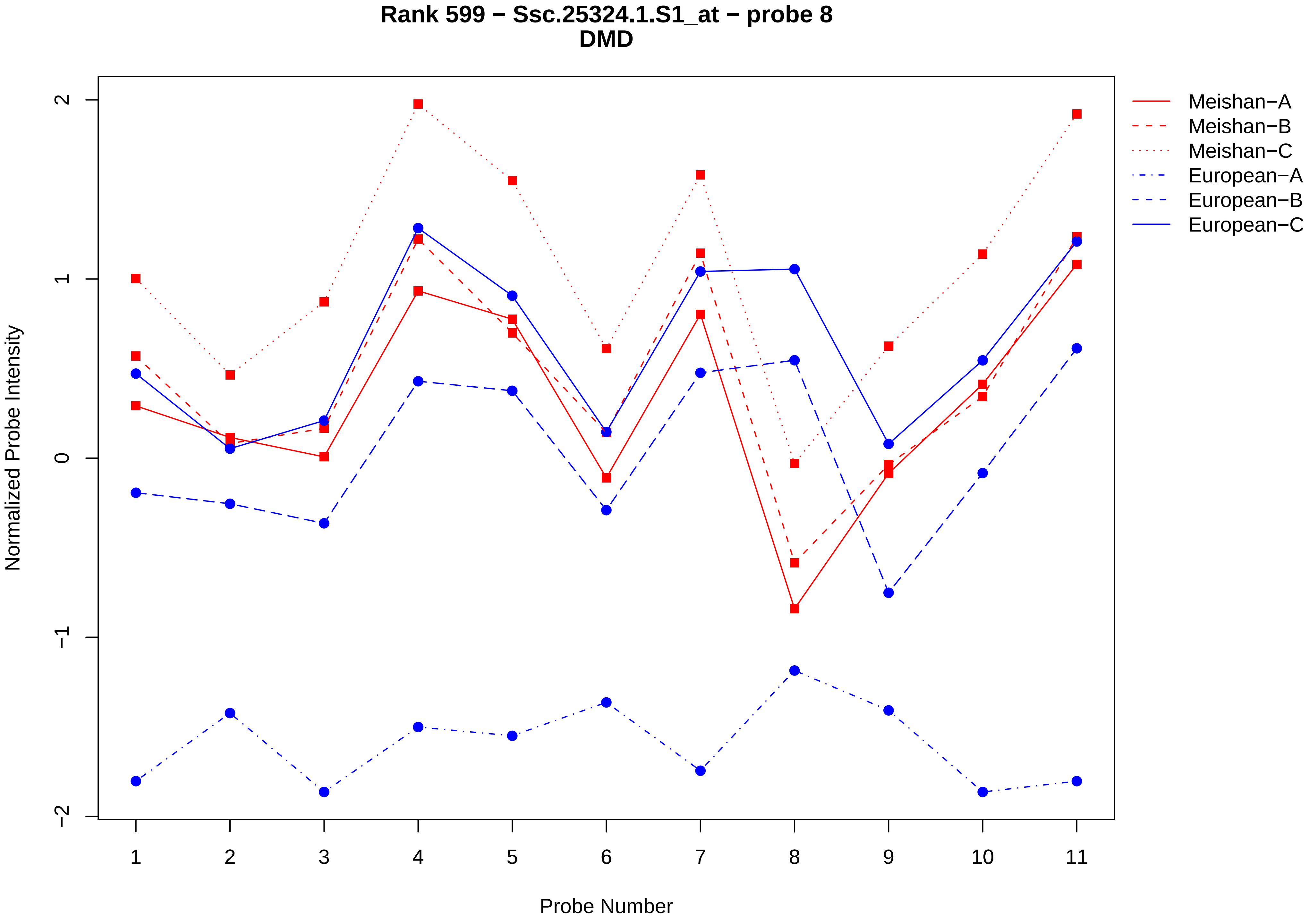

Rank 599 − Ssc.25324.1.S1_at − probe 8
DMD
Normalized Probe Intensity
Probe Number
−2
−1
0
1
2
1
2
3
4
5
6
7
8
9
10
11
Meishan−A
Meishan−B
Meishan−C
European−A
European−B
European−C

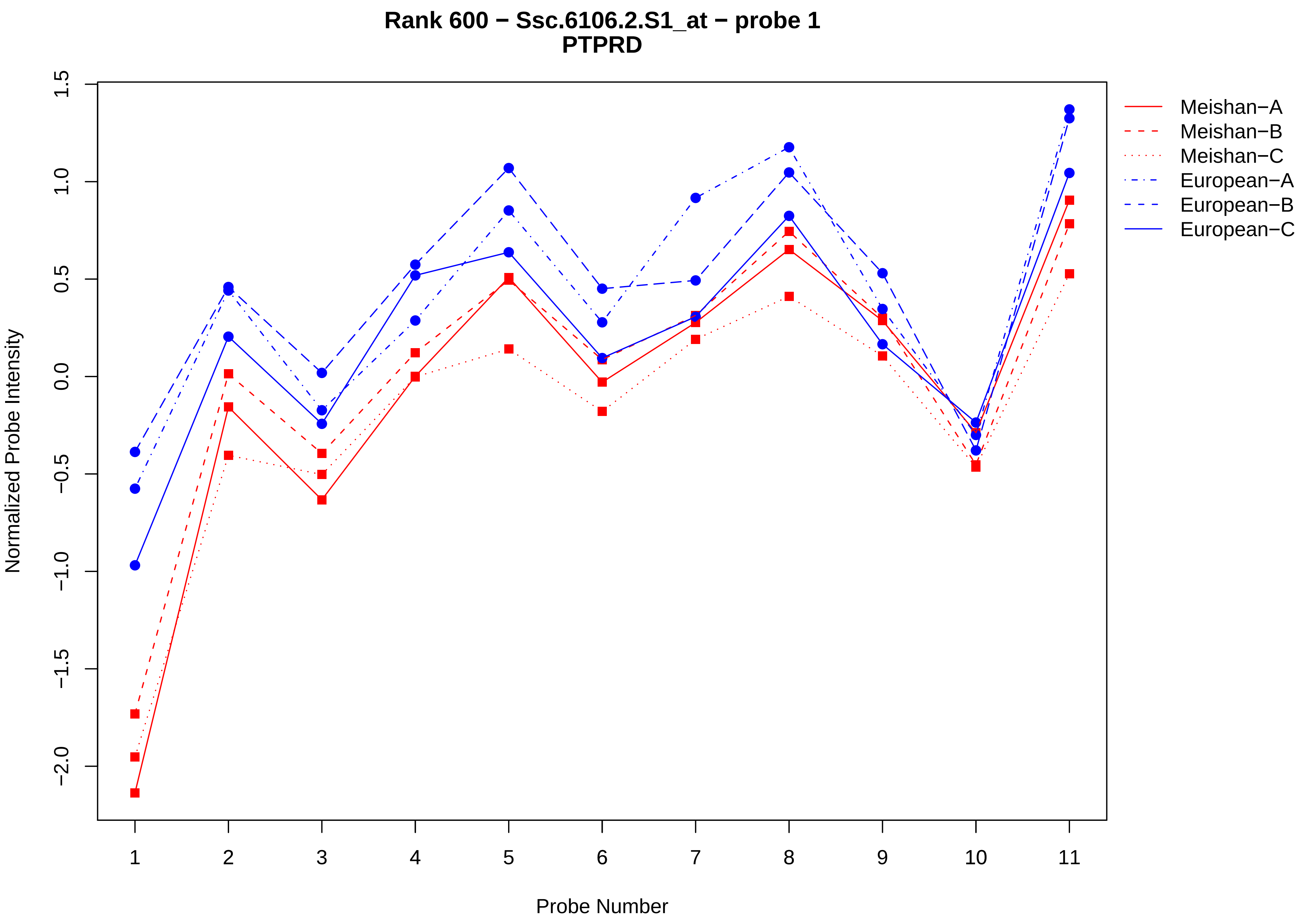

Rank 600 − Ssc.6106.2.S1_at − probe 1
PTPRD
Normalized Probe Intensity
1.5
1.0
0.5
0.0
−0.5
−1.0
−1.5
−2.0
Probe Number
1
2
3
4
5
6
7
8
9
10
11
Meishan−A
Meishan−B
Meishan−C
European−A
European−B
European−C

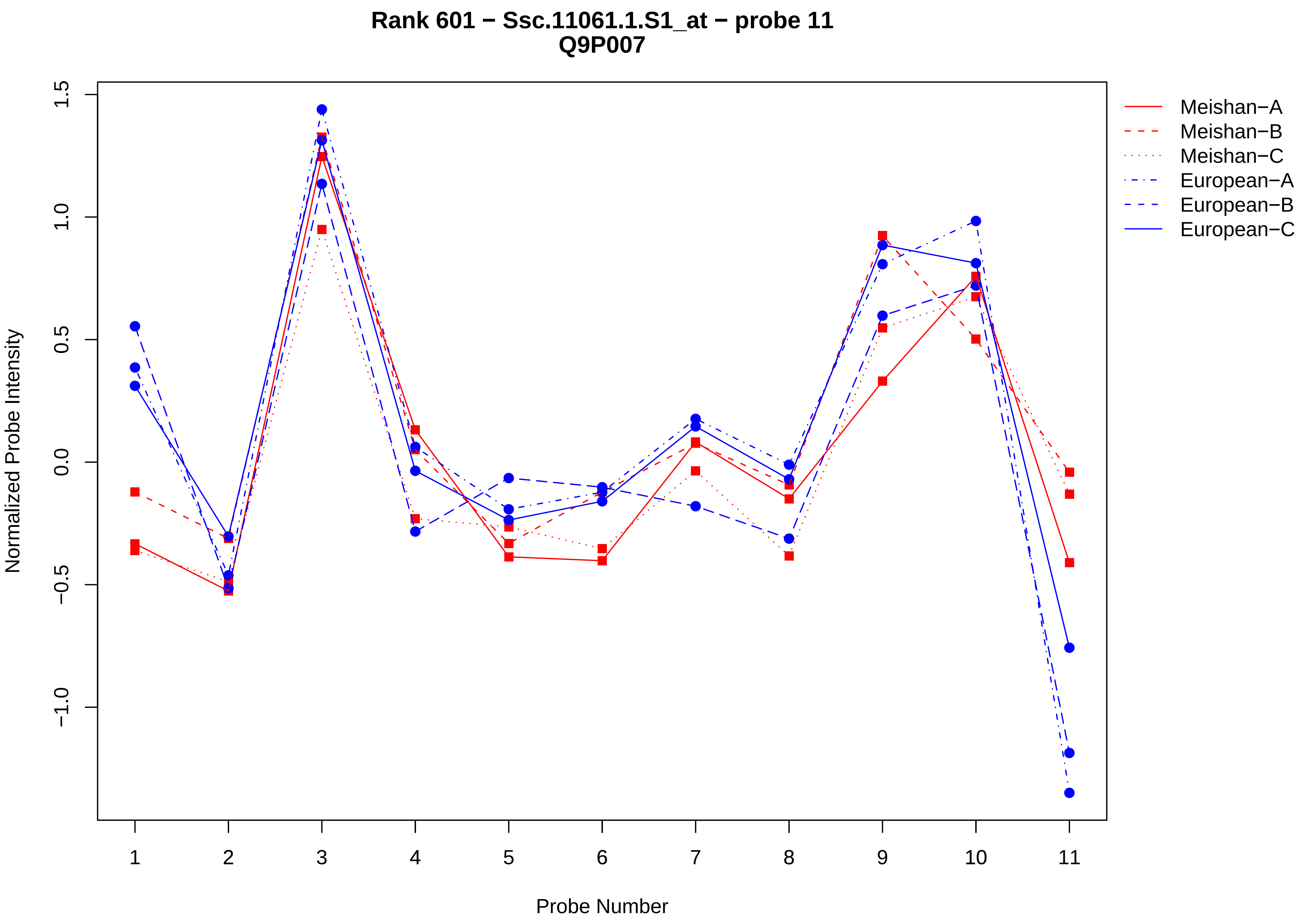

Rank 601 − Ssc.11061.1.S1_at − probe 11
Q9P007
Normalized Probe Intensity
Probe Number
1.5
1.0
0.5
0.0
−0.5
−1.0
1
2
3
4
5
6
7
8
9
10
11
Meishan−A
Meishan−B
Meishan−C
European−A
European−B
European−C

Rank 602 − Ssc.28497.1.S1_at − probe 1
C6orf211

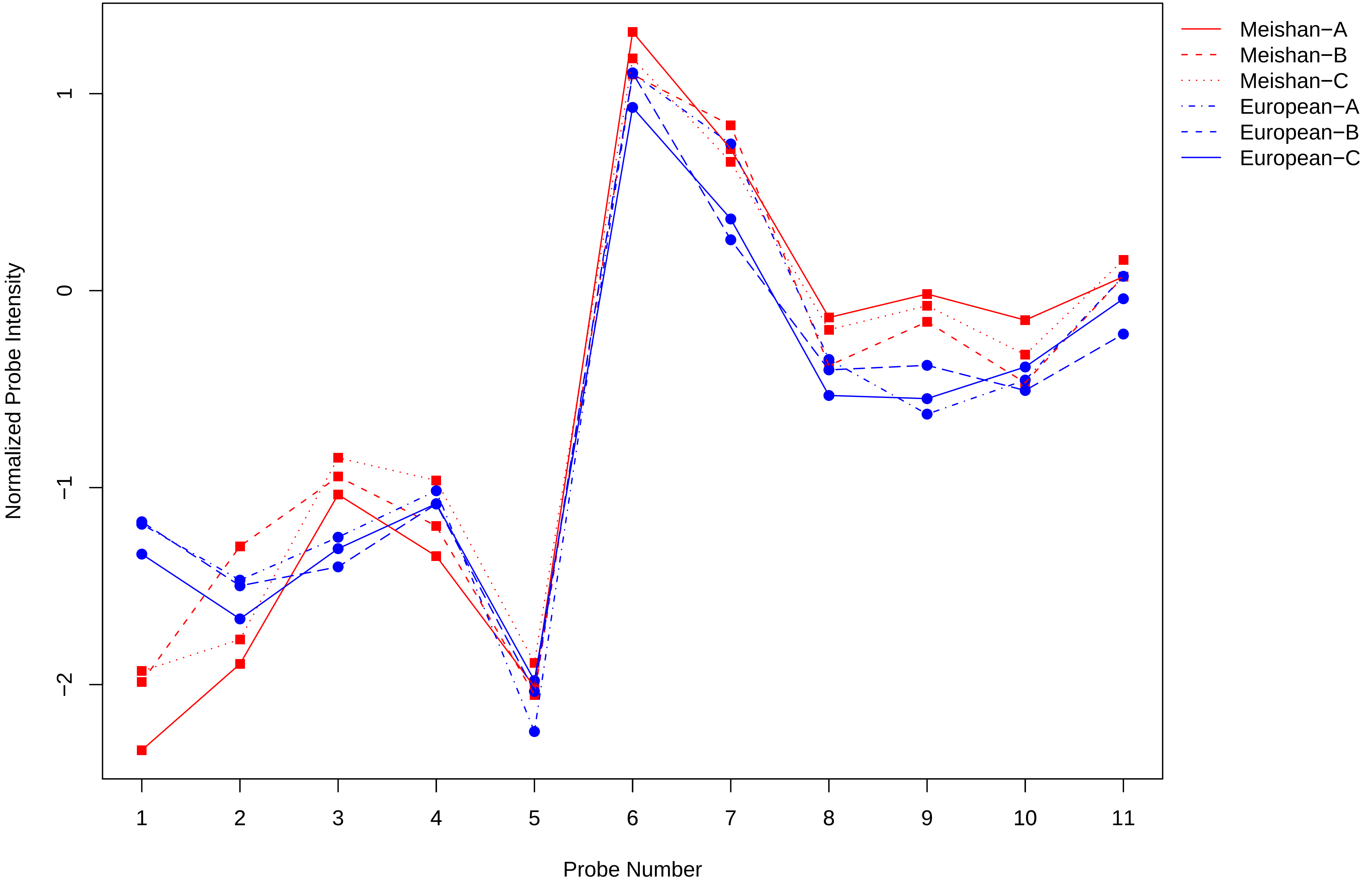

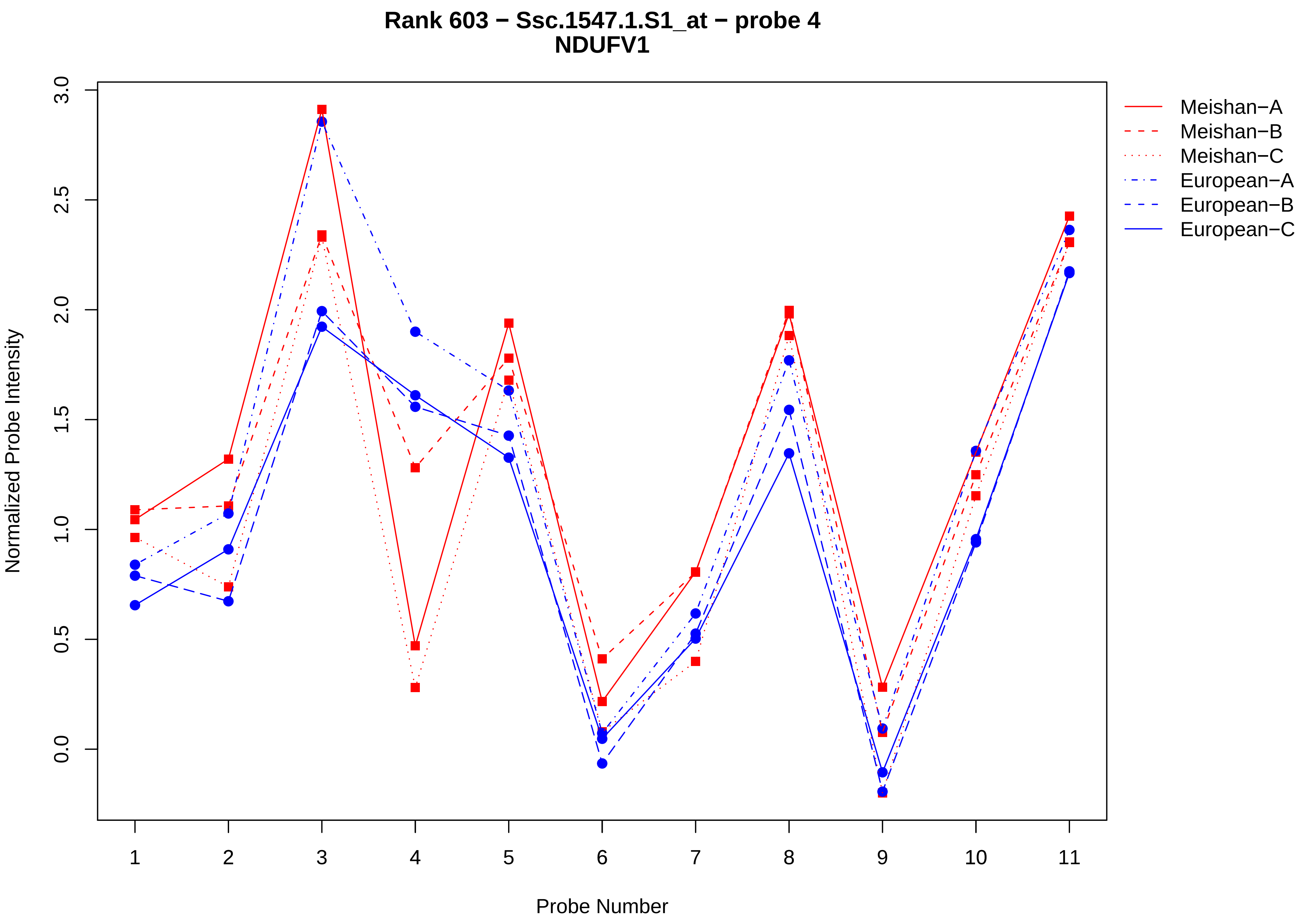
Rank 603 − Ssc.1547.1.S1_at − probe 4
NDUFV1
Normalized Probe Intensity
Probe Number
0.0
0.5
1.0
1.5
2.0
2.5
3.0
1
2
3
4
5
6
7
8
9
10
11
Meishan−A
Meishan−B
Meishan−C
European−A
European−B
European−C

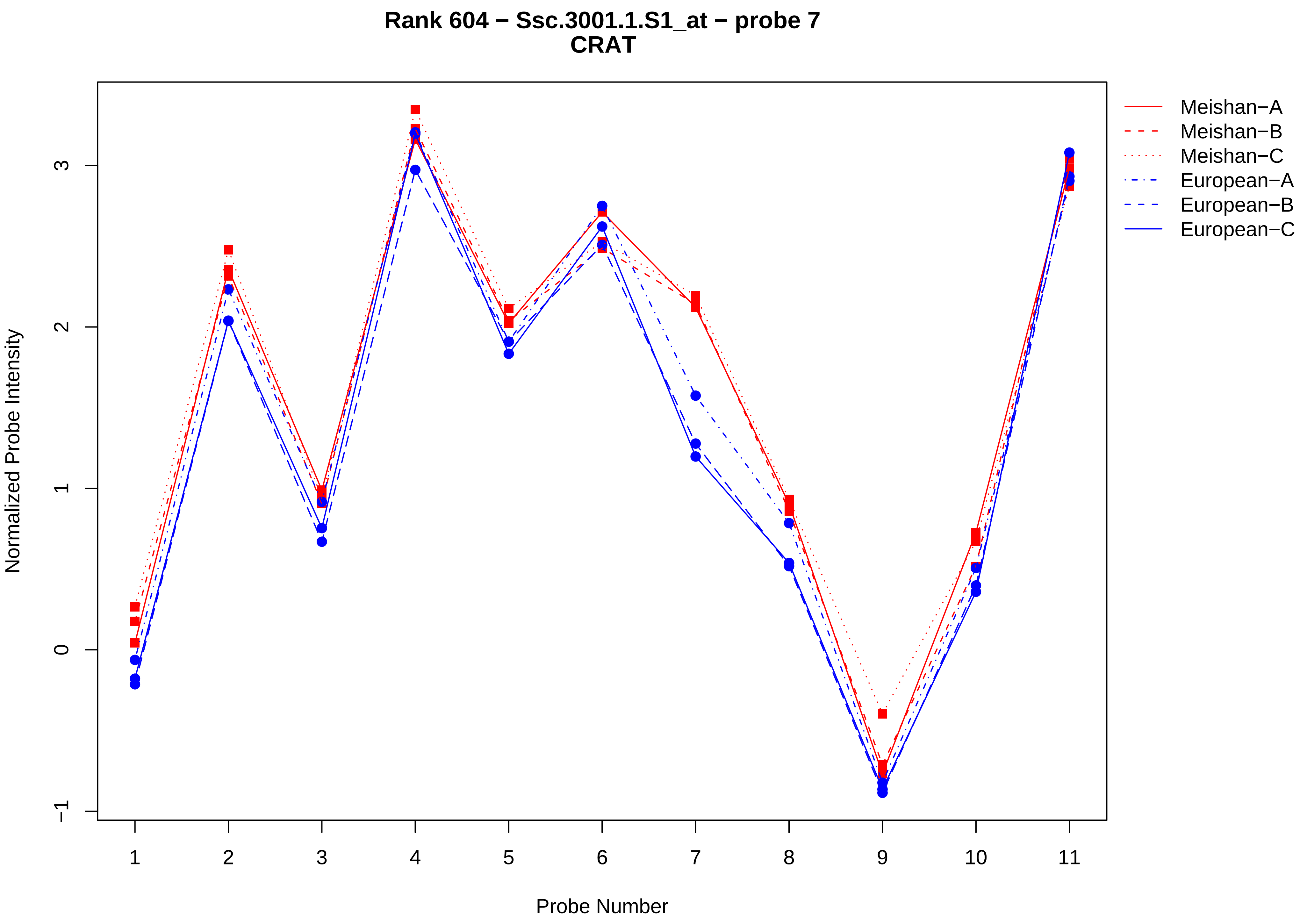
Rank 604 − Ssc.3001.1.S1_at − probe 7
CRAT
Normalized Probe Intensity
Probe Number
−1
0
1
2
3
1
2
3
4
5
6
7
8
9
10
11
Meishan−A
Meishan−B
Meishan−C
European−A
European−B
European−C

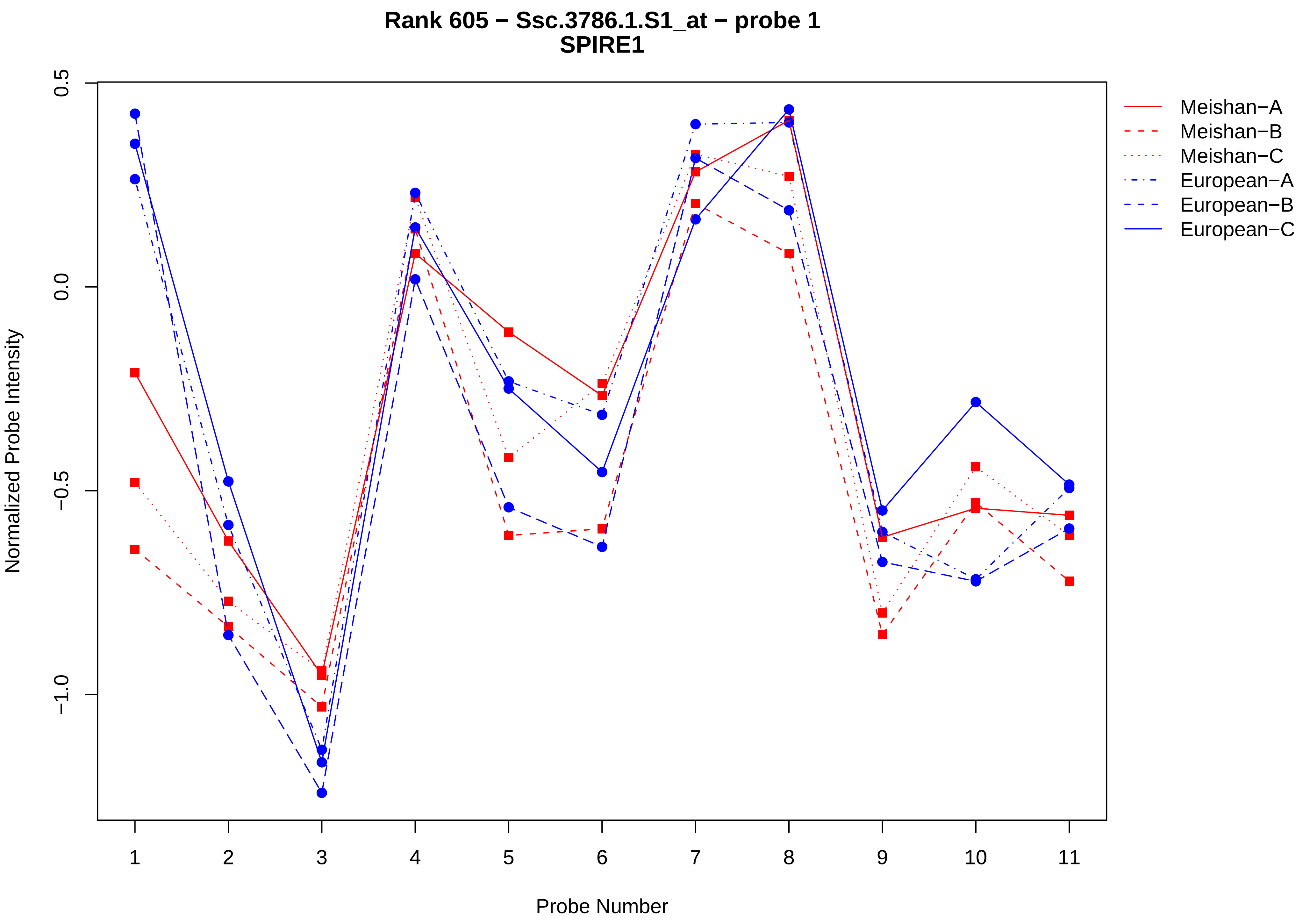

Rank 605 − Ssc.3786.1.S1_at − probe 1
SPIRE1
Normalized Probe Intensity
Probe Number
0.5
0.0
−0.5
−1.0
1
2
3
4
5
6
7
8
9
10
11
Meishan−A
Meishan−B
Meishan−C
European−A
European−B
European−C

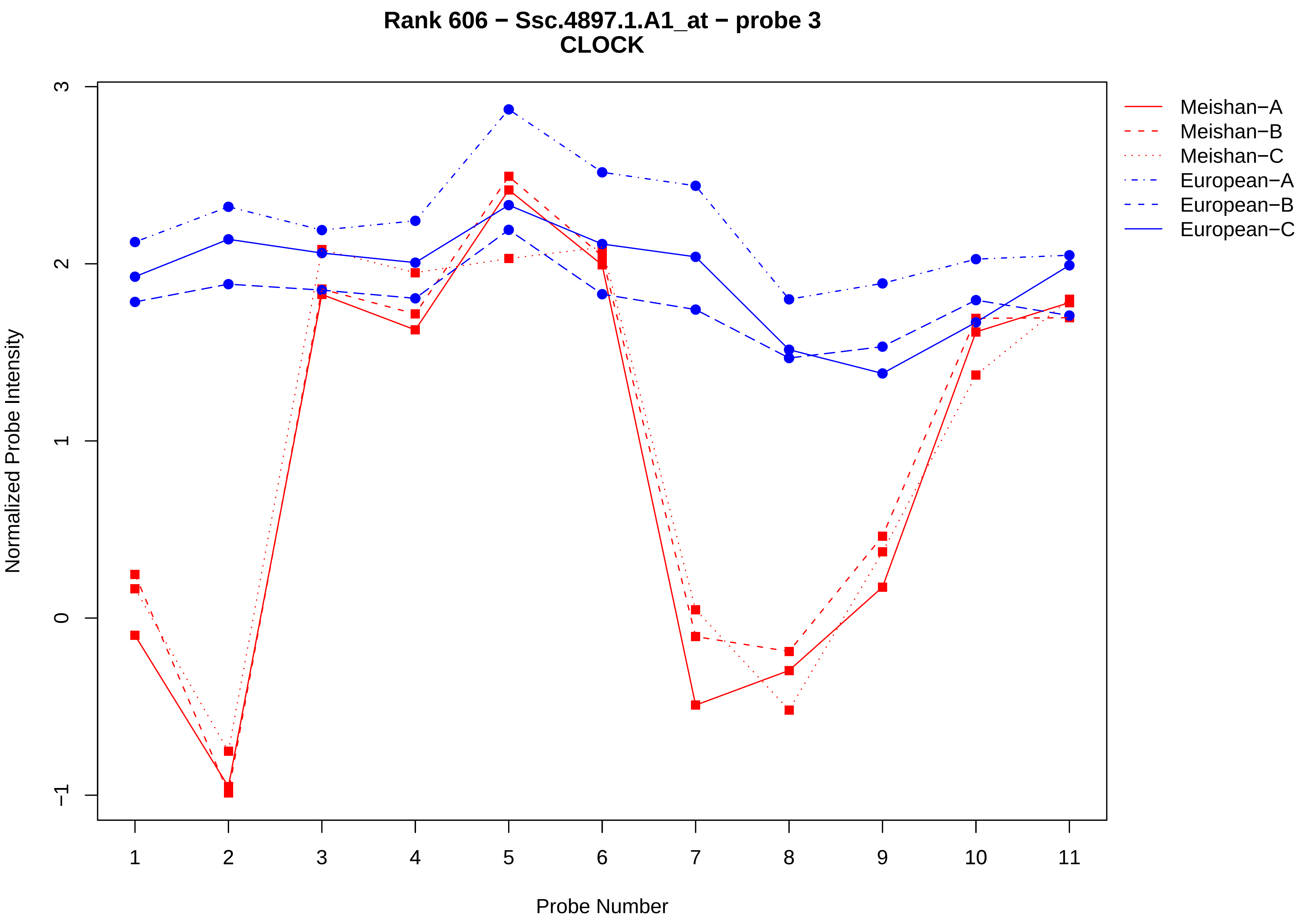

Rank 606 − Ssc.4897.1.A1_at − probe 3
CLOCK
Normalized Probe Intensity
Probe Number
−1
0
1
2
3
1
2
3
4
5
6
7
8
9
10
11
Meishan−A
Meishan−B
Meishan−C
European−A
European−B
European−C

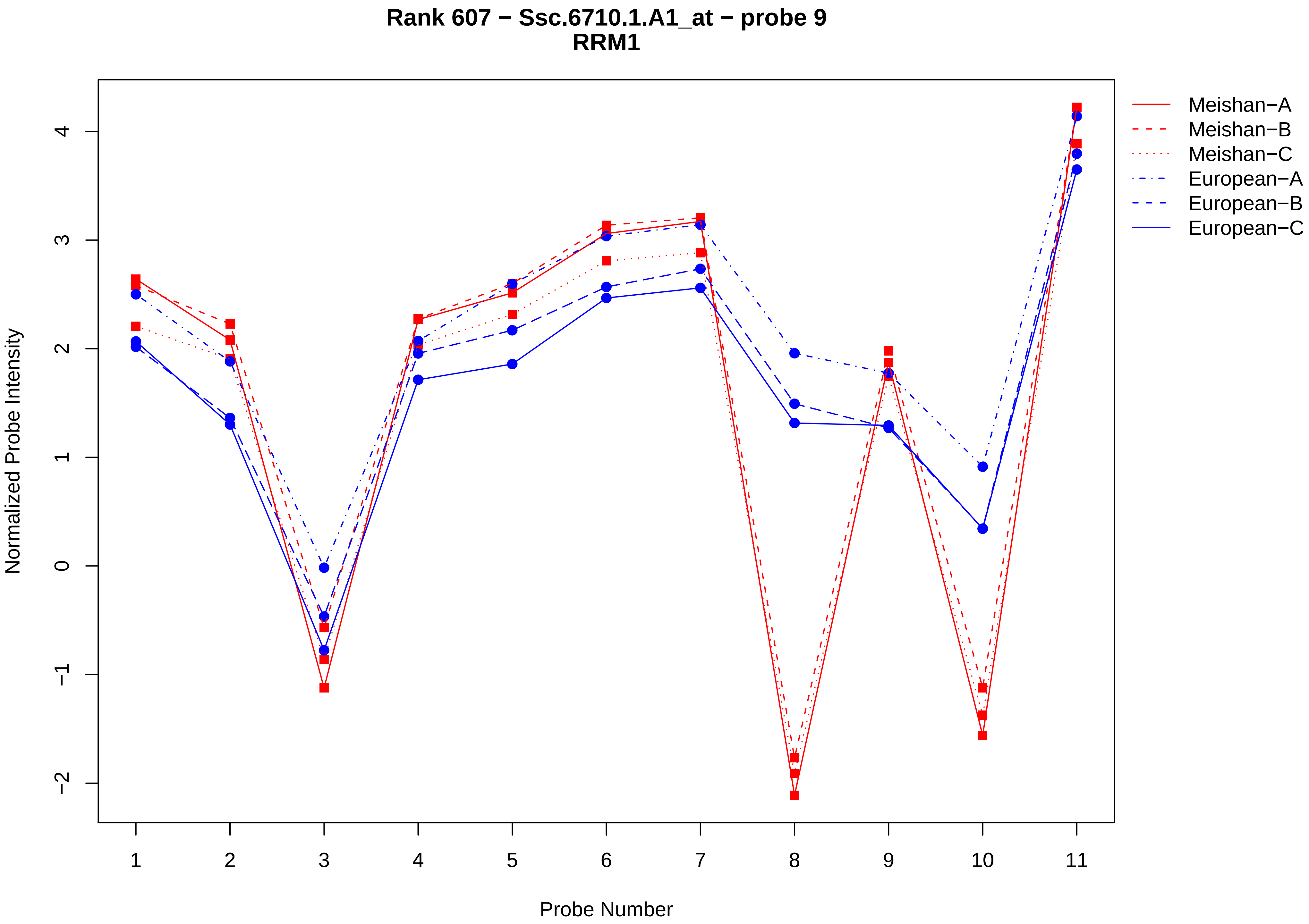

Rank 607 − Ssc.6710.1.A1_at − probe 9
RRM1
Normalized Probe Intensity
Probe Number
−2
−1
0
1
2
3
4
1
2
3
4
5
6
7
8
9
10
11
Meishan−A
Meishan−B
Meishan−C
European−A
European−B
European−C

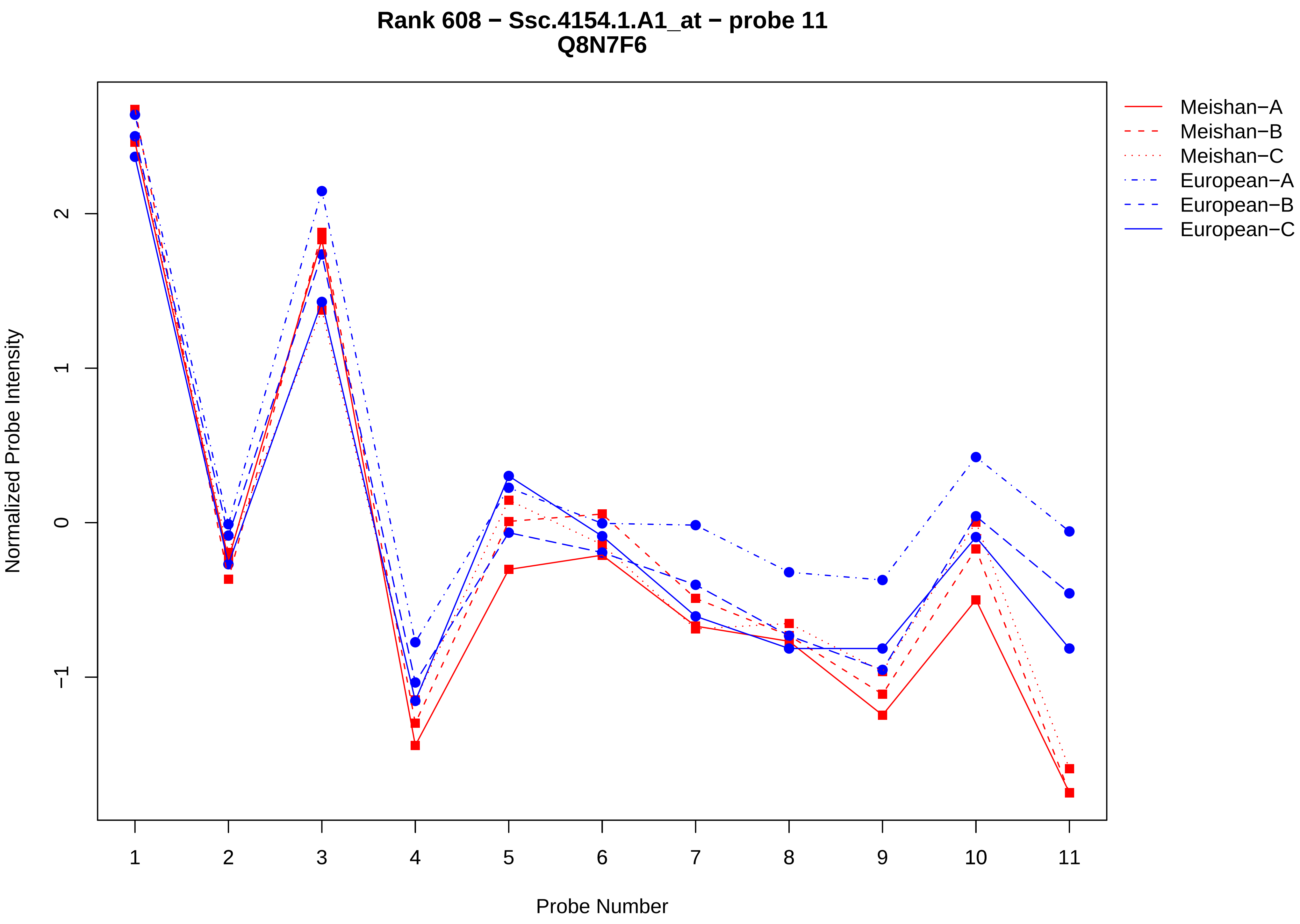

Rank 608 − Ssc.4154.1.A1_at − probe 11
Q8N7F6
Normalized Probe Intensity
Probe Number
Meishan−A
Meishan−B
Meishan−C
European−A
European−B
European−C
−1
0
1
2
1
2
3
4
5
6
7
8
9
10
11

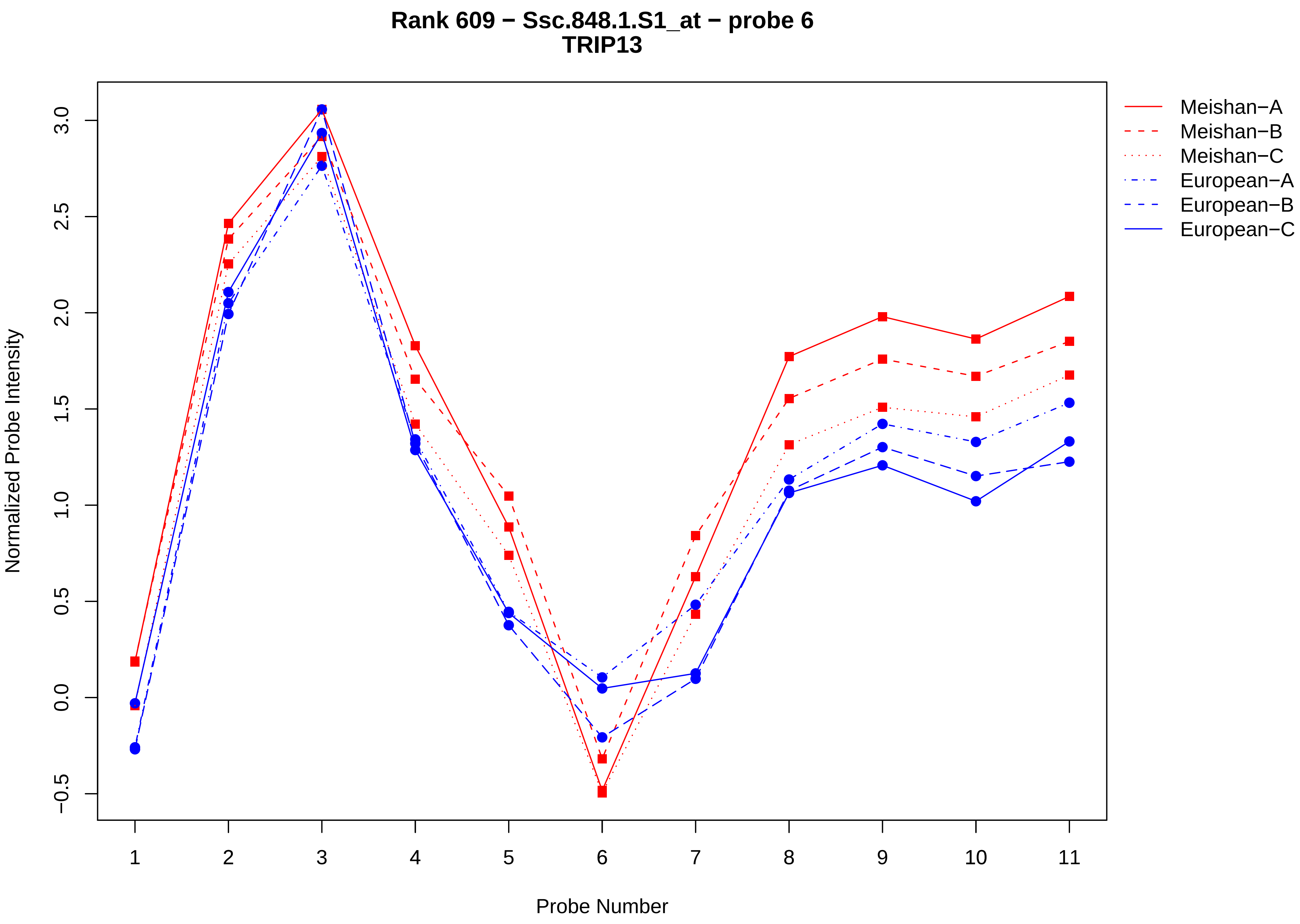

Rank 609 − Ssc.848.1.S1_at − probe 6
TRIP13
Normalized Probe Intensity
Probe Number
Meishan−A
Meishan−B
Meishan−C
European−A
European−B
European−C
−0.5
0.0
0.5
1.0
1.5
2.0
2.5
3.0
1
2
3
4
5
6
7
8
9
10
11

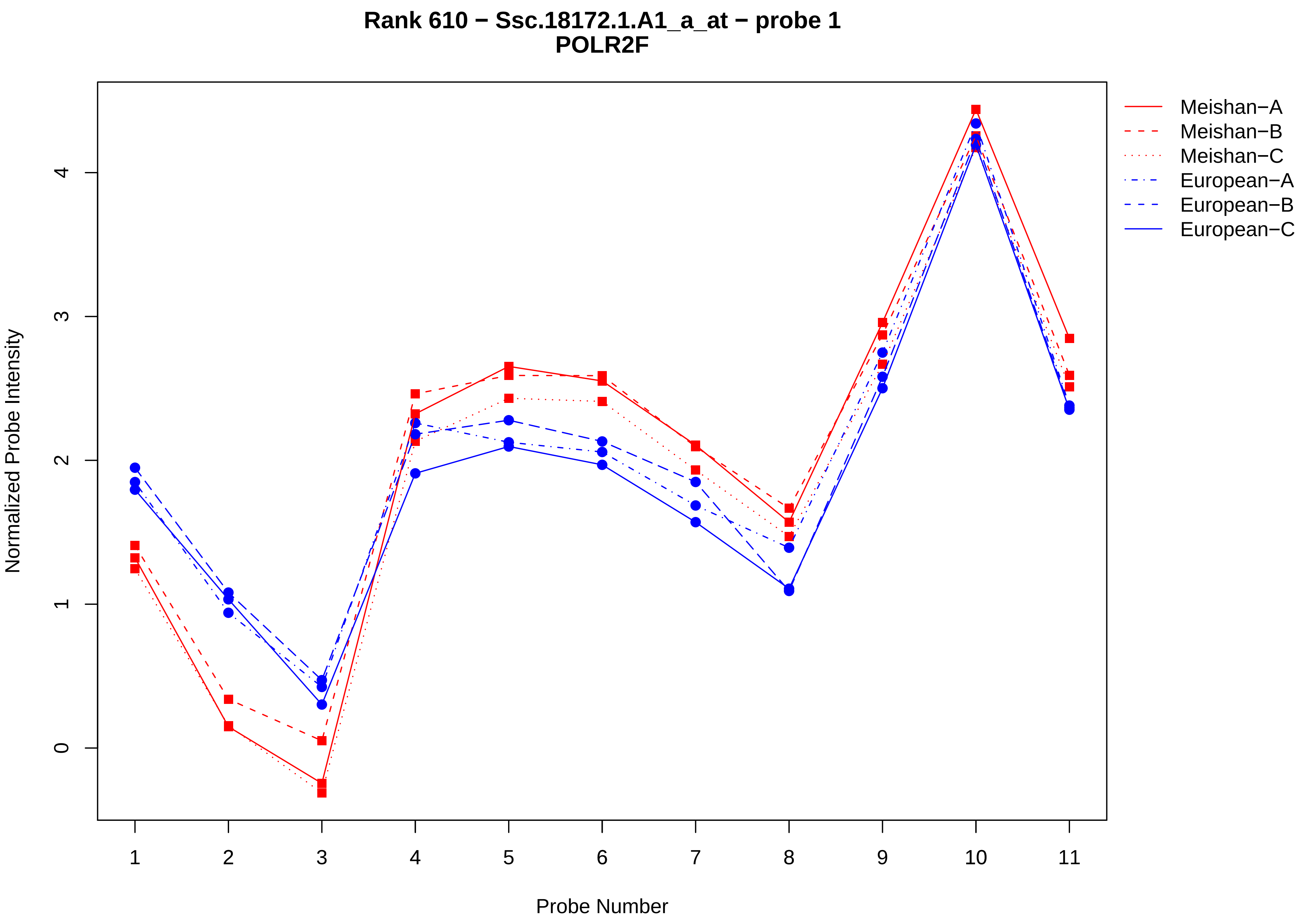

Rank 610 − Ssc.18172.1.A1_a_at − probe 1
POLR2F
Normalized Probe Intensity
Probe Number
0
1
2
3
4
1
2
3
4
5
6
7
8
9
10
11
Meishan−A
Meishan−B
Meishan−C
European−A
European−B
European−C

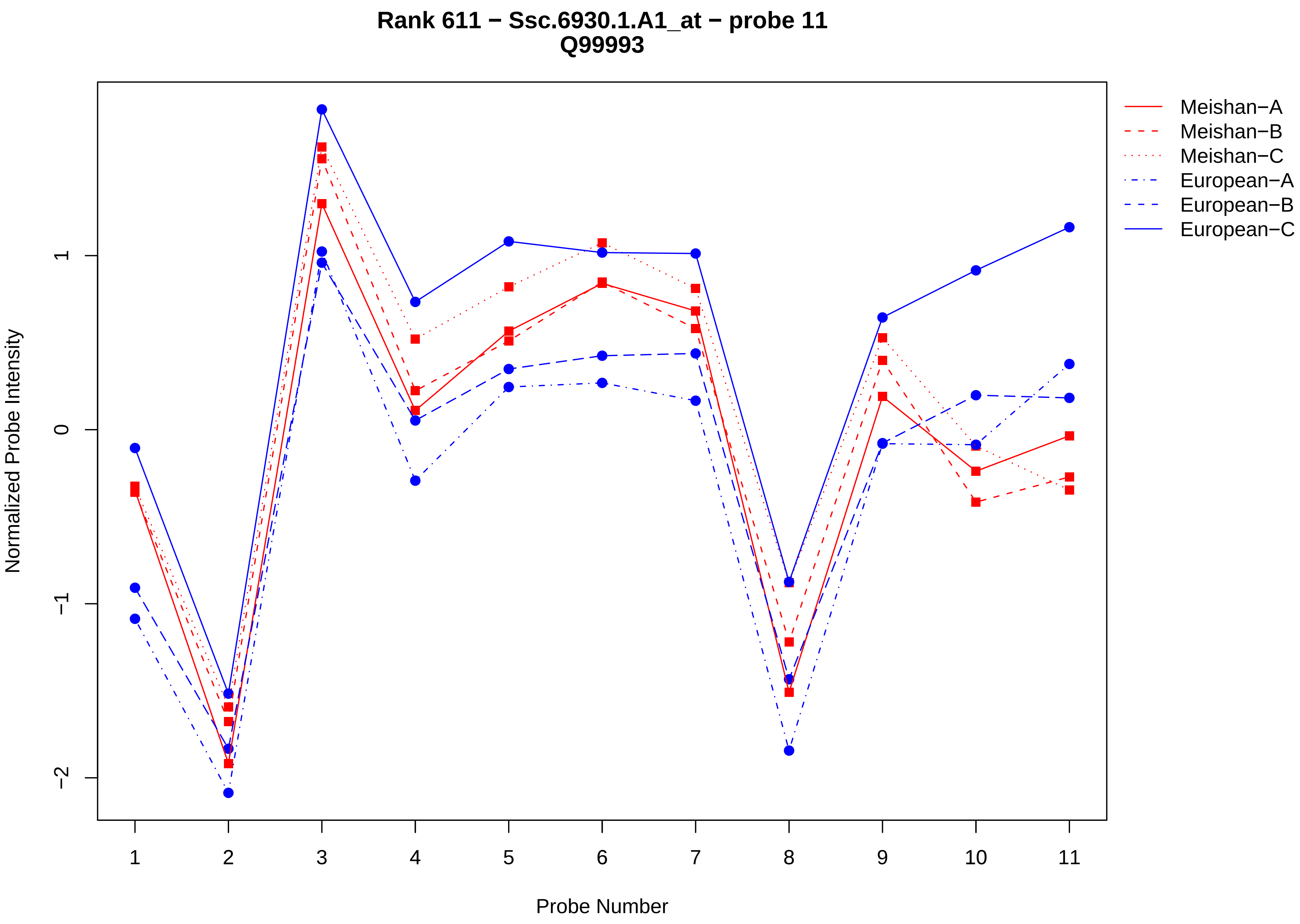

Rank 611 − Ssc.6930.1.A1_at − probe 11
Q99993
Normalized Probe Intensity
Probe Number
Meishan−A
Meishan−B
Meishan−C
European−A
European−B
European−C

Rank 612 − Ssc.11603.1.S1_at − probe 3
AEBP1

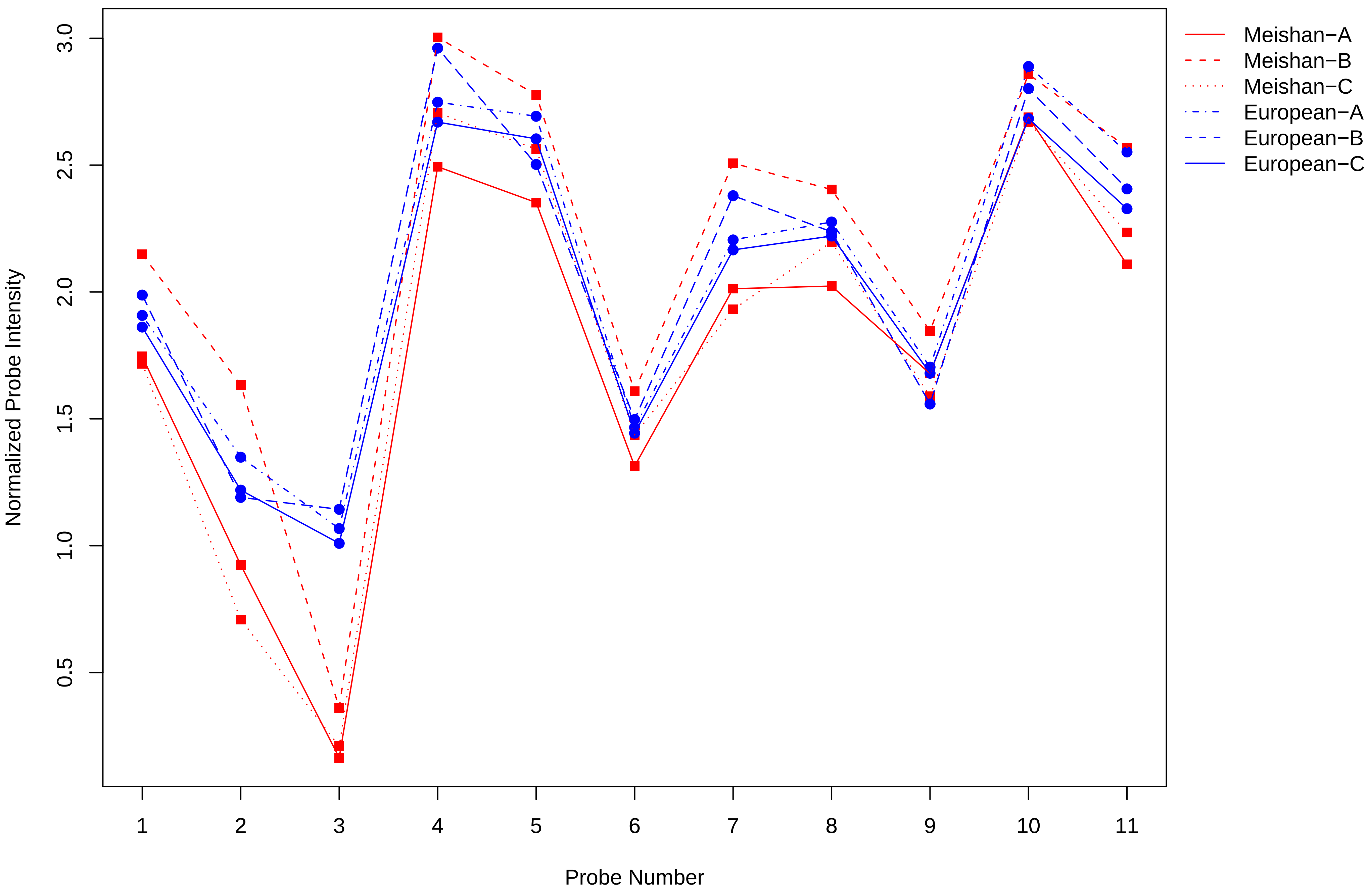

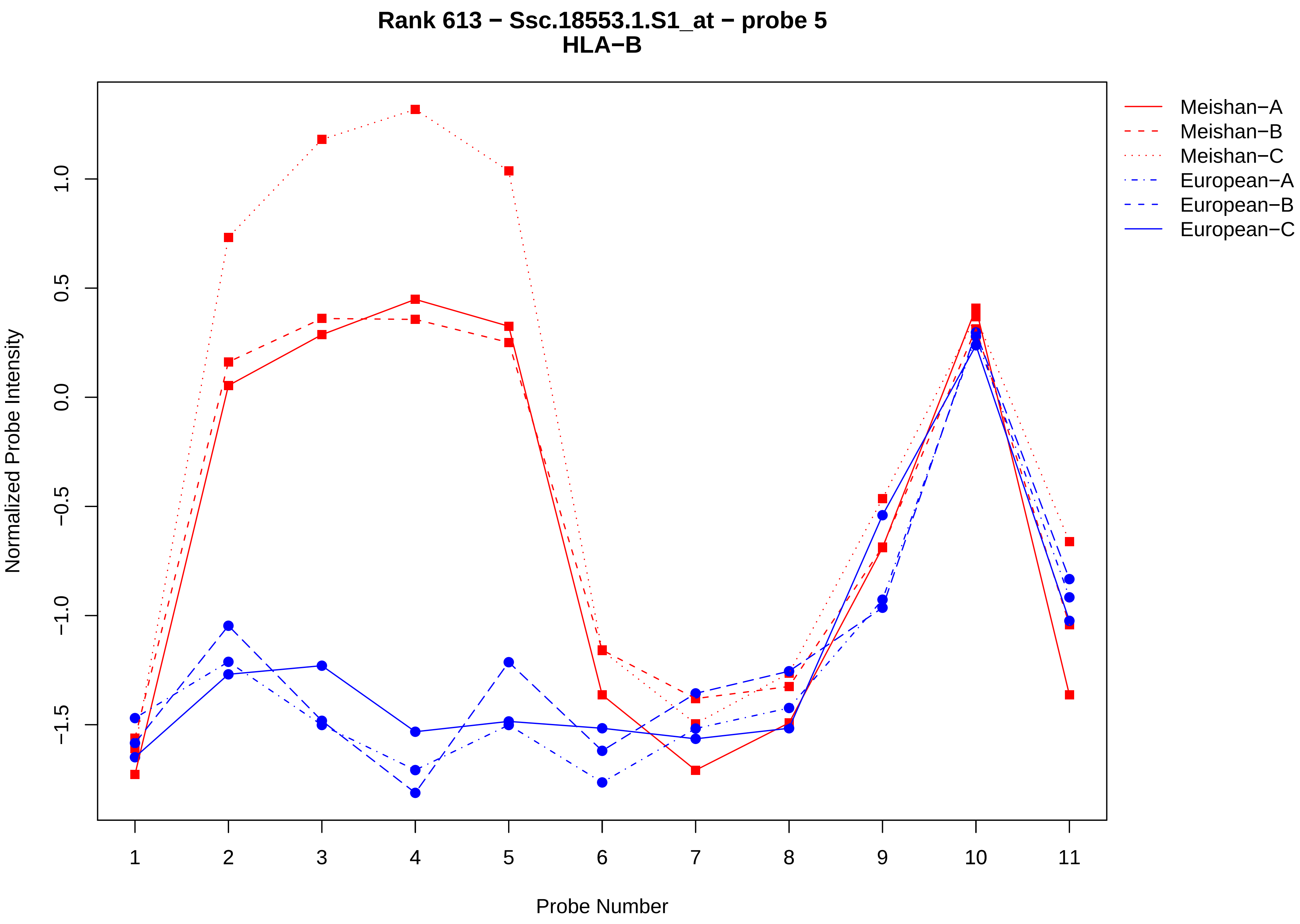

Rank 613 − Ssc.18553.1.S1_at − probe 5
HLA−B
Normalized Probe Intensity
Probe Number
1.0
0.5
0.0
−0.5
−1.0
−1.5
1
2
3
4
5
6
7
8
9
10
11
Meishan−A
Meishan−B
Meishan−C
European−A
European−B
European−C

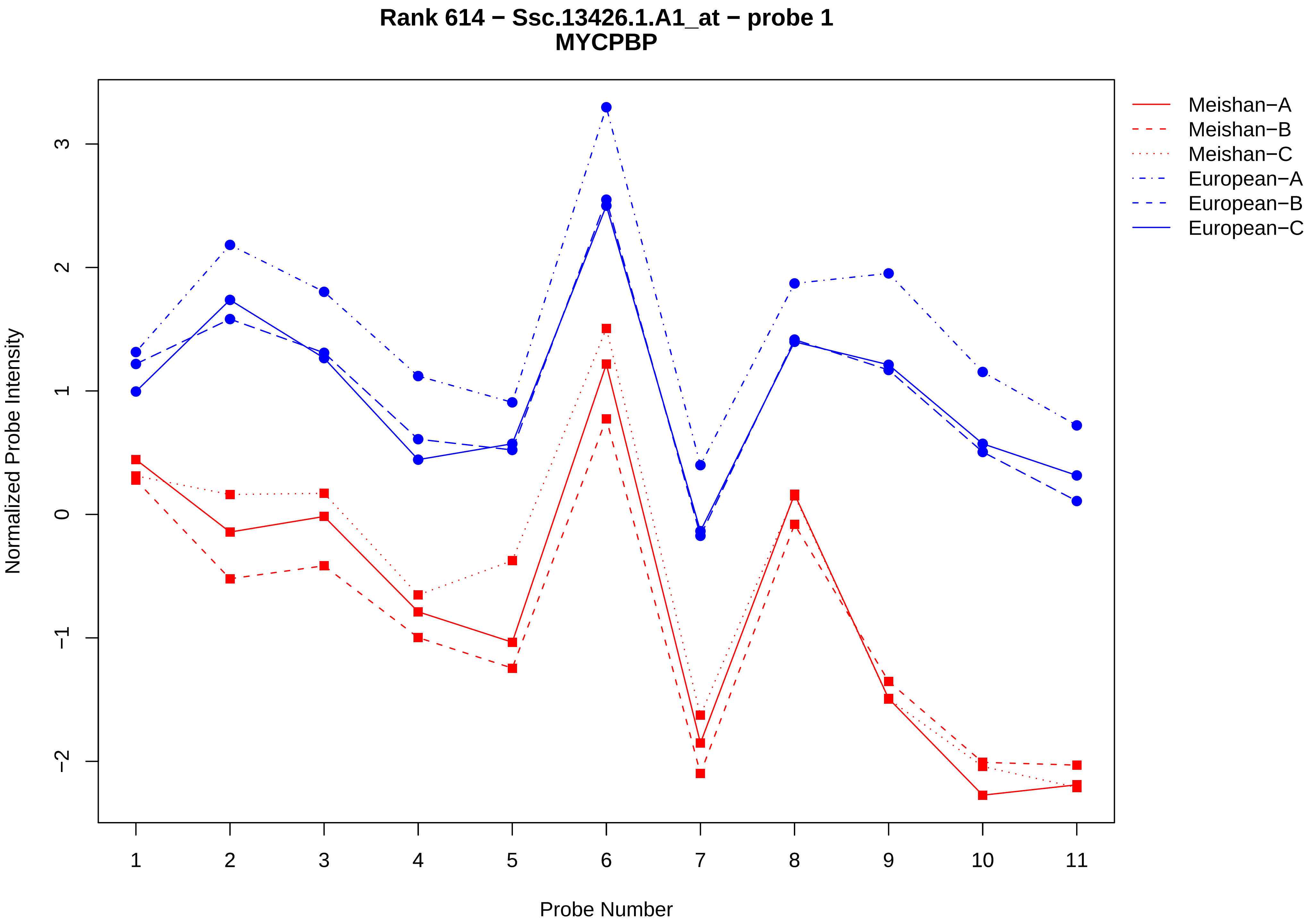
Rank 614 − Ssc.13426.1.A1_at − probe 1
MYCPBP
Normalized Probe Intensity
Probe Number
−2
−1
0
1
2
3
1
2
3
4
5
6
7
8
9
10
11
Meishan−A
Meishan−B
Meishan−C
European−A
European−B
European−C

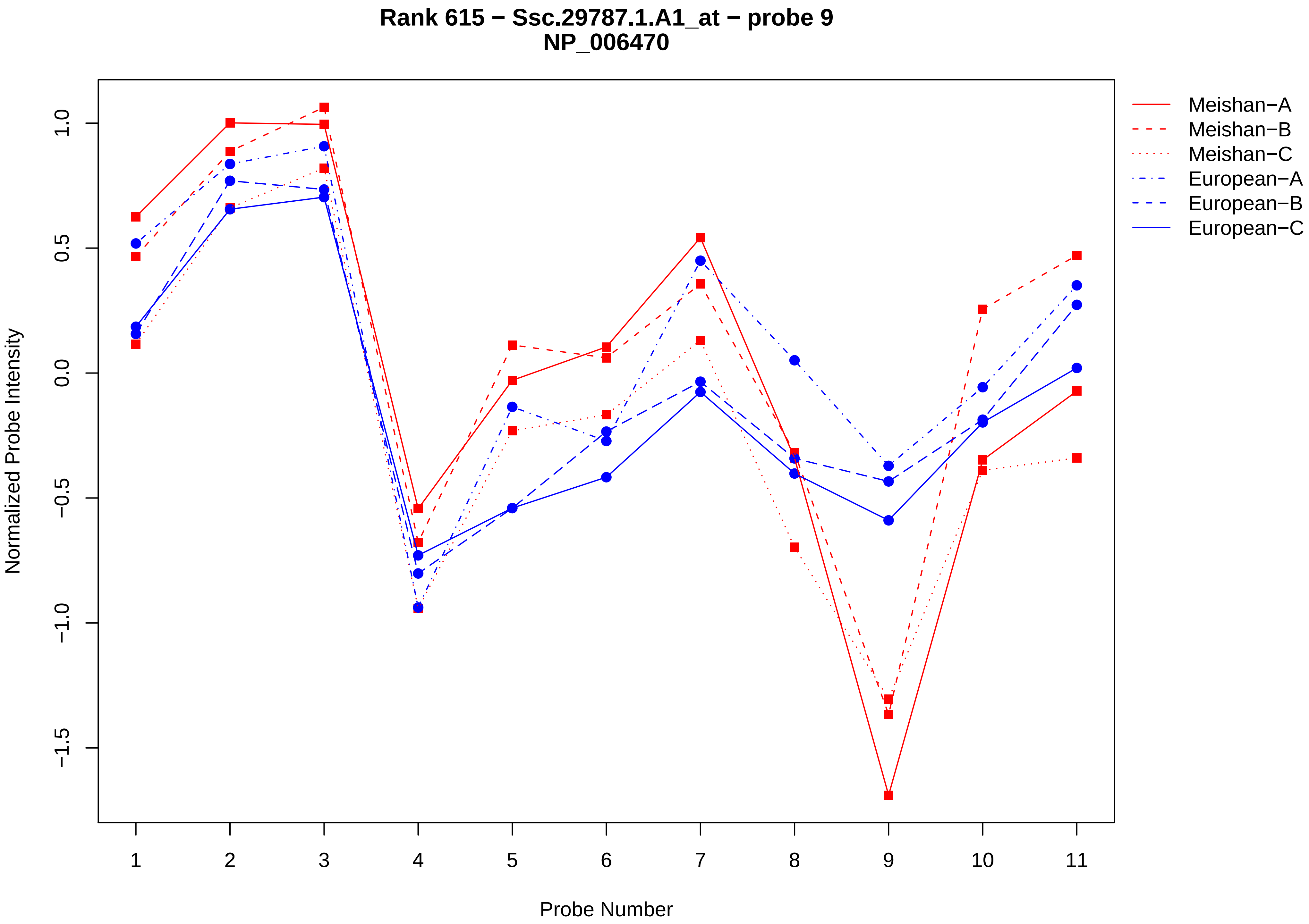

Rank 615 − Ssc.29787.1.A1_at − probe 9
NP_006470
Normalized Probe Intensity
Probe Number
1.0
0.5
0.0
−0.5
−1.0
−1.5
1
2
3
4
5
6
7
8
9
10
11
Meishan−A
Meishan−B
Meishan−C
European−A
European−B
European−C

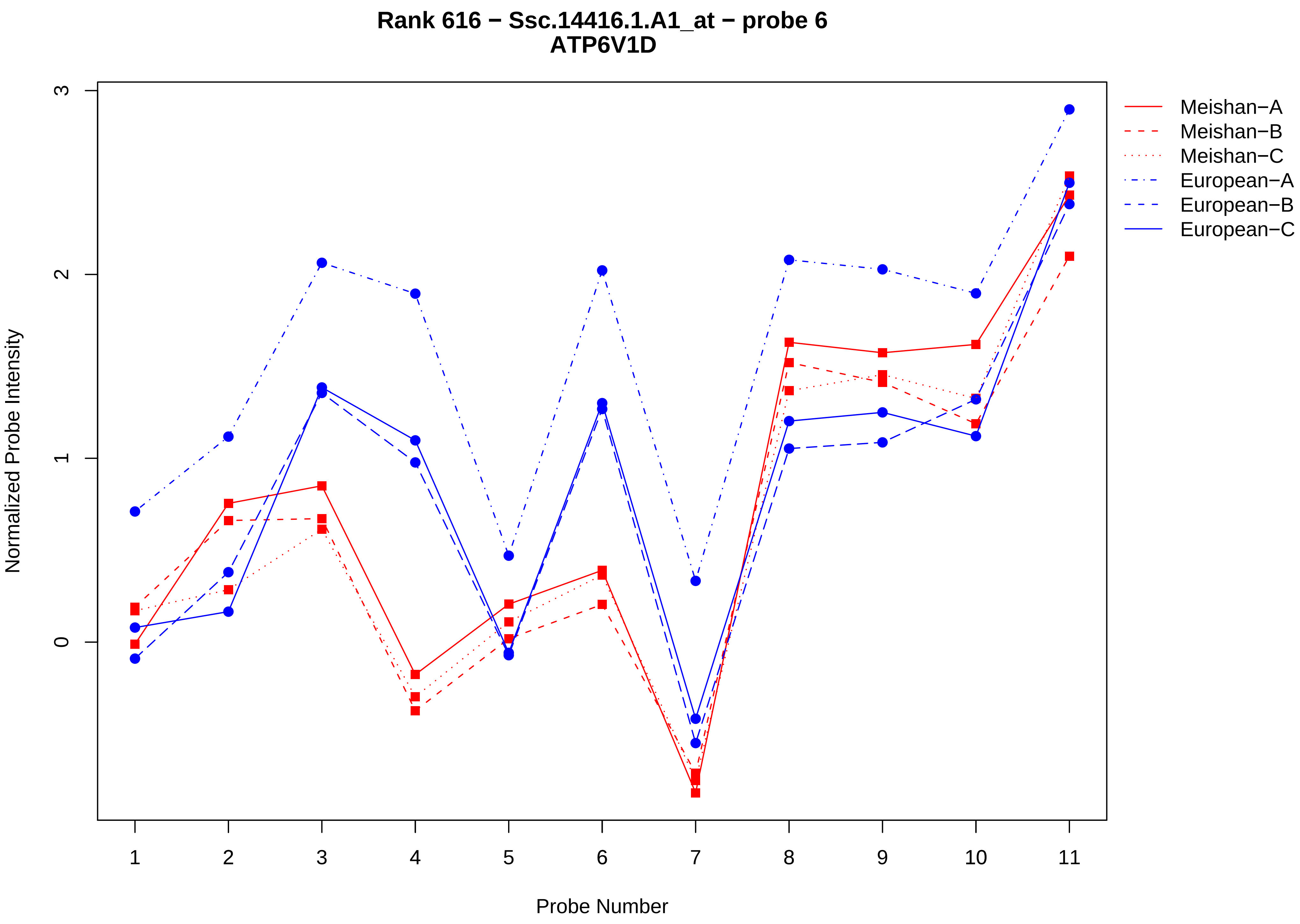

Rank 616 − Ssc.14416.1.A1_at − probe 6
ATP6V1D
Normalized Probe Intensity
Probe Number
0
1
2
3
1
2
3
4
5
6
7
8
9
10
11
Meishan−A
Meishan−B
Meishan−C
European−A
European−B
European−C

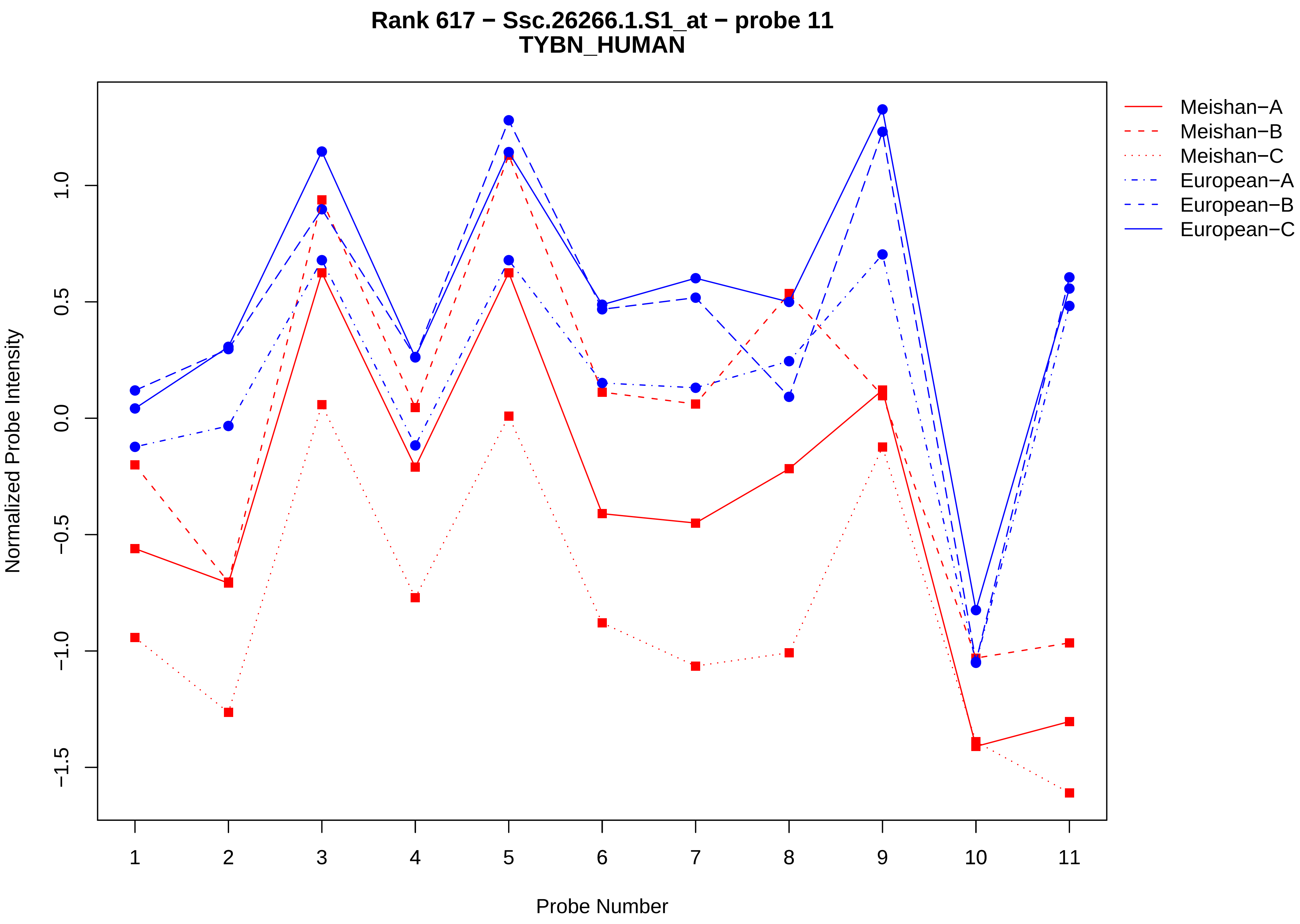

Rank 617 − Ssc.26266.1.S1_at − probe 11
TYBN_HUMAN
Normalized Probe Intensity
Probe Number
1.0
0.5
0.0
−0.5
−1.0
−1.5
1
2
3
4
5
6
7
8
9
10
11
Meishan−A
Meishan−B
Meishan−C
European−A
European−B
European−C

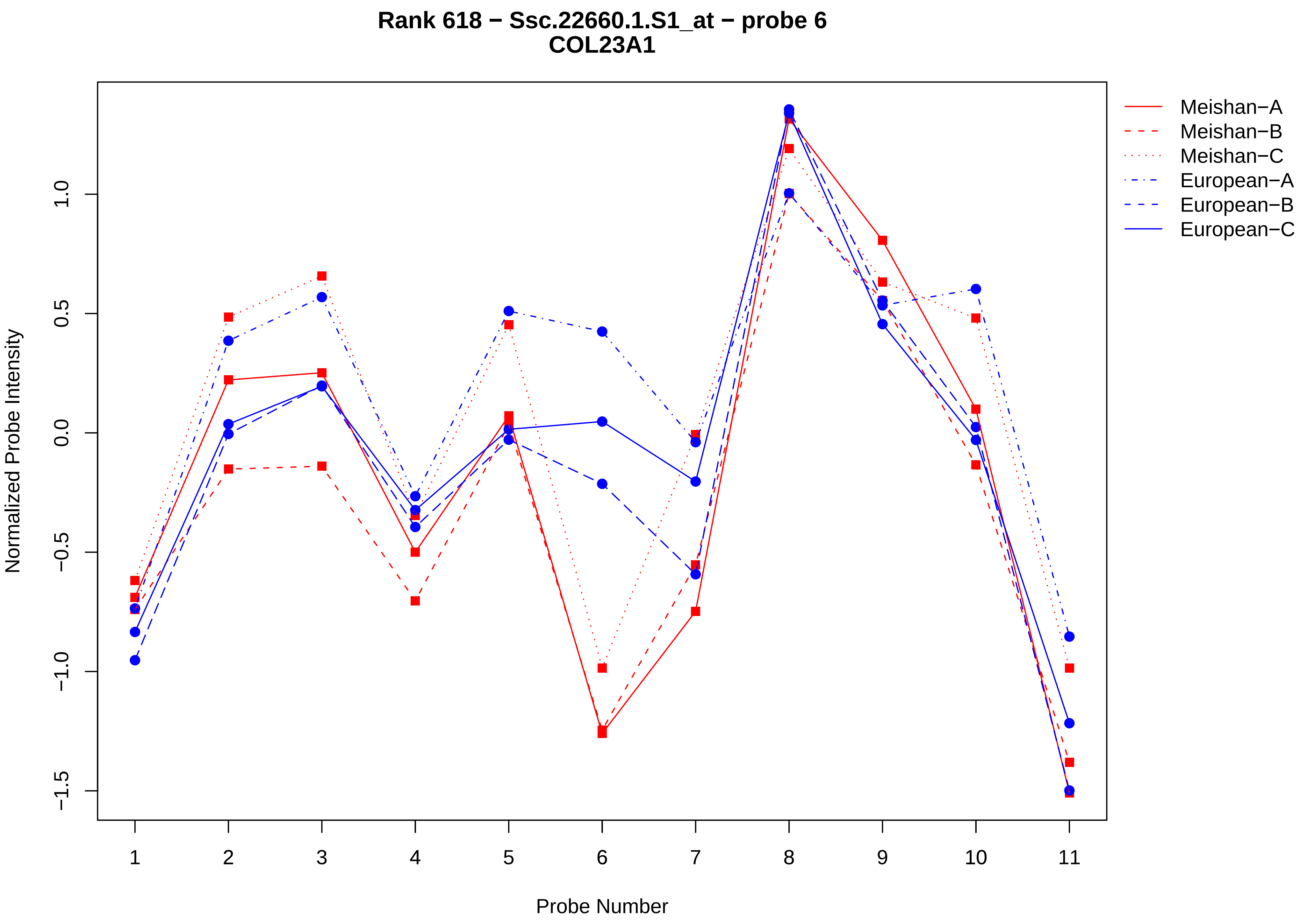

Rank 618 − Ssc.22660.1.S1_at − probe 6
COL23A1
Normalized Probe Intensity
Probe Number
−1.5
−1.0
−0.5
0.0
0.5
1.0
1
2
3
4
5
6
7
8
9
10
11
Meishan−A
Meishan−B
Meishan−C
European−A
European−B
European−C

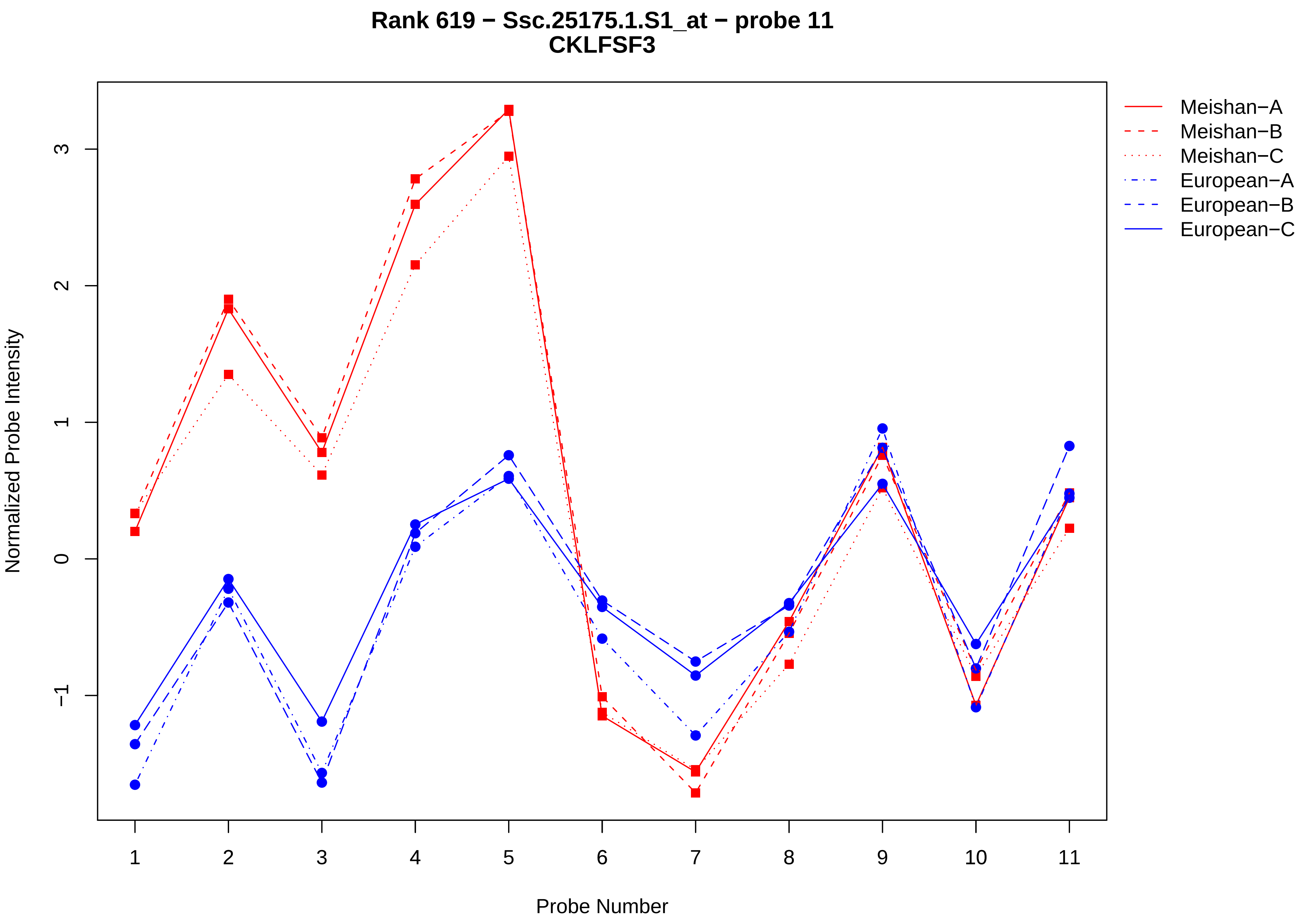

Rank 619 − Ssc.25175.1.S1_at − probe 11
CKLFSF3
Normalized Probe Intensity
Probe Number
Meishan−A
Meishan−B
Meishan−C
European−A
European−B
European−C
−1
0
1
2
3
1
2
3
4
5
6
7
8
9
10
11

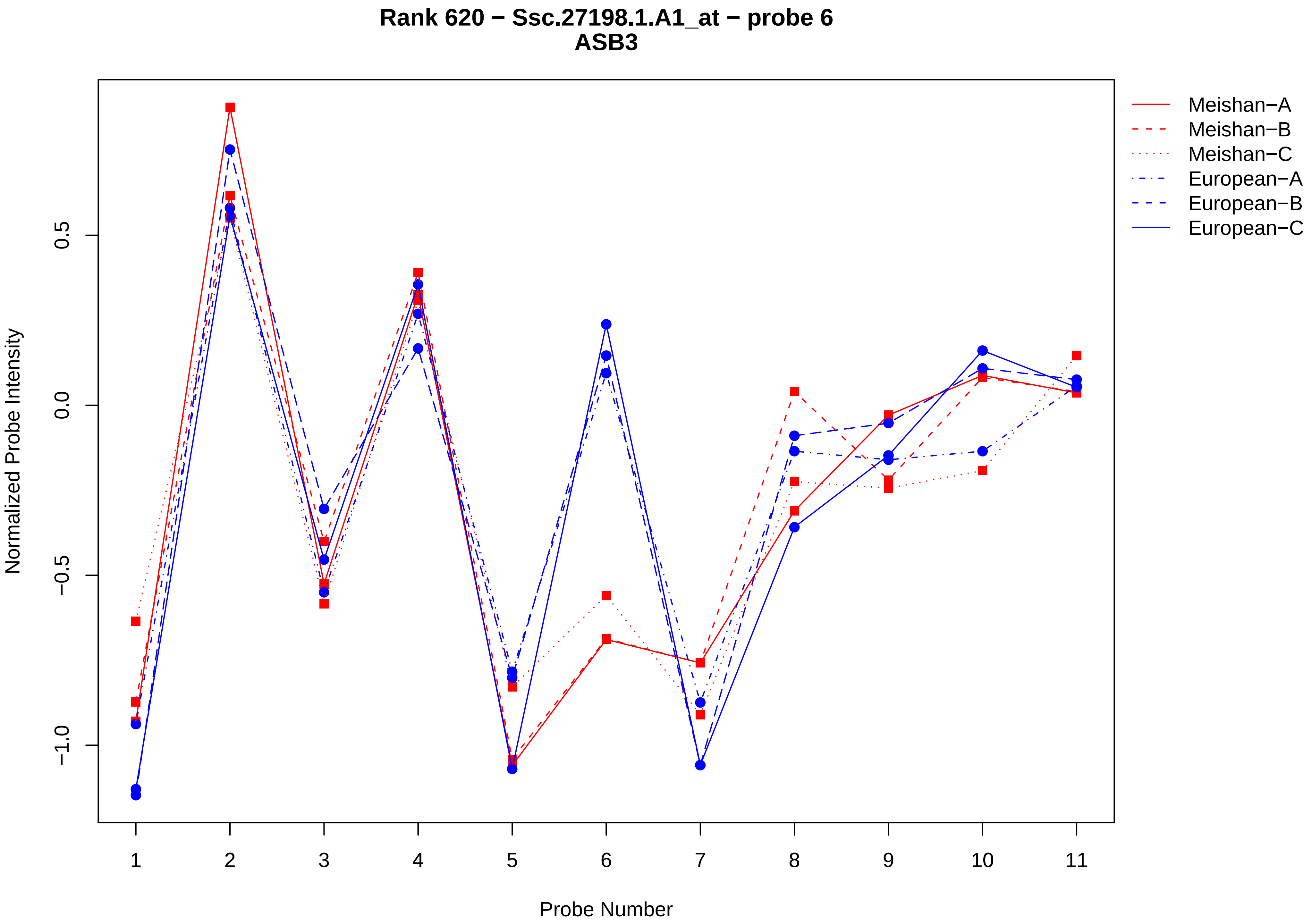

Rank 620 − Ssc.27198.1.A1_at − probe 6
ASB3
Normalized Probe Intensity
Probe Number
−1.0
−0.5
0.0
0.5
1
2
3
4
5
6
7
8
9
10
11
Meishan−A
Meishan−B
Meishan−C
European−A
European−B
European−C

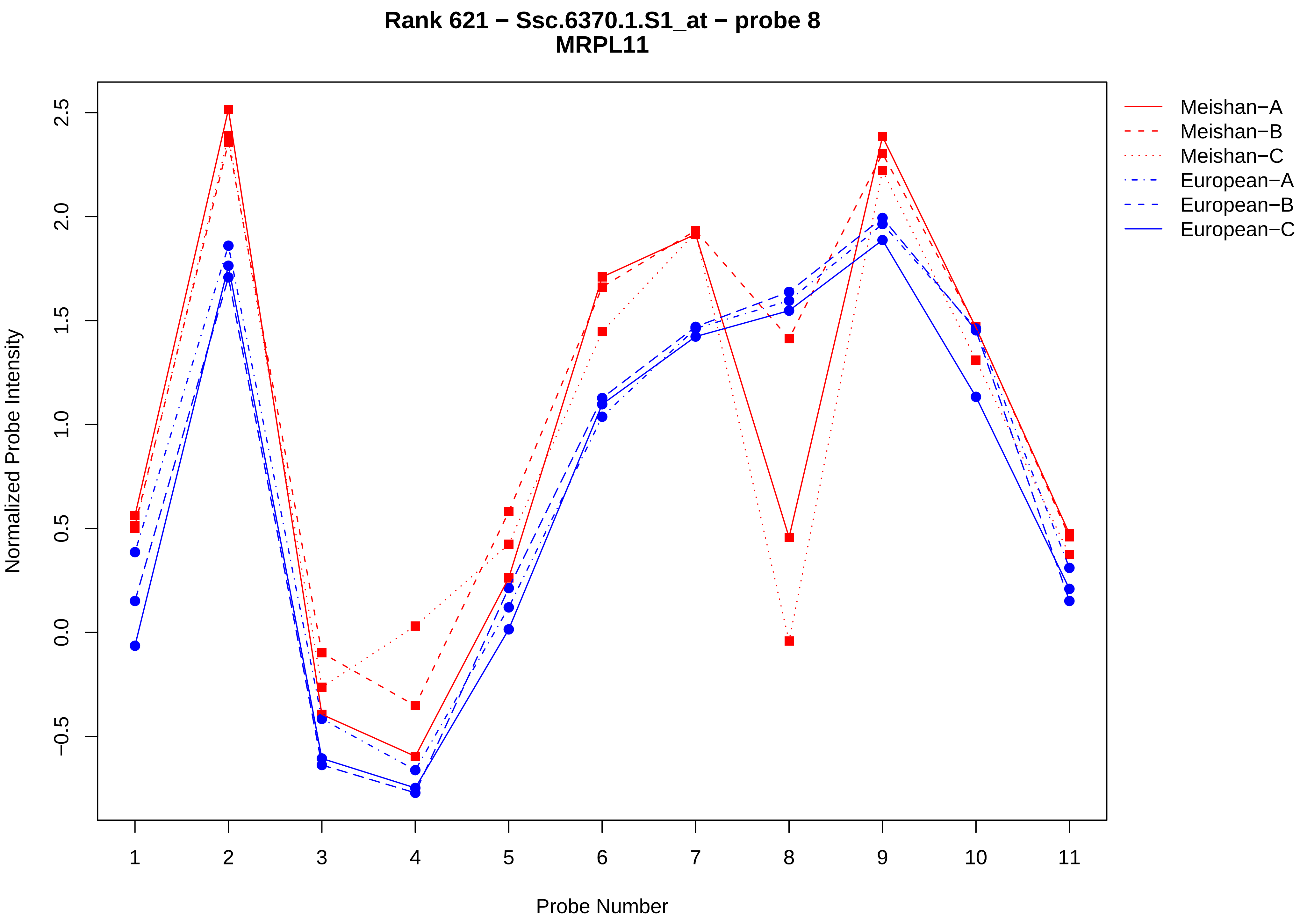

Rank 621 − Ssc.6370.1.S1_at − probe 8
MRPL11
Normalized Probe Intensity
Probe Number
Meishan−A
Meishan−B
Meishan−C
European−A
European−B
European−C

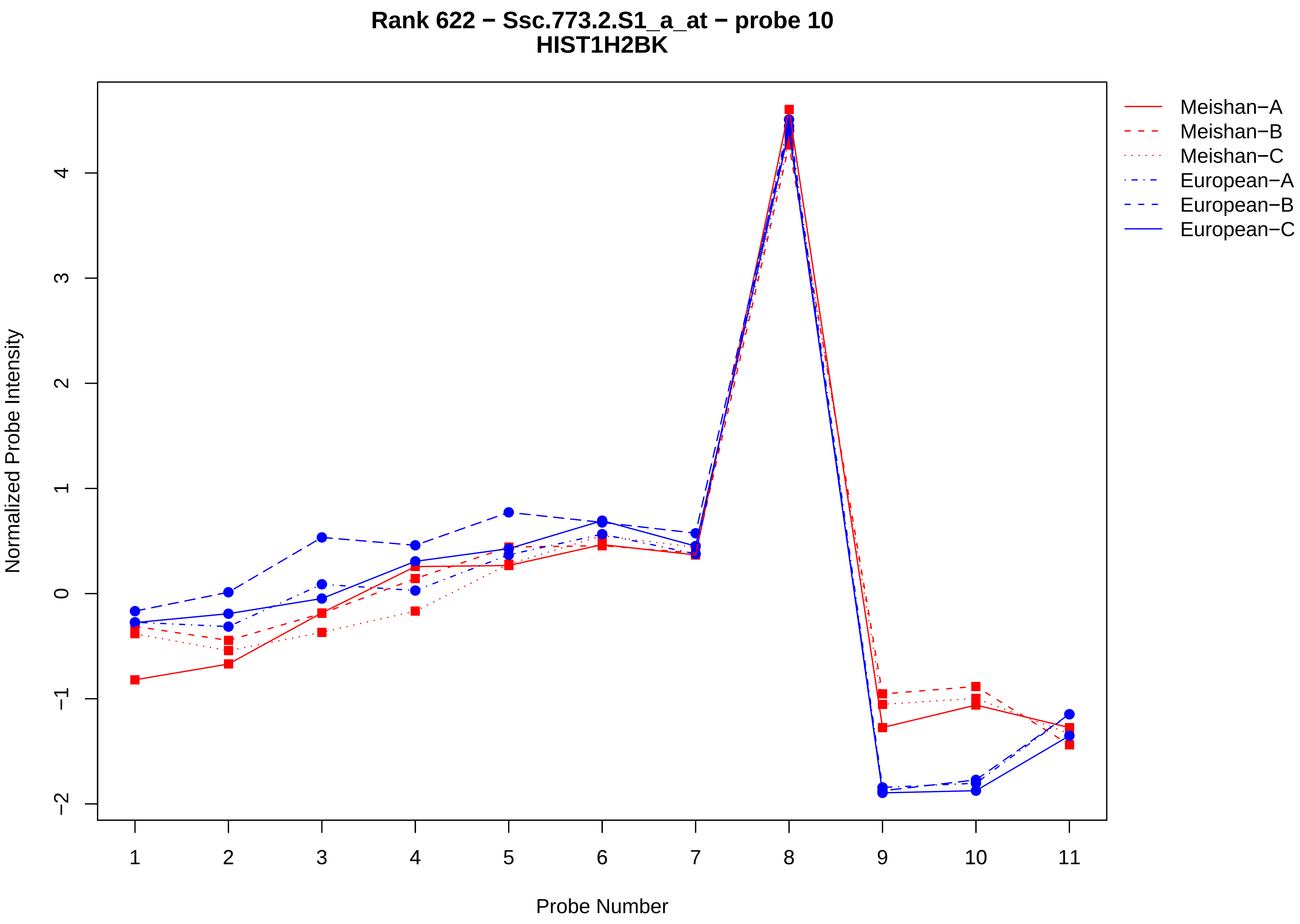
Rank 622 − Ssc.773.2.S1_a_at − probe 10
HIST1H2BK
Normalized Probe Intensity
Probe Number
−2
−1
0
1
2
3
4
1
2
3
4
5
6
7
8
9
10
11
Meishan−A
Meishan−B
Meishan−C
European−A
European−B
European−C

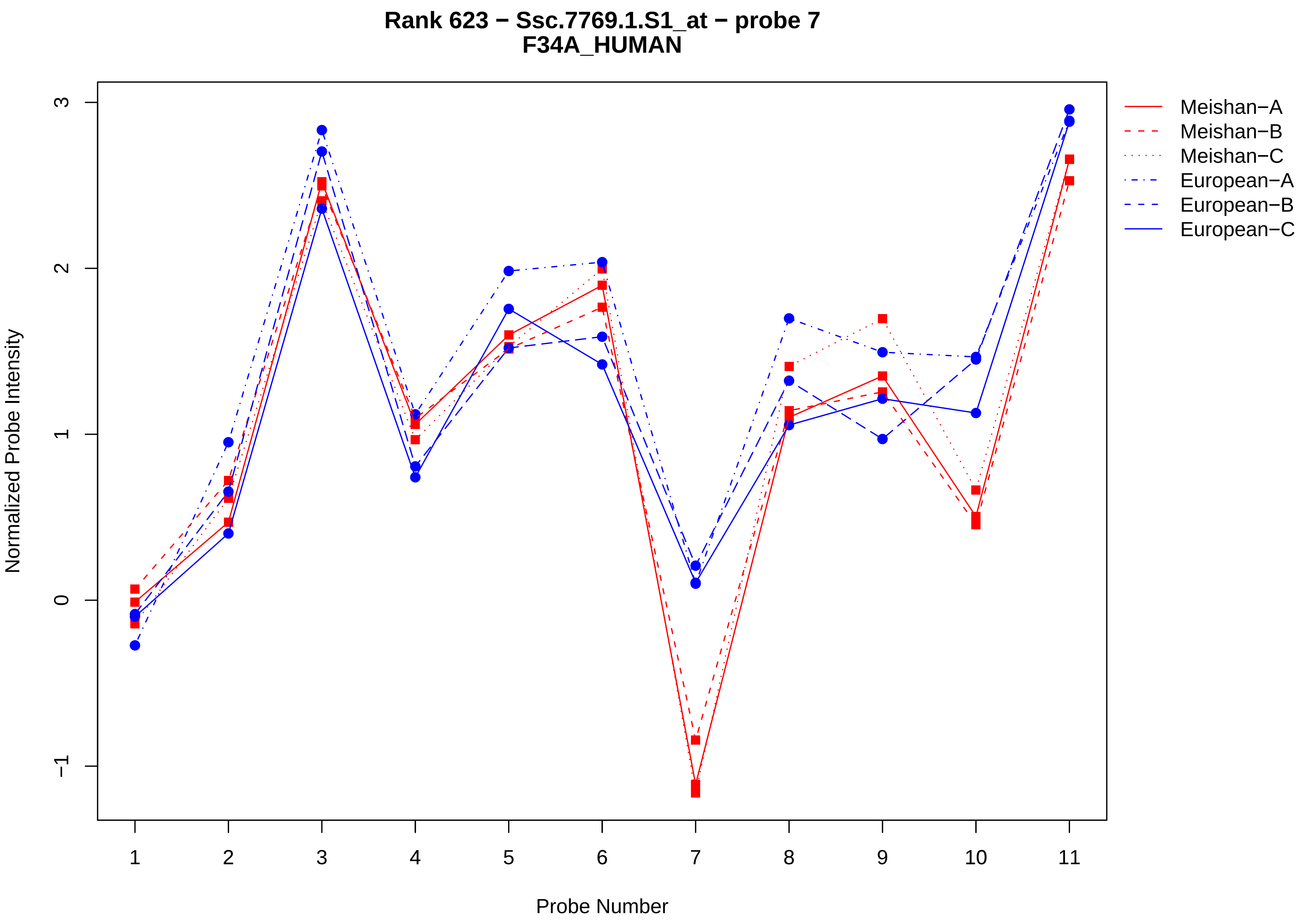

Rank 623 − Ssc.7769.1.S1_at − probe 7
F34A_HUMAN
Normalized Probe Intensity
Probe Number
Meishan−A
Meishan−B
Meishan−C
European−A
European−B
European−C
−1
0
1
2
3
1
2
3
4
5
6
7
8
9
10
11

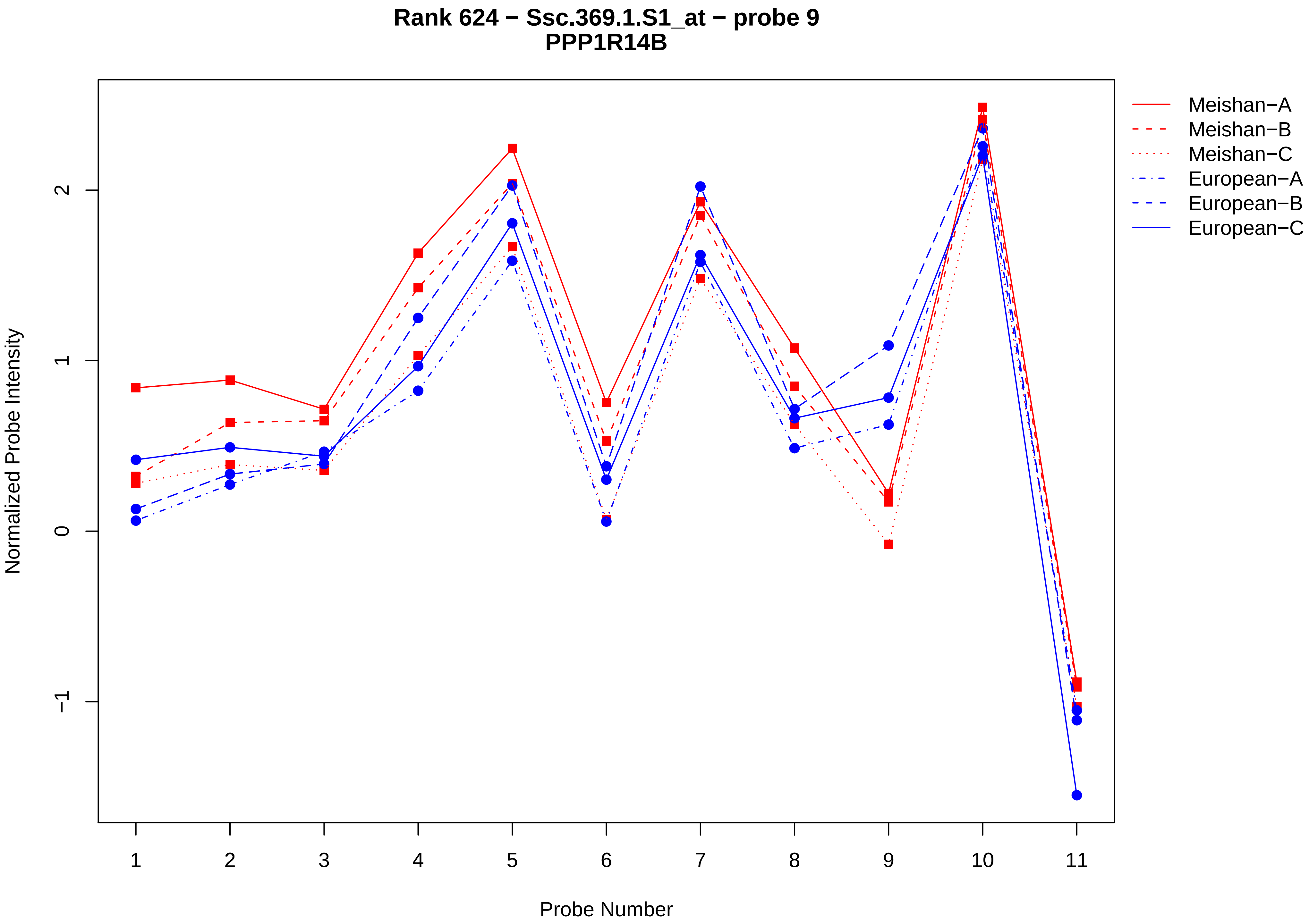

Rank 624 − Ssc.369.1.S1_at − probe 9
PPP1R14B
Normalized Probe Intensity
Probe Number
Meishan−A
Meishan−B
Meishan−C
European−A
European−B
European−C
−1
0
1
2
1
2
3
4
5
6
7
8
9
10
11

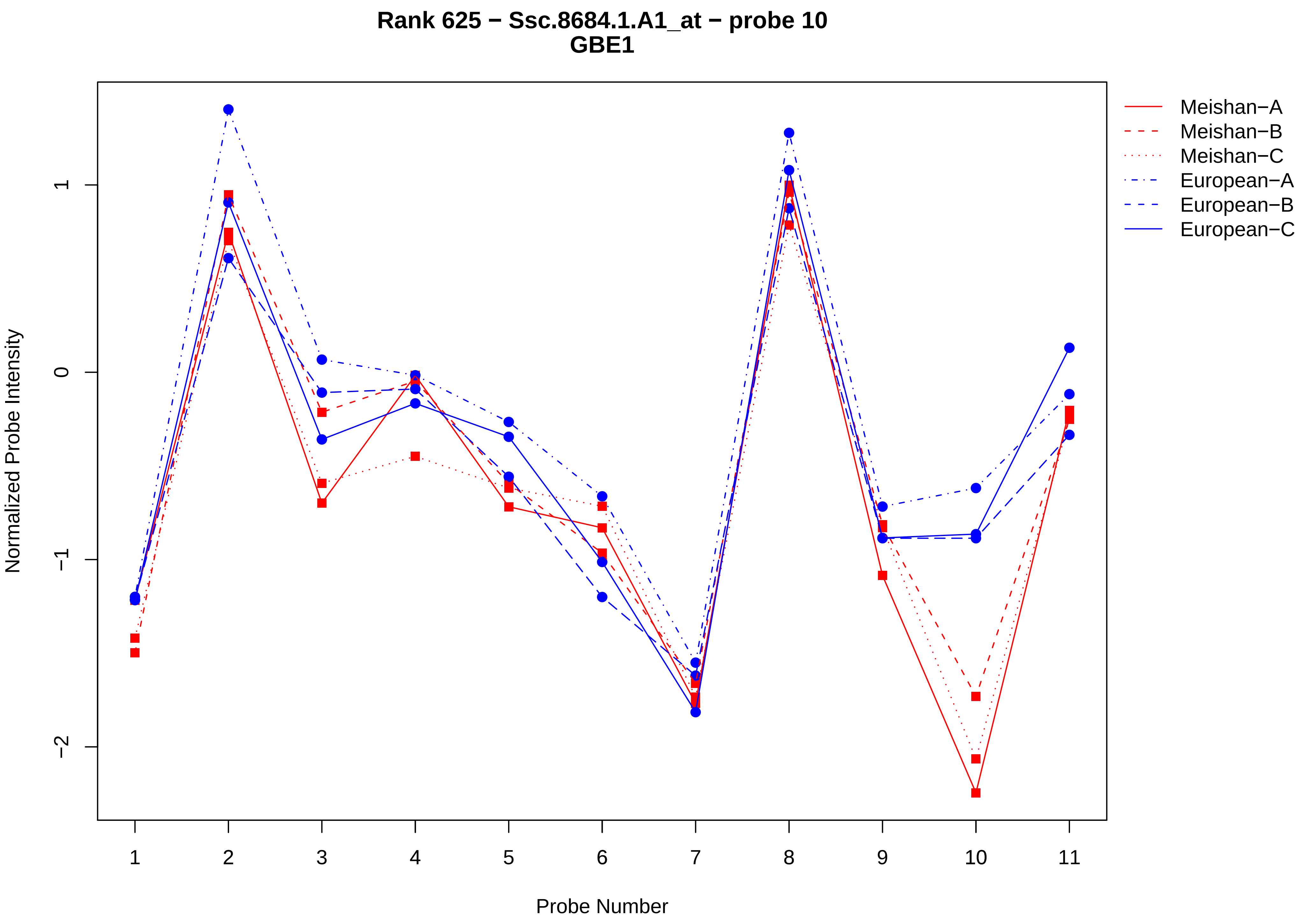

Rank 625 − Ssc.8684.1.A1_at − probe 10
GBE1
Normalized Probe Intensity
Probe Number
Meishan−A
Meishan−B
Meishan−C
European−A
European−B
European−C

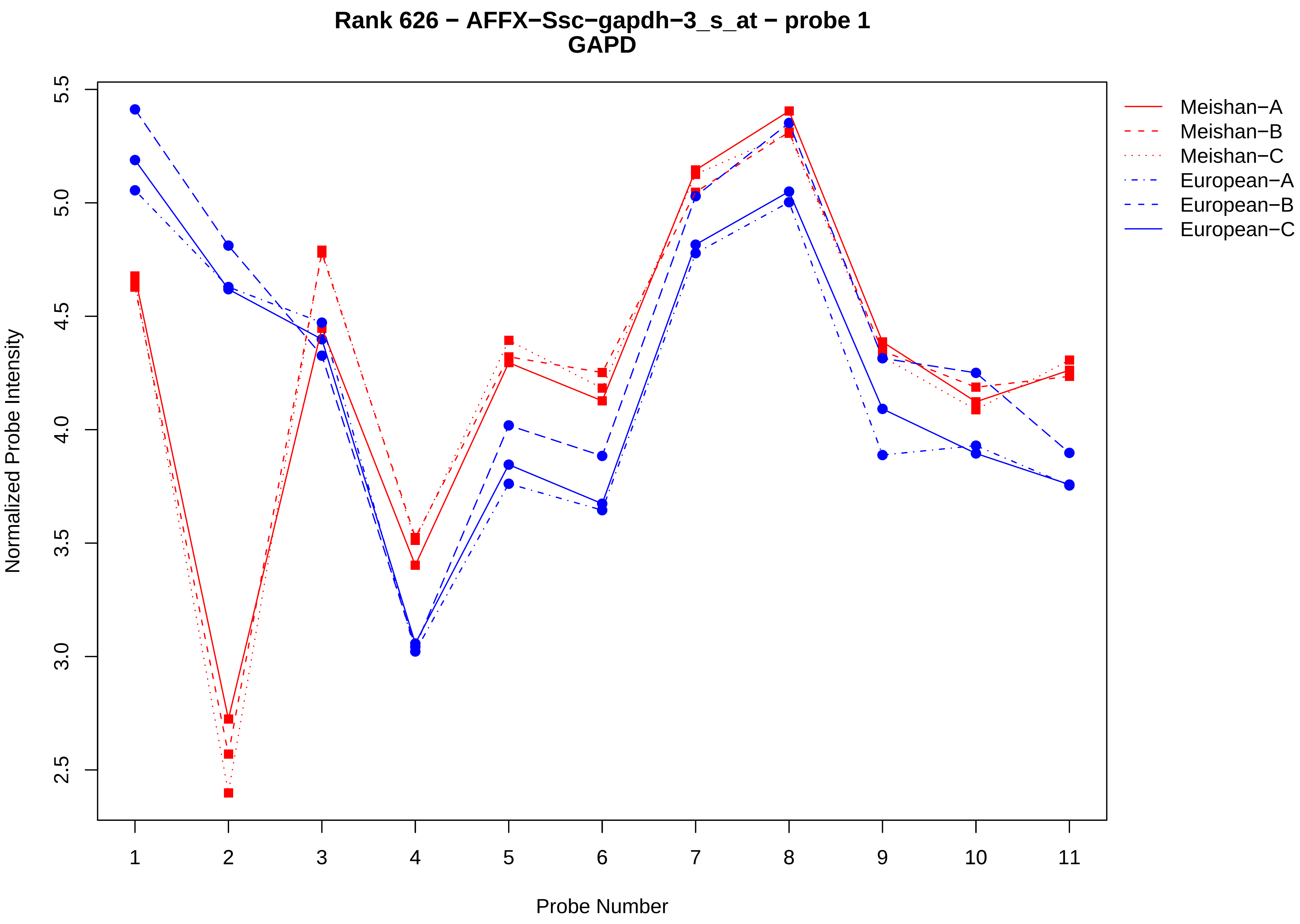
Rank 626 − AFFX−Ssc−gapdh−3_s_at − probe 1
GAPD
Normalized Probe Intensity
5.5
5.0
4.5
4.0
3.5
3.0
2.5
1
2
3
4
5
6
7
8
9
10
11
Probe Number
Meishan−A
Meishan−B
Meishan−C
European−A
European−B
European−C

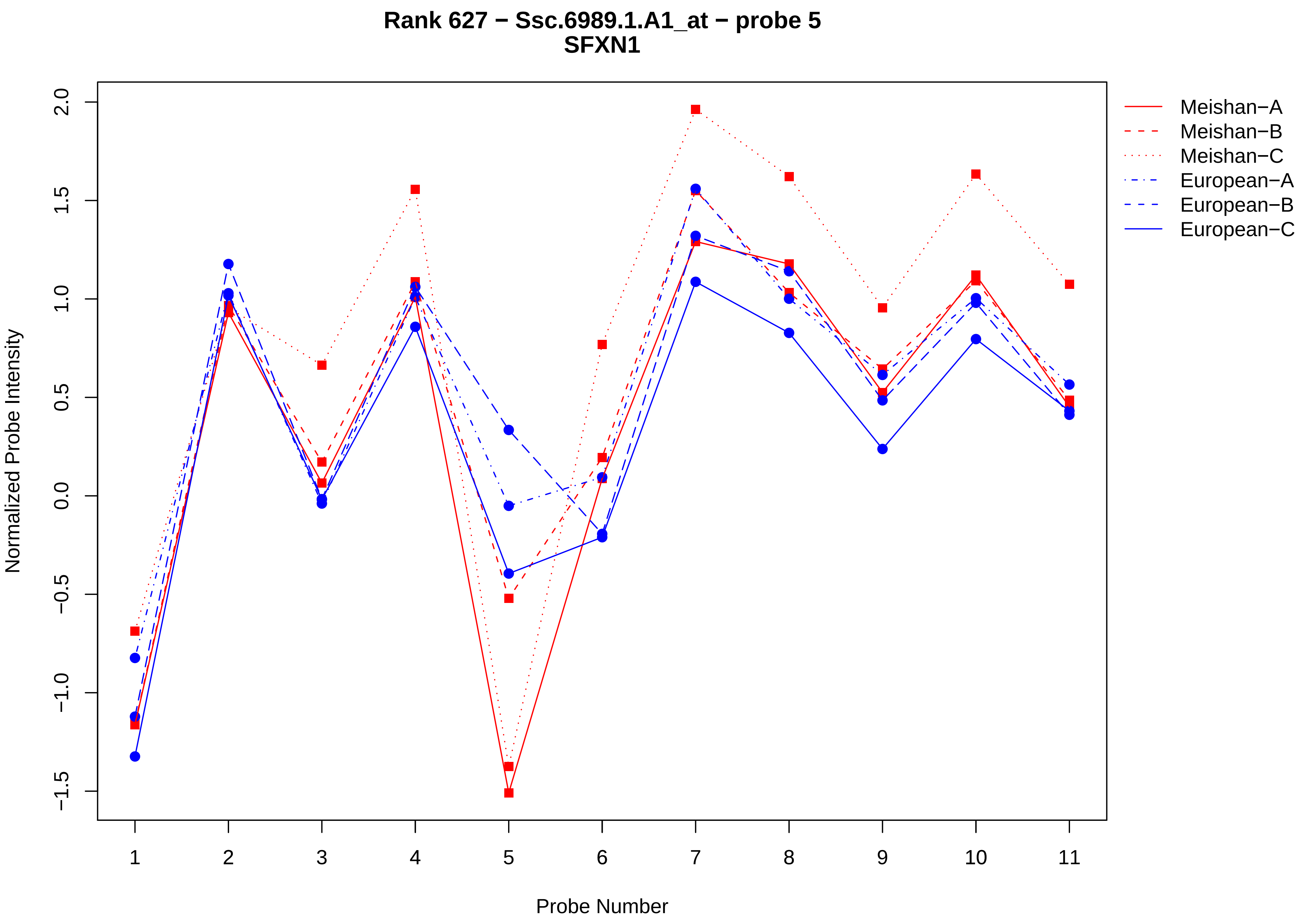

Rank 627 − Ssc.6989.1.A1_at − probe 5
SFXN1
Normalized Probe Intensity
Probe Number
Meishan−A
Meishan−B
Meishan−C
European−A
European−B
European−C

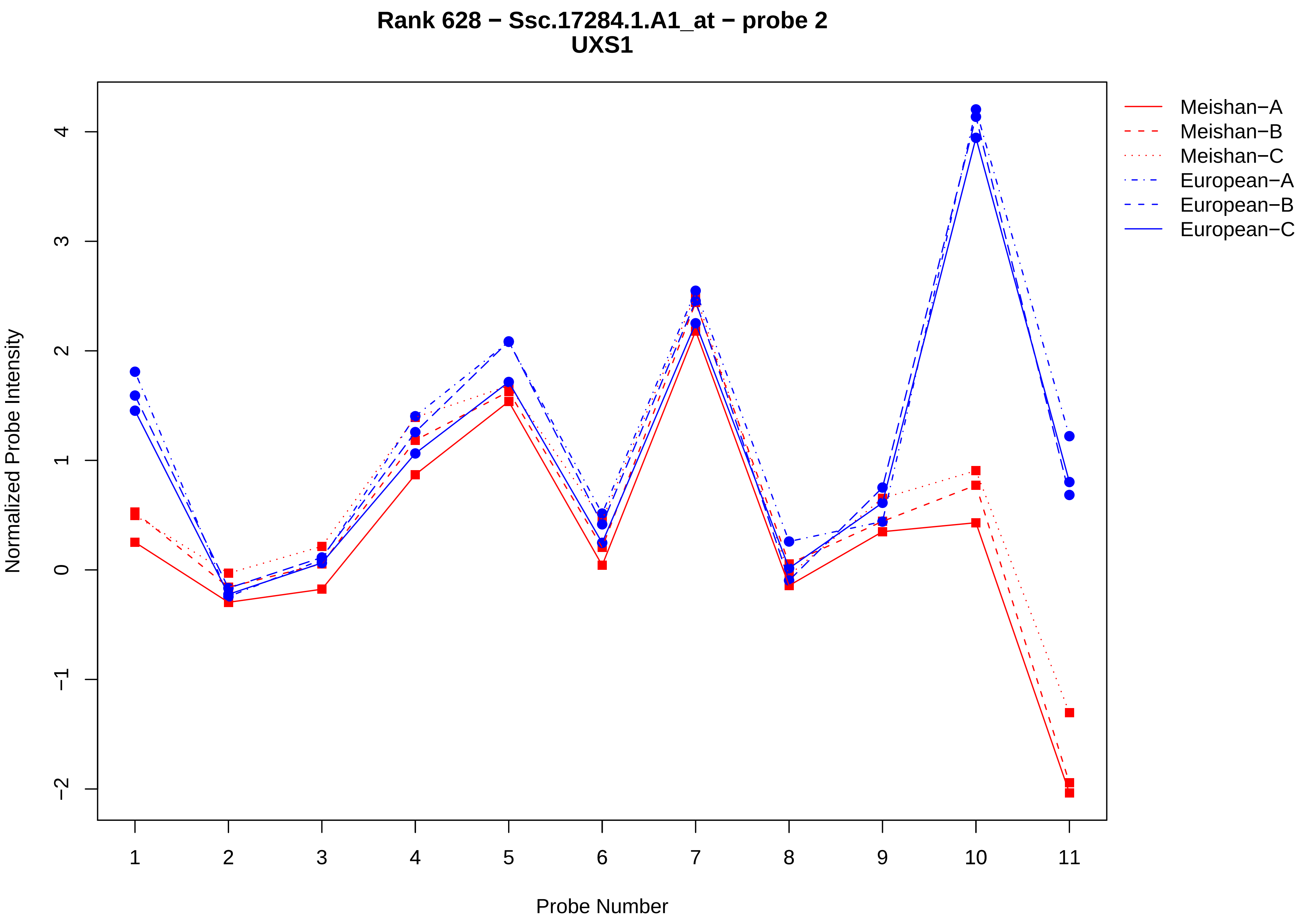

Rank 628 − Ssc.17284.1.A1_at − probe 2
UXS1
Normalized Probe Intensity
Probe Number
Meishan−A
Meishan−B
Meishan−C
European−A
European−B
European−C
−2
−1
0
1
2
3
4
1
2
3
4
5
6
7
8
9
10
11

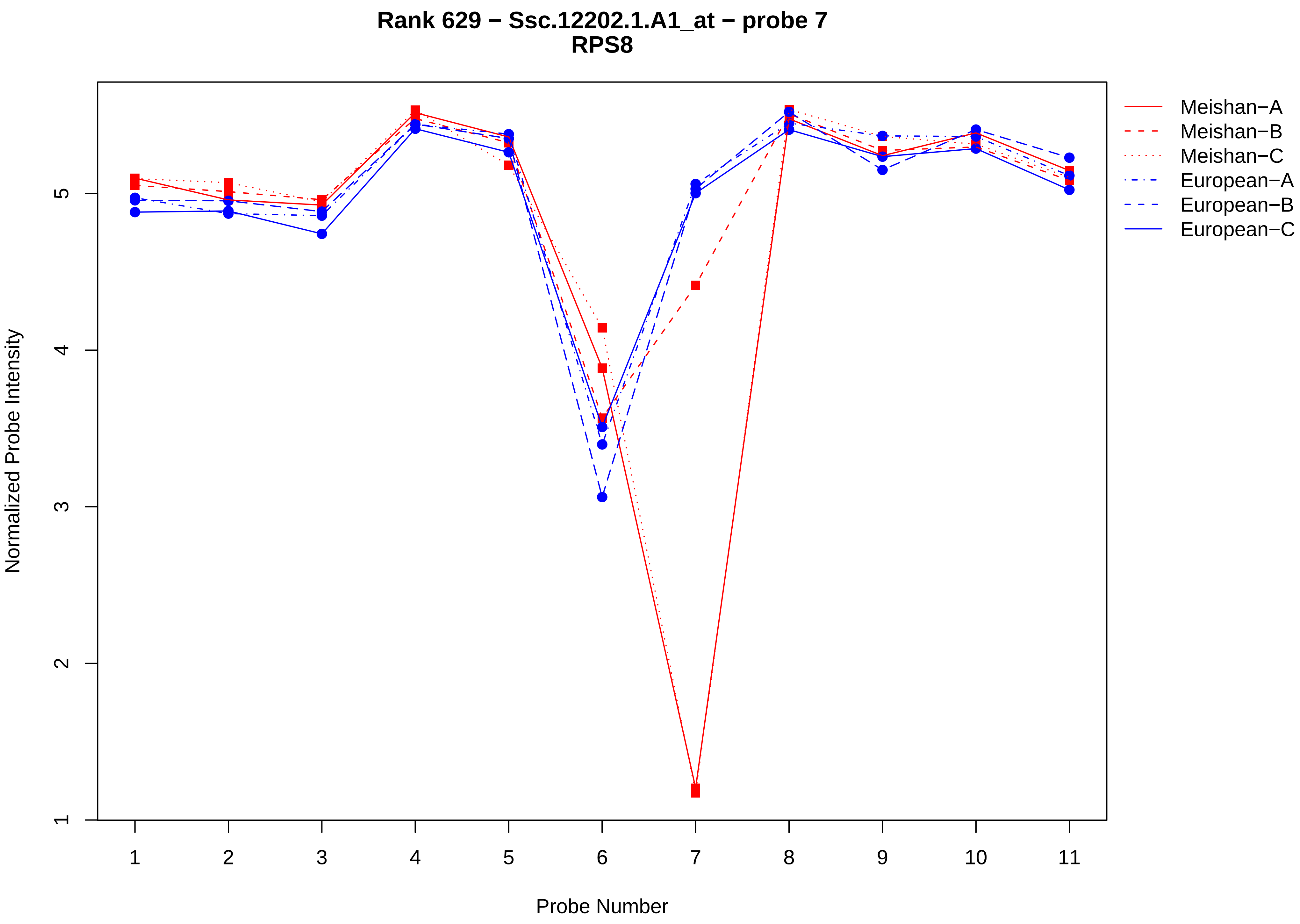

Rank 629 − Ssc.12202.1.A1_at − probe 7
RPS8
Normalized Probe Intensity
Probe Number
1
2
3
4
5
6
7
8
9
10
11
Meishan−A
Meishan−B
Meishan−C
European−A
European−B
European−C

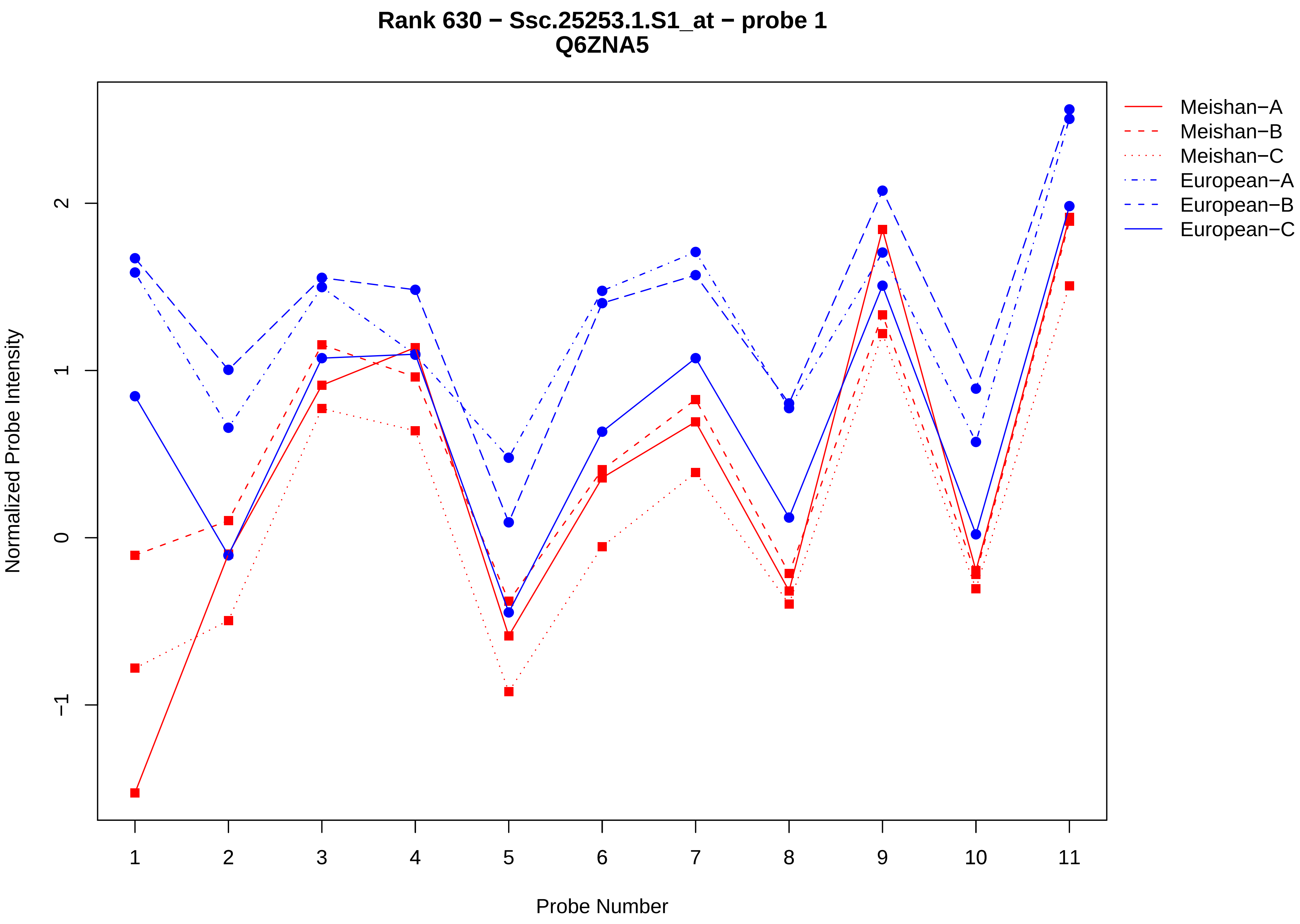

Rank 630 − Ssc.25253.1.S1_at − probe 1
Q6ZNA5
Normalized Probe Intensity
Probe Number
−1
0
1
2
1
2
3
4
5
6
7
8
9
10
11
Meishan−A
Meishan−B
Meishan−C
European−A
European−B
European−C

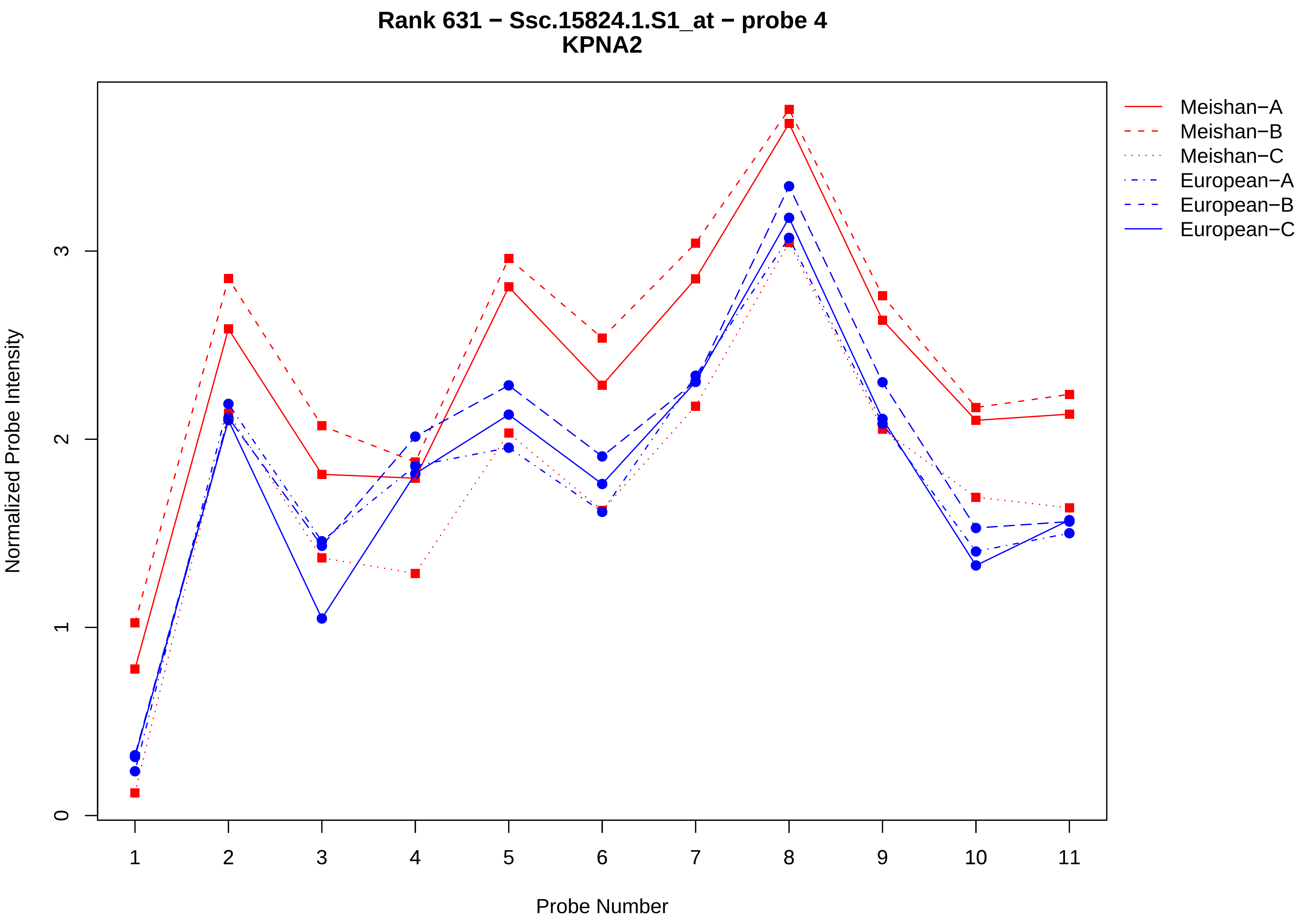
Rank 631 − Ssc.15824.1.S1_at − probe 4
KPNA2
Normalized Probe Intensity
Probe Number
0
1
2
3
1
2
3
4
5
6
7
8
9
10
11
Meishan−A
Meishan−B
Meishan−C
European−A
European−B
European−C

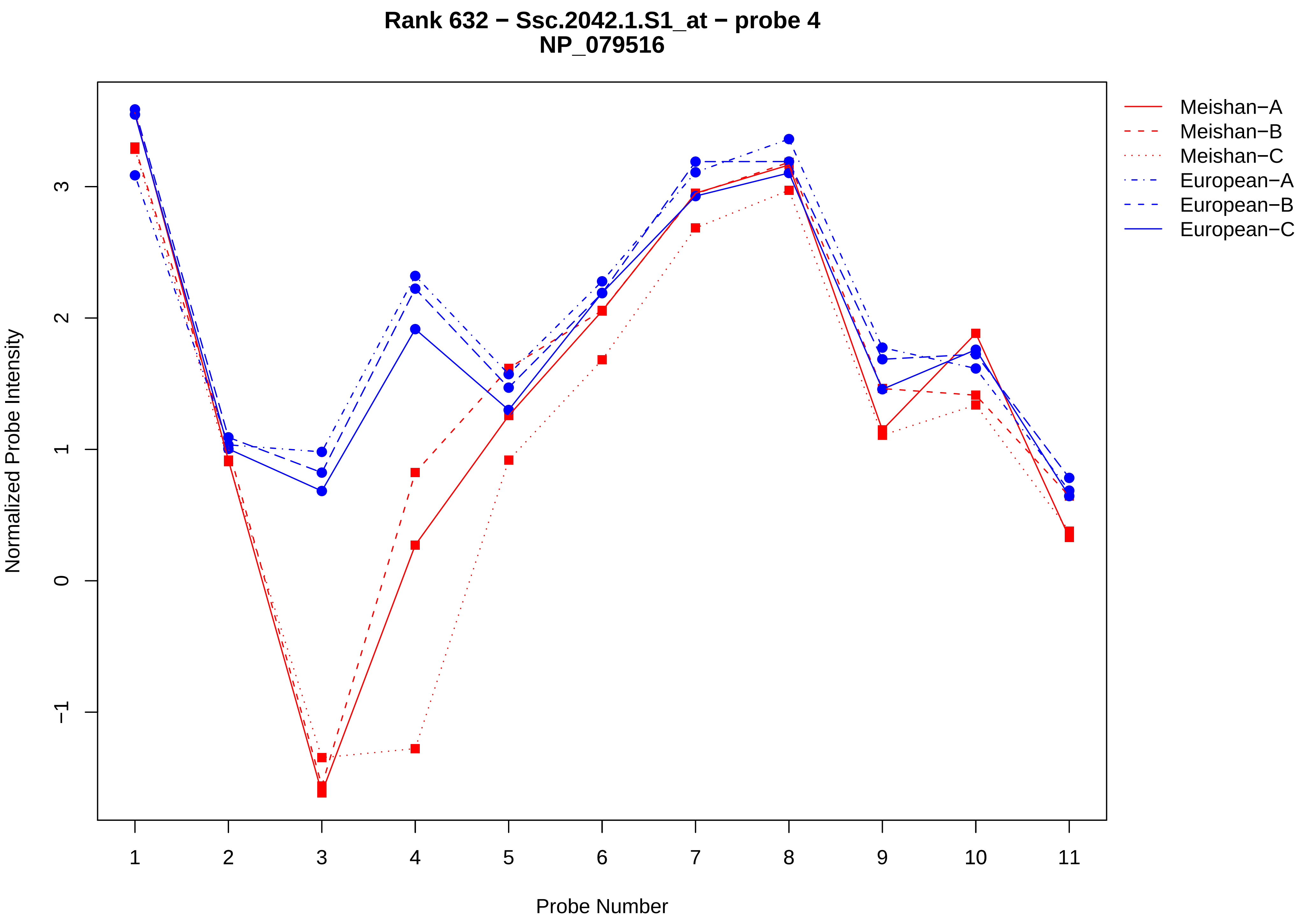

Rank 632 − Ssc.2042.1.S1_at − probe 4
NP_079516
Normalized Probe Intensity
Probe Number
−1
0
1
2
3
1
2
3
4
5
6
7
8
9
10
11
Meishan−A
Meishan−B
Meishan−C
European−A
European−B
European−C

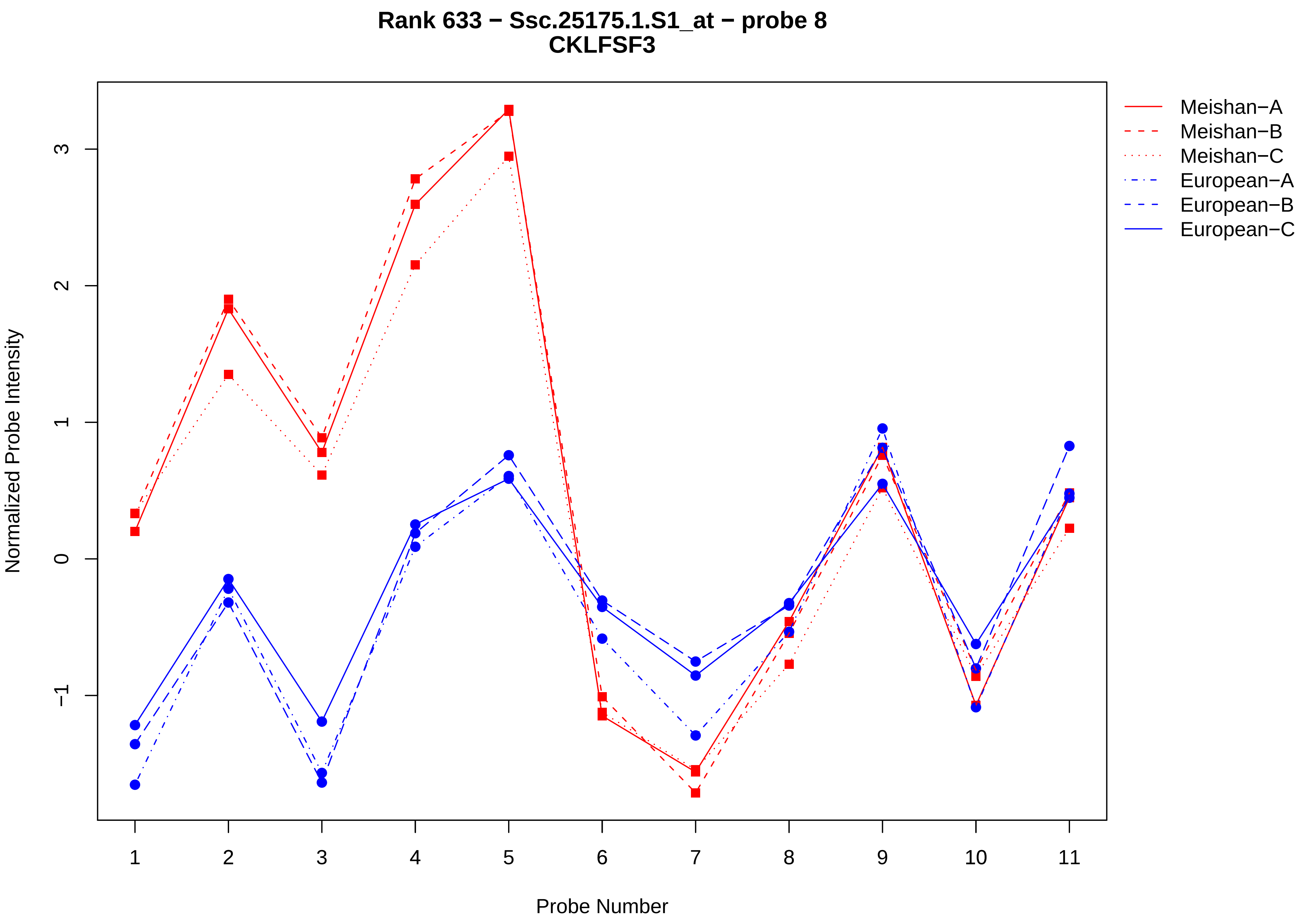

Rank 633 − Ssc.25175.1.S1_at − probe 8
CKLFSF3
Normalized Probe Intensity
Probe Number
Meishan−A
Meishan−B
Meishan−C
European−A
European−B
European−C
−1
0
1
2
3
1
2
3
4
5
6
7
8
9
10
11

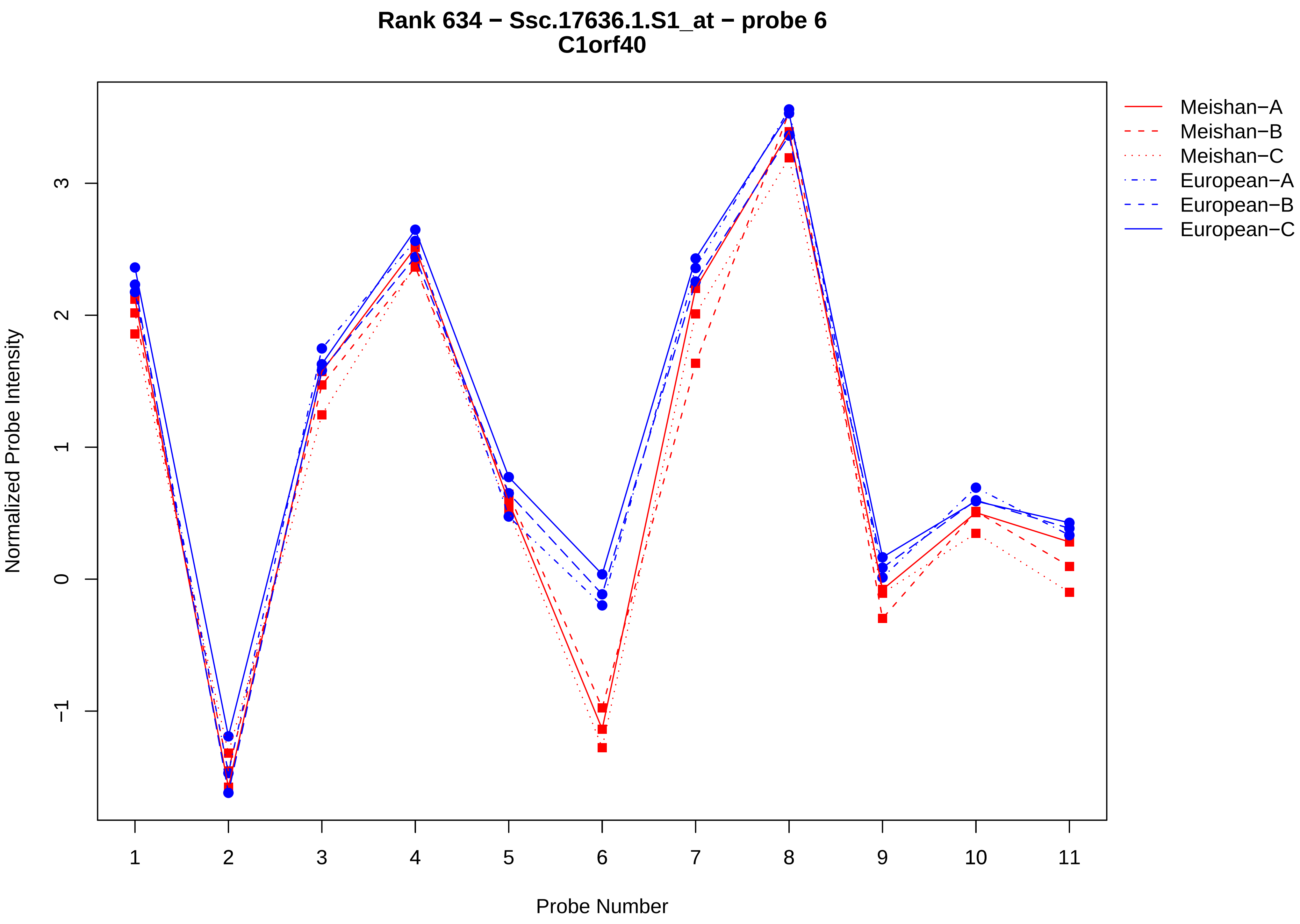
Rank 634 − Ssc.17636.1.S1_at − probe 6
C1orf40
Normalized Probe Intensity
Probe Number
−1
0
1
2
3
1
2
3
4
5
6
7
8
9
10
11
Meishan−A
Meishan−B
Meishan−C
European−A
European−B
European−C

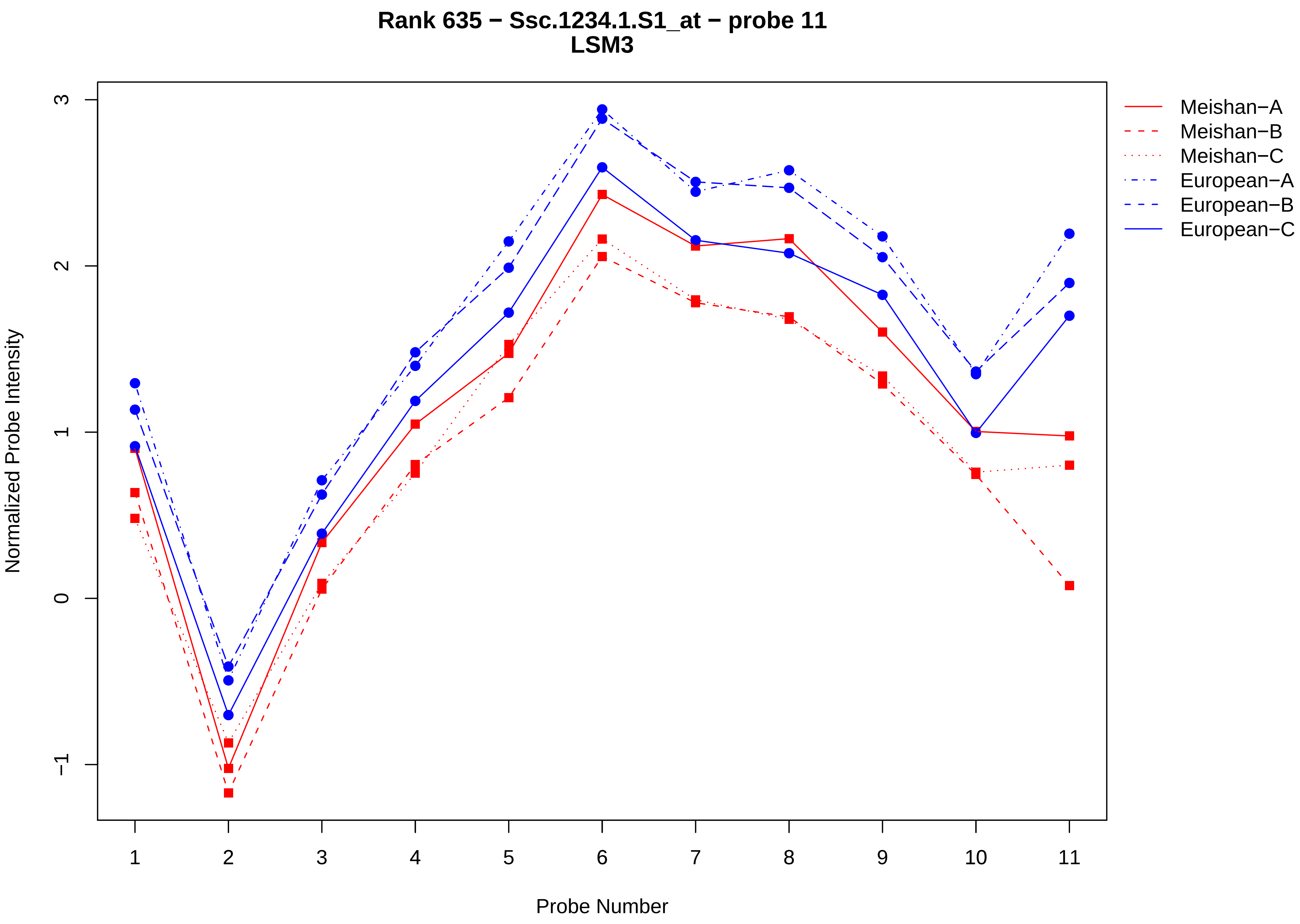

Rank 635 − Ssc.1234.1.S1_at − probe 11
LSM3
Normalized Probe Intensity
Probe Number
−1
0
1
2
3
1
2
3
4
5
6
7
8
9
10
11
Meishan−A
Meishan−B
Meishan−C
European−A
European−B
European−C

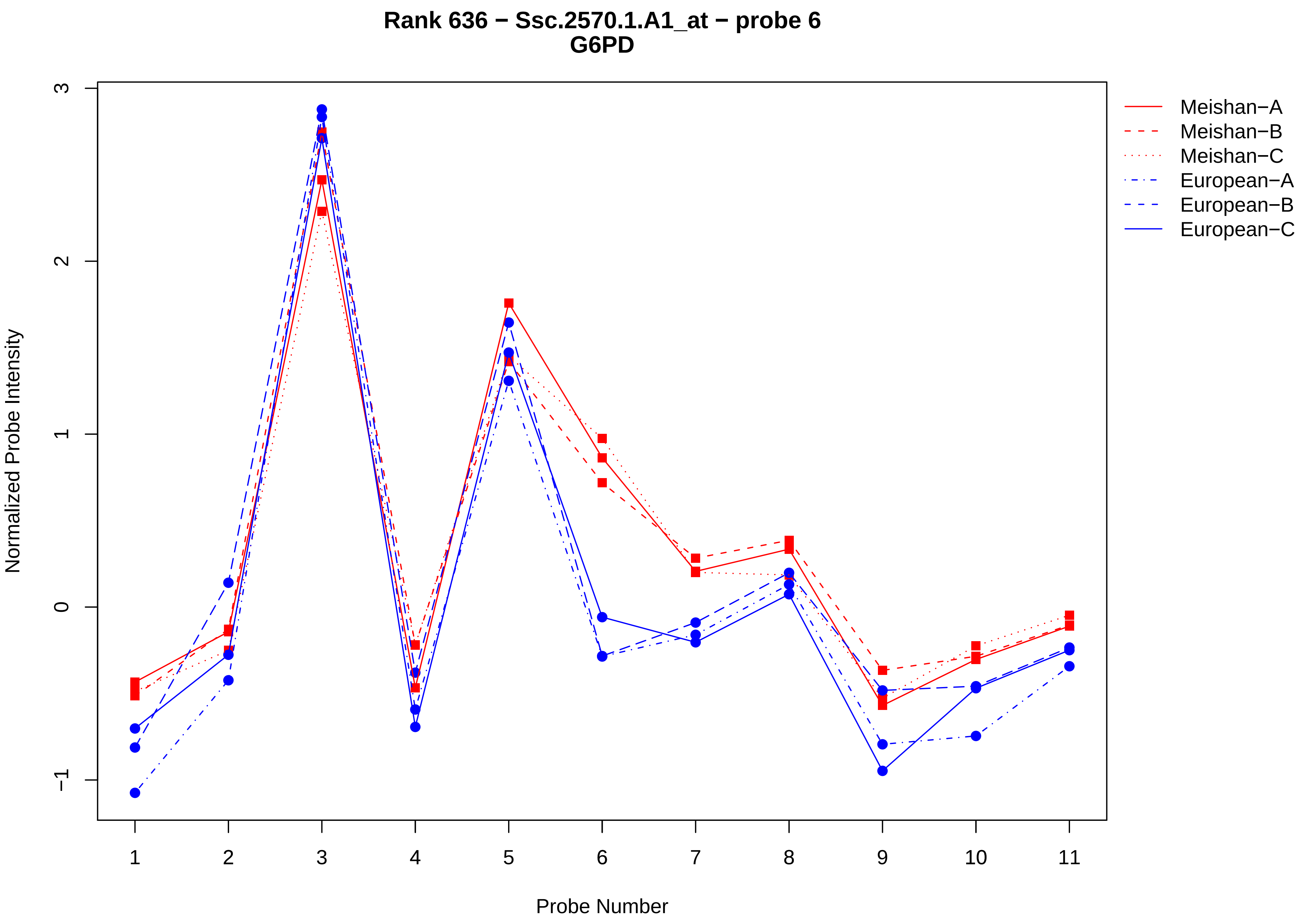
Rank 636 − Ssc.2570.1.A1_at − probe 6
G6PD
Normalized Probe Intensity
Probe Number
Meishan−A
Meishan−B
Meishan−C
European−A
European−B
European−C
−1
0
1
2
3
1
2
3
4
5
6
7
8
9
10
11

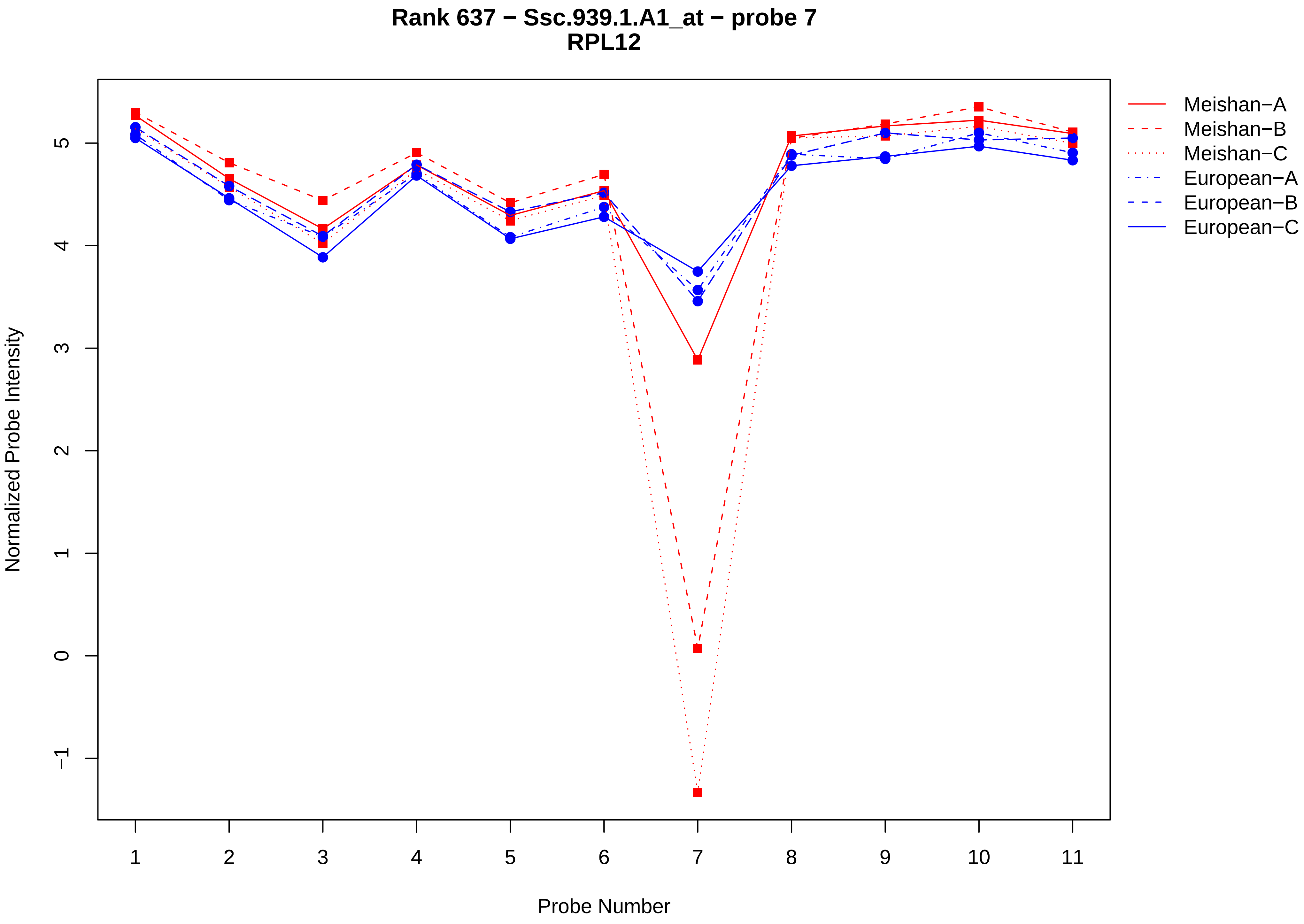

Rank 637 − Ssc.939.1.A1_at − probe 7
RPL12
Normalized Probe Intensity
Probe Number
−1
0
1
2
3
4
5
1
2
3
4
5
6
7
8
9
10
11
Meishan−A
Meishan−B
Meishan−C
European−A
European−B
European−C

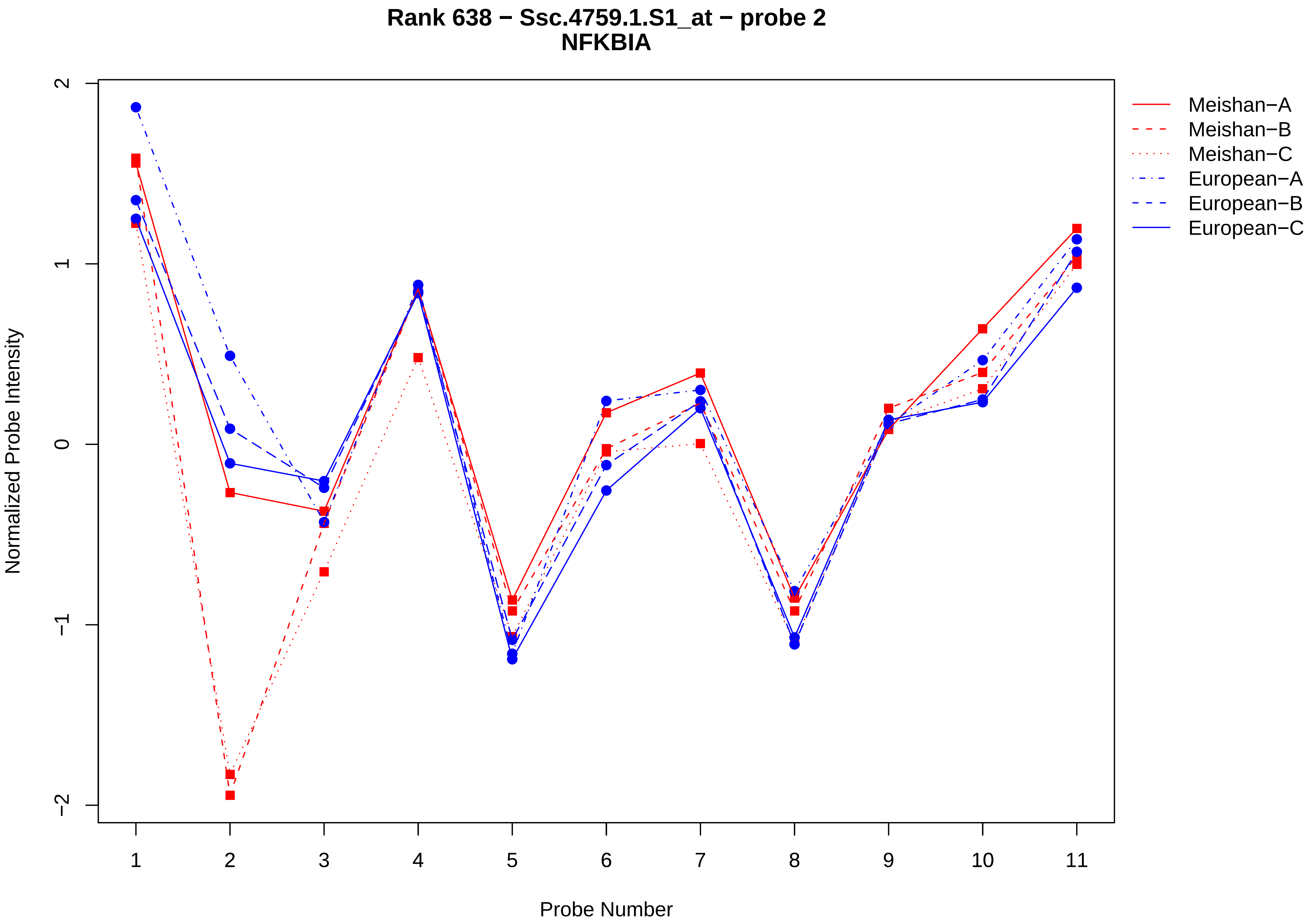
Rank 638 − Ssc.4759.1.S1_at − probe 2
NFKBIA
Normalized Probe Intensity
Probe Number
2
1
0
−1
−2
1
2
3
4
5
6
7
8
9
10
11
Meishan−A
Meishan−B
Meishan−C
European−A
European−B
European−C

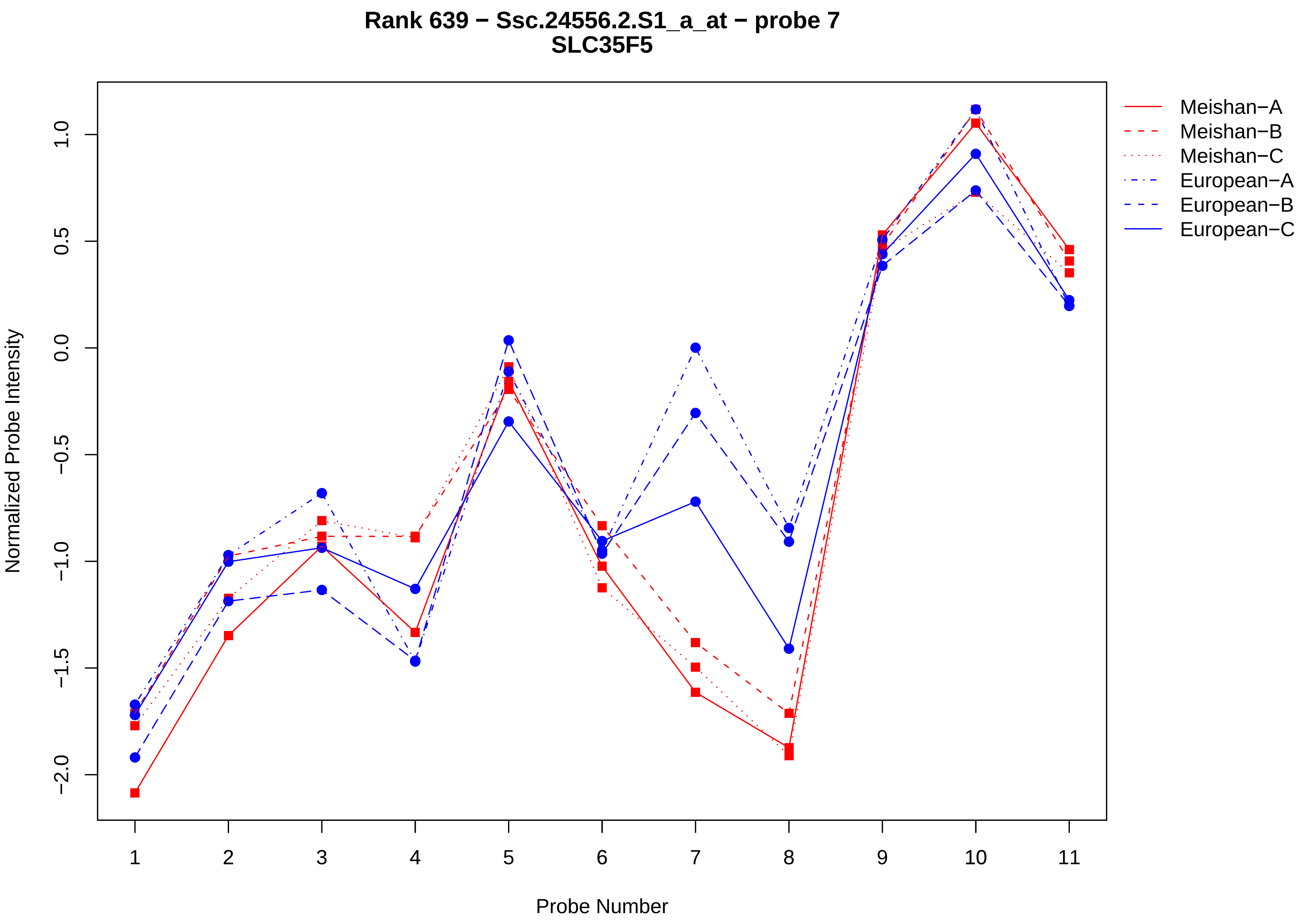

Rank 639 − Ssc.24556.2.S1_a_at − probe 7
SLC35F5
Normalized Probe Intensity
Probe Number
−2.0
−1.5
−1.0
−0.5
0.0
0.5
1.0
1
2
3
4
5
6
7
8
9
10
11
Meishan−A
Meishan−B
Meishan−C
European−A
European−B
European−C

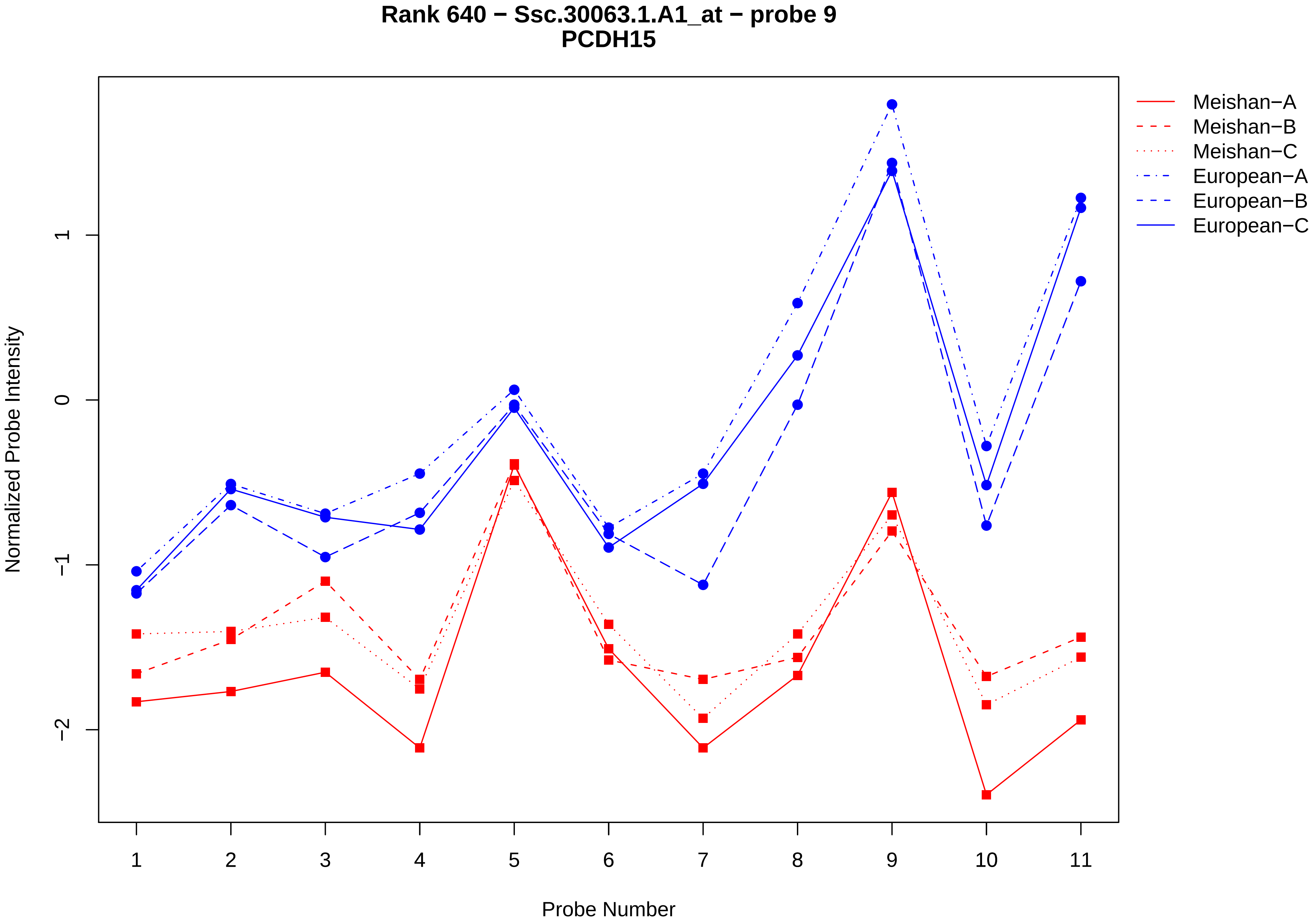
Rank 640 − Ssc.30063.1.A1_at − probe 9
PCDH15
Normalized Probe Intensity
Probe Number
1
0
−1
−2
1
2
3
4
5
6
7
8
9
10
11
Meishan−A
Meishan−B
Meishan−C
European−A
European−B
European−C

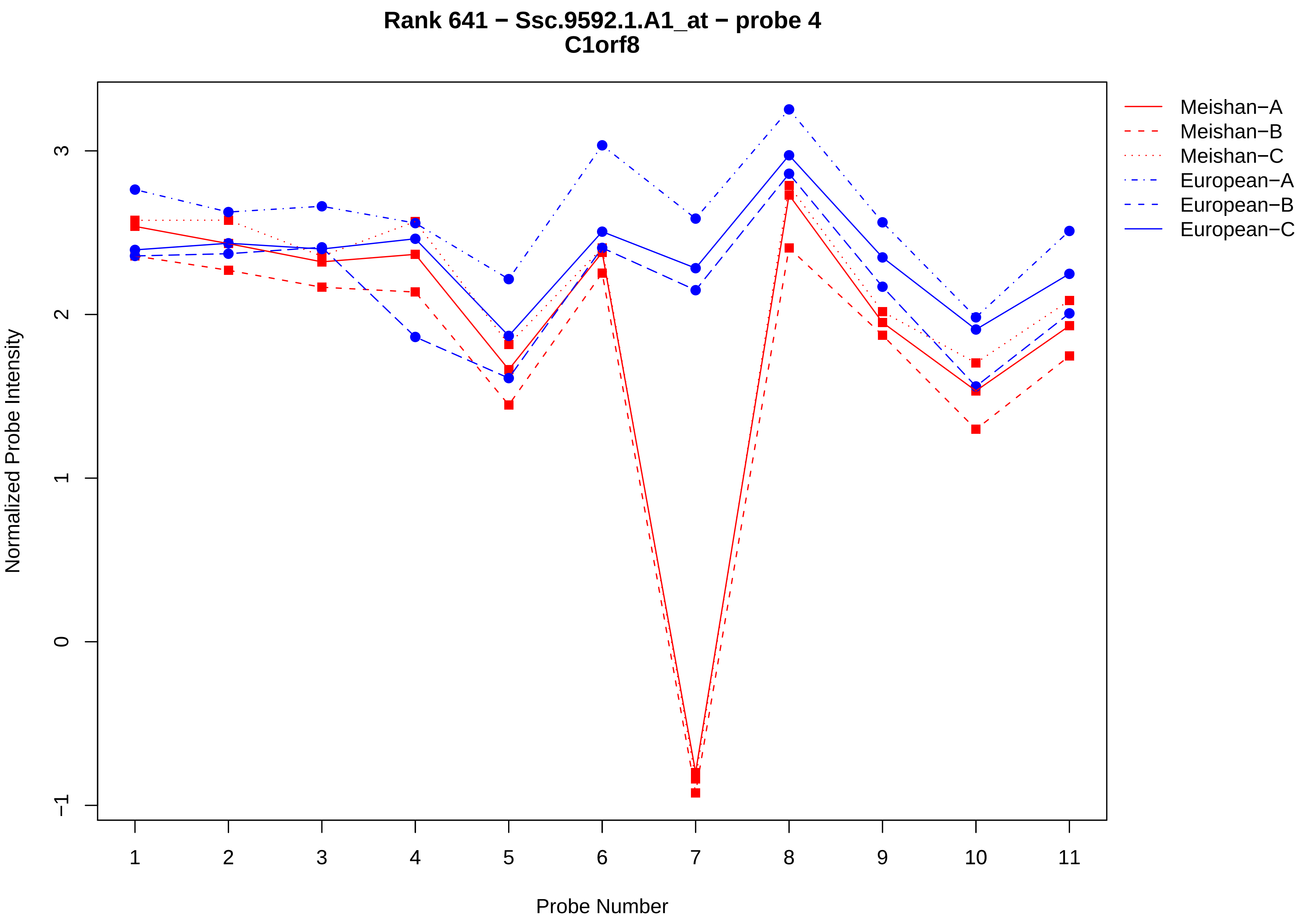
Rank 641 − Ssc.9592.1.A1_at − probe 4
C1orf8
Normalized Probe Intensity
Probe Number
−1
0
1
2
3
1
2
3
4
5
6
7
8
9
10
11
Meishan−A
Meishan−B
Meishan−C
European−A
European−B
European−C

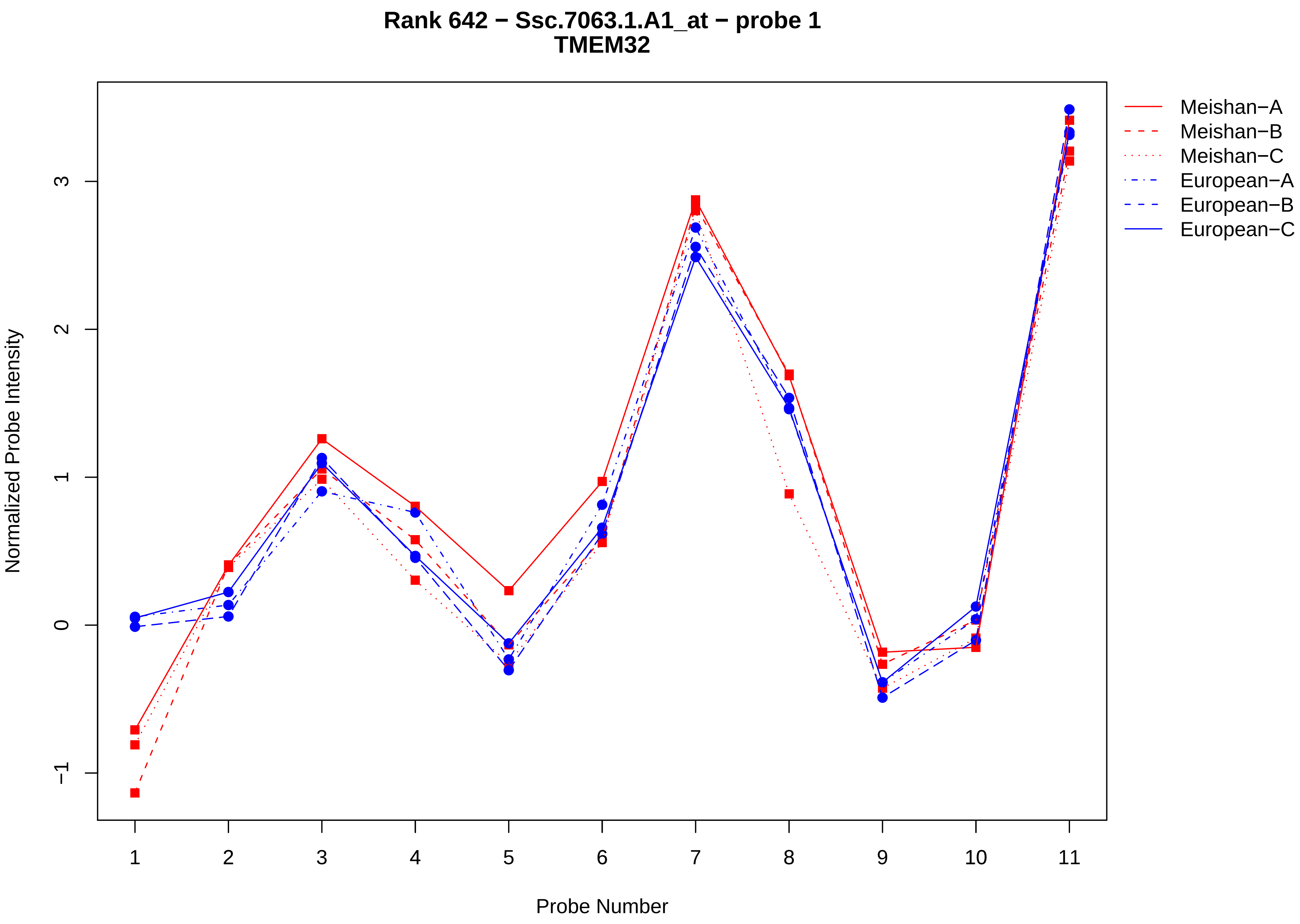

Rank 642 − Ssc.7063.1.A1_at − probe 1
TMEM32
Normalized Probe Intensity
Probe Number
−1
0
1
2
3
1
2
3
4
5
6
7
8
9
10
11
Meishan−A
Meishan−B
Meishan−C
European−A
European−B
European−C

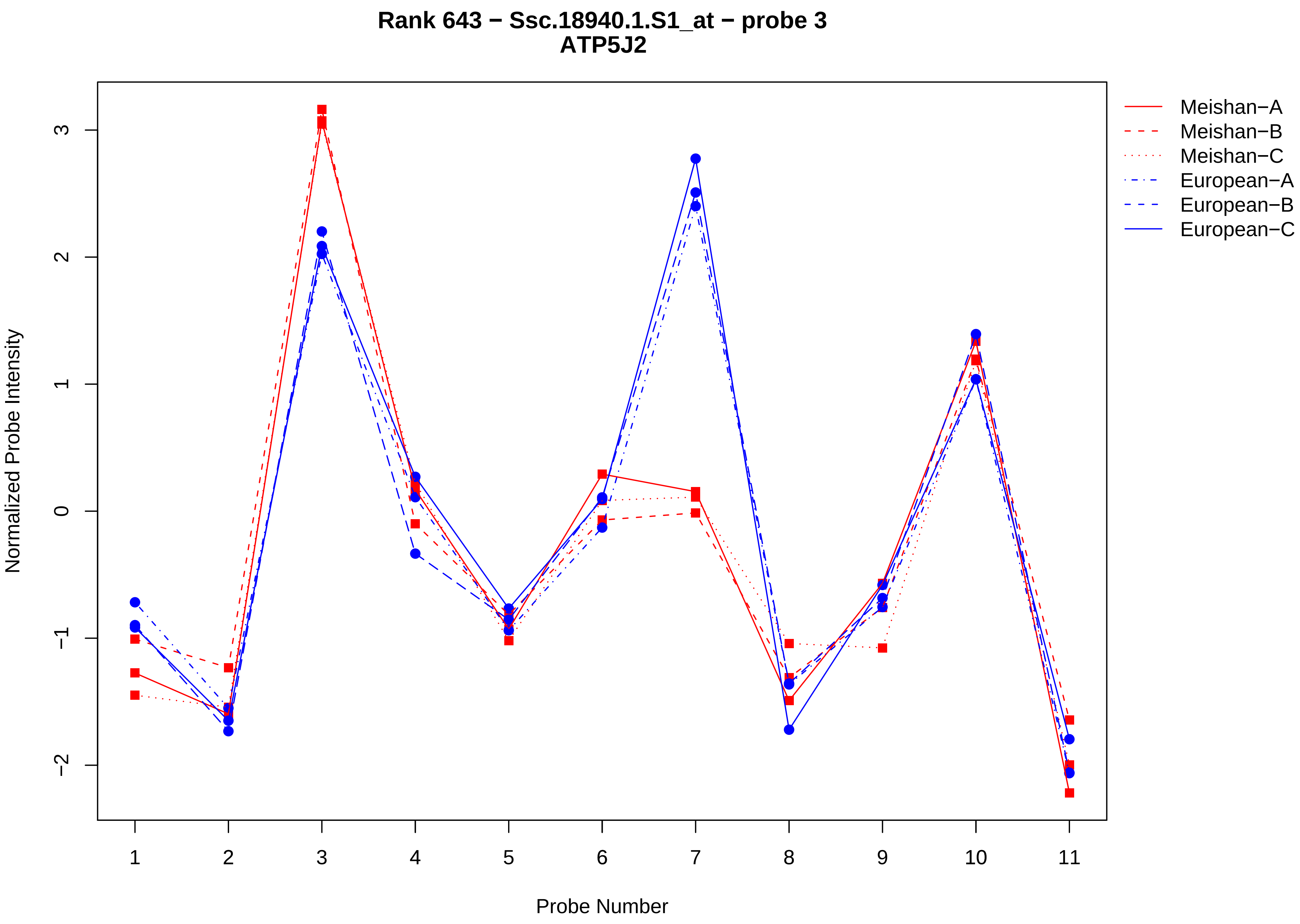

Rank 643 − Ssc.18940.1.S1_at − probe 3
ATP5J2
Normalized Probe Intensity
Probe Number
Meishan−A
Meishan−B
Meishan−C
European−A
European−B
European−C
−2
−1
0
1
2
3
1
2
3
4
5
6
7
8
9
10
11

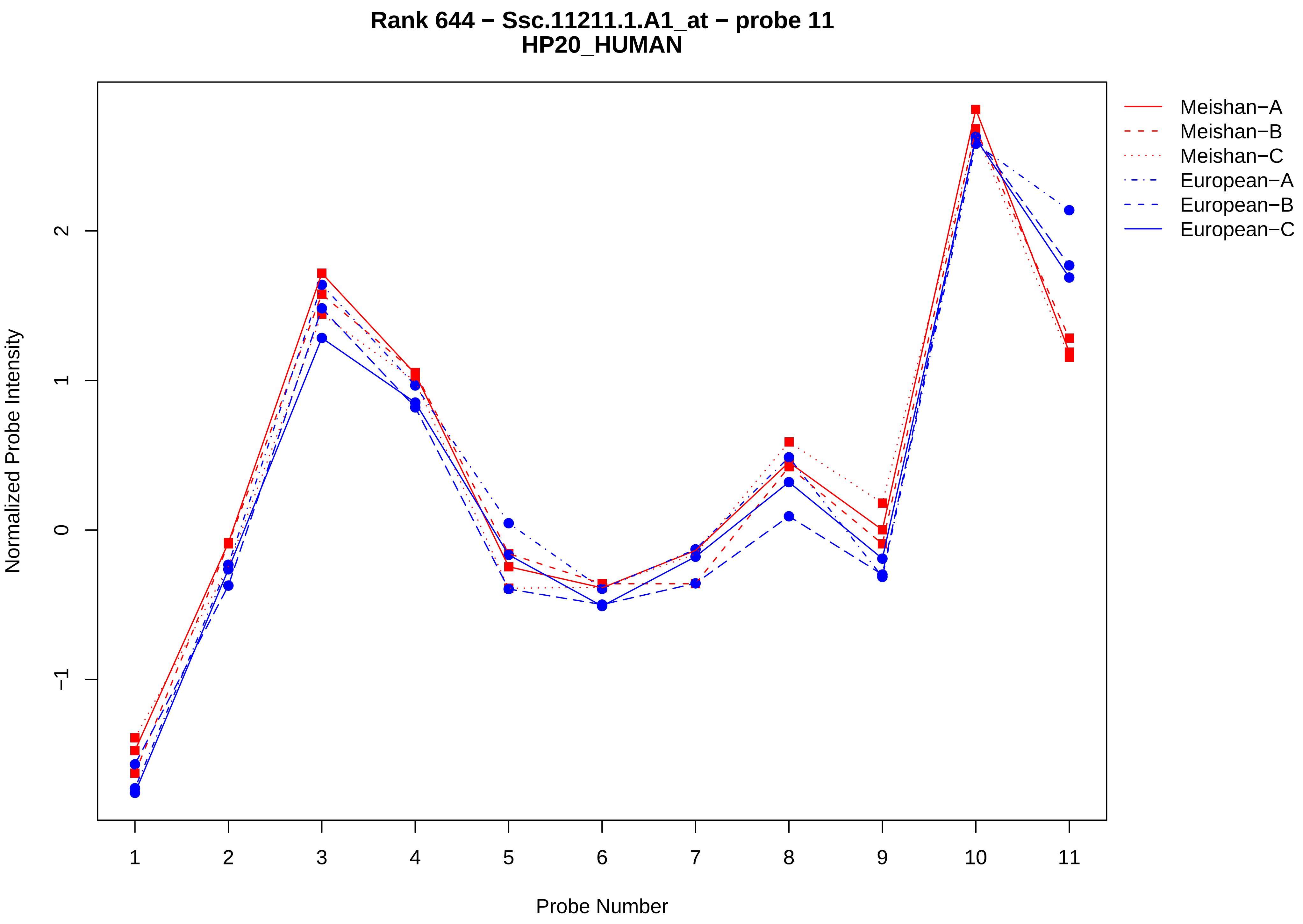
Rank 644 − Ssc.11211.1.A1_at − probe 11
HP20_HUMAN
Normalized Probe Intensity
Probe Number
Meishan−A
Meishan−B
Meishan−C
European−A
European−B
European−C

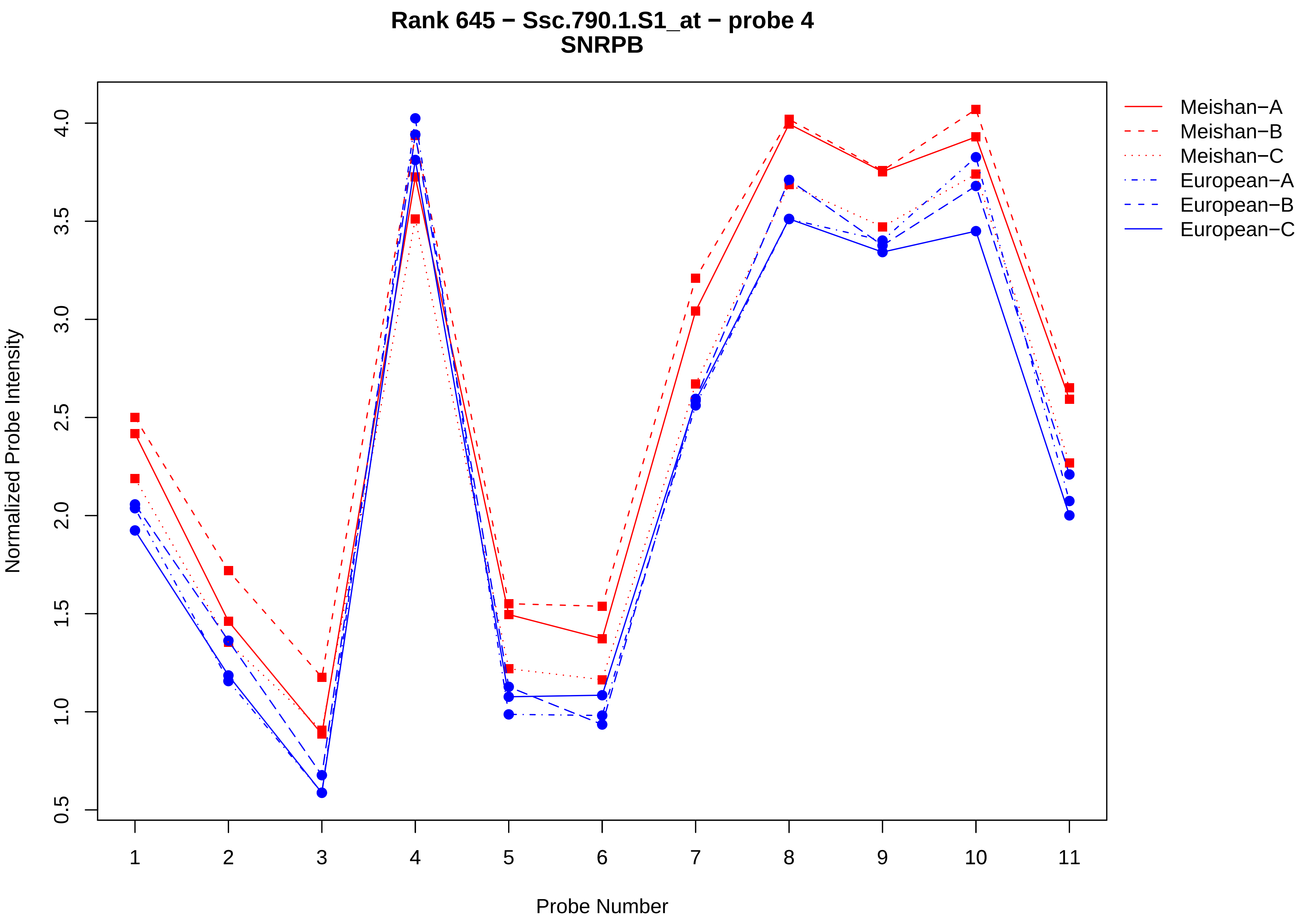

Rank 645 − Ssc.790.1.S1_at − probe 4
SNRPB
Normalized Probe Intensity
Probe Number
Meishan−A
Meishan−B
Meishan−C
European−A
European−B
European−C
0.5
1.0
1.5
2.0
2.5
3.0
3.5
4.0
1
2
3
4
5
6
7
8
9
10
11

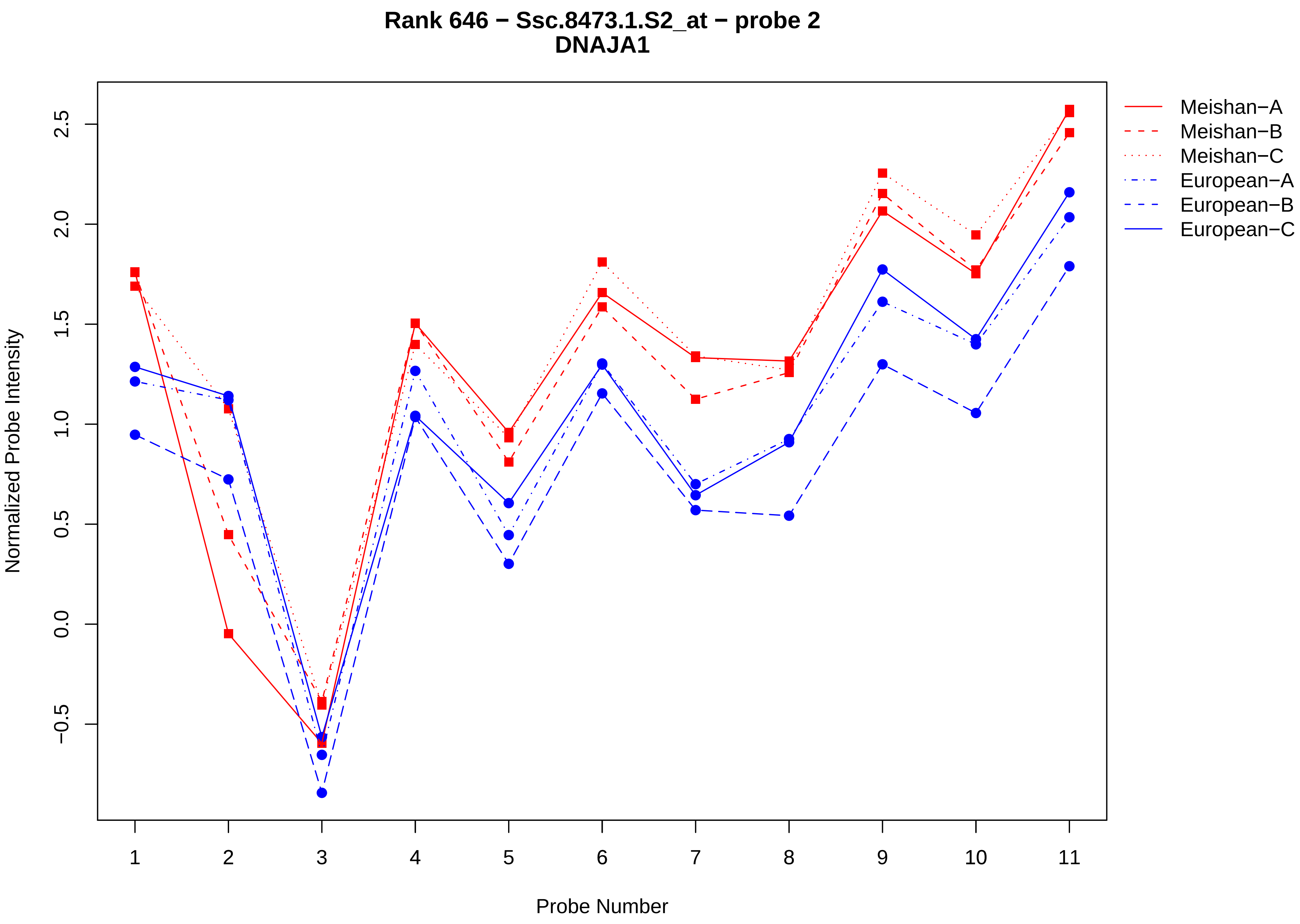

Rank 646 − Ssc.8473.1.S2_at − probe 2
DNAJA1
Normalized Probe Intensity
Probe Number
−0.5
0.0
0.5
1.0
1.5
2.0
2.5
1
2
3
4
5
6
7
8
9
10
11
Meishan−A
Meishan−B
Meishan−C
European−A
European−B
European−C

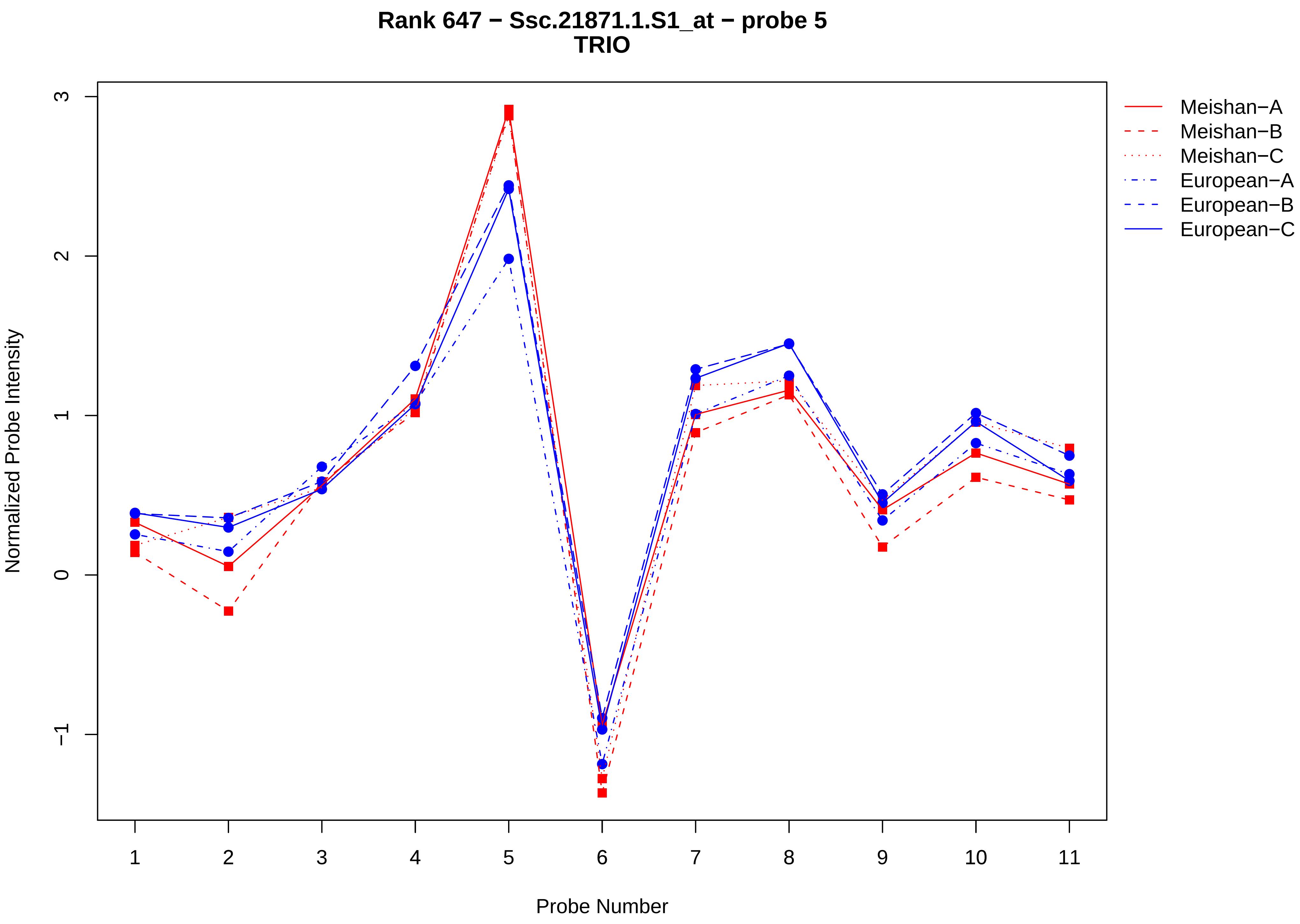

Rank 647 − Ssc.21871.1.S1_at − probe 5
TRIO
Normalized Probe Intensity
Probe Number
−1
0
1
2
3
1
2
3
4
5
6
7
8
9
10
11
Meishan−A
Meishan−B
Meishan−C
European−A
European−B
European−C

Rank 648 − Ssc.24981.1.S1_at − probe 3
Q8TE89

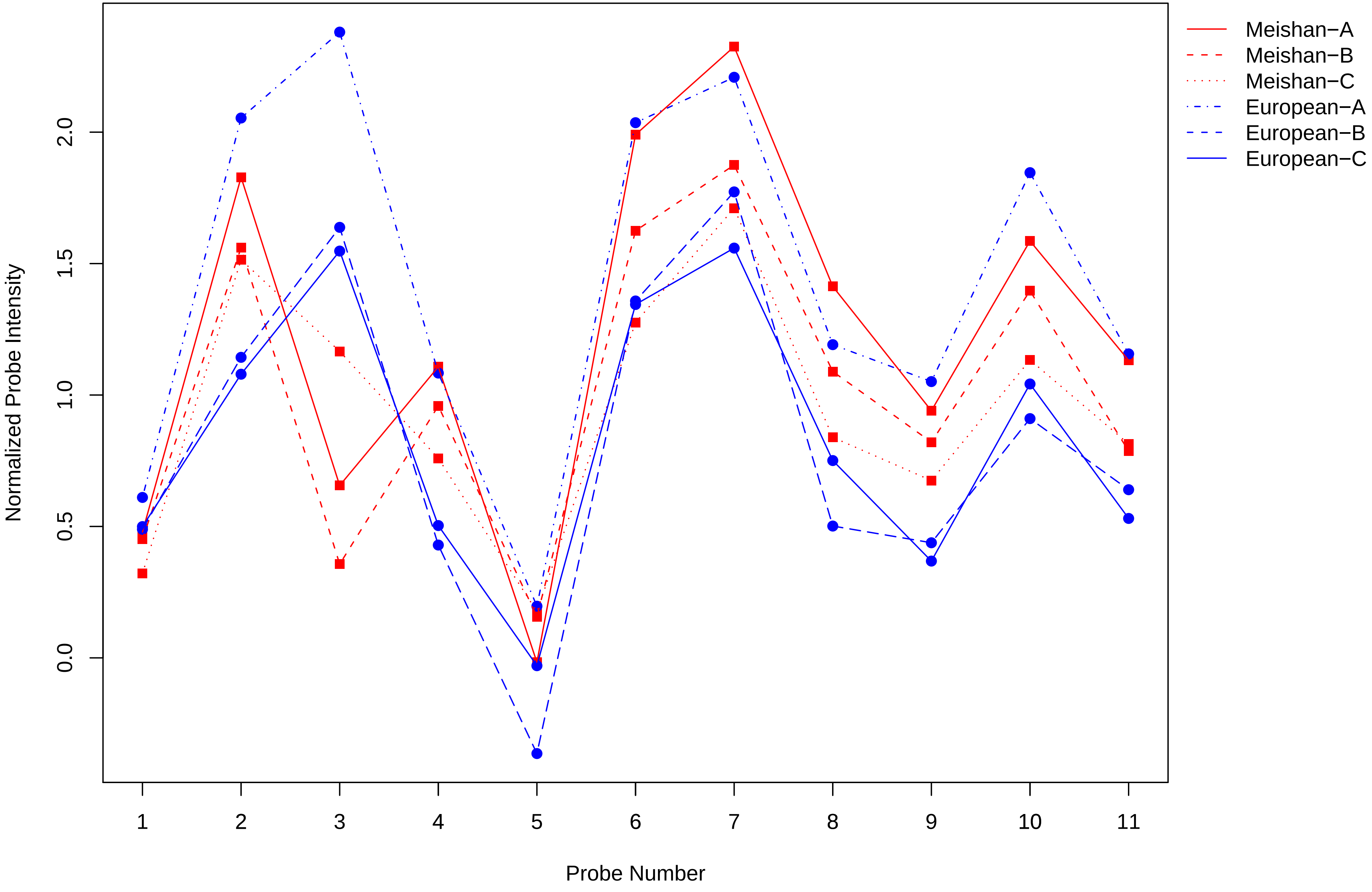

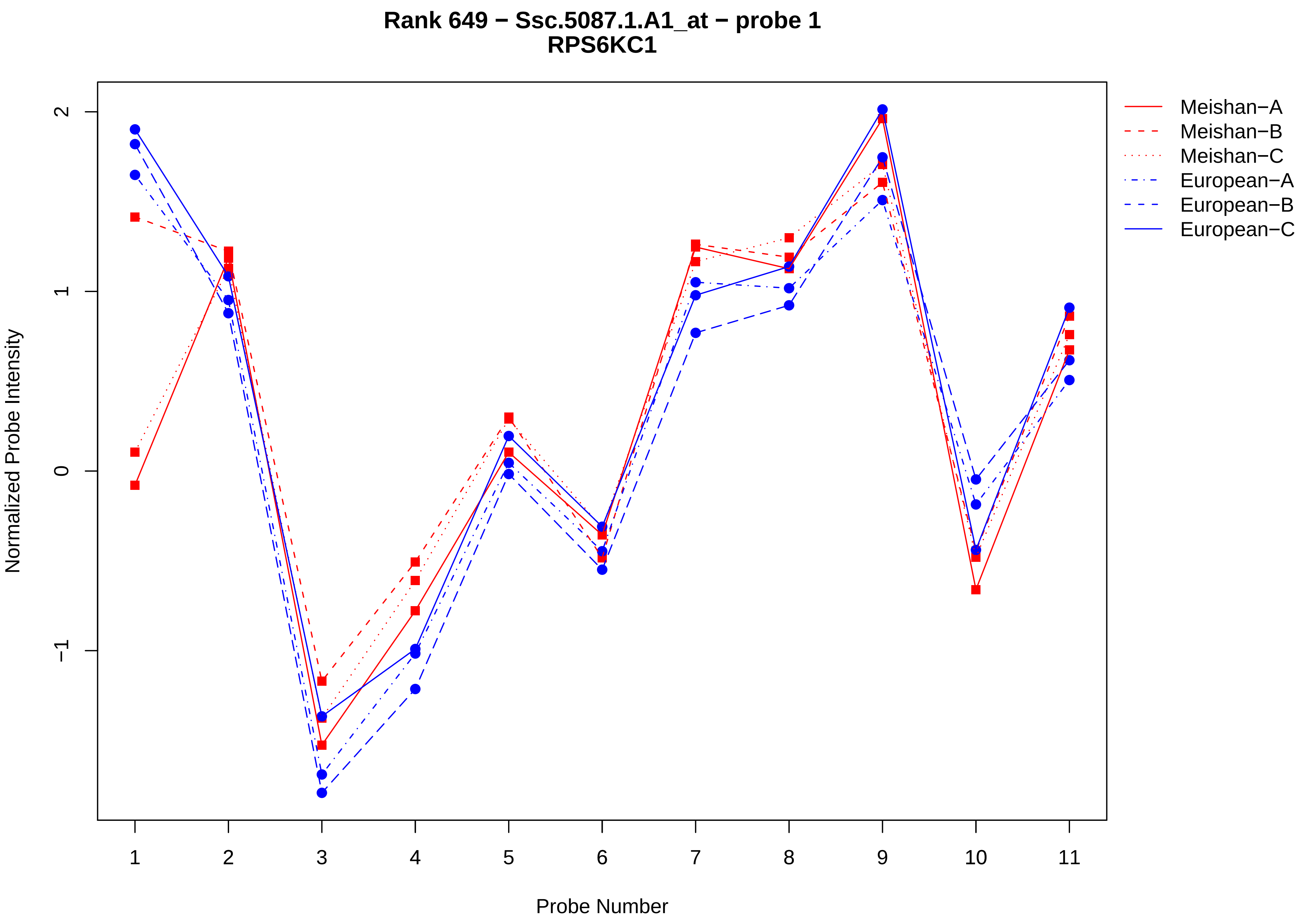

Rank 649 − Ssc.5087.1.A1_at − probe 1
RPS6KC1
Normalized Probe Intensity
Probe Number
−1
0
1
2
1
2
3
4
5
6
7
8
9
10
11
Meishan−A
Meishan−B
Meishan−C
European−A
European−B
European−C

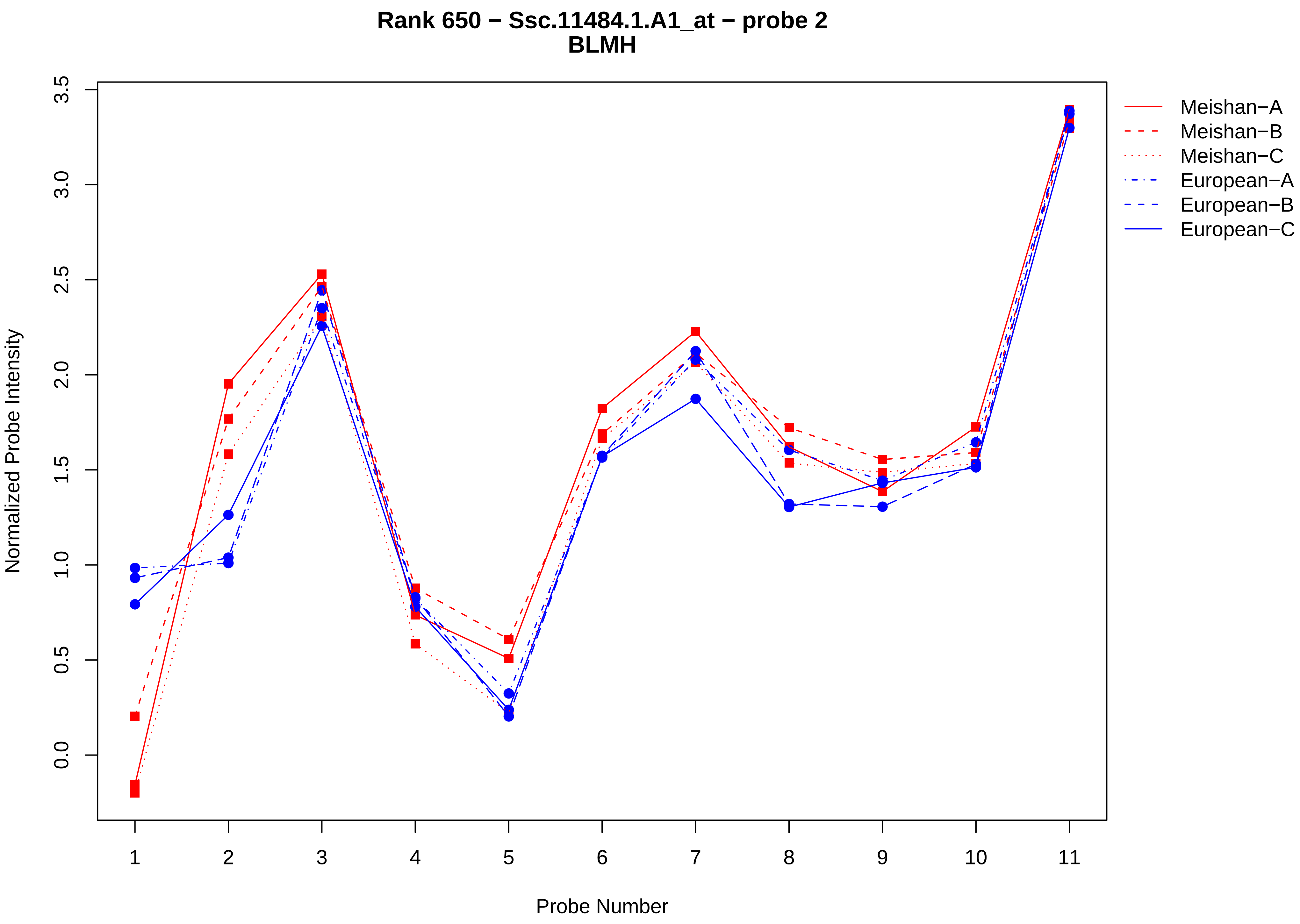

Rank 650 − Ssc.11484.1.A1_at − probe 2
BLMH
Normalized Probe Intensity
Probe Number
0.0
0.5
1.0
1.5
2.0
2.5
3.0
3.5
1
2
3
4
5
6
7
8
9
10
11
Meishan−A
Meishan−B
Meishan−C
European−A
European−B
European−C

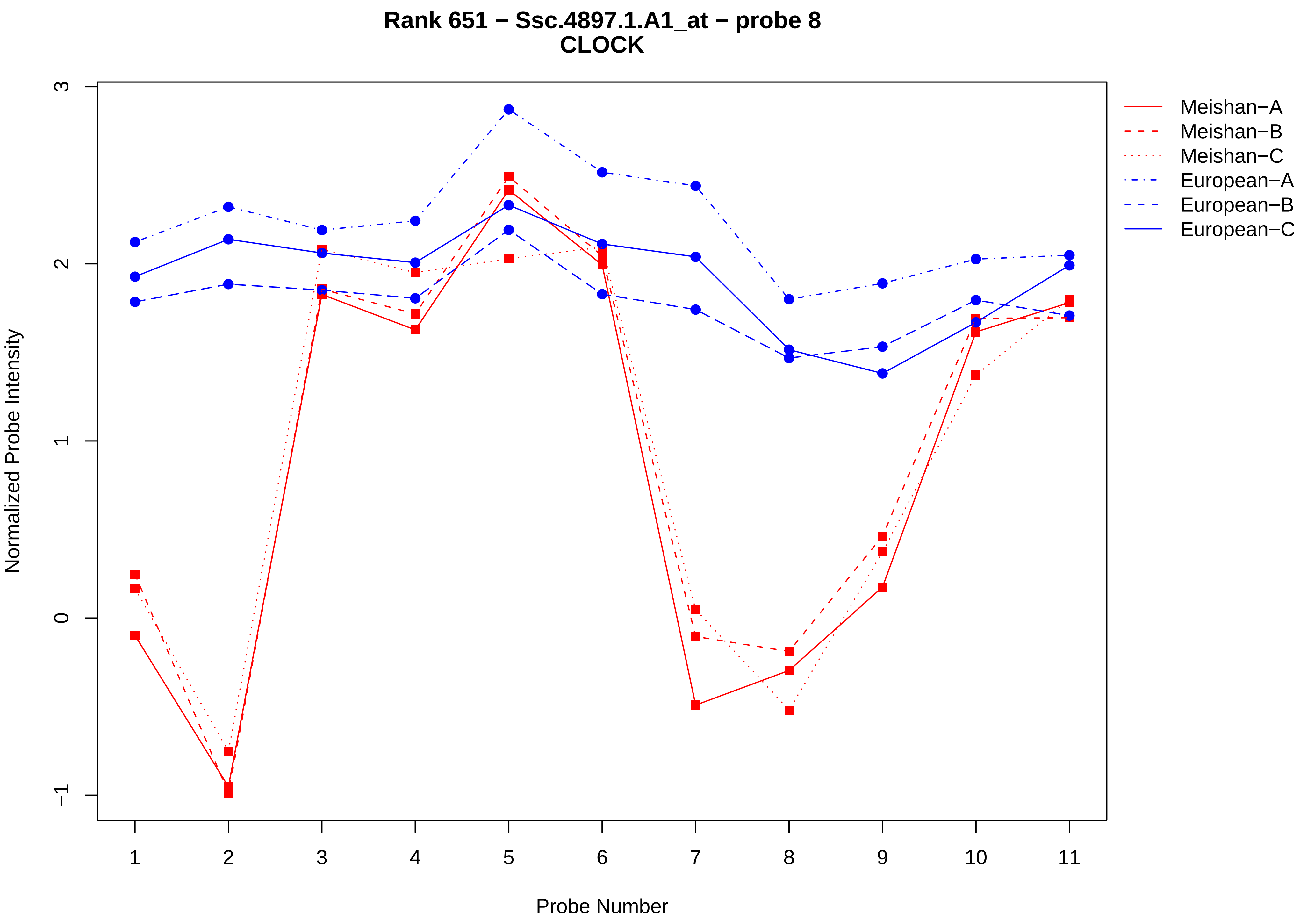

Rank 651 − Ssc.4897.1.A1_at − probe 8
CLOCK
Normalized Probe Intensity
Probe Number
−1
0
1
2
3
1
2
3
4
5
6
7
8
9
10
11
Meishan−A
Meishan−B
Meishan−C
European−A
European−B
European−C

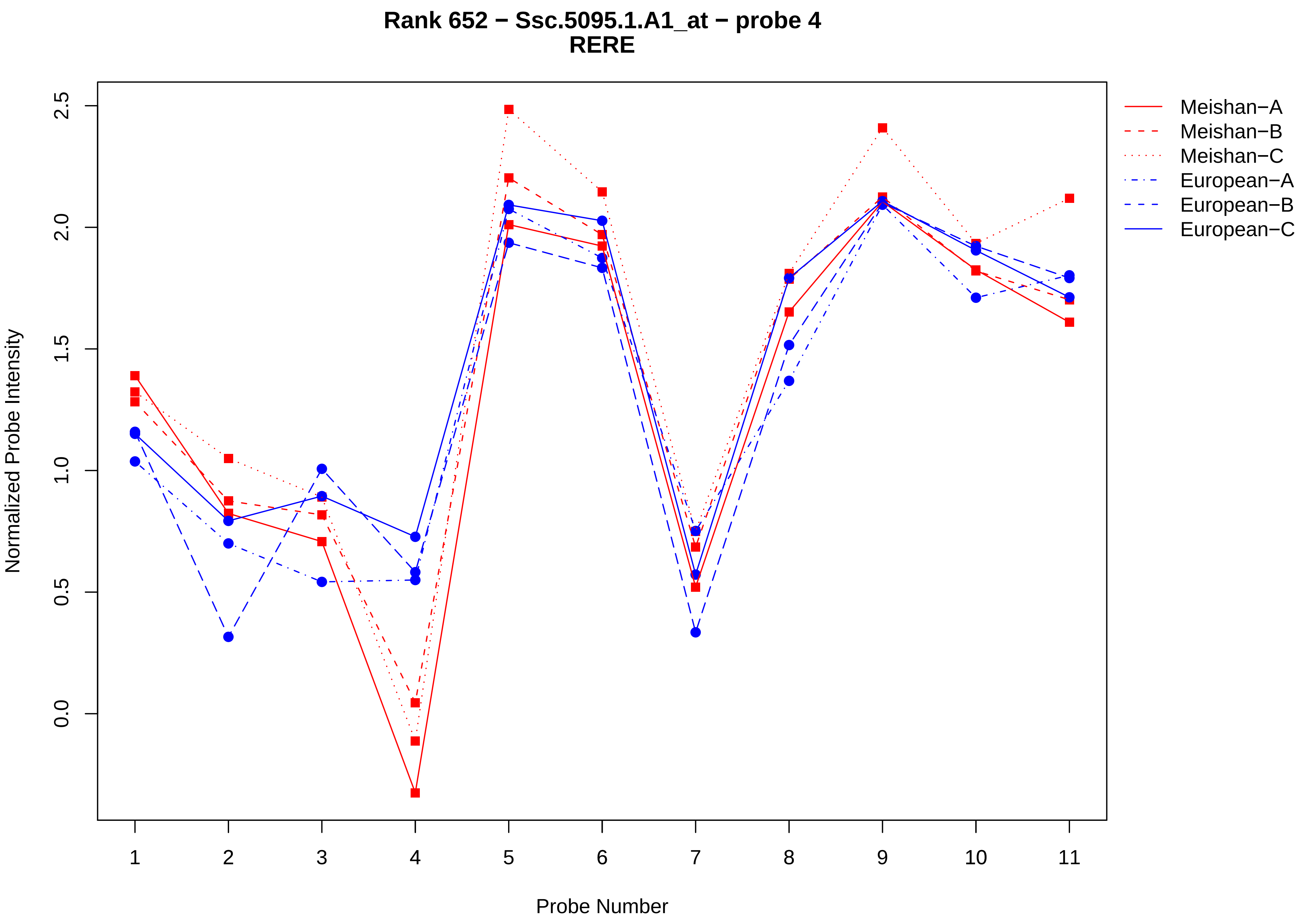

Rank 652 − Ssc.5095.1.A1_at − probe 4
RERE
Normalized Probe Intensity
Probe Number
0.0
0.5
1.0
1.5
2.0
2.5
1
2
3
4
5
6
7
8
9
10
11
Meishan−A
Meishan−B
Meishan−C
European−A
European−B
European−C

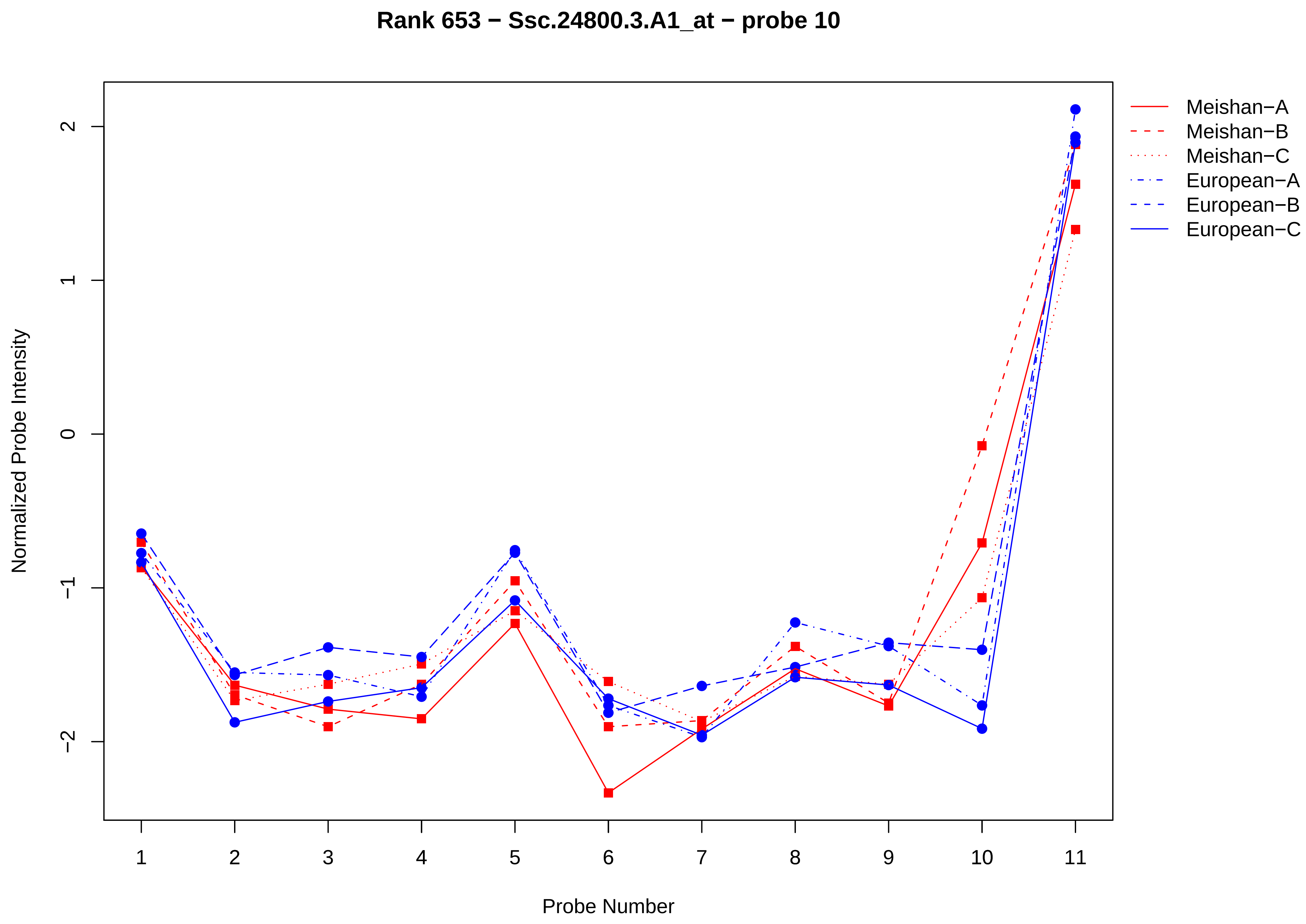

Rank 653 − Ssc.24800.3.A1_at − probe 10
Normalized Probe Intensity
Probe Number
−2
−1
0
1
2
1
2
3
4
5
6
7
8
9
10
11
Meishan−A
Meishan−B
Meishan−C
European−A
European−B
European−C

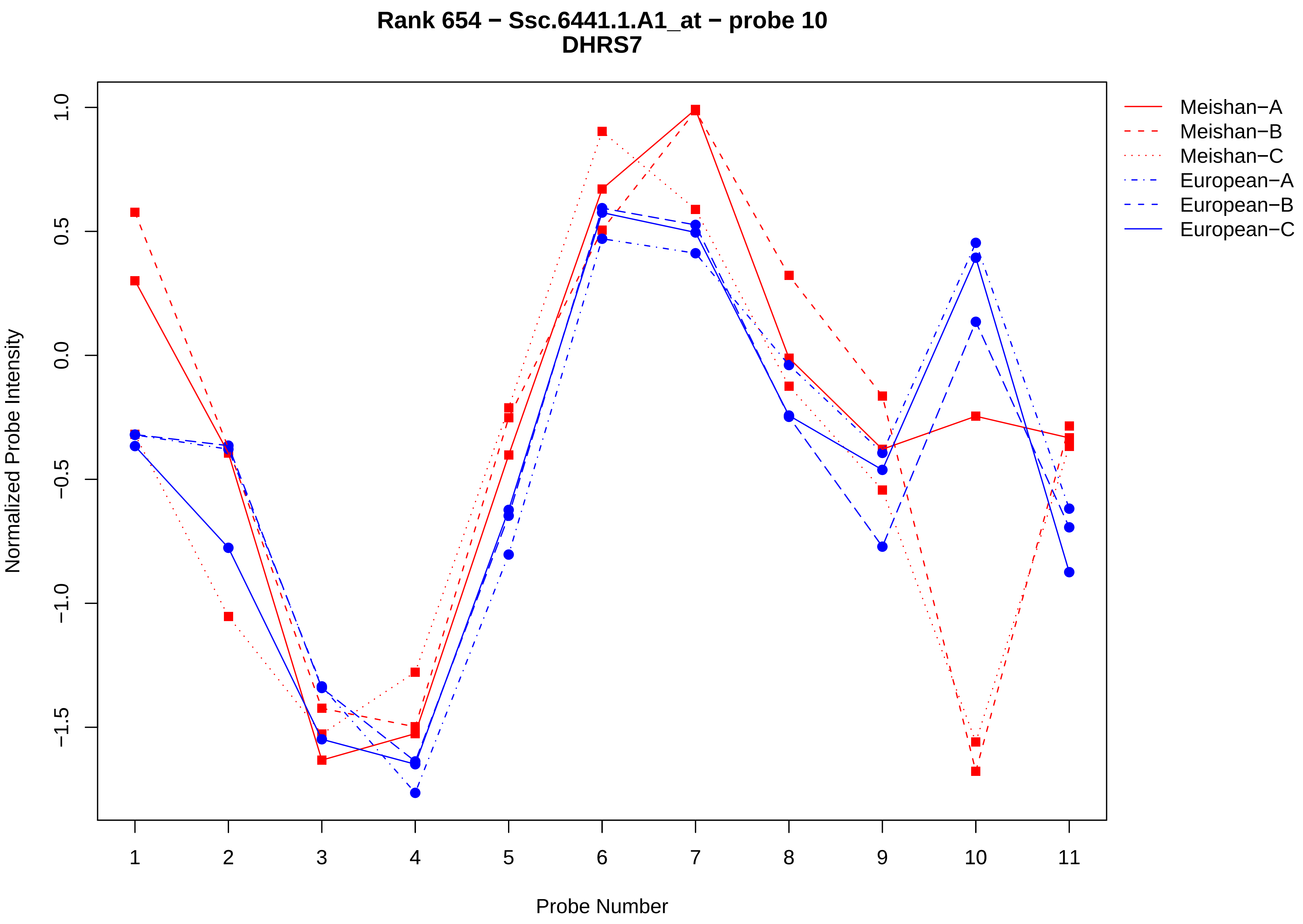
Rank 654 − Ssc.6441.1.A1_at − probe 10
DHRS7
Normalized Probe Intensity
Probe Number
1.0
0.5
0.0
−0.5
−1.0
−1.5
1
2
3
4
5
6
7
8
9
10
11
Meishan−A
Meishan−B
Meishan−C
European−A
European−B
European−C

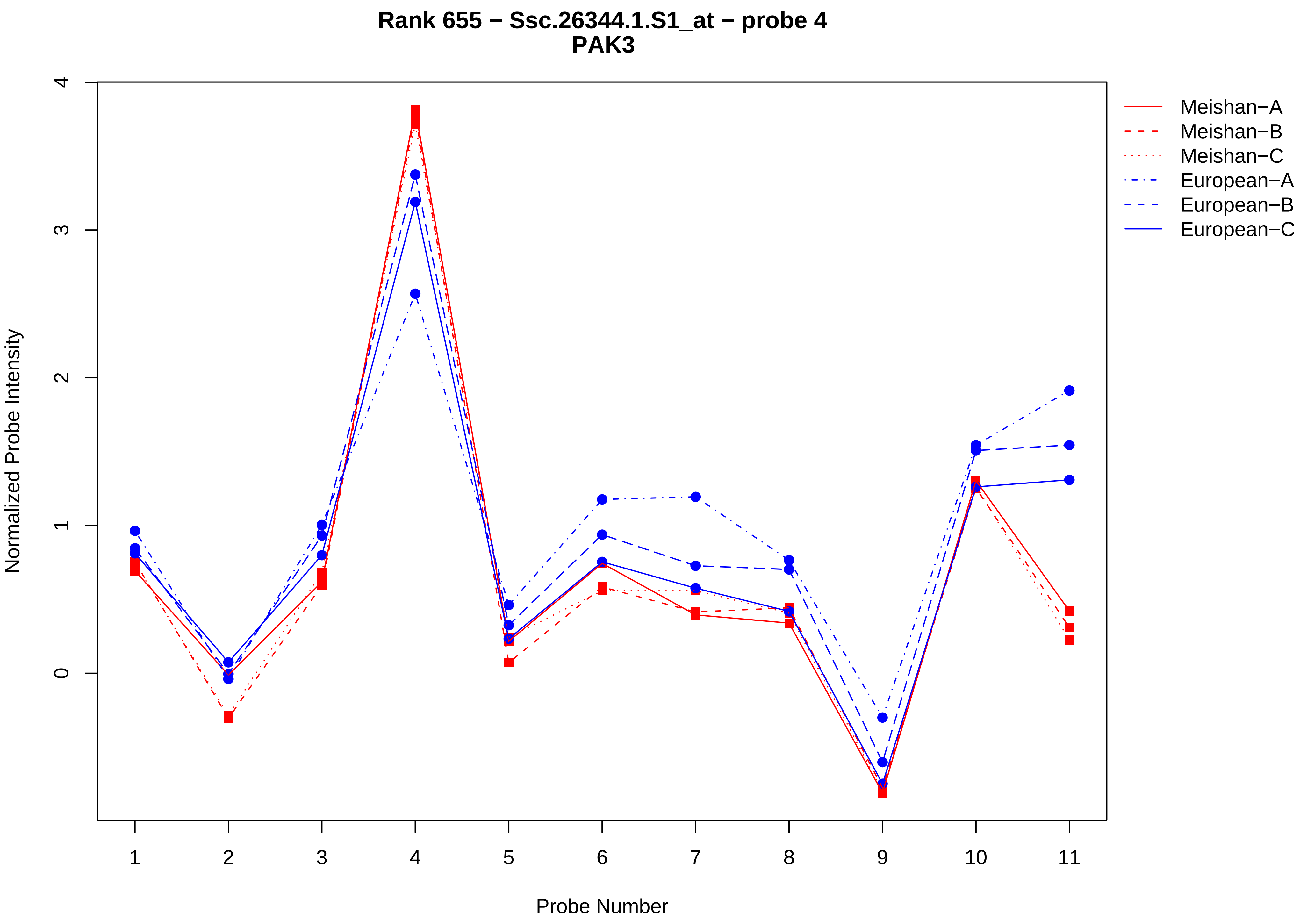
Rank 655 − Ssc.26344.1.S1_at − probe 4
PAK3
Normalized Probe Intensity
Probe Number
0
1
2
3
4
1
2
3
4
5
6
7
8
9
10
11
Meishan−A
Meishan−B
Meishan−C
European−A
European−B
European−C

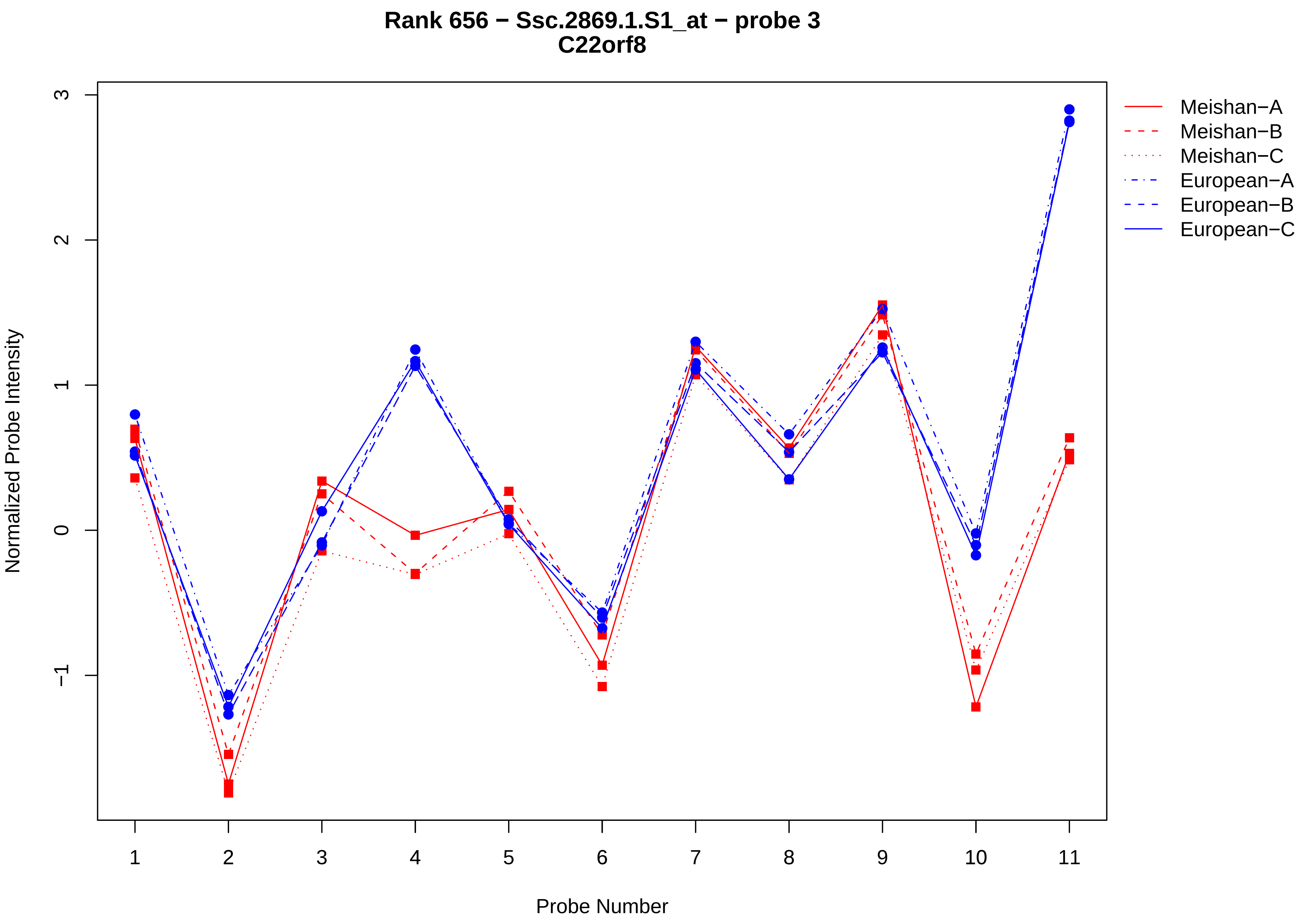

Rank 656 − Ssc.2869.1.S1_at − probe 3
C22orf8
Normalized Probe Intensity
Probe Number
−1
0
1
2
3
1
2
3
4
5
6
7
8
9
10
11
Meishan−A
Meishan−B
Meishan−C
European−A
European−B
European−C

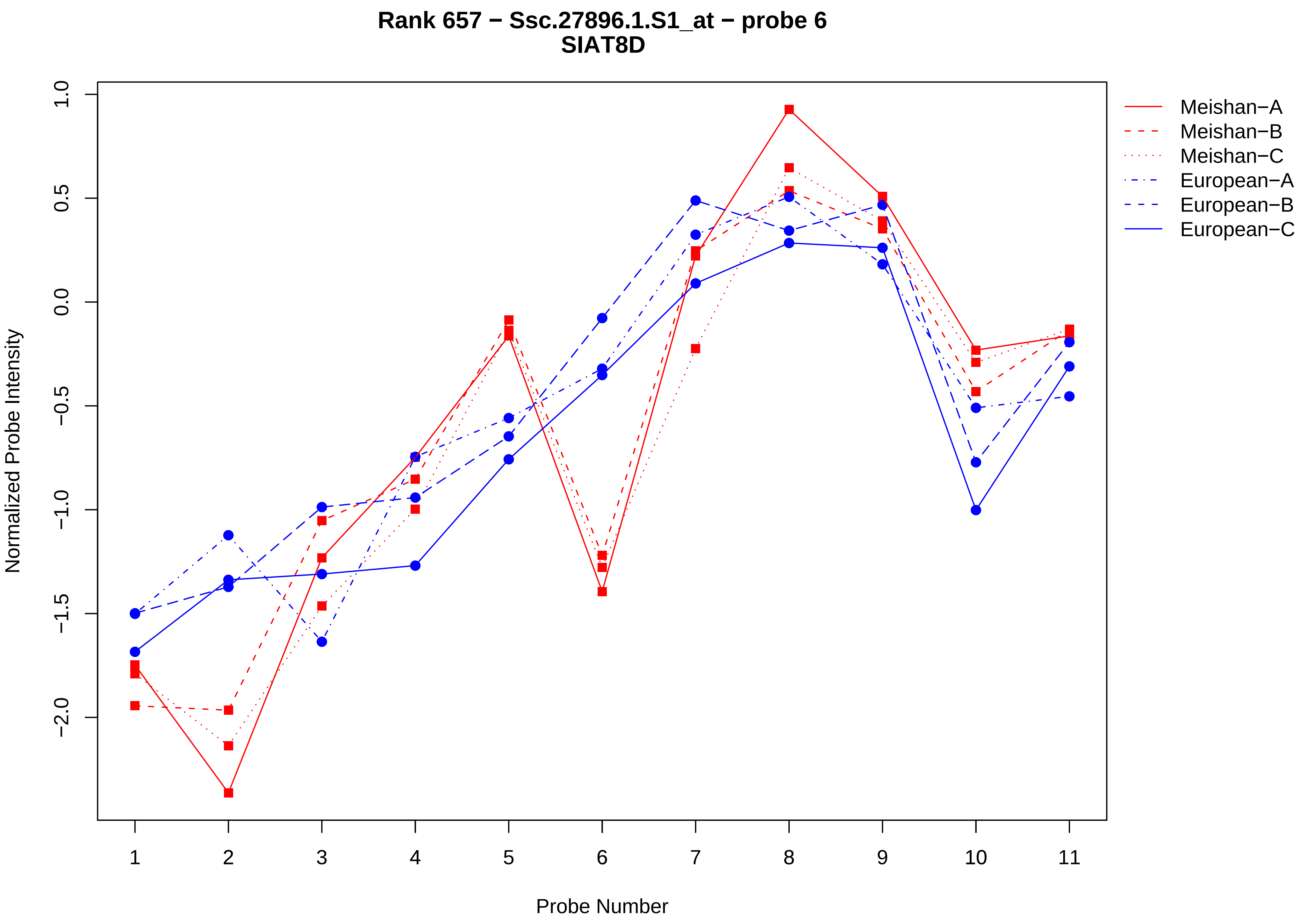
Rank 657 − Ssc.27896.1.S1_at − probe 6
SIAT8D
Normalized Probe Intensity
Probe Number
Meishan−A
Meishan−B
Meishan−C
European−A
European−B
European−C
1.0
0.5
0.0
−0.5
−1.0
−1.5
−2.0
1
2
3
4
5
6
7
8
9
10
11

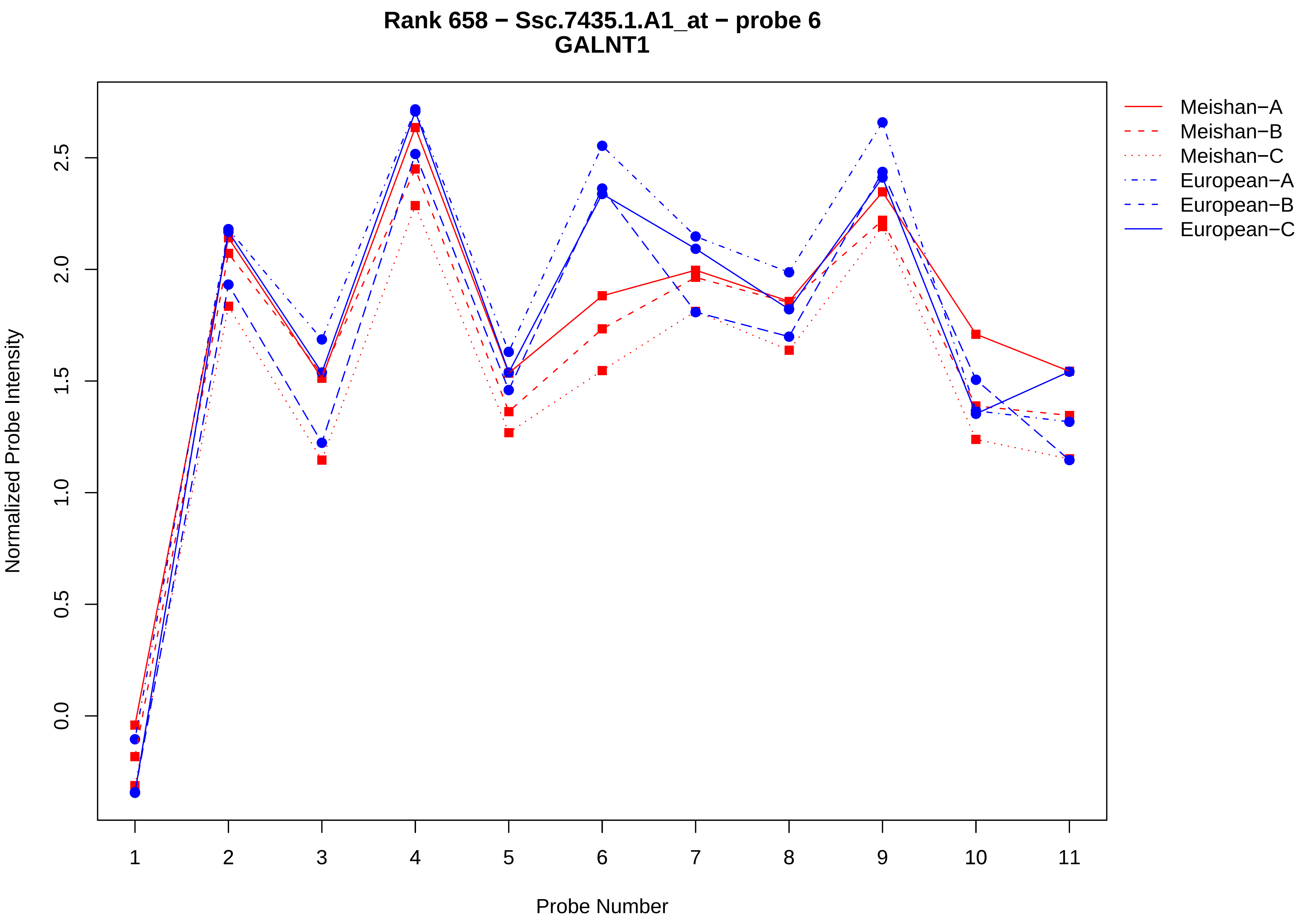

Rank 658 − Ssc.7435.1.A1_at − probe 6
GALNT1
Normalized Probe Intensity
Probe Number
Meishan−A
Meishan−B
Meishan−C
European−A
European−B
European−C
0.0
0.5
1.0
1.5
2.0
2.5
1
2
3
4
5
6
7
8
9
10
11

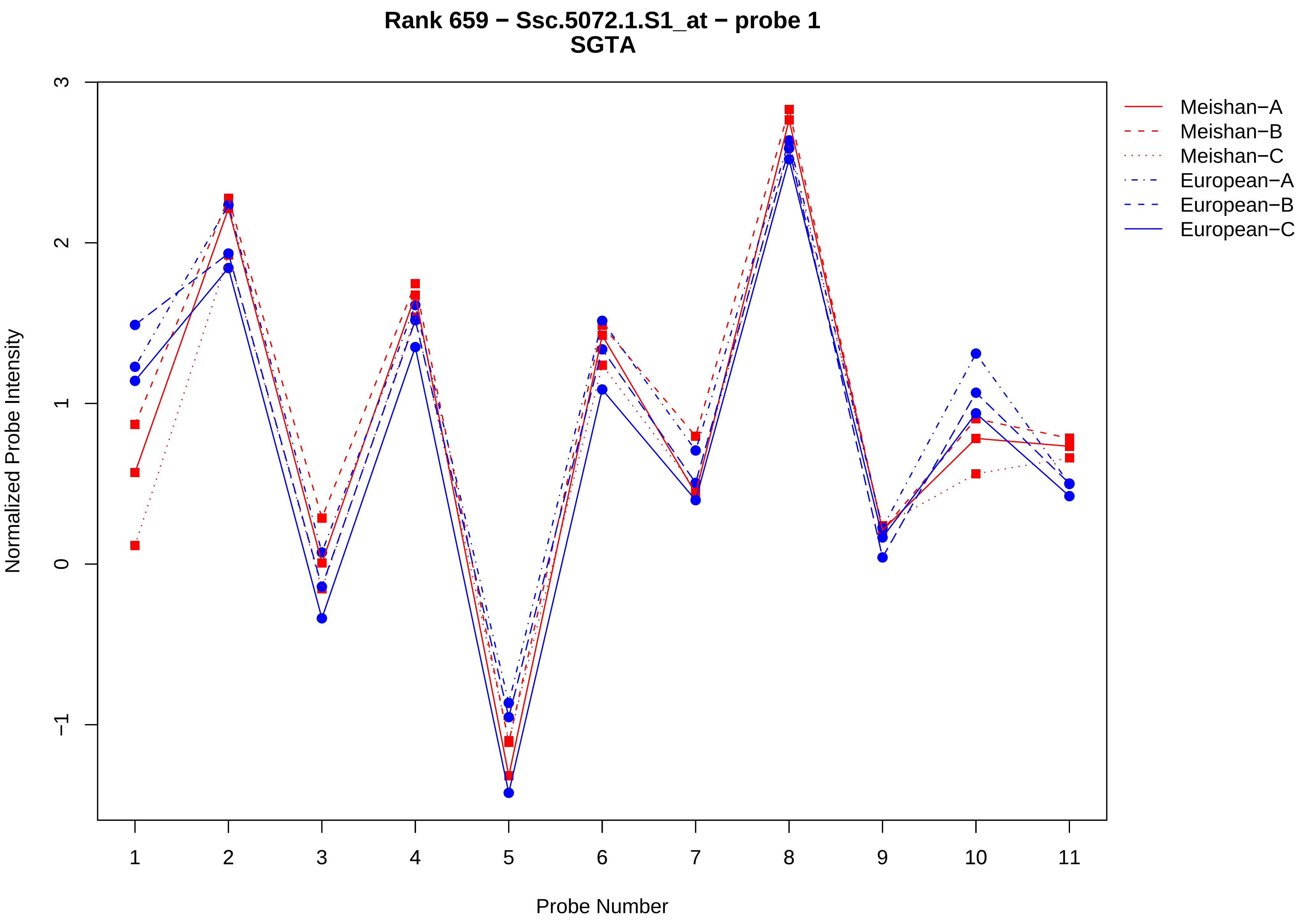

Rank 659 − Ssc.5072.1.S1_at − probe 1
SGTA
Normalized Probe Intensity
Probe Number
Meishan−A
Meishan−B
Meishan−C
European−A
European−B
European−C

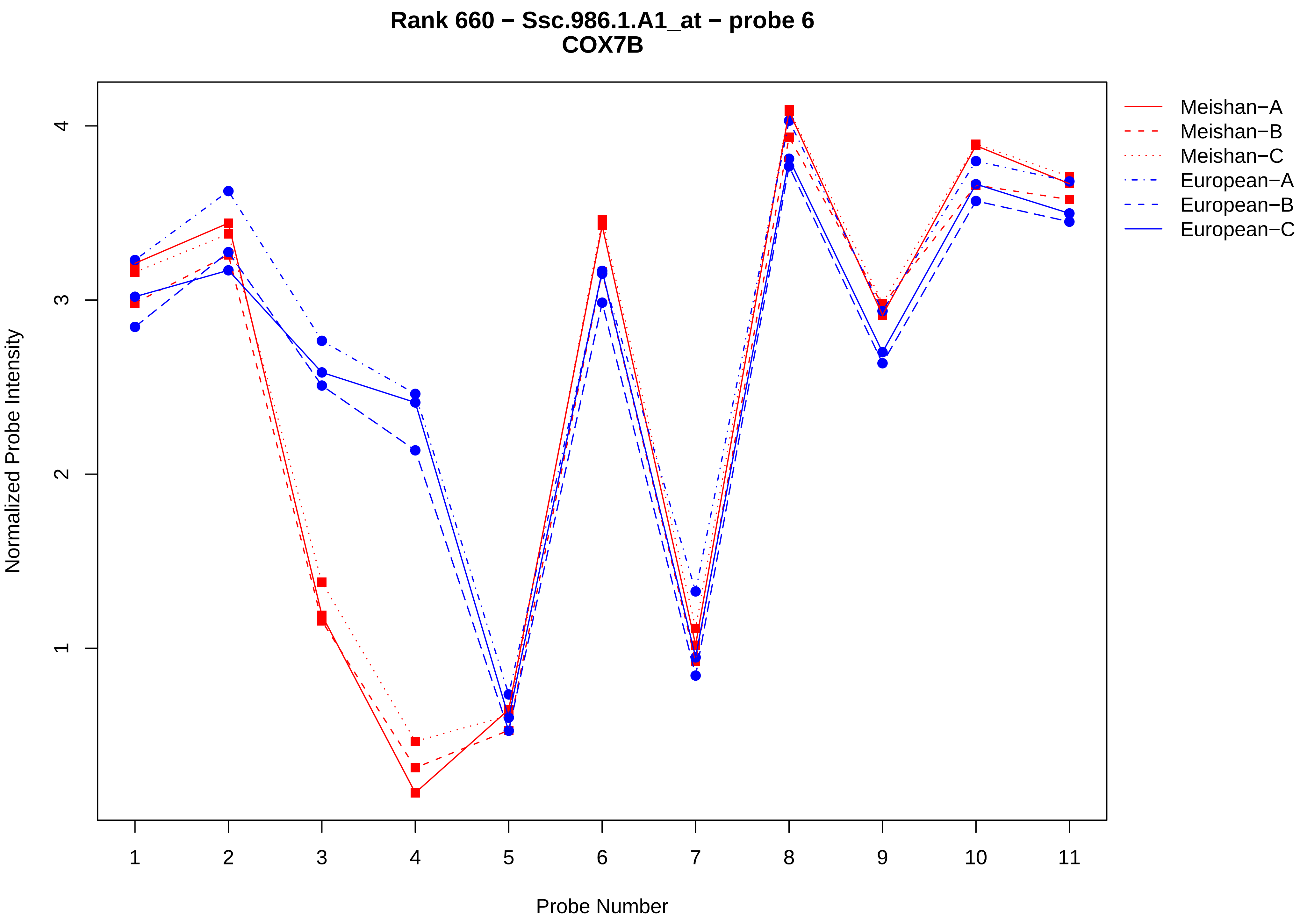

Rank 660 − Ssc.986.1.A1_at − probe 6
COX7B
Normalized Probe Intensity
Probe Number
Meishan−A
Meishan−B
Meishan−C
European−A
European−B
European−C
1
2
3
4
5
6
7
8
9
10
11

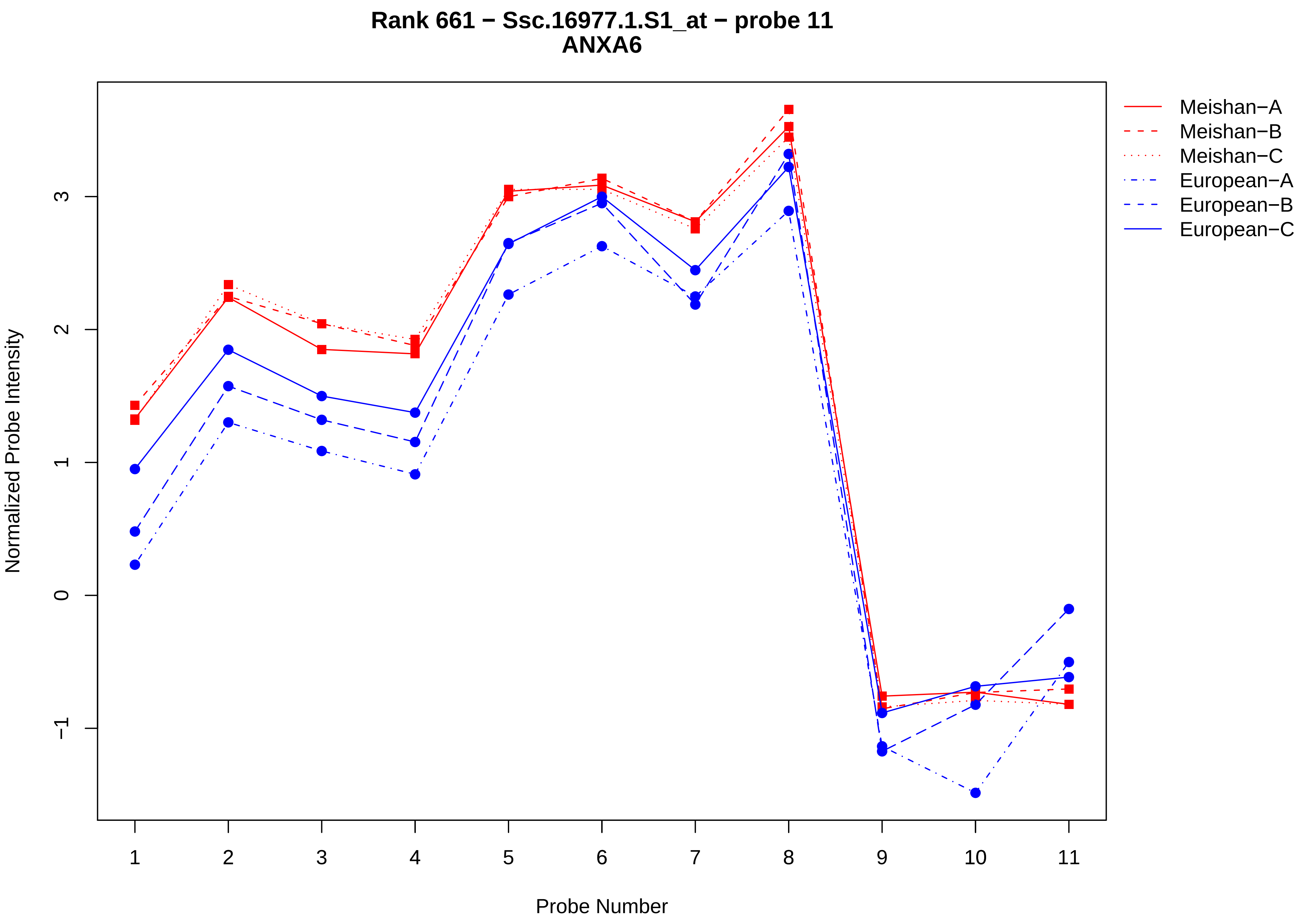

Rank 661 − Ssc.16977.1.S1_at − probe 11
ANXA6
Normalized Probe Intensity
Probe Number
−1
0
1
2
3
1
2
3
4
5
6
7
8
9
10
11
Meishan−A
Meishan−B
Meishan−C
European−A
European−B
European−C

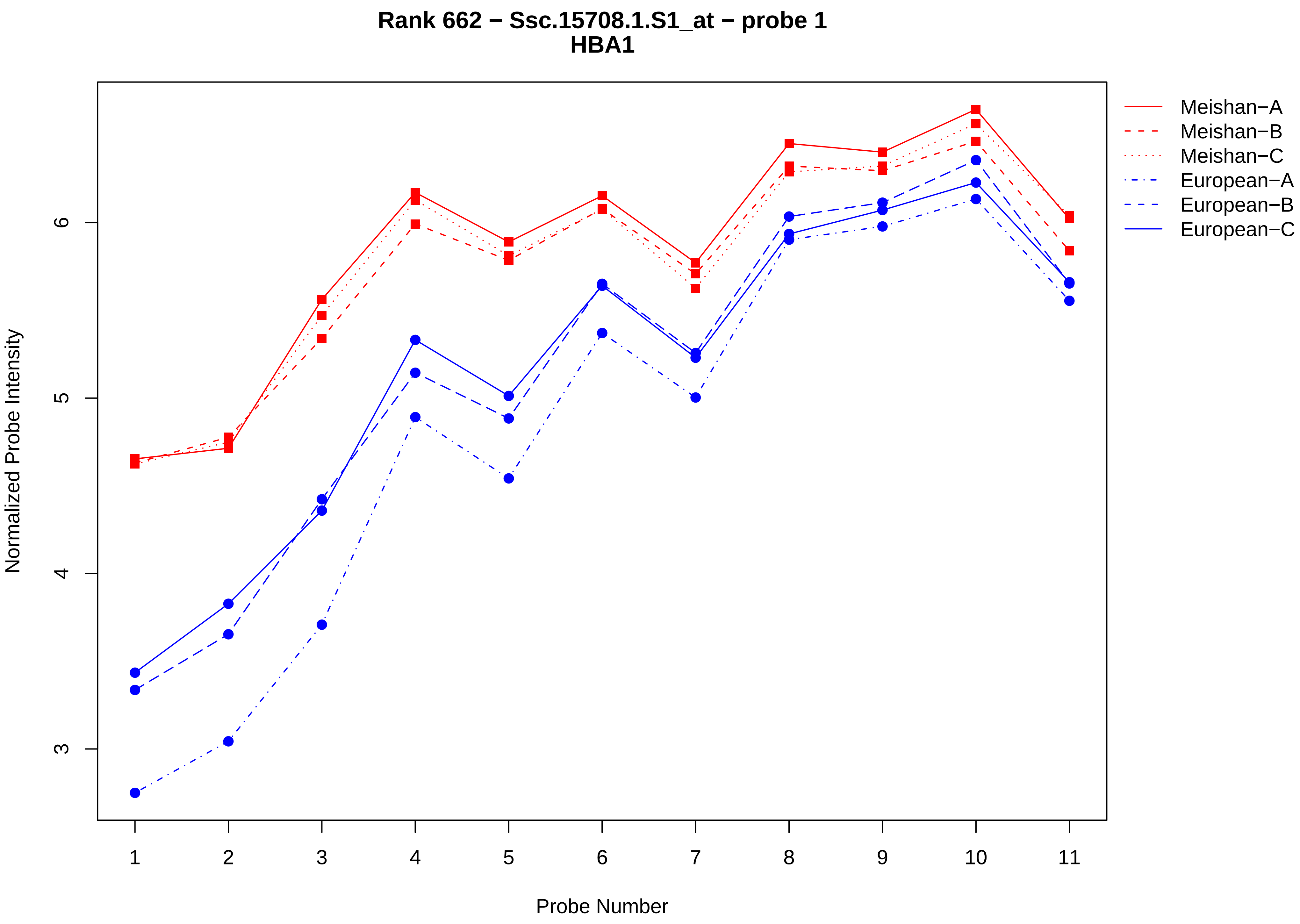
Rank 662 − Ssc.15708.1.S1_at − probe 1
HBA1
Normalized Probe Intensity
Probe Number
Meishan−A
Meishan−B
Meishan−C
European−A
European−B
European−C
3
4
5
6
1
2
3
4
5
6
7
8
9
10
11

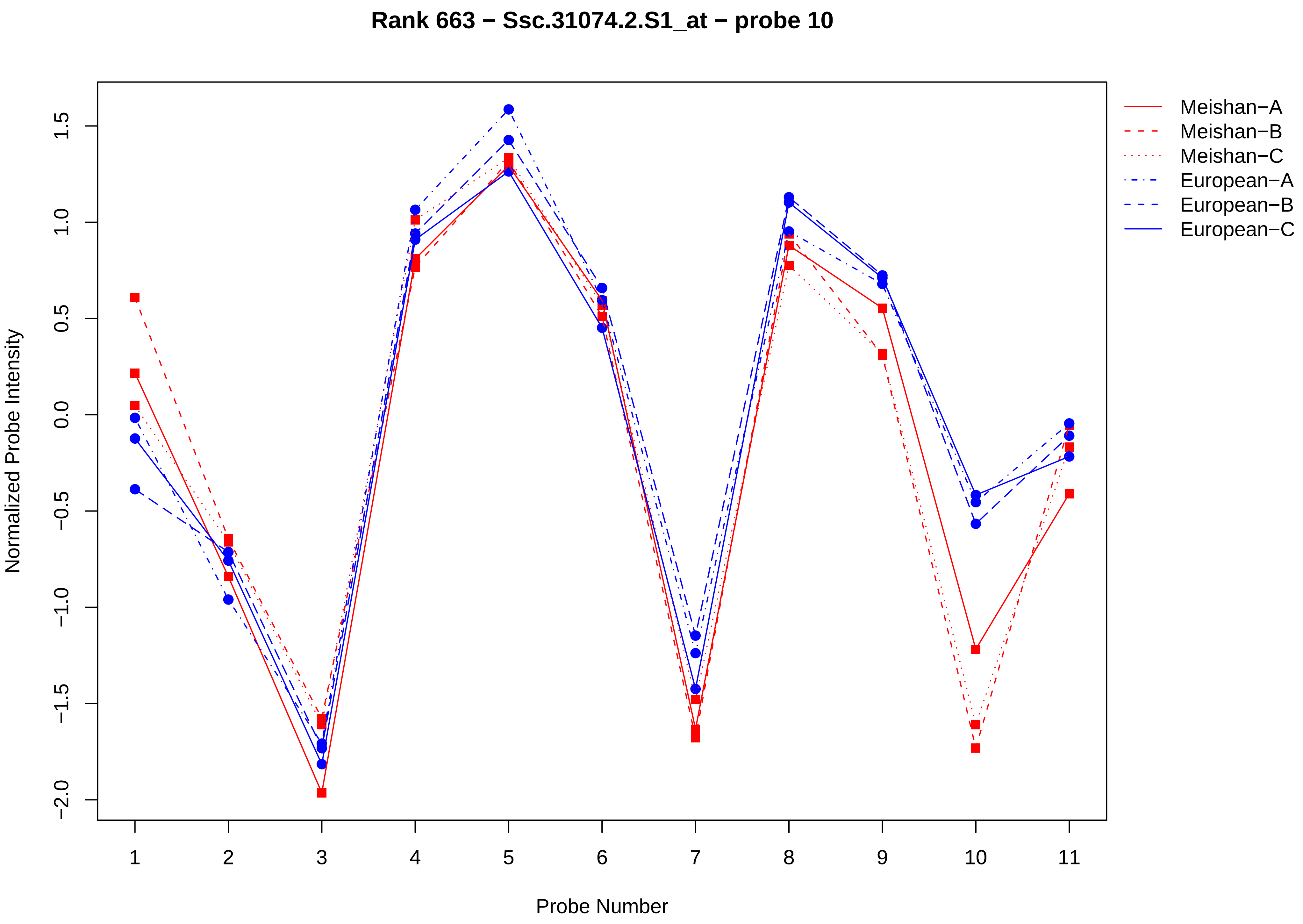

Rank 663 − Ssc.31074.2.S1_at − probe 10
Normalized Probe Intensity
Probe Number
1.5
1.0
0.5
0.0
−0.5
−1.0
−1.5
−2.0
1
2
3
4
5
6
7
8
9
10
11
Meishan−A
Meishan−B
Meishan−C
European−A
European−B
European−C

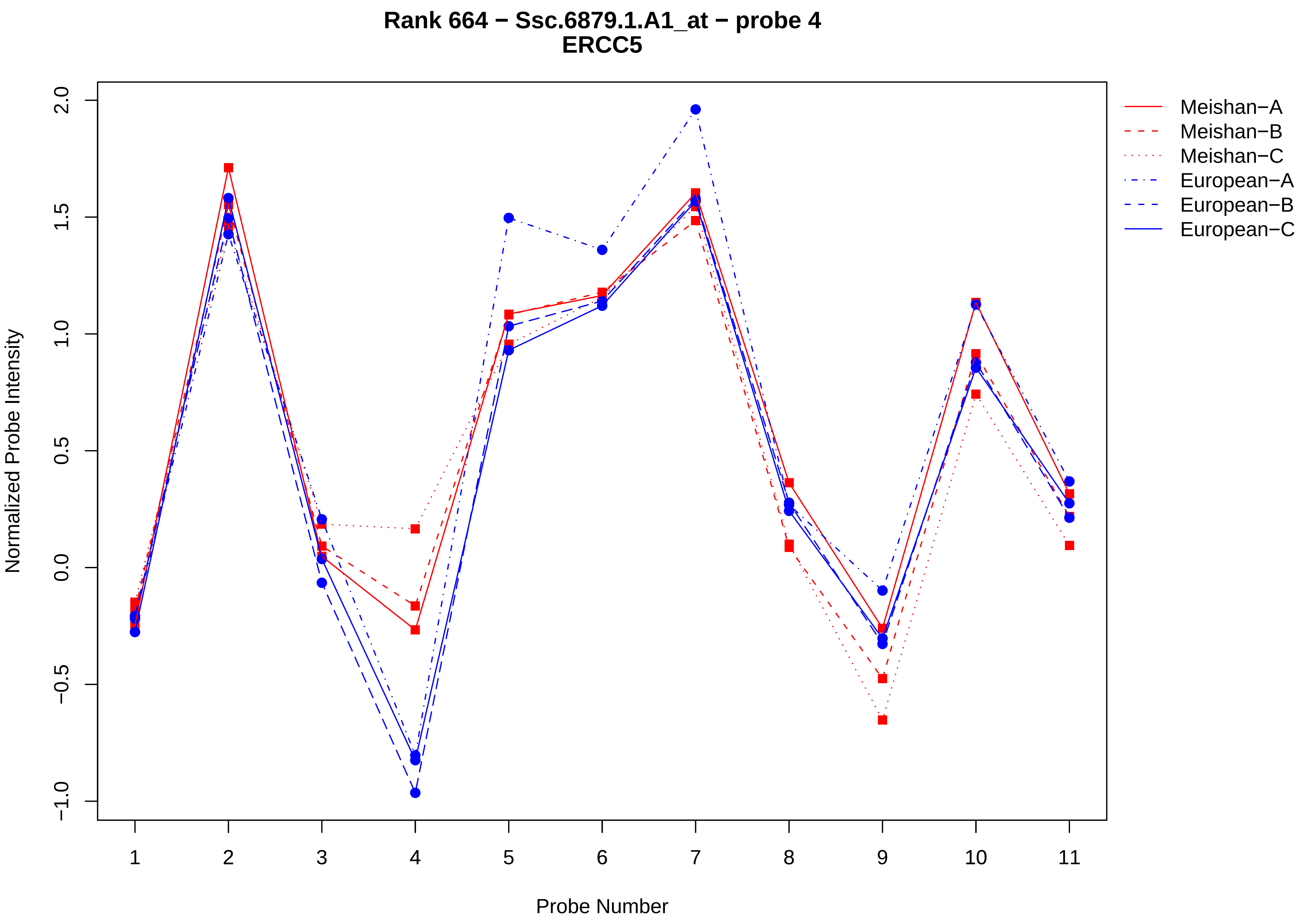

Rank 664 − Ssc.6879.1.A1_at − probe 4
ERCC5
Normalized Probe Intensity
Probe Number
−1.0
−0.5
0.0
0.5
1.0
1.5
2.0
1
2
3
4
5
6
7
8
9
10
11
Meishan−A
Meishan−B
Meishan−C
European−A
European−B
European−C

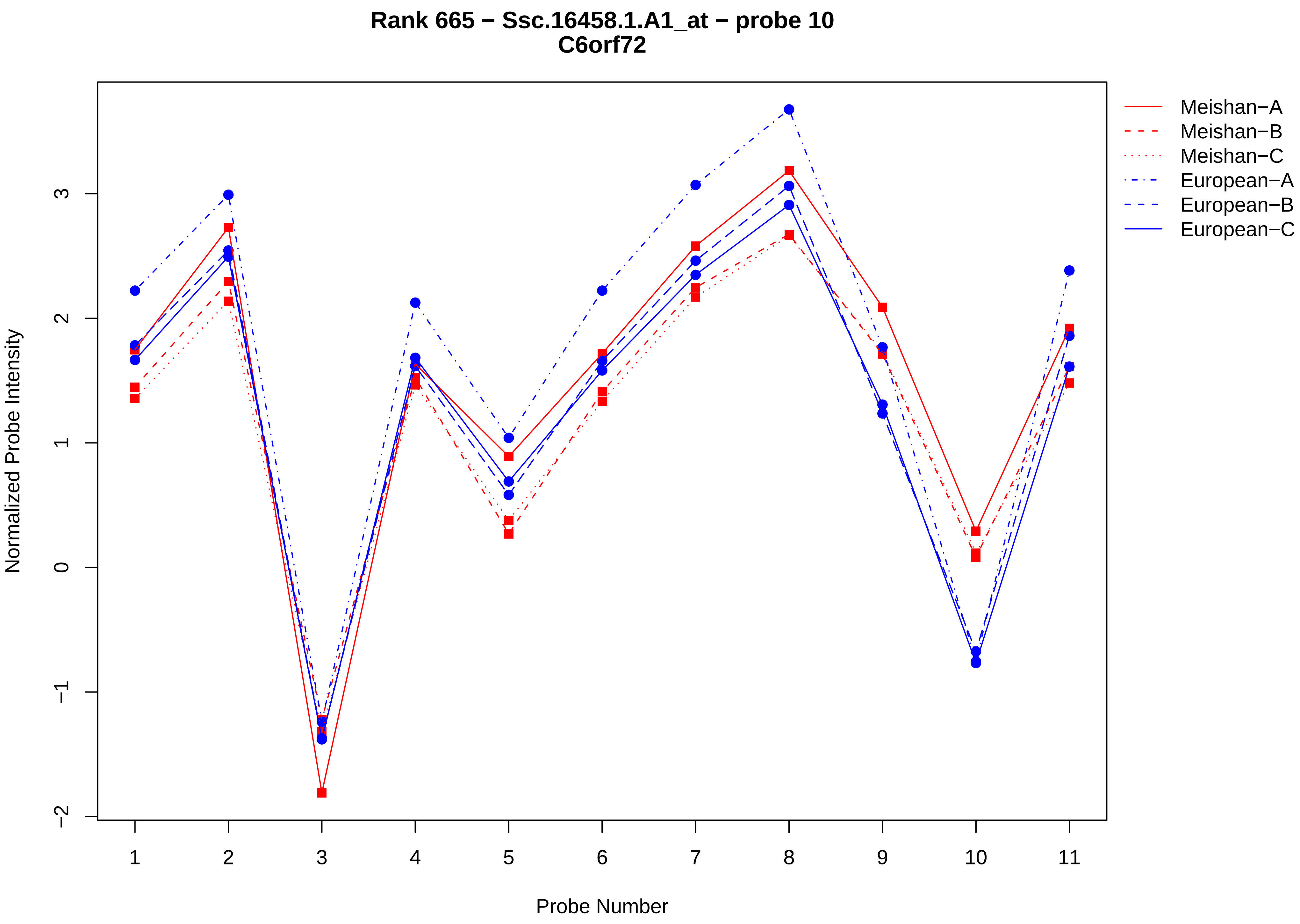

Rank 665 − Ssc.16458.1.A1_at − probe 10
C6orf72
Normalized Probe Intensity
Probe Number
−2
−1
0
1
2
3
1
2
3
4
5
6
7
8
9
10
11
Meishan−A
Meishan−B
Meishan−C
European−A
European−B
European−C

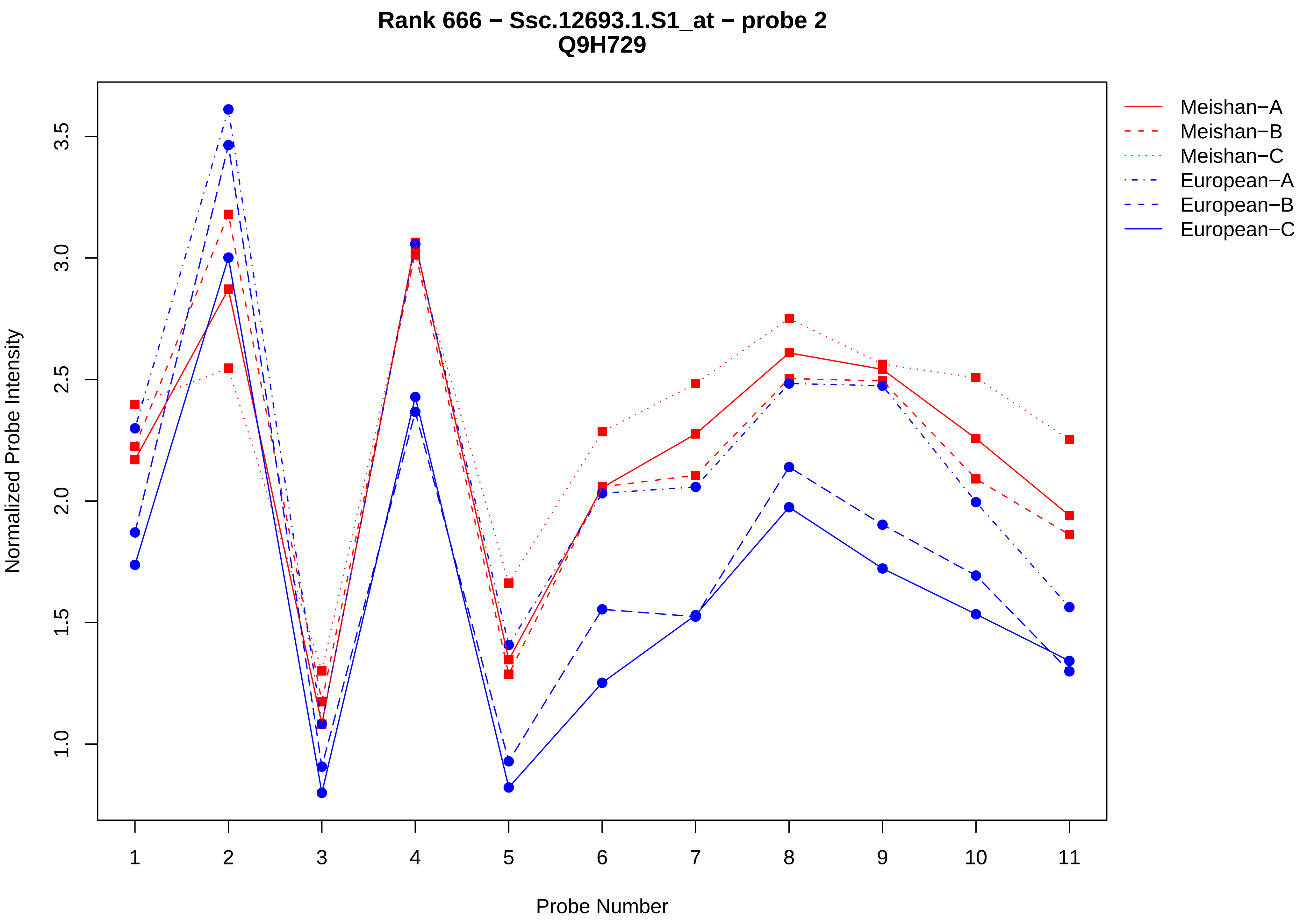

Rank 666 − Ssc.12693.1.S1_at − probe 2
Q9H729
Normalized Probe Intensity
1.0
1.5
2.0
2.5
3.0
3.5
1
2
3
4
5
6
7
8
9
10
11
Probe Number
Meishan−A
Meishan−B
Meishan−C
European−A
European−B
European−C

Rank 667 − Ssc.20409.1.S1_at − probe 11
NP_114111

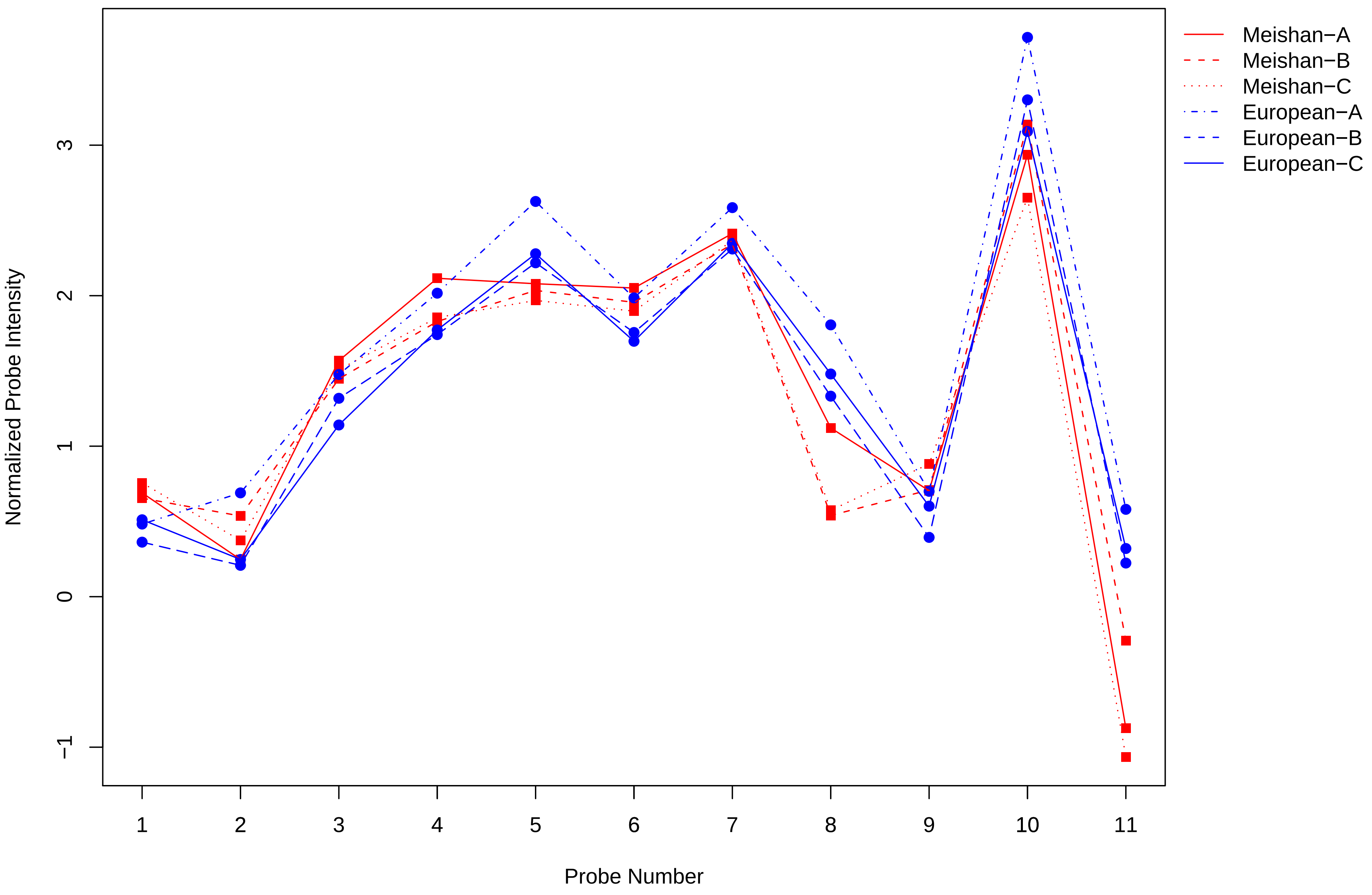

Rank 668 − Ssc.6595.1.A1_at − probe 6
TM7SF1

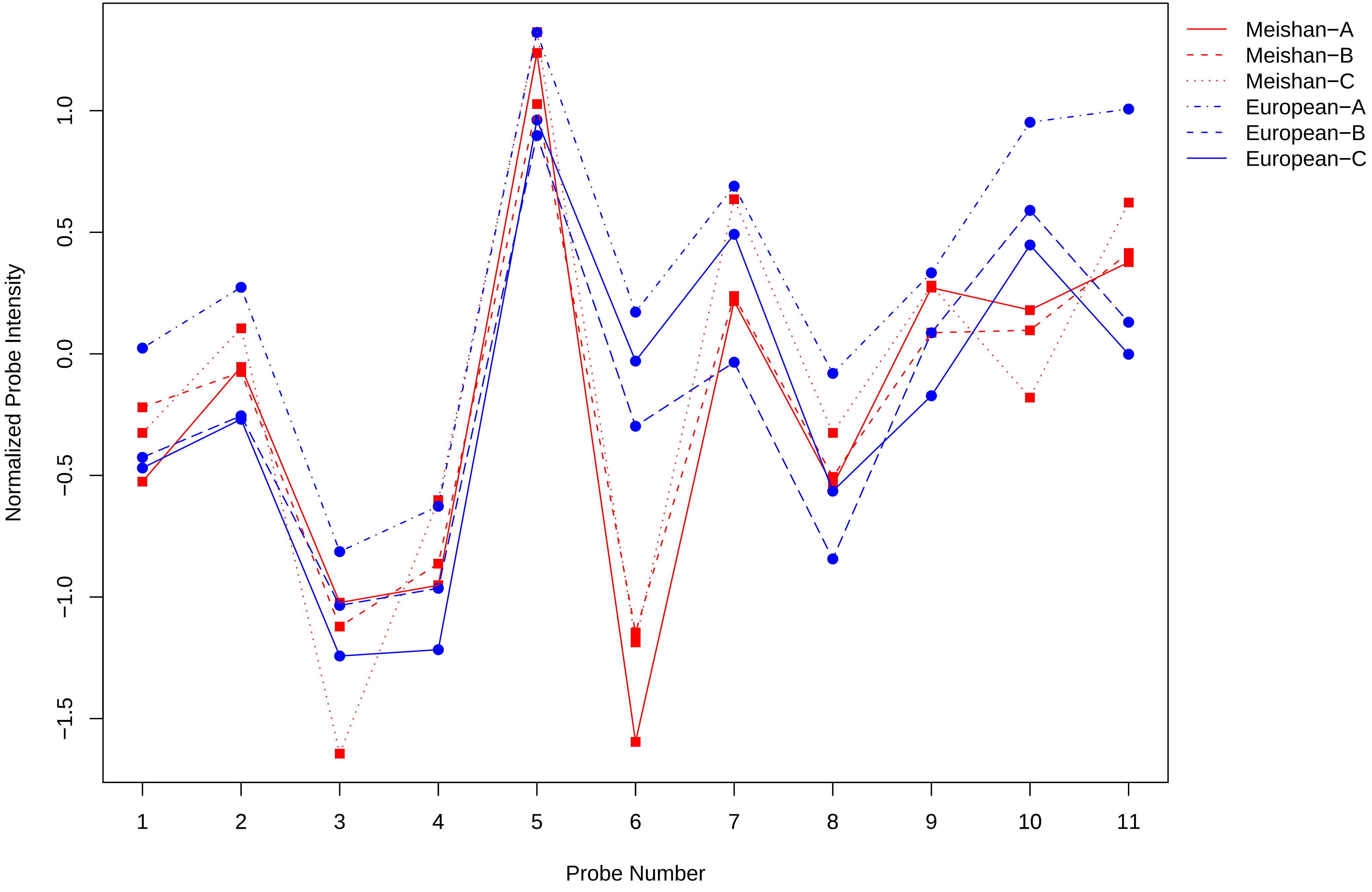

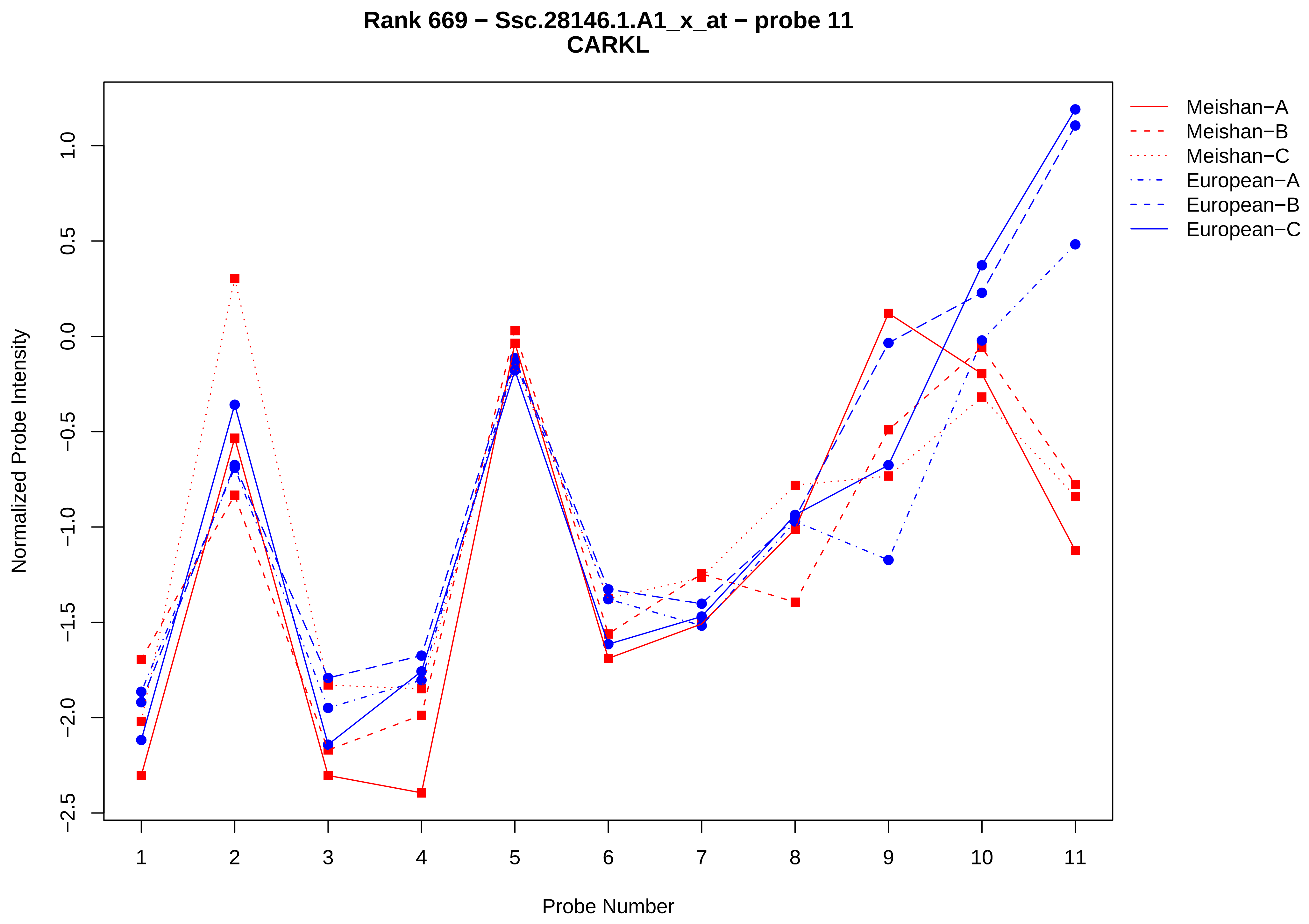

Rank 669 − Ssc.28146.1.A1_x_at − probe 11
CARKL
Normalized Probe Intensity
Probe Number
−2.5
−2.0
−1.5
−1.0
−0.5
0.0
0.5
1.0
1
2
3
4
5
6
7
8
9
10
11
Meishan−A
Meishan−B
Meishan−C
European−A
European−B
European−C

Rank 670 − Ssc.1574.1.S1_at − probe 6
SMARCA1

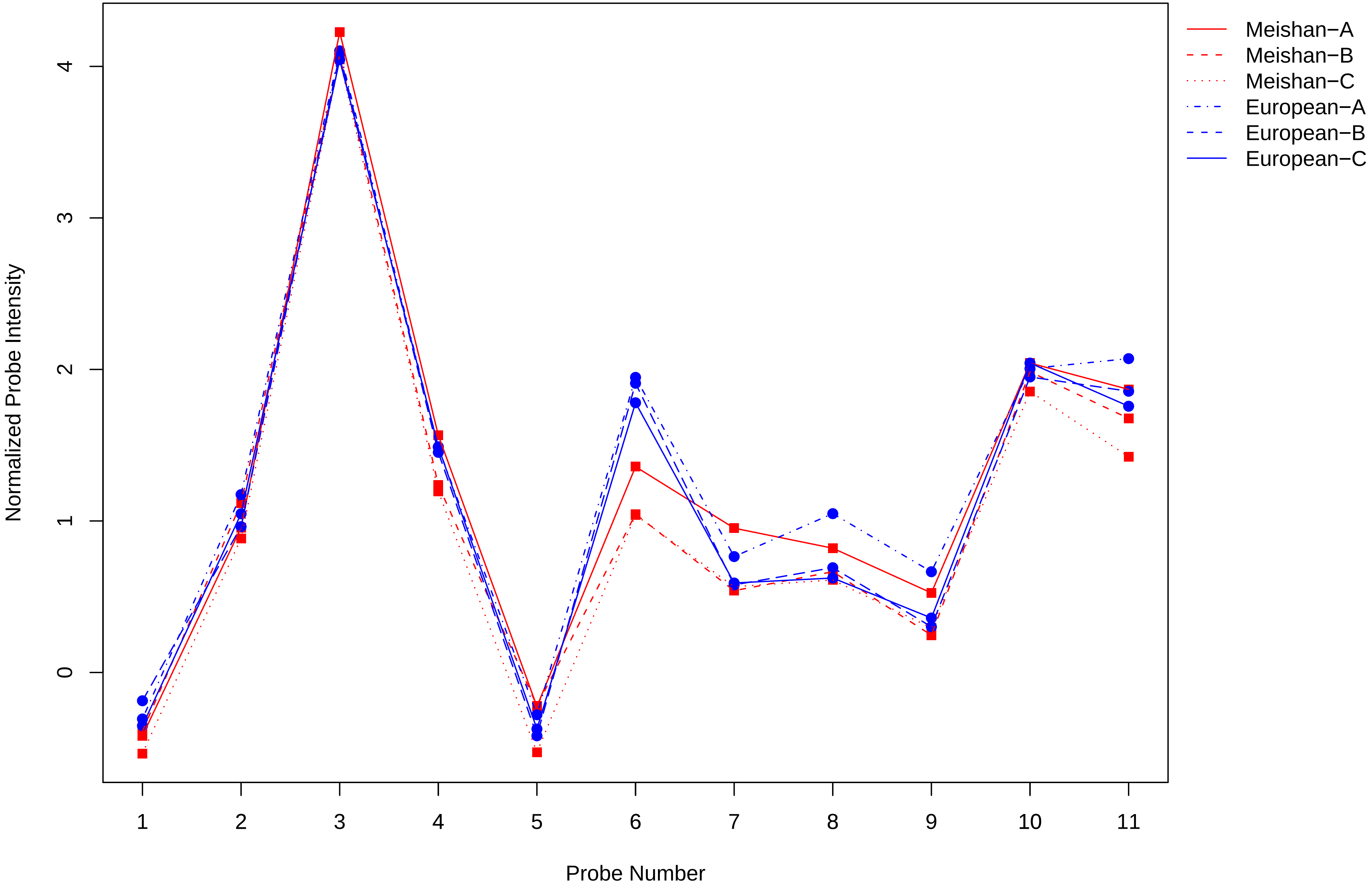

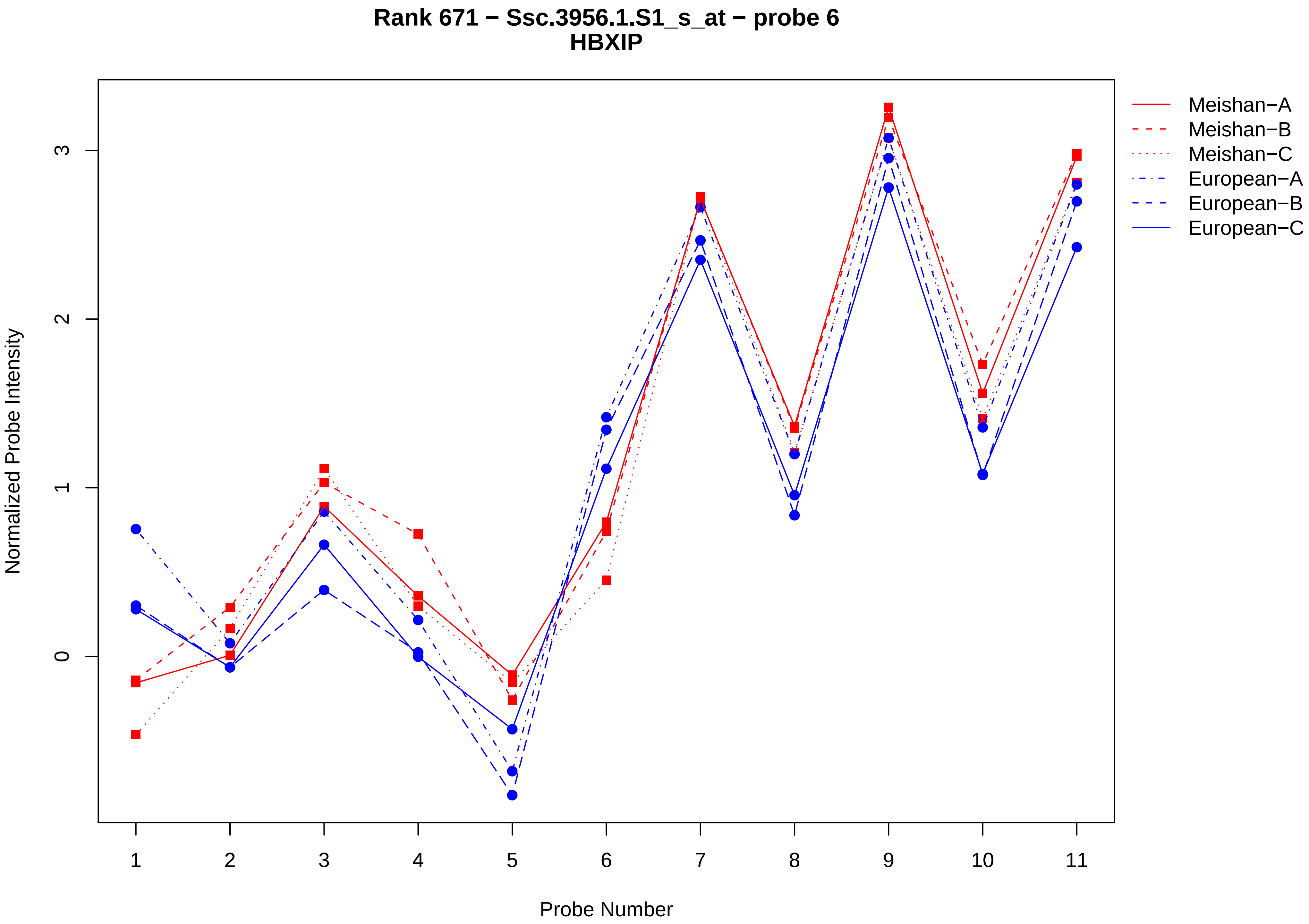
Rank 671 − Ssc.3956.1.S1_s_at − probe 6
HBXIP
Normalized Probe Intensity
Probe Number
0
1
2
3
1
2
3
4
5
6
7
8
9
10
11
Meishan−A
Meishan−B
Meishan−C
European−A
European−B
European−C

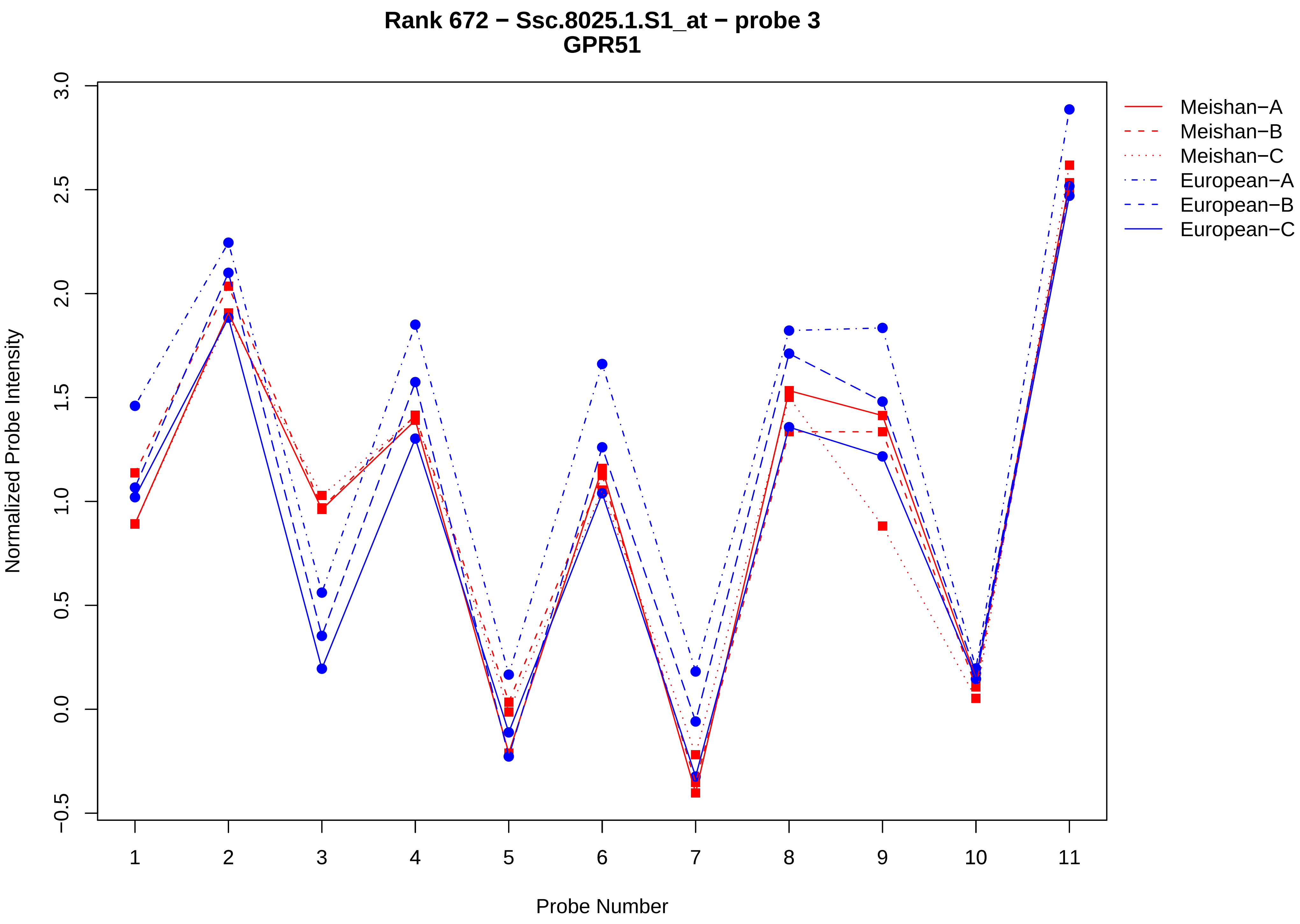

Rank 672 − Ssc.8025.1.S1_at − probe 3
GPR51
Normalized Probe Intensity
Probe Number
−0.5
0.0
0.5
1.0
1.5
2.0
2.5
3.0
1
2
3
4
5
6
7
8
9
10
11
Meishan−A
Meishan−B
Meishan−C
European−A
European−B
European−C

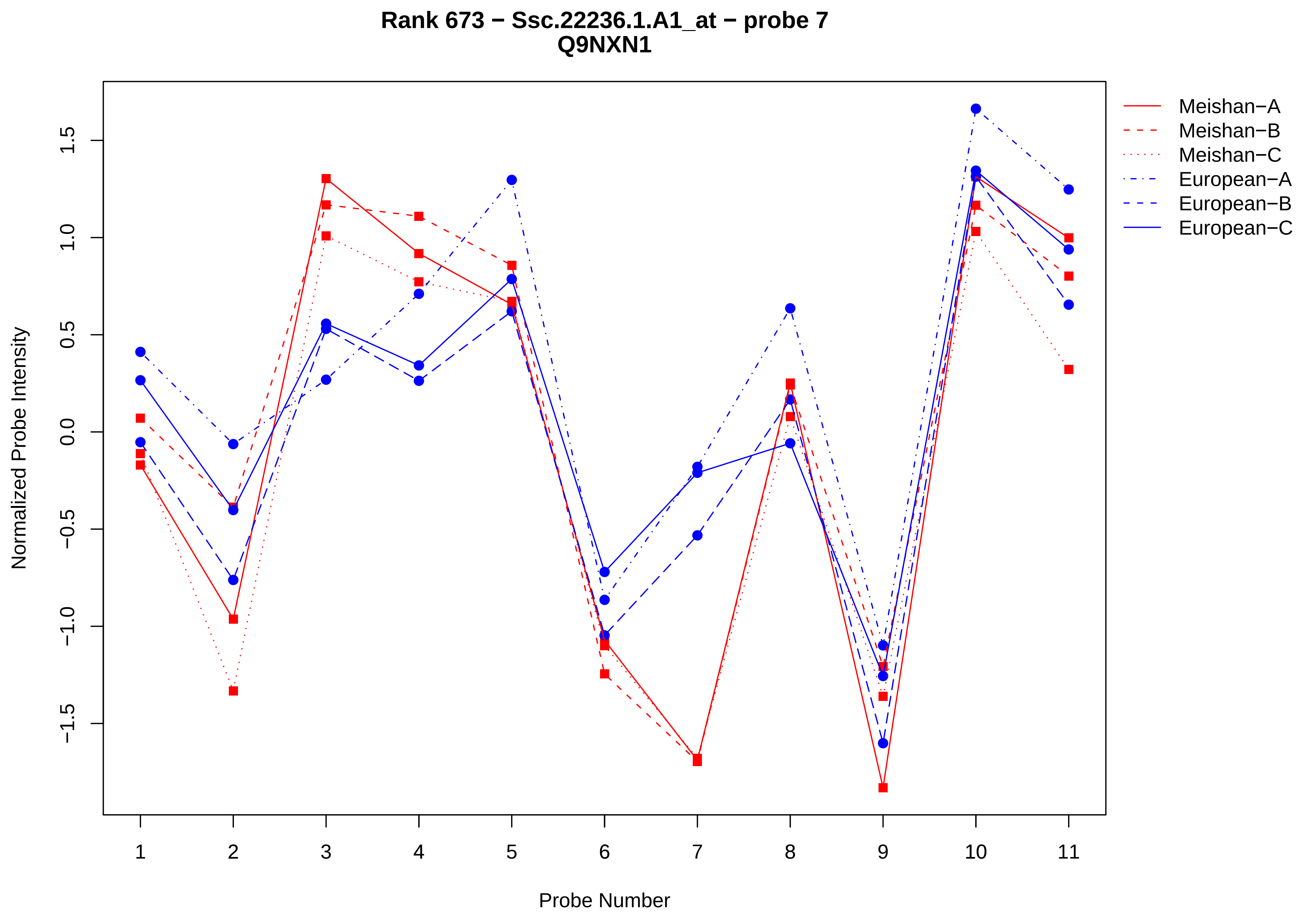
Rank 673 − Ssc.22236.1.A1_at − probe 7
Q9NXN1
Normalized Probe Intensity
Probe Number
Meishan−A
Meishan−B
Meishan−C
European−A
European−B
European−C
1.5
1.0
0.5
0.0
−0.5
−1.0
−1.5
1
2
3
4
5
6
7
8
9
10
11

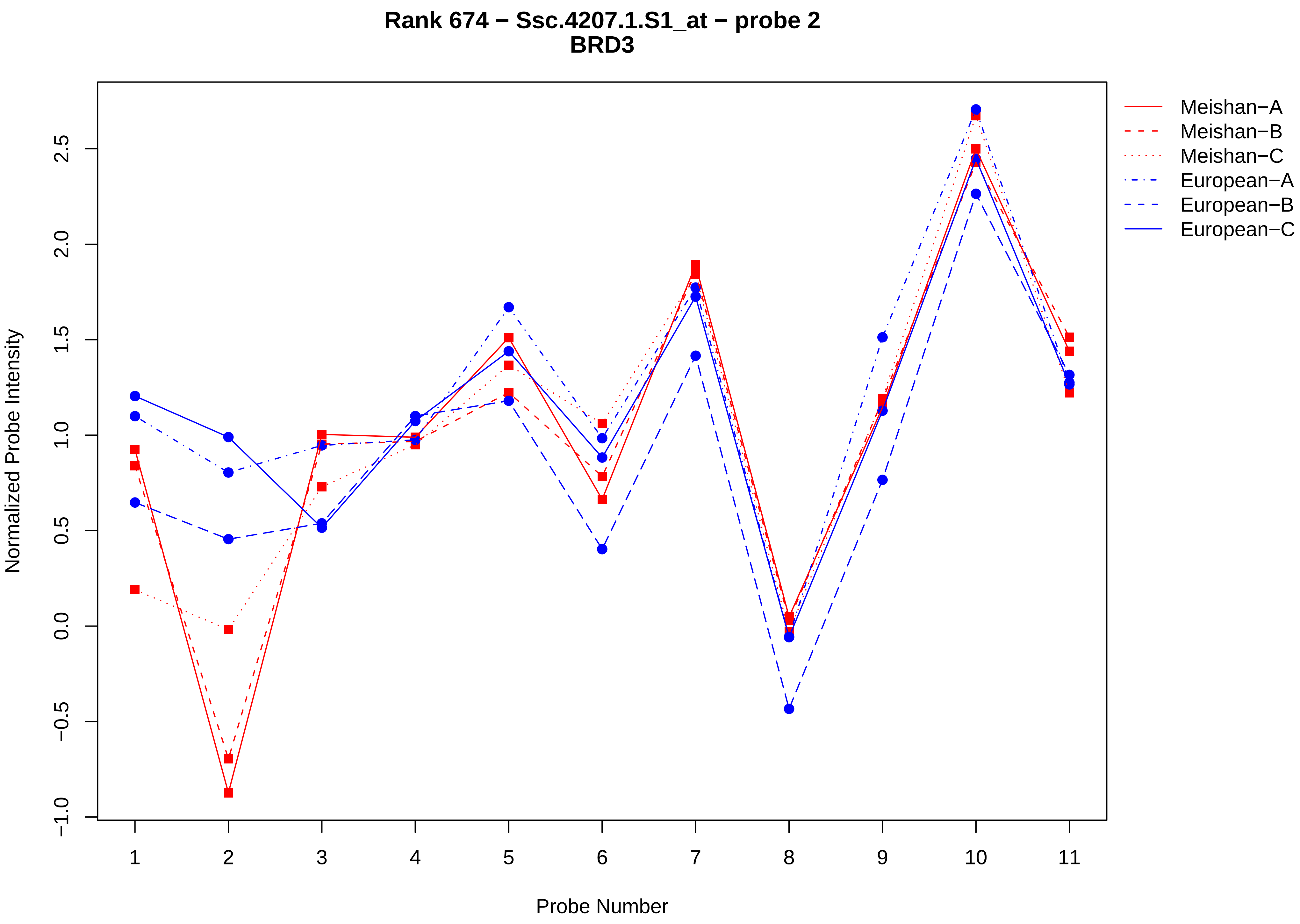
Rank 674 − Ssc.4207.1.S1_at − probe 2
BRD3
Normalized Probe Intensity
Probe Number
−1.0
−0.5
0.0
0.5
1.0
1.5
2.0
2.5
1
2
3
4
5
6
7
8
9
10
11
Meishan−A
Meishan−B
Meishan−C
European−A
European−B
European−C

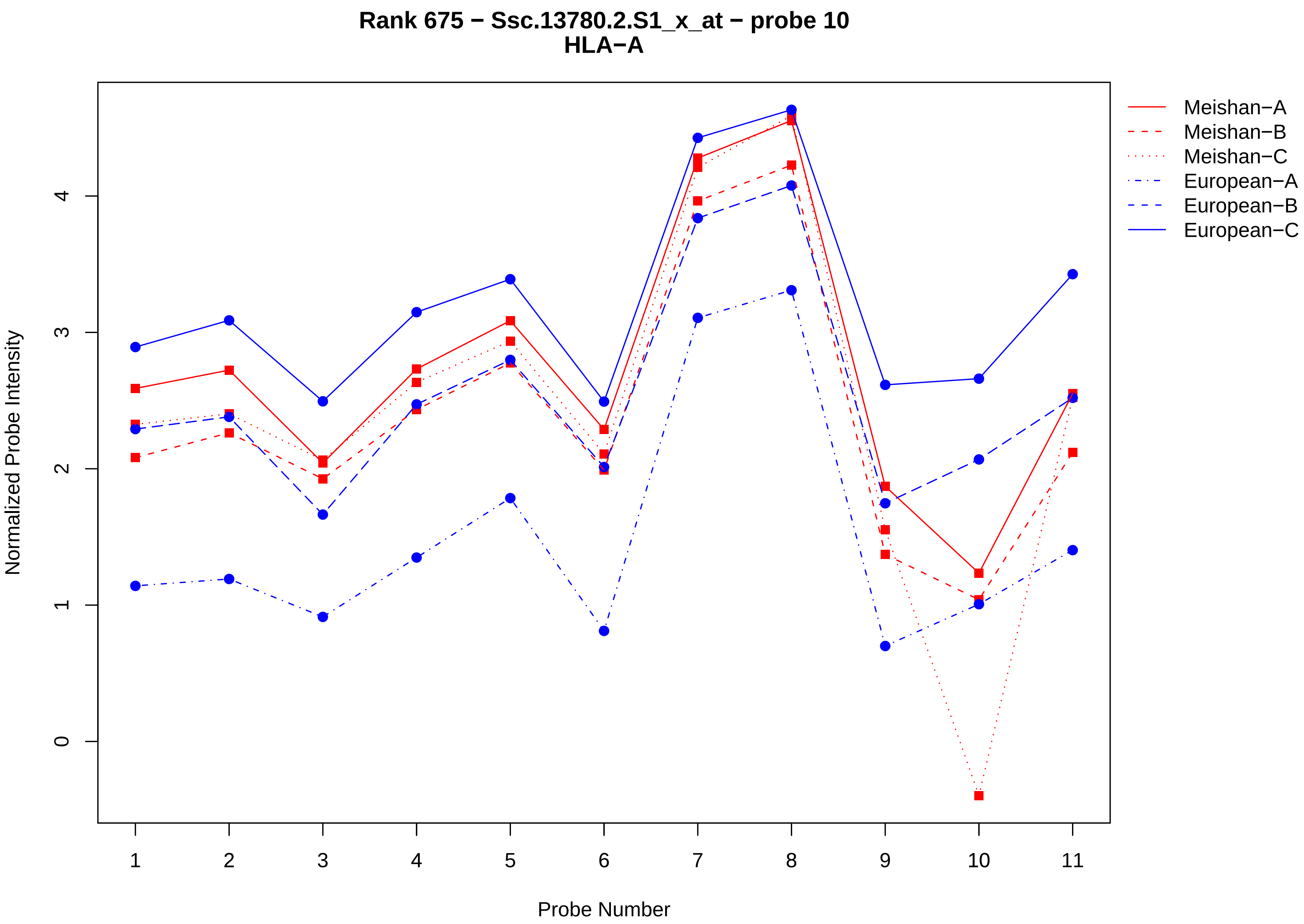

Rank 675 − Ssc.13780.2.S1_x_at − probe 10
HLA−A
Normalized Probe Intensity
Probe Number
0
1
2
3
4
1
2
3
4
5
6
7
8
9
10
11
Meishan−A
Meishan−B
Meishan−C
European−A
European−B
European−C

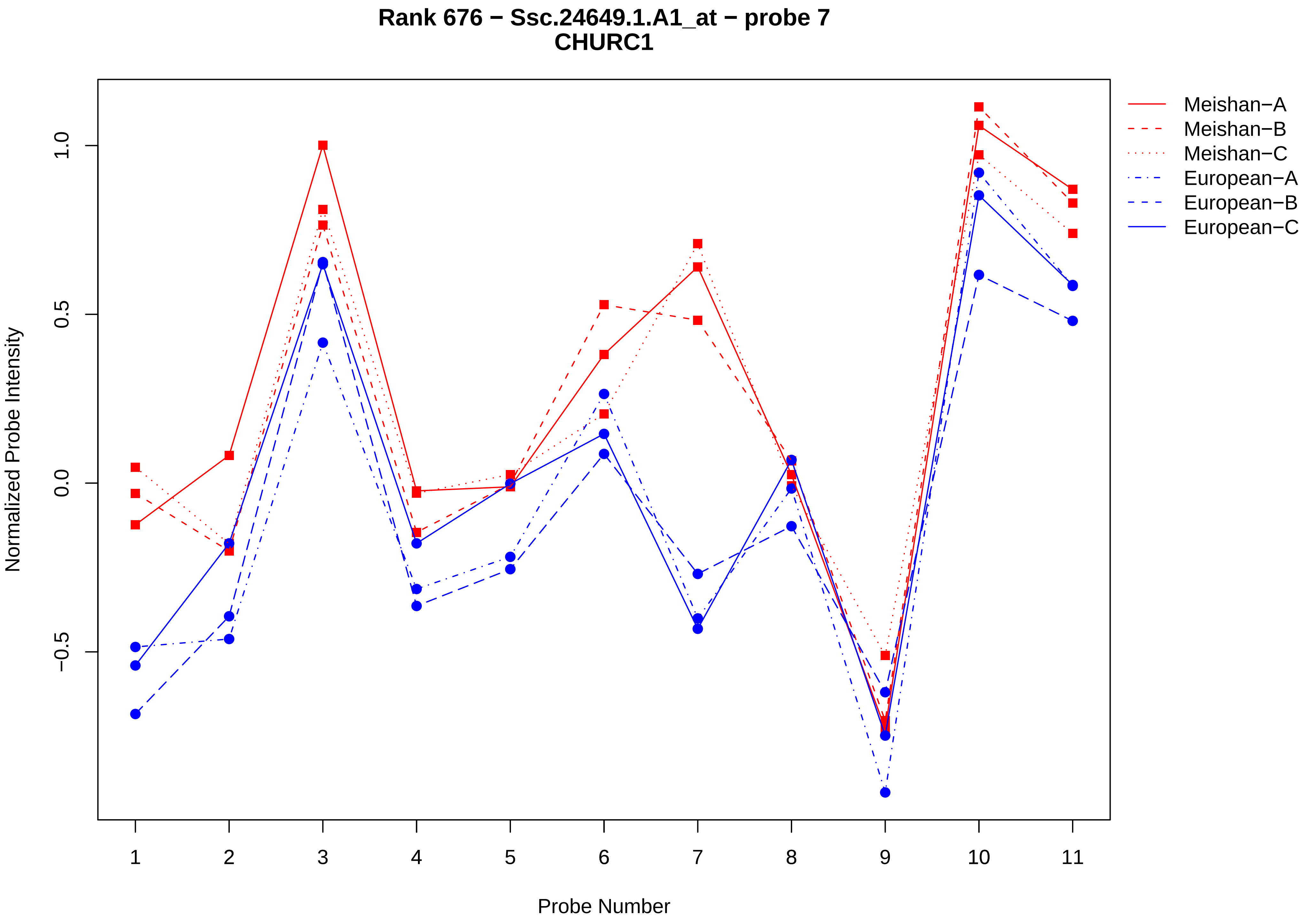
Rank 676 − Ssc.24649.1.A1_at − probe 7
CHURC1
Normalized Probe Intensity
Probe Number
−0.5
0.0
0.5
1.0
1
2
3
4
5
6
7
8
9
10
11
Meishan−A
Meishan−B
Meishan−C
European−A
European−B
European−C

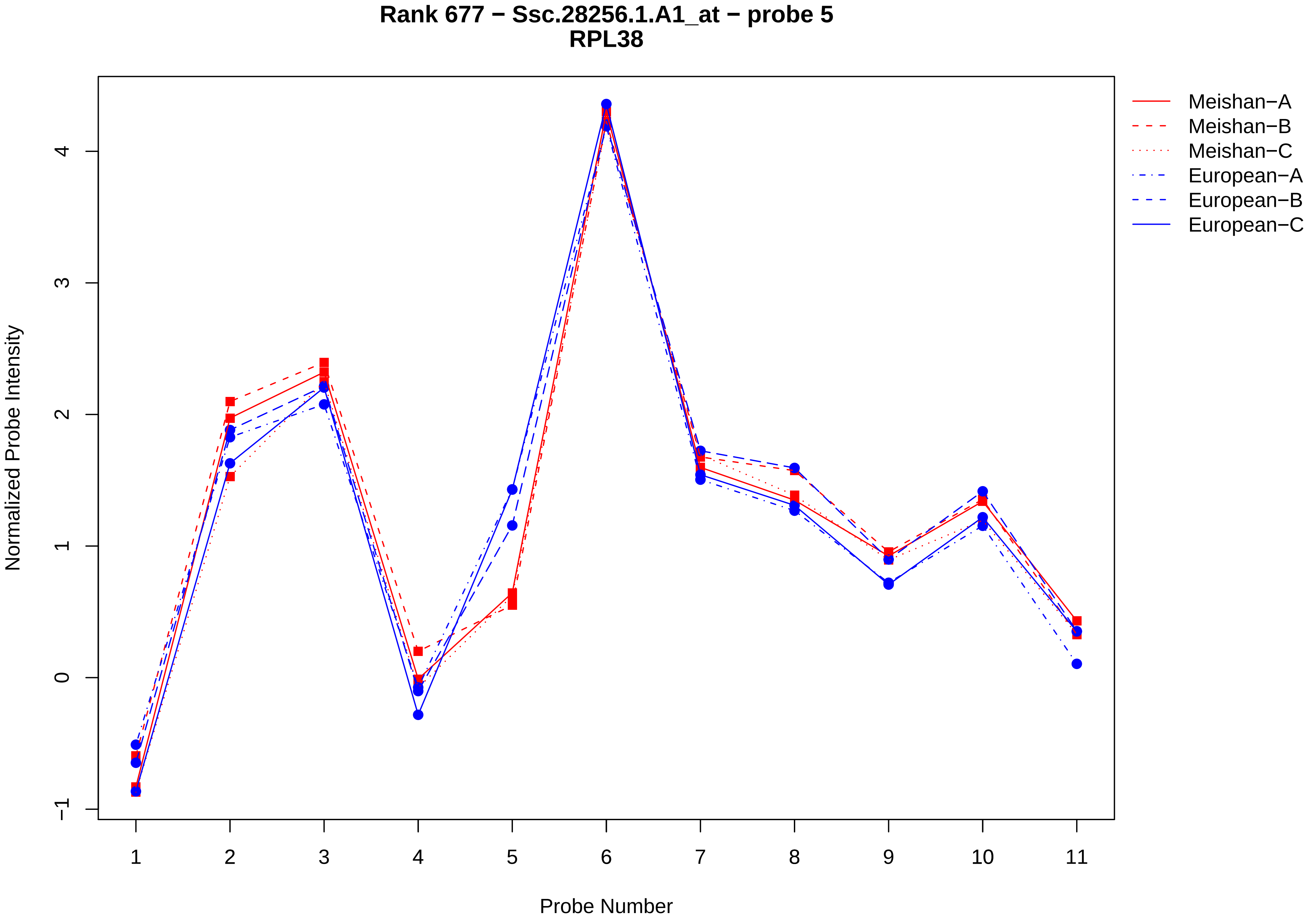

Rank 677 − Ssc.28256.1.A1_at − probe 5
RPL38
Normalized Probe Intensity
Probe Number
−1
0
1
2
3
4
1
2
3
4
5
6
7
8
9
10
11
Meishan−A
Meishan−B
Meishan−C
European−A
European−B
European−C

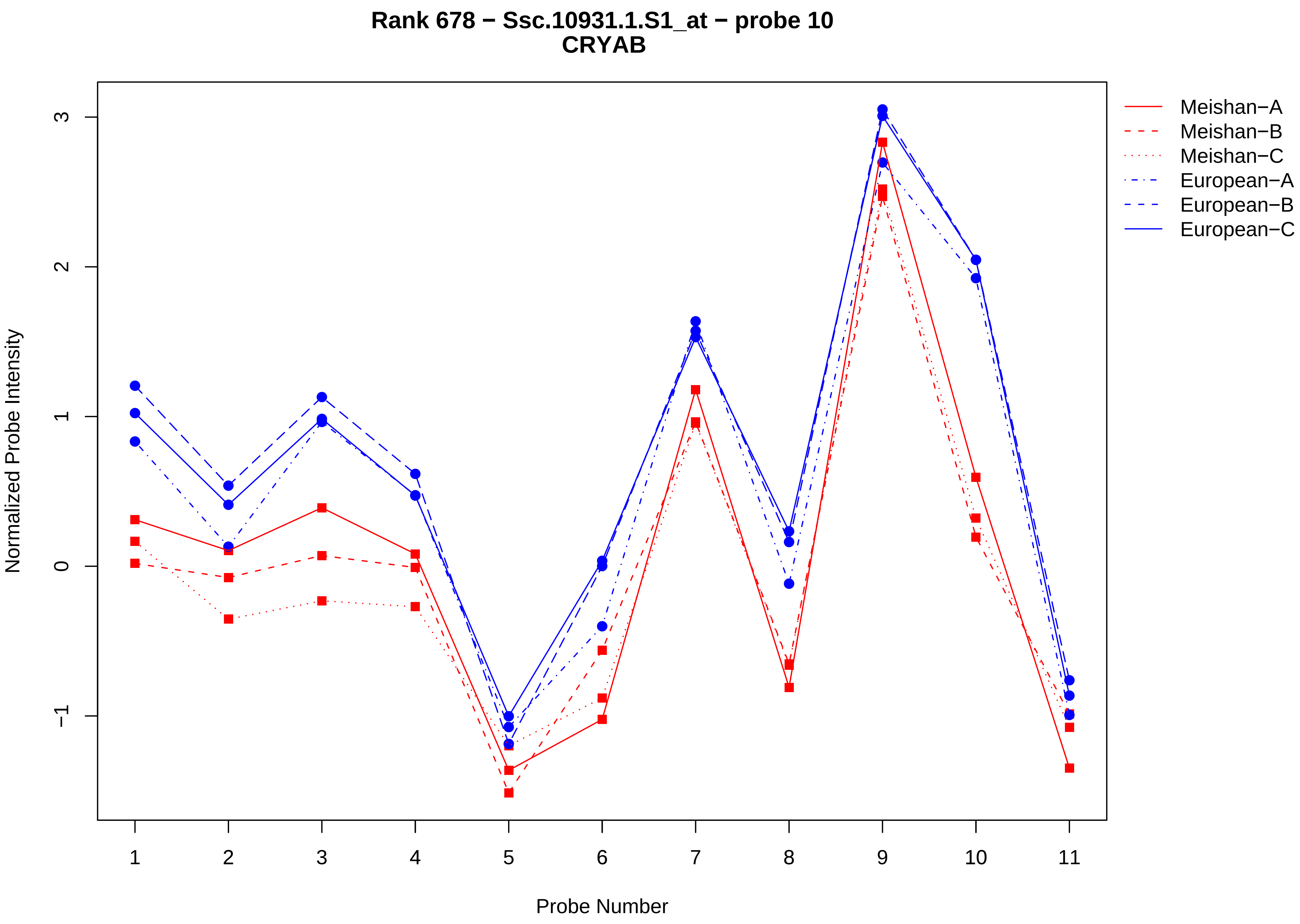
Rank 678 − Ssc.10931.1.S1_at − probe 10
CRYAB
Normalized Probe Intensity
Probe Number
−1
0
1
2
3
1
2
3
4
5
6
7
8
9
10
11
Meishan−A
Meishan−B
Meishan−C
European−A
European−B
European−C

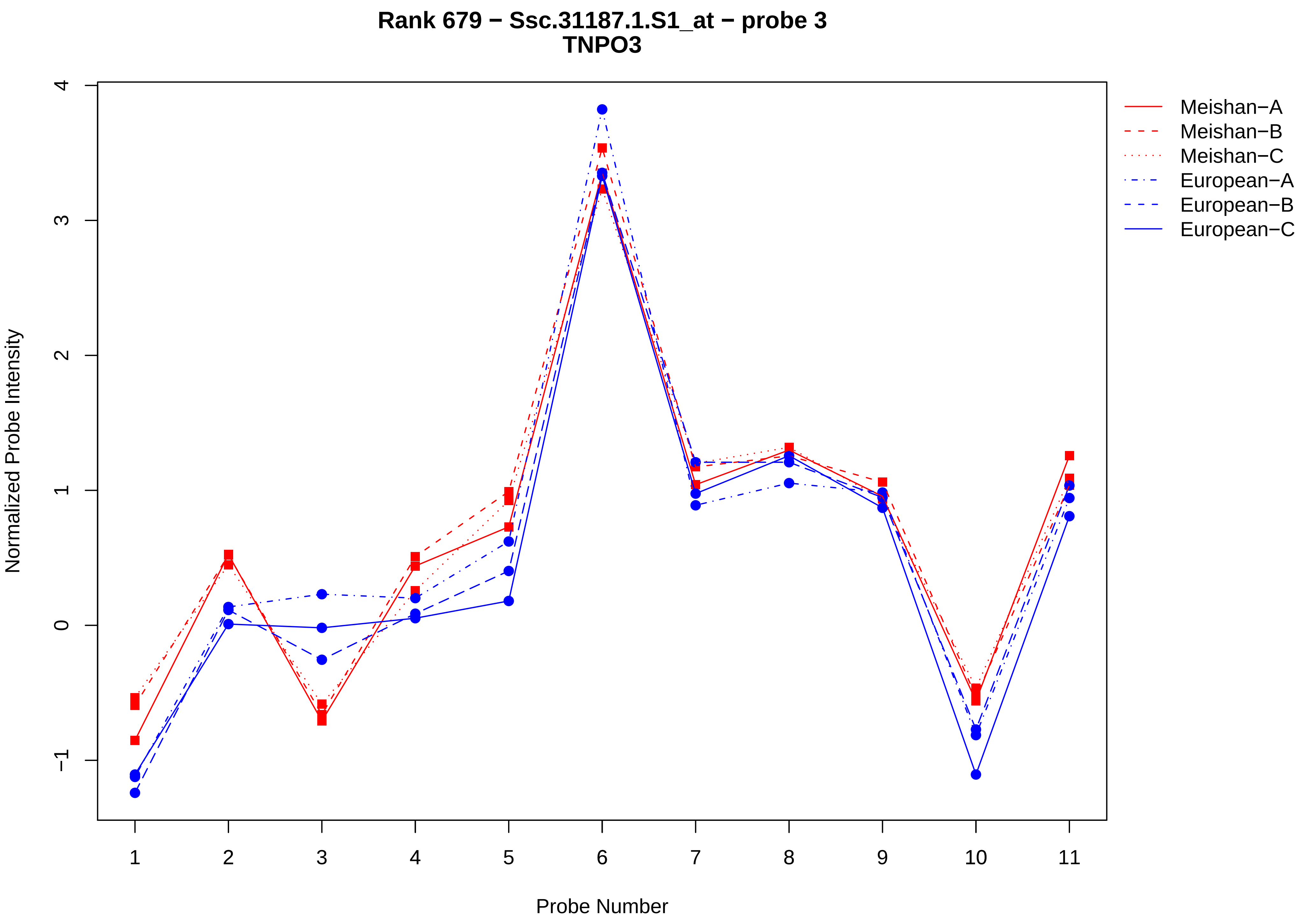

Rank 679 − Ssc.31187.1.S1_at − probe 3
TNPO3
Normalized Probe Intensity
Probe Number
Meishan−A
Meishan−B
Meishan−C
European−A
European−B
European−C
−1
0
1
2
3
4
1
2
3
4
5
6
7
8
9
10
11

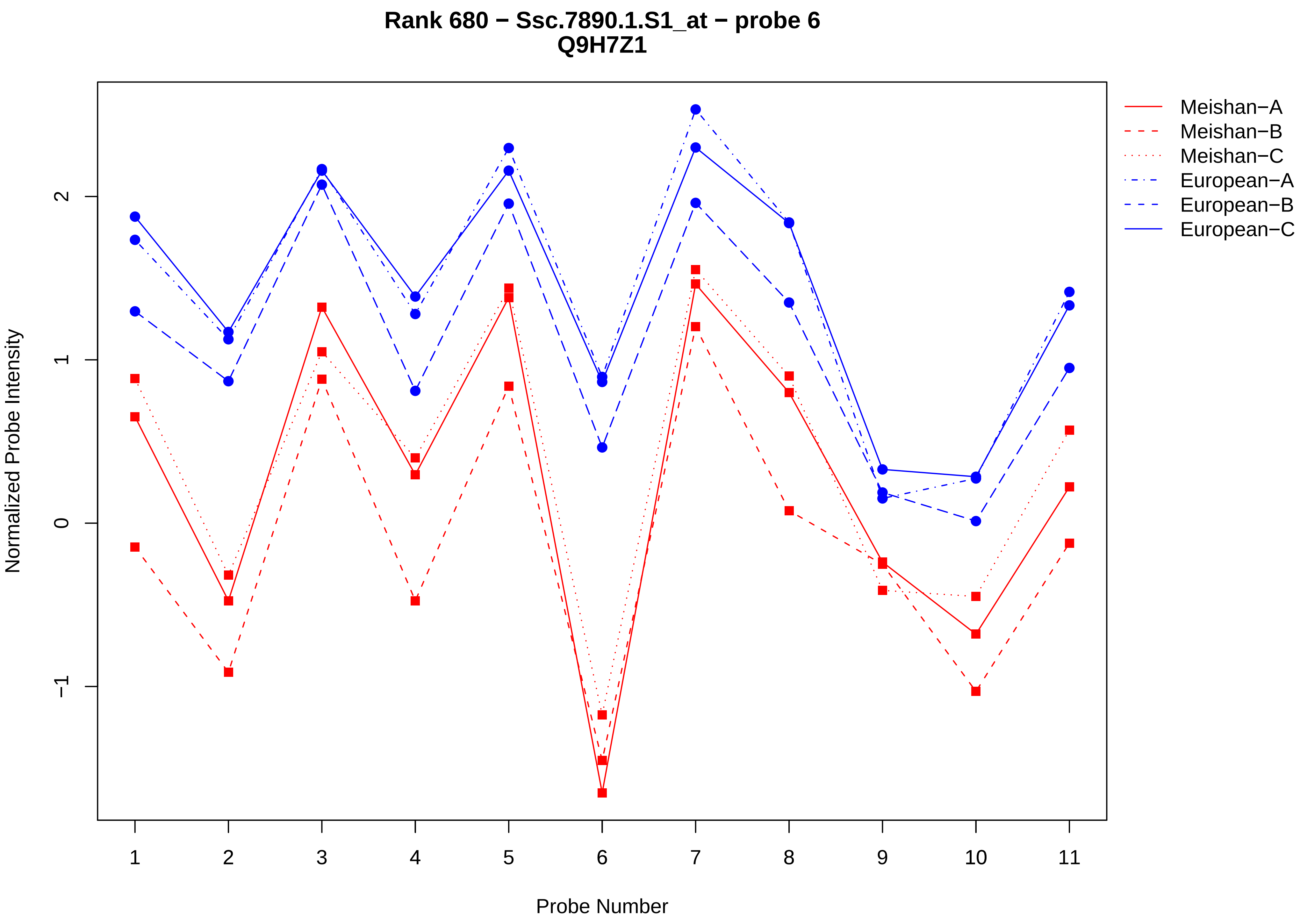

Rank 680 − Ssc.7890.1.S1_at − probe 6
Q9H7Z1
Normalized Probe Intensity
−1
0
1
2
1
2
3
4
5
6
7
8
9
10
11
Probe Number
Meishan−A
Meishan−B
Meishan−C
European−A
European−B
European−C

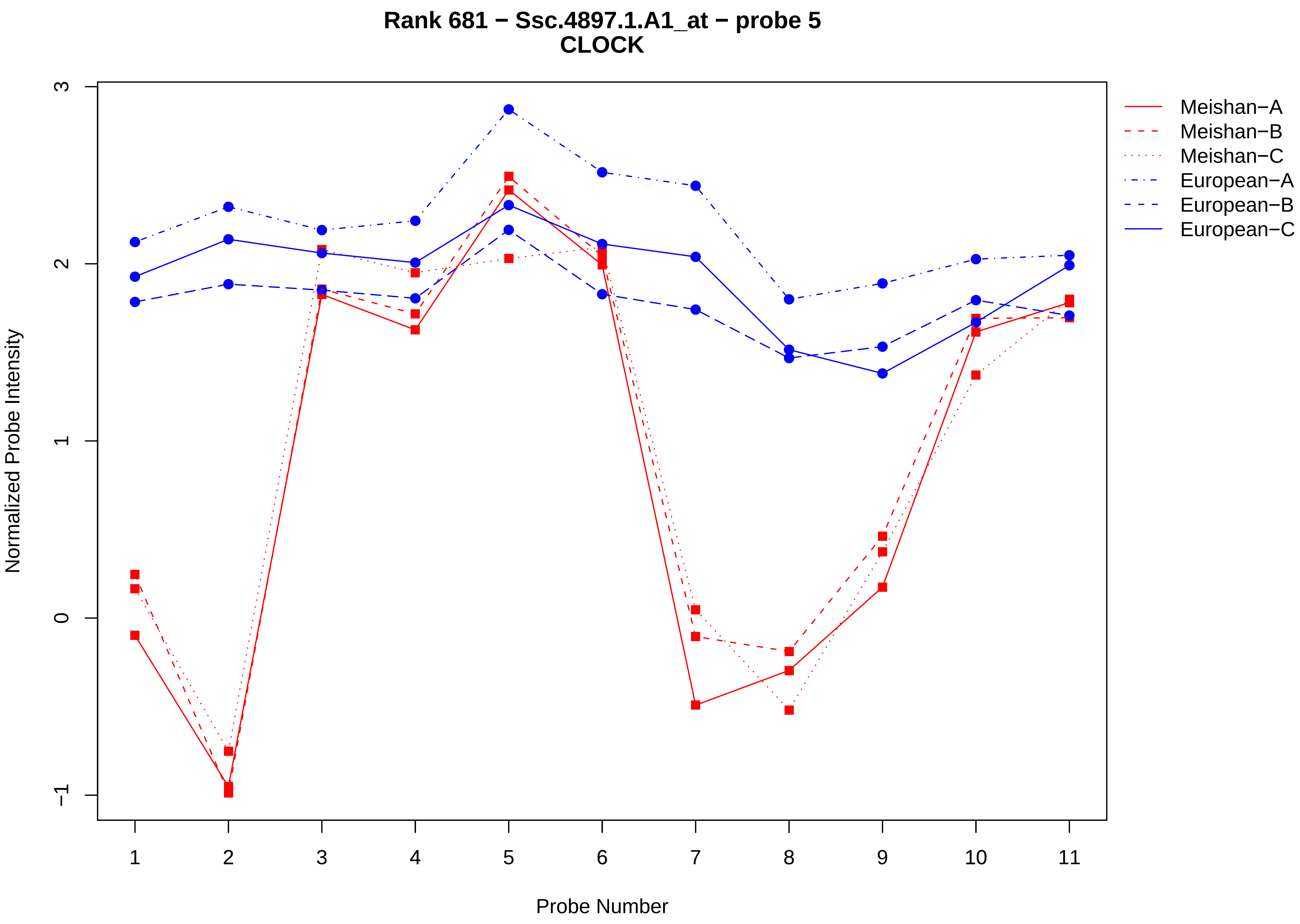

Rank 681 − Ssc.4897.1.A1_at − probe 5
CLOCK
Normalized Probe Intensity
Probe Number
−1
0
1
2
3
1
2
3
4
5
6
7
8
9
10
11
Meishan−A
Meishan−B
Meishan−C
European−A
European−B
European−C

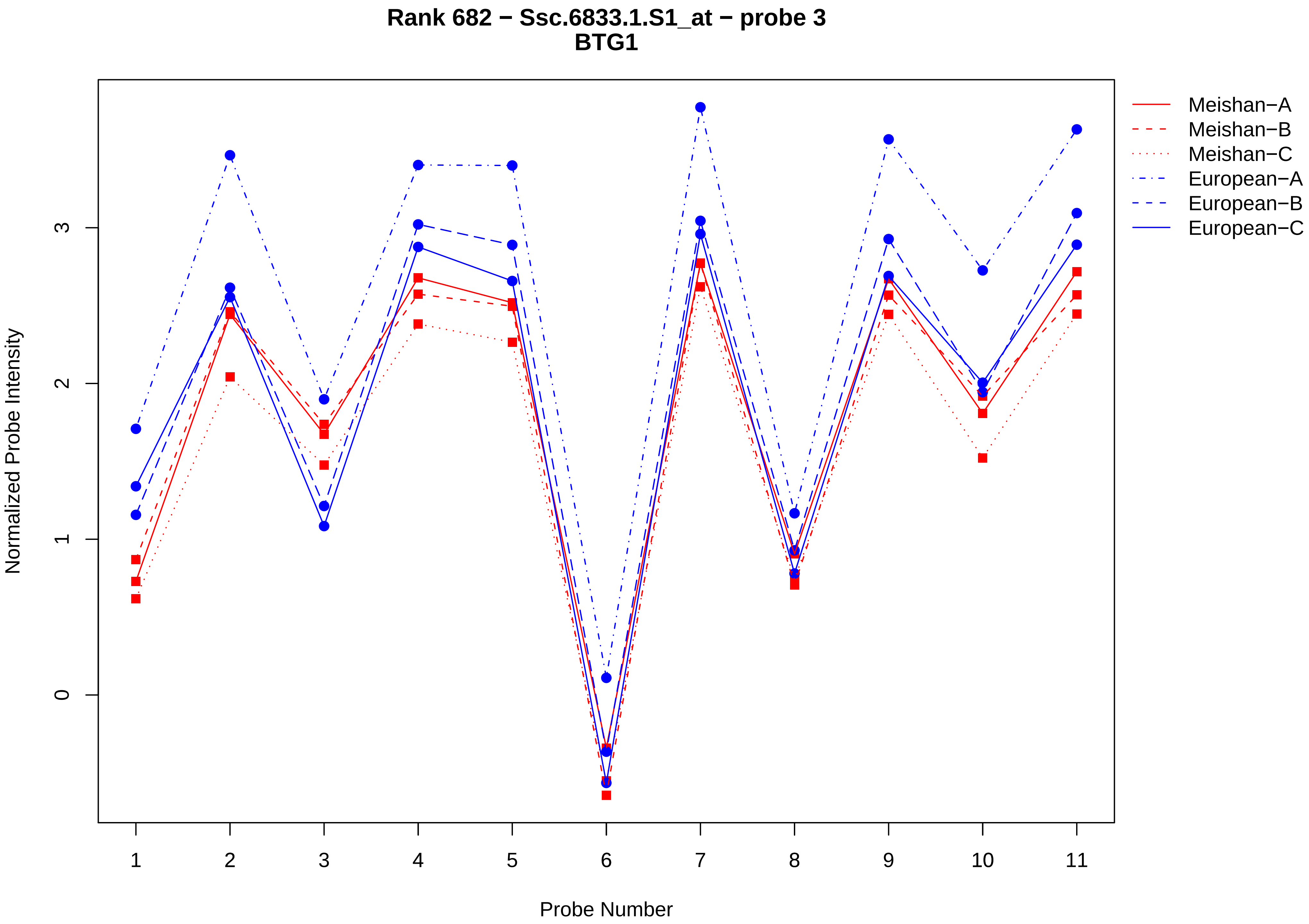

Rank 682 − Ssc.6833.1.S1_at − probe 3
BTG1
Normalized Probe Intensity
Probe Number
0
1
2
3
1
2
3
4
5
6
7
8
9
10
11
Meishan−A
Meishan−B
Meishan−C
European−A
European−B
European−C

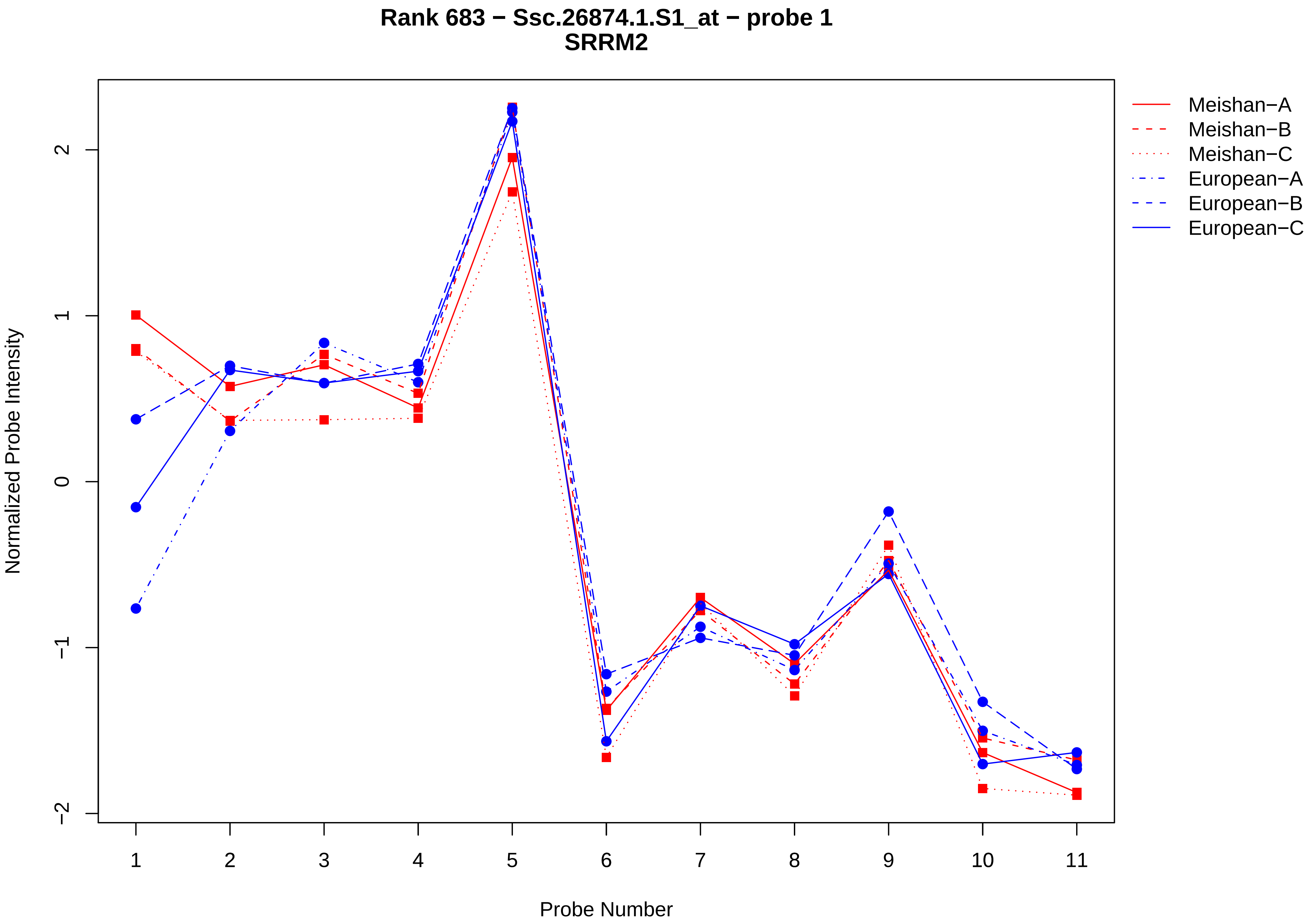

Rank 683 − Ssc.26874.1.S1_at − probe 1
SRRM2
Normalized Probe Intensity
Probe Number
−2
−1
0
1
2
1
2
3
4
5
6
7
8
9
10
11
Meishan−A
Meishan−B
Meishan−C
European−A
European−B
European−C

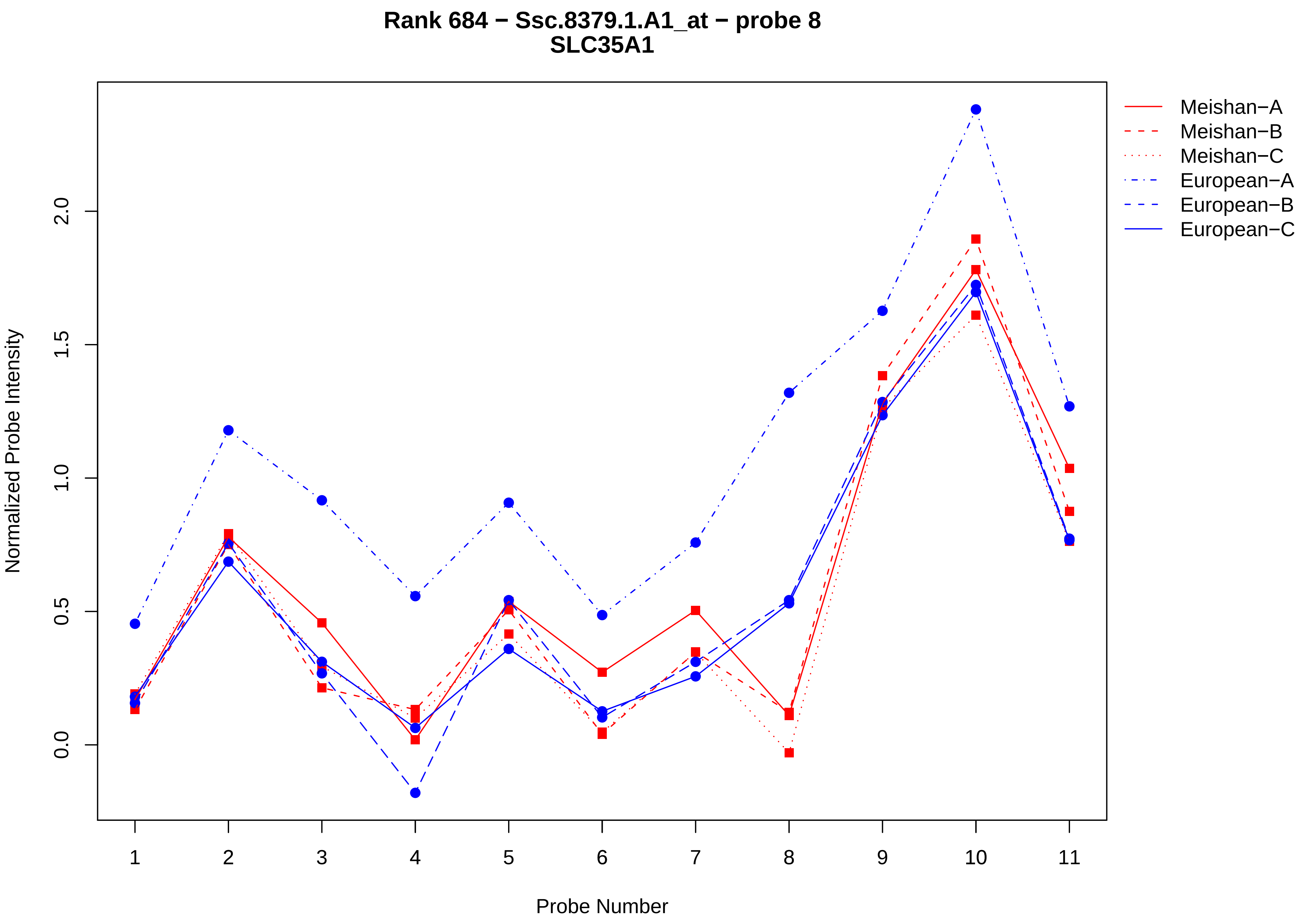

Rank 684 − Ssc.8379.1.A1_at − probe 8
SLC35A1
Normalized Probe Intensity
Probe Number
0.0
0.5
1.0
1.5
2.0
1
2
3
4
5
6
7
8
9
10
11
Meishan−A
Meishan−B
Meishan−C
European−A
European−B
European−C

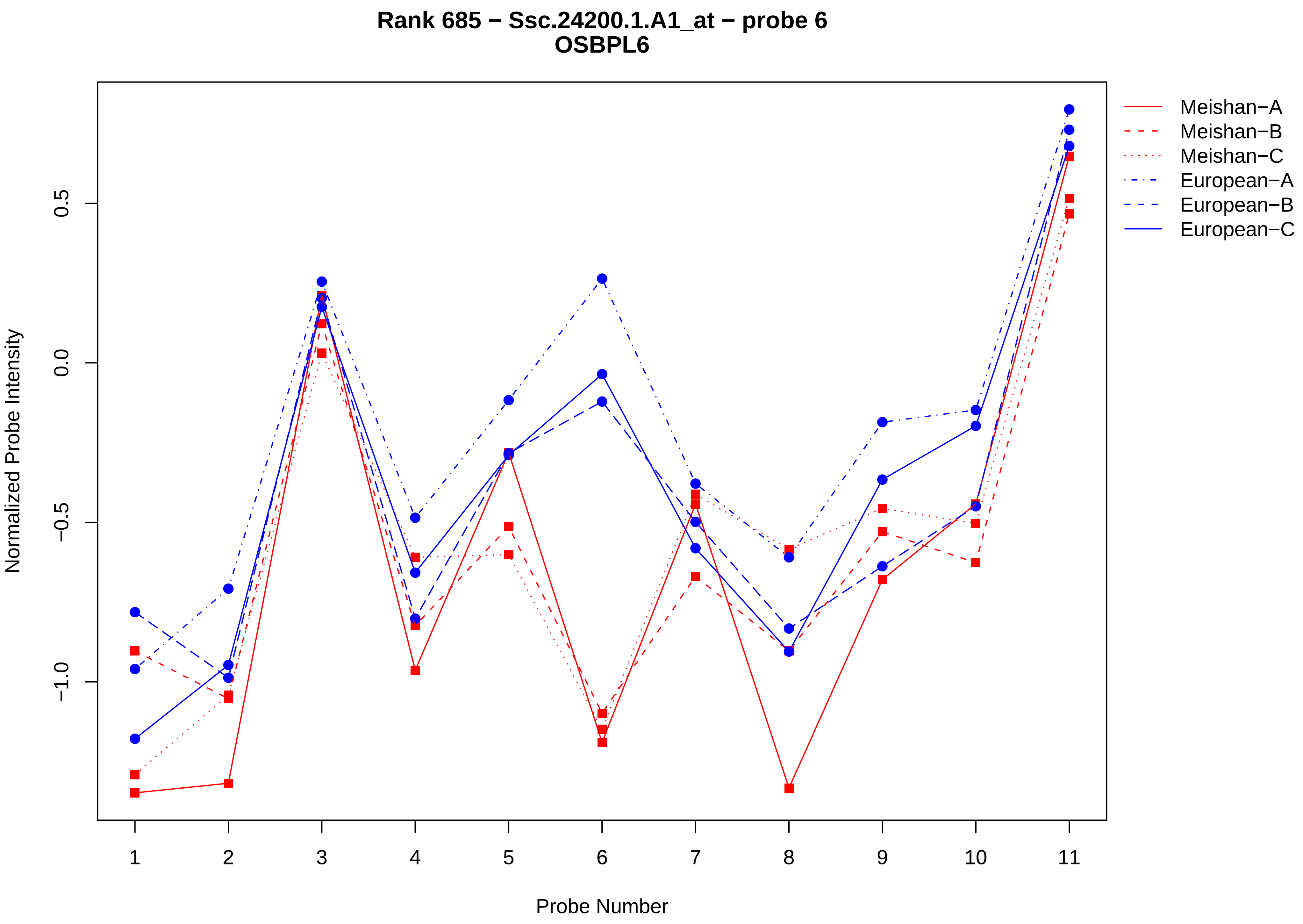

Rank 685 − Ssc.24200.1.A1_at − probe 6
OSBPL6
Normalized Probe Intensity
Probe Number
−1.0
−0.5
0.0
0.5
1
2
3
4
5
6
7
8
9
10
11
Meishan−A
Meishan−B
Meishan−C
European−A
European−B
European−C

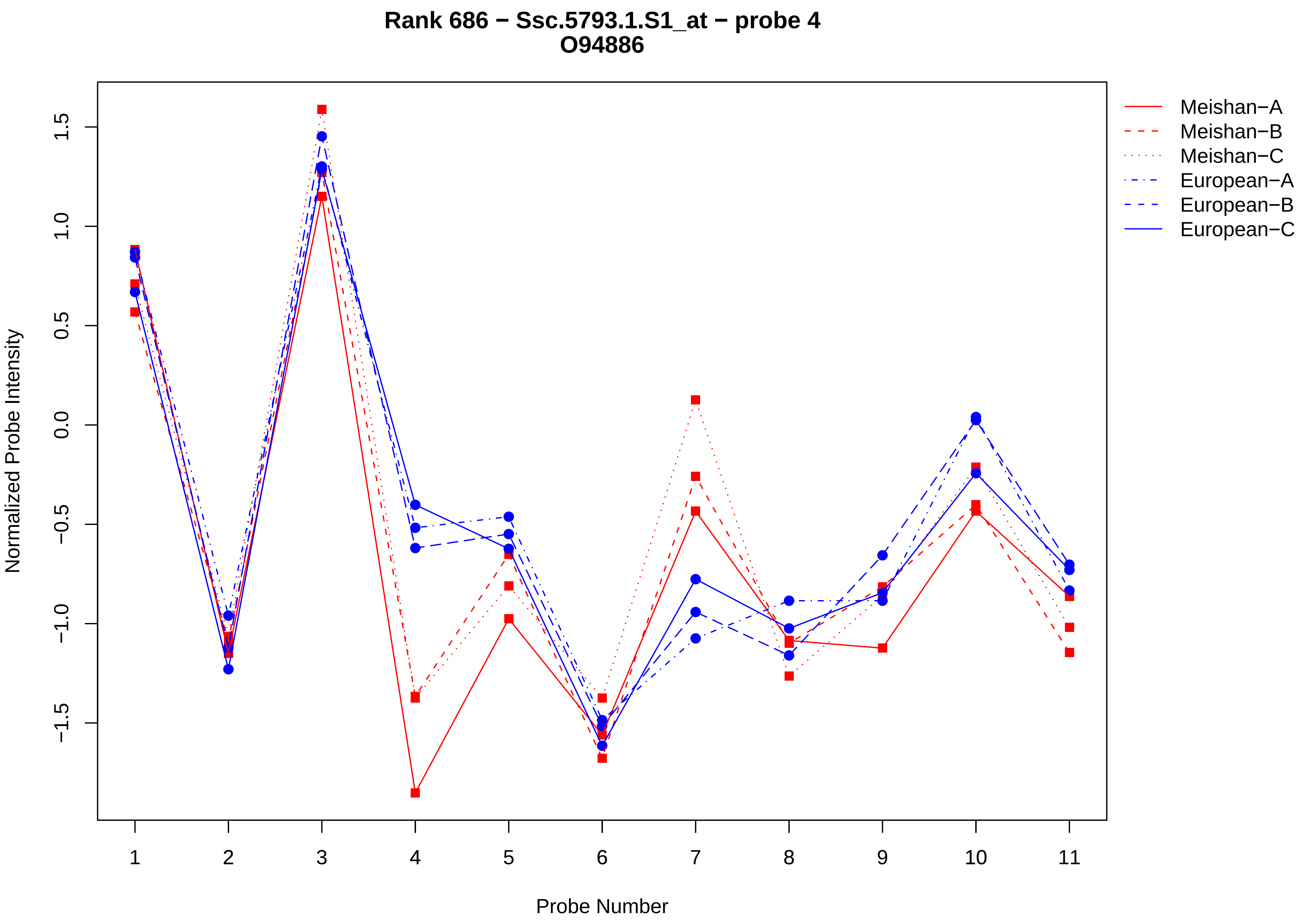

Rank 686 − Ssc.5793.1.S1_at − probe 4
O94886
Normalized Probe Intensity
Probe Number
Meishan−A
Meishan−B
Meishan−C
European−A
European−B
European−C
1.5
1.0
0.5
0.0
−0.5
−1.0
−1.5
1
2
3
4
5
6
7
8
9
10
11

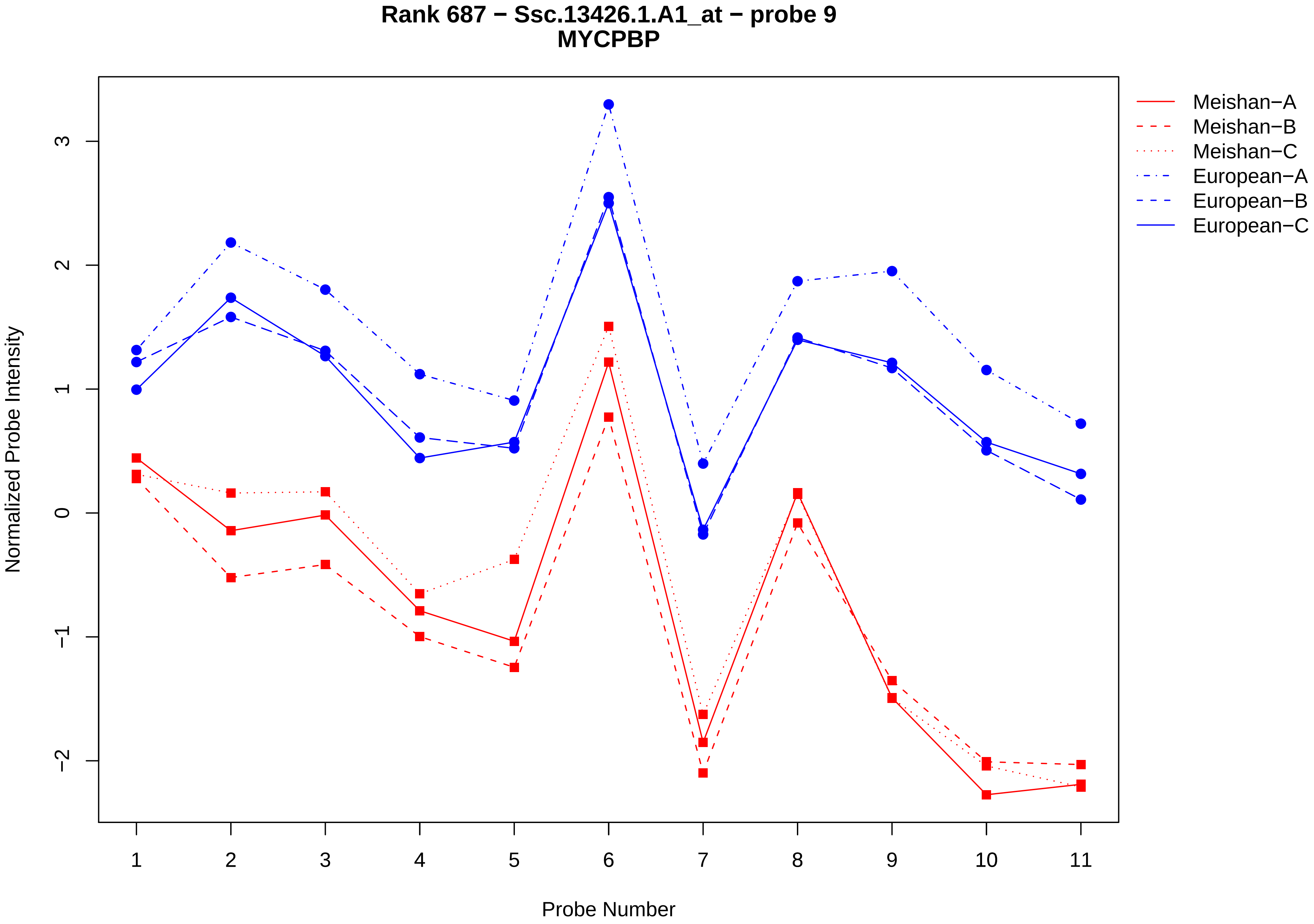

Rank 687 − Ssc.13426.1.A1_at − probe 9
MYCPBP
Normalized Probe Intensity
Probe Number
Meishan−A
Meishan−B
Meishan−C
European−A
European−B
European−C

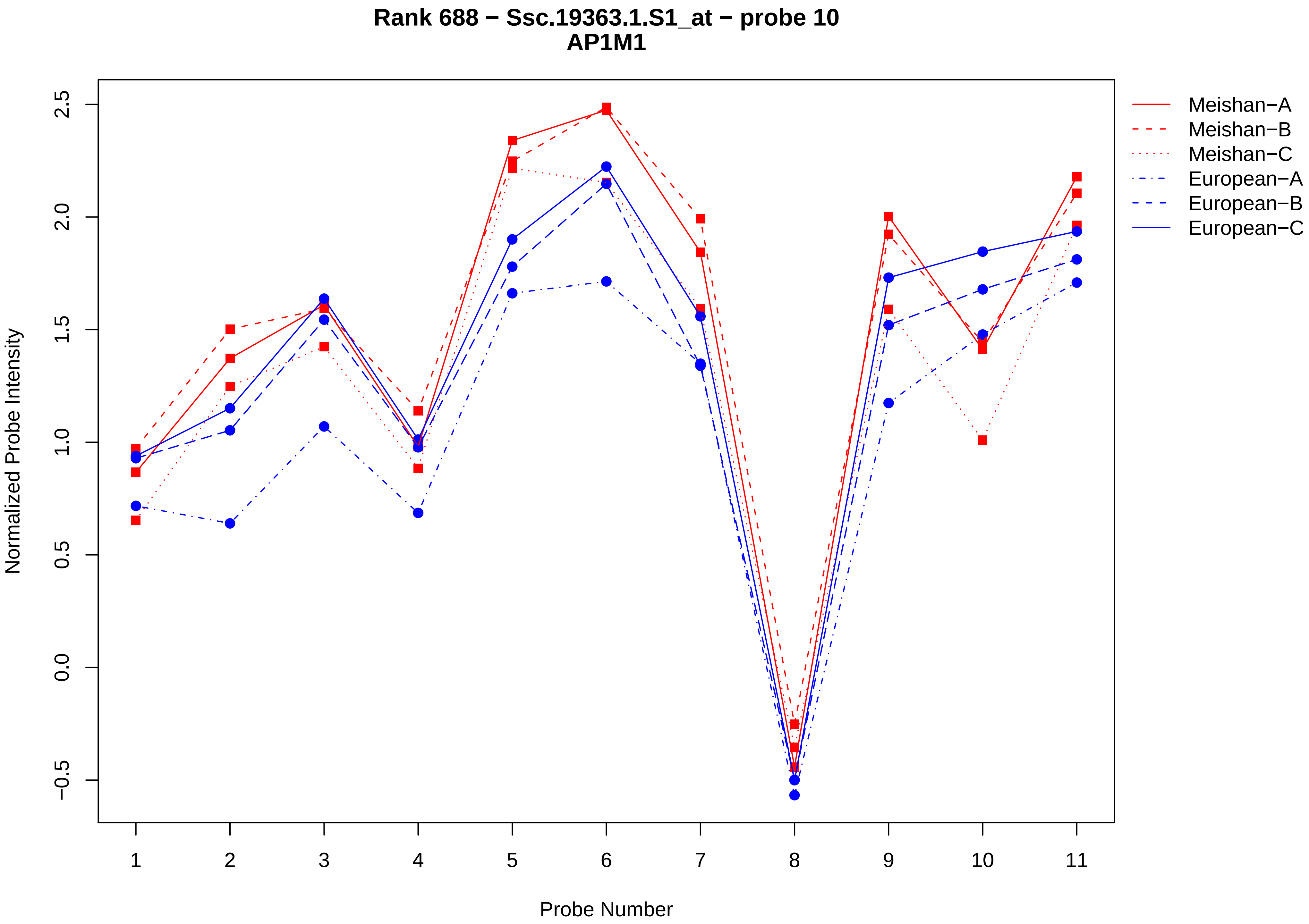

Rank 688 − Ssc.19363.1.S1_at − probe 10
AP1M1
Normalized Probe Intensity
Probe Number
Meishan−A
Meishan−B
Meishan−C
European−A
European−B
European−C
−0.5
0.0
0.5
1.0
1.5
2.0
2.5
1
2
3
4
5
6
7
8
9
10
11

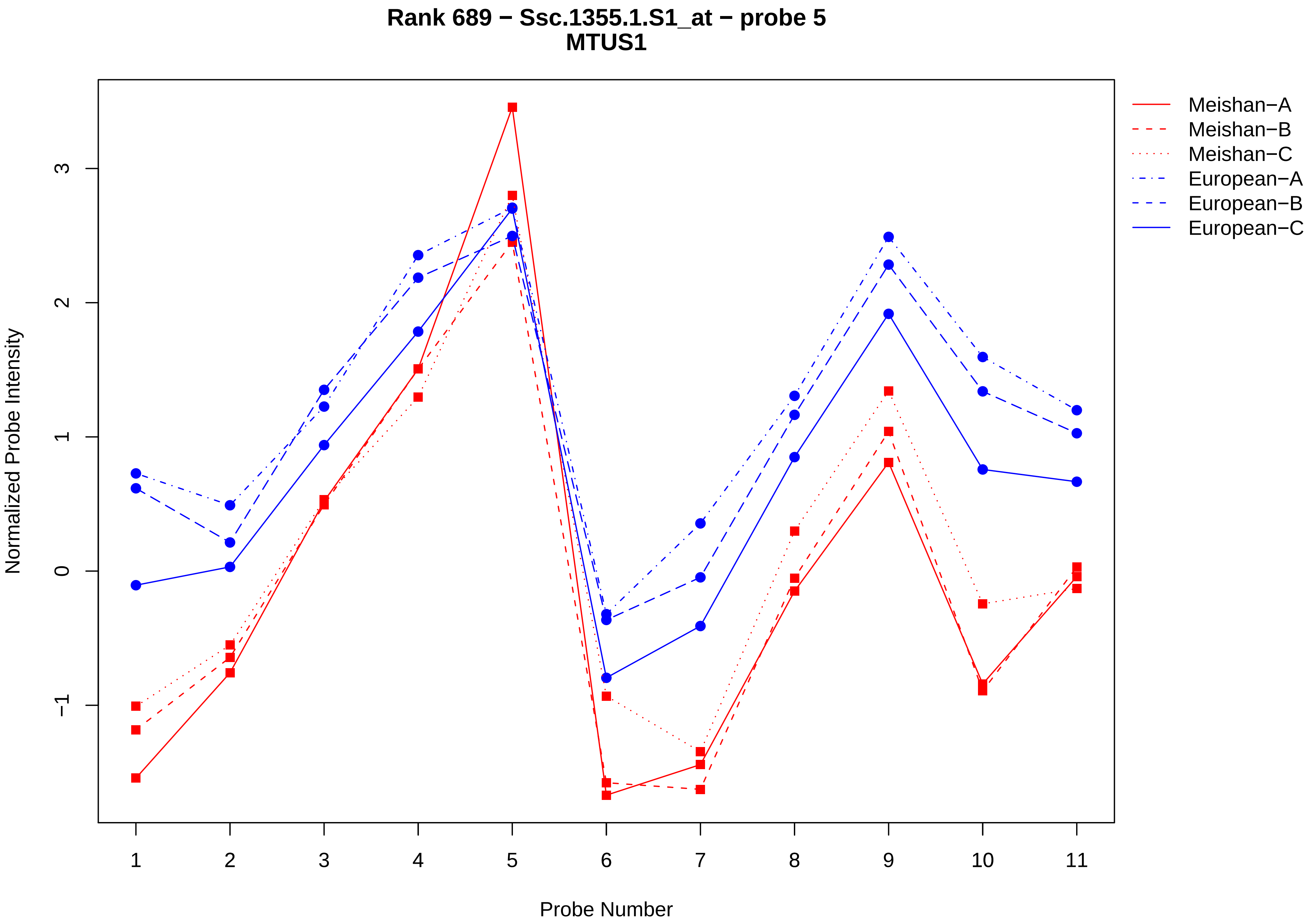

Rank 689 − Ssc.1355.1.S1_at − probe 5
MTUS1
Normalized Probe Intensity
Probe Number
Meishan−A
Meishan−B
Meishan−C
European−A
European−B
European−C
−1
0
1
2
3
1
2
3
4
5
6
7
8
9
10
11

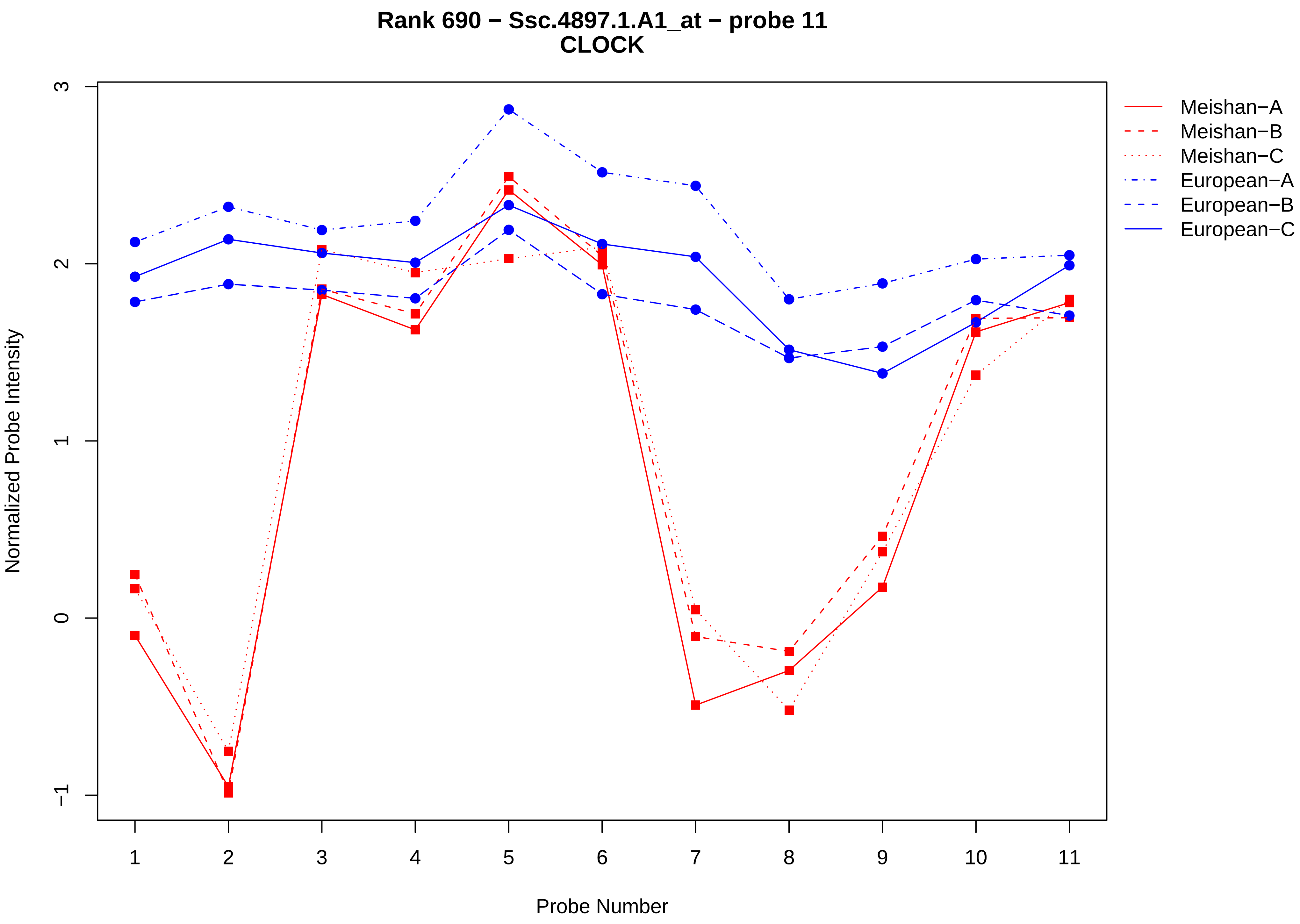

Rank 690 − Ssc.4897.1.A1_at − probe 11
CLOCK
Normalized Probe Intensity
Probe Number
Meishan−A
Meishan−B
Meishan−C
European−A
European−B
European−C
−1
0
1
2
3
1
2
3
4
5
6
7
8
9
10
11

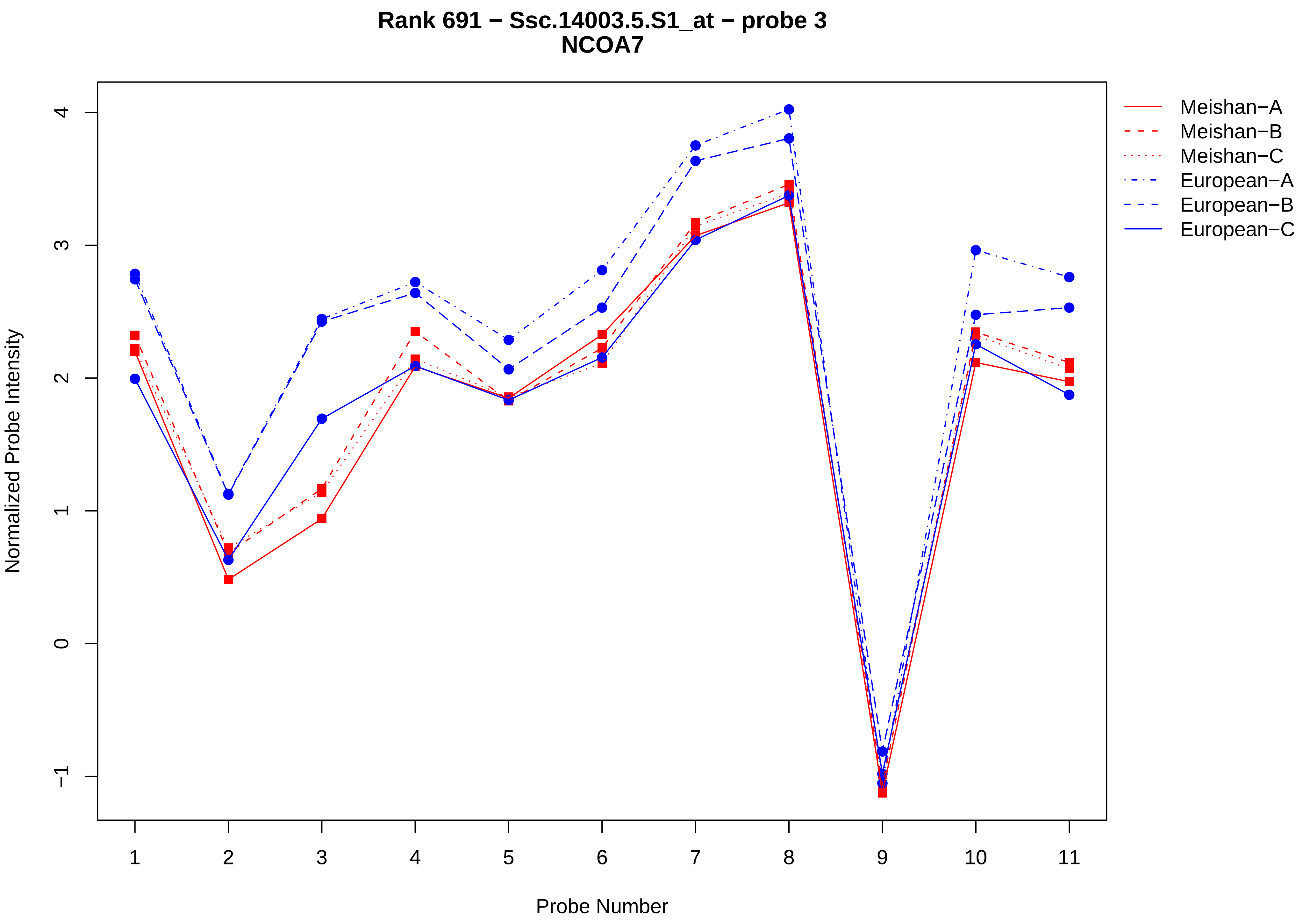
Rank 691 − Ssc.14003.5.S1_at − probe 3
NCOA7
Normalized Probe Intensity
Probe Number
4
3
2
1
0
−1
1
2
3
4
5
6
7
8
9
10
11
Meishan−A
Meishan−B
Meishan−C
European−A
European−B
European−C

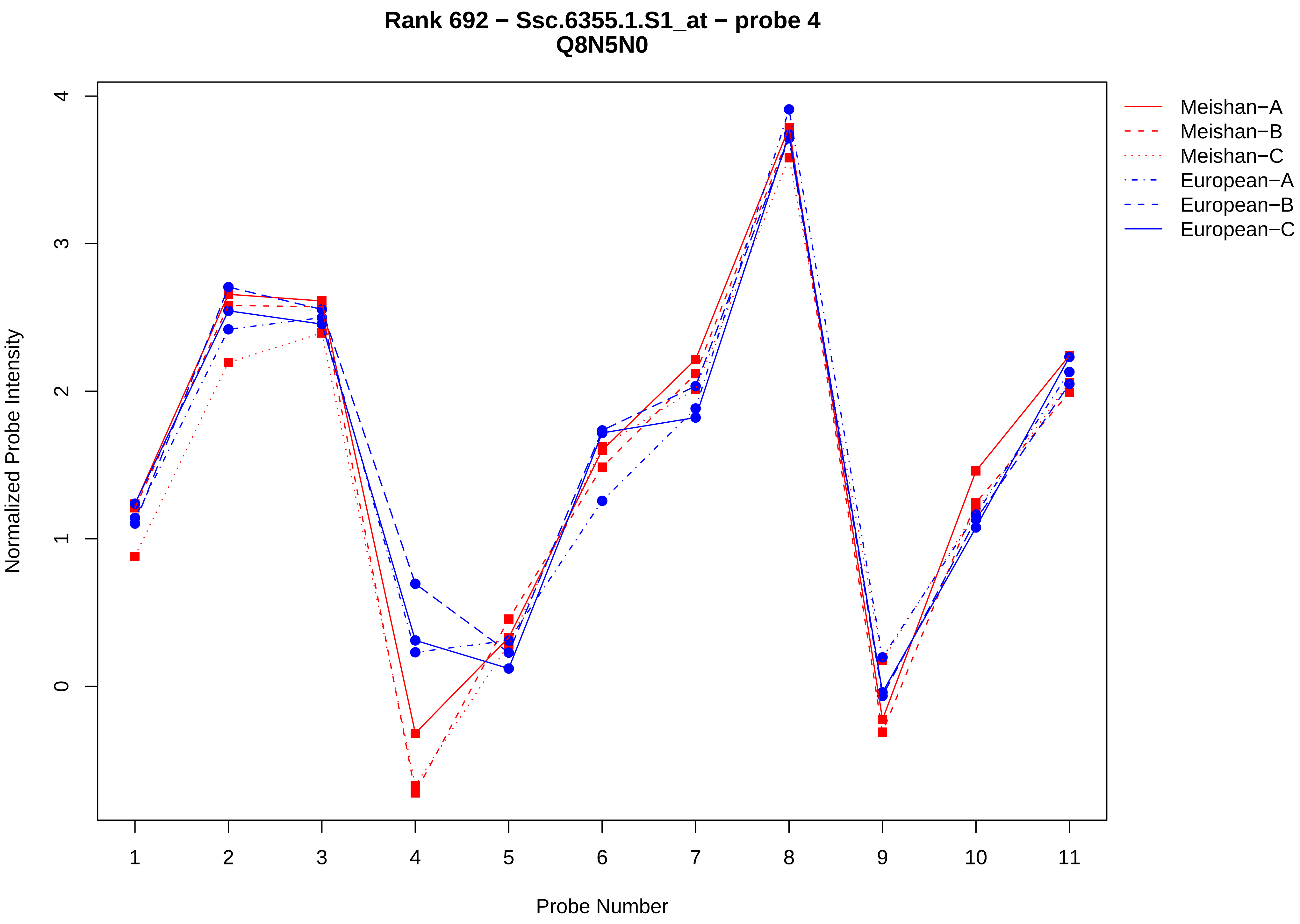

Rank 692 − Ssc.6355.1.S1_at − probe 4
Q8N5N0
Normalized Probe Intensity
Probe Number
0
1
2
3
4
1
2
3
4
5
6
7
8
9
10
11
Meishan−A
Meishan−B
Meishan−C
European−A
European−B
European−C

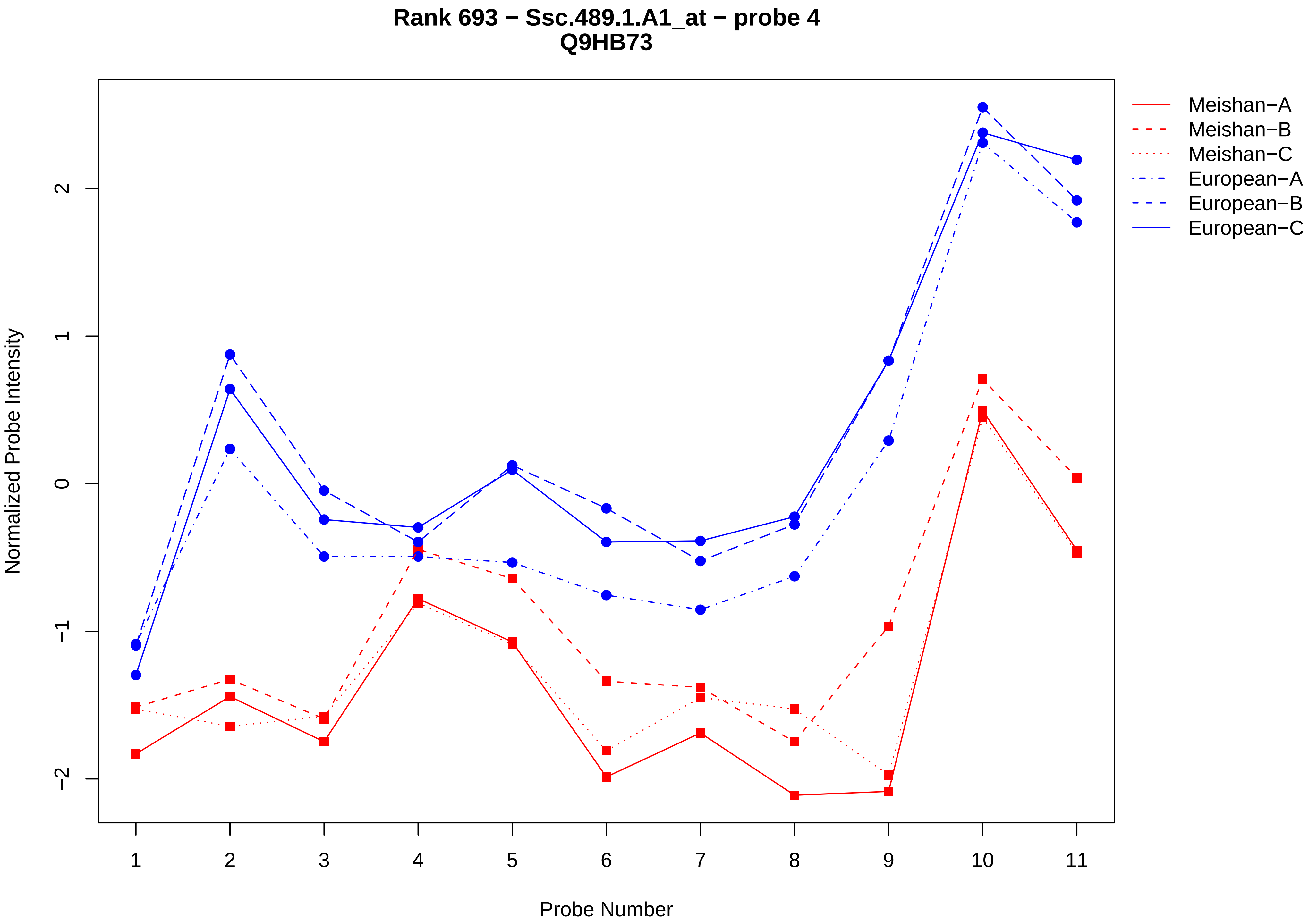

Rank 693 − Ssc.489.1.A1_at − probe 4
Q9HB73
Normalized Probe Intensity
Probe Number
−2
−1
0
1
2
1
2
3
4
5
6
7
8
9
10
11
Meishan−A
Meishan−B
Meishan−C
European−A
European−B
European−C

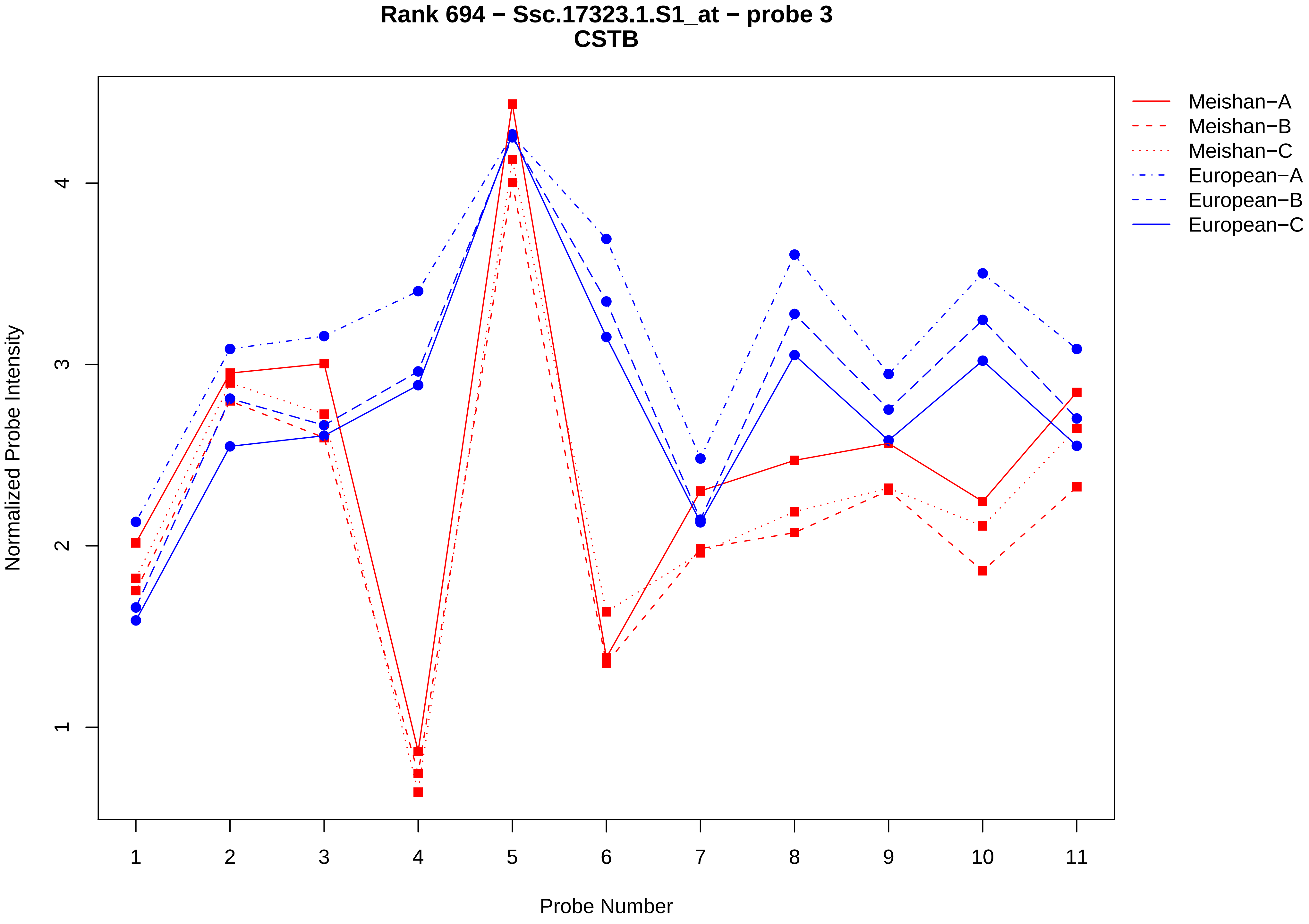
Rank 694 − Ssc.17323.1.S1_at − probe 3
CSTB
Normalized Probe Intensity
Probe Number
1
2
3
4
1
2
3
4
5
6
7
8
9
10
11
Meishan−A
Meishan−B
Meishan−C
European−A
European−B
European−C

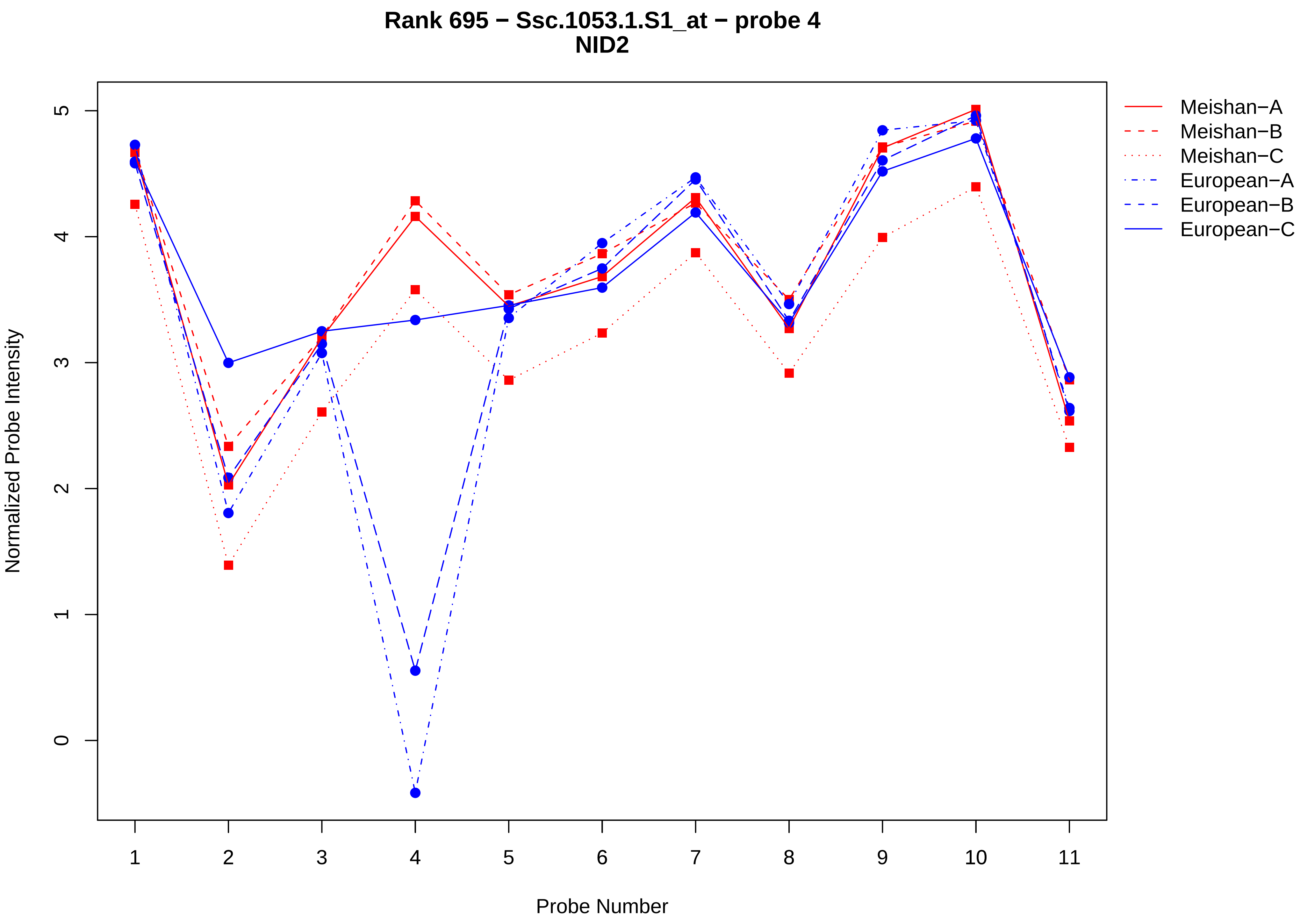
Rank 695 − Ssc.1053.1.S1_at − probe 4
NID2
Normalized Probe Intensity
Probe Number
0
1
2
3
4
5
1
2
3
4
5
6
7
8
9
10
11
Meishan−A
Meishan−B
Meishan−C
European−A
European−B
European−C

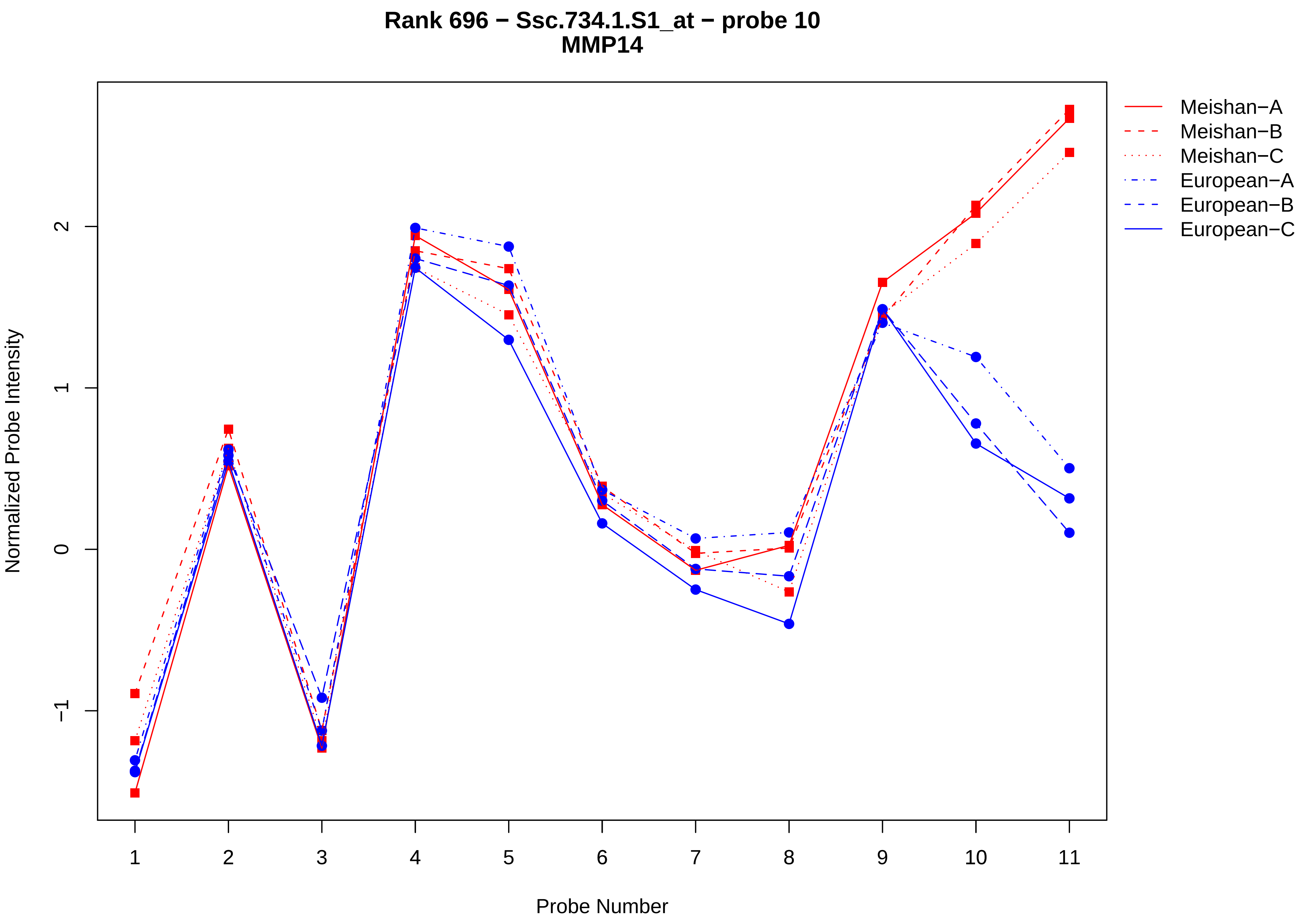

Rank 696 − Ssc.734.1.S1_at − probe 10
MMP14
Normalized Probe Intensity
Probe Number
Meishan−A
Meishan−B
Meishan−C
European−A
European−B
European−C
−1
0
1
2
1
2
3
4
5
6
7
8
9
10
11

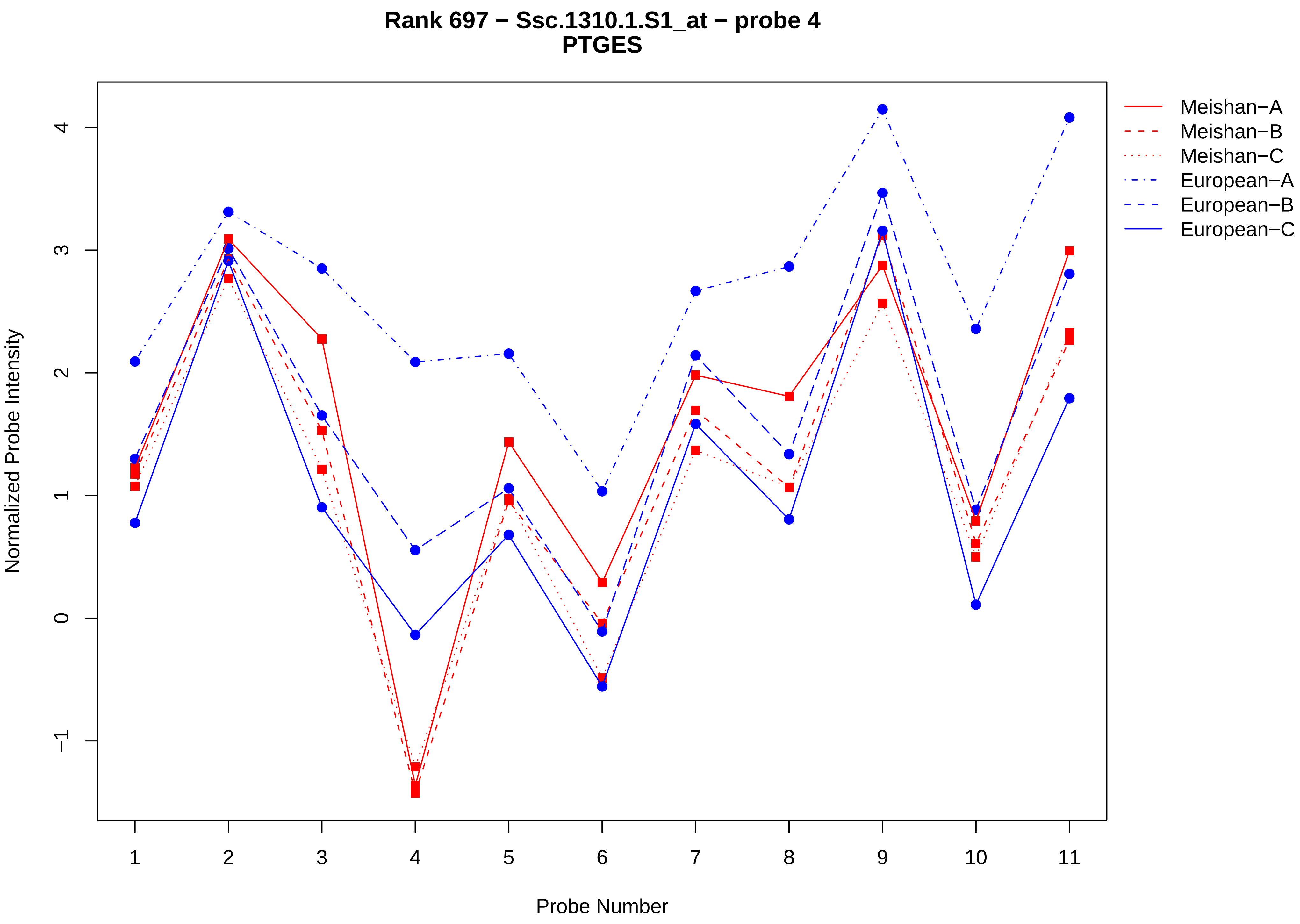
Rank 697 − Ssc.1310.1.S1_at − probe 4
PTGES
Normalized Probe Intensity
Probe Number
−1
0
1
2
3
4
1
2
3
4
5
6
7
8
9
10
11
Meishan−A
Meishan−B
Meishan−C
European−A
European−B
European−C

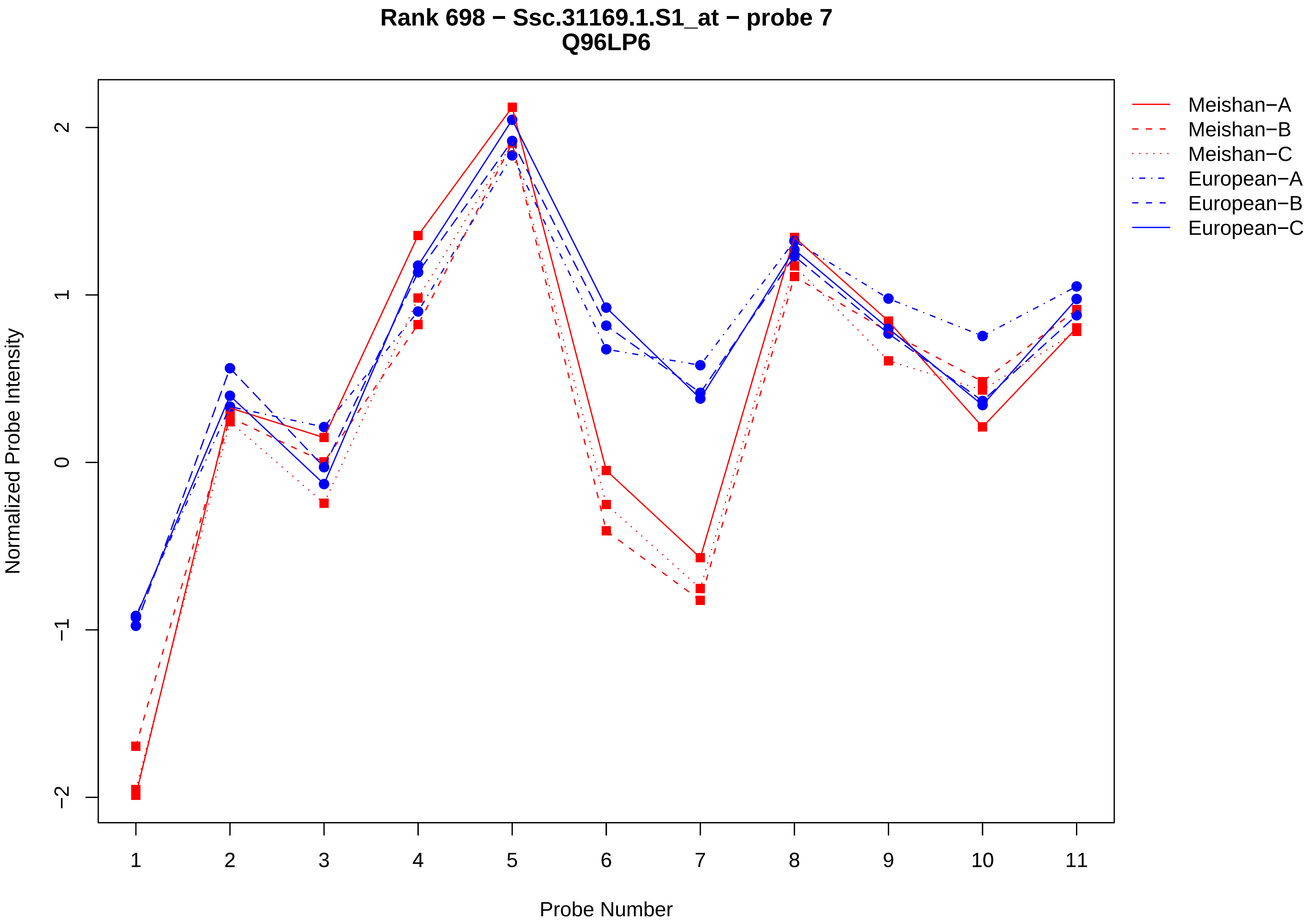
Rank 698 − Ssc.31169.1.S1_at − probe 7
Q96LP6
Normalized Probe Intensity
Probe Number
Meishan−A
Meishan−B
Meishan−C
European−A
European−B
European−C
−2
−1
0
1
2
1
2
3
4
5
6
7
8
9
10
11

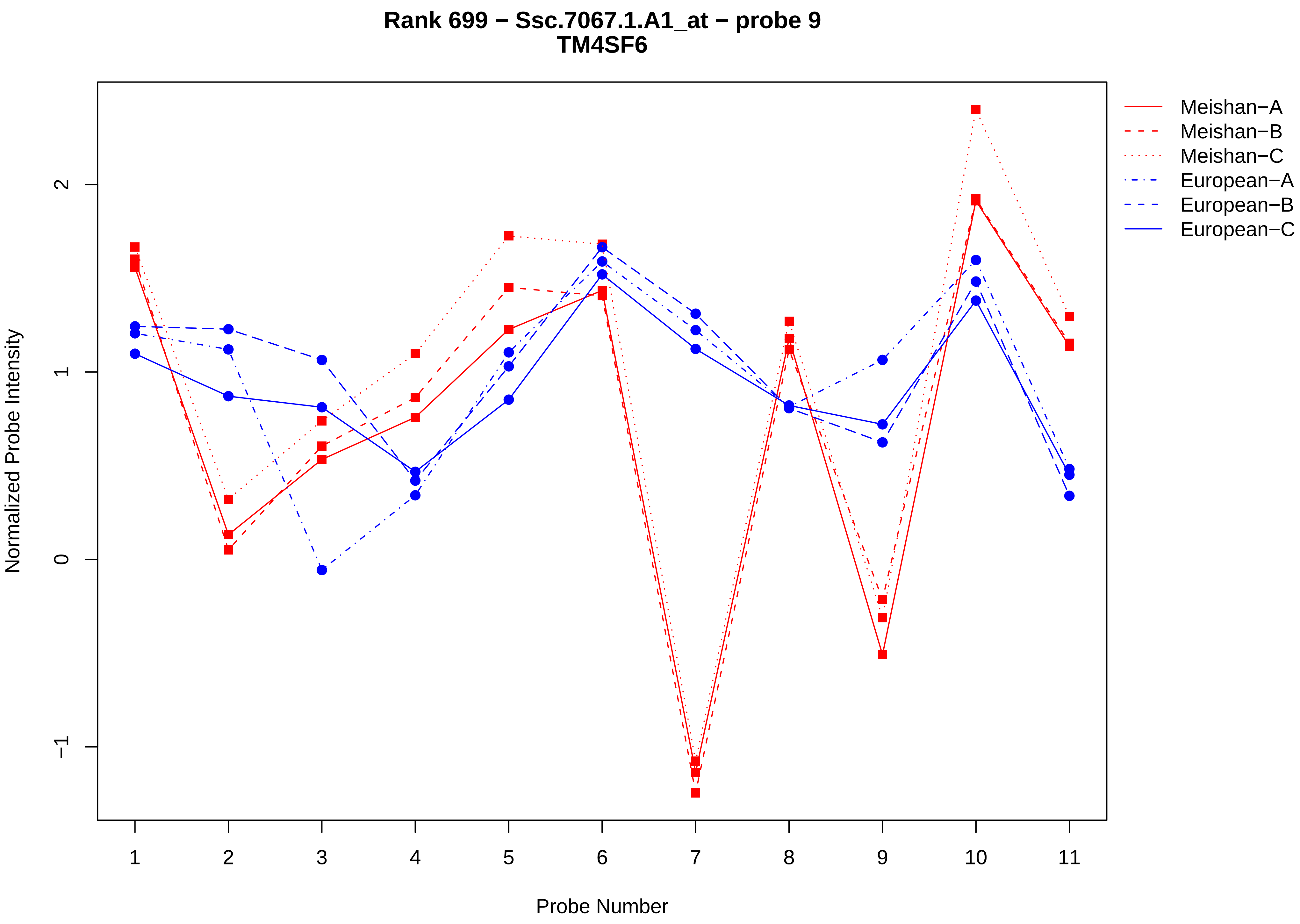

Rank 699 − Ssc.7067.1.A1_at − probe 9
TM4SF6
Normalized Probe Intensity
Probe Number
−1
0
1
2
1
2
3
4
5
6
7
8
9
10
11
Meishan−A
Meishan−B
Meishan−C
European−A
European−B
European−C

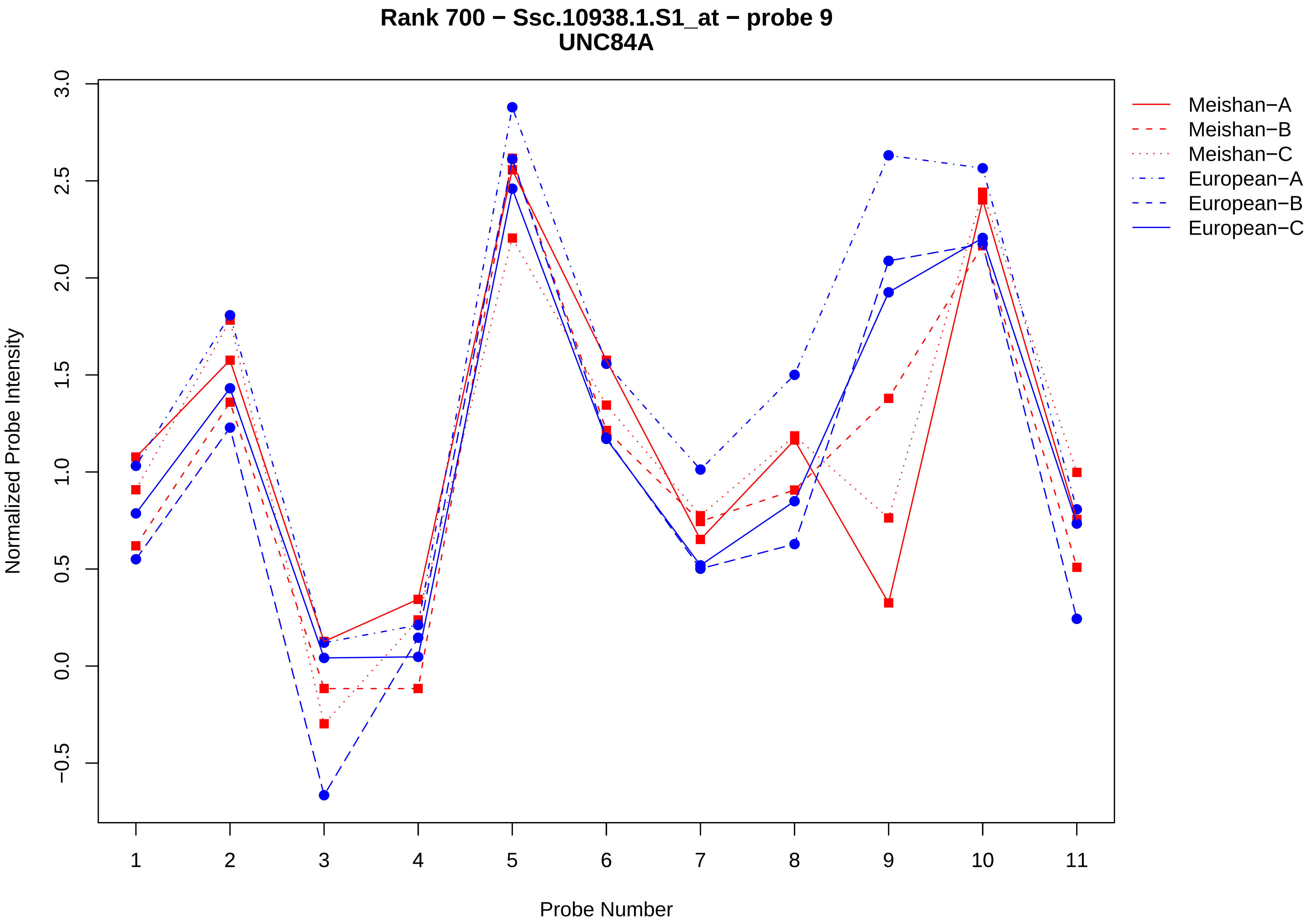

Rank 700 − Ssc.10938.1.S1_at − probe 9
UNC84A
Normalized Probe Intensity
Probe Number
Meishan−A
Meishan−B
Meishan−C
European−A
European−B
European−C

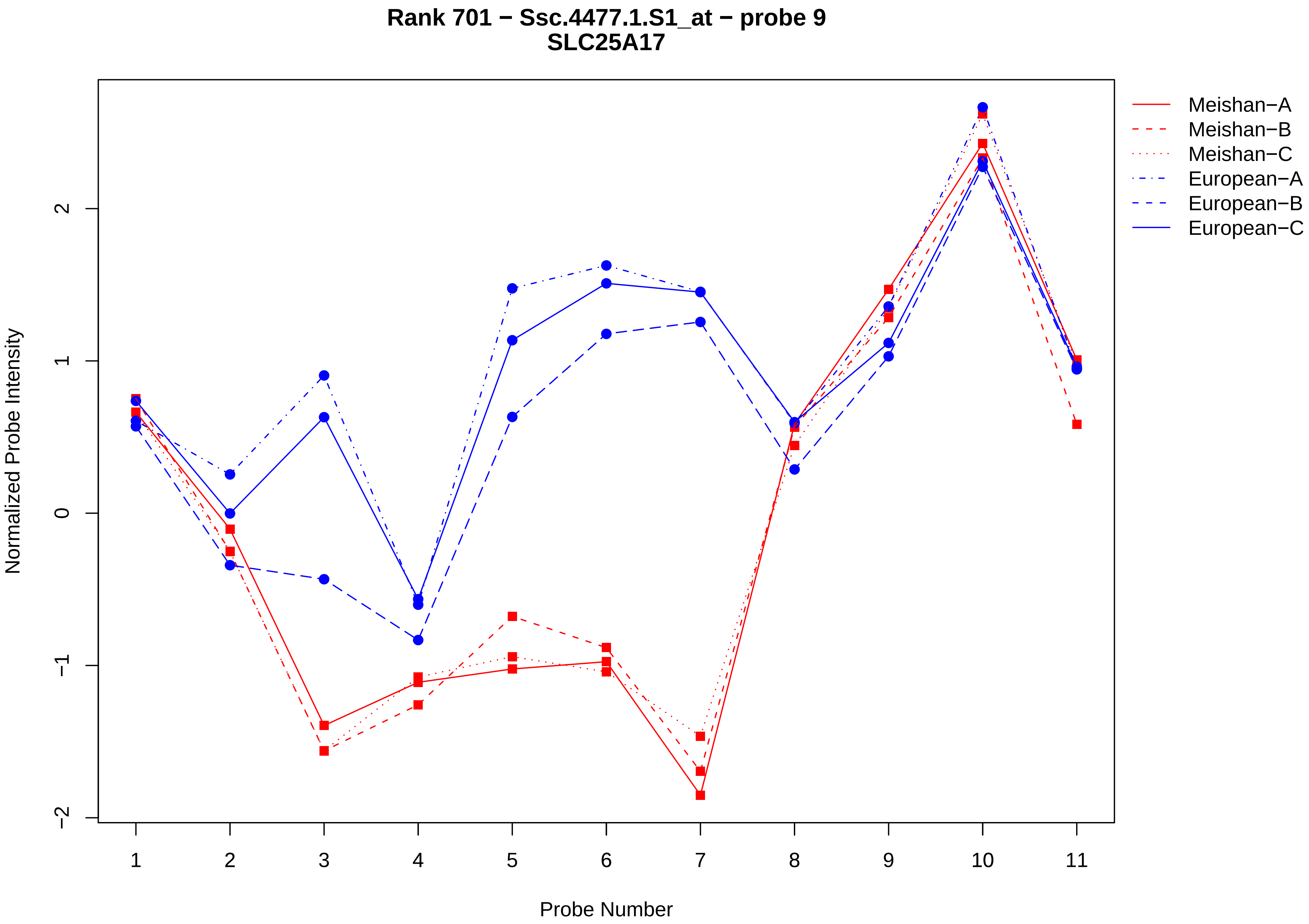

Rank 701 − Ssc.4477.1.S1_at − probe 9
SLC25A17
Normalized Probe Intensity
Probe Number
Meishan−A
Meishan−B
Meishan−C
European−A
European−B
European−C
−2
−1
0
1
2
1
2
3
4
5
6
7
8
9
10
11

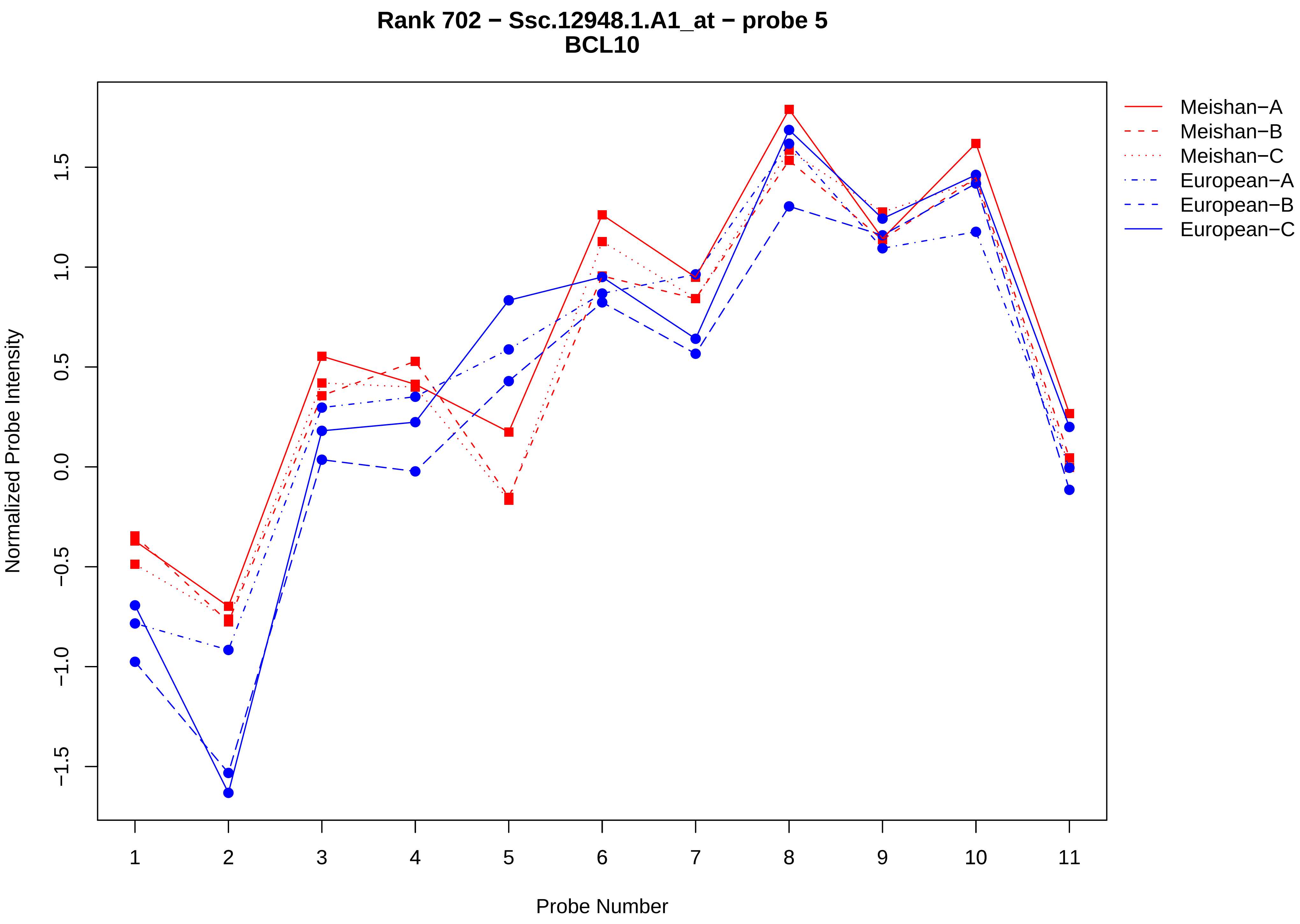

Rank 702 − Ssc.12948.1.A1_at − probe 5
BCL10
Normalized Probe Intensity
Probe Number
−1.5
−1.0
−0.5
0.0
0.5
1.0
1.5
1
2
3
4
5
6
7
8
9
10
11
Meishan−A
Meishan−B
Meishan−C
European−A
European−B
European−C

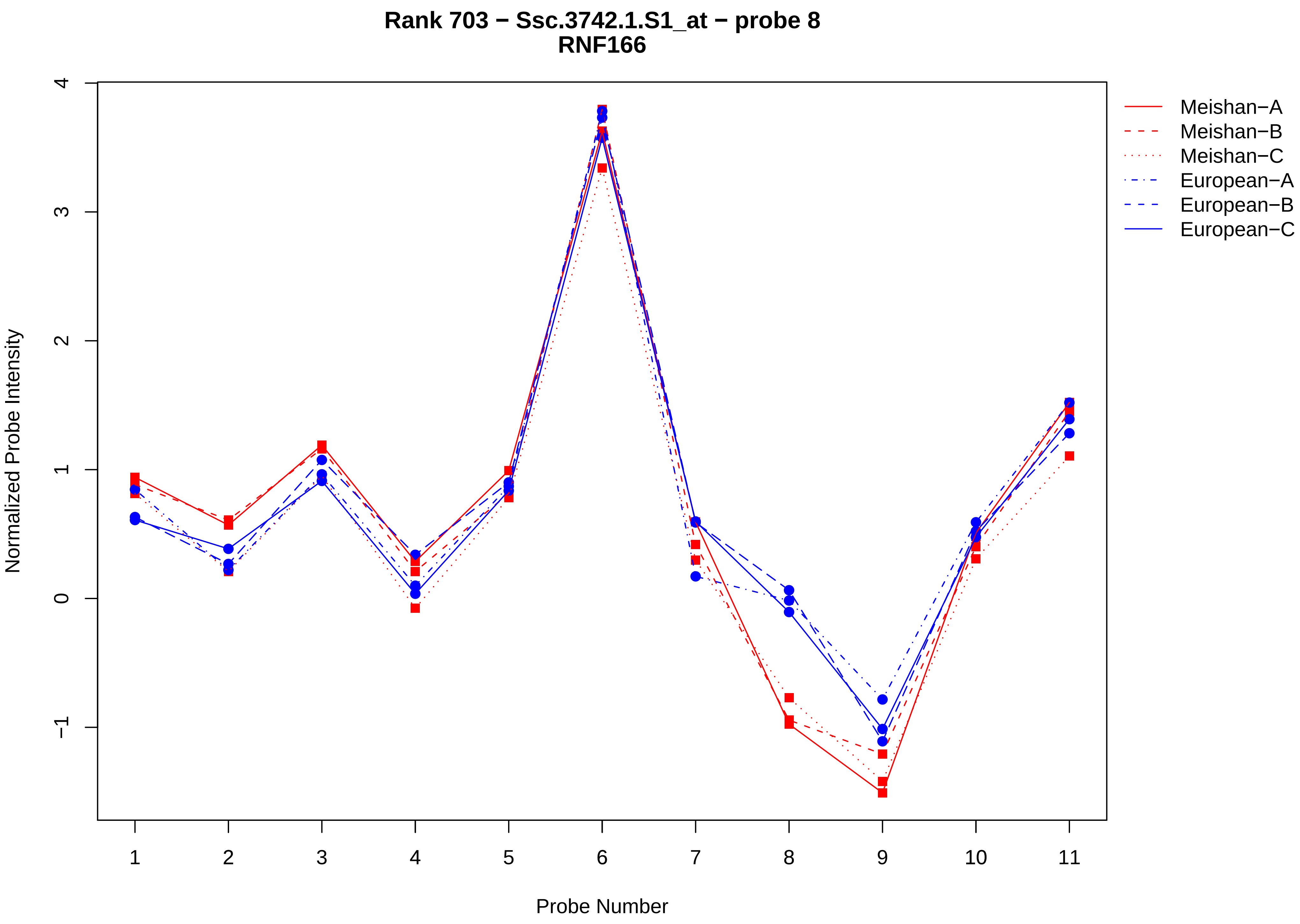

Rank 703 − Ssc.3742.1.S1_at − probe 8
RNF166
Normalized Probe Intensity
Probe Number
Meishan−A
Meishan−B
Meishan−C
European−A
European−B
European−C

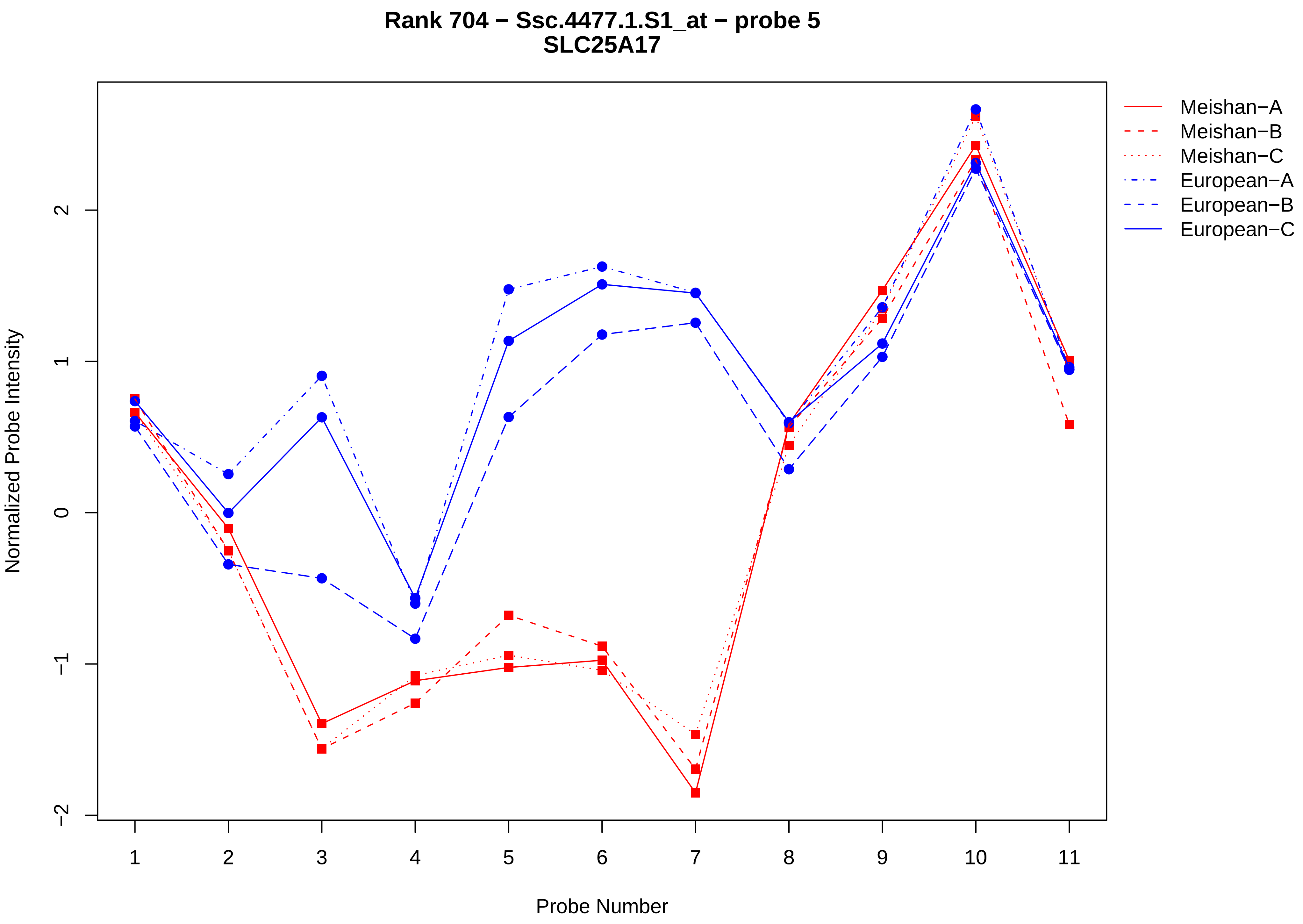

Rank 704 − Ssc.4477.1.S1_at − probe 5
SLC25A17
Normalized Probe Intensity
Probe Number
−2
−1
0
1
2
1
2
3
4
5
6
7
8
9
10
11
Meishan−A
Meishan−B
Meishan−C
European−A
European−B
European−C

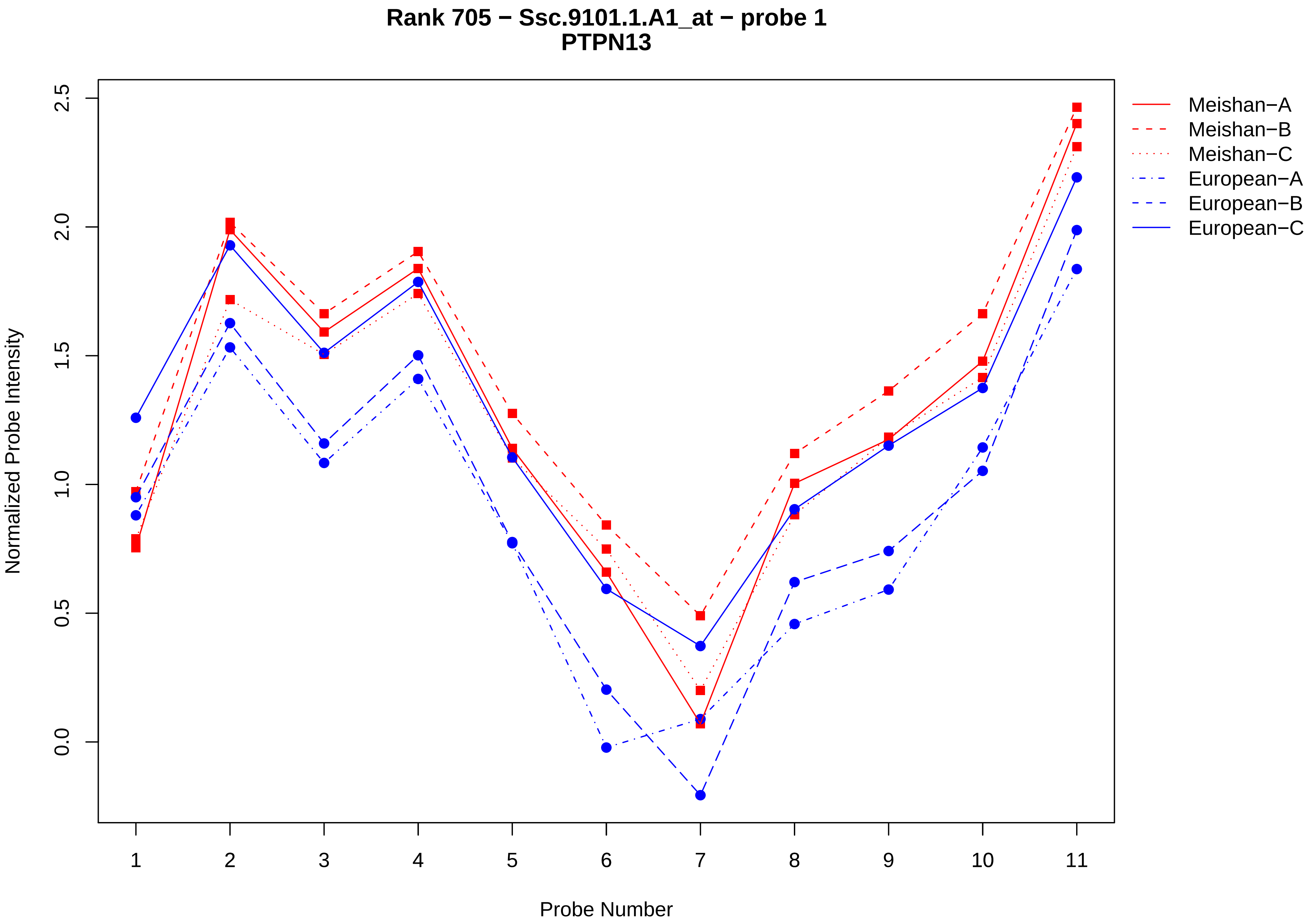
Rank 705 − Ssc.9101.1.A1_at − probe 1
PTPN13
Normalized Probe Intensity
Probe Number
0.0
0.5
1.0
1.5
2.0
2.5
1
2
3
4
5
6
7
8
9
10
11
Meishan−A
Meishan−B
Meishan−C
European−A
European−B
European−C

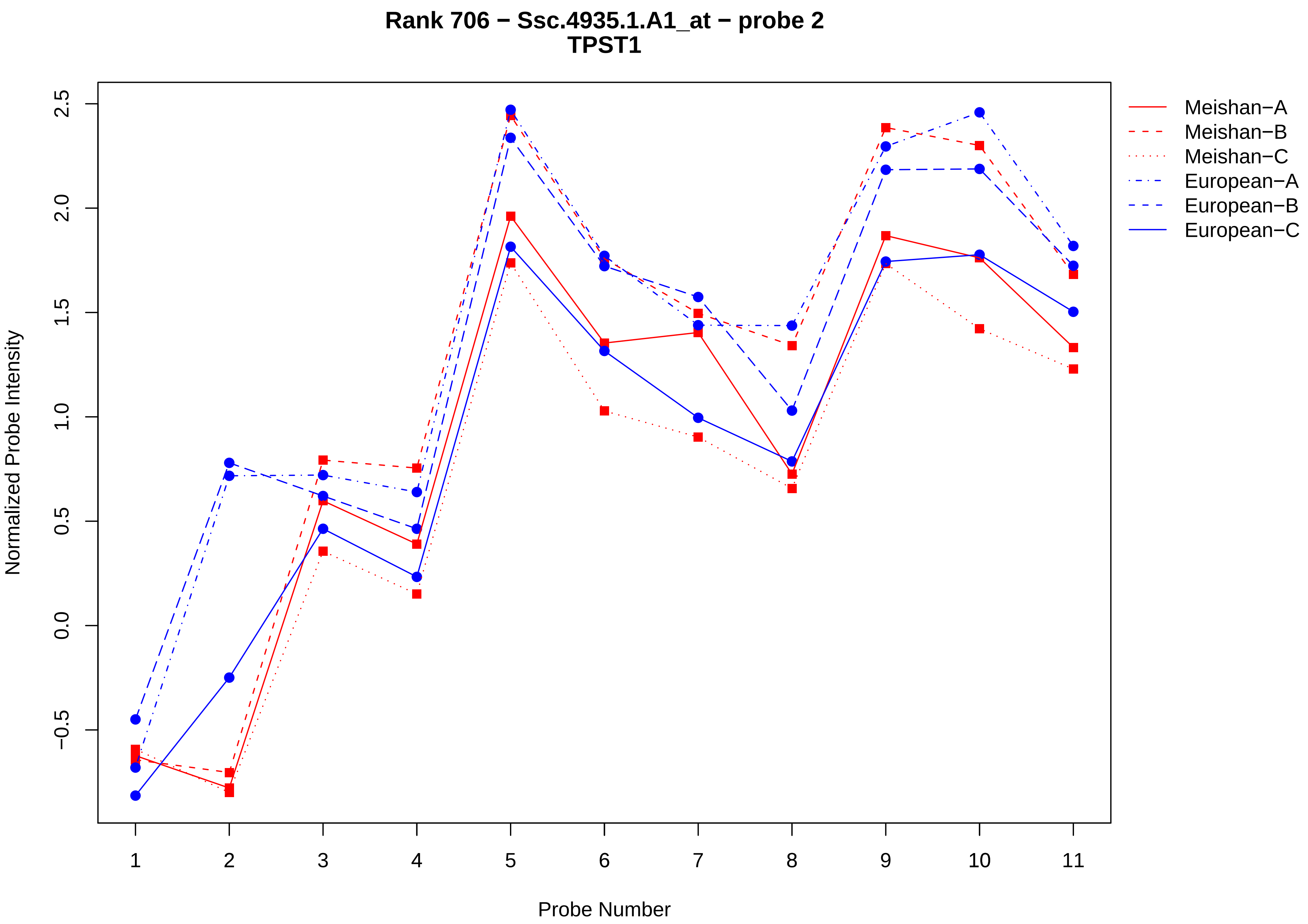
Rank 706 − Ssc.4935.1.A1_at − probe 2
TPST1
Normalized Probe Intensity
Probe Number
−0.5
0.0
0.5
1.0
1.5
2.0
2.5
1
2
3
4
5
6
7
8
9
10
11
Meishan−A
Meishan−B
Meishan−C
European−A
European−B
European−C

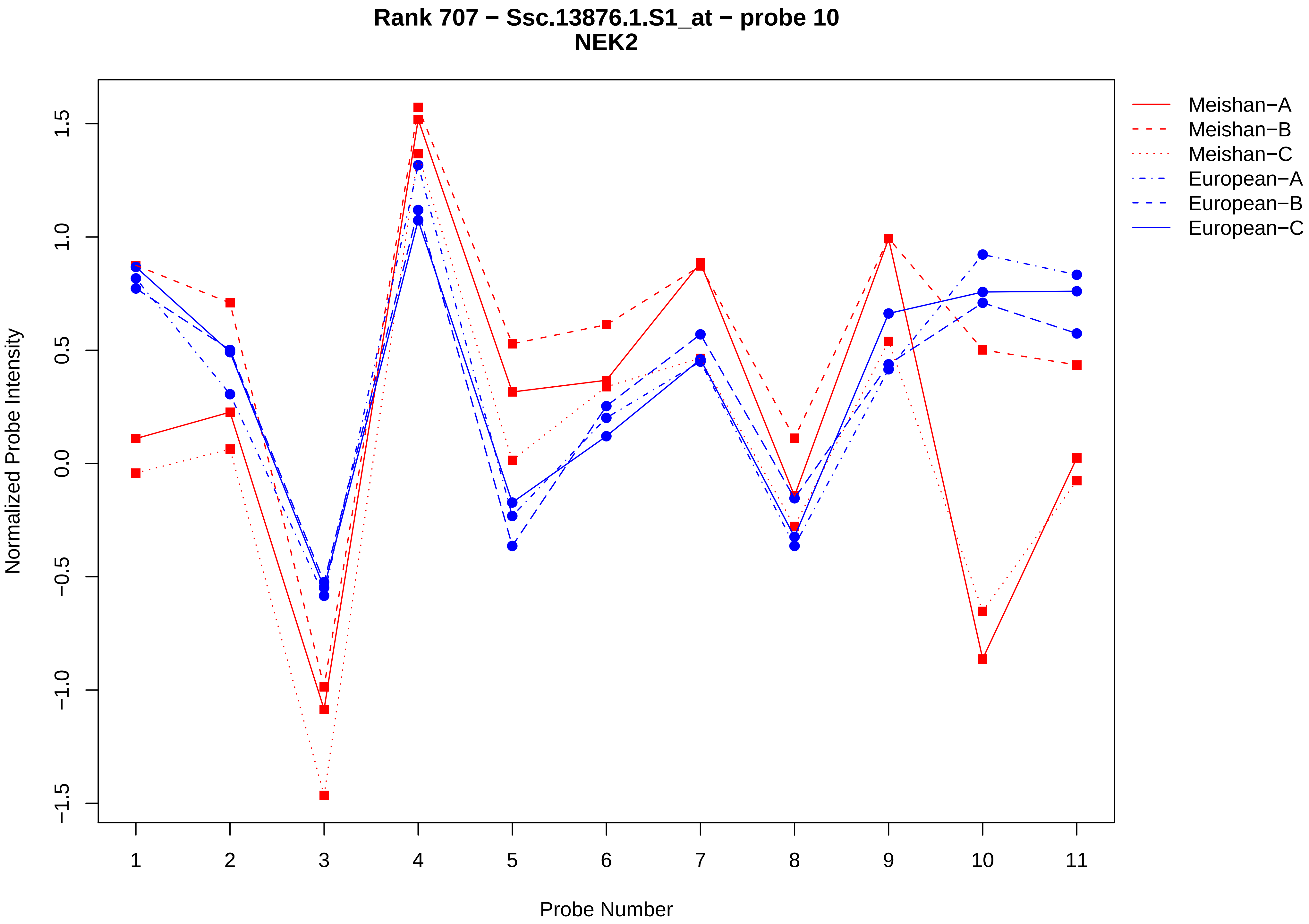

Rank 707 − Ssc.13876.1.S1_at − probe 10
NEK2
Normalized Probe Intensity
Probe Number
1.5
1.0
0.5
0.0
−0.5
−1.0
−1.5
1
2
3
4
5
6
7
8
9
10
11
Meishan−A
Meishan−B
Meishan−C
European−A
European−B
European−C

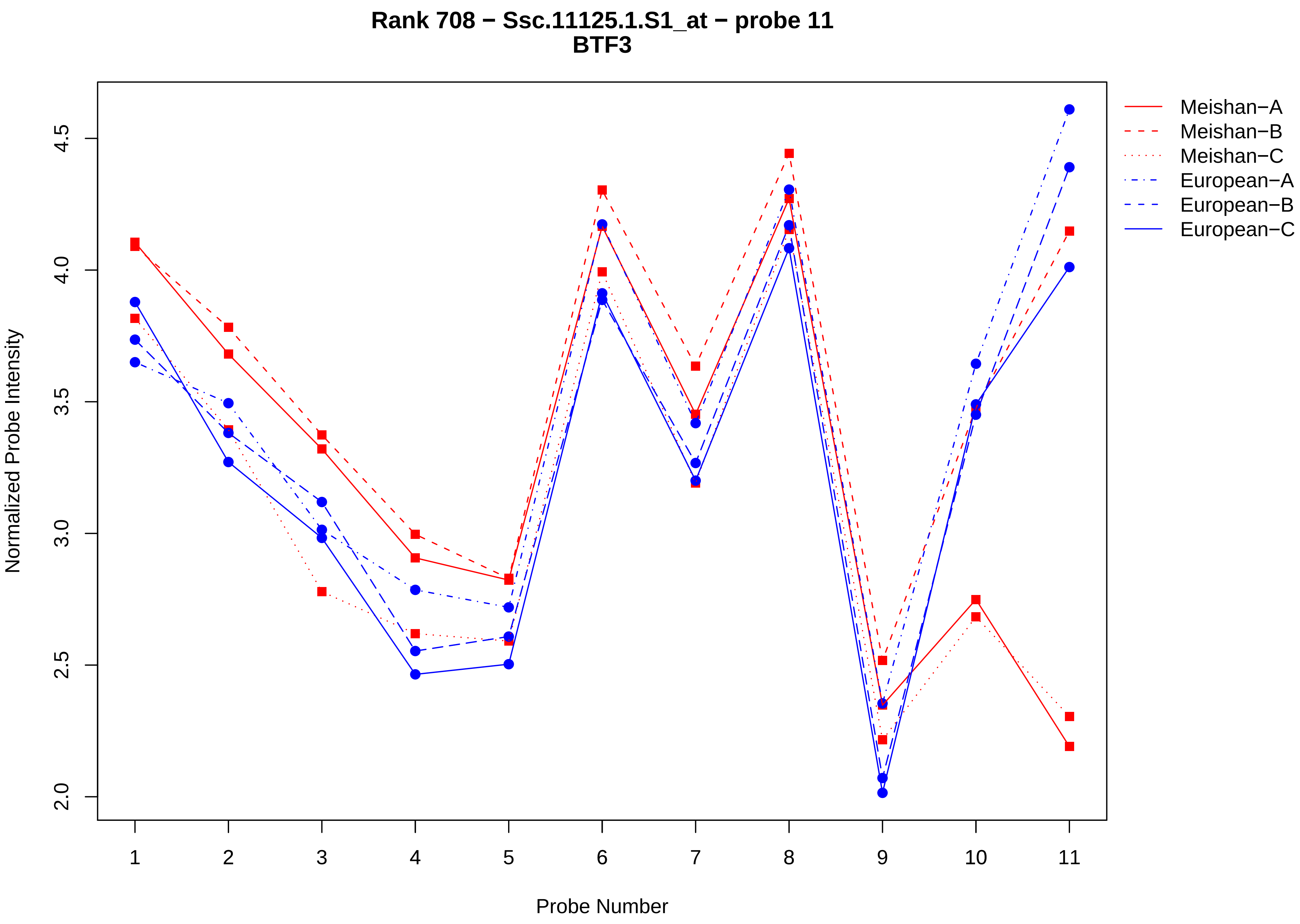
Rank 708 − Ssc.11125.1.S1_at − probe 11
BTF3
Normalized Probe Intensity
Probe Number
2.0
2.5
3.0
3.5
4.0
4.5
1
2
3
4
5
6
7
8
9
10
11
Meishan−A
Meishan−B
Meishan−C
European−A
European−B
European−C

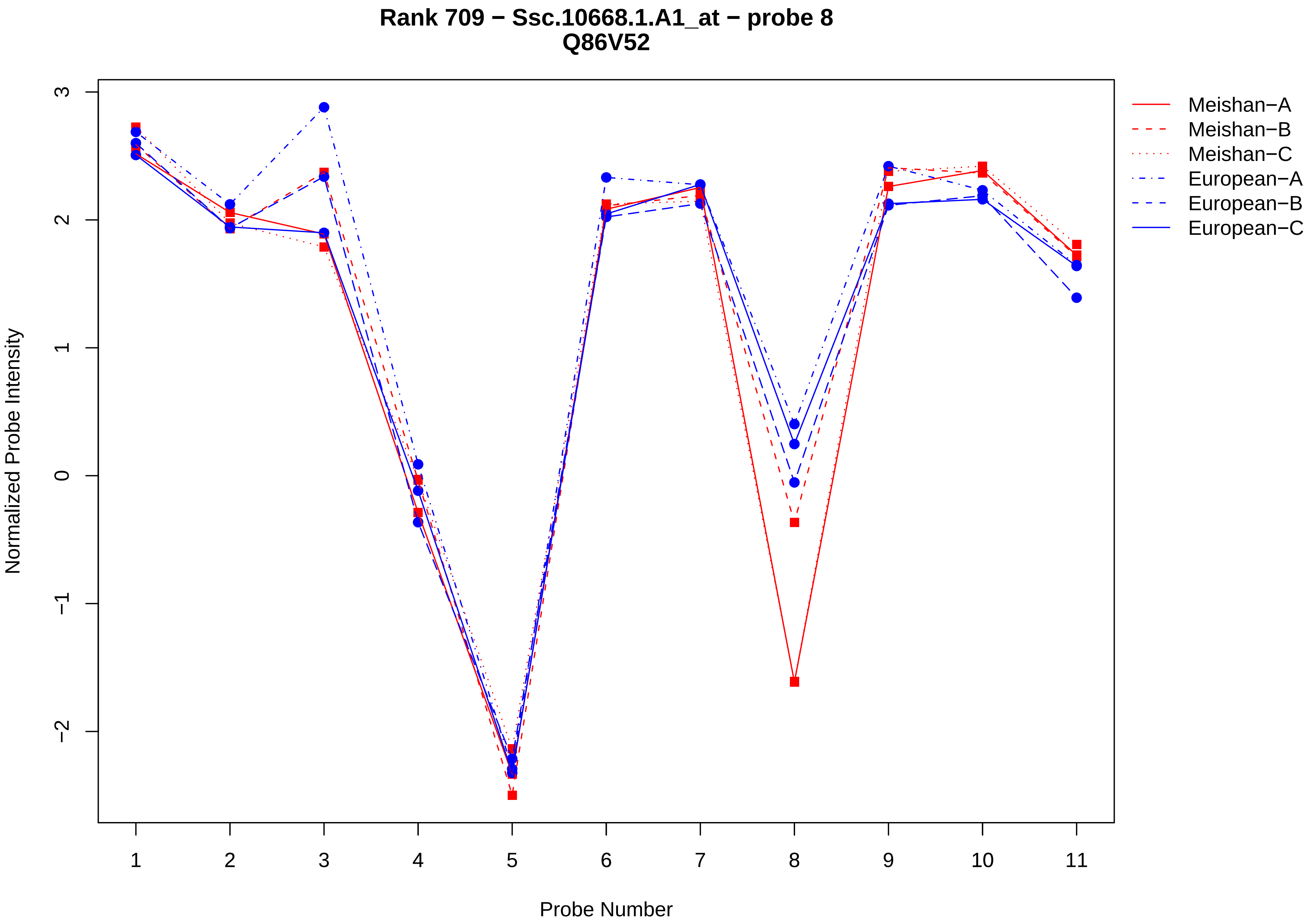

Rank 709 − Ssc.10668.1.A1_at − probe 8
Q86V52
Normalized Probe Intensity
Probe Number
−2
−1
0
1
2
3
1
2
3
4
5
6
7
8
9
10
11
Meishan−A
Meishan−B
Meishan−C
European−A
European−B
European−C

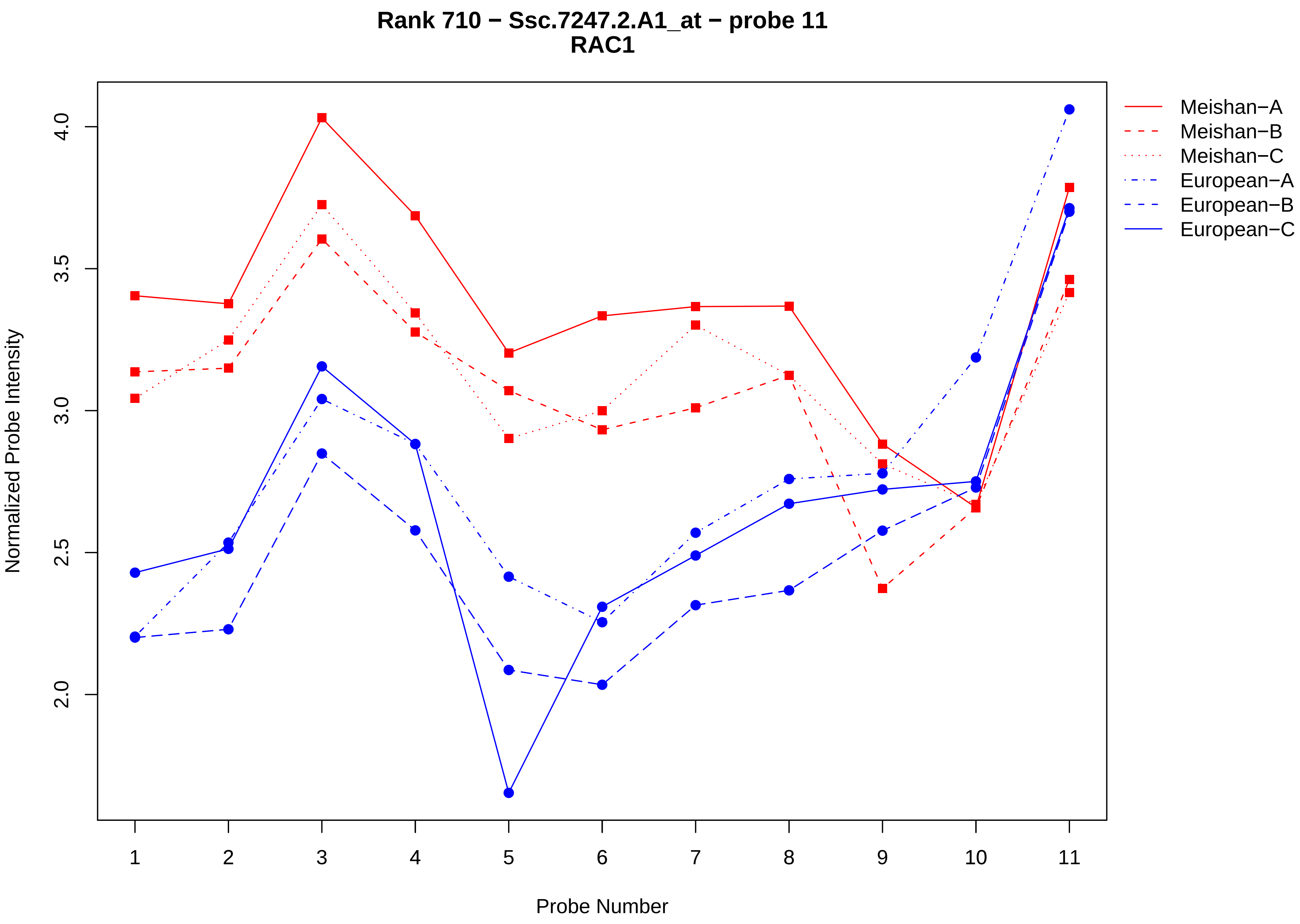

Rank 710 − Ssc.7247.2.A1_at − probe 11
RAC1
Normalized Probe Intensity
Probe Number
2.0
2.5
3.0
3.5
4.0
1
2
3
4
5
6
7
8
9
10
11
Meishan−A
Meishan−B
Meishan−C
European−A
European−B
European−C

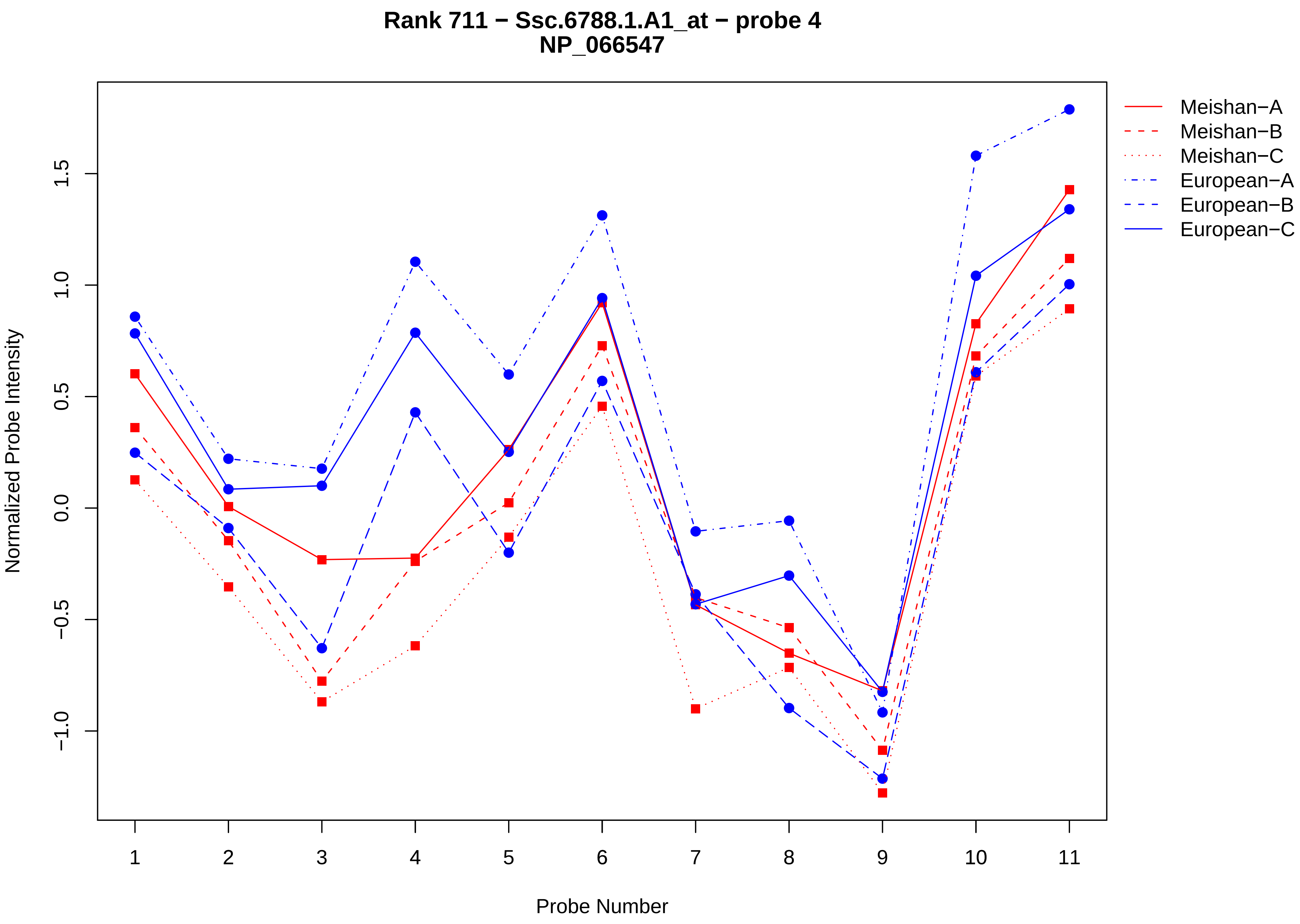

Rank 711 − Ssc.6788.1.A1_at − probe 4
NP_066547
Normalized Probe Intensity
Probe Number
−1.0
−0.5
0.0
0.5
1.0
1.5
1
2
3
4
5
6
7
8
9
10
11
Meishan−A
Meishan−B
Meishan−C
European−A
European−B
European−C

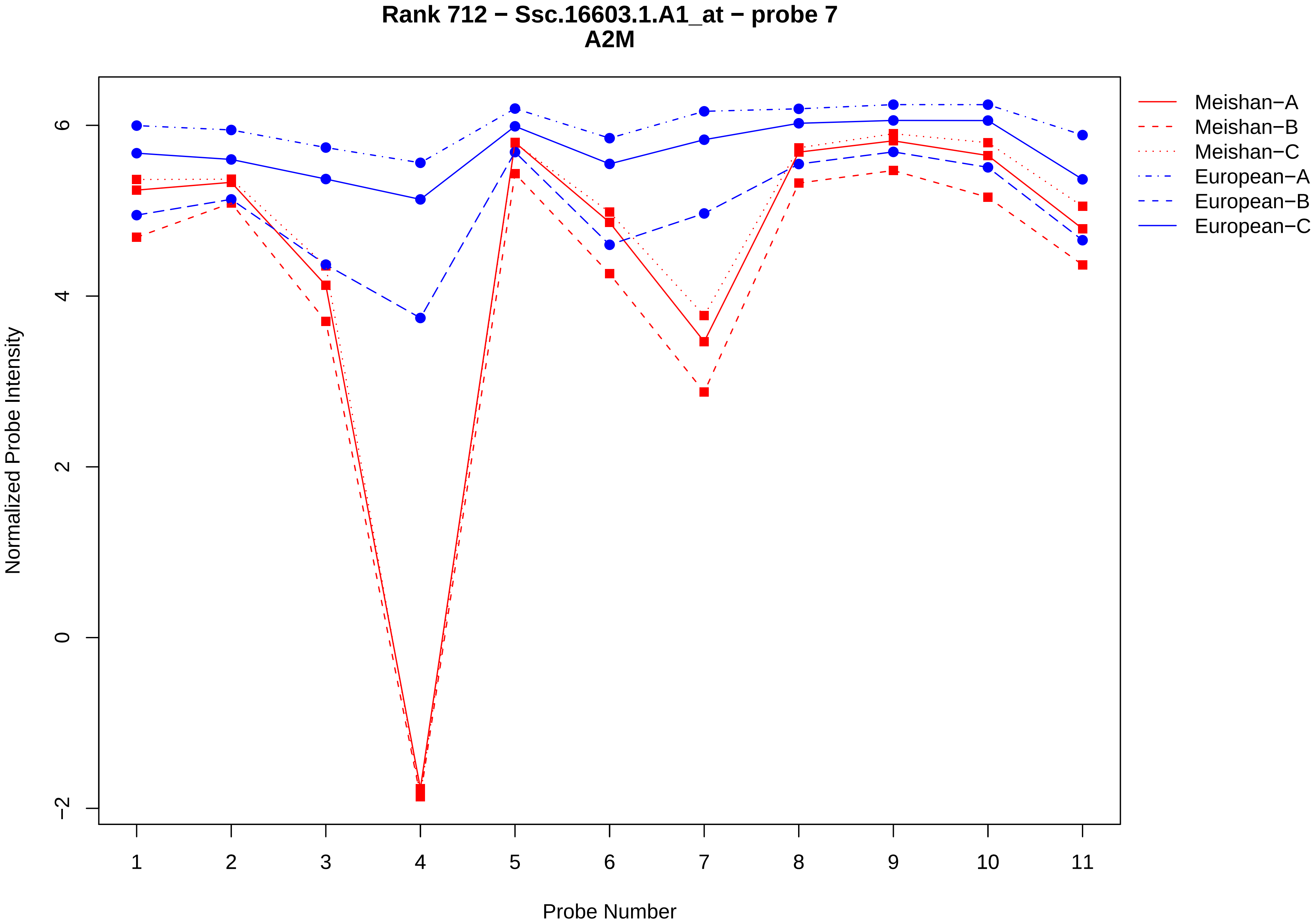

Rank 712 − Ssc.16603.1.A1_at − probe 7
A2M
Normalized Probe Intensity
Probe Number
−2
0
2
4
6
1
2
3
4
5
6
7
8
9
10
11
Meishan−A
Meishan−B
Meishan−C
European−A
European−B
European−C

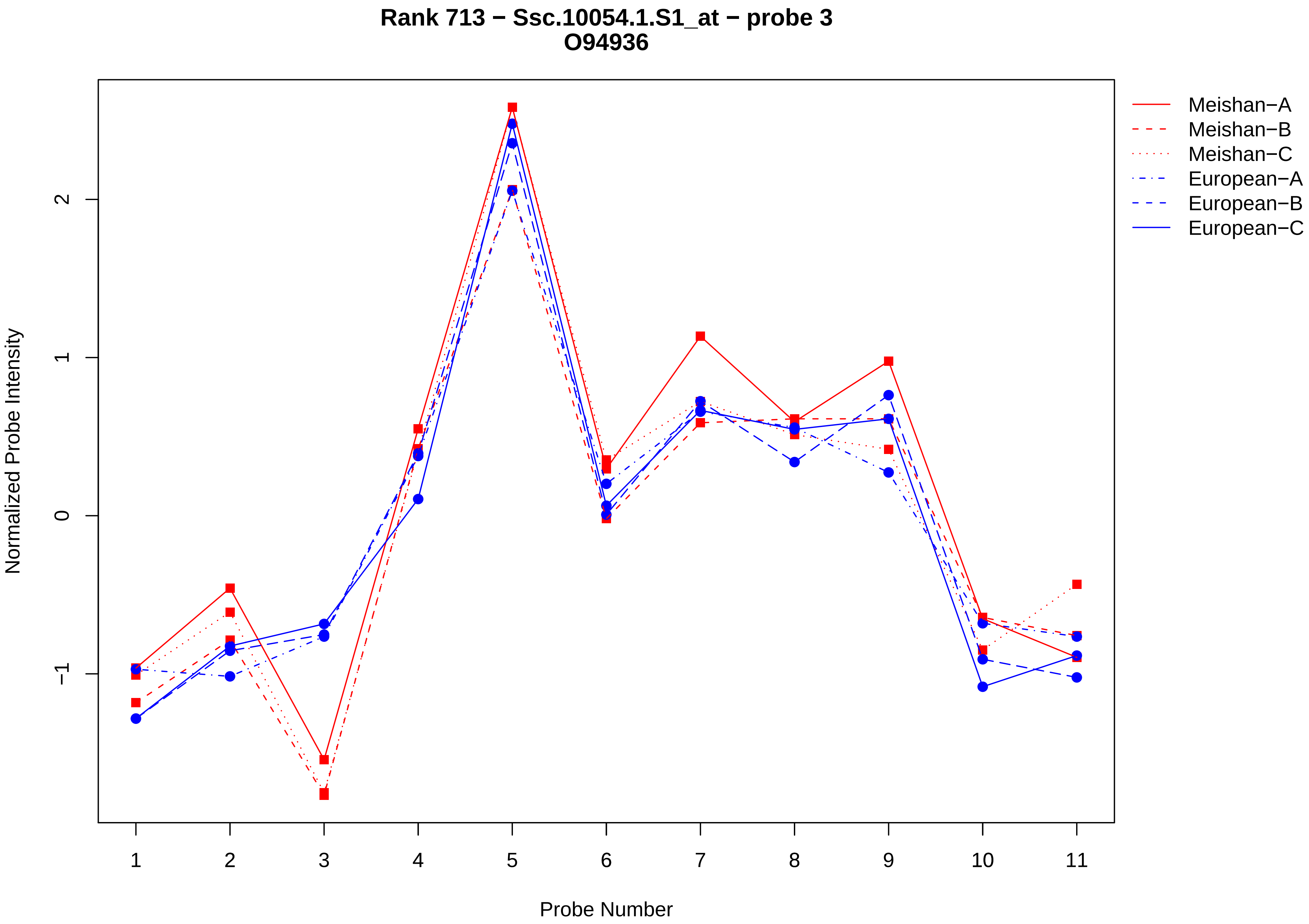
Rank 713 − Ssc.10054.1.S1_at − probe 3
O94936
Normalized Probe Intensity
Probe Number
Meishan−A
Meishan−B
Meishan−C
European−A
European−B
European−C
−1
0
1
2
1
2
3
4
5
6
7
8
9
10
11

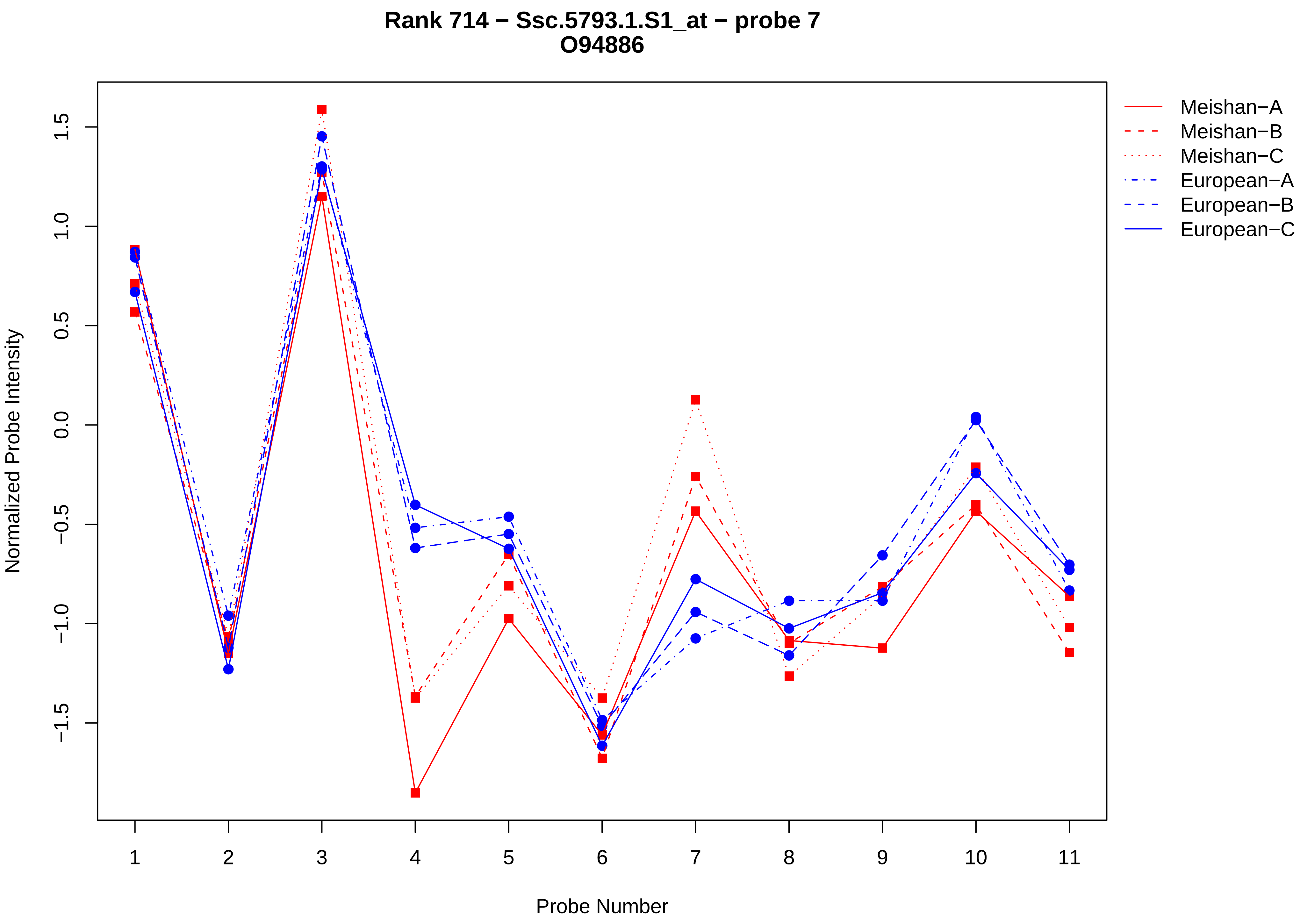

Rank 714 − Ssc.5793.1.S1_at − probe 7
O94886
Normalized Probe Intensity
Probe Number
Meishan−A
Meishan−B
Meishan−C
European−A
European−B
European−C
−1.5
−1.0
−0.5
0.0
0.5
1.0
1.5
1
2
3
4
5
6
7
8
9
10
11

Rank 715 − Ssc.29318.1.A1_at − probe 8
MTMR1

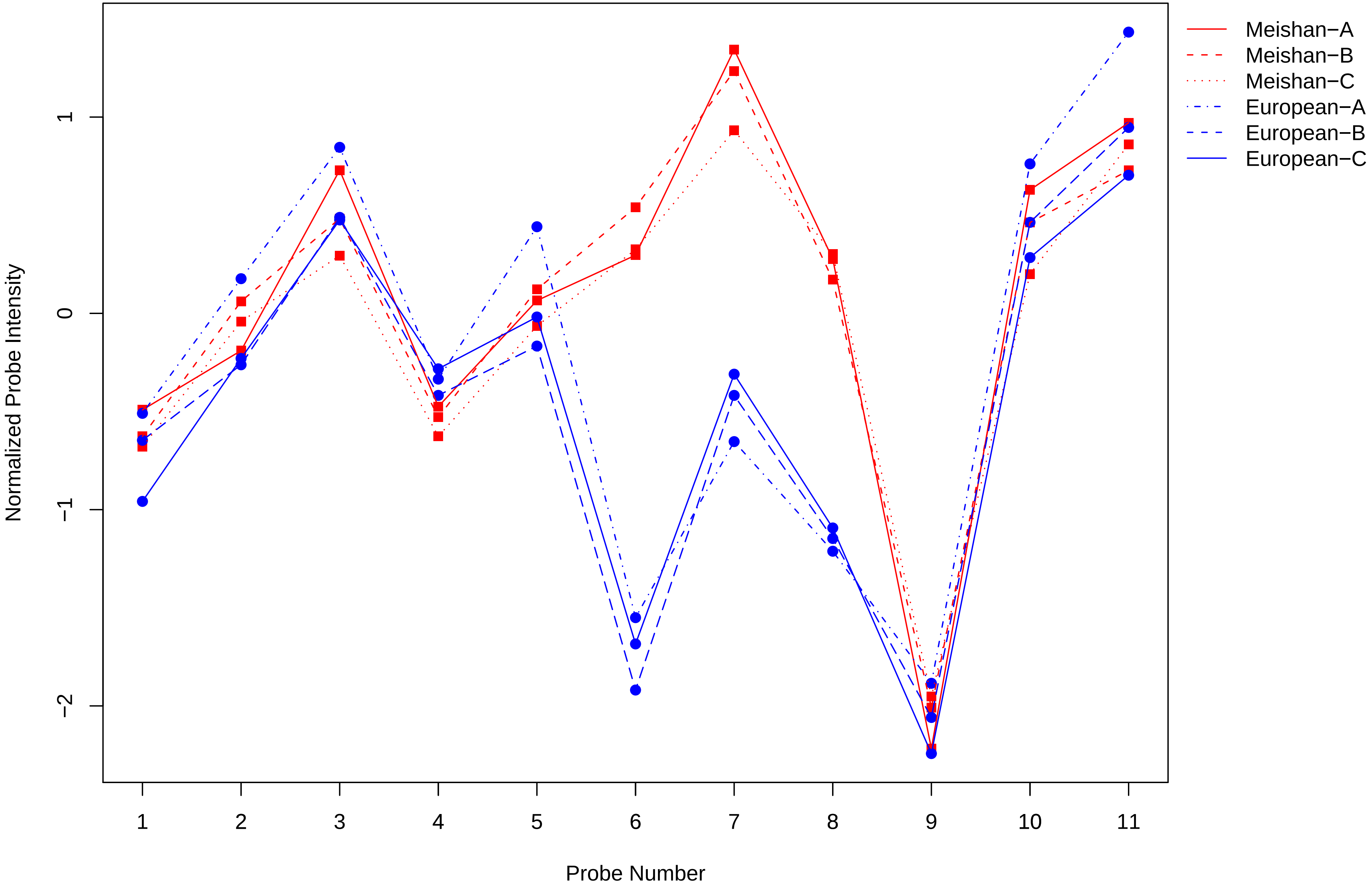

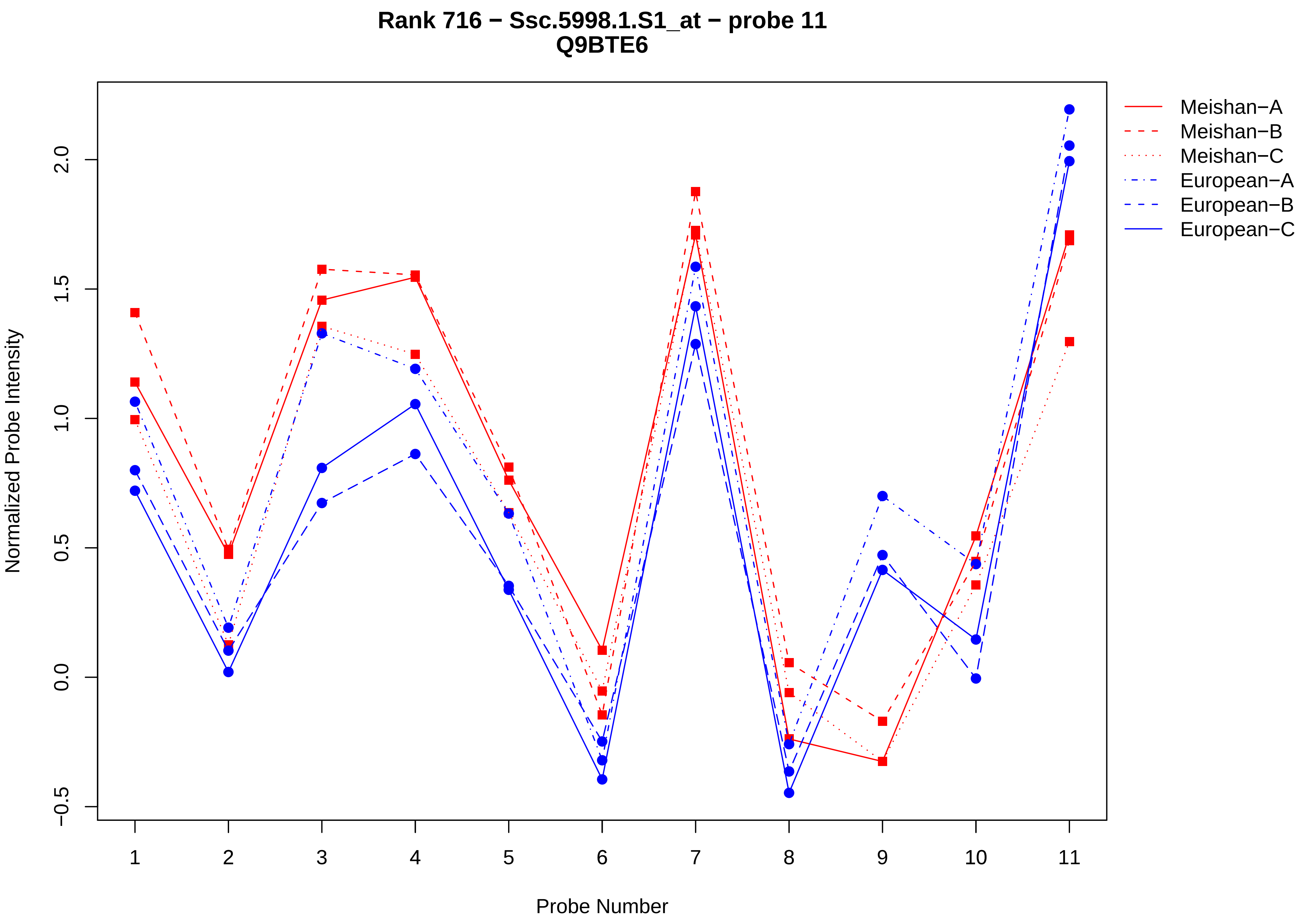

Rank 716 − Ssc.5998.1.S1_at − probe 11
Q9BTE6
Normalized Probe Intensity
Probe Number
−0.5
0.0
0.5
1.0
1.5
2.0
1
2
3
4
5
6
7
8
9
10
11
Meishan−A
Meishan−B
Meishan−C
European−A
European−B
European−C

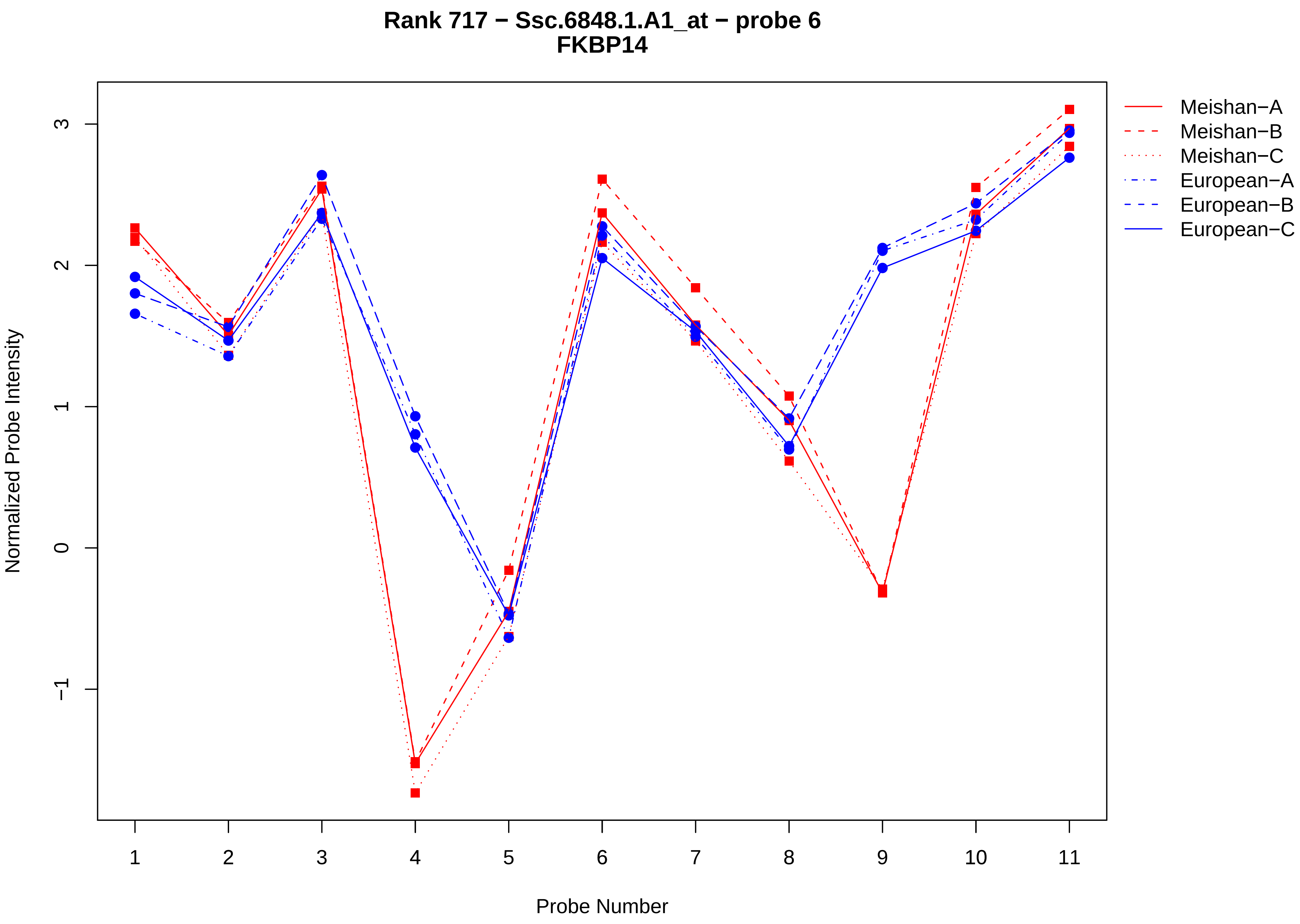

Rank 717 − Ssc.6848.1.A1_at − probe 6
FKBP14
Normalized Probe Intensity
Probe Number
Meishan−A
Meishan−B
Meishan−C
European−A
European−B
European−C
−1
0
1
2
3
1
2
3
4
5
6
7
8
9
10
11

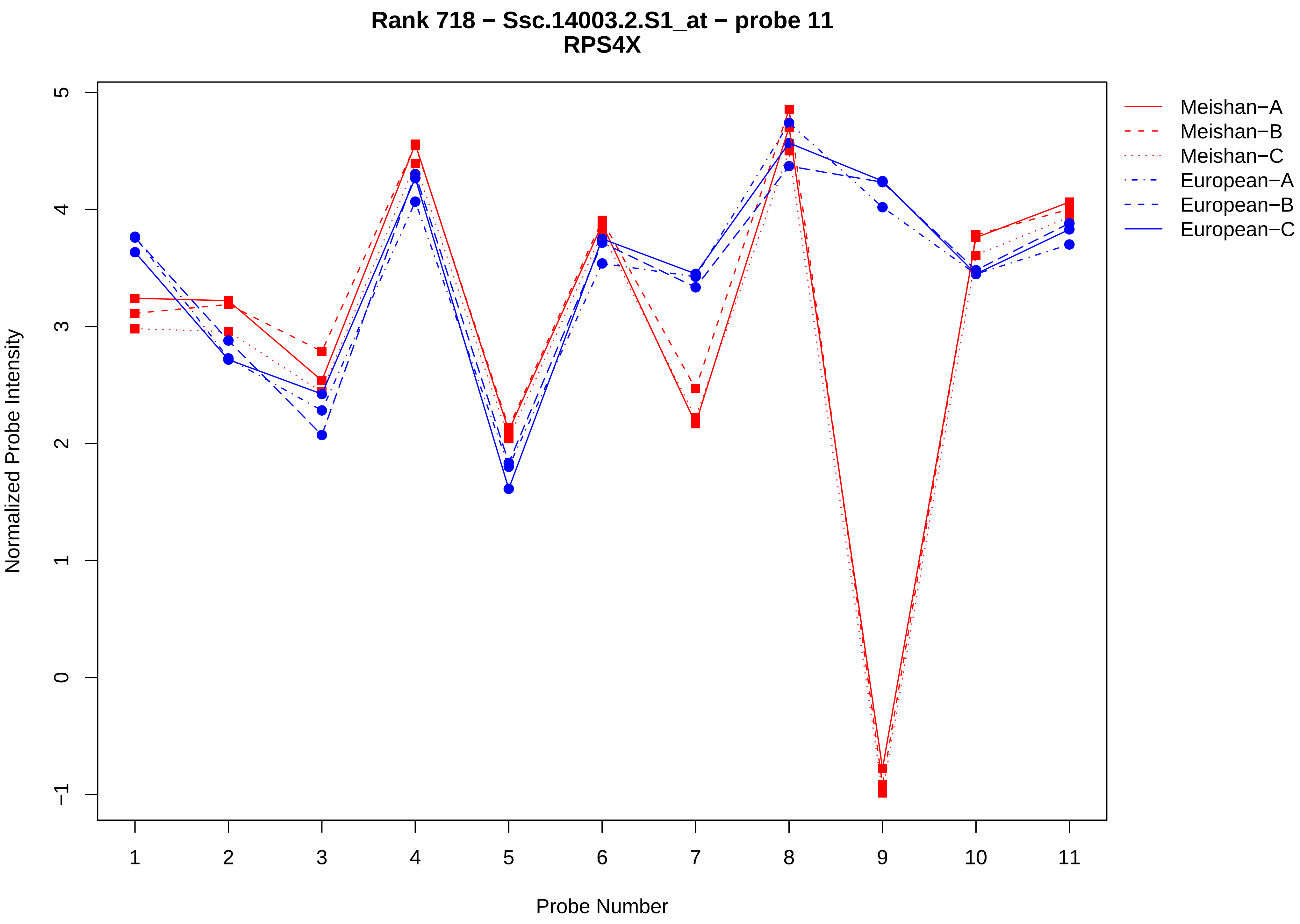

Rank 718 − Ssc.14003.2.S1_at − probe 11
RPS4X
Normalized Probe Intensity
Probe Number
Meishan−A
Meishan−B
Meishan−C
European−A
European−B
European−C
−1
0
1
2
3
4
5
1
2
3
4
5
6
7
8
9
10
11

Rank 719 − Ssc.1742.1.S1_at − probe 11
Q15453

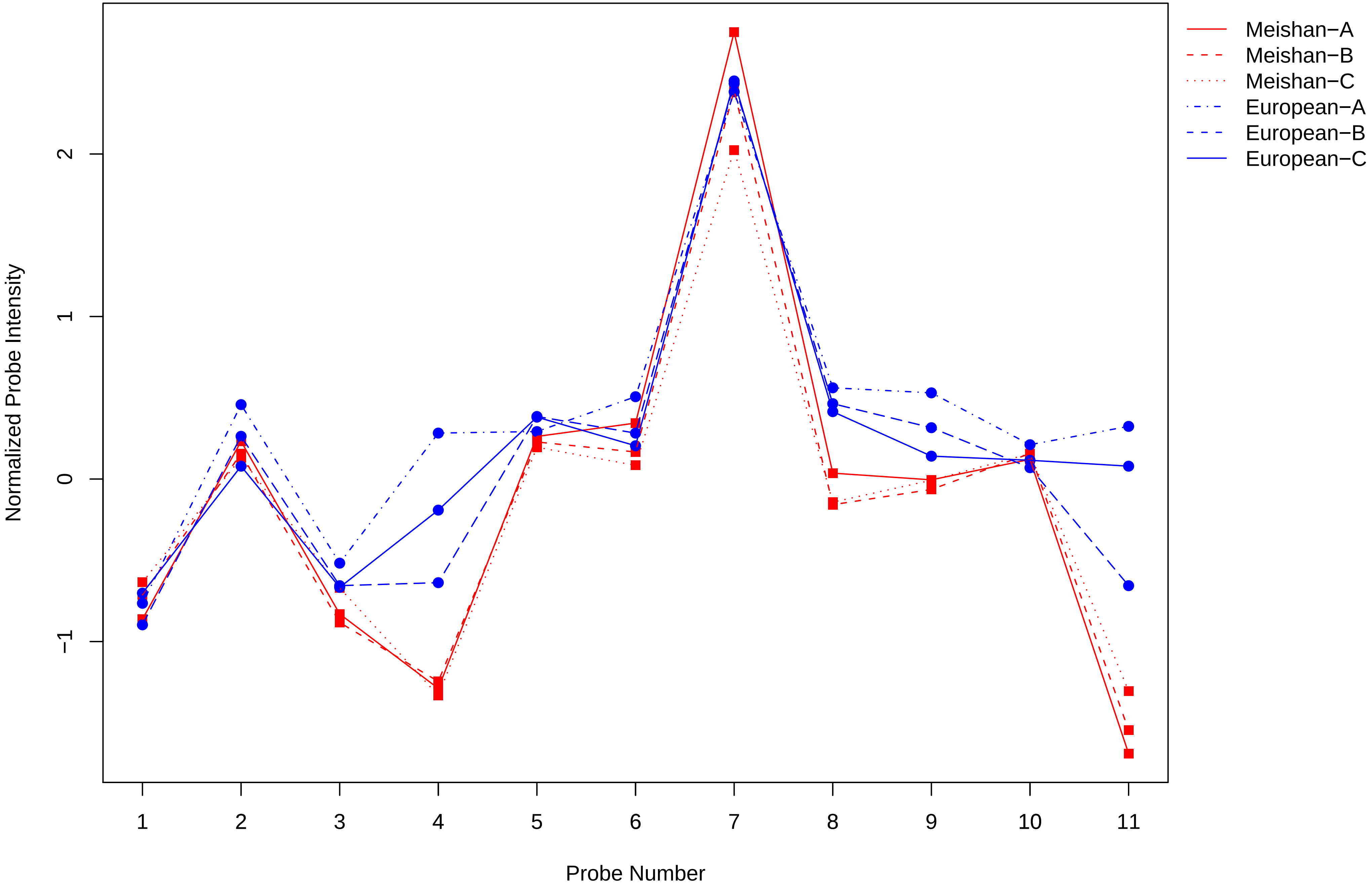

Rank 720 − Ssc.23883.1.A1_at − probe 8
Q8N4K7

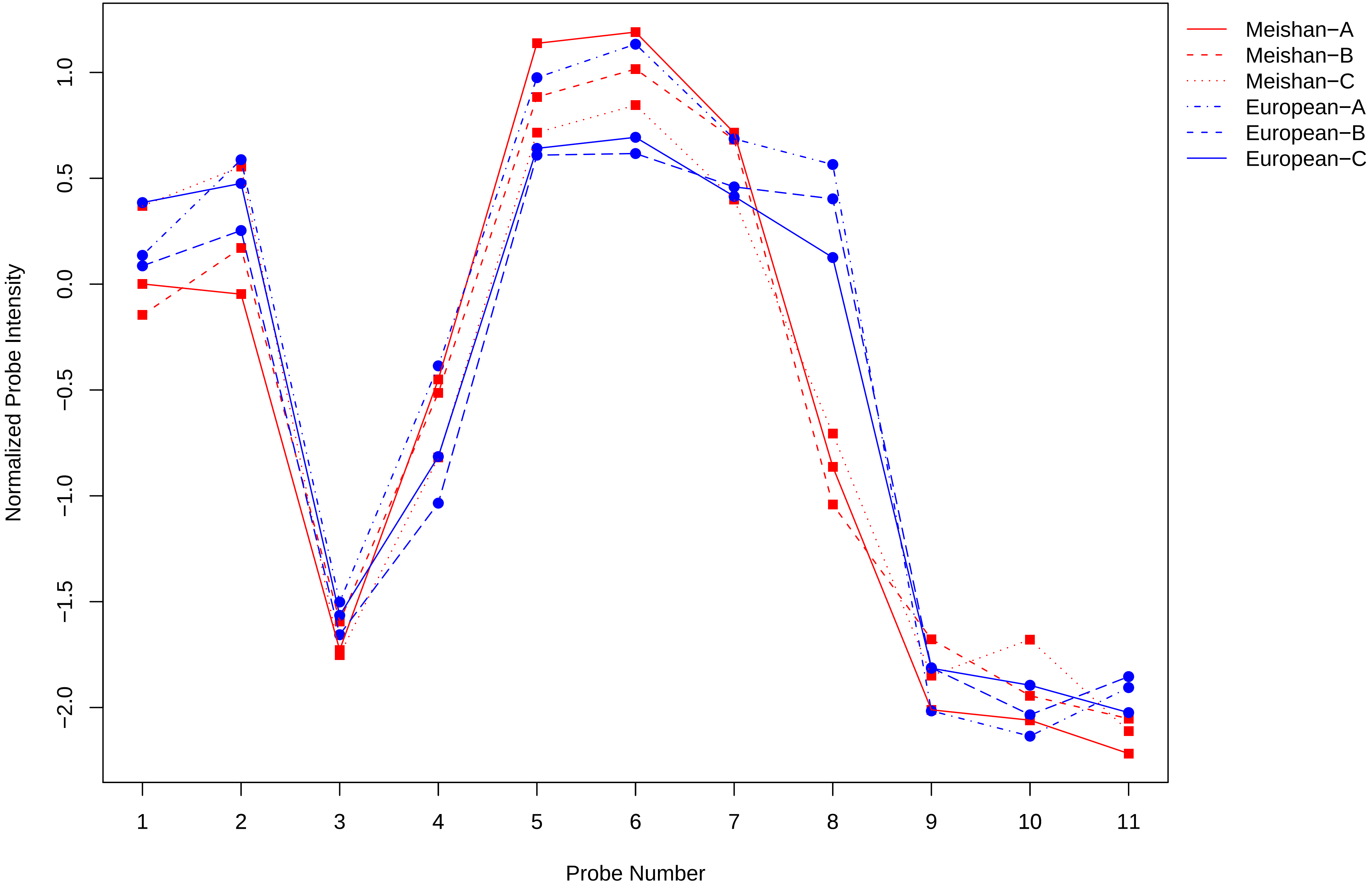

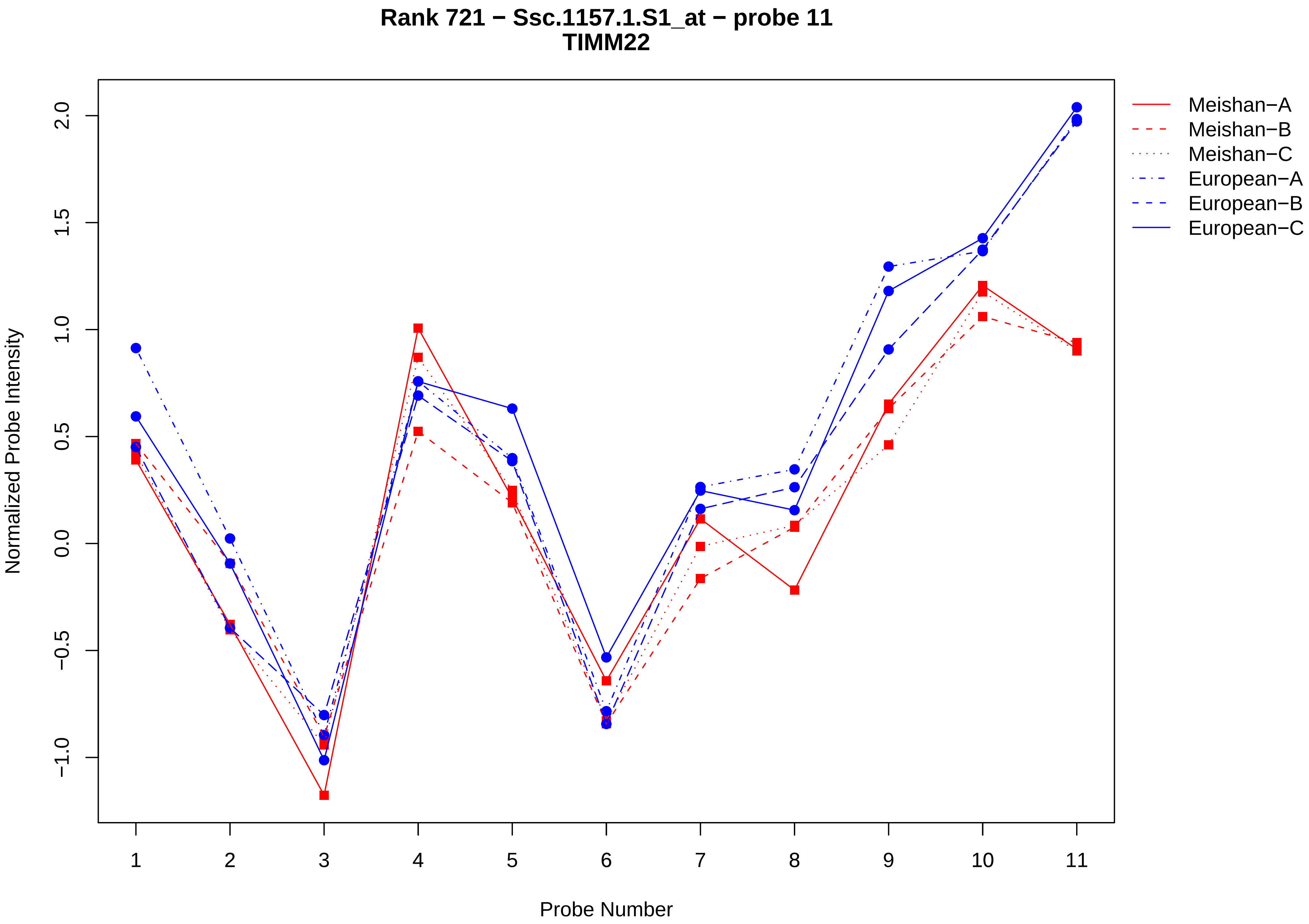
Rank 721 − Ssc.1157.1.S1_at − probe 11
TIMM22
Normalized Probe Intensity
Probe Number
−1.0
−0.5
0.0
0.5
1.0
1.5
2.0
1
2
3
4
5
6
7
8
9
10
11
Meishan−A
Meishan−B
Meishan−C
European−A
European−B
European−C

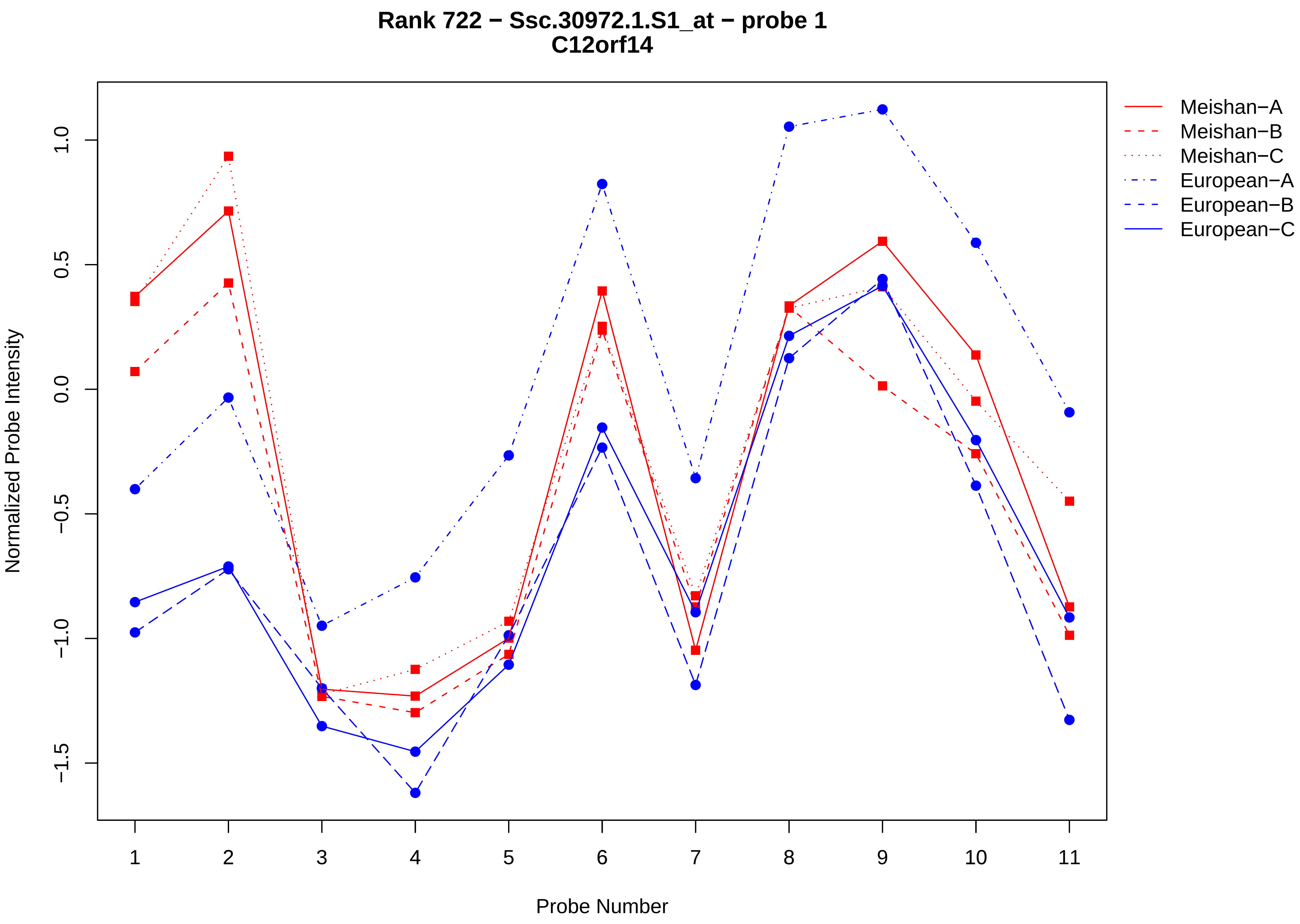

Rank 722 − Ssc.30972.1.S1_at − probe 1
C12orf14
Normalized Probe Intensity
Probe Number
1.0
0.5
0.0
−0.5
−1.0
−1.5
1
2
3
4
5
6
7
8
9
10
11
Meishan−A
Meishan−B
Meishan−C
European−A
European−B
European−C

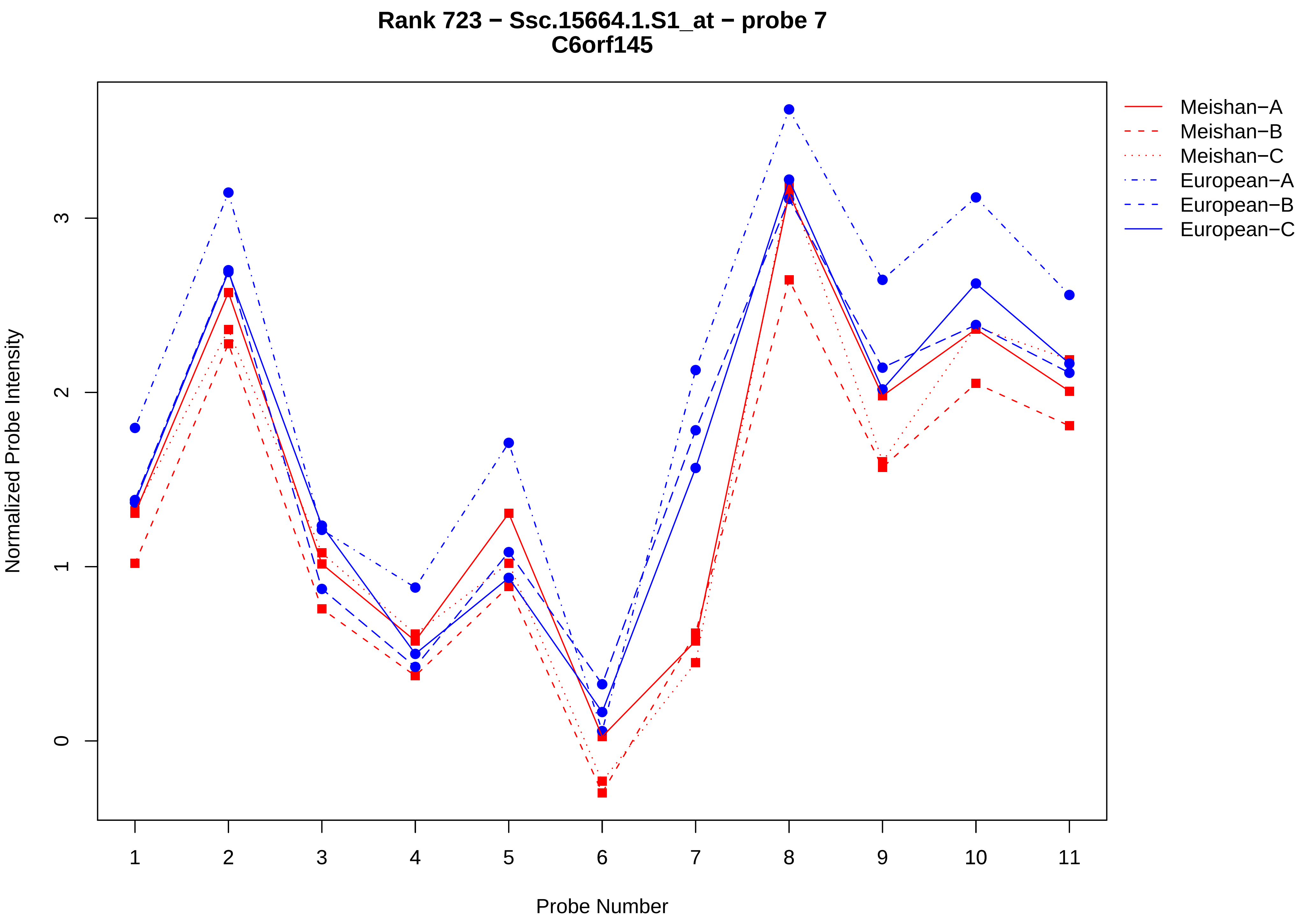

Rank 723 − Ssc.15664.1.S1_at − probe 7
C6orf145
Normalized Probe Intensity
Probe Number
0
1
2
3
1
2
3
4
5
6
7
8
9
10
11
Meishan−A
Meishan−B
Meishan−C
European−A
European−B
European−C

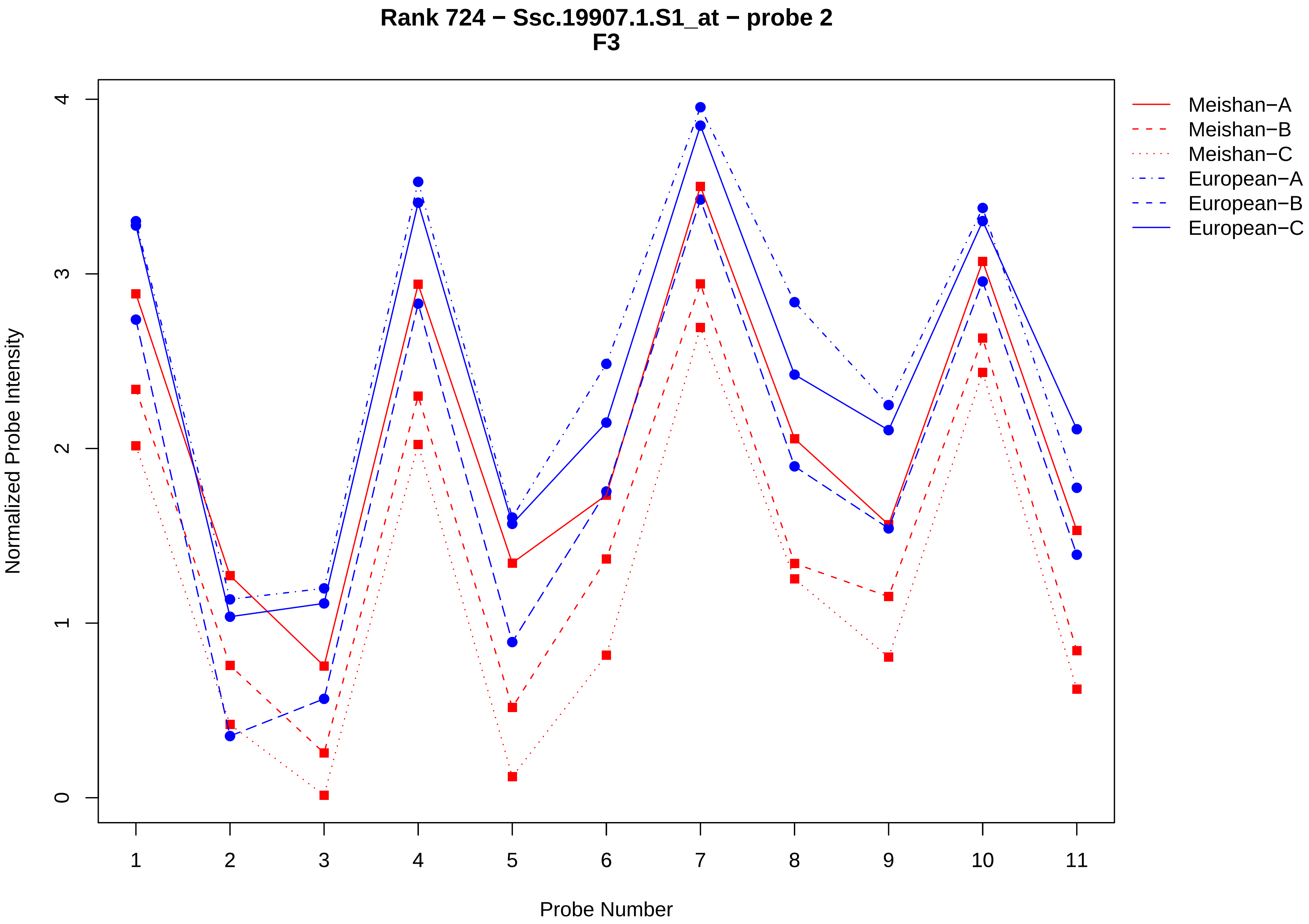
Rank 724 − Ssc.19907.1.S1_at − probe 2
F3
Normalized Probe Intensity
Probe Number
0
1
2
3
4
1
2
3
4
5
6
7
8
9
10
11
Meishan−A
Meishan−B
Meishan−C
European−A
European−B
European−C

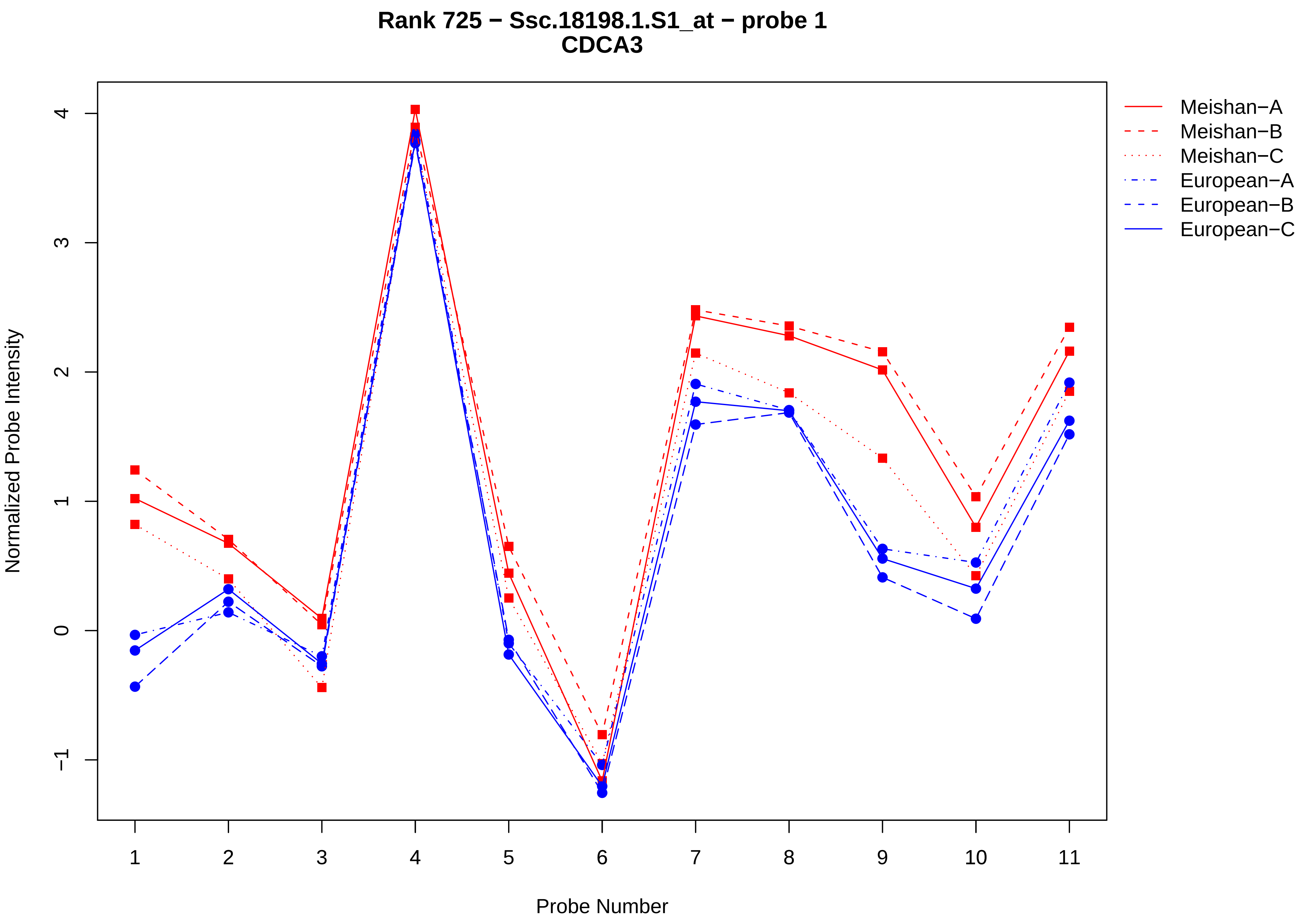
Rank 725 − Ssc.18198.1.S1_at − probe 1
CDCA3
Normalized Probe Intensity
Probe Number
−1
0
1
2
3
4
1
2
3
4
5
6
7
8
9
10
11
Meishan−A
Meishan−B
Meishan−C
European−A
European−B
European−C

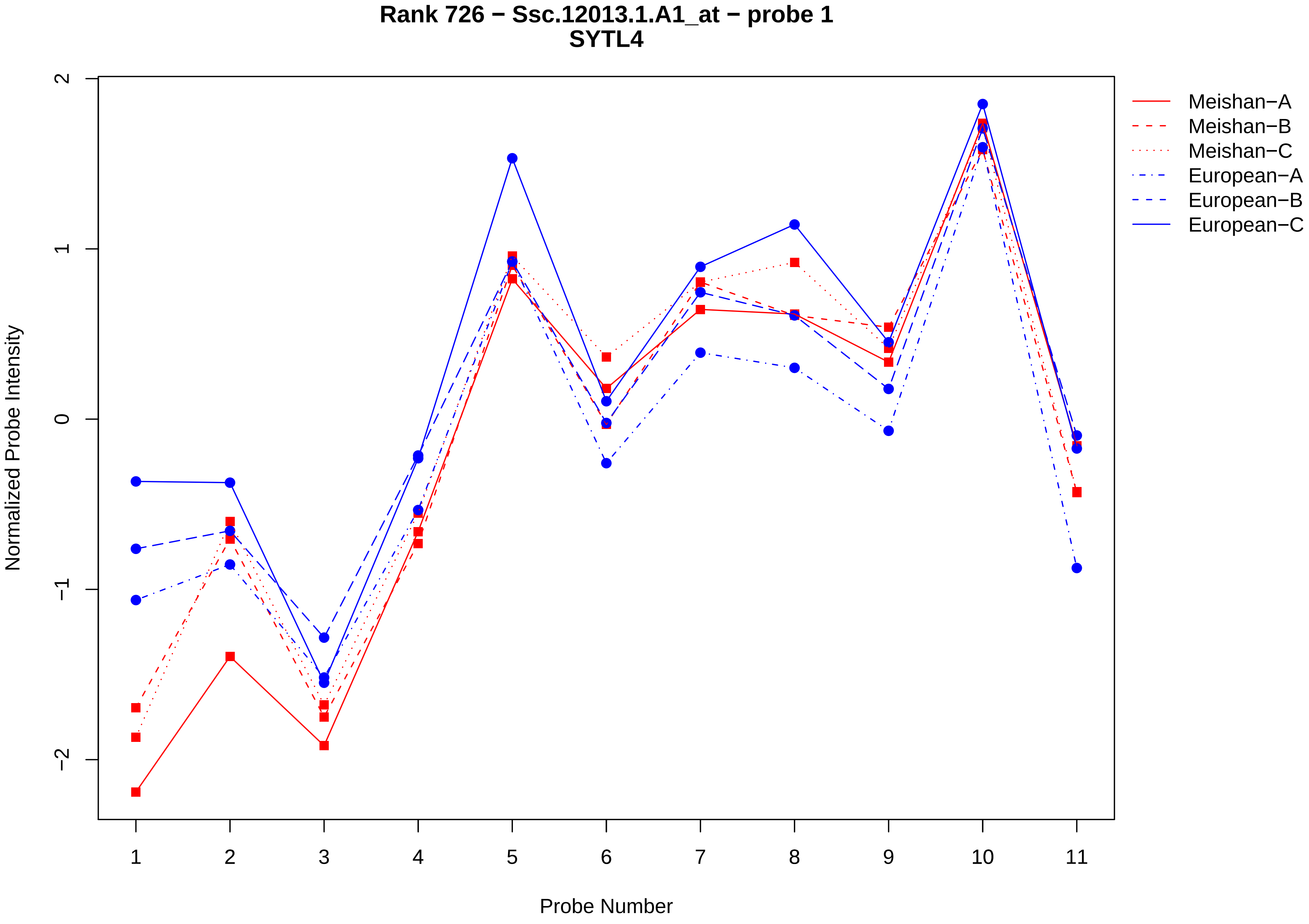

Rank 726 − Ssc.12013.1.A1_at − probe 1
SYTL4
Normalized Probe Intensity
Probe Number
2
1
0
−1
−2
1
2
3
4
5
6
7
8
9
10
11
Meishan−A
Meishan−B
Meishan−C
European−A
European−B
European−C

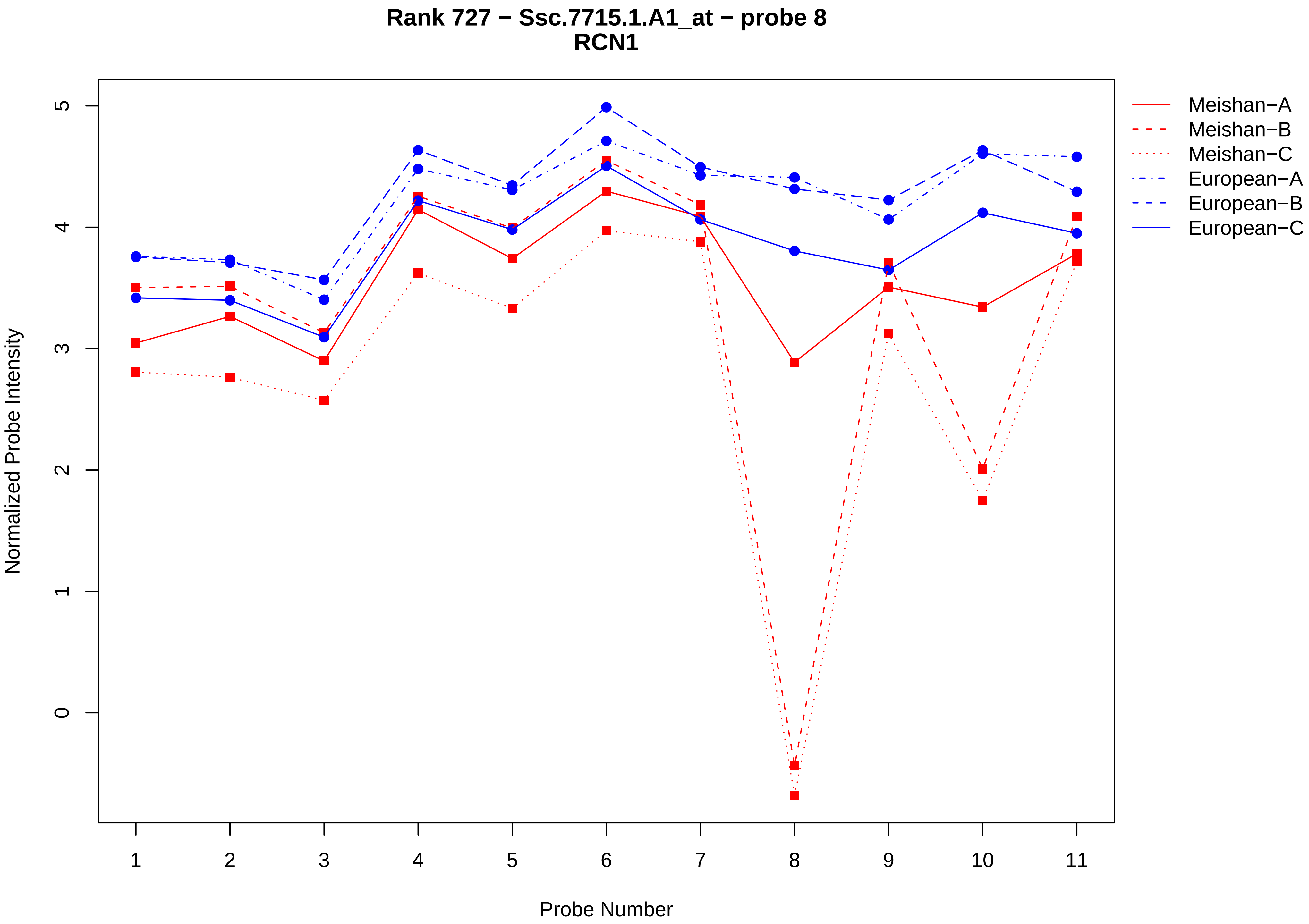

Rank 727 − Ssc.7715.1.A1_at − probe 8
RCN1
Normalized Probe Intensity
Probe Number
0
1
2
3
4
5
1
2
3
4
5
6
7
8
9
10
11
Meishan−A
Meishan−B
Meishan−C
European−A
European−B
European−C

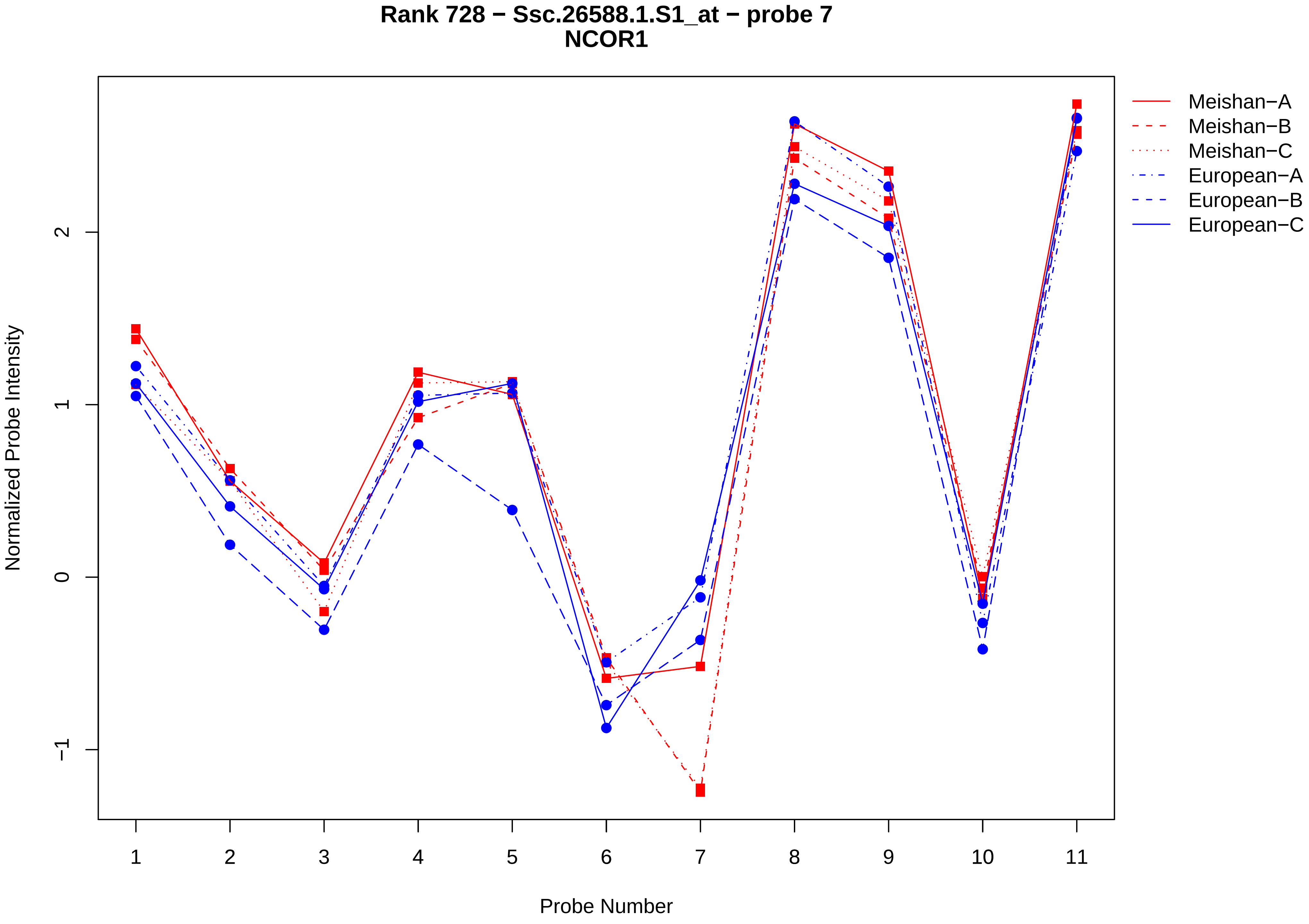

Rank 728 − Ssc.26588.1.S1_at − probe 7
NCOR1
Normalized Probe Intensity
Probe Number
−1
0
1
2
1
2
3
4
5
6
7
8
9
10
11
Meishan−A
Meishan−B
Meishan−C
European−A
European−B
European−C

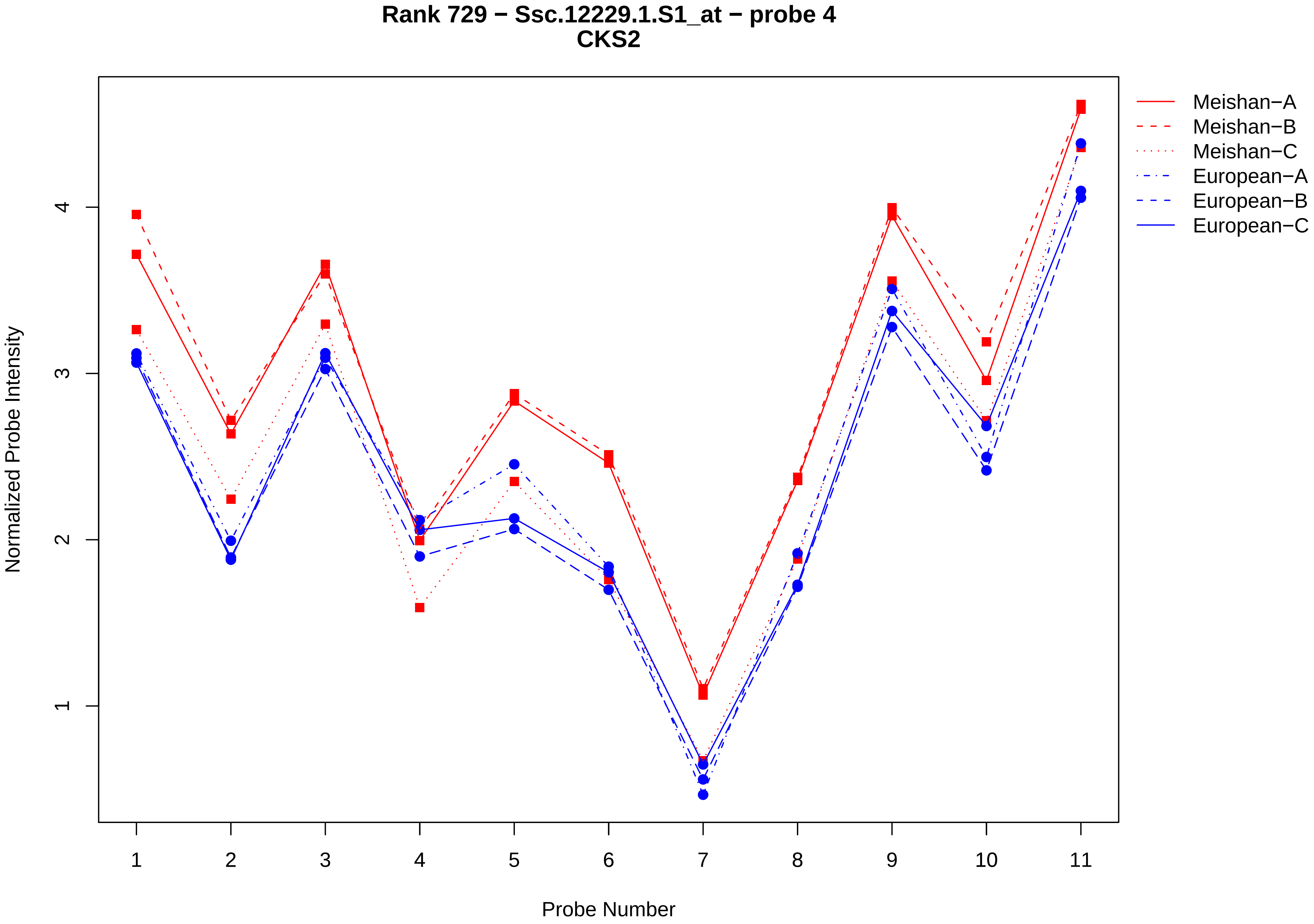
Rank 729 − Ssc.12229.1.S1_at − probe 4
CKS2
Normalized Probe Intensity
Probe Number
1
2
3
4
1
2
3
4
5
6
7
8
9
10
11
Meishan−A
Meishan−B
Meishan−C
European−A
European−B
European−C

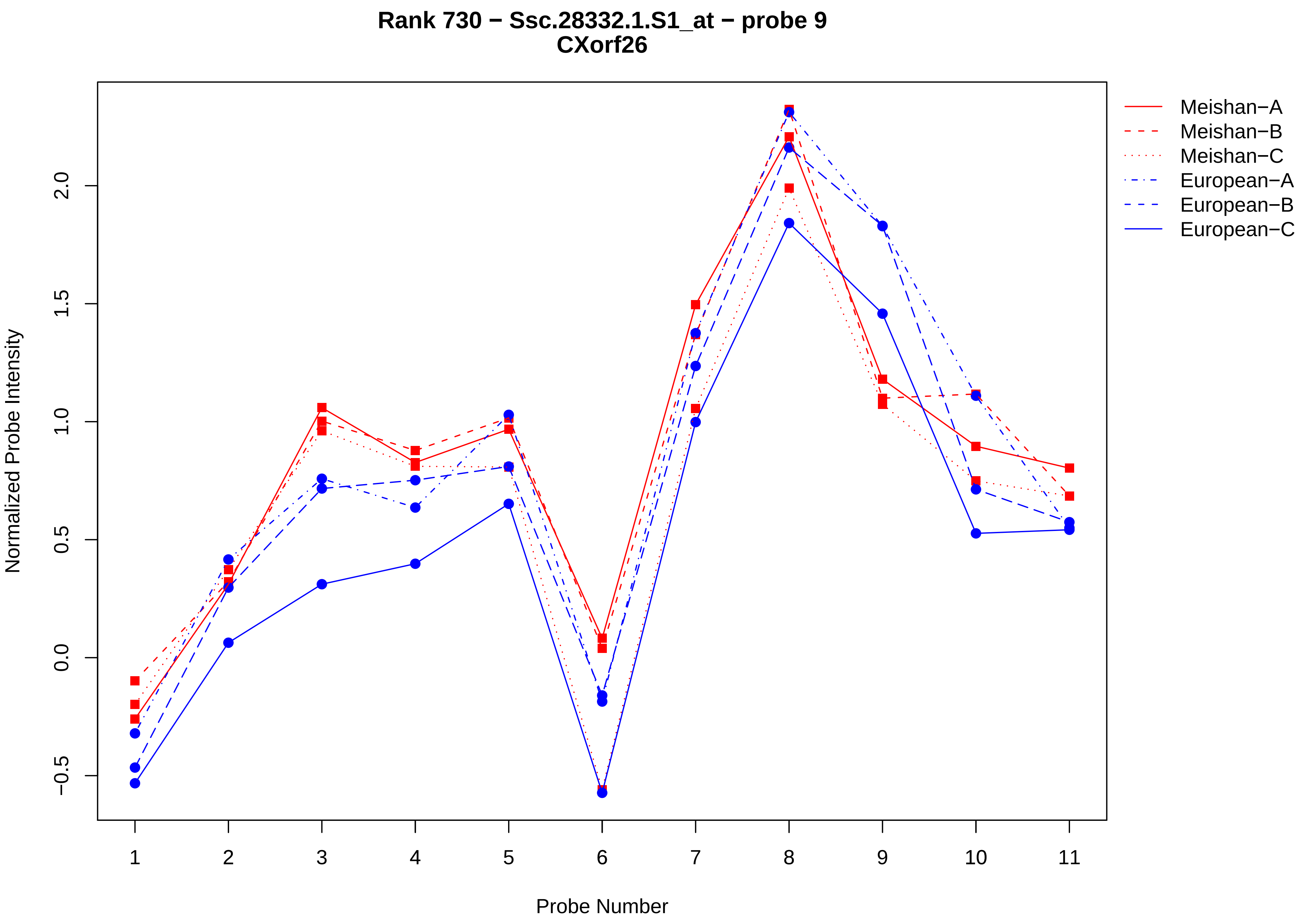

Rank 730 − Ssc.28332.1.S1_at − probe 9
CXorf26
Normalized Probe Intensity
Probe Number
−0.5
0.0
0.5
1.0
1.5
2.0
1
2
3
4
5
6
7
8
9
10
11
Meishan−A
Meishan−B
Meishan−C
European−A
European−B
European−C

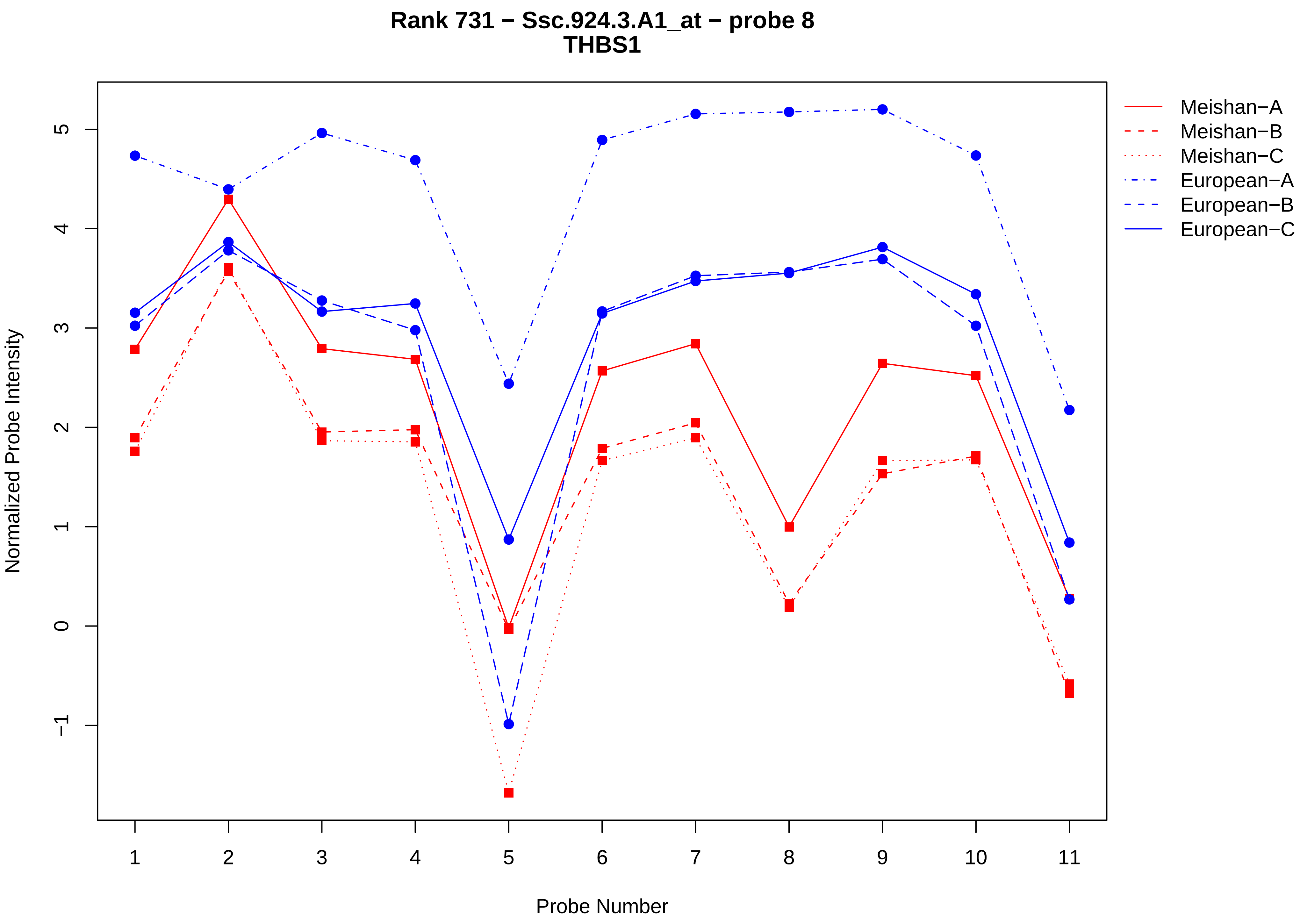

Rank 731 − Ssc.924.3.A1_at − probe 8
THBS1
Normalized Probe Intensity
Probe Number
Meishan−A
Meishan−B
Meishan−C
European−A
European−B
European−C

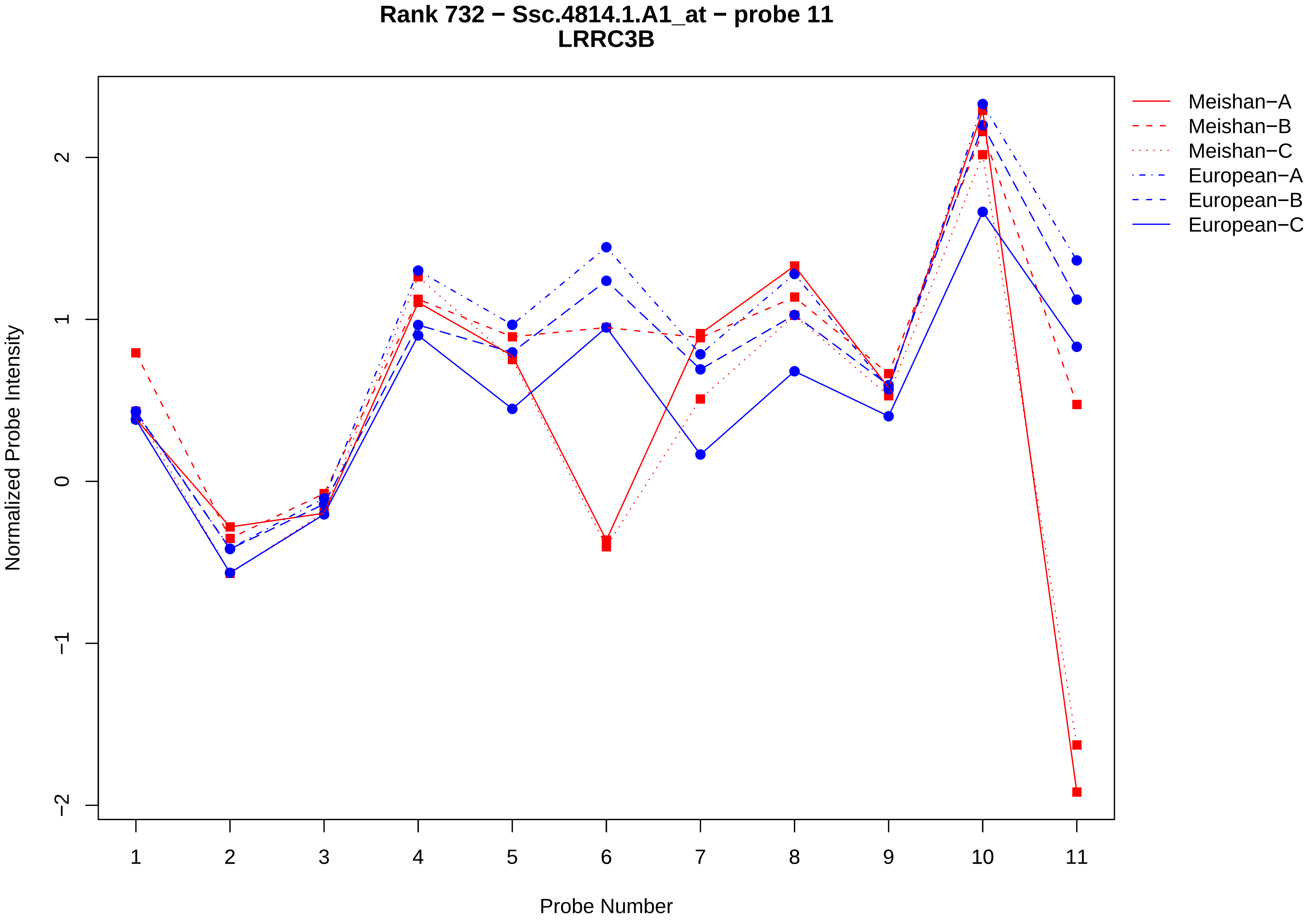
Rank 732 − Ssc.4814.1.A1_at − probe 11
LRRC3B
Meishan−A
Meishan−B
Meishan−C
European−A
European−B
European−C
Normalized Probe Intensity
Probe Number
−2
−1
0
1
2
1
2
3
4
5
6
7
8
9
10
11

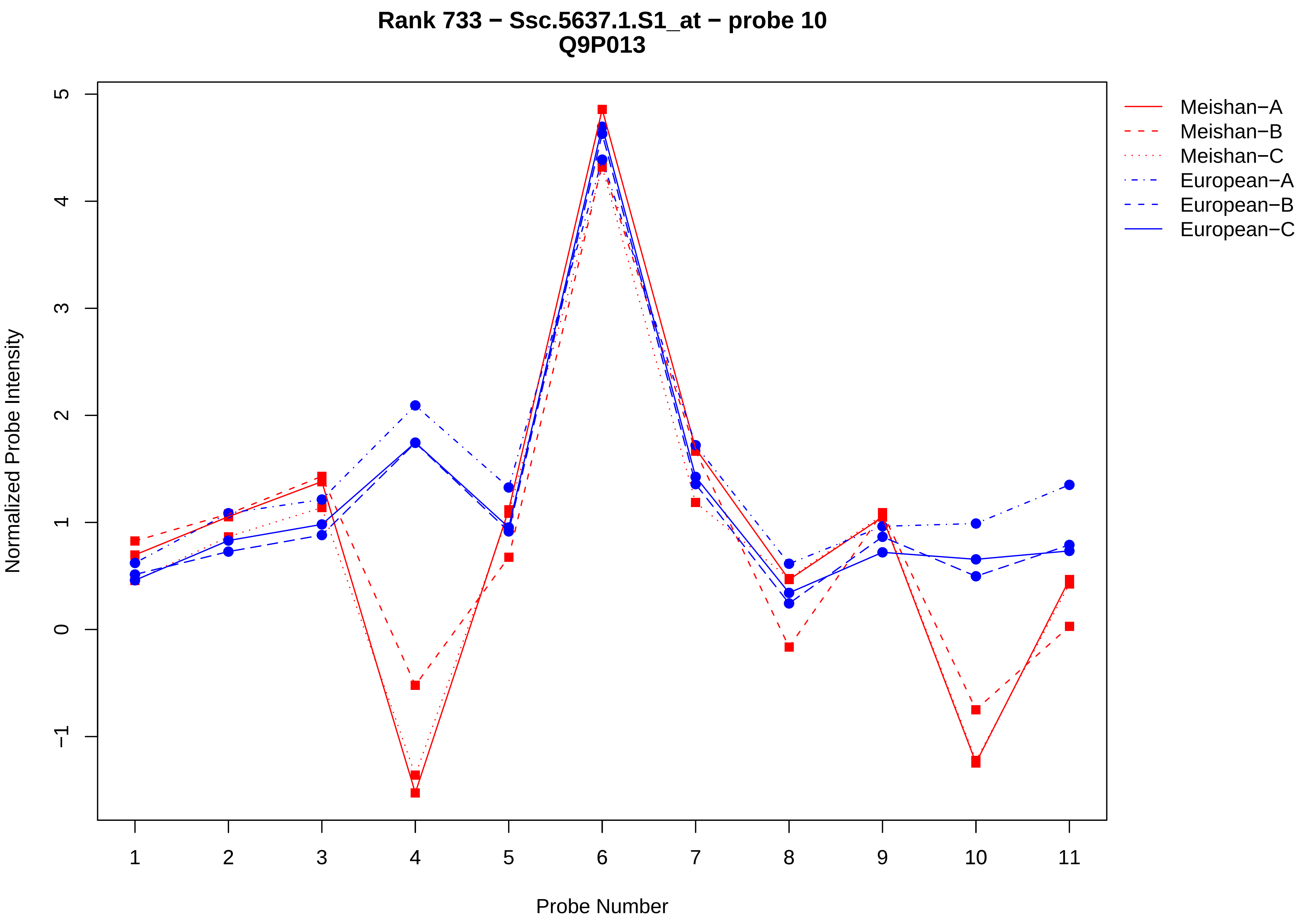

Rank 733 − Ssc.5637.1.S1_at − probe 10
Q9P013
Normalized Probe Intensity
Probe Number
Meishan−A
Meishan−B
Meishan−C
European−A
European−B
European−C

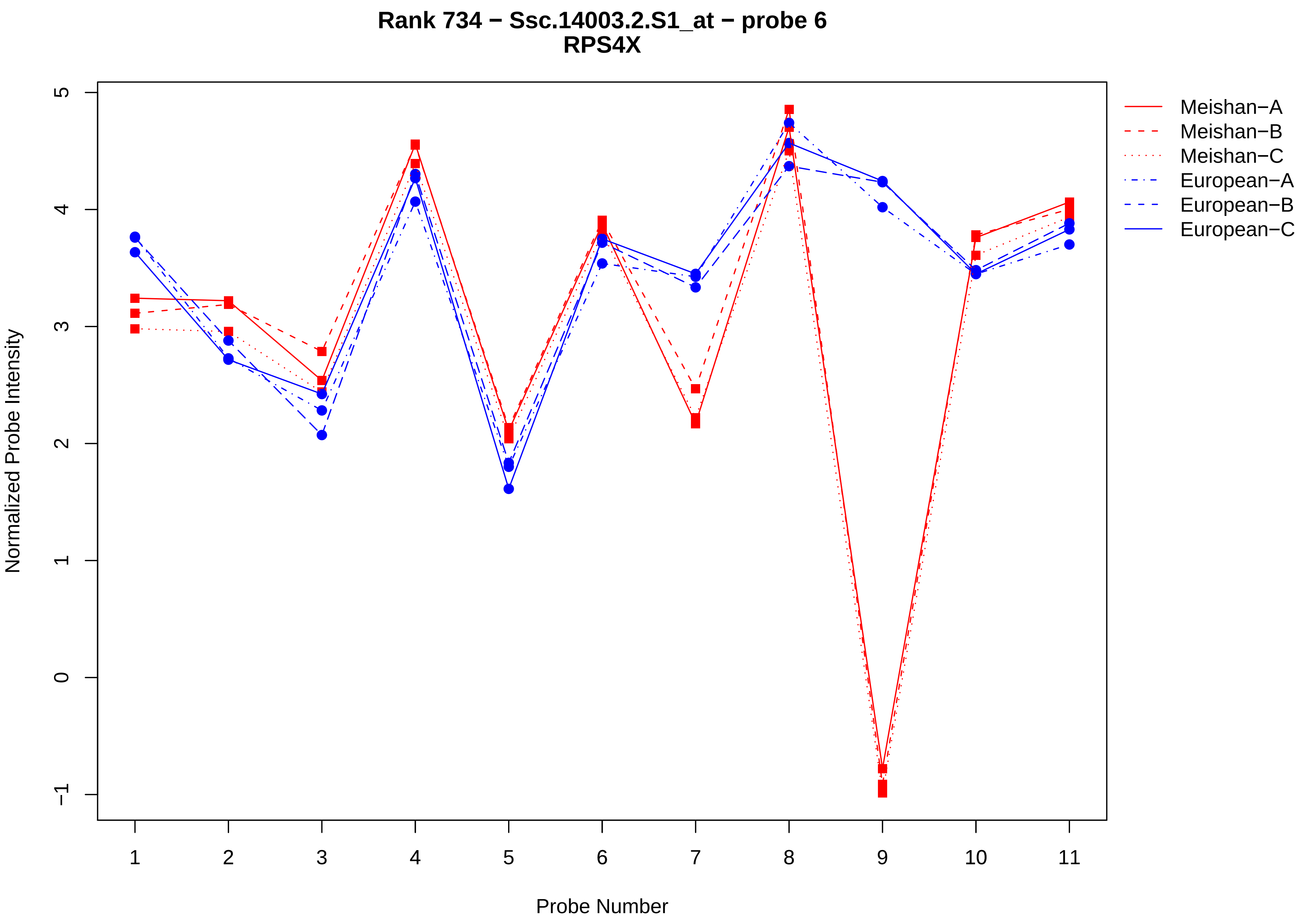

Rank 734 − Ssc.14003.2.S1_at − probe 6
RPS4X
Normalized Probe Intensity
Probe Number
Meishan−A
Meishan−B
Meishan−C
European−A
European−B
European−C
−1
0
1
2
3
4
5
1
2
3
4
5
6
7
8
9
10
11

Rank 735 − Ssc.9673.1.A1_at − probe 2
CASP2

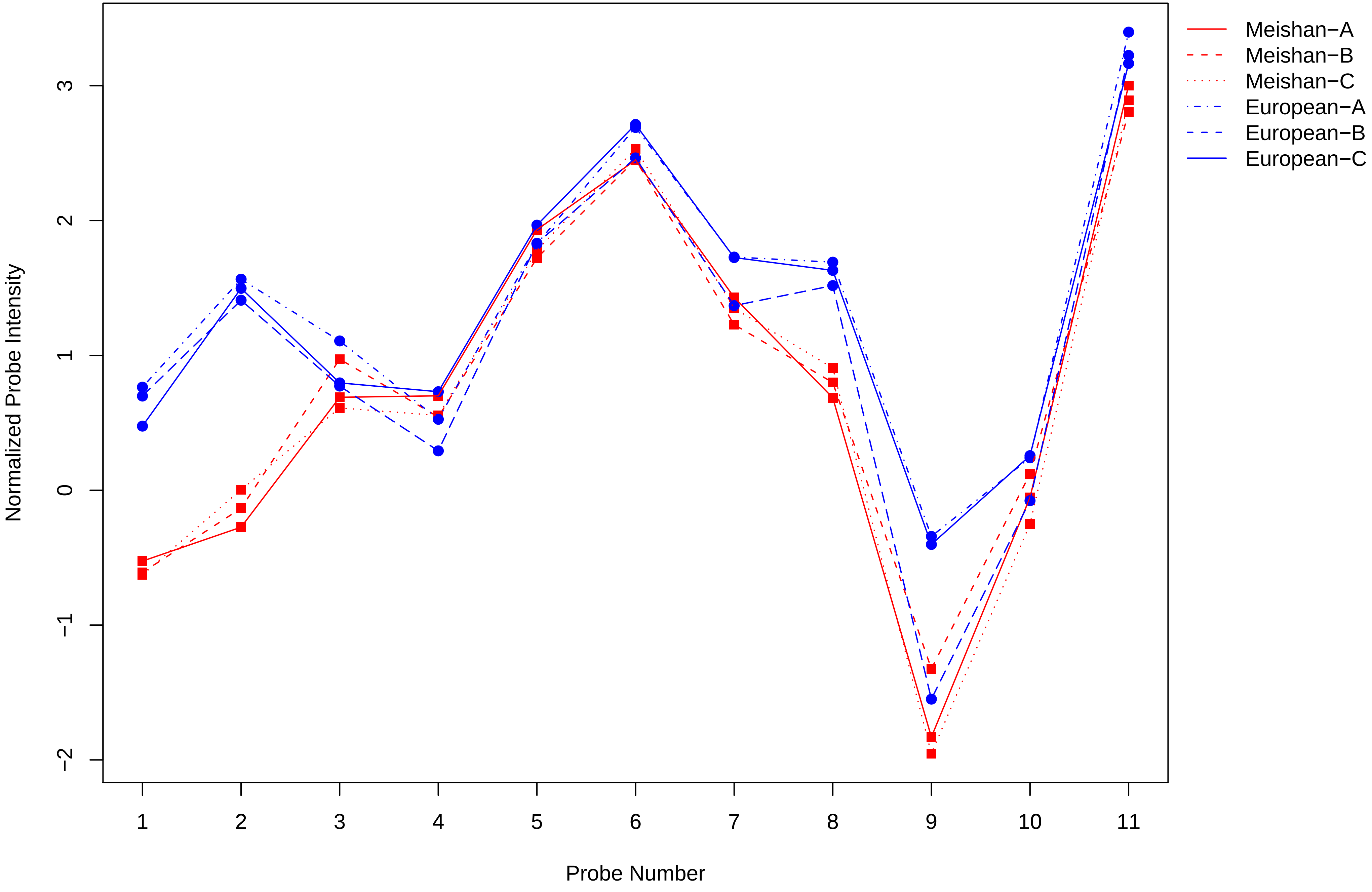

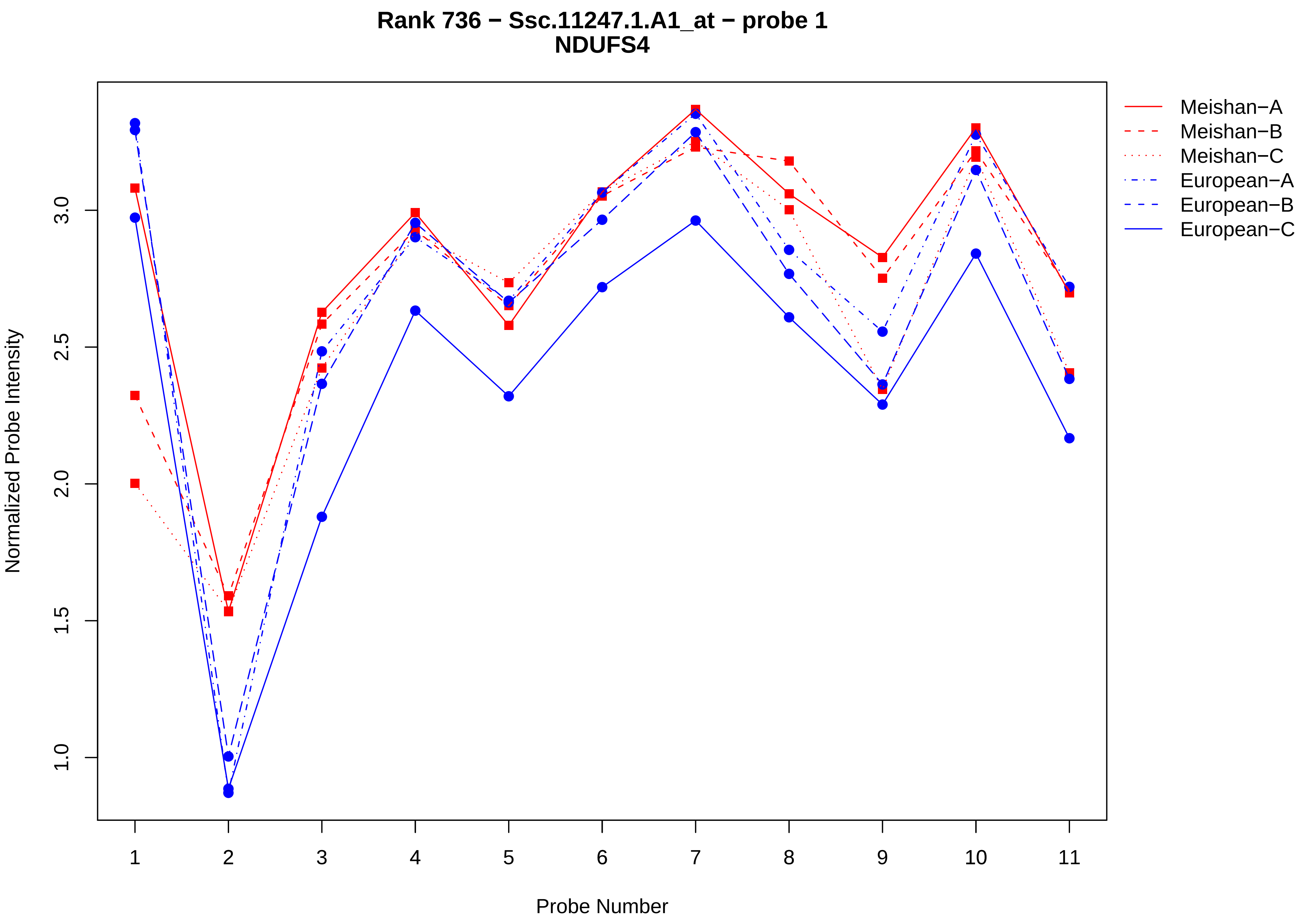

Rank 736 − Ssc.11247.1.A1_at − probe 1
NDUFS4
Normalized Probe Intensity
Probe Number
1.0
1.5
2.0
2.5
3.0
1
2
3
4
5
6
7
8
9
10
11
Meishan−A
Meishan−B
Meishan−C
European−A
European−B
European−C

Rank 737 − Ssc.4035.2.S1_at − probe 9
C10orf68

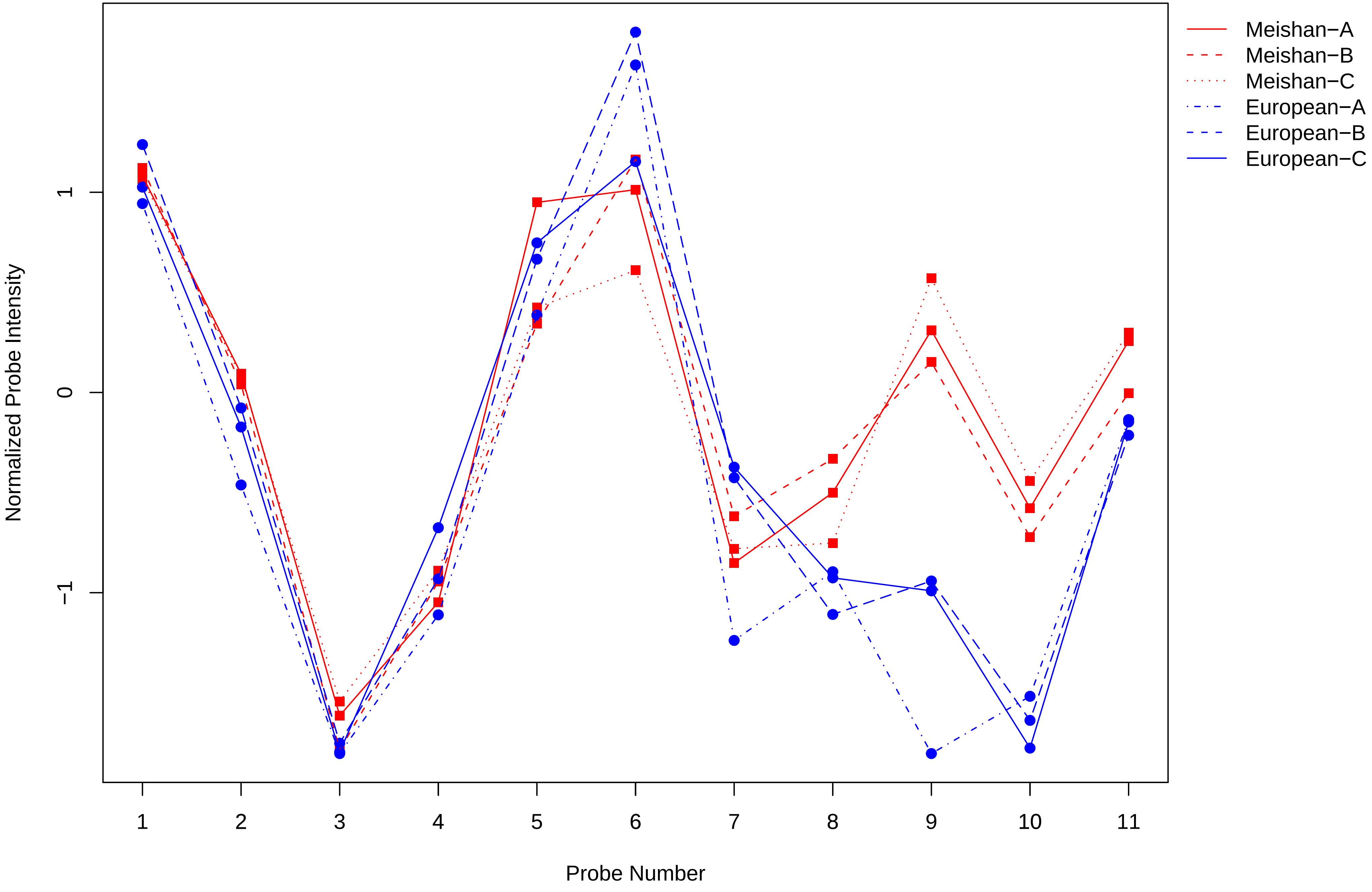

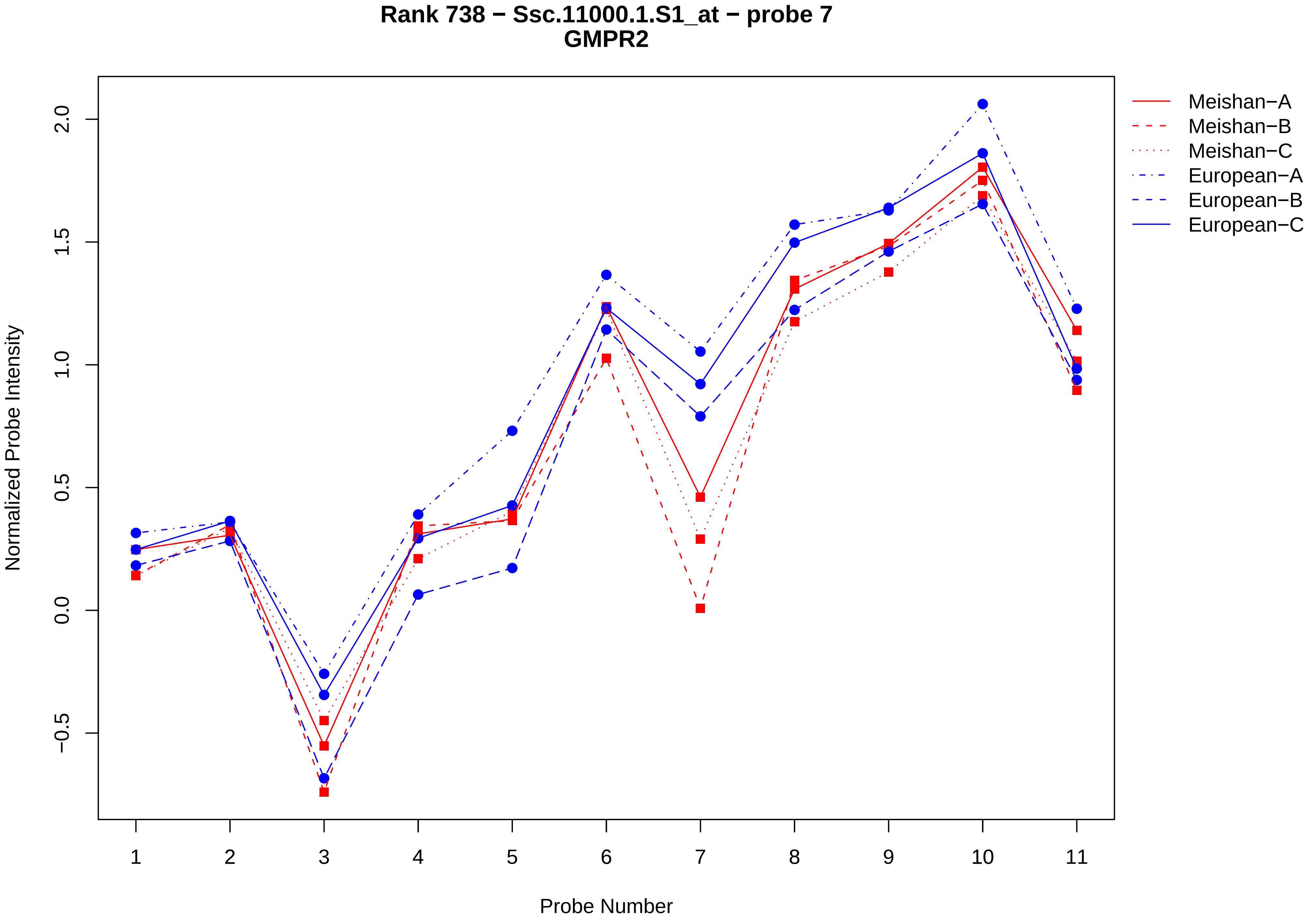
Rank 738 − Ssc.11000.1.S1_at − probe 7
GMPR2
Normalized Probe Intensity
Probe Number
−0.5
0.0
0.5
1.0
1.5
2.0
1
2
3
4
5
6
7
8
9
10
11
Meishan−A
Meishan−B
Meishan−C
European−A
European−B
European−C

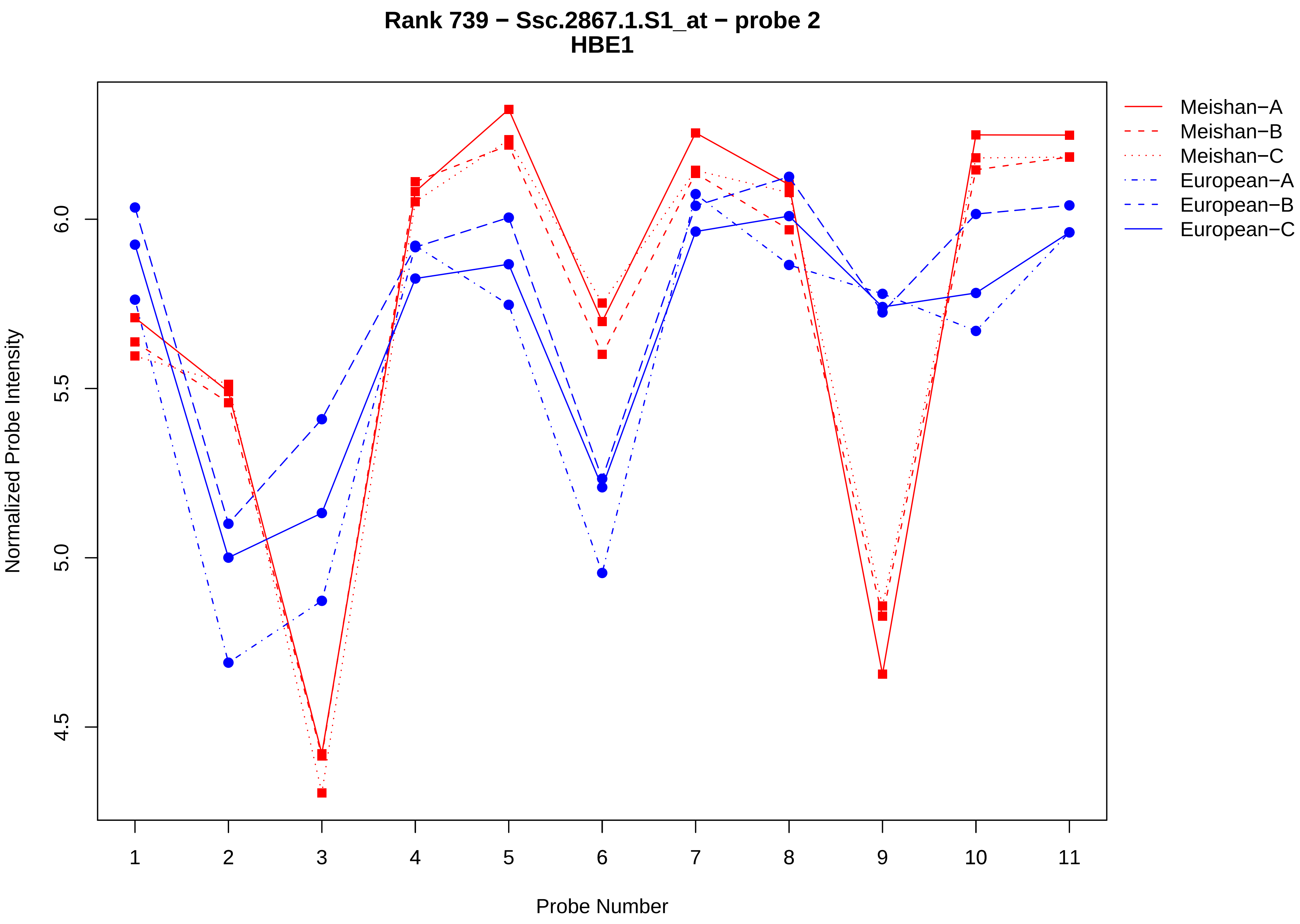

Rank 739 − Ssc.2867.1.S1_at − probe 2
HBE1
Normalized Probe Intensity
Probe Number
4.5
5.0
5.5
6.0
1
2
3
4
5
6
7
8
9
10
11
Meishan−A
Meishan−B
Meishan−C
European−A
European−B
European−C

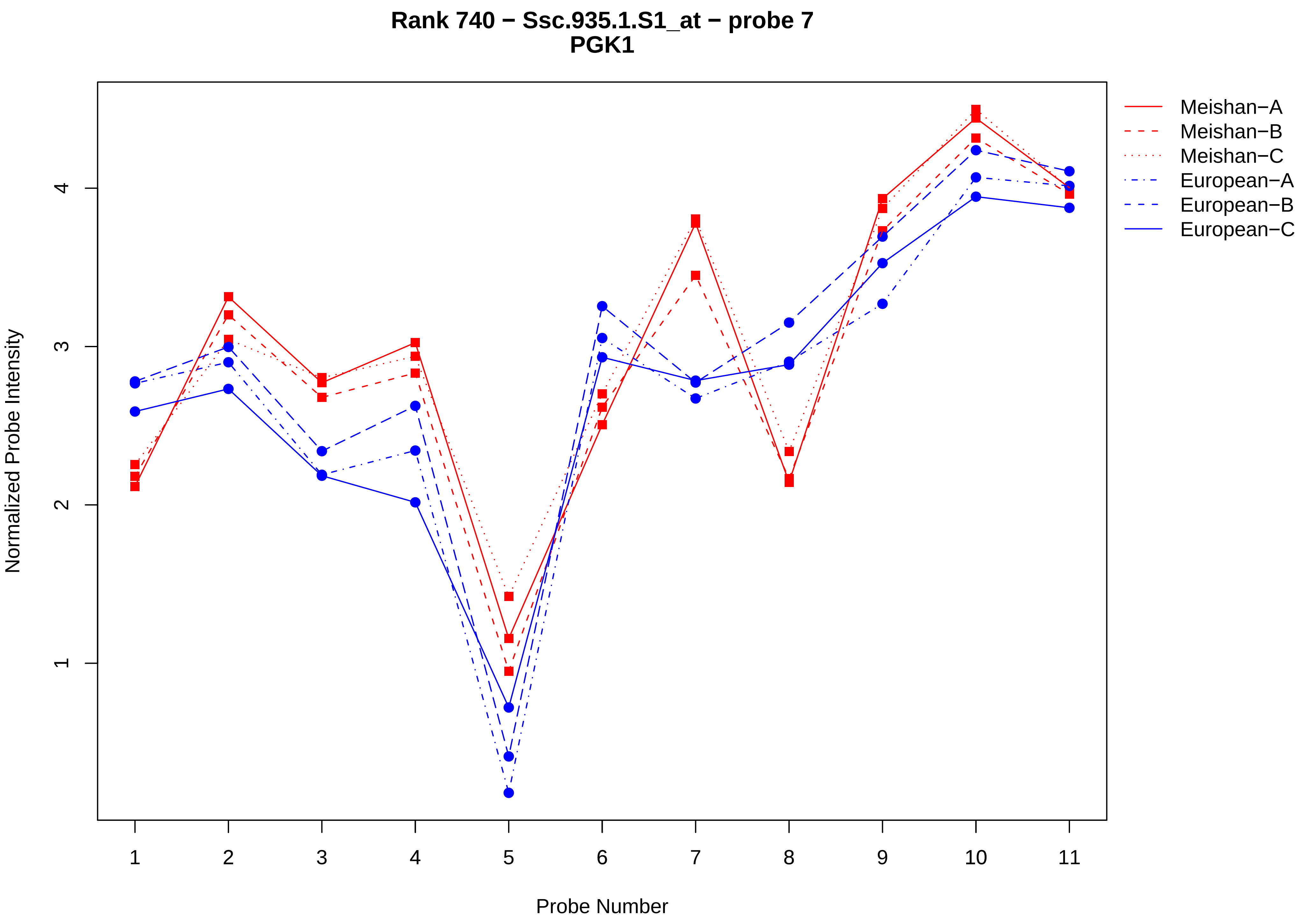

Rank 740 − Ssc.935.1.S1_at − probe 7
PGK1
Normalized Probe Intensity
Probe Number
1
2
3
4
1
2
3
4
5
6
7
8
9
10
11
Meishan−A
Meishan−B
Meishan−C
European−A
European−B
European−C

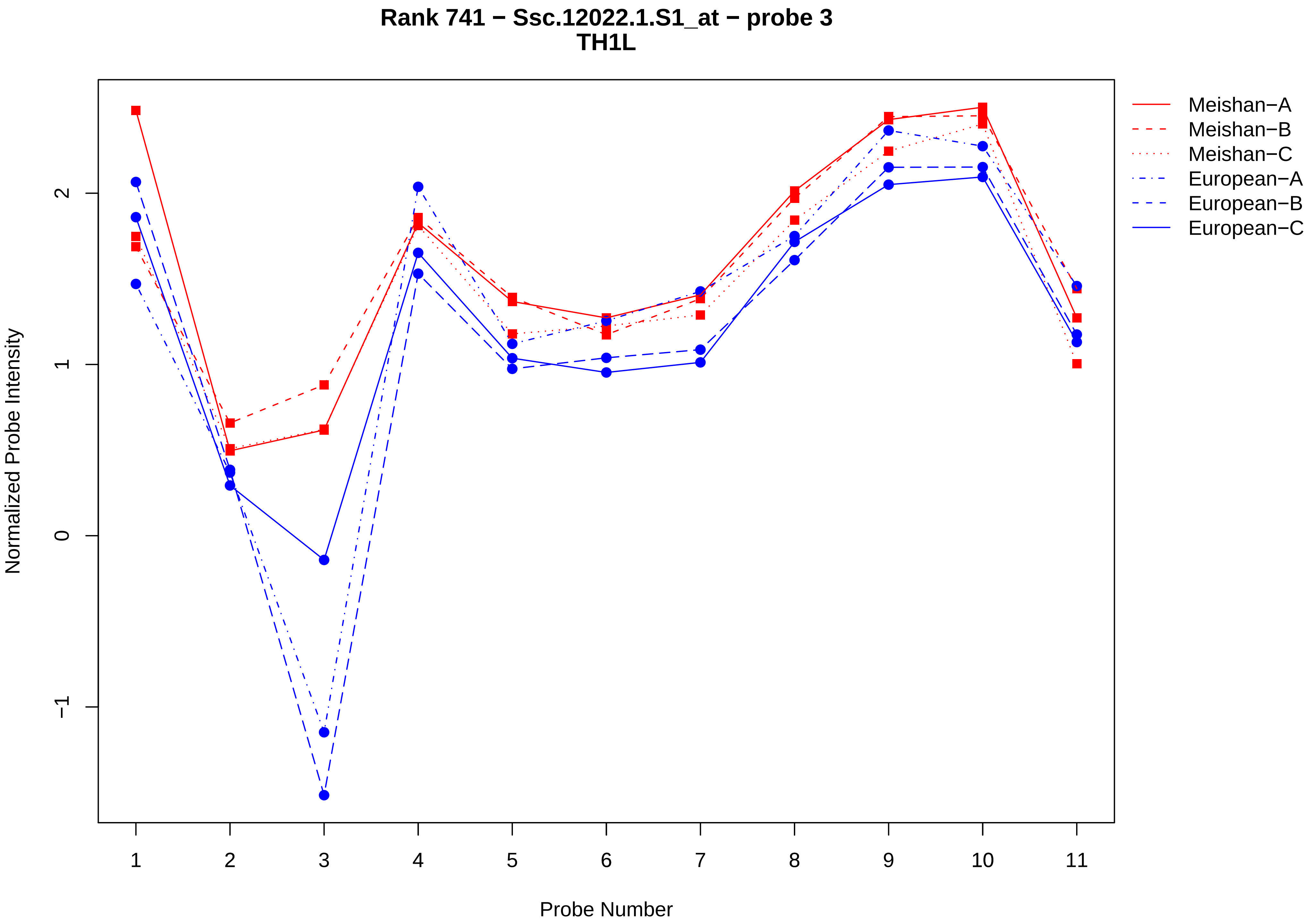

Rank 741 − Ssc.12022.1.S1_at − probe 3
TH1L
Normalized Probe Intensity
Probe Number
−1
0
1
2
1
2
3
4
5
6
7
8
9
10
11
Meishan−A
Meishan−B
Meishan−C
European−A
European−B
European−C

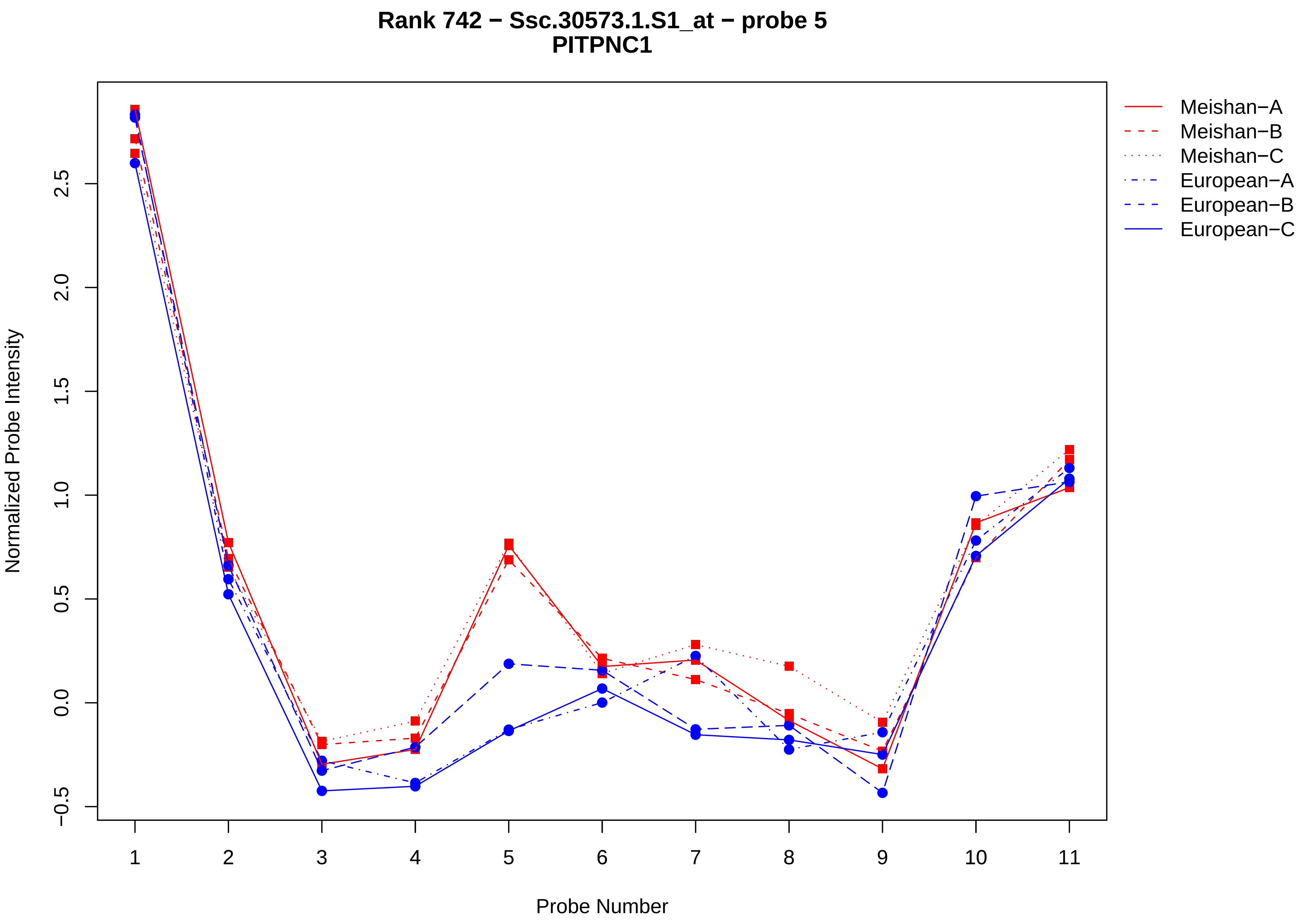
Rank 742 − Ssc.30573.1.S1_at − probe 5
PITPNC1
Normalized Probe Intensity
Probe Number
Meishan−A
Meishan−B
Meishan−C
European−A
European−B
European−C
−0.5
0.0
0.5
1.0
1.5
2.0
2.5
1
2
3
4
5
6
7
8
9
10
11

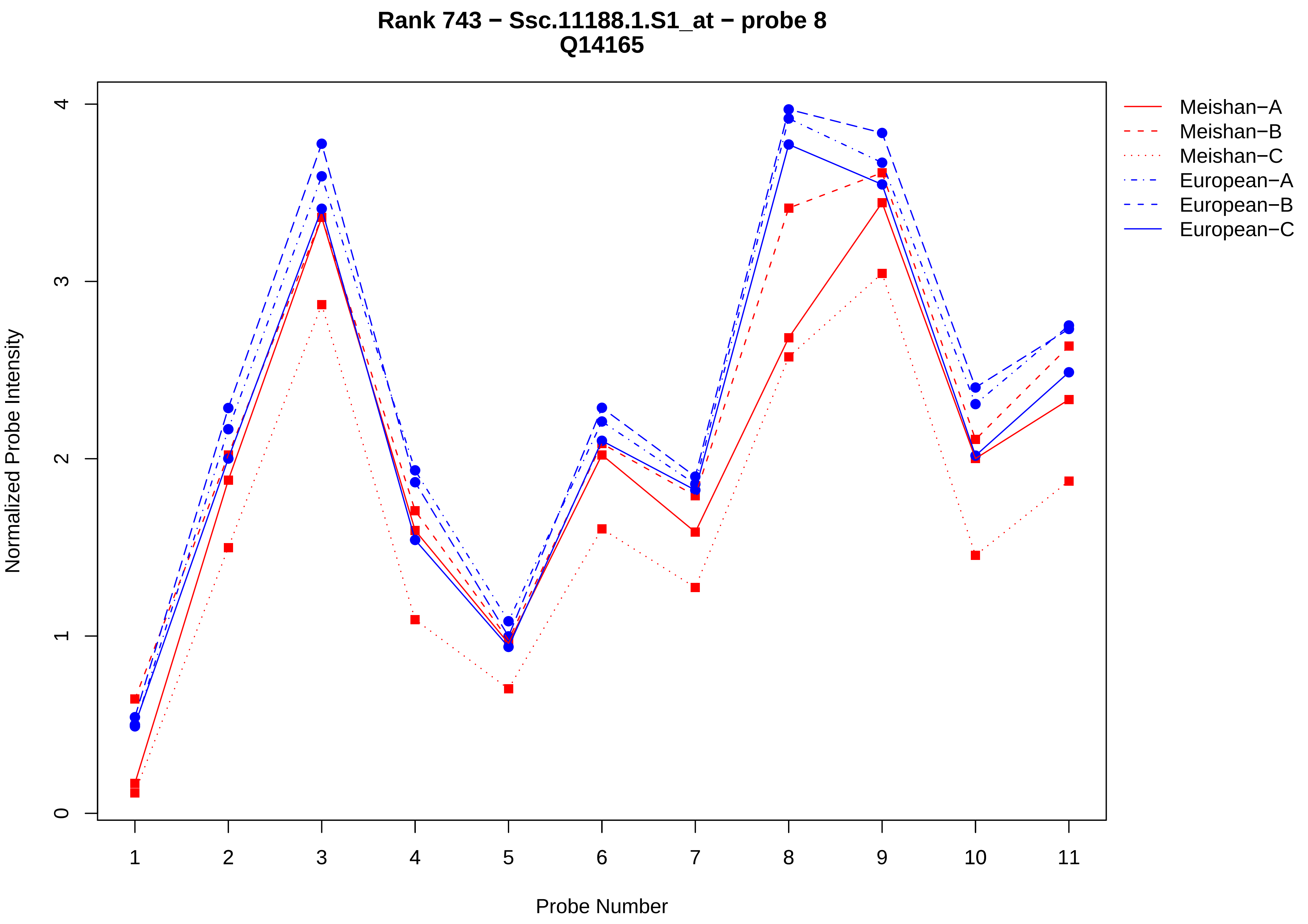

Rank 743 − Ssc.11188.1.S1_at − probe 8
Q14165
Normalized Probe Intensity
Probe Number
0
1
2
3
4
1
2
3
4
5
6
7
8
9
10
11
Meishan−A
Meishan−B
Meishan−C
European−A
European−B
European−C

Rank 744 − Ssc.23802.1.S1_at − probe 9
FUB3_HUMAN

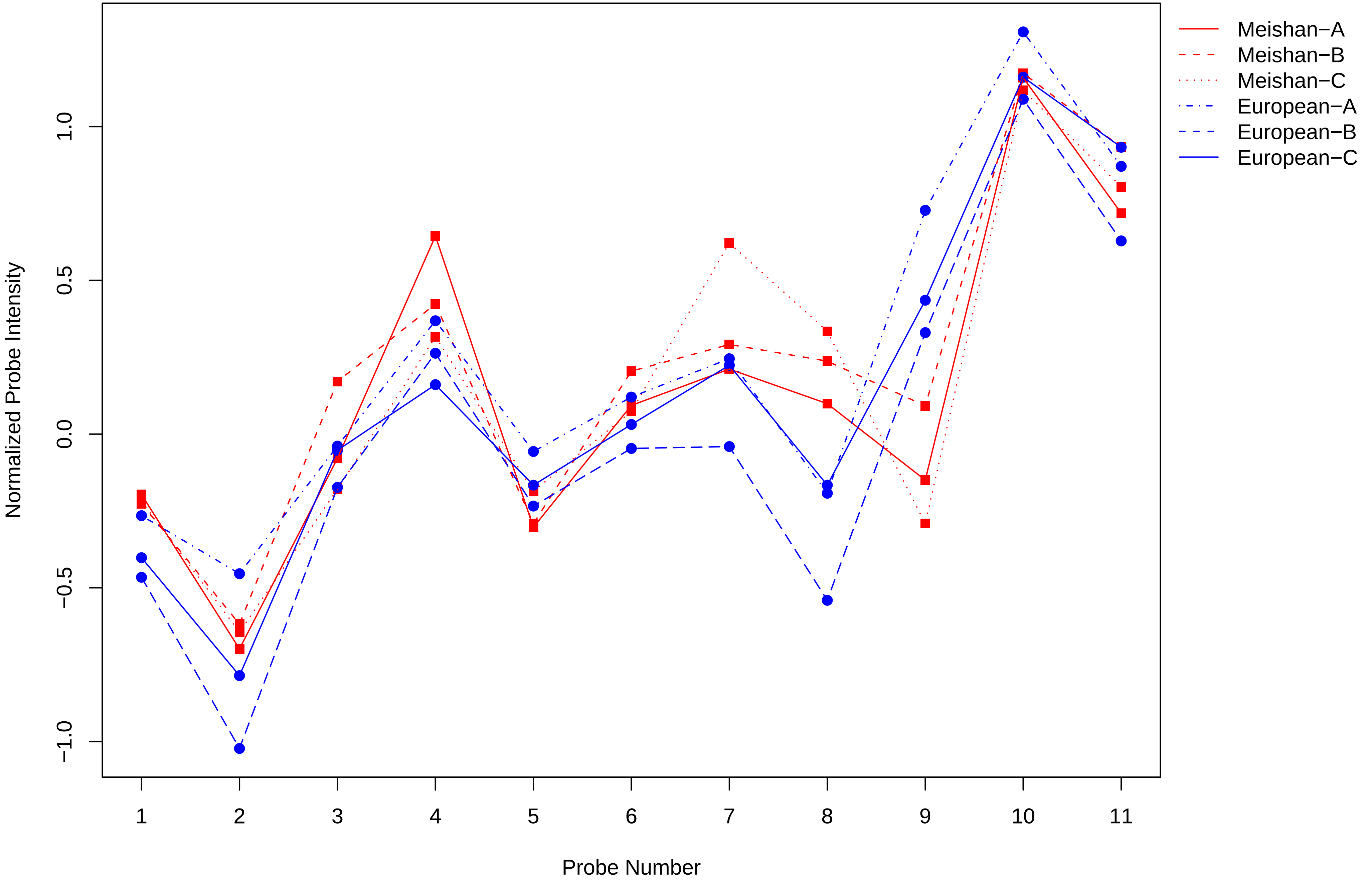

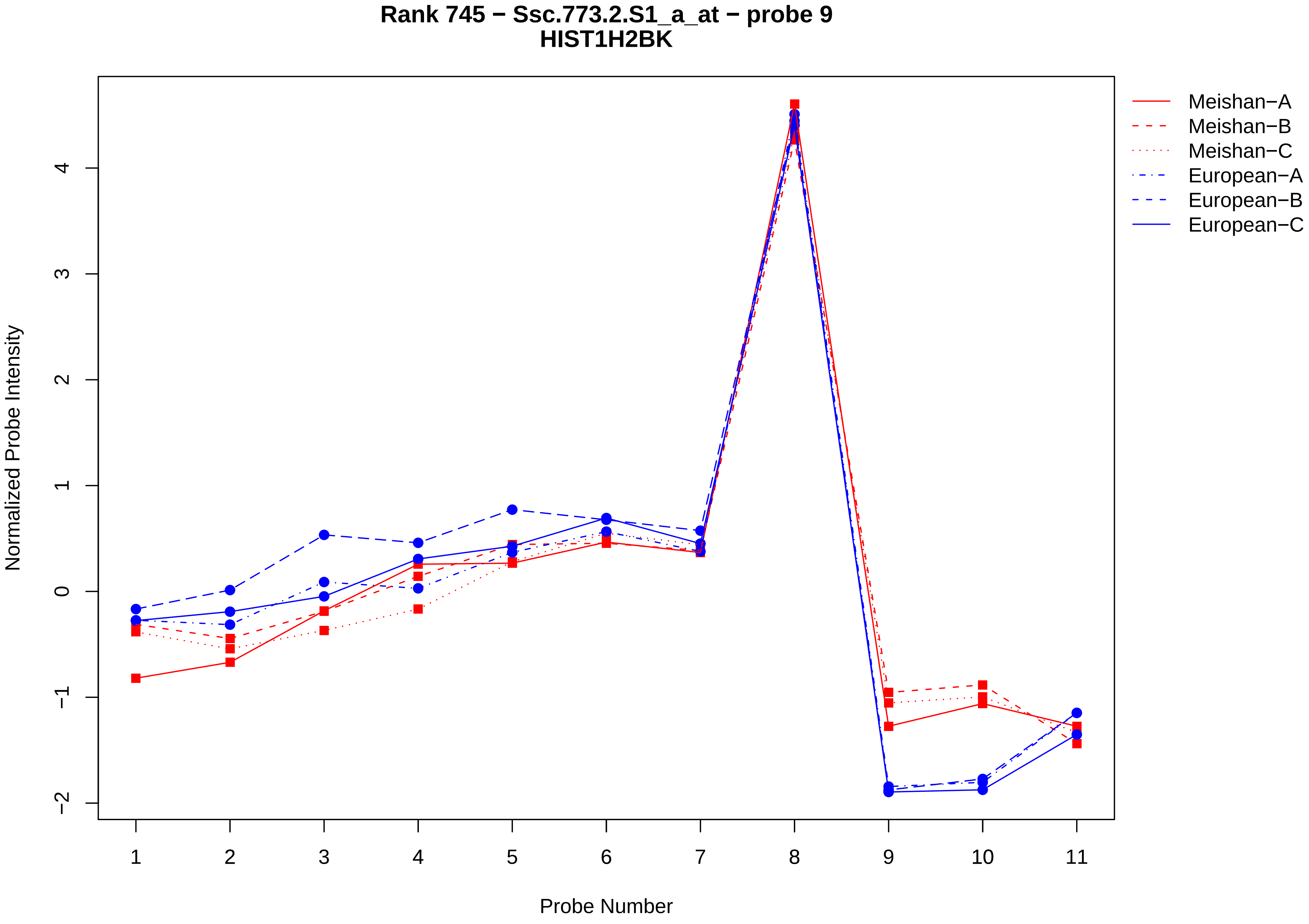

Rank 745 − Ssc.773.2.S1_a_at − probe 9
HIST1H2BK
Normalized Probe Intensity
Probe Number
−2
−1
0
1
2
3
4
1
2
3
4
5
6
7
8
9
10
11
Meishan−A
Meishan−B
Meishan−C
European−A
European−B
European−C

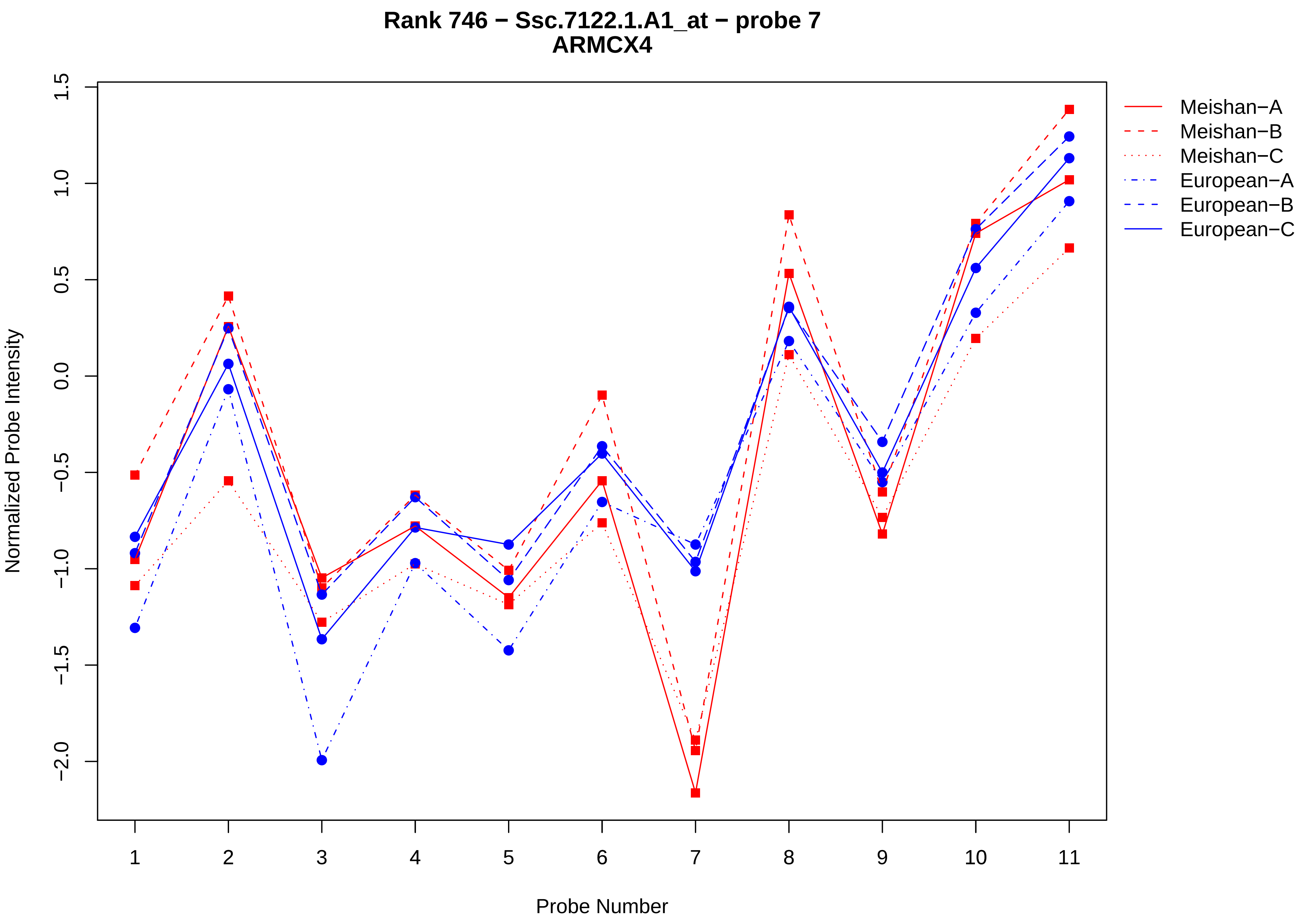

Rank 746 − Ssc.7122.1.A1_at − probe 7
ARMCX4
Normalized Probe Intensity
Probe Number
Meishan−A
Meishan−B
Meishan−C
European−A
European−B
European−C

Rank 747 − Ssc.927.1.S1_at − probe 3
EYA3

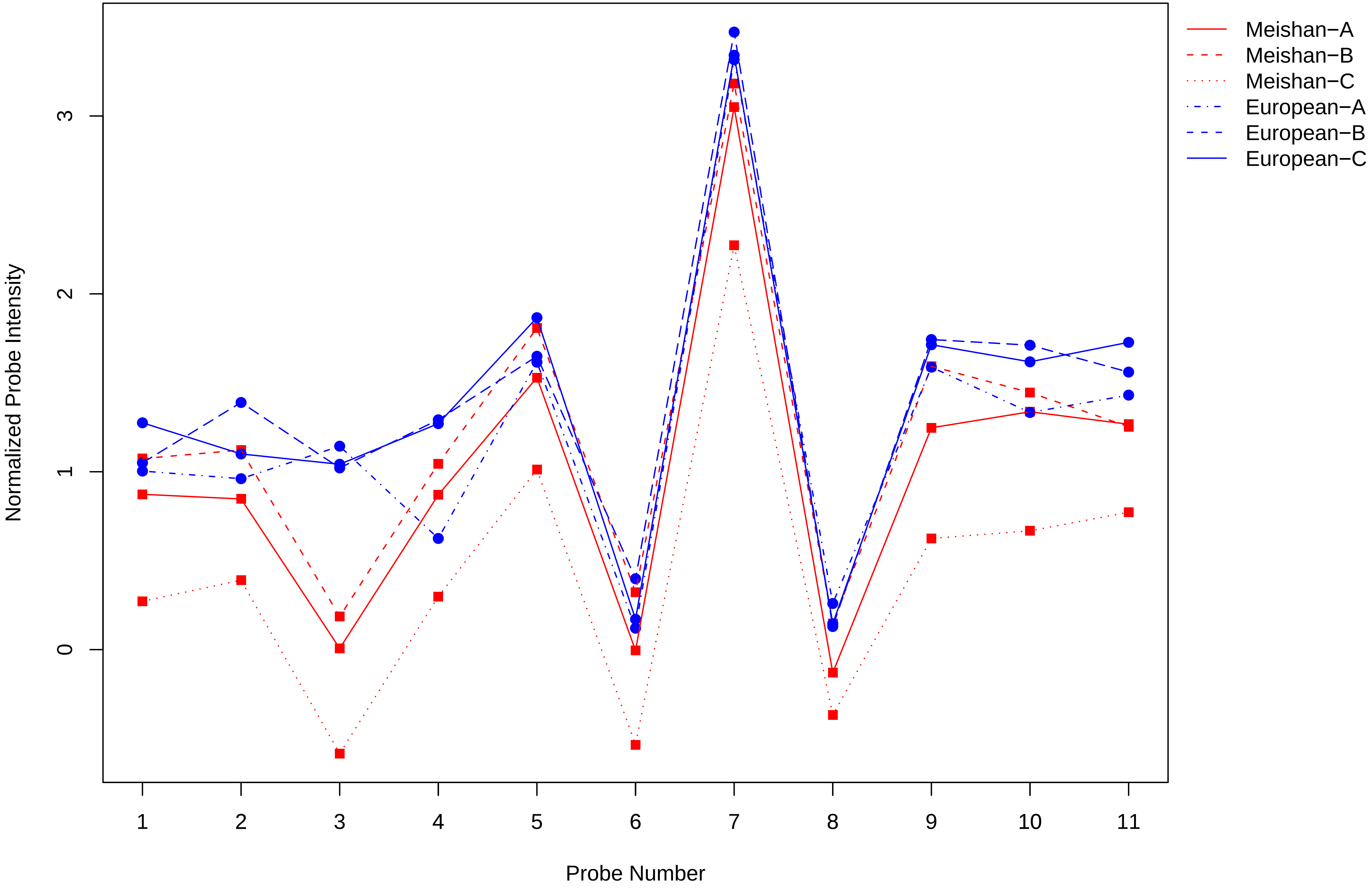

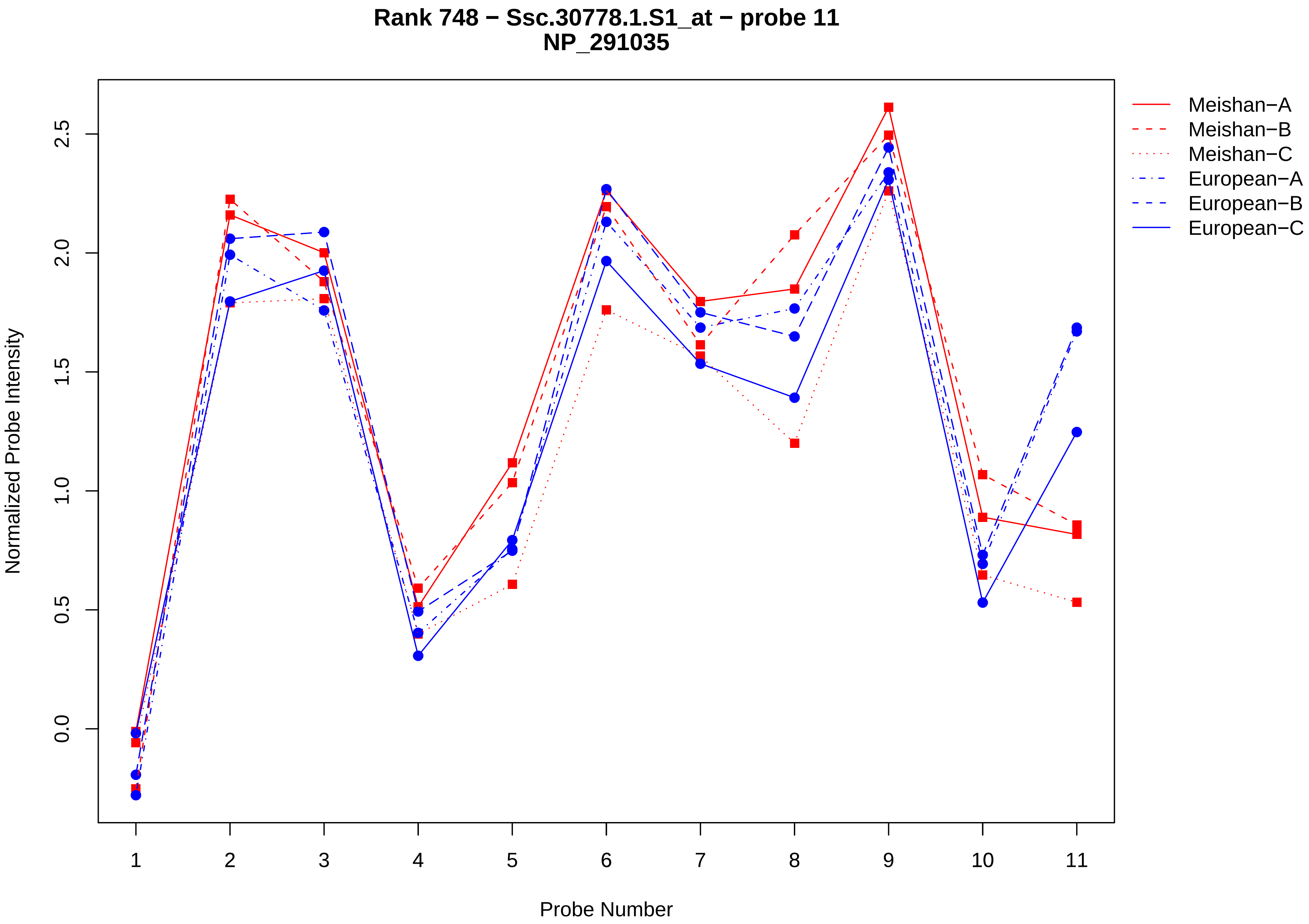

Rank 748 − Ssc.30778.1.S1_at − probe 11
NP_291035
Normalized Probe Intensity
Probe Number
0.0
0.5
1.0
1.5
2.0
2.5
1
2
3
4
5
6
7
8
9
10
11
Meishan−A
Meishan−B
Meishan−C
European−A
European−B
European−C

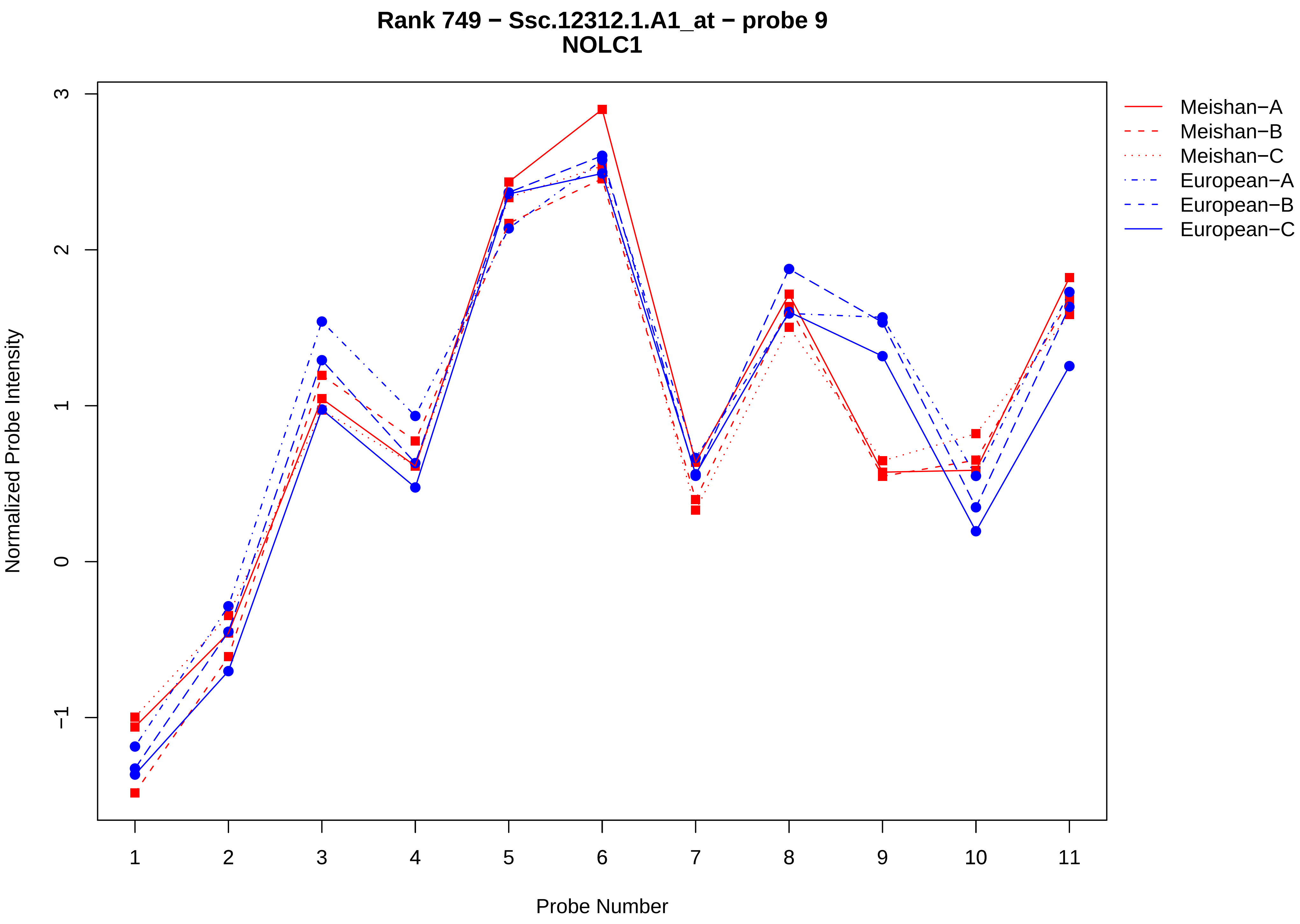
Rank 749 − Ssc.12312.1.A1_at − probe 9
NOLC1
Normalized Probe Intensity
Probe Number
−1
0
1
2
3
1
2
3
4
5
6
7
8
9
10
11
Meishan−A
Meishan−B
Meishan−C
European−A
European−B
European−C

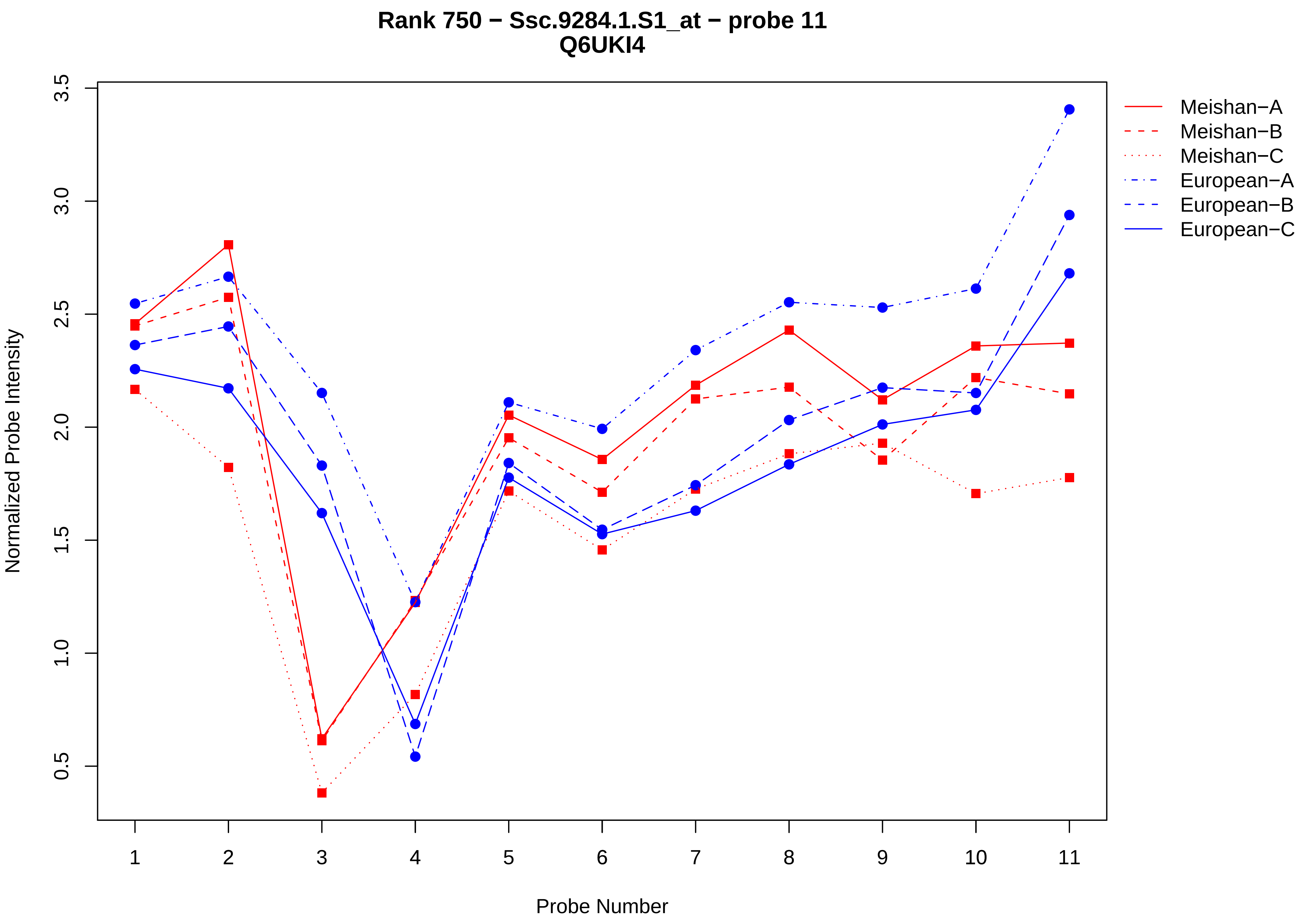
Rank 750 − Ssc.9284.1.S1_at − probe 11
Q6UKI4
Normalized Probe Intensity
Probe Number
0.5
1.0
1.5
2.0
2.5
3.0
3.5
1
2
3
4
5
6
7
8
9
10
11
Meishan−A
Meishan−B
Meishan−C
European−A
European−B
European−C

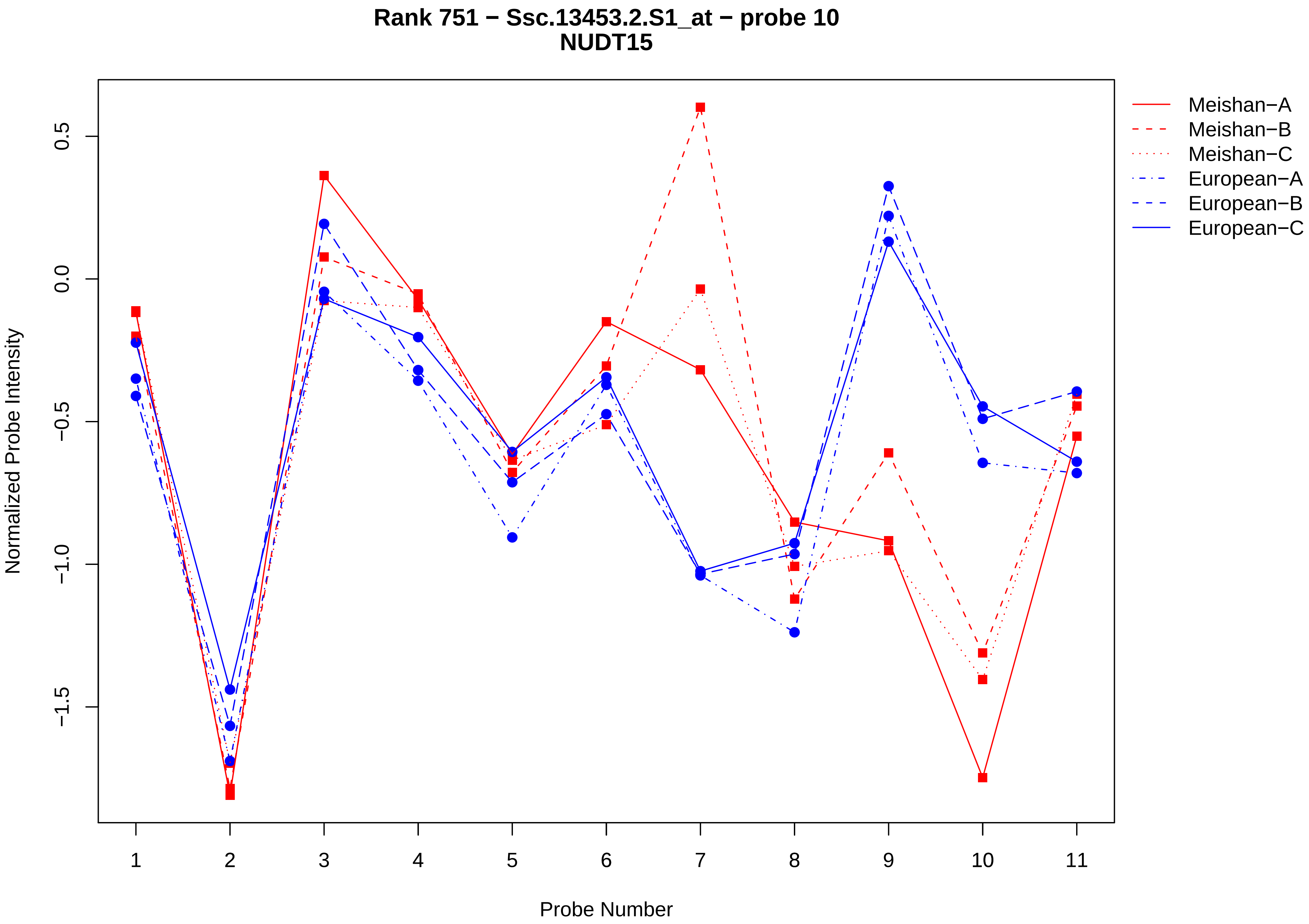

Rank 751 − Ssc.13453.2.S1_at − probe 10
NUDT15
Normalized Probe Intensity
Probe Number
0.5
0.0
−0.5
−1.0
−1.5
1
2
3
4
5
6
7
8
9
10
11
Meishan−A
Meishan−B
Meishan−C
European−A
European−B
European−C

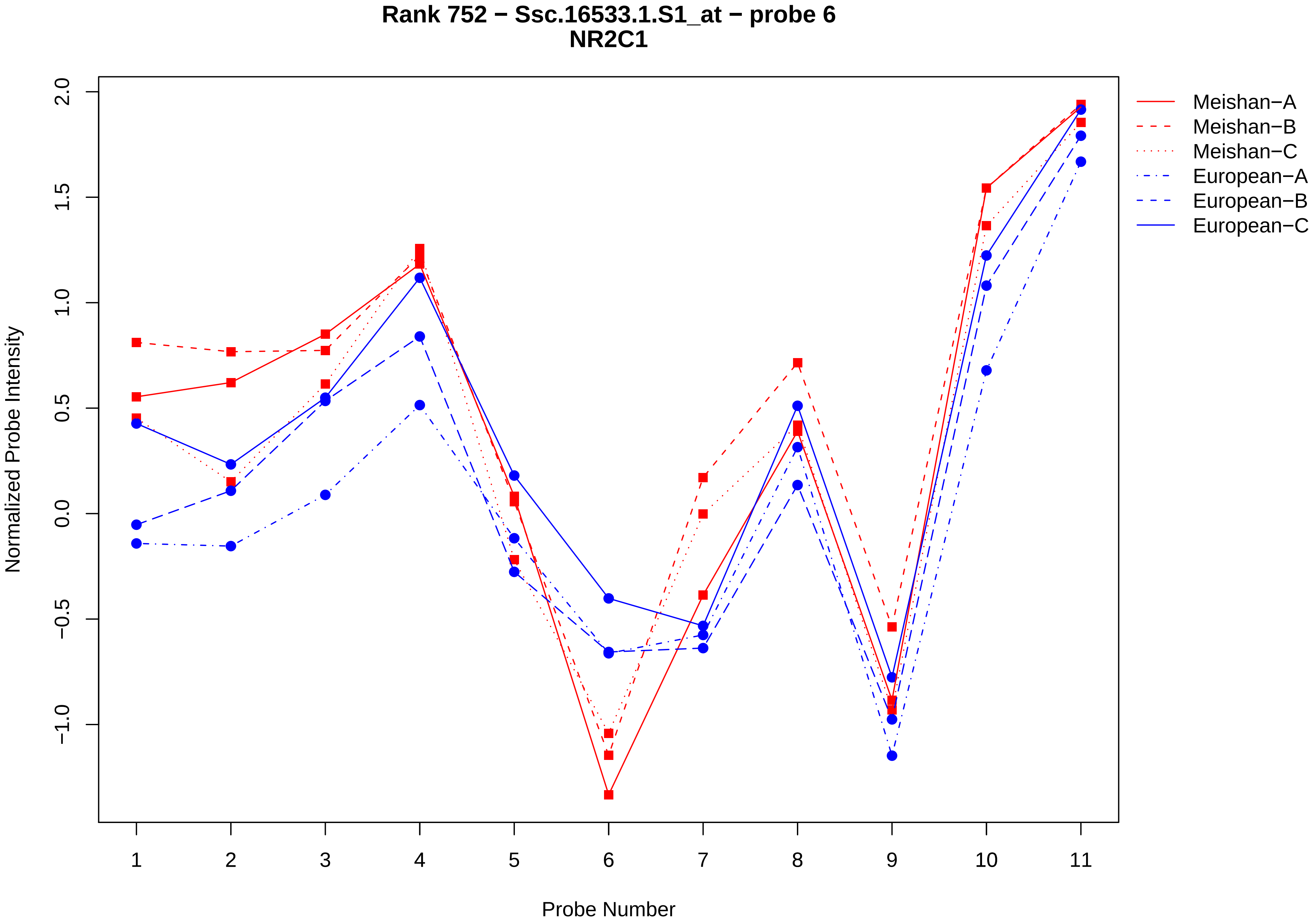

Rank 752 − Ssc.16533.1.S1_at − probe 6
NR2C1
Normalized Probe Intensity
Probe Number
−1.0
−0.5
0.0
0.5
1.0
1.5
2.0
1
2
3
4
5
6
7
8
9
10
11
Meishan−A
Meishan−B
Meishan−C
European−A
European−B
European−C

Rank 753 − Ssc.1236.1.S1_at − probe 5
Q9Y548

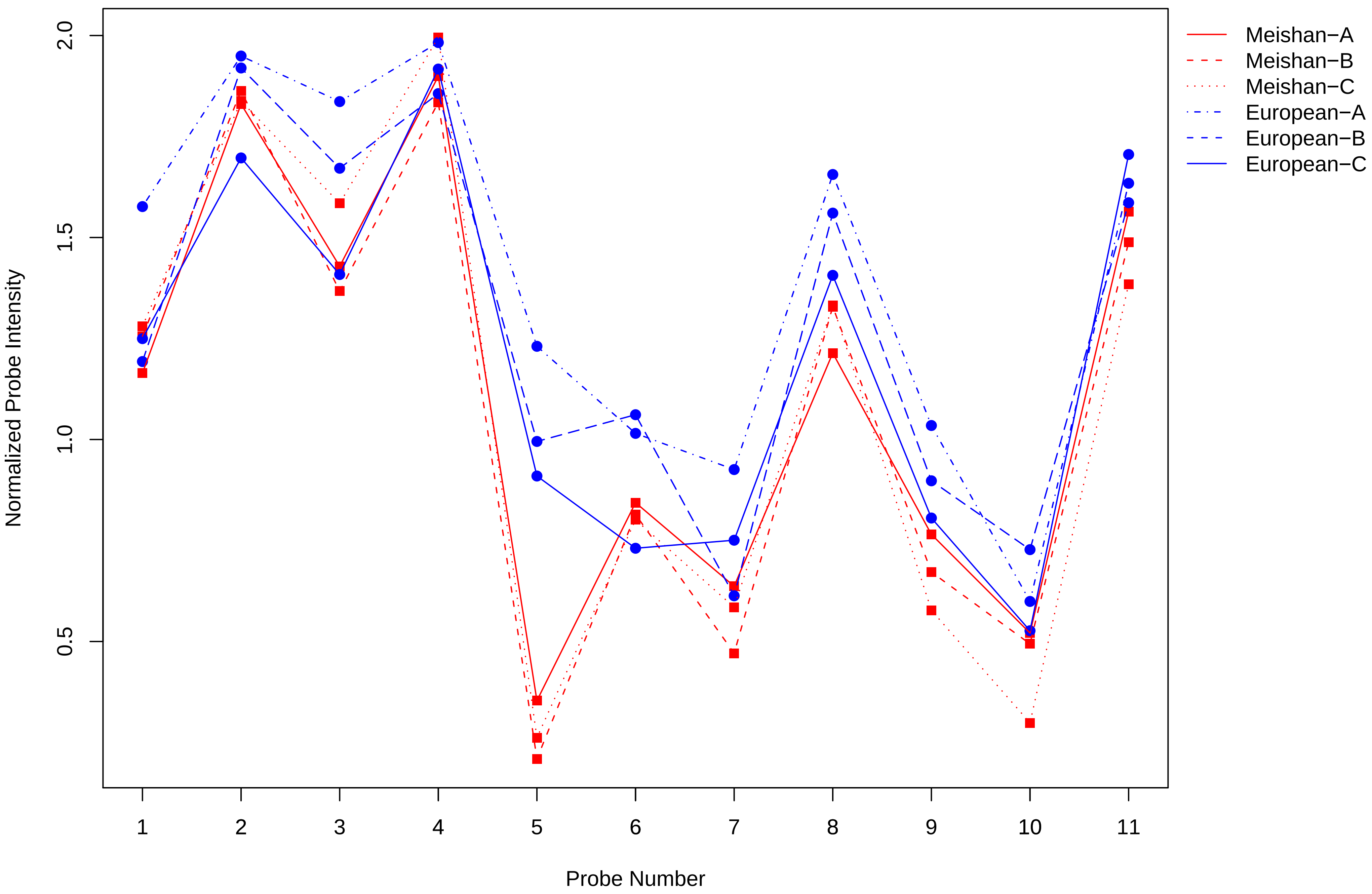

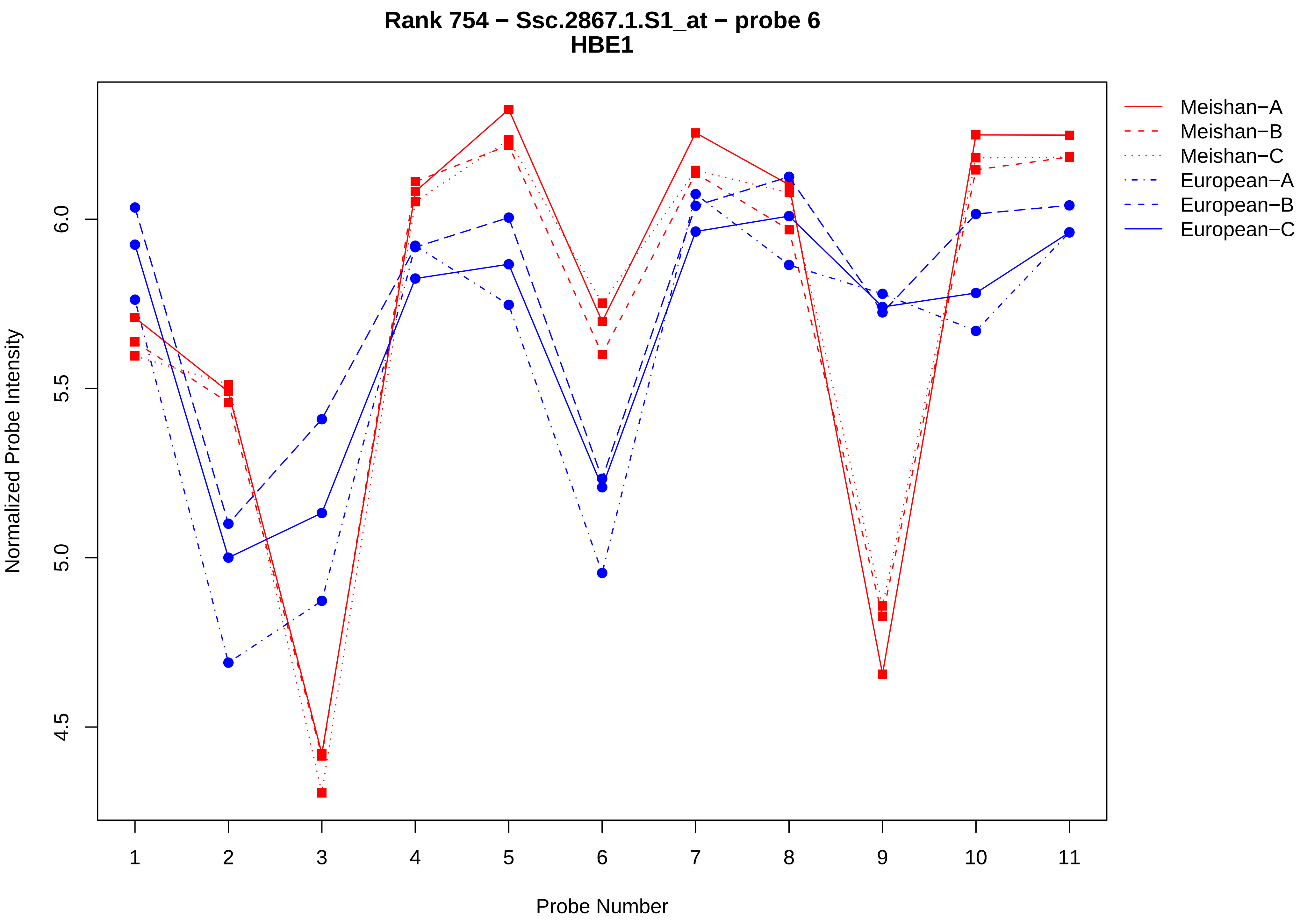
Rank 754 − Ssc.2867.1.S1_at − probe 6
HBE1
Normalized Probe Intensity
Probe Number
4.5
5.0
5.5
6.0
1
2
3
4
5
6
7
8
9
10
11
Meishan−A
Meishan−B
Meishan−C
European−A
European−B
European−C

Rank 755 − Ssc.13426.1.A1_at − probe 10
MYCPBP

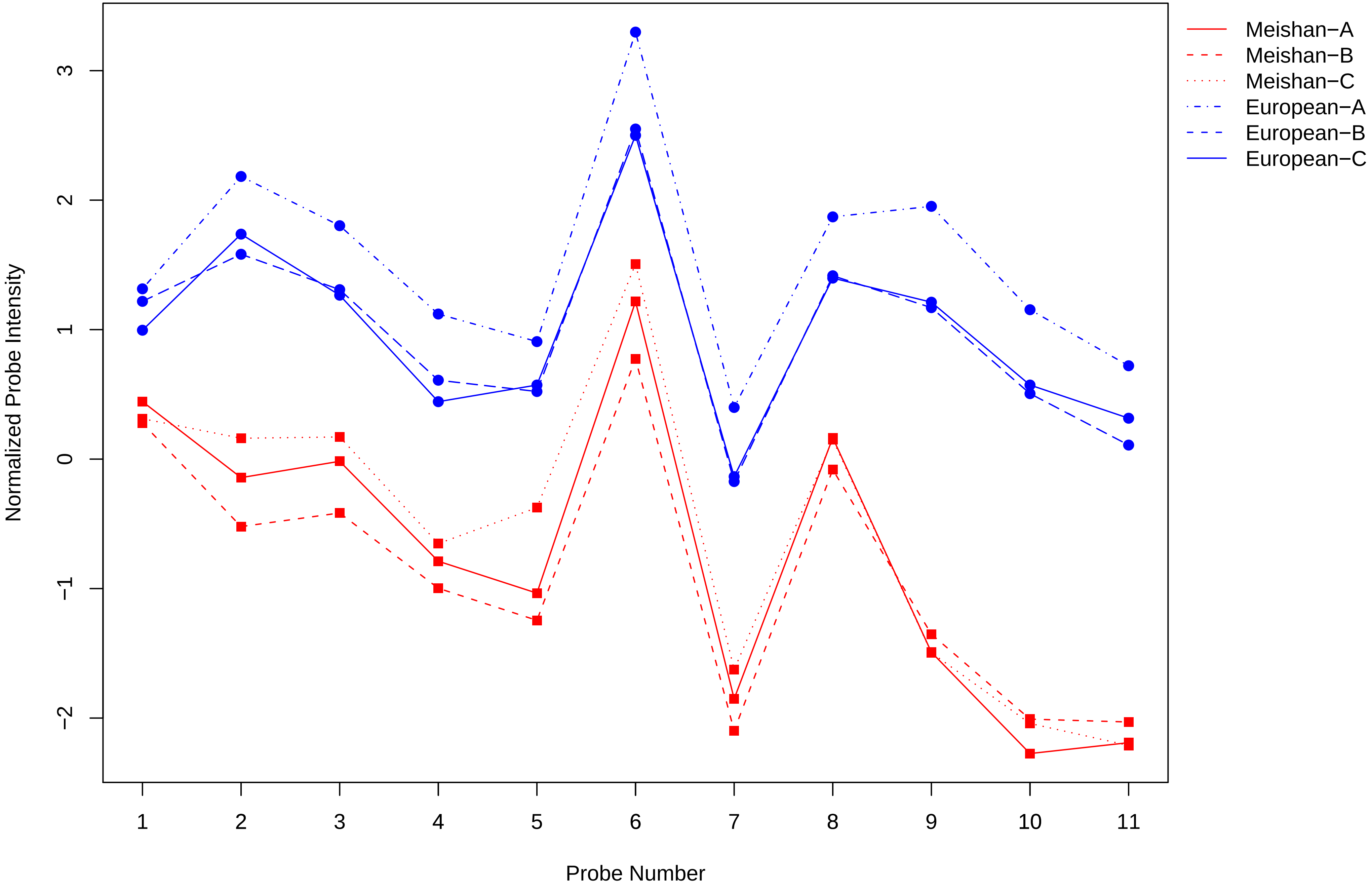

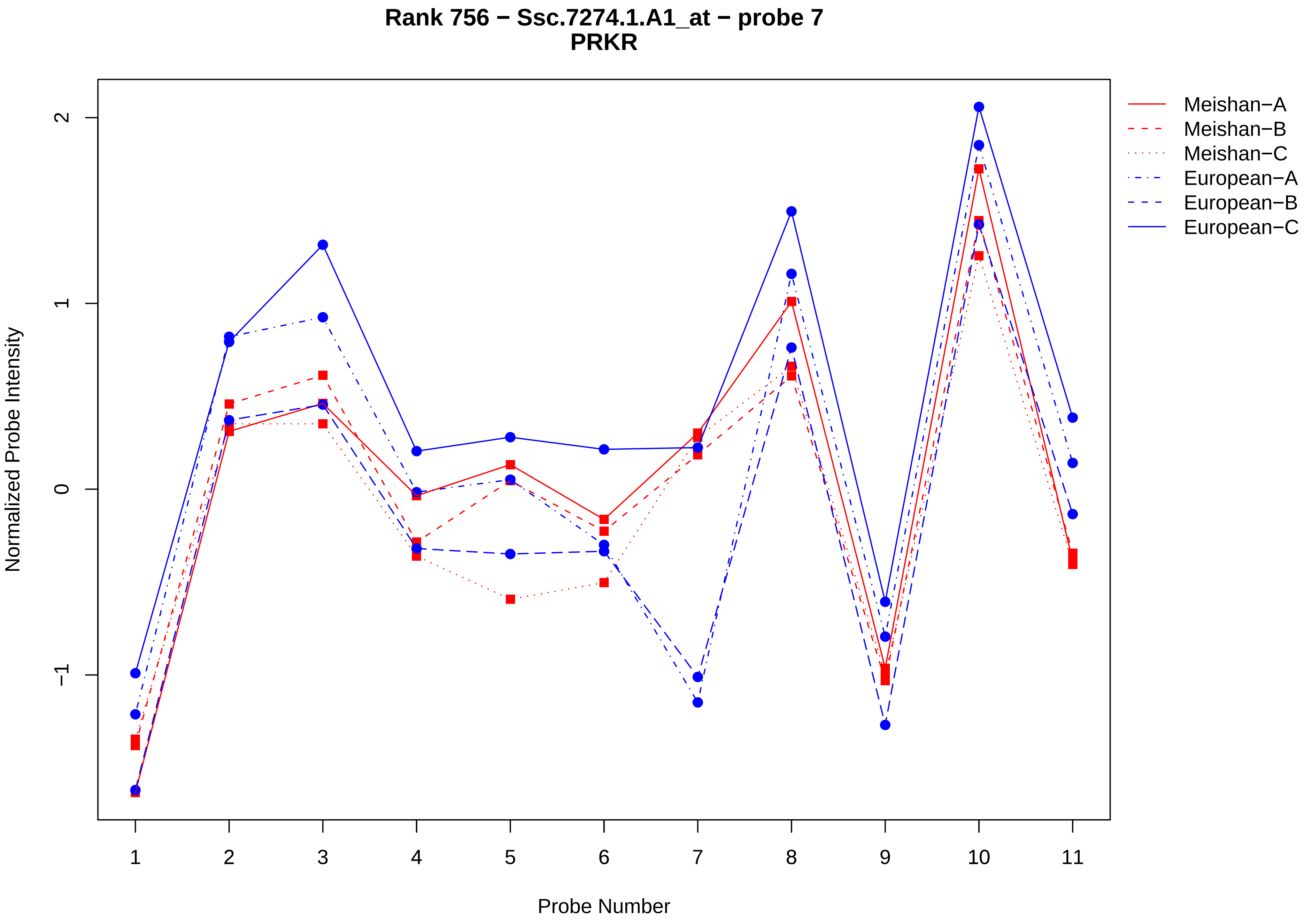

Rank 756 − Ssc.7274.1.A1_at − probe 7
PRKR
Normalized Probe Intensity
Probe Number
Meishan−A
Meishan−B
Meishan−C
European−A
European−B
European−C
−1
0
1
2
1
2
3
4
5
6
7
8
9
10
11

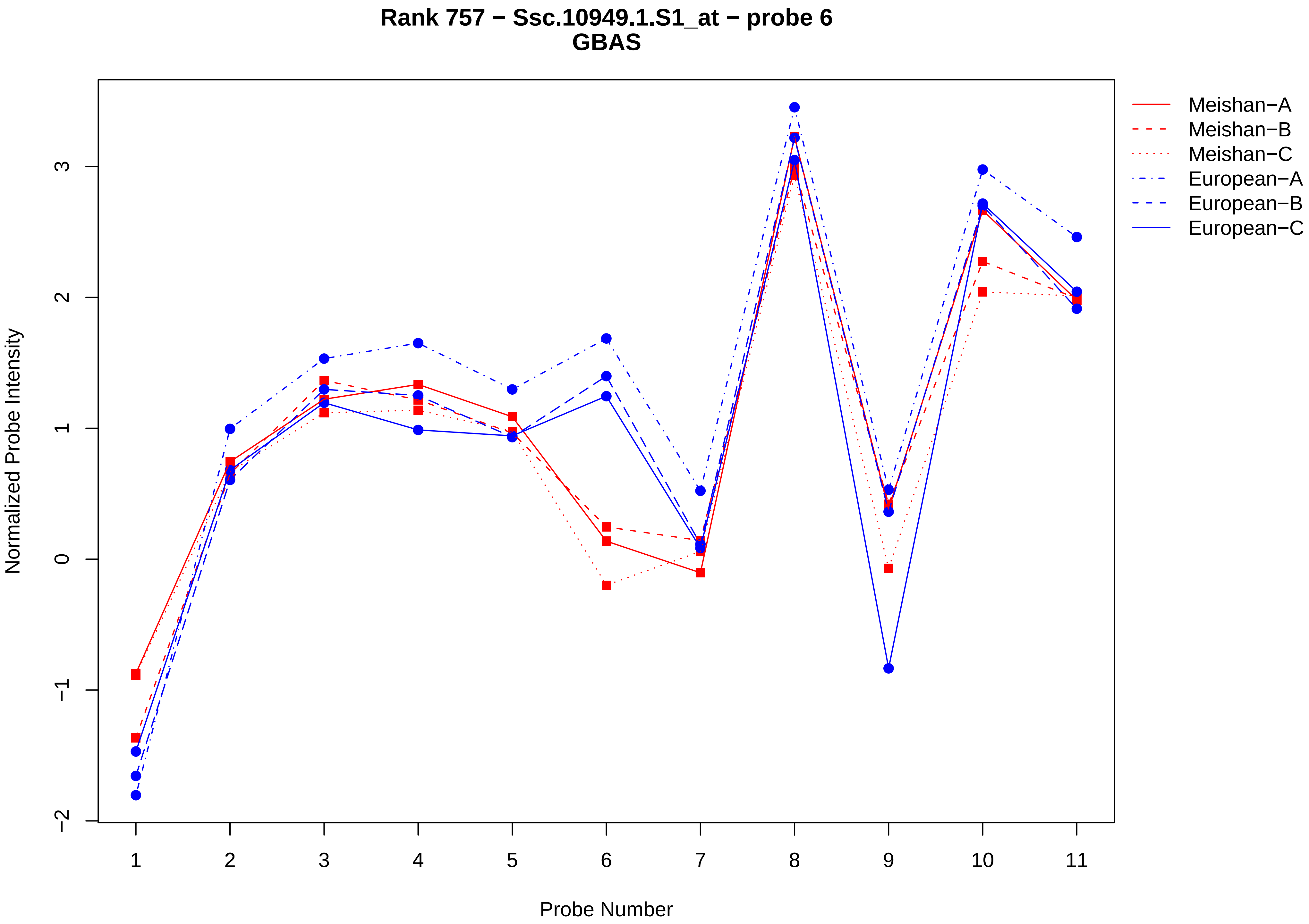

Rank 757 − Ssc.10949.1.S1_at − probe 6
GBAS
Normalized Probe Intensity
Probe Number
−2
−1
0
1
2
3
1
2
3
4
5
6
7
8
9
10
11
Meishan−A
Meishan−B
Meishan−C
European−A
European−B
European−C

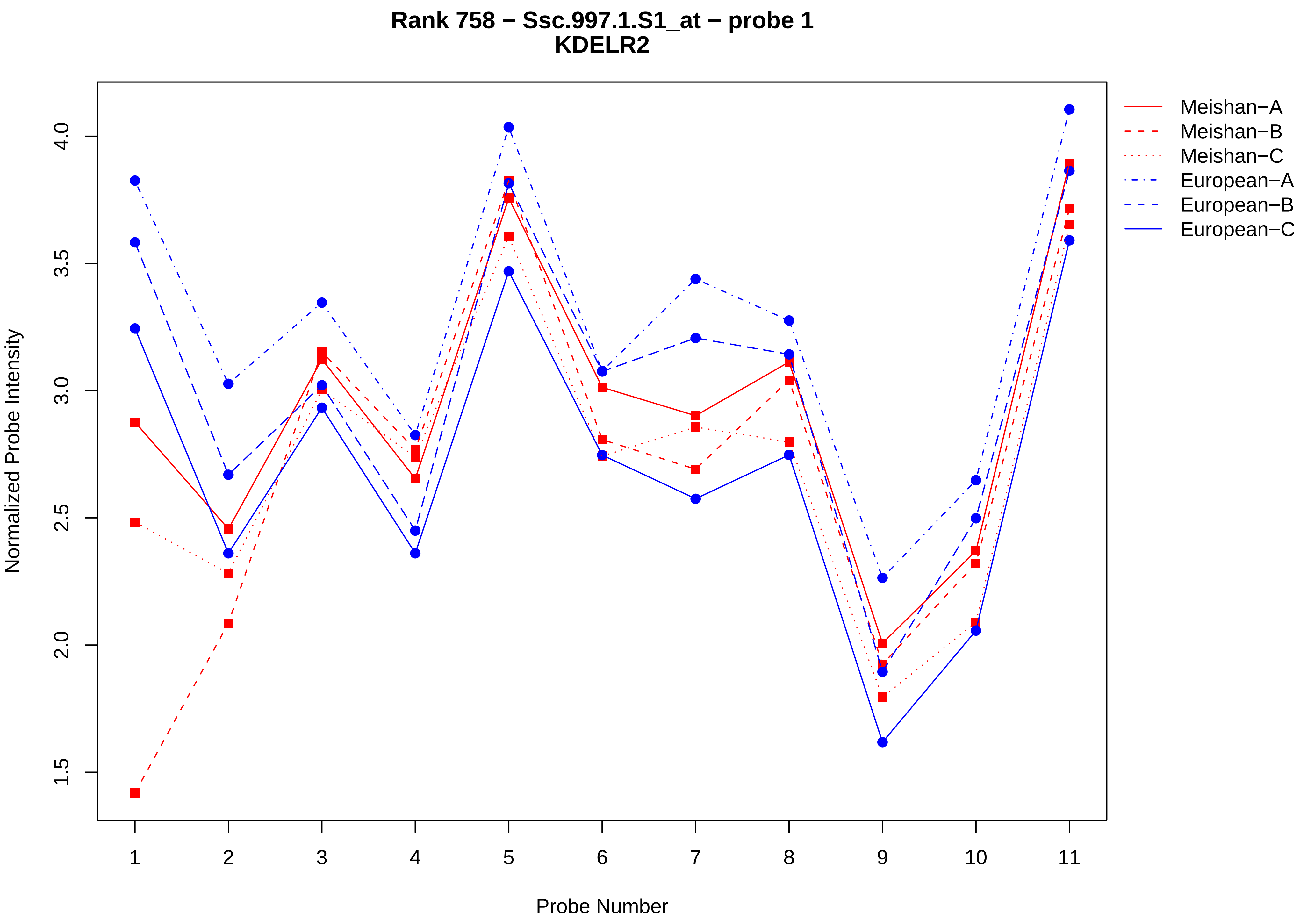

Rank 758 − Ssc.997.1.S1_at − probe 1
KDELR2
Normalized Probe Intensity
Probe Number
1.5
2.0
2.5
3.0
3.5
4.0
1
2
3
4
5
6
7
8
9
10
11
Meishan−A
Meishan−B
Meishan−C
European−A
European−B
European−C

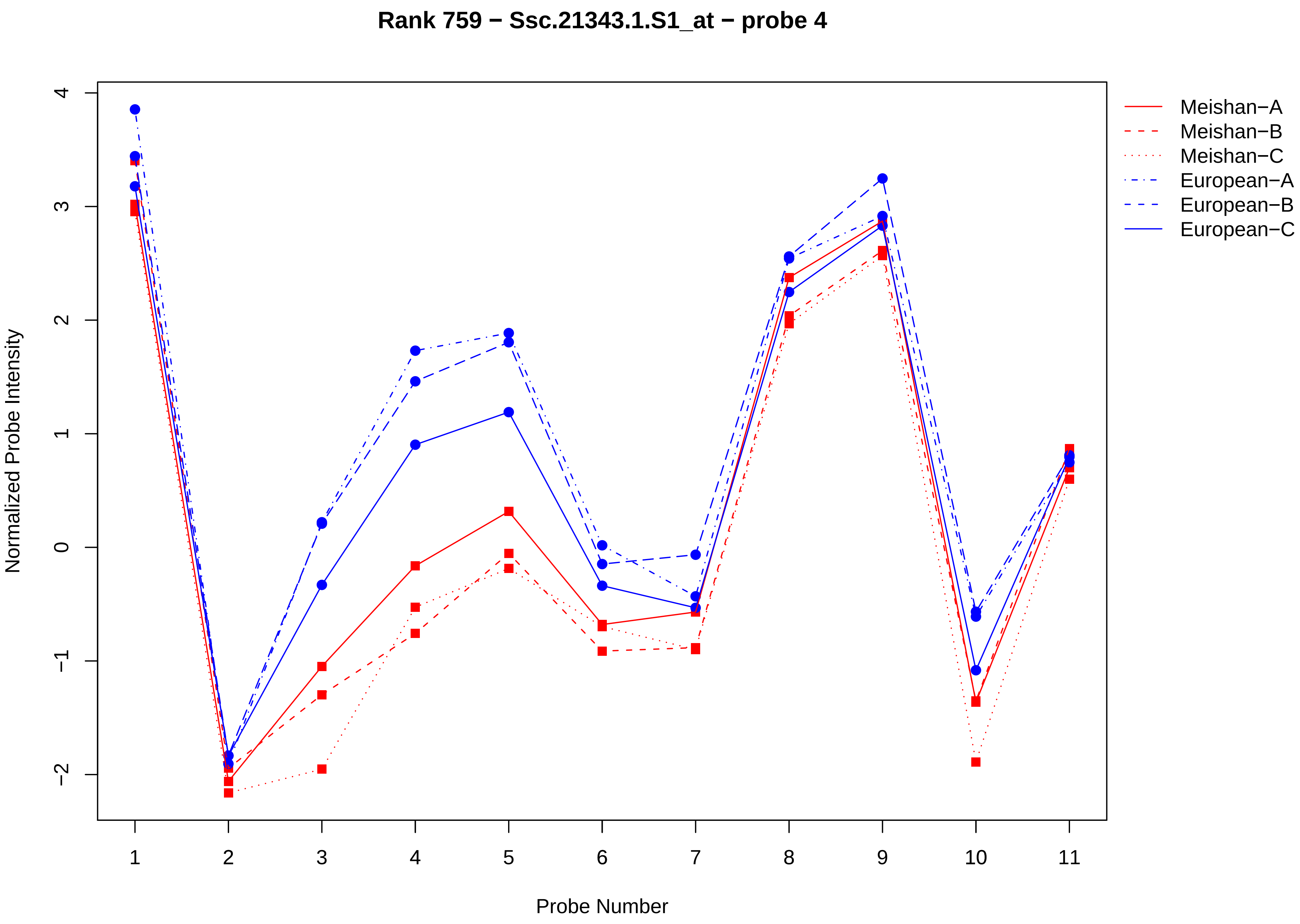
Rank 759 − Ssc.21343.1.S1_at − probe 4
Normalized Probe Intensity
Probe Number
Meishan−A
Meishan−B
Meishan−C
European−A
European−B
European−C
4
3
2
1
0
−1
−2
1
2
3
4
5
6
7
8
9
10
11

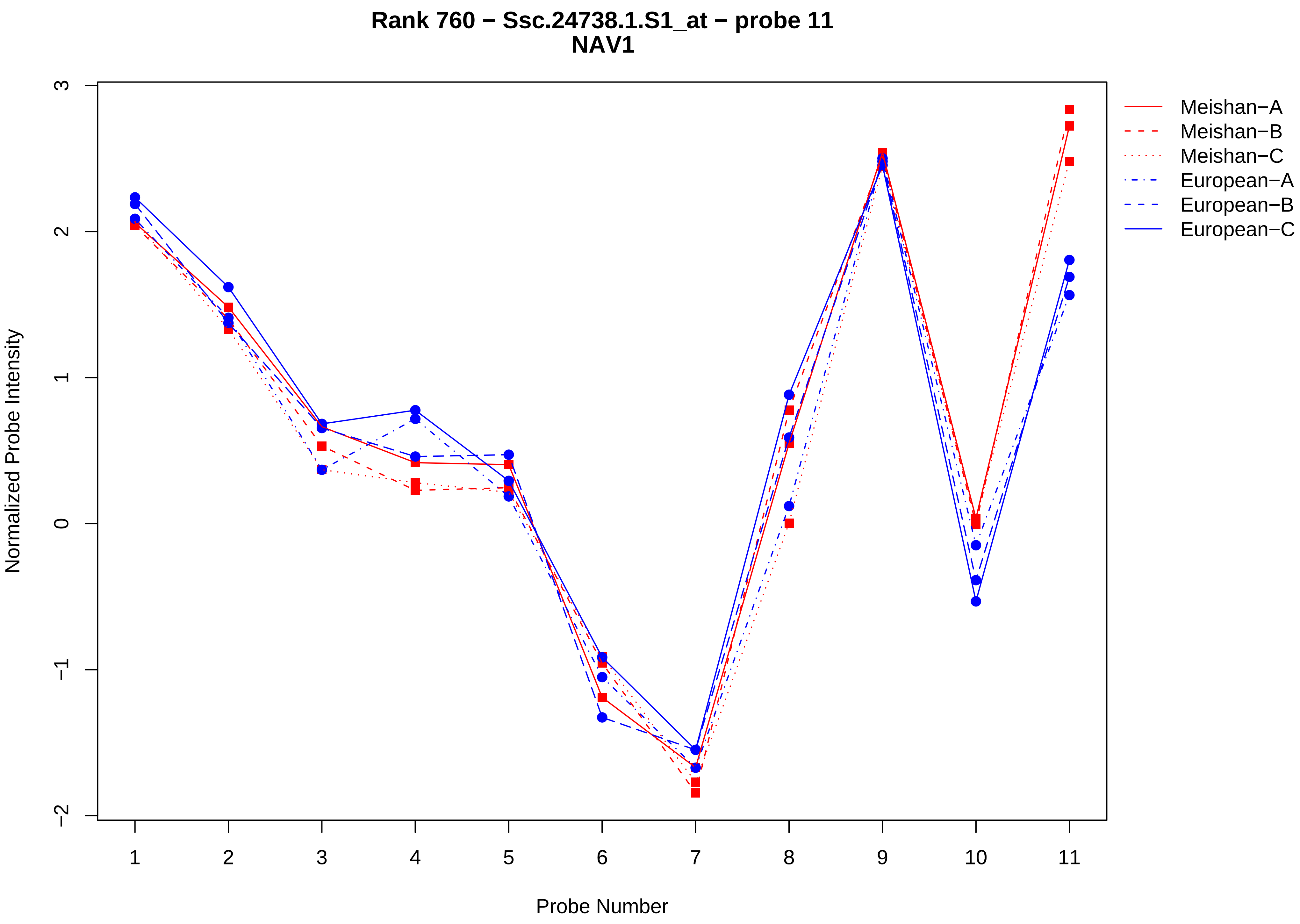

Rank 760 − Ssc.24738.1.S1_at − probe 11
NAV1
Normalized Probe Intensity
Probe Number
3
2
1
0
−1
−2
1
2
3
4
5
6
7
8
9
10
11
Meishan−A
Meishan−B
Meishan−C
European−A
European−B
European−C

Rank 761 − Ssc.5052.1.S1_at − probe 4
KIAA0674

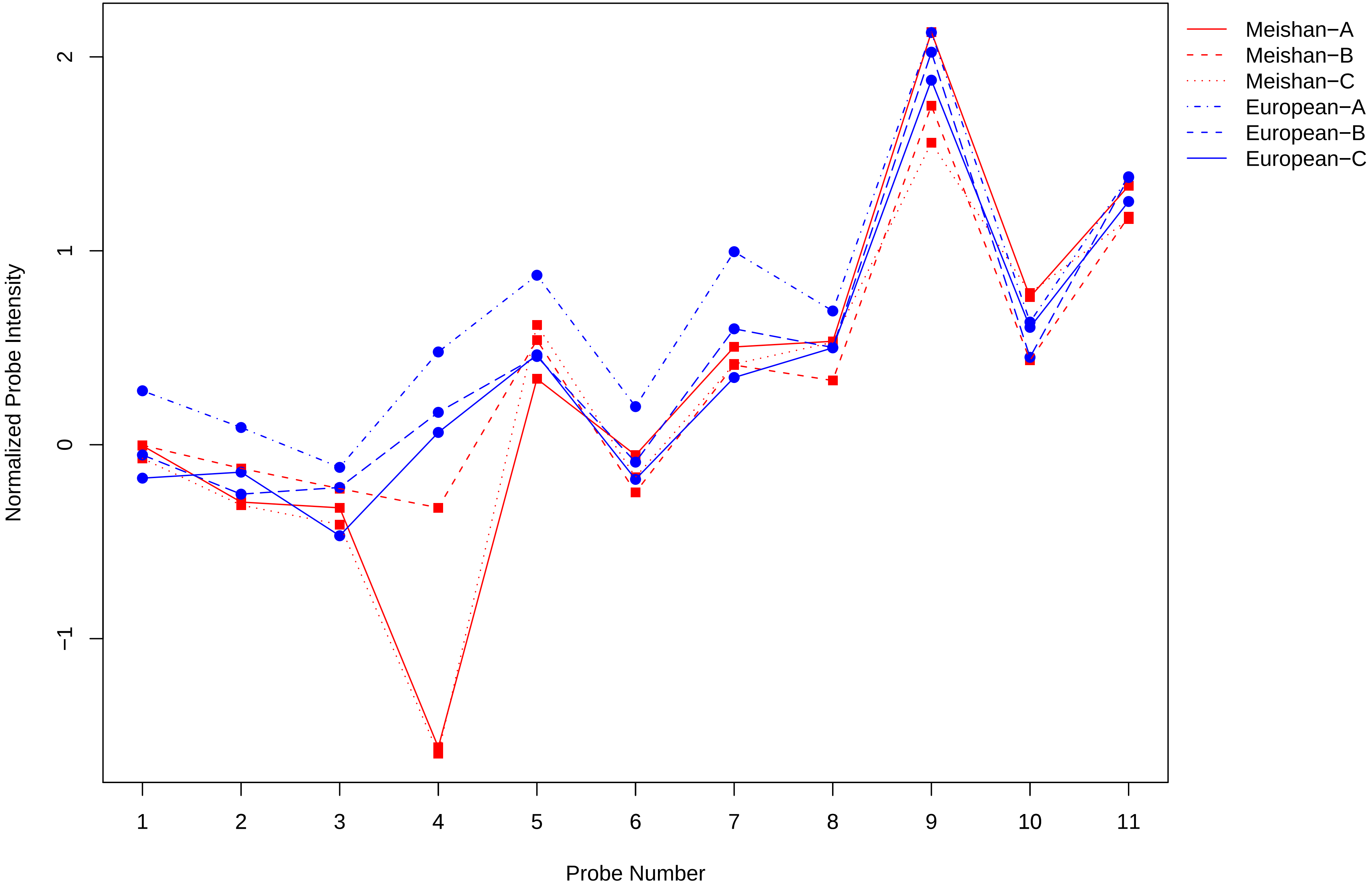

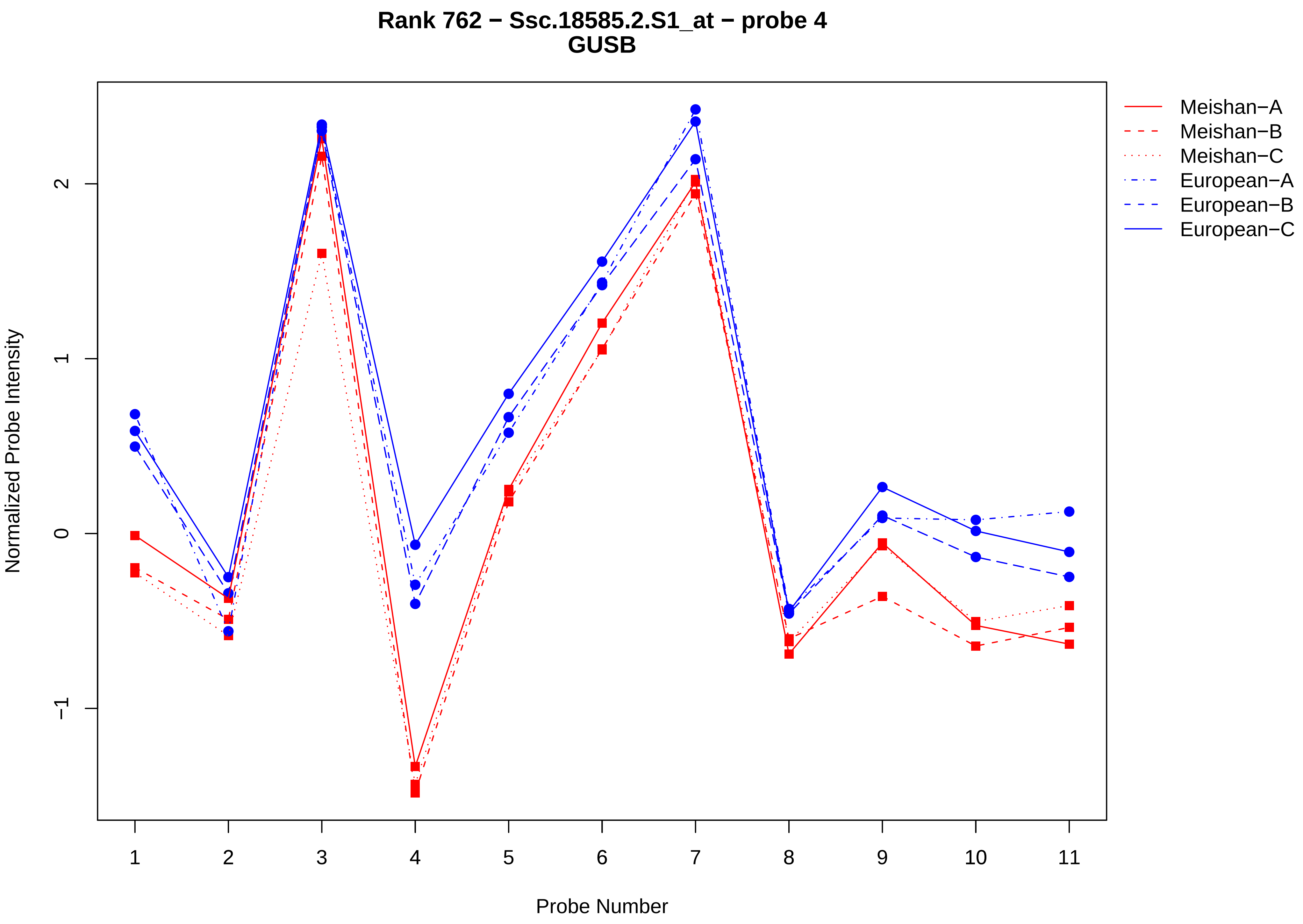

Rank 762 − Ssc.18585.2.S1_at − probe 4
GUSB
Normalized Probe Intensity
Probe Number
−1
0
1
2
1
2
3
4
5
6
7
8
9
10
11
Meishan−A
Meishan−B
Meishan−C
European−A
European−B
European−C

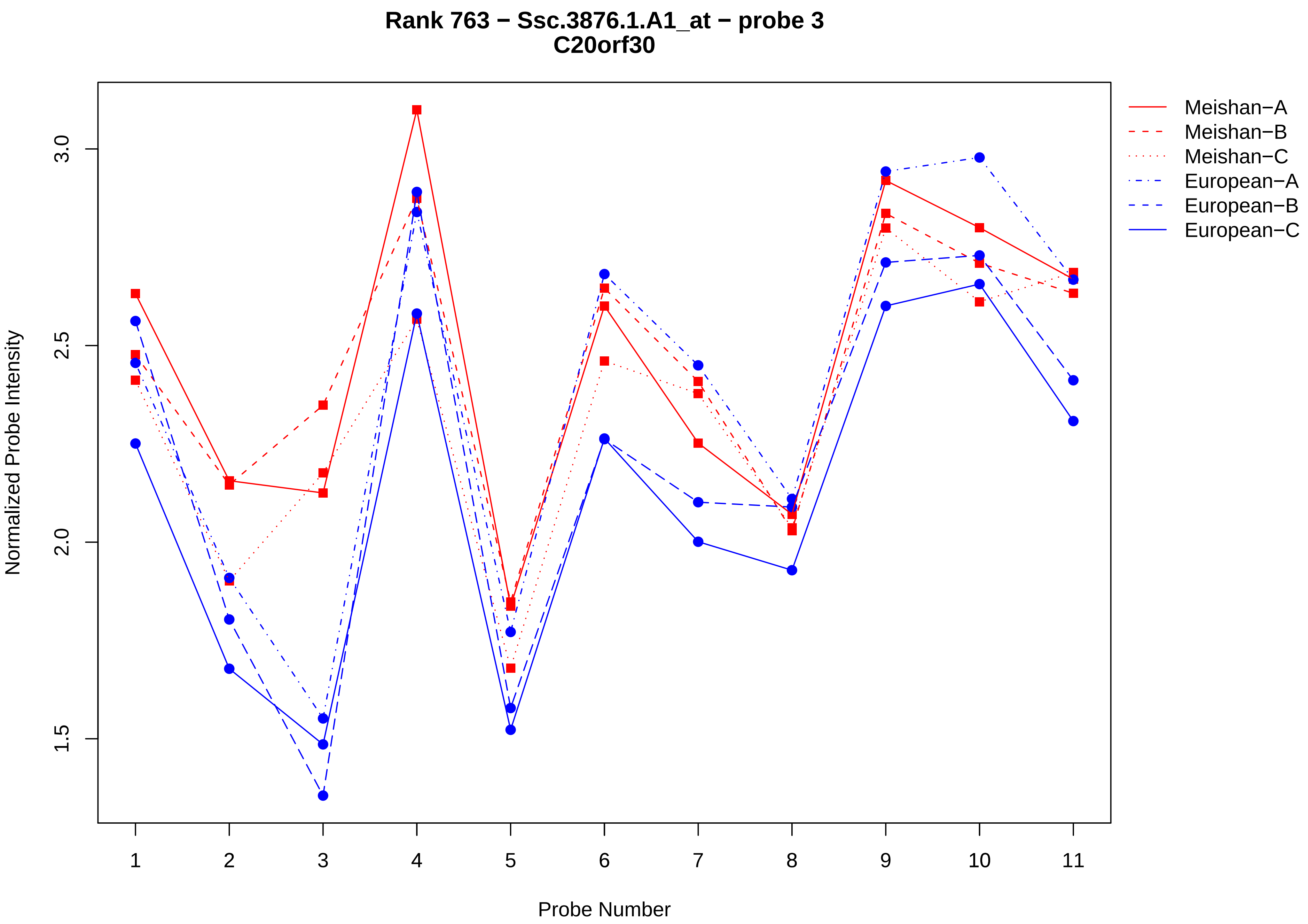
Rank 763 − Ssc.3876.1.A1_at − probe 3
C20orf30
Normalized Probe Intensity
1.5
2.0
2.5
3.0
1
2
3
4
5
6
7
8
9
10
11
Probe Number
Meishan−A
Meishan−B
Meishan−C
European−A
European−B
European−C

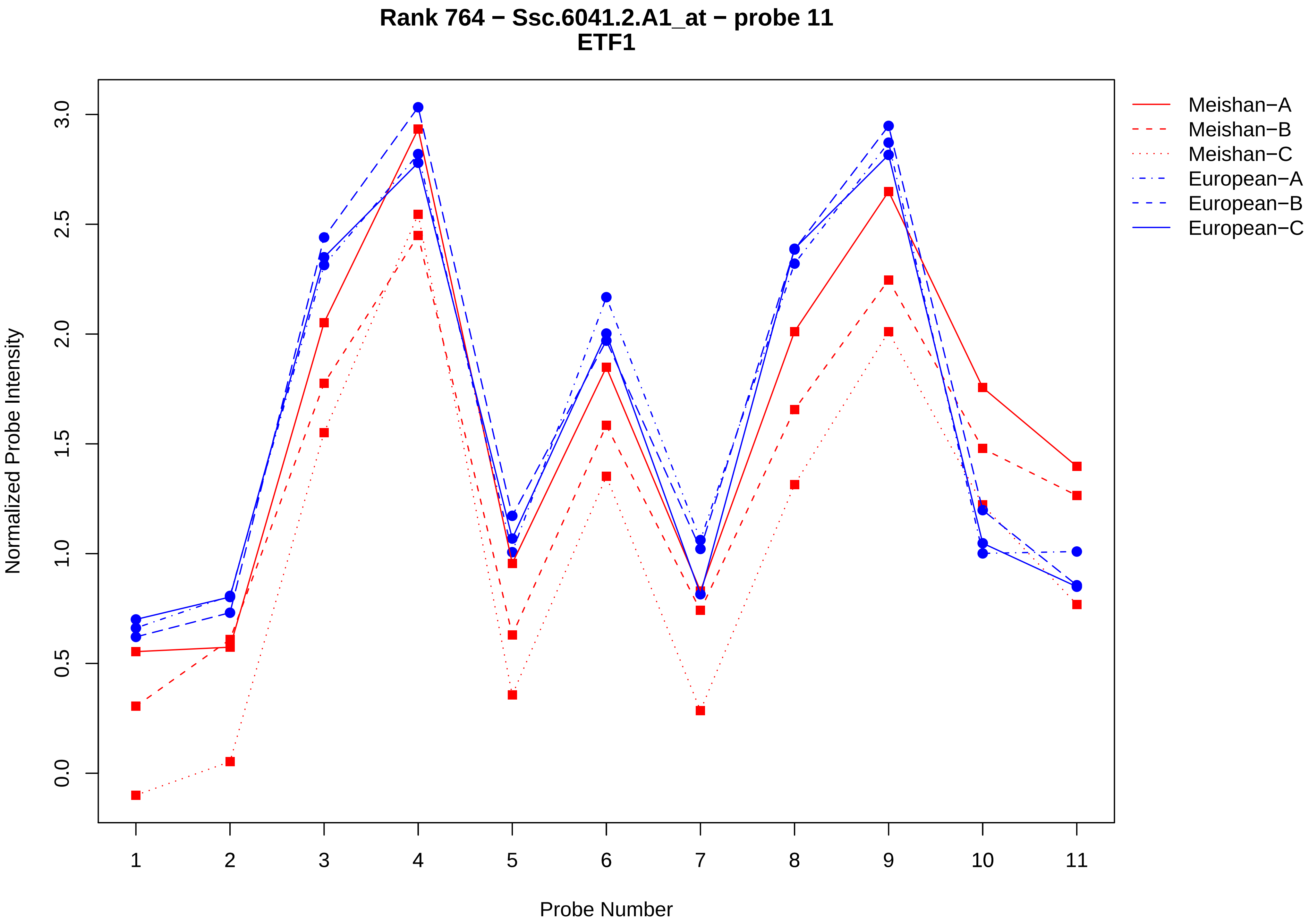
Rank 764 − Ssc.6041.2.A1_at − probe 11
ETF1
Normalized Probe Intensity
Probe Number
0.0
0.5
1.0
1.5
2.0
2.5
3.0
1
2
3
4
5
6
7
8
9
10
11
Meishan−A
Meishan−B
Meishan−C
European−A
European−B
European−C

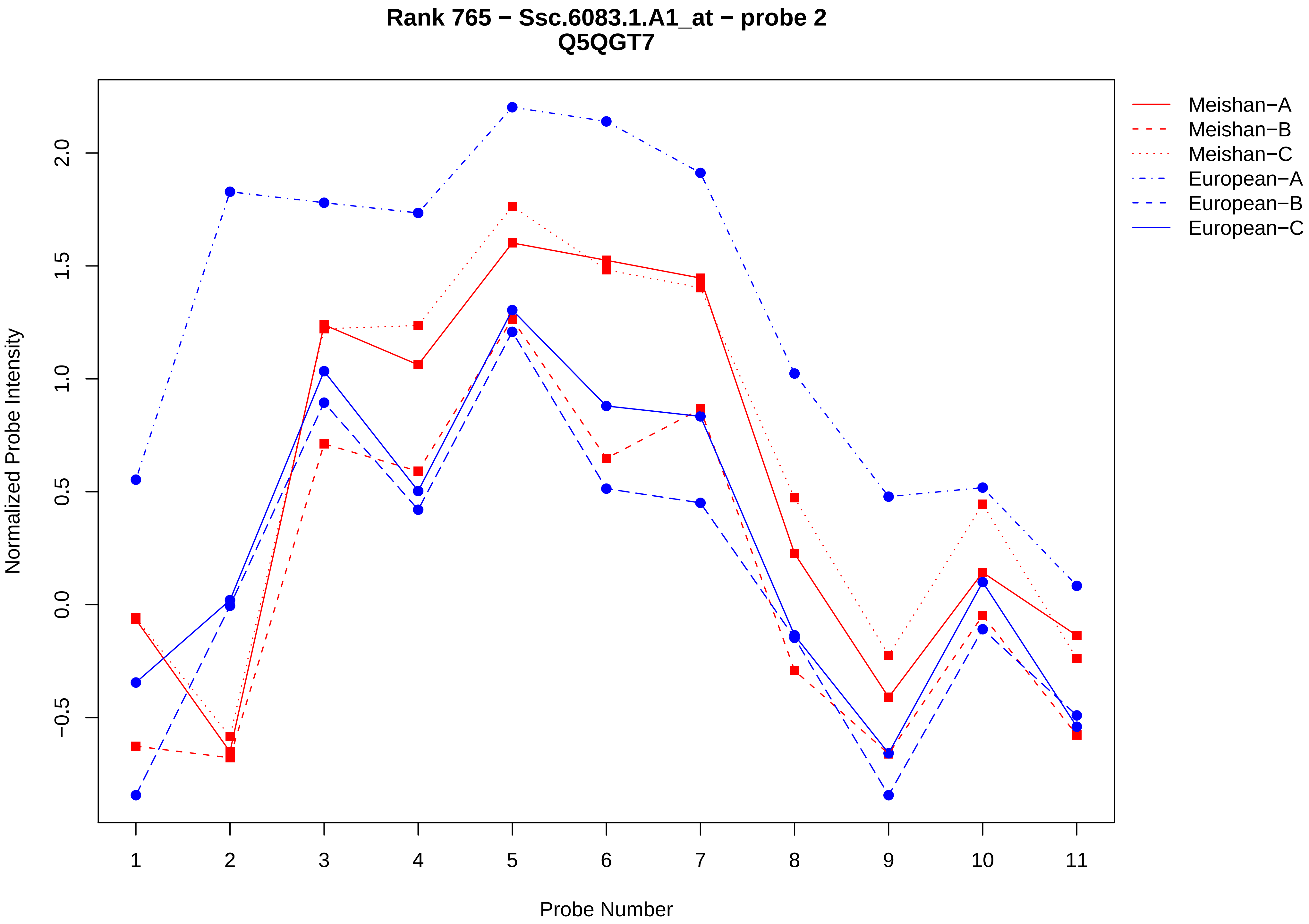

Rank 765 − Ssc.6083.1.A1_at − probe 2
Q5QGT7
Normalized Probe Intensity
Probe Number
−0.5
0.0
0.5
1.0
1.5
2.0
1
2
3
4
5
6
7
8
9
10
11
Meishan−A
Meishan−B
Meishan−C
European−A
European−B
European−C

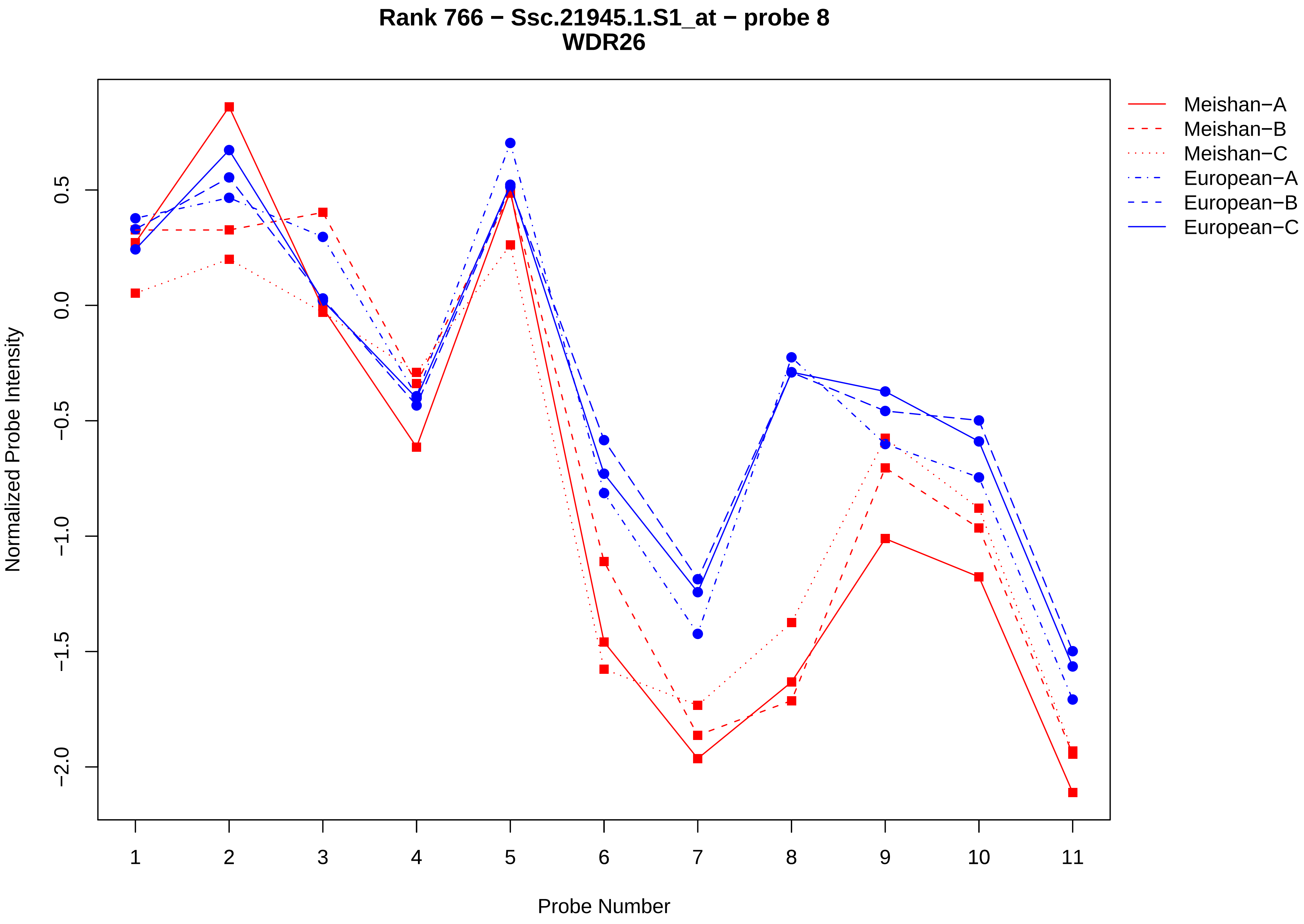

Rank 766 − Ssc.21945.1.S1_at − probe 8
WDR26
Normalized Probe Intensity
Probe Number
0.5
0.0
−0.5
−1.0
−1.5
−2.0
1
2
3
4
5
6
7
8
9
10
11
Meishan−A
Meishan−B
Meishan−C
European−A
European−B
European−C

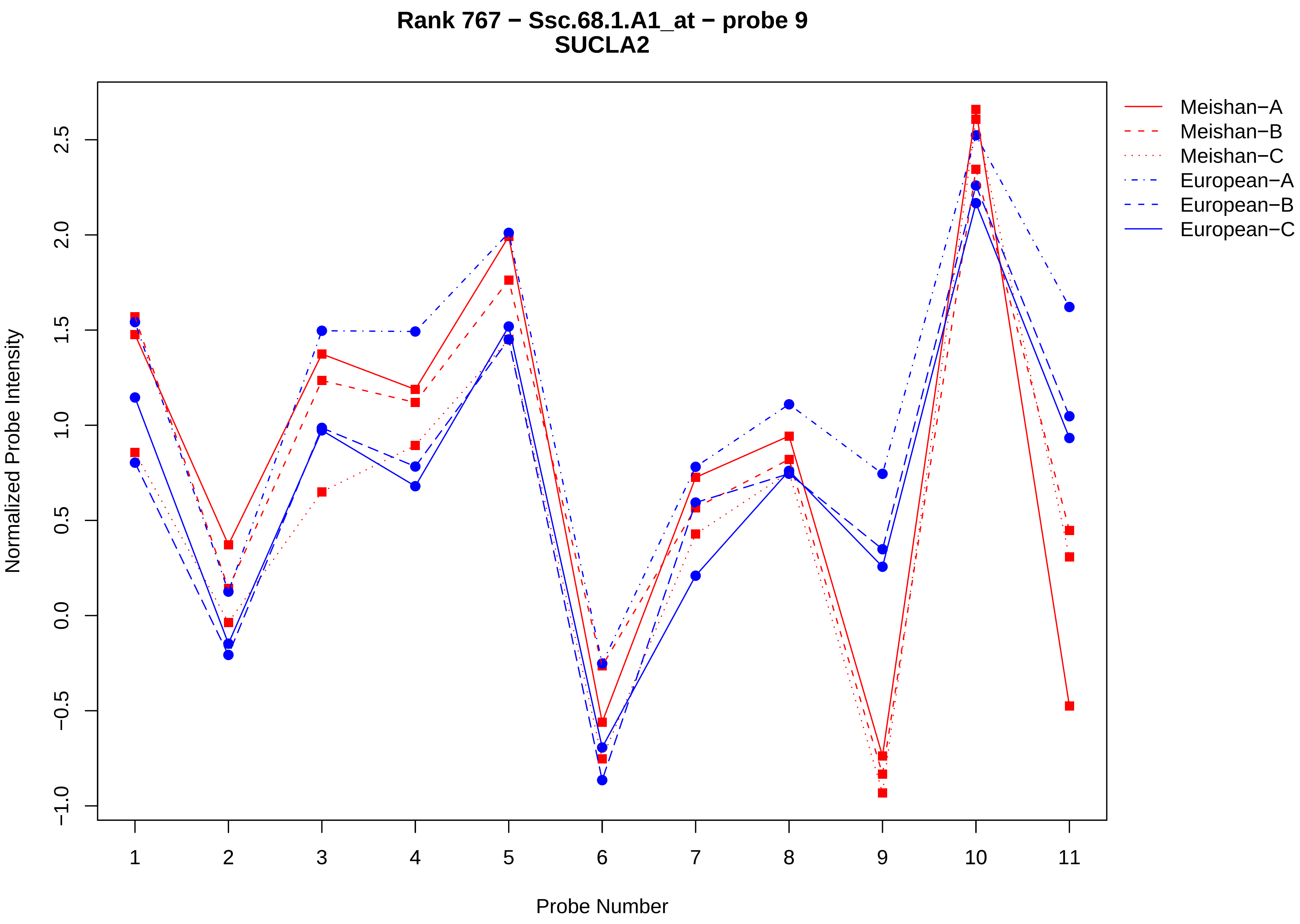

Rank 767 − Ssc.68.1.A1_at − probe 9
SUCLA2
Normalized Probe Intensity
Probe Number
Meishan−A
Meishan−B
Meishan−C
European−A
European−B
European−C
−1.0
−0.5
0.0
0.5
1.0
1.5
2.0
2.5
1
2
3
4
5
6
7
8
9
10
11

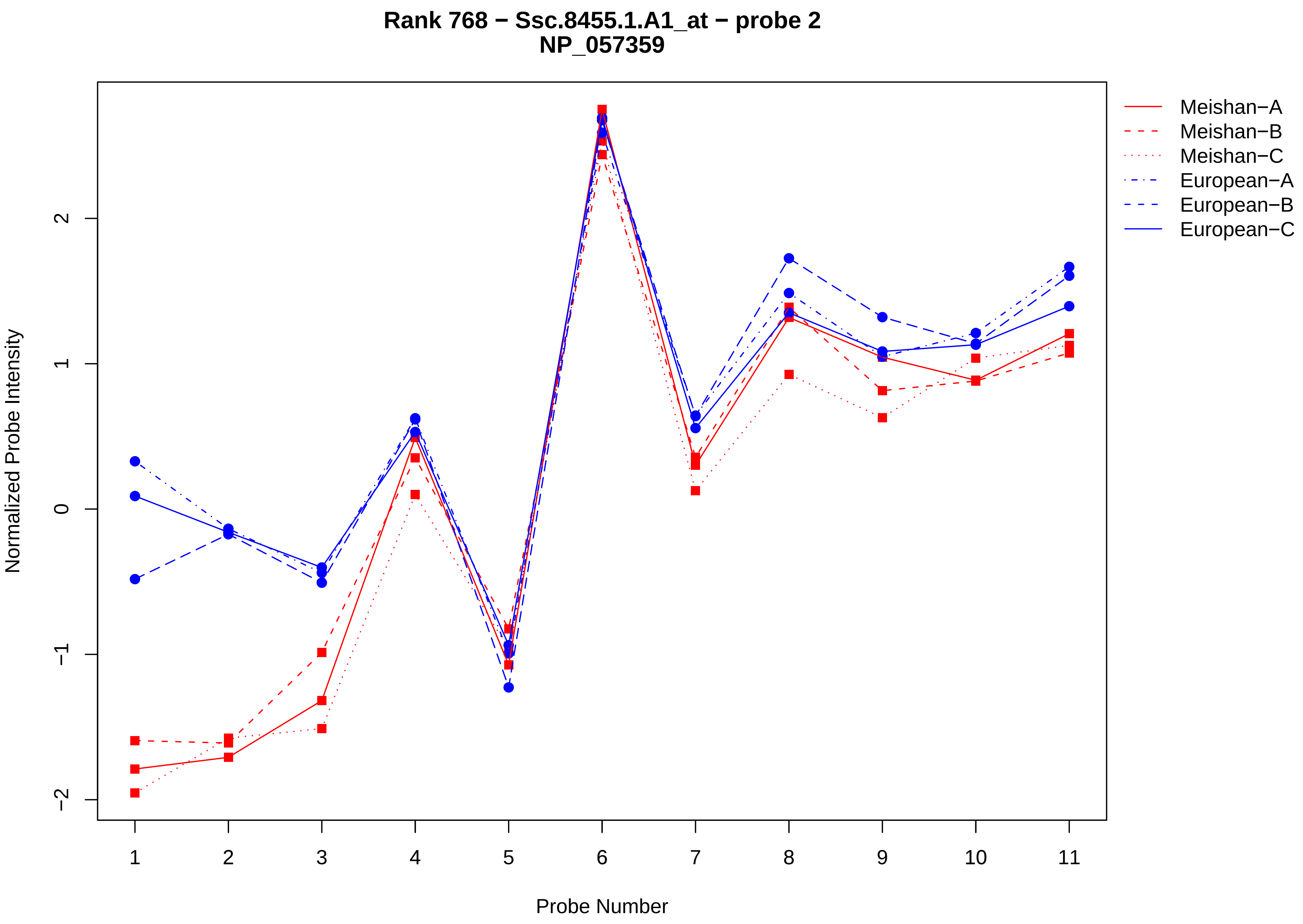
Rank 768 − Ssc.8455.1.A1_at − probe 2
NP_057359
Normalized Probe Intensity
Probe Number
−2
−1
0
1
2
1
2
3
4
5
6
7
8
9
10
11
Meishan−A
Meishan−B
Meishan−C
European−A
European−B
European−C

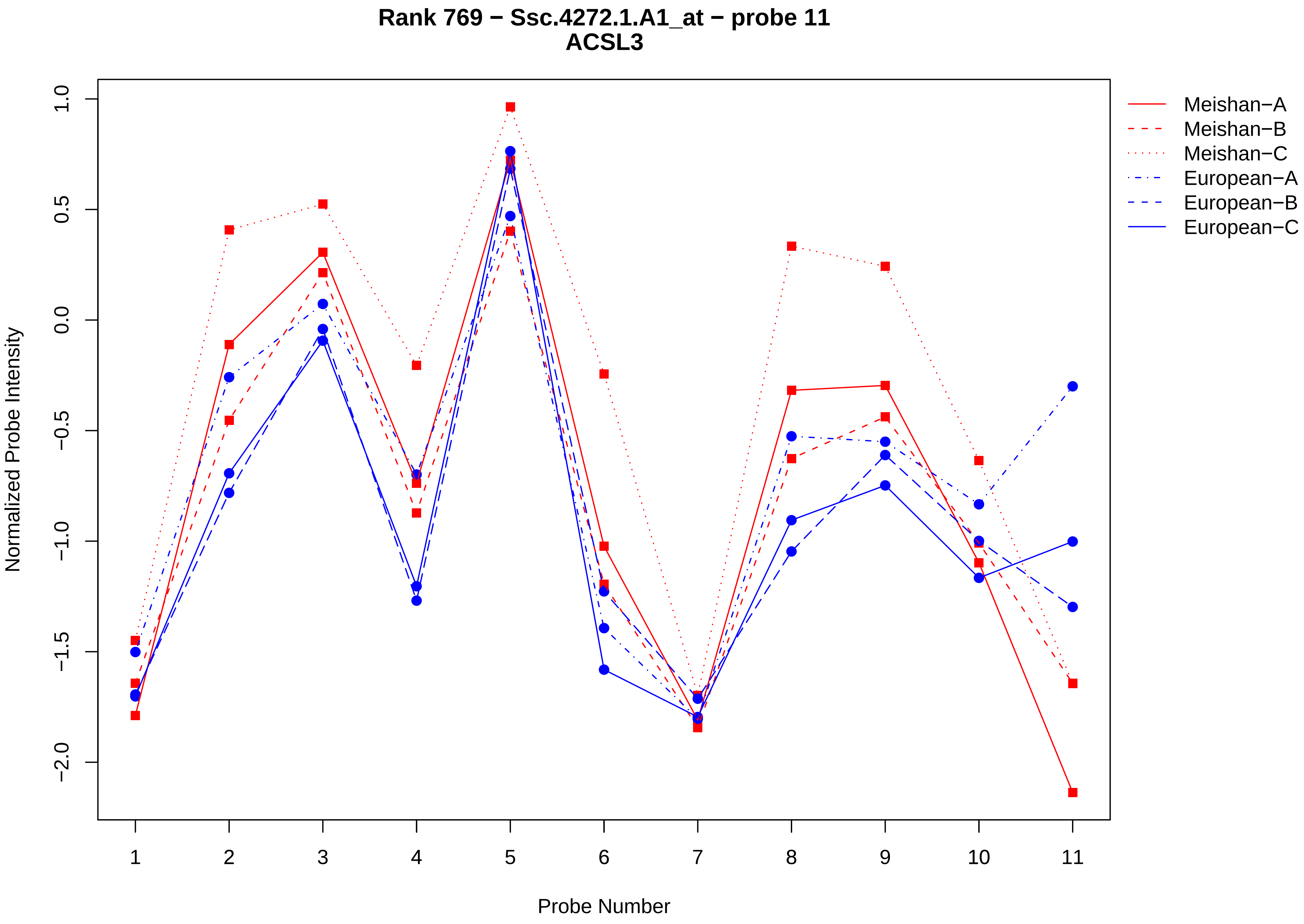

Rank 769 − Ssc.4272.1.A1_at − probe 11
ACSL3
Normalized Probe Intensity
Probe Number
1.0
0.5
0.0
−0.5
−1.0
−1.5
−2.0
1
2
3
4
5
6
7
8
9
10
11
Meishan−A
Meishan−B
Meishan−C
European−A
European−B
European−C

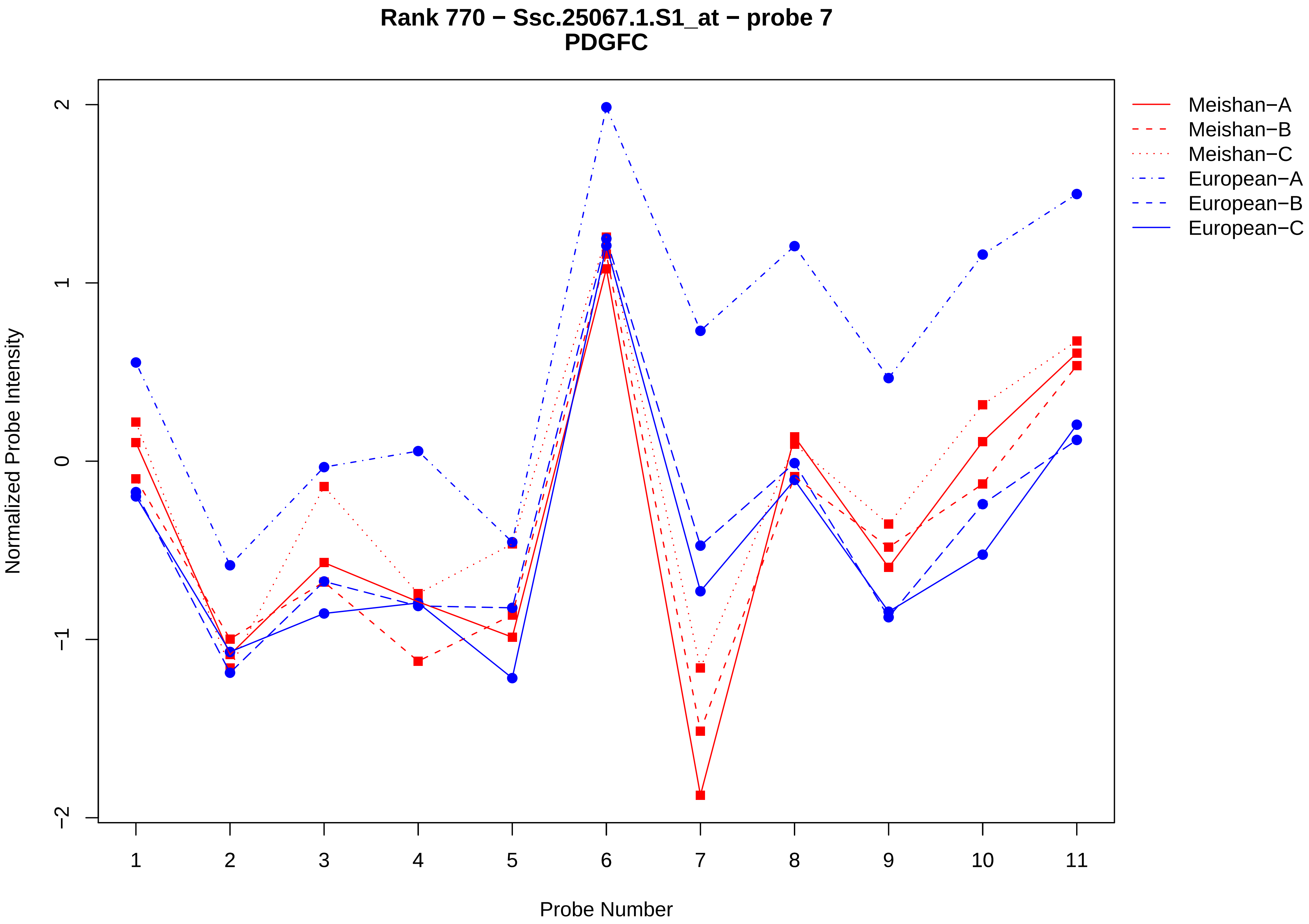

Rank 770 − Ssc.25067.1.S1_at − probe 7
PDGFC
Normalized Probe Intensity
Probe Number
Meishan−A
Meishan−B
Meishan−C
European−A
European−B
European−C
2
1
0
−1
−2
1
2
3
4
5
6
7
8
9
10
11

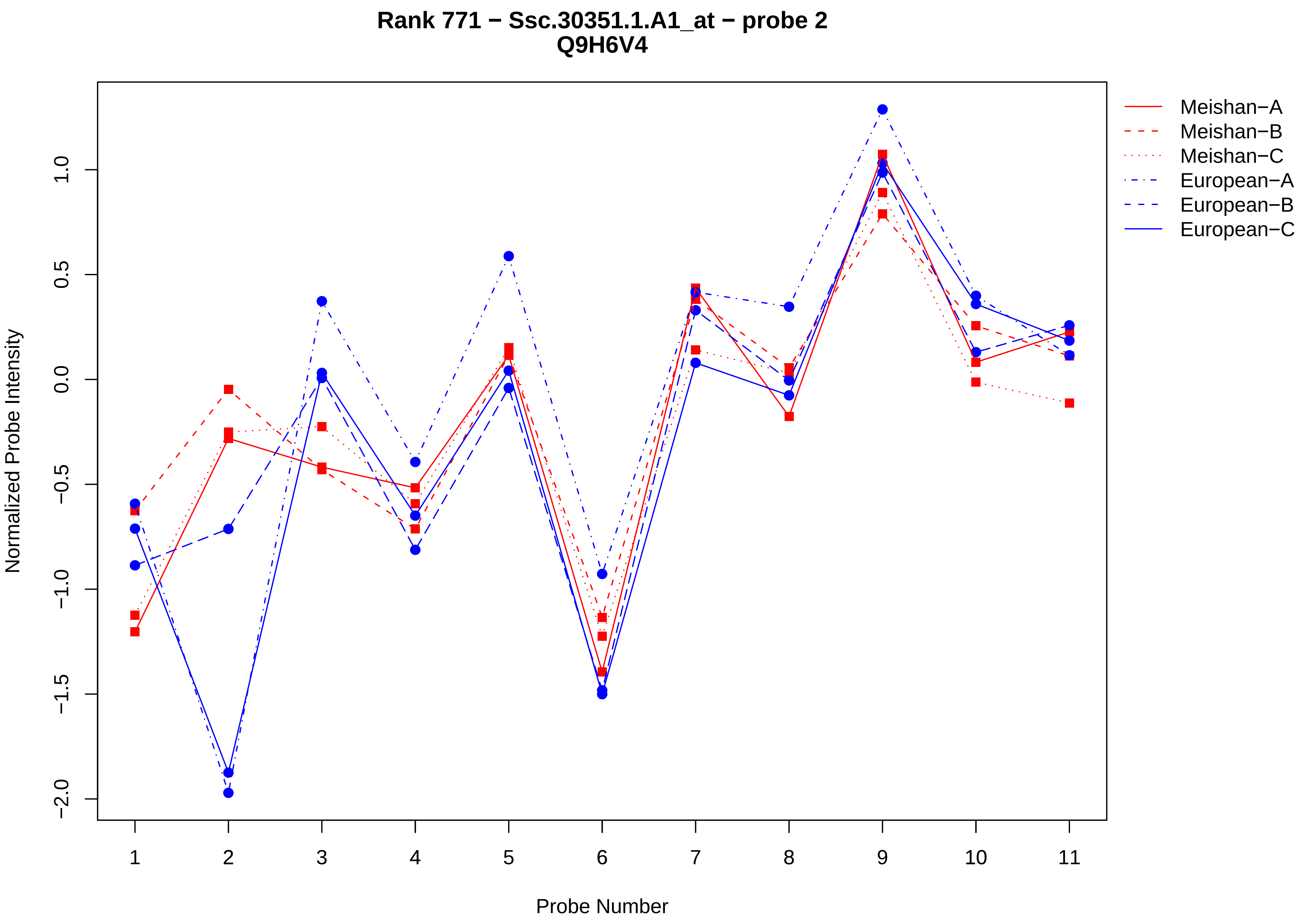

Rank 771 − Ssc.30351.1.A1_at − probe 2
Q9H6V4
Normalized Probe Intensity
Probe Number
Meishan−A
Meishan−B
Meishan−C
European−A
European−B
European−C

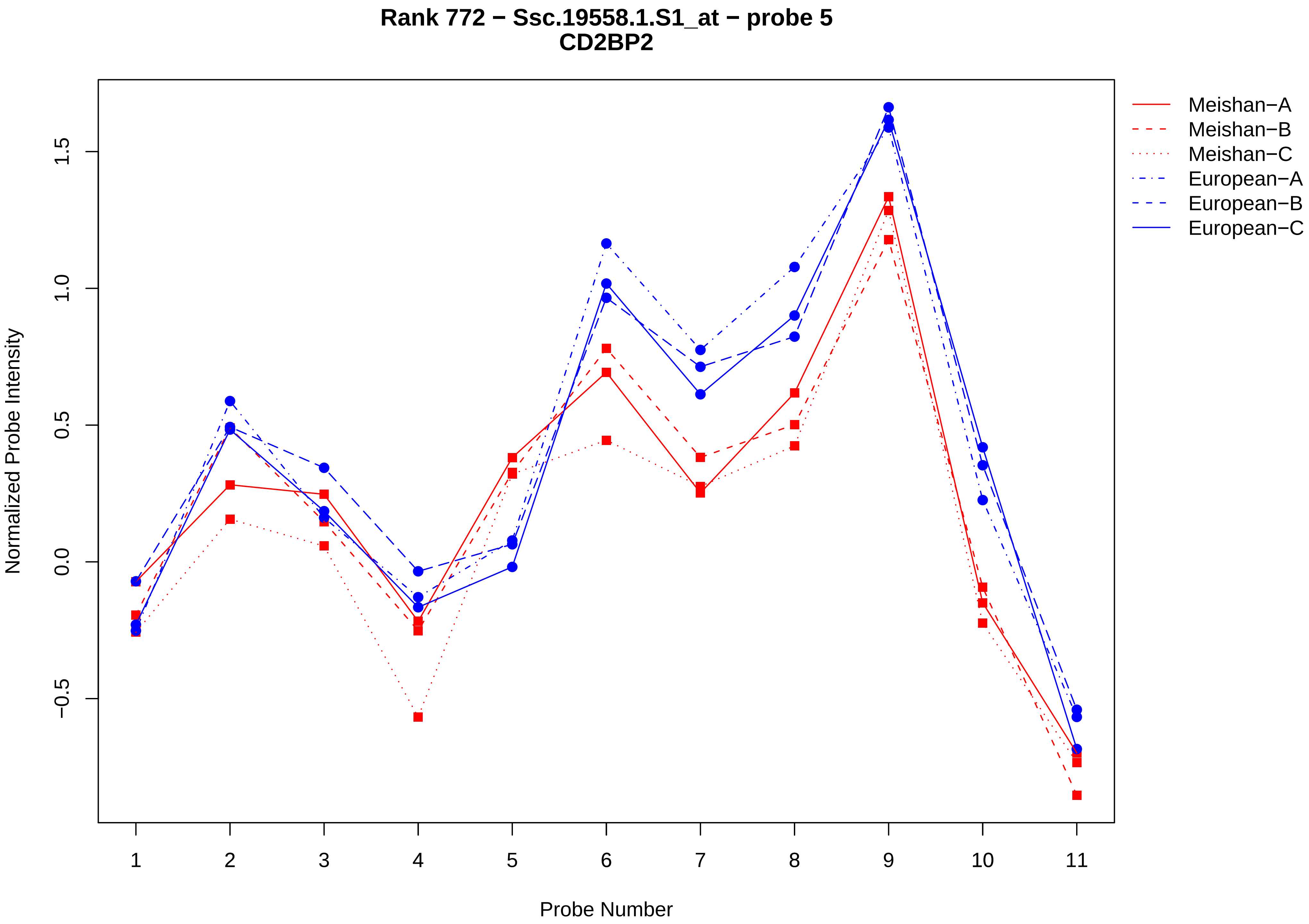
Rank 772 − Ssc.19558.1.S1_at − probe 5
CD2BP2
Normalized Probe Intensity
Probe Number
−0.5
0.0
0.5
1.0
1.5
1
2
3
4
5
6
7
8
9
10
11
Meishan−A
Meishan−B
Meishan−C
European−A
European−B
European−C

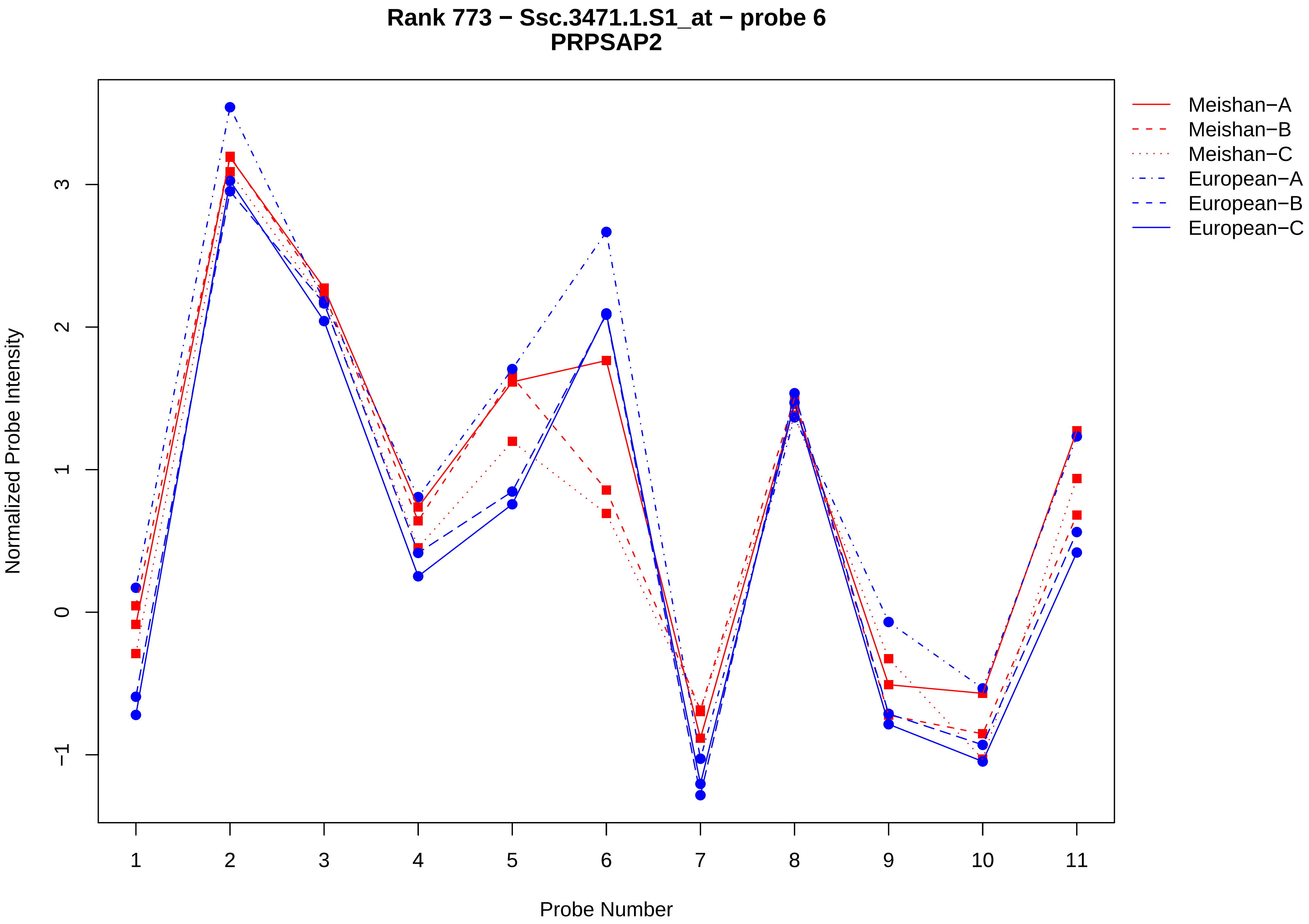

Rank 773 − Ssc.3471.1.S1_at − probe 6
PRPSAP2
Normalized Probe Intensity
Probe Number
−1
0
1
2
3
1
2
3
4
5
6
7
8
9
10
11
Meishan−A
Meishan−B
Meishan−C
European−A
European−B
European−C

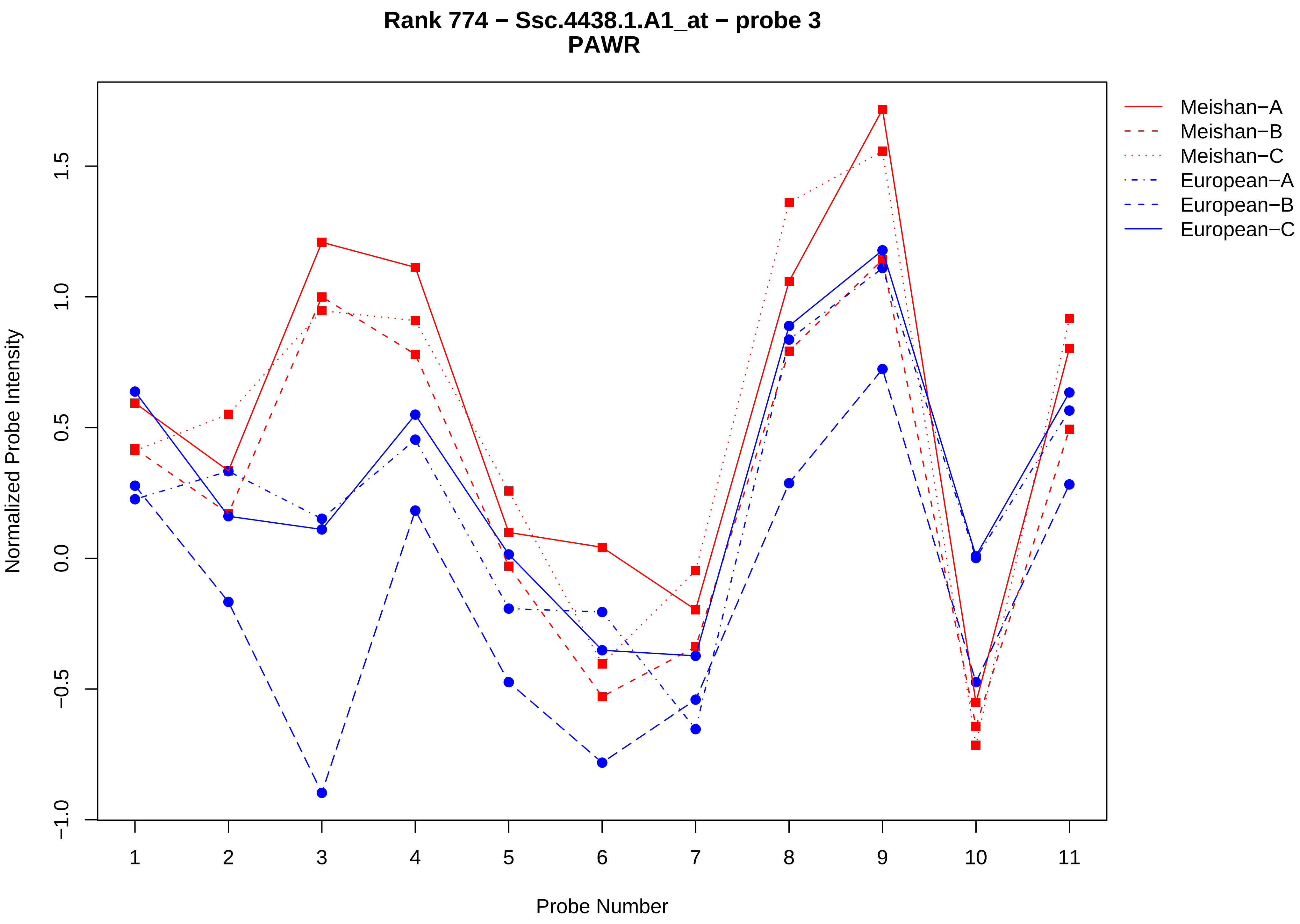
Rank 774 − Ssc.4438.1.A1_at − probe 3
PAWR
Normalized Probe Intensity
Probe Number
−1.0
−0.5
0.0
0.5
1.0
1.5
1
2
3
4
5
6
7
8
9
10
11
Meishan−A
Meishan−B
Meishan−C
European−A
European−B
European−C

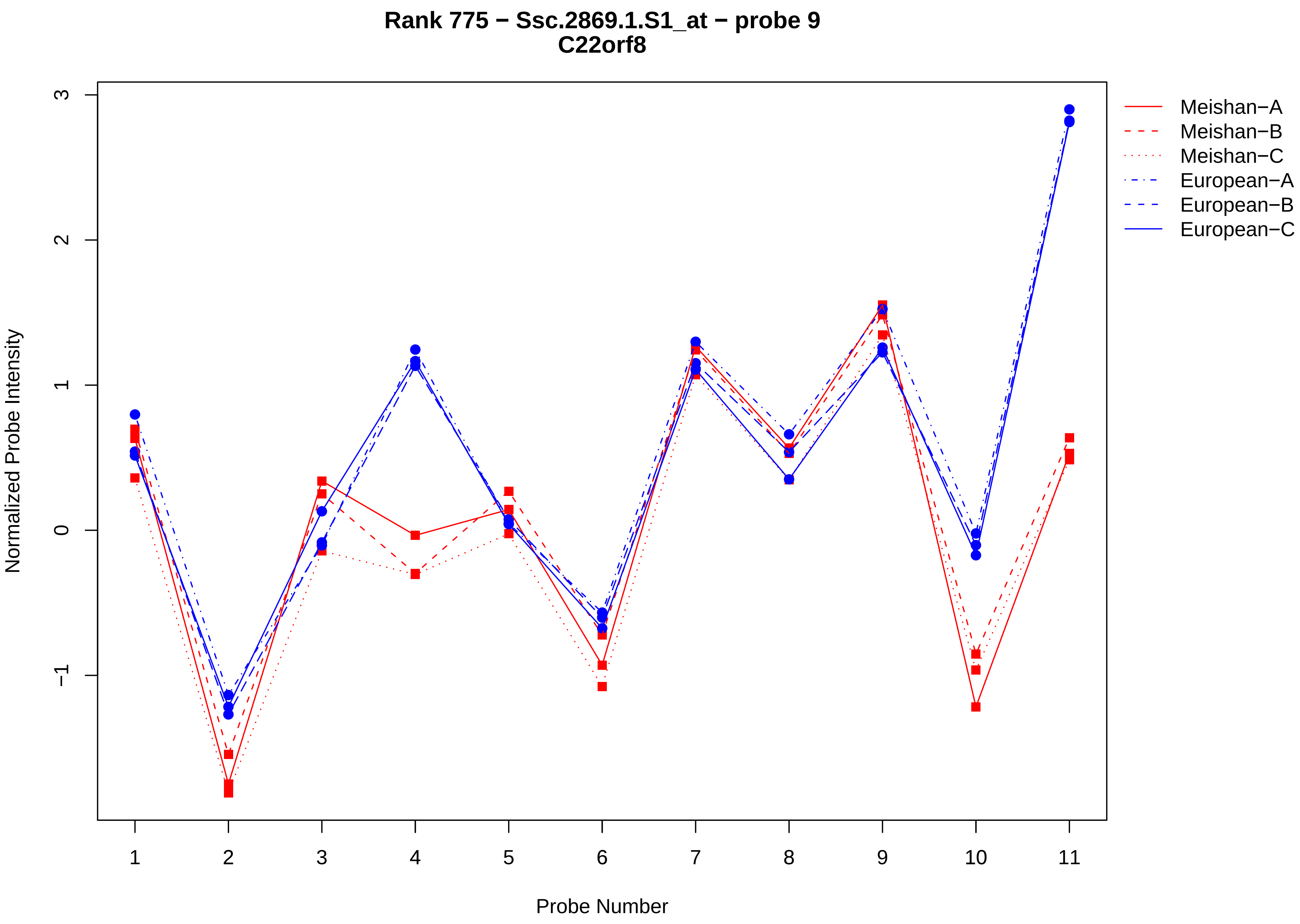

Rank 775 − Ssc.2869.1.S1_at − probe 9
C22orf8
Normalized Probe Intensity
Probe Number
−1
0
1
2
3
1
2
3
4
5
6
7
8
9
10
11
Meishan−A
Meishan−B
Meishan−C
European−A
European−B
European−C

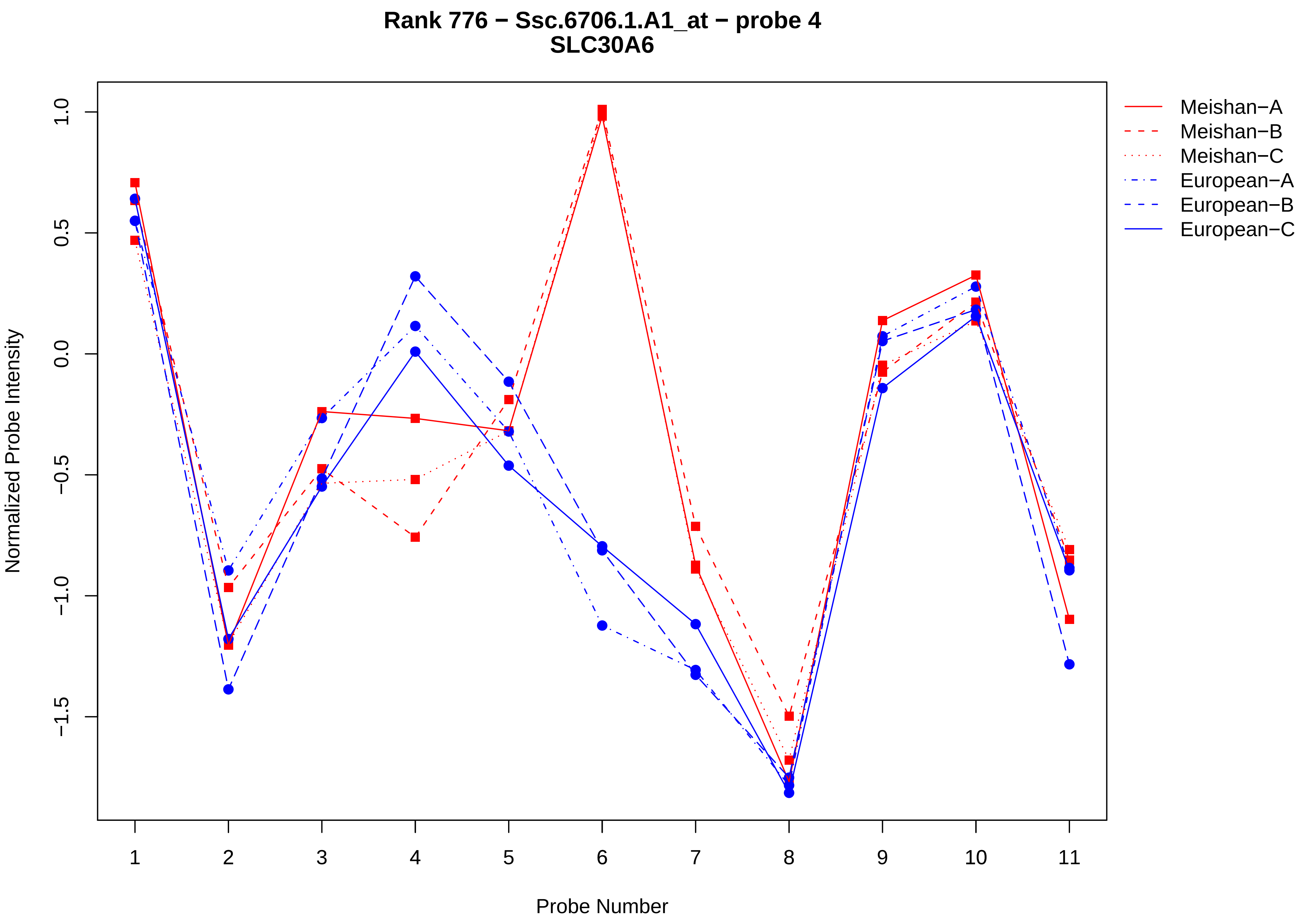

Rank 776 − Ssc.6706.1.A1_at − probe 4
SLC30A6
Normalized Probe Intensity
Probe Number
Meishan−A
Meishan−B
Meishan−C
European−A
European−B
European−C
1.0
0.5
0.0
−0.5
−1.0
−1.5
1
2
3
4
5
6
7
8
9
10
11

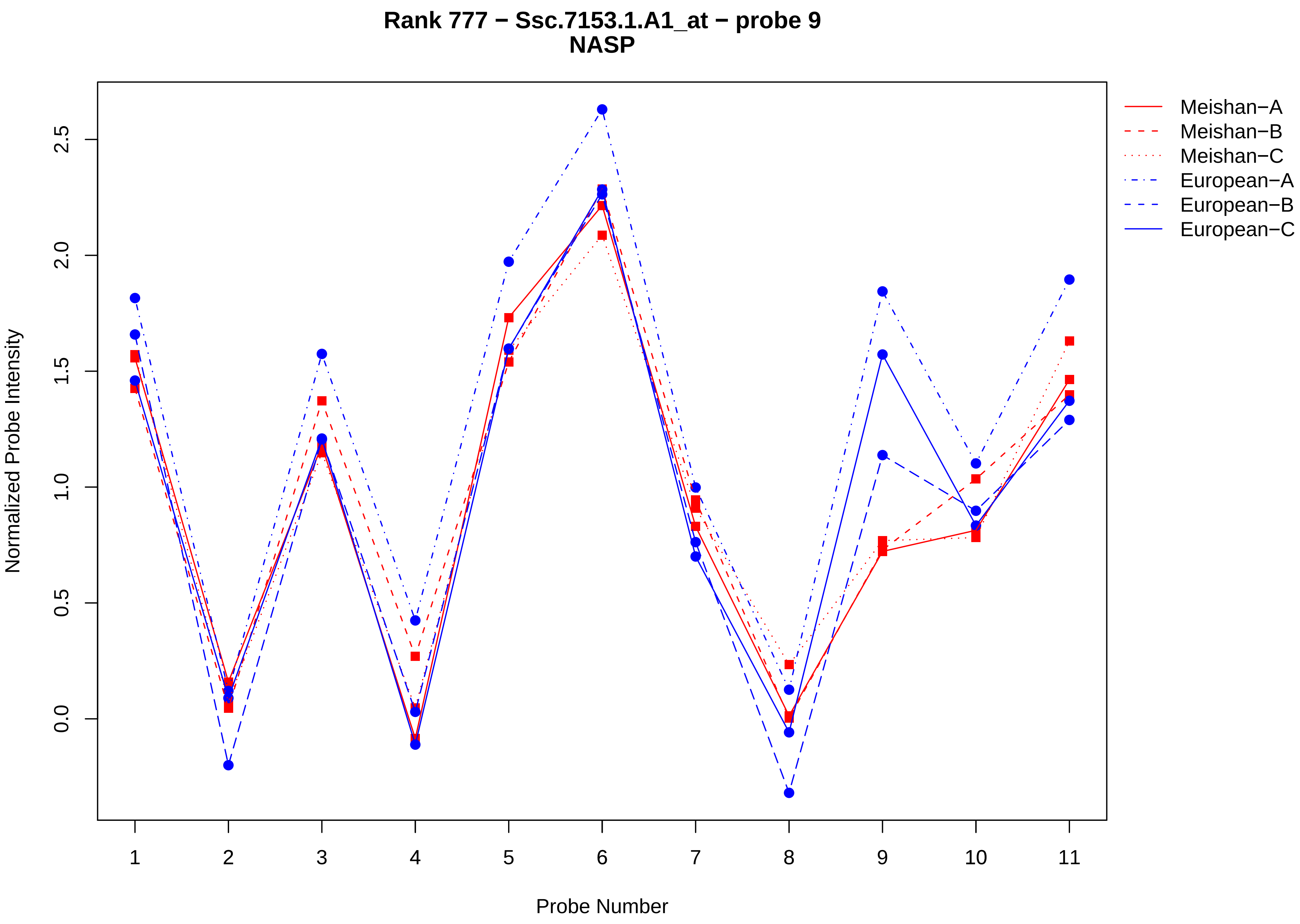

Rank 777 − Ssc.7153.1.A1_at − probe 9
NASP
Normalized Probe Intensity
Probe Number
0.0
0.5
1.0
1.5
2.0
2.5
1
2
3
4
5
6
7
8
9
10
11
Meishan−A
Meishan−B
Meishan−C
European−A
European−B
European−C

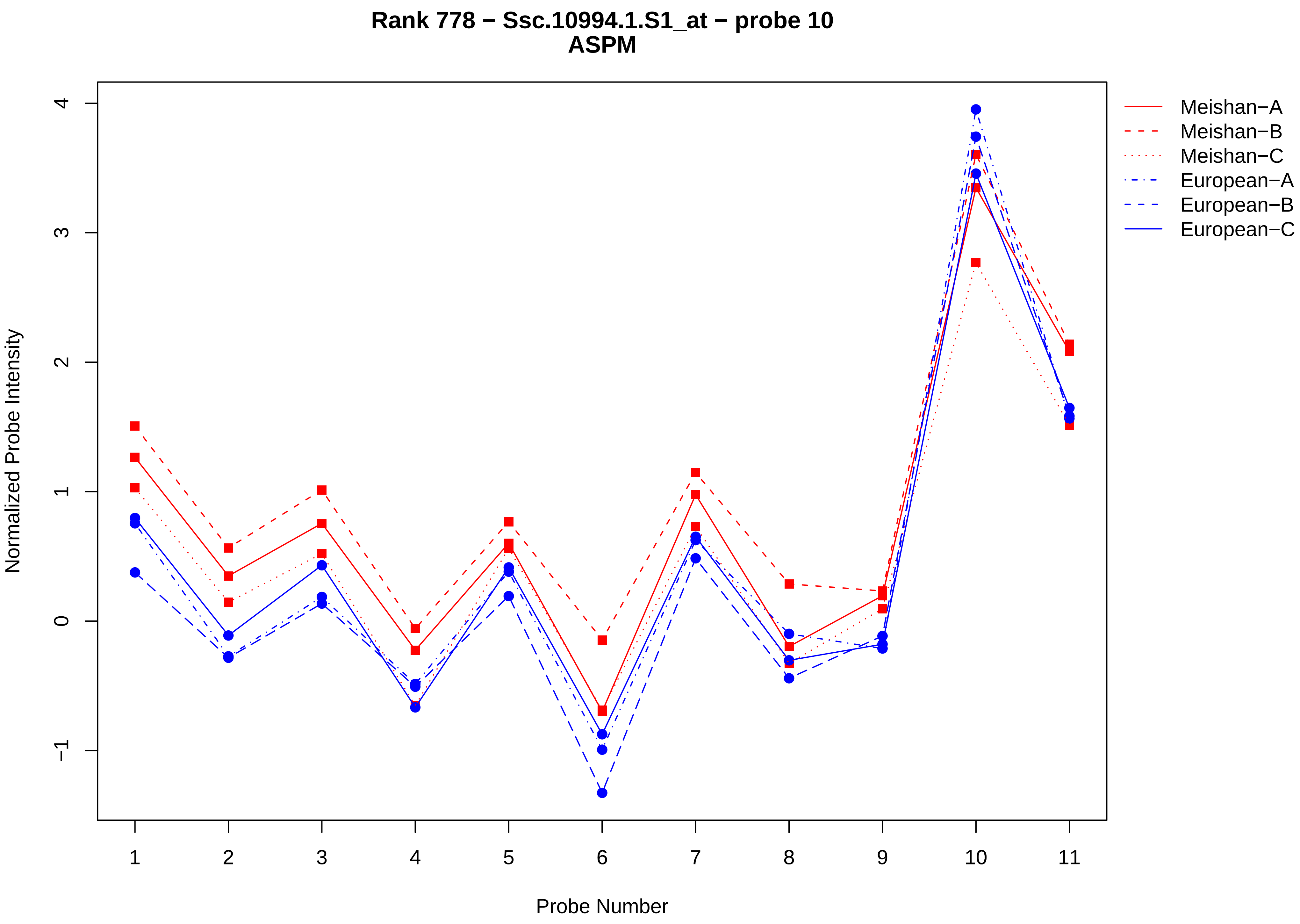
Rank 778 − Ssc.10994.1.S1_at − probe 10
ASPM
Normalized Probe Intensity
Probe Number
Meishan−A
Meishan−B
Meishan−C
European−A
European−B
European−C
−1
0
1
2
3
4
1
2
3
4
5
6
7
8
9
10
11

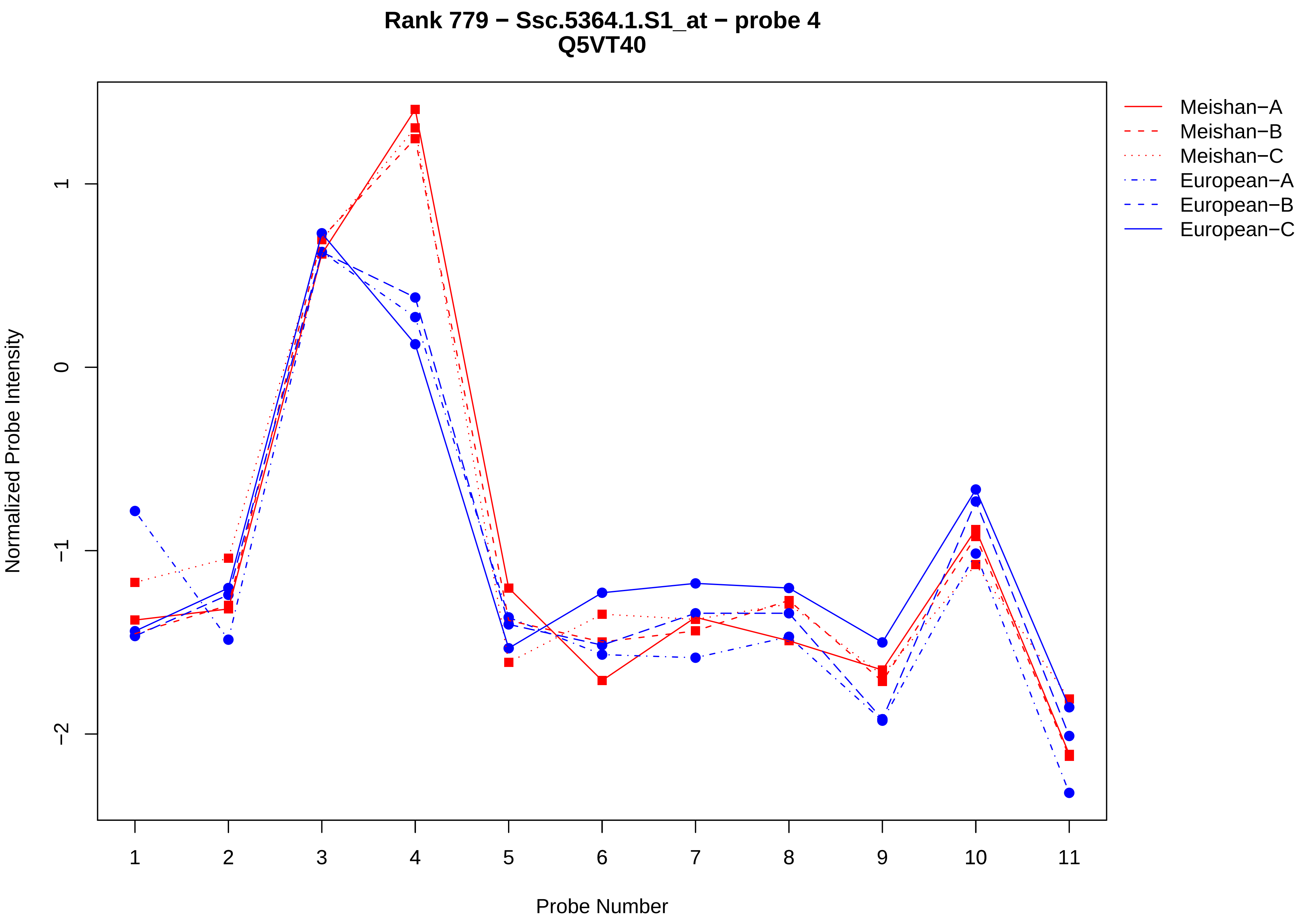

Rank 779 − Ssc.5364.1.S1_at − probe 4
Q5VT40
Normalized Probe Intensity
Probe Number
1
0
−1
−2
1
2
3
4
5
6
7
8
9
10
11
Meishan−A
Meishan−B
Meishan−C
European−A
European−B
European−C

Rank 780 − Ssc.5676.1.S1_at − probe 10
NP_056455

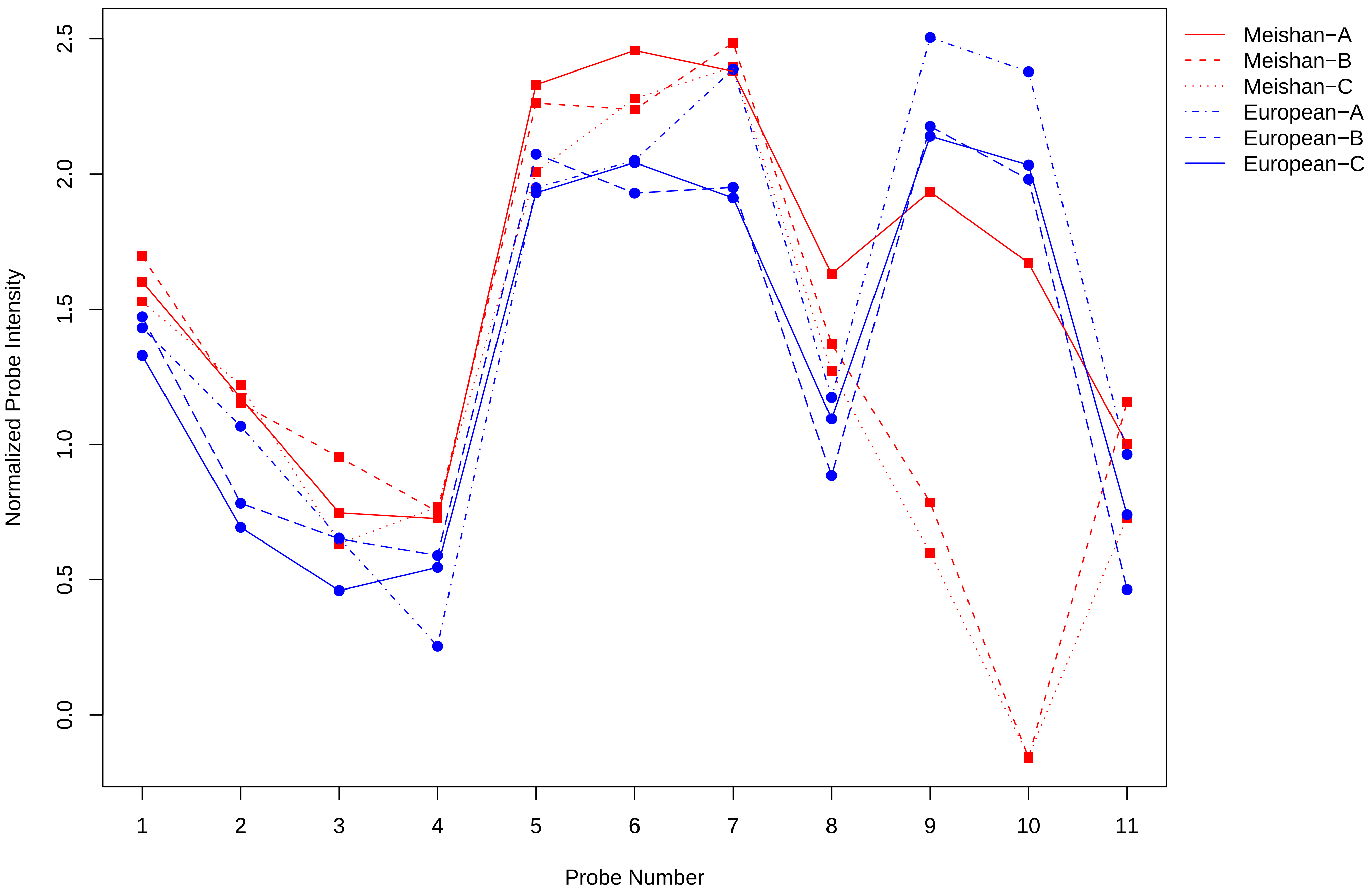

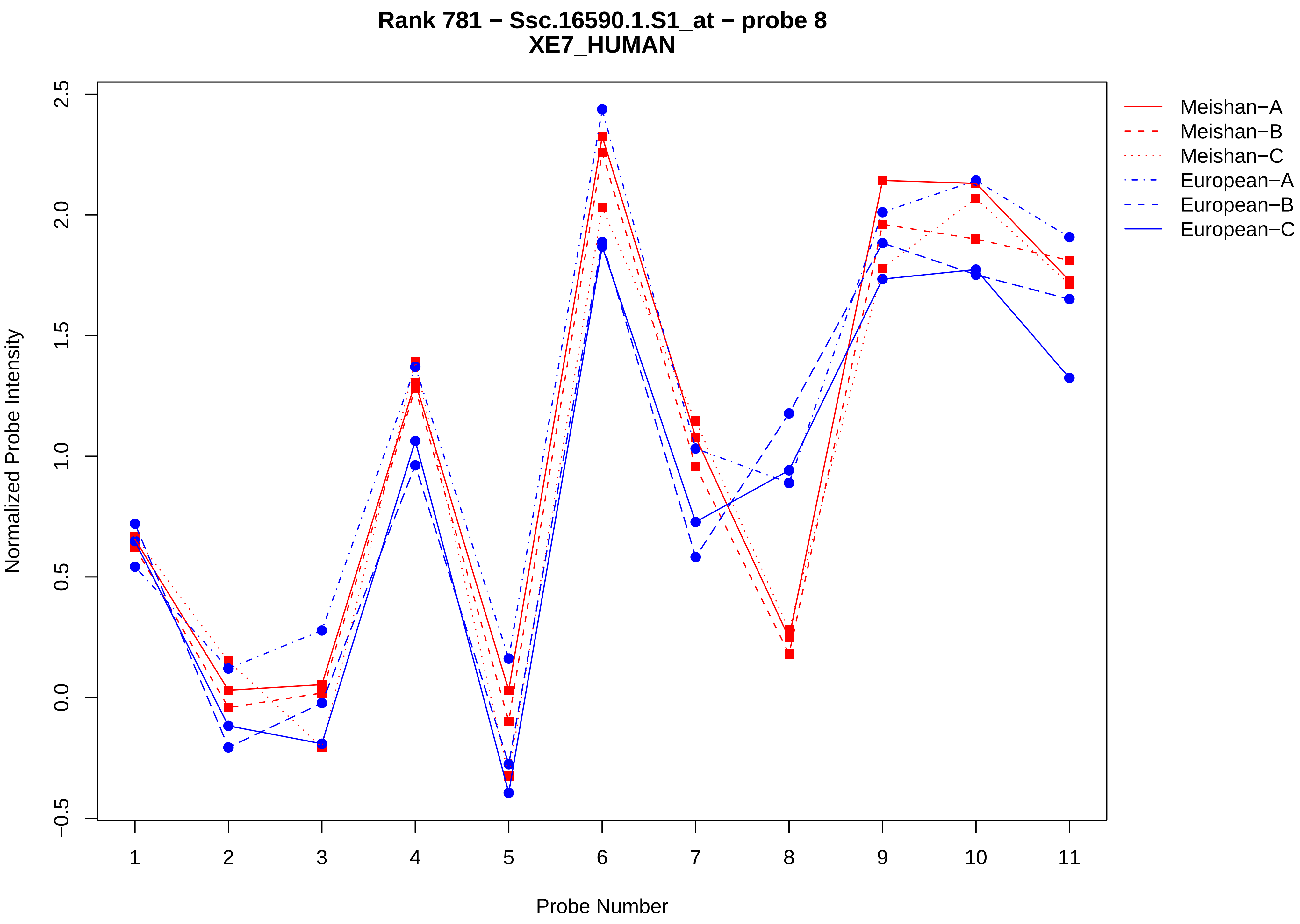
Rank 781 − Ssc.16590.1.S1_at − probe 8
XE7_HUMAN
Normalized Probe Intensity
Probe Number
−0.5
0.0
0.5
1.0
1.5
2.0
2.5
1
2
3
4
5
6
7
8
9
10
11
Meishan−A
Meishan−B
Meishan−C
European−A
European−B
European−C

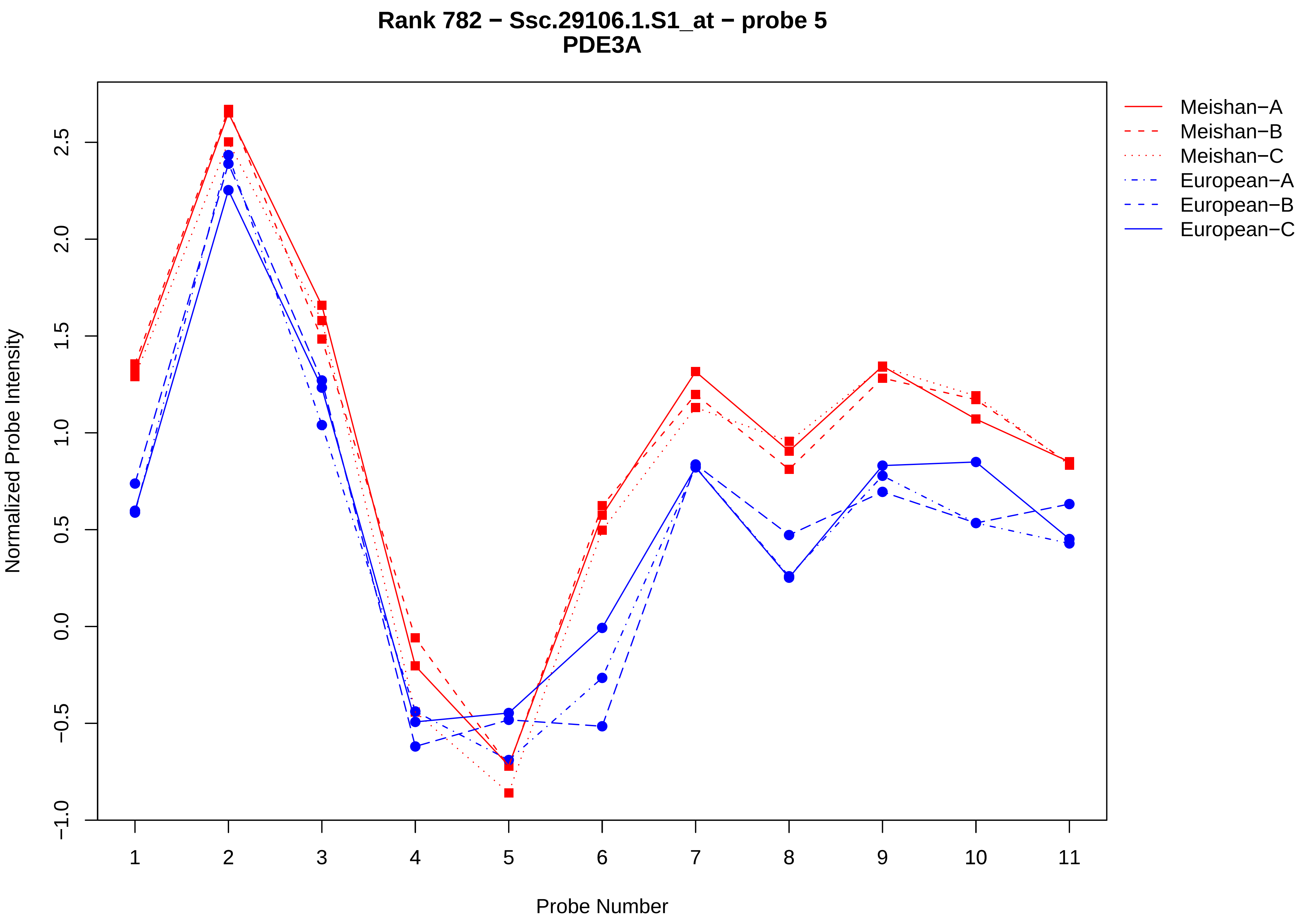
Rank 782 − Ssc.29106.1.S1_at − probe 5
PDE3A
Normalized Probe Intensity
Probe Number
−1.0
−0.5
0.0
0.5
1.0
1.5
2.0
2.5
1
2
3
4
5
6
7
8
9
10
11
Meishan−A
Meishan−B
Meishan−C
European−A
European−B
European−C

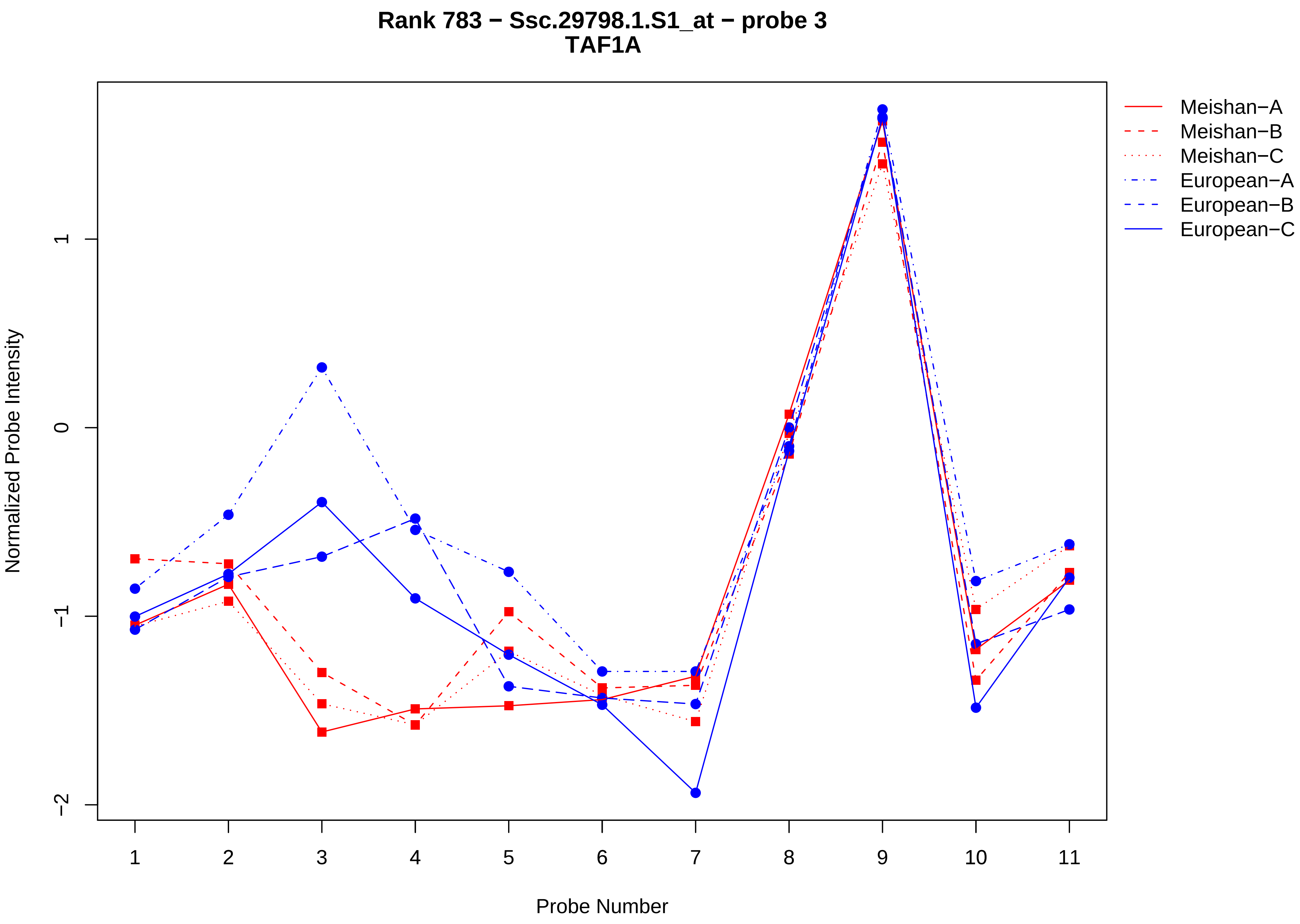

Rank 783 − Ssc.29798.1.S1_at − probe 3
TAF1A
Normalized Probe Intensity
Probe Number
−2
−1
0
1
1
2
3
4
5
6
7
8
9
10
11
Meishan−A
Meishan−B
Meishan−C
European−A
European−B
European−C

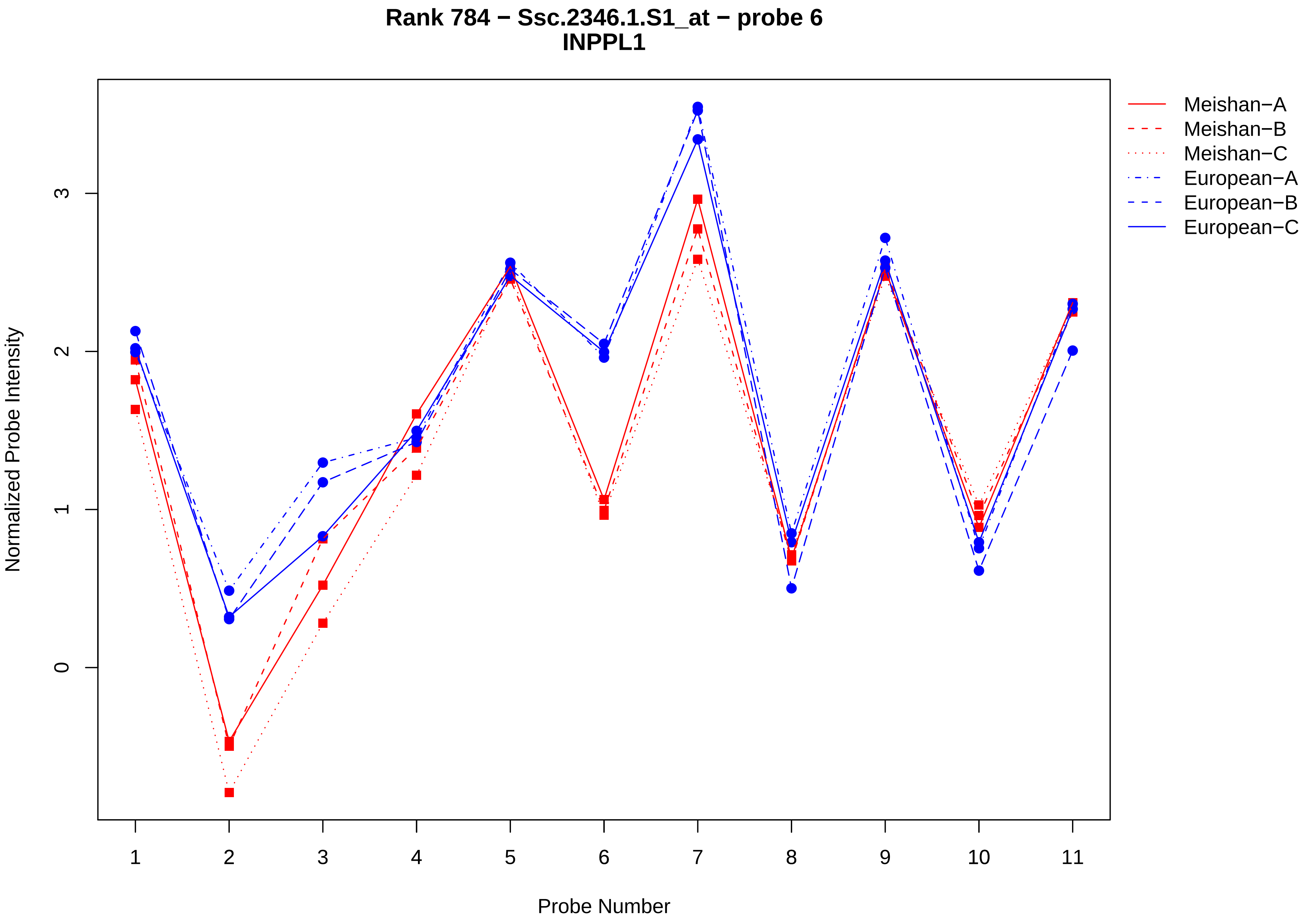

Rank 784 − Ssc.2346.1.S1_at − probe 6
INPPL1
Normalized Probe Intensity
0
1
2
3
1
2
3
4
5
6
7
8
9
10
11
Probe Number
Meishan−A
Meishan−B
Meishan−C
European−A
European−B
European−C

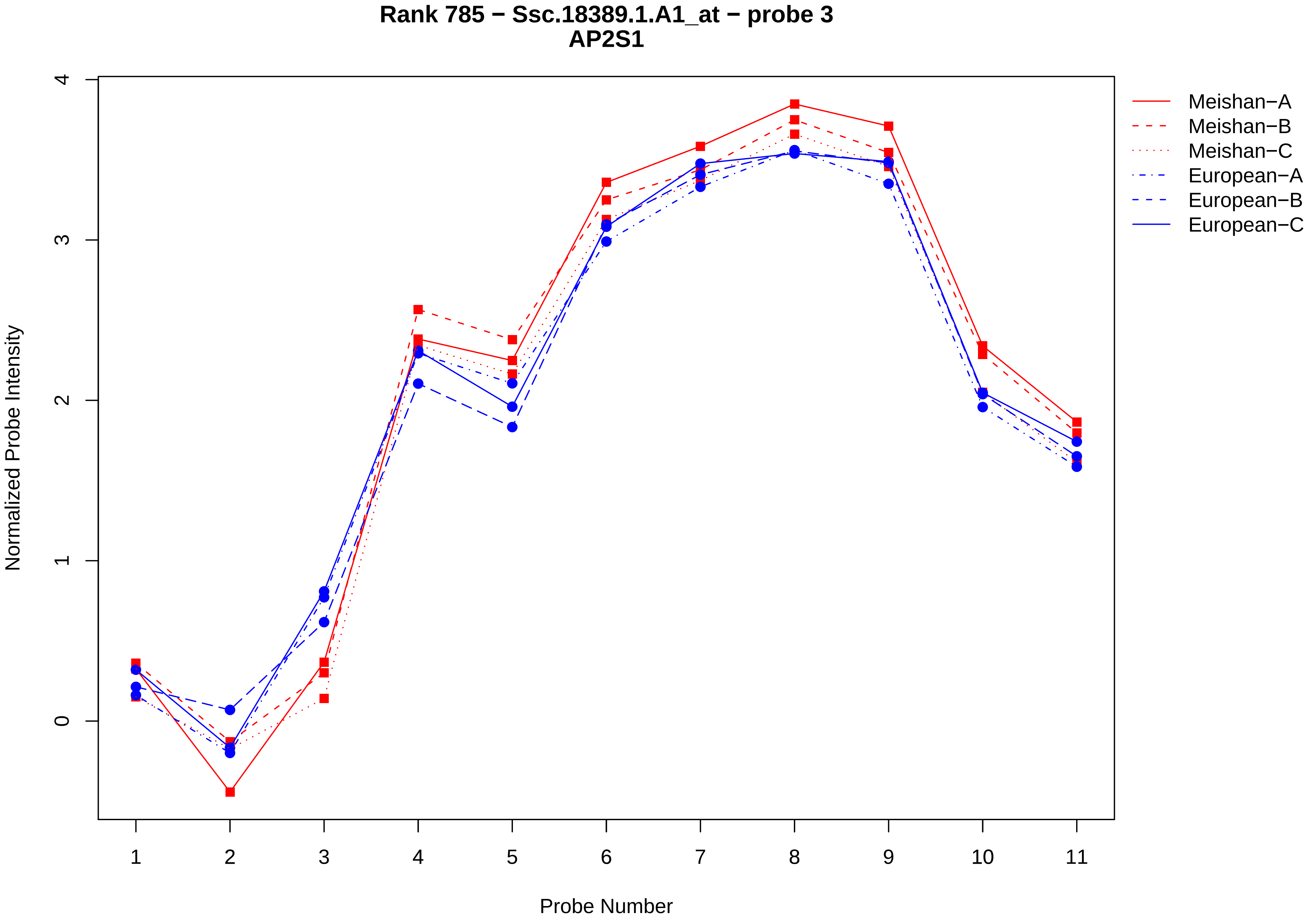
Rank 785 − Ssc.18389.1.A1_at − probe 3
AP2S1
Normalized Probe Intensity
Probe Number
0
1
2
3
4
1
2
3
4
5
6
7
8
9
10
11
Meishan−A
Meishan−B
Meishan−C
European−A
European−B
European−C

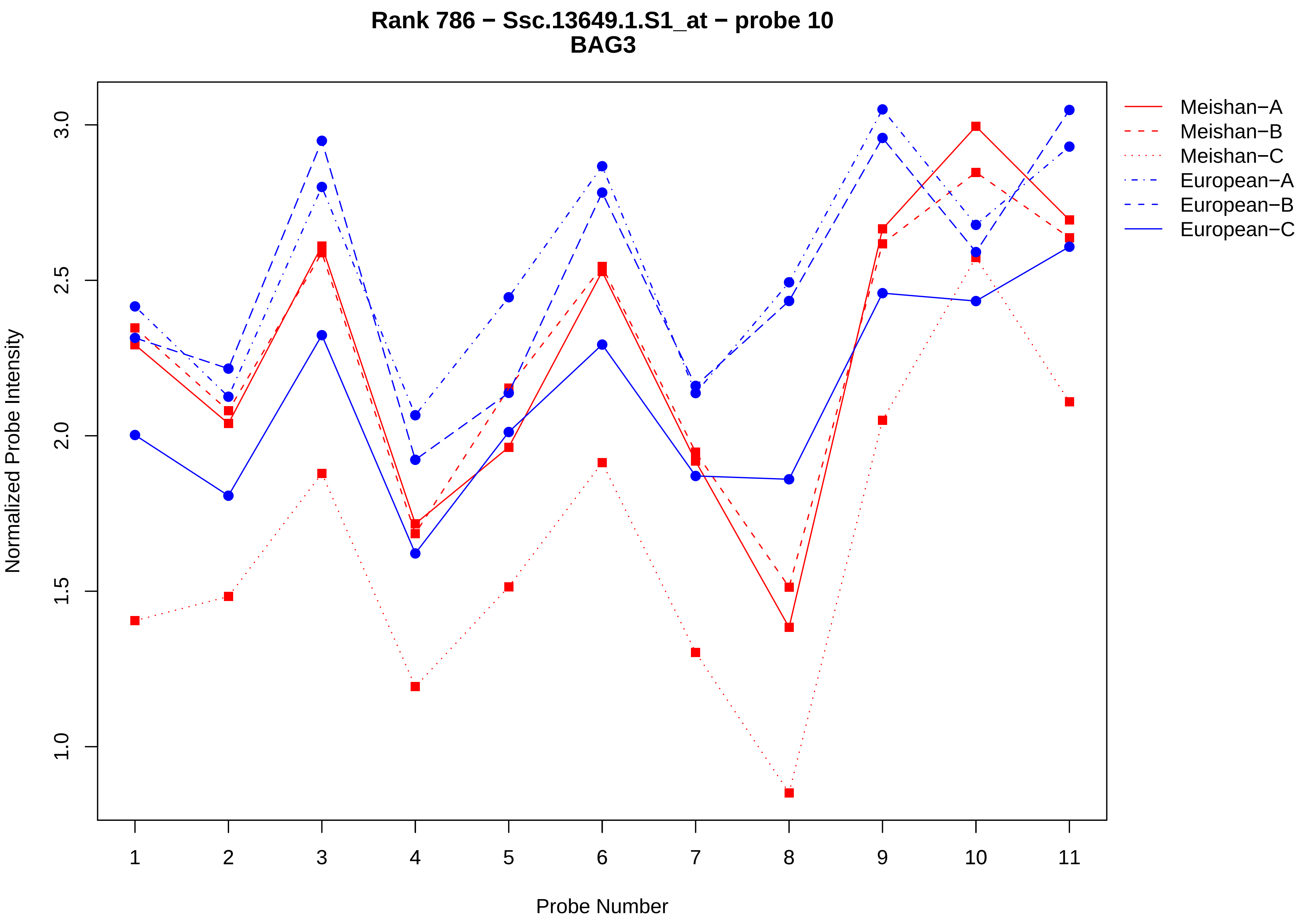

Rank 786 − Ssc.13649.1.S1_at − probe 10
BAG3
Normalized Probe Intensity
Probe Number
1.0
1.5
2.0
2.5
3.0
1
2
3
4
5
6
7
8
9
10
11
Meishan−A
Meishan−B
Meishan−C
European−A
European−B
European−C

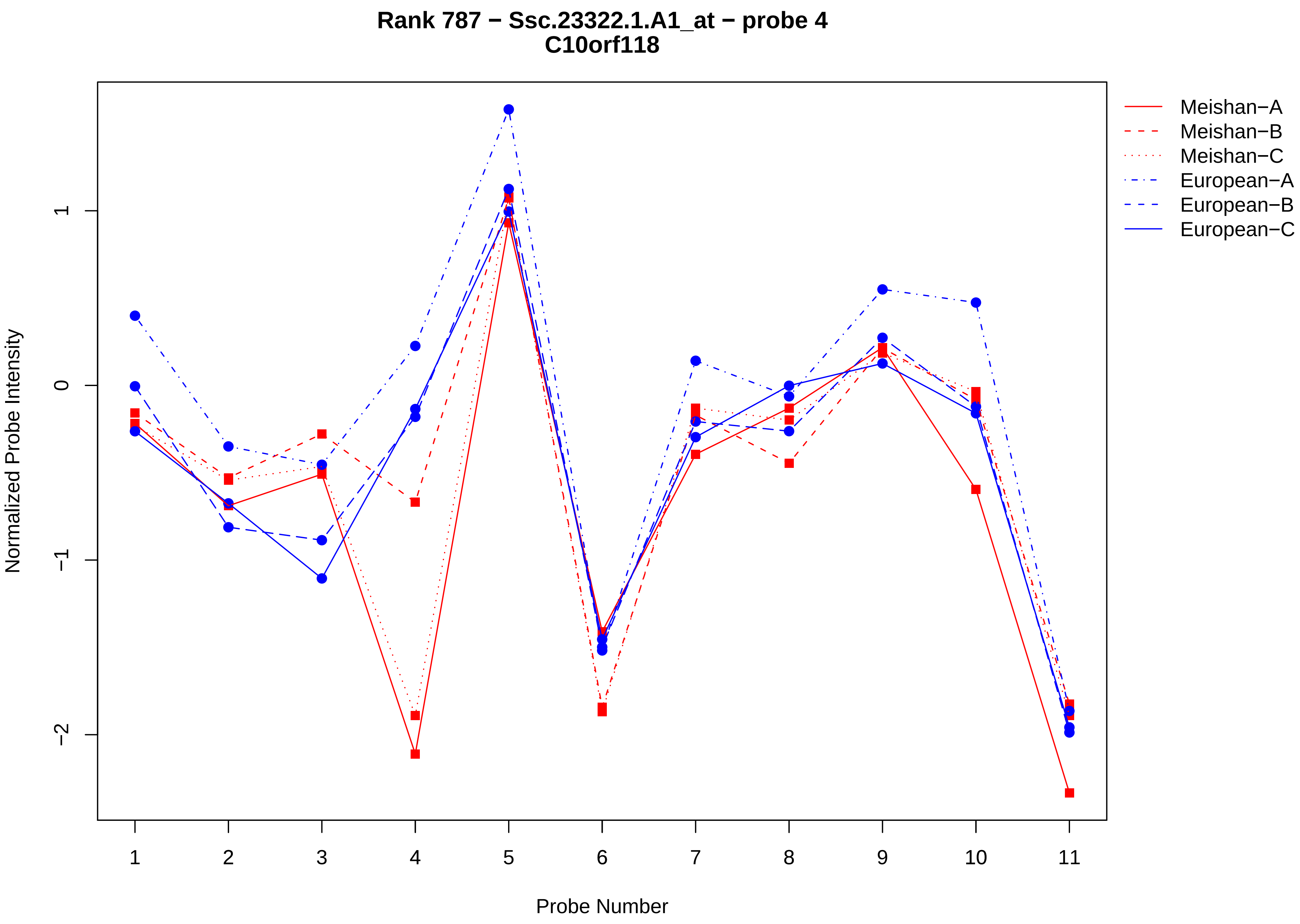
Rank 787 − Ssc.23322.1.A1_at − probe 4
C10orf118
Normalized Probe Intensity
Probe Number
−2
−1
0
1
1
2
3
4
5
6
7
8
9
10
11
Meishan−A
Meishan−B
Meishan−C
European−A
European−B
European−C

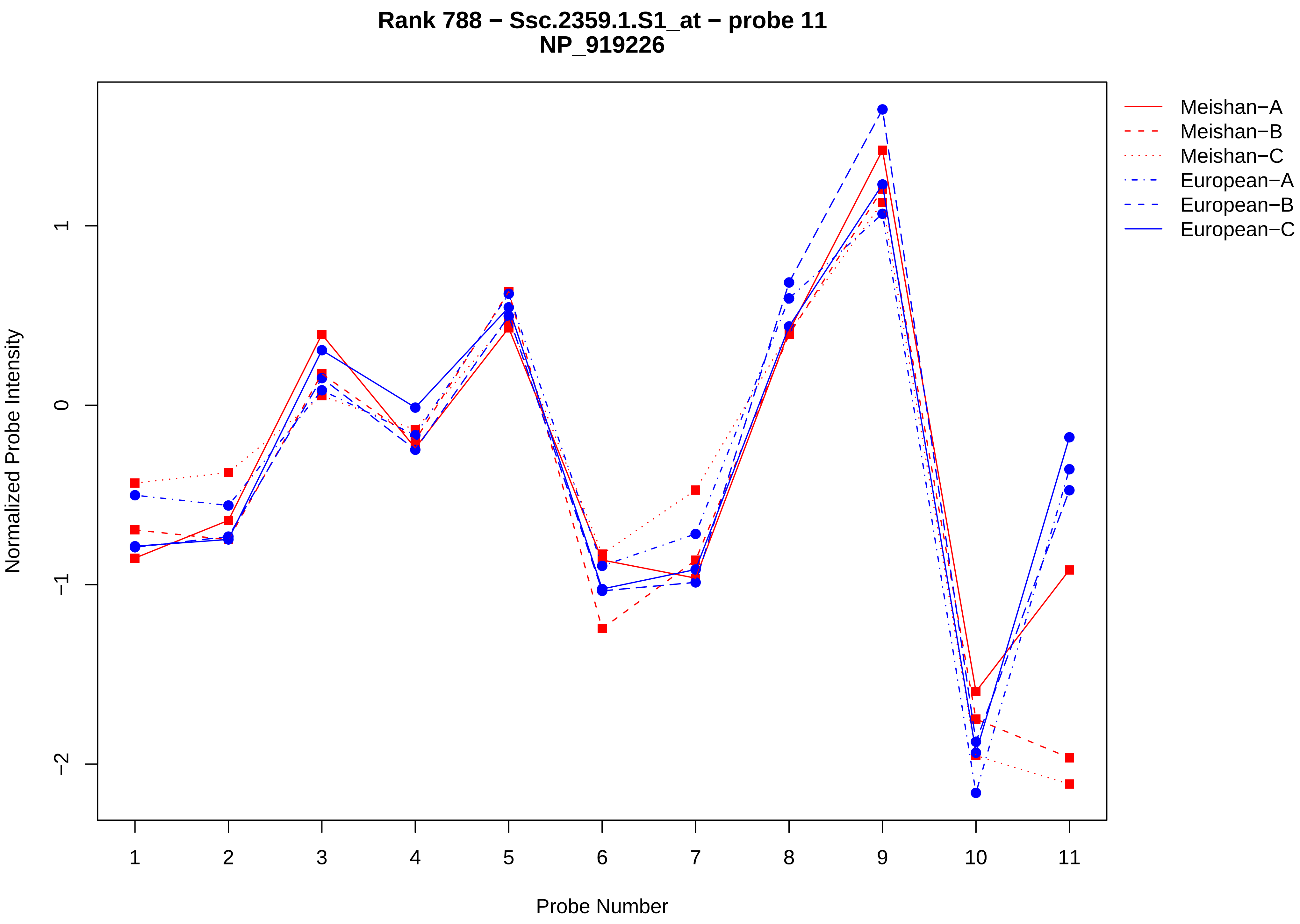

Rank 788 − Ssc.2359.1.S1_at − probe 11
NP_919226
Normalized Probe Intensity
Probe Number
Meishan−A
Meishan−B
Meishan−C
European−A
European−B
European−C
1
0
−1
−2
1
2
3
4
5
6
7
8
9
10
11

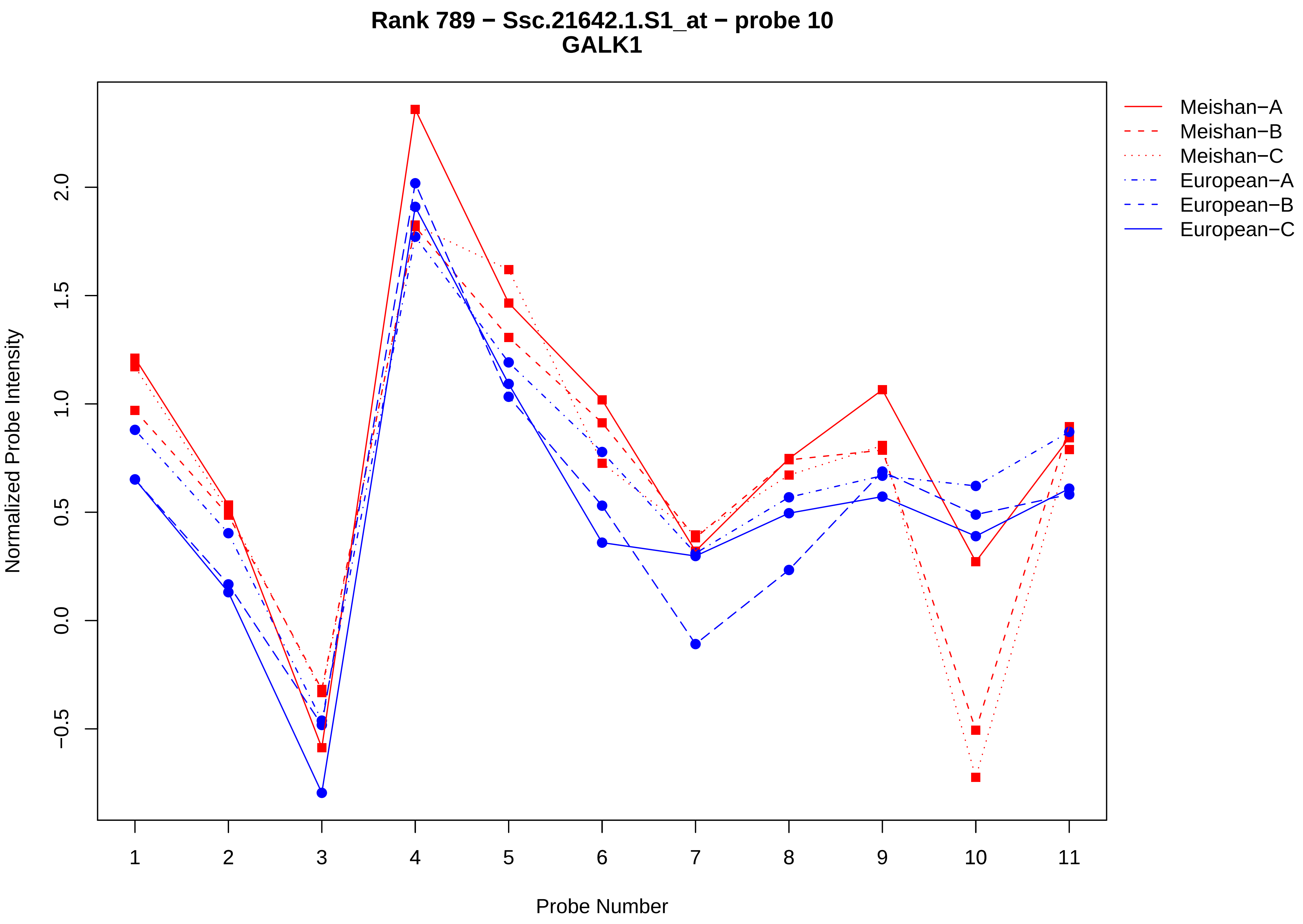

Rank 789 − Ssc.21642.1.S1_at − probe 10
GALK1
Normalized Probe Intensity
Probe Number
−0.5
0.0
0.5
1.0
1.5
2.0
1
2
3
4
5
6
7
8
9
10
11
Meishan−A
Meishan−B
Meishan−C
European−A
European−B
European−C

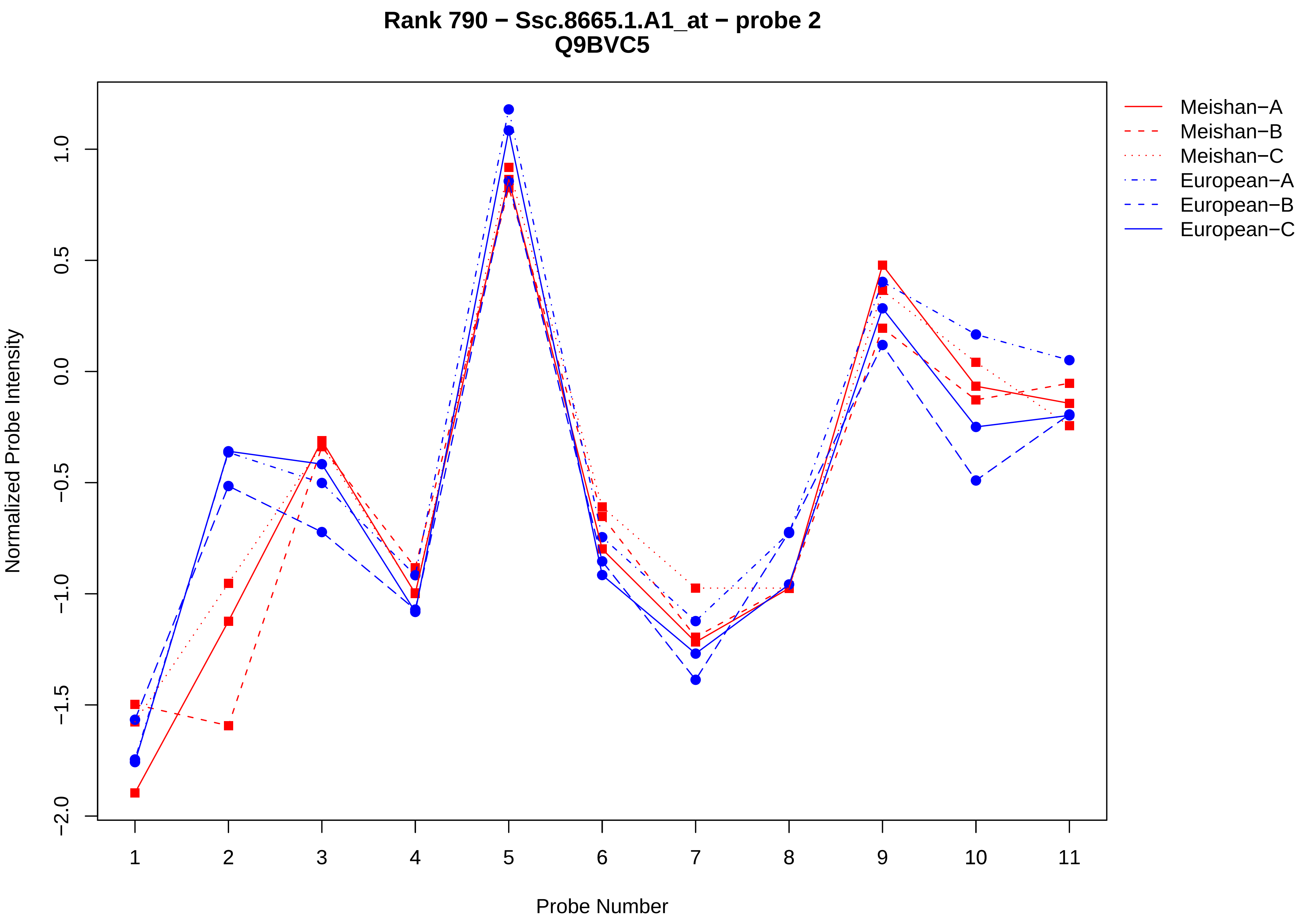

Rank 790 − Ssc.8665.1.A1_at − probe 2
Q9BVC5
Normalized Probe Intensity
Probe Number
1.0
0.5
0.0
−0.5
−1.0
−1.5
−2.0
1
2
3
4
5
6
7
8
9
10
11
Meishan−A
Meishan−B
Meishan−C
European−A
European−B
European−C

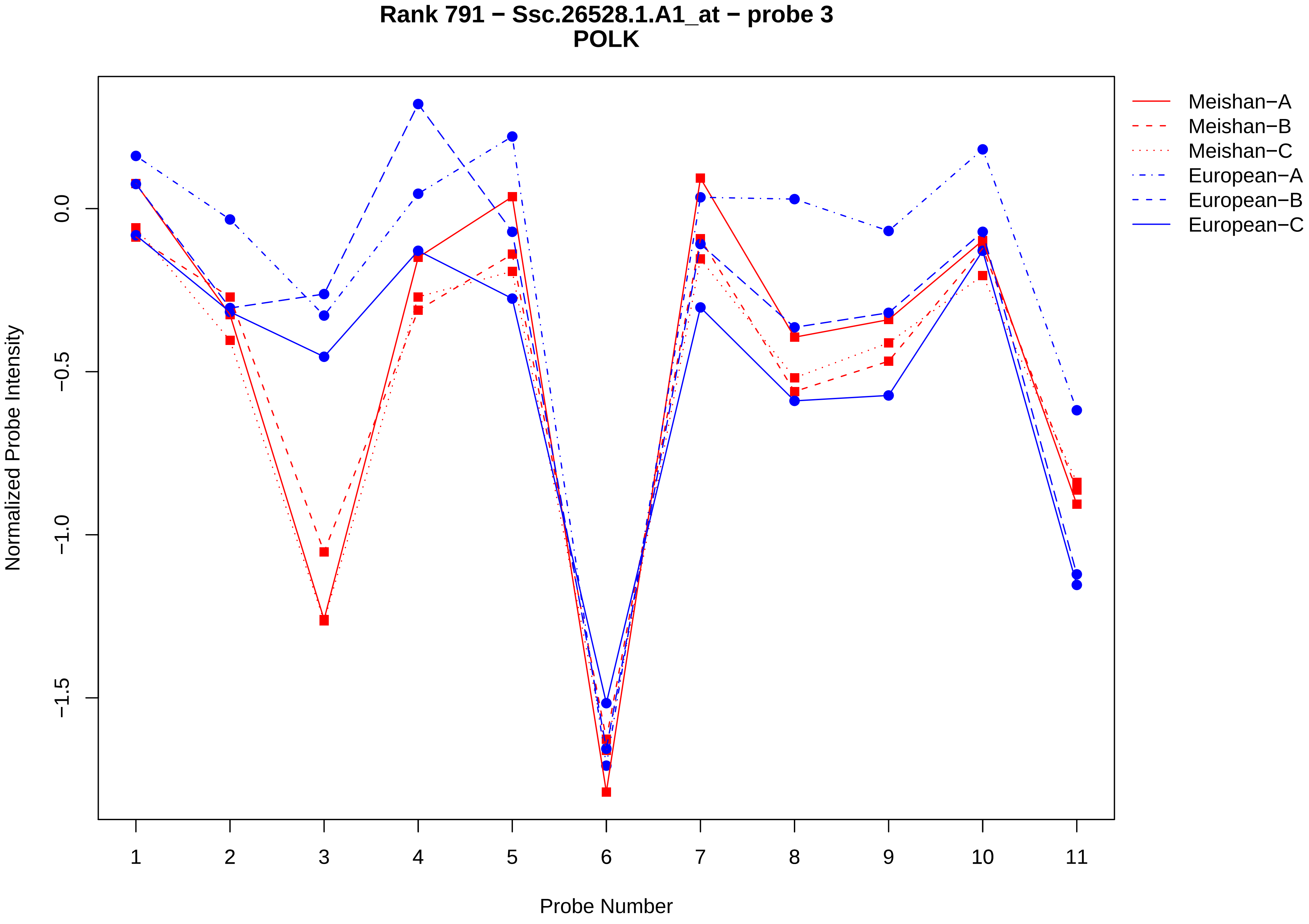
Rank 791 − Ssc.26528.1.A1_at − probe 3
POLK
Normalized Probe Intensity
Probe Number
0.0
−0.5
−1.0
−1.5
1
2
3
4
5
6
7
8
9
10
11
Meishan−A
Meishan−B
Meishan−C
European−A
European−B
European−C

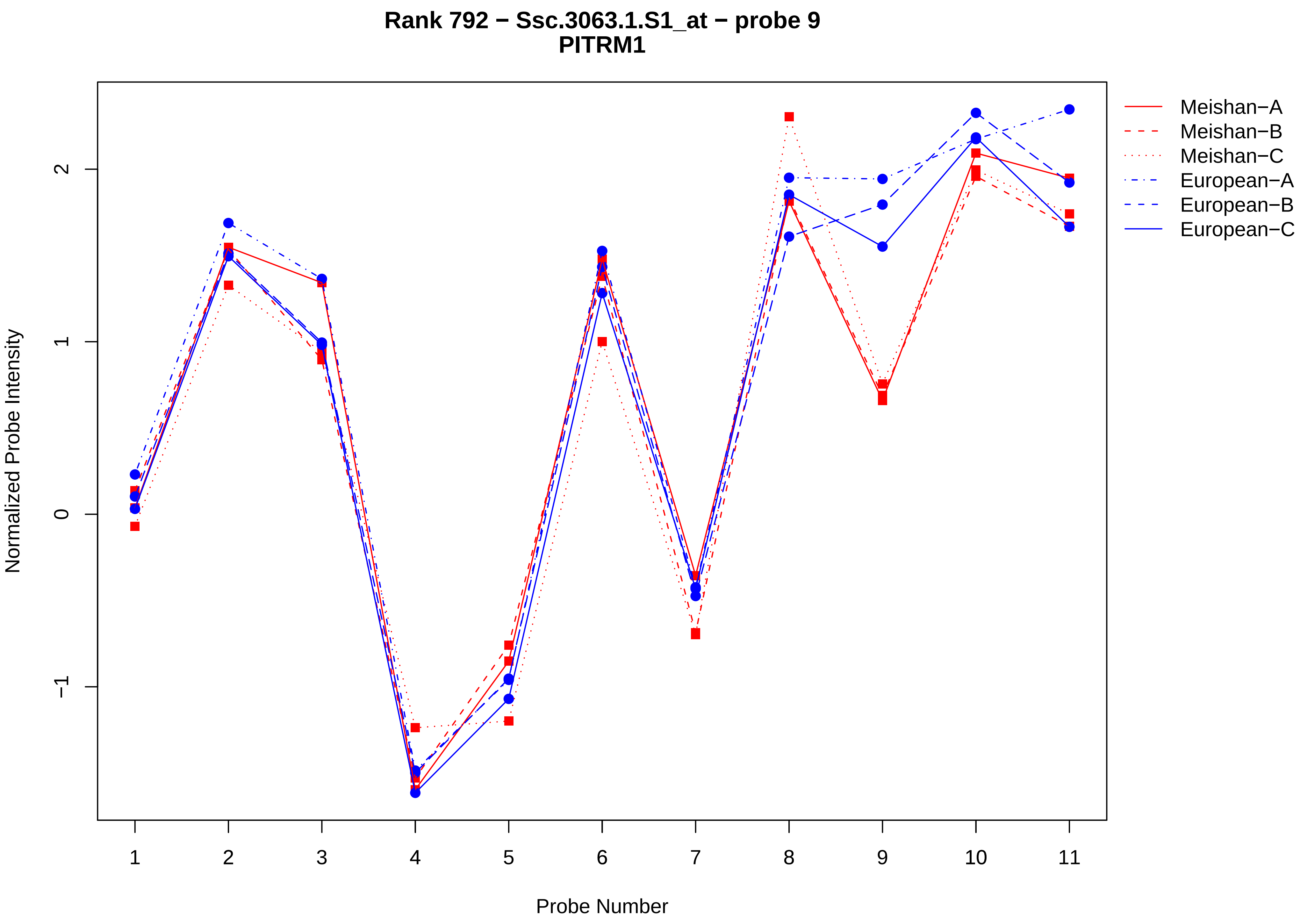

Rank 792 − Ssc.3063.1.S1_at − probe 9
PITRM1
Normalized Probe Intensity
Probe Number
−1
0
1
2
1
2
3
4
5
6
7
8
9
10
11
Meishan−A
Meishan−B
Meishan−C
European−A
European−B
European−C

Rank 793 − Ssc.810.1.S2_at − probe 3
SCARB2

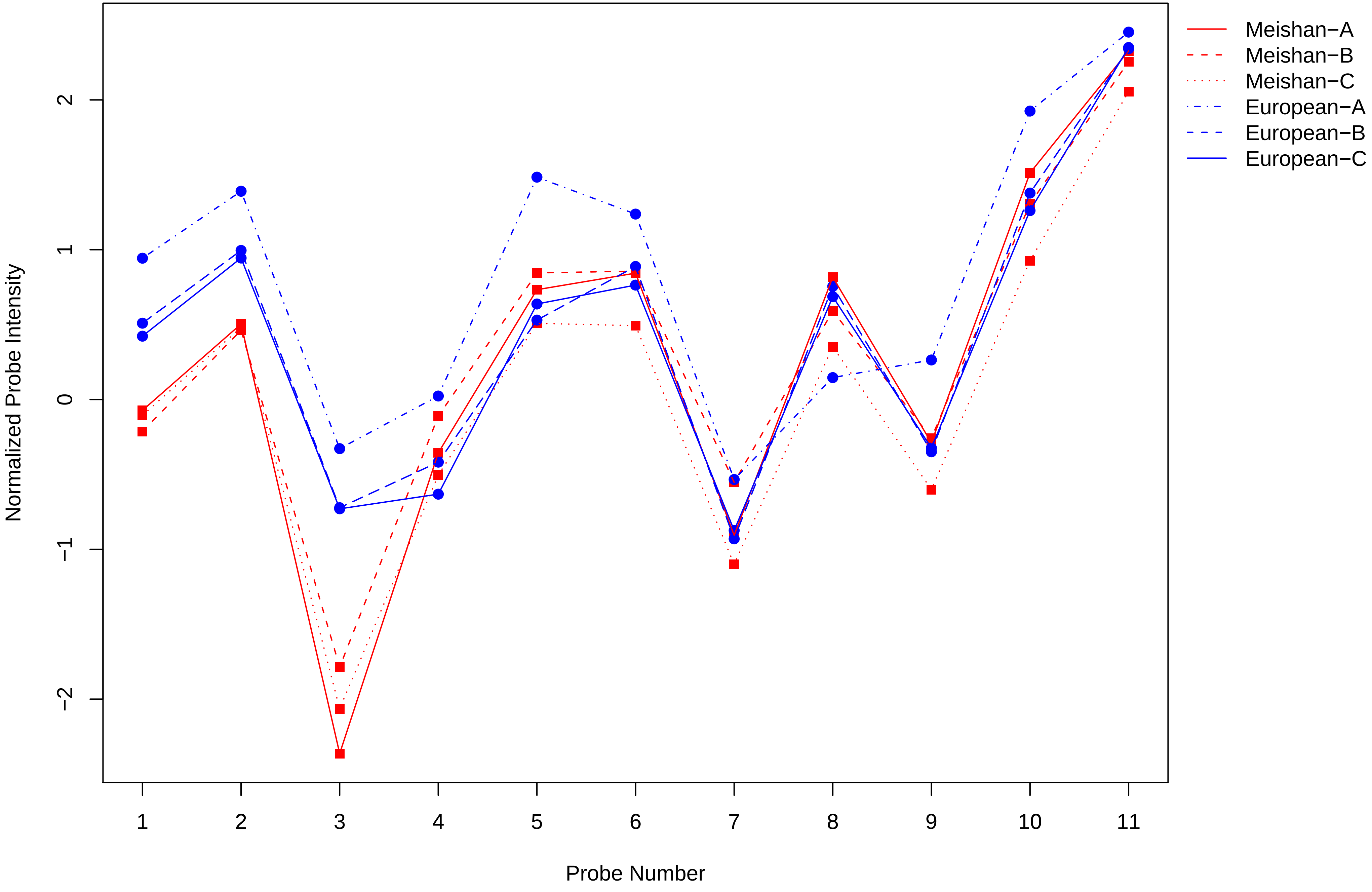

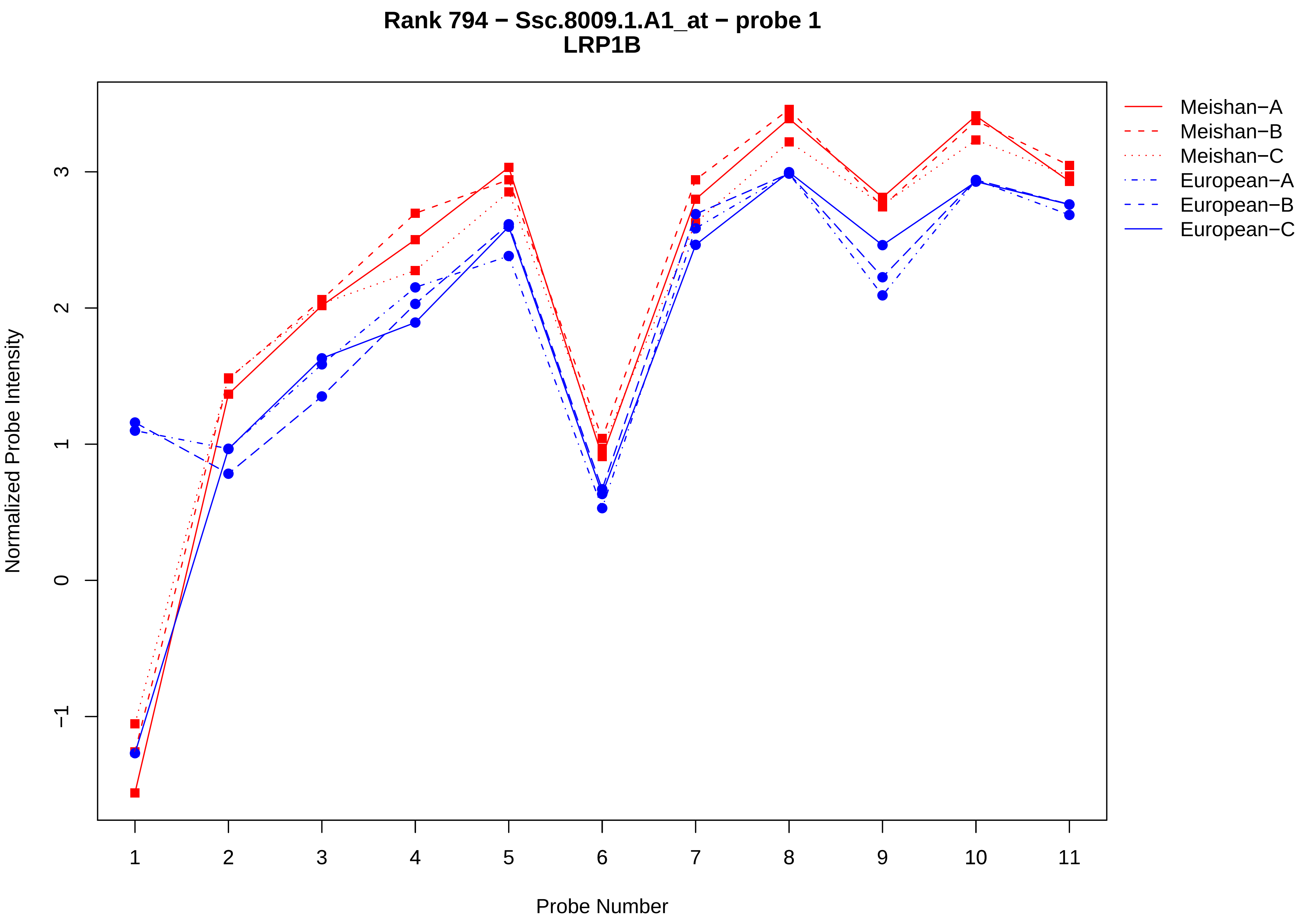
Rank 794 − Ssc.8009.1.A1_at − probe 1
LRP1B
Normalized Probe Intensity
Probe Number
Meishan−A
Meishan−B
Meishan−C
European−A
European−B
European−C
−1
0
1
2
3
1
2
3
4
5
6
7
8
9
10
11

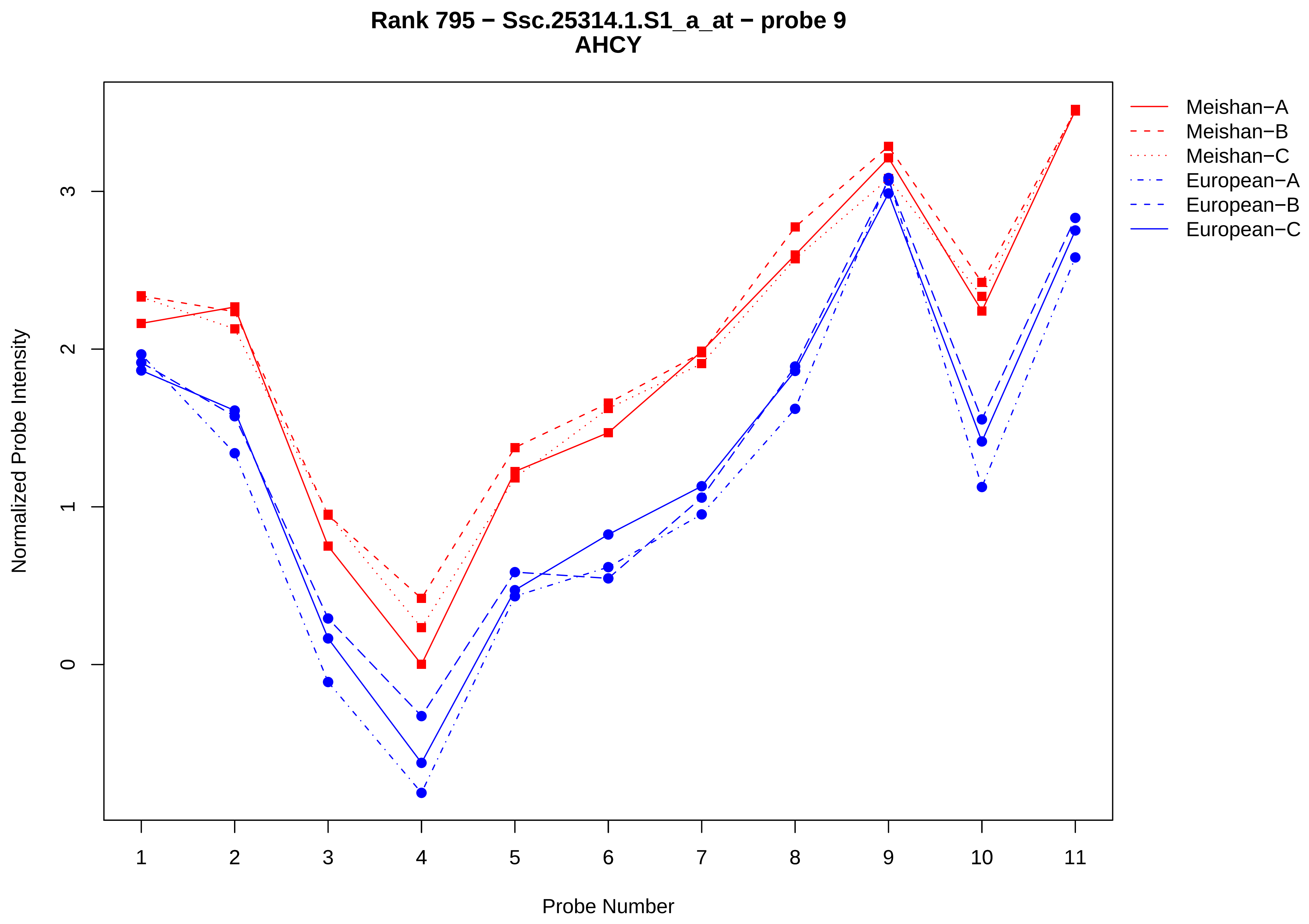

Rank 795 − Ssc.25314.1.S1_a_at − probe 9
AHCY
Normalized Probe Intensity
Probe Number
0
1
2
3
1
2
3
4
5
6
7
8
9
10
11
Meishan−A
Meishan−B
Meishan−C
European−A
European−B
European−C

Rank 796 − Ssc.15463.1.S1_at − probe 11
BCLAF1

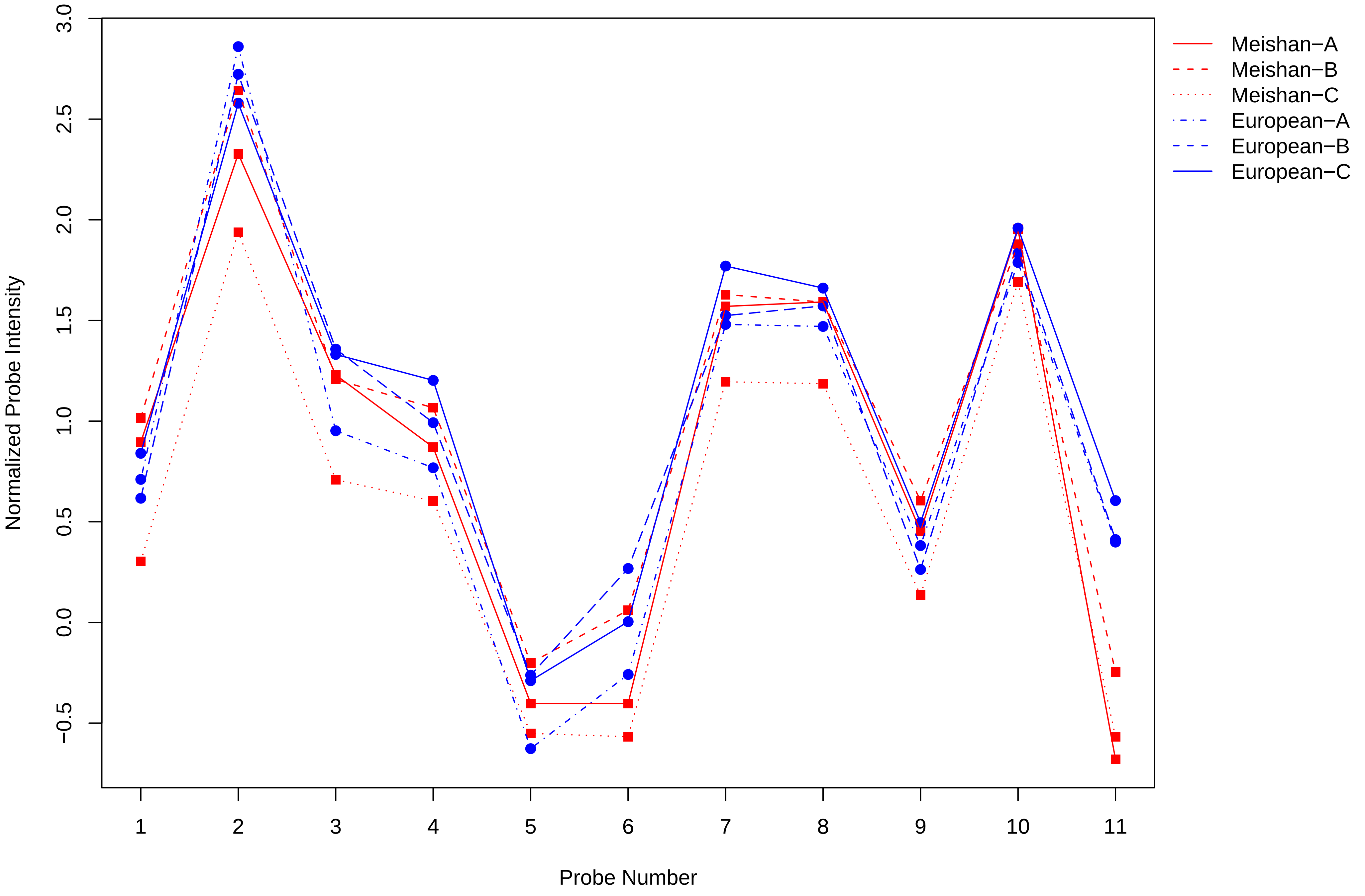

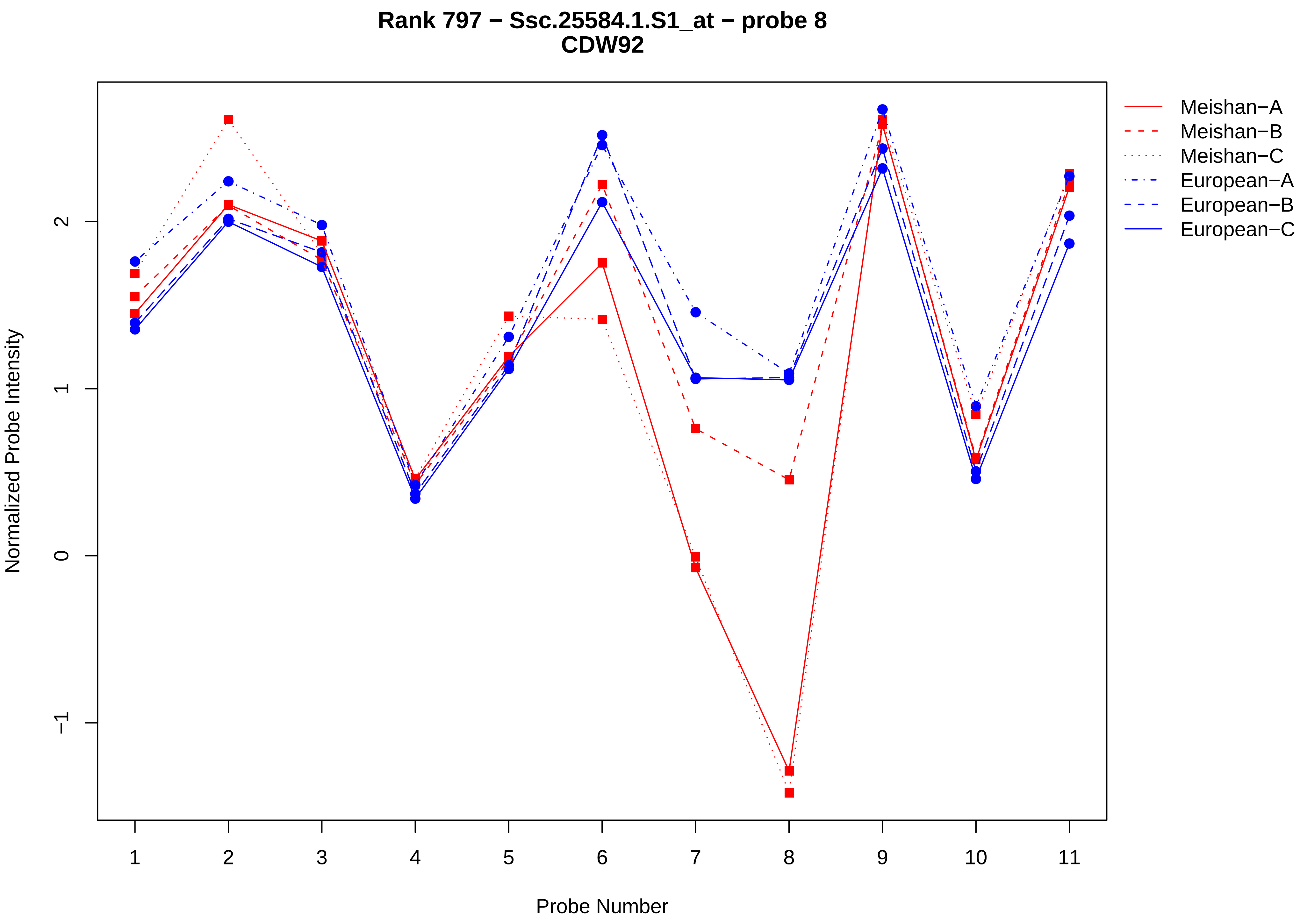

Rank 797 − Ssc.25584.1.S1_at − probe 8
CDW92
Normalized Probe Intensity
Probe Number
−1
0
1
2
1
2
3
4
5
6
7
8
9
10
11
Meishan−A
Meishan−B
Meishan−C
European−A
European−B
European−C

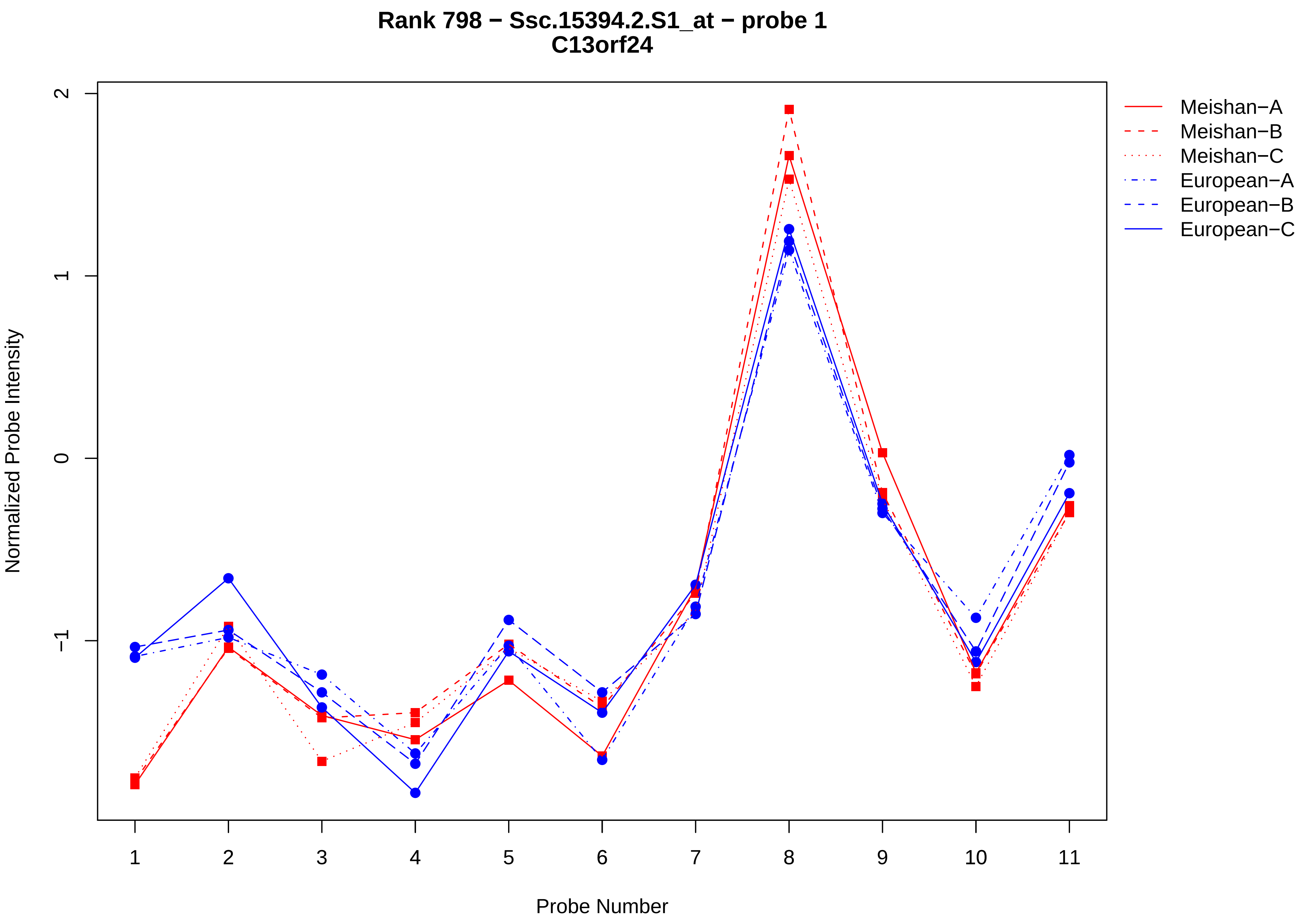

Rank 798 − Ssc.15394.2.S1_at − probe 1
C13orf24
Normalized Probe Intensity
Probe Number
2
1
0
−1
1
2
3
4
5
6
7
8
9
10
11
Meishan−A
Meishan−B
Meishan−C
European−A
European−B
European−C

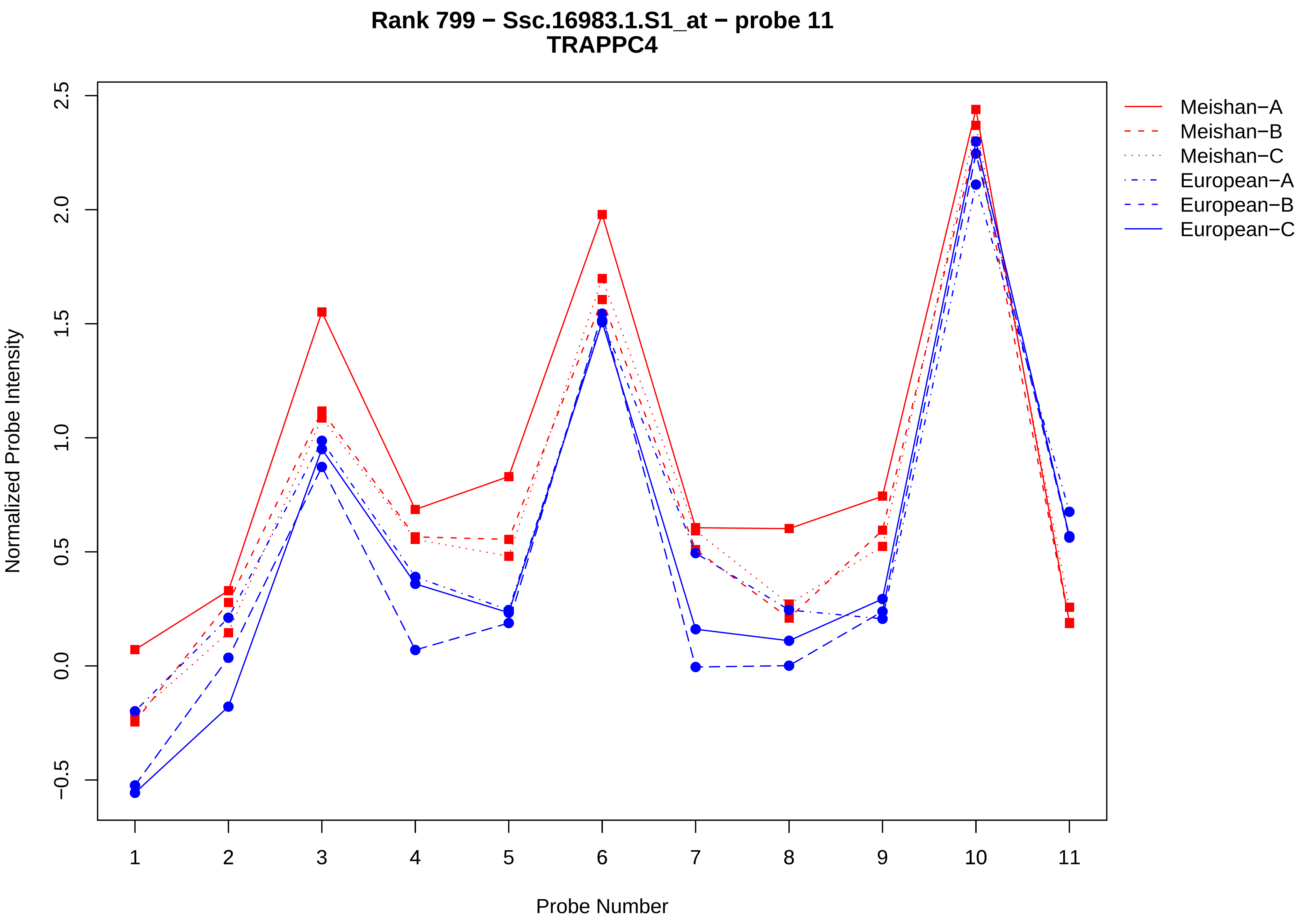
Rank 799 − Ssc.16983.1.S1_at − probe 11
TRAPPC4
Normalized Probe Intensity
Probe Number
−0.5
0.0
0.5
1.0
1.5
2.0
2.5
1
2
3
4
5
6
7
8
9
10
11
Meishan−A
Meishan−B
Meishan−C
European−A
European−B
European−C

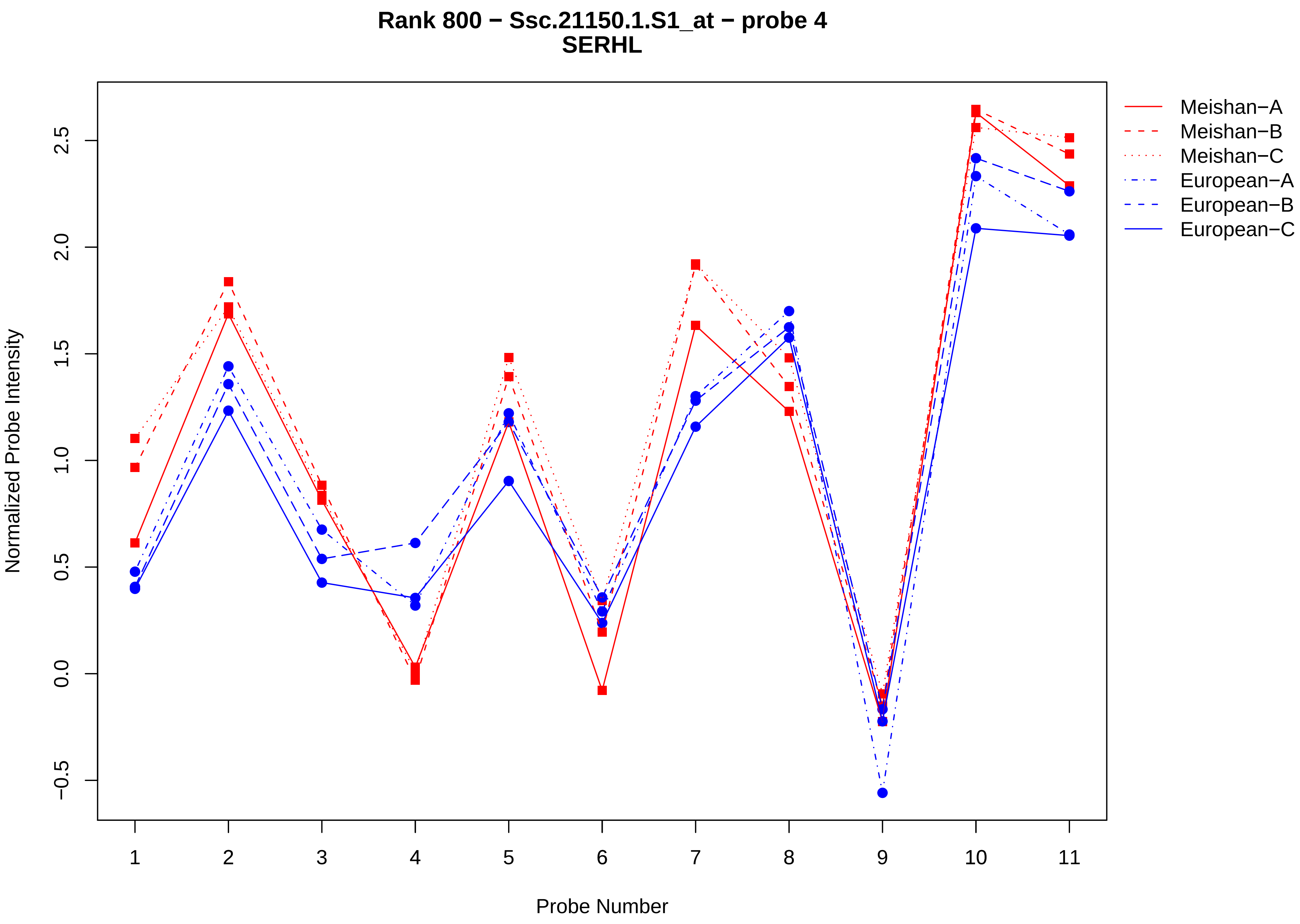

Rank 800 − Ssc.21150.1.S1_at − probe 4
SERHL
Normalized Probe Intensity
Probe Number
Meishan−A
Meishan−B
Meishan−C
European−A
European−B
European−C
−0.5
0.0
0.5
1.0
1.5
2.0
2.5
1
2
3
4
5
6
7
8
9
10
11

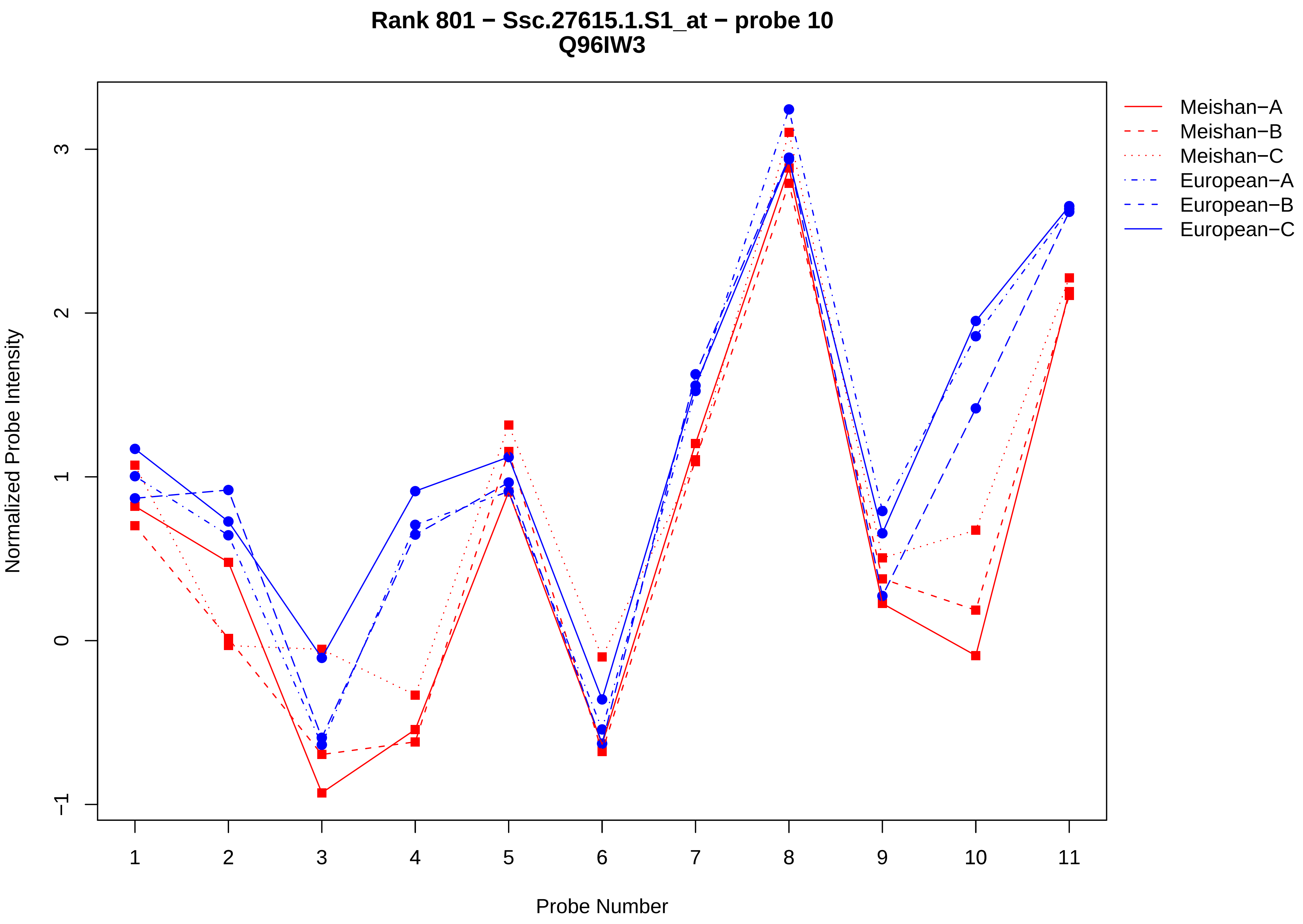

Rank 801 − Ssc.27615.1.S1_at − probe 10
Q96IW3
Normalized Probe Intensity
Probe Number
−1
0
1
2
3
1
2
3
4
5
6
7
8
9
10
11
Meishan−A
Meishan−B
Meishan−C
European−A
European−B
European−C

Rank 802 − Ssc.17323.1.S1_at − probe 5
CSTB

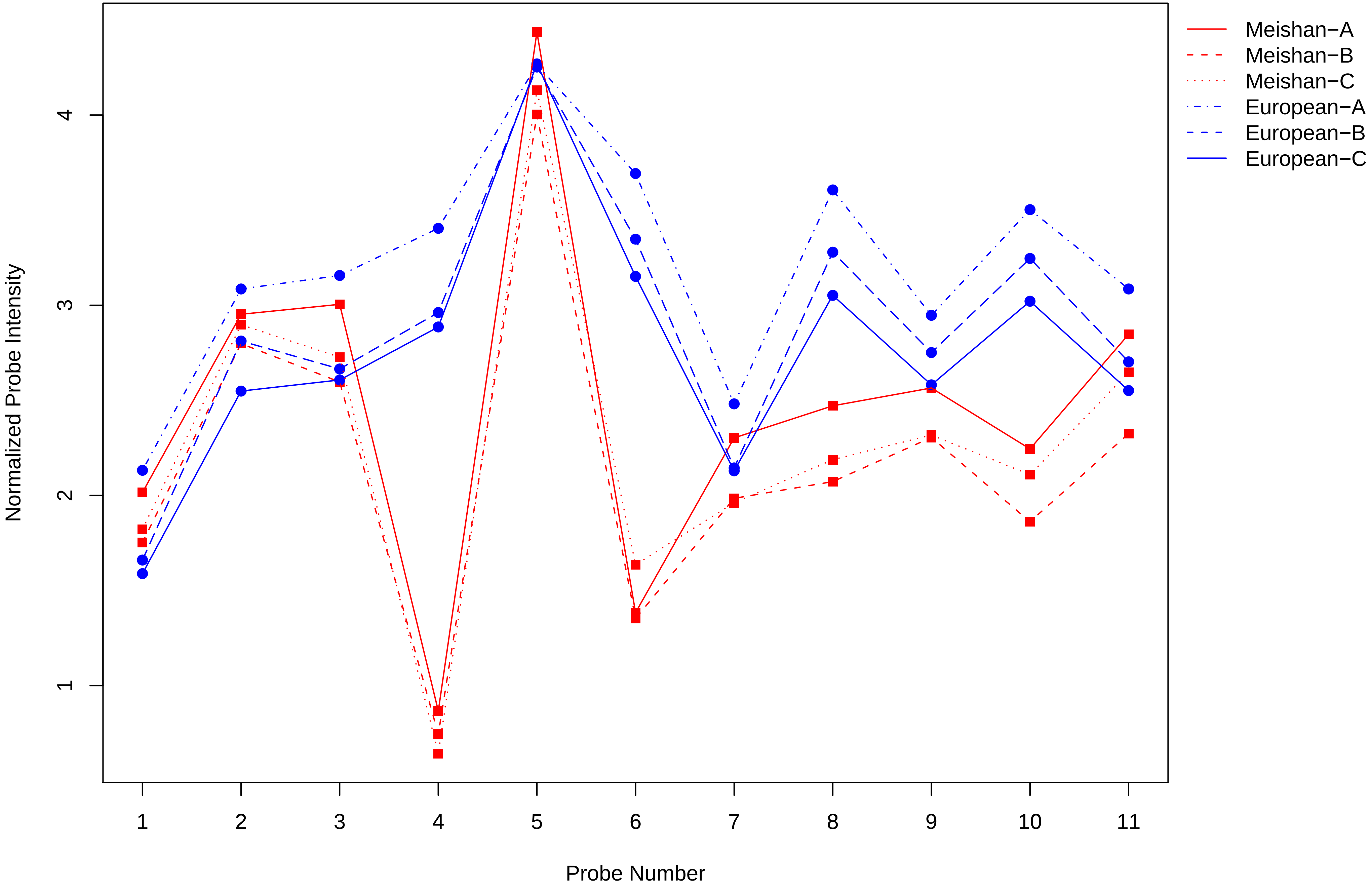

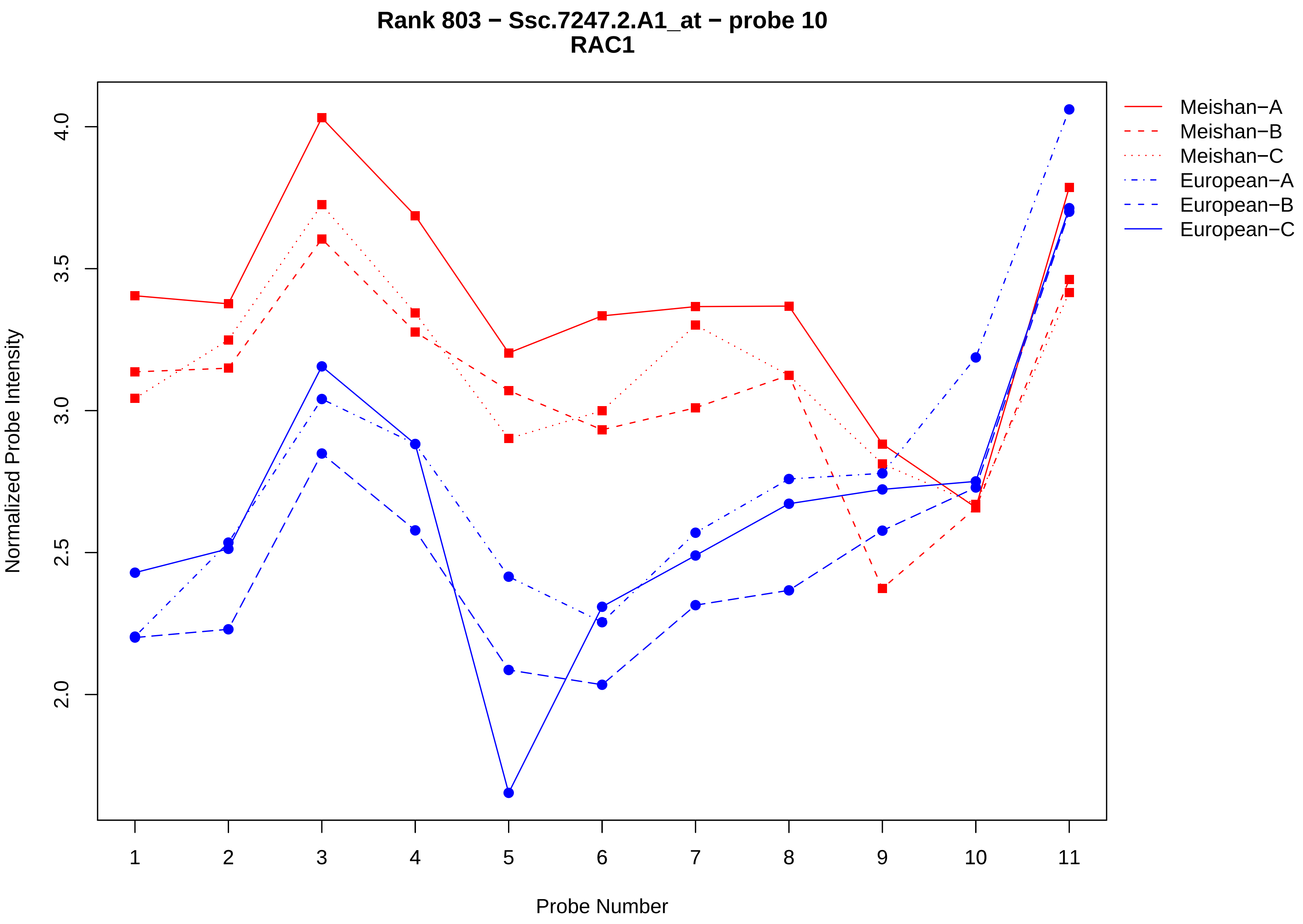

Rank 803 − Ssc.7247.2.A1_at − probe 10
RAC1
Normalized Probe Intensity
Probe Number
2.0
2.5
3.0
3.5
4.0
1
2
3
4
5
6
7
8
9
10
11
Meishan−A
Meishan−B
Meishan−C
European−A
European−B
European−C

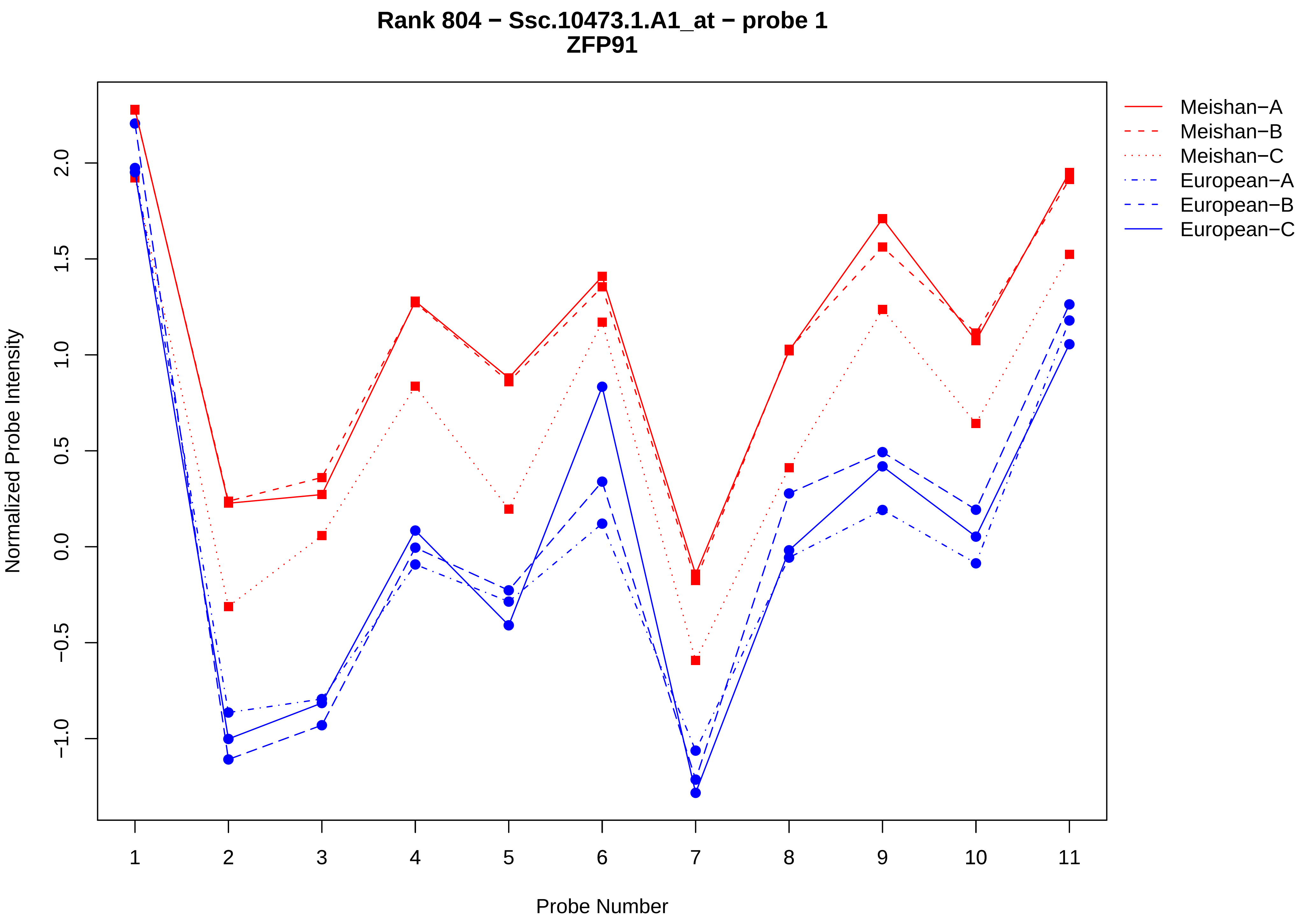

Rank 804 − Ssc.10473.1.A1_at − probe 1
ZFP91
Normalized Probe Intensity
Probe Number
−1.0
−0.5
0.0
0.5
1.0
1.5
2.0
1
2
3
4
5
6
7
8
9
10
11
Meishan−A
Meishan−B
Meishan−C
European−A
European−B
European−C

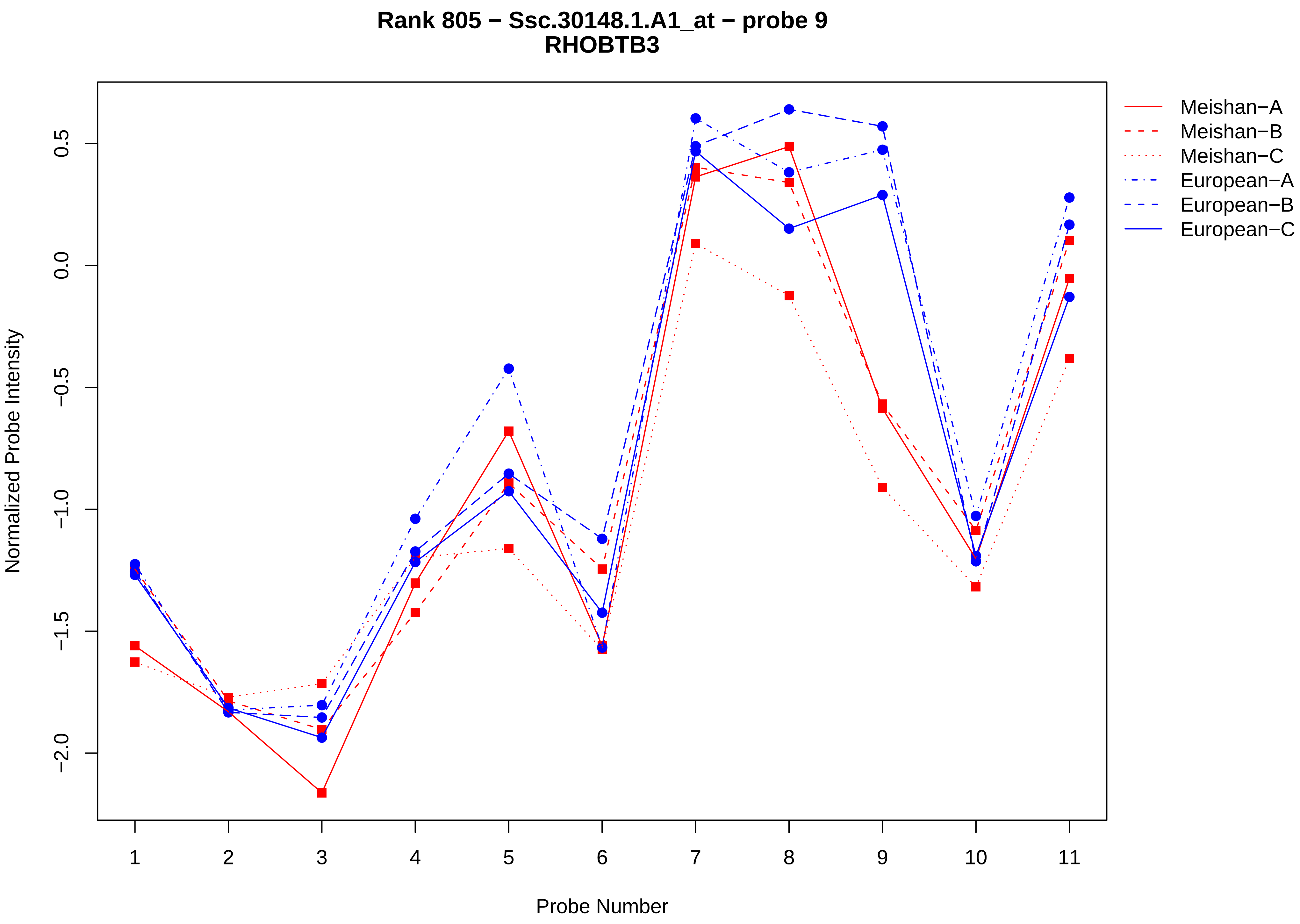
Rank 805 − Ssc.30148.1.A1_at − probe 9
RHOBTB3
Normalized Probe Intensity
Probe Number
Meishan−A
Meishan−B
Meishan−C
European−A
European−B
European−C
0.5
0.0
−0.5
−1.0
−1.5
−2.0
1
2
3
4
5
6
7
8
9
10
11

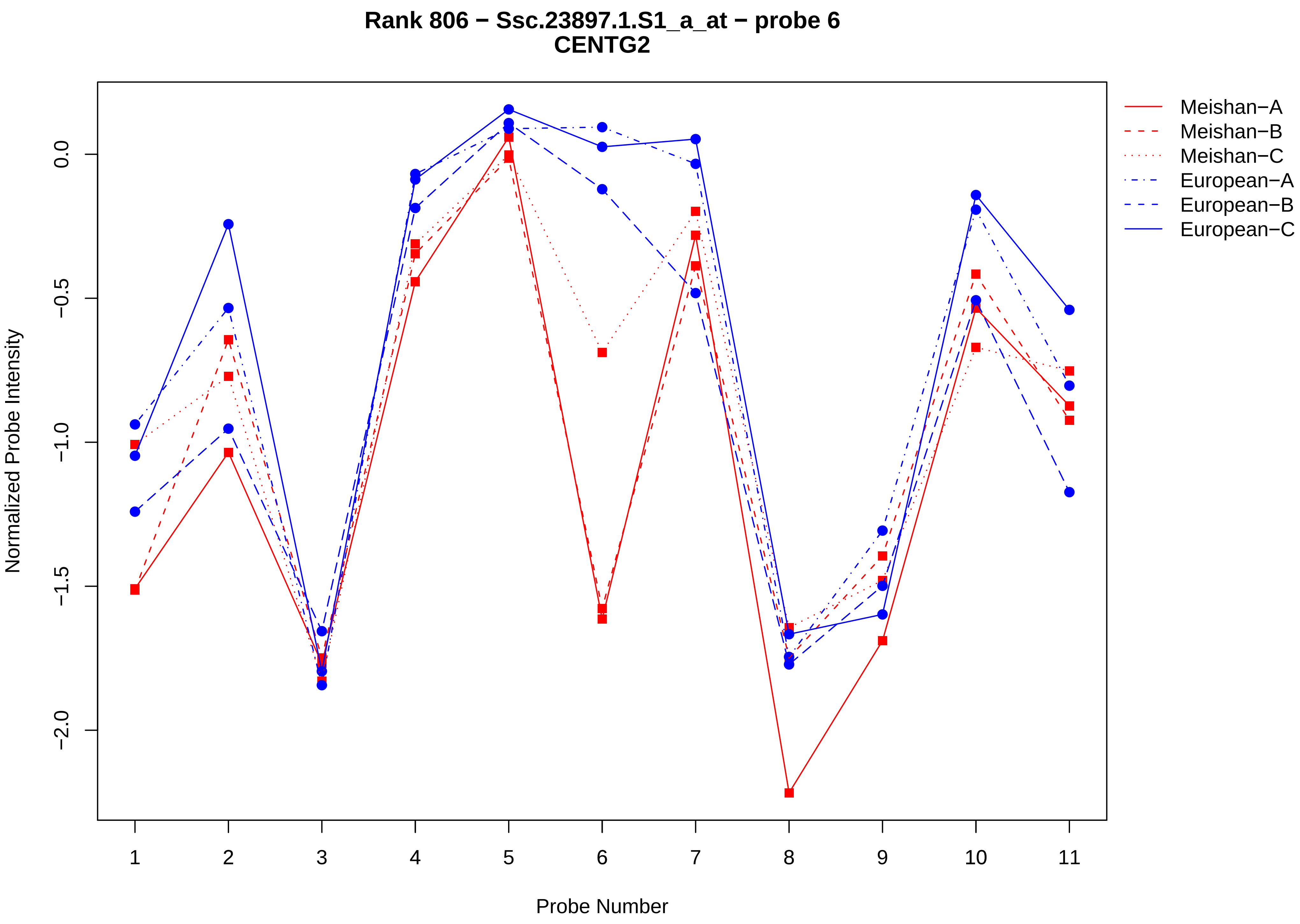

Rank 806 − Ssc.23897.1.S1_a_at − probe 6
CENTG2
Normalized Probe Intensity
0.0
−0.5
−1.0
−1.5
−2.0
1
2
3
4
5
6
7
8
9
10
11
Probe Number
Meishan−A
Meishan−B
Meishan−C
European−A
European−B
European−C

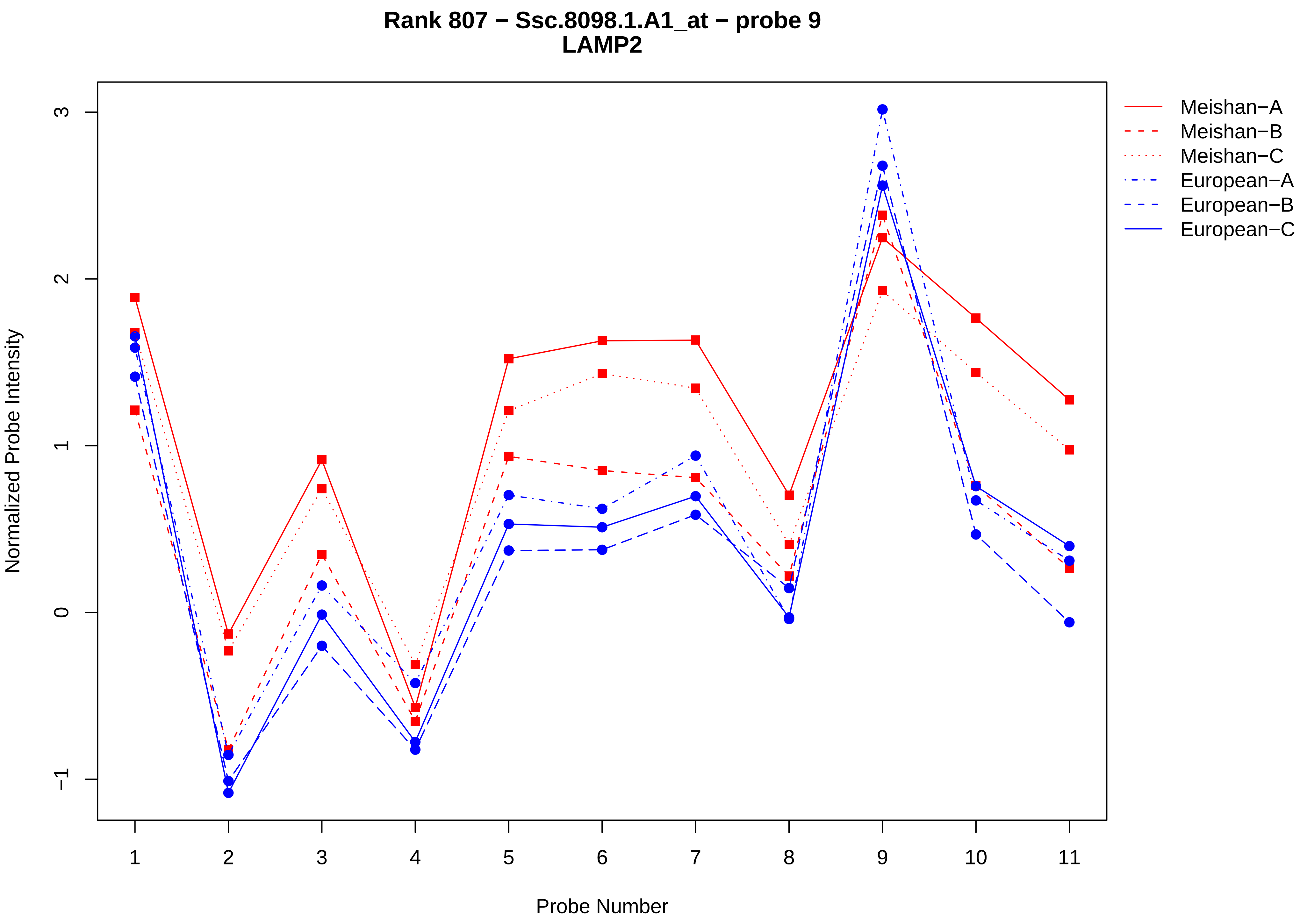

Rank 807 − Ssc.8098.1.A1_at − probe 9
LAMP2
Normalized Probe Intensity
Probe Number
−1
0
1
2
3
1
2
3
4
5
6
7
8
9
10
11
Meishan−A
Meishan−B
Meishan−C
European−A
European−B
European−C

Rank 808 − Ssc.14003.4.S1_at − probe 8
NP_060739

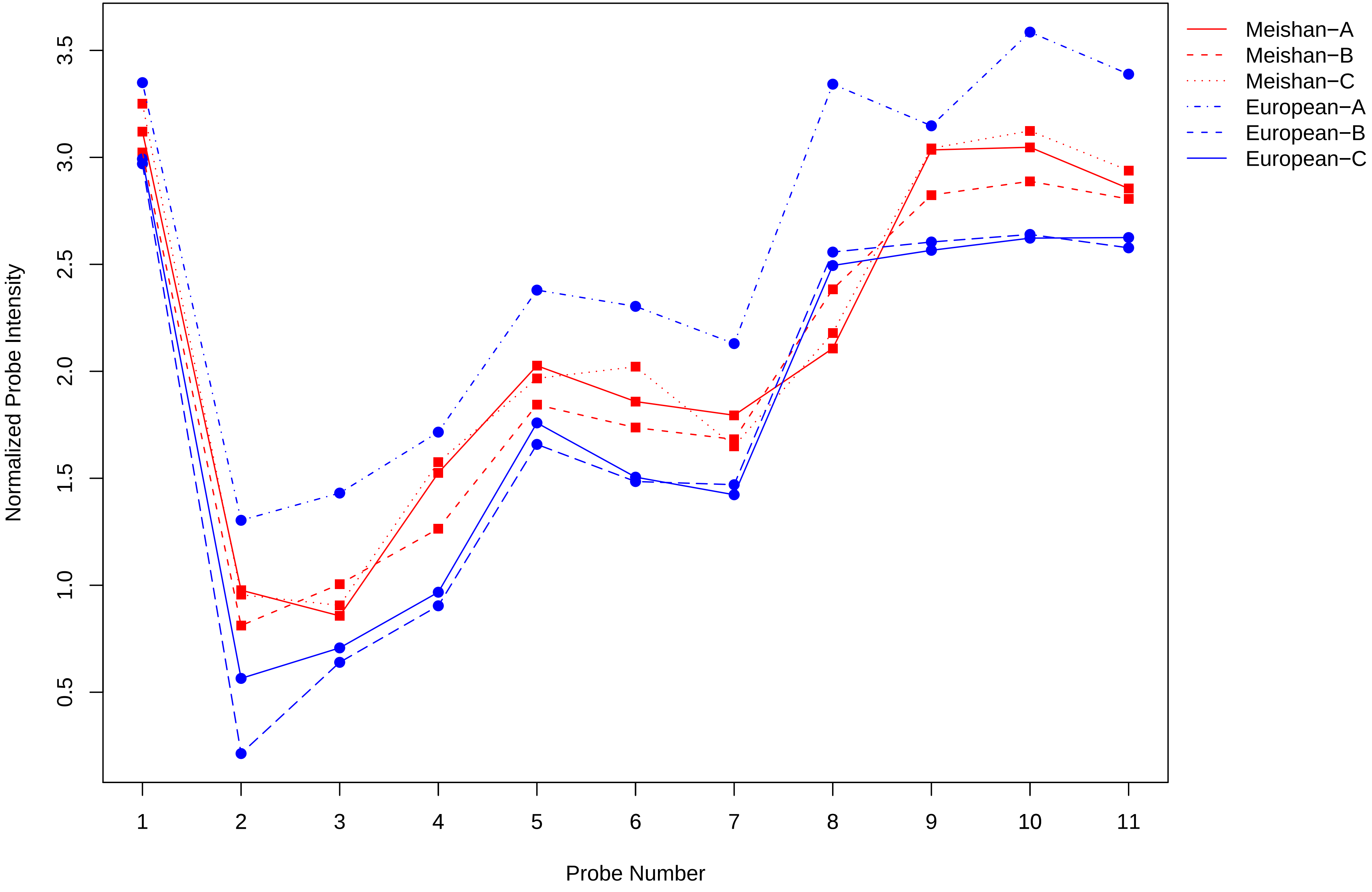

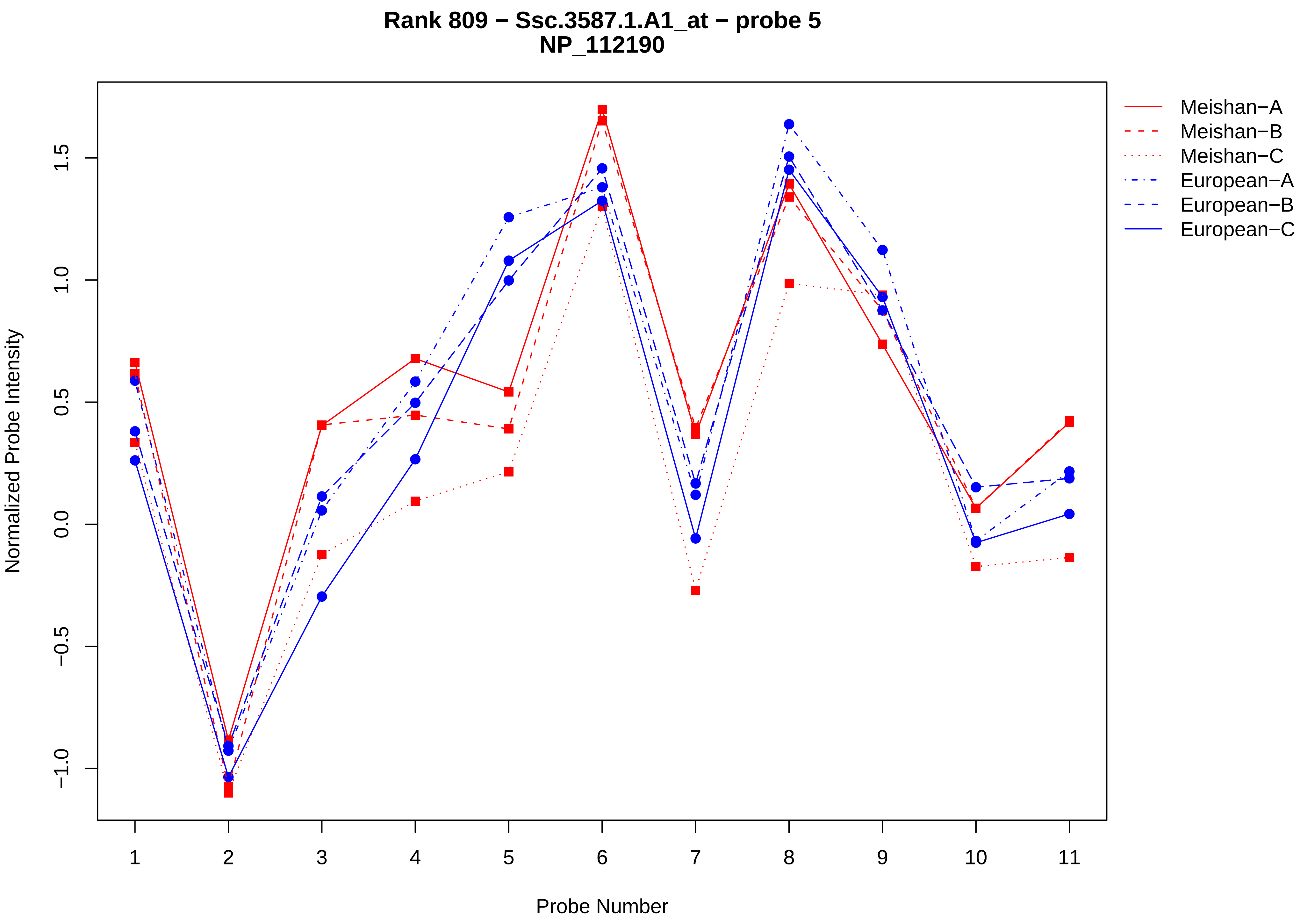

Rank 809 − Ssc.3587.1.A1_at − probe 5
NP_112190
Normalized Probe Intensity
Probe Number
−1.0
−0.5
0.0
0.5
1.0
1.5
1
2
3
4
5
6
7
8
9
10
11
Meishan−A
Meishan−B
Meishan−C
European−A
European−B
European−C

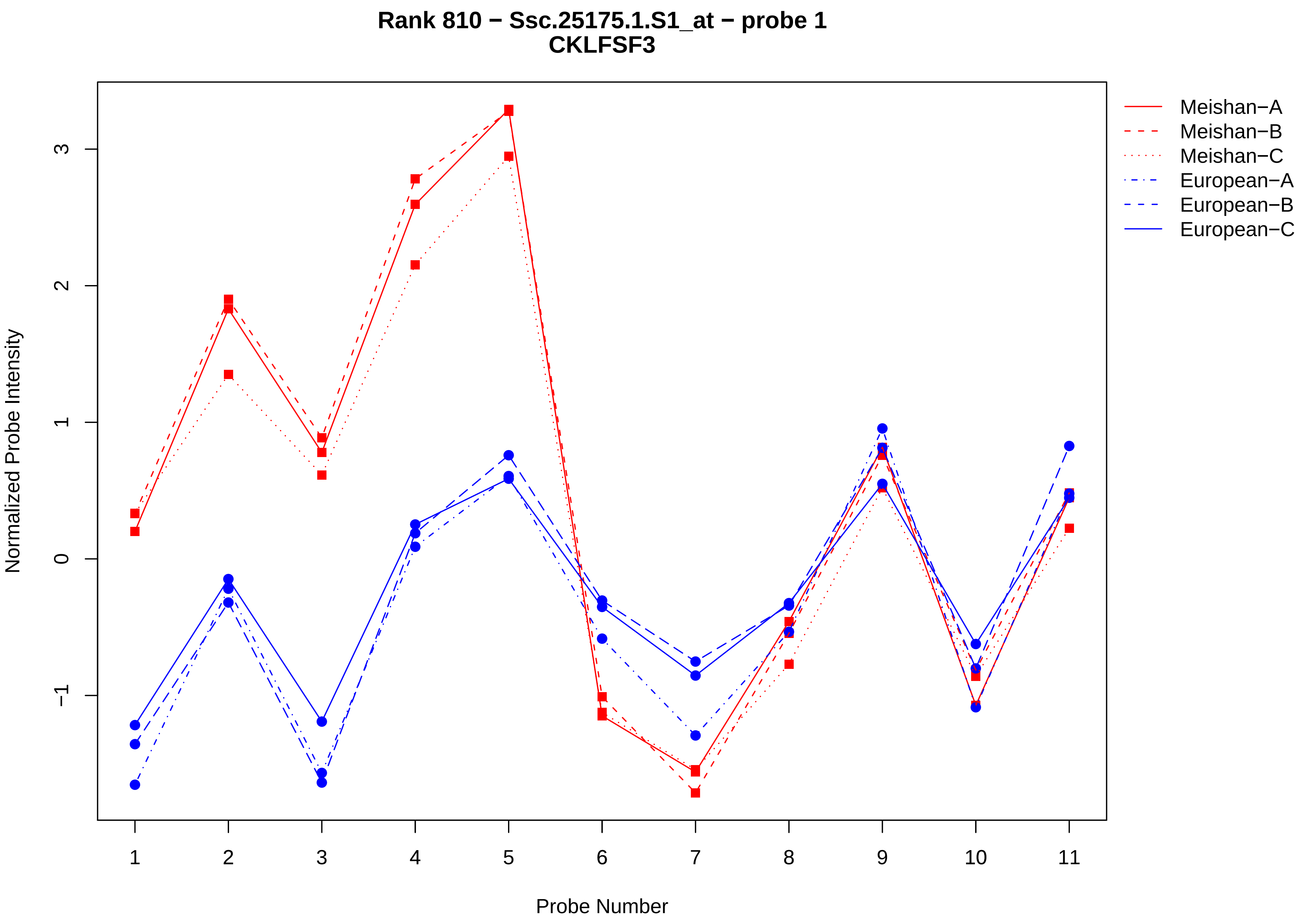

Rank 810 − Ssc.25175.1.S1_at − probe 1
CKLFSF3
Normalized Probe Intensity
Probe Number
Meishan−A
Meishan−B
Meishan−C
European−A
European−B
European−C
−1
0
1
2
3
1
2
3
4
5
6
7
8
9
10
11

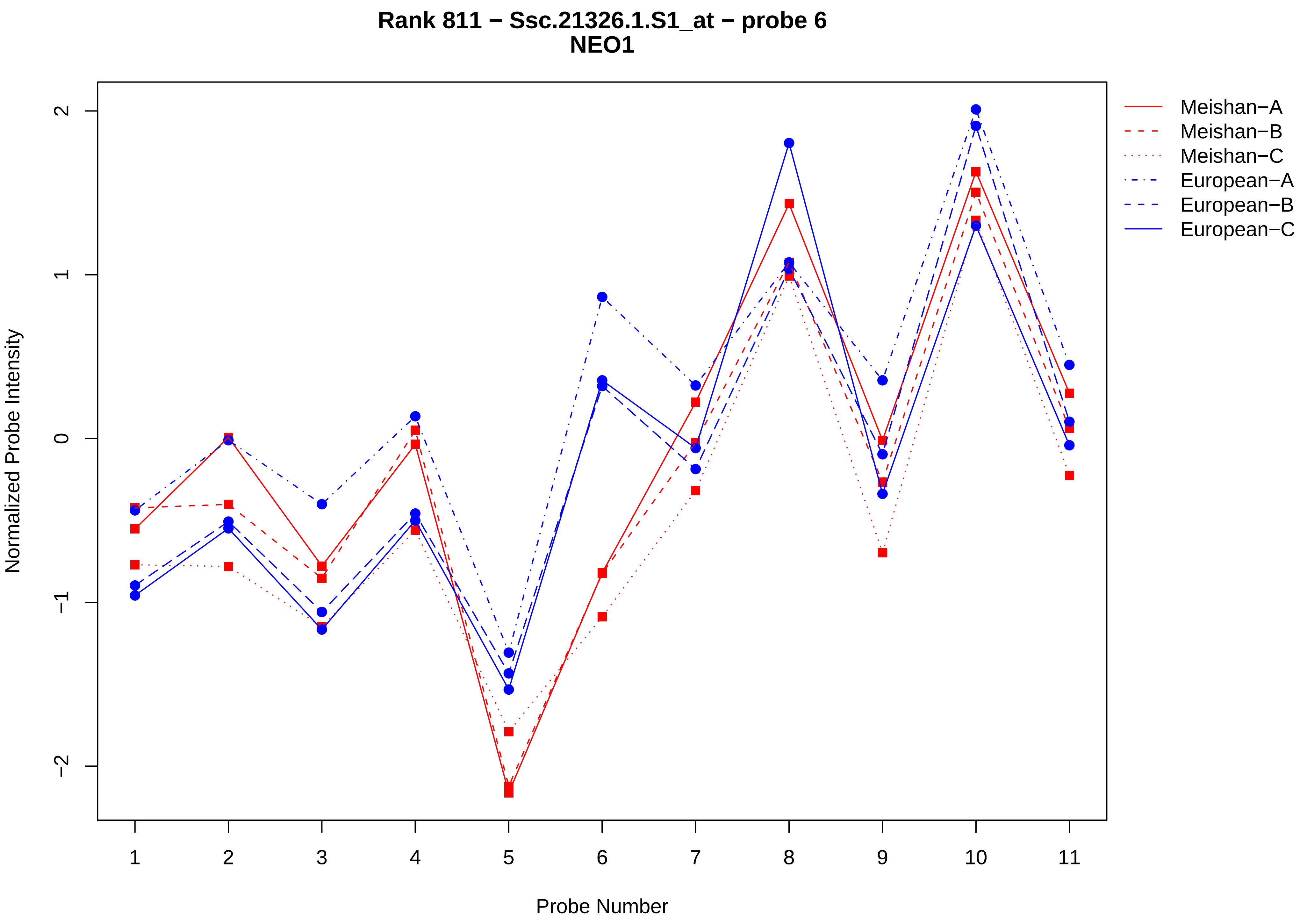

Rank 811 − Ssc.21326.1.S1_at − probe 6
NEO1
Normalized Probe Intensity
Probe Number
−2
−1
0
1
2
1
2
3
4
5
6
7
8
9
10
11
Meishan−A
Meishan−B
Meishan−C
European−A
European−B
European−C

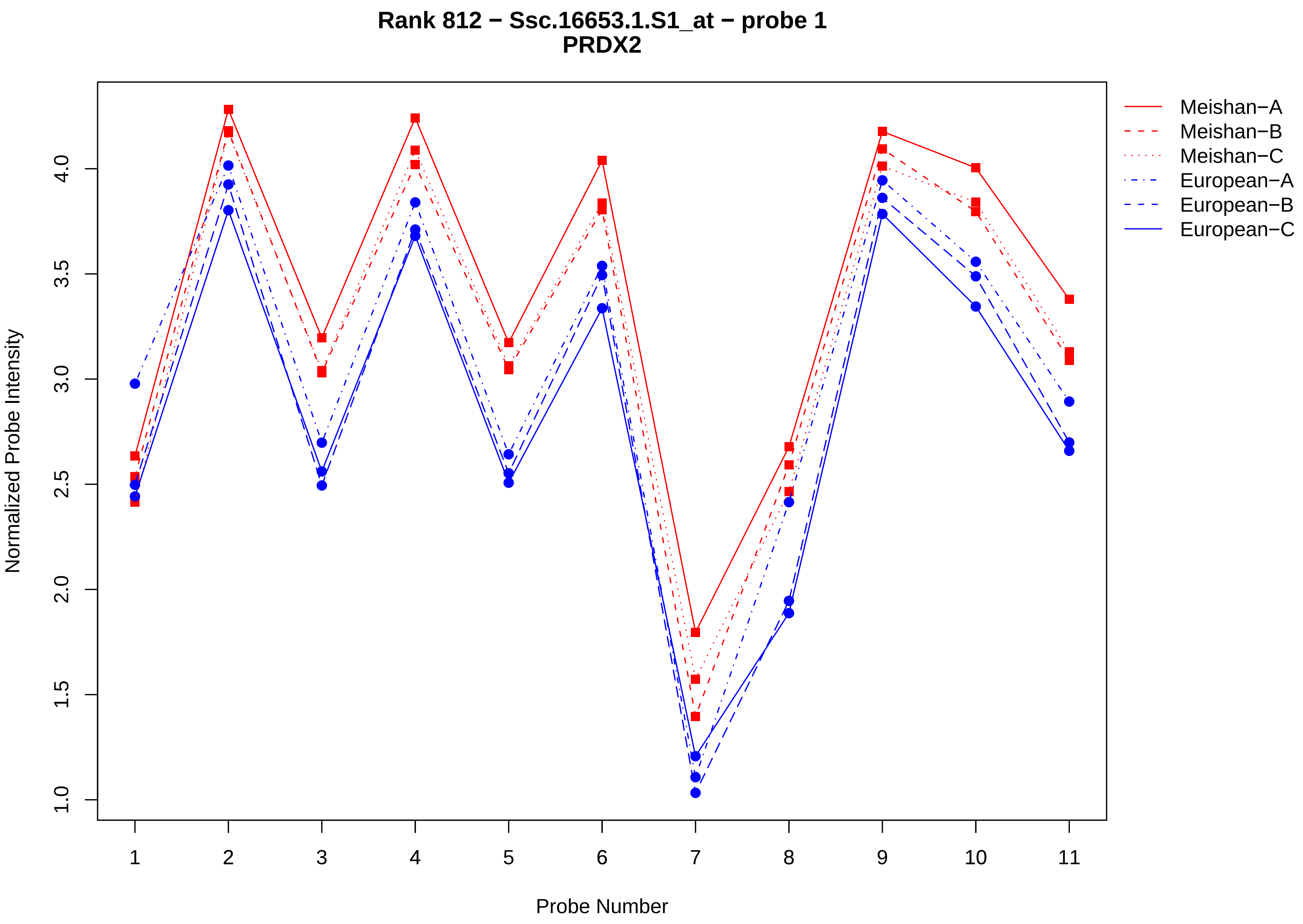

Rank 812 − Ssc.16653.1.S1_at − probe 1
PRDX2
Normalized Probe Intensity
Probe Number
1.0
1.5
2.0
2.5
3.0
3.5
4.0
1
2
3
4
5
6
7
8
9
10
11
Meishan−A
Meishan−B
Meishan−C
European−A
European−B
European−C

Rank 813 − Ssc.1046.1.A1_at − probe 6
Q96C30

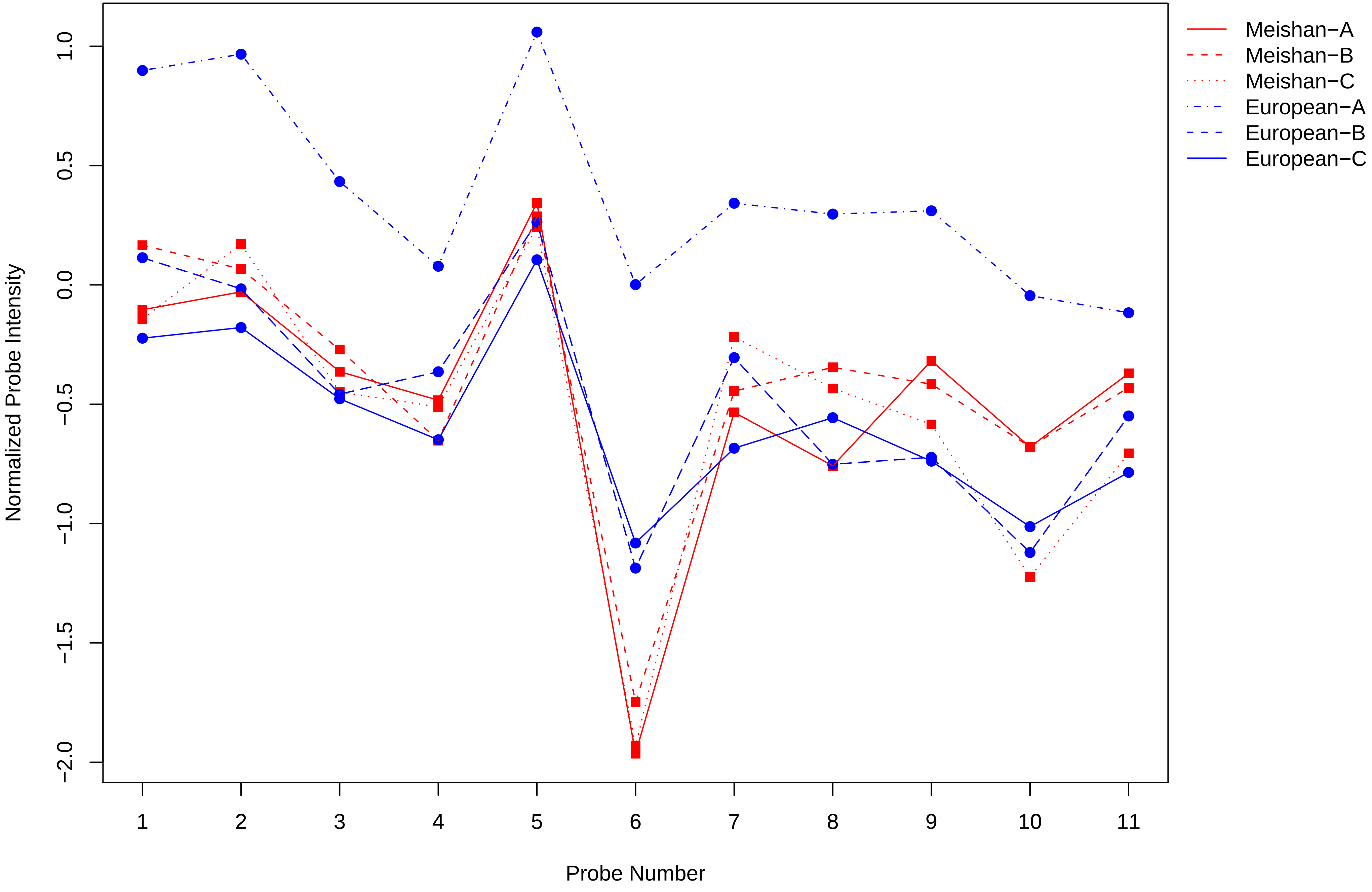

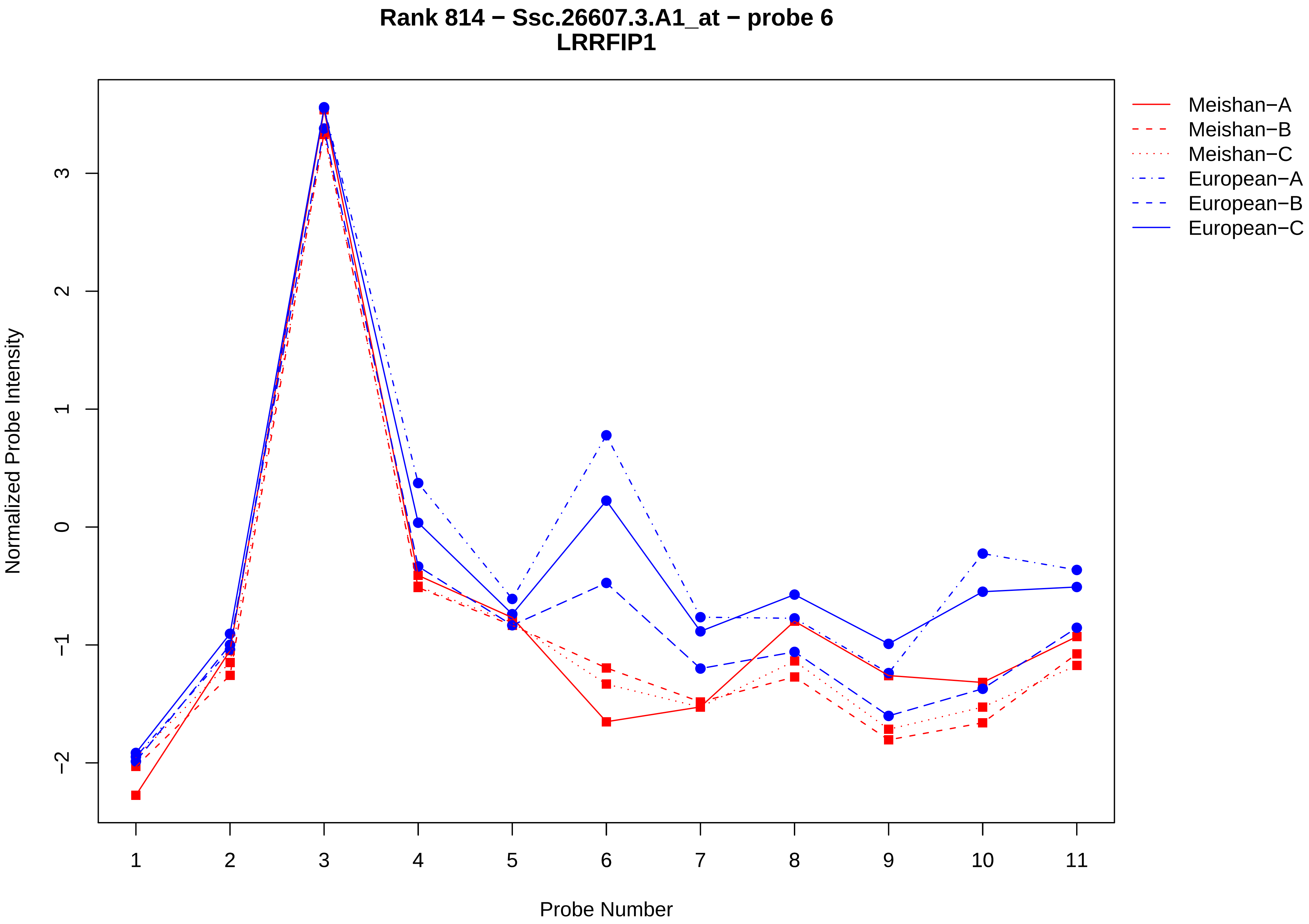

Rank 814 − Ssc.26607.3.A1_at − probe 6
LRRFIP1
Normalized Probe Intensity
Probe Number
Meishan−A
Meishan−B
Meishan−C
European−A
European−B
European−C

Rank 815 − Ssc.10716.1.A1_at − probe 4
Q9P2I7

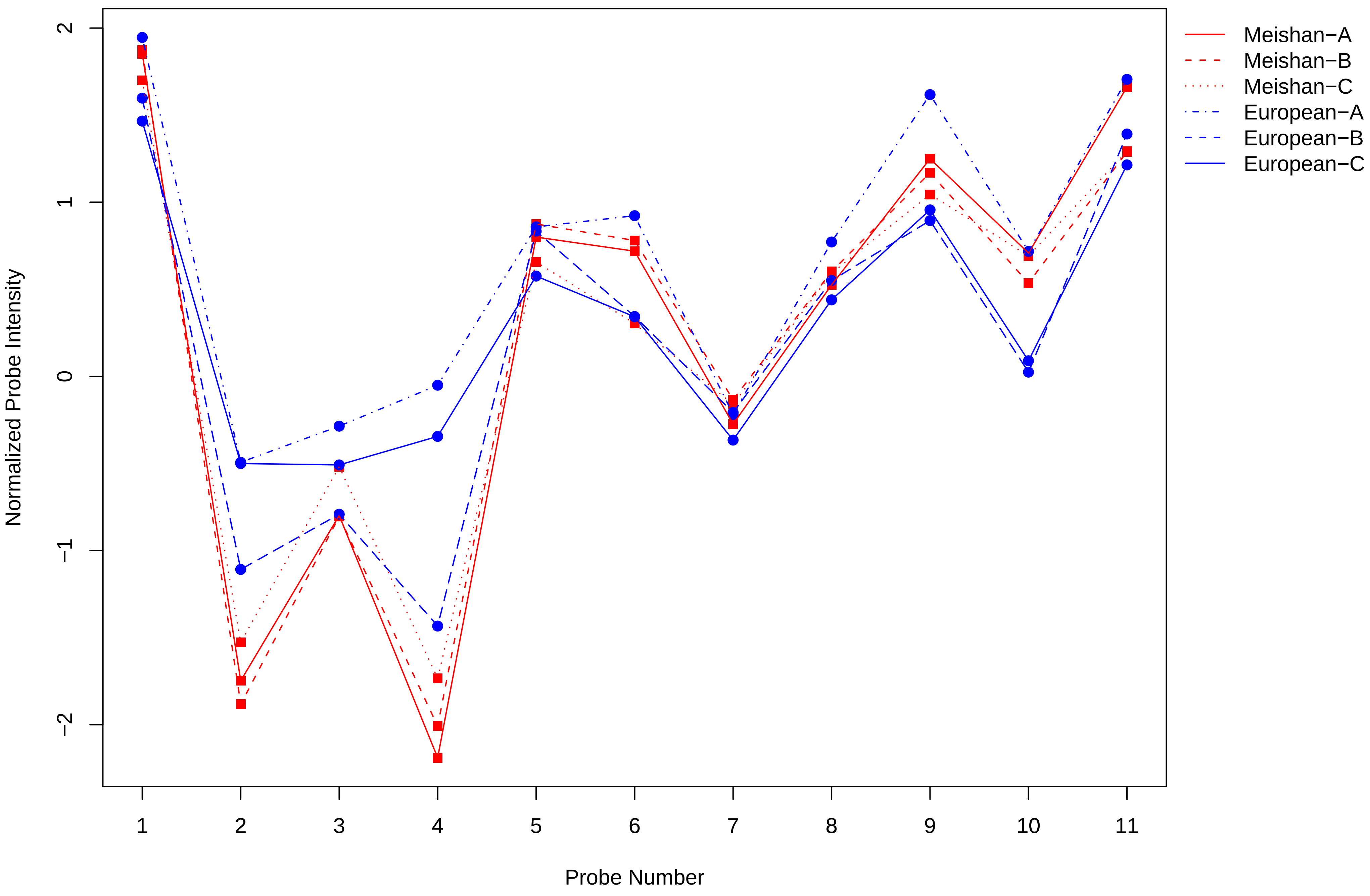

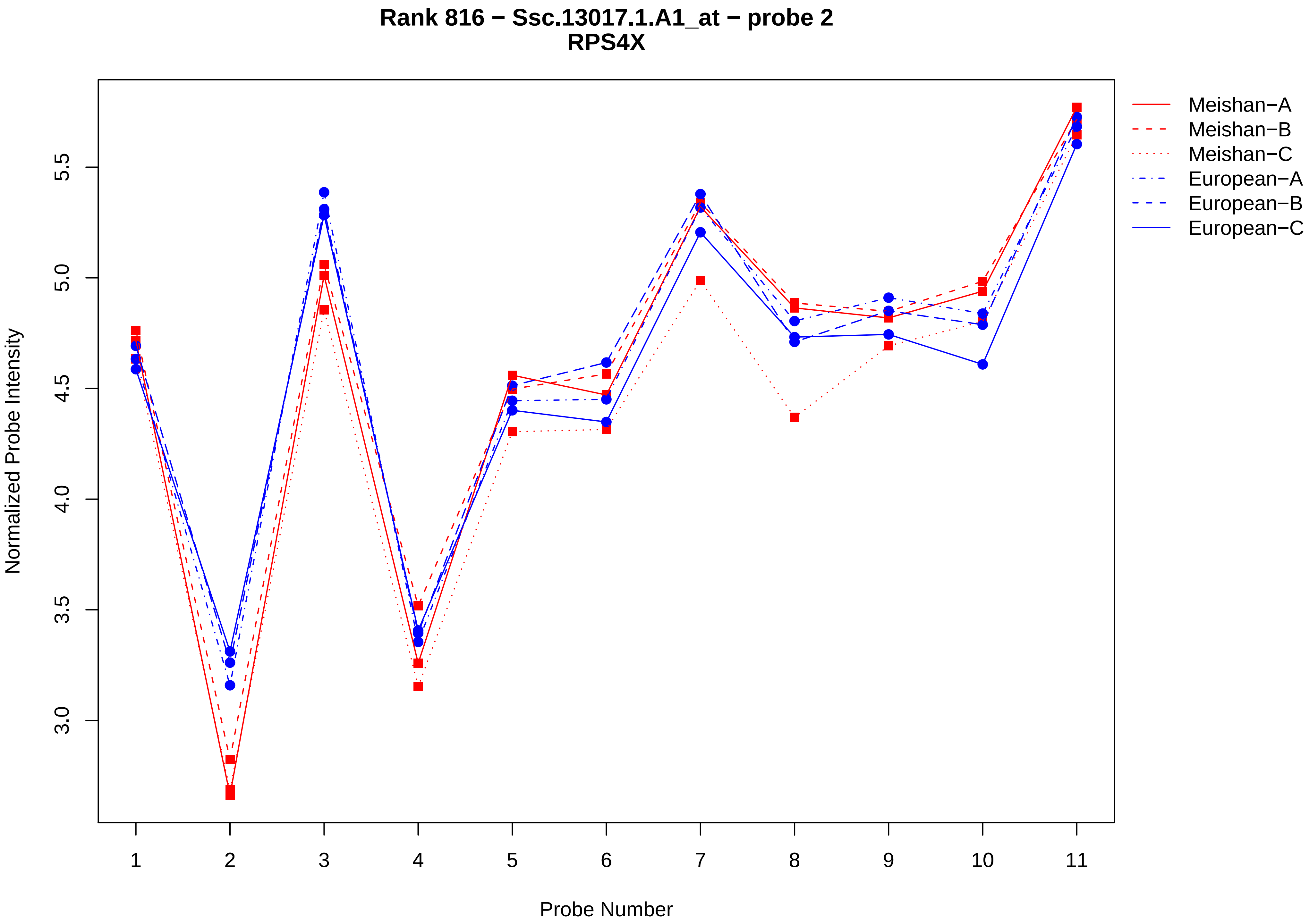
Rank 816 − Ssc.13017.1.A1_at − probe 2
RPS4X
Normalized Probe Intensity
Probe Number
3.0
3.5
4.0
4.5
5.0
5.5
1
2
3
4
5
6
7
8
9
10
11
Meishan−A
Meishan−B
Meishan−C
European−A
European−B
European−C

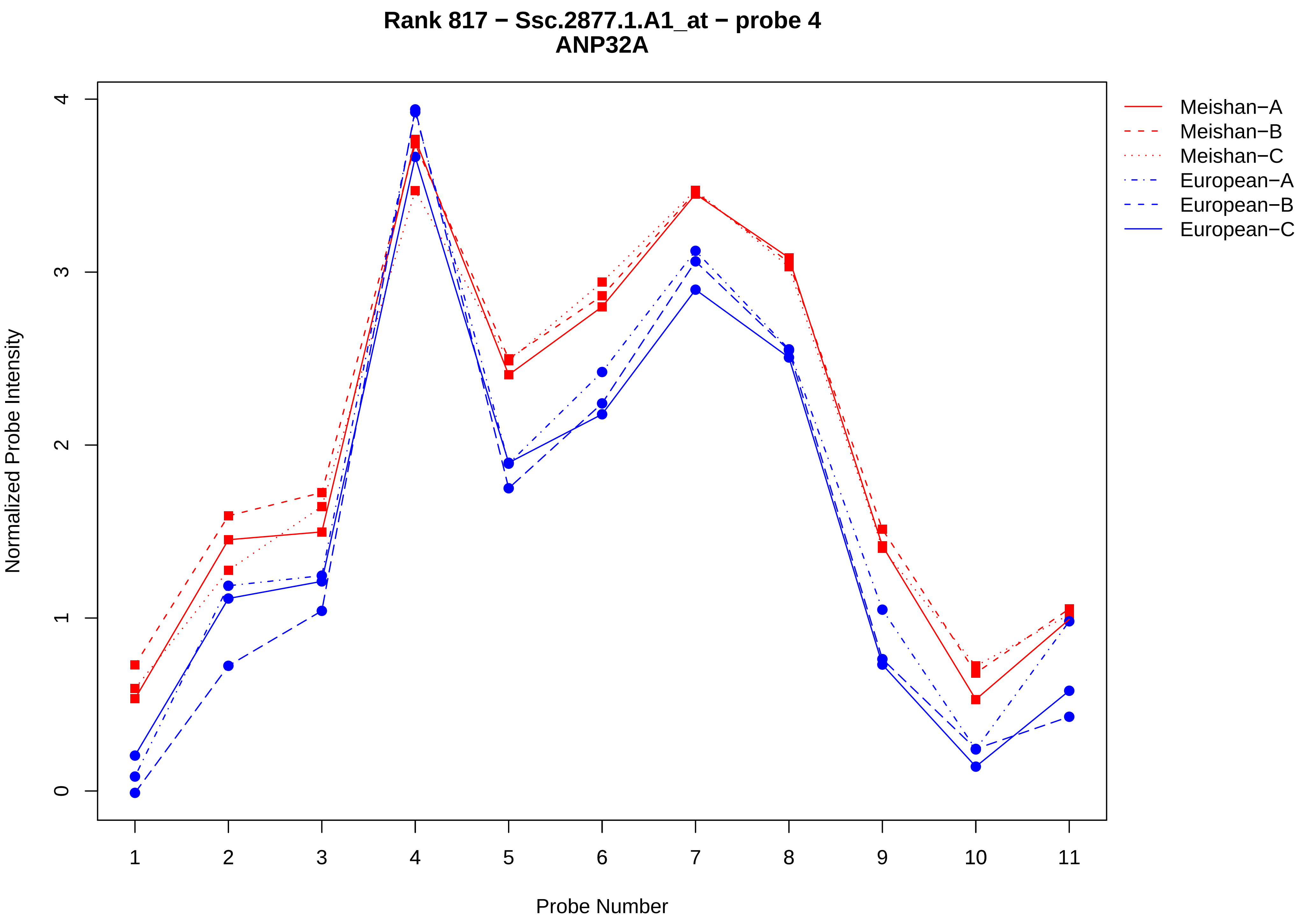

Rank 817 − Ssc.2877.1.A1_at − probe 4
ANP32A
Normalized Probe Intensity
Probe Number
0
1
2
3
4
1
2
3
4
5
6
7
8
9
10
11
Meishan−A
Meishan−B
Meishan−C
European−A
European−B
European−C

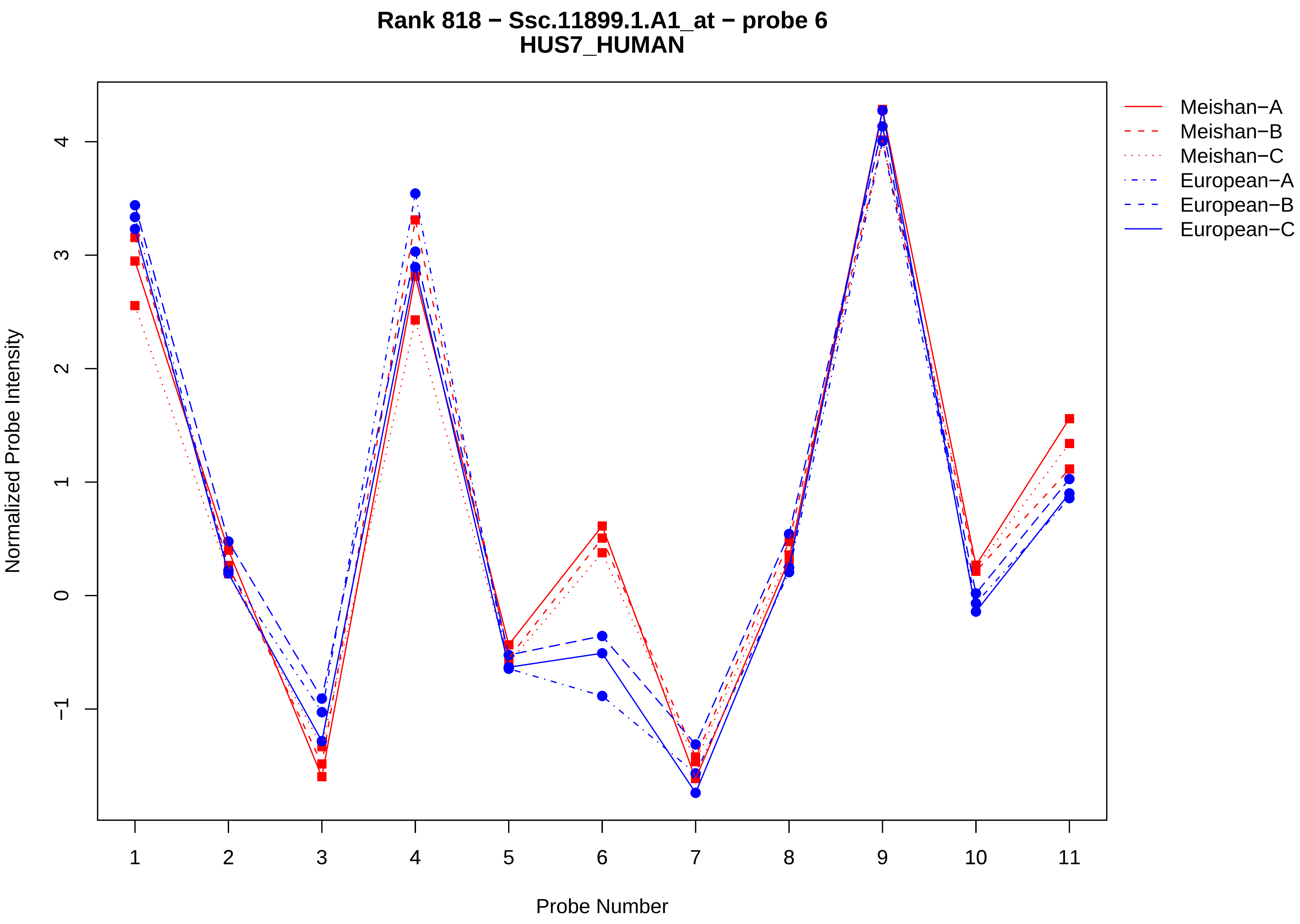

Rank 818 − Ssc.11899.1.A1_at − probe 6
HUS7_HUMAN
Normalized Probe Intensity
Probe Number
−1
0
1
2
3
4
1
2
3
4
5
6
7
8
9
10
11
Meishan−A
Meishan−B
Meishan−C
European−A
European−B
European−C

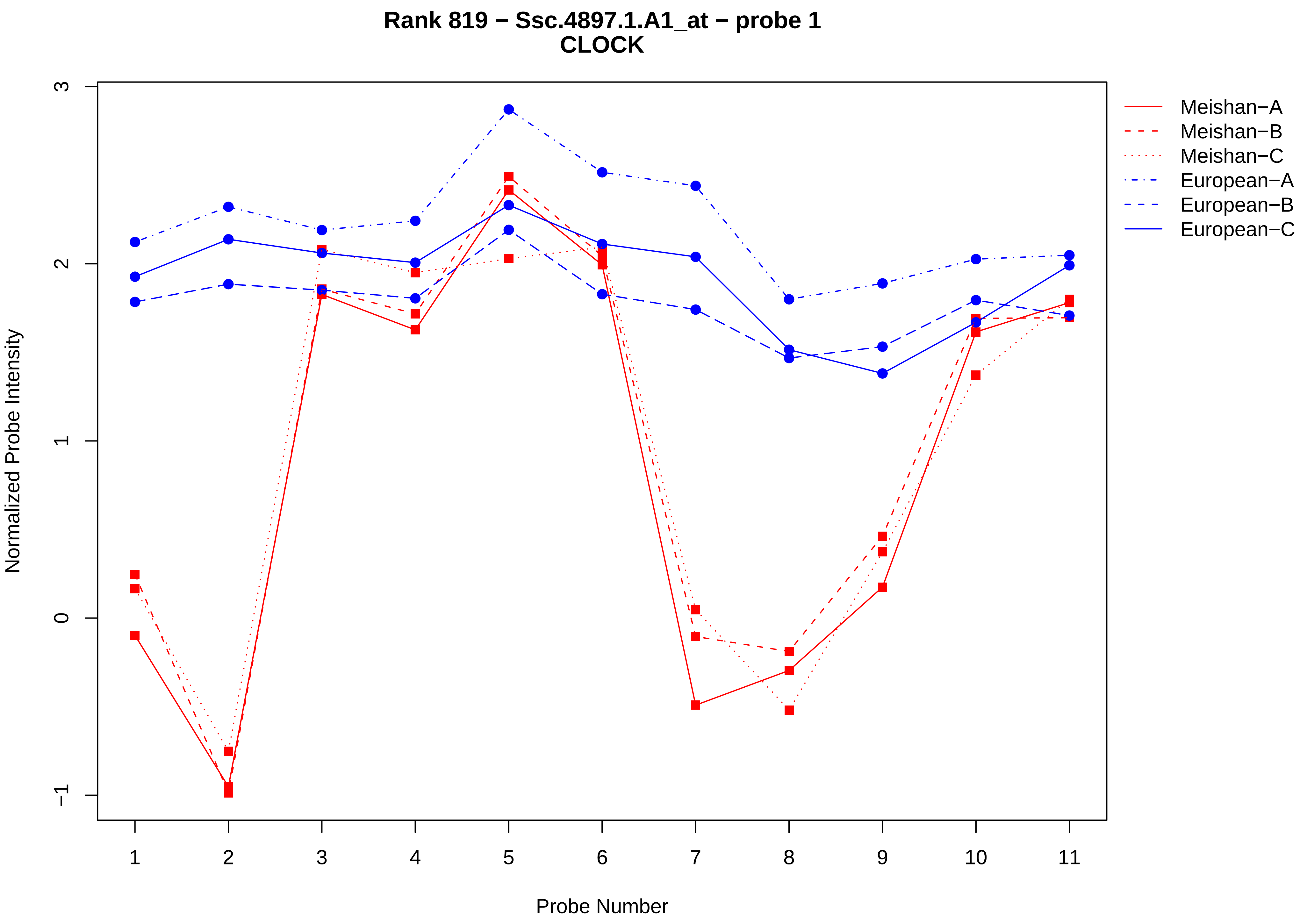

Rank 819 − Ssc.4897.1.A1_at − probe 1
CLOCK
Normalized Probe Intensity
Probe Number
−1
0
1
2
3
1
2
3
4
5
6
7
8
9
10
11
Meishan−A
Meishan−B
Meishan−C
European−A
European−B
European−C

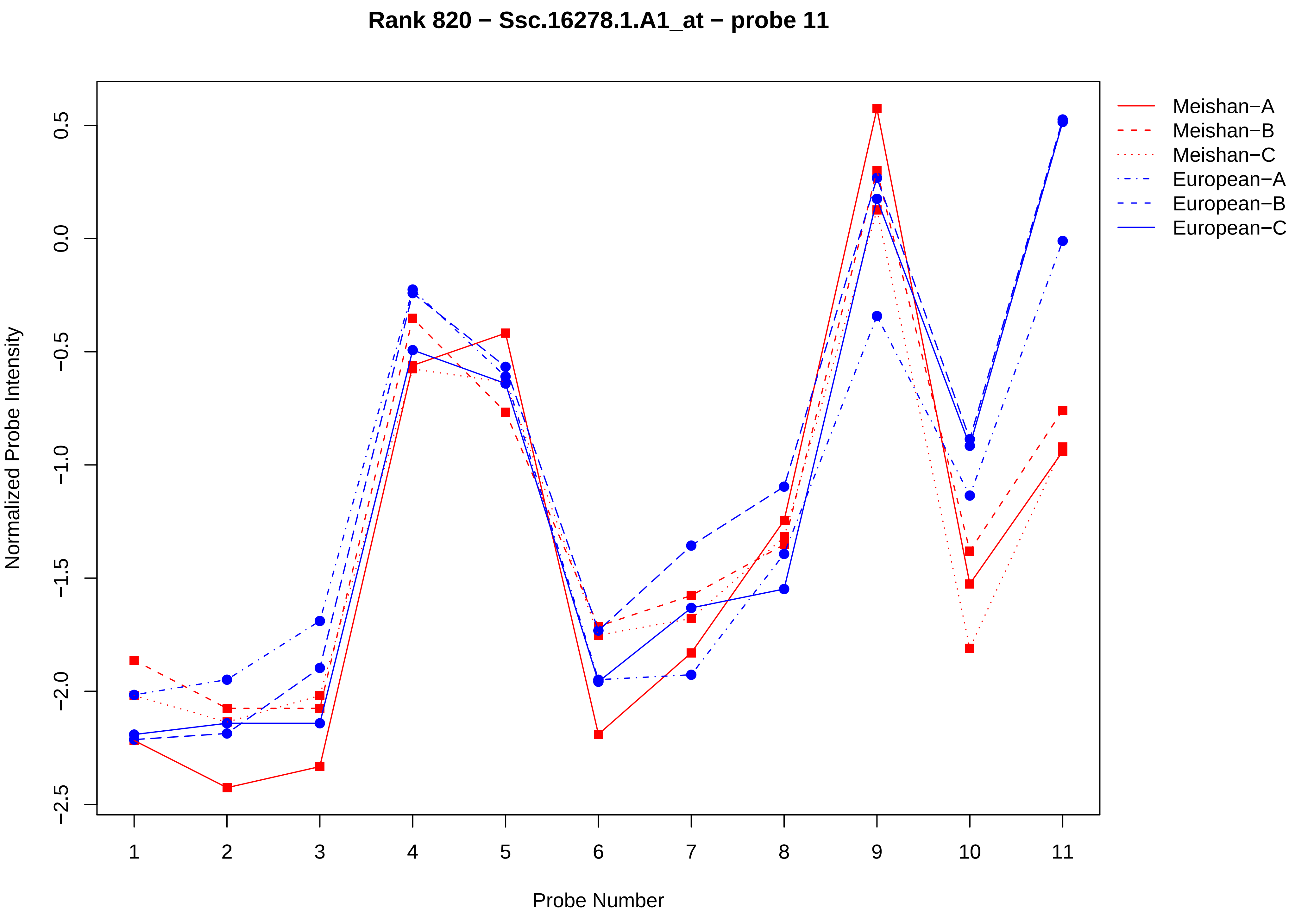

Rank 820 − Ssc.16278.1.A1_at − probe 11
Normalized Probe Intensity
Probe Number
0.5
0.0
−0.5
−1.0
−1.5
−2.0
−2.5
1
2
3
4
5
6
7
8
9
10
11
Meishan−A
Meishan−B
Meishan−C
European−A
European−B
European−C

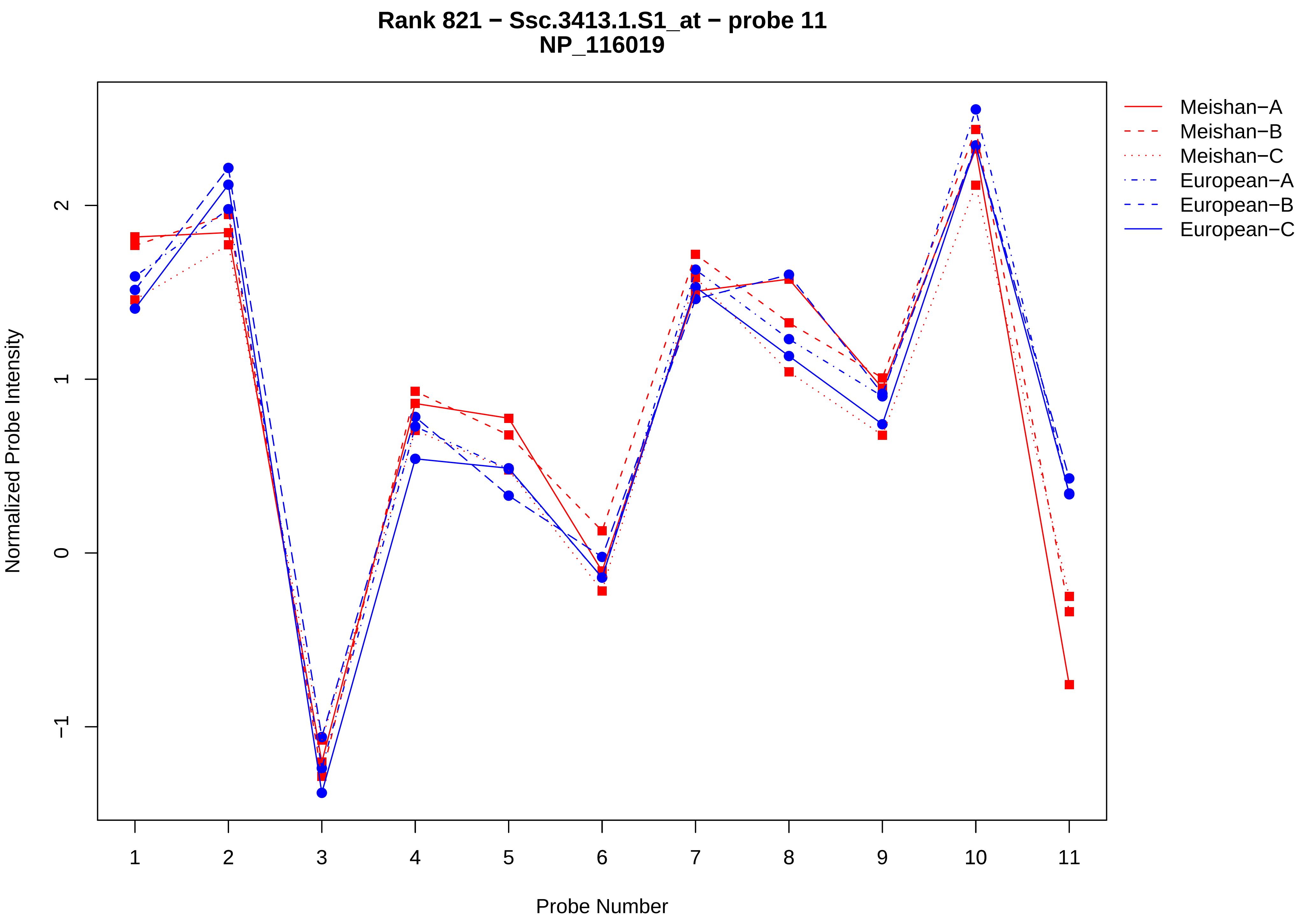

Rank 821 − Ssc.3413.1.S1_at − probe 11
NP_116019
Normalized Probe Intensity
Probe Number
−1
0
1
2
1
2
3
4
5
6
7
8
9
10
11
Meishan−A
Meishan−B
Meishan−C
European−A
European−B
European−C

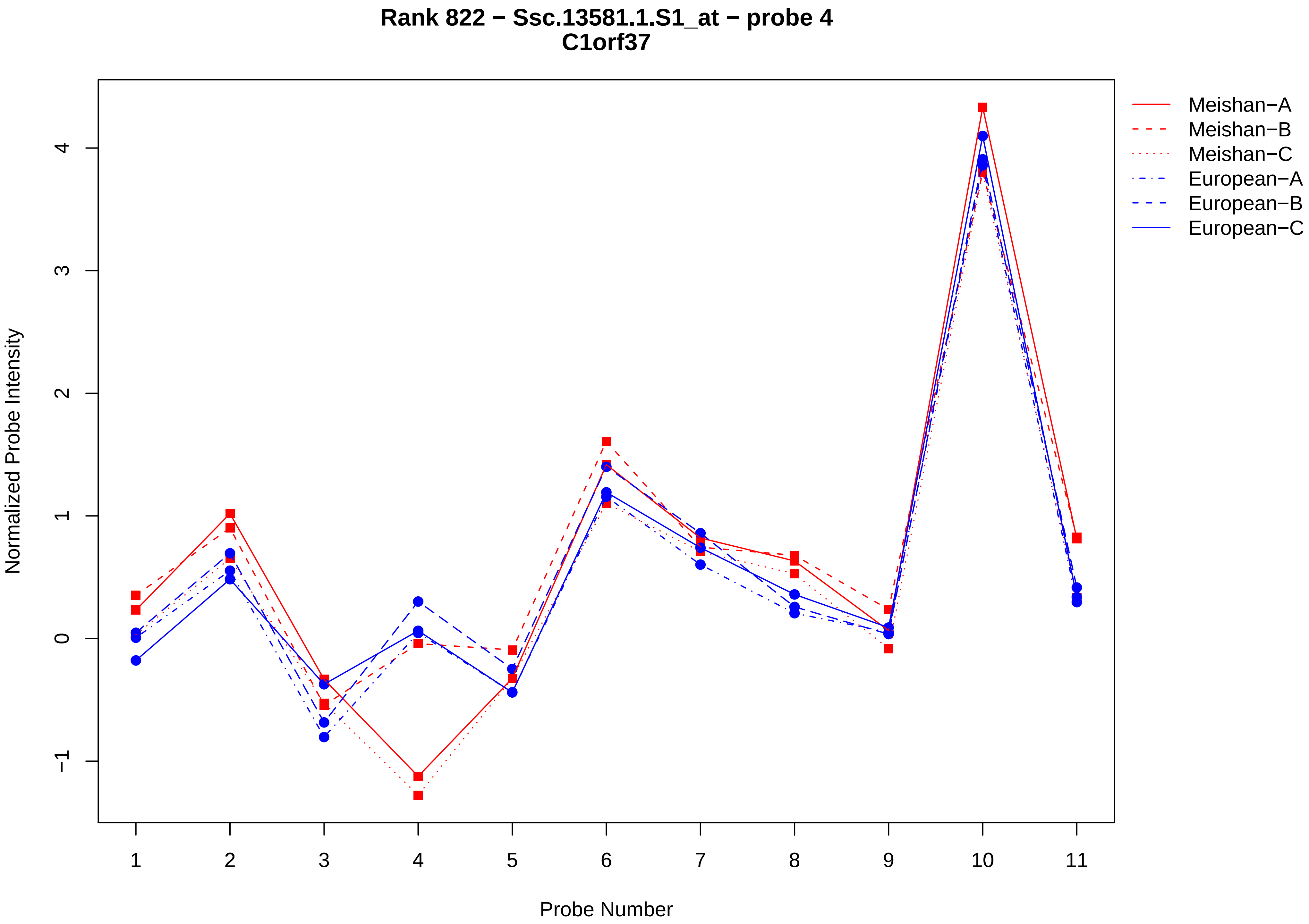

Rank 822 − Ssc.13581.1.S1_at − probe 4
C1orf37
Normalized Probe Intensity
Probe Number
−1
0
1
2
3
4
1
2
3
4
5
6
7
8
9
10
11
Meishan−A
Meishan−B
Meishan−C
European−A
European−B
European−C

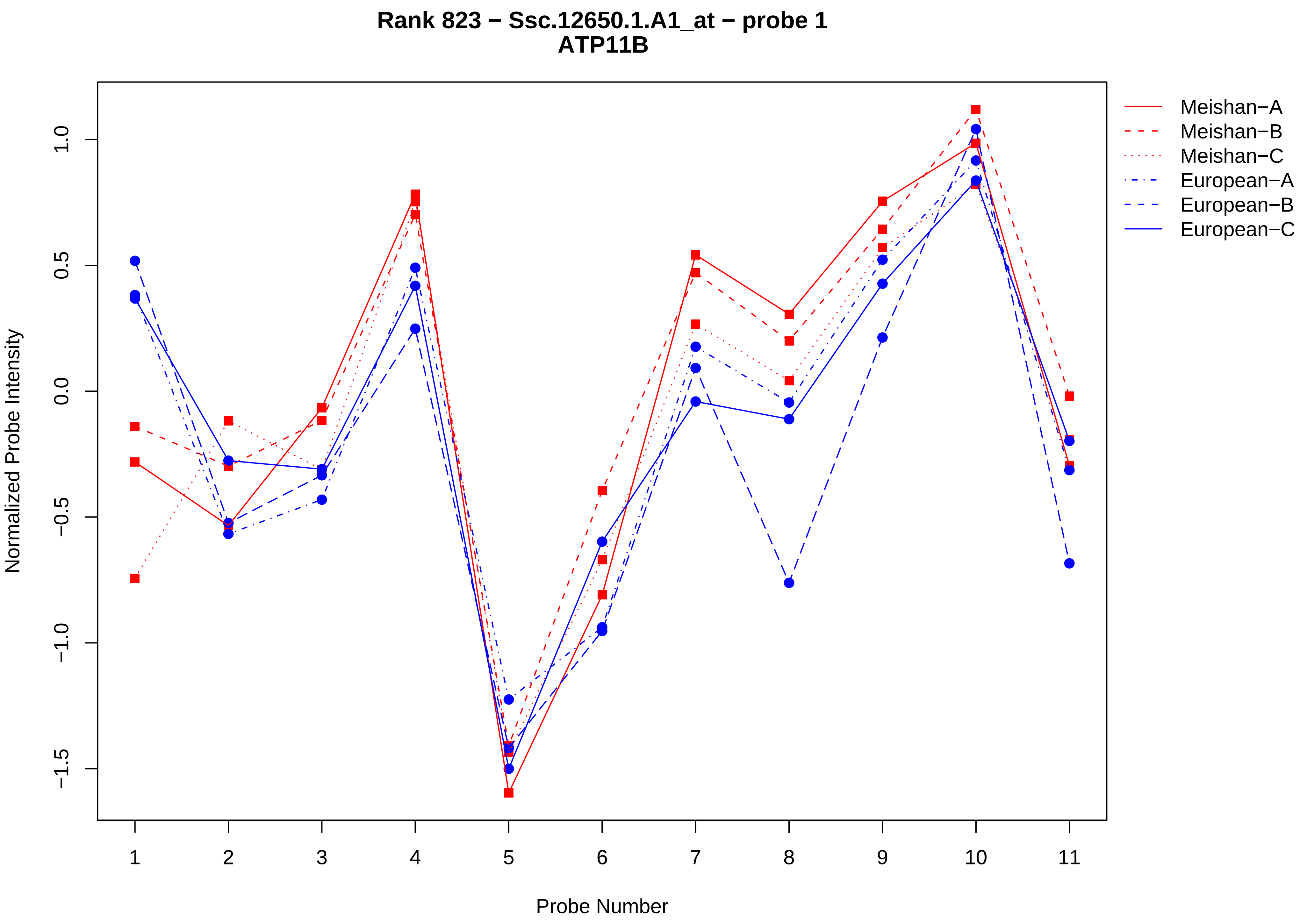
Rank 823 − Ssc.12650.1.A1_at − probe 1
ATP11B
Normalized Probe Intensity
Probe Number
Meishan−A
Meishan−B
Meishan−C
European−A
European−B
European−C
1.0
0.5
0.0
−0.5
−1.0
−1.5
1
2
3
4
5
6
7
8
9
10
11

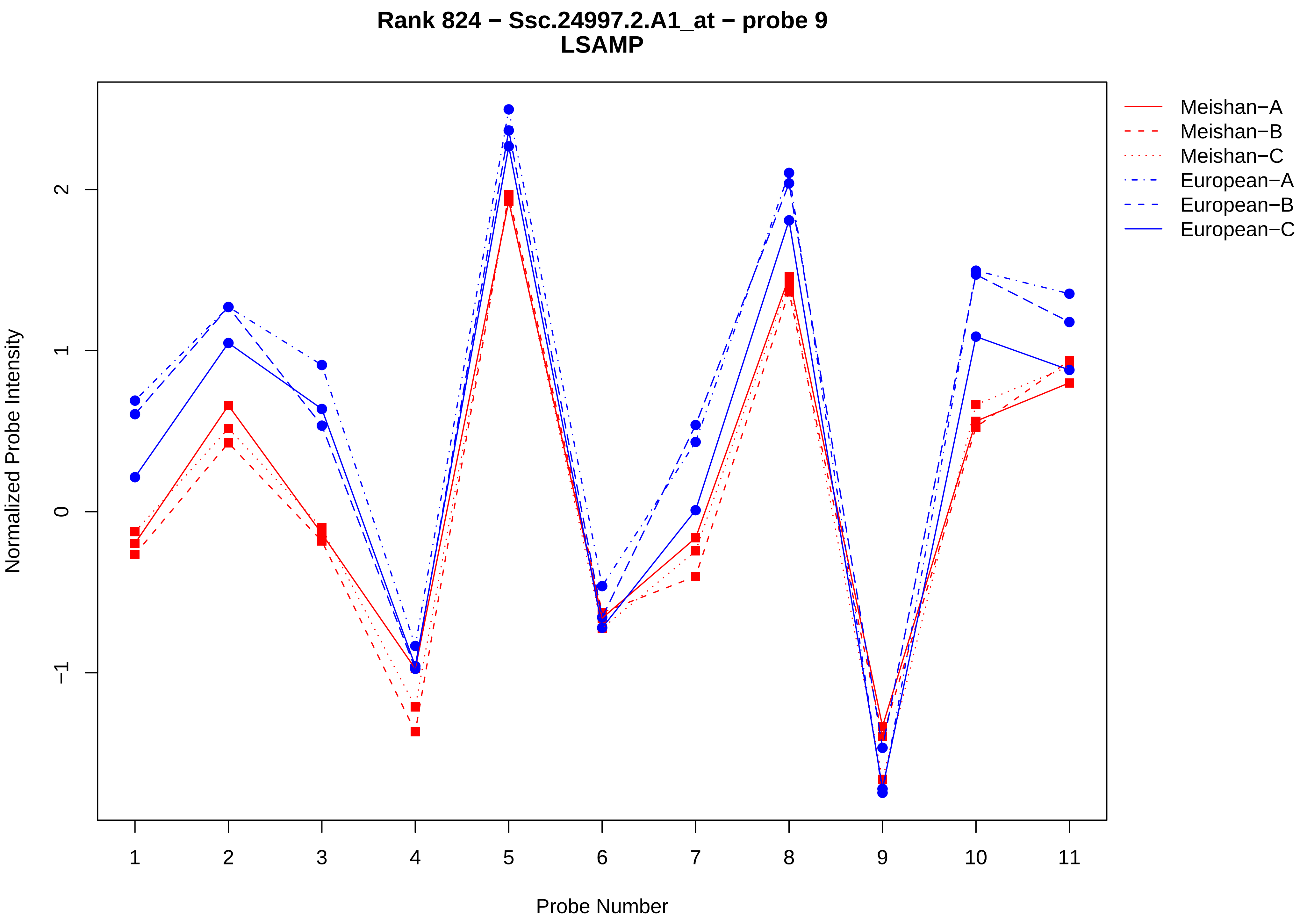

Rank 824 − Ssc.24997.2.A1_at − probe 9
LSAMP
Normalized Probe Intensity
Probe Number
−1
0
1
2
1
2
3
4
5
6
7
8
9
10
11
Meishan−A
Meishan−B
Meishan−C
European−A
European−B
European−C

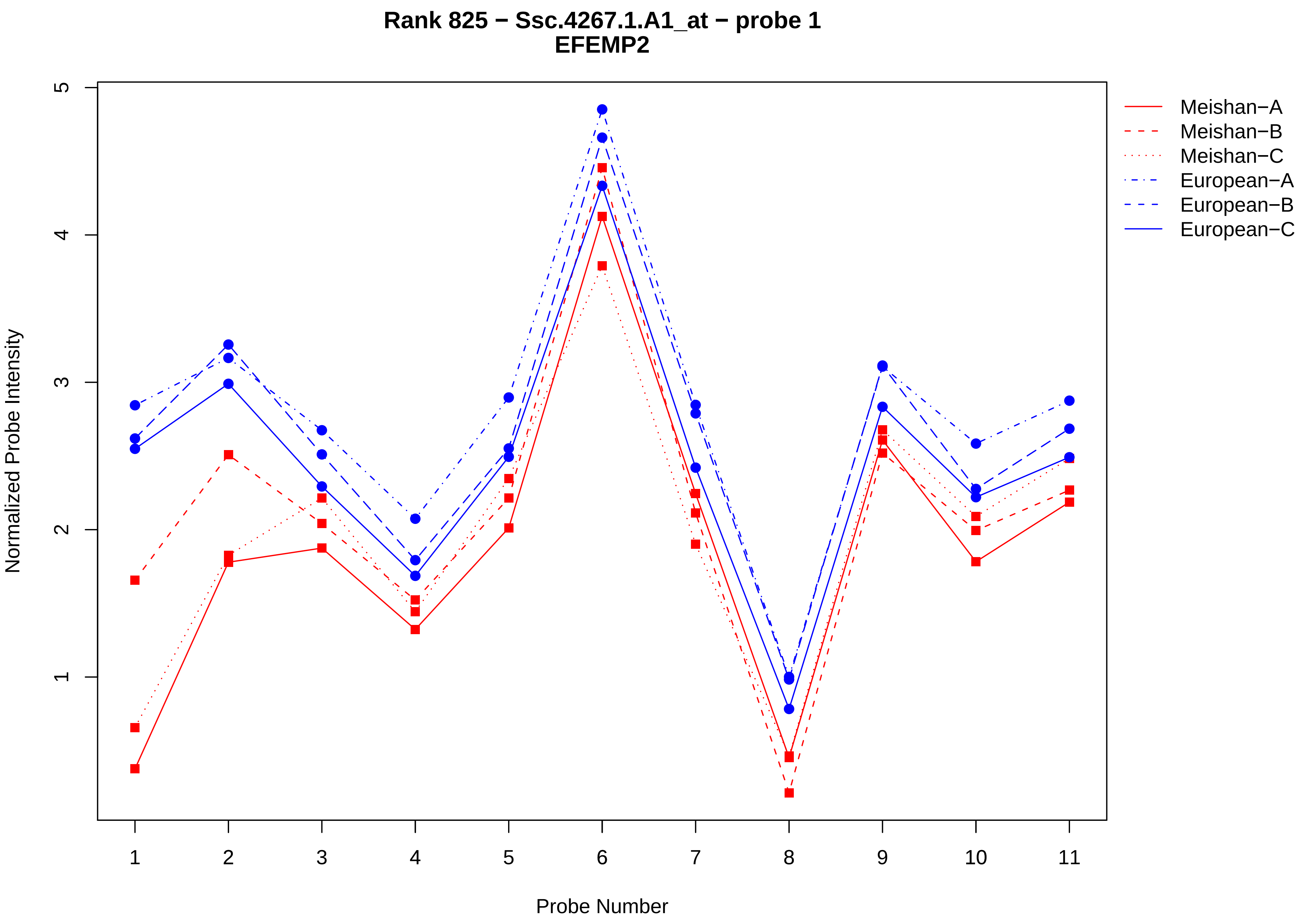
Rank 825 − Ssc.4267.1.A1_at − probe 1
EFEMP2
Normalized Probe Intensity
Probe Number
1
2
3
4
5
6
7
8
9
10
11
Meishan−A
Meishan−B
Meishan−C
European−A
European−B
European−C

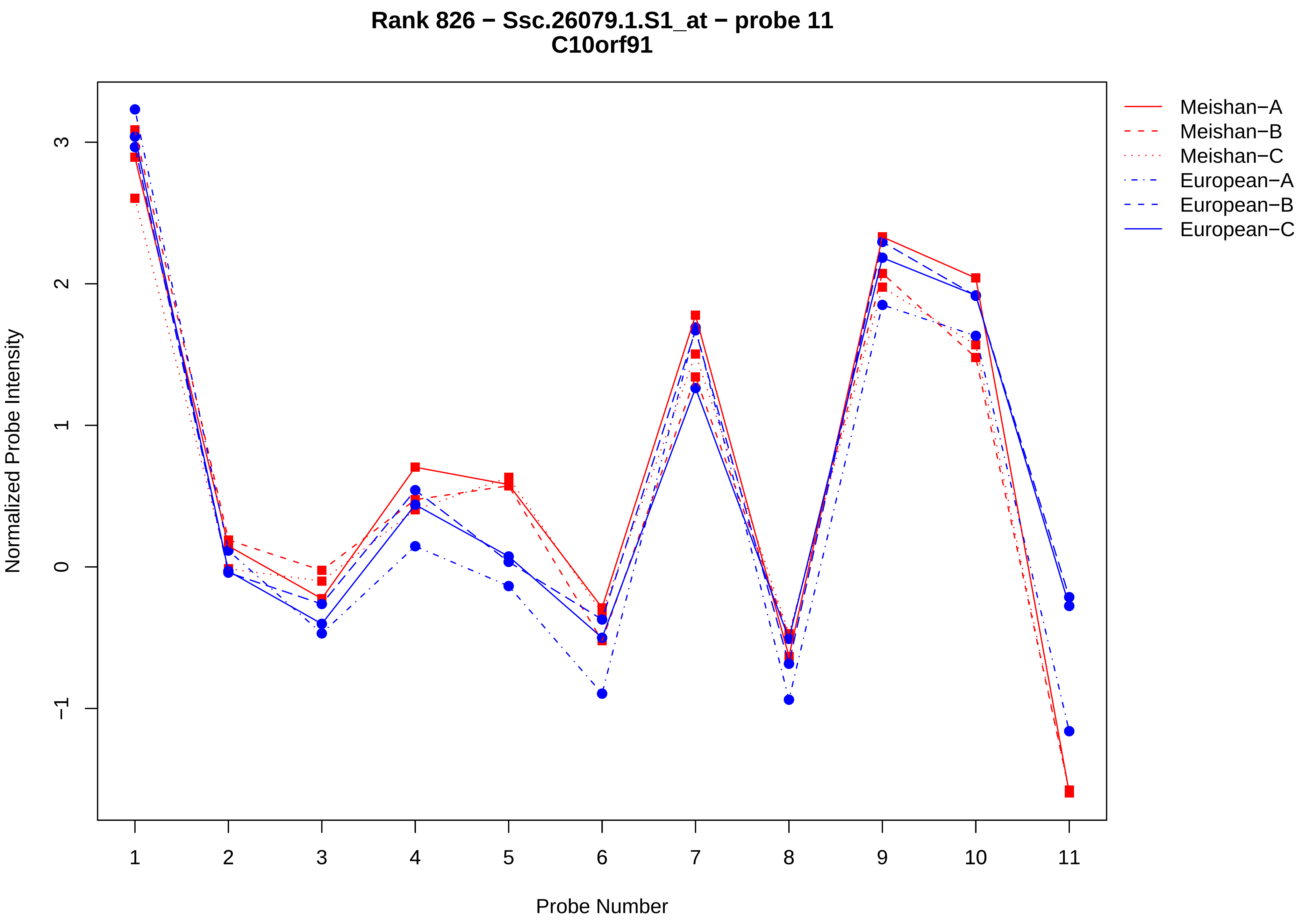

Rank 826 − Ssc.26079.1.S1_at − probe 11
C10orf91
Normalized Probe Intensity
Probe Number
−1
0
1
2
3
1
2
3
4
5
6
7
8
9
10
11
Meishan−A
Meishan−B
Meishan−C
European−A
European−B
European−C

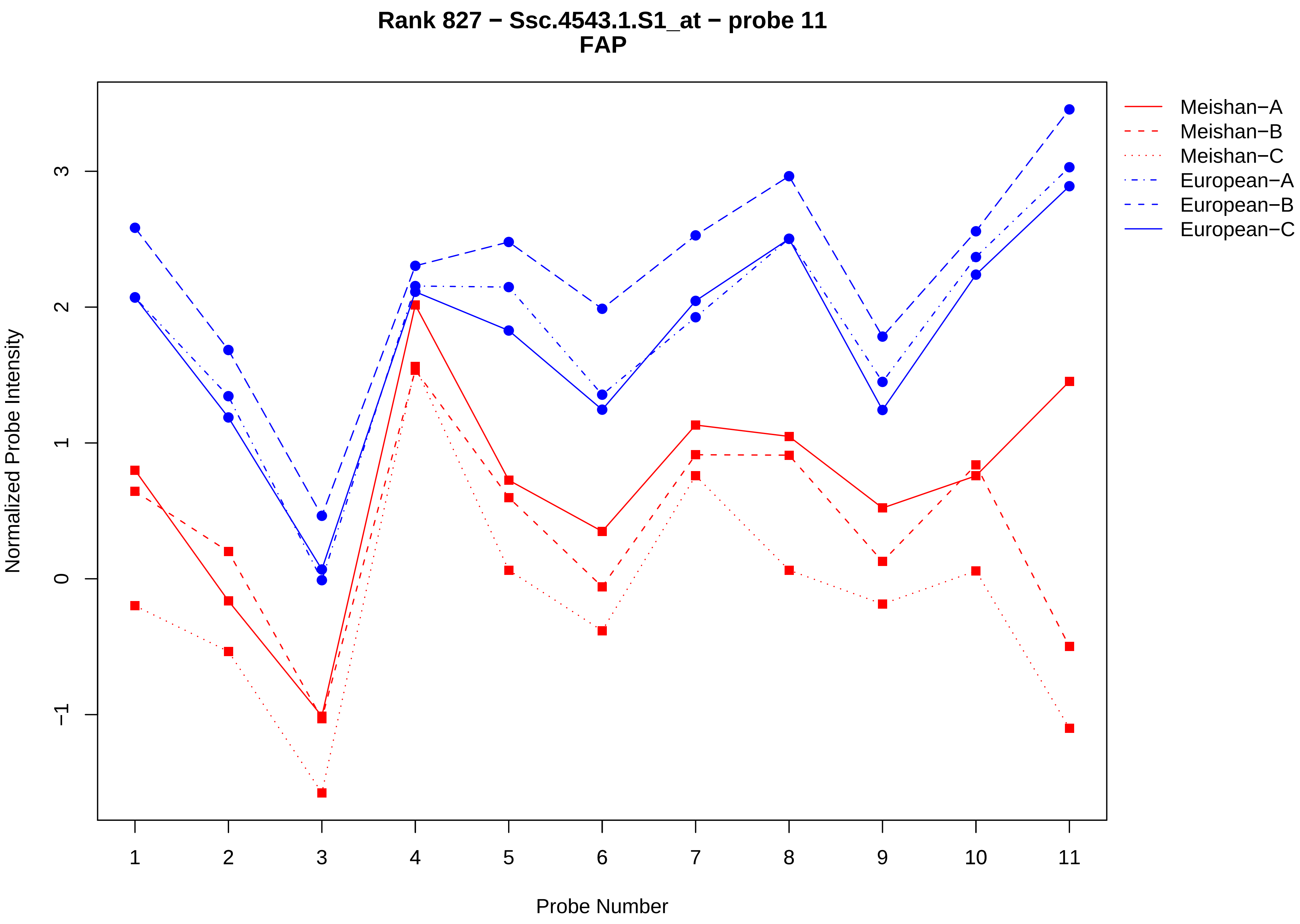
Rank 827 − Ssc.4543.1.S1_at − probe 11
FAP
Normalized Probe Intensity
Probe Number
−1
0
1
2
3
1
2
3
4
5
6
7
8
9
10
11
Meishan−A
Meishan−B
Meishan−C
European−A
European−B
European−C

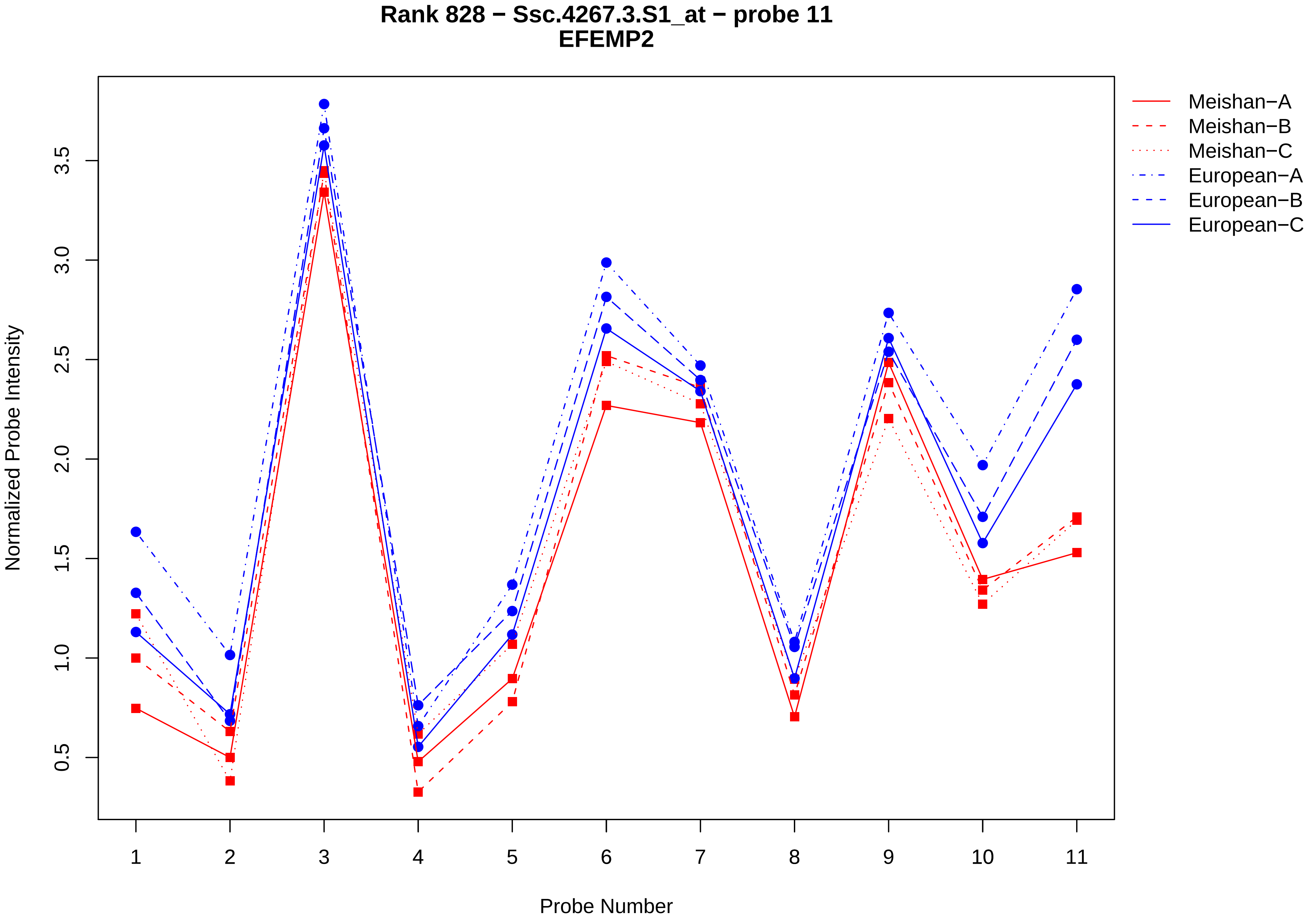

Rank 828 − Ssc.4267.3.S1_at − probe 11
EFEMP2
Normalized Probe Intensity
Probe Number
0.5
1.0
1.5
2.0
2.5
3.0
3.5
1
2
3
4
5
6
7
8
9
10
11
Meishan−A
Meishan−B
Meishan−C
European−A
European−B
European−C

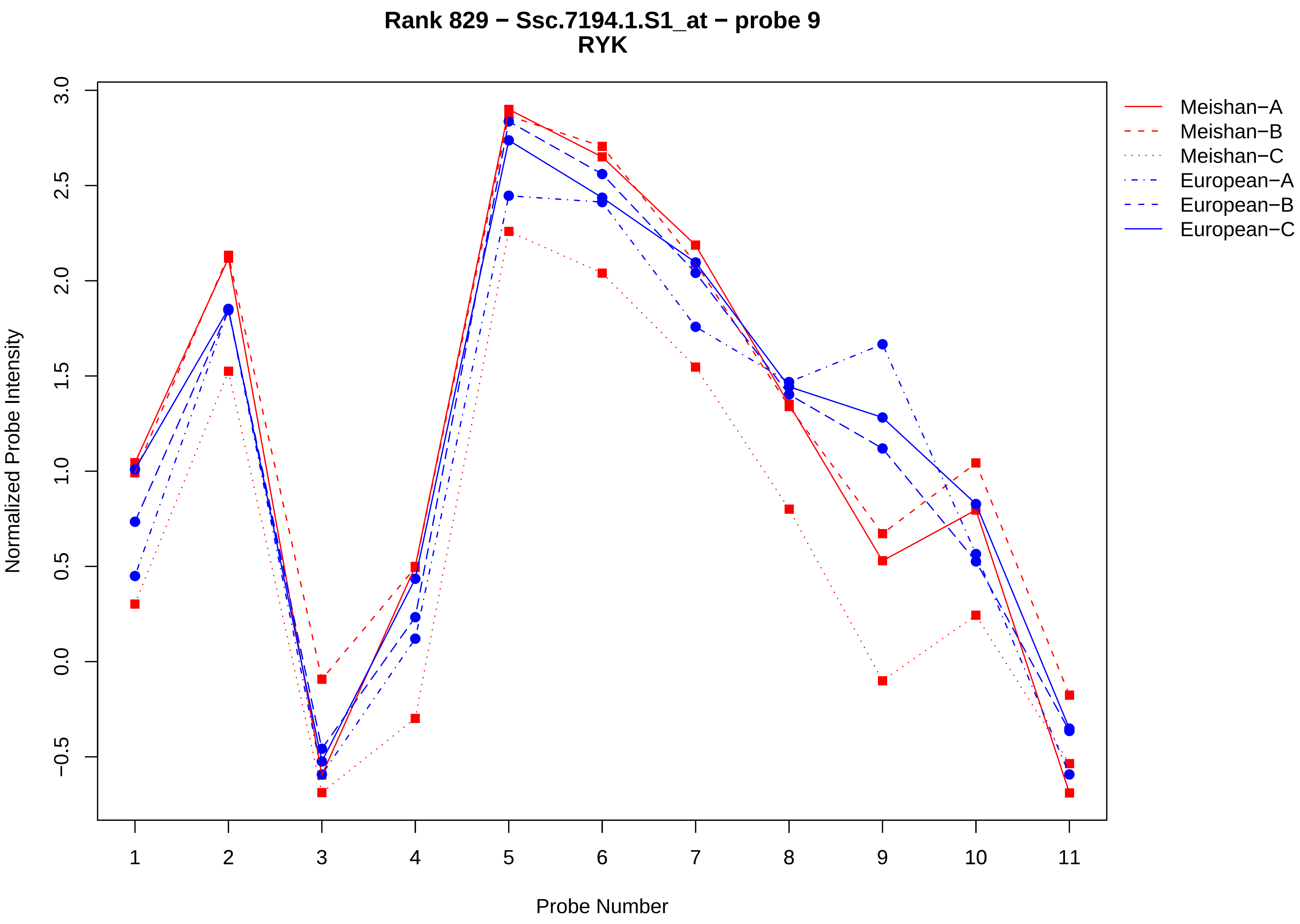

Rank 829 − Ssc.7194.1.S1_at − probe 9
RYK
Normalized Probe Intensity
Probe Number
Meishan−A
Meishan−B
Meishan−C
European−A
European−B
European−C

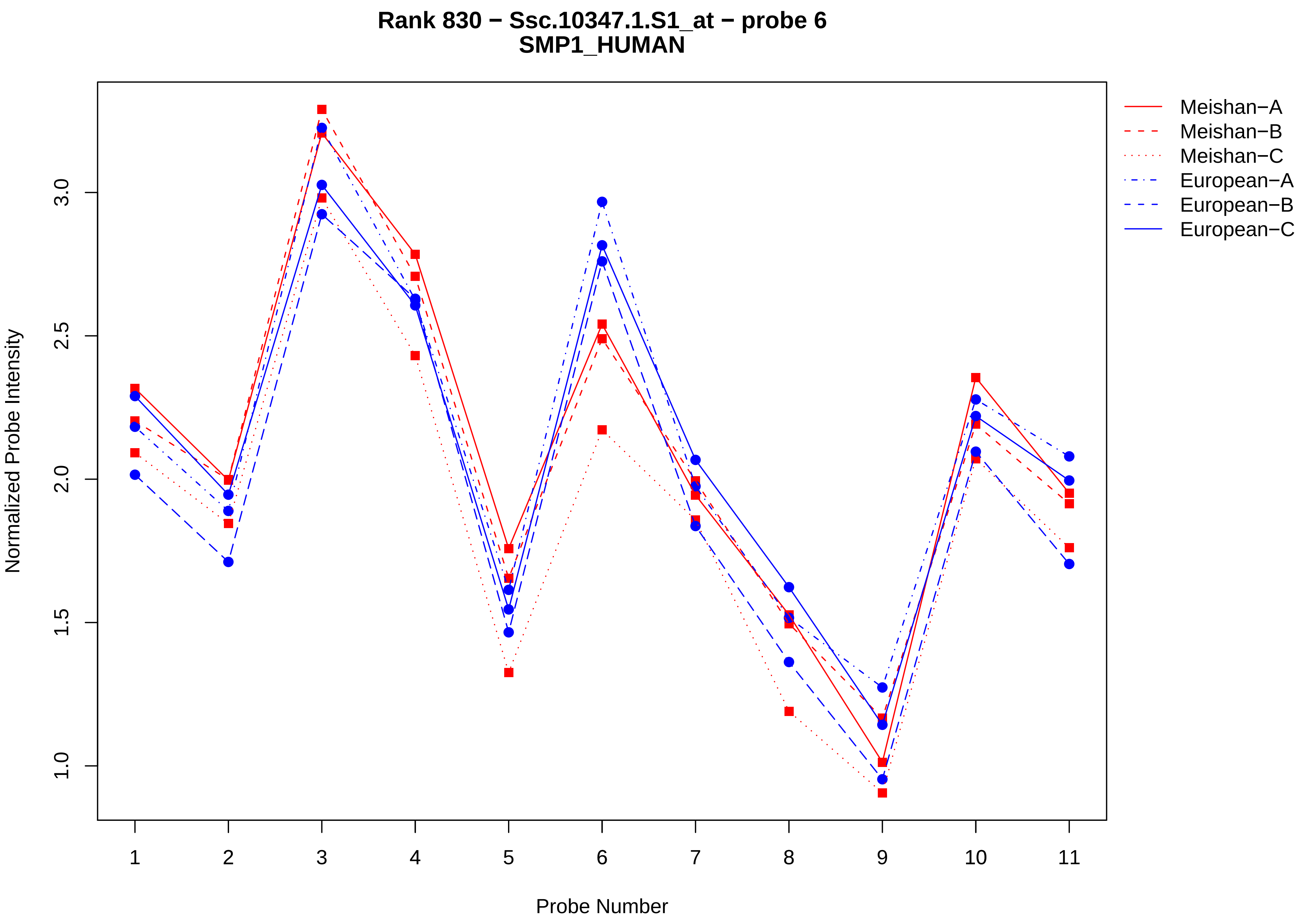

Rank 830 − Ssc.10347.1.S1_at − probe 6
SMP1_HUMAN
Normalized Probe Intensity
Probe Number
Meishan−A
Meishan−B
Meishan−C
European−A
European−B
European−C
1.0
1.5
2.0
2.5
3.0
1
2
3
4
5
6
7
8
9
10
11

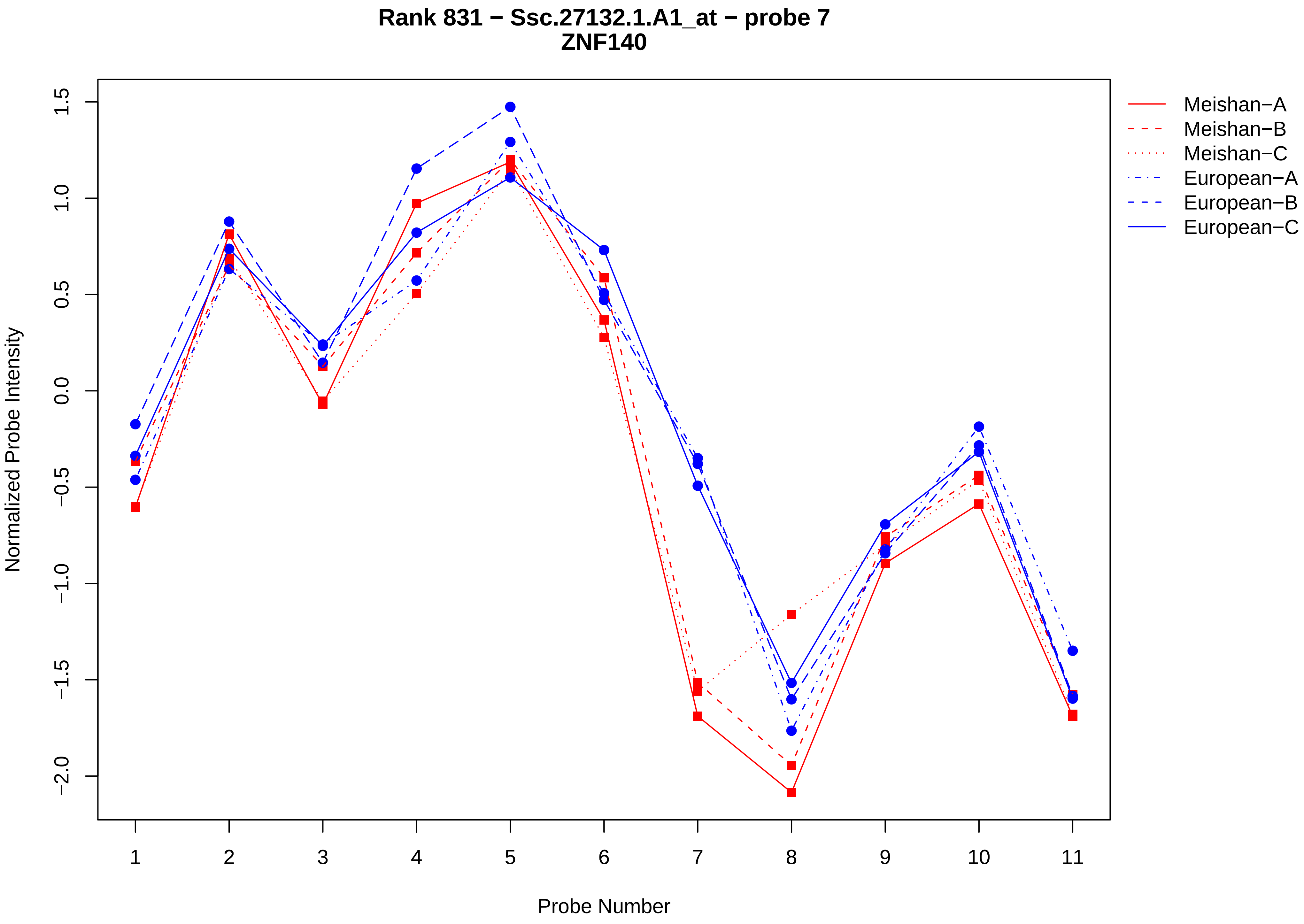

Rank 831 − Ssc.27132.1.A1_at − probe 7
ZNF140
Normalized Probe Intensity
Probe Number
1.5
1.0
0.5
0.0
−0.5
−1.0
−1.5
−2.0
1
2
3
4
5
6
7
8
9
10
11
Meishan−A
Meishan−B
Meishan−C
European−A
European−B
European−C

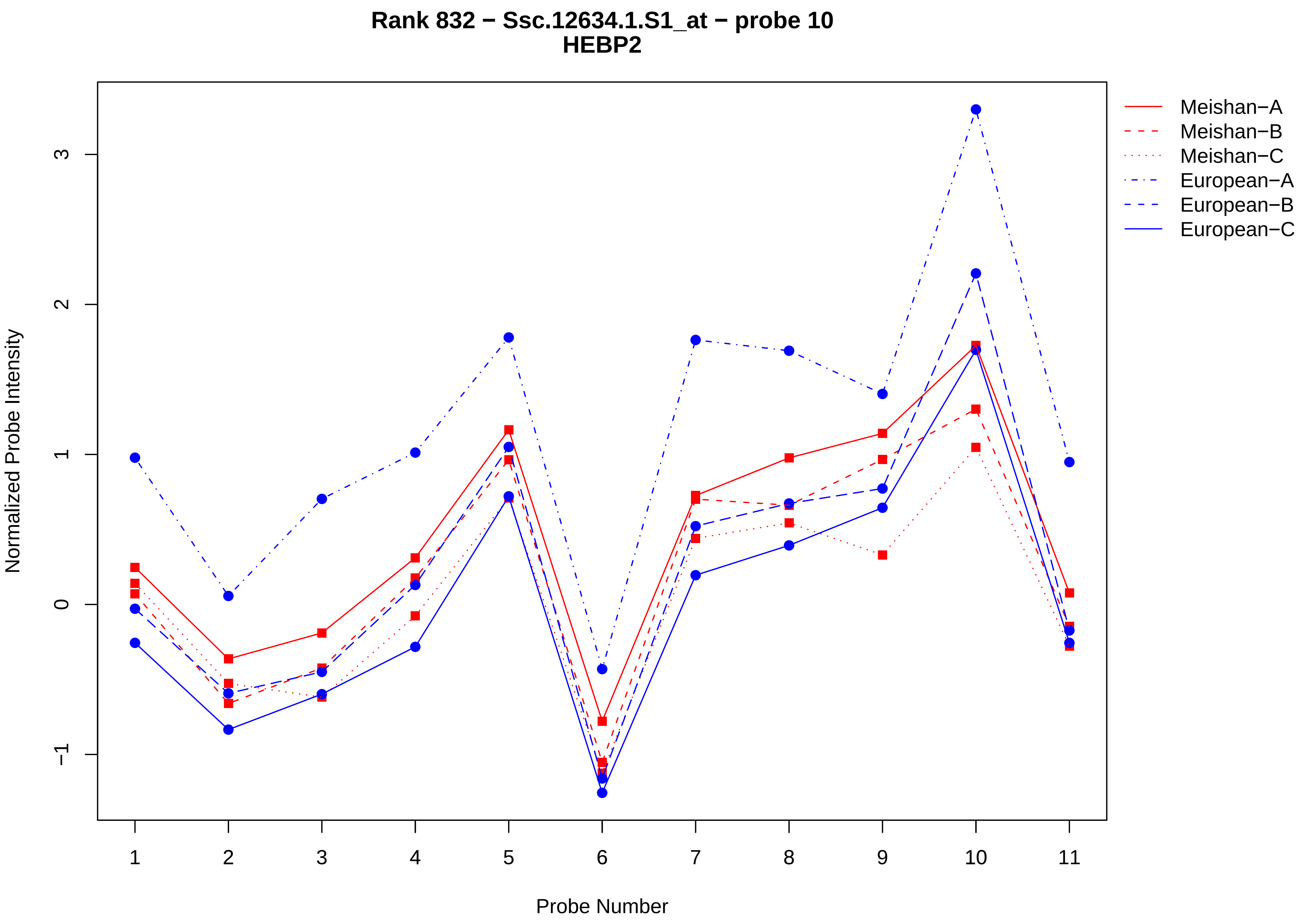

Rank 832 − Ssc.12634.1.S1_at − probe 10
HEBP2
Normalized Probe Intensity
Probe Number
−1
0
1
2
3
1
2
3
4
5
6
7
8
9
10
11
Meishan−A
Meishan−B
Meishan−C
European−A
European−B
European−C

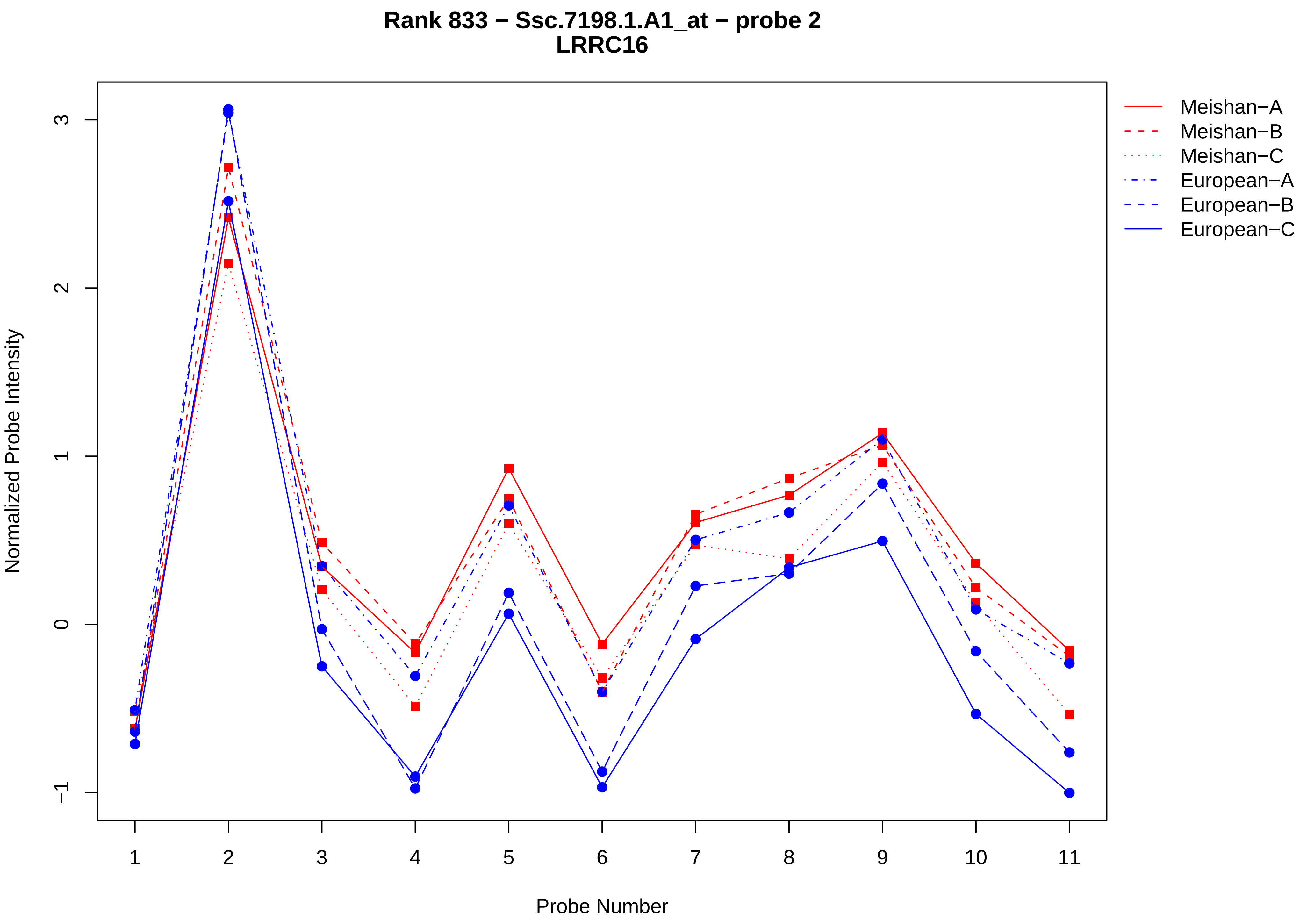

Rank 833 − Ssc.7198.1.A1_at − probe 2
LRRC16
Normalized Probe Intensity
Probe Number
−1
0
1
2
3
1
2
3
4
5
6
7
8
9
10
11
Meishan−A
Meishan−B
Meishan−C
European−A
European−B
European−C

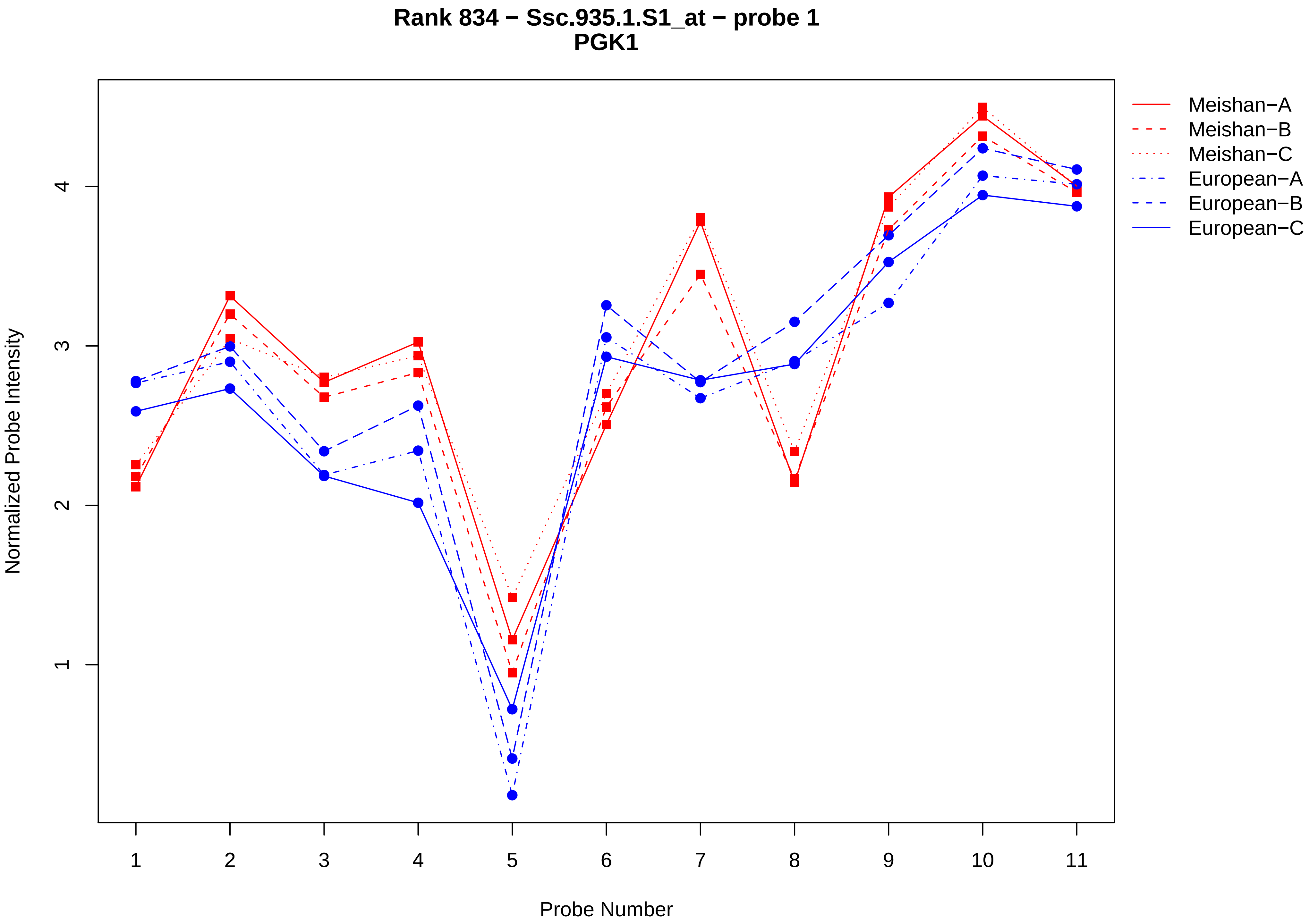

Rank 834 − Ssc.935.1.S1_at − probe 1
PGK1
Normalized Probe Intensity
1
2
3
4
Probe Number
1
2
3
4
5
6
7
8
9
10
11
Meishan−A
Meishan−B
Meishan−C
European−A
European−B
European−C

Rank 835 − Ssc.10727.1.A1_at − probe 3
RBL2

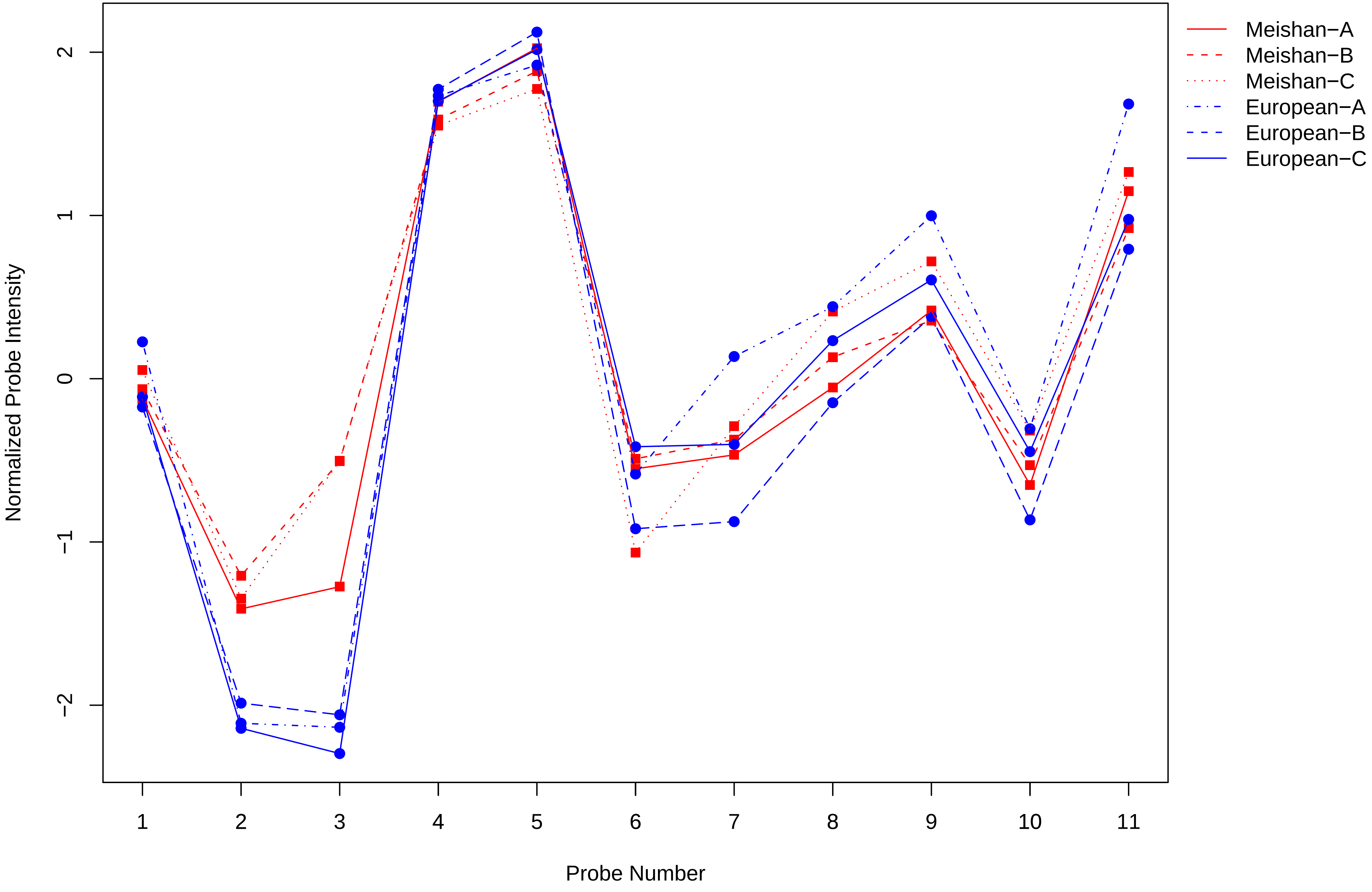

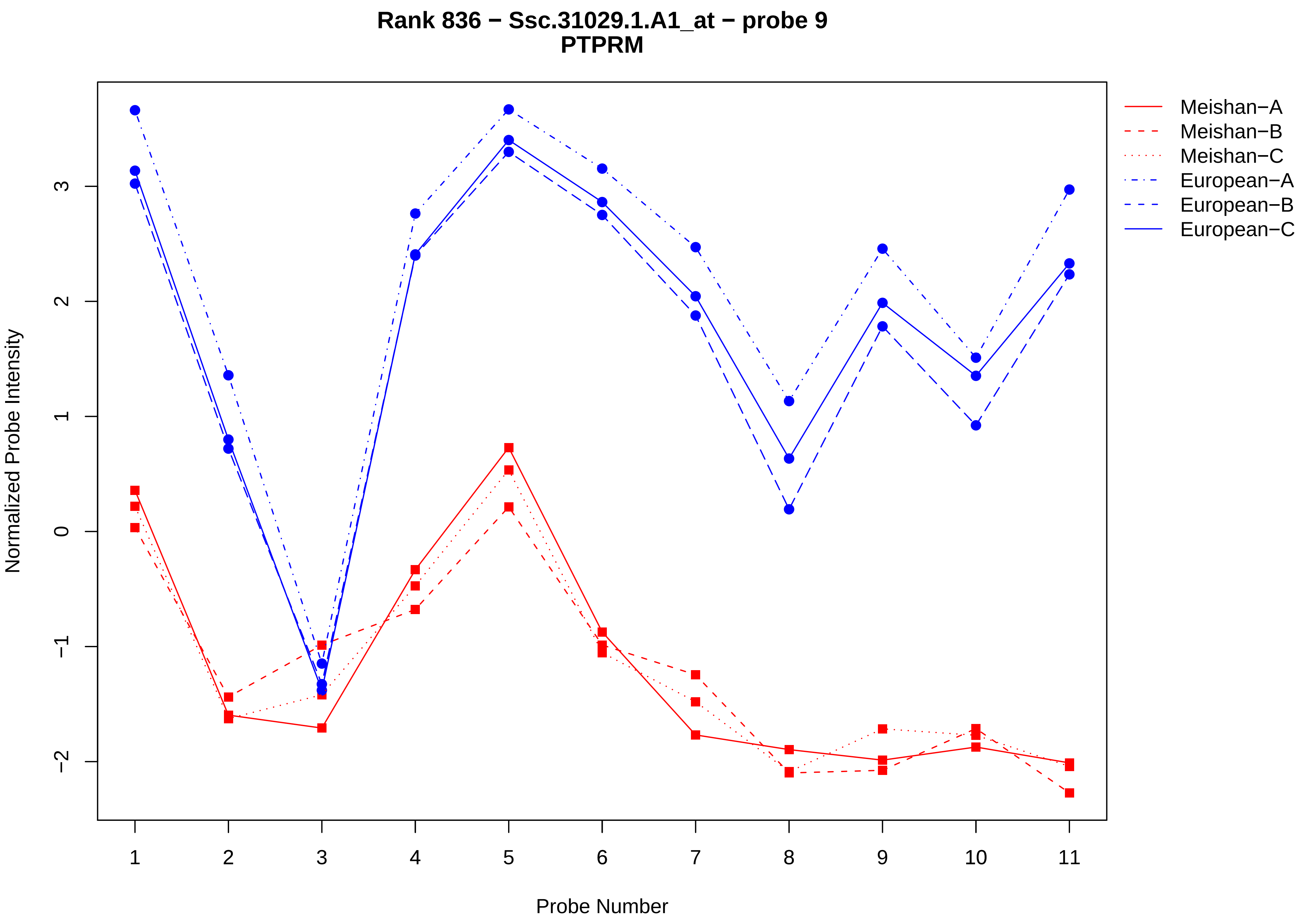
Rank 836 − Ssc.31029.1.A1_at − probe 9
PTPRM
Normalized Probe Intensity
Probe Number
Meishan−A
Meishan−B
Meishan−C
European−A
European−B
European−C
−2
−1
0
1
2
3
1
2
3
4
5
6
7
8
9
10
11

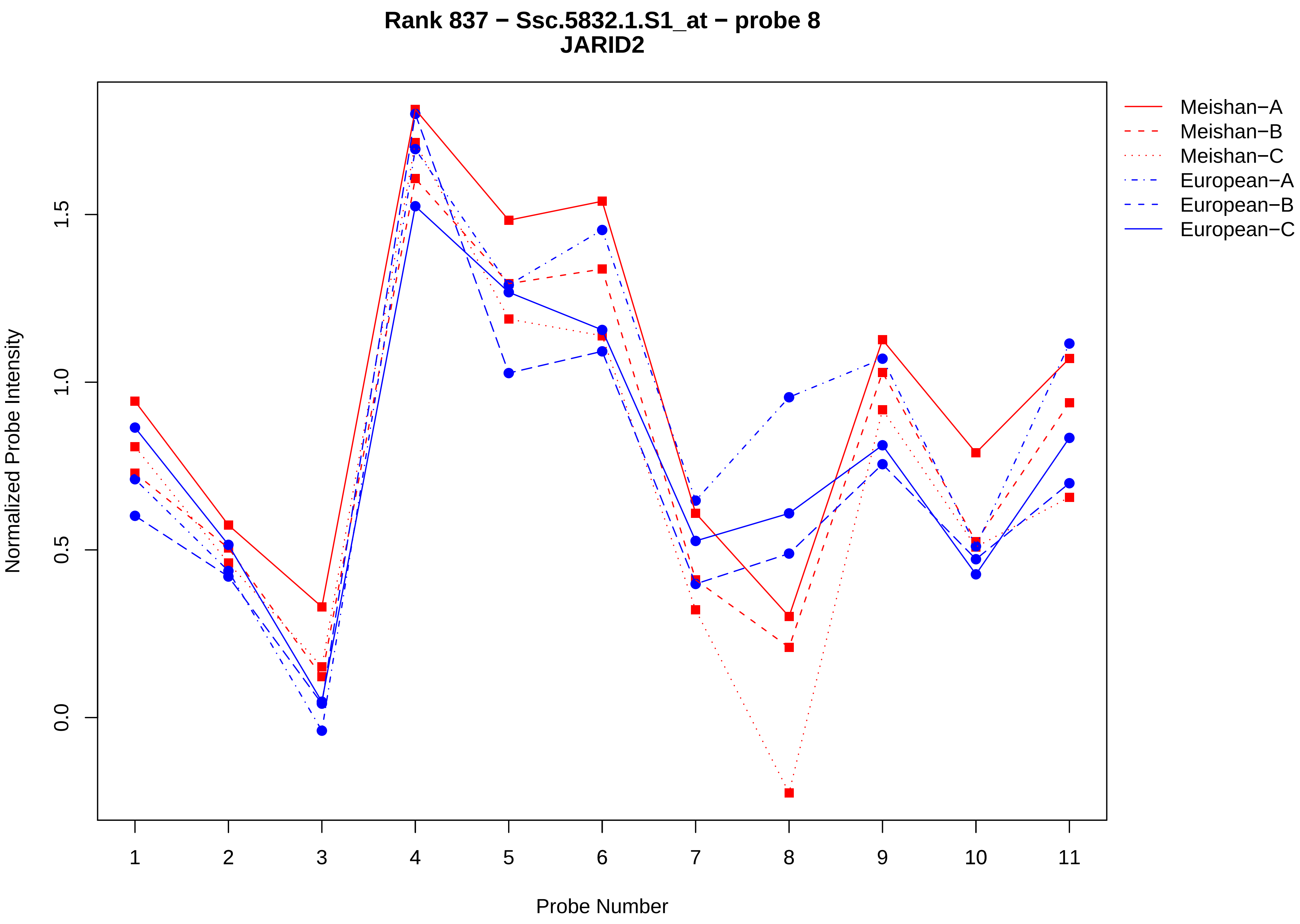
Rank 837 − Ssc.5832.1.S1_at − probe 8
JARID2
Normalized Probe Intensity
0.0
0.5
1.0
1.5
1
2
3
4
5
6
7
8
9
10
11
Probe Number
Meishan−A
Meishan−B
Meishan−C
European−A
European−B
European−C

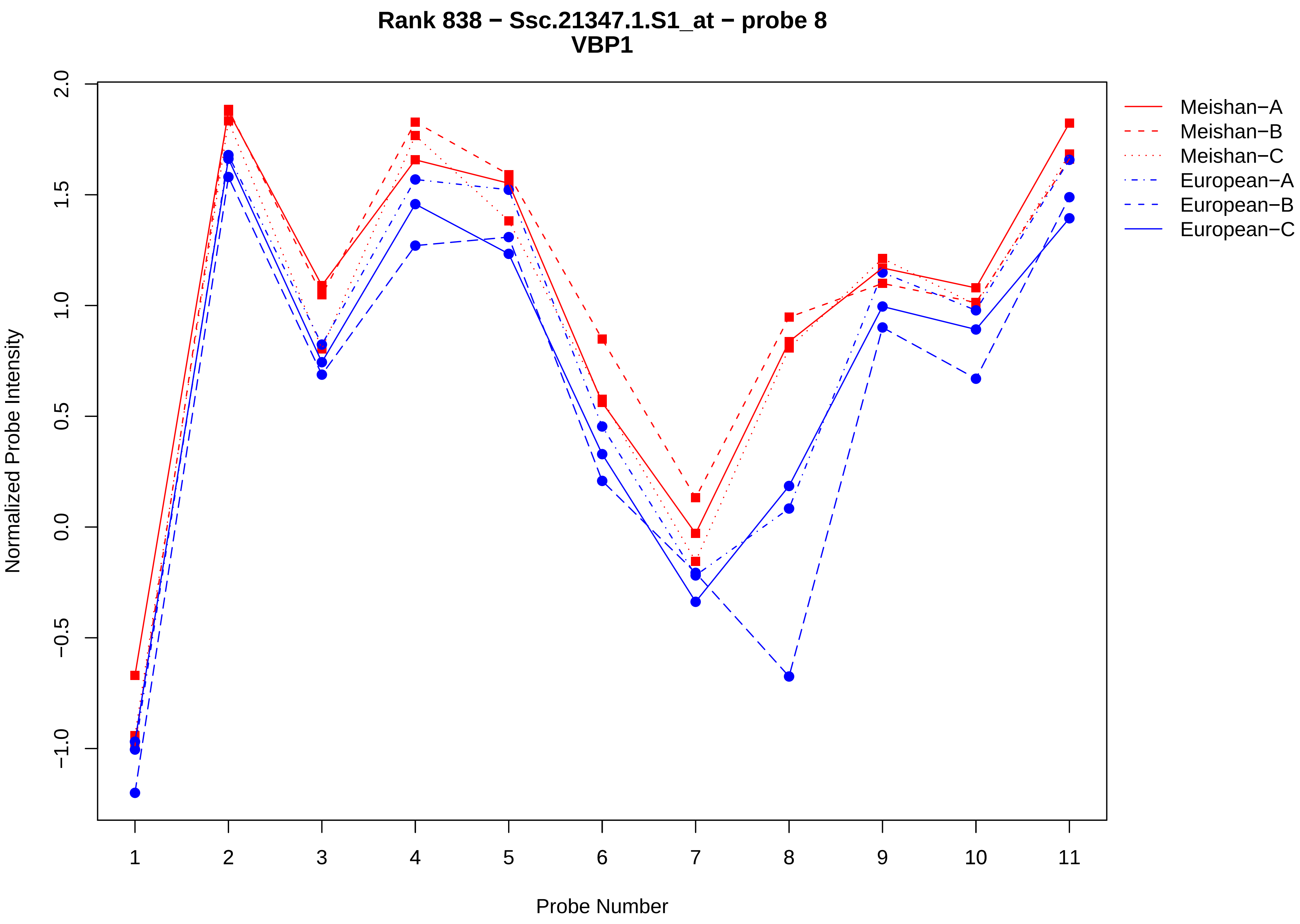

Rank 838 − Ssc.21347.1.S1_at − probe 8
VBP1
Normalized Probe Intensity
Probe Number
Meishan−A
Meishan−B
Meishan−C
European−A
European−B
European−C

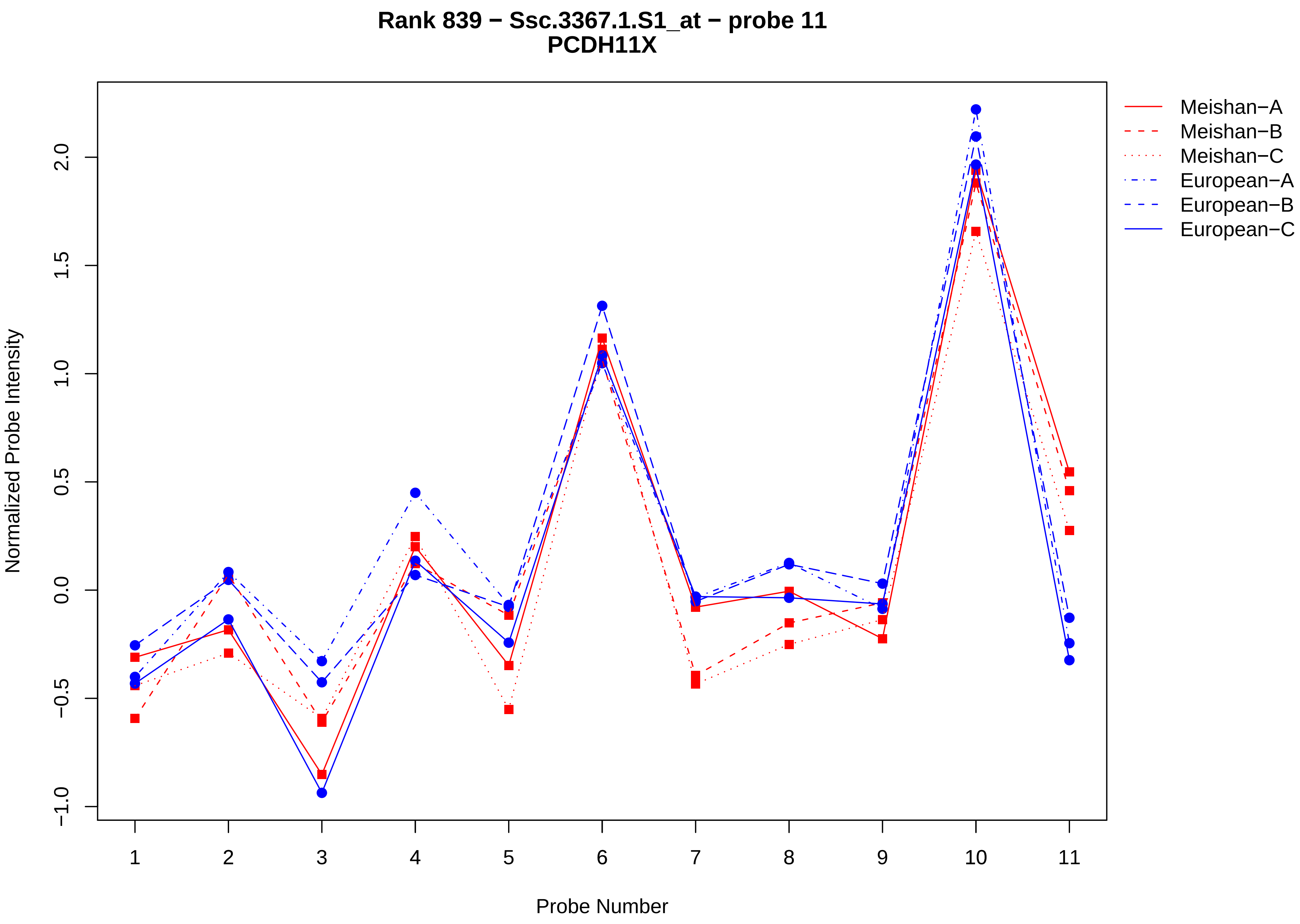
Rank 839 − Ssc.3367.1.S1_at − probe 11
PCDH11X
Normalized Probe Intensity
Probe Number
−1.0
−0.5
0.0
0.5
1.0
1.5
2.0
1
2
3
4
5
6
7
8
9
10
11
Meishan−A
Meishan−B
Meishan−C
European−A
European−B
European−C

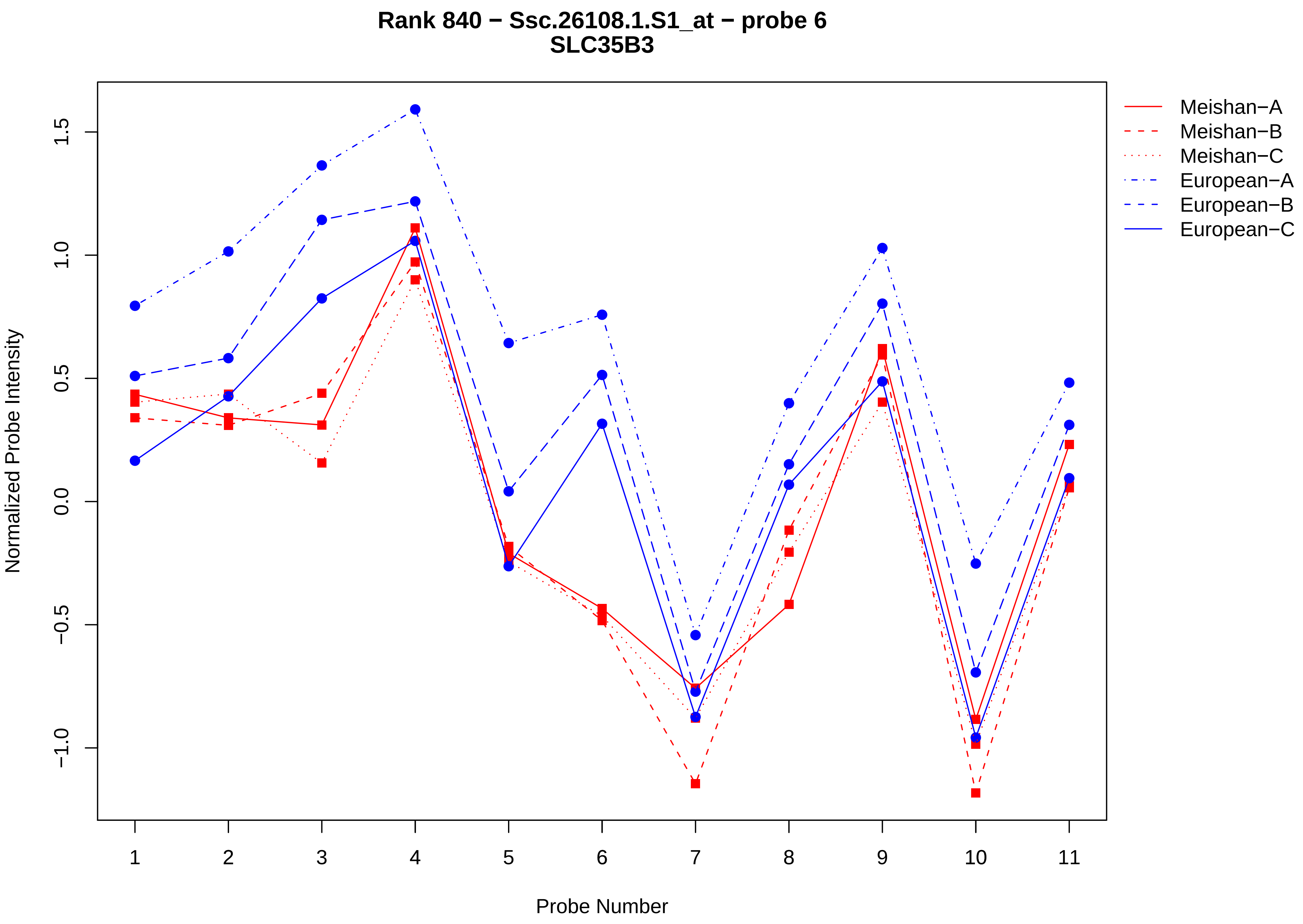
Rank 840 − Ssc.26108.1.S1_at − probe 6
SLC35B3
Normalized Probe Intensity
Probe Number
1.5
1.0
0.5
0.0
−0.5
−1.0
1
2
3
4
5
6
7
8
9
10
11
Meishan−A
Meishan−B
Meishan−C
European−A
European−B
European−C

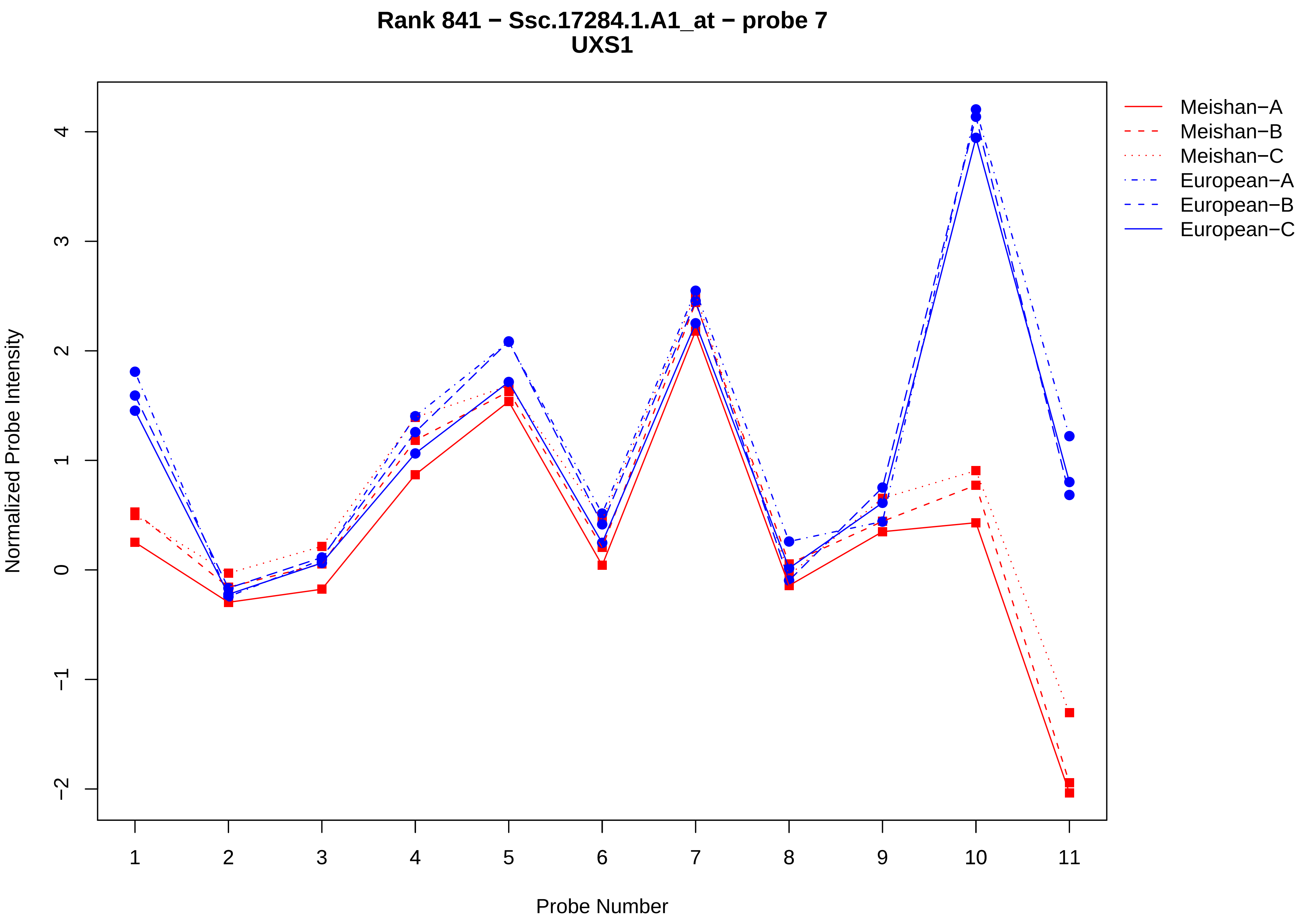

Rank 841 − Ssc.17284.1.A1_at − probe 7
UXS1
Normalized Probe Intensity
Probe Number
−2
−1
0
1
2
3
4
1
2
3
4
5
6
7
8
9
10
11
Meishan−A
Meishan−B
Meishan−C
European−A
European−B
European−C

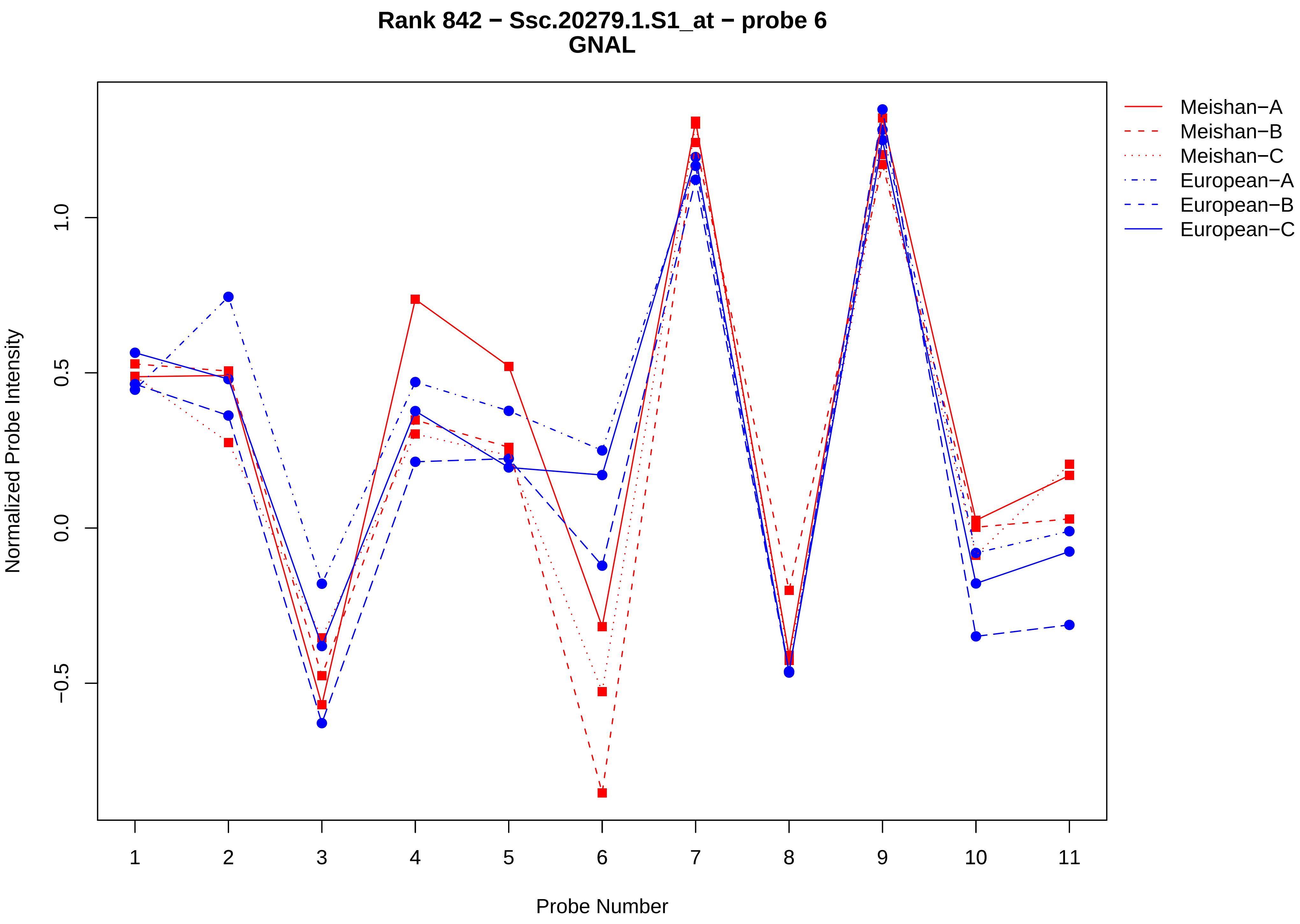

Rank 842 − Ssc.20279.1.S1_at − probe 6
GNAL
Normalized Probe Intensity
−0.5
0.0
0.5
1.0
1
2
3
4
5
6
7
8
9
10
11
Probe Number
Meishan−A
Meishan−B
Meishan−C
European−A
European−B
European−C

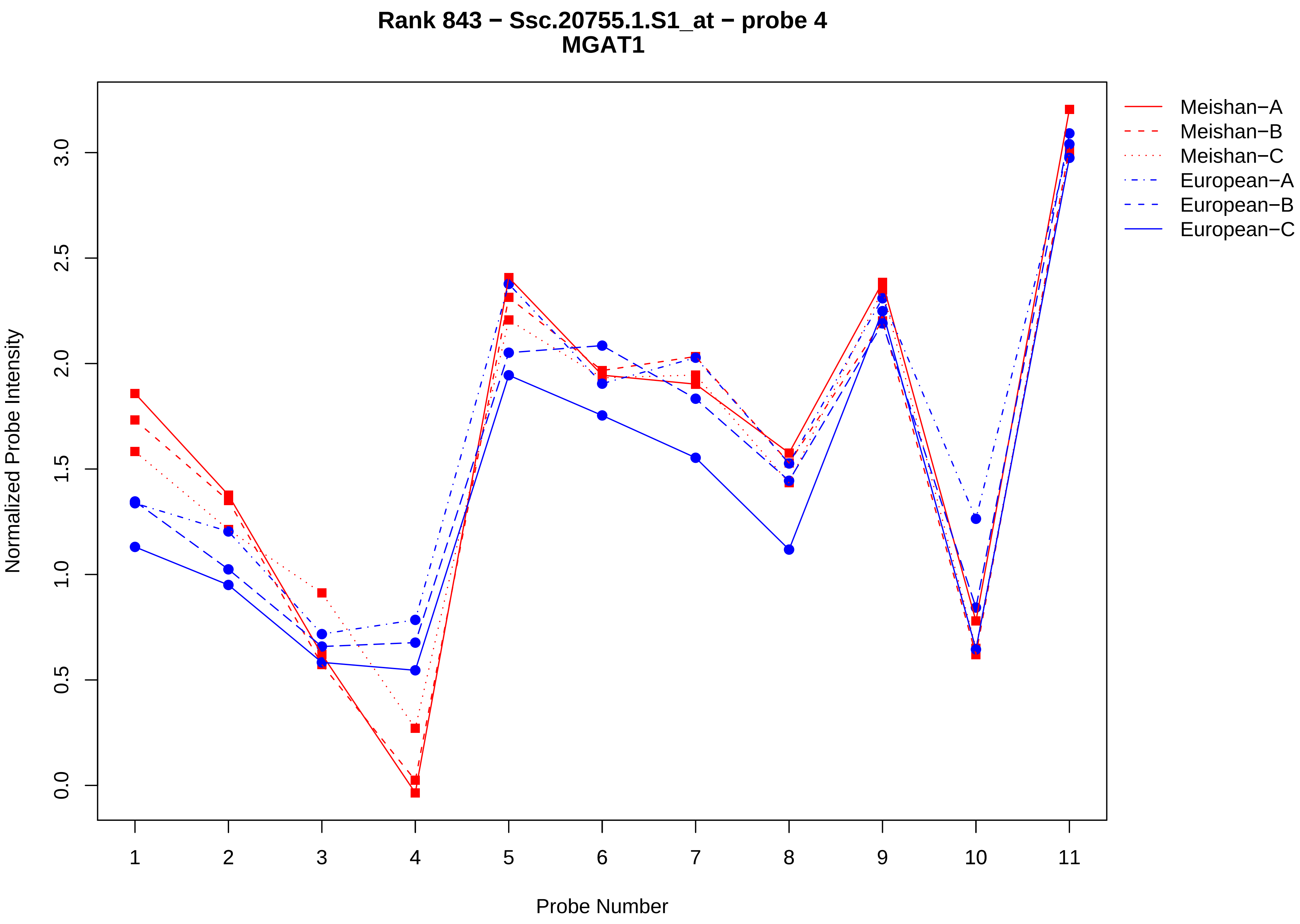

Rank 843 − Ssc.20755.1.S1_at − probe 4
MGAT1
Normalized Probe Intensity
0.0
0.5
1.0
1.5
2.0
2.5
3.0
Probe Number
1
2
3
4
5
6
7
8
9
10
11
Meishan−A
Meishan−B
Meishan−C
European−A
European−B
European−C

Rank 844 − Ssc.25175.1.S1_at − probe 10
CKLFSF3

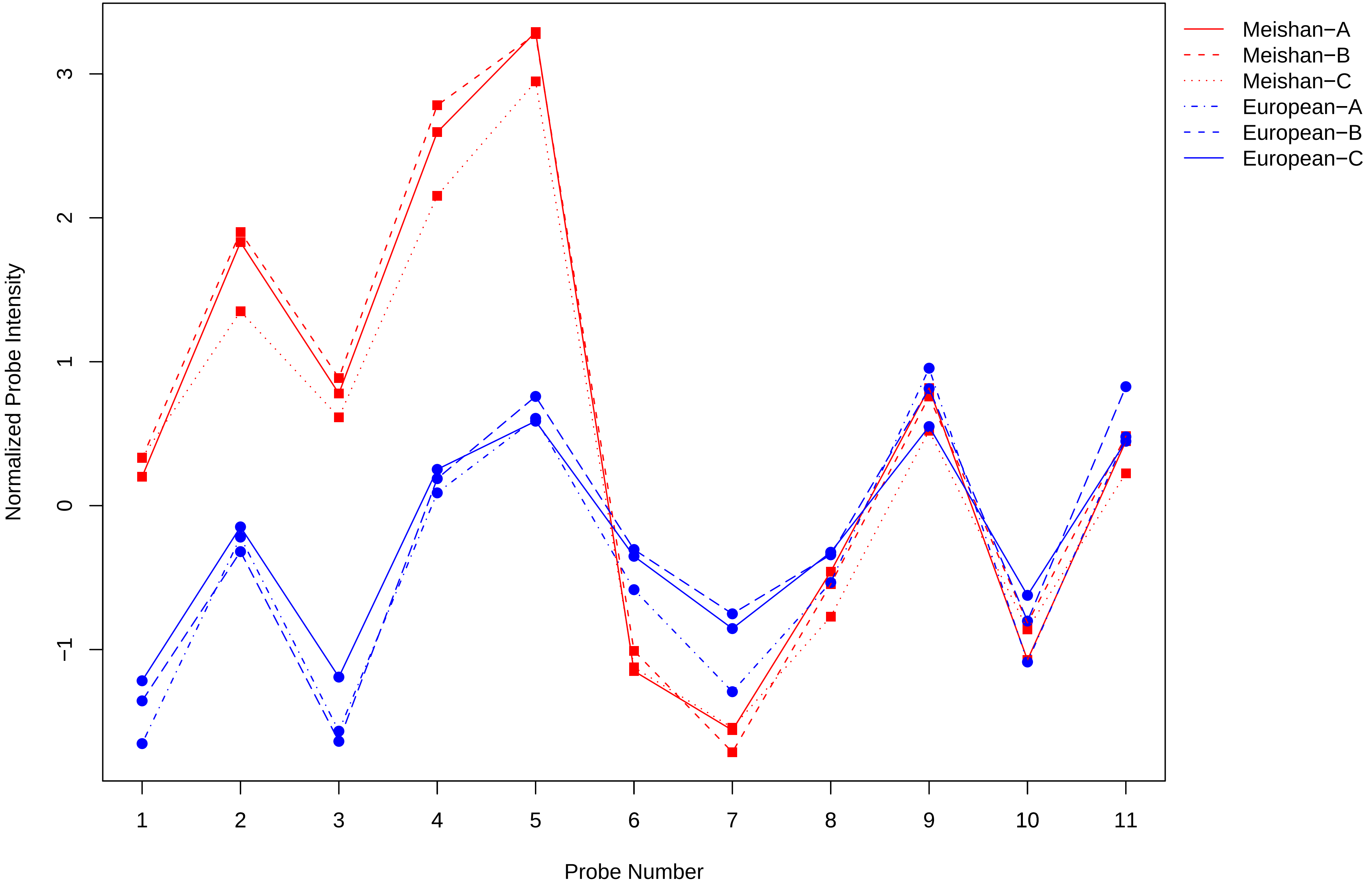

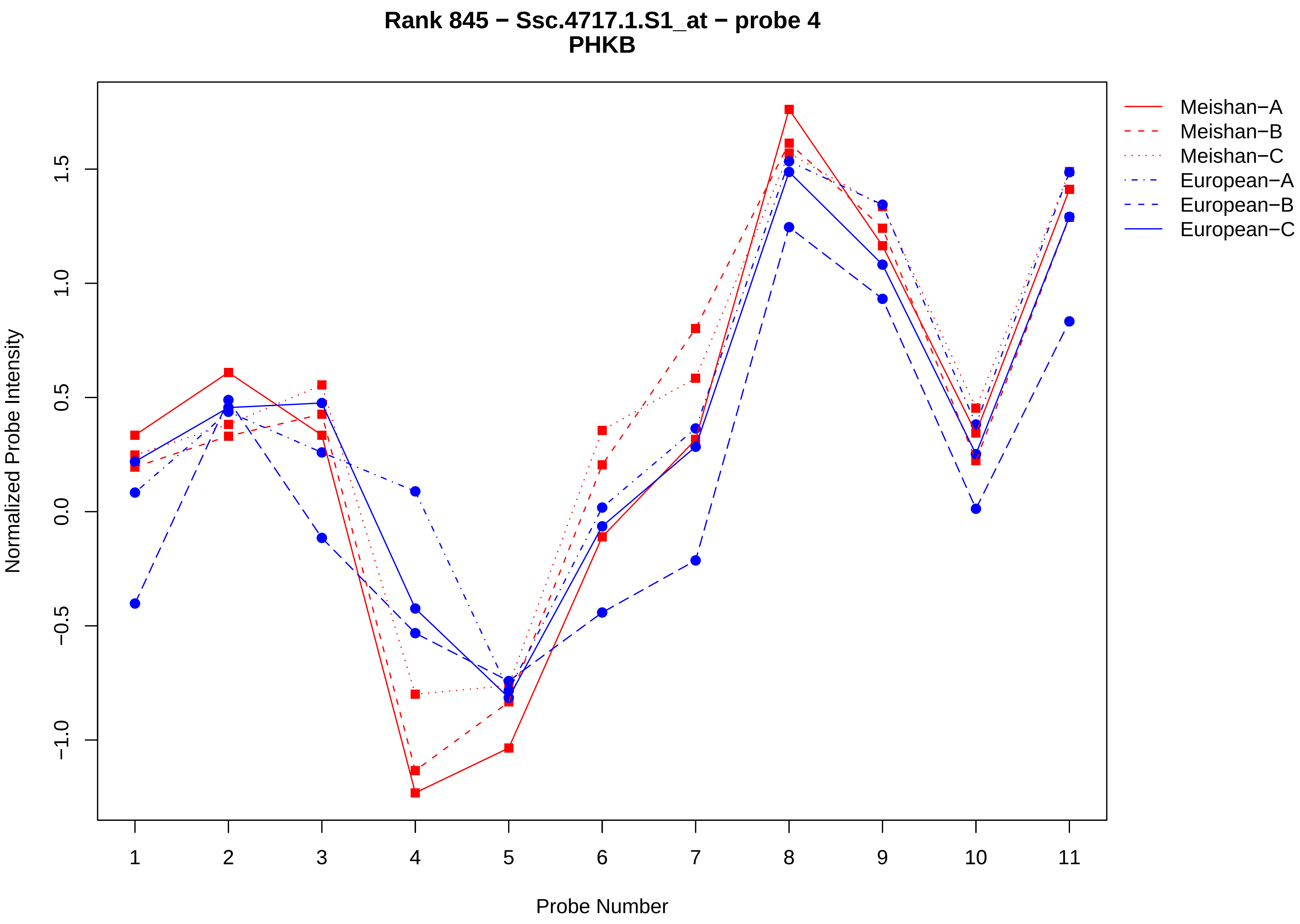

Rank 845 − Ssc.4717.1.S1_at − probe 4
PHKB
Normalized Probe Intensity
Probe Number
Meishan−A
Meishan−B
Meishan−C
European−A
European−B
European−C
−1.0
−0.5
0.0
0.5
1.0
1.5
1
2
3
4
5
6
7
8
9
10
11

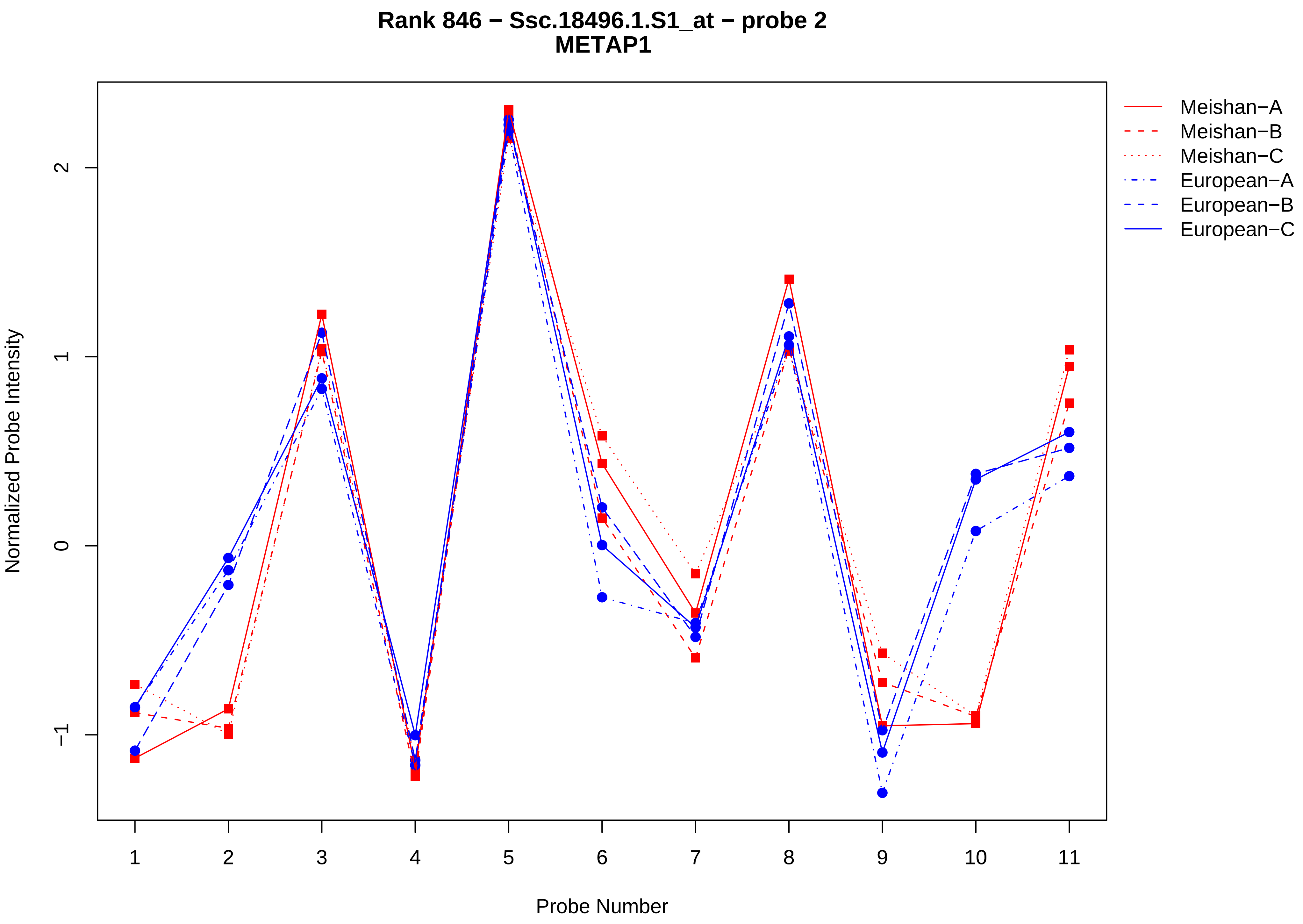

Rank 846 − Ssc.18496.1.S1_at − probe 2
METAP1
Normalized Probe Intensity
Probe Number
−1
0
1
2
1
2
3
4
5
6
7
8
9
10
11
Meishan−A
Meishan−B
Meishan−C
European−A
European−B
European−C

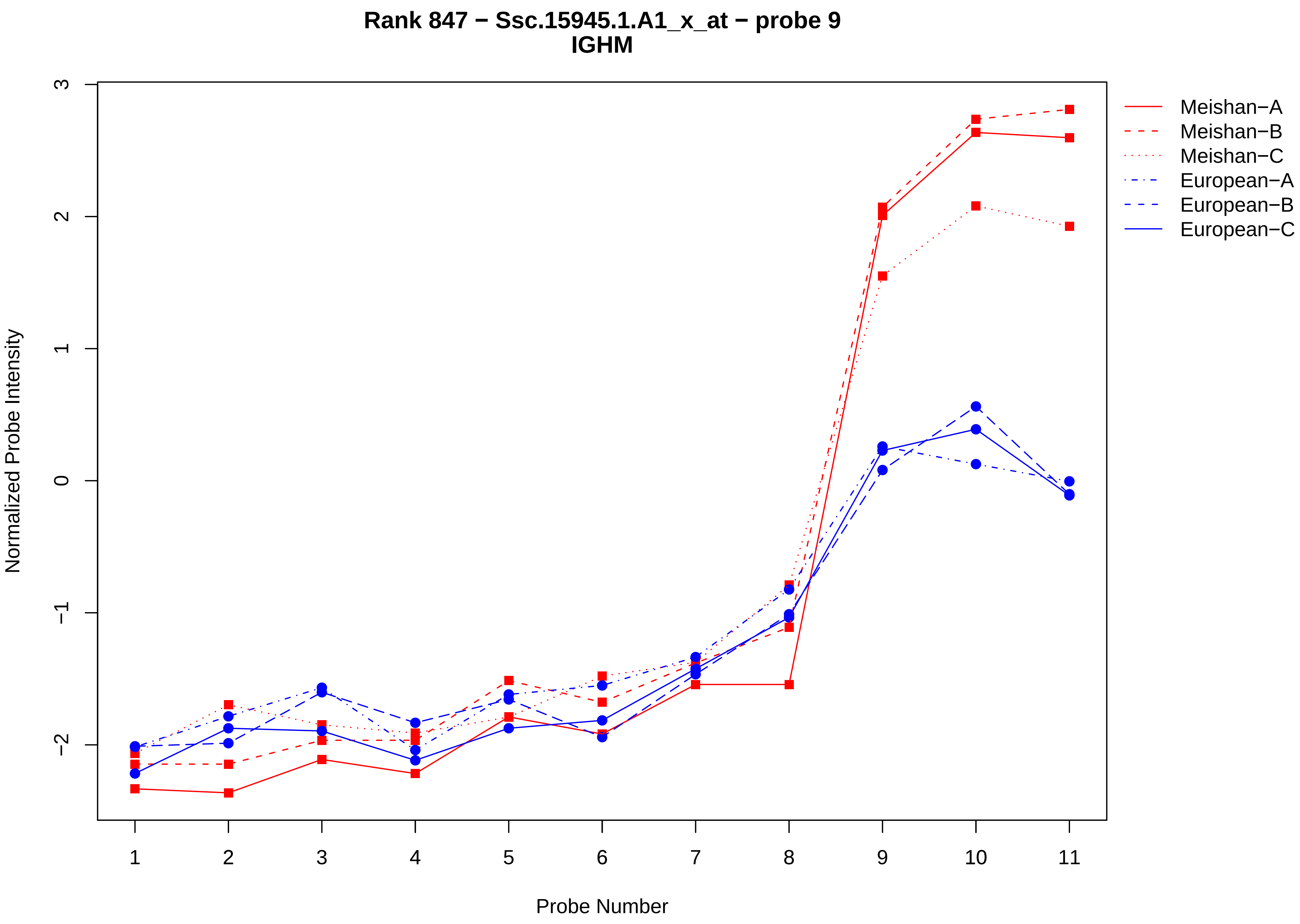
Rank 847 − Ssc.15945.1.A1_x_at − probe 9
IGHM
Normalized Probe Intensity
Probe Number
Meishan−A
Meishan−B
Meishan−C
European−A
European−B
European−C
−2
−1
0
1
2
3
1
2
3
4
5
6
7
8
9
10
11

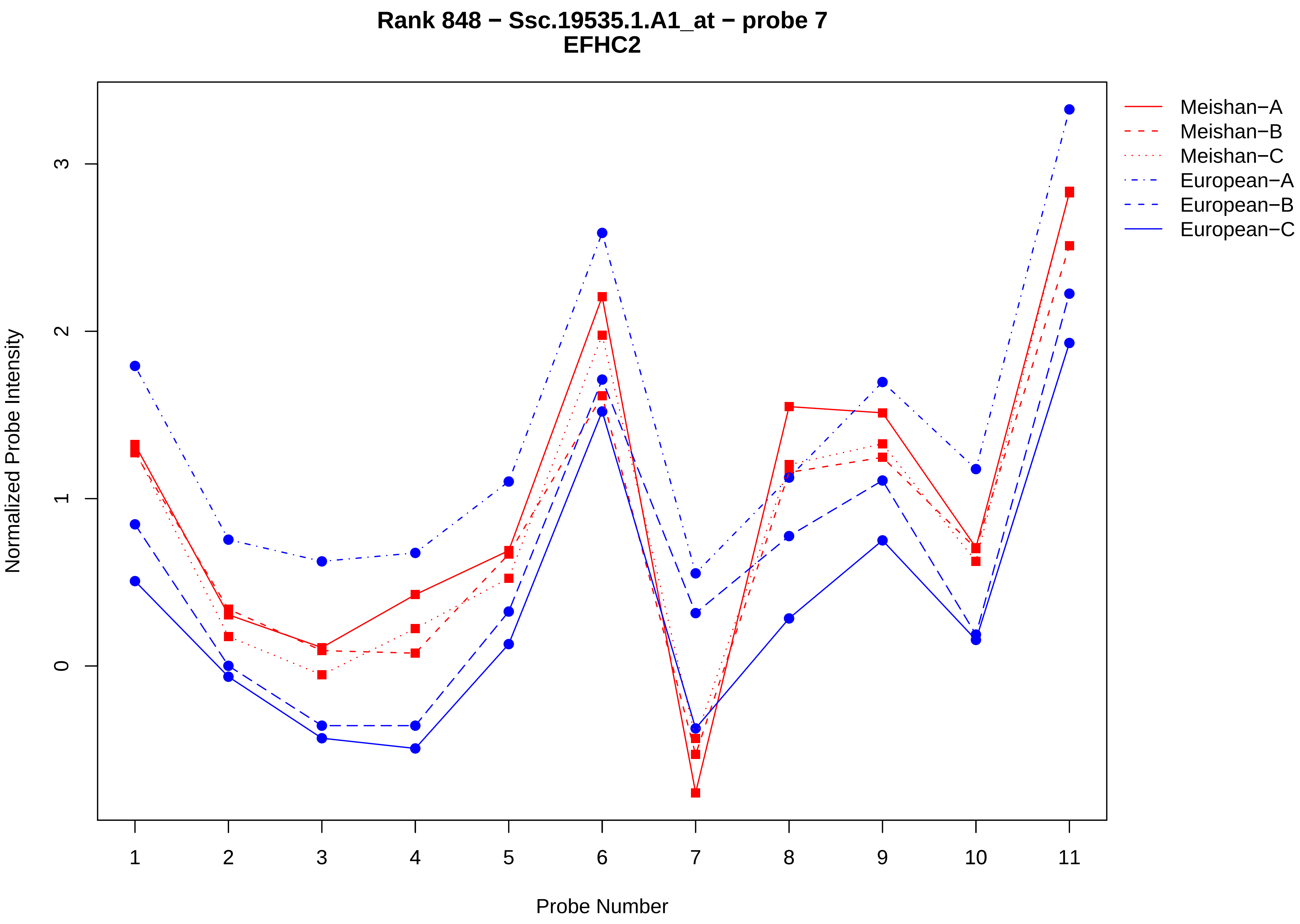
Rank 848 − Ssc.19535.1.A1_at − probe 7
EFHC2
Normalized Probe Intensity
Probe Number
0
1
2
3
1
2
3
4
5
6
7
8
9
10
11
Meishan−A
Meishan−B
Meishan−C
European−A
European−B
European−C

Rank 849 − Ssc.19542.1.S1_at − probe 7
GOLGA3

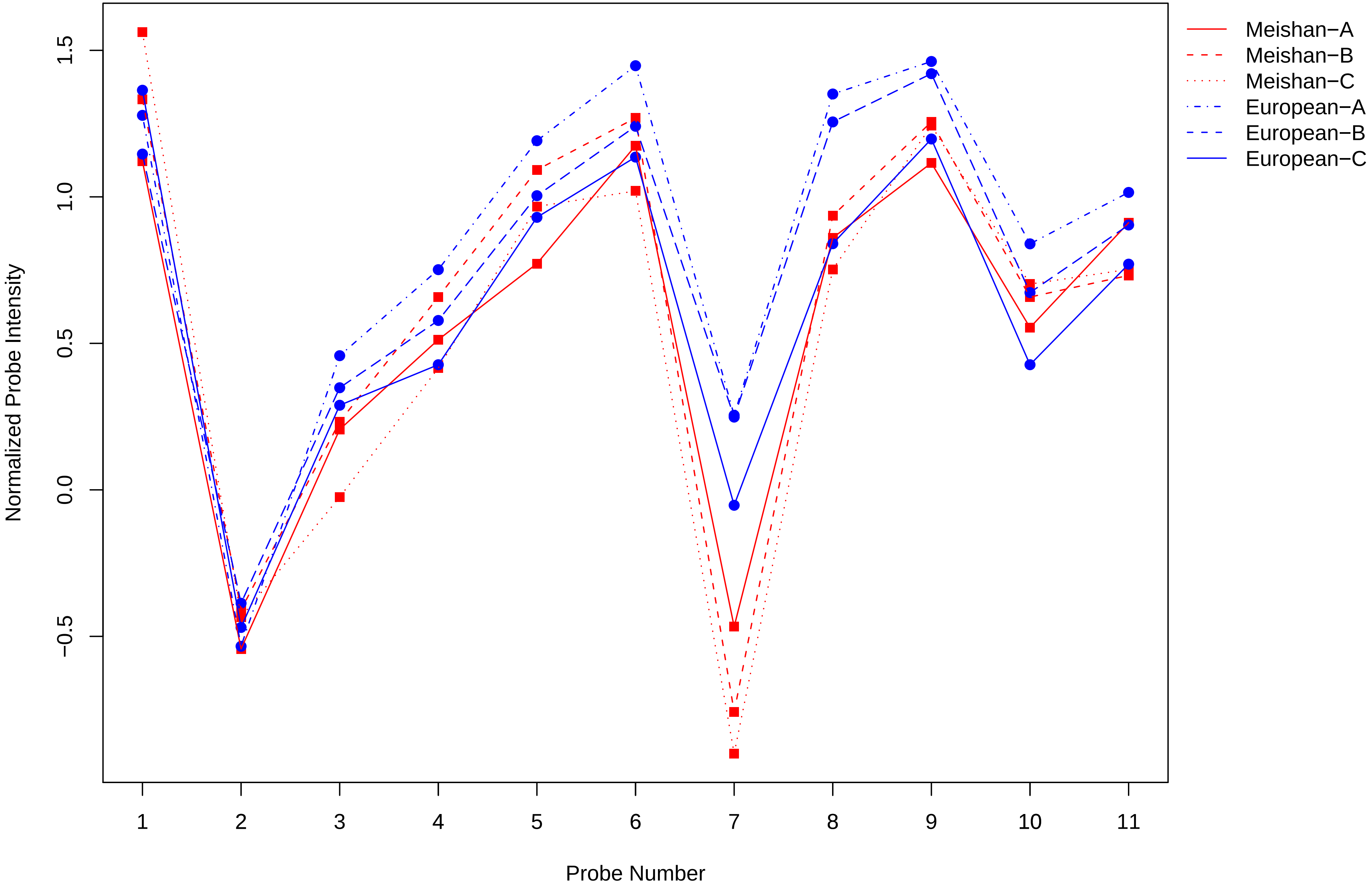

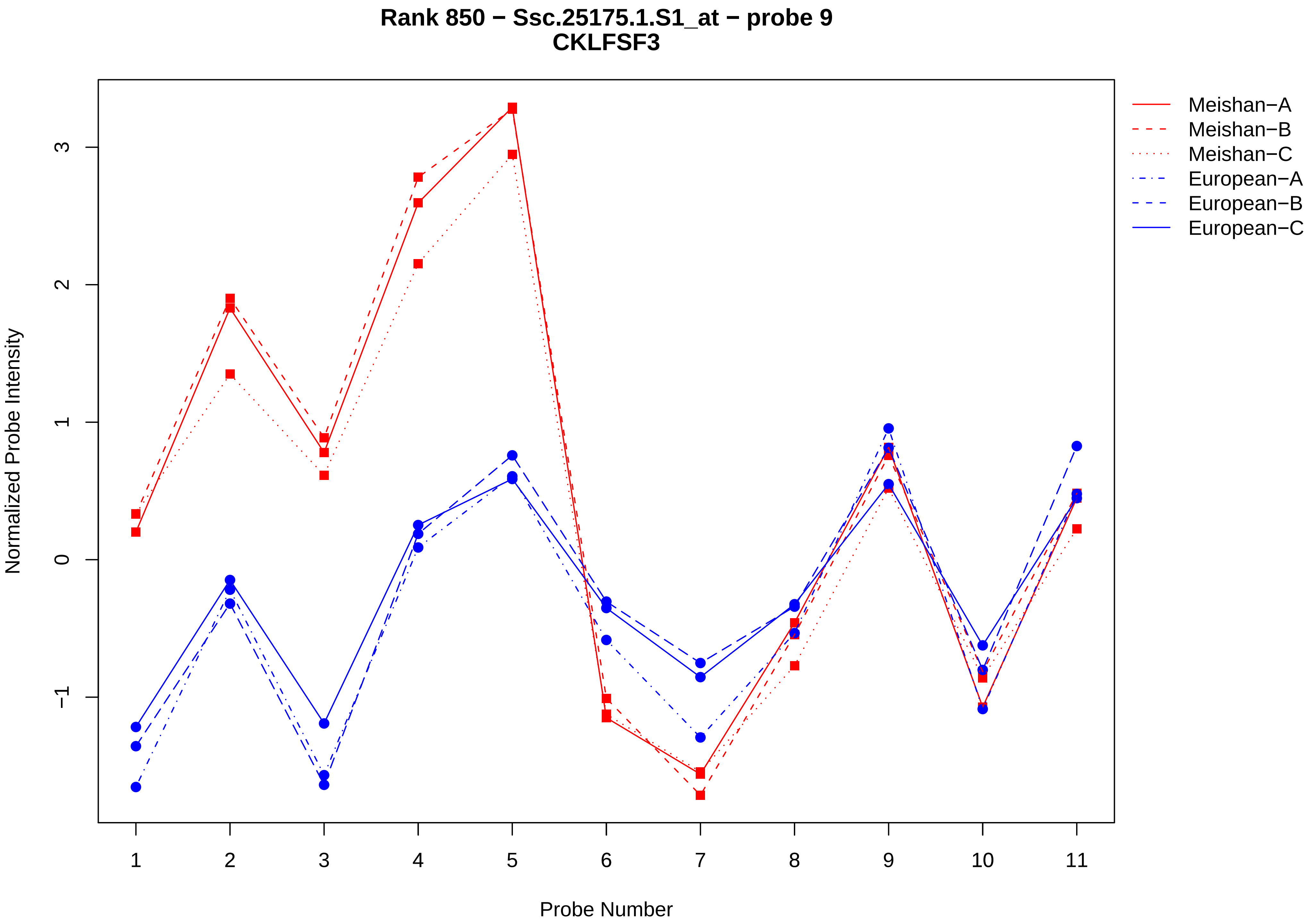
Rank 850 − Ssc.25175.1.S1_at − probe 9
CKLFSF3
Normalized Probe Intensity
Probe Number
−1
0
1
2
3
1
2
3
4
5
6
7
8
9
10
11
Meishan−A
Meishan−B
Meishan−C
European−A
European−B
European−C

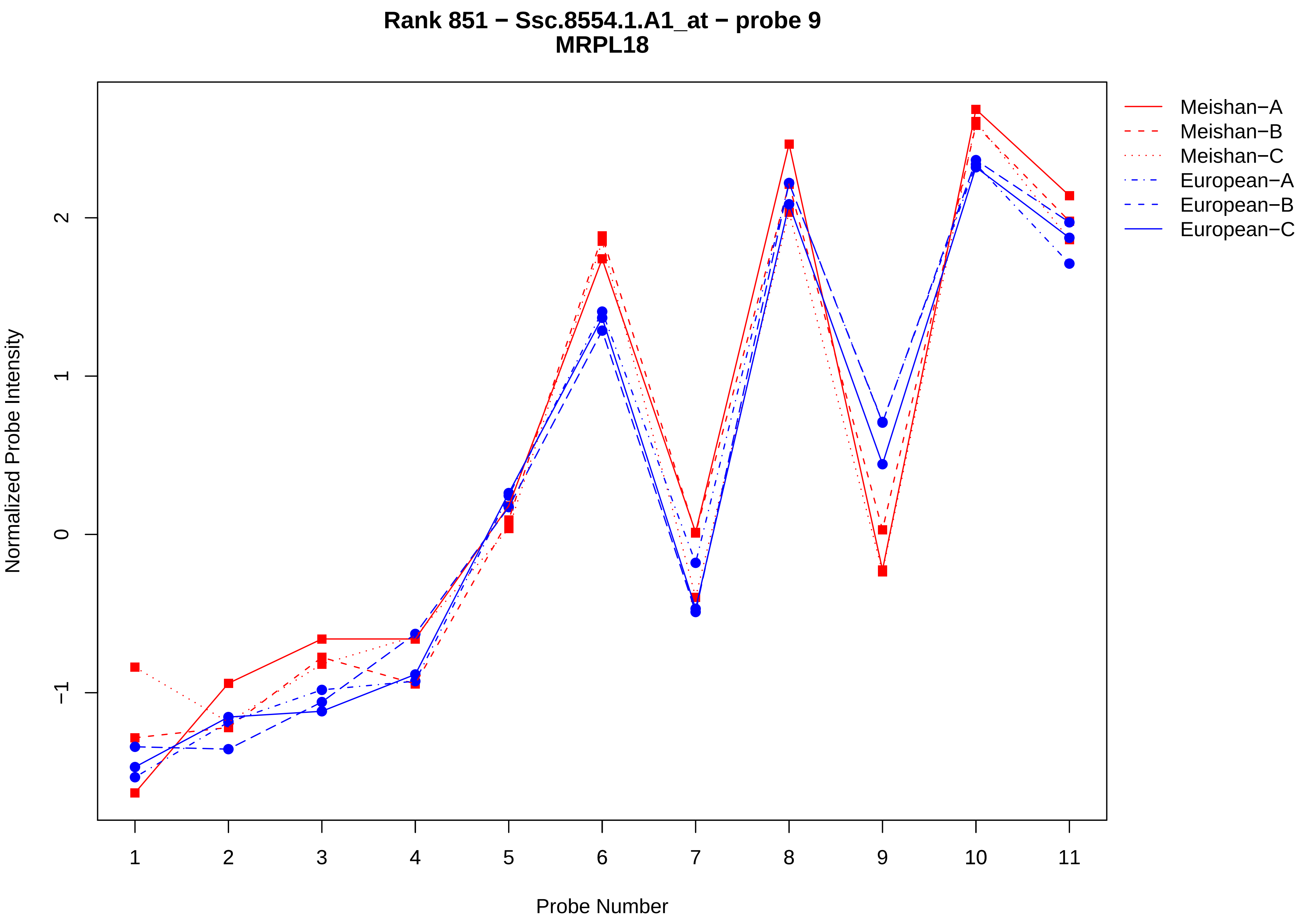
Rank 851 − Ssc.8554.1.A1_at − probe 9
MRPL18
Normalized Probe Intensity
Probe Number
Meishan−A
Meishan−B
Meishan−C
European−A
European−B
European−C
−1
0
1
2
1
2
3
4
5
6
7
8
9
10
11

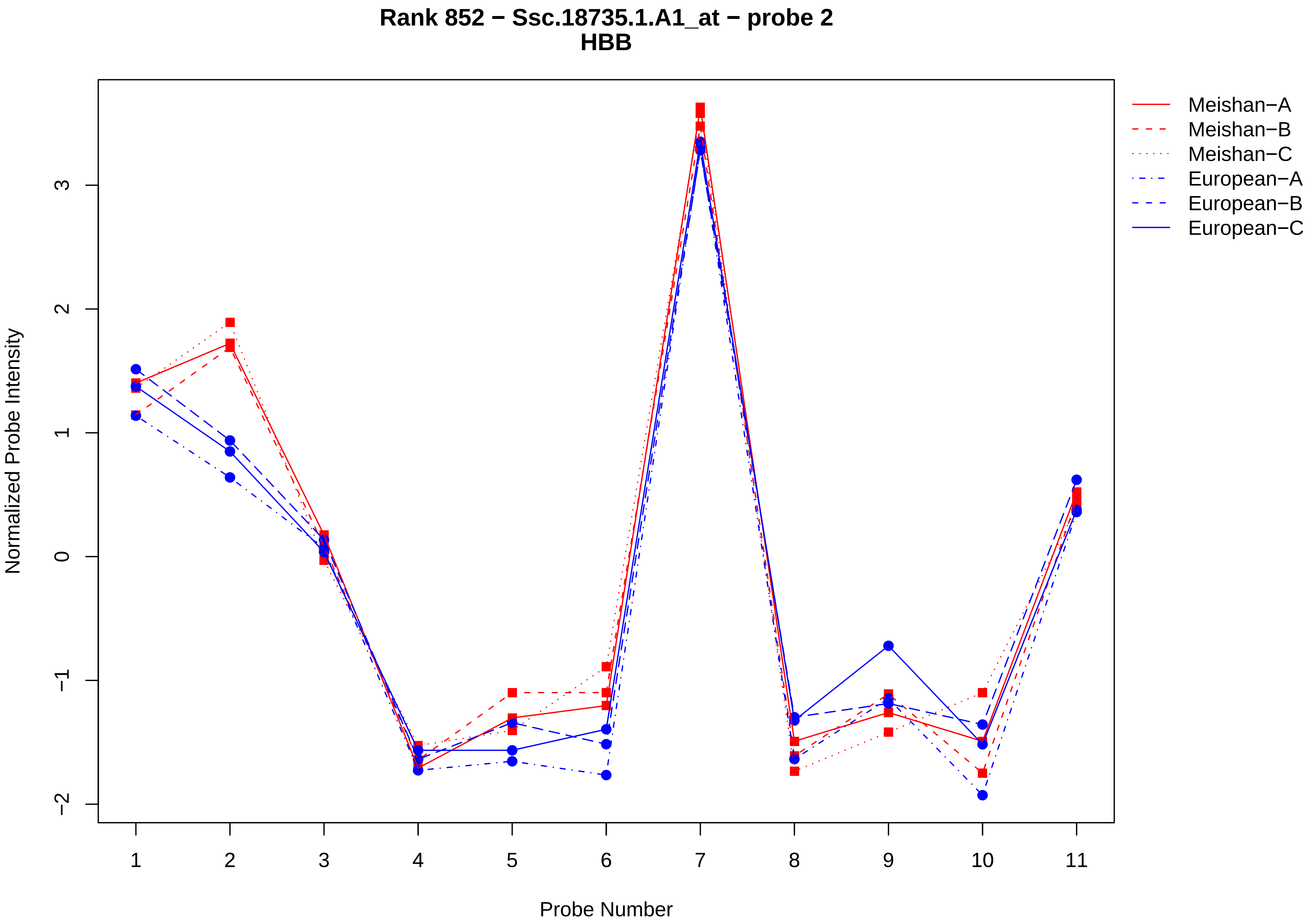

Rank 852 − Ssc.18735.1.A1_at − probe 2
HBB
Normalized Probe Intensity
Probe Number
−2
−1
0
1
2
3
1
2
3
4
5
6
7
8
9
10
11
Meishan−A
Meishan−B
Meishan−C
European−A
European−B
European−C

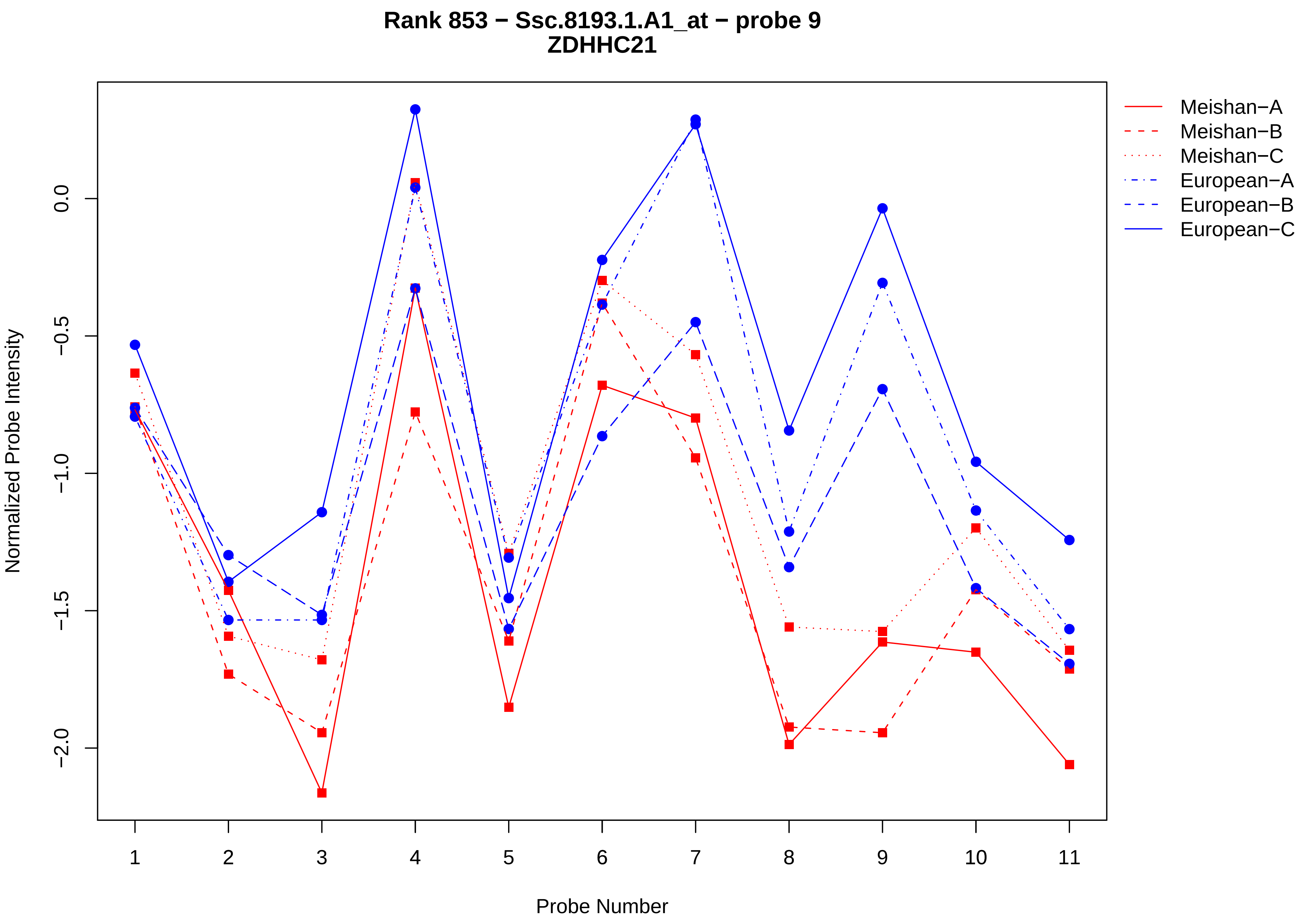

Rank 853 − Ssc.8193.1.A1_at − probe 9
ZDHHC21
Normalized Probe Intensity
Probe Number
0.0
−0.5
−1.0
−1.5
−2.0
1
2
3
4
5
6
7
8
9
10
11
Meishan−A
Meishan−B
Meishan−C
European−A
European−B
European−C

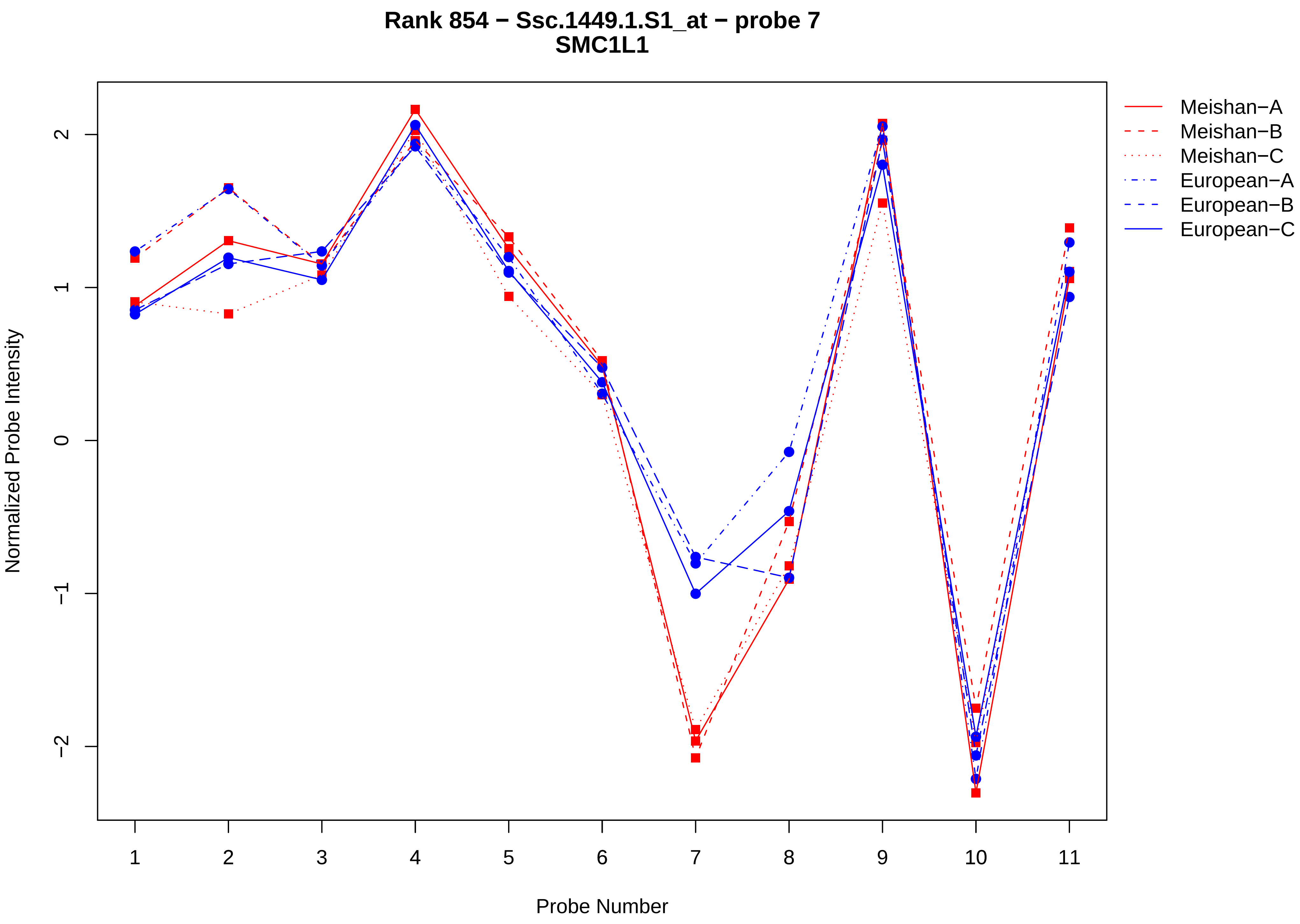
Rank 854 − Ssc.1449.1.S1_at − probe 7
SMC1L1
Normalized Probe Intensity
Probe Number
Meishan−A
Meishan−B
Meishan−C
European−A
European−B
European−C
−2
−1
0
1
2
1
2
3
4
5
6
7
8
9
10
11

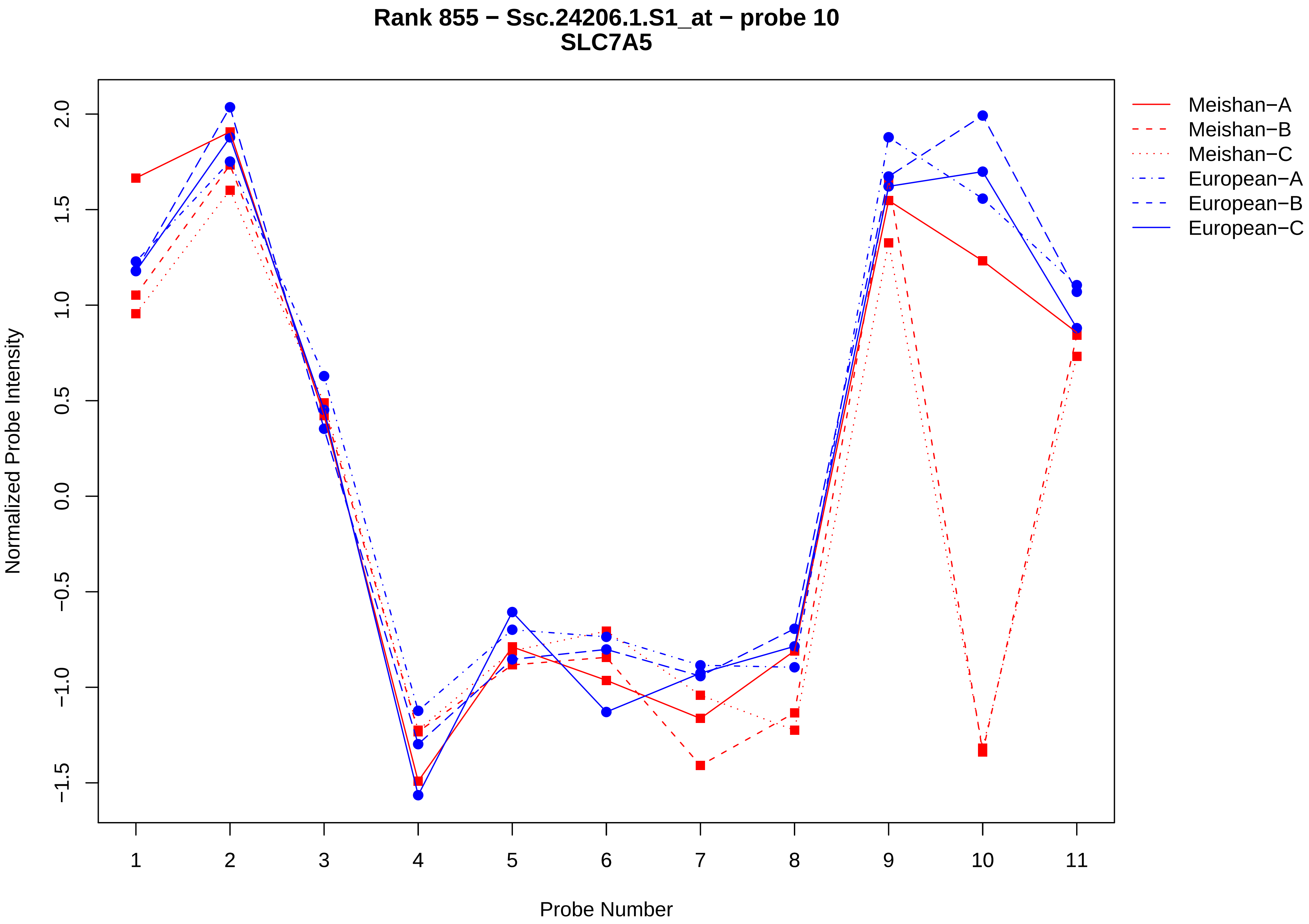

Rank 855 − Ssc.24206.1.S1_at − probe 10
SLC7A5
Normalized Probe Intensity
Probe Number
−1.5
−1.0
−0.5
0.0
0.5
1.0
1.5
2.0
1
2
3
4
5
6
7
8
9
10
11
Meishan−A
Meishan−B
Meishan−C
European−A
European−B
European−C

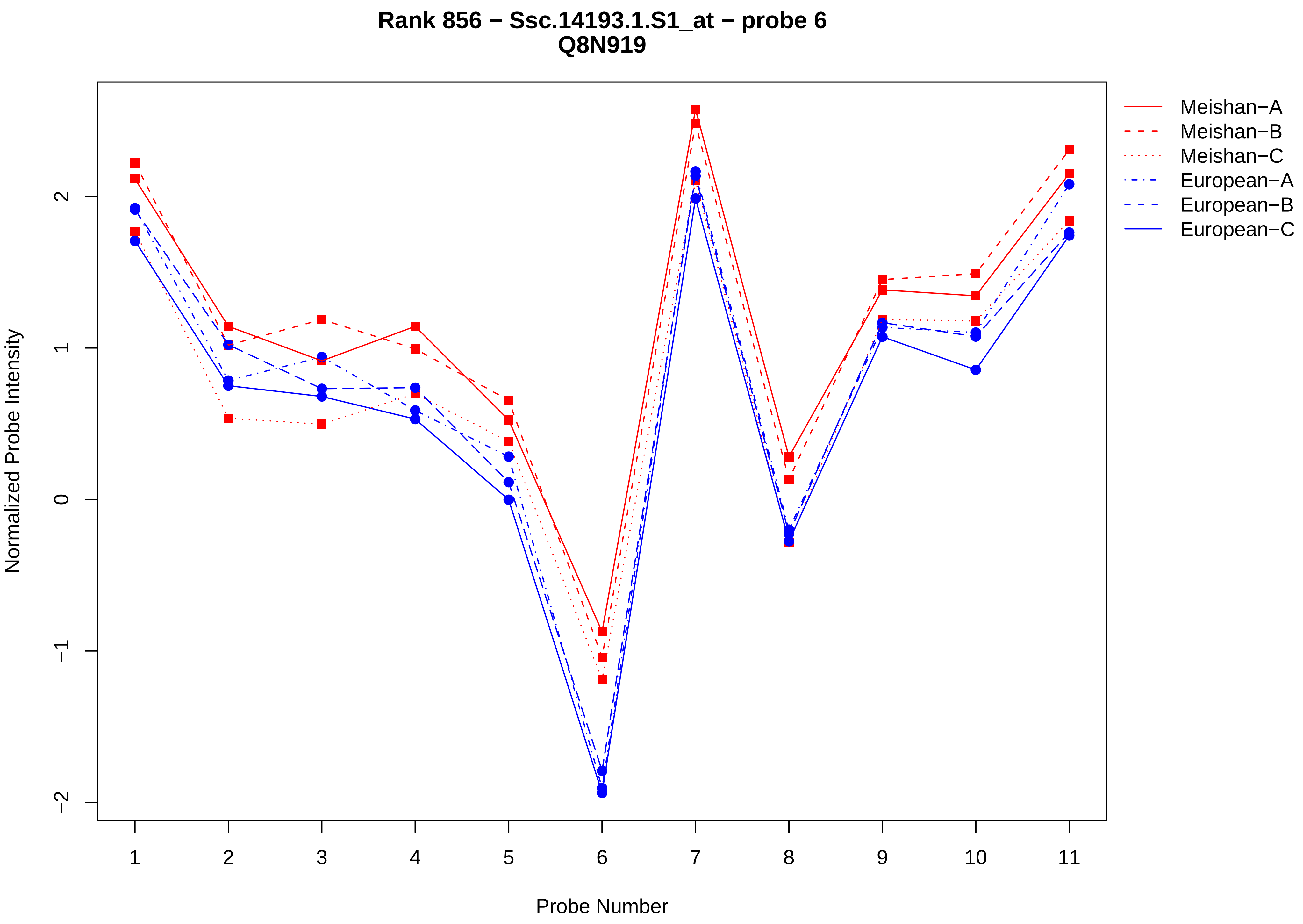
Rank 856 − Ssc.14193.1.S1_at − probe 6
Q8N919
Normalized Probe Intensity
Probe Number
−2
−1
0
1
2
1
2
3
4
5
6
7
8
9
10
11
Meishan−A
Meishan−B
Meishan−C
European−A
European−B
European−C

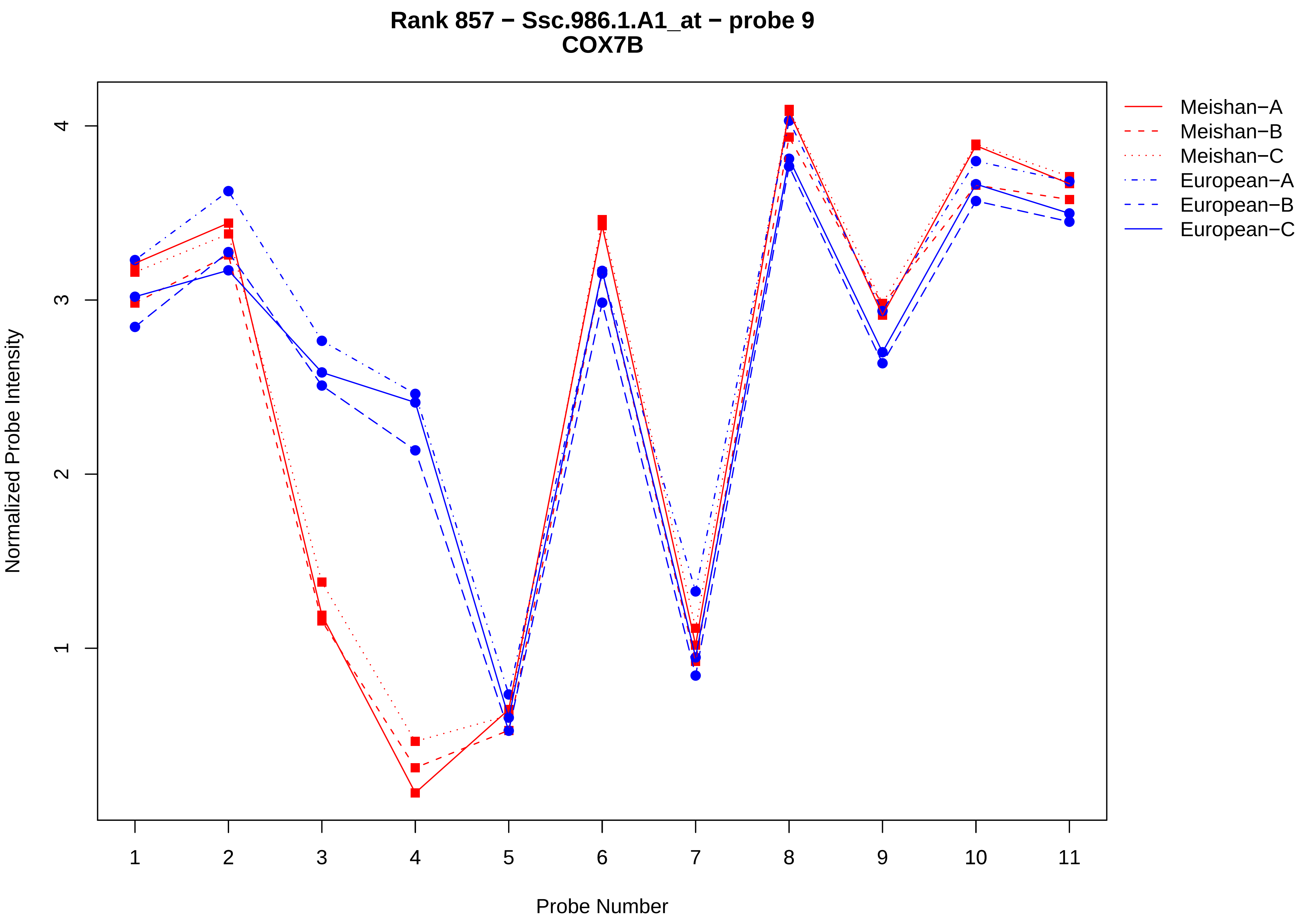

Rank 857 − Ssc.986.1.A1_at − probe 9
COX7B
Normalized Probe Intensity
Probe Number
1
2
3
4
5
6
7
8
9
10
11
Meishan−A
Meishan−B
Meishan−C
European−A
European−B
European−C

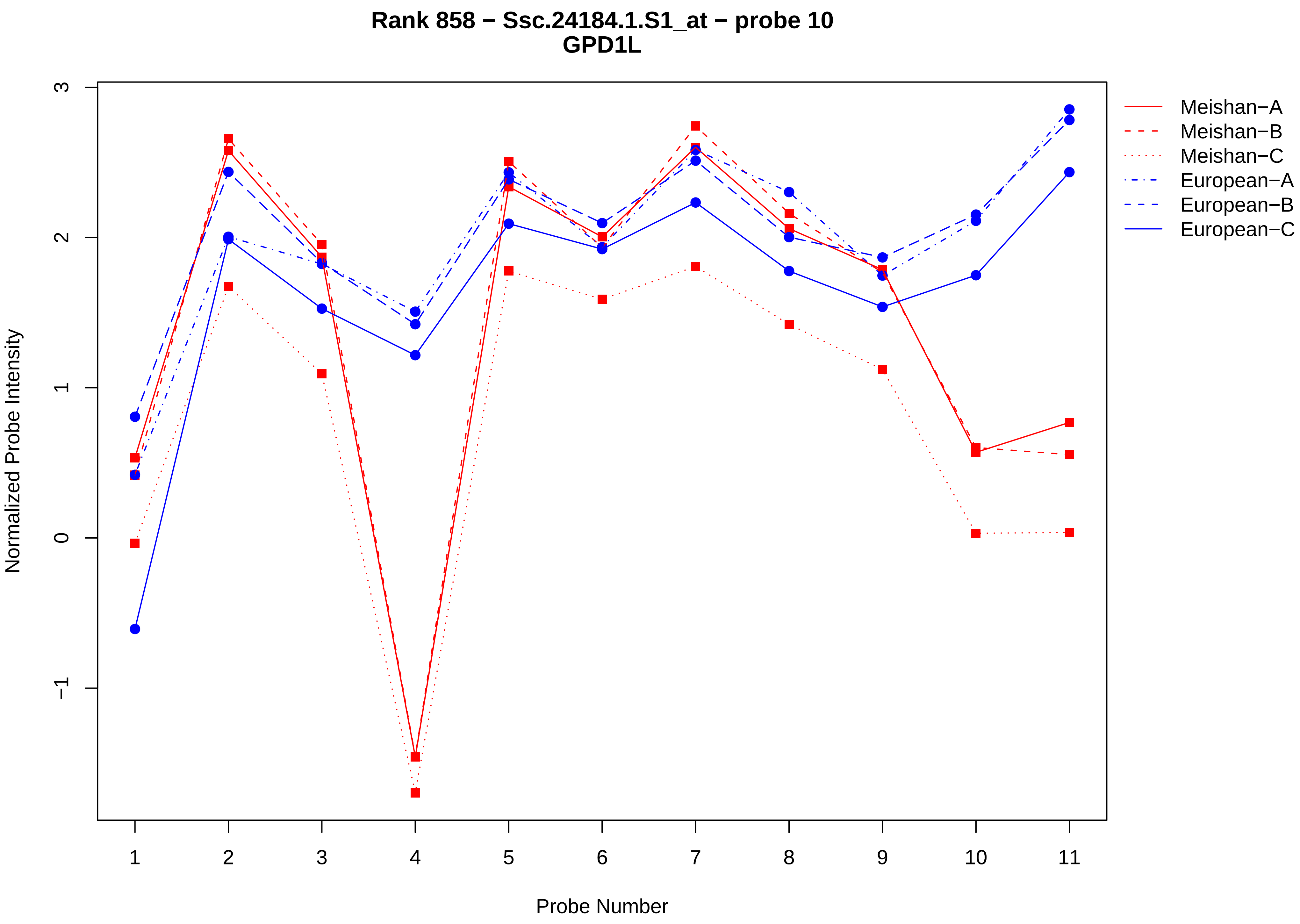

Rank 858 − Ssc.24184.1.S1_at − probe 10
GPD1L
Normalized Probe Intensity
Probe Number
3
2
1
0
−1
1
2
3
4
5
6
7
8
9
10
11
Meishan−A
Meishan−B
Meishan−C
European−A
European−B
European−C

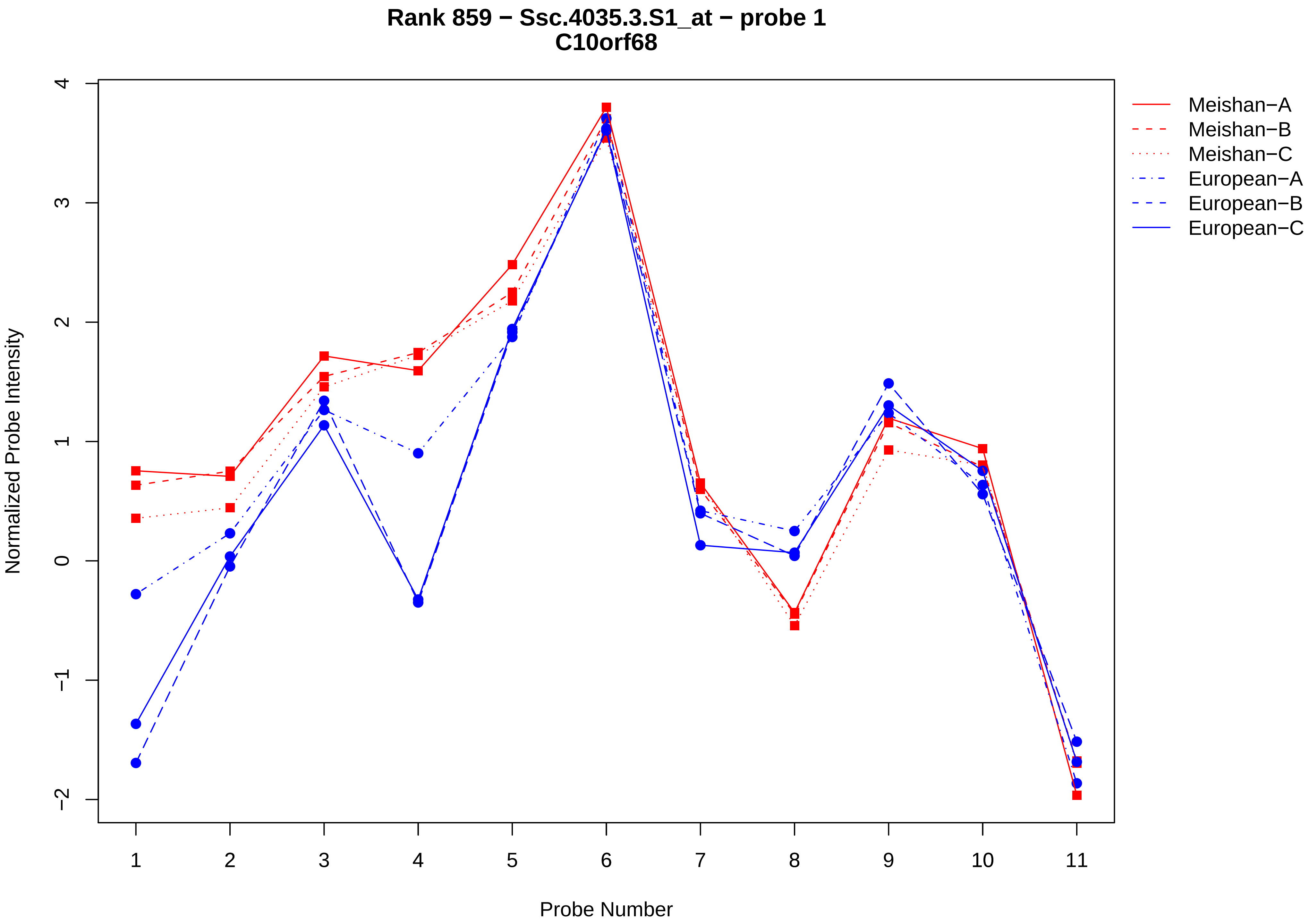

Rank 859 − Ssc.4035.3.S1_at − probe 1
C10orf68
Normalized Probe Intensity
Probe Number
Meishan−A
Meishan−B
Meishan−C
European−A
European−B
European−C
−2
−1
0
1
2
3
4
1
2
3
4
5
6
7
8
9
10
11

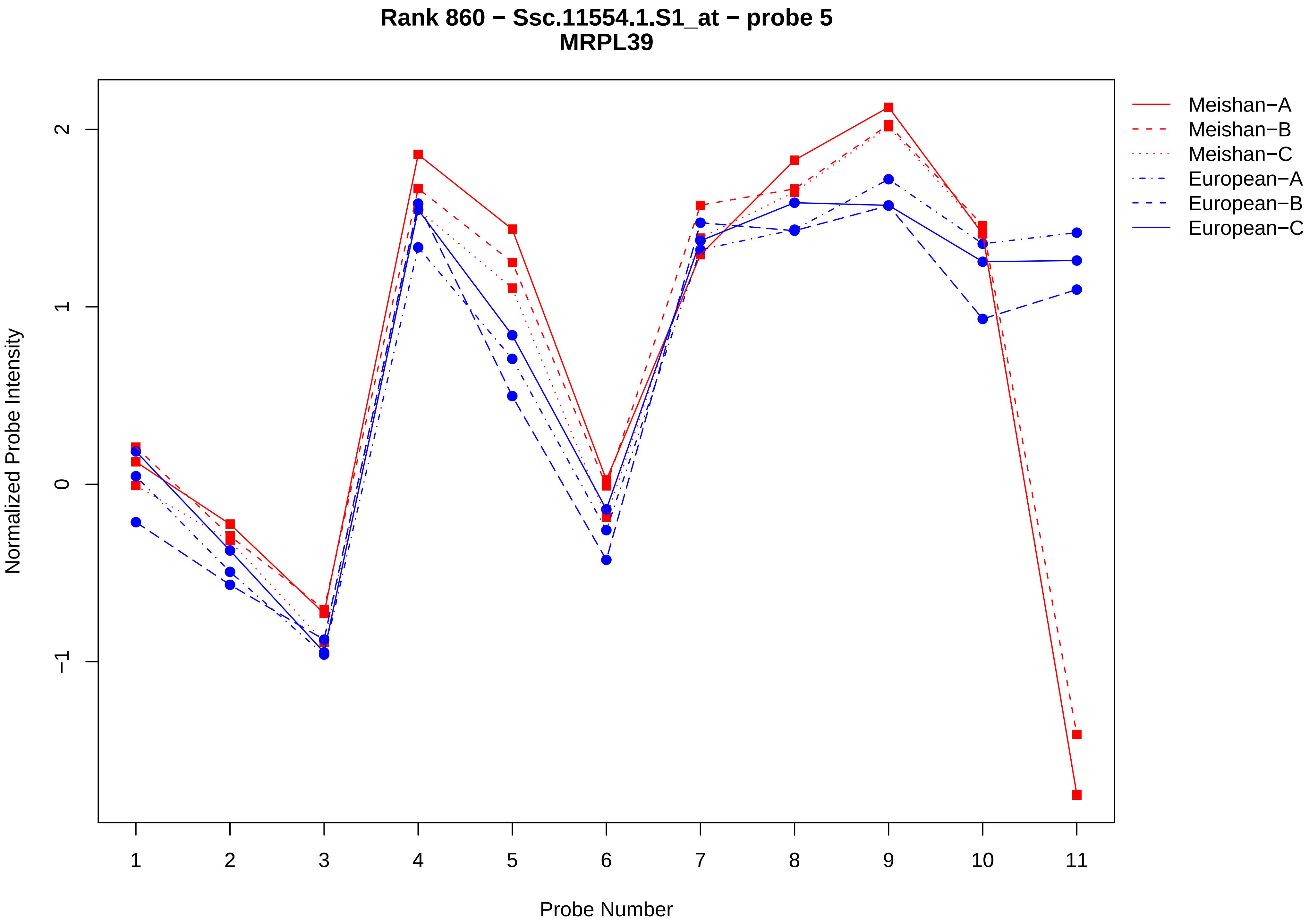

Rank 860 − Ssc.11554.1.S1_at − probe 5
MRPL39
Normalized Probe Intensity
Probe Number
Meishan−A
Meishan−B
Meishan−C
European−A
European−B
European−C
−1
0
1
2
1
2
3
4
5
6
7
8
9
10
11

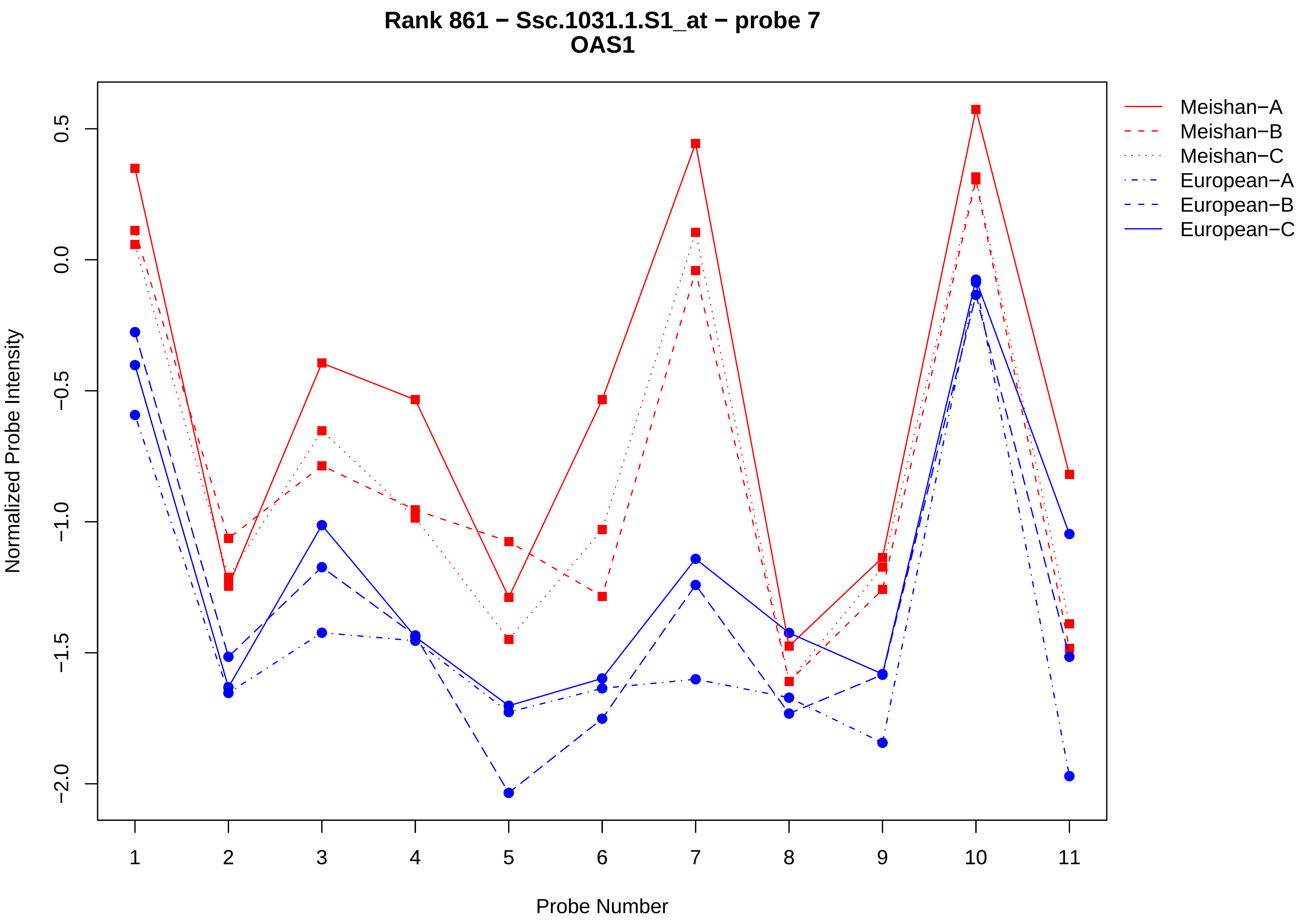
Rank 861 − Ssc.1031.1.S1_at − probe 7
OAS1
Normalized Probe Intensity
Probe Number
Meishan−A
Meishan−B
Meishan−C
European−A
European−B
European−C
0.5
0.0
−0.5
−1.0
−1.5
−2.0
1
2
3
4
5
6
7
8
9
10
11

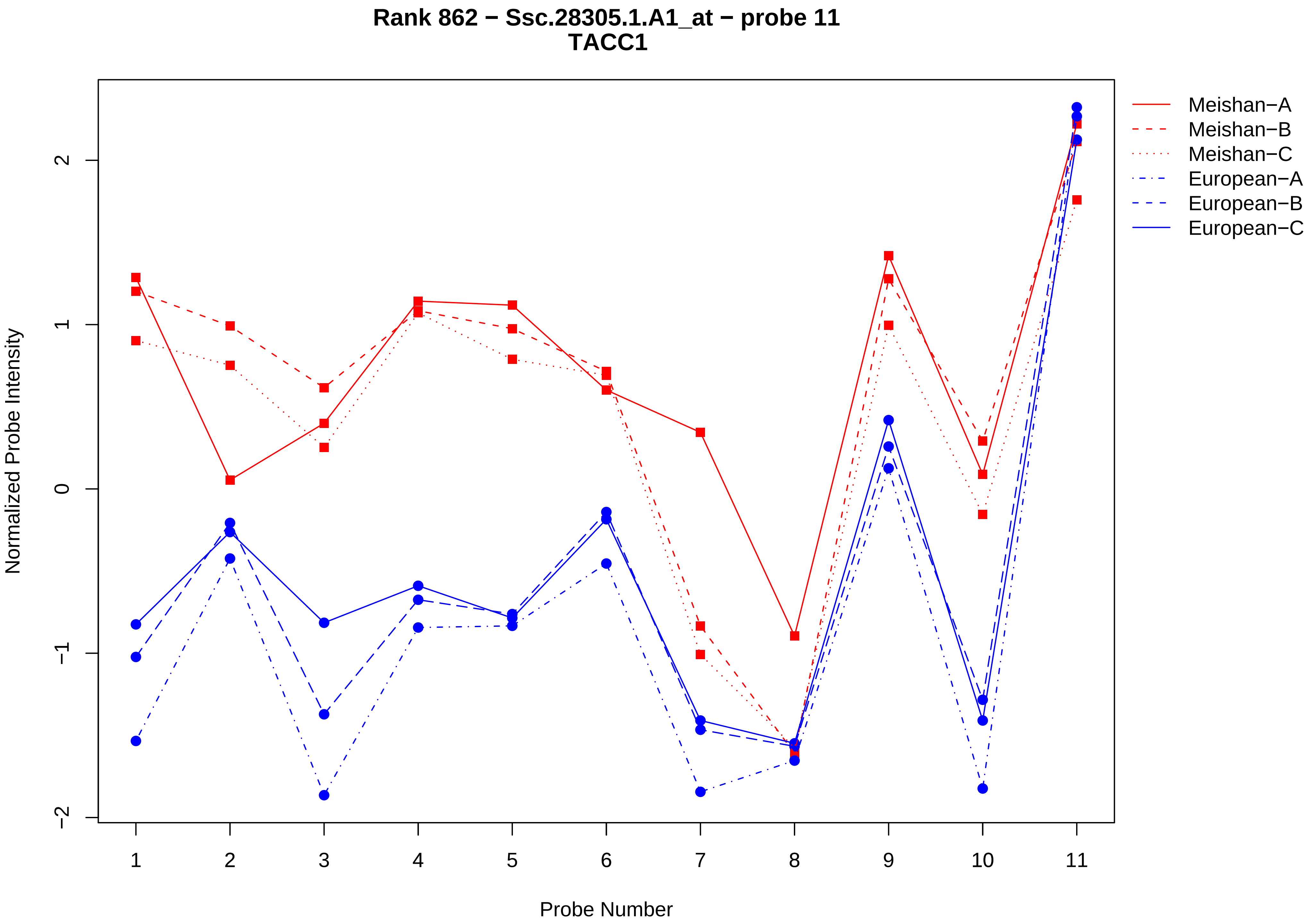

Rank 862 − Ssc.28305.1.A1_at − probe 11
TACC1
Normalized Probe Intensity
Probe Number
−2
−1
0
1
2
1
2
3
4
5
6
7
8
9
10
11
Meishan−A
Meishan−B
Meishan−C
European−A
European−B
European−C

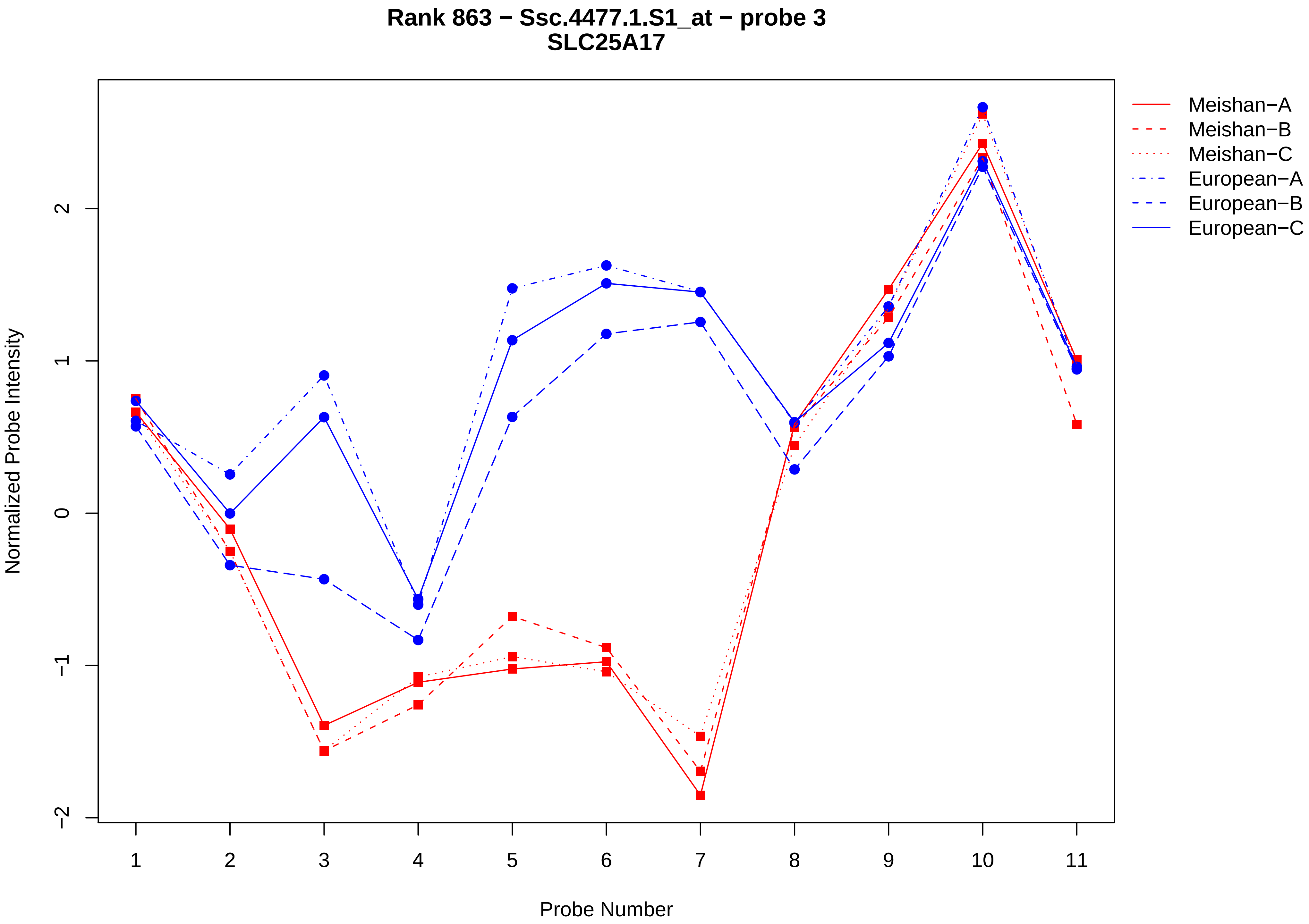
Rank 863 − Ssc.4477.1.S1_at − probe 3
SLC25A17
Normalized Probe Intensity
Probe Number
Meishan−A
Meishan−B
Meishan−C
European−A
European−B
European−C
−2
−1
0
1
2
1
2
3
4
5
6
7
8
9
10
11

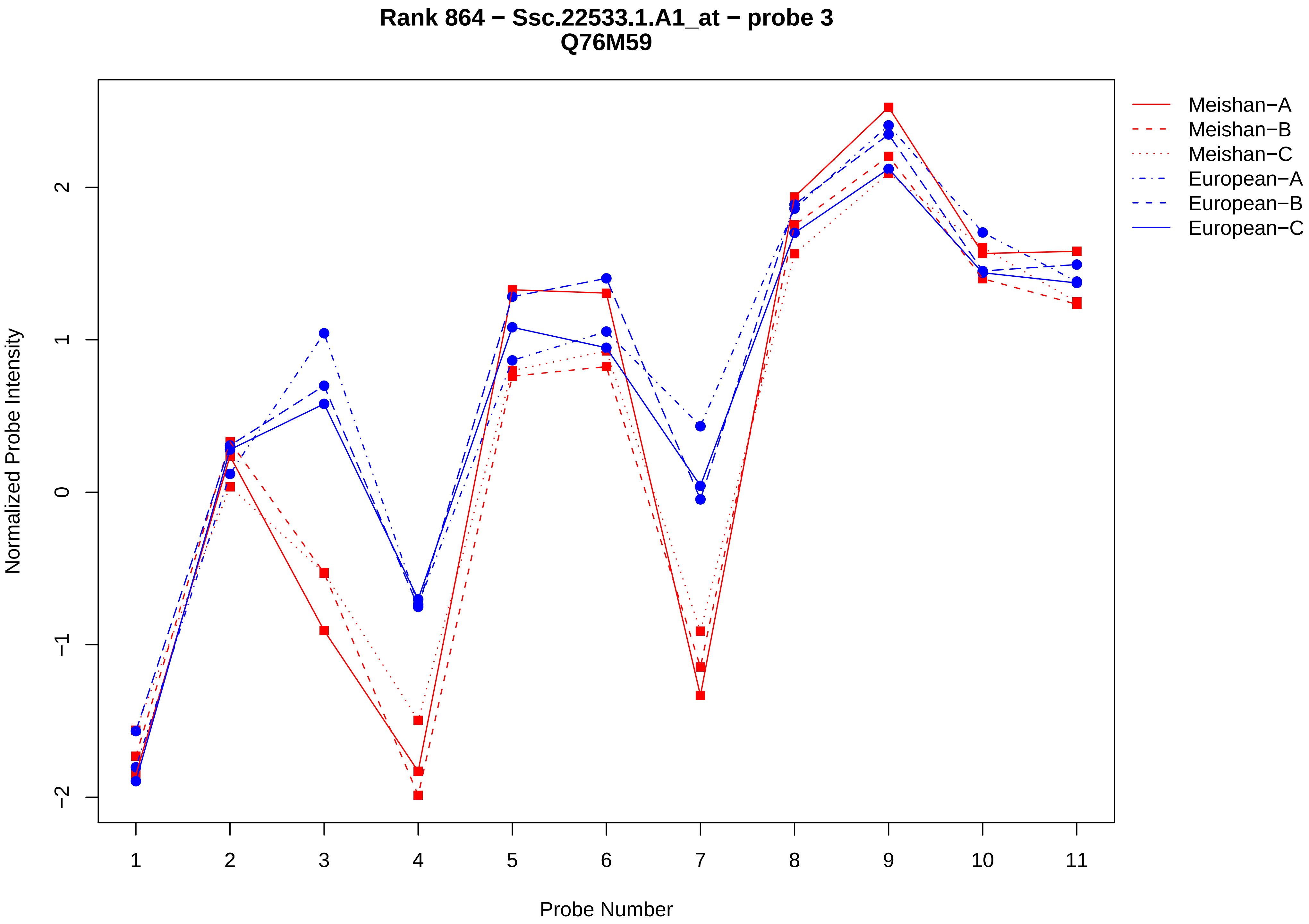

Rank 864 − Ssc.22533.1.A1_at − probe 3
Q76M59
Normalized Probe Intensity
Probe Number
−2
−1
0
1
2
1
2
3
4
5
6
7
8
9
10
11
Meishan−A
Meishan−B
Meishan−C
European−A
European−B
European−C

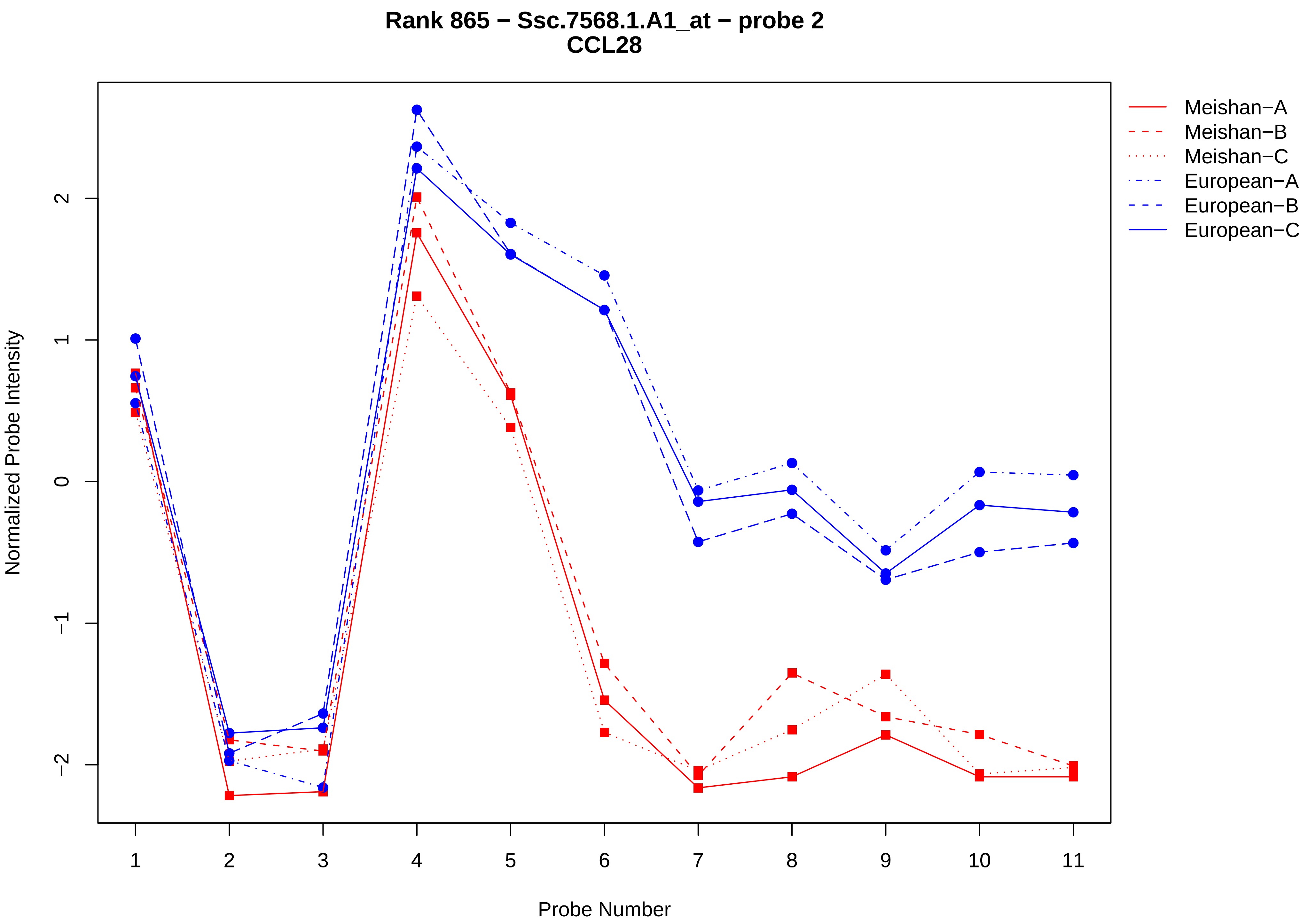

Rank 865 − Ssc.7568.1.A1_at − probe 2
CCL28
Normalized Probe Intensity
Probe Number
−2
−1
0
1
2
1
2
3
4
5
6
7
8
9
10
11
Meishan−A
Meishan−B
Meishan−C
European−A
European−B
European−C

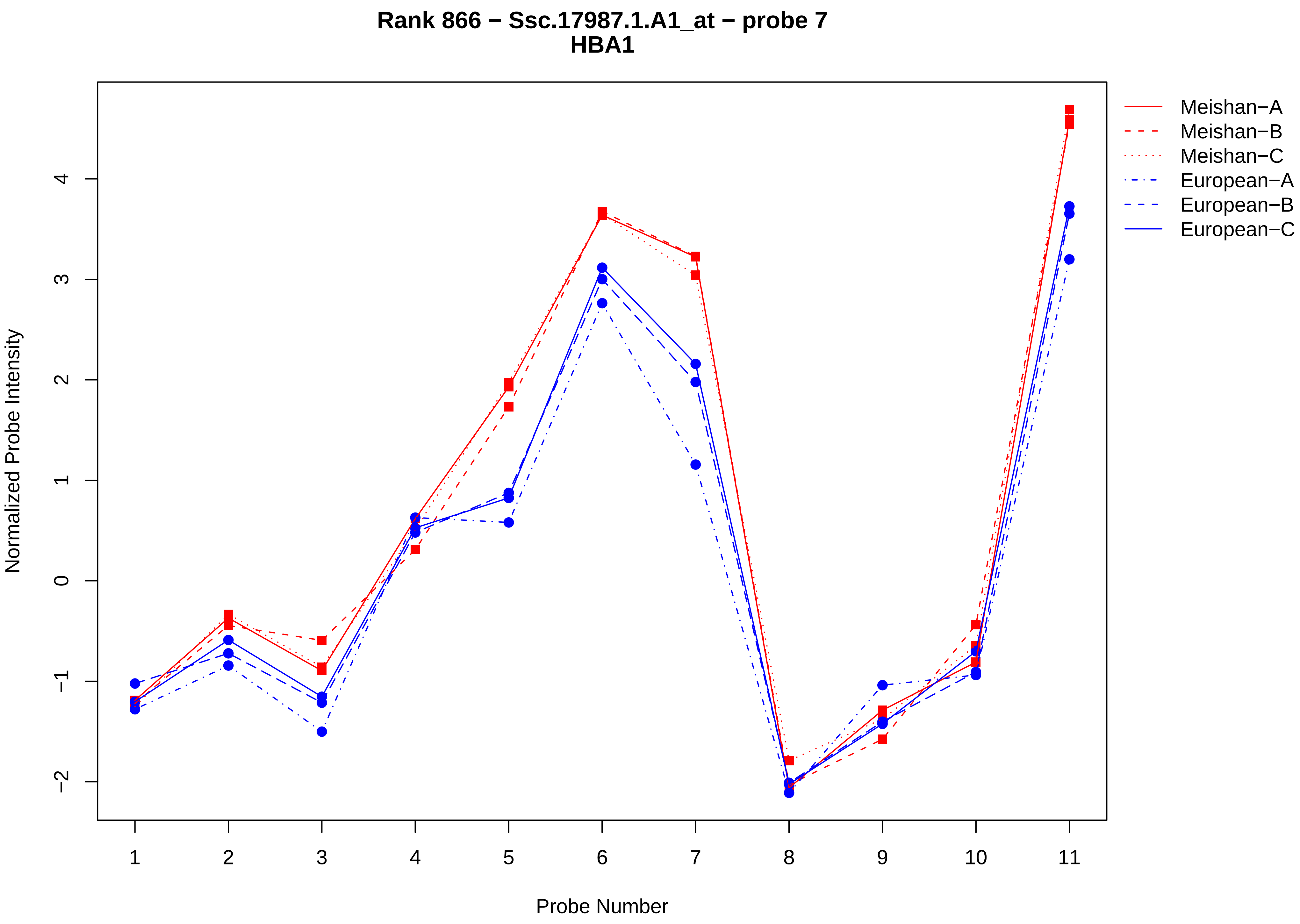

Rank 866 − Ssc.17987.1.A1_at − probe 7
HBA1
Normalized Probe Intensity
Probe Number
−2
−1
0
1
2
3
4
1
2
3
4
5
6
7
8
9
10
11
Meishan−A
Meishan−B
Meishan−C
European−A
European−B
European−C

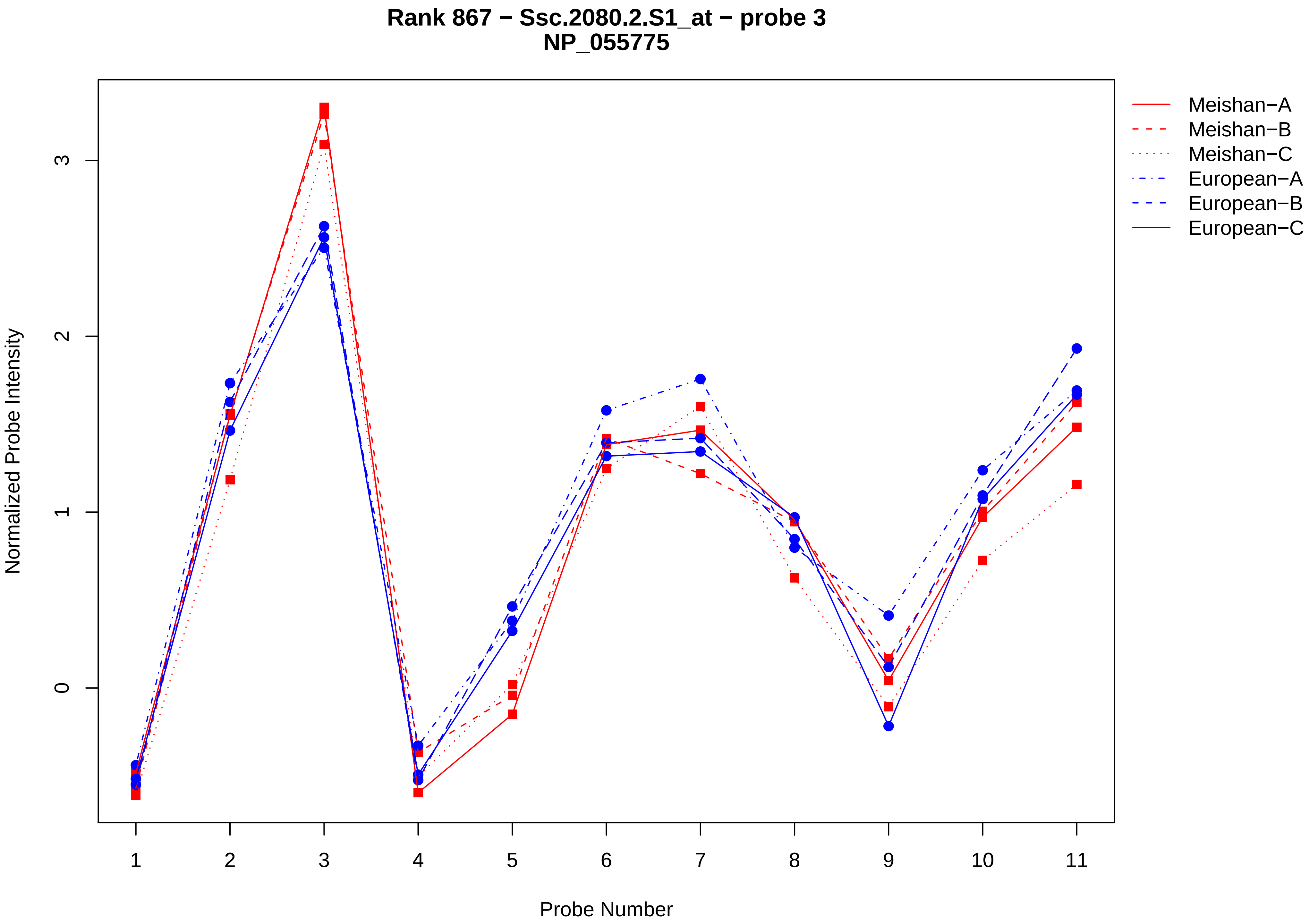

Rank 867 − Ssc.2080.2.S1_at − probe 3
NP_055775
Normalized Probe Intensity
Probe Number
0
1
2
3
1
2
3
4
5
6
7
8
9
10
11
Meishan−A
Meishan−B
Meishan−C
European−A
European−B
European−C

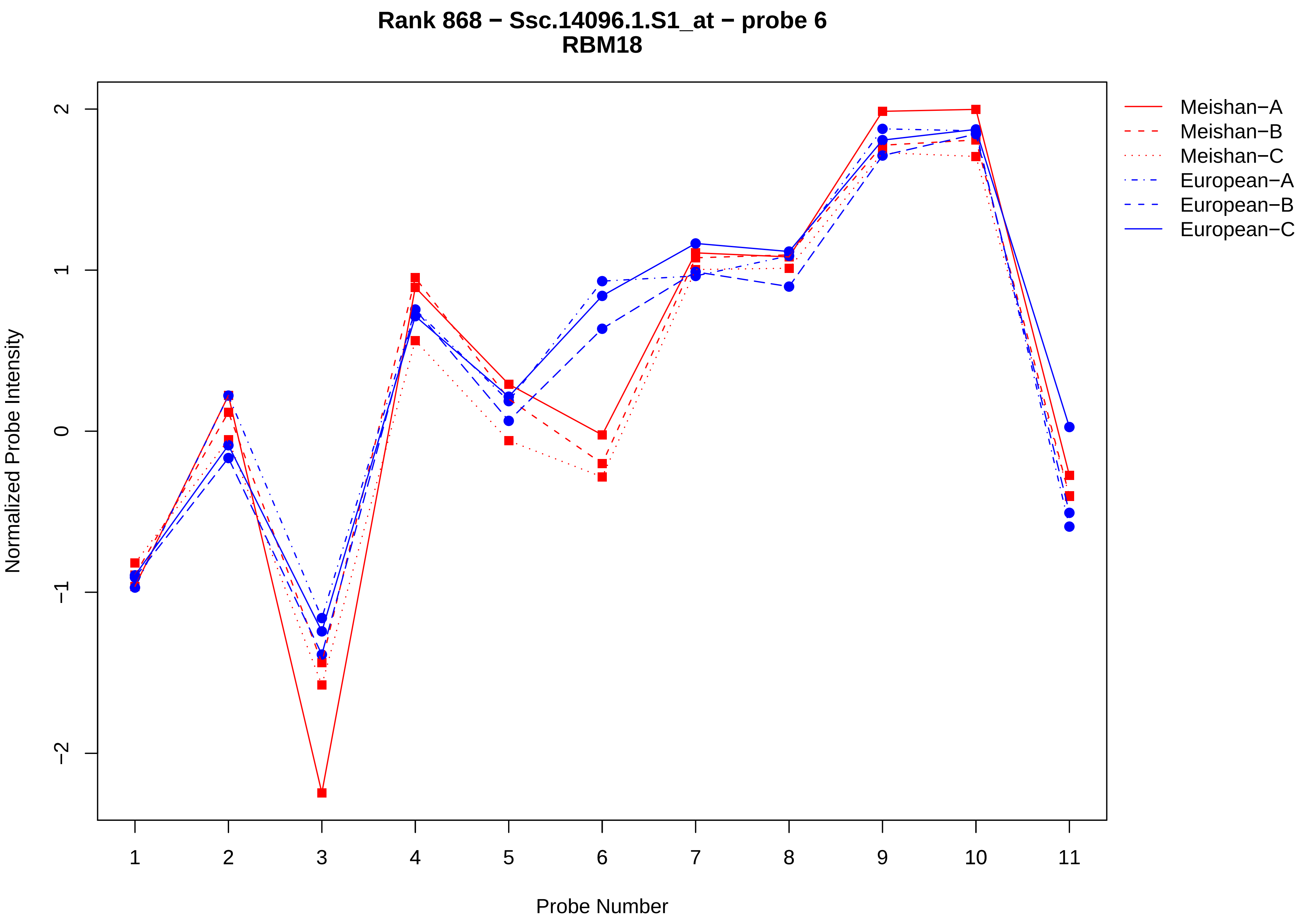

Rank 868 − Ssc.14096.1.S1_at − probe 6
RBM18
Normalized Probe Intensity
Probe Number
−2
−1
0
1
2
1
2
3
4
5
6
7
8
9
10
11
Meishan−A
Meishan−B
Meishan−C
European−A
European−B
European−C

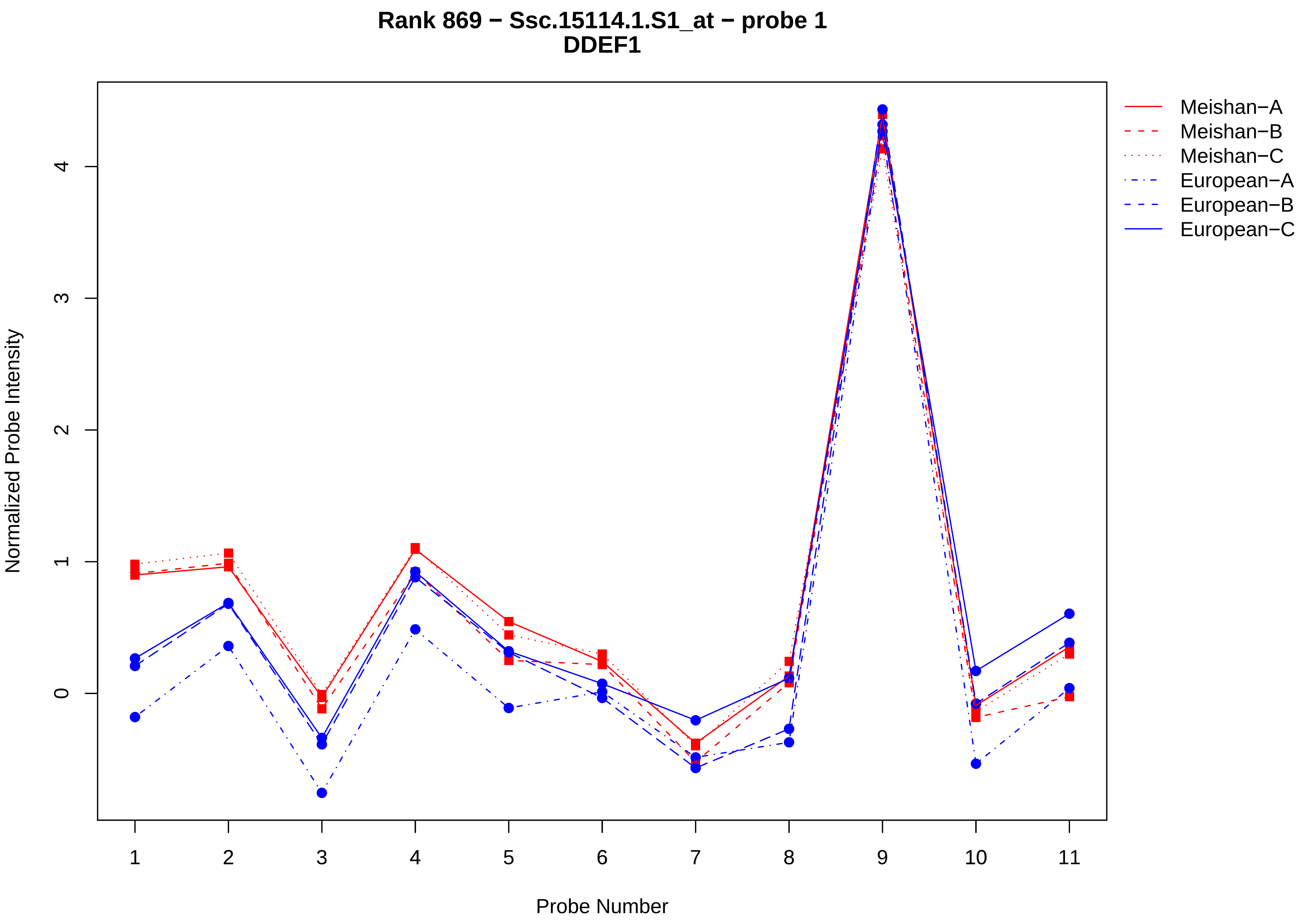

Rank 869 − Ssc.15114.1.S1_at − probe 1
DDEF1
Normalized Probe Intensity
Probe Number
0
1
2
3
4
1
2
3
4
5
6
7
8
9
10
11
Meishan−A
Meishan−B
Meishan−C
European−A
European−B
European−C

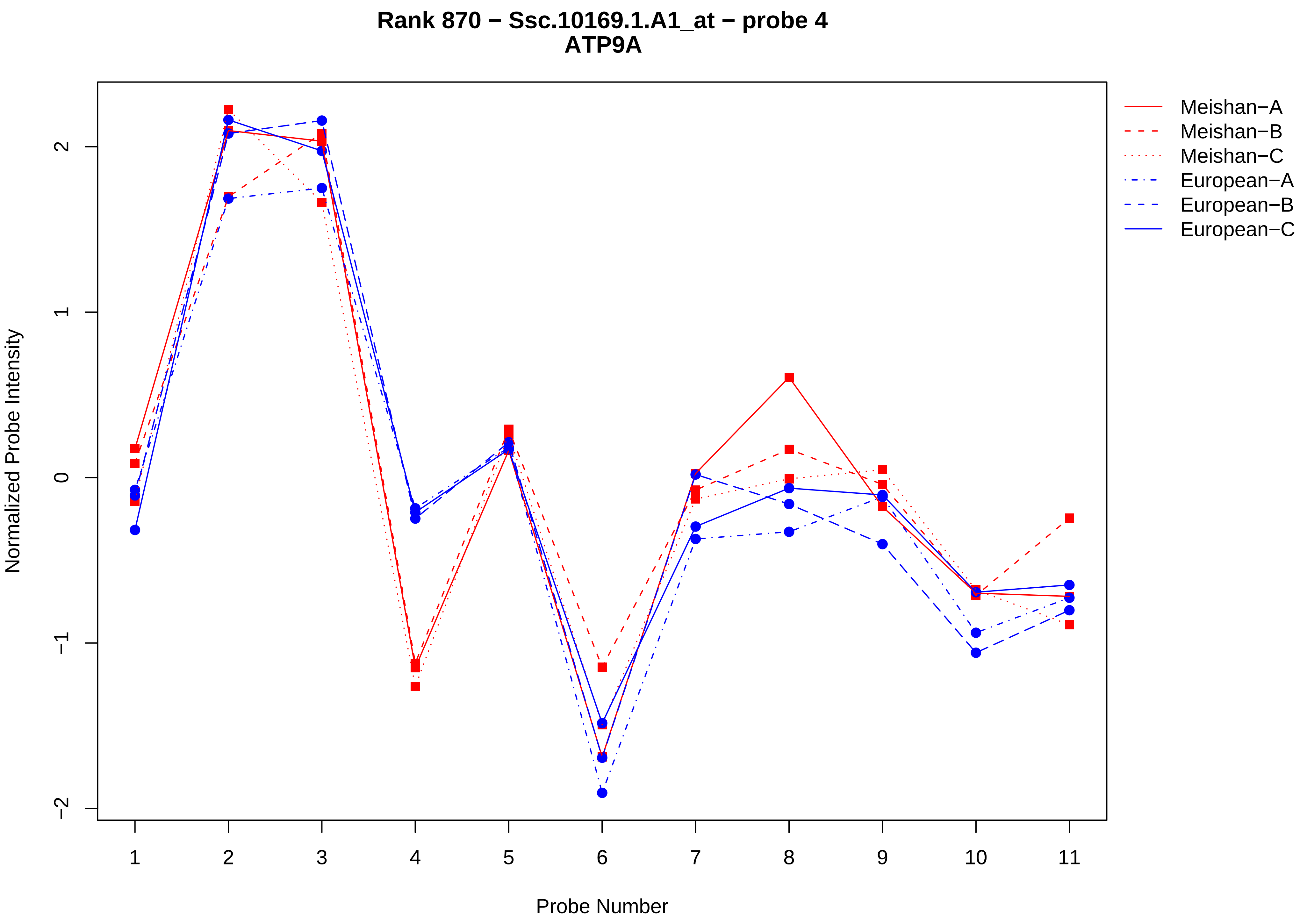
Rank 870 − Ssc.10169.1.A1_at − probe 4
ATP9A
Normalized Probe Intensity
Probe Number
Meishan−A
Meishan−B
Meishan−C
European−A
European−B
European−C
−2
−1
0
1
2
1
2
3
4
5
6
7
8
9
10
11

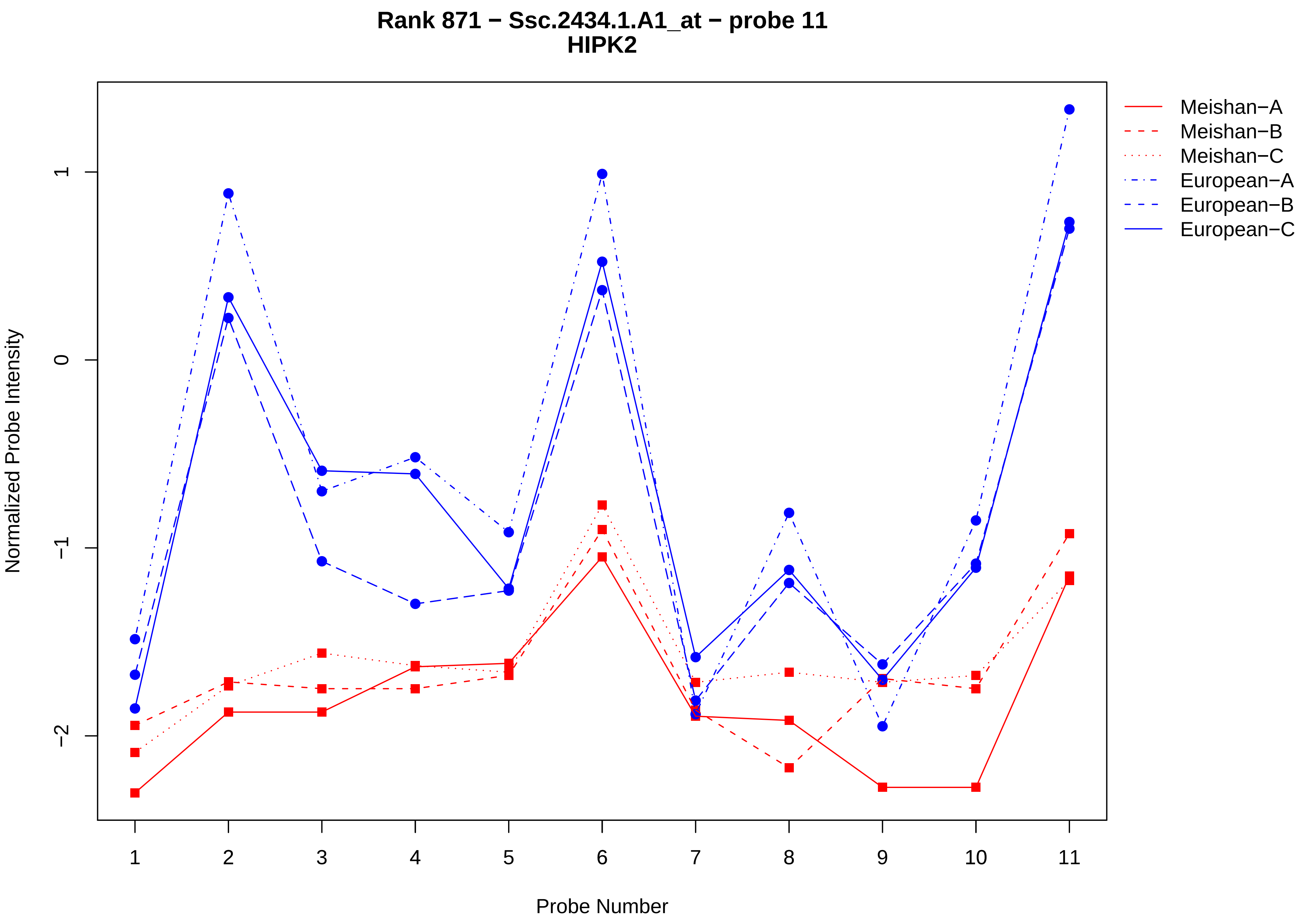
Rank 871 − Ssc.2434.1.A1_at − probe 11
HIPK2
Normalized Probe Intensity
Probe Number
Meishan−A
Meishan−B
Meishan−C
European−A
European−B
European−C
1
0
−1
−2
1
2
3
4
5
6
7
8
9
10
11

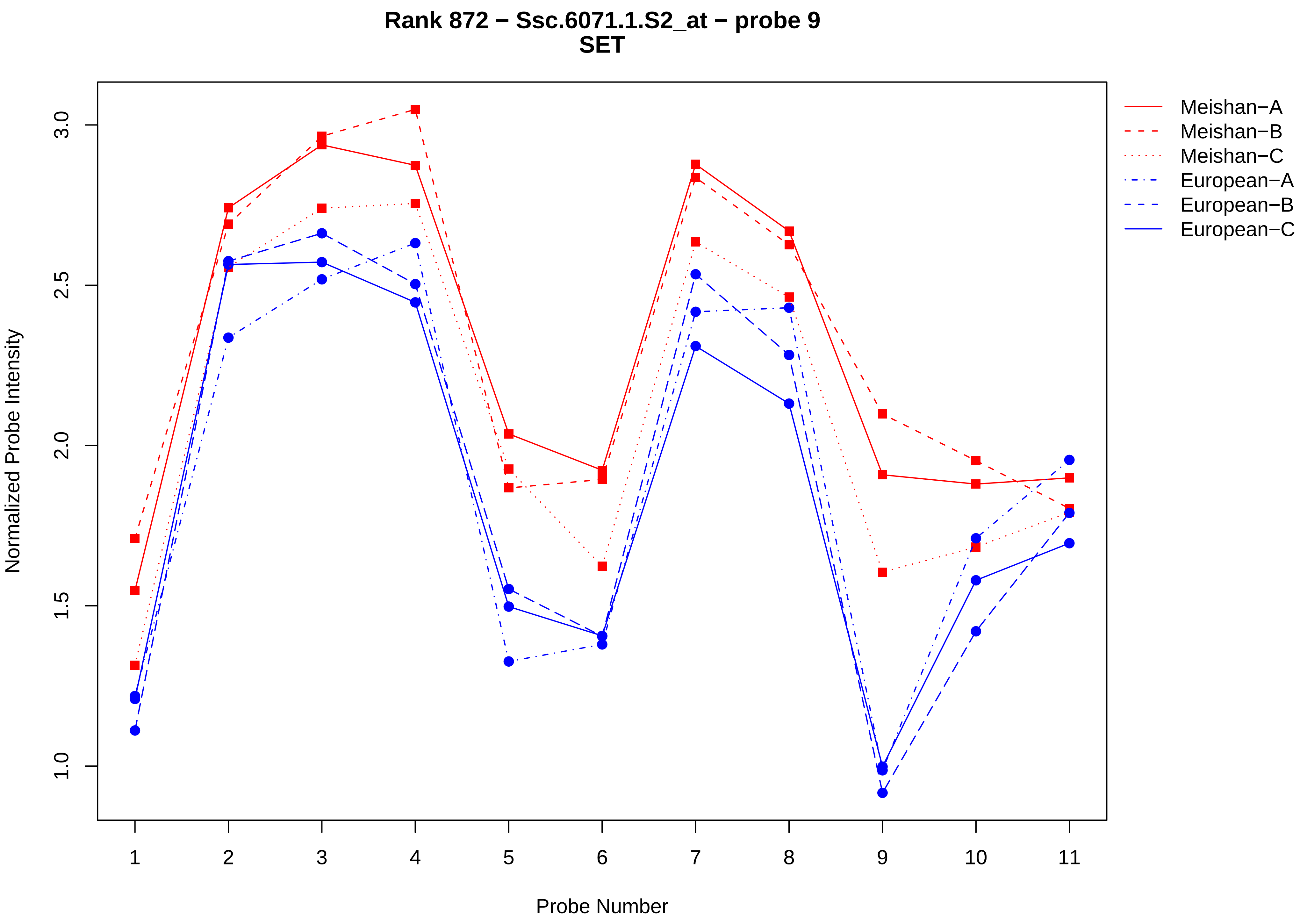
Rank 872 − Ssc.6071.1.S2_at − probe 9
SET
Normalized Probe Intensity
Probe Number
1.0
1.5
2.0
2.5
3.0
1
2
3
4
5
6
7
8
9
10
11
Meishan−A
Meishan−B
Meishan−C
European−A
European−B
European−C

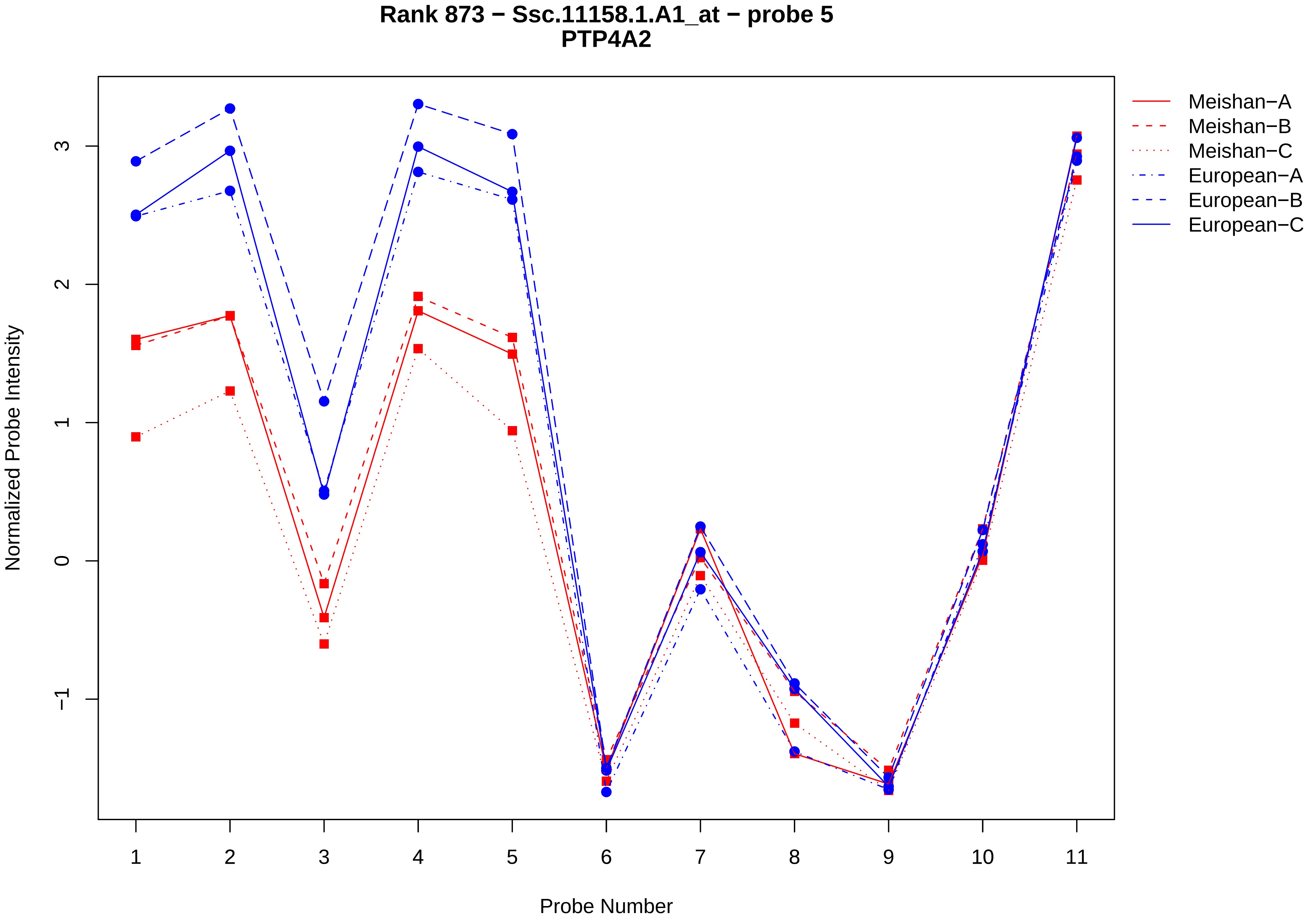

Rank 873 − Ssc.11158.1.A1_at − probe 5
PTP4A2
Normalized Probe Intensity
Probe Number
−1
0
1
2
3
1
2
3
4
5
6
7
8
9
10
11
Meishan−A
Meishan−B
Meishan−C
European−A
European−B
European−C

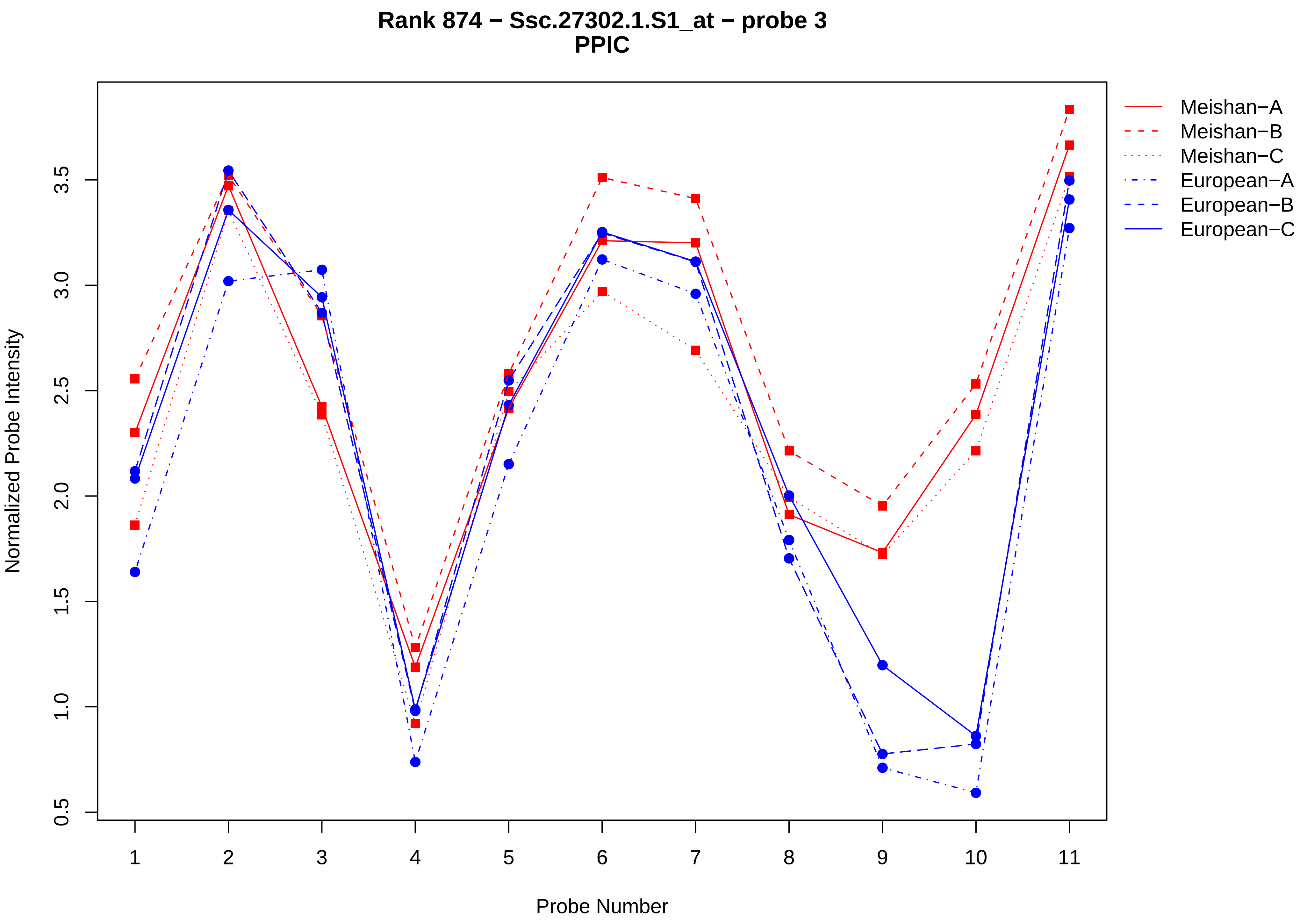

Rank 874 − Ssc.27302.1.S1_at − probe 3
PPIC
Normalized Probe Intensity
Probe Number
0.5
1.0
1.5
2.0
2.5
3.0
3.5
1
2
3
4
5
6
7
8
9
10
11
Meishan−A
Meishan−B
Meishan−C
European−A
European−B
European−C

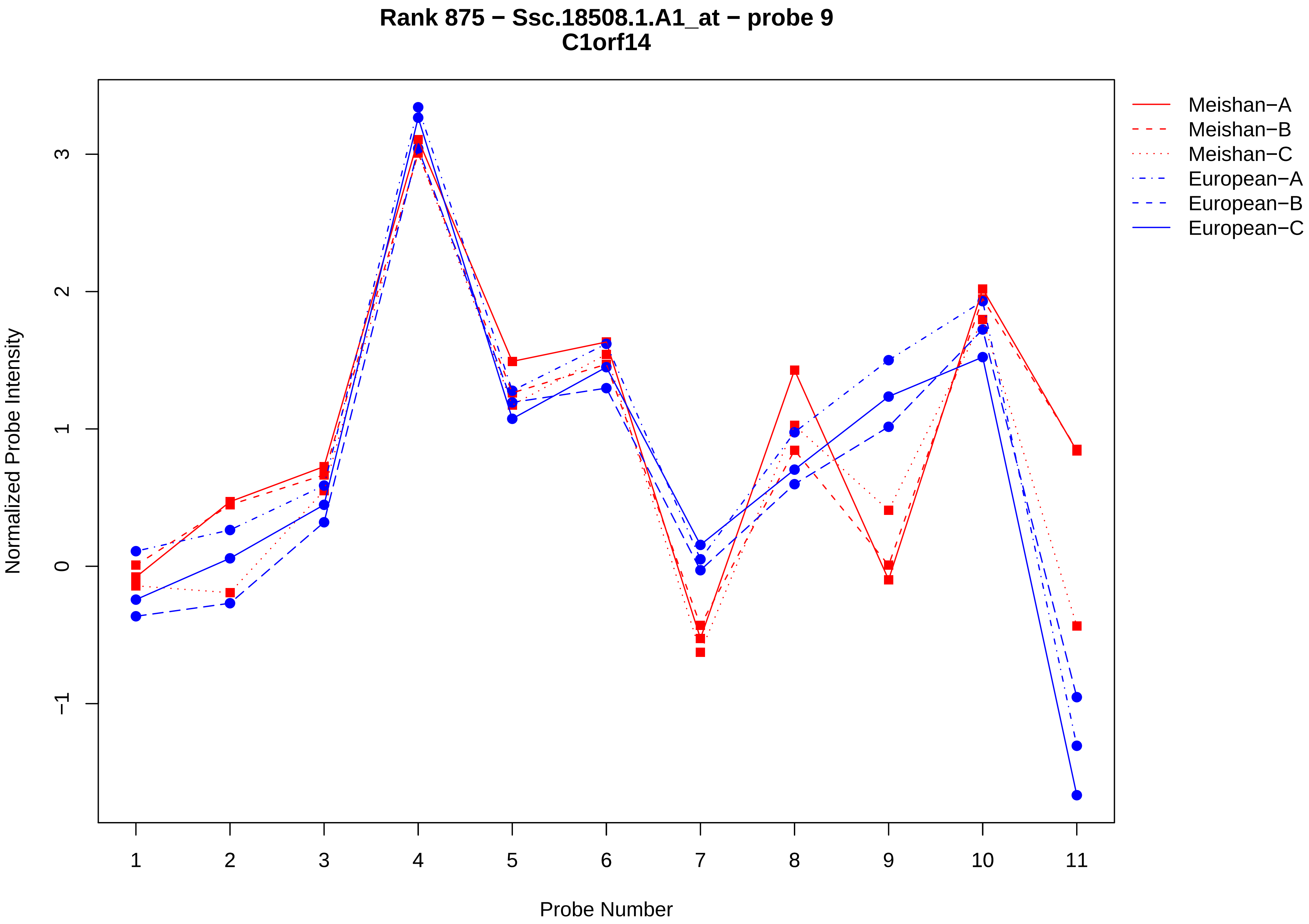
Rank 875 − Ssc.18508.1.A1_at − probe 9
C1orf14
Normalized Probe Intensity
Probe Number
−1
0
1
2
3
1
2
3
4
5
6
7
8
9
10
11
Meishan−A
Meishan−B
Meishan−C
European−A
European−B
European−C

Rank 876 − Ssc.19192.2.A1_at − probe 1
Q96KR6

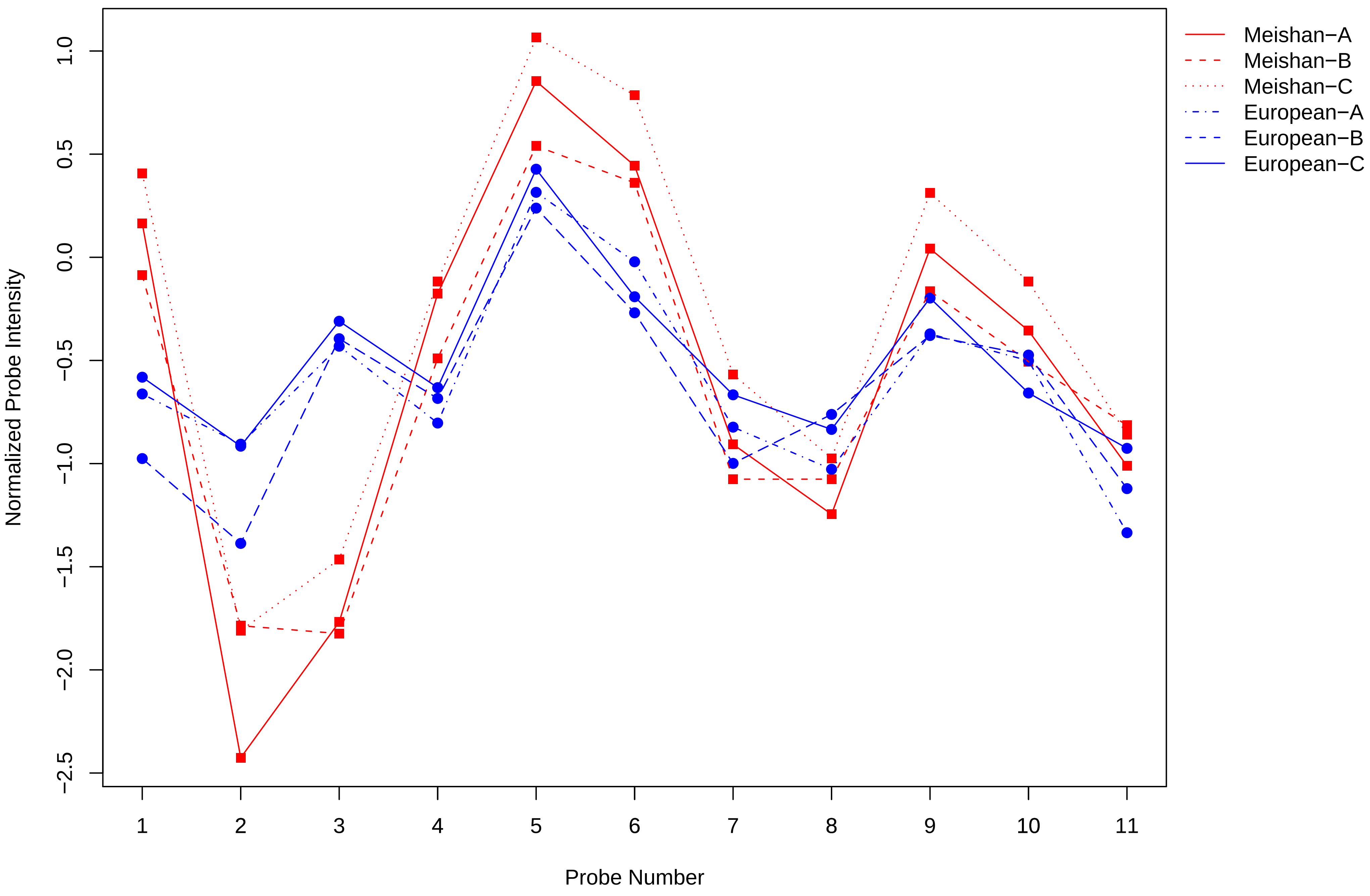

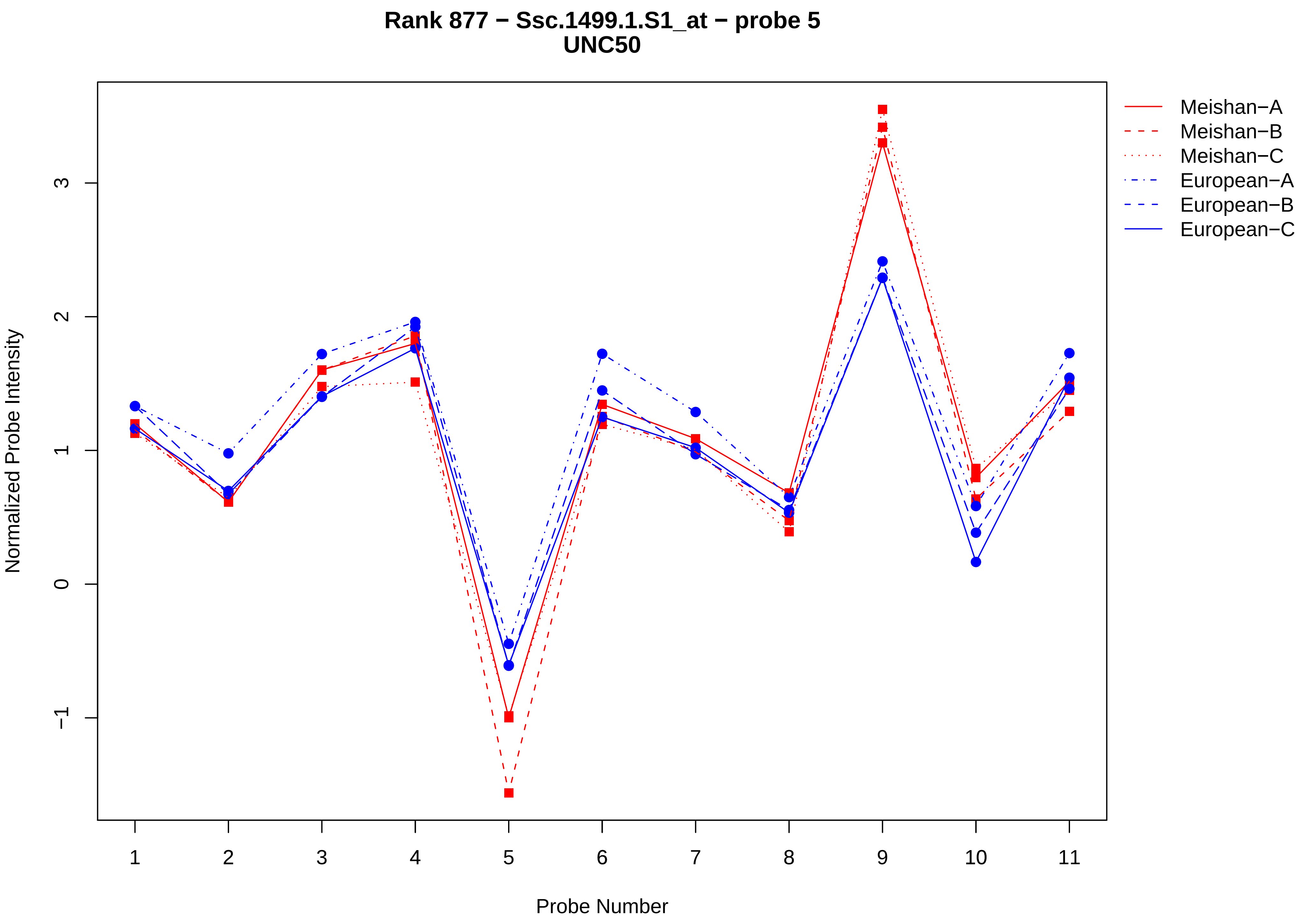
Rank 877 − Ssc.1499.1.S1_at − probe 5
UNC50
Normalized Probe Intensity
Probe Number
Meishan−A
Meishan−B
Meishan−C
European−A
European−B
European−C
−1
0
1
2
3
1
2
3
4
5
6
7
8
9
10
11

Rank 878 − Ssc.24006.1.S1_at − probe 6
PPP2R5E

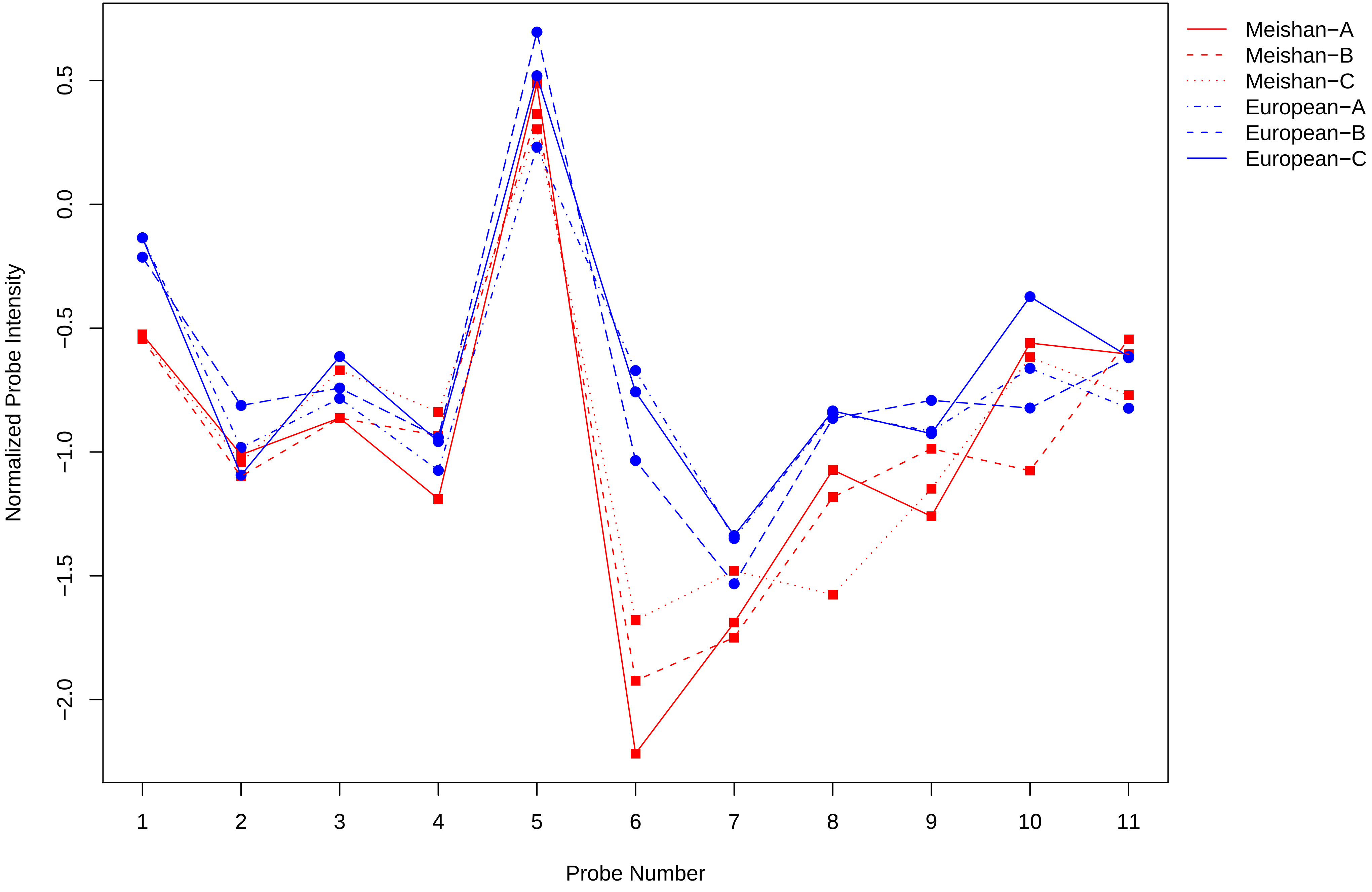

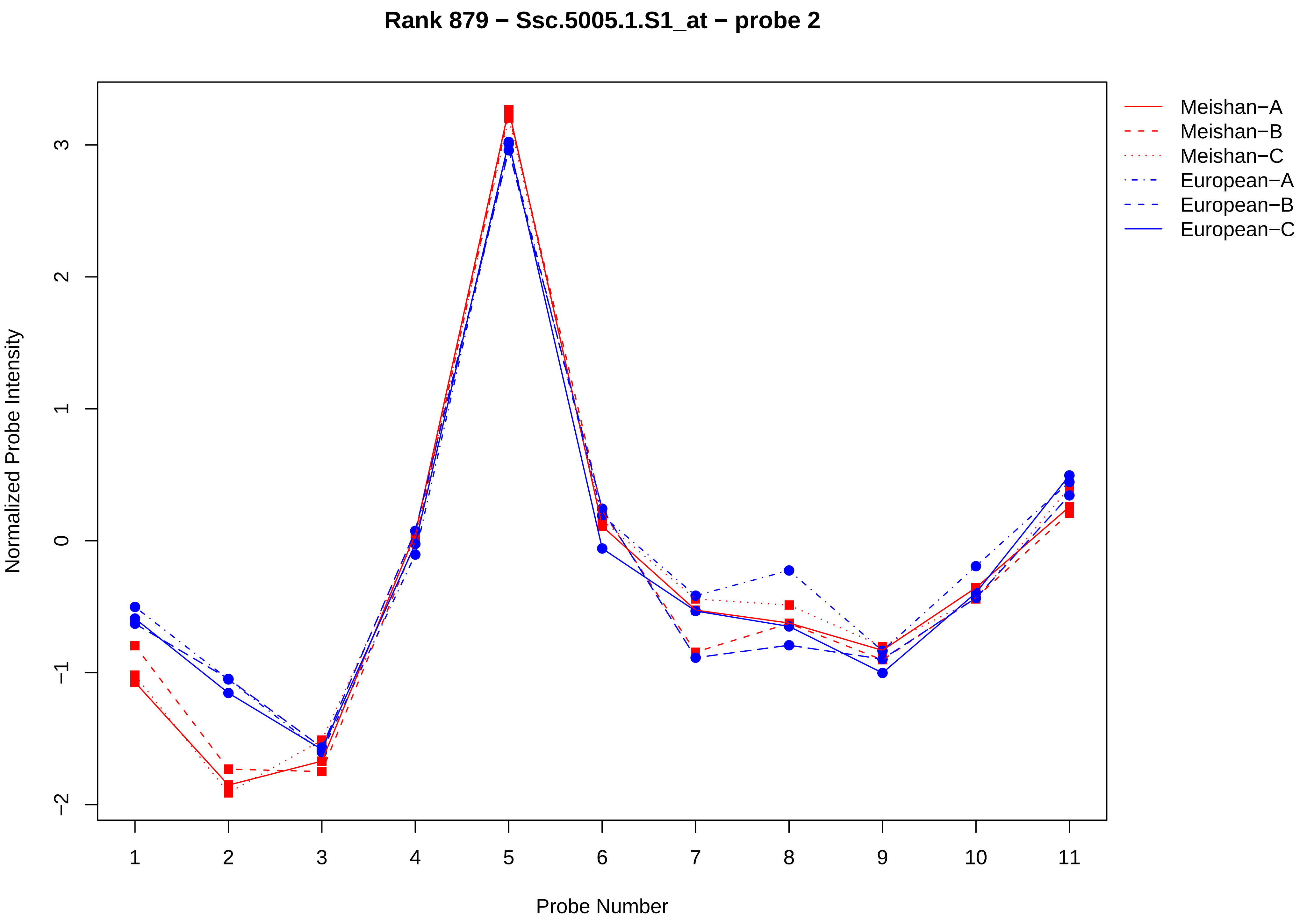
Rank 879 − Ssc.5005.1.S1_at − probe 2
Normalized Probe Intensity
Probe Number
−2
−1
0
1
2
3
1
2
3
4
5
6
7
8
9
10
11
Meishan−A
Meishan−B
Meishan−C
European−A
European−B
European−C

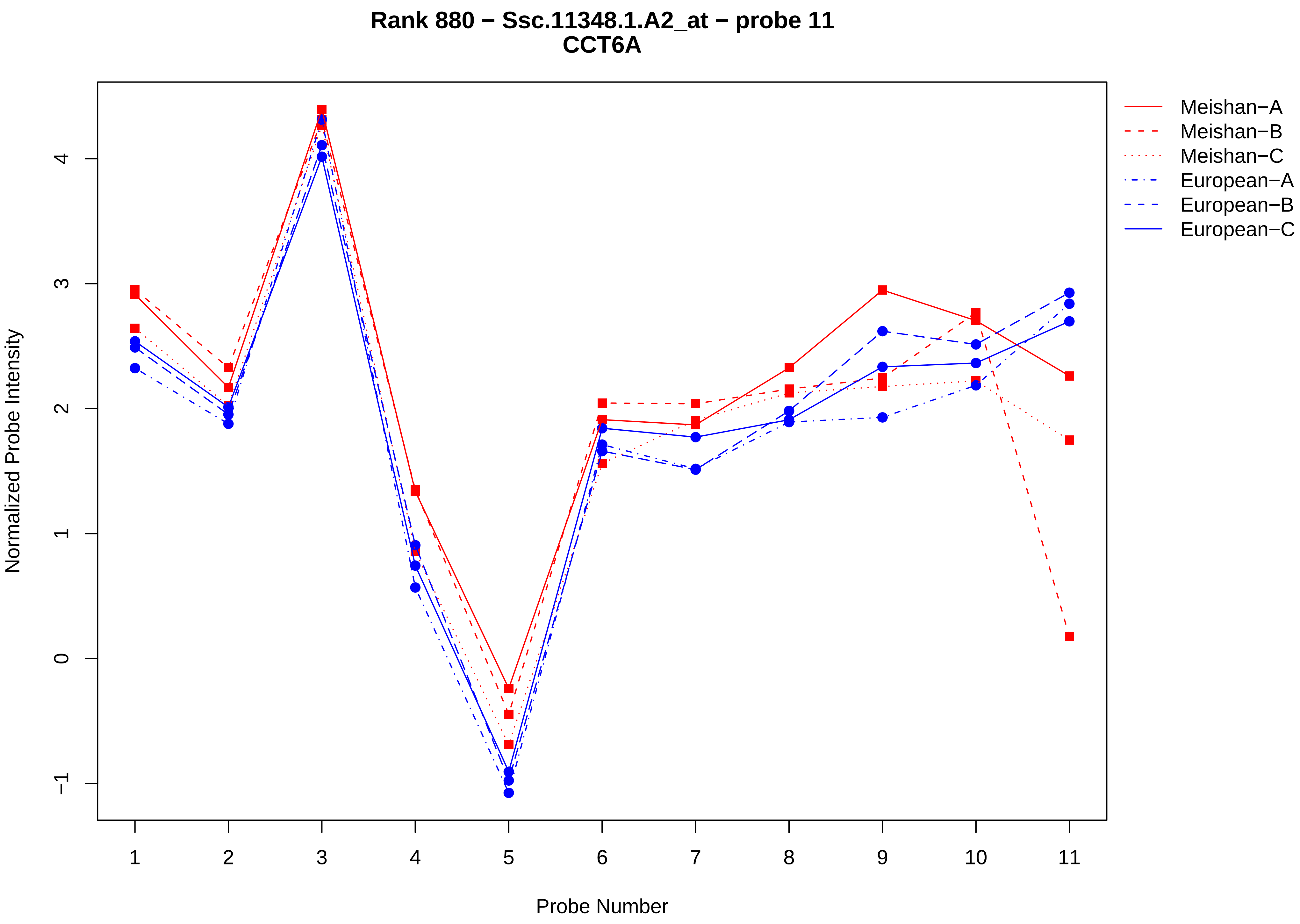

Rank 880 − Ssc.11348.1.A2_at − probe 11
CCT6A
Normalized Probe Intensity
Probe Number
−1
0
1
2
3
4
1
2
3
4
5
6
7
8
9
10
11
Meishan−A
Meishan−B
Meishan−C
European−A
European−B
European−C

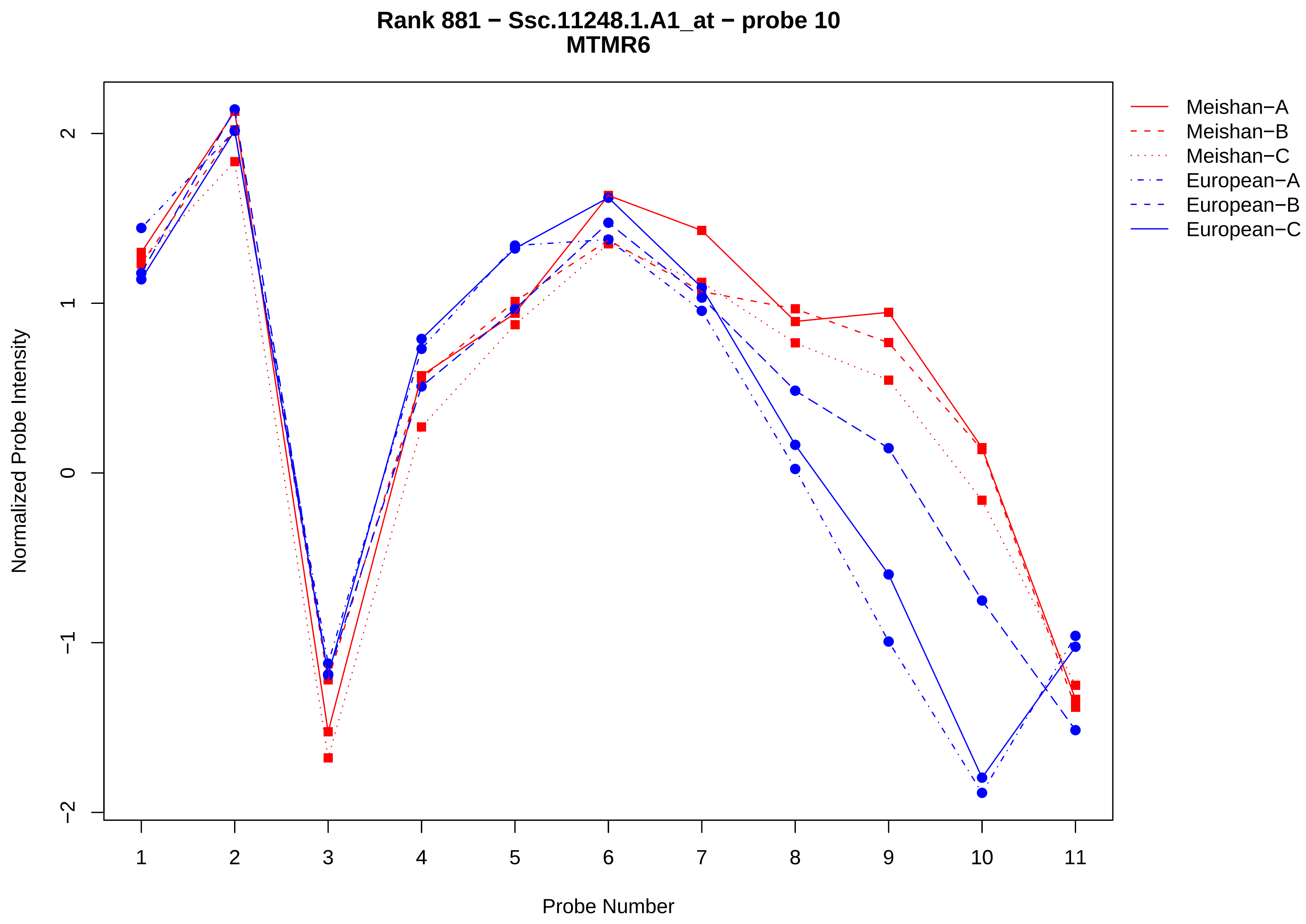

Rank 881 − Ssc.11248.1.A1_at − probe 10
MTMR6
Normalized Probe Intensity
Probe Number
−2
−1
0
1
2
1
2
3
4
5
6
7
8
9
10
11
Meishan−A
Meishan−B
Meishan−C
European−A
European−B
European−C

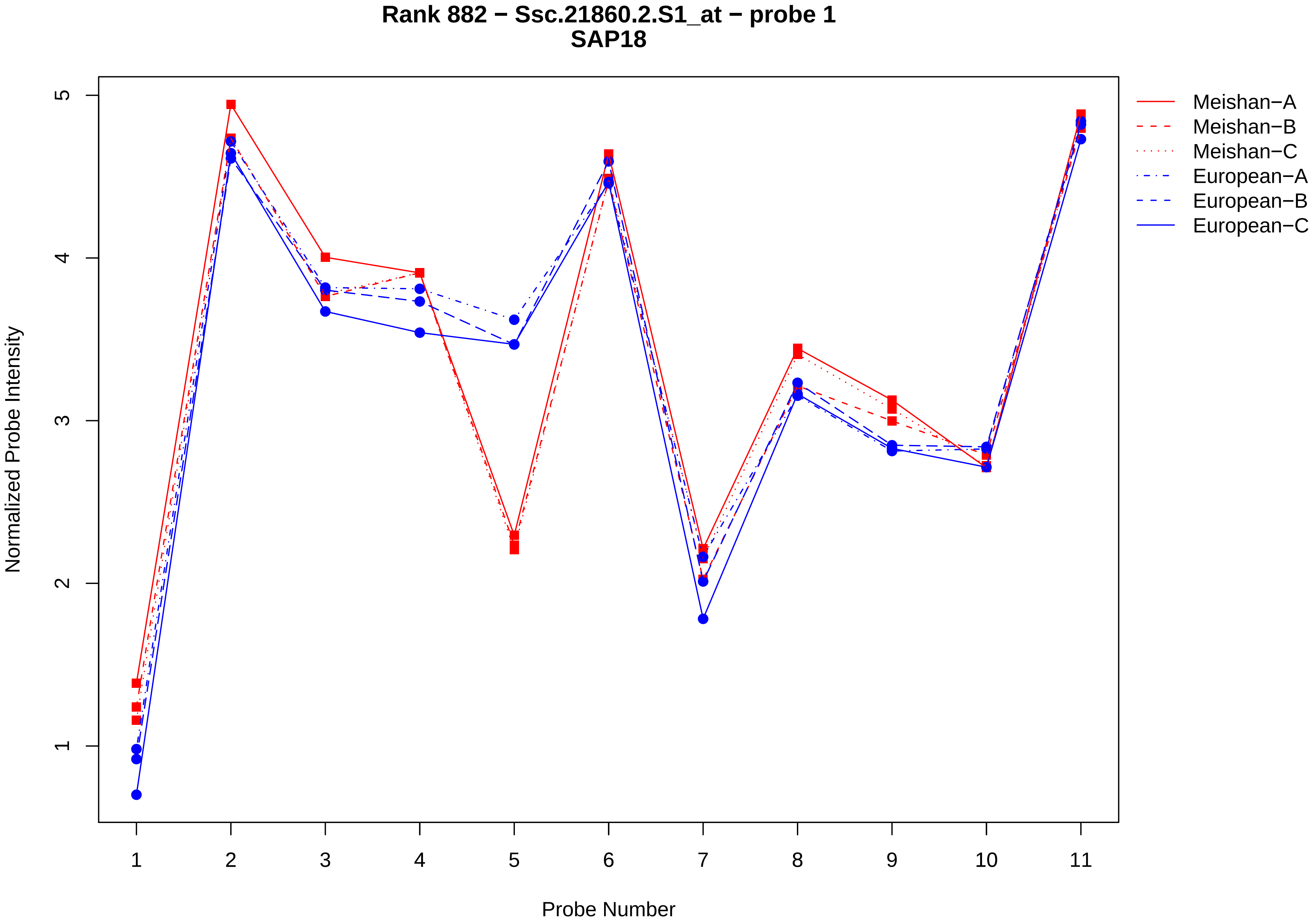

Rank 882 − Ssc.21860.2.S1_at − probe 1
SAP18
Normalized Probe Intensity
Probe Number
1
2
3
4
5
1
2
3
4
5
6
7
8
9
10
11
Meishan−A
Meishan−B
Meishan−C
European−A
European−B
European−C

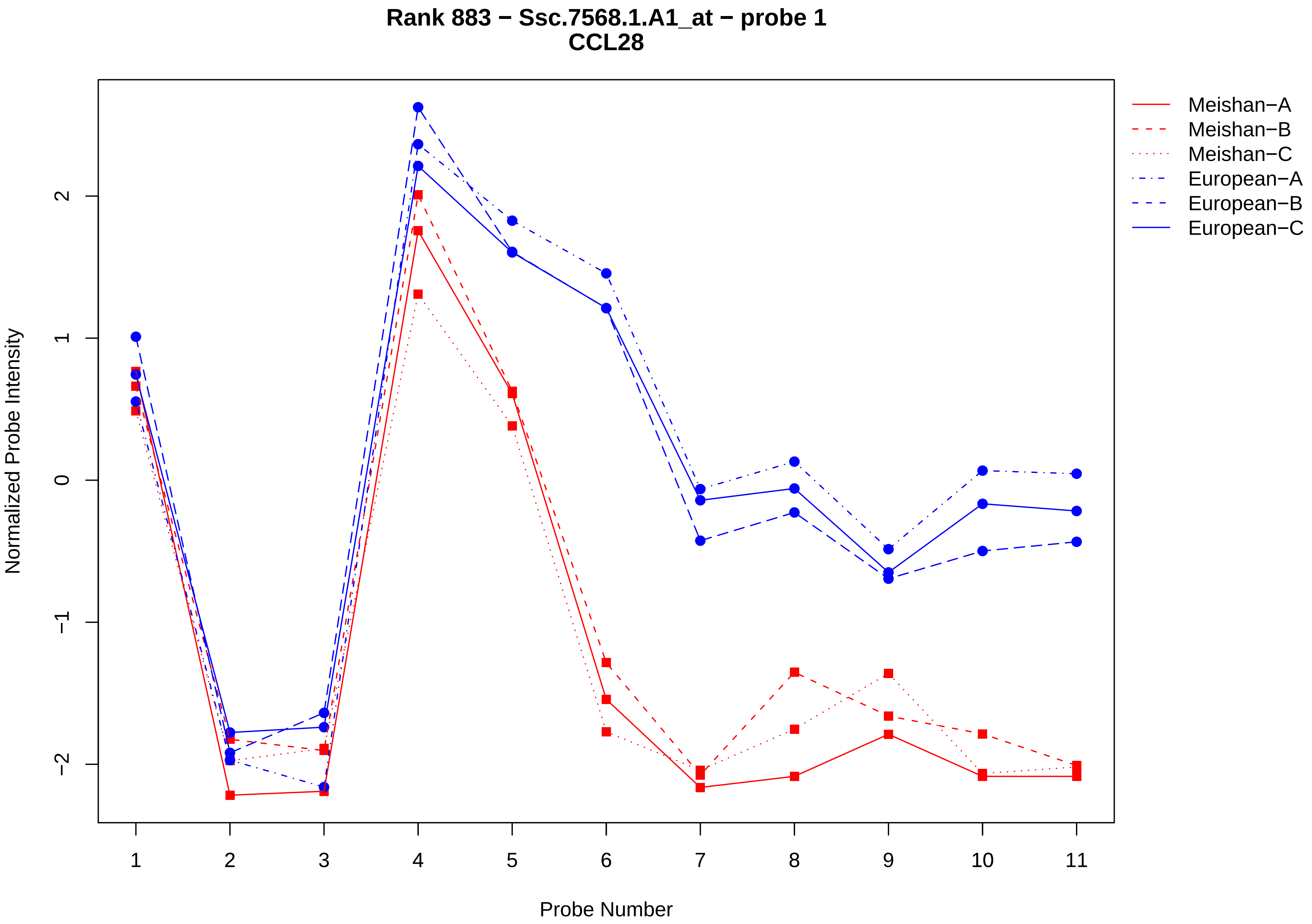

Rank 883 − Ssc.7568.1.A1_at − probe 1
CCL28
Normalized Probe Intensity
Probe Number
Meishan−A
Meishan−B
Meishan−C
European−A
European−B
European−C
−2
−1
0
1
2
1
2
3
4
5
6
7
8
9
10
11

Rank 884 − Ssc.1490.1.S1_at − probe 7
PRDX3

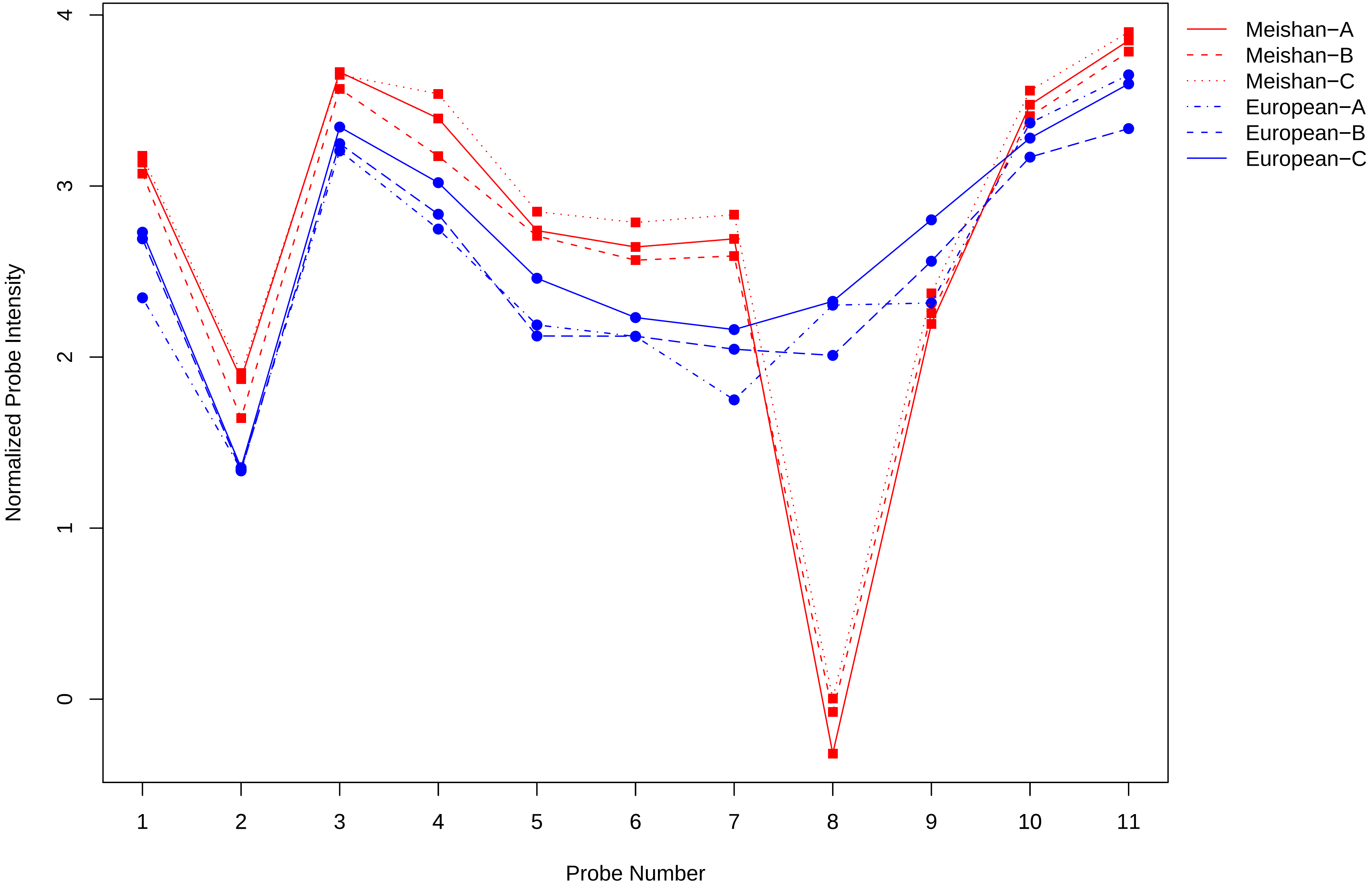

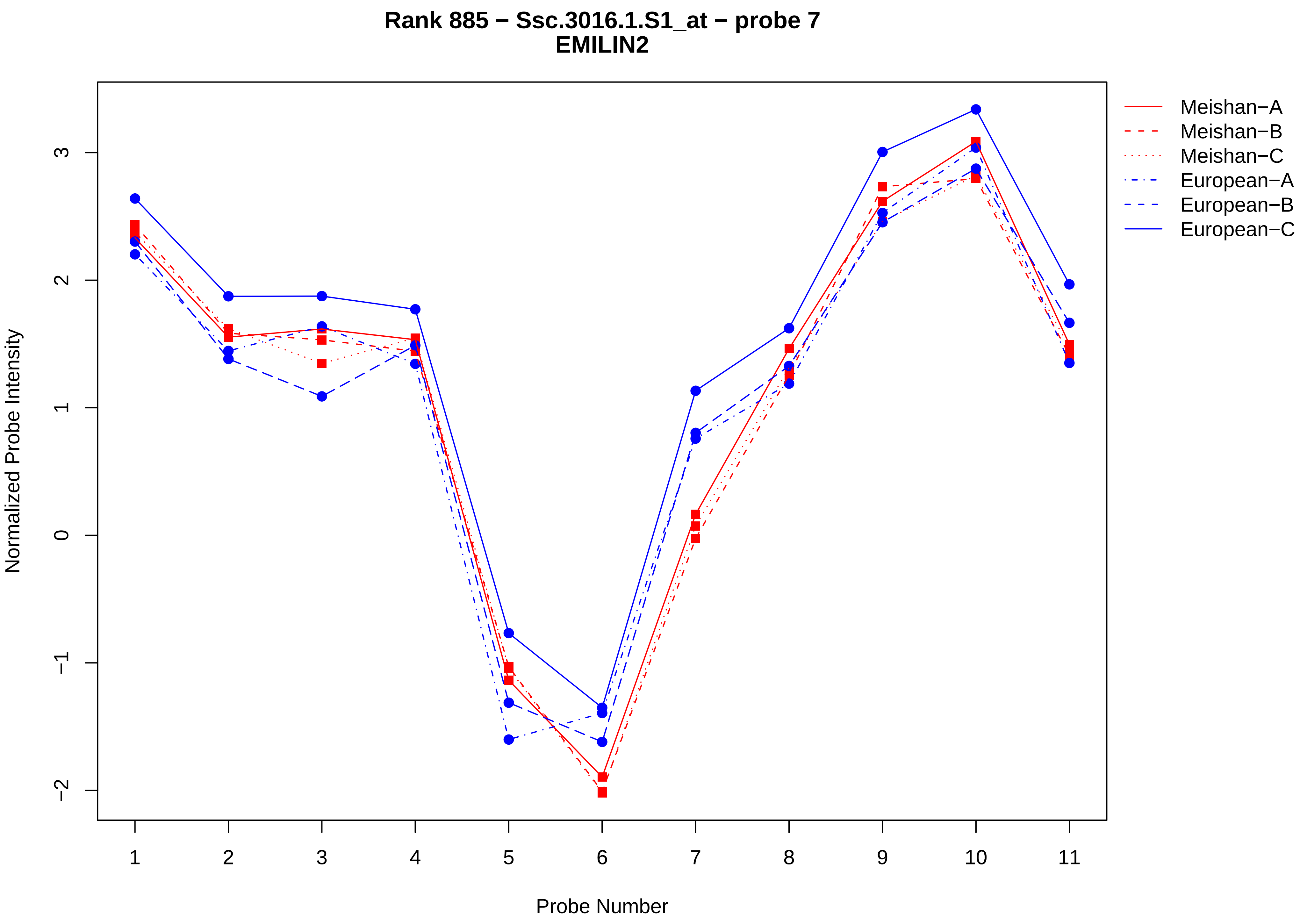

Rank 885 − Ssc.3016.1.S1_at − probe 7
EMILIN2
Normalized Probe Intensity
Probe Number
−2
−1
0
1
2
3
1
2
3
4
5
6
7
8
9
10
11
Meishan−A
Meishan−B
Meishan−C
European−A
European−B
European−C

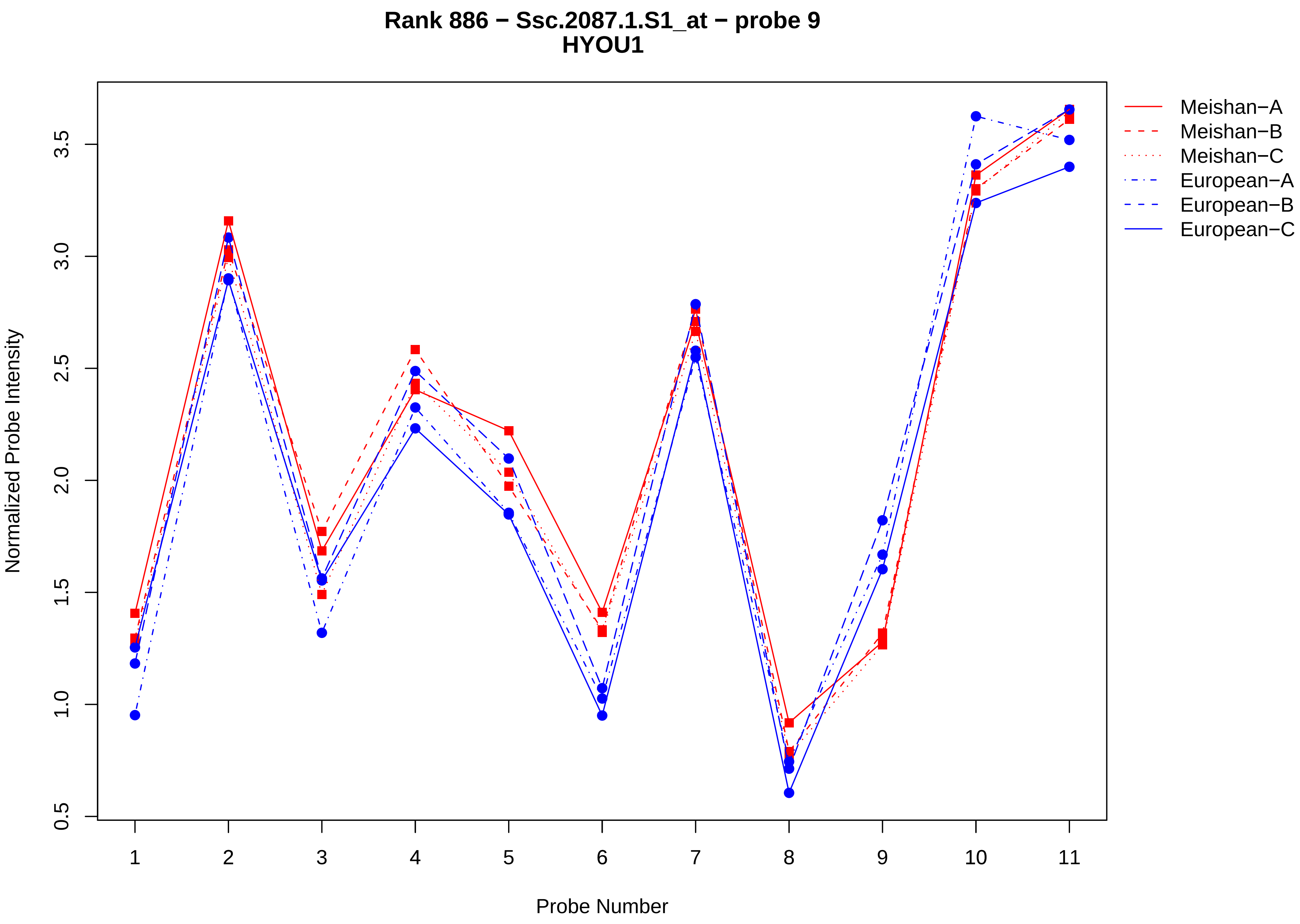

Rank 886 − Ssc.2087.1.S1_at − probe 9
HYOU1
Normalized Probe Intensity
Probe Number
0.5
1.0
1.5
2.0
2.5
3.0
3.5
1
2
3
4
5
6
7
8
9
10
11
Meishan−A
Meishan−B
Meishan−C
European−A
European−B
European−C

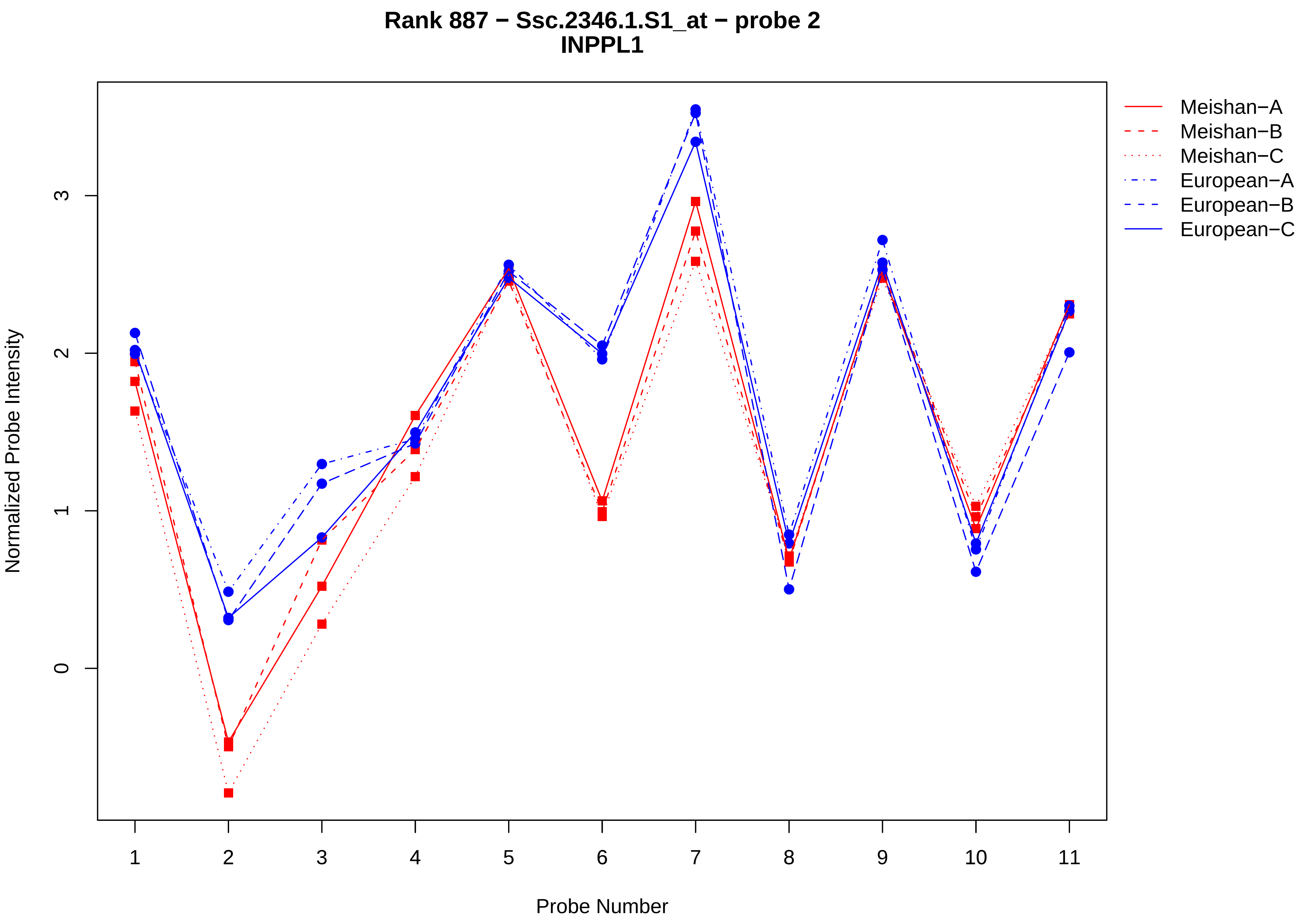

Rank 887 − Ssc.2346.1.S1_at − probe 2
INPPL1
Normalized Probe Intensity
Probe Number
0
1
2
3
1
2
3
4
5
6
7
8
9
10
11
Meishan−A
Meishan−B
Meishan−C
European−A
European−B
European−C

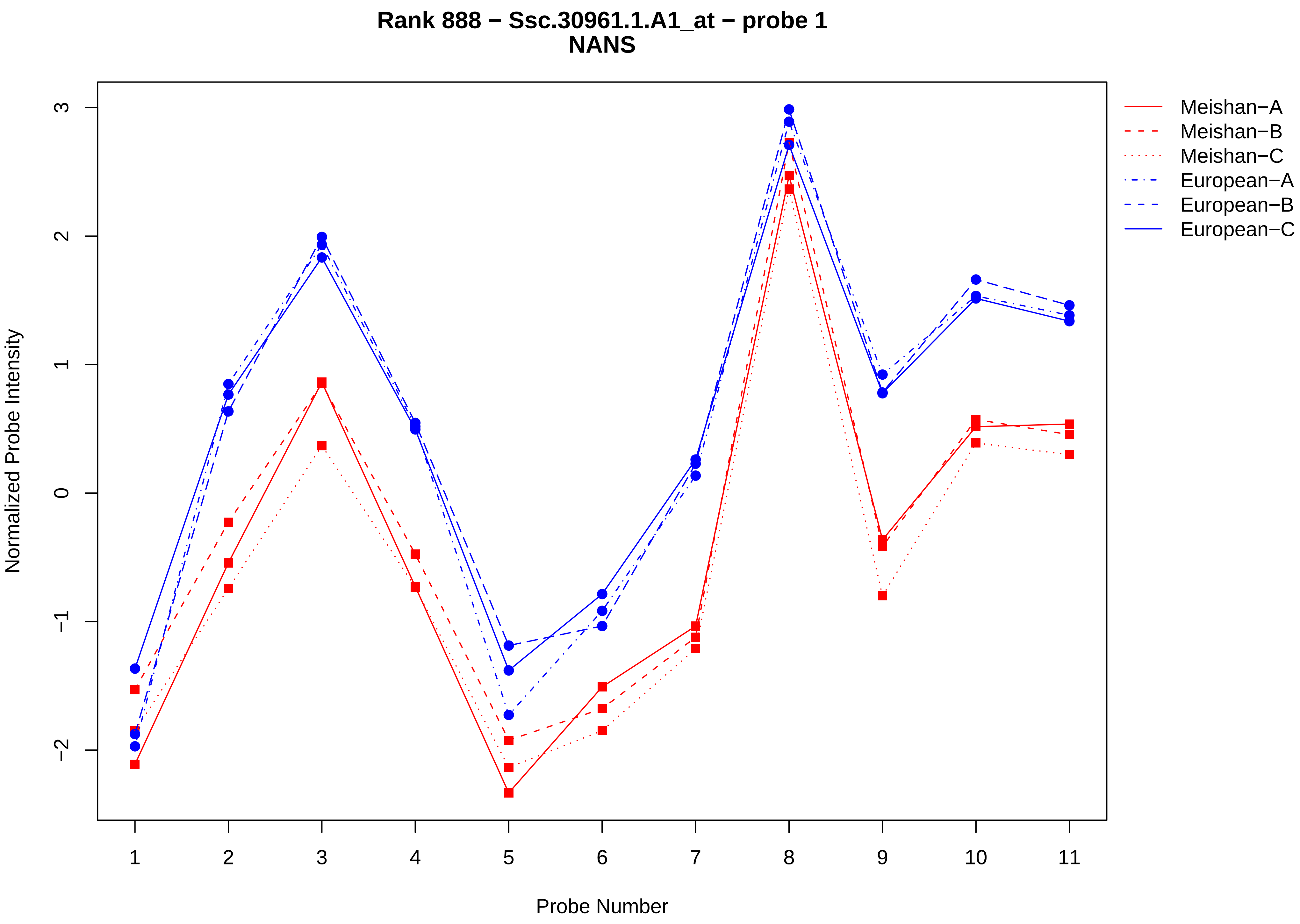

Rank 888 − Ssc.30961.1.A1_at − probe 1
NANS
Normalized Probe Intensity
Probe Number
Meishan−A
Meishan−B
Meishan−C
European−A
European−B
European−C

Rank 889 − Ssc.7190.1.S1_at − probe 1
BUB1B

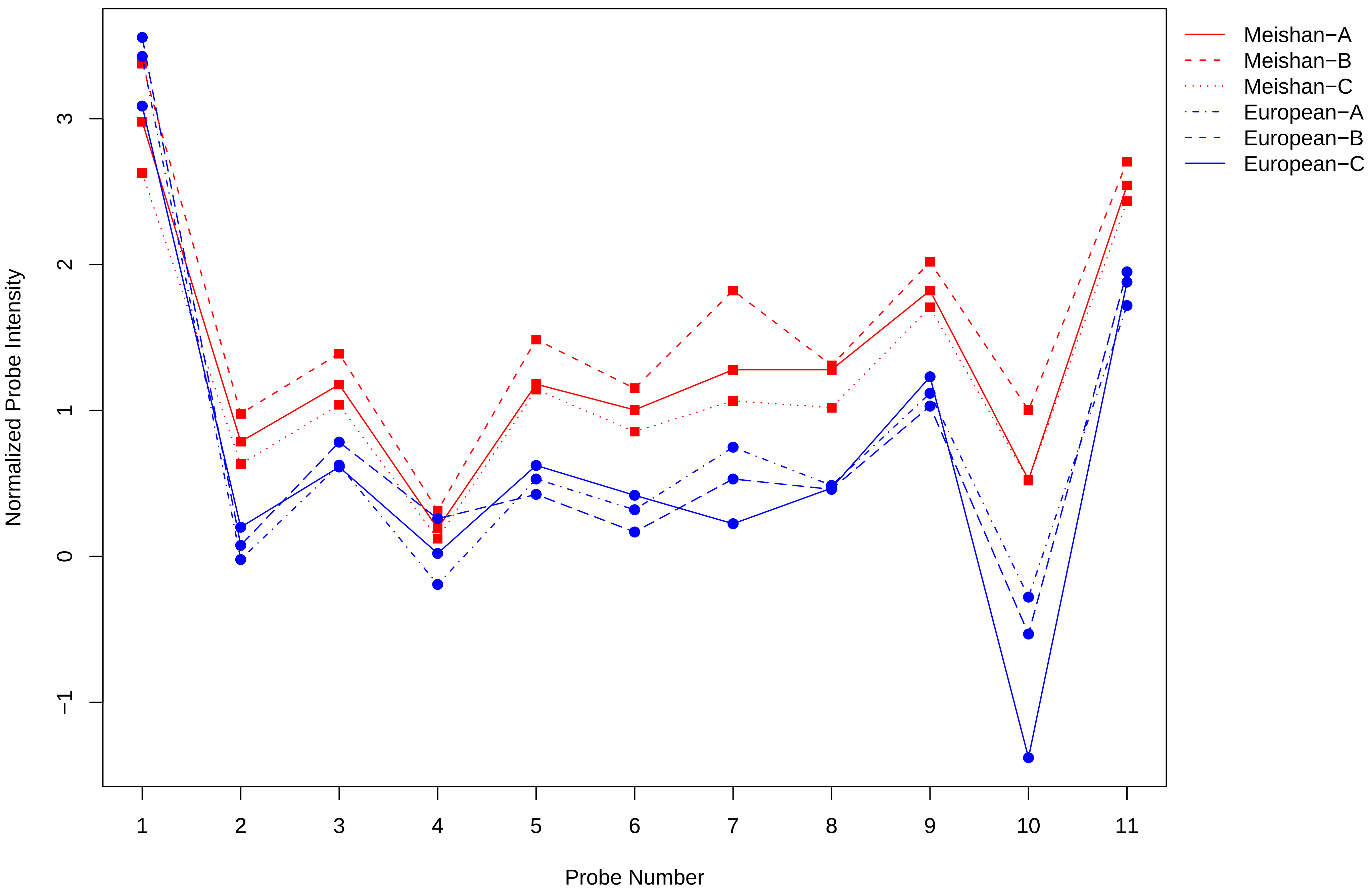

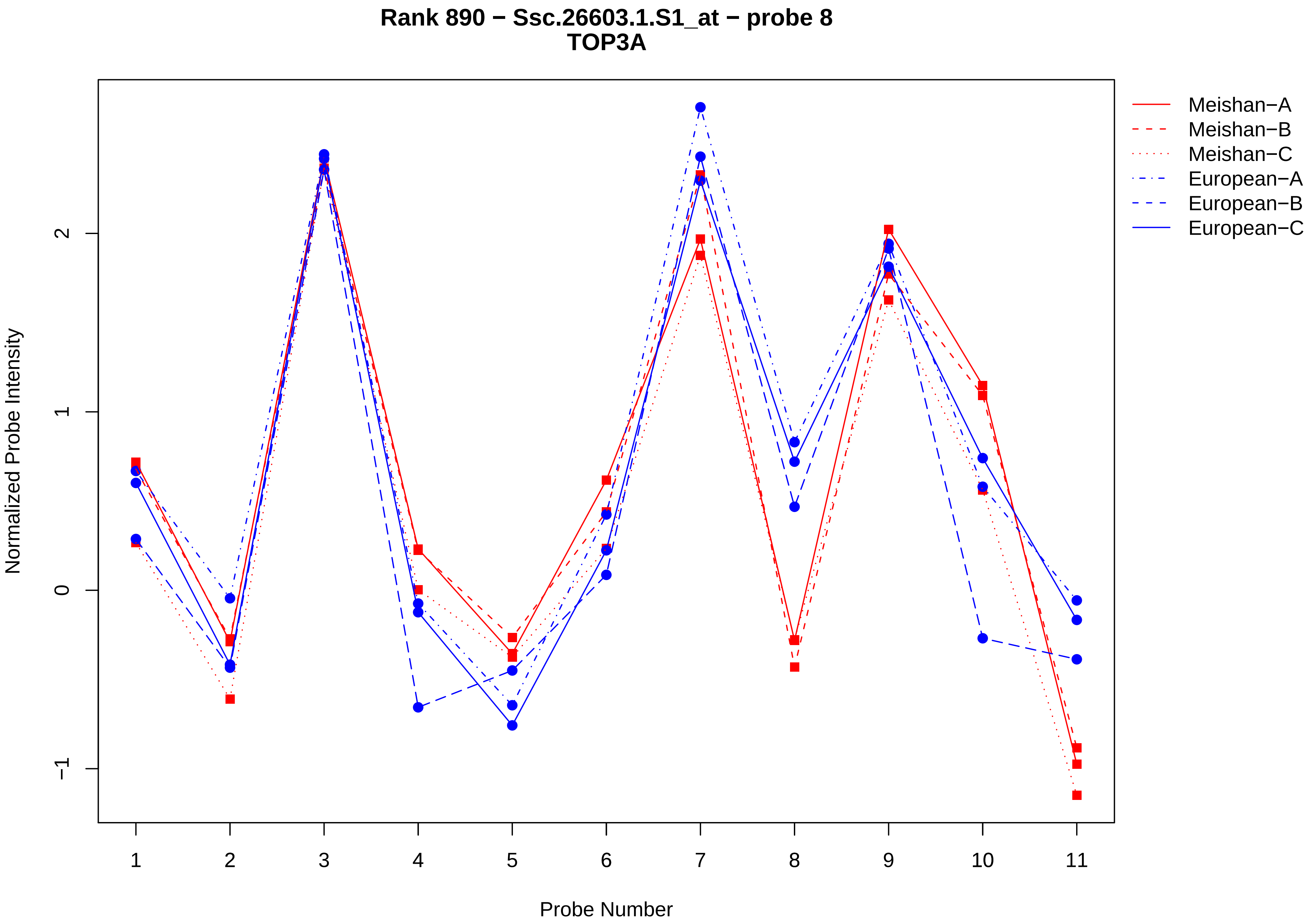
Rank 890 − Ssc.26603.1.S1_at − probe 8
TOP3A
Normalized Probe Intensity
Probe Number
−1
0
1
2
1
2
3
4
5
6
7
8
9
10
11
Meishan−A
Meishan−B
Meishan−C
European−A
European−B
European−C

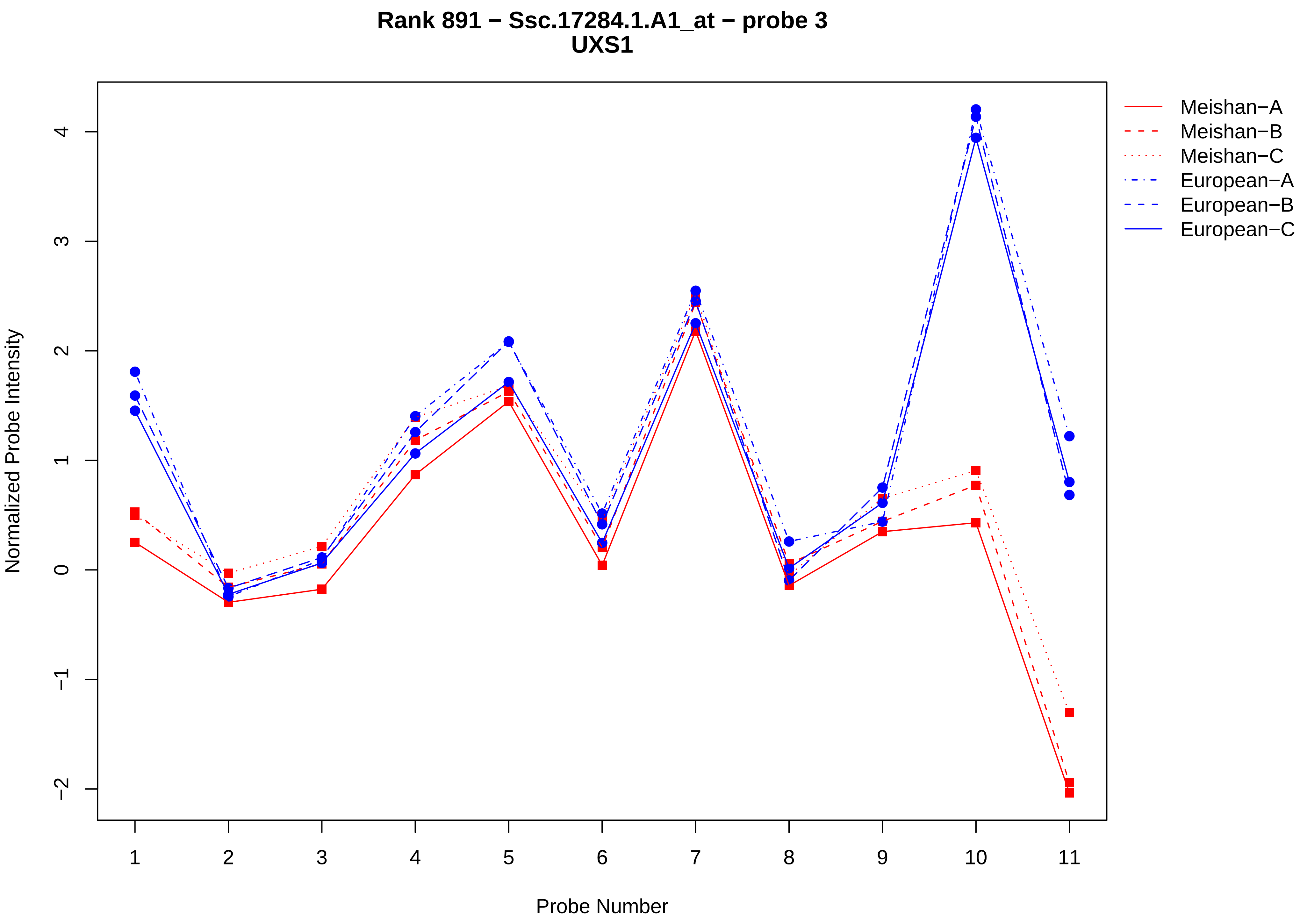
Rank 891 − Ssc.17284.1.A1_at − probe 3
UXS1
Normalized Probe Intensity
Probe Number
Meishan−A
Meishan−B
Meishan−C
European−A
European−B
European−C
−2
−1
0
1
2
3
4
1
2
3
4
5
6
7
8
9
10
11

Rank 892 − Ssc.17449.1.S1_at − probe 6
OLFM1

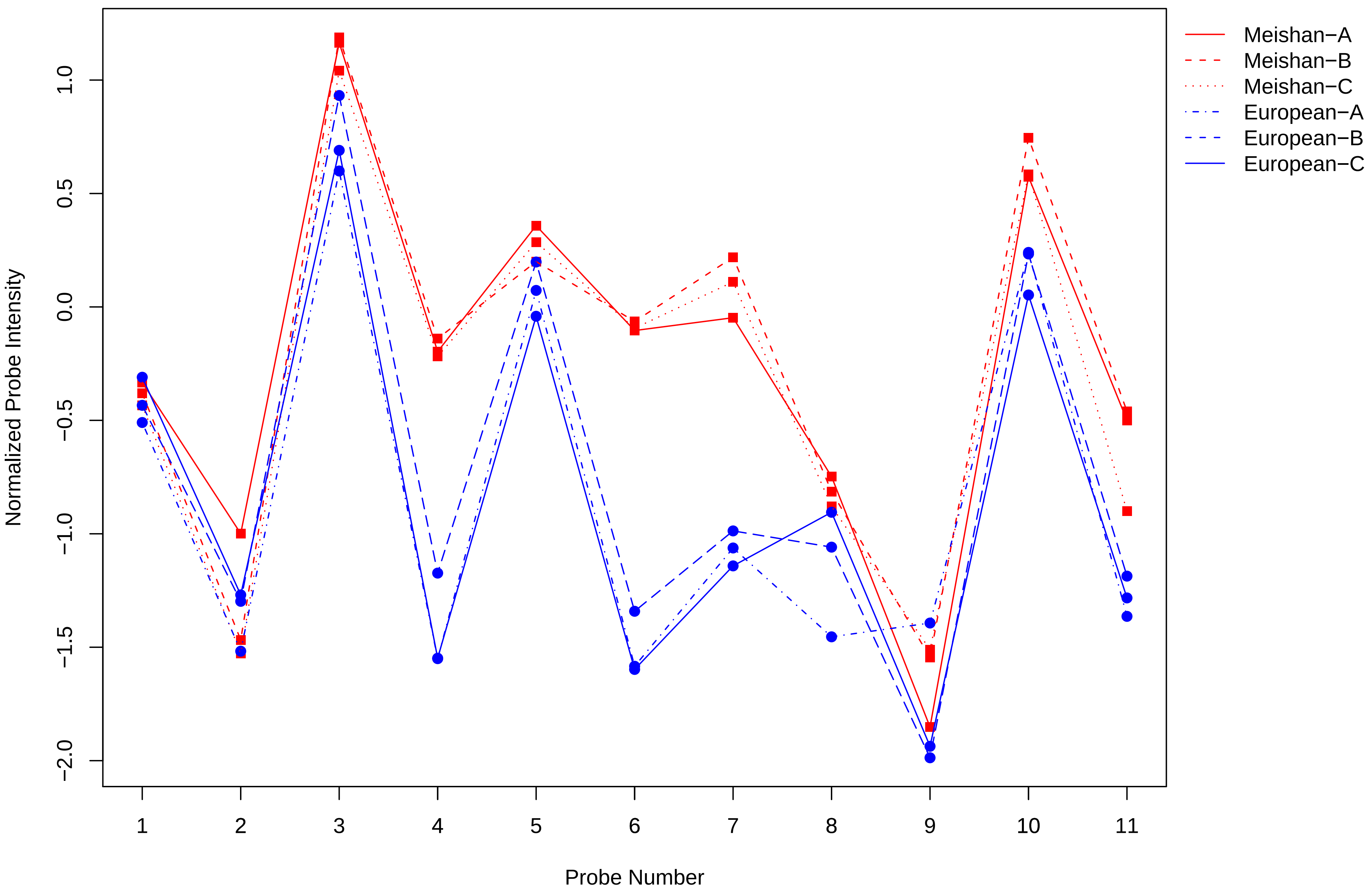

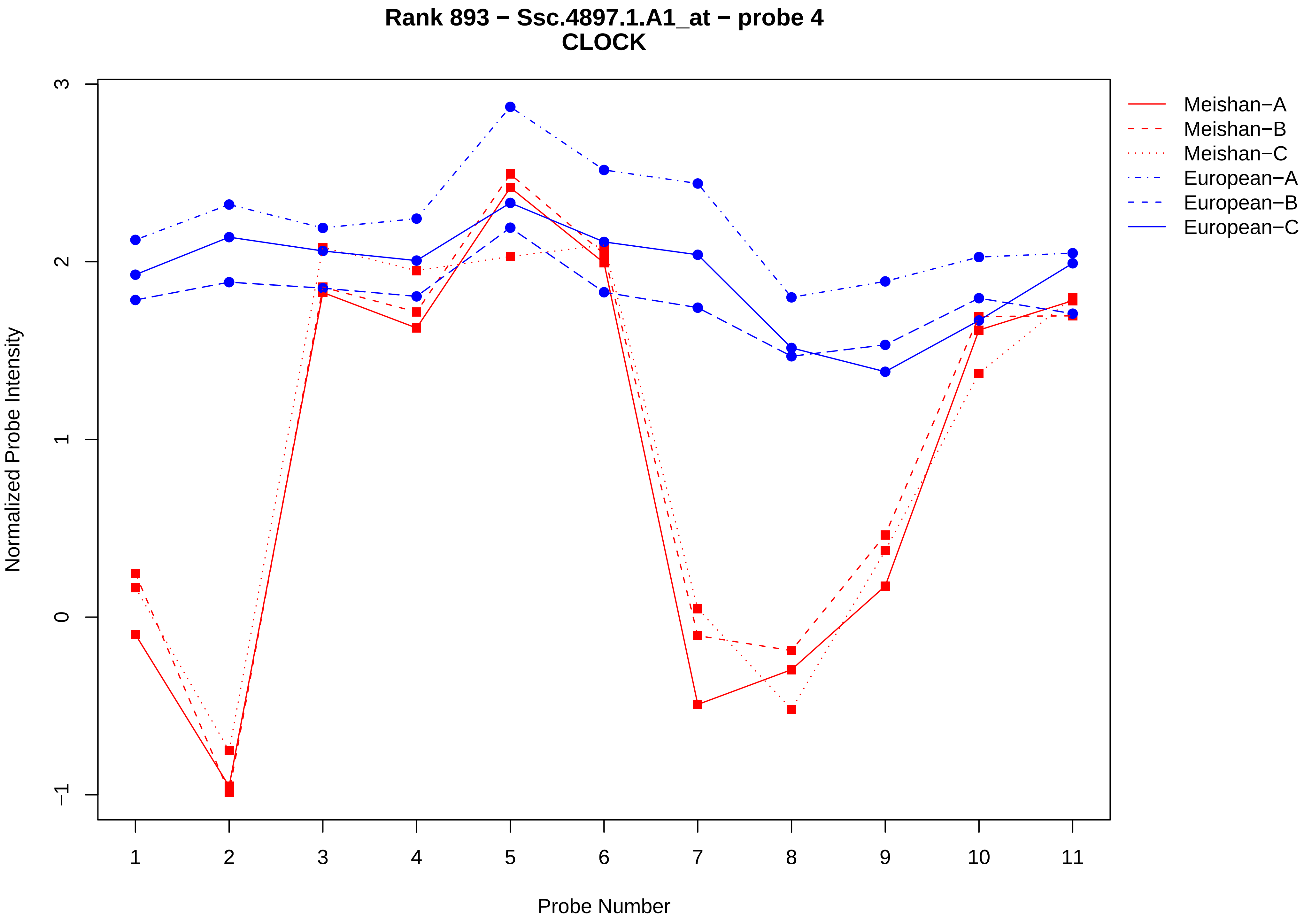
Rank 893 − Ssc.4897.1.A1_at − probe 4
CLOCK
Normalized Probe Intensity
Probe Number
−1
0
1
2
3
1
2
3
4
5
6
7
8
9
10
11
Meishan−A
Meishan−B
Meishan−C
European−A
European−B
European−C

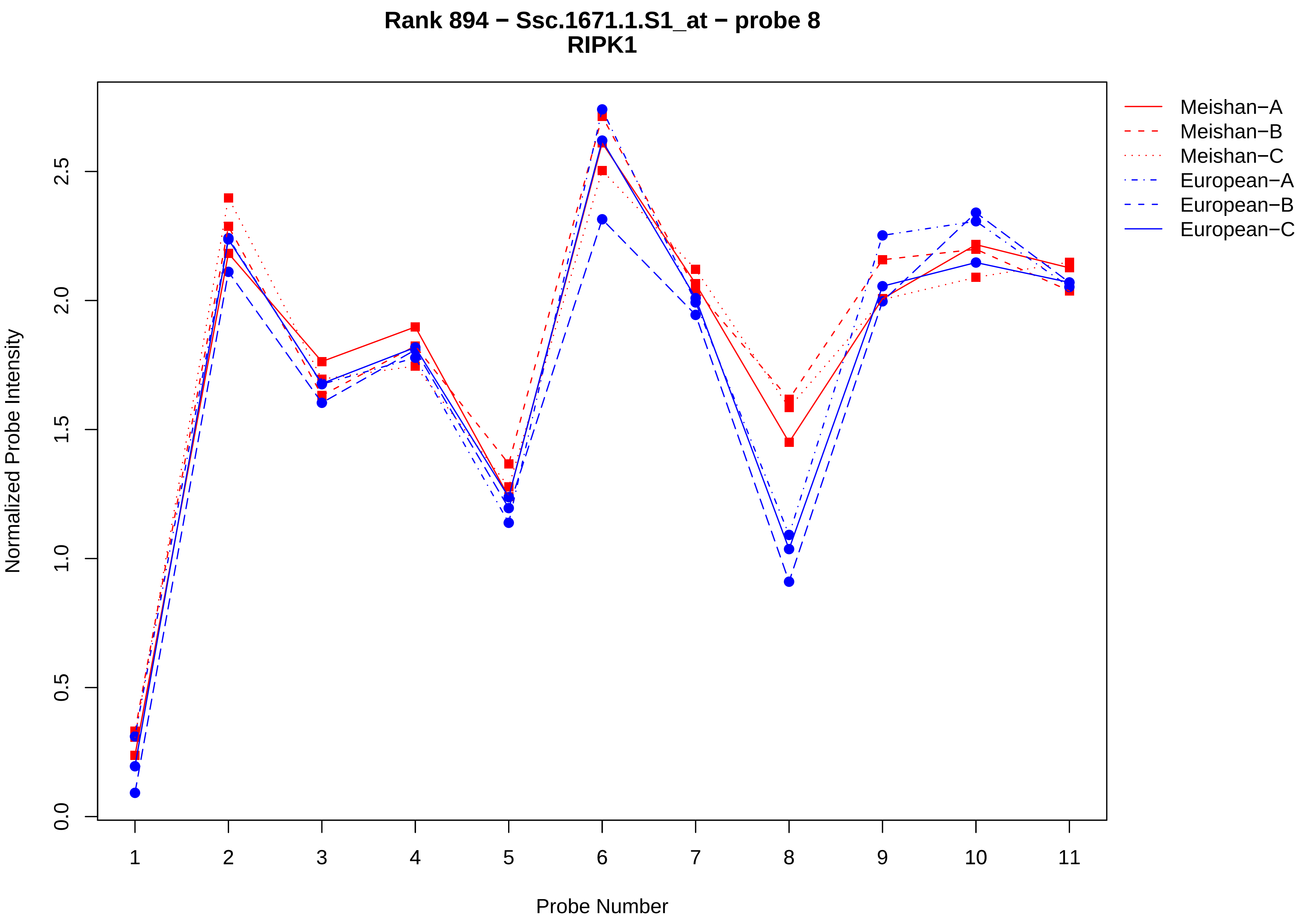

Rank 894 − Ssc.1671.1.S1_at − probe 8
RIPK1
Normalized Probe Intensity
0.0
0.5
1.0
1.5
2.0
2.5
1
2
3
4
5
6
7
8
9
10
11
Probe Number
Meishan−A
Meishan−B
Meishan−C
European−A
European−B
European−C

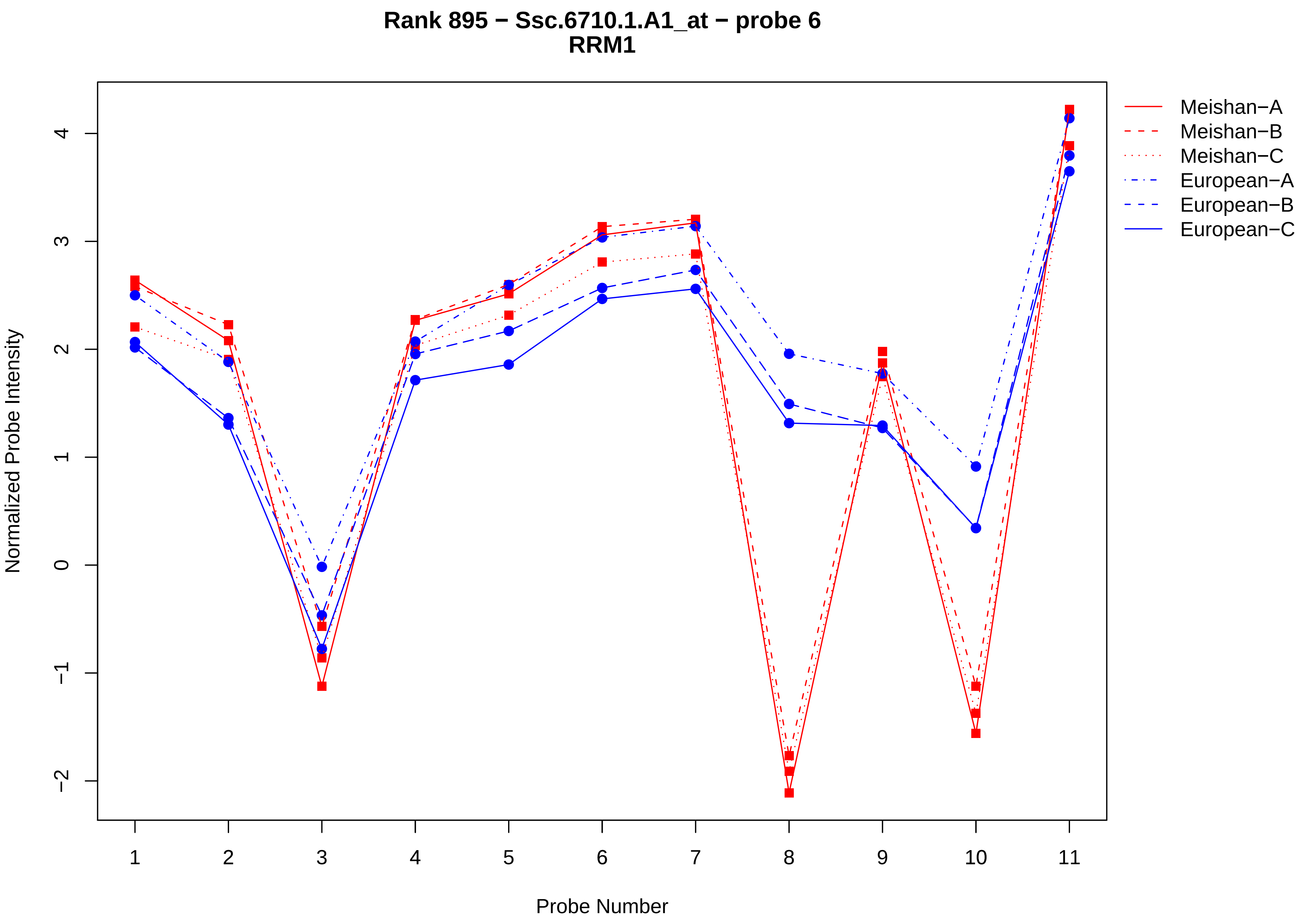

Rank 895 − Ssc.6710.1.A1_at − probe 6
RRM1
Normalized Probe Intensity
Probe Number
Meishan−A
Meishan−B
Meishan−C
European−A
European−B
European−C
−2
−1
0
1
2
3
4
1
2
3
4
5
6
7
8
9
10
11

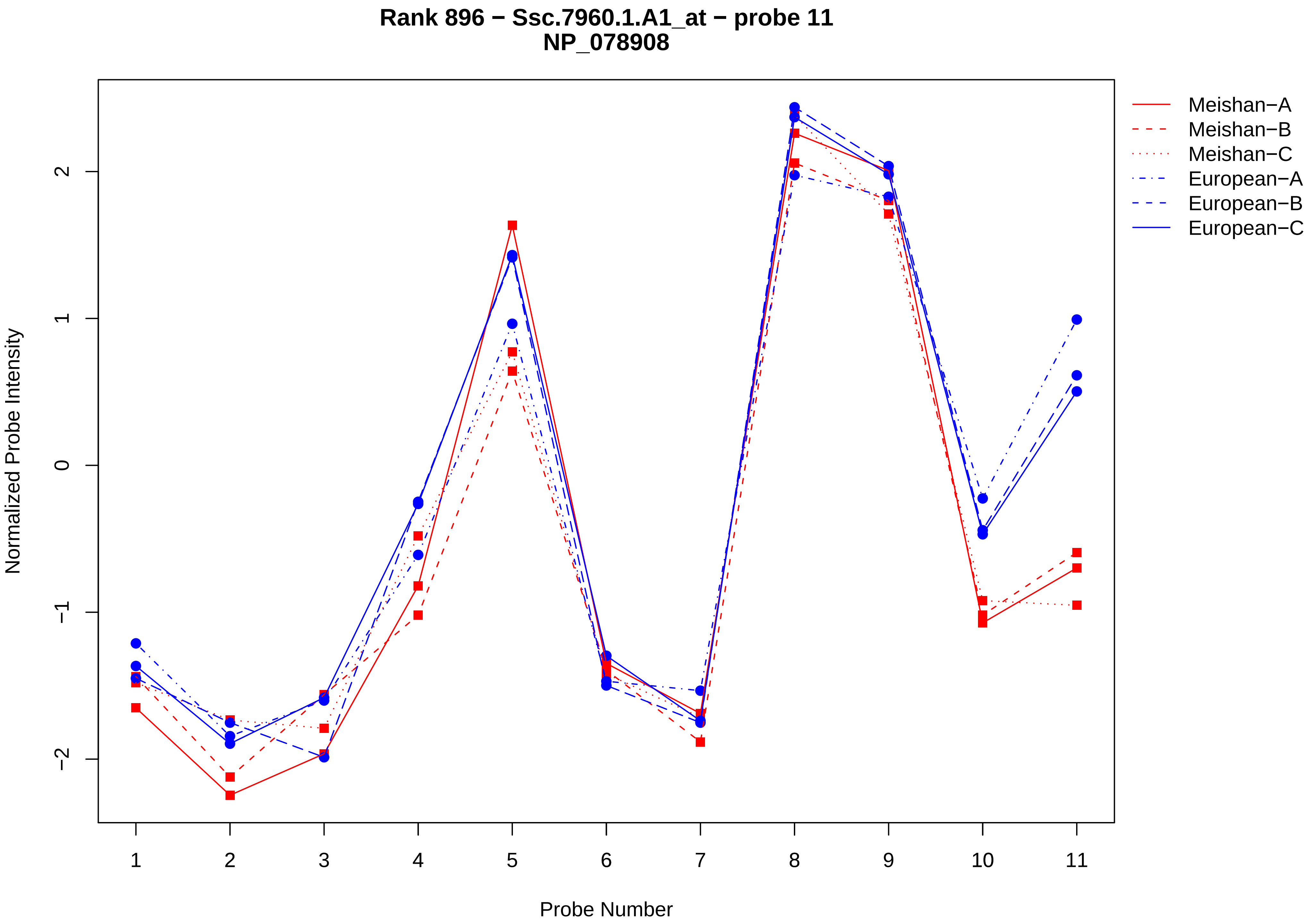

Rank 896 − Ssc.7960.1.A1_at − probe 11
NP_078908
Normalized Probe Intensity
Probe Number
−2
−1
0
1
2
1
2
3
4
5
6
7
8
9
10
11
Meishan−A
Meishan−B
Meishan−C
European−A
European−B
European−C

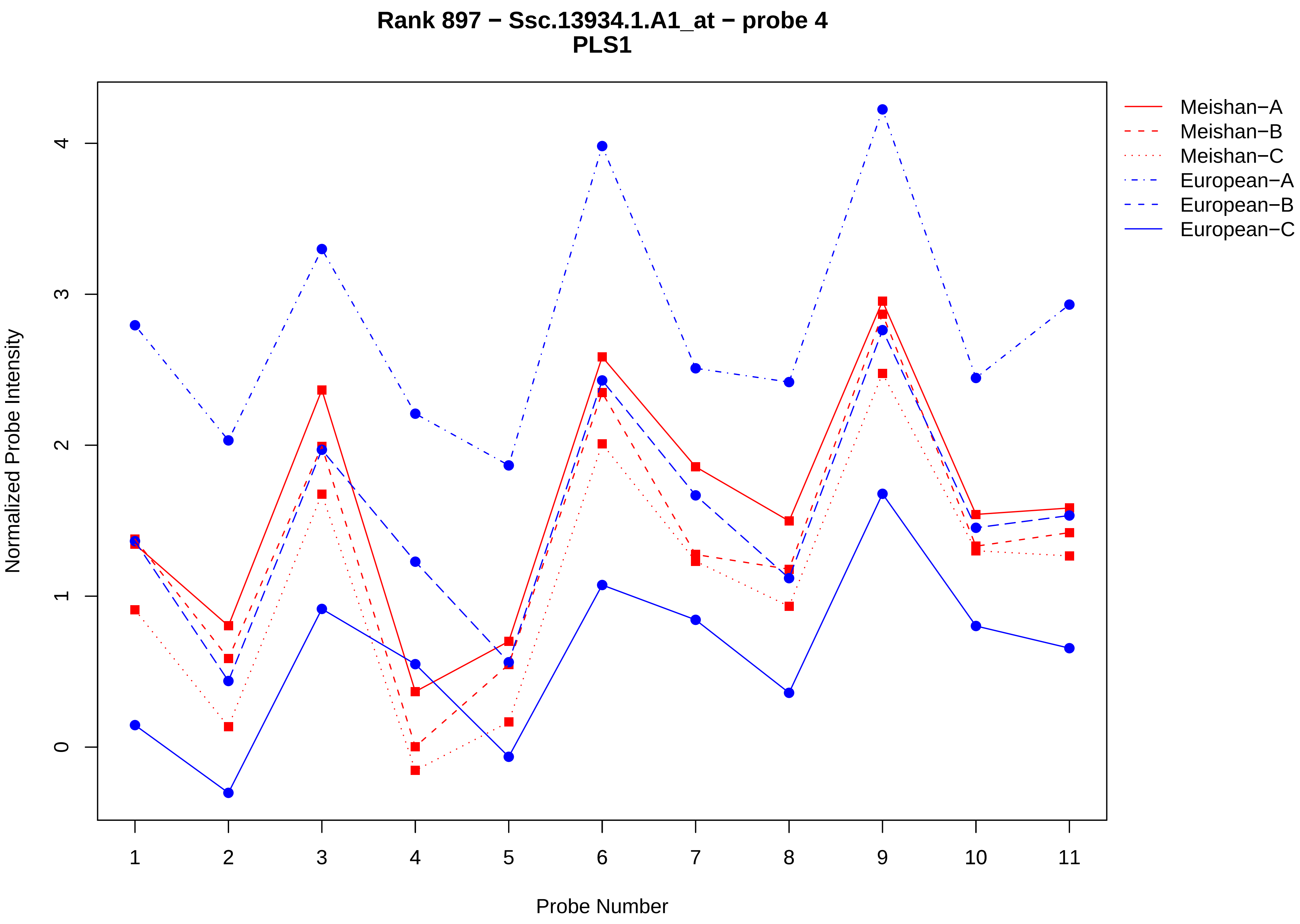

Rank 897 − Ssc.13934.1.A1_at − probe 4
PLS1
Normalized Probe Intensity
Probe Number
0
1
2
3
4
1
2
3
4
5
6
7
8
9
10
11
Meishan−A
Meishan−B
Meishan−C
European−A
European−B
European−C

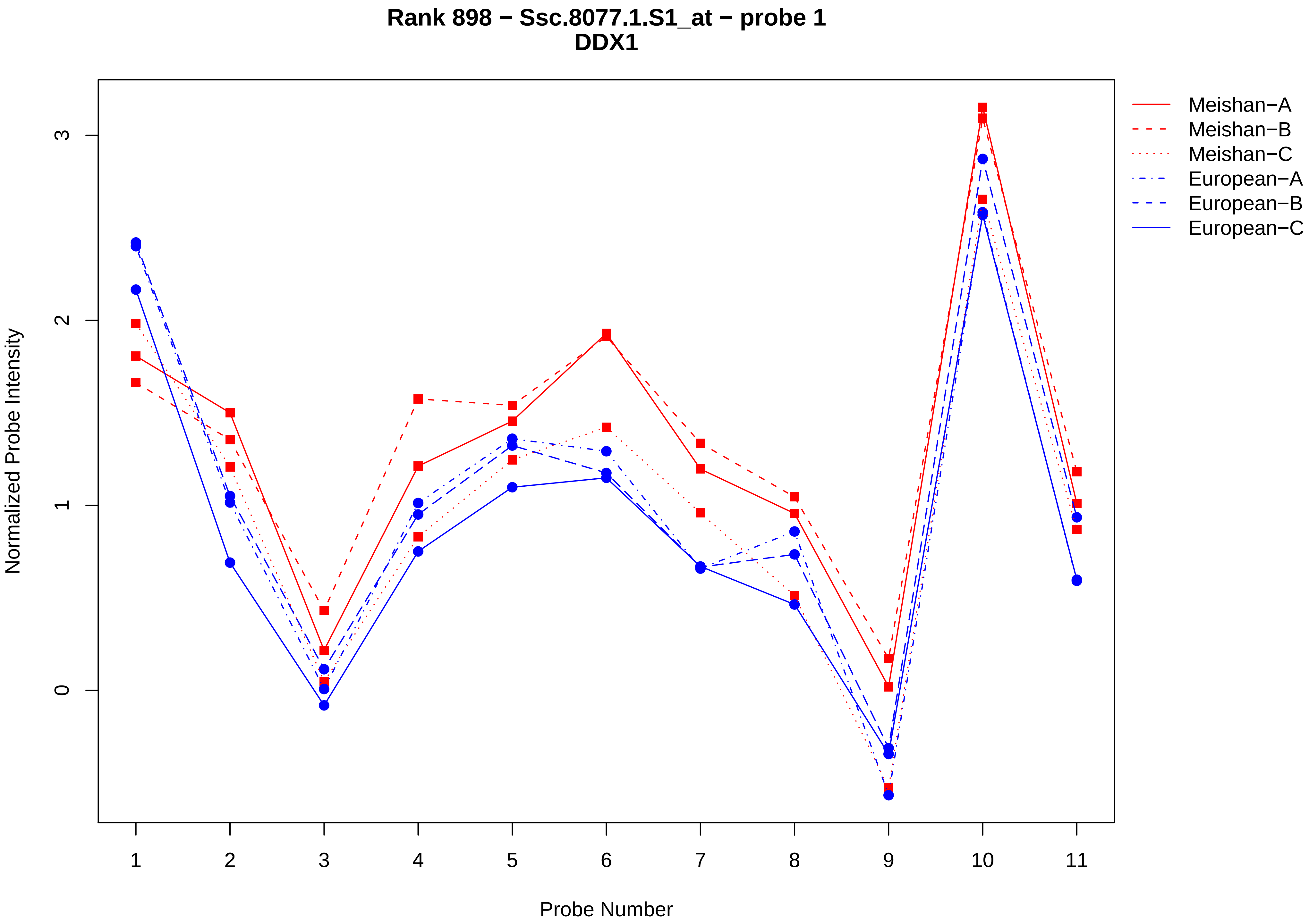

Rank 898 − Ssc.8077.1.S1_at − probe 1
DDX1
Normalized Probe Intensity
Probe Number
0
1
2
3
1
2
3
4
5
6
7
8
9
10
11
Meishan−A
Meishan−B
Meishan−C
European−A
European−B
European−C

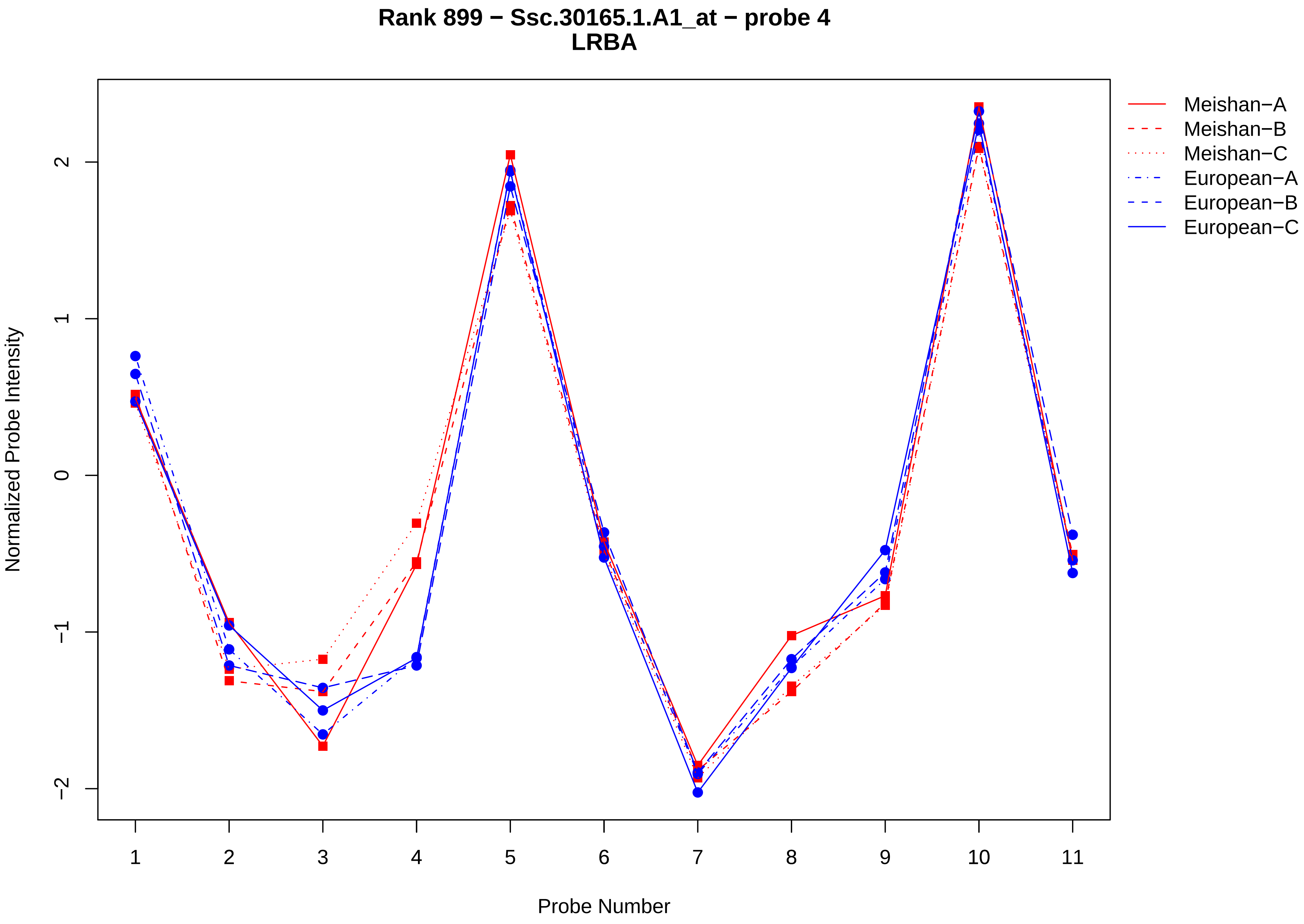

Rank 899 − Ssc.30165.1.A1_at − probe 4
LRBA
Normalized Probe Intensity
Probe Number
−2
−1
0
1
2
1
2
3
4
5
6
7
8
9
10
11
Meishan−A
Meishan−B
Meishan−C
European−A
European−B
European−C

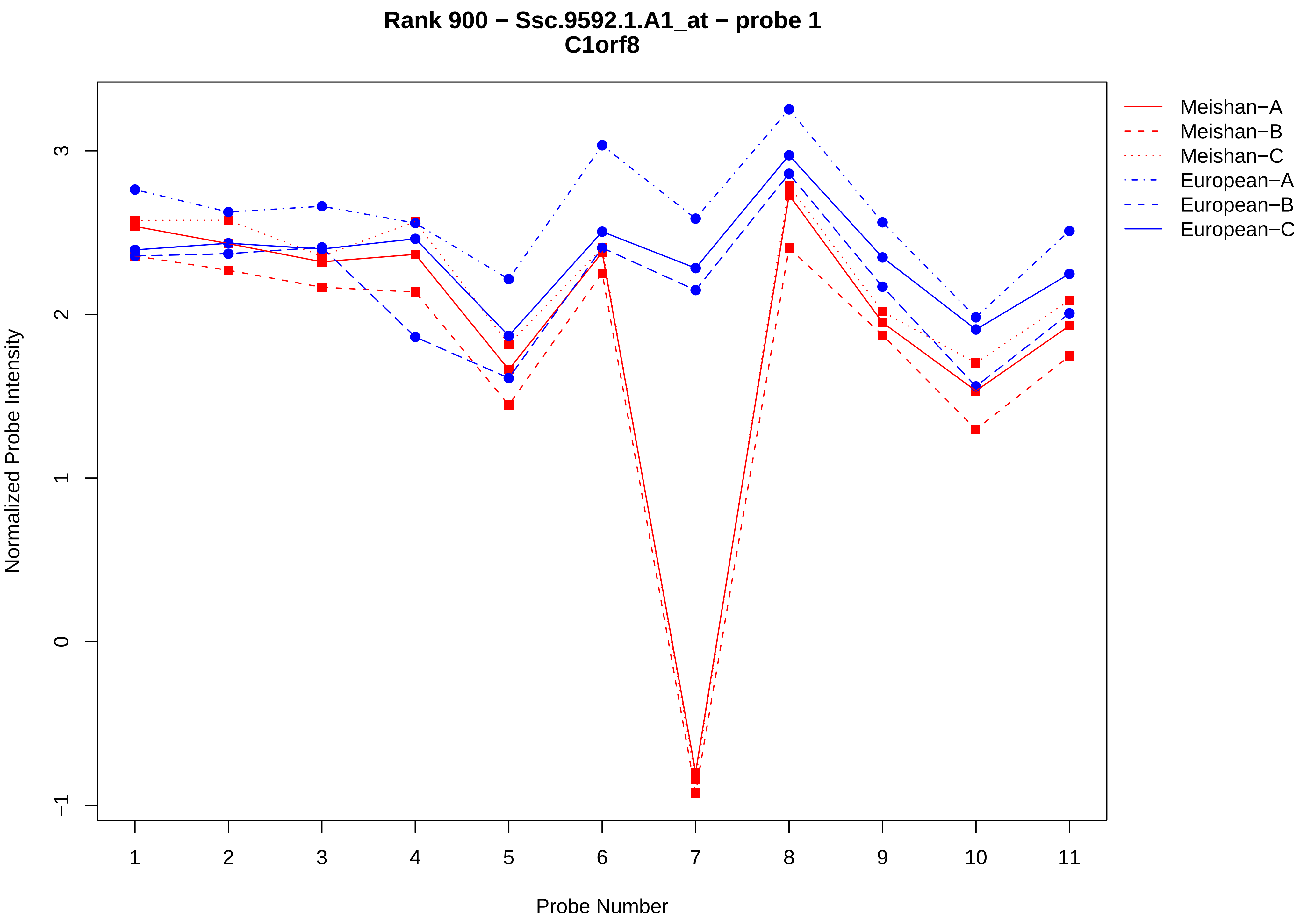

Rank 900 − Ssc.9592.1.A1_at − probe 1
C1orf8
Normalized Probe Intensity
Probe Number
Meishan−A
Meishan−B
Meishan−C
European−A
European−B
European−C

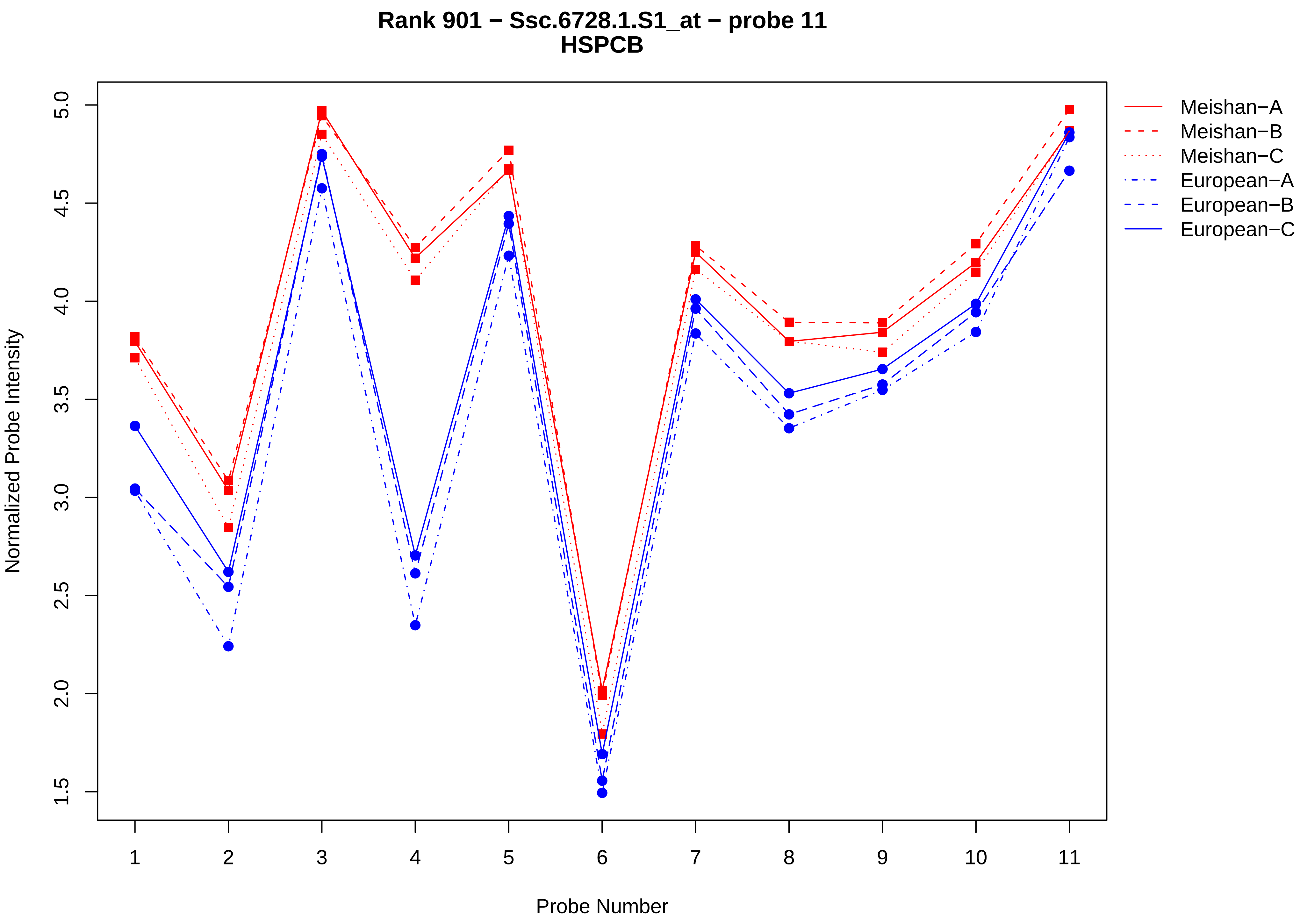
Rank 901 − Ssc.6728.1.S1_at − probe 11
HSPCB
Normalized Probe Intensity
Probe Number
Meishan−A
Meishan−B
Meishan−C
European−A
European−B
European−C
1.5
2.0
2.5
3.0
3.5
4.0
4.5
5.0
1
2
3
4
5
6
7
8
9
10
11

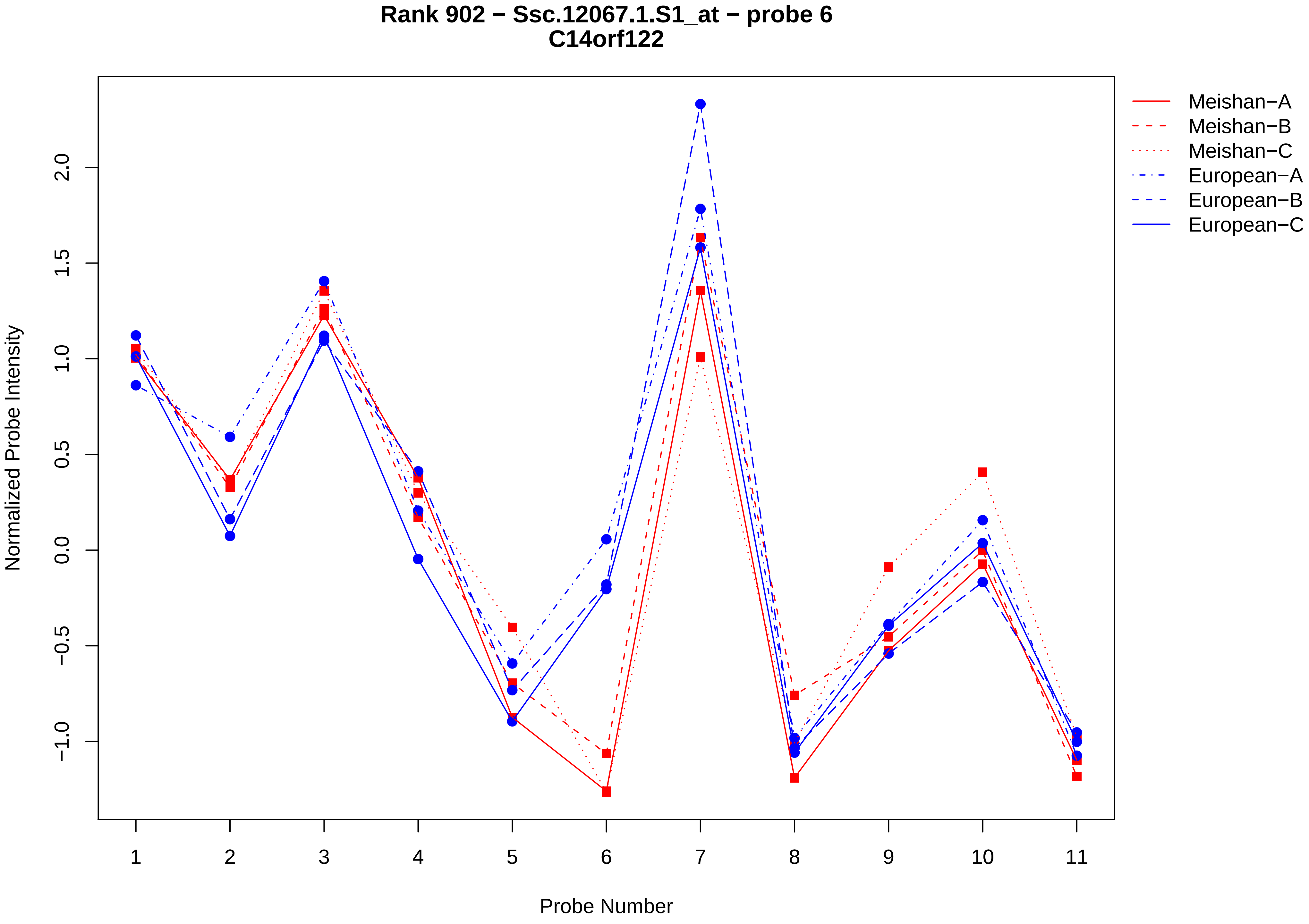

Rank 902 − Ssc.12067.1.S1_at − probe 6
C14orf122
Normalized Probe Intensity
Probe Number
−1.0
−0.5
0.0
0.5
1.0
1.5
2.0
1
2
3
4
5
6
7
8
9
10
11
Meishan−A
Meishan−B
Meishan−C
European−A
European−B
European−C

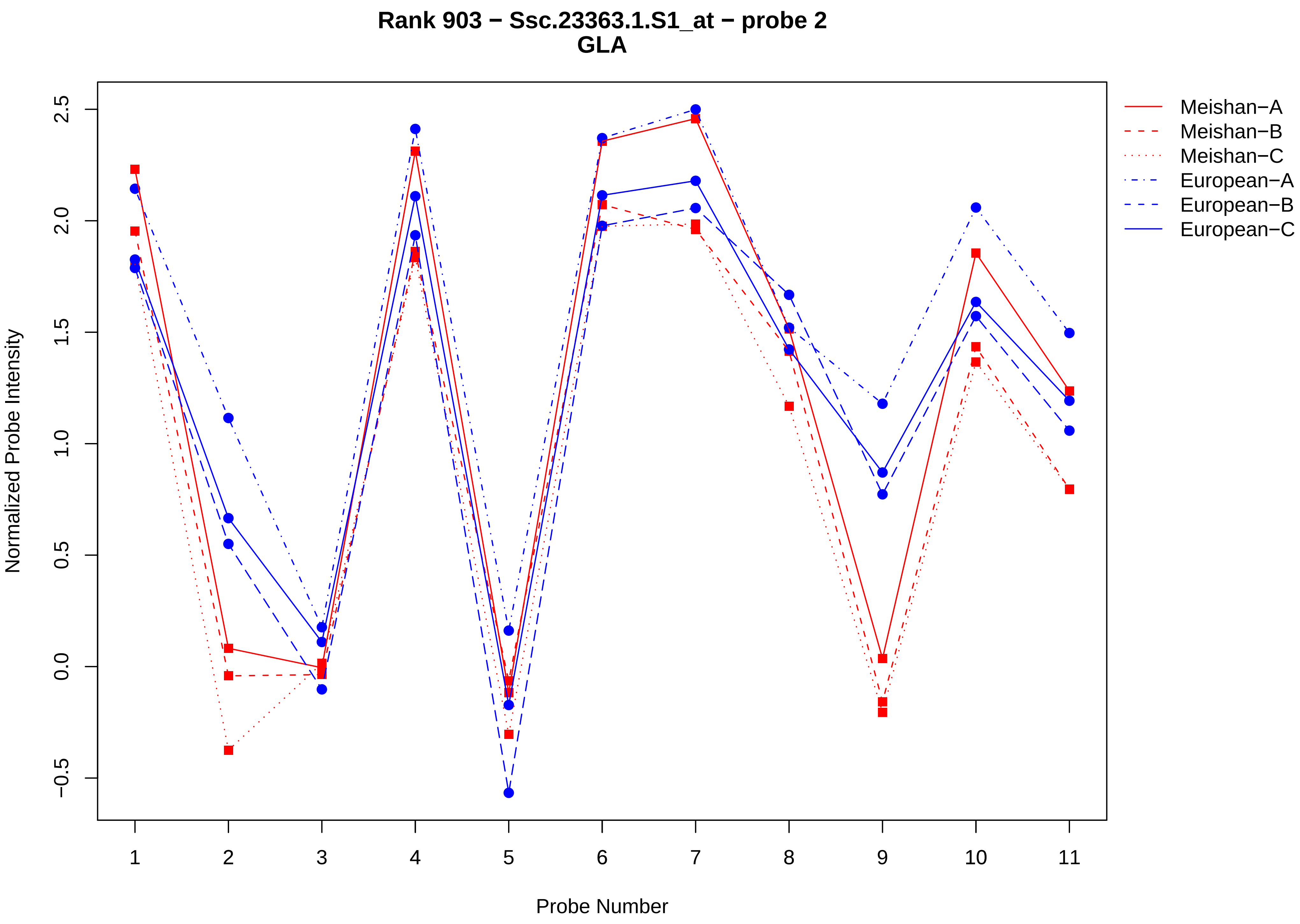

Rank 903 − Ssc.23363.1.S1_at − probe 2
GLA
Normalized Probe Intensity
Probe Number
−0.5
0.0
0.5
1.0
1.5
2.0
2.5
1
2
3
4
5
6
7
8
9
10
11
Meishan−A
Meishan−B
Meishan−C
European−A
European−B
European−C

Rank 904 − Ssc.16980.1.S1_at − probe 5
SLC35B1

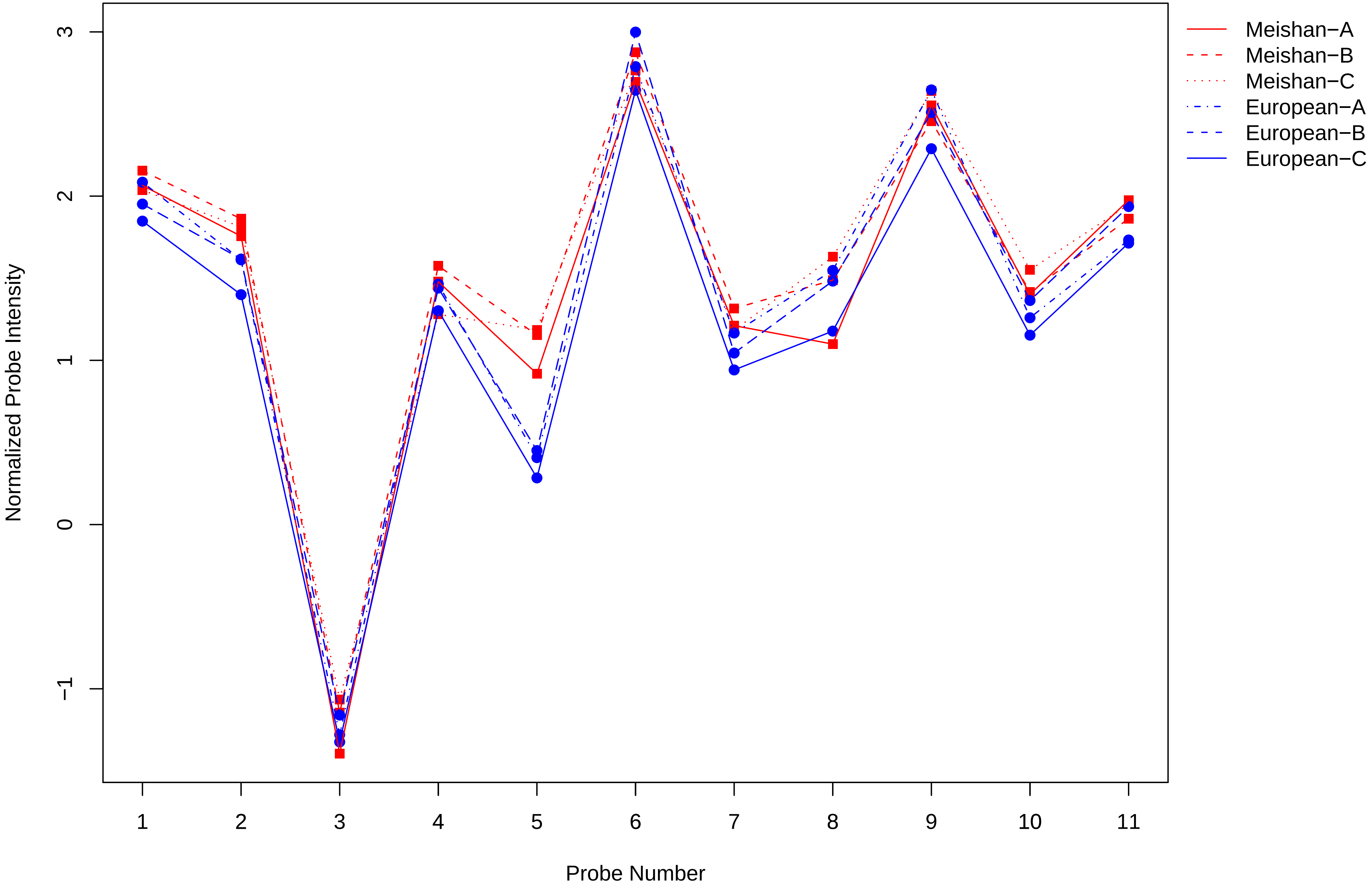

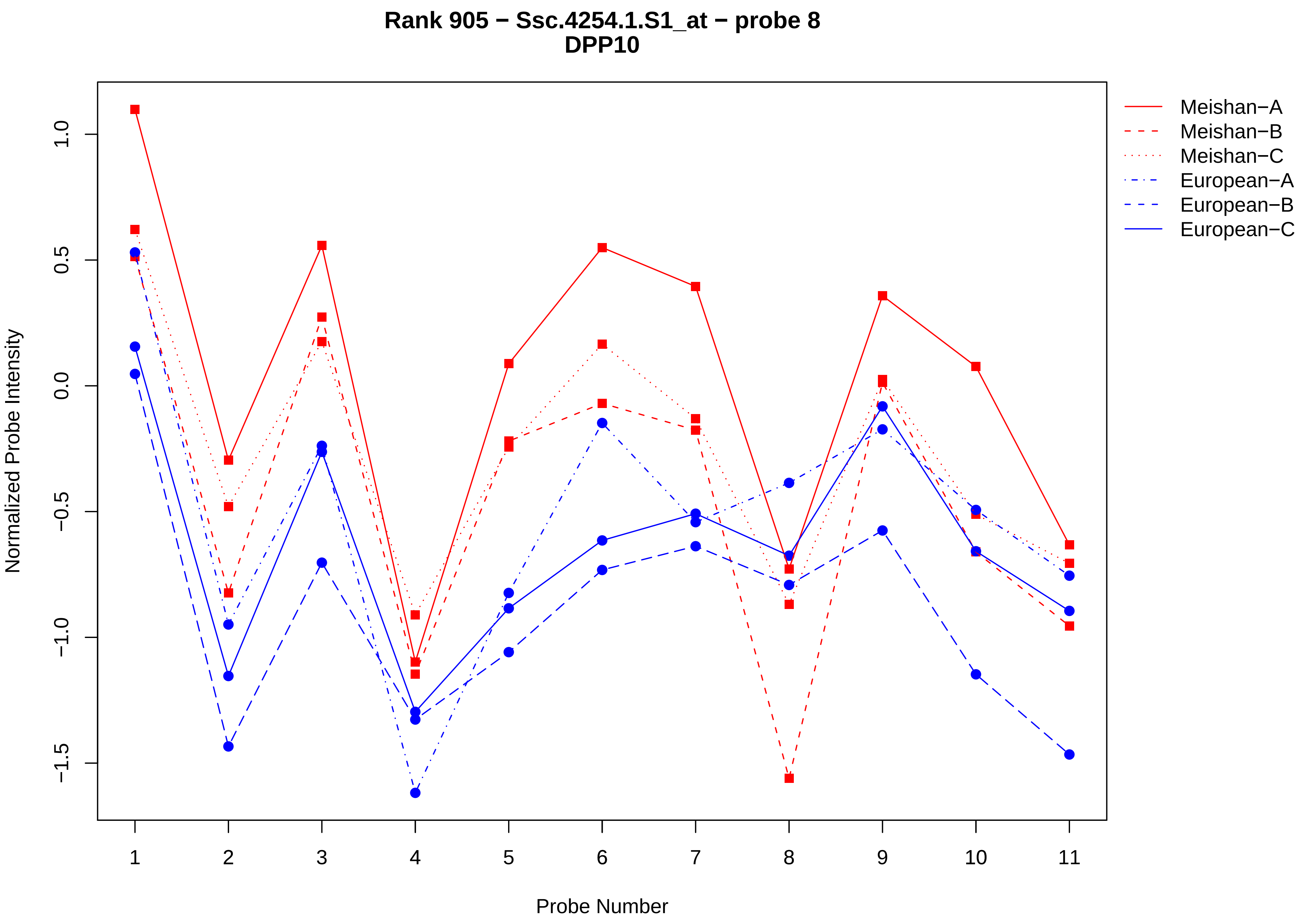

Rank 905 − Ssc.4254.1.S1_at − probe 8
DPP10
Normalized Probe Intensity
Probe Number
1.0
0.5
0.0
−0.5
−1.0
−1.5
1
2
3
4
5
6
7
8
9
10
11
Meishan−A
Meishan−B
Meishan−C
European−A
European−B
European−C

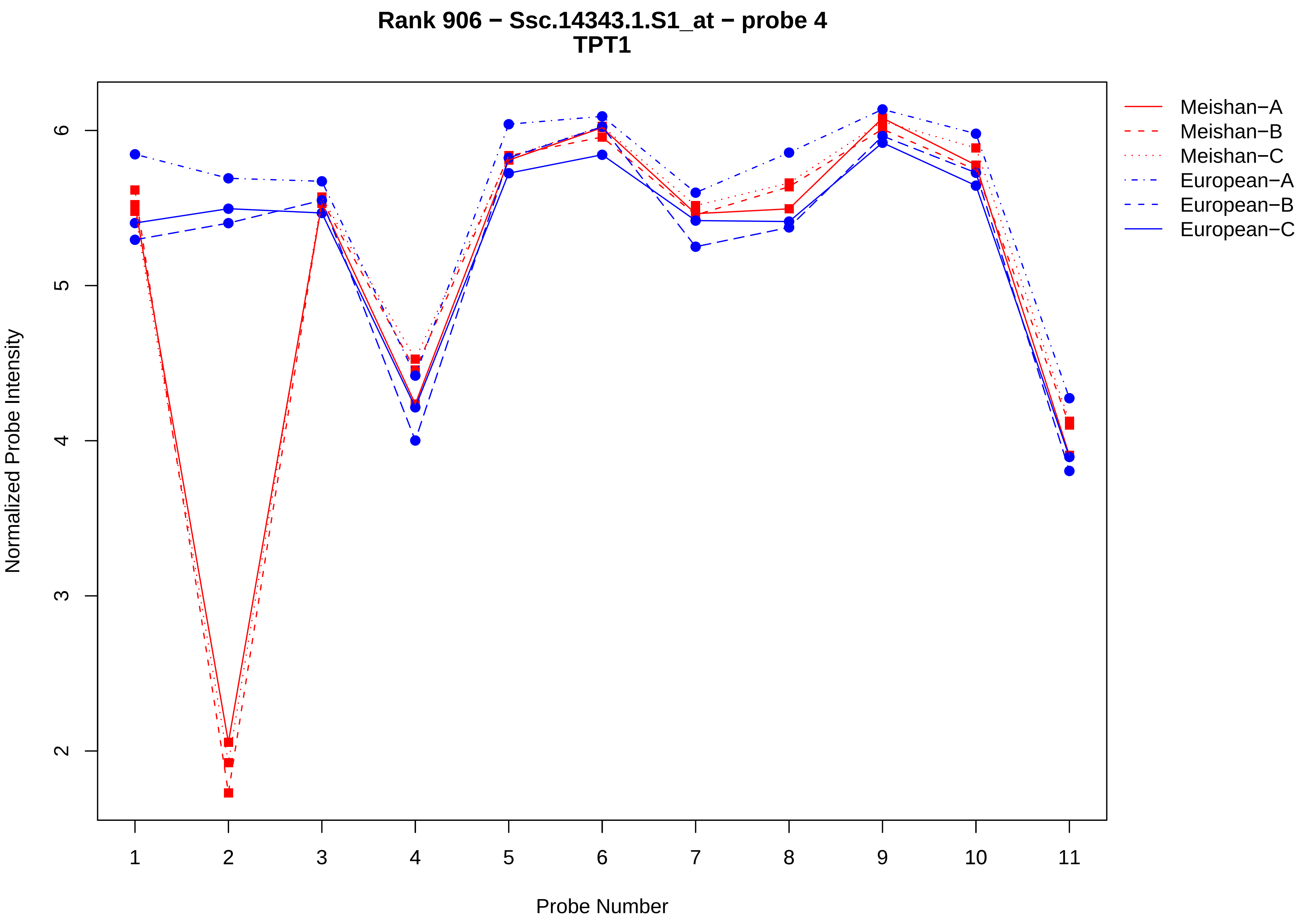
Rank 906 − Ssc.14343.1.S1_at − probe 4
TPT1
Normalized Probe Intensity
Probe Number
2
3
4
5
6
1
2
3
4
5
6
7
8
9
10
11
Meishan−A
Meishan−B
Meishan−C
European−A
European−B
European−C

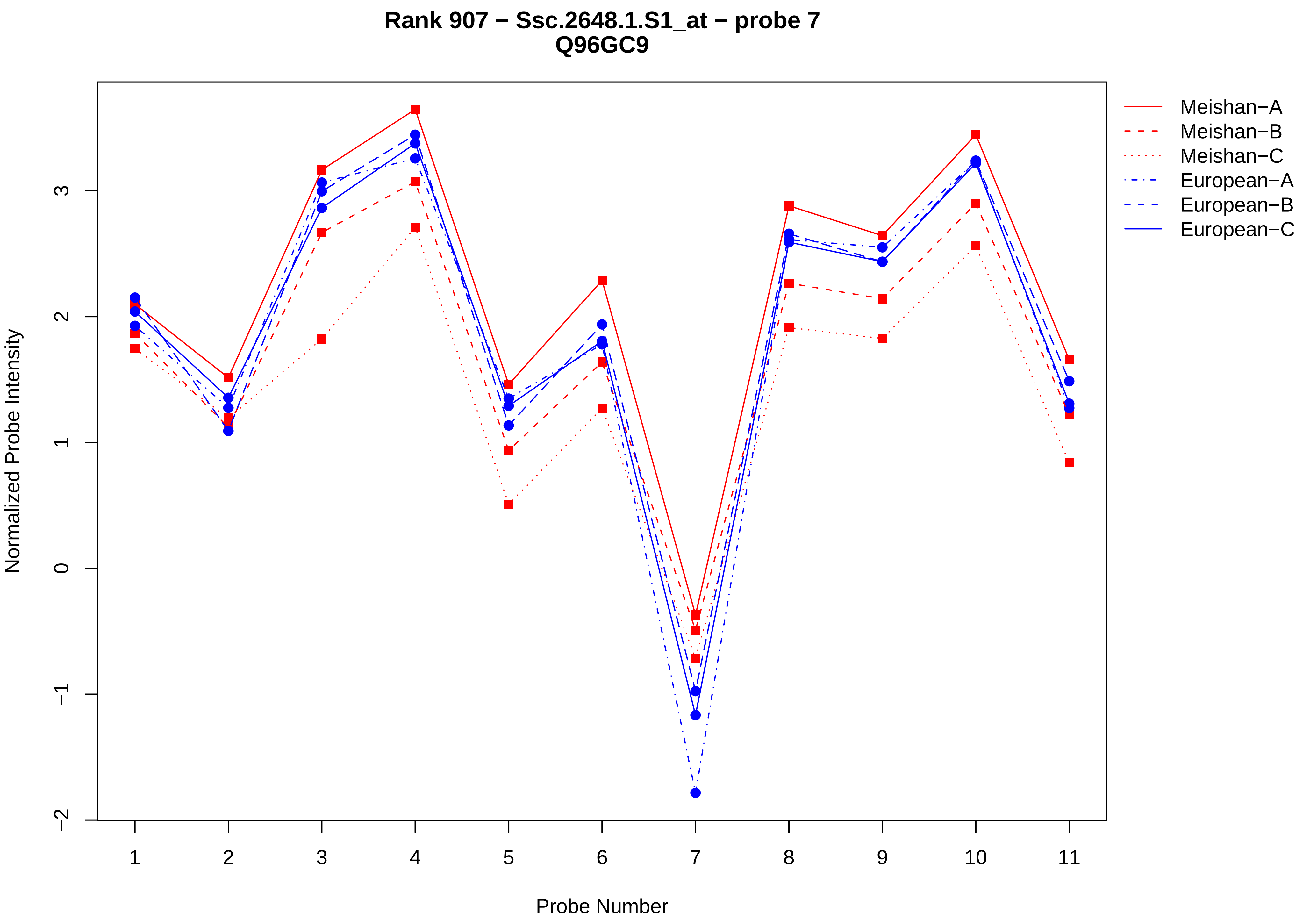

Rank 907 − Ssc.2648.1.S1_at − probe 7
Q96GC9
Normalized Probe Intensity
Probe Number
−2
−1
0
1
2
3
1
2
3
4
5
6
7
8
9
10
11
Meishan−A
Meishan−B
Meishan−C
European−A
European−B
European−C

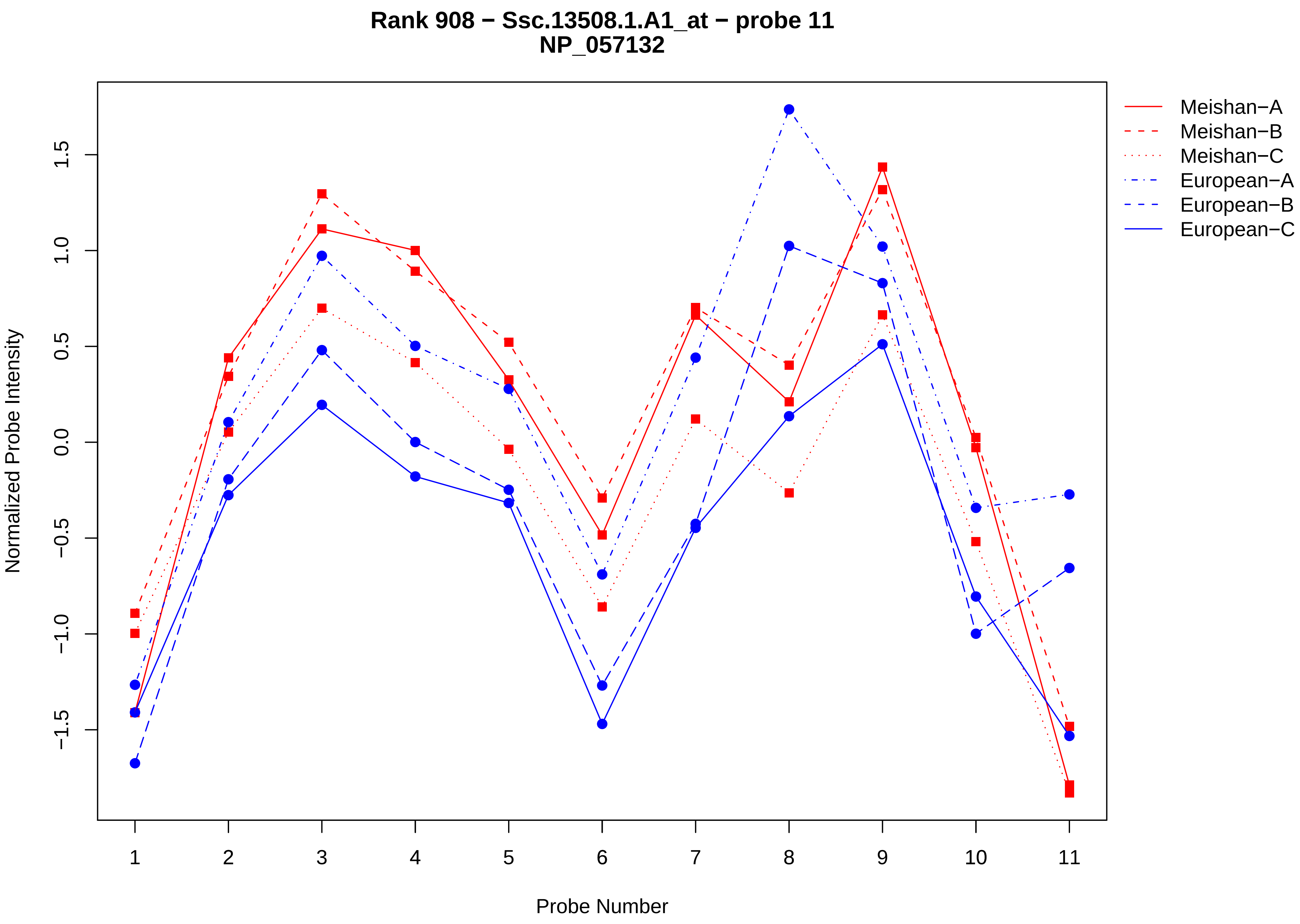
Rank 908 − Ssc.13508.1.A1_at − probe 11
NP_057132
Normalized Probe Intensity
Probe Number
1.5
1.0
0.5
0.0
−0.5
−1.0
−1.5
1
2
3
4
5
6
7
8
9
10
11
Meishan−A
Meishan−B
Meishan−C
European−A
European−B
European−C

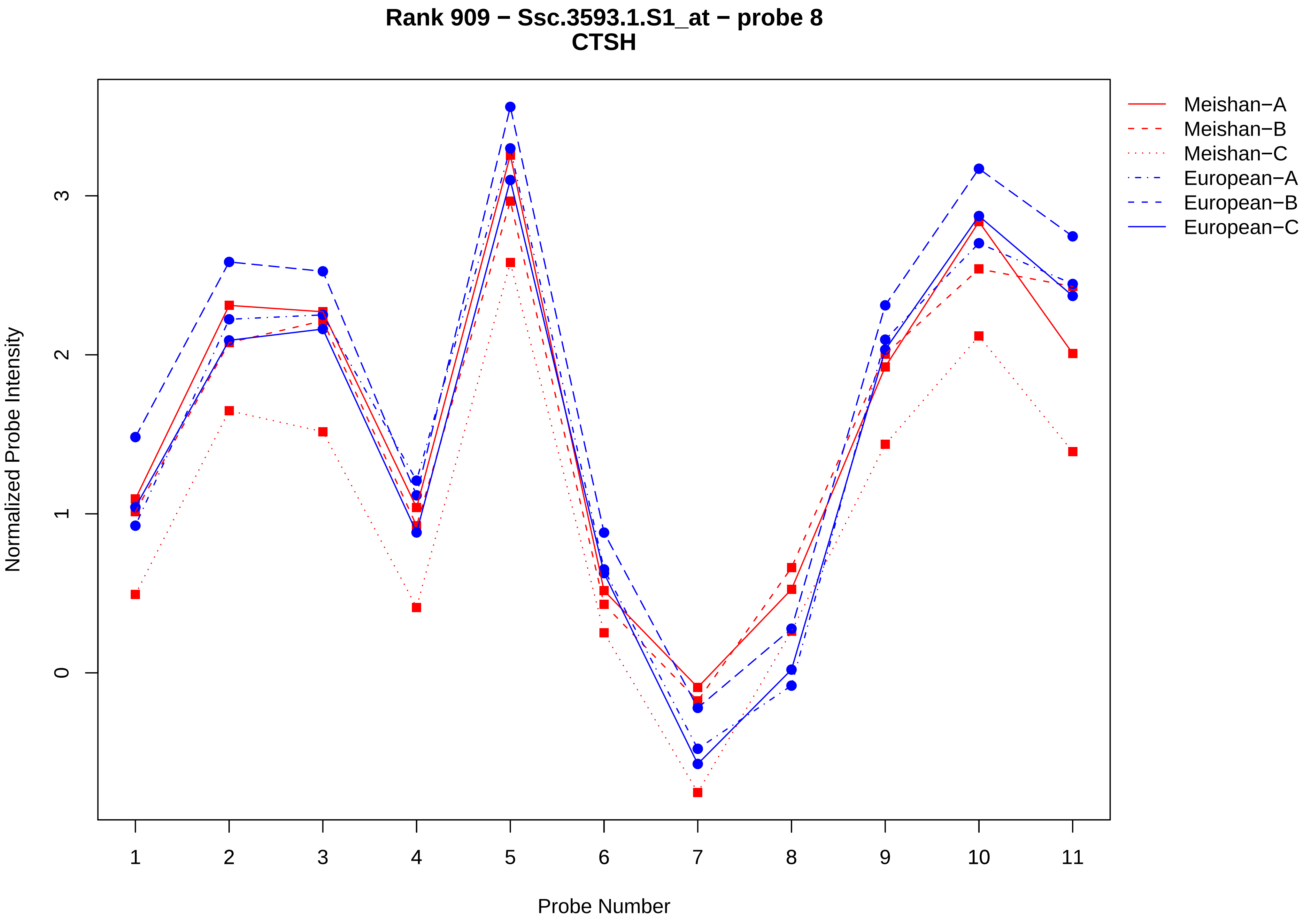

Rank 909 − Ssc.3593.1.S1_at − probe 8
CTSH
Normalized Probe Intensity
Probe Number
0
1
2
3
1
2
3
4
5
6
7
8
9
10
11
Meishan−A
Meishan−B
Meishan−C
European−A
European−B
European−C

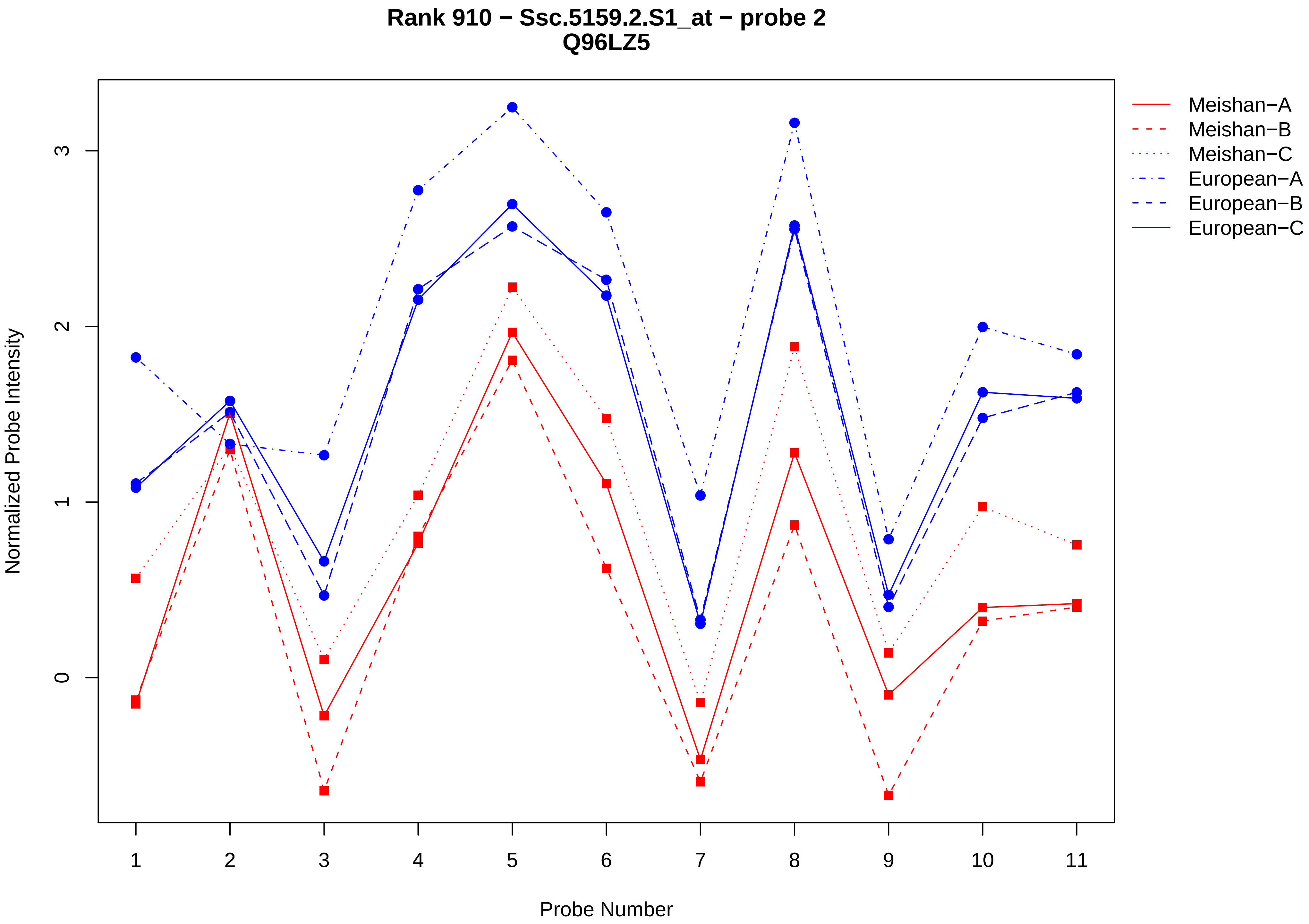
Rank 910 − Ssc.5159.2.S1_at − probe 2
Q96LZ5
Normalized Probe Intensity
Probe Number
0
1
2
3
1
2
3
4
5
6
7
8
9
10
11
Meishan−A
Meishan−B
Meishan−C
European−A
European−B
European−C

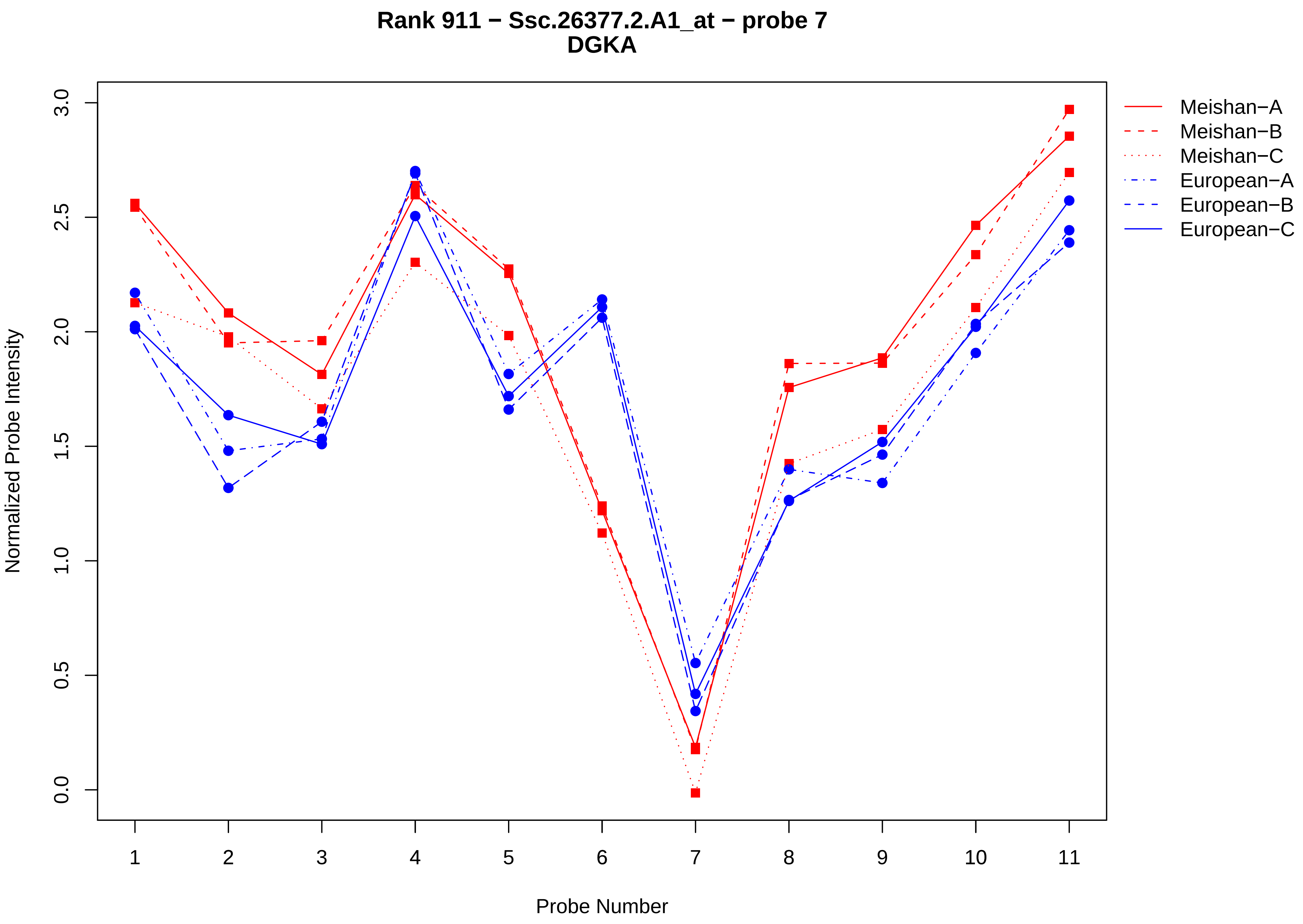

Rank 911 − Ssc.26377.2.A1_at − probe 7
DGKA
Normalized Probe Intensity
Probe Number
0.0
0.5
1.0
1.5
2.0
2.5
3.0
1
2
3
4
5
6
7
8
9
10
11
Meishan−A
Meishan−B
Meishan−C
European−A
European−B
European−C

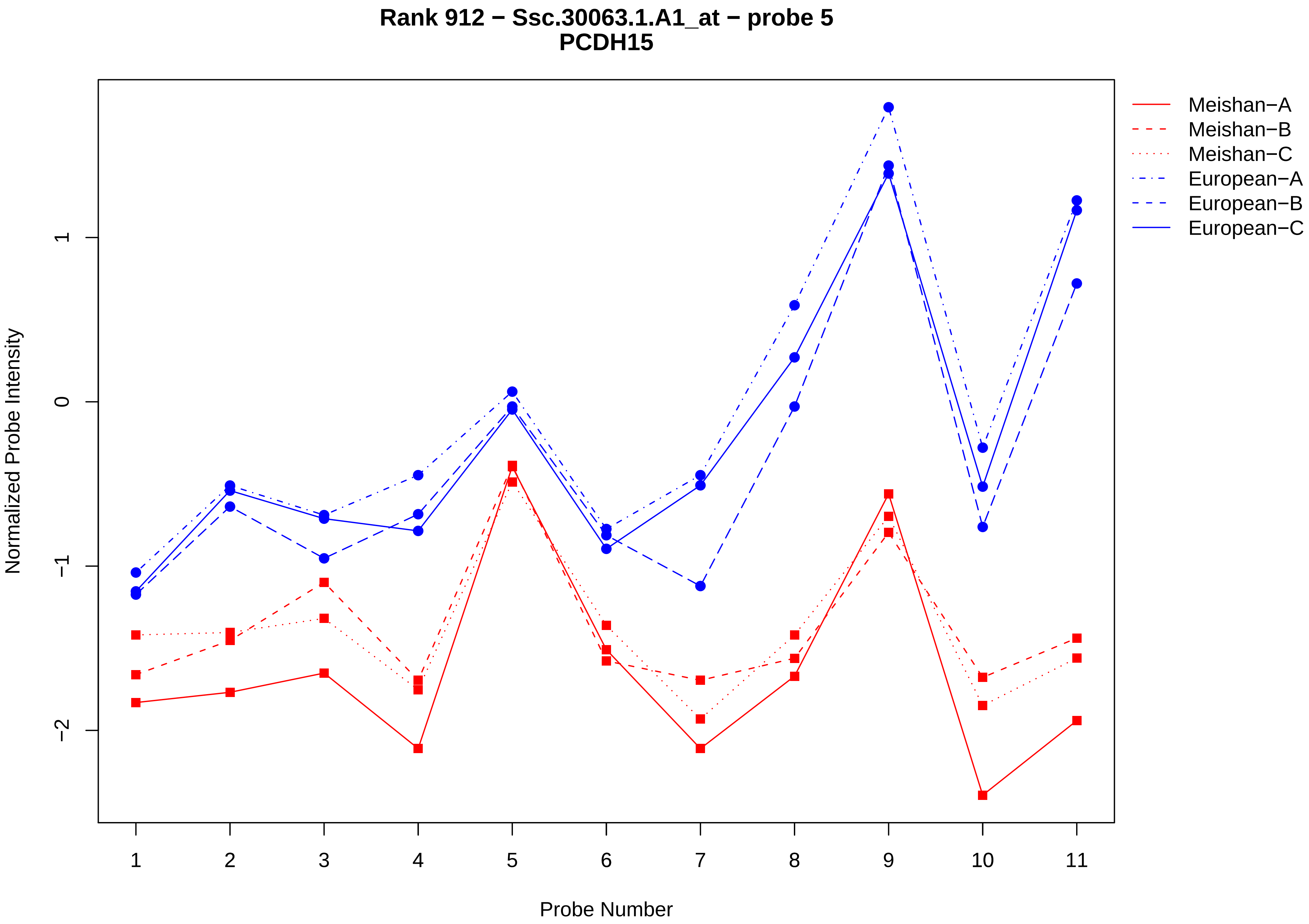

Rank 912 − Ssc.30063.1.A1_at − probe 5
PCDH15
Normalized Probe Intensity
Probe Number
−2
−1
0
1
1
2
3
4
5
6
7
8
9
10
11
Meishan−A
Meishan−B
Meishan−C
European−A
European−B
European−C

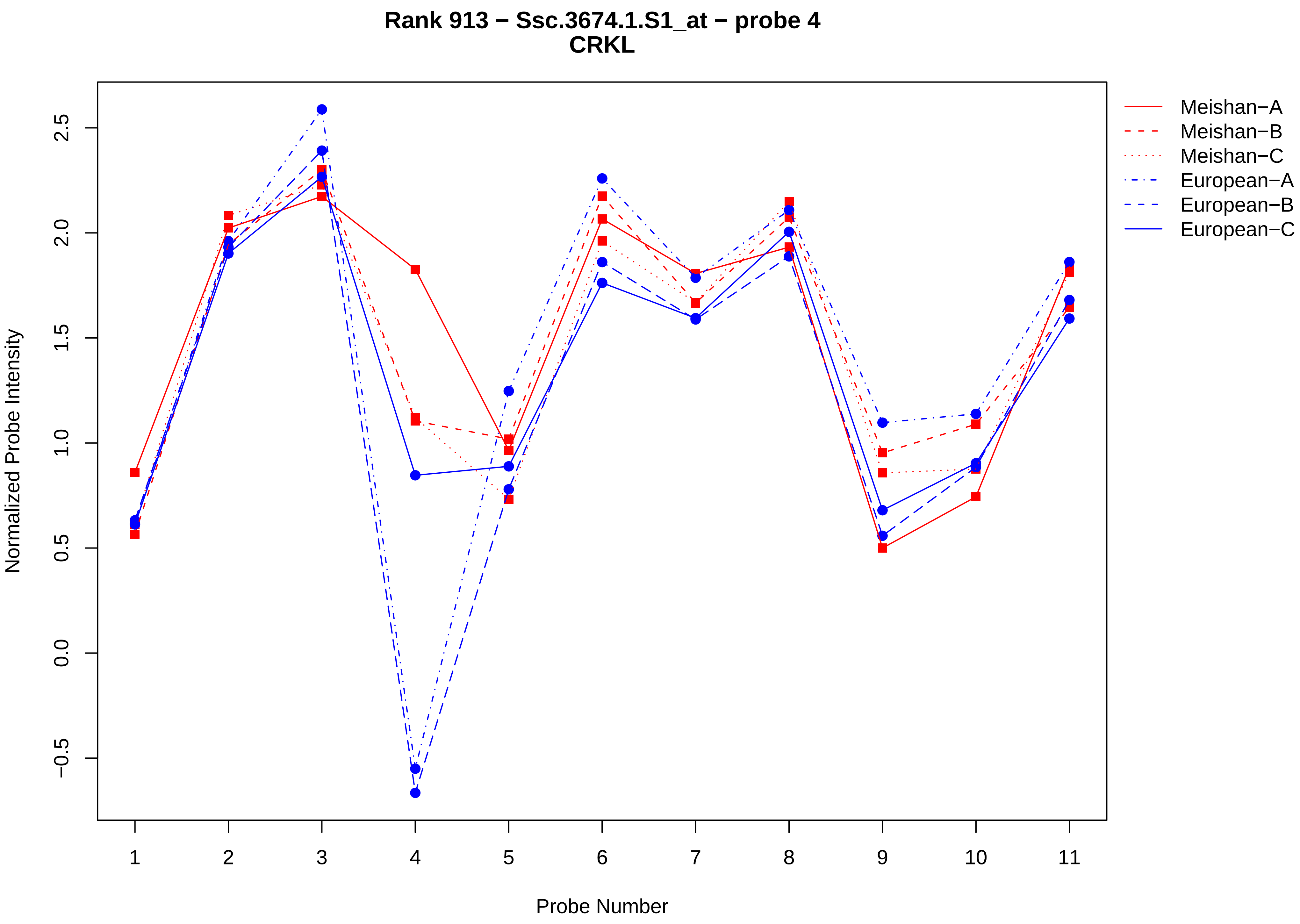

Rank 913 − Ssc.3674.1.S1_at − probe 4
CRKL
Normalized Probe Intensity
Probe Number
−0.5
0.0
0.5
1.0
1.5
2.0
2.5
1
2
3
4
5
6
7
8
9
10
11
Meishan−A
Meishan−B
Meishan−C
European−A
European−B
European−C

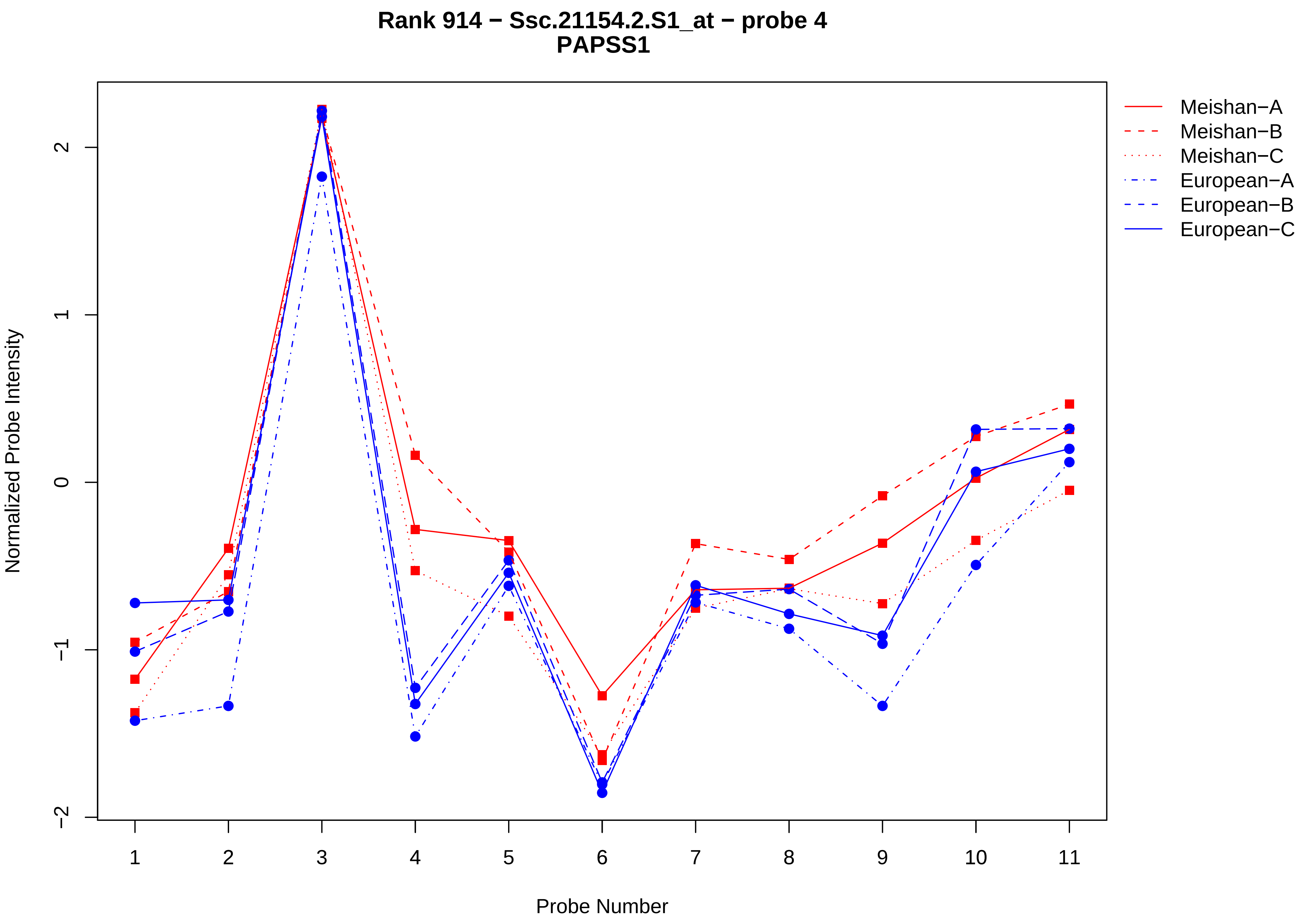
Rank 914 − Ssc.21154.2.S1_at − probe 4
PAPSS1
Normalized Probe Intensity
Probe Number
Meishan−A
Meishan−B
Meishan−C
European−A
European−B
European−C
−2
−1
0
1
2
1
2
3
4
5
6
7
8
9
10
11

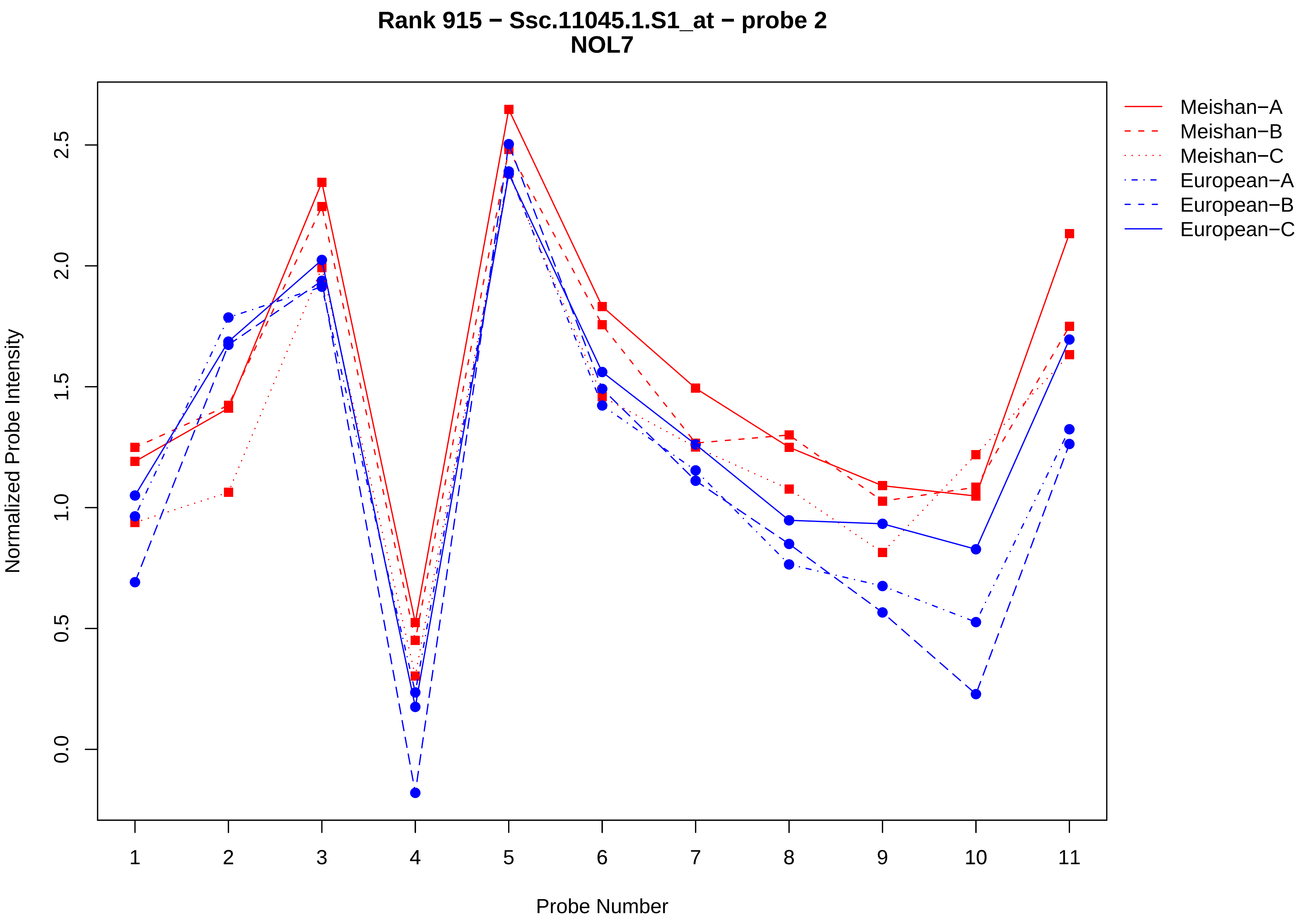

Rank 915 − Ssc.11045.1.S1_at − probe 2
NOL7
Normalized Probe Intensity
Probe Number
0.0
0.5
1.0
1.5
2.0
2.5
1
2
3
4
5
6
7
8
9
10
11
Meishan−A
Meishan−B
Meishan−C
European−A
European−B
European−C

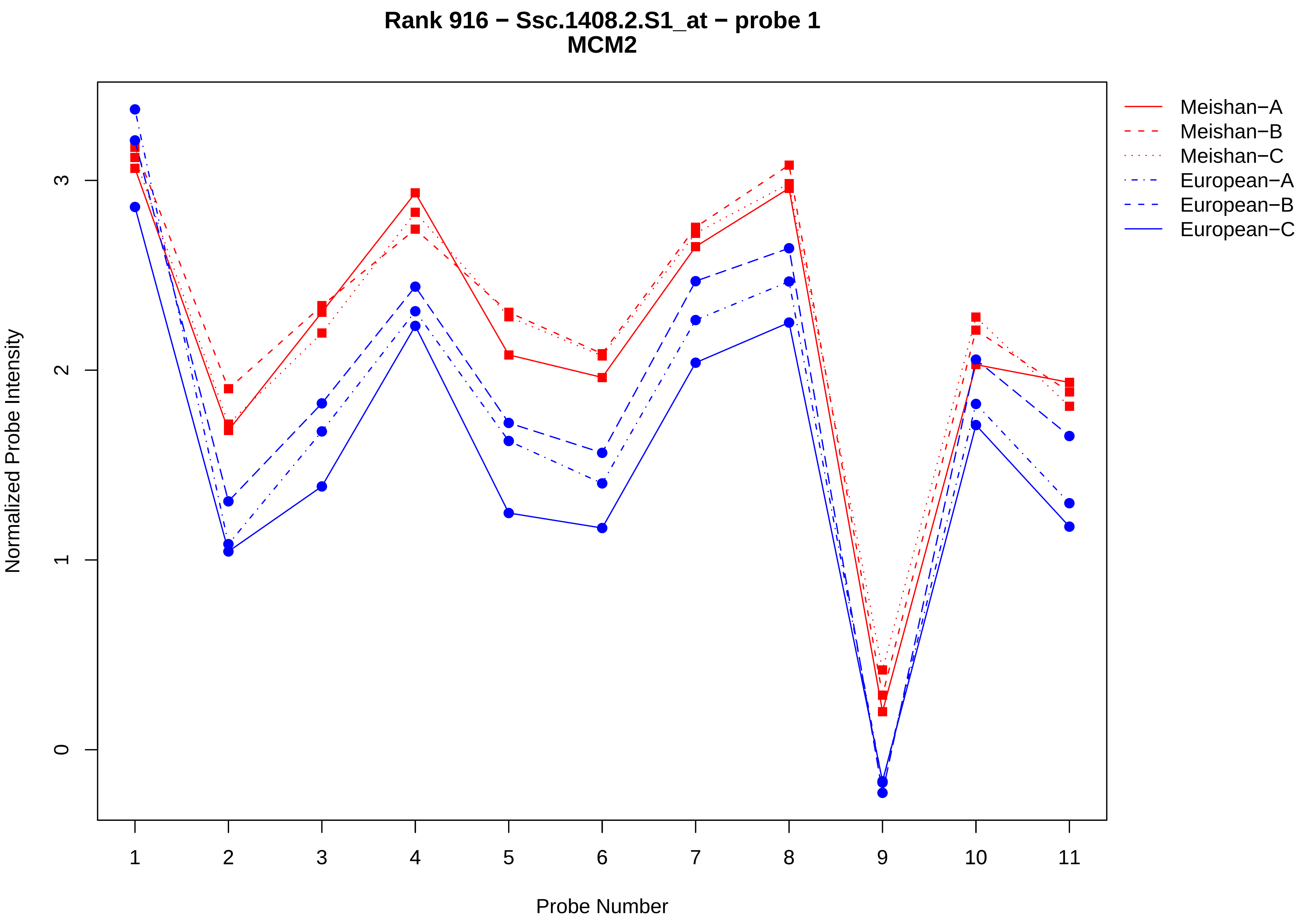

Rank 916 − Ssc.1408.2.S1_at − probe 1
MCM2
Normalized Probe Intensity
Probe Number
0
1
2
3
1
2
3
4
5
6
7
8
9
10
11
Meishan−A
Meishan−B
Meishan−C
European−A
European−B
European−C

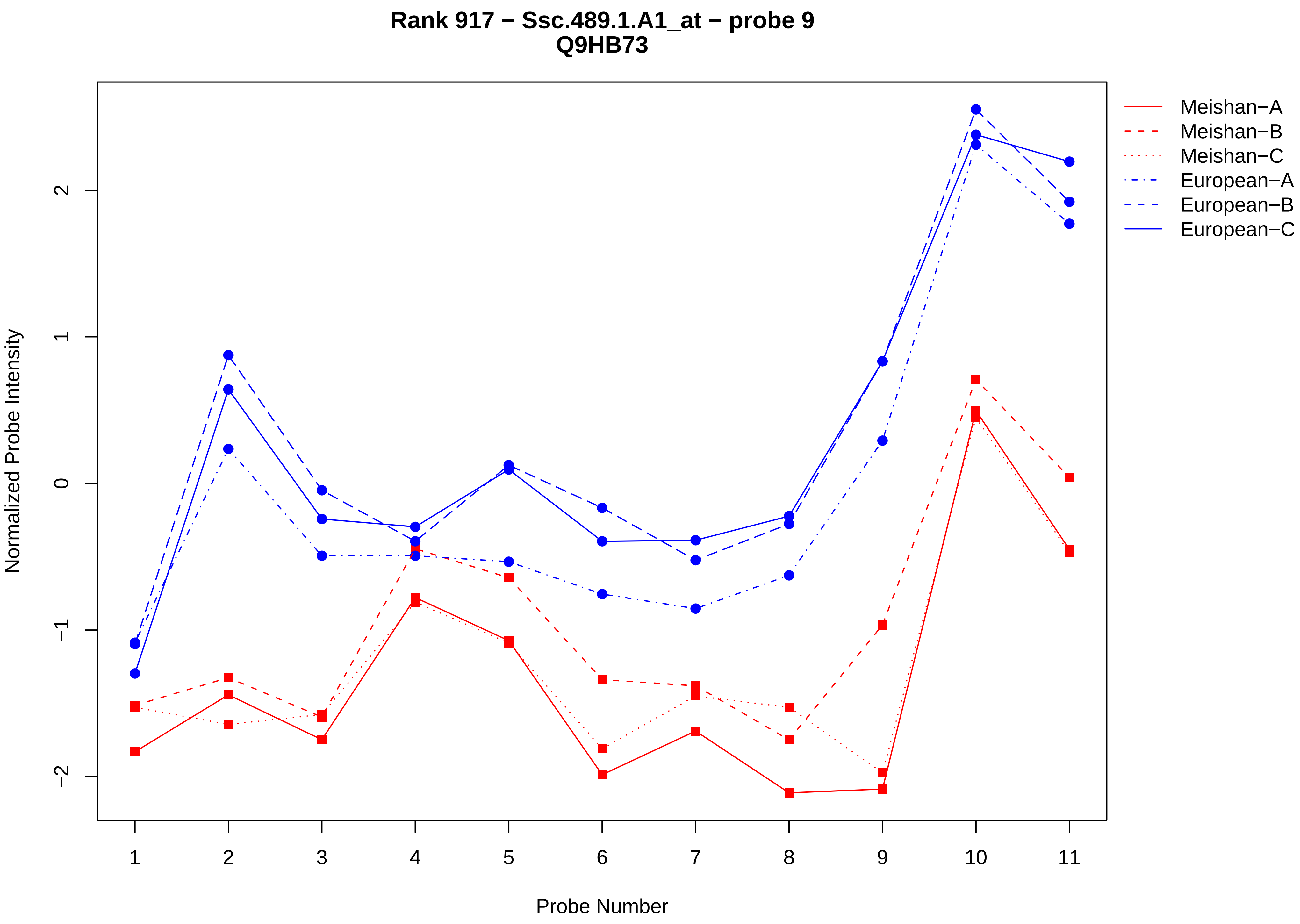

Rank 917 − Ssc.489.1.A1_at − probe 9
Q9HB73
Normalized Probe Intensity
Probe Number
−2
−1
0
1
2
1
2
3
4
5
6
7
8
9
10
11
Meishan−A
Meishan−B
Meishan−C
European−A
European−B
European−C

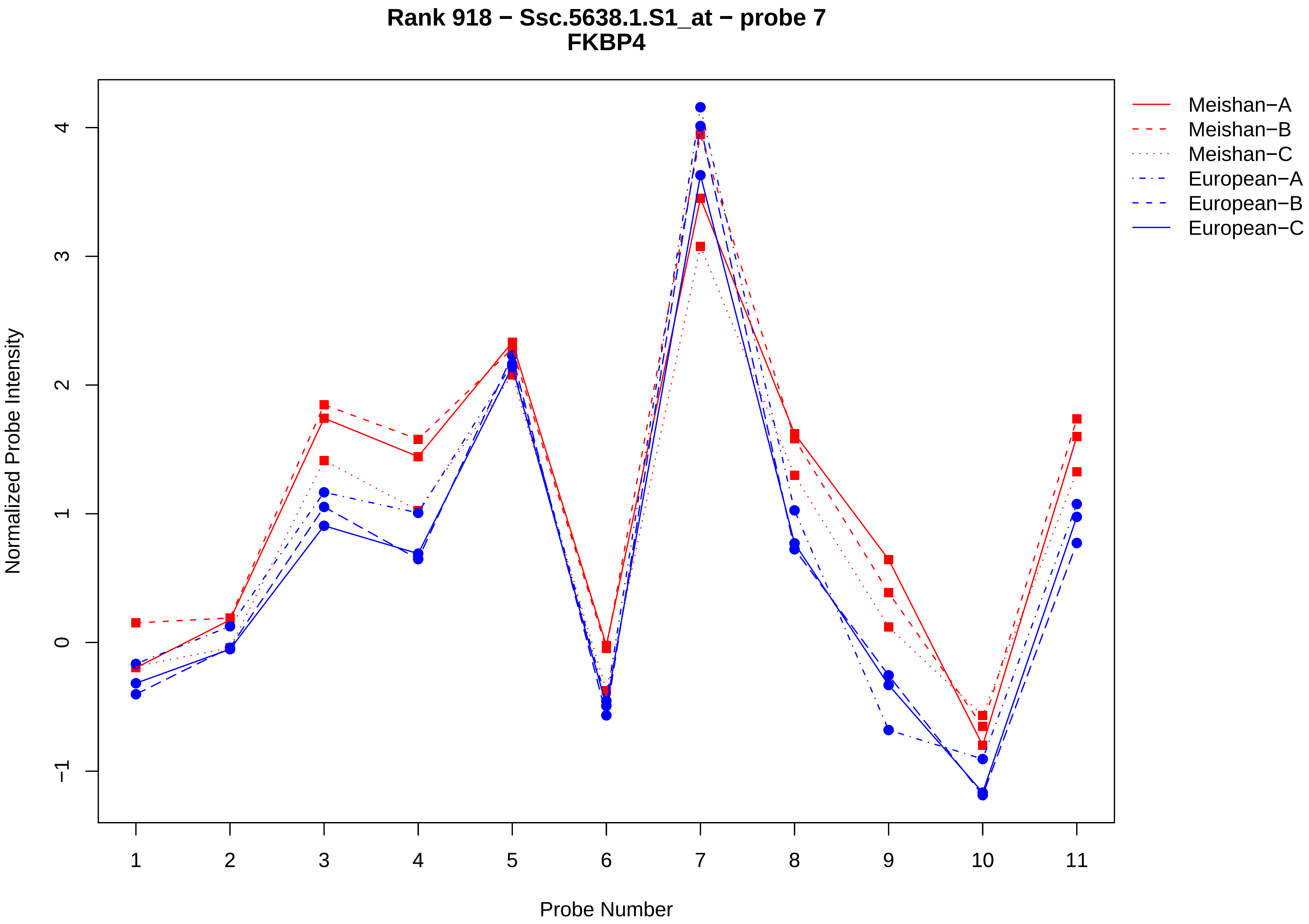
Rank 918 − Ssc.5638.1.S1_at − probe 7
FKBP4
Normalized Probe Intensity
Probe Number
−1
0
1
2
3
4
1
2
3
4
5
6
7
8
9
10
11
Meishan−A
Meishan−B
Meishan−C
European−A
European−B
European−C

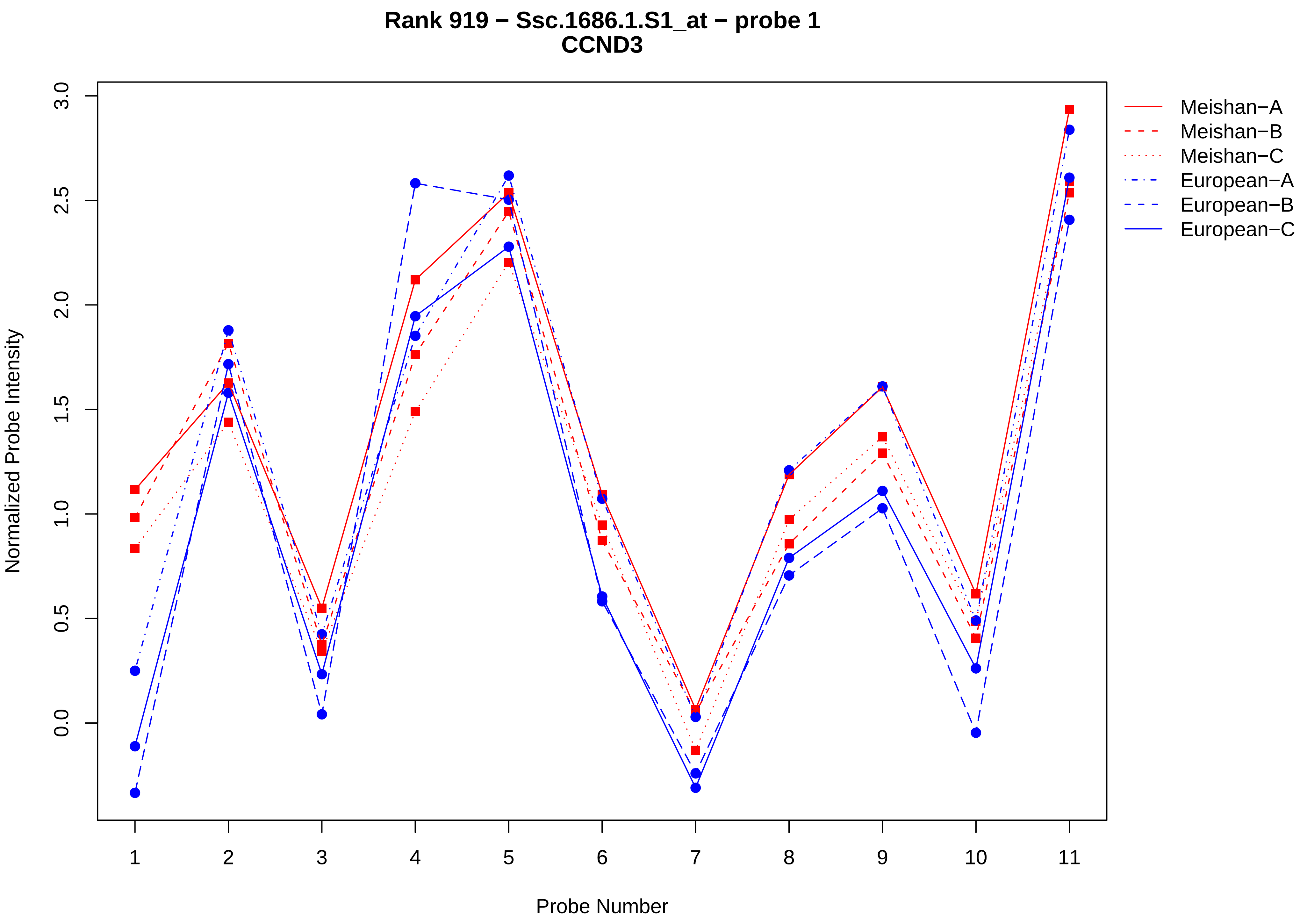

Rank 919 − Ssc.1686.1.S1_at − probe 1
CCND3
Normalized Probe Intensity
Probe Number
0.0
0.5
1.0
1.5
2.0
2.5
3.0
1
2
3
4
5
6
7
8
9
10
11
Meishan−A
Meishan−B
Meishan−C
European−A
European−B
European−C

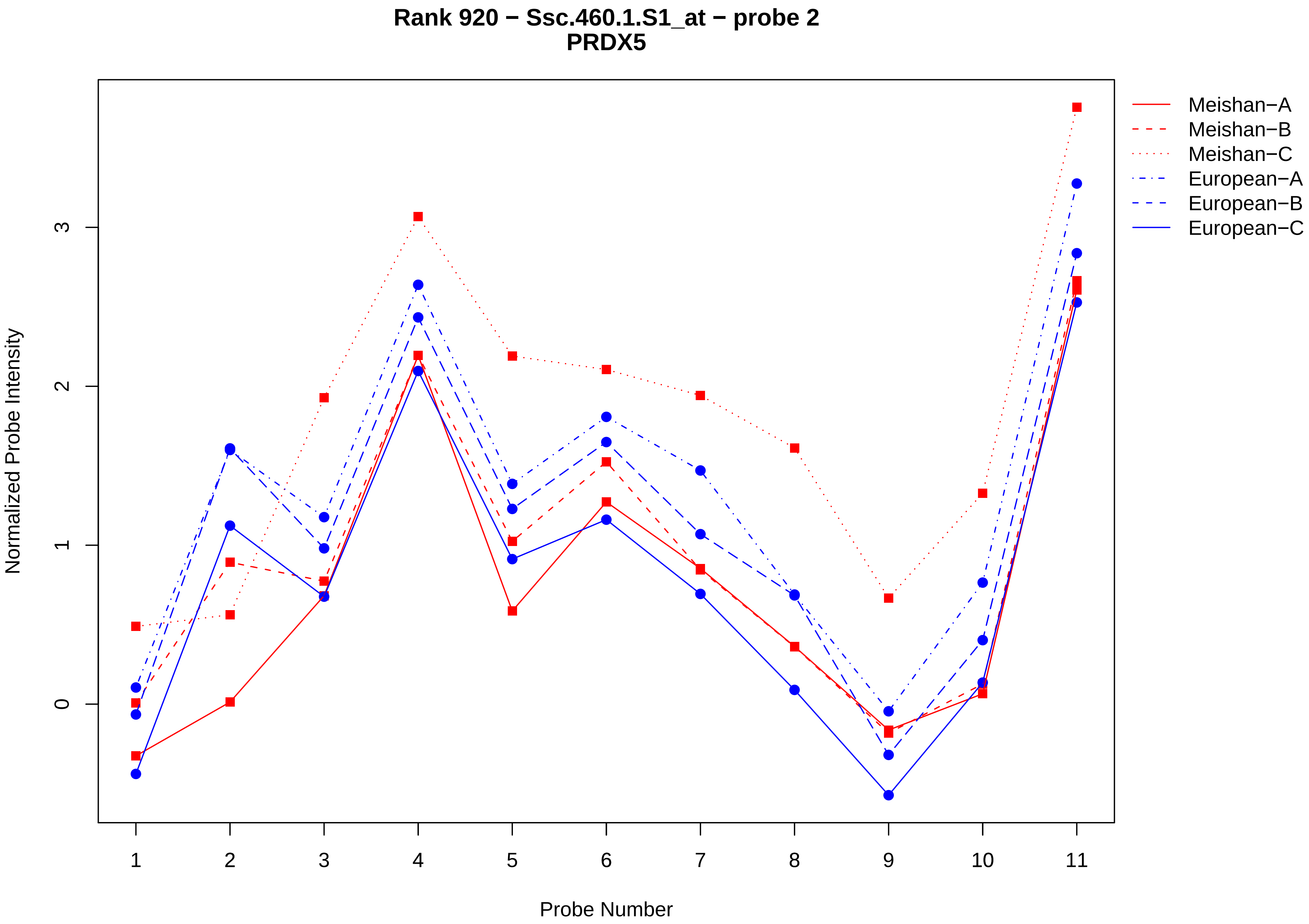

Rank 920 − Ssc.460.1.S1_at − probe 2
PRDX5
Normalized Probe Intensity
Probe Number
0
1
2
3
1
2
3
4
5
6
7
8
9
10
11
Meishan−A
Meishan−B
Meishan−C
European−A
European−B
European−C

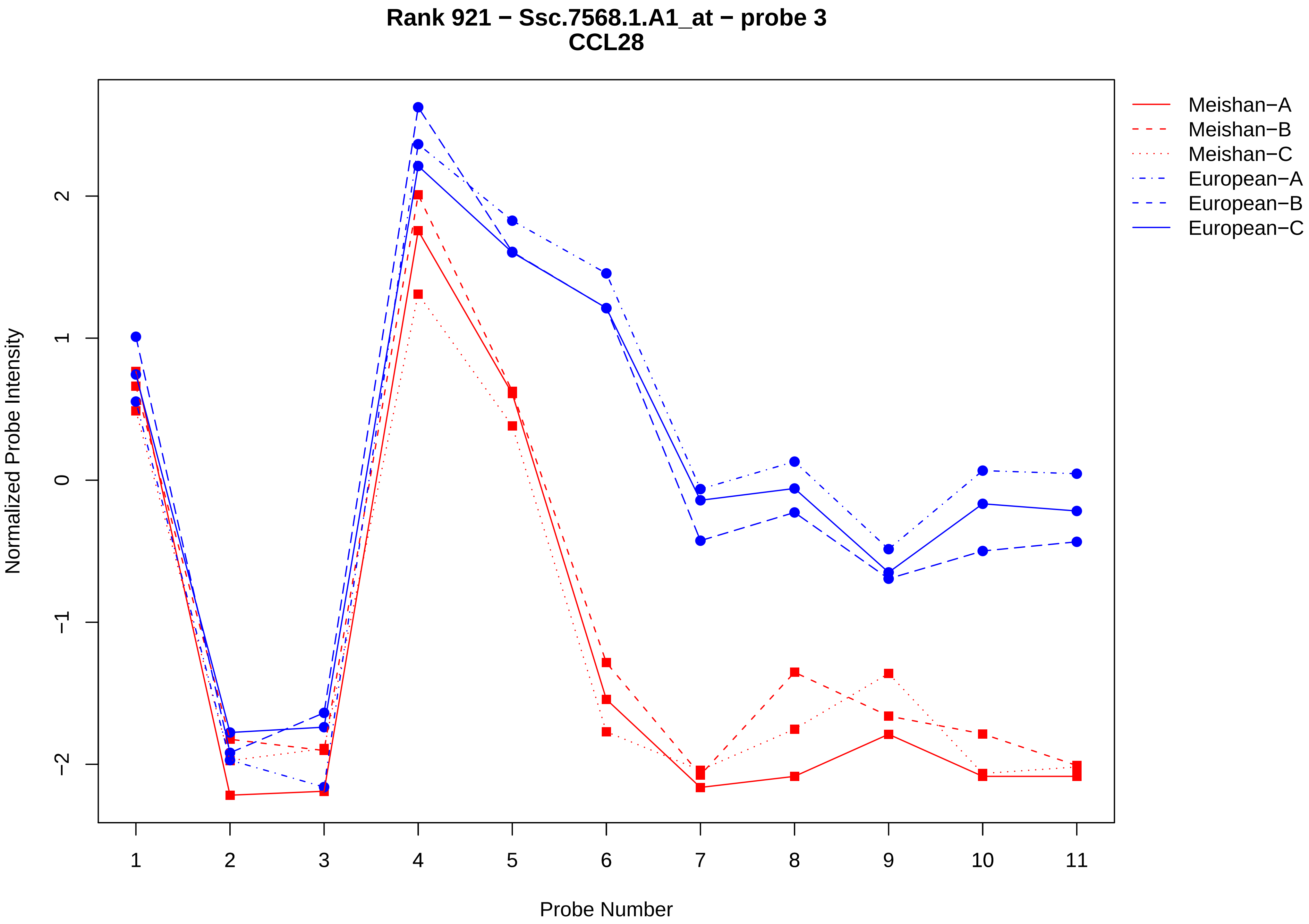

Rank 921 − Ssc.7568.1.A1_at − probe 3
CCL28
Normalized Probe Intensity
Probe Number
Meishan−A
Meishan−B
Meishan−C
European−A
European−B
European−C
−2
−1
0
1
2
1
2
3
4
5
6
7
8
9
10
11

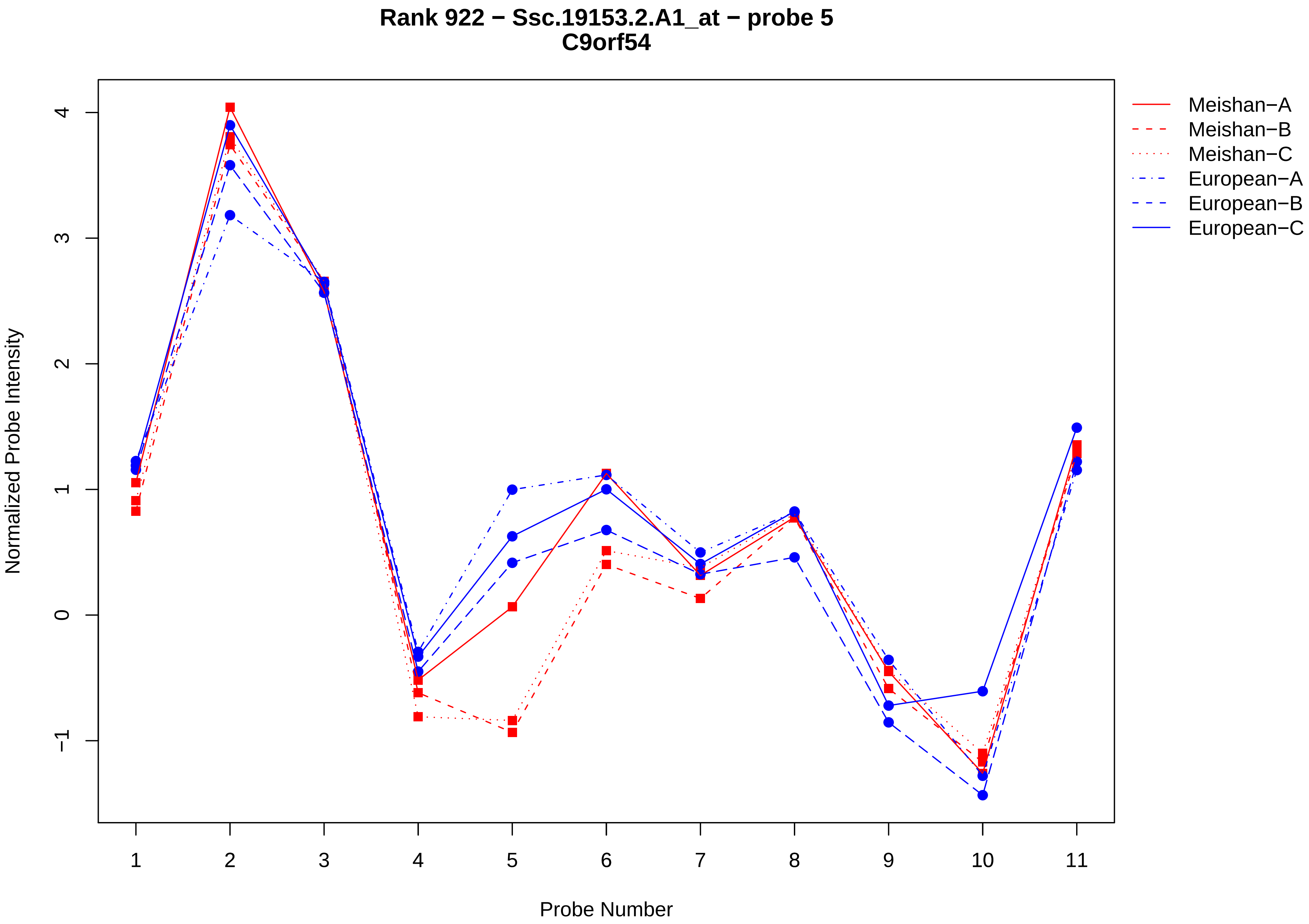

Rank 922 − Ssc.19153.2.A1_at − probe 5
C9orf54
Normalized Probe Intensity
Probe Number
Meishan−A
Meishan−B
Meishan−C
European−A
European−B
European−C
−1
0
1
2
3
4
1
2
3
4
5
6
7
8
9
10
11

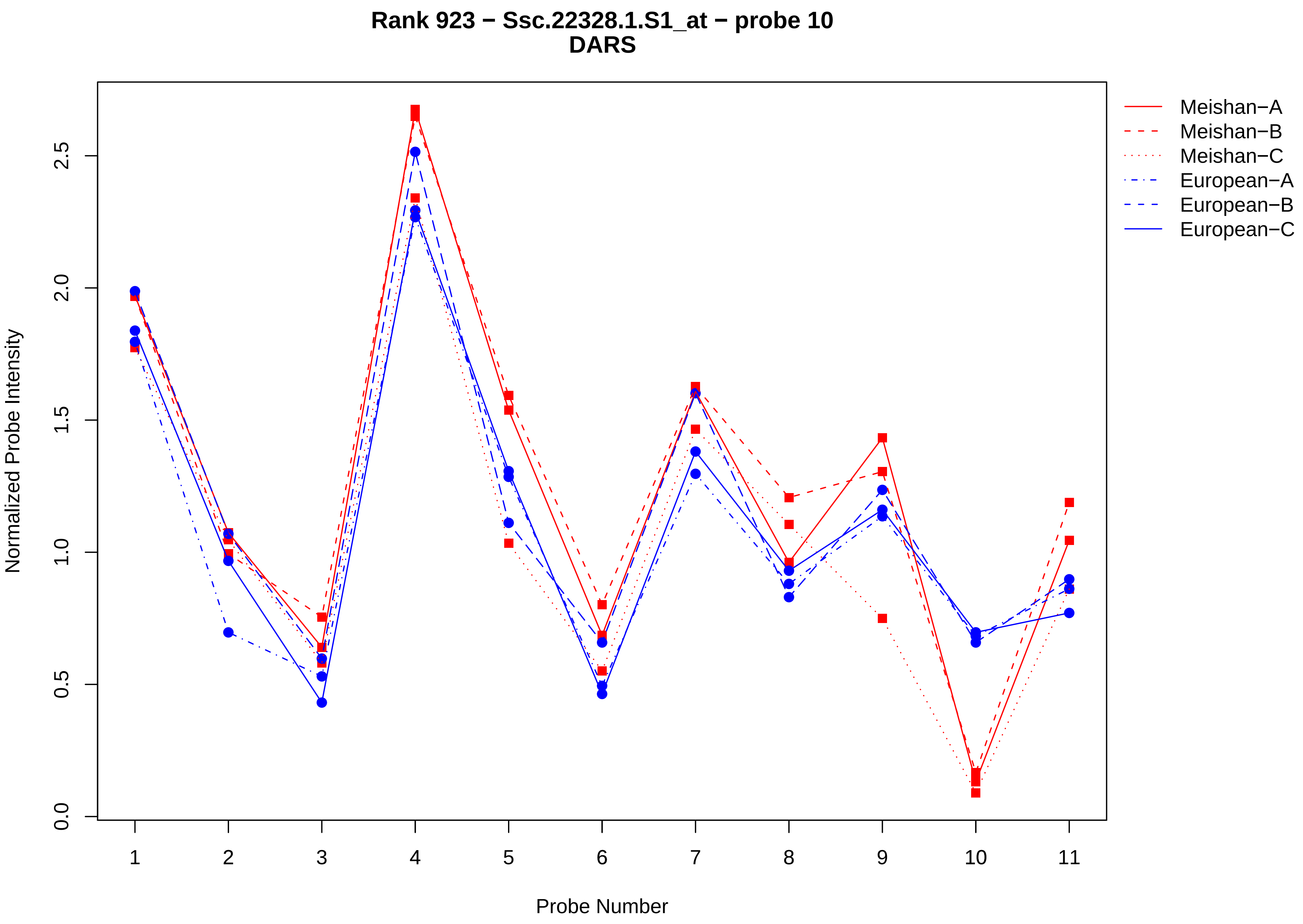
Rank 923 − Ssc.22328.1.S1_at − probe 10
DARS
Normalized Probe Intensity
0.0
0.5
1.0
1.5
2.0
2.5
Probe Number
1
2
3
4
5
6
7
8
9
10
11
Meishan−A
Meishan−B
Meishan−C
European−A
European−B
European−C

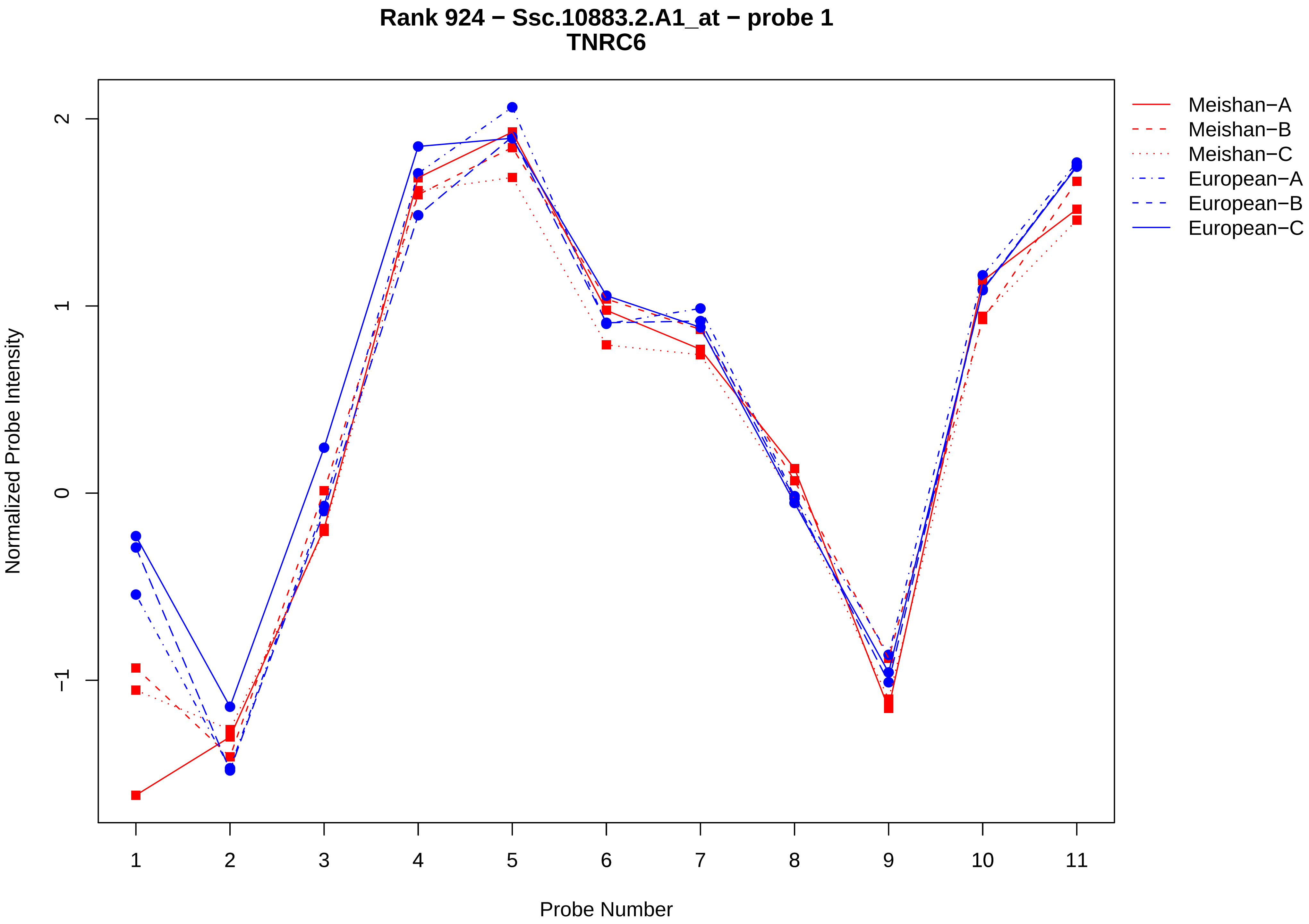

Rank 924 − Ssc.10883.2.A1_at − probe 1
TNRC6
Normalized Probe Intensity
Probe Number
2
1
0
−1
1
2
3
4
5
6
7
8
9
10
11
Meishan−A
Meishan−B
Meishan−C
European−A
European−B
European−C

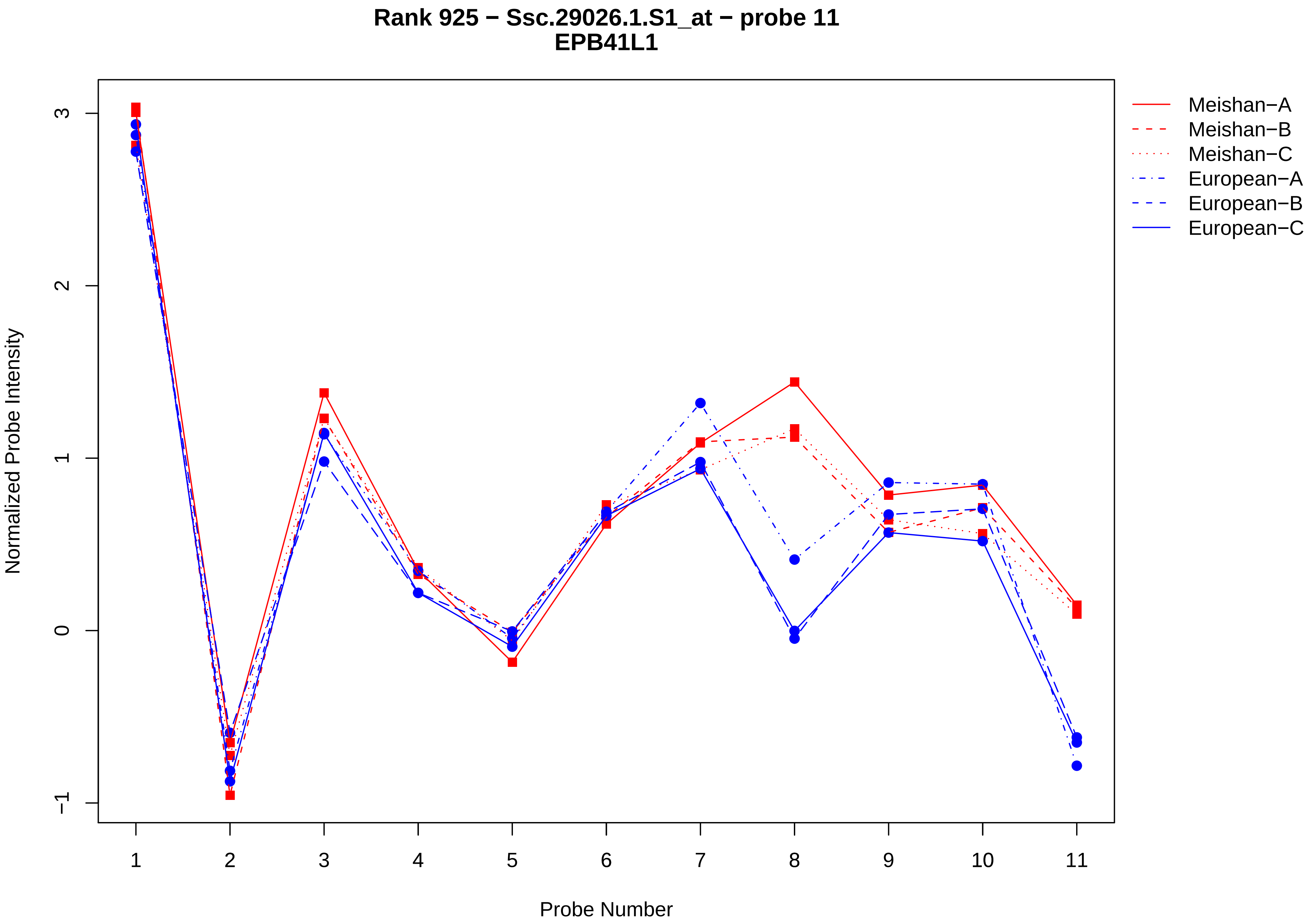

Rank 925 − Ssc.29026.1.S1_at − probe 11
EPB41L1
Normalized Probe Intensity
Probe Number
−1
0
1
2
3
1
2
3
4
5
6
7
8
9
10
11
Meishan−A
Meishan−B
Meishan−C
European−A
European−B
European−C

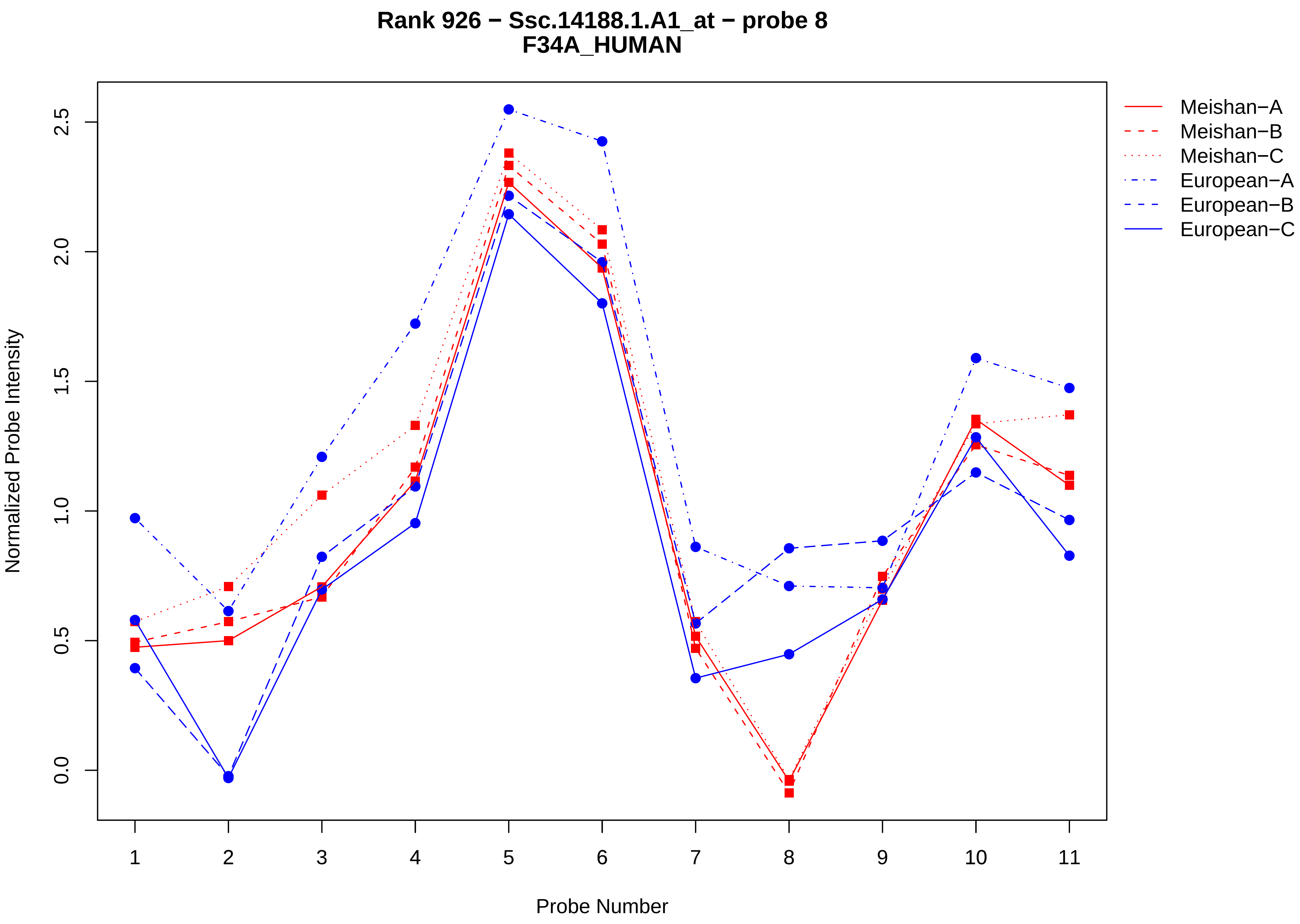

Rank 926 − Ssc.14188.1.A1_at − probe 8
F34A_HUMAN
Normalized Probe Intensity
Probe Number
0.0
0.5
1.0
1.5
2.0
2.5
1
2
3
4
5
6
7
8
9
10
11
Meishan−A
Meishan−B
Meishan−C
European−A
European−B
European−C

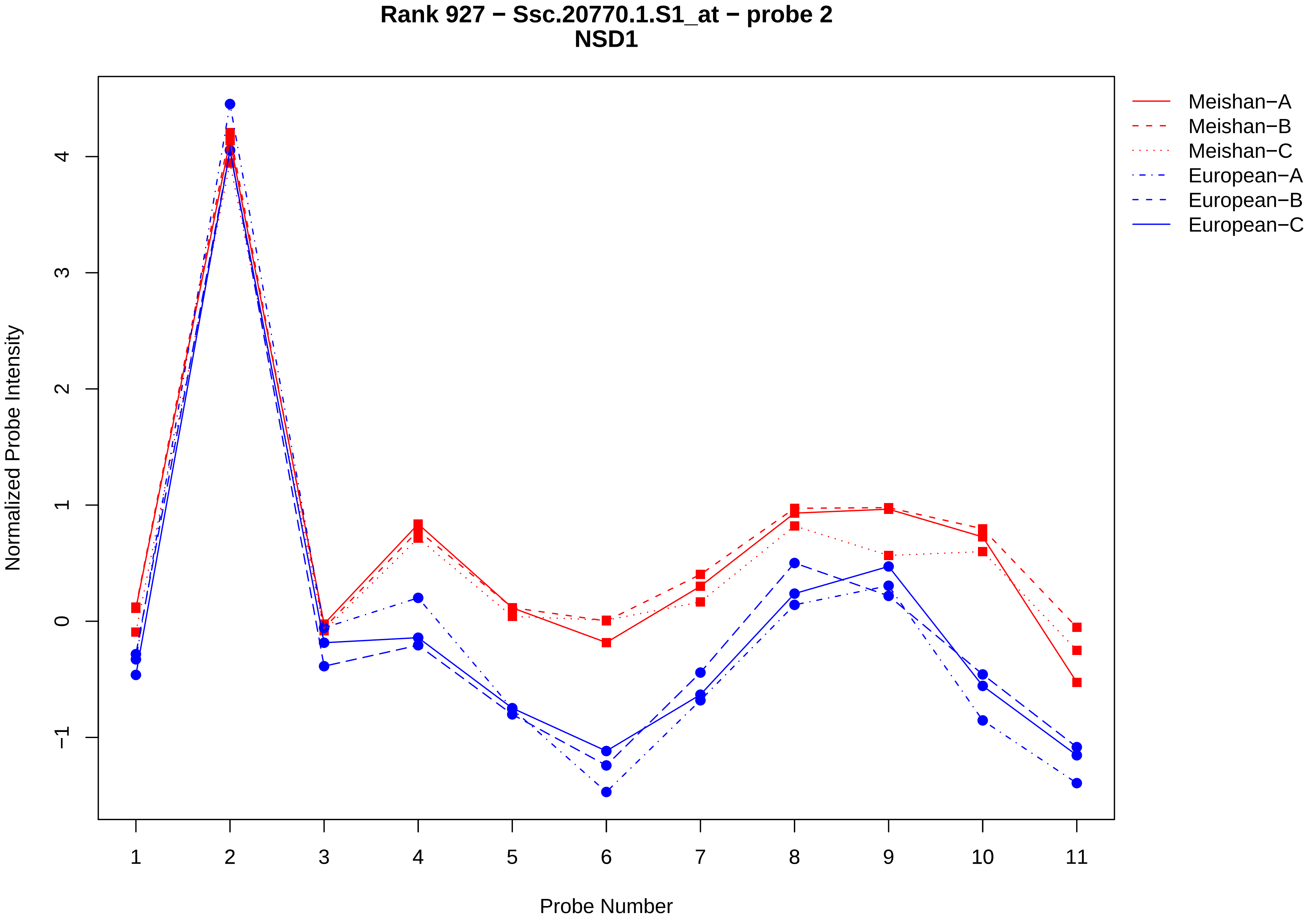

Rank 927 − Ssc.20770.1.S1_at − probe 2
NSD1
Normalized Probe Intensity
Probe Number
−1
0
1
2
3
4
1
2
3
4
5
6
7
8
9
10
11
Meishan−A
Meishan−B
Meishan−C
European−A
European−B
European−C

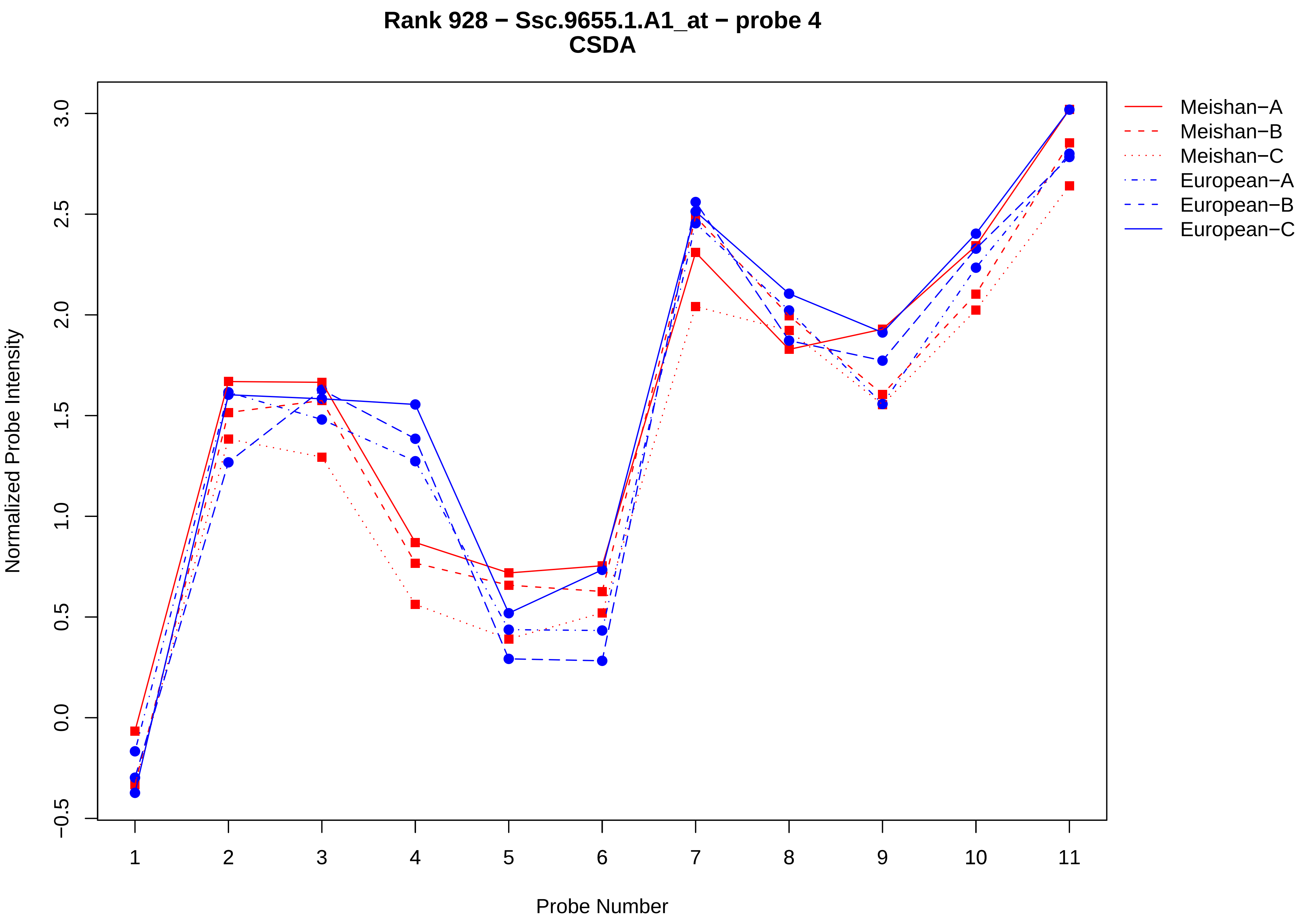
Rank 928 − Ssc.9655.1.A1_at − probe 4
CSDA
Normalized Probe Intensity
Probe Number
Meishan−A
Meishan−B
Meishan−C
European−A
European−B
European−C

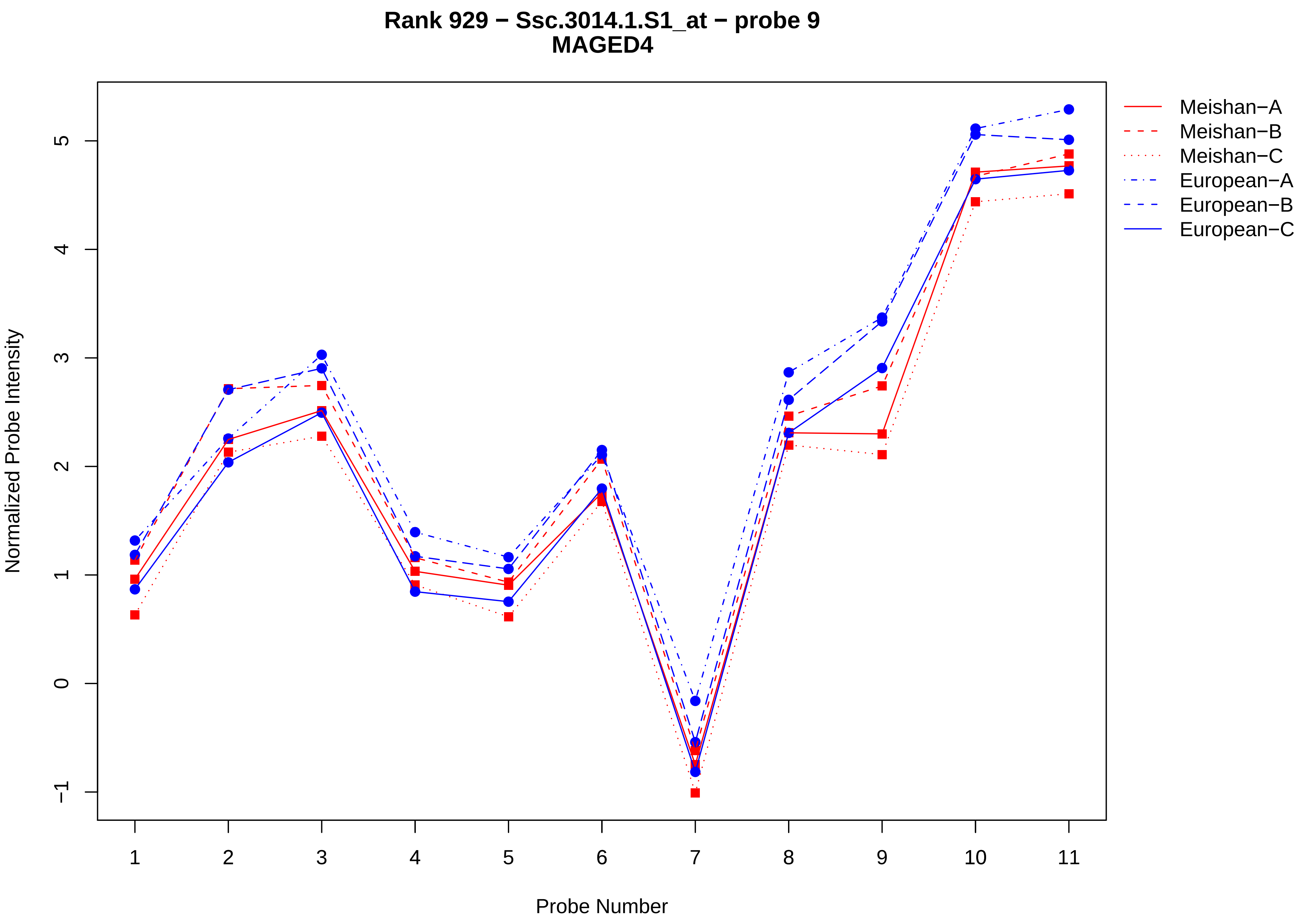

Rank 929 − Ssc.3014.1.S1_at − probe 9
MAGED4
Normalized Probe Intensity
Probe Number
−1
0
1
2
3
4
5
1
2
3
4
5
6
7
8
9
10
11
Meishan−A
Meishan−B
Meishan−C
European−A
European−B
European−C

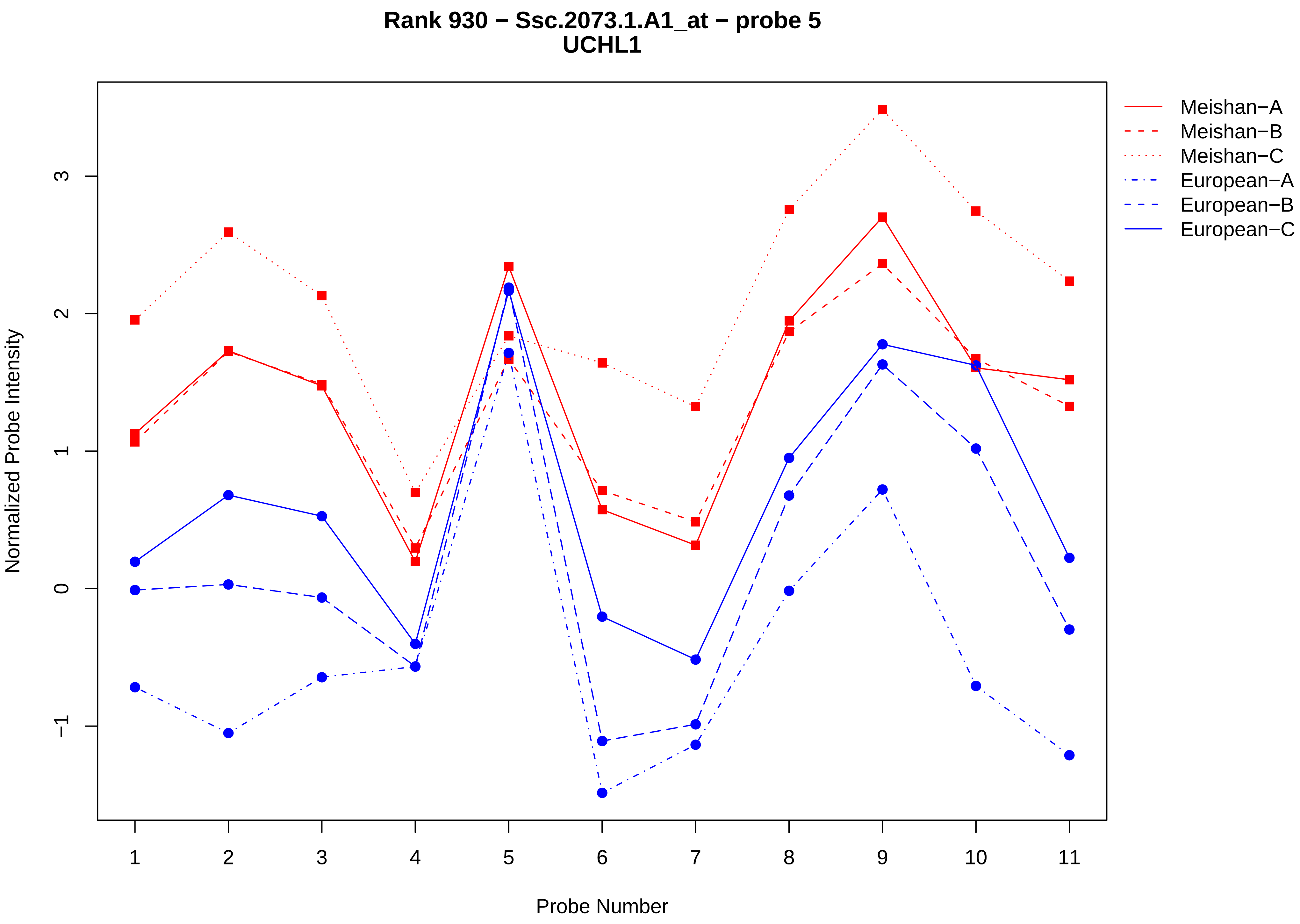
Rank 930 − Ssc.2073.1.A1_at − probe 5
UCHL1
Normalized Probe Intensity
Probe Number
Meishan−A
Meishan−B
Meishan−C
European−A
European−B
European−C
−1
0
1
2
3
1
2
3
4
5
6
7
8
9
10
11

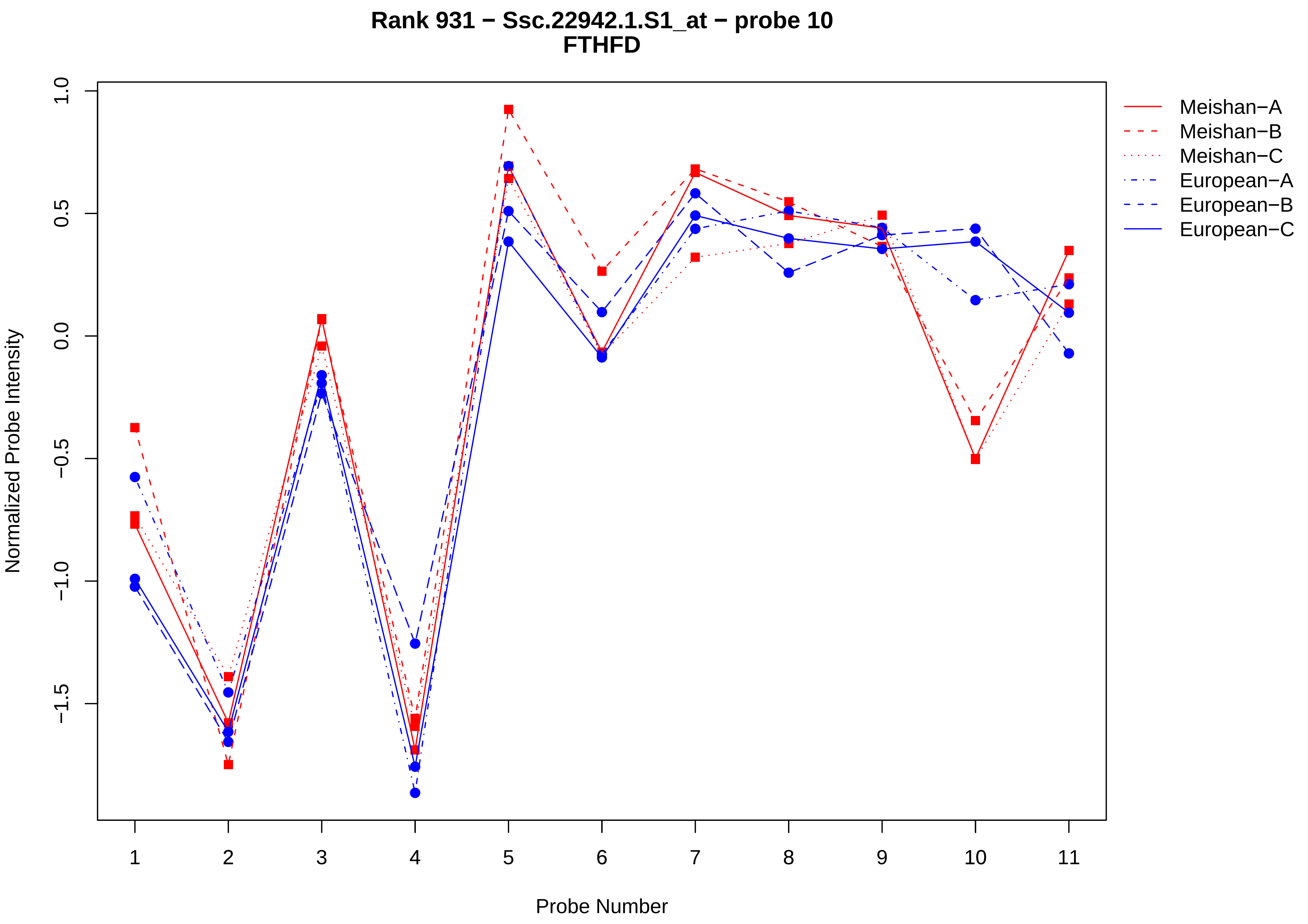

Rank 931 − Ssc.22942.1.S1_at − probe 10
FTHFD
Normalized Probe Intensity
Probe Number
1.0
0.5
0.0
−0.5
−1.0
−1.5
1
2
3
4
5
6
7
8
9
10
11
Meishan−A
Meishan−B
Meishan−C
European−A
European−B
European−C

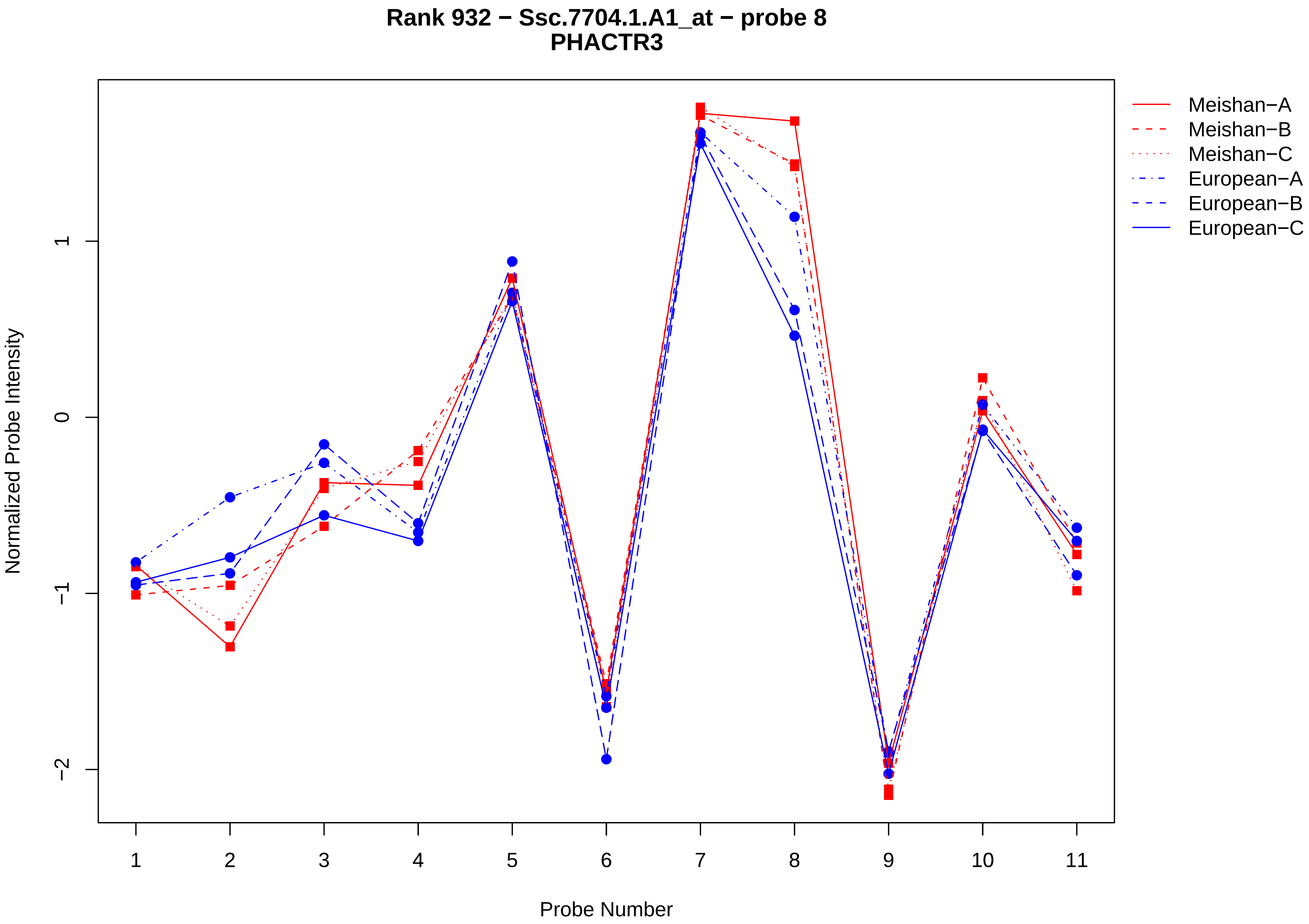

Rank 932 − Ssc.7704.1.A1_at − probe 8
PHACTR3
Normalized Probe Intensity
Probe Number
−2
−1
0
1
1
2
3
4
5
6
7
8
9
10
11
Meishan−A
Meishan−B
Meishan−C
European−A
European−B
European−C

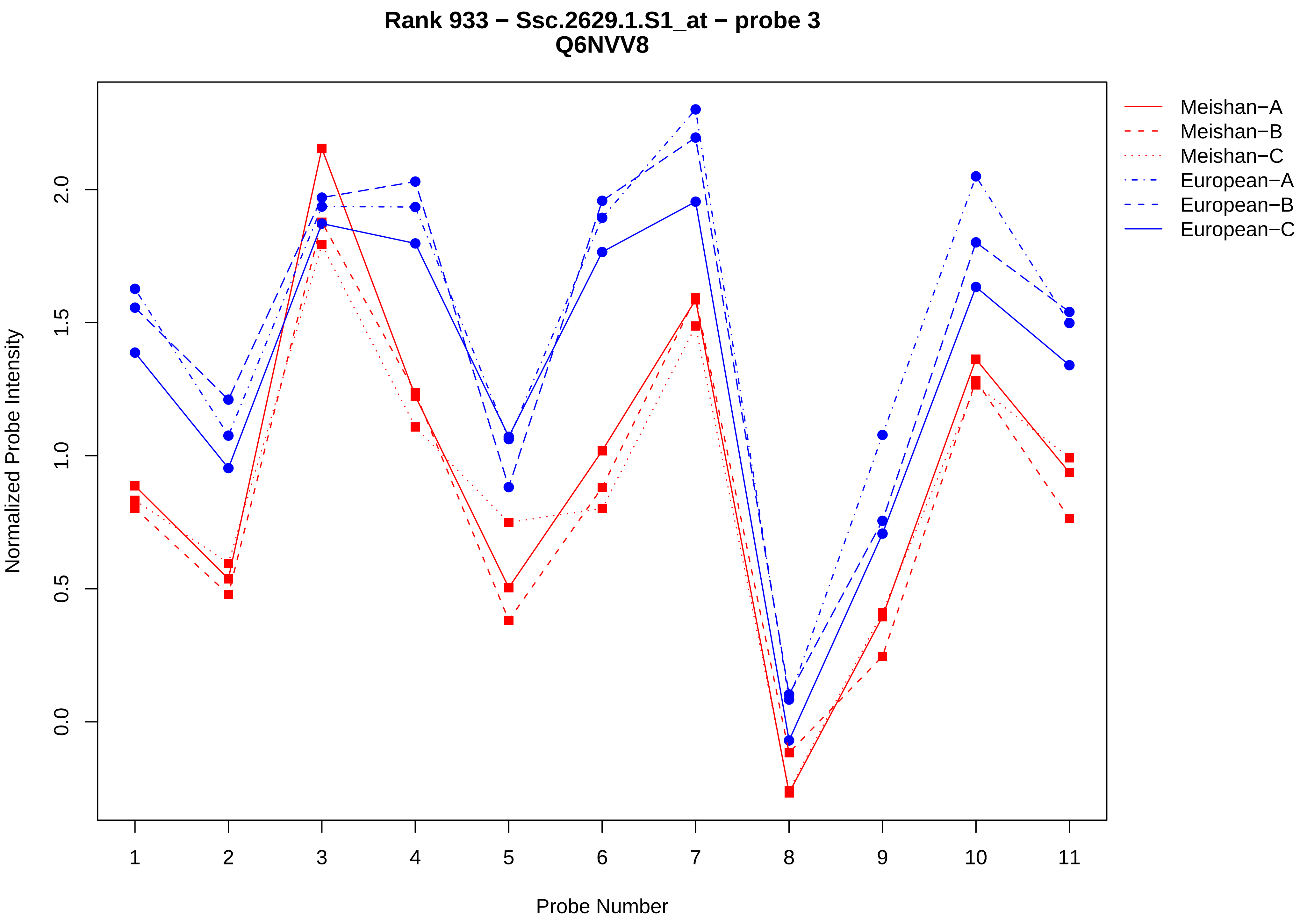

Rank 933 − Ssc.2629.1.S1_at − probe 3
Q6NVV8
Normalized Probe Intensity
Probe Number
0.0
0.5
1.0
1.5
2.0
1
2
3
4
5
6
7
8
9
10
11
Meishan−A
Meishan−B
Meishan−C
European−A
European−B
European−C

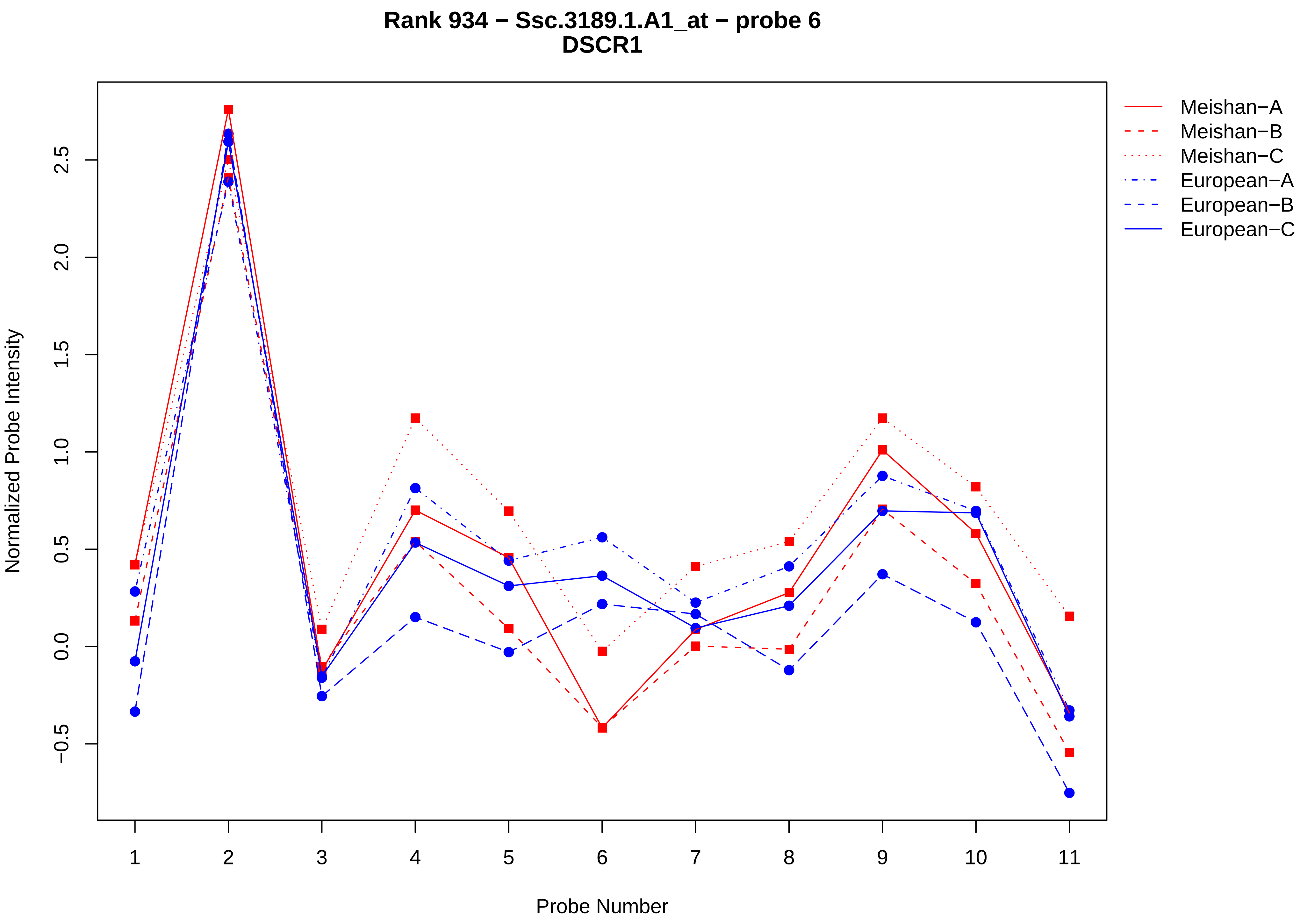

Rank 934 − Ssc.3189.1.A1_at − probe 6
DSCR1
Normalized Probe Intensity
Probe Number
−0.5
0.0
0.5
1.0
1.5
2.0
2.5
1
2
3
4
5
6
7
8
9
10
11
Meishan−A
Meishan−B
Meishan−C
European−A
European−B
European−C

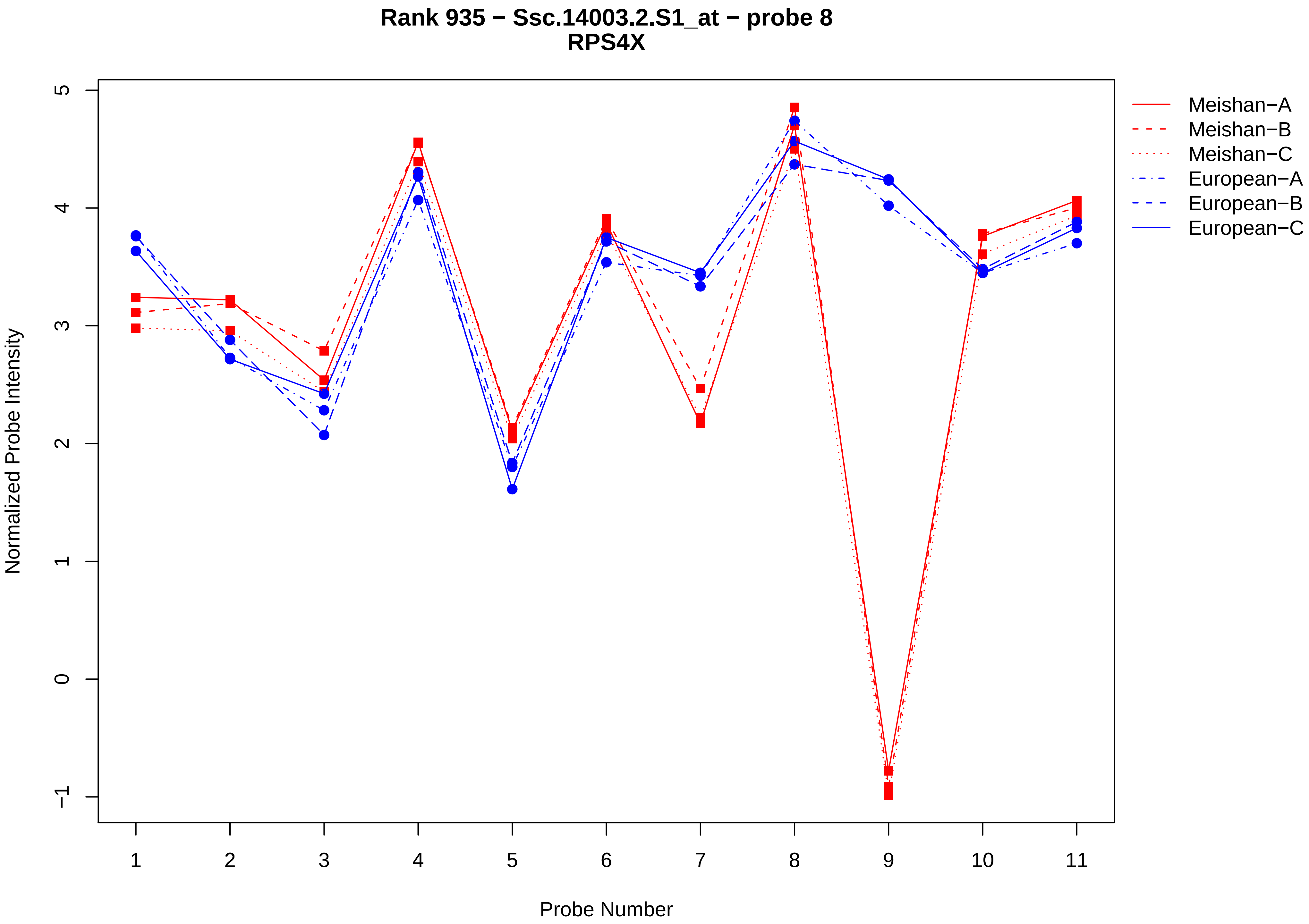

Rank 935 − Ssc.14003.2.S1_at − probe 8
RPS4X
Normalized Probe Intensity
Probe Number
−1
0
1
2
3
4
5
1
2
3
4
5
6
7
8
9
10
11
Meishan−A
Meishan−B
Meishan−C
European−A
European−B
European−C

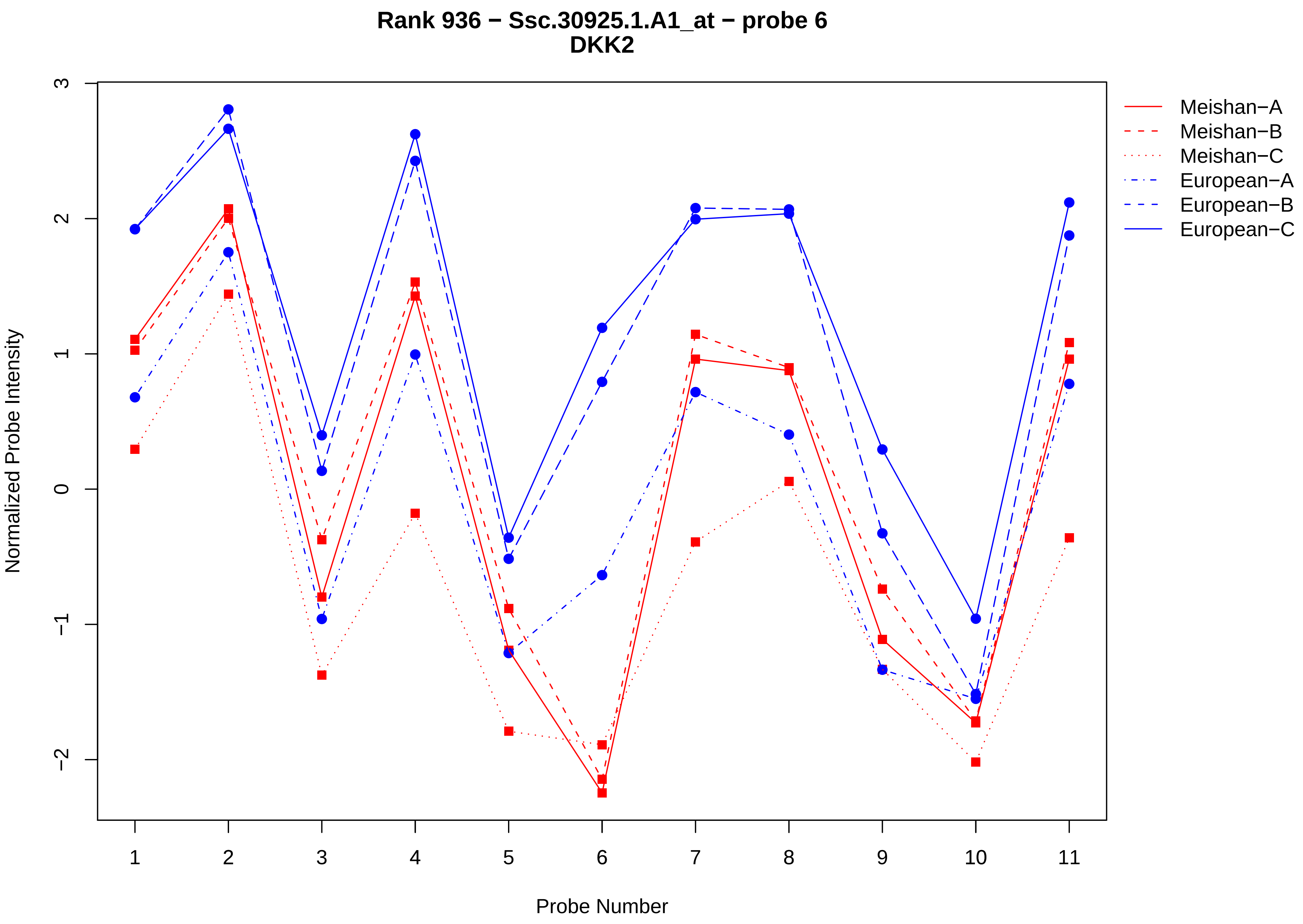
Rank 936 − Ssc.30925.1.A1_at − probe 6
DKK2
Normalized Probe Intensity
Probe Number
Meishan−A
Meishan−B
Meishan−C
European−A
European−B
European−C
−2
−1
0
1
2
3
1
2
3
4
5
6
7
8
9
10
11

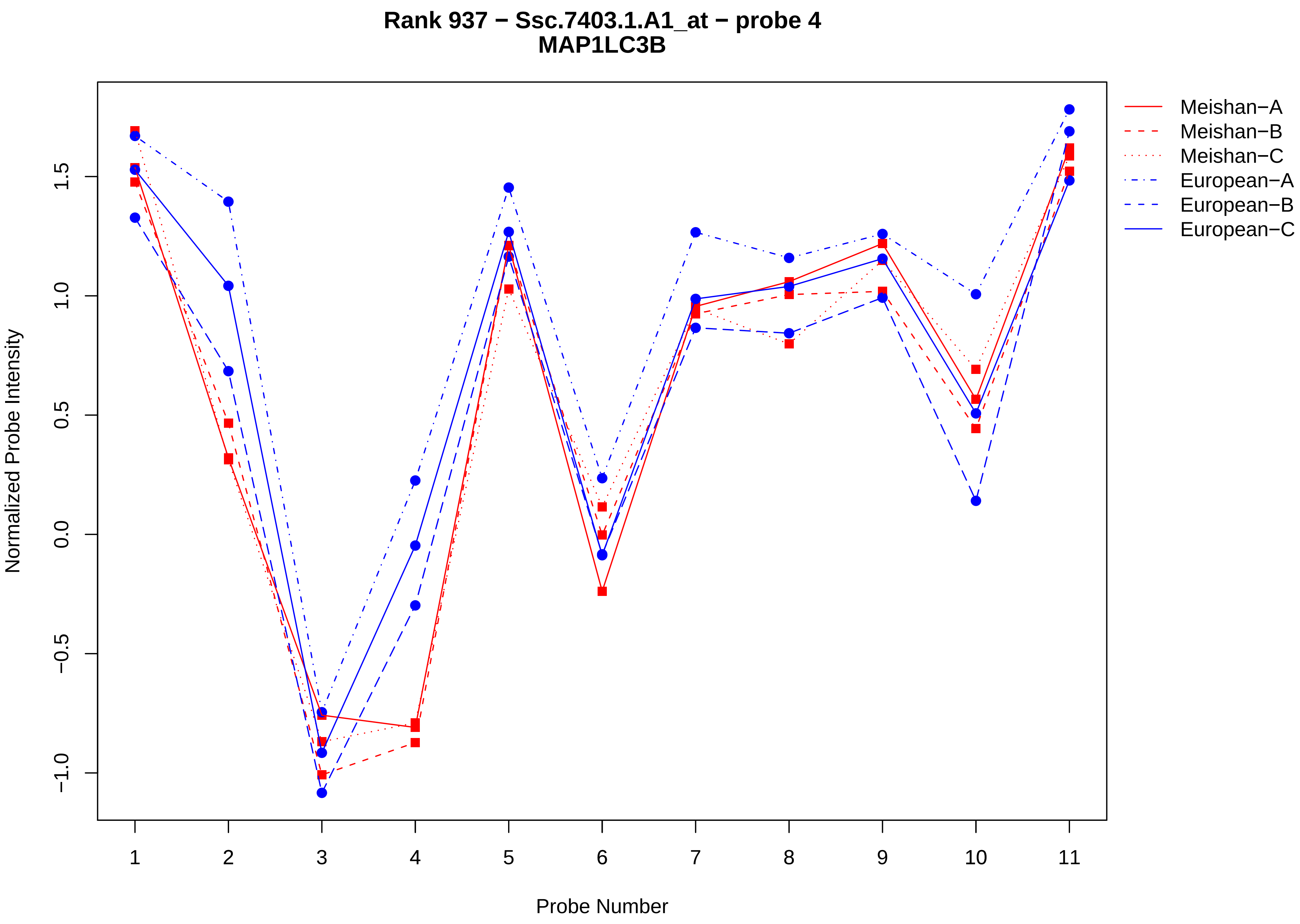

Rank 937 − Ssc.7403.1.A1_at − probe 4
MAP1LC3B
Normalized Probe Intensity
Probe Number
−1.0
−0.5
0.0
0.5
1.0
1.5
1
2
3
4
5
6
7
8
9
10
11
Meishan−A
Meishan−B
Meishan−C
European−A
European−B
European−C

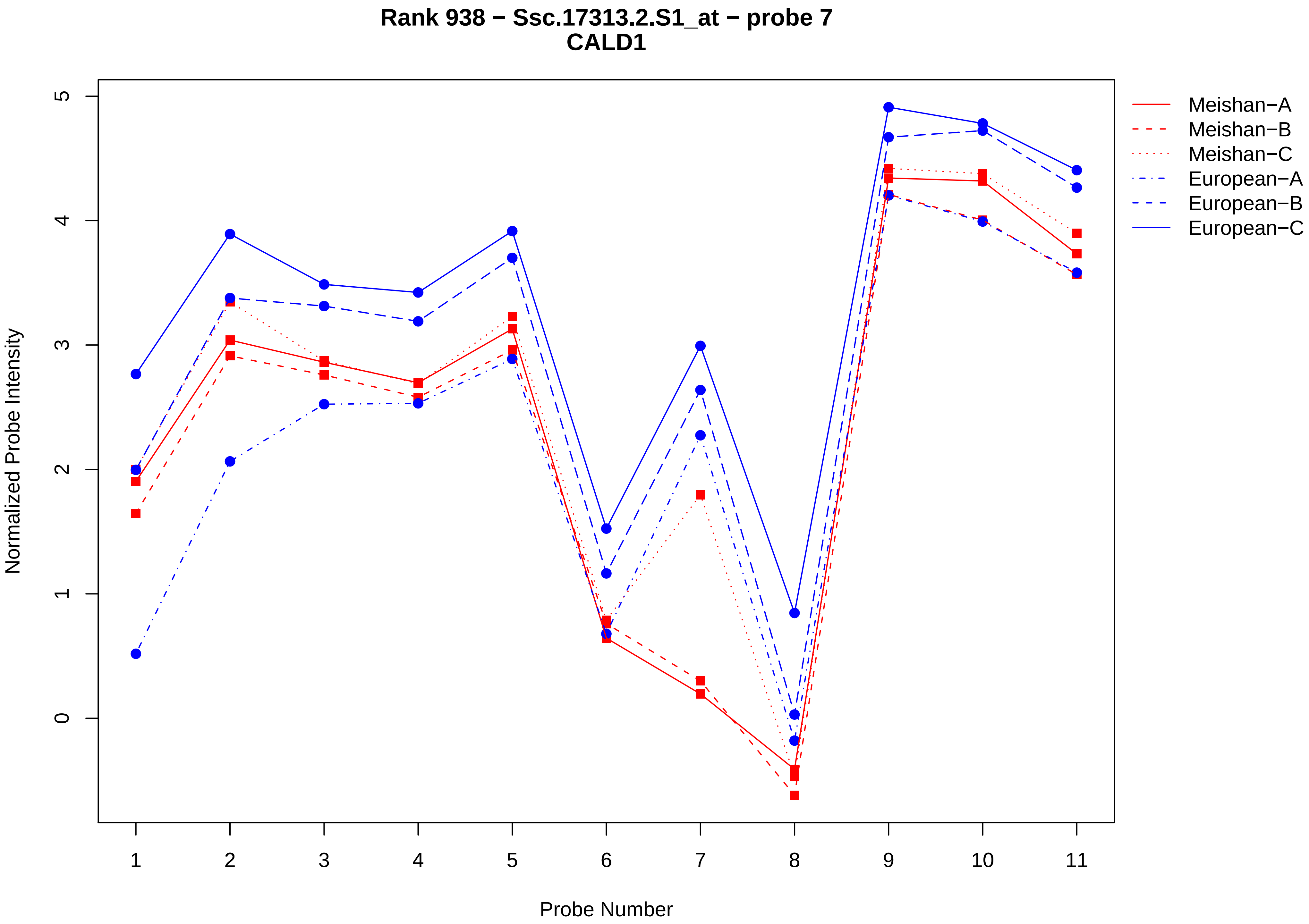

Rank 938 − Ssc.17313.2.S1_at − probe 7
CALD1
Normalized Probe Intensity
Probe Number
0
1
2
3
4
5
1
2
3
4
5
6
7
8
9
10
11
Meishan−A
Meishan−B
Meishan−C
European−A
European−B
European−C

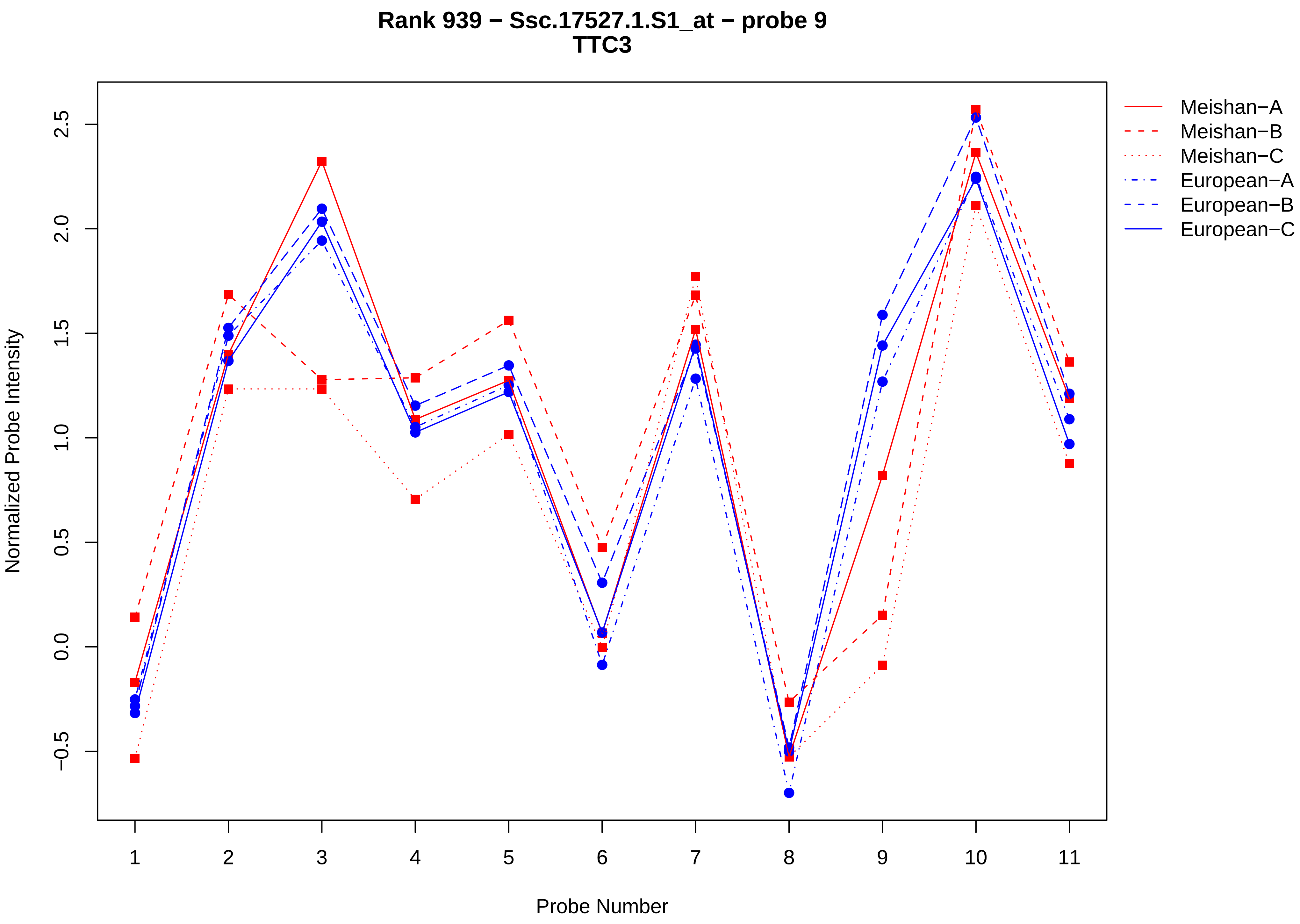
Rank 939 − Ssc.17527.1.S1_at − probe 9
TTC3
Normalized Probe Intensity
Probe Number
−0.5
0.0
0.5
1.0
1.5
2.0
2.5
1
2
3
4
5
6
7
8
9
10
11
Meishan−A
Meishan−B
Meishan−C
European−A
European−B
European−C

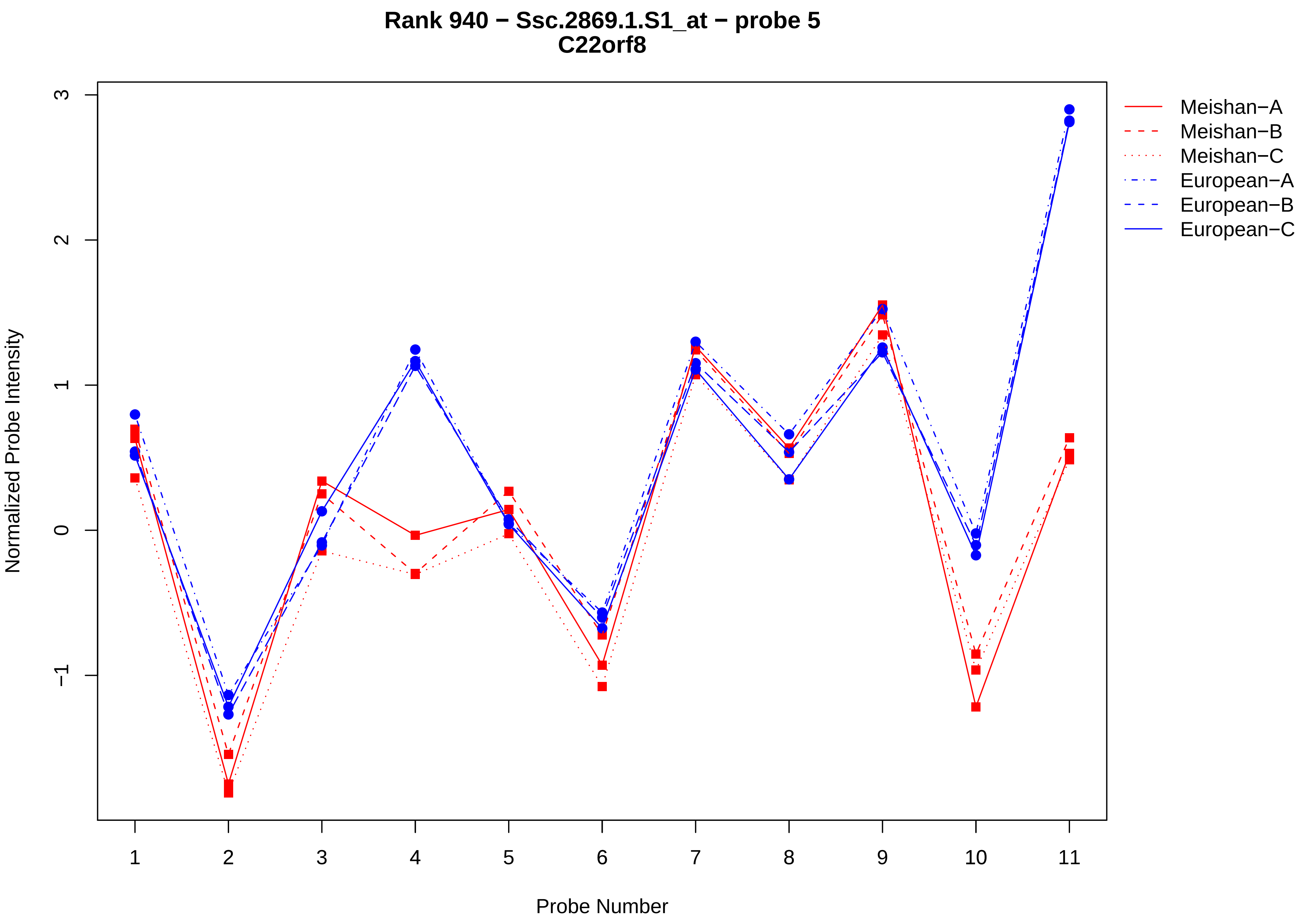

Rank 940 − Ssc.2869.1.S1_at − probe 5
C22orf8
Normalized Probe Intensity
Probe Number
Meishan−A
Meishan−B
Meishan−C
European−A
European−B
European−C
−1
0
1
2
3
1
2
3
4
5
6
7
8
9
10
11

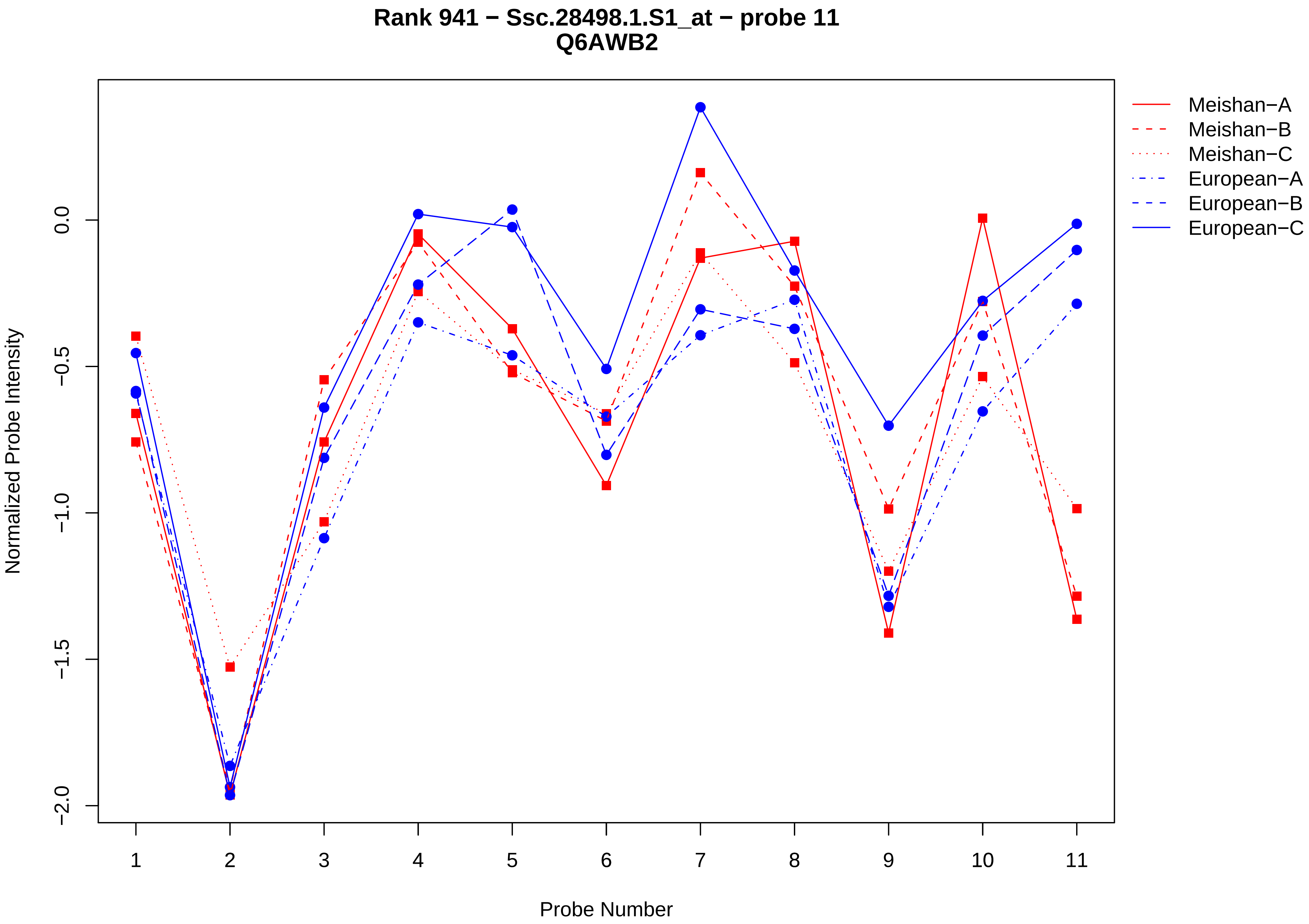

Rank 941 − Ssc.28498.1.S1_at − probe 11
Q6AWB2
Normalized Probe Intensity
Probe Number
0.0
−0.5
−1.0
−1.5
−2.0
1
2
3
4
5
6
7
8
9
10
11
Meishan−A
Meishan−B
Meishan−C
European−A
European−B
European−C

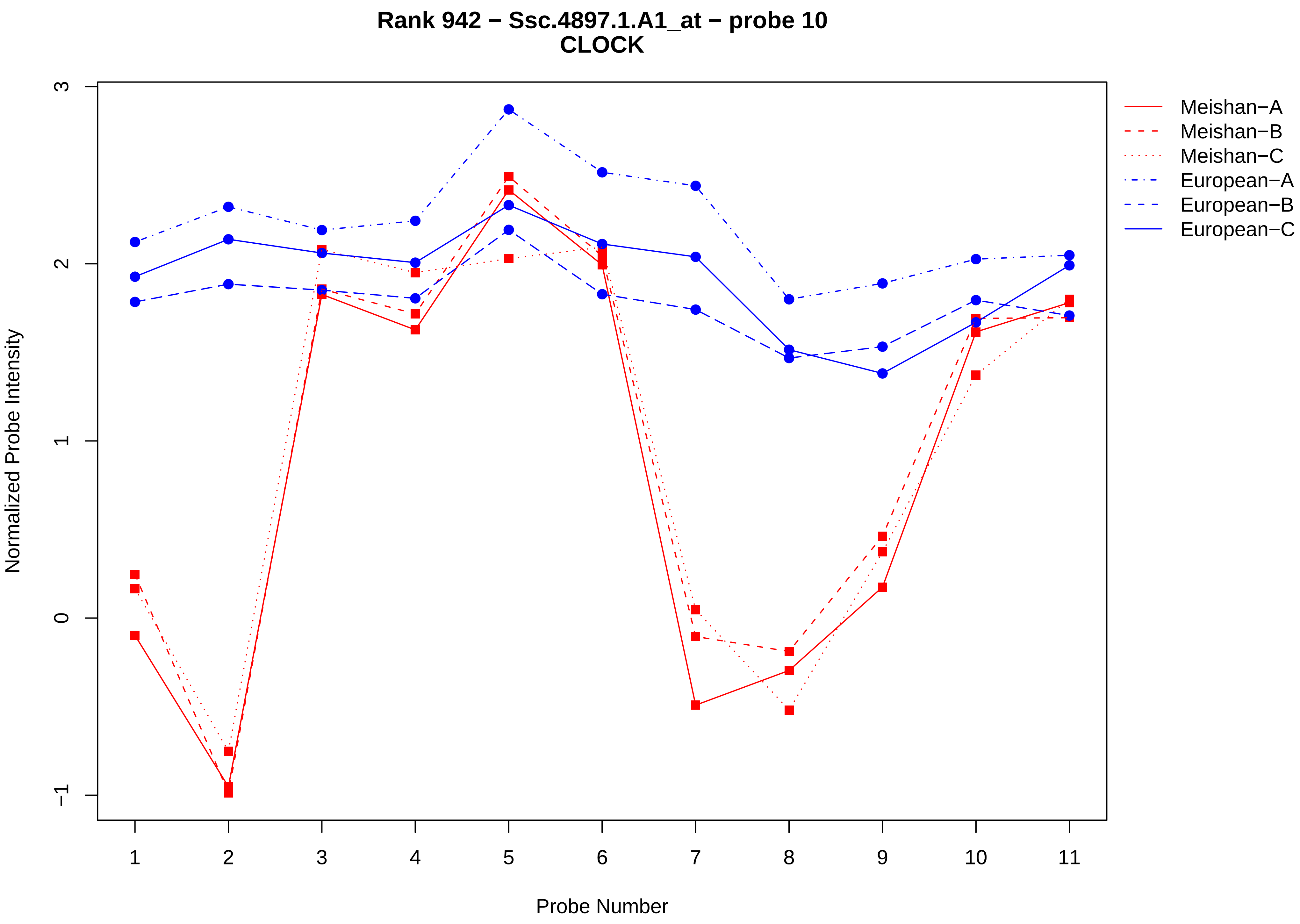

Rank 942 − Ssc.4897.1.A1_at − probe 10
CLOCK
Normalized Probe Intensity
Probe Number
−1
0
1
2
3
1
2
3
4
5
6
7
8
9
10
11
Meishan−A
Meishan−B
Meishan−C
European−A
European−B
European−C

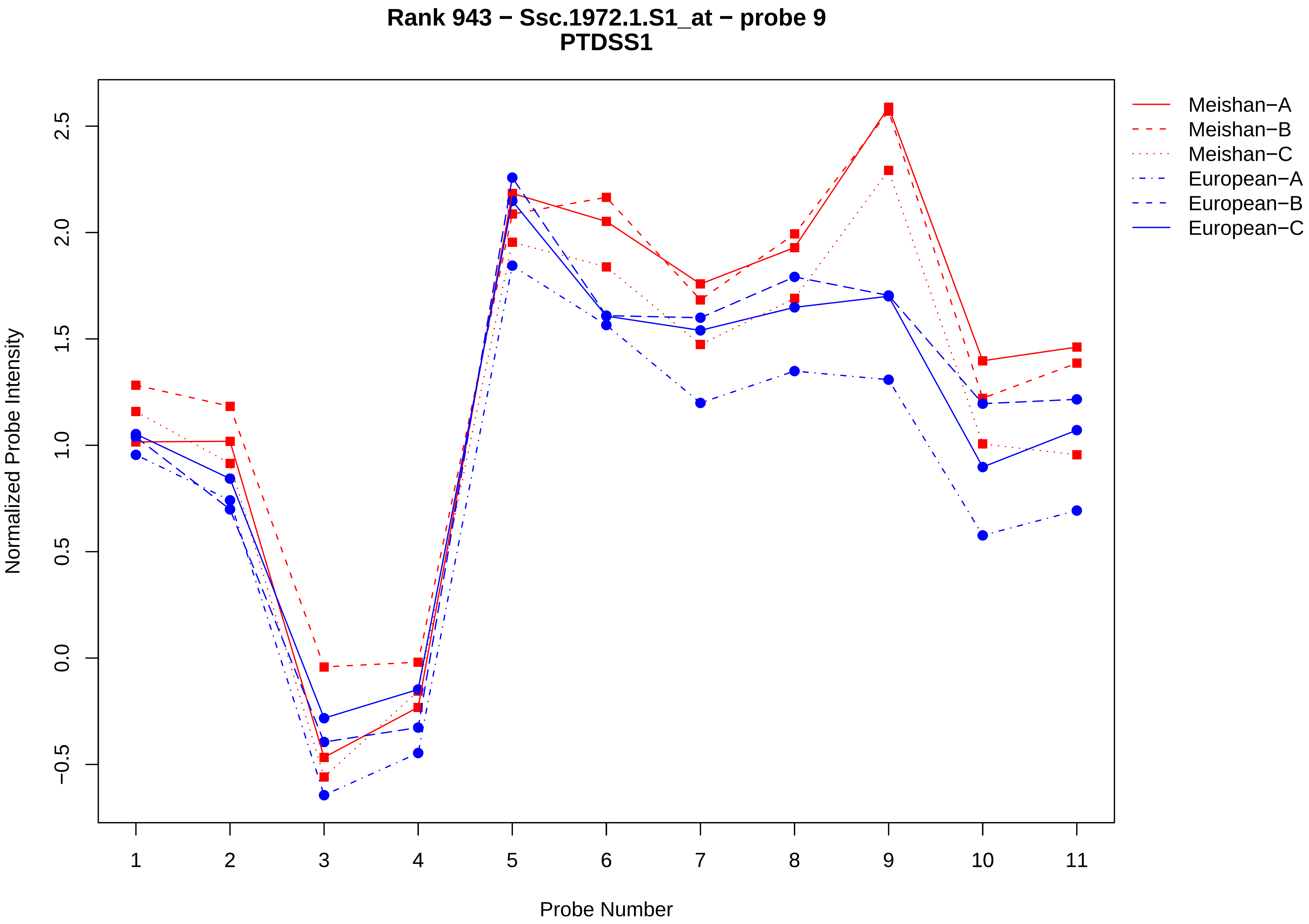

Rank 943 − Ssc.1972.1.S1_at − probe 9
PTDSS1
Normalized Probe Intensity
Probe Number
Meishan−A
Meishan−B
Meishan−C
European−A
European−B
European−C

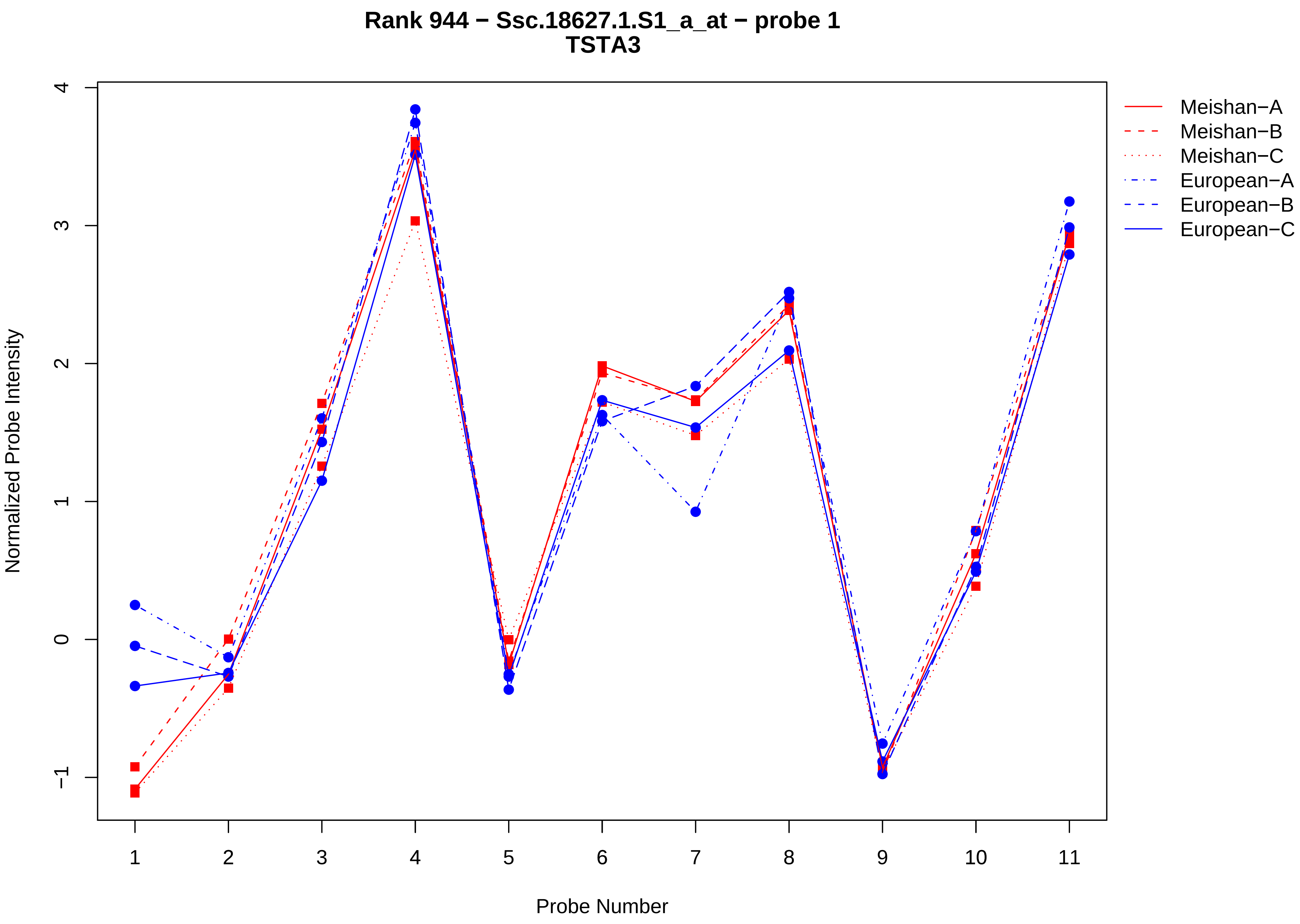

Rank 944 − Ssc.18627.1.S1_a_at − probe 1
TSTA3
Normalized Probe Intensity
Probe Number
Meishan−A
Meishan−B
Meishan−C
European−A
European−B
European−C

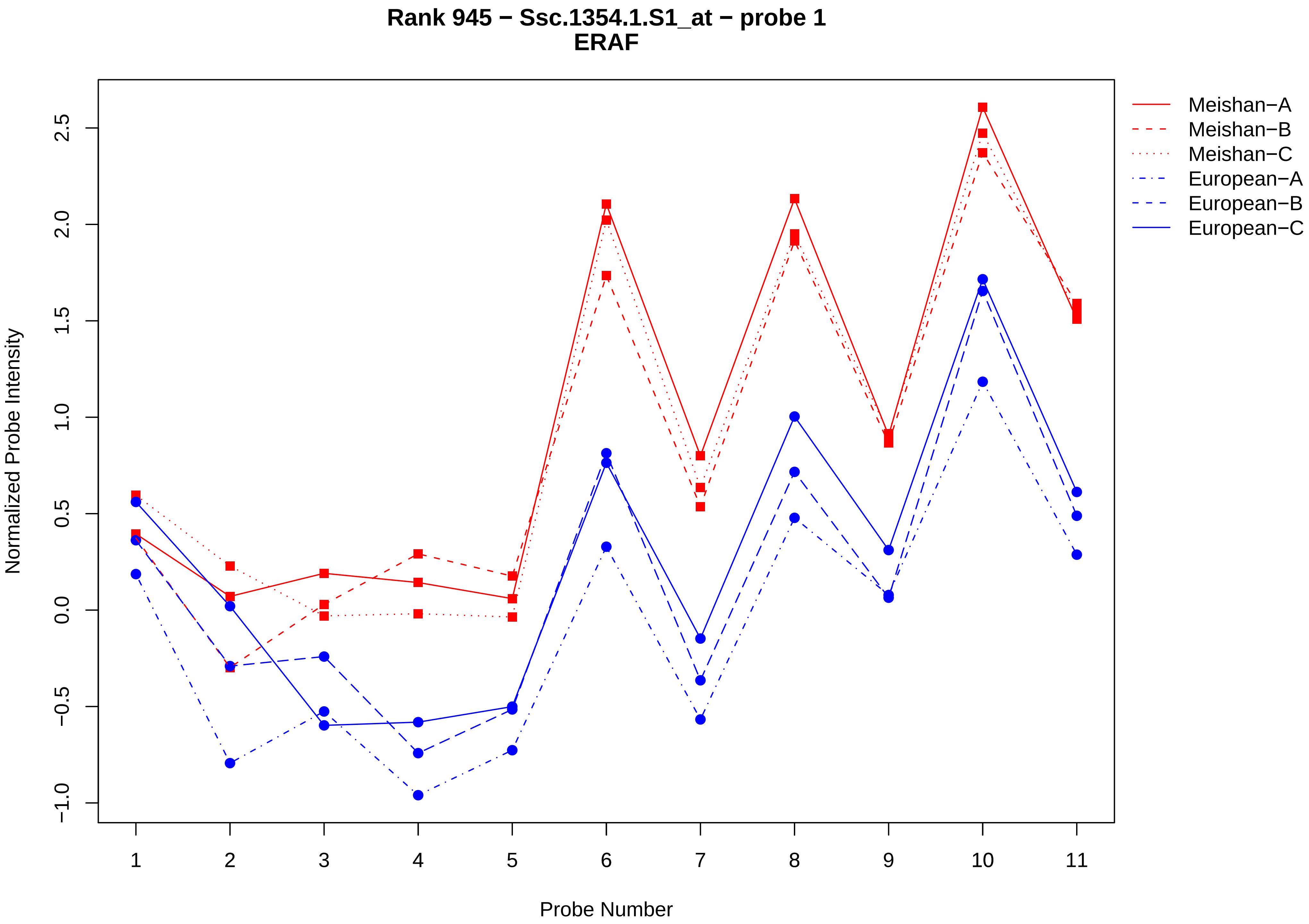

Rank 945 − Ssc.1354.1.S1_at − probe 1
ERAF
Normalized Probe Intensity
Probe Number
Meishan−A
Meishan−B
Meishan−C
European−A
European−B
European−C
−1.0
−0.5
0.0
0.5
1.0
1.5
2.0
2.5
1
2
3
4
5
6
7
8
9
10
11

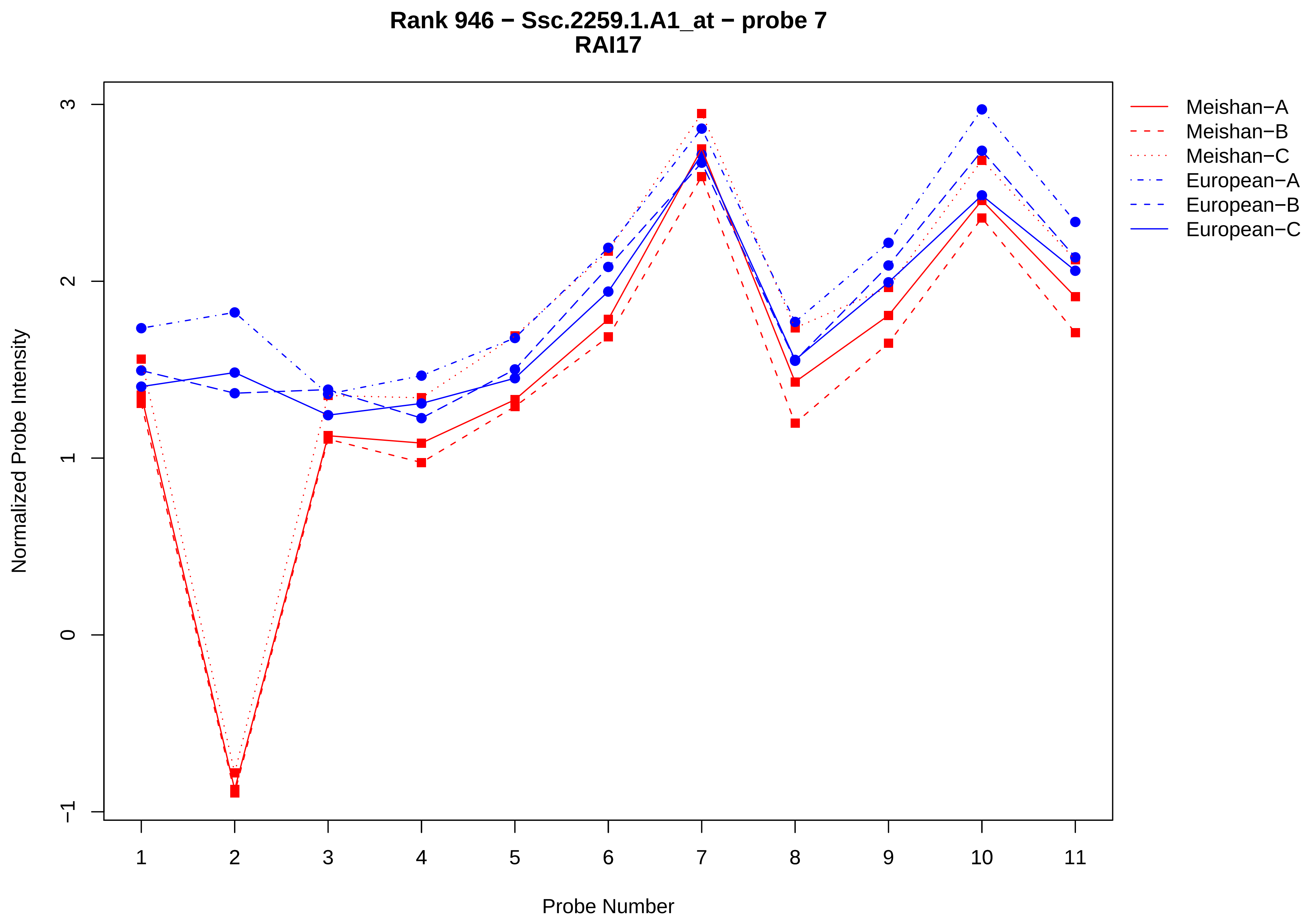

Rank 946 − Ssc.2259.1.A1_at − probe 7
RAI17
Normalized Probe Intensity
Probe Number
−1
0
1
2
3
1
2
3
4
5
6
7
8
9
10
11
Meishan−A
Meishan−B
Meishan−C
European−A
European−B
European−C

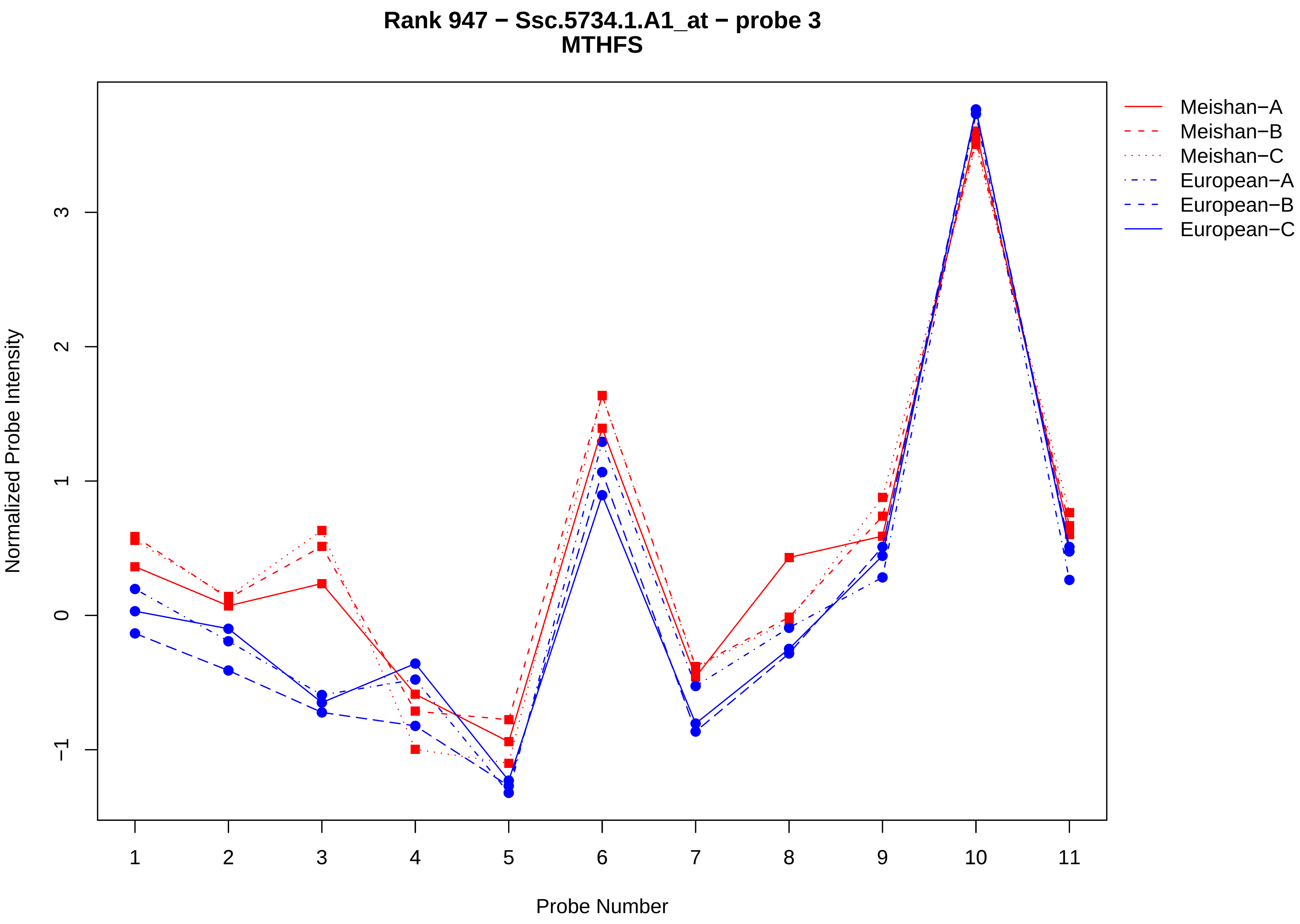

Rank 947 − Ssc.5734.1.A1_at − probe 3
MTHFS
Normalized Probe Intensity
Probe Number
−1
0
1
2
3
1
2
3
4
5
6
7
8
9
10
11
Meishan−A
Meishan−B
Meishan−C
European−A
European−B
European−C

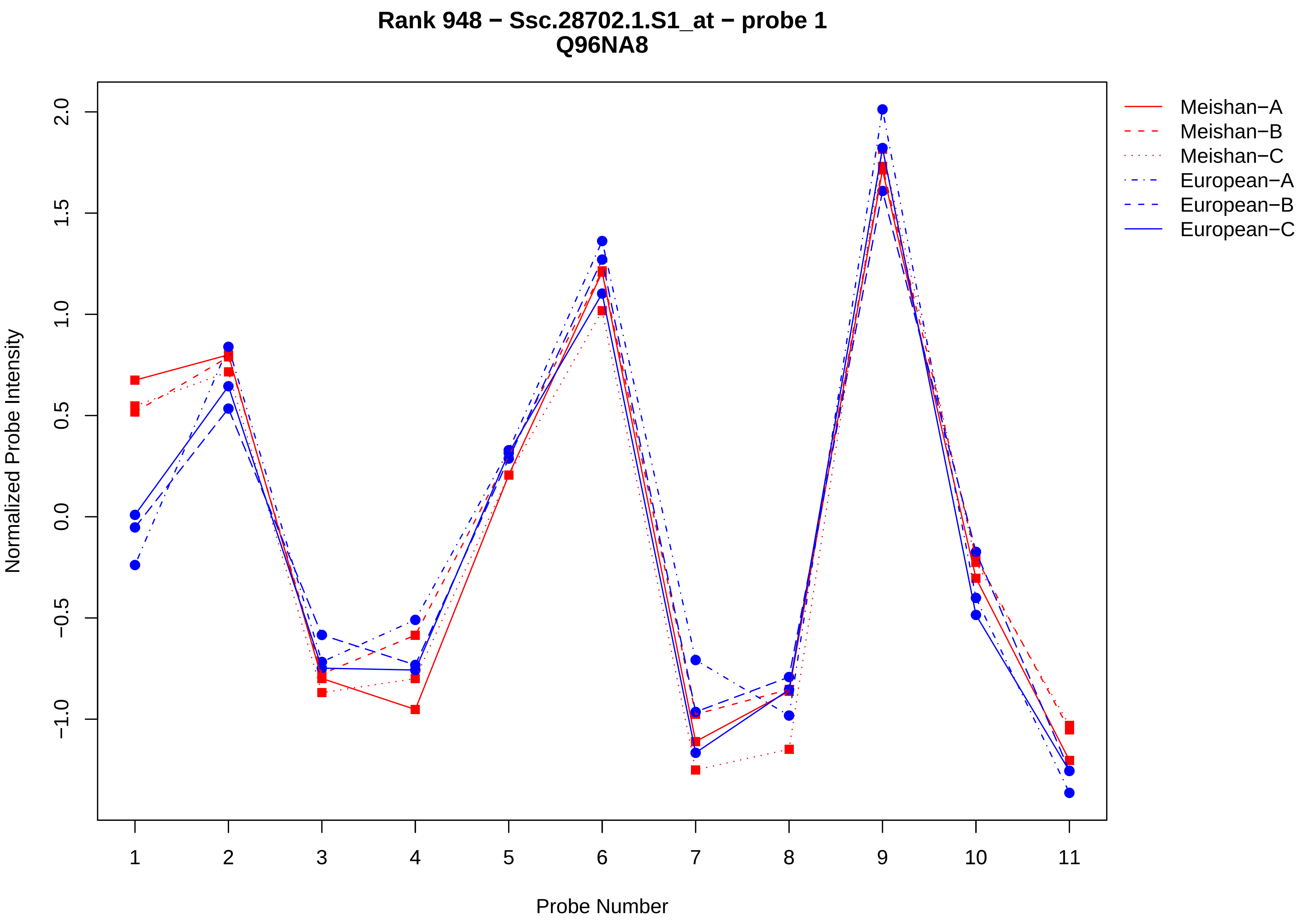

Rank 948 − Ssc.28702.1.S1_at − probe 1
Q96NA8
Normalized Probe Intensity
Probe Number
2.0
1.5
1.0
0.5
0.0
−0.5
−1.0
1
2
3
4
5
6
7
8
9
10
11
Meishan−A
Meishan−B
Meishan−C
European−A
European−B
European−C

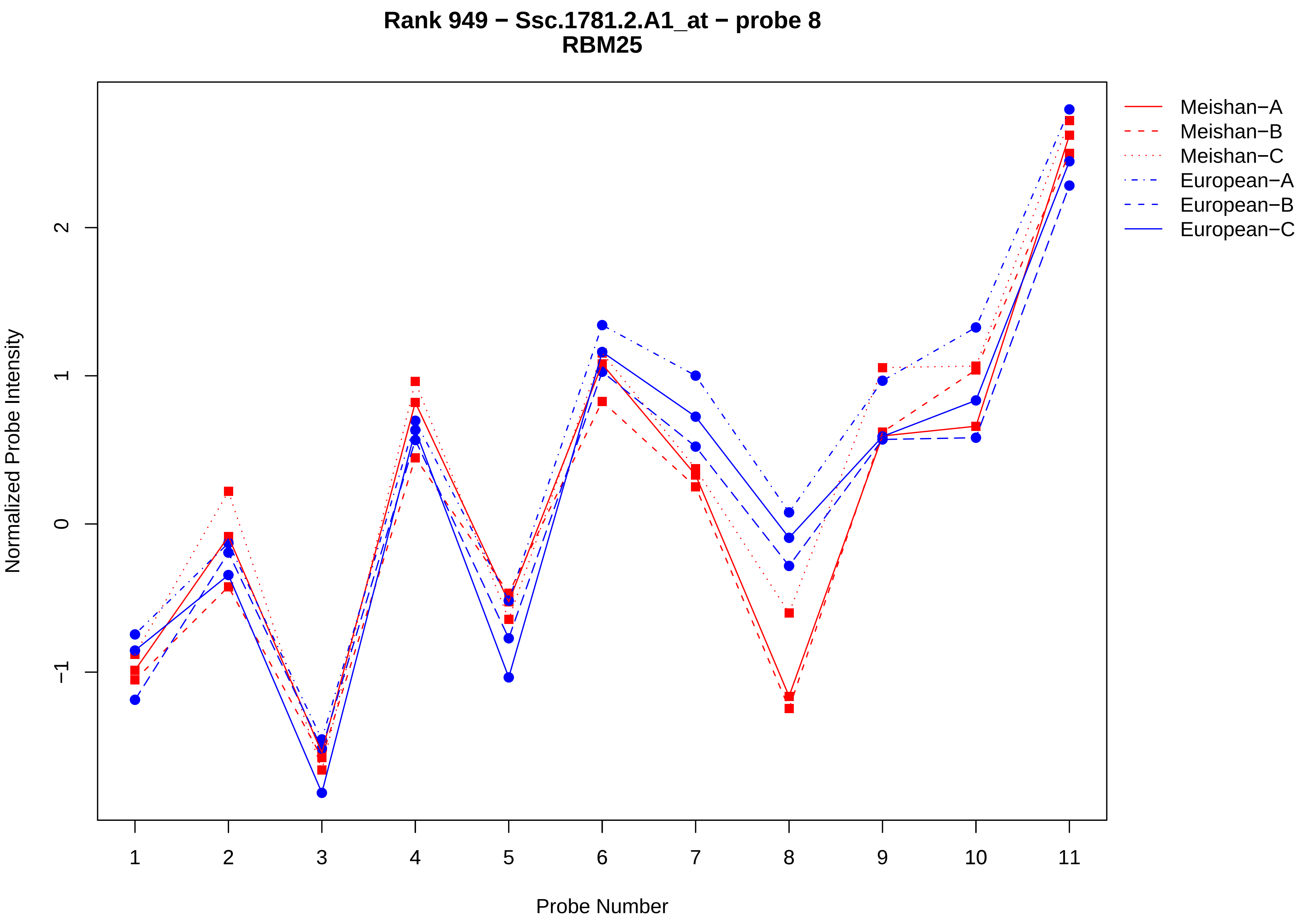

Rank 949 − Ssc.1781.2.A1_at − probe 8
RBM25
Normalized Probe Intensity
Probe Number
−1
0
1
2
1
2
3
4
5
6
7
8
9
10
11
Meishan−A
Meishan−B
Meishan−C
European−A
European−B
European−C

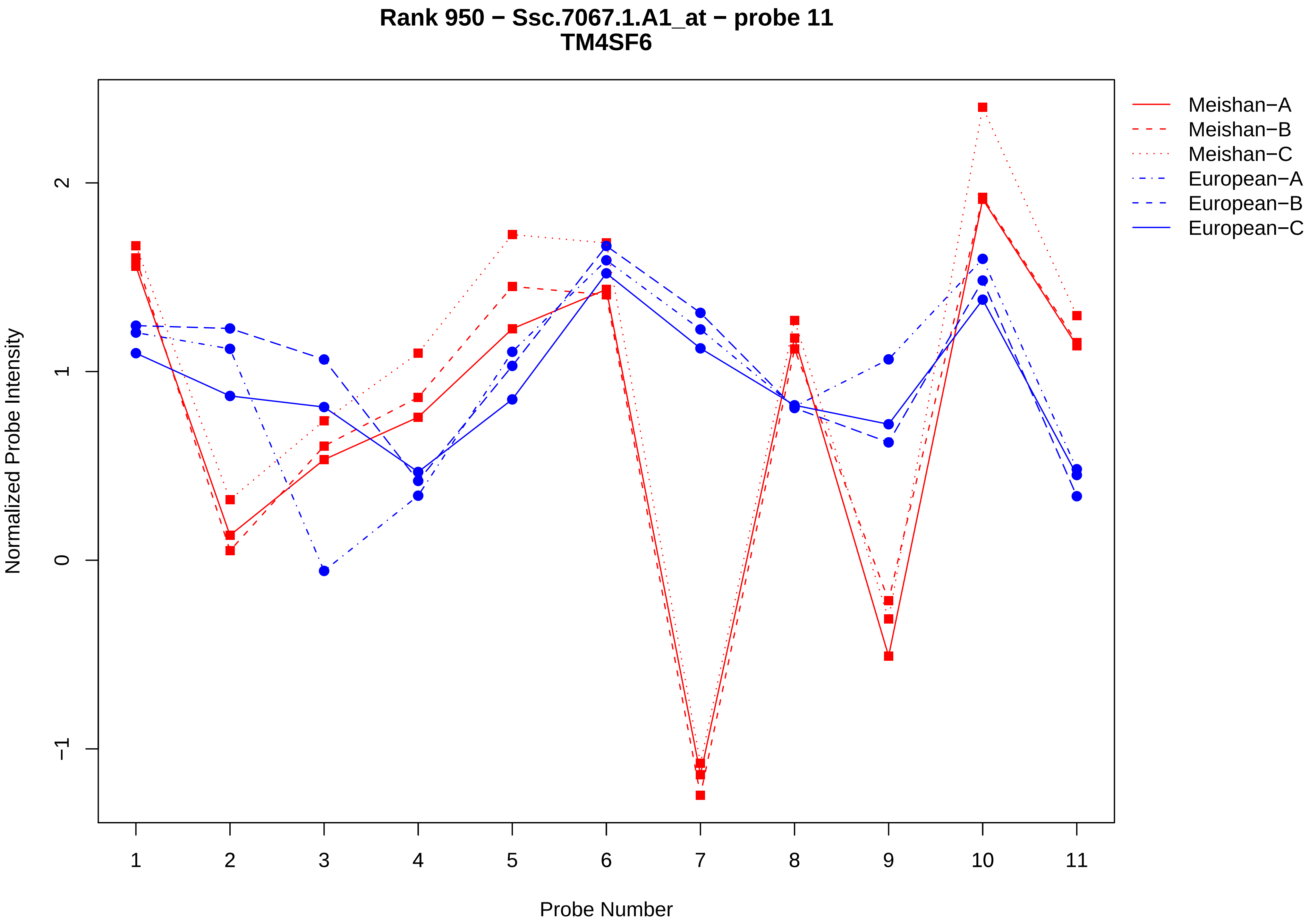

Rank 950 − Ssc.7067.1.A1_at − probe 11
TM4SF6
Normalized Probe Intensity
Probe Number
−1
0
1
2
1
2
3
4
5
6
7
8
9
10
11
Meishan−A
Meishan−B
Meishan−C
European−A
European−B
European−C

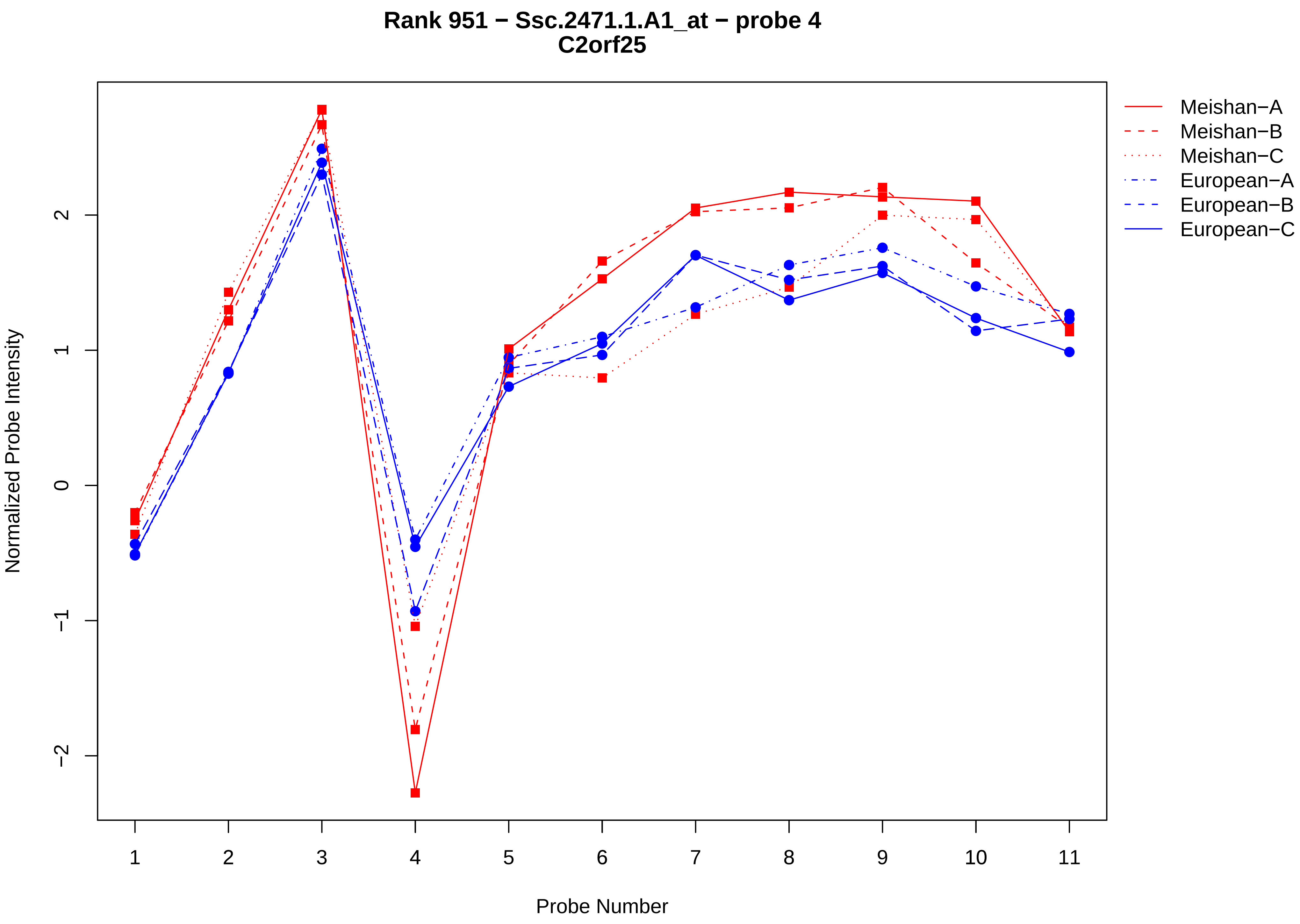

Rank 951 − Ssc.2471.1.A1_at − probe 4
C2orf25
Normalized Probe Intensity
Probe Number
−2
−1
0
1
2
1
2
3
4
5
6
7
8
9
10
11
Meishan−A
Meishan−B
Meishan−C
European−A
European−B
European−C

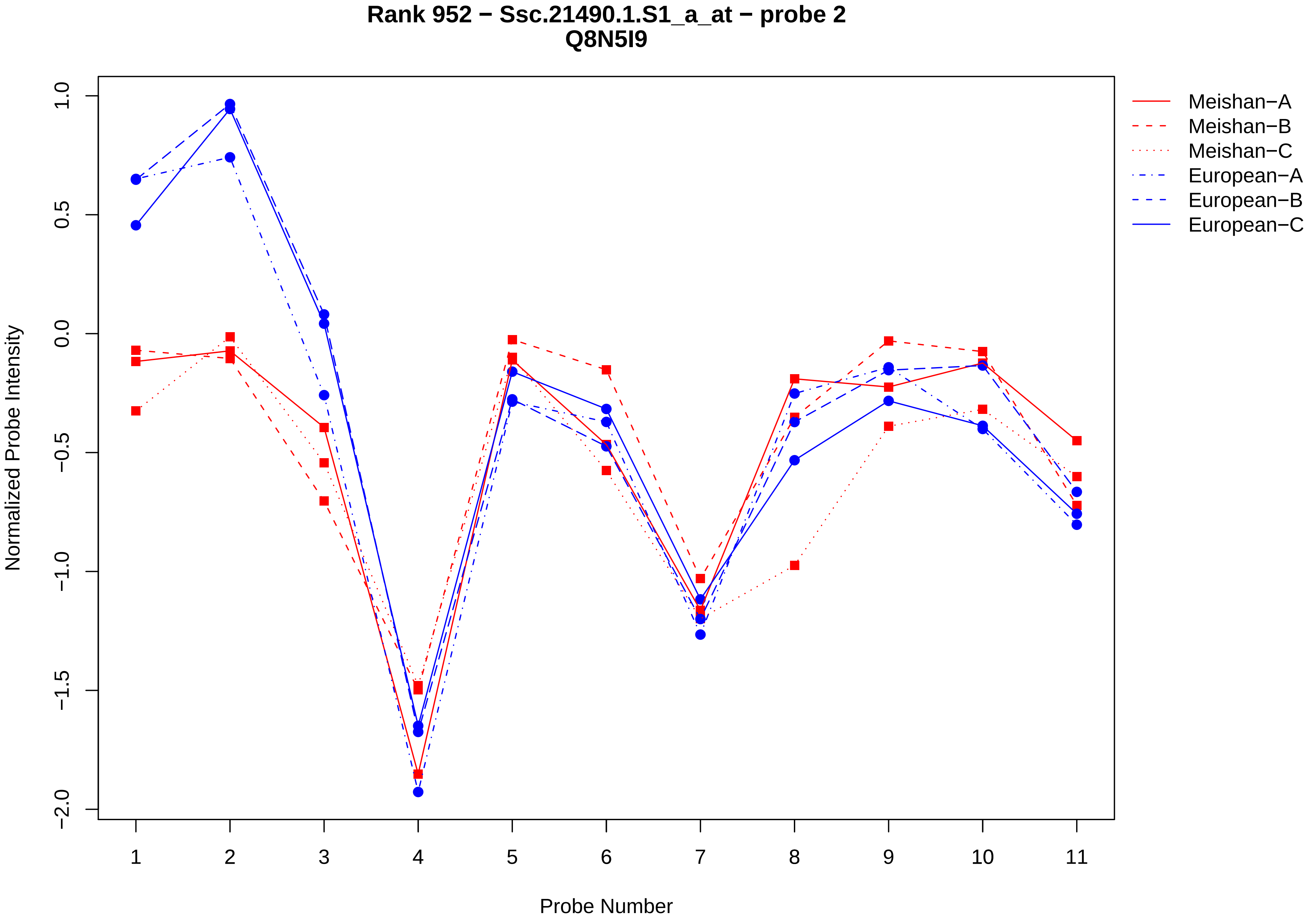

Rank 952 − Ssc.21490.1.S1_a_at − probe 2
Q8N5I9
Normalized Probe Intensity
Probe Number
1.0
0.5
0.0
−0.5
−1.0
−1.5
−2.0
1
2
3
4
5
6
7
8
9
10
11
Meishan−A
Meishan−B
Meishan−C
European−A
European−B
European−C

Rank 953 − Ssc.2114.1.S1_at − probe 2
TNRC6C

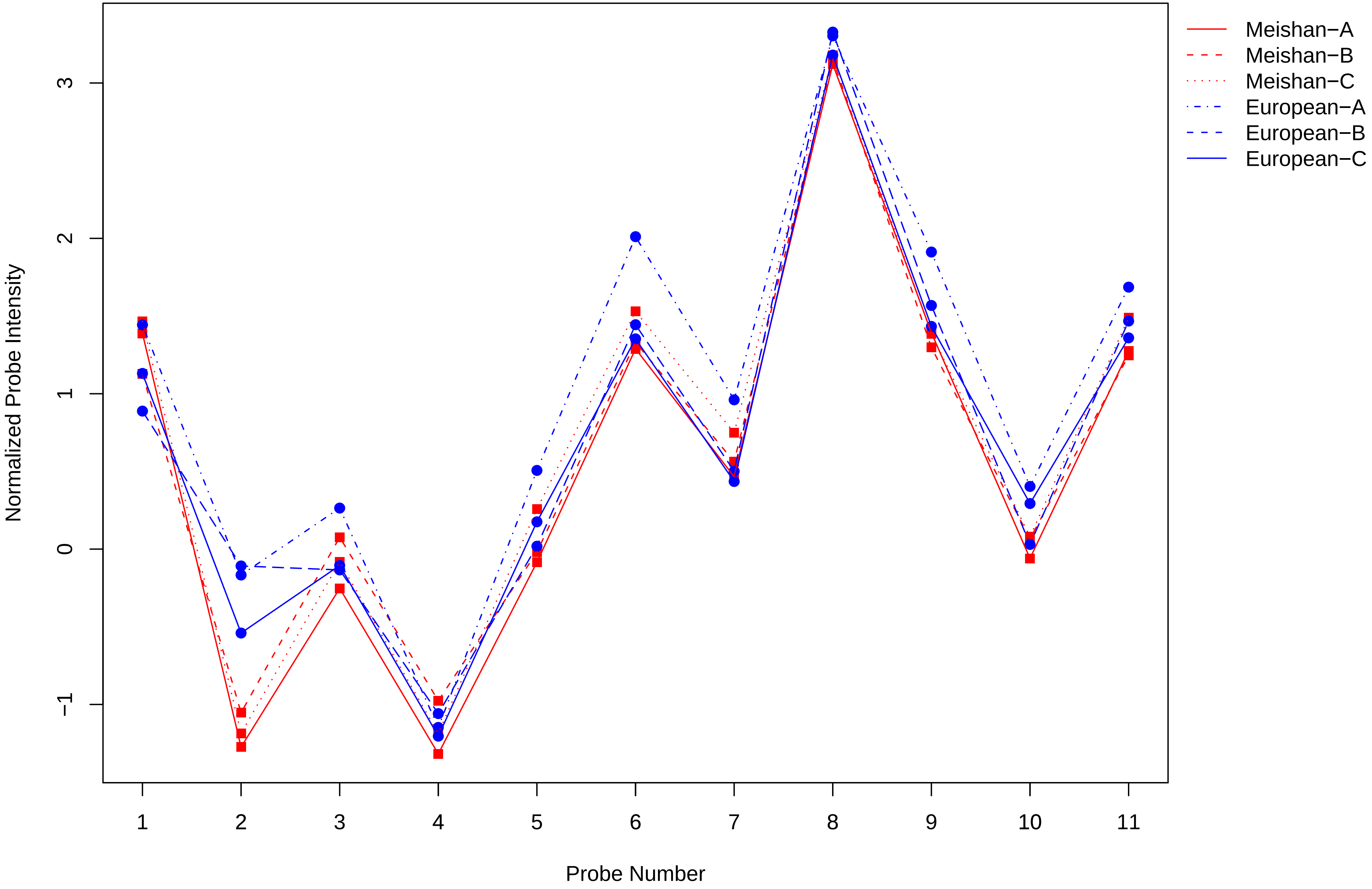

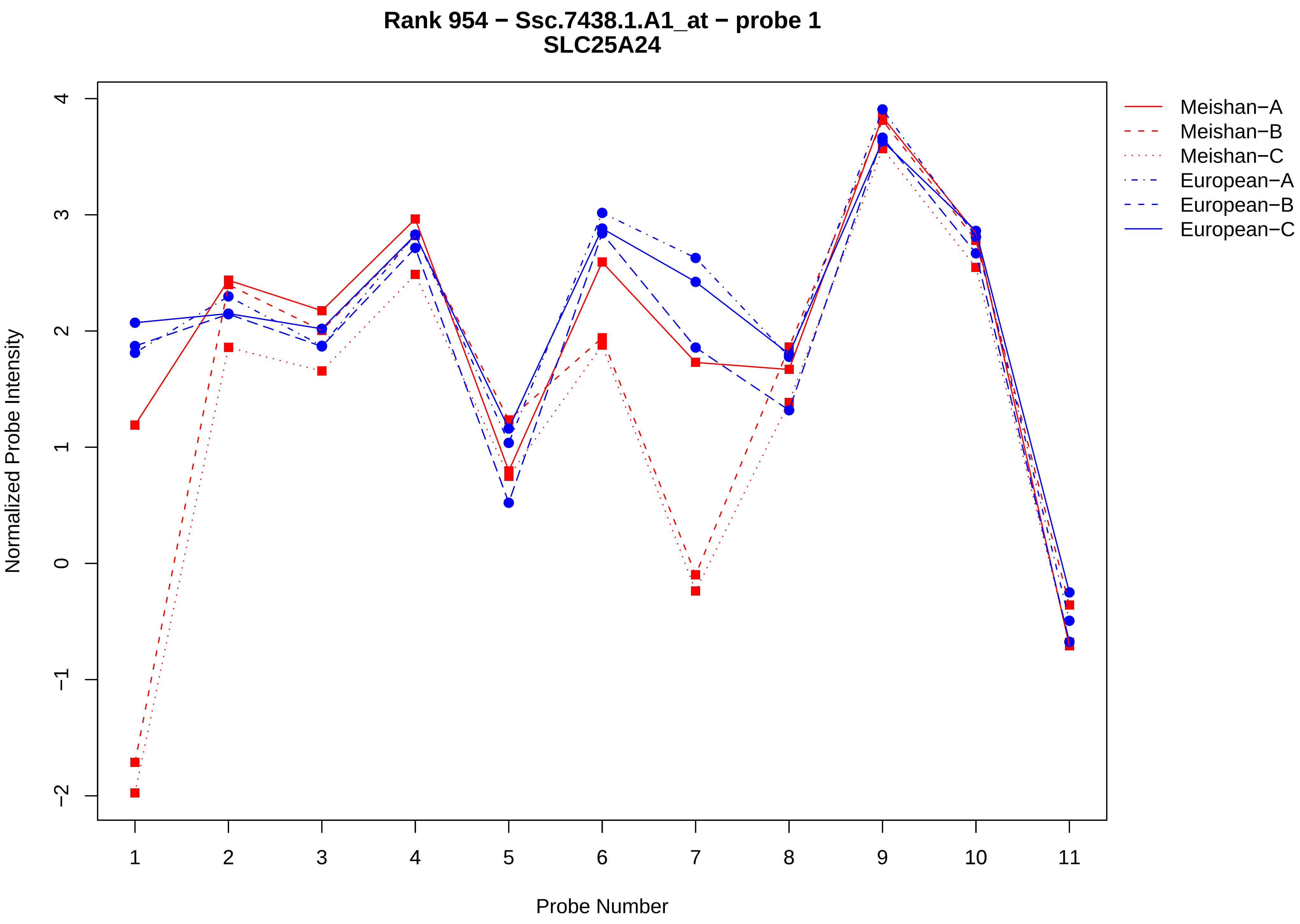

Rank 954 − Ssc.7438.1.A1_at − probe 1
SLC25A24
Normalized Probe Intensity
Probe Number
−2
−1
0
1
2
3
4
1
2
3
4
5
6
7
8
9
10
11
Meishan−A
Meishan−B
Meishan−C
European−A
European−B
European−C

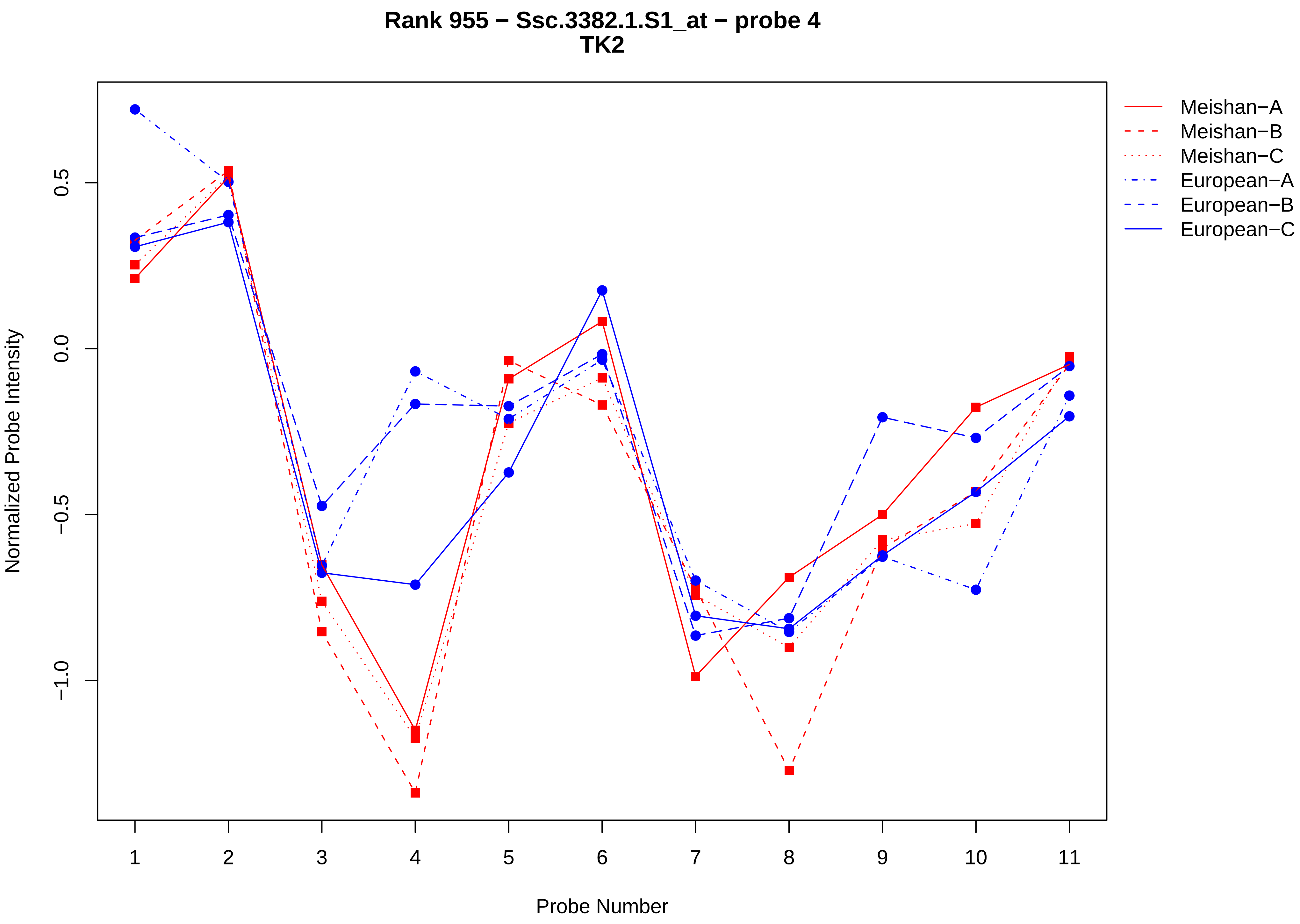

Rank 955 − Ssc.3382.1.S1_at − probe 4
TK2
Normalized Probe Intensity
Probe Number
0.5
0.0
−0.5
−1.0
1
2
3
4
5
6
7
8
9
10
11
Meishan−A
Meishan−B
Meishan−C
European−A
European−B
European−C

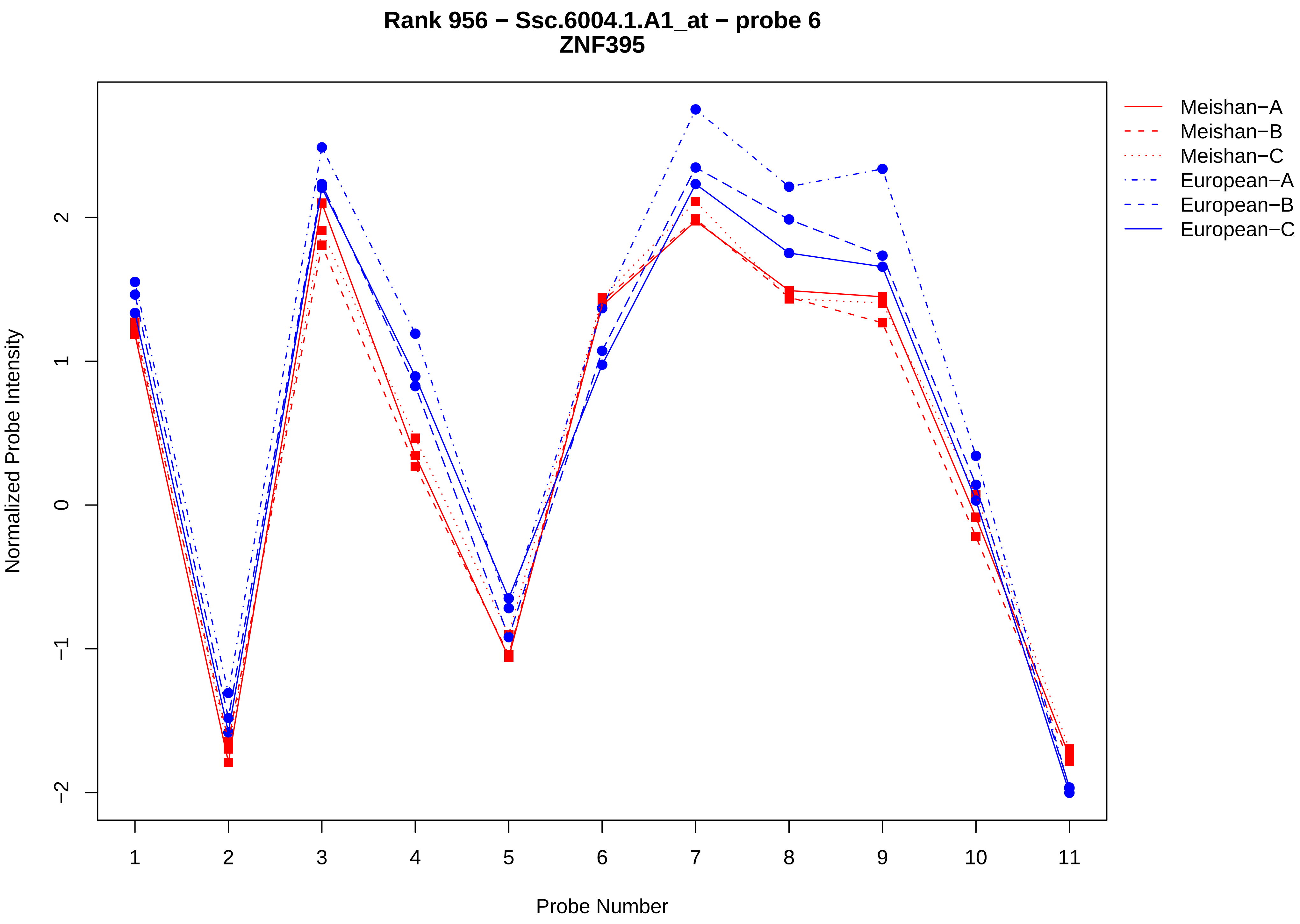

Rank 956 − Ssc.6004.1.A1_at − probe 6
ZNF395
Normalized Probe Intensity
Probe Number
−2
−1
0
1
2
1
2
3
4
5
6
7
8
9
10
11
Meishan−A
Meishan−B
Meishan−C
European−A
European−B
European−C

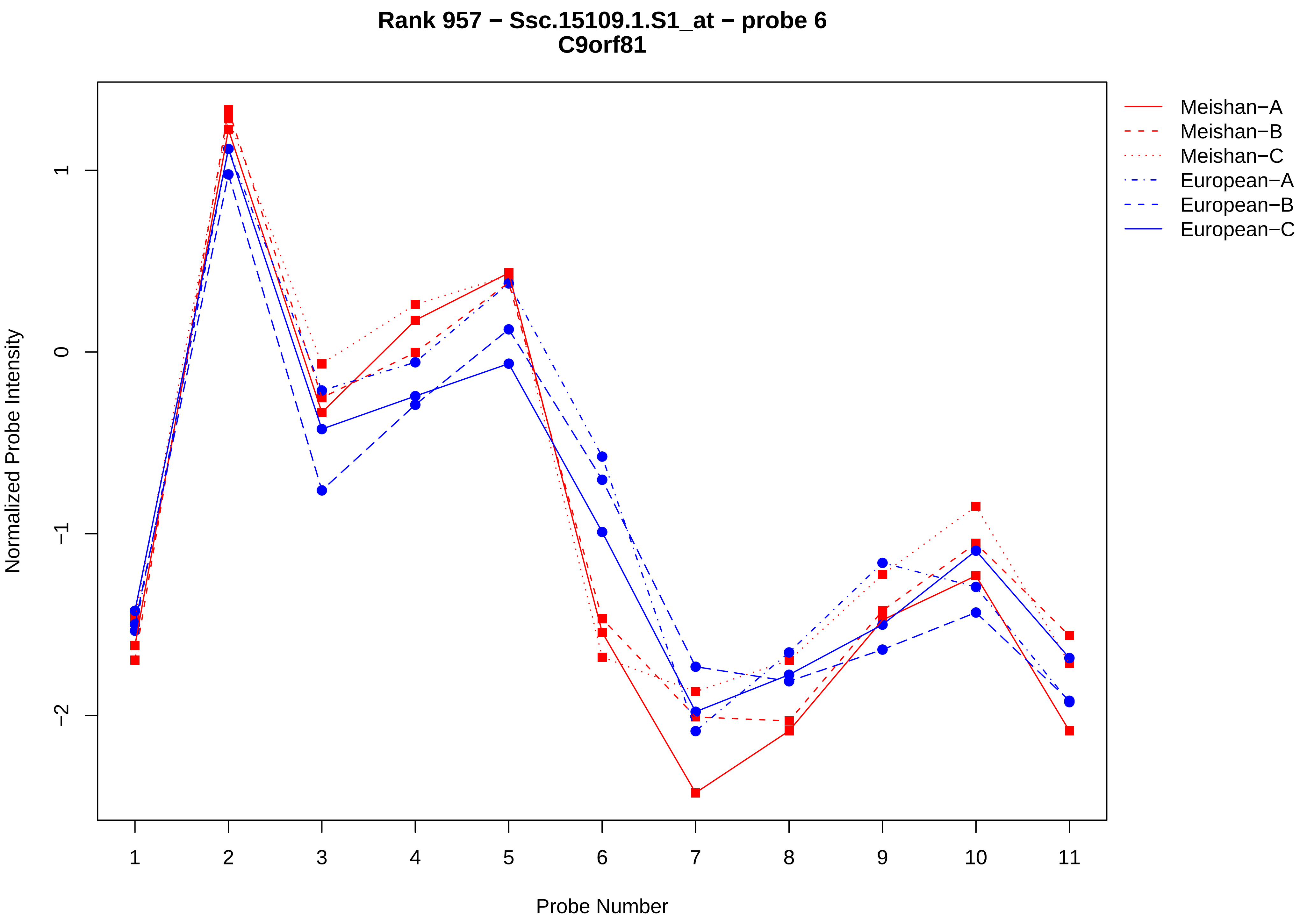

Rank 957 − Ssc.15109.1.S1_at − probe 6
C9orf81
Normalized Probe Intensity
Probe Number
1
0
−1
−2
1
2
3
4
5
6
7
8
9
10
11
Meishan−A
Meishan−B
Meishan−C
European−A
European−B
European−C

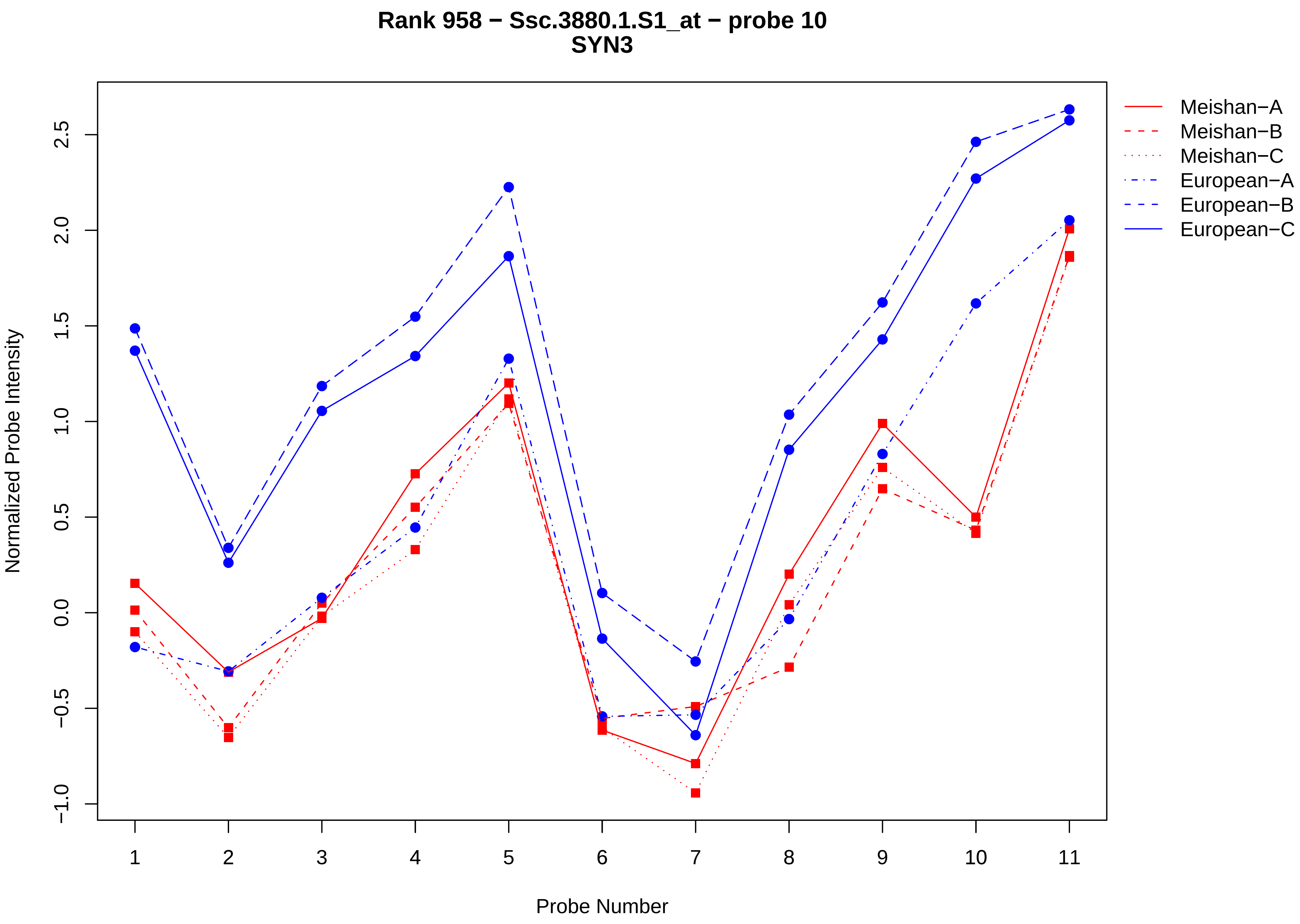

Rank 958 − Ssc.3880.1.S1_at − probe 10
SYN3
Normalized Probe Intensity
Probe Number
Meishan−A
Meishan−B
Meishan−C
European−A
European−B
European−C

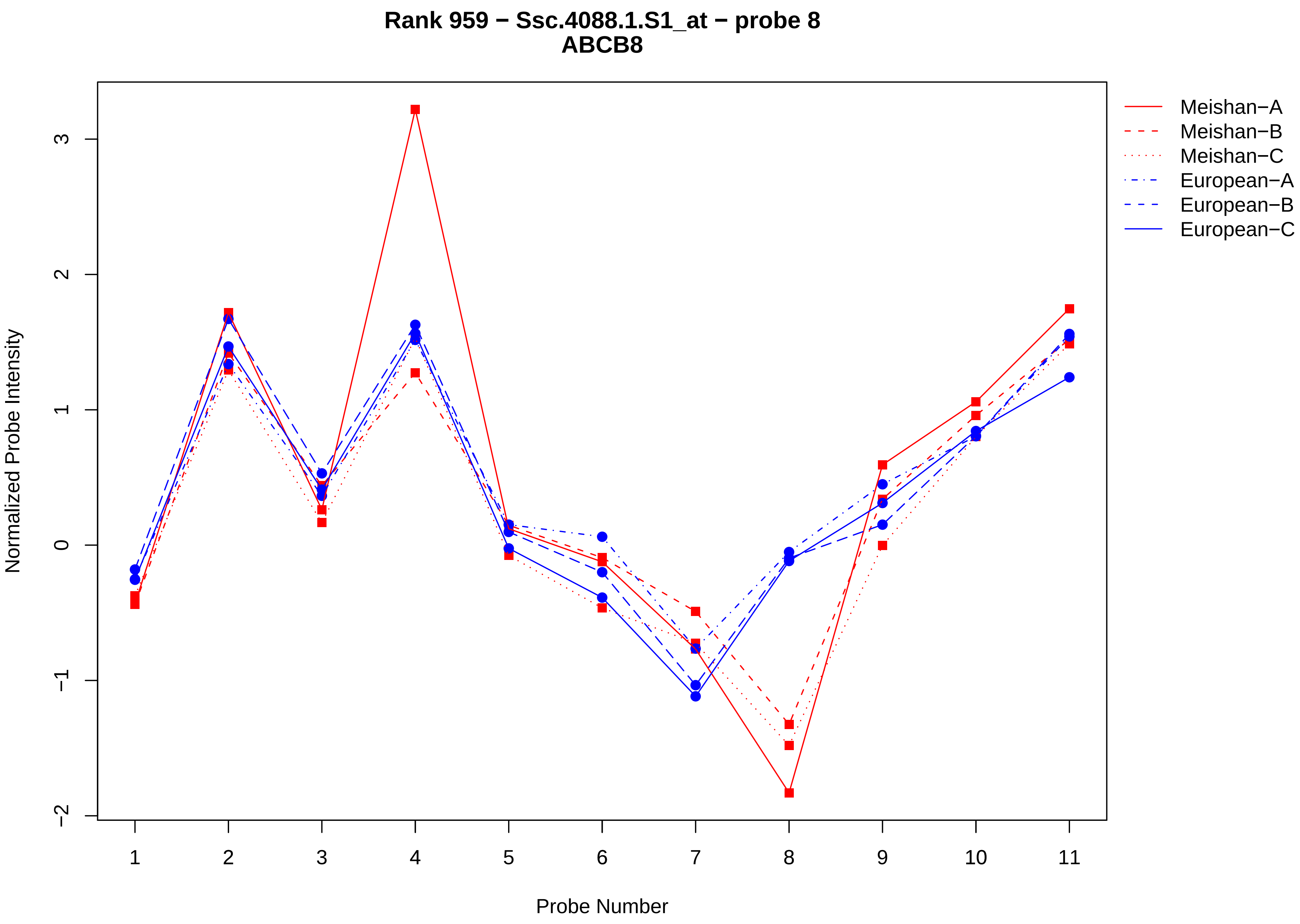

Rank 959 − Ssc.4088.1.S1_at − probe 8
ABCB8
Normalized Probe Intensity
Probe Number
Meishan−A
Meishan−B
Meishan−C
European−A
European−B
European−C

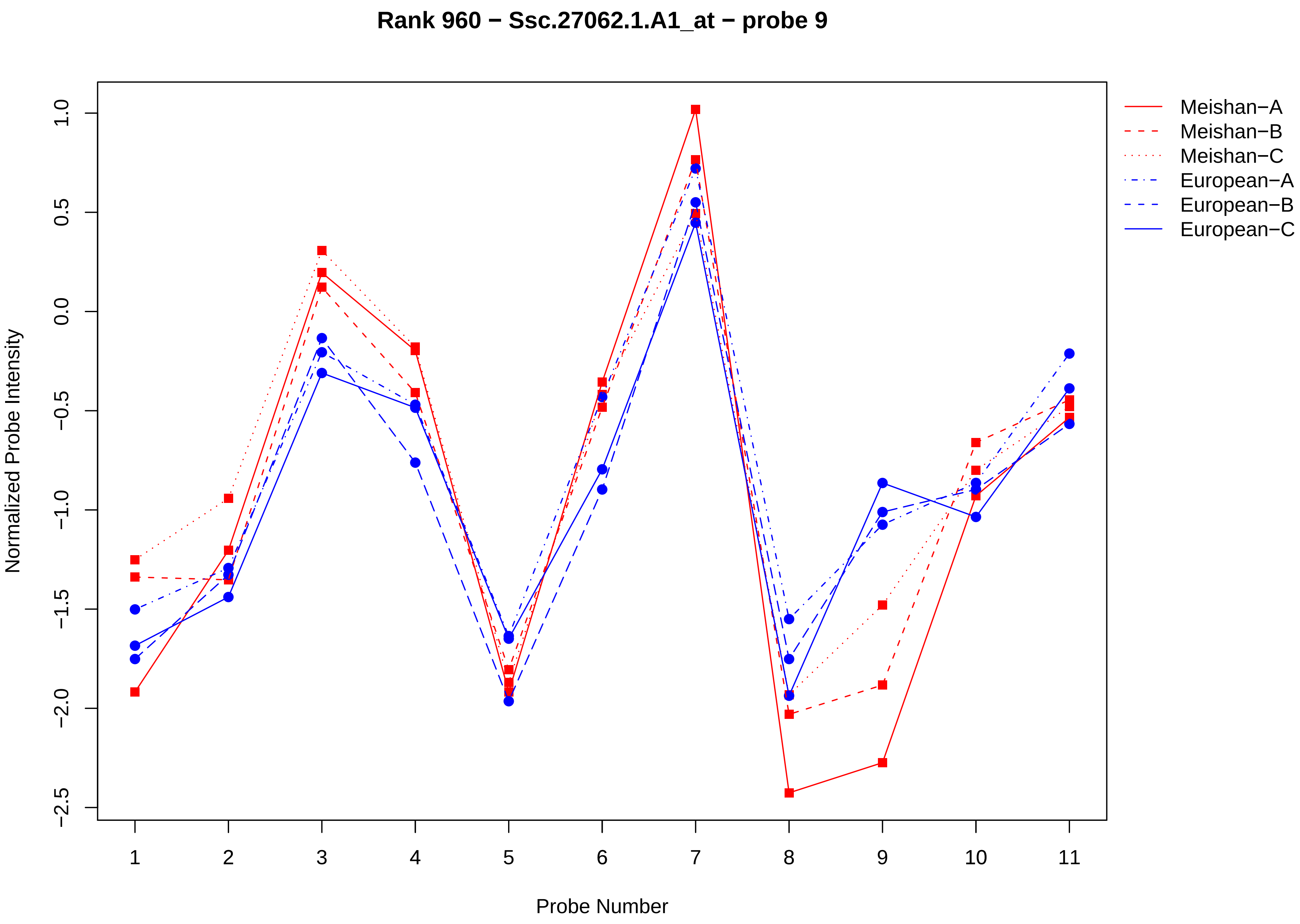

Rank 960 − Ssc.27062.1.A1_at − probe 9
Normalized Probe Intensity
Probe Number
1.0
0.5
0.0
−0.5
−1.0
−1.5
−2.0
−2.5
1
2
3
4
5
6
7
8
9
10
11
Meishan−A
Meishan−B
Meishan−C
European−A
European−B
European−C

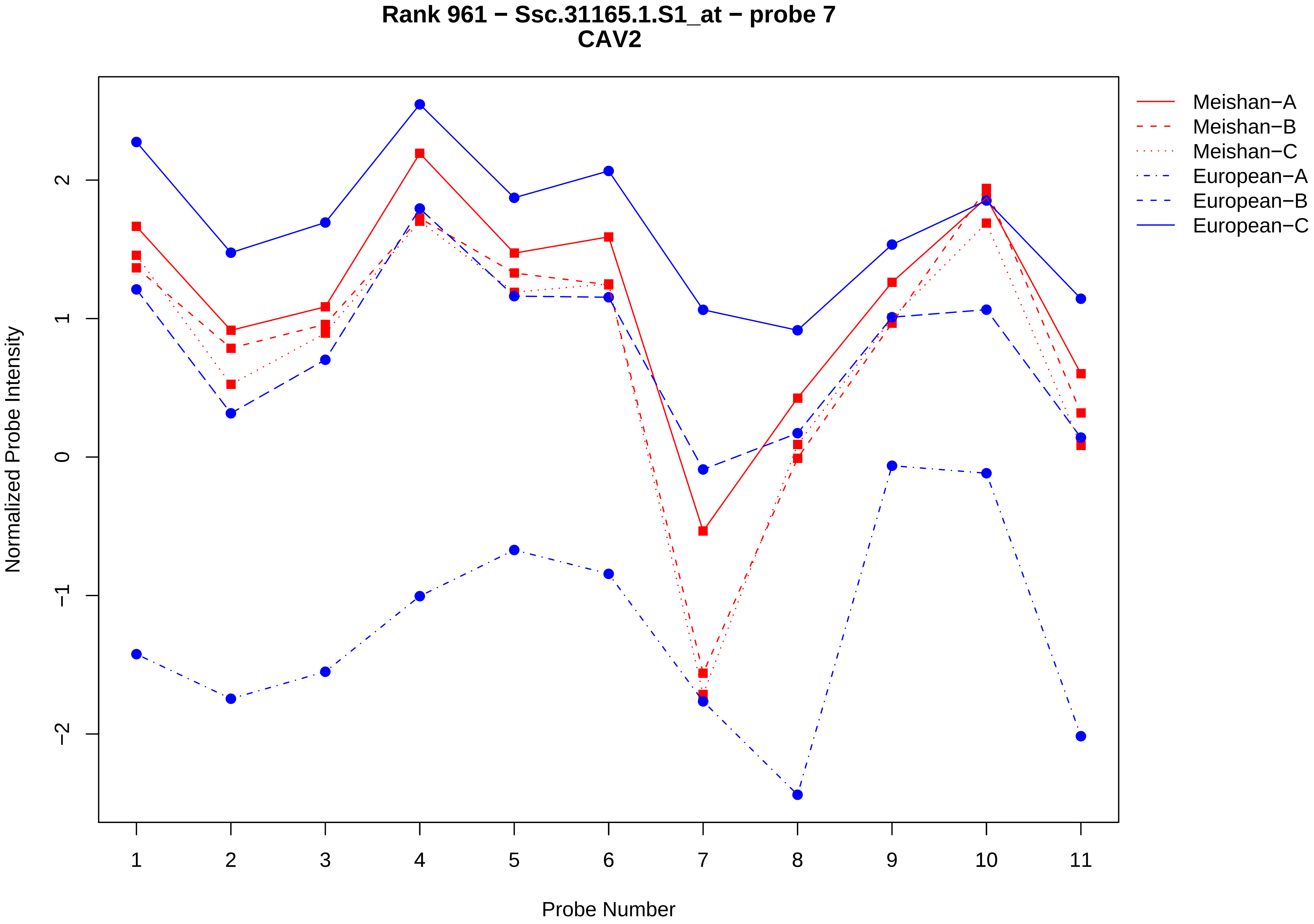

Rank 961 − Ssc.31165.1.S1_at − probe 7
CAV2
Normalized Probe Intensity
Probe Number
−2
−1
0
1
2
1
2
3
4
5
6
7
8
9
10
11
Meishan−A
Meishan−B
Meishan−C
European−A
European−B
European−C

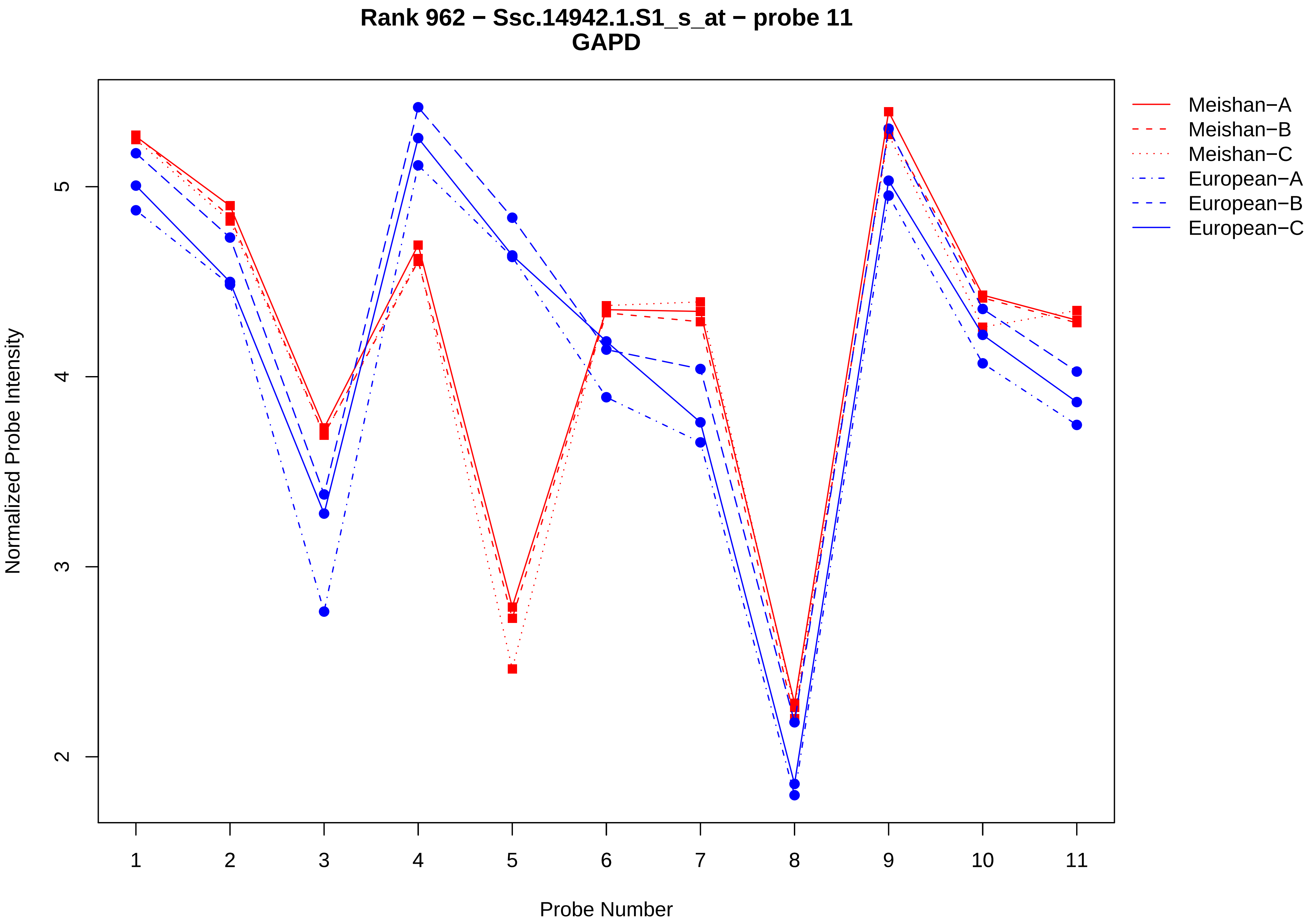

Rank 962 − Ssc.14942.1.S1_s_at − probe 11
GAPD
Normalized Probe Intensity
Probe Number
2
3
4
5
1
2
3
4
5
6
7
8
9
10
11
Meishan−A
Meishan−B
Meishan−C
European−A
European−B
European−C

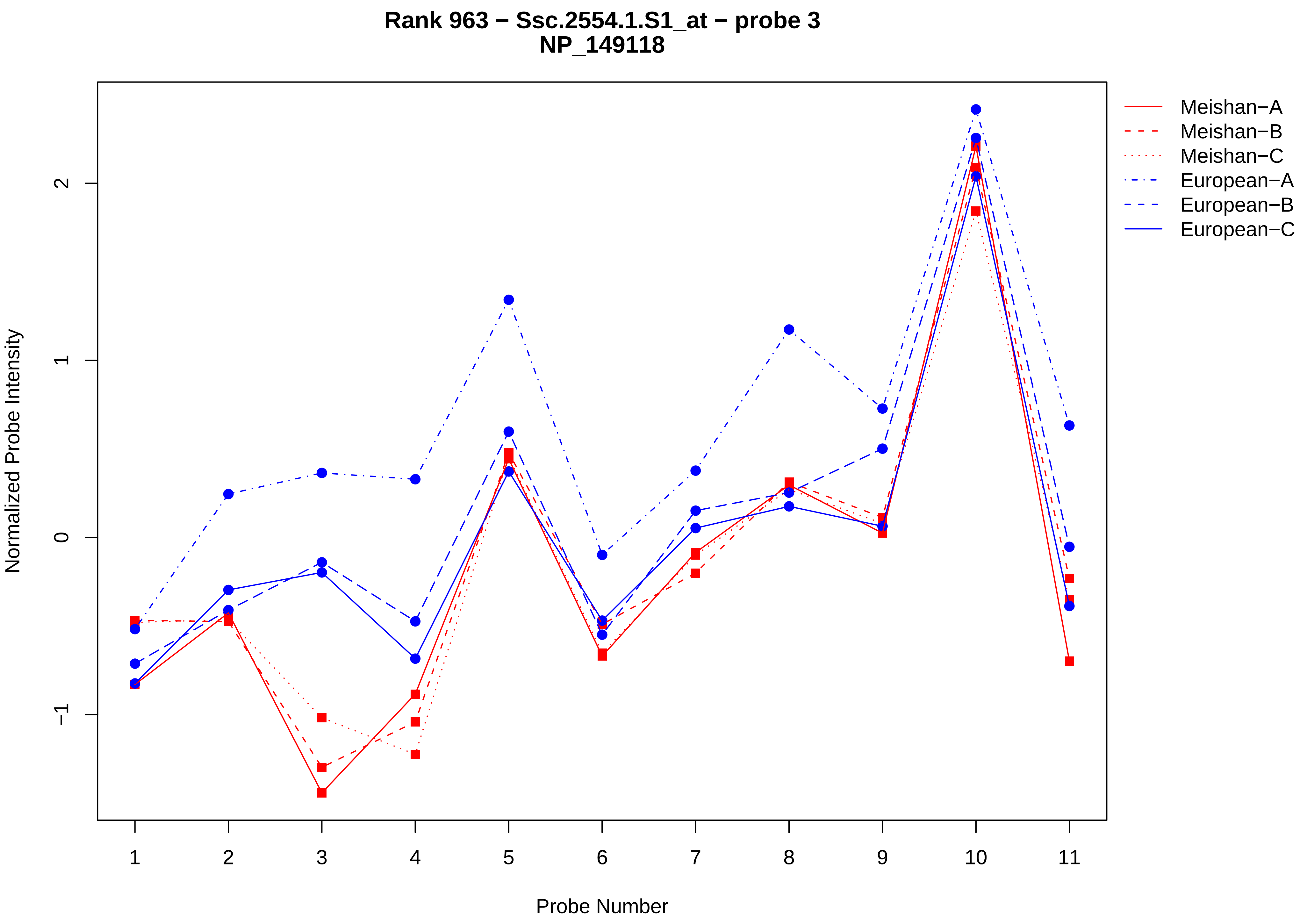

Rank 963 − Ssc.2554.1.S1_at − probe 3
NP_149118
Normalized Probe Intensity
Probe Number
−1
0
1
2
1
2
3
4
5
6
7
8
9
10
11
Meishan−A
Meishan−B
Meishan−C
European−A
European−B
European−C

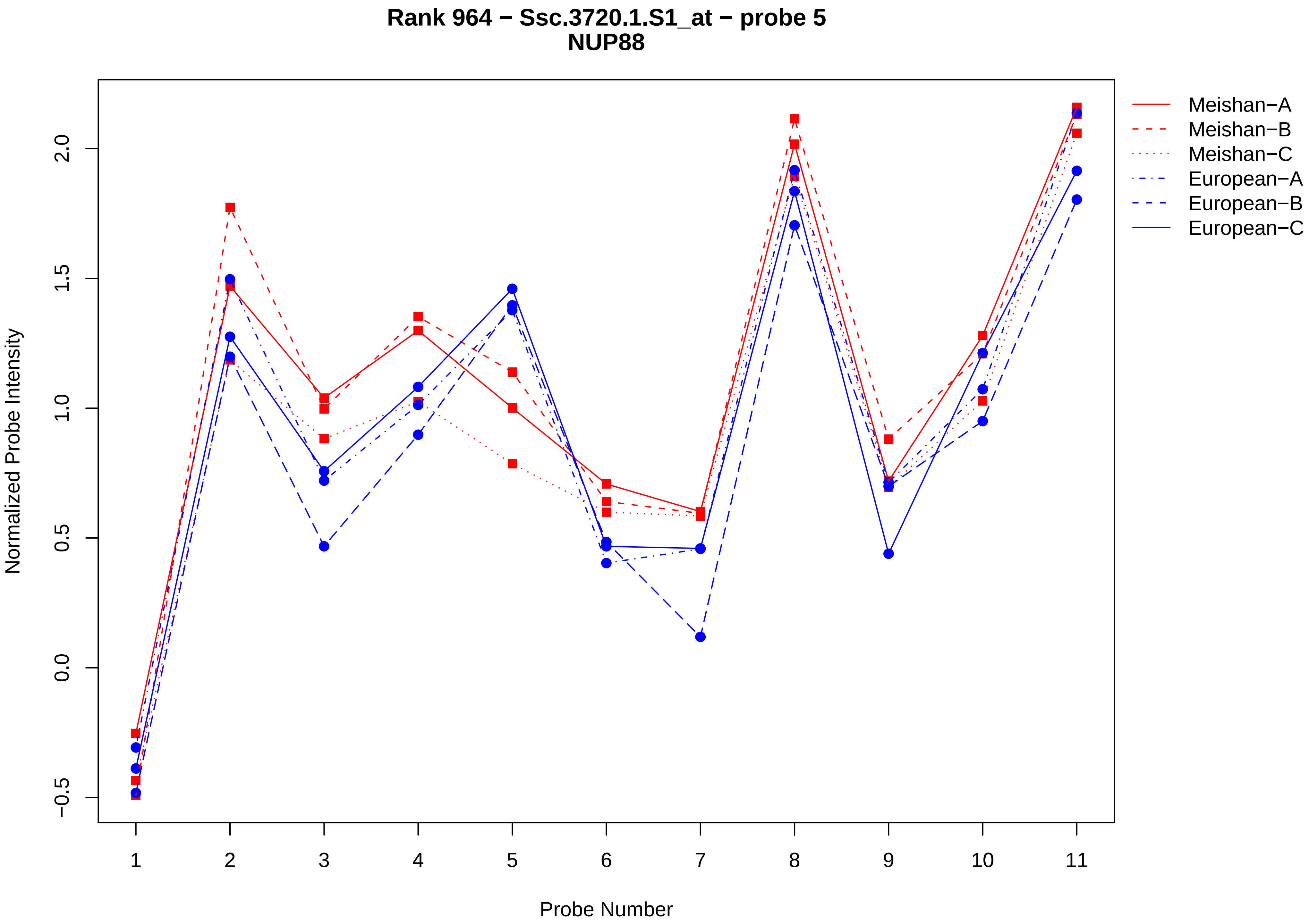

Rank 964 − Ssc.3720.1.S1_at − probe 5
NUP88
Normalized Probe Intensity
Probe Number
−0.5
0.0
0.5
1.0
1.5
2.0
1
2
3
4
5
6
7
8
9
10
11
Meishan−A
Meishan−B
Meishan−C
European−A
European−B
European−C

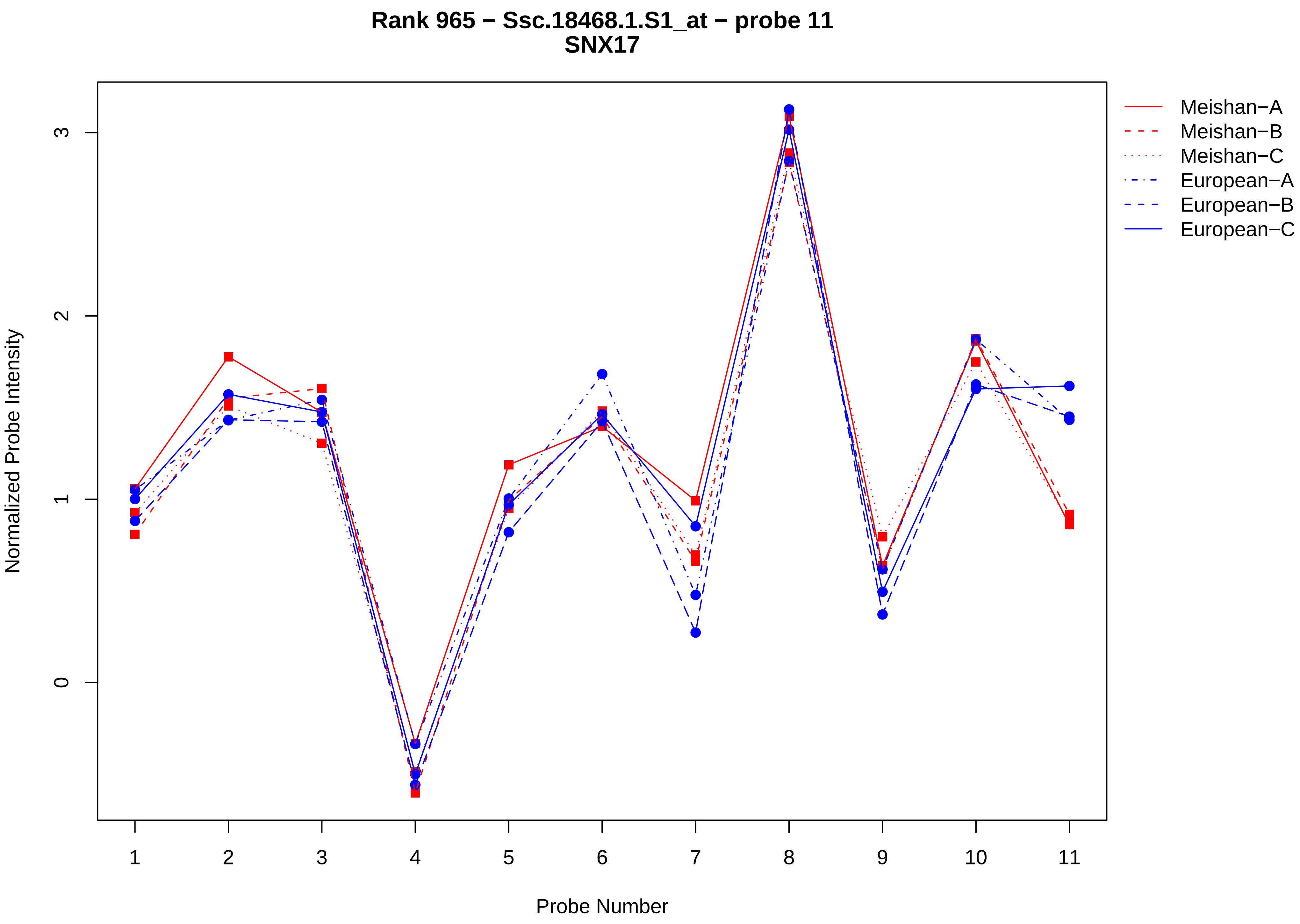

Rank 965 − Ssc.18468.1.S1_at − probe 11
SNX17
Normalized Probe Intensity
Probe Number
0
1
2
3
1
2
3
4
5
6
7
8
9
10
11
Meishan−A
Meishan−B
Meishan−C
European−A
European−B
European−C

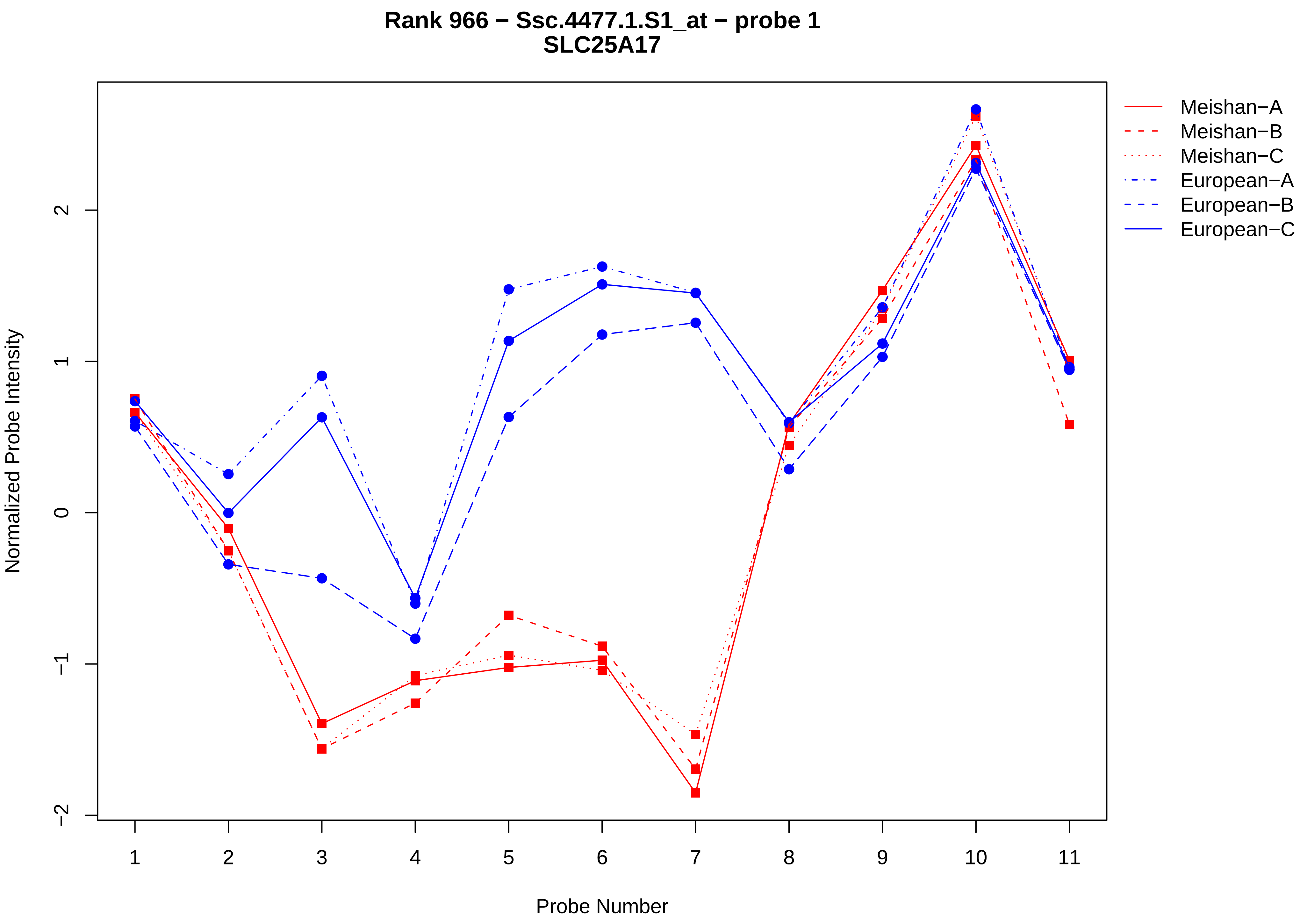
Rank 966 − Ssc.4477.1.S1_at − probe 1
SLC25A17
Normalized Probe Intensity
Probe Number
−2
−1
0
1
2
1
2
3
4
5
6
7
8
9
10
11
Meishan−A
Meishan−B
Meishan−C
European−A
European−B
European−C

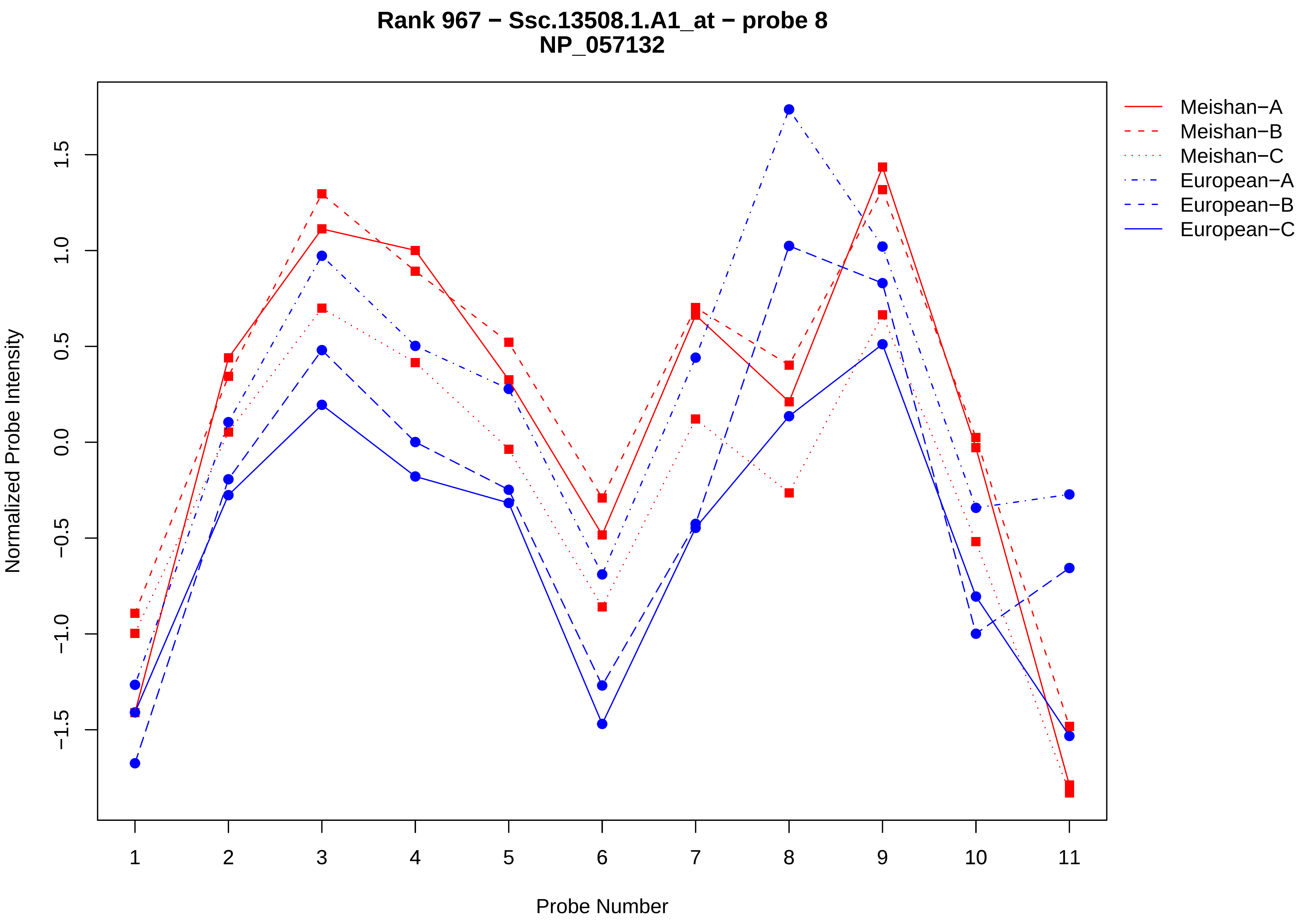

Rank 967 − Ssc.13508.1.A1_at − probe 8
NP_057132
Normalized Probe Intensity
−1.5
−1.0
−0.5
0.0
0.5
1.0
1.5
1
2
3
4
5
6
7
8
9
10
11
Probe Number
Meishan−A
Meishan−B
Meishan−C
European−A
European−B
European−C

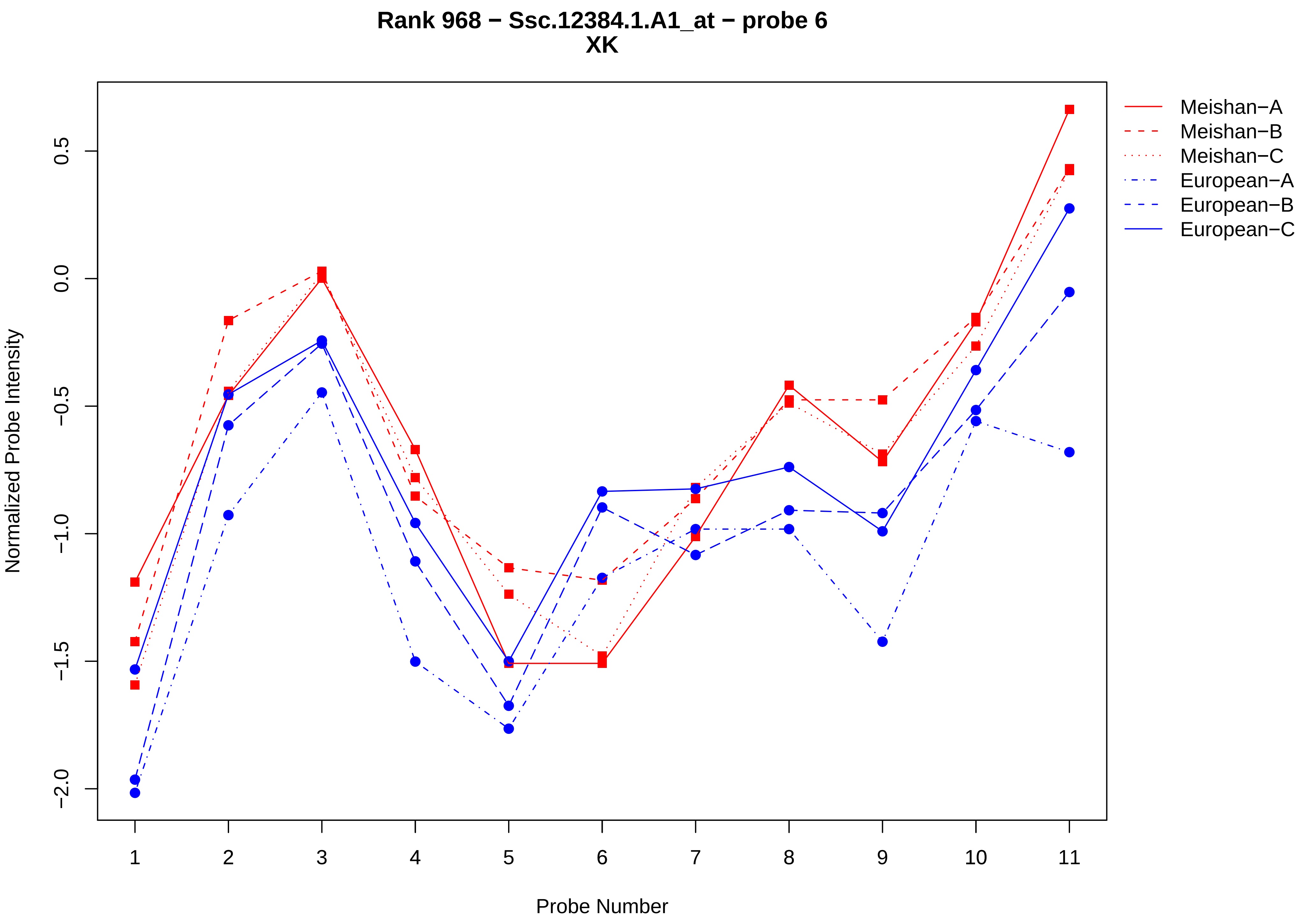

Rank 968 − Ssc.12384.1.A1_at − probe 6
XK
Normalized Probe Intensity
Probe Number
0.5
0.0
−0.5
−1.0
−1.5
−2.0
1
2
3
4
5
6
7
8
9
10
11
Meishan−A
Meishan−B
Meishan−C
European−A
European−B
European−C

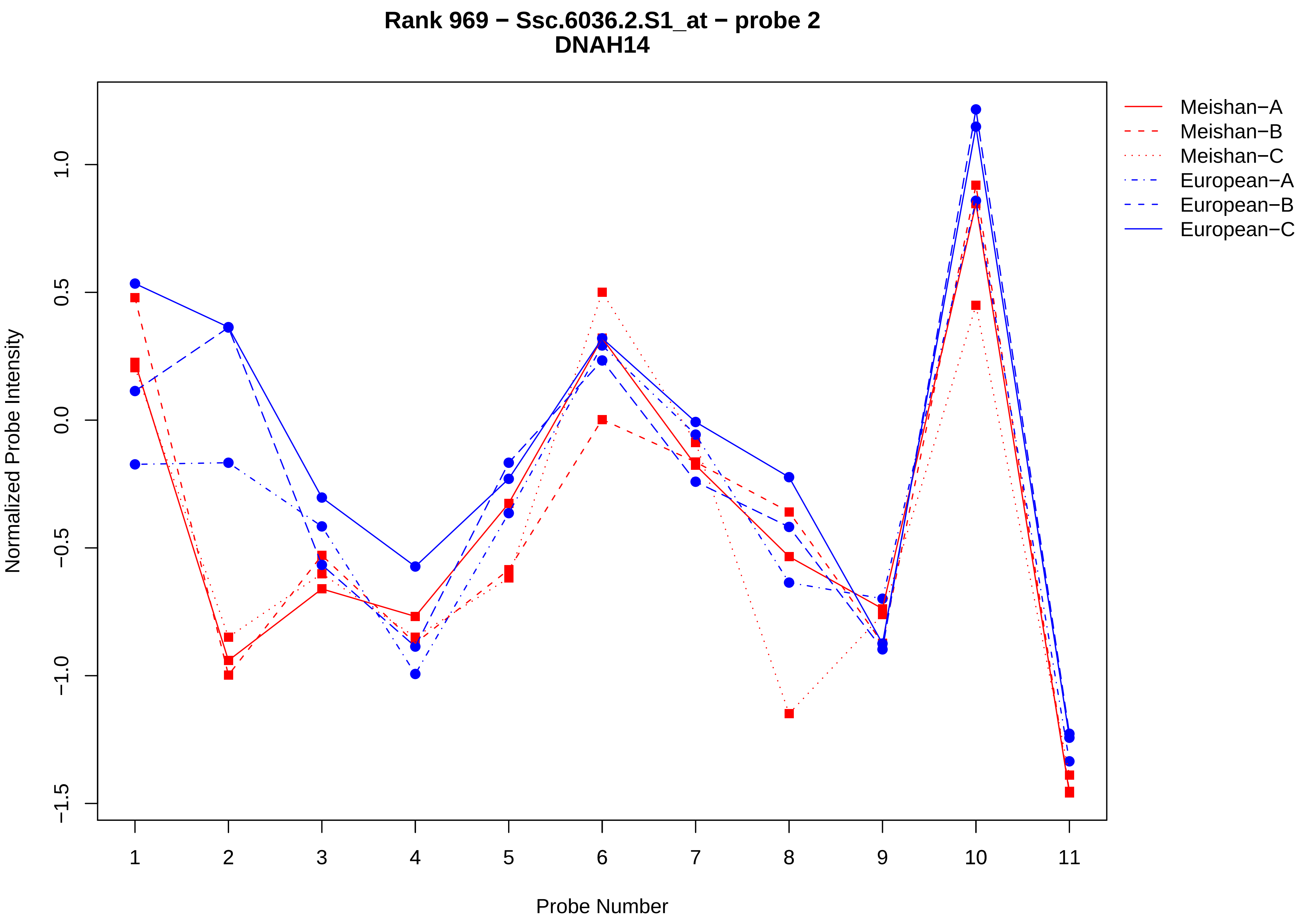

Rank 969 − Ssc.6036.2.S1_at − probe 2
DNAH14
Normalized Probe Intensity
Probe Number
1.0
0.5
0.0
−0.5
−1.0
−1.5
1
2
3
4
5
6
7
8
9
10
11
Meishan−A
Meishan−B
Meishan−C
European−A
European−B
European−C

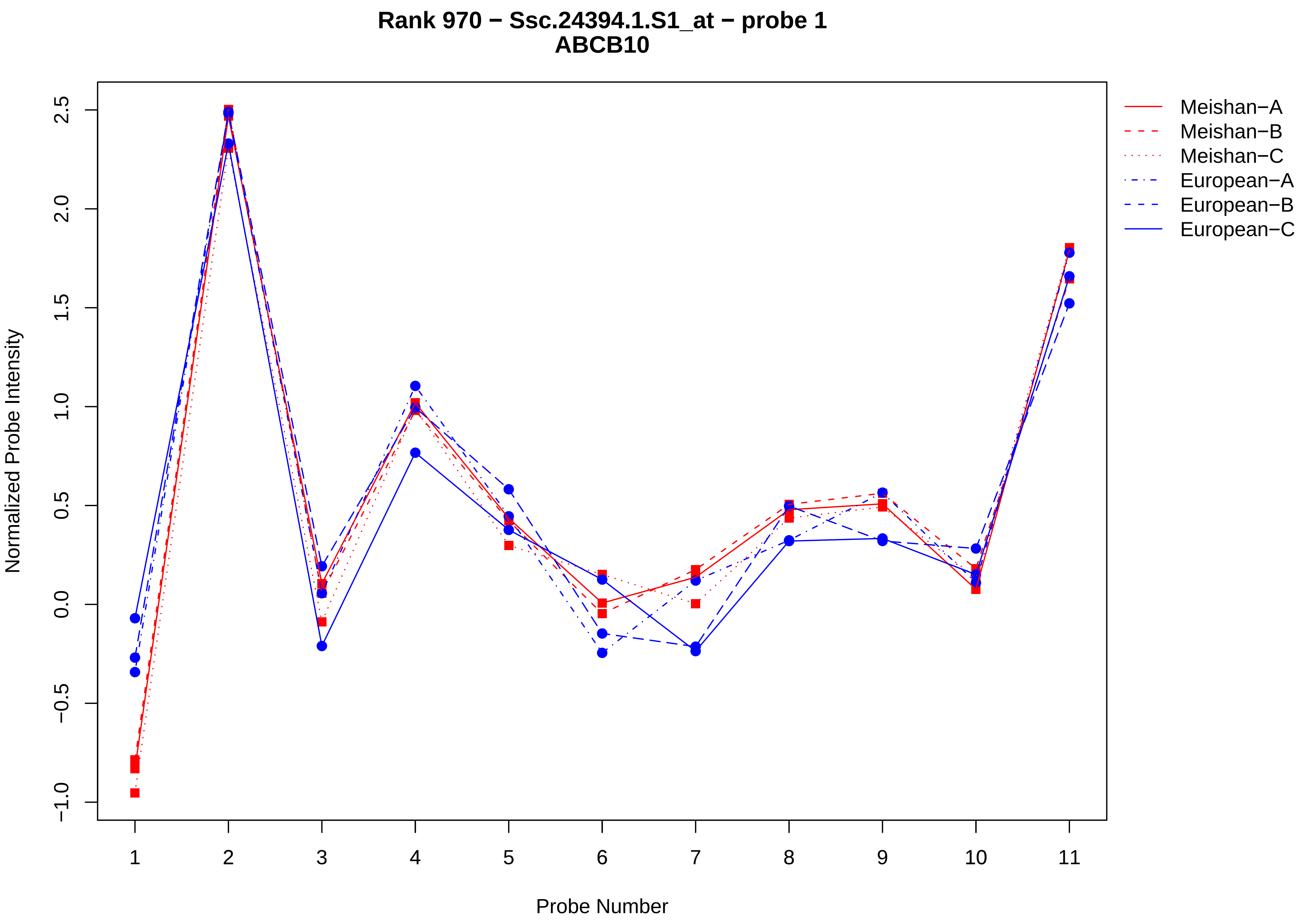

Rank 970 − Ssc.24394.1.S1_at − probe 1
ABCB10
Normalized Probe Intensity
Probe Number
Meishan−A
Meishan−B
Meishan−C
European−A
European−B
European−C
−1.0
−0.5
0.0
0.5
1.0
1.5
2.0
2.5
1
2
3
4
5
6
7
8
9
10
11

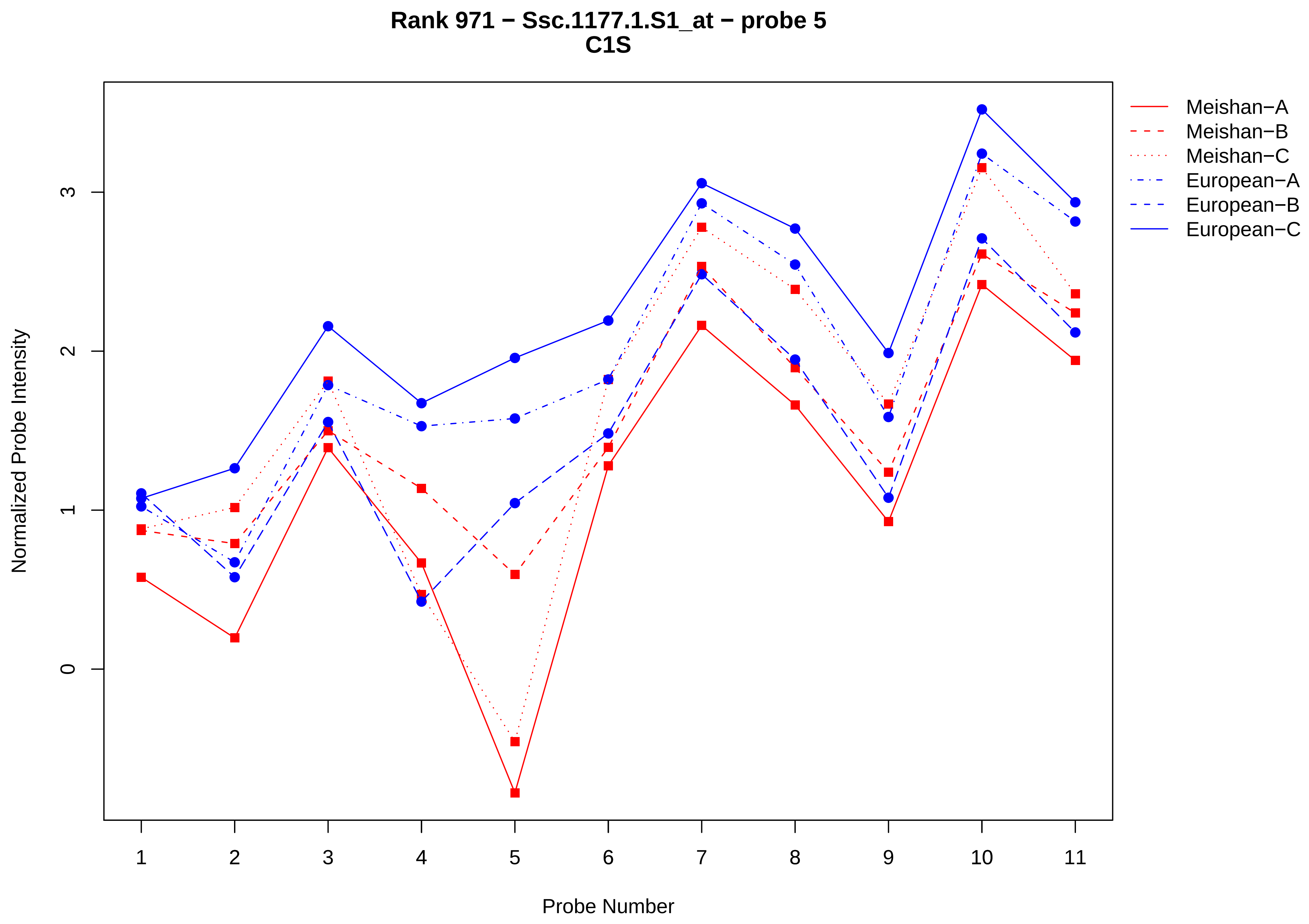

Rank 971 − Ssc.1177.1.S1_at − probe 5
C1S
Normalized Probe Intensity
Probe Number
0
1
2
3
1
2
3
4
5
6
7
8
9
10
11
Meishan−A
Meishan−B
Meishan−C
European−A
European−B
European−C

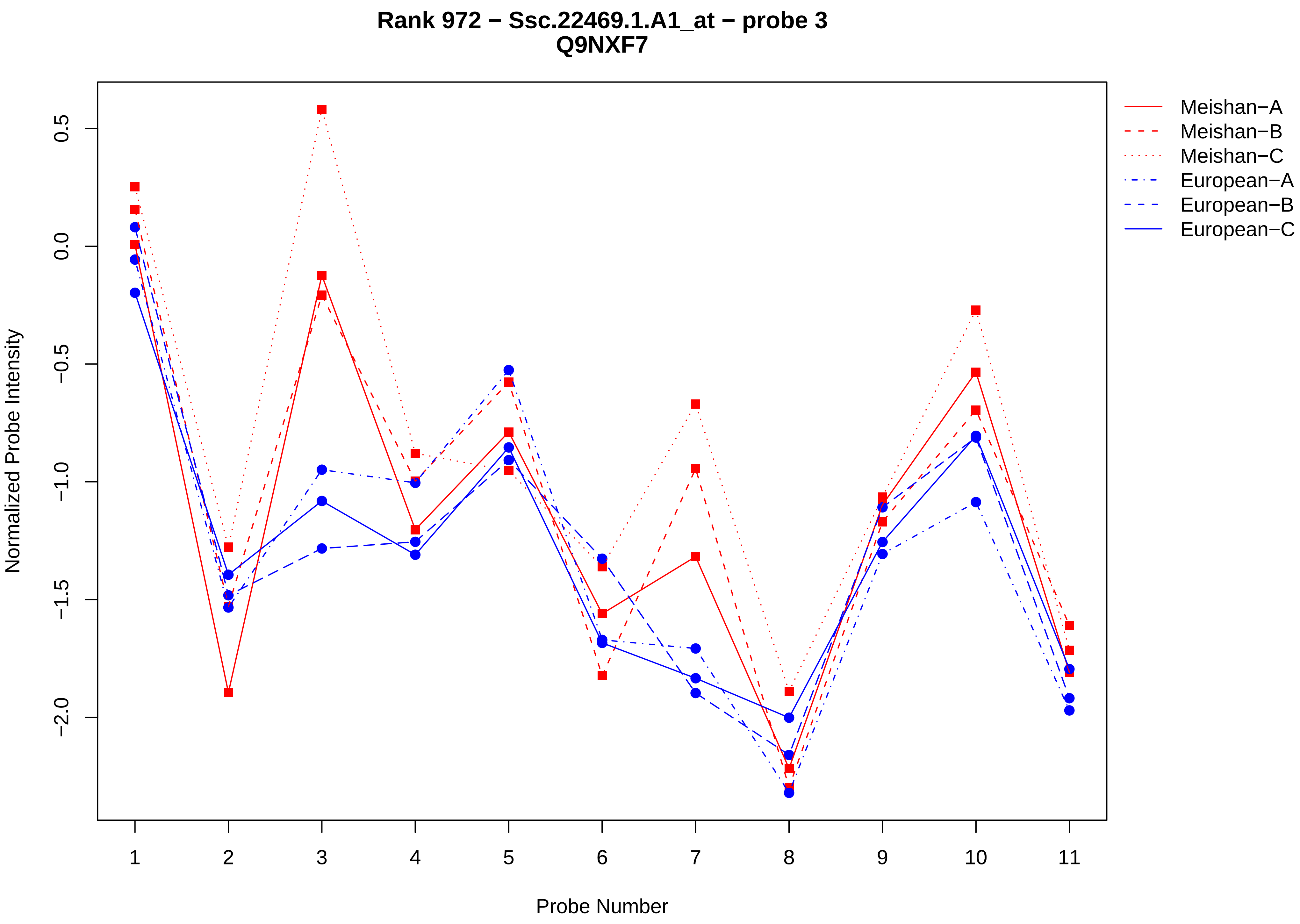
Rank 972 − Ssc.22469.1.A1_at − probe 3
Q9NXF7
Normalized Probe Intensity
Probe Number
0.5
0.0
−0.5
−1.0
−1.5
−2.0
1
2
3
4
5
6
7
8
9
10
11
Meishan−A
Meishan−B
Meishan−C
European−A
European−B
European−C

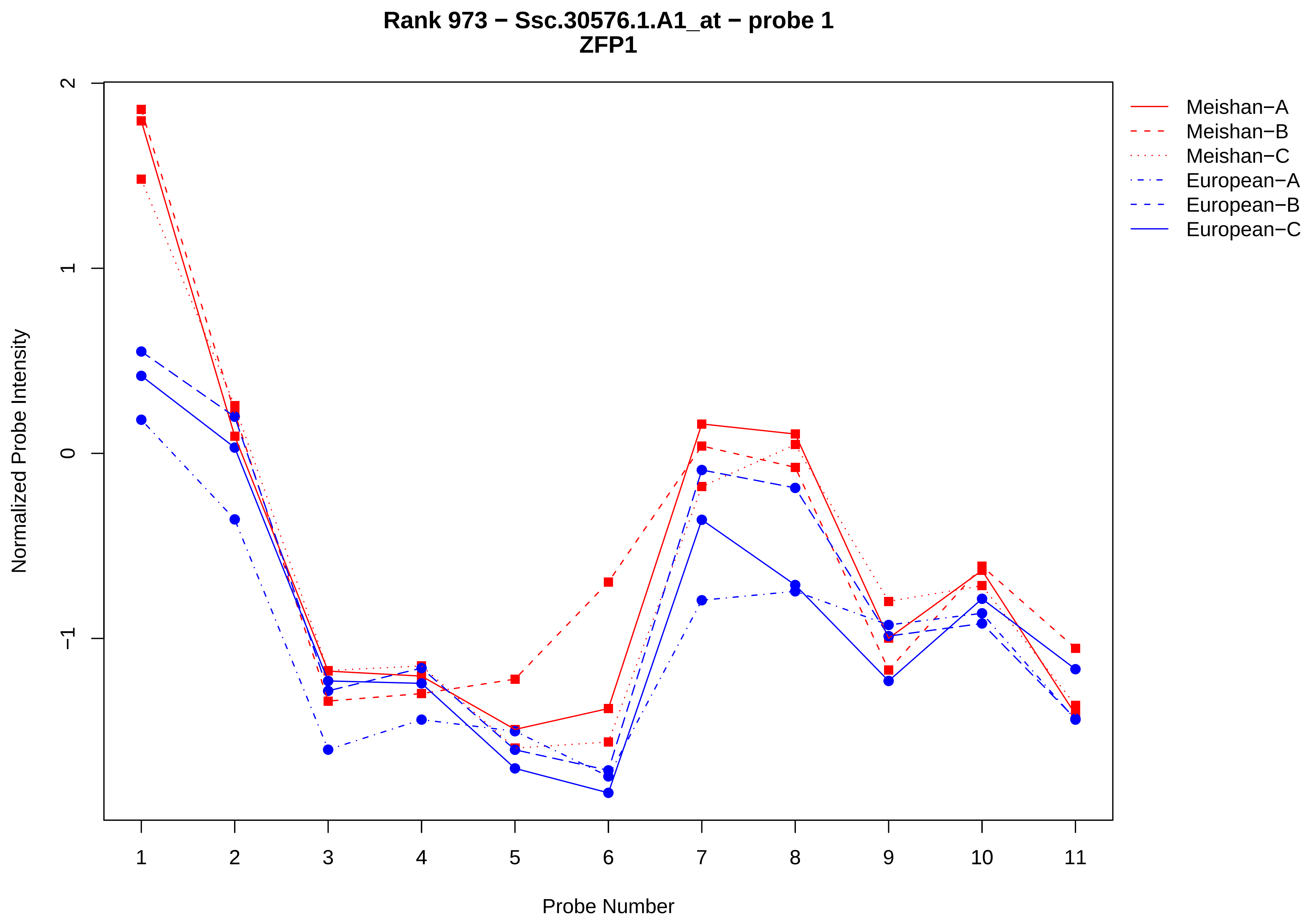
Rank 973 − Ssc.30576.1.A1_at − probe 1
ZFP1
Normalized Probe Intensity
Probe Number
Meishan−A
Meishan−B
Meishan−C
European−A
European−B
European−C

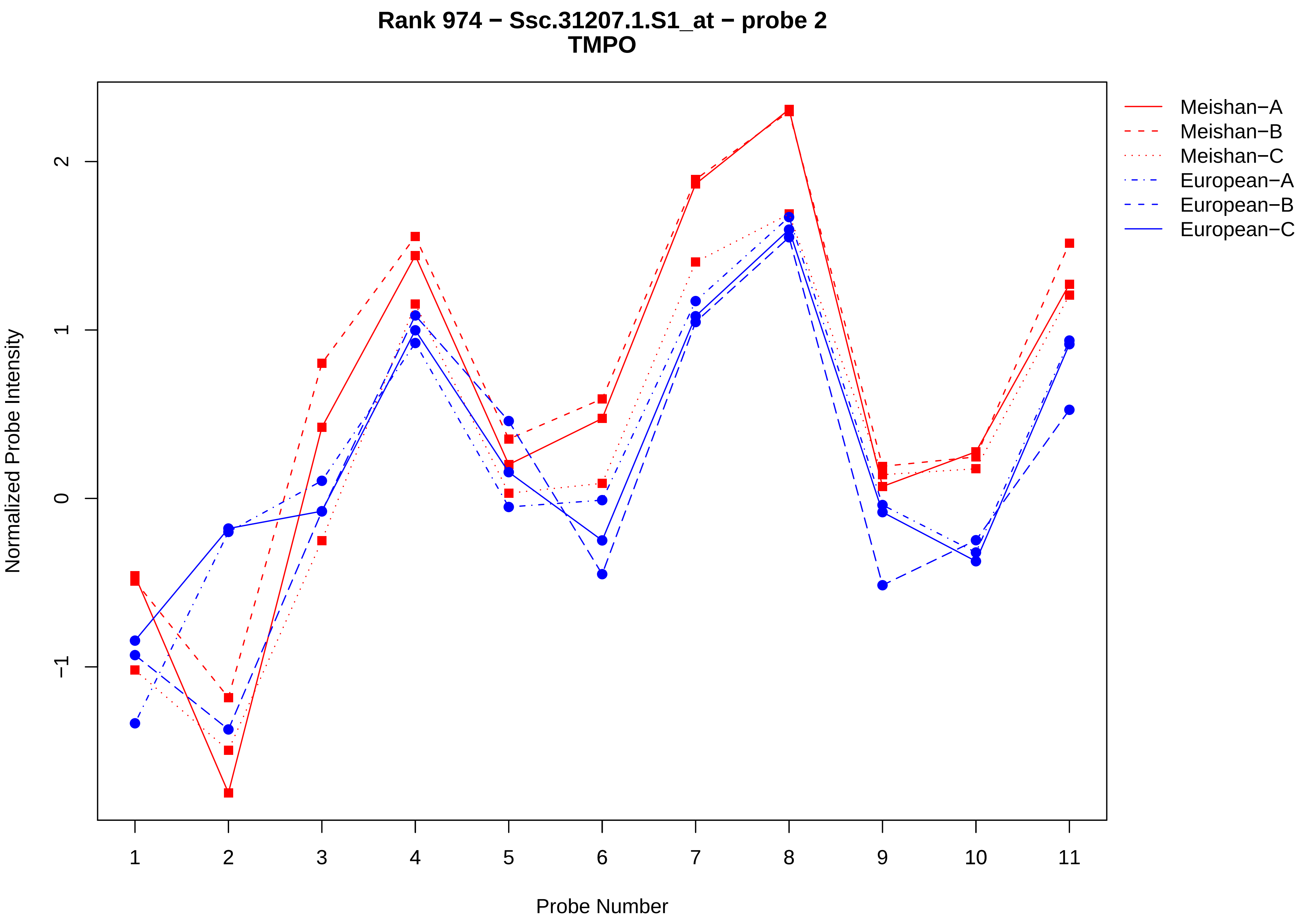

Rank 974 − Ssc.31207.1.S1_at − probe 2
TMPO
Normalized Probe Intensity
Probe Number
−1
0
1
2
1
2
3
4
5
6
7
8
9
10
11
Meishan−A
Meishan−B
Meishan−C
European−A
European−B
European−C

Rank 975 − Ssc.16603.1.A1_at − probe 9
A2M

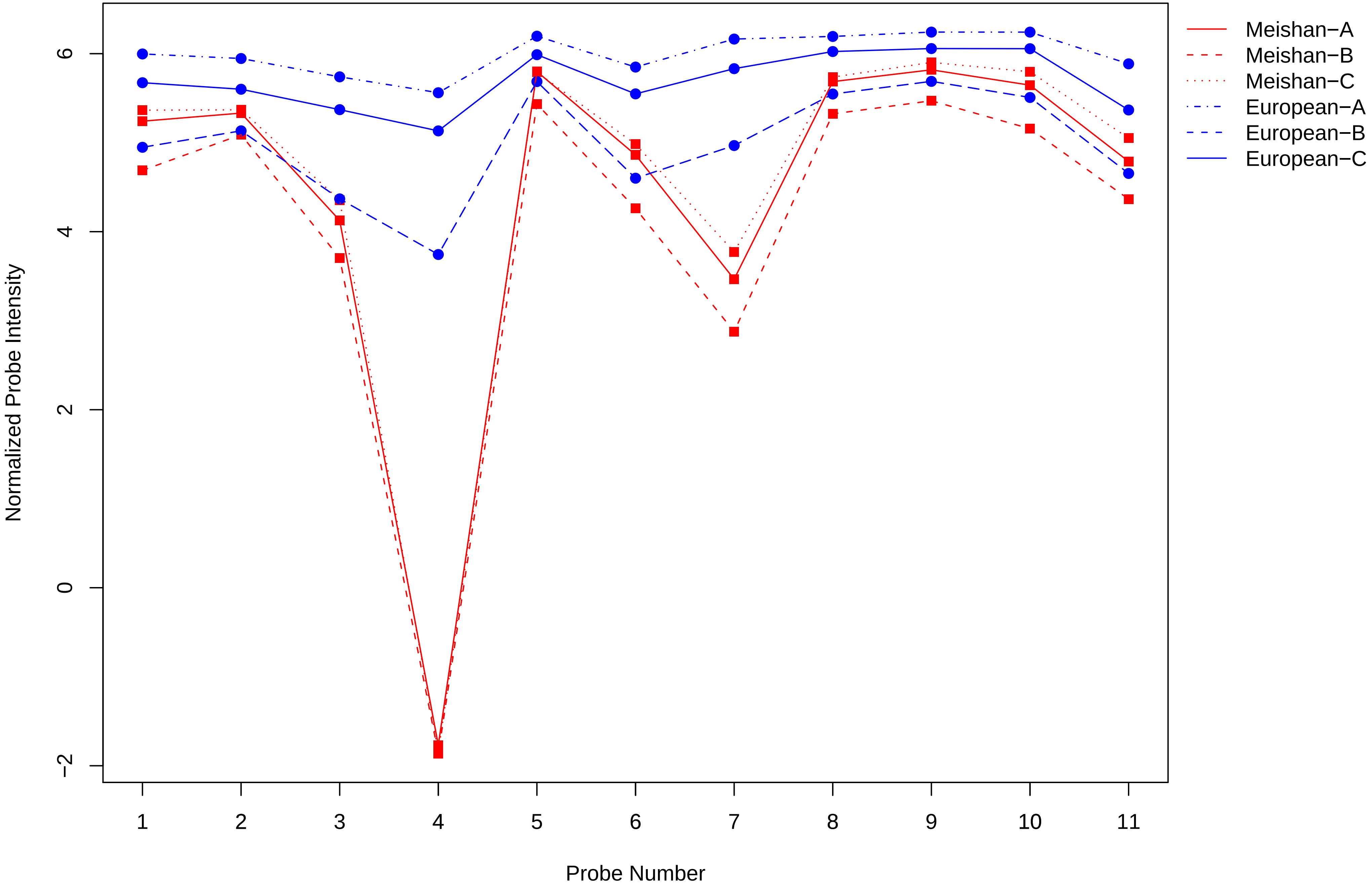

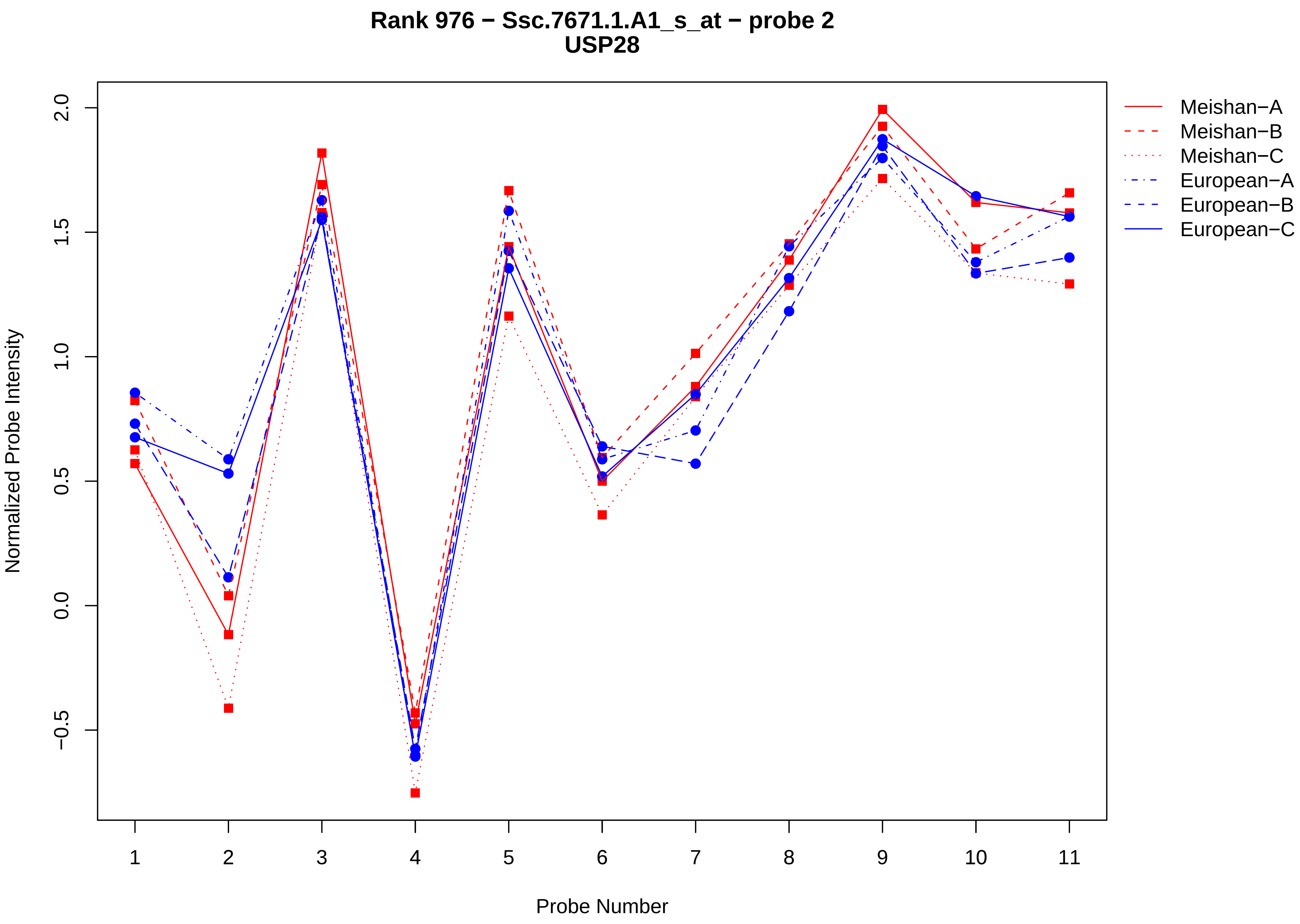

Rank 976 − Ssc.7671.1.A1_s_at − probe 2
USP28
Normalized Probe Intensity
Probe Number
−0.5
0.0
0.5
1.0
1.5
2.0
1
2
3
4
5
6
7
8
9
10
11
Meishan−A
Meishan−B
Meishan−C
European−A
European−B
European−C

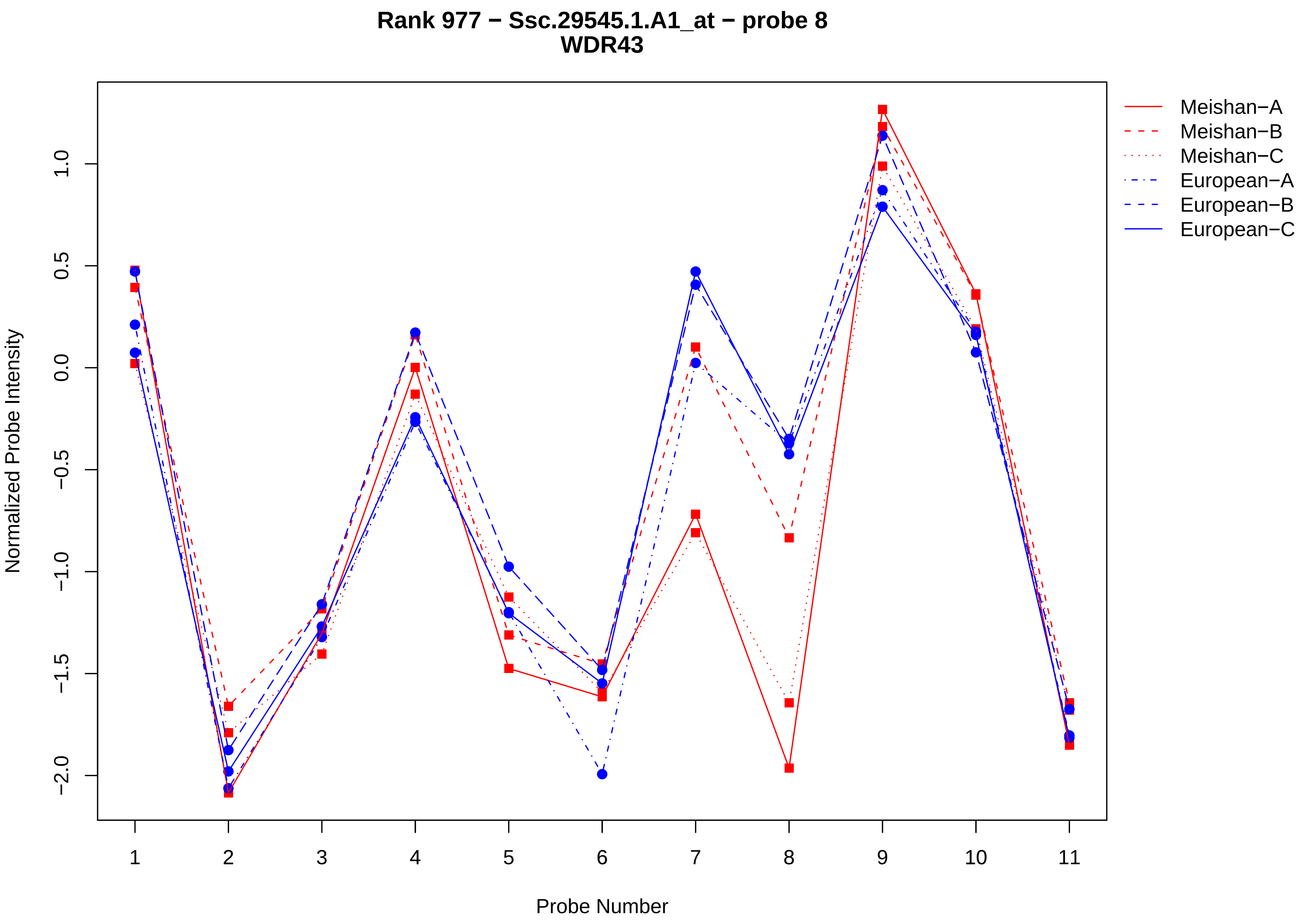

Rank 977 − Ssc.29545.1.A1_at − probe 8
WDR43
Normalized Probe Intensity
Probe Number
−2.0
−1.5
−1.0
−0.5
0.0
0.5
1.0
1
2
3
4
5
6
7
8
9
10
11
Meishan−A
Meishan−B
Meishan−C
European−A
European−B
European−C

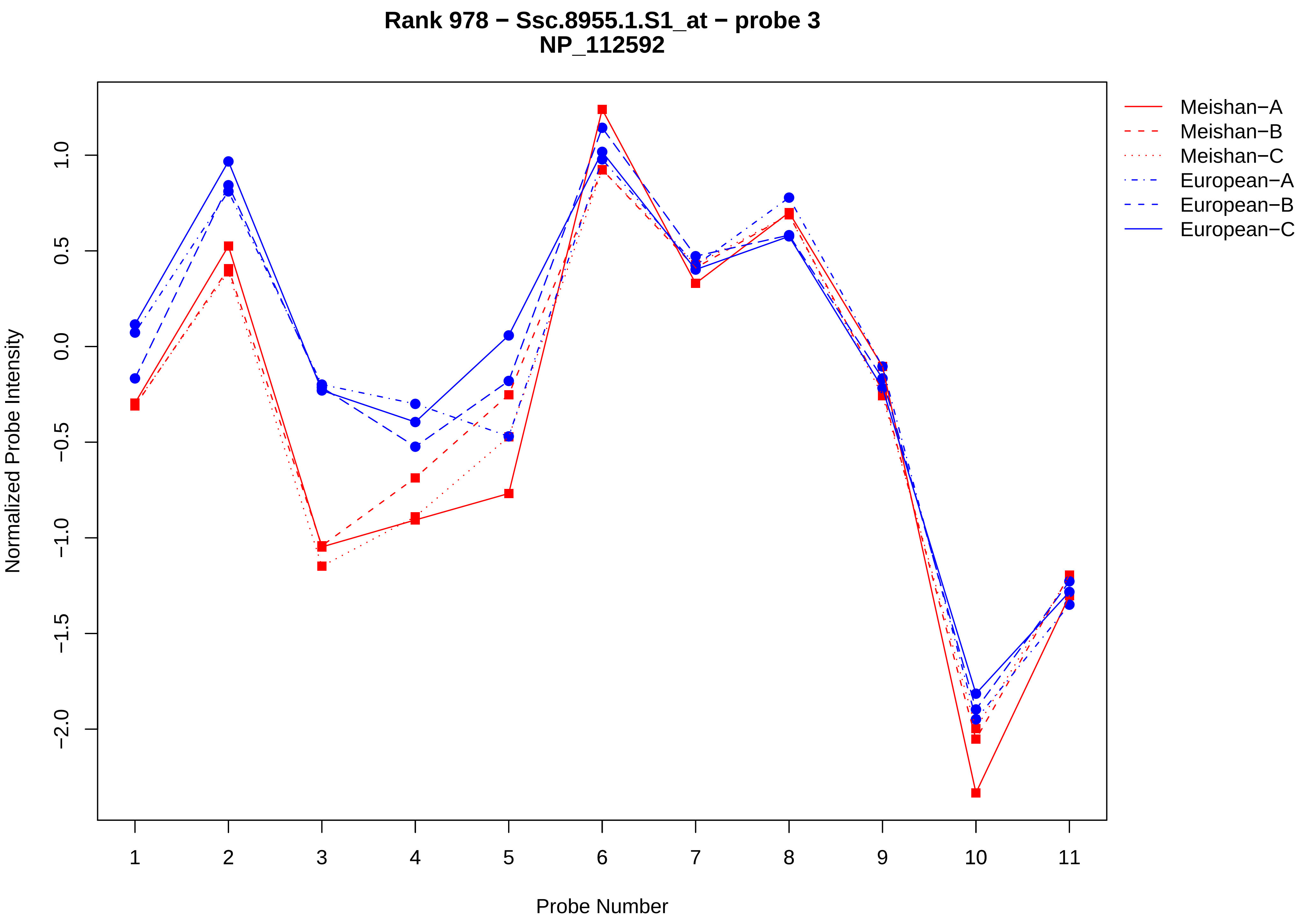

Rank 978 − Ssc.8955.1.S1_at − probe 3
NP_112592
Normalized Probe Intensity
Probe Number
Meishan−A
Meishan−B
Meishan−C
European−A
European−B
European−C

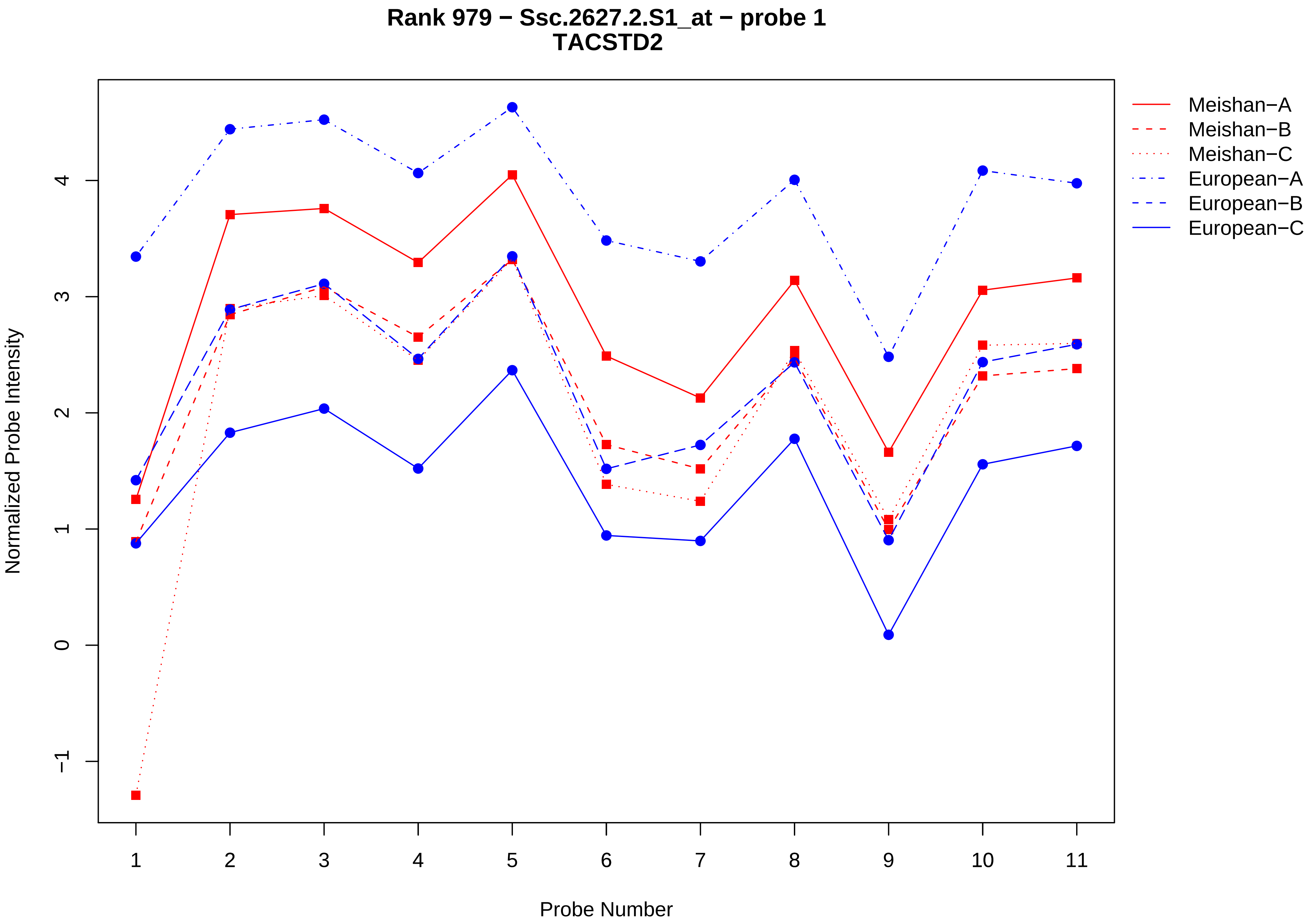

Rank 979 − Ssc.2627.2.S1_at − probe 1
TACSTD2
Normalized Probe Intensity
Probe Number
Meishan−A
Meishan−B
Meishan−C
European−A
European−B
European−C
−1
0
1
2
3
4
1
2
3
4
5
6
7
8
9
10
11

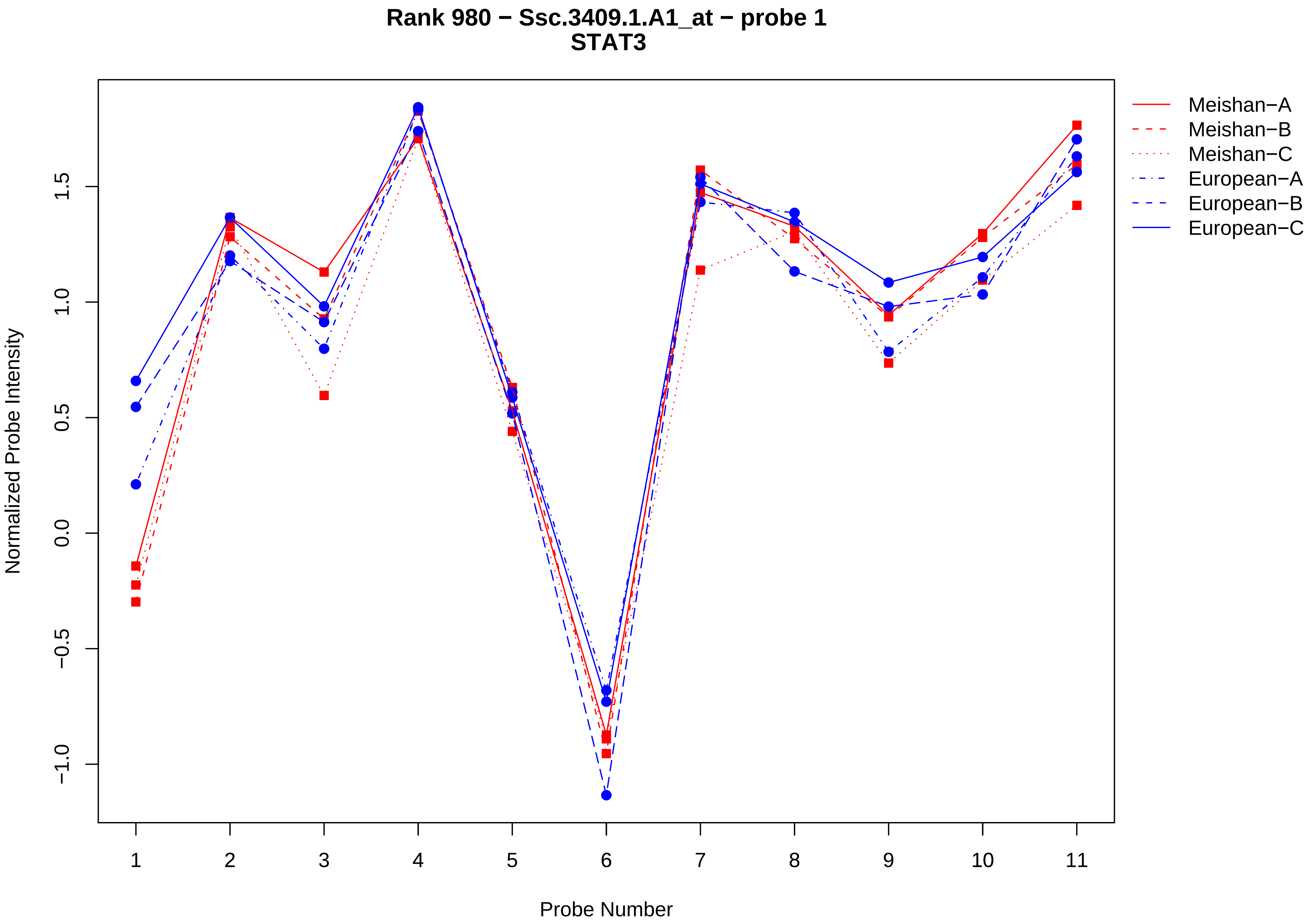

Rank 980 − Ssc.3409.1.A1_at − probe 1
STAT3
Normalized Probe Intensity
Probe Number
−1.0
−0.5
0.0
0.5
1.0
1.5
1
2
3
4
5
6
7
8
9
10
11
Meishan−A
Meishan−B
Meishan−C
European−A
European−B
European−C

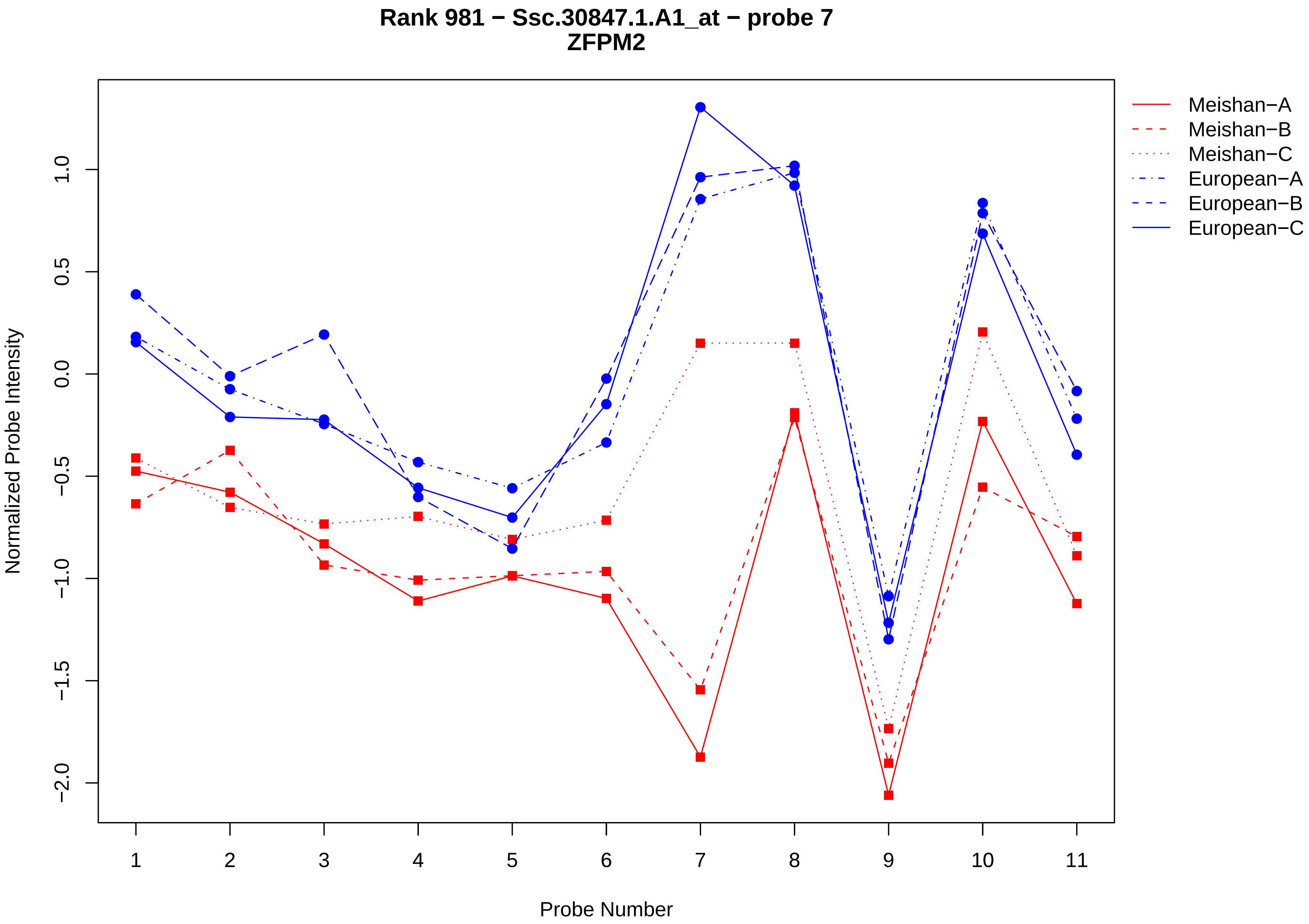
Rank 981 − Ssc.30847.1.A1_at − probe 7
ZFPM2
Normalized Probe Intensity
Probe Number
−2.0
−1.5
−1.0
−0.5
0.0
0.5
1.0
1
2
3
4
5
6
7
8
9
10
11
Meishan−A
Meishan−B
Meishan−C
European−A
European−B
European−C

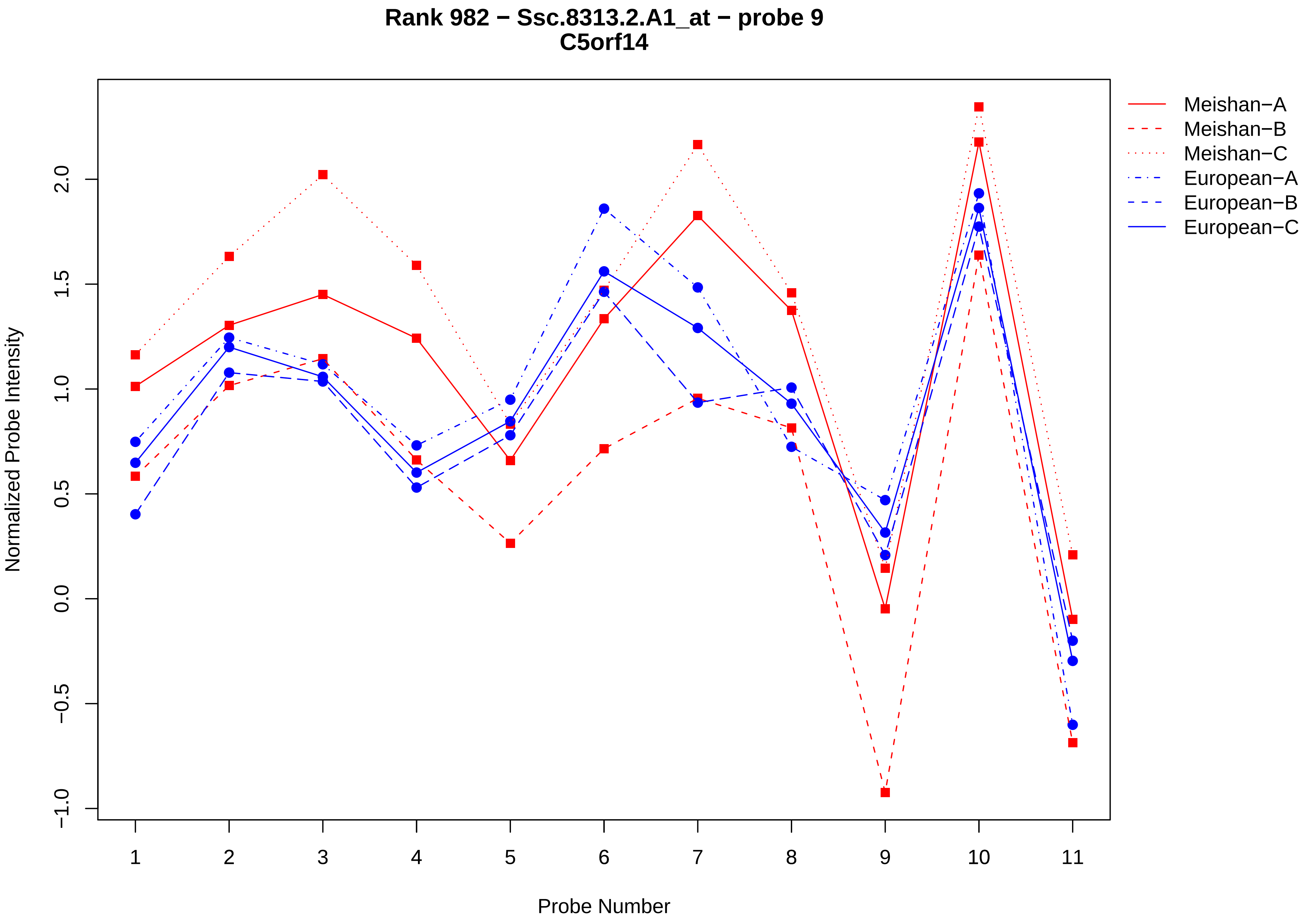

Rank 982 − Ssc.8313.2.A1_at − probe 9
C5orf14
Normalized Probe Intensity
Probe Number
−1.0
−0.5
0.0
0.5
1.0
1.5
2.0
1
2
3
4
5
6
7
8
9
10
11
Meishan−A
Meishan−B
Meishan−C
European−A
European−B
European−C

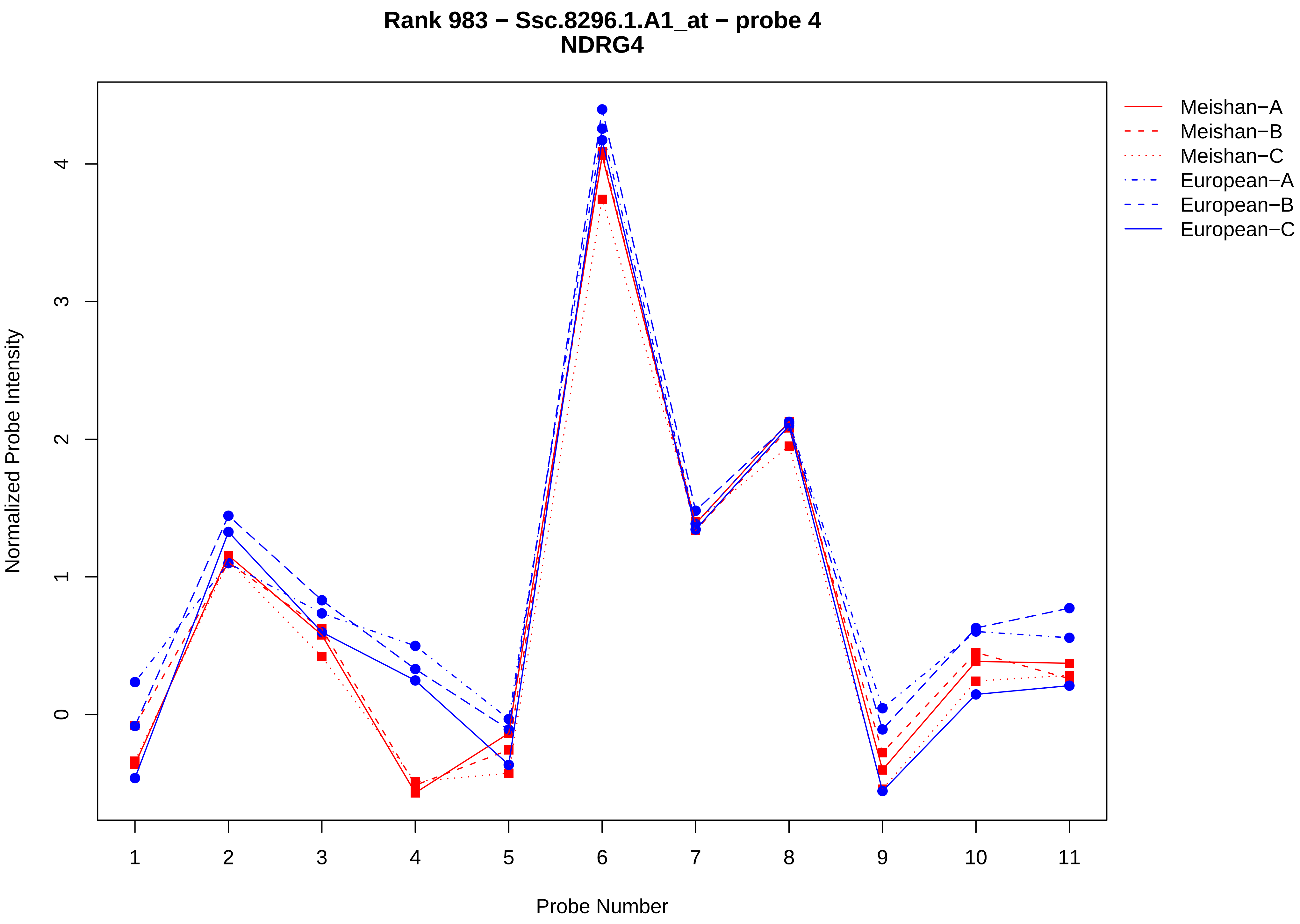

Rank 983 − Ssc.8296.1.A1_at − probe 4
NDRG4
Normalized Probe Intensity
Probe Number
0
1
2
3
4
1
2
3
4
5
6
7
8
9
10
11
Meishan−A
Meishan−B
Meishan−C
European−A
European−B
European−C

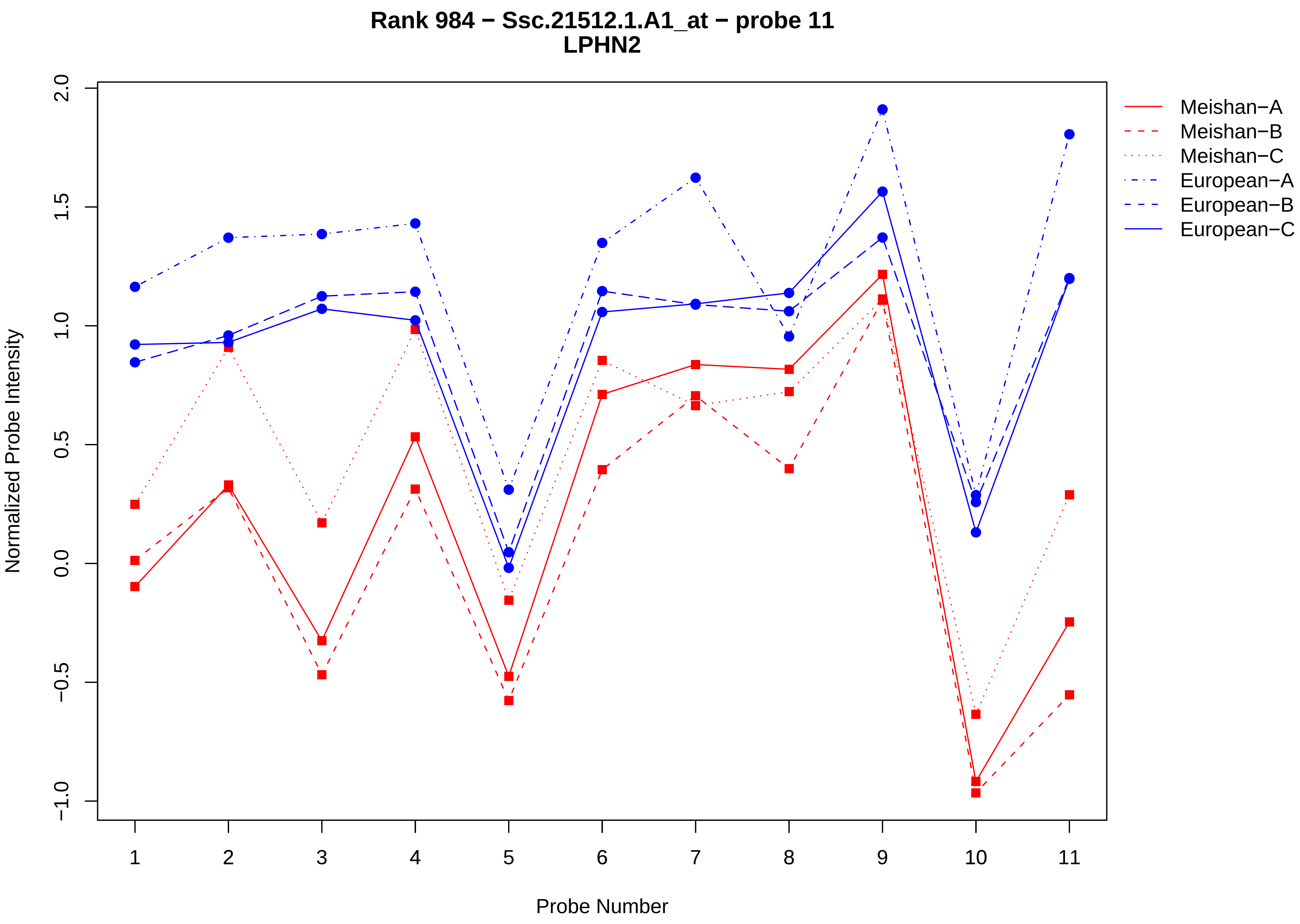

Rank 984 − Ssc.21512.1.A1_at − probe 11
LPHN2
Normalized Probe Intensity
−1.0
−0.5
0.0
0.5
1.0
1.5
2.0
1
2
3
4
5
6
7
8
9
10
11
Probe Number
Meishan−A
Meishan−B
Meishan−C
European−A
European−B
European−C

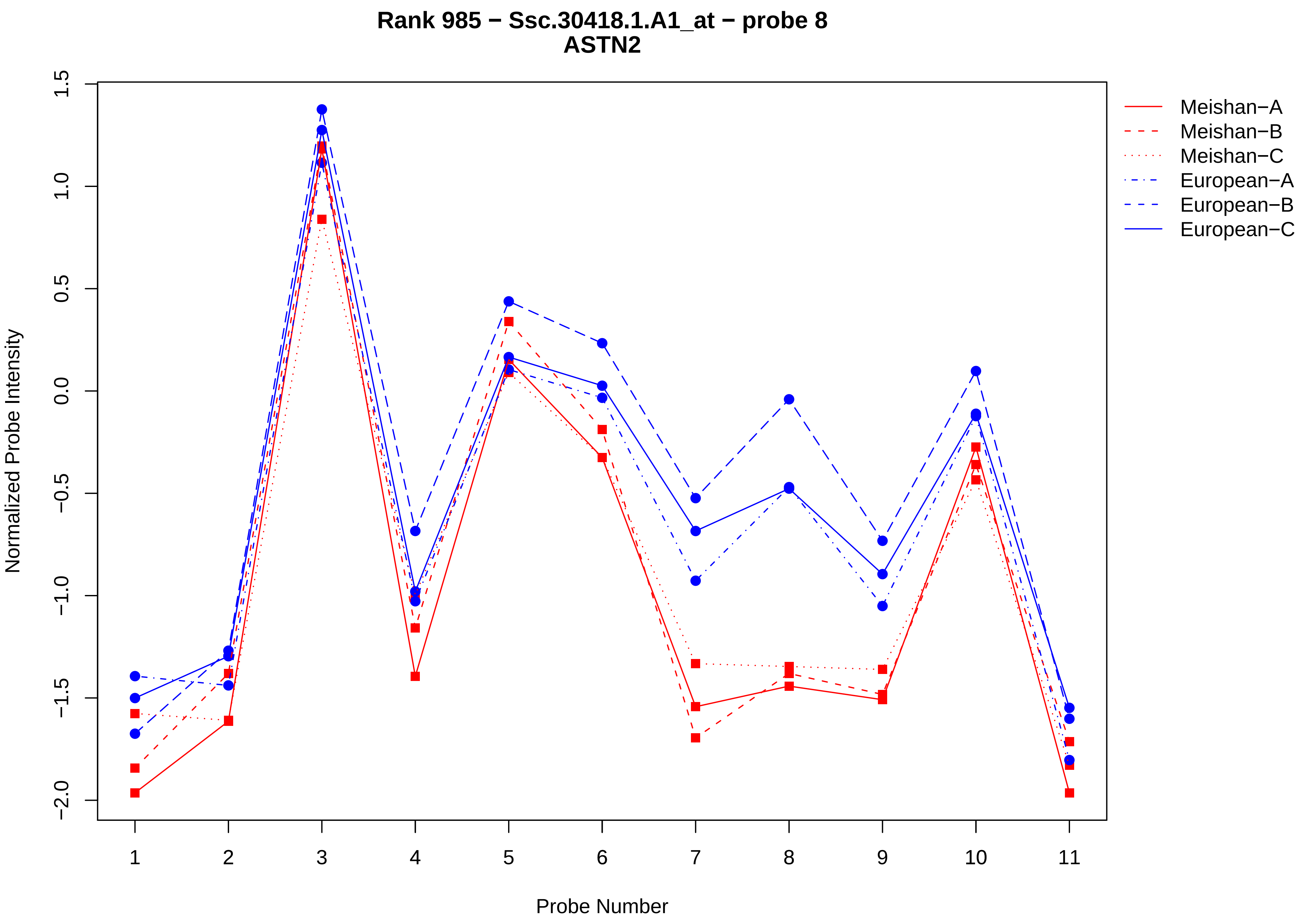

Rank 985 − Ssc.30418.1.A1_at − probe 8
ASTN2
Normalized Probe Intensity
Probe Number
1.5
1.0
0.5
0.0
−0.5
−1.0
−1.5
−2.0
1
2
3
4
5
6
7
8
9
10
11
Meishan−A
Meishan−B
Meishan−C
European−A
European−B
European−C

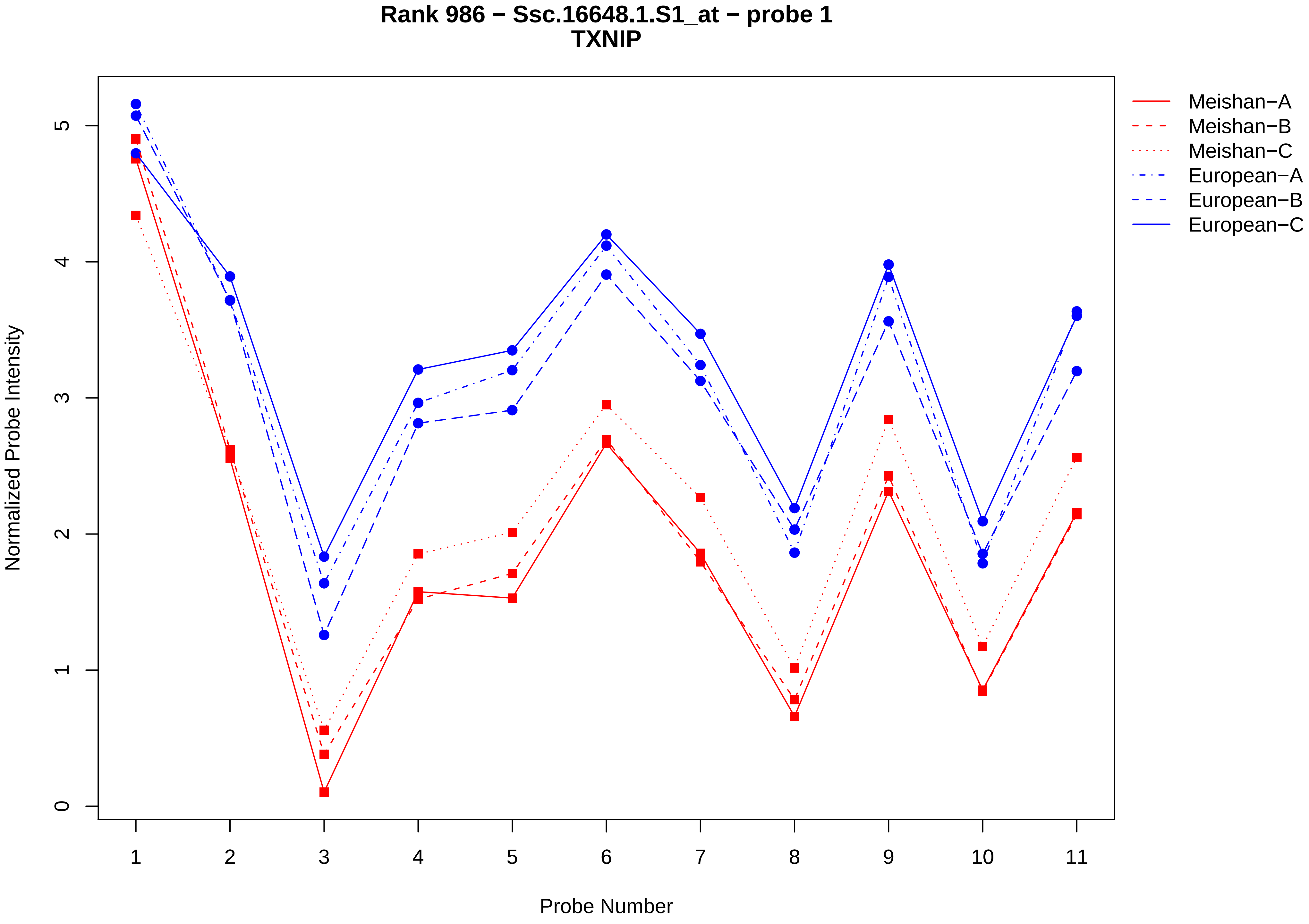

Rank 986 − Ssc.16648.1.S1_at − probe 1
TXNIP
Normalized Probe Intensity
Probe Number
Meishan−A
Meishan−B
Meishan−C
European−A
European−B
European−C

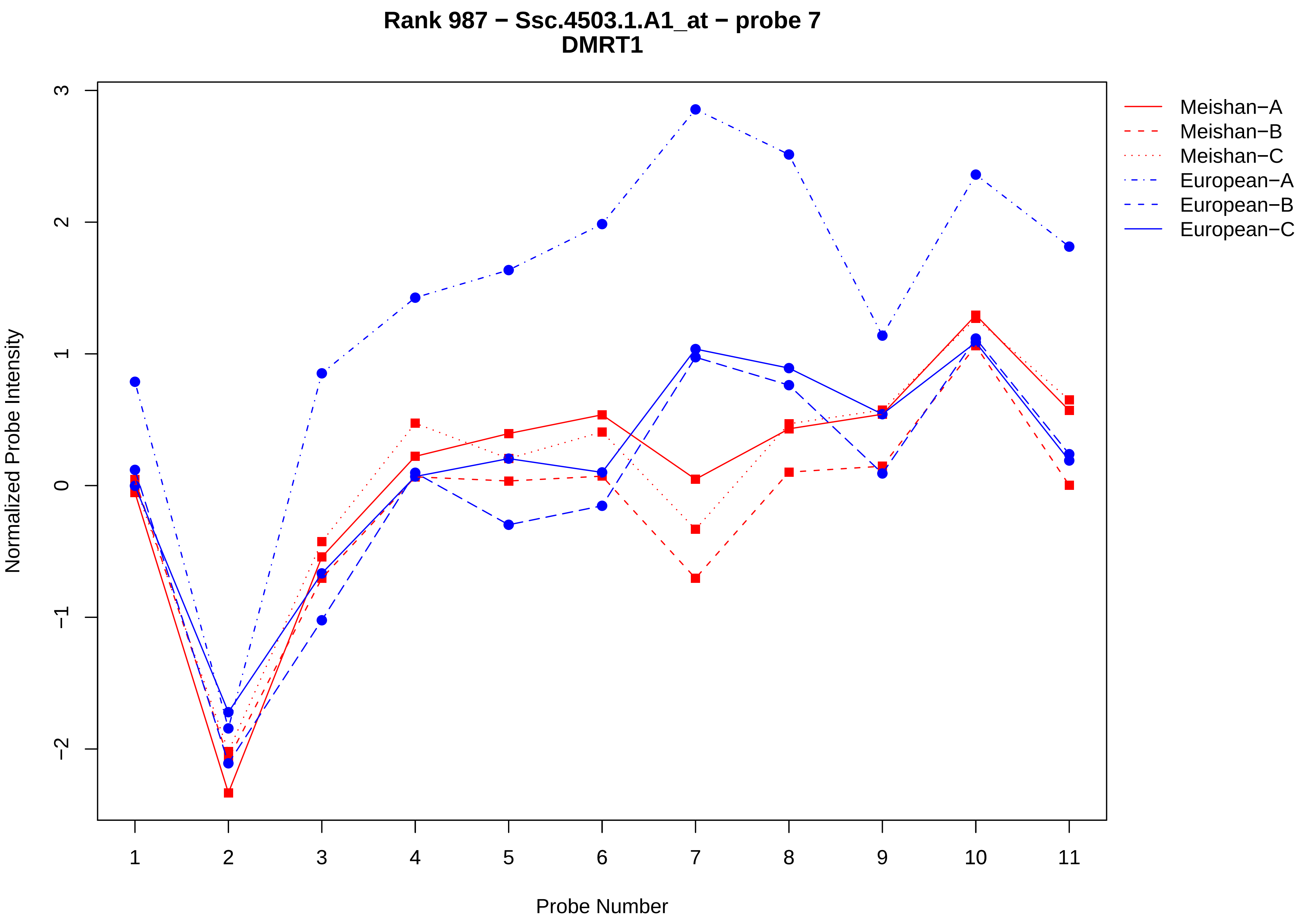

Rank 987 − Ssc.4503.1.A1_at − probe 7
DMRT1
Normalized Probe Intensity
Probe Number
Meishan−A
Meishan−B
Meishan−C
European−A
European−B
European−C
−2
−1
0
1
2
3
1
2
3
4
5
6
7
8
9
10
11

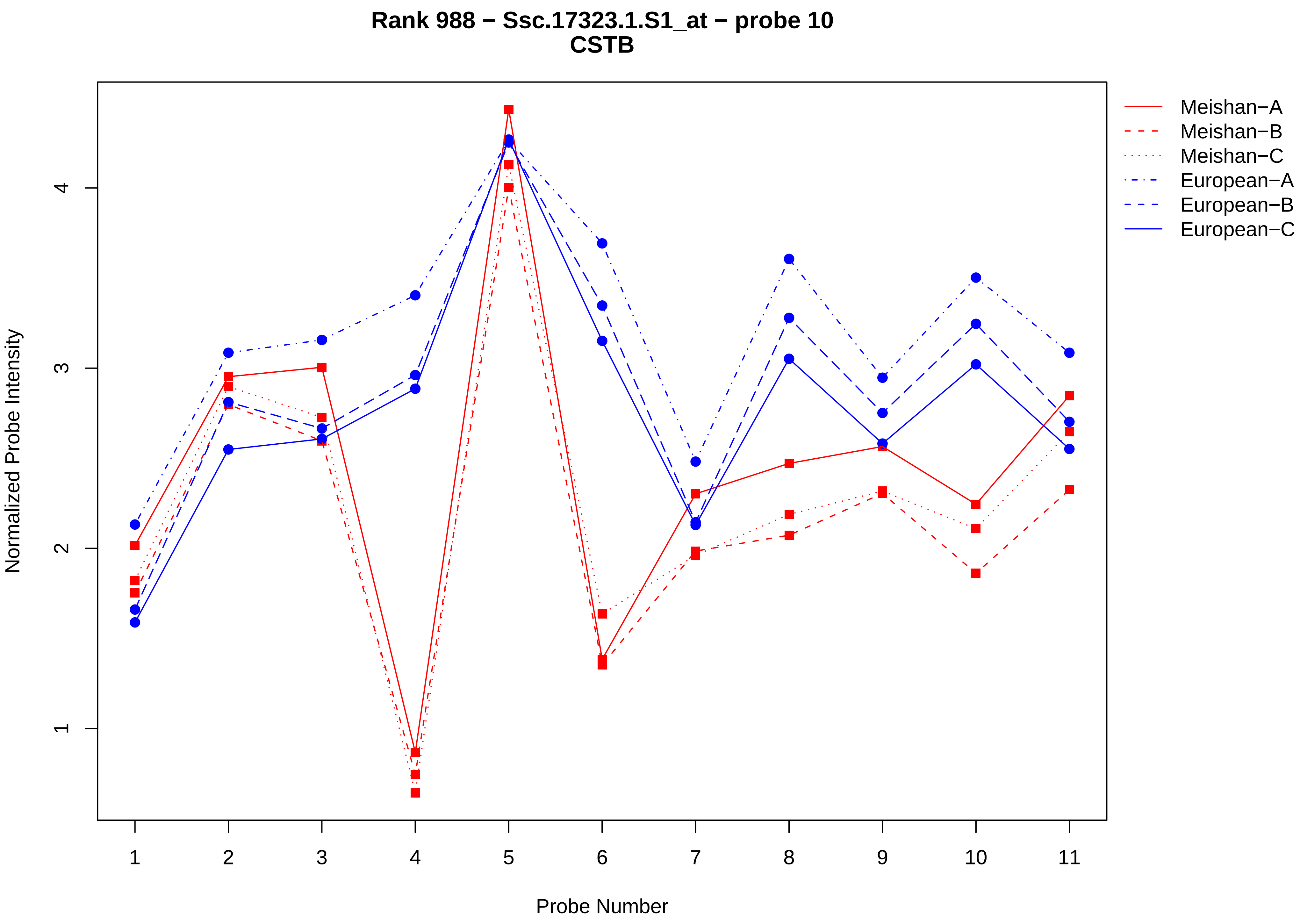
Rank 988 − Ssc.17323.1.S1_at − probe 10
CSTB
Normalized Probe Intensity
Probe Number
1
2
3
4
1
2
3
4
5
6
7
8
9
10
11
Meishan−A
Meishan−B
Meishan−C
European−A
European−B
European−C

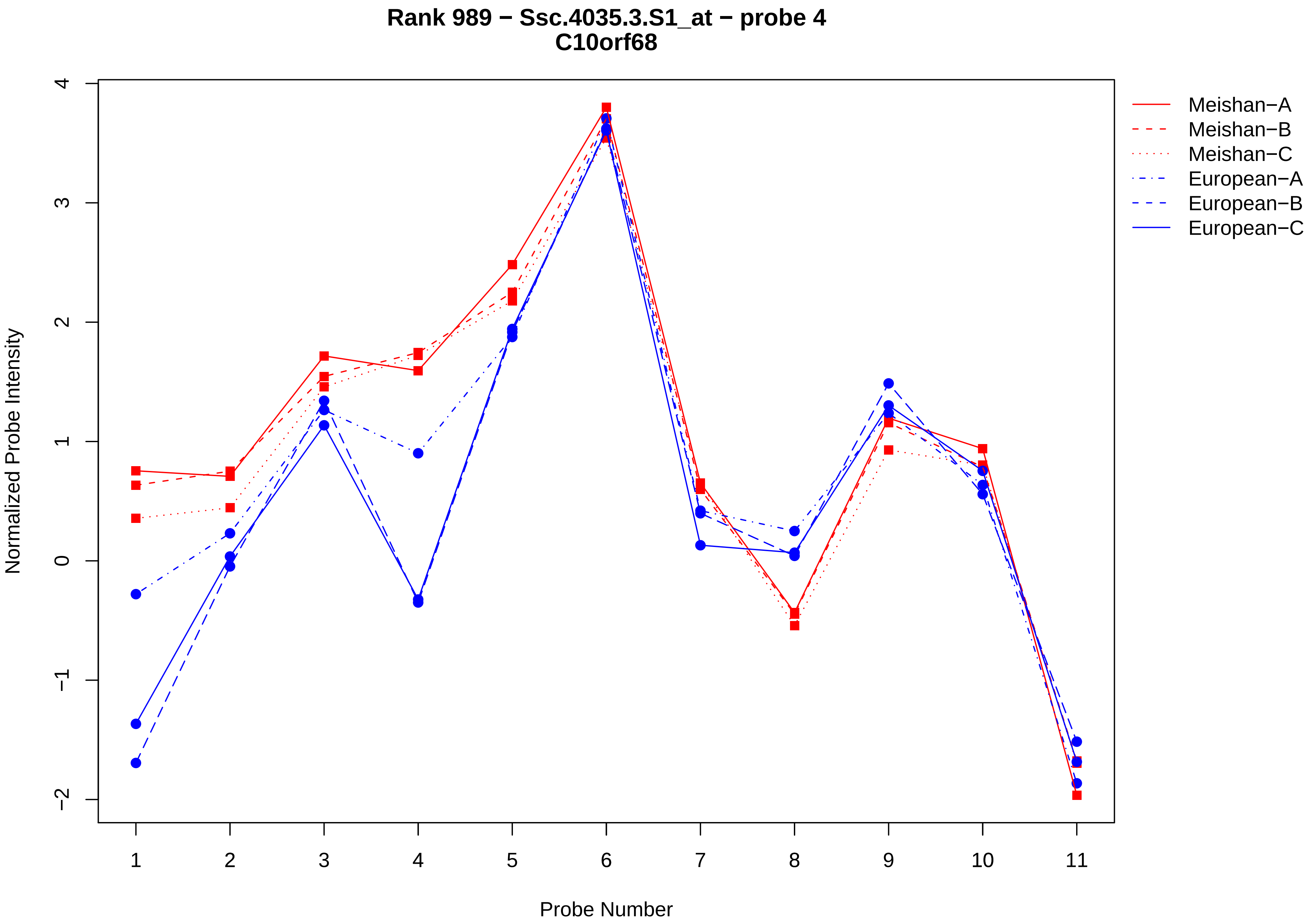

Rank 989 − Ssc.4035.3.S1_at − probe 4
C10orf68
Normalized Probe Intensity
Probe Number
−2
−1
0
1
2
3
4
1
2
3
4
5
6
7
8
9
10
11
Meishan−A
Meishan−B
Meishan−C
European−A
European−B
European−C

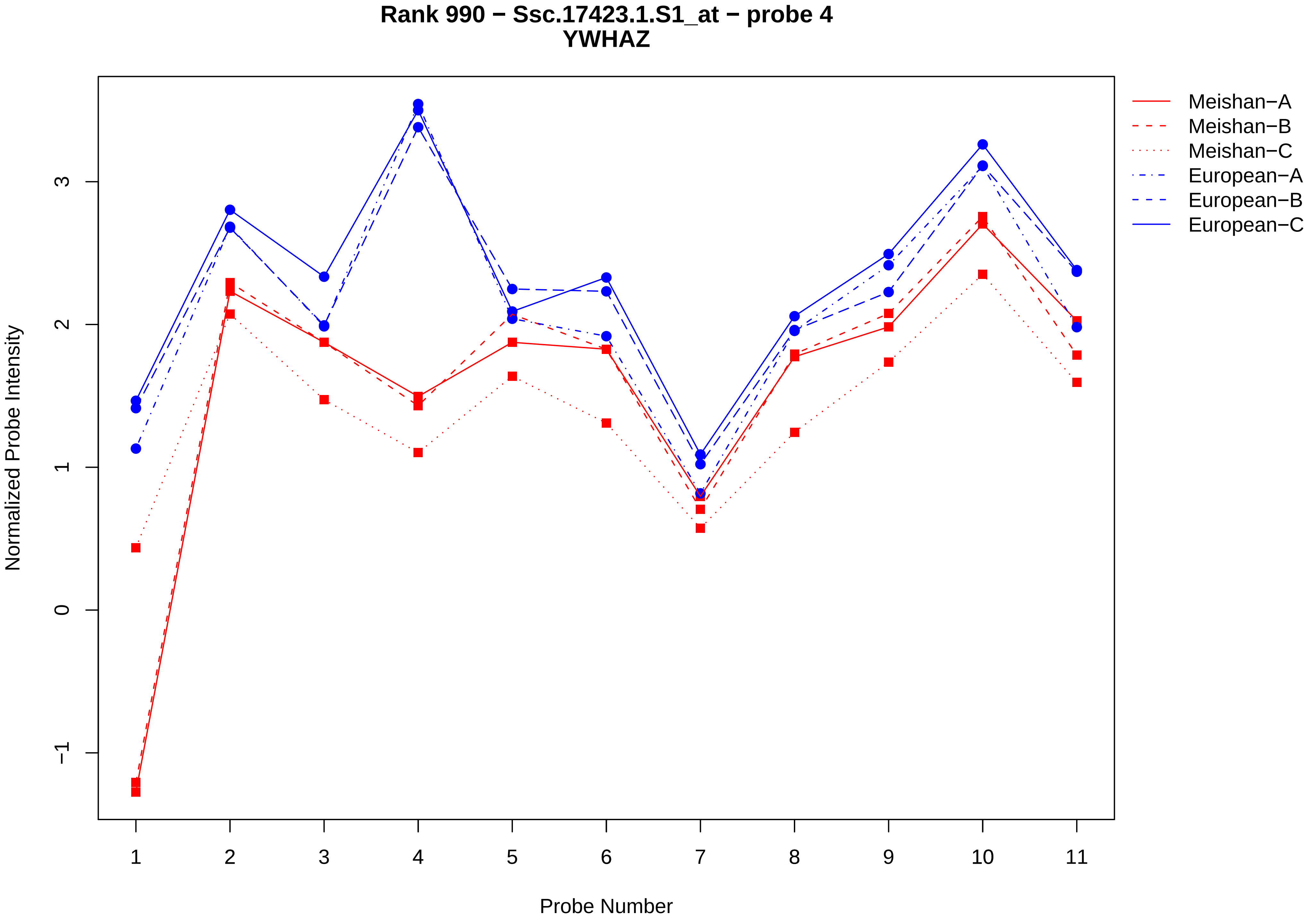

Rank 990 − Ssc.17423.1.S1_at − probe 4
YWHAZ
Normalized Probe Intensity
Probe Number
−1
0
1
2
3
1
2
3
4
5
6
7
8
9
10
11
Meishan−A
Meishan−B
Meishan−C
European−A
European−B
European−C

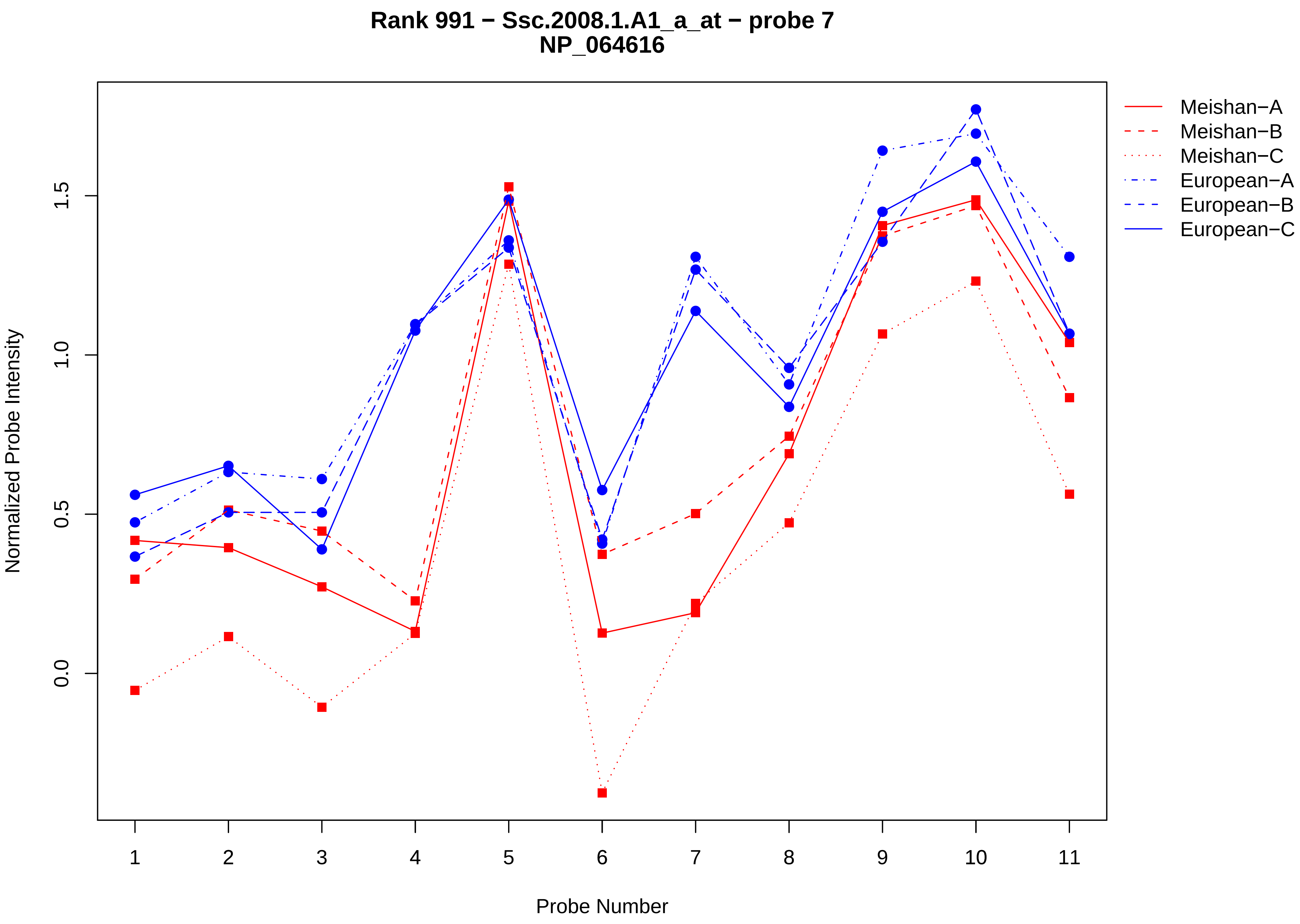

Rank 991 − Ssc.2008.1.A1_a_at − probe 7
NP_064616
Normalized Probe Intensity
Probe Number
0.0
0.5
1.0
1.5
1
2
3
4
5
6
7
8
9
10
11
Meishan−A
Meishan−B
Meishan−C
European−A
European−B
European−C

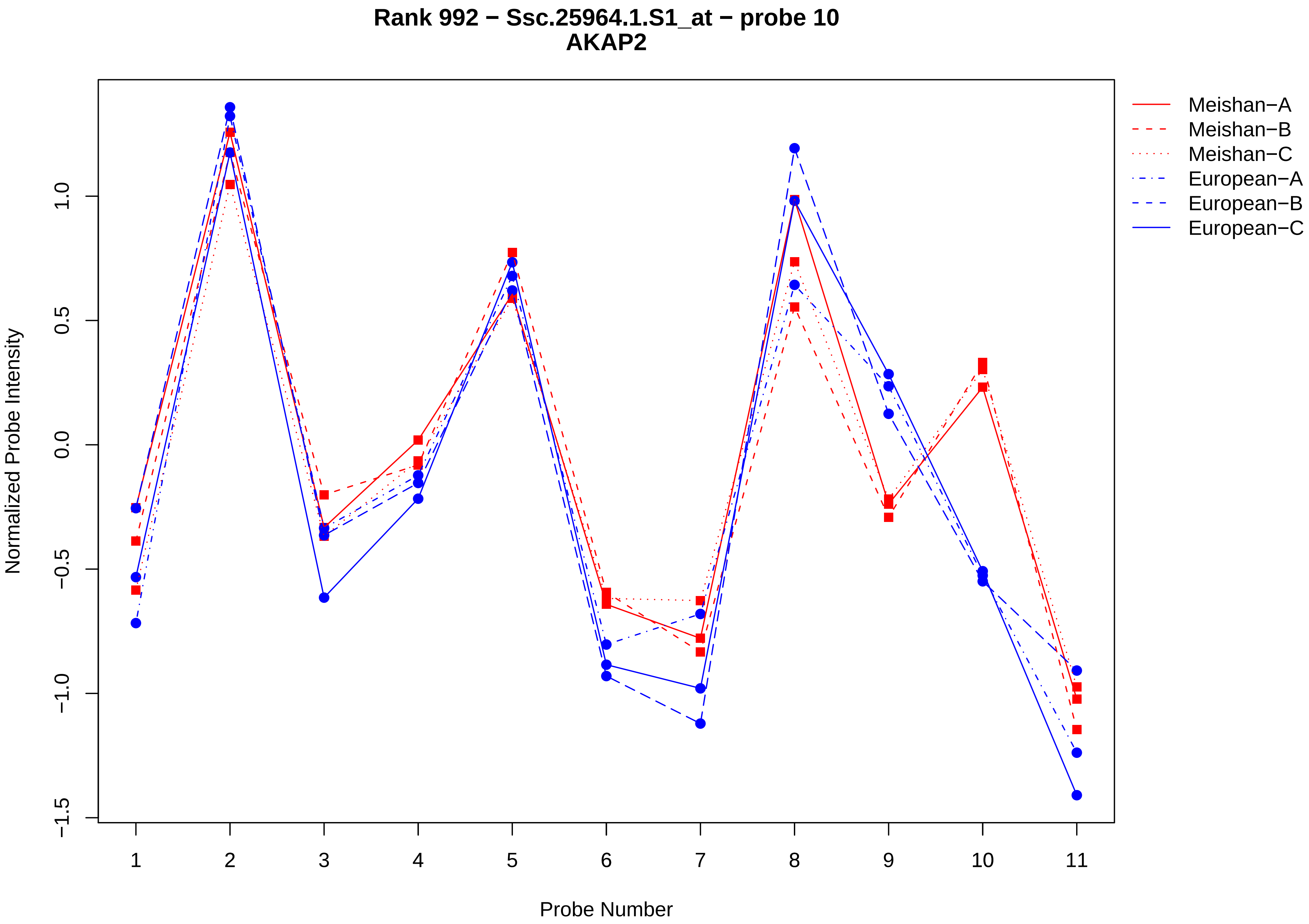

Rank 992 − Ssc.25964.1.S1_at − probe 10
AKAP2
Normalized Probe Intensity
Probe Number
Meishan−A
Meishan−B
Meishan−C
European−A
European−B
European−C
1.0
0.5
0.0
−0.5
−1.0
−1.5
1
2
3
4
5
6
7
8
9
10
11

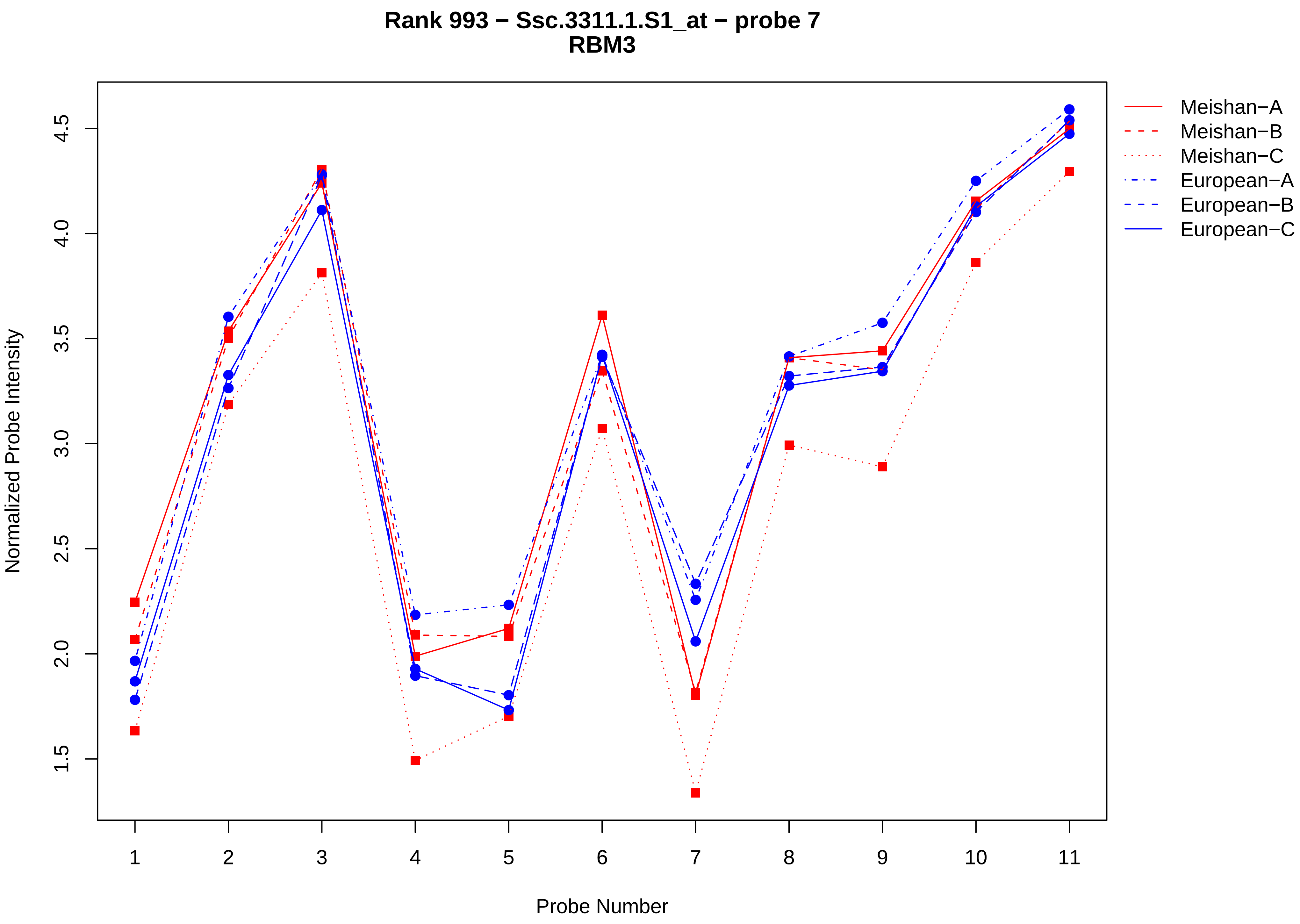

Rank 993 − Ssc.3311.1.S1_at − probe 7
RBM3
Normalized Probe Intensity
1.5
2.0
2.5
3.0
3.5
4.0
4.5
1
2
3
4
5
6
7
8
9
10
11
Probe Number
Meishan−A
Meishan−B
Meishan−C
European−A
European−B
European−C

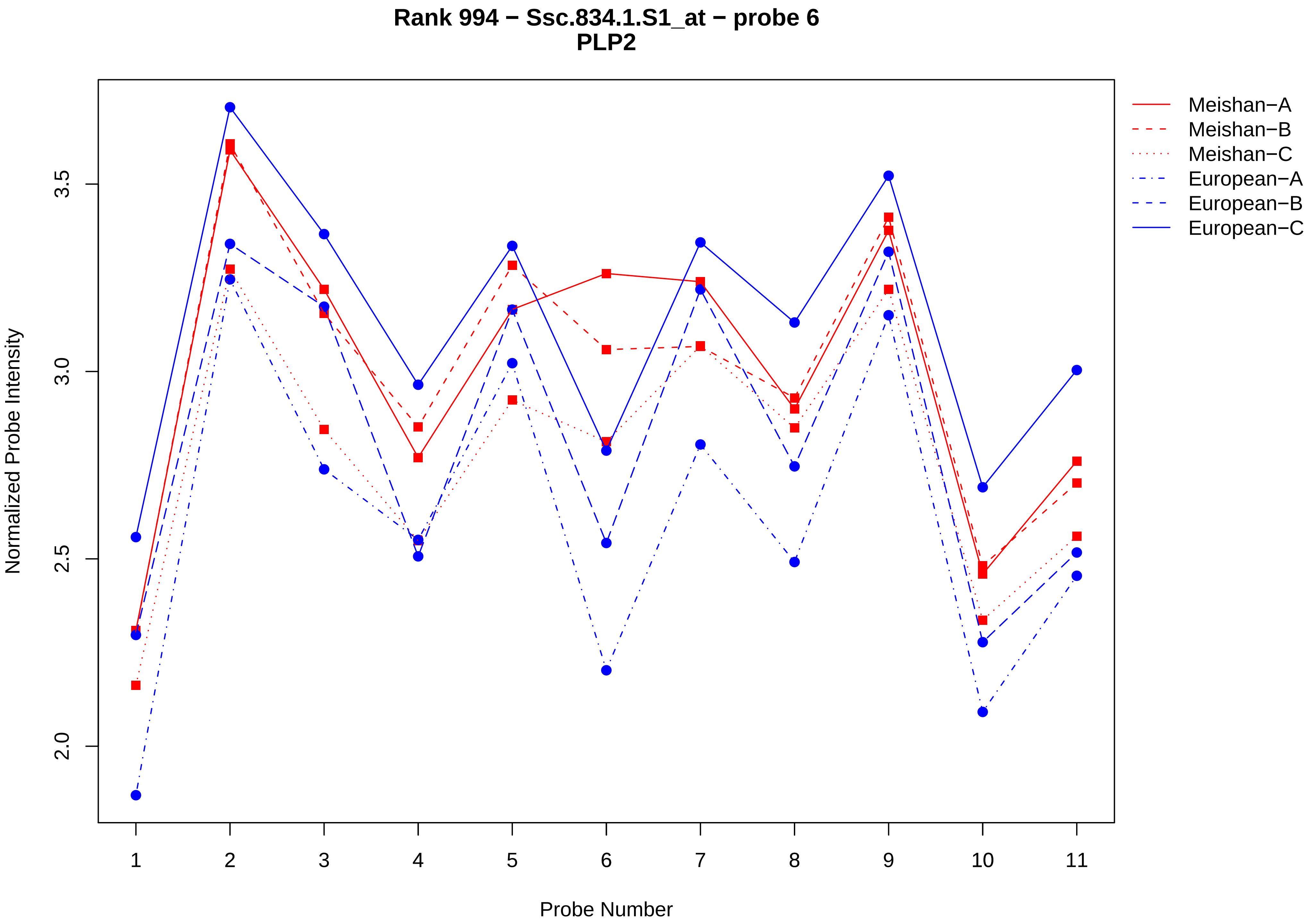
Rank 994 − Ssc.834.1.S1_at − probe 6
PLP2
Normalized Probe Intensity
Probe Number
2.0
2.5
3.0
3.5
1
2
3
4
5
6
7
8
9
10
11
Meishan−A
Meishan−B
Meishan−C
European−A
European−B
European−C

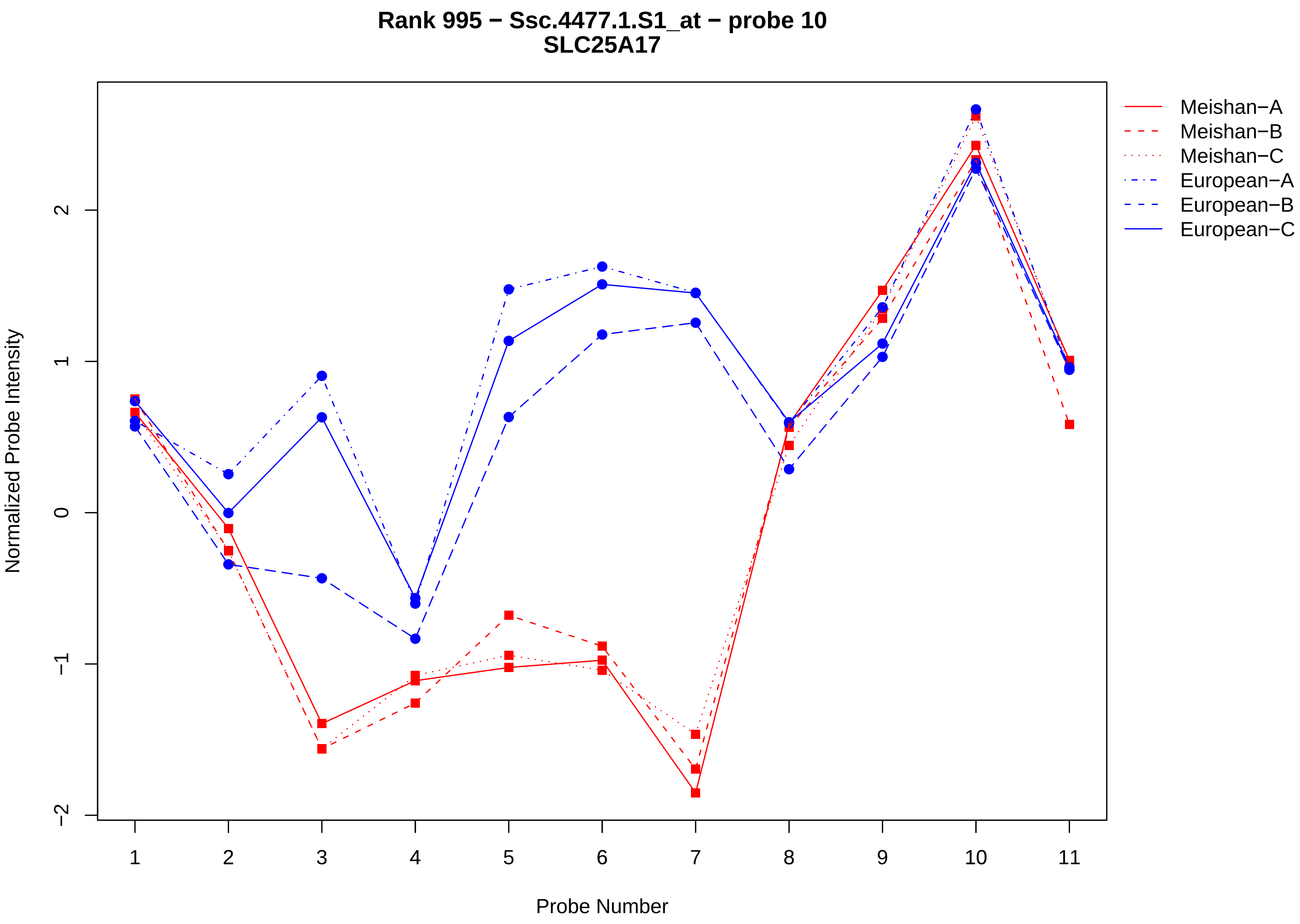

Rank 995 − Ssc.4477.1.S1_at − probe 10
SLC25A17
Normalized Probe Intensity
Probe Number
−2
−1
0
1
2
1
2
3
4
5
6
7
8
9
10
11
Meishan−A
Meishan−B
Meishan−C
European−A
European−B
European−C

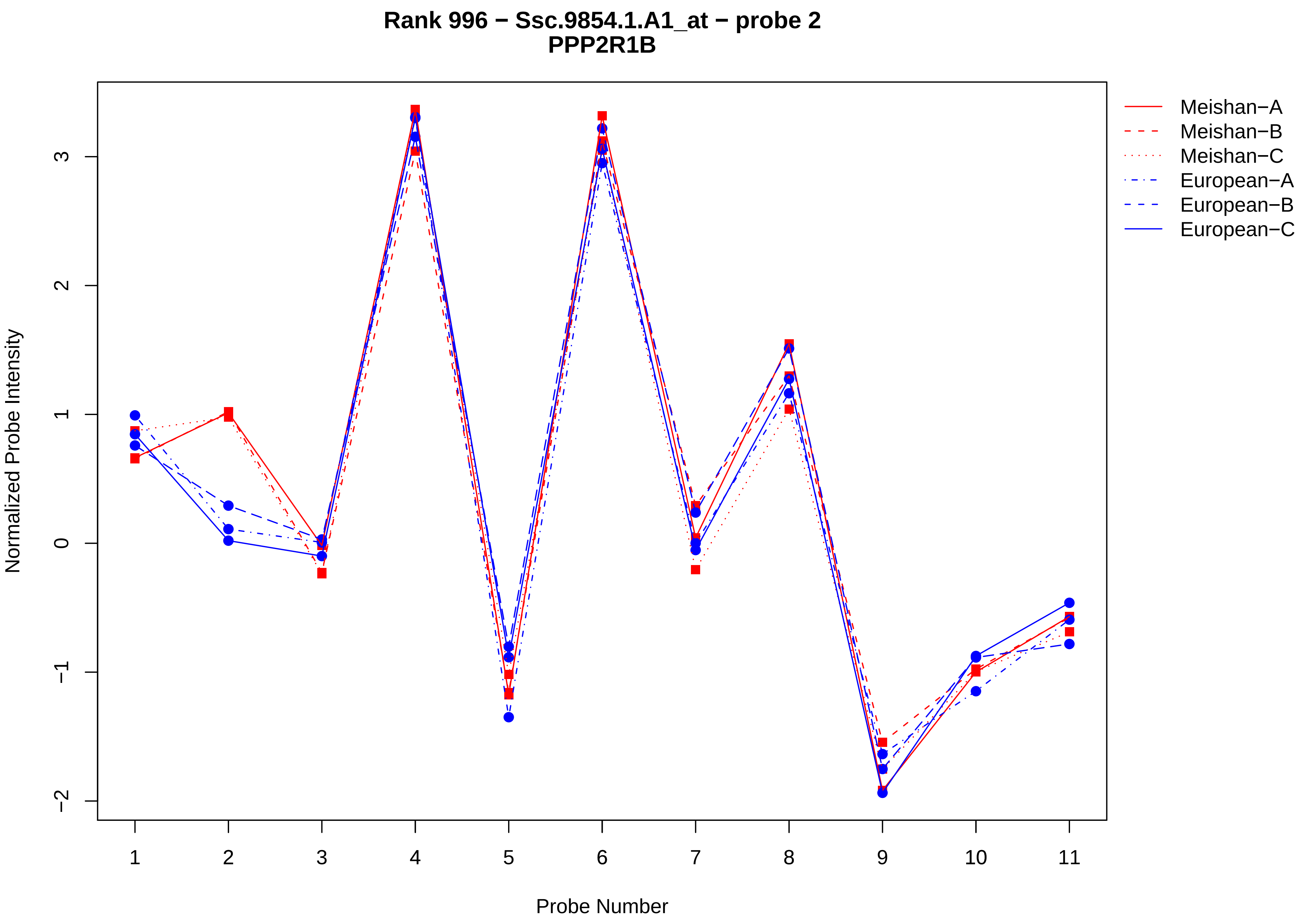
Rank 996 − Ssc.9854.1.A1_at − probe 2
PPP2R1B
Normalized Probe Intensity
Probe Number
−2
−1
0
1
2
3
1
2
3
4
5
6
7
8
9
10
11
Meishan−A
Meishan−B
Meishan−C
European−A
European−B
European−C

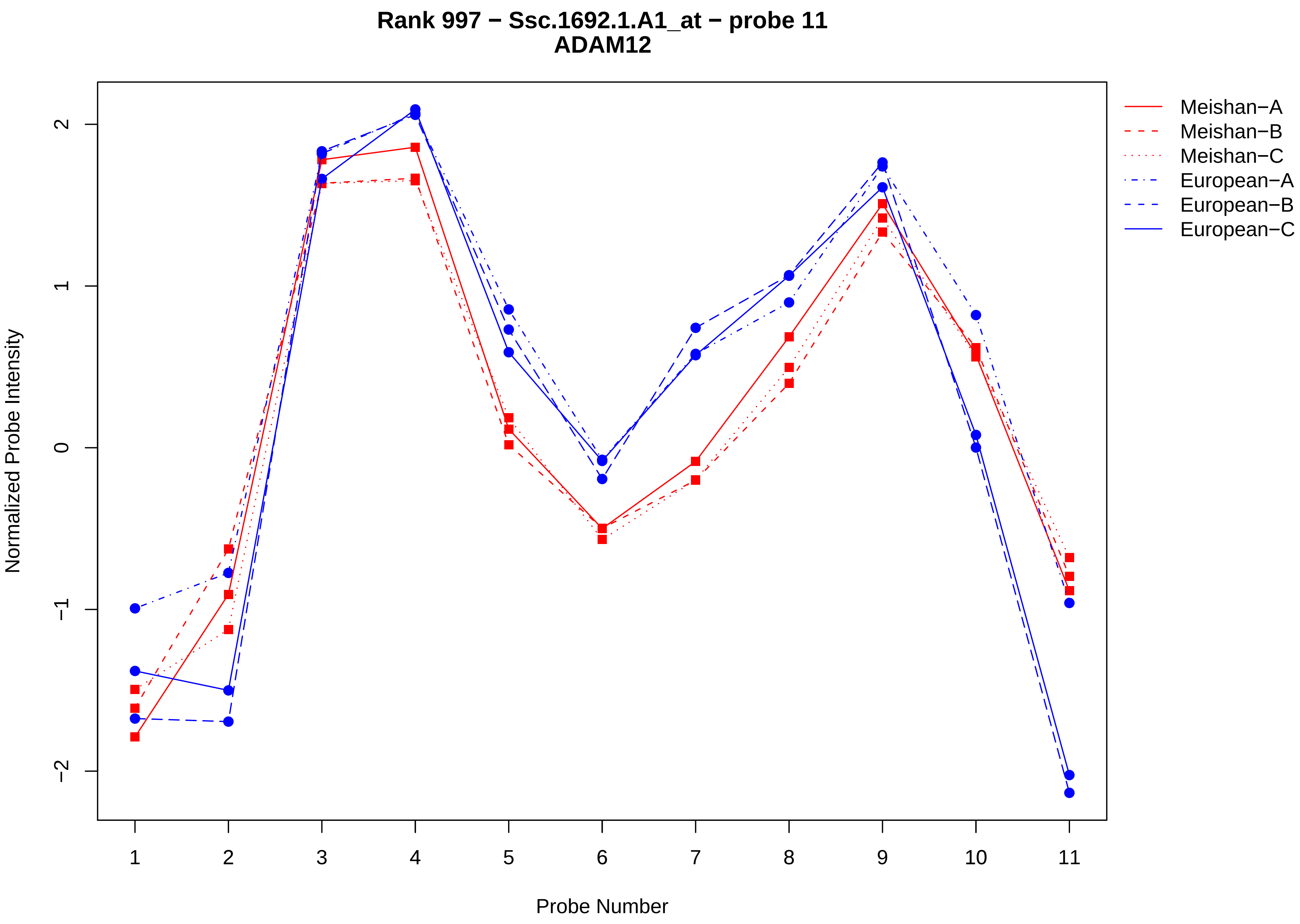

Rank 997 − Ssc.1692.1.A1_at − probe 11
ADAM12
Normalized Probe Intensity
Probe Number
−2
−1
0
1
2
1
2
3
4
5
6
7
8
9
10
11
Meishan−A
Meishan−B
Meishan−C
European−A
European−B
European−C

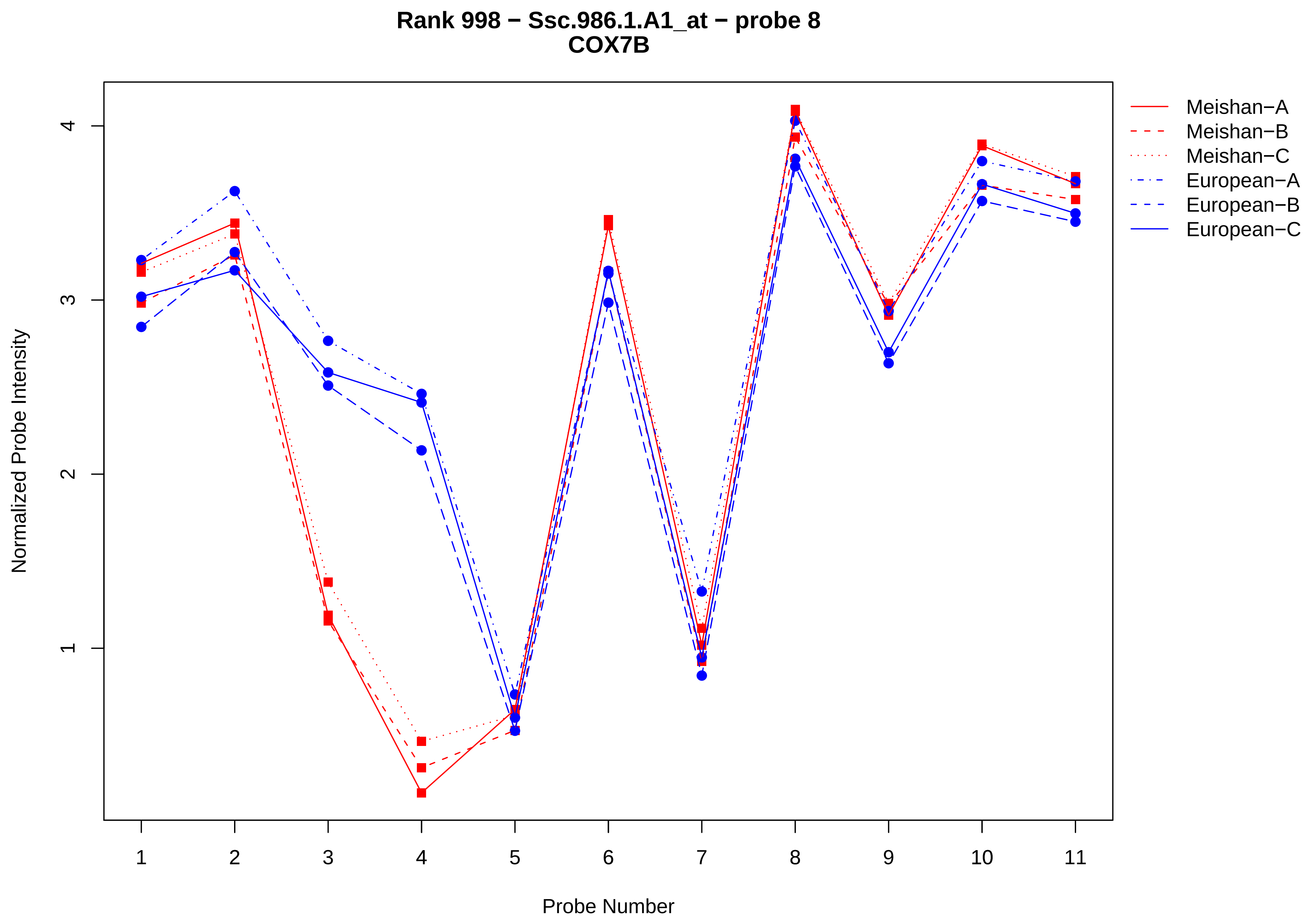

Rank 998 − Ssc.986.1.A1_at − probe 8
COX7B
Normalized Probe Intensity
Probe Number
1
2
3
4
1
2
3
4
5
6
7
8
9
10
11
Meishan−A
Meishan−B
Meishan−C
European−A
European−B
European−C

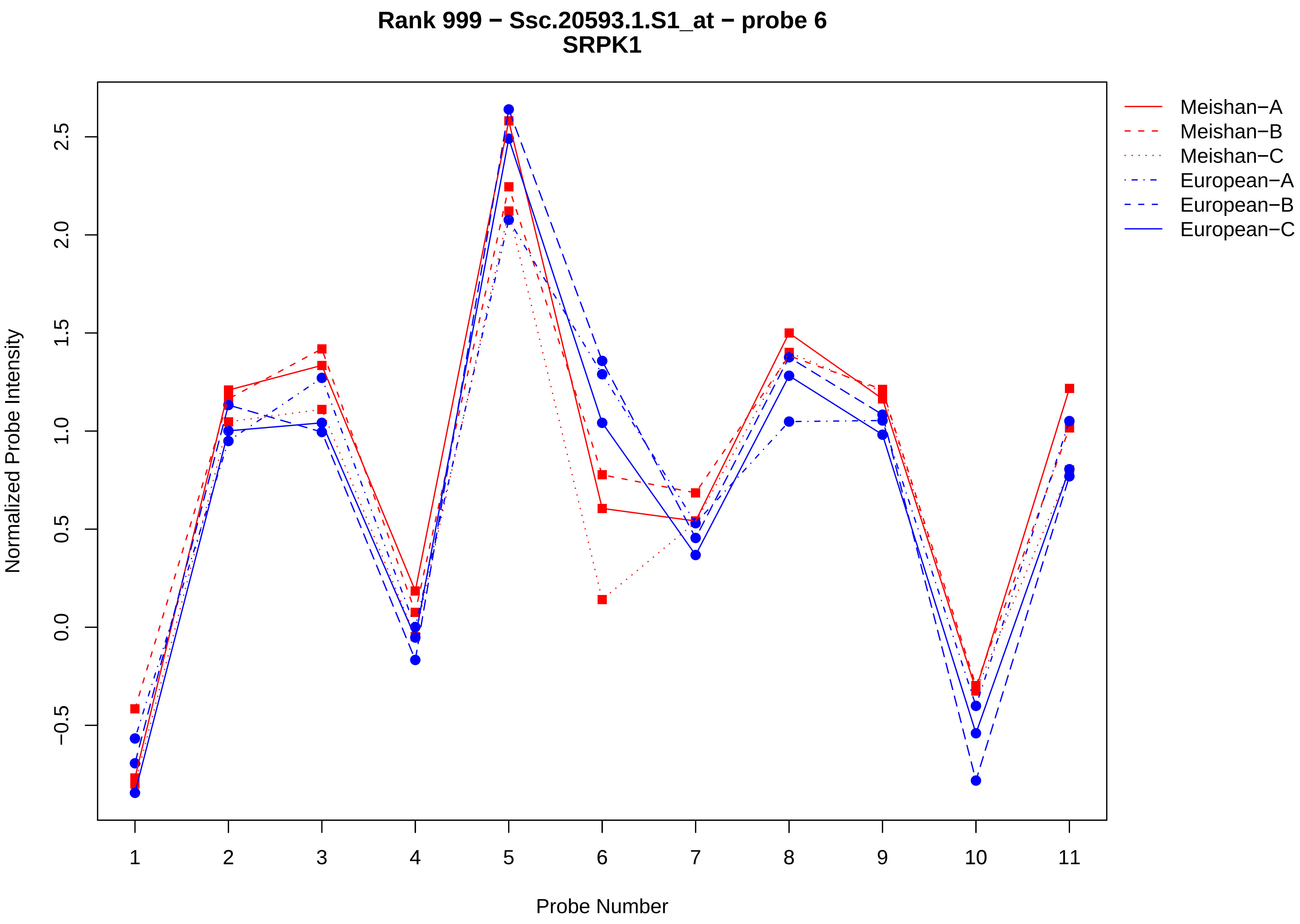
Rank 999 − Ssc.20593.1.S1_at − probe 6
SRPK1
Normalized Probe Intensity
−0.5
0.0
0.5
1.0
1.5
2.0
2.5
1
2
3
4
5
6
7
8
9
10
11
Probe Number
Meishan−A
Meishan−B
Meishan−C
European−A
European−B
European−C

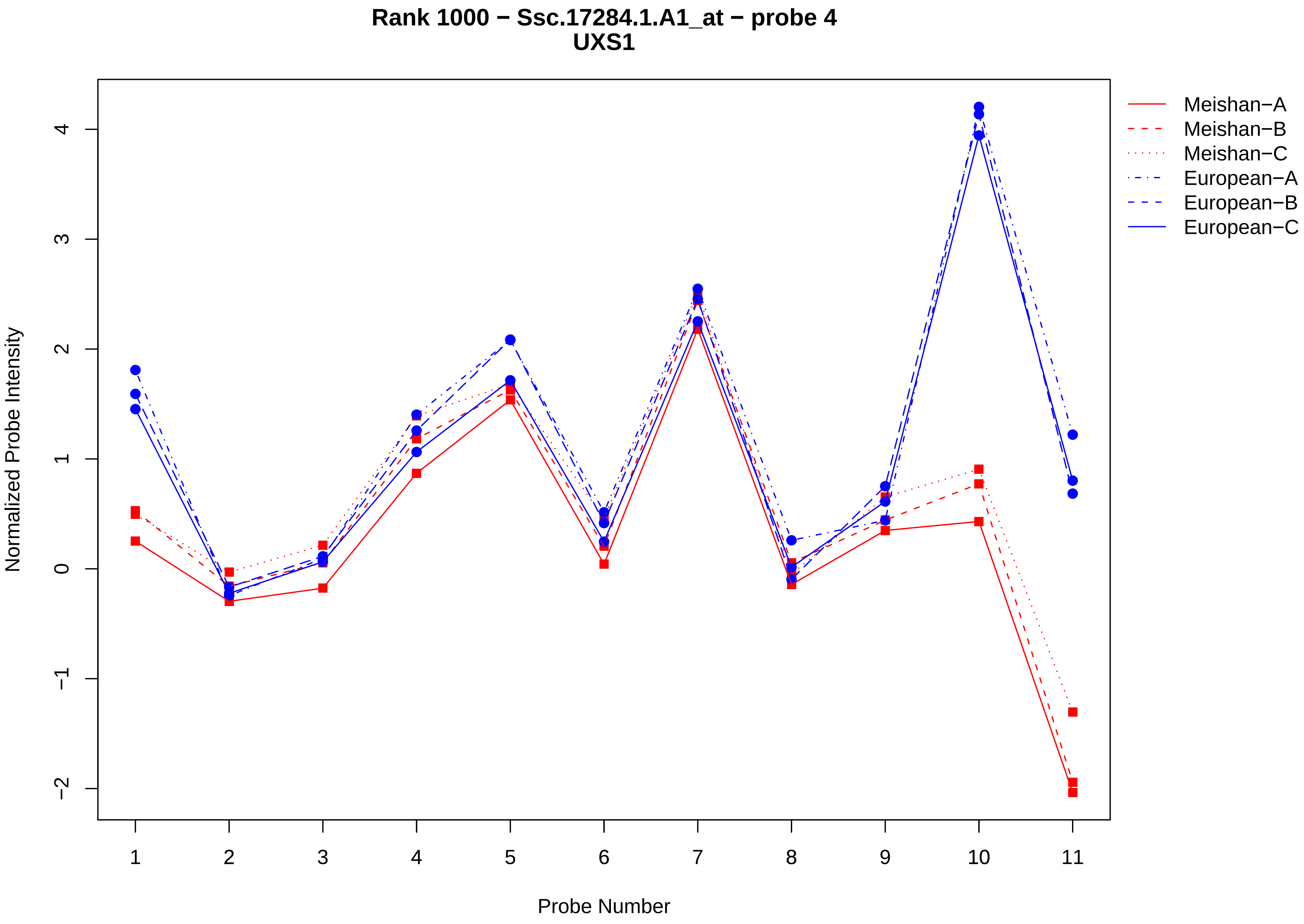

Rank 1000 − Ssc.17284.1.A1_at − probe 4
UXS1
Normalized Probe Intensity
Probe Number
−2
−1
0
1
2
3
4
1
2
3
4
5
6
7
8
9
10
11
Meishan−A
Meishan−B
Meishan−C
European−A
European−B
European−C

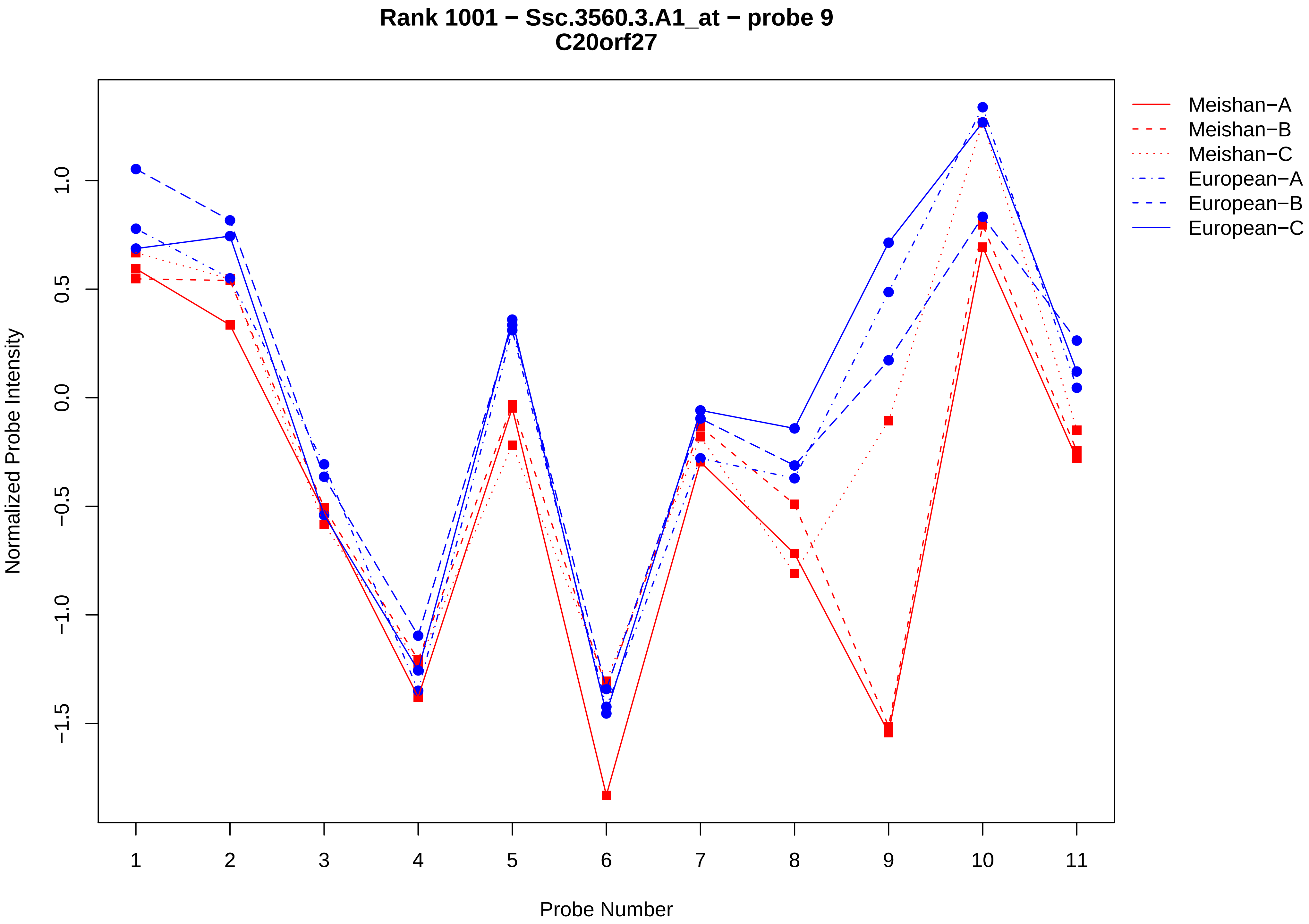
Rank 1001 − Ssc.3560.3.A1_at − probe 9
C20orf27
Normalized Probe Intensity
1.0
0.5
0.0
−0.5
−1.0
−1.5
1
2
3
4
5
6
7
8
9
10
11
Probe Number
Meishan−A
Meishan−B
Meishan−C
European−A
European−B
European−C

Rank 1002 − Ssc.1296.1.S1_at − probe 3
YWHAZ

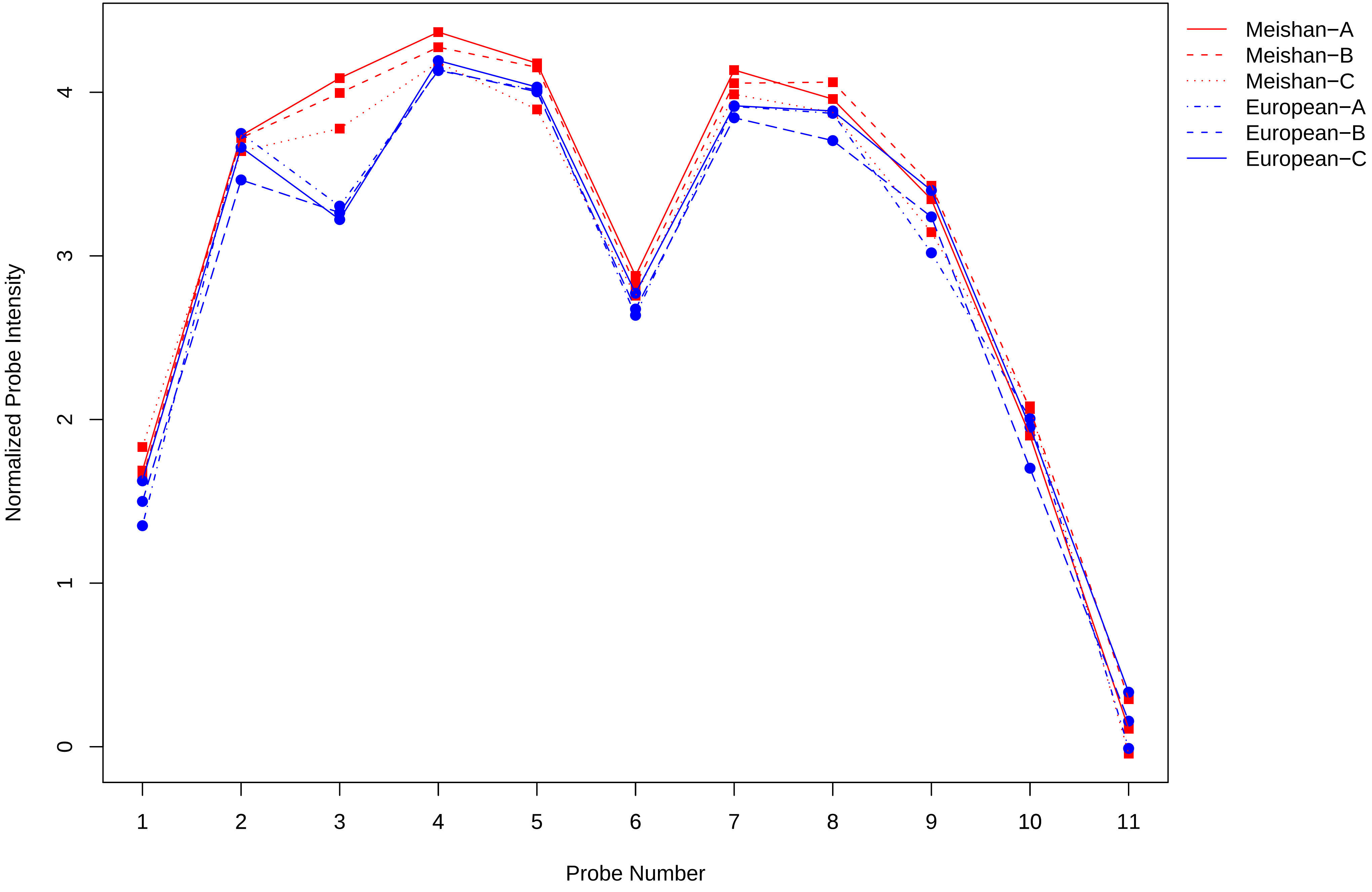

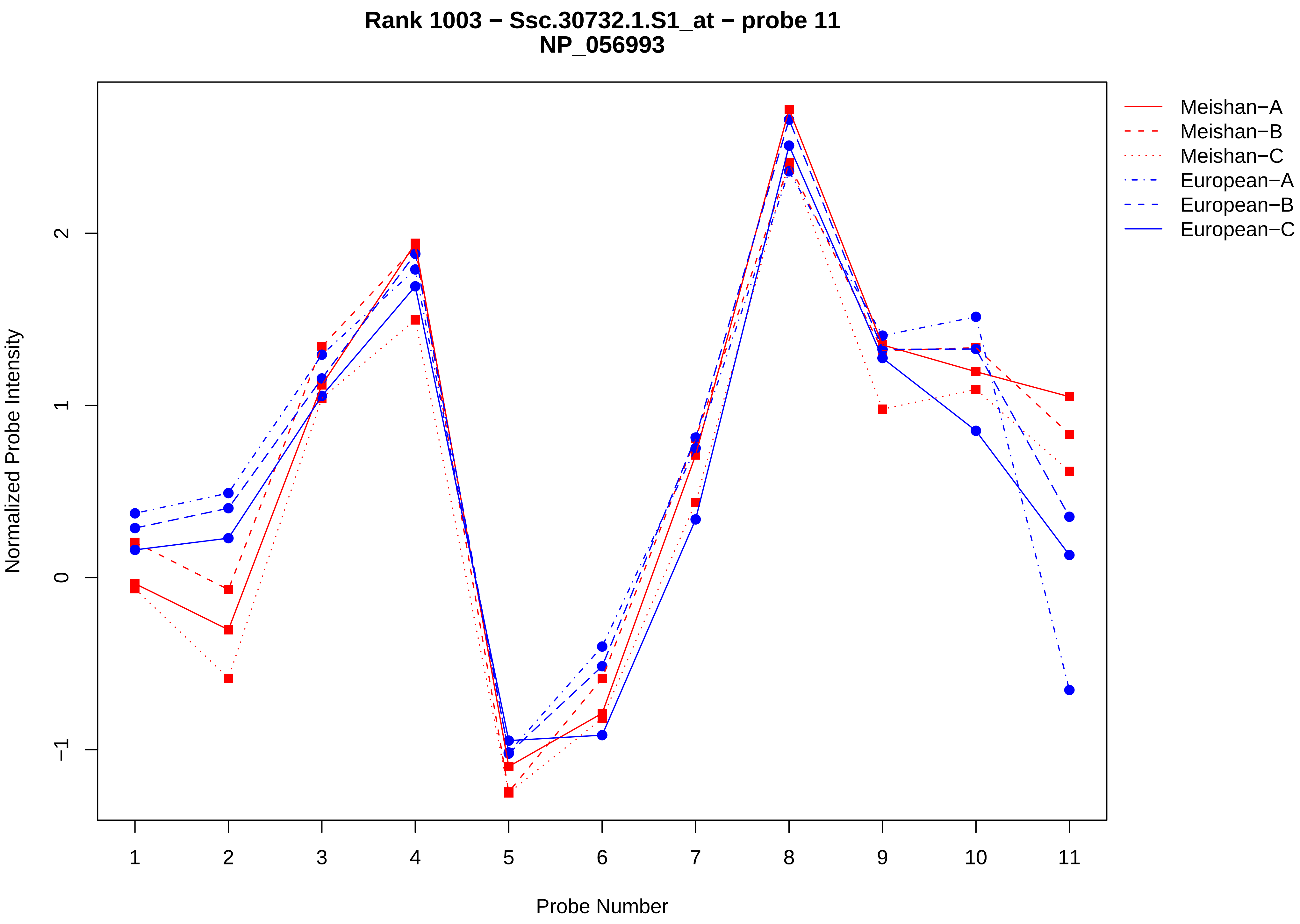

Rank 1003 − Ssc.30732.1.S1_at − probe 11
NP_056993
Normalized Probe Intensity
Probe Number
Meishan−A
Meishan−B
Meishan−C
European−A
European−B
European−C
−1
0
1
2
1
2
3
4
5
6
7
8
9
10
11

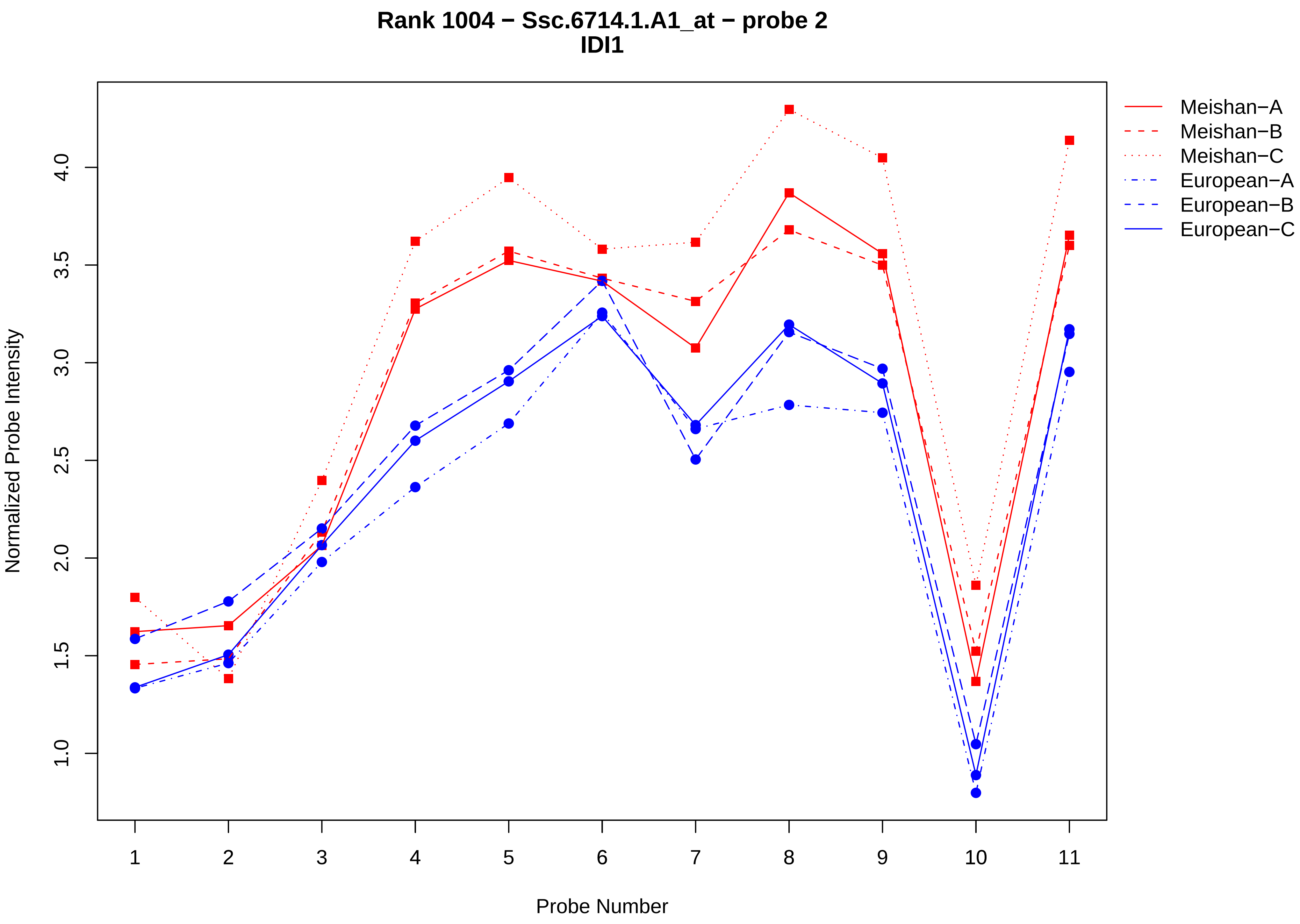
Rank 1004 − Ssc.6714.1.A1_at − probe 2
IDI1
Normalized Probe Intensity
Probe Number
1.0
1.5
2.0
2.5
3.0
3.5
4.0
1
2
3
4
5
6
7
8
9
10
11
Meishan−A
Meishan−B
Meishan−C
European−A
European−B
European−C

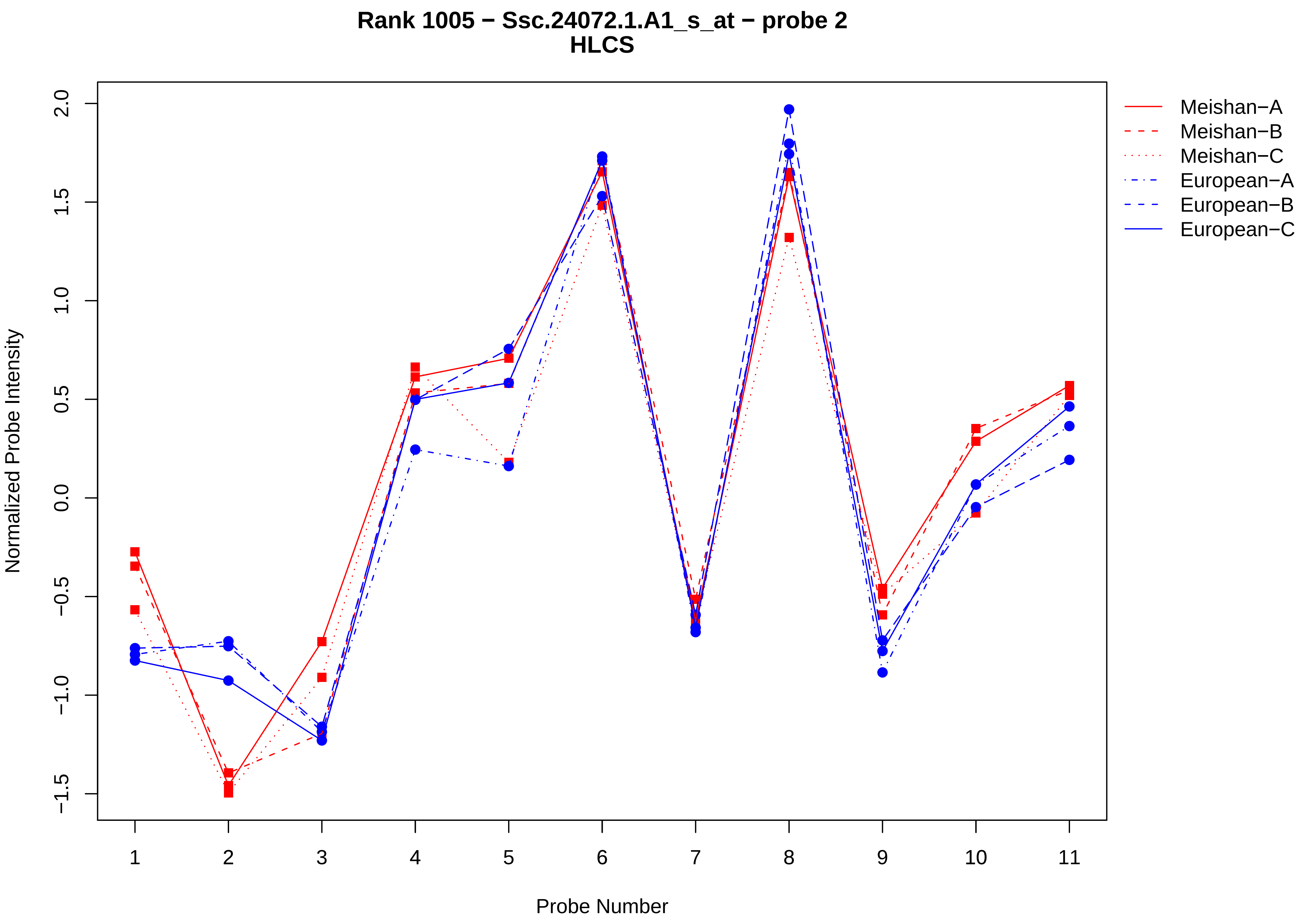

Rank 1005 − Ssc.24072.1.A1_s_at − probe 2
HLCS
Normalized Probe Intensity
Probe Number
−1.5
−1.0
−0.5
0.0
0.5
1.0
1.5
2.0
1
2
3
4
5
6
7
8
9
10
11
Meishan−A
Meishan−B
Meishan−C
European−A
European−B
European−C

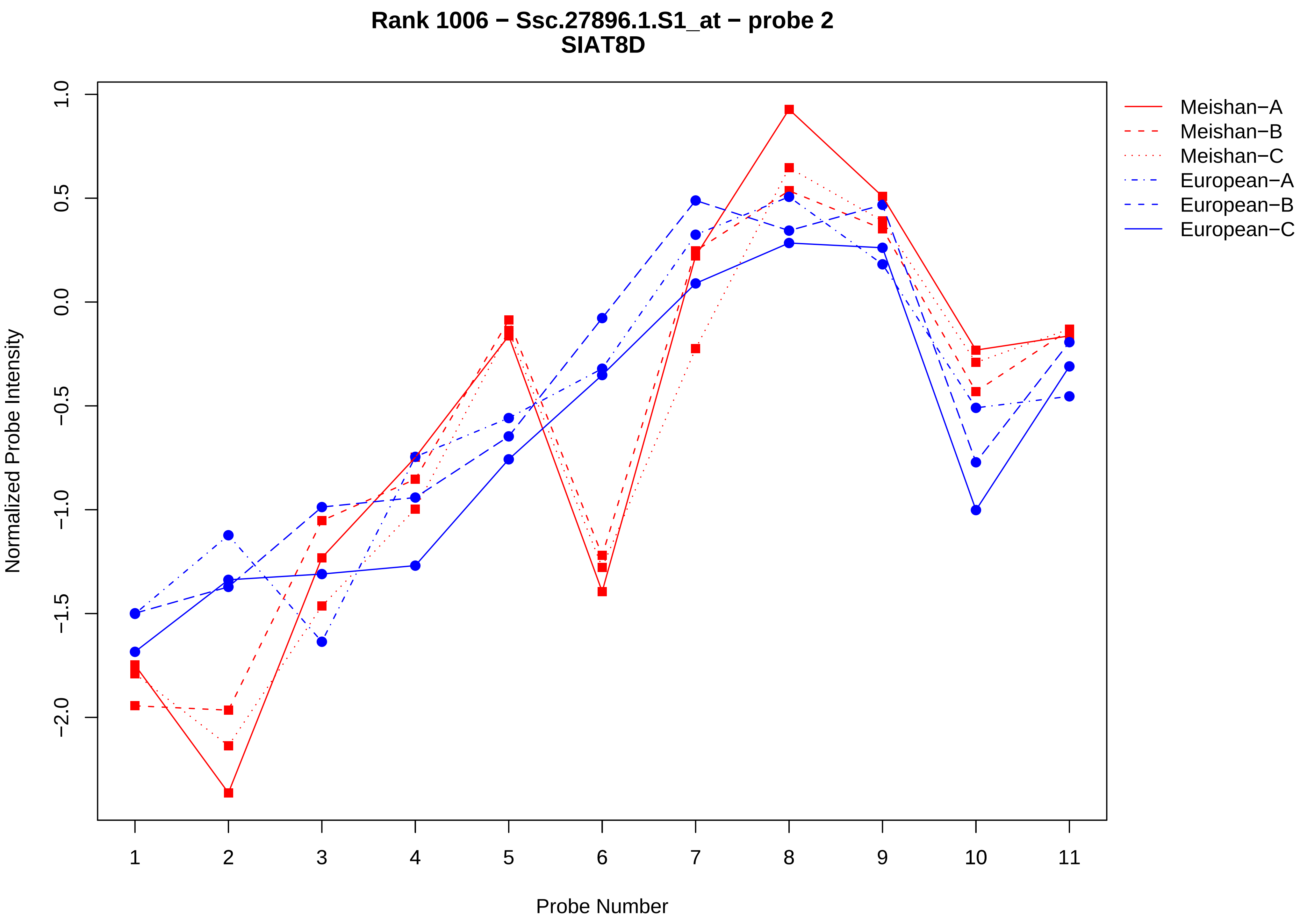

Rank 1006 − Ssc.27896.1.S1_at − probe 2
SIAT8D
Normalized Probe Intensity
Probe Number
1.0
0.5
0.0
−0.5
−1.0
−1.5
−2.0
1
2
3
4
5
6
7
8
9
10
11
Meishan−A
Meishan−B
Meishan−C
European−A
European−B
European−C

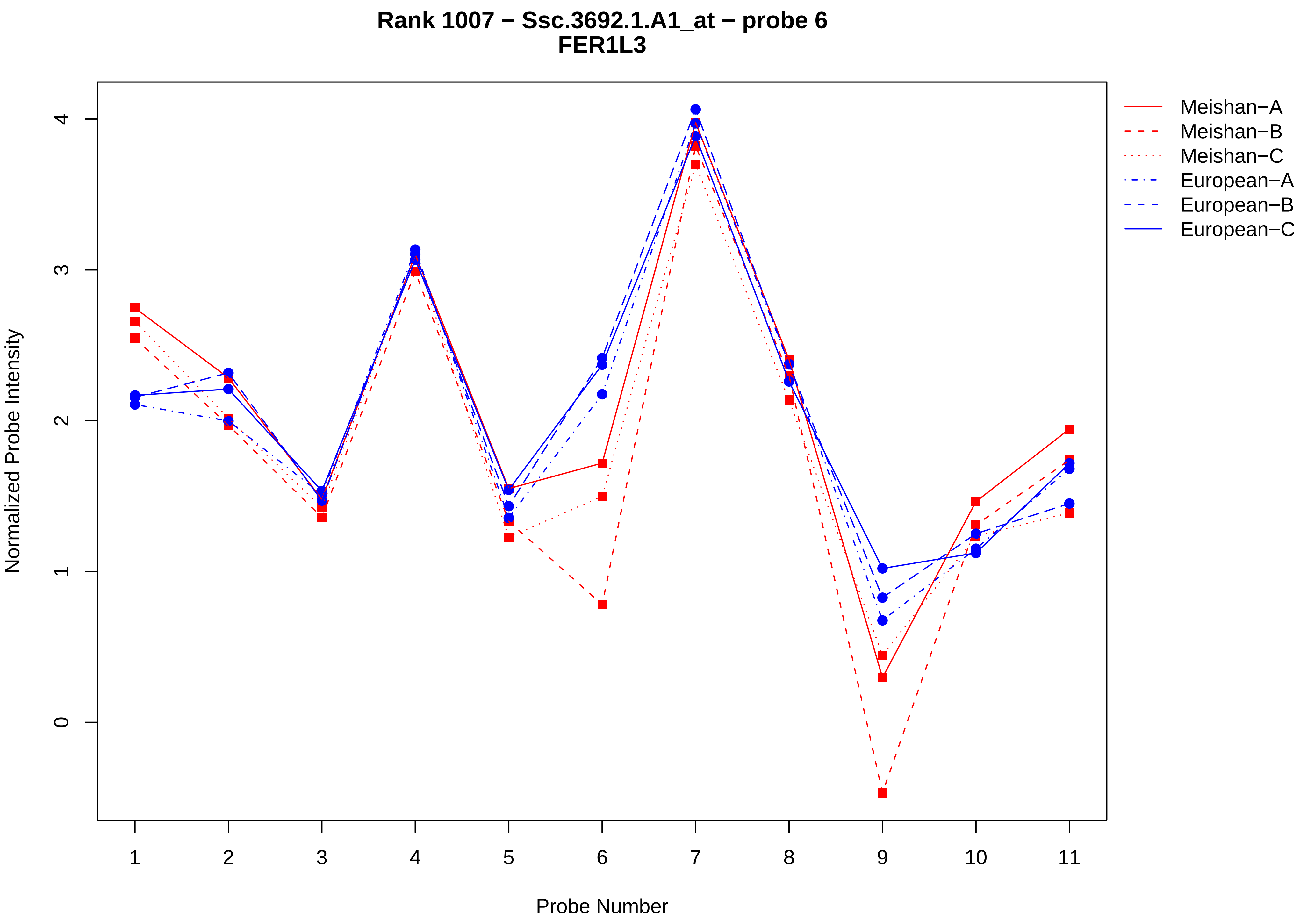
Rank 1007 − Ssc.3692.1.A1_at − probe 6
FER1L3
Normalized Probe Intensity
Probe Number
Meishan−A
Meishan−B
Meishan−C
European−A
European−B
European−C
0
1
2
3
4
1
2
3
4
5
6
7
8
9
10
11

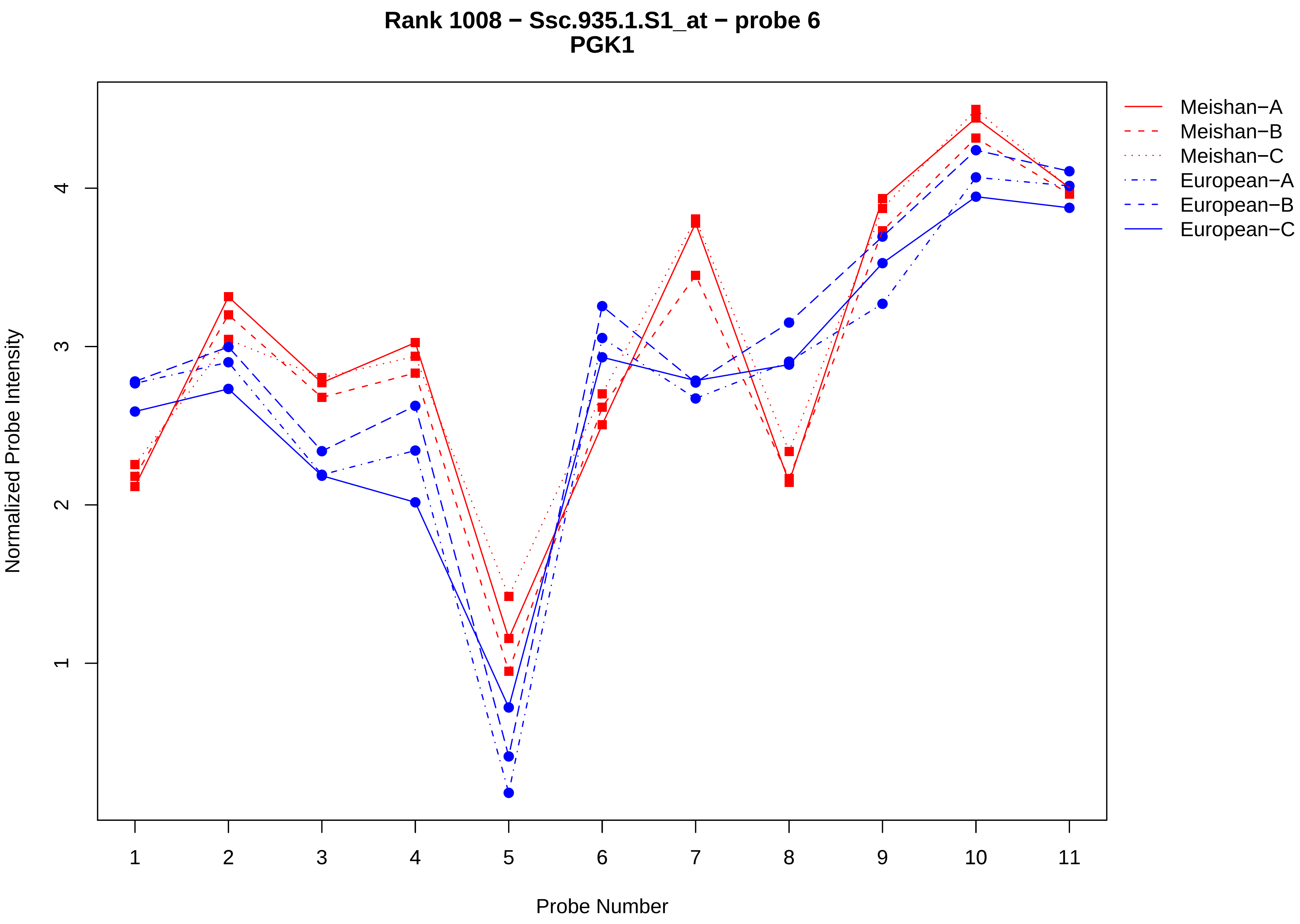

Rank 1008 − Ssc.935.1.S1_at − probe 6
PGK1
Normalized Probe Intensity
Probe Number
1
2
3
4
1
2
3
4
5
6
7
8
9
10
11
Meishan−A
Meishan−B
Meishan−C
European−A
European−B
European−C

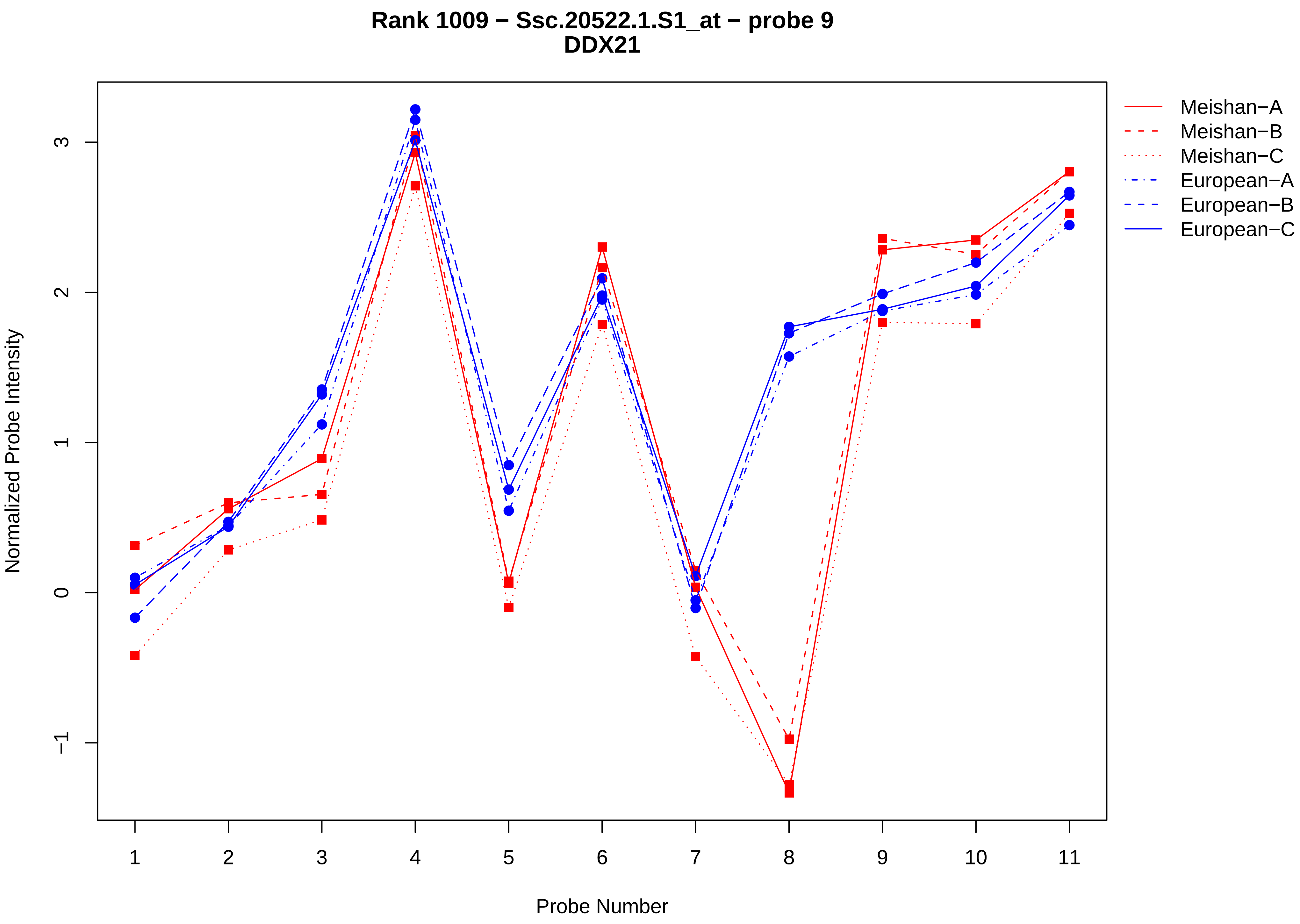

Rank 1009 − Ssc.20522.1.S1_at − probe 9
DDX21
Normalized Probe Intensity
Probe Number
−1
0
1
2
3
1
2
3
4
5
6
7
8
9
10
11
Meishan−A
Meishan−B
Meishan−C
European−A
European−B
European−C

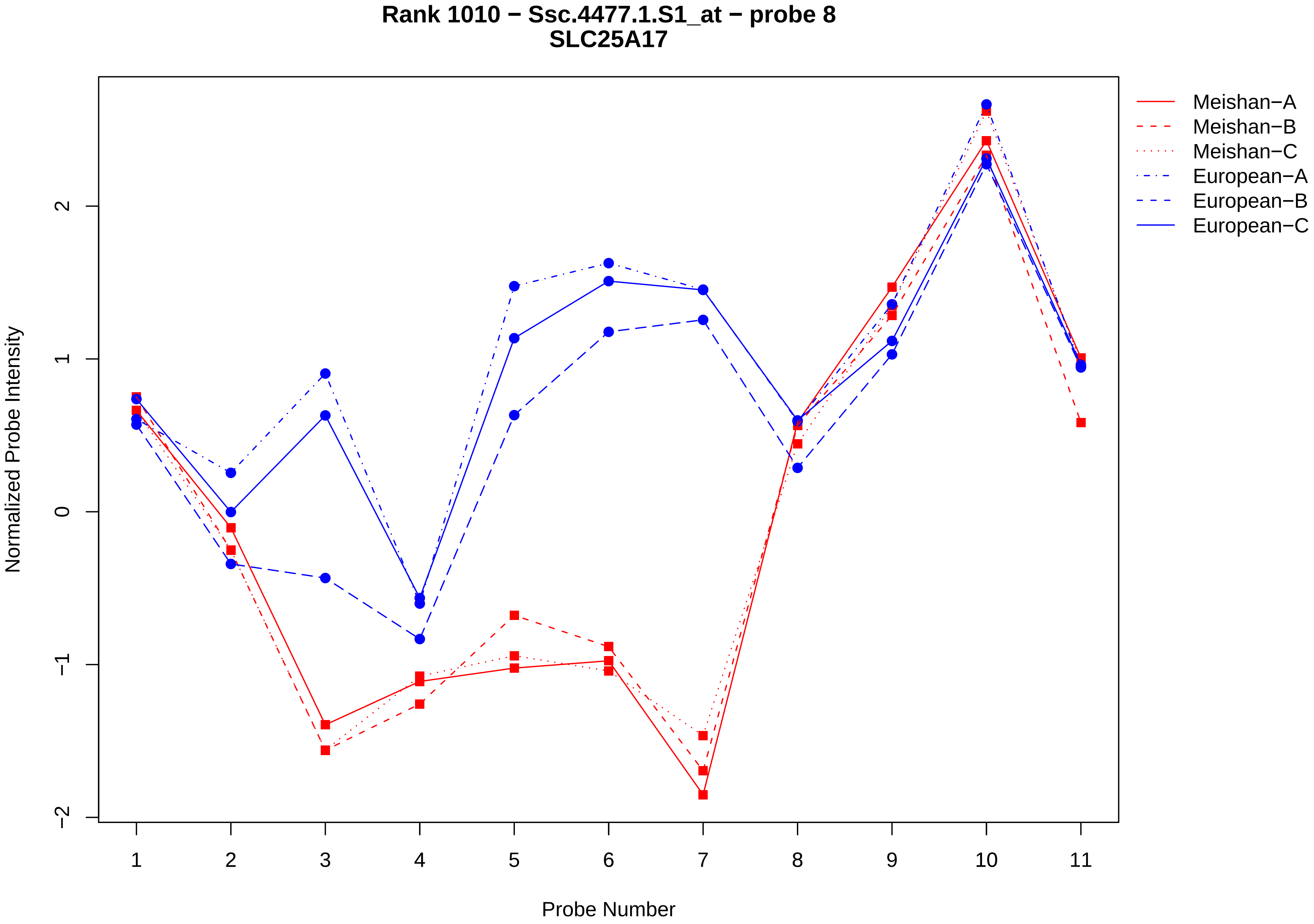

Rank 1010 − Ssc.4477.1.S1_at − probe 8
SLC25A17
Normalized Probe Intensity
Probe Number
−2
−1
0
1
2
1
2
3
4
5
6
7
8
9
10
11
Meishan−A
Meishan−B
Meishan−C
European−A
European−B
European−C

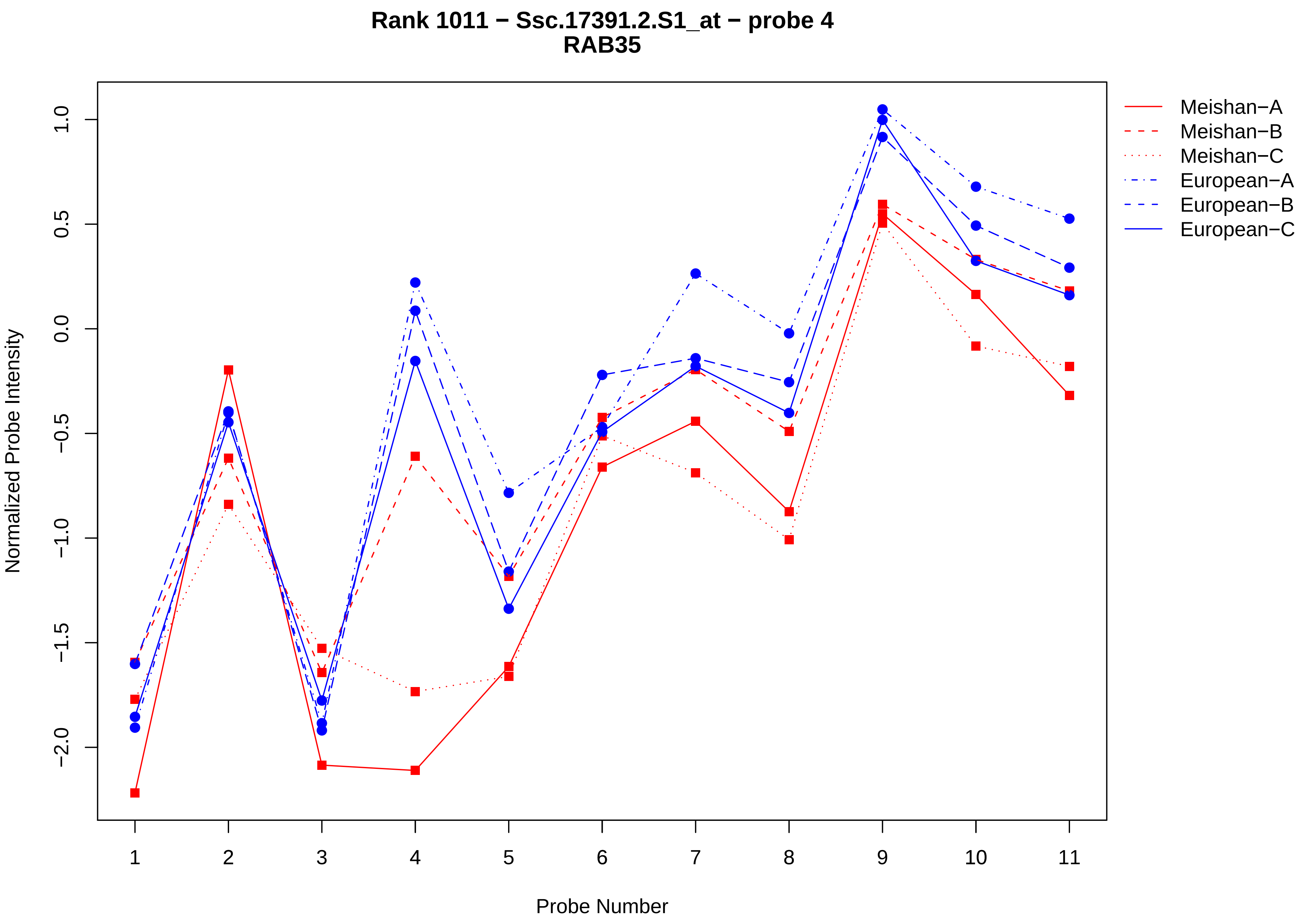

Rank 1011 − Ssc.17391.2.S1_at − probe 4
RAB35
Normalized Probe Intensity
Probe Number
Meishan−A
Meishan−B
Meishan−C
European−A
European−B
European−C
1.0
0.5
0.0
−0.5
−1.0
−1.5
−2.0
1
2
3
4
5
6
7
8
9
10
11

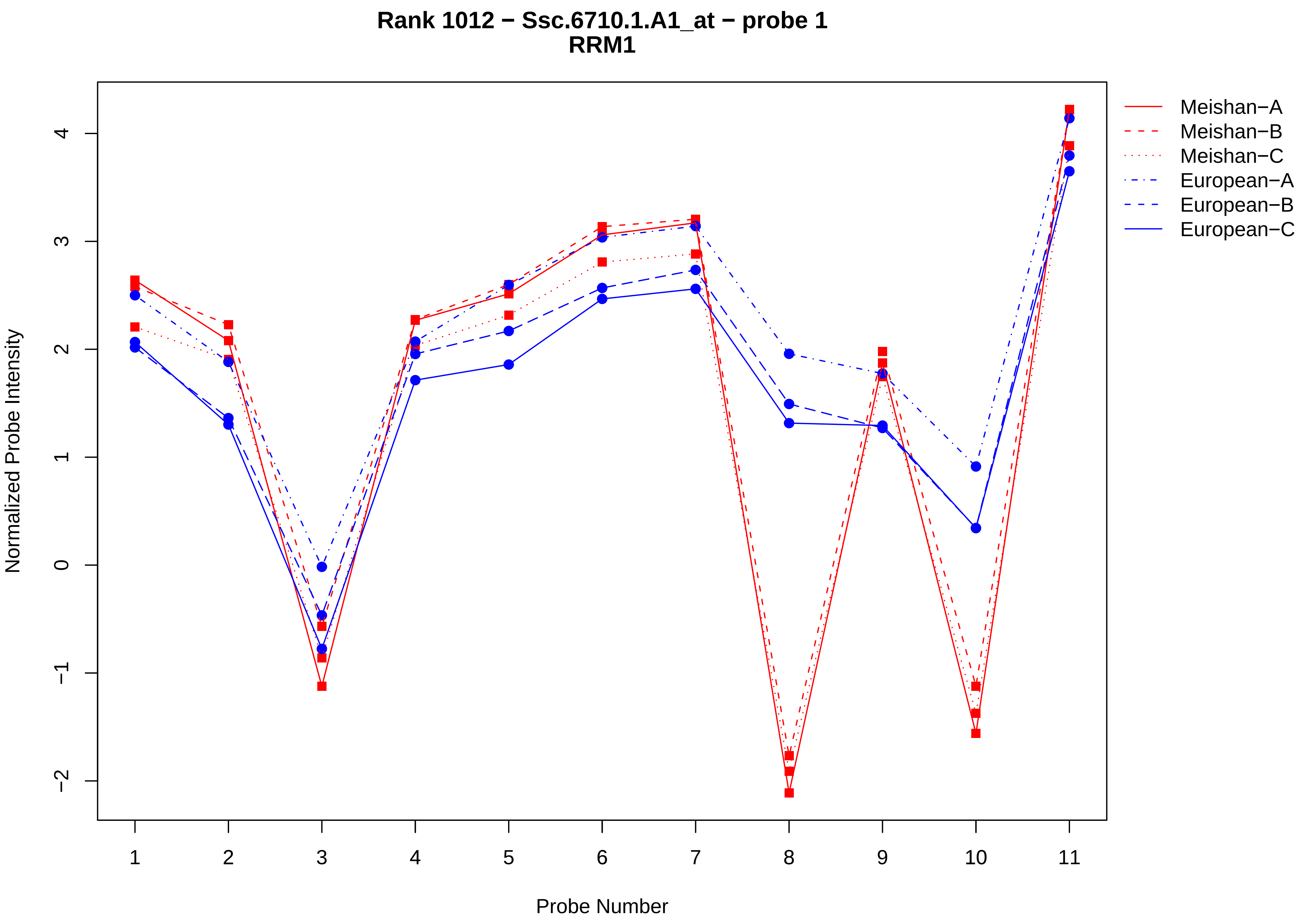

Rank 1012 − Ssc.6710.1.A1_at − probe 1
RRM1
Normalized Probe Intensity
Probe Number
−2
−1
0
1
2
3
4
1
2
3
4
5
6
7
8
9
10
11
Meishan−A
Meishan−B
Meishan−C
European−A
European−B
European−C

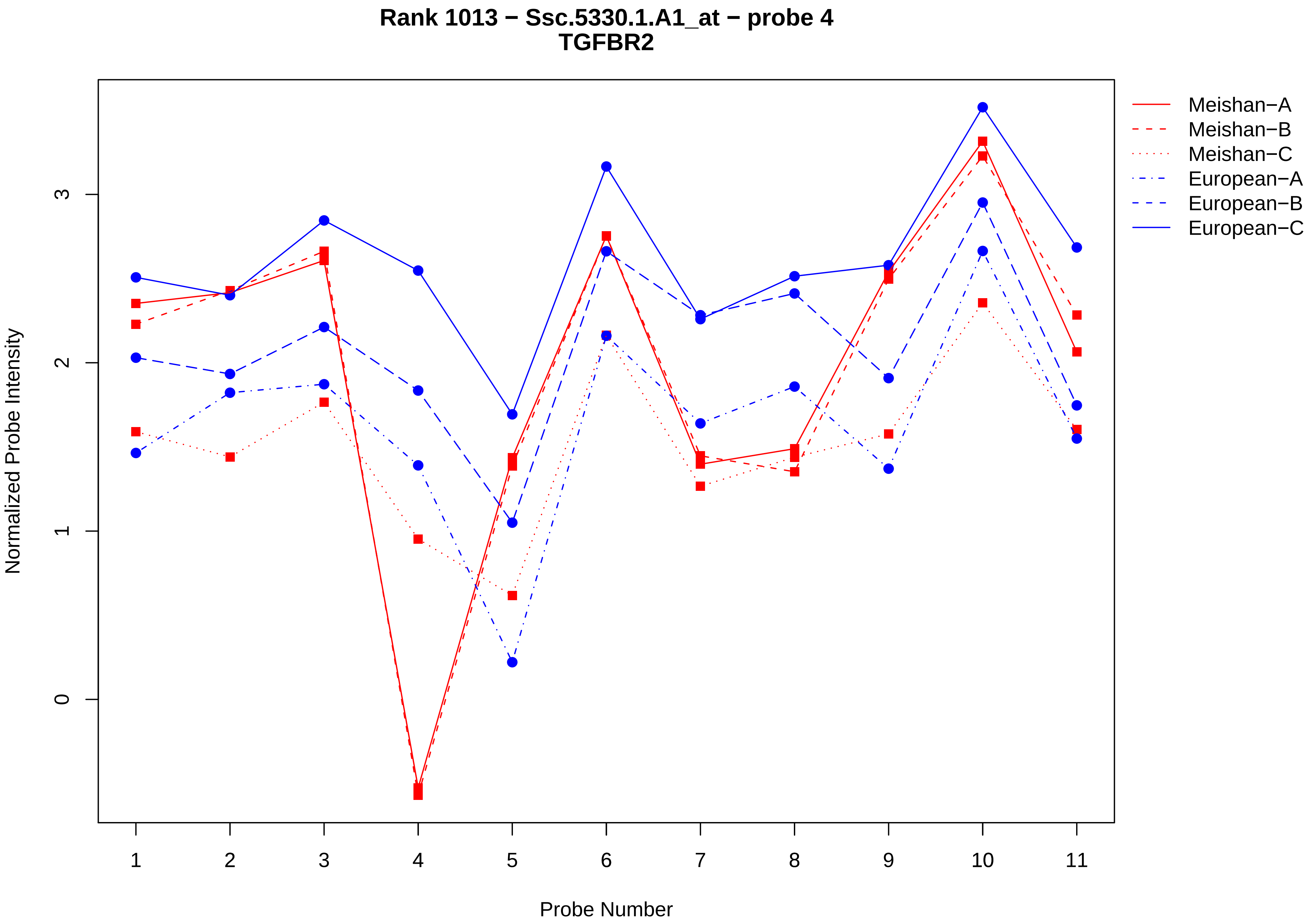

Rank 1013 − Ssc.5330.1.A1_at − probe 4
TGFBR2
Normalized Probe Intensity
Probe Number
0
1
2
3
1
2
3
4
5
6
7
8
9
10
11
Meishan−A
Meishan−B
Meishan−C
European−A
European−B
European−C

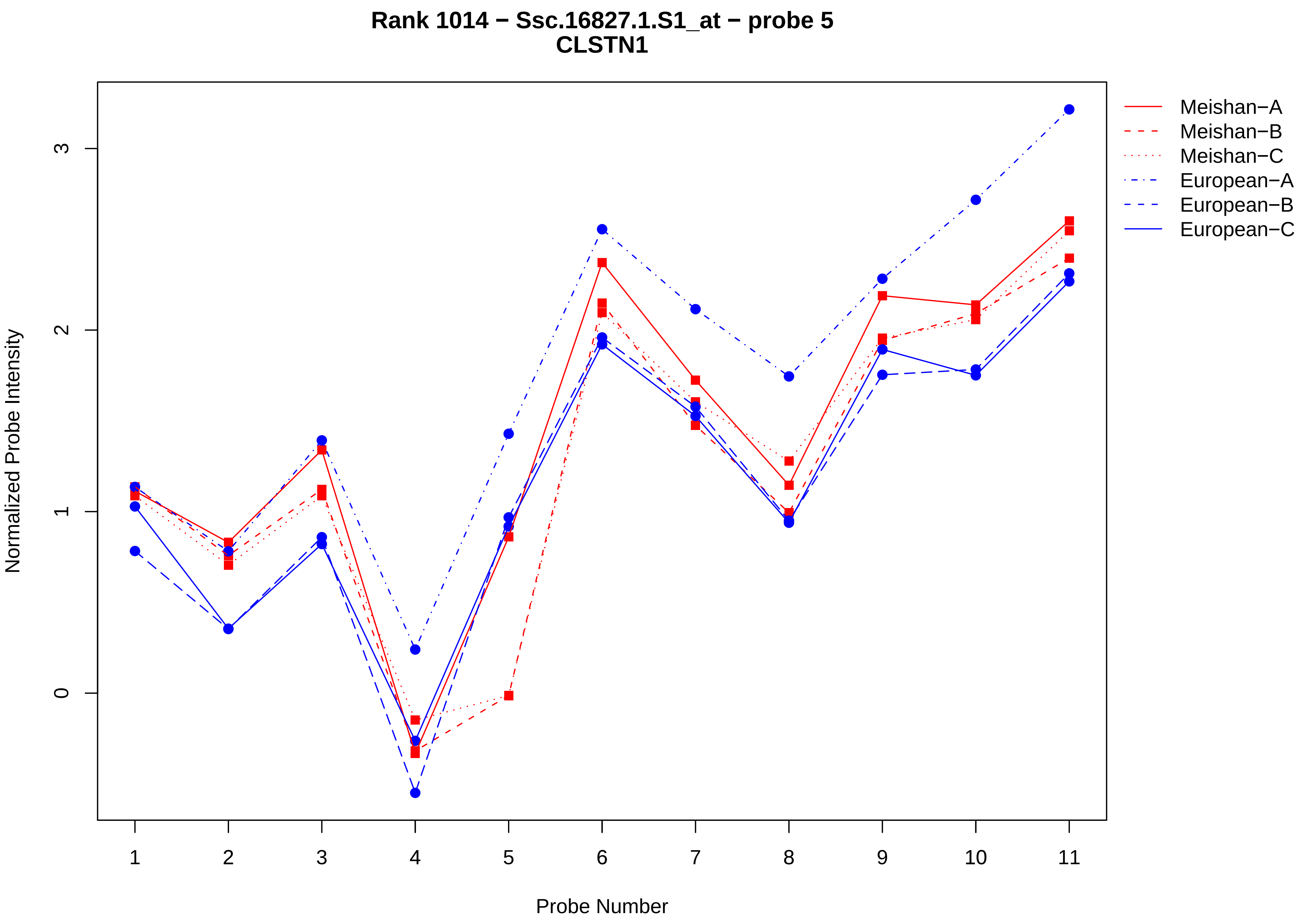

Rank 1014 − Ssc.16827.1.S1_at − probe 5
CLSTN1
Normalized Probe Intensity
Probe Number
0
1
2
3
1
2
3
4
5
6
7
8
9
10
11
Meishan−A
Meishan−B
Meishan−C
European−A
European−B
European−C

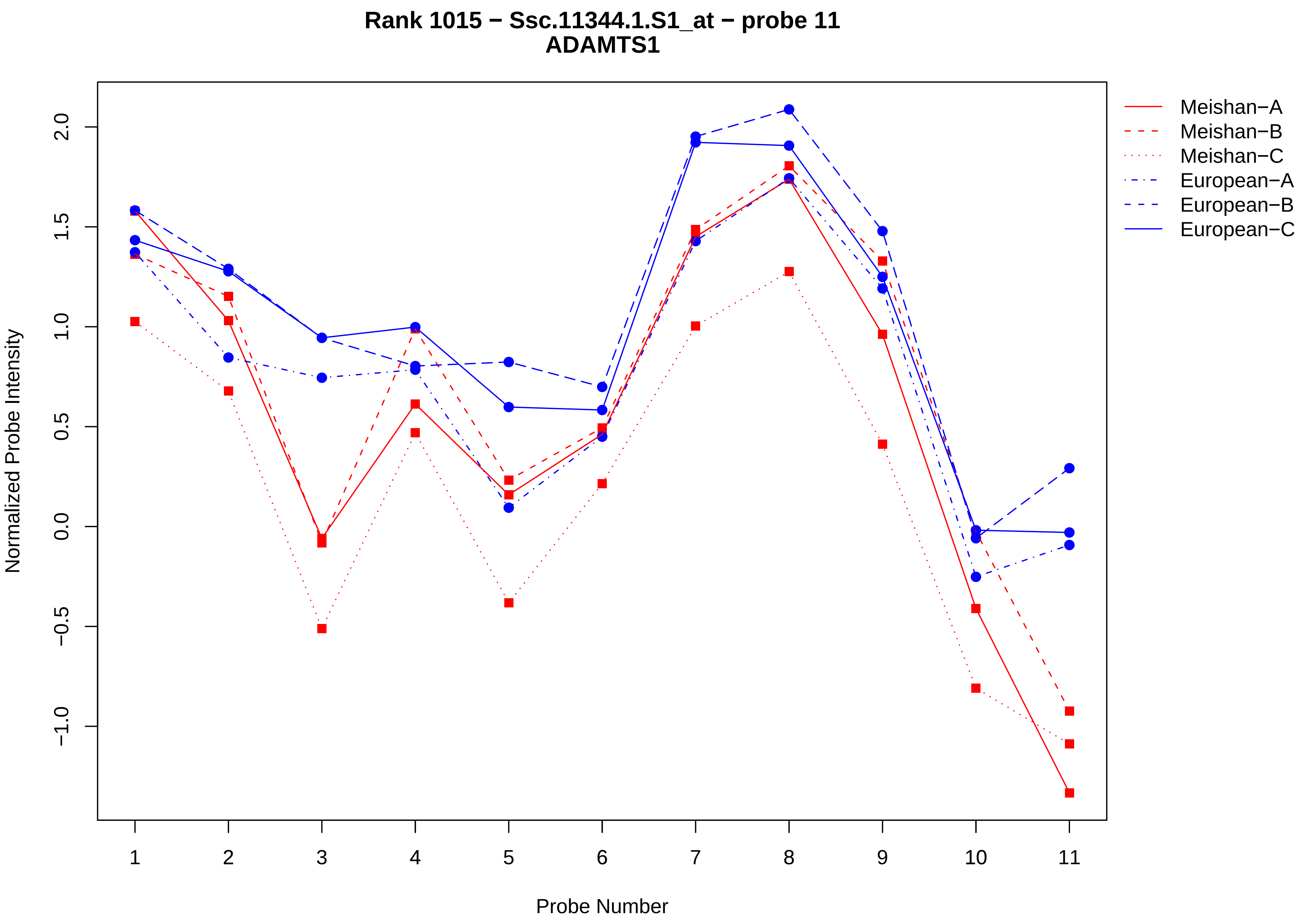

Rank 1015 − Ssc.11344.1.S1_at − probe 11
ADAMTS1
Normalized Probe Intensity
Probe Number
Meishan−A
Meishan−B
Meishan−C
European−A
European−B
European−C

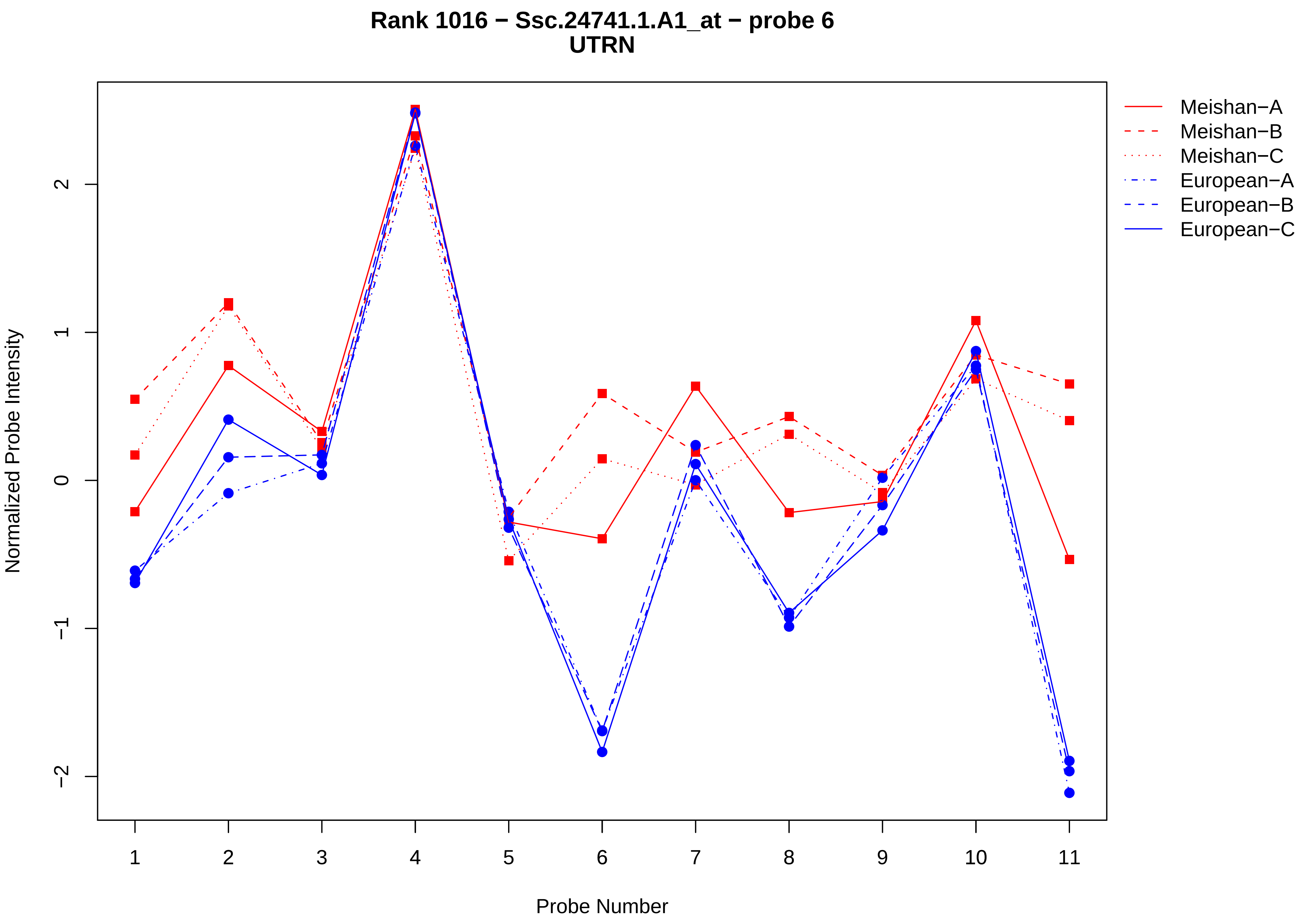

Rank 1016 − Ssc.24741.1.A1_at − probe 6
UTRN
Normalized Probe Intensity
Probe Number
−2
−1
0
1
2
1
2
3
4
5
6
7
8
9
10
11
Meishan−A
Meishan−B
Meishan−C
European−A
European−B
European−C

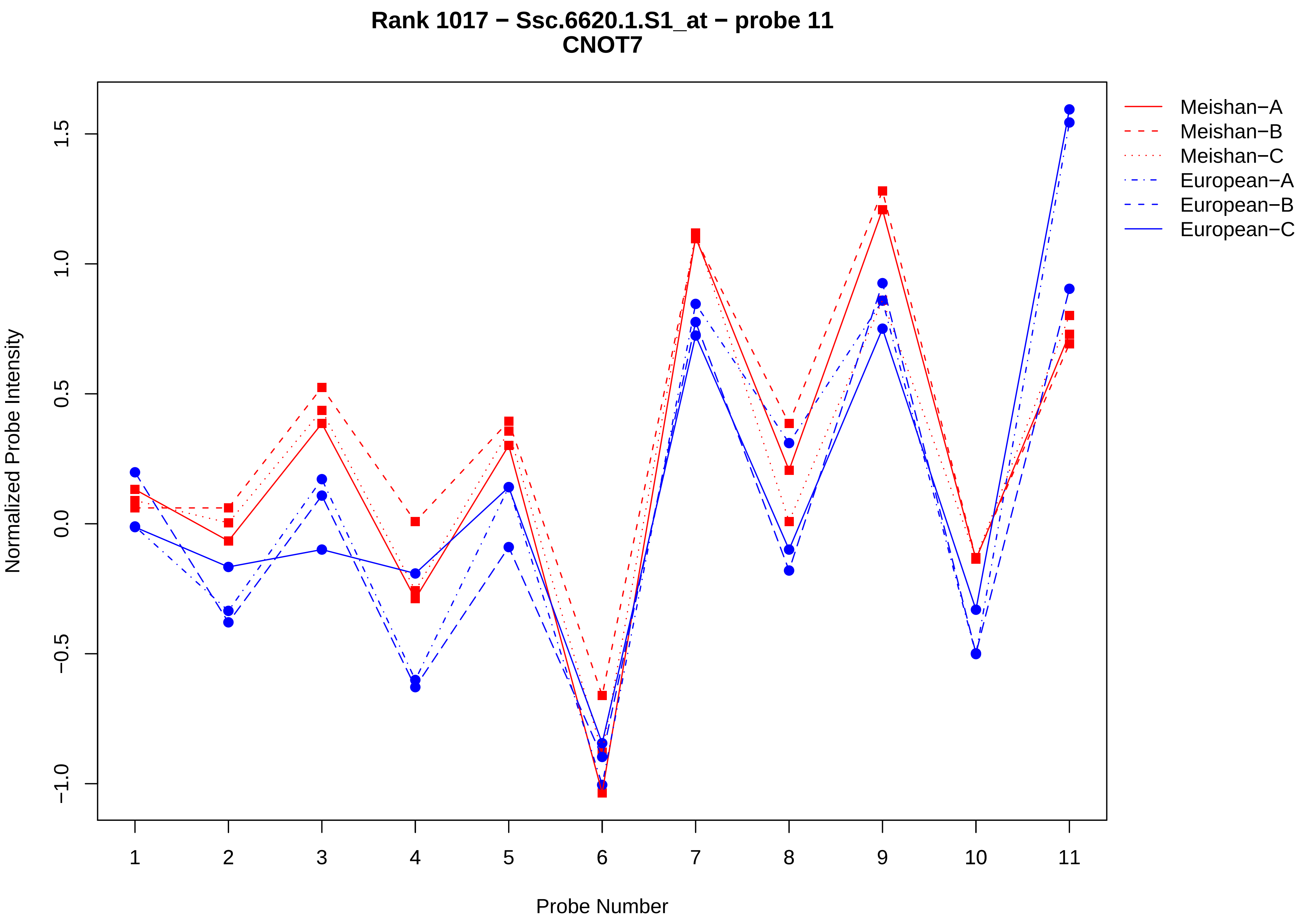
Rank 1017 − Ssc.6620.1.S1_at − probe 11
CNOT7
Normalized Probe Intensity
Probe Number
−1.0
−0.5
0.0
0.5
1.0
1.5
1
2
3
4
5
6
7
8
9
10
11
Meishan−A
Meishan−B
Meishan−C
European−A
European−B
European−C

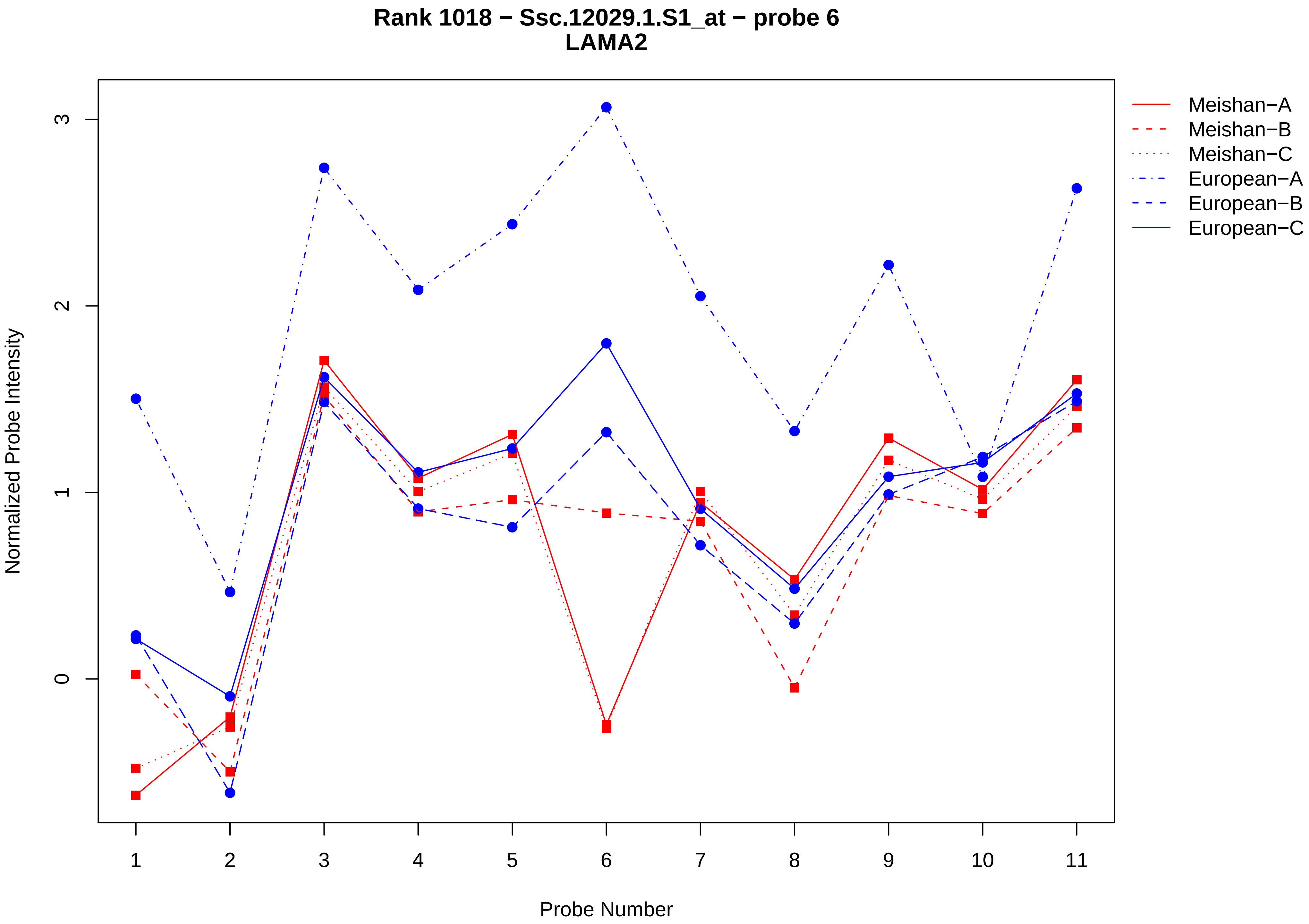

Rank 1018 − Ssc.12029.1.S1_at − probe 6
LAMA2
Normalized Probe Intensity
Probe Number
0
1
2
3
1
2
3
4
5
6
7
8
9
10
11
Meishan−A
Meishan−B
Meishan−C
European−A
European−B
European−C

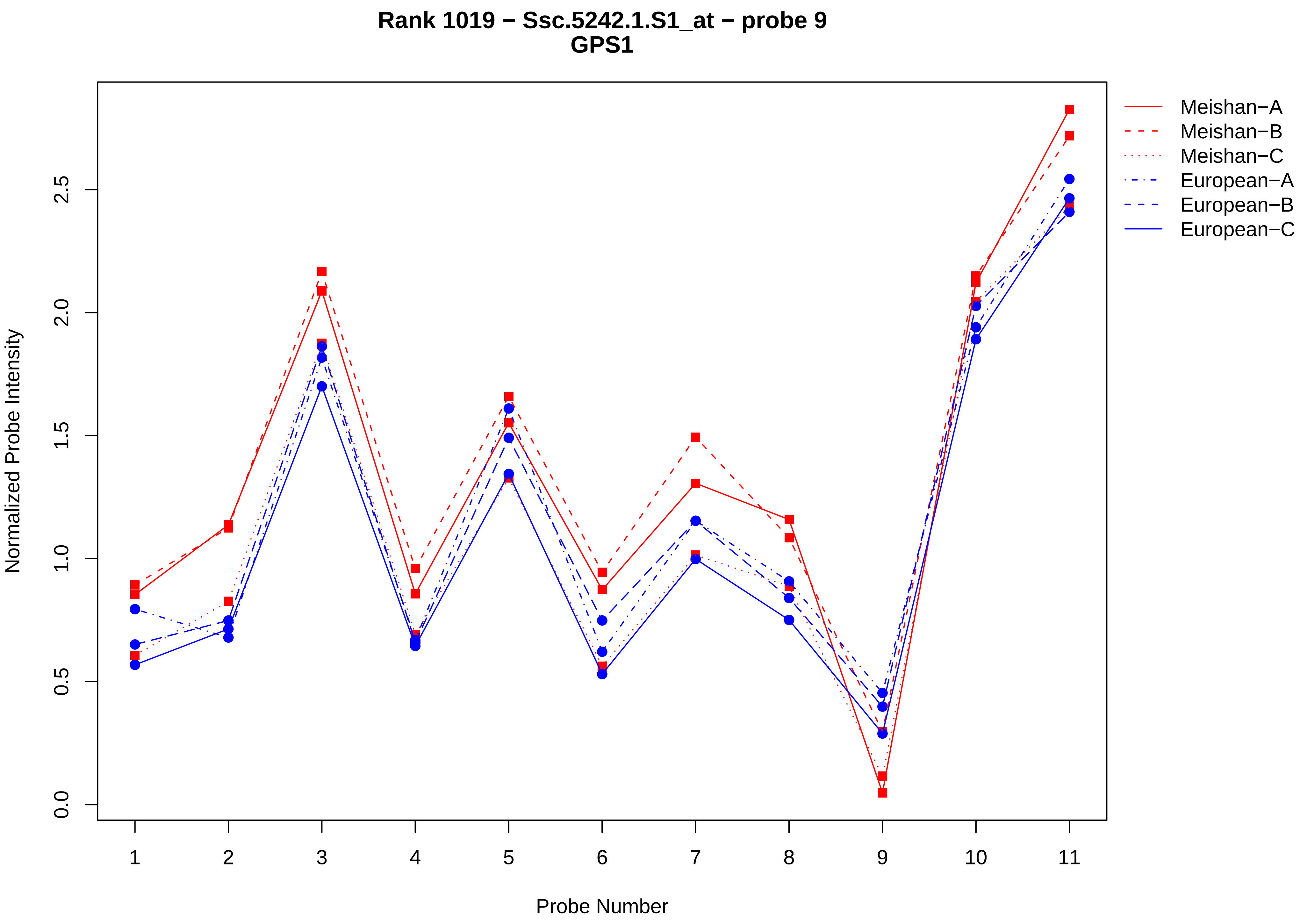
Rank 1019 − Ssc.5242.1.S1_at − probe 9
GPS1
Meishan−A
Meishan−B
Meishan−C
European−A
European−B
European−C
Normalized Probe Intensity
0.0
0.5
1.0
1.5
2.0
2.5
1
2
3
4
5
6
7
8
9
10
11
Probe Number

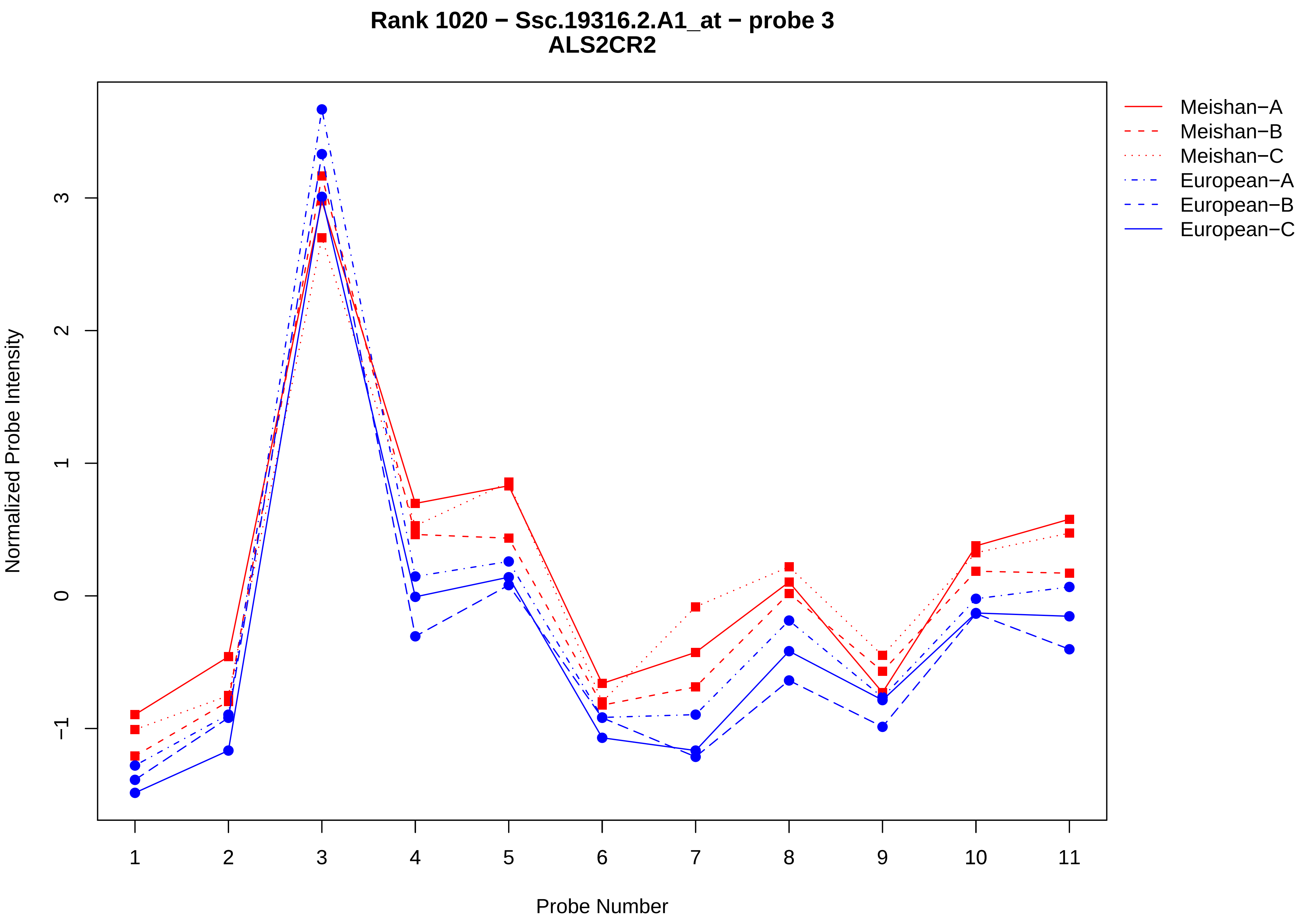

Rank 1020 − Ssc.19316.2.A1_at − probe 3
ALS2CR2
Normalized Probe Intensity
Probe Number
−1
0
1
2
3
1
2
3
4
5
6
7
8
9
10
11
Meishan−A
Meishan−B
Meishan−C
European−A
European−B
European−C

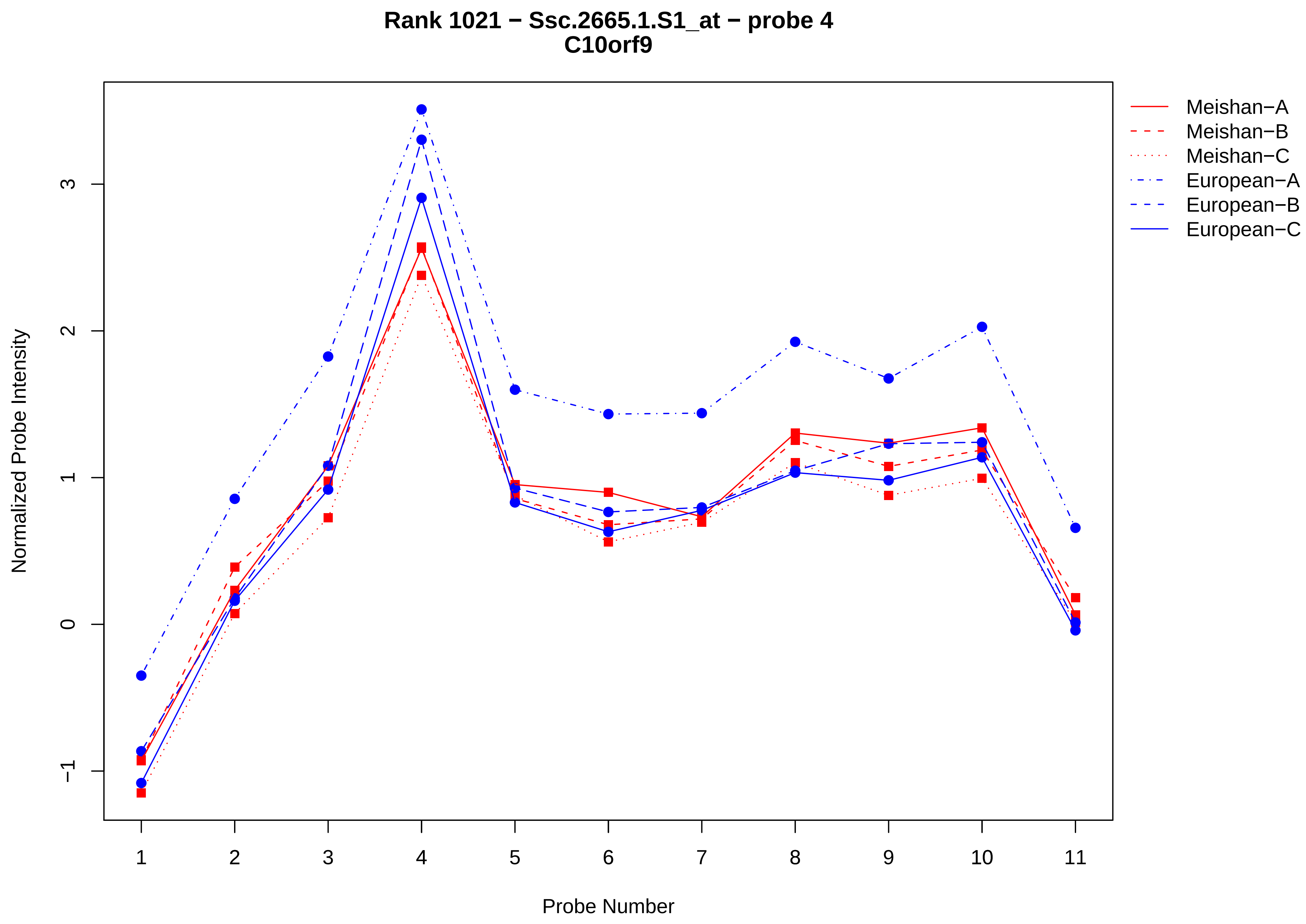

Rank 1021 − Ssc.2665.1.S1_at − probe 4
C10orf9
Normalized Probe Intensity
Probe Number
−1
0
1
2
3
1
2
3
4
5
6
7
8
9
10
11
Meishan−A
Meishan−B
Meishan−C
European−A
European−B
European−C

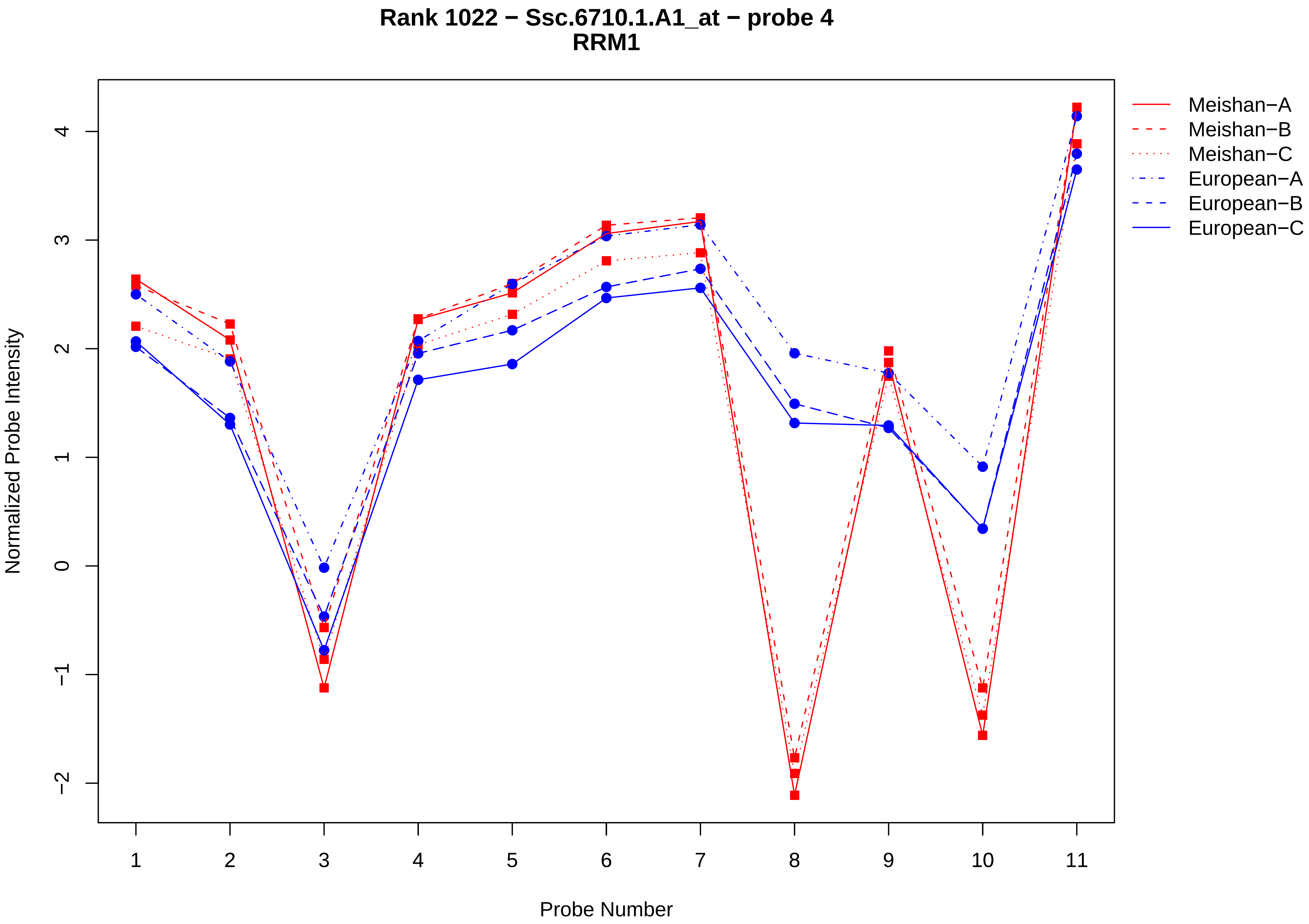

Rank 1022 − Ssc.6710.1.A1_at − probe 4
RRM1
Normalized Probe Intensity
Probe Number
−2
−1
0
1
2
3
4
1
2
3
4
5
6
7
8
9
10
11
Meishan−A
Meishan−B
Meishan−C
European−A
European−B
European−C

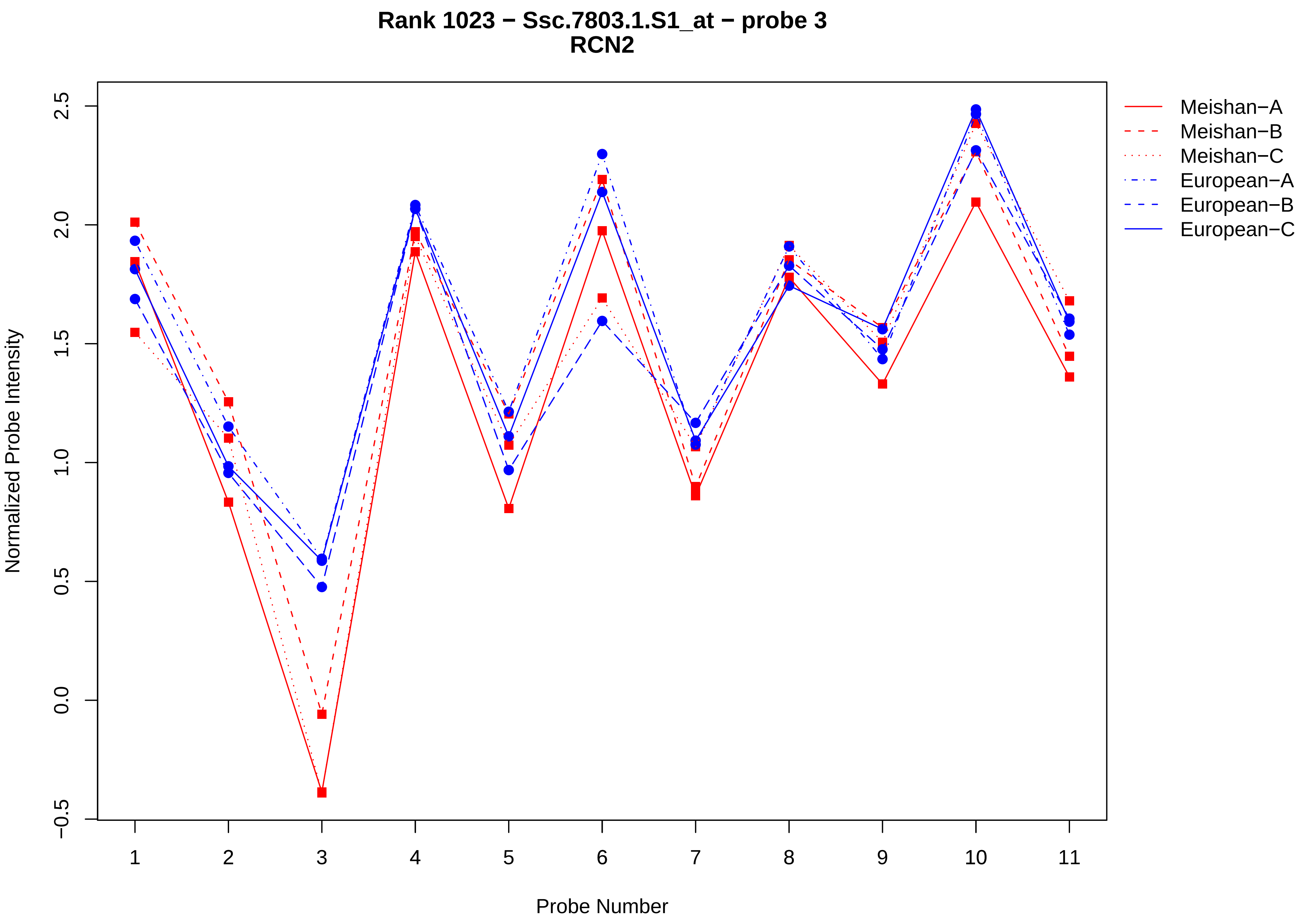

Rank 1023 − Ssc.7803.1.S1_at − probe 3
RCN2
Normalized Probe Intensity
−0.5
0.0
0.5
1.0
1.5
2.0
2.5
1
2
3
4
5
6
7
8
9
10
11
Probe Number
Meishan−A
Meishan−B
Meishan−C
European−A
European−B
European−C

Rank 1024 − Ssc.8208.1.A1_at − probe 11
GPAM

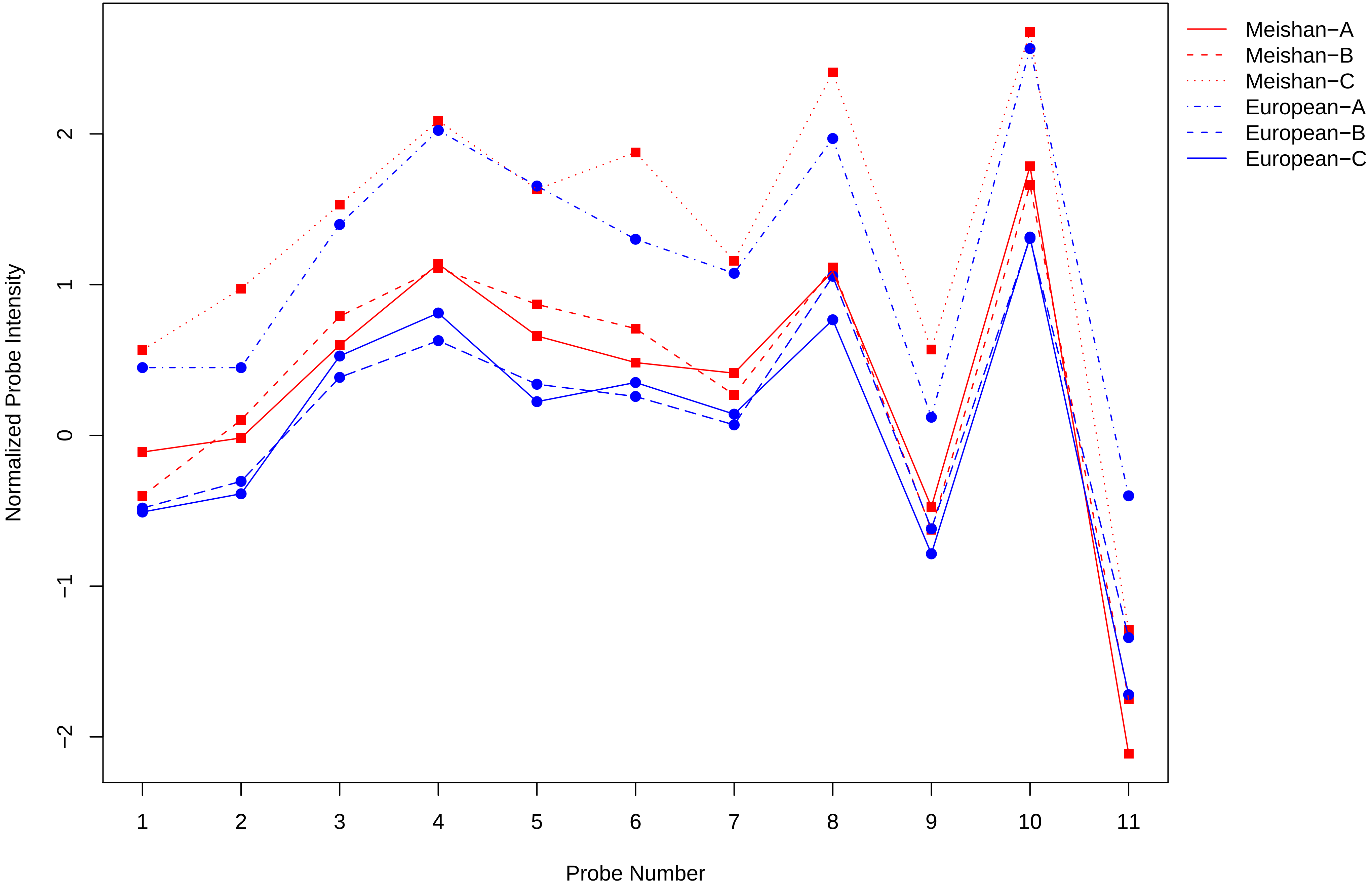

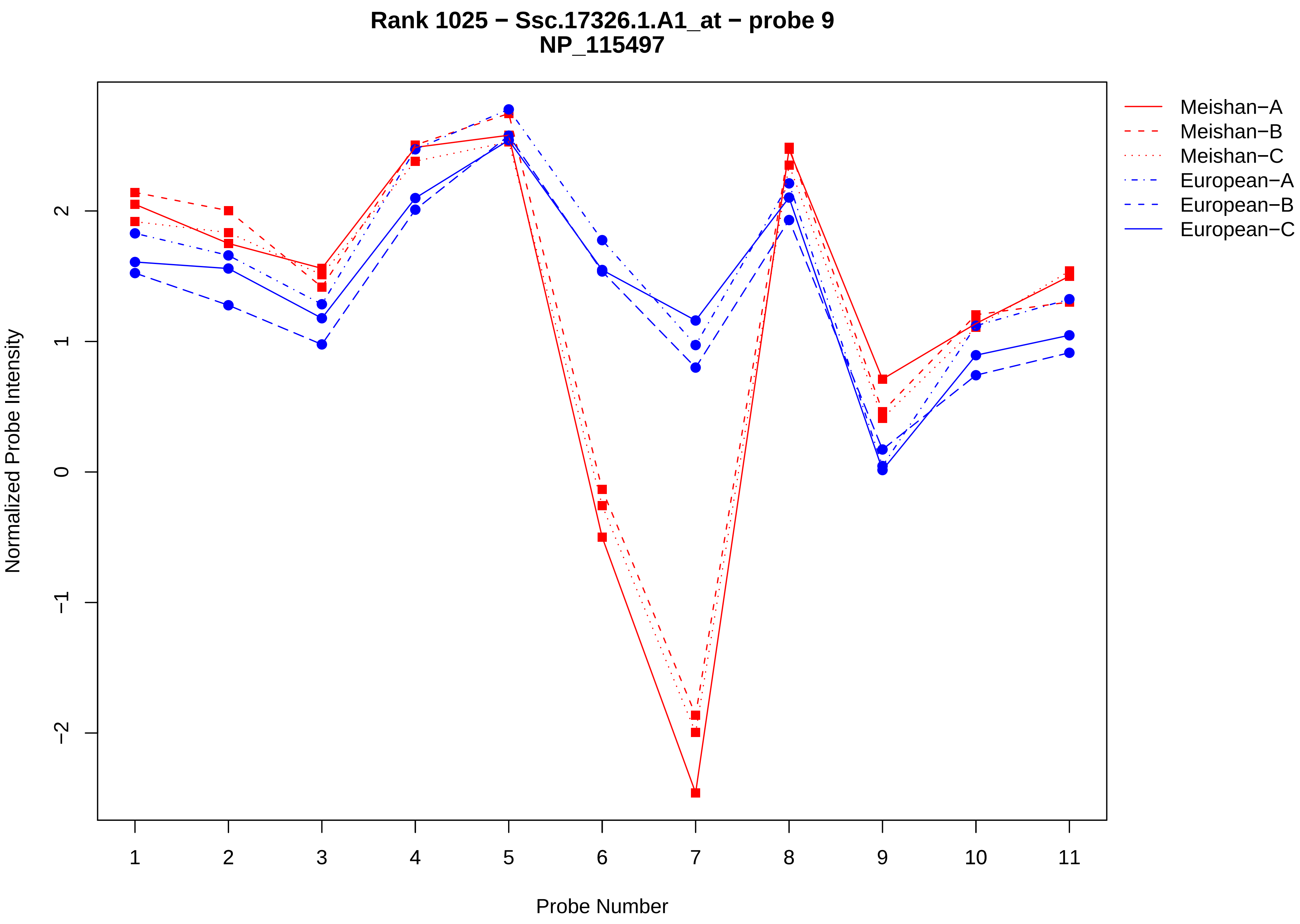

Rank 1025 − Ssc.17326.1.A1_at − probe 9
NP_115497
Normalized Probe Intensity
−2
−1
0
1
2
Probe Number
1
2
3
4
5
6
7
8
9
10
11
Meishan−A
Meishan−B
Meishan−C
European−A
European−B
European−C

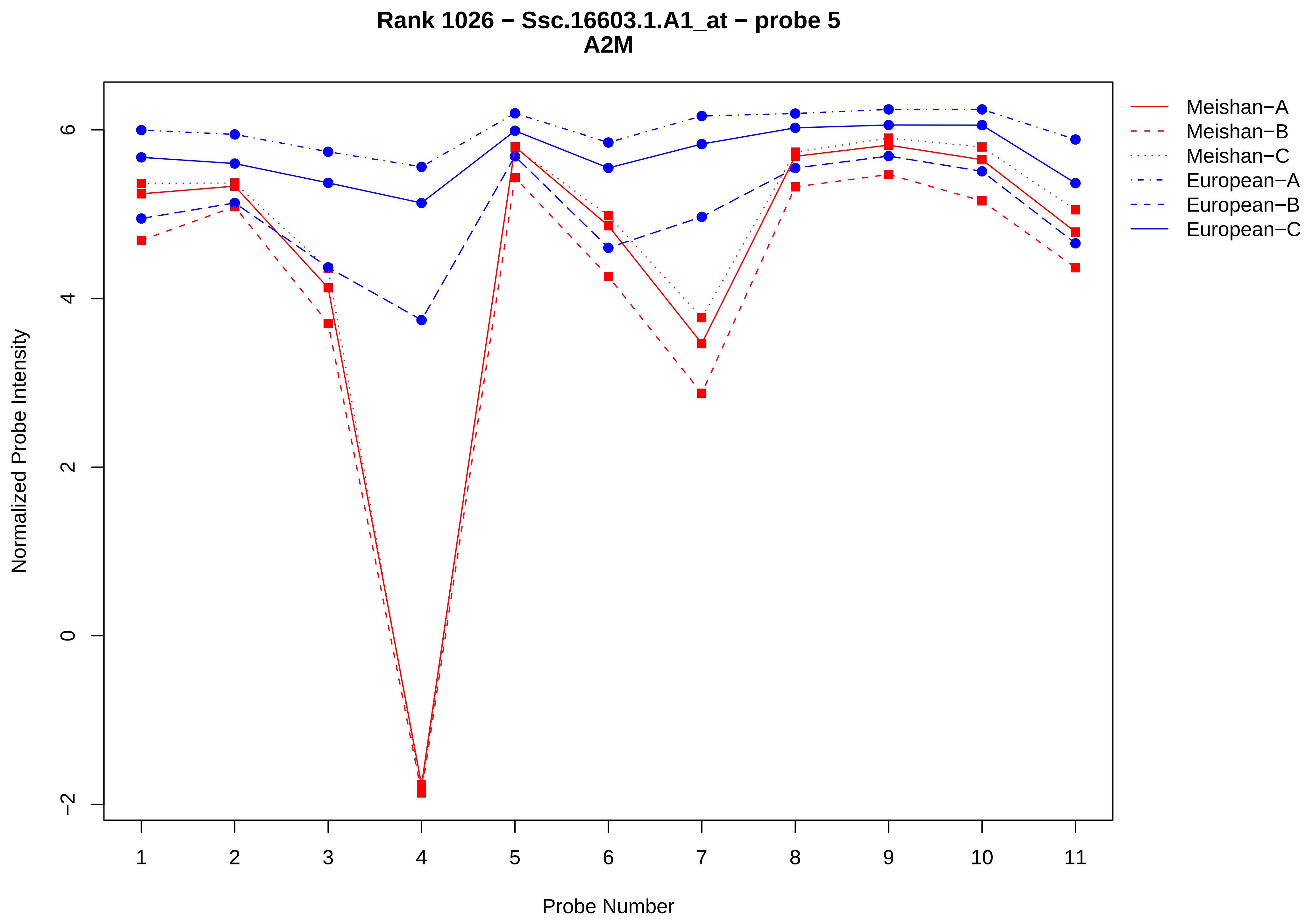
Rank 1026 − Ssc.16603.1.A1_at − probe 5
A2M
Normalized Probe Intensity
Probe Number
−2
0
2
4
6
1
2
3
4
5
6
7
8
9
10
11
Meishan−A
Meishan−B
Meishan−C
European−A
European−B
European−C

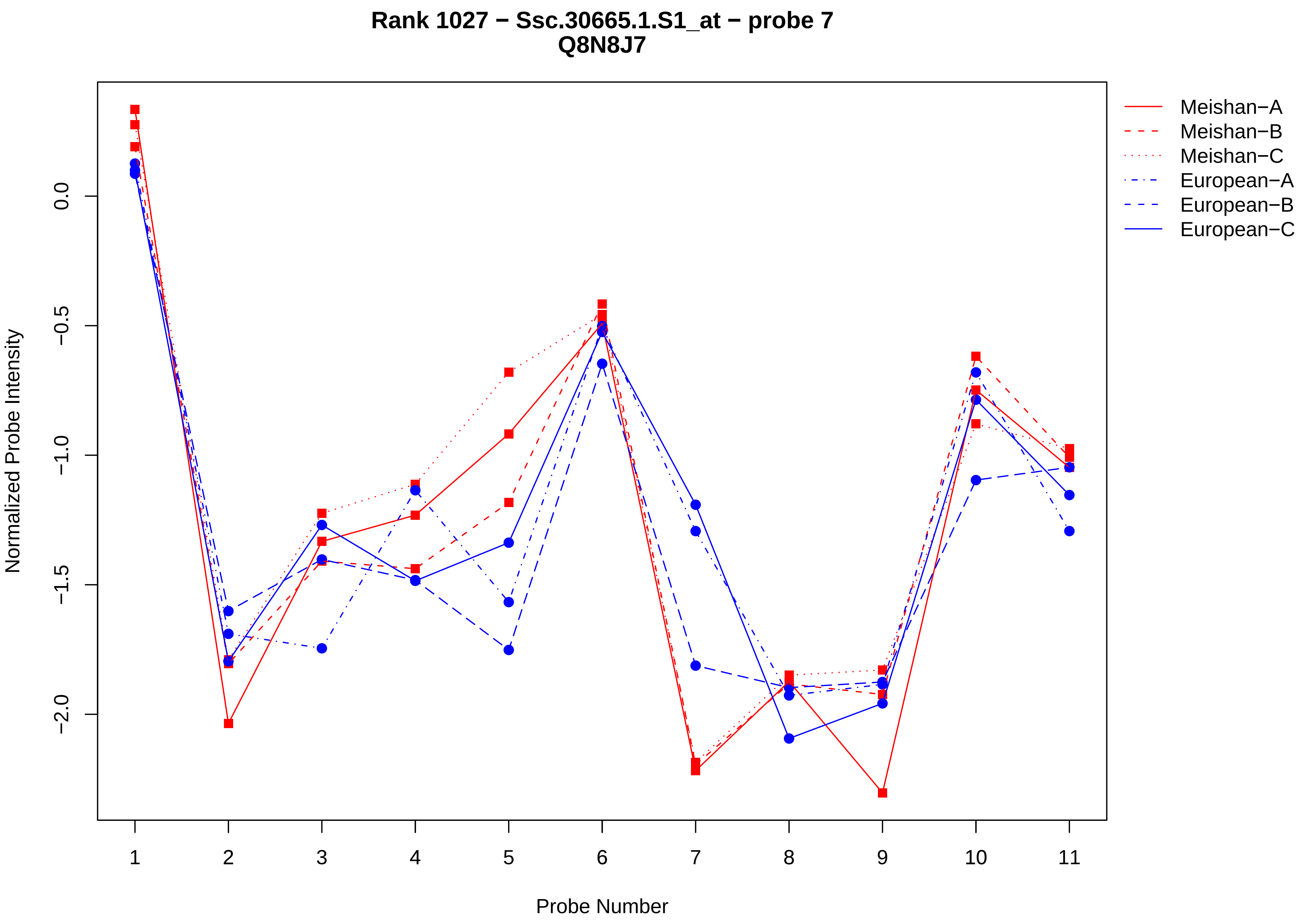

Rank 1027 − Ssc.30665.1.S1_at − probe 7
Q8N8J7
Normalized Probe Intensity
Probe Number
0.0
−0.5
−1.0
−1.5
−2.0
1
2
3
4
5
6
7
8
9
10
11
Meishan−A
Meishan−B
Meishan−C
European−A
European−B
European−C

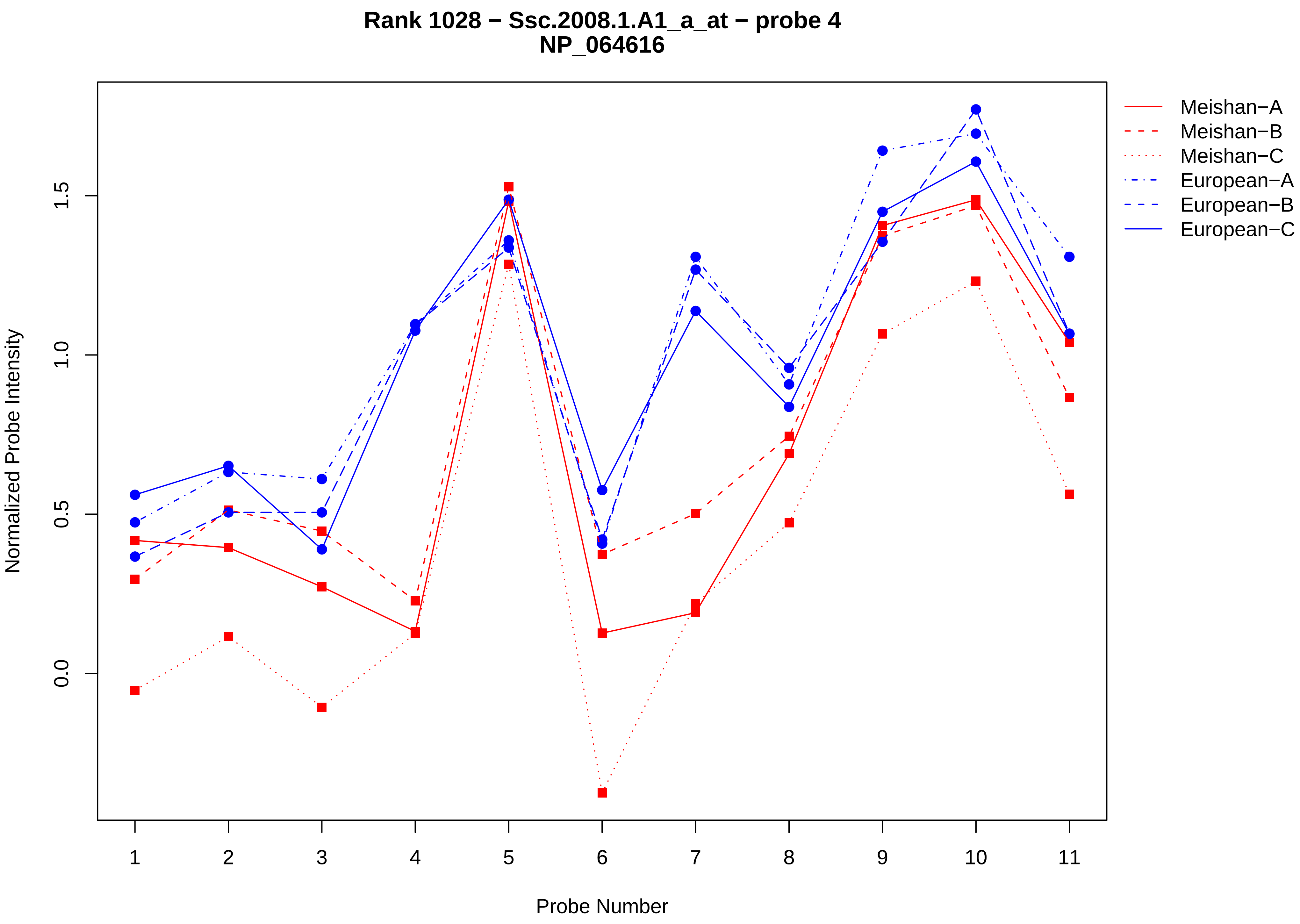
Rank 1028 − Ssc.2008.1.A1_a_at − probe 4
NP_064616
Normalized Probe Intensity
0.0
0.5
1.0
1.5
Probe Number
1
2
3
4
5
6
7
8
9
10
11
Meishan−A
Meishan−B
Meishan−C
European−A
European−B
European−C

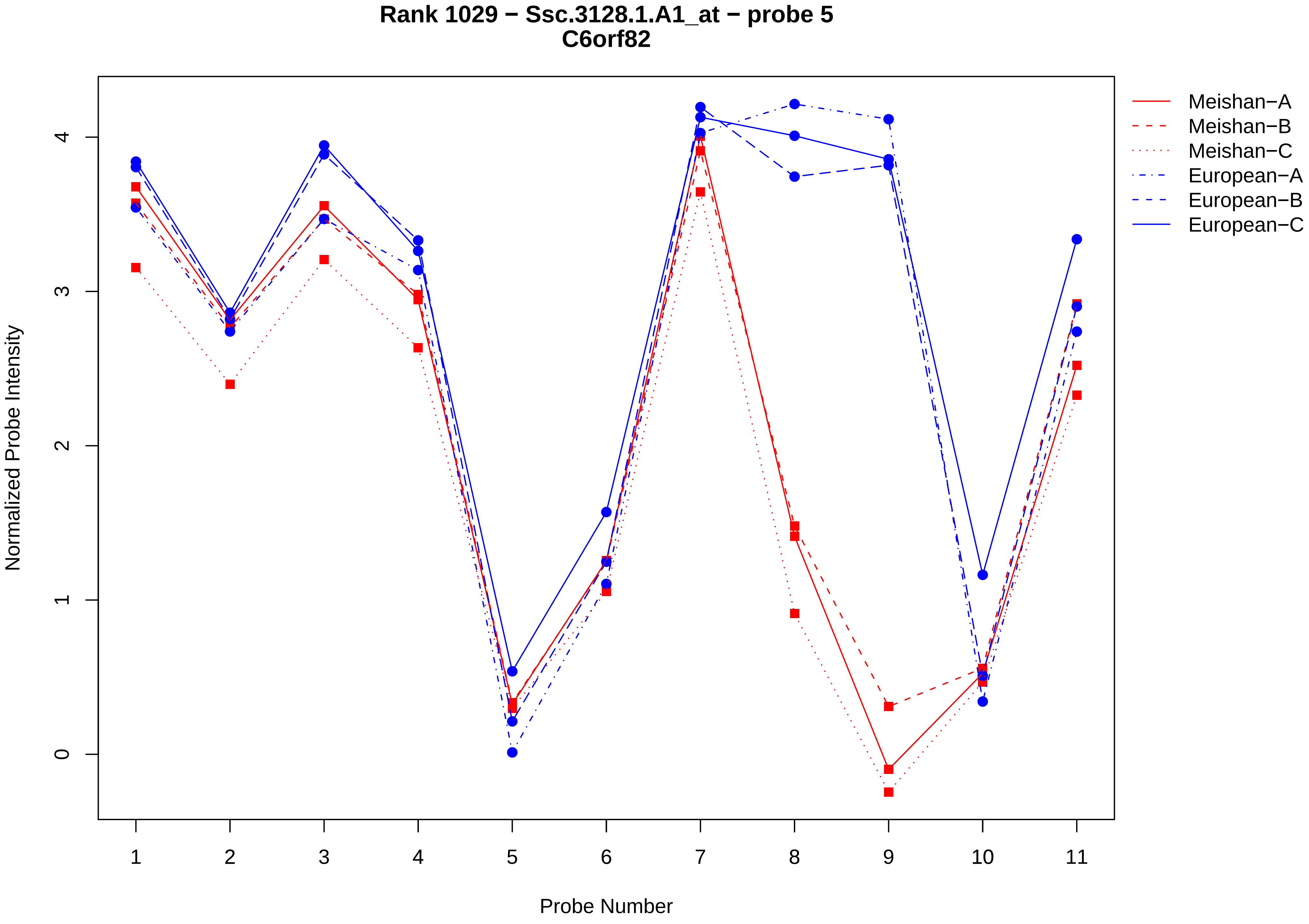

Rank 1029 − Ssc.3128.1.A1_at − probe 5
C6orf82
Normalized Probe Intensity
Probe Number
0
1
2
3
4
1
2
3
4
5
6
7
8
9
10
11
Meishan−A
Meishan−B
Meishan−C
European−A
European−B
European−C

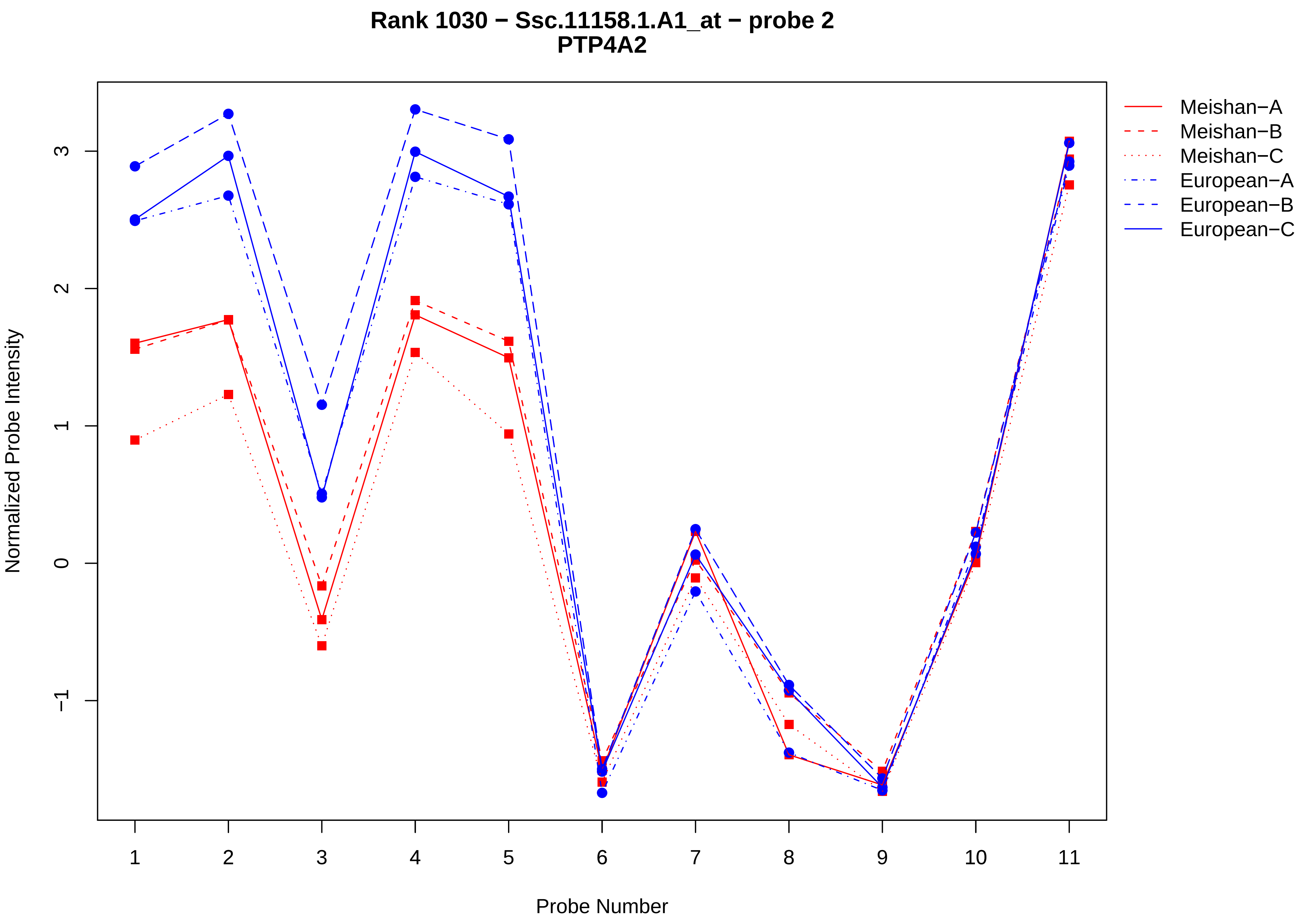

Rank 1030 − Ssc.11158.1.A1_at − probe 2
PTP4A2
Normalized Probe Intensity
Probe Number
Meishan−A
Meishan−B
Meishan−C
European−A
European−B
European−C
−1
0
1
2
3
1
2
3
4
5
6
7
8
9
10
11

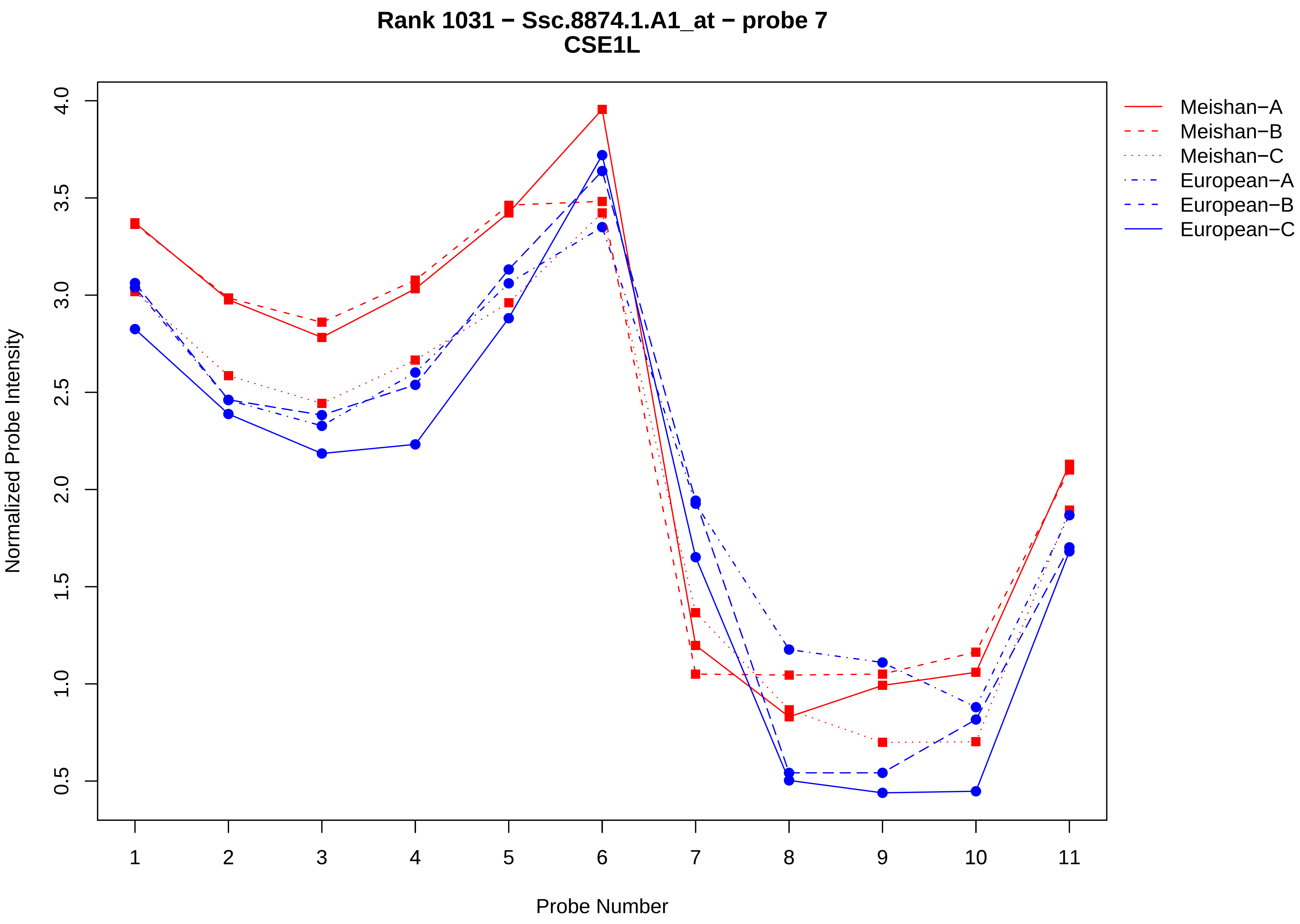

Rank 1031 − Ssc.8874.1.A1_at − probe 7
CSE1L
Normalized Probe Intensity
0.5
1.0
1.5
2.0
2.5
3.0
3.5
4.0
1
2
3
4
5
6
7
8
9
10
11
Probe Number
Meishan−A
Meishan−B
Meishan−C
European−A
European−B
European−C

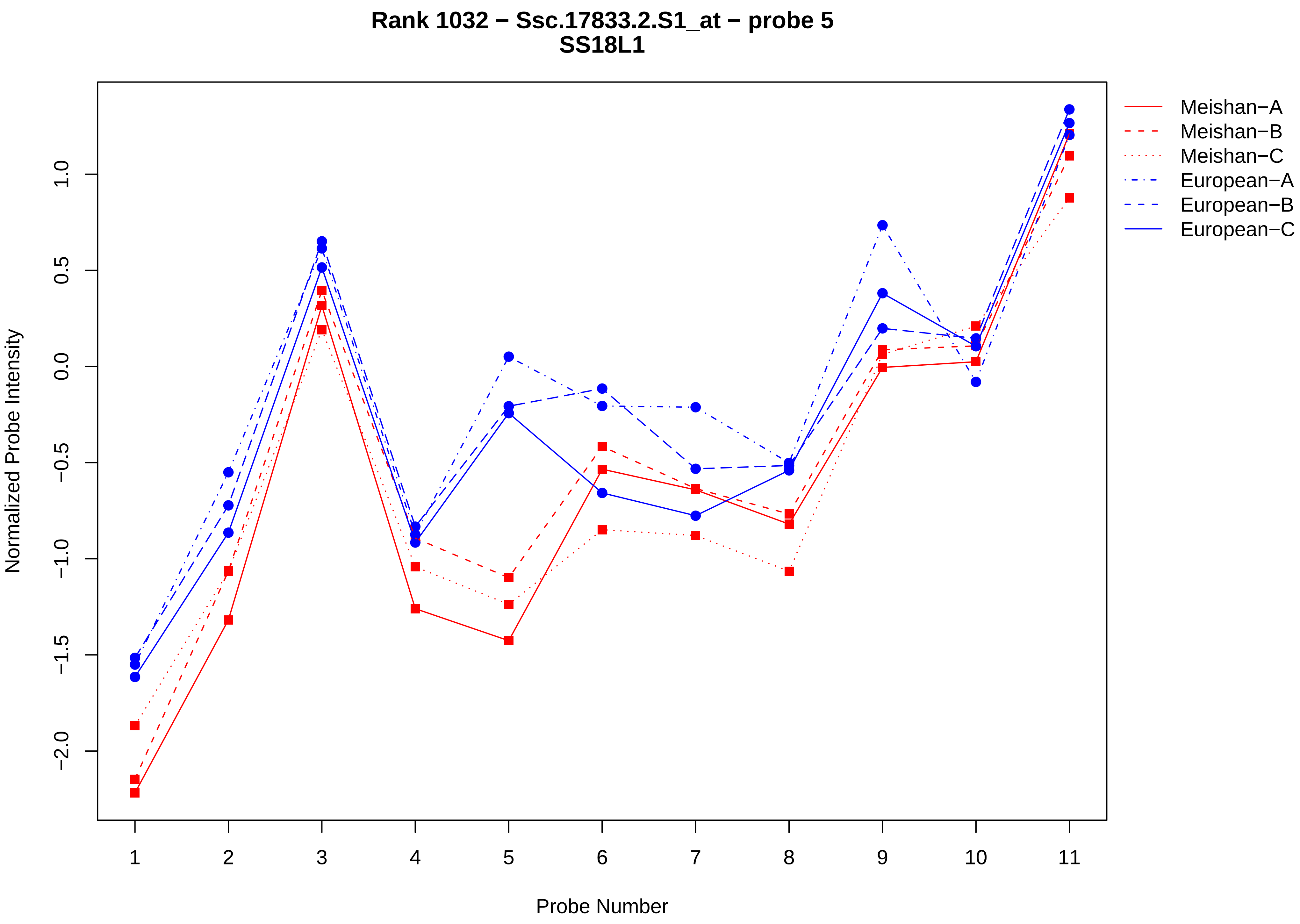

Rank 1032 − Ssc.17833.2.S1_at − probe 5
SS18L1
Normalized Probe Intensity
Probe Number
Meishan−A
Meishan−B
Meishan−C
European−A
European−B
European−C

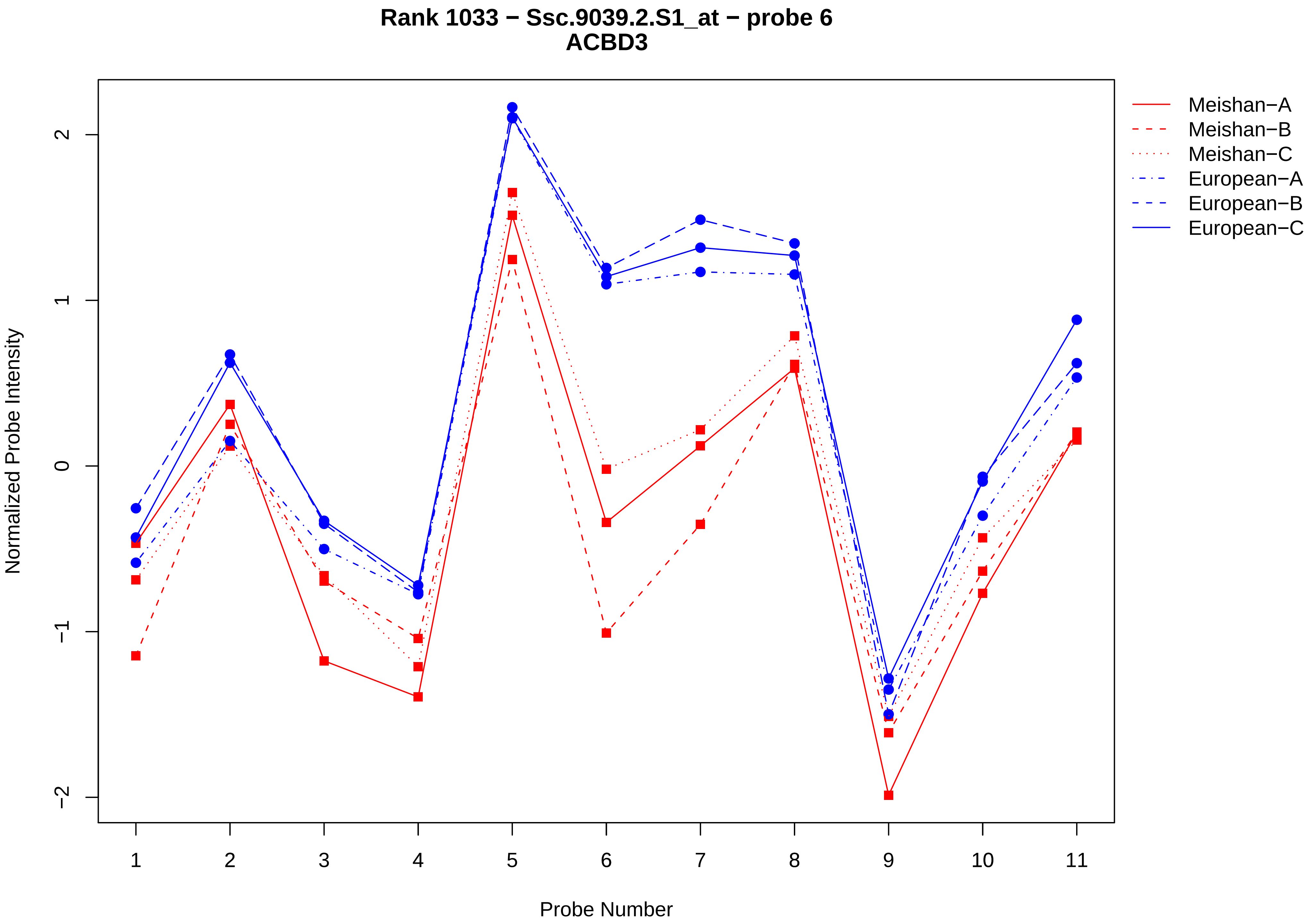
Rank 1033 − Ssc.9039.2.S1_at − probe 6
ACBD3
Normalized Probe Intensity
Probe Number
−2
−1
0
1
2
1
2
3
4
5
6
7
8
9
10
11
Meishan−A
Meishan−B
Meishan−C
European−A
European−B
European−C

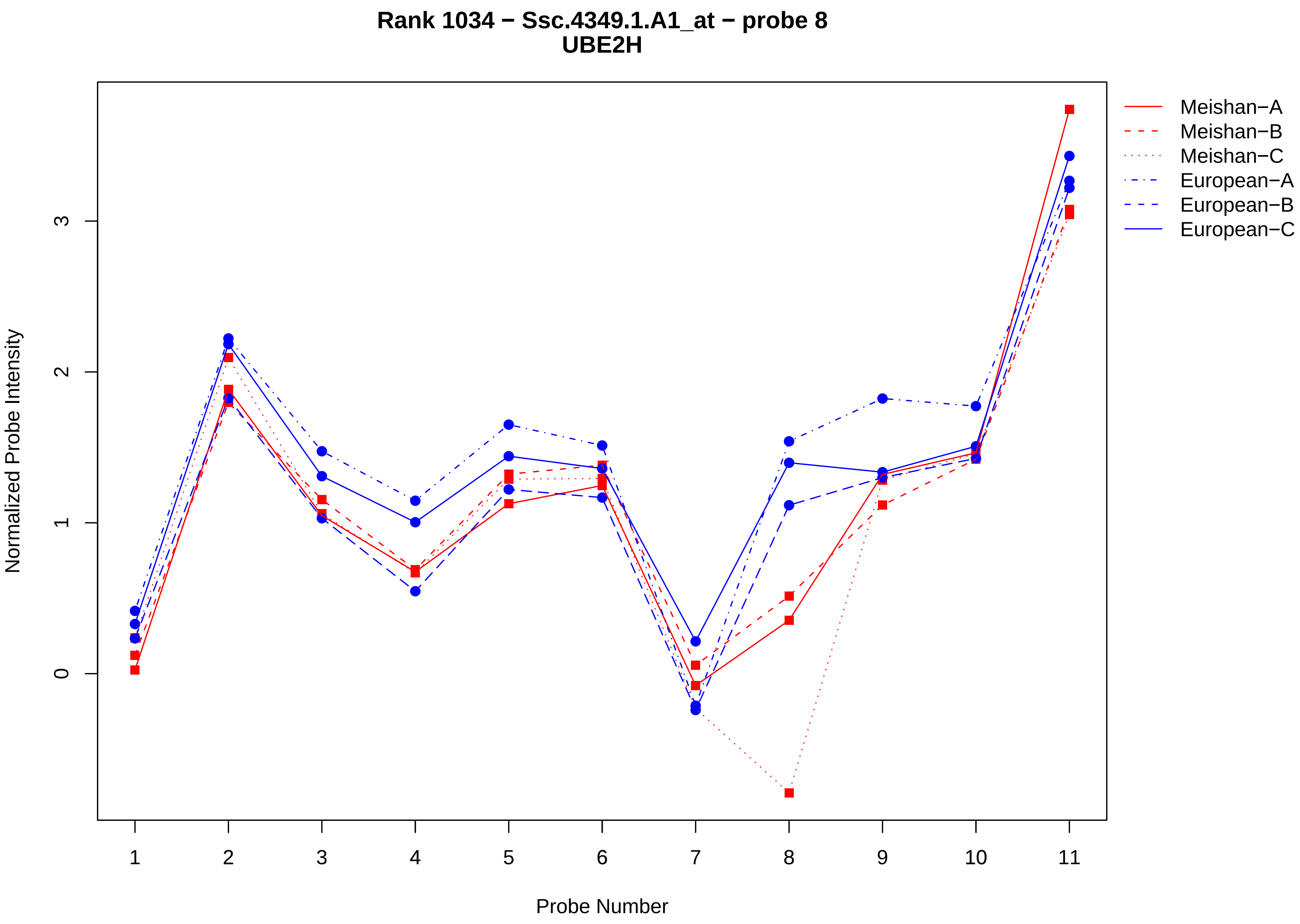

Rank 1034 − Ssc.4349.1.A1_at − probe 8
UBE2H
Normalized Probe Intensity
Probe Number
0
1
2
3
1
2
3
4
5
6
7
8
9
10
11
Meishan−A
Meishan−B
Meishan−C
European−A
European−B
European−C

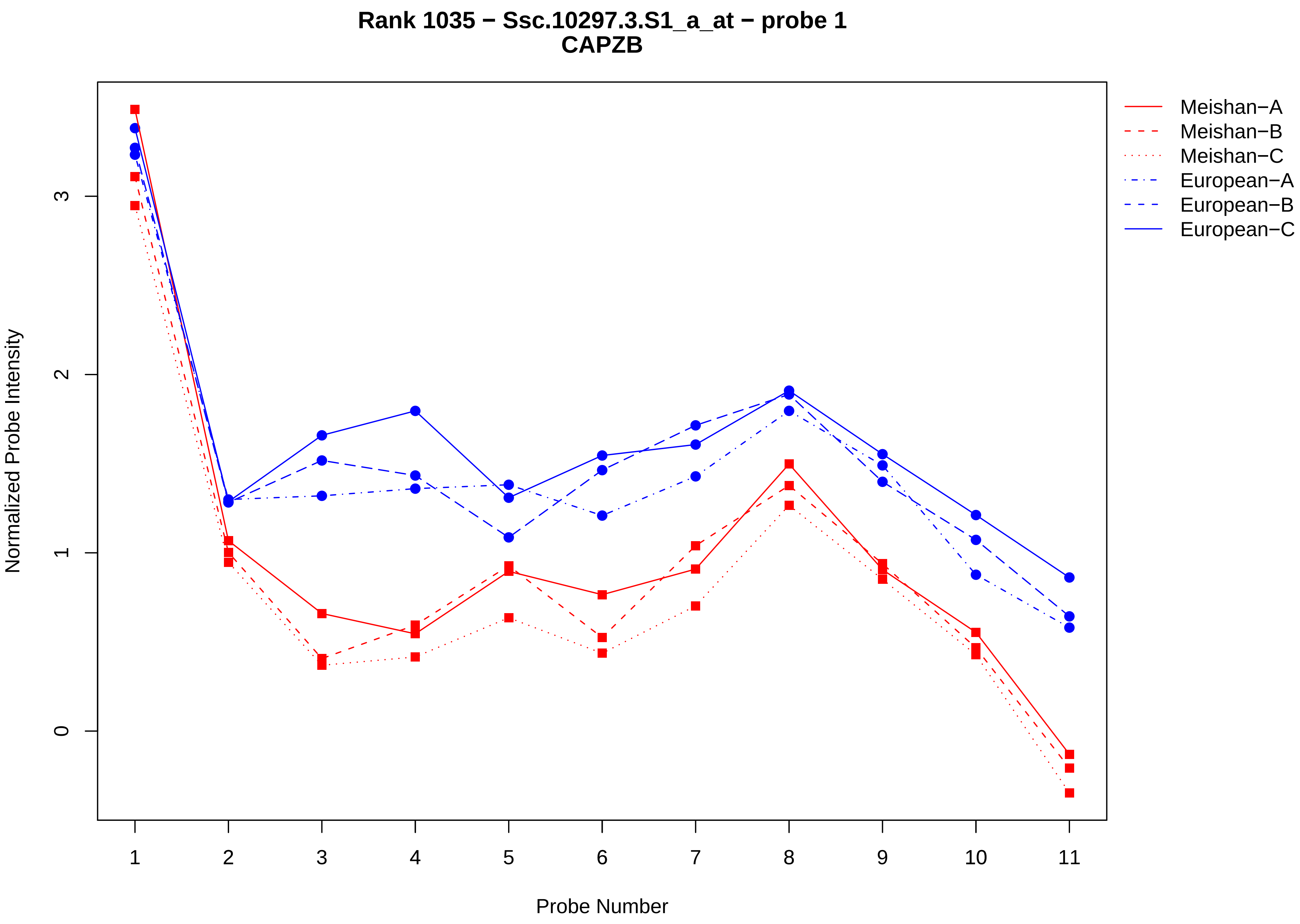

Rank 1035 − Ssc.10297.3.S1_a_at − probe 1
CAPZB
Normalized Probe Intensity
Probe Number
0
1
2
3
1
2
3
4
5
6
7
8
9
10
11
Meishan−A
Meishan−B
Meishan−C
European−A
European−B
European−C

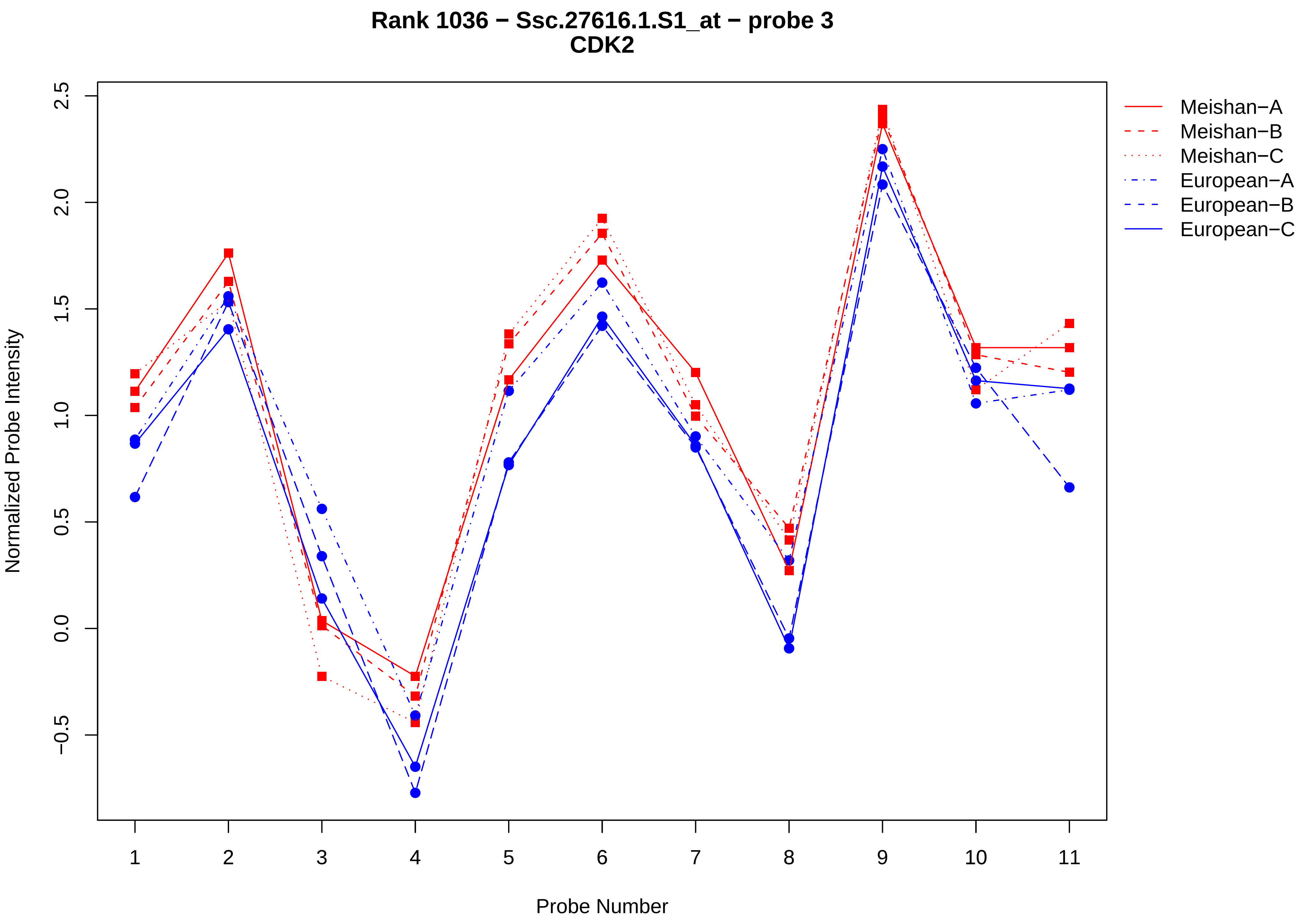
Rank 1036 − Ssc.27616.1.S1_at − probe 3
CDK2
Normalized Probe Intensity
Probe Number
−0.5
0.0
0.5
1.0
1.5
2.0
2.5
1
2
3
4
5
6
7
8
9
10
11
Meishan−A
Meishan−B
Meishan−C
European−A
European−B
European−C

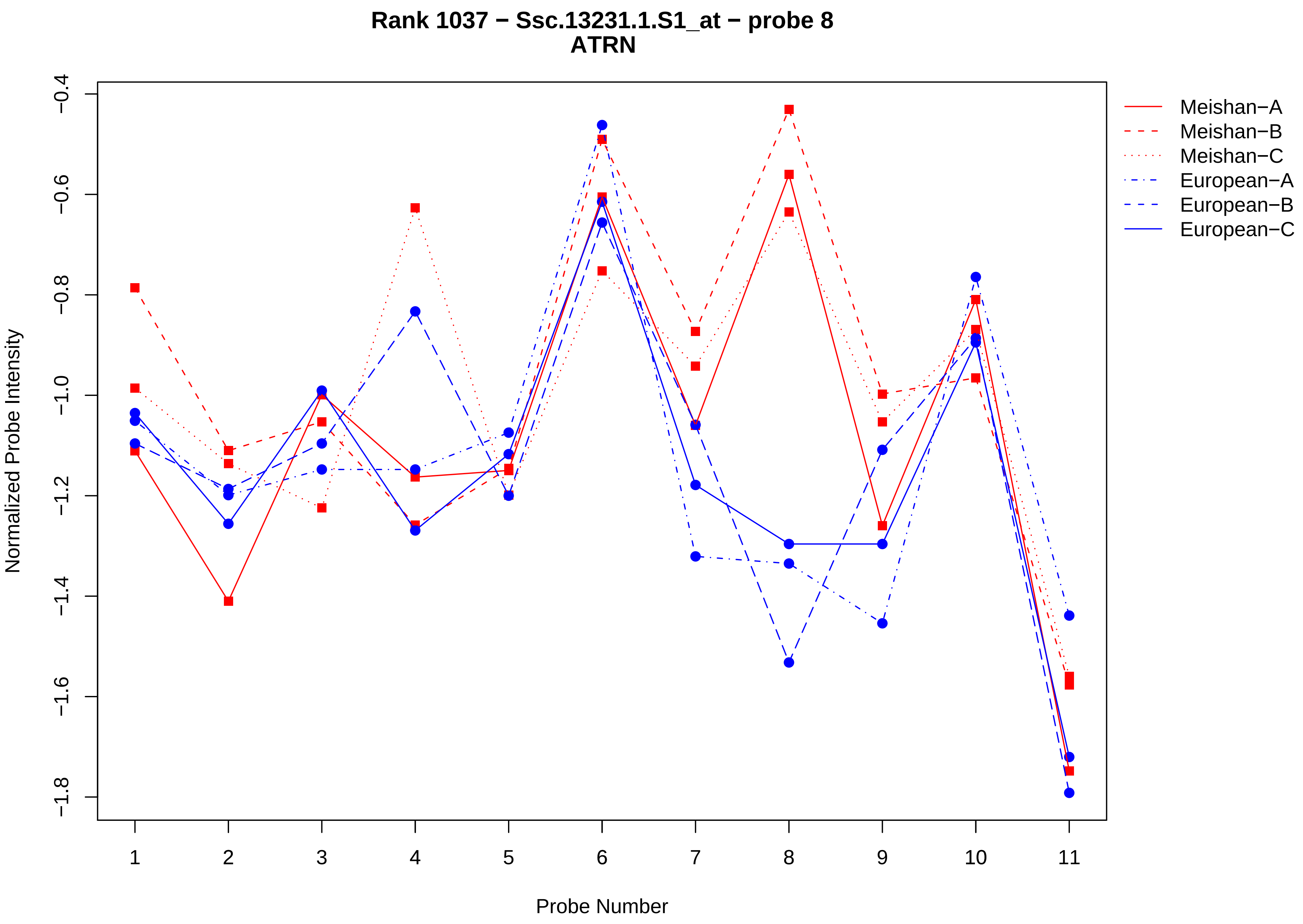
Rank 1037 − Ssc.13231.1.S1_at − probe 8
ATRN
Normalized Probe Intensity
Probe Number
−0.4
−0.6
−0.8
−1.0
−1.2
−1.4
−1.6
−1.8
1
2
3
4
5
6
7
8
9
10
11
Meishan−A
Meishan−B
Meishan−C
European−A
European−B
European−C

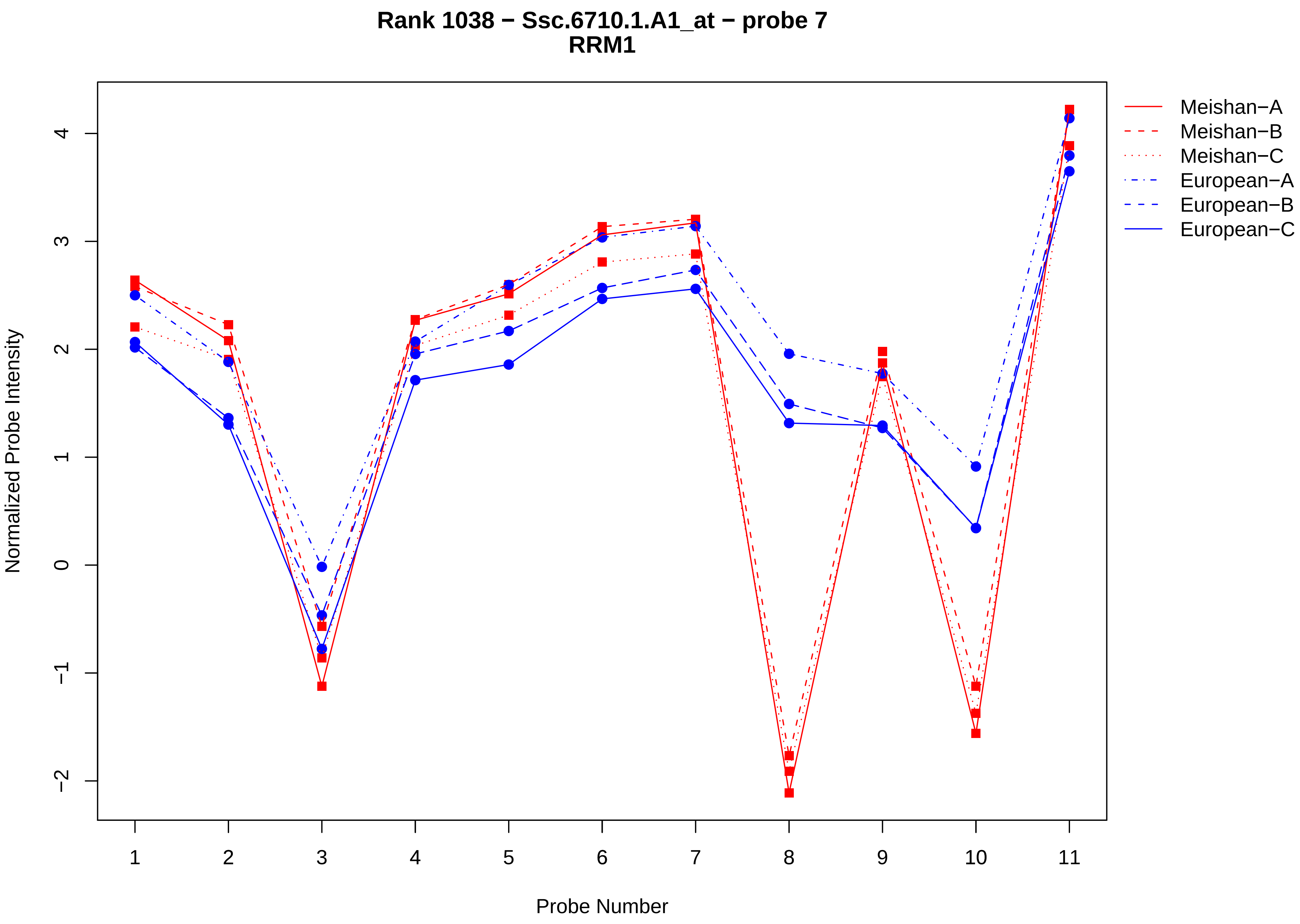

Rank 1038 − Ssc.6710.1.A1_at − probe 7
RRM1
Meishan−A
Meishan−B
Meishan−C
European−A
European−B
European−C
Normalized Probe Intensity
Probe Number
−2
−1
0
1
2
3
4
1
2
3
4
5
6
7
8
9
10
11

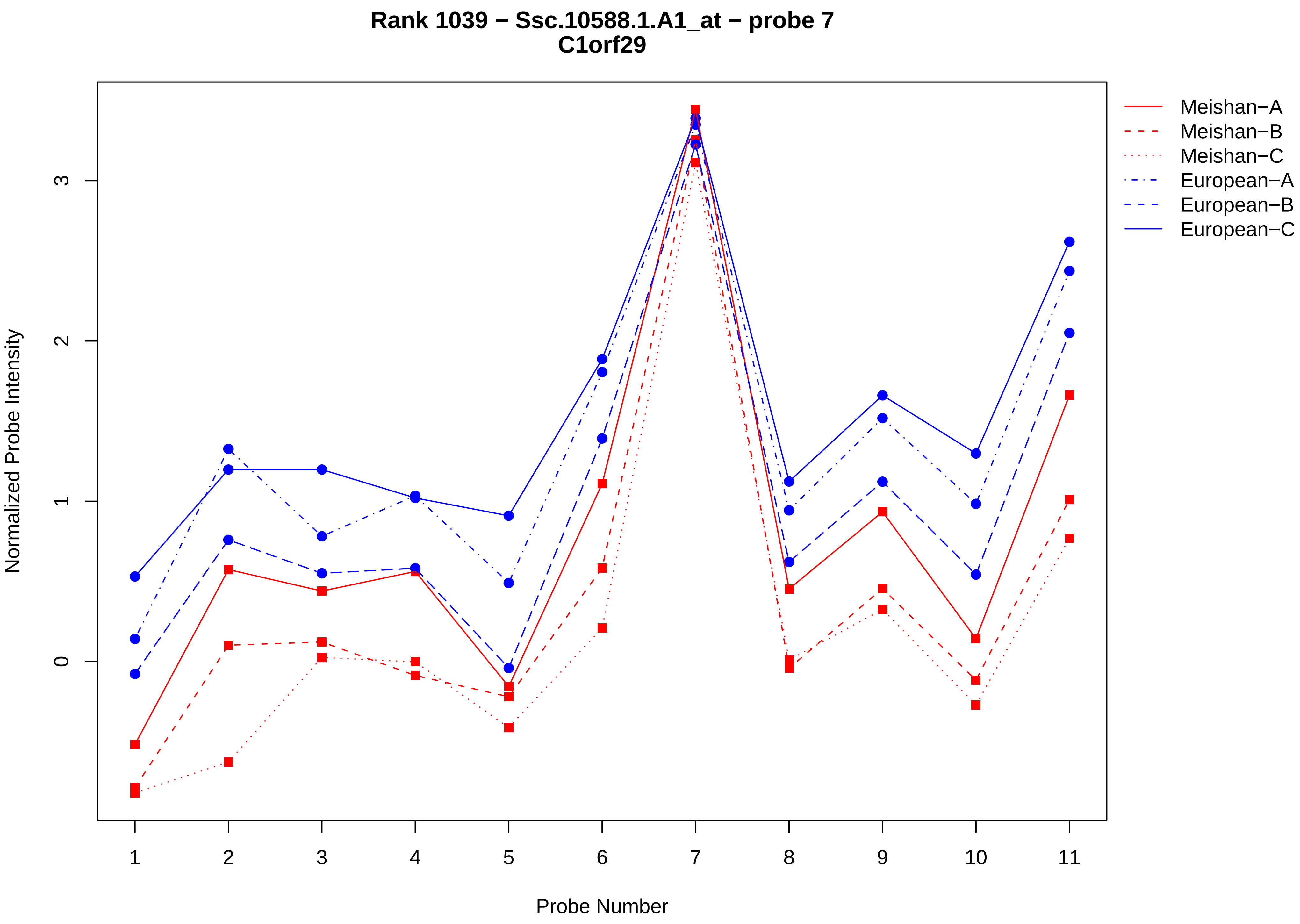

Rank 1039 − Ssc.10588.1.A1_at − probe 7
C1orf29
Normalized Probe Intensity
Probe Number
0
1
2
3
1
2
3
4
5
6
7
8
9
10
11
Meishan−A
Meishan−B
Meishan−C
European−A
European−B
European−C

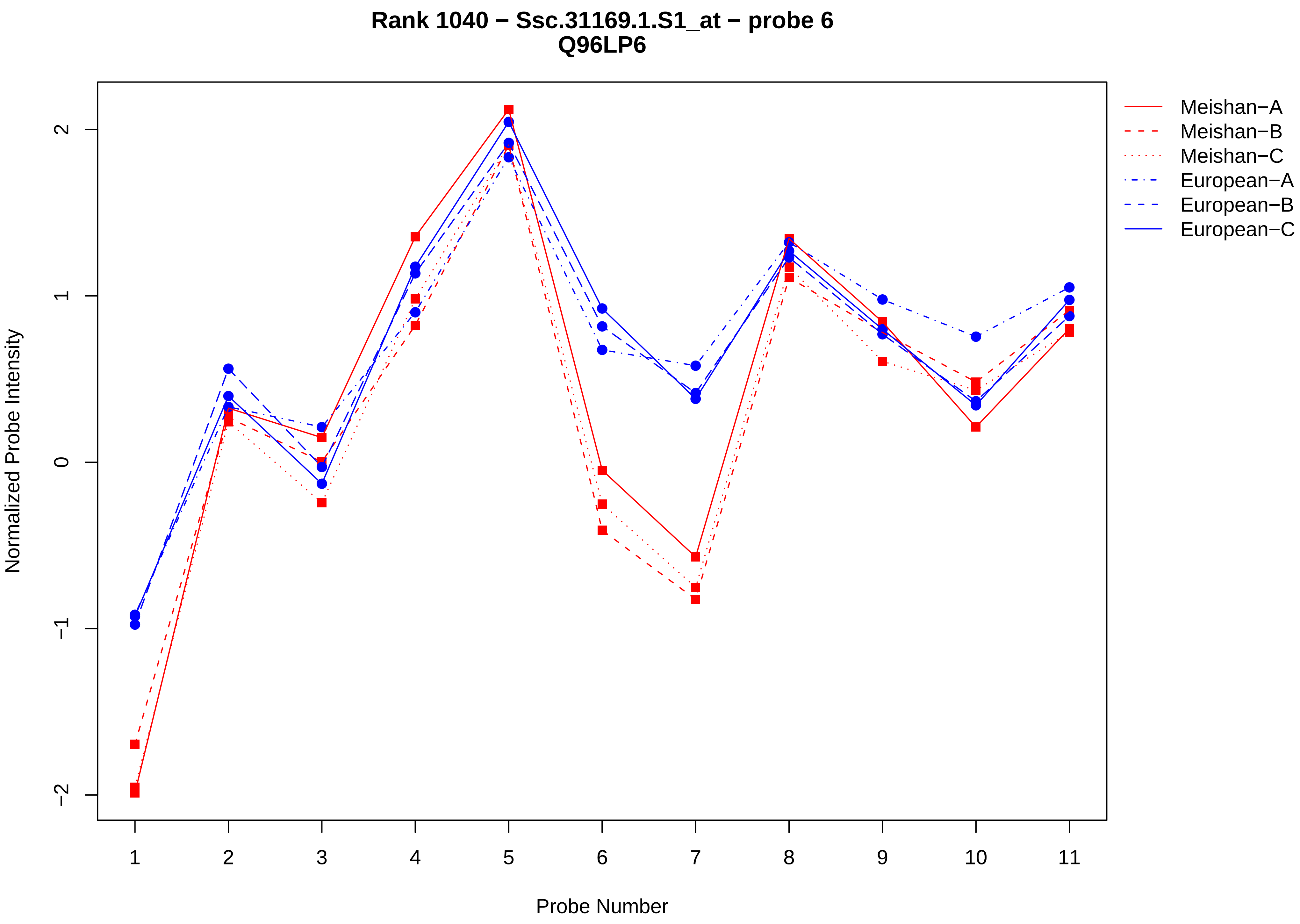
Rank 1040 − Ssc.31169.1.S1_at − probe 6
Q96LP6
Normalized Probe Intensity
−2
−1
0
1
2
1
2
3
4
5
6
7
8
9
10
11
Probe Number
Meishan−A
Meishan−B
Meishan−C
European−A
European−B
European−C

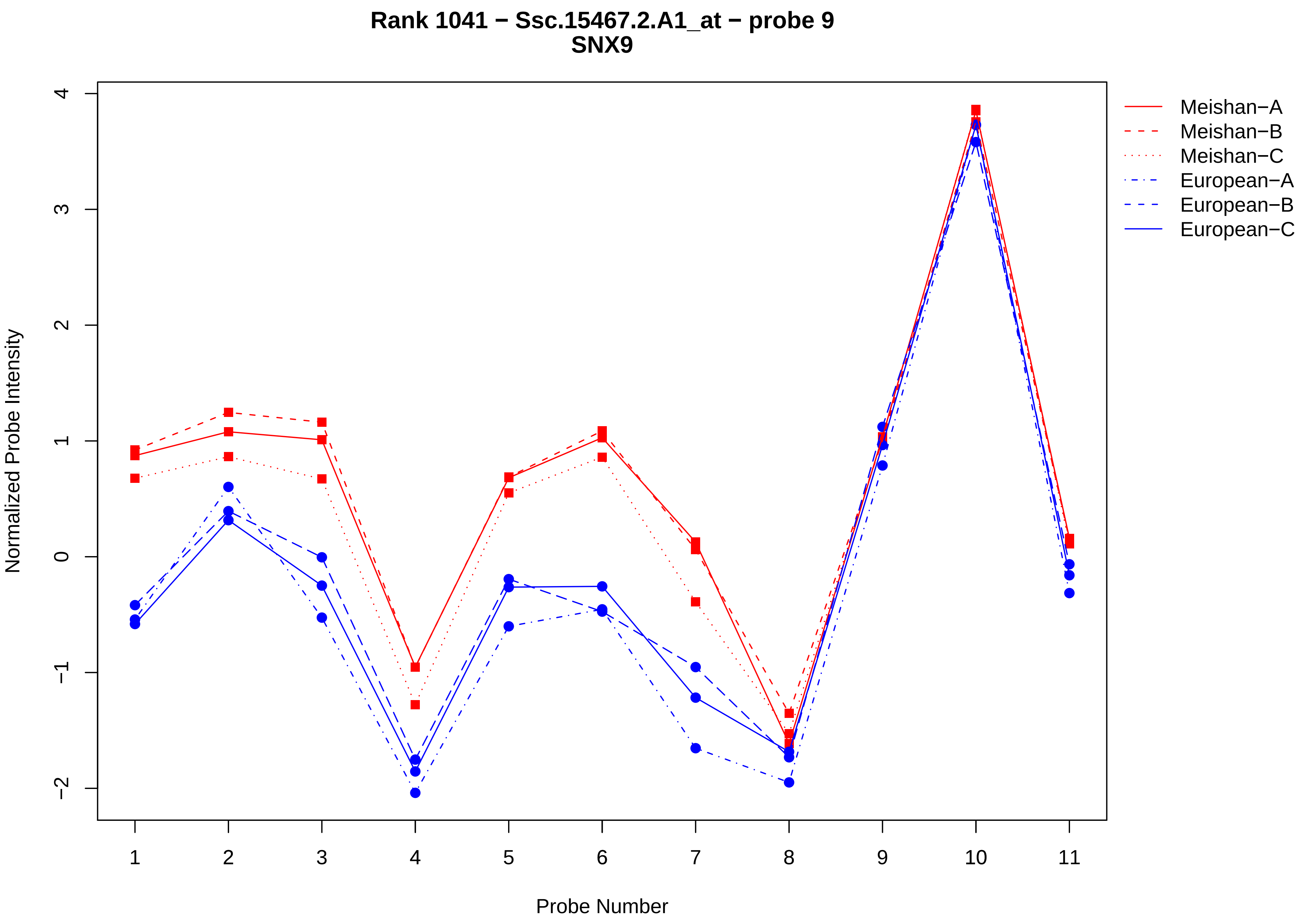

Rank 1041 − Ssc.15467.2.A1_at − probe 9
SNX9
Normalized Probe Intensity
Probe Number
−2
−1
0
1
2
3
4
1
2
3
4
5
6
7
8
9
10
11
Meishan−A
Meishan−B
Meishan−C
European−A
European−B
European−C

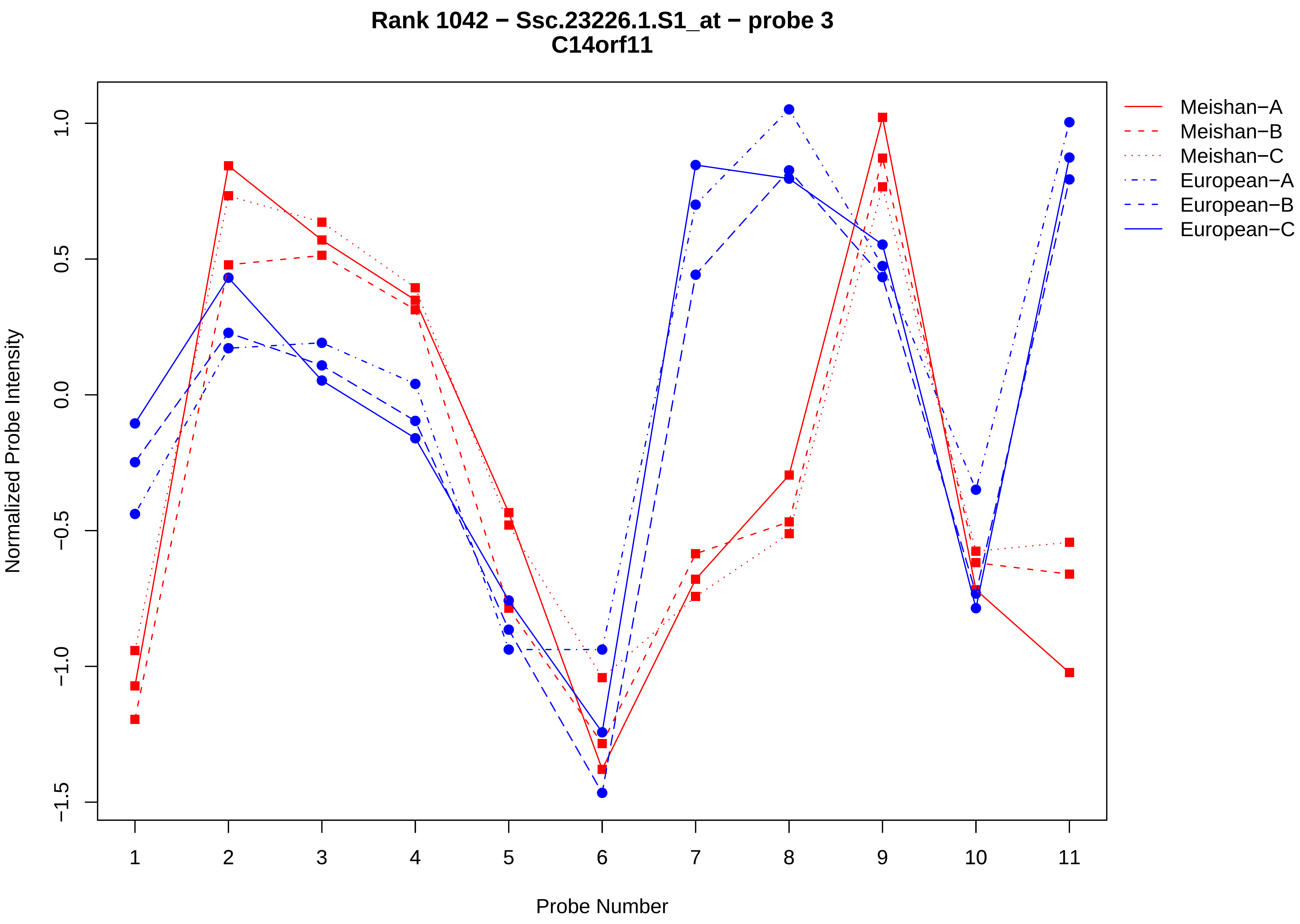

Rank 1042 − Ssc.23226.1.S1_at − probe 3
C14orf11
Normalized Probe Intensity
Probe Number
1.0
0.5
0.0
−0.5
−1.0
−1.5
1
2
3
4
5
6
7
8
9
10
11
Meishan−A
Meishan−B
Meishan−C
European−A
European−B
European−C

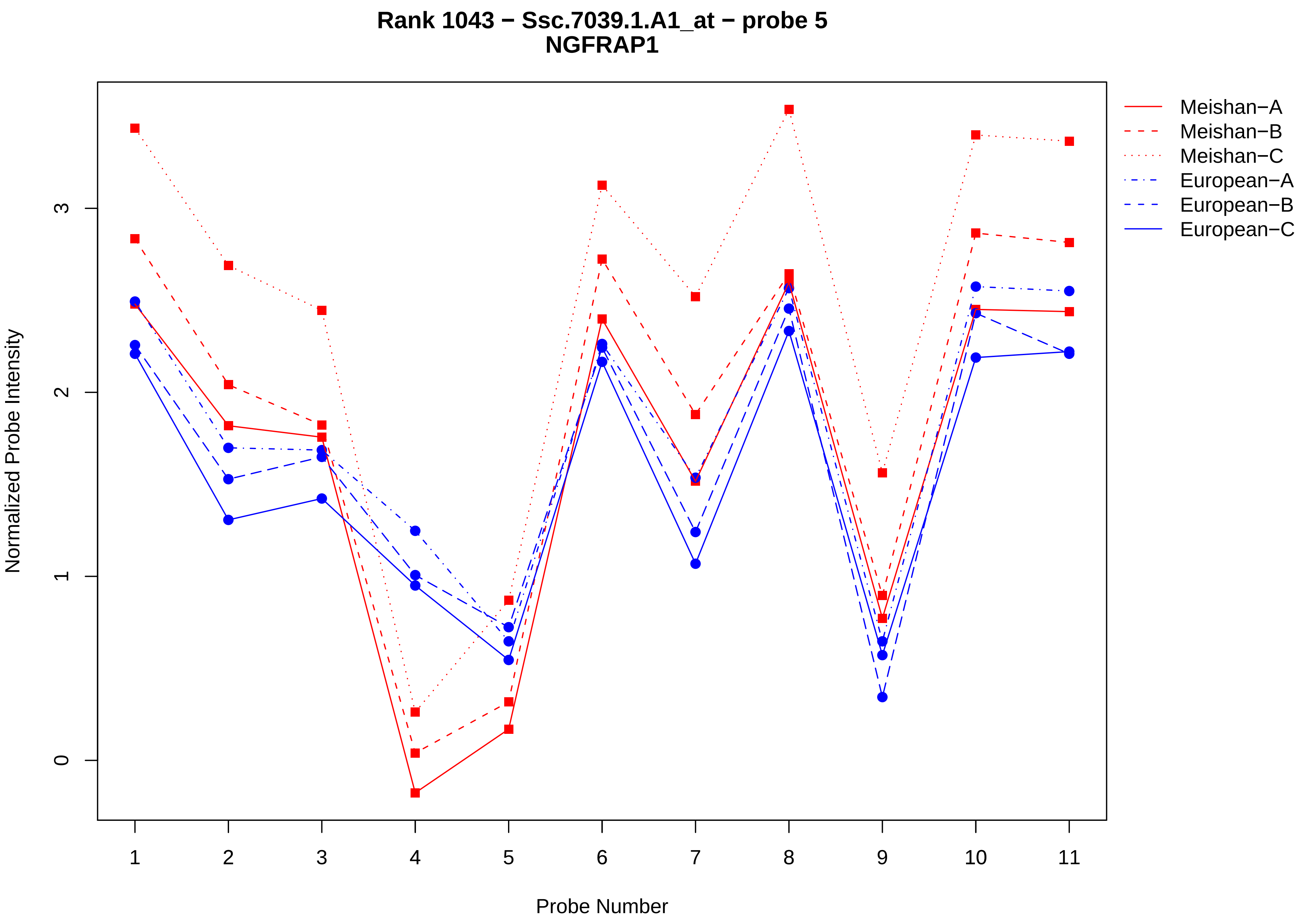
Rank 1043 − Ssc.7039.1.A1_at − probe 5
NGFRAP1
Normalized Probe Intensity
0
1
2
3
1
2
3
4
5
6
7
8
9
10
11
Probe Number
Meishan−A
Meishan−B
Meishan−C
European−A
European−B
European−C

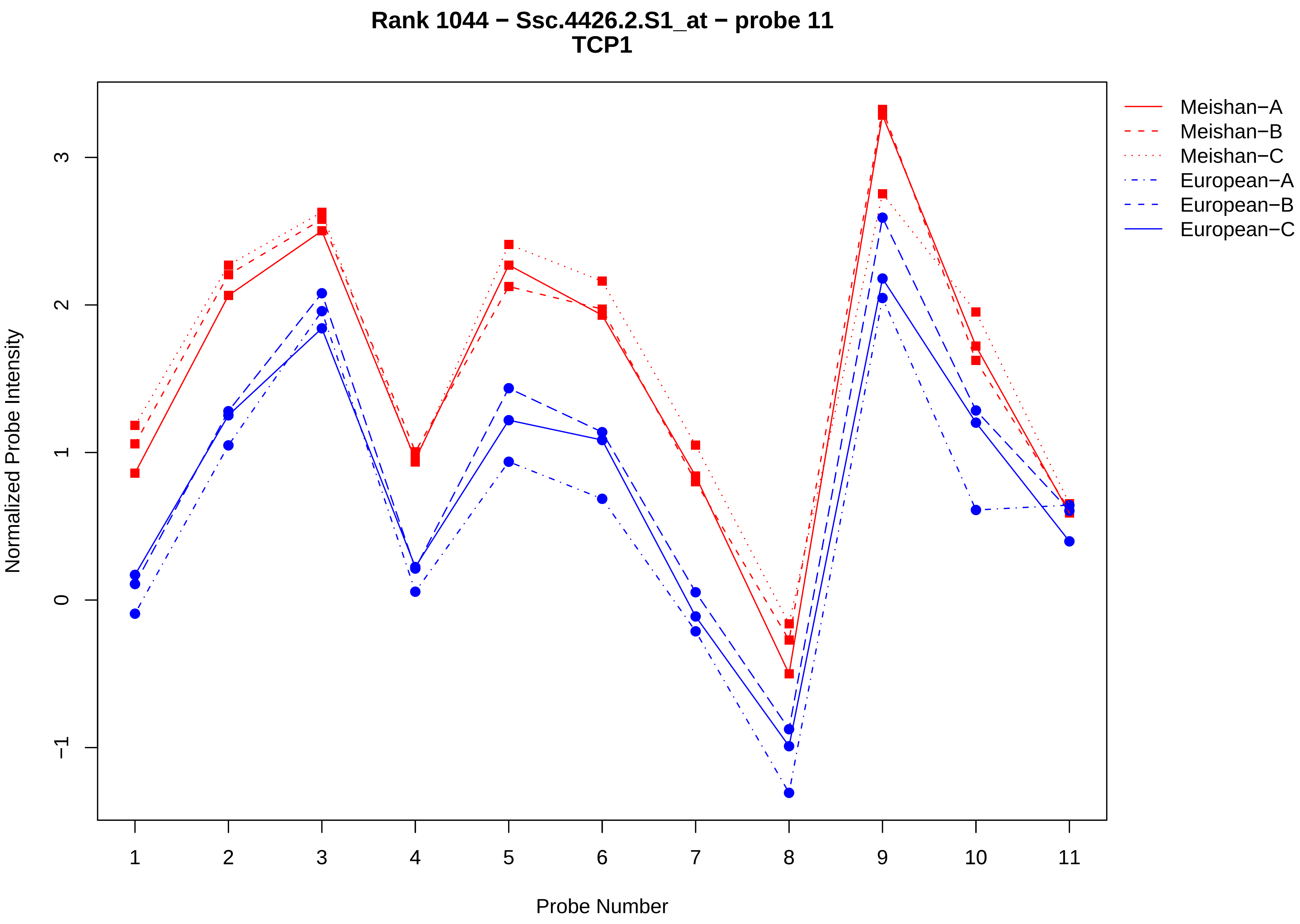
Rank 1044 − Ssc.4426.2.S1_at − probe 11
TCP1
Normalized Probe Intensity
Probe Number
Meishan−A
Meishan−B
Meishan−C
European−A
European−B
European−C

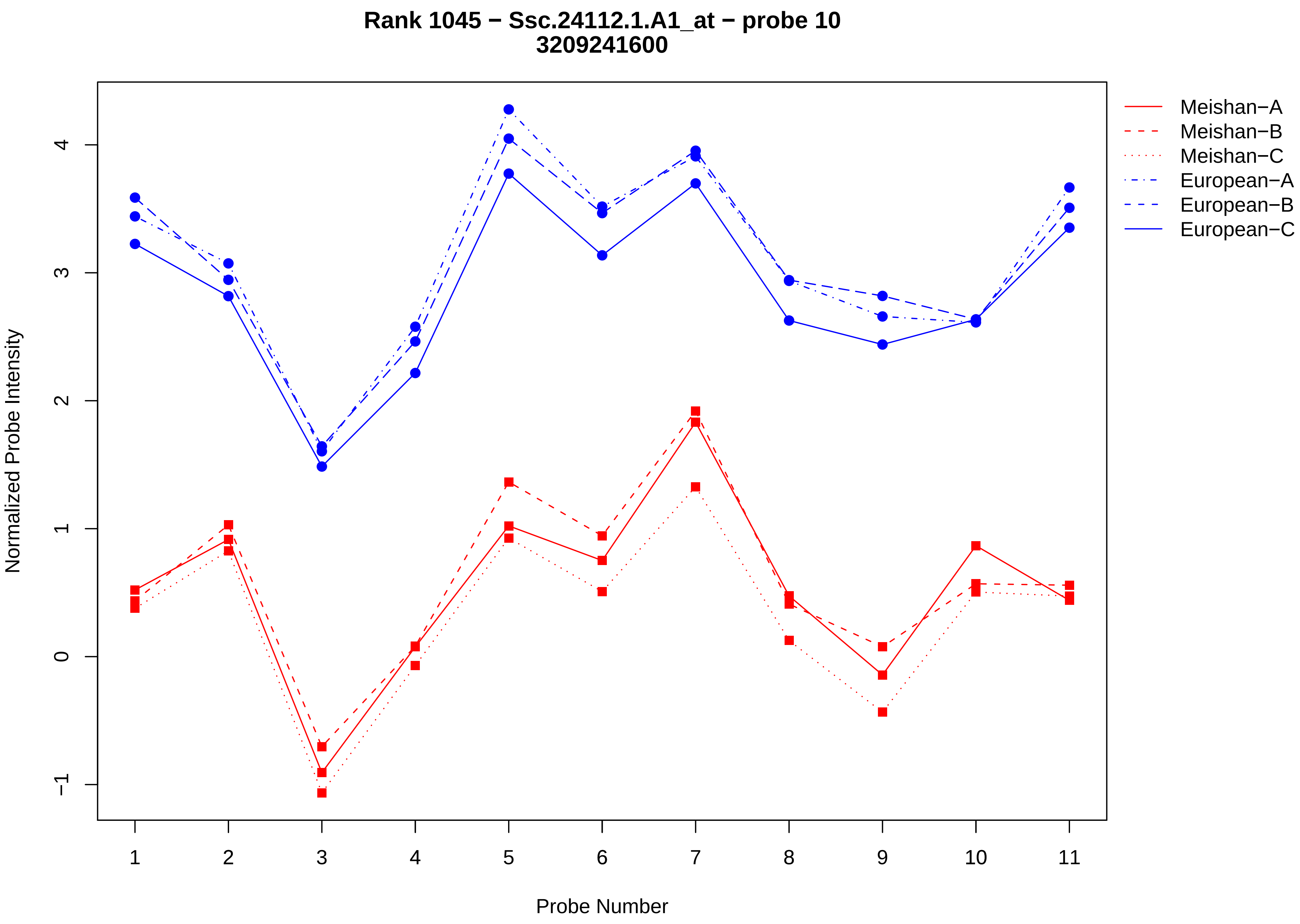

Rank 1045 − Ssc.24112.1.A1_at − probe 10
3209241600
Normalized Probe Intensity
Probe Number
Meishan−A
Meishan−B
Meishan−C
European−A
European−B
European−C
−1
0
1
2
3
4
1
2
3
4
5
6
7
8
9
10
11

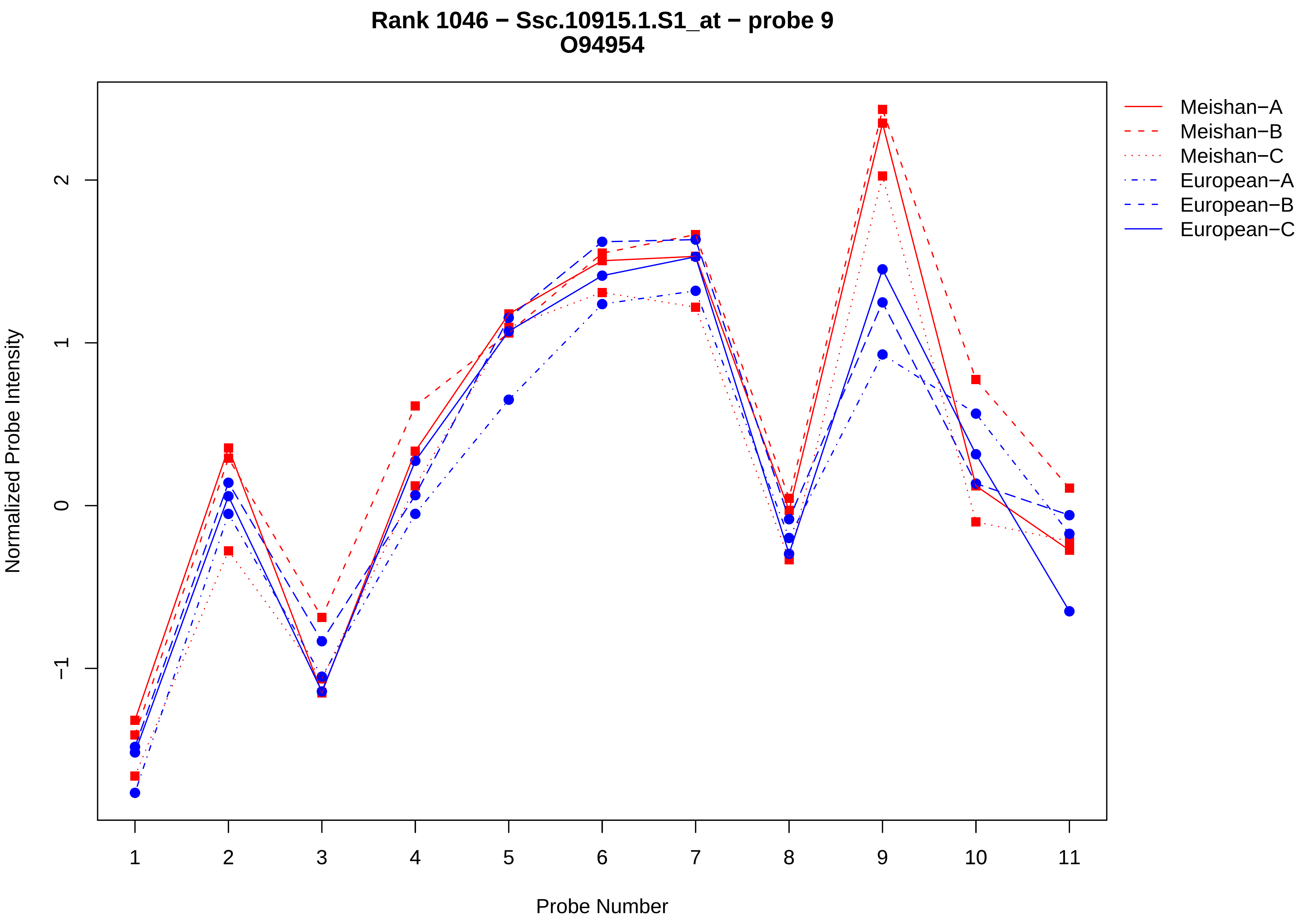

Rank 1046 − Ssc.10915.1.S1_at − probe 9
O94954
Normalized Probe Intensity
Probe Number
−1
0
1
2
1
2
3
4
5
6
7
8
9
10
11
Meishan−A
Meishan−B
Meishan−C
European−A
European−B
European−C

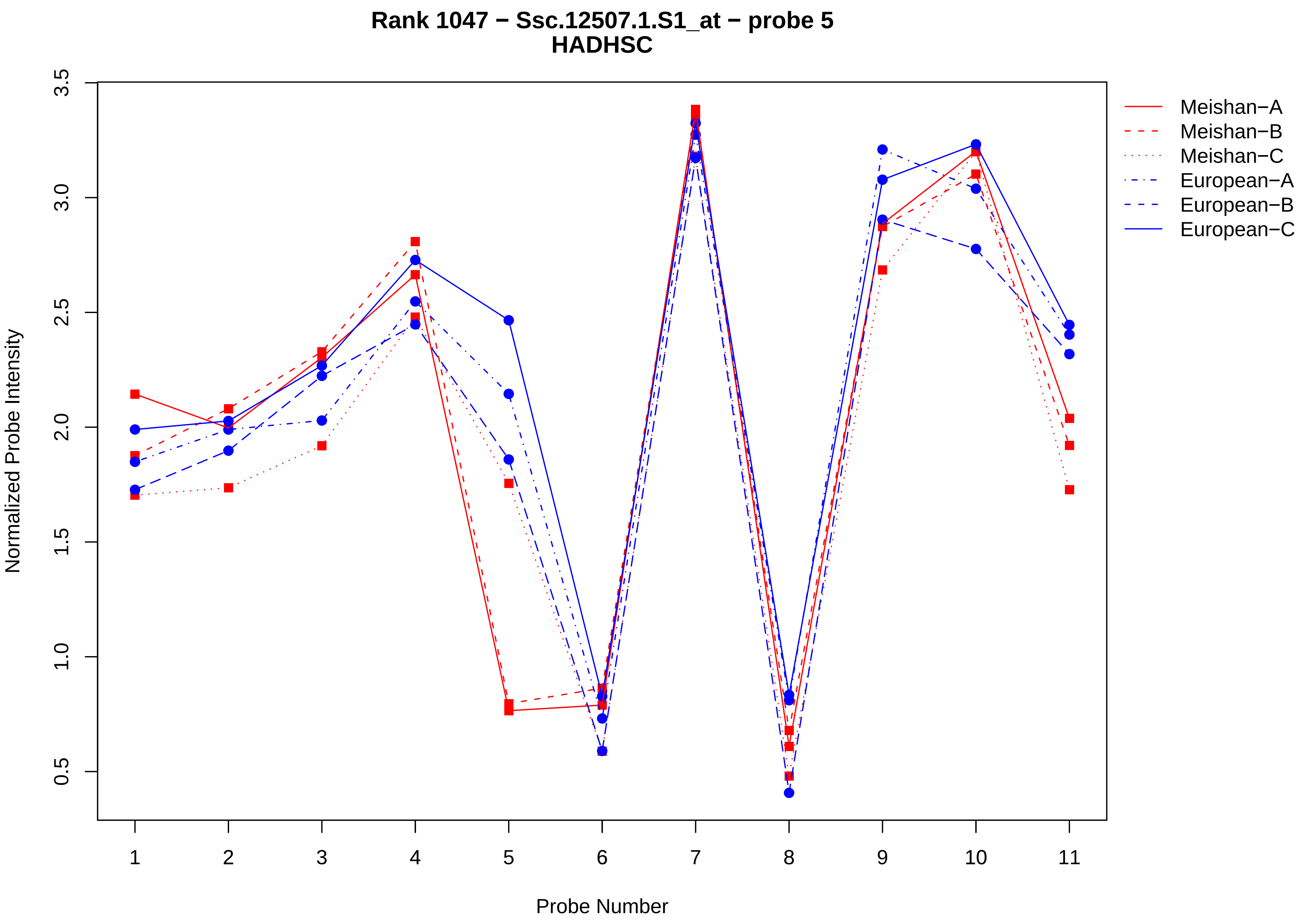

Rank 1047 − Ssc.12507.1.S1_at − probe 5
HADHSC
Normalized Probe Intensity
0.5
1.0
1.5
2.0
2.5
3.0
3.5
Probe Number
1
2
3
4
5
6
7
8
9
10
11
Meishan−A
Meishan−B
Meishan−C
European−A
European−B
European−C

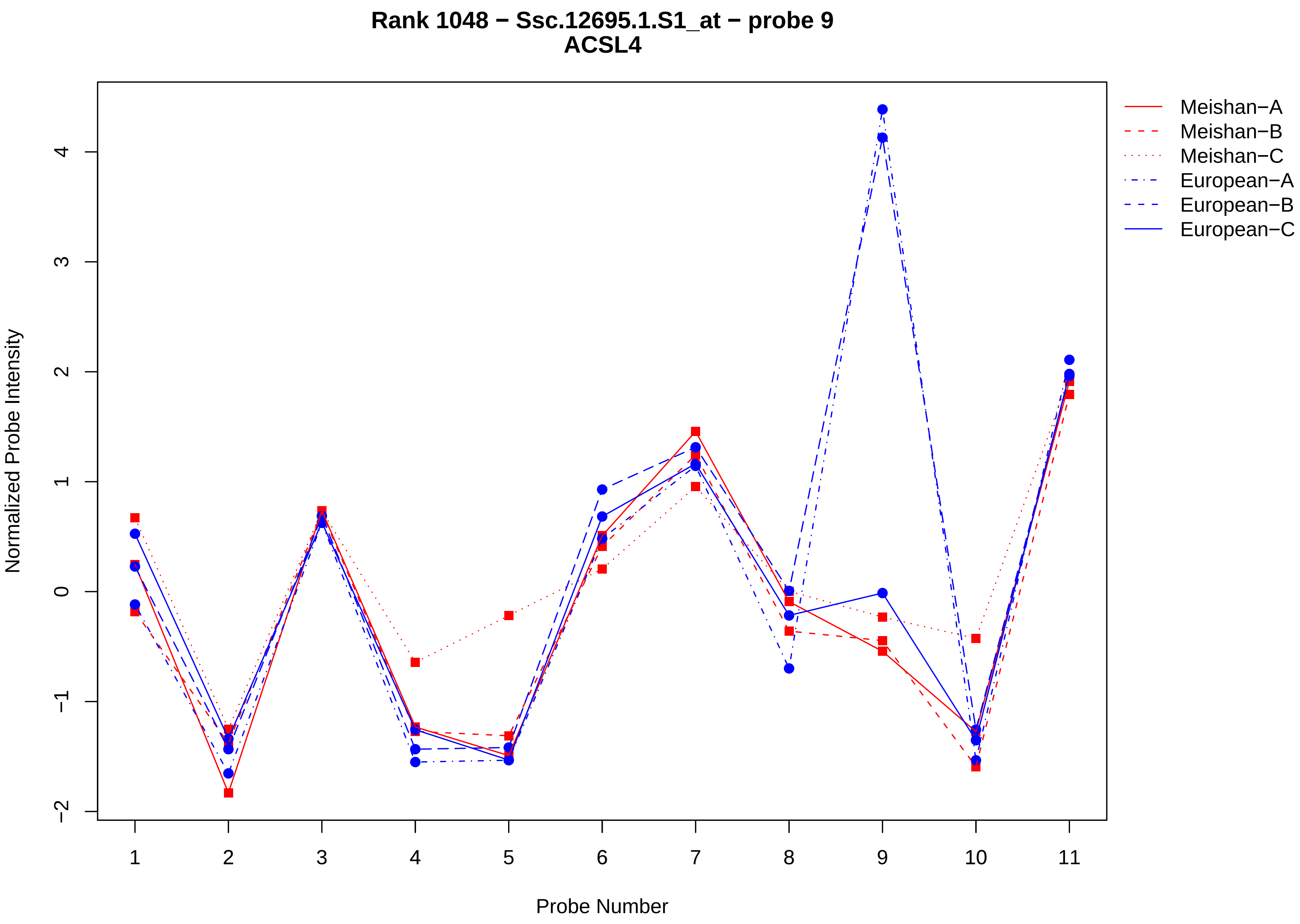

Rank 1048 − Ssc.12695.1.S1_at − probe 9
ACSL4
Normalized Probe Intensity
Probe Number
−2
−1
0
1
2
3
4
1
2
3
4
5
6
7
8
9
10
11
Meishan−A
Meishan−B
Meishan−C
European−A
European−B
European−C

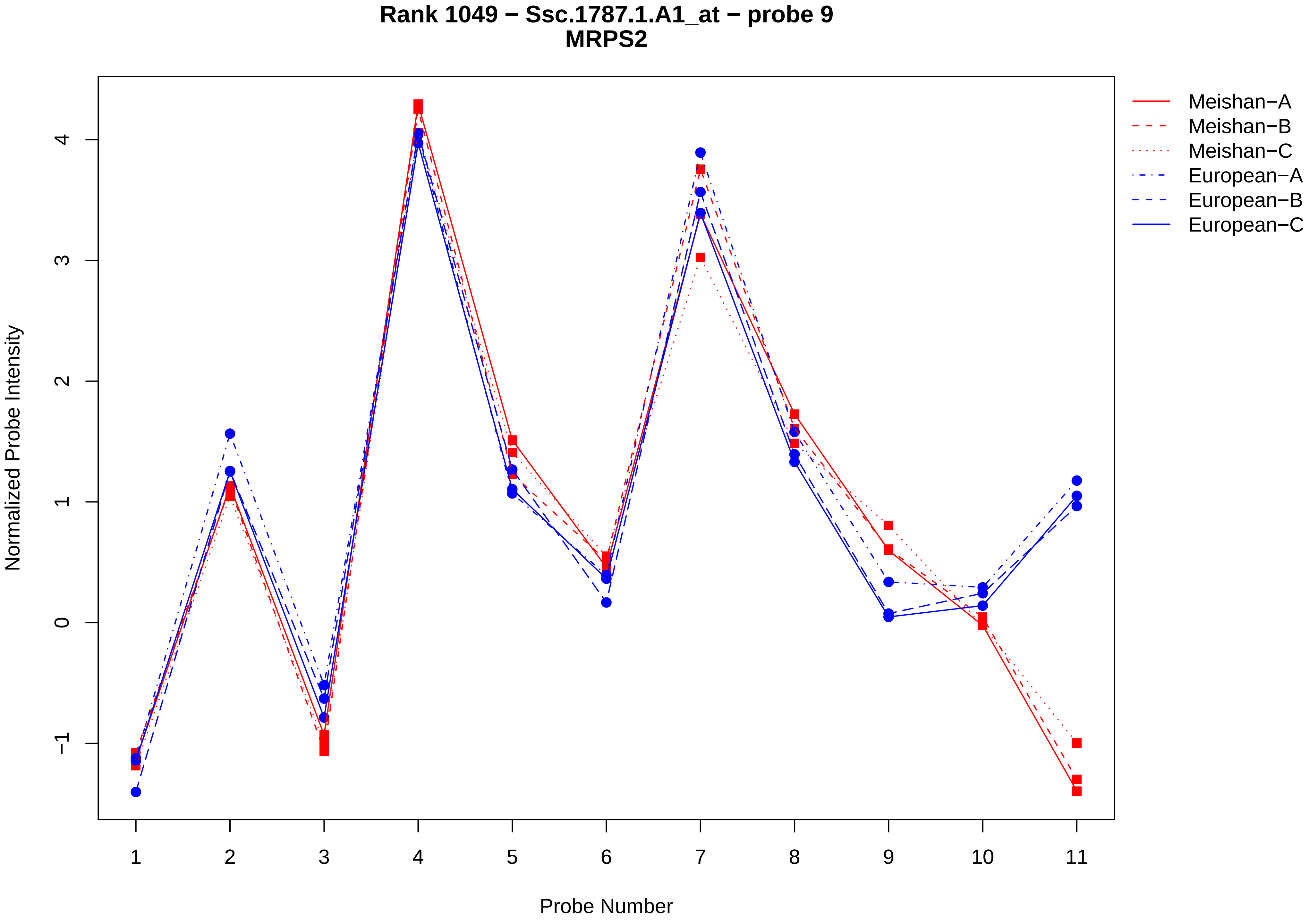

Rank 1049 − Ssc.1787.1.A1_at − probe 9
MRPS2
Normalized Probe Intensity
Probe Number
Meishan−A
Meishan−B
Meishan−C
European−A
European−B
European−C

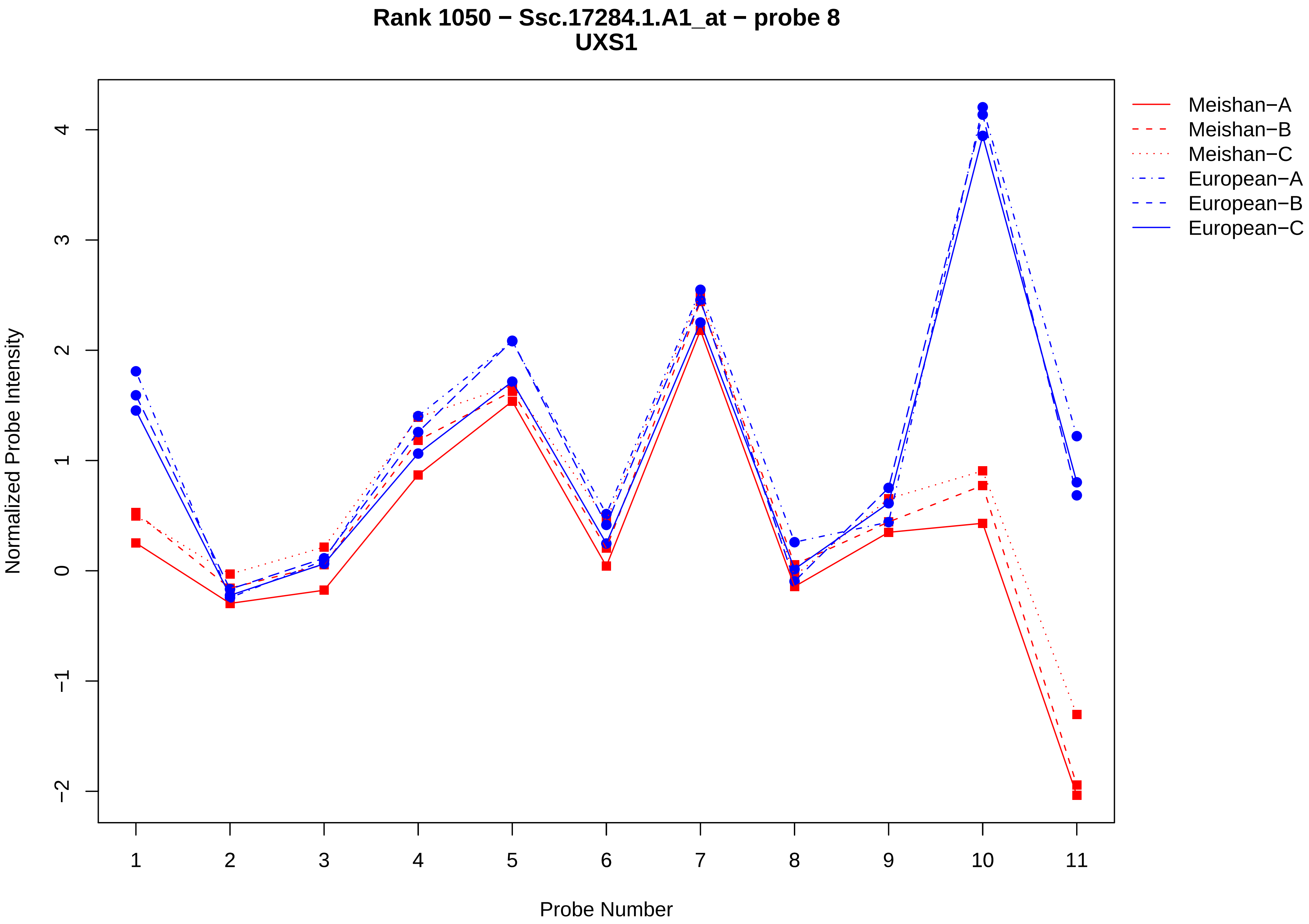
Rank 1050 − Ssc.17284.1.A1_at − probe 8
UXS1
Normalized Probe Intensity
Probe Number
Meishan−A
Meishan−B
Meishan−C
European−A
European−B
European−C
−2
−1
0
1
2
3
4
1
2
3
4
5
6
7
8
9
10
11

Rank 1051 − Ssc.489.1.A1_at − probe 1
Q9HB73

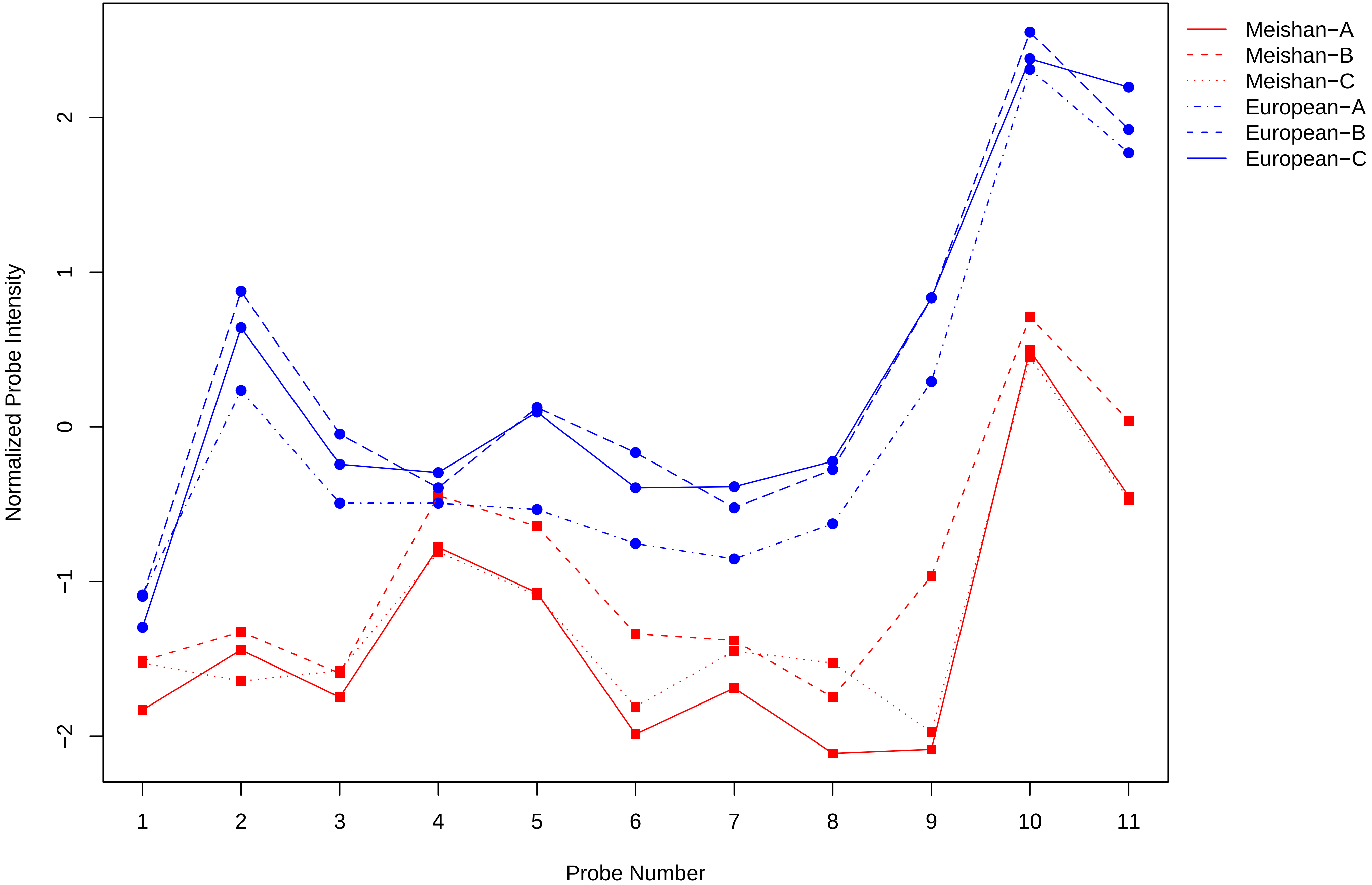

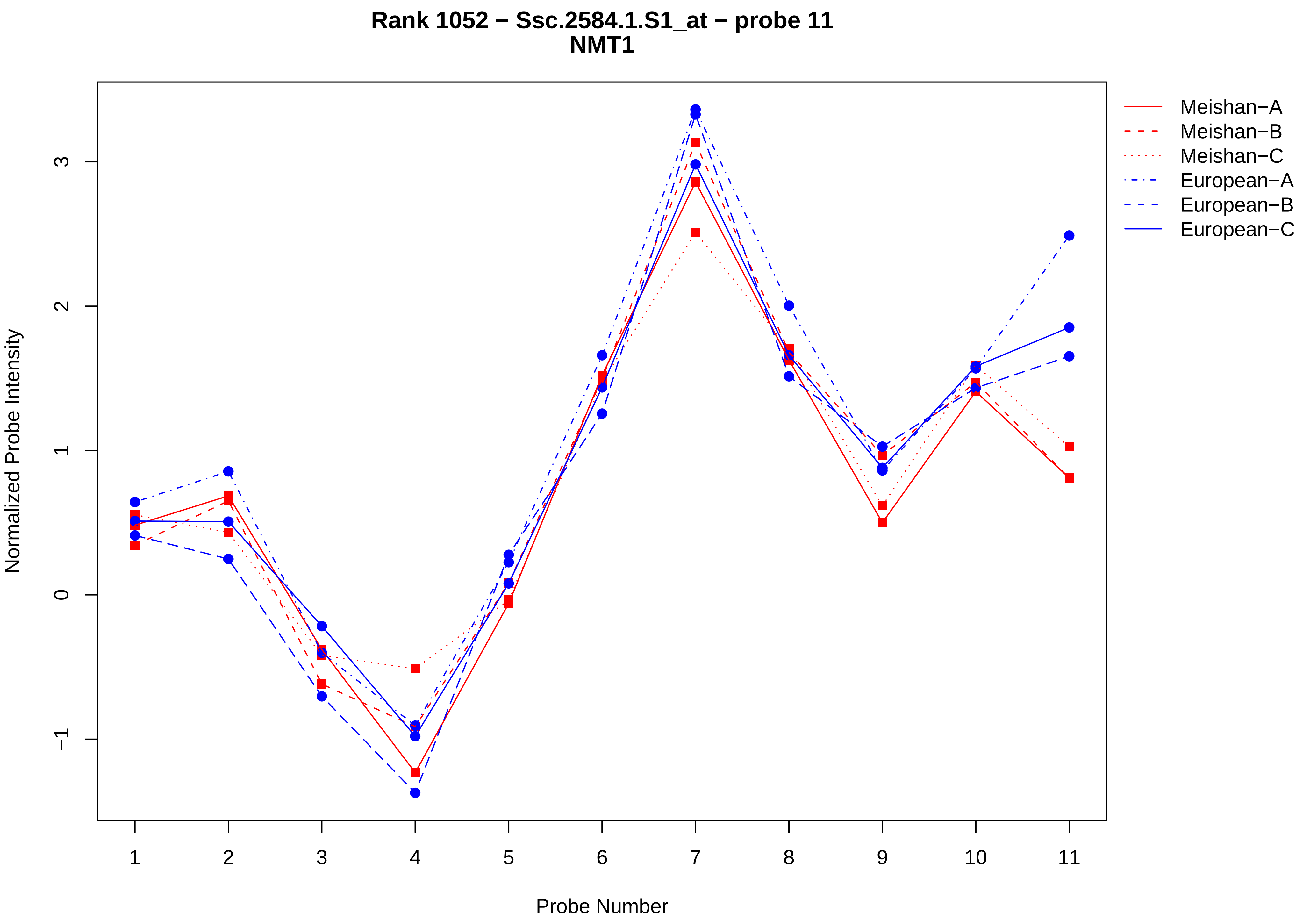

Rank 1052 − Ssc.2584.1.S1_at − probe 11
NMT1
Normalized Probe Intensity
Probe Number
−1
0
1
2
3
1
2
3
4
5
6
7
8
9
10
11
Meishan−A
Meishan−B
Meishan−C
European−A
European−B
European−C

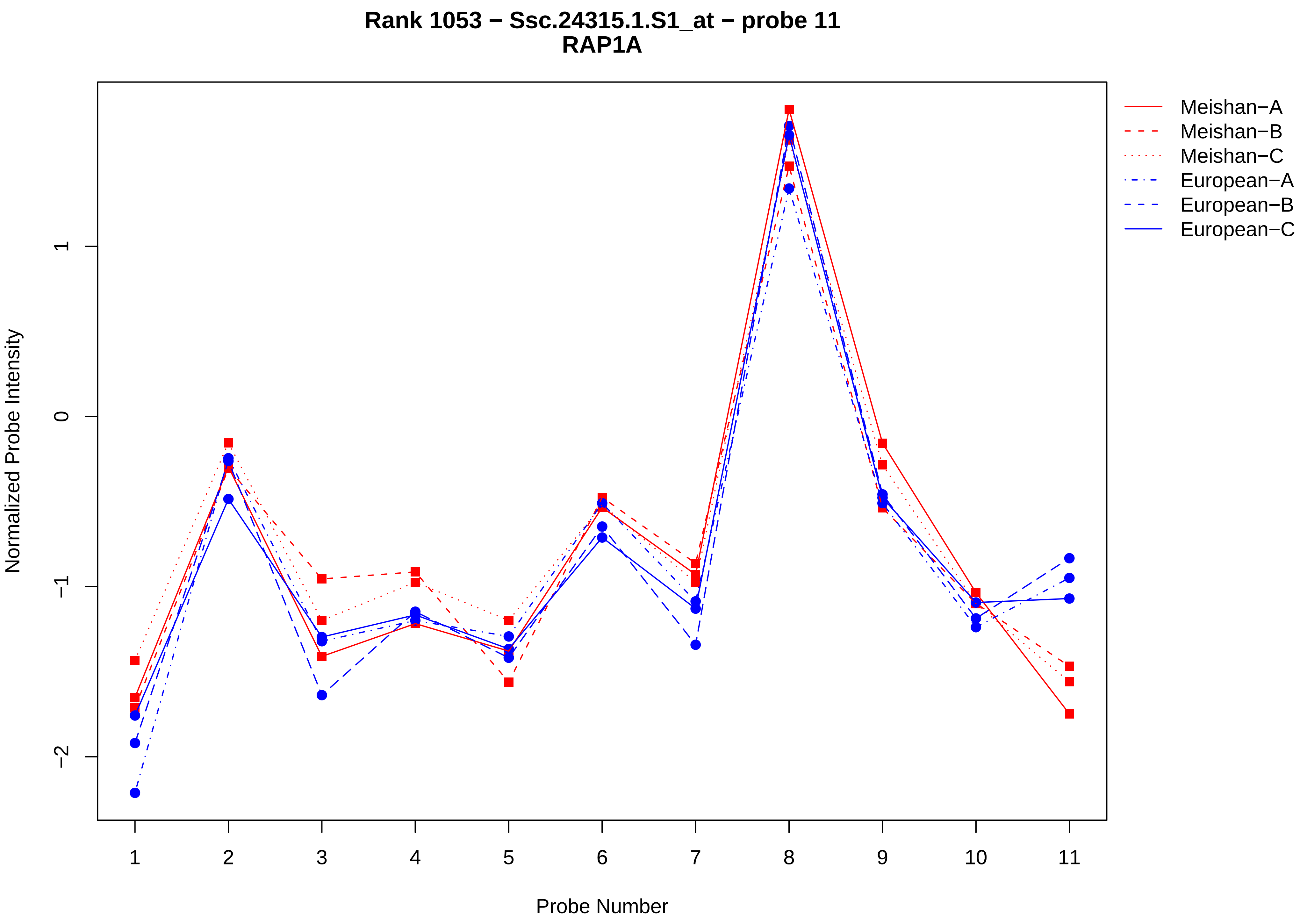

Rank 1053 − Ssc.24315.1.S1_at − probe 11
RAP1A
Normalized Probe Intensity
Probe Number
−2
−1
0
1
1
2
3
4
5
6
7
8
9
10
11
Meishan−A
Meishan−B
Meishan−C
European−A
European−B
European−C

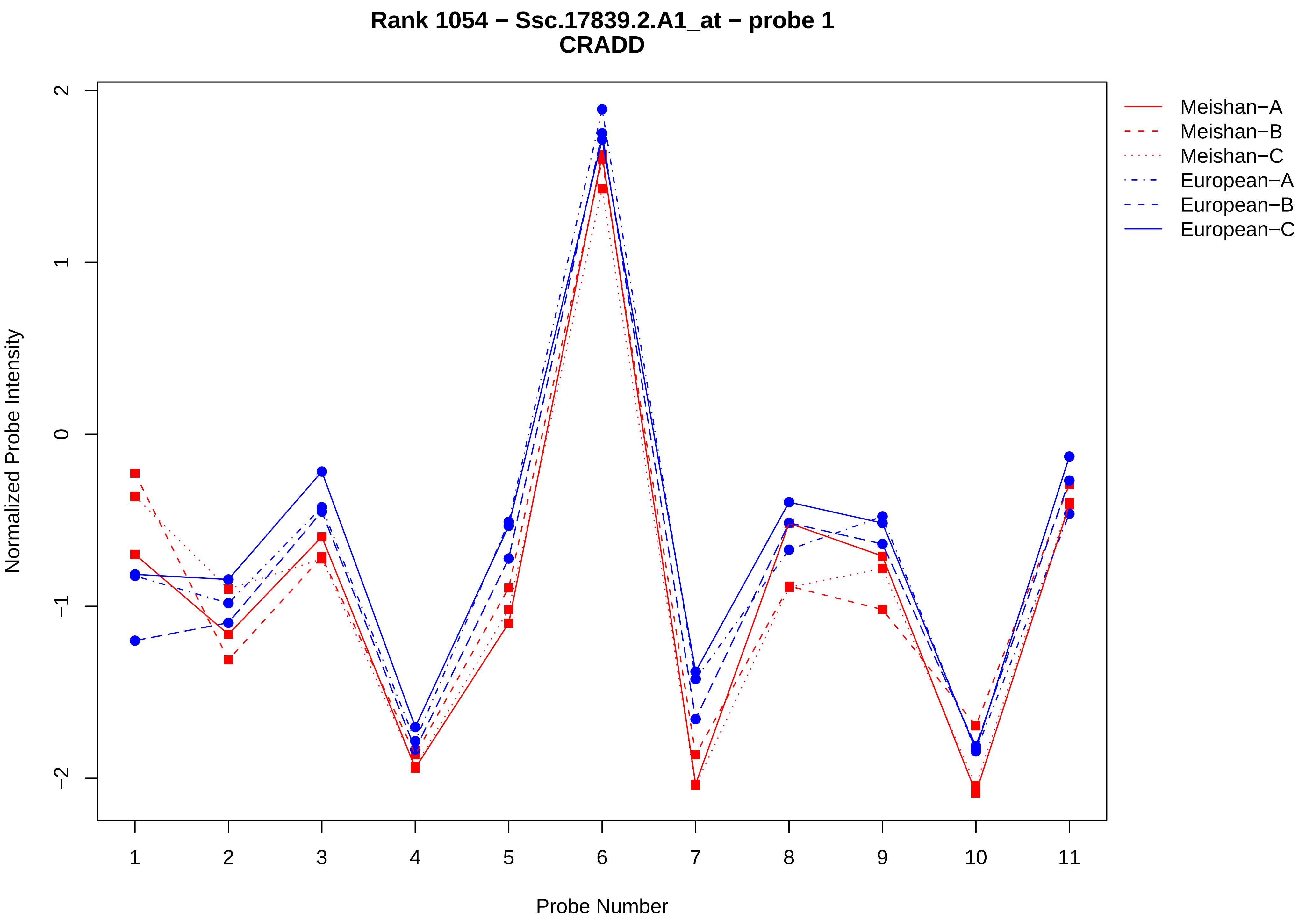

Rank 1054 − Ssc.17839.2.A1_at − probe 1
CRADD
Normalized Probe Intensity
Probe Number
Meishan−A
Meishan−B
Meishan−C
European−A
European−B
European−C

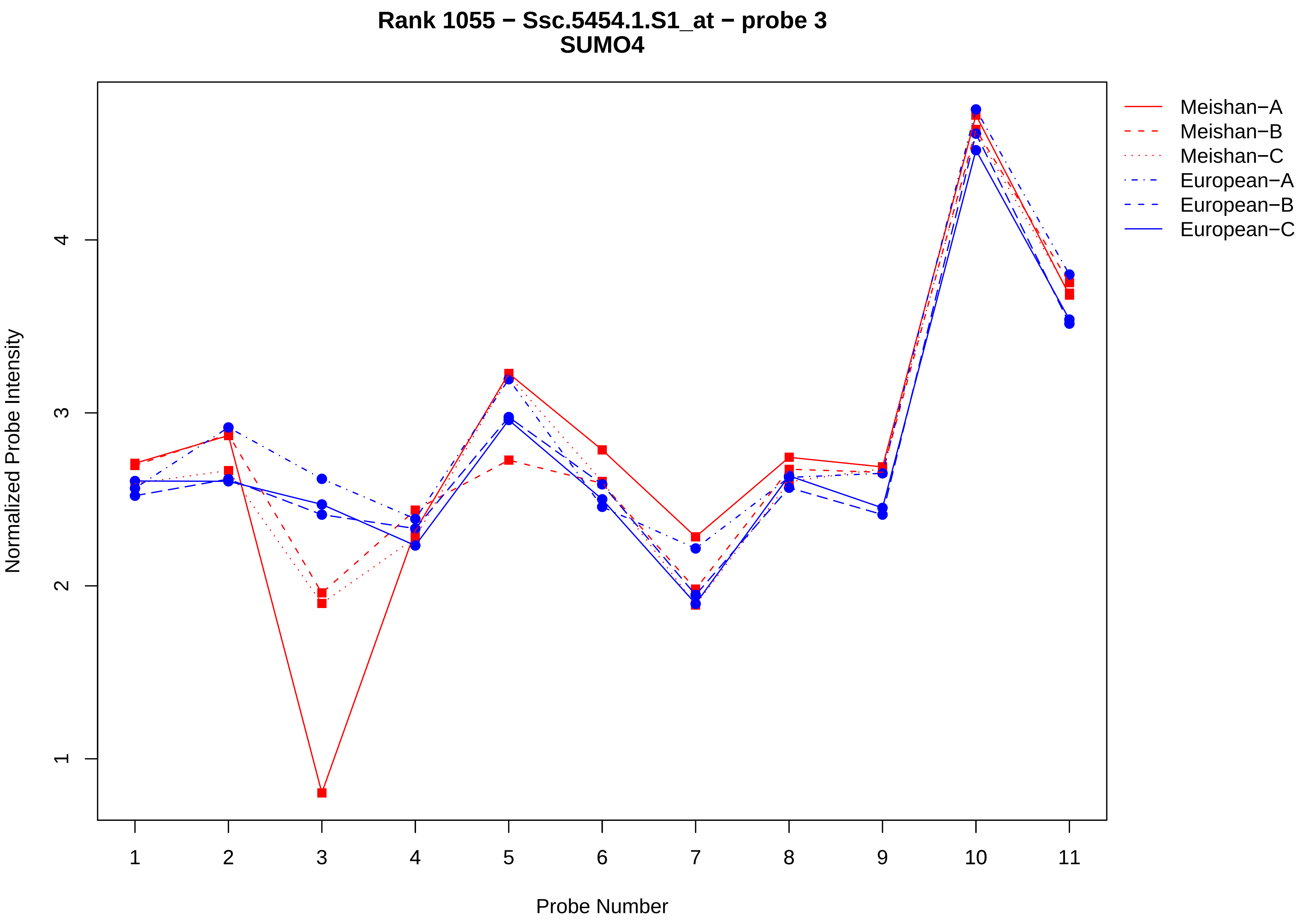

Rank 1055 − Ssc.5454.1.S1_at − probe 3
SUMO4
Normalized Probe Intensity
Probe Number
1
2
3
4
1
2
3
4
5
6
7
8
9
10
11
Meishan−A
Meishan−B
Meishan−C
European−A
European−B
European−C

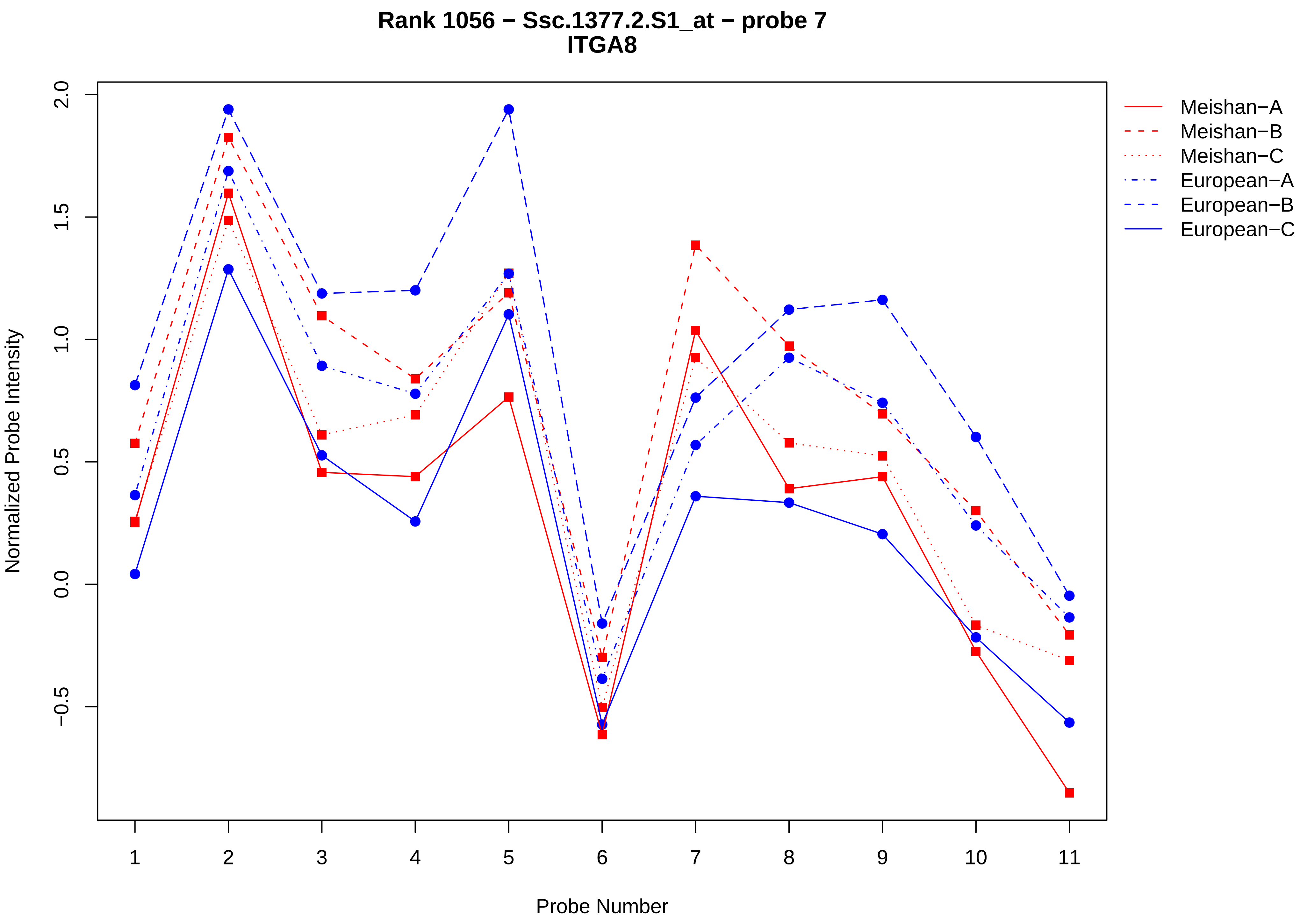

Rank 1056 − Ssc.1377.2.S1_at − probe 7
ITGA8
Normalized Probe Intensity
Probe Number
−0.5
0.0
0.5
1.0
1.5
2.0
1
2
3
4
5
6
7
8
9
10
11
Meishan−A
Meishan−B
Meishan−C
European−A
European−B
European−C

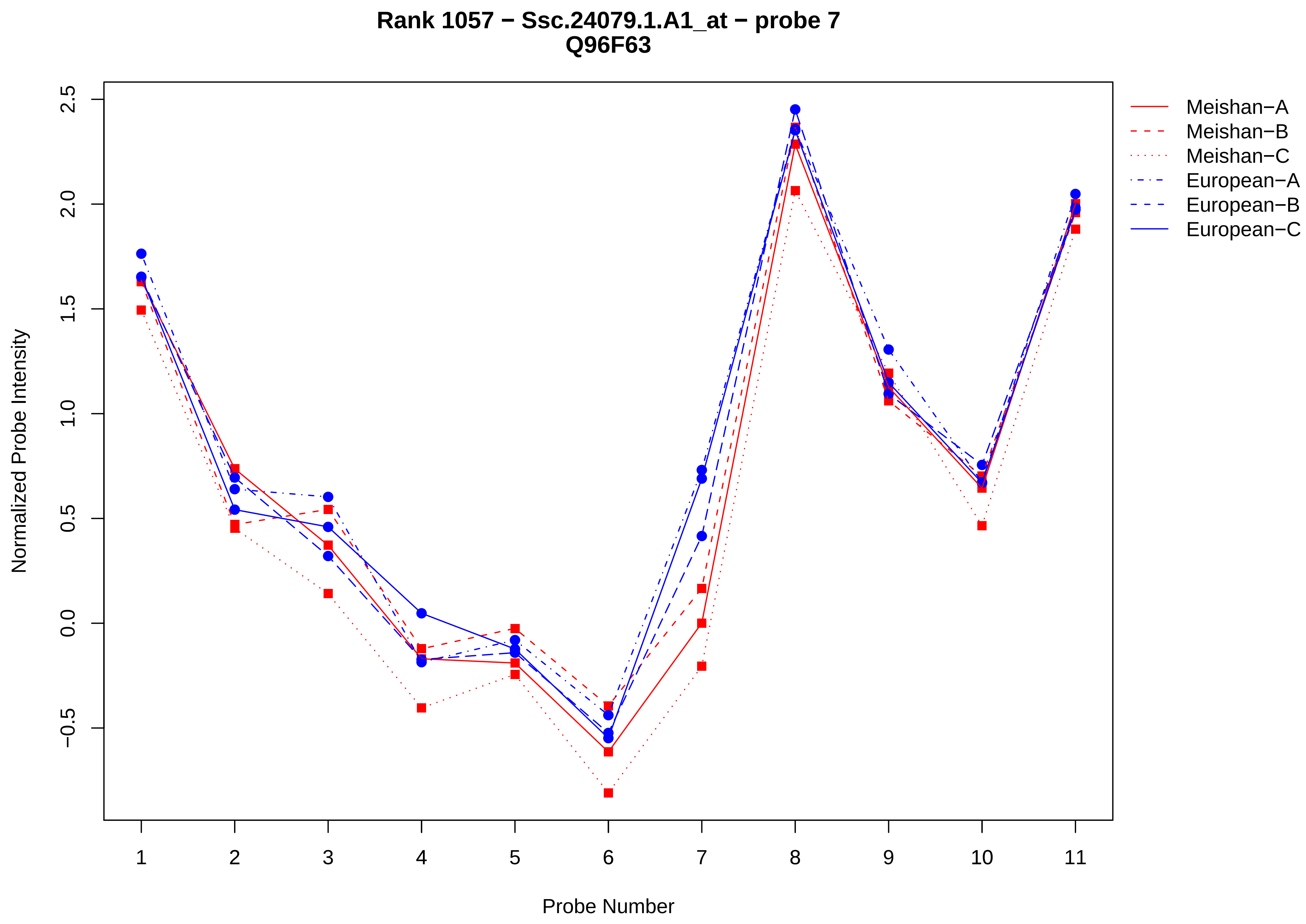

Rank 1057 − Ssc.24079.1.A1_at − probe 7
Q96F63
Normalized Probe Intensity
Probe Number
−0.5
0.0
0.5
1.0
1.5
2.0
2.5
1
2
3
4
5
6
7
8
9
10
11
Meishan−A
Meishan−B
Meishan−C
European−A
European−B
European−C

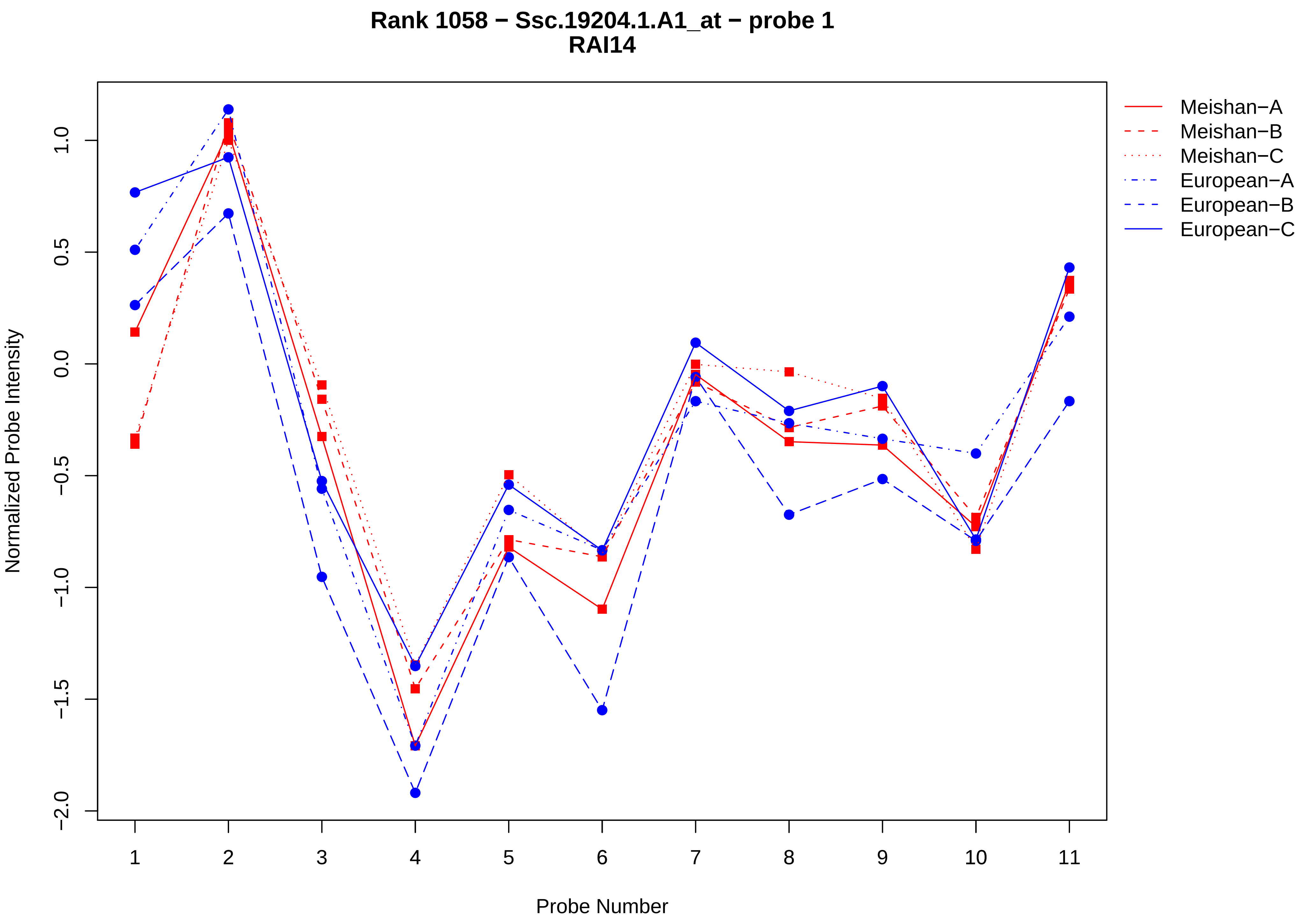
Rank 1058 − Ssc.19204.1.A1_at − probe 1
RAI14
Normalized Probe Intensity
Probe Number
1.0
0.5
0.0
−0.5
−1.0
−1.5
−2.0
1
2
3
4
5
6
7
8
9
10
11
Meishan−A
Meishan−B
Meishan−C
European−A
European−B
European−C

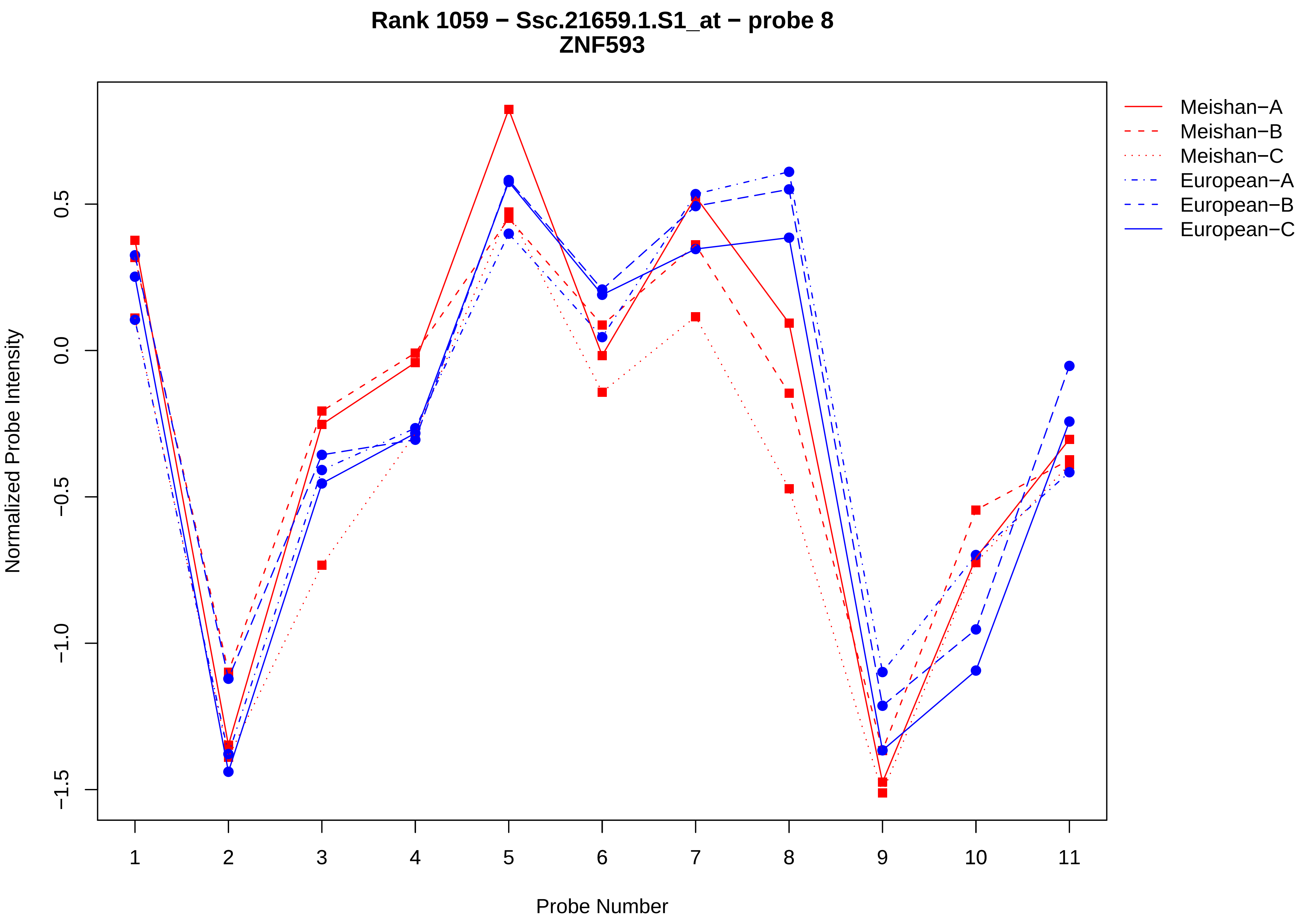

Rank 1059 − Ssc.21659.1.S1_at − probe 8
ZNF593
Normalized Probe Intensity
Probe Number
−1.5
−1.0
−0.5
0.0
0.5
1
2
3
4
5
6
7
8
9
10
11
Meishan−A
Meishan−B
Meishan−C
European−A
European−B
European−C

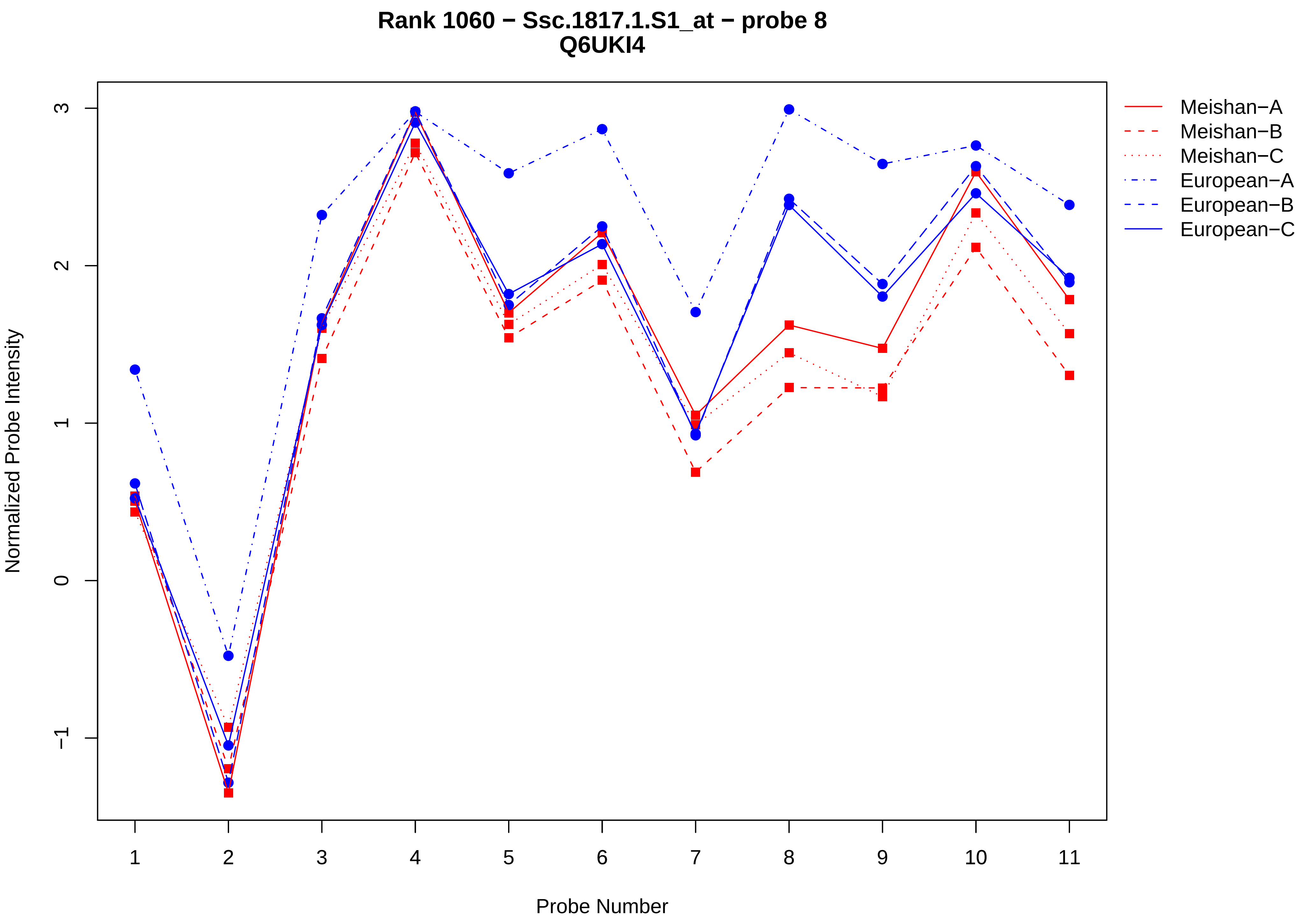

Rank 1060 − Ssc.1817.1.S1_at − probe 8
Q6UKI4
Normalized Probe Intensity
Probe Number
−1
0
1
2
3
1
2
3
4
5
6
7
8
9
10
11
Meishan−A
Meishan−B
Meishan−C
European−A
European−B
European−C

Rank 1061 − Ssc.3897.1.S1_at − probe 3
GPSN2

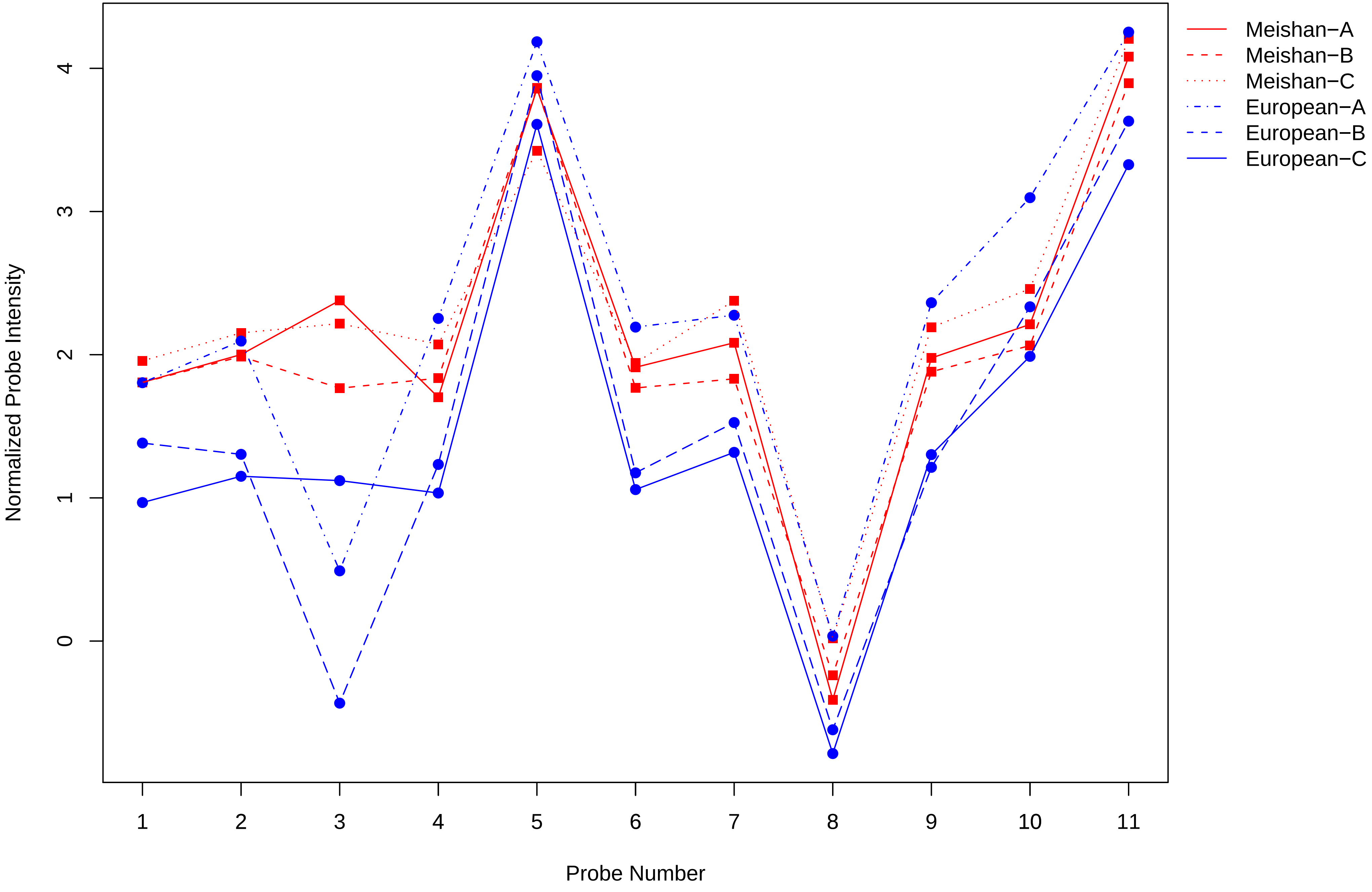

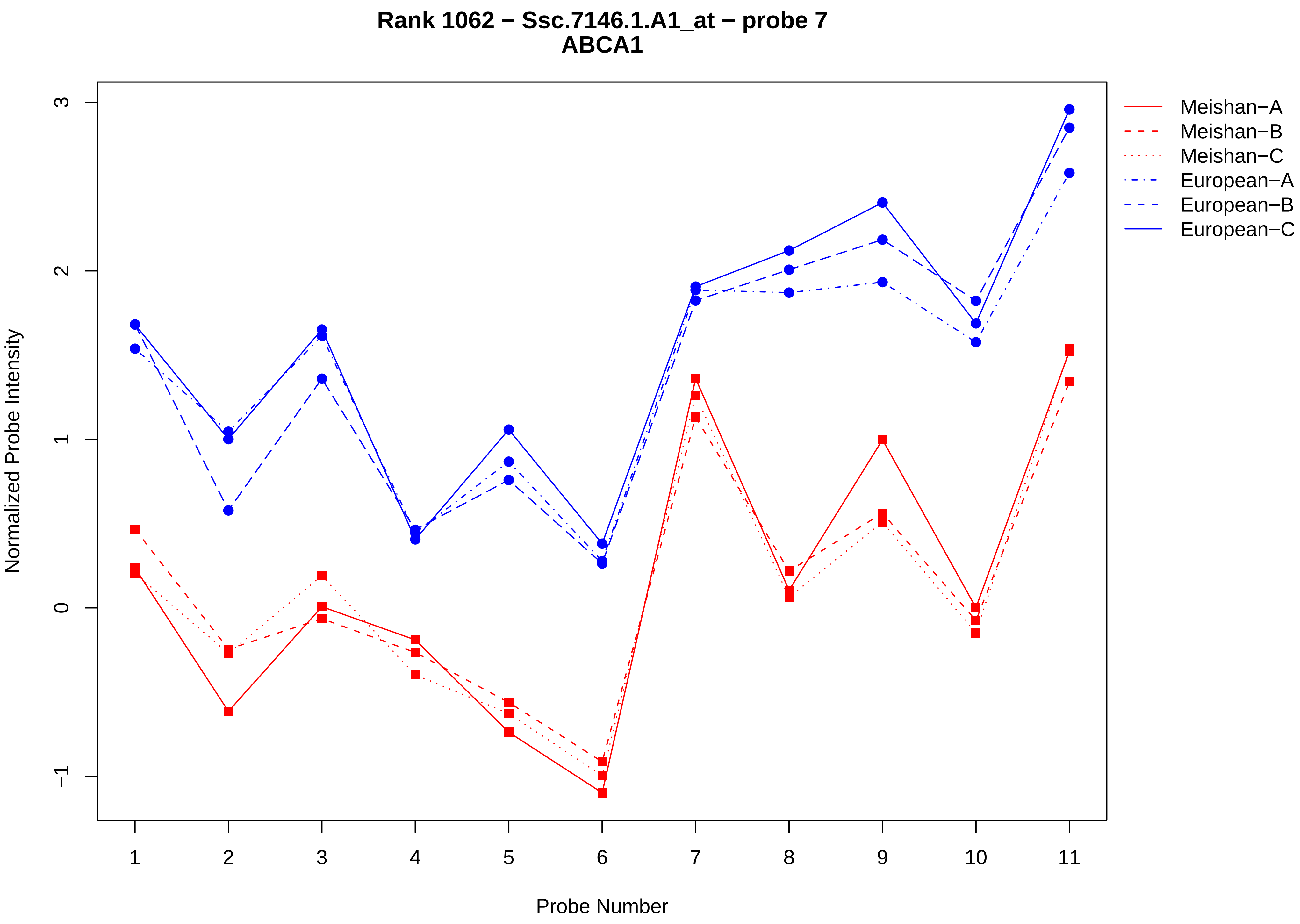

Rank 1062 − Ssc.7146.1.A1_at − probe 7
ABCA1
Normalized Probe Intensity
Probe Number
−1
0
1
2
3
1
2
3
4
5
6
7
8
9
10
11
Meishan−A
Meishan−B
Meishan−C
European−A
European−B
European−C

Rank 1063 − Ssc.16603.1.A1_at − probe 2
A2M

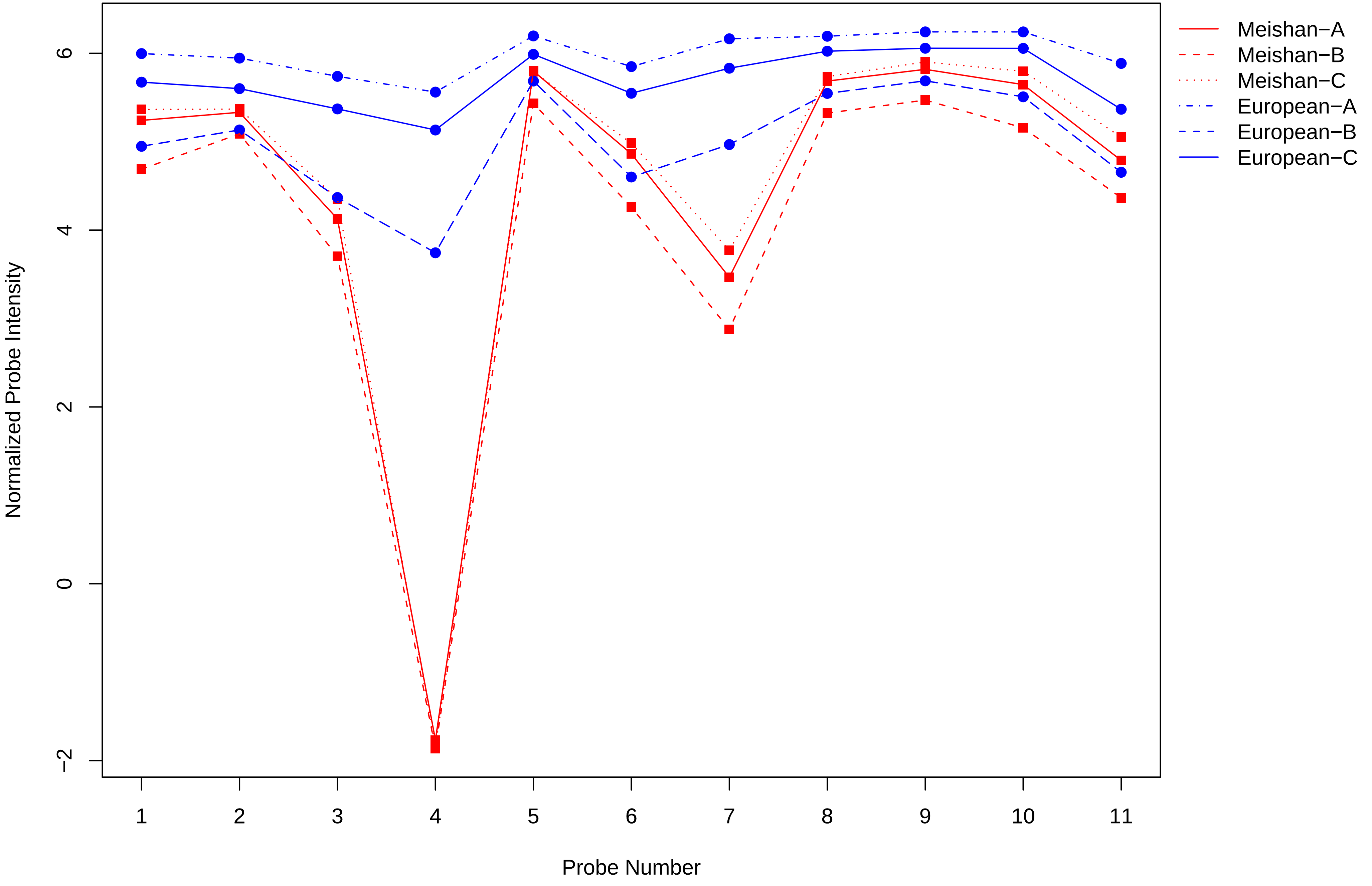

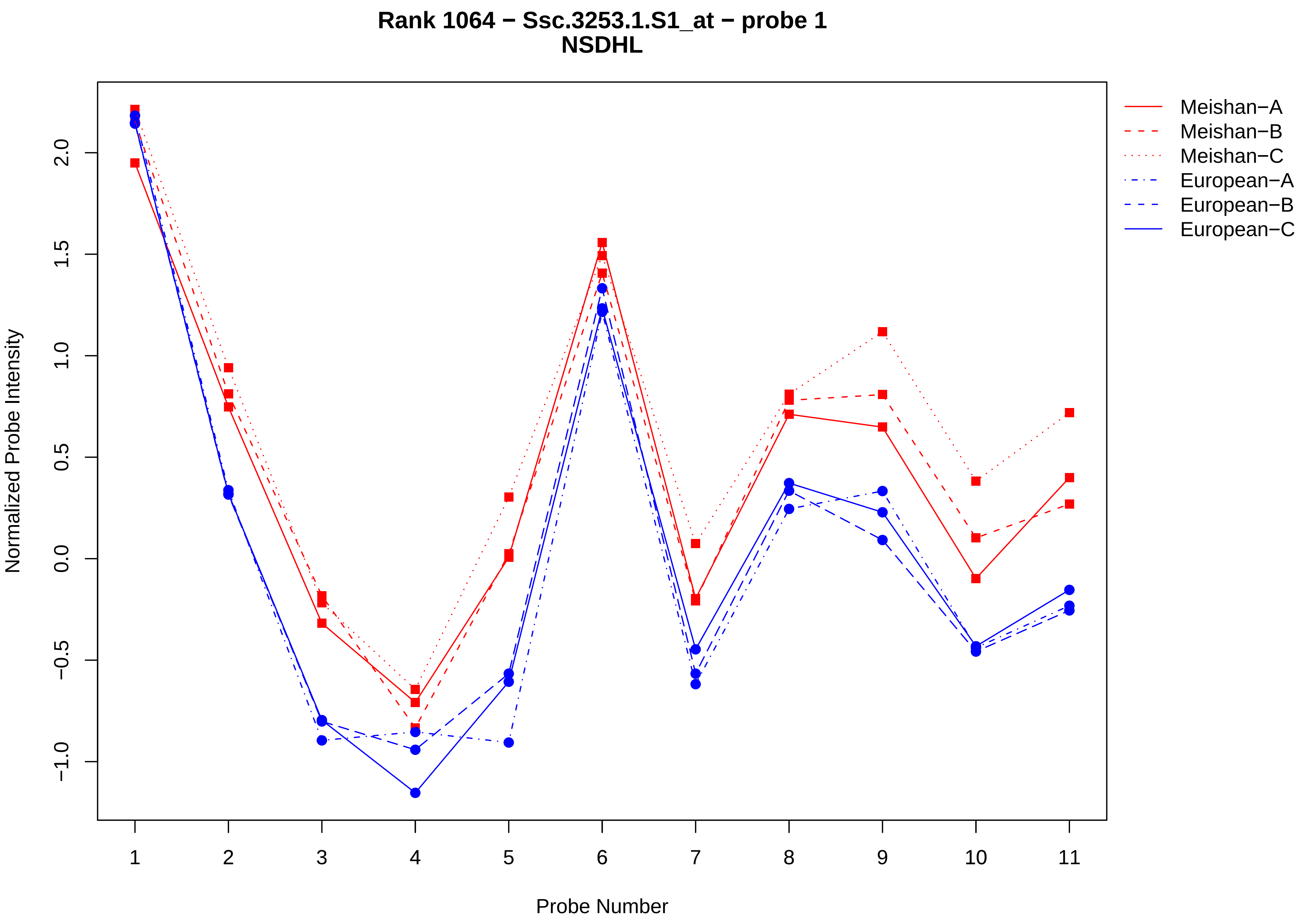

Rank 1064 − Ssc.3253.1.S1_at − probe 1
NSDHL
Normalized Probe Intensity
Probe Number
Meishan−A
Meishan−B
Meishan−C
European−A
European−B
European−C
−1.0
−0.5
0.0
0.5
1.0
1.5
2.0
1
2
3
4
5
6
7
8
9
10
11

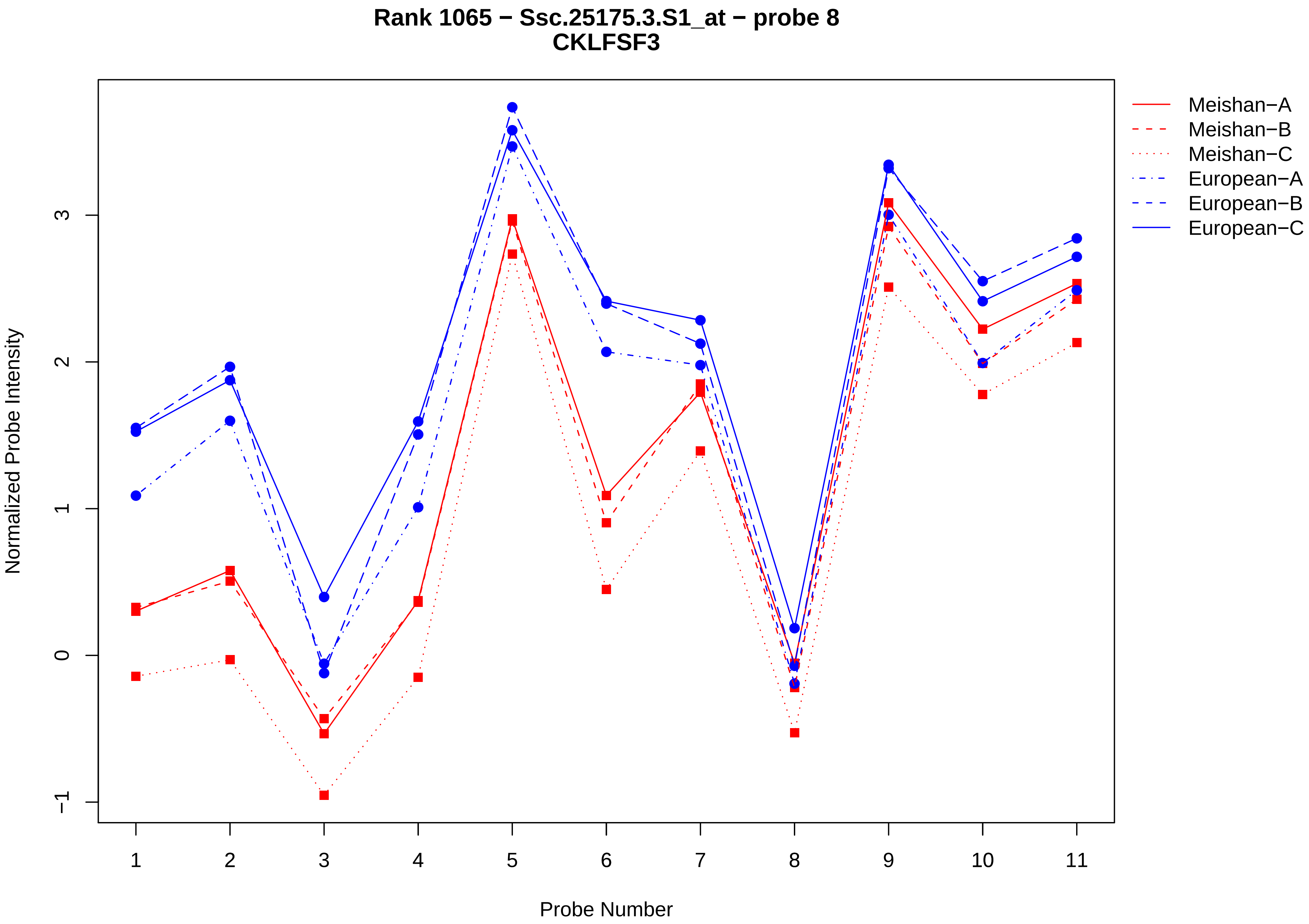

Rank 1065 − Ssc.25175.3.S1_at − probe 8
CKLFSF3
Normalized Probe Intensity
Probe Number
−1
0
1
2
3
1
2
3
4
5
6
7
8
9
10
11
Meishan−A
Meishan−B
Meishan−C
European−A
European−B
European−C

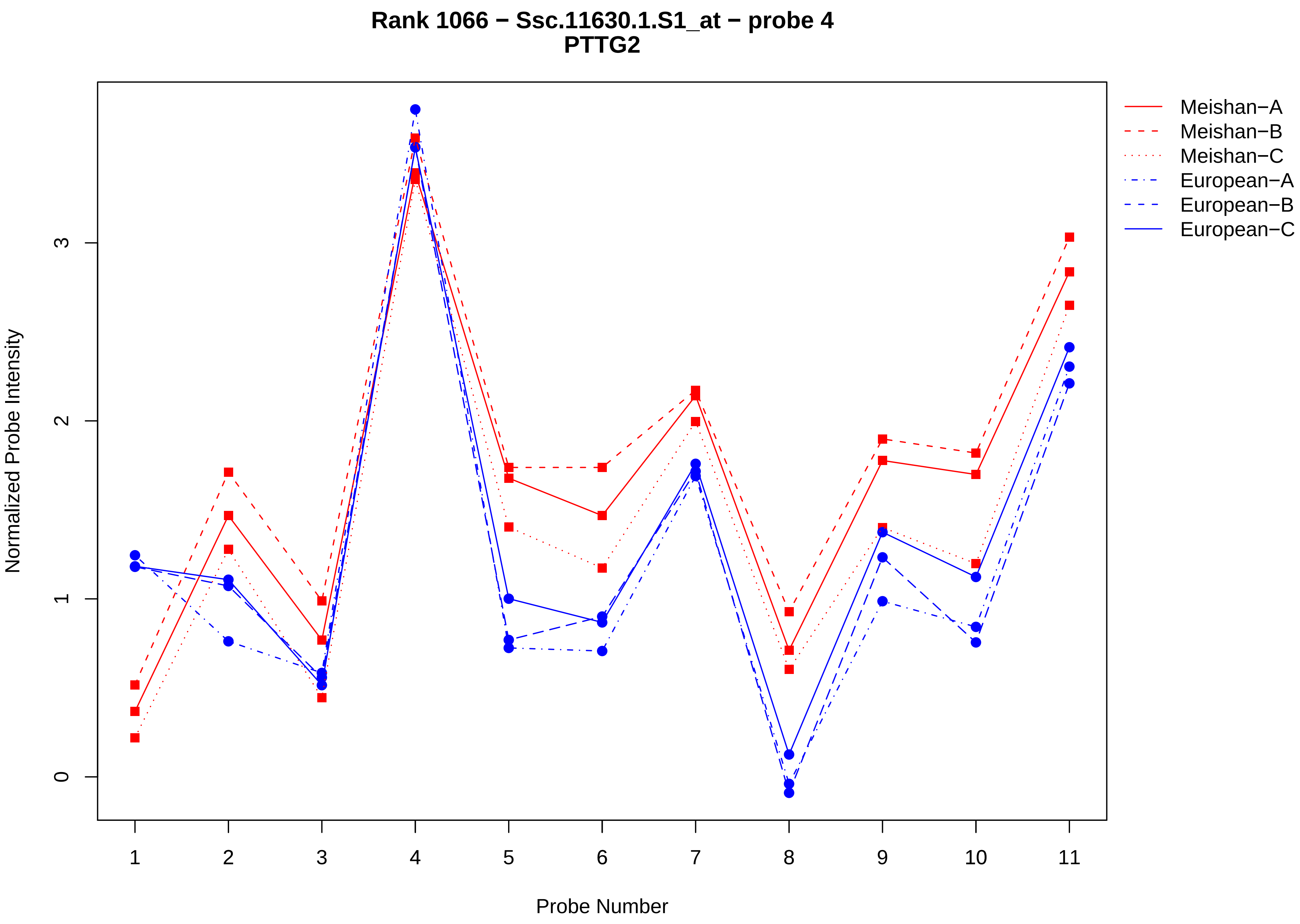

Rank 1066 − Ssc.11630.1.S1_at − probe 4
PTTG2
Normalized Probe Intensity
Probe Number
0
1
2
3
1
2
3
4
5
6
7
8
9
10
11
Meishan−A
Meishan−B
Meishan−C
European−A
European−B
European−C

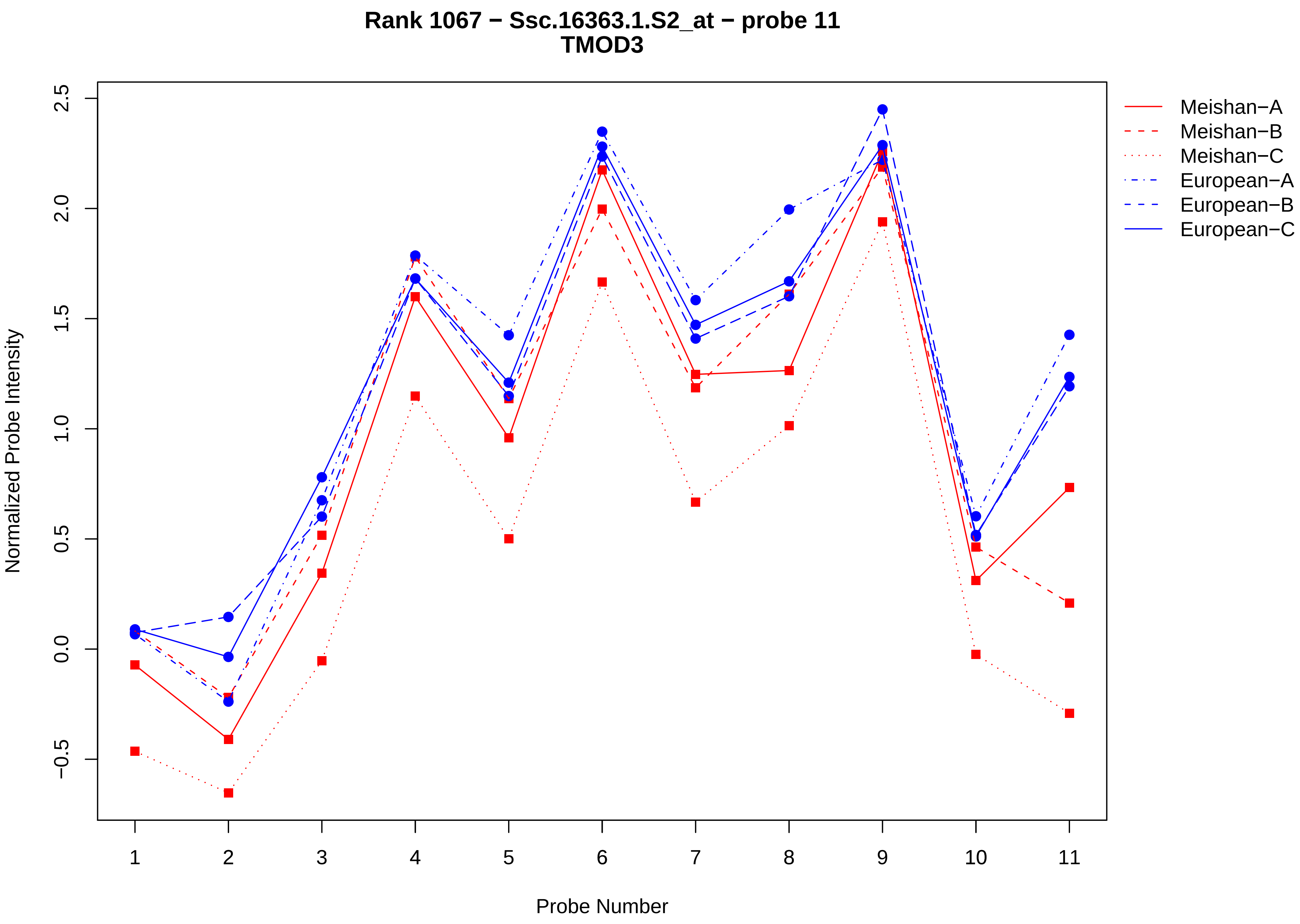

Rank 1067 − Ssc.16363.1.S2_at − probe 11
TMOD3
Normalized Probe Intensity
Probe Number
Meishan−A
Meishan−B
Meishan−C
European−A
European−B
European−C
−0.5
0.0
0.5
1.0
1.5
2.0
2.5
1
2
3
4
5
6
7
8
9
10
11

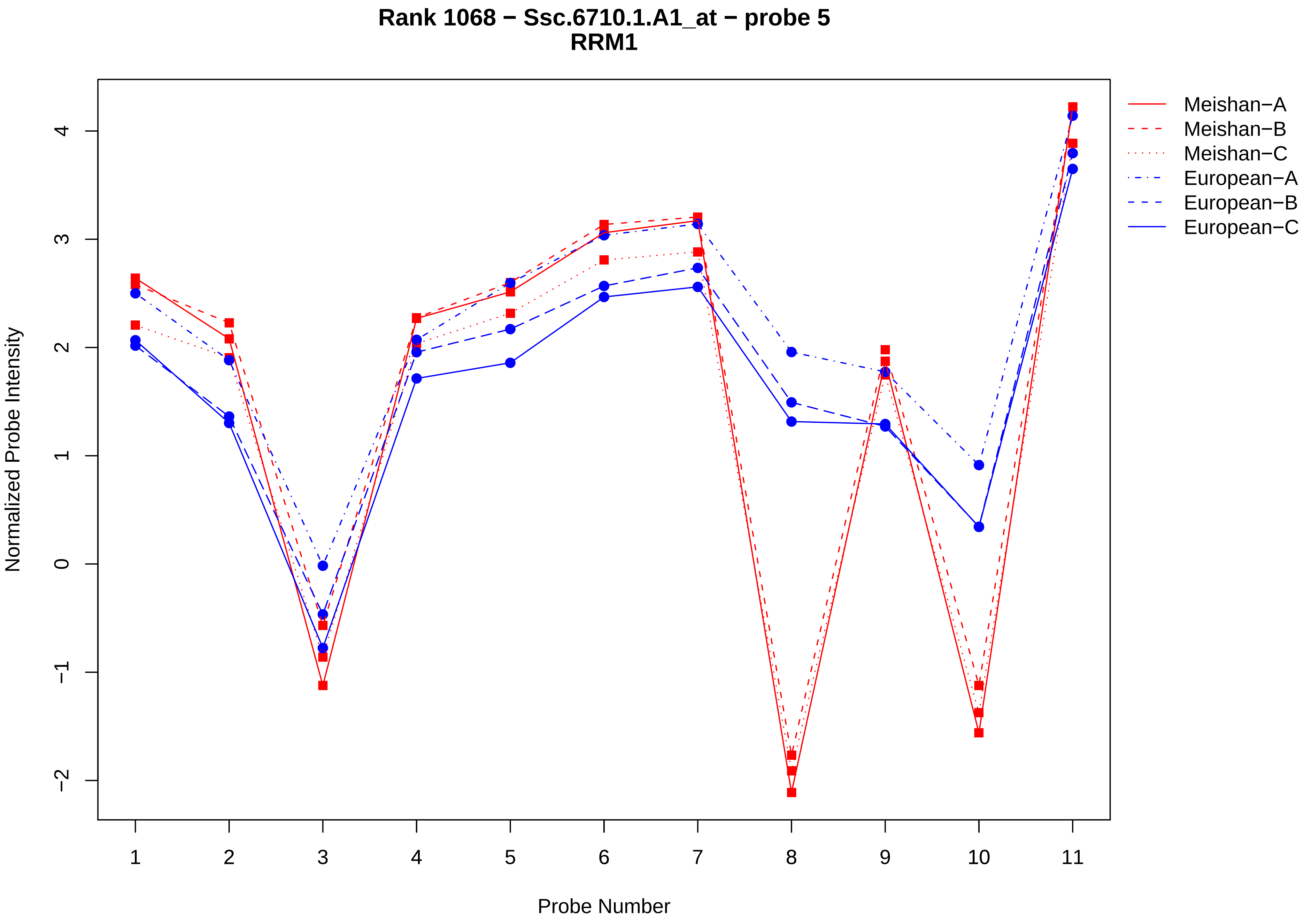

Rank 1068 − Ssc.6710.1.A1_at − probe 5
RRM1
Normalized Probe Intensity
Probe Number
−2
−1
0
1
2
3
4
1
2
3
4
5
6
7
8
9
10
11
Meishan−A
Meishan−B
Meishan−C
European−A
European−B
European−C

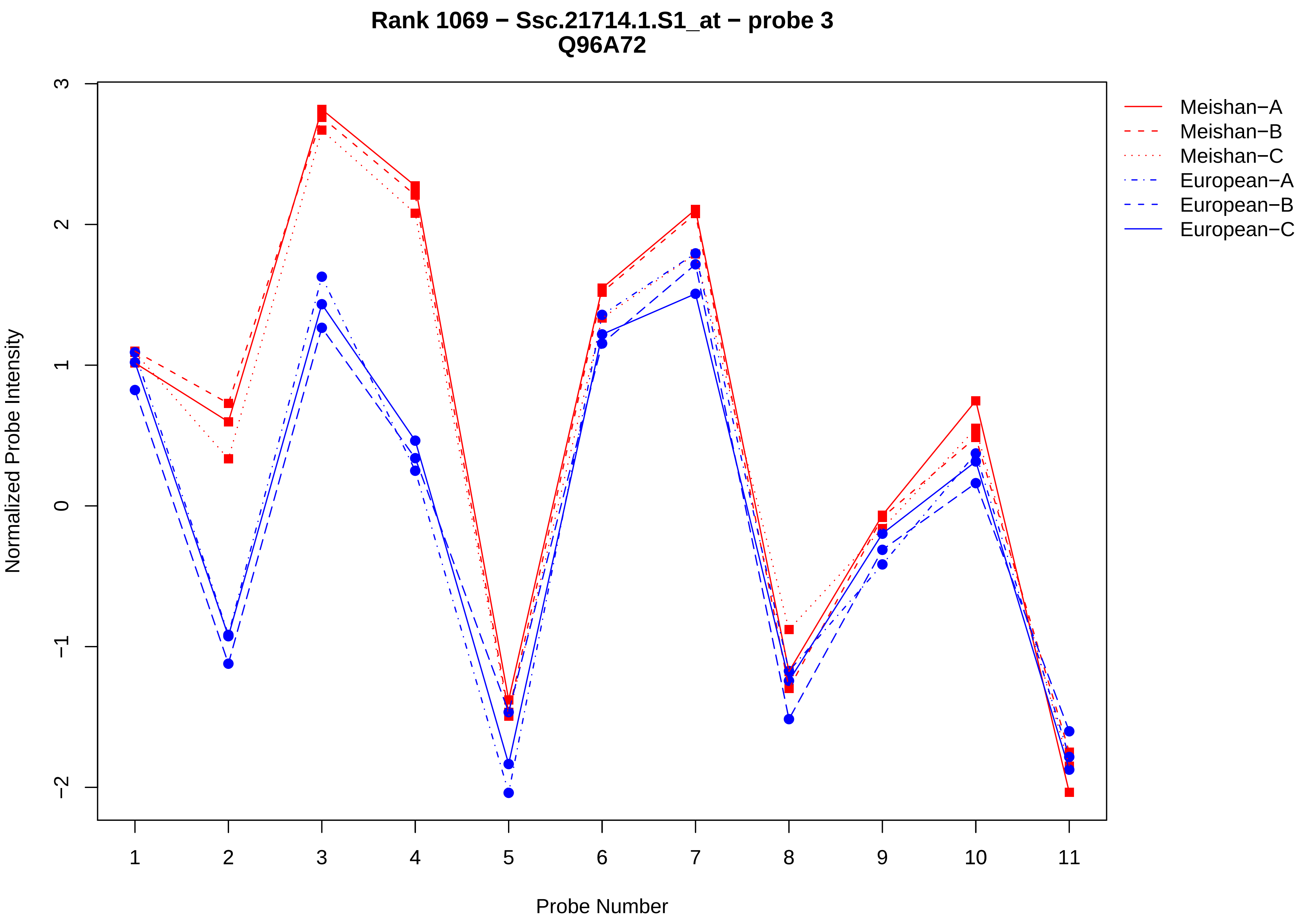
Rank 1069 − Ssc.21714.1.S1_at − probe 3
Q96A72
Normalized Probe Intensity
Probe Number
Meishan−A
Meishan−B
Meishan−C
European−A
European−B
European−C
−2
−1
0
1
2
3
1
2
3
4
5
6
7
8
9
10
11

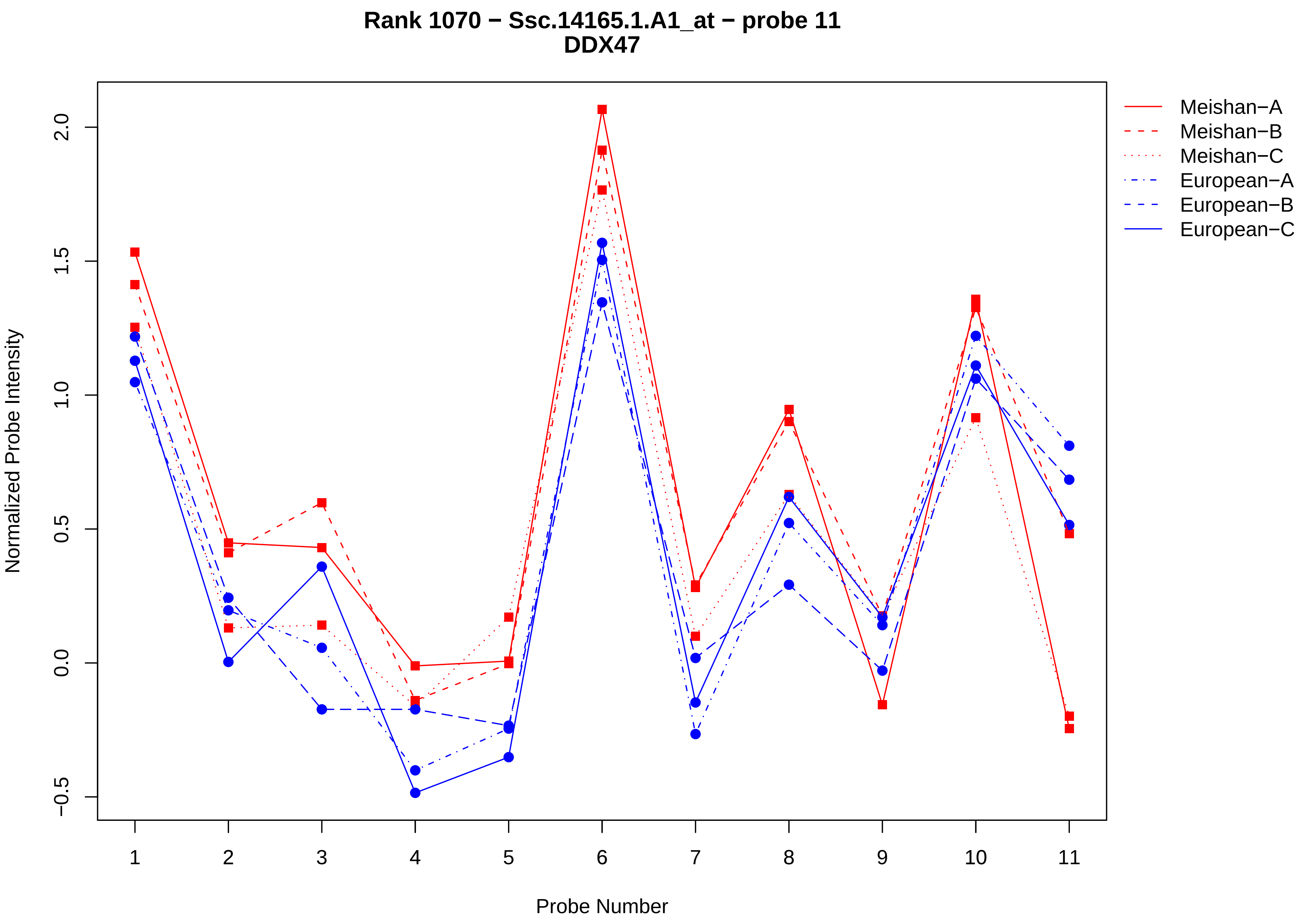

Rank 1070 − Ssc.14165.1.A1_at − probe 11
DDX47
Normalized Probe Intensity
Probe Number
−0.5
0.0
0.5
1.0
1.5
2.0
1
2
3
4
5
6
7
8
9
10
11
Meishan−A
Meishan−B
Meishan−C
European−A
European−B
European−C

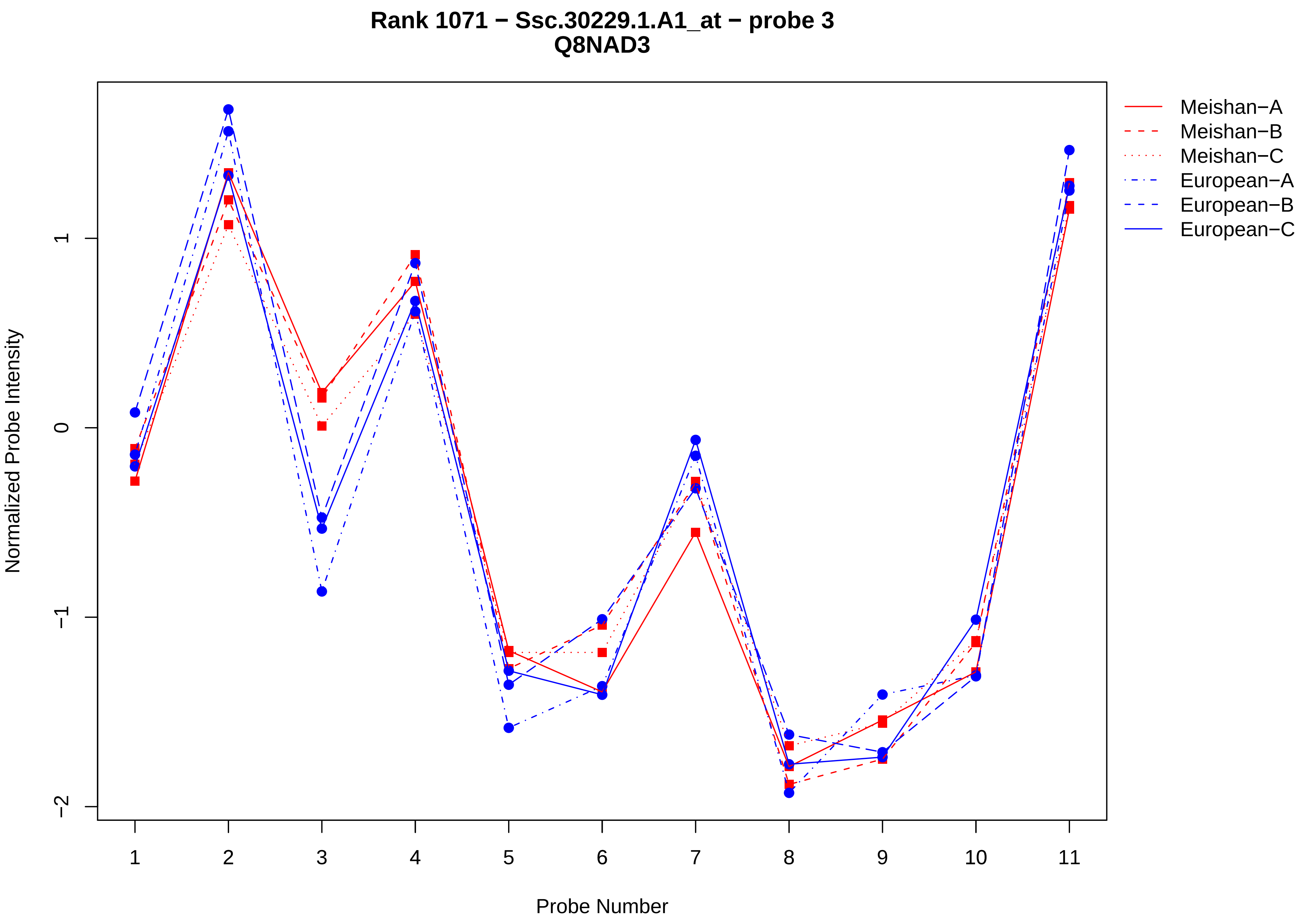

Rank 1071 − Ssc.30229.1.A1_at − probe 3
Q8NAD3
Normalized Probe Intensity
−2
−1
0
1
1
2
3
4
5
6
7
8
9
10
11
Probe Number
Meishan−A
Meishan−B
Meishan−C
European−A
European−B
European−C

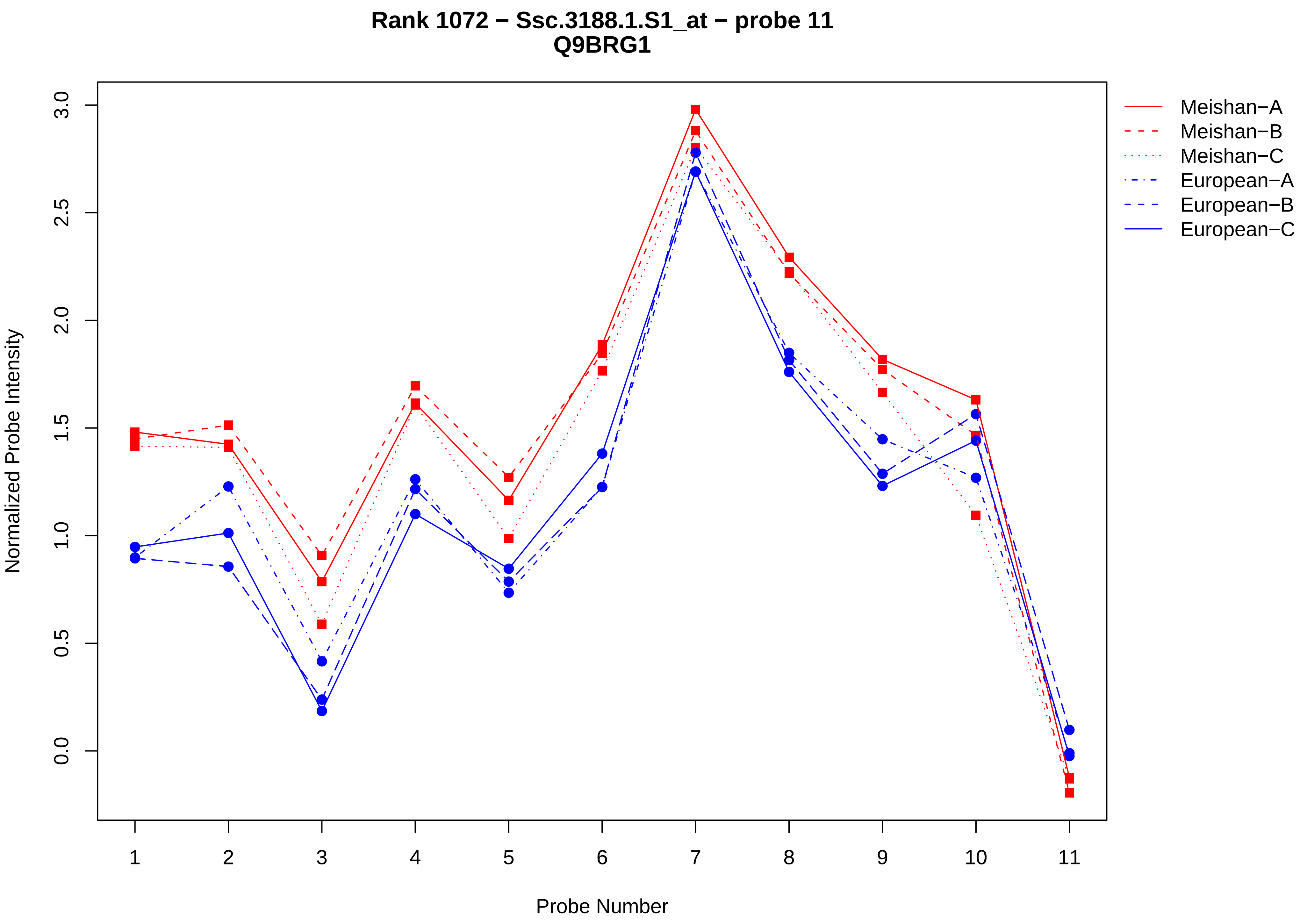

Rank 1072 − Ssc.3188.1.S1_at − probe 11
Q9BRG1
Normalized Probe Intensity
Probe Number
0.0
0.5
1.0
1.5
2.0
2.5
3.0
1
2
3
4
5
6
7
8
9
10
11
Meishan−A
Meishan−B
Meishan−C
European−A
European−B
European−C

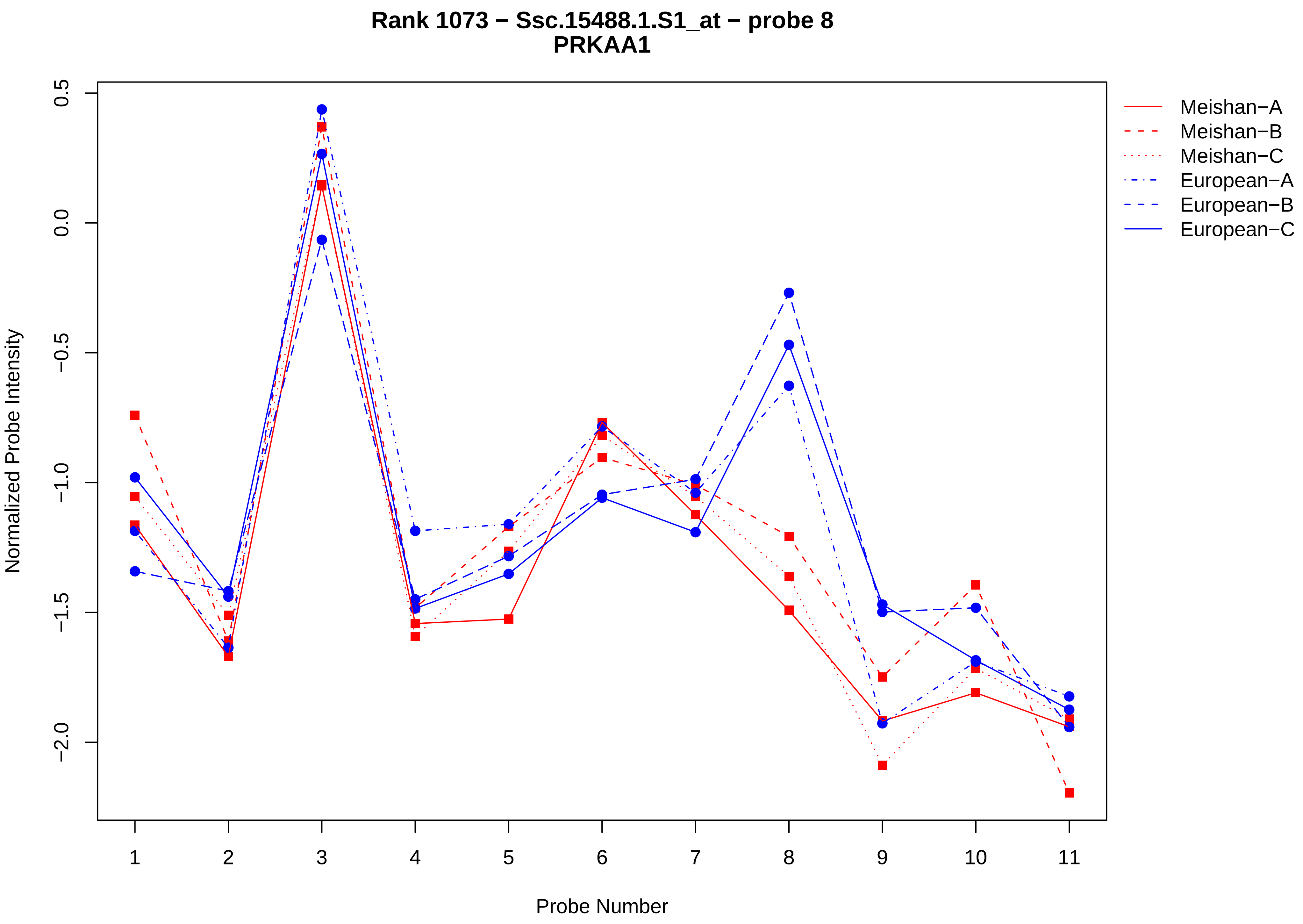

Rank 1073 − Ssc.15488.1.S1_at − probe 8
PRKAA1
Normalized Probe Intensity
Probe Number
0.5
0.0
−0.5
−1.0
−1.5
−2.0
1
2
3
4
5
6
7
8
9
10
11
Meishan−A
Meishan−B
Meishan−C
European−A
European−B
European−C

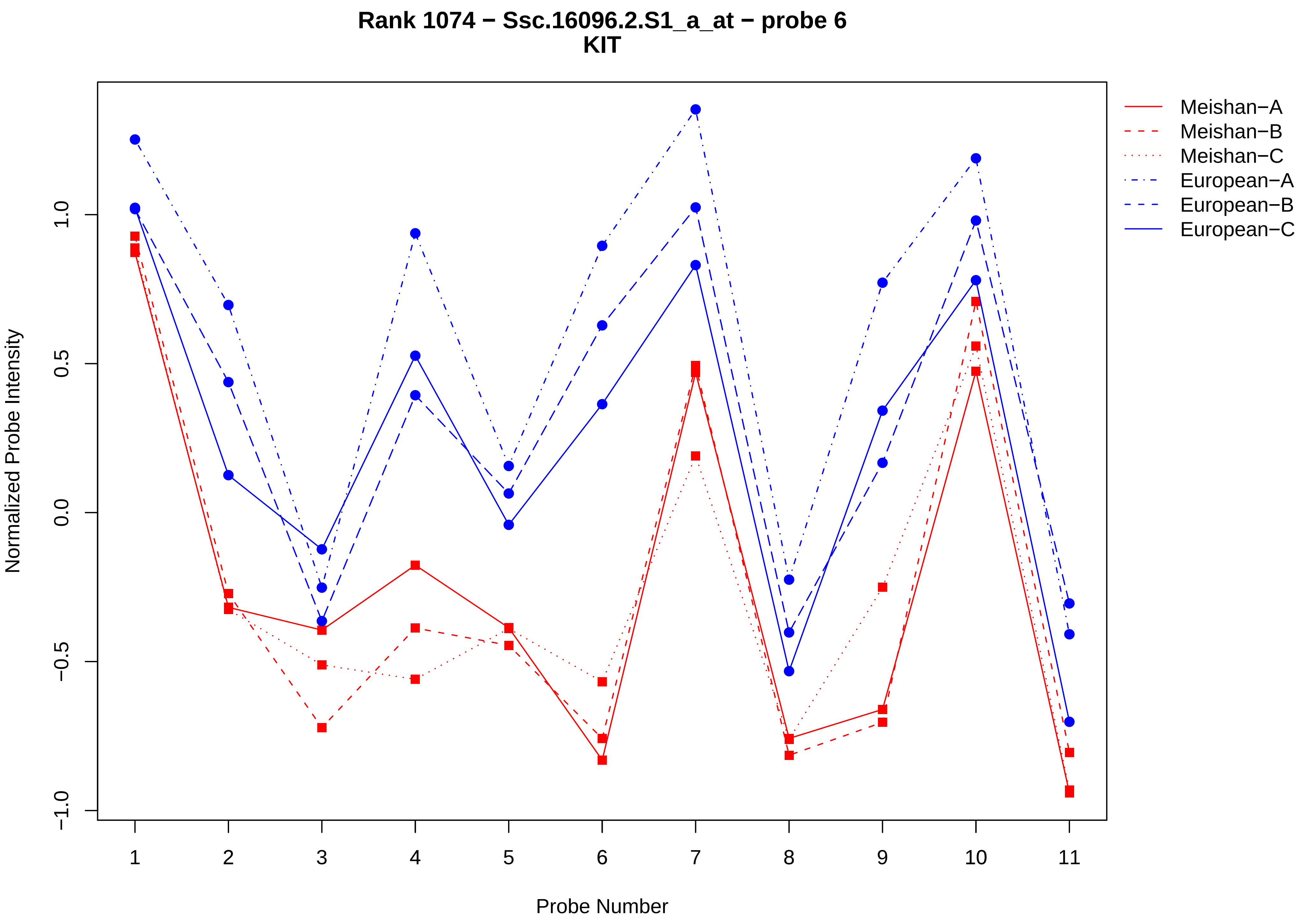

Rank 1074 − Ssc.16096.2.S1_a_at − probe 6
KIT
Normalized Probe Intensity
Probe Number
−1.0
−0.5
0.0
0.5
1.0
1
2
3
4
5
6
7
8
9
10
11
Meishan−A
Meishan−B
Meishan−C
European−A
European−B
European−C

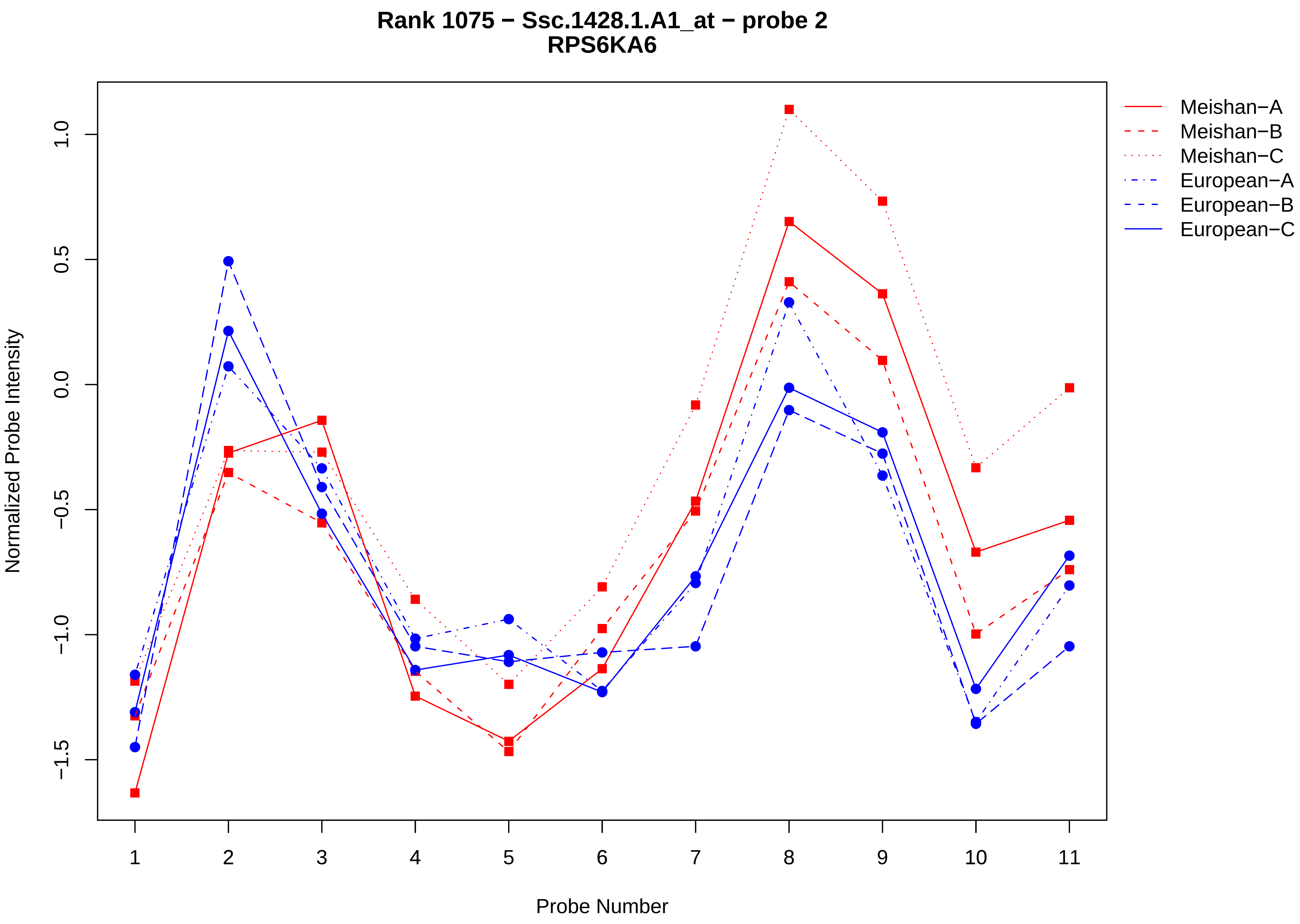

Rank 1075 − Ssc.1428.1.A1_at − probe 2
RPS6KA6
Normalized Probe Intensity
Probe Number
1.0
0.5
0.0
−0.5
−1.0
−1.5
1
2
3
4
5
6
7
8
9
10
11
Meishan−A
Meishan−B
Meishan−C
European−A
European−B
European−C

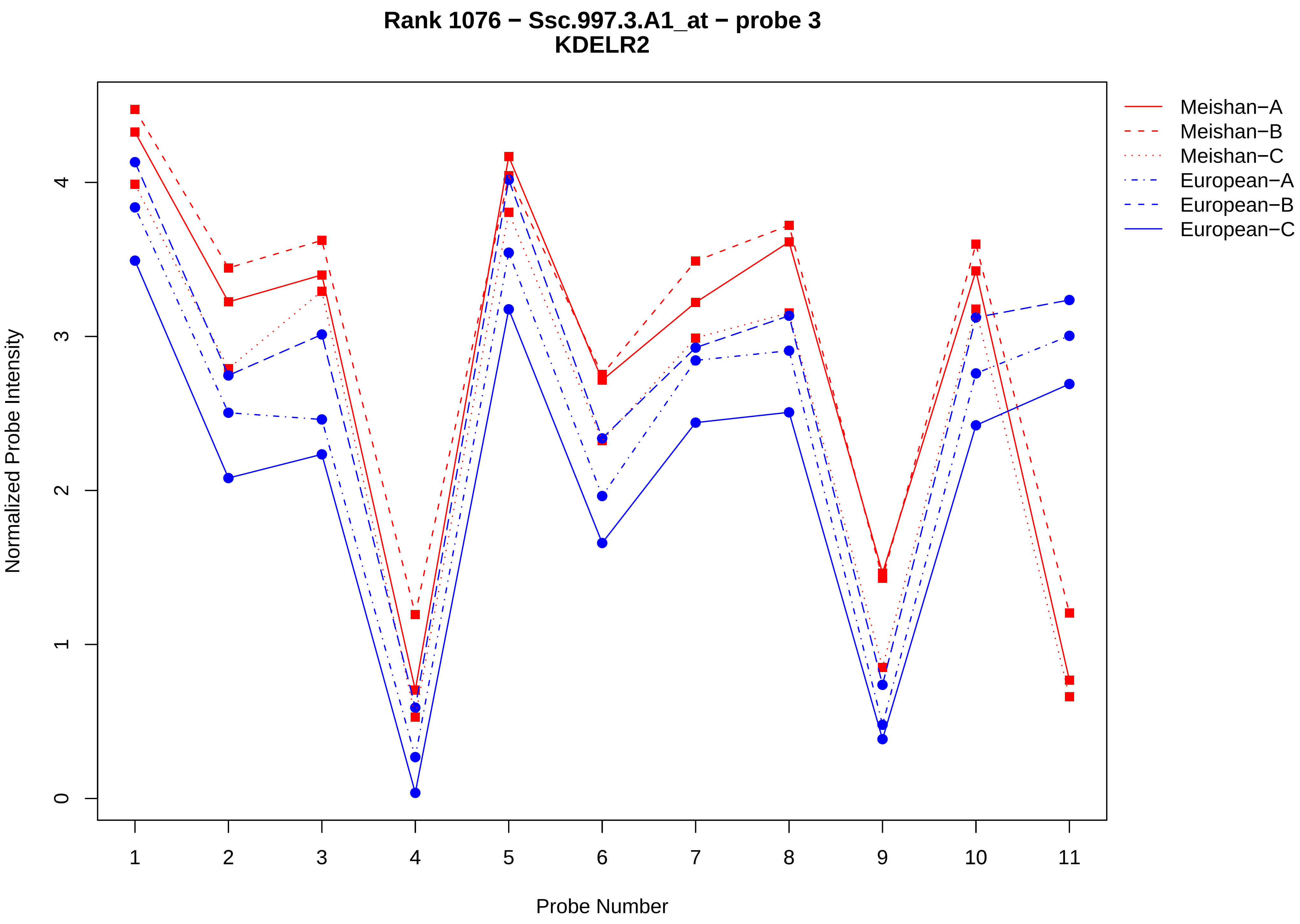

Rank 1076 − Ssc.997.3.A1_at − probe 3
KDELR2
Normalized Probe Intensity
Probe Number
0
1
2
3
4
1
2
3
4
5
6
7
8
9
10
11
Meishan−A
Meishan−B
Meishan−C
European−A
European−B
European−C

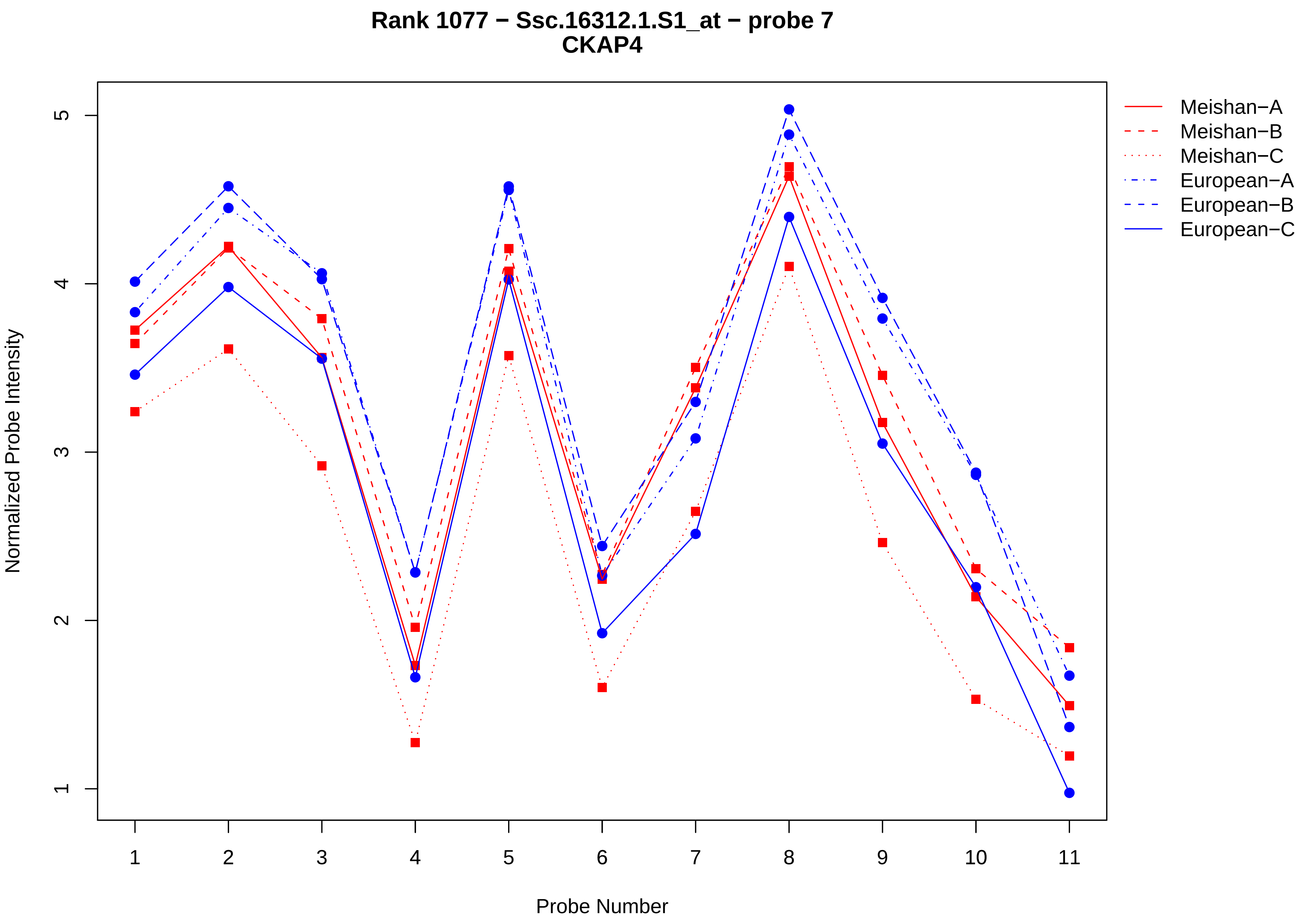

Rank 1077 − Ssc.16312.1.S1_at − probe 7
CKAP4
Normalized Probe Intensity
Probe Number
1
2
3
4
5
1
2
3
4
5
6
7
8
9
10
11
Meishan−A
Meishan−B
Meishan−C
European−A
European−B
European−C

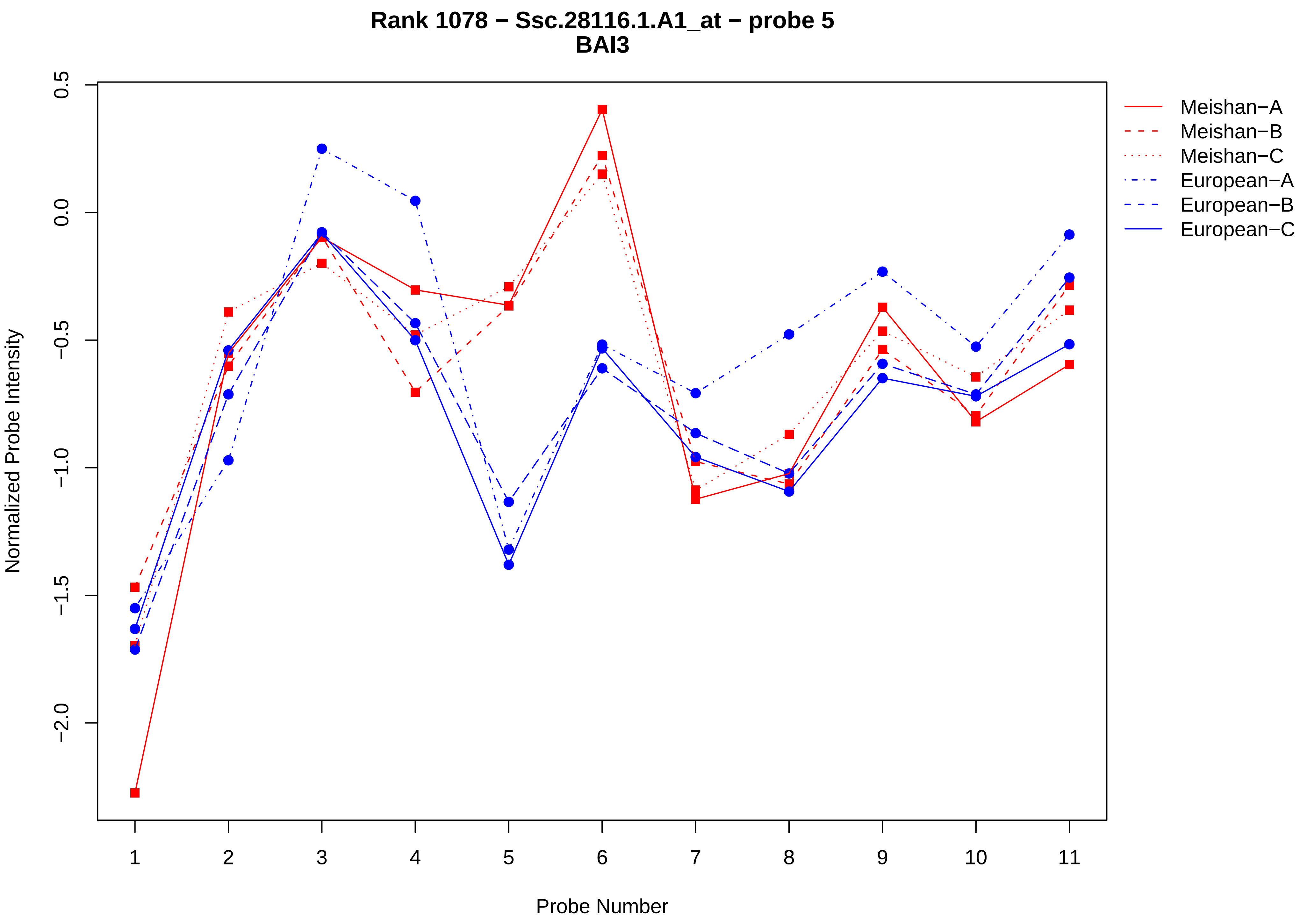
Rank 1078 − Ssc.28116.1.A1_at − probe 5
BAI3
Normalized Probe Intensity
Probe Number
0.5
0.0
−0.5
−1.0
−1.5
−2.0
1
2
3
4
5
6
7
8
9
10
11
Meishan−A
Meishan−B
Meishan−C
European−A
European−B
European−C

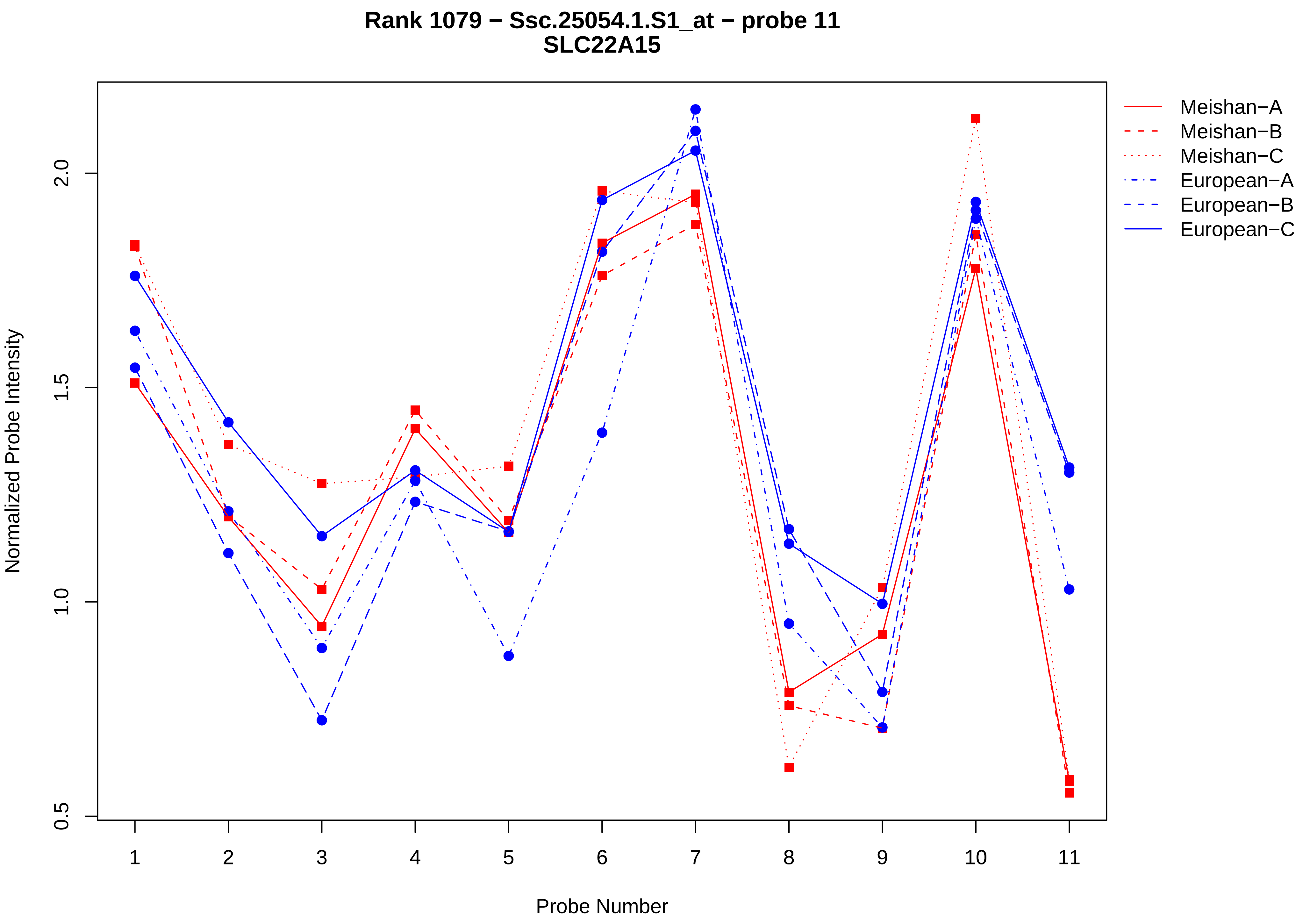
Rank 1079 − Ssc.25054.1.S1_at − probe 11
SLC22A15
Normalized Probe Intensity
Probe Number
0.5
1.0
1.5
2.0
1
2
3
4
5
6
7
8
9
10
11
Meishan−A
Meishan−B
Meishan−C
European−A
European−B
European−C

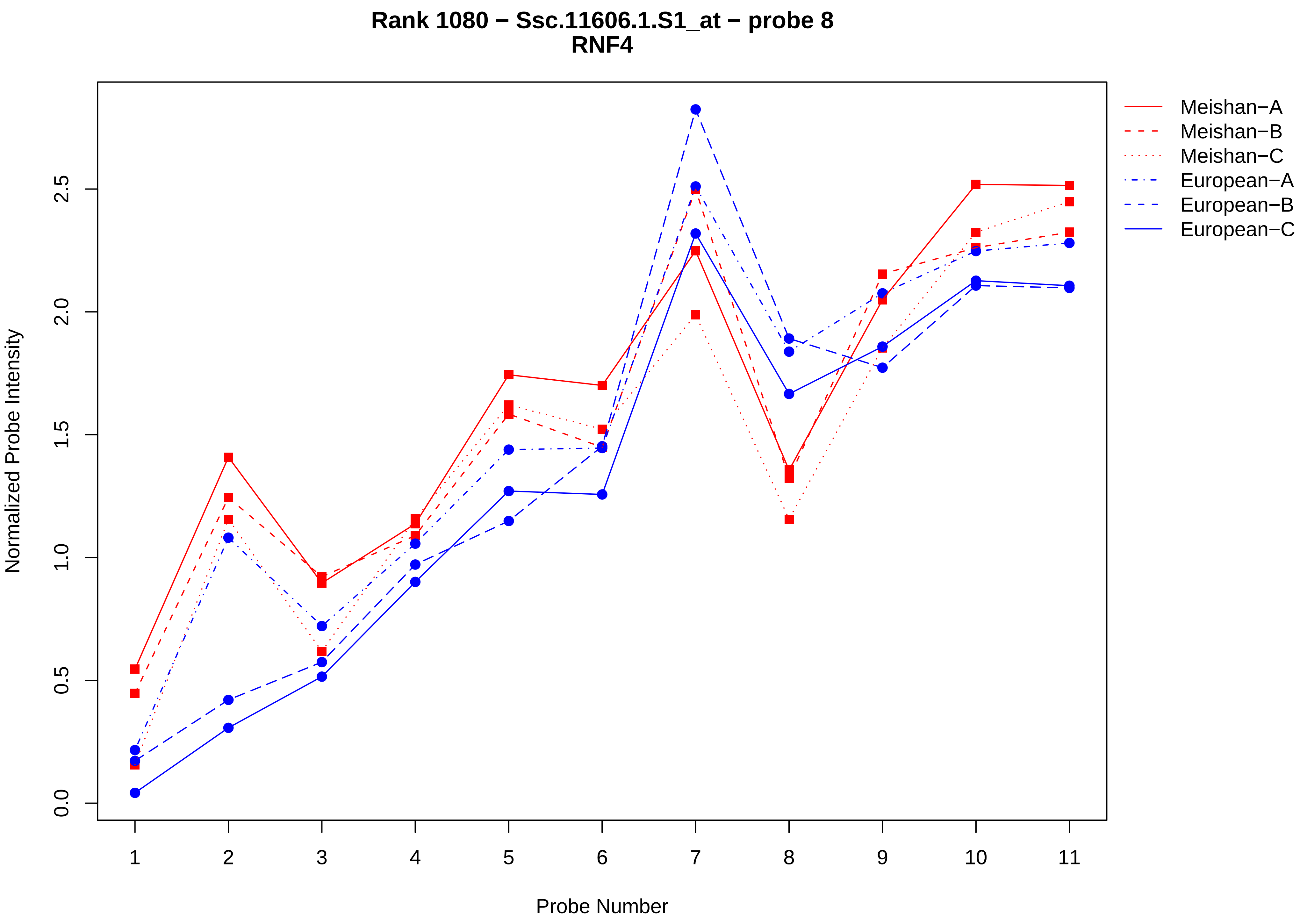

Rank 1080 − Ssc.11606.1.S1_at − probe 8
RNF4
Normalized Probe Intensity
0.0
0.5
1.0
1.5
2.0
2.5
1
2
3
4
5
6
7
8
9
10
11
Probe Number
Meishan−A
Meishan−B
Meishan−C
European−A
European−B
European−C

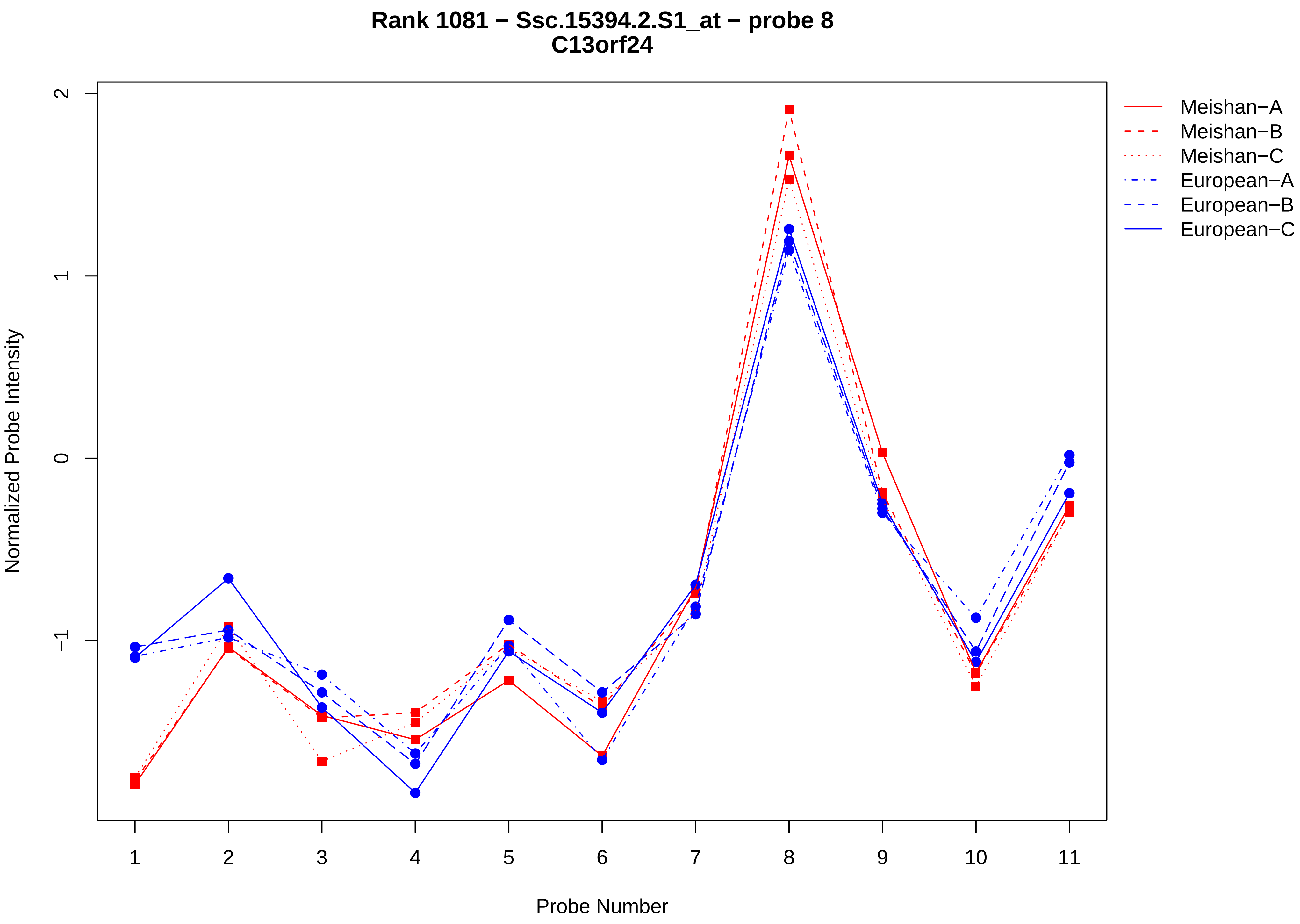

Rank 1081 − Ssc.15394.2.S1_at − probe 8
C13orf24
Normalized Probe Intensity
Probe Number
−1
0
1
2
1
2
3
4
5
6
7
8
9
10
11
Meishan−A
Meishan−B
Meishan−C
European−A
European−B
European−C

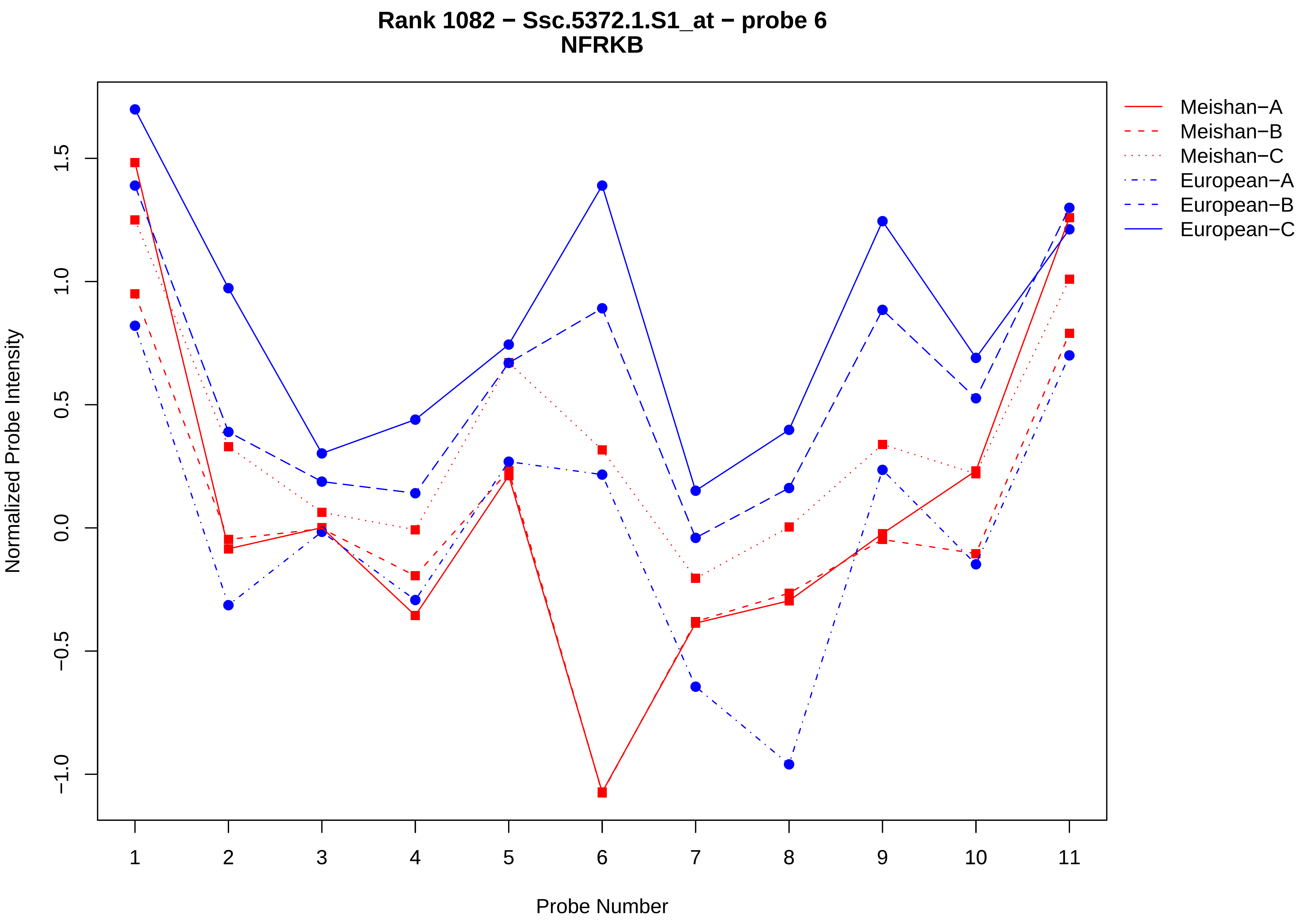

Rank 1082 − Ssc.5372.1.S1_at − probe 6
NFRKB
Normalized Probe Intensity
Probe Number
−1.0
−0.5
0.0
0.5
1.0
1.5
1
2
3
4
5
6
7
8
9
10
11
Meishan−A
Meishan−B
Meishan−C
European−A
European−B
European−C

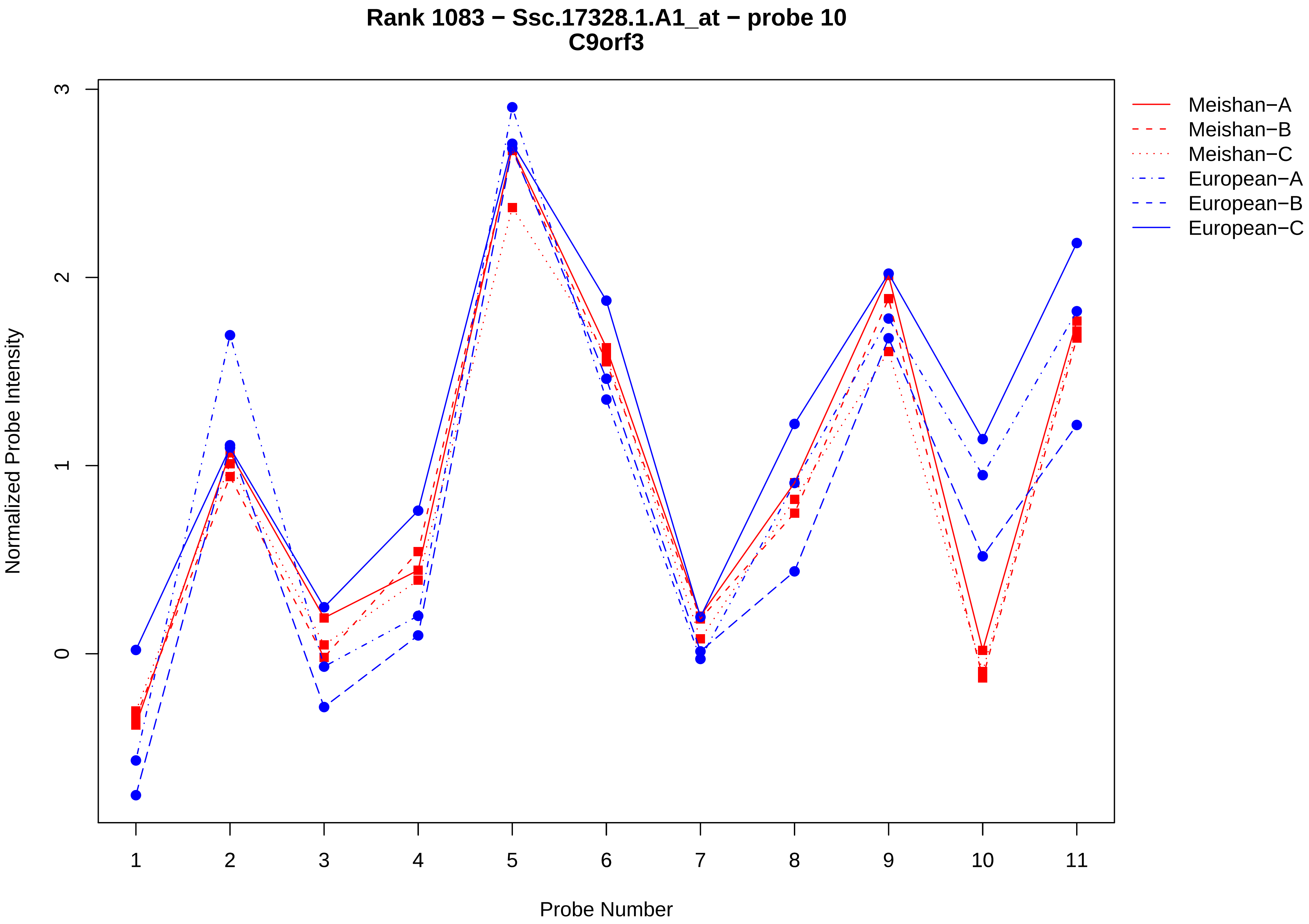

Rank 1083 − Ssc.17328.1.A1_at − probe 10
C9orf3
Normalized Probe Intensity
Probe Number
0
1
2
3
1
2
3
4
5
6
7
8
9
10
11
Meishan−A
Meishan−B
Meishan−C
European−A
European−B
European−C

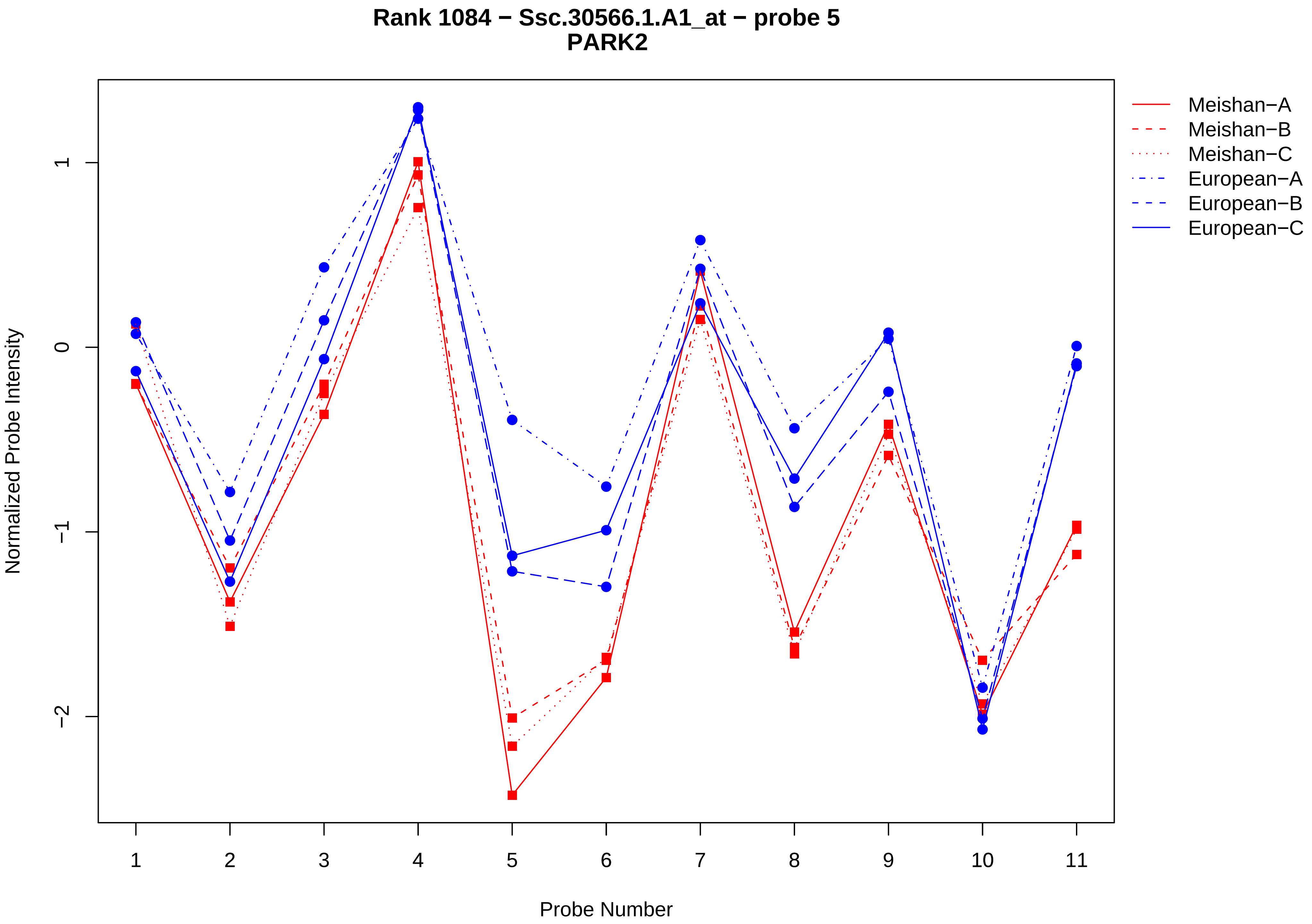

Rank 1084 − Ssc.30566.1.A1_at − probe 5
PARK2
Normalized Probe Intensity
Probe Number
1
0
−1
−2
1
2
3
4
5
6
7
8
9
10
11
Meishan−A
Meishan−B
Meishan−C
European−A
European−B
European−C

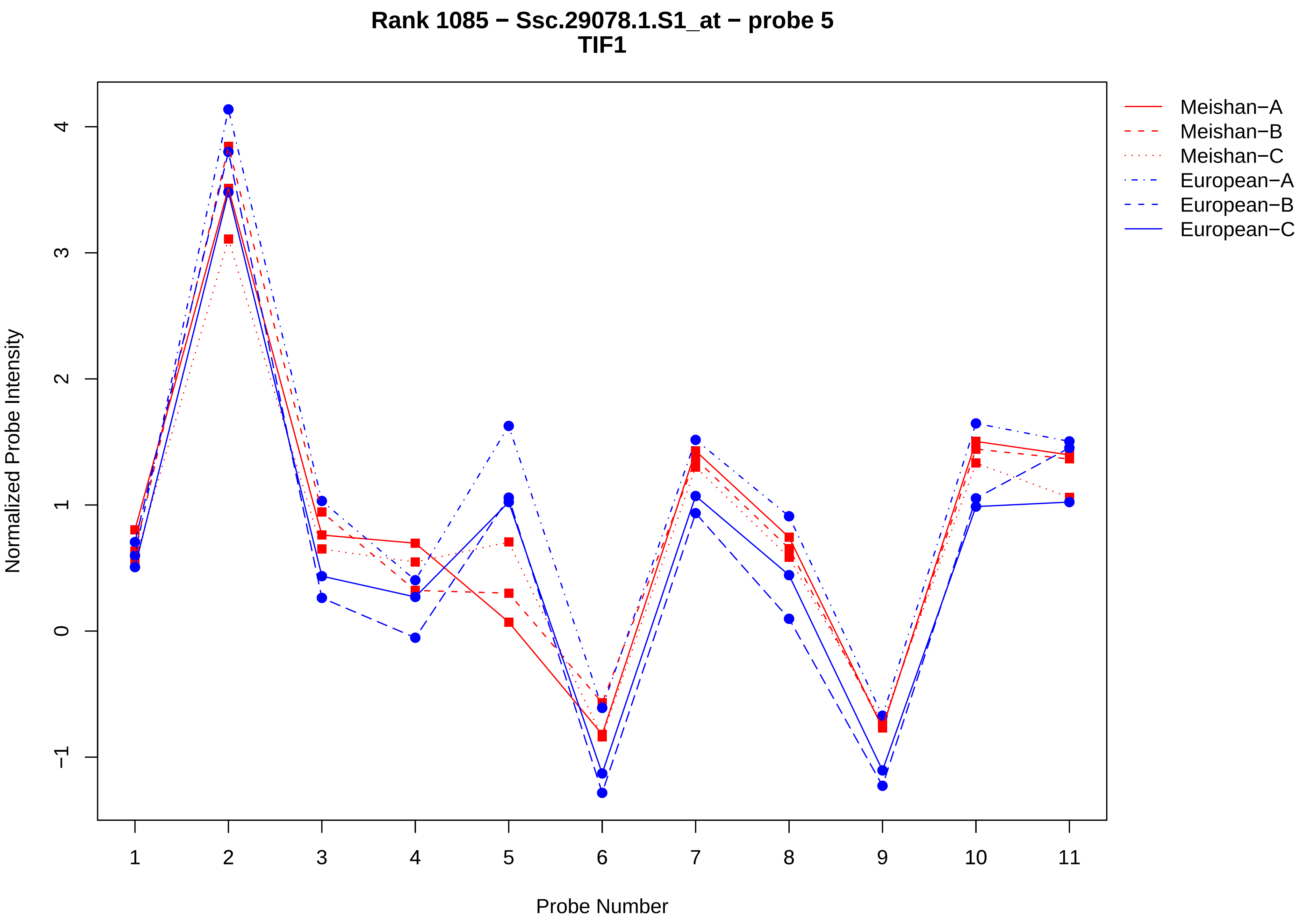

Rank 1085 − Ssc.29078.1.S1_at − probe 5
TIF1
Normalized Probe Intensity
Probe Number
−1
0
1
2
3
4
1
2
3
4
5
6
7
8
9
10
11
Meishan−A
Meishan−B
Meishan−C
European−A
European−B
European−C

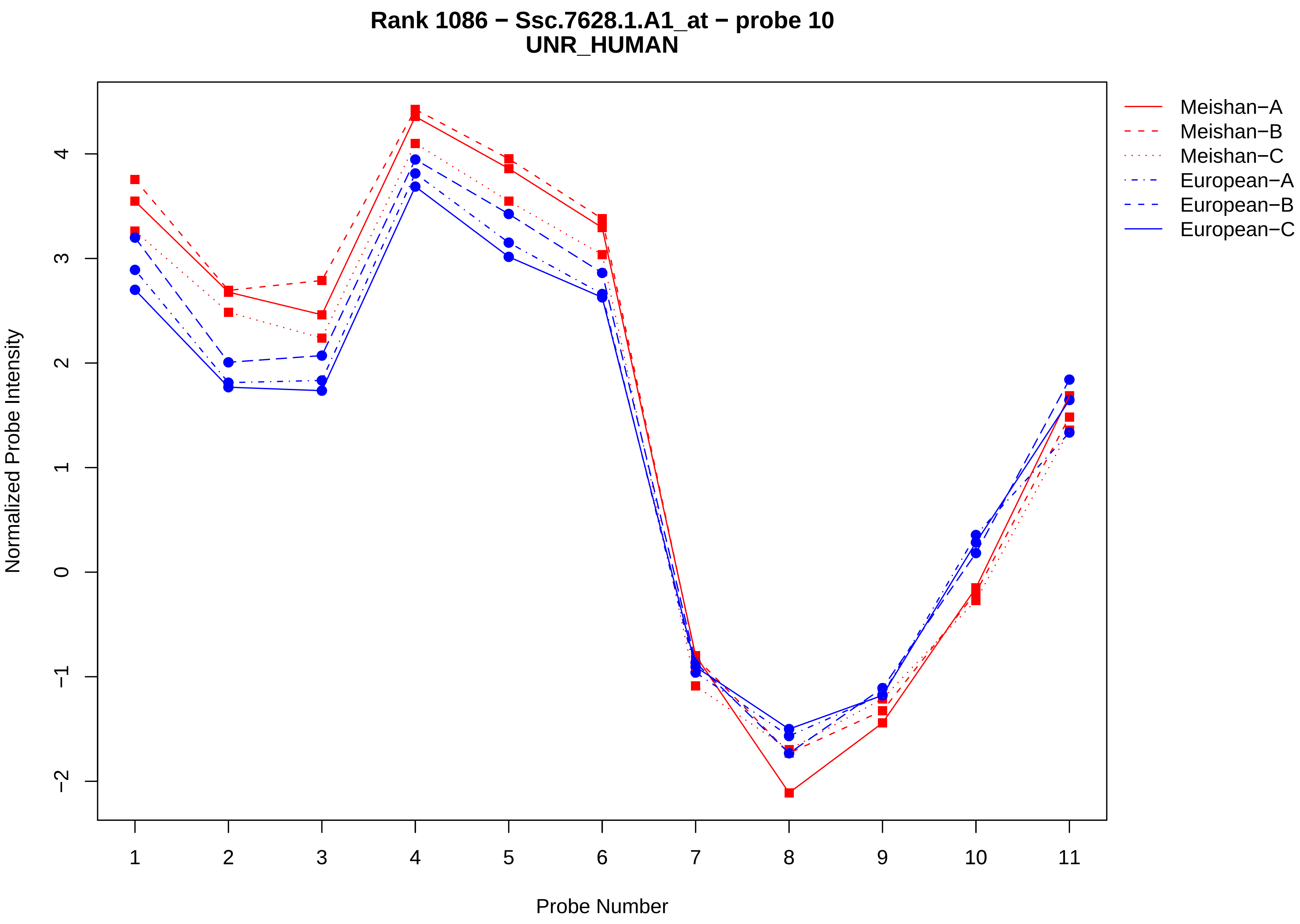

Rank 1086 − Ssc.7628.1.A1_at − probe 10
UNR_HUMAN
Normalized Probe Intensity
Probe Number
Meishan−A
Meishan−B
Meishan−C
European−A
European−B
European−C
−2
−1
0
1
2
3
4
1
2
3
4
5
6
7
8
9
10
11

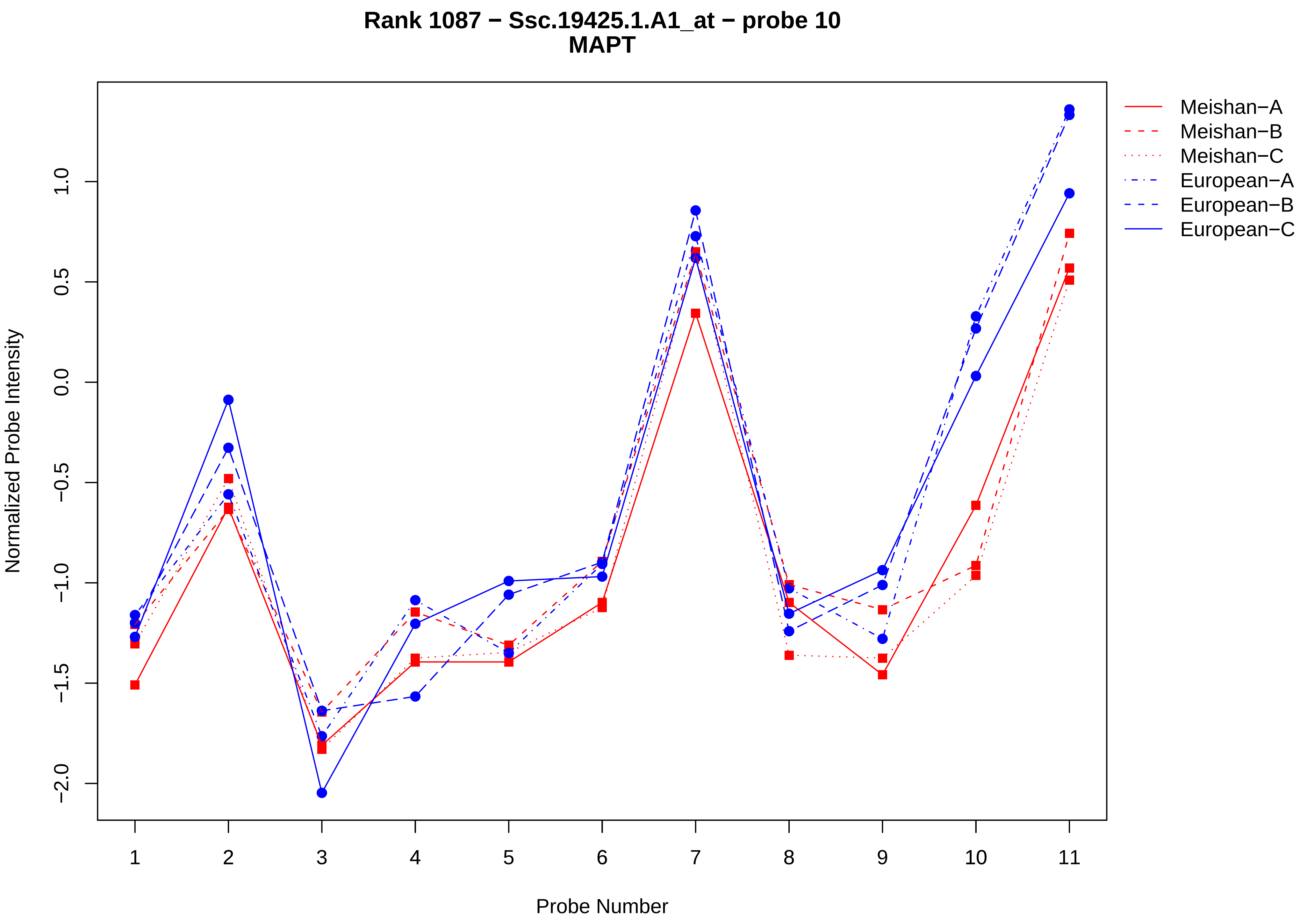
Rank 1087 − Ssc.19425.1.A1_at − probe 10
MAPT
Normalized Probe Intensity
Probe Number
Meishan−A
Meishan−B
Meishan−C
European−A
European−B
European−C
−2.0
−1.5
−1.0
−0.5
0.0
0.5
1.0
1
2
3
4
5
6
7
8
9
10
11

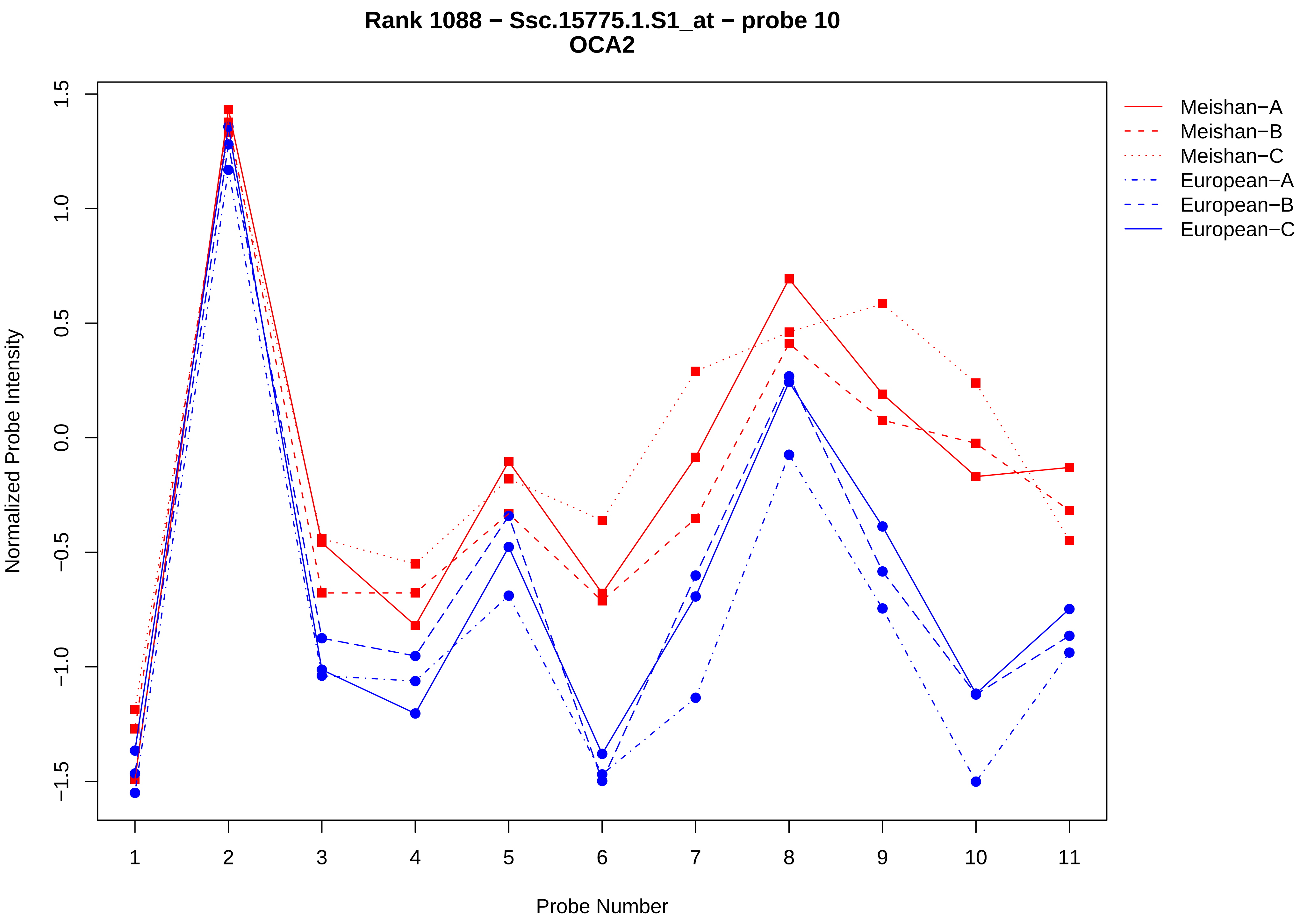

Rank 1088 − Ssc.15775.1.S1_at − probe 10
OCA2
Normalized Probe Intensity
Probe Number
Meishan−A
Meishan−B
Meishan−C
European−A
European−B
European−C

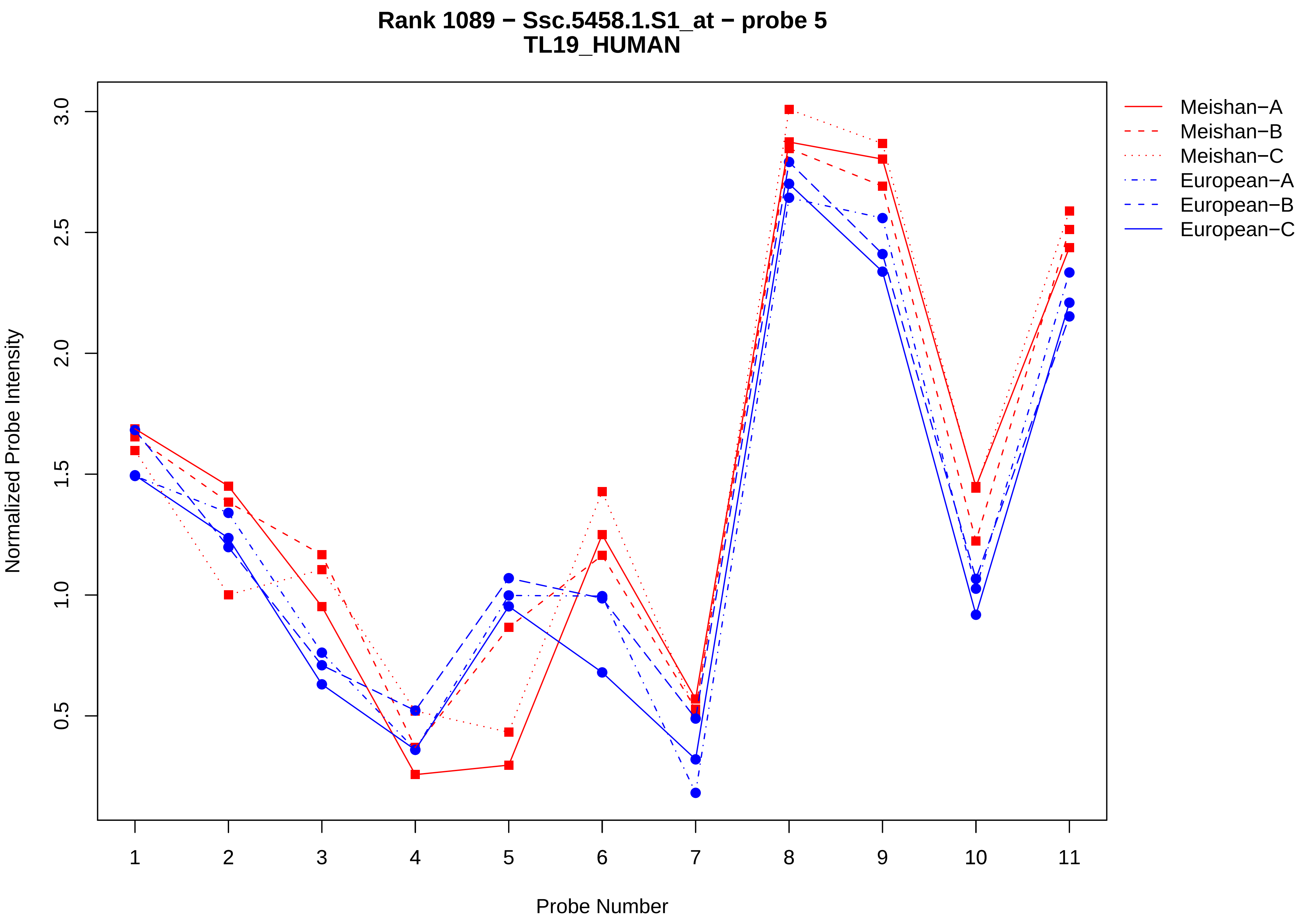
Rank 1089 − Ssc.5458.1.S1_at − probe 5
TL19_HUMAN
Normalized Probe Intensity
0.5
1.0
1.5
2.0
2.5
3.0
Probe Number
1
2
3
4
5
6
7
8
9
10
11
Meishan−A
Meishan−B
Meishan−C
European−A
European−B
European−C

Rank 1090 − Ssc.6848.1.A1_at − probe 5
FKBP14

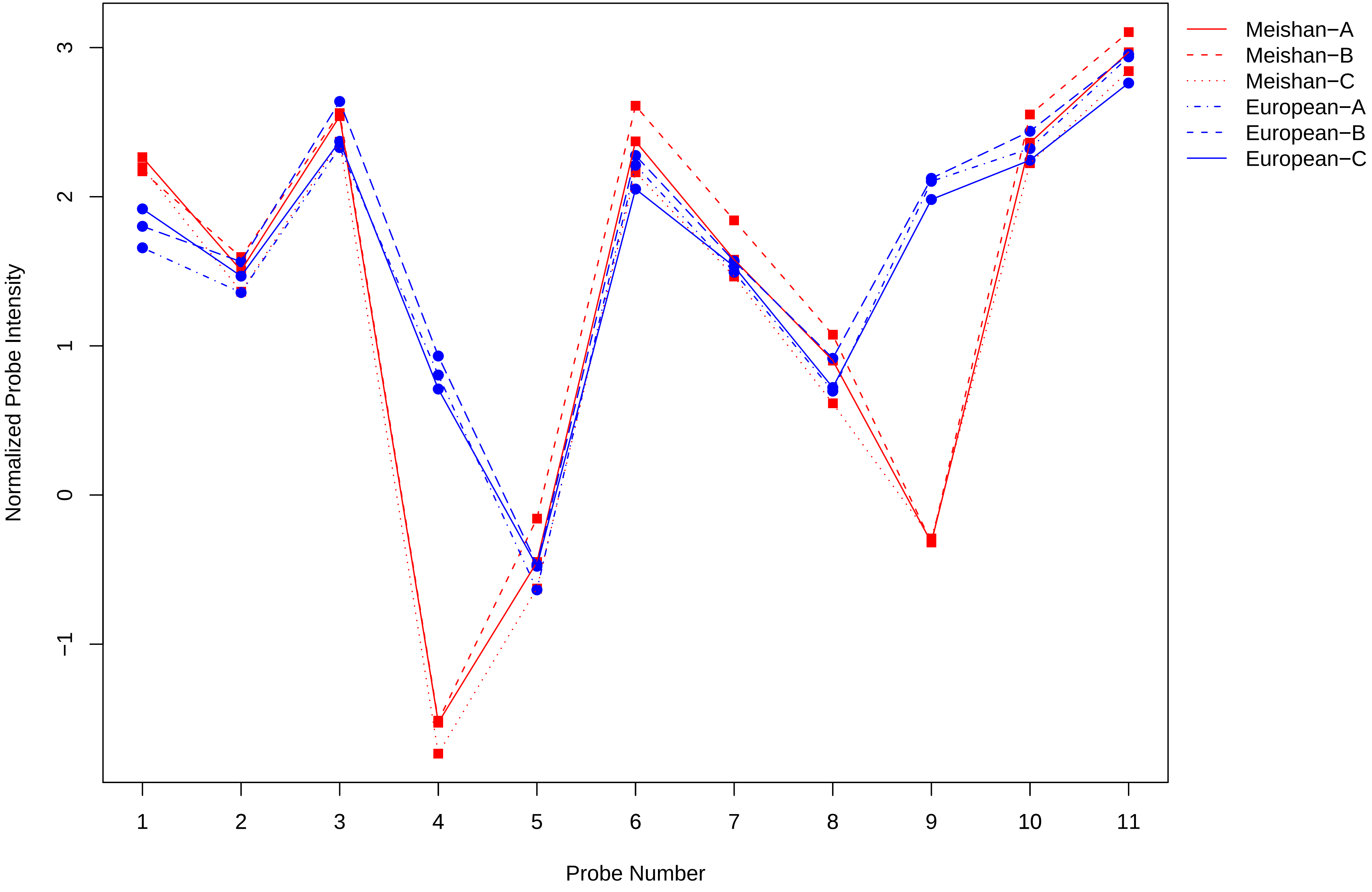

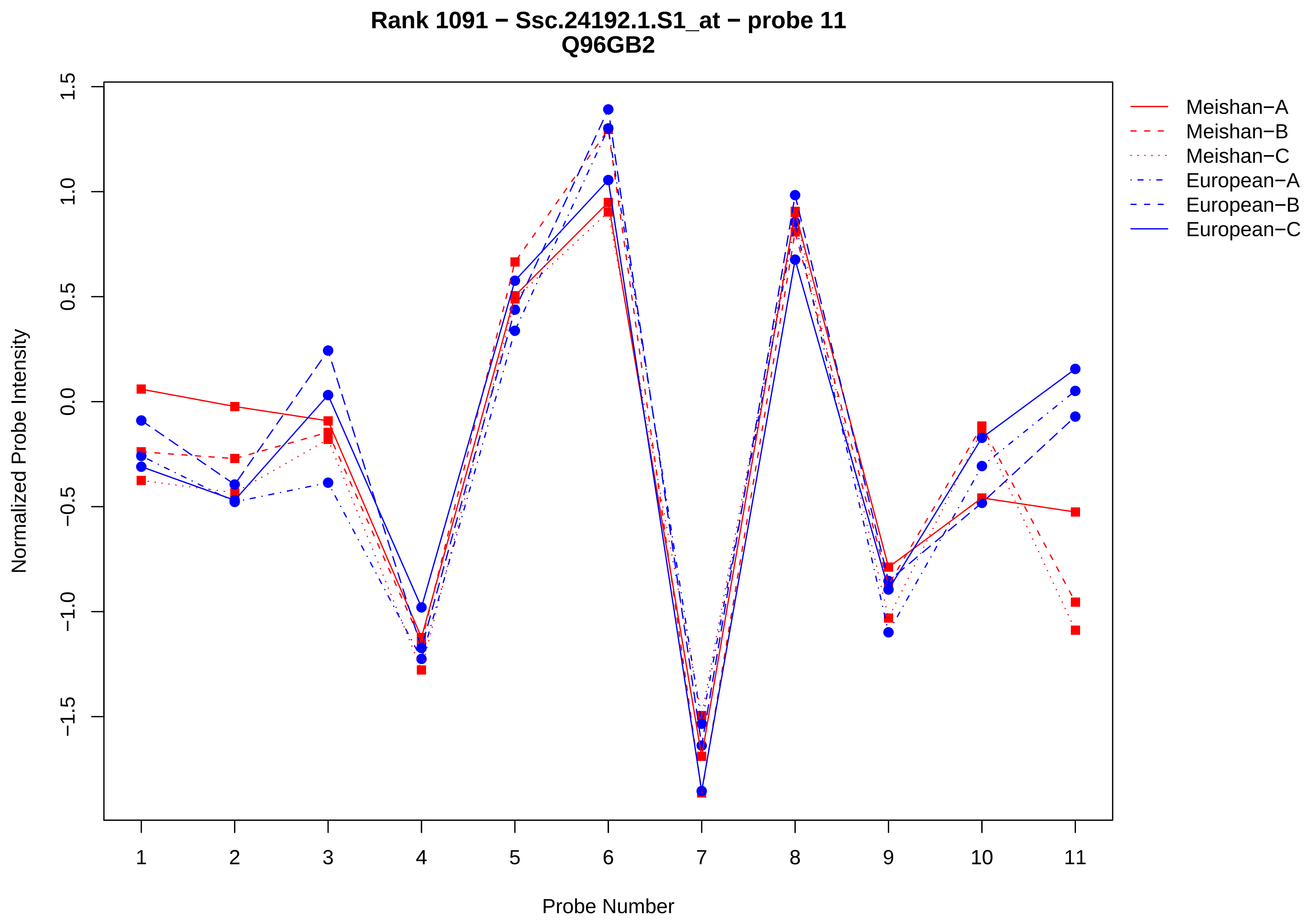

Rank 1091 − Ssc.24192.1.S1_at − probe 11
Q96GB2
Normalized Probe Intensity
Probe Number
1.5
1.0
0.5
0.0
−0.5
−1.0
−1.5
1
2
3
4
5
6
7
8
9
10
11
Meishan−A
Meishan−B
Meishan−C
European−A
European−B
European−C

Rank 1092 − Ssc.2230.2.S1_at − probe 8
C10orf117

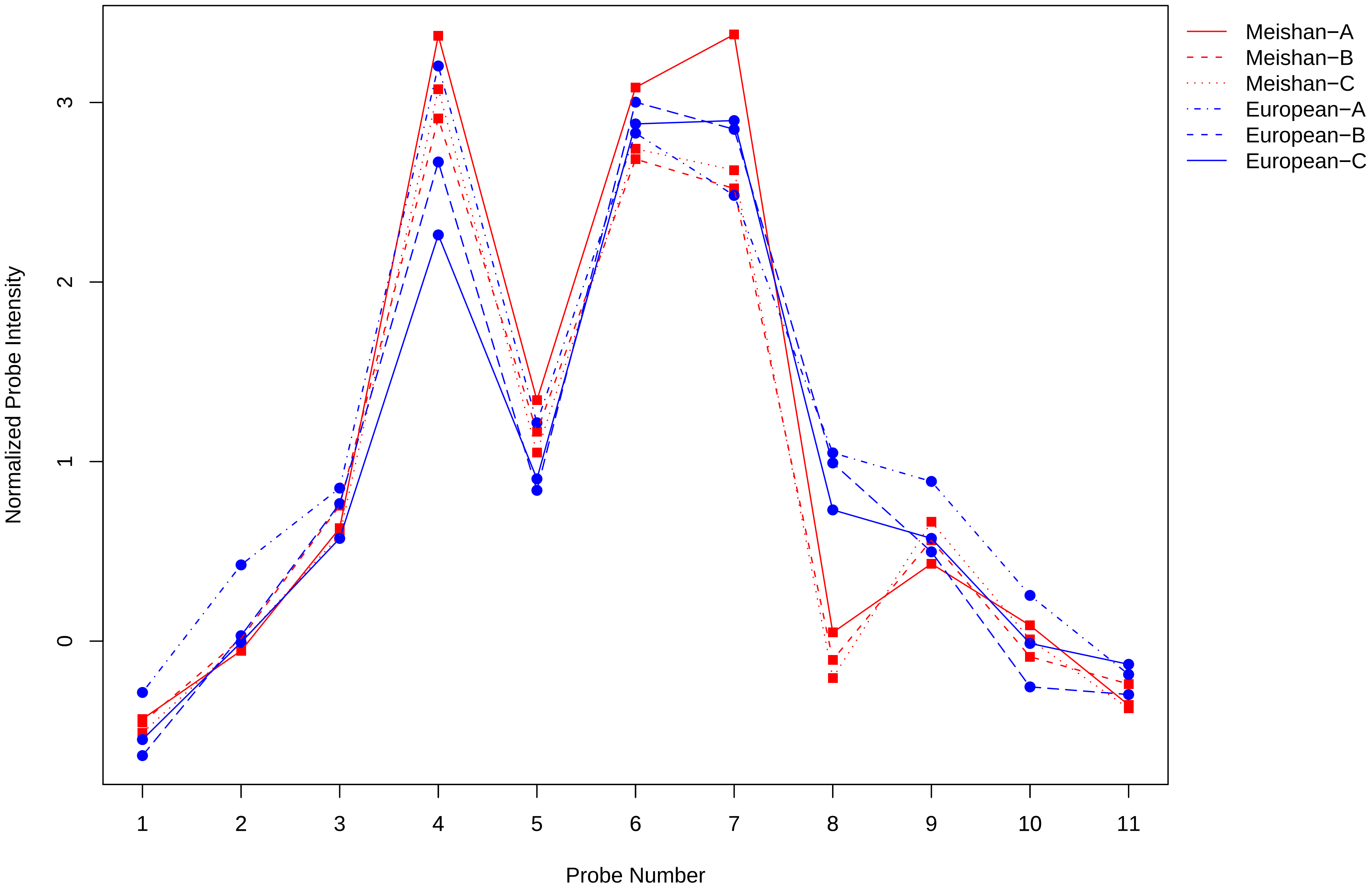

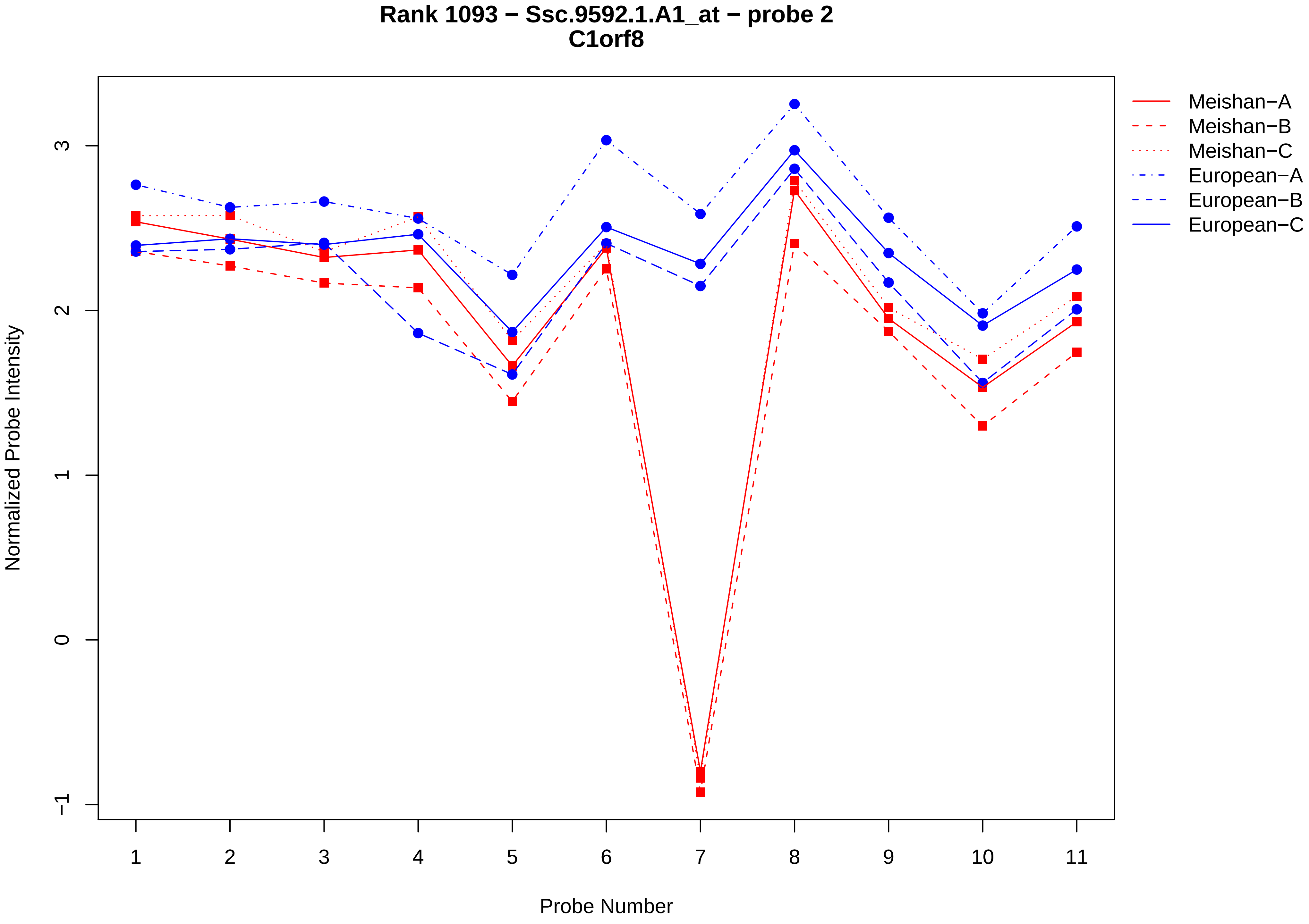

Rank 1093 − Ssc.9592.1.A1_at − probe 2
C1orf8
Normalized Probe Intensity
Probe Number
−1
0
1
2
3
1
2
3
4
5
6
7
8
9
10
11
Meishan−A
Meishan−B
Meishan−C
European−A
European−B
European−C

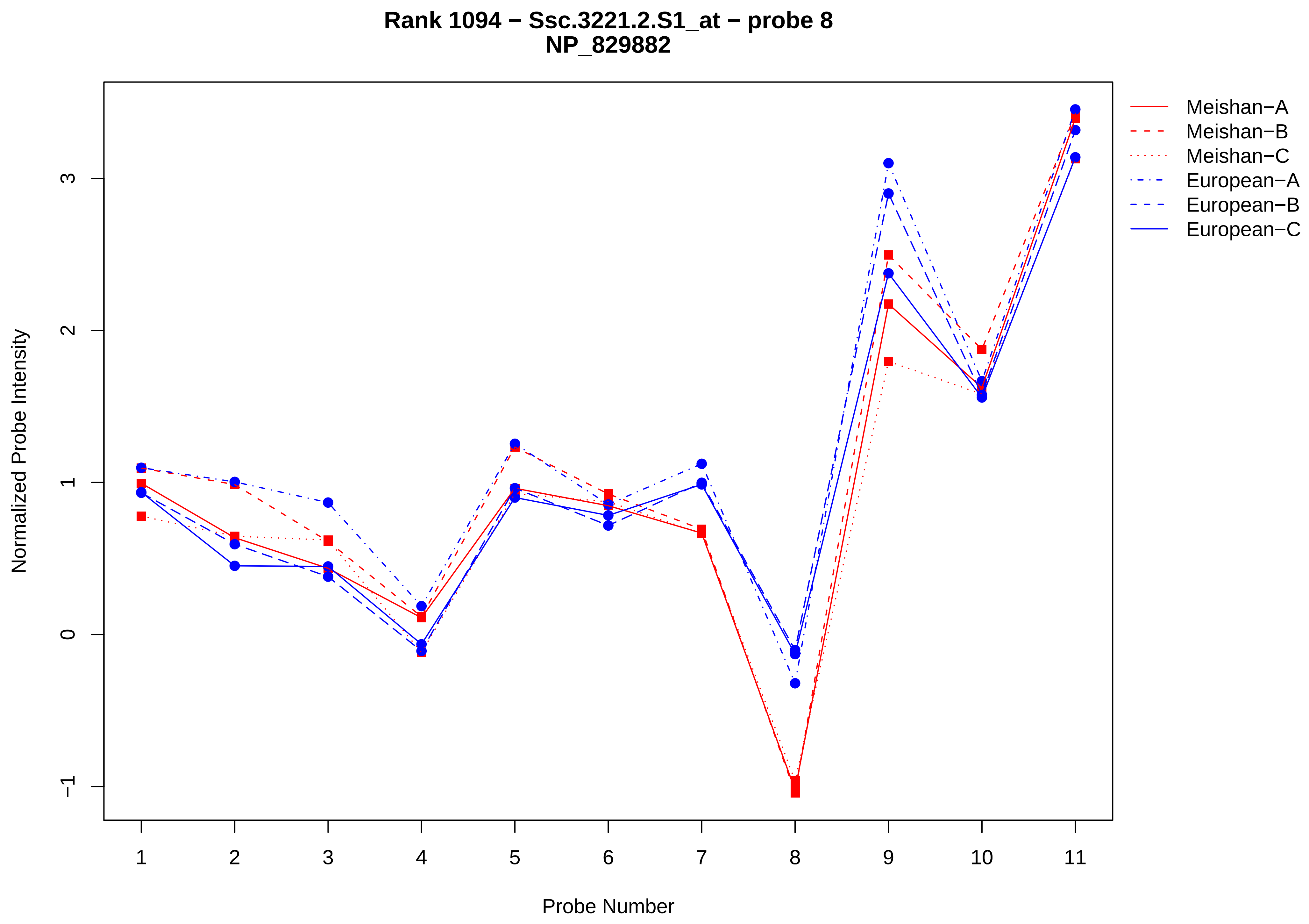

Rank 1094 − Ssc.3221.2.S1_at − probe 8
NP_829882
Normalized Probe Intensity
Probe Number
Meishan−A
Meishan−B
Meishan−C
European−A
European−B
European−C

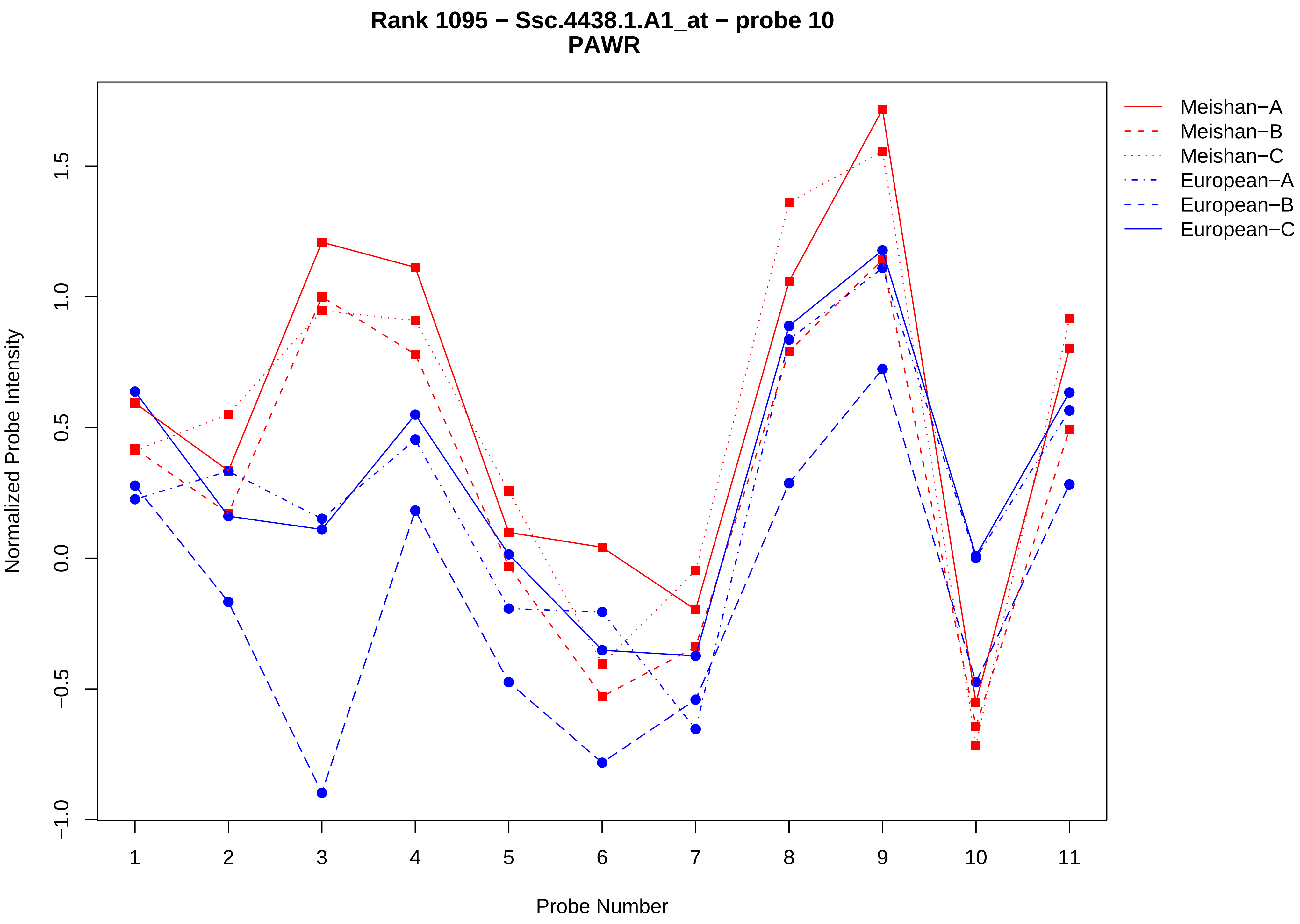

Rank 1095 − Ssc.4438.1.A1_at − probe 10
PAWR
Normalized Probe Intensity
Probe Number
−1.0
−0.5
0.0
0.5
1.0
1.5
1
2
3
4
5
6
7
8
9
10
11
Meishan−A
Meishan−B
Meishan−C
European−A
European−B
European−C

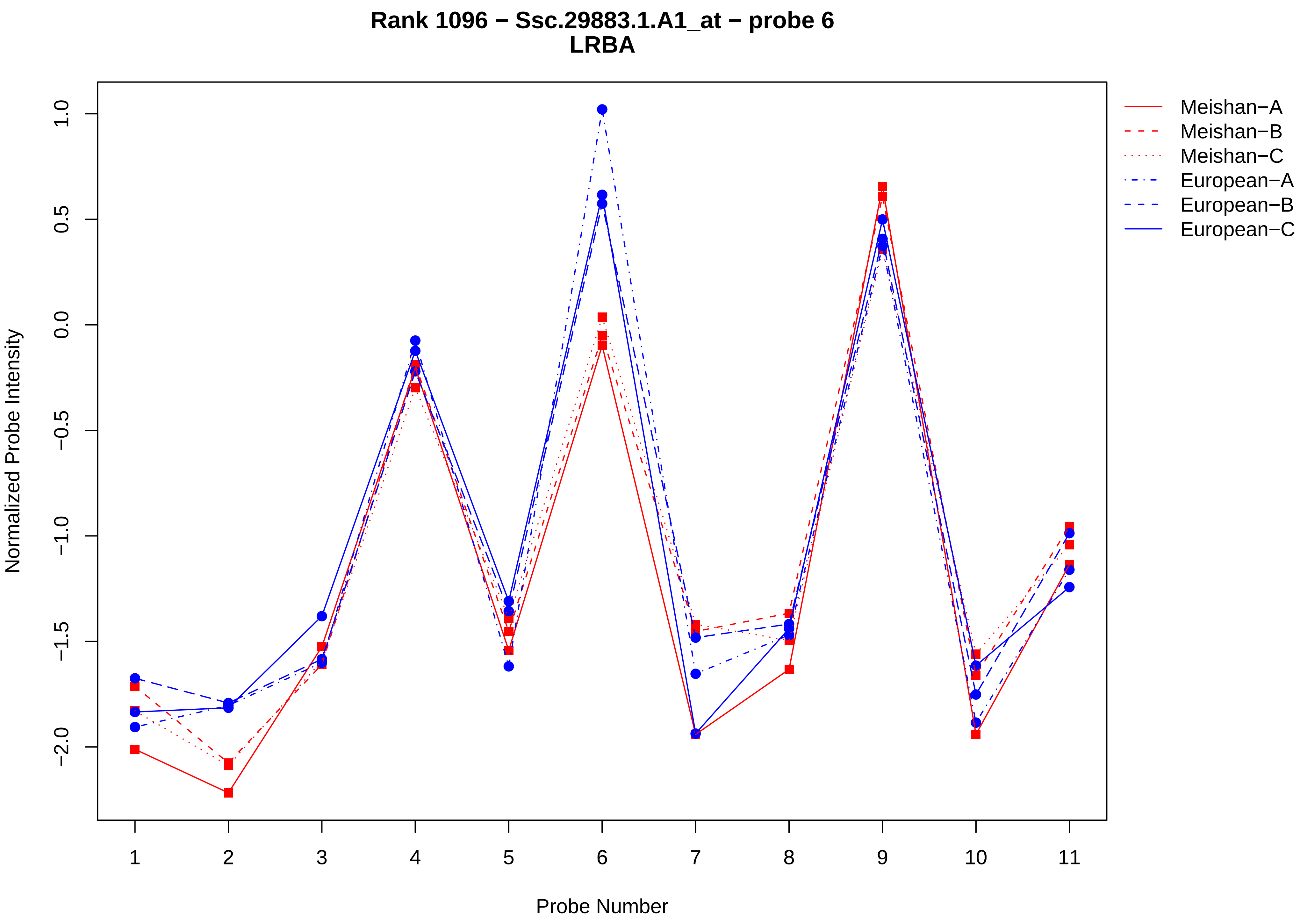
Rank 1096 − Ssc.29883.1.A1_at − probe 6
LRBA
Normalized Probe Intensity
Probe Number
1.0
0.5
0.0
−0.5
−1.0
−1.5
−2.0
1
2
3
4
5
6
7
8
9
10
11
Meishan−A
Meishan−B
Meishan−C
European−A
European−B
European−C

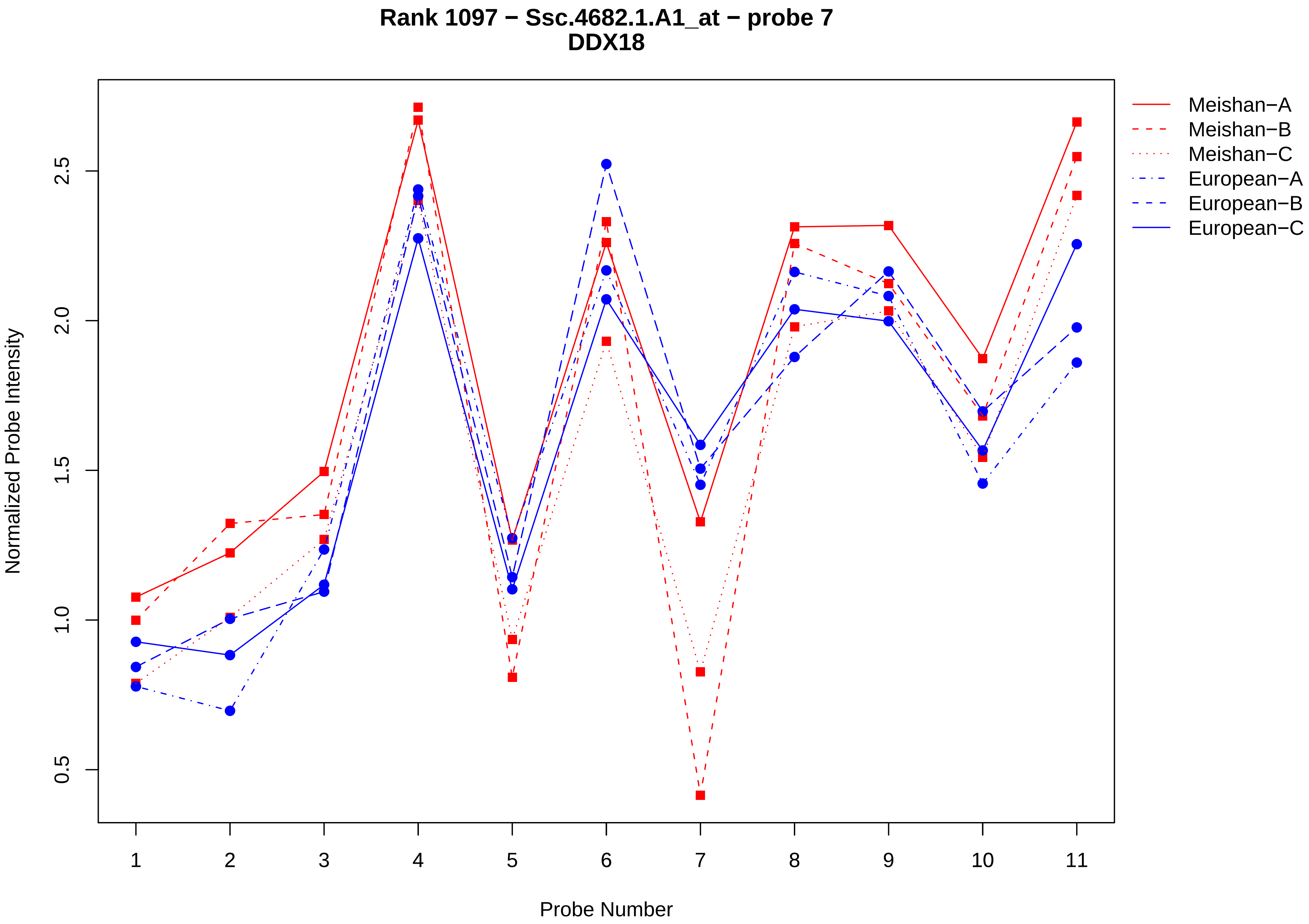

Rank 1097 − Ssc.4682.1.A1_at − probe 7
DDX18
Normalized Probe Intensity
0.5
1.0
1.5
2.0
2.5
1
2
3
4
5
6
7
8
9
10
11
Probe Number
Meishan−A
Meishan−B
Meishan−C
European−A
European−B
European−C

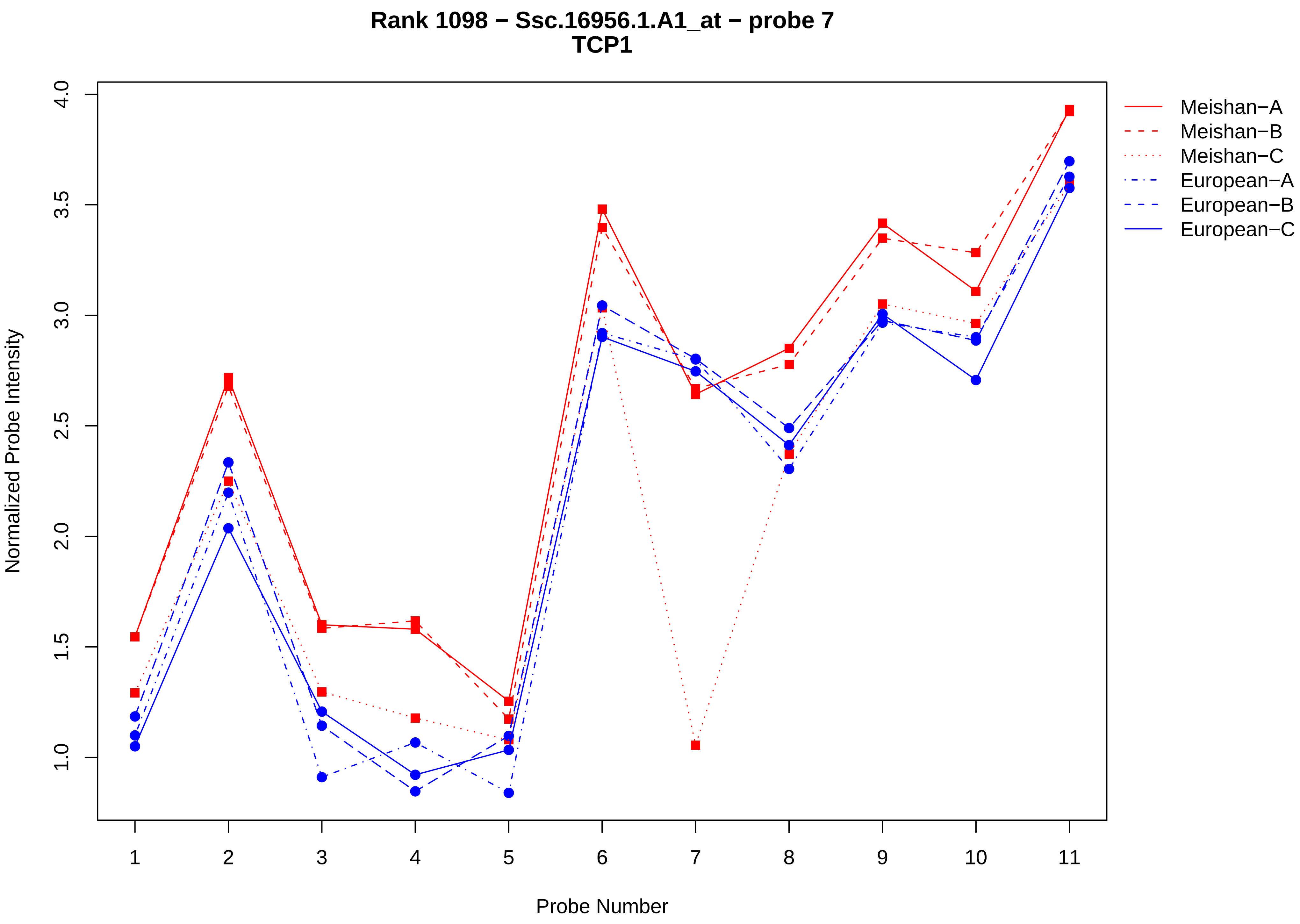

Rank 1098 − Ssc.16956.1.A1_at − probe 7
TCP1
Normalized Probe Intensity
1.0
1.5
2.0
2.5
3.0
3.5
4.0
1
2
3
4
5
6
7
8
9
10
11
Probe Number
Meishan−A
Meishan−B
Meishan−C
European−A
European−B
European−C

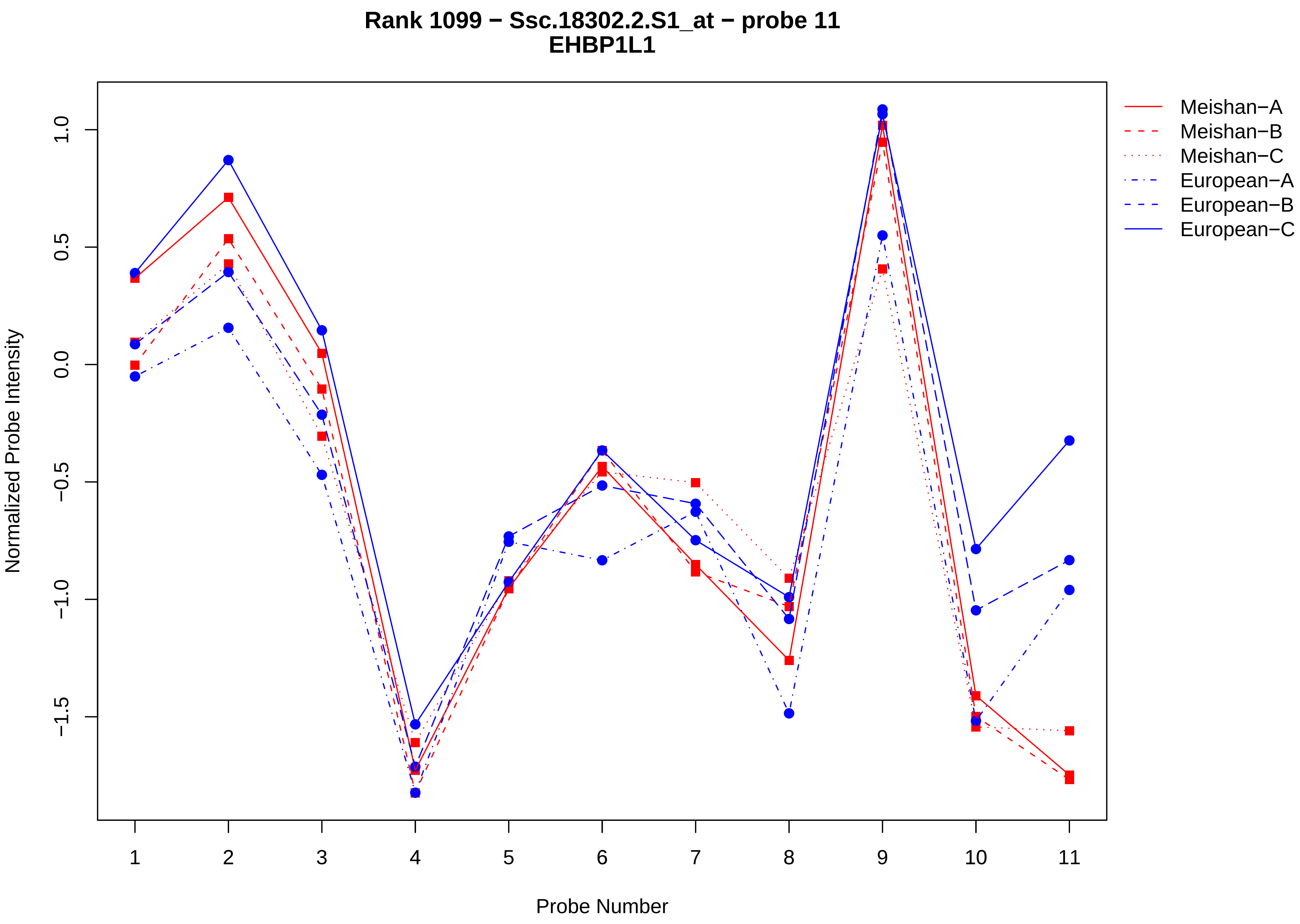

Rank 1099 − Ssc.18302.2.S1_at − probe 11
EHBP1L1
Normalized Probe Intensity
Probe Number
1.0
0.5
0.0
−0.5
−1.0
−1.5
1
2
3
4
5
6
7
8
9
10
11
Meishan−A
Meishan−B
Meishan−C
European−A
European−B
European−C

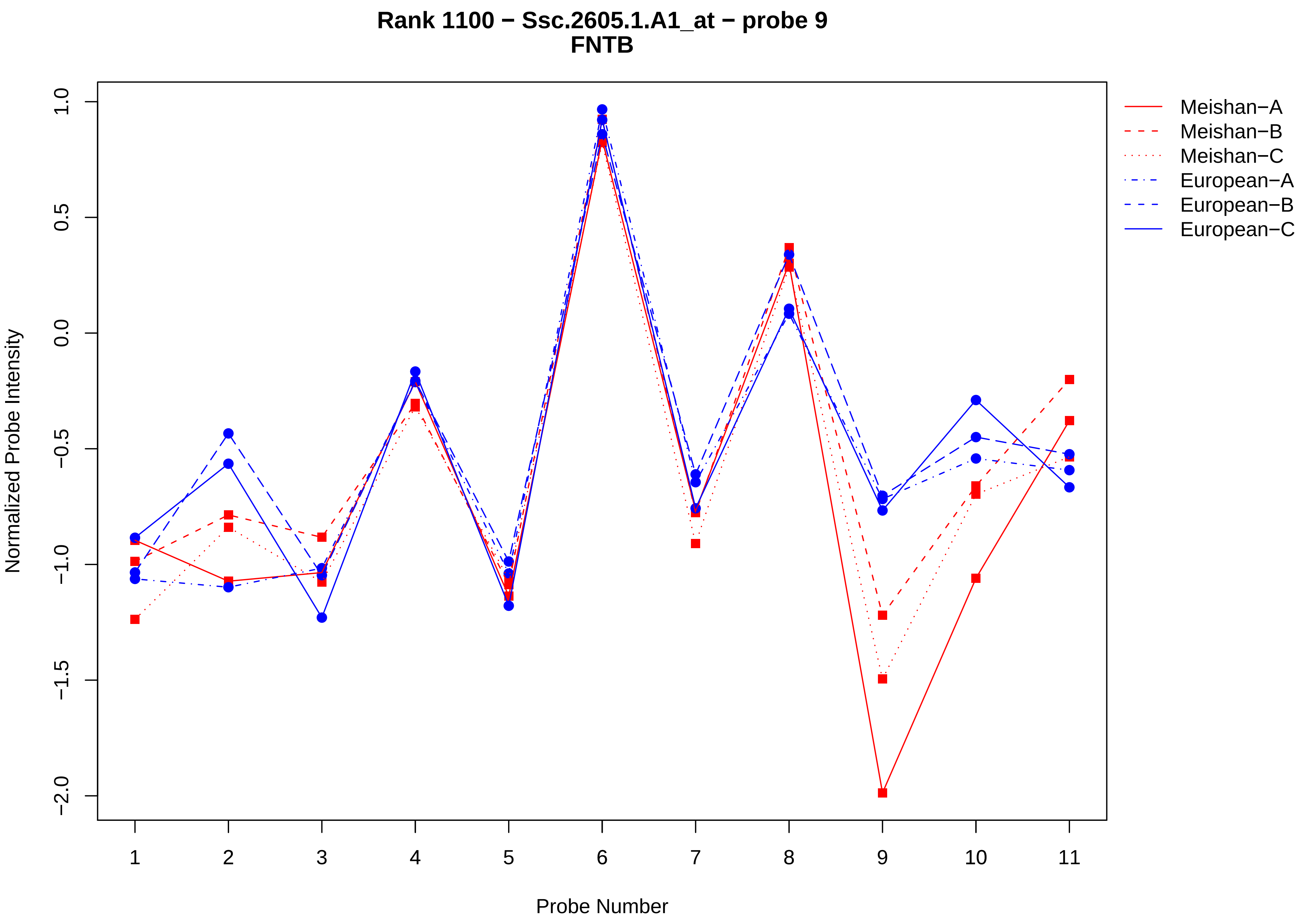

Rank 1100 − Ssc.2605.1.A1_at − probe 9
FNTB
Normalized Probe Intensity
Probe Number
1.0
0.5
0.0
−0.5
−1.0
−1.5
−2.0
1
2
3
4
5
6
7
8
9
10
11
Meishan−A
Meishan−B
Meishan−C
European−A
European−B
European−C

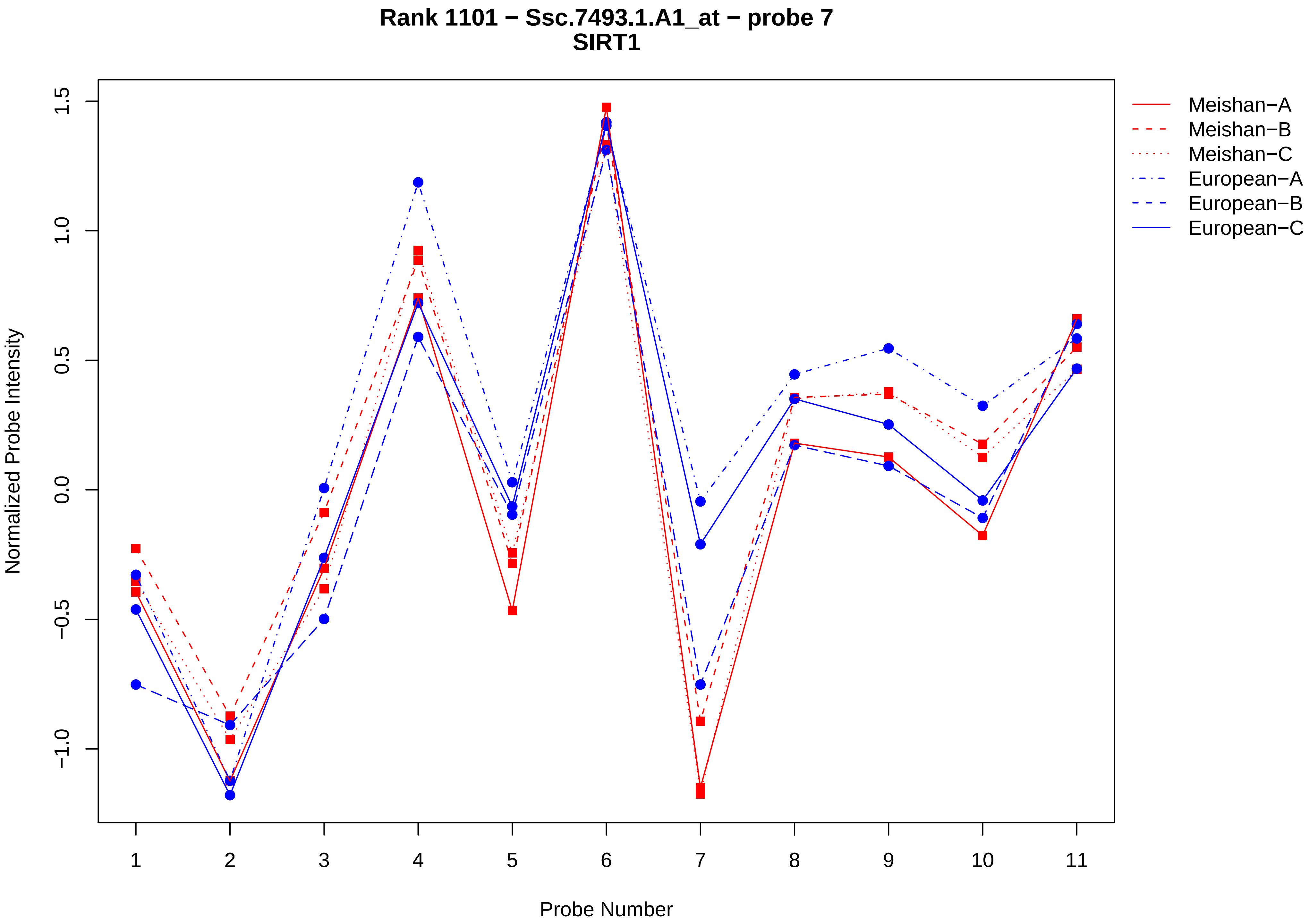
Rank 1101 − Ssc.7493.1.A1_at − probe 7
SIRT1
Normalized Probe Intensity
Probe Number
1.5
1.0
0.5
0.0
−0.5
−1.0
1
2
3
4
5
6
7
8
9
10
11
Meishan−A
Meishan−B
Meishan−C
European−A
European−B
European−C

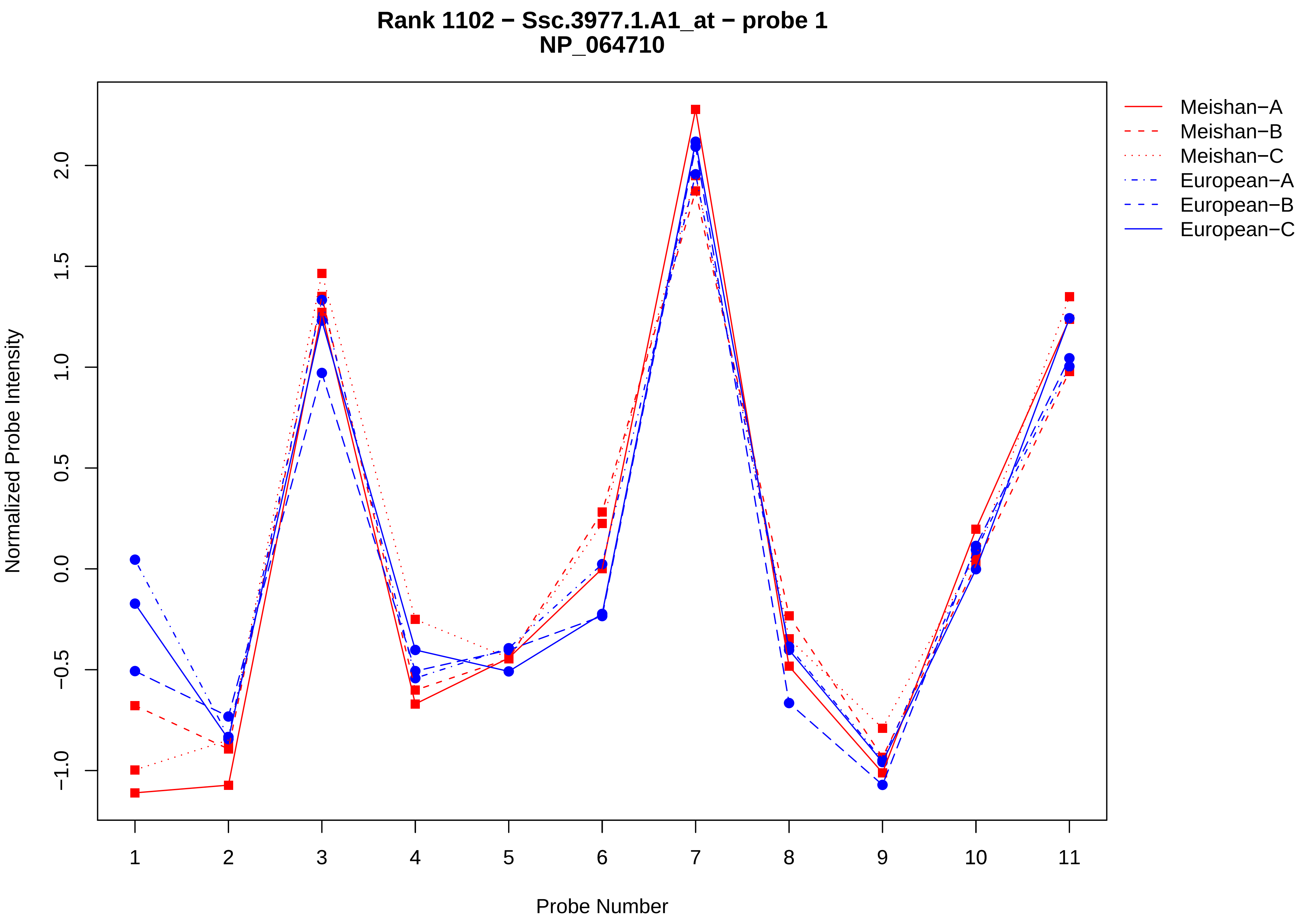

Rank 1102 − Ssc.3977.1.A1_at − probe 1
NP_064710
Normalized Probe Intensity
Probe Number
−1.0
−0.5
0.0
0.5
1.0
1.5
2.0
1
2
3
4
5
6
7
8
9
10
11
Meishan−A
Meishan−B
Meishan−C
European−A
European−B
European−C

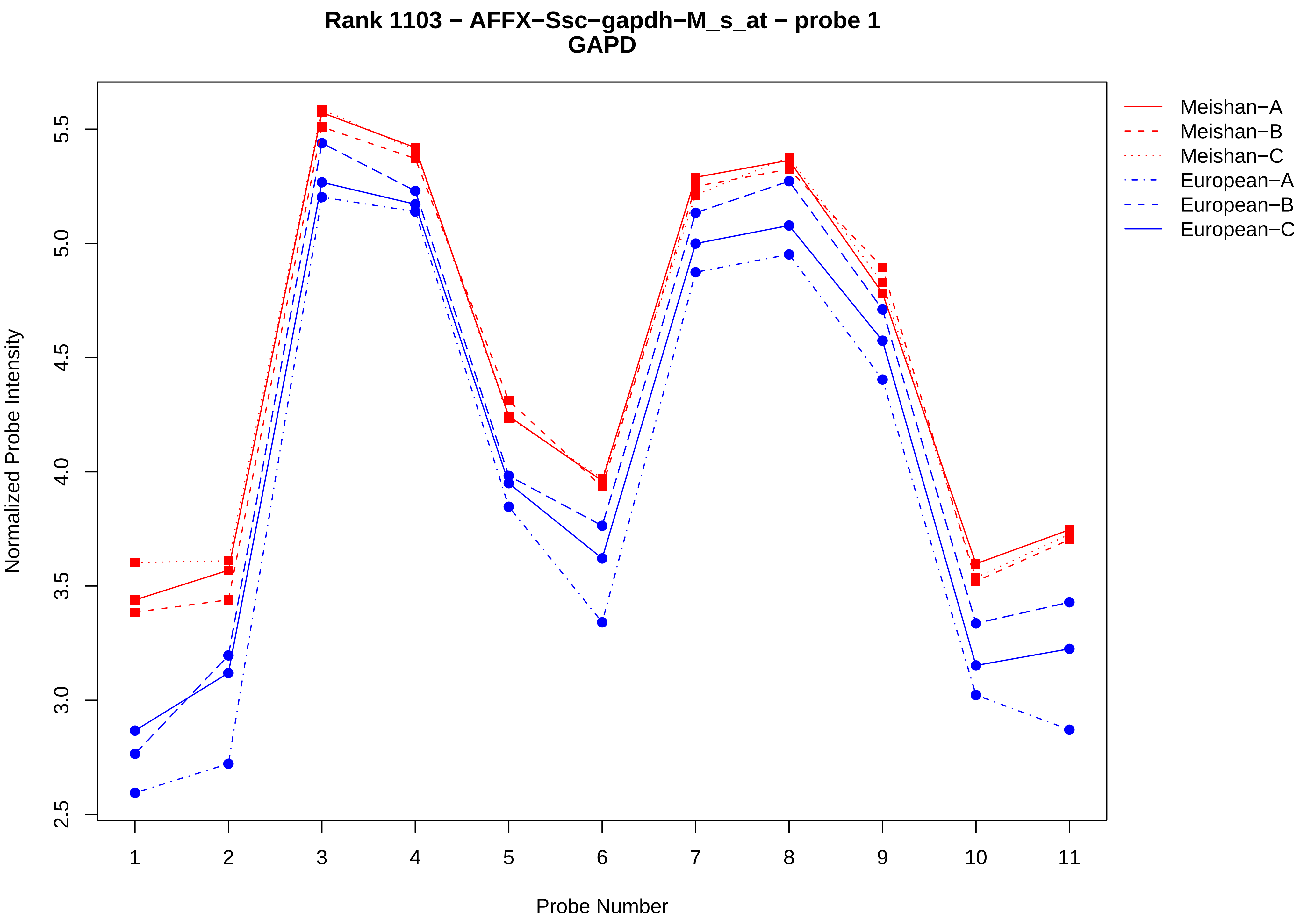
Rank 1103 − AFFX−Ssc−gapdh−M_s_at − probe 1
GAPD
Normalized Probe Intensity
Probe Number
2.5
3.0
3.5
4.0
4.5
5.0
5.5
1
2
3
4
5
6
7
8
9
10
11
Meishan−A
Meishan−B
Meishan−C
European−A
European−B
European−C

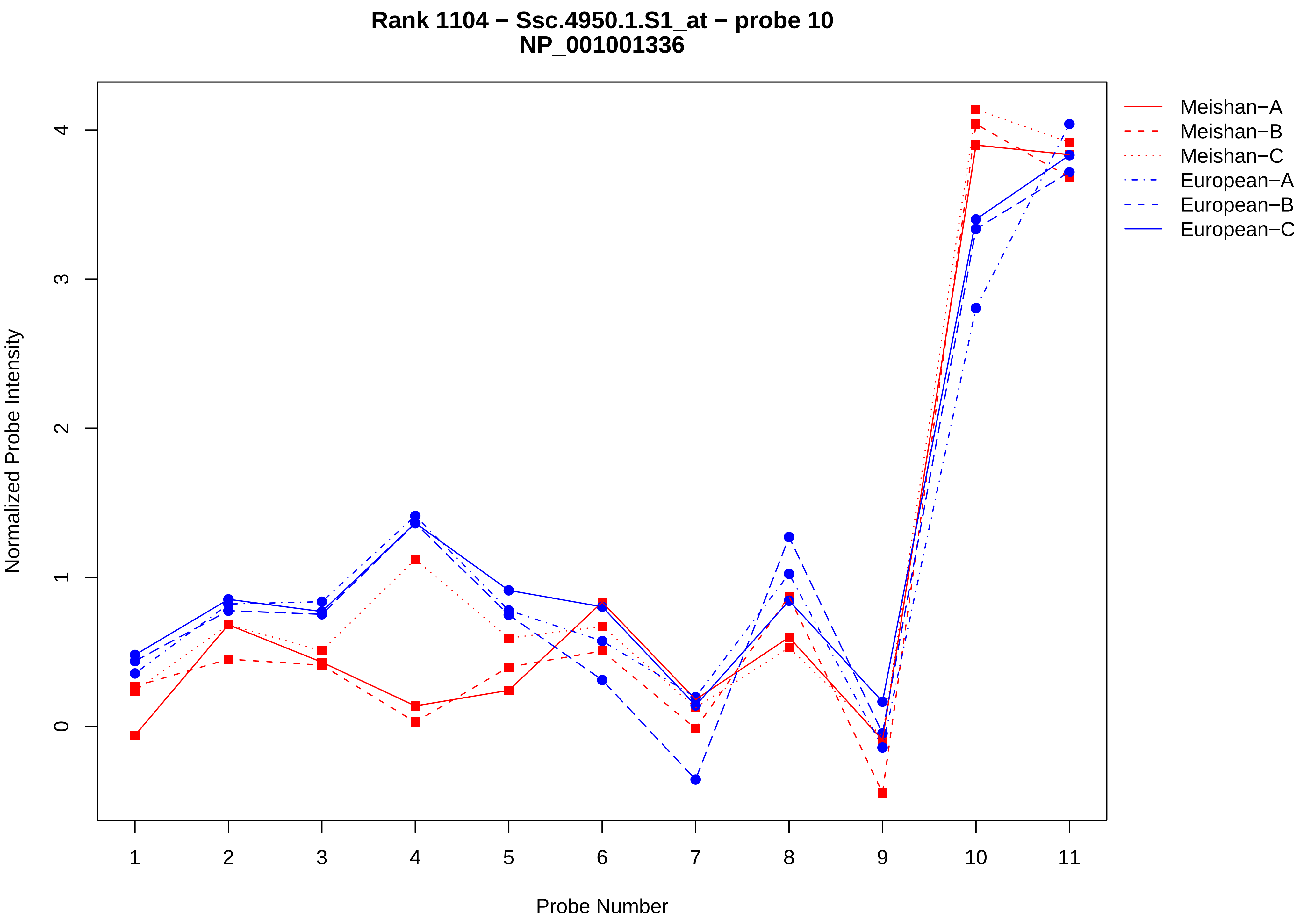

Rank 1104 − Ssc.4950.1.S1_at − probe 10
NP_001001336
Normalized Probe Intensity
Probe Number
0
1
2
3
4
1
2
3
4
5
6
7
8
9
10
11
Meishan−A
Meishan−B
Meishan−C
European−A
European−B
European−C

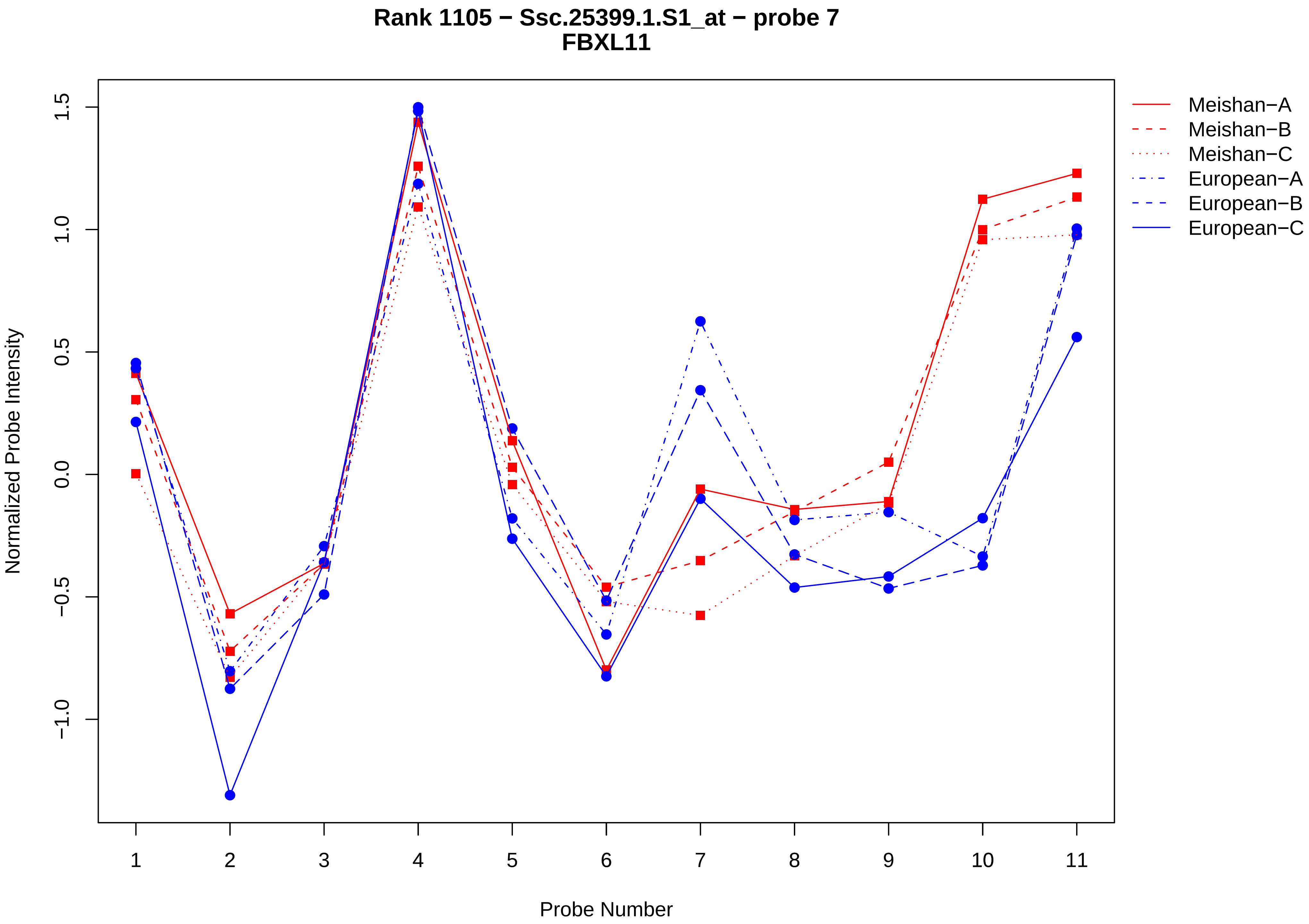
Rank 1105 − Ssc.25399.1.S1_at − probe 7
FBXL11
Normalized Probe Intensity
Probe Number
Meishan−A
Meishan−B
Meishan−C
European−A
European−B
European−C
−1.0
−0.5
0.0
0.5
1.0
1.5
1
2
3
4
5
6
7
8
9
10
11

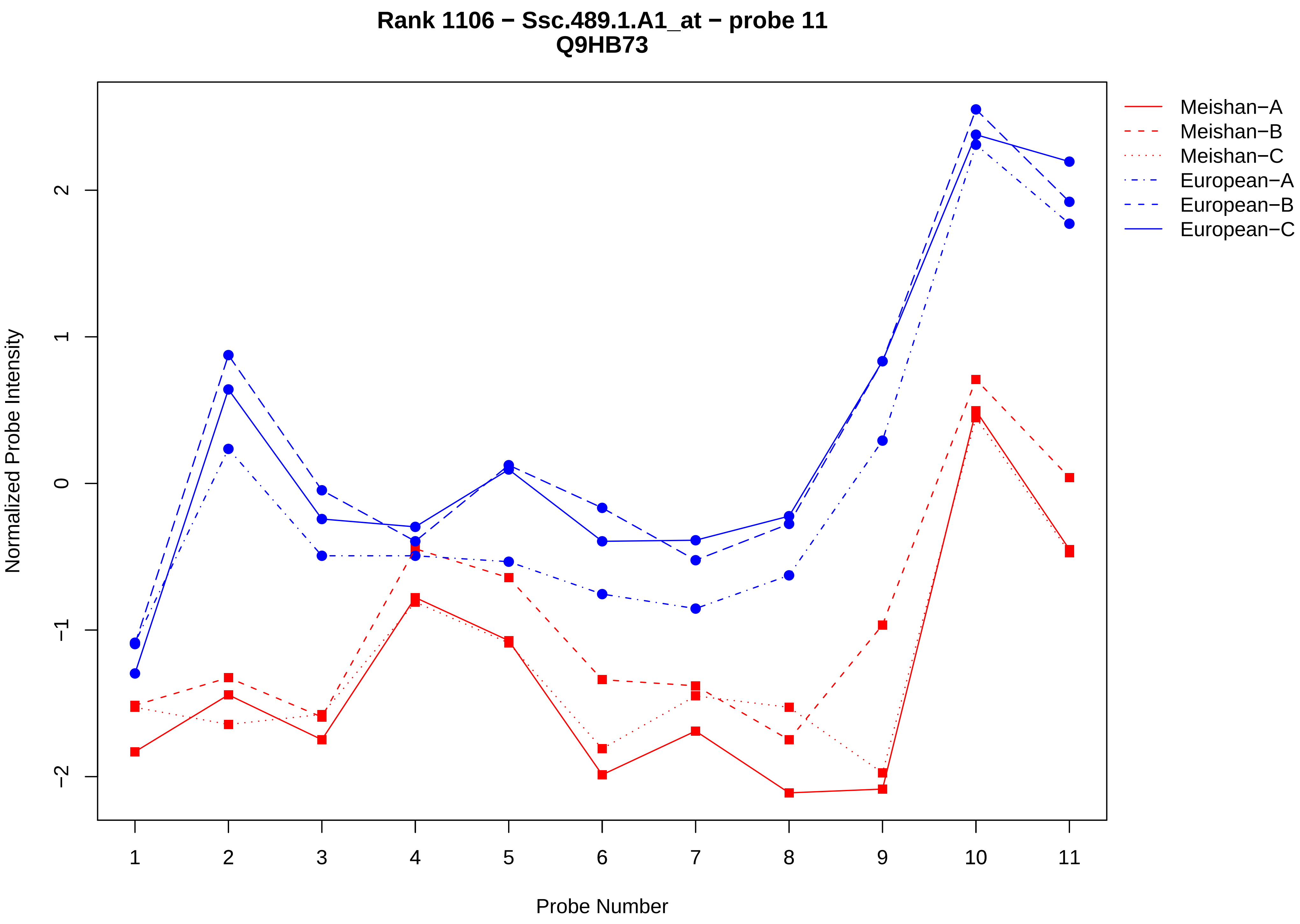
Rank 1106 − Ssc.489.1.A1_at − probe 11
Q9HB73
Normalized Probe Intensity
Probe Number
−2
−1
0
1
2
1
2
3
4
5
6
7
8
9
10
11
Meishan−A
Meishan−B
Meishan−C
European−A
European−B
European−C

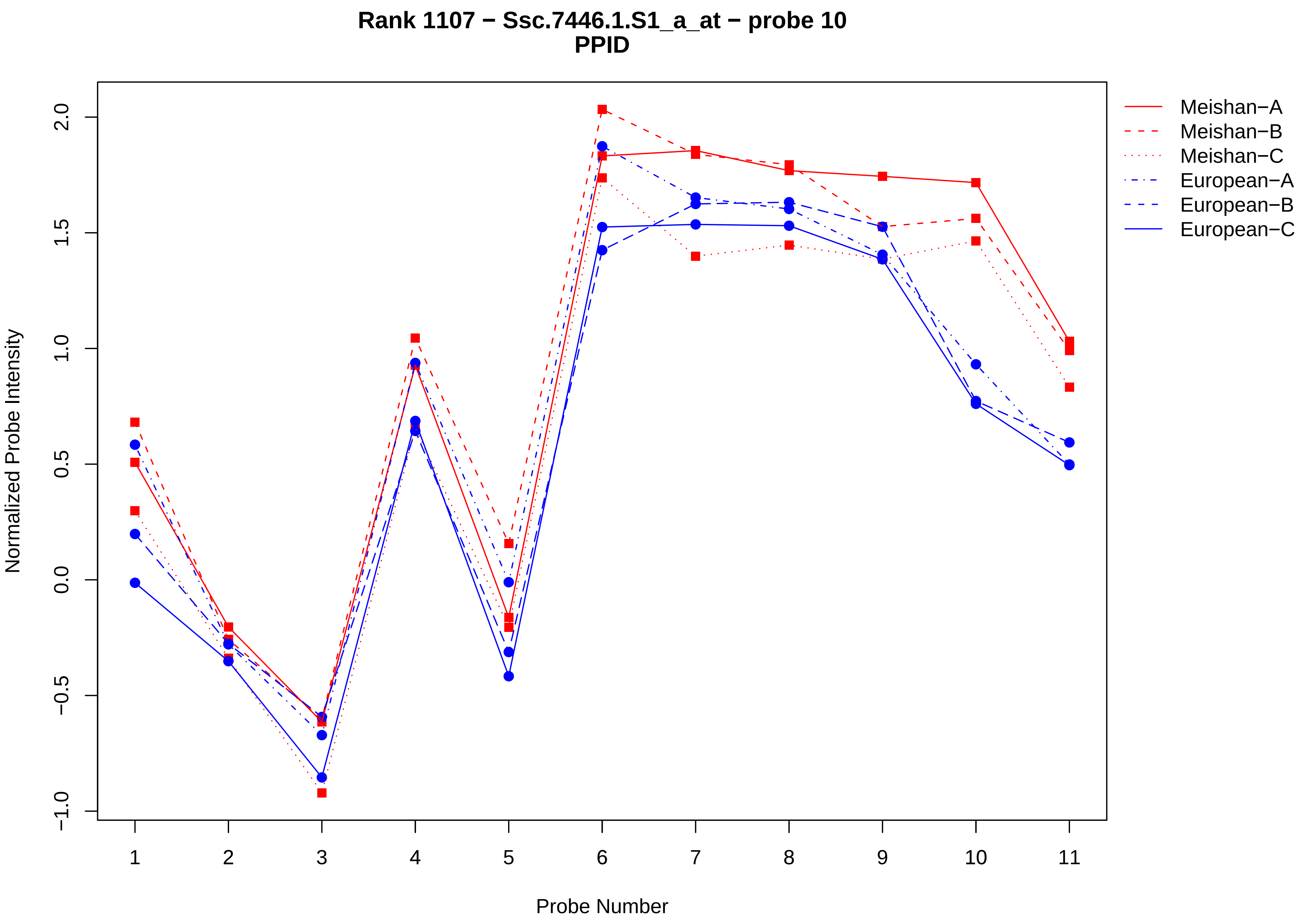

Rank 1107 − Ssc.7446.1.S1_a_at − probe 10
PPID
Normalized Probe Intensity
Probe Number
−1.0
−0.5
0.0
0.5
1.0
1.5
2.0
1
2
3
4
5
6
7
8
9
10
11
Meishan−A
Meishan−B
Meishan−C
European−A
European−B
European−C

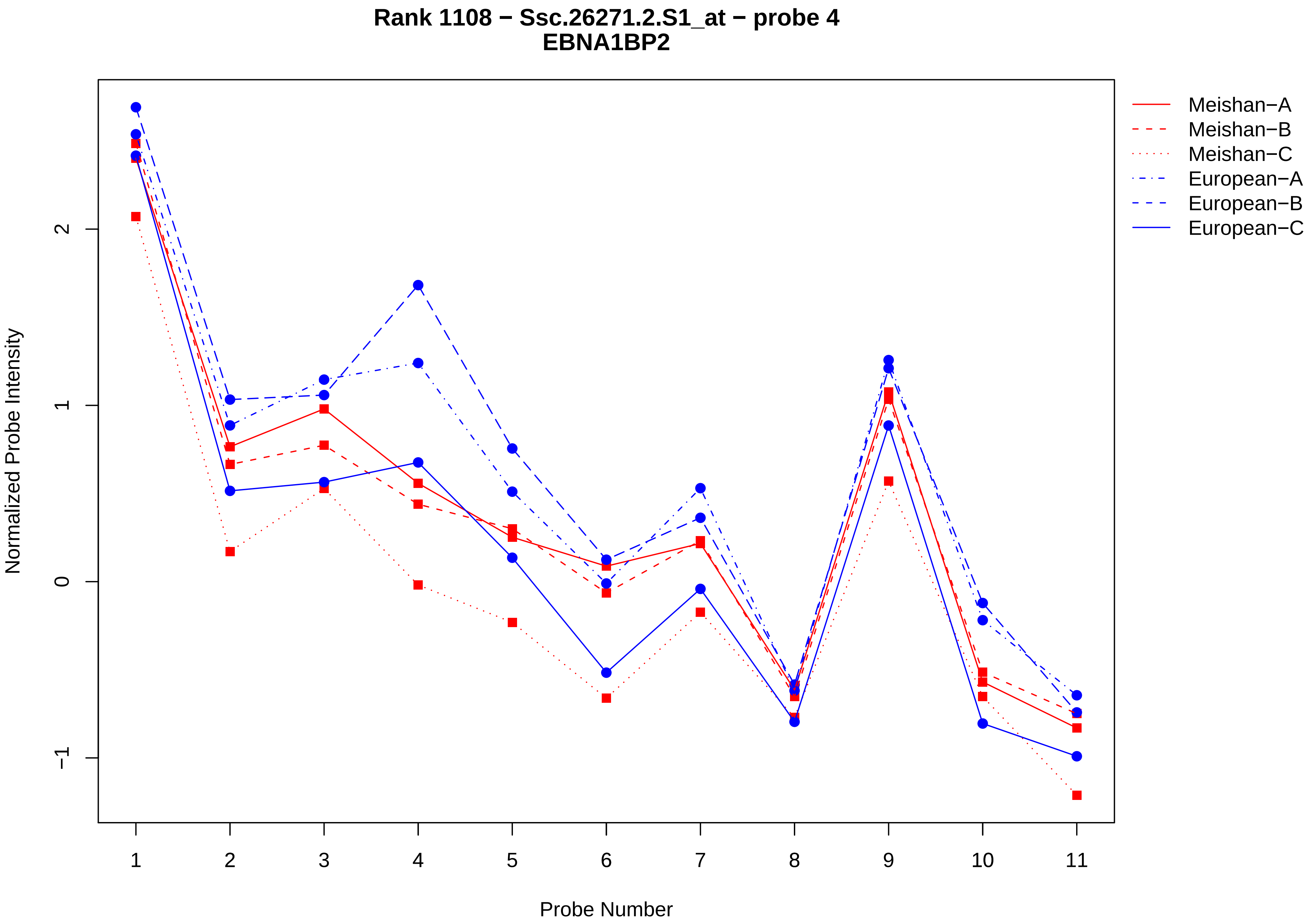

Rank 1108 − Ssc.26271.2.S1_at − probe 4
EBNA1BP2
Normalized Probe Intensity
Probe Number
−1
0
1
2
1
2
3
4
5
6
7
8
9
10
11
Meishan−A
Meishan−B
Meishan−C
European−A
European−B
European−C

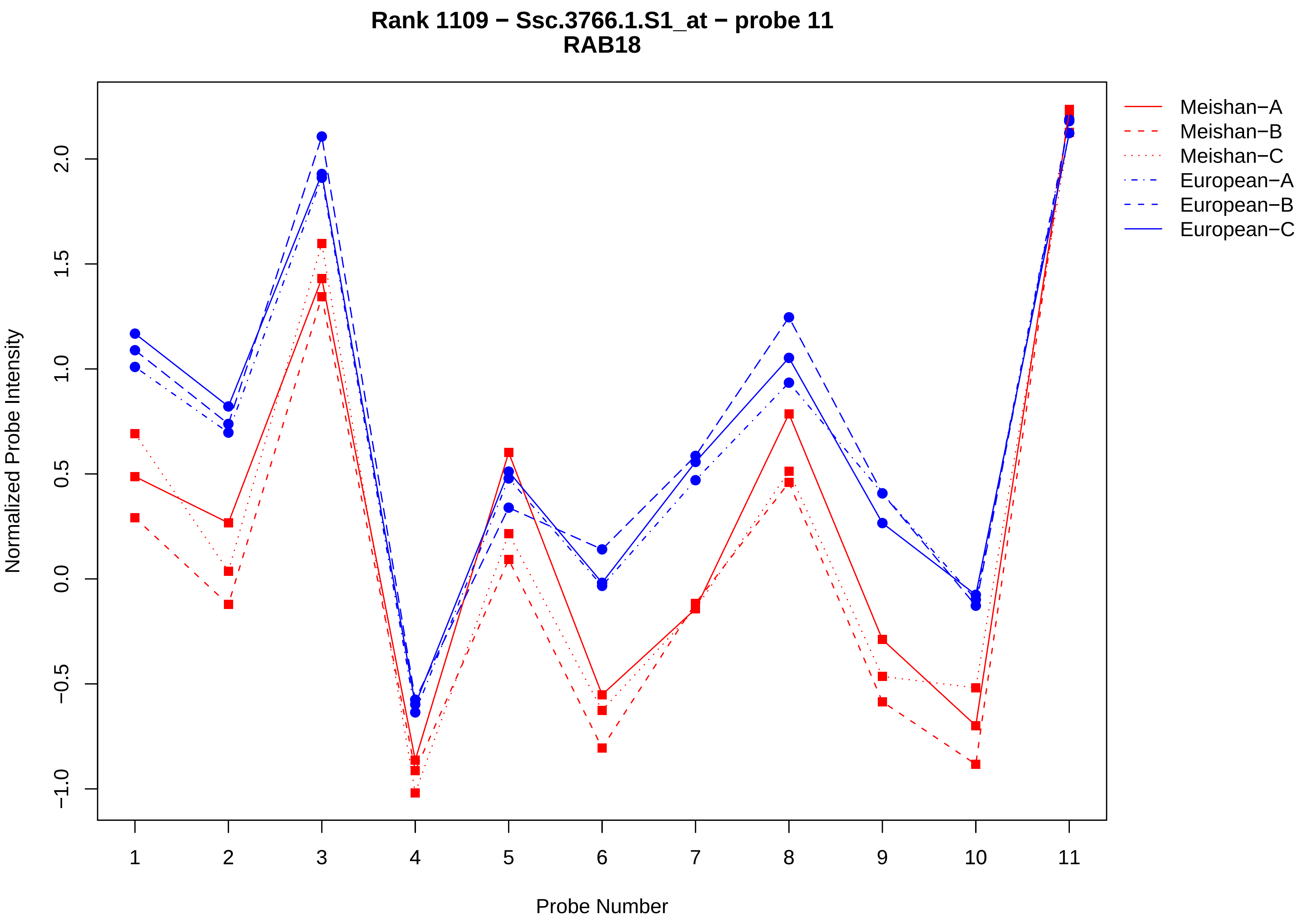

Rank 1109 − Ssc.3766.1.S1_at − probe 11
RAB18
Normalized Probe Intensity
Probe Number
−1.0
−0.5
0.0
0.5
1.0
1.5
2.0
1
2
3
4
5
6
7
8
9
10
11
Meishan−A
Meishan−B
Meishan−C
European−A
European−B
European−C

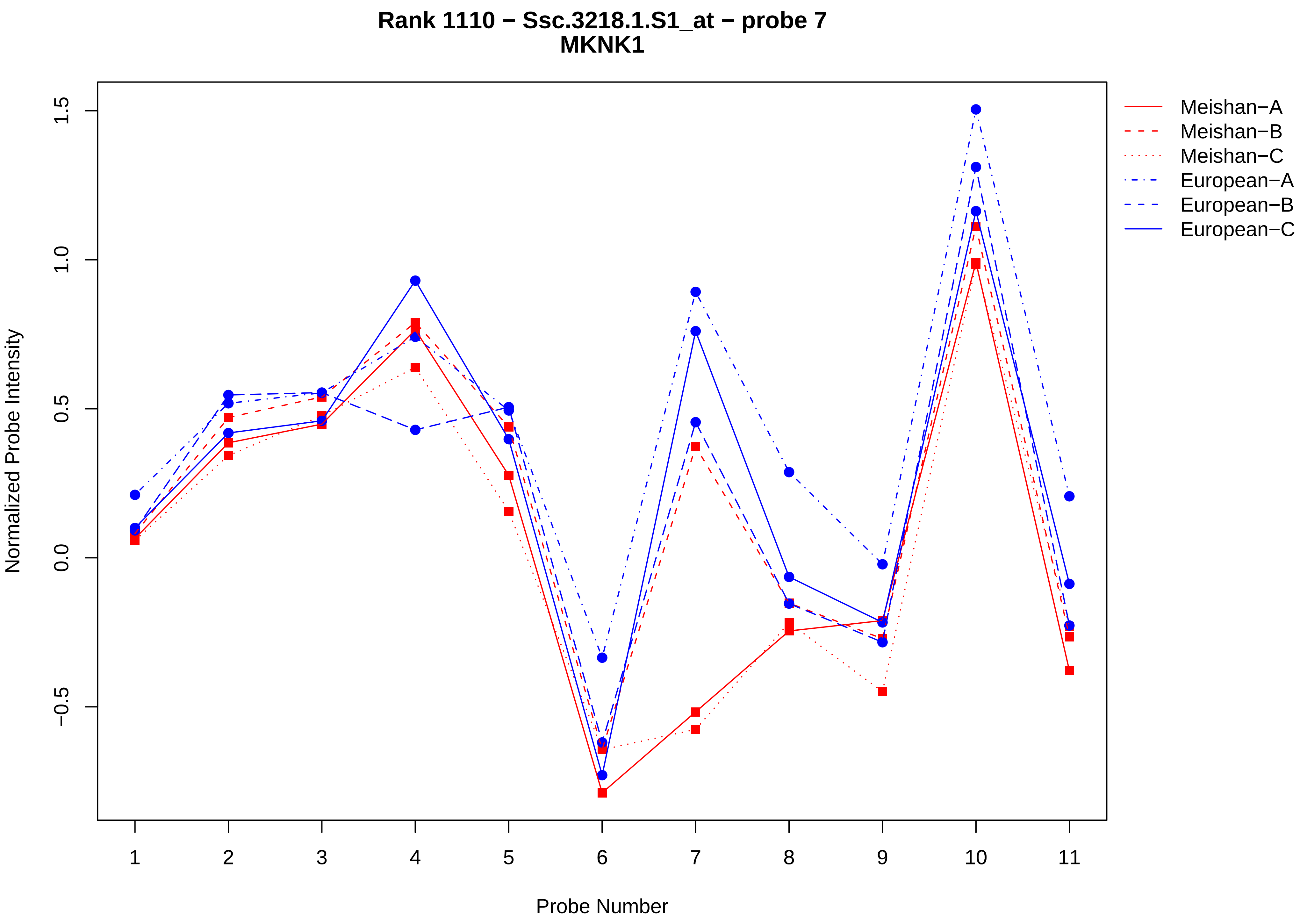

Rank 1110 − Ssc.3218.1.S1_at − probe 7
MKNK1
Normalized Probe Intensity
Probe Number
−0.5
0.0
0.5
1.0
1.5
1
2
3
4
5
6
7
8
9
10
11
Meishan−A
Meishan−B
Meishan−C
European−A
European−B
European−C

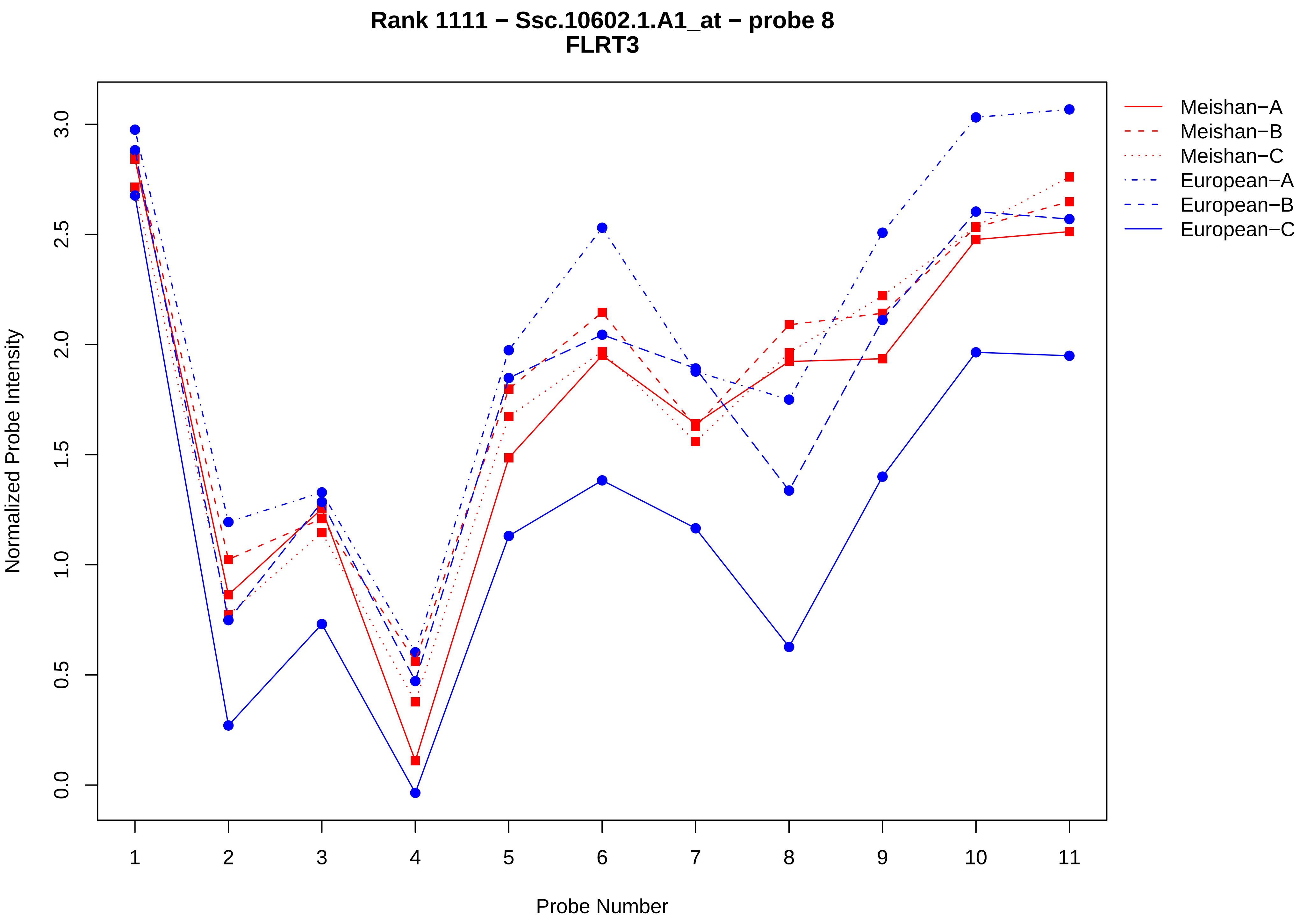

Rank 1111 − Ssc.10602.1.A1_at − probe 8
FLRT3
Normalized Probe Intensity
0.0
0.5
1.0
1.5
2.0
2.5
3.0
1
2
3
4
5
6
7
8
9
10
11
Probe Number
Meishan−A
Meishan−B
Meishan−C
European−A
European−B
European−C

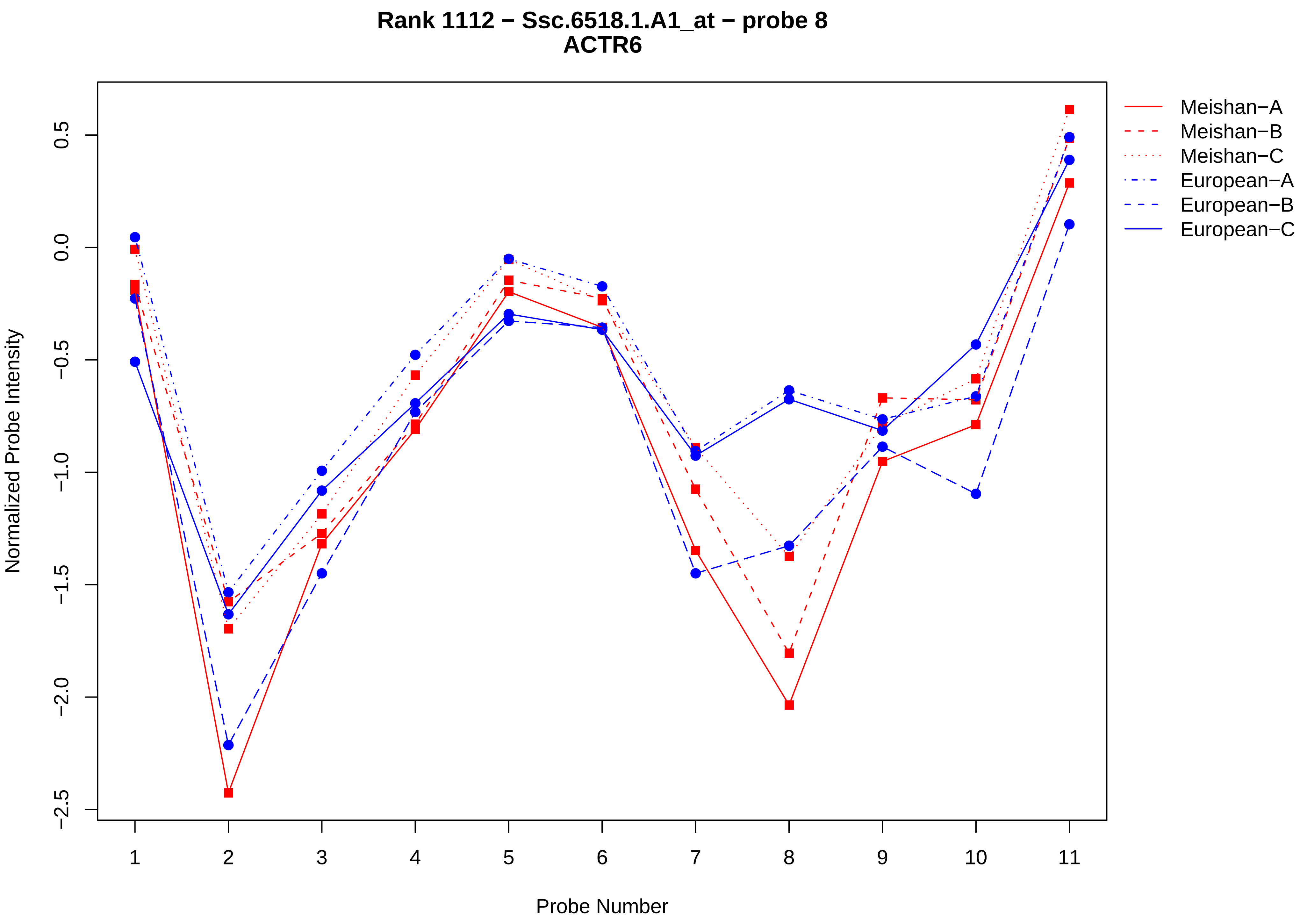

Rank 1112 − Ssc.6518.1.A1_at − probe 8
ACTR6
Normalized Probe Intensity
Probe Number
Meishan−A
Meishan−B
Meishan−C
European−A
European−B
European−C
0.5
0.0
−0.5
−1.0
−1.5
−2.0
−2.5
1
2
3
4
5
6
7
8
9
10
11

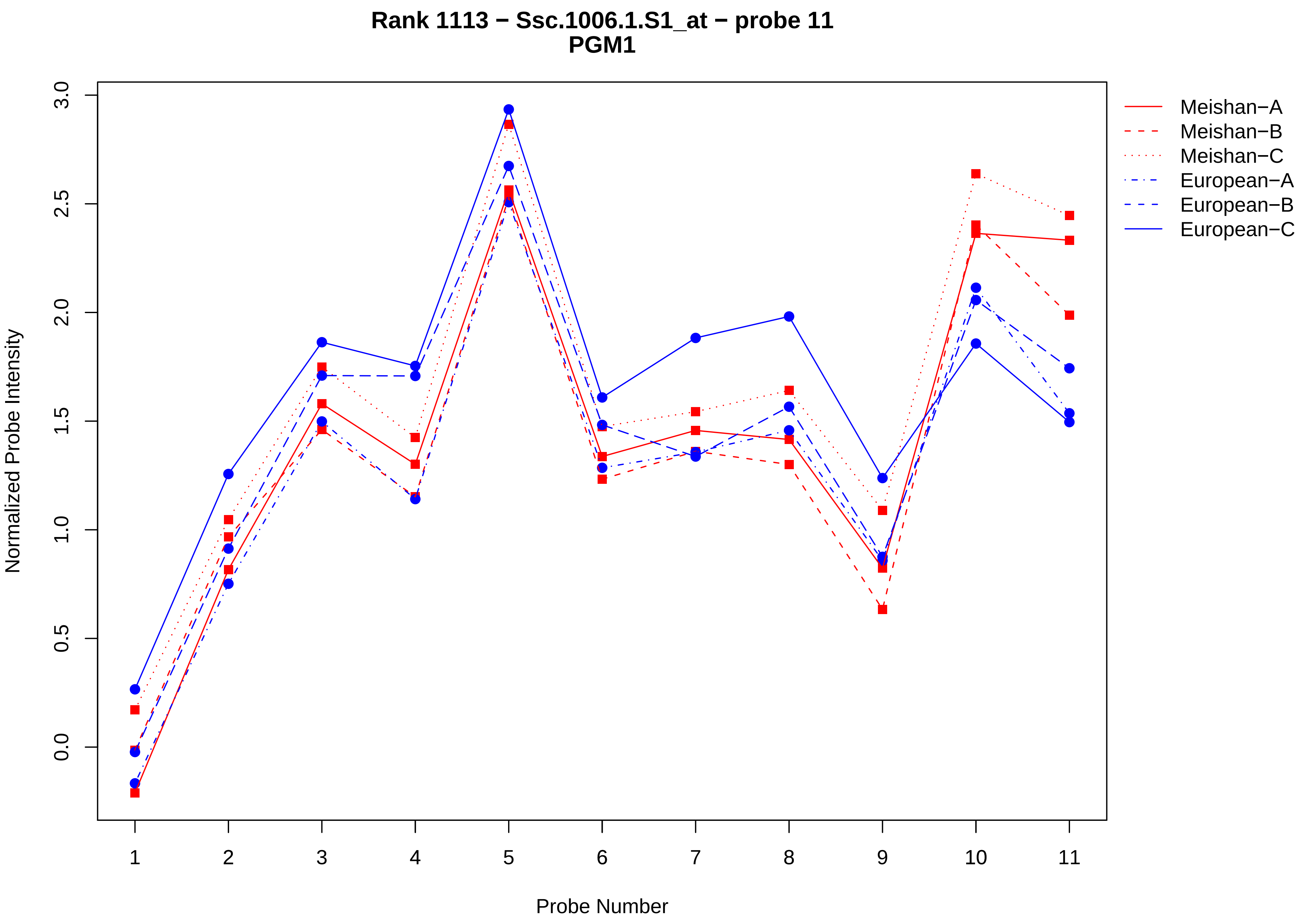

Rank 1113 − Ssc.1006.1.S1_at − probe 11
PGM1
Normalized Probe Intensity
Probe Number
0.0
0.5
1.0
1.5
2.0
2.5
3.0
1
2
3
4
5
6
7
8
9
10
11
Meishan−A
Meishan−B
Meishan−C
European−A
European−B
European−C

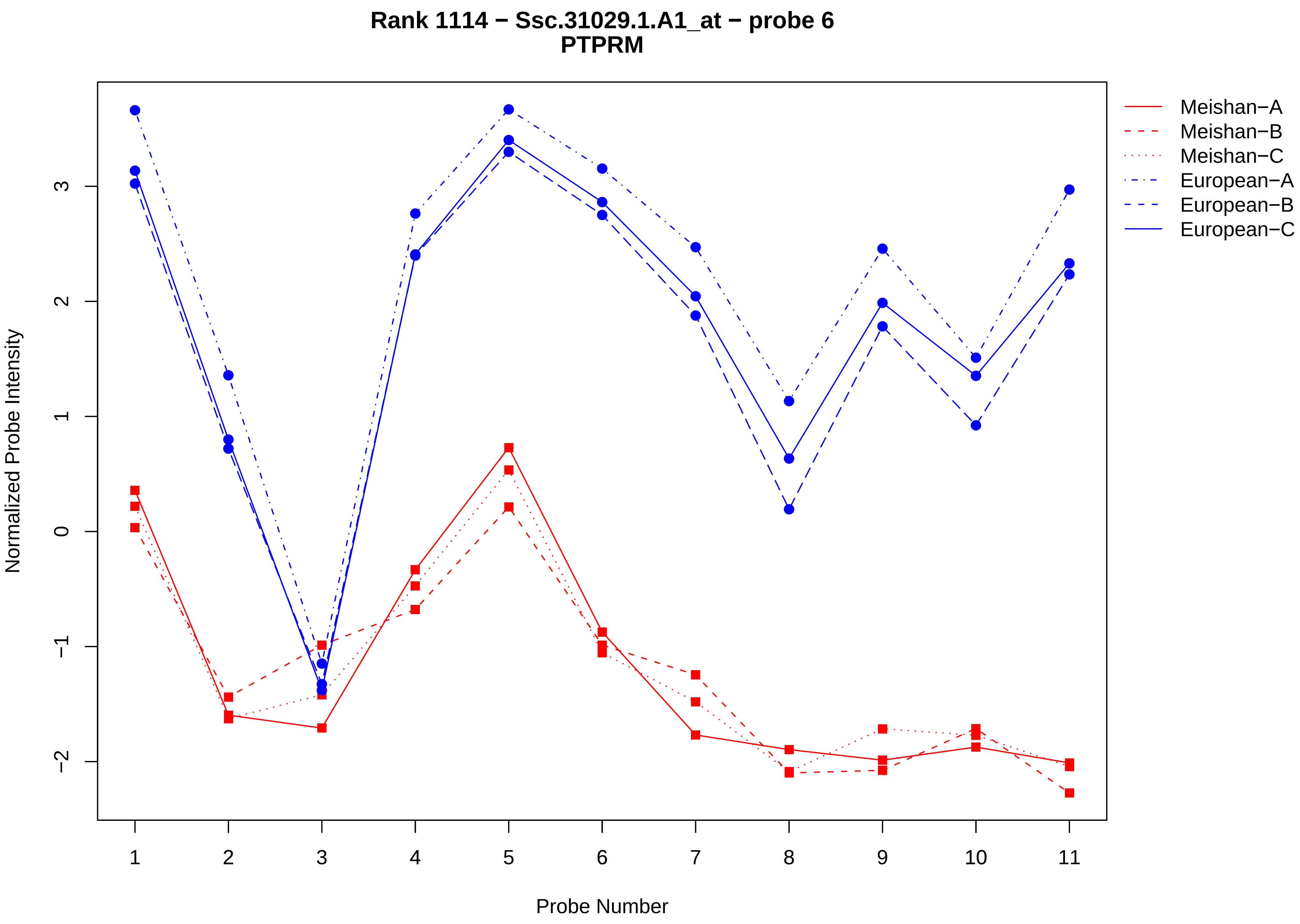

Rank 1114 − Ssc.31029.1.A1_at − probe 6
PTPRM
Normalized Probe Intensity
Probe Number
−2
−1
0
1
2
3
1
2
3
4
5
6
7
8
9
10
11
Meishan−A
Meishan−B
Meishan−C
European−A
European−B
European−C

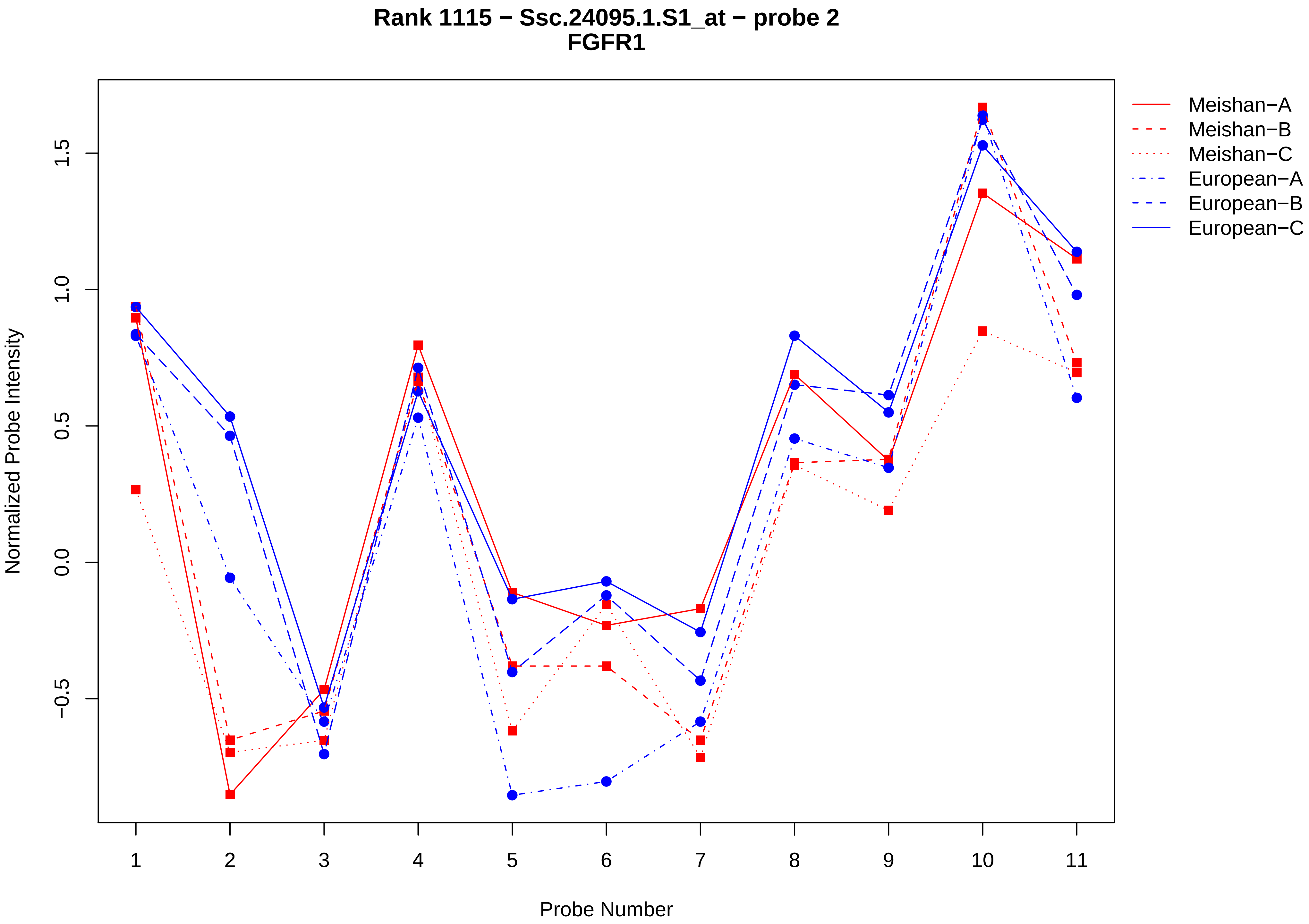

Rank 1115 − Ssc.24095.1.S1_at − probe 2
FGFR1
Normalized Probe Intensity
Probe Number
−0.5
0.0
0.5
1.0
1.5
1
2
3
4
5
6
7
8
9
10
11
Meishan−A
Meishan−B
Meishan−C
European−A
European−B
European−C

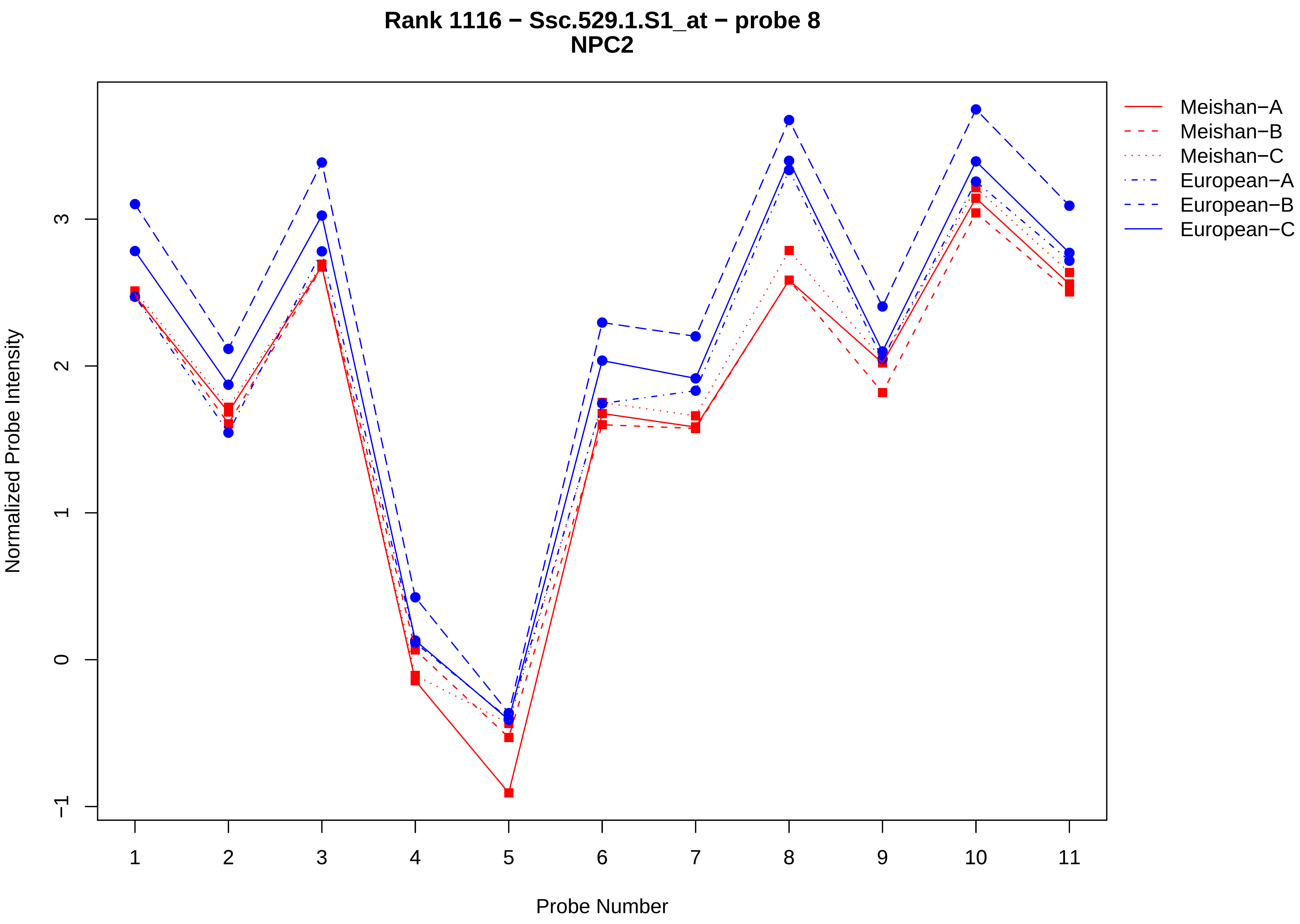

Rank 1116 − Ssc.529.1.S1_at − probe 8
NPC2
Normalized Probe Intensity
Probe Number
−1
0
1
2
3
1
2
3
4
5
6
7
8
9
10
11
Meishan−A
Meishan−B
Meishan−C
European−A
European−B
European−C

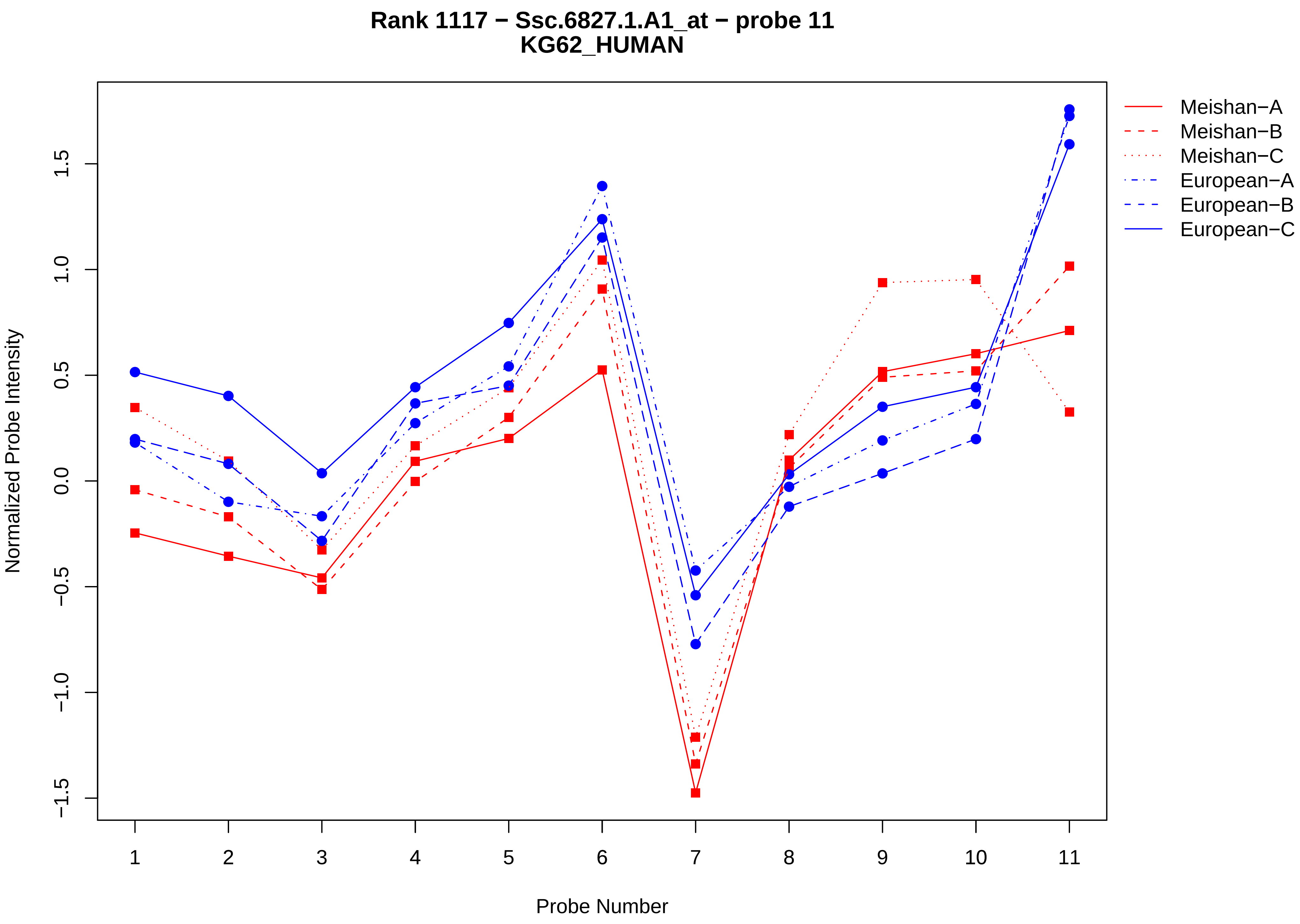

Rank 1117 − Ssc.6827.1.A1_at − probe 11
KG62_HUMAN
Normalized Probe Intensity
Probe Number
Meishan−A
Meishan−B
Meishan−C
European−A
European−B
European−C
−1.5
−1.0
−0.5
0.0
0.5
1.0
1.5
1
2
3
4
5
6
7
8
9
10
11

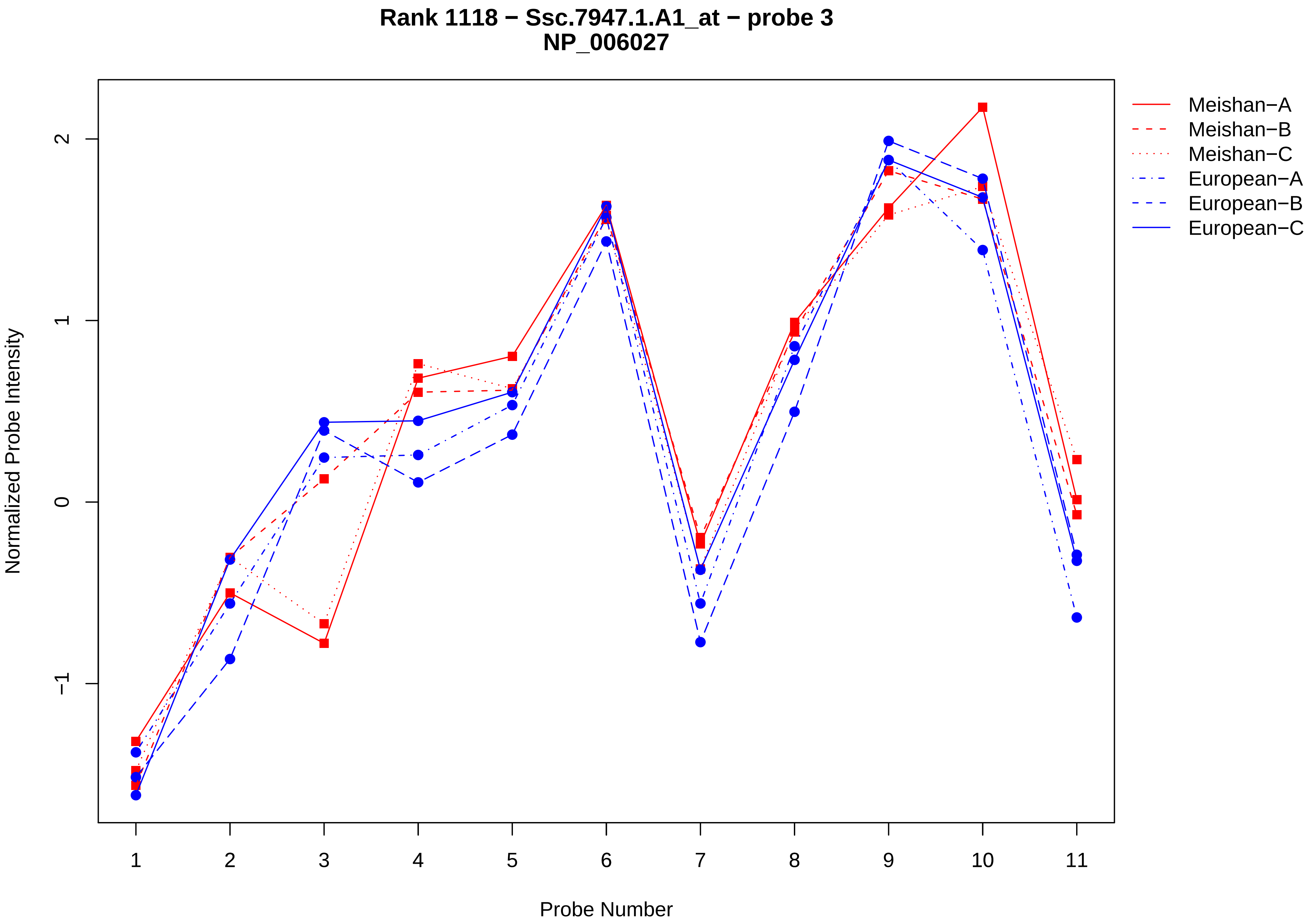

Rank 1118 − Ssc.7947.1.A1_at − probe 3
NP_006027
Normalized Probe Intensity
Probe Number
−1
0
1
2
1
2
3
4
5
6
7
8
9
10
11
Meishan−A
Meishan−B
Meishan−C
European−A
European−B
European−C

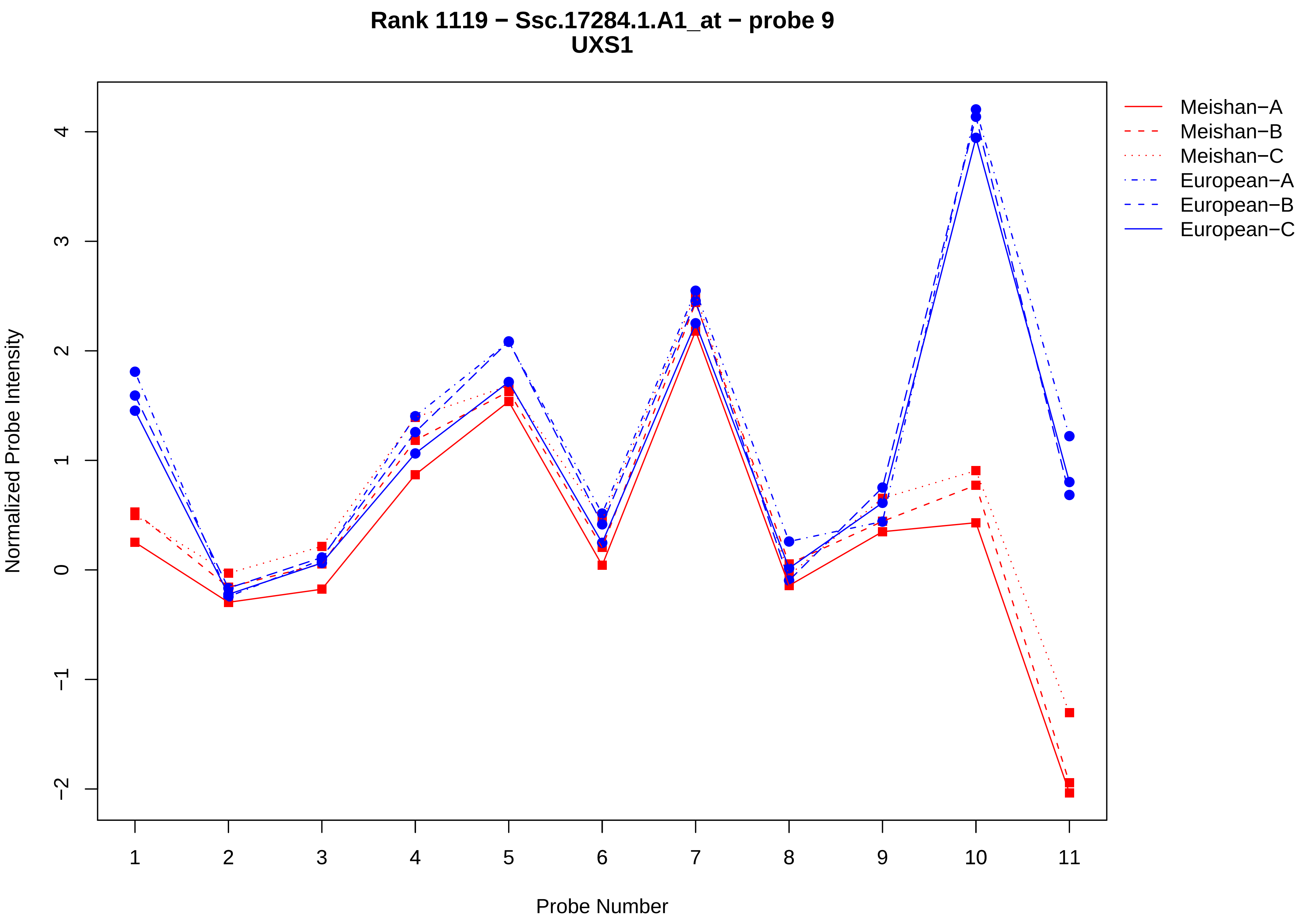
Rank 1119 − Ssc.17284.1.A1_at − probe 9
UXS1
Normalized Probe Intensity
Probe Number
Meishan−A
Meishan−B
Meishan−C
European−A
European−B
European−C
−2
−1
0
1
2
3
4
1
2
3
4
5
6
7
8
9
10
11

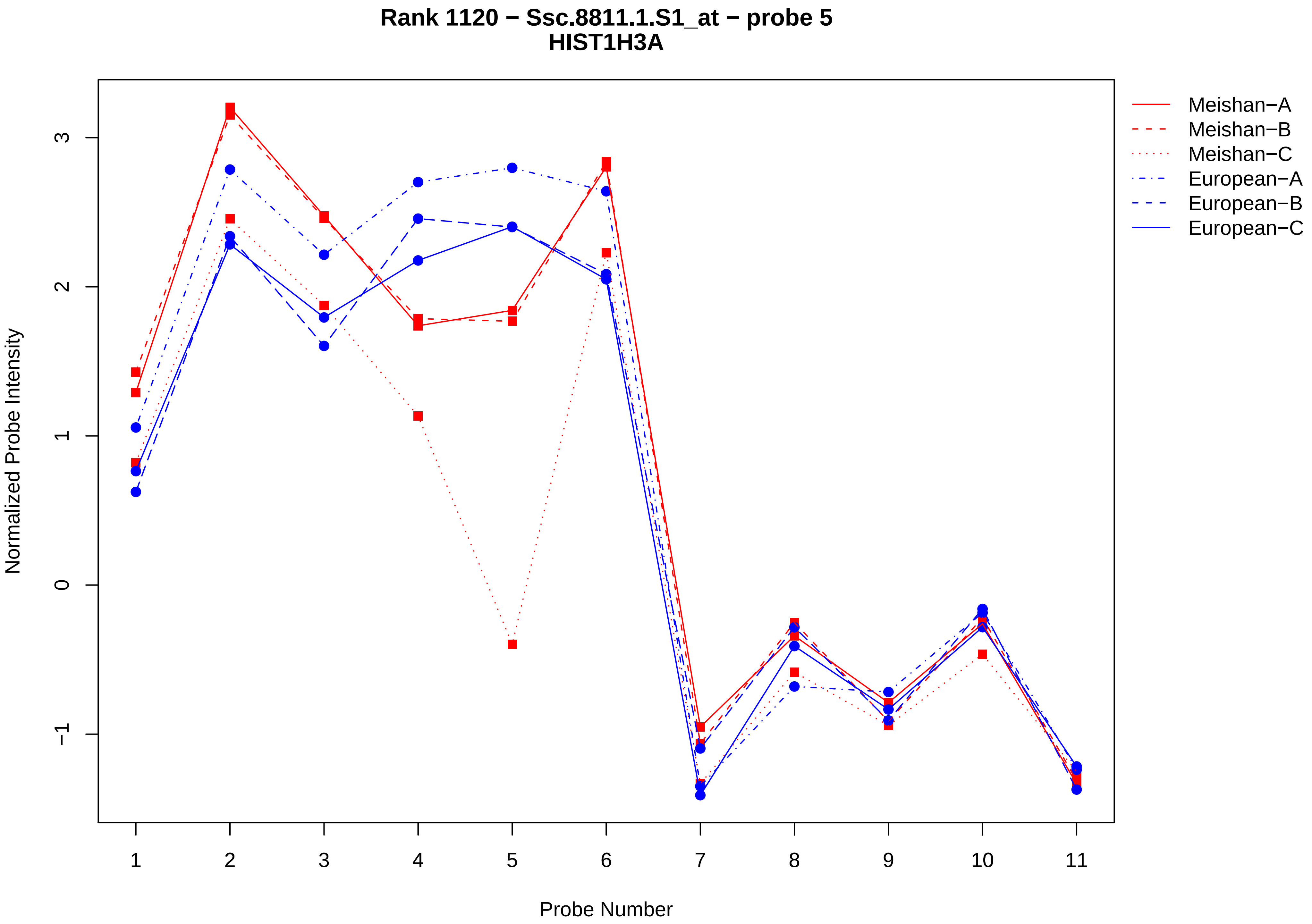

Rank 1120 − Ssc.8811.1.S1_at − probe 5
HIST1H3A
Normalized Probe Intensity
Probe Number
−1
0
1
2
3
1
2
3
4
5
6
7
8
9
10
11
Meishan−A
Meishan−B
Meishan−C
European−A
European−B
European−C

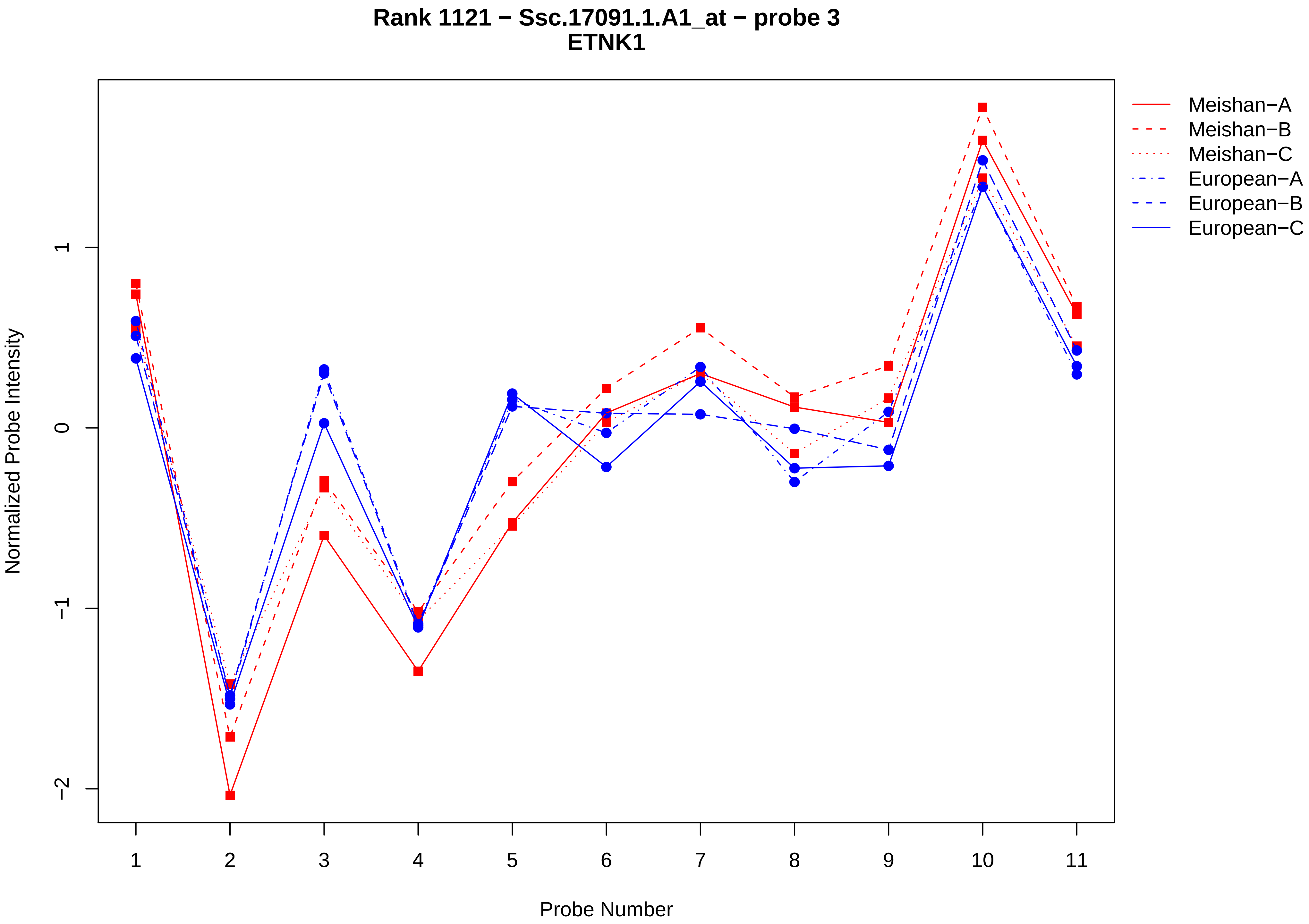

Rank 1121 − Ssc.17091.1.A1_at − probe 3
ETNK1
Normalized Probe Intensity
Probe Number
1
0
−1
−2
1
2
3
4
5
6
7
8
9
10
11
Meishan−A
Meishan−B
Meishan−C
European−A
European−B
European−C

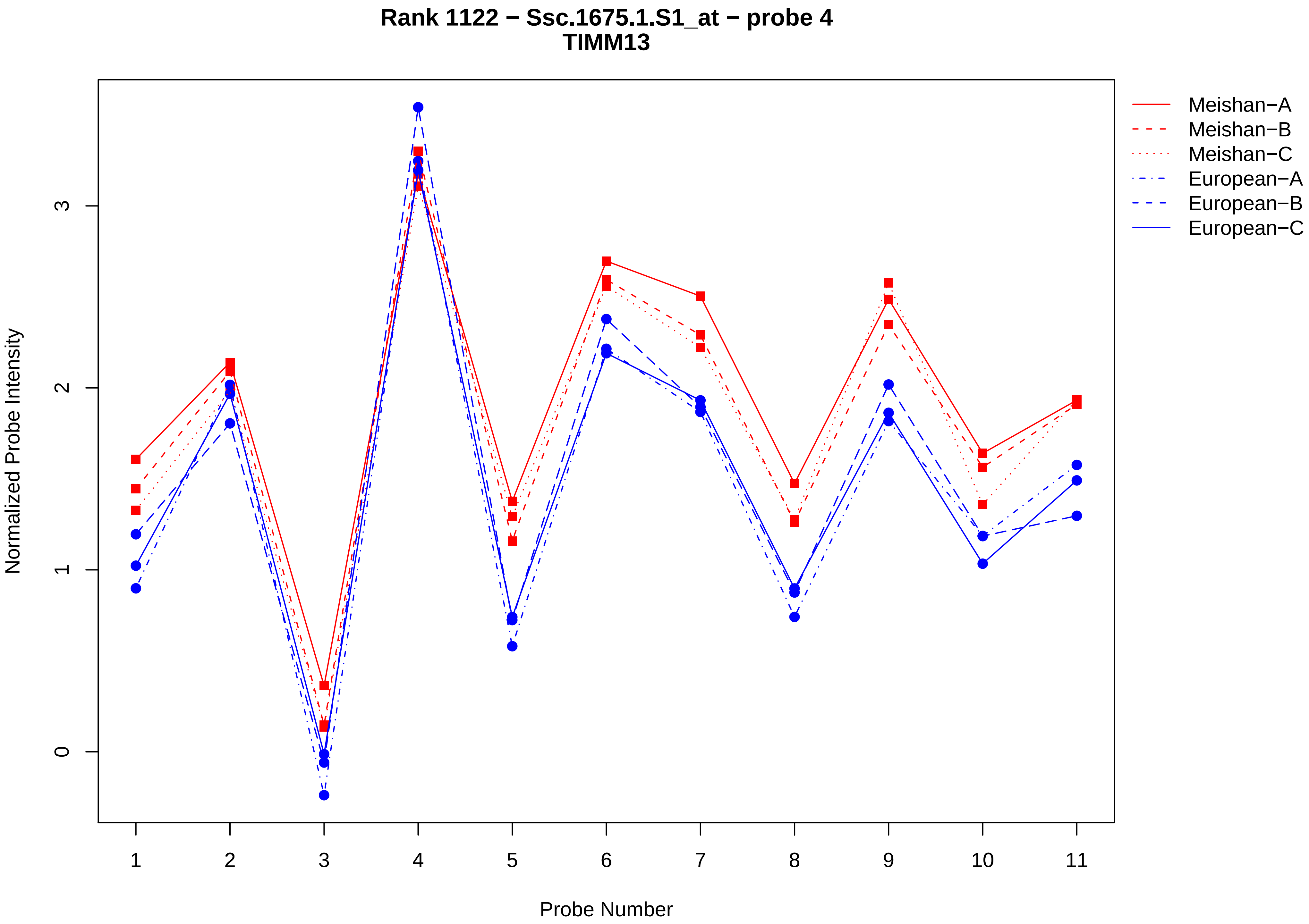

Rank 1122 − Ssc.1675.1.S1_at − probe 4
TIMM13
Normalized Probe Intensity
Probe Number
0
1
2
3
1
2
3
4
5
6
7
8
9
10
11
Meishan−A
Meishan−B
Meishan−C
European−A
European−B
European−C

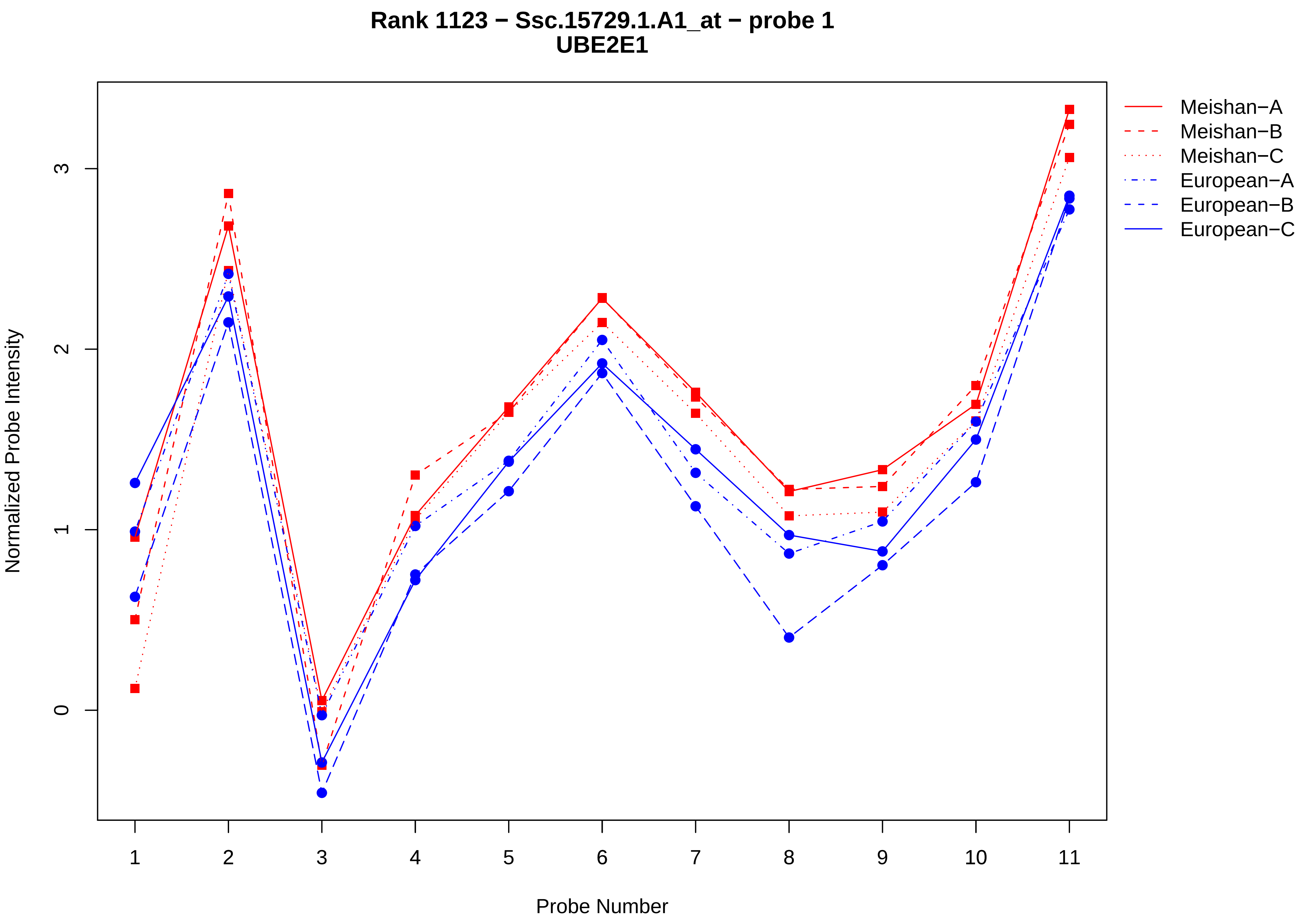
Rank 1123 − Ssc.15729.1.A1_at − probe 1
UBE2E1
Normalized Probe Intensity
Probe Number
0
1
2
3
1
2
3
4
5
6
7
8
9
10
11
Meishan−A
Meishan−B
Meishan−C
European−A
European−B
European−C

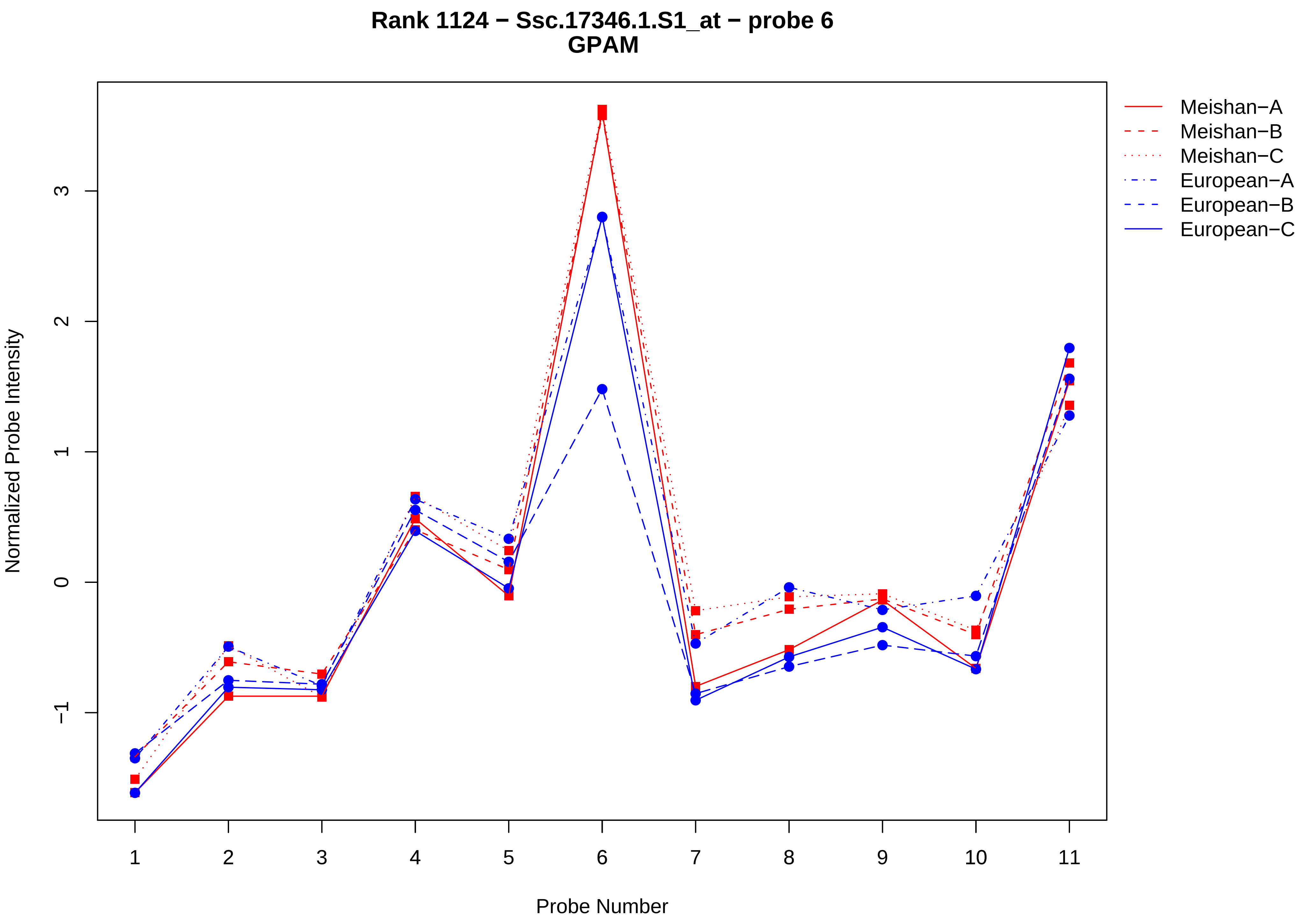

Rank 1124 − Ssc.17346.1.S1_at − probe 6
GPAM
Normalized Probe Intensity
Probe Number
−1
0
1
2
3
1
2
3
4
5
6
7
8
9
10
11
Meishan−A
Meishan−B
Meishan−C
European−A
European−B
European−C

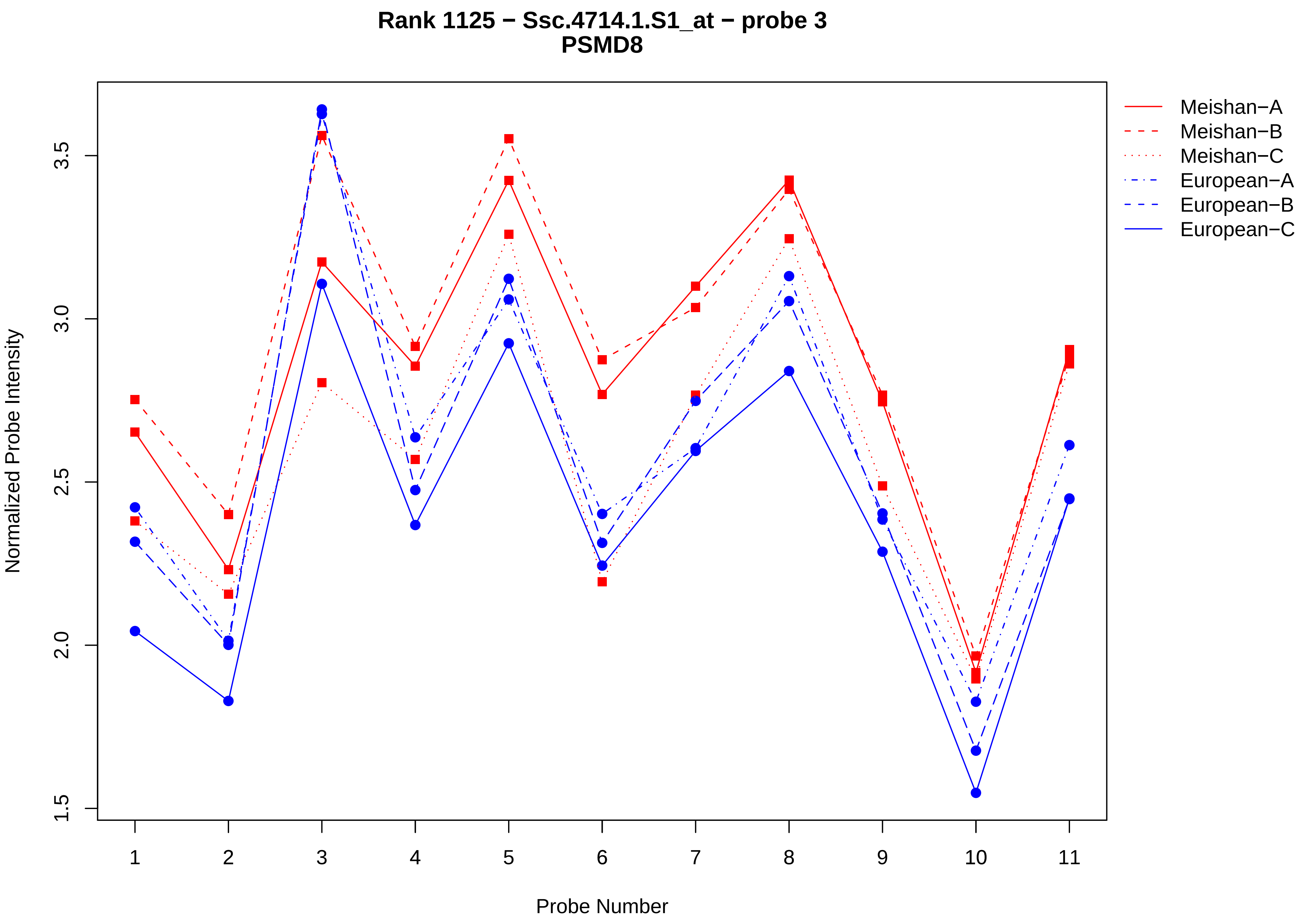

Rank 1125 − Ssc.4714.1.S1_at − probe 3
PSMD8
Normalized Probe Intensity
Probe Number
1.5
2.0
2.5
3.0
3.5
1
2
3
4
5
6
7
8
9
10
11
Meishan−A
Meishan−B
Meishan−C
European−A
European−B
European−C

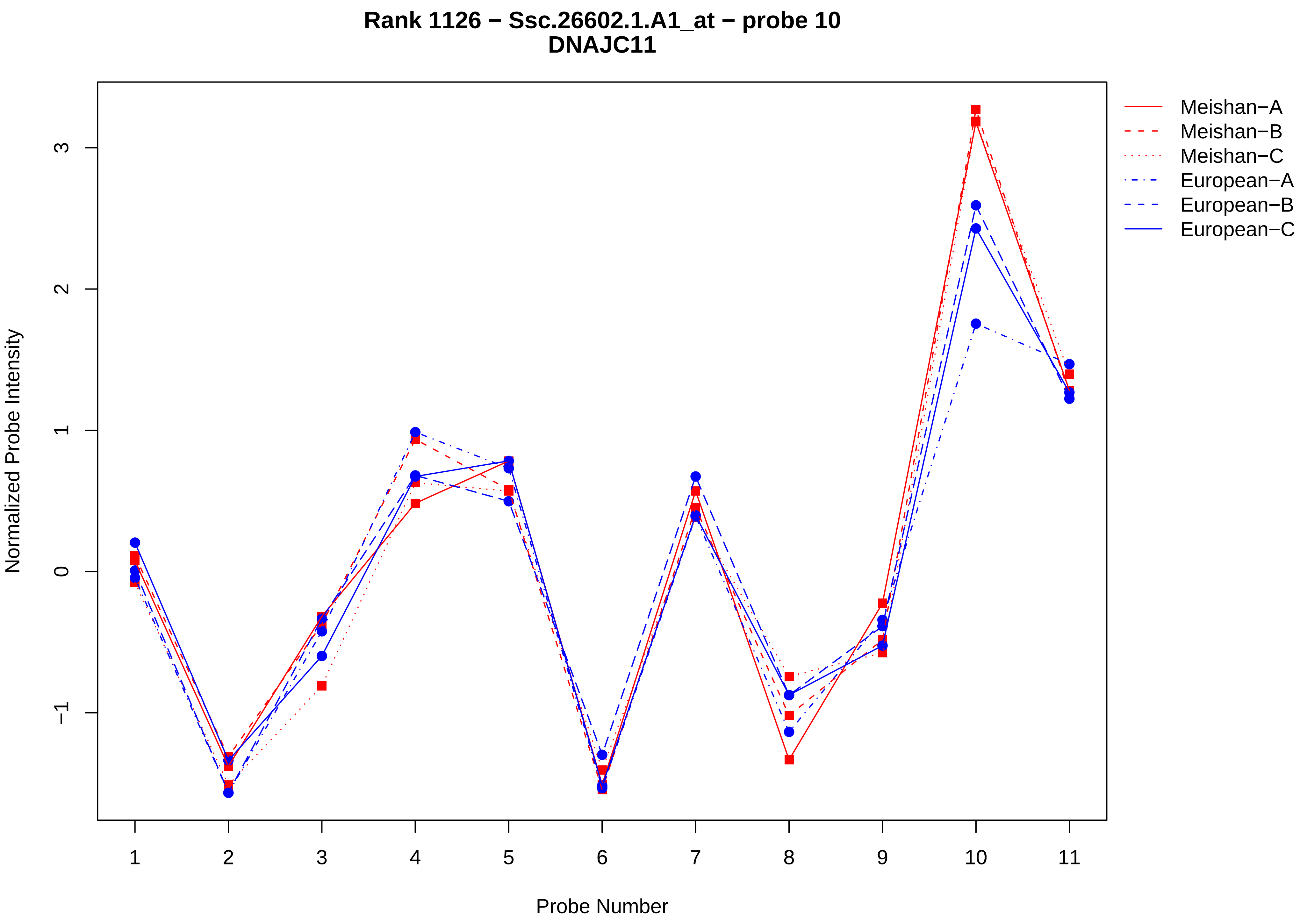

Rank 1126 − Ssc.26602.1.A1_at − probe 10
DNAJC11
Normalized Probe Intensity
Probe Number
−1
0
1
2
3
1
2
3
4
5
6
7
8
9
10
11
Meishan−A
Meishan−B
Meishan−C
European−A
European−B
European−C

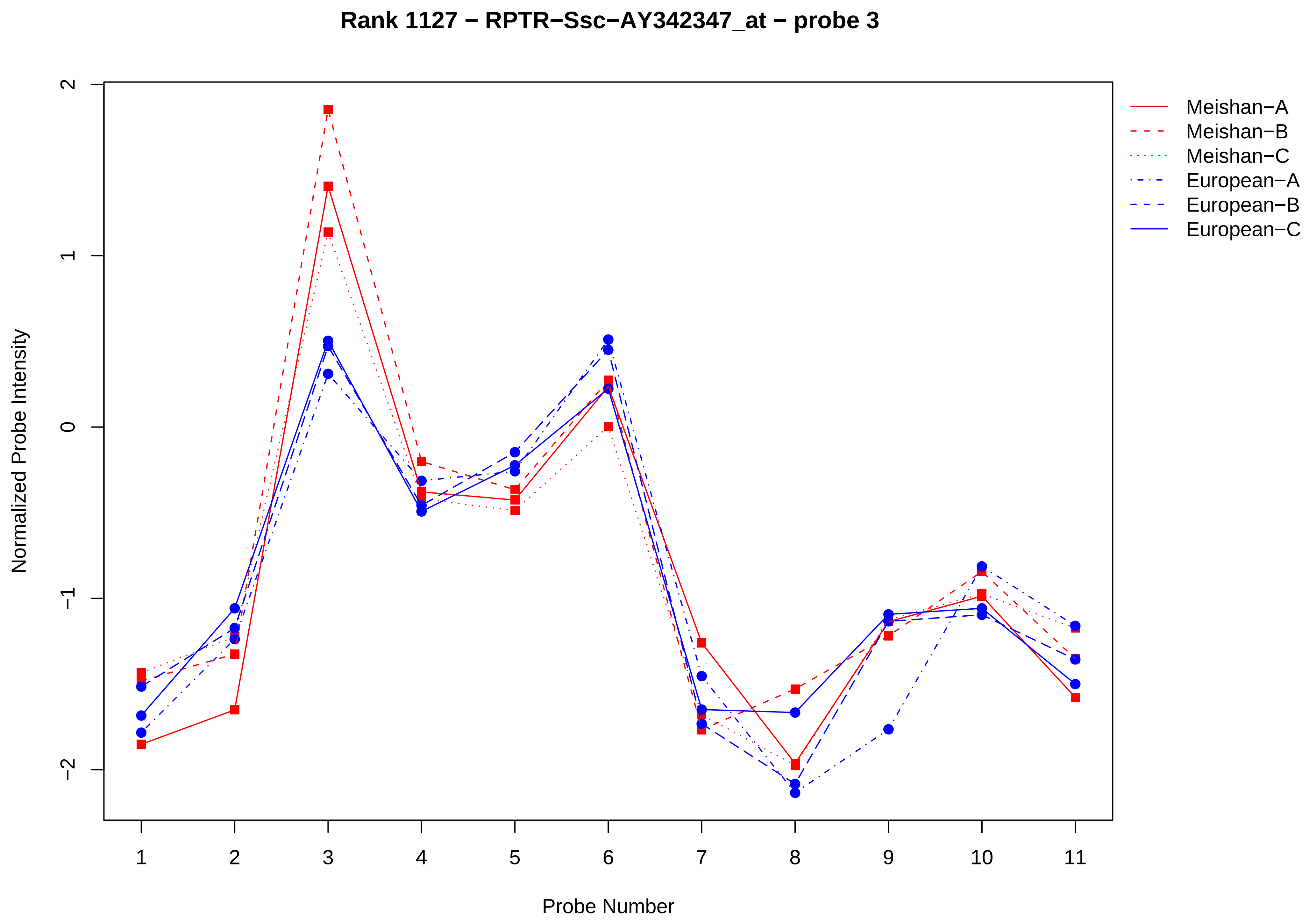

Rank 1127 − RPTR−Ssc−AY342347_at − probe 3
Normalized Probe Intensity
Probe Number
2
1
0
−1
−2
1
2
3
4
5
6
7
8
9
10
11
Meishan−A
Meishan−B
Meishan−C
European−A
European−B
European−C

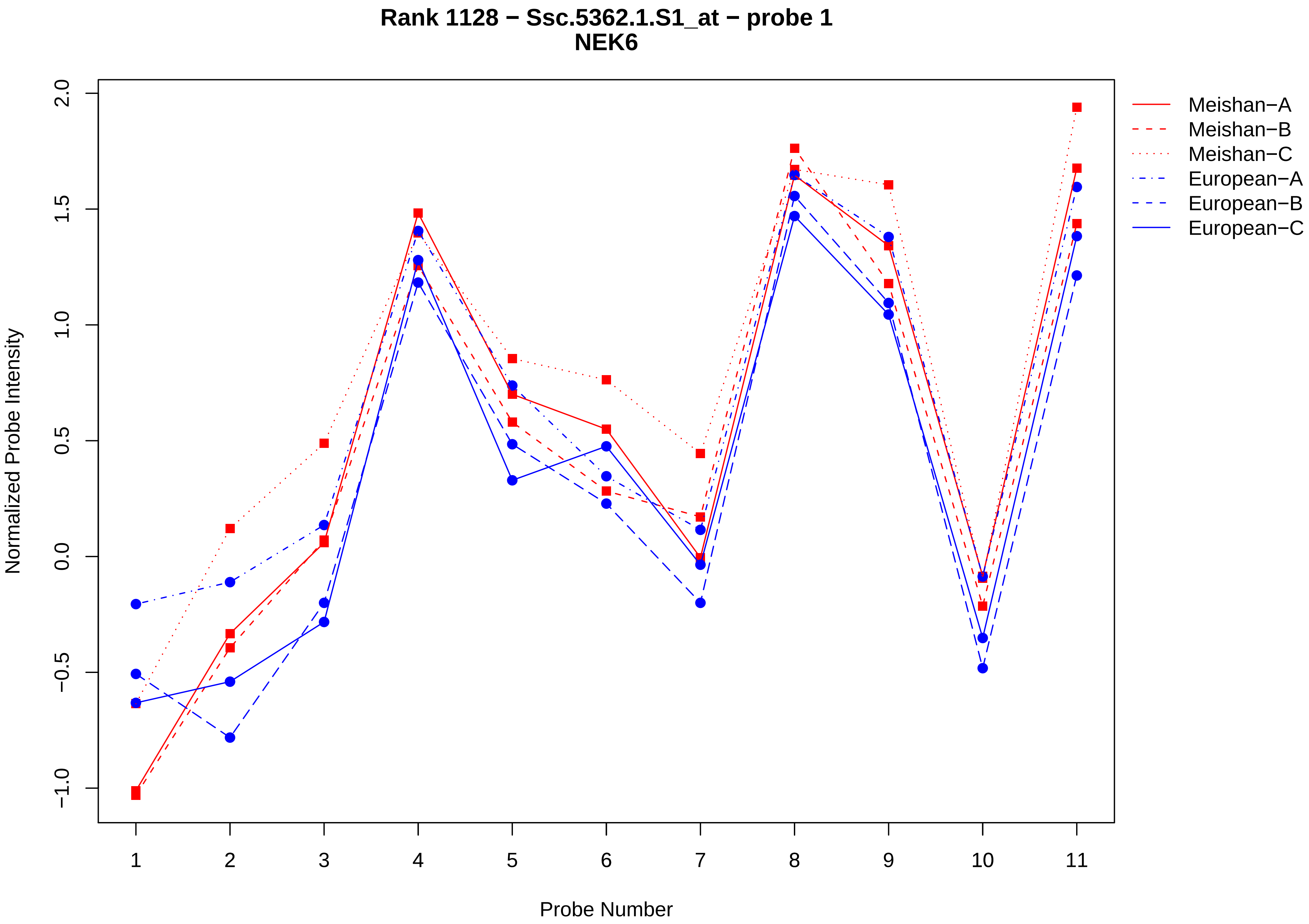

Rank 1128 − Ssc.5362.1.S1_at − probe 1
NEK6
Normalized Probe Intensity
Probe Number
Meishan−A
Meishan−B
Meishan−C
European−A
European−B
European−C

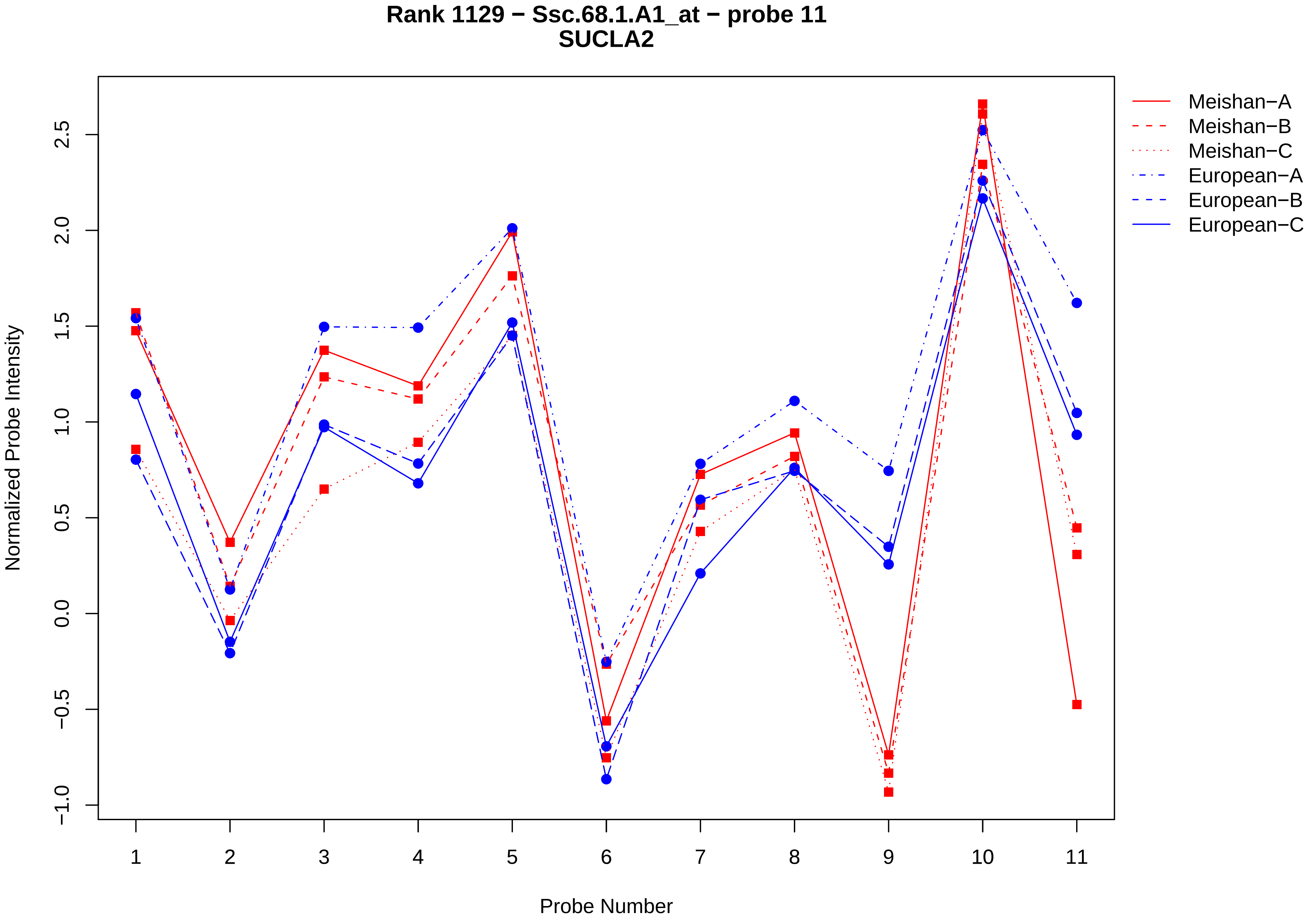

Rank 1129 − Ssc.68.1.A1_at − probe 11
SUCLA2
Normalized Probe Intensity
Probe Number
Meishan−A
Meishan−B
Meishan−C
European−A
European−B
European−C
−1.0
−0.5
0.0
0.5
1.0
1.5
2.0
2.5
1
2
3
4
5
6
7
8
9
10
11

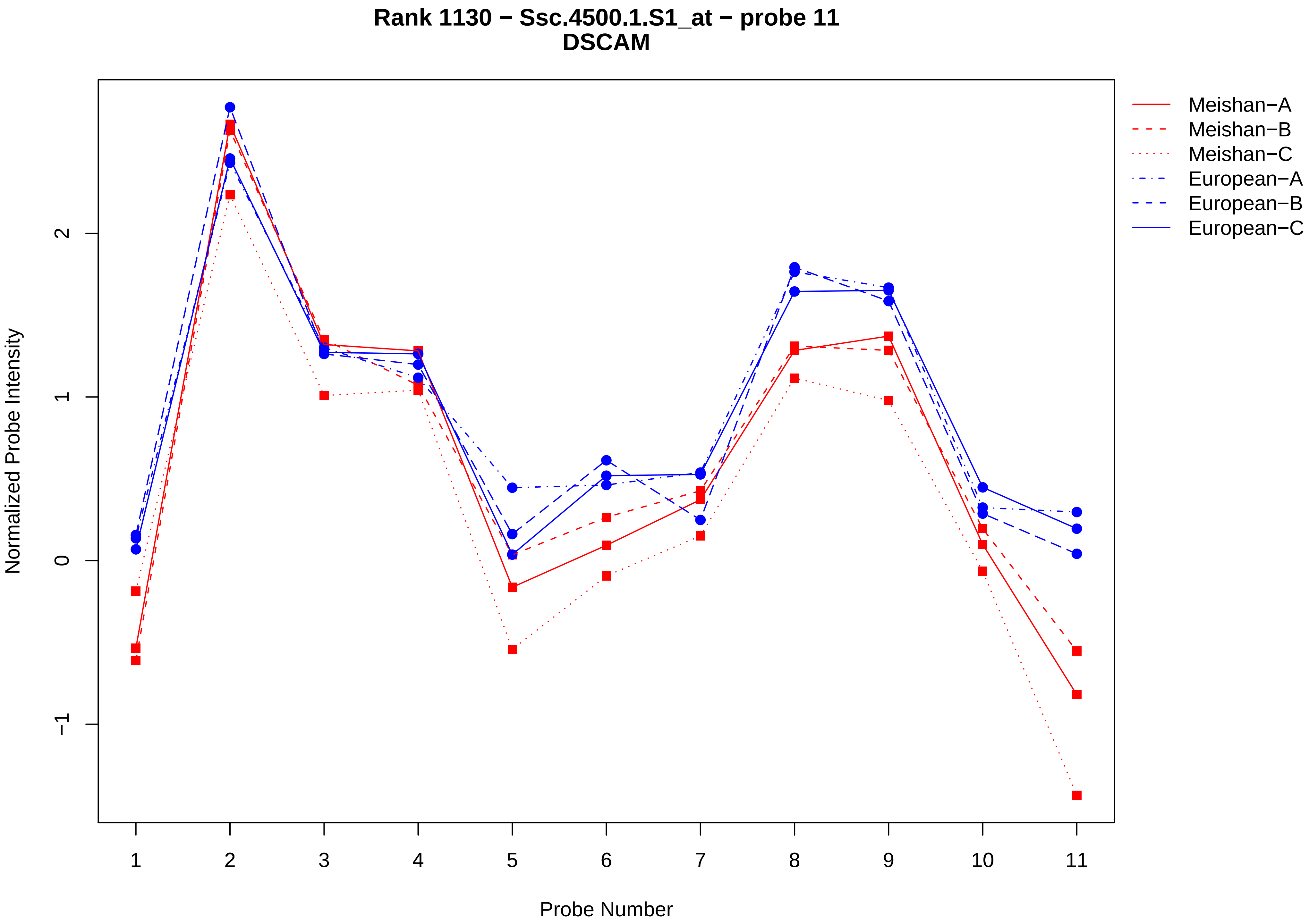

Rank 1130 − Ssc.4500.1.S1_at − probe 11
DSCAM
Normalized Probe Intensity
Probe Number
−1
0
1
2
1
2
3
4
5
6
7
8
9
10
11
Meishan−A
Meishan−B
Meishan−C
European−A
European−B
European−C

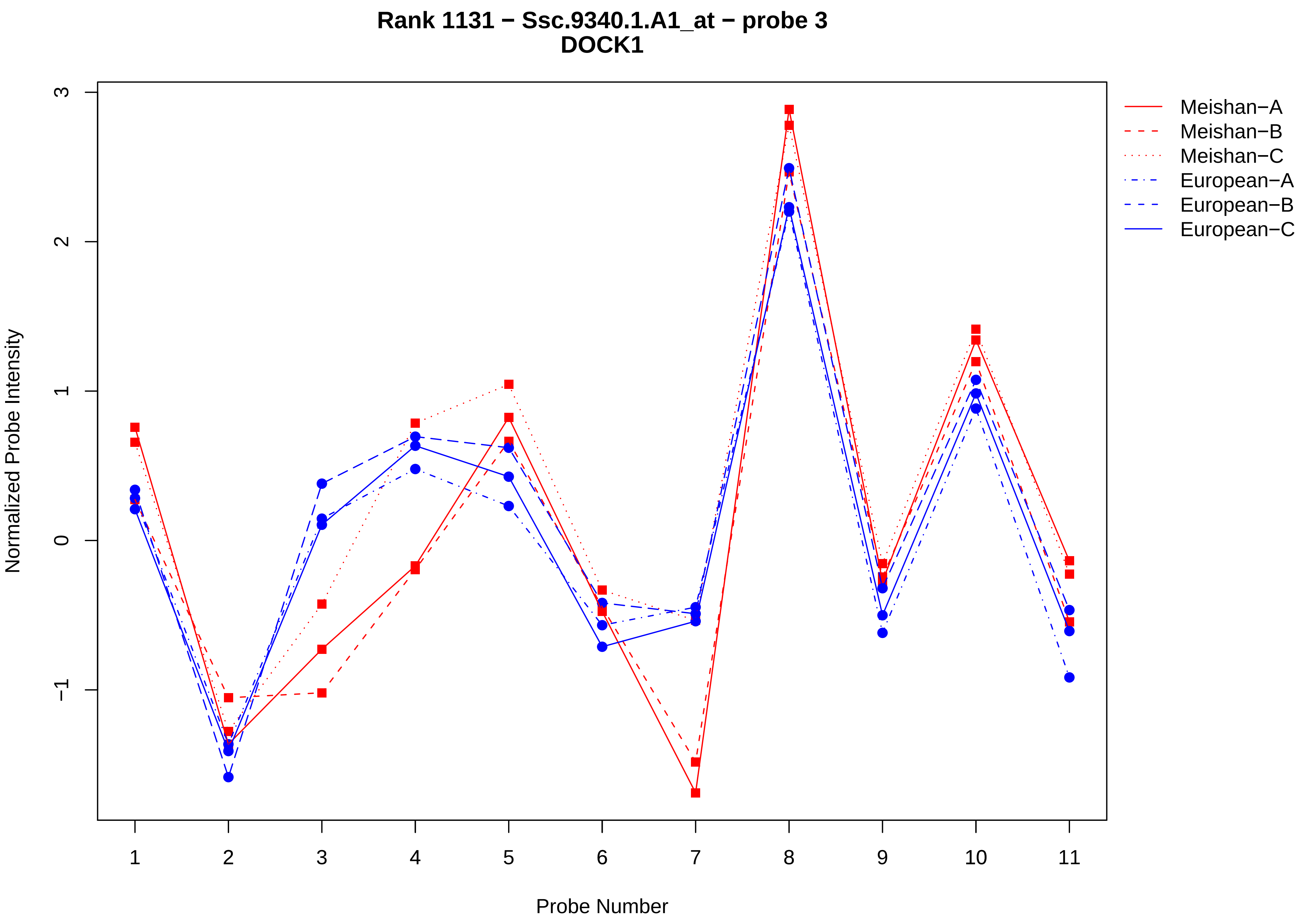

Rank 1131 − Ssc.9340.1.A1_at − probe 3
DOCK1
Normalized Probe Intensity
Probe Number
Meishan−A
Meishan−B
Meishan−C
European−A
European−B
European−C

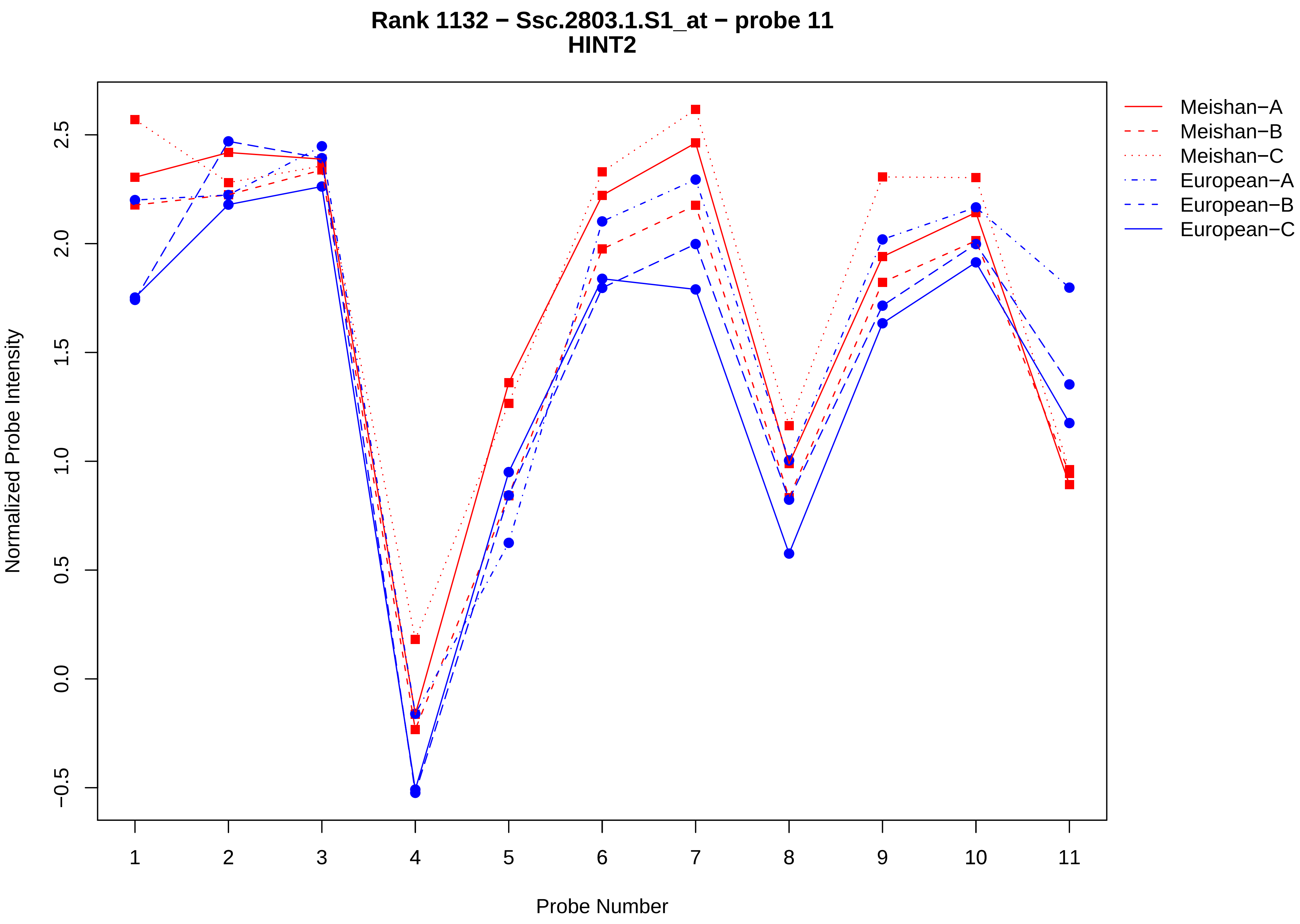

Rank 1132 − Ssc.2803.1.S1_at − probe 11
HINT2
Normalized Probe Intensity
Probe Number
Meishan−A
Meishan−B
Meishan−C
European−A
European−B
European−C
−0.5
0.0
0.5
1.0
1.5
2.0
2.5
1
2
3
4
5
6
7
8
9
10
11

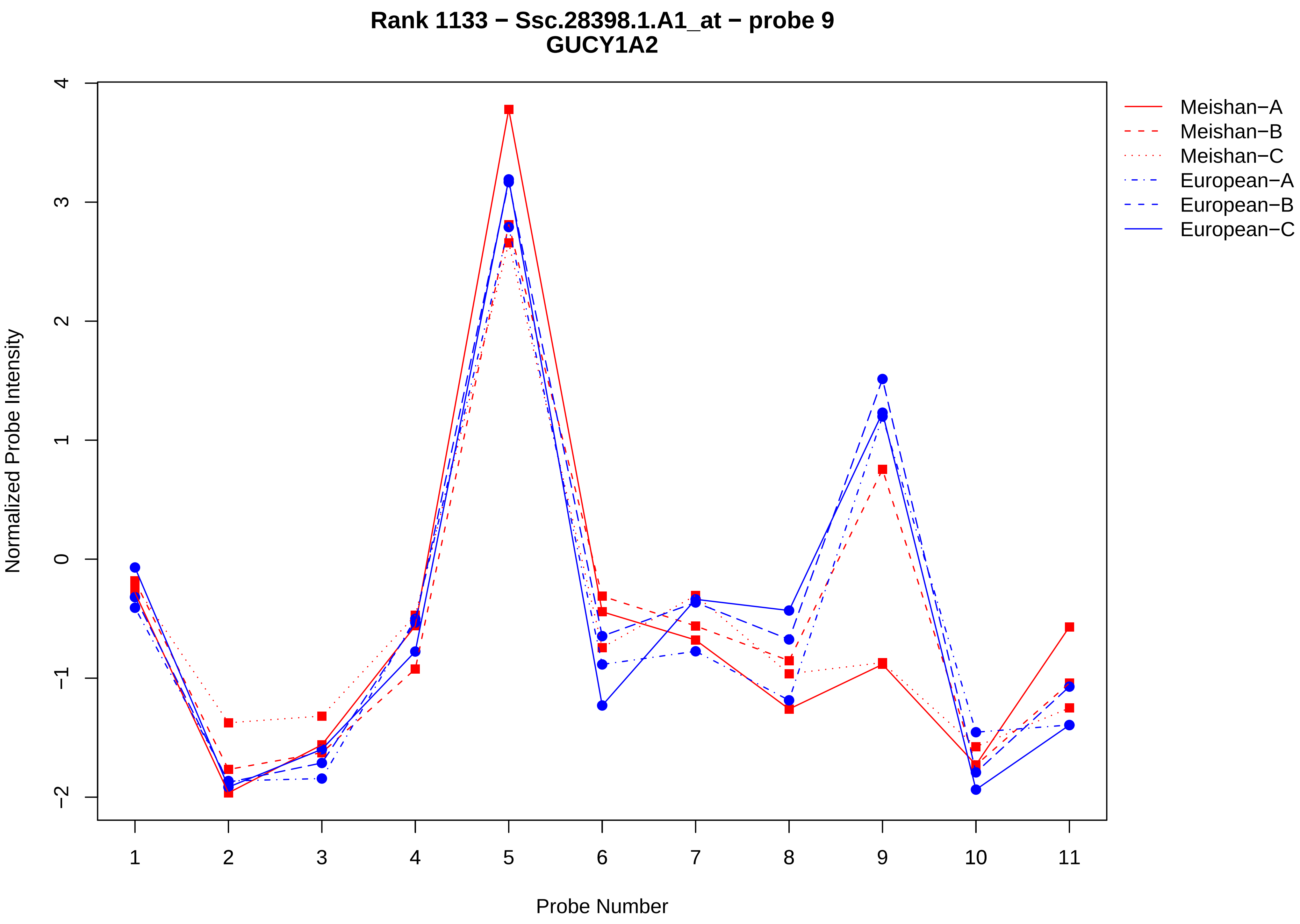

Rank 1133 − Ssc.28398.1.A1_at − probe 9
GUCY1A2
Normalized Probe Intensity
Probe Number
Meishan−A
Meishan−B
Meishan−C
European−A
European−B
European−C

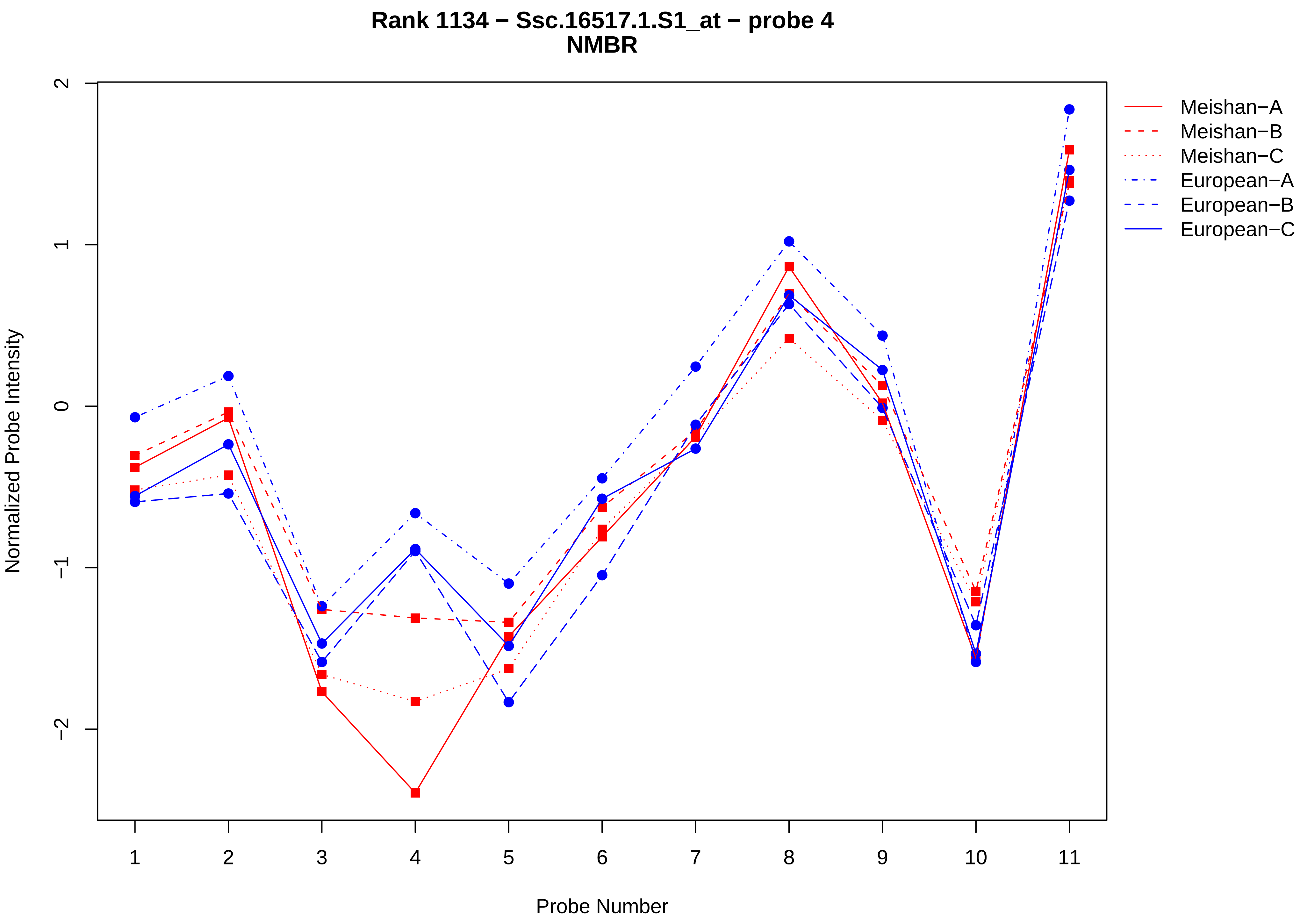

Rank 1134 − Ssc.16517.1.S1_at − probe 4
NMBR
Normalized Probe Intensity
Probe Number
Meishan−A
Meishan−B
Meishan−C
European−A
European−B
European−C
2
1
0
−1
−2
1
2
3
4
5
6
7
8
9
10
11

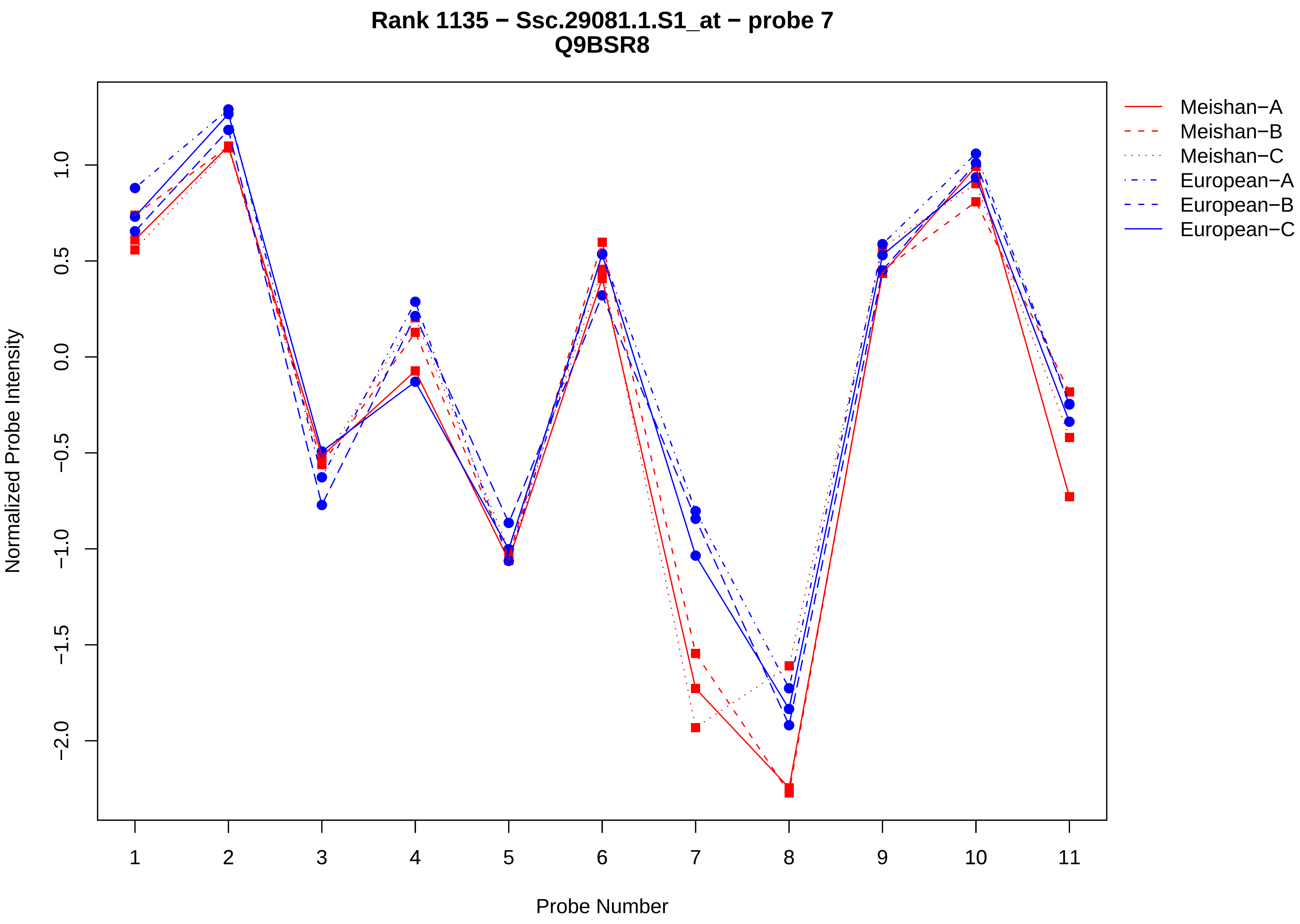
Rank 1135 − Ssc.29081.1.S1_at − probe 7
Q9BSR8
Normalized Probe Intensity
Probe Number
Meishan−A
Meishan−B
Meishan−C
European−A
European−B
European−C
−2.0
−1.5
−1.0
−0.5
0.0
0.5
1.0
1
2
3
4
5
6
7
8
9
10
11

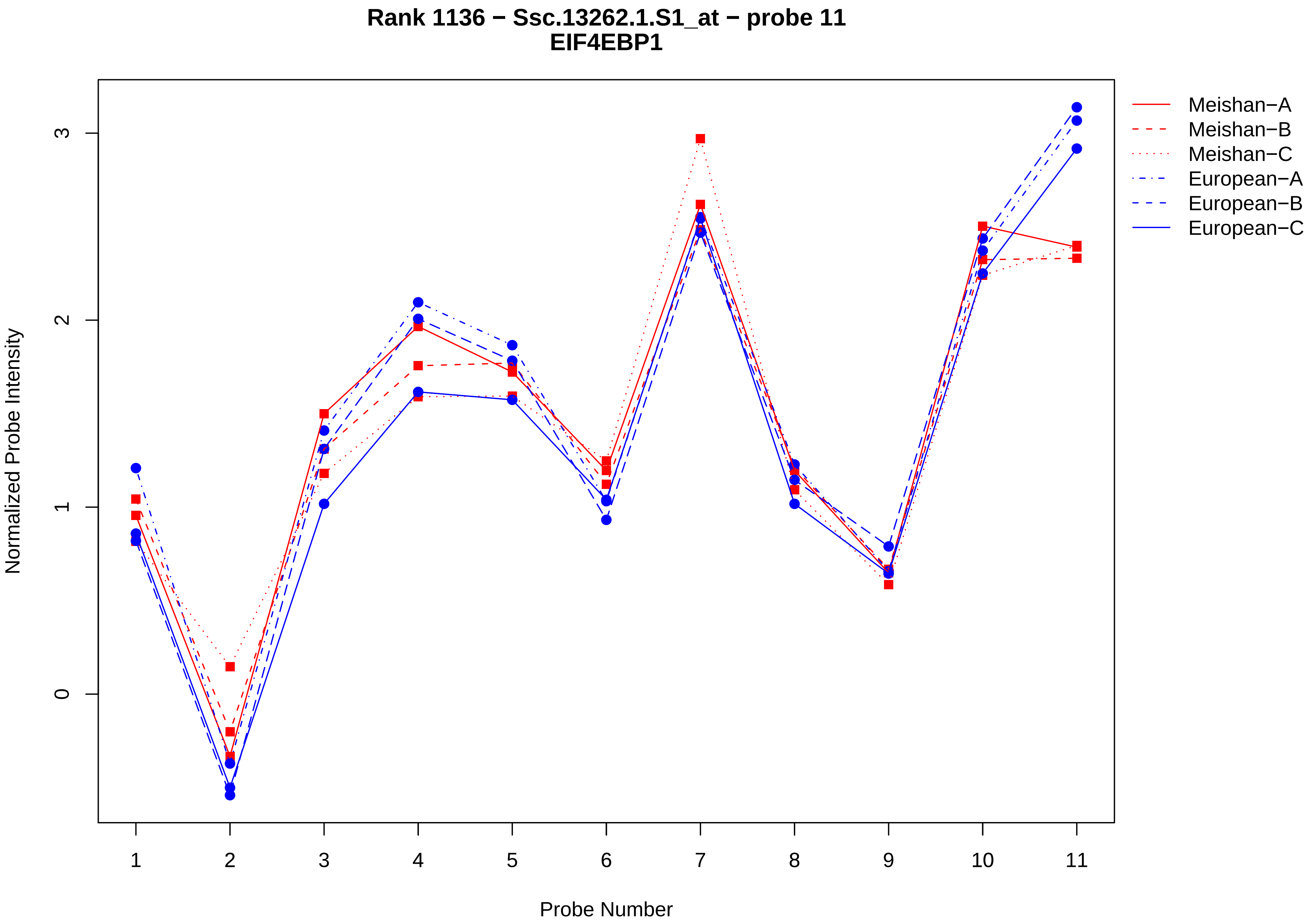
Rank 1136 − Ssc.13262.1.S1_at − probe 11
EIF4EBP1
Normalized Probe Intensity
0
1
2
3
1
2
3
4
5
6
7
8
9
10
11
Probe Number
Meishan−A
Meishan−B
Meishan−C
European−A
European−B
European−C

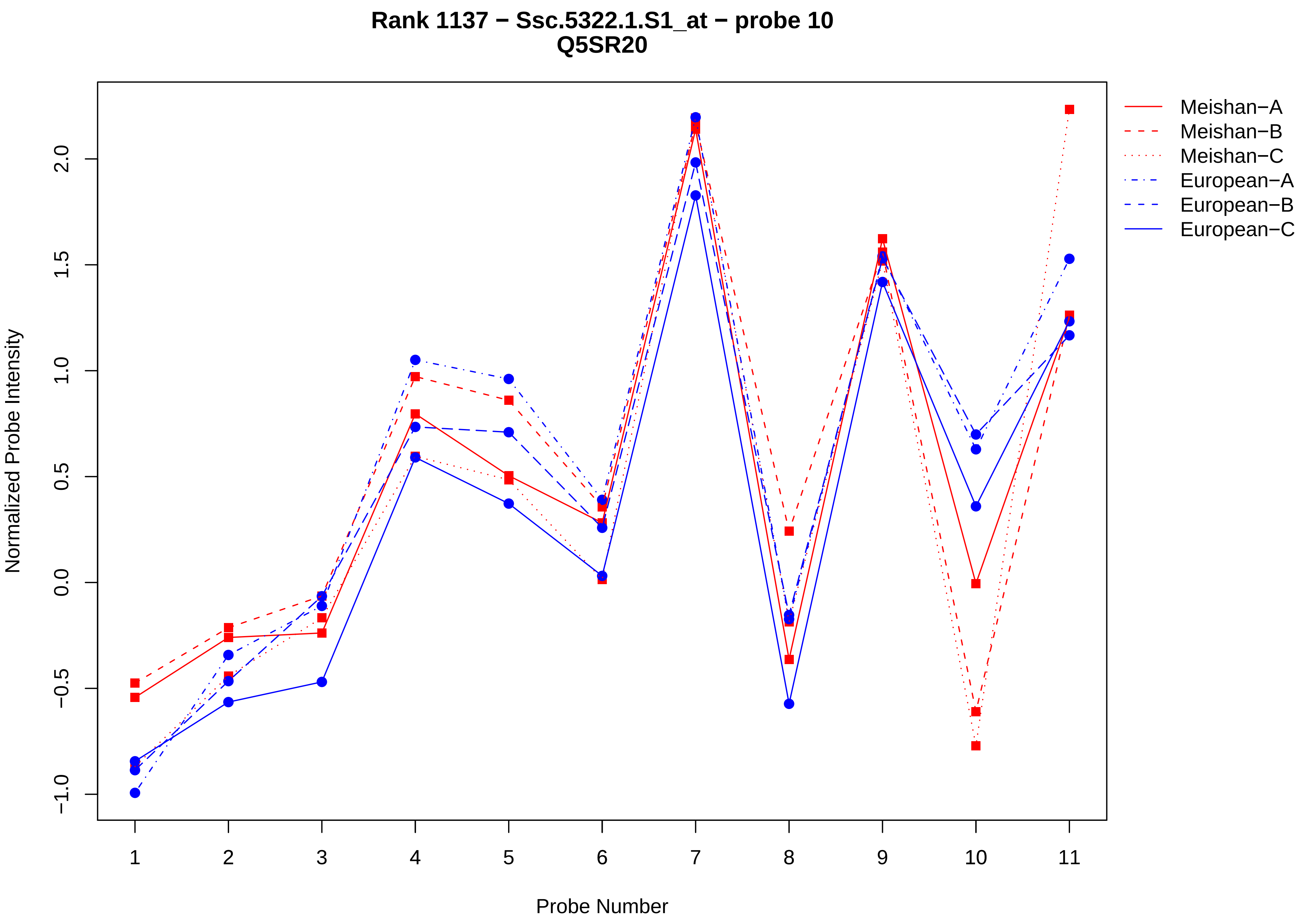

Rank 1137 − Ssc.5322.1.S1_at − probe 10
Q5SR20
Normalized Probe Intensity
Probe Number
Meishan−A
Meishan−B
Meishan−C
European−A
European−B
European−C

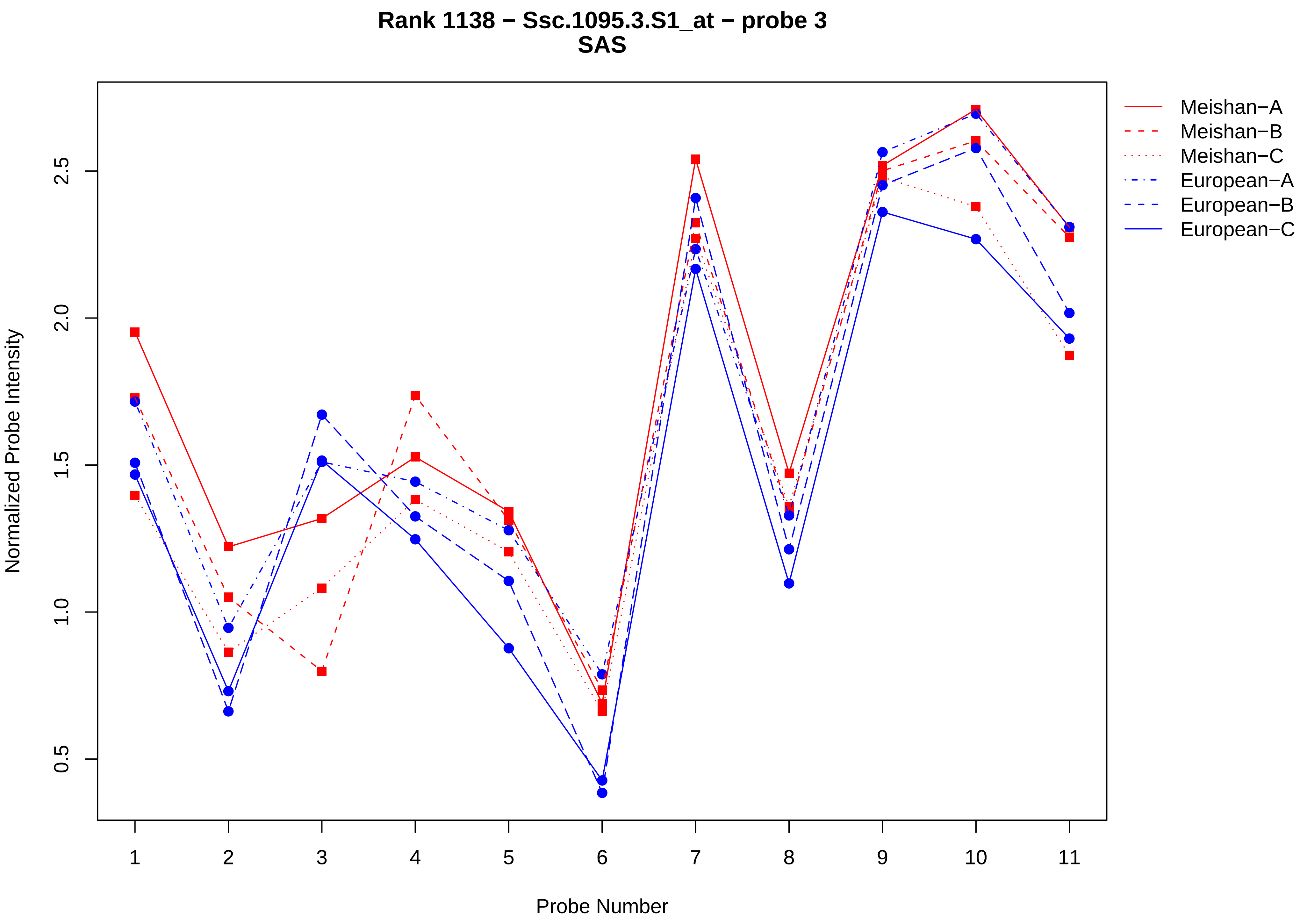
Rank 1138 − Ssc.1095.3.S1_at − probe 3
SAS
Normalized Probe Intensity
Probe Number
0.5
1.0
1.5
2.0
2.5
1
2
3
4
5
6
7
8
9
10
11
Meishan−A
Meishan−B
Meishan−C
European−A
European−B
European−C

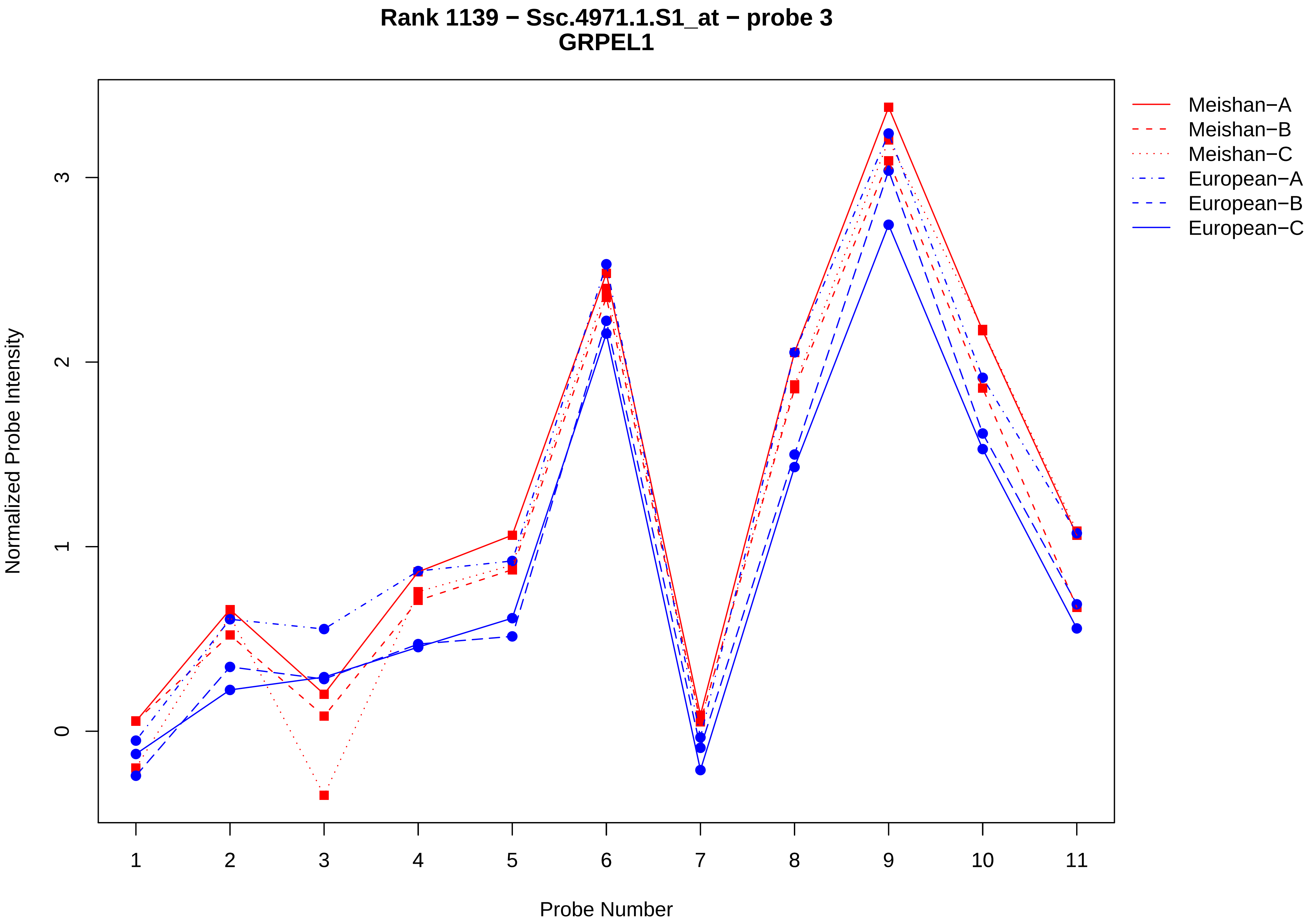

Rank 1139 − Ssc.4971.1.S1_at − probe 3
GRPEL1
Normalized Probe Intensity
Probe Number
0
1
2
3
1
2
3
4
5
6
7
8
9
10
11
Meishan−A
Meishan−B
Meishan−C
European−A
European−B
European−C

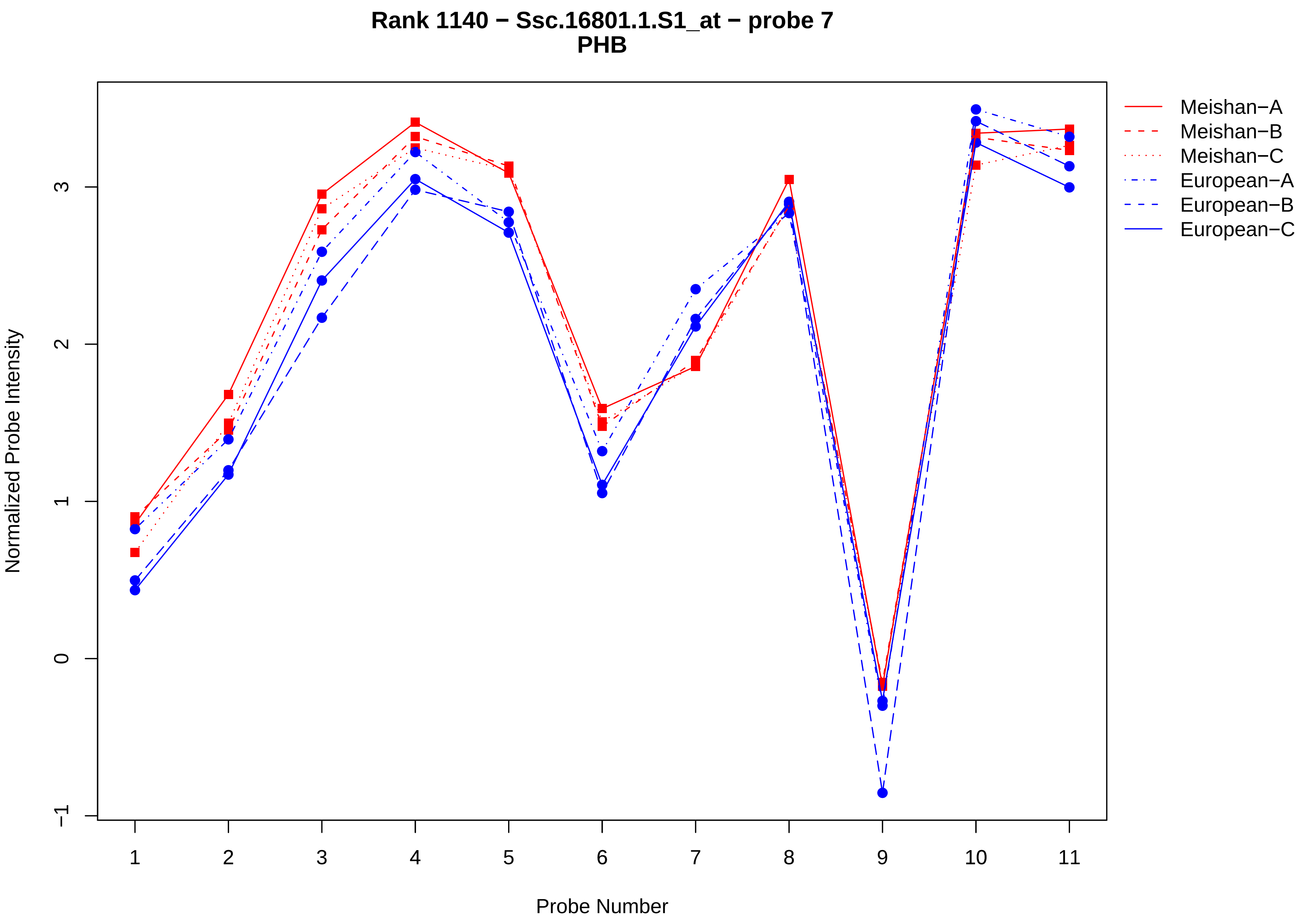

Rank 1140 − Ssc.16801.1.S1_at − probe 7
PHB
Normalized Probe Intensity
Probe Number
−1
0
1
2
3
1
2
3
4
5
6
7
8
9
10
11
Meishan−A
Meishan−B
Meishan−C
European−A
European−B
European−C

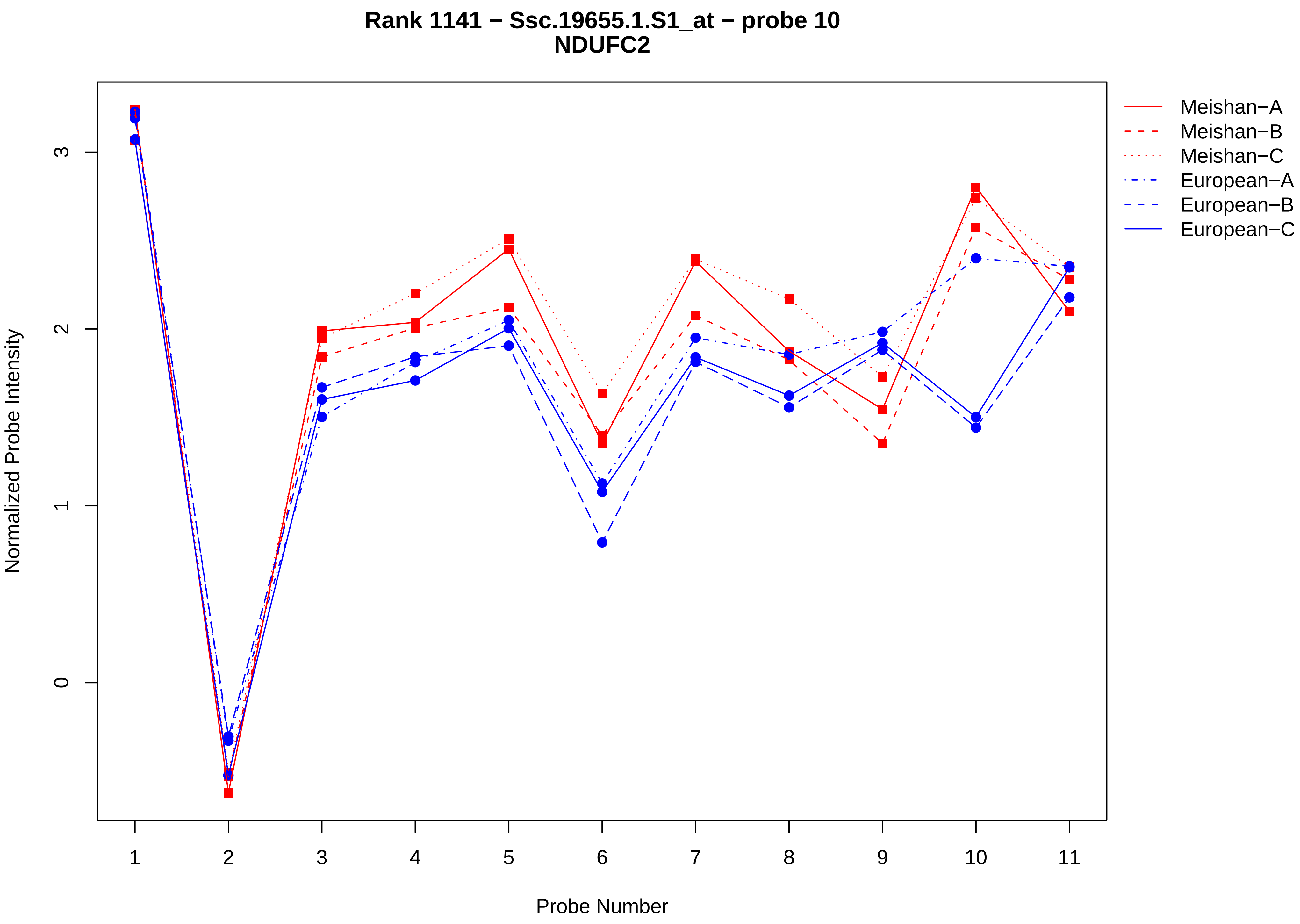
Rank 1141 − Ssc.19655.1.S1_at − probe 10
NDUFC2
Normalized Probe Intensity
Probe Number
0
1
2
3
1
2
3
4
5
6
7
8
9
10
11
Meishan−A
Meishan−B
Meishan−C
European−A
European−B
European−C

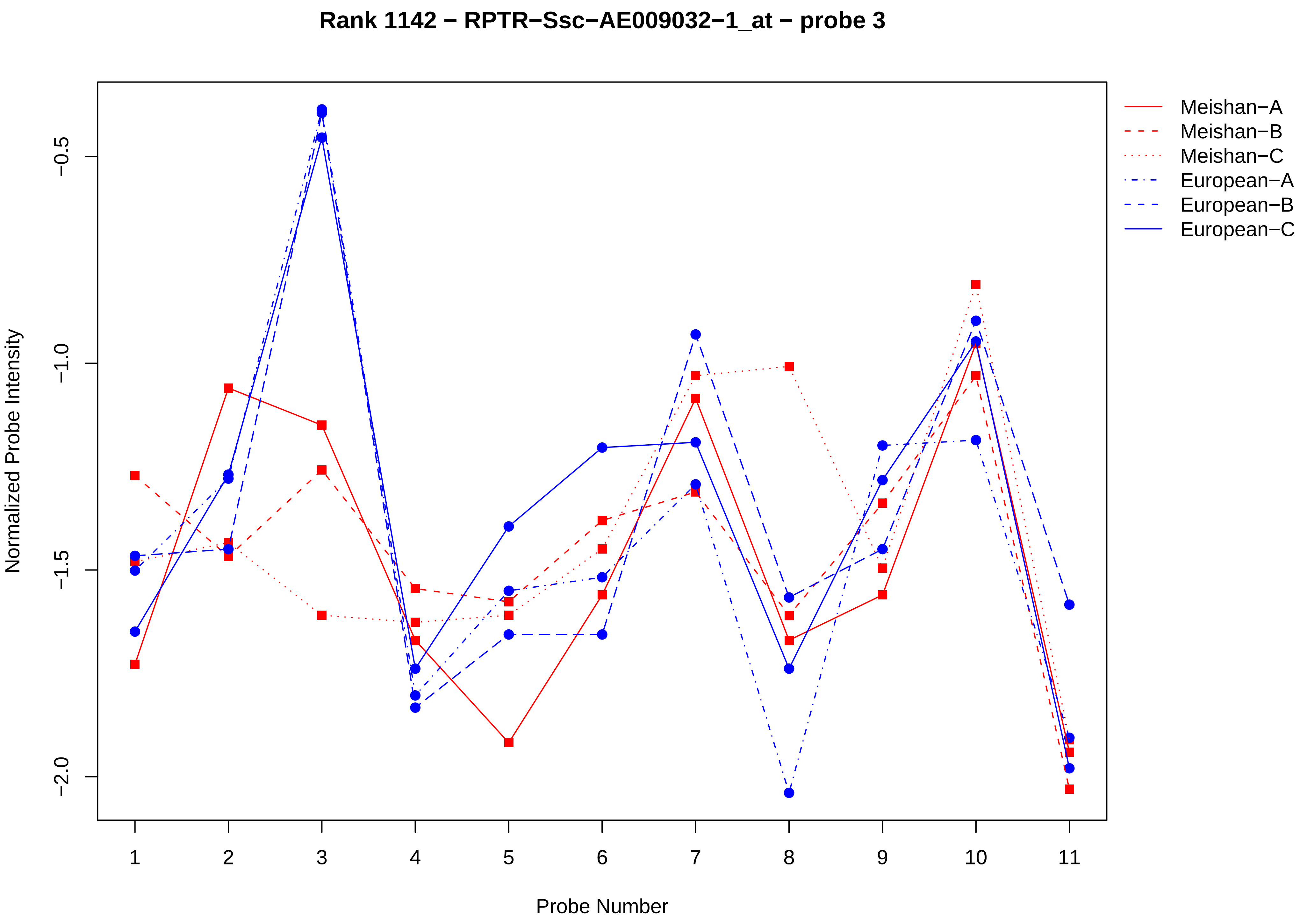

Rank 1142 − RPTR−Ssc−AE009032−1_at − probe 3
Normalized Probe Intensity
Probe Number
−0.5
−1.0
−1.5
−2.0
1
2
3
4
5
6
7
8
9
10
11
Meishan−A
Meishan−B
Meishan−C
European−A
European−B
European−C

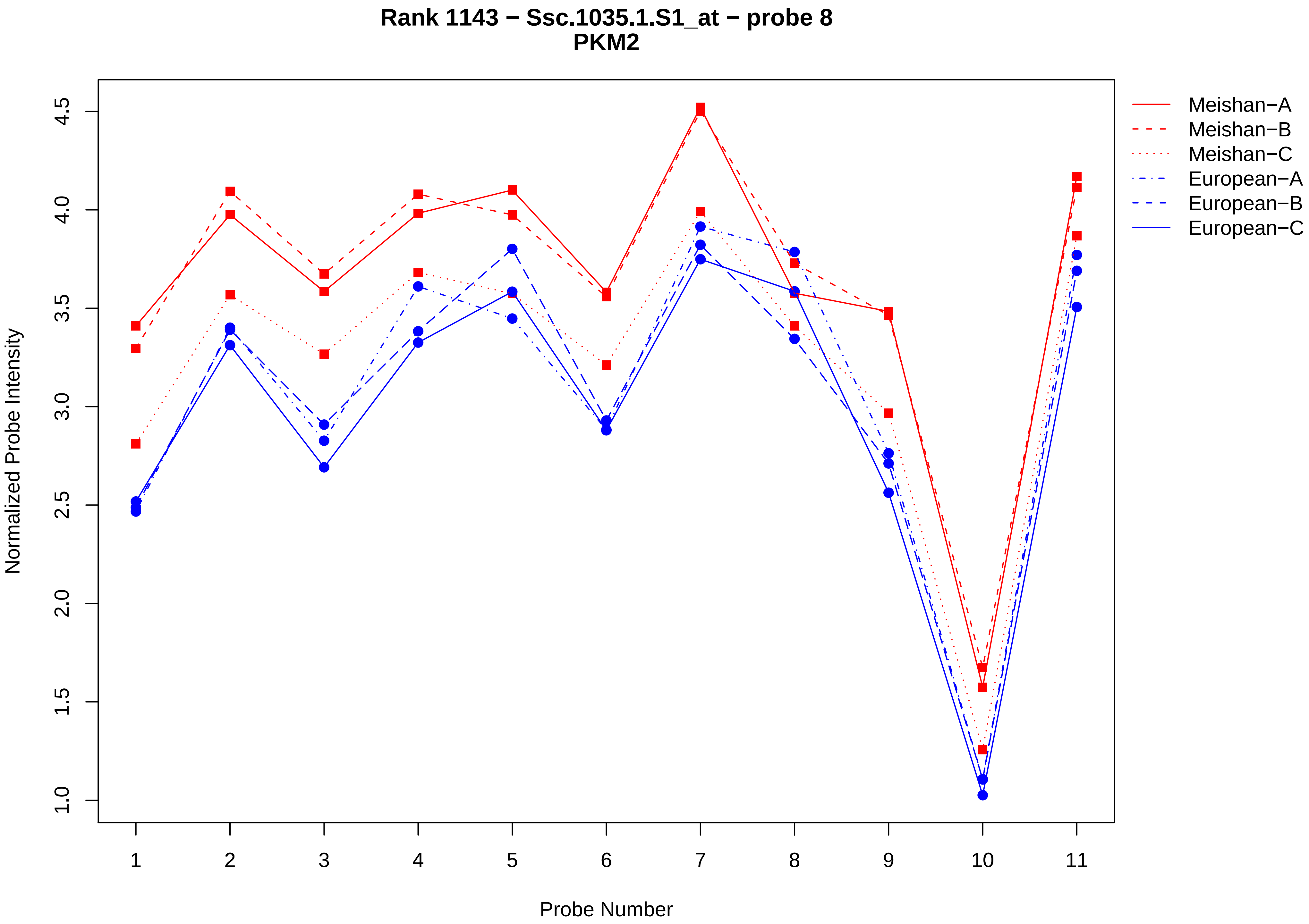

Rank 1143 − Ssc.1035.1.S1_at − probe 8
PKM2
Normalized Probe Intensity
1.0
1.5
2.0
2.5
3.0
3.5
4.0
4.5
1
2
3
4
5
6
7
8
9
10
11
Probe Number
Meishan−A
Meishan−B
Meishan−C
European−A
European−B
European−C

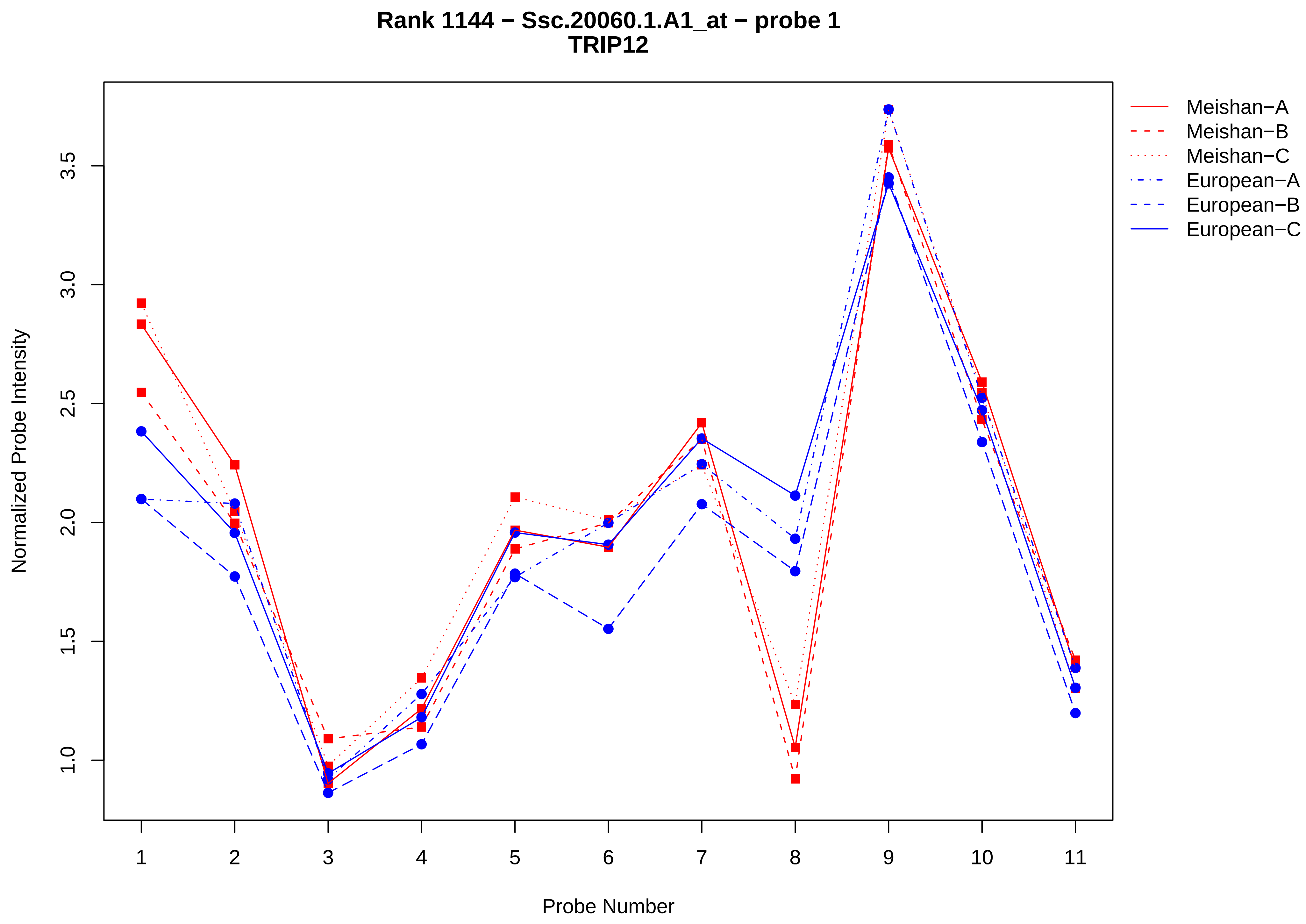
Rank 1144 − Ssc.20060.1.A1_at − probe 1
TRIP12
Normalized Probe Intensity
Probe Number
1.0
1.5
2.0
2.5
3.0
3.5
1
2
3
4
5
6
7
8
9
10
11
Meishan−A
Meishan−B
Meishan−C
European−A
European−B
European−C

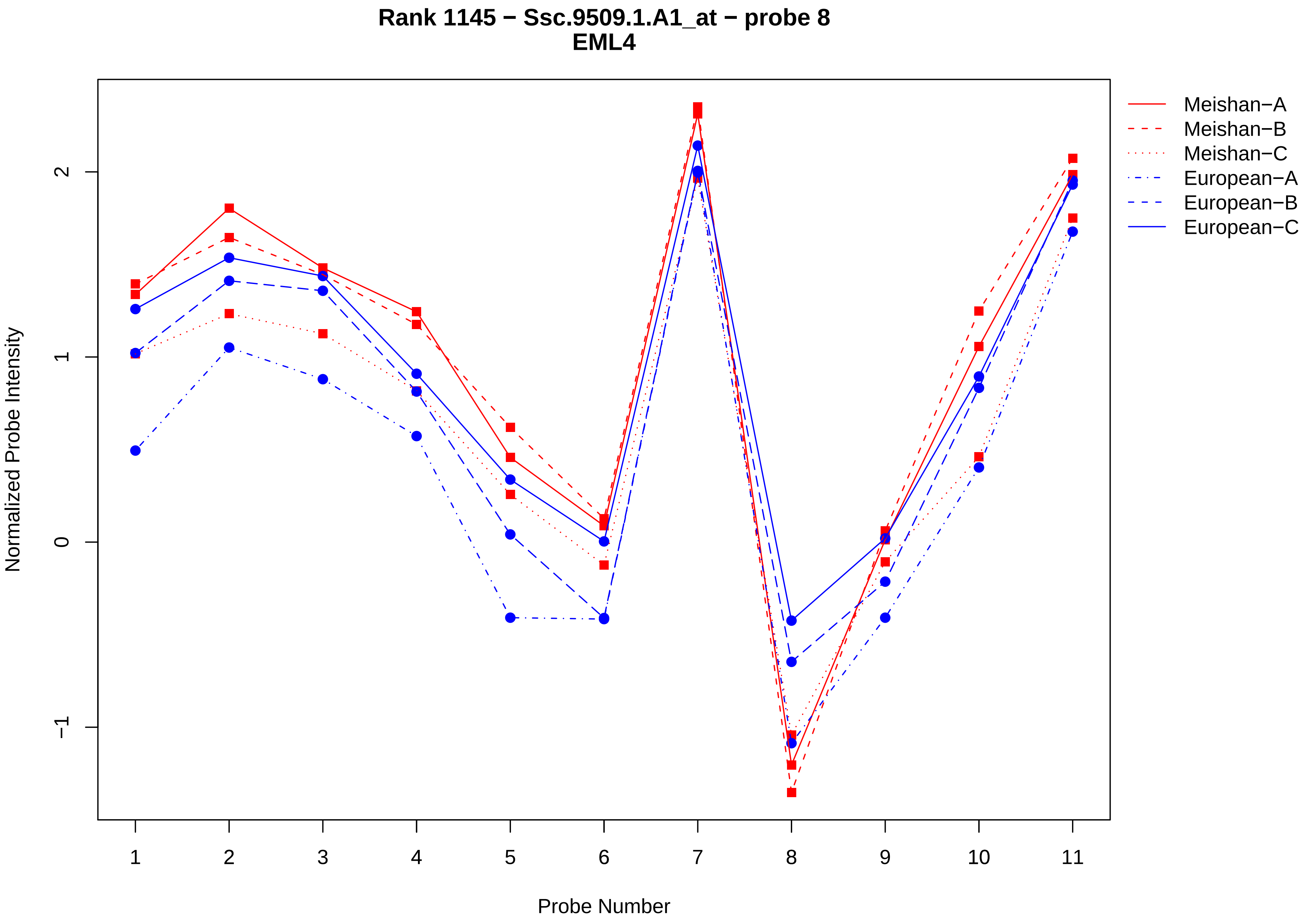

Rank 1145 − Ssc.9509.1.A1_at − probe 8
EML4
Normalized Probe Intensity
Probe Number
−1
0
1
2
1
2
3
4
5
6
7
8
9
10
11
Meishan−A
Meishan−B
Meishan−C
European−A
European−B
European−C

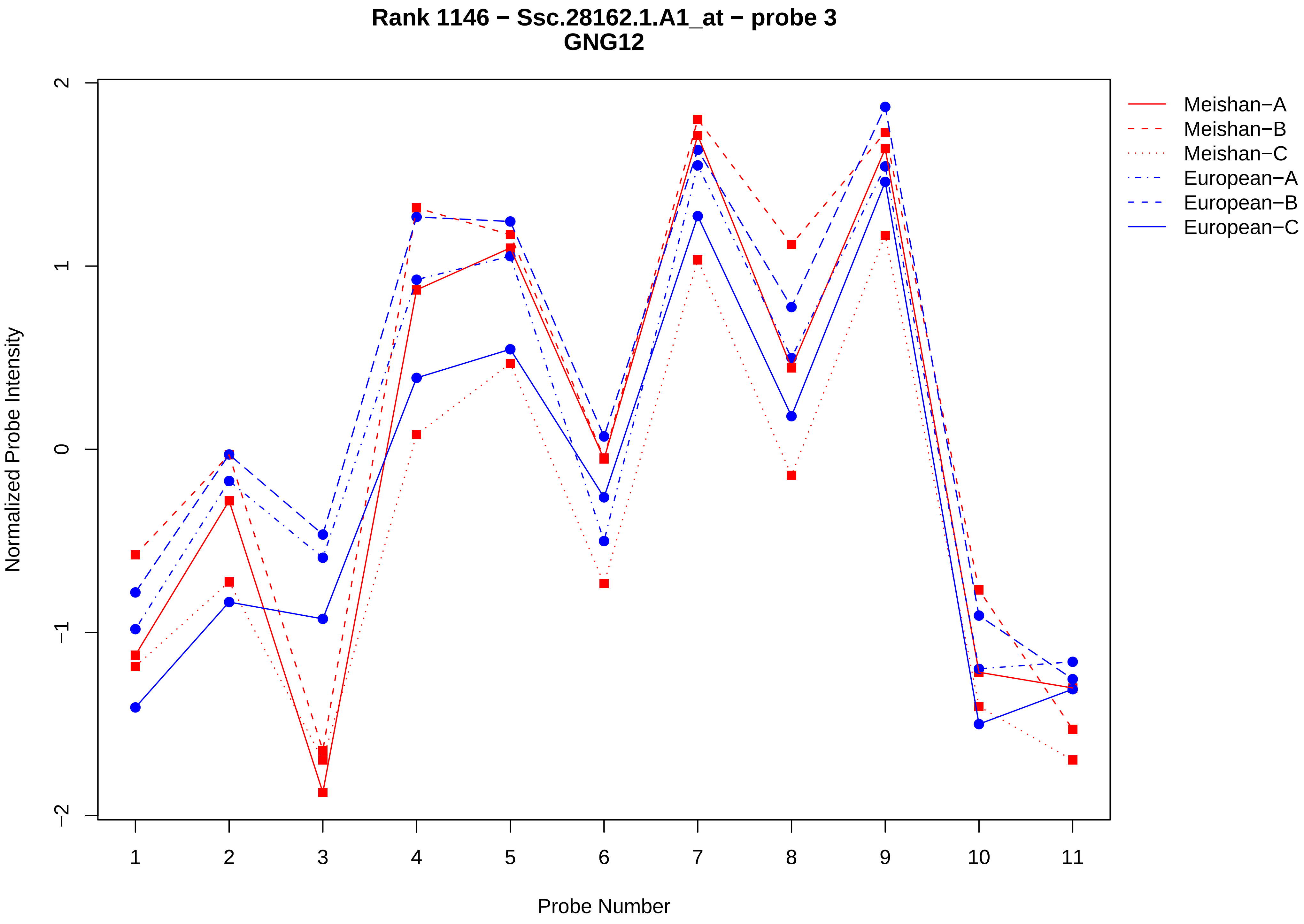
Rank 1146 − Ssc.28162.1.A1_at − probe 3
GNG12
Normalized Probe Intensity
Probe Number
Meishan−A
Meishan−B
Meishan−C
European−A
European−B
European−C
2
1
0
−1
−2
1
2
3
4
5
6
7
8
9
10
11

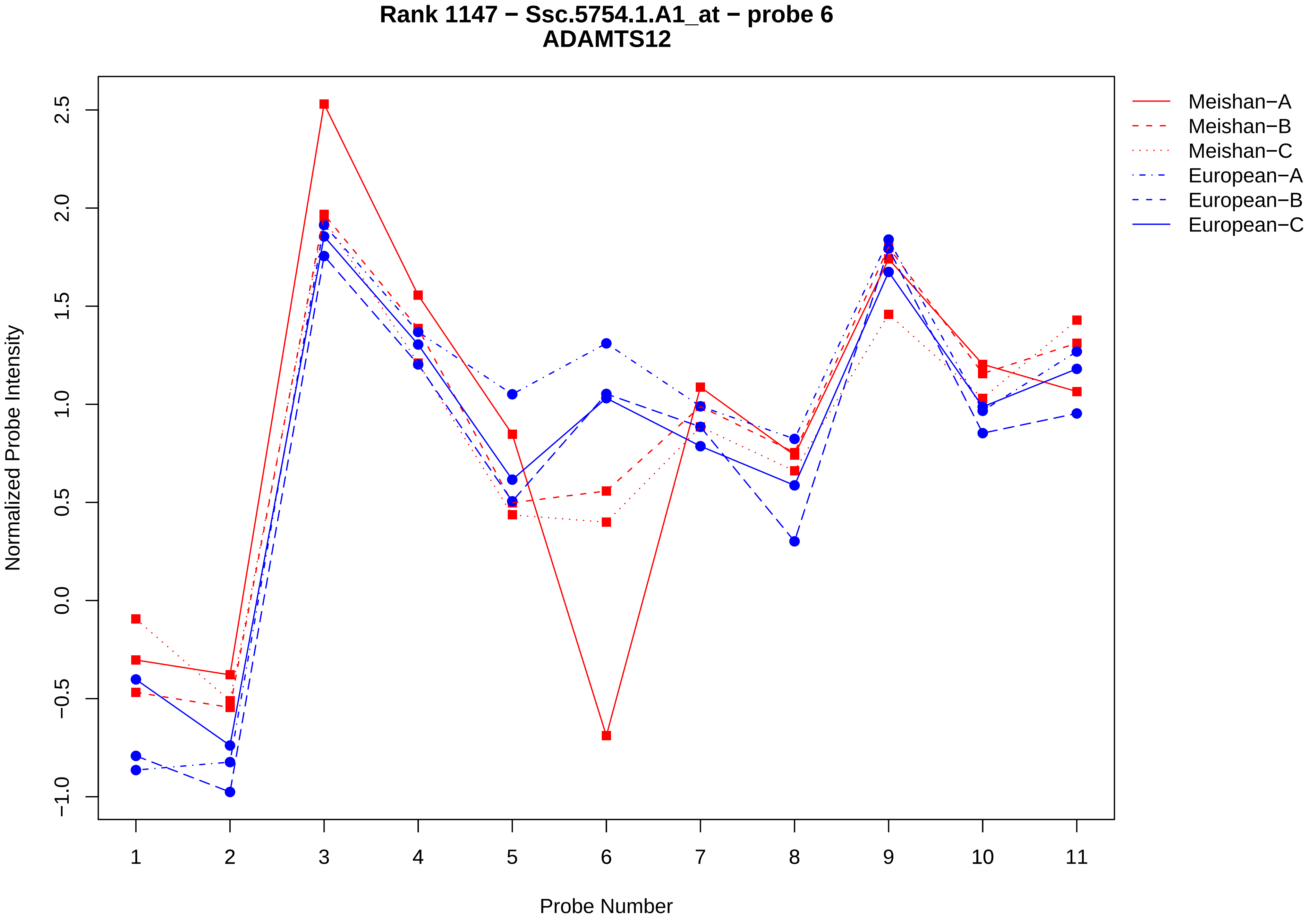

Rank 1147 − Ssc.5754.1.A1_at − probe 6
ADAMTS12
Normalized Probe Intensity
Probe Number
−1.0
−0.5
0.0
0.5
1.0
1.5
2.0
2.5
1
2
3
4
5
6
7
8
9
10
11
Meishan−A
Meishan−B
Meishan−C
European−A
European−B
European−C

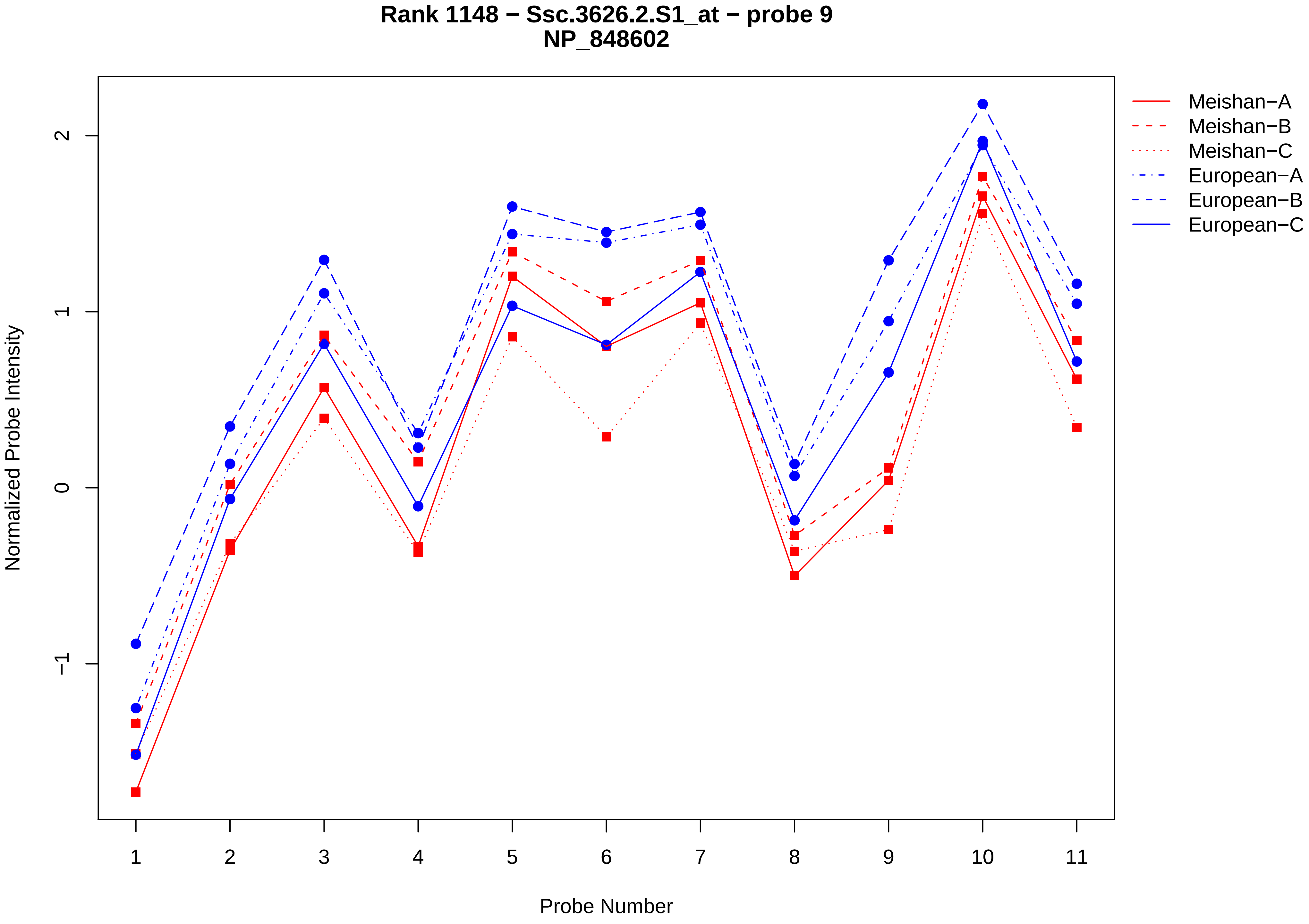

Rank 1148 − Ssc.3626.2.S1_at − probe 9
NP_848602
Normalized Probe Intensity
Probe Number
−1
0
1
2
1
2
3
4
5
6
7
8
9
10
11
Meishan−A
Meishan−B
Meishan−C
European−A
European−B
European−C

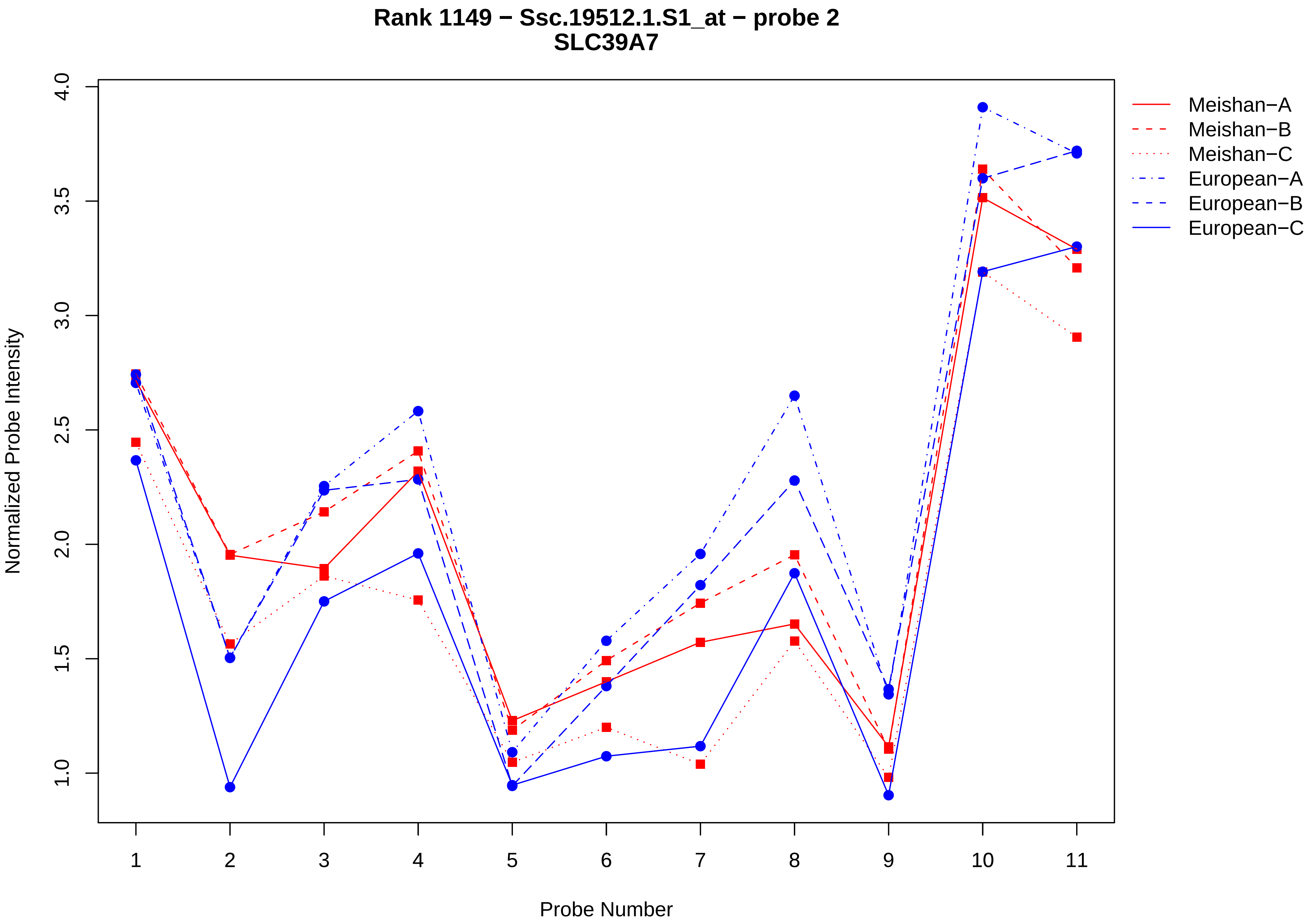

Rank 1149 − Ssc.19512.1.S1_at − probe 2
SLC39A7
Normalized Probe Intensity
1.0
1.5
2.0
2.5
3.0
3.5
4.0
Probe Number
1
2
3
4
5
6
7
8
9
10
11
Meishan−A
Meishan−B
Meishan−C
European−A
European−B
European−C

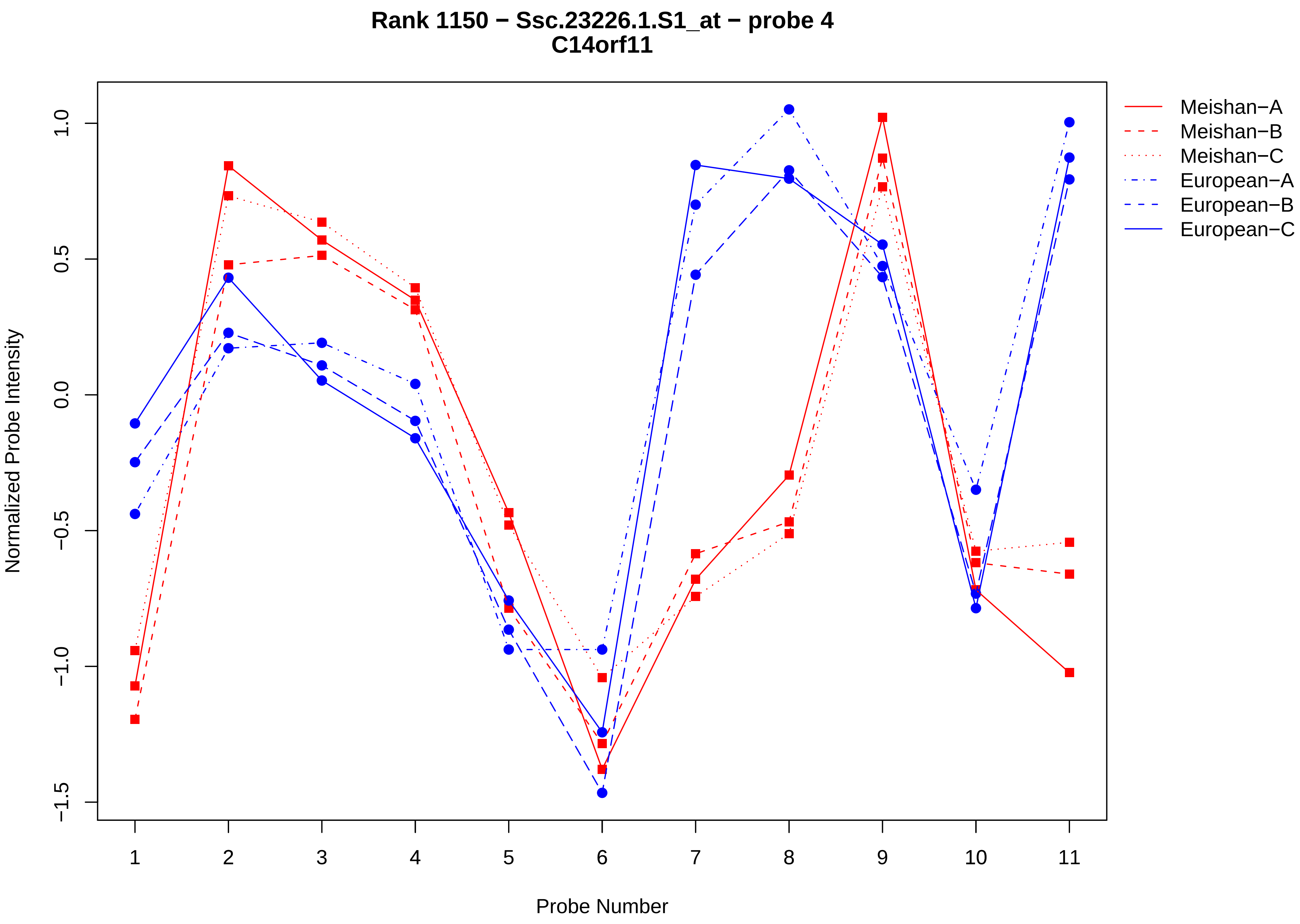
Rank 1150 − Ssc.23226.1.S1_at − probe 4
C14orf11
Normalized Probe Intensity
Probe Number
1.0
0.5
0.0
−0.5
−1.0
−1.5
1
2
3
4
5
6
7
8
9
10
11
Meishan−A
Meishan−B
Meishan−C
European−A
European−B
European−C

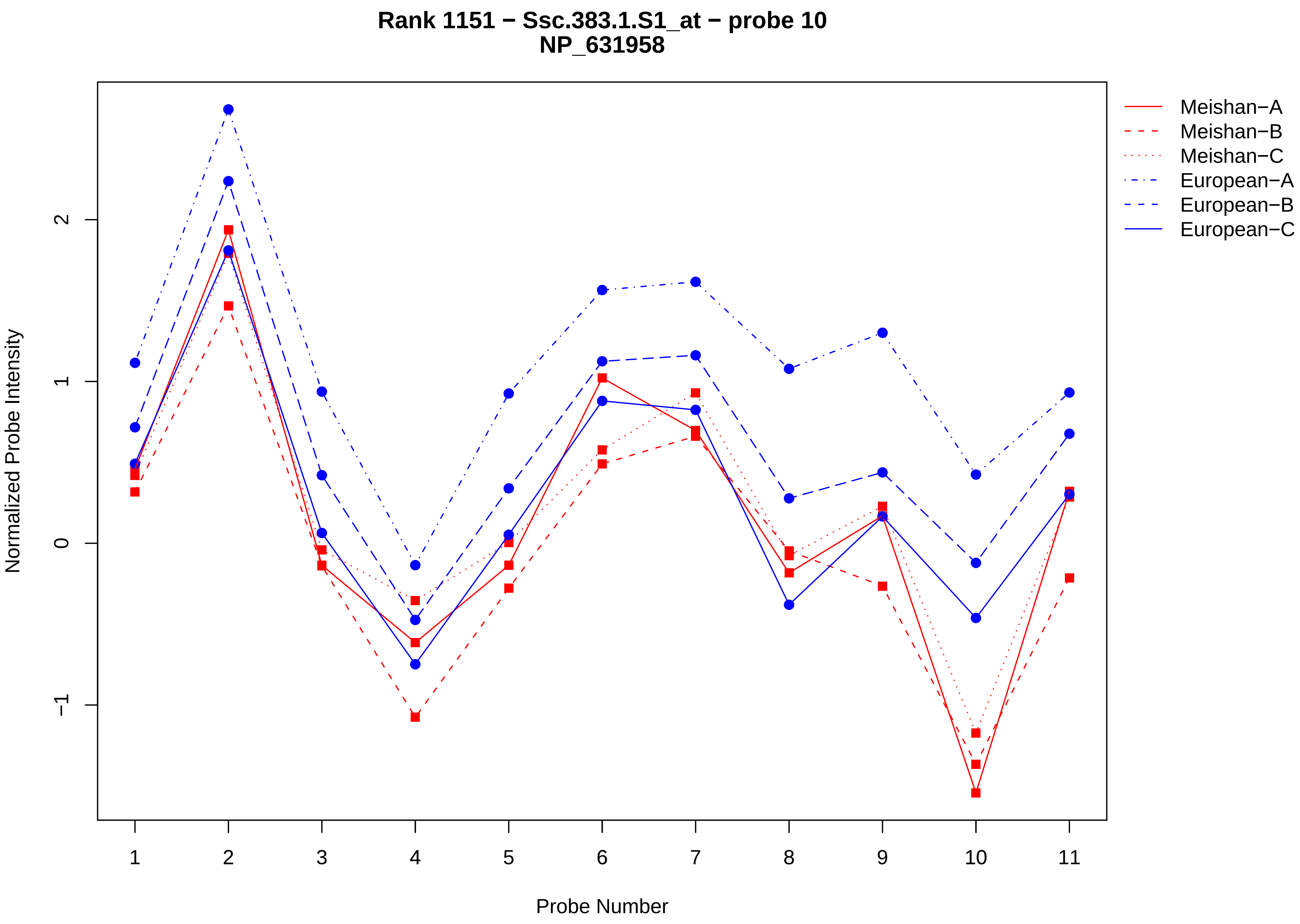

Rank 1151 − Ssc.383.1.S1_at − probe 10
NP_631958
Normalized Probe Intensity
−1
0
1
2
1
2
3
4
5
6
7
8
9
10
11
Probe Number
Meishan−A
Meishan−B
Meishan−C
European−A
European−B
European−C

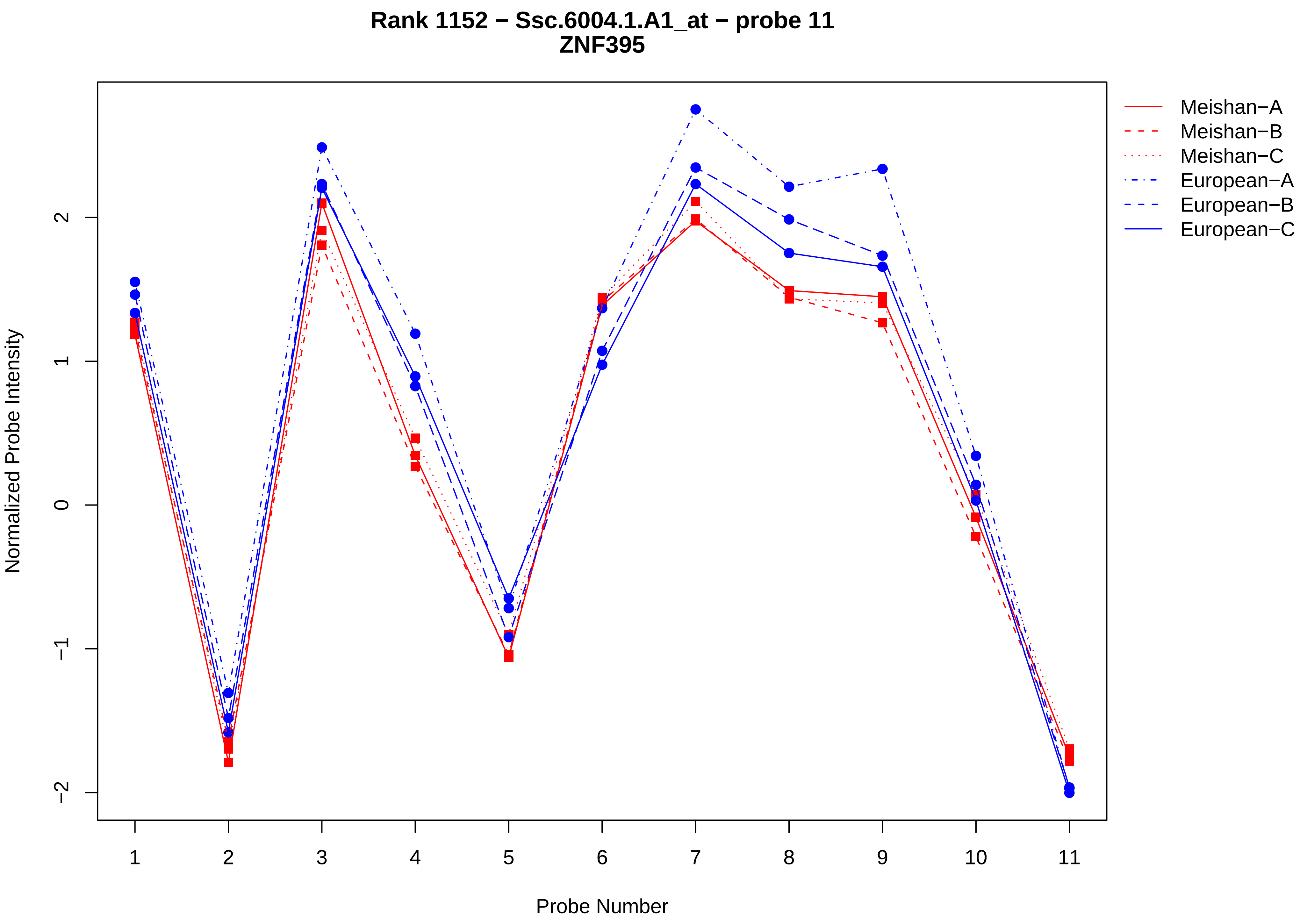

Rank 1152 − Ssc.6004.1.A1_at − probe 11
ZNF395
Normalized Probe Intensity
Probe Number
−2
−1
0
1
2
1
2
3
4
5
6
7
8
9
10
11
Meishan−A
Meishan−B
Meishan−C
European−A
European−B
European−C

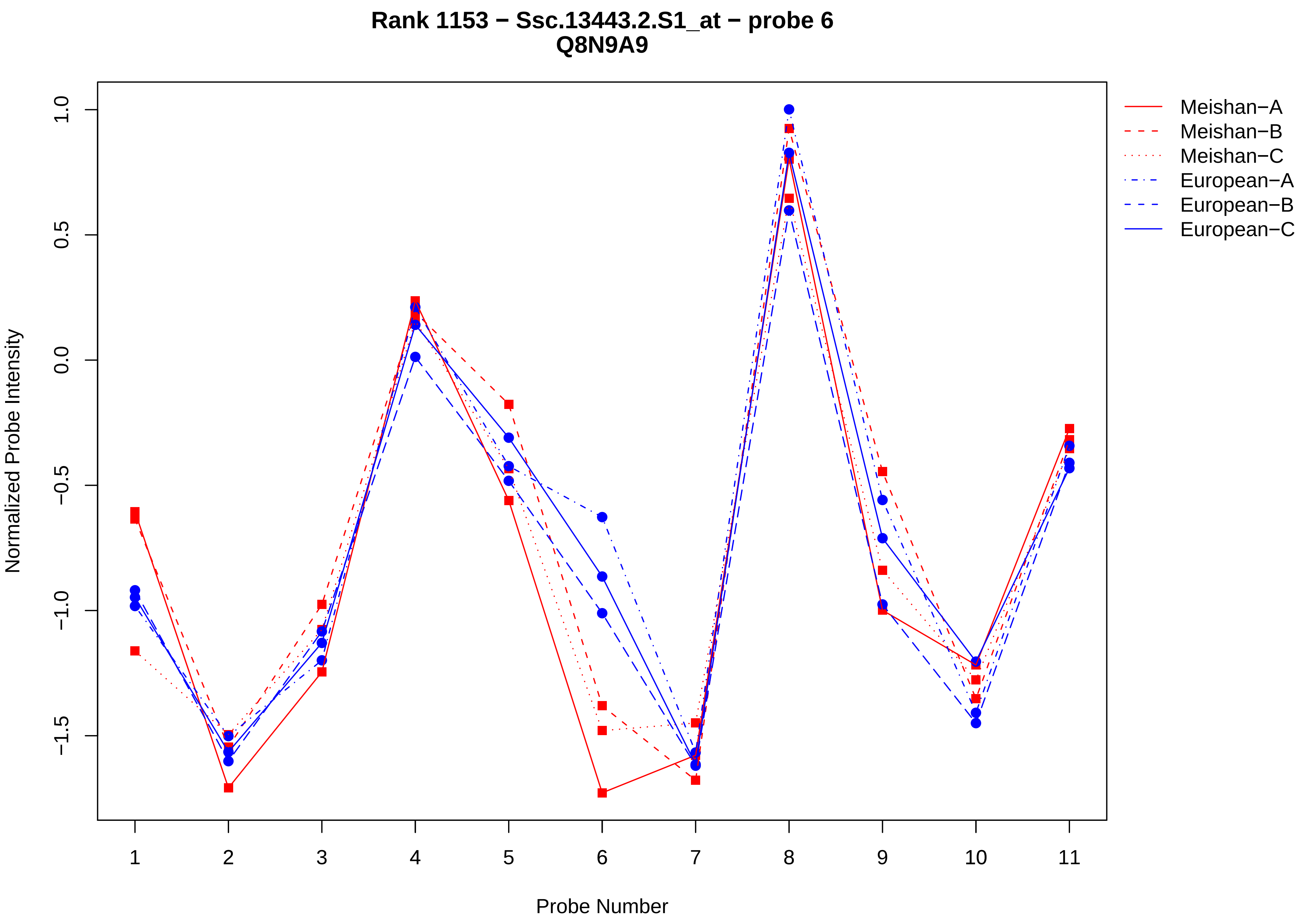

Rank 1153 − Ssc.13443.2.S1_at − probe 6
Q8N9A9
Normalized Probe Intensity
Probe Number
1.0
0.5
0.0
−0.5
−1.0
−1.5
1
2
3
4
5
6
7
8
9
10
11
Meishan−A
Meishan−B
Meishan−C
European−A
European−B
European−C

Rank 1154 − Ssc.30452.1.A1_at − probe 10
CADPS2

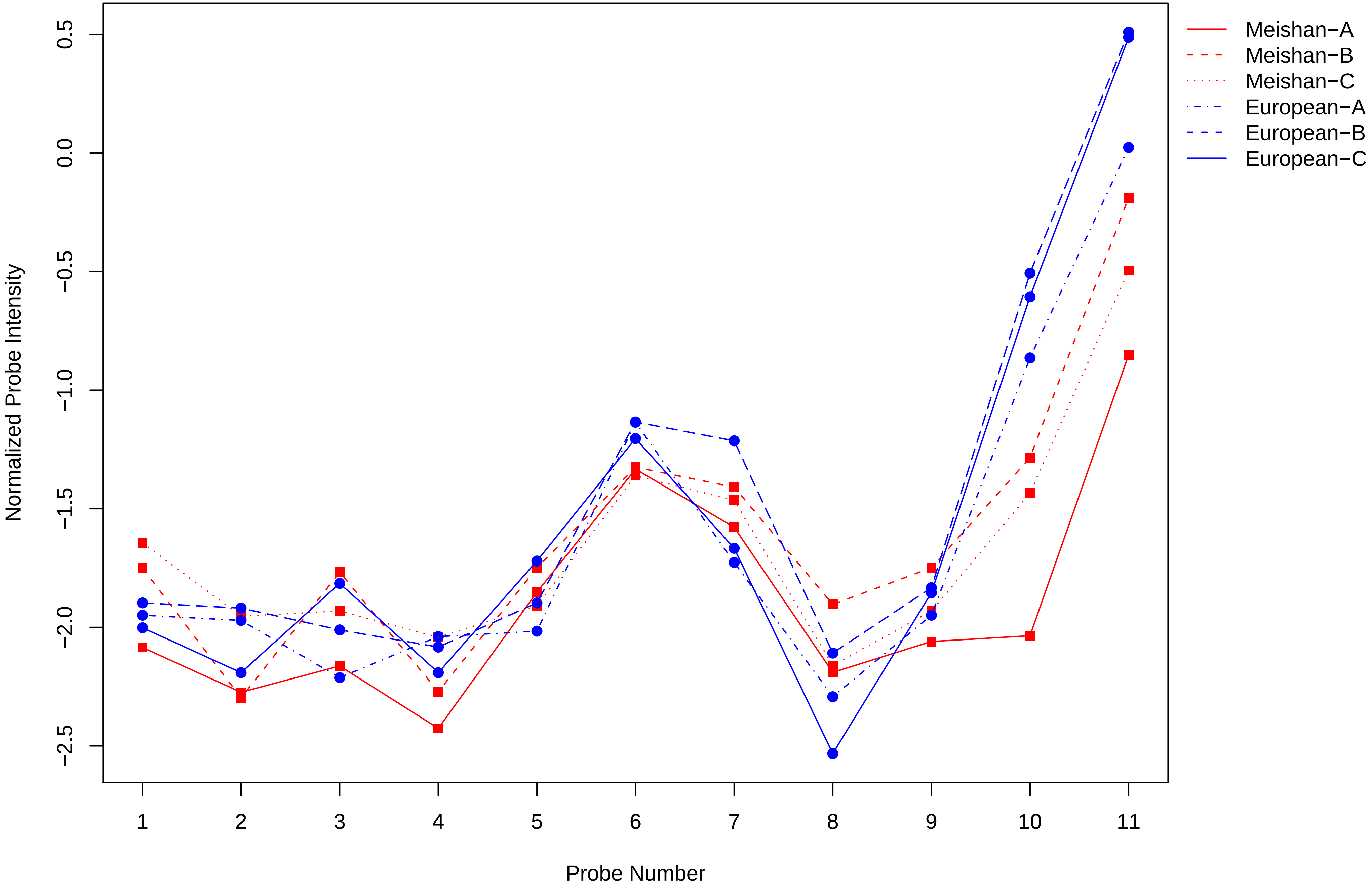

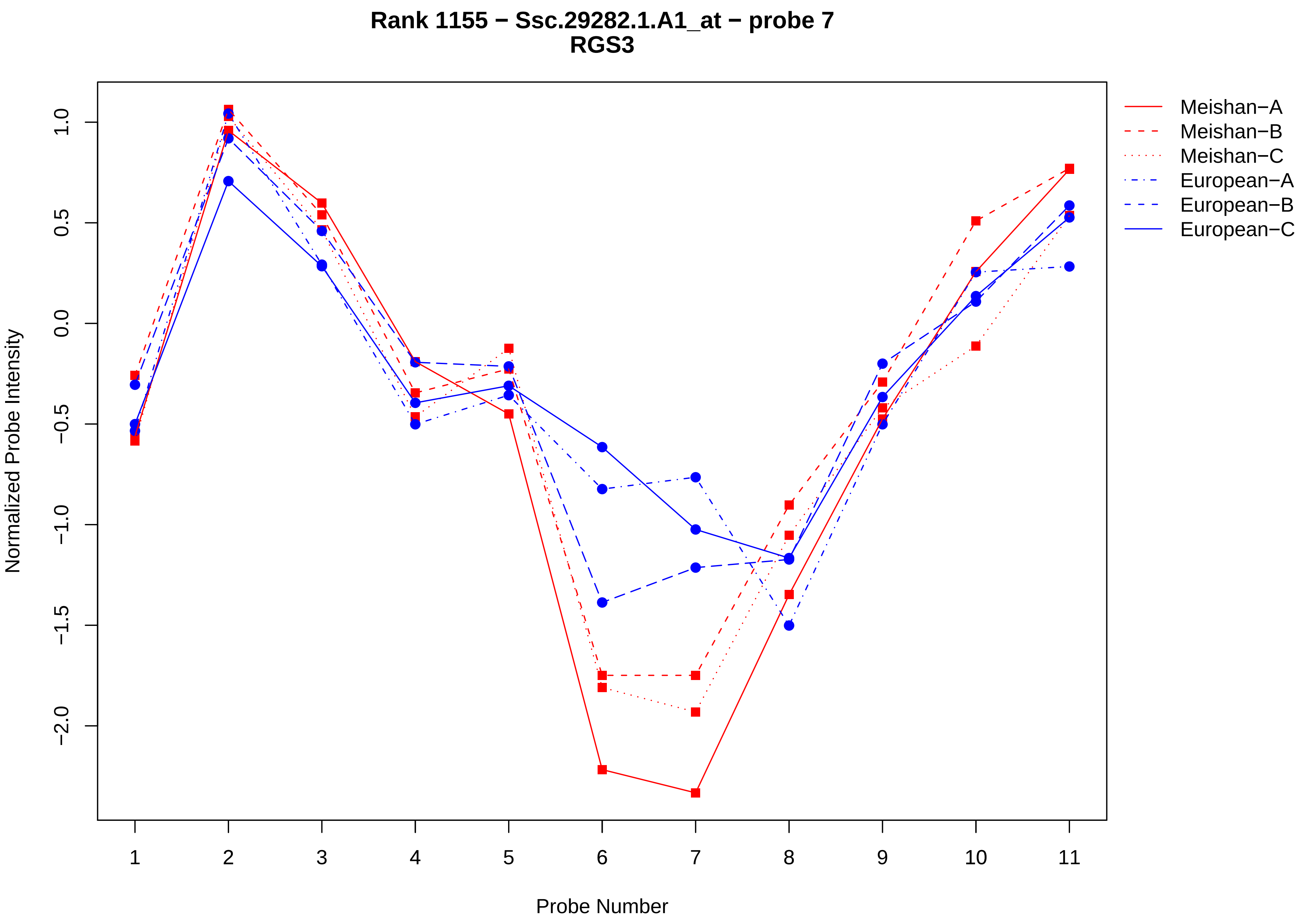

Rank 1155 − Ssc.29282.1.A1_at − probe 7
RGS3
Normalized Probe Intensity
Probe Number
1.0
0.5
0.0
−0.5
−1.0
−1.5
−2.0
1
2
3
4
5
6
7
8
9
10
11
Meishan−A
Meishan−B
Meishan−C
European−A
European−B
European−C

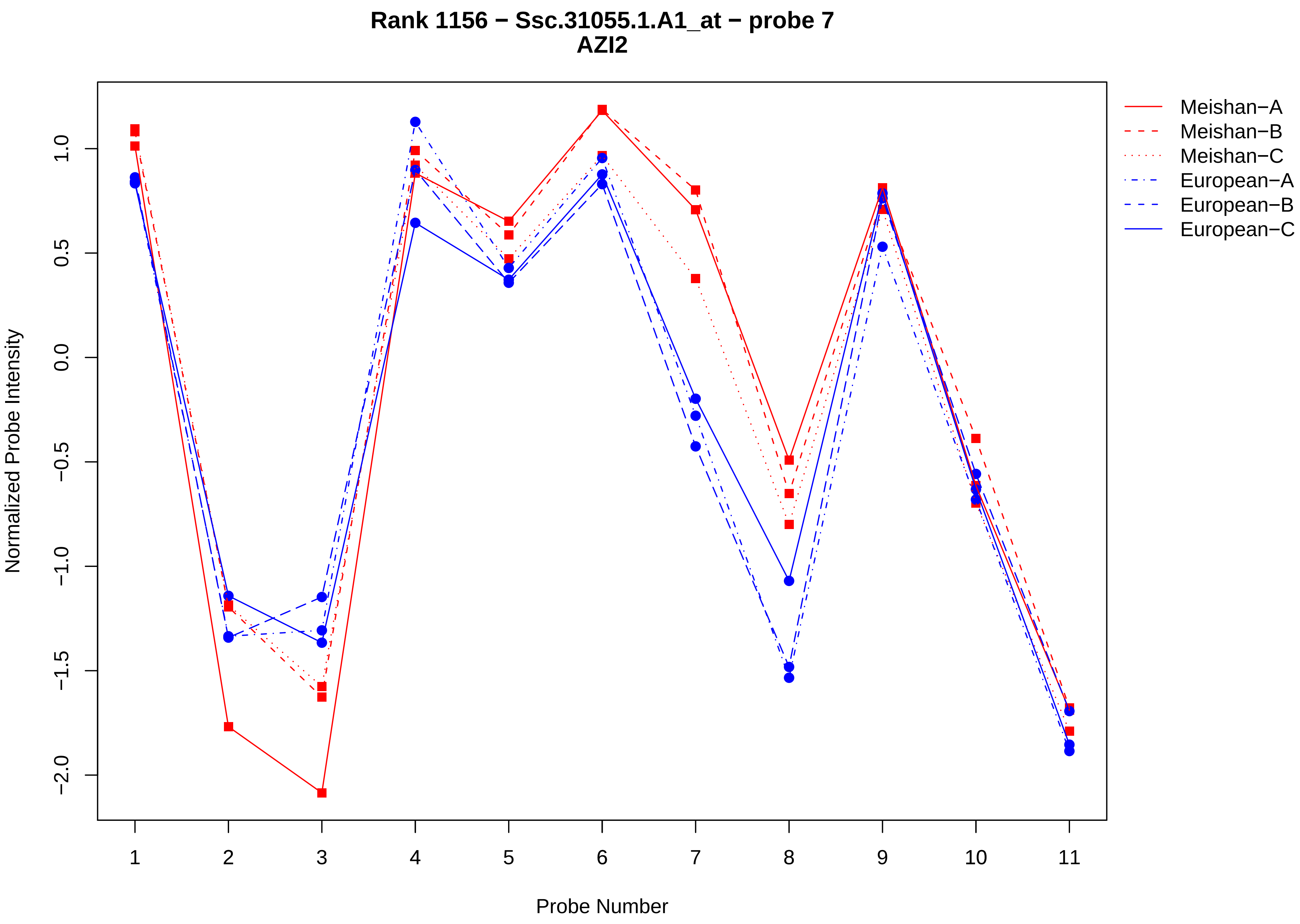
Rank 1156 − Ssc.31055.1.A1_at − probe 7
AZI2
Normalized Probe Intensity
Probe Number
1.0
0.5
0.0
−0.5
−1.0
−1.5
−2.0
1
2
3
4
5
6
7
8
9
10
11
Meishan−A
Meishan−B
Meishan−C
European−A
European−B
European−C

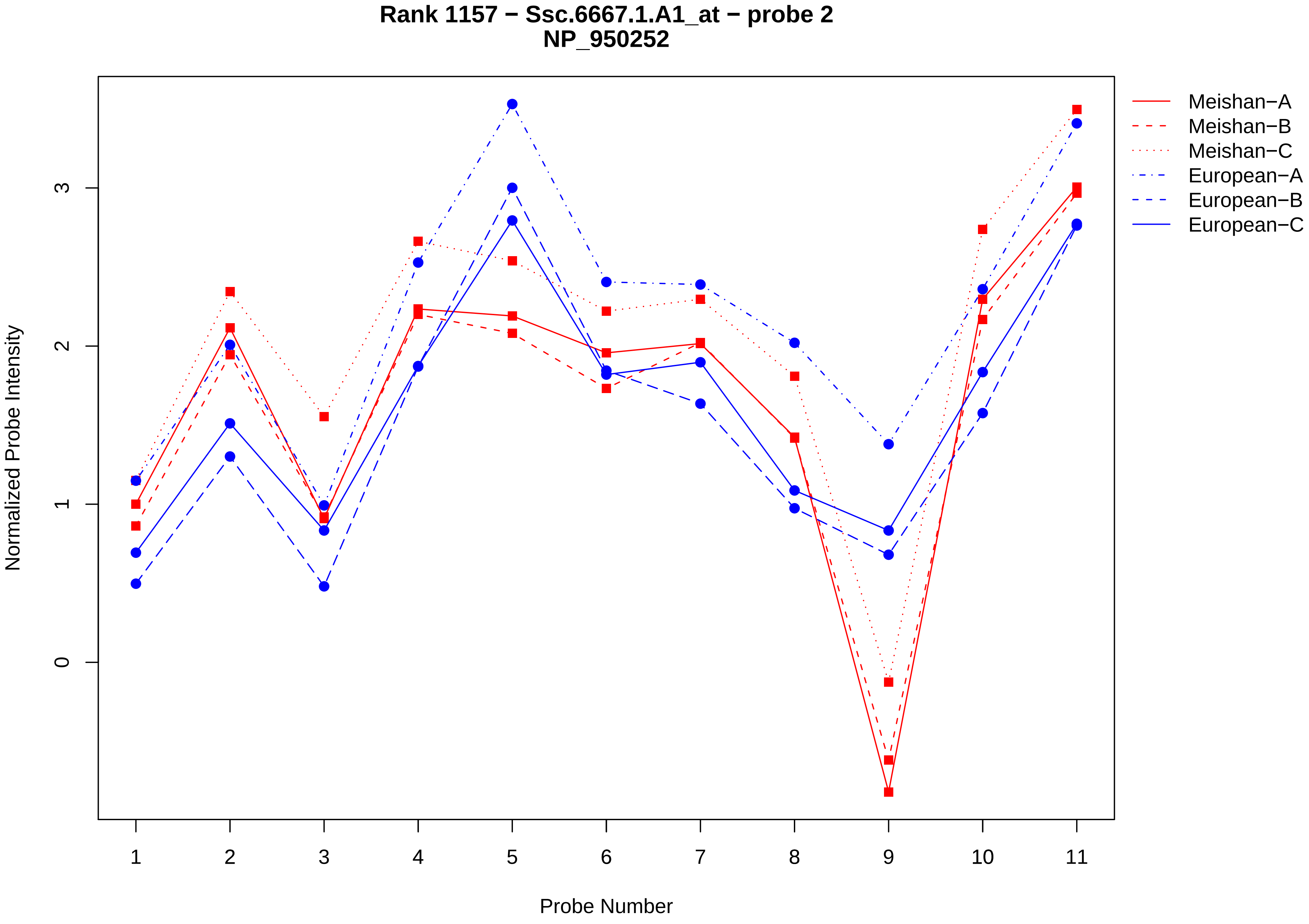
Rank 1157 − Ssc.6667.1.A1_at − probe 2
NP_950252
Normalized Probe Intensity
Probe Number
0
1
2
3
1
2
3
4
5
6
7
8
9
10
11
Meishan−A
Meishan−B
Meishan−C
European−A
European−B
European−C

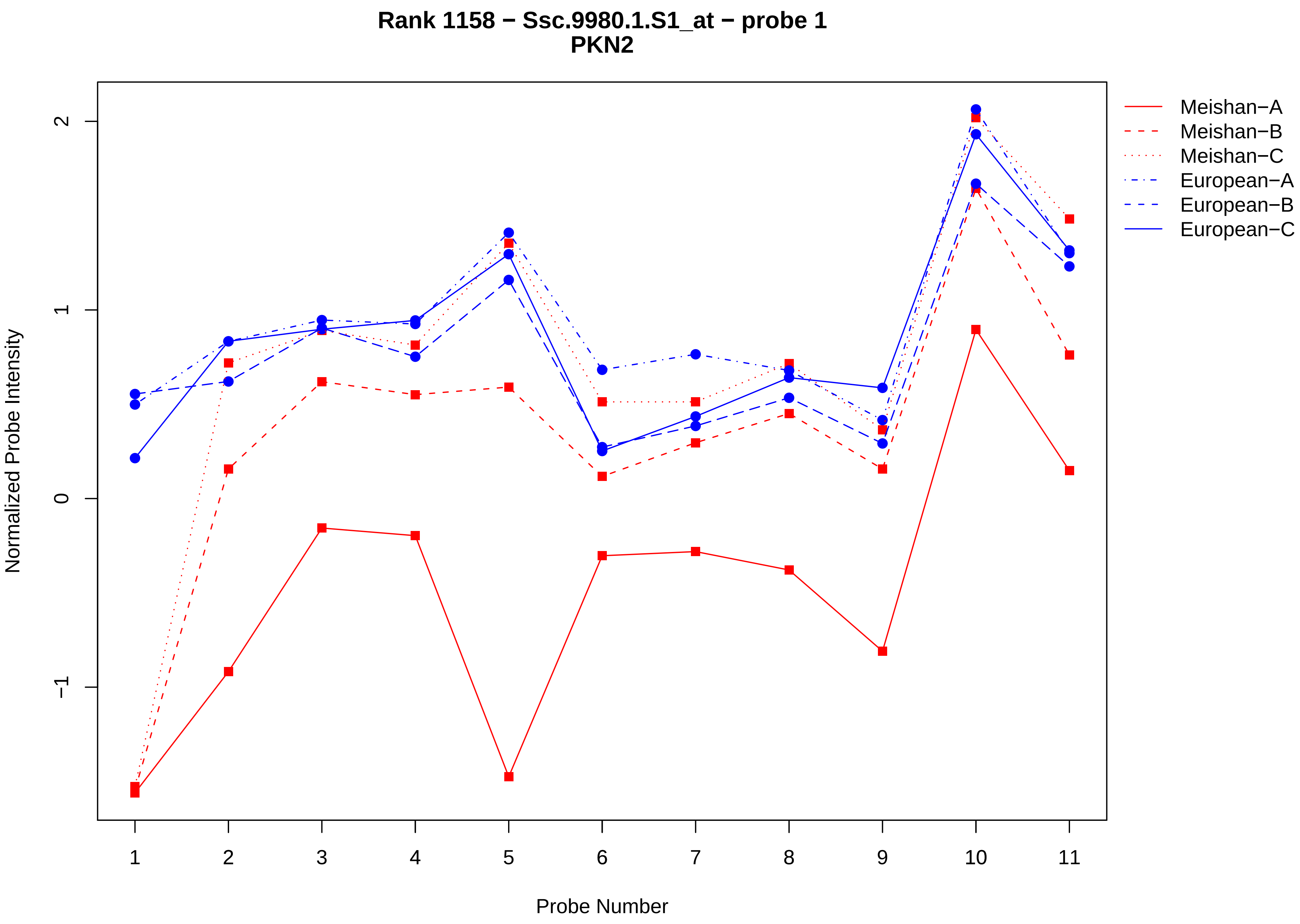
Rank 1158 − Ssc.9980.1.S1_at − probe 1
PKN2
Normalized Probe Intensity
Probe Number
−1
0
1
2
1
2
3
4
5
6
7
8
9
10
11
Meishan−A
Meishan−B
Meishan−C
European−A
European−B
European−C

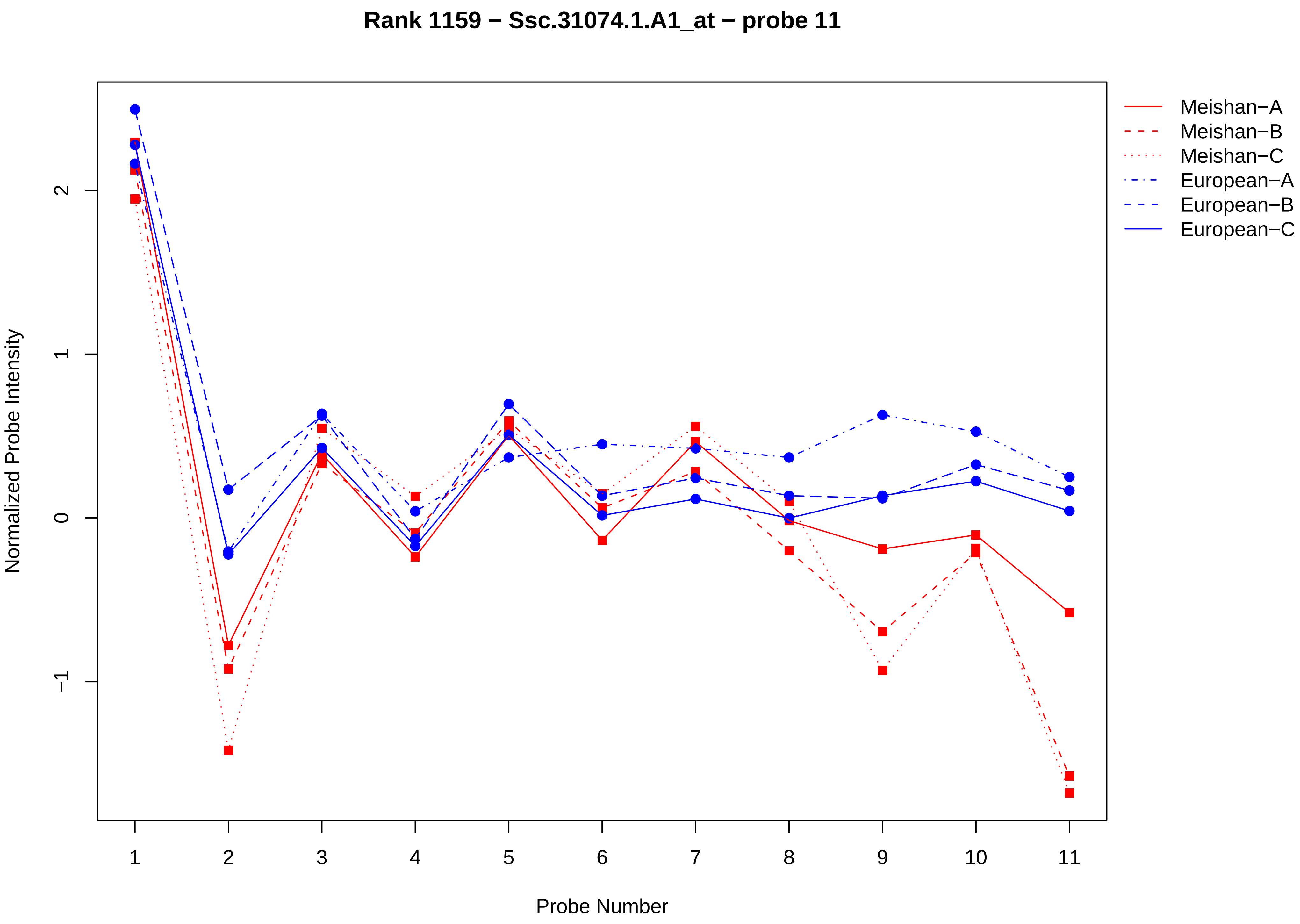

Rank 1159 − Ssc.31074.1.A1_at − probe 11
Normalized Probe Intensity
Probe Number
−1
0
1
2
1
2
3
4
5
6
7
8
9
10
11
Meishan−A
Meishan−B
Meishan−C
European−A
European−B
European−C

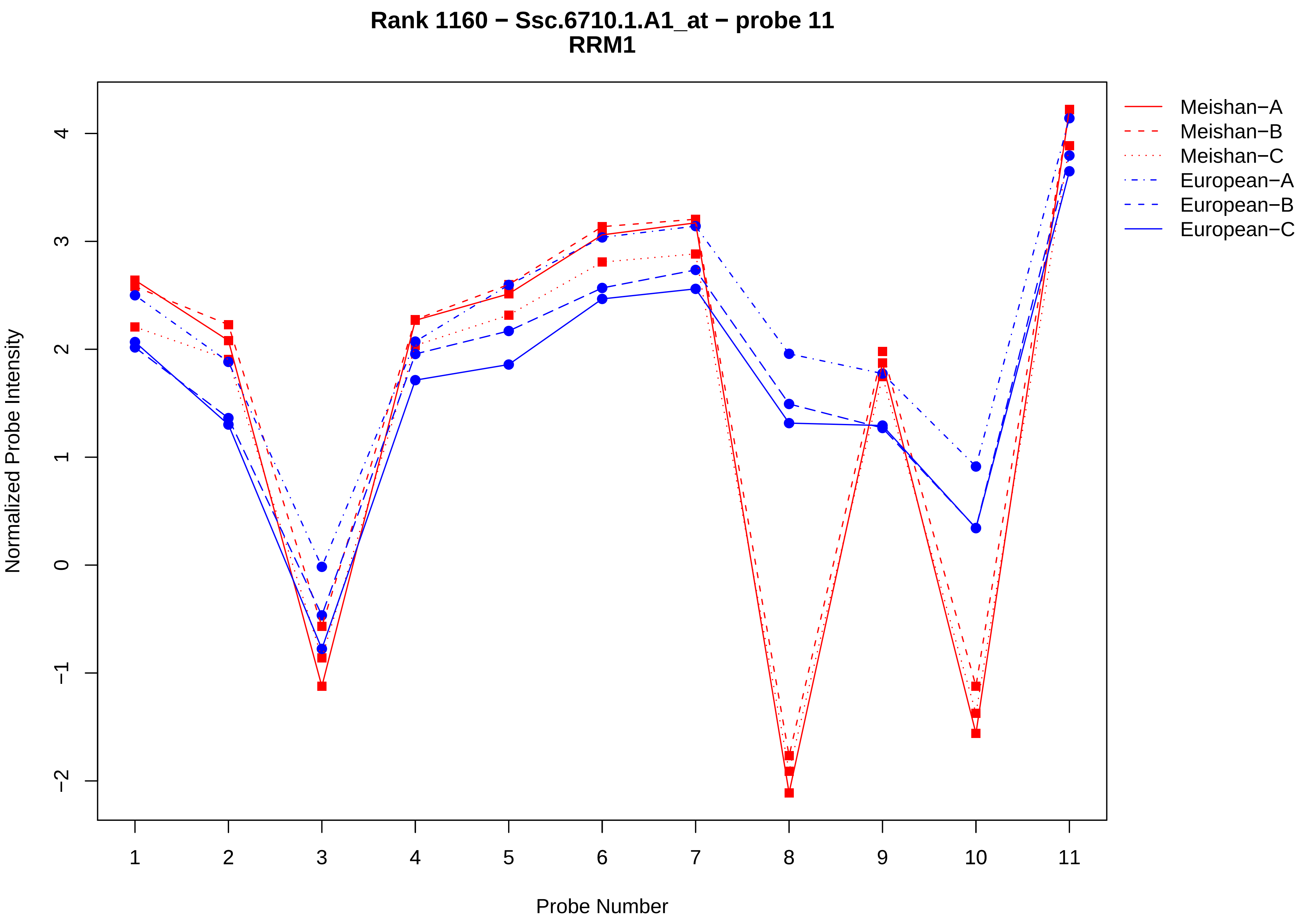

Rank 1160 − Ssc.6710.1.A1_at − probe 11
RRM1
Normalized Probe Intensity
Probe Number
−2
−1
0
1
2
3
4
1
2
3
4
5
6
7
8
9
10
11
Meishan−A
Meishan−B
Meishan−C
European−A
European−B
European−C

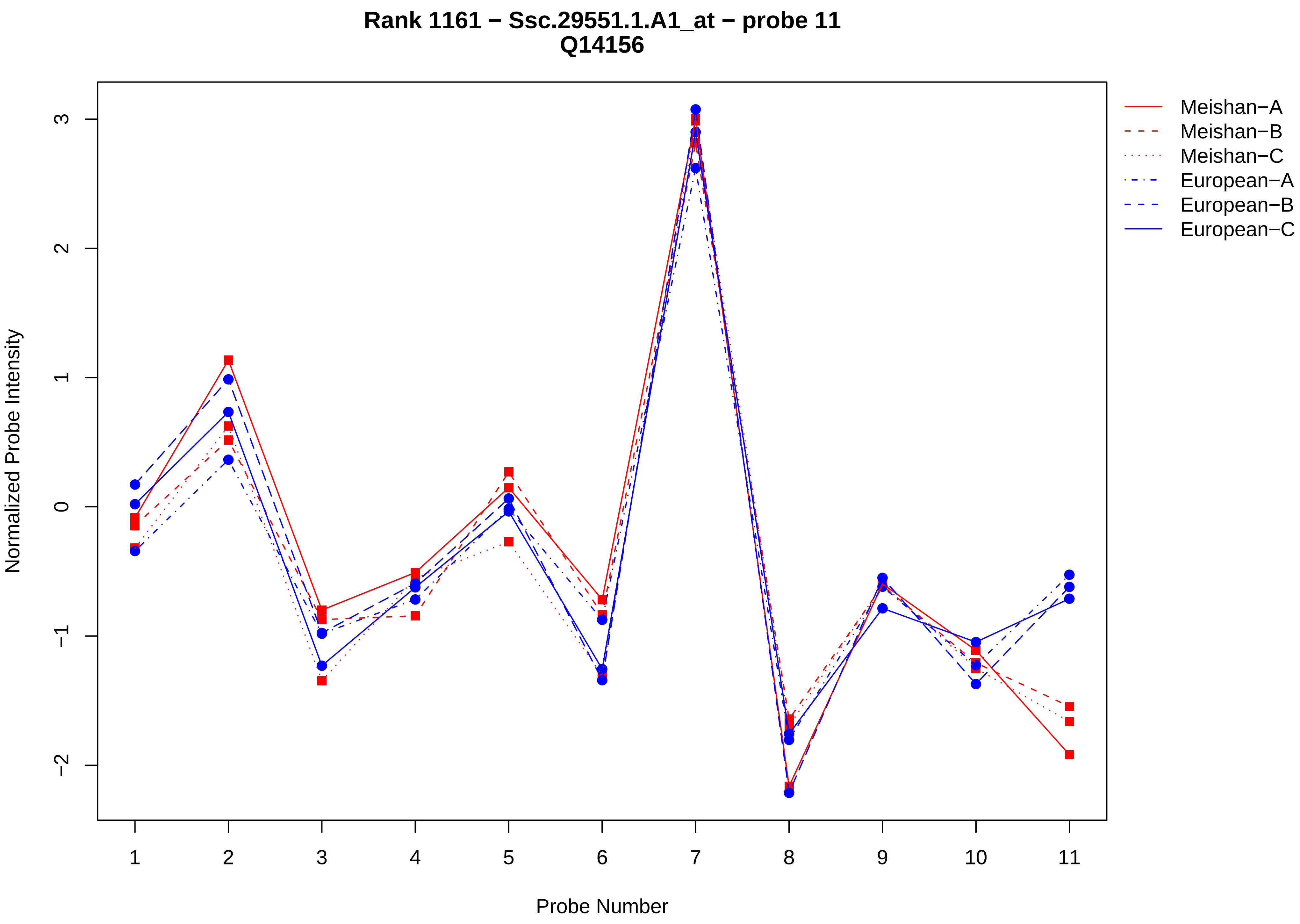
Rank 1161 − Ssc.29551.1.A1_at − probe 11
Q14156
Normalized Probe Intensity
Probe Number
3
2
1
0
−1
−2
1
2
3
4
5
6
7
8
9
10
11
Meishan−A
Meishan−B
Meishan−C
European−A
European−B
European−C

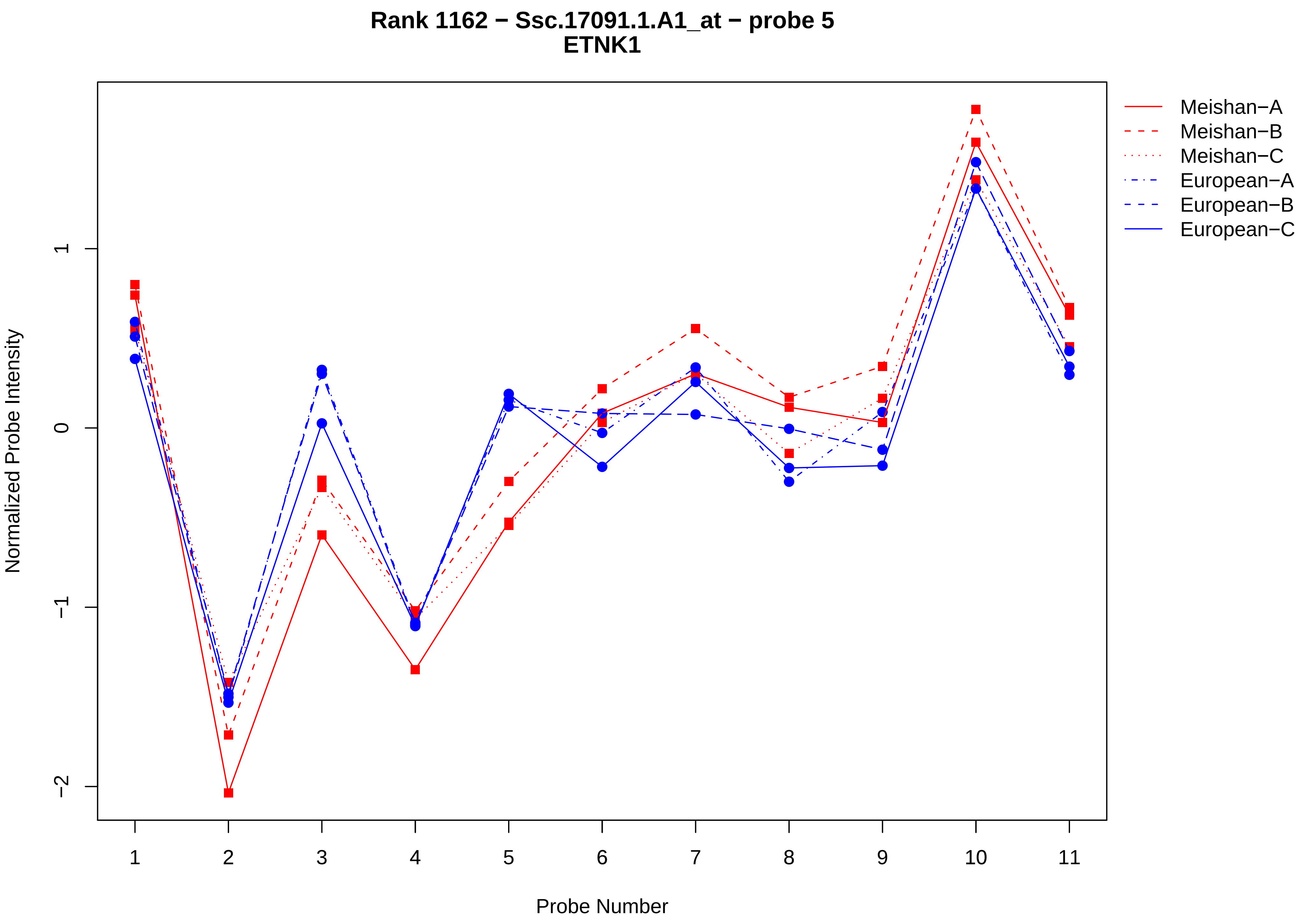

Rank 1162 − Ssc.17091.1.A1_at − probe 5
ETNK1
Normalized Probe Intensity
Probe Number
Meishan−A
Meishan−B
Meishan−C
European−A
European−B
European−C

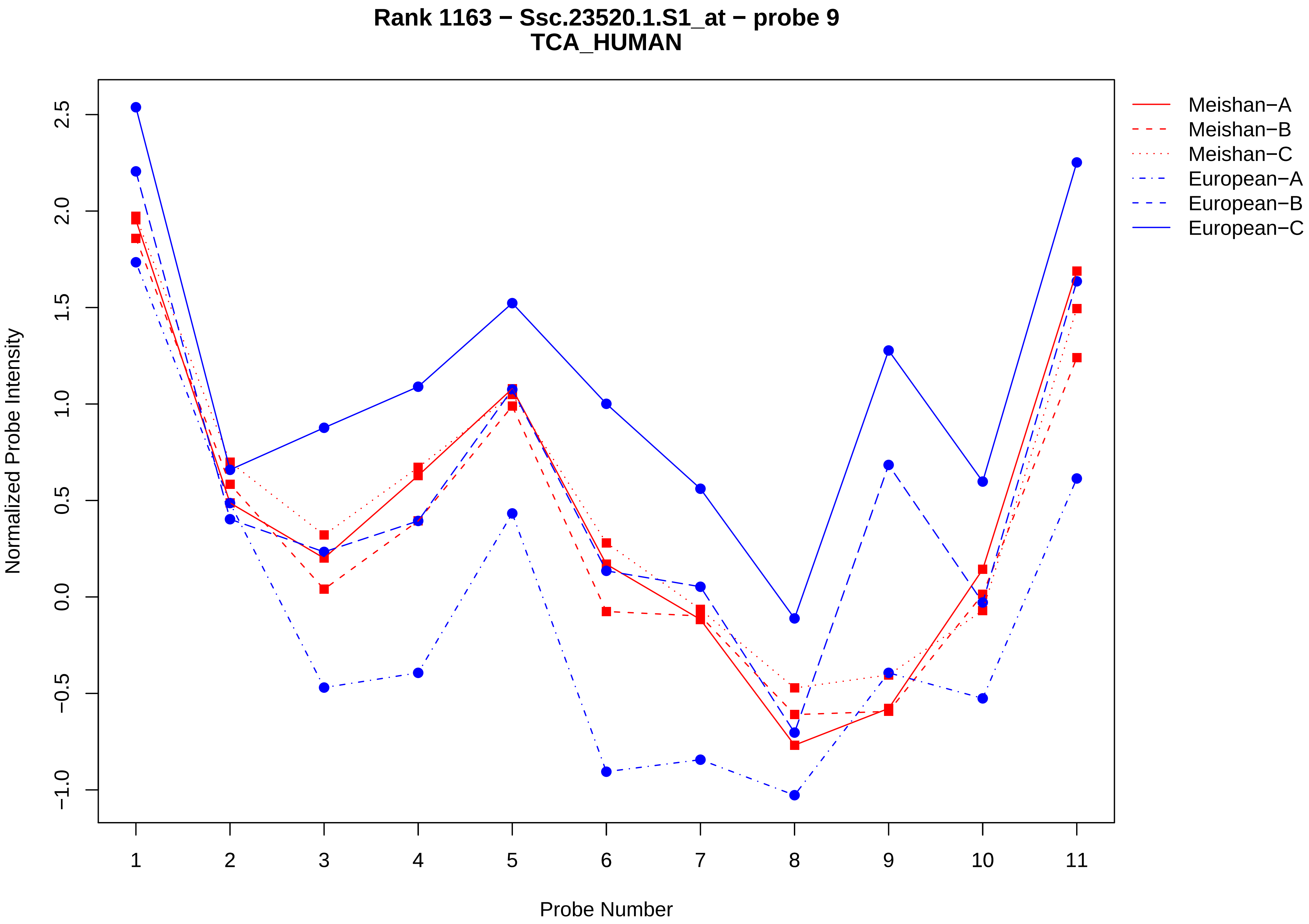

Rank 1163 − Ssc.23520.1.S1_at − probe 9
TCA_HUMAN
Normalized Probe Intensity
Probe Number
Meishan−A
Meishan−B
Meishan−C
European−A
European−B
European−C
−1.0
−0.5
0.0
0.5
1.0
1.5
2.0
2.5
1
2
3
4
5
6
7
8
9
10
11

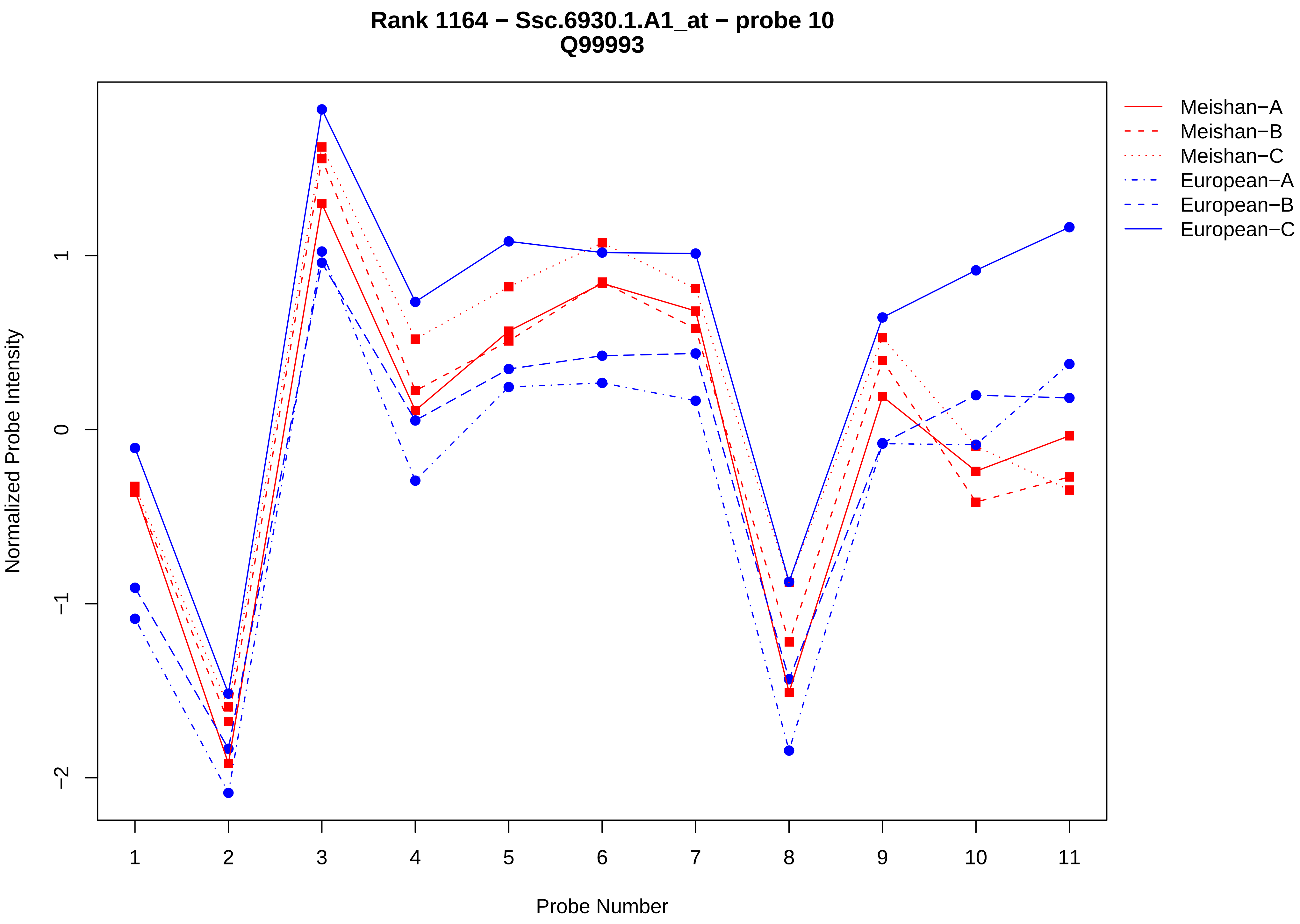
Rank 1164 − Ssc.6930.1.A1_at − probe 10
Q99993
Normalized Probe Intensity
Probe Number
−2
−1
0
1
1
2
3
4
5
6
7
8
9
10
11
Meishan−A
Meishan−B
Meishan−C
European−A
European−B
European−C

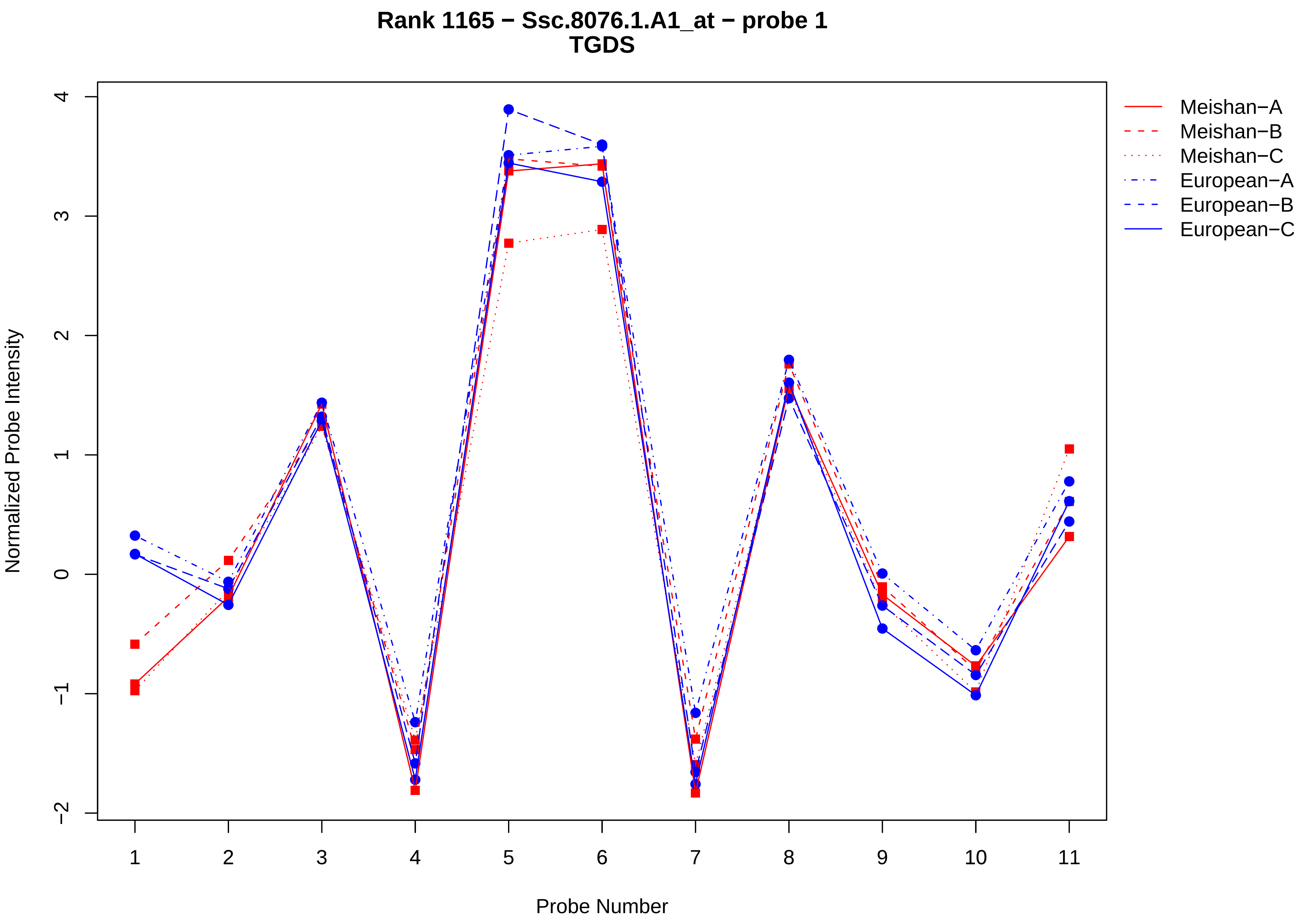

Rank 1165 − Ssc.8076.1.A1_at − probe 1
TGDS
Normalized Probe Intensity
Probe Number
−2
−1
0
1
2
3
4
1
2
3
4
5
6
7
8
9
10
11
Meishan−A
Meishan−B
Meishan−C
European−A
European−B
European−C

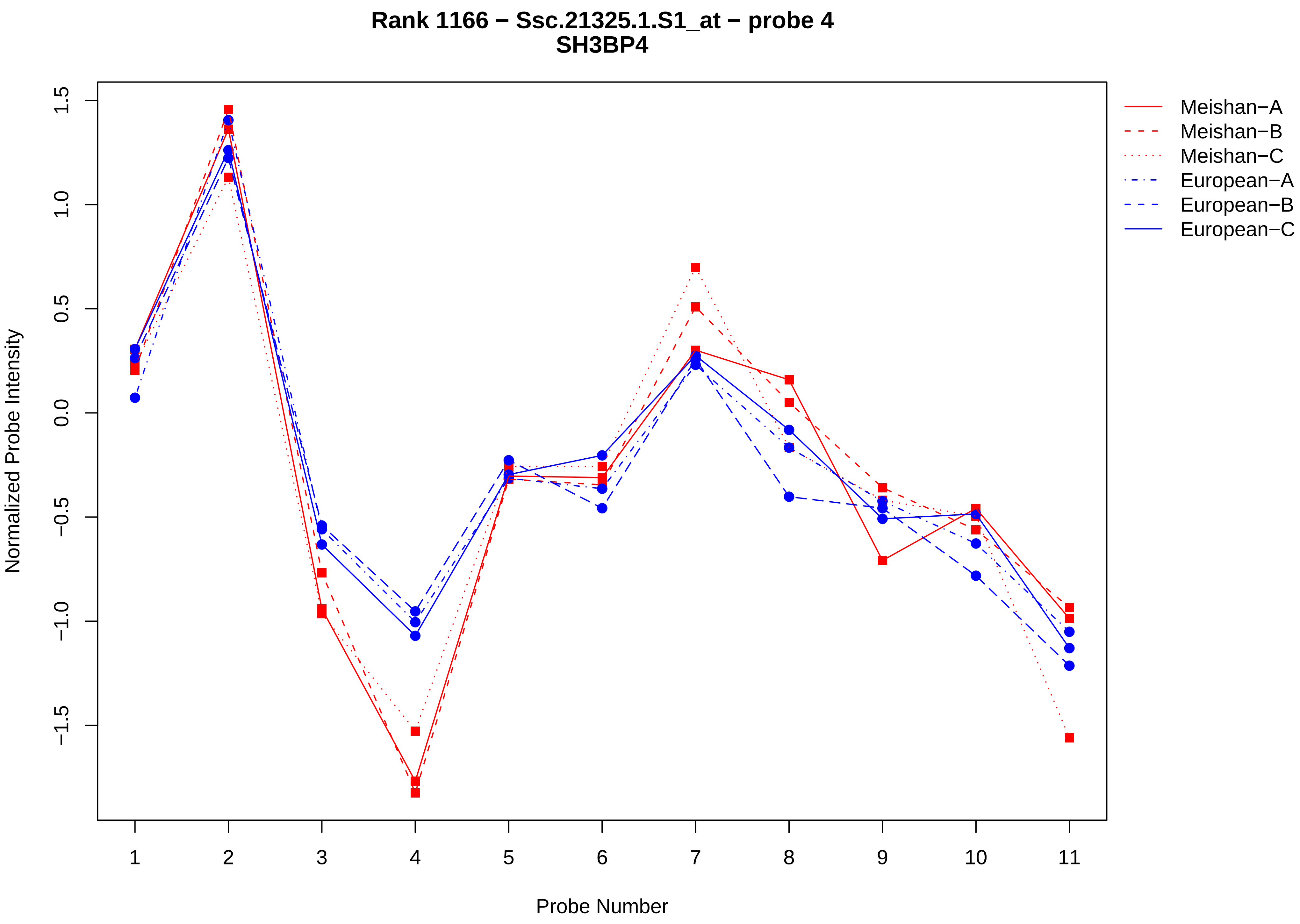
Rank 1166 − Ssc.21325.1.S1_at − probe 4
SH3BP4
Normalized Probe Intensity
Probe Number
Meishan−A
Meishan−B
Meishan−C
European−A
European−B
European−C
1.5
1.0
0.5
0.0
−0.5
−1.0
−1.5
1
2
3
4
5
6
7
8
9
10
11

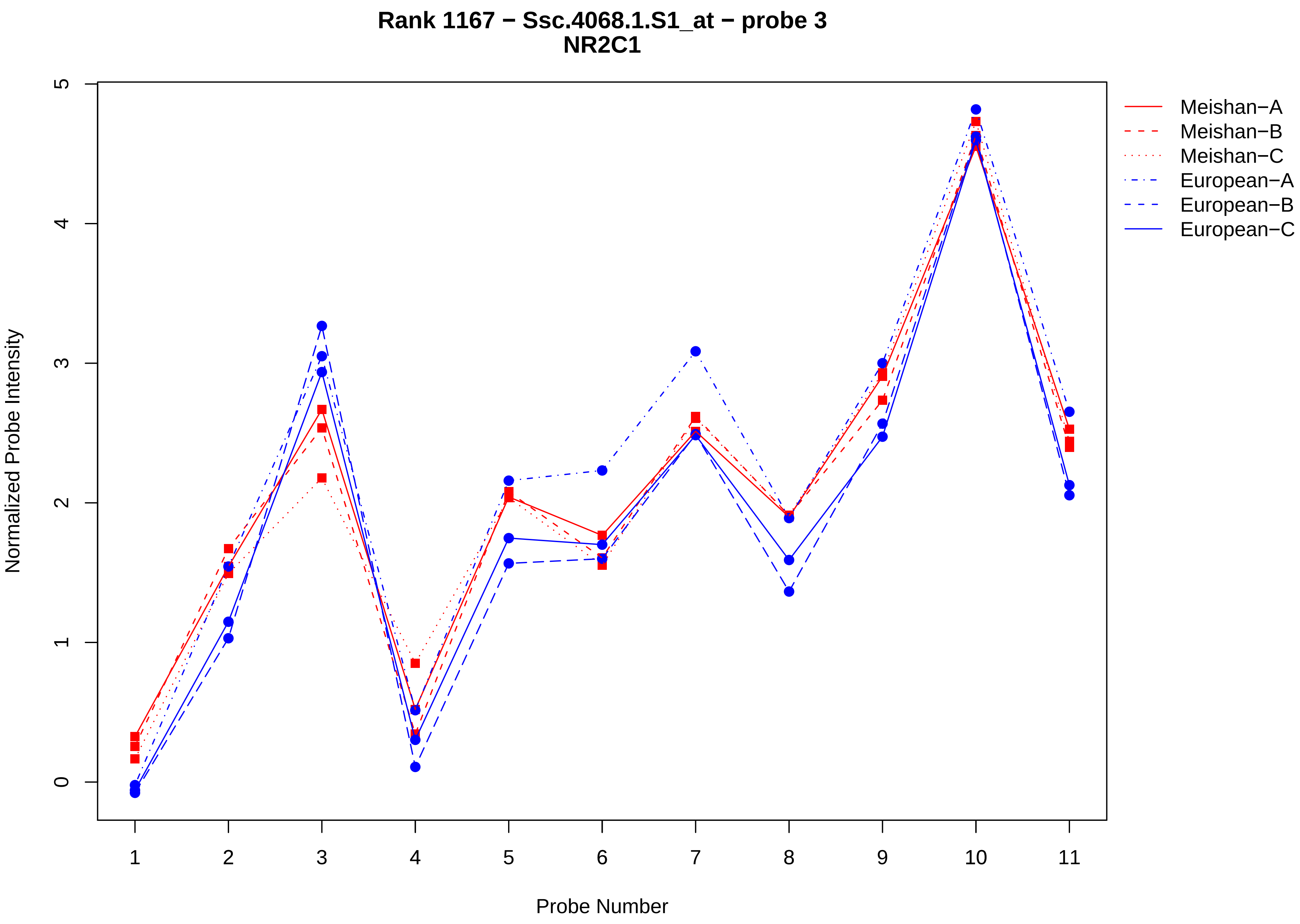

Rank 1167 − Ssc.4068.1.S1_at − probe 3
NR2C1
Normalized Probe Intensity
Probe Number
0
1
2
3
4
5
1
2
3
4
5
6
7
8
9
10
11
Meishan−A
Meishan−B
Meishan−C
European−A
European−B
European−C

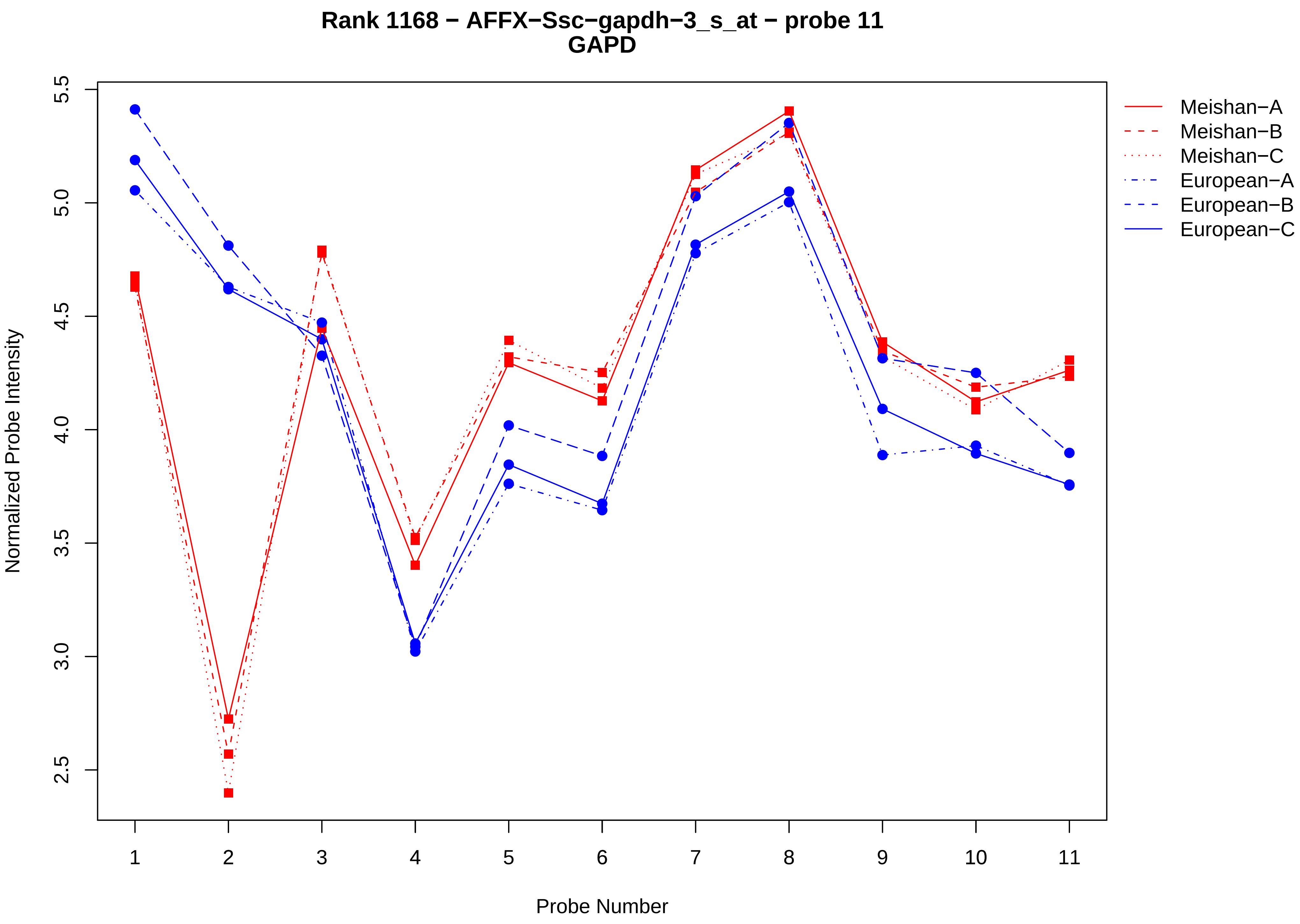
Rank 1168 − AFFX−Ssc−gapdh−3_s_at − probe 11
GAPD
Normalized Probe Intensity
Probe Number
2.5
3.0
3.5
4.0
4.5
5.0
5.5
1
2
3
4
5
6
7
8
9
10
11
Meishan−A
Meishan−B
Meishan−C
European−A
European−B
European−C

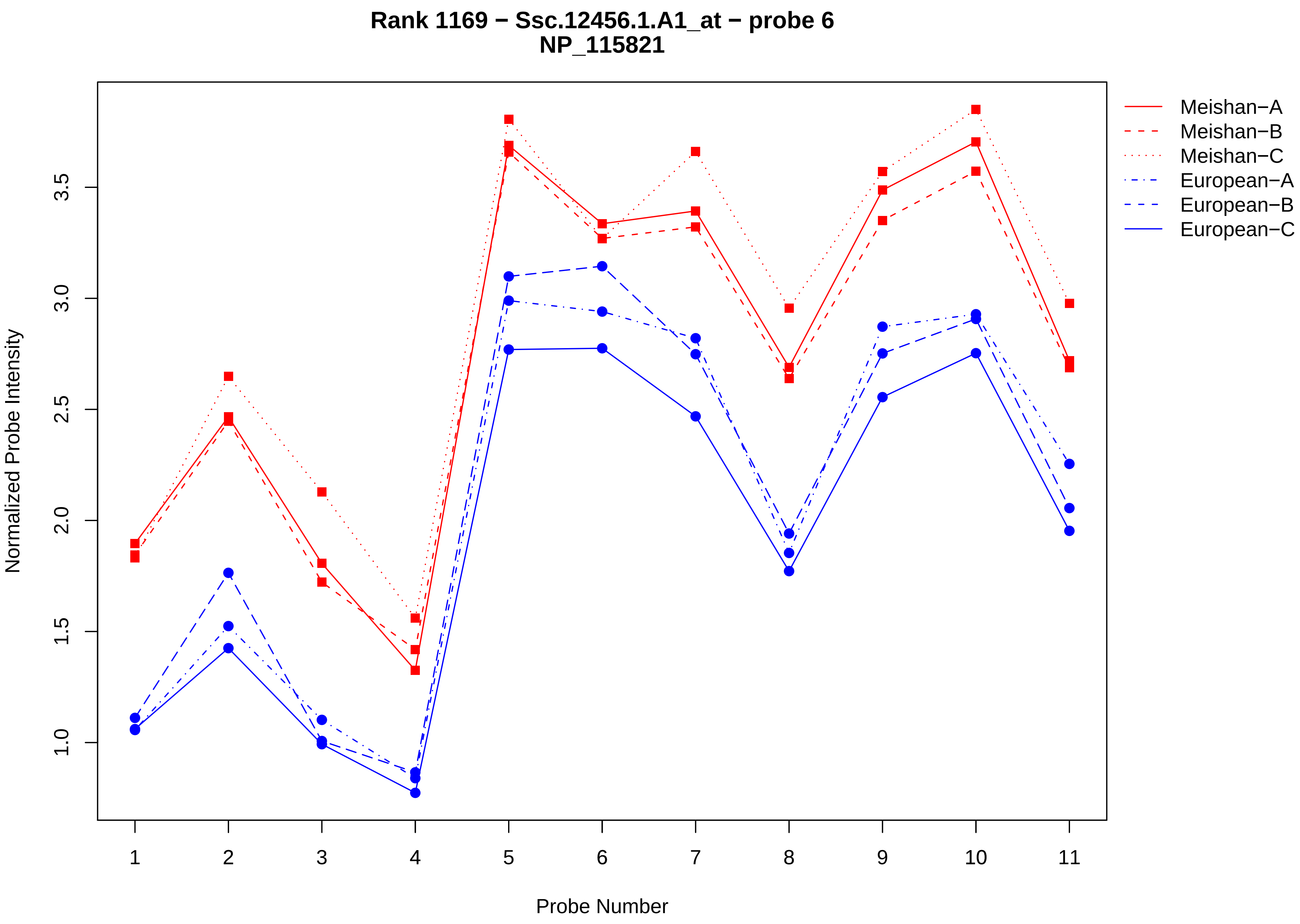
Rank 1169 − Ssc.12456.1.A1_at − probe 6
NP_115821
Normalized Probe Intensity
1.0
1.5
2.0
2.5
3.0
3.5
Probe Number
1
2
3
4
5
6
7
8
9
10
11
Meishan−A
Meishan−B
Meishan−C
European−A
European−B
European−C

Rank 1170 − Ssc.13434.1.A1_at − probe 10
NDST4

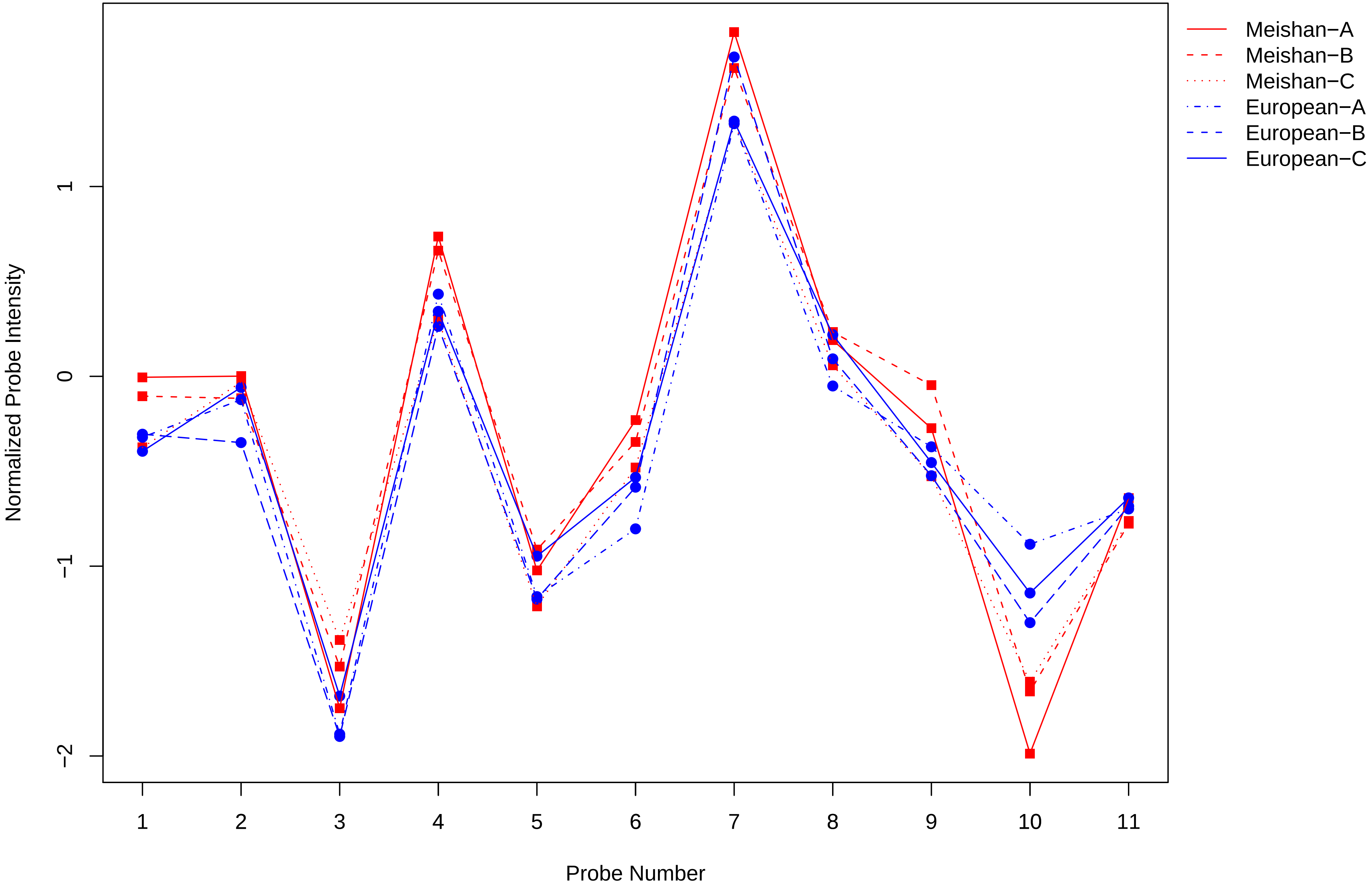

Rank 1171 − Ssc.21968.1.S1_at − probe 4
CLNS1A

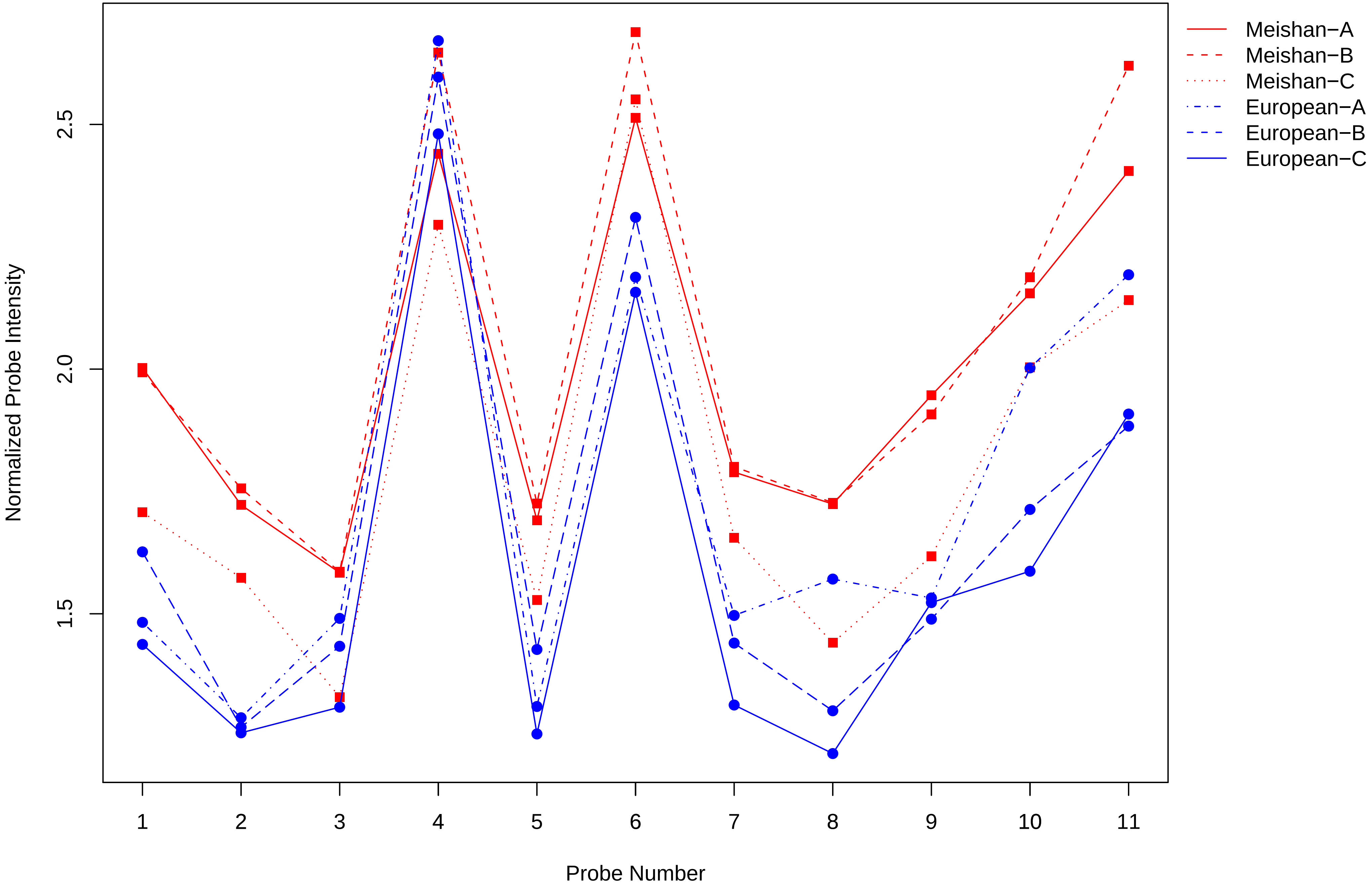

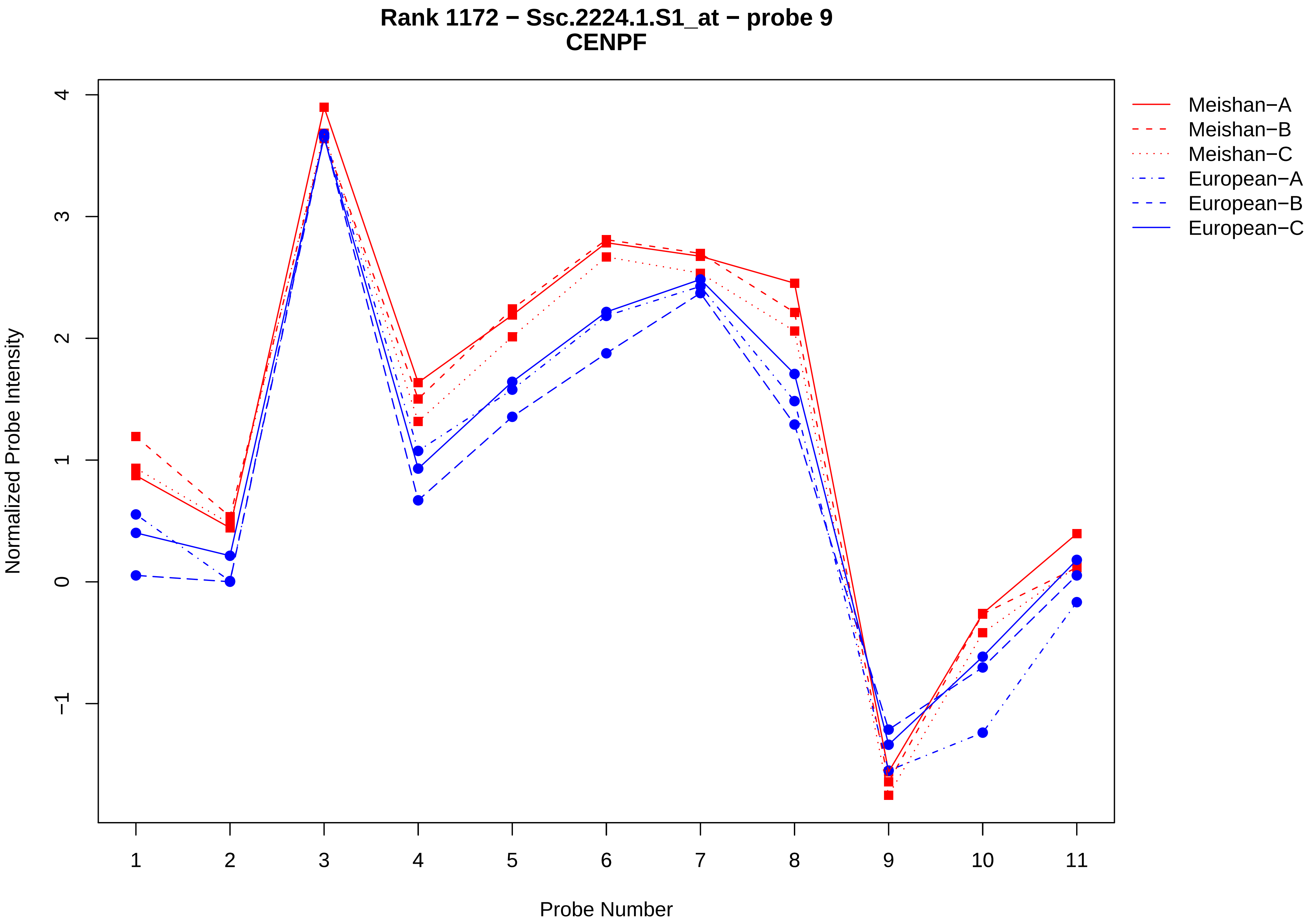
Rank 1172 − Ssc.2224.1.S1_at − probe 9
CENPF
Normalized Probe Intensity
Probe Number
4
3
2
1
0
−1
1
2
3
4
5
6
7
8
9
10
11
Meishan−A
Meishan−B
Meishan−C
European−A
European−B
European−C

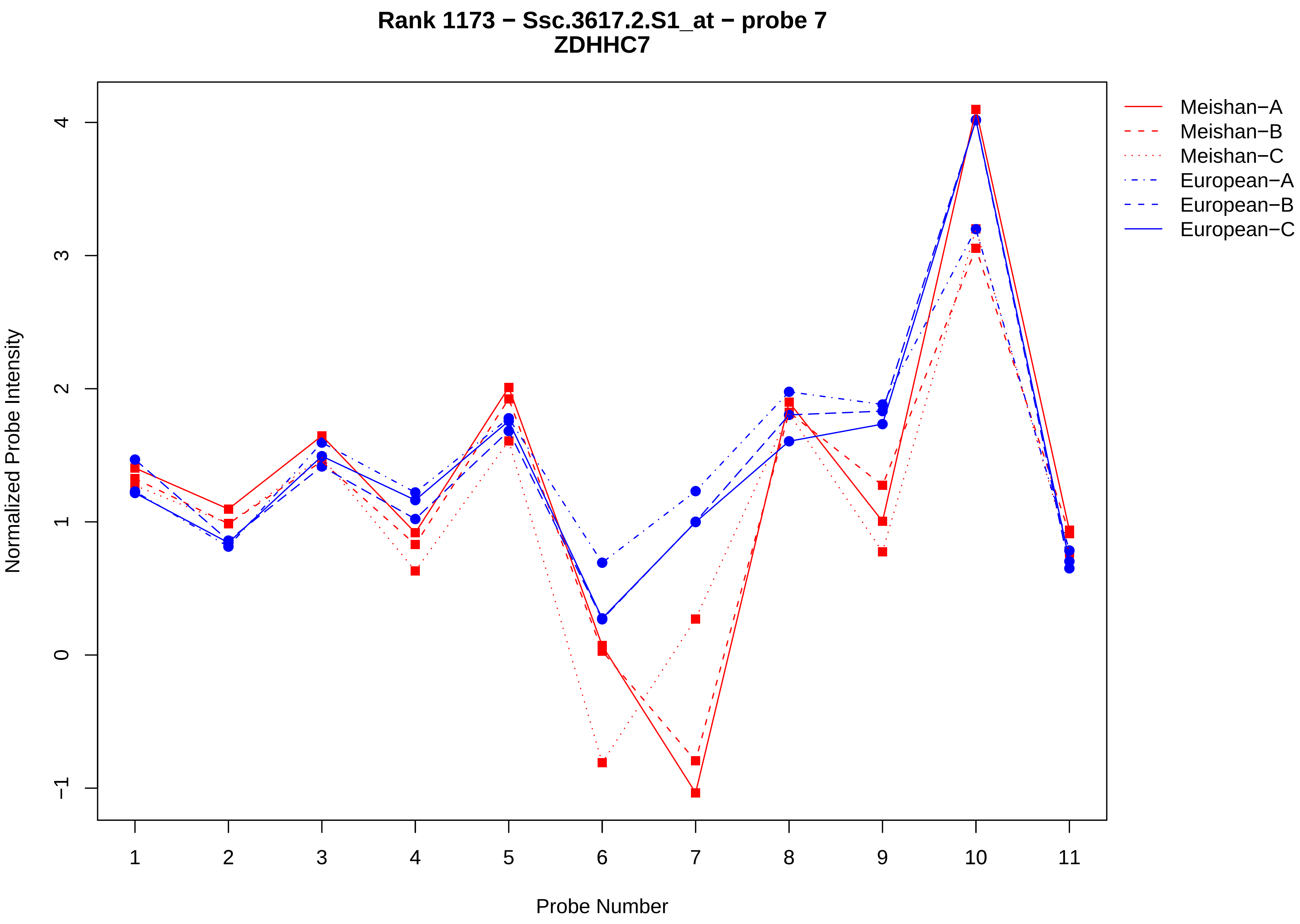

Rank 1173 − Ssc.3617.2.S1_at − probe 7
ZDHHC7
Normalized Probe Intensity
Probe Number
−1
0
1
2
3
4
1
2
3
4
5
6
7
8
9
10
11
Meishan−A
Meishan−B
Meishan−C
European−A
European−B
European−C

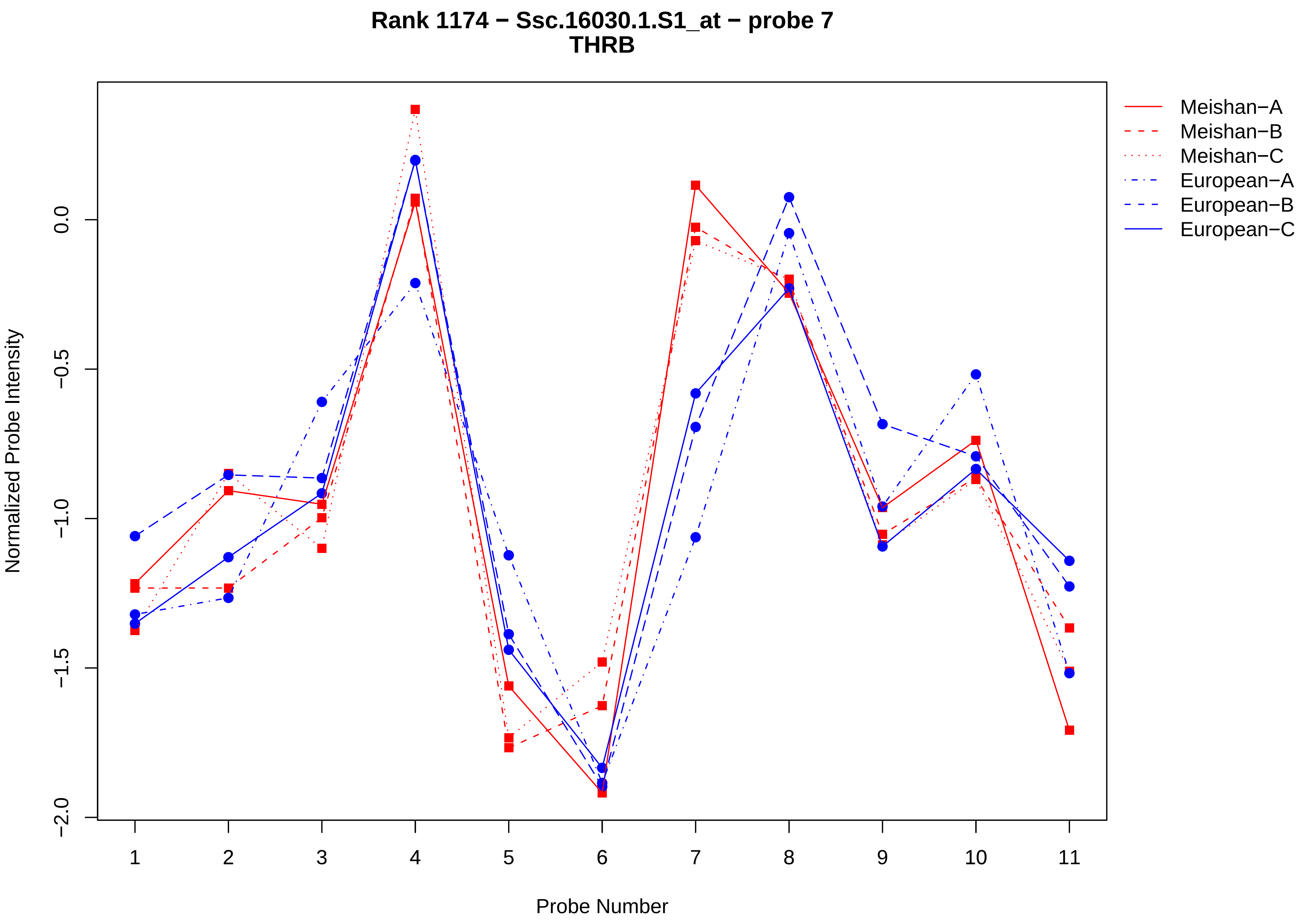

Rank 1174 − Ssc.16030.1.S1_at − probe 7
THRB
Normalized Probe Intensity
Probe Number
0.0
−0.5
−1.0
−1.5
−2.0
1
2
3
4
5
6
7
8
9
10
11
Meishan−A
Meishan−B
Meishan−C
European−A
European−B
European−C

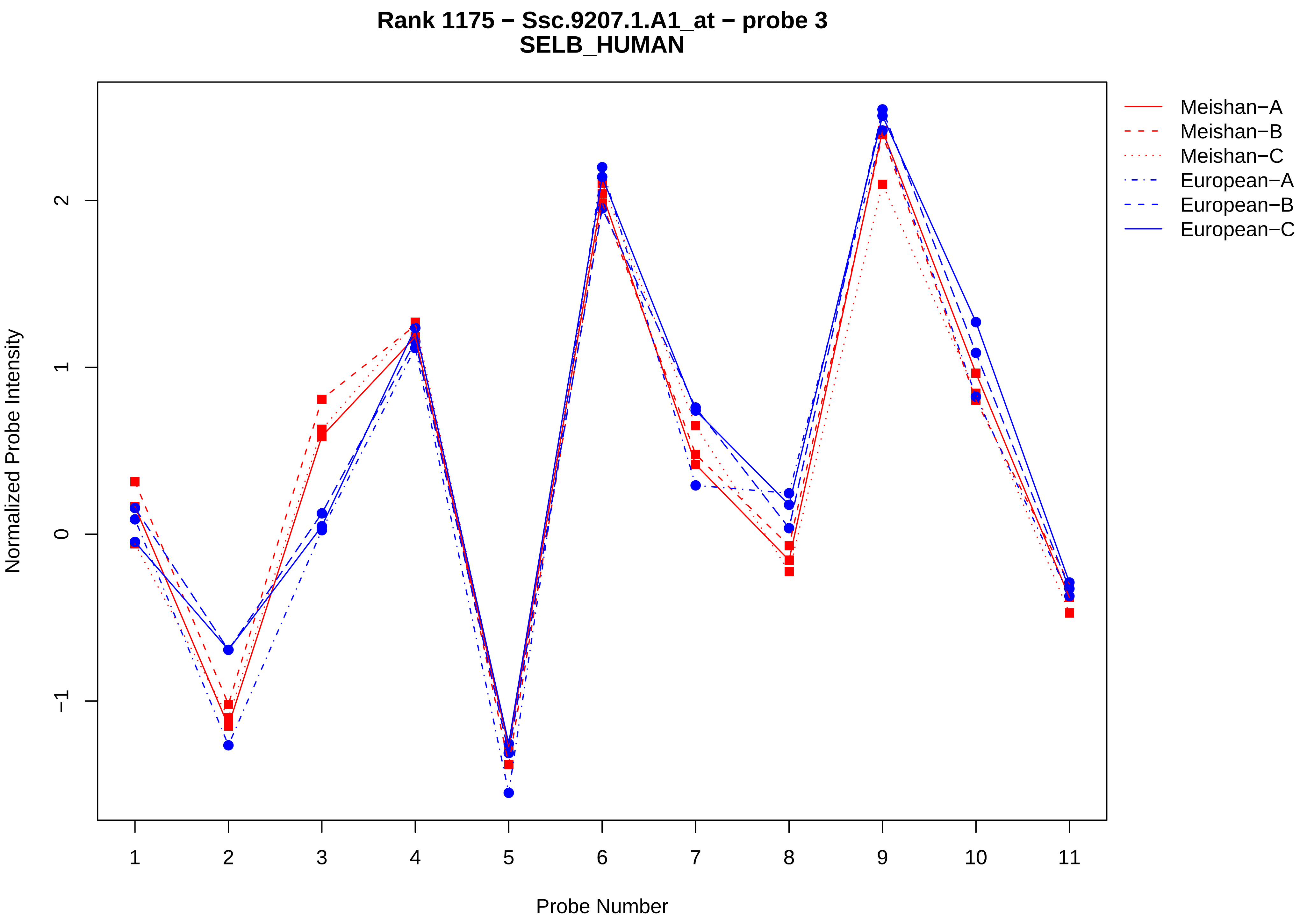

Rank 1175 − Ssc.9207.1.A1_at − probe 3
SELB_HUMAN
Normalized Probe Intensity
Probe Number
−1
0
1
2
1
2
3
4
5
6
7
8
9
10
11
Meishan−A
Meishan−B
Meishan−C
European−A
European−B
European−C

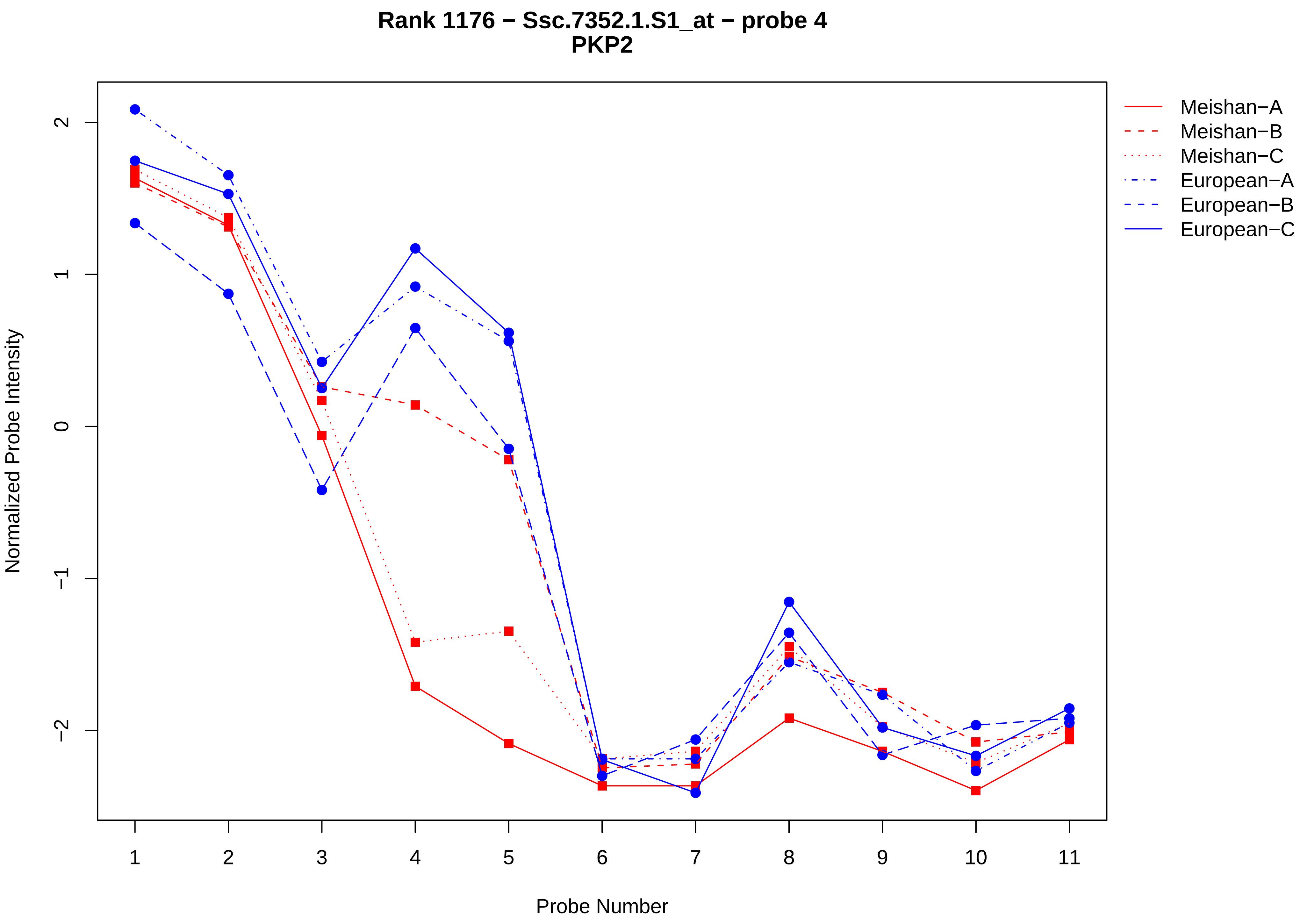

Rank 1176 − Ssc.7352.1.S1_at − probe 4
PKP2
Normalized Probe Intensity
Probe Number
Meishan−A
Meishan−B
Meishan−C
European−A
European−B
European−C
2
1
0
−1
−2
1
2
3
4
5
6
7
8
9
10
11

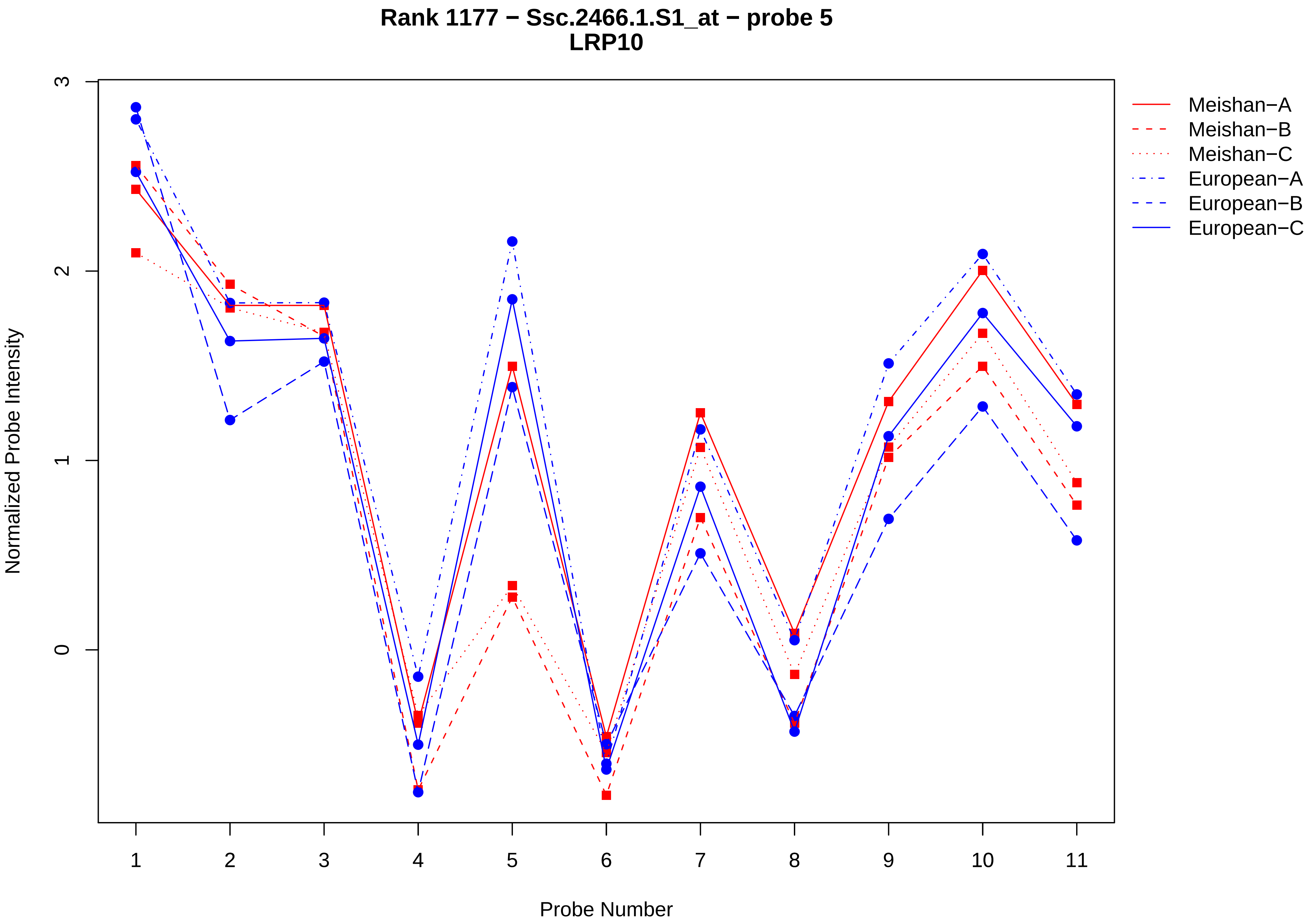

Rank 1177 − Ssc.2466.1.S1_at − probe 5
LRP10
Normalized Probe Intensity
Probe Number
0
1
2
3
1
2
3
4
5
6
7
8
9
10
11
Meishan−A
Meishan−B
Meishan−C
European−A
European−B
European−C

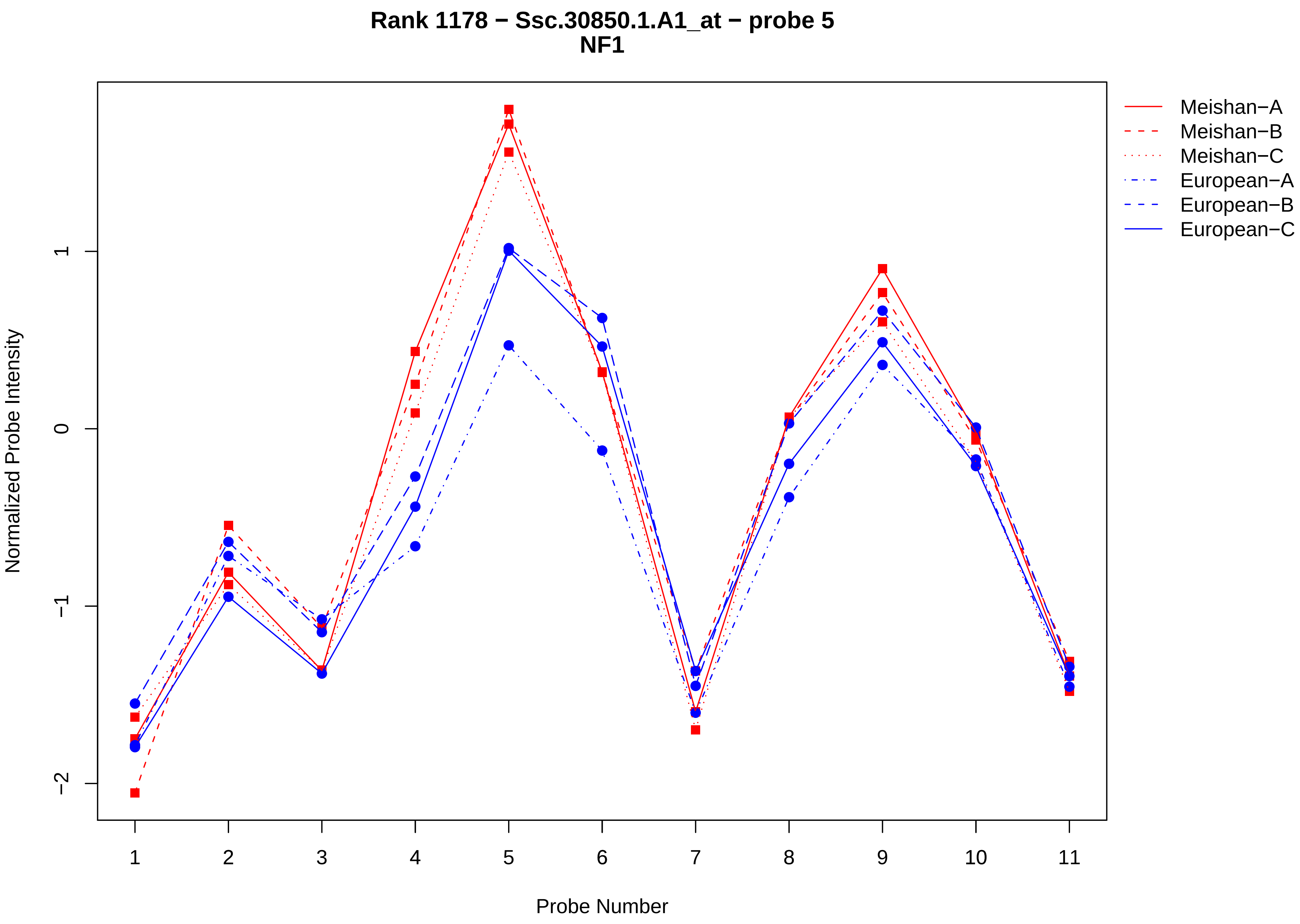

Rank 1178 − Ssc.30850.1.A1_at − probe 5
NF1
Normalized Probe Intensity
Probe Number
−2
−1
0
1
1
2
3
4
5
6
7
8
9
10
11
Meishan−A
Meishan−B
Meishan−C
European−A
European−B
European−C

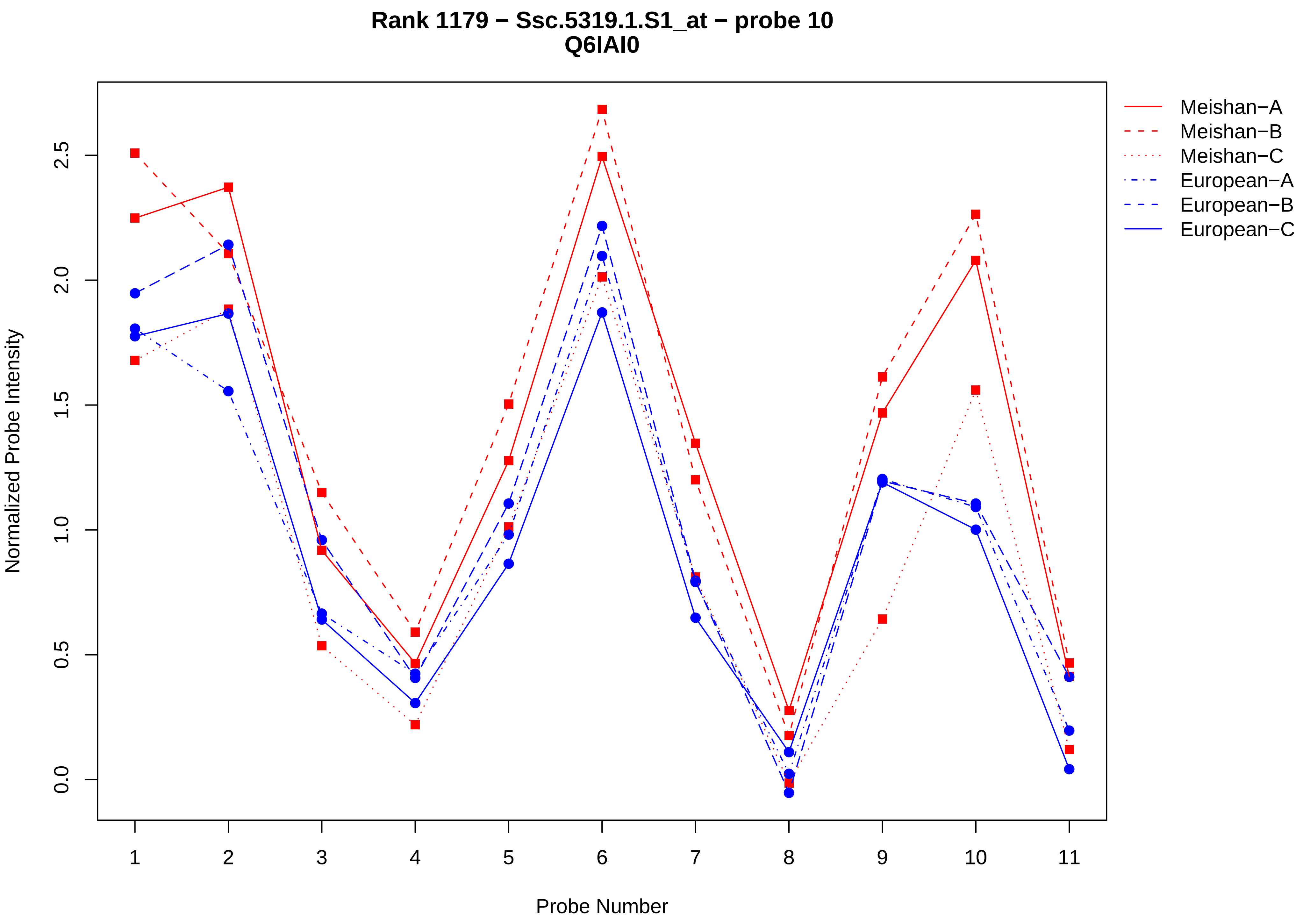

Rank 1179 − Ssc.5319.1.S1_at − probe 10
Q6IAI0
Normalized Probe Intensity
0.0
0.5
1.0
1.5
2.0
2.5
1
2
3
4
5
6
7
8
9
10
11
Probe Number
Meishan−A
Meishan−B
Meishan−C
European−A
European−B
European−C

Rank 1180 − Ssc.16603.1.A1_at − probe 8
A2M

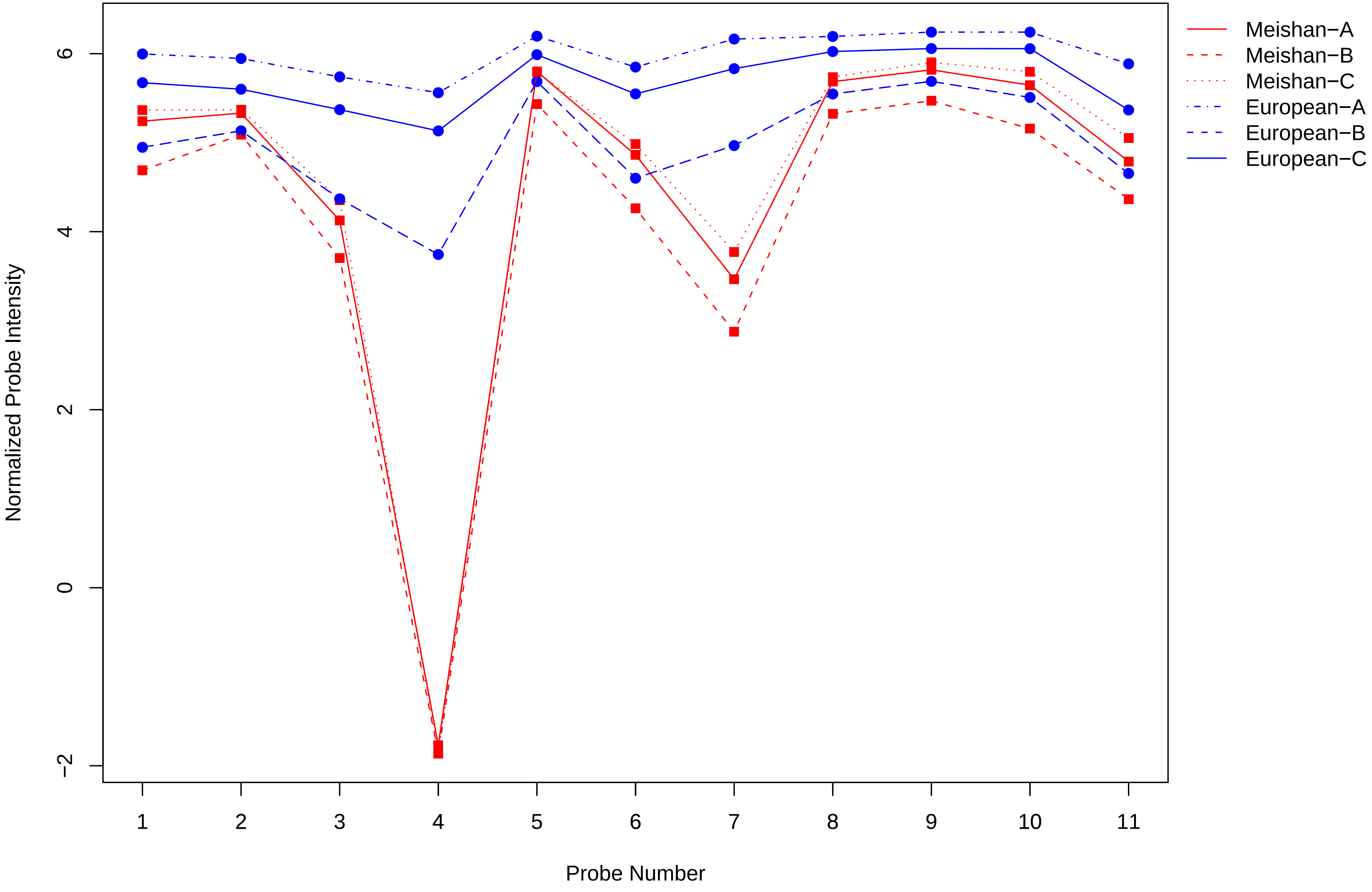

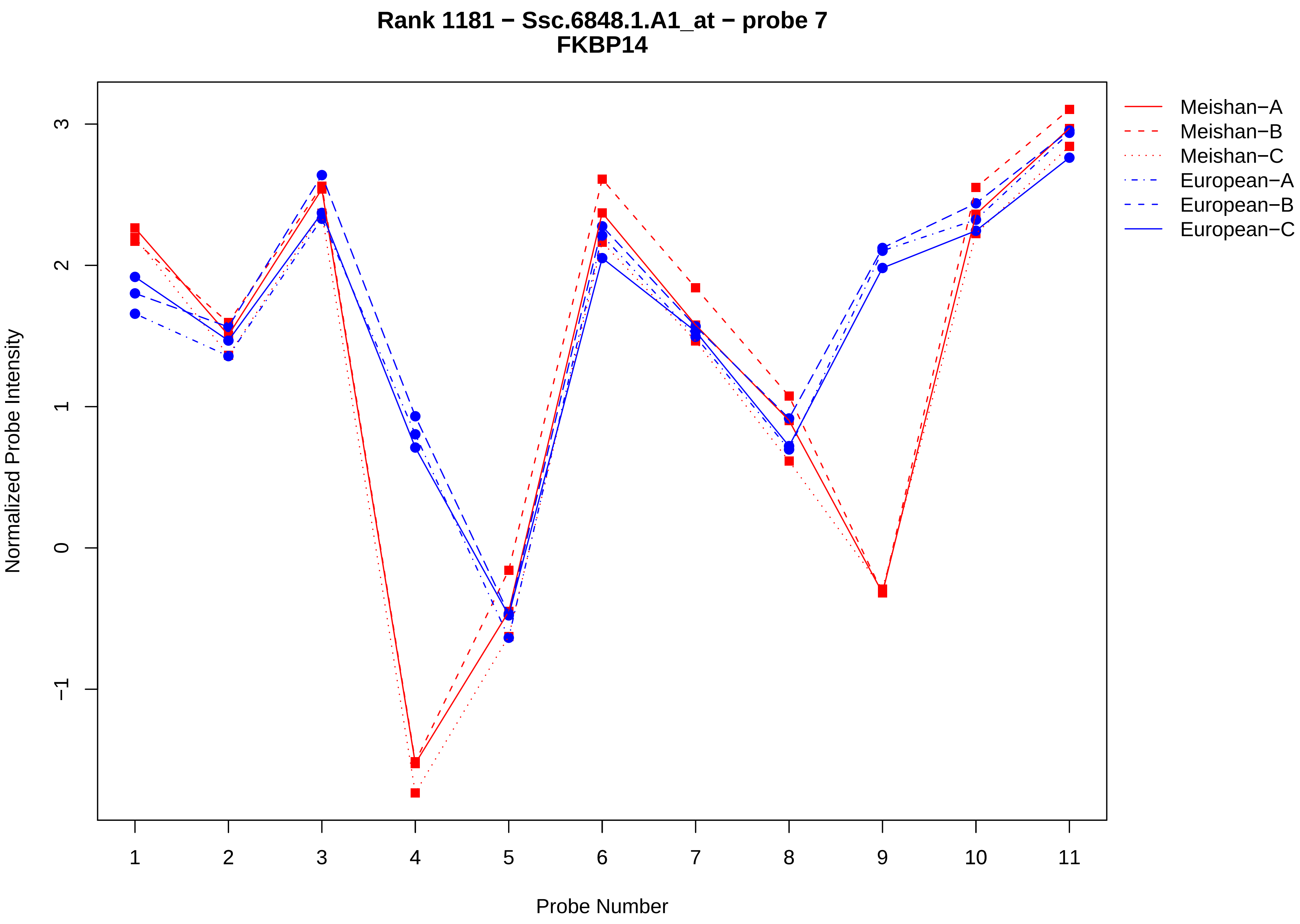

Rank 1181 − Ssc.6848.1.A1_at − probe 7
FKBP14
Normalized Probe Intensity
Probe Number
−1
0
1
2
3
1
2
3
4
5
6
7
8
9
10
11
Meishan−A
Meishan−B
Meishan−C
European−A
European−B
European−C

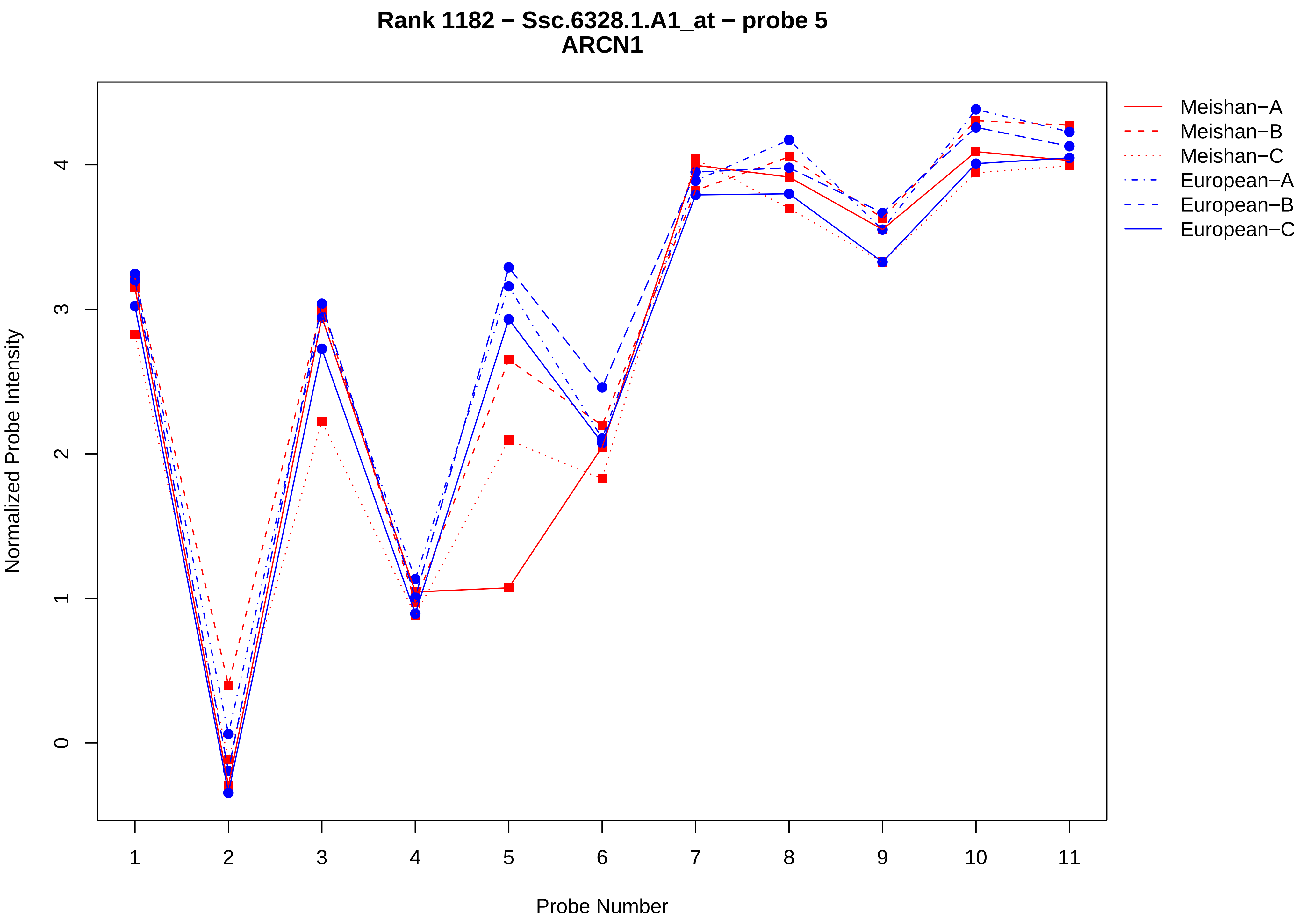

Rank 1182 − Ssc.6328.1.A1_at − probe 5
ARCN1
Normalized Probe Intensity
Probe Number
0
1
2
3
4
1
2
3
4
5
6
7
8
9
10
11
Meishan−A
Meishan−B
Meishan−C
European−A
European−B
European−C

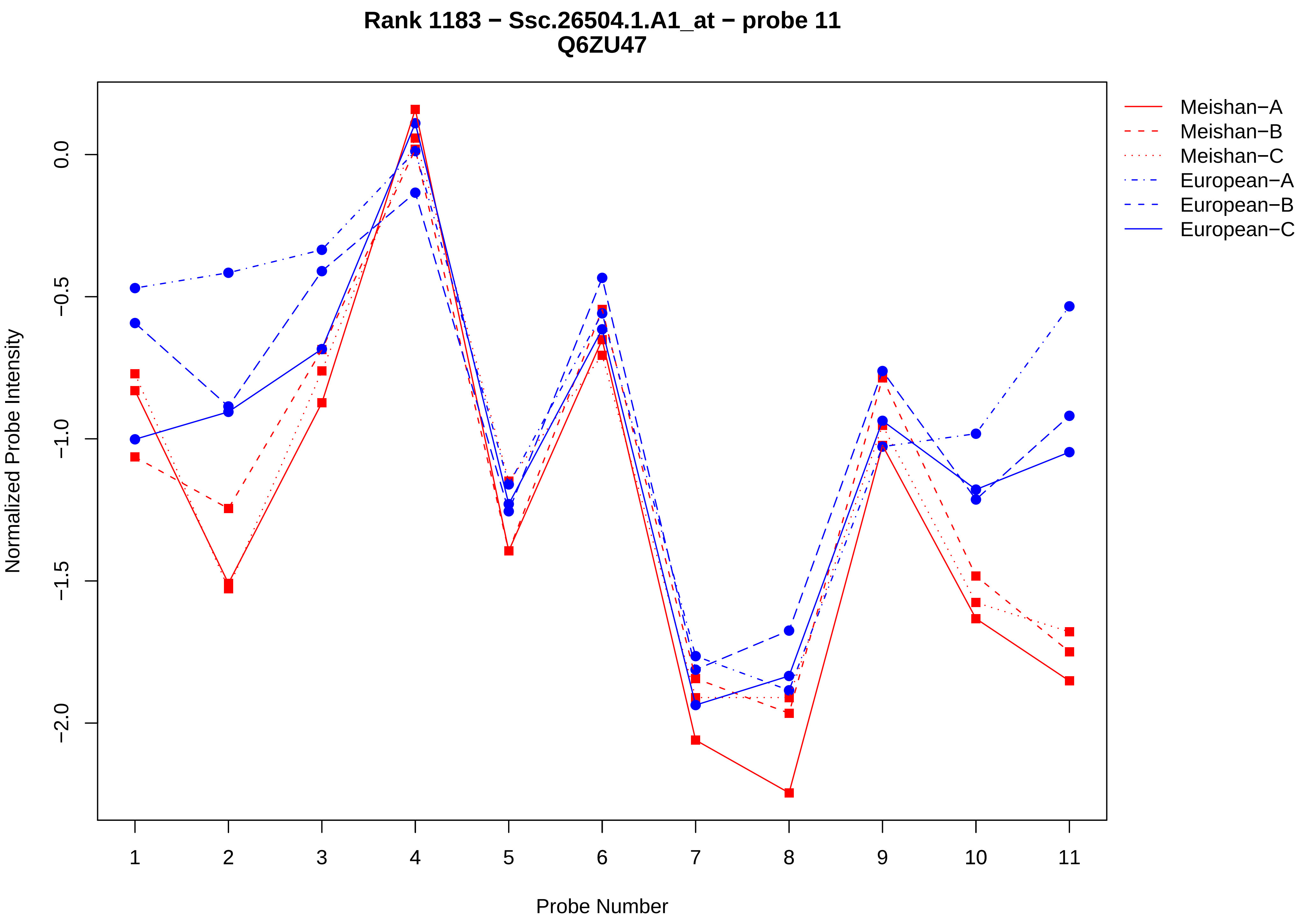
Rank 1183 − Ssc.26504.1.A1_at − probe 11
Q6ZU47
Normalized Probe Intensity
Probe Number
0.0
−0.5
−1.0
−1.5
−2.0
1
2
3
4
5
6
7
8
9
10
11
Meishan−A
Meishan−B
Meishan−C
European−A
European−B
European−C

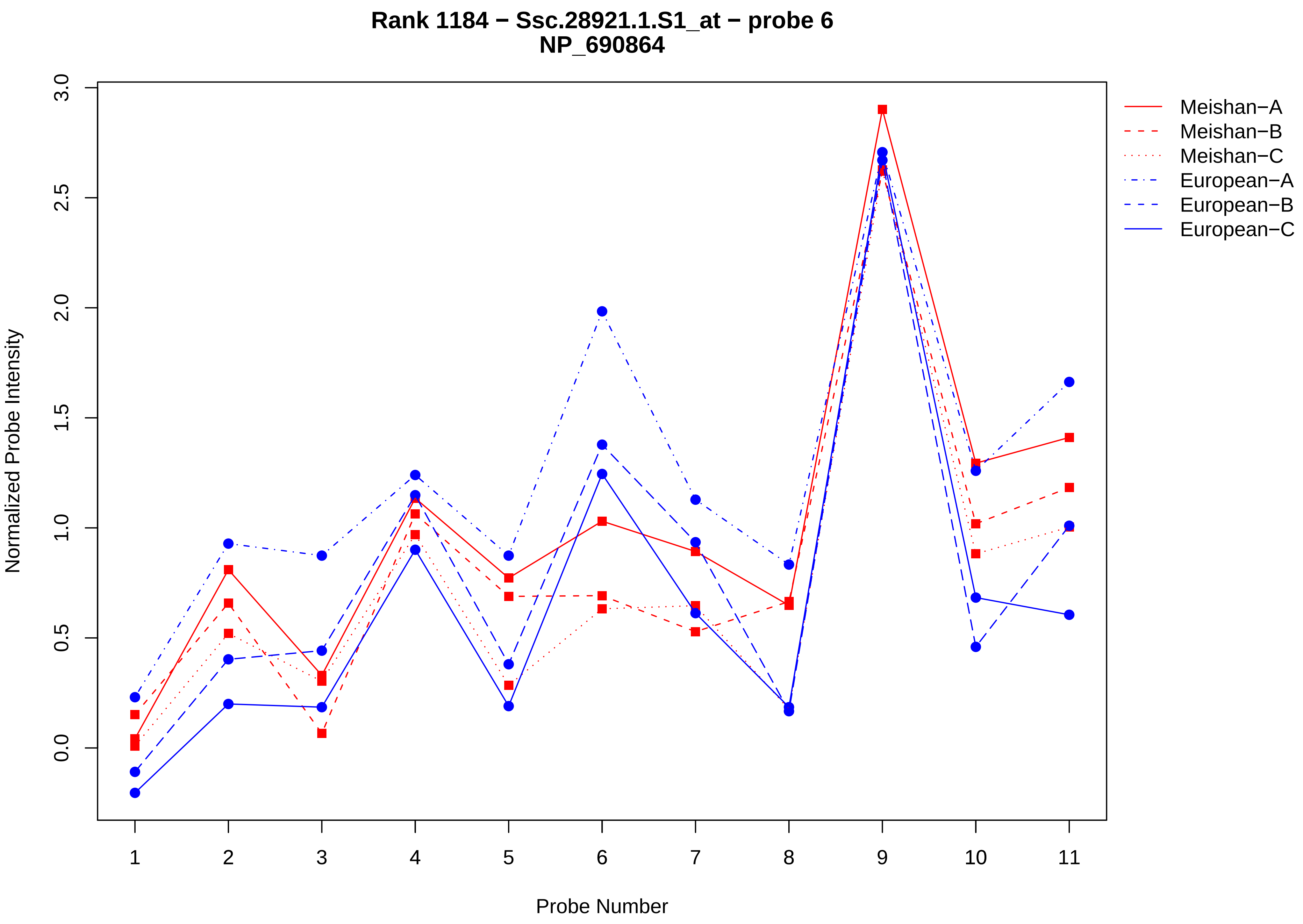
Rank 1184 − Ssc.28921.1.S1_at − probe 6
NP_690864
Normalized Probe Intensity
Probe Number
0.0
0.5
1.0
1.5
2.0
2.5
3.0
1
2
3
4
5
6
7
8
9
10
11
Meishan−A
Meishan−B
Meishan−C
European−A
European−B
European−C

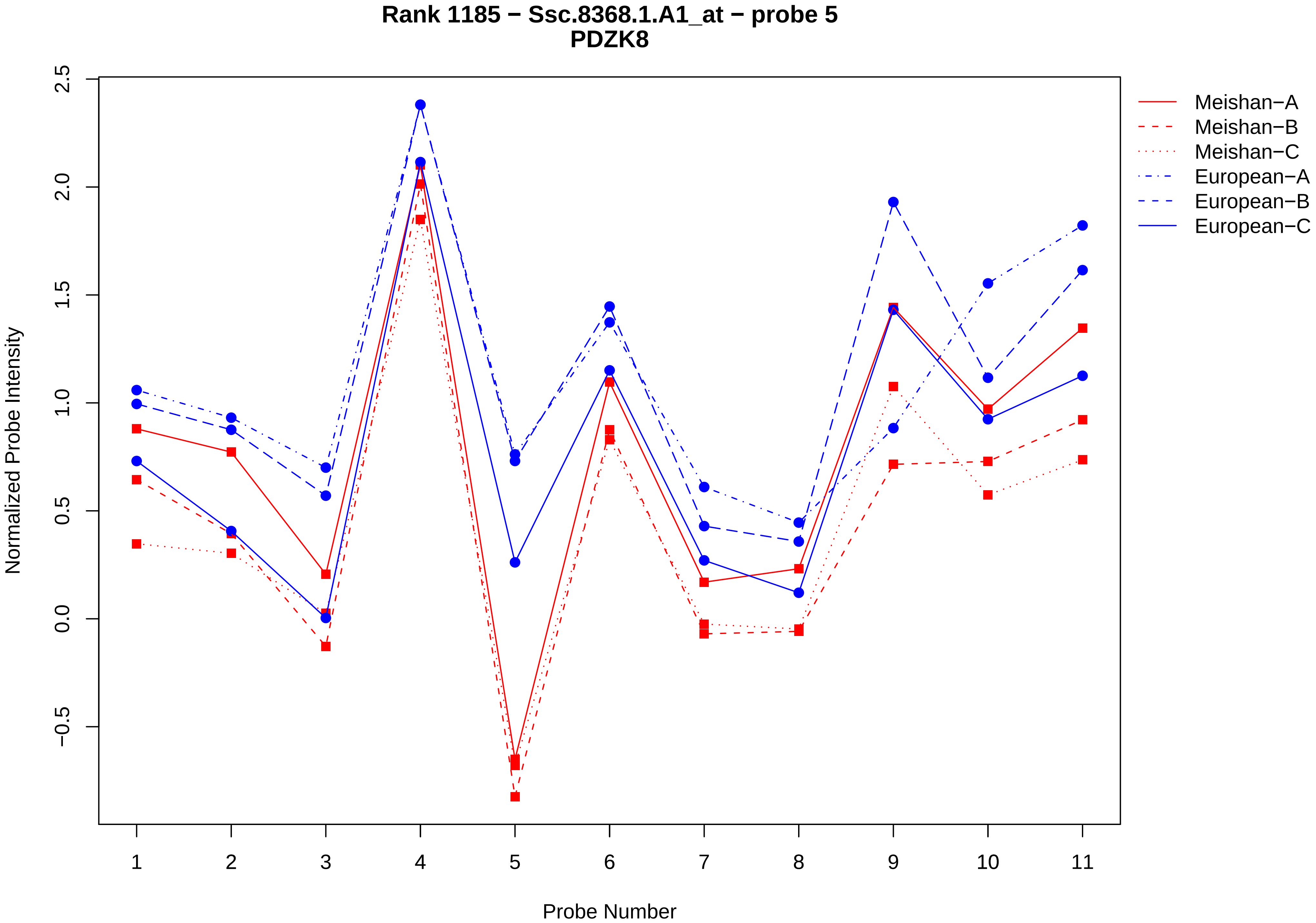

Rank 1185 − Ssc.8368.1.A1_at − probe 5
PDZK8
Normalized Probe Intensity
Probe Number
−0.5
0.0
0.5
1.0
1.5
2.0
2.5
1
2
3
4
5
6
7
8
9
10
11
Meishan−A
Meishan−B
Meishan−C
European−A
European−B
European−C

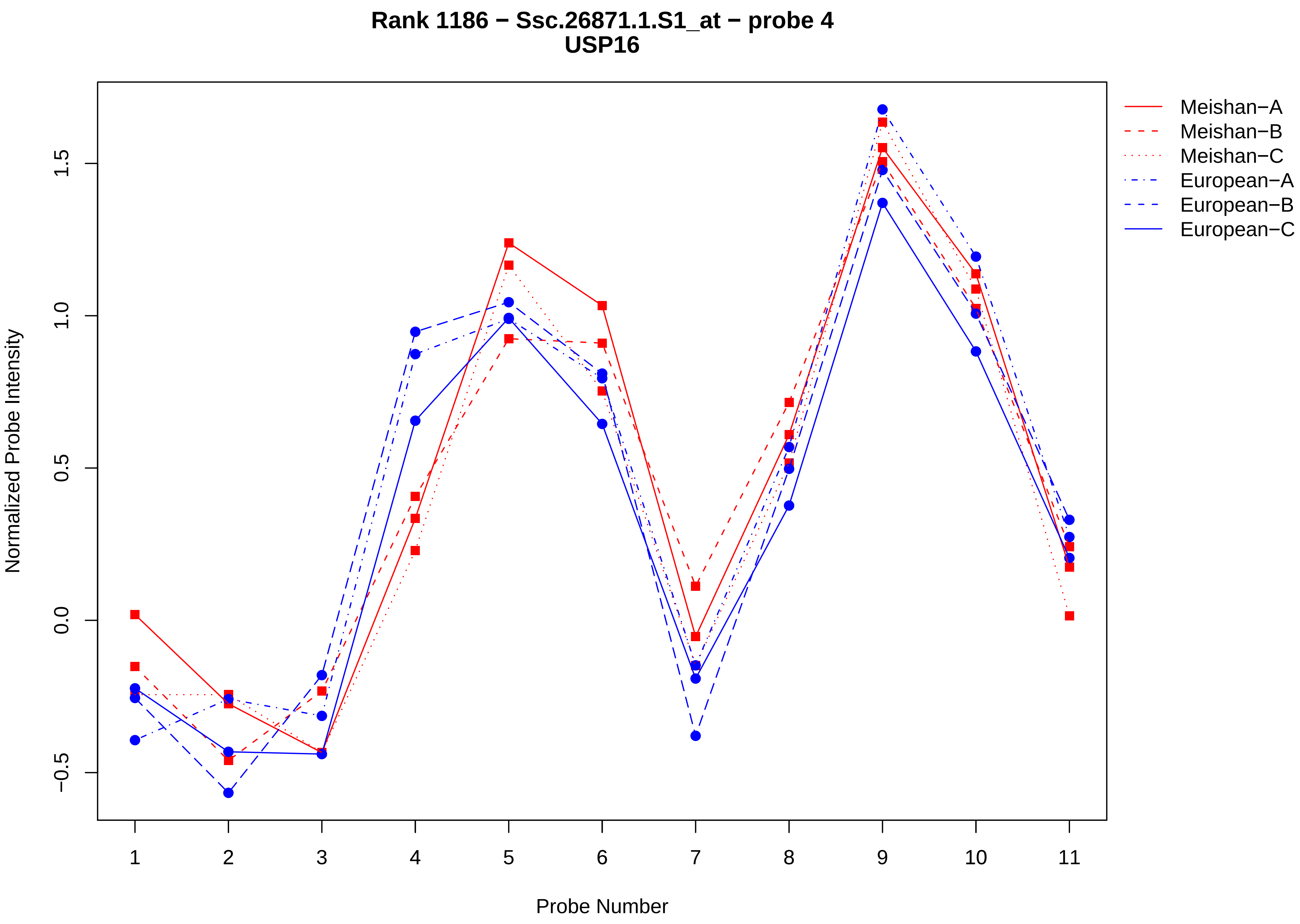

Rank 1186 − Ssc.26871.1.S1_at − probe 4
USP16
Normalized Probe Intensity
−0.5
0.0
0.5
1.0
1.5
1
2
3
4
5
6
7
8
9
10
11
Probe Number
Meishan−A
Meishan−B
Meishan−C
European−A
European−B
European−C

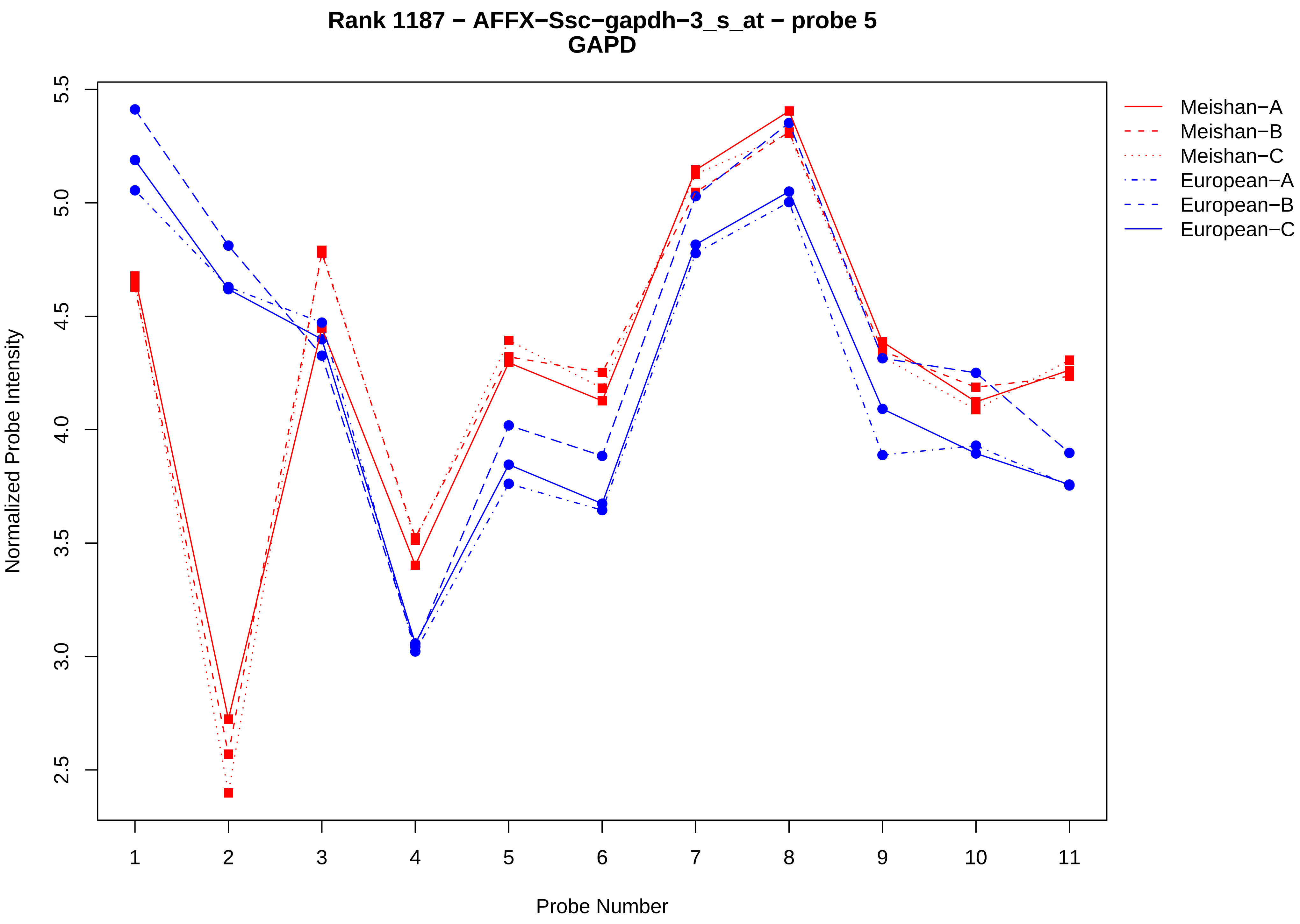

Rank 1187 − AFFX−Ssc−gapdh−3_s_at − probe 5
GAPD
Normalized Probe Intensity
2.5
3.0
3.5
4.0
4.5
5.0
5.5
1
2
3
4
5
6
7
8
9
10
11
Probe Number
Meishan−A
Meishan−B
Meishan−C
European−A
European−B
European−C

Rank 1188 − Ssc.309.1.S1_at − probe 10
VTN

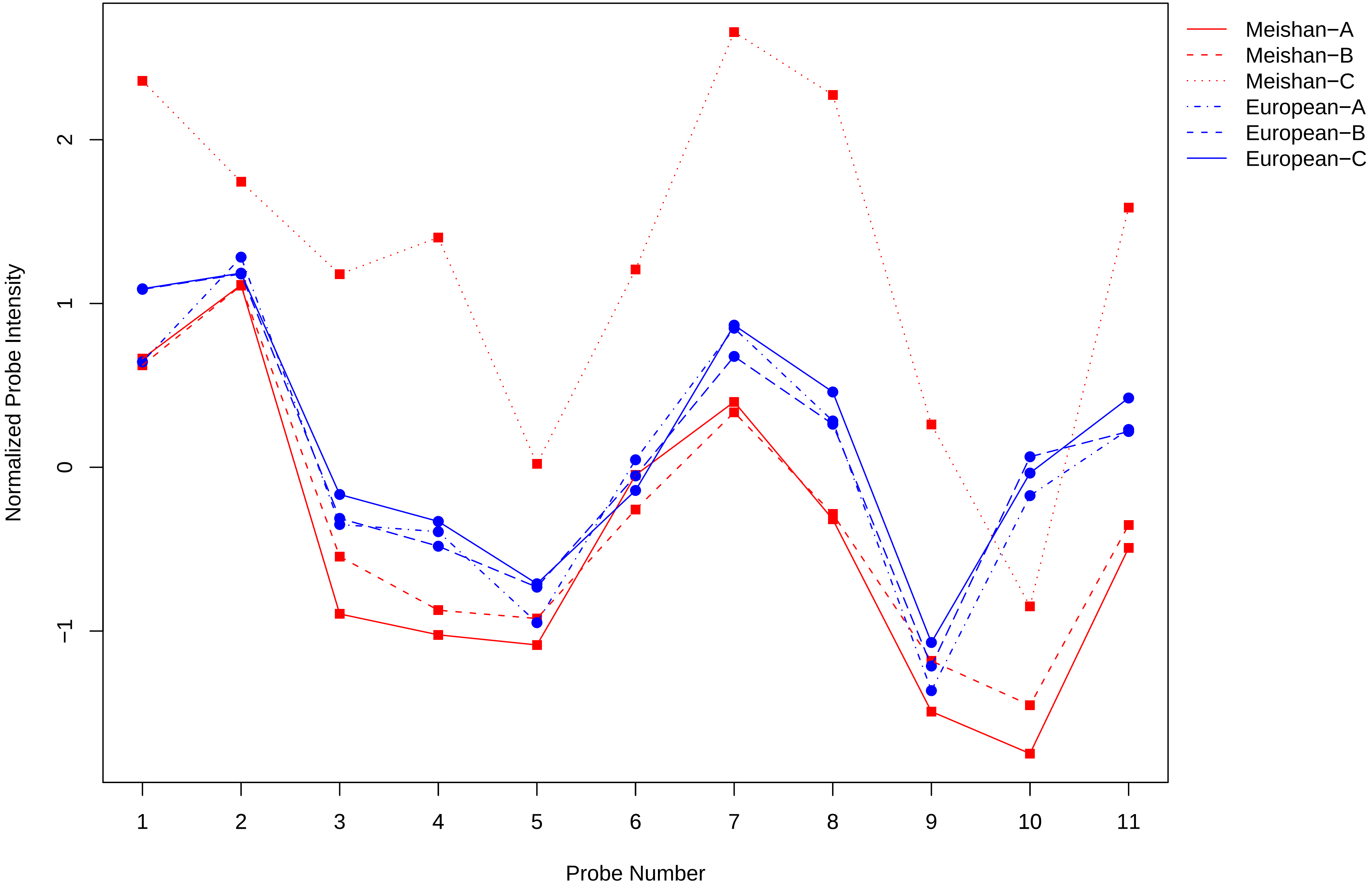

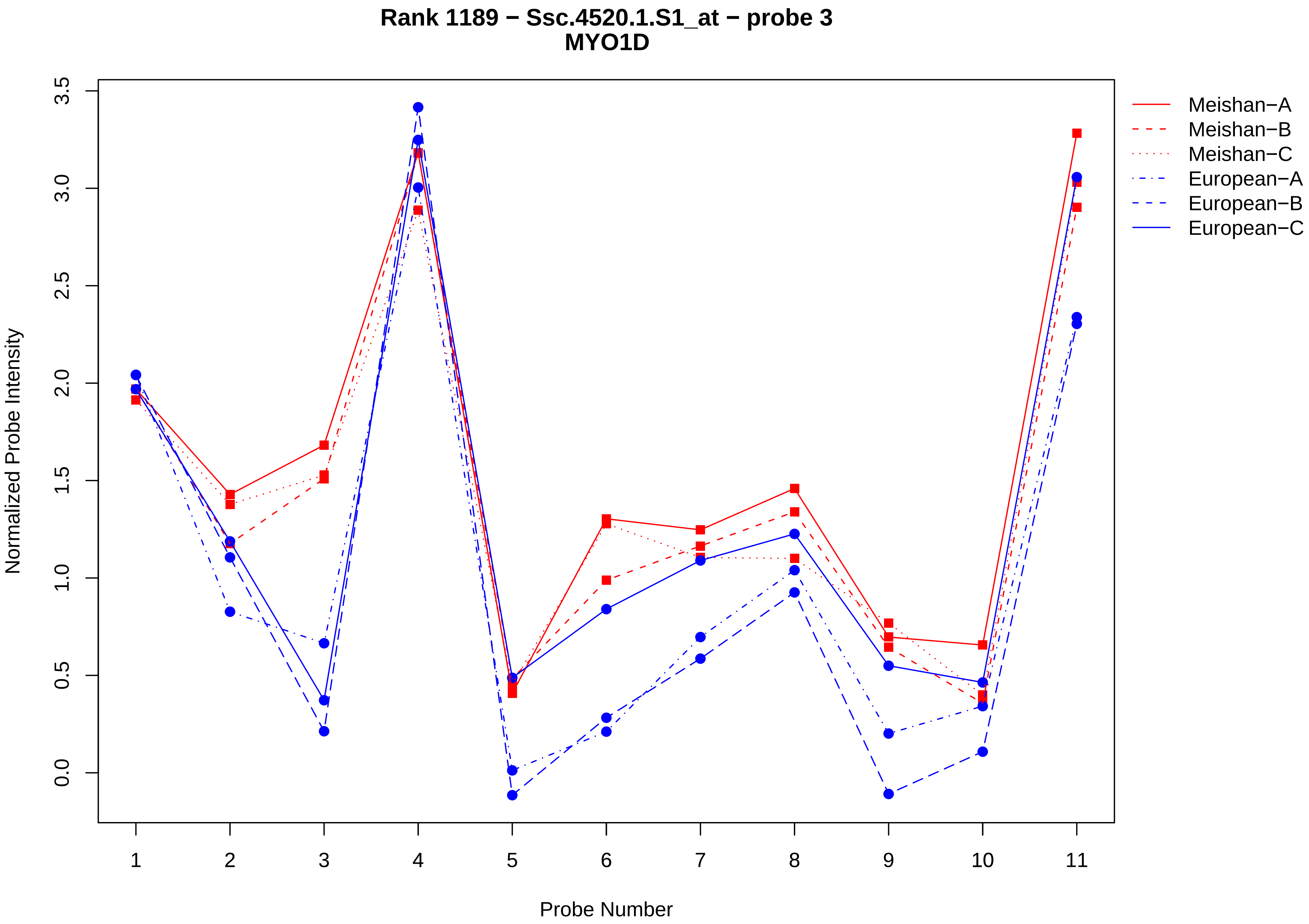

Rank 1189 − Ssc.4520.1.S1_at − probe 3
MYO1D
Normalized Probe Intensity
0.0
0.5
1.0
1.5
2.0
2.5
3.0
3.5
1
2
3
4
5
6
7
8
9
10
11
Probe Number
Meishan−A
Meishan−B
Meishan−C
European−A
European−B
European−C

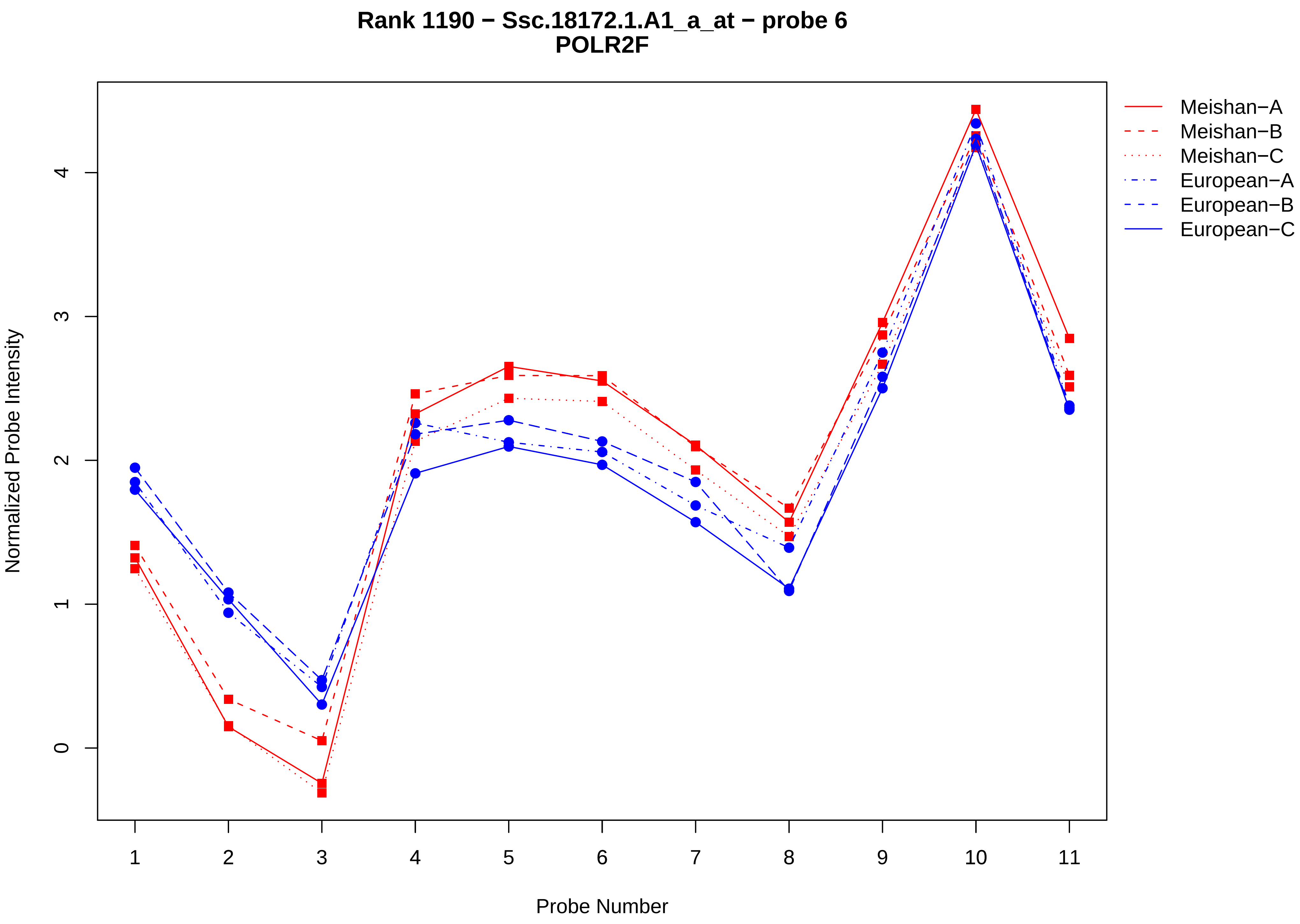

Rank 1190 − Ssc.18172.1.A1_a_at − probe 6
POLR2F
Normalized Probe Intensity
Probe Number
0
1
2
3
4
1
2
3
4
5
6
7
8
9
10
11
Meishan−A
Meishan−B
Meishan−C
European−A
European−B
European−C

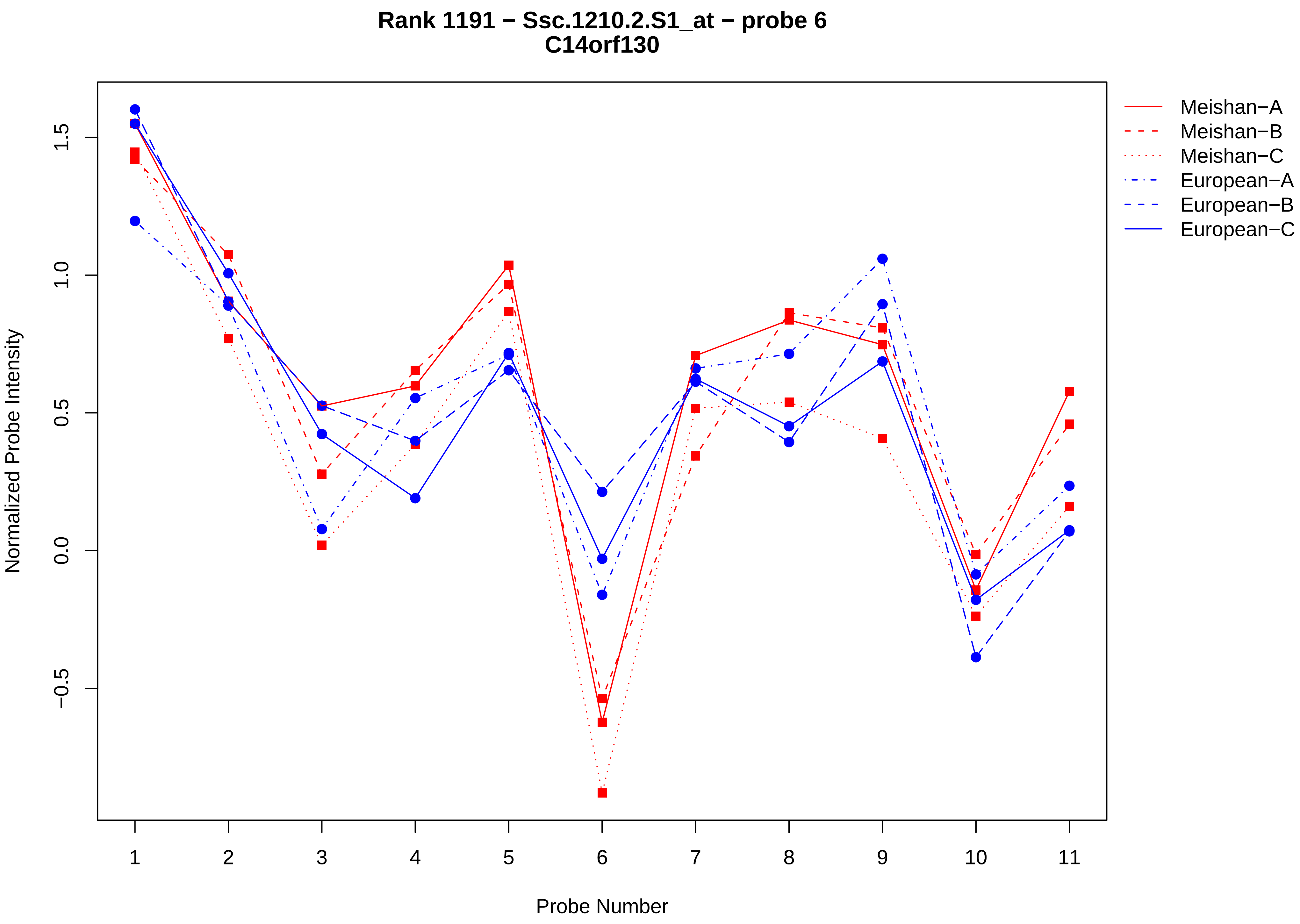

Rank 1191 − Ssc.1210.2.S1_at − probe 6
C14orf130
Normalized Probe Intensity
Probe Number
−0.5
0.0
0.5
1.0
1.5
1
2
3
4
5
6
7
8
9
10
11
Meishan−A
Meishan−B
Meishan−C
European−A
European−B
European−C

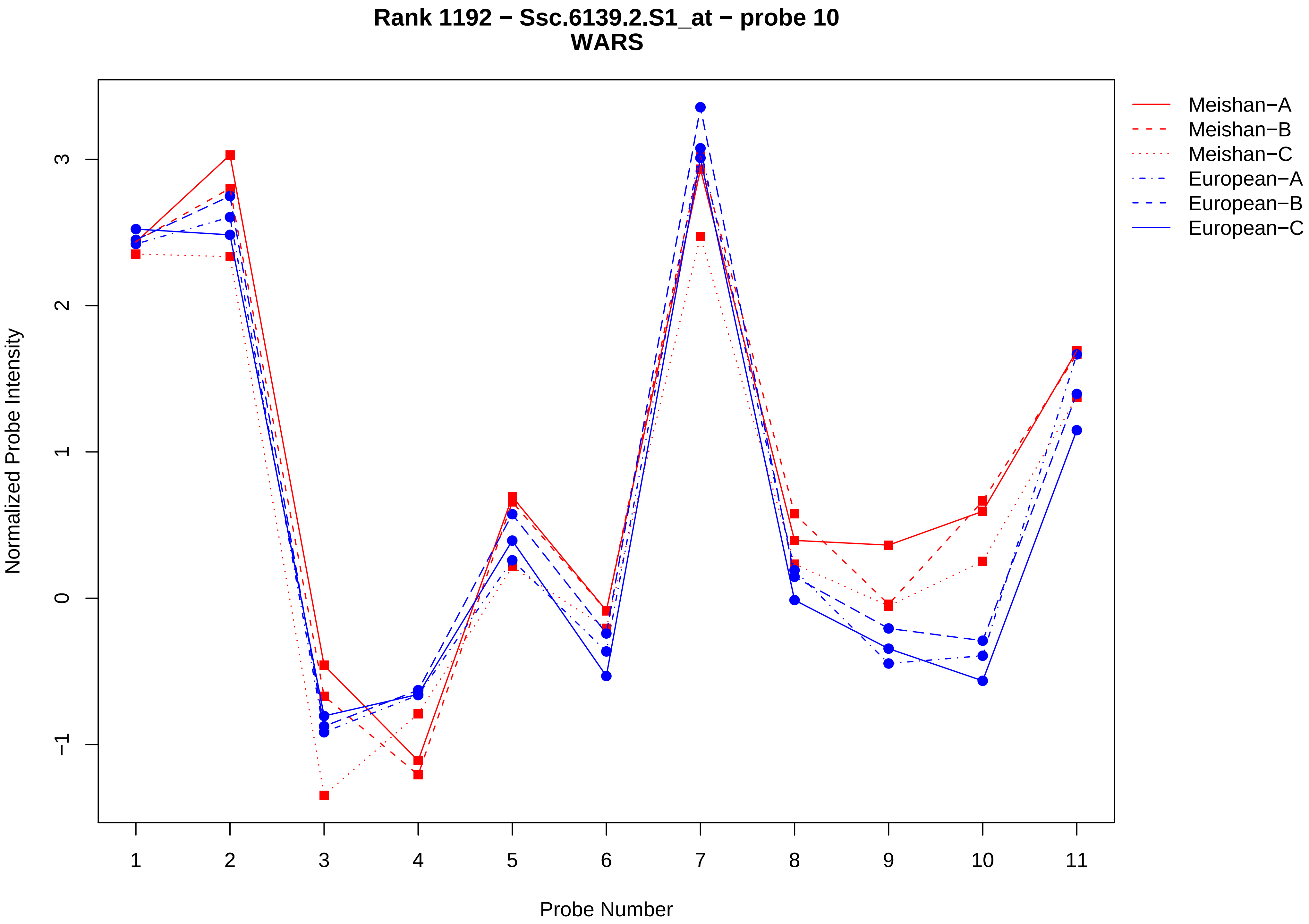
Rank 1192 − Ssc.6139.2.S1_at − probe 10
WARS
Normalized Probe Intensity
Probe Number
−1
0
1
2
3
1
2
3
4
5
6
7
8
9
10
11
Meishan−A
Meishan−B
Meishan−C
European−A
European−B
European−C

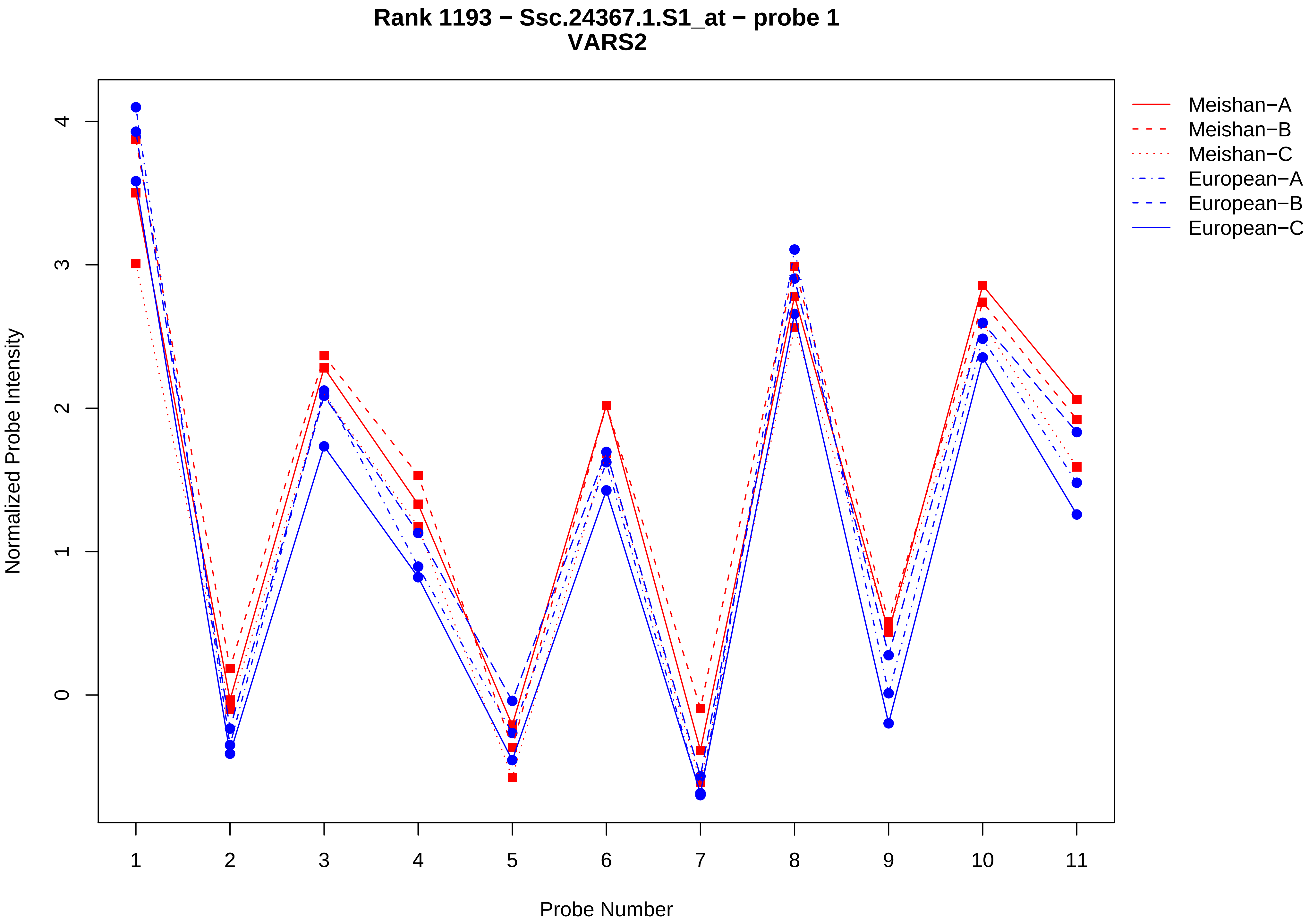

Rank 1193 − Ssc.24367.1.S1_at − probe 1
VARS2
Normalized Probe Intensity
0
1
2
3
4
1
2
3
4
5
6
7
8
9
10
11
Probe Number
Meishan−A
Meishan−B
Meishan−C
European−A
European−B
European−C

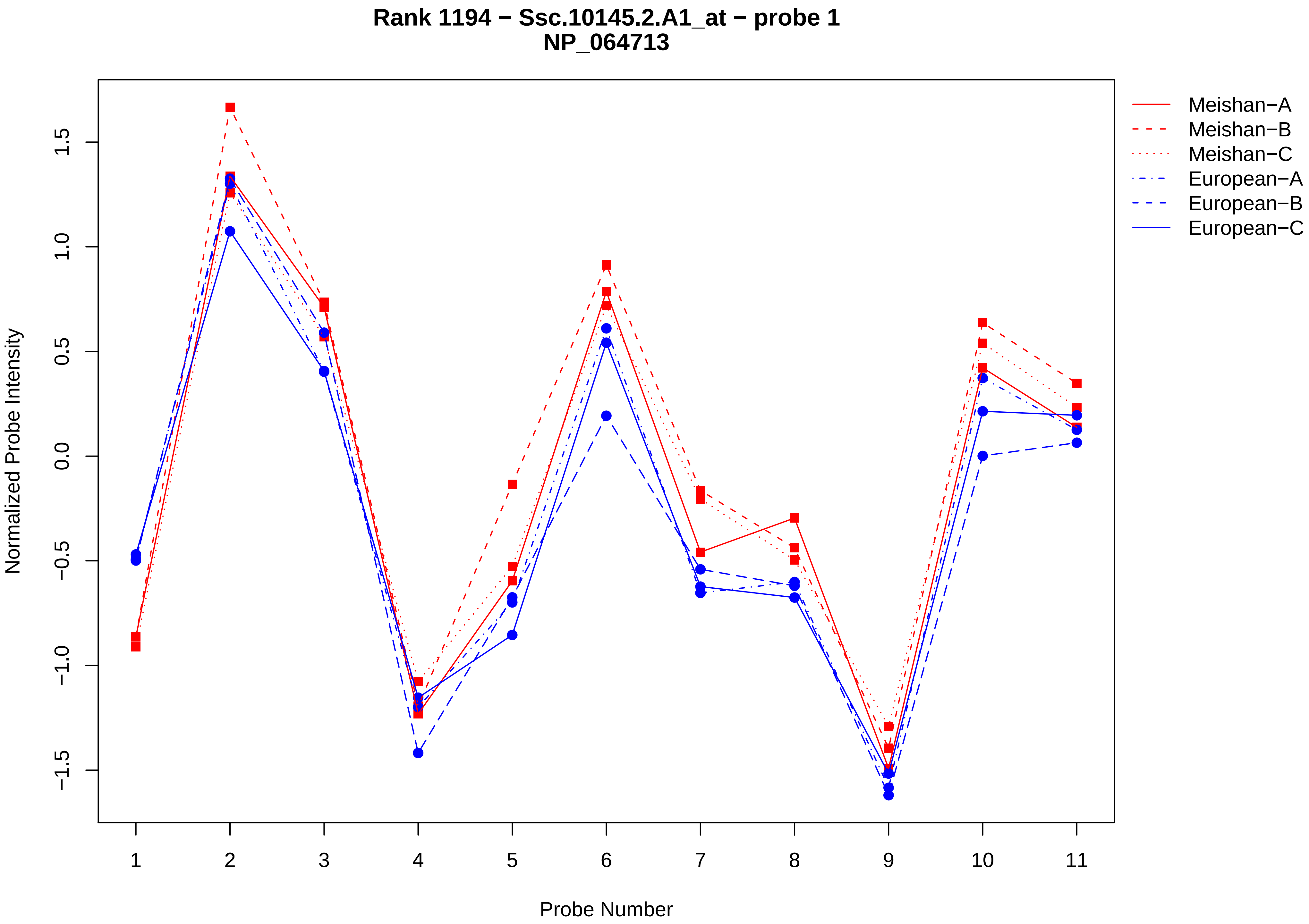

Rank 1194 − Ssc.10145.2.A1_at − probe 1
NP_064713
Normalized Probe Intensity
Probe Number
1.5
1.0
0.5
0.0
−0.5
−1.0
−1.5
1
2
3
4
5
6
7
8
9
10
11
Meishan−A
Meishan−B
Meishan−C
European−A
European−B
European−C

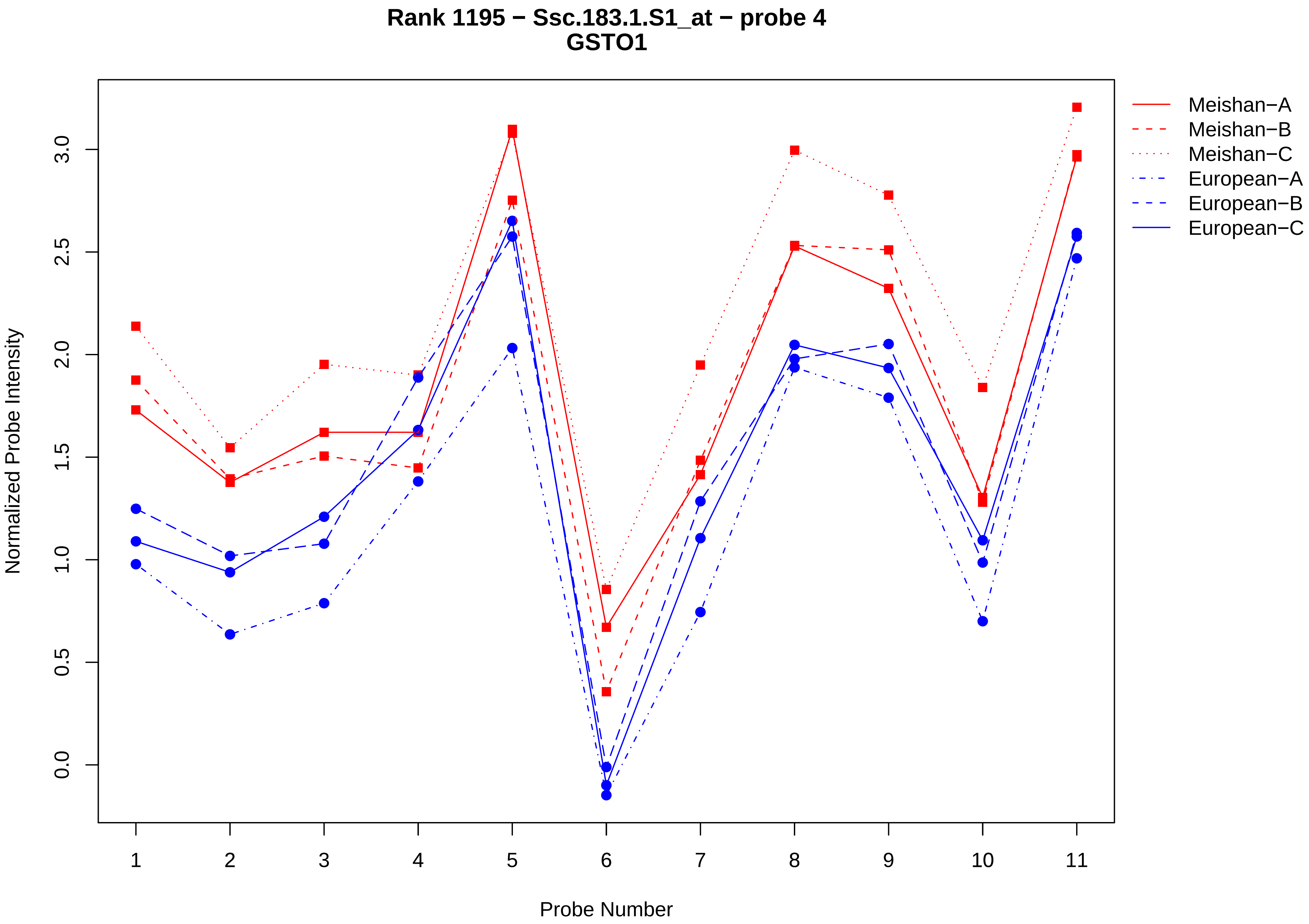

Rank 1195 − Ssc.183.1.S1_at − probe 4
GSTO1
Normalized Probe Intensity
0.0
0.5
1.0
1.5
2.0
2.5
3.0
1
2
3
4
5
6
7
8
9
10
11
Probe Number
Meishan−A
Meishan−B
Meishan−C
European−A
European−B
European−C

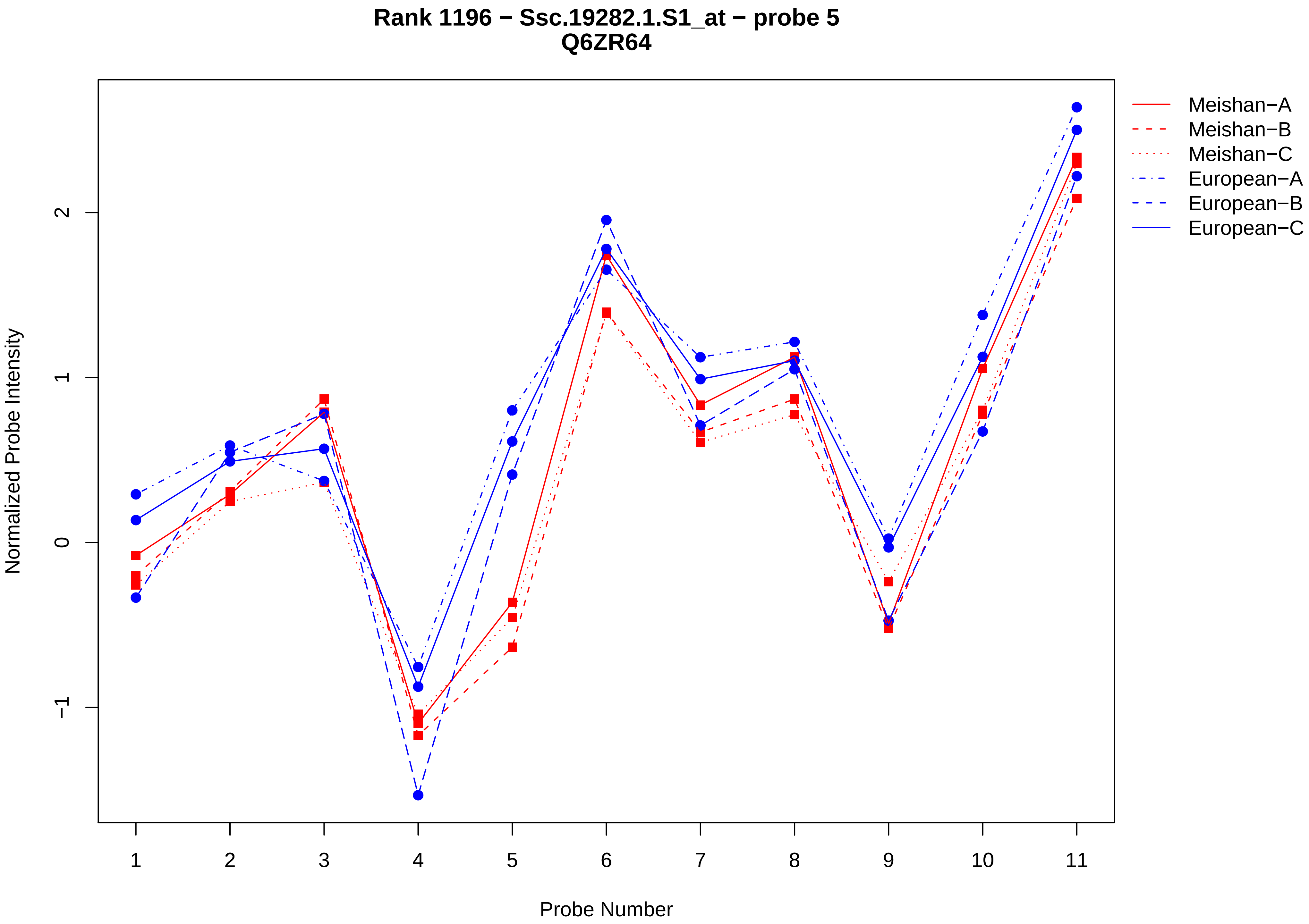

Rank 1196 − Ssc.19282.1.S1_at − probe 5
Q6ZR64
Normalized Probe Intensity
Probe Number
−1
0
1
2
1
2
3
4
5
6
7
8
9
10
11
Meishan−A
Meishan−B
Meishan−C
European−A
European−B
European−C

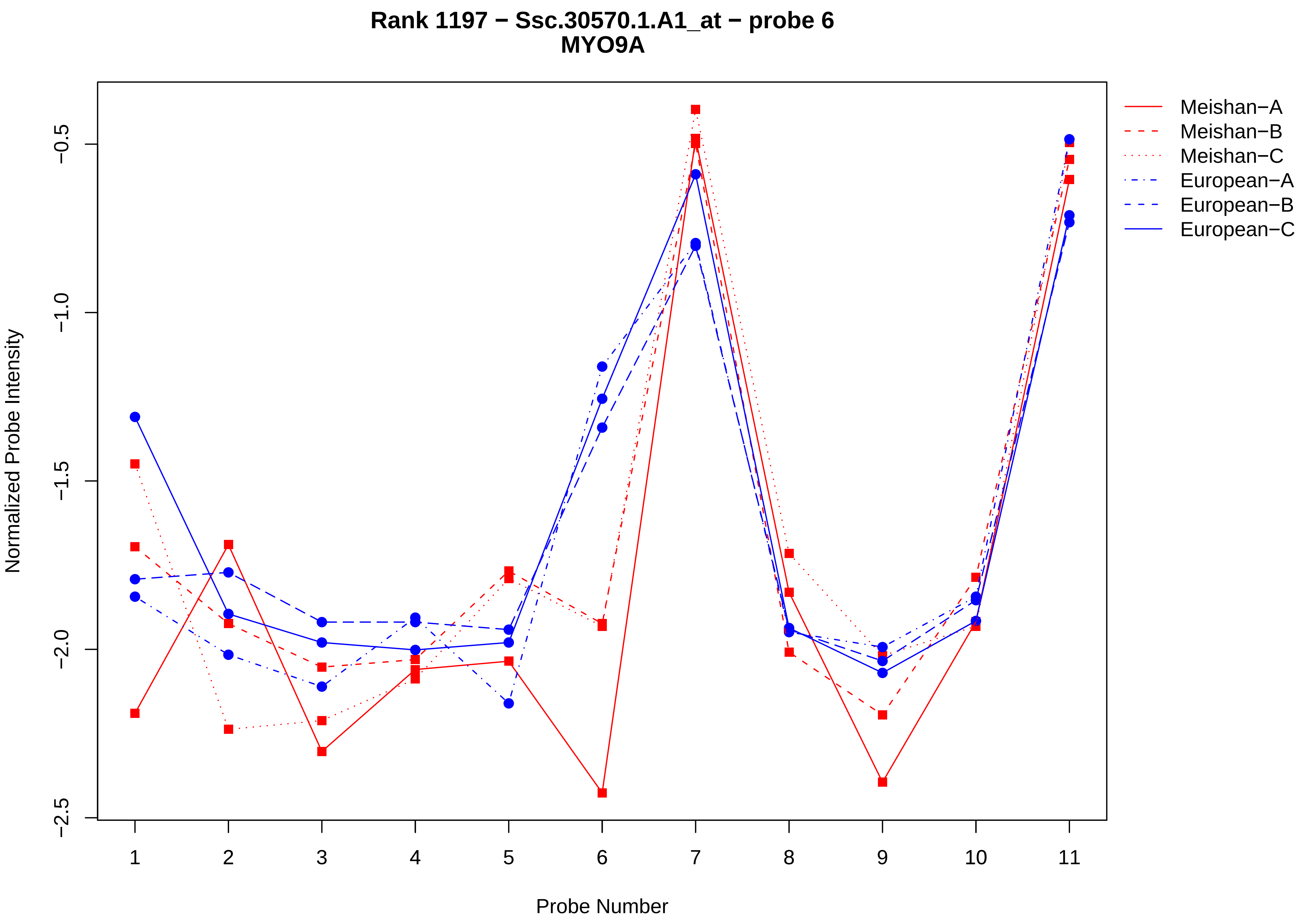

Rank 1197 − Ssc.30570.1.A1_at − probe 6
MYO9A
Normalized Probe Intensity
−0.5
−1.0
−1.5
−2.0
−2.5
1
2
3
4
5
6
7
8
9
10
11
Probe Number
Meishan−A
Meishan−B
Meishan−C
European−A
European−B
European−C

Rank 1198 − Ssc.25147.1.A1_at − probe 3
O43303

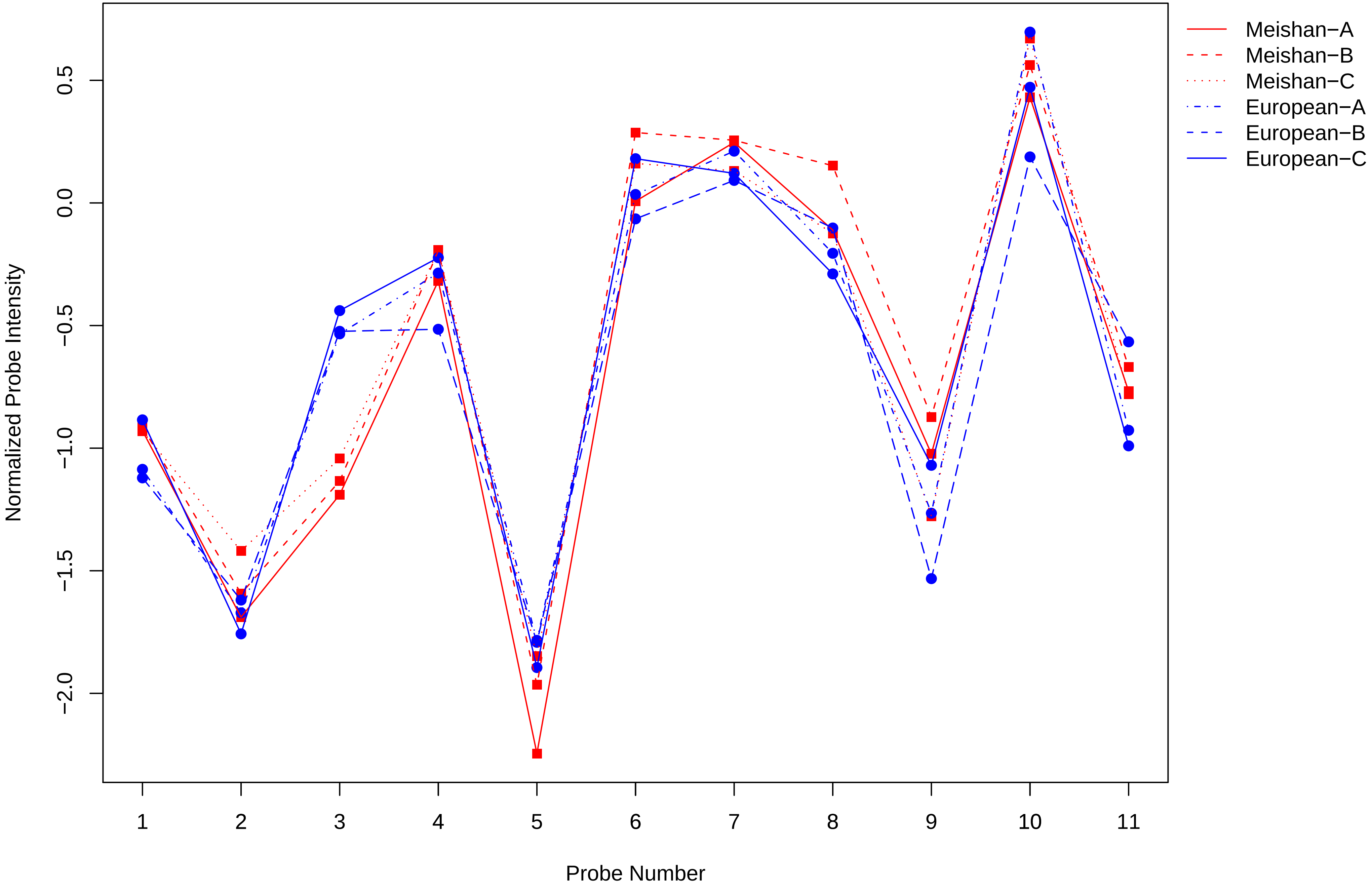

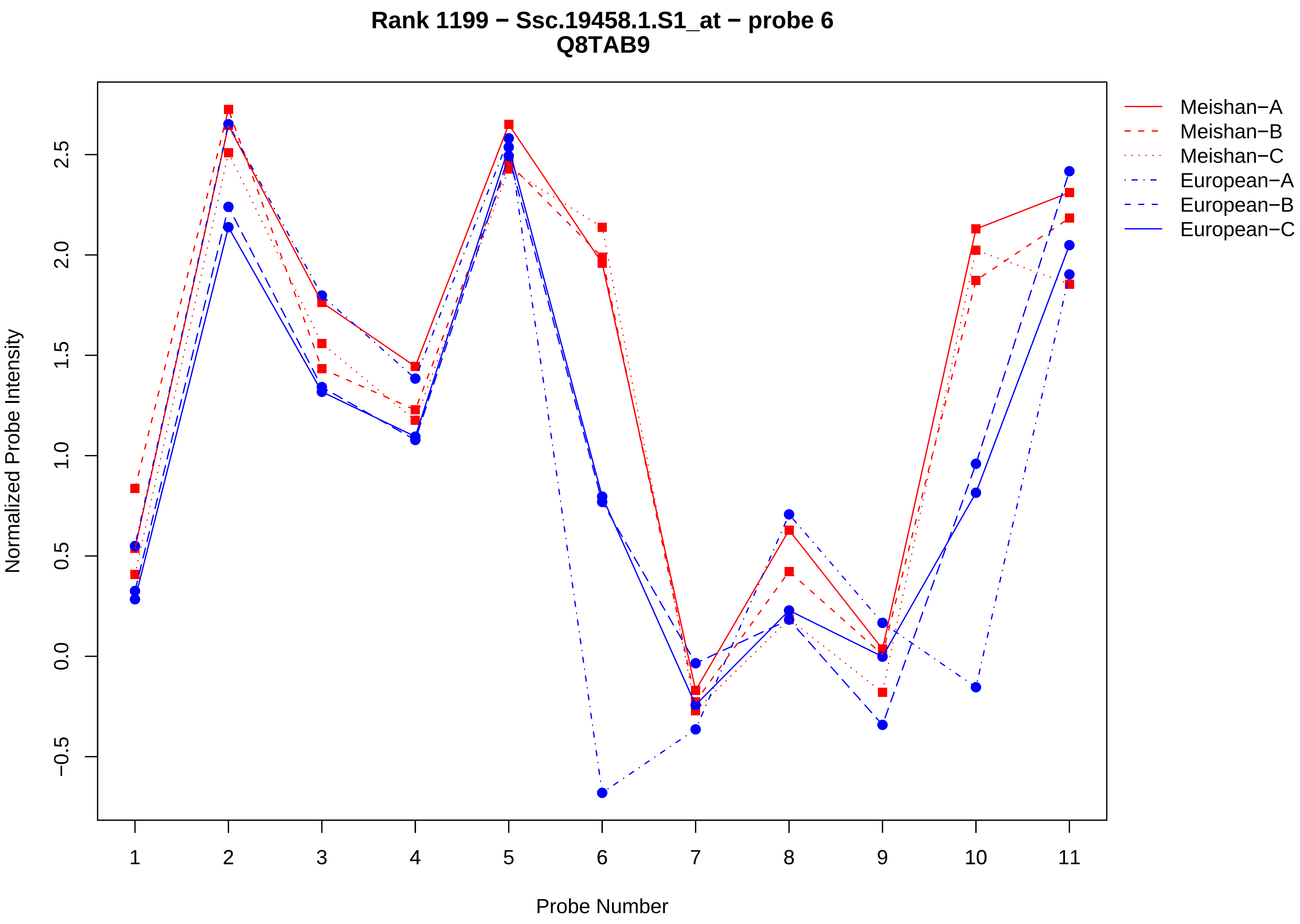

Rank 1199 − Ssc.19458.1.S1_at − probe 6
Q8TAB9
Normalized Probe Intensity
Probe Number
−0.5
0.0
0.5
1.0
1.5
2.0
2.5
1
2
3
4
5
6
7
8
9
10
11
Meishan−A
Meishan−B
Meishan−C
European−A
European−B
European−C

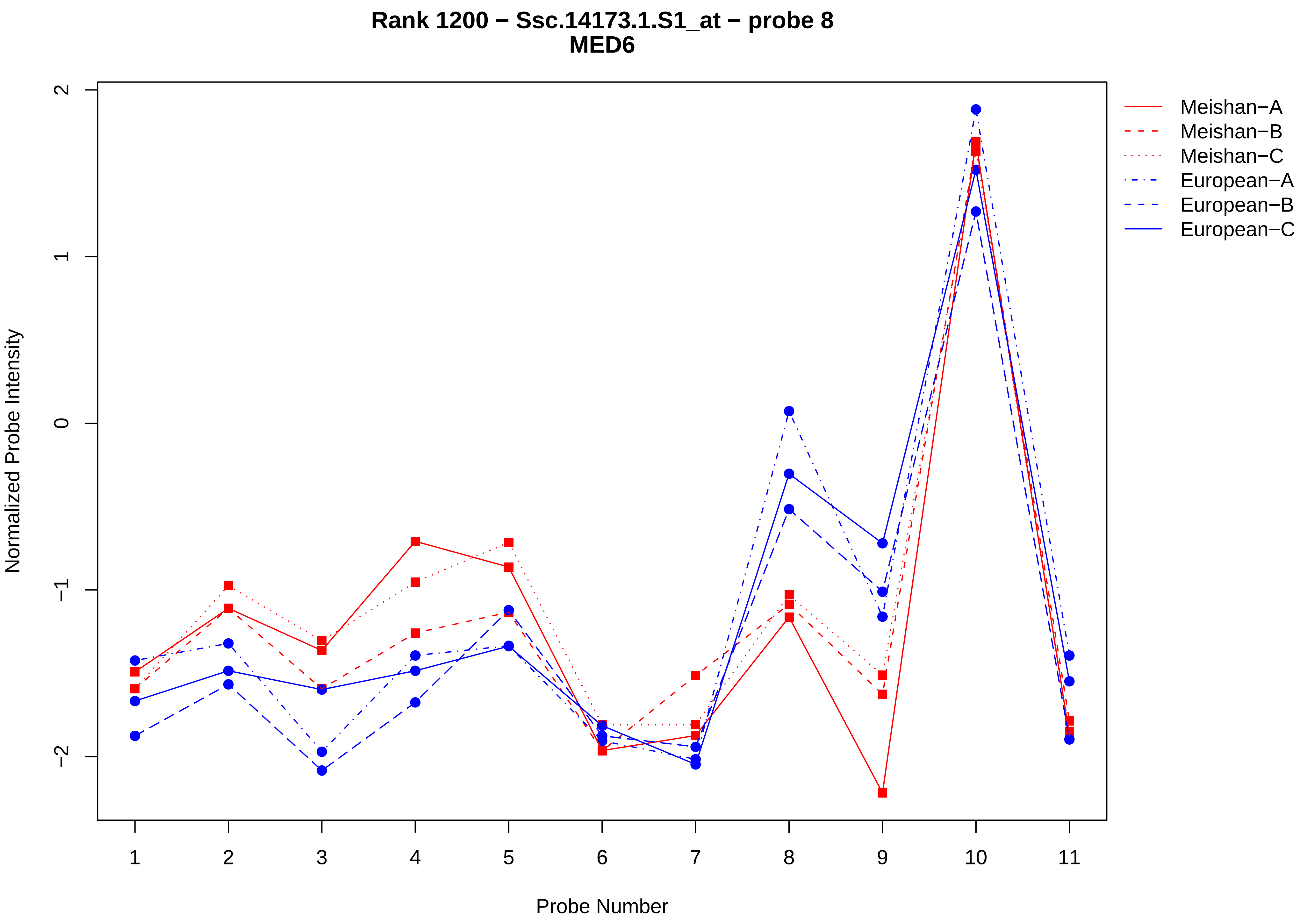
Rank 1200 − Ssc.14173.1.S1_at − probe 8
MED6
Normalized Probe Intensity
Probe Number
−2
−1
0
1
2
1
2
3
4
5
6
7
8
9
10
11
Meishan−A
Meishan−B
Meishan−C
European−A
European−B
European−C

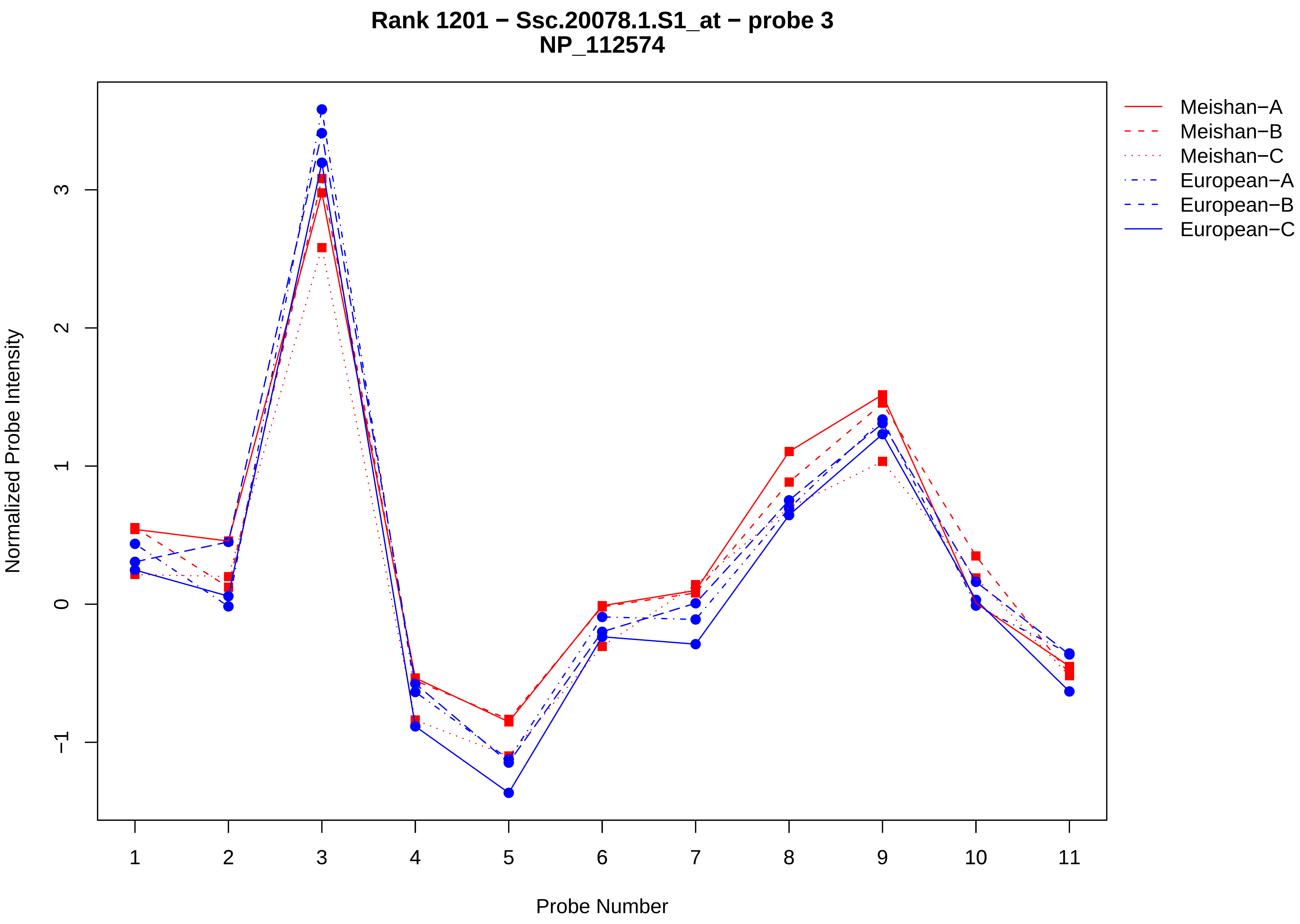

Rank 1201 − Ssc.20078.1.S1_at − probe 3
NP_112574
Normalized Probe Intensity
Probe Number
−1
0
1
2
3
1
2
3
4
5
6
7
8
9
10
11
Meishan−A
Meishan−B
Meishan−C
European−A
European−B
European−C

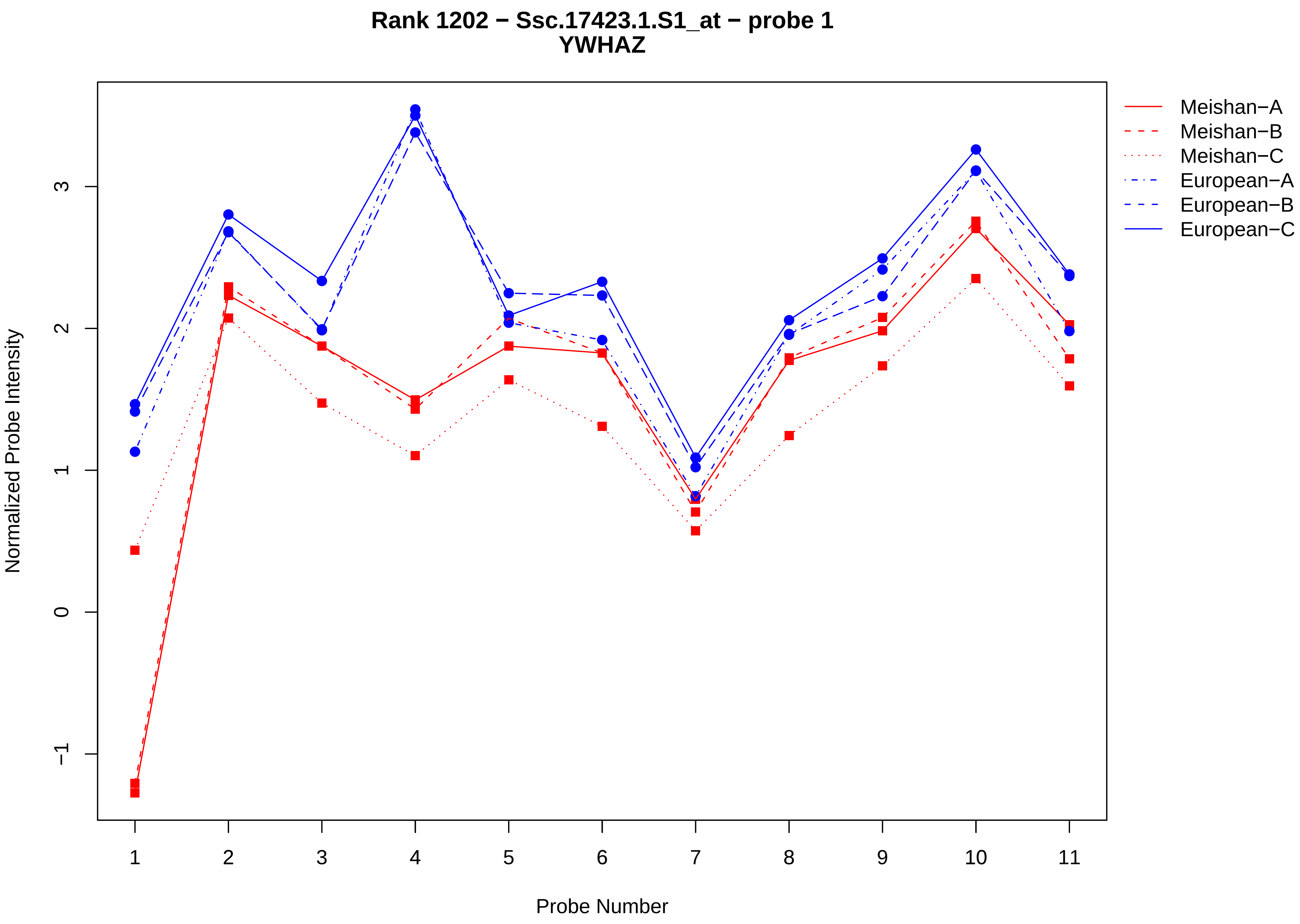

Rank 1202 − Ssc.17423.1.S1_at − probe 1
YWHAZ
Normalized Probe Intensity
−1
0
1
2
3
1
2
3
4
5
6
7
8
9
10
11
Probe Number
Meishan−A
Meishan−B
Meishan−C
European−A
European−B
European−C

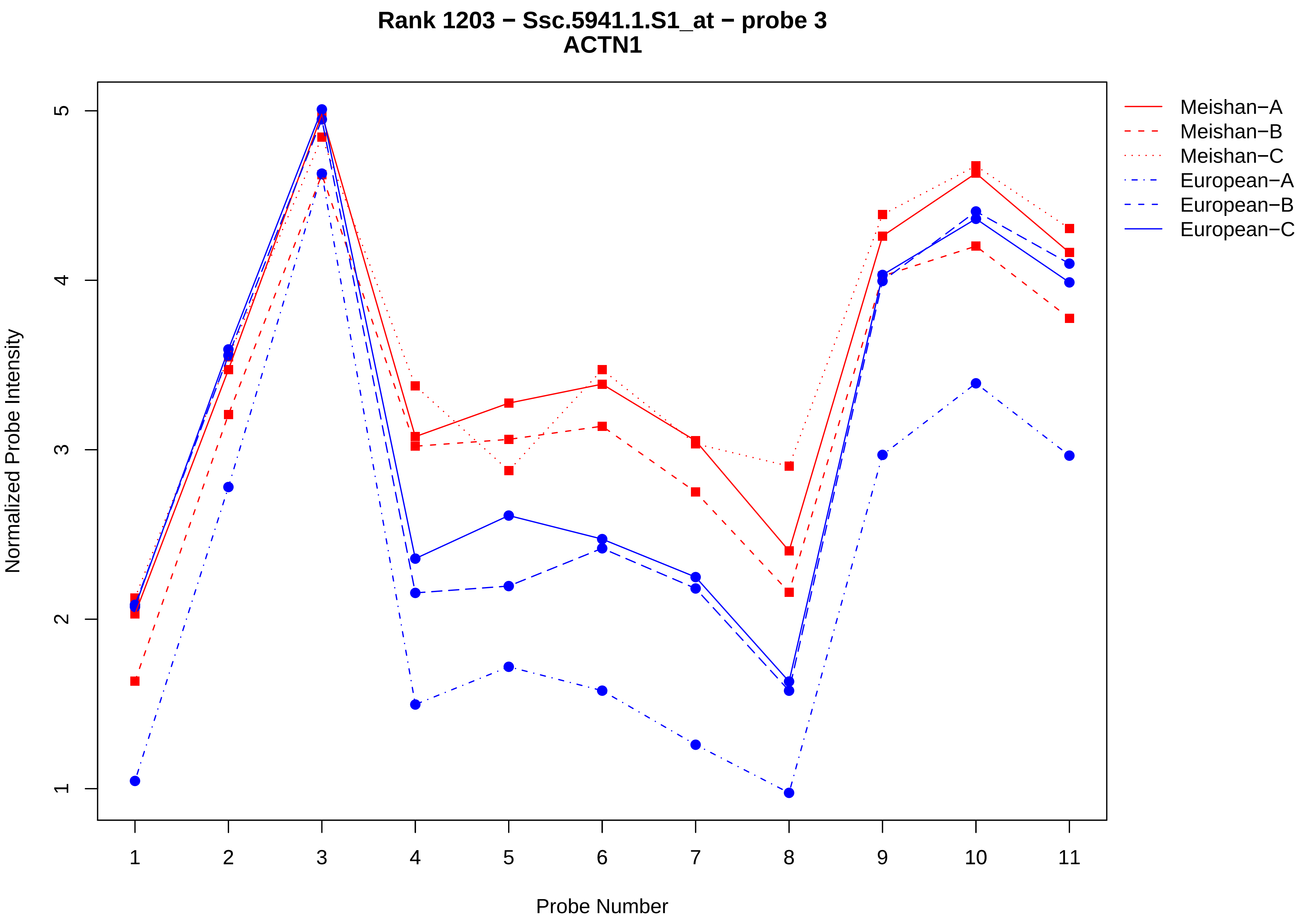

Rank 1203 − Ssc.5941.1.S1_at − probe 3
ACTN1
Normalized Probe Intensity
Probe Number
1
2
3
4
5
1
2
3
4
5
6
7
8
9
10
11
Meishan−A
Meishan−B
Meishan−C
European−A
European−B
European−C

Rank 1204 − Ssc.3604.1.S1_at − probe 9
HDHD1A

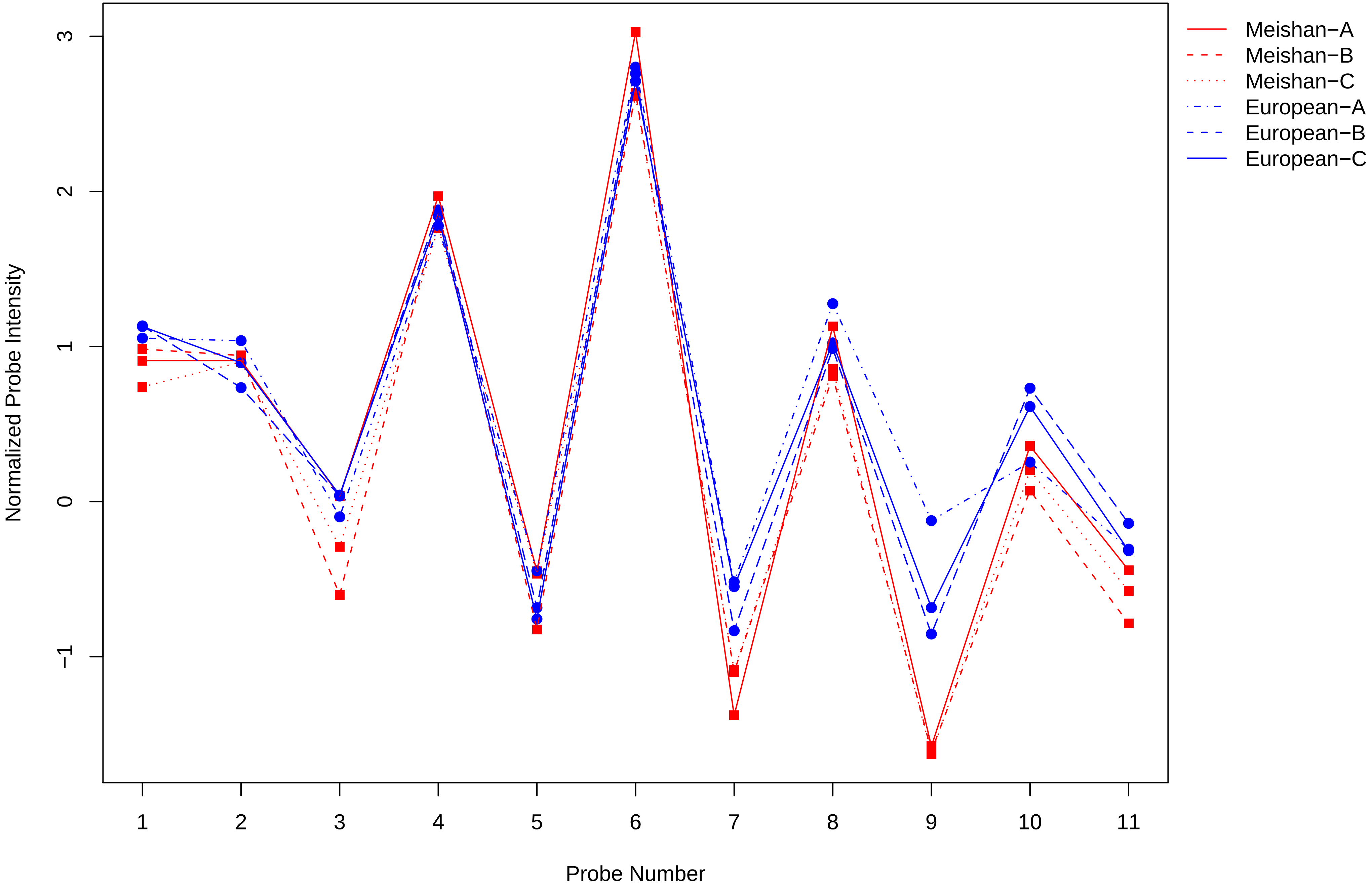

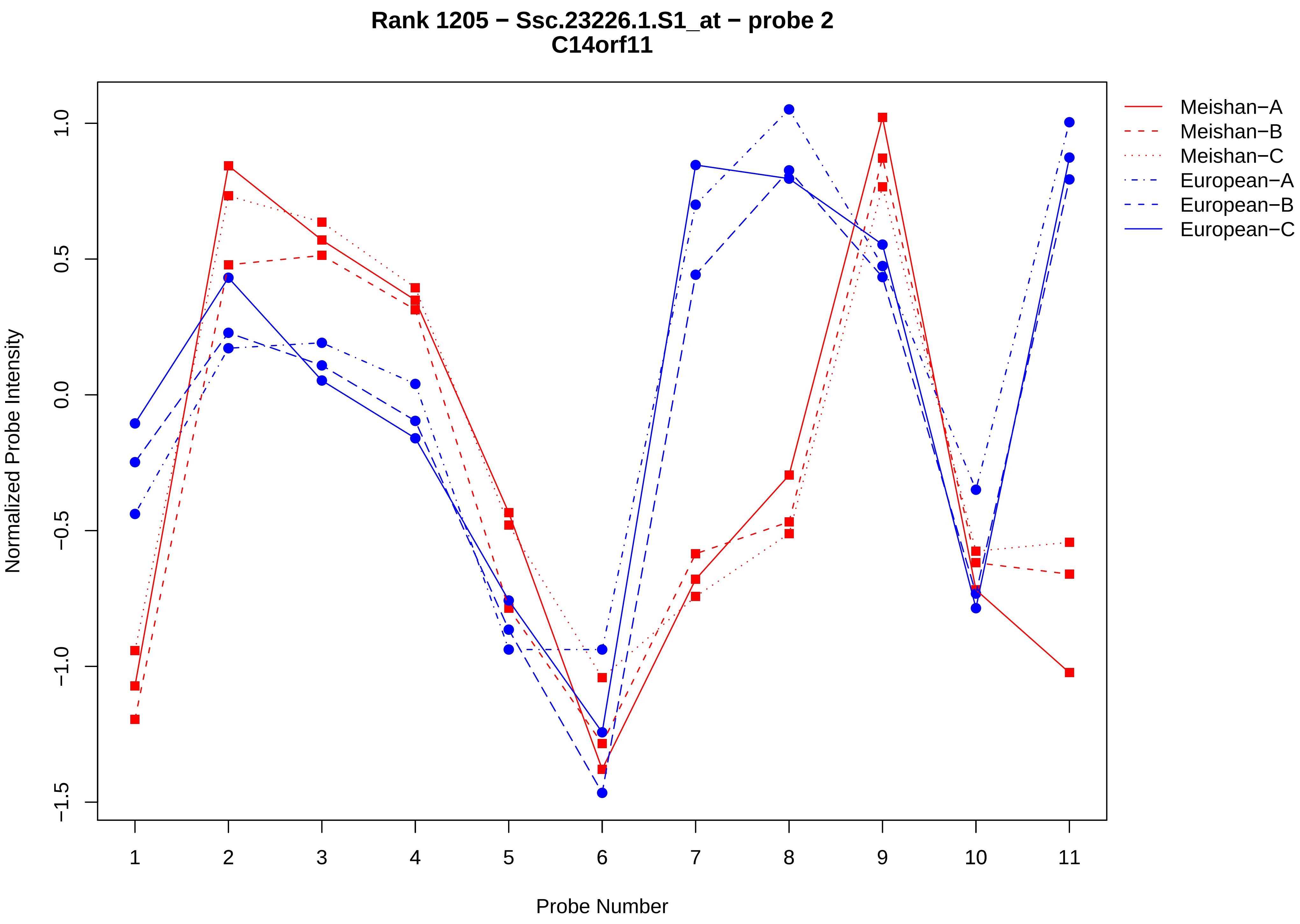
Rank 1205 − Ssc.23226.1.S1_at − probe 2
C14orf11
Normalized Probe Intensity
Probe Number
1.0
0.5
0.0
−0.5
−1.0
−1.5
1
2
3
4
5
6
7
8
9
10
11
Meishan−A
Meishan−B
Meishan−C
European−A
European−B
European−C

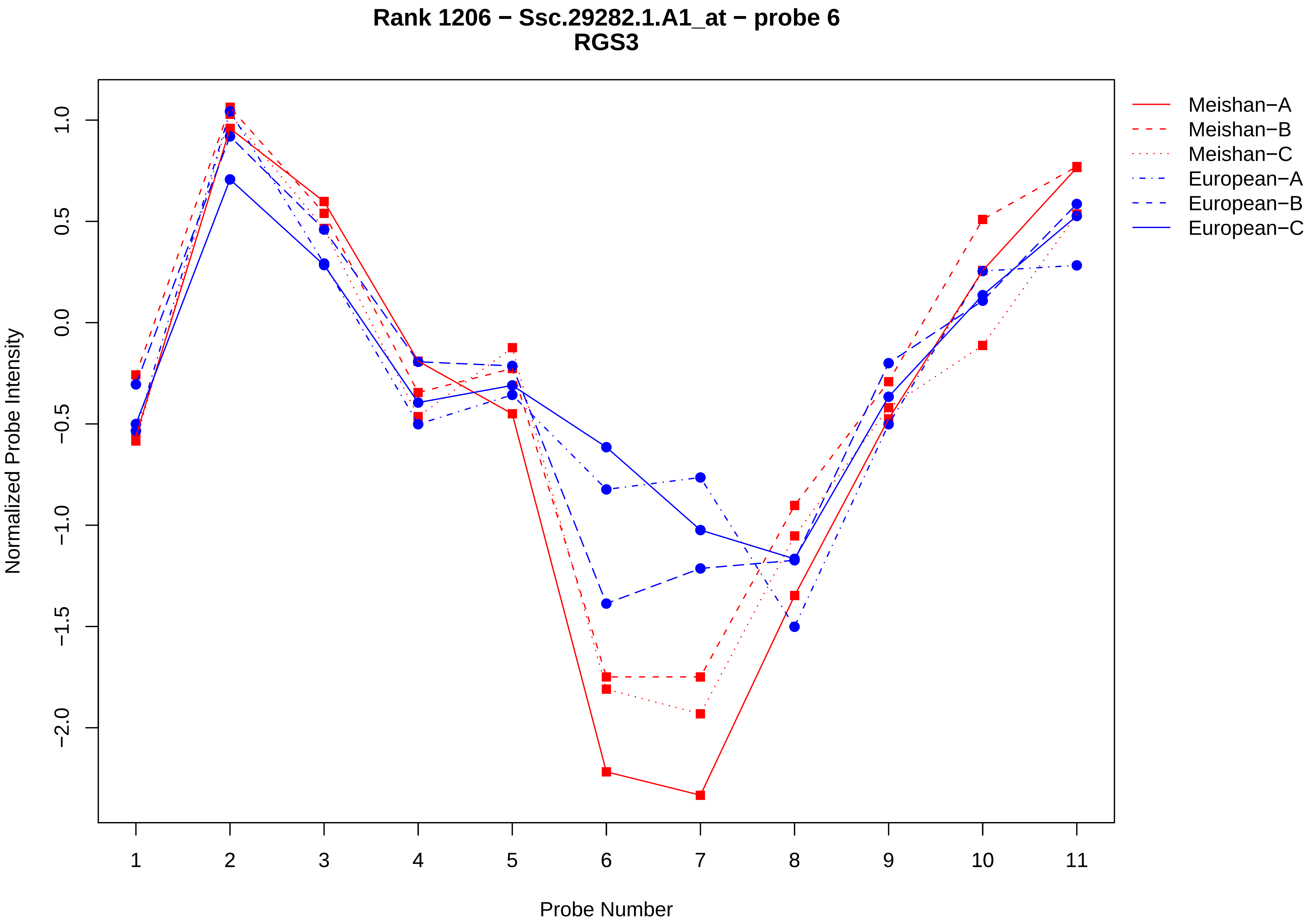

Rank 1206 − Ssc.29282.1.A1_at − probe 6
RGS3
Normalized Probe Intensity
Probe Number
1.0
0.5
0.0
−0.5
−1.0
−1.5
−2.0
1
2
3
4
5
6
7
8
9
10
11
Meishan−A
Meishan−B
Meishan−C
European−A
European−B
European−C

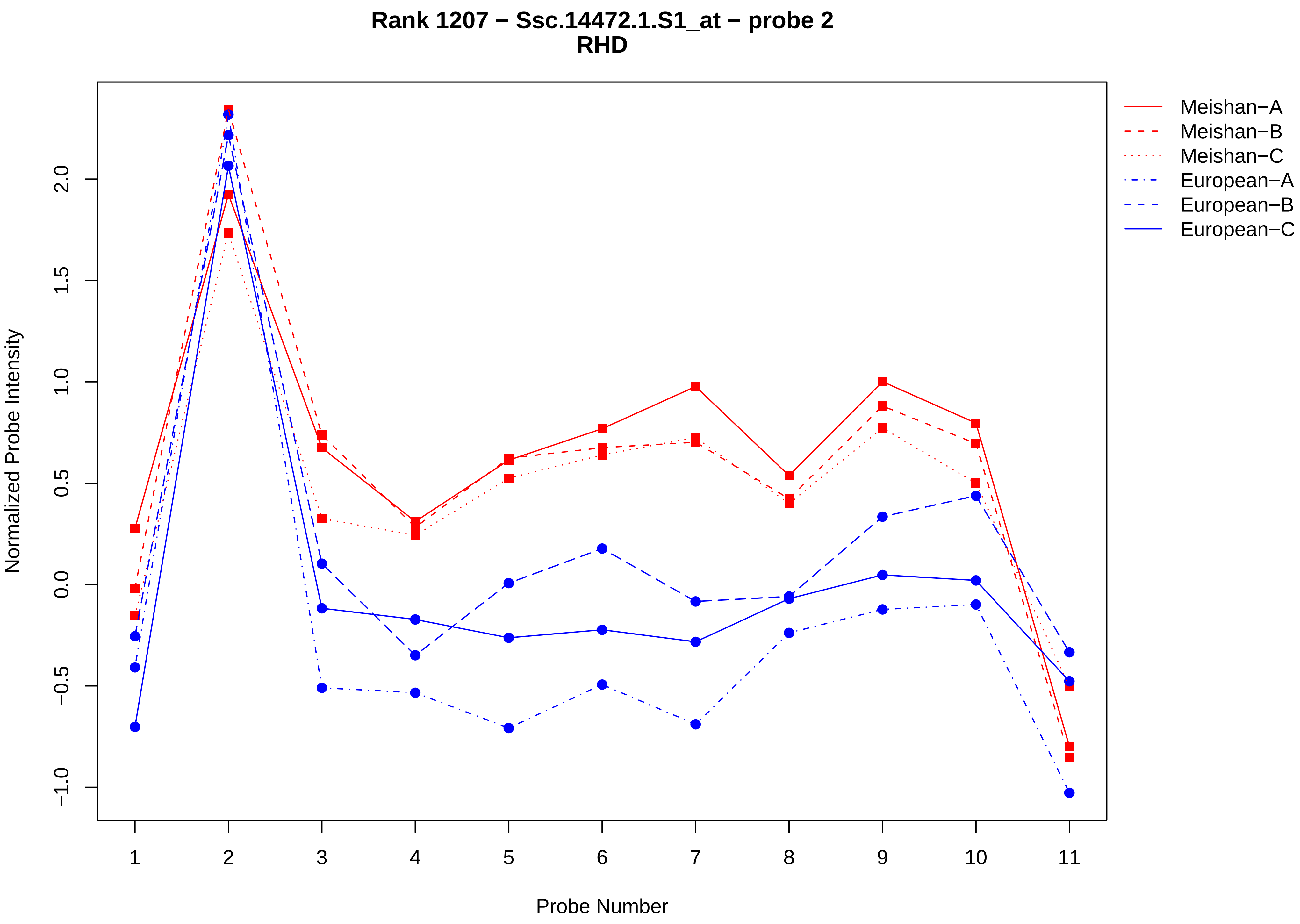

Rank 1207 − Ssc.14472.1.S1_at − probe 2
RHD
Meishan−A
Meishan−B
Meishan−C
European−A
European−B
European−C
Normalized Probe Intensity
−1.0
−0.5
0.0
0.5
1.0
1.5
2.0
1
2
3
4
5
6
7
8
9
10
11
Probe Number

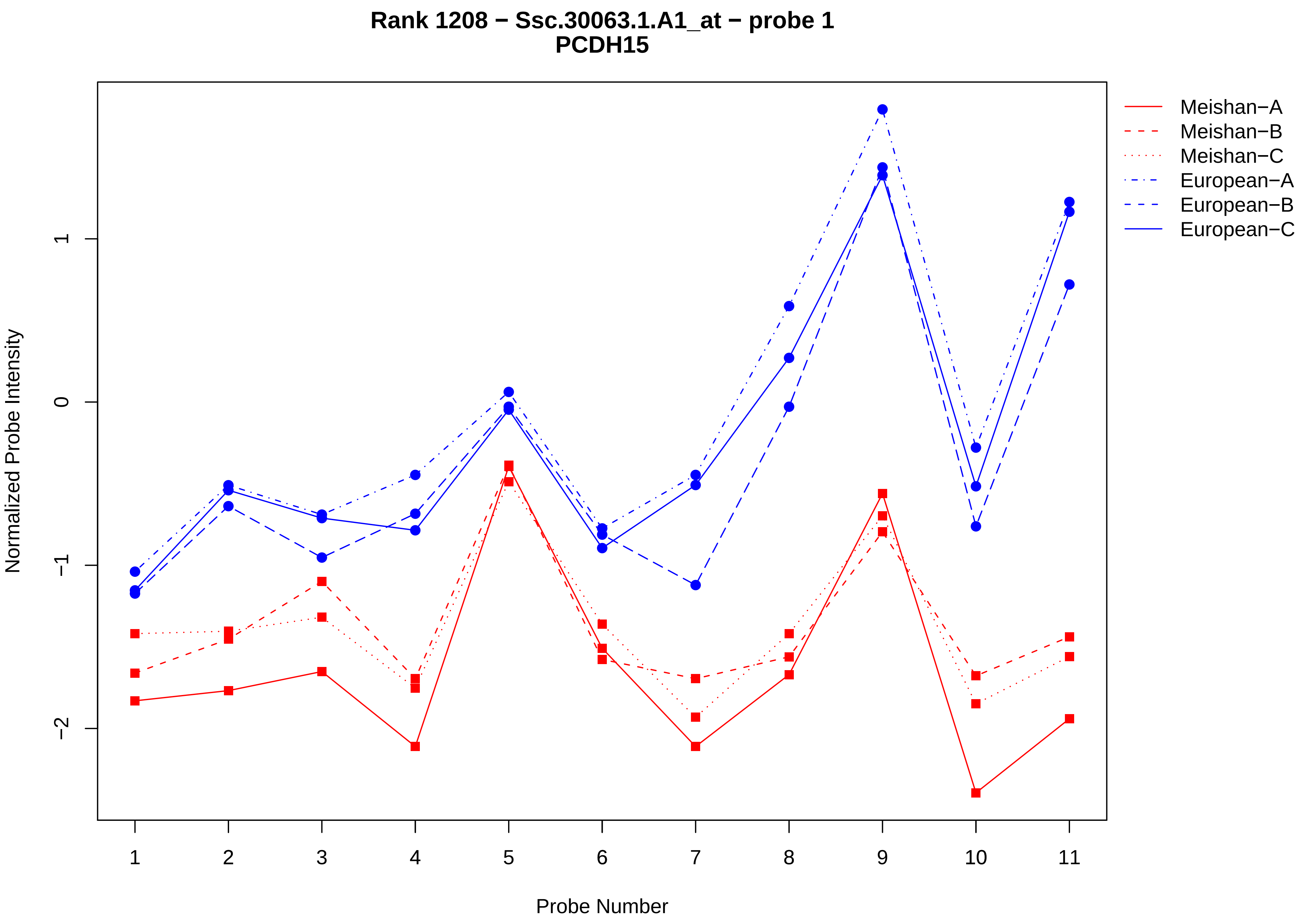

Rank 1208 − Ssc.30063.1.A1_at − probe 1
PCDH15
Normalized Probe Intensity
Probe Number
−2
−1
0
1
1
2
3
4
5
6
7
8
9
10
11
Meishan−A
Meishan−B
Meishan−C
European−A
European−B
European−C

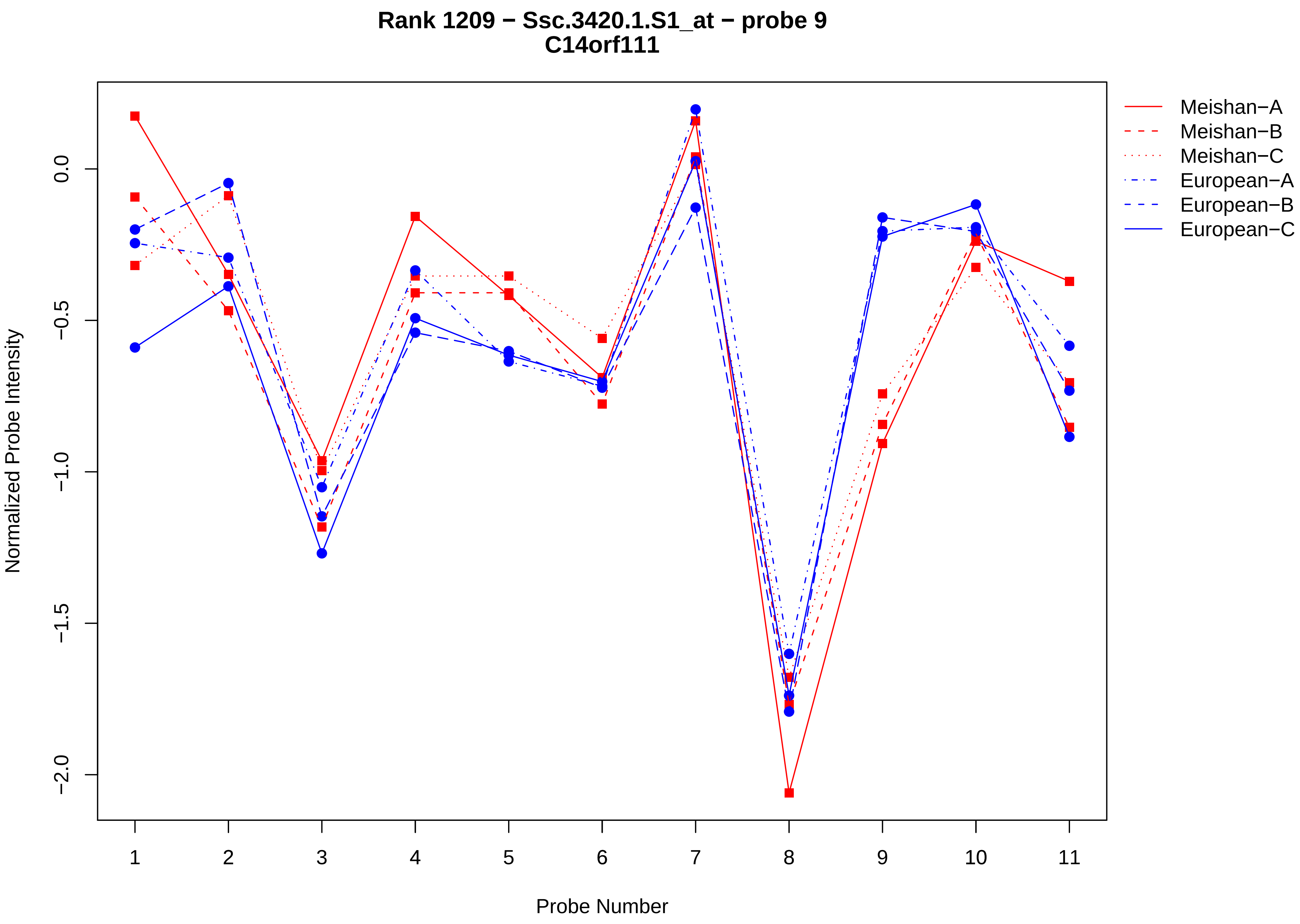
Rank 1209 − Ssc.3420.1.S1_at − probe 9
C14orf111
Normalized Probe Intensity
0.0
−0.5
−1.0
−1.5
−2.0
1
2
3
4
5
6
7
8
9
10
11
Probe Number
Meishan−A
Meishan−B
Meishan−C
European−A
European−B
European−C

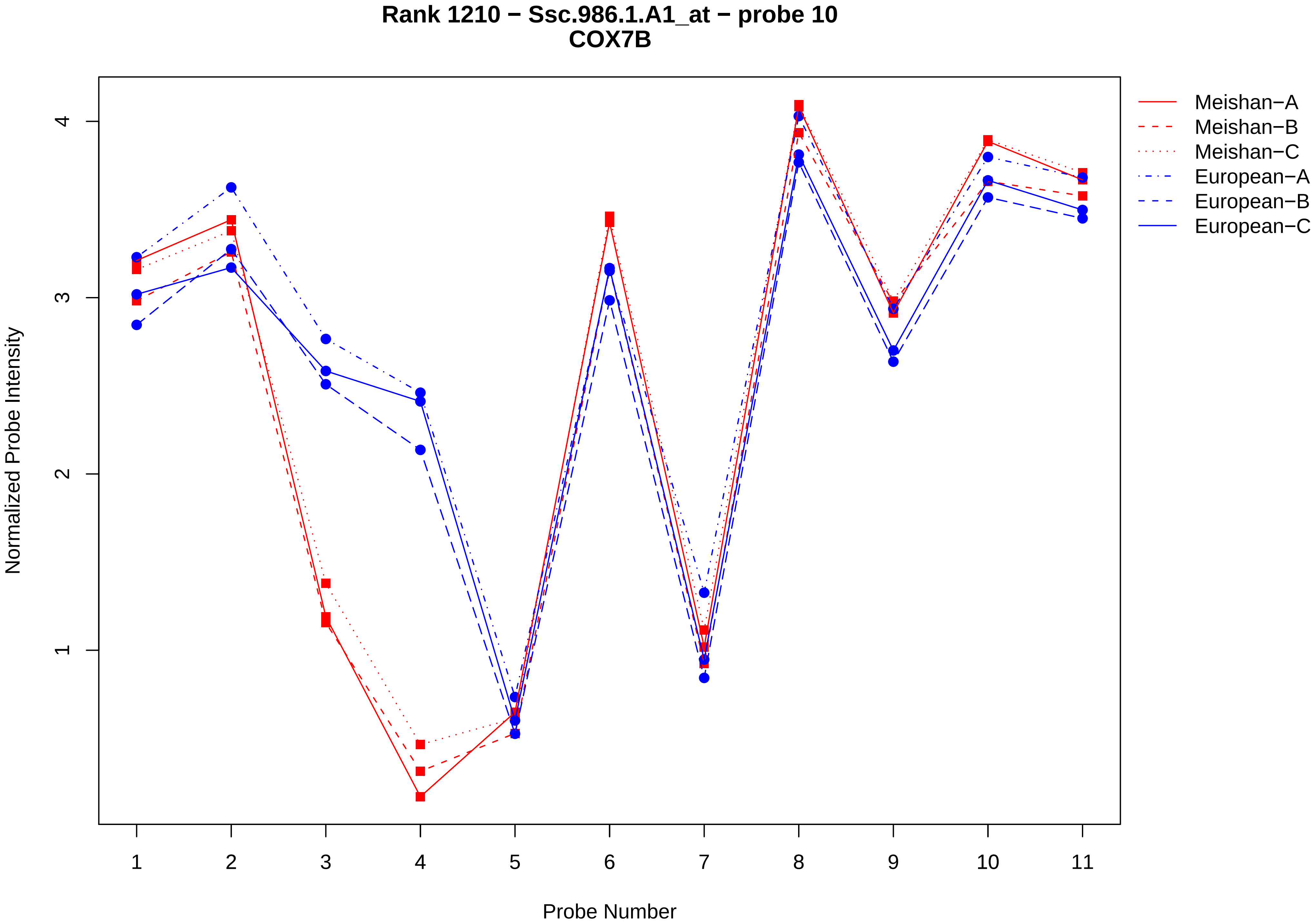

Rank 1210 − Ssc.986.1.A1_at − probe 10
COX7B
Normalized Probe Intensity
Probe Number
1
2
3
4
1
2
3
4
5
6
7
8
9
10
11
Meishan−A
Meishan−B
Meishan−C
European−A
European−B
European−C

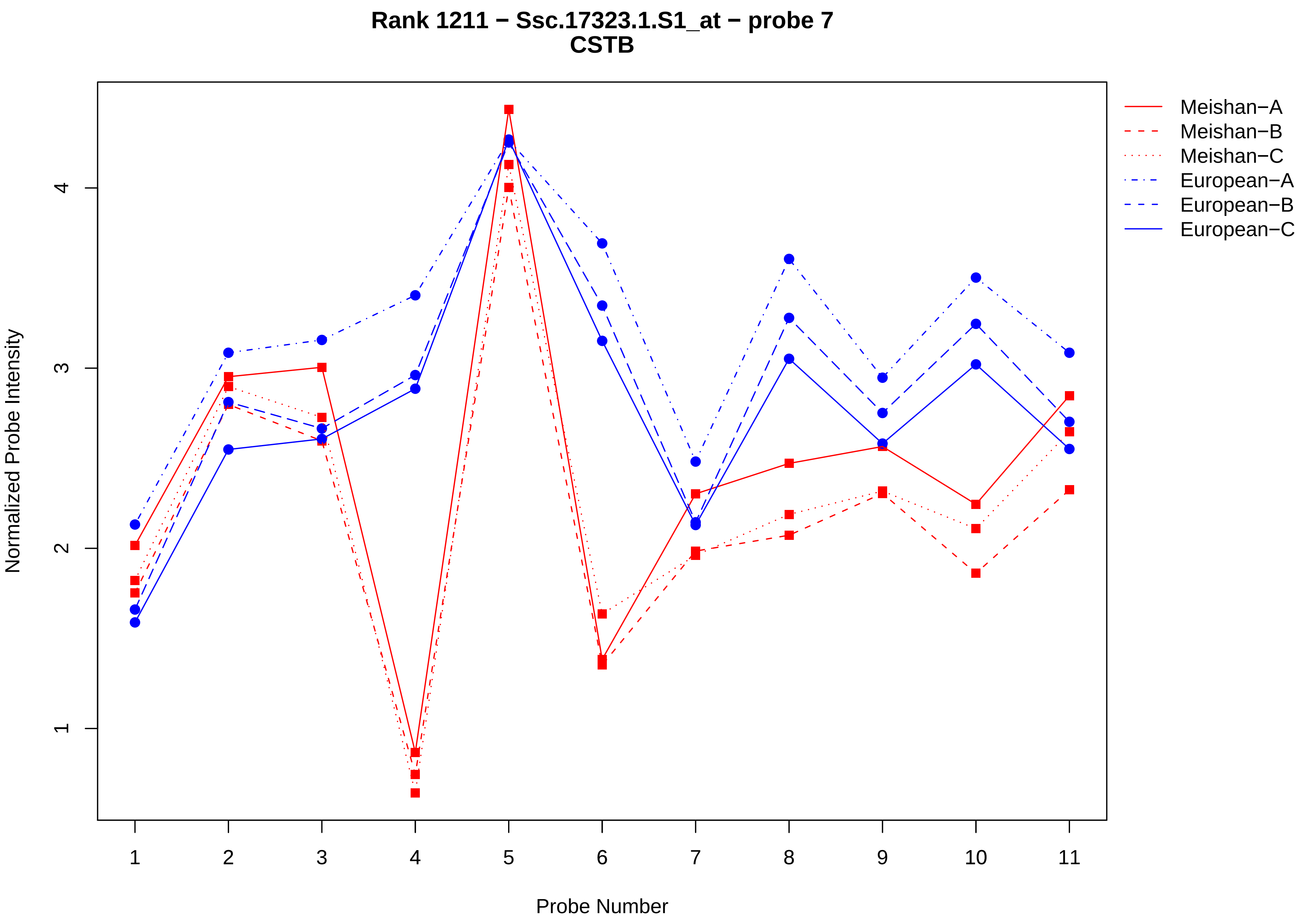

Rank 1211 − Ssc.17323.1.S1_at − probe 7
CSTB
Normalized Probe Intensity
Probe Number
1
2
3
4
5
6
7
8
9
10
11
Meishan−A
Meishan−B
Meishan−C
European−A
European−B
European−C

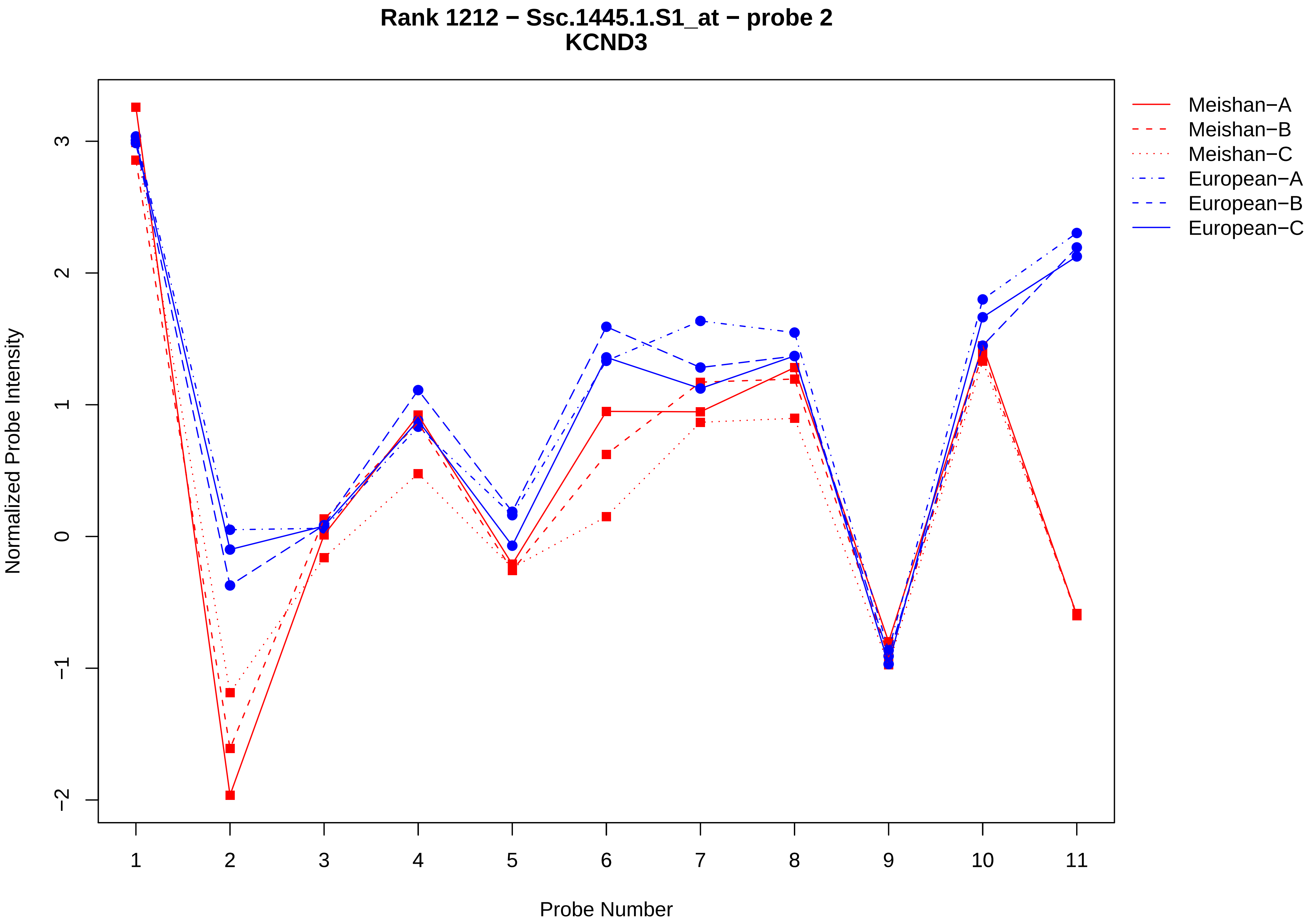

Rank 1212 − Ssc.1445.1.S1_at − probe 2
KCND3
Meishan−A
Meishan−B
Meishan−C
European−A
European−B
European−C
Normalized Probe Intensity
Probe Number
−2
−1
0
1
2
3
1
2
3
4
5
6
7
8
9
10
11

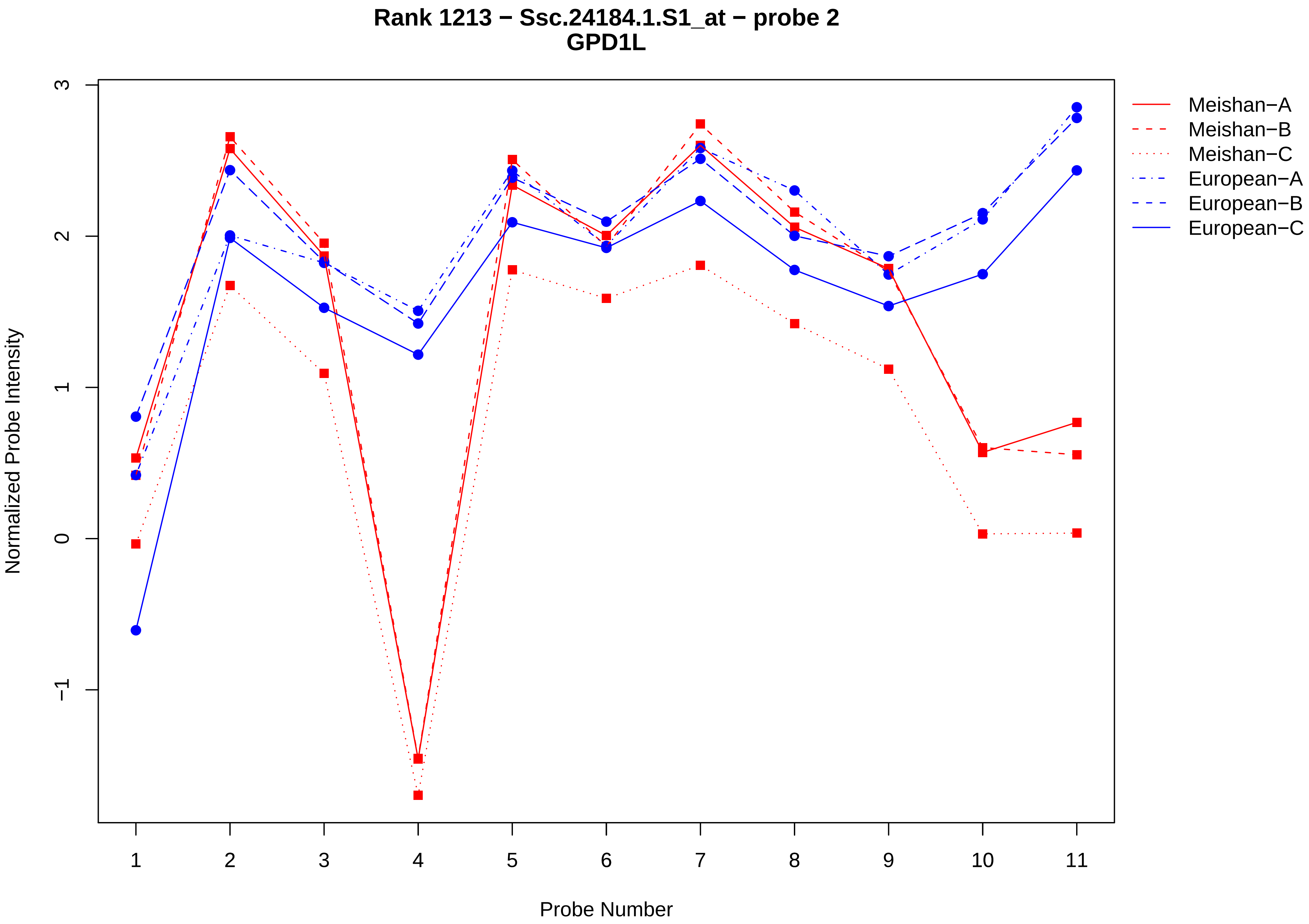

Rank 1213 − Ssc.24184.1.S1_at − probe 2
GPD1L
Normalized Probe Intensity
Probe Number
−1
0
1
2
3
1
2
3
4
5
6
7
8
9
10
11
Meishan−A
Meishan−B
Meishan−C
European−A
European−B
European−C

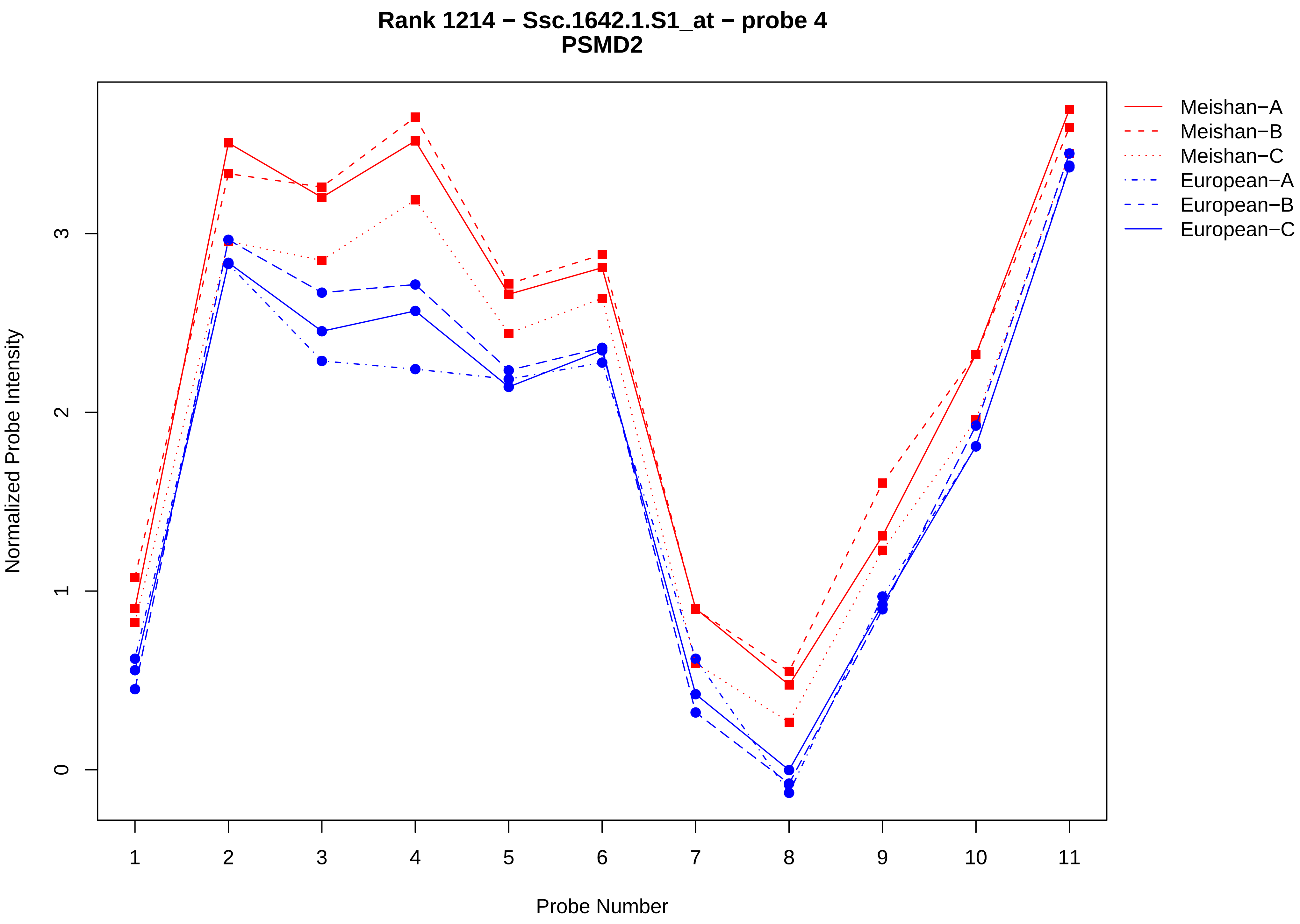

Rank 1214 − Ssc.1642.1.S1_at − probe 4
PSMD2
Normalized Probe Intensity
Probe Number
0
1
2
3
1
2
3
4
5
6
7
8
9
10
11
Meishan−A
Meishan−B
Meishan−C
European−A
European−B
European−C

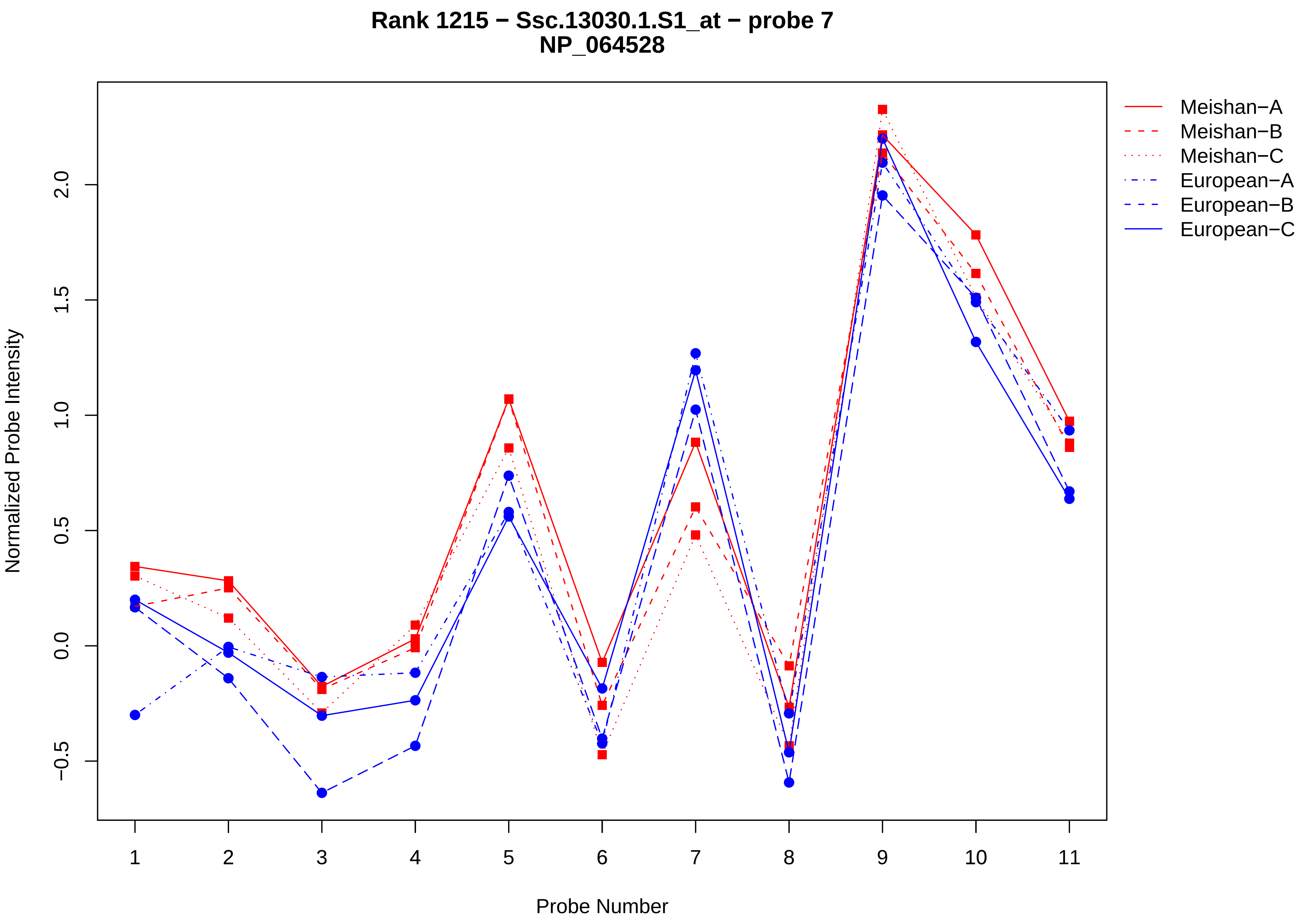

Rank 1215 − Ssc.13030.1.S1_at − probe 7
NP_064528
Normalized Probe Intensity
Probe Number
−0.5
0.0
0.5
1.0
1.5
2.0
1
2
3
4
5
6
7
8
9
10
11
Meishan−A
Meishan−B
Meishan−C
European−A
European−B
European−C

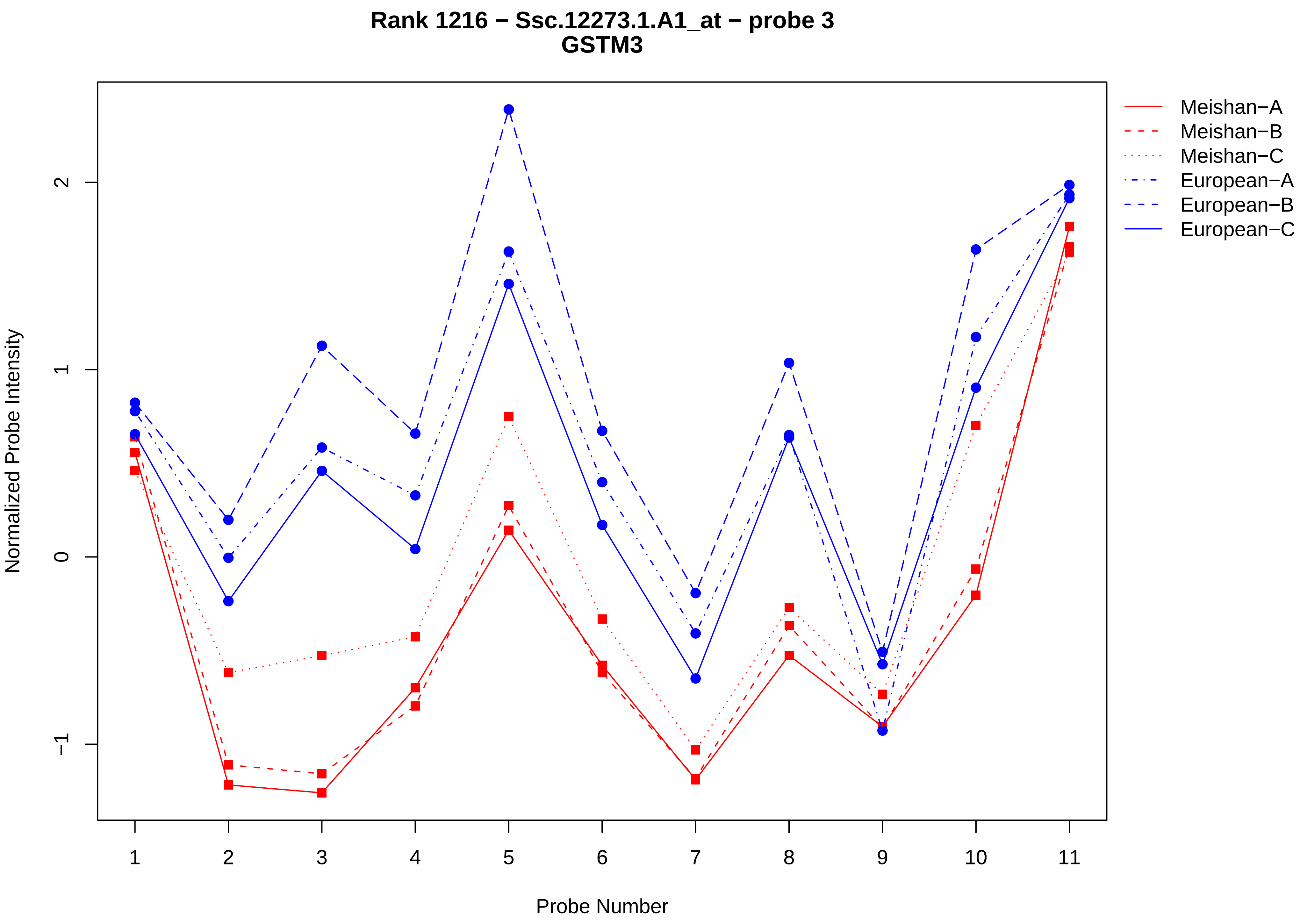

Rank 1216 − Ssc.12273.1.A1_at − probe 3
GSTM3
Normalized Probe Intensity
Probe Number
−1
0
1
2
1
2
3
4
5
6
7
8
9
10
11
Meishan−A
Meishan−B
Meishan−C
European−A
European−B
European−C

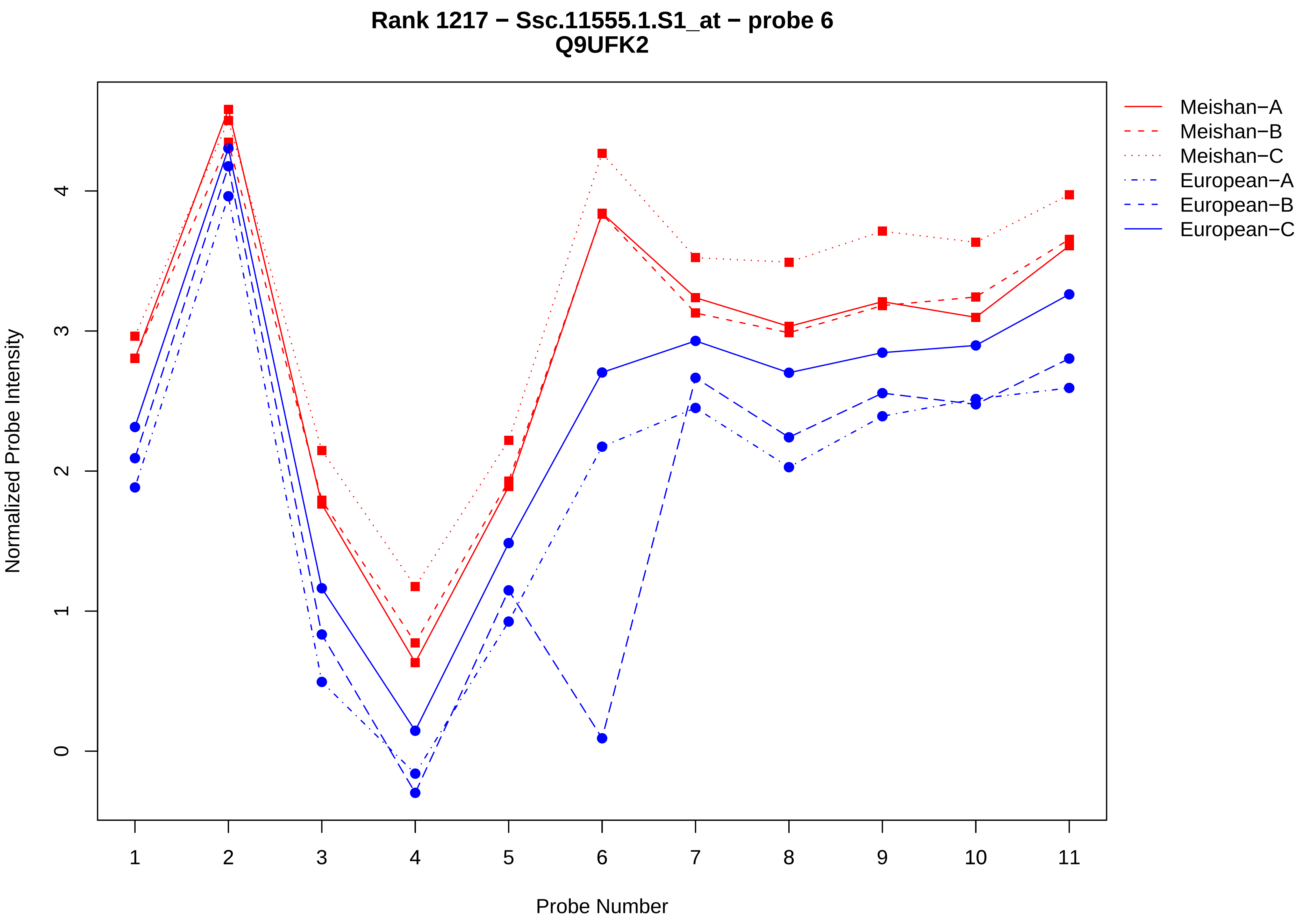

Rank 1217 − Ssc.11555.1.S1_at − probe 6
Q9UFK2
Normalized Probe Intensity
Probe Number
0
1
2
3
4
1
2
3
4
5
6
7
8
9
10
11
Meishan−A
Meishan−B
Meishan−C
European−A
European−B
European−C

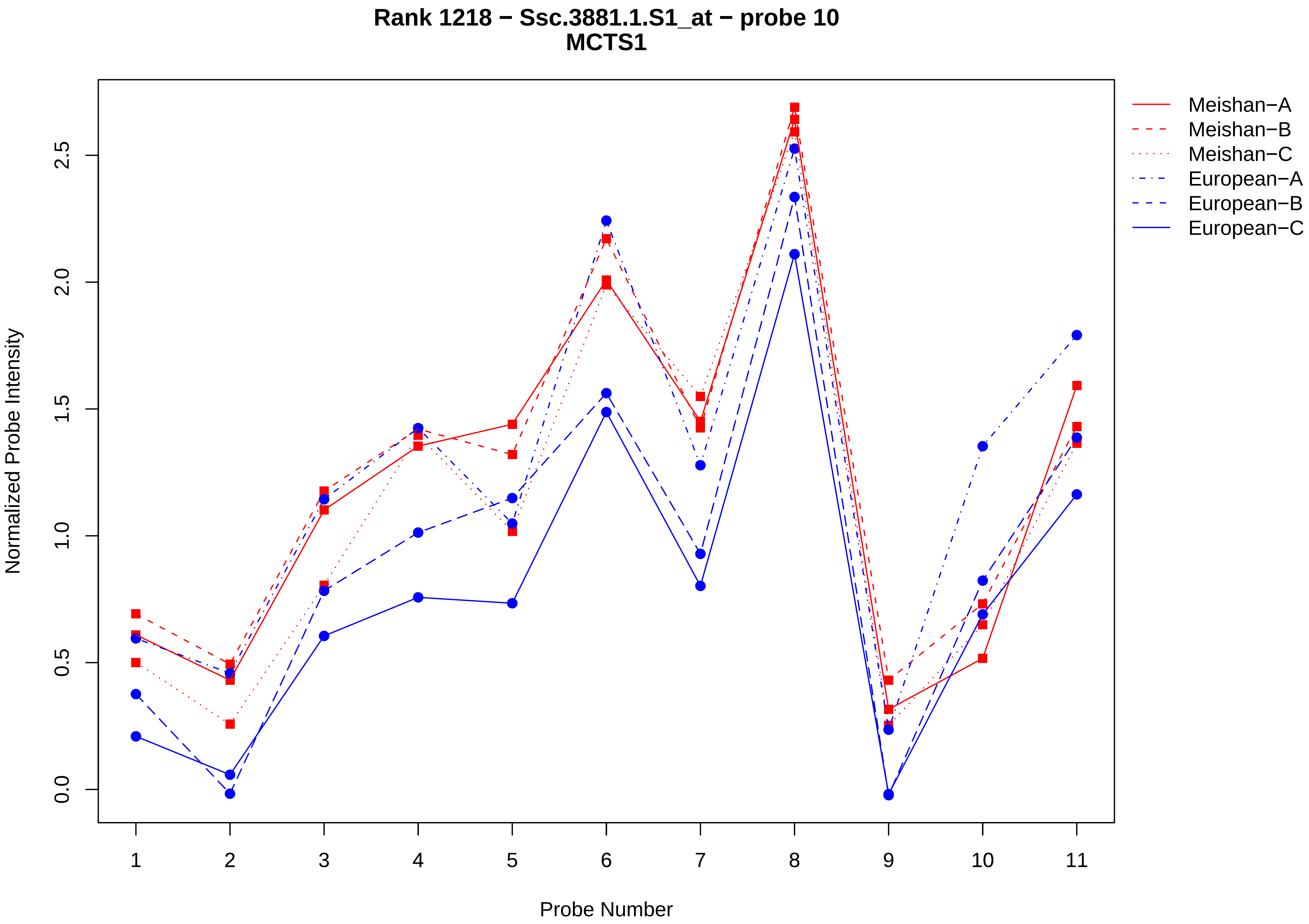

Rank 1218 − Ssc.3881.1.S1_at − probe 10
MCTS1
Normalized Probe Intensity
0.0
0.5
1.0
1.5
2.0
2.5
1
2
3
4
5
6
7
8
9
10
11
Probe Number
Meishan−A
Meishan−B
Meishan−C
European−A
European−B
European−C

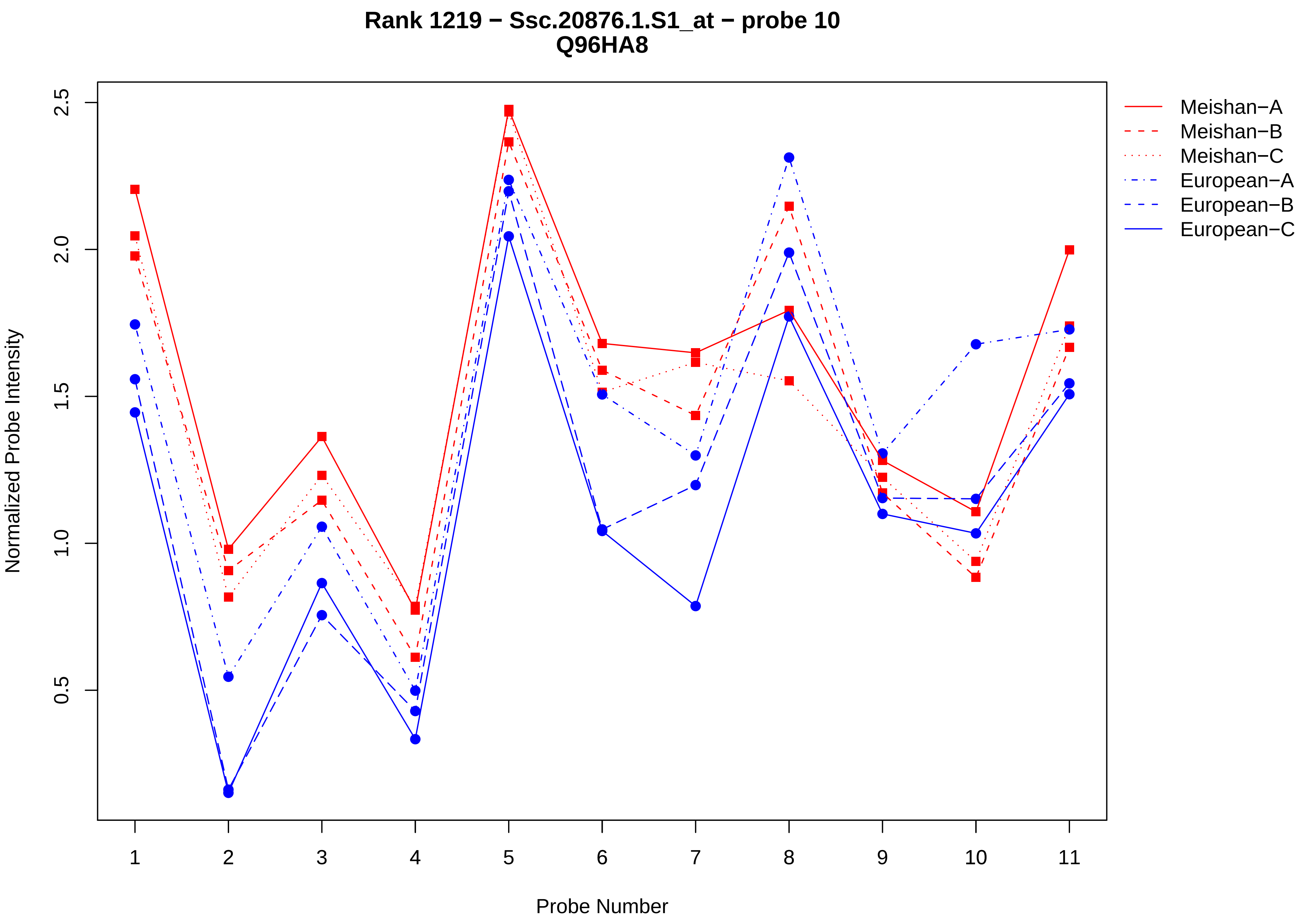

Rank 1219 − Ssc.20876.1.S1_at − probe 10
Q96HA8
Normalized Probe Intensity
Probe Number
0.5
1.0
1.5
2.0
2.5
1
2
3
4
5
6
7
8
9
10
11
Meishan−A
Meishan−B
Meishan−C
European−A
European−B
European−C

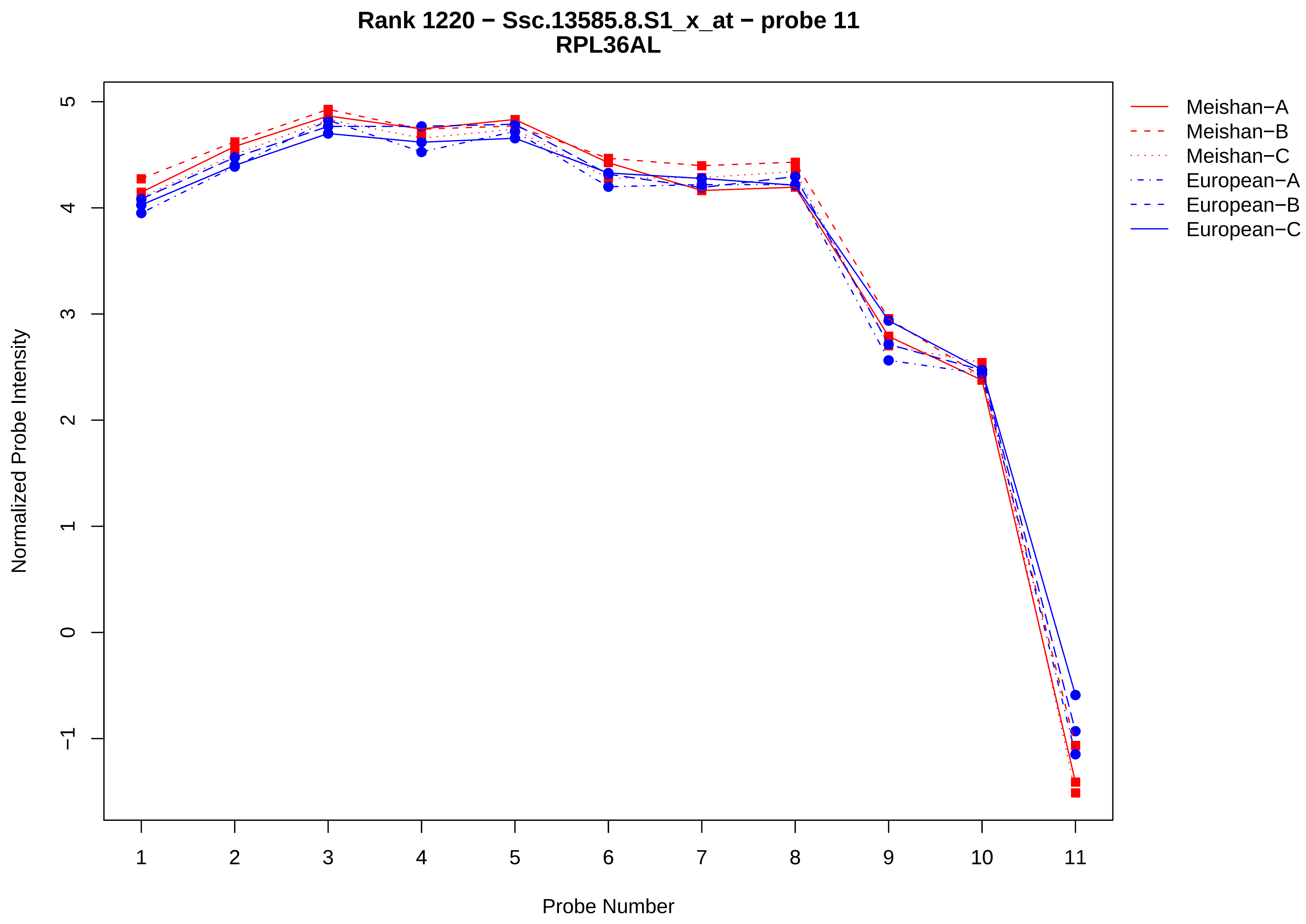

Rank 1220 − Ssc.13585.8.S1_x_at − probe 11
RPL36AL
Normalized Probe Intensity
Probe Number
Meishan−A
Meishan−B
Meishan−C
European−A
European−B
European−C

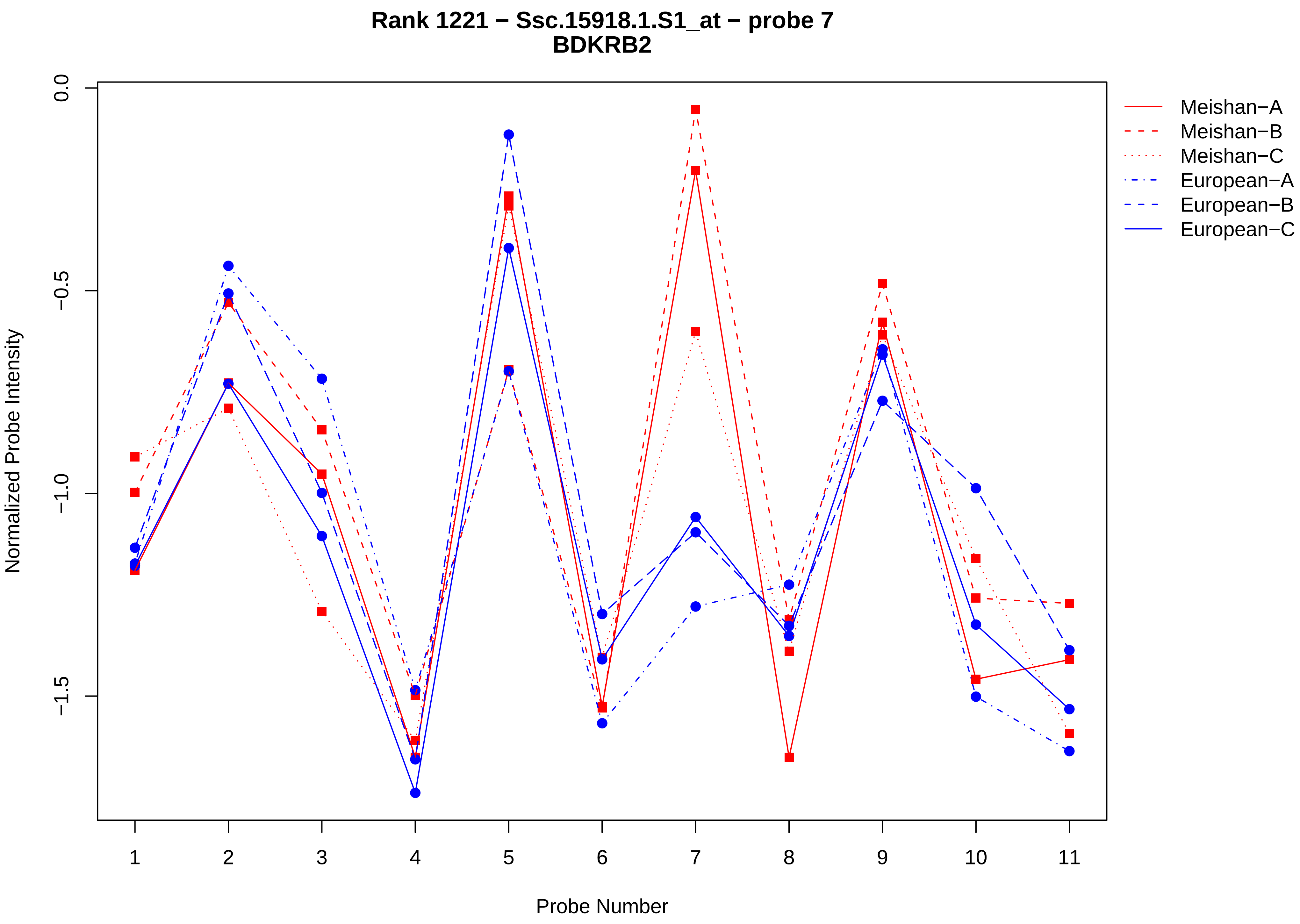

Rank 1221 − Ssc.15918.1.S1_at − probe 7
BDKRB2
Normalized Probe Intensity
0.0
−0.5
−1.0
−1.5
1
2
3
4
5
6
7
8
9
10
11
Probe Number
Meishan−A
Meishan−B
Meishan−C
European−A
European−B
European−C

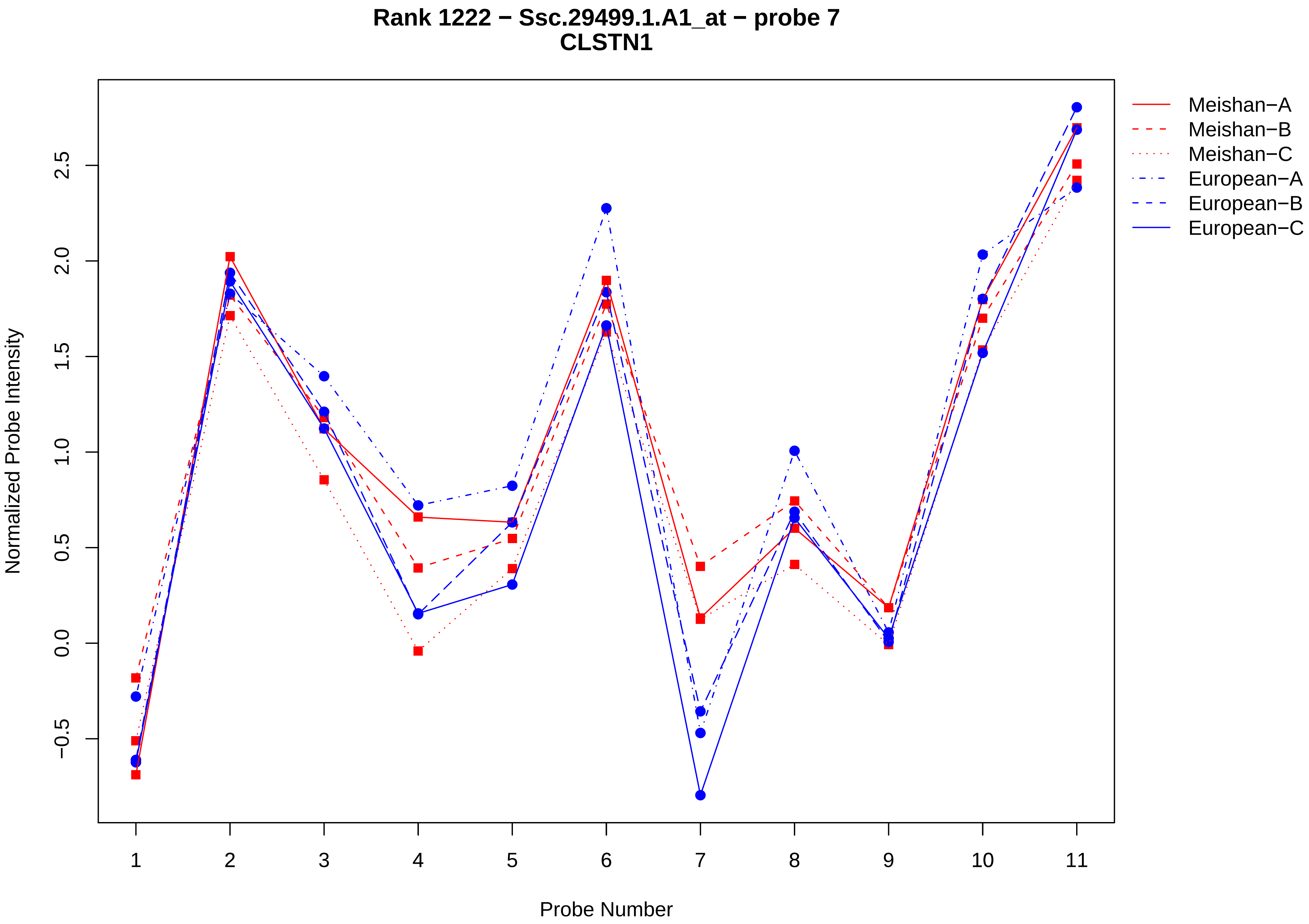

Rank 1222 − Ssc.29499.1.A1_at − probe 7
CLSTN1
Normalized Probe Intensity
Probe Number
−0.5
0.0
0.5
1.0
1.5
2.0
2.5
1
2
3
4
5
6
7
8
9
10
11
Meishan−A
Meishan−B
Meishan−C
European−A
European−B
European−C

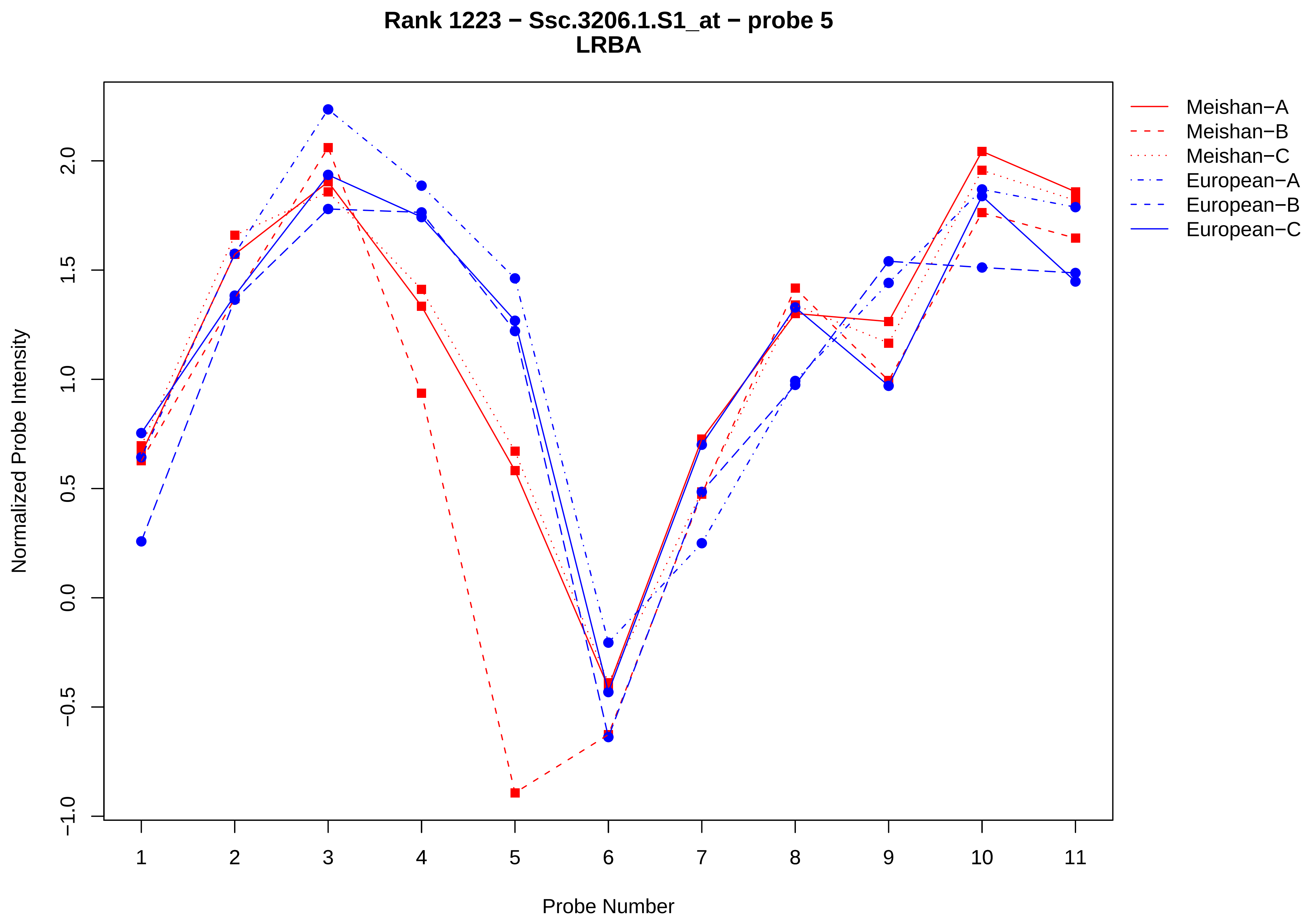
Rank 1223 − Ssc.3206.1.S1_at − probe 5
LRBA
Normalized Probe Intensity
Probe Number
−1.0
−0.5
0.0
0.5
1.0
1.5
2.0
1
2
3
4
5
6
7
8
9
10
11
Meishan−A
Meishan−B
Meishan−C
European−A
European−B
European−C

Rank 1224 − Ssc.6457.1.S1_at − probe 5
NP_996898

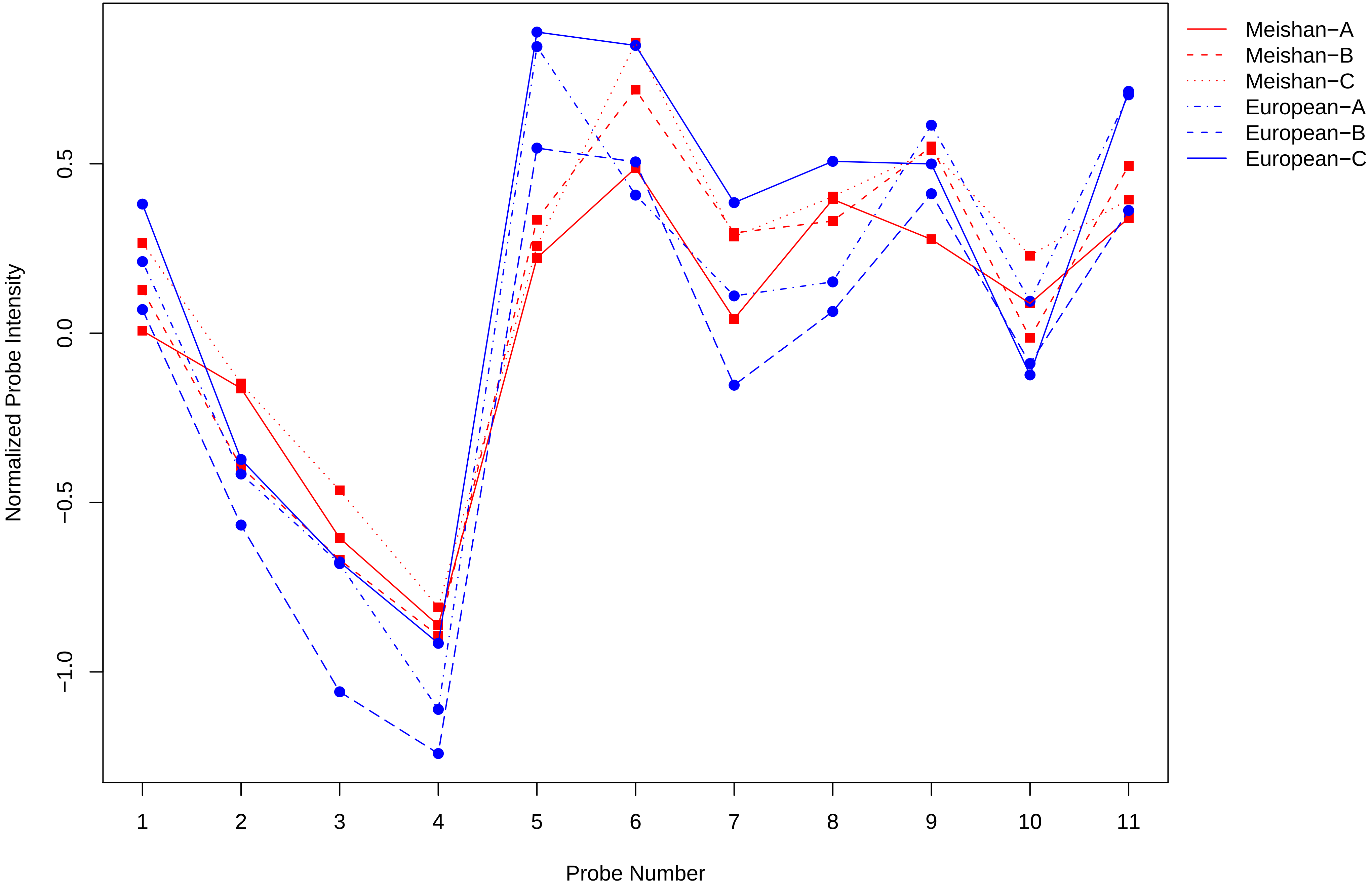

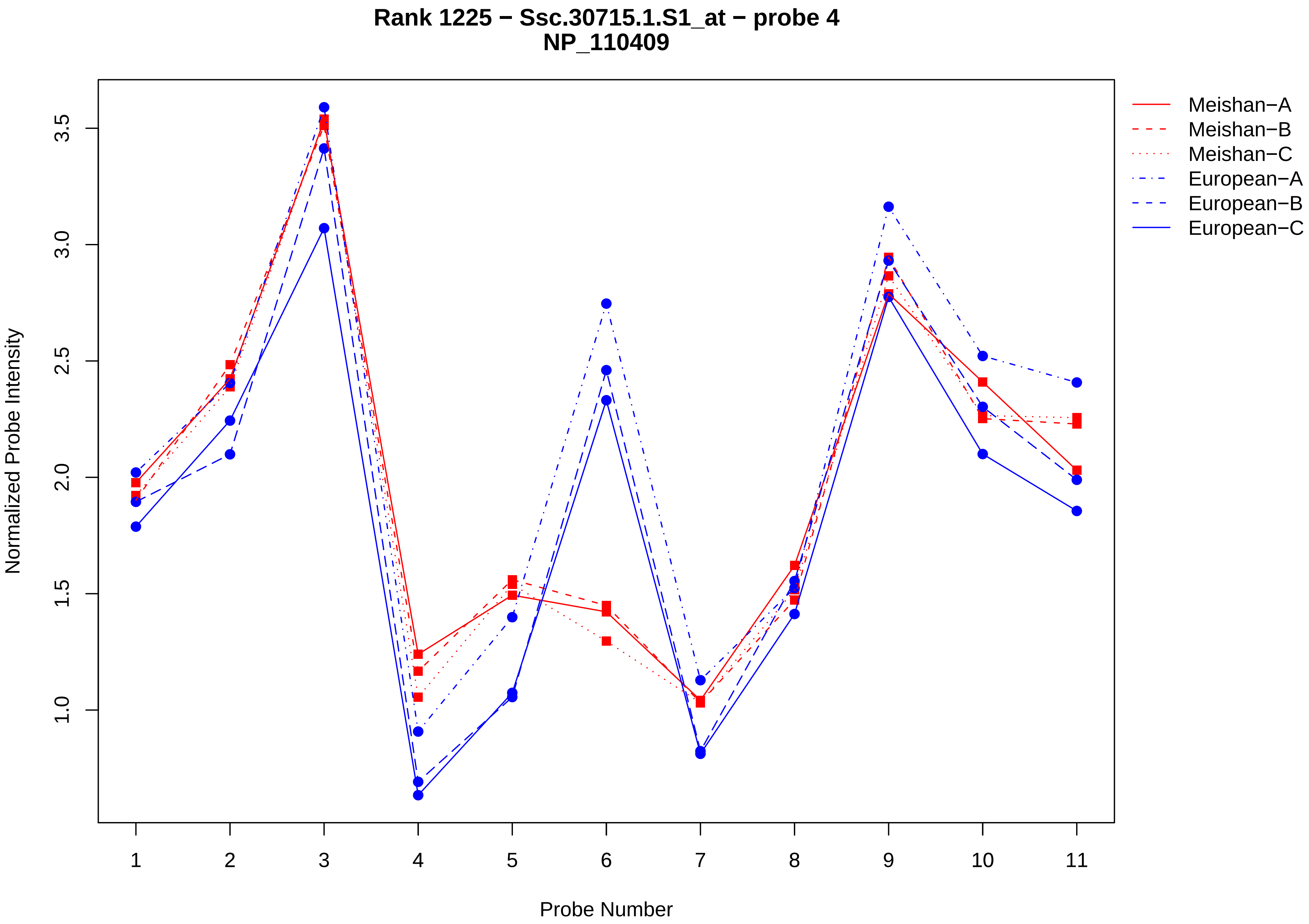

Rank 1225 − Ssc.30715.1.S1_at − probe 4
NP_110409
Normalized Probe Intensity
Probe Number
1.0
1.5
2.0
2.5
3.0
3.5
1
2
3
4
5
6
7
8
9
10
11
Meishan−A
Meishan−B
Meishan−C
European−A
European−B
European−C

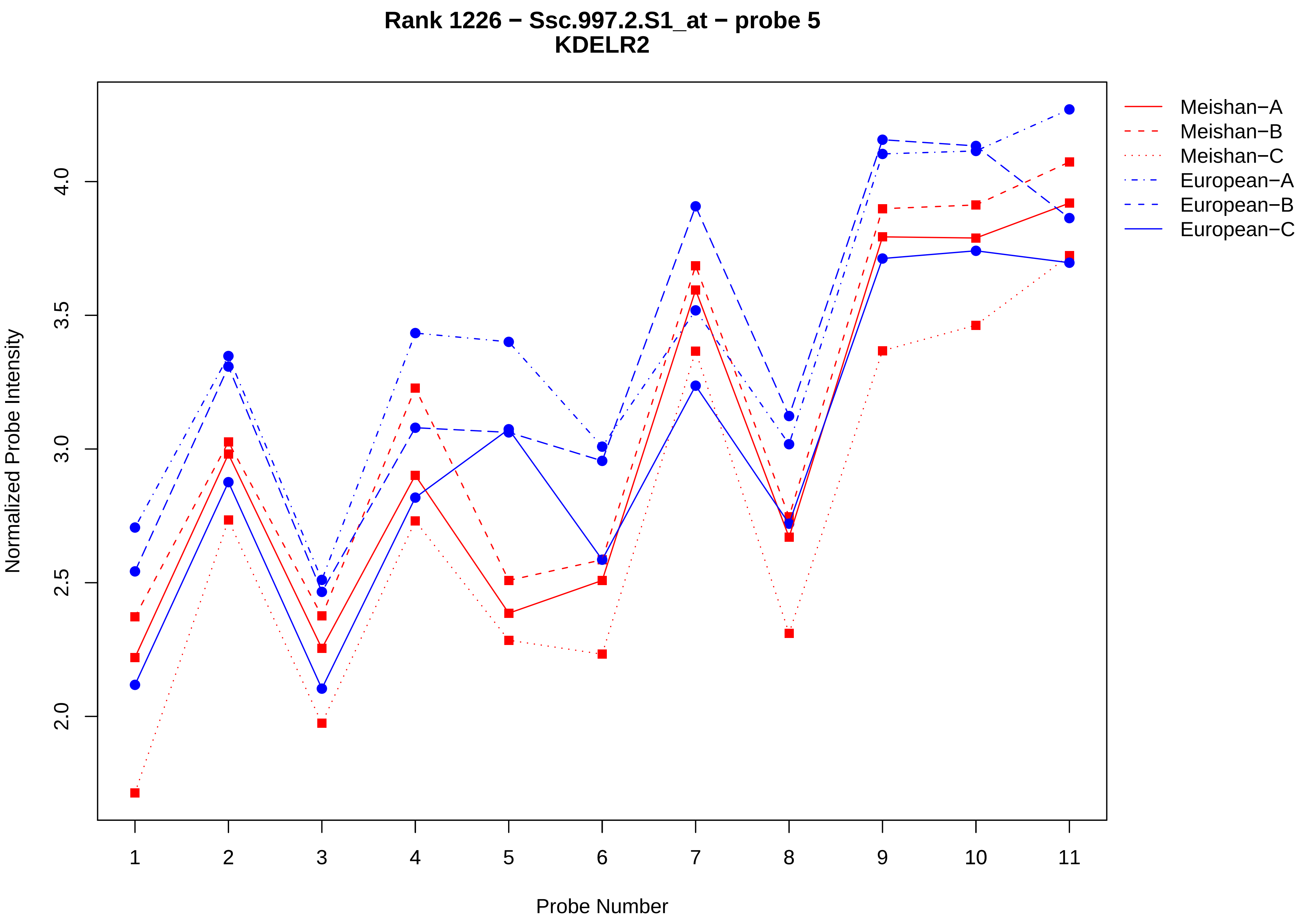

Rank 1226 − Ssc.997.2.S1_at − probe 5
KDELR2
Normalized Probe Intensity
Probe Number
2.0
2.5
3.0
3.5
4.0
1
2
3
4
5
6
7
8
9
10
11
Meishan−A
Meishan−B
Meishan−C
European−A
European−B
European−C

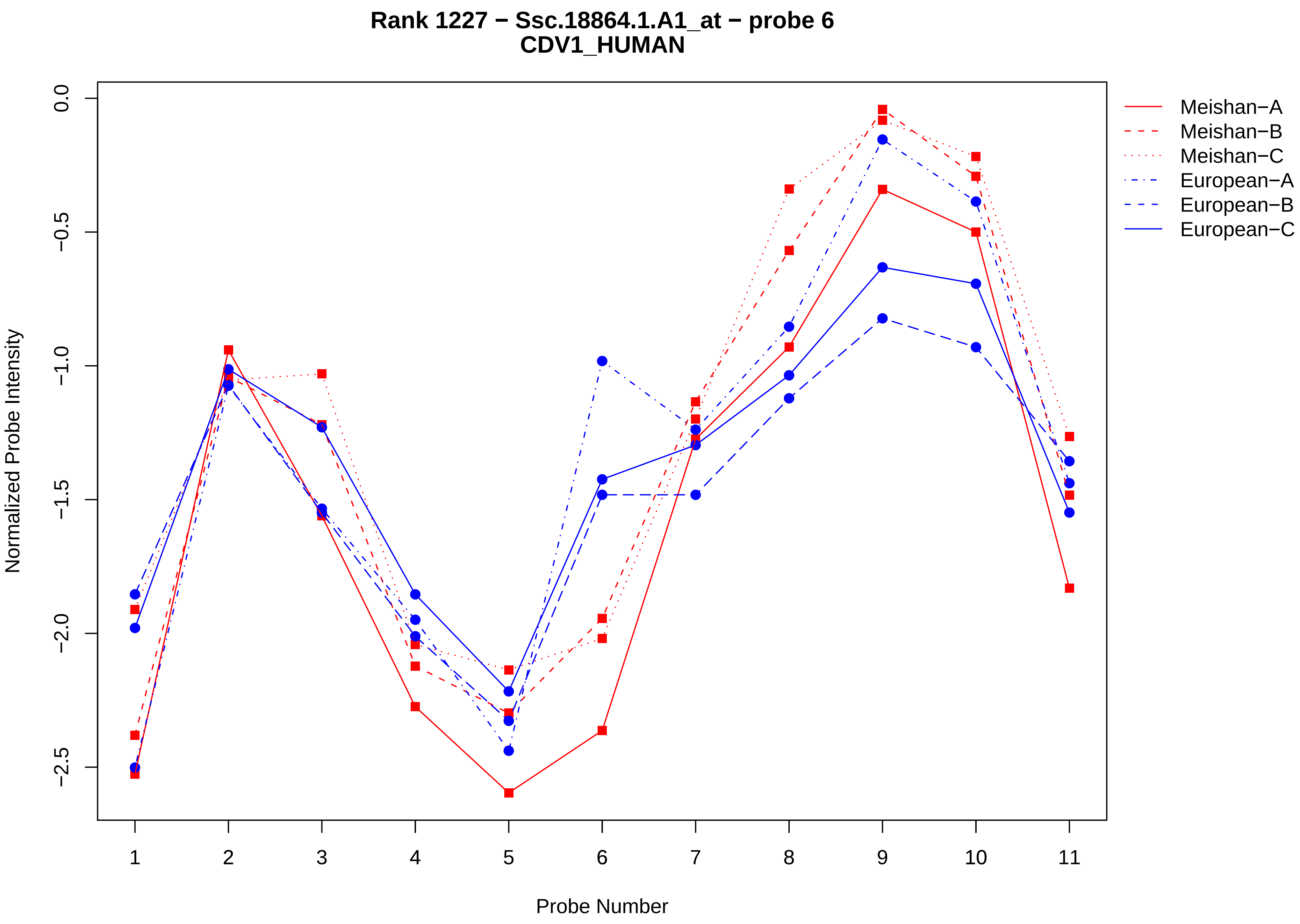
Rank 1227 − Ssc.18864.1.A1_at − probe 6
CDV1_HUMAN
Normalized Probe Intensity
Probe Number
0.0
−0.5
−1.0
−1.5
−2.0
−2.5
1
2
3
4
5
6
7
8
9
10
11
Meishan−A
Meishan−B
Meishan−C
European−A
European−B
European−C

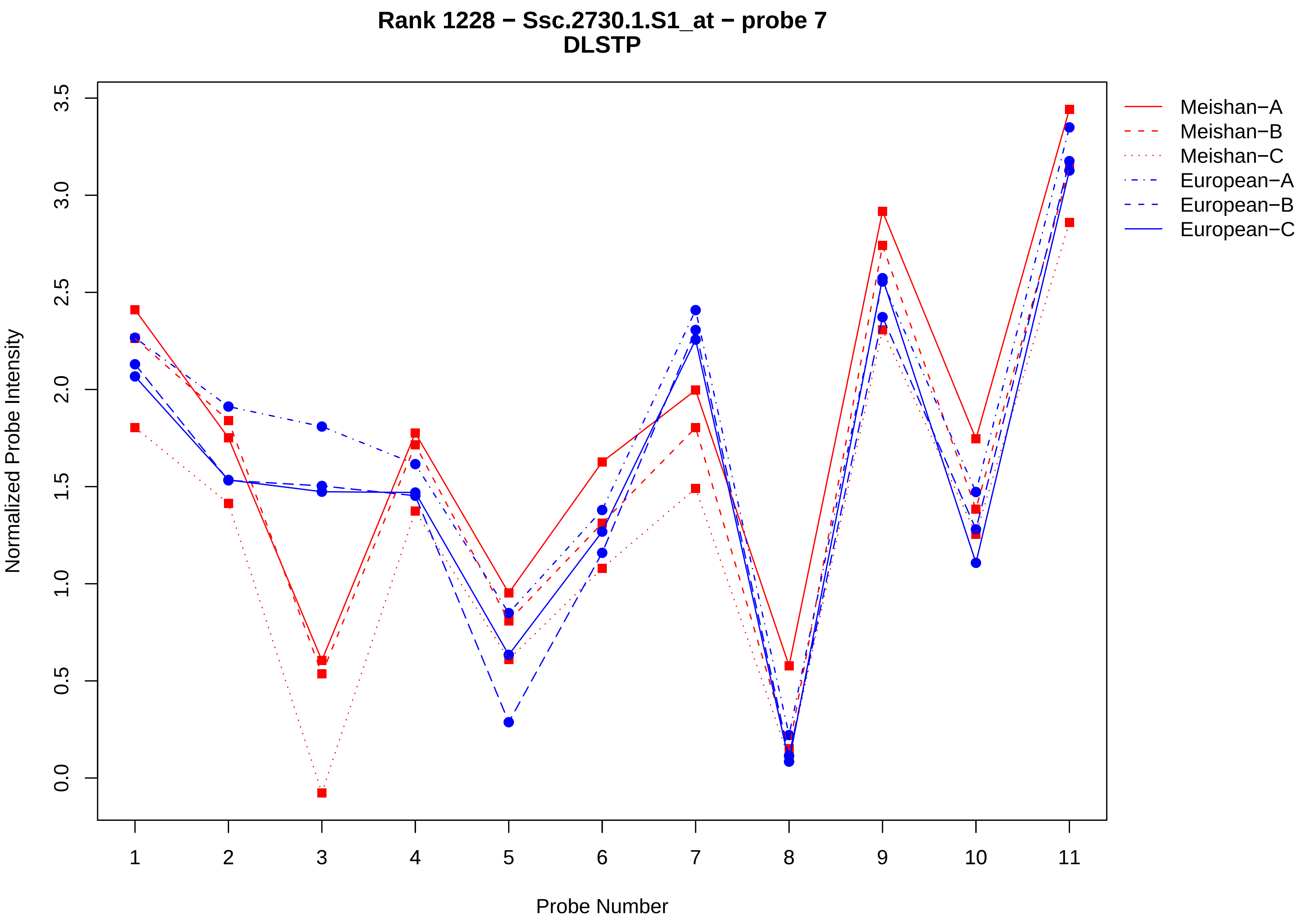

Rank 1228 − Ssc.2730.1.S1_at − probe 7
DLSTP
Normalized Probe Intensity
0.0
0.5
1.0
1.5
2.0
2.5
3.0
3.5
1
2
3
4
5
6
7
8
9
10
11
Probe Number
Meishan−A
Meishan−B
Meishan−C
European−A
European−B
European−C

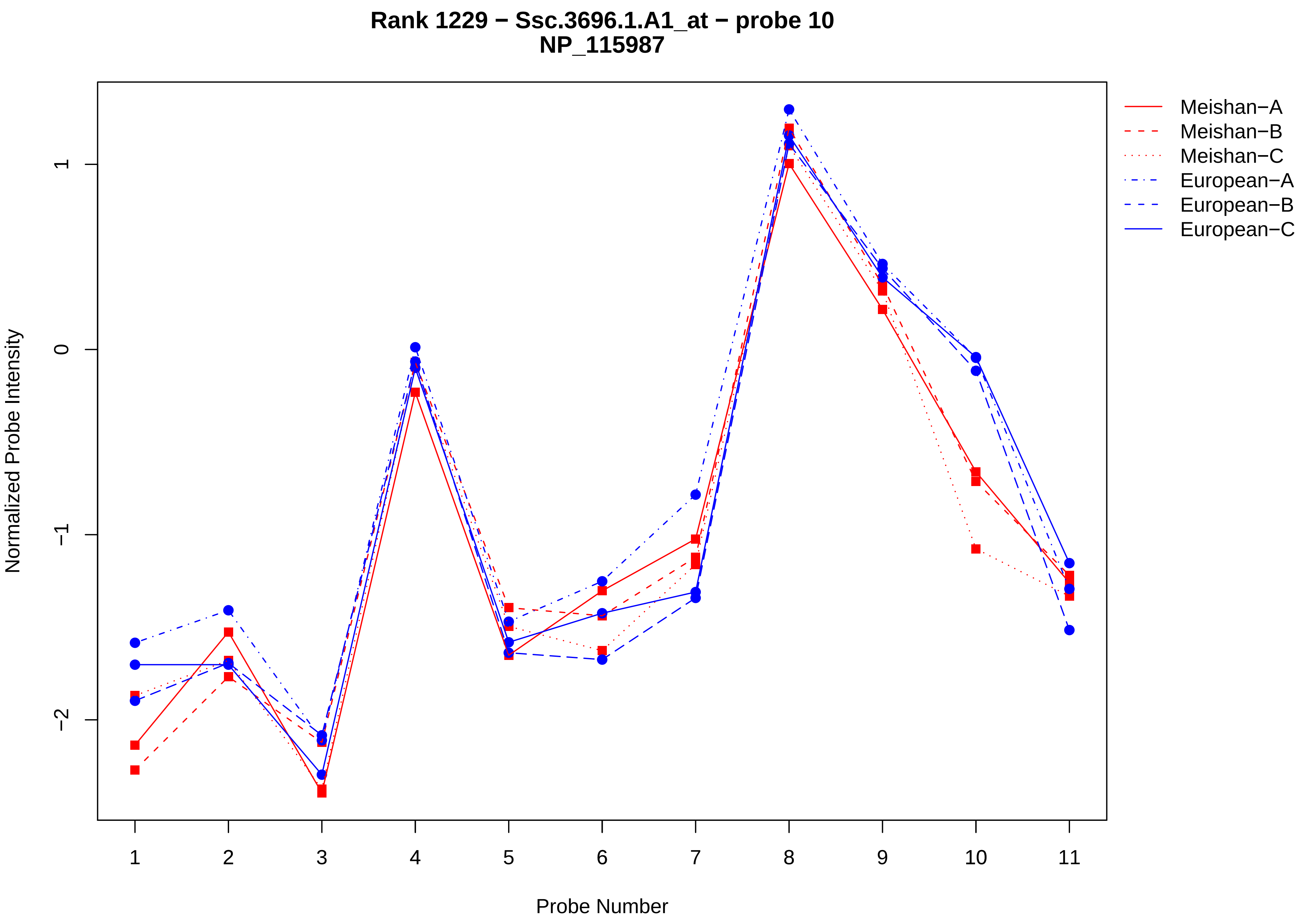

Rank 1229 − Ssc.3696.1.A1_at − probe 10
NP_115987
Normalized Probe Intensity
Probe Number
1
0
−1
−2
1
2
3
4
5
6
7
8
9
10
11
Meishan−A
Meishan−B
Meishan−C
European−A
European−B
European−C

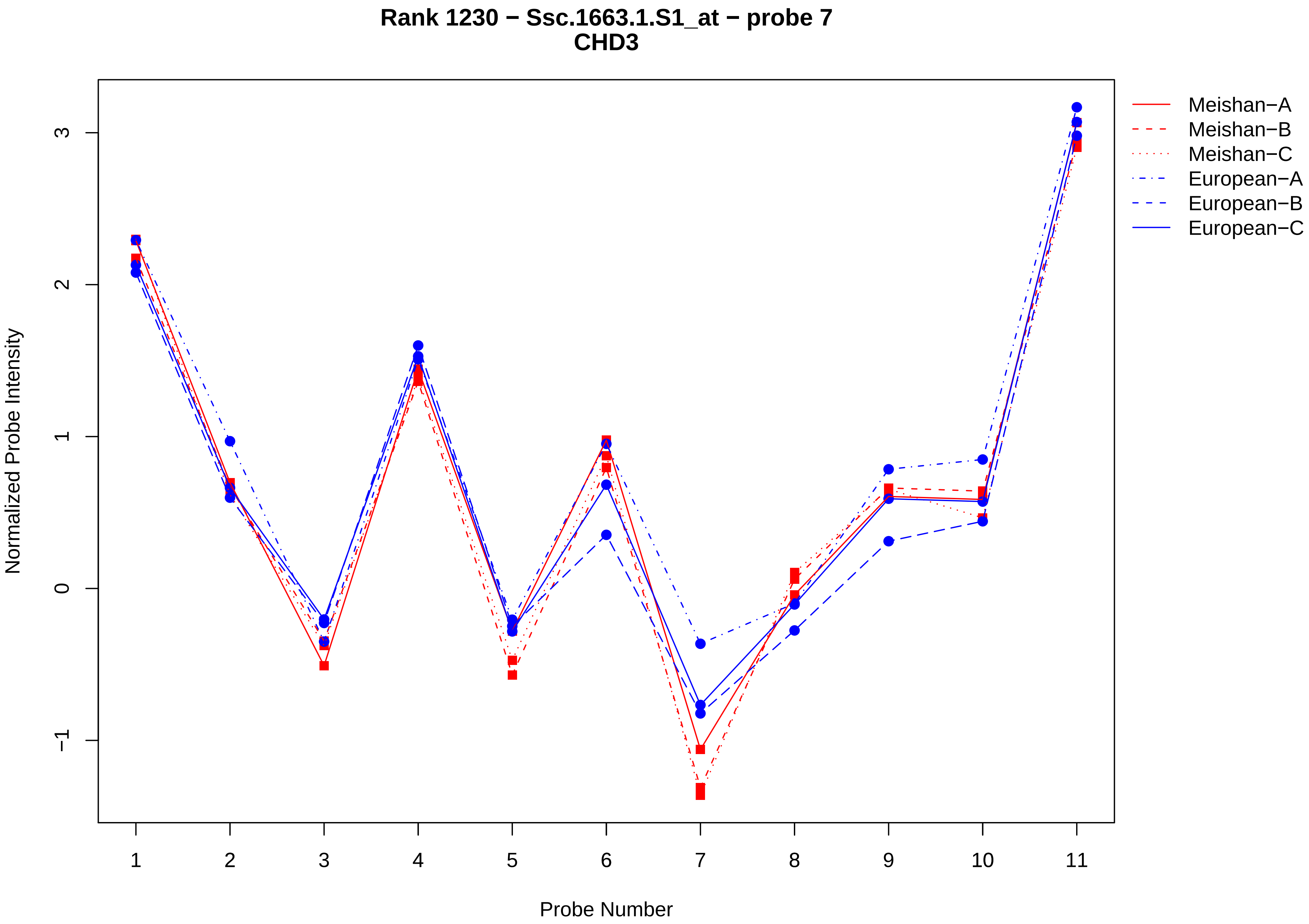

Rank 1230 − Ssc.1663.1.S1_at − probe 7
CHD3
Normalized Probe Intensity
Probe Number
−1
0
1
2
3
1
2
3
4
5
6
7
8
9
10
11
Meishan−A
Meishan−B
Meishan−C
European−A
European−B
European−C

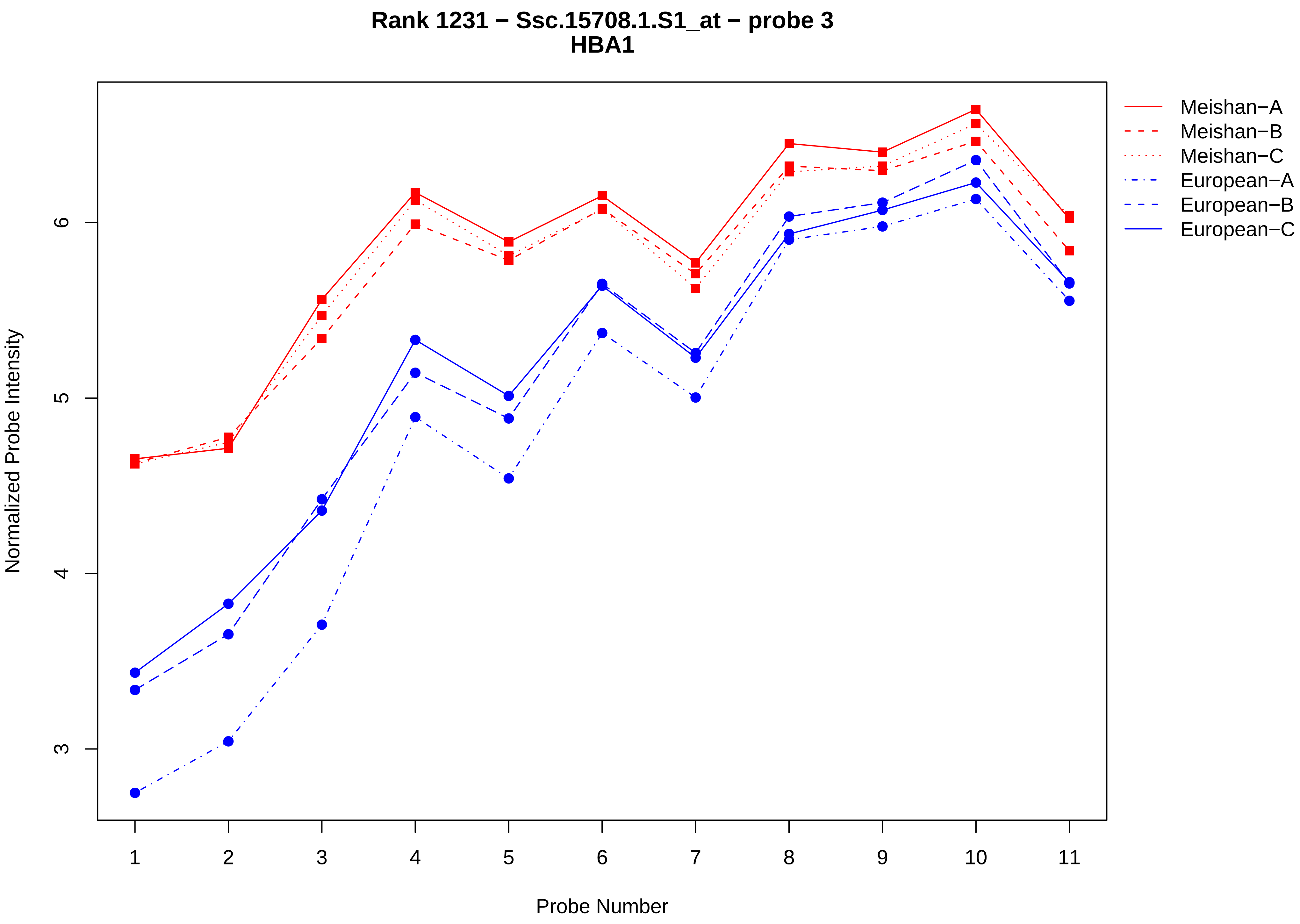

Rank 1231 − Ssc.15708.1.S1_at − probe 3
HBA1
Normalized Probe Intensity
Probe Number
3
4
5
6
1
2
3
4
5
6
7
8
9
10
11
Meishan−A
Meishan−B
Meishan−C
European−A
European−B
European−C

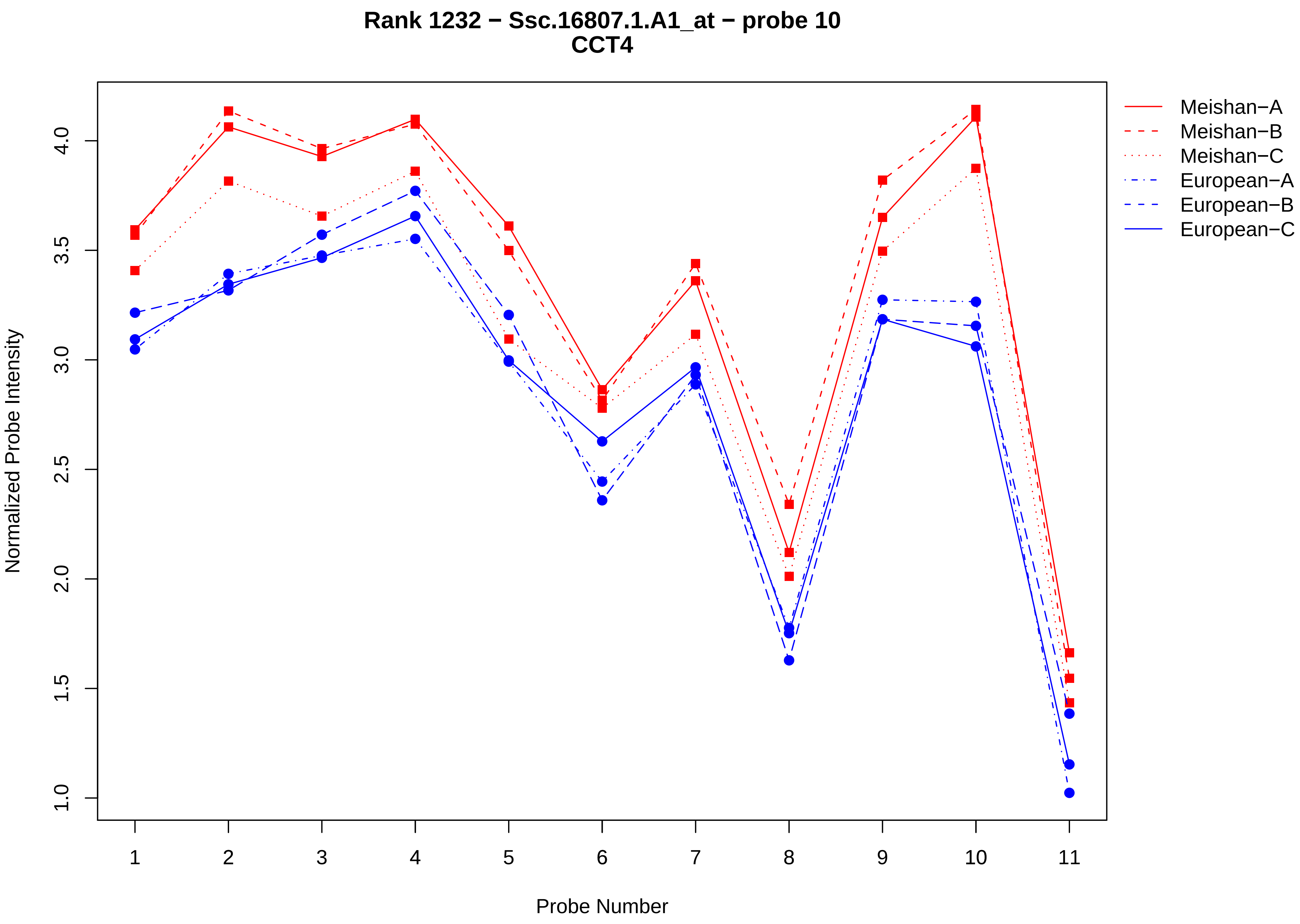

Rank 1232 − Ssc.16807.1.A1_at − probe 10
CCT4
Normalized Probe Intensity
1.0
1.5
2.0
2.5
3.0
3.5
4.0
Probe Number
1
2
3
4
5
6
7
8
9
10
11
Meishan−A
Meishan−B
Meishan−C
European−A
European−B
European−C

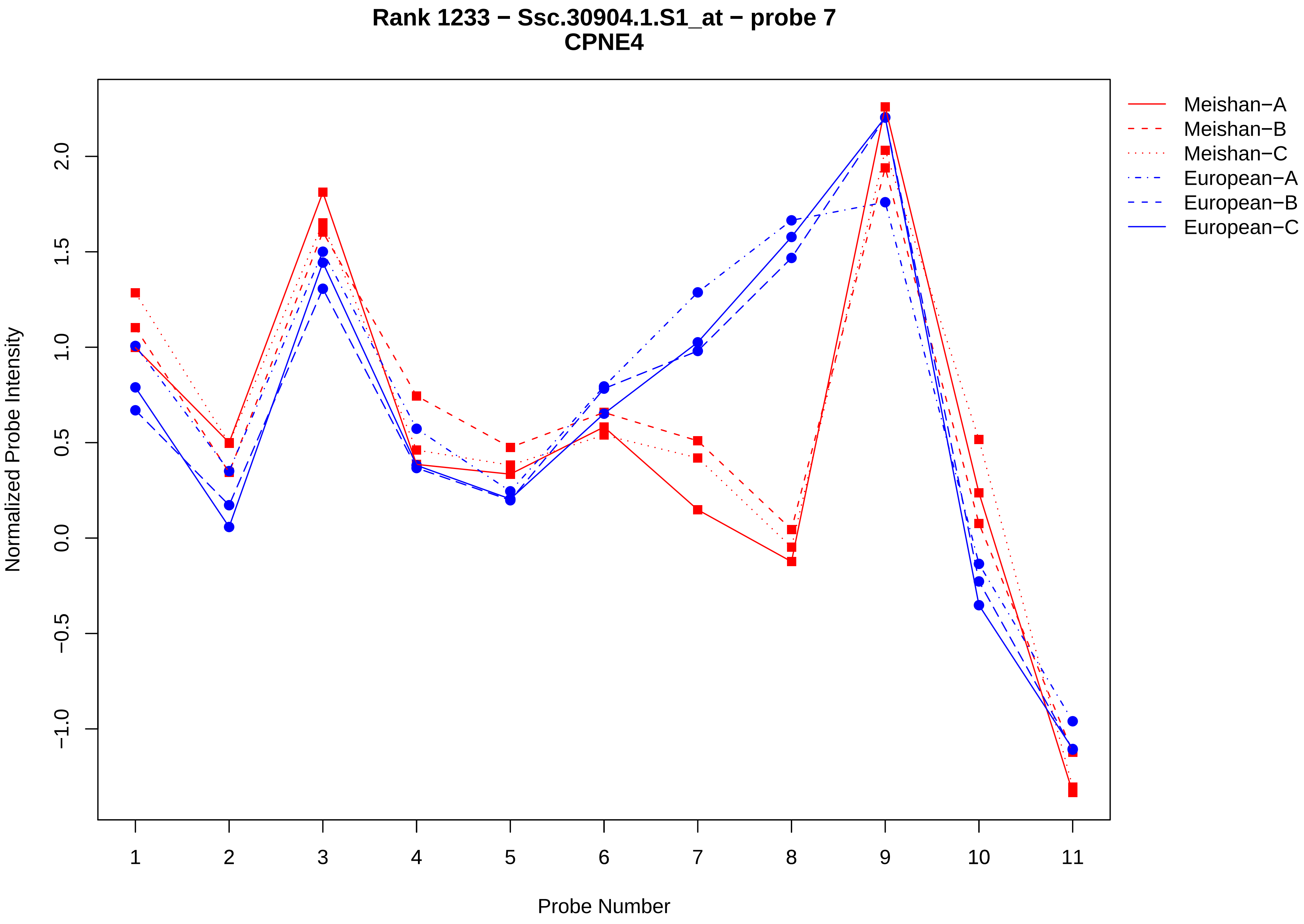

Rank 1233 − Ssc.30904.1.S1_at − probe 7
CPNE4
Normalized Probe Intensity
Probe Number
Meishan−A
Meishan−B
Meishan−C
European−A
European−B
European−C
−1.0
−0.5
0.0
0.5
1.0
1.5
2.0
1
2
3
4
5
6
7
8
9
10
11

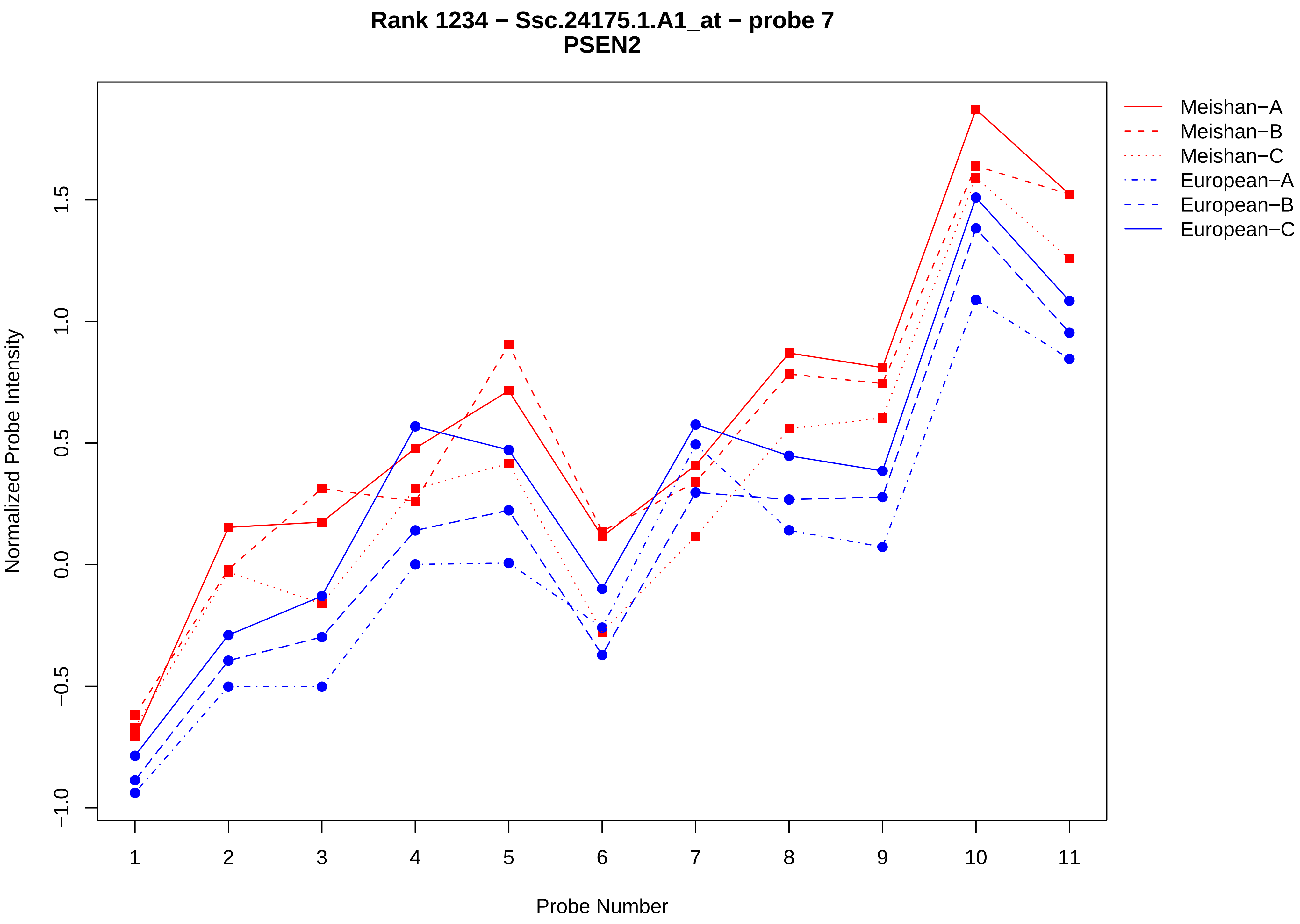

Rank 1234 − Ssc.24175.1.A1_at − probe 7
PSEN2
Normalized Probe Intensity
Probe Number
−1.0
−0.5
0.0
0.5
1.0
1.5
1
2
3
4
5
6
7
8
9
10
11
Meishan−A
Meishan−B
Meishan−C
European−A
European−B
European−C

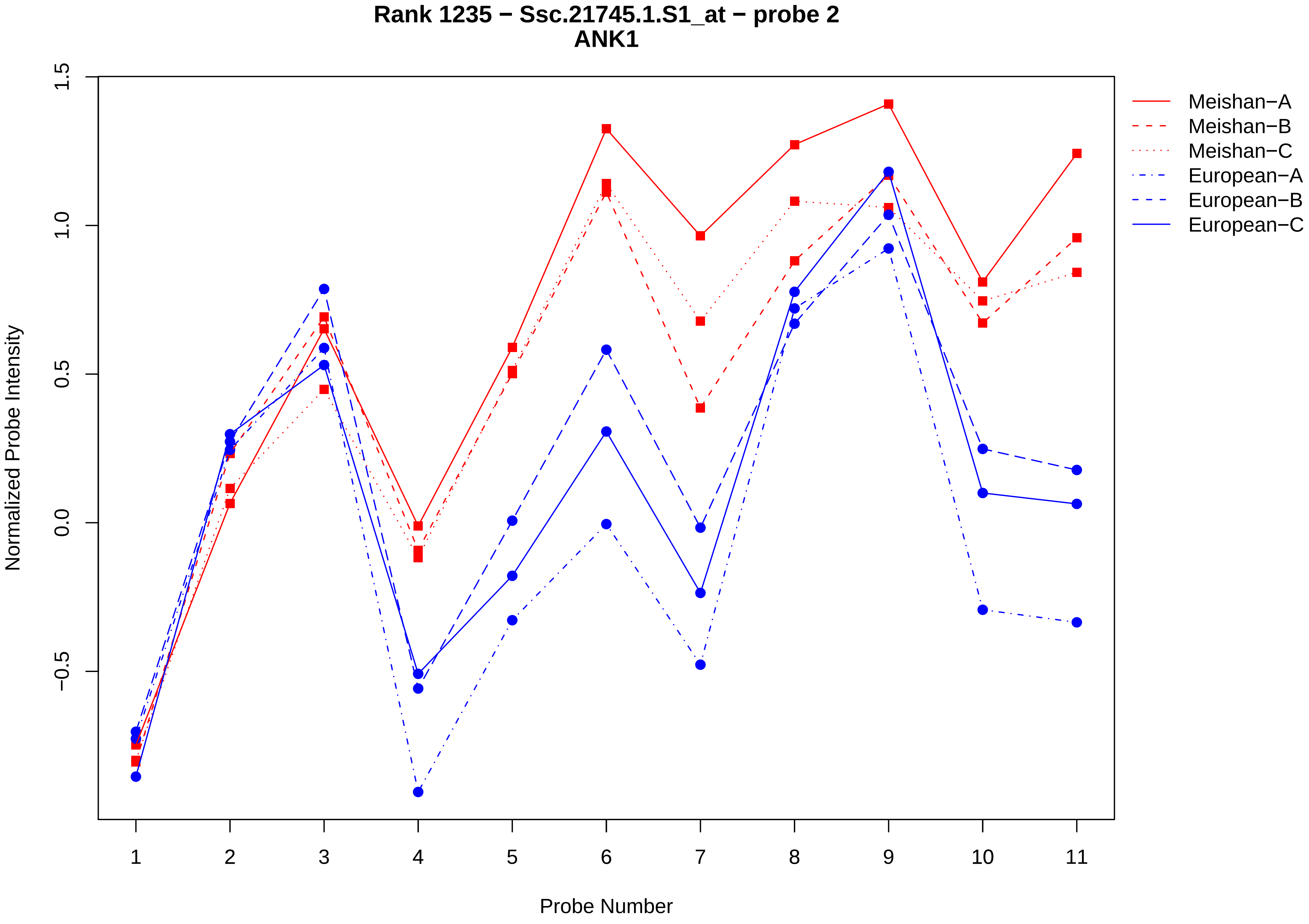

Rank 1235 − Ssc.21745.1.S1_at − probe 2
ANK1
Normalized Probe Intensity
Probe Number
1.5
1.0
0.5
0.0
−0.5
1
2
3
4
5
6
7
8
9
10
11
Meishan−A
Meishan−B
Meishan−C
European−A
European−B
European−C

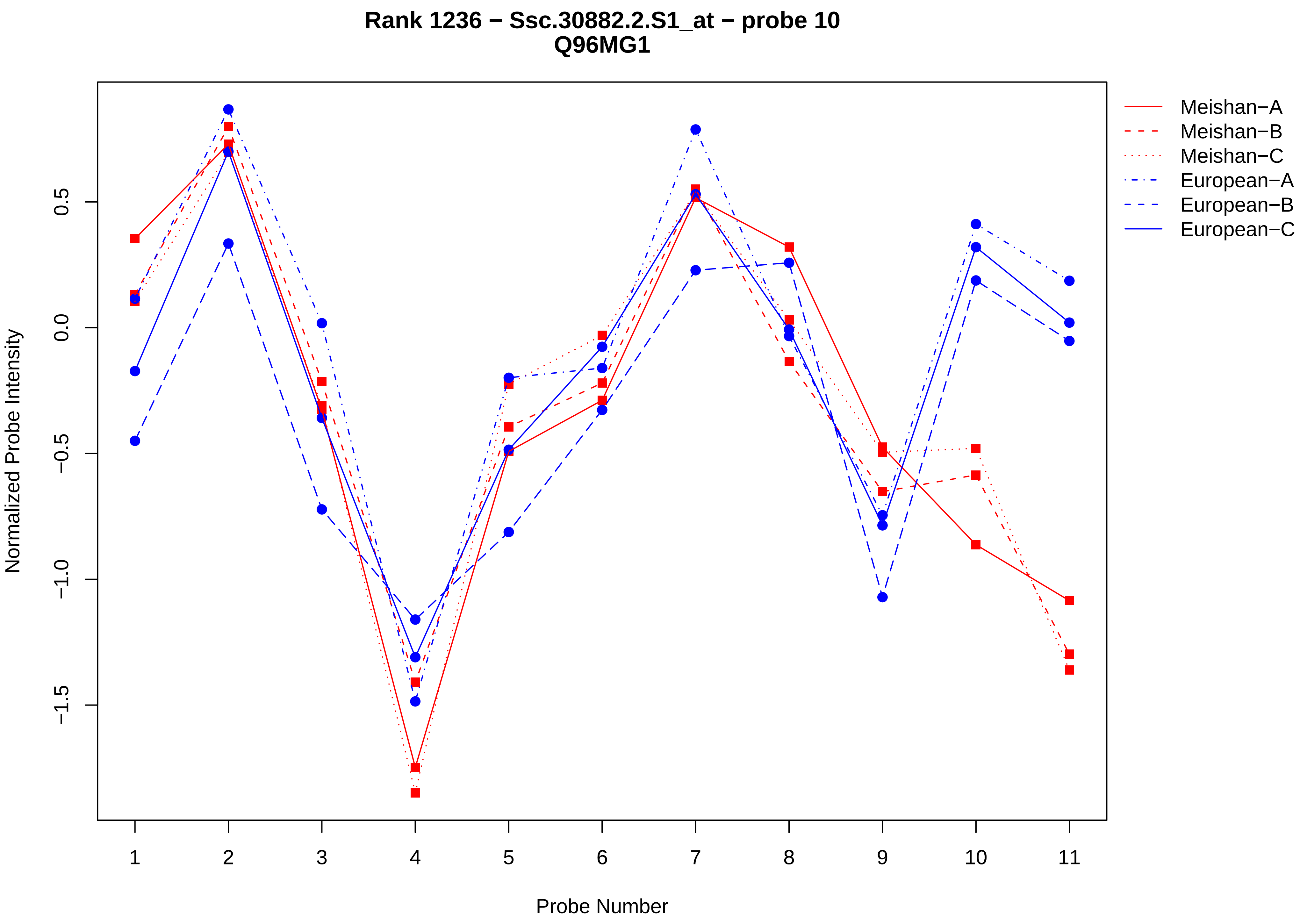
Rank 1236 − Ssc.30882.2.S1_at − probe 10
Q96MG1
Normalized Probe Intensity
Probe Number
Meishan−A
Meishan−B
Meishan−C
European−A
European−B
European−C
0.5
0.0
−0.5
−1.0
−1.5
1
2
3
4
5
6
7
8
9
10
11

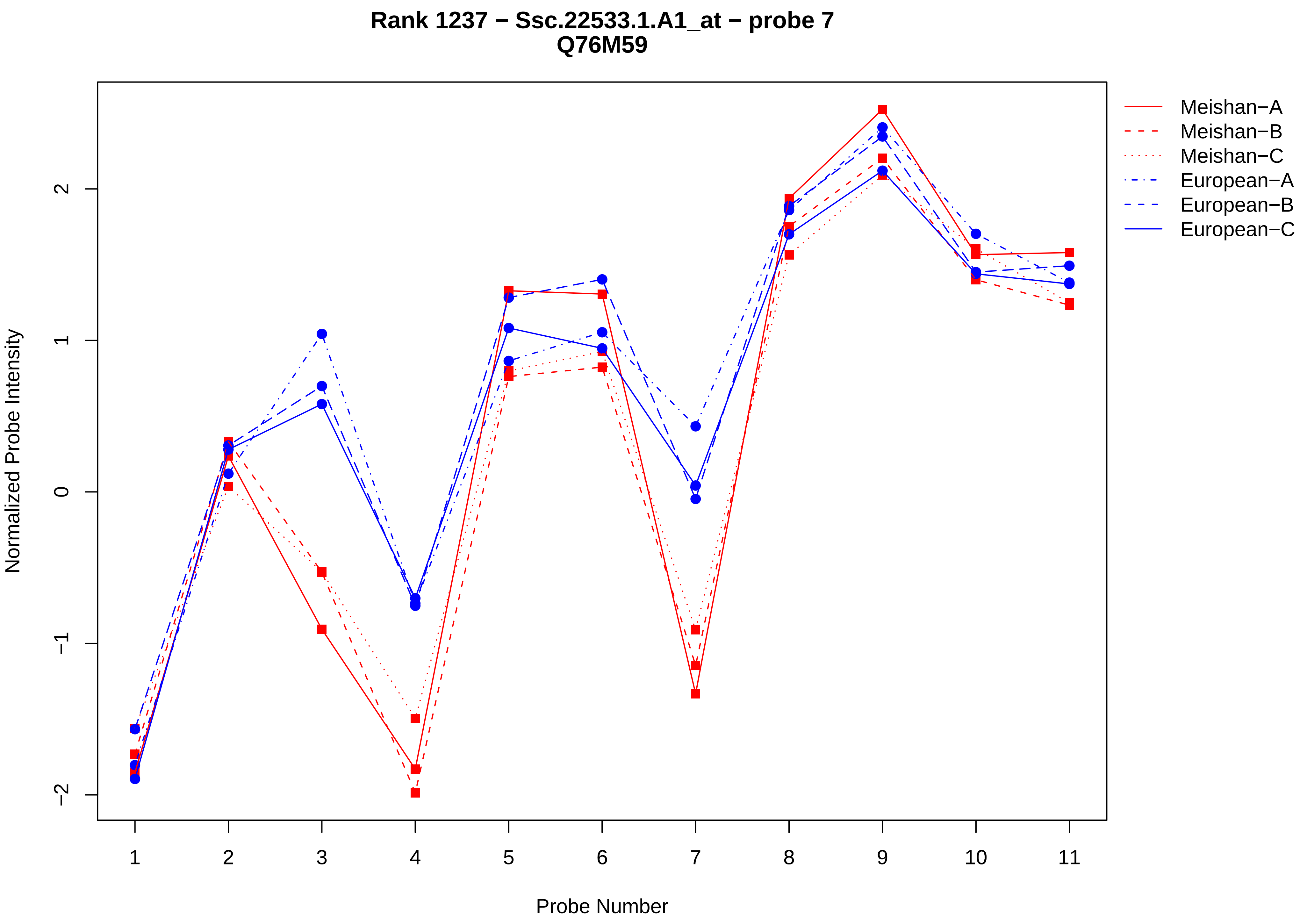
Rank 1237 − Ssc.22533.1.A1_at − probe 7
Q76M59
Normalized Probe Intensity
−2
−1
0
1
2
Probe Number
1
2
3
4
5
6
7
8
9
10
11
Meishan−A
Meishan−B
Meishan−C
European−A
European−B
European−C

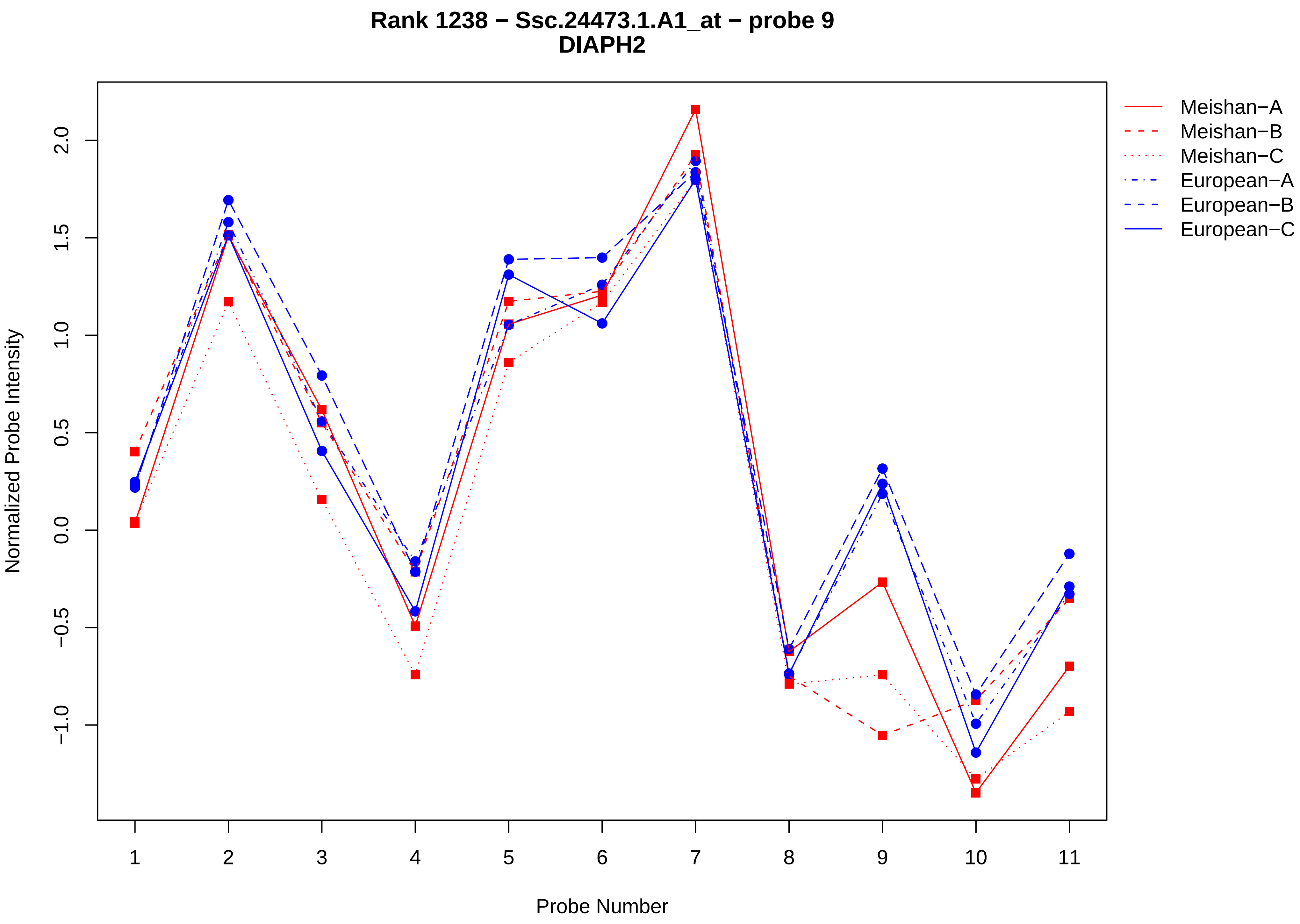
Rank 1238 − Ssc.24473.1.A1_at − probe 9
DIAPH2
Normalized Probe Intensity
Probe Number
Meishan−A
Meishan−B
Meishan−C
European−A
European−B
European−C
−1.0
−0.5
0.0
0.5
1.0
1.5
2.0
1
2
3
4
5
6
7
8
9
10
11

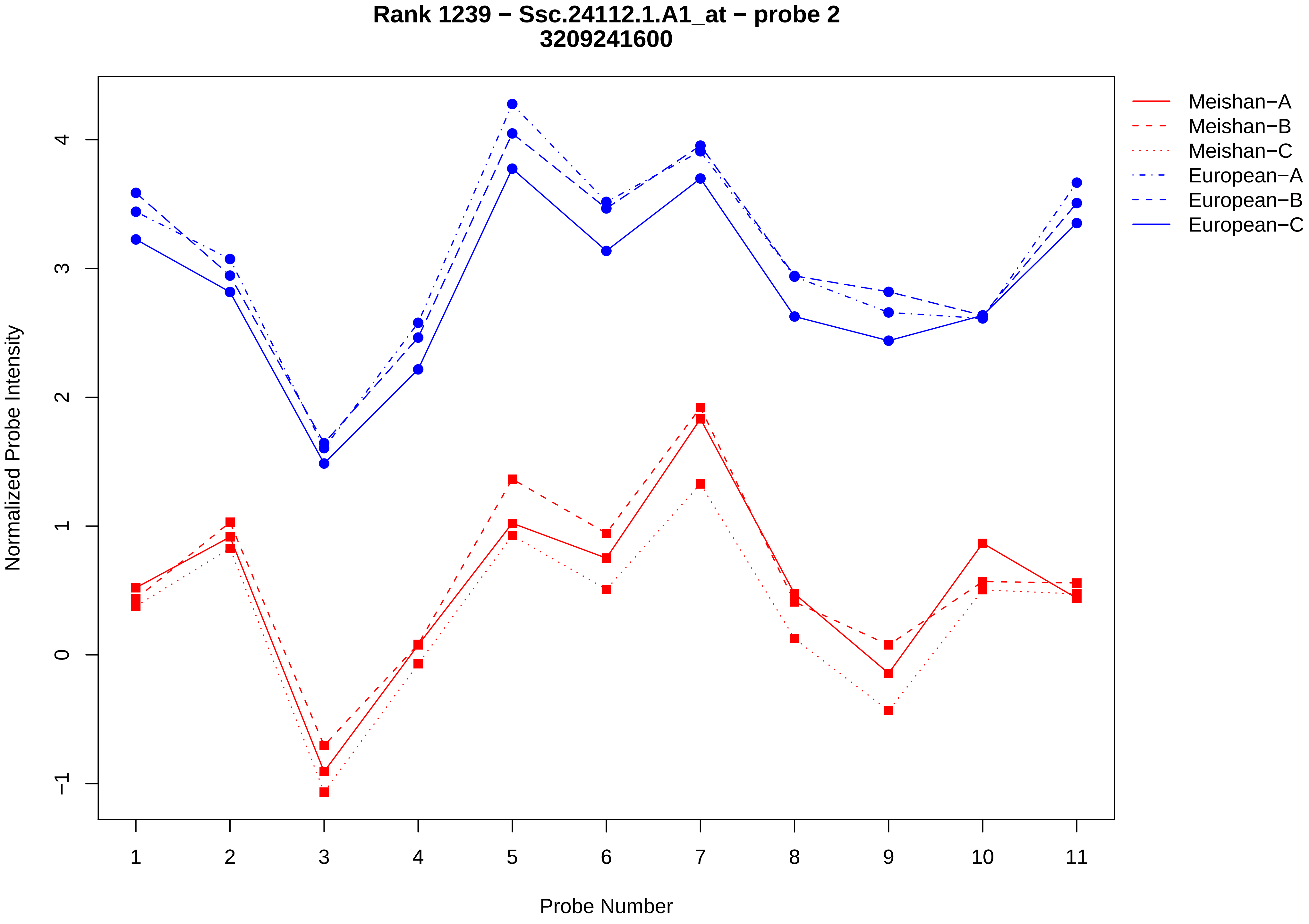
Rank 1239 − Ssc.24112.1.A1_at − probe 2
3209241600
Normalized Probe Intensity
Probe Number
Meishan−A
Meishan−B
Meishan−C
European−A
European−B
European−C
−1
0
1
2
3
4
1
2
3
4
5
6
7
8
9
10
11

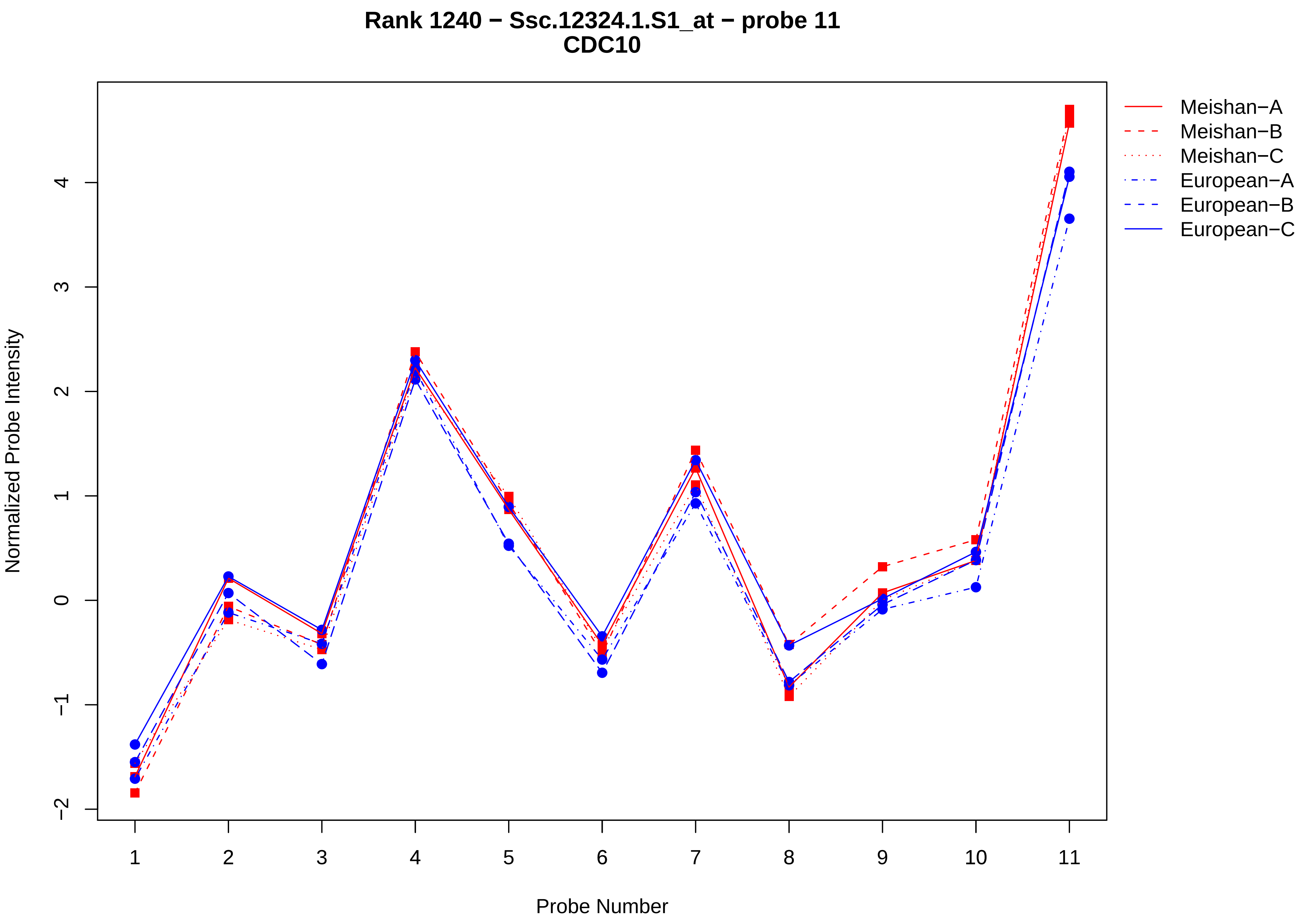
Rank 1240 − Ssc.12324.1.S1_at − probe 11
CDC10
Normalized Probe Intensity
Probe Number
−2
−1
0
1
2
3
4
1
2
3
4
5
6
7
8
9
10
11
Meishan−A
Meishan−B
Meishan−C
European−A
European−B
European−C

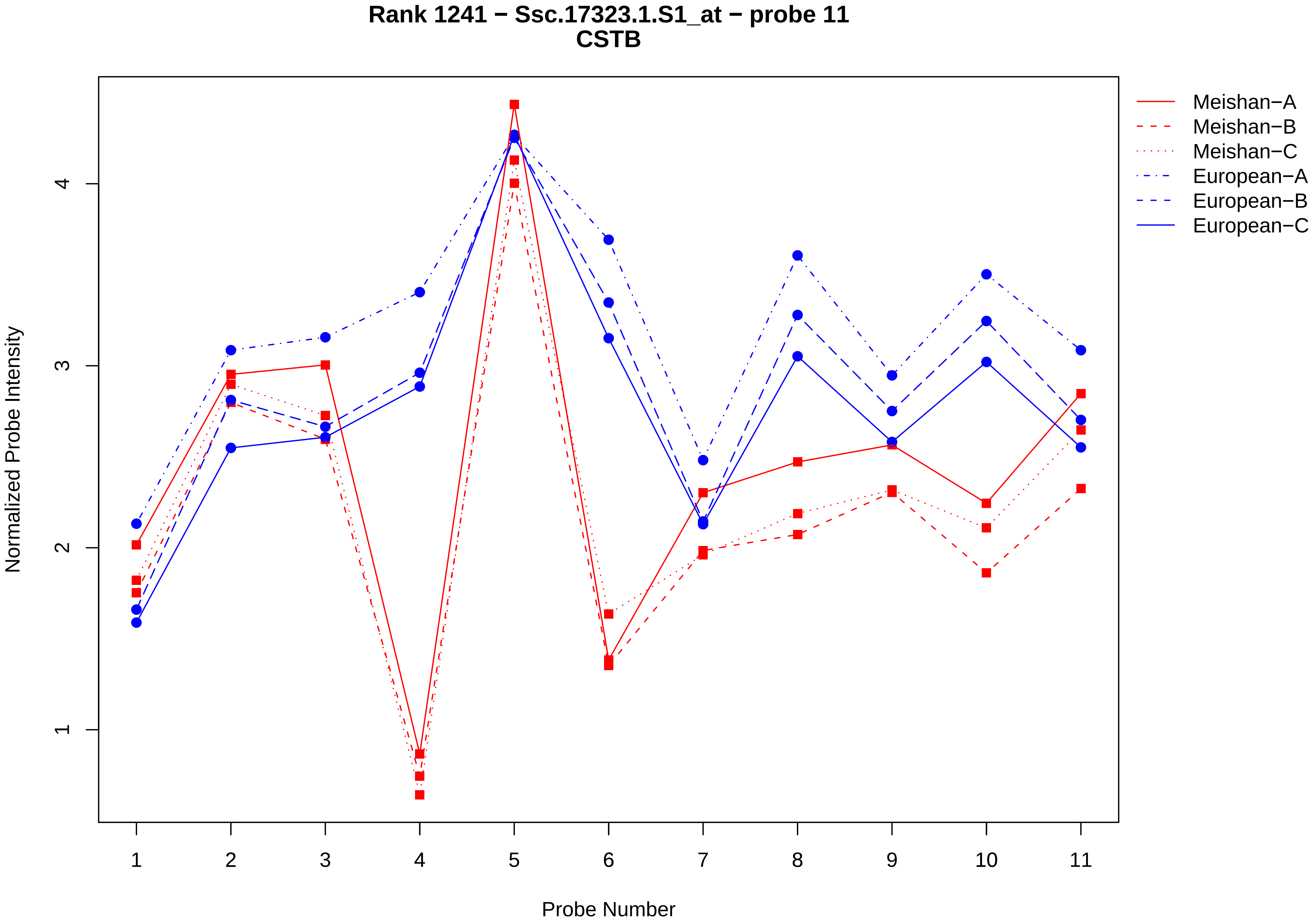

Rank 1241 − Ssc.17323.1.S1_at − probe 11
CSTB
Normalized Probe Intensity
1
2
3
4
1
2
3
4
5
6
7
8
9
10
11
Probe Number
Meishan−A
Meishan−B
Meishan−C
European−A
European−B
European−C

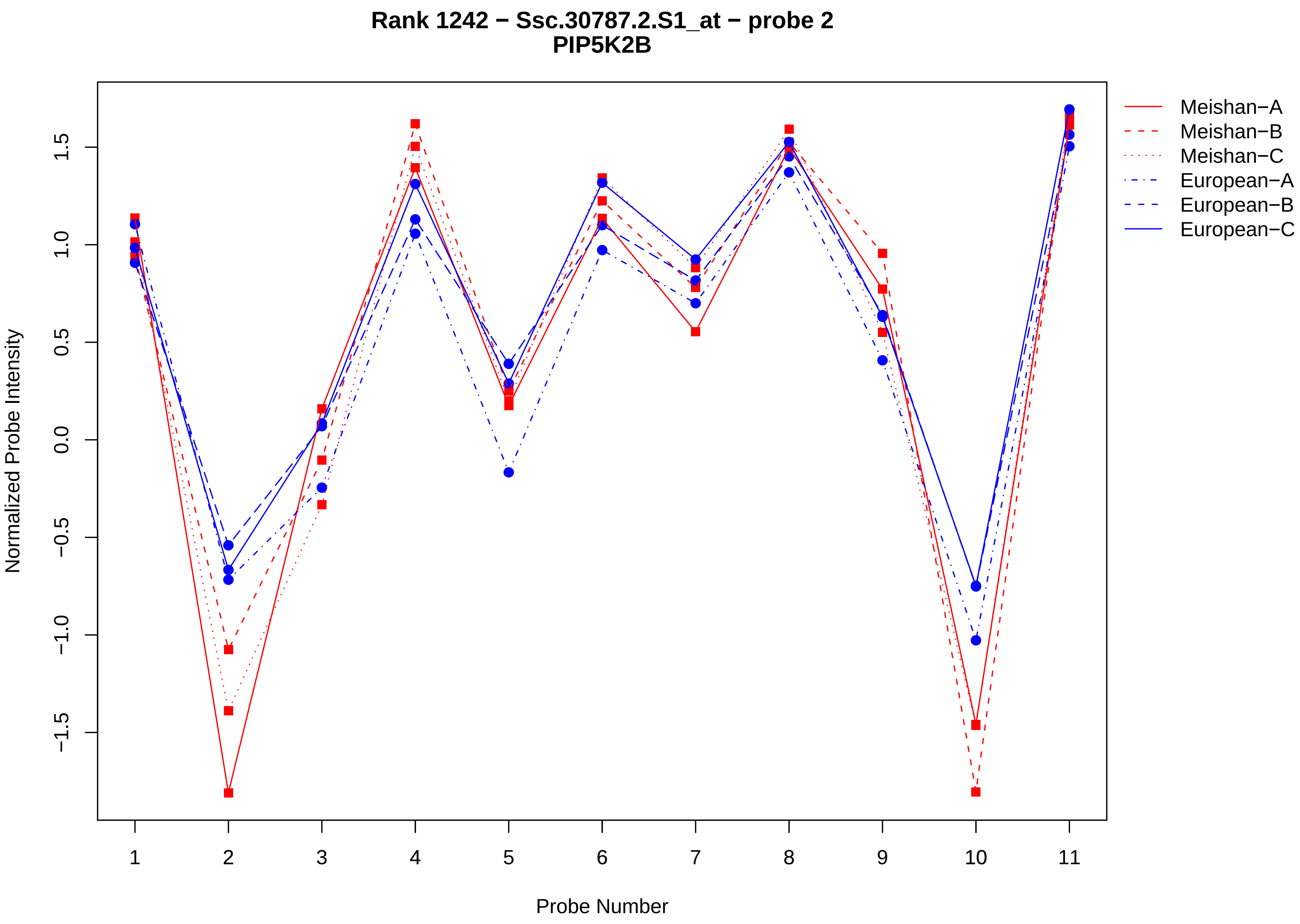

Rank 1242 − Ssc.30787.2.S1_at − probe 2
PIP5K2B
Normalized Probe Intensity
Probe Number
1.5
1.0
0.5
0.0
−0.5
−1.0
−1.5
1
2
3
4
5
6
7
8
9
10
11
Meishan−A
Meishan−B
Meishan−C
European−A
European−B
European−C

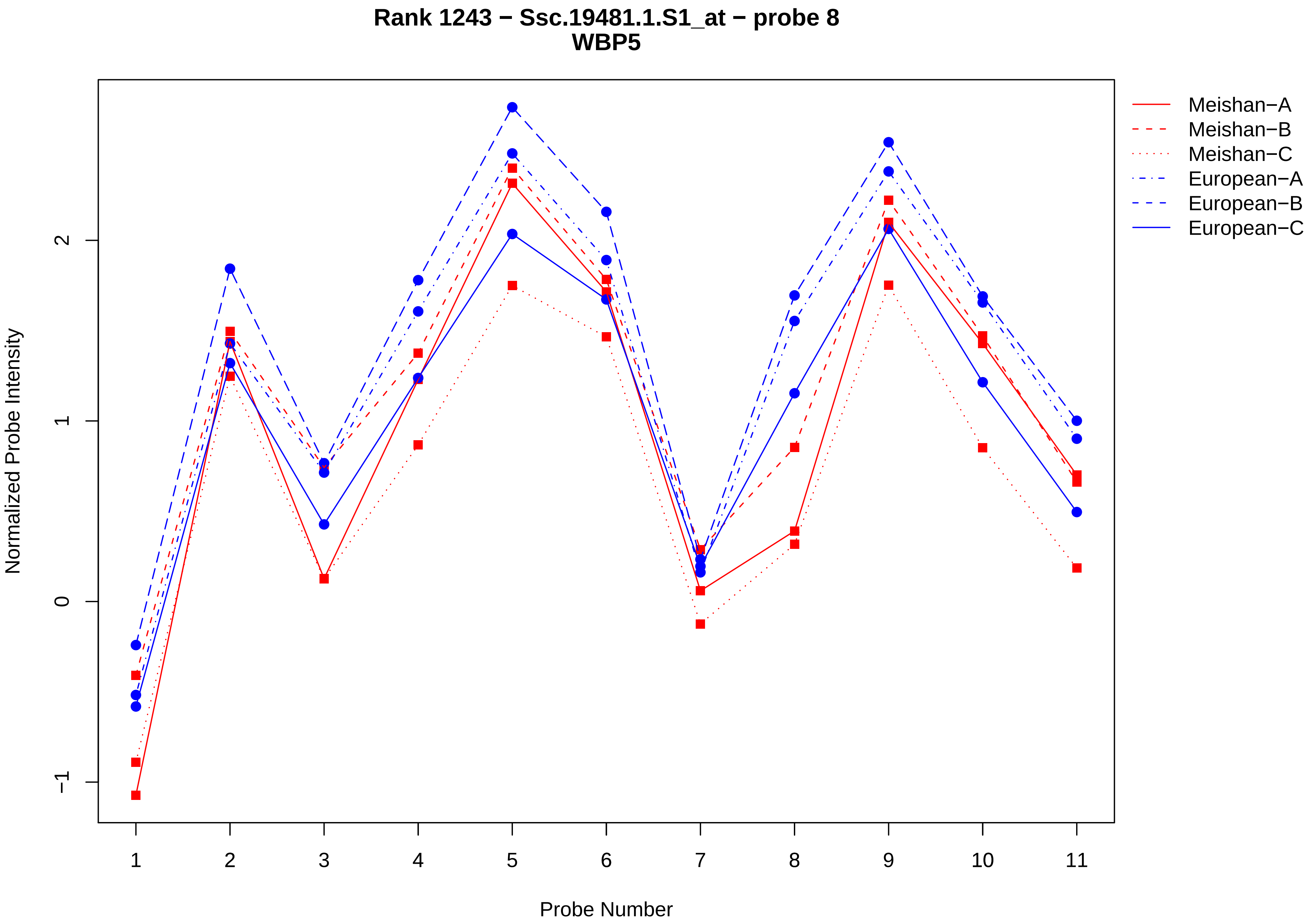

Rank 1243 − Ssc.19481.1.S1_at − probe 8
WBP5
Normalized Probe Intensity
Probe Number
−1
0
1
2
1
2
3
4
5
6
7
8
9
10
11
Meishan−A
Meishan−B
Meishan−C
European−A
European−B
European−C

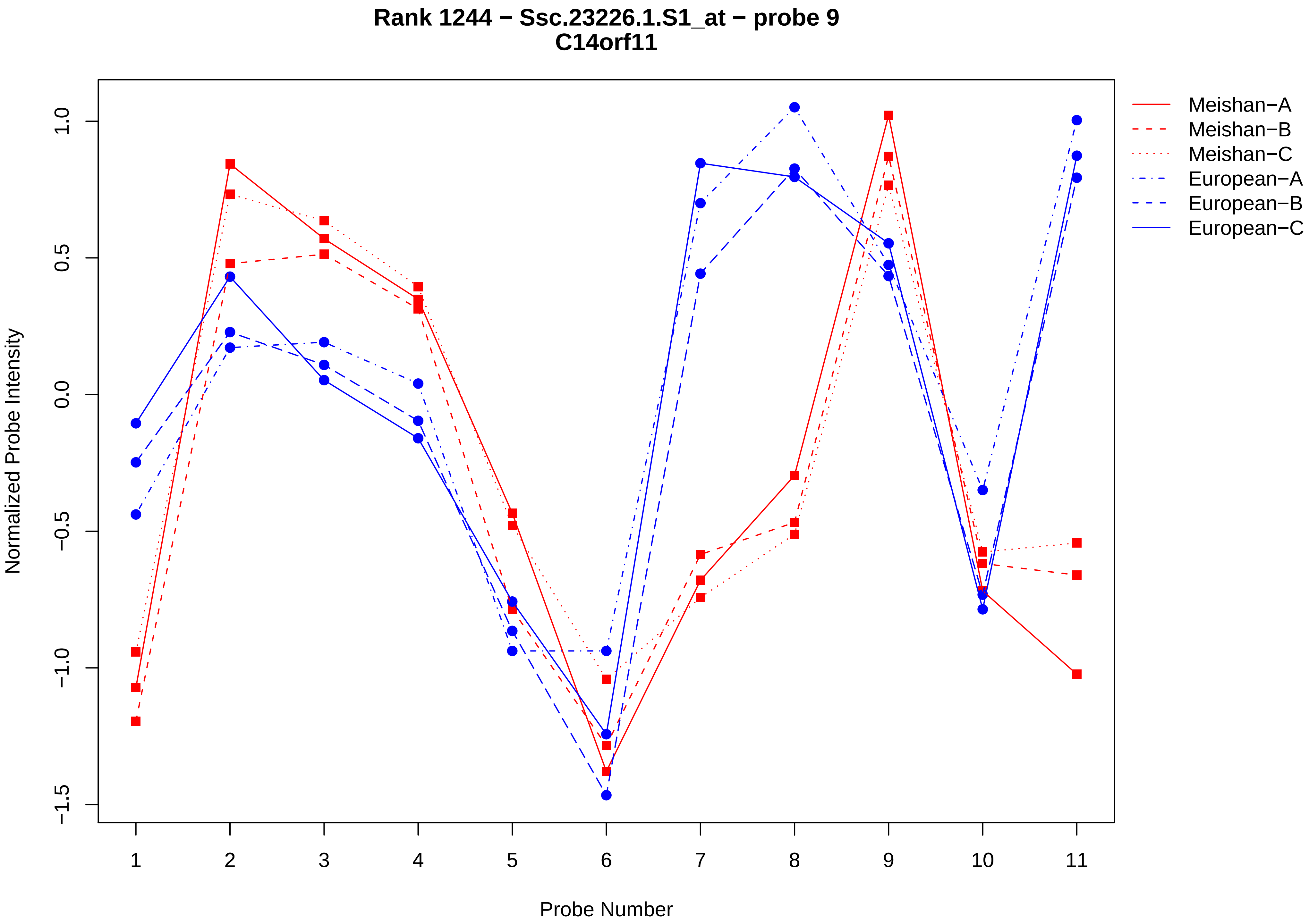

Rank 1244 − Ssc.23226.1.S1_at − probe 9
C14orf11
Normalized Probe Intensity
Probe Number
Meishan−A
Meishan−B
Meishan−C
European−A
European−B
European−C
1.0
0.5
0.0
−0.5
−1.0
−1.5
1
2
3
4
5
6
7
8
9
10
11

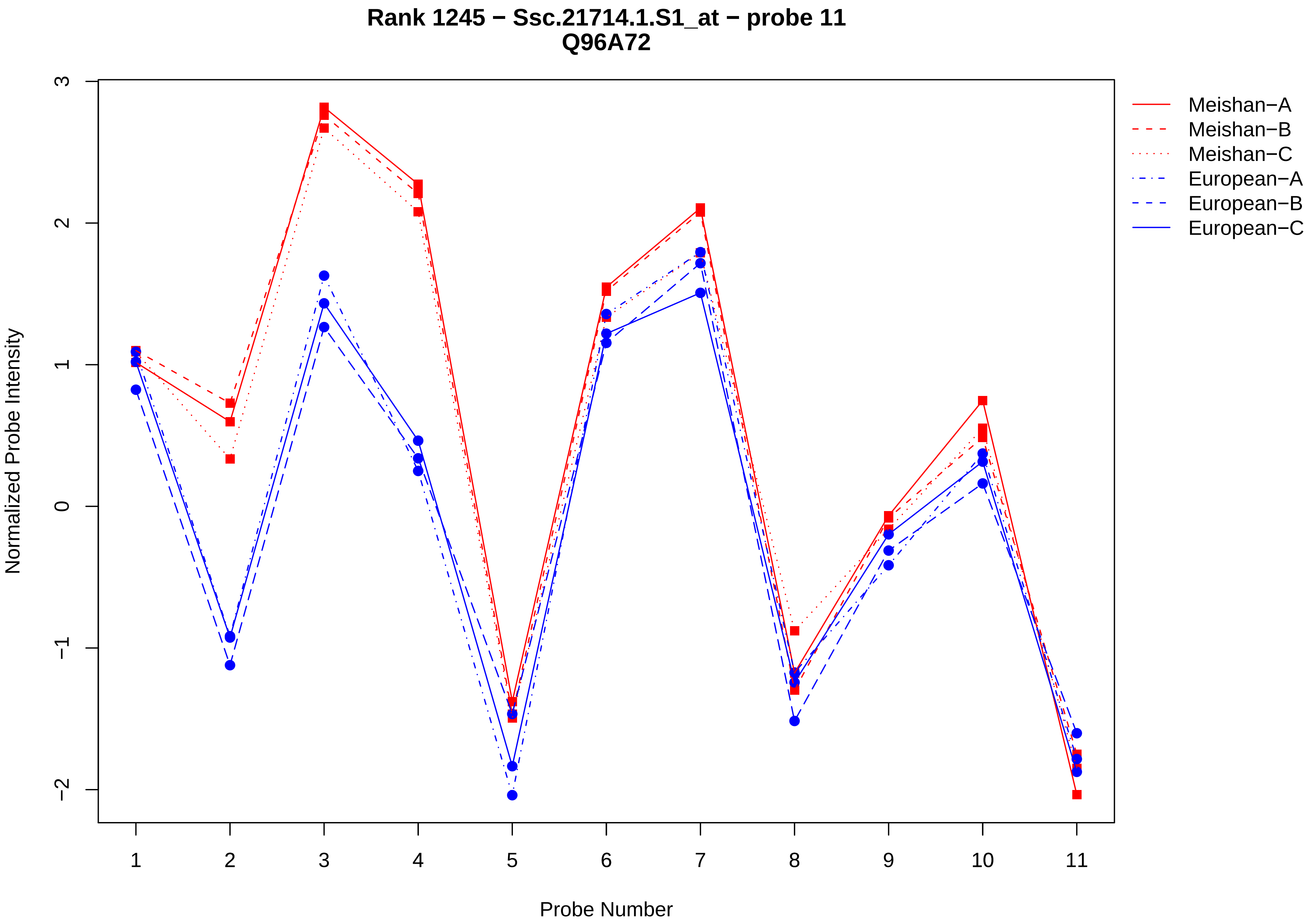

Rank 1245 − Ssc.21714.1.S1_at − probe 11
Q96A72
Normalized Probe Intensity
Probe Number
3
2
1
0
−1
−2
1
2
3
4
5
6
7
8
9
10
11
Meishan−A
Meishan−B
Meishan−C
European−A
European−B
European−C

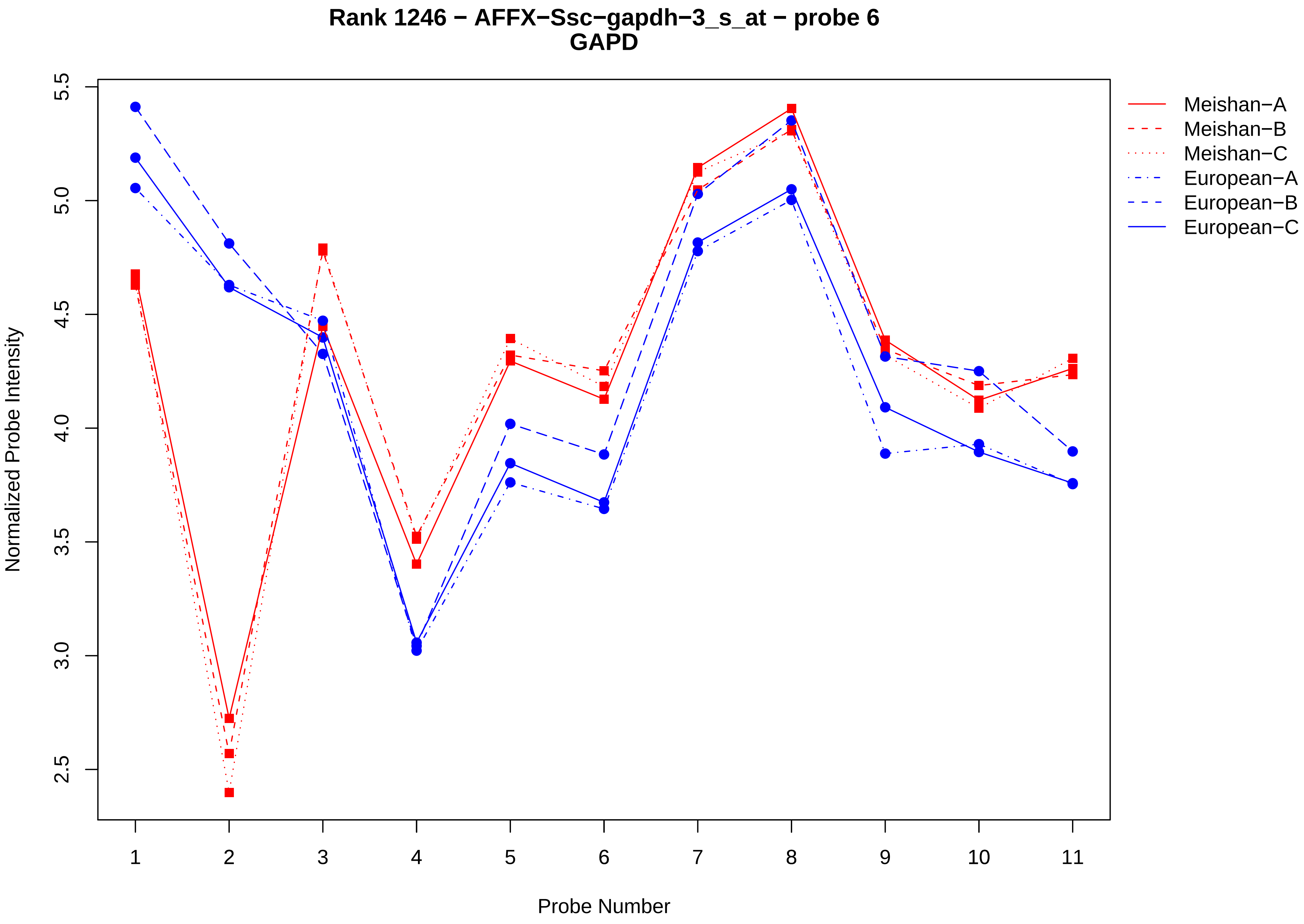
Rank 1246 − AFFX−Ssc−gapdh−3_s_at − probe 6
GAPD
Normalized Probe Intensity
Probe Number
2.5
3.0
3.5
4.0
4.5
5.0
5.5
1
2
3
4
5
6
7
8
9
10
11
Meishan−A
Meishan−B
Meishan−C
European−A
European−B
European−C

Rank 1247 − Ssc.16442.1.S1_at − probe 9
C10orf72

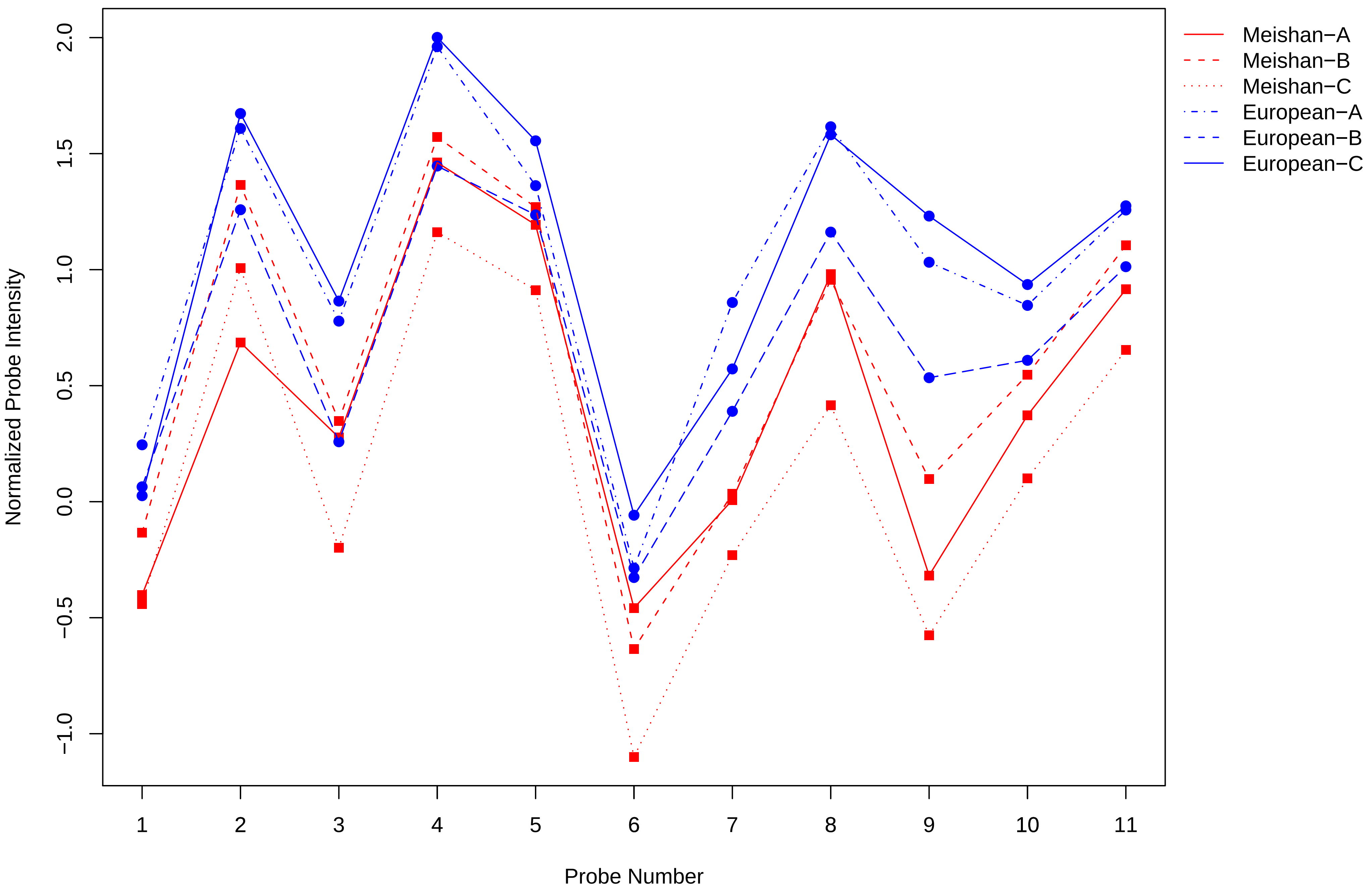

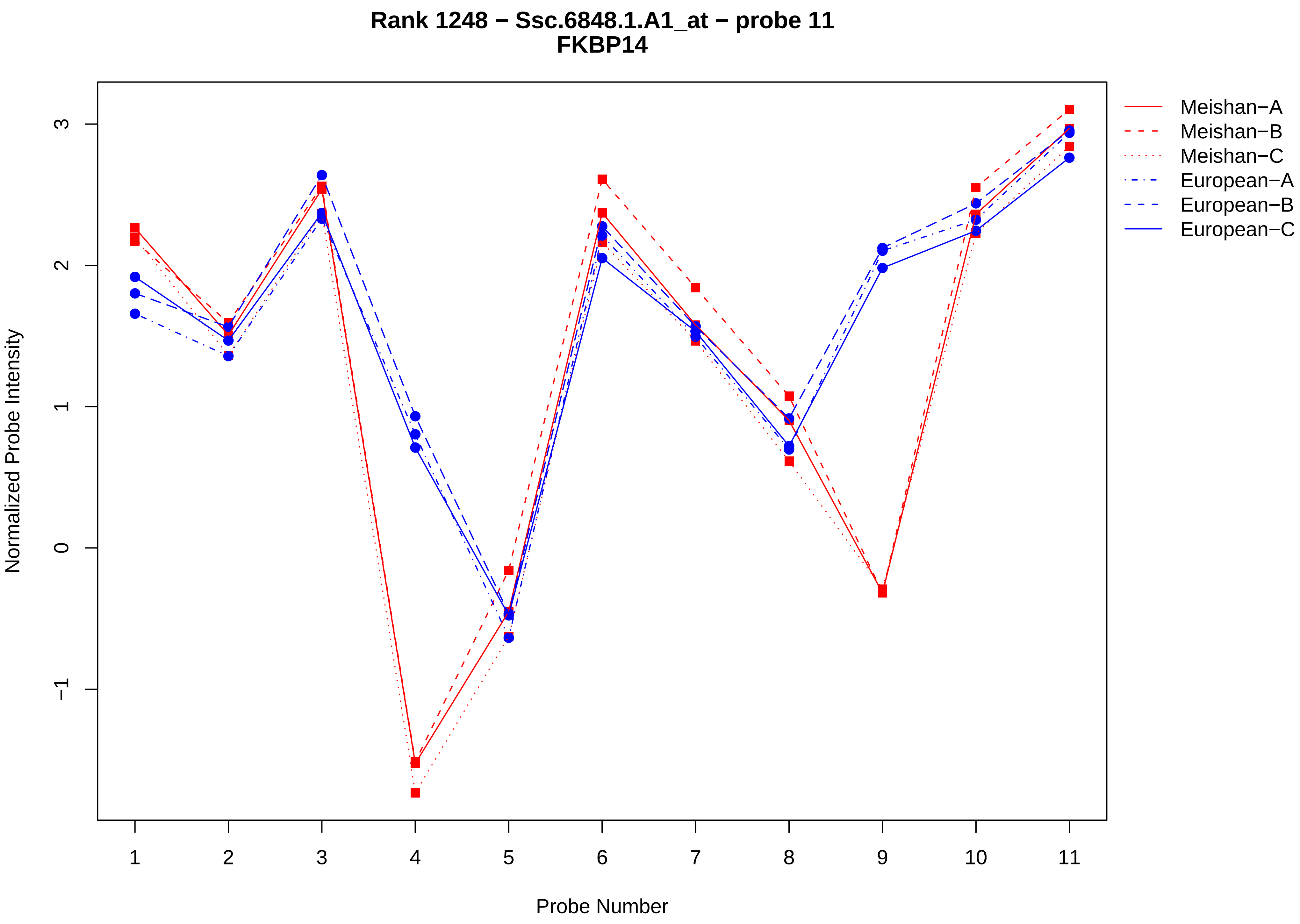
Rank 1248 − Ssc.6848.1.A1_at − probe 11
FKBP14
Normalized Probe Intensity
Probe Number
−1
0
1
2
3
1
2
3
4
5
6
7
8
9
10
11
Meishan−A
Meishan−B
Meishan−C
European−A
European−B
European−C

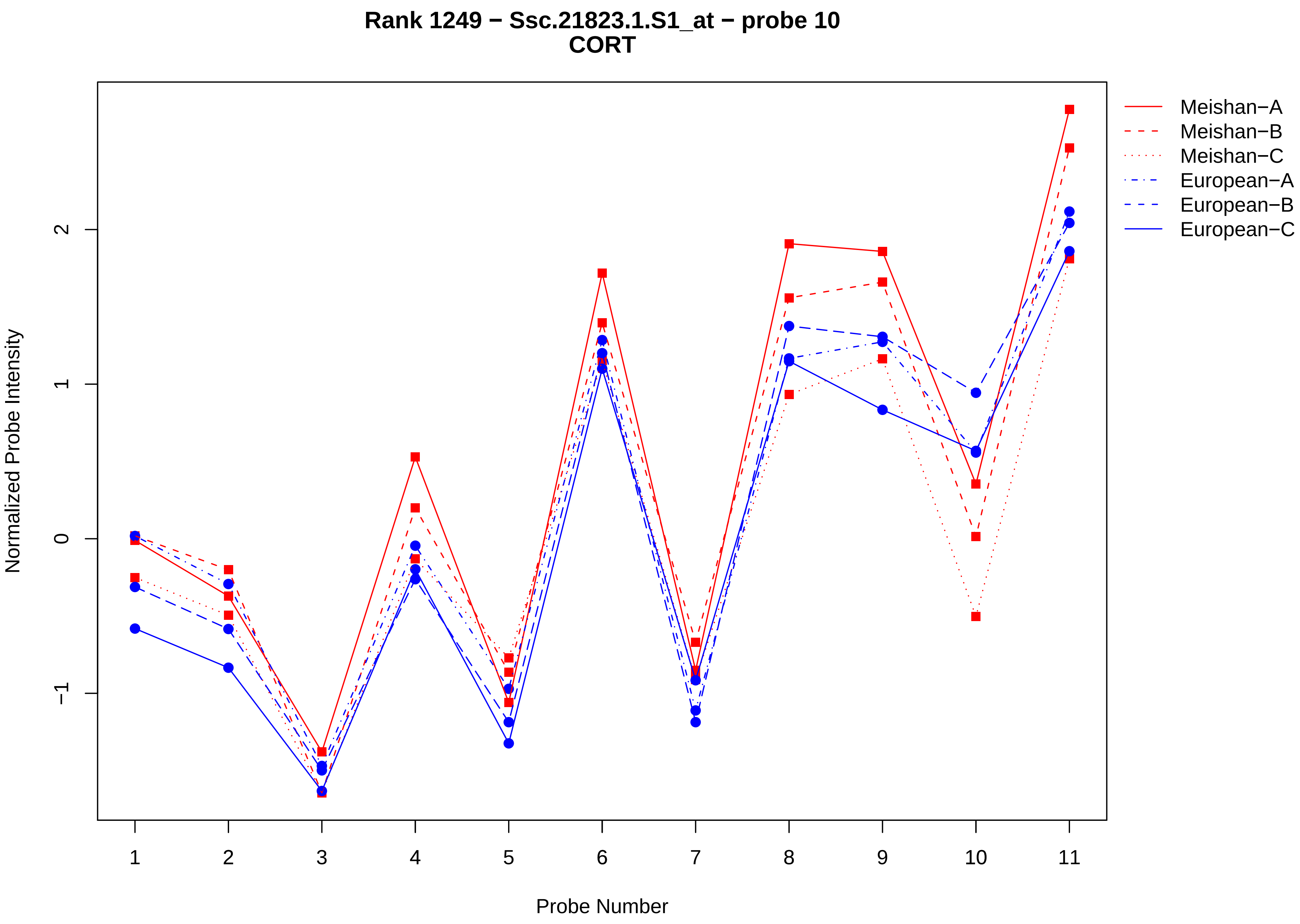
Rank 1249 − Ssc.21823.1.S1_at − probe 10
CORT
Normalized Probe Intensity
Probe Number
Meishan−A
Meishan−B
Meishan−C
European−A
European−B
European−C
−1
0
1
2
1
2
3
4
5
6
7
8
9
10
11

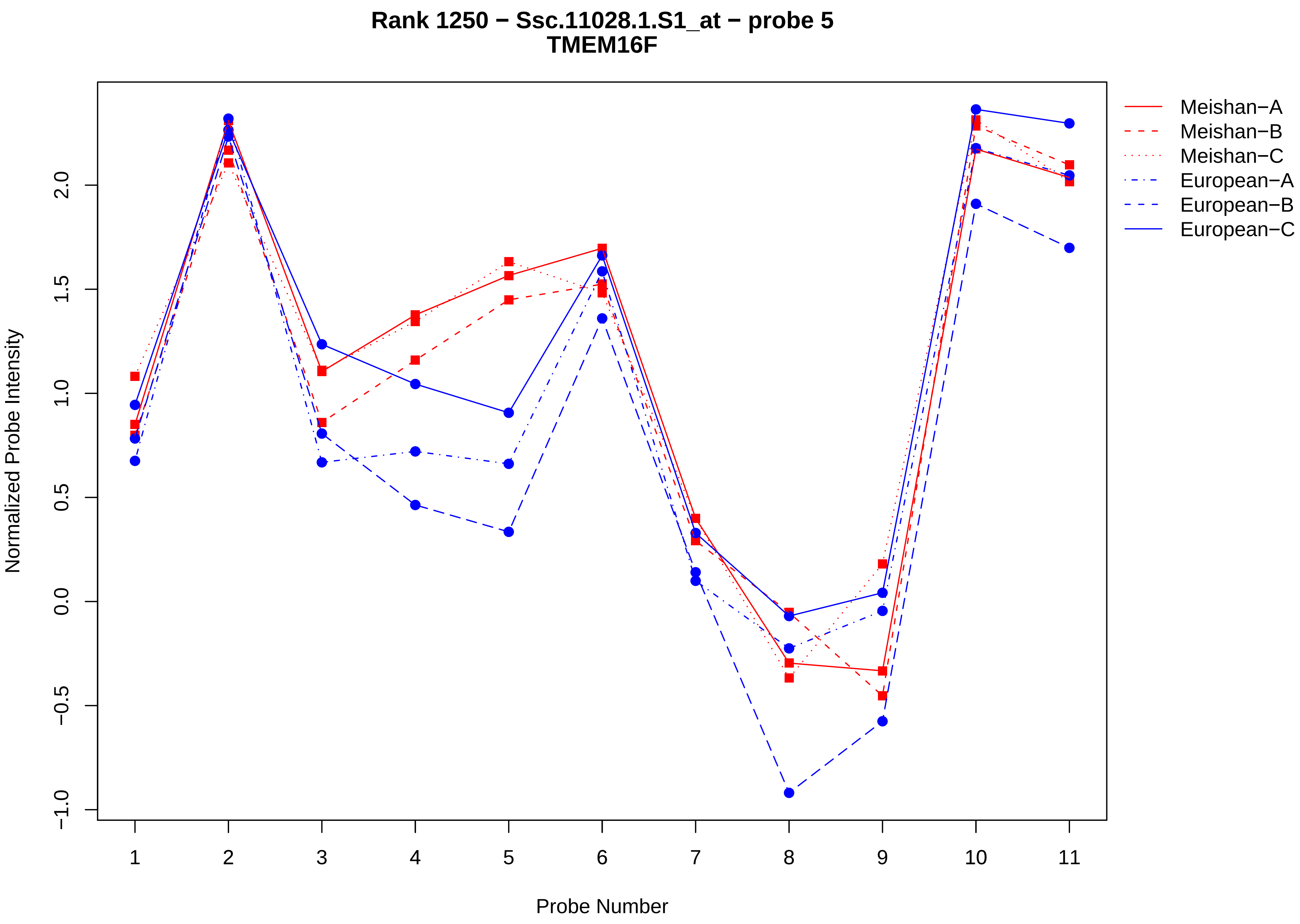

Rank 1250 − Ssc.11028.1.S1_at − probe 5
TMEM16F
Normalized Probe Intensity
Probe Number
Meishan−A
Meishan−B
Meishan−C
European−A
European−B
European−C
−1.0
−0.5
0.0
0.5
1.0
1.5
2.0
1
2
3
4
5
6
7
8
9
10
11

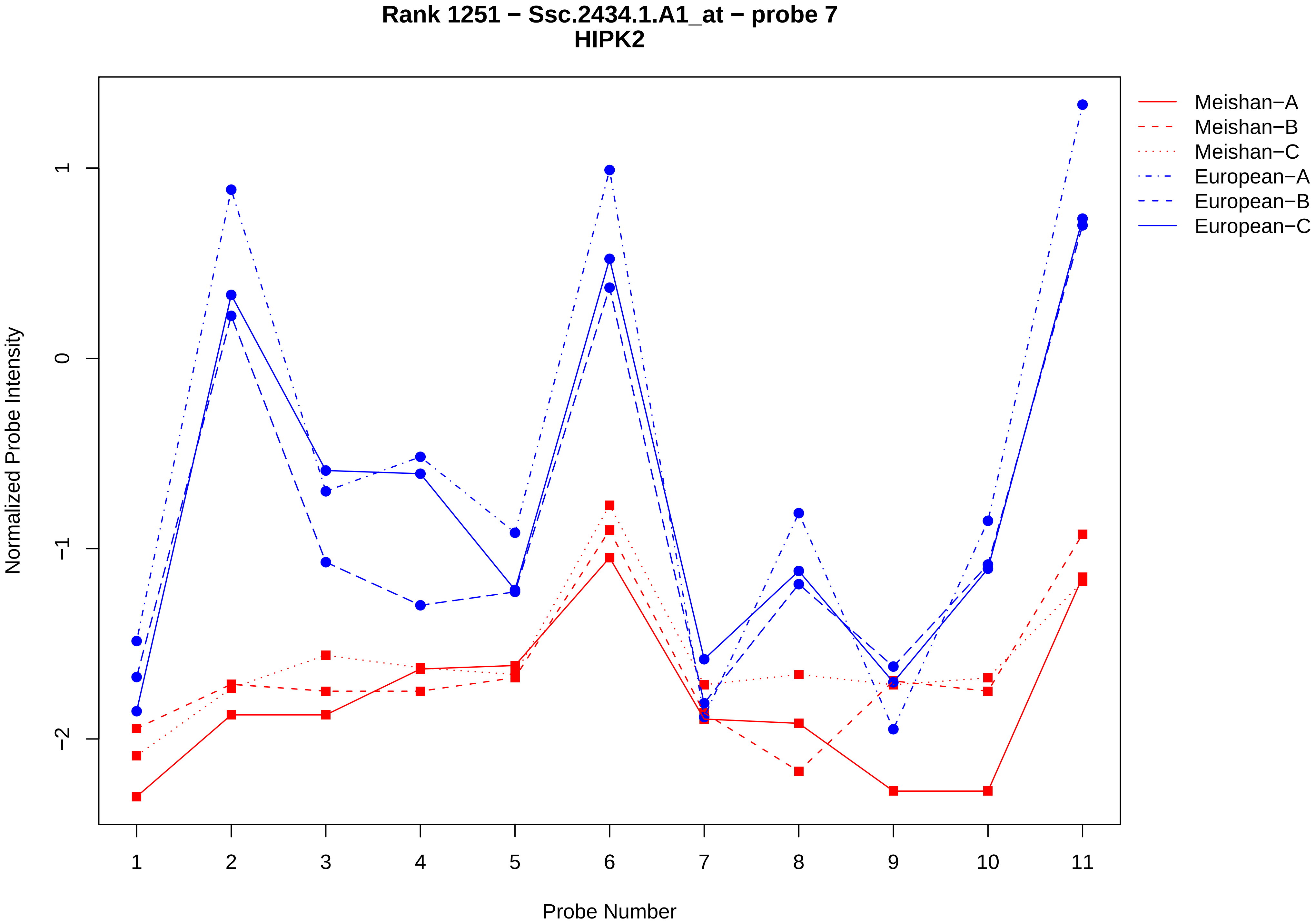

Rank 1251 − Ssc.2434.1.A1_at − probe 7
HIPK2
Normalized Probe Intensity
Probe Number
1
0
−1
−2
1
2
3
4
5
6
7
8
9
10
11
Meishan−A
Meishan−B
Meishan−C
European−A
European−B
European−C

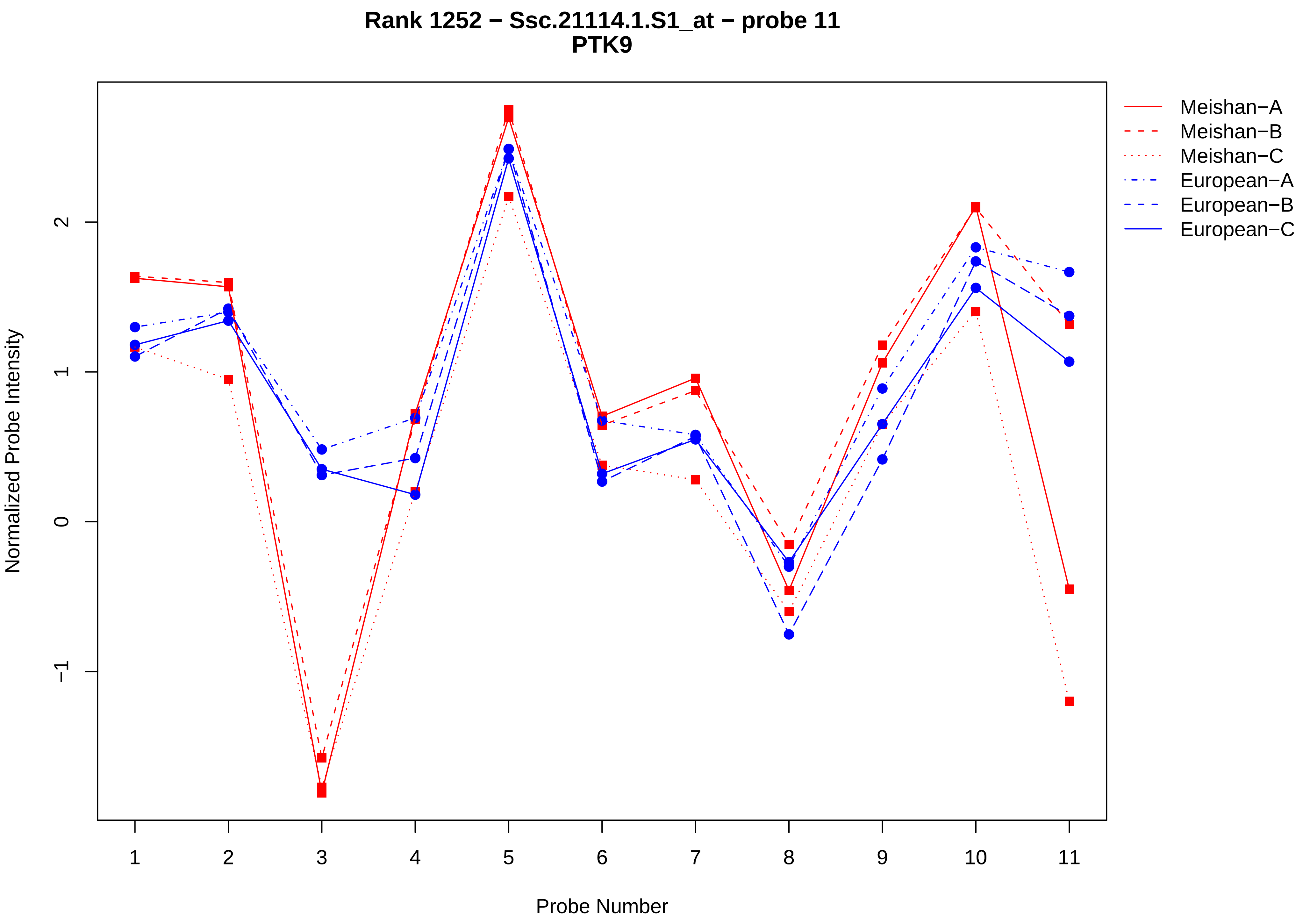

Rank 1252 − Ssc.21114.1.S1_at − probe 11
PTK9
Normalized Probe Intensity
Probe Number
Meishan−A
Meishan−B
Meishan−C
European−A
European−B
European−C
−1
0
1
2
1
2
3
4
5
6
7
8
9
10
11

Rank 1253 − Ssc.6848.1.A1_at − probe 8
FKBP14

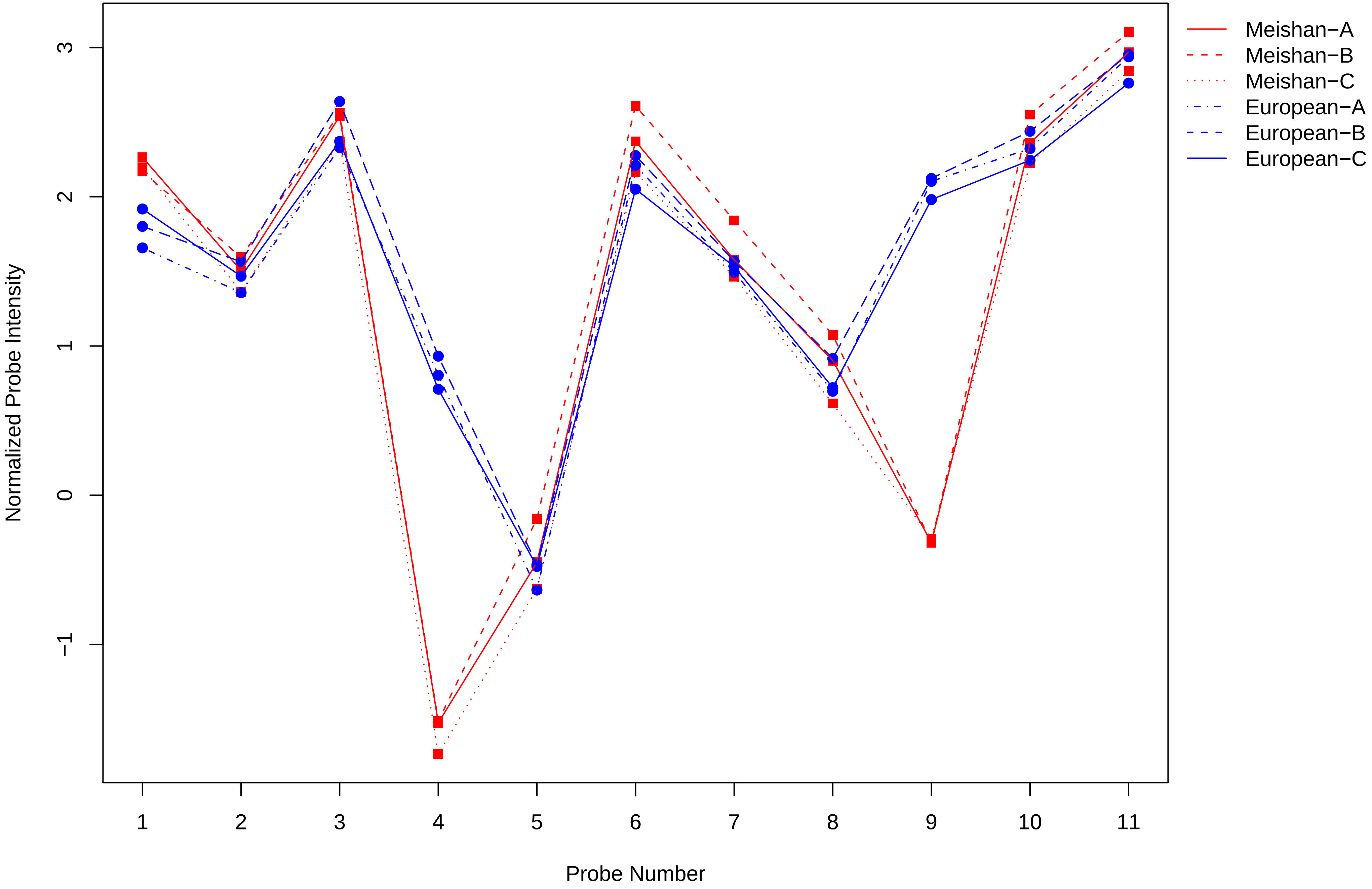

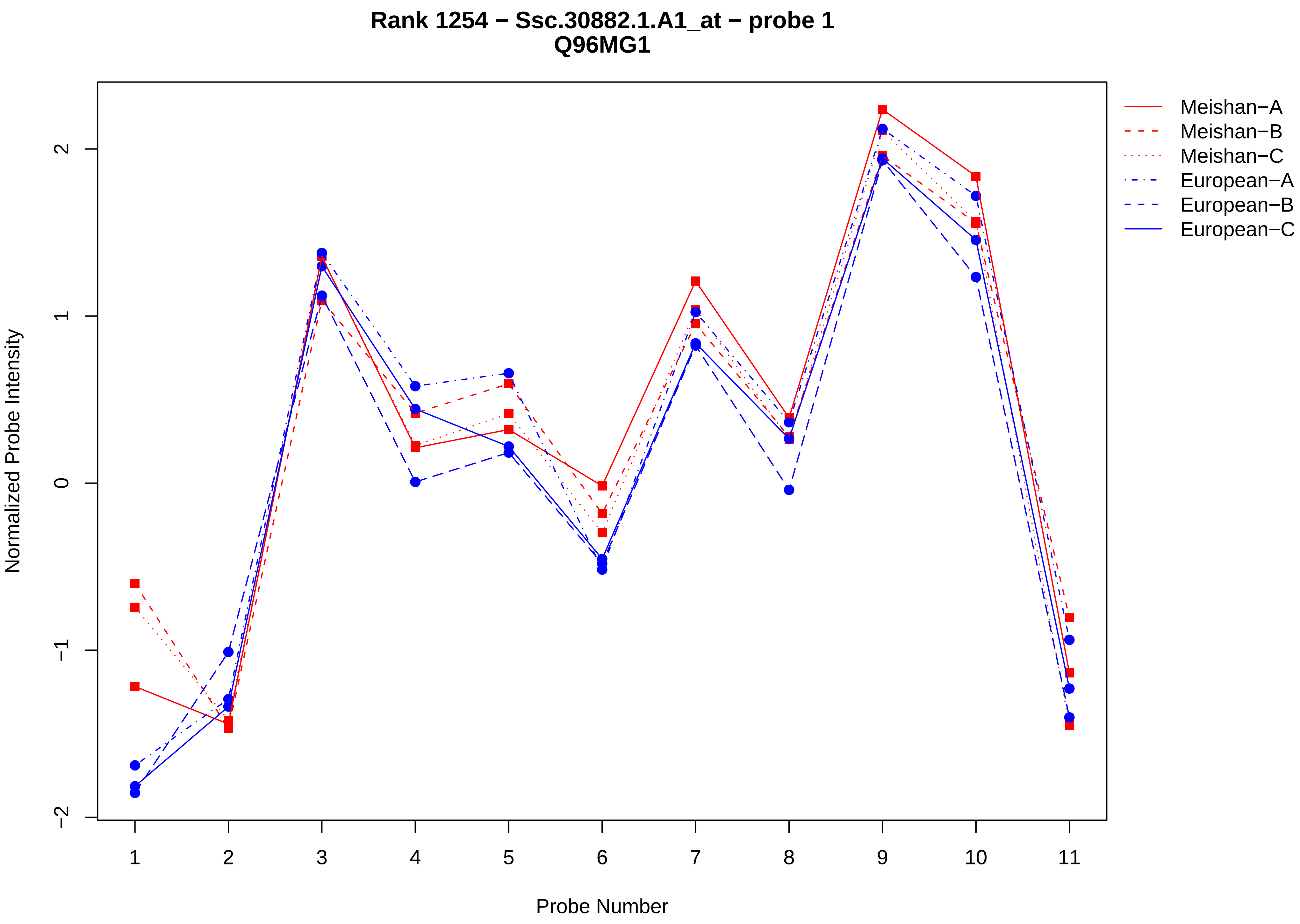

Rank 1254 − Ssc.30882.1.A1_at − probe 1
Q96MG1
Normalized Probe Intensity
Probe Number
Meishan−A
Meishan−B
Meishan−C
European−A
European−B
European−C

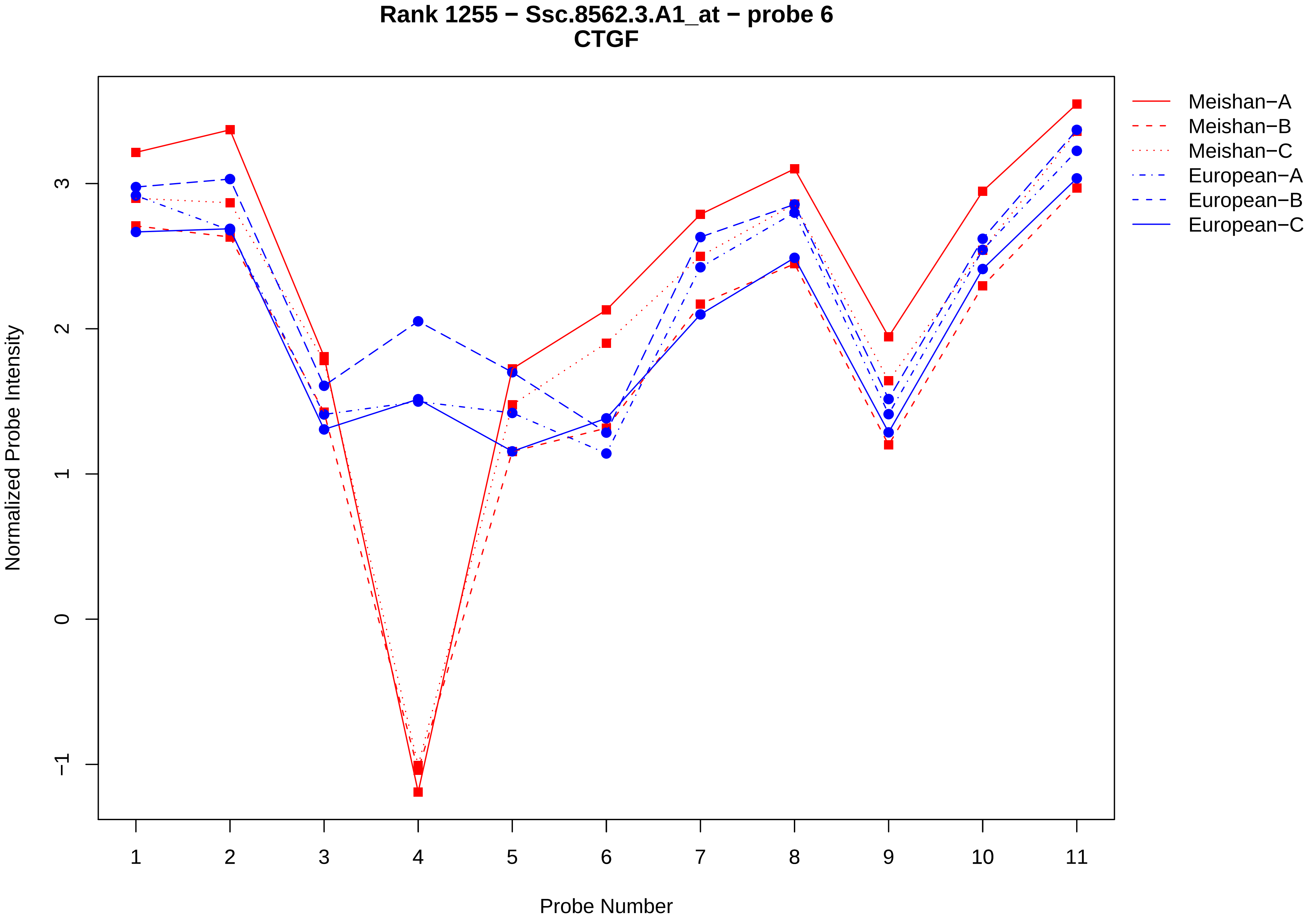

Rank 1255 − Ssc.8562.3.A1_at − probe 6
CTGF
Normalized Probe Intensity
Probe Number
−1
0
1
2
3
1
2
3
4
5
6
7
8
9
10
11
Meishan−A
Meishan−B
Meishan−C
European−A
European−B
European−C

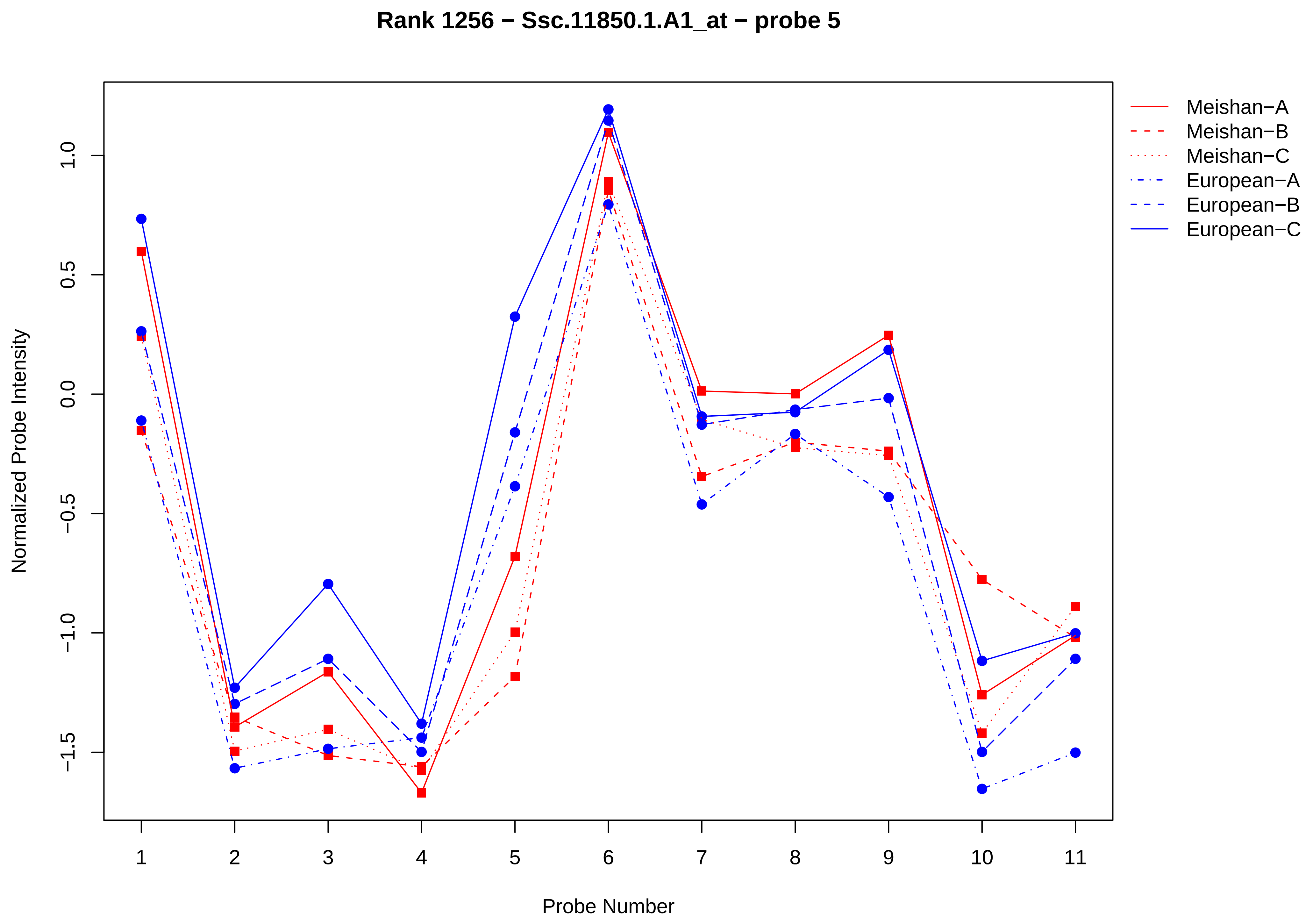

Rank 1256 − Ssc.11850.1.A1_at − probe 5
Normalized Probe Intensity
Probe Number
1.0
0.5
0.0
−0.5
−1.0
−1.5
1
2
3
4
5
6
7
8
9
10
11
Meishan−A
Meishan−B
Meishan−C
European−A
European−B
European−C

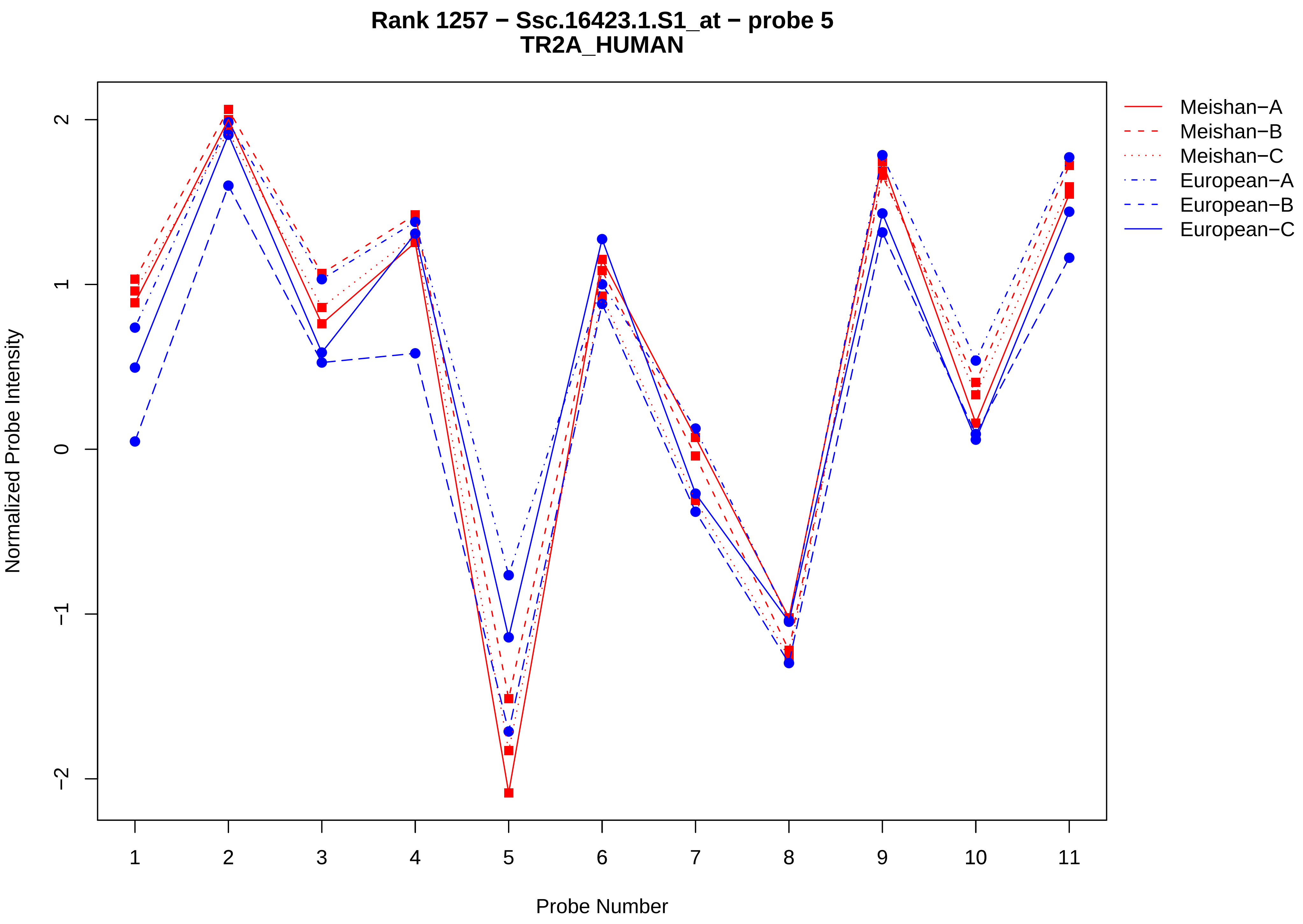

Rank 1257 − Ssc.16423.1.S1_at − probe 5
TR2A_HUMAN
Normalized Probe Intensity
Probe Number
−2
−1
0
1
2
1
2
3
4
5
6
7
8
9
10
11
Meishan−A
Meishan−B
Meishan−C
European−A
European−B
European−C

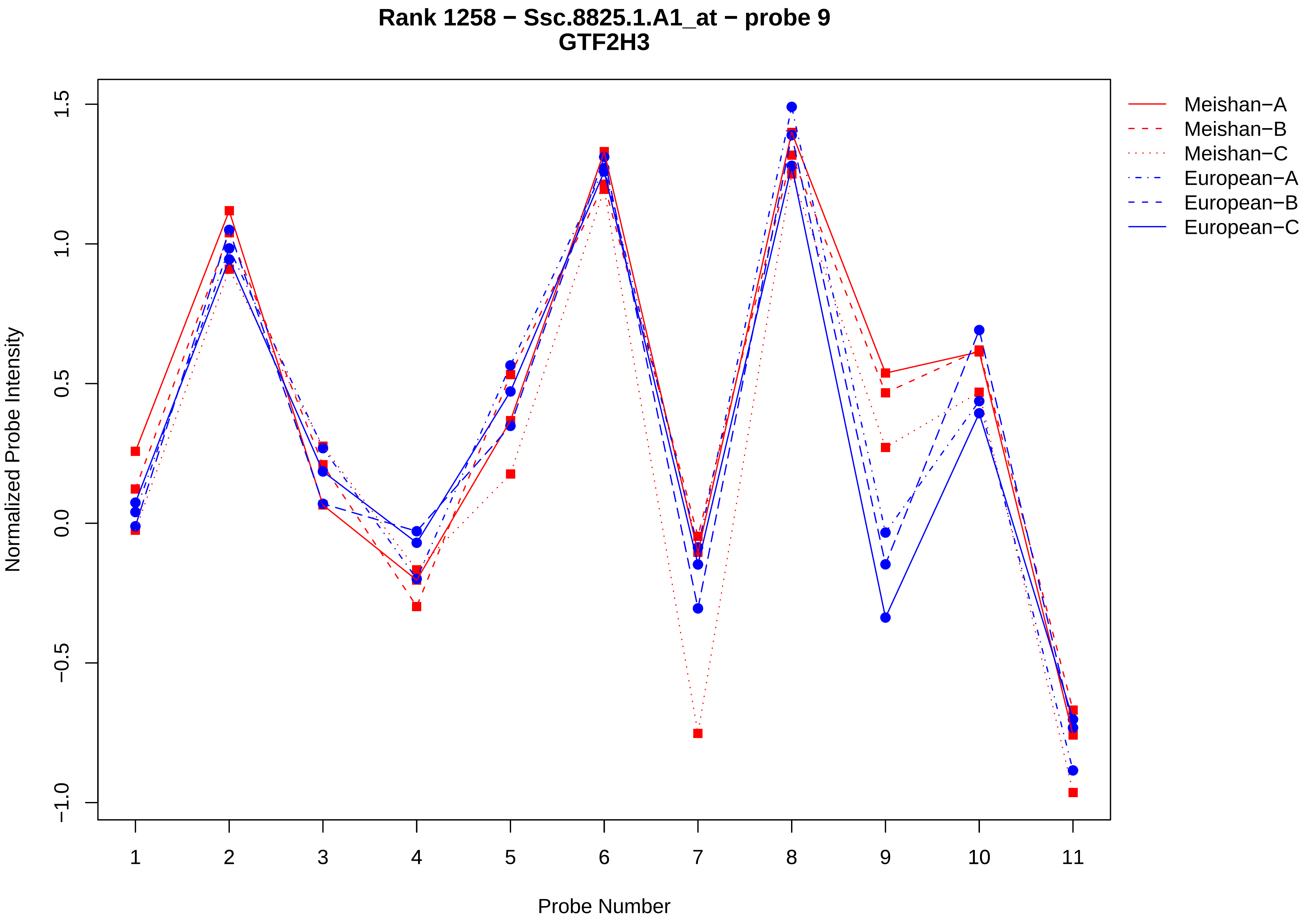

Rank 1258 − Ssc.8825.1.A1_at − probe 9
GTF2H3
Normalized Probe Intensity
Probe Number
Meishan−A
Meishan−B
Meishan−C
European−A
European−B
European−C
1.5
1.0
0.5
0.0
−0.5
−1.0
1
2
3
4
5
6
7
8
9
10
11

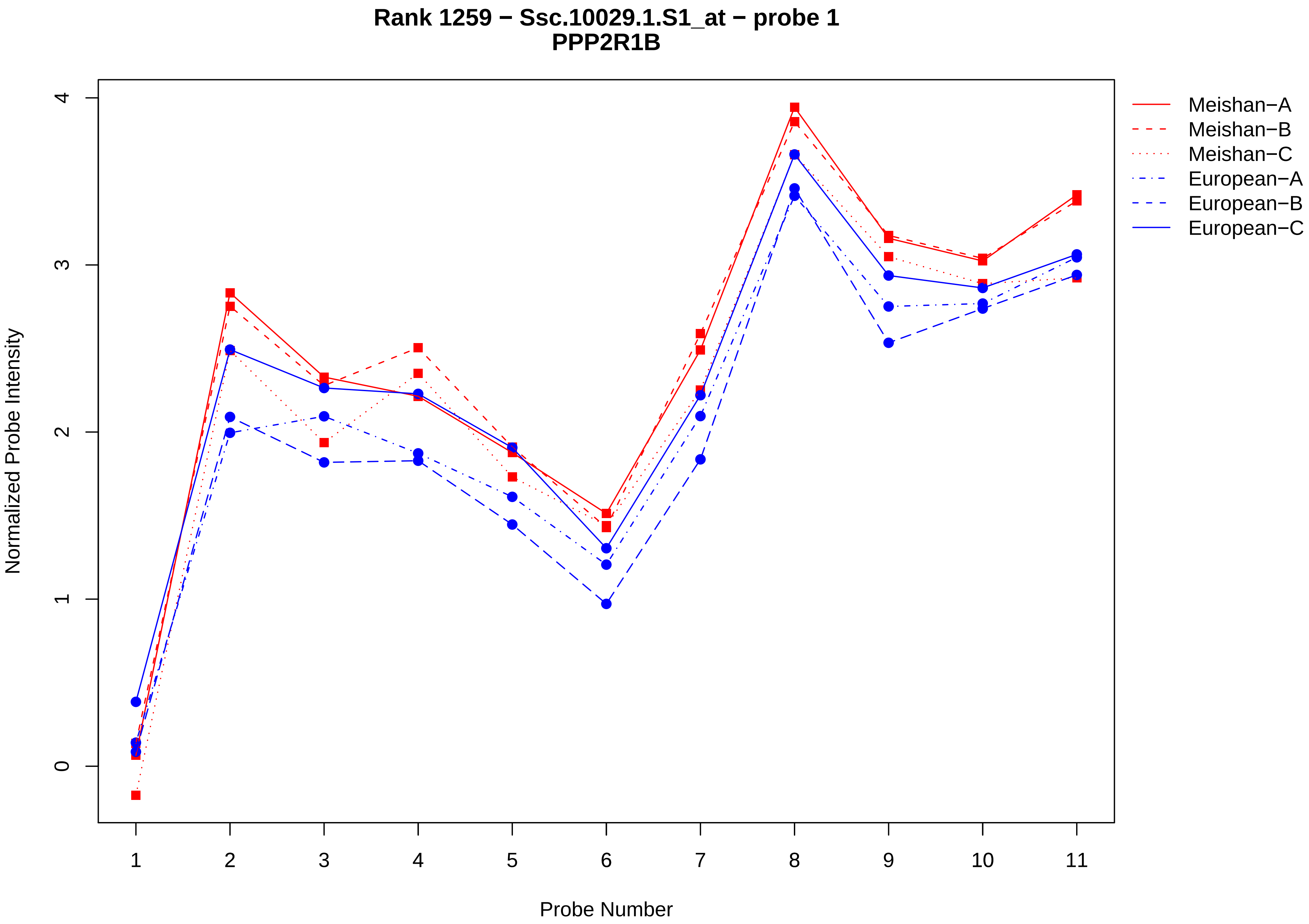

Rank 1259 − Ssc.10029.1.S1_at − probe 1
PPP2R1B
Normalized Probe Intensity
0
1
2
3
4
1
2
3
4
5
6
7
8
9
10
11
Probe Number
Meishan−A
Meishan−B
Meishan−C
European−A
European−B
European−C

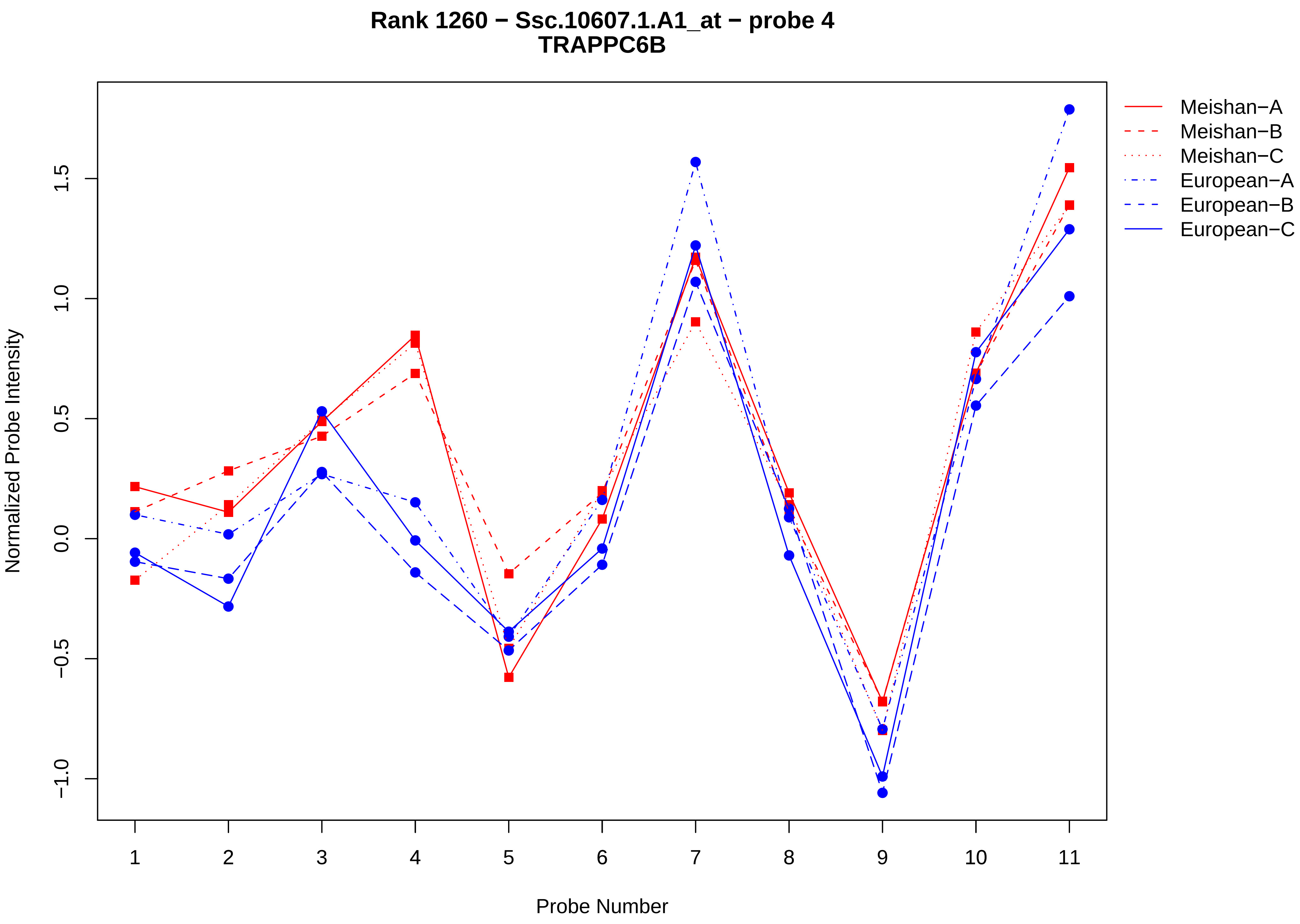
Rank 1260 − Ssc.10607.1.A1_at − probe 4
TRAPPC6B
Normalized Probe Intensity
Probe Number
Meishan−A
Meishan−B
Meishan−C
European−A
European−B
European−C
−1.0
−0.5
0.0
0.5
1.0
1.5
1
2
3
4
5
6
7
8
9
10
11

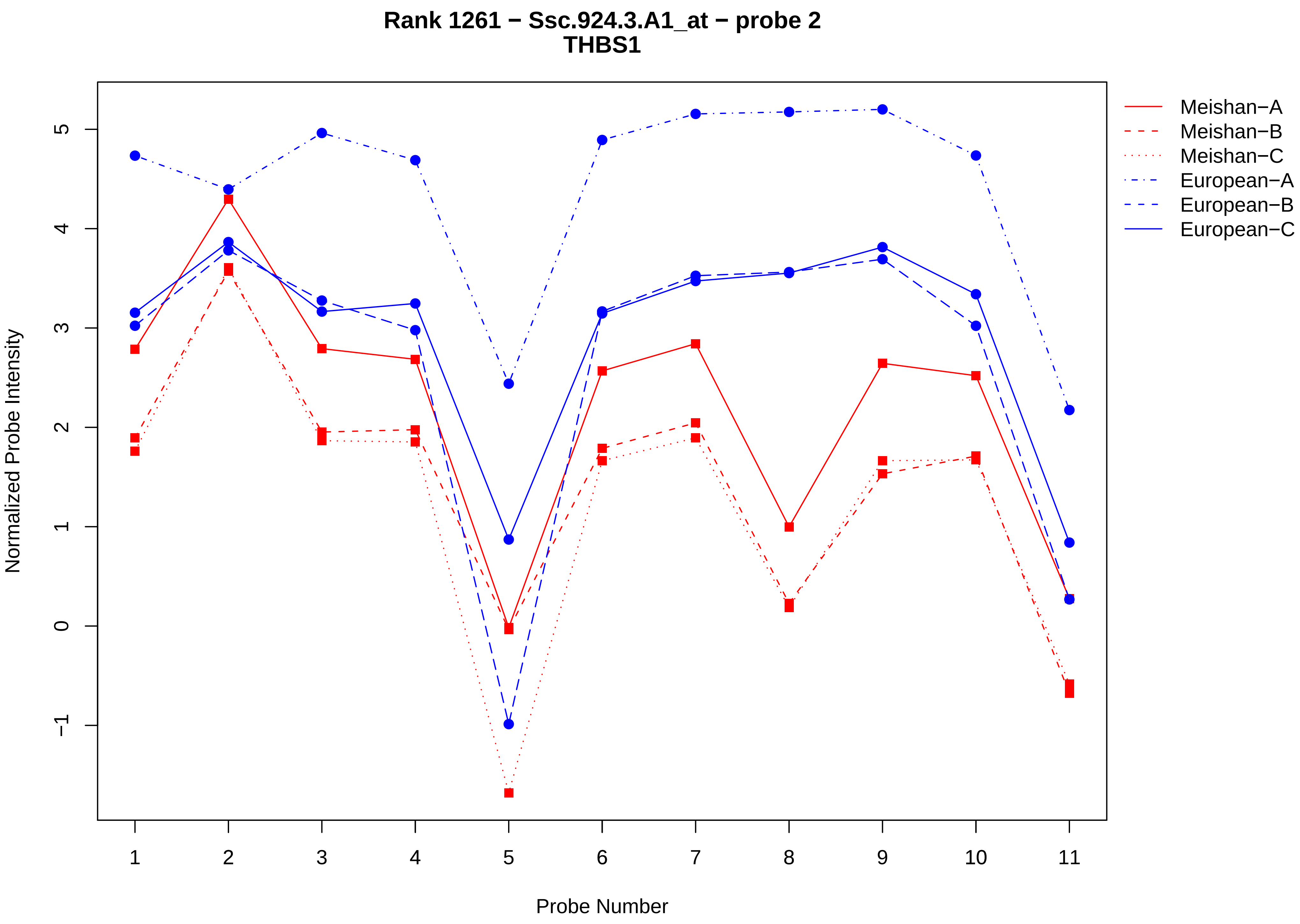

Rank 1261 − Ssc.924.3.A1_at − probe 2
THBS1
Normalized Probe Intensity
Probe Number
Meishan−A
Meishan−B
Meishan−C
European−A
European−B
European−C

Rank 1262 − Ssc.1010.1.A1_at − probe 3
Q9NUY8

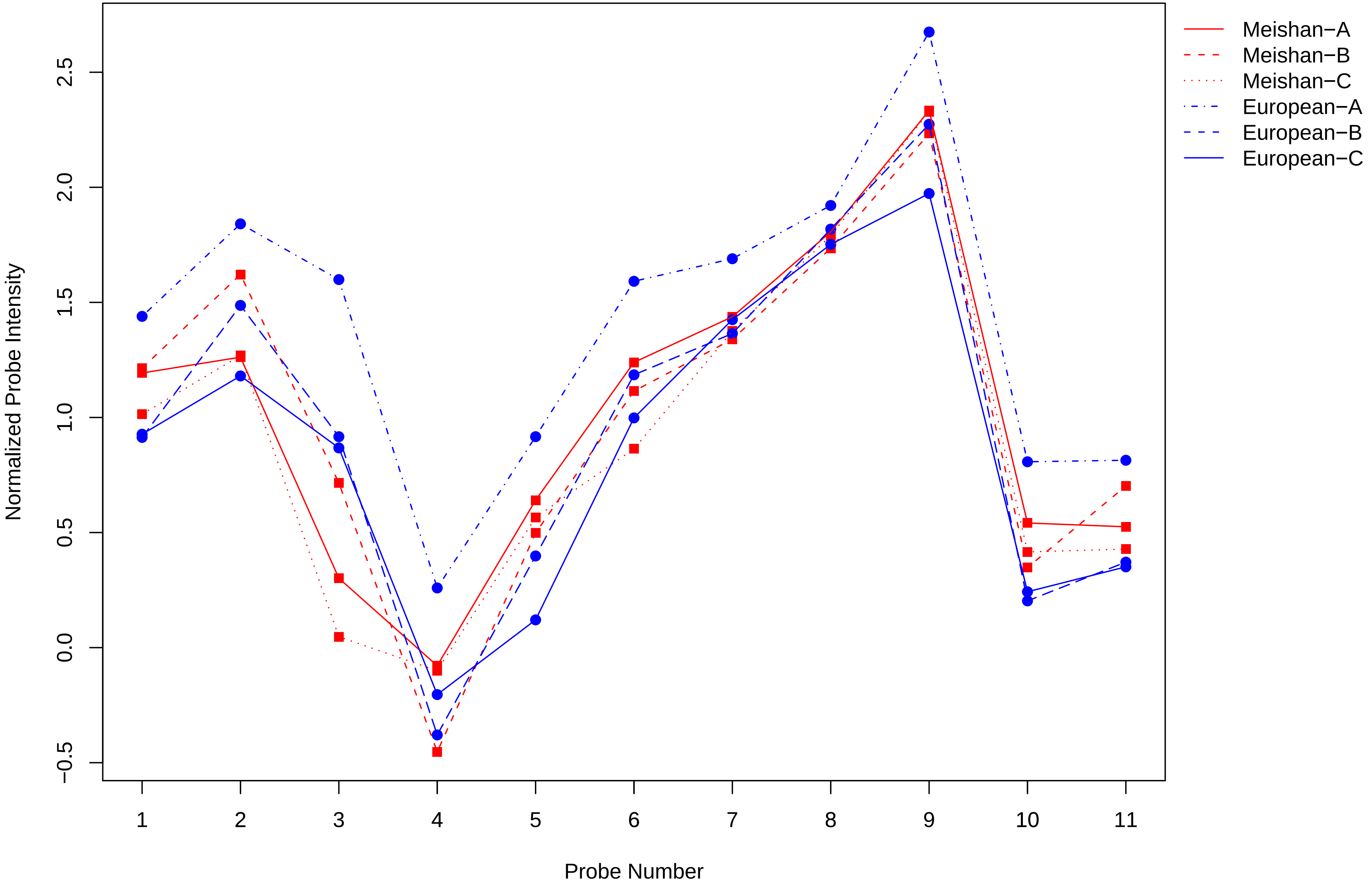

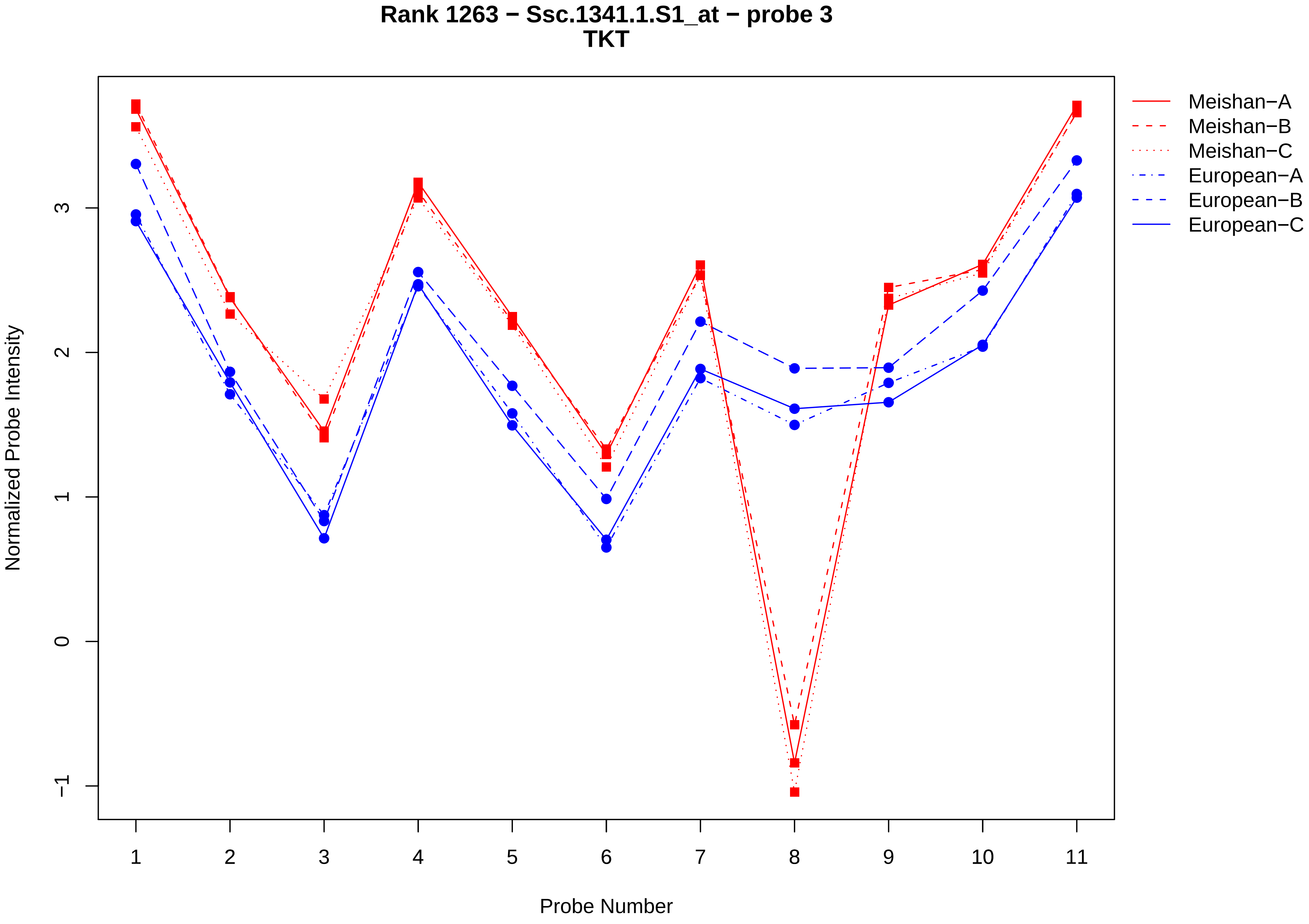
Rank 1263 − Ssc.1341.1.S1_at − probe 3
TKT
Normalized Probe Intensity
Probe Number
−1
0
1
2
3
1
2
3
4
5
6
7
8
9
10
11
Meishan−A
Meishan−B
Meishan−C
European−A
European−B
European−C

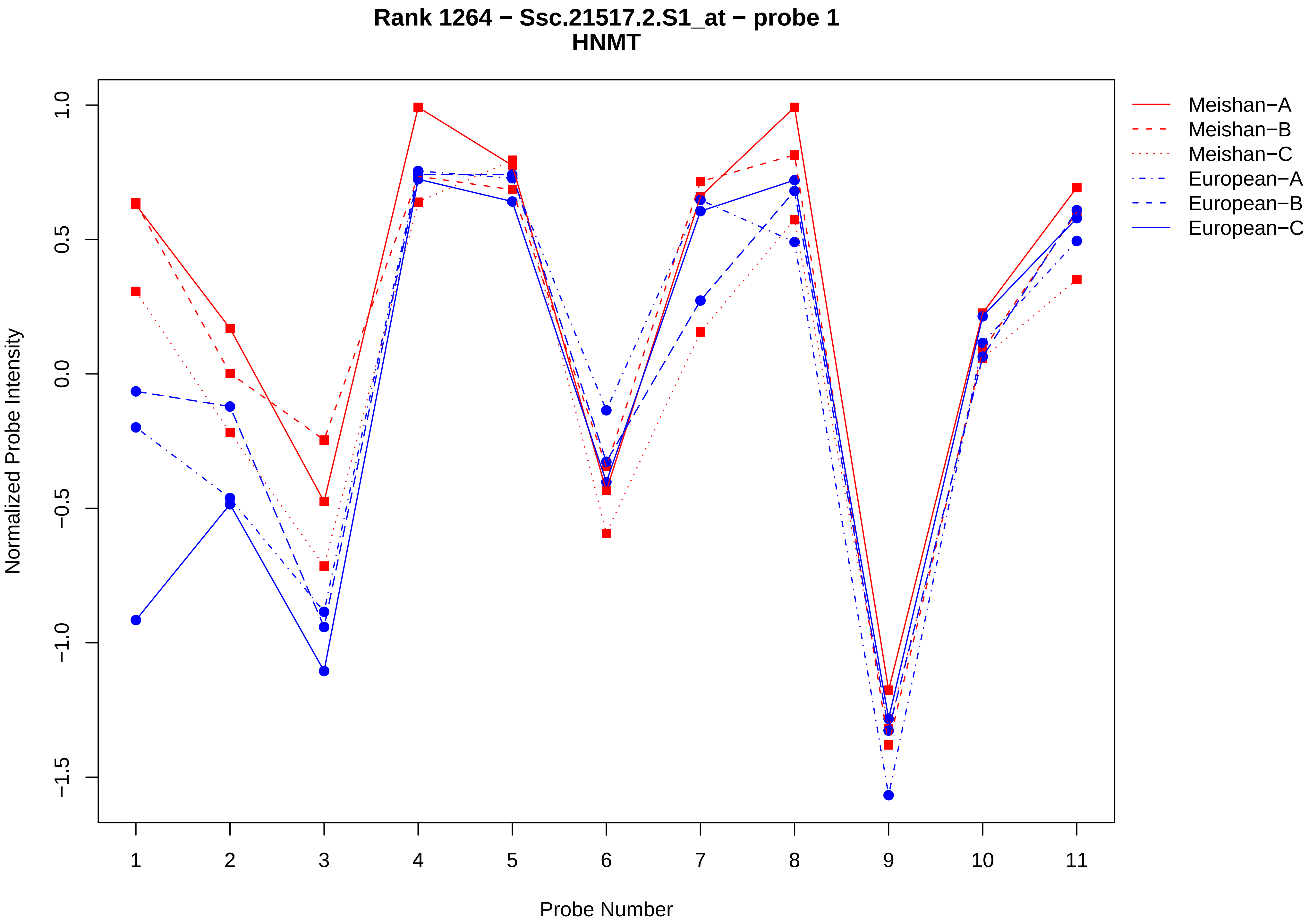

Rank 1264 − Ssc.21517.2.S1_at − probe 1
HNMT
Normalized Probe Intensity
Probe Number
1.0
0.5
0.0
−0.5
−1.0
−1.5
1
2
3
4
5
6
7
8
9
10
11
Meishan−A
Meishan−B
Meishan−C
European−A
European−B
European−C

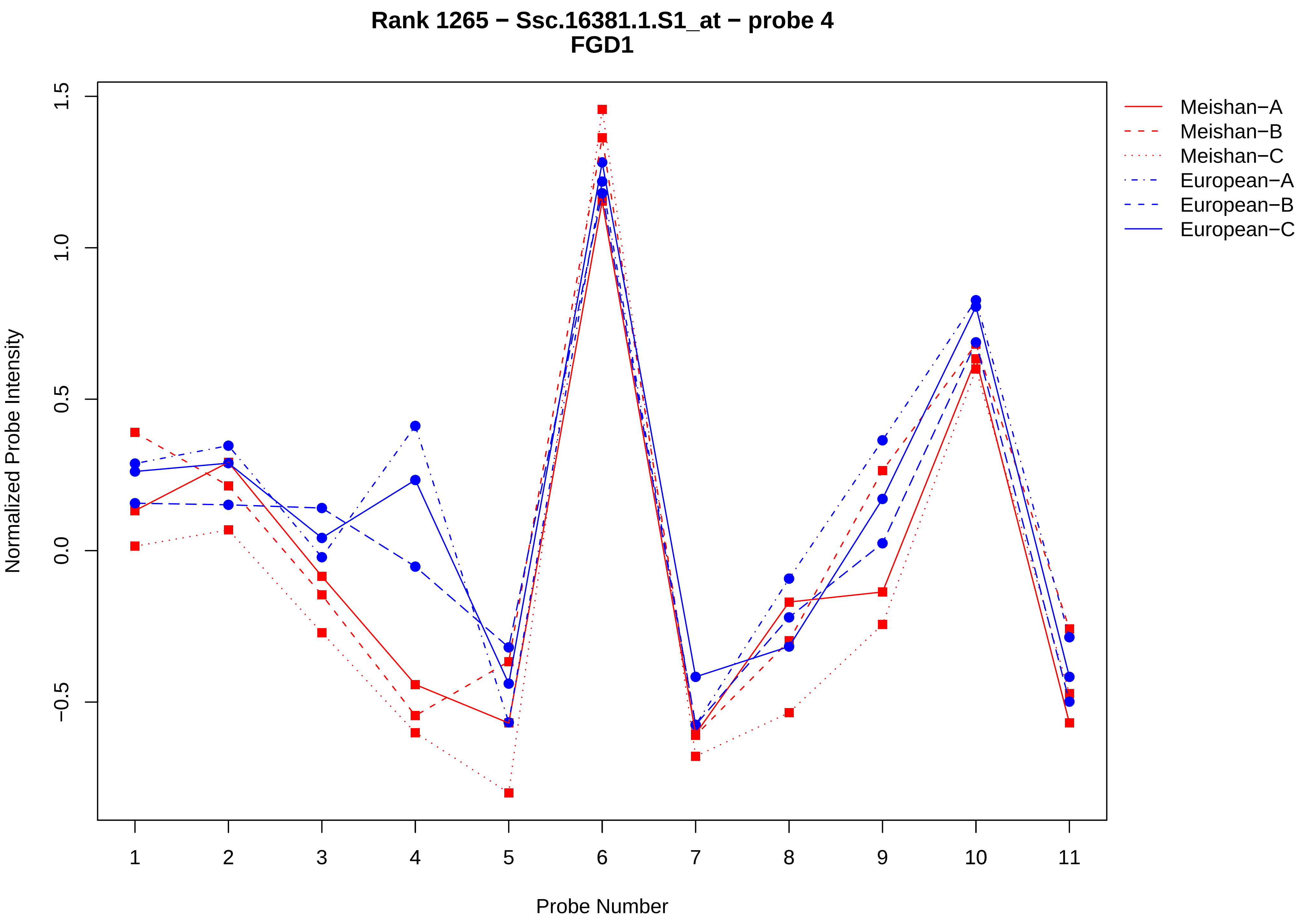

Rank 1265 − Ssc.16381.1.S1_at − probe 4
FGD1
Normalized Probe Intensity
Probe Number
1.5
1.0
0.5
0.0
−0.5
1
2
3
4
5
6
7
8
9
10
11
Meishan−A
Meishan−B
Meishan−C
European−A
European−B
European−C

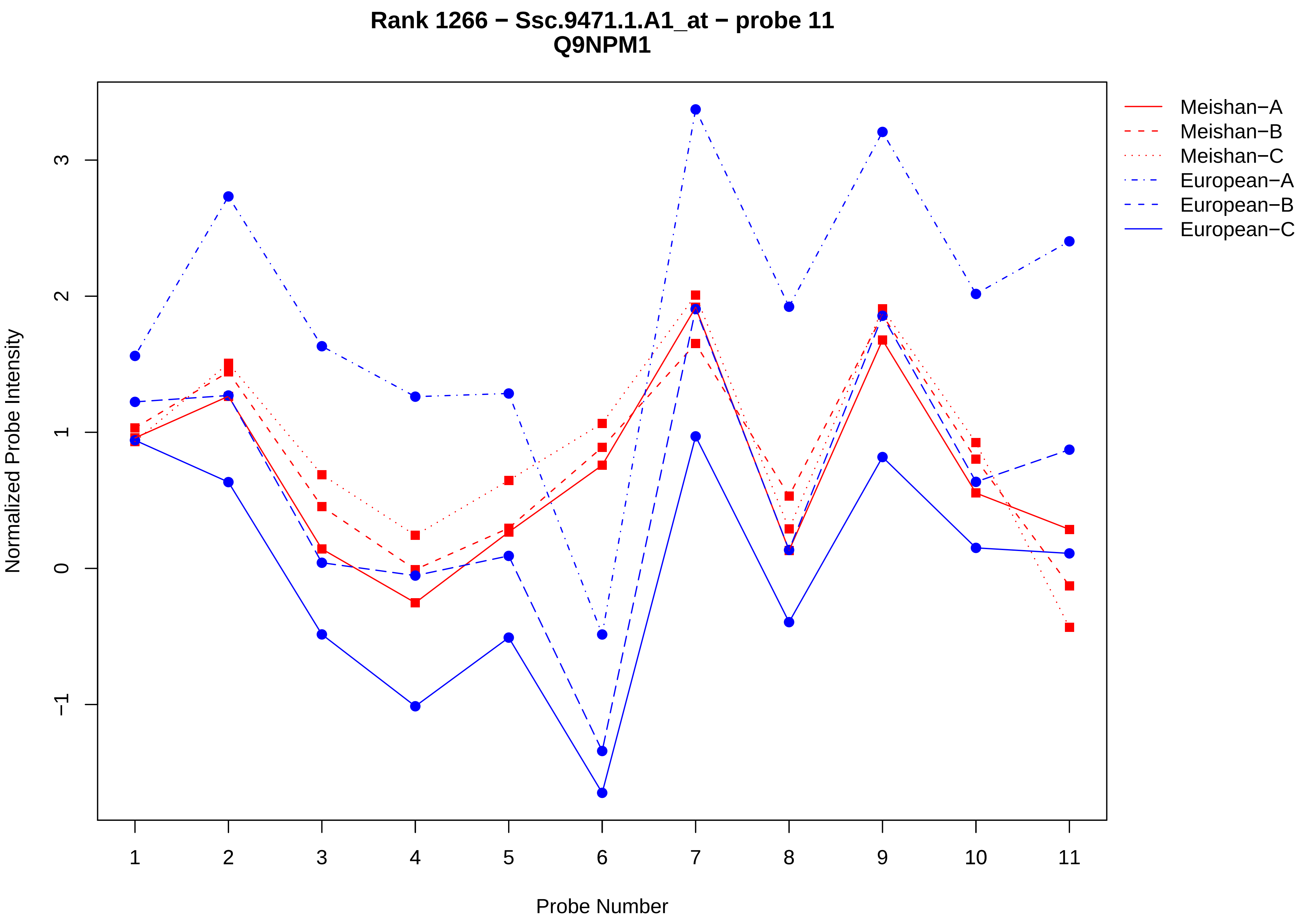

Rank 1266 − Ssc.9471.1.A1_at − probe 11
Q9NPM1
Normalized Probe Intensity
Probe Number
Meishan−A
Meishan−B
Meishan−C
European−A
European−B
European−C
−1
0
1
2
3
1
2
3
4
5
6
7
8
9
10
11

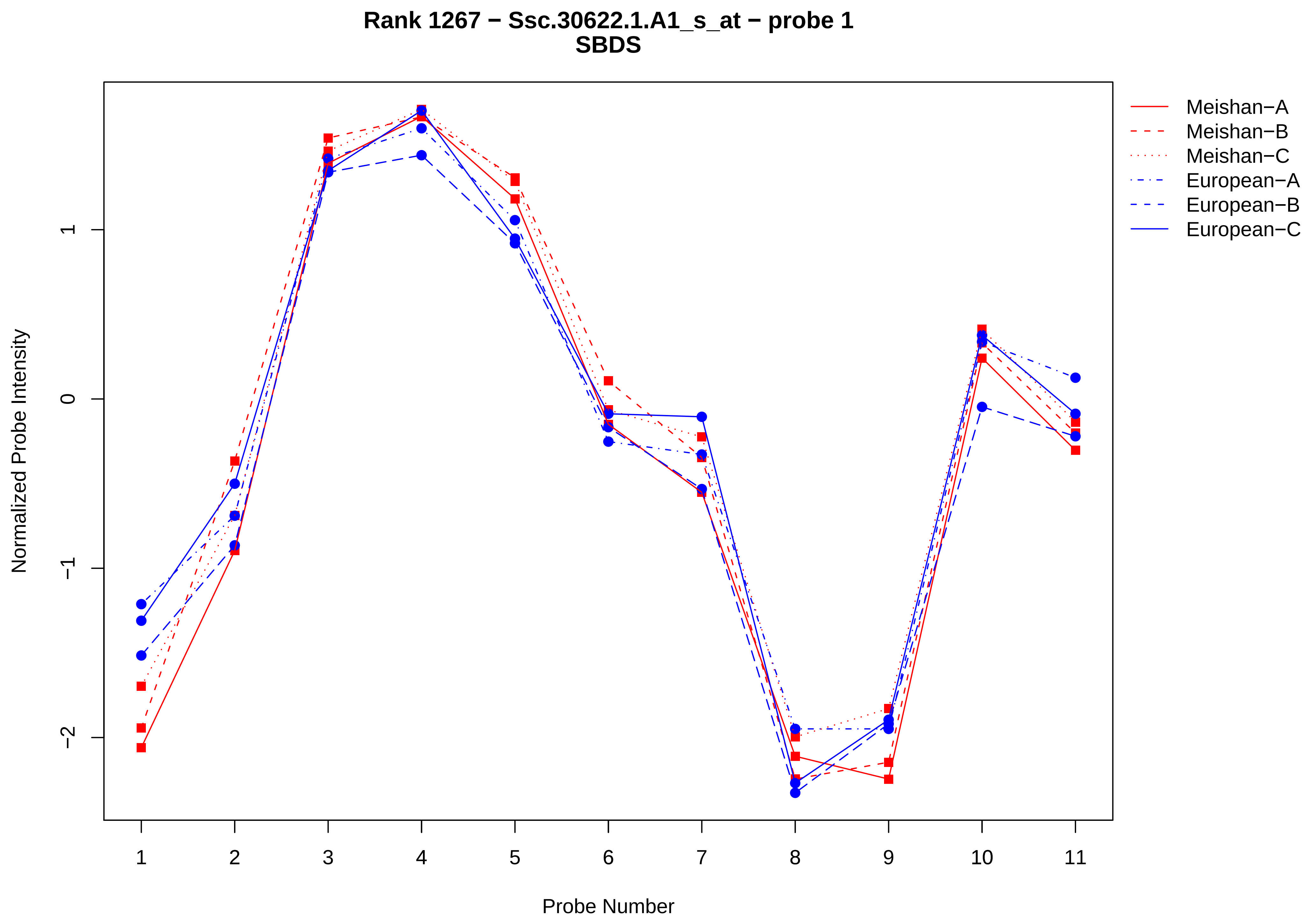
Rank 1267 − Ssc.30622.1.A1_s_at − probe 1
SBDS
Normalized Probe Intensity
Probe Number
Meishan−A
Meishan−B
Meishan−C
European−A
European−B
European−C
−2
−1
0
1
1
2
3
4
5
6
7
8
9
10
11

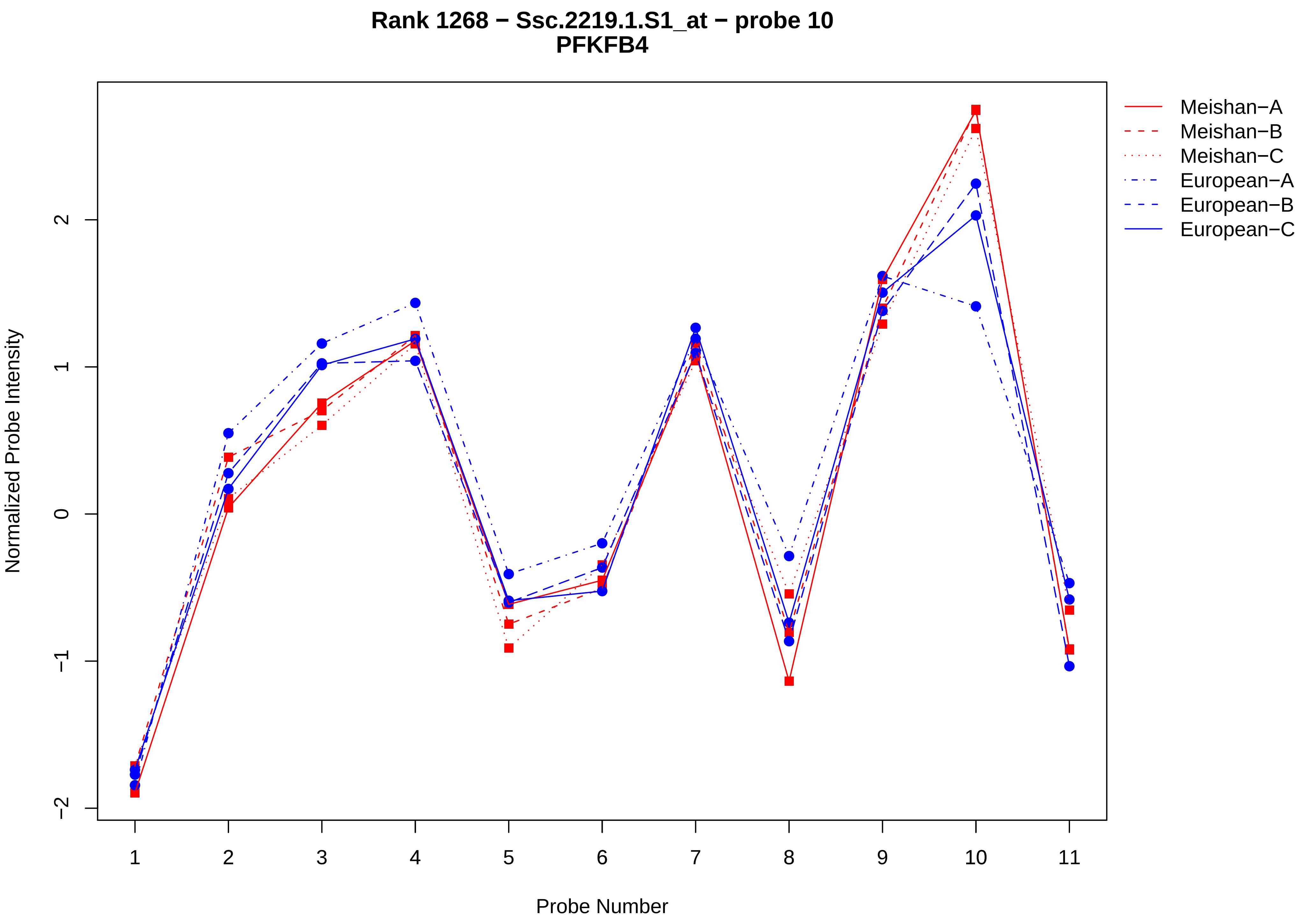

Rank 1268 − Ssc.2219.1.S1_at − probe 10
PFKFB4
Normalized Probe Intensity
Probe Number
−2
−1
0
1
2
1
2
3
4
5
6
7
8
9
10
11
Meishan−A
Meishan−B
Meishan−C
European−A
European−B
European−C

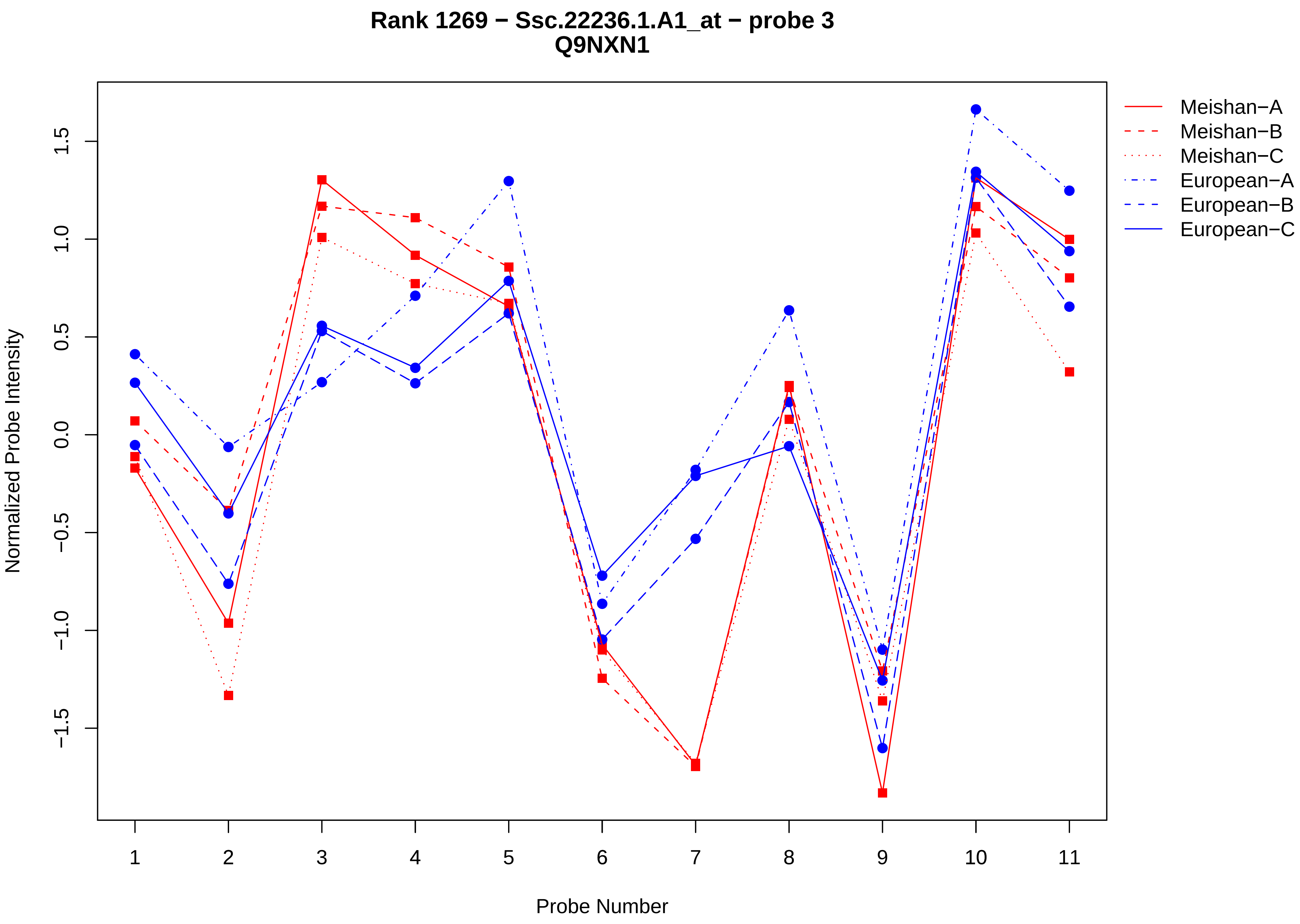
Rank 1269 − Ssc.22236.1.A1_at − probe 3
Q9NXN1
Normalized Probe Intensity
Probe Number
−1.5
−1.0
−0.5
0.0
0.5
1.0
1.5
1
2
3
4
5
6
7
8
9
10
11
Meishan−A
Meishan−B
Meishan−C
European−A
European−B
European−C

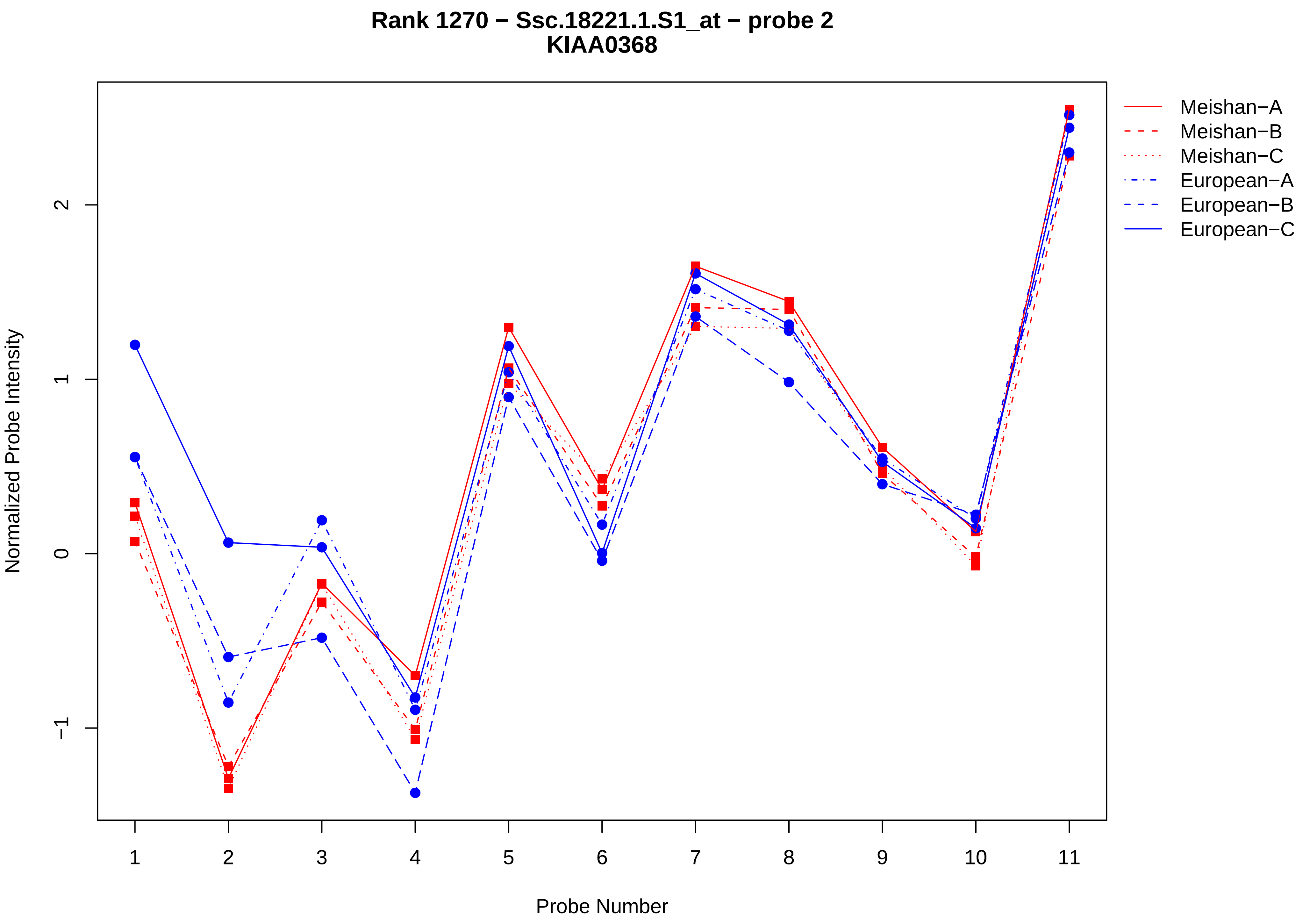

Rank 1270 − Ssc.18221.1.S1_at − probe 2
KIAA0368
Normalized Probe Intensity
Probe Number
−1
0
1
2
1
2
3
4
5
6
7
8
9
10
11
Meishan−A
Meishan−B
Meishan−C
European−A
European−B
European−C

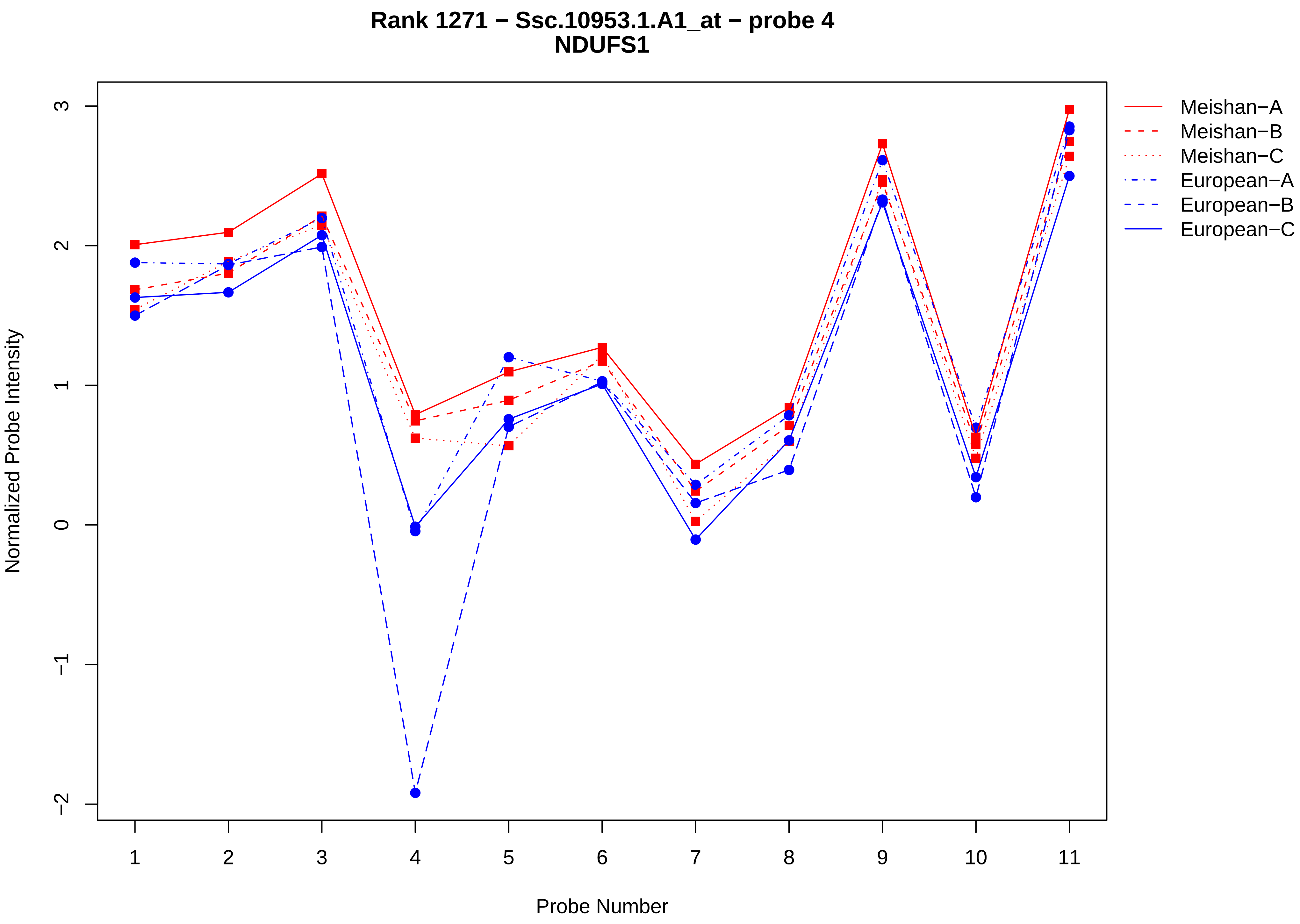

Rank 1271 − Ssc.10953.1.A1_at − probe 4
NDUFS1
Normalized Probe Intensity
Probe Number
−2
−1
0
1
2
3
1
2
3
4
5
6
7
8
9
10
11
Meishan−A
Meishan−B
Meishan−C
European−A
European−B
European−C

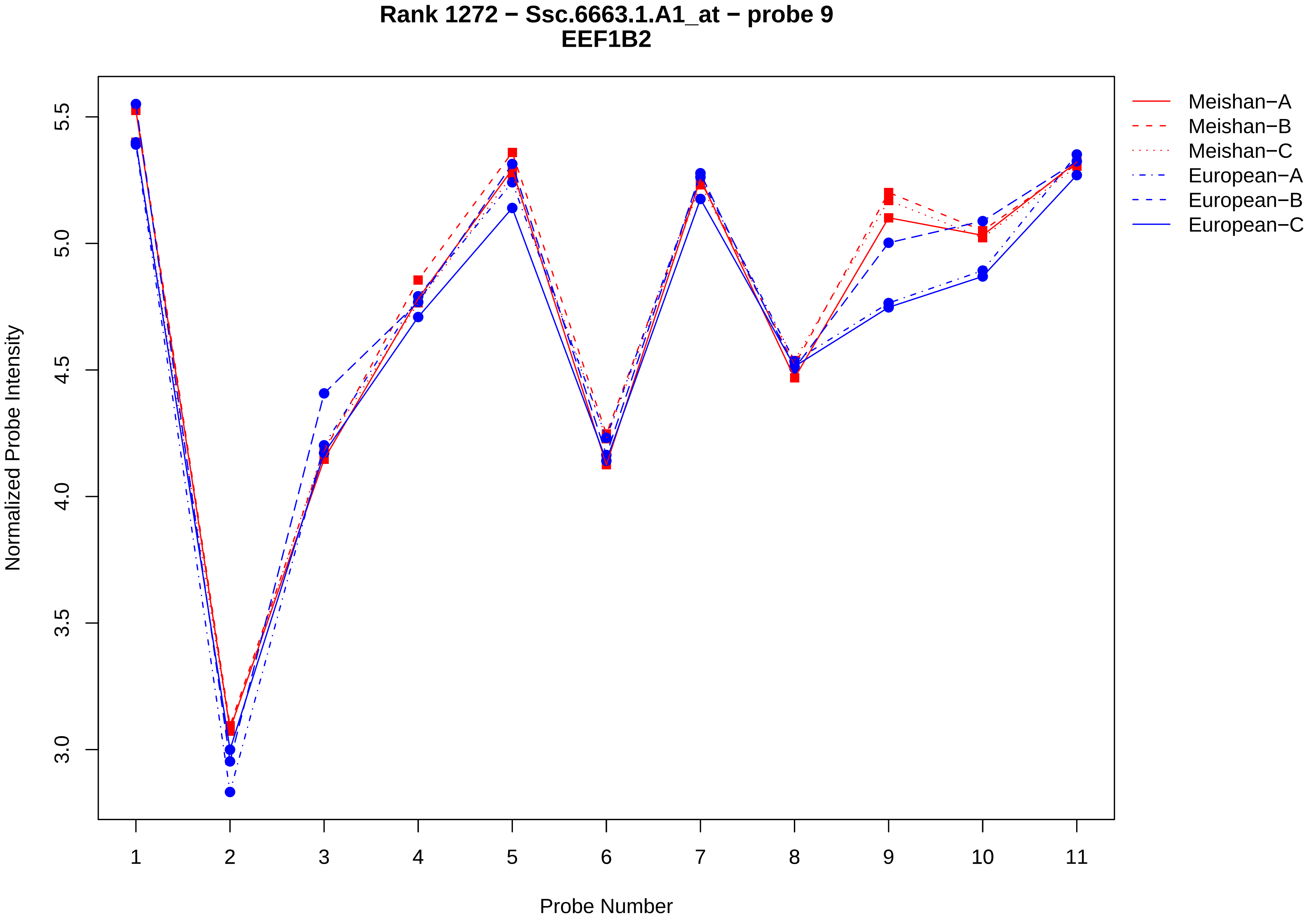

Rank 1272 − Ssc.6663.1.A1_at − probe 9
EEF1B2
Normalized Probe Intensity
Probe Number
Meishan−A
Meishan−B
Meishan−C
European−A
European−B
European−C
5.5
5.0
4.5
4.0
3.5
3.0
1
2
3
4
5
6
7
8
9
10
11

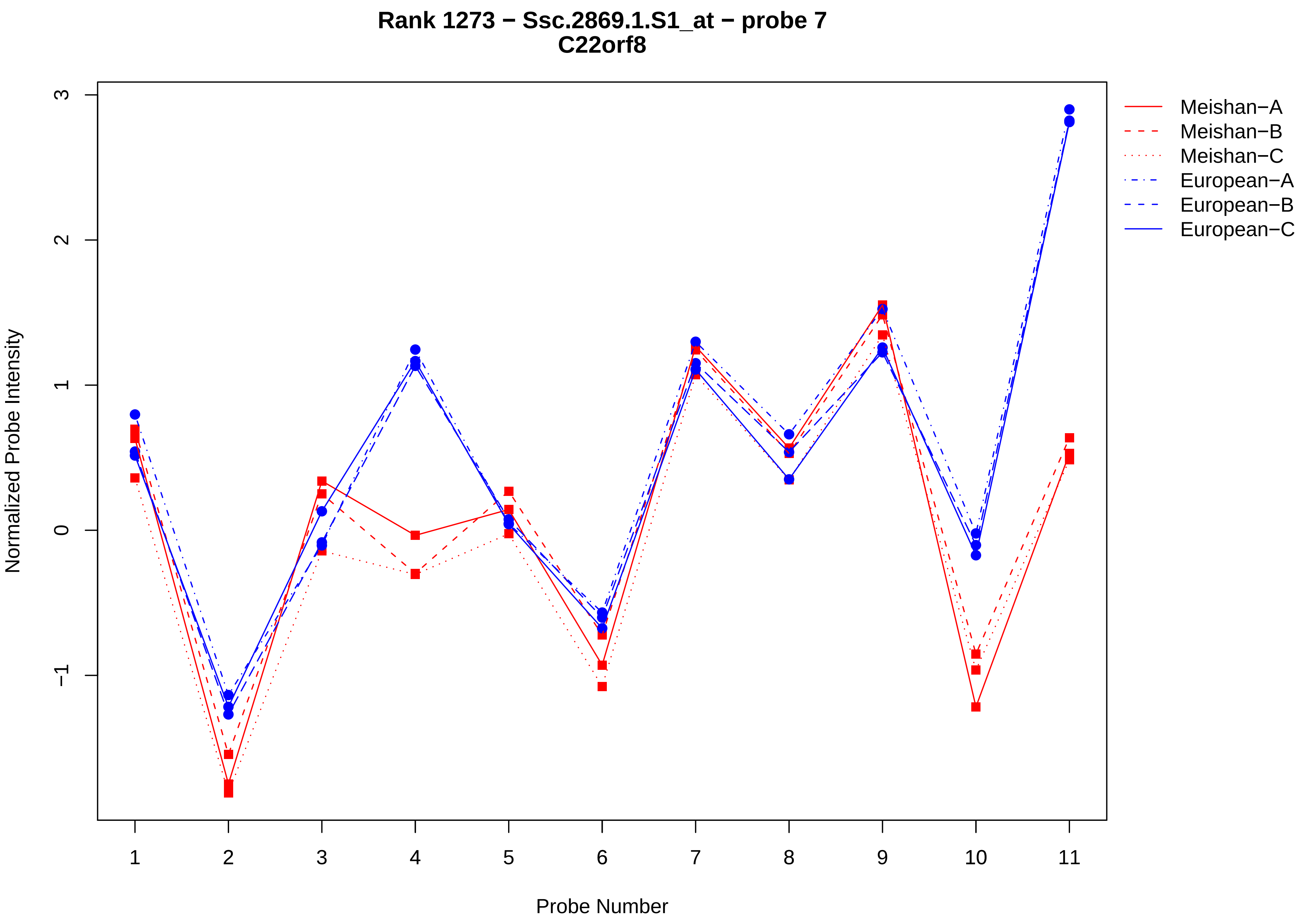

Rank 1273 − Ssc.2869.1.S1_at − probe 7
C22orf8
Normalized Probe Intensity
Probe Number
Meishan−A
Meishan−B
Meishan−C
European−A
European−B
European−C
3
2
1
0
−1
1
2
3
4
5
6
7
8
9
10
11

Rank 1274 − Ssc.18487.1.S1_a_at − probe 10
FBXO31

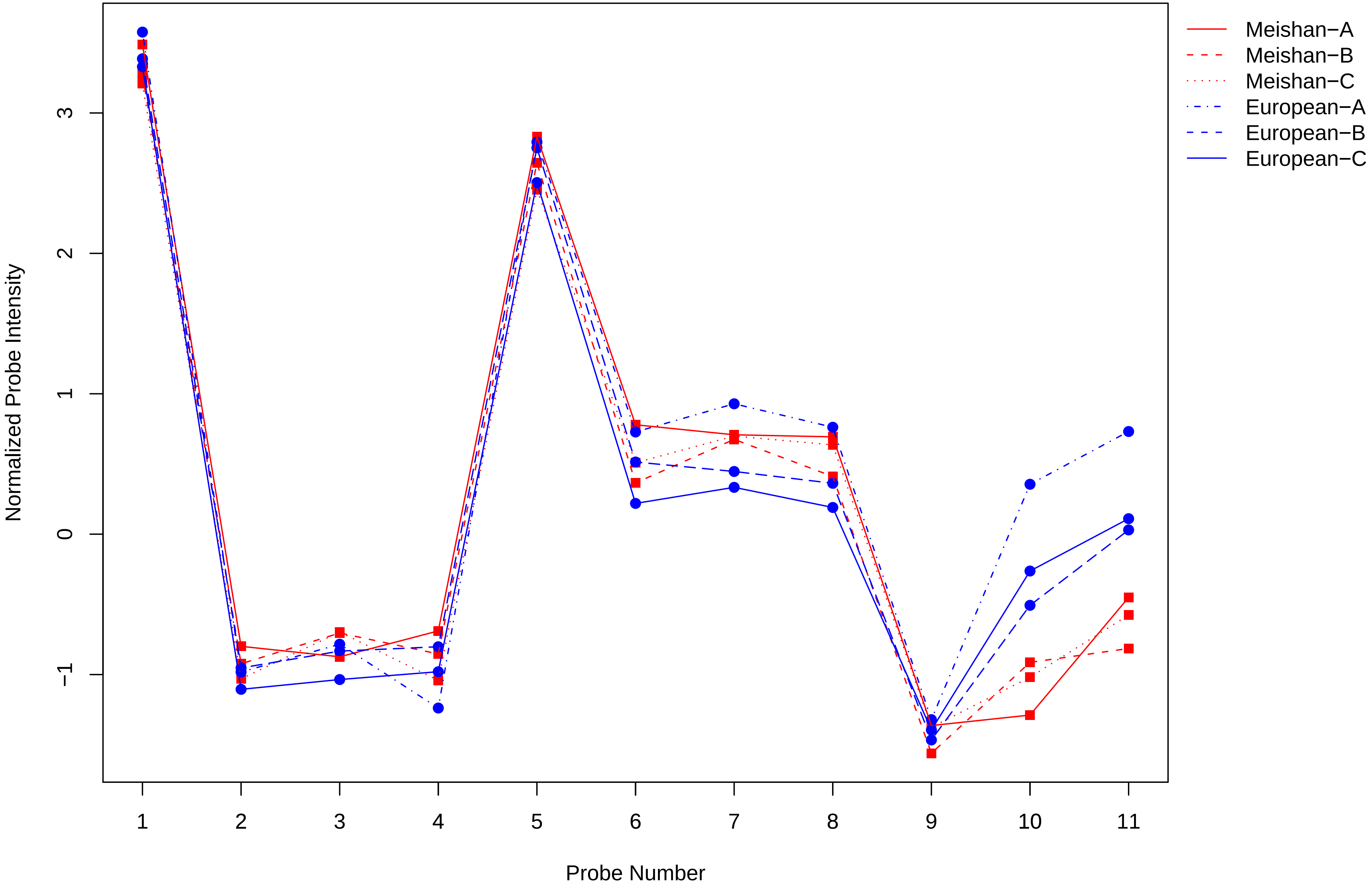

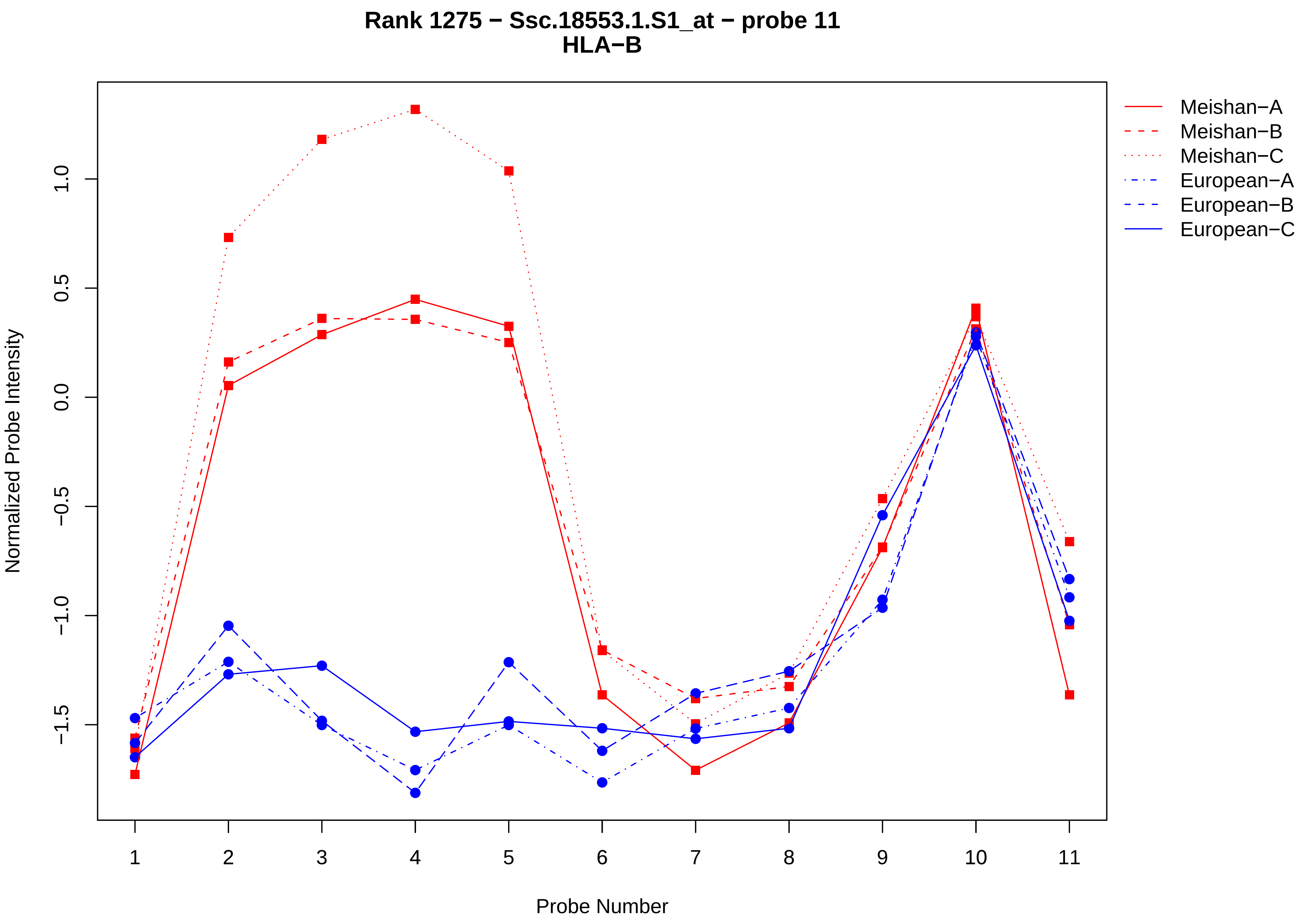

Rank 1275 − Ssc.18553.1.S1_at − probe 11
HLA−B
Normalized Probe Intensity
Probe Number
−1.5
−1.0
−0.5
0.0
0.5
1.0
1
2
3
4
5
6
7
8
9
10
11
Meishan−A
Meishan−B
Meishan−C
European−A
European−B
European−C

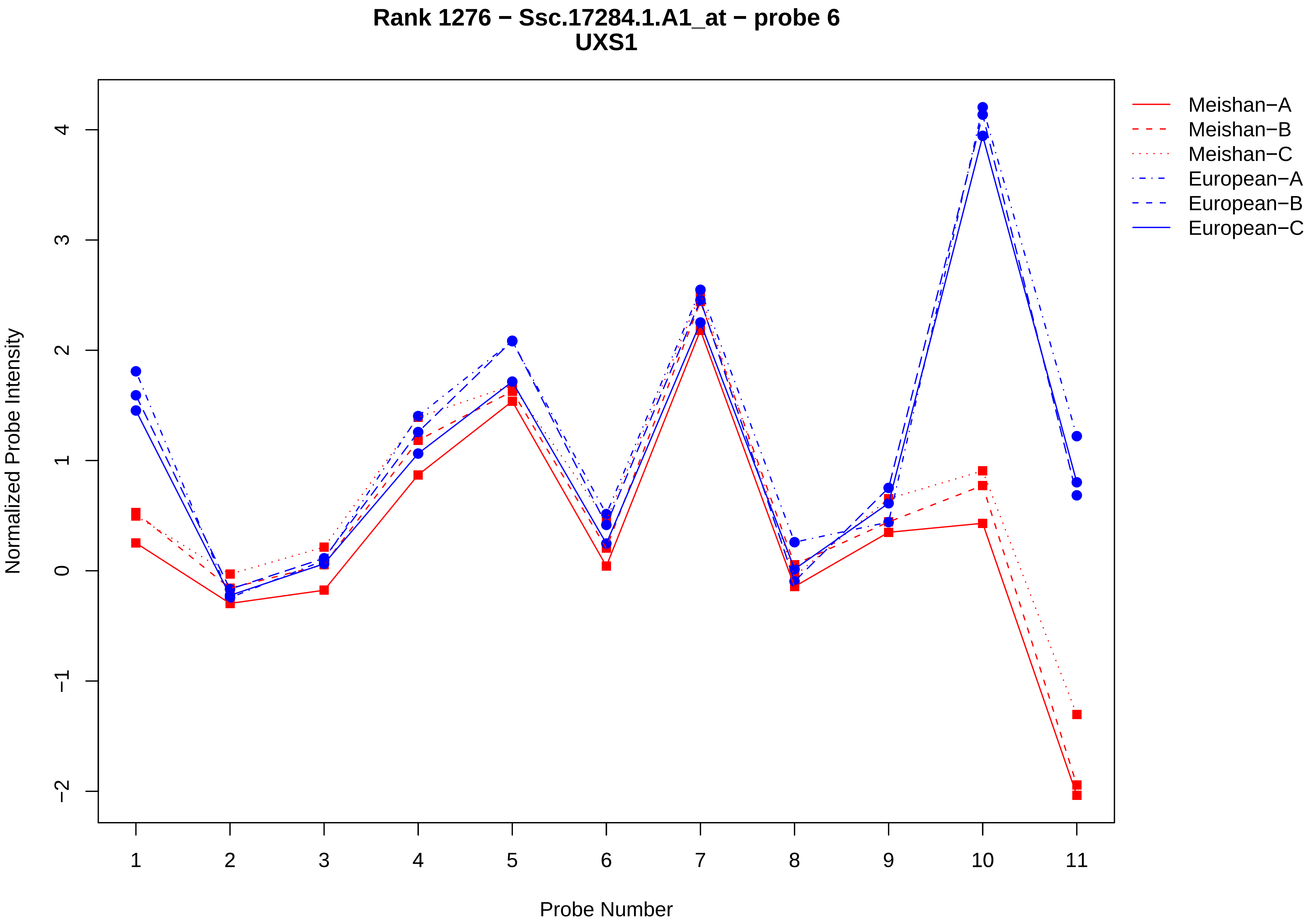

Rank 1276 − Ssc.17284.1.A1_at − probe 6
UXS1
Normalized Probe Intensity
Probe Number
Meishan−A
Meishan−B
Meishan−C
European−A
European−B
European−C
−2
−1
0
1
2
3
4
1
2
3
4
5
6
7
8
9
10
11

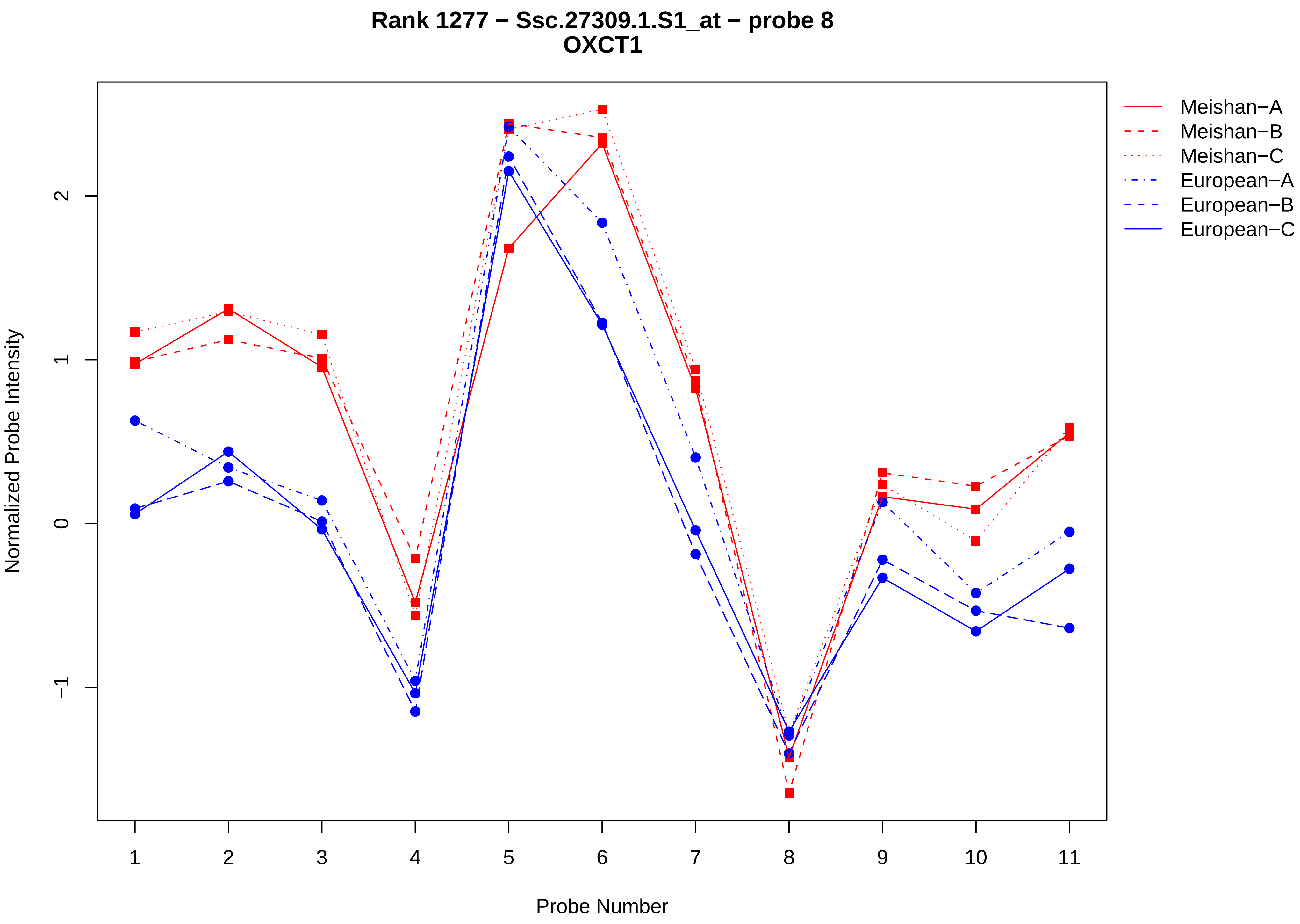
Rank 1277 − Ssc.27309.1.S1_at − probe 8
OXCT1
Normalized Probe Intensity
Probe Number
−1
0
1
2
1
2
3
4
5
6
7
8
9
10
11
Meishan−A
Meishan−B
Meishan−C
European−A
European−B
European−C

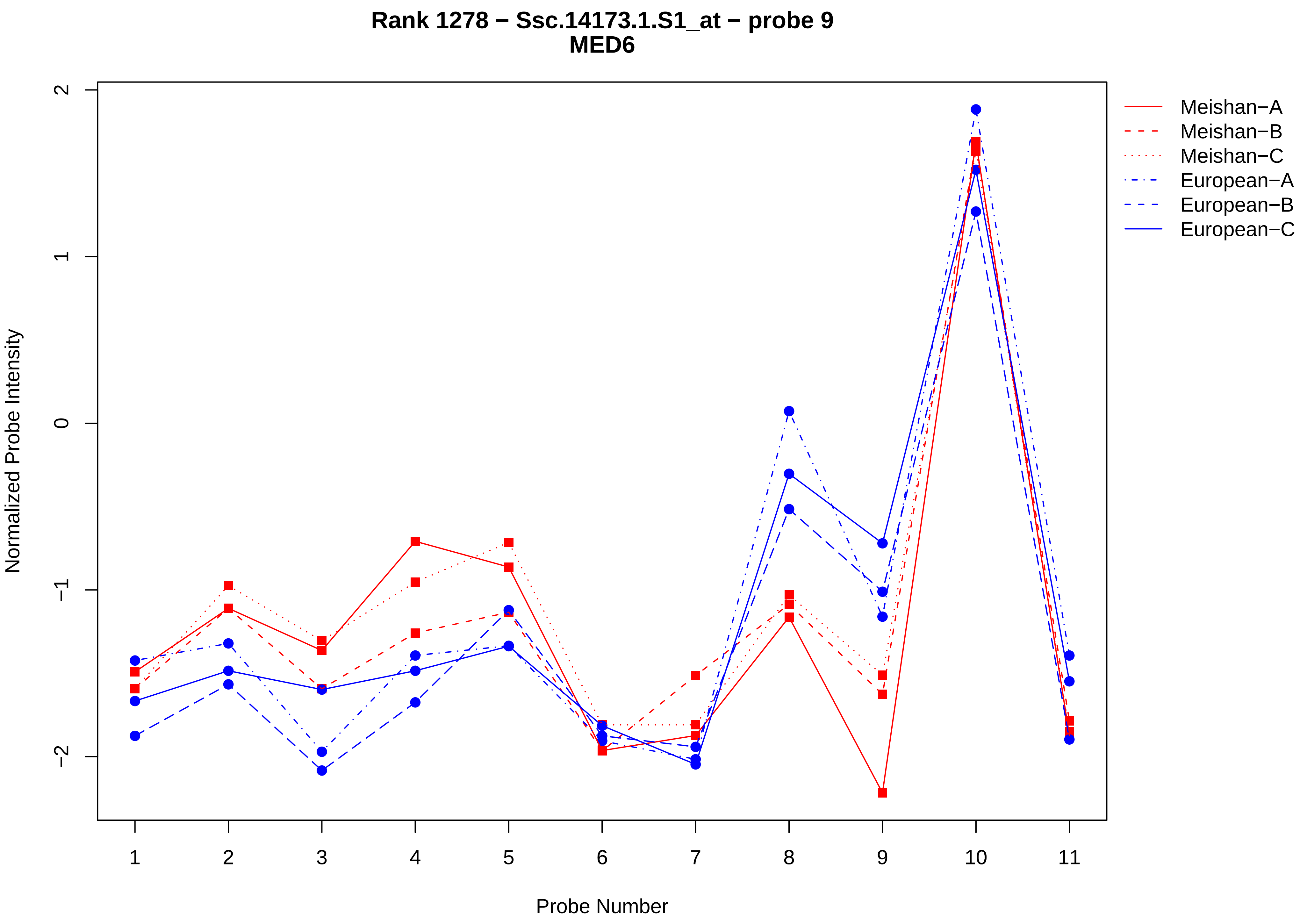

Rank 1278 − Ssc.14173.1.S1_at − probe 9
MED6
Normalized Probe Intensity
Probe Number
−2
−1
0
1
2
1
2
3
4
5
6
7
8
9
10
11
Meishan−A
Meishan−B
Meishan−C
European−A
European−B
European−C

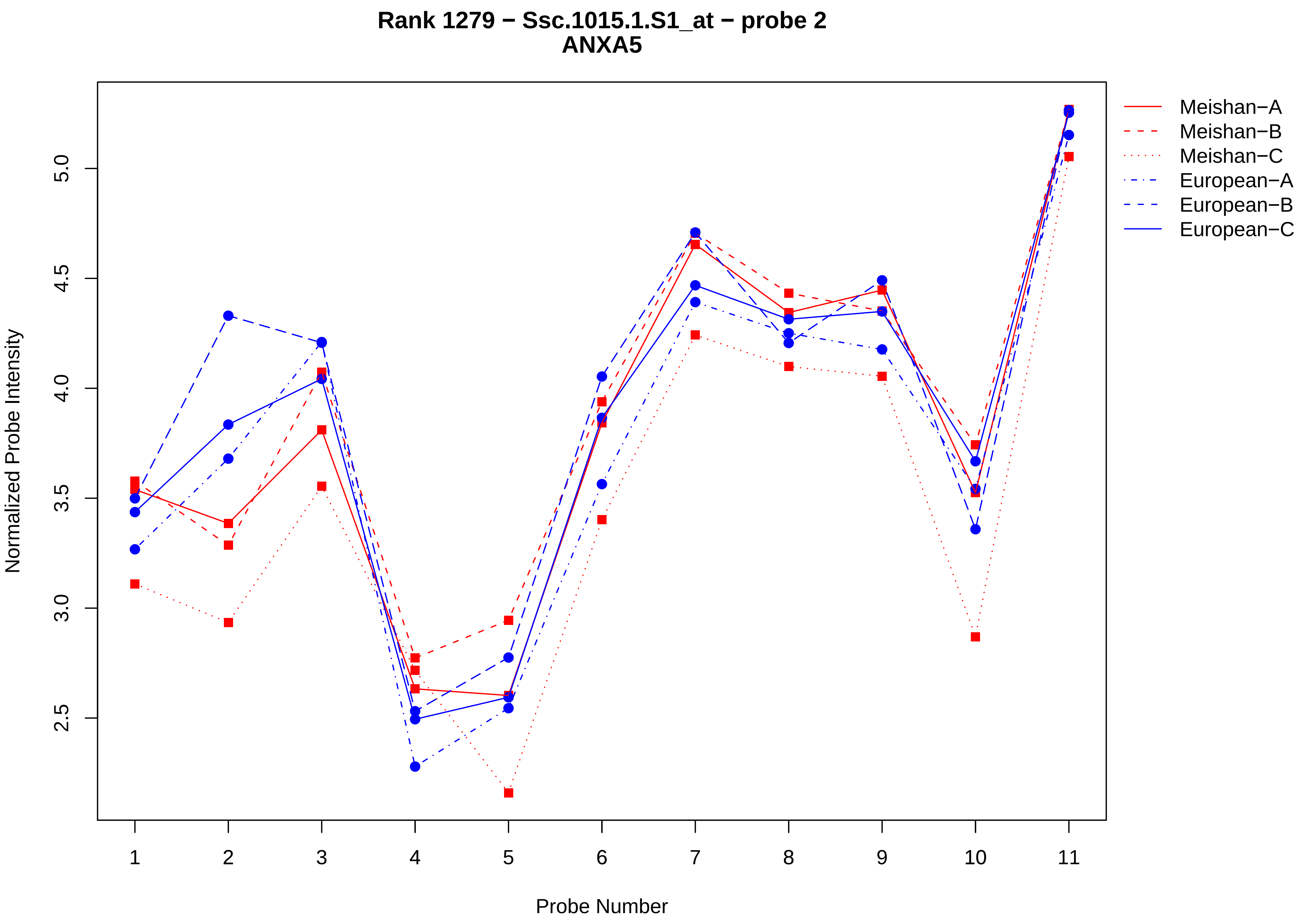

Rank 1279 − Ssc.1015.1.S1_at − probe 2
ANXA5
Normalized Probe Intensity
Probe Number
2.5
3.0
3.5
4.0
4.5
5.0
1
2
3
4
5
6
7
8
9
10
11
Meishan−A
Meishan−B
Meishan−C
European−A
European−B
European−C

Rank 1280 − Ssc.7464.1.S1_at − probe 1
POLR2E

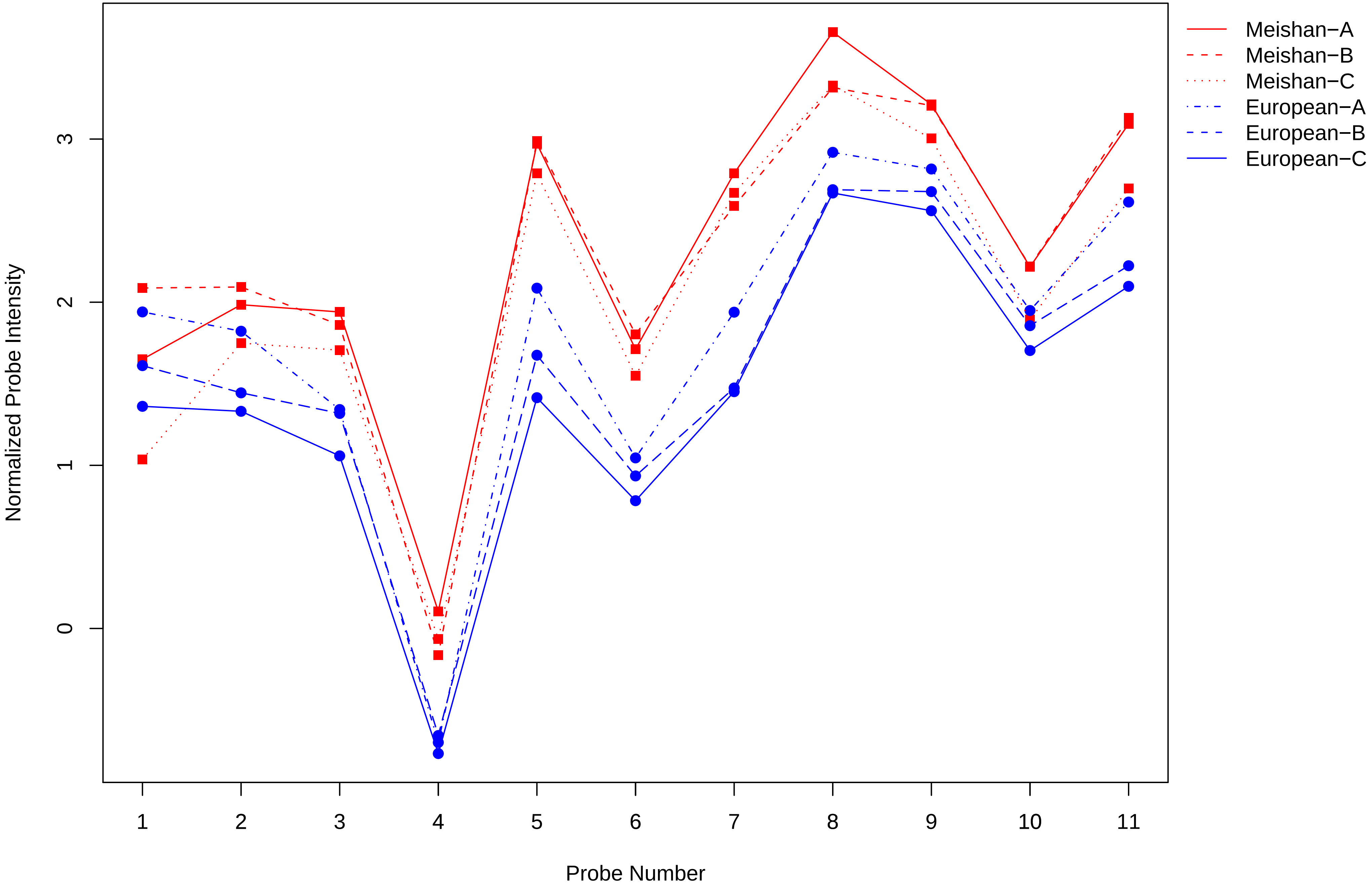

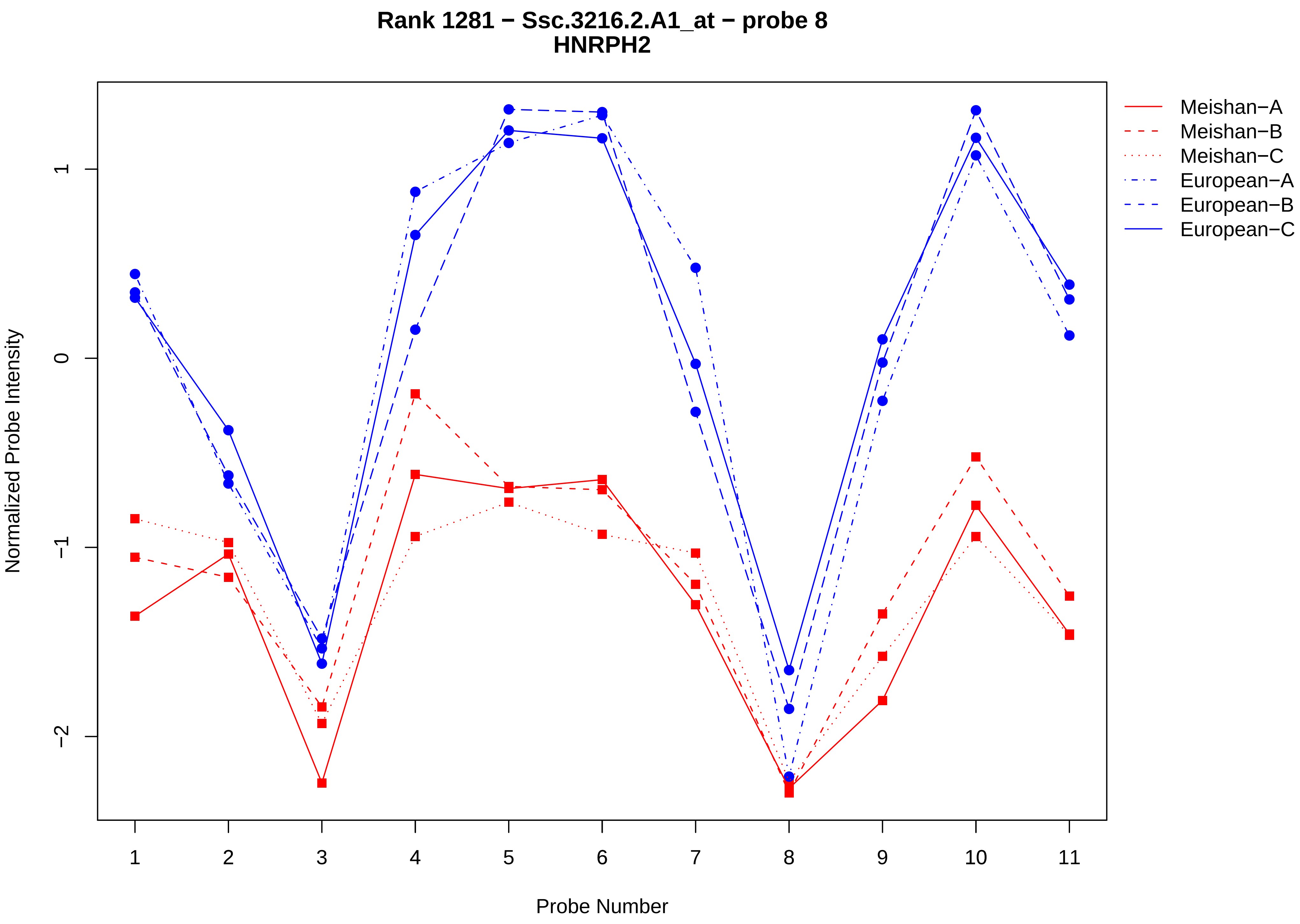
Rank 1281 − Ssc.3216.2.A1_at − probe 8
HNRPH2
Normalized Probe Intensity
Probe Number
−2
−1
0
1
1
2
3
4
5
6
7
8
9
10
11
Meishan−A
Meishan−B
Meishan−C
European−A
European−B
European−C

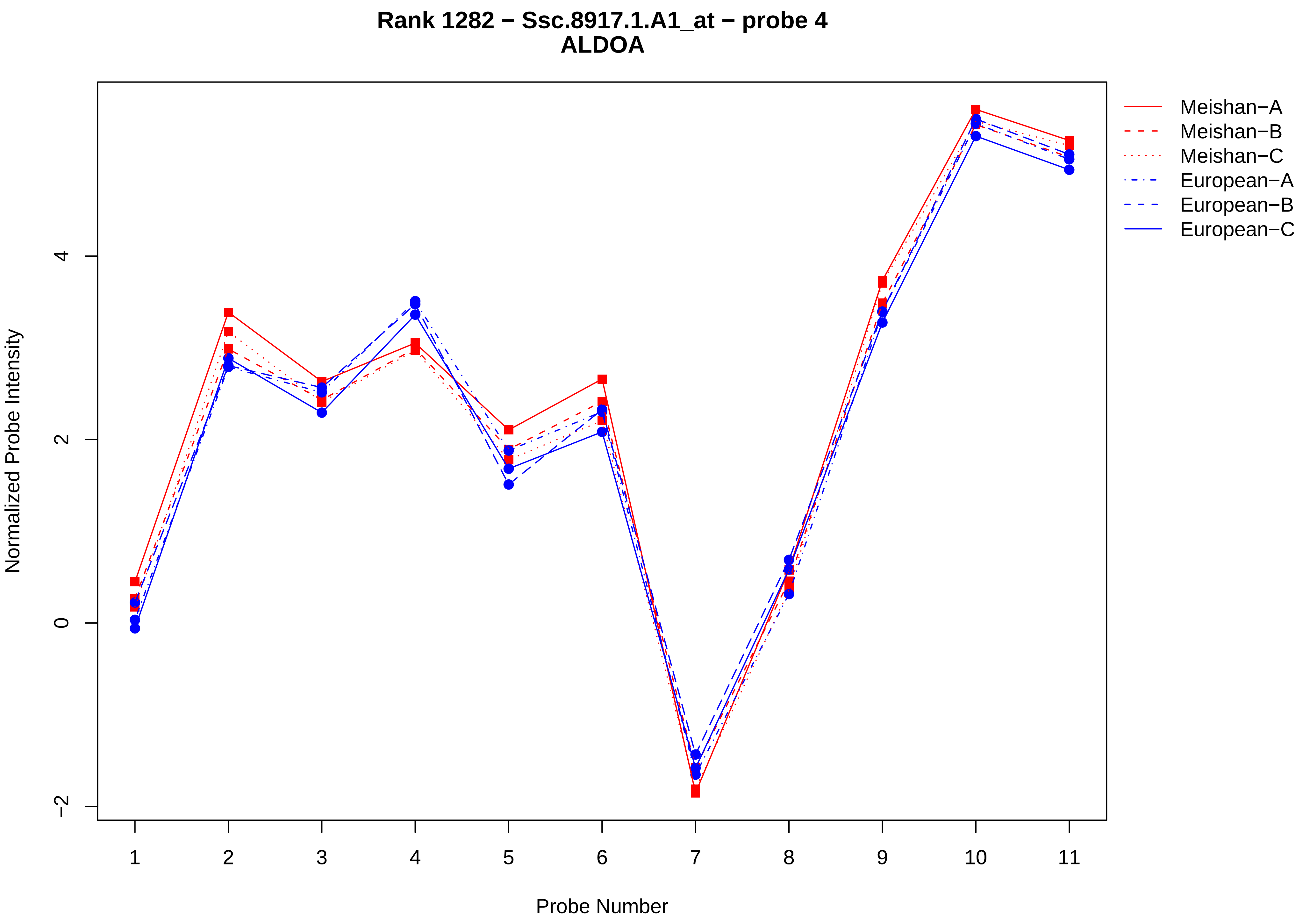

Rank 1282 − Ssc.8917.1.A1_at − probe 4
ALDOA
Normalized Probe Intensity
−2
0
2
4
1
2
3
4
5
6
7
8
9
10
11
Probe Number
Meishan−A
Meishan−B
Meishan−C
European−A
European−B
European−C

Rank 1283 − Ssc.24440.1.A1_at − probe 5
CAB39L

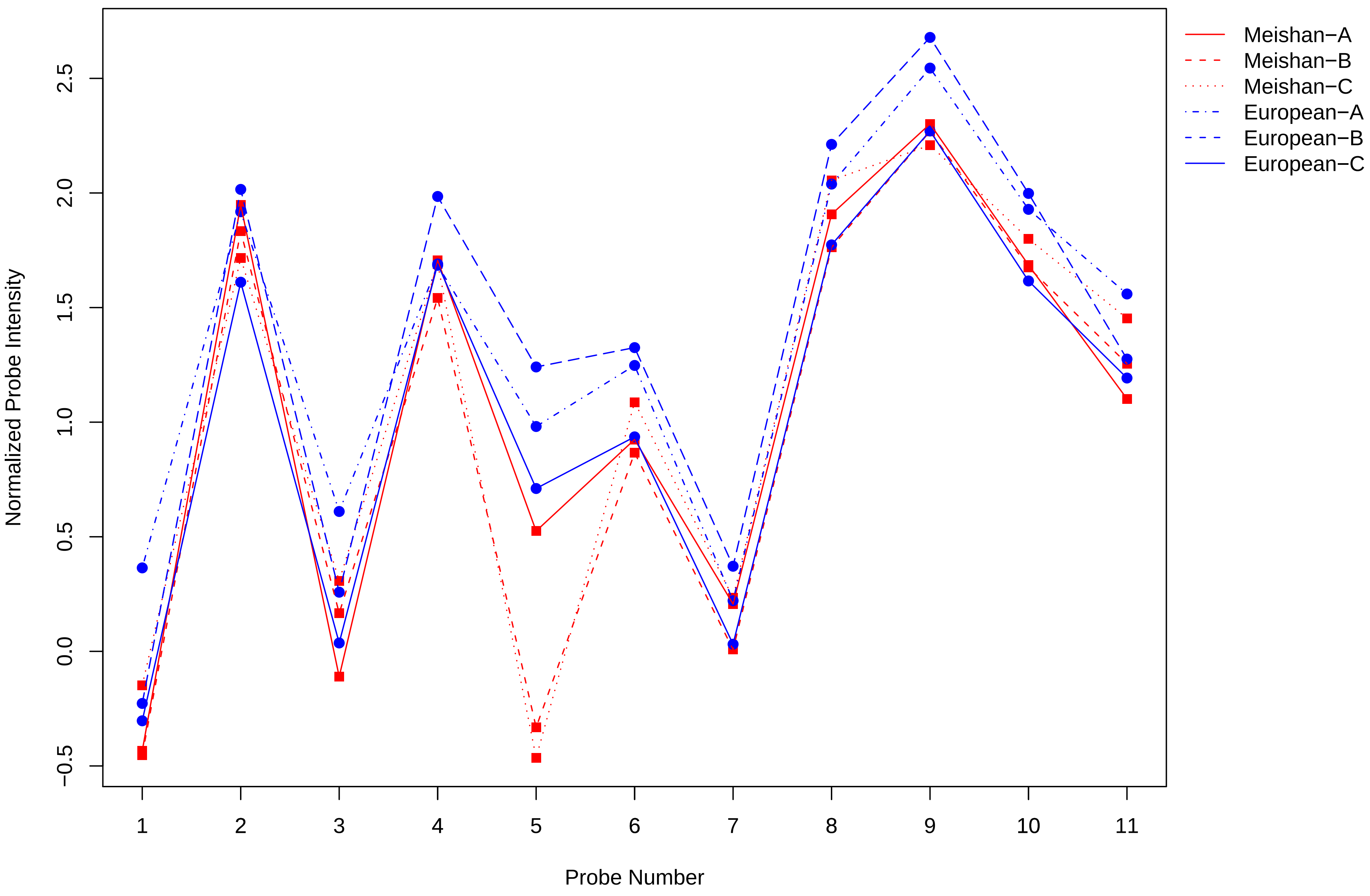

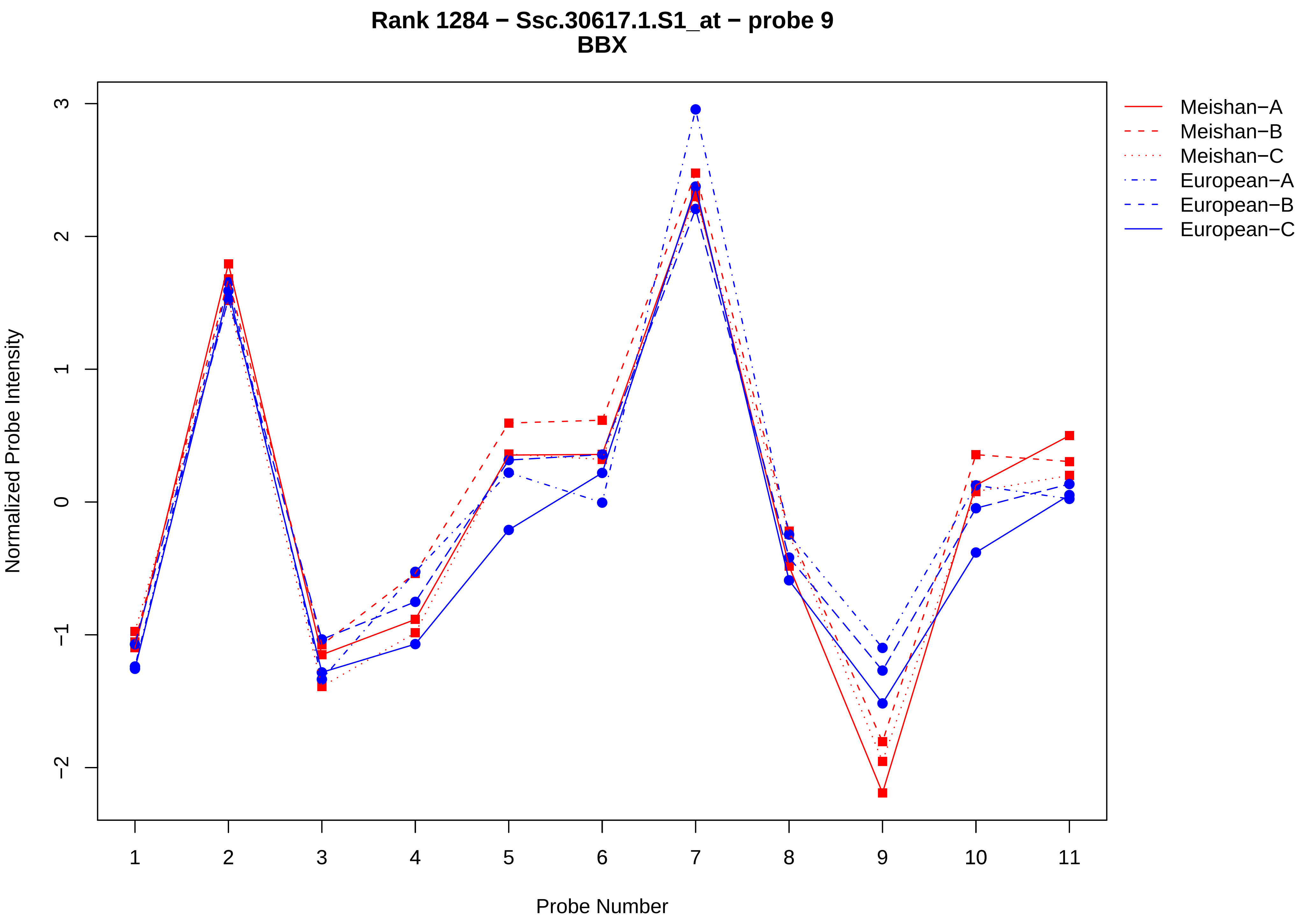
Rank 1284 − Ssc.30617.1.S1_at − probe 9
BBX
Normalized Probe Intensity
Probe Number
Meishan−A
Meishan−B
Meishan−C
European−A
European−B
European−C
−2
−1
0
1
2
3
1
2
3
4
5
6
7
8
9
10
11

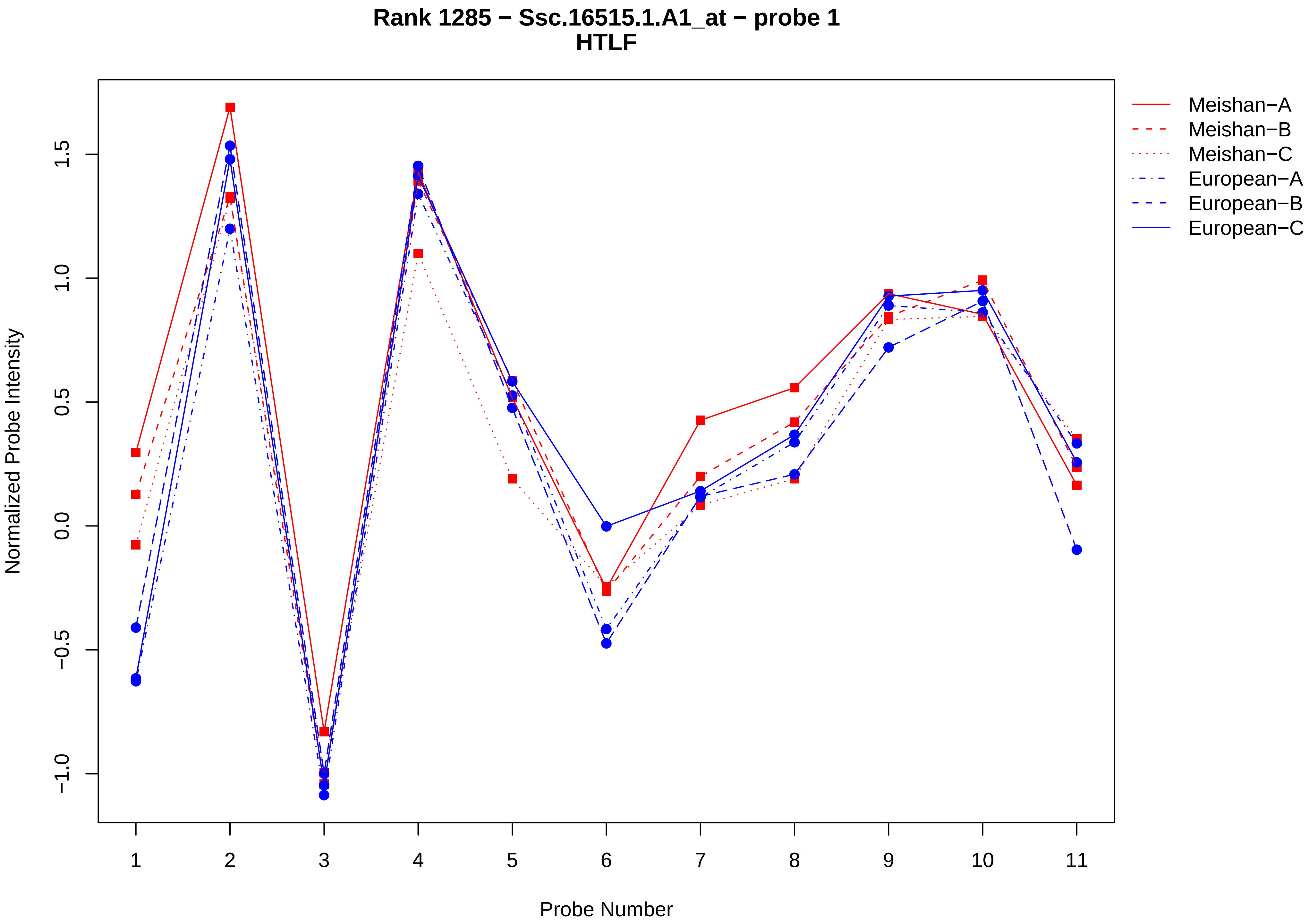

Rank 1285 − Ssc.16515.1.A1_at − probe 1
HTLF
Normalized Probe Intensity
Probe Number
1.5
1.0
0.5
0.0
−0.5
−1.0
1
2
3
4
5
6
7
8
9
10
11
Meishan−A
Meishan−B
Meishan−C
European−A
European−B
European−C

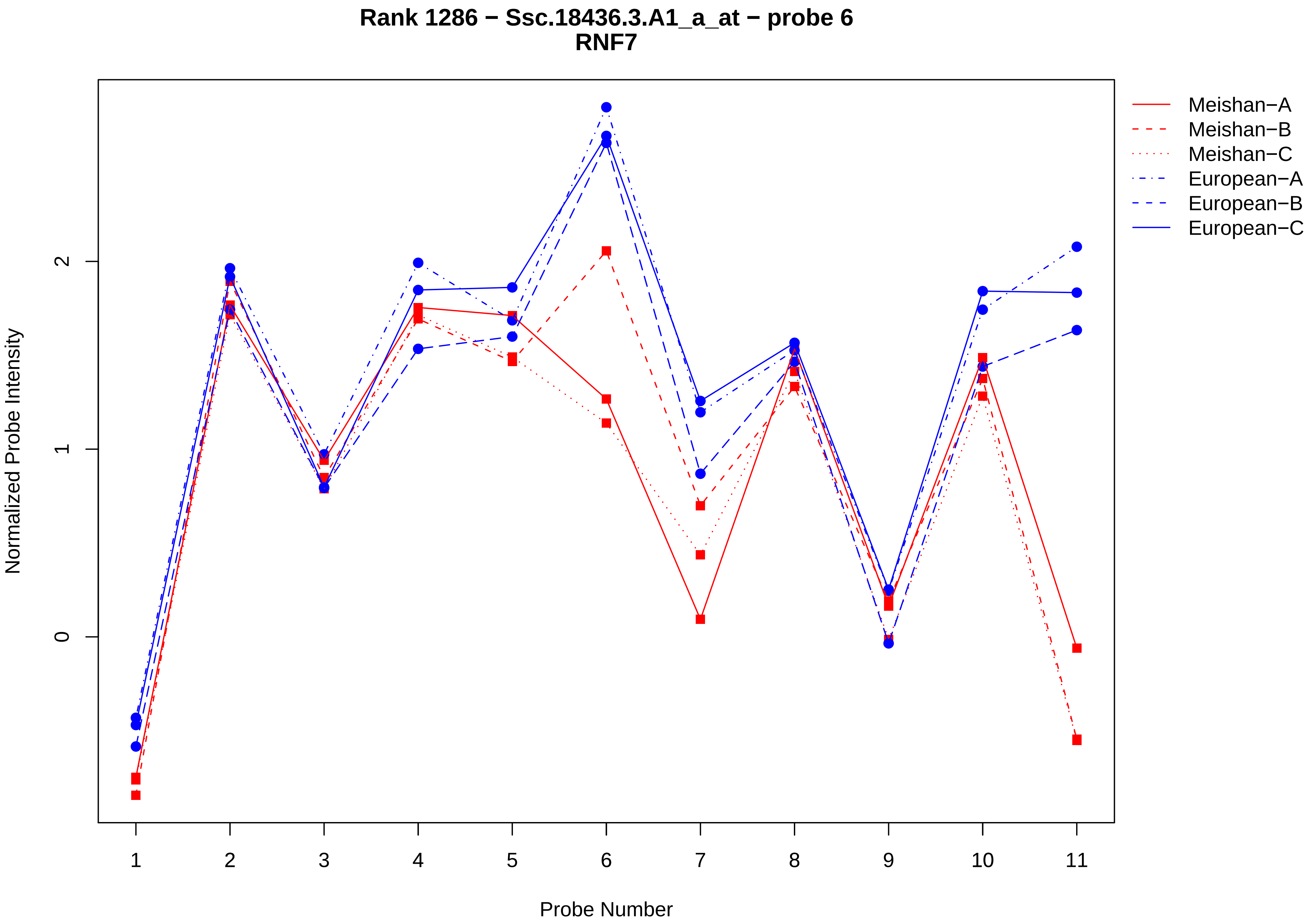

Rank 1286 − Ssc.18436.3.A1_a_at − probe 6
RNF7
Normalized Probe Intensity
Probe Number
0
1
2
1
2
3
4
5
6
7
8
9
10
11
Meishan−A
Meishan−B
Meishan−C
European−A
European−B
European−C

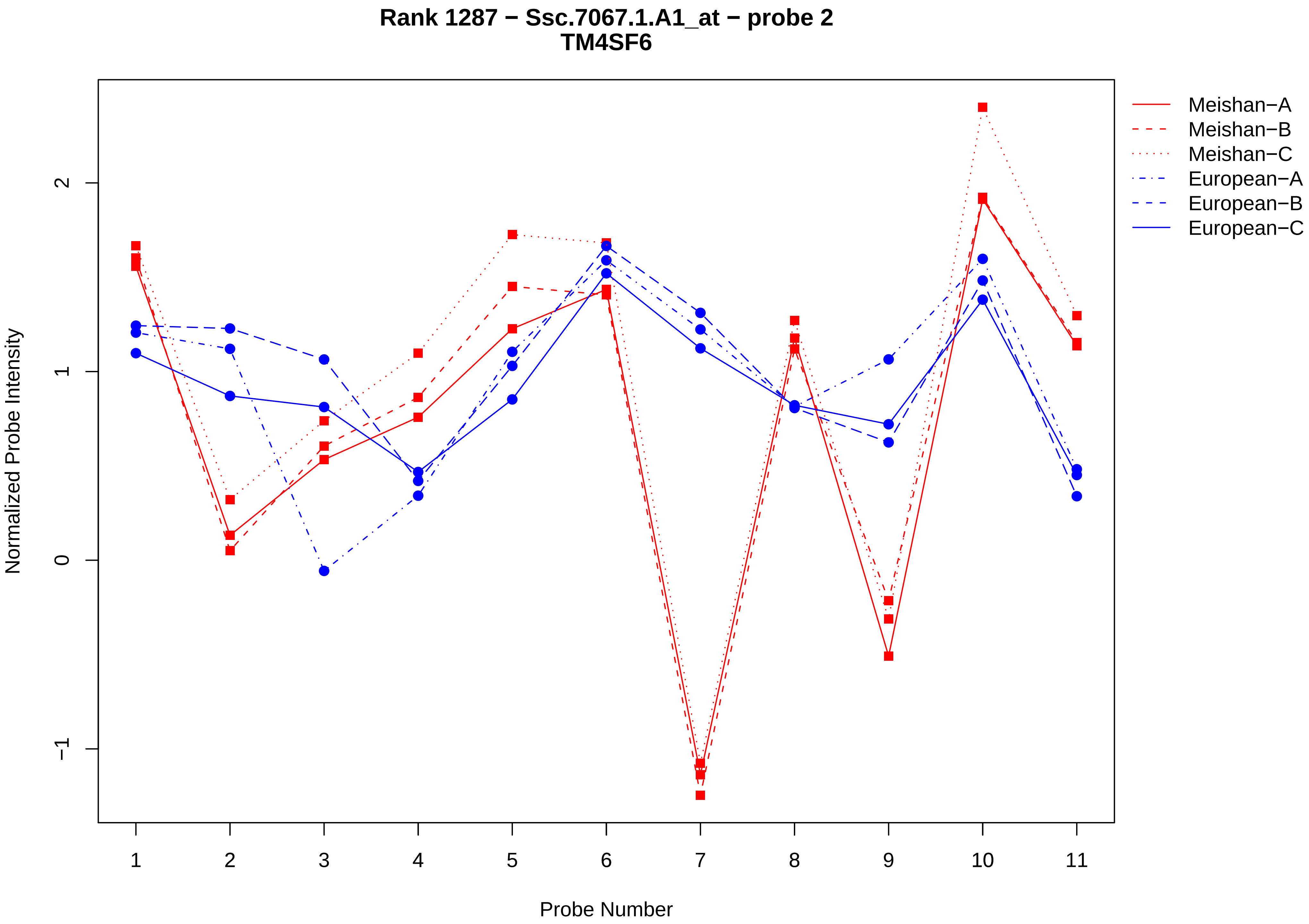
Rank 1287 − Ssc.7067.1.A1_at − probe 2
TM4SF6
Normalized Probe Intensity
Probe Number
−1
0
1
2
1
2
3
4
5
6
7
8
9
10
11
Meishan−A
Meishan−B
Meishan−C
European−A
European−B
European−C

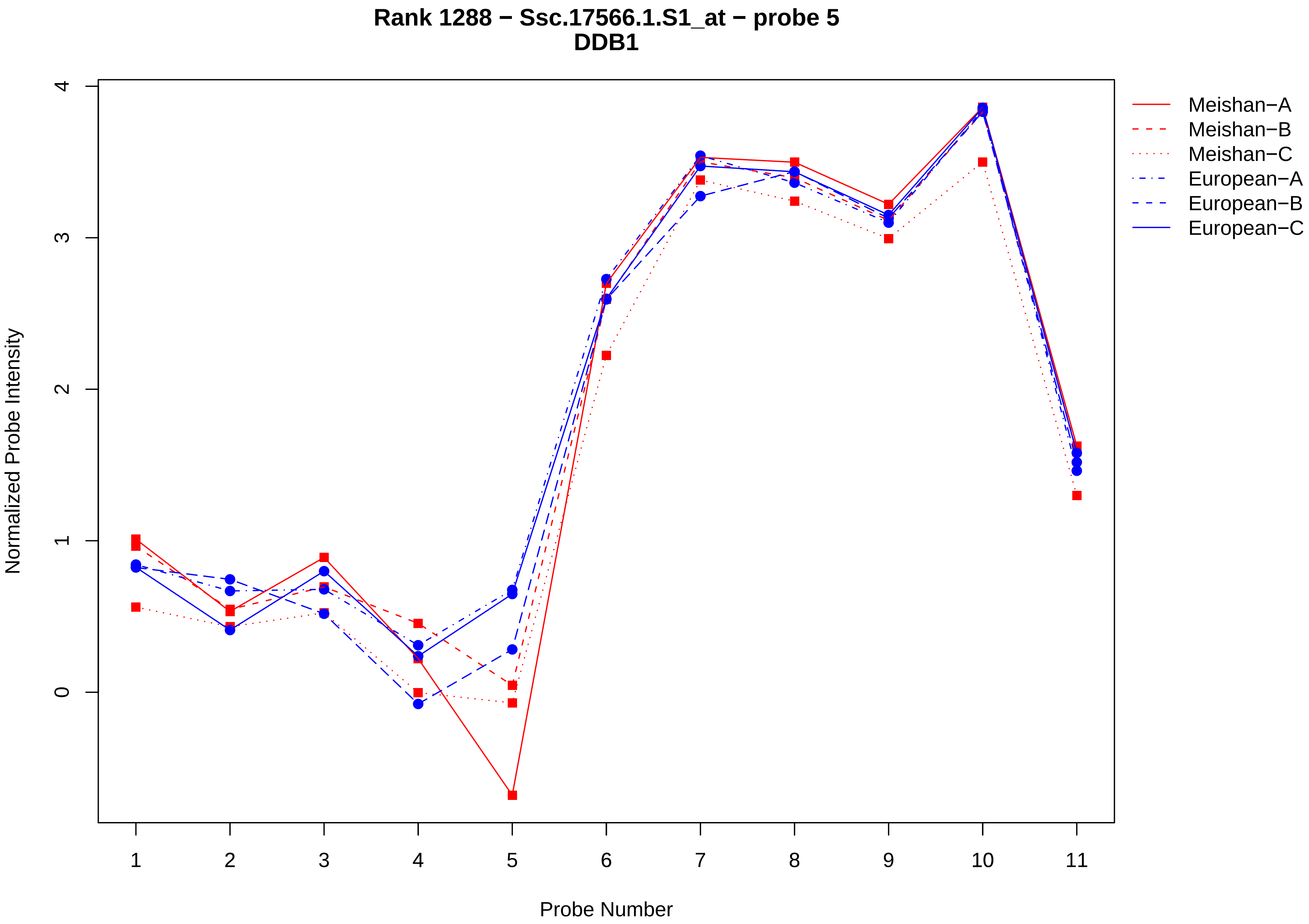

Rank 1288 − Ssc.17566.1.S1_at − probe 5
DDB1
Normalized Probe Intensity
Probe Number
0
1
2
3
4
1
2
3
4
5
6
7
8
9
10
11
Meishan−A
Meishan−B
Meishan−C
European−A
European−B
European−C

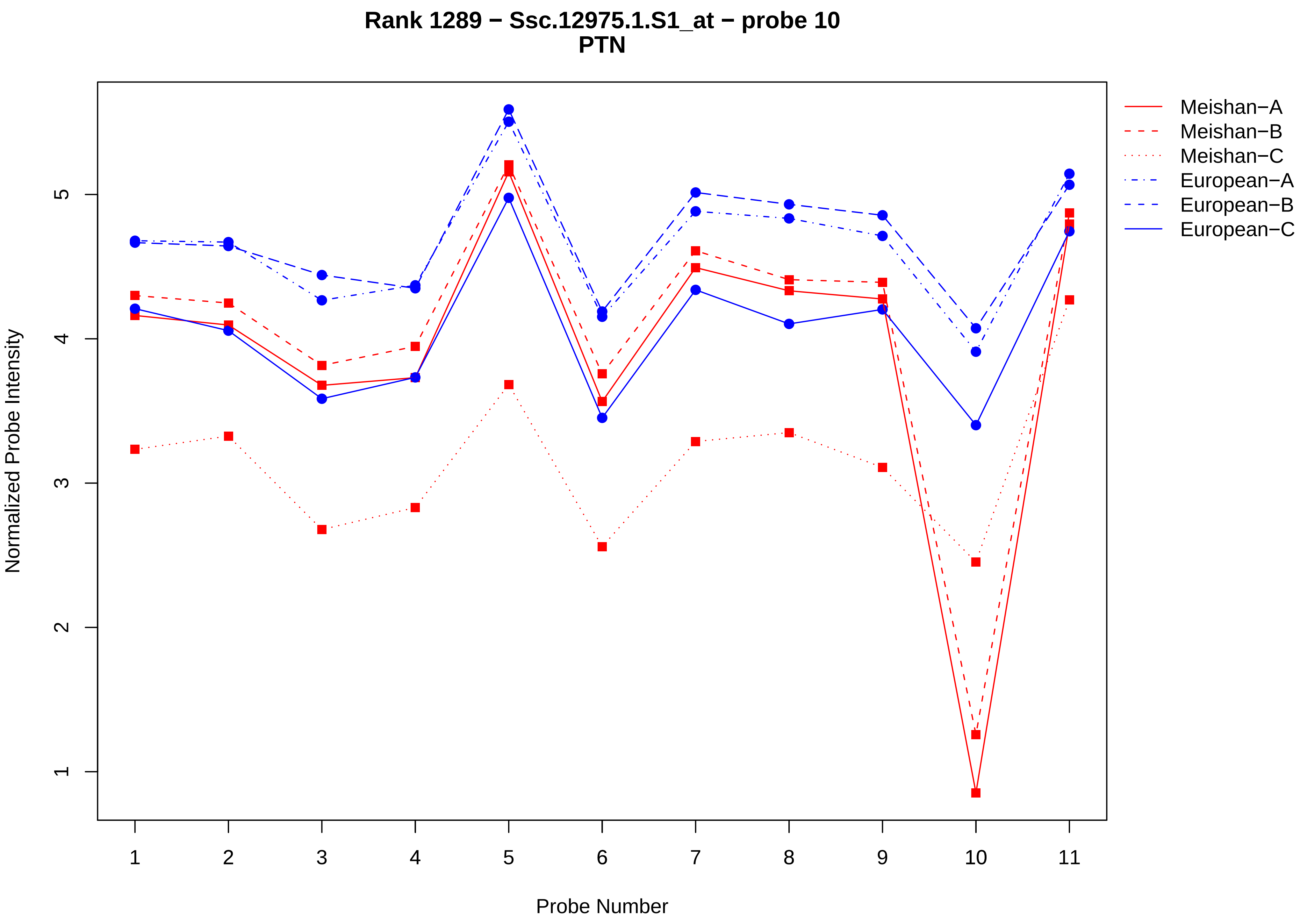

Rank 1289 − Ssc.12975.1.S1_at − probe 10
PTN
Normalized Probe Intensity
Probe Number
1
2
3
4
5
1
2
3
4
5
6
7
8
9
10
11
Meishan−A
Meishan−B
Meishan−C
European−A
European−B
European−C

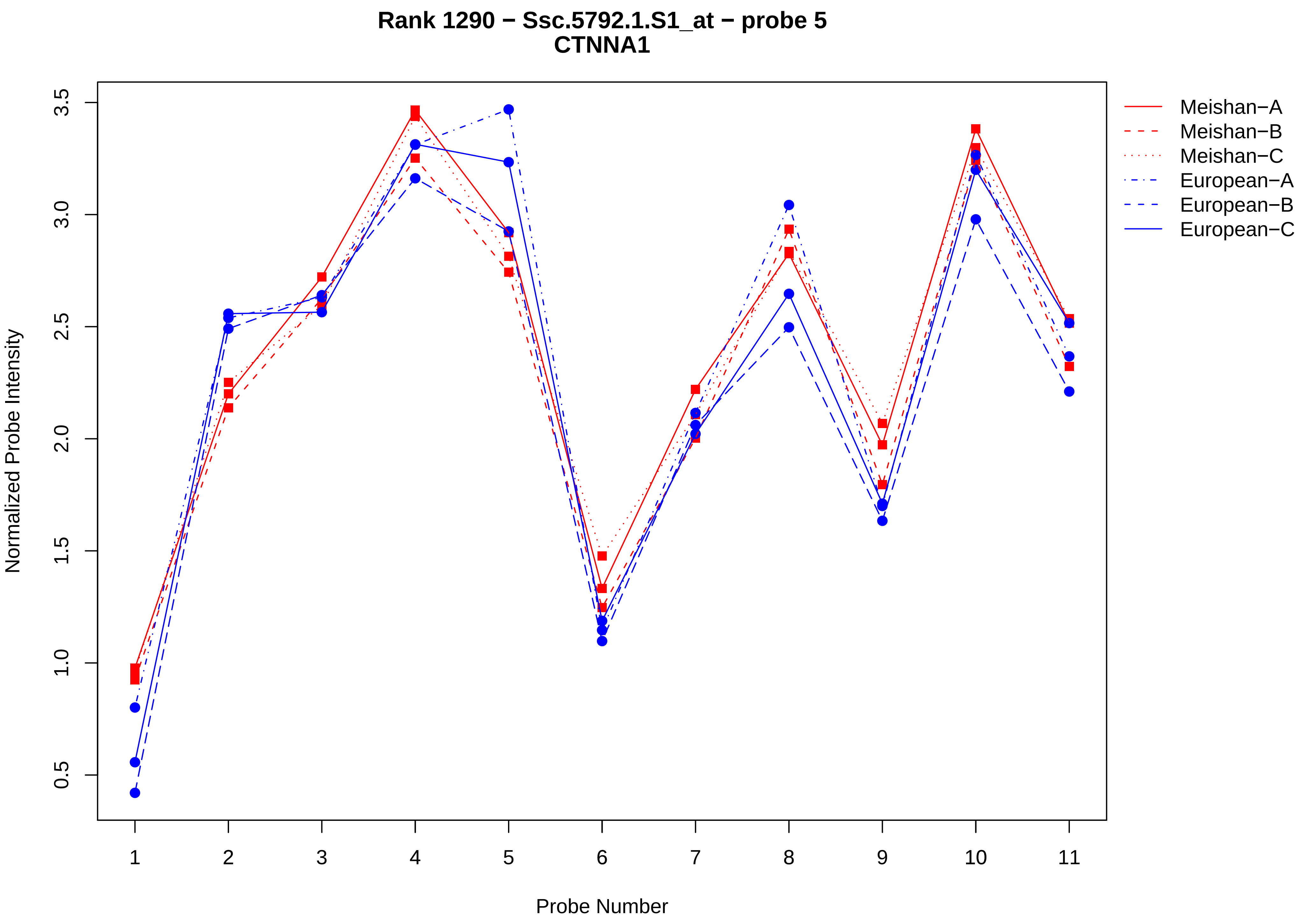

Rank 1290 − Ssc.5792.1.S1_at − probe 5
CTNNA1
Normalized Probe Intensity
Probe Number
0.5
1.0
1.5
2.0
2.5
3.0
3.5
1
2
3
4
5
6
7
8
9
10
11
Meishan−A
Meishan−B
Meishan−C
European−A
European−B
European−C

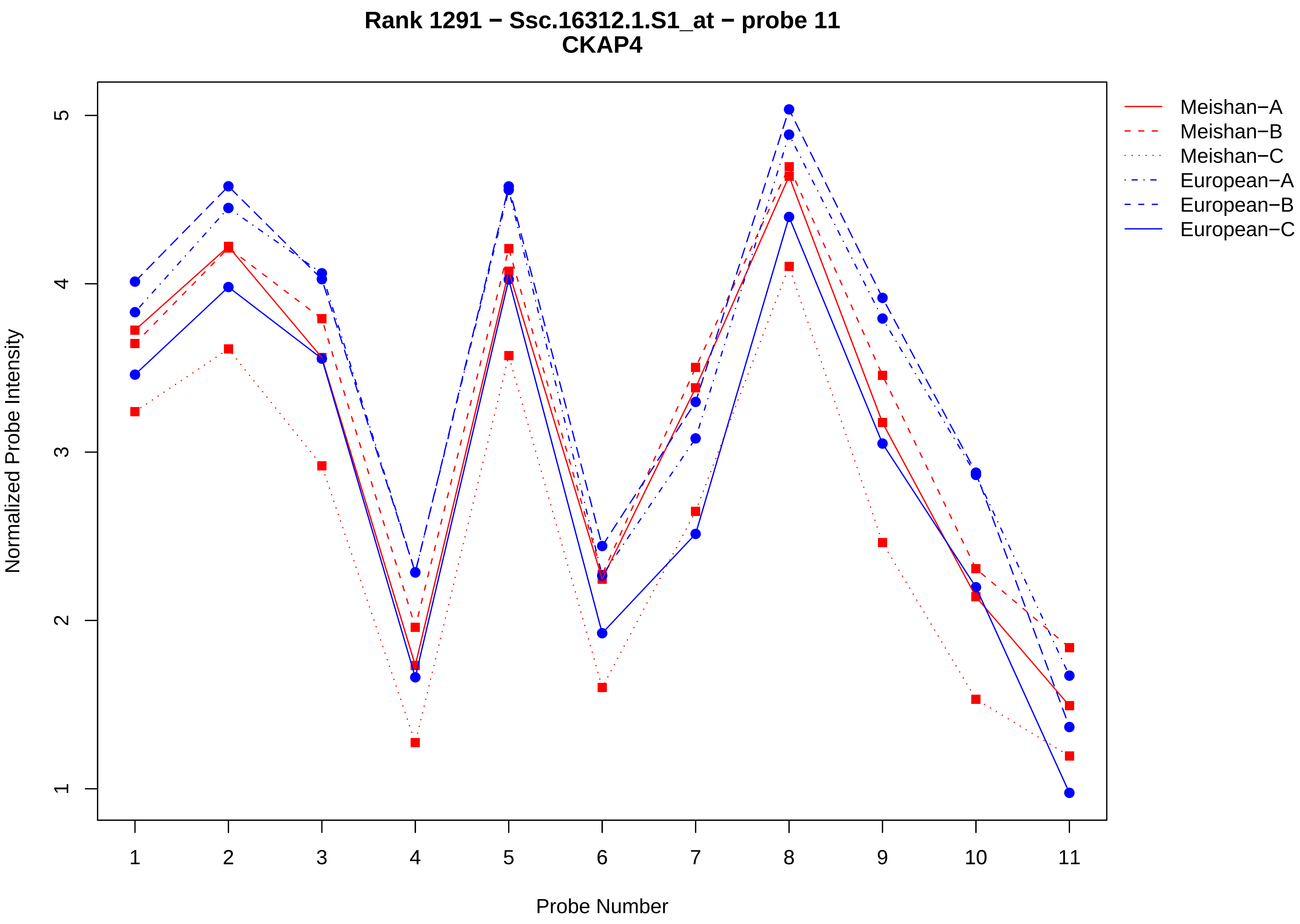
Rank 1291 − Ssc.16312.1.S1_at − probe 11
CKAP4
Normalized Probe Intensity
Probe Number
Meishan−A
Meishan−B
Meishan−C
European−A
European−B
European−C
1
2
3
4
5
6
7
8
9
10
11

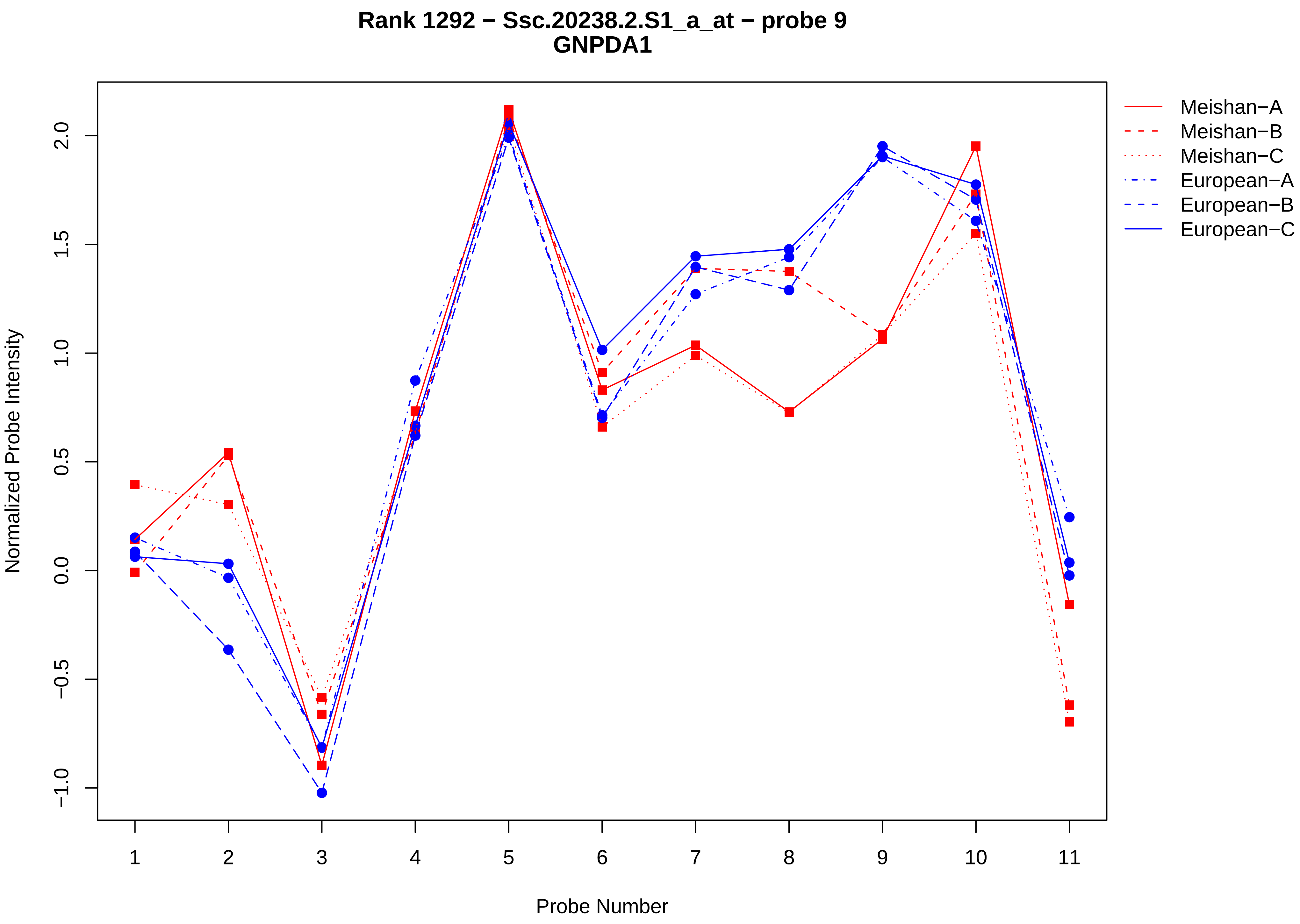

Rank 1292 − Ssc.20238.2.S1_a_at − probe 9
GNPDA1
Normalized Probe Intensity
Probe Number
Meishan−A
Meishan−B
Meishan−C
European−A
European−B
European−C
−1.0
−0.5
0.0
0.5
1.0
1.5
2.0
1
2
3
4
5
6
7
8
9
10
11

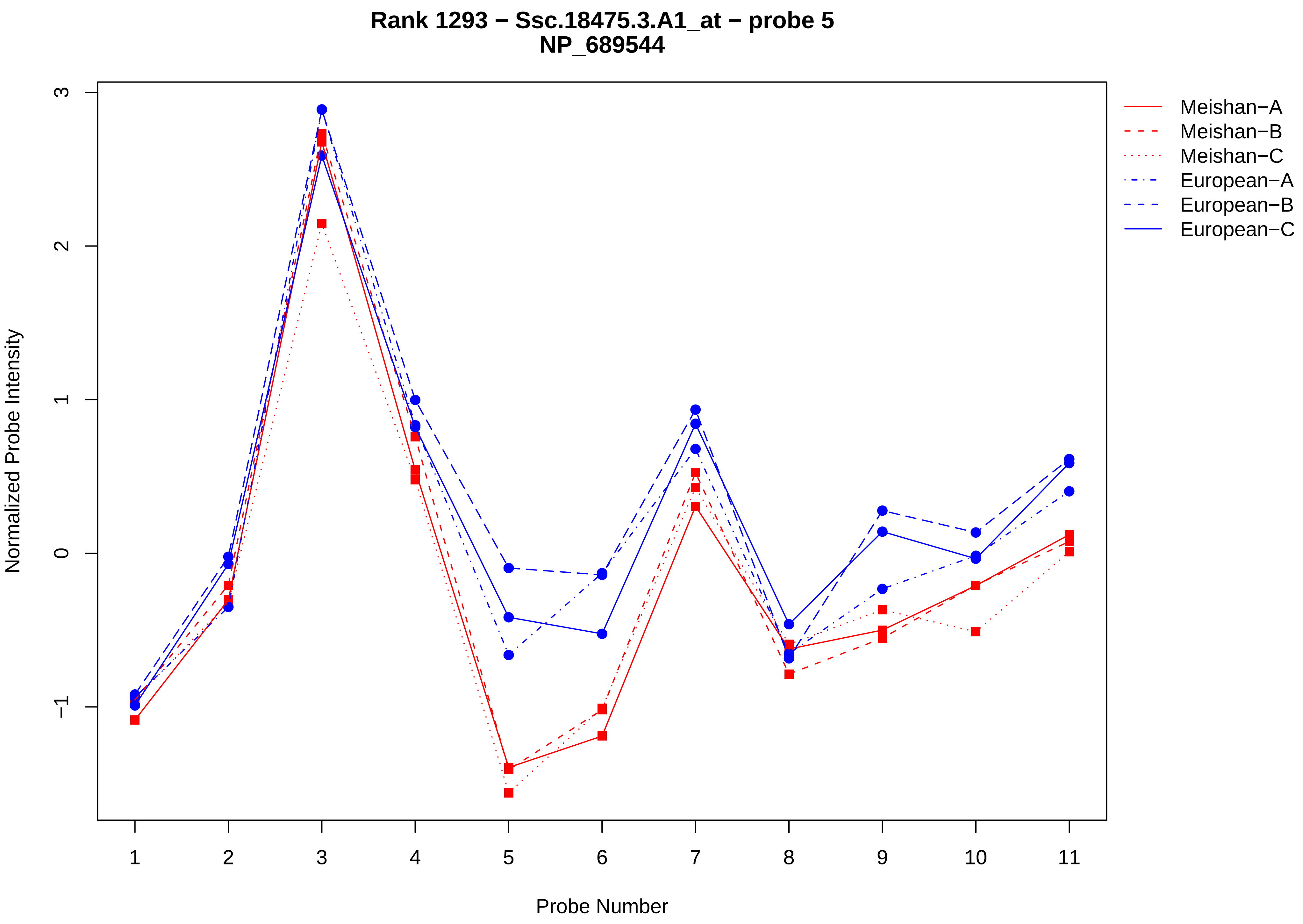

Rank 1293 − Ssc.18475.3.A1_at − probe 5
NP_689544
Normalized Probe Intensity
Probe Number
Meishan−A
Meishan−B
Meishan−C
European−A
European−B
European−C

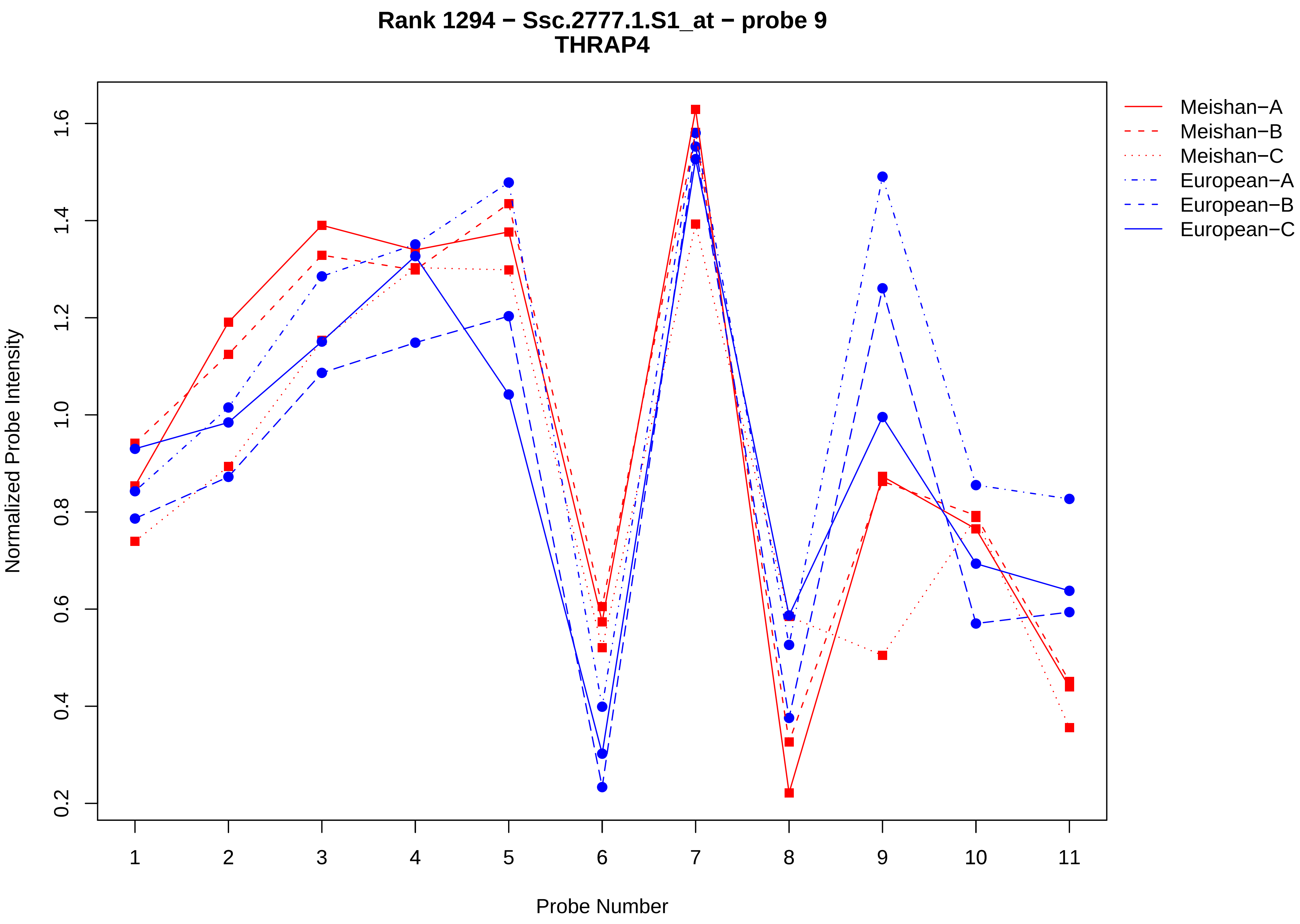

Rank 1294 − Ssc.2777.1.S1_at − probe 9
THRAP4
Normalized Probe Intensity
0.2
0.4
0.6
0.8
1.0
1.2
1.4
1.6
1
2
3
4
5
6
7
8
9
10
11
Probe Number
Meishan−A
Meishan−B
Meishan−C
European−A
European−B
European−C

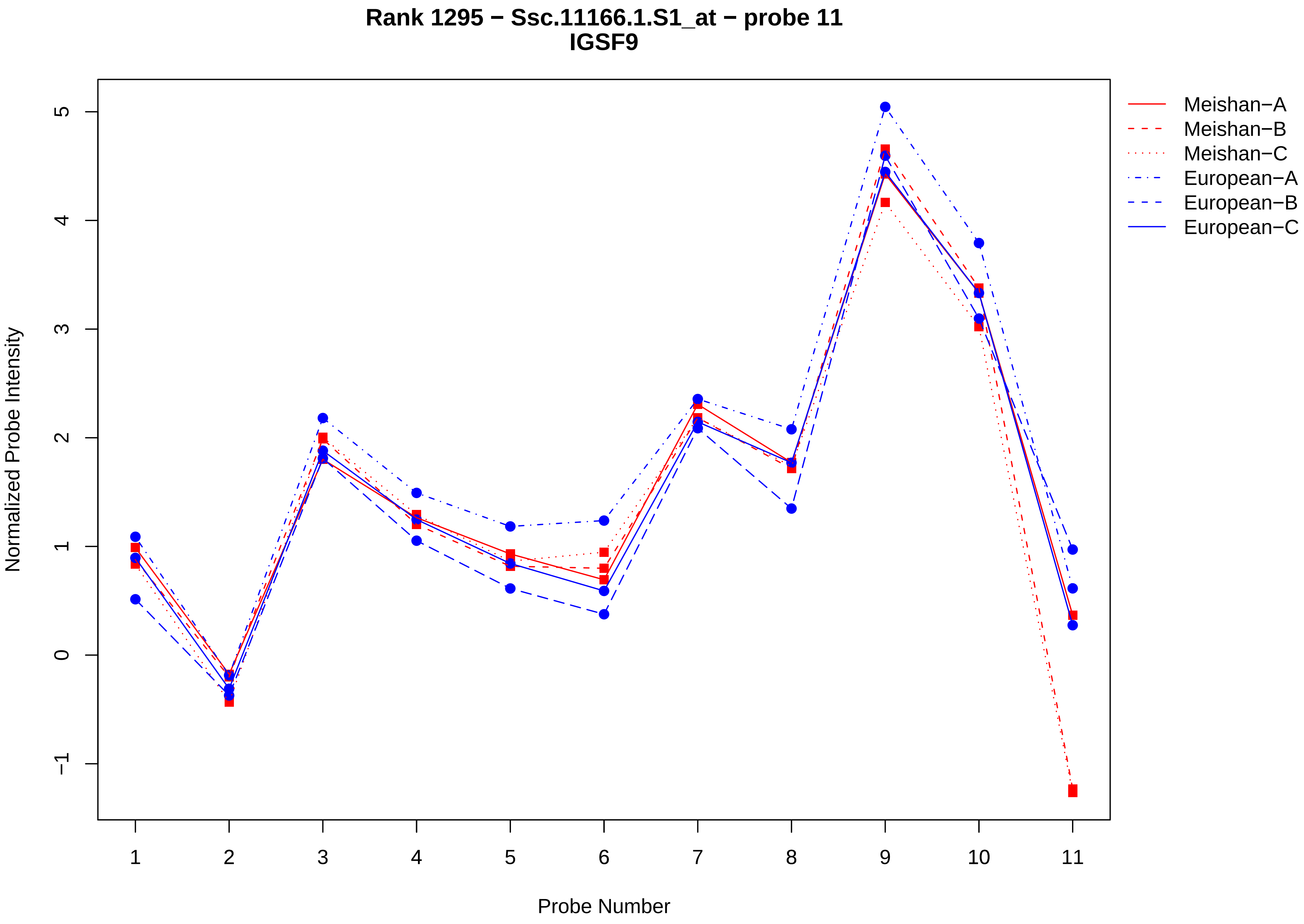
Rank 1295 − Ssc.11166.1.S1_at − probe 11
IGSF9
Normalized Probe Intensity
Probe Number
Meishan−A
Meishan−B
Meishan−C
European−A
European−B
European−C
−1
0
1
2
3
4
5
1
2
3
4
5
6
7
8
9
10
11

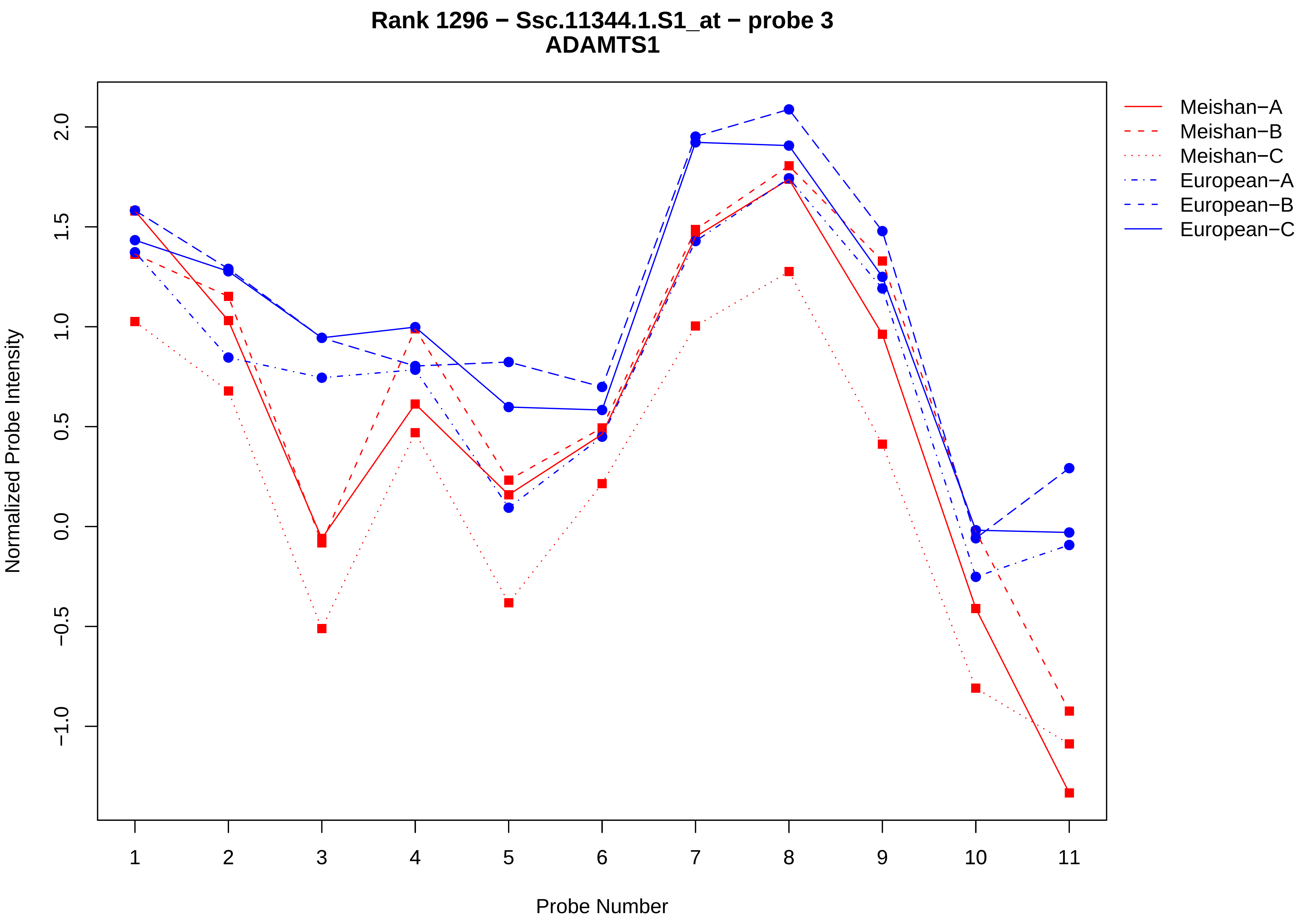
Rank 1296 − Ssc.11344.1.S1_at − probe 3
ADAMTS1
Normalized Probe Intensity
Probe Number
Meishan−A
Meishan−B
Meishan−C
European−A
European−B
European−C

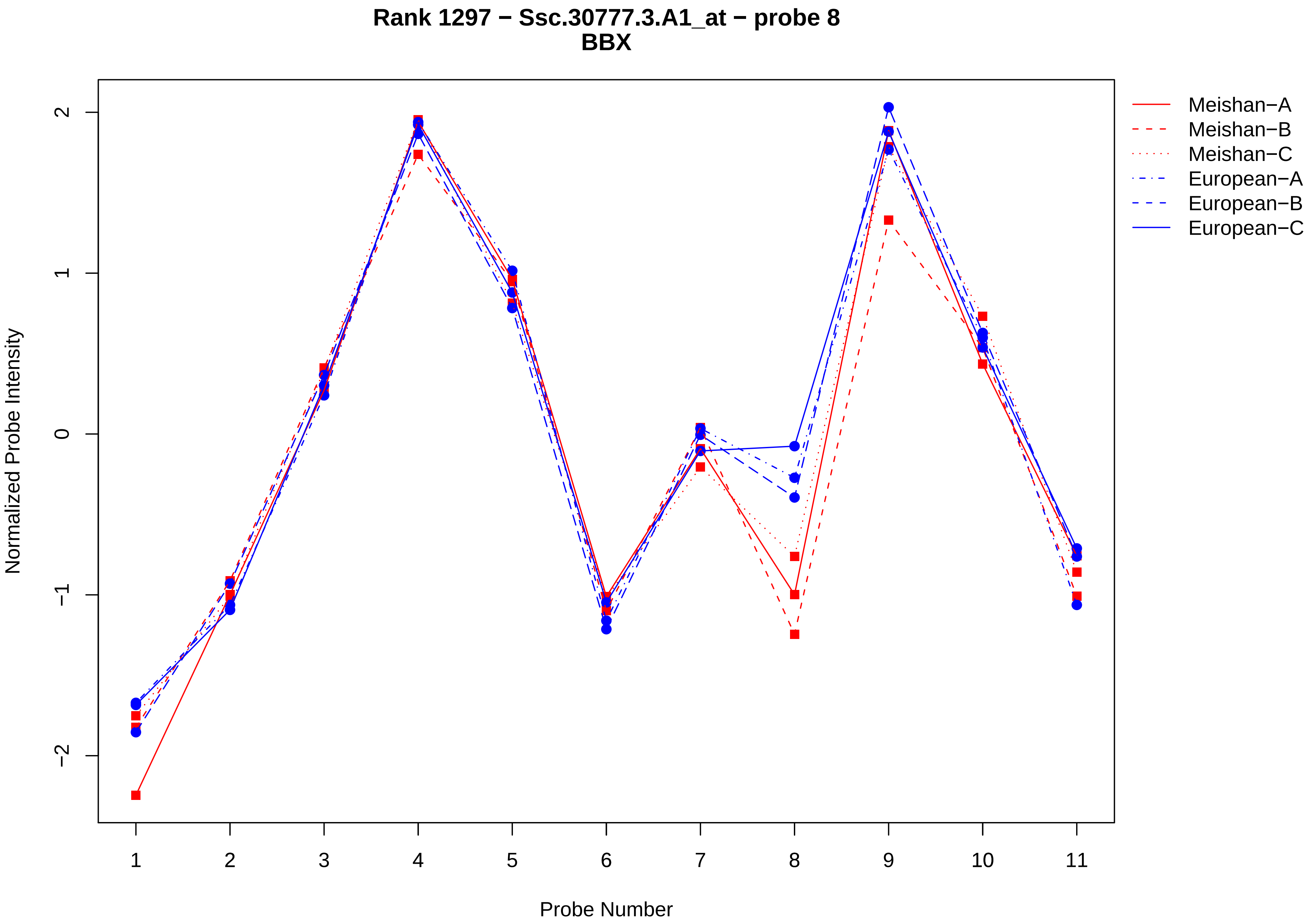
Rank 1297 − Ssc.30777.3.A1_at − probe 8
BBX
Normalized Probe Intensity
−2
−1
0
1
2
1
2
3
4
5
6
7
8
9
10
11
Probe Number
Meishan−A
Meishan−B
Meishan−C
European−A
European−B
European−C

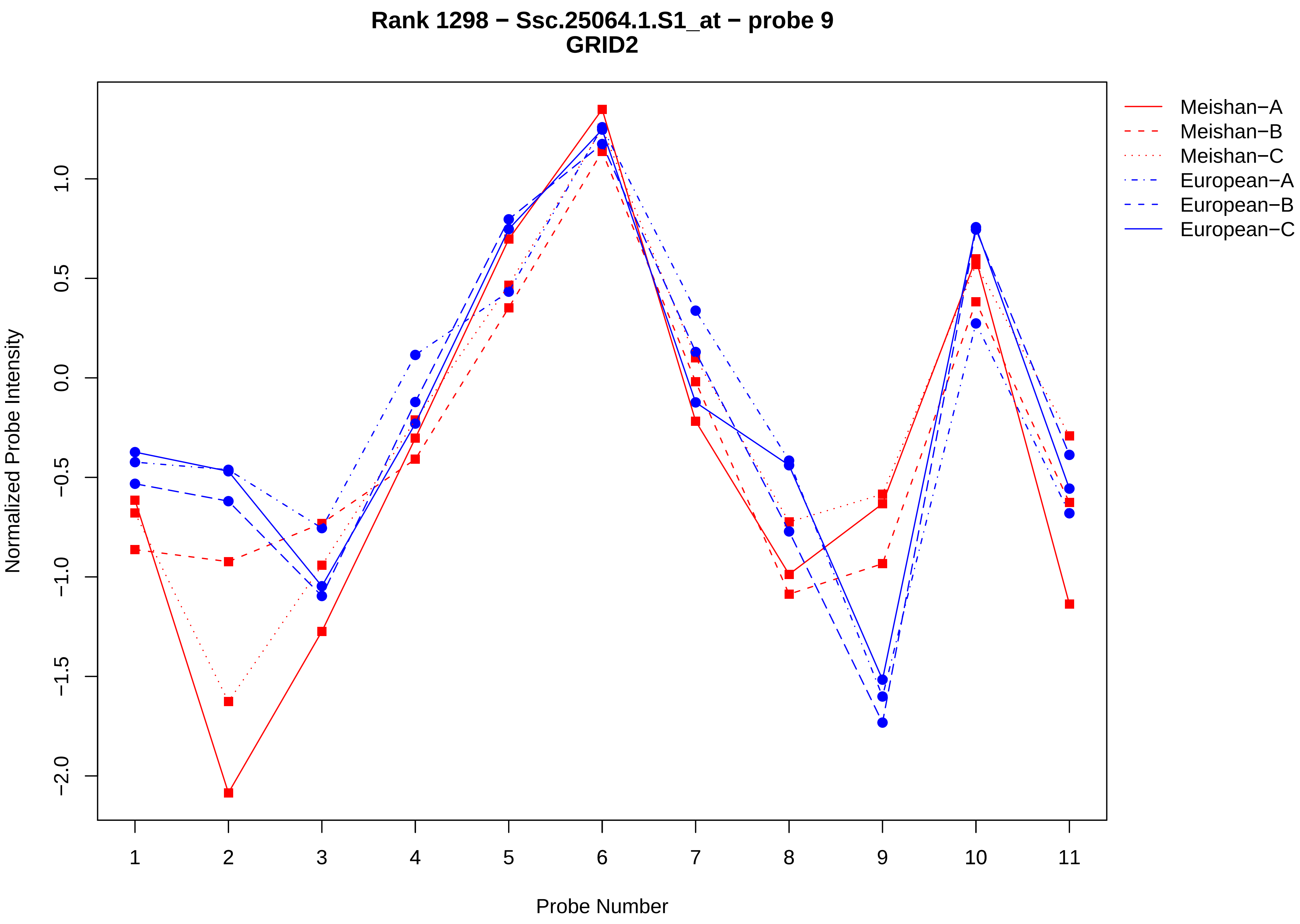

Rank 1298 − Ssc.25064.1.S1_at − probe 9
GRID2
Normalized Probe Intensity
Probe Number
Meishan−A
Meishan−B
Meishan−C
European−A
European−B
European−C
1.0
0.5
0.0
−0.5
−1.0
−1.5
−2.0
1
2
3
4
5
6
7
8
9
10
11

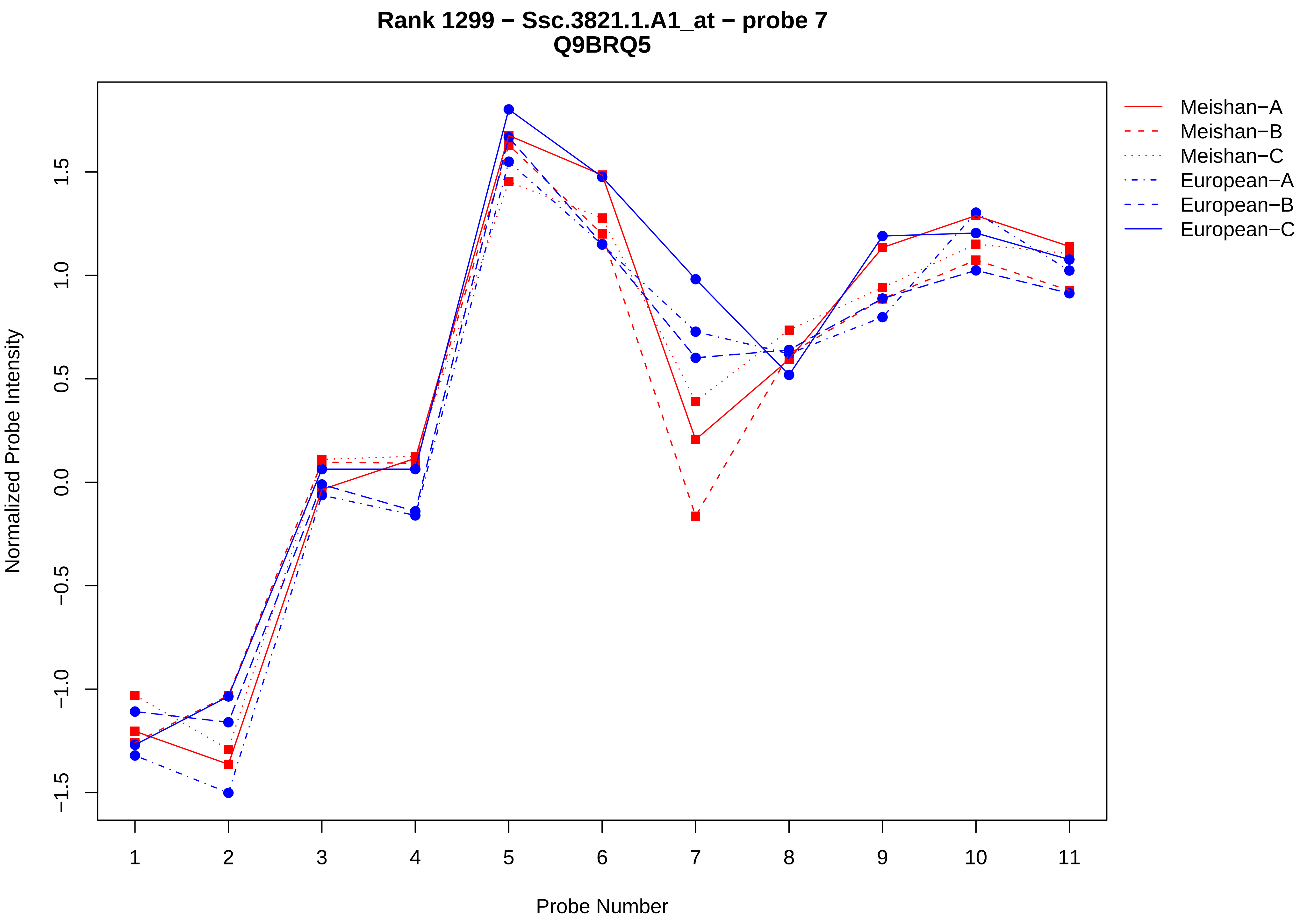
Rank 1299 − Ssc.3821.1.A1_at − probe 7
Q9BRQ5
Normalized Probe Intensity
−1.5
−1.0
−0.5
0.0
0.5
1.0
1.5
1
2
3
4
5
6
7
8
9
10
11
Probe Number
Meishan−A
Meishan−B
Meishan−C
European−A
European−B
European−C

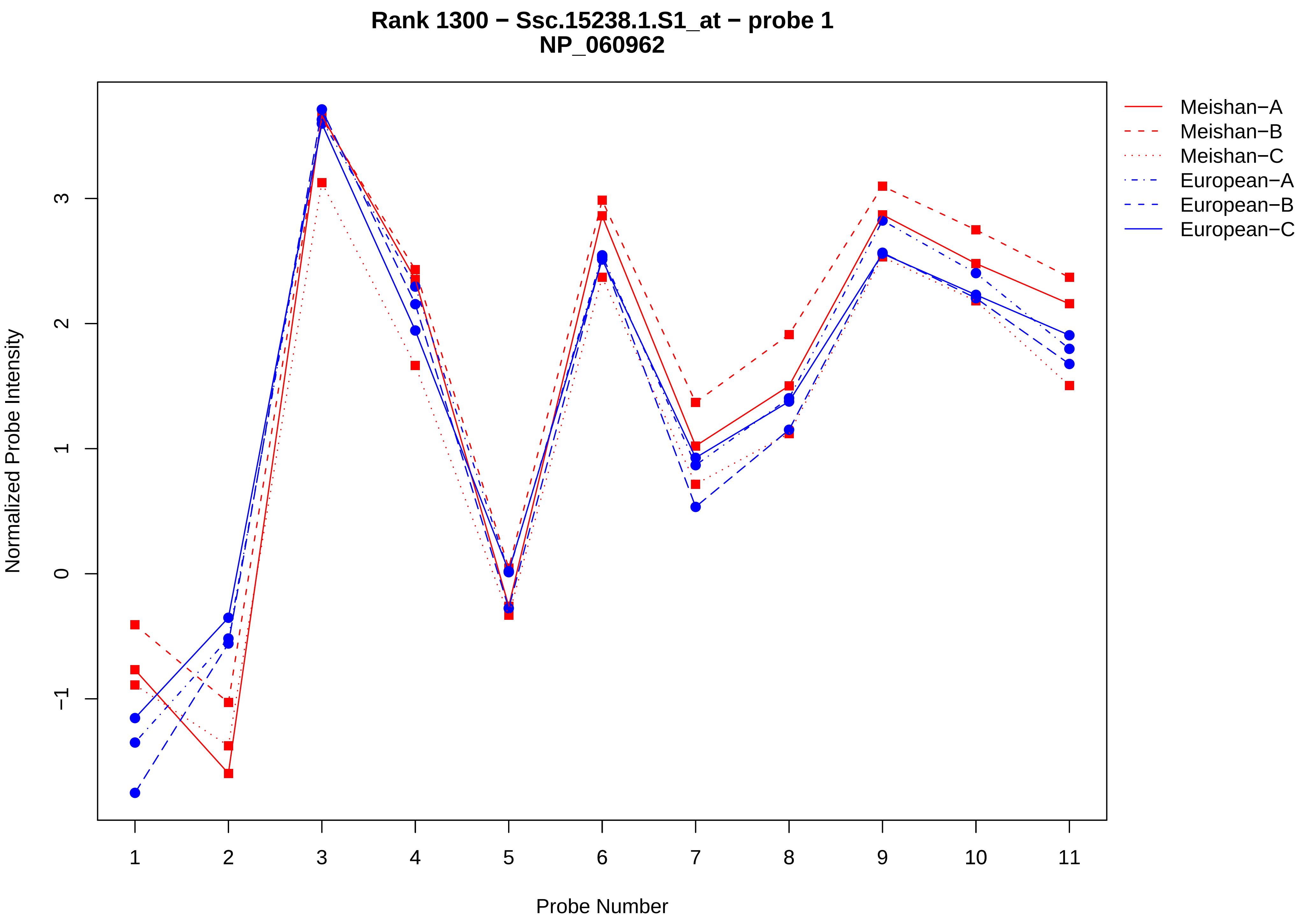

Rank 1300 − Ssc.15238.1.S1_at − probe 1
NP_060962
Normalized Probe Intensity
Probe Number
Meishan−A
Meishan−B
Meishan−C
European−A
European−B
European−C
−1
0
1
2
3
1
2
3
4
5
6
7
8
9
10
11

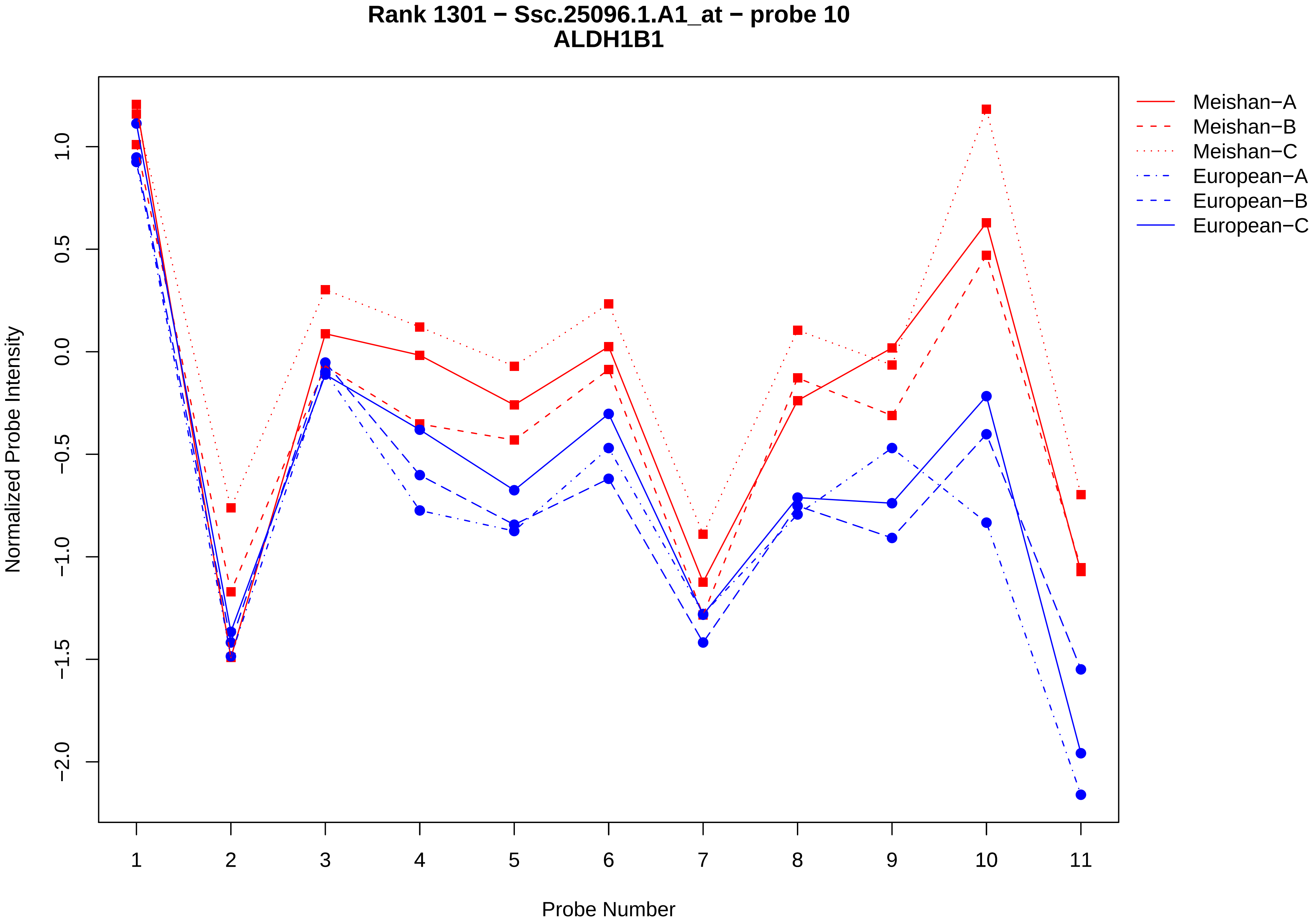

Rank 1301 − Ssc.25096.1.A1_at − probe 10
ALDH1B1
Normalized Probe Intensity
Probe Number
Meishan−A
Meishan−B
Meishan−C
European−A
European−B
European−C
1.0
0.5
0.0
−0.5
−1.0
−1.5
−2.0
1
2
3
4
5
6
7
8
9
10
11

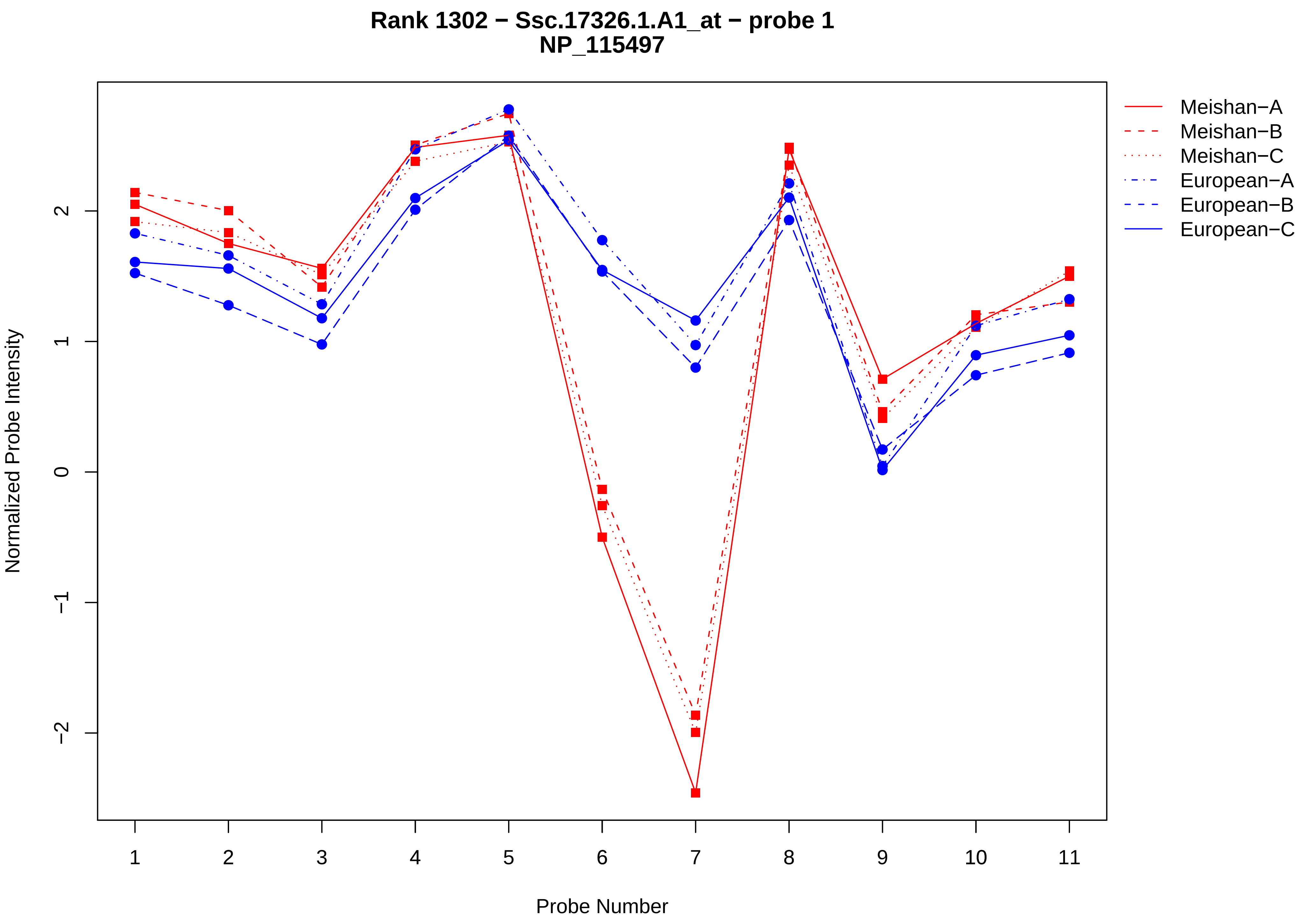

Rank 1302 − Ssc.17326.1.A1_at − probe 1
NP_115497
Normalized Probe Intensity
Probe Number
−2
−1
0
1
2
1
2
3
4
5
6
7
8
9
10
11
Meishan−A
Meishan−B
Meishan−C
European−A
European−B
European−C

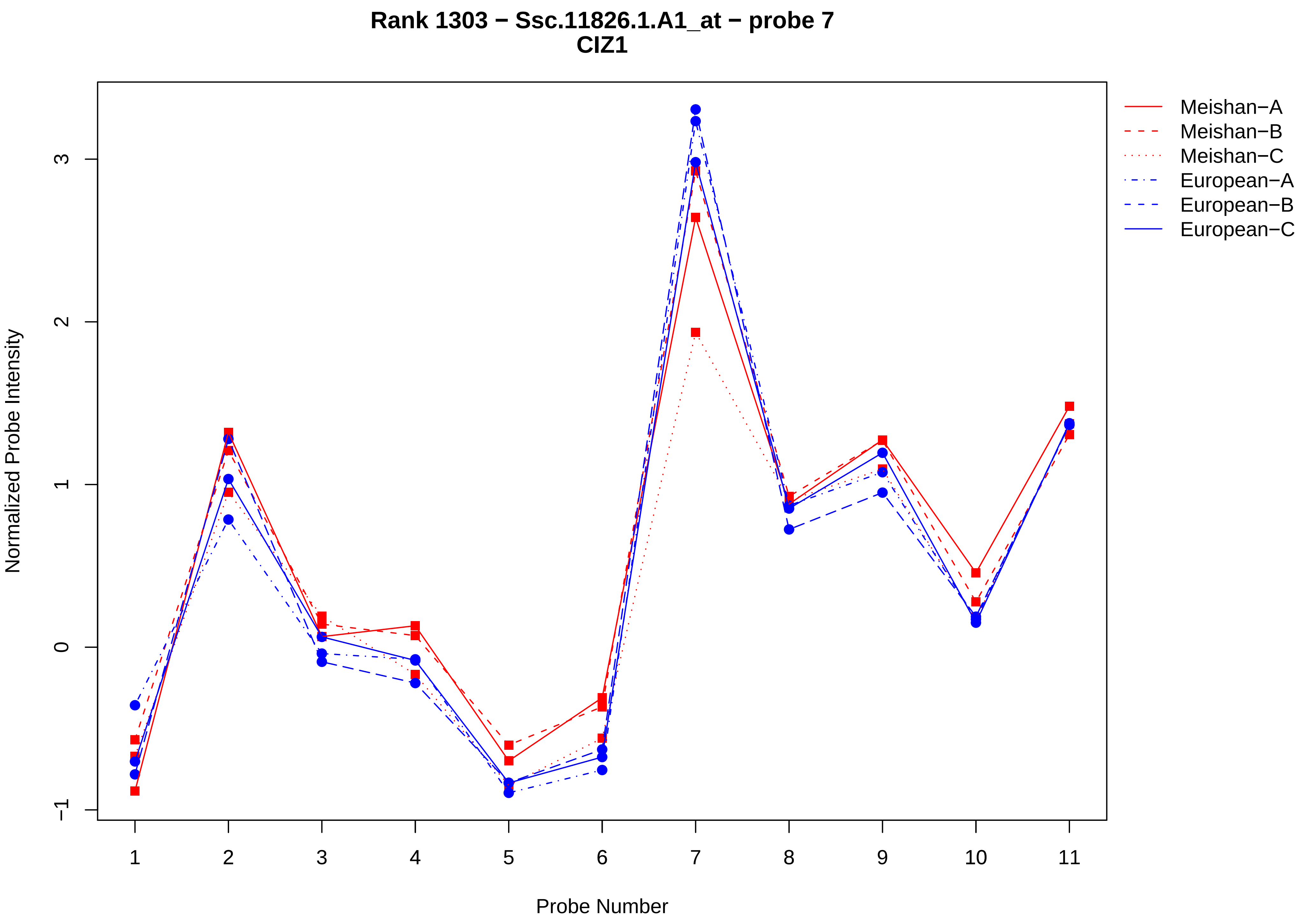

Rank 1303 − Ssc.11826.1.A1_at − probe 7
CIZ1
Normalized Probe Intensity
Probe Number
−1
0
1
2
3
1
2
3
4
5
6
7
8
9
10
11
Meishan−A
Meishan−B
Meishan−C
European−A
European−B
European−C

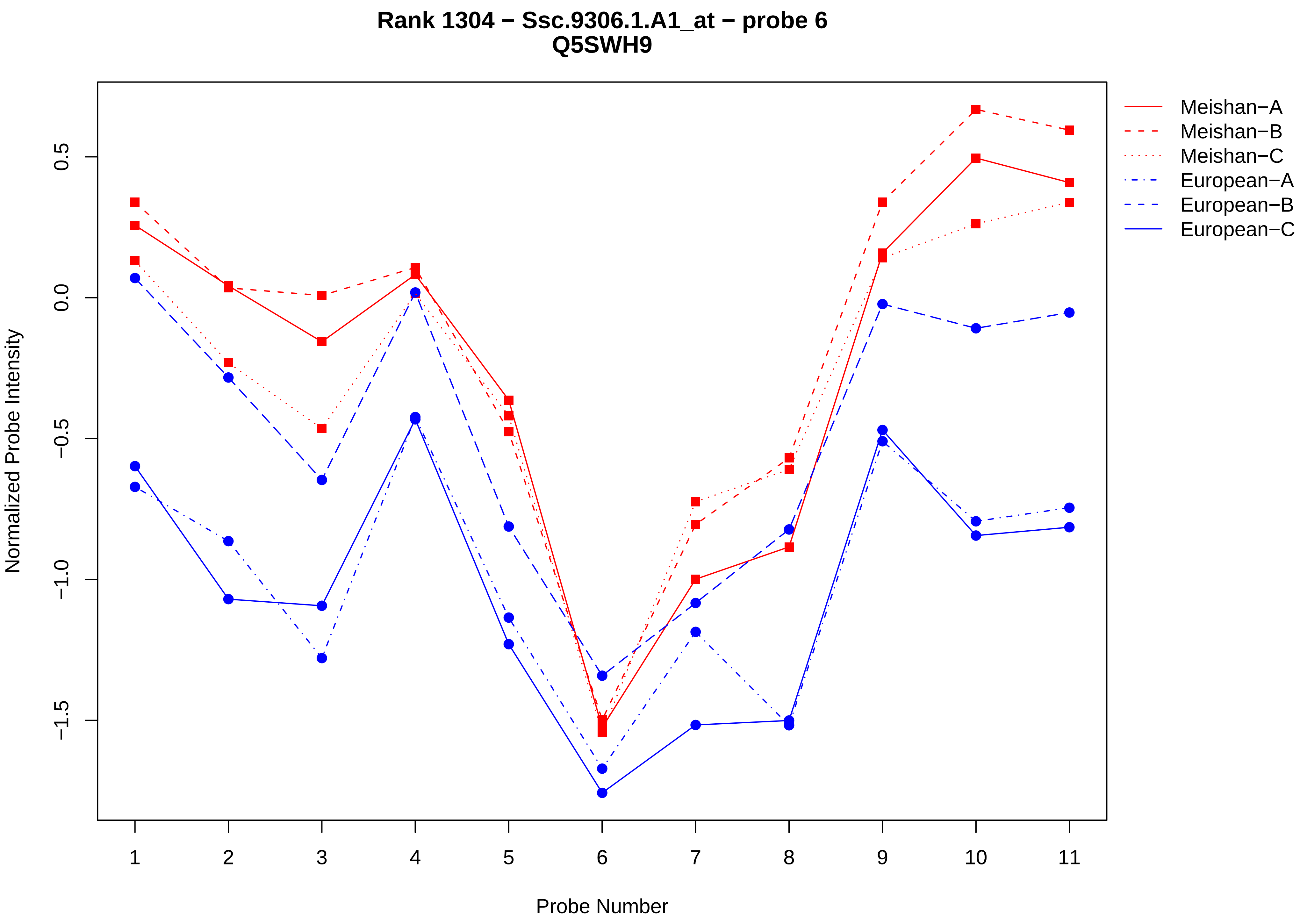

Rank 1304 − Ssc.9306.1.A1_at − probe 6
Q5SWH9
Normalized Probe Intensity
Probe Number
Meishan−A
Meishan−B
Meishan−C
European−A
European−B
European−C

Rank 1305 − Ssc.10088.1.A1_at − probe 11
Q7Z3D4

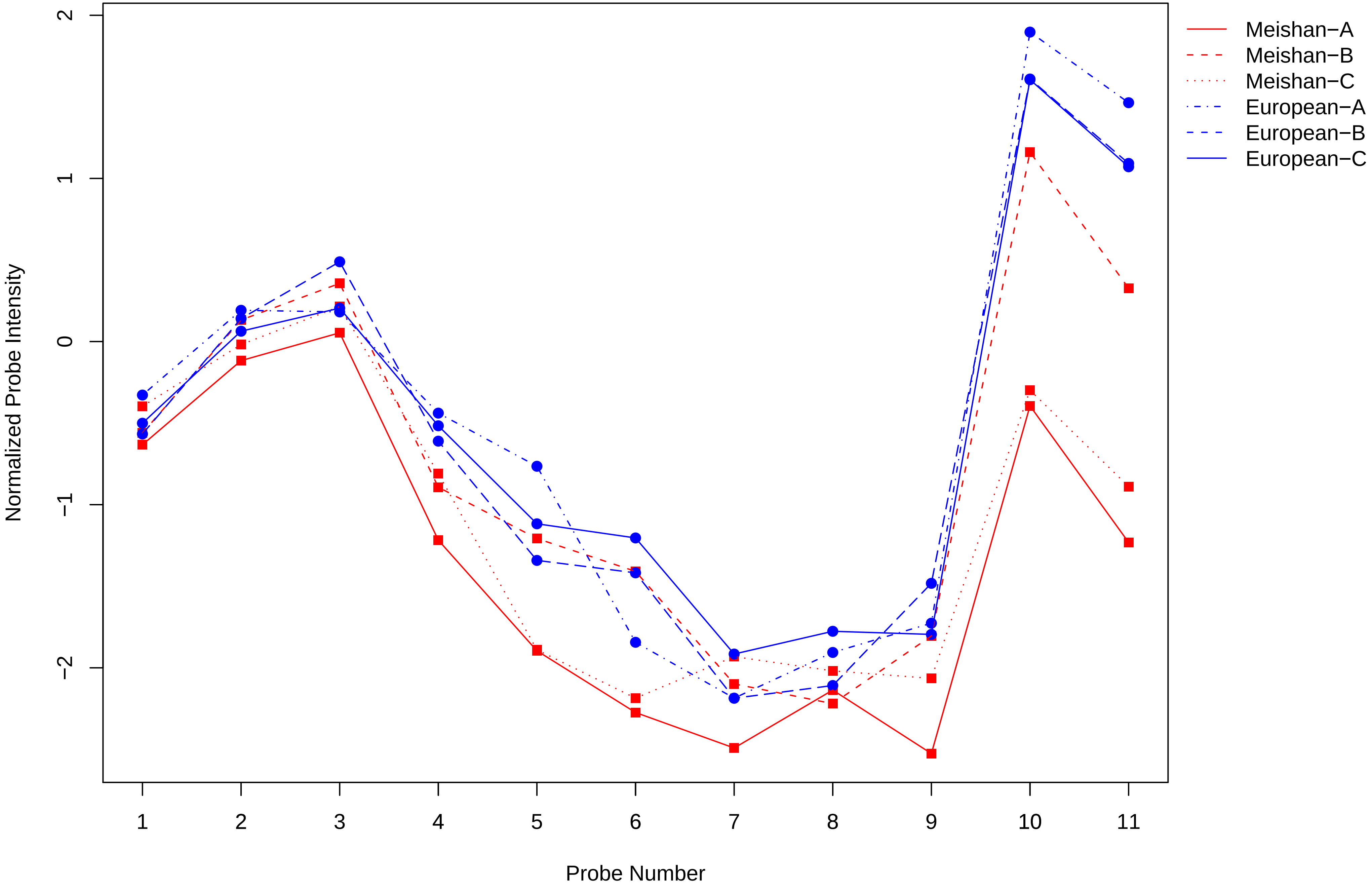

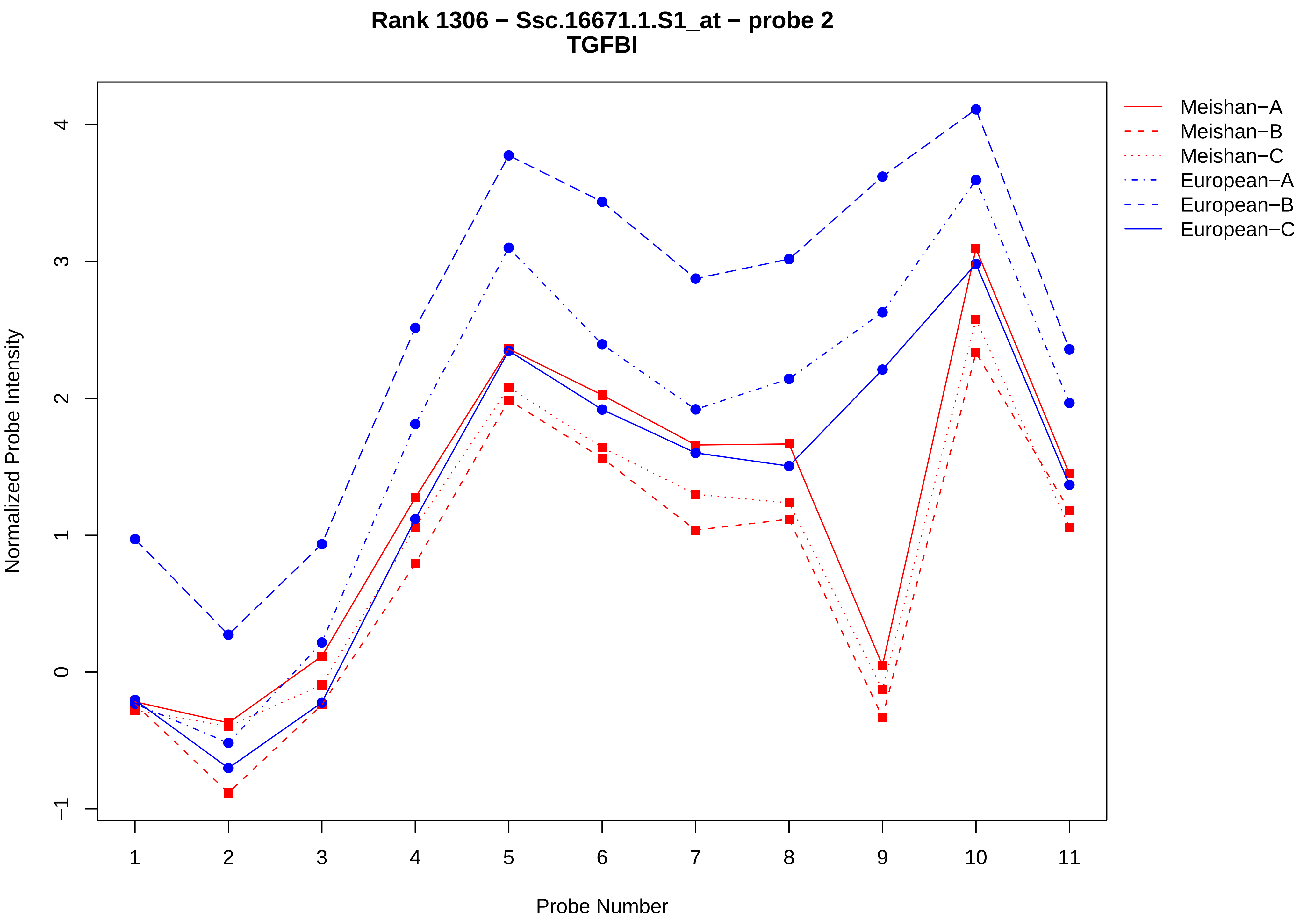

Rank 1306 − Ssc.16671.1.S1_at − probe 2
TGFBI
Normalized Probe Intensity
Probe Number
−1
0
1
2
3
4
1
2
3
4
5
6
7
8
9
10
11
Meishan−A
Meishan−B
Meishan−C
European−A
European−B
European−C

Rank 1307 − Ssc.26686.1.S1_at − probe 8
RBM19

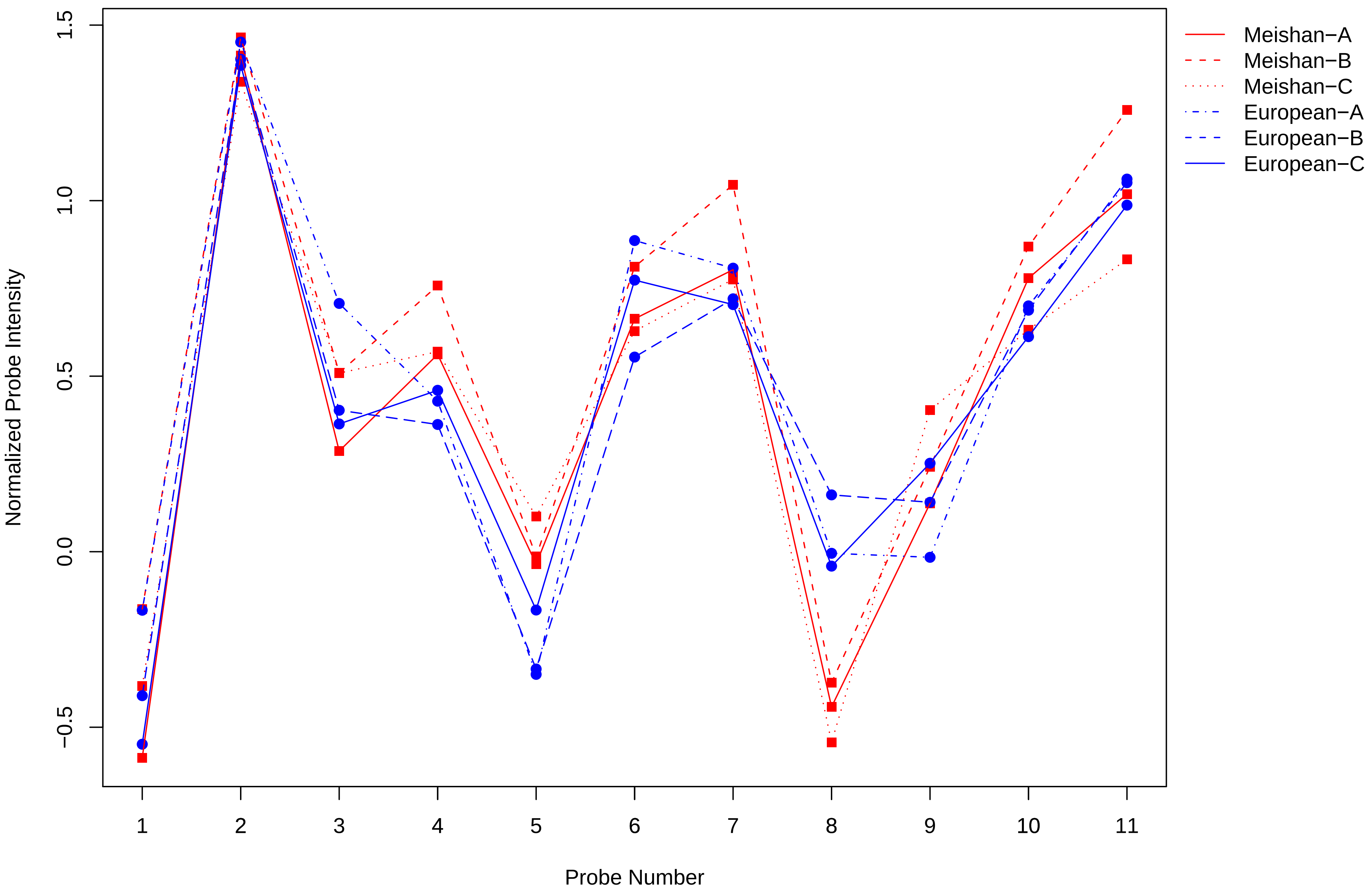

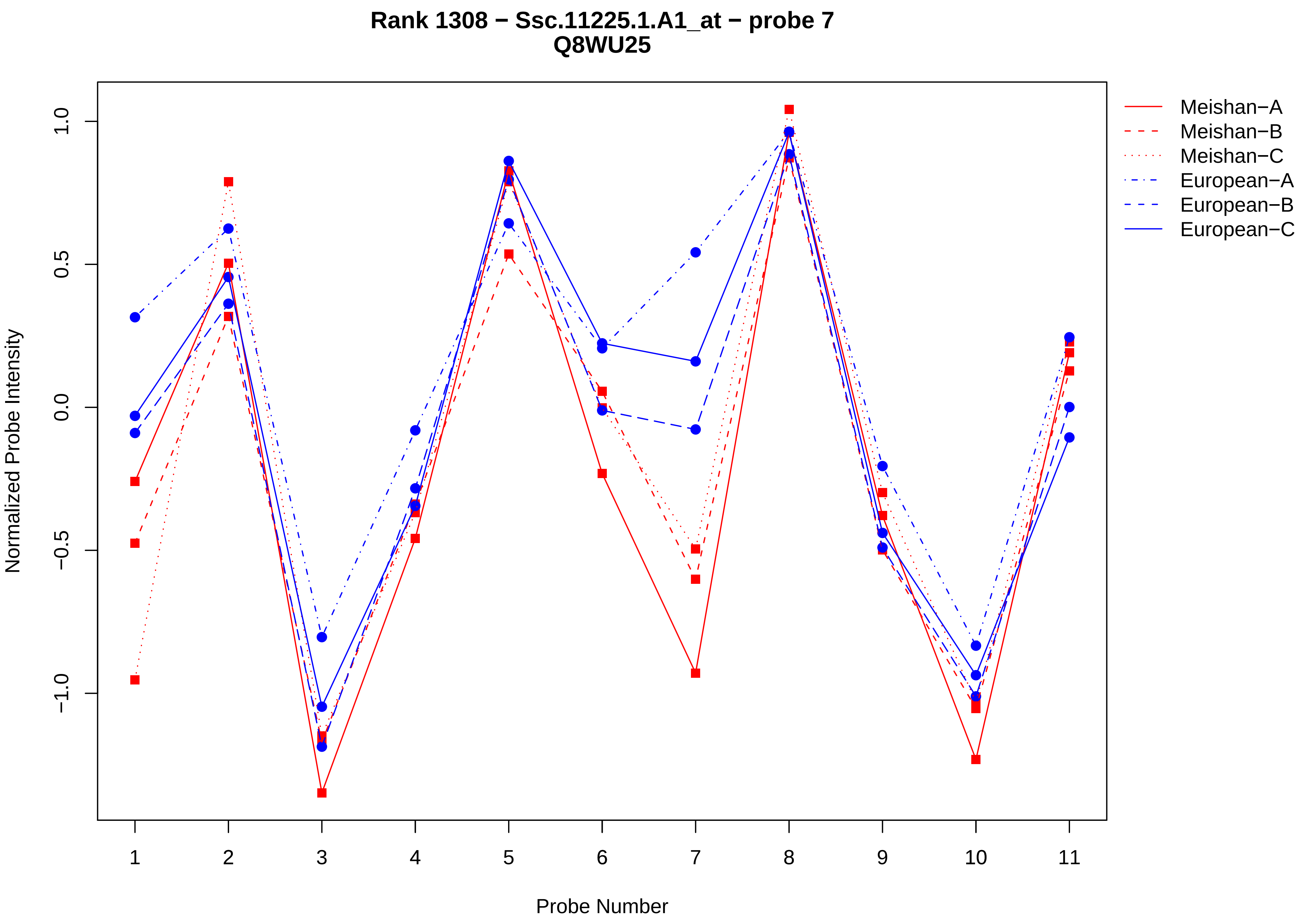

Rank 1308 − Ssc.11225.1.A1_at − probe 7
Q8WU25
Normalized Probe Intensity
Probe Number
1.0
0.5
0.0
−0.5
−1.0
1
2
3
4
5
6
7
8
9
10
11
Meishan−A
Meishan−B
Meishan−C
European−A
European−B
European−C

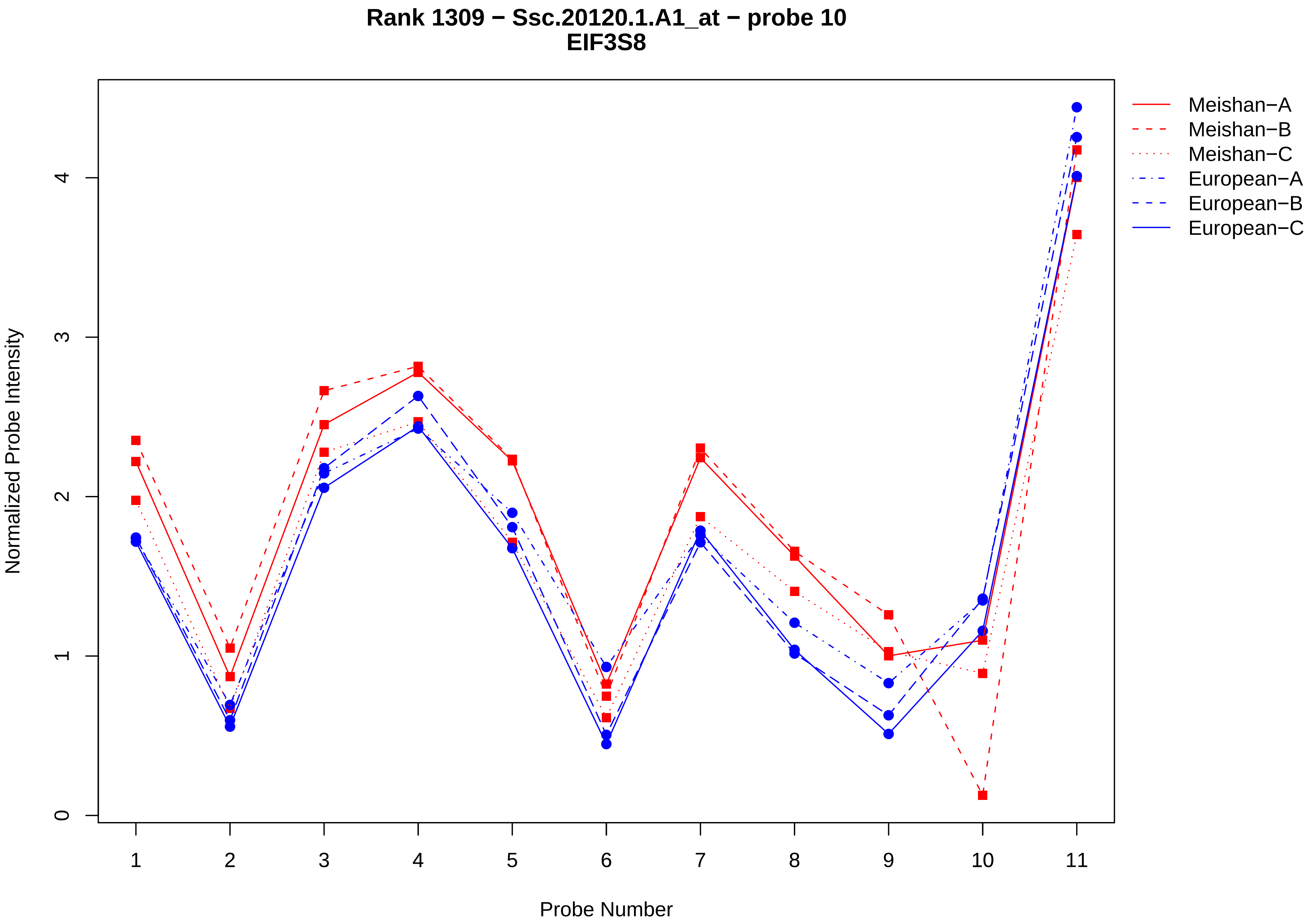

Rank 1309 − Ssc.20120.1.A1_at − probe 10
EIF3S8
Normalized Probe Intensity
Probe Number
0
1
2
3
4
1
2
3
4
5
6
7
8
9
10
11
Meishan−A
Meishan−B
Meishan−C
European−A
European−B
European−C

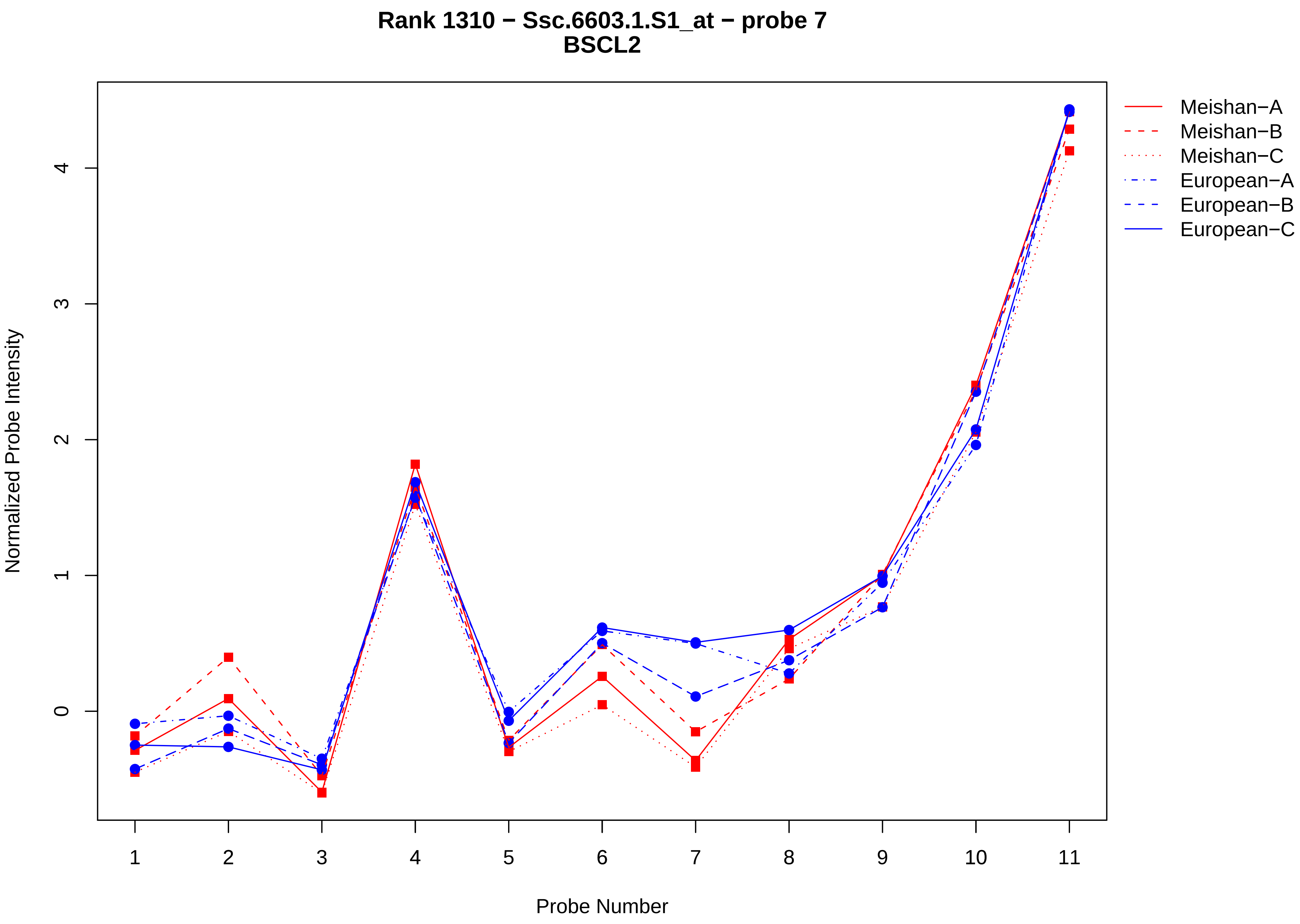

Rank 1310 − Ssc.6603.1.S1_at − probe 7
BSCL2
Normalized Probe Intensity
0
1
2
3
4
1
2
3
4
5
6
7
8
9
10
11
Probe Number
Meishan−A
Meishan−B
Meishan−C
European−A
European−B
European−C

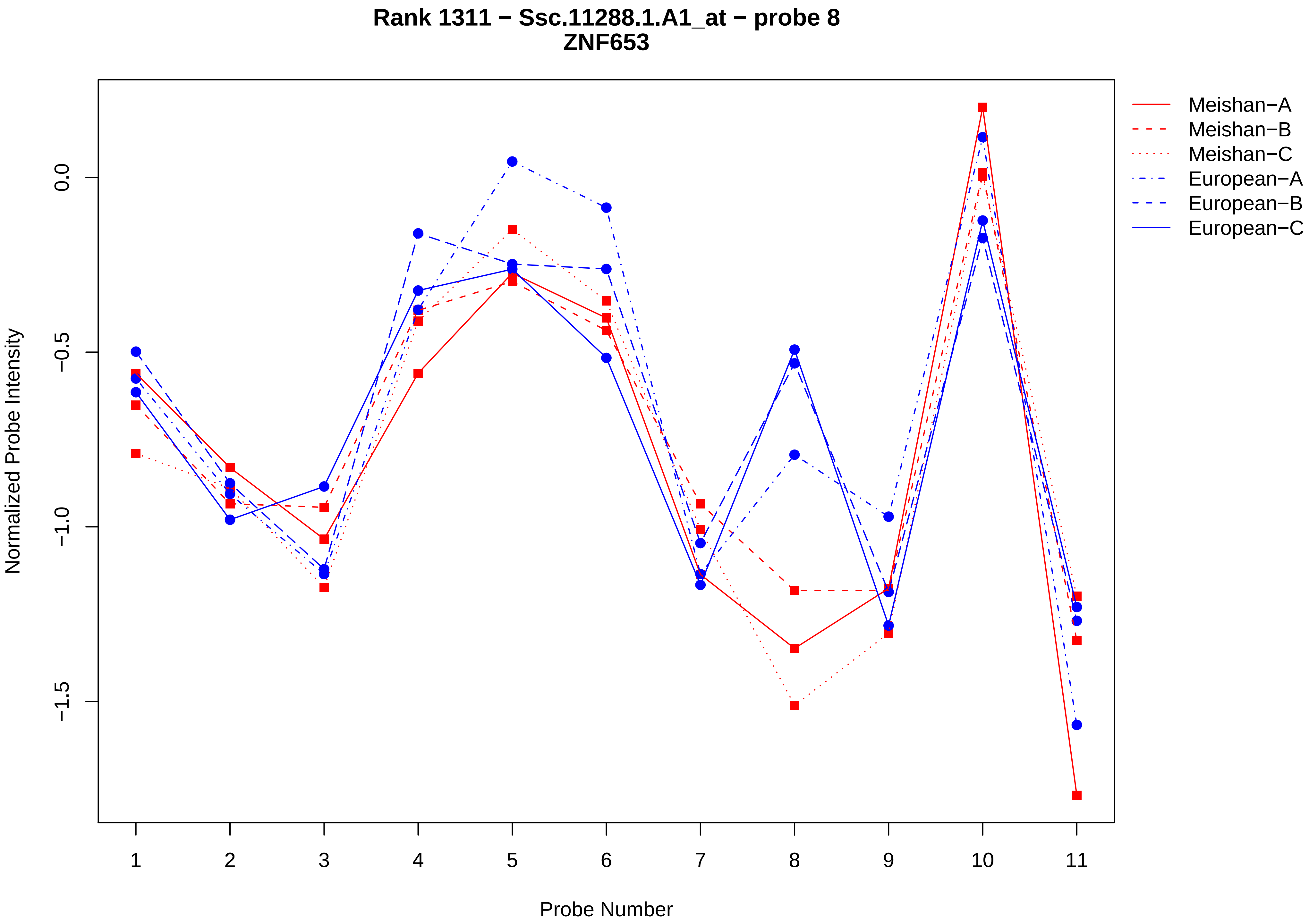
Rank 1311 − Ssc.11288.1.A1_at − probe 8
ZNF653
Normalized Probe Intensity
Probe Number
0.0
−0.5
−1.0
−1.5
1
2
3
4
5
6
7
8
9
10
11
Meishan−A
Meishan−B
Meishan−C
European−A
European−B
European−C

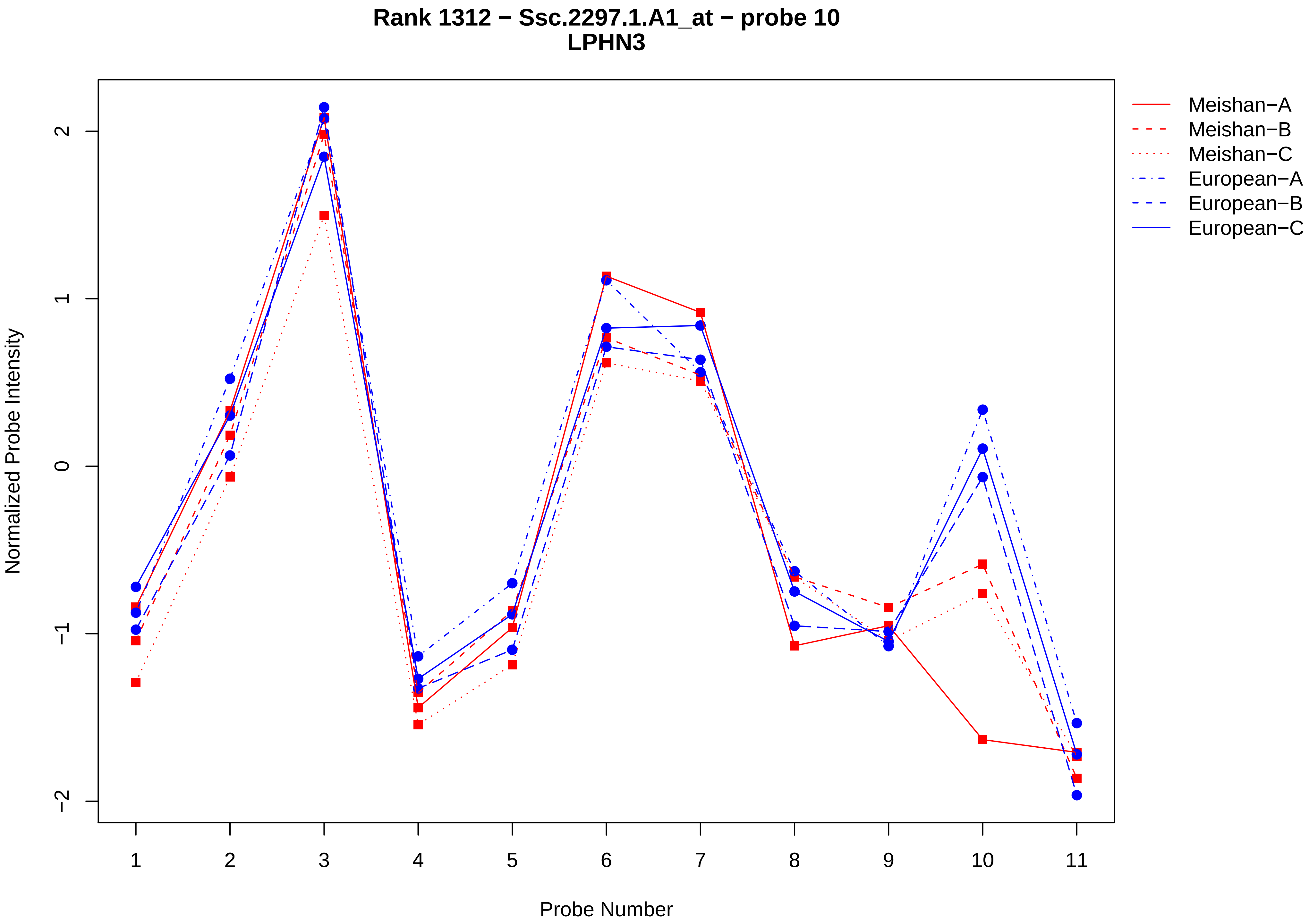
Rank 1312 − Ssc.2297.1.A1_at − probe 10
LPHN3
Normalized Probe Intensity
Probe Number
Meishan−A
Meishan−B
Meishan−C
European−A
European−B
European−C
−2
−1
0
1
2
1
2
3
4
5
6
7
8
9
10
11

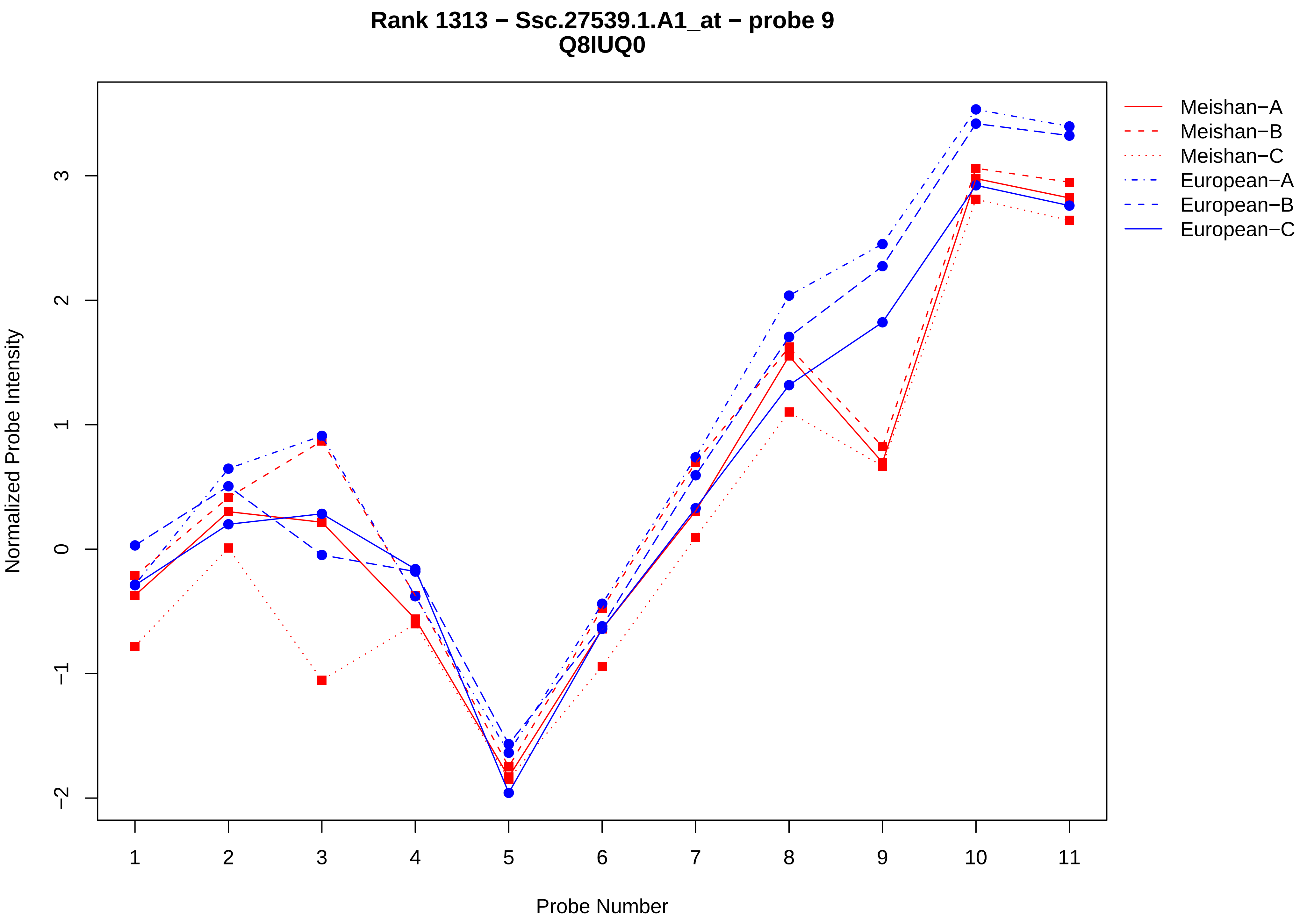

Rank 1313 − Ssc.27539.1.A1_at − probe 9
Q8IUQ0
Normalized Probe Intensity
Probe Number
−2
−1
0
1
2
3
1
2
3
4
5
6
7
8
9
10
11
Meishan−A
Meishan−B
Meishan−C
European−A
European−B
European−C

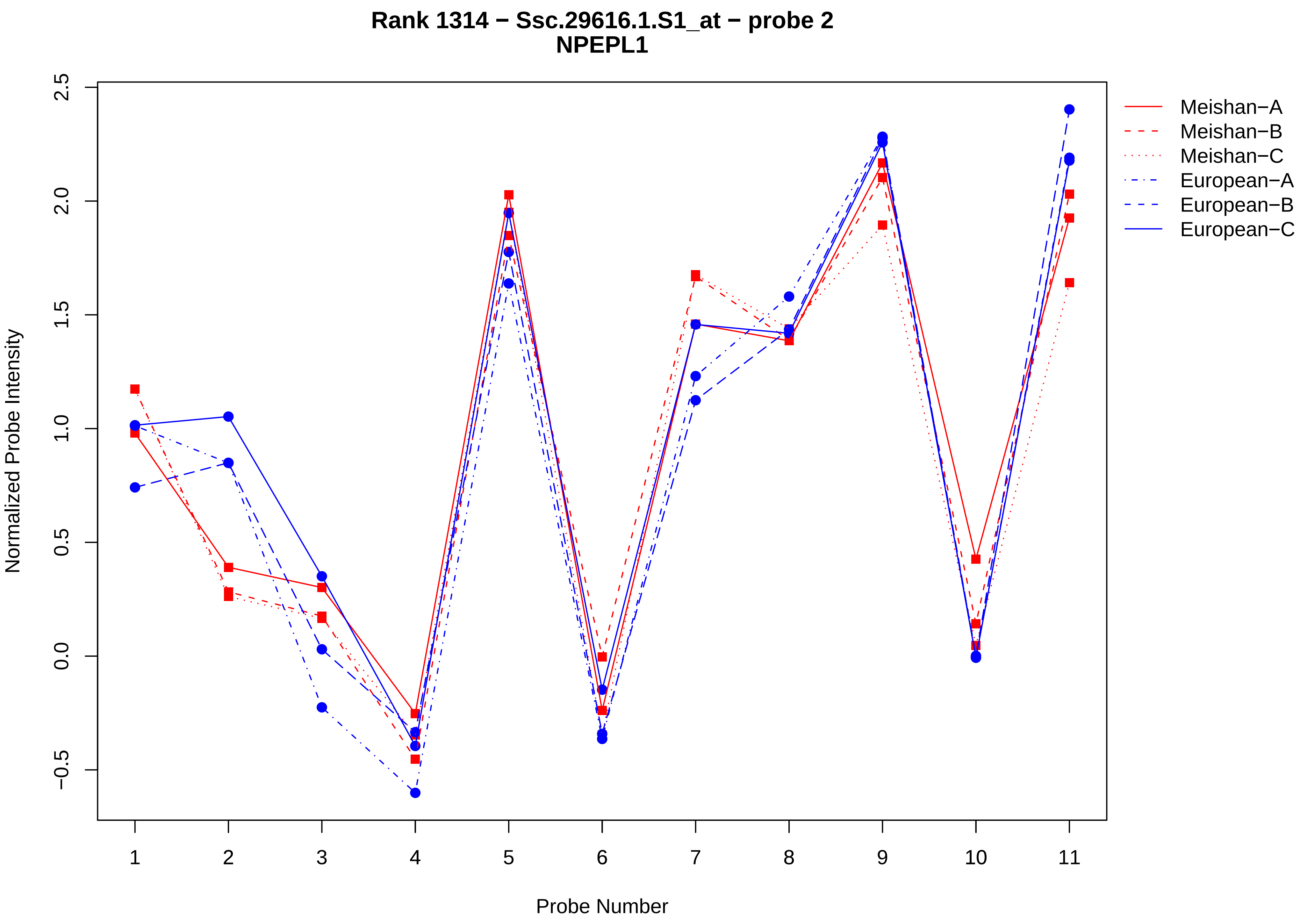

Rank 1314 − Ssc.29616.1.S1_at − probe 2
NPEPL1
Normalized Probe Intensity
Probe Number
−0.5
0.0
0.5
1.0
1.5
2.0
2.5
1
2
3
4
5
6
7
8
9
10
11
Meishan−A
Meishan−B
Meishan−C
European−A
European−B
European−C

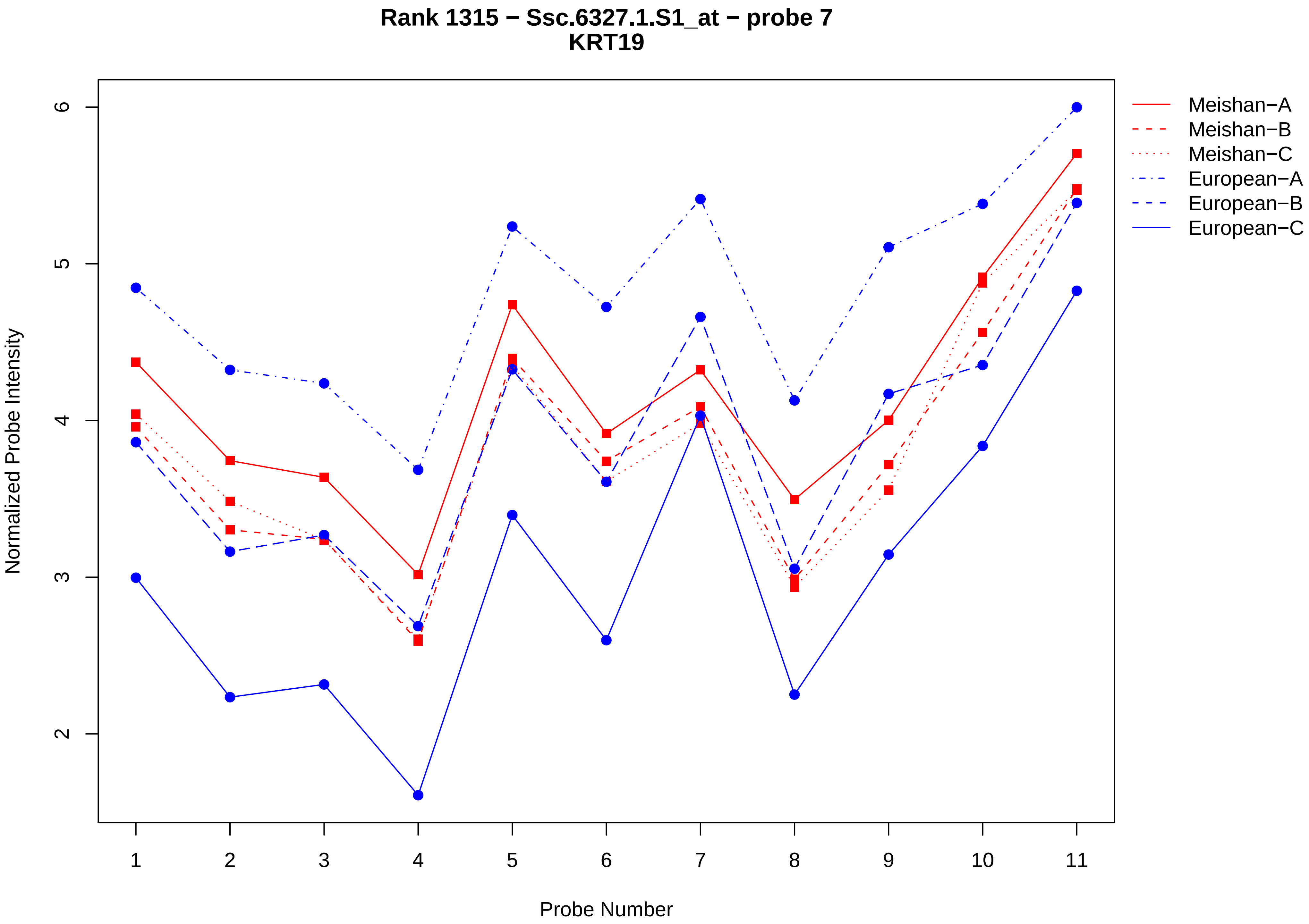
Rank 1315 − Ssc.6327.1.S1_at − probe 7
KRT19
Normalized Probe Intensity
Probe Number
Meishan−A
Meishan−B
Meishan−C
European−A
European−B
European−C
2
3
4
5
6
1
2
3
4
5
6
7
8
9
10
11

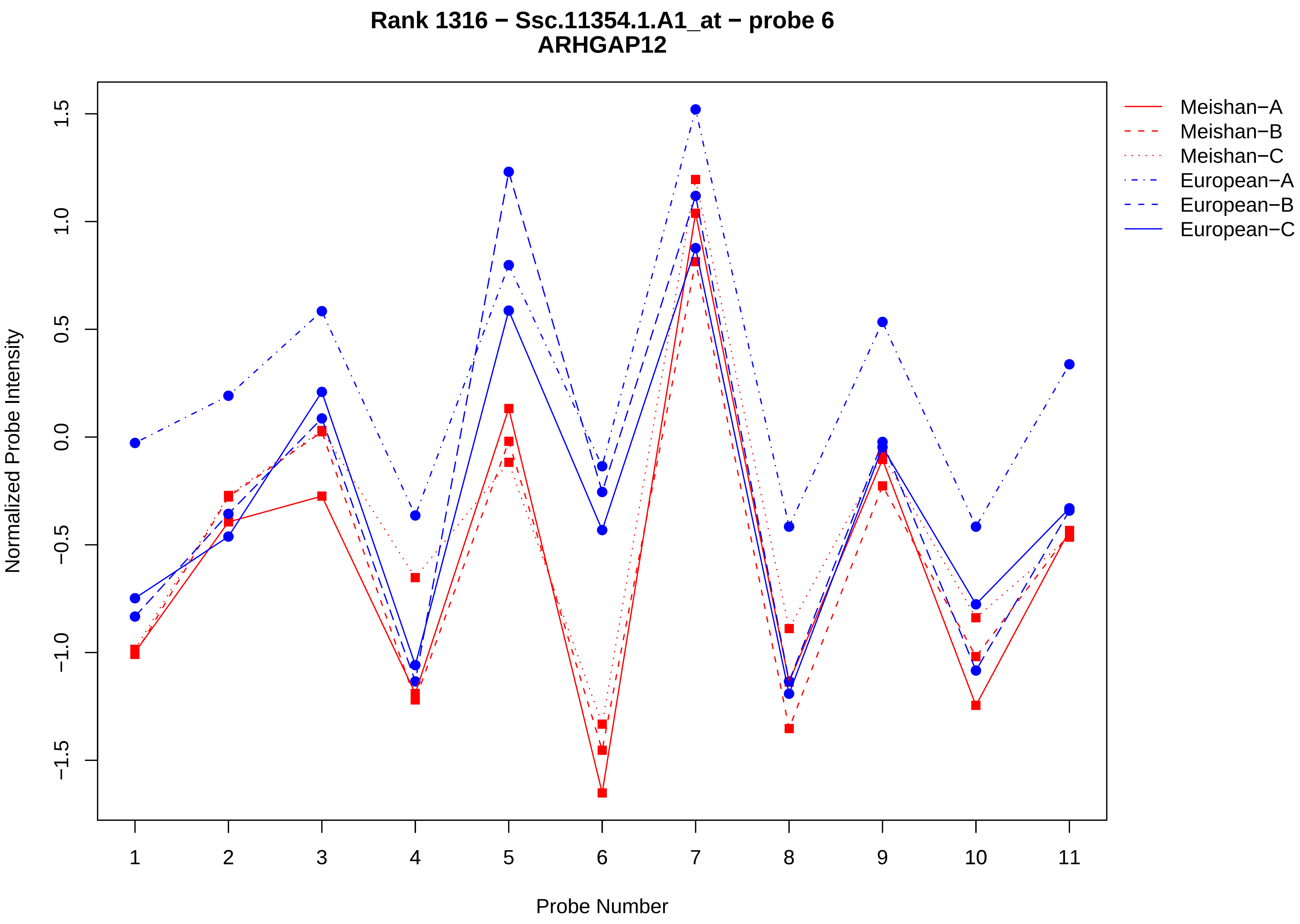

Rank 1316 − Ssc.11354.1.A1_at − probe 6
ARHGAP12
Normalized Probe Intensity
Probe Number
1.5
1.0
0.5
0.0
−0.5
−1.0
−1.5
1
2
3
4
5
6
7
8
9
10
11
Meishan−A
Meishan−B
Meishan−C
European−A
European−B
European−C

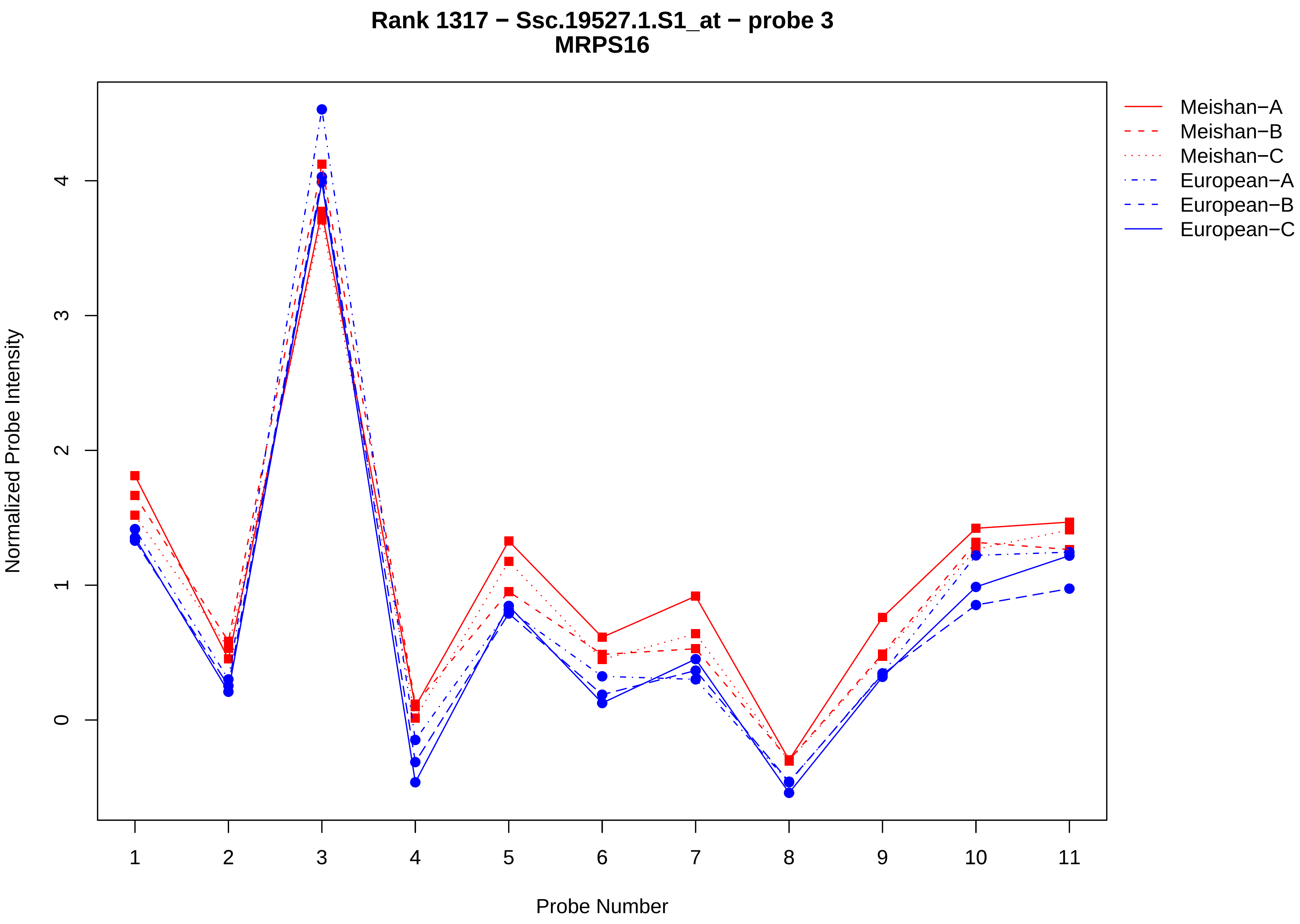

Rank 1317 − Ssc.19527.1.S1_at − probe 3
MRPS16
Normalized Probe Intensity
Probe Number
0
1
2
3
4
1
2
3
4
5
6
7
8
9
10
11
Meishan−A
Meishan−B
Meishan−C
European−A
European−B
European−C

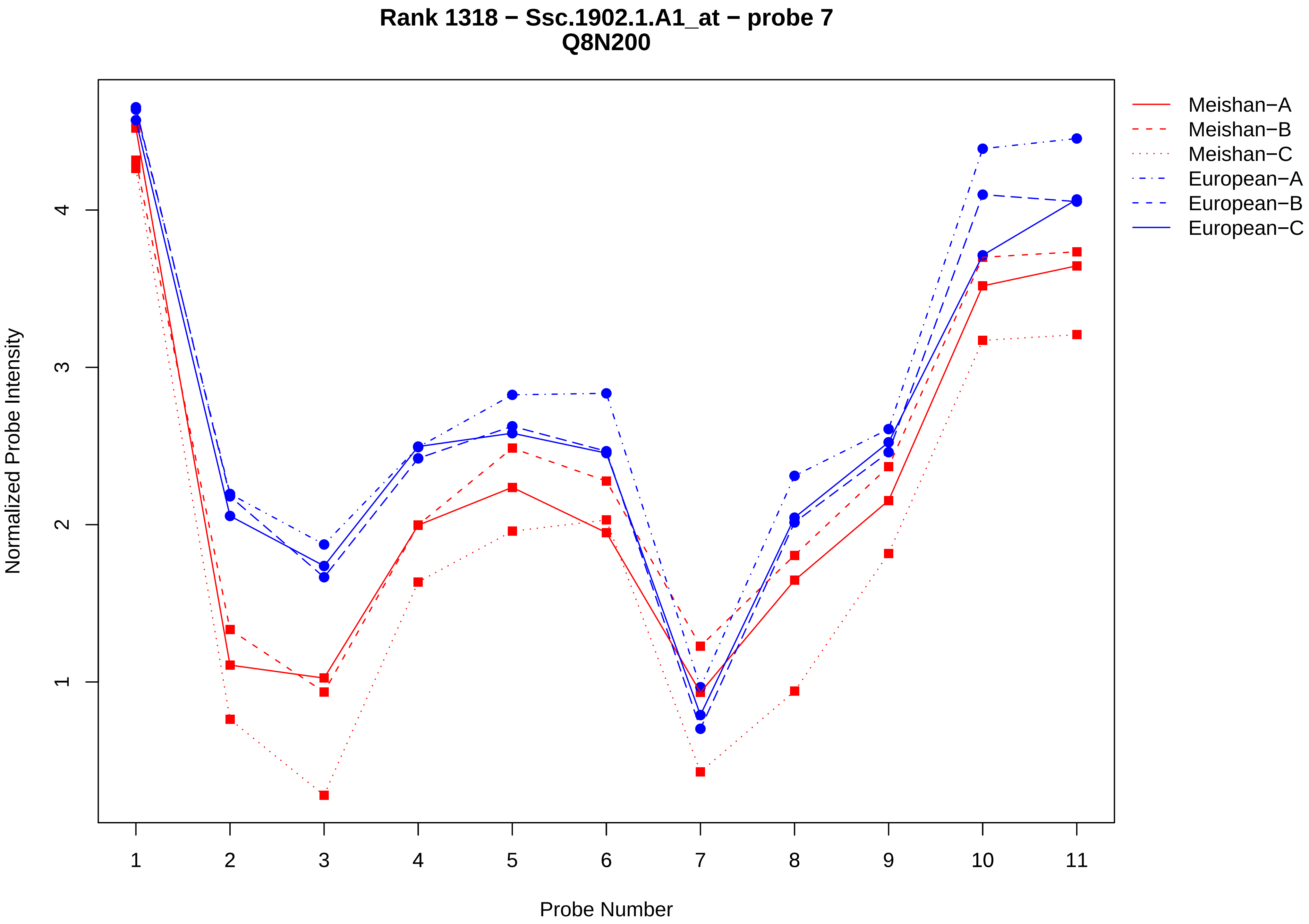

Rank 1318 − Ssc.1902.1.A1_at − probe 7
Q8N200
Normalized Probe Intensity
Probe Number
1
2
3
4
1
2
3
4
5
6
7
8
9
10
11
Meishan−A
Meishan−B
Meishan−C
European−A
European−B
European−C

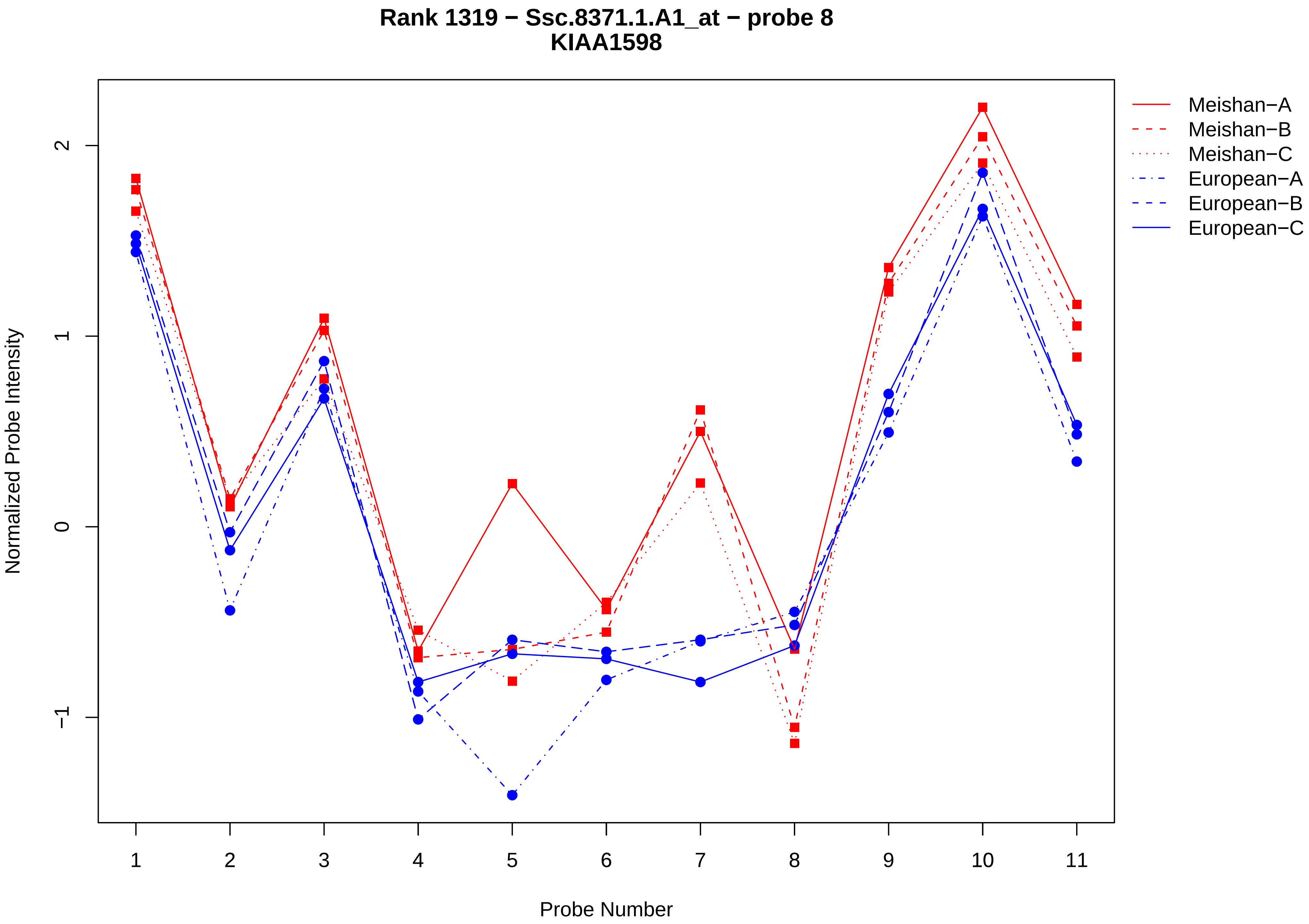
Rank 1319 − Ssc.8371.1.A1_at − probe 8
KIAA1598
Normalized Probe Intensity
Probe Number
−1
0
1
2
1
2
3
4
5
6
7
8
9
10
11
Meishan−A
Meishan−B
Meishan−C
European−A
European−B
European−C

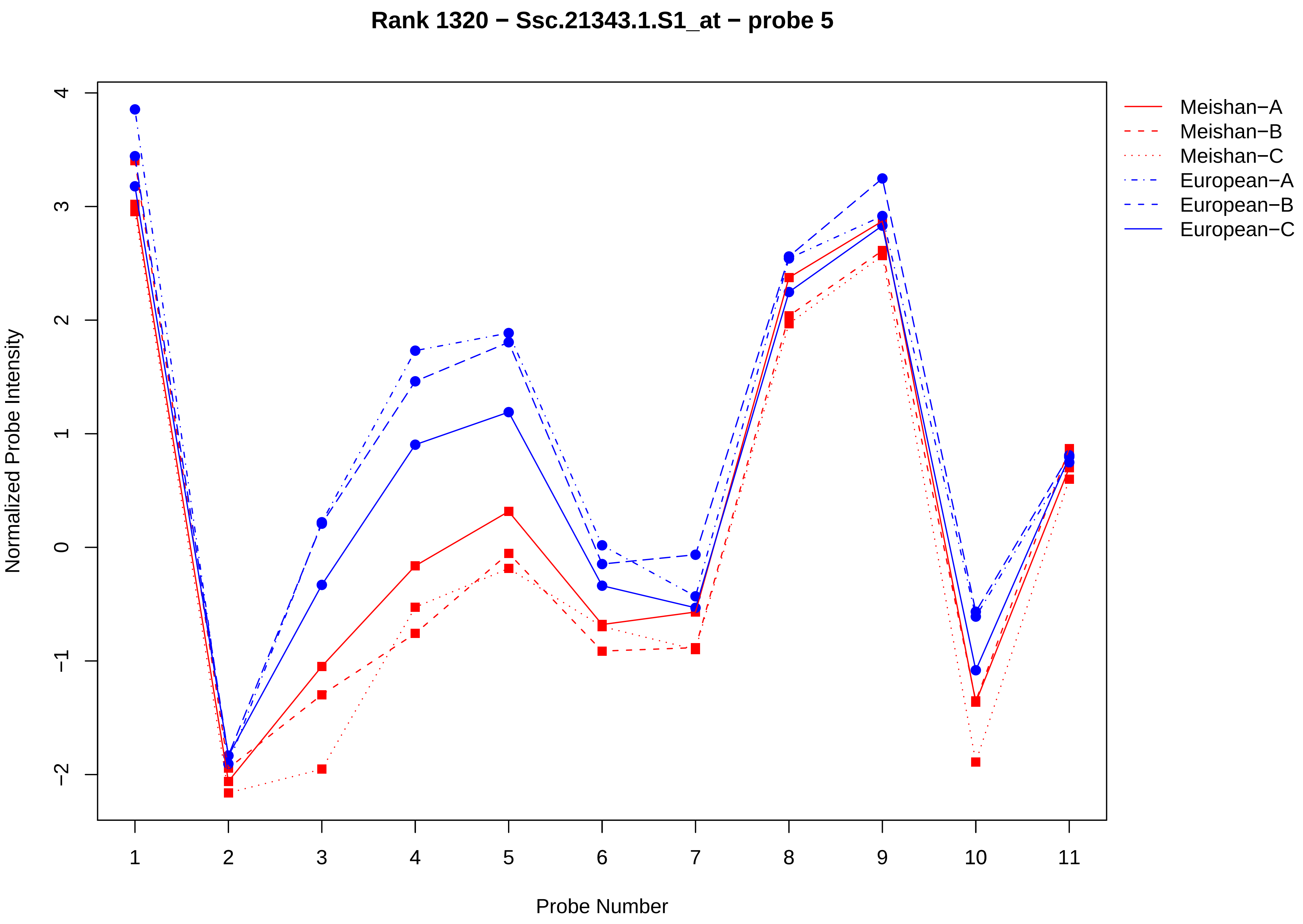

Rank 1320 − Ssc.21343.1.S1_at − probe 5
Normalized Probe Intensity
Probe Number
Meishan−A
Meishan−B
Meishan−C
European−A
European−B
European−C

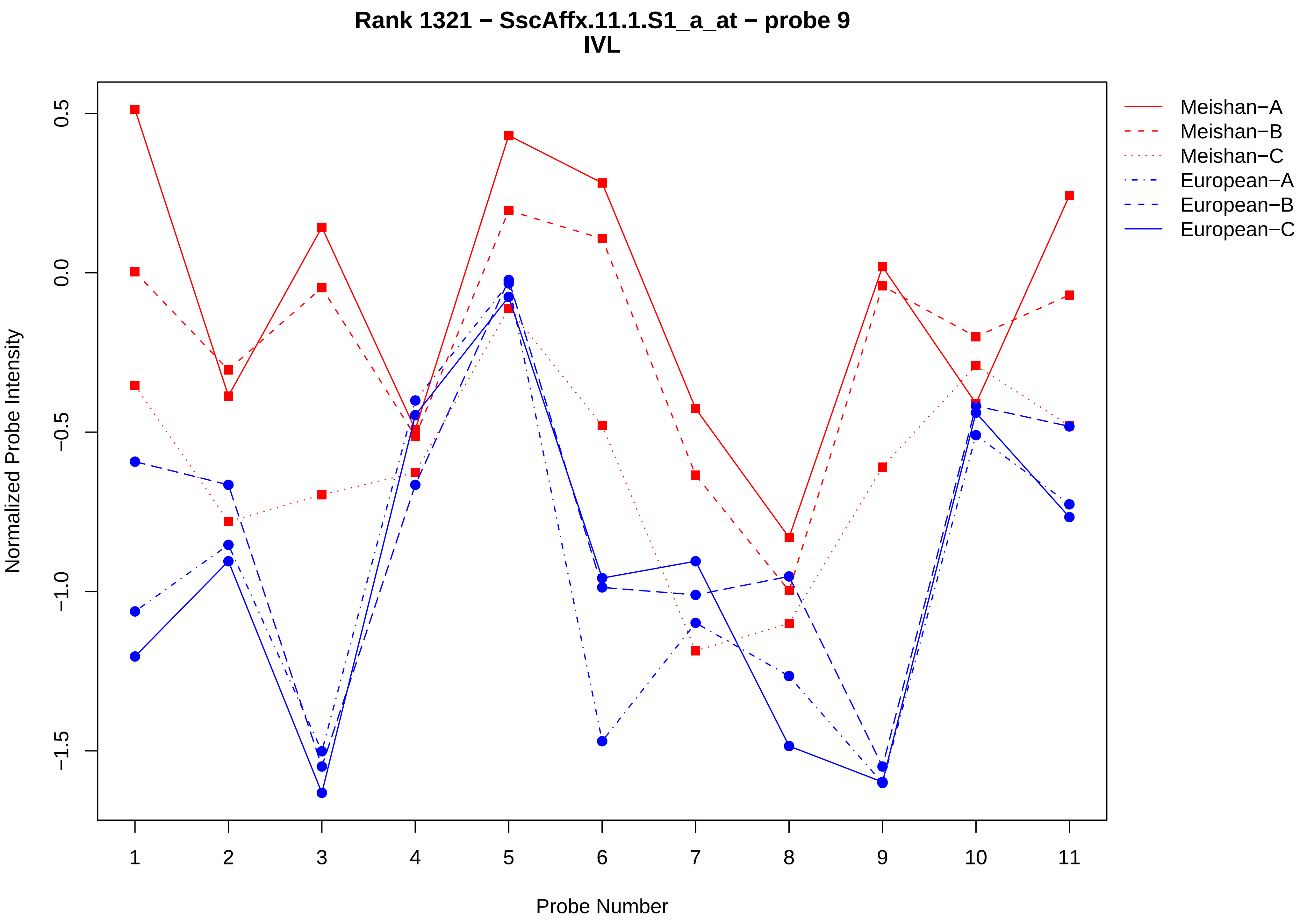

Rank 1321 − SscAffx.11.1.S1_a_at − probe 9
IVL
Normalized Probe Intensity
Probe Number
0.5
0.0
−0.5
−1.0
−1.5
1
2
3
4
5
6
7
8
9
10
11
Meishan−A
Meishan−B
Meishan−C
European−A
European−B
European−C

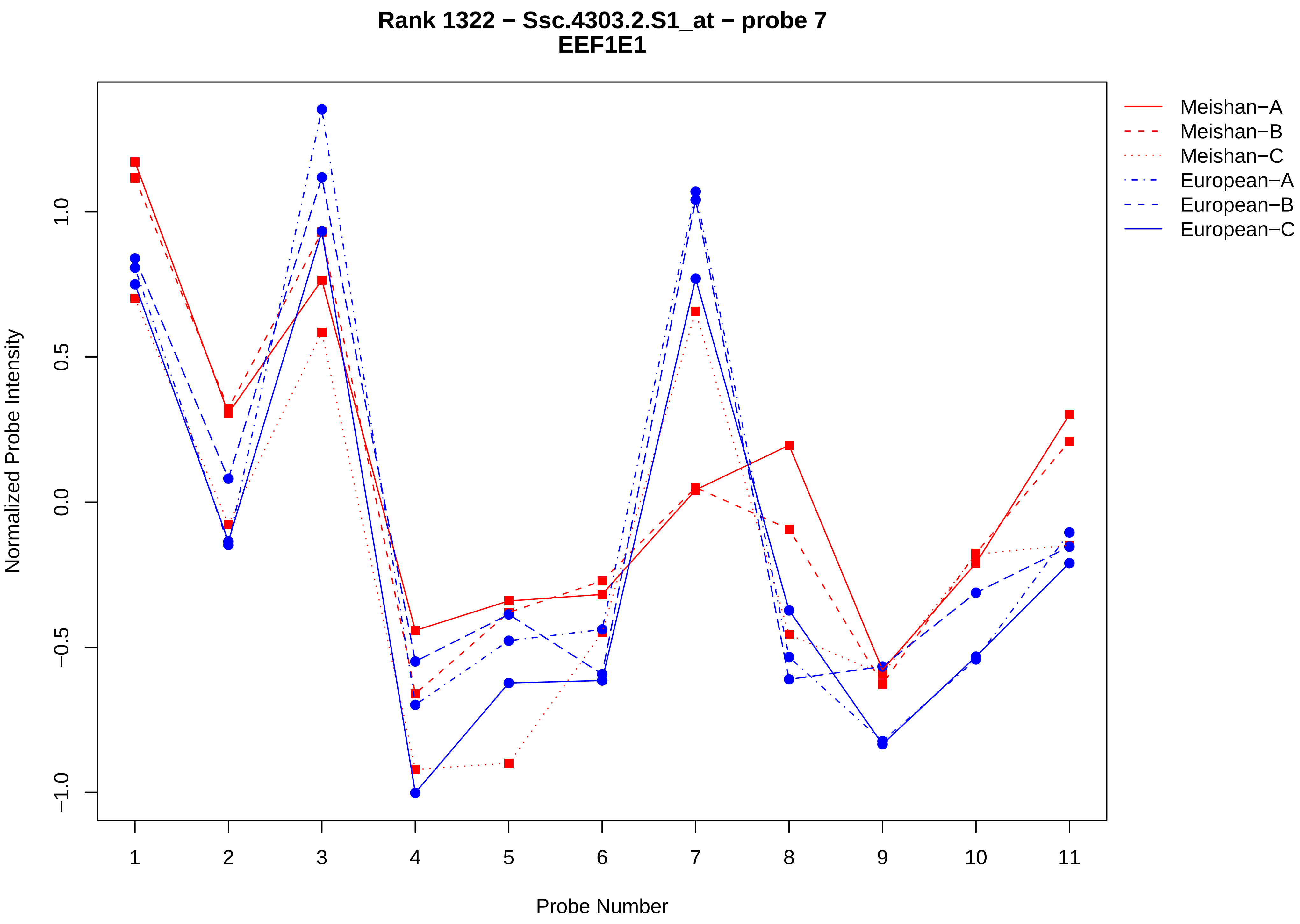

Rank 1322 − Ssc.4303.2.S1_at − probe 7
EEF1E1
Normalized Probe Intensity
Probe Number
−1.0
−0.5
0.0
0.5
1.0
1
2
3
4
5
6
7
8
9
10
11
Meishan−A
Meishan−B
Meishan−C
European−A
European−B
European−C

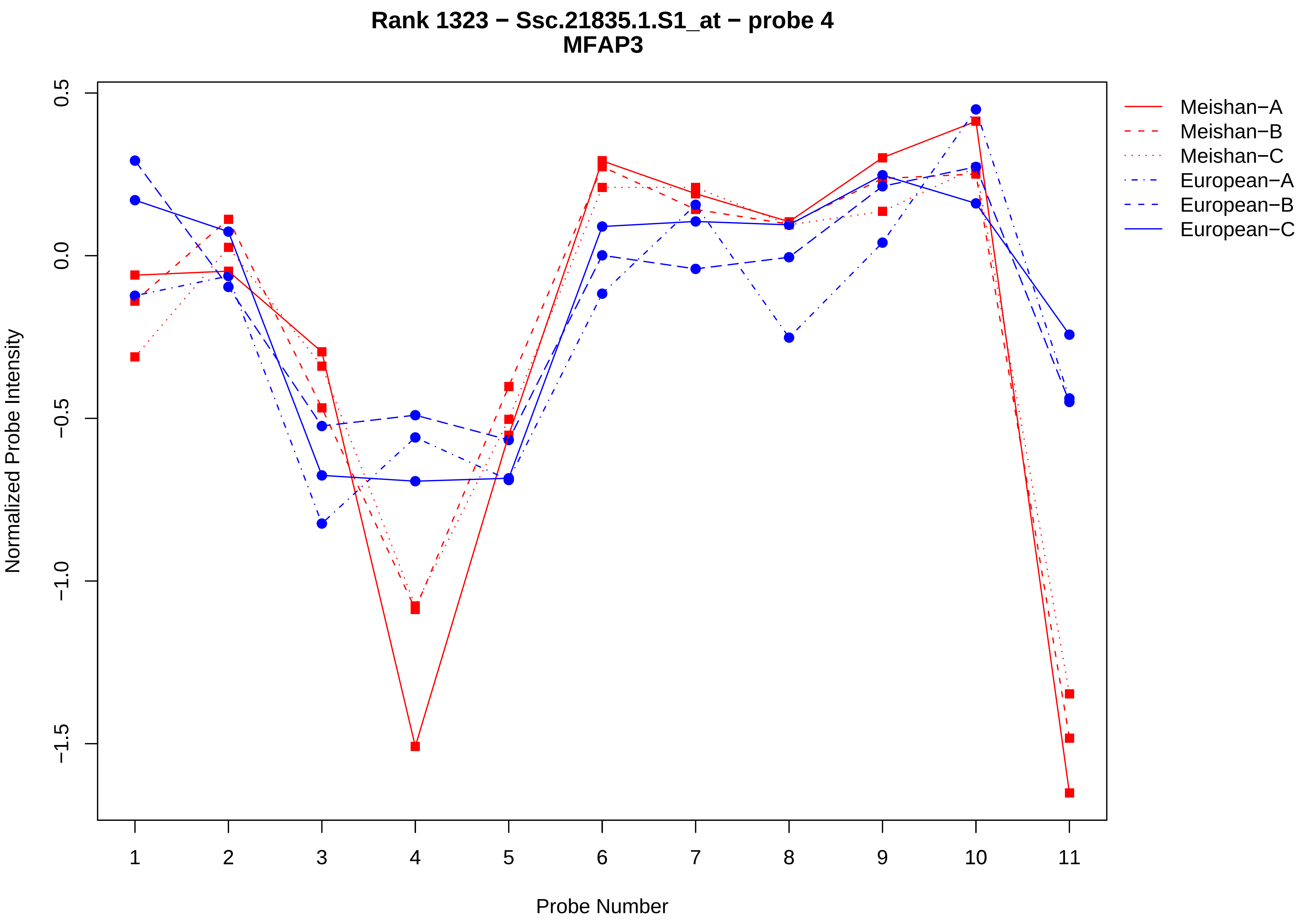

Rank 1323 − Ssc.21835.1.S1_at − probe 4
MFAP3
Normalized Probe Intensity
Probe Number
0.5
0.0
−0.5
−1.0
−1.5
1
2
3
4
5
6
7
8
9
10
11
Meishan−A
Meishan−B
Meishan−C
European−A
European−B
European−C

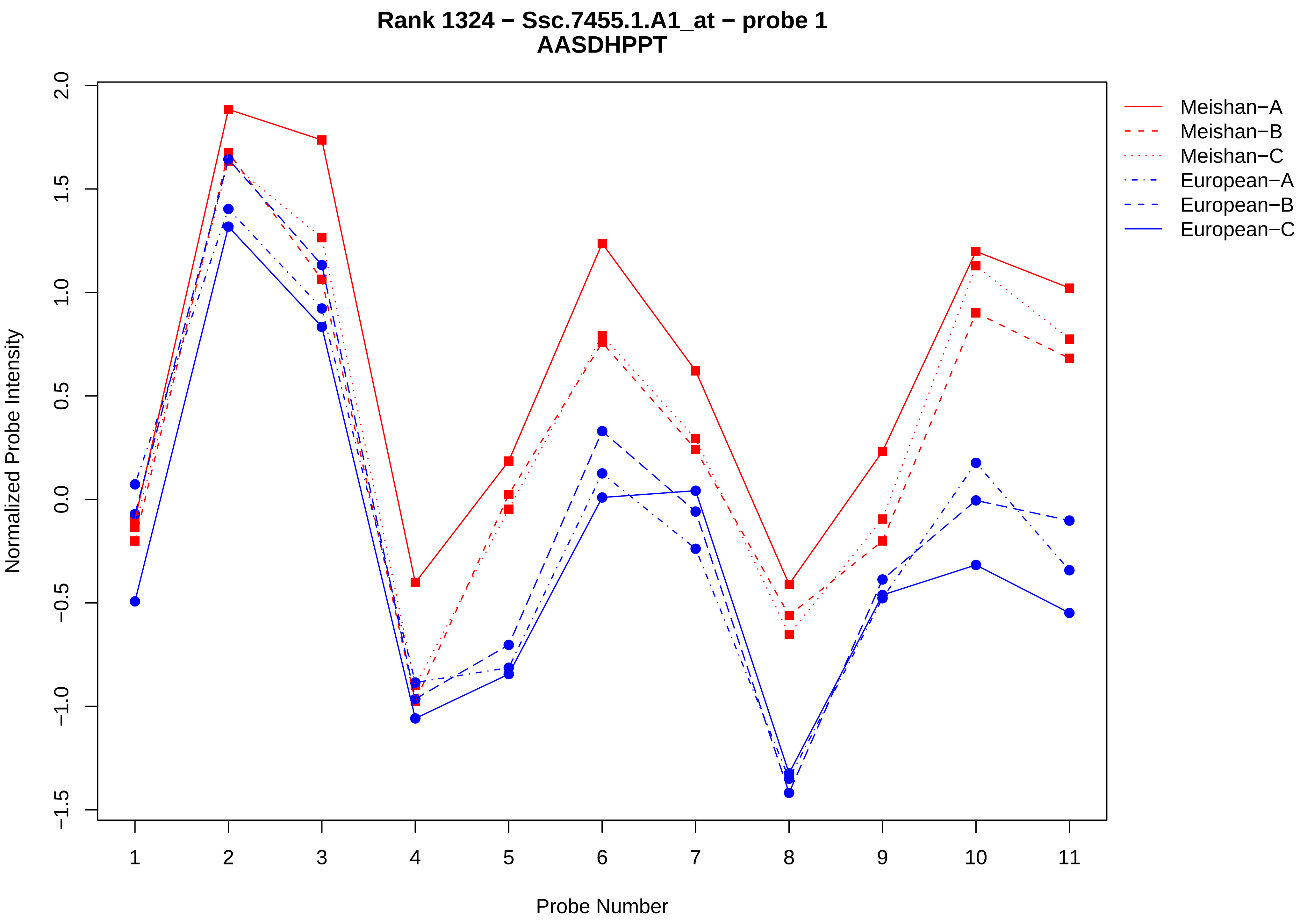
Rank 1324 − Ssc.7455.1.A1_at − probe 1
AASDHPPT
Normalized Probe Intensity
Probe Number
Meishan−A
Meishan−B
Meishan−C
European−A
European−B
European−C
2.0
1.5
1.0
0.5
0.0
−0.5
−1.0
−1.5
1
2
3
4
5
6
7
8
9
10
11

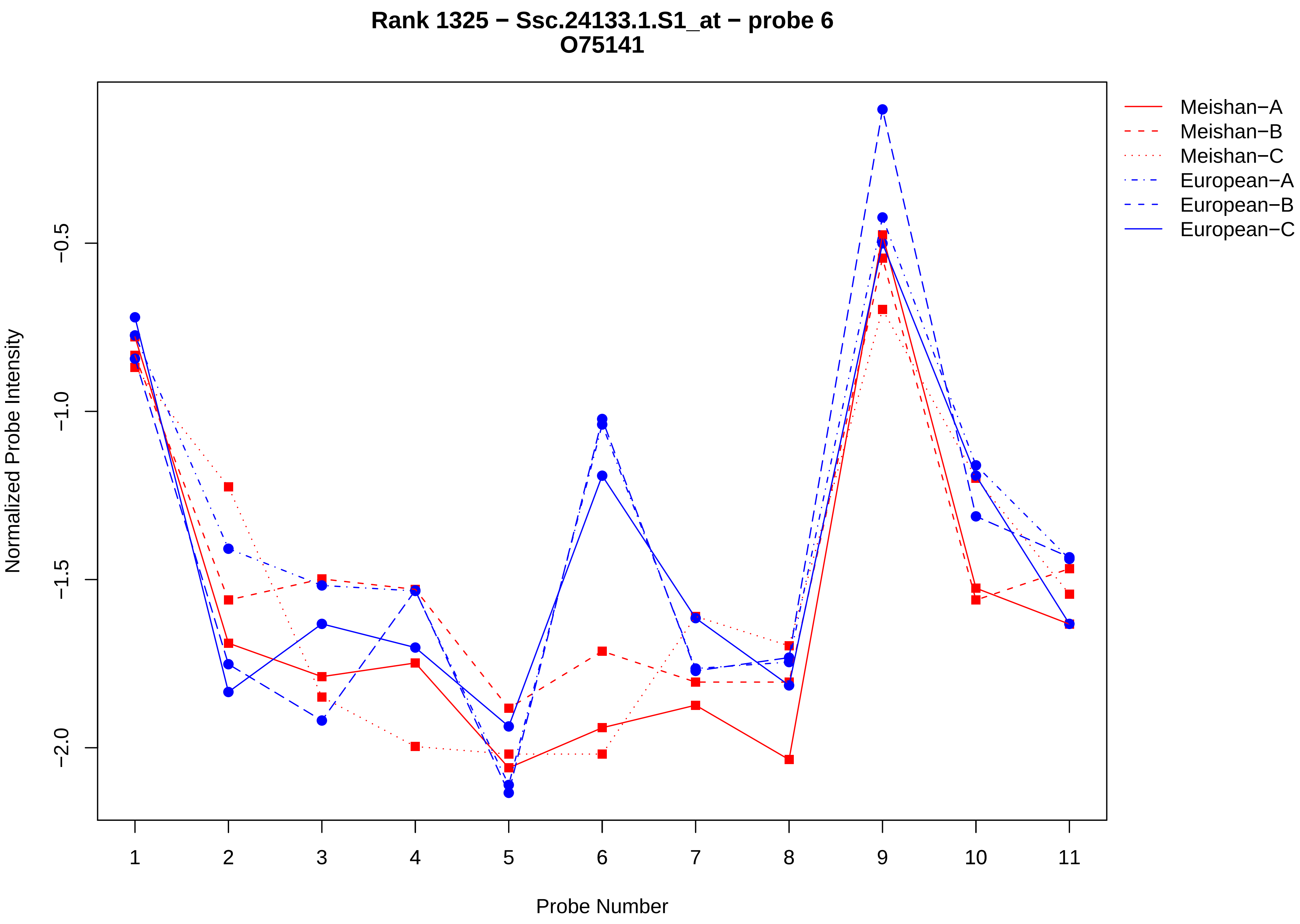

Rank 1325 − Ssc.24133.1.S1_at − probe 6
O75141
Normalized Probe Intensity
−0.5
−1.0
−1.5
−2.0
Probe Number
1
2
3
4
5
6
7
8
9
10
11
Meishan−A
Meishan−B
Meishan−C
European−A
European−B
European−C

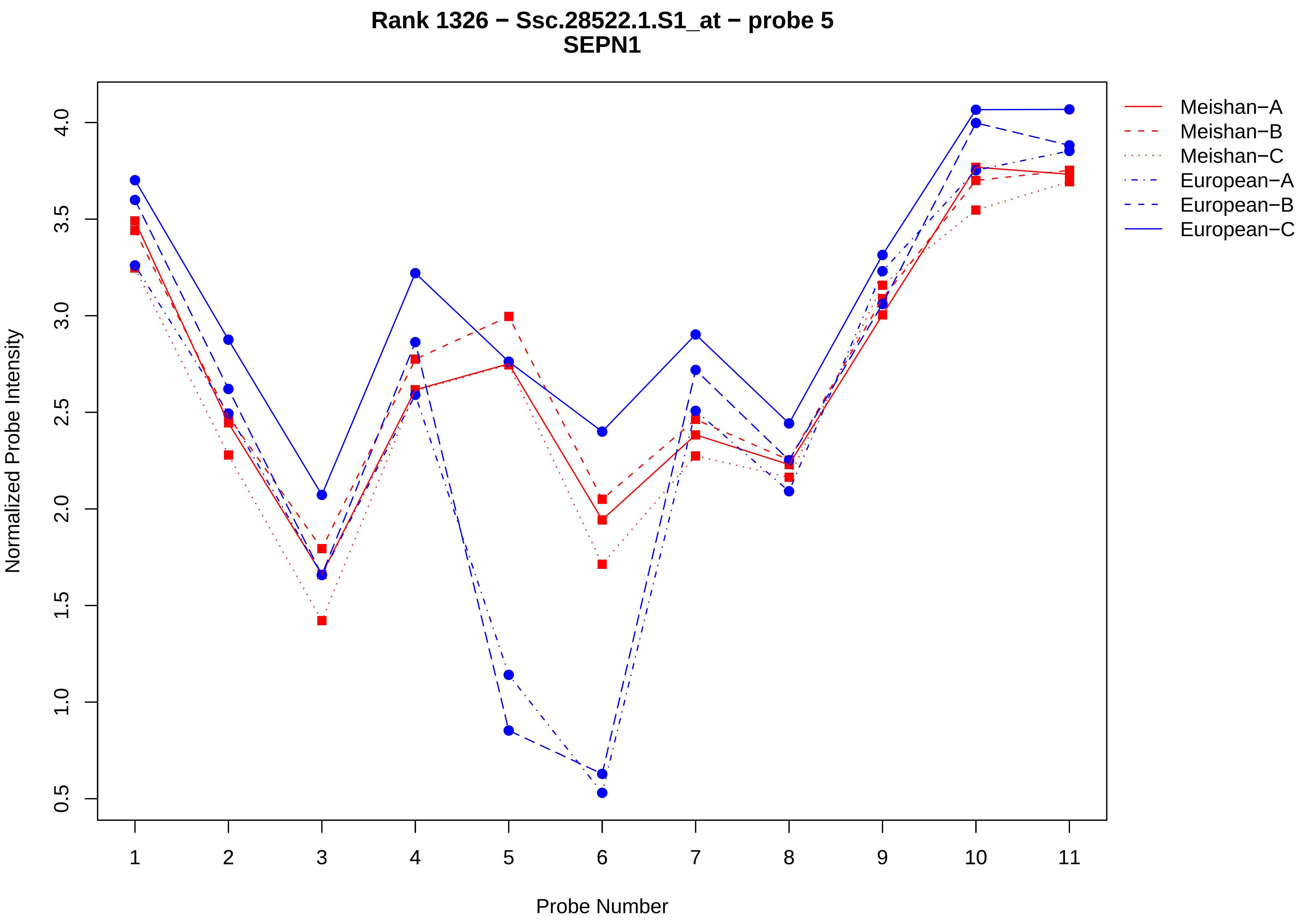

Rank 1326 − Ssc.28522.1.S1_at − probe 5
SEPN1
Normalized Probe Intensity
0.5
1.0
1.5
2.0
2.5
3.0
3.5
4.0
Probe Number
1
2
3
4
5
6
7
8
9
10
11
Meishan−A
Meishan−B
Meishan−C
European−A
European−B
European−C

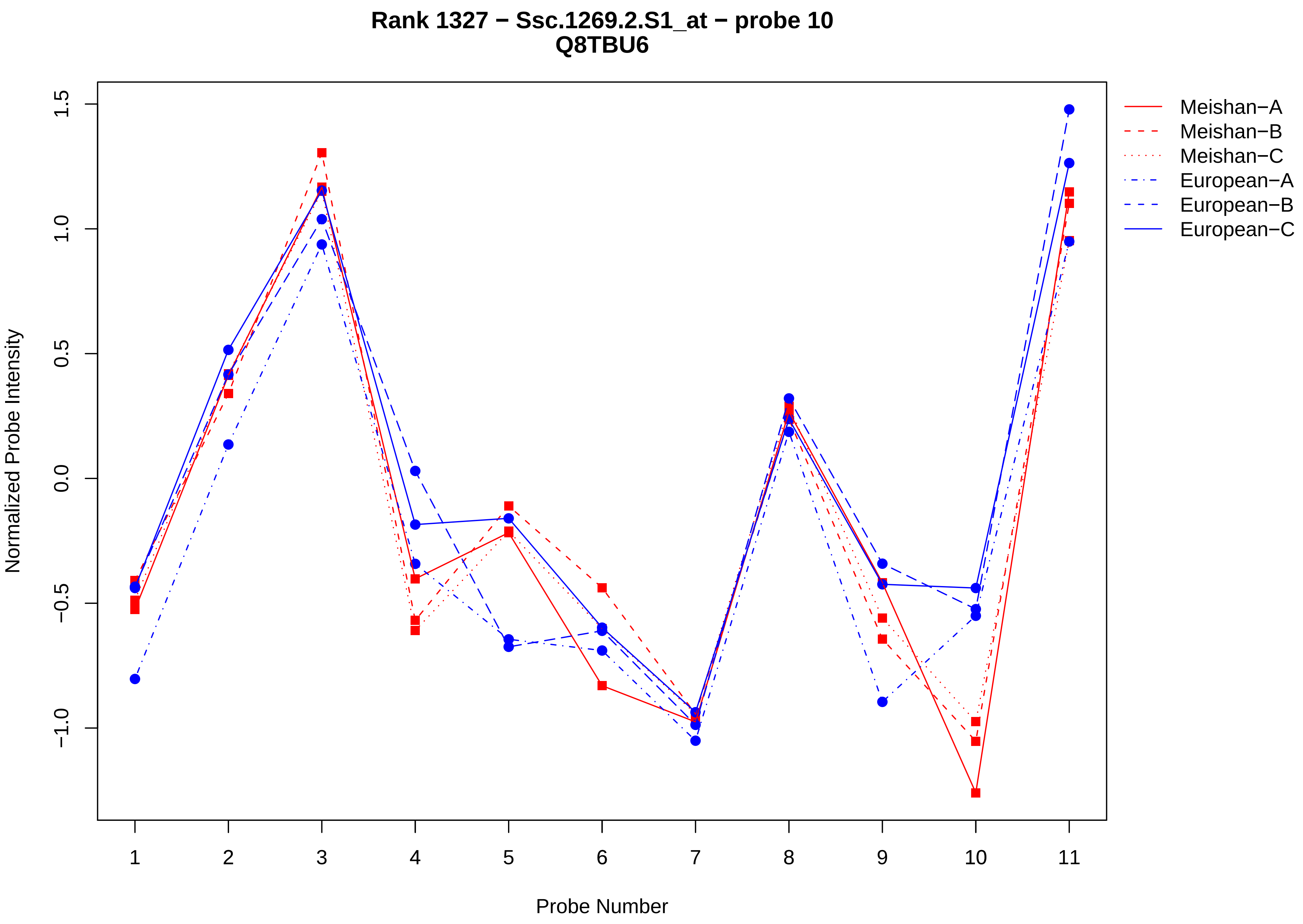

Rank 1327 − Ssc.1269.2.S1_at − probe 10
Q8TBU6
Normalized Probe Intensity
Probe Number
Meishan−A
Meishan−B
Meishan−C
European−A
European−B
European−C

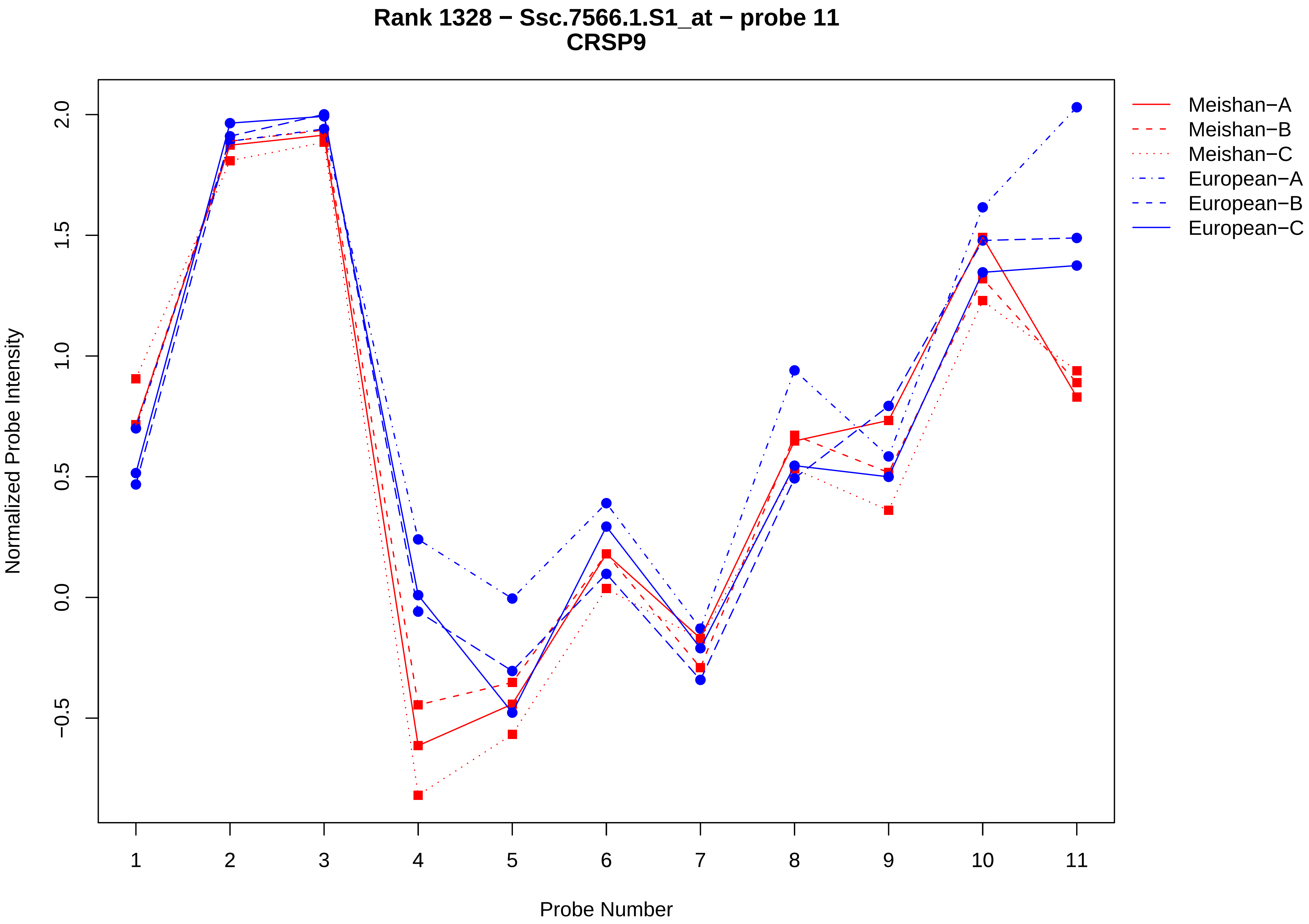

Rank 1328 − Ssc.7566.1.S1_at − probe 11
CRSP9
Normalized Probe Intensity
Probe Number
−0.5
0.0
0.5
1.0
1.5
2.0
1
2
3
4
5
6
7
8
9
10
11
Meishan−A
Meishan−B
Meishan−C
European−A
European−B
European−C

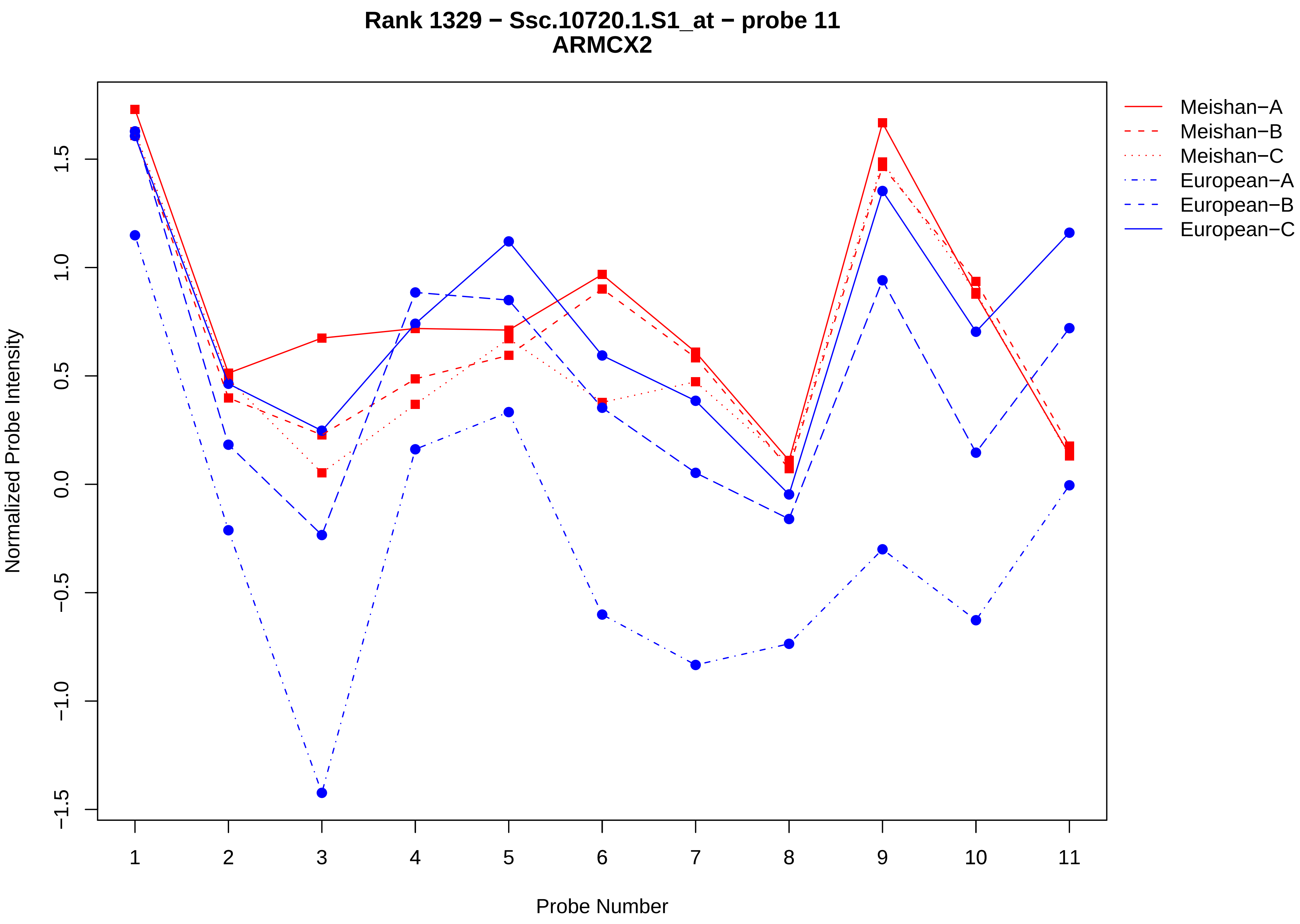
Rank 1329 − Ssc.10720.1.S1_at − probe 11
ARMCX2
Normalized Probe Intensity
Probe Number
Meishan−A
Meishan−B
Meishan−C
European−A
European−B
European−C
−1.5
−1.0
−0.5
0.0
0.5
1.0
1.5
1
2
3
4
5
6
7
8
9
10
11

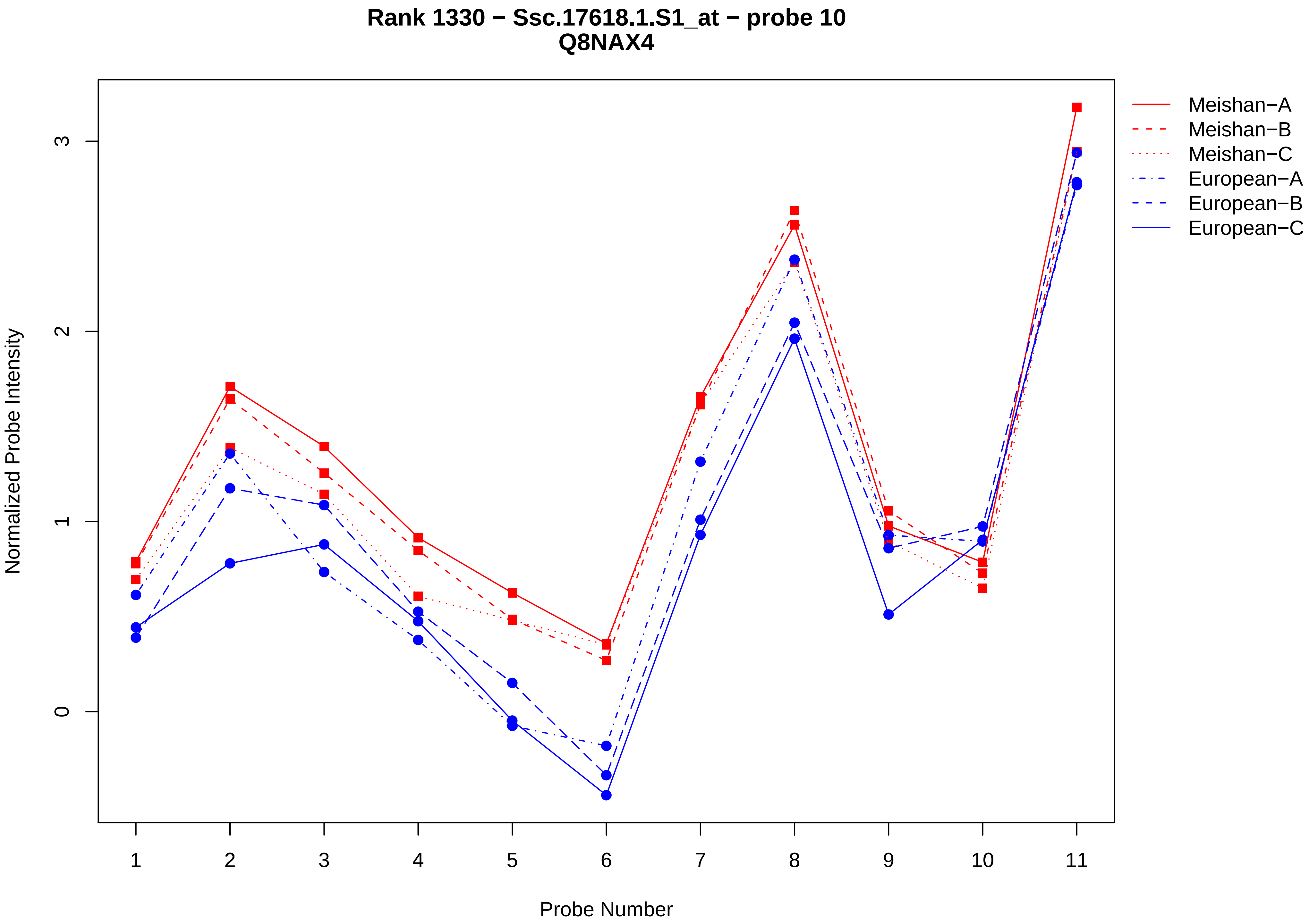
Rank 1330 − Ssc.17618.1.S1_at − probe 10
Q8NAX4
Normalized Probe Intensity
Probe Number
0
1
2
3
1
2
3
4
5
6
7
8
9
10
11
Meishan−A
Meishan−B
Meishan−C
European−A
European−B
European−C

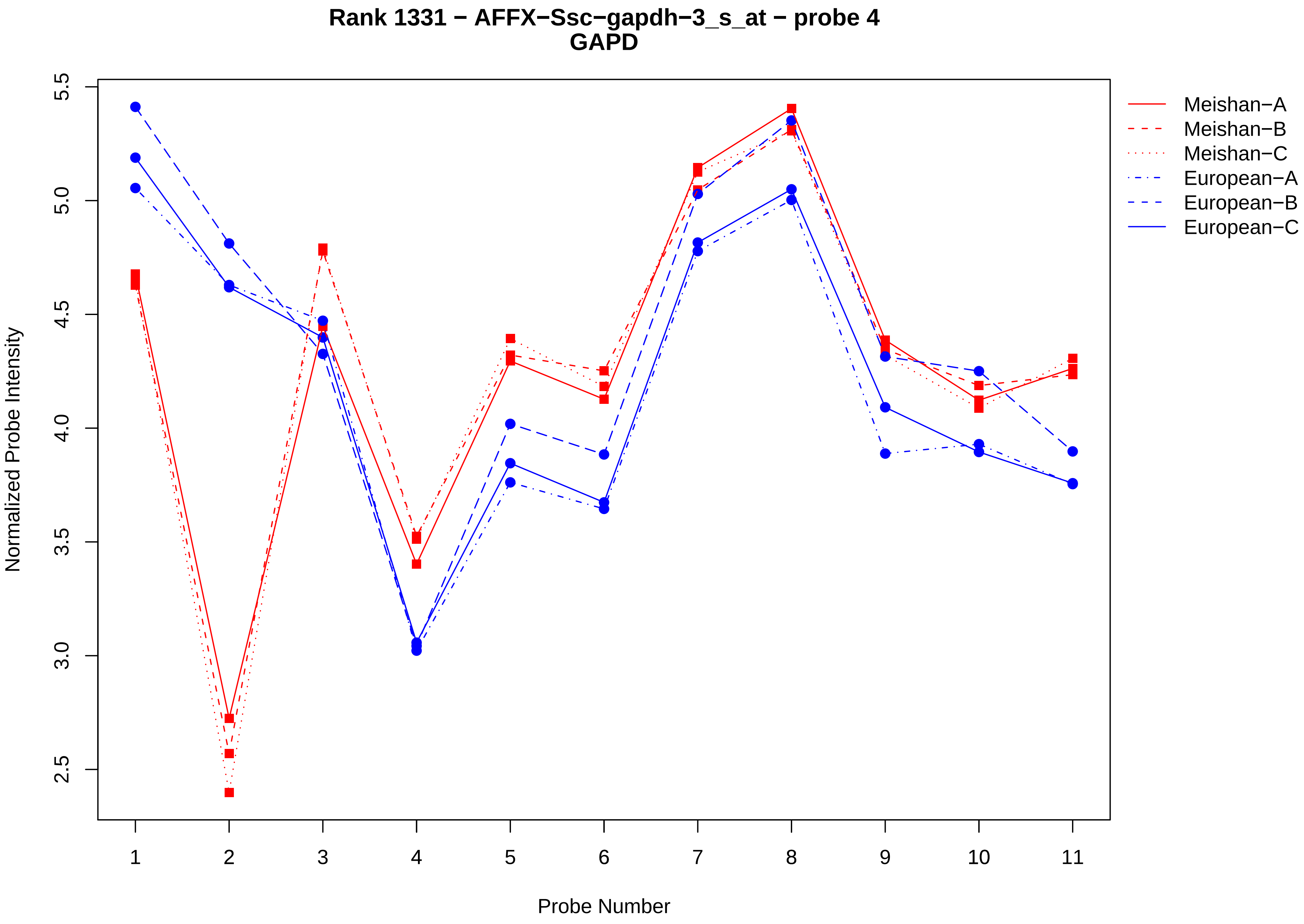

Rank 1331 − AFFX−Ssc−gapdh−3_s_at − probe 4
GAPD
Normalized Probe Intensity
Probe Number
2.5
3.0
3.5
4.0
4.5
5.0
5.5
1
2
3
4
5
6
7
8
9
10
11
Meishan−A
Meishan−B
Meishan−C
European−A
European−B
European−C

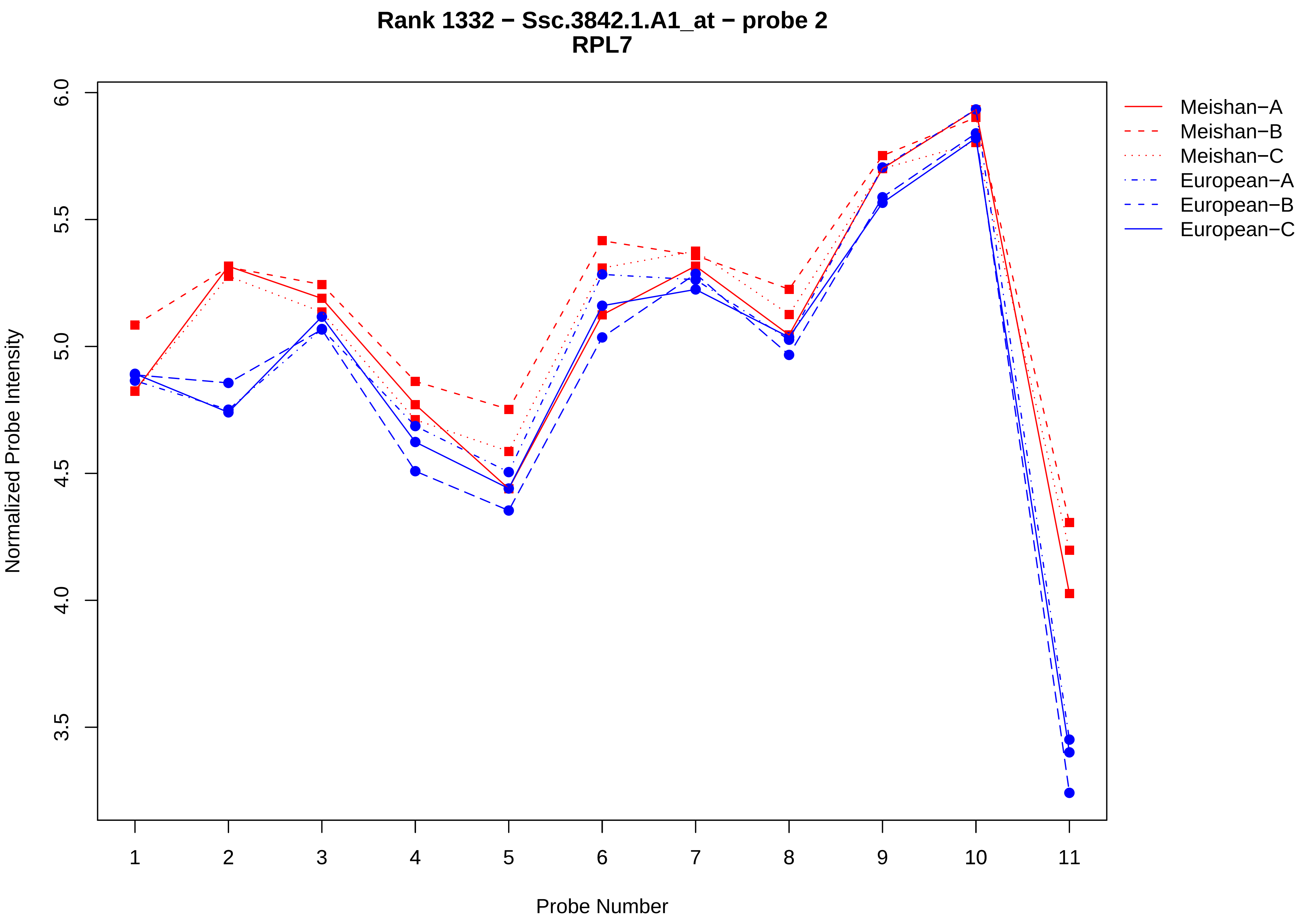

Rank 1332 − Ssc.3842.1.A1_at − probe 2
RPL7
Normalized Probe Intensity
Probe Number
6.0
5.5
5.0
4.5
4.0
3.5
1
2
3
4
5
6
7
8
9
10
11
Meishan−A
Meishan−B
Meishan−C
European−A
European−B
European−C

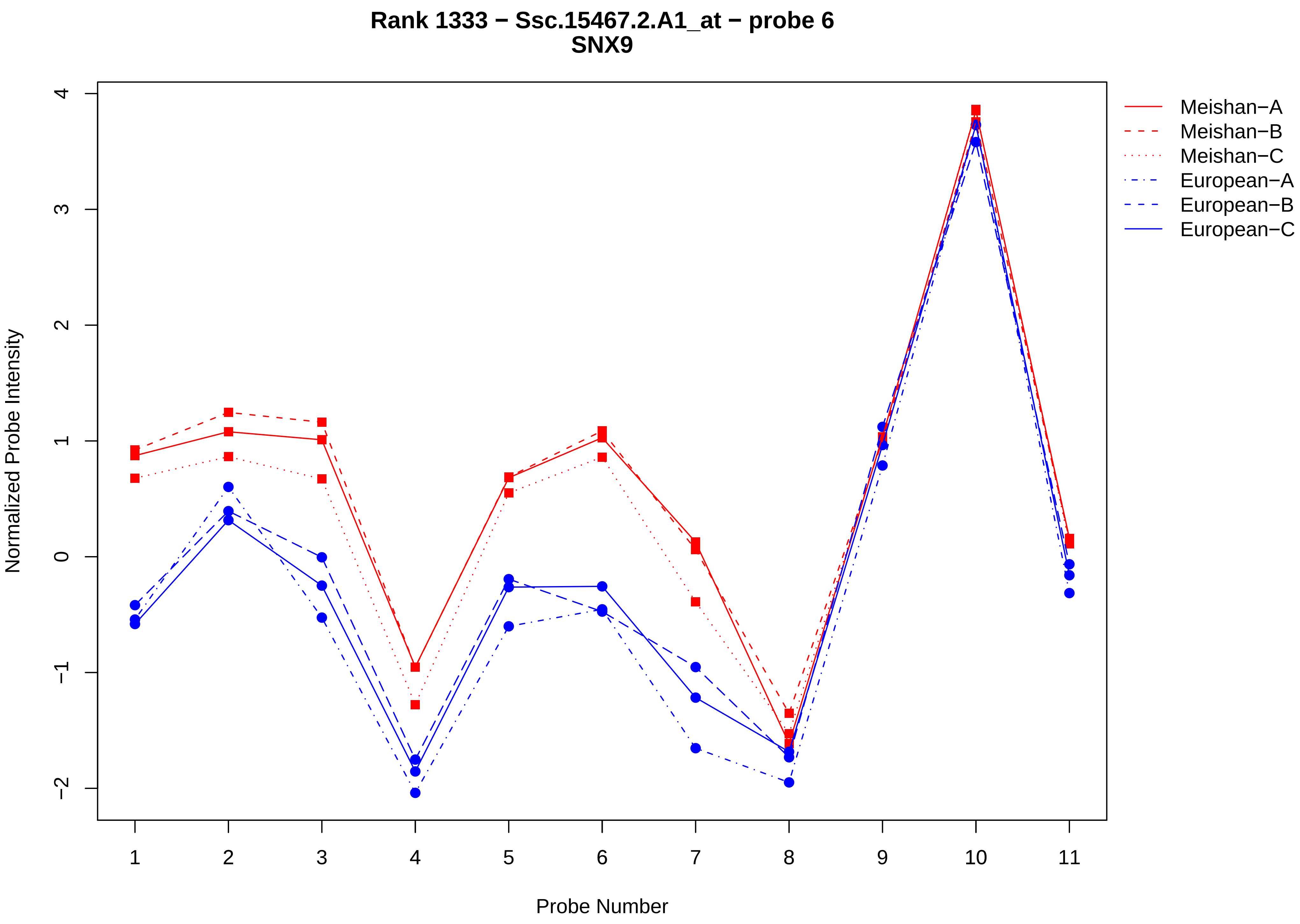

Rank 1333 − Ssc.15467.2.A1_at − probe 6
SNX9
Normalized Probe Intensity
Probe Number
−2
−1
0
1
2
3
4
1
2
3
4
5
6
7
8
9
10
11
Meishan−A
Meishan−B
Meishan−C
European−A
European−B
European−C

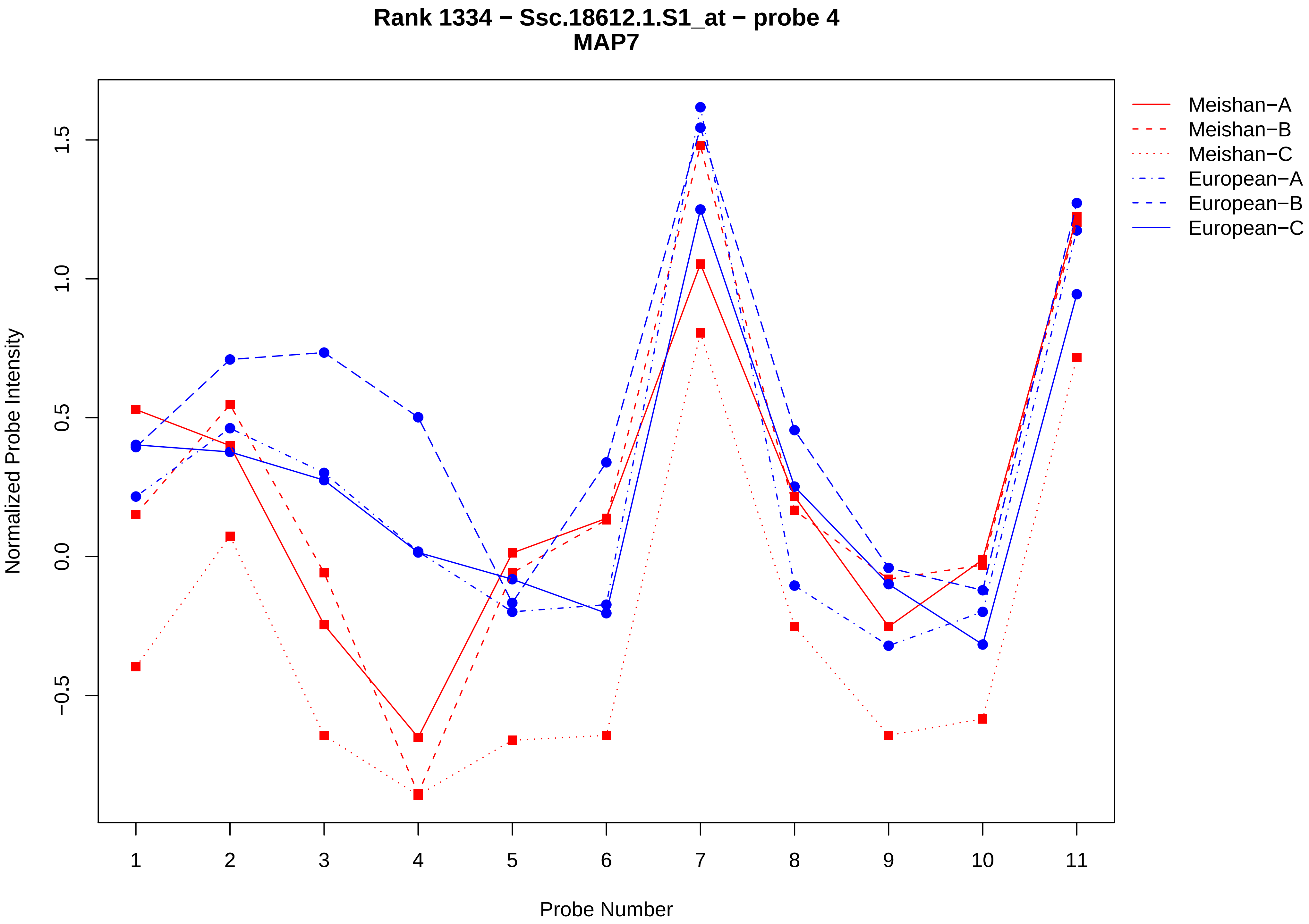
Rank 1334 − Ssc.18612.1.S1_at − probe 4
MAP7
Normalized Probe Intensity
Probe Number
−0.5
0.0
0.5
1.0
1.5
1
2
3
4
5
6
7
8
9
10
11
Meishan−A
Meishan−B
Meishan−C
European−A
European−B
European−C

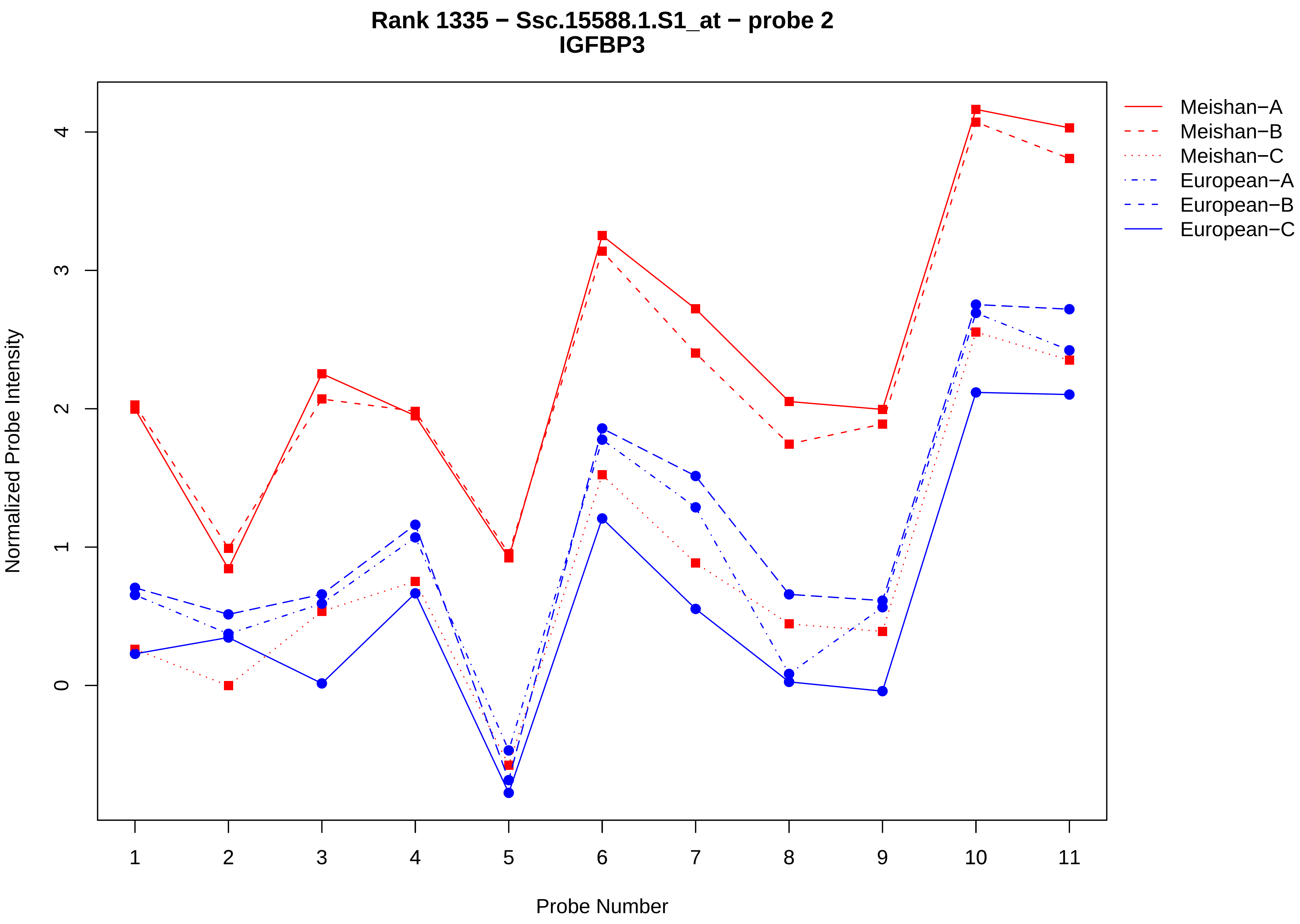

Rank 1335 − Ssc.15588.1.S1_at − probe 2
IGFBP3
Normalized Probe Intensity
0
1
2
3
4
1
2
3
4
5
6
7
8
9
10
11
Probe Number
Meishan−A
Meishan−B
Meishan−C
European−A
European−B
European−C

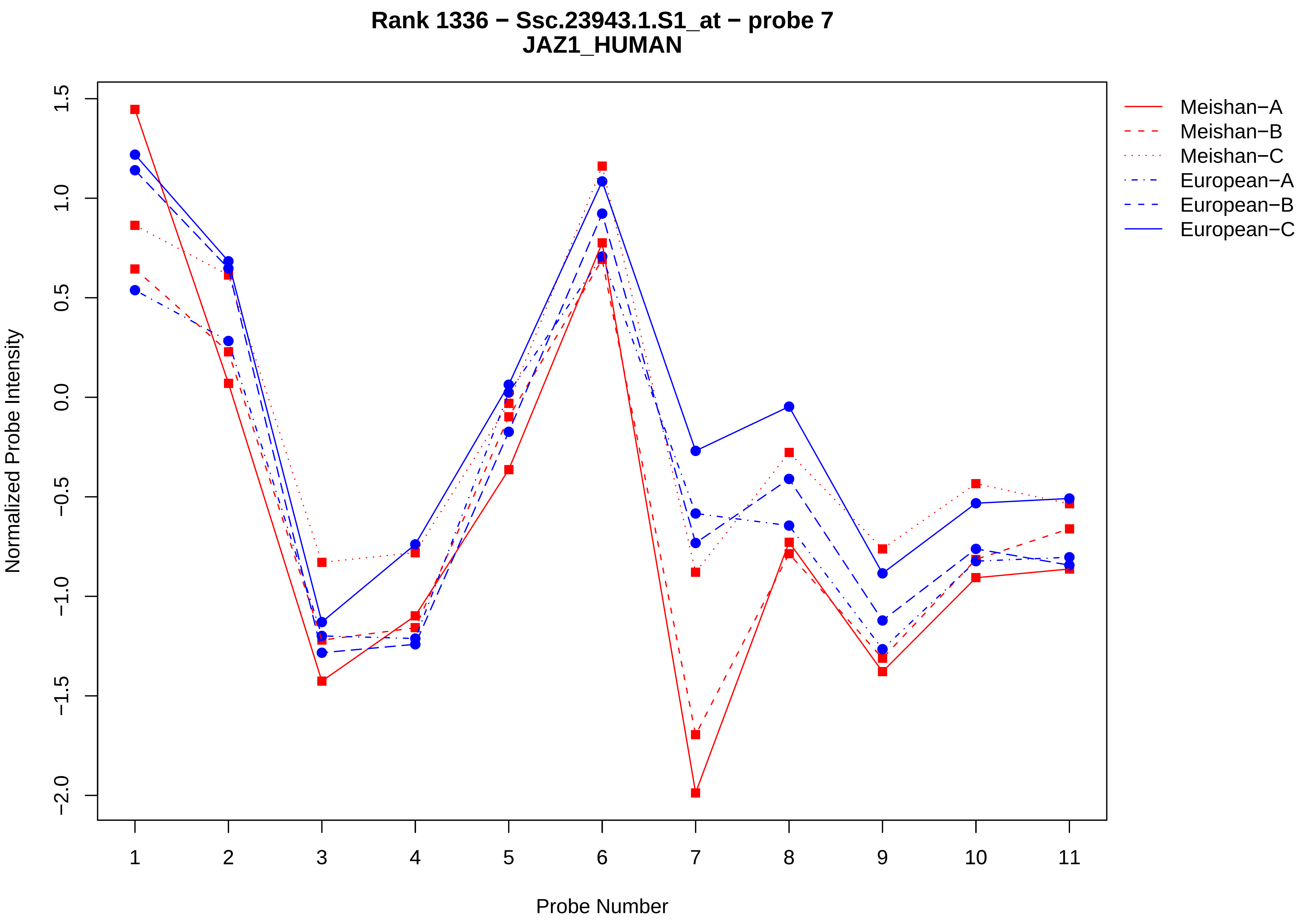

Rank 1336 − Ssc.23943.1.S1_at − probe 7
JAZ1_HUMAN
Normalized Probe Intensity
Probe Number
1.5
1.0
0.5
0.0
−0.5
−1.0
−1.5
−2.0
1
2
3
4
5
6
7
8
9
10
11
Meishan−A
Meishan−B
Meishan−C
European−A
European−B
European−C

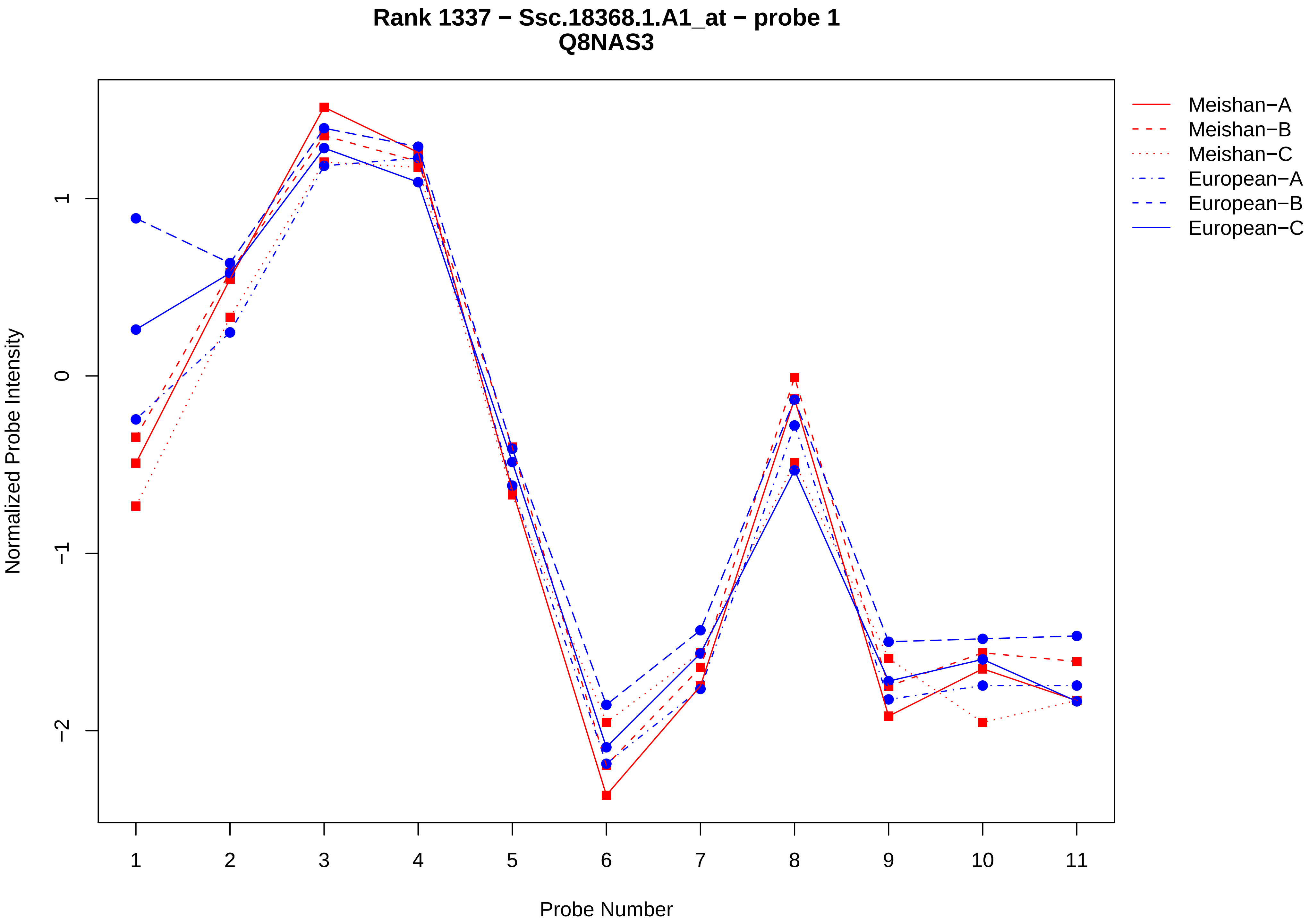

Rank 1337 − Ssc.18368.1.A1_at − probe 1
Q8NAS3
Normalized Probe Intensity
Probe Number
Meishan−A
Meishan−B
Meishan−C
European−A
European−B
European−C
−2
−1
0
1
1
2
3
4
5
6
7
8
9
10
11

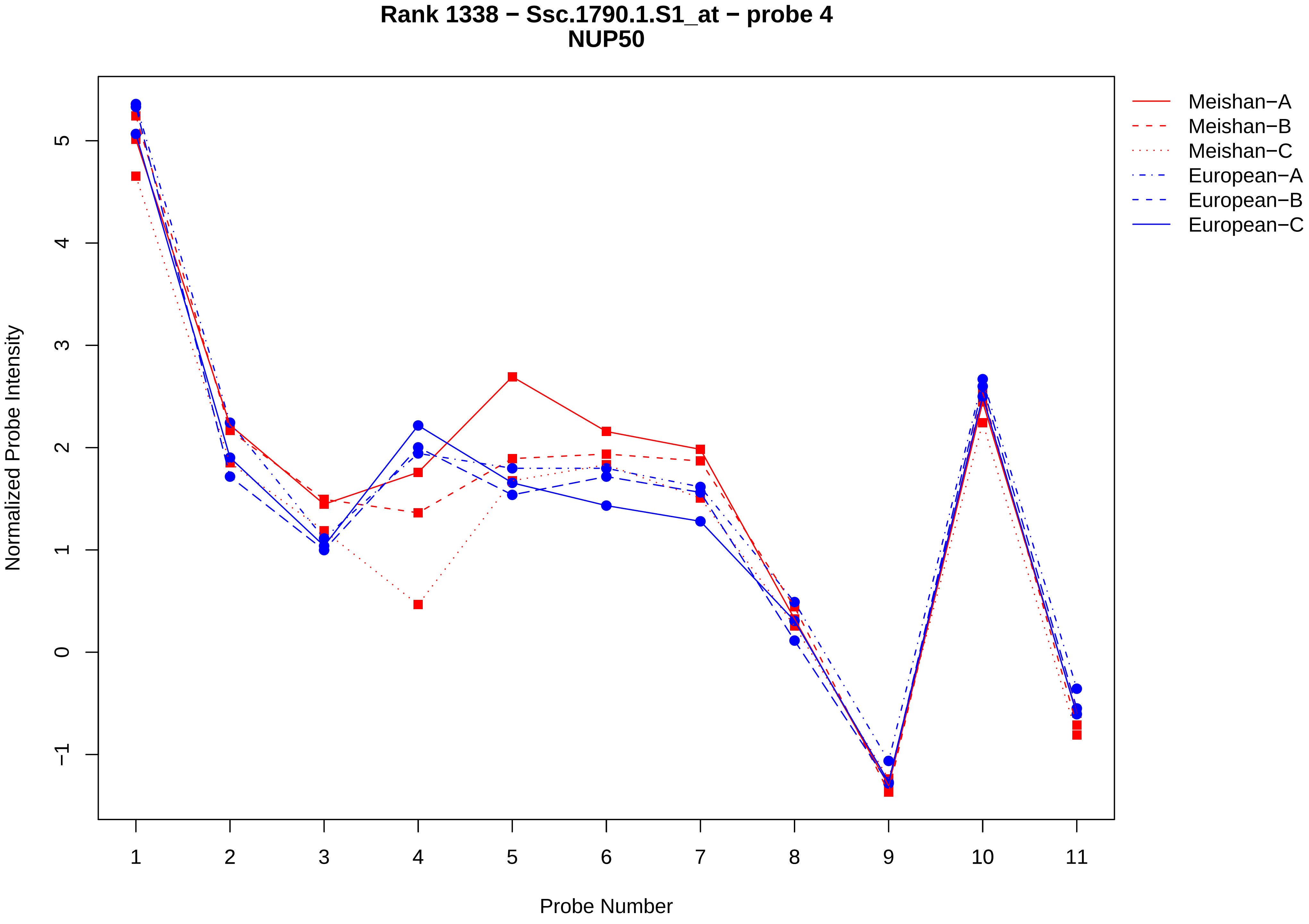

Rank 1338 − Ssc.1790.1.S1_at − probe 4
NUP50
Normalized Probe Intensity
Probe Number
Meishan−A
Meishan−B
Meishan−C
European−A
European−B
European−C

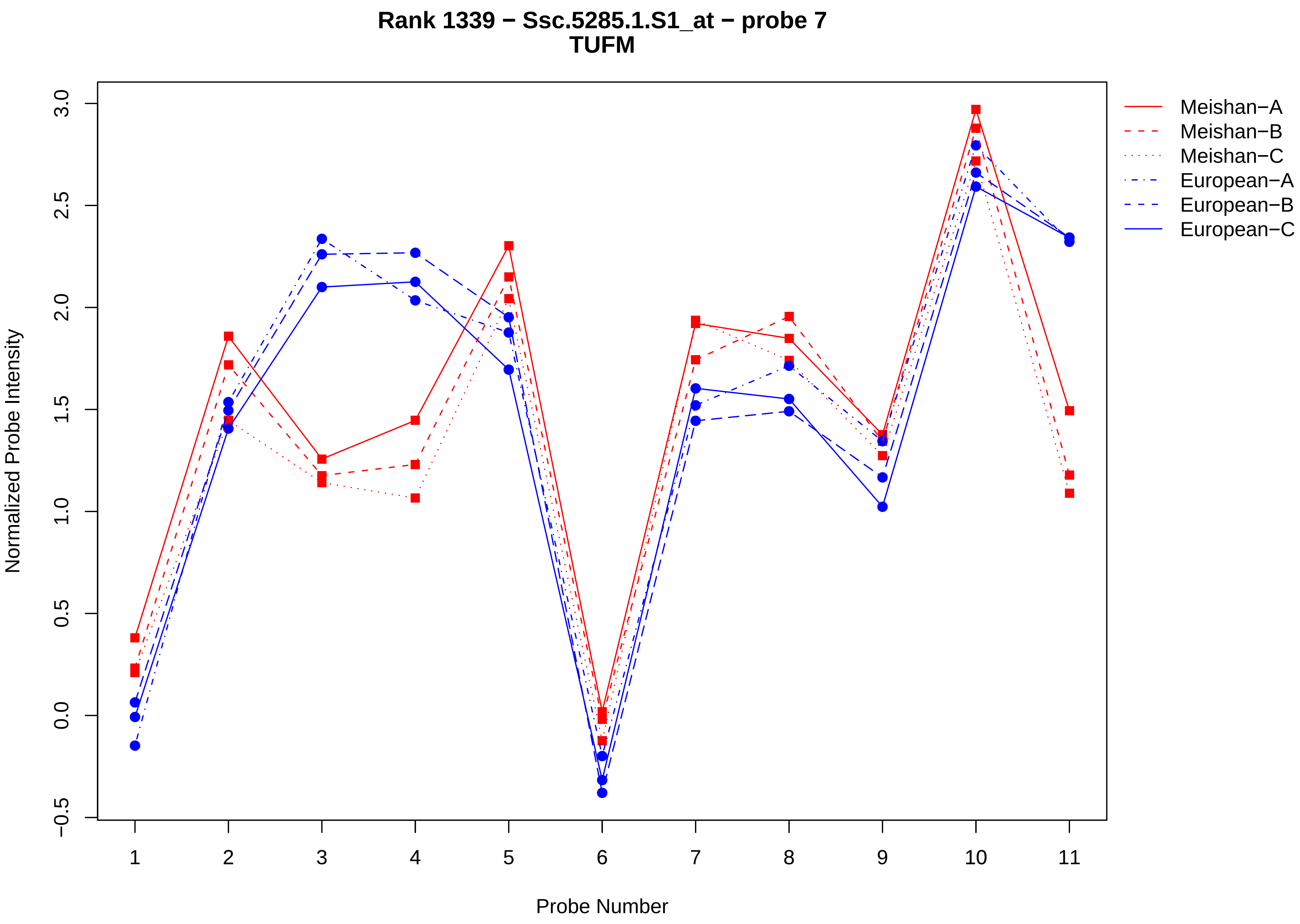
Rank 1339 − Ssc.5285.1.S1_at − probe 7
TUFM
Normalized Probe Intensity
Probe Number
−0.5
0.0
0.5
1.0
1.5
2.0
2.5
3.0
1
2
3
4
5
6
7
8
9
10
11
Meishan−A
Meishan−B
Meishan−C
European−A
European−B
European−C

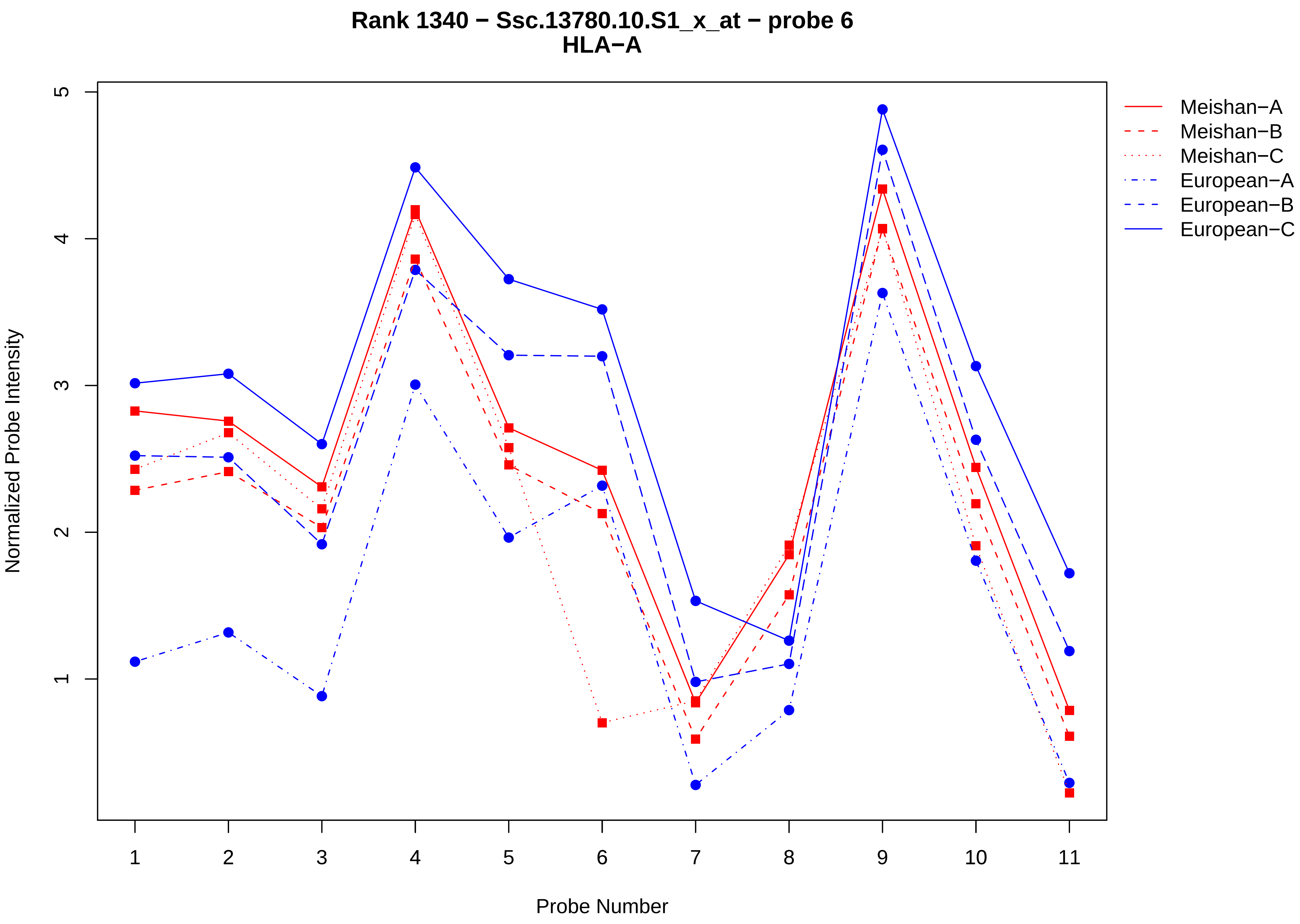

Rank 1340 − Ssc.13780.10.S1_x_at − probe 6
HLA−A
Normalized Probe Intensity
Probe Number
1
2
3
4
5
1
2
3
4
5
6
7
8
9
10
11
Meishan−A
Meishan−B
Meishan−C
European−A
European−B
European−C

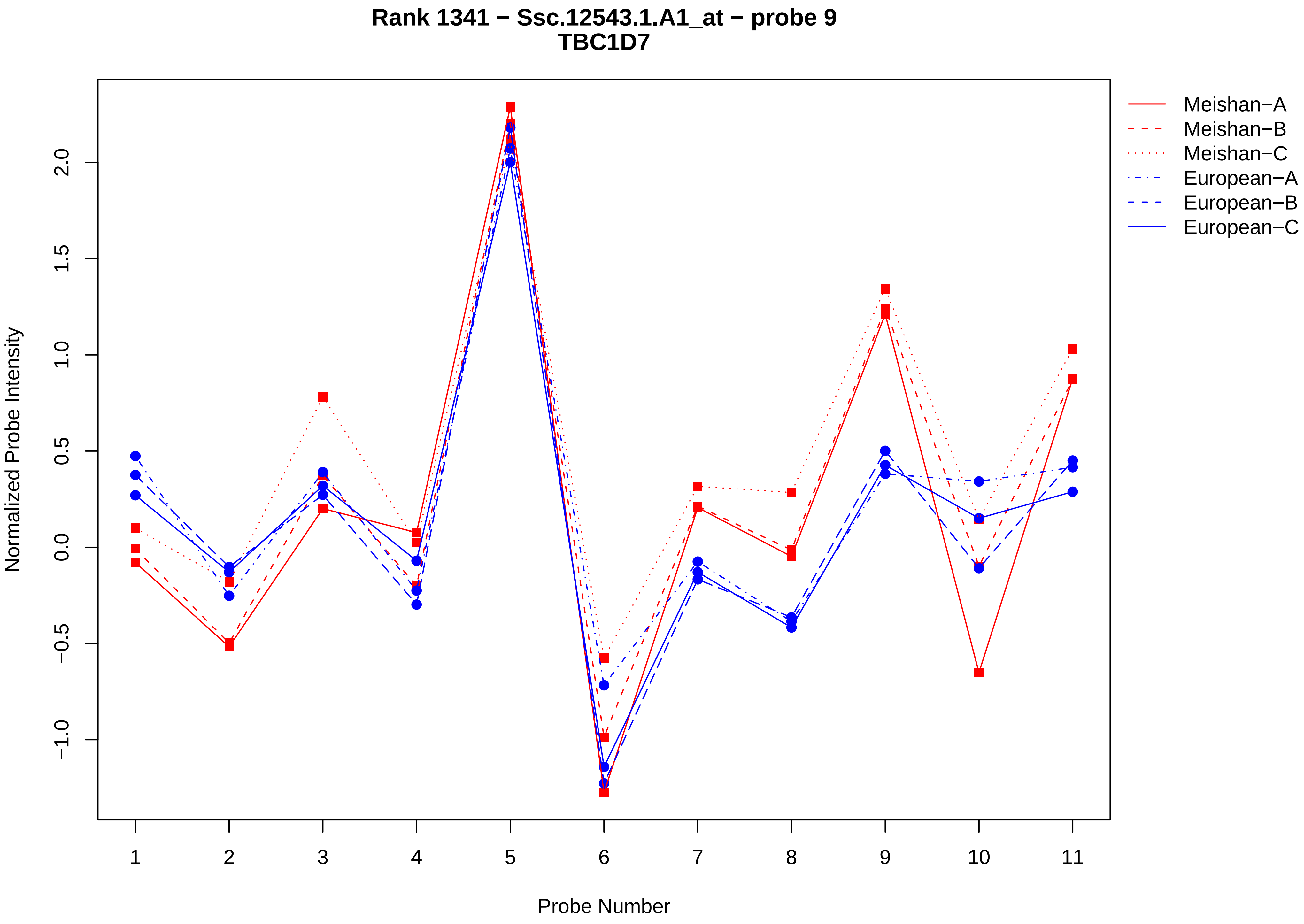

Rank 1341 − Ssc.12543.1.A1_at − probe 9
TBC1D7
Normalized Probe Intensity
−1.0
−0.5
0.0
0.5
1.0
1.5
2.0
1
2
3
4
5
6
7
8
9
10
11
Probe Number
Meishan−A
Meishan−B
Meishan−C
European−A
European−B
European−C

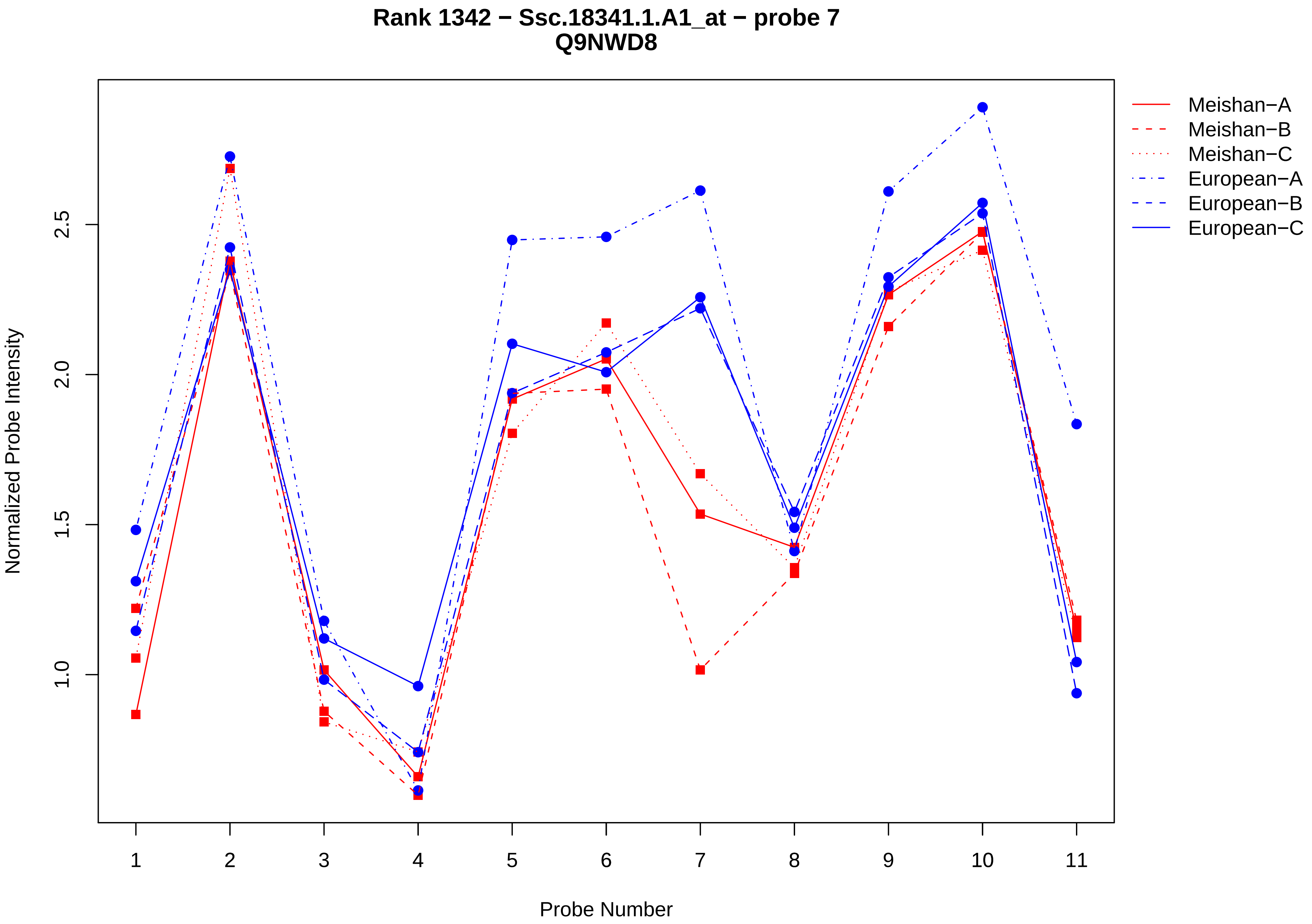

Rank 1342 − Ssc.18341.1.A1_at − probe 7
Q9NWD8
Normalized Probe Intensity
Probe Number
1.0
1.5
2.0
2.5
1
2
3
4
5
6
7
8
9
10
11
Meishan−A
Meishan−B
Meishan−C
European−A
European−B
European−C

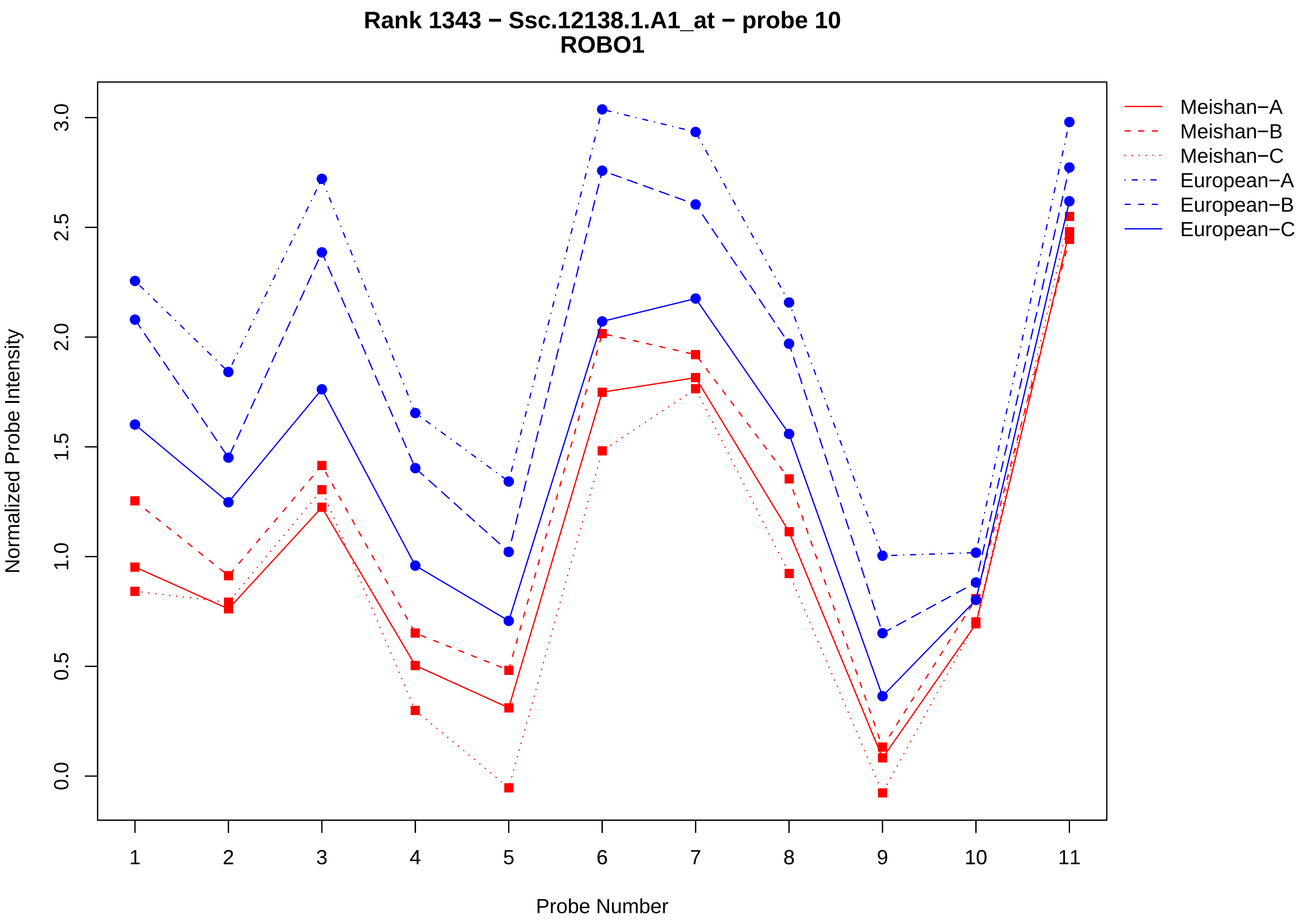

Rank 1343 − Ssc.12138.1.A1_at − probe 10
ROBO1
Normalized Probe Intensity
Probe Number
0.0
0.5
1.0
1.5
2.0
2.5
3.0
1
2
3
4
5
6
7
8
9
10
11
Meishan−A
Meishan−B
Meishan−C
European−A
European−B
European−C

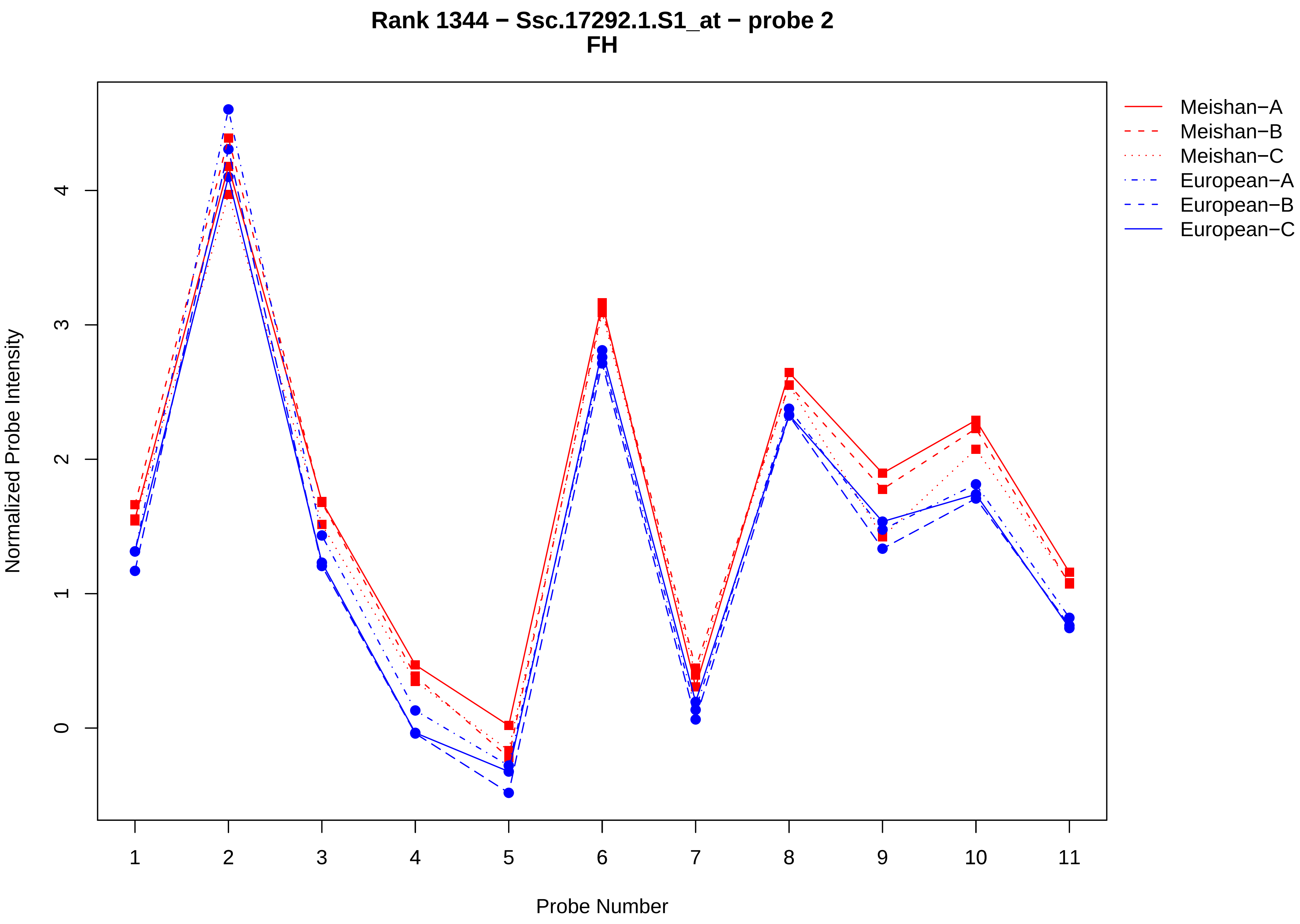

Rank 1344 − Ssc.17292.1.S1_at − probe 2
FH
Normalized Probe Intensity
Probe Number
0
1
2
3
4
1
2
3
4
5
6
7
8
9
10
11
Meishan−A
Meishan−B
Meishan−C
European−A
European−B
European−C

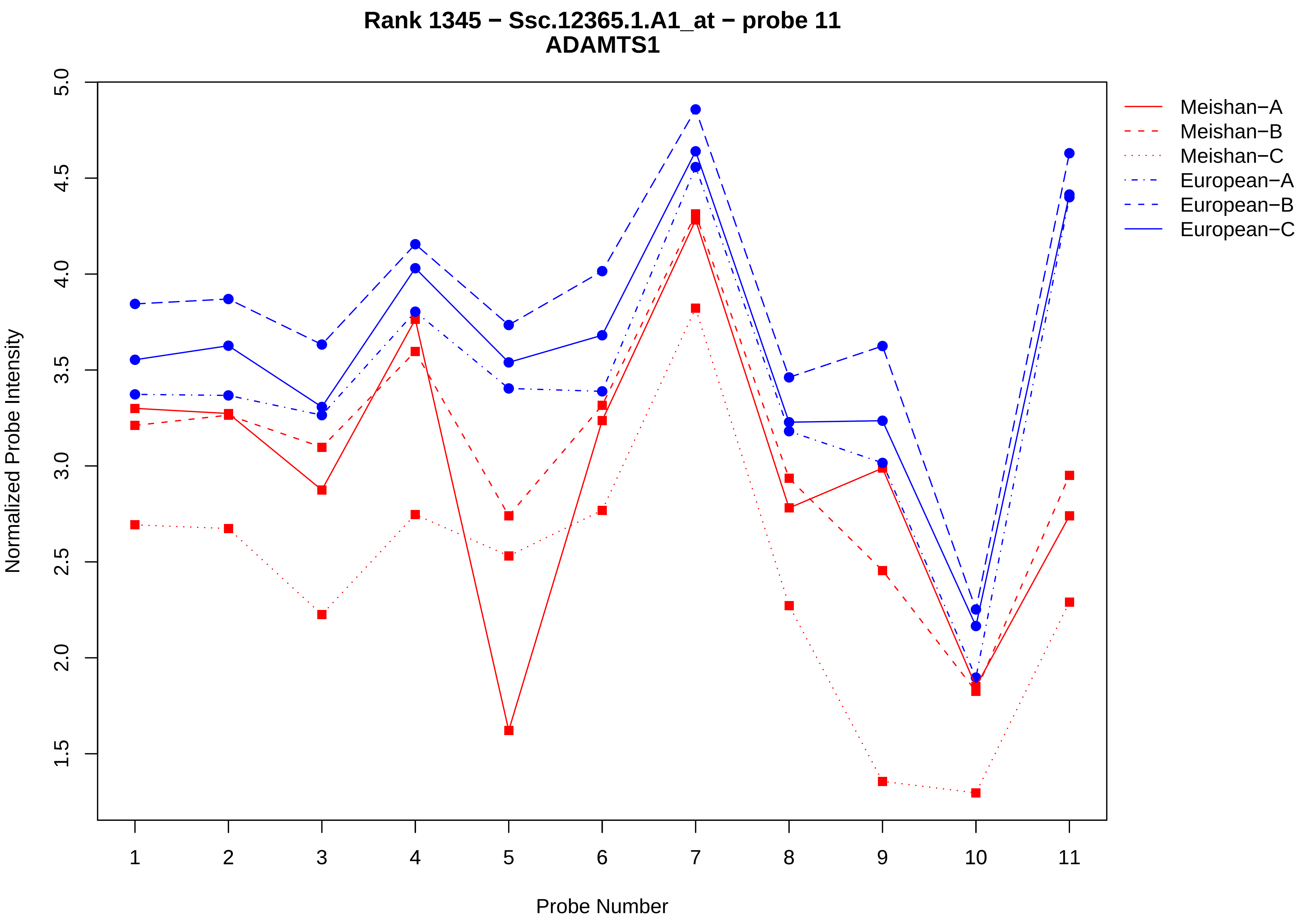

Rank 1345 − Ssc.12365.1.A1_at − probe 11
ADAMTS1
Normalized Probe Intensity
Probe Number
5.0
4.5
4.0
3.5
3.0
2.5
2.0
1.5
1
2
3
4
5
6
7
8
9
10
11
Meishan−A
Meishan−B
Meishan−C
European−A
European−B
European−C

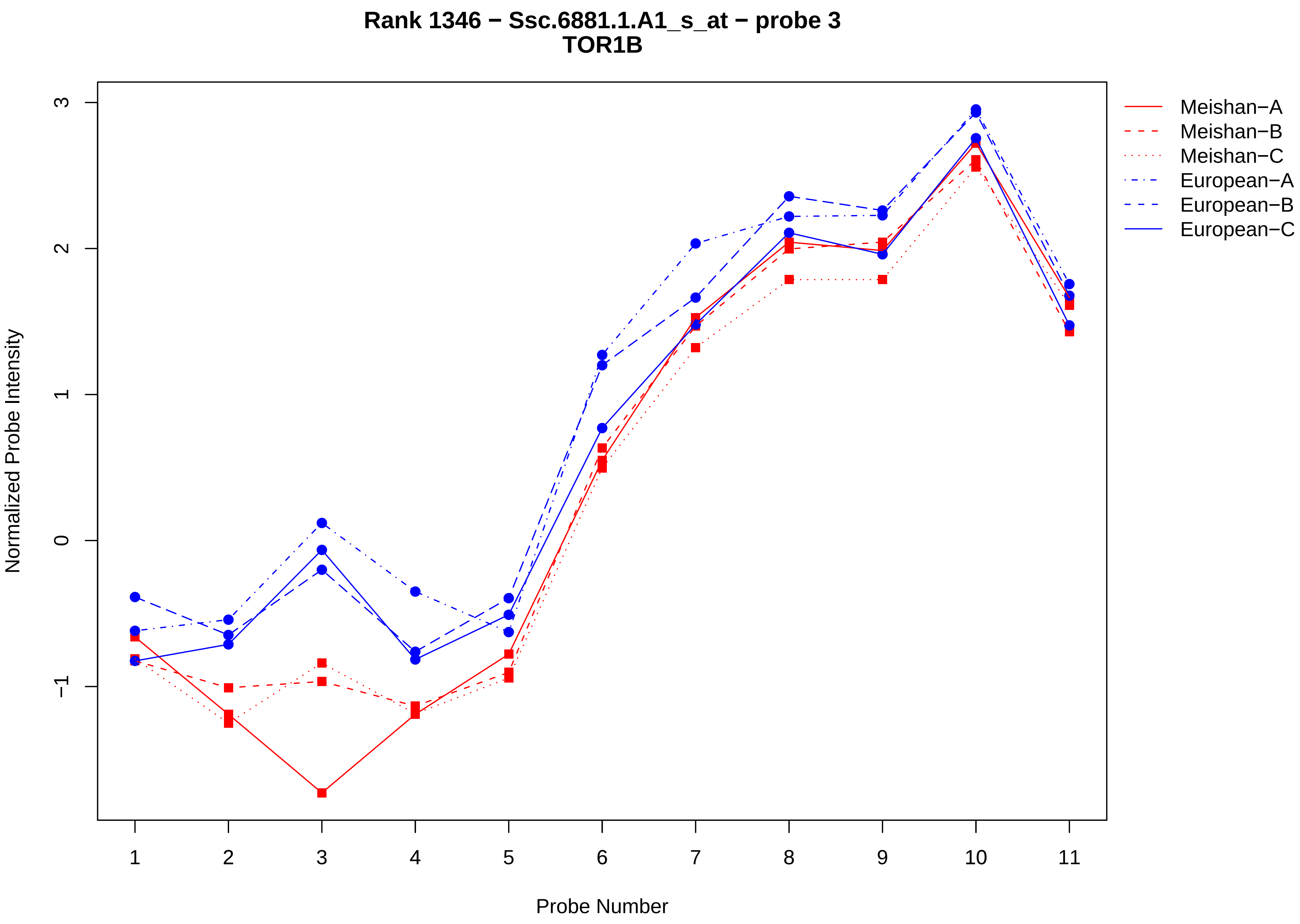

Rank 1346 − Ssc.6881.1.A1_s_at − probe 3
TOR1B
Normalized Probe Intensity
Probe Number
−1
0
1
2
3
1
2
3
4
5
6
7
8
9
10
11
Meishan−A
Meishan−B
Meishan−C
European−A
European−B
European−C

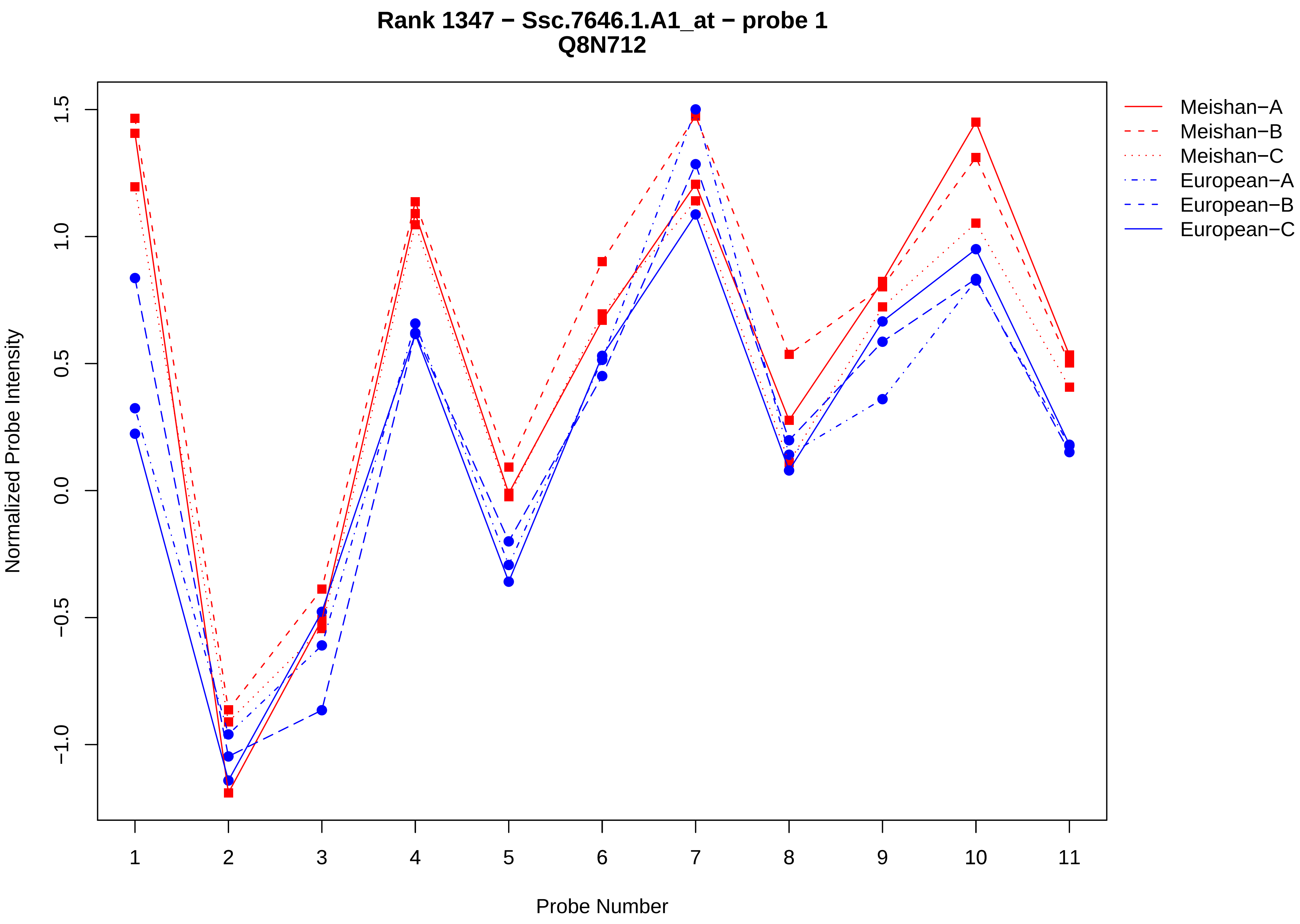

Rank 1347 − Ssc.7646.1.A1_at − probe 1
Q8N712
Normalized Probe Intensity
Probe Number
Meishan−A
Meishan−B
Meishan−C
European−A
European−B
European−C

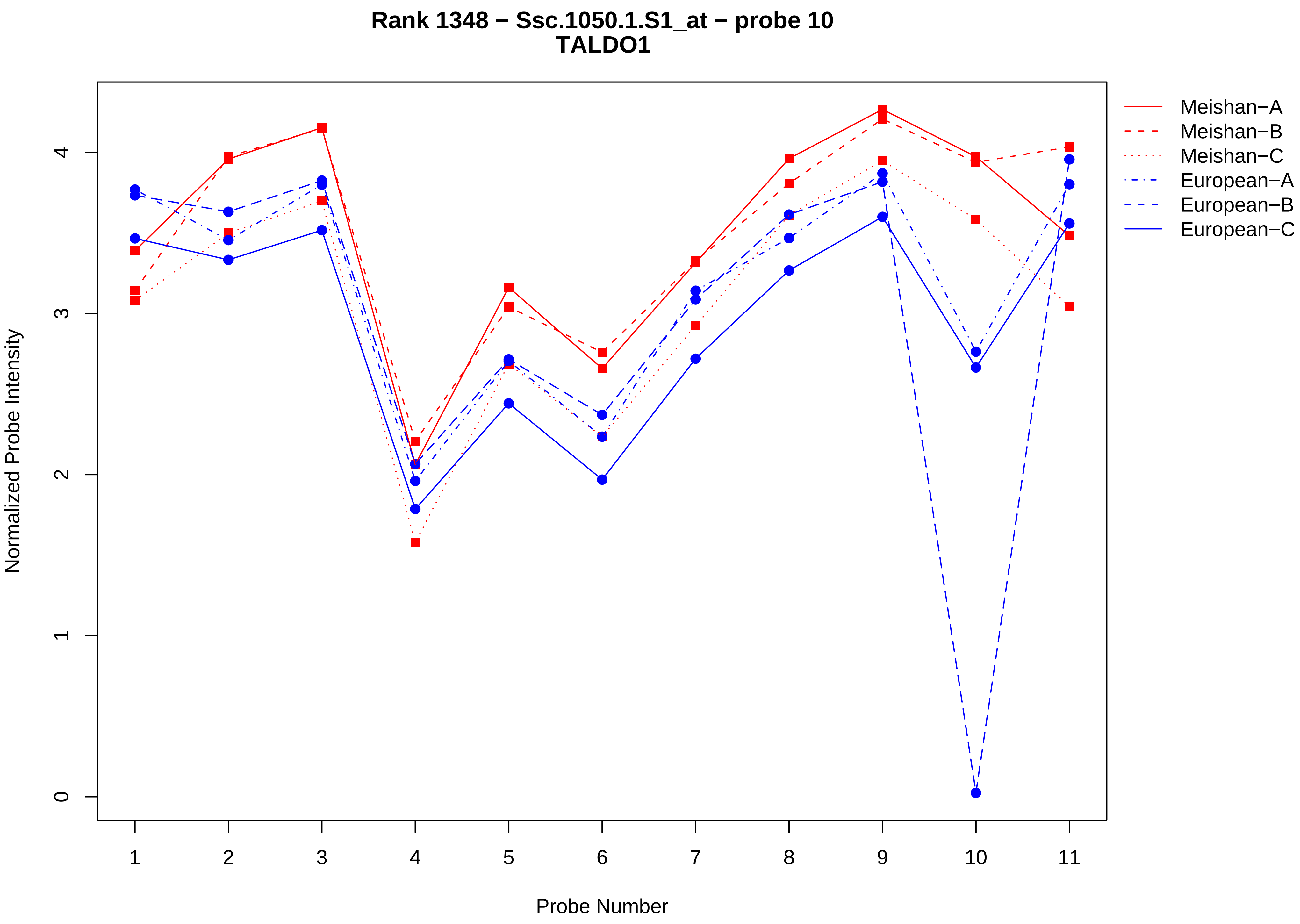
Rank 1348 − Ssc.1050.1.S1_at − probe 10
TALDO1
Normalized Probe Intensity
Probe Number
0
1
2
3
4
1
2
3
4
5
6
7
8
9
10
11
Meishan−A
Meishan−B
Meishan−C
European−A
European−B
European−C

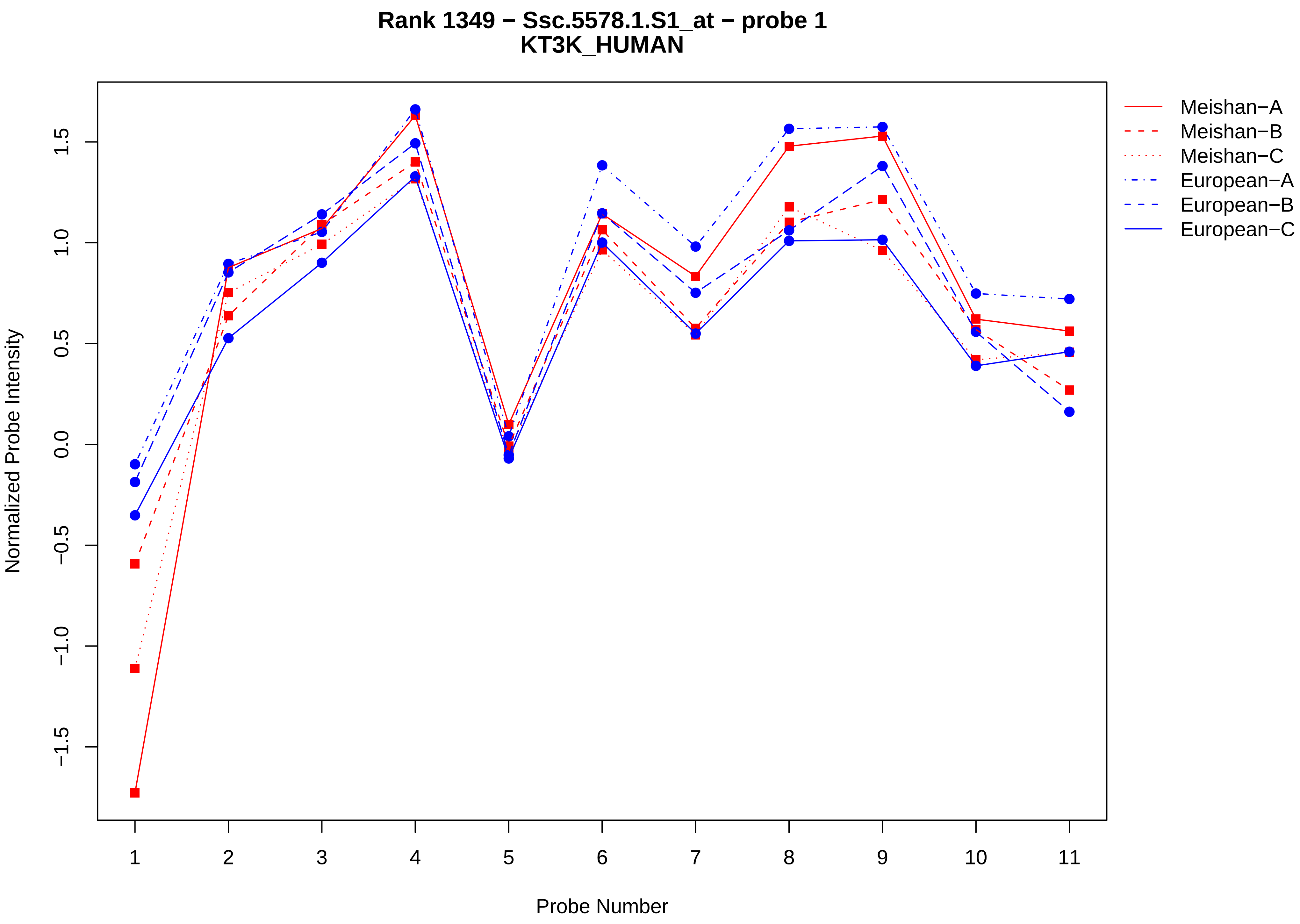

Rank 1349 − Ssc.5578.1.S1_at − probe 1
KT3K_HUMAN
Normalized Probe Intensity
Probe Number
−1.5
−1.0
−0.5
0.0
0.5
1.0
1.5
1
2
3
4
5
6
7
8
9
10
11
Meishan−A
Meishan−B
Meishan−C
European−A
European−B
European−C

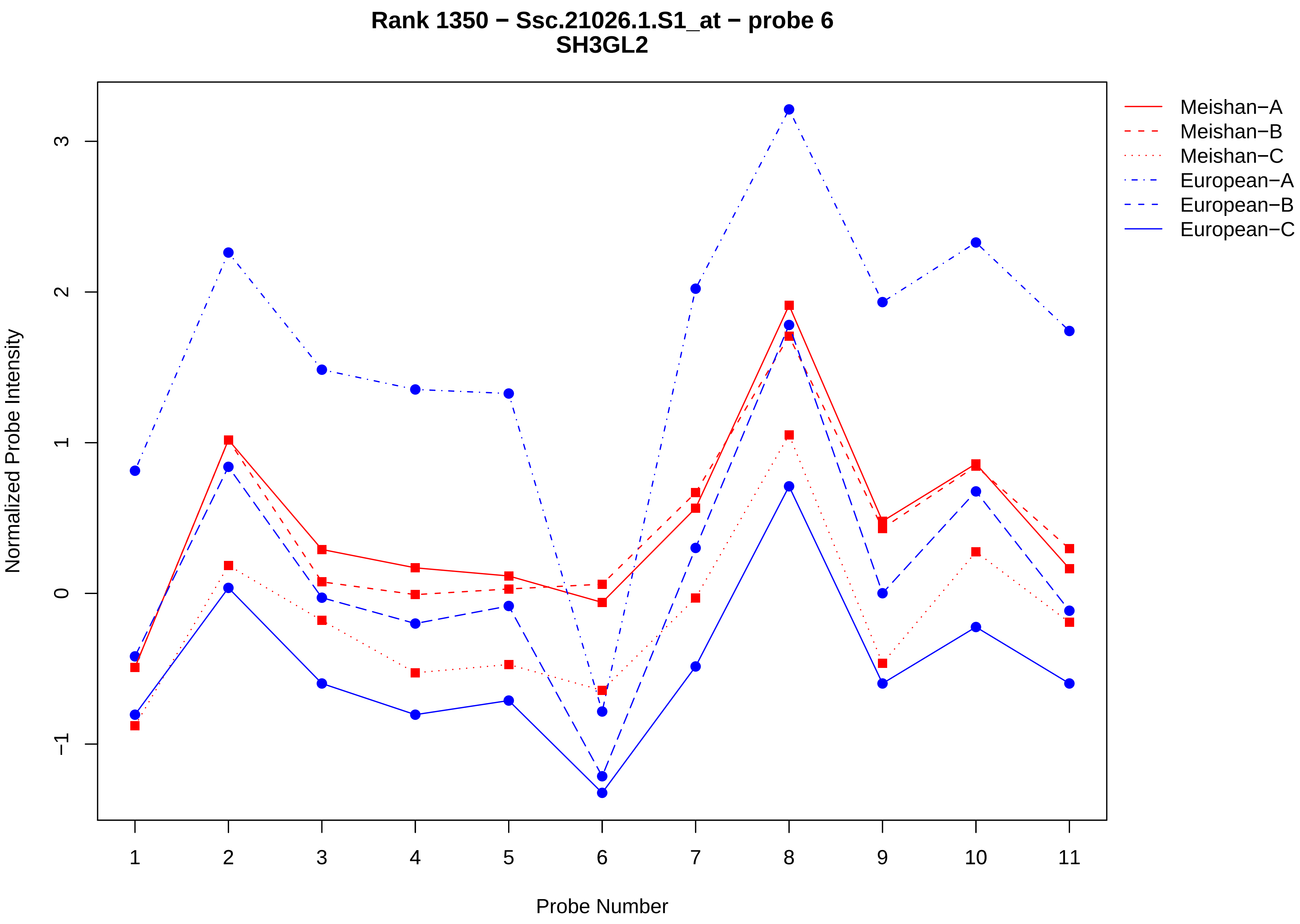

Rank 1350 − Ssc.21026.1.S1_at − probe 6
SH3GL2
Normalized Probe Intensity
Probe Number
−1
0
1
2
3
1
2
3
4
5
6
7
8
9
10
11
Meishan−A
Meishan−B
Meishan−C
European−A
European−B
European−C

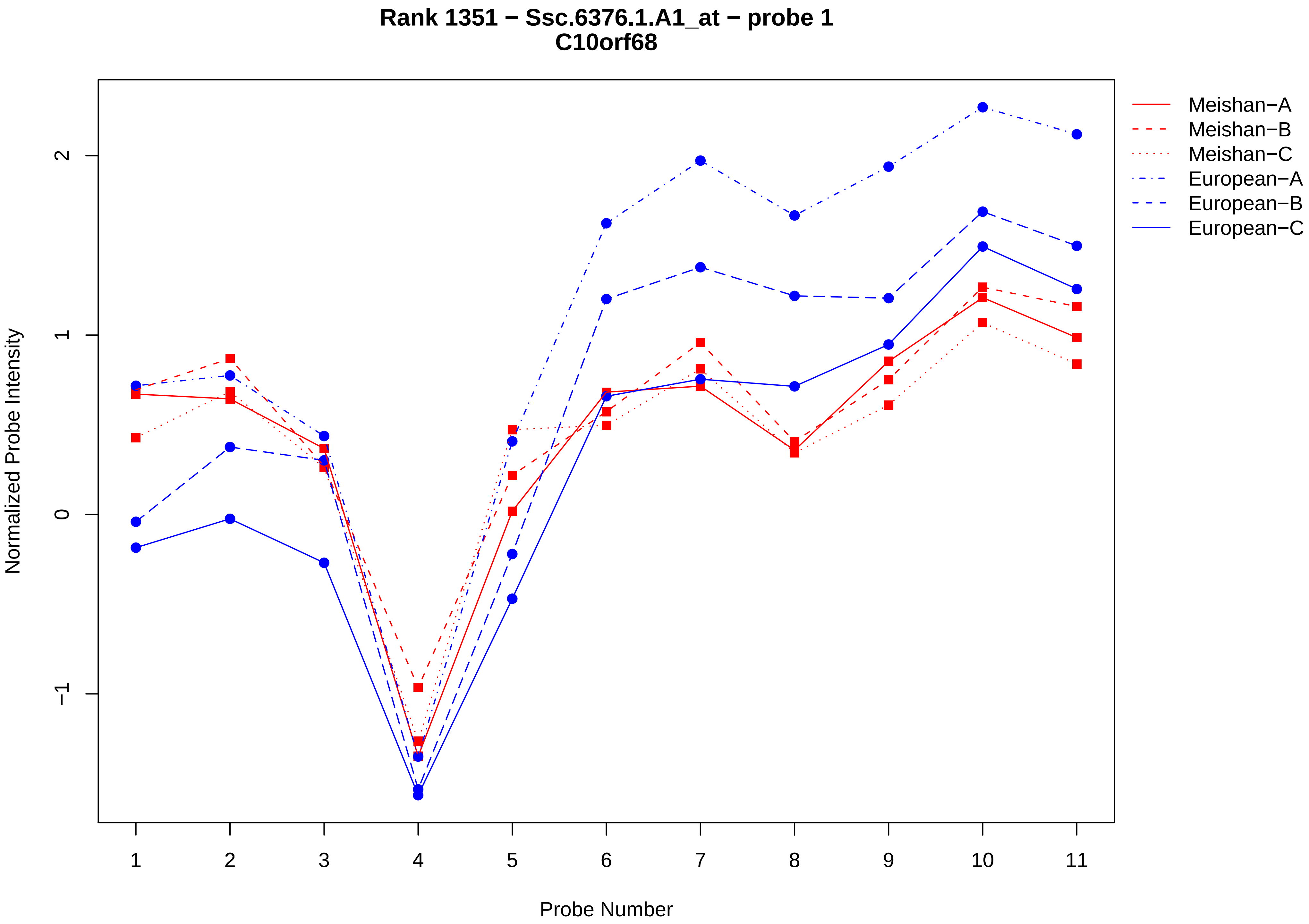

Rank 1351 − Ssc.6376.1.A1_at − probe 1
C10orf68
Normalized Probe Intensity
Probe Number
−1
0
1
2
1
2
3
4
5
6
7
8
9
10
11
Meishan−A
Meishan−B
Meishan−C
European−A
European−B
European−C

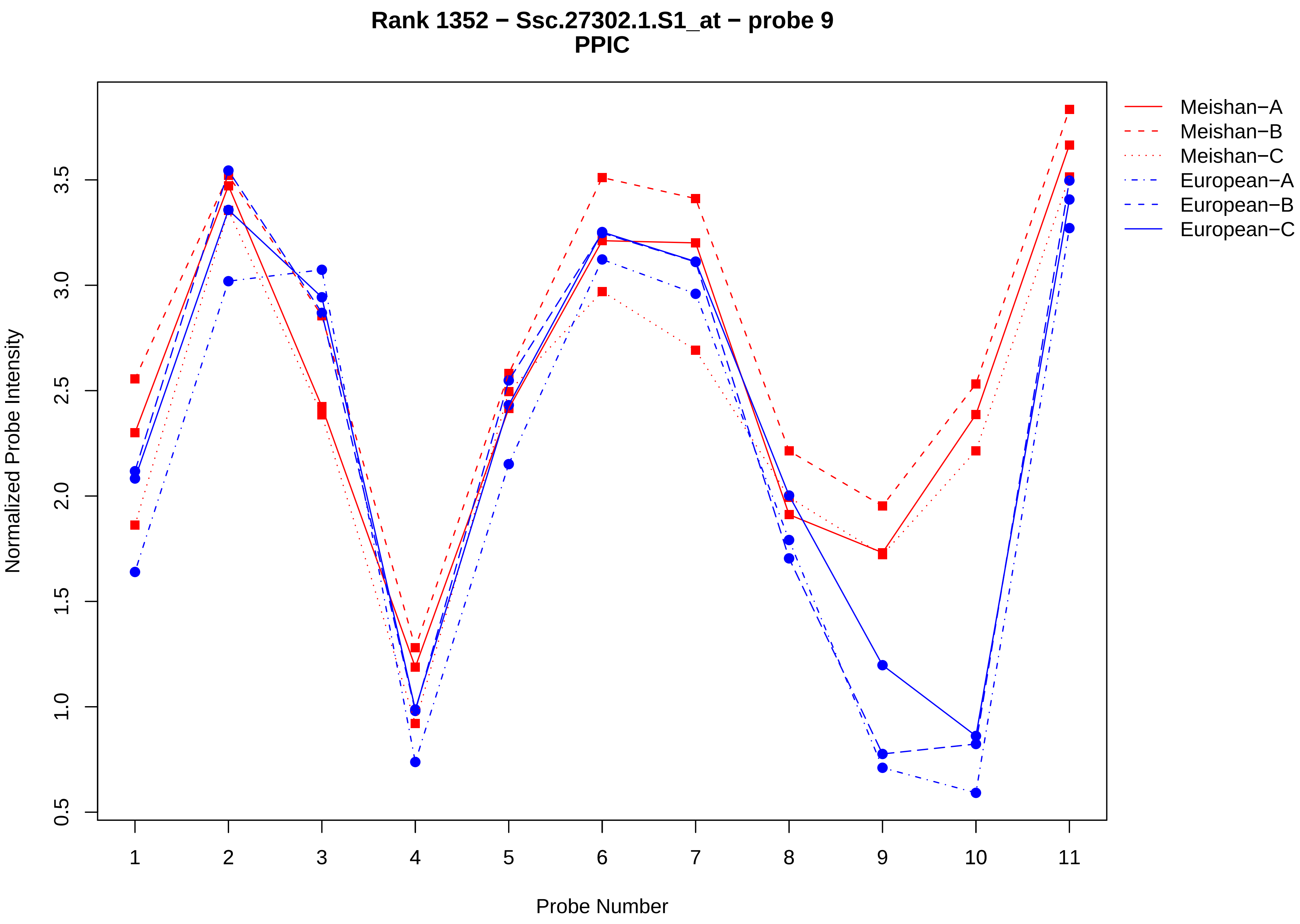
Rank 1352 − Ssc.27302.1.S1_at − probe 9
PPIC
Normalized Probe Intensity
Probe Number
0.5
1.0
1.5
2.0
2.5
3.0
3.5
1
2
3
4
5
6
7
8
9
10
11
Meishan−A
Meishan−B
Meishan−C
European−A
European−B
European−C

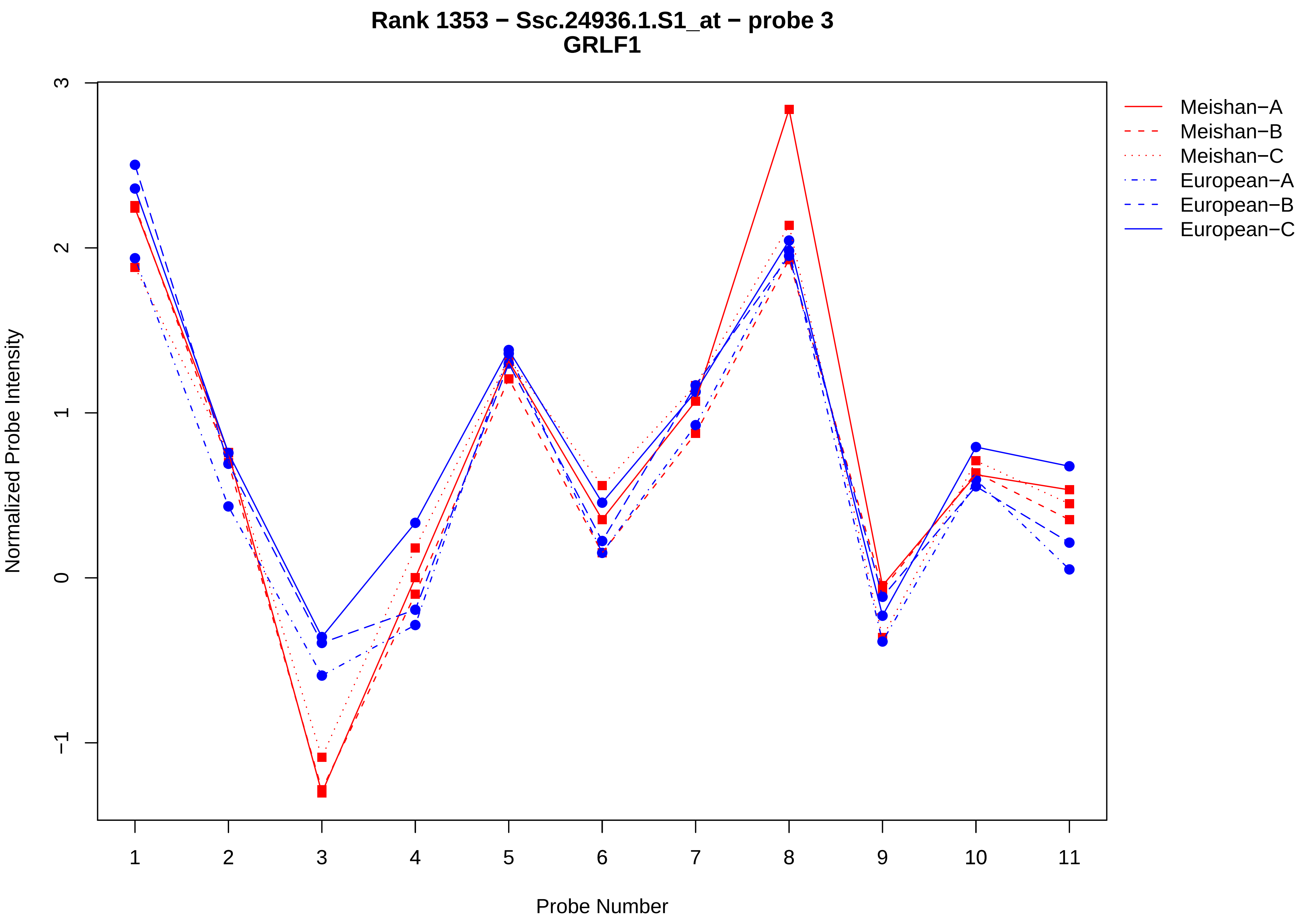

Rank 1353 − Ssc.24936.1.S1_at − probe 3
GRLF1
Normalized Probe Intensity
Probe Number
−1
0
1
2
3
1
2
3
4
5
6
7
8
9
10
11
Meishan−A
Meishan−B
Meishan−C
European−A
European−B
European−C

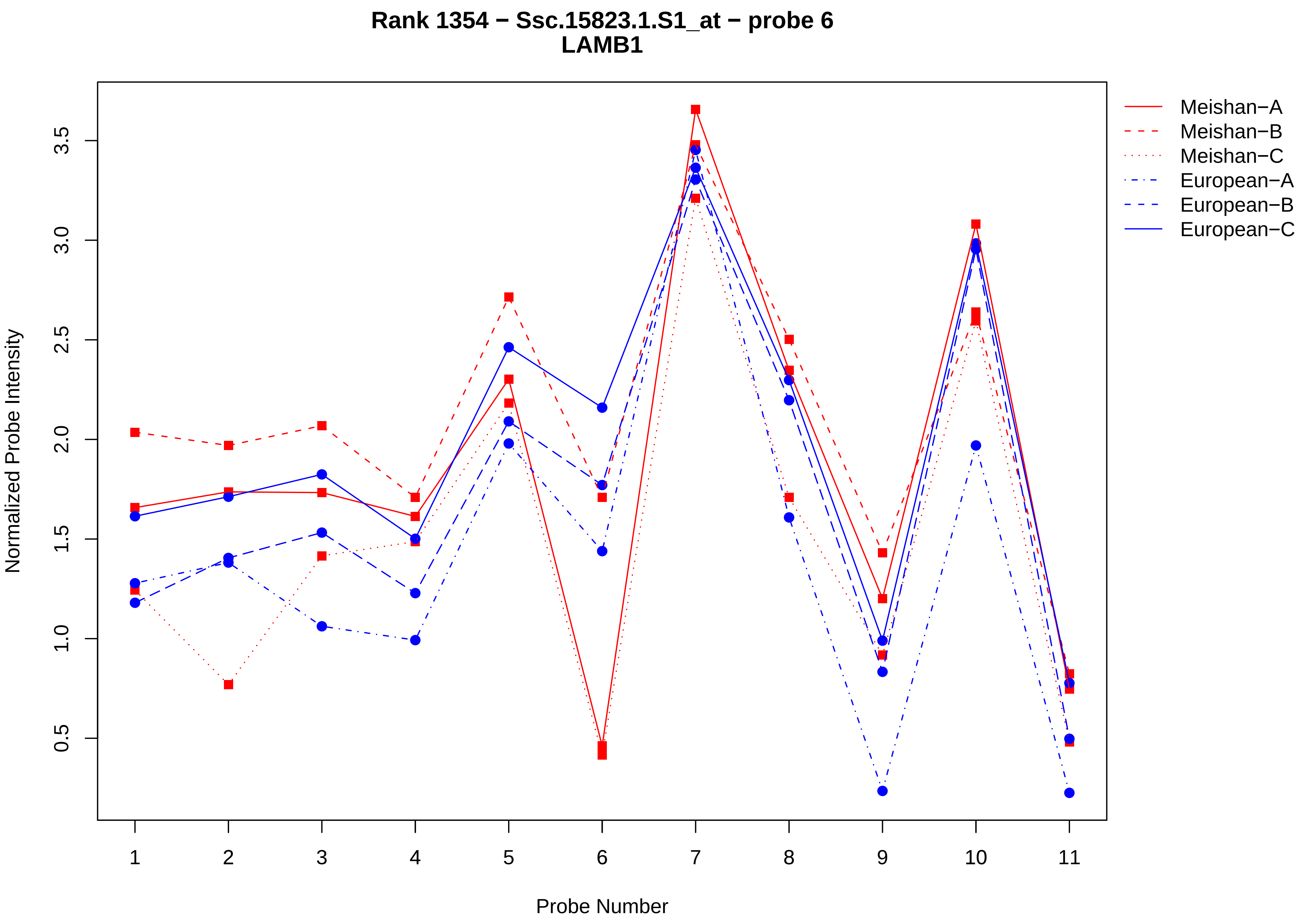

Rank 1354 − Ssc.15823.1.S1_at − probe 6
LAMB1
Normalized Probe Intensity
Probe Number
0.5
1.0
1.5
2.0
2.5
3.0
3.5
1
2
3
4
5
6
7
8
9
10
11
Meishan−A
Meishan−B
Meishan−C
European−A
European−B
European−C

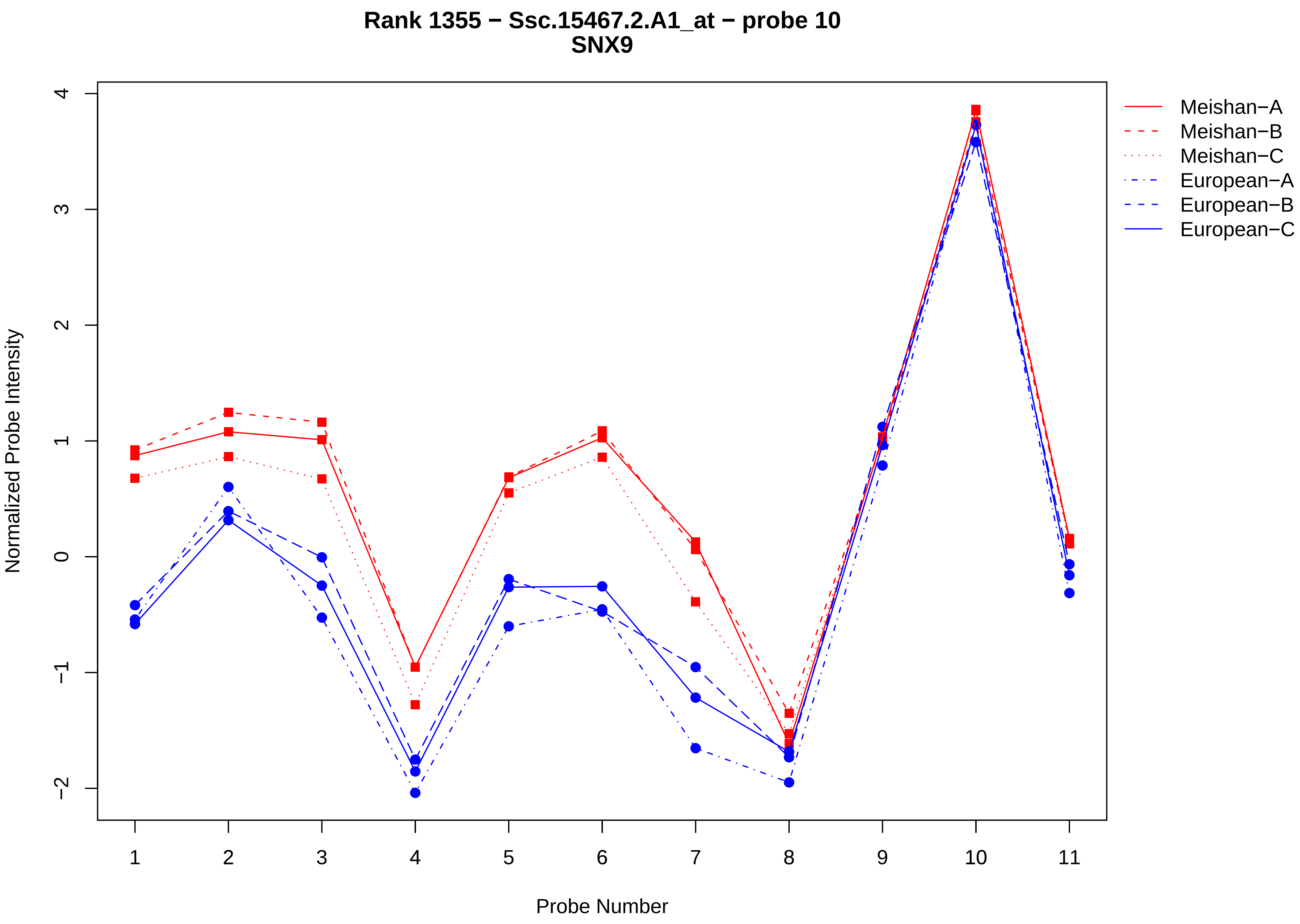

Rank 1355 − Ssc.15467.2.A1_at − probe 10
SNX9
Normalized Probe Intensity
Probe Number
−2
−1
0
1
2
3
4
1
2
3
4
5
6
7
8
9
10
11
Meishan−A
Meishan−B
Meishan−C
European−A
European−B
European−C

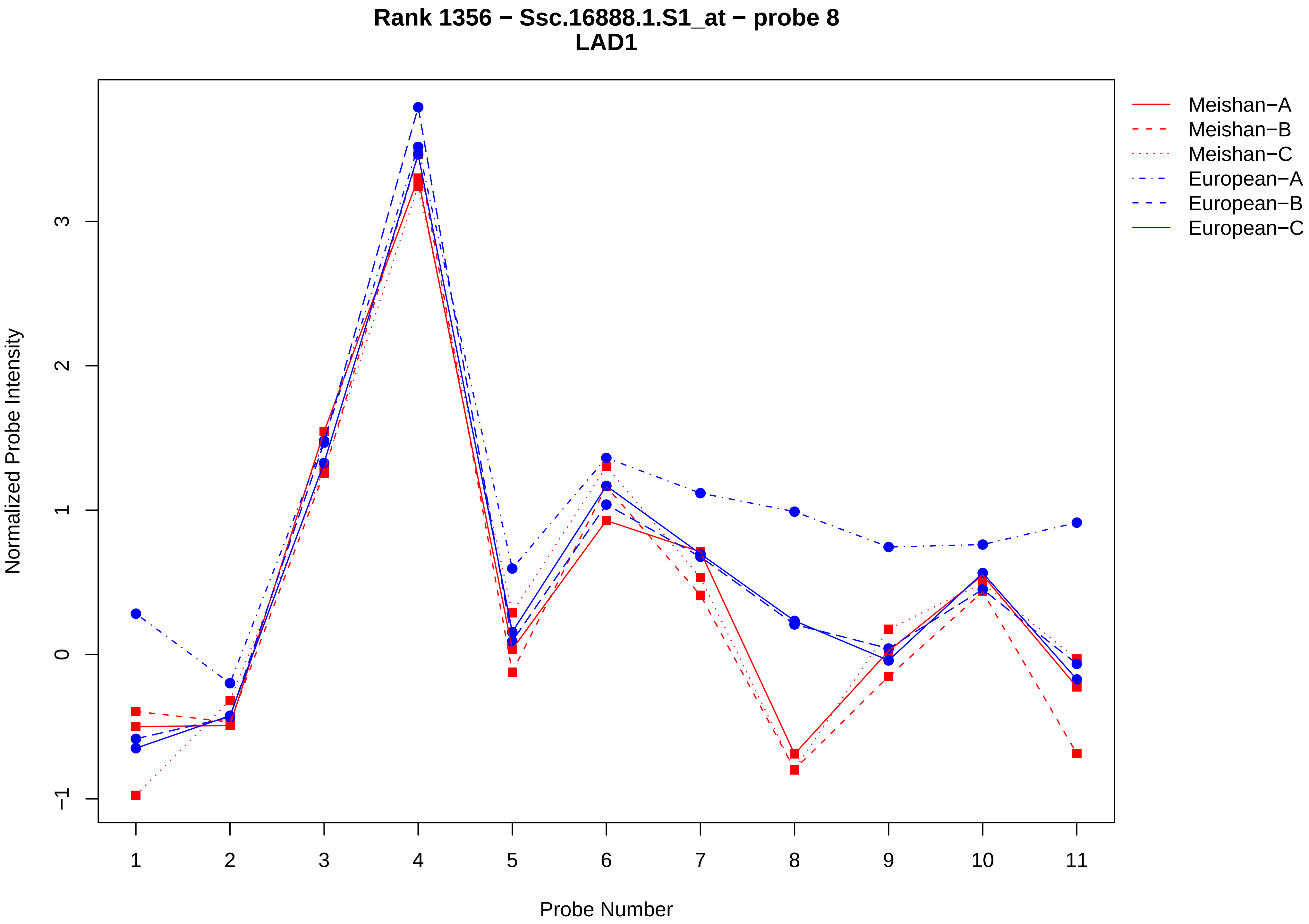
Rank 1356 − Ssc.16888.1.S1_at − probe 8
LAD1
Normalized Probe Intensity
Probe Number
−1
0
1
2
3
1
2
3
4
5
6
7
8
9
10
11
Meishan−A
Meishan−B
Meishan−C
European−A
European−B
European−C

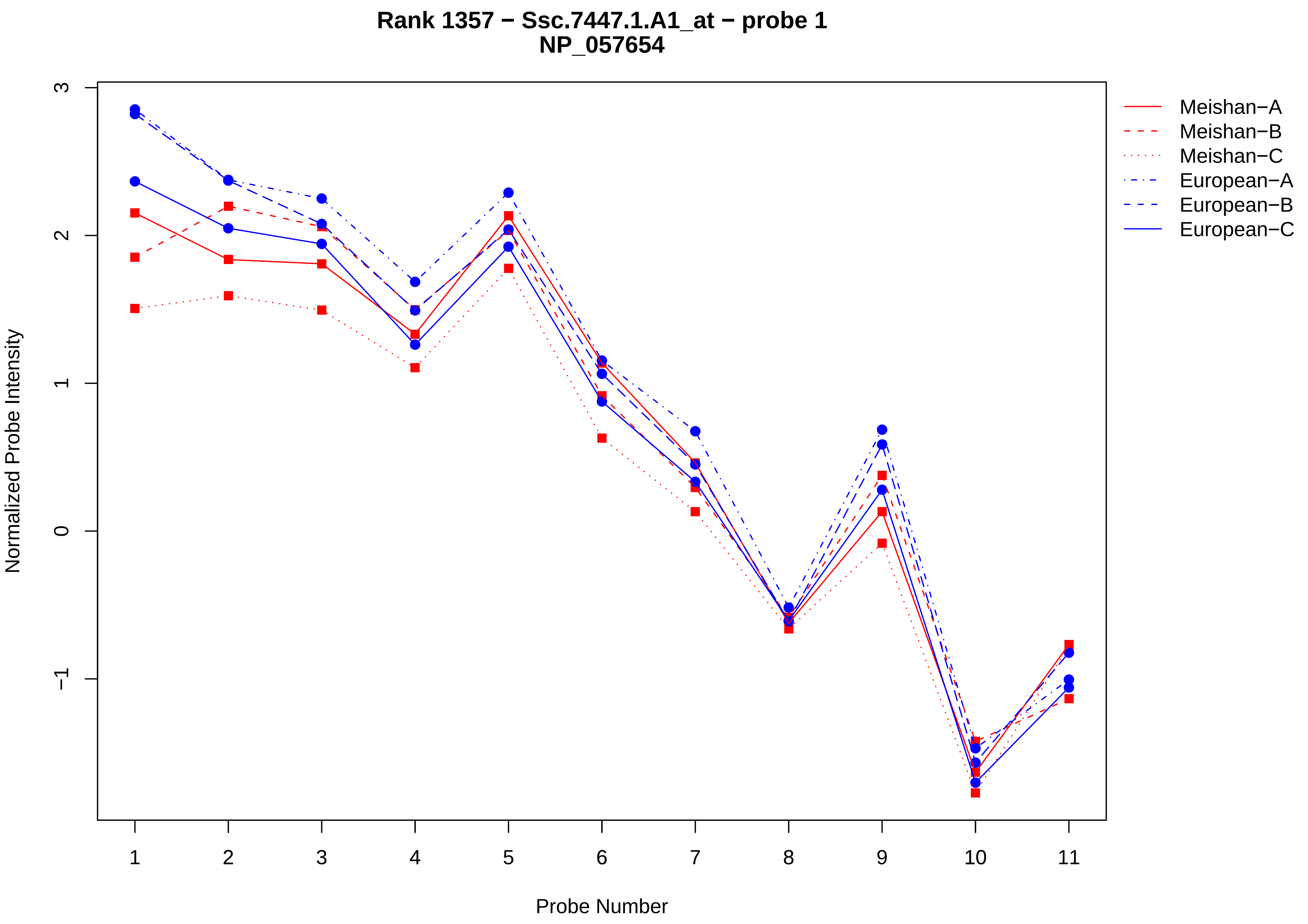
Rank 1357 − Ssc.7447.1.A1_at − probe 1
NP_057654
Normalized Probe Intensity
Probe Number
3
2
1
0
−1
1
2
3
4
5
6
7
8
9
10
11
Meishan−A
Meishan−B
Meishan−C
European−A
European−B
European−C

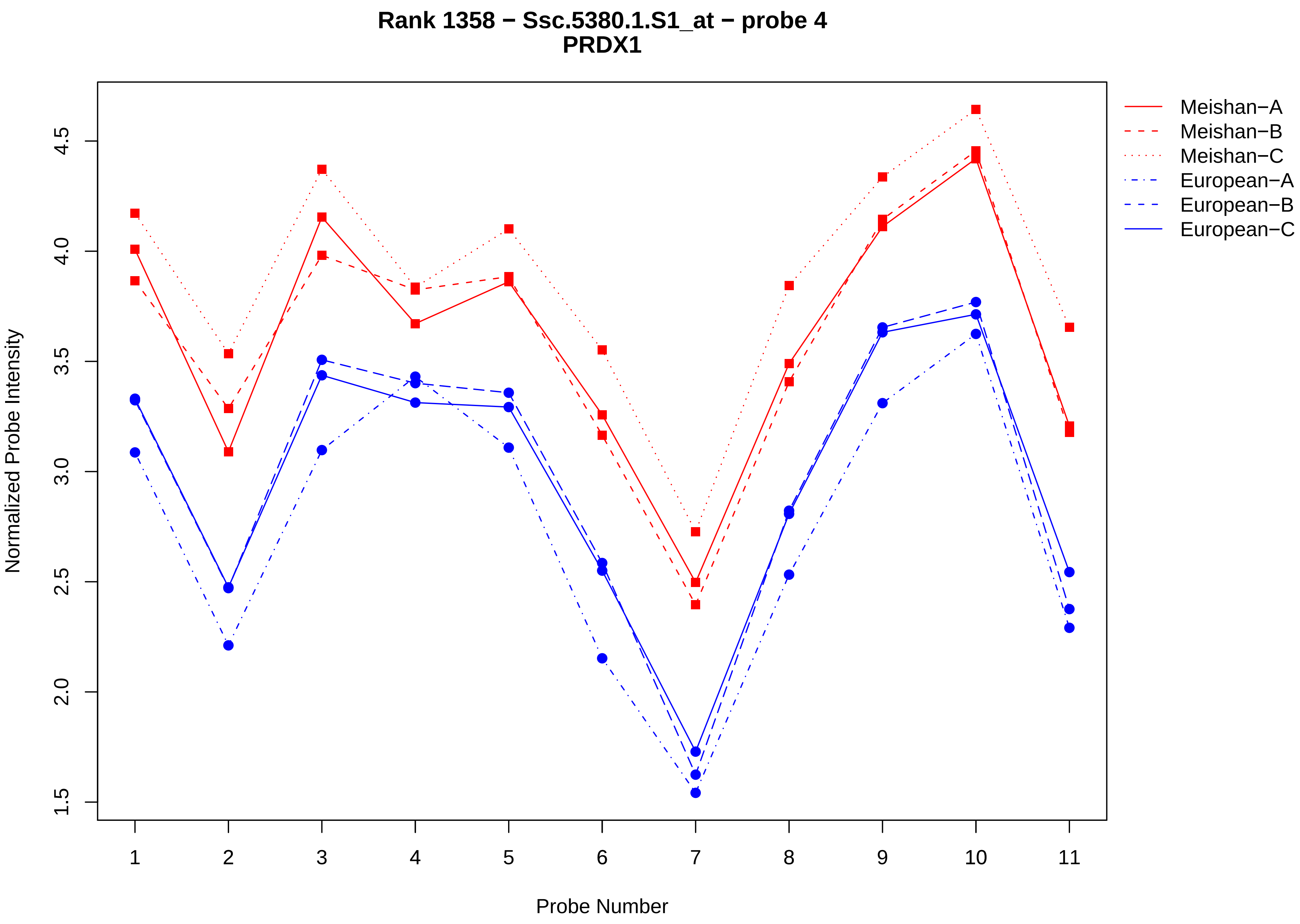
Rank 1358 − Ssc.5380.1.S1_at − probe 4
PRDX1
Normalized Probe Intensity
Probe Number
1.5
2.0
2.5
3.0
3.5
4.0
4.5
1
2
3
4
5
6
7
8
9
10
11
Meishan−A
Meishan−B
Meishan−C
European−A
European−B
European−C

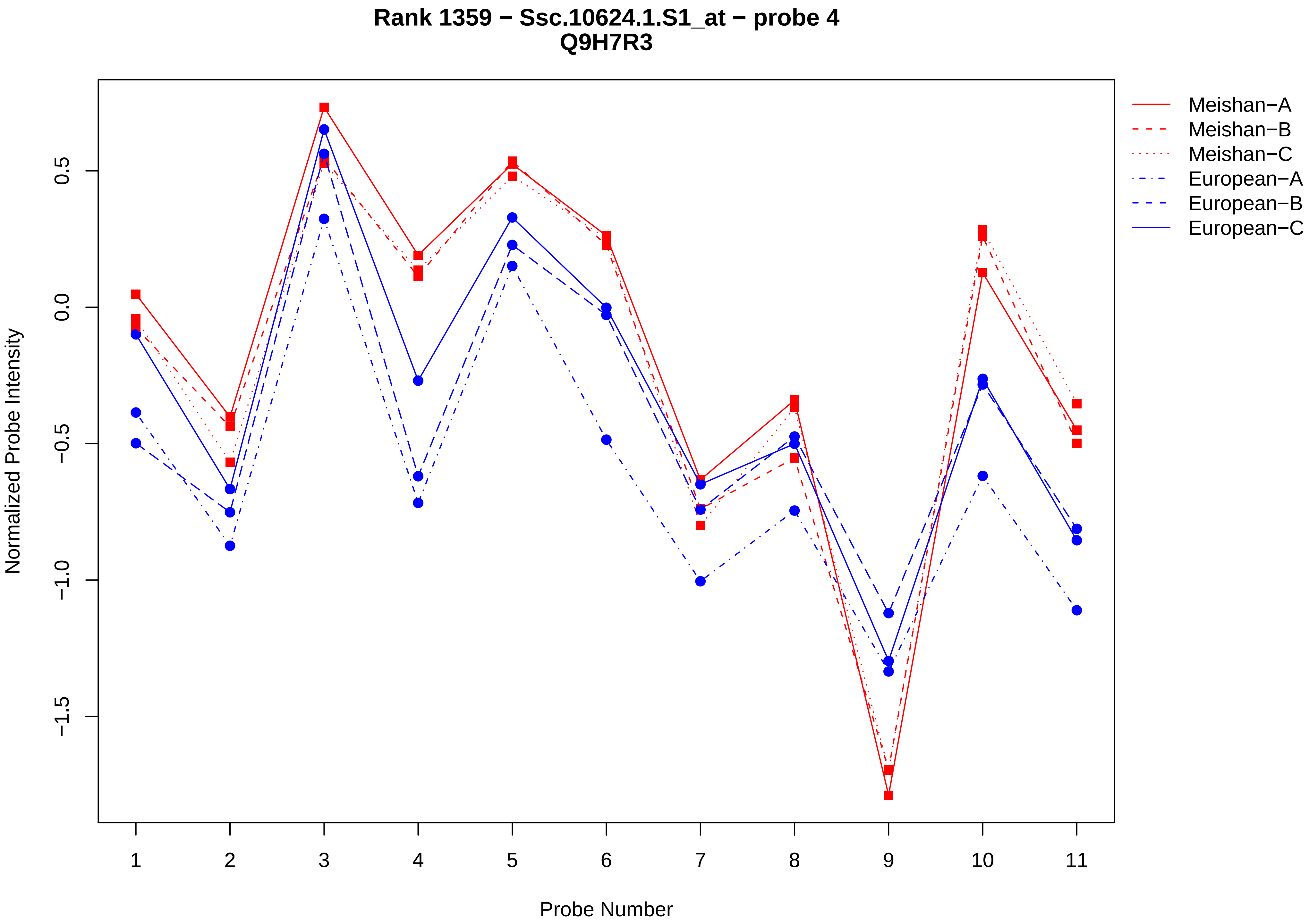

Rank 1359 − Ssc.10624.1.S1_at − probe 4
Q9H7R3
Normalized Probe Intensity
Probe Number
0.5
0.0
−0.5
−1.0
−1.5
1
2
3
4
5
6
7
8
9
10
11
Meishan−A
Meishan−B
Meishan−C
European−A
European−B
European−C

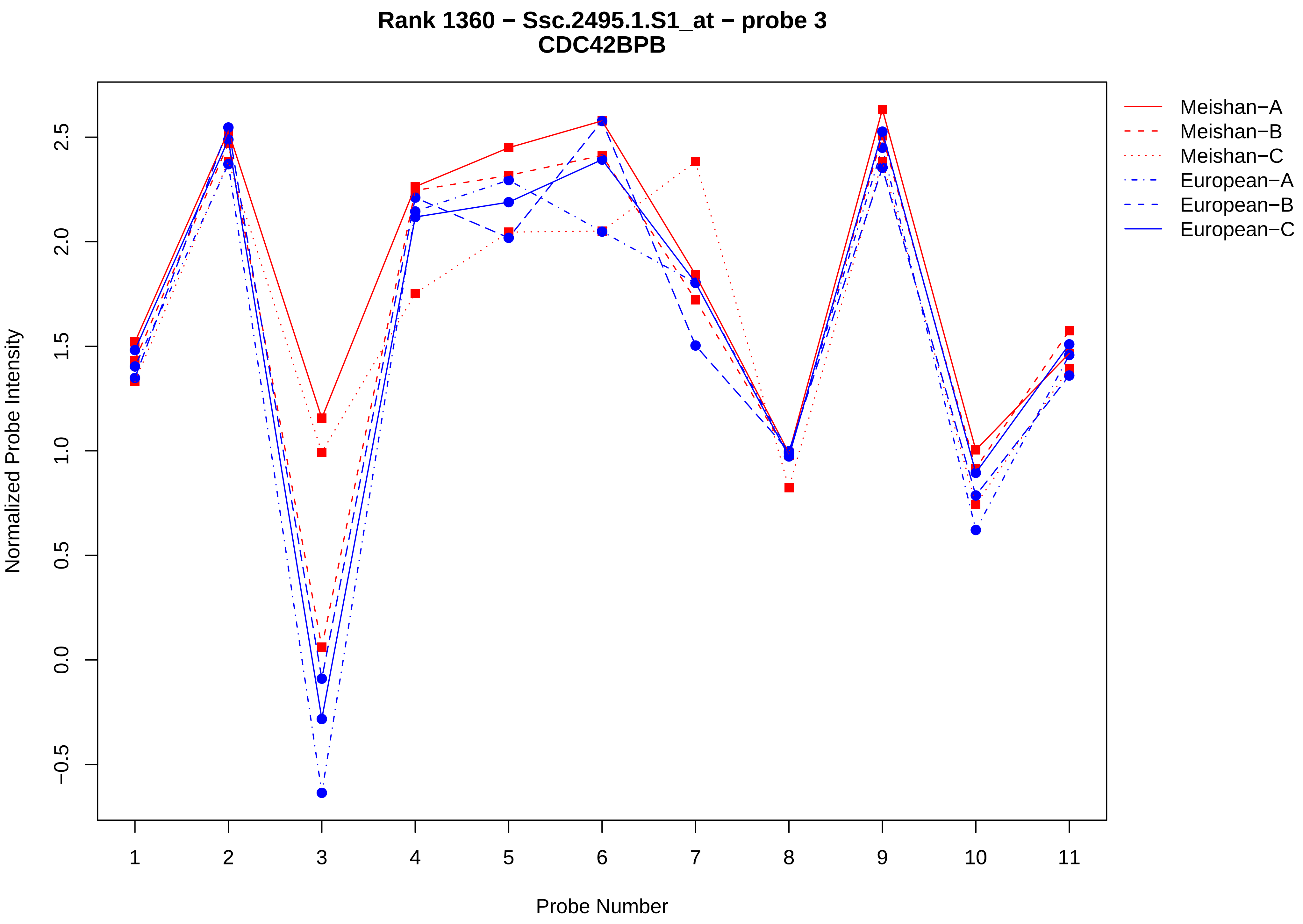

Rank 1360 − Ssc.2495.1.S1_at − probe 3
CDC42BPB
Normalized Probe Intensity
Probe Number
−0.5
0.0
0.5
1.0
1.5
2.0
2.5
1
2
3
4
5
6
7
8
9
10
11
Meishan−A
Meishan−B
Meishan−C
European−A
European−B
European−C

Rank 1361 − Ssc.6119.1.A1_at − probe 9
RAB18

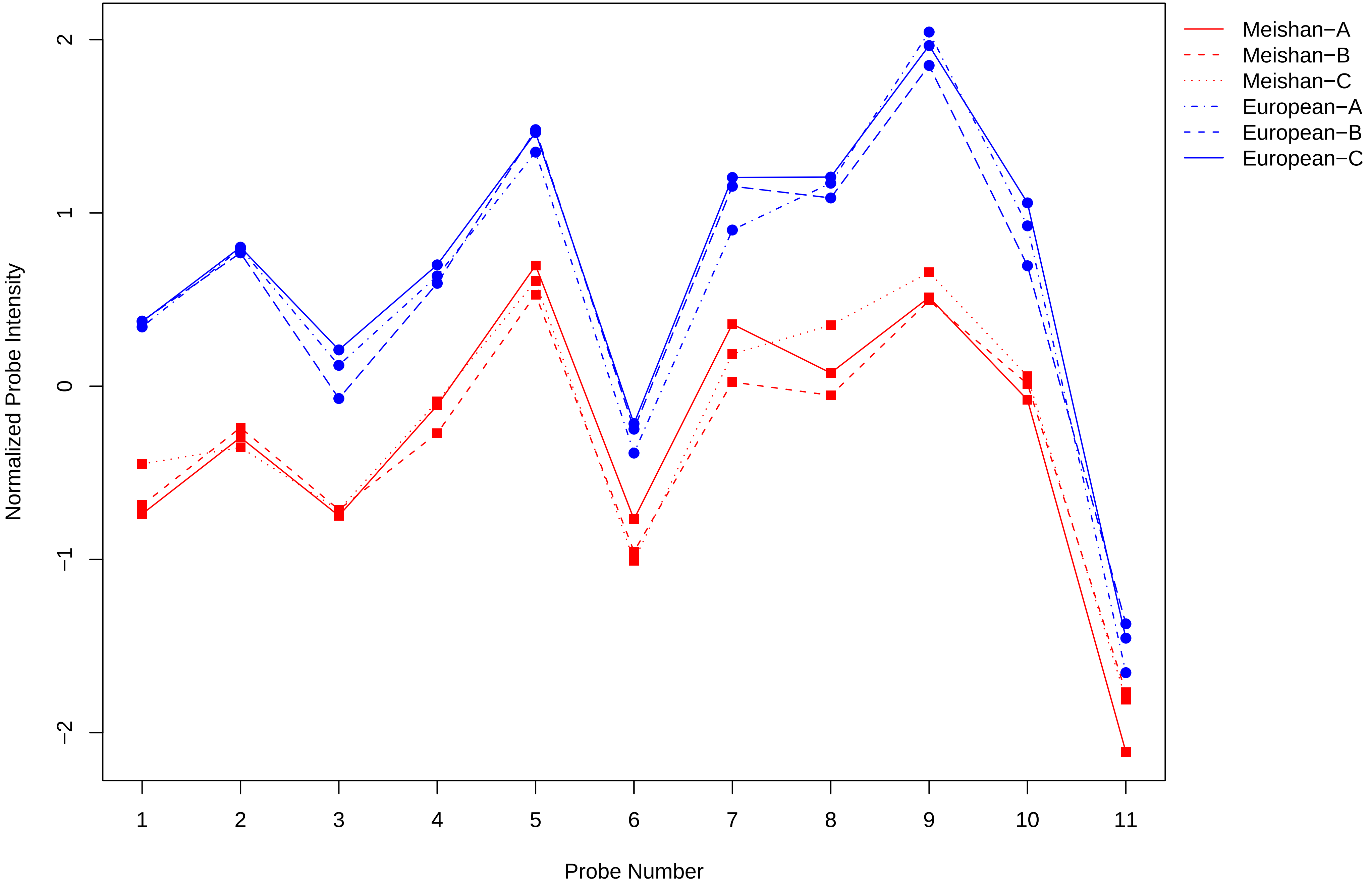

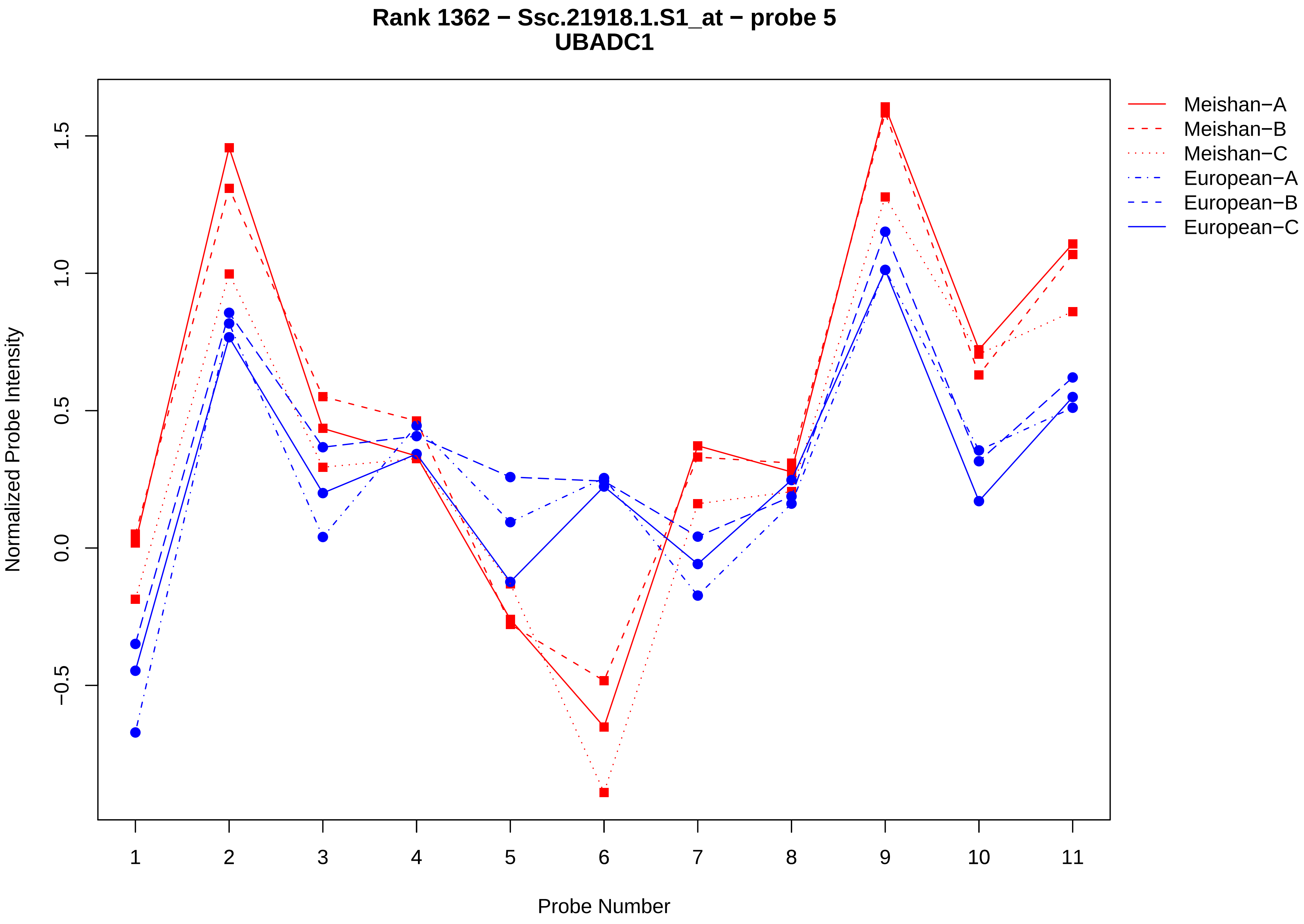

Rank 1362 − Ssc.21918.1.S1_at − probe 5
UBADC1
Normalized Probe Intensity
Probe Number
−0.5
0.0
0.5
1.0
1.5
1
2
3
4
5
6
7
8
9
10
11
Meishan−A
Meishan−B
Meishan−C
European−A
European−B
European−C

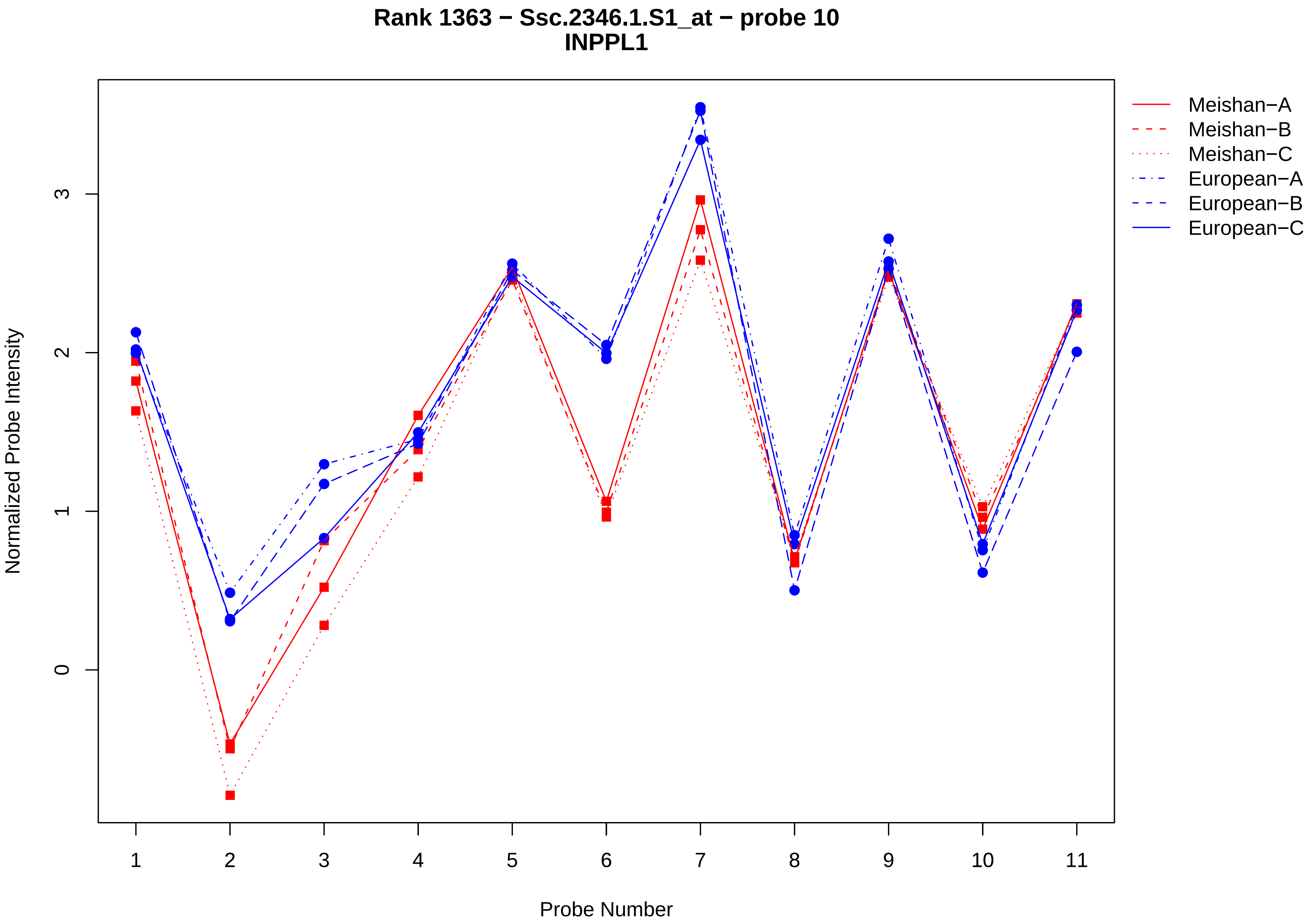
Rank 1363 − Ssc.2346.1.S1_at − probe 10
INPPL1
Normalized Probe Intensity
Probe Number
Meishan−A
Meishan−B
Meishan−C
European−A
European−B
European−C
0
1
2
3
1
2
3
4
5
6
7
8
9
10
11

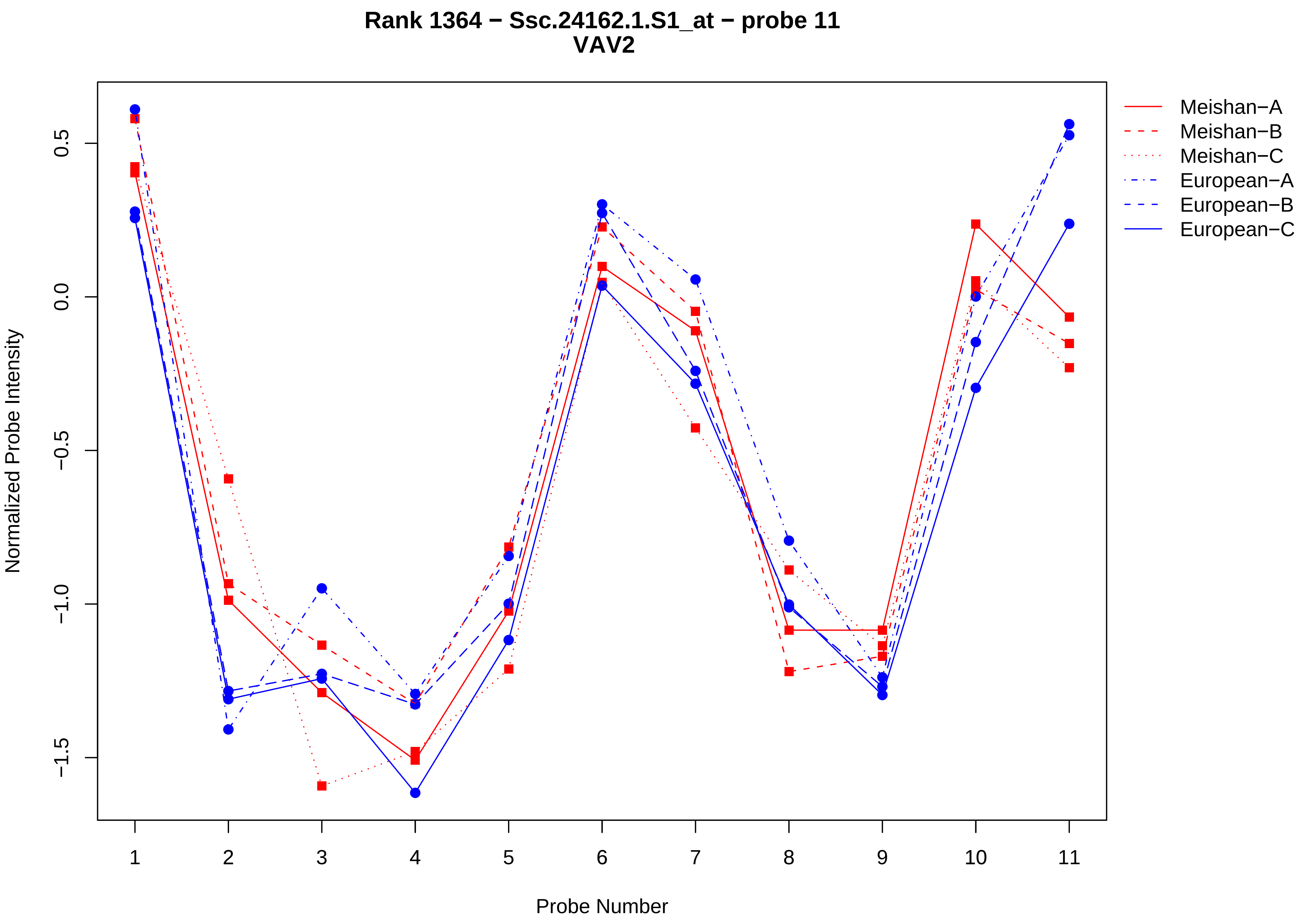

Rank 1364 − Ssc.24162.1.S1_at − probe 11
VAV2
Normalized Probe Intensity
Probe Number
0.5
0.0
−0.5
−1.0
−1.5
1
2
3
4
5
6
7
8
9
10
11
Meishan−A
Meishan−B
Meishan−C
European−A
European−B
European−C

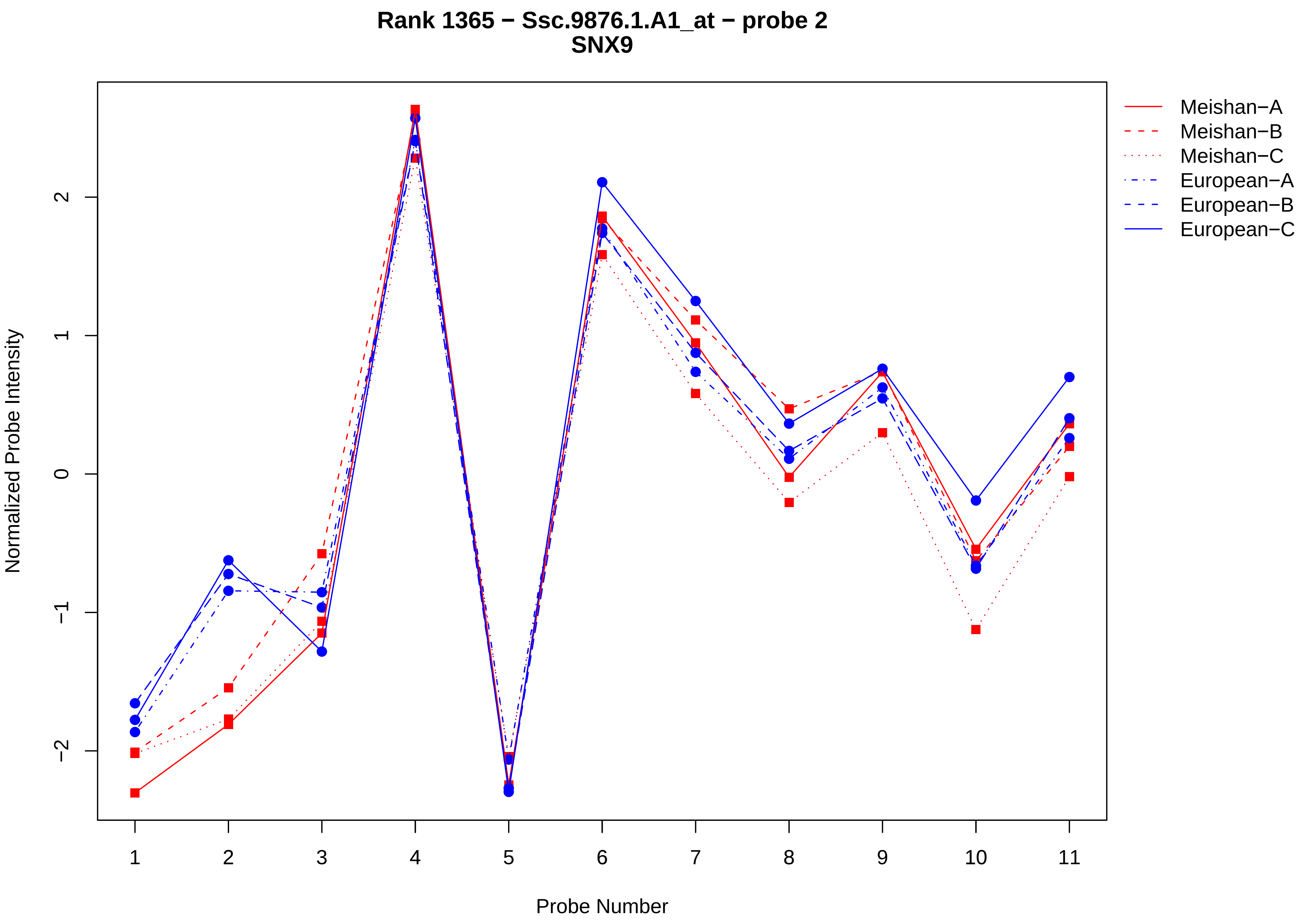

Rank 1365 − Ssc.9876.1.A1_at − probe 2
SNX9
Normalized Probe Intensity
Probe Number
−2
−1
0
1
2
1
2
3
4
5
6
7
8
9
10
11
Meishan−A
Meishan−B
Meishan−C
European−A
European−B
European−C

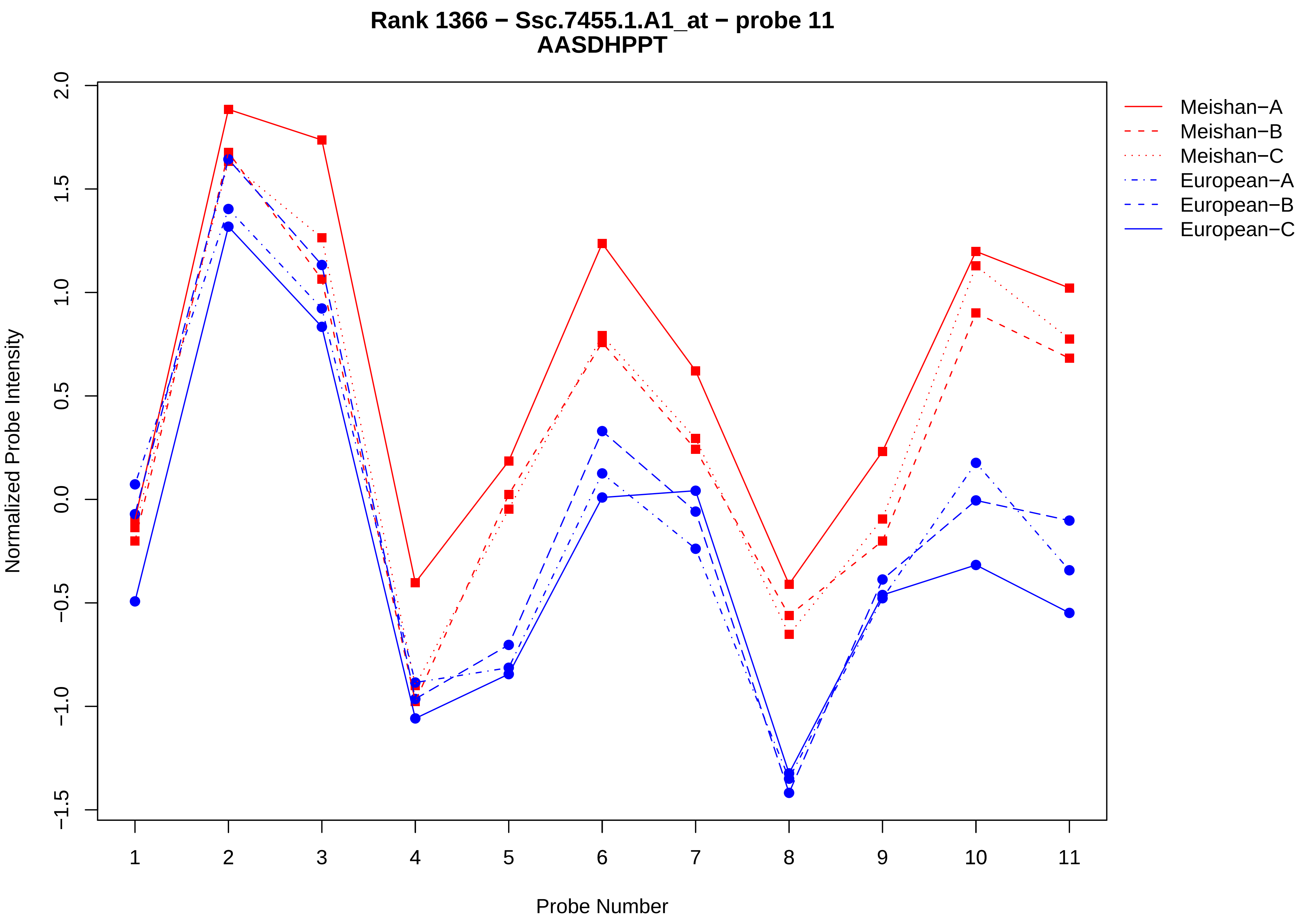
Rank 1366 − Ssc.7455.1.A1_at − probe 11
AASDHPPT
Normalized Probe Intensity
Probe Number
Meishan−A
Meishan−B
Meishan−C
European−A
European−B
European−C
2.0
1.5
1.0
0.5
0.0
−0.5
−1.0
−1.5
1
2
3
4
5
6
7
8
9
10
11

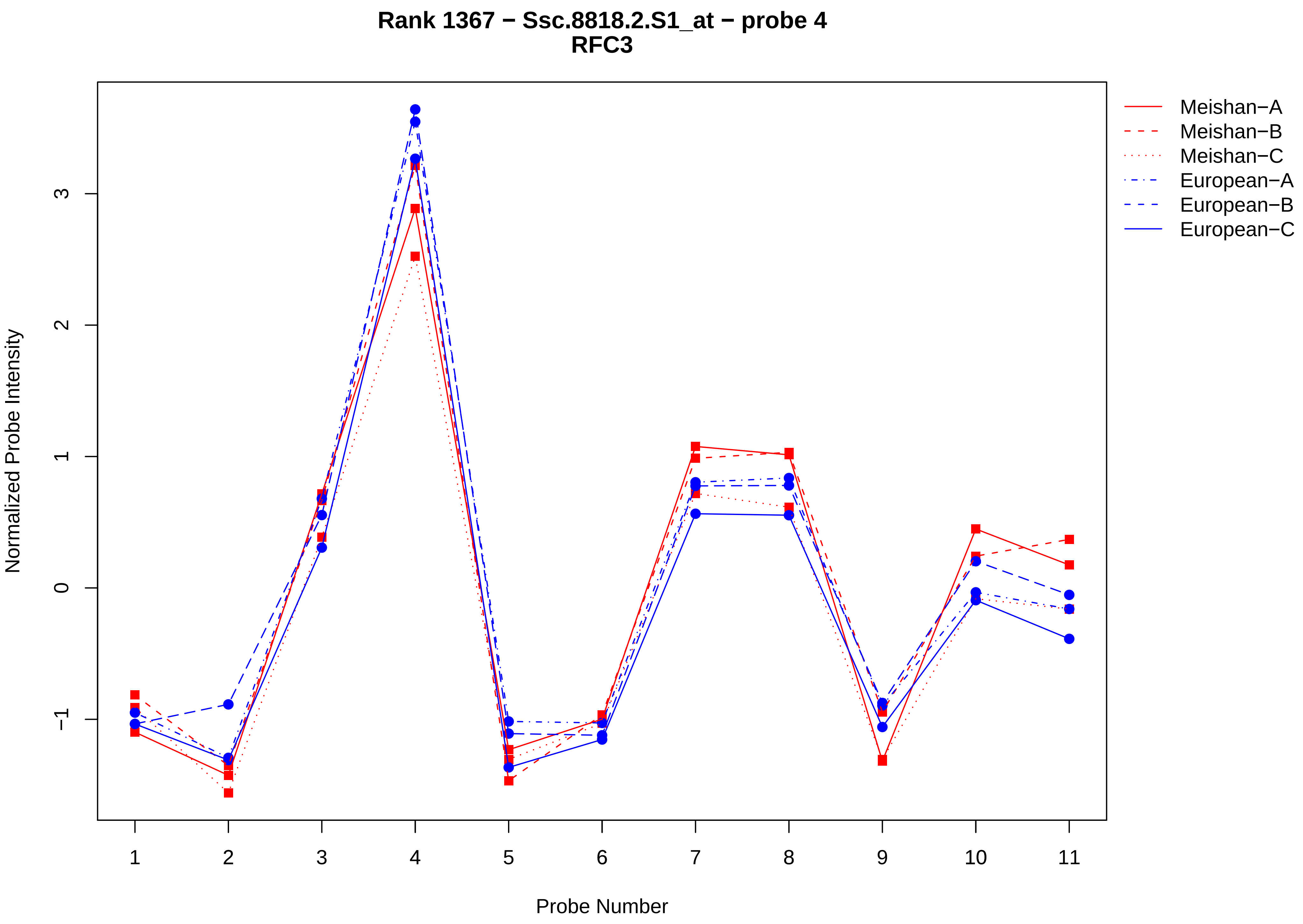

Rank 1367 − Ssc.8818.2.S1_at − probe 4
RFC3
Normalized Probe Intensity
Probe Number
−1
0
1
2
3
1
2
3
4
5
6
7
8
9
10
11
Meishan−A
Meishan−B
Meishan−C
European−A
European−B
European−C

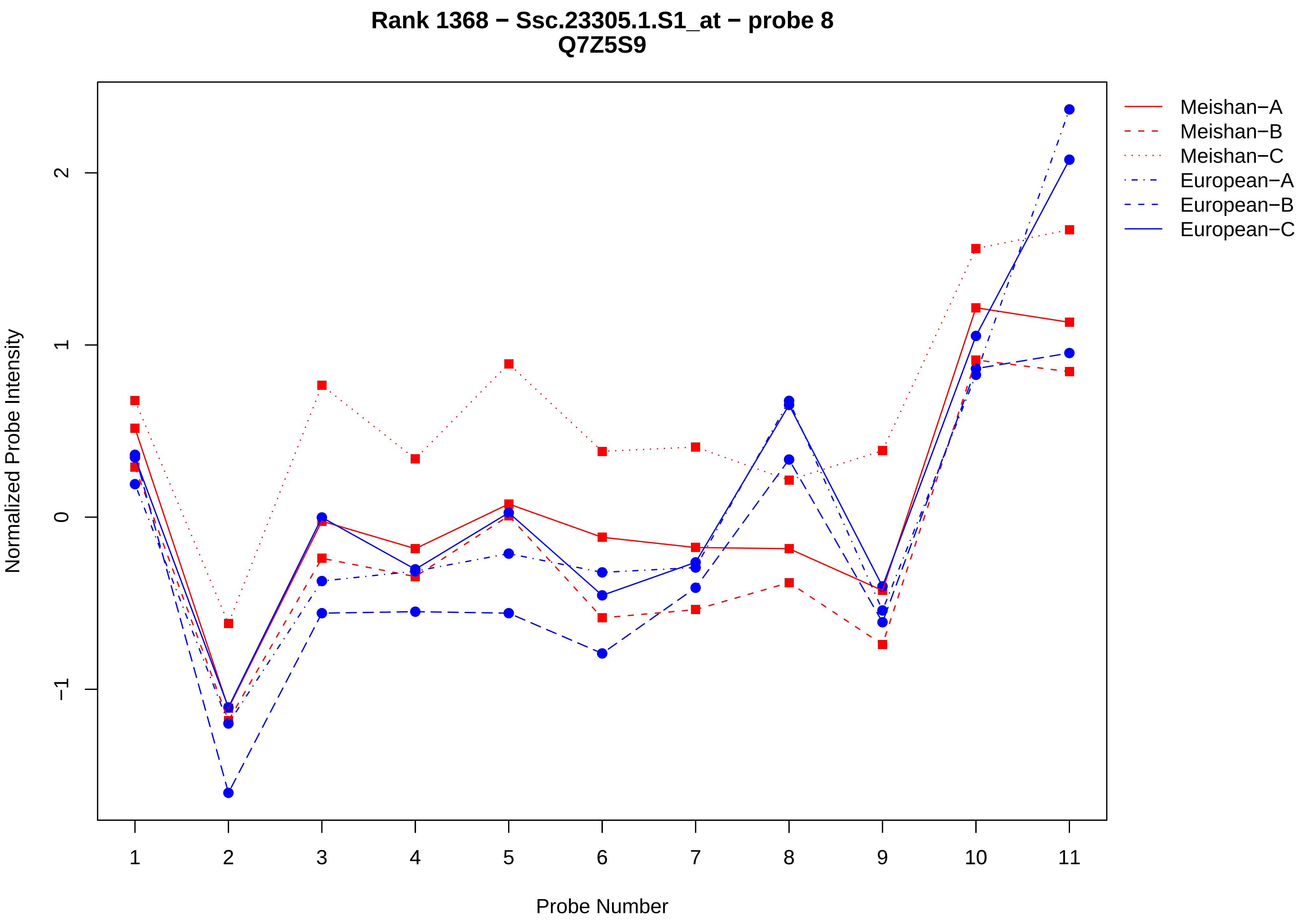

Rank 1368 − Ssc.23305.1.S1_at − probe 8
Q7Z5S9
Normalized Probe Intensity
Probe Number
−1
0
1
2
1
2
3
4
5
6
7
8
9
10
11
Meishan−A
Meishan−B
Meishan−C
European−A
European−B
European−C

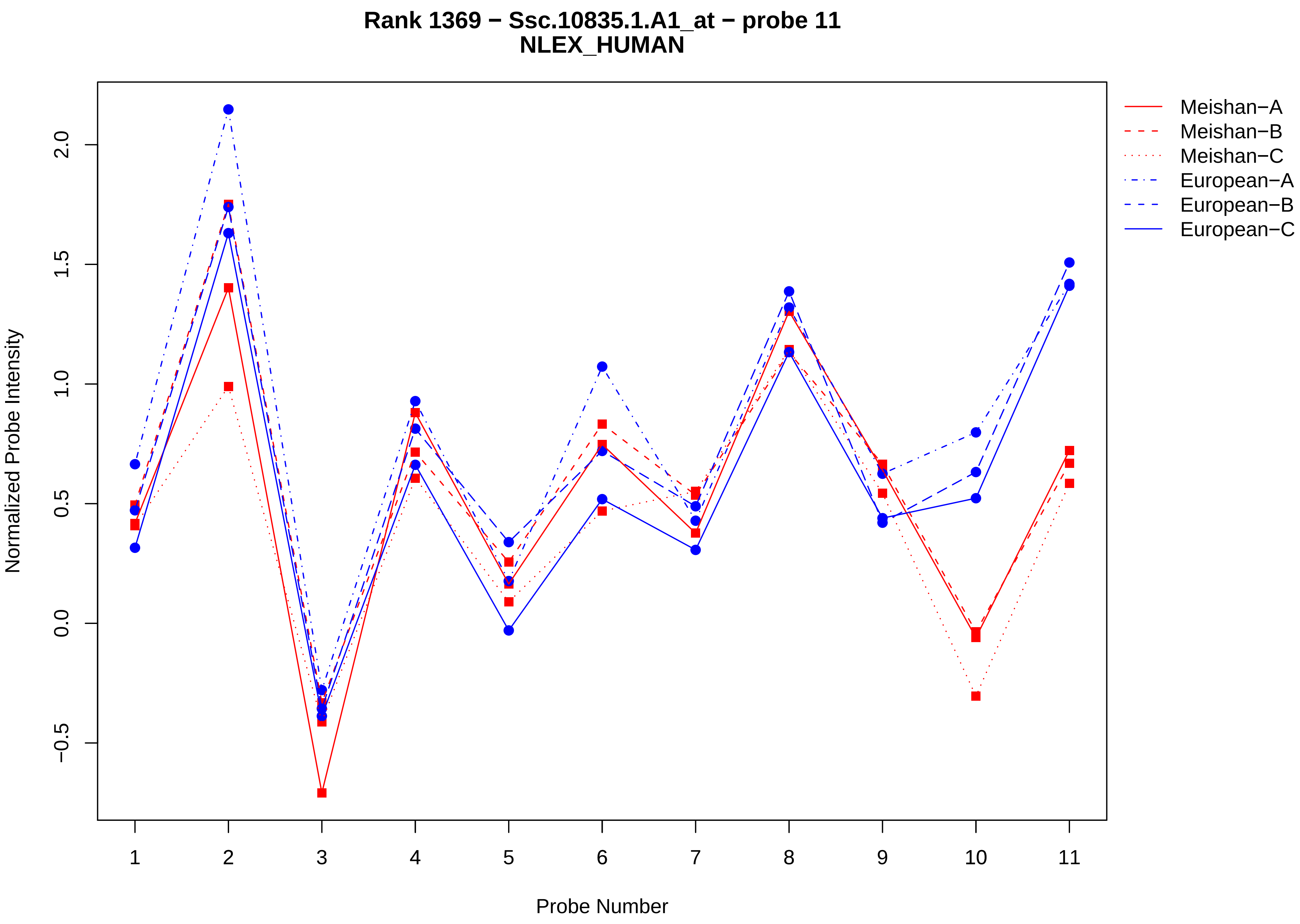

Rank 1369 − Ssc.10835.1.A1_at − probe 11
NLEX_HUMAN
Normalized Probe Intensity
Probe Number
−0.5
0.0
0.5
1.0
1.5
2.0
1
2
3
4
5
6
7
8
9
10
11
Meishan−A
Meishan−B
Meishan−C
European−A
European−B
European−C

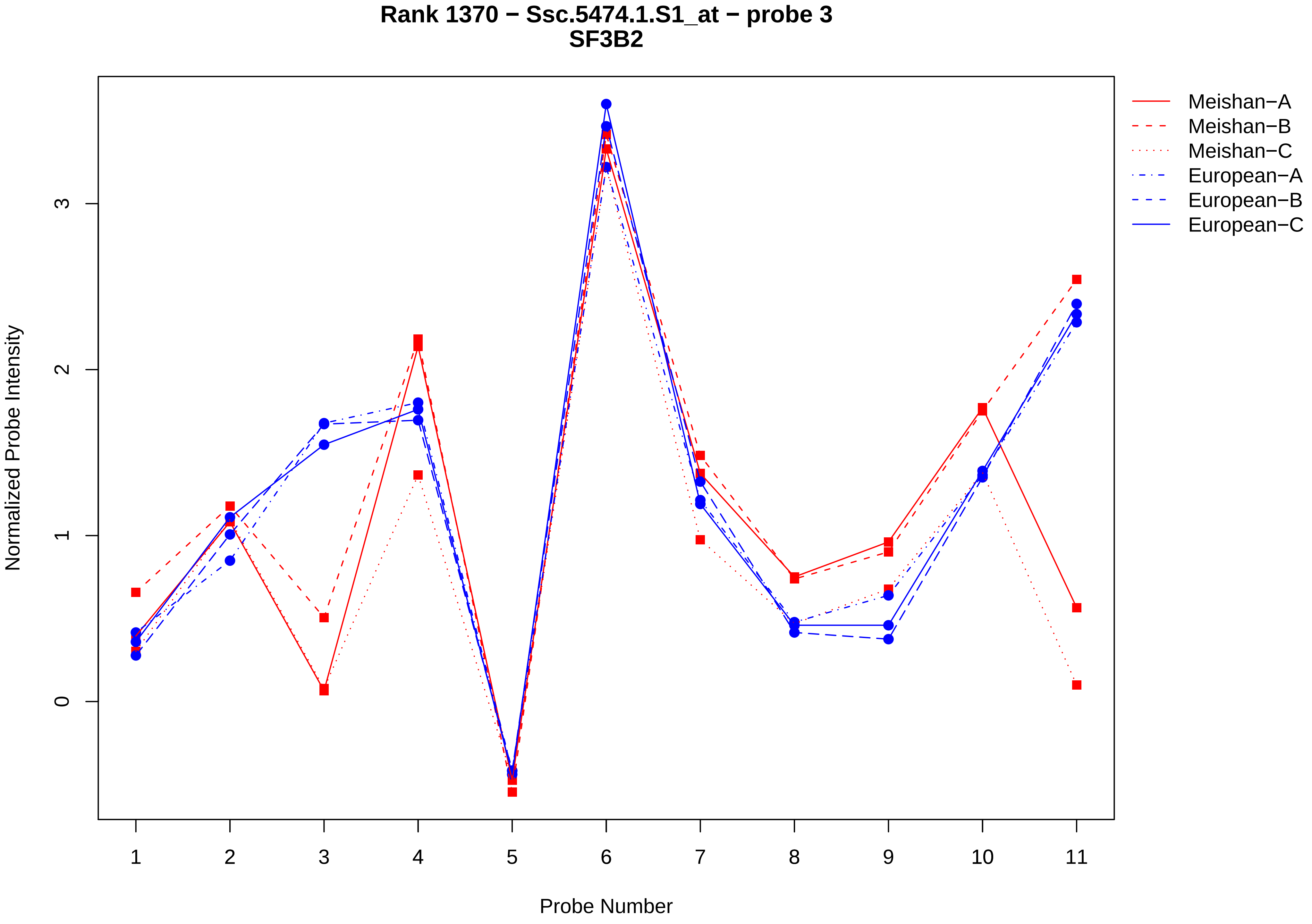

Rank 1370 − Ssc.5474.1.S1_at − probe 3
SF3B2
Normalized Probe Intensity
Probe Number
0
1
2
3
1
2
3
4
5
6
7
8
9
10
11
Meishan−A
Meishan−B
Meishan−C
European−A
European−B
European−C

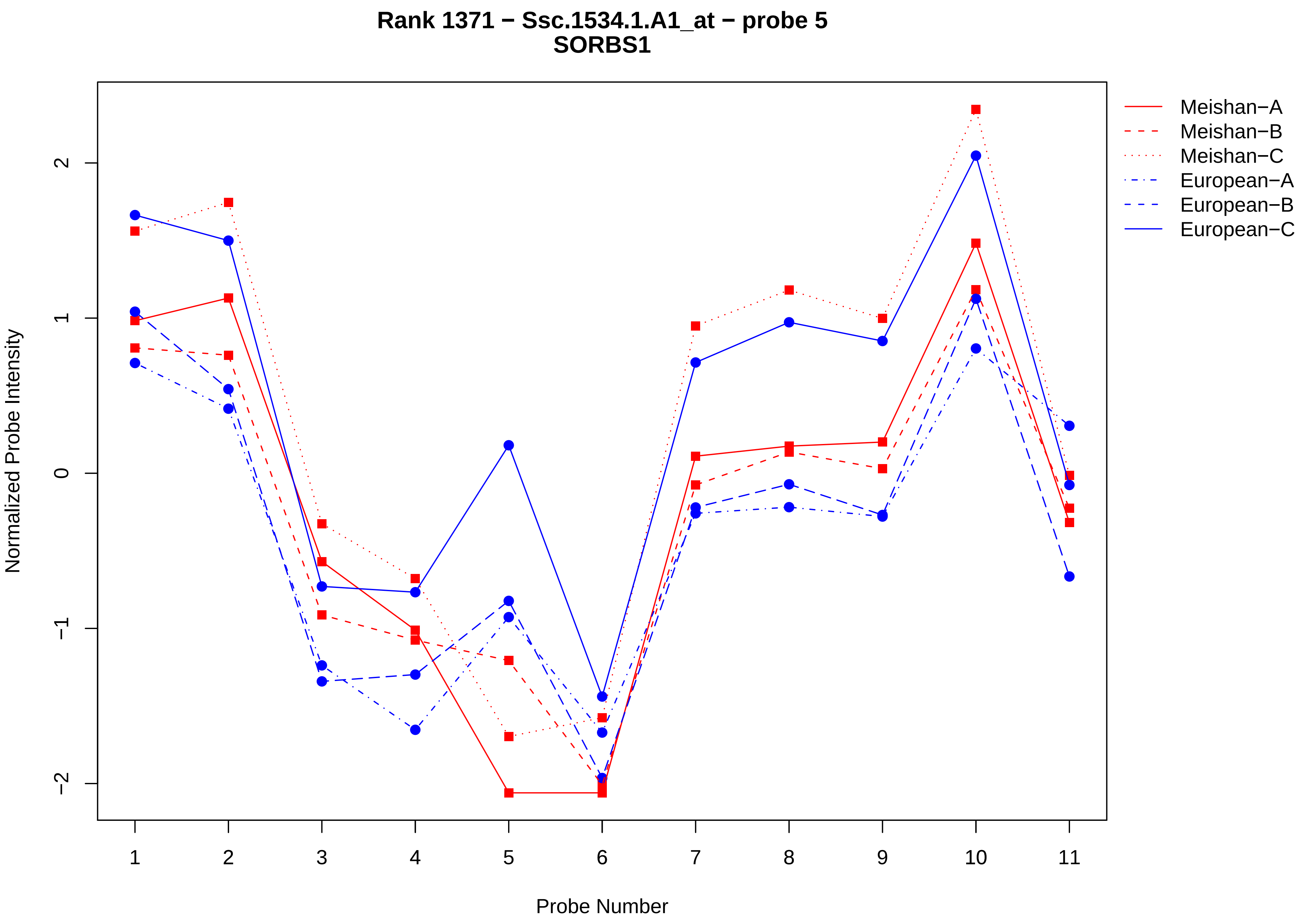
Rank 1371 − Ssc.1534.1.A1_at − probe 5
SORBS1
Normalized Probe Intensity
Probe Number
−2
−1
0
1
2
1
2
3
4
5
6
7
8
9
10
11
Meishan−A
Meishan−B
Meishan−C
European−A
European−B
European−C

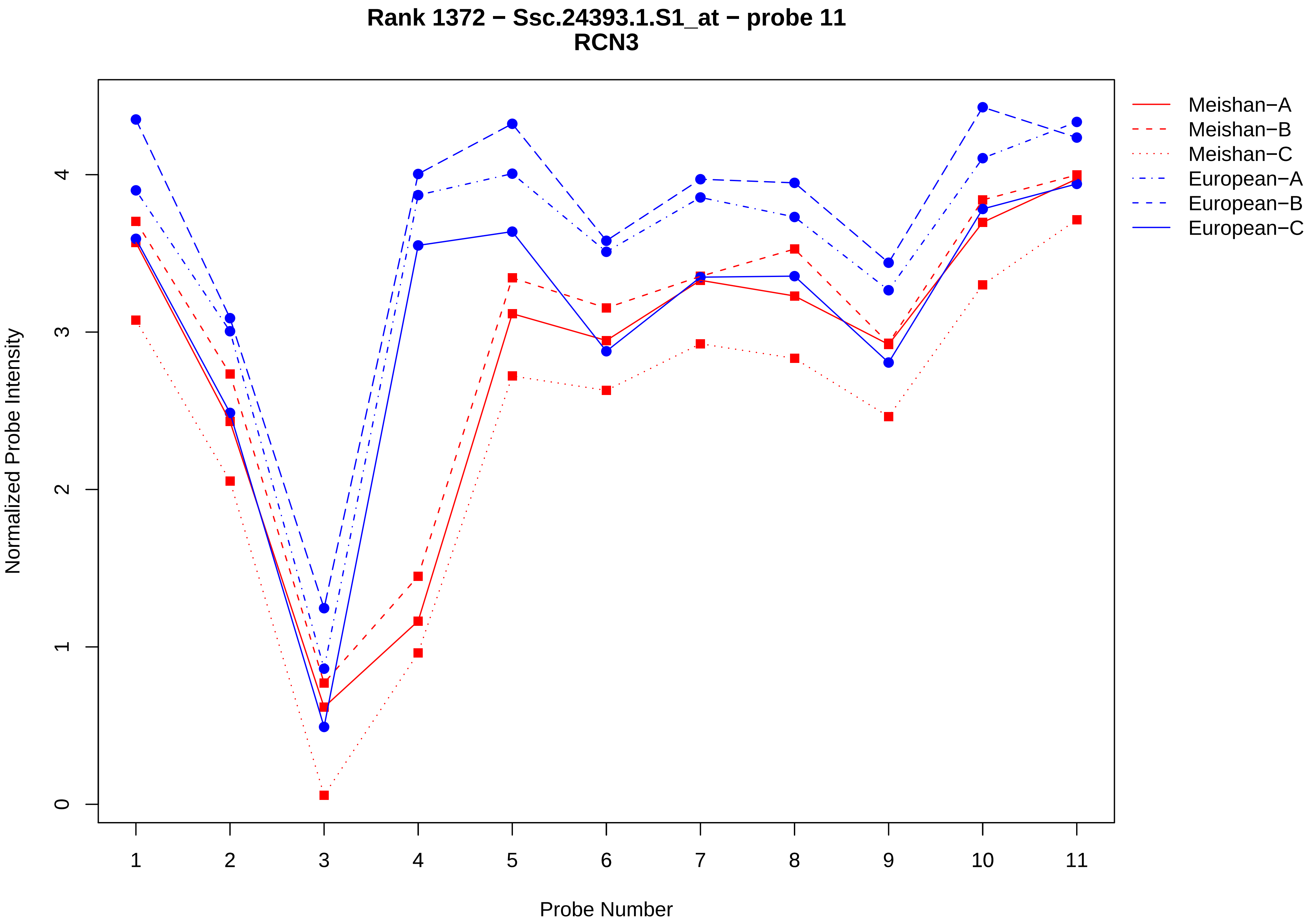

Rank 1372 − Ssc.24393.1.S1_at − probe 11
RCN3
Normalized Probe Intensity
Probe Number
0
1
2
3
4
1
2
3
4
5
6
7
8
9
10
11
Meishan−A
Meishan−B
Meishan−C
European−A
European−B
European−C

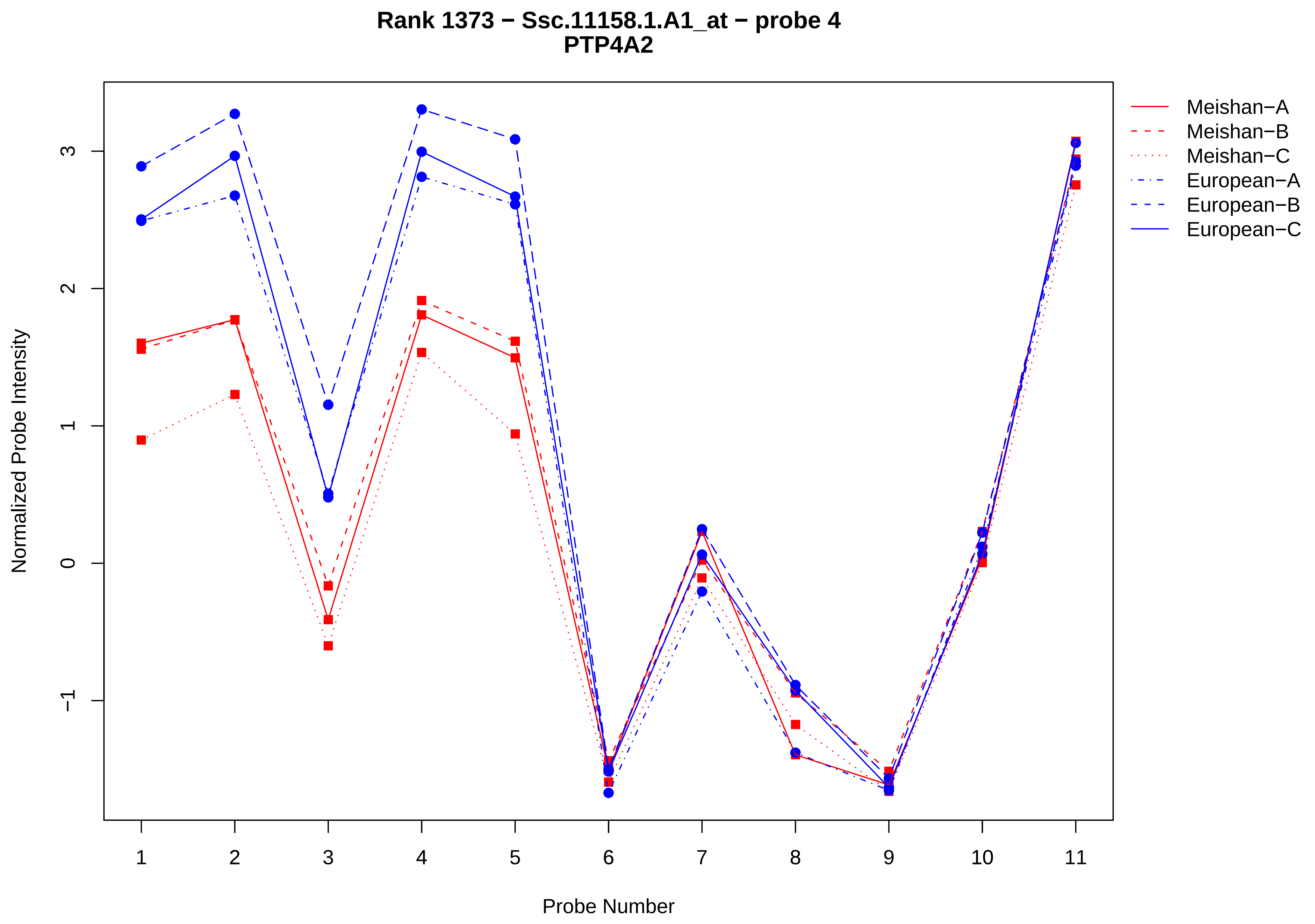

Rank 1373 − Ssc.11158.1.A1_at − probe 4
PTP4A2
Normalized Probe Intensity
Probe Number
Meishan−A
Meishan−B
Meishan−C
European−A
European−B
European−C

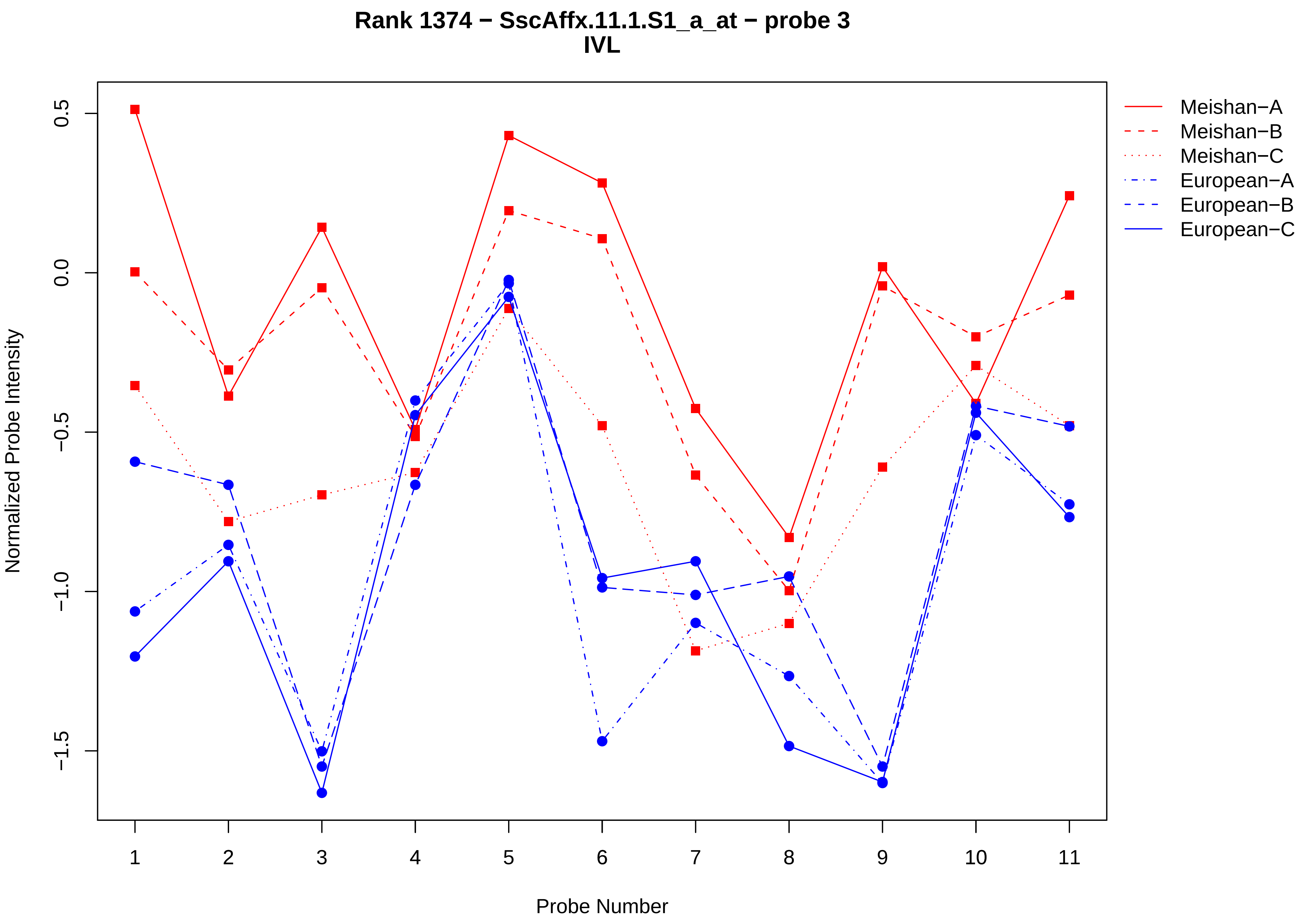

Rank 1374 − SscAffx.11.1.S1_a_at − probe 3
IVL
Normalized Probe Intensity
Probe Number
0.5
0.0
−0.5
−1.0
−1.5
1
2
3
4
5
6
7
8
9
10
11
Meishan−A
Meishan−B
Meishan−C
European−A
European−B
European−C

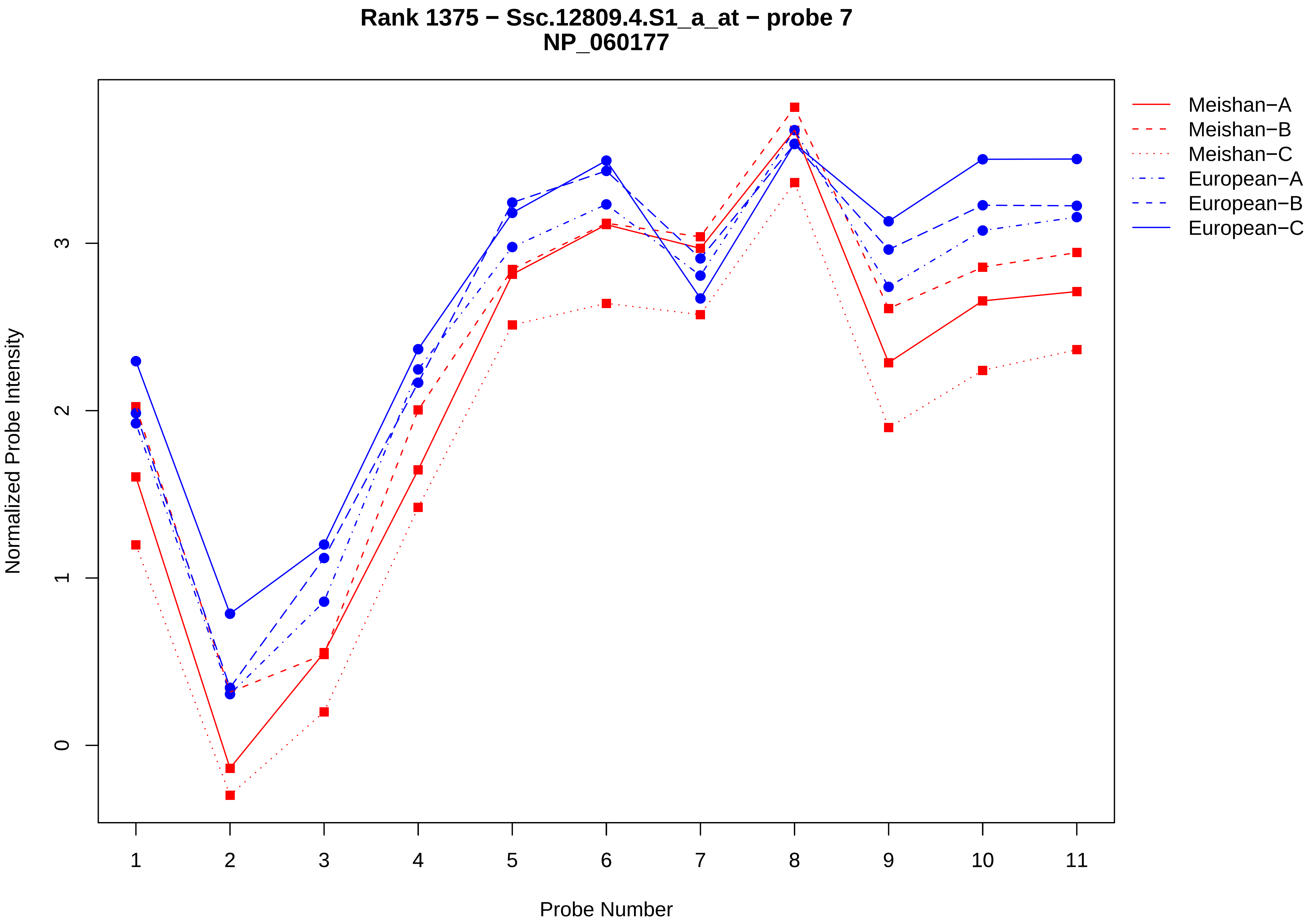

Rank 1375 − Ssc.12809.4.S1_a_at − probe 7
NP_060177
Normalized Probe Intensity
Probe Number
0
1
2
3
1
2
3
4
5
6
7
8
9
10
11
Meishan−A
Meishan−B
Meishan−C
European−A
European−B
European−C

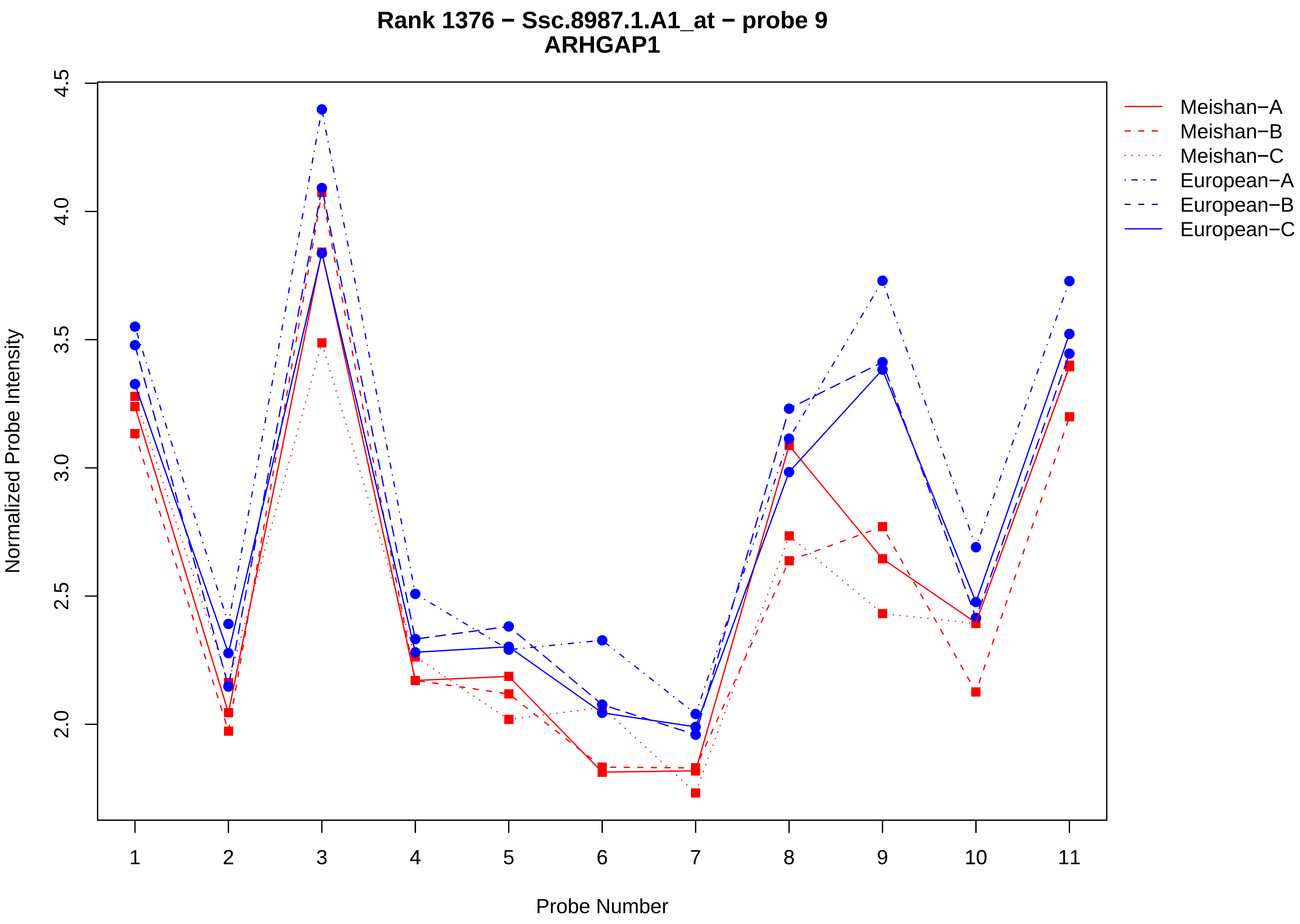

Rank 1376 − Ssc.8987.1.A1_at − probe 9
ARHGAP1
Normalized Probe Intensity
Probe Number
4.5
4.0
3.5
3.0
2.5
2.0
1
2
3
4
5
6
7
8
9
10
11
Meishan−A
Meishan−B
Meishan−C
European−A
European−B
European−C

Rank 1377 − Ssc.27882.1.A1_at − probe 3
TCEAL4

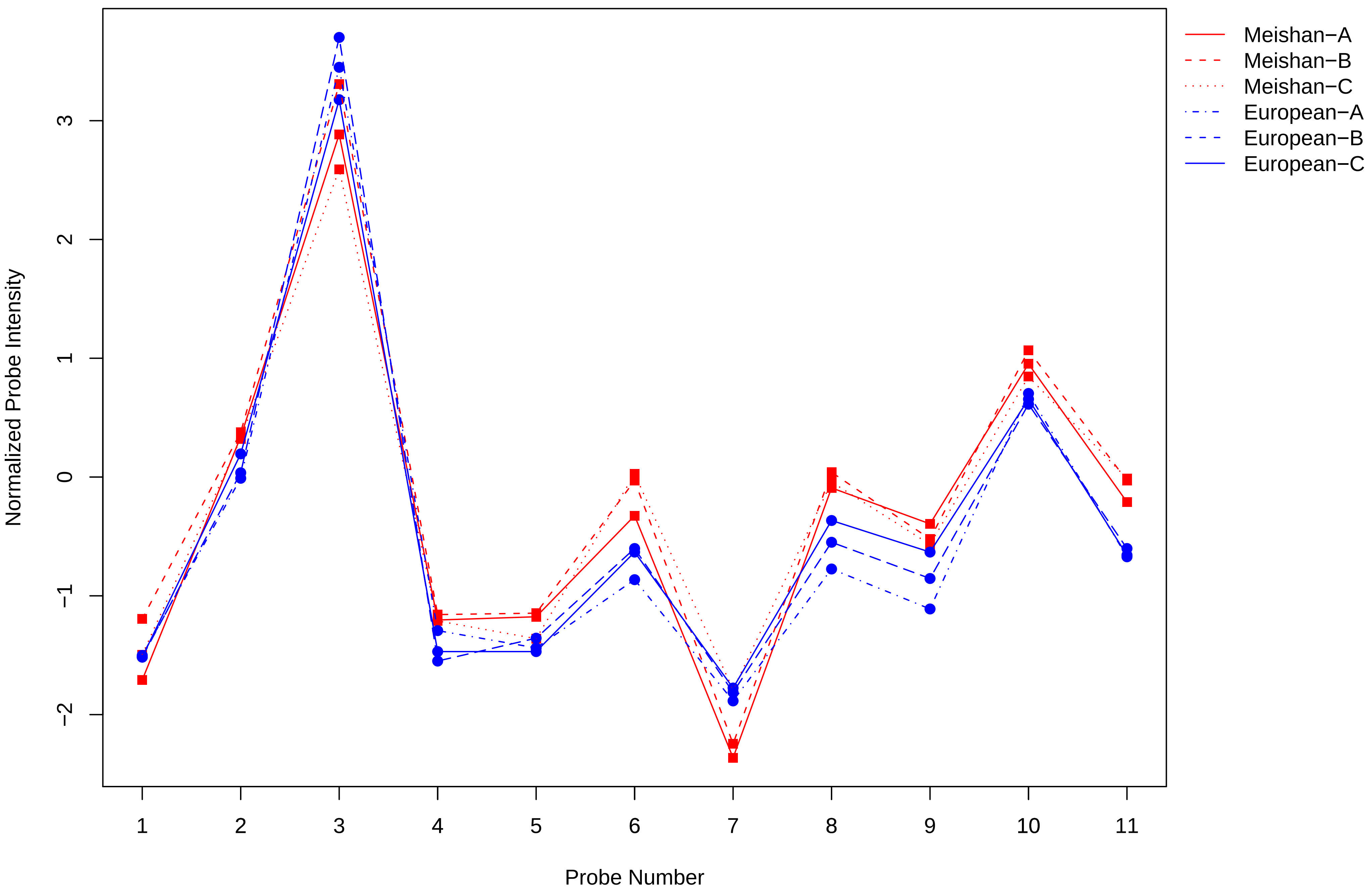

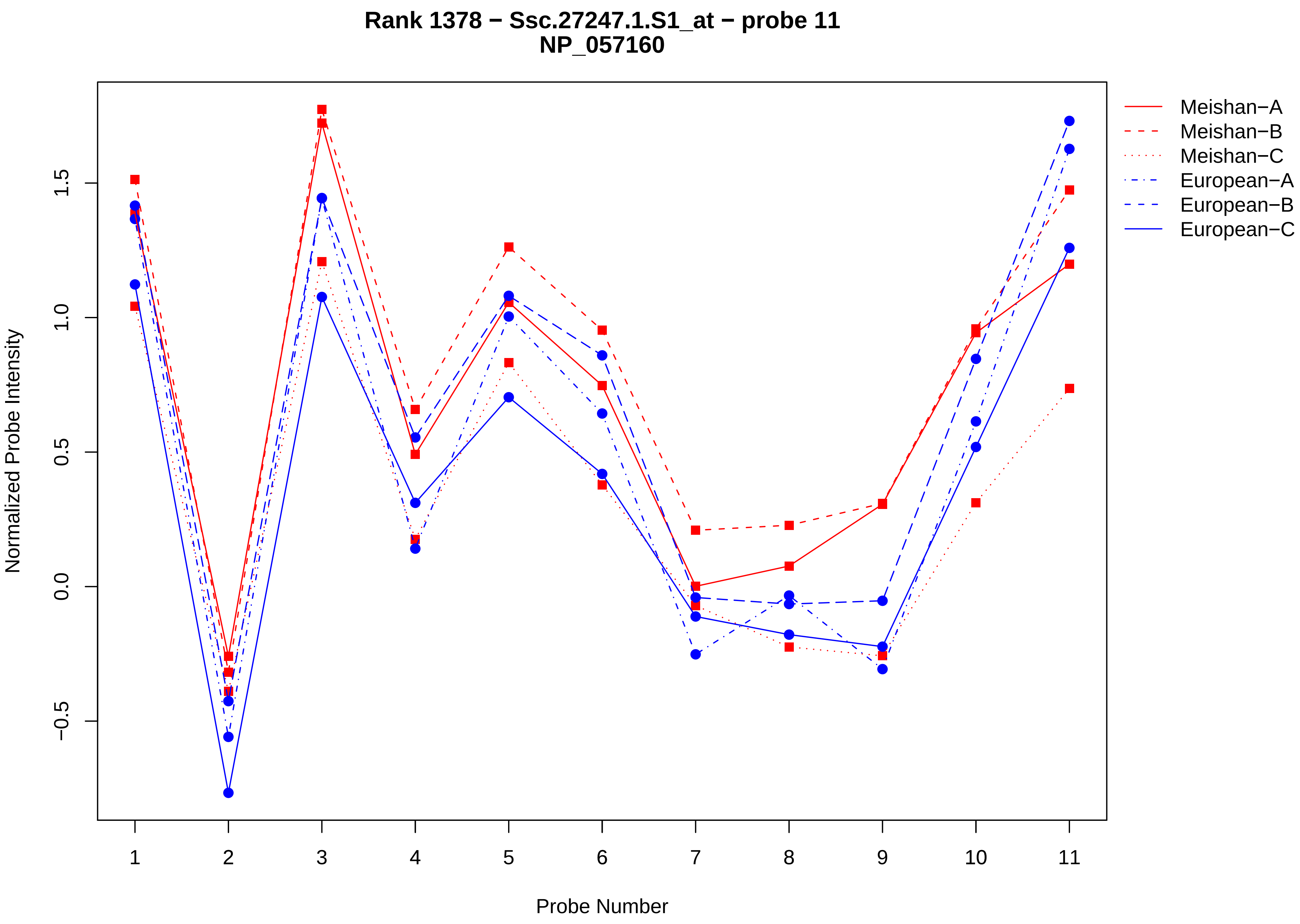

Rank 1378 − Ssc.27247.1.S1_at − probe 11
NP_057160
Normalized Probe Intensity
Probe Number
−0.5
0.0
0.5
1.0
1.5
1
2
3
4
5
6
7
8
9
10
11
Meishan−A
Meishan−B
Meishan−C
European−A
European−B
European−C

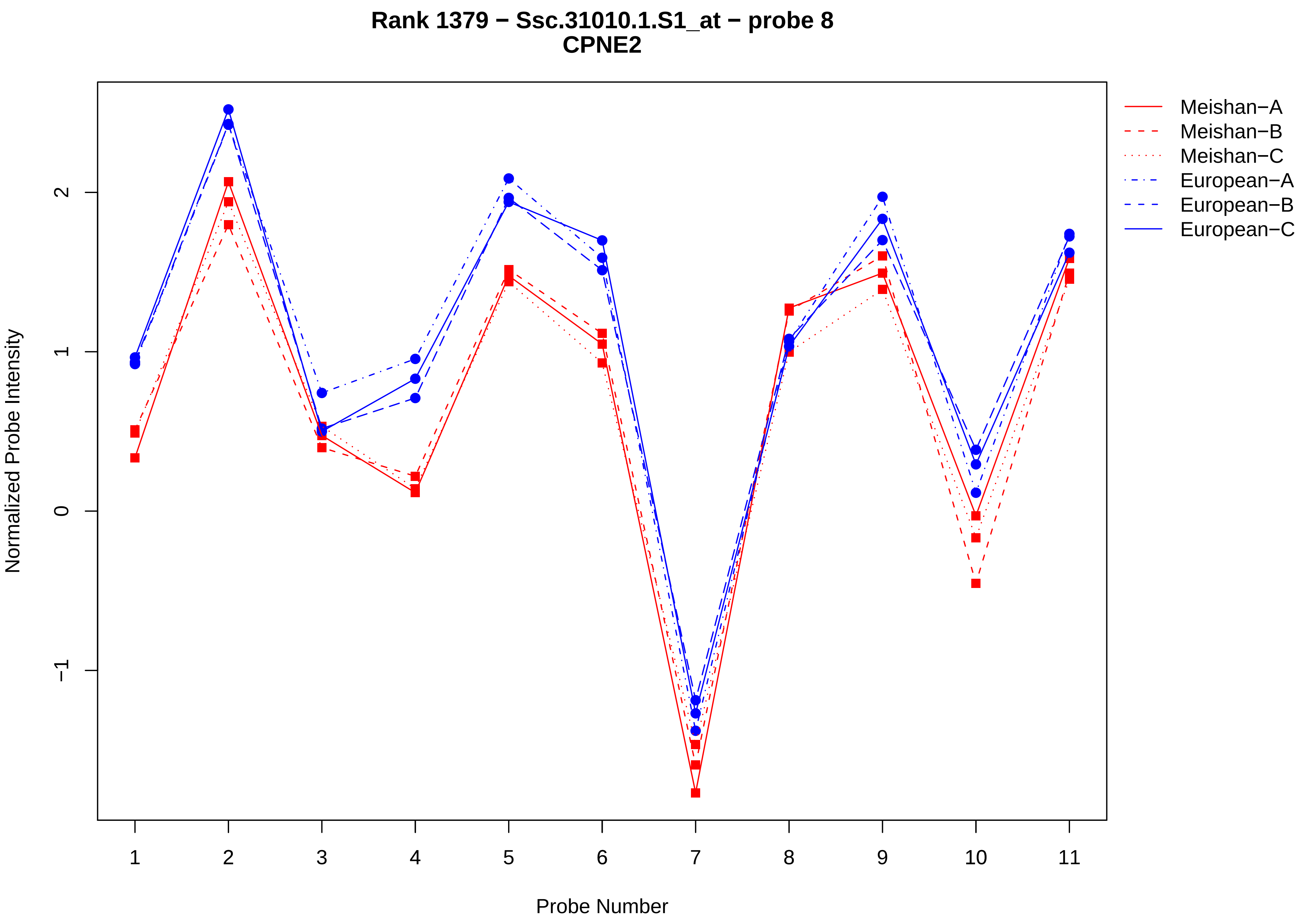

Rank 1379 − Ssc.31010.1.S1_at − probe 8
CPNE2
Normalized Probe Intensity
Probe Number
−1
0
1
2
1
2
3
4
5
6
7
8
9
10
11
Meishan−A
Meishan−B
Meishan−C
European−A
European−B
European−C

Rank 1380 − Ssc.30904.1.S1_at − probe 10
CPNE4

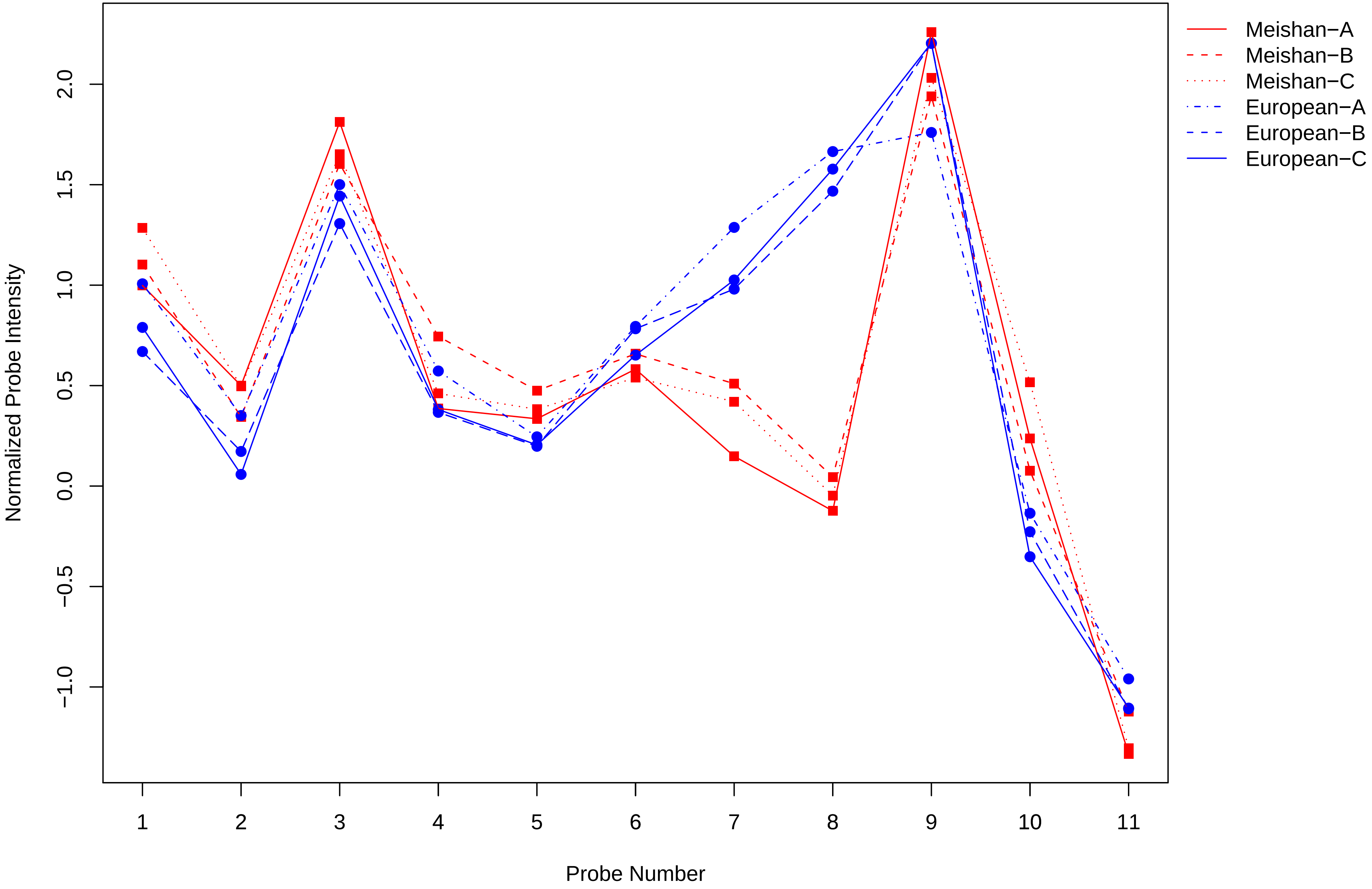

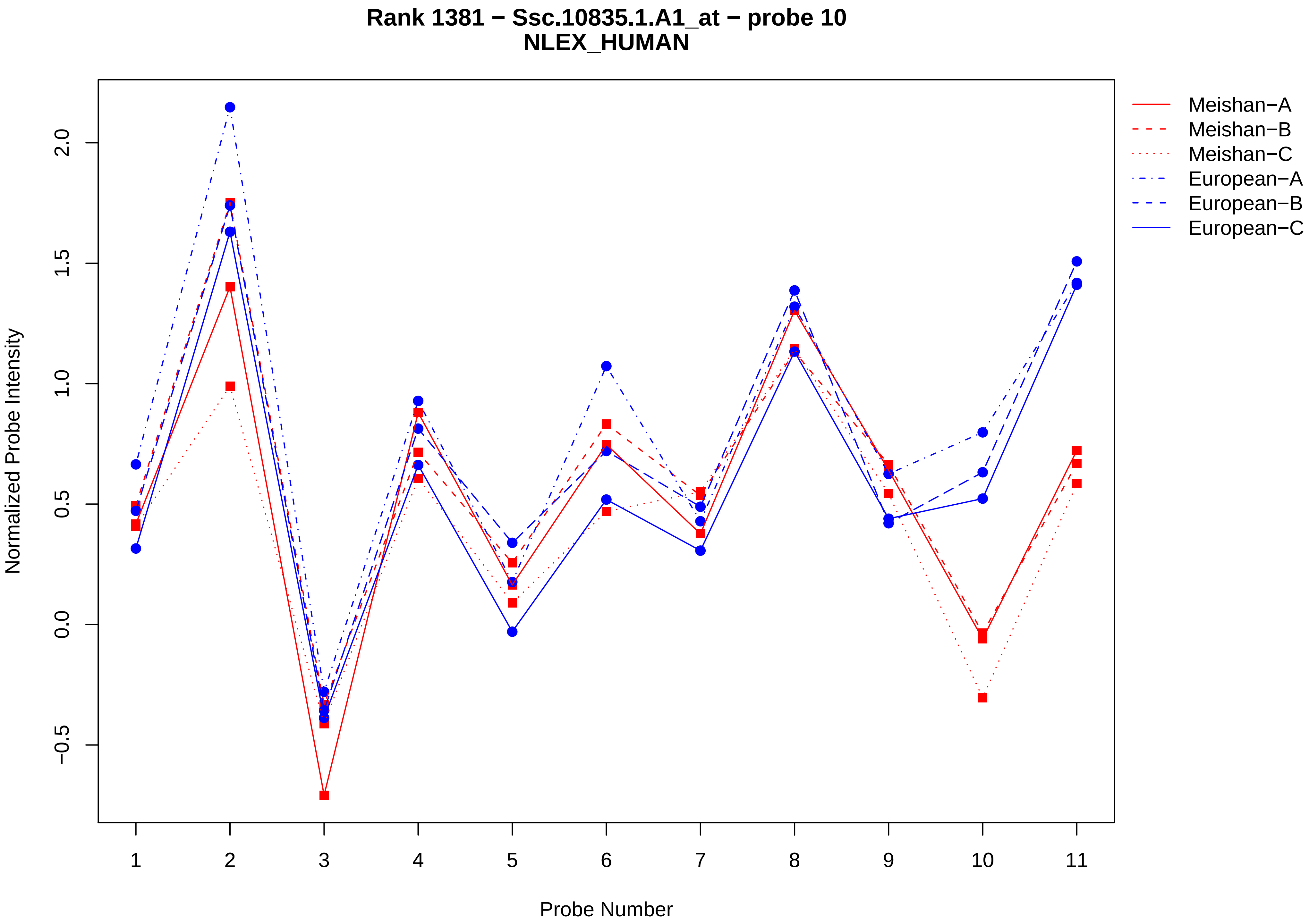
Rank 1381 − Ssc.10835.1.A1_at − probe 10
NLEX_HUMAN
Normalized Probe Intensity
Probe Number
Meishan−A
Meishan−B
Meishan−C
European−A
European−B
European−C
−0.5
0.0
0.5
1.0
1.5
2.0
1
2
3
4
5
6
7
8
9
10
11

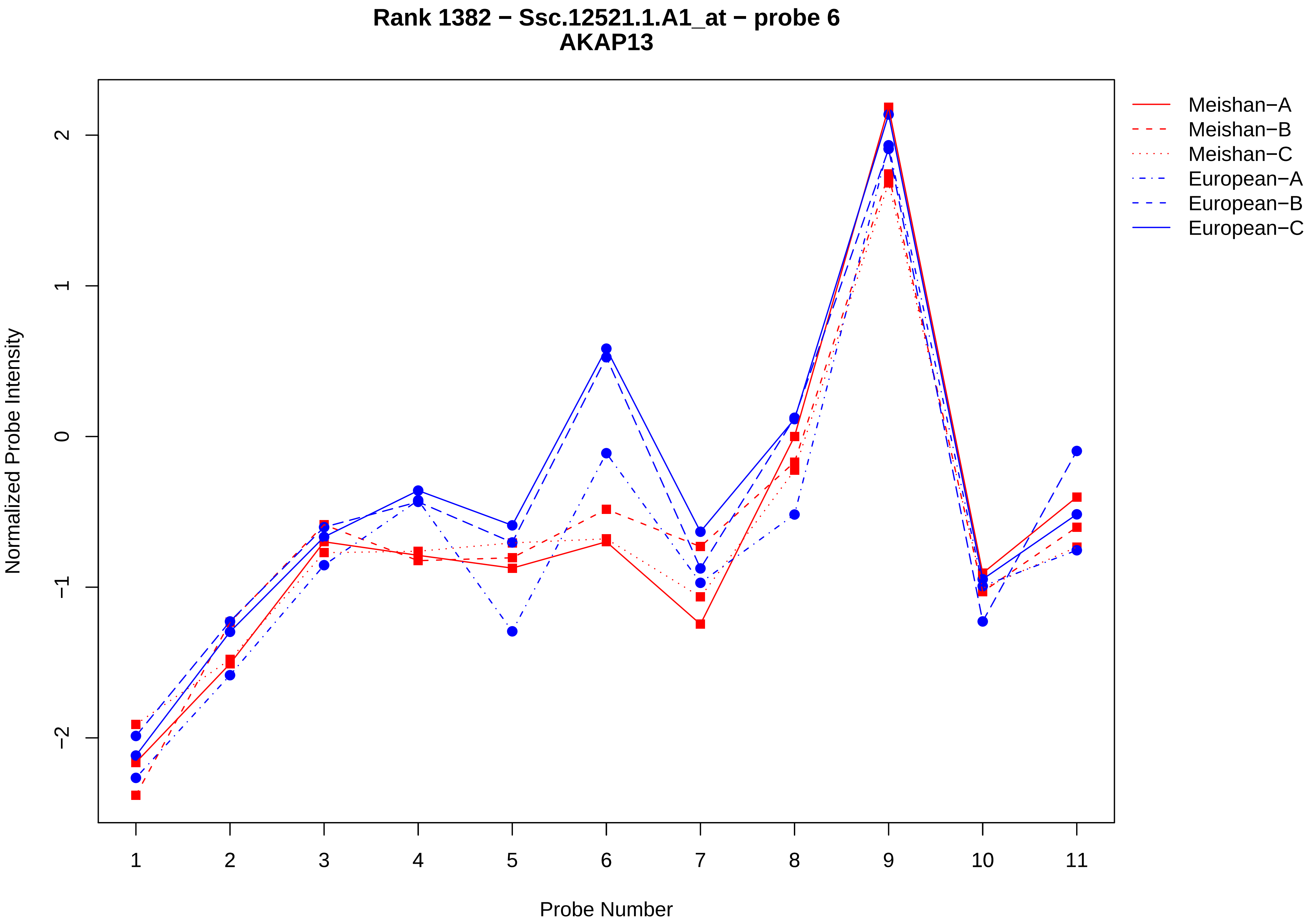

Rank 1382 − Ssc.12521.1.A1_at − probe 6
AKAP13
Normalized Probe Intensity
Probe Number
−2
−1
0
1
2
1
2
3
4
5
6
7
8
9
10
11
Meishan−A
Meishan−B
Meishan−C
European−A
European−B
European−C

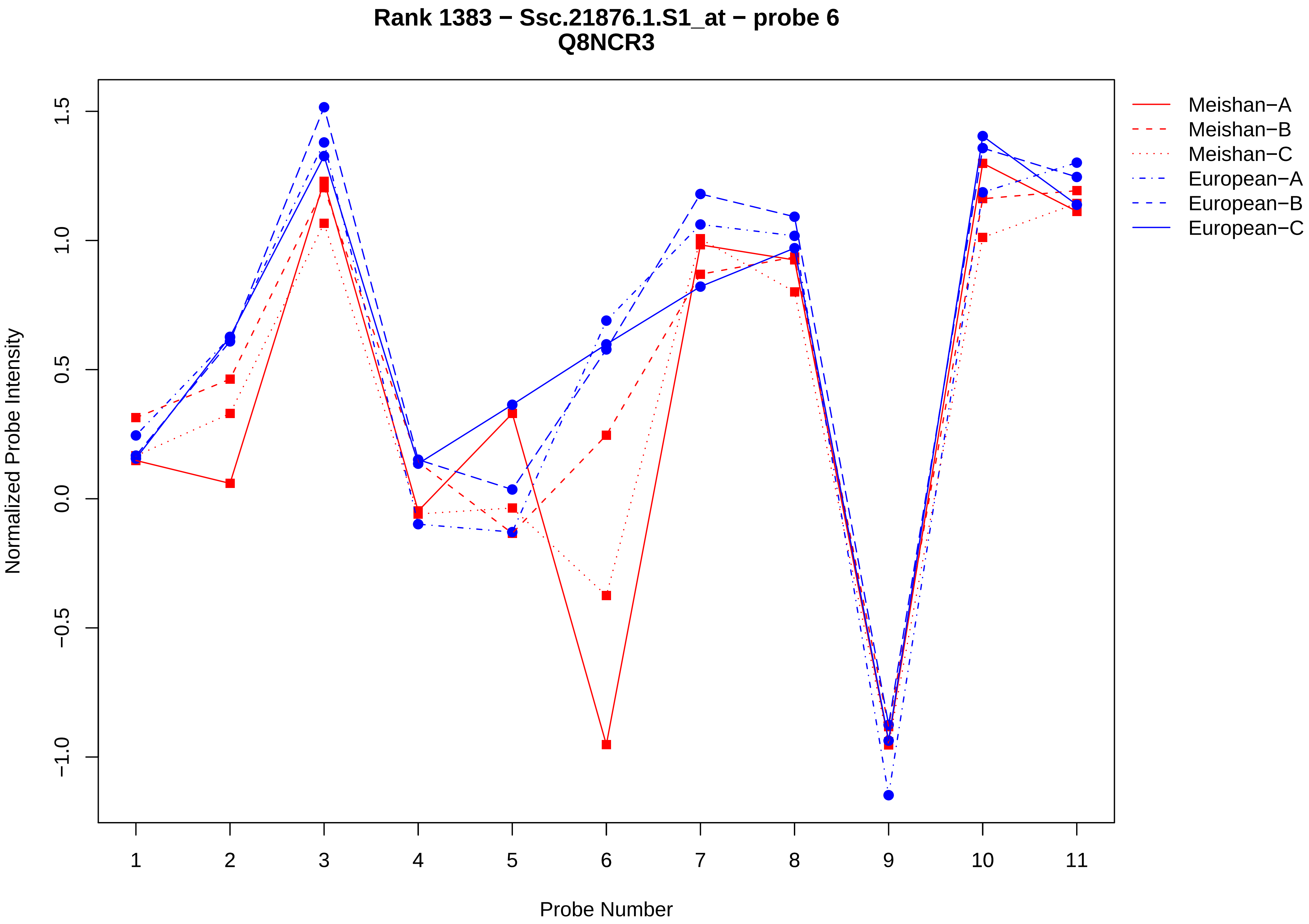

Rank 1383 − Ssc.21876.1.S1_at − probe 6
Q8NCR3
Normalized Probe Intensity
Probe Number
1.5
1.0
0.5
0.0
−0.5
−1.0
1
2
3
4
5
6
7
8
9
10
11
Meishan−A
Meishan−B
Meishan−C
European−A
European−B
European−C

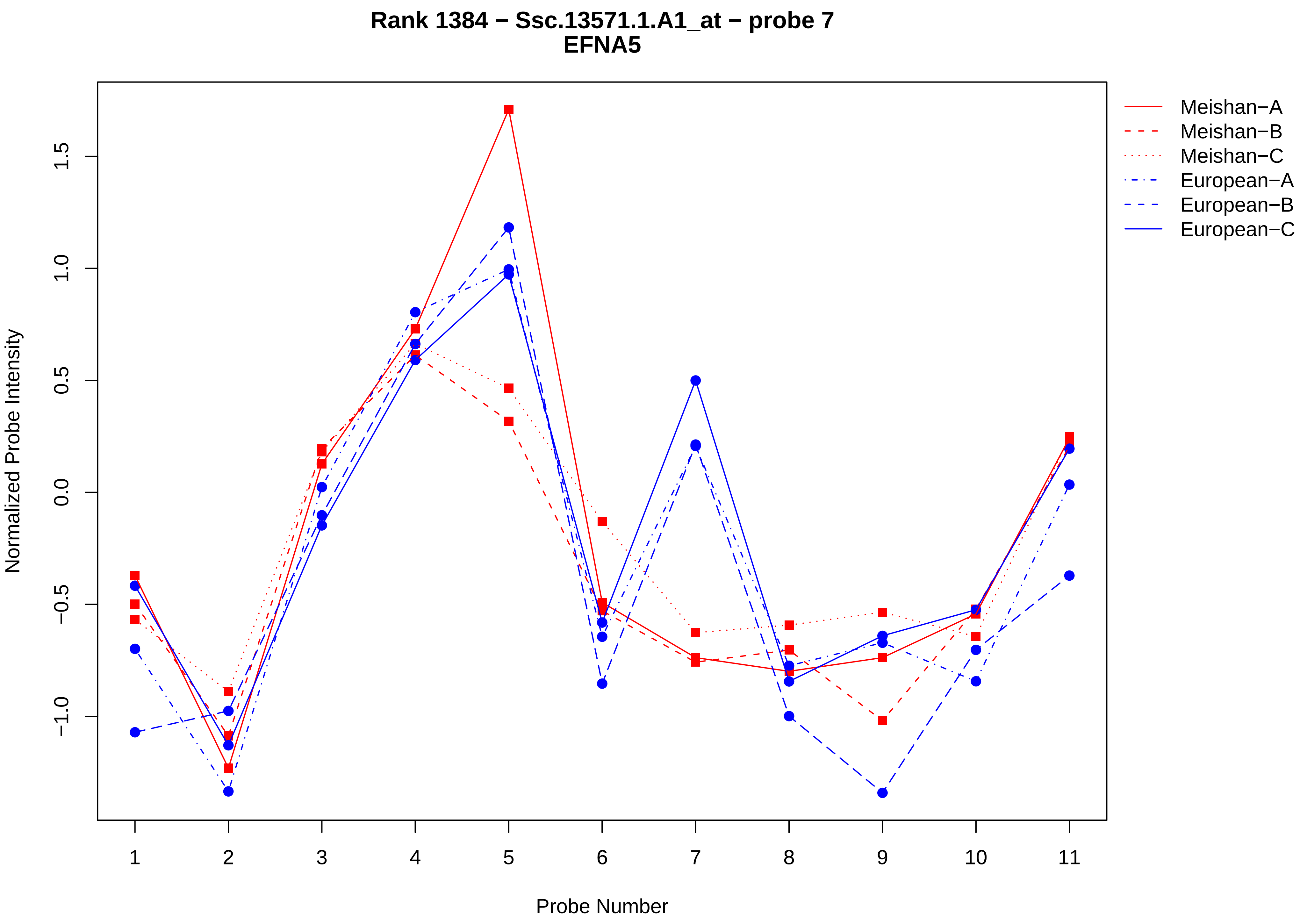

Rank 1384 − Ssc.13571.1.A1_at − probe 7
EFNA5
Normalized Probe Intensity
Probe Number
Meishan−A
Meishan−B
Meishan−C
European−A
European−B
European−C

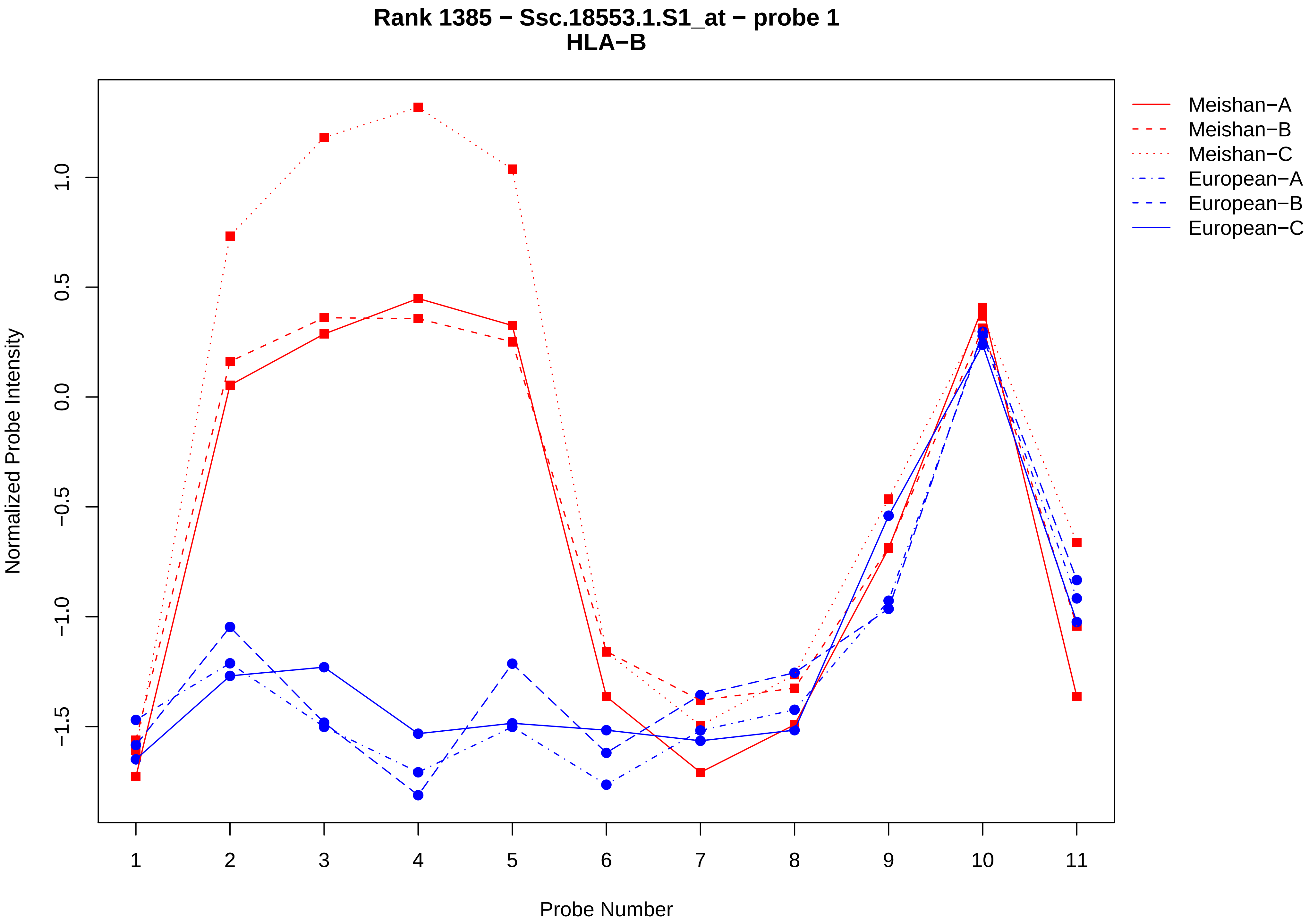

Rank 1385 − Ssc.18553.1.S1_at − probe 1
HLA−B
Normalized Probe Intensity
Probe Number
1.0
0.5
0.0
−0.5
−1.0
−1.5
1
2
3
4
5
6
7
8
9
10
11
Meishan−A
Meishan−B
Meishan−C
European−A
European−B
European−C

Rank 1386 − Ssc.12996.1.A1_at − probe 7
RBMS3

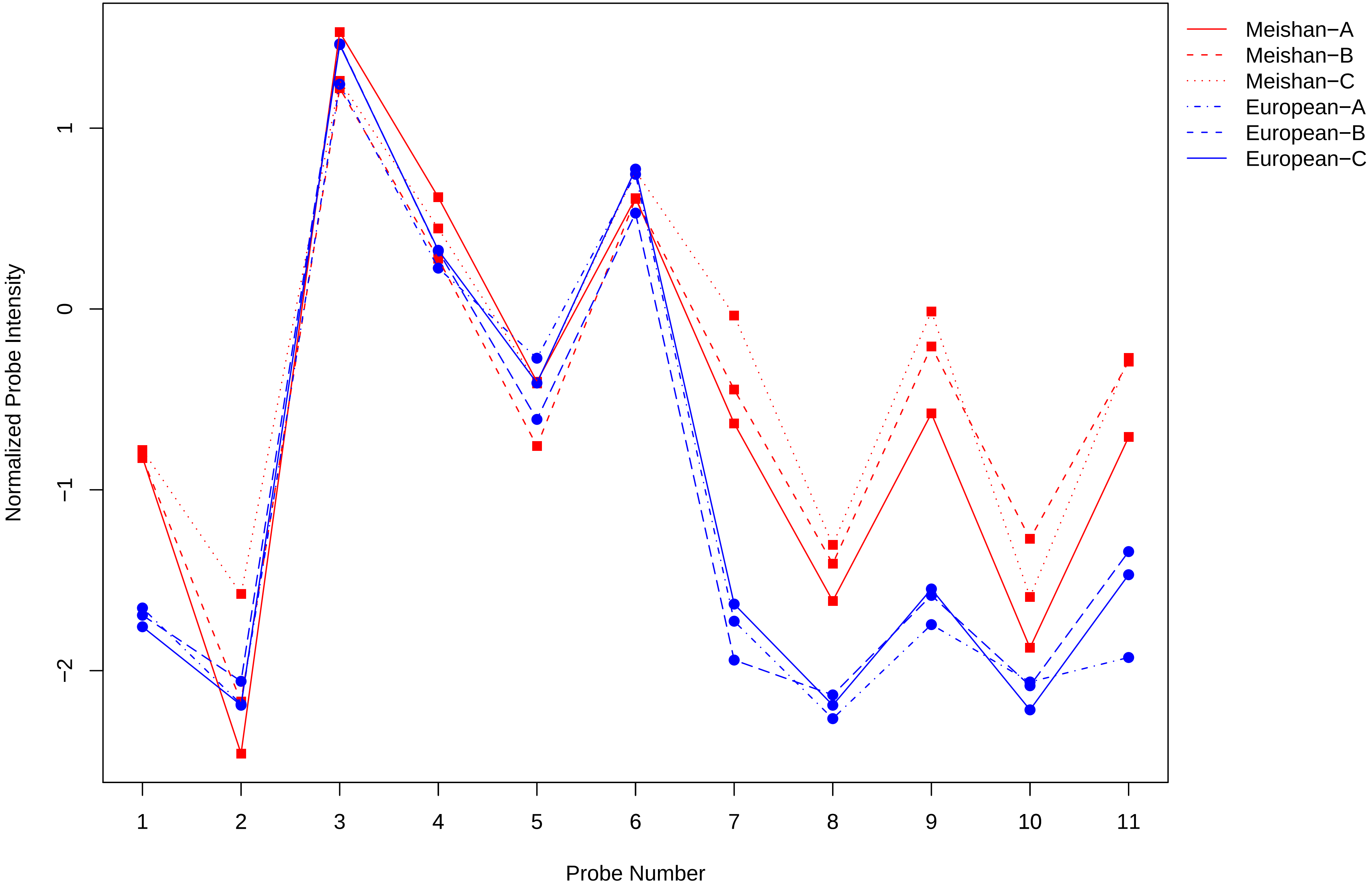

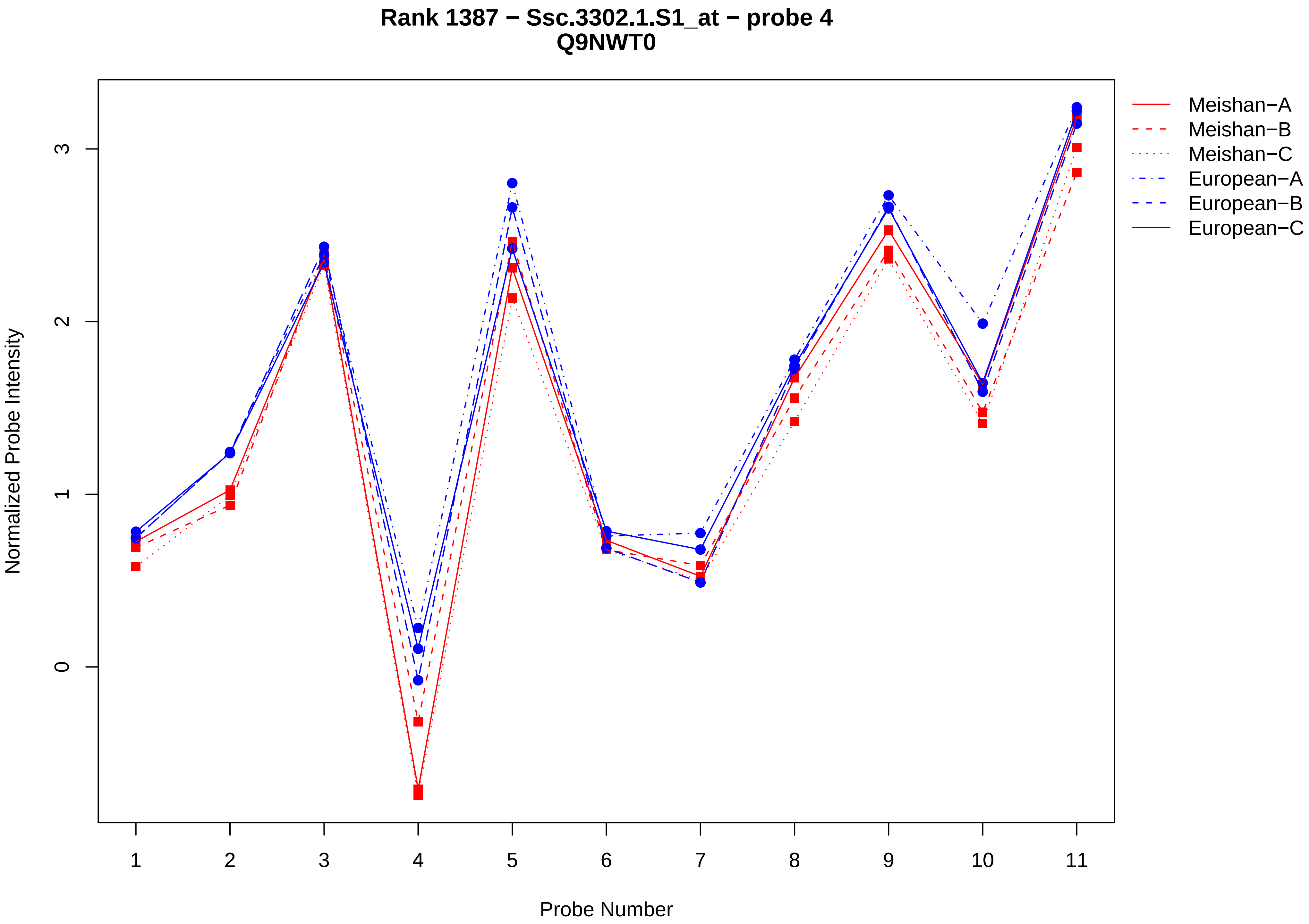

Rank 1387 − Ssc.3302.1.S1_at − probe 4
Q9NWT0
Normalized Probe Intensity
Probe Number
0
1
2
3
1
2
3
4
5
6
7
8
9
10
11
Meishan−A
Meishan−B
Meishan−C
European−A
European−B
European−C

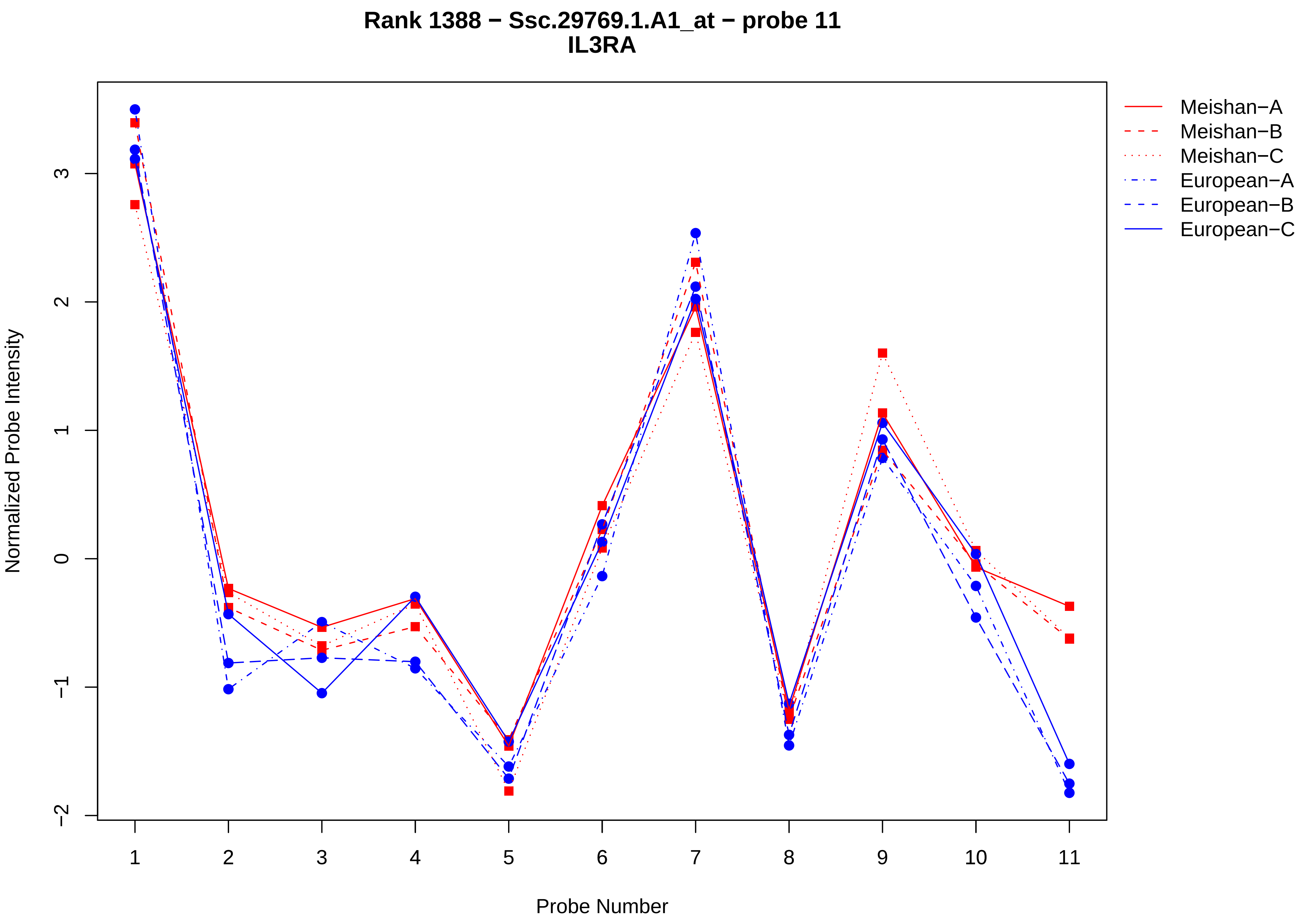

Rank 1388 − Ssc.29769.1.A1_at − probe 11
IL3RA
Normalized Probe Intensity
Probe Number
−2
−1
0
1
2
3
1
2
3
4
5
6
7
8
9
10
11
Meishan−A
Meishan−B
Meishan−C
European−A
European−B
European−C

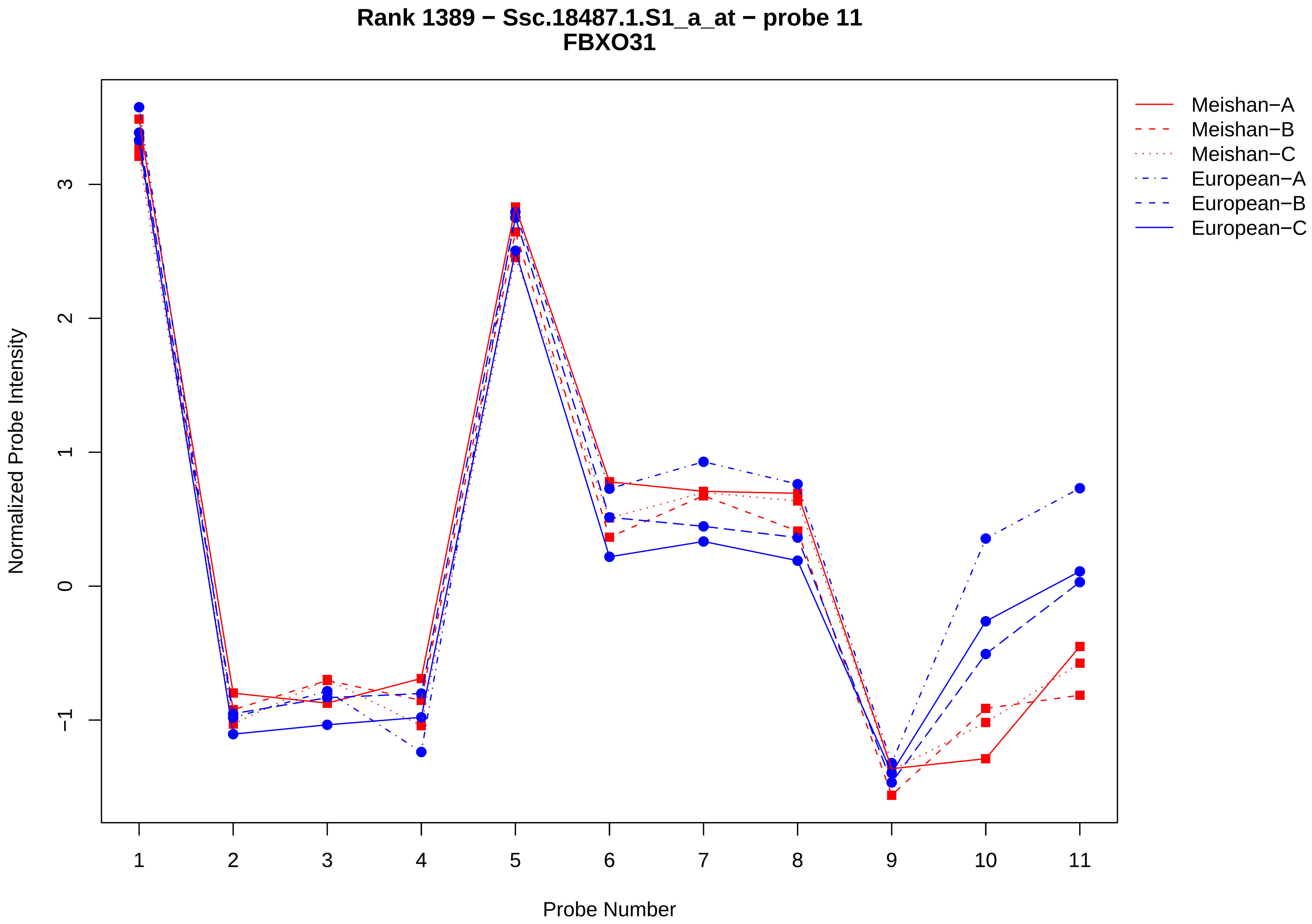

Rank 1389 − Ssc.18487.1.S1_a_at − probe 11
FBXO31
Normalized Probe Intensity
Probe Number
Meishan−A
Meishan−B
Meishan−C
European−A
European−B
European−C
−1
0
1
2
3
1
2
3
4
5
6
7
8
9
10
11

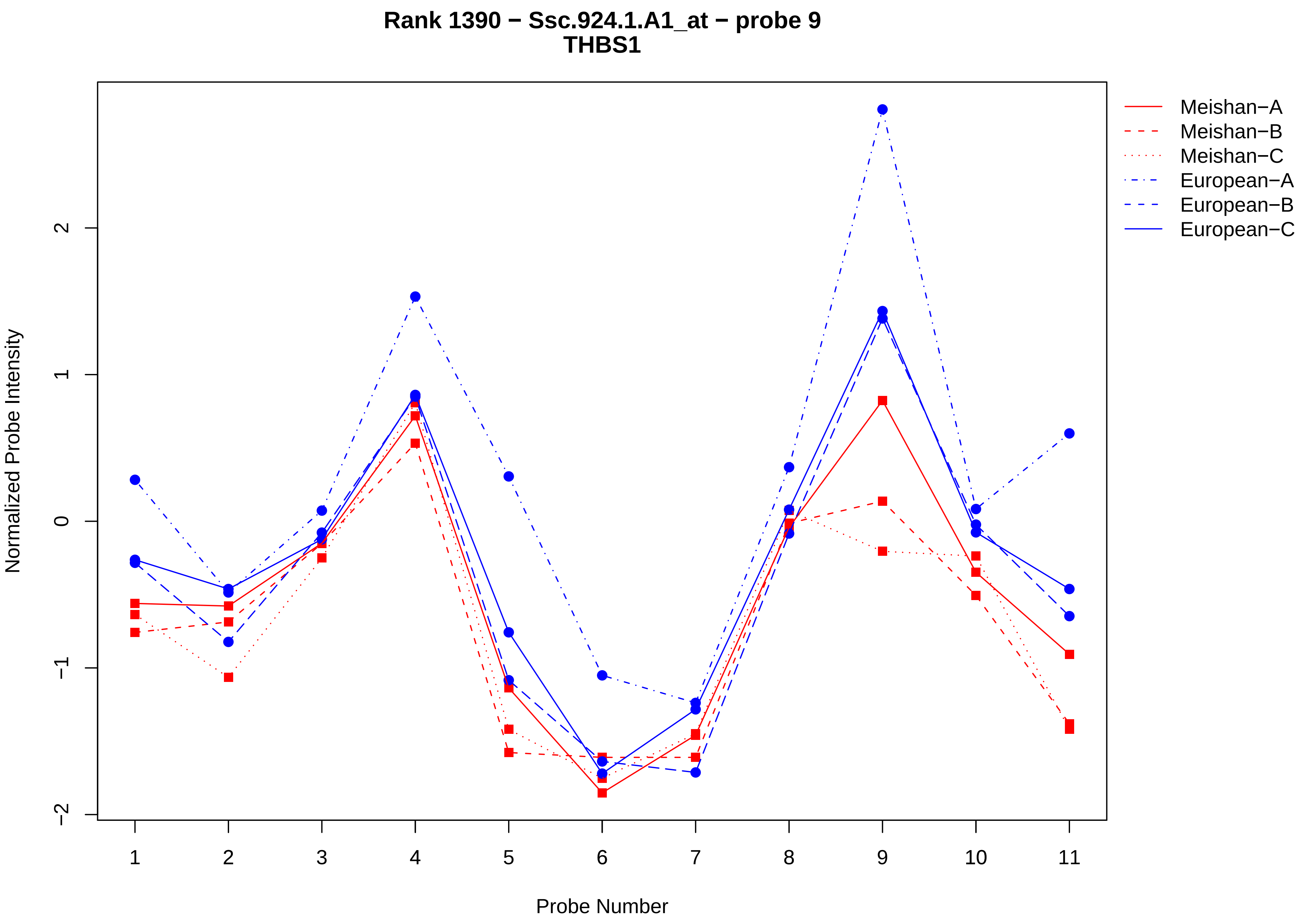

Rank 1390 − Ssc.924.1.A1_at − probe 9
THBS1
Meishan−A
Meishan−B
Meishan−C
European−A
European−B
European−C
Normalized Probe Intensity
Probe Number
−2
−1
0
1
2
1
2
3
4
5
6
7
8
9
10
11

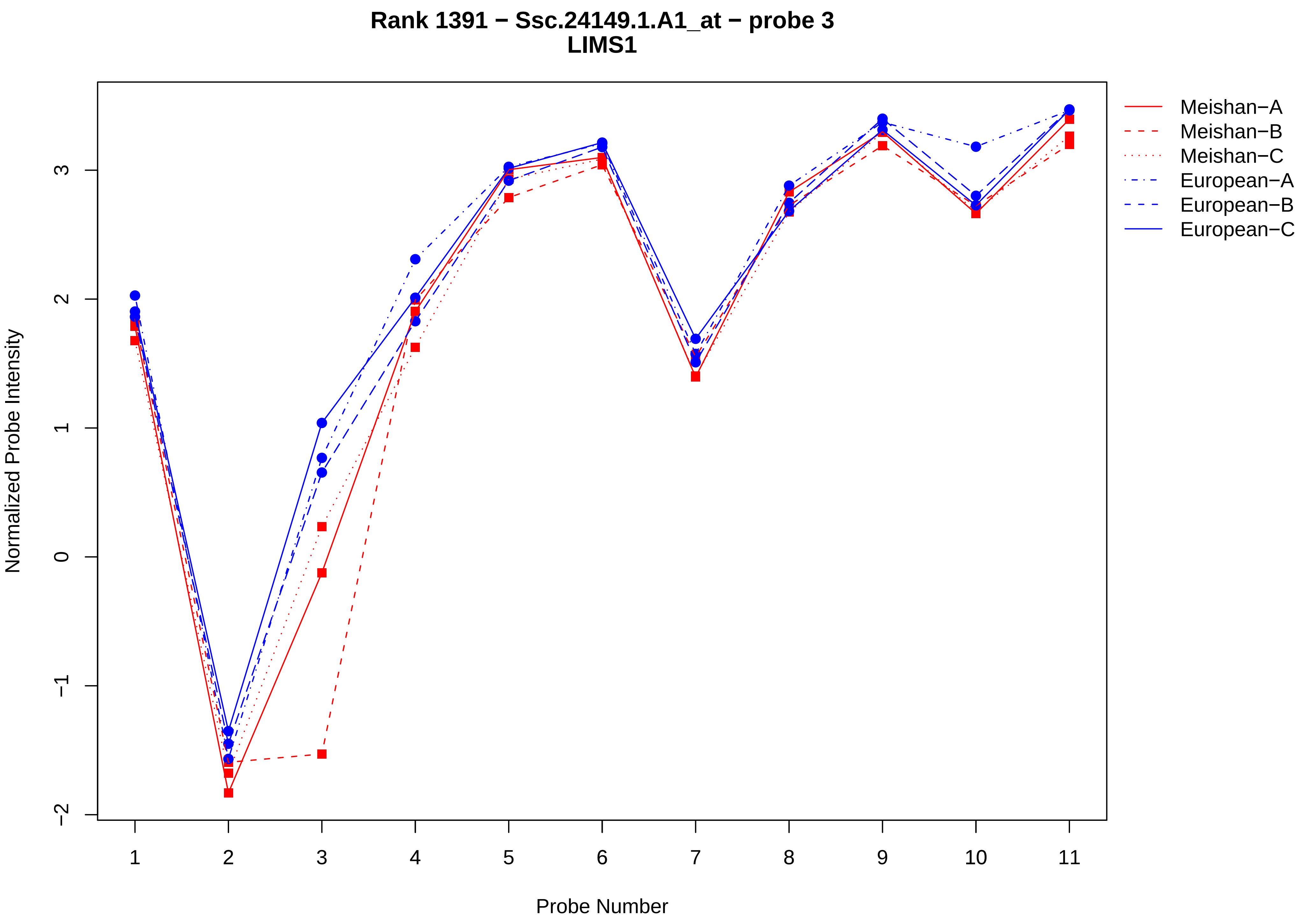

Rank 1391 − Ssc.24149.1.A1_at − probe 3
LIMS1
Normalized Probe Intensity
Probe Number
−2
−1
0
1
2
3
1
2
3
4
5
6
7
8
9
10
11
Meishan−A
Meishan−B
Meishan−C
European−A
European−B
European−C

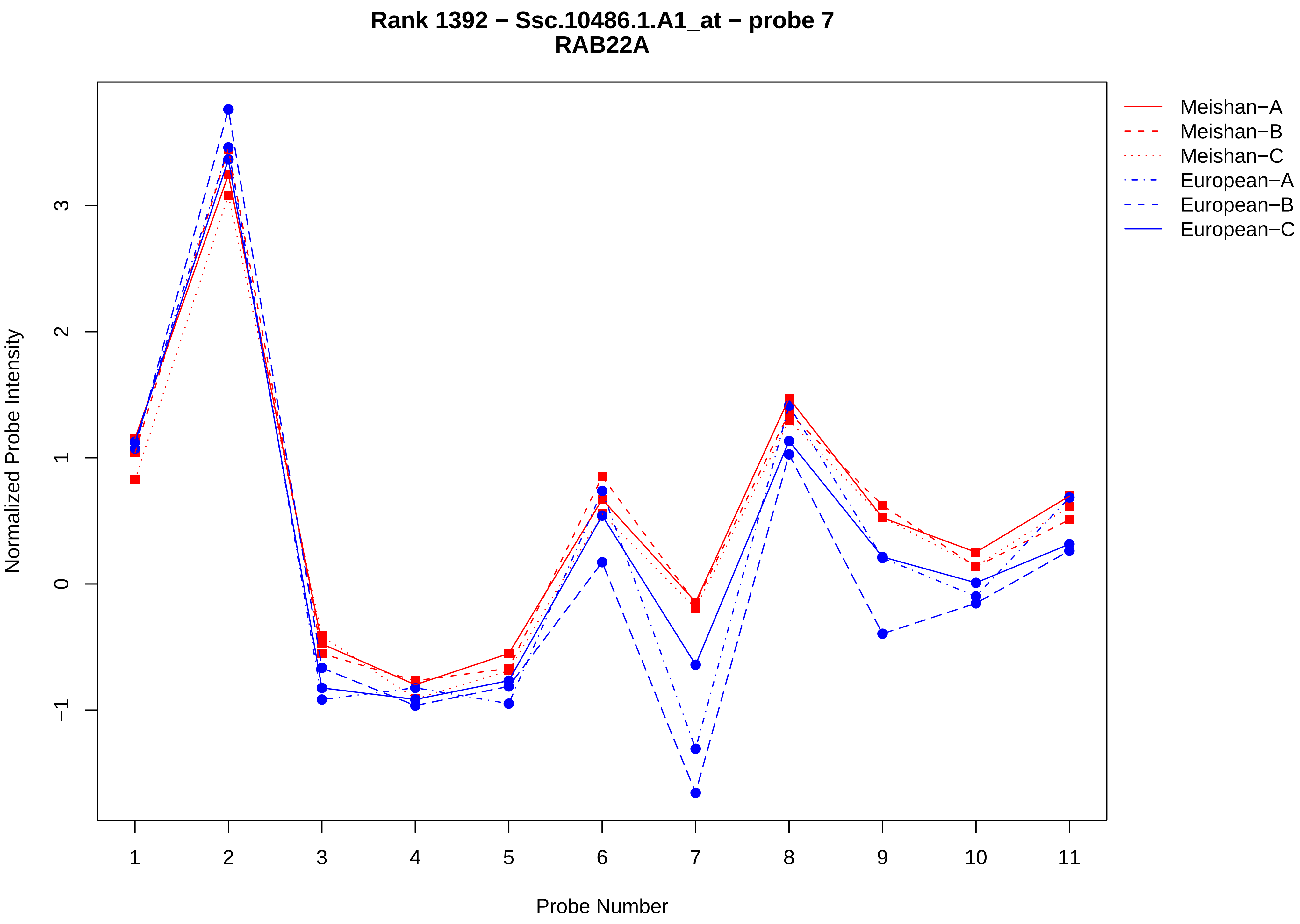

Rank 1392 − Ssc.10486.1.A1_at − probe 7
RAB22A
Normalized Probe Intensity
Probe Number
−1
0
1
2
3
1
2
3
4
5
6
7
8
9
10
11
Meishan−A
Meishan−B
Meishan−C
European−A
European−B
European−C

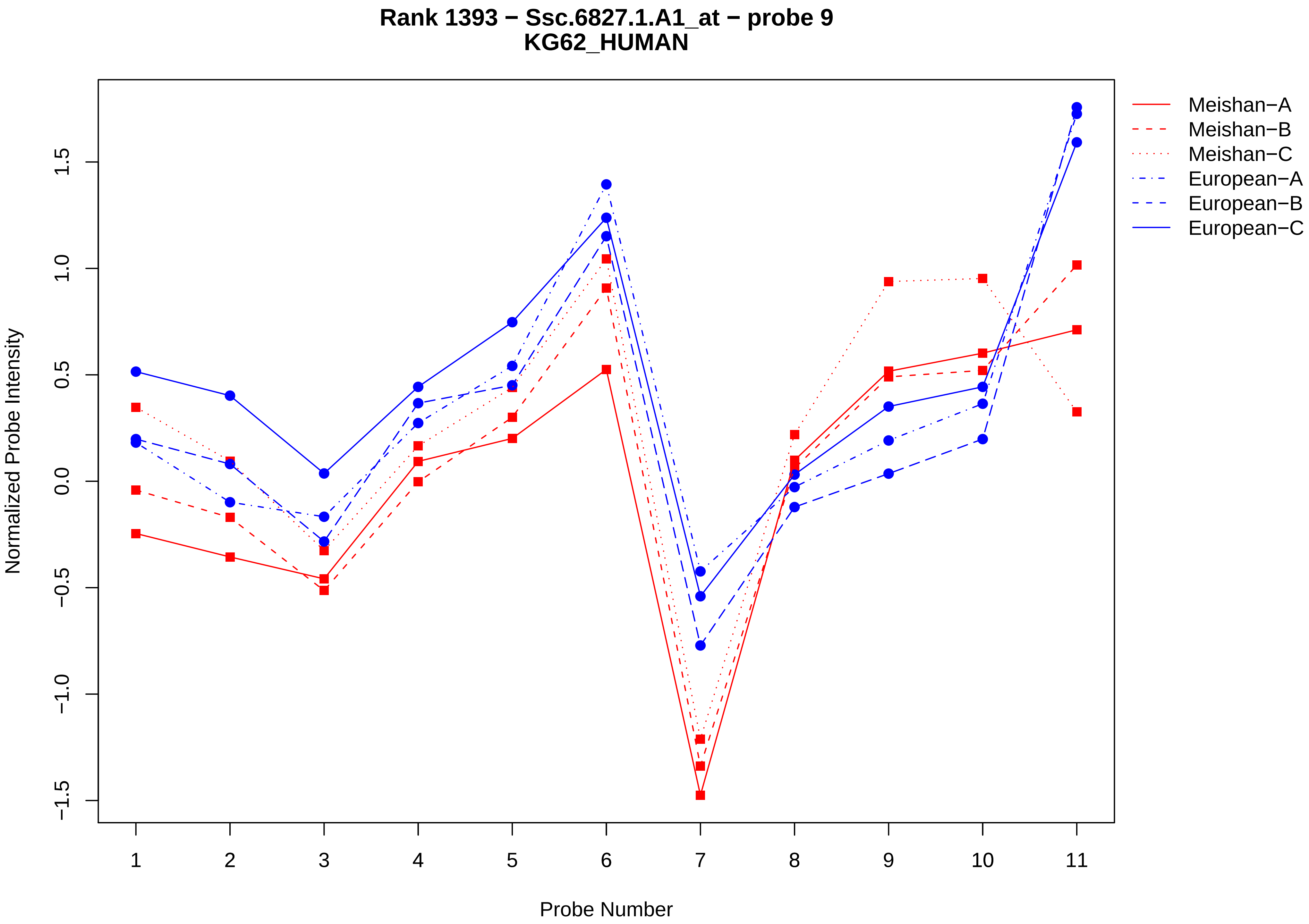

Rank 1393 − Ssc.6827.1.A1_at − probe 9
KG62_HUMAN
Normalized Probe Intensity
Probe Number
−1.5
−1.0
−0.5
0.0
0.5
1.0
1.5
1
2
3
4
5
6
7
8
9
10
11
Meishan−A
Meishan−B
Meishan−C
European−A
European−B
European−C

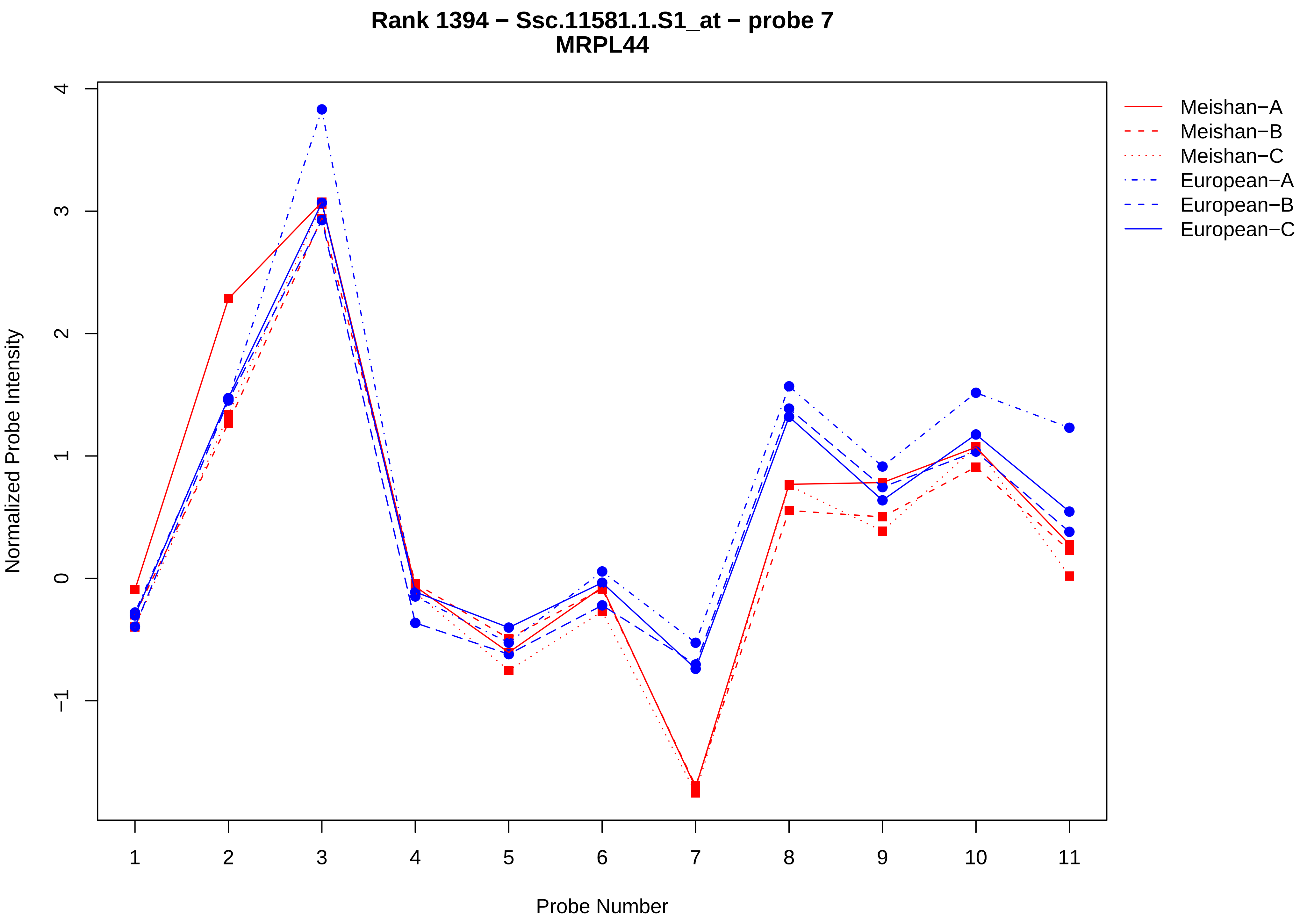

Rank 1394 − Ssc.11581.1.S1_at − probe 7
MRPL44
Normalized Probe Intensity
Probe Number
4
3
2
1
0
−1
1
2
3
4
5
6
7
8
9
10
11
Meishan−A
Meishan−B
Meishan−C
European−A
European−B
European−C

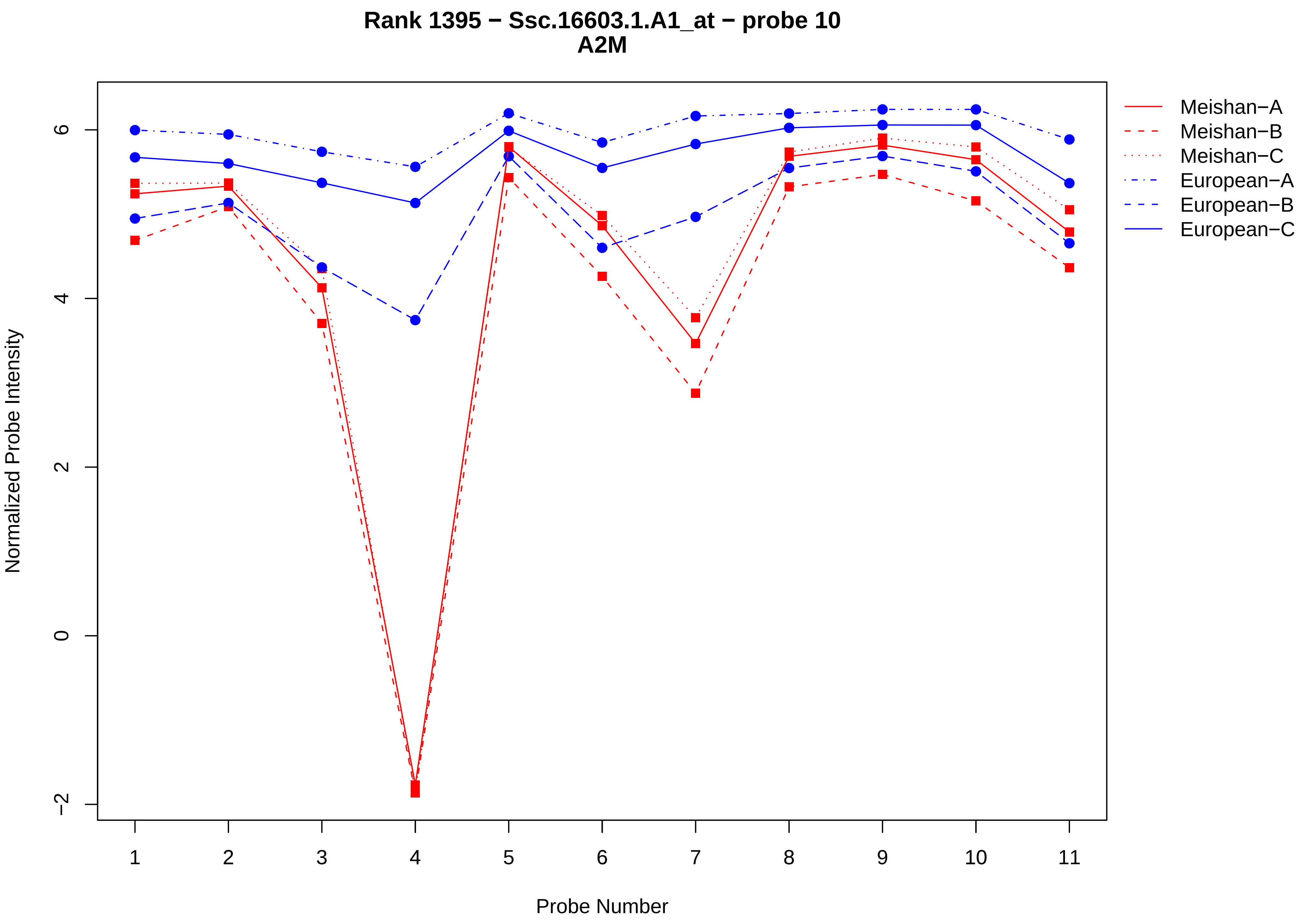

Rank 1395 − Ssc.16603.1.A1_at − probe 10
A2M
Normalized Probe Intensity
Probe Number
−2
0
2
4
6
1
2
3
4
5
6
7
8
9
10
11
Meishan−A
Meishan−B
Meishan−C
European−A
European−B
European−C

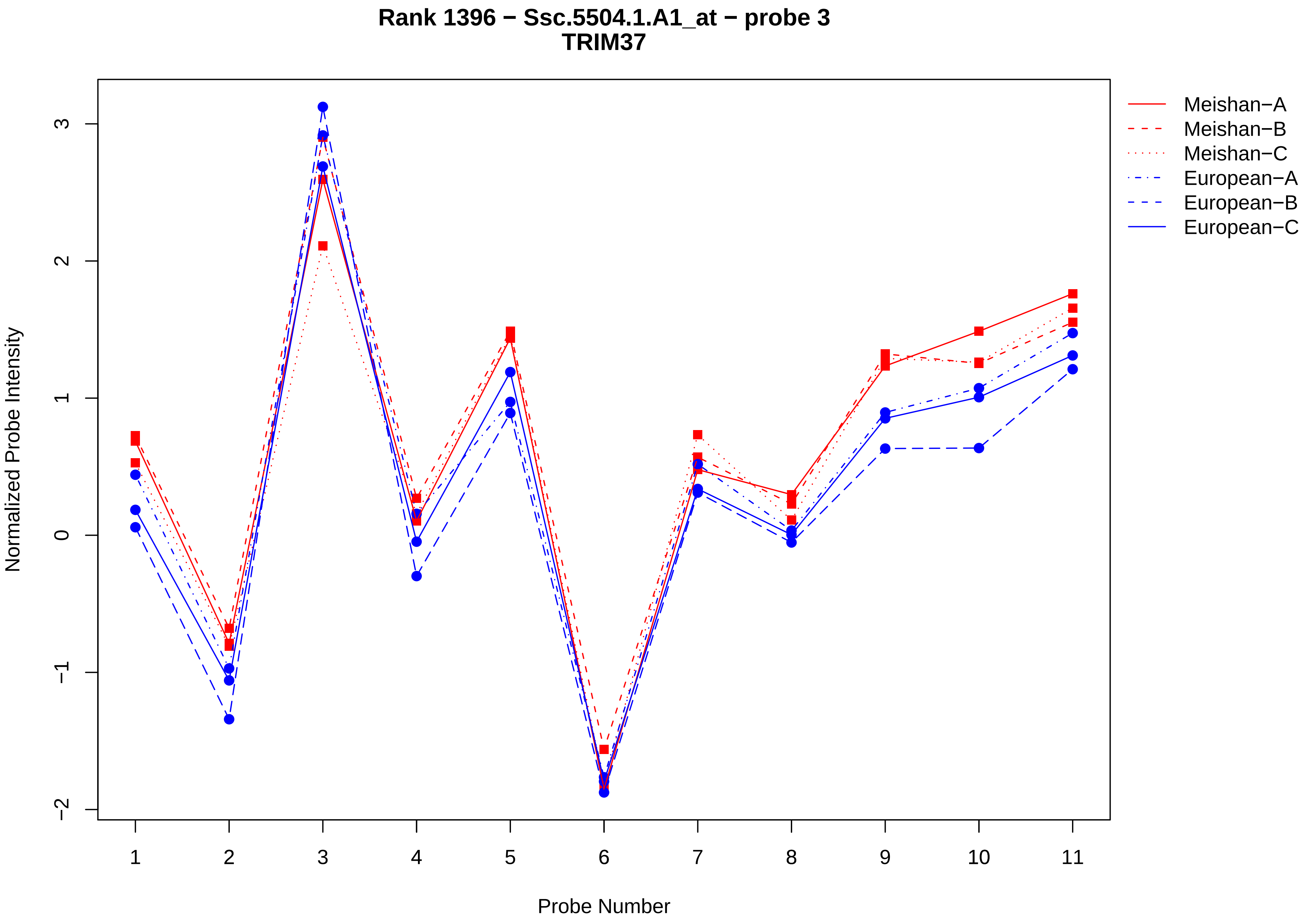

Rank 1396 − Ssc.5504.1.A1_at − probe 3
TRIM37
Normalized Probe Intensity
Probe Number
−2
−1
0
1
2
3
1
2
3
4
5
6
7
8
9
10
11
Meishan−A
Meishan−B
Meishan−C
European−A
European−B
European−C

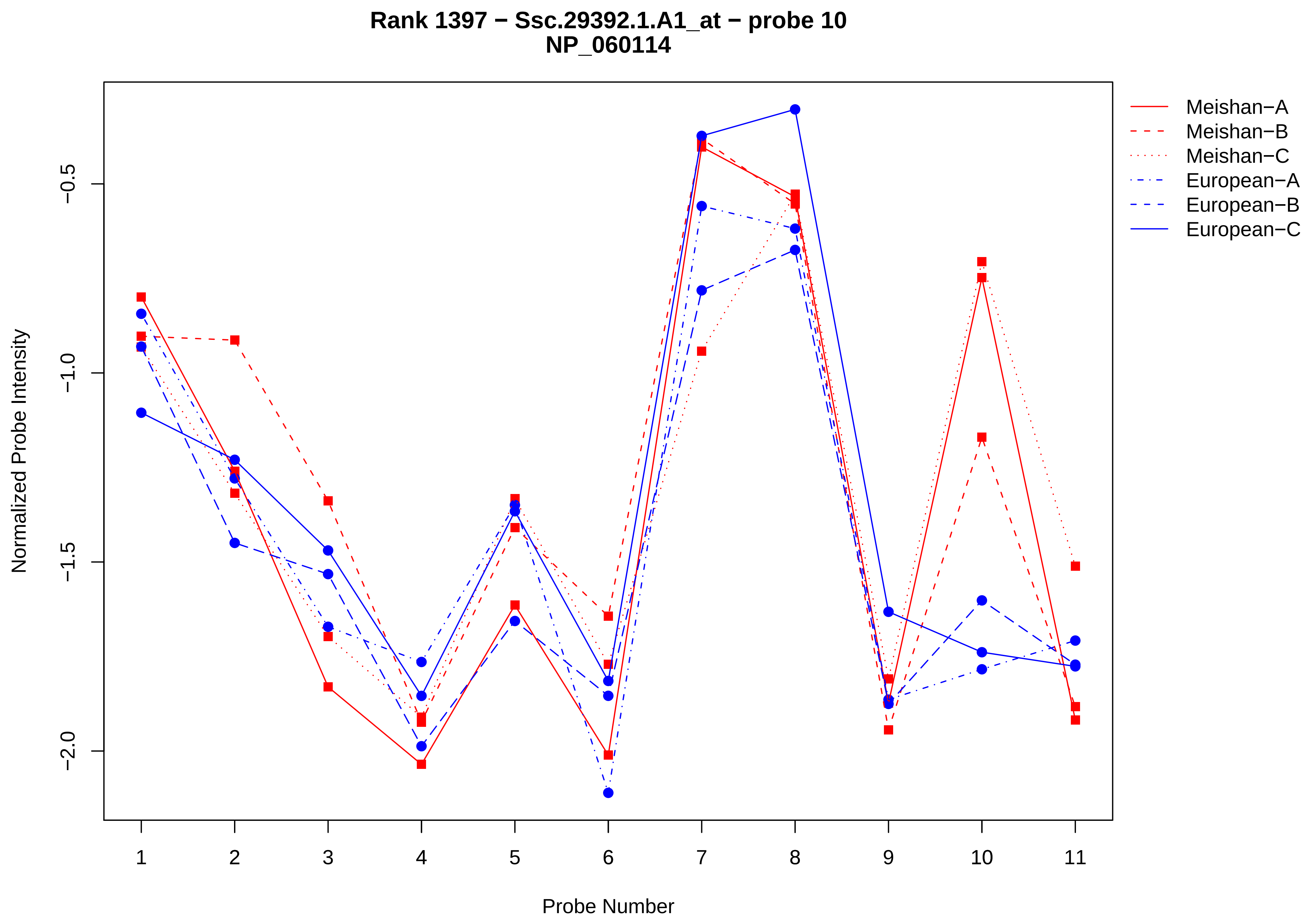

Rank 1397 − Ssc.29392.1.A1_at − probe 10
NP_060114
Normalized Probe Intensity
Probe Number
−0.5
−1.0
−1.5
−2.0
1
2
3
4
5
6
7
8
9
10
11
Meishan−A
Meishan−B
Meishan−C
European−A
European−B
European−C

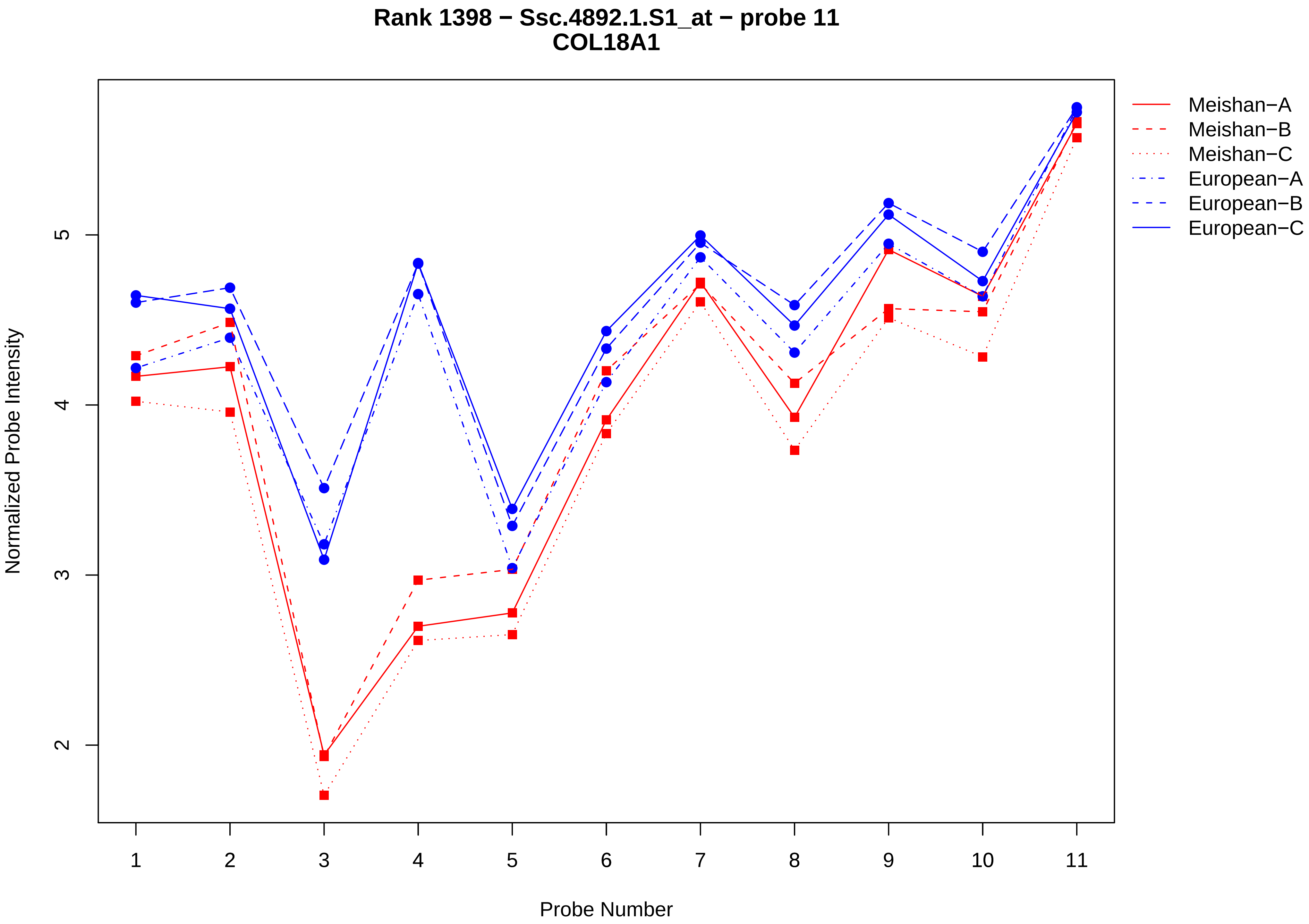

Rank 1398 − Ssc.4892.1.S1_at − probe 11
COL18A1
Normalized Probe Intensity
Probe Number
2
3
4
5
1
2
3
4
5
6
7
8
9
10
11
Meishan−A
Meishan−B
Meishan−C
European−A
European−B
European−C

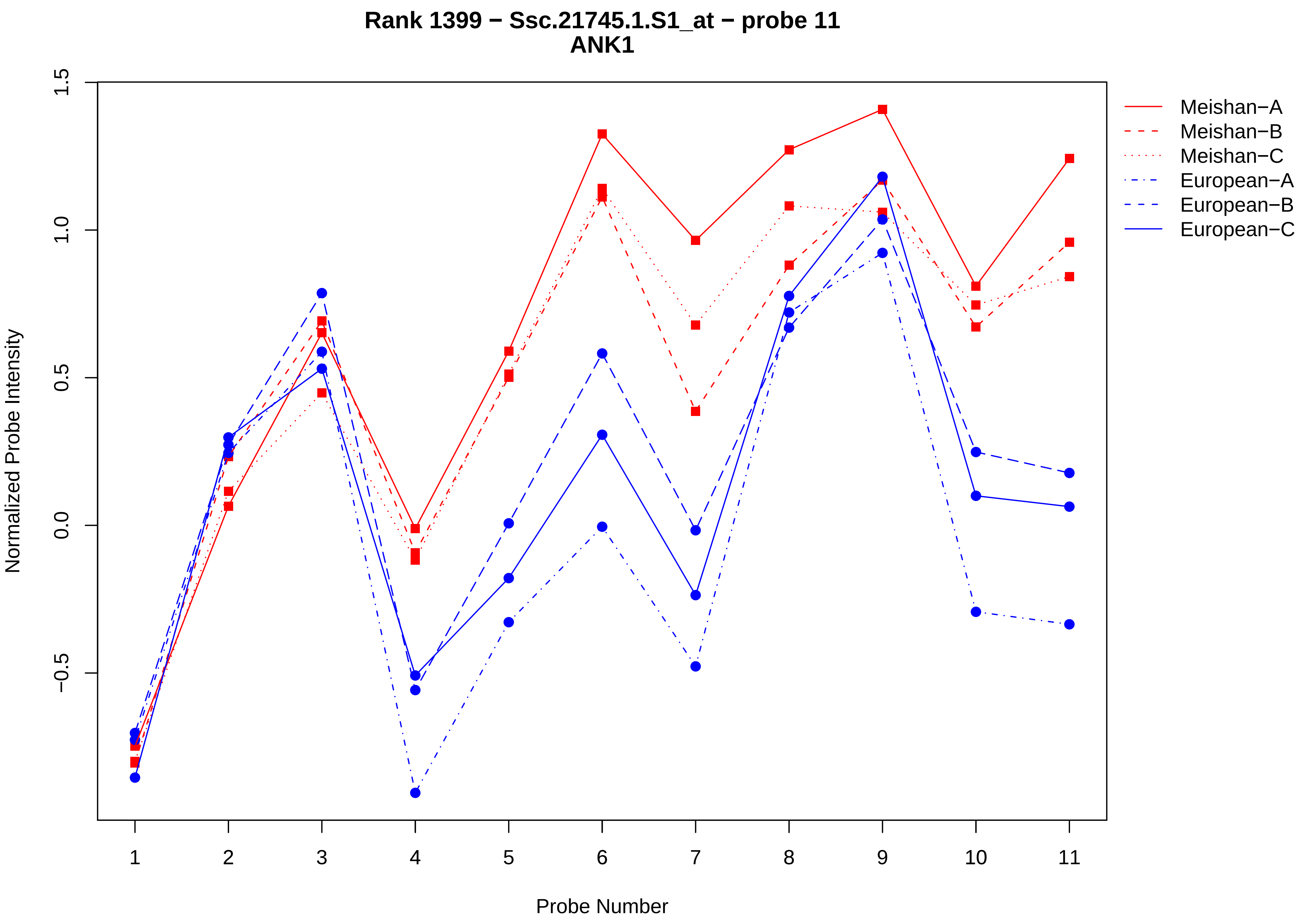

Rank 1399 − Ssc.21745.1.S1_at − probe 11
ANK1
Normalized Probe Intensity
Probe Number
−0.5
0.0
0.5
1.0
1.5
1
2
3
4
5
6
7
8
9
10
11
Meishan−A
Meishan−B
Meishan−C
European−A
European−B
European−C

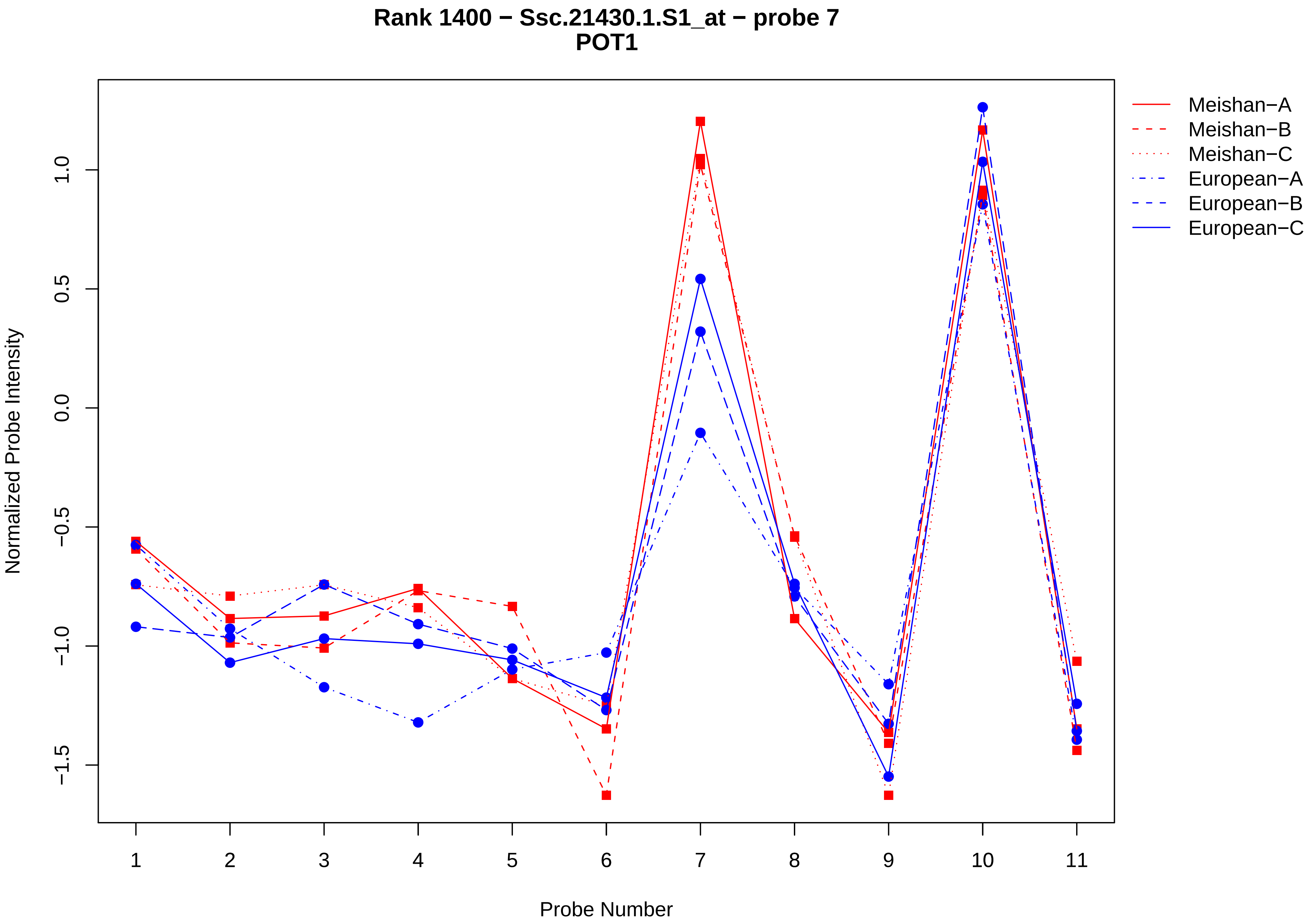

Rank 1400 − Ssc.21430.1.S1_at − probe 7
POT1
Normalized Probe Intensity
Probe Number
−1.5
−1.0
−0.5
0.0
0.5
1.0
1
2
3
4
5
6
7
8
9
10
11
Meishan−A
Meishan−B
Meishan−C
European−A
European−B
European−C

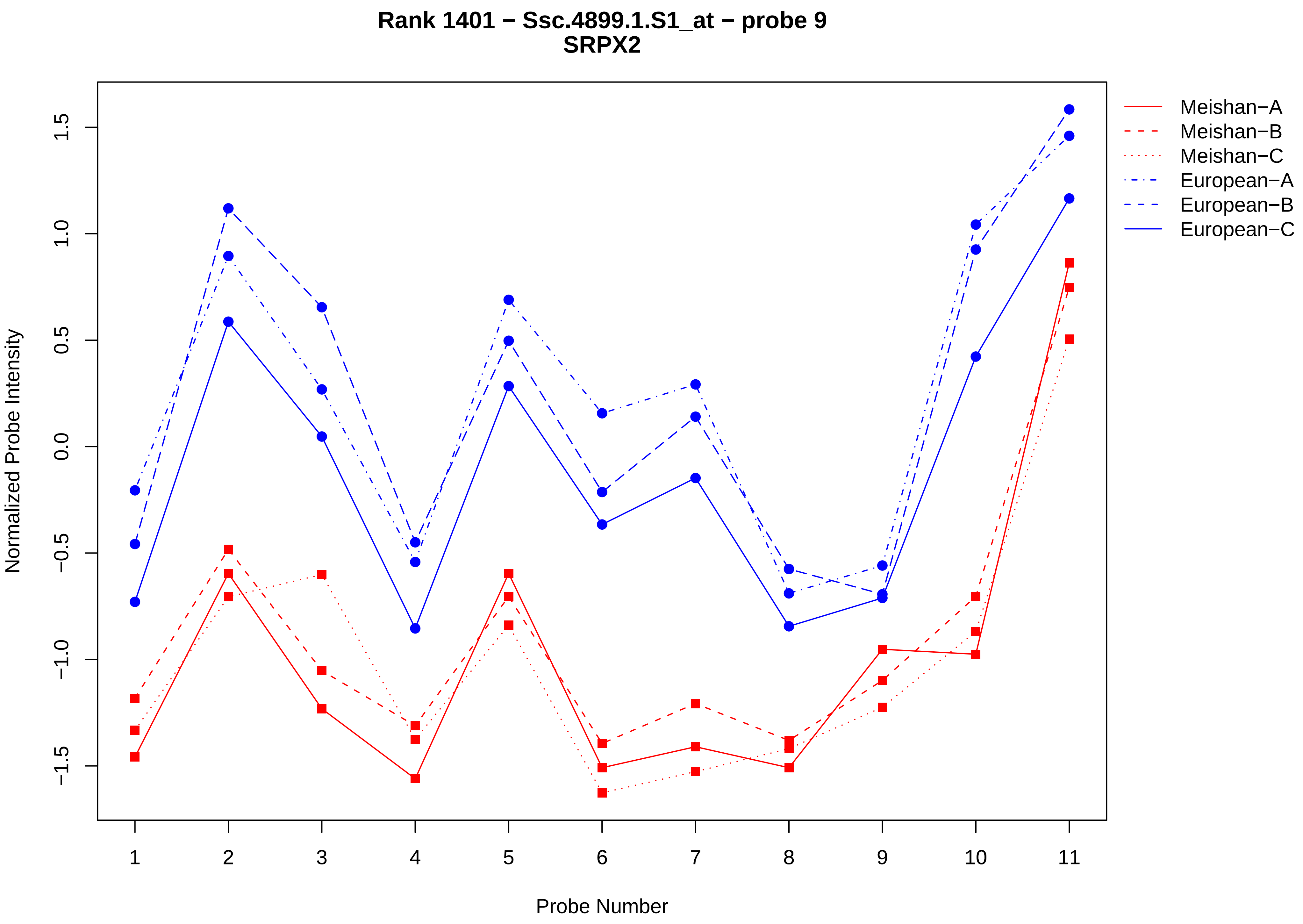

Rank 1401 − Ssc.4899.1.S1_at − probe 9
SRPX2
Normalized Probe Intensity
Probe Number
Meishan−A
Meishan−B
Meishan−C
European−A
European−B
European−C

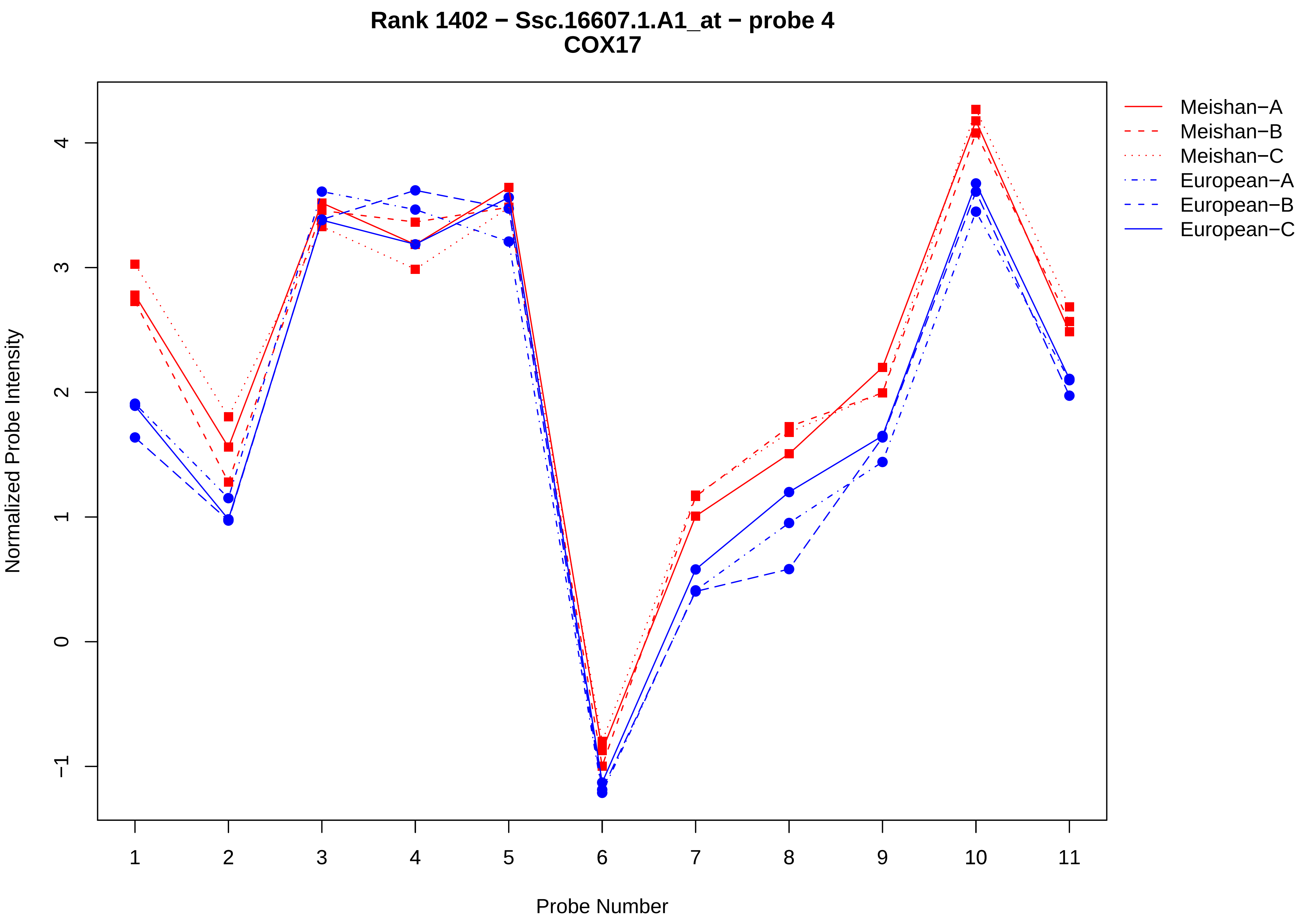
Rank 1402 − Ssc.16607.1.A1_at − probe 4
COX17
Normalized Probe Intensity
Probe Number
Meishan−A
Meishan−B
Meishan−C
European−A
European−B
European−C
−1
0
1
2
3
4
1
2
3
4
5
6
7
8
9
10
11

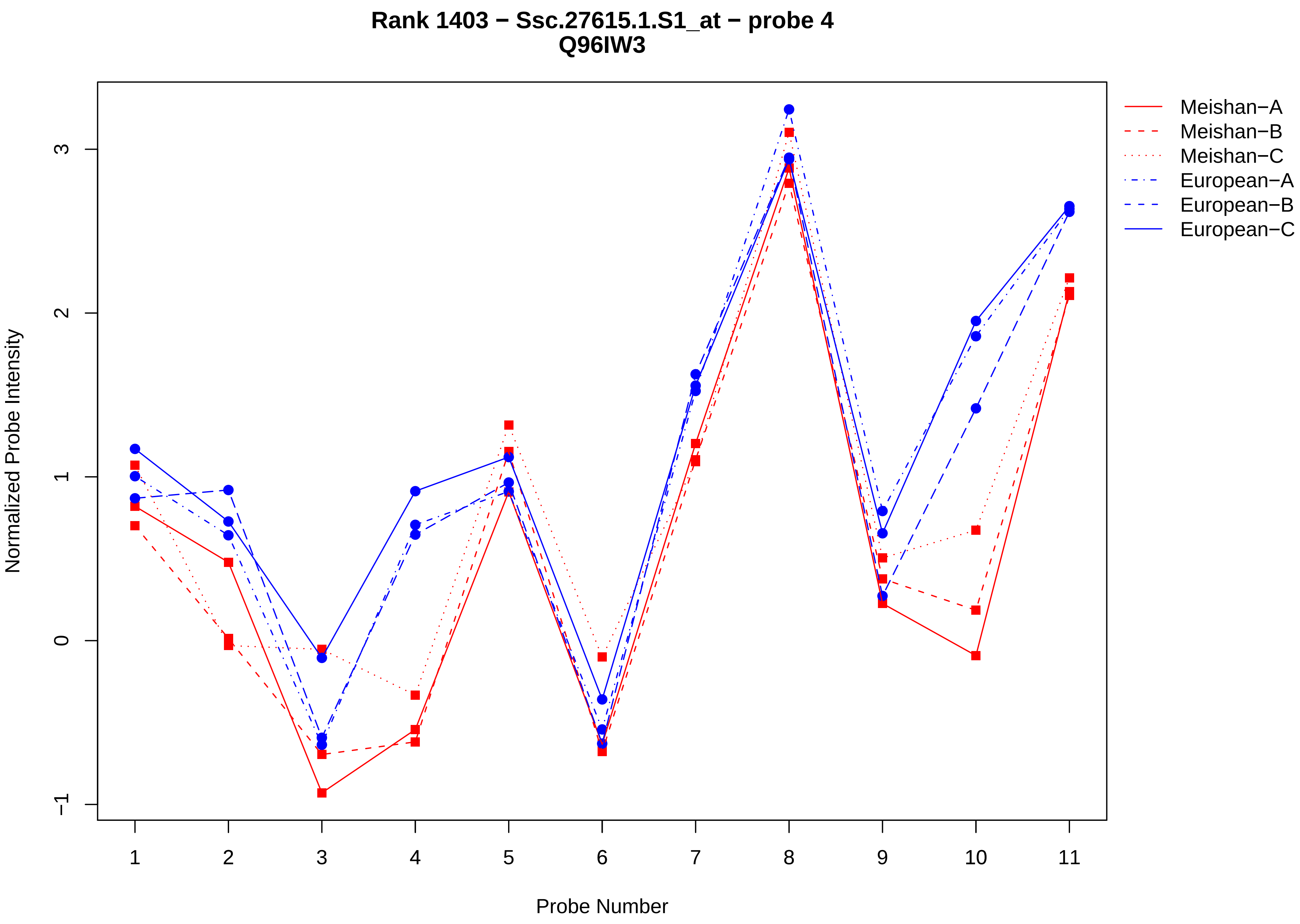

Rank 1403 − Ssc.27615.1.S1_at − probe 4
Q96IW3
Normalized Probe Intensity
Probe Number
Meishan−A
Meishan−B
Meishan−C
European−A
European−B
European−C
−1
0
1
2
3
1
2
3
4
5
6
7
8
9
10
11

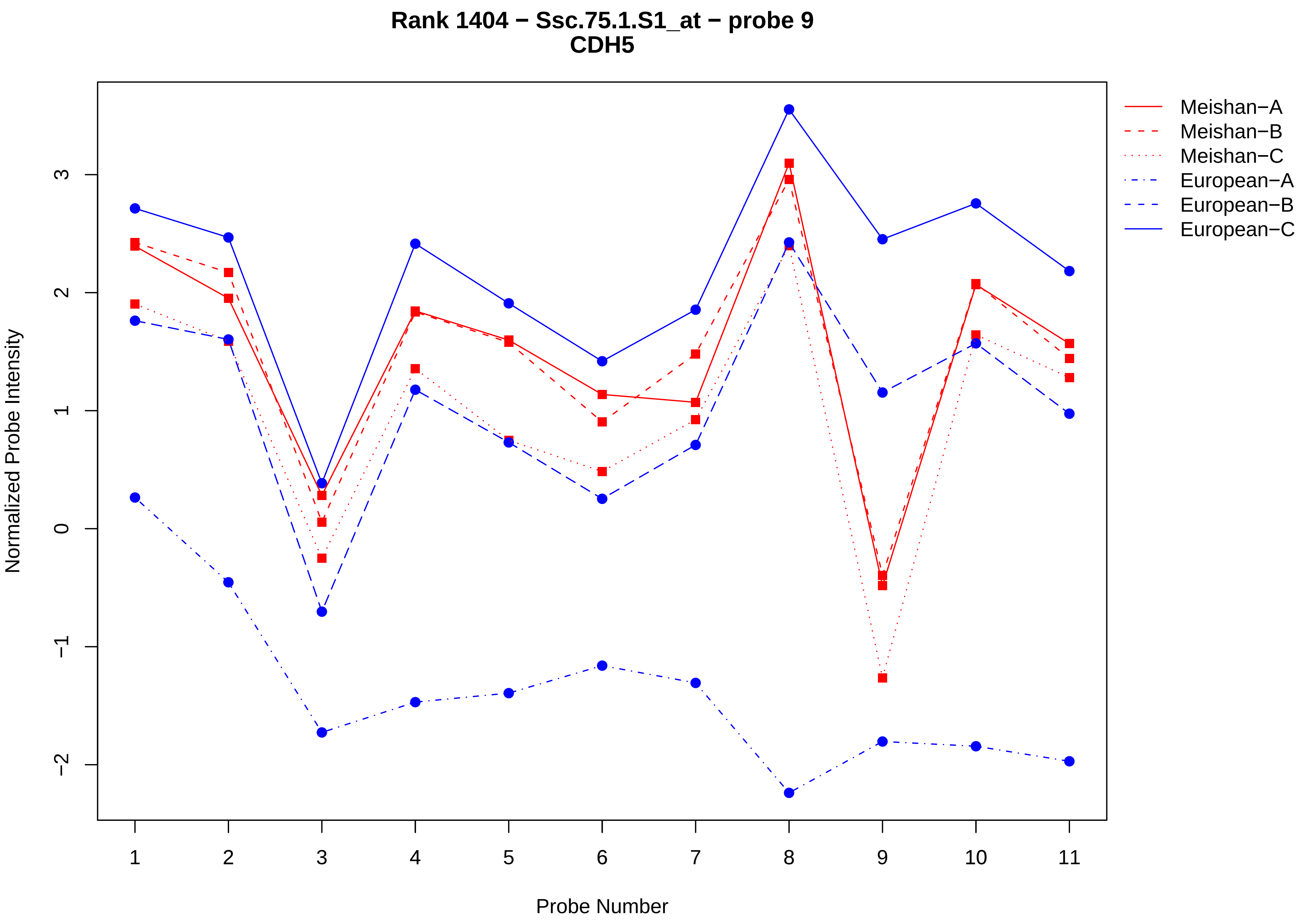

Rank 1404 − Ssc.75.1.S1_at − probe 9
CDH5
Normalized Probe Intensity
Probe Number
Meishan−A
Meishan−B
Meishan−C
European−A
European−B
European−C

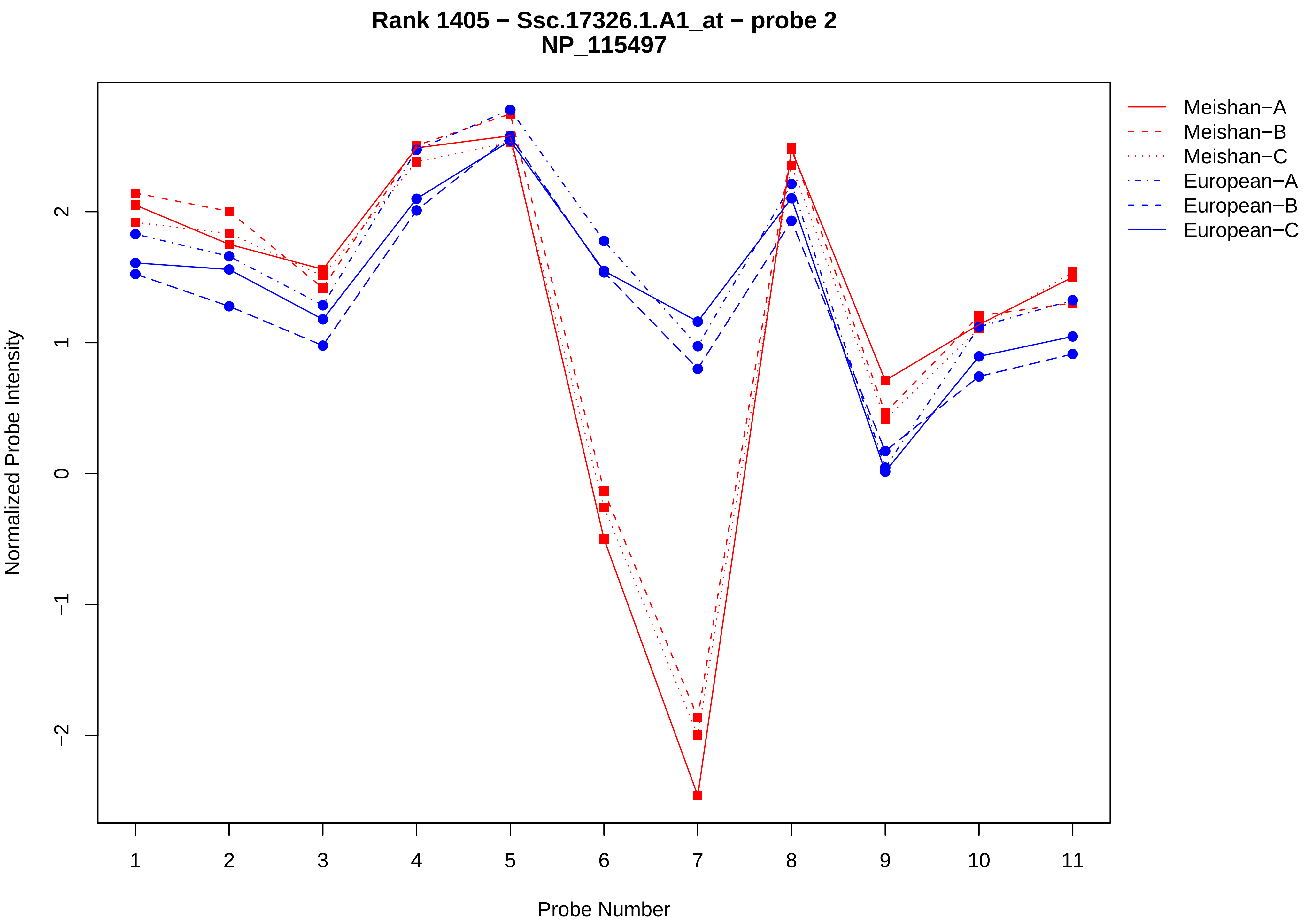

Rank 1405 − Ssc.17326.1.A1_at − probe 2
NP_115497
Normalized Probe Intensity
Probe Number
−2
−1
0
1
2
1
2
3
4
5
6
7
8
9
10
11
Meishan−A
Meishan−B
Meishan−C
European−A
European−B
European−C

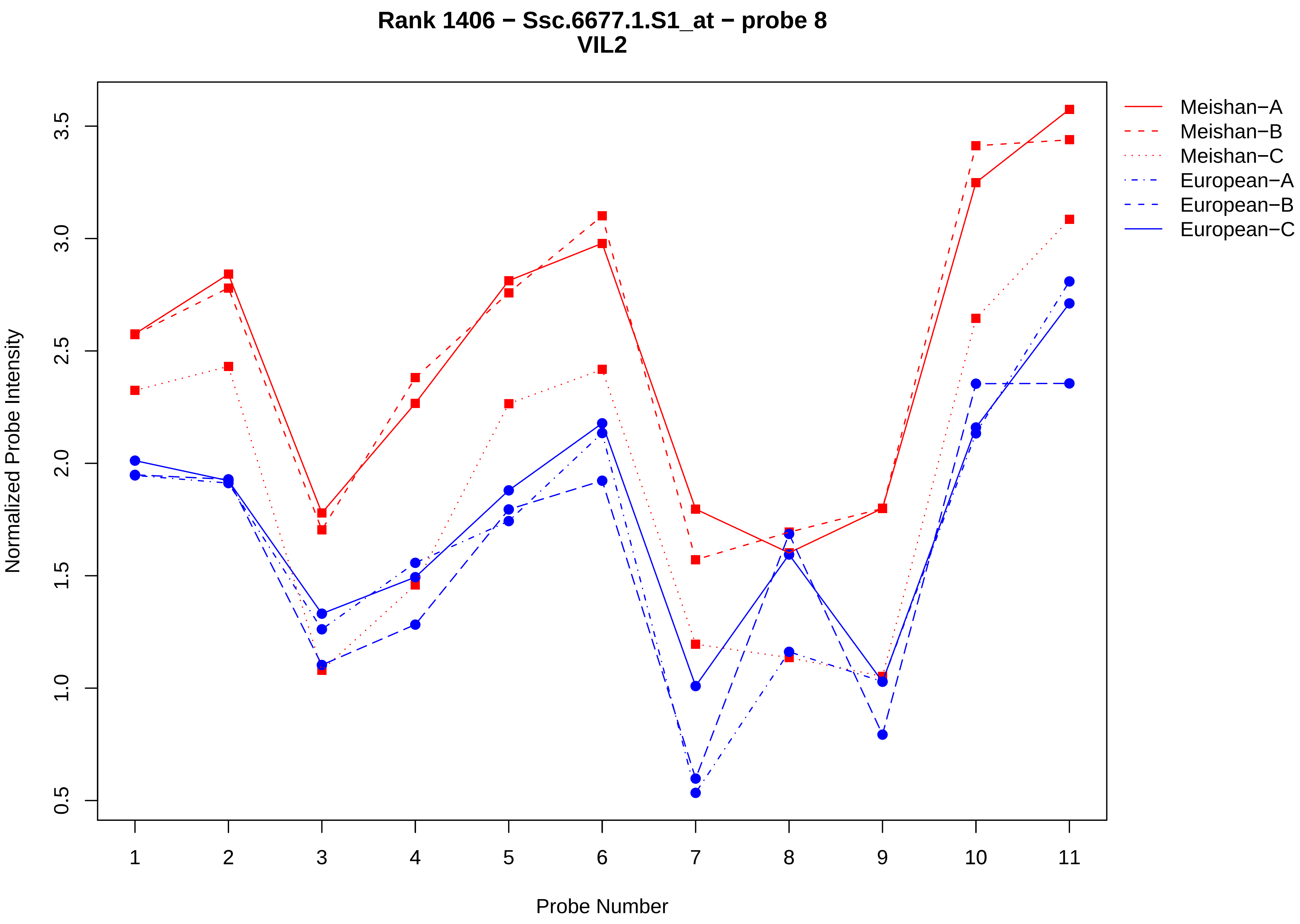

Rank 1406 − Ssc.6677.1.S1_at − probe 8
VIL2
Normalized Probe Intensity
0.5
1.0
1.5
2.0
2.5
3.0
3.5
1
2
3
4
5
6
7
8
9
10
11
Probe Number
Meishan−A
Meishan−B
Meishan−C
European−A
European−B
European−C

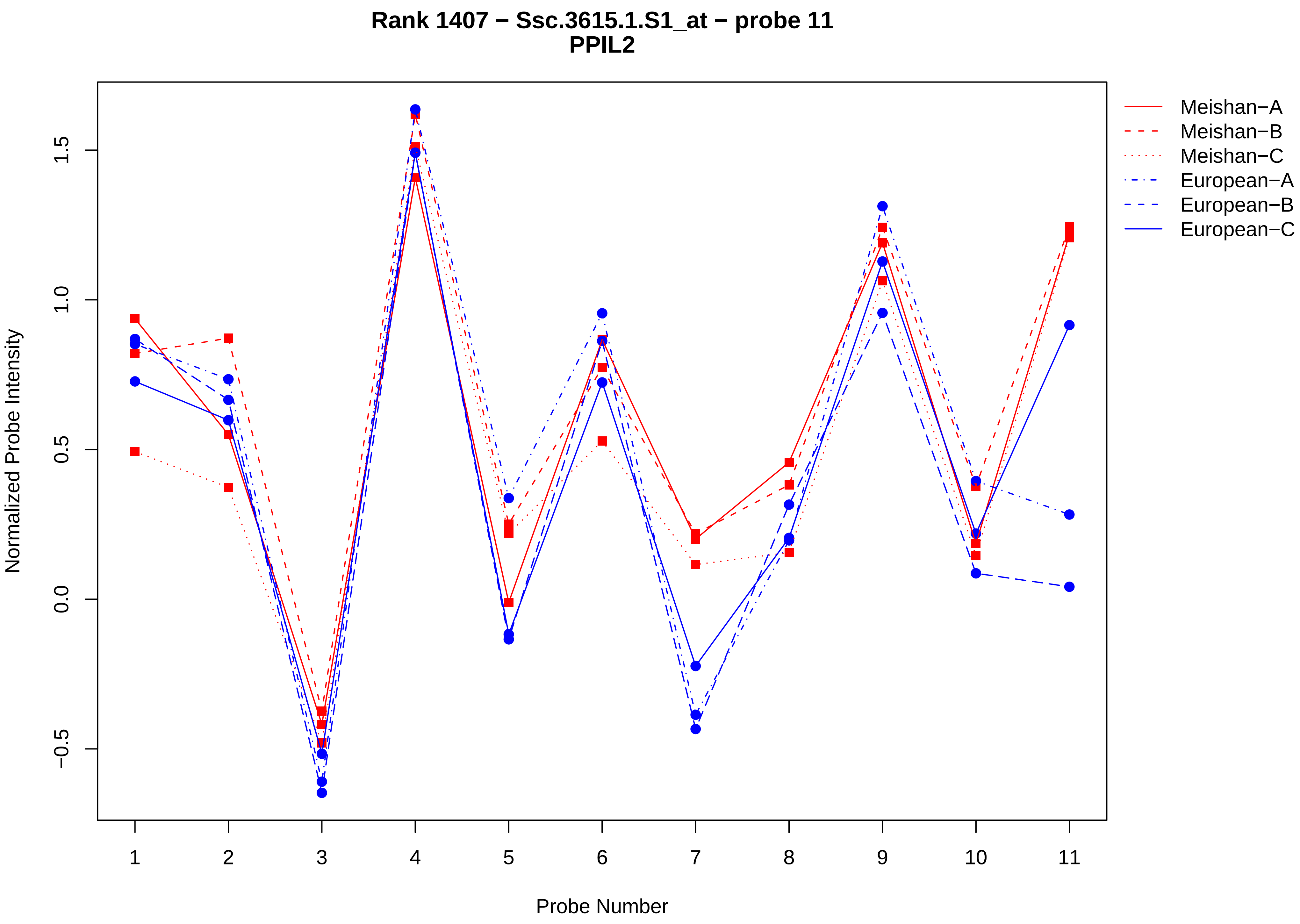
Rank 1407 − Ssc.3615.1.S1_at − probe 11
PPIL2
Normalized Probe Intensity
Probe Number
−0.5
0.0
0.5
1.0
1.5
1
2
3
4
5
6
7
8
9
10
11
Meishan−A
Meishan−B
Meishan−C
European−A
European−B
European−C

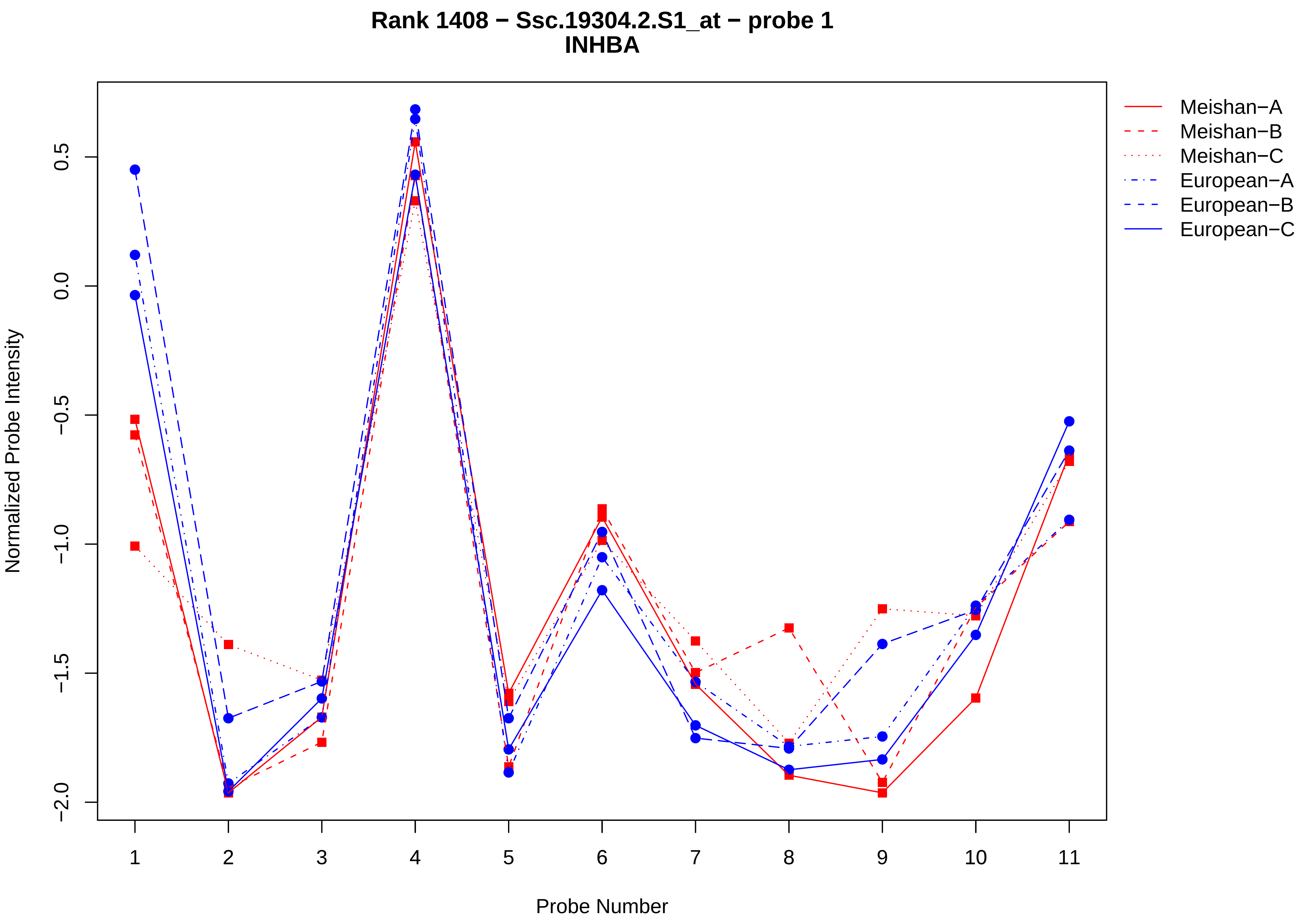

Rank 1408 − Ssc.19304.2.S1_at − probe 1
INHBA
Normalized Probe Intensity
Probe Number
0.5
0.0
−0.5
−1.0
−1.5
−2.0
1
2
3
4
5
6
7
8
9
10
11
Meishan−A
Meishan−B
Meishan−C
European−A
European−B
European−C

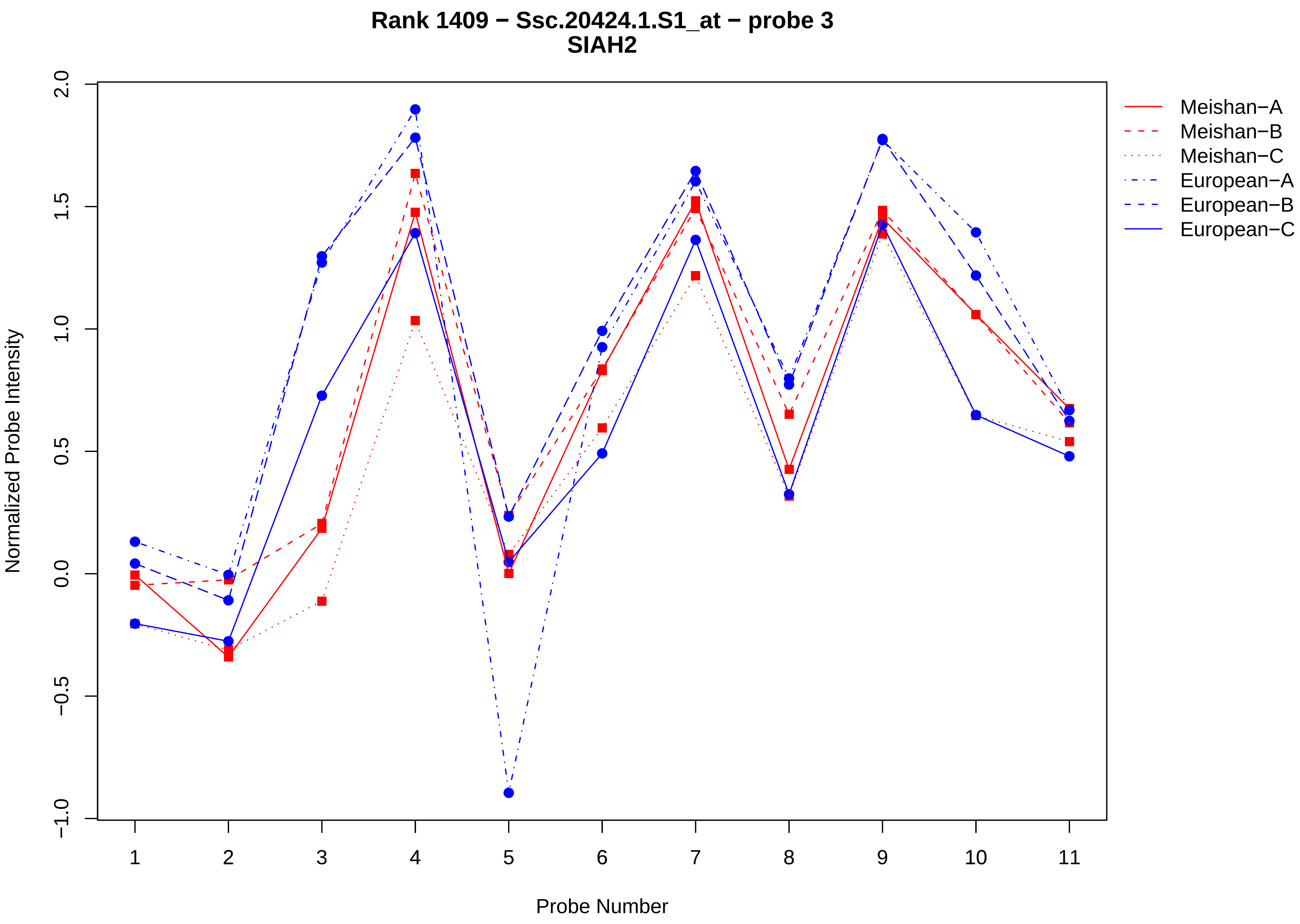

Rank 1409 − Ssc.20424.1.S1_at − probe 3
SIAH2
Normalized Probe Intensity
Probe Number
-1.0
-0.5
0.0
0.5
1.0
1.5
2.0
1
2
3
4
5
6
7
8
9
10
11
Meishan−A
Meishan−B
Meishan−C
European−A
European−B
European−C

Rank 1410 − Ssc.11170.1.S1_at − probe 2
C21orf124

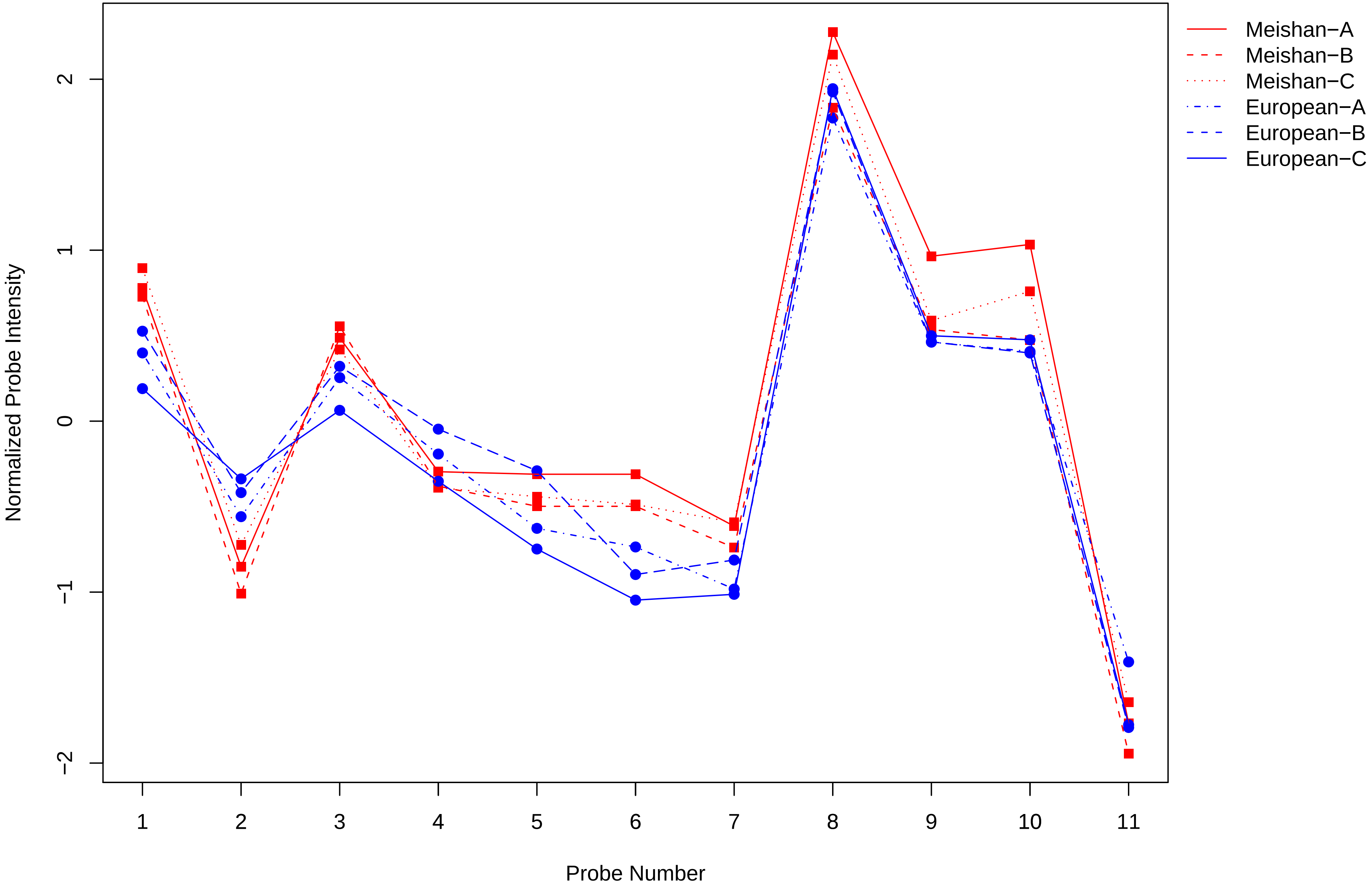

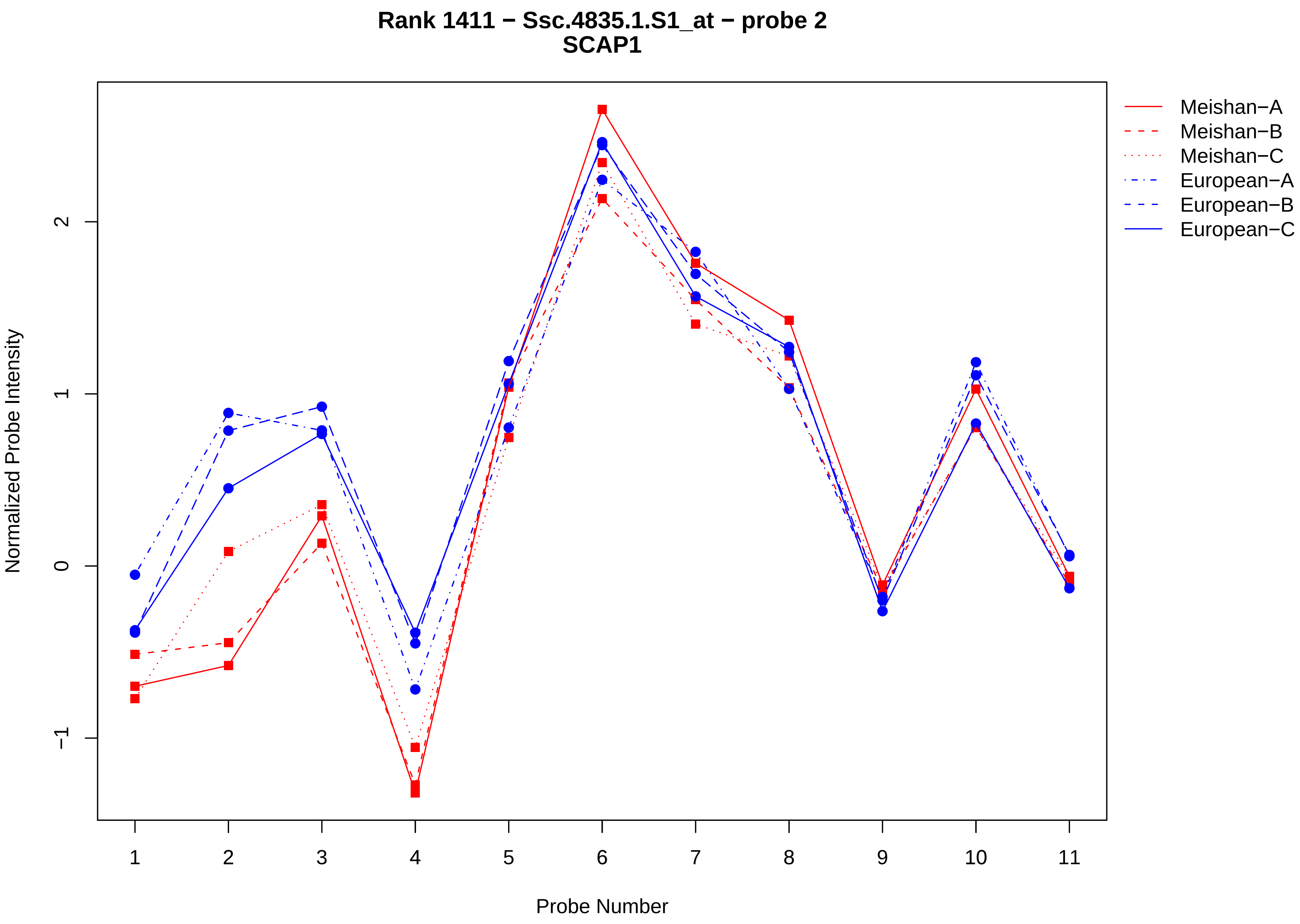

Rank 1411 − Ssc.4835.1.S1_at − probe 2
SCAP1
Normalized Probe Intensity
Probe Number
−1
0
1
2
1
2
3
4
5
6
7
8
9
10
11
Meishan−A
Meishan−B
Meishan−C
European−A
European−B
European−C

Rank 1412 − Ssc.11360.1.S1_at − probe 4
Q96BJ3

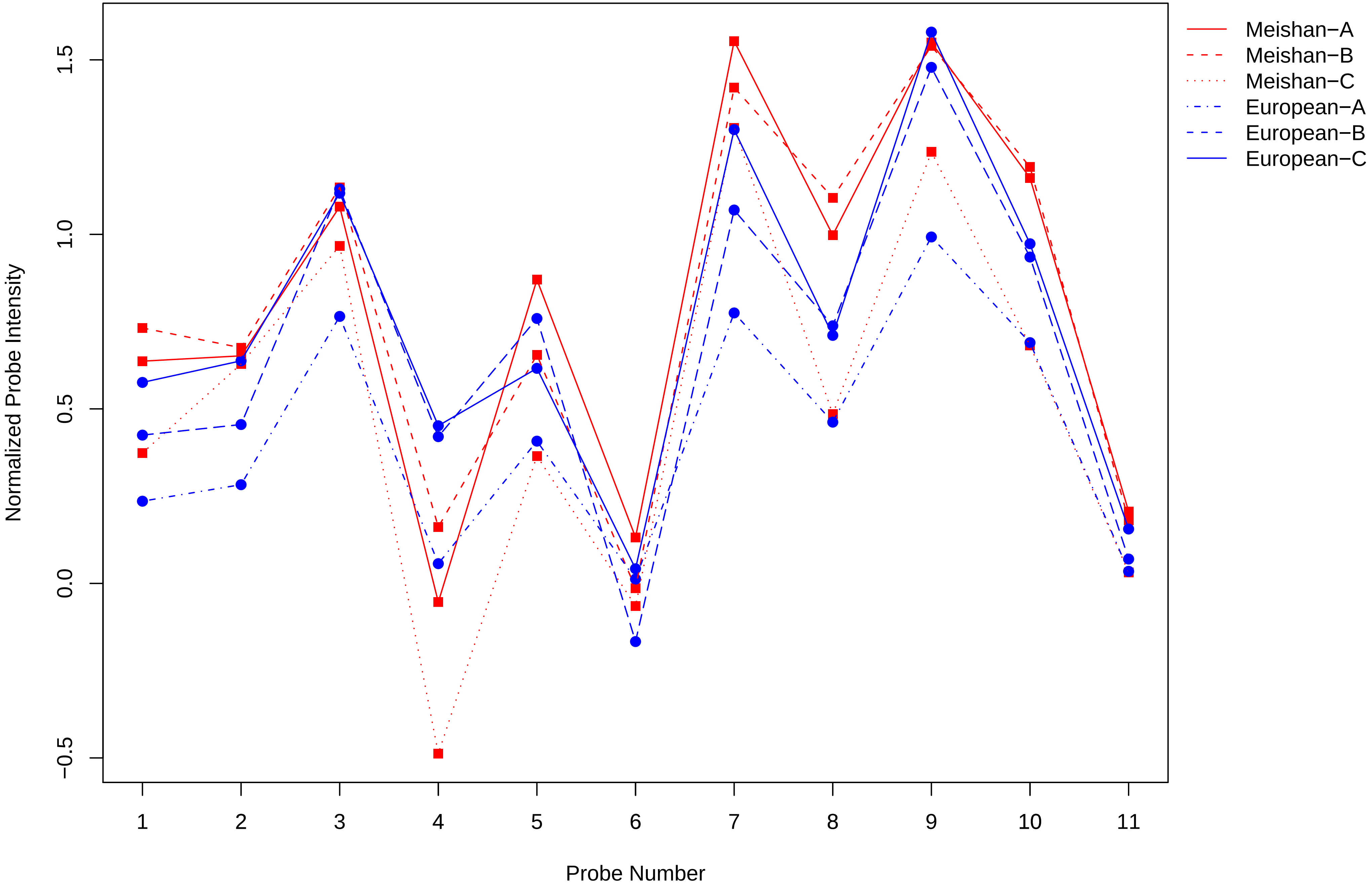

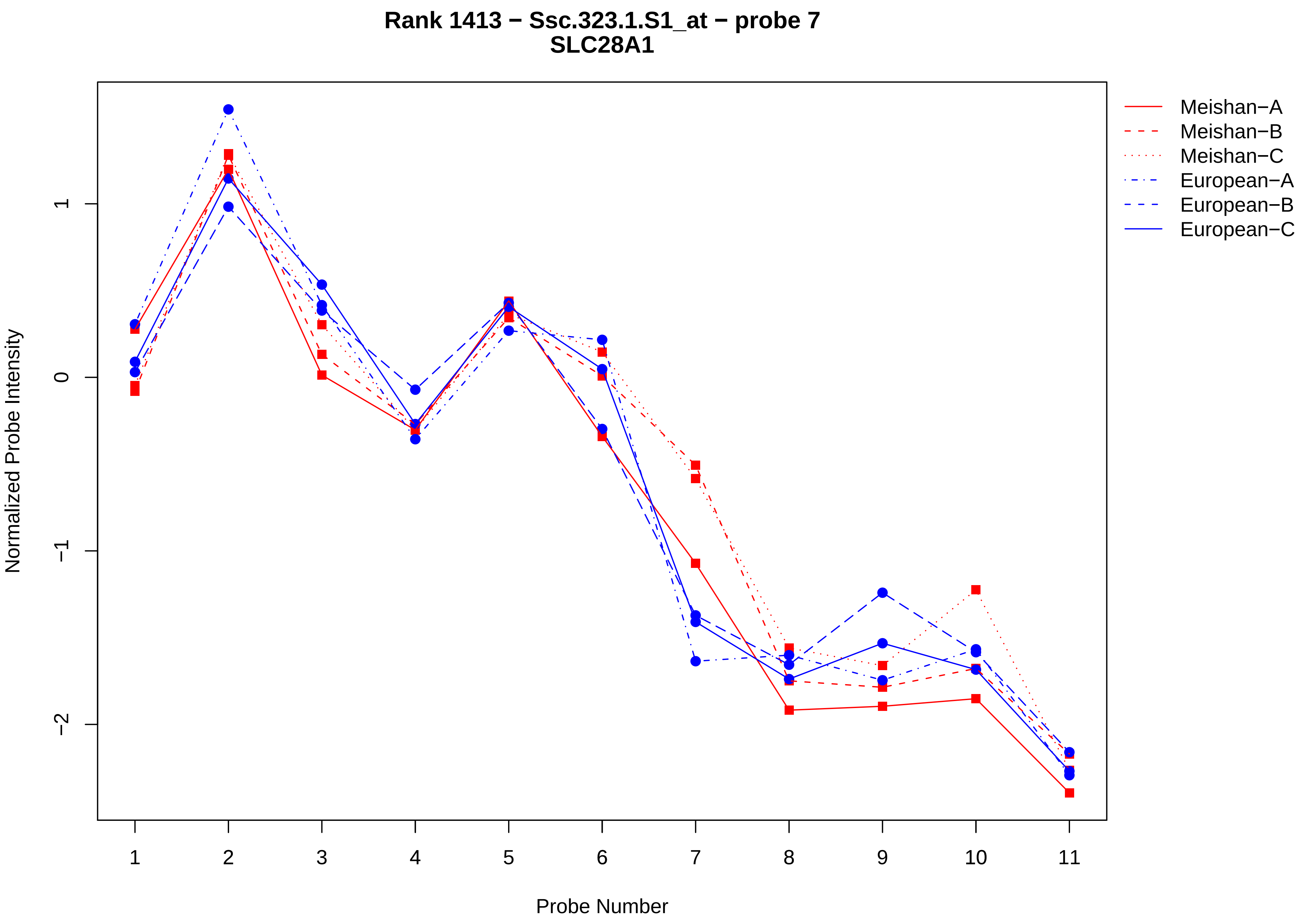

Rank 1413 − Ssc.323.1.S1_at − probe 7
SLC28A1
Normalized Probe Intensity
Probe Number
Meishan−A
Meishan−B
Meishan−C
European−A
European−B
European−C

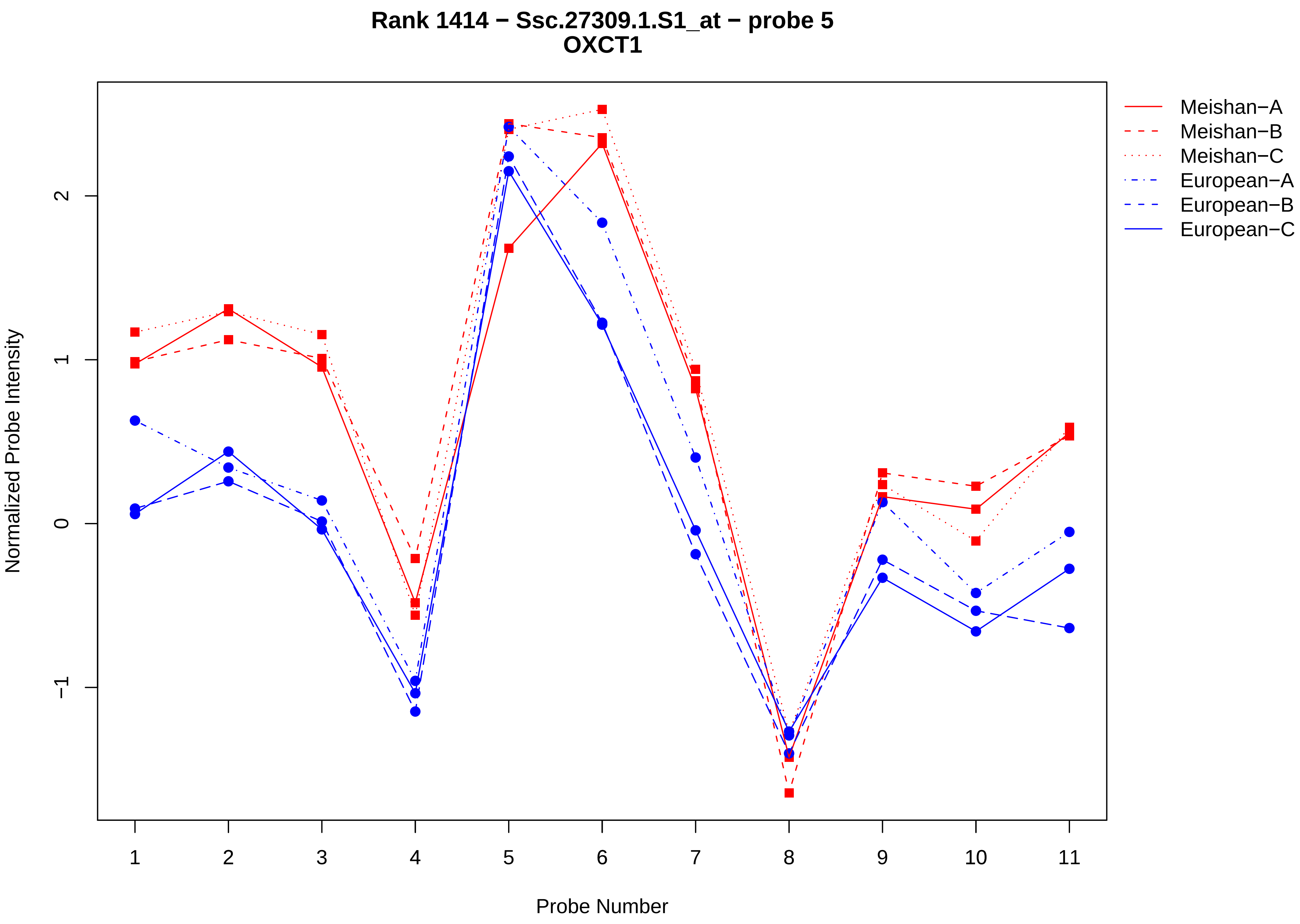
Rank 1414 − Ssc.27309.1.S1_at − probe 5
OXCT1
Normalized Probe Intensity
Probe Number
−1
0
1
2
1
2
3
4
5
6
7
8
9
10
11
Meishan−A
Meishan−B
Meishan−C
European−A
European−B
European−C

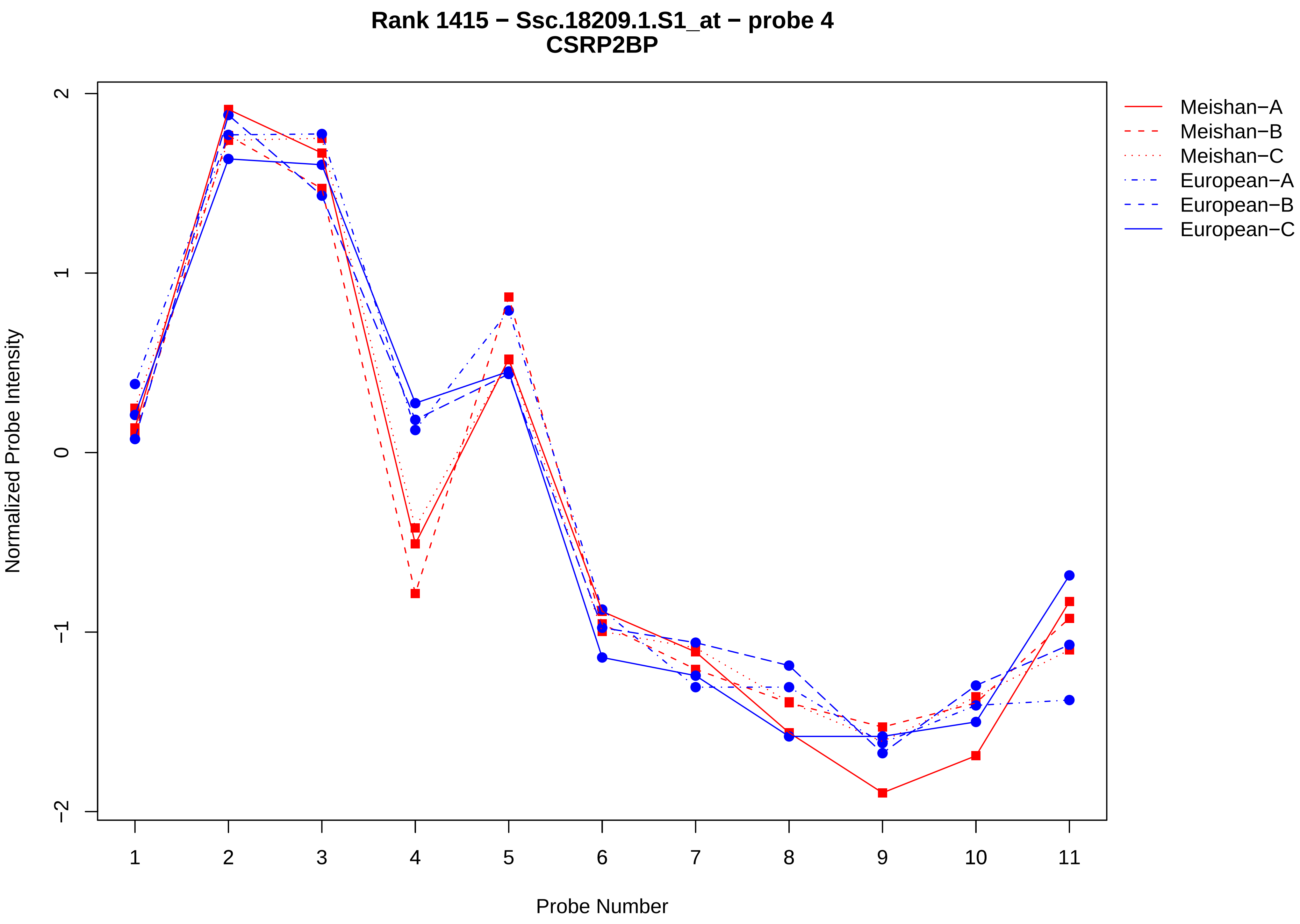

Rank 1415 − Ssc.18209.1.S1_at − probe 4
CSRP2BP
Normalized Probe Intensity
Probe Number
2
1
0
−1
−2
1
2
3
4
5
6
7
8
9
10
11
Meishan−A
Meishan−B
Meishan−C
European−A
European−B
European−C

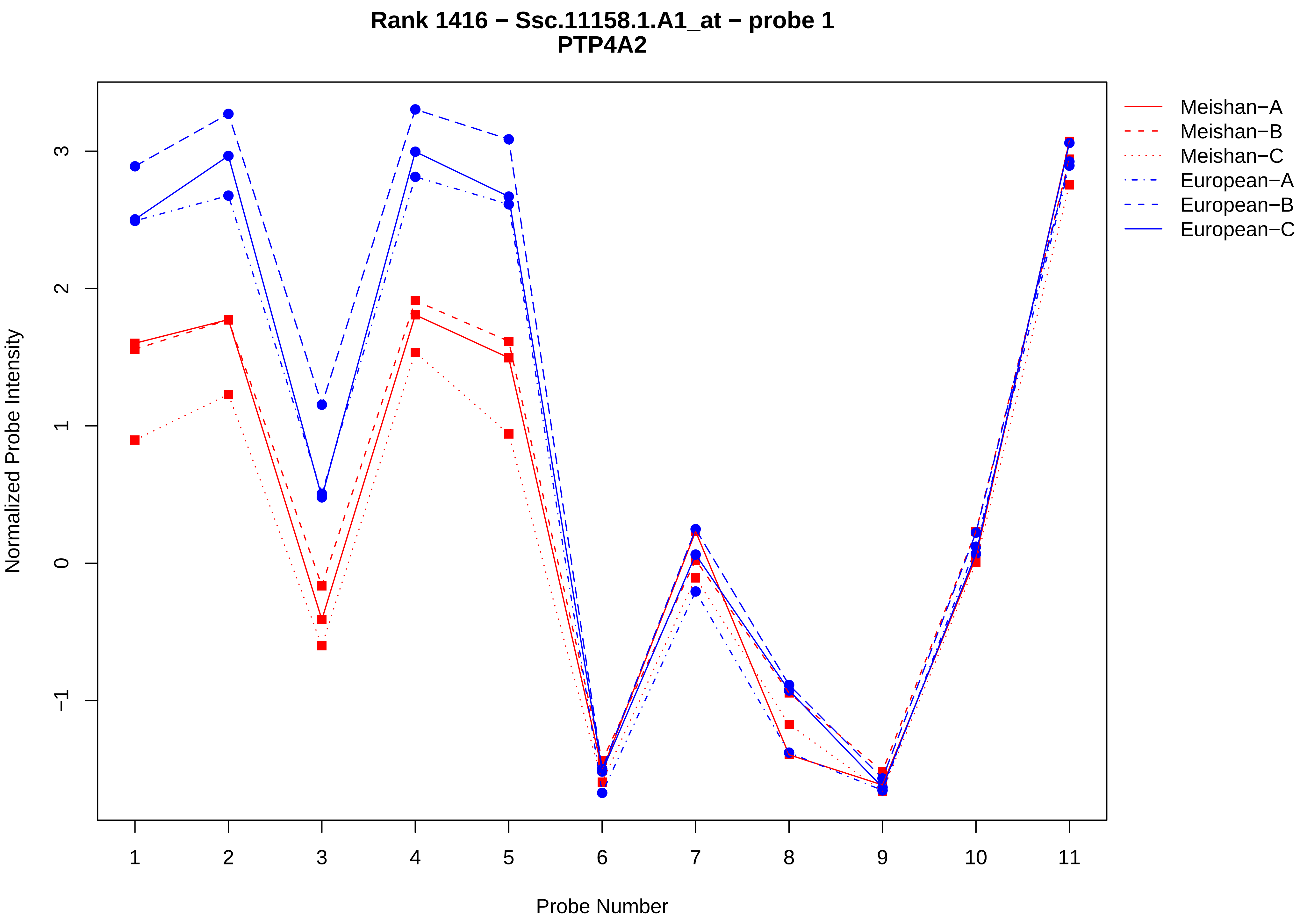
Rank 1416 − Ssc.11158.1.A1_at − probe 1
PTP4A2
Normalized Probe Intensity
Probe Number
Meishan−A
Meishan−B
Meishan−C
European−A
European−B
European−C

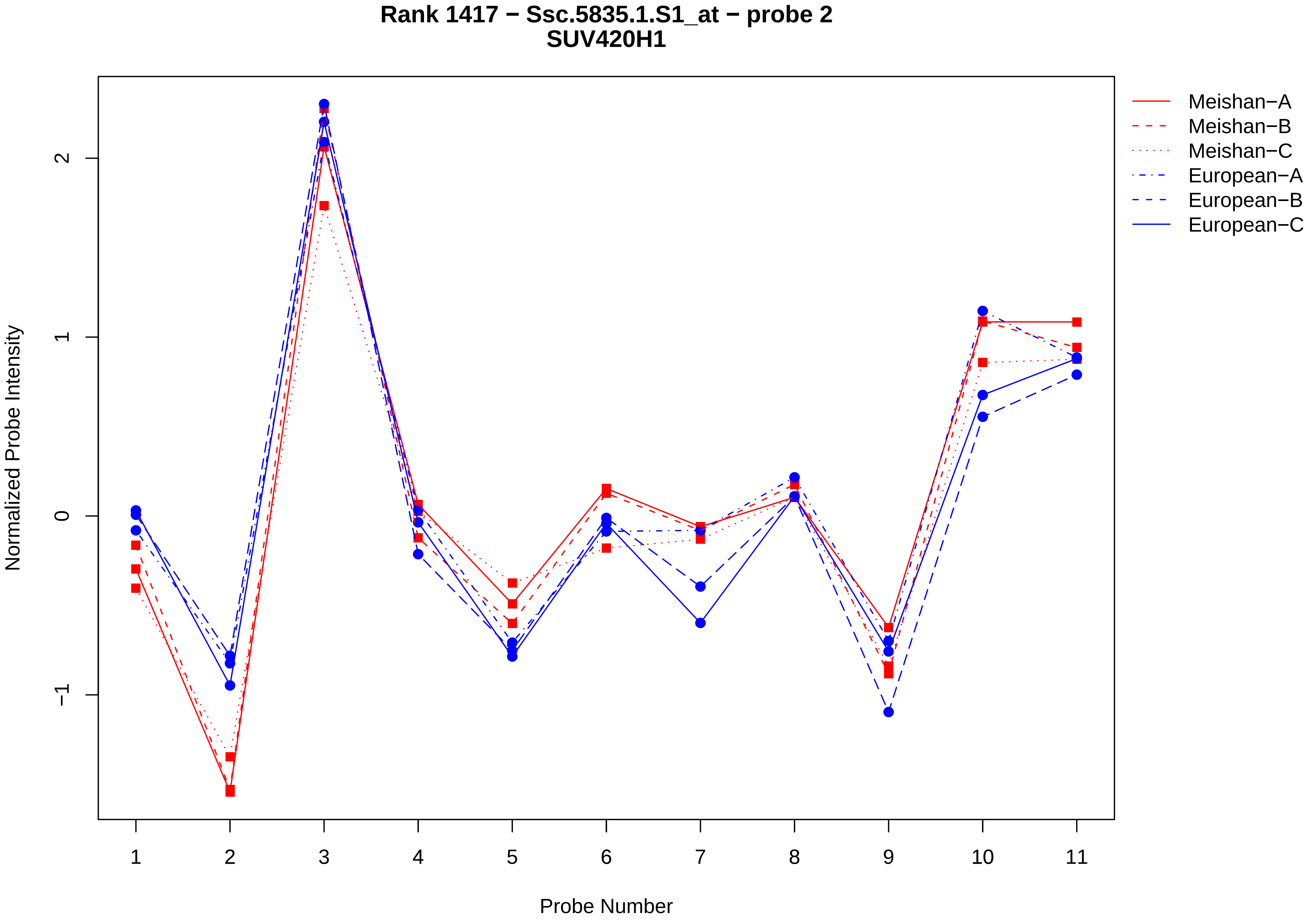

Rank 1417 − Ssc.5835.1.S1_at − probe 2
SUV420H1
Normalized Probe Intensity
Probe Number
−1
0
1
2
1
2
3
4
5
6
7
8
9
10
11
Meishan−A
Meishan−B
Meishan−C
European−A
European−B
European−C

Rank 1418 − Ssc.135.1.S1_a_at − probe 9
GHRHR

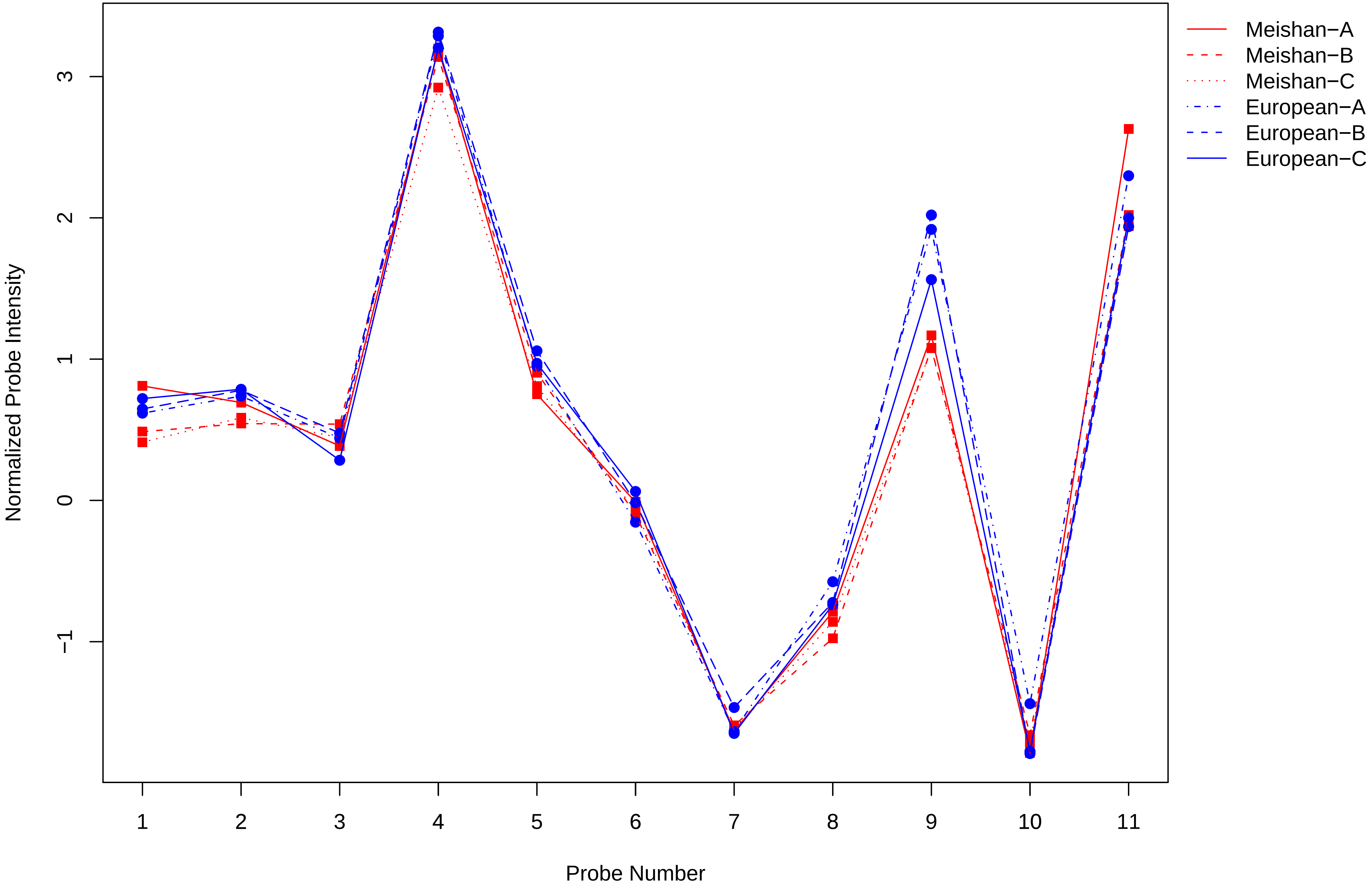

Rank 1419 − Ssc.26230.1.S1_at − probe 5
RRAD

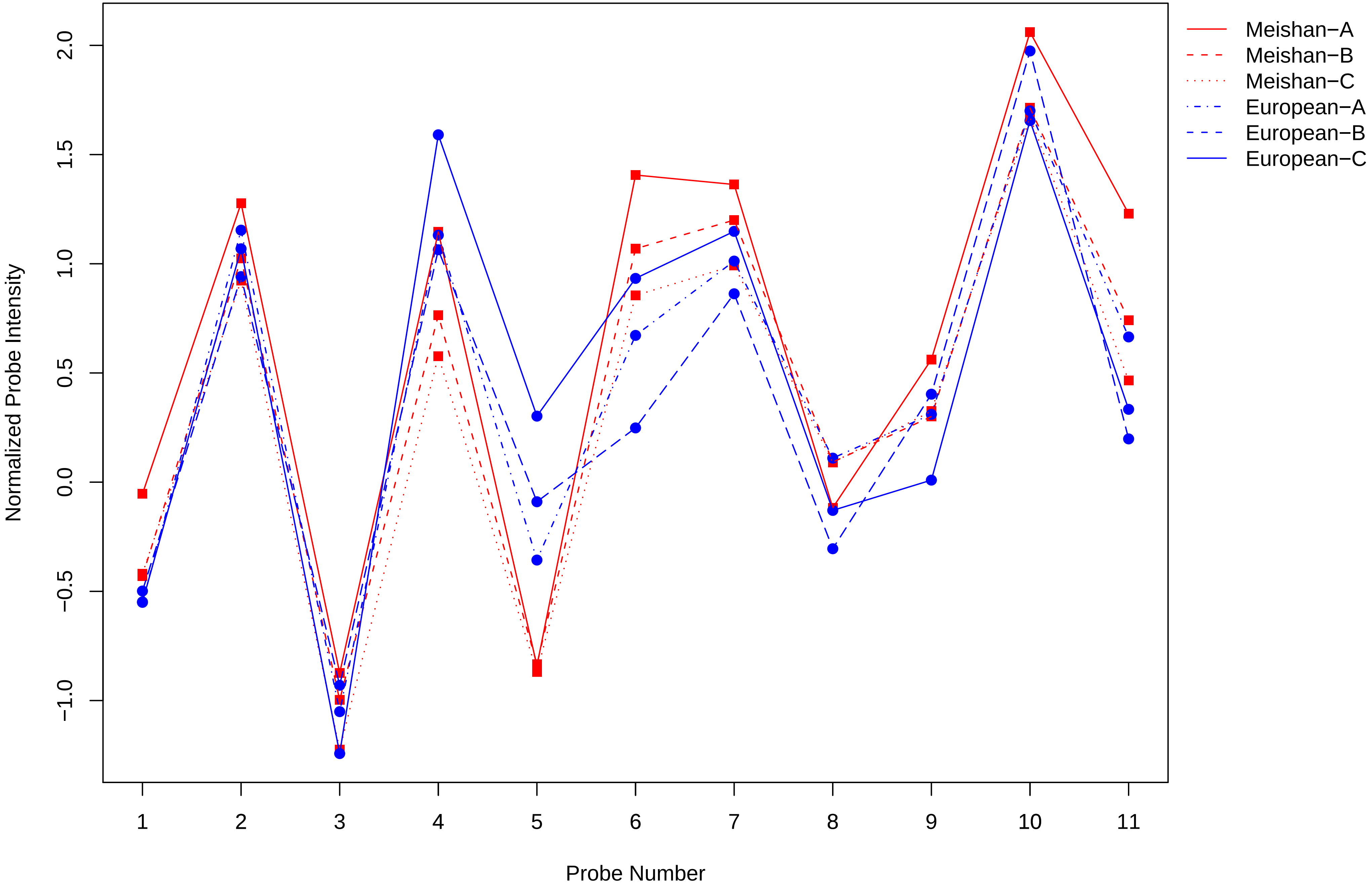

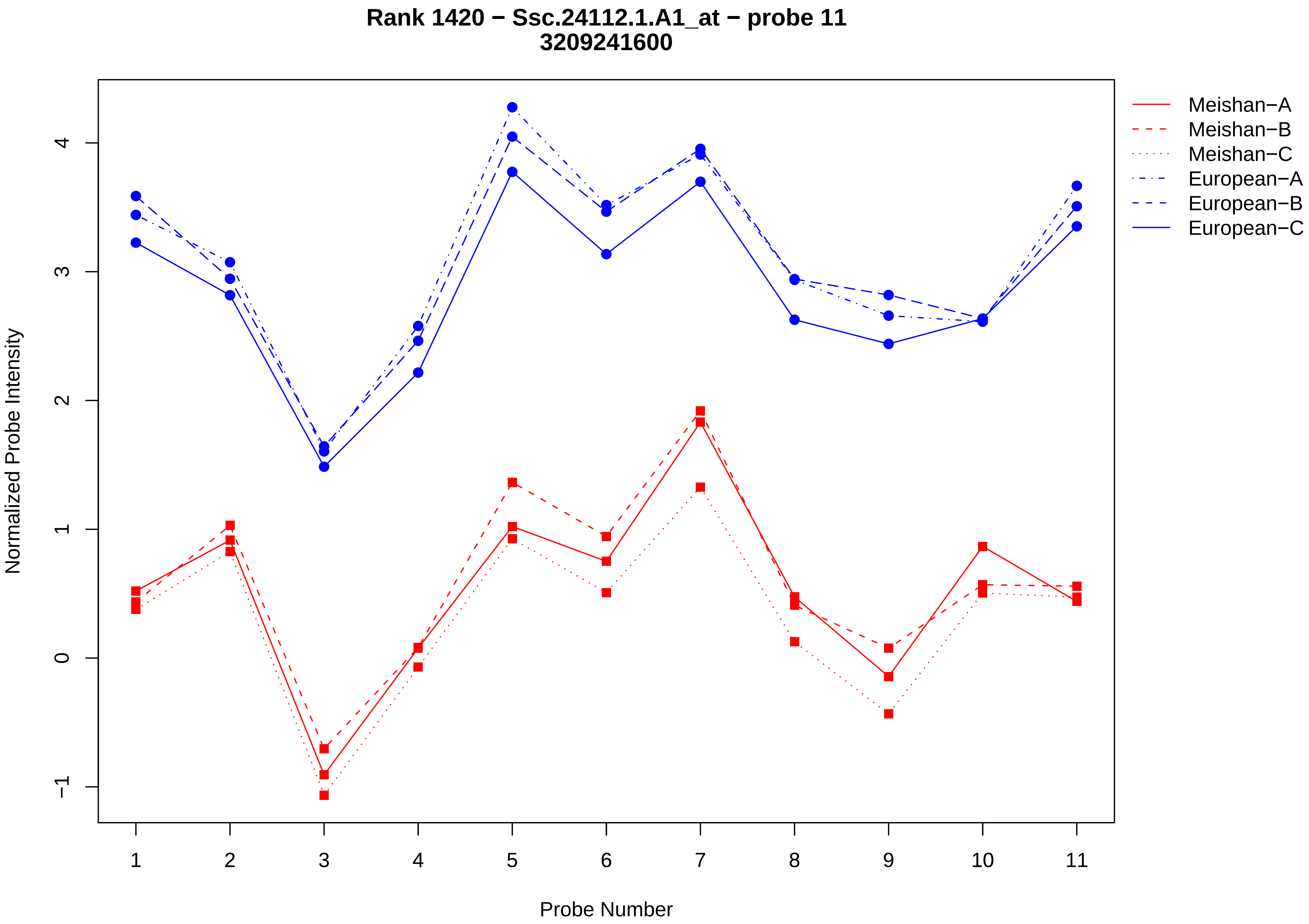

Rank 1420 − Ssc.24112.1.A1_at − probe 11
3209241600
Normalized Probe Intensity
Probe Number
−1
0
1
2
3
4
1
2
3
4
5
6
7
8
9
10
11
Meishan−A
Meishan−B
Meishan−C
European−A
European−B
European−C

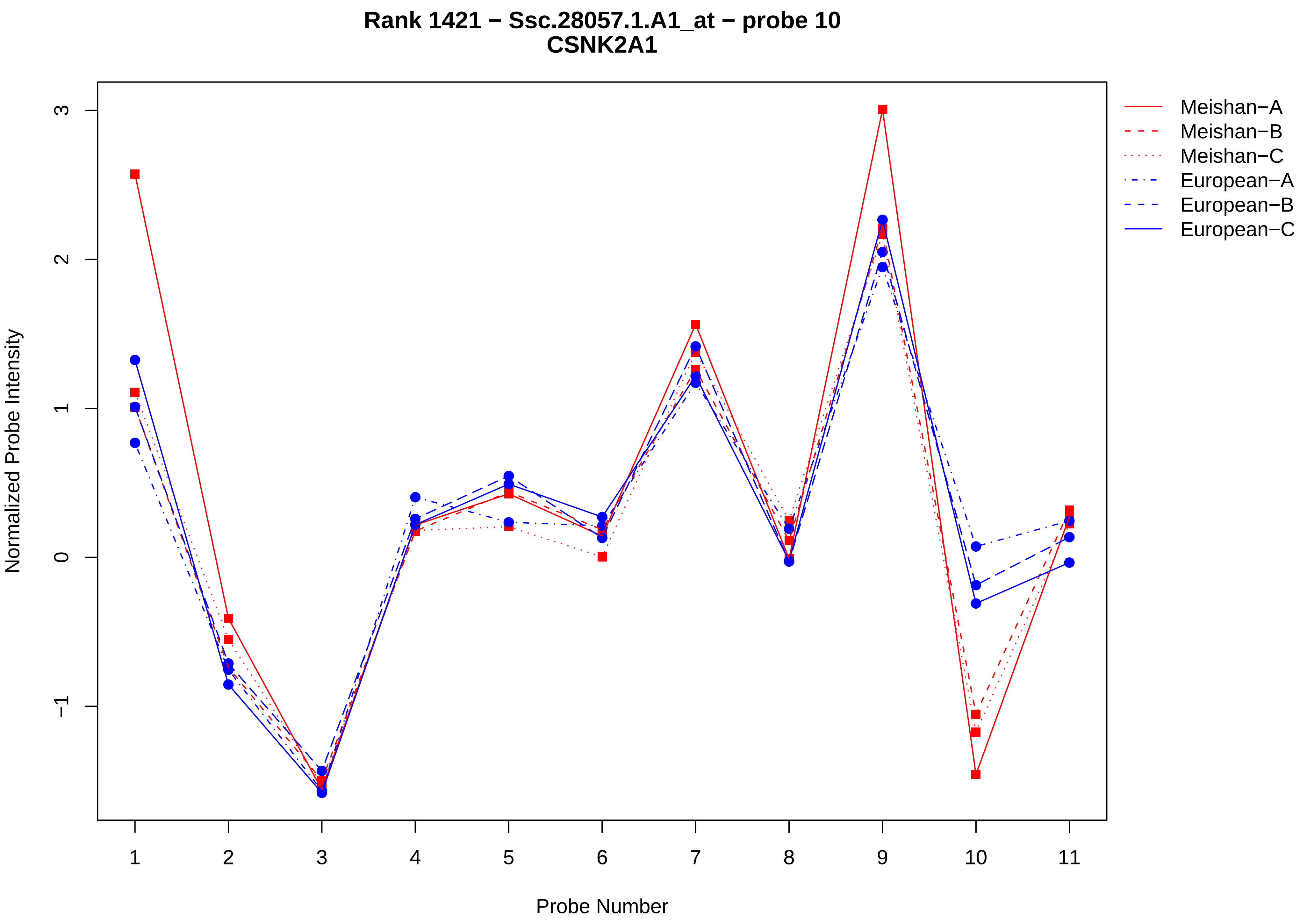
Rank 1421 − Ssc.28057.1.A1_at − probe 10
CSNK2A1
Normalized Probe Intensity
Probe Number
Meishan−A
Meishan−B
Meishan−C
European−A
European−B
European−C
−1
0
1
2
3
1
2
3
4
5
6
7
8
9
10
11

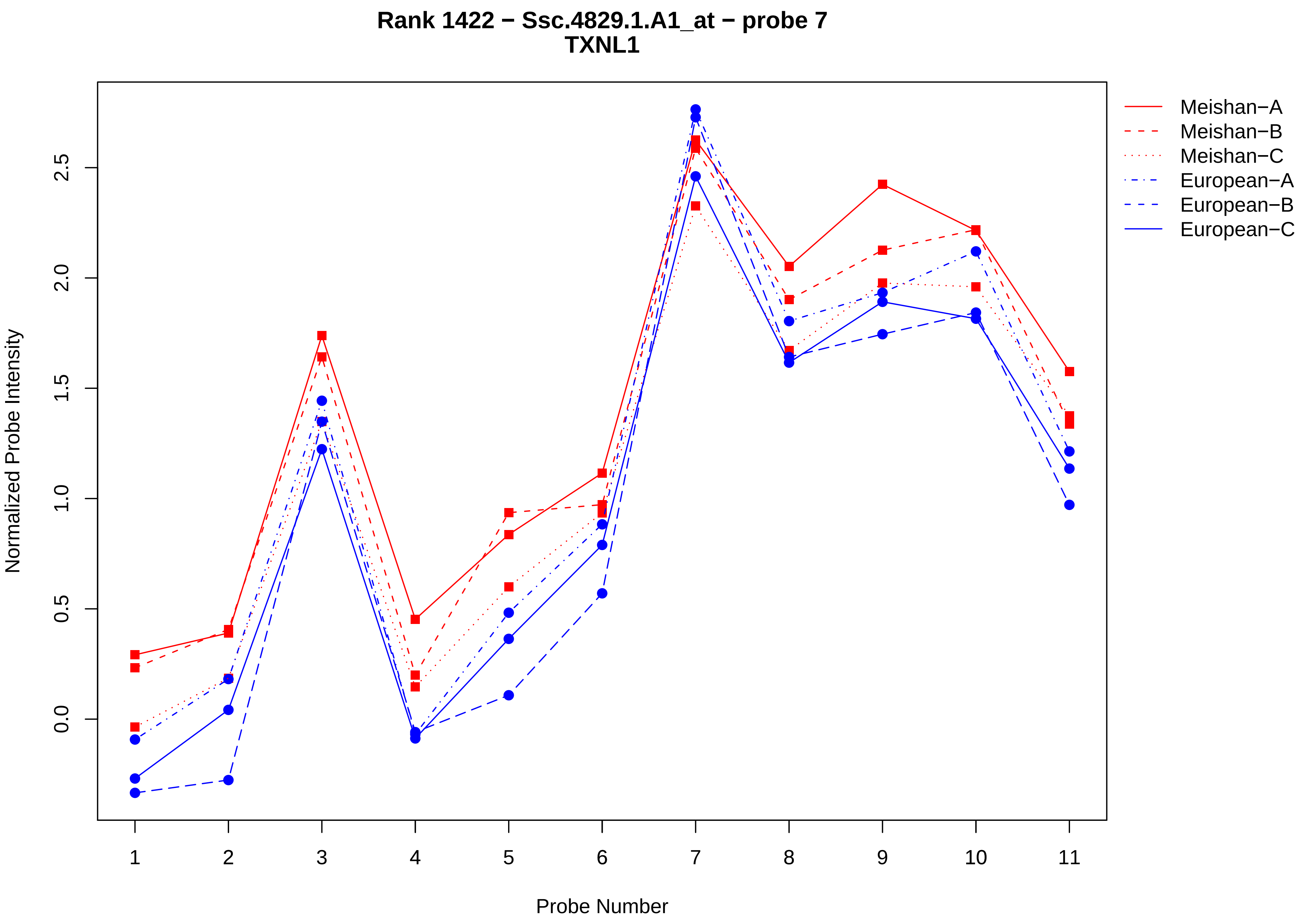

Rank 1422 − Ssc.4829.1.A1_at − probe 7
TXNL1
Normalized Probe Intensity
0.0
0.5
1.0
1.5
2.0
2.5
1
2
3
4
5
6
7
8
9
10
11
Probe Number
Meishan−A
Meishan−B
Meishan−C
European−A
European−B
European−C

Rank 1423 − Ssc.20371.1.A1_at − probe 9
COL4A3BP

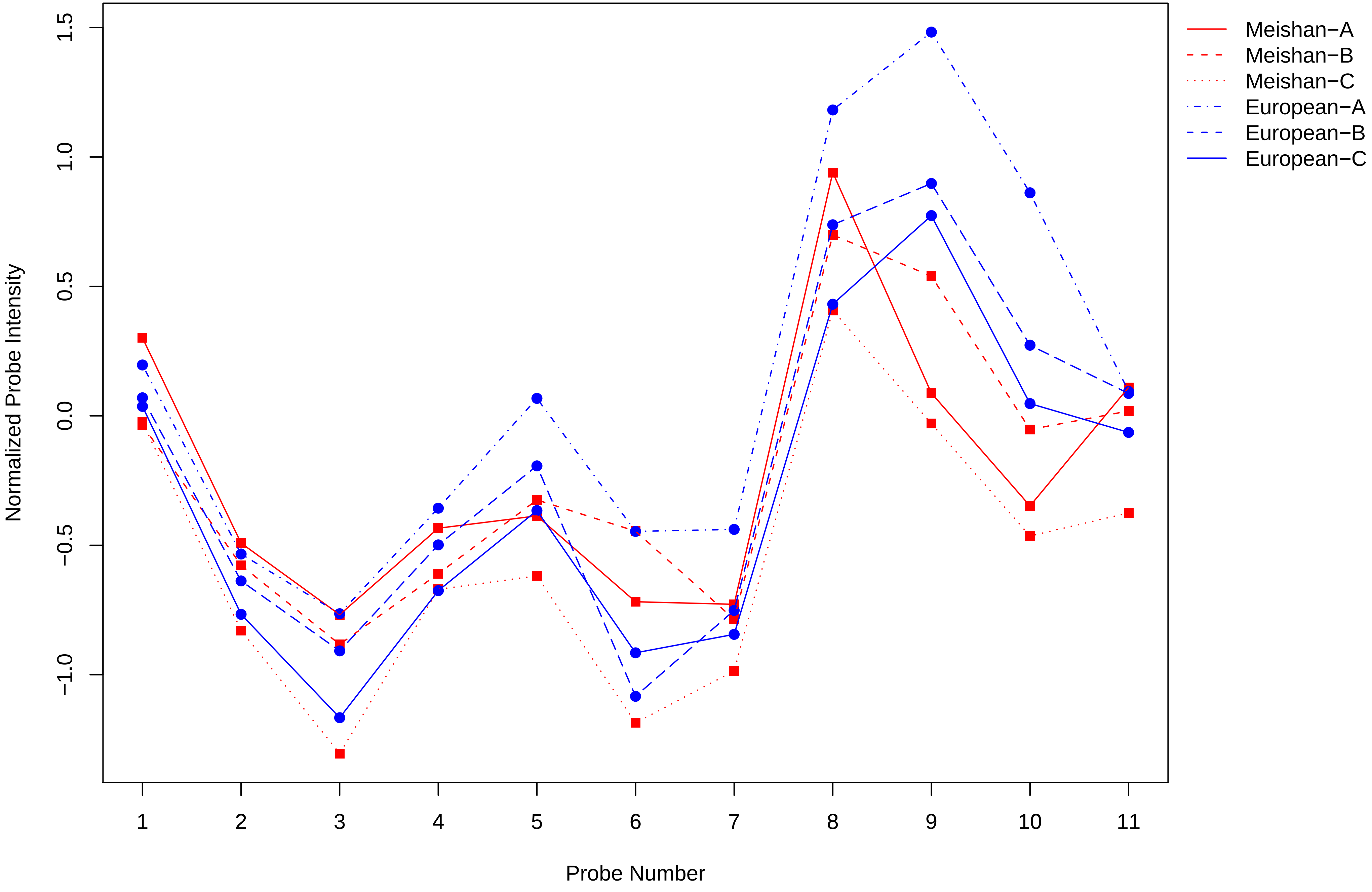

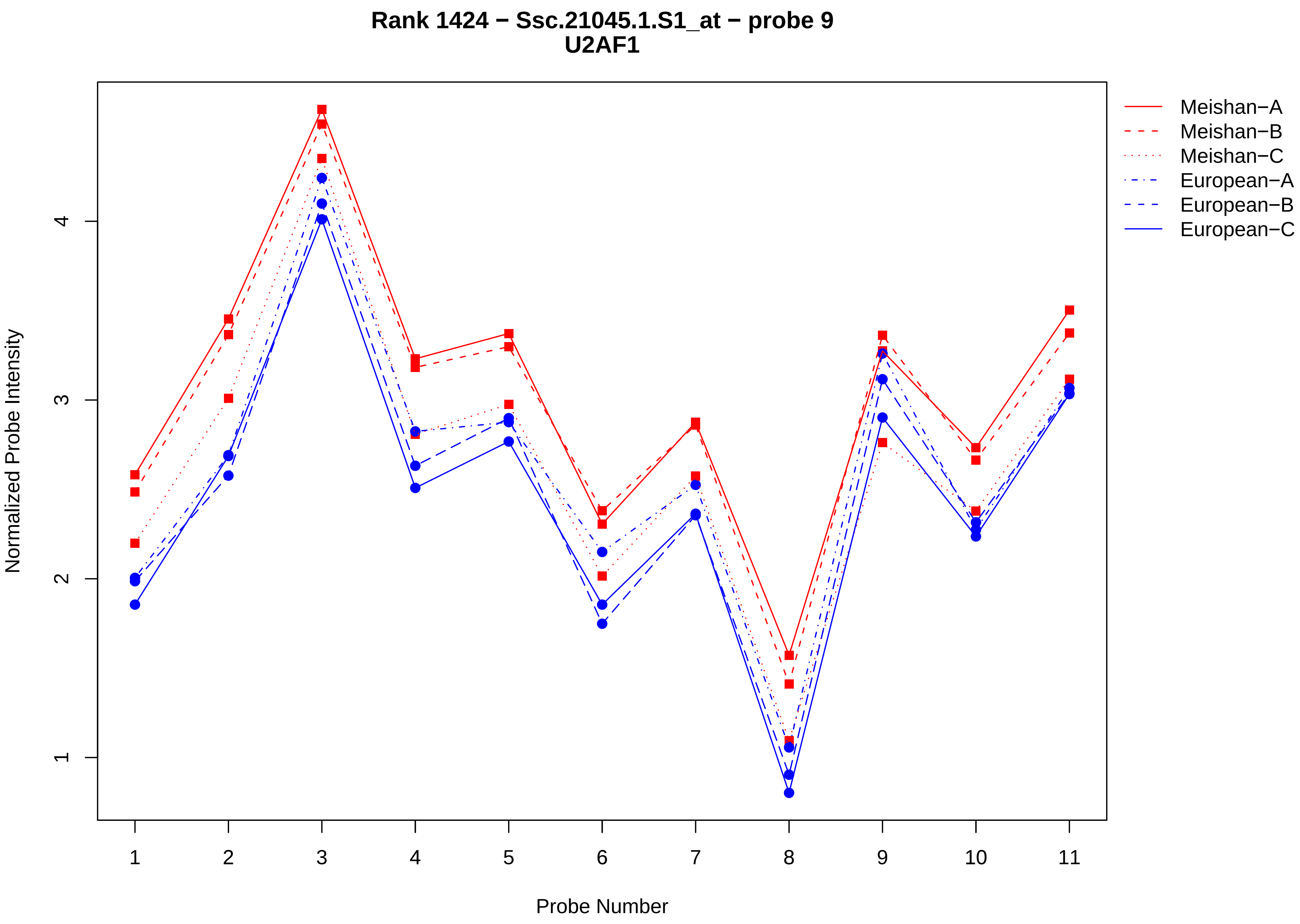

Rank 1424 − Ssc.21045.1.S1_at − probe 9
U2AF1
Normalized Probe Intensity
Probe Number
Meishan−A
Meishan−B
Meishan−C
European−A
European−B
European−C

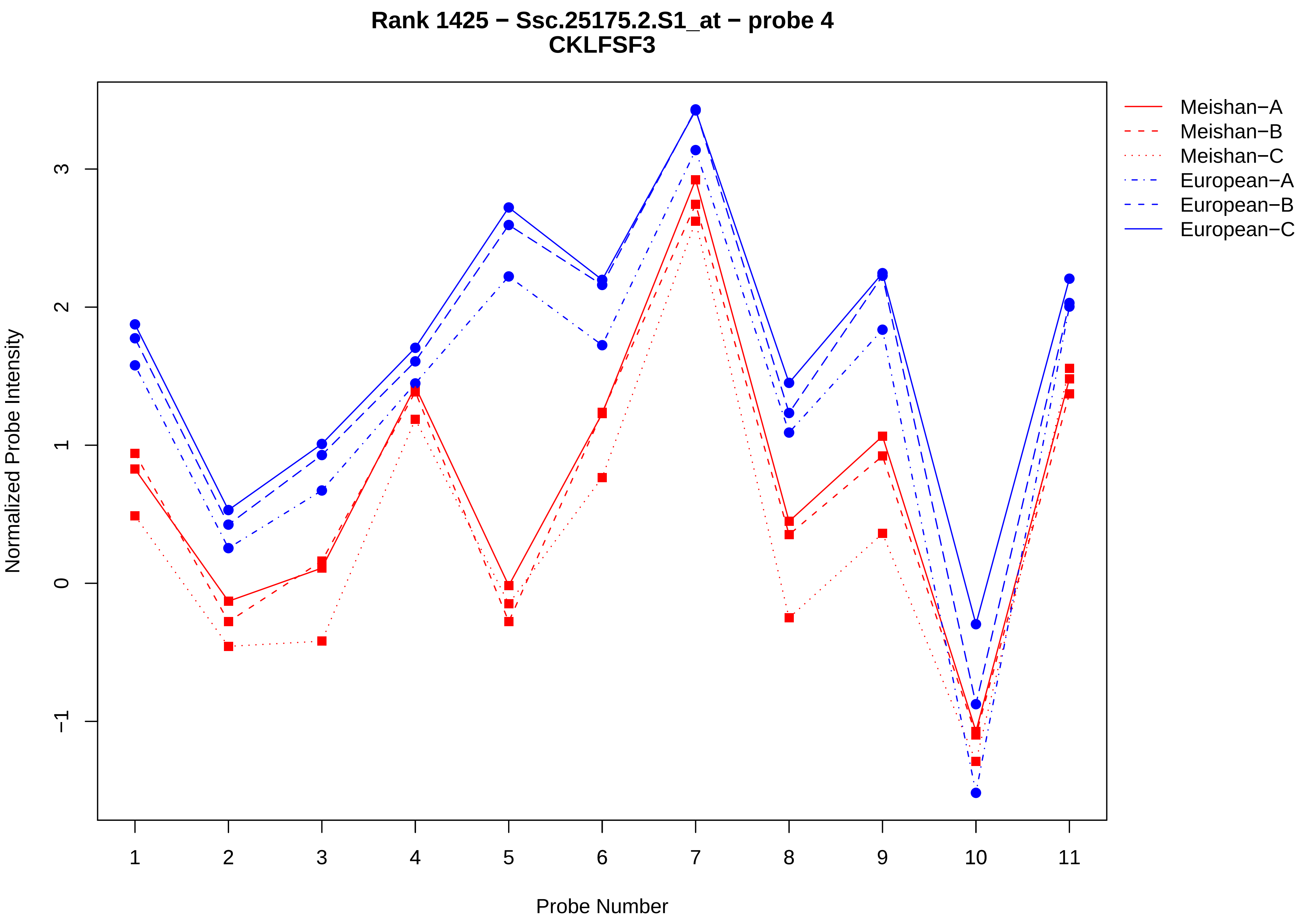
Rank 1425 − Ssc.25175.2.S1_at − probe 4
CKLFSF3
Normalized Probe Intensity
Probe Number
−1
0
1
2
3
1
2
3
4
5
6
7
8
9
10
11
Meishan−A
Meishan−B
Meishan−C
European−A
European−B
European−C

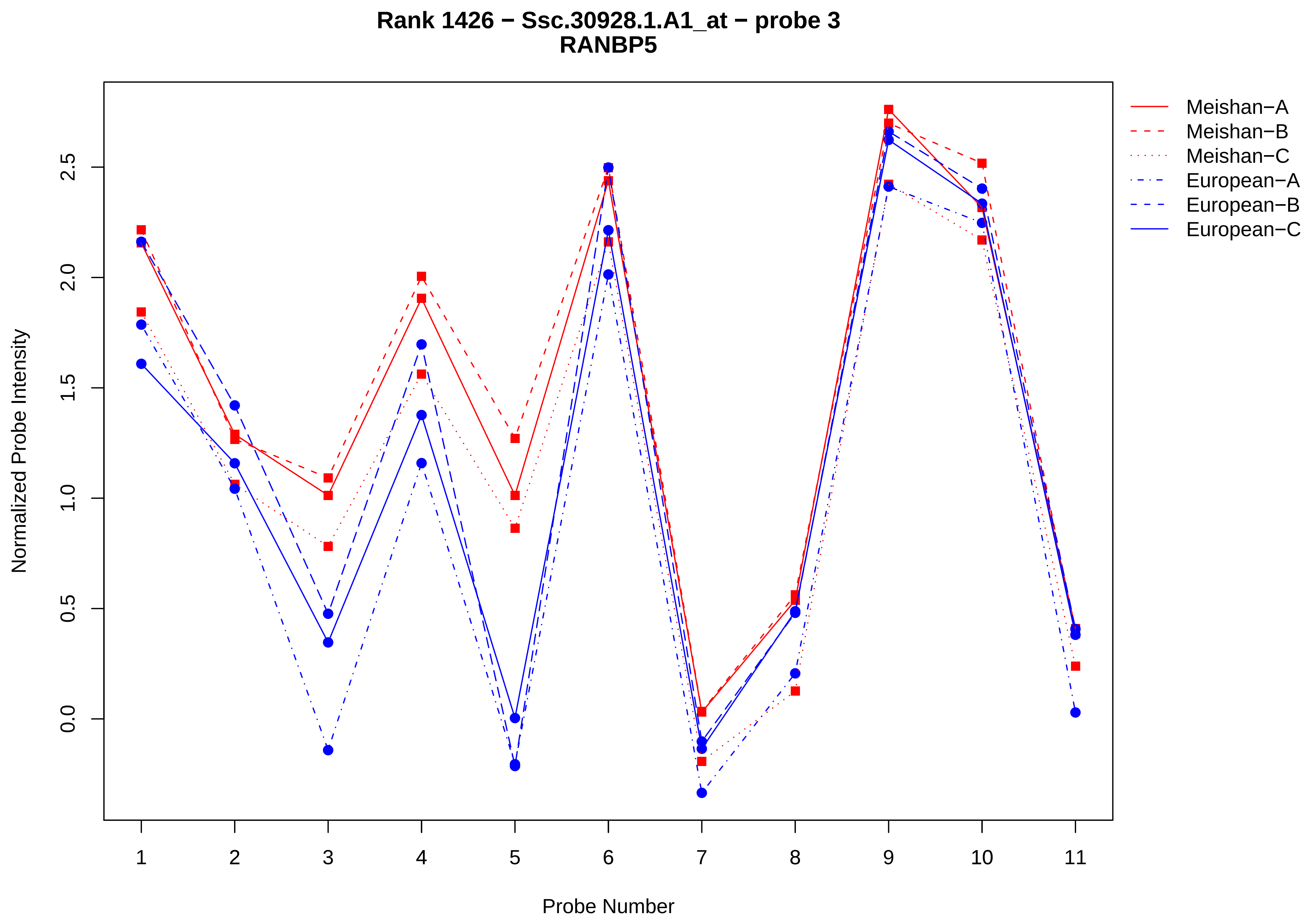

Rank 1426 − Ssc.30928.1.A1_at − probe 3
RANBP5
Normalized Probe Intensity
Probe Number
0.0
0.5
1.0
1.5
2.0
2.5
1
2
3
4
5
6
7
8
9
10
11
Meishan−A
Meishan−B
Meishan−C
European−A
European−B
European−C

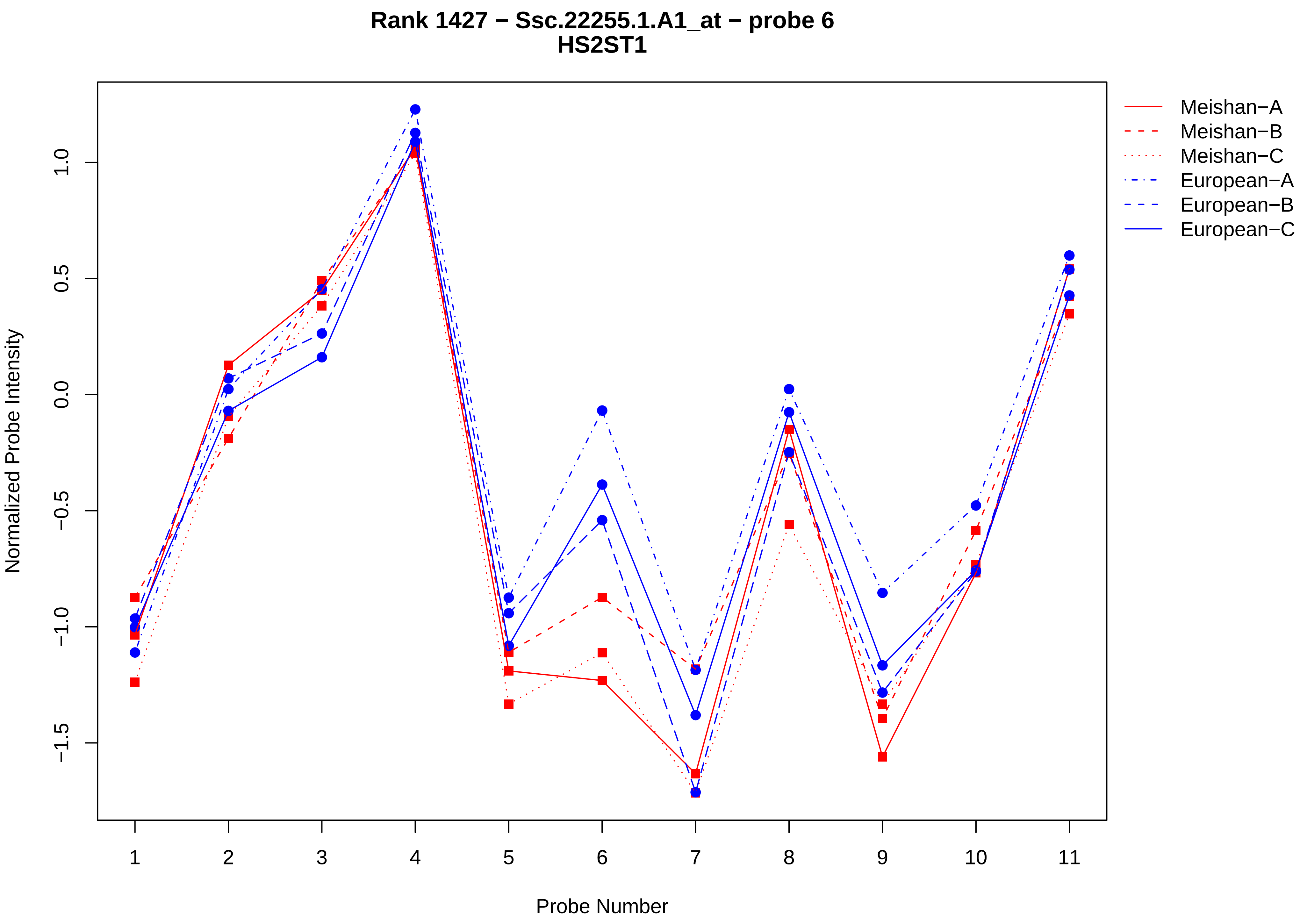

Rank 1427 − Ssc.22255.1.A1_at − probe 6
HS2ST1
Normalized Probe Intensity
Probe Number
1.0
0.5
0.0
−0.5
−1.0
−1.5
1
2
3
4
5
6
7
8
9
10
11
Meishan−A
Meishan−B
Meishan−C
European−A
European−B
European−C

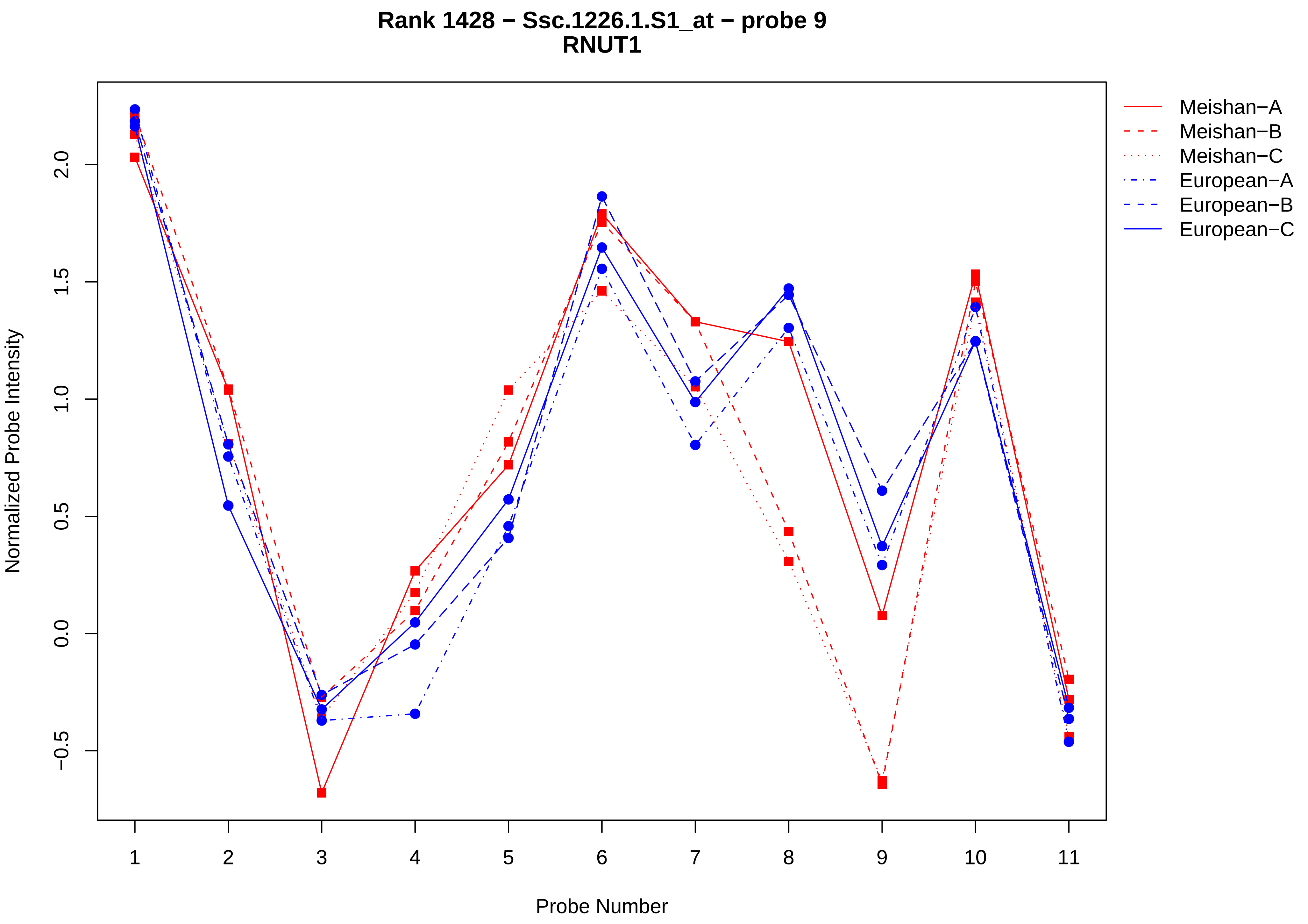
Rank 1428 − Ssc.1226.1.S1_at − probe 9
RNUT1
Normalized Probe Intensity
Probe Number
Meishan−A
Meishan−B
Meishan−C
European−A
European−B
European−C
−0.5
0.0
0.5
1.0
1.5
2.0
1
2
3
4
5
6
7
8
9
10
11

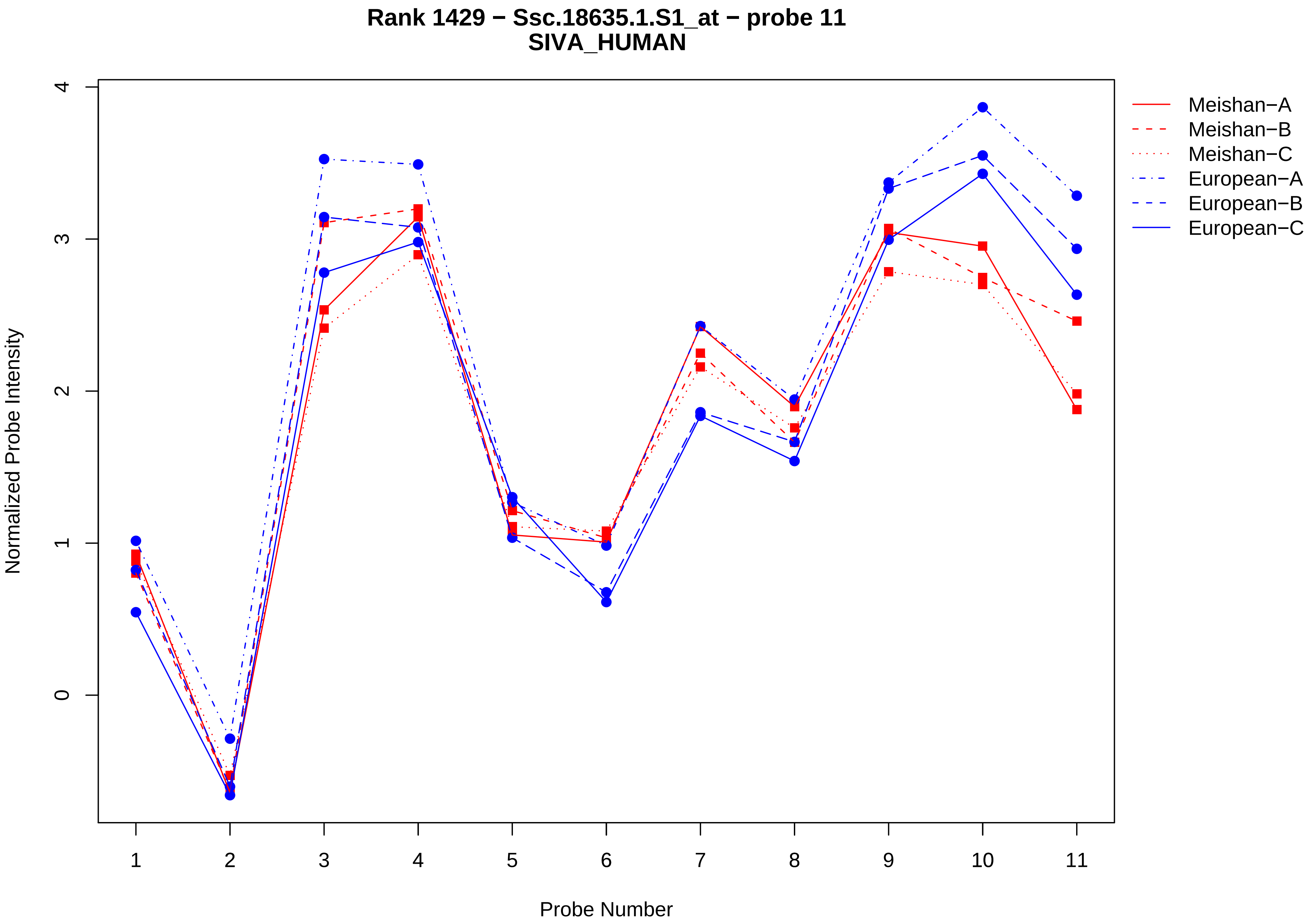

Rank 1429 − Ssc.18635.1.S1_at − probe 11
SIVA_HUMAN
Normalized Probe Intensity
Probe Number
Meishan−A
Meishan−B
Meishan−C
European−A
European−B
European−C
0
1
2
3
4
1
2
3
4
5
6
7
8
9
10
11

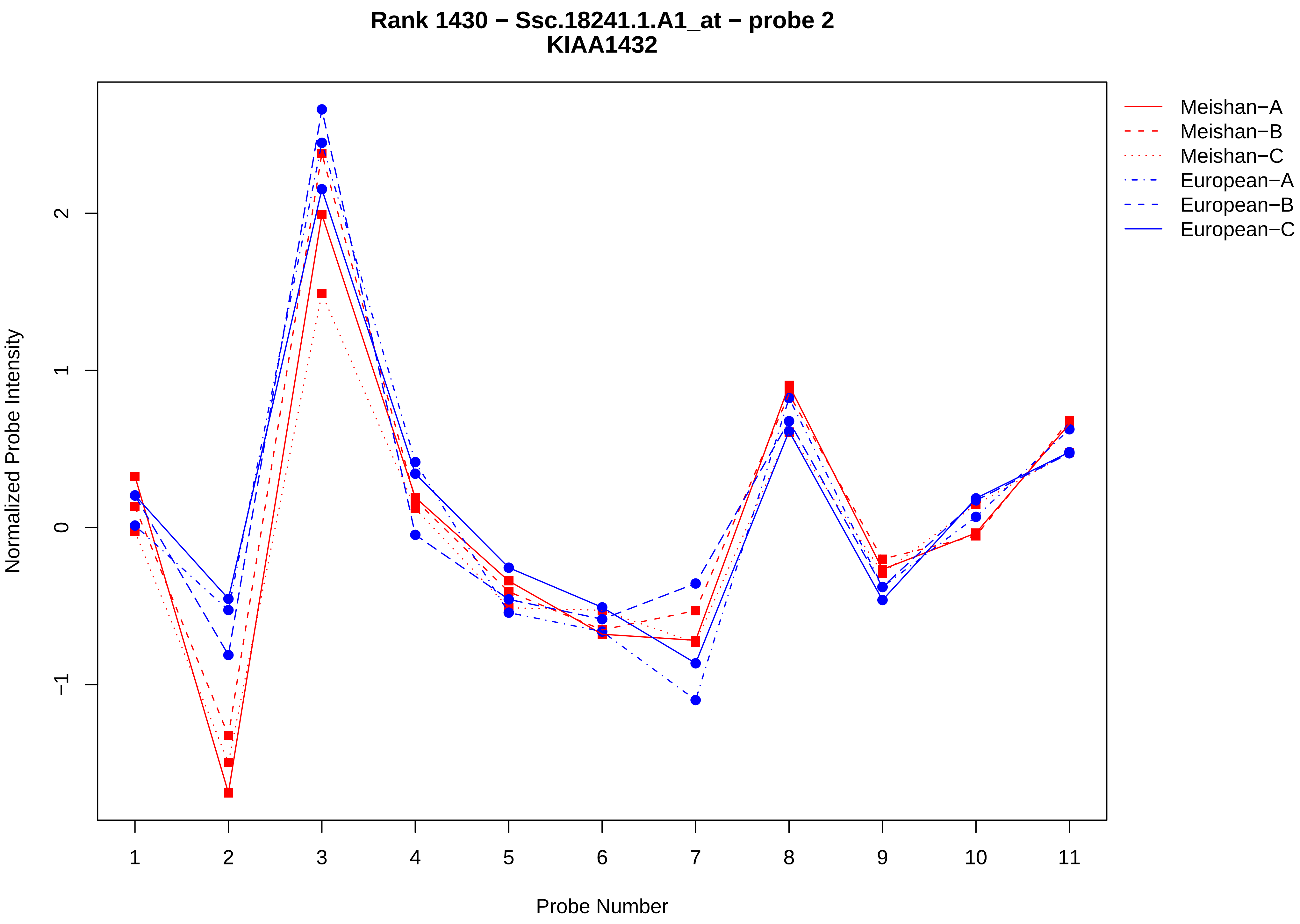

Rank 1430 − Ssc.18241.1.A1_at − probe 2
KIAA1432
Normalized Probe Intensity
Probe Number
−1
0
1
2
1
2
3
4
5
6
7
8
9
10
11
Meishan−A
Meishan−B
Meishan−C
European−A
European−B
European−C

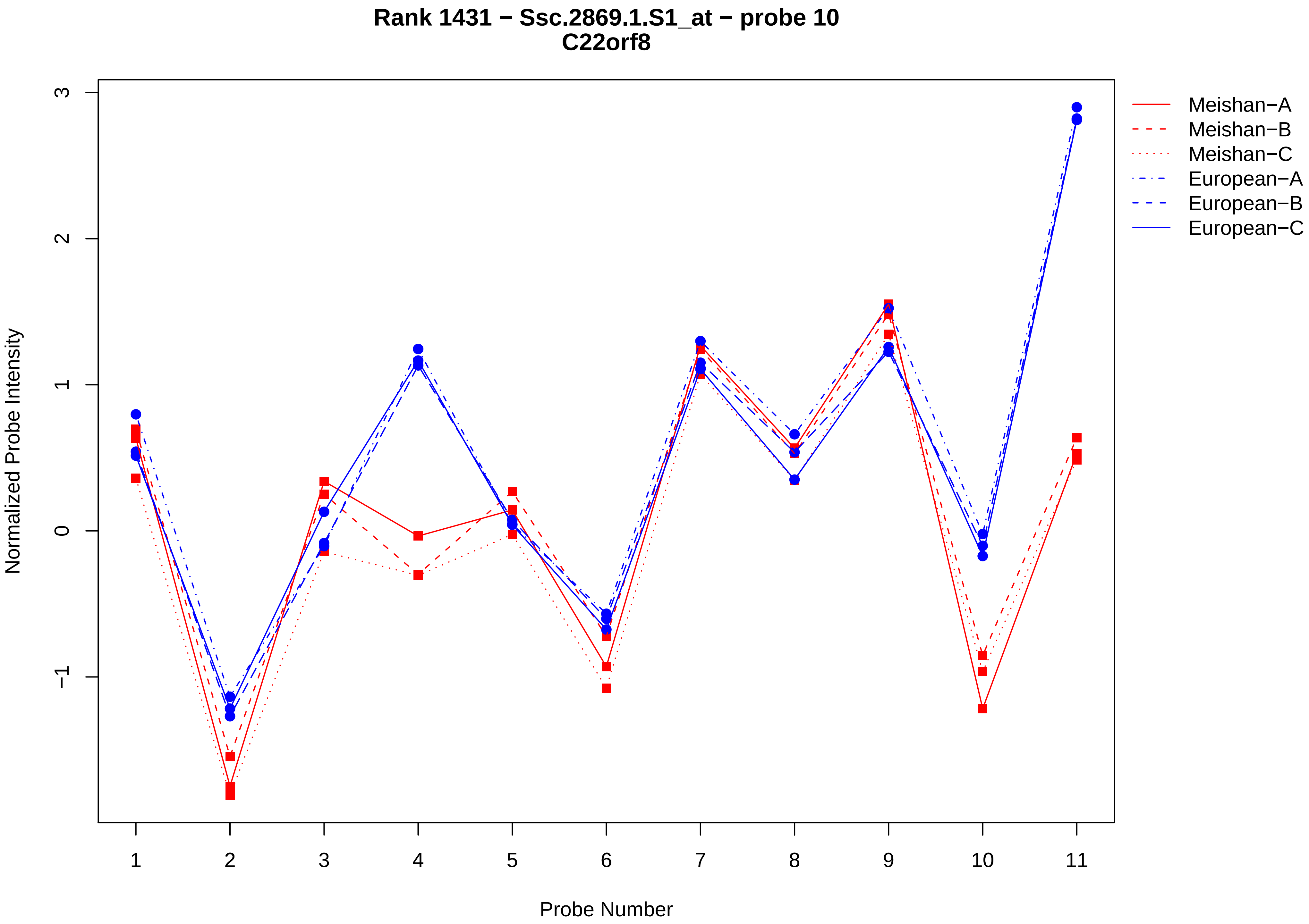

Rank 1431 − Ssc.2869.1.S1_at − probe 10
C22orf8
Normalized Probe Intensity
Probe Number
−1
0
1
2
3
1
2
3
4
5
6
7
8
9
10
11
Meishan−A
Meishan−B
Meishan−C
European−A
European−B
European−C

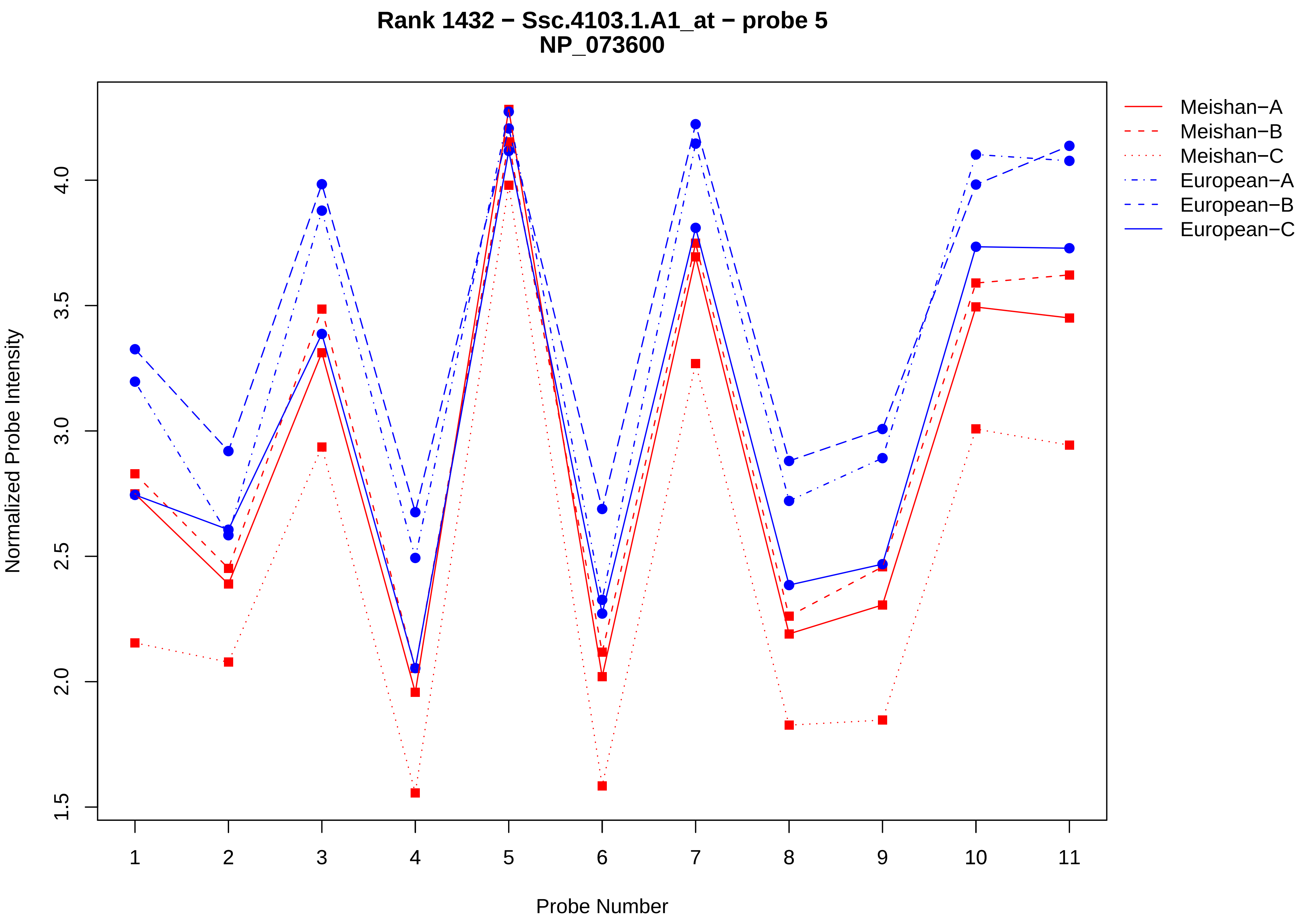

Rank 1432 − Ssc.4103.1.A1_at − probe 5
NP_073600
Normalized Probe Intensity
Probe Number
1.5
2.0
2.5
3.0
3.5
4.0
1
2
3
4
5
6
7
8
9
10
11
Meishan−A
Meishan−B
Meishan−C
European−A
European−B
European−C

Rank 1433 − Ssc.21290.2.A1_at − probe 6
Q6PI50

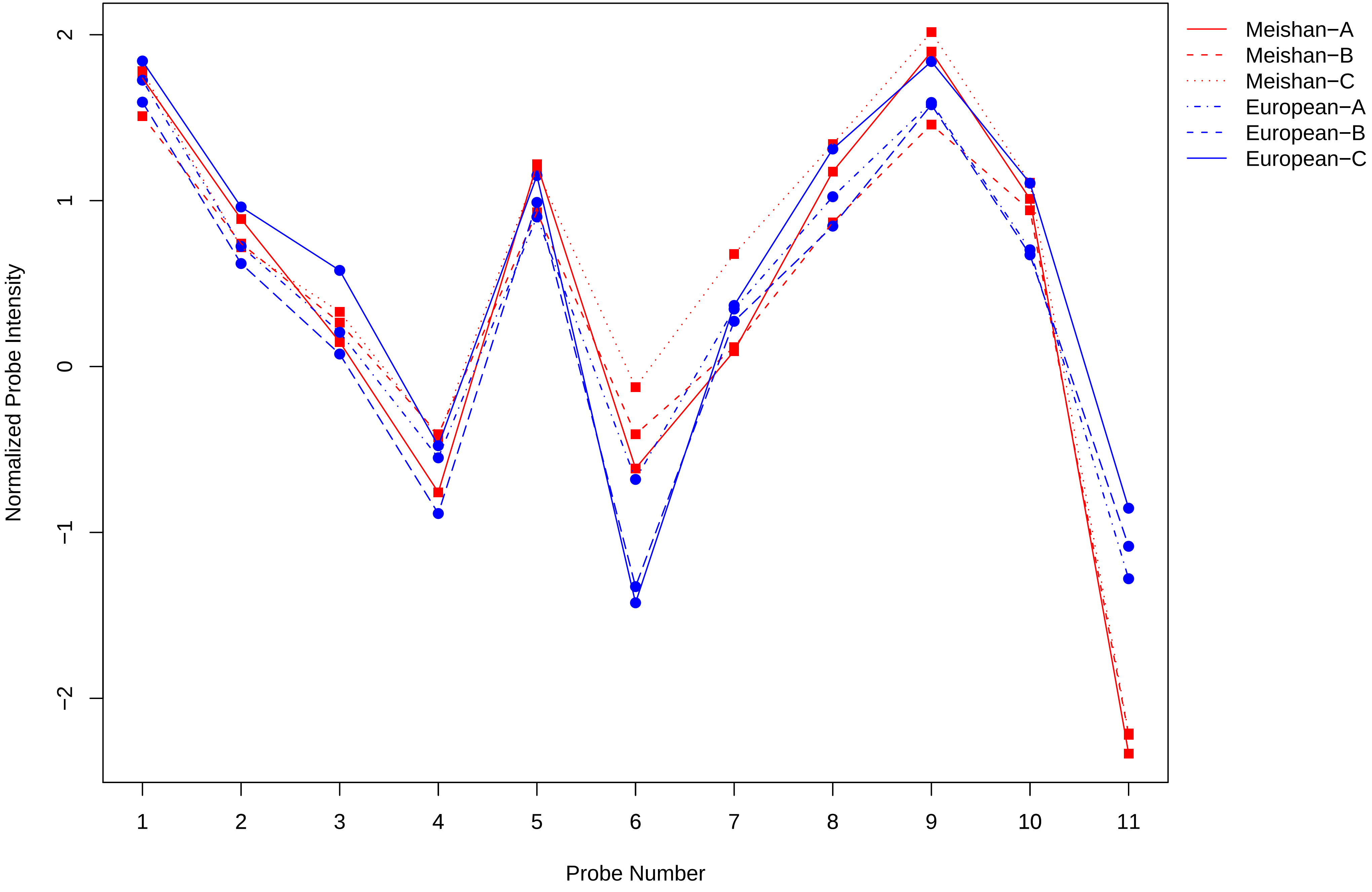

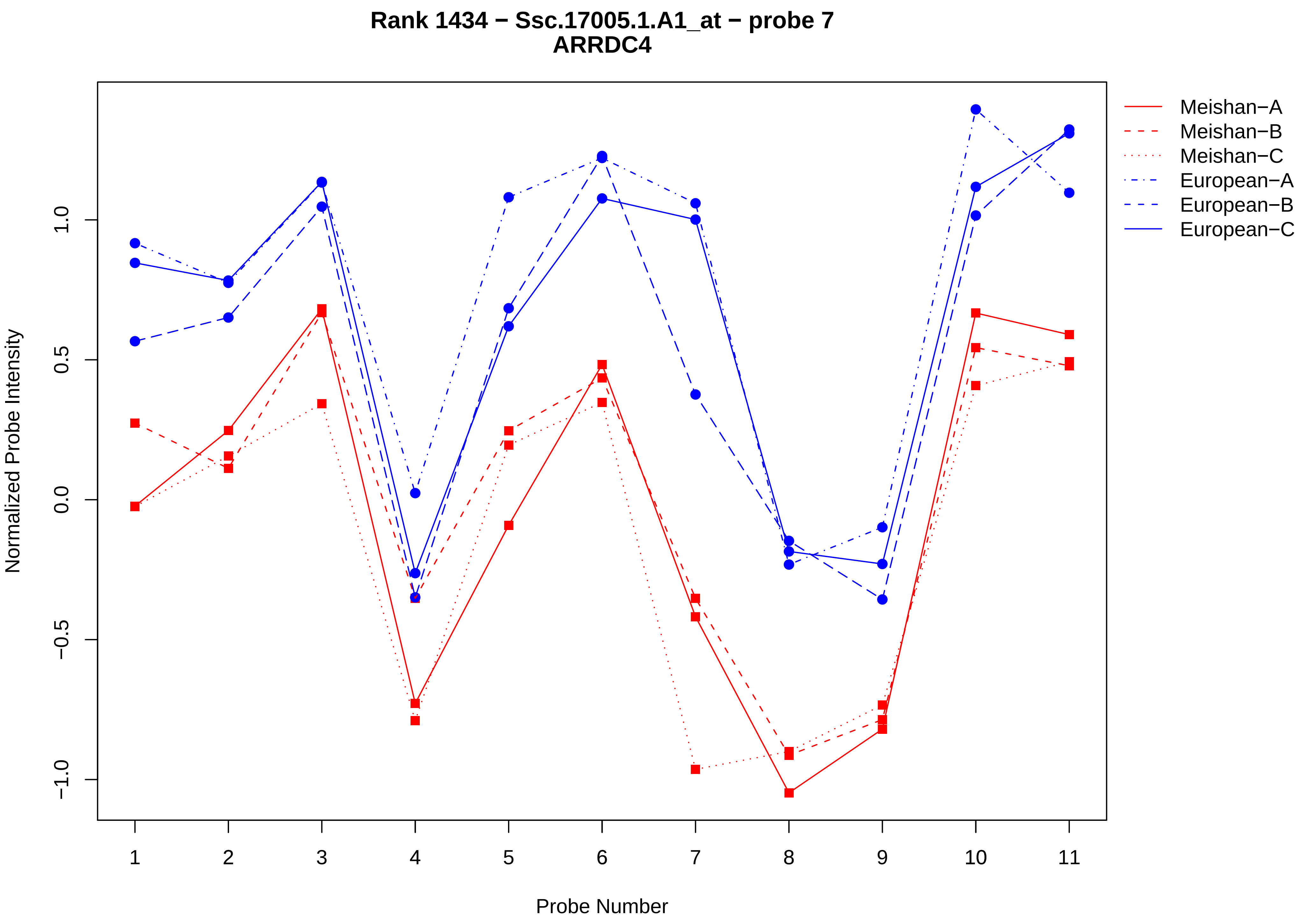

Rank 1434 − Ssc.17005.1.A1_at − probe 7
ARRDC4
Normalized Probe Intensity
Probe Number
−1.0
−0.5
0.0
0.5
1.0
1
2
3
4
5
6
7
8
9
10
11
Meishan−A
Meishan−B
Meishan−C
European−A
European−B
European−C

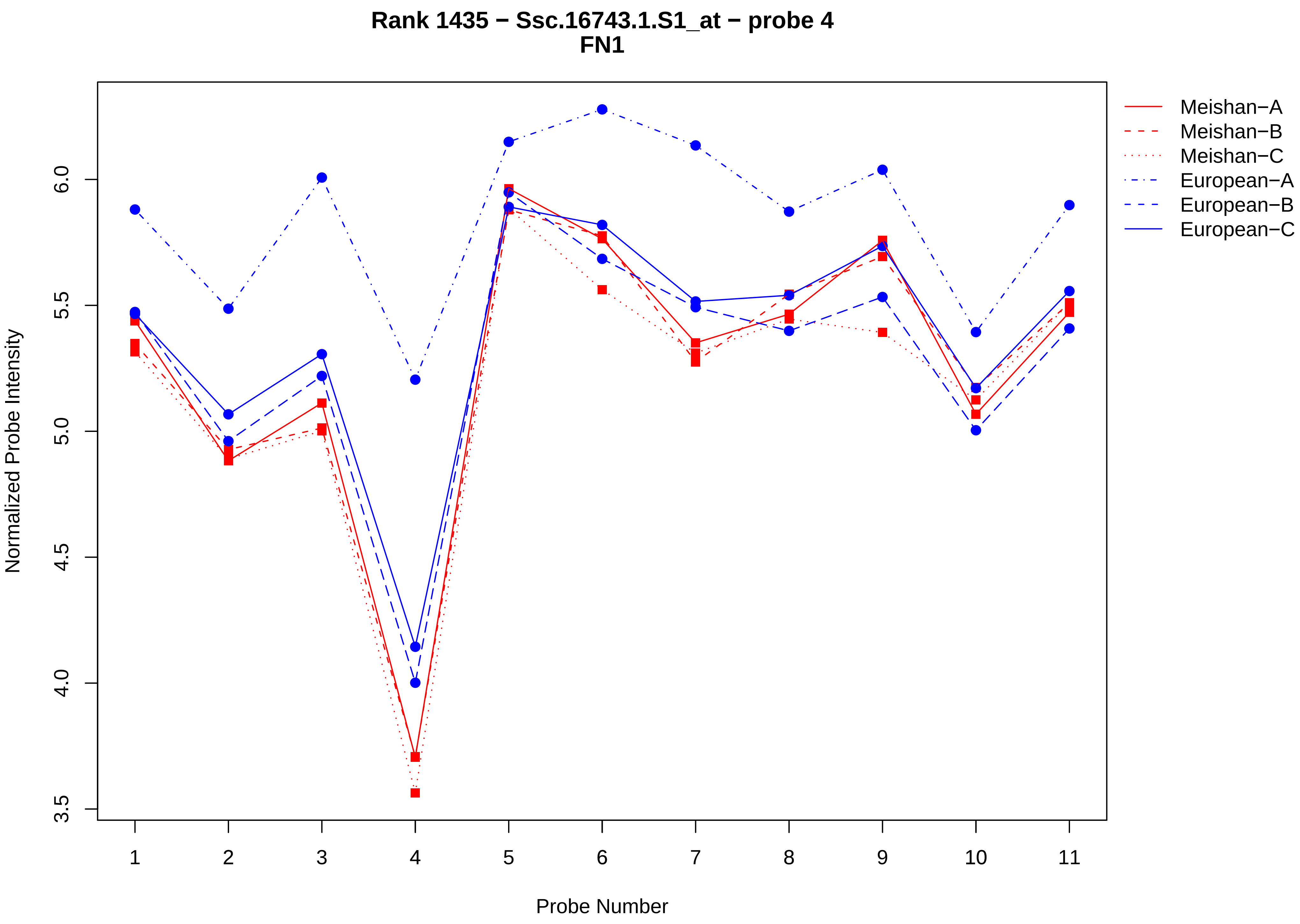

Rank 1435 − Ssc.16743.1.S1_at − probe 4
FN1
Normalized Probe Intensity
Probe Number
3.5
4.0
4.5
5.0
5.5
6.0
1
2
3
4
5
6
7
8
9
10
11
Meishan−A
Meishan−B
Meishan−C
European−A
European−B
European−C

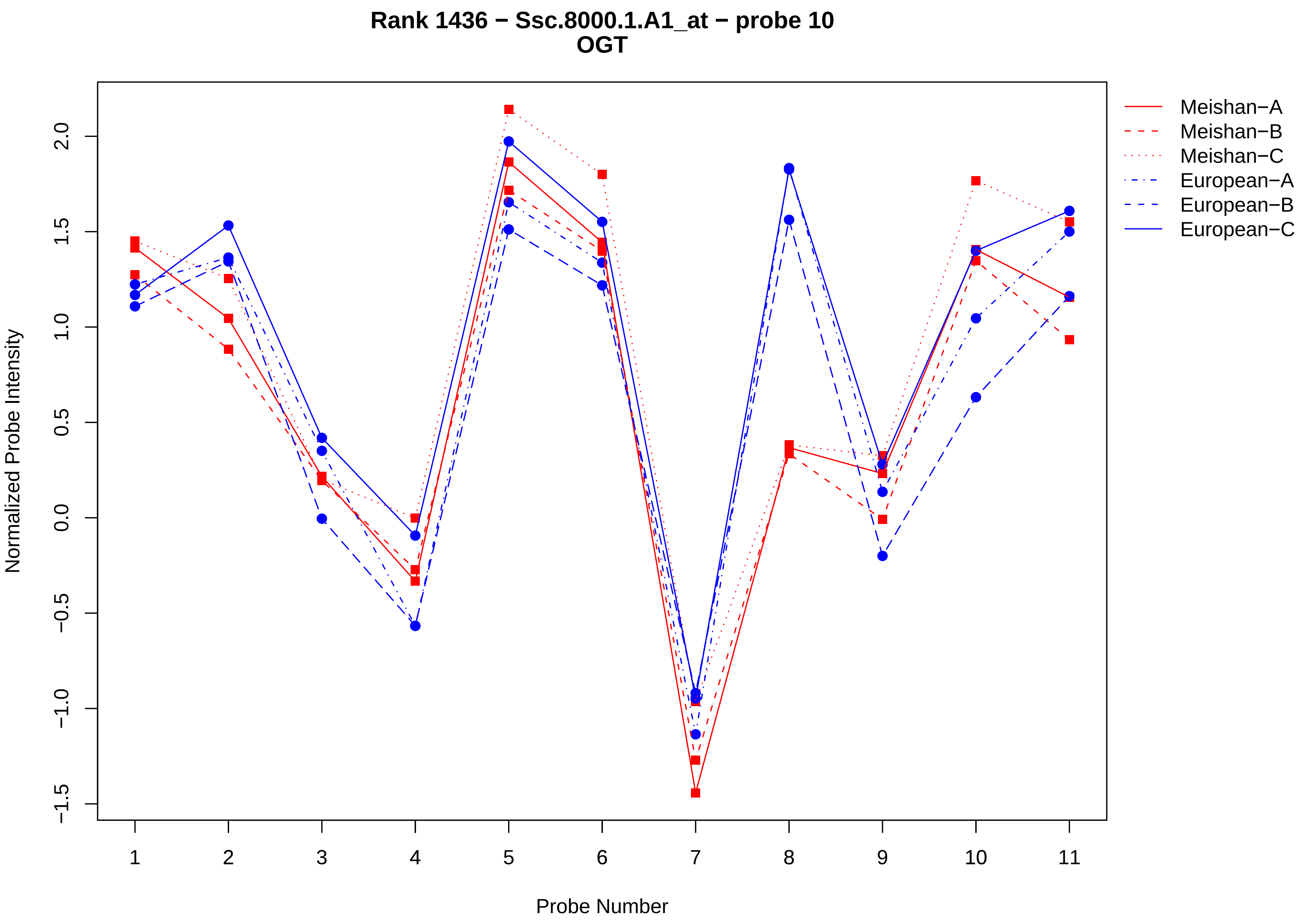

Rank 1436 − Ssc.8000.1.A1_at − probe 10
OGT
Normalized Probe Intensity
Probe Number
−1.5
−1.0
−0.5
0.0
0.5
1.0
1.5
2.0
1
2
3
4
5
6
7
8
9
10
11
Meishan−A
Meishan−B
Meishan−C
European−A
European−B
European−C

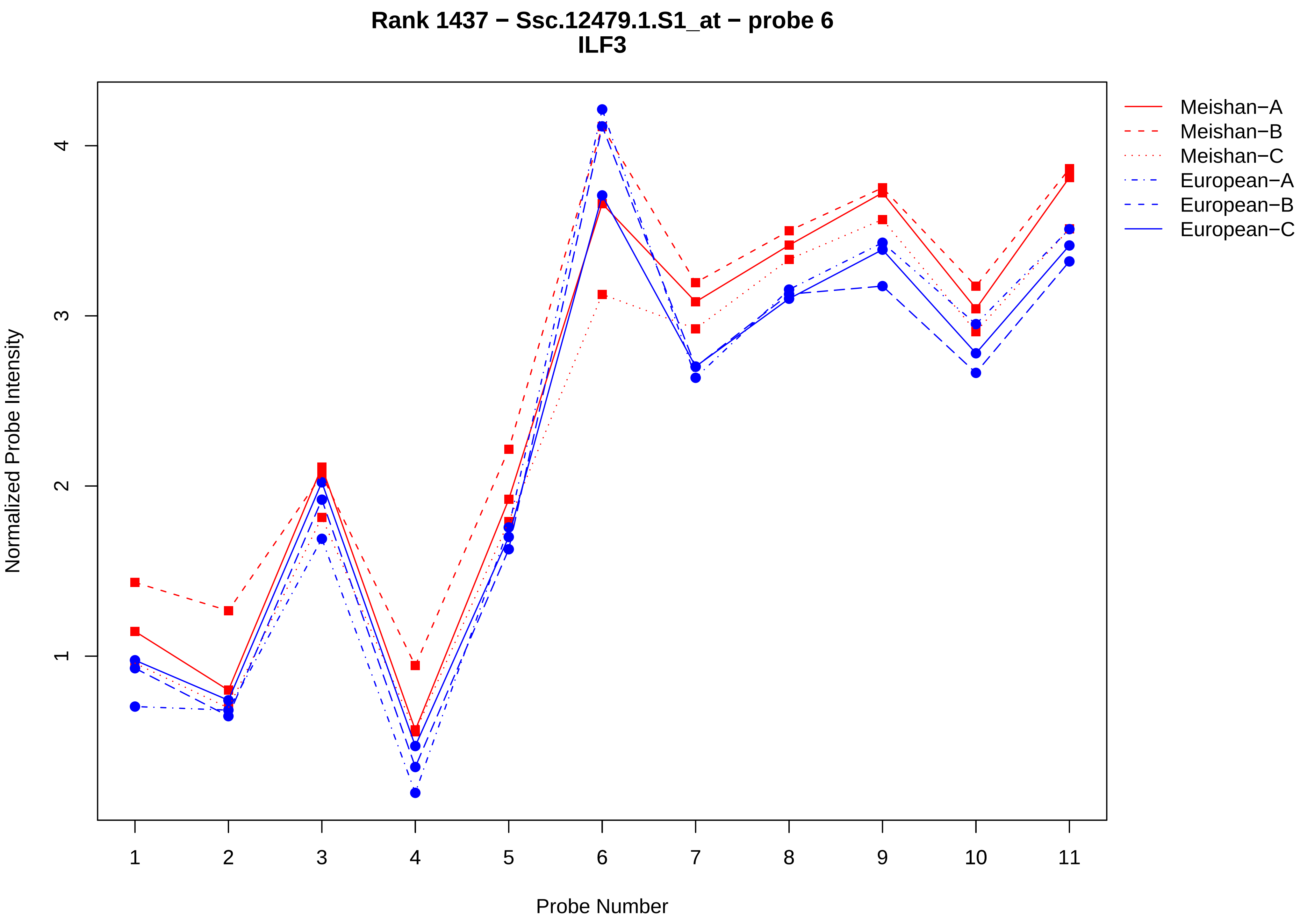

Rank 1437 − Ssc.12479.1.S1_at − probe 6
ILF3
Normalized Probe Intensity
Probe Number
1
2
3
4
5
6
7
8
9
10
11
Meishan−A
Meishan−B
Meishan−C
European−A
European−B
European−C

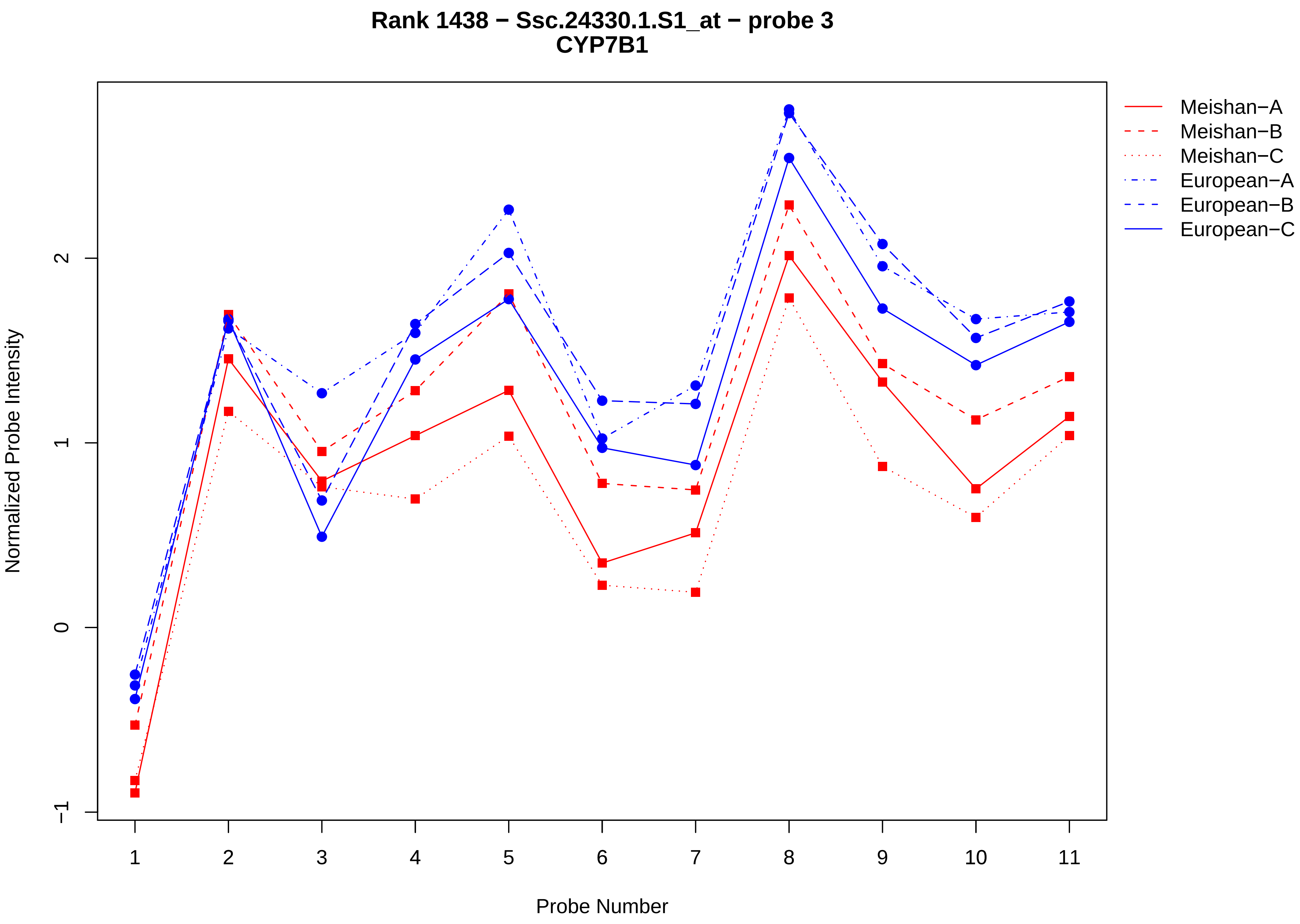

Rank 1438 − Ssc.24330.1.S1_at − probe 3
CYP7B1
Normalized Probe Intensity
Probe Number
−1
0
1
2
1
2
3
4
5
6
7
8
9
10
11
Meishan−A
Meishan−B
Meishan−C
European−A
European−B
European−C

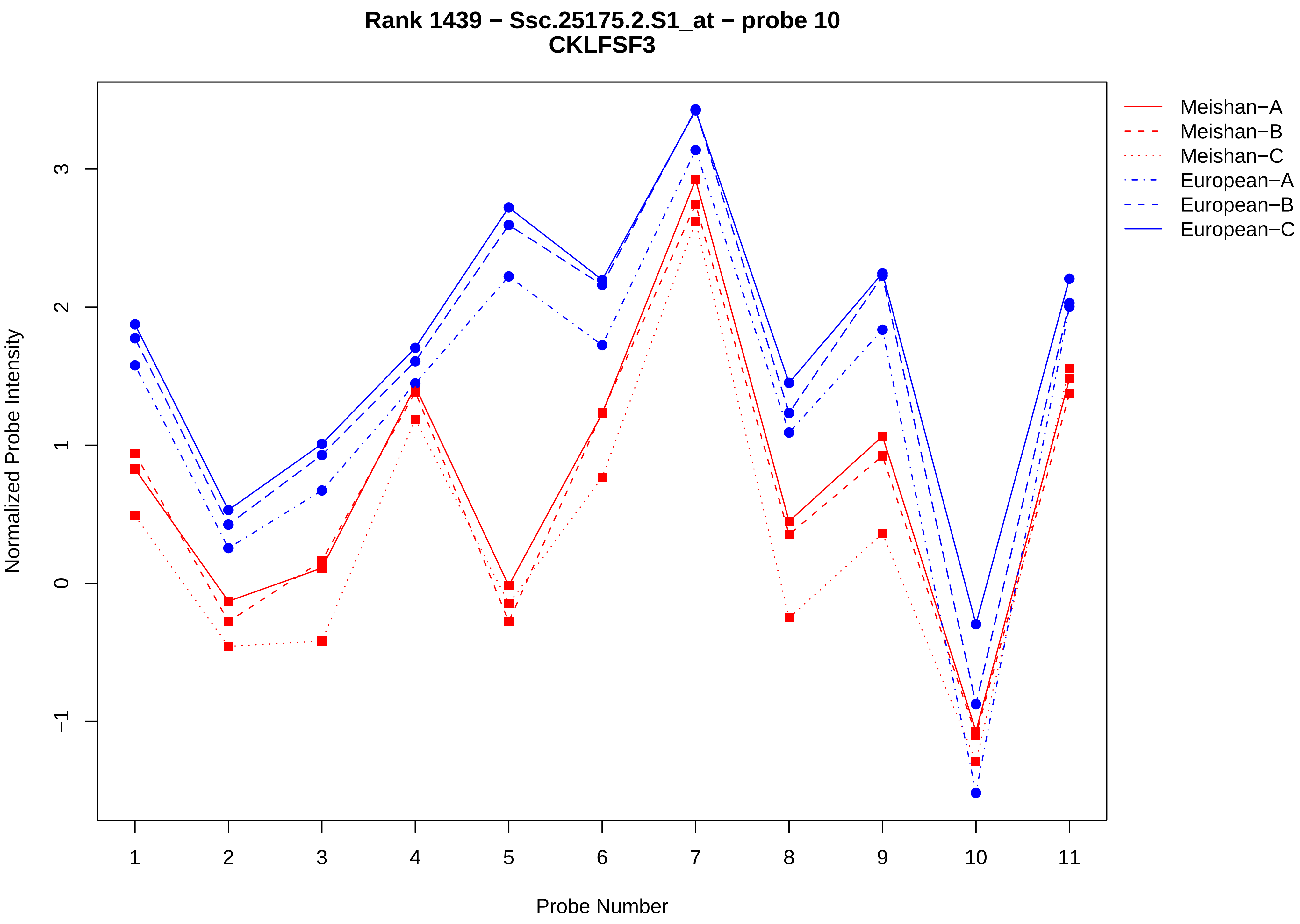
Rank 1439 − Ssc.25175.2.S1_at − probe 10
CKLFSF3
Normalized Probe Intensity
Probe Number
−1
0
1
2
3
1
2
3
4
5
6
7
8
9
10
11
Meishan−A
Meishan−B
Meishan−C
European−A
European−B
European−C

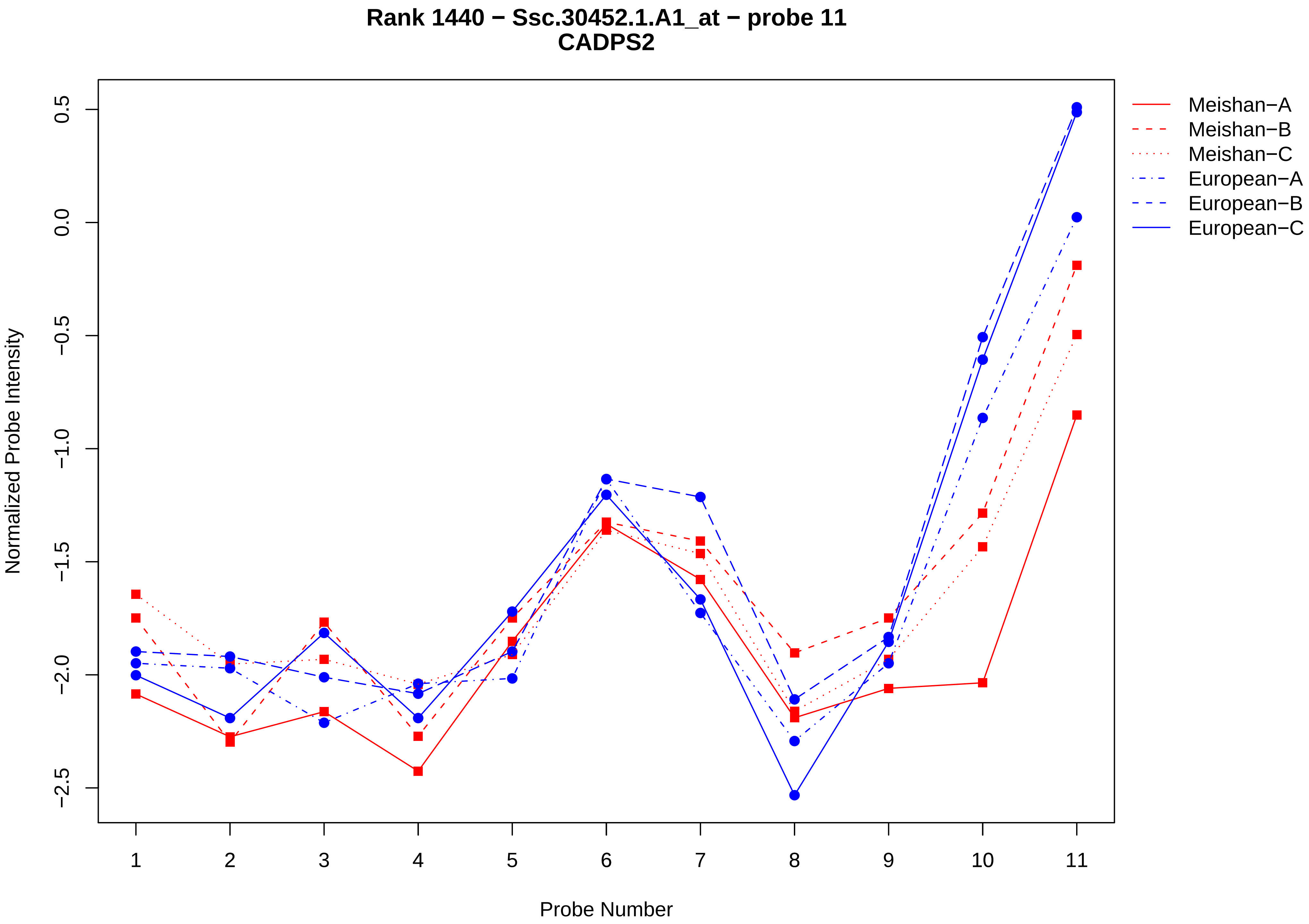

Rank 1440 − Ssc.30452.1.A1_at − probe 11
CADPS2
Normalized Probe Intensity
Probe Number
0.5
0.0
−0.5
−1.0
−1.5
−2.0
−2.5
1
2
3
4
5
6
7
8
9
10
11
Meishan−A
Meishan−B
Meishan−C
European−A
European−B
European−C

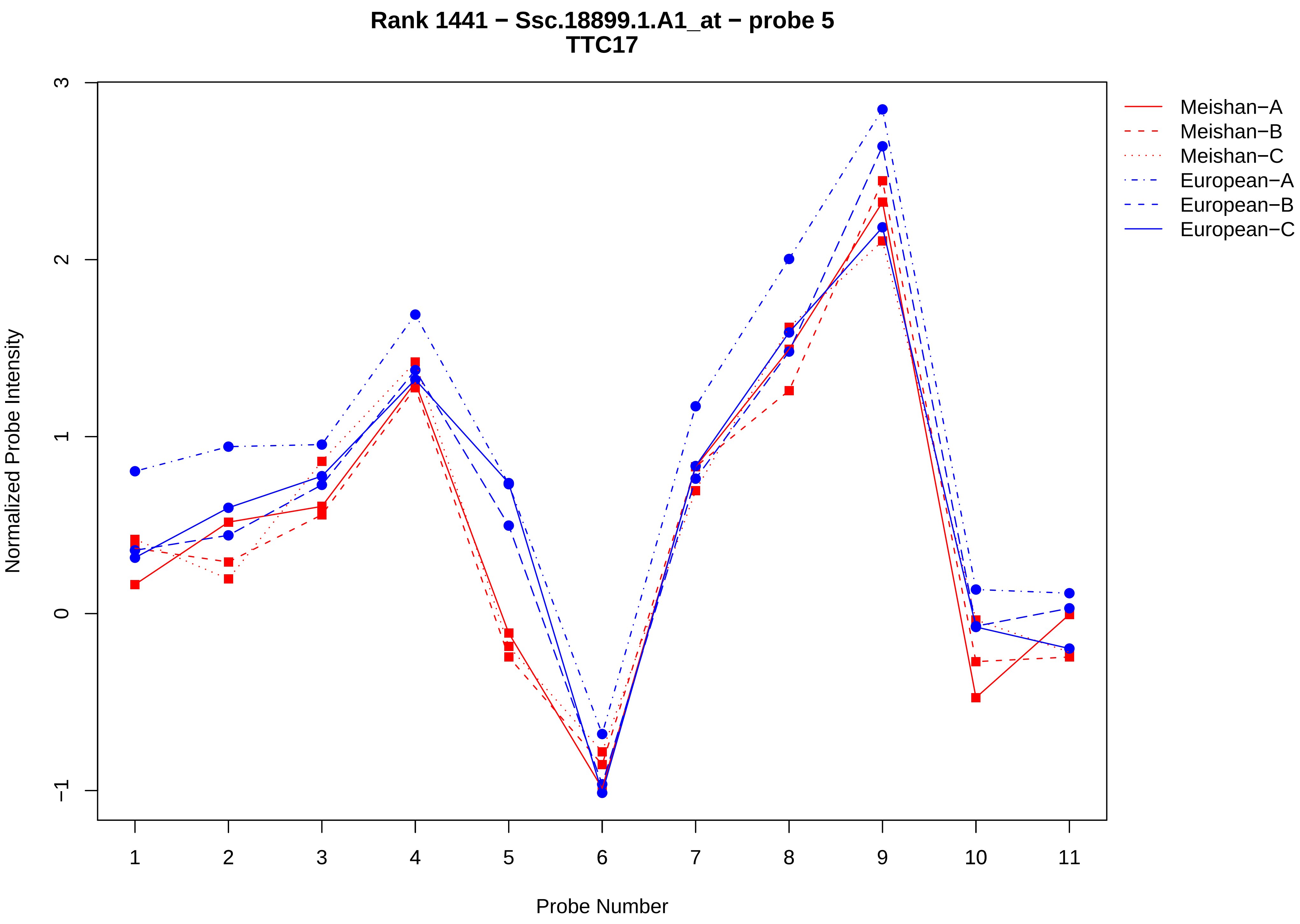

Rank 1441 − Ssc.18899.1.A1_at − probe 5
TTC17
Normalized Probe Intensity
Probe Number
−1
0
1
2
3
1
2
3
4
5
6
7
8
9
10
11
Meishan−A
Meishan−B
Meishan−C
European−A
European−B
European−C

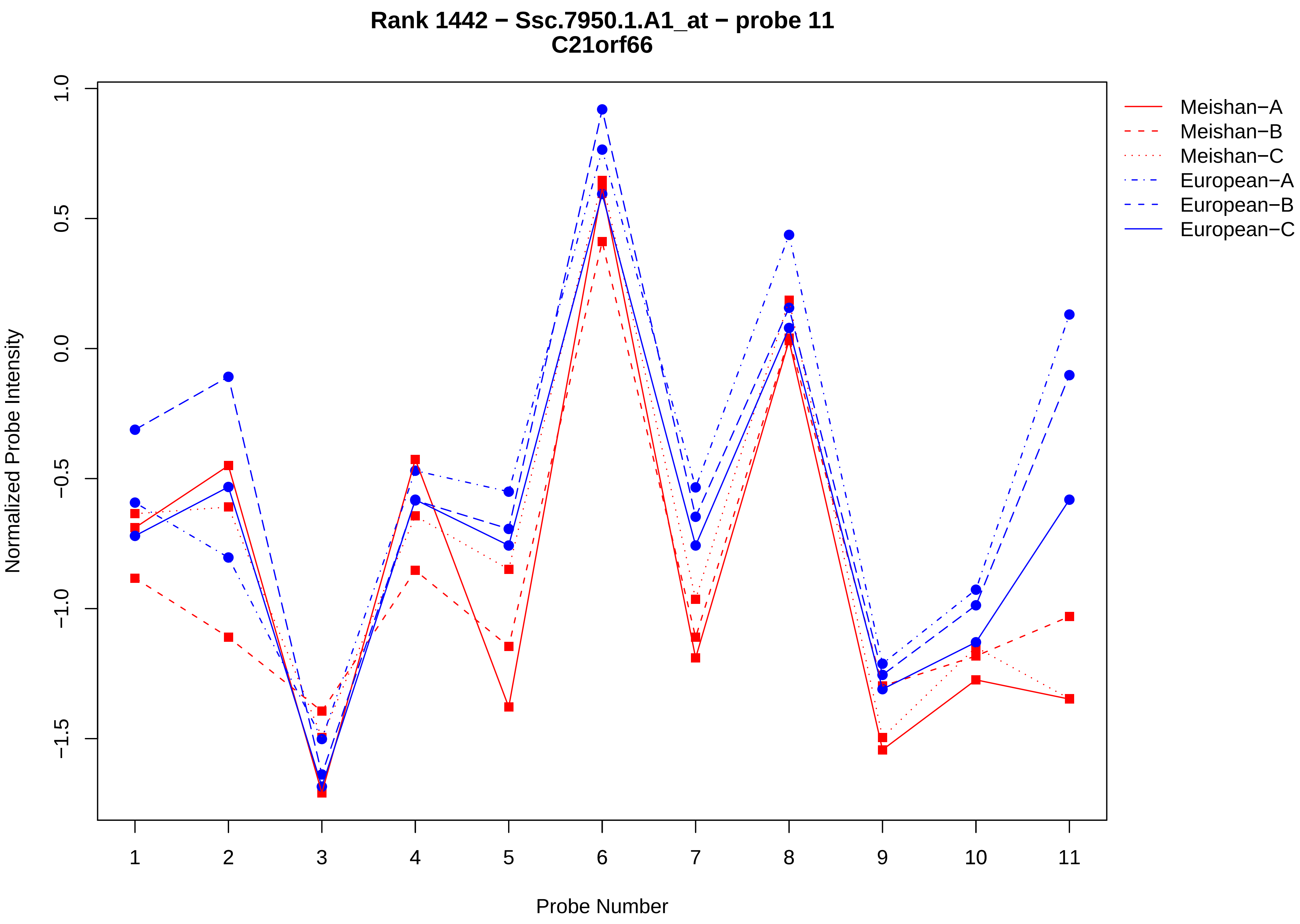

Rank 1442 − Ssc.7950.1.A1_at − probe 11
C21orf66
Normalized Probe Intensity
Probe Number
1.0
0.5
0.0
−0.5
−1.0
−1.5
1
2
3
4
5
6
7
8
9
10
11
Meishan−A
Meishan−B
Meishan−C
European−A
European−B
European−C

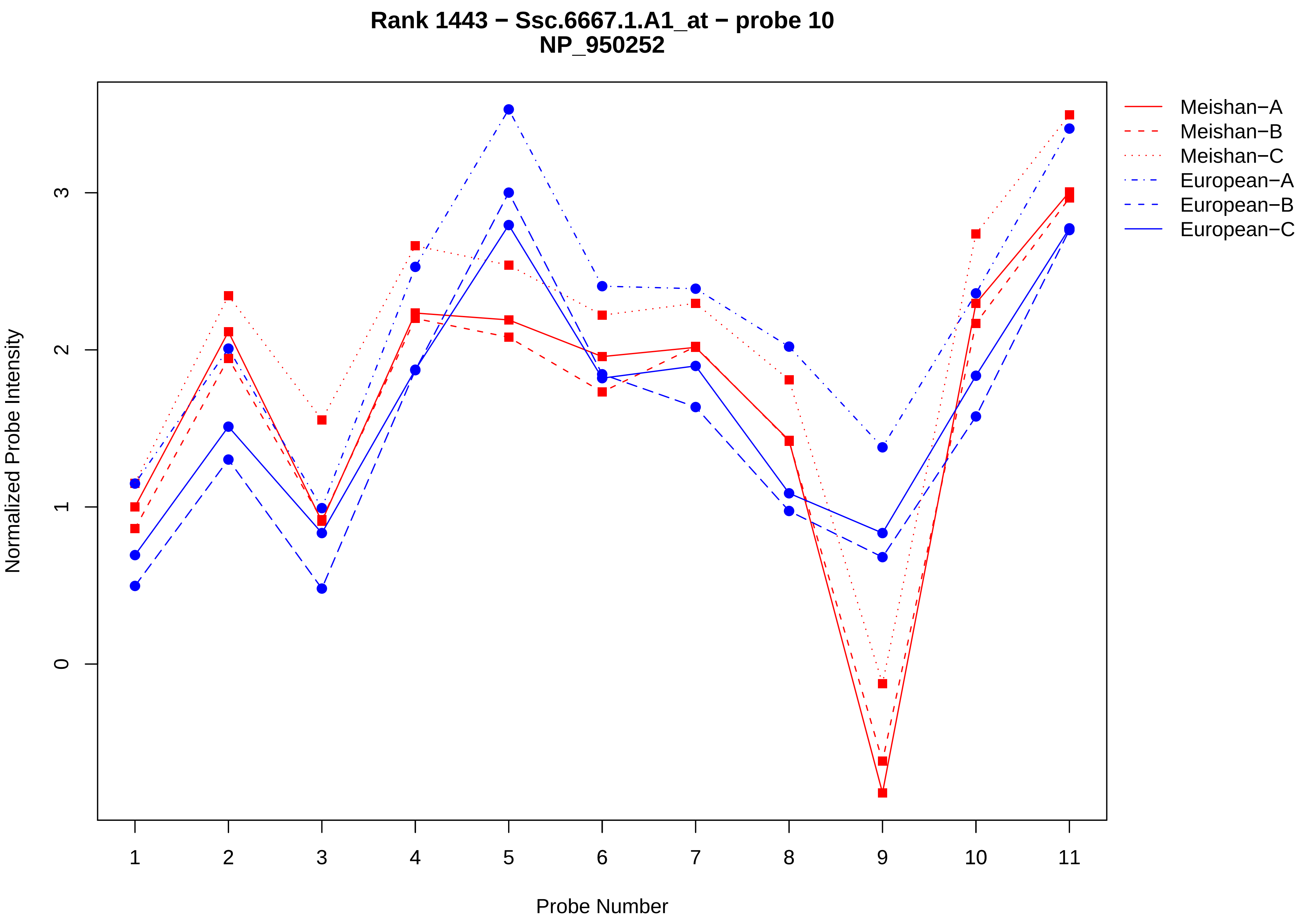
Rank 1443 − Ssc.6667.1.A1_at − probe 10
NP_950252
Normalized Probe Intensity
0
1
2
3
Probe Number
1
2
3
4
5
6
7
8
9
10
11
Meishan−A
Meishan−B
Meishan−C
European−A
European−B
European−C

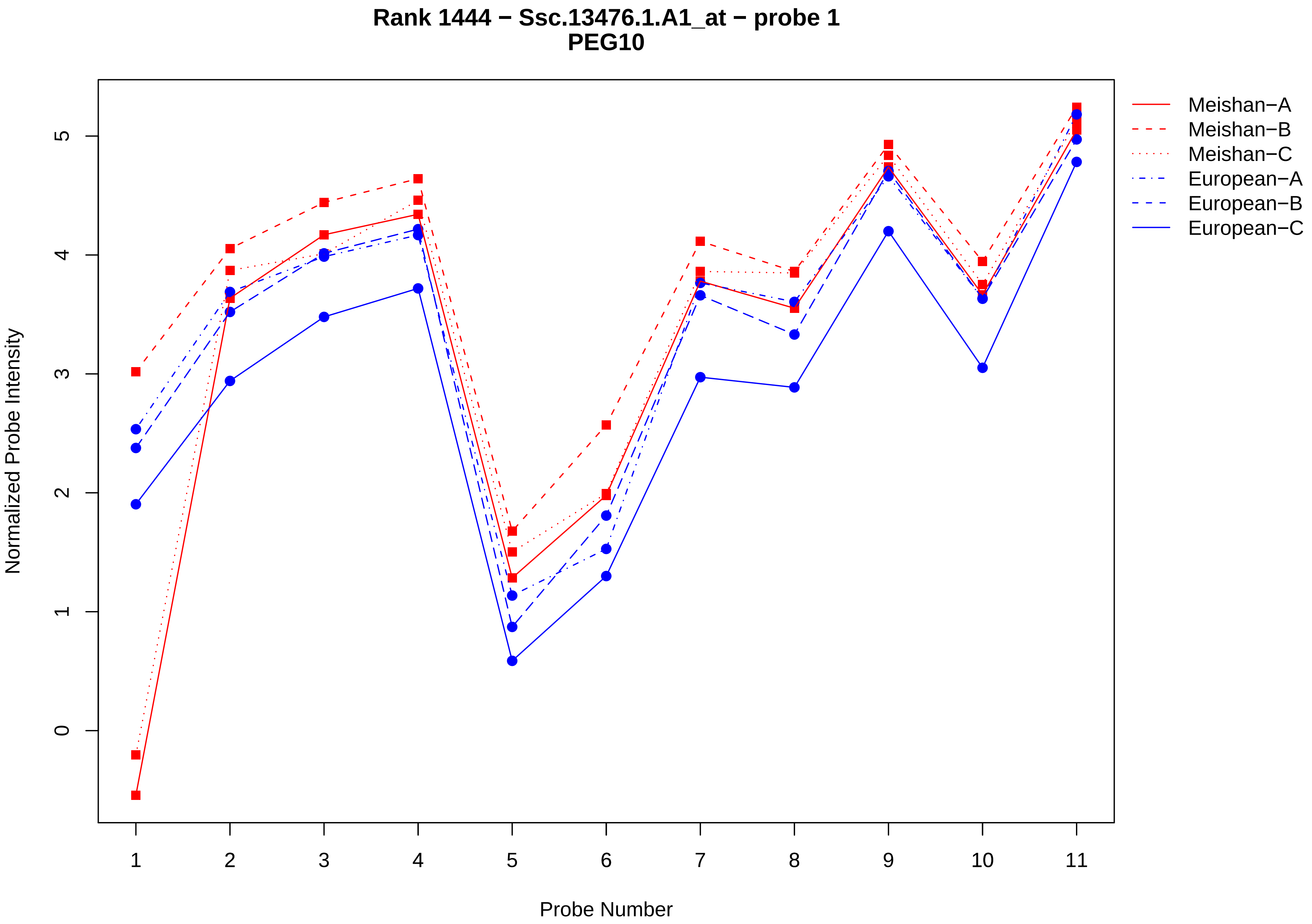
Rank 1444 − Ssc.13476.1.A1_at − probe 1
PEG10
Normalized Probe Intensity
Probe Number
0
1
2
3
4
5
1
2
3
4
5
6
7
8
9
10
11
Meishan−A
Meishan−B
Meishan−C
European−A
European−B
European−C

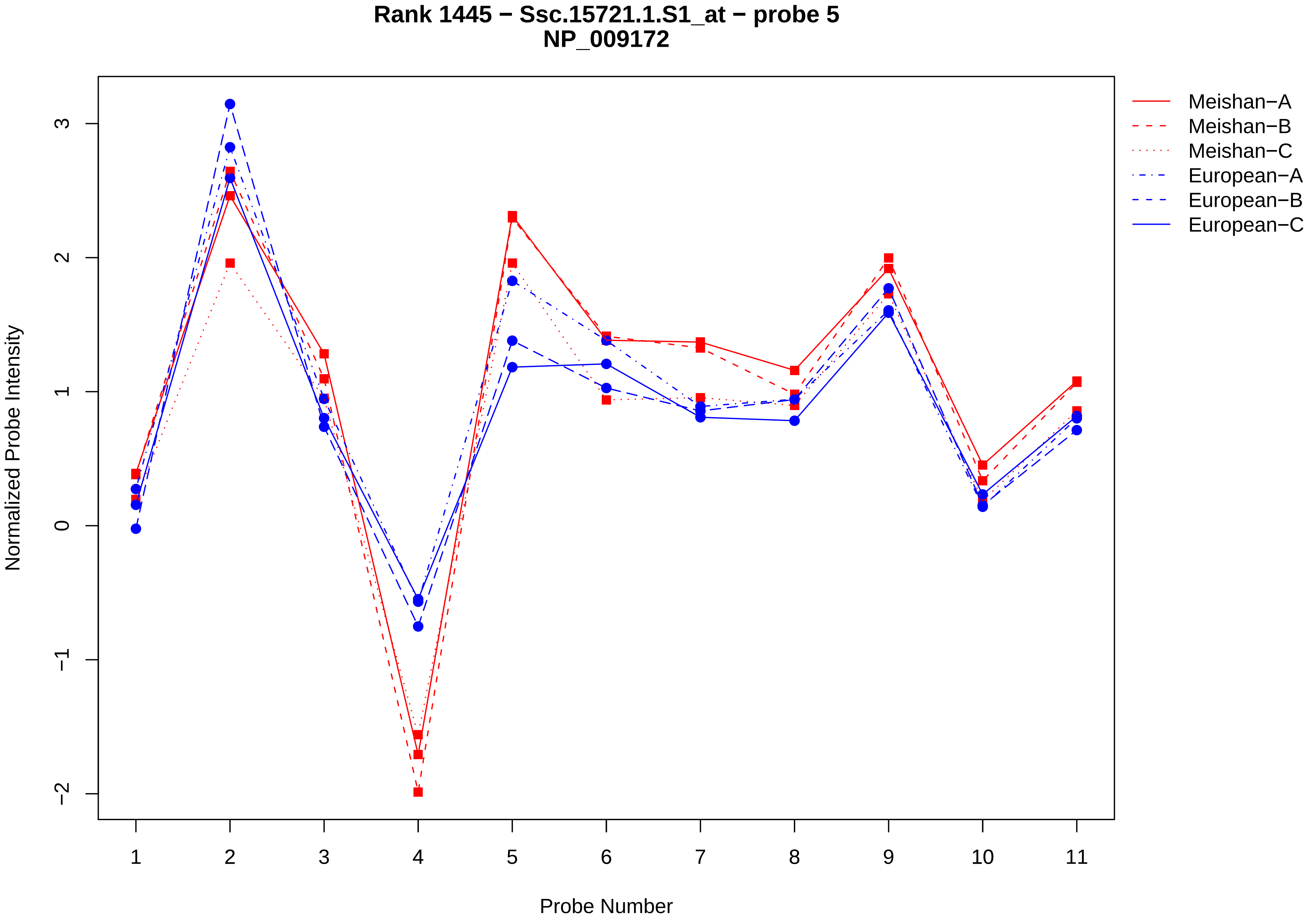

Rank 1445 − Ssc.15721.1.S1_at − probe 5
NP_009172
Normalized Probe Intensity
Probe Number
−2
−1
0
1
2
3
1
2
3
4
5
6
7
8
9
10
11
Meishan−A
Meishan−B
Meishan−C
European−A
European−B
European−C

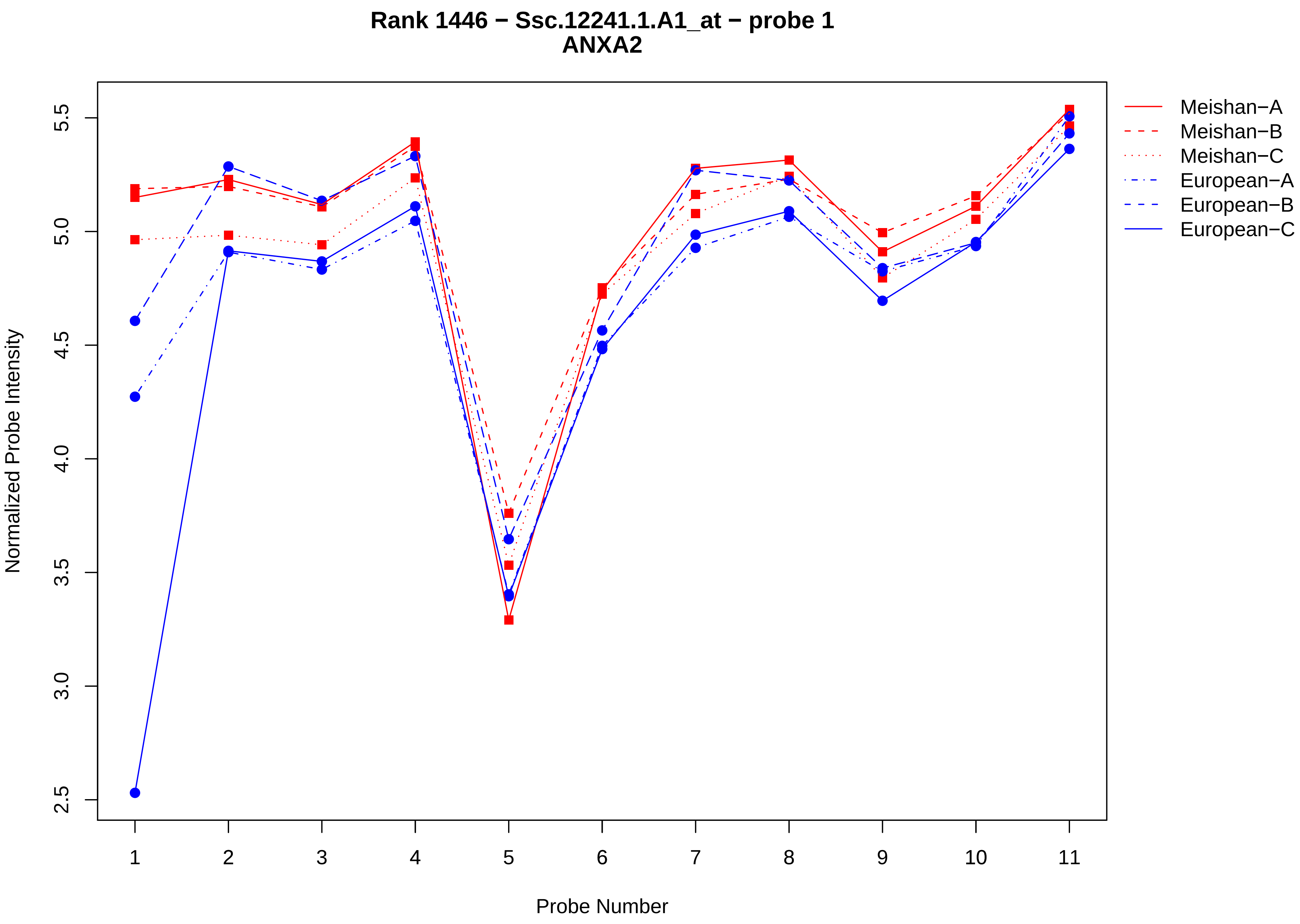

Rank 1446 − Ssc.12241.1.A1_at − probe 1
ANXA2
Normalized Probe Intensity
Probe Number
Meishan−A
Meishan−B
Meishan−C
European−A
European−B
European−C
2.5
3.0
3.5
4.0
4.5
5.0
5.5
1
2
3
4
5
6
7
8
9
10
11

Rank 1447 − Ssc.3408.2.S1_at − probe 4
SSBP3

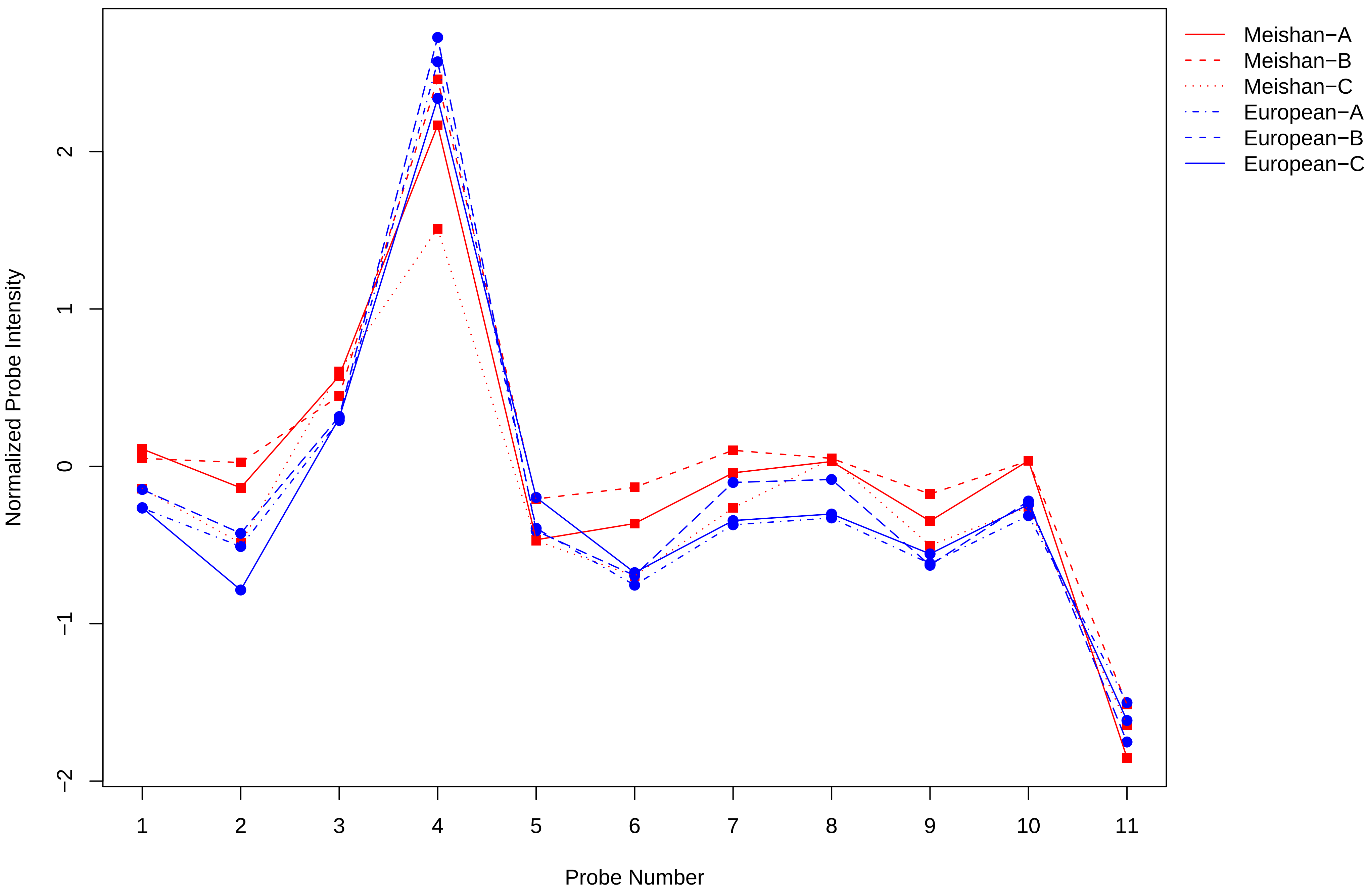

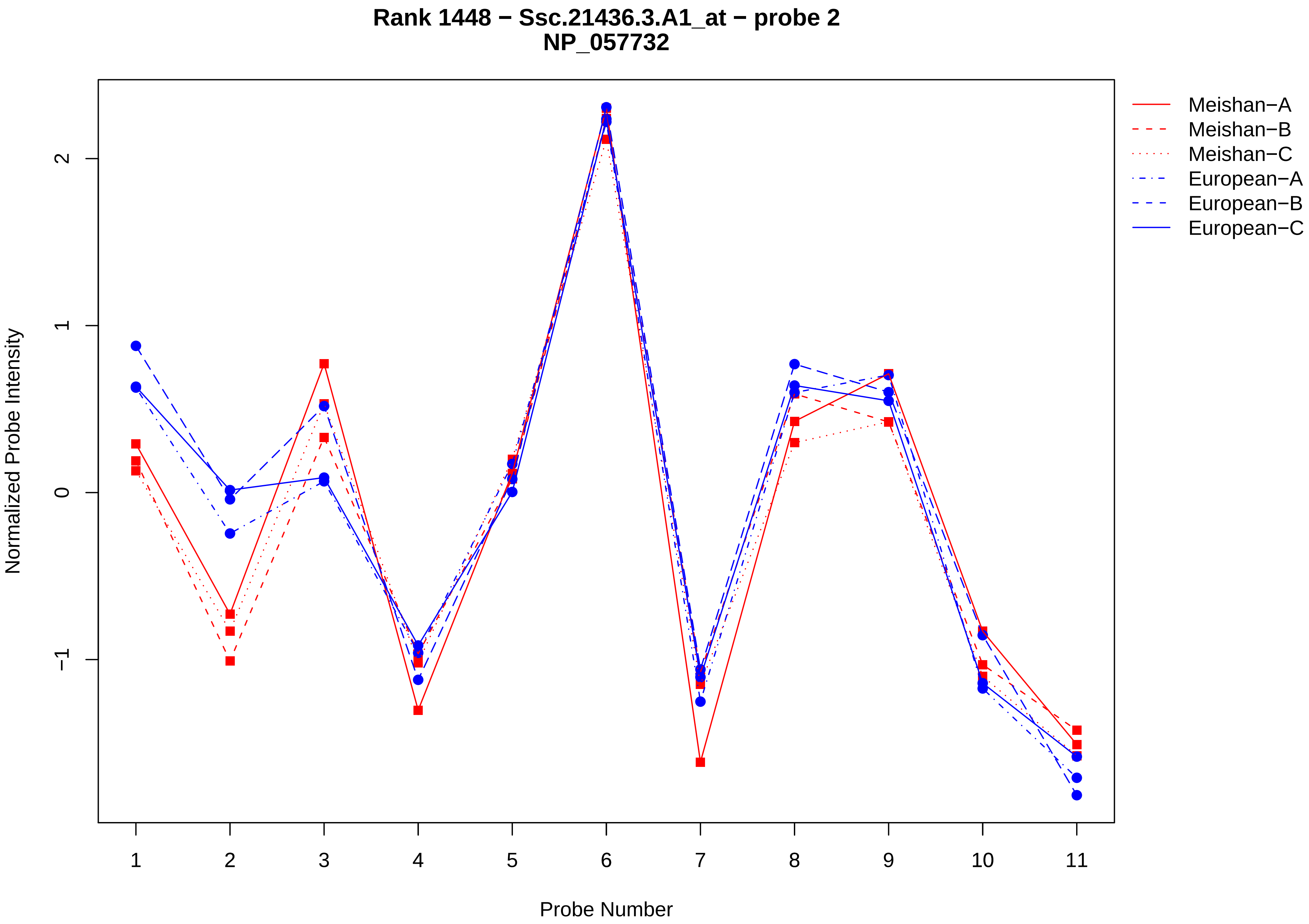

Rank 1448 − Ssc.21436.3.A1_at − probe 2
NP_057732
Normalized Probe Intensity
−1
0
1
2
1
2
3
4
5
6
7
8
9
10
11
Probe Number
Meishan−A
Meishan−B
Meishan−C
European−A
European−B
European−C

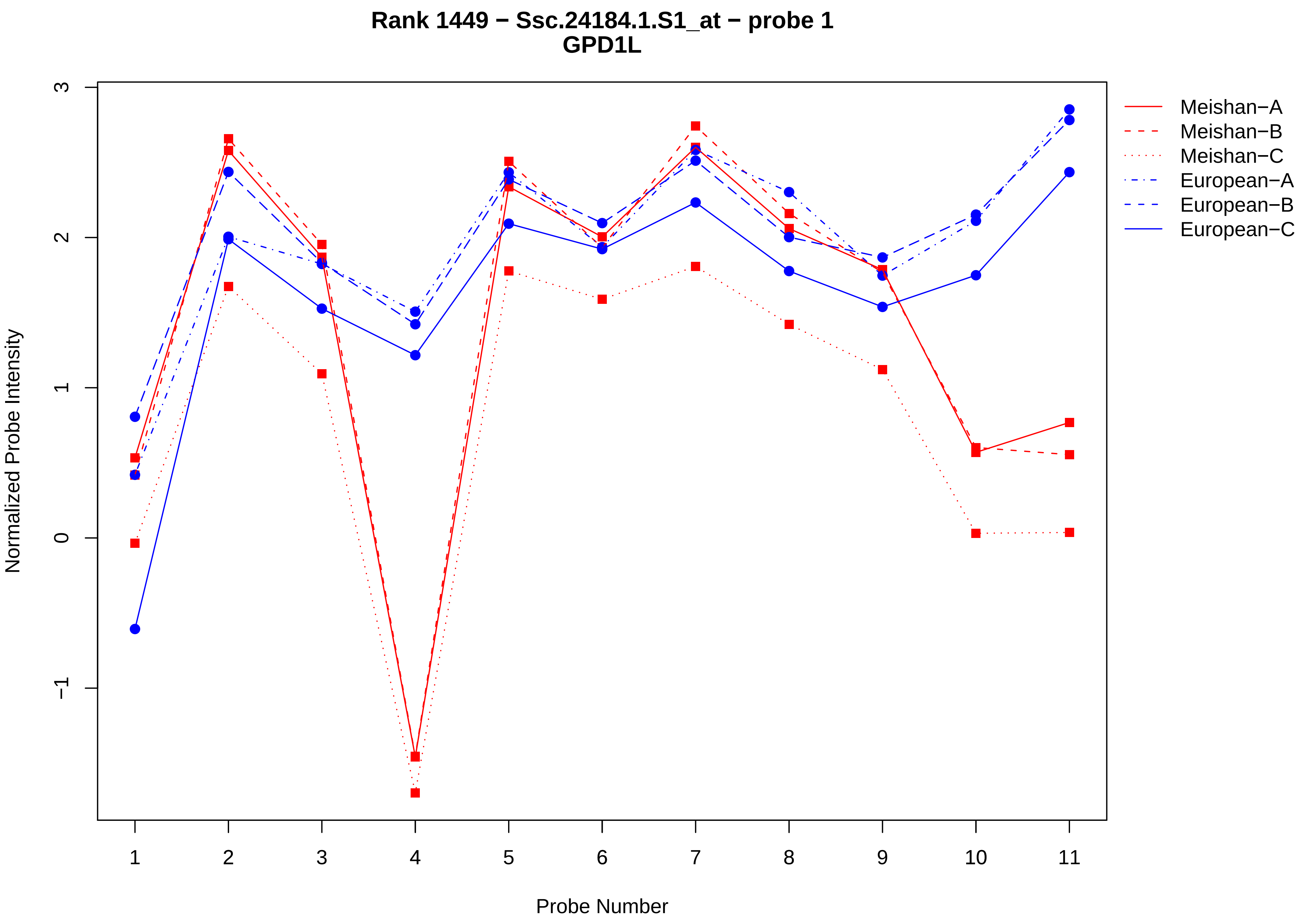

Rank 1449 − Ssc.24184.1.S1_at − probe 1
GPD1L
Normalized Probe Intensity
Probe Number
−1
0
1
2
3
1
2
3
4
5
6
7
8
9
10
11
Meishan−A
Meishan−B
Meishan−C
European−A
European−B
European−C

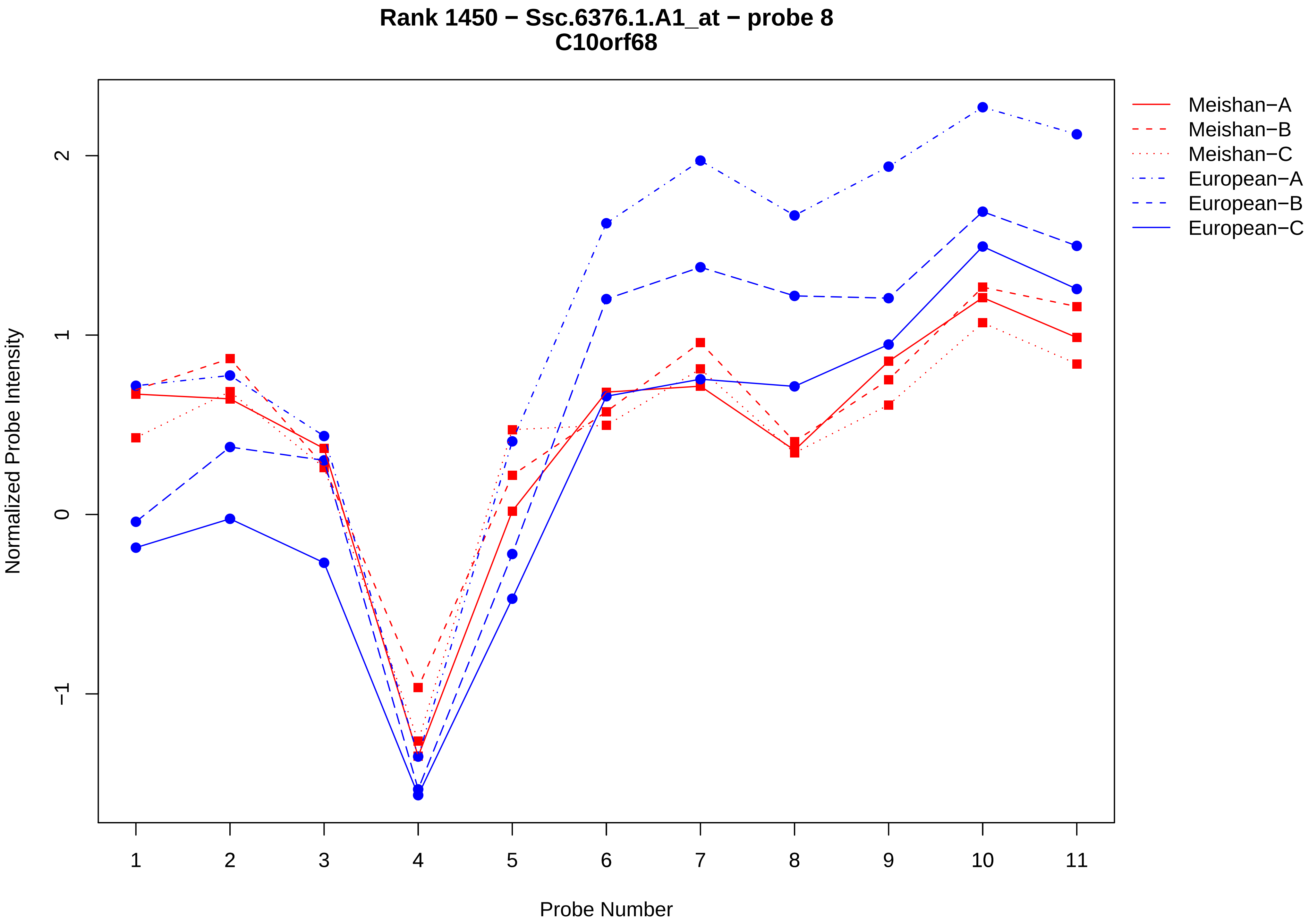

Rank 1450 − Ssc.6376.1.A1_at − probe 8
C10orf68
Normalized Probe Intensity
Probe Number
Meishan−A
Meishan−B
Meishan−C
European−A
European−B
European−C
−1
0
1
2
1
2
3
4
5
6
7
8
9
10
11

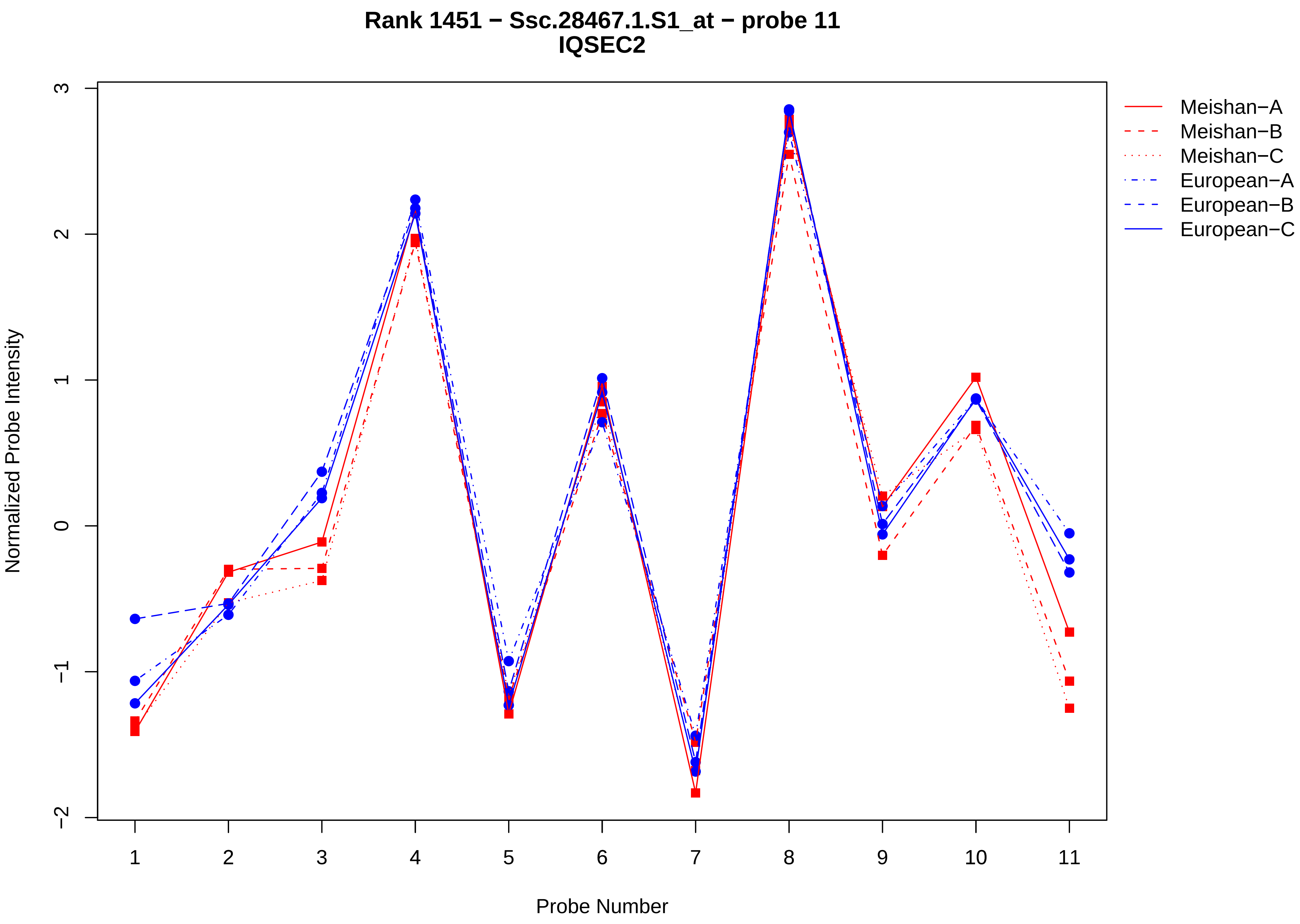

Rank 1451 − Ssc.28467.1.S1_at − probe 11
IQSEC2
Normalized Probe Intensity
Probe Number
−2
−1
0
1
2
3
1
2
3
4
5
6
7
8
9
10
11
Meishan−A
Meishan−B
Meishan−C
European−A
European−B
European−C

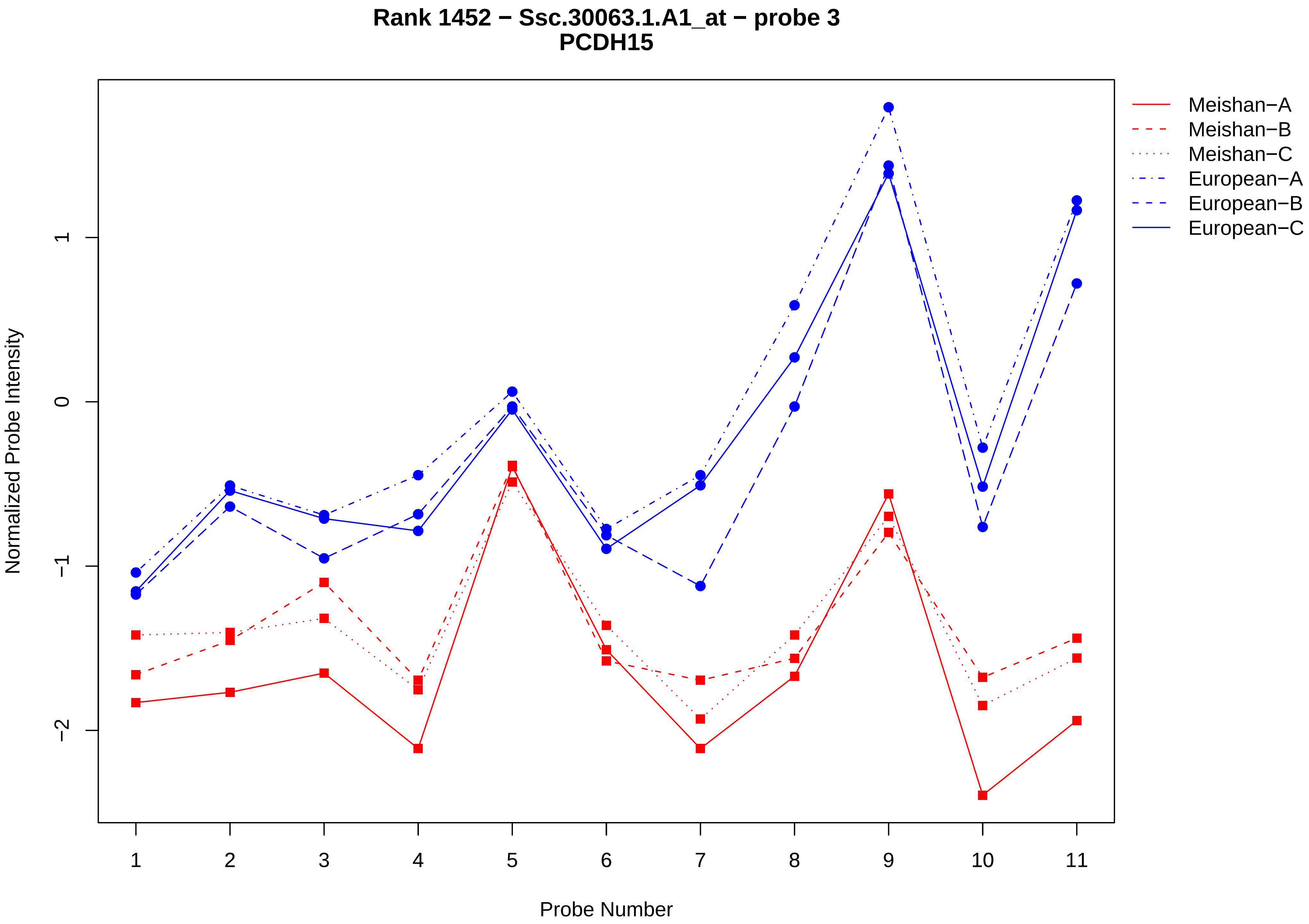

Rank 1452 − Ssc.30063.1.A1_at − probe 3
PCDH15
Normalized Probe Intensity
Probe Number
Meishan−A
Meishan−B
Meishan−C
European−A
European−B
European−C

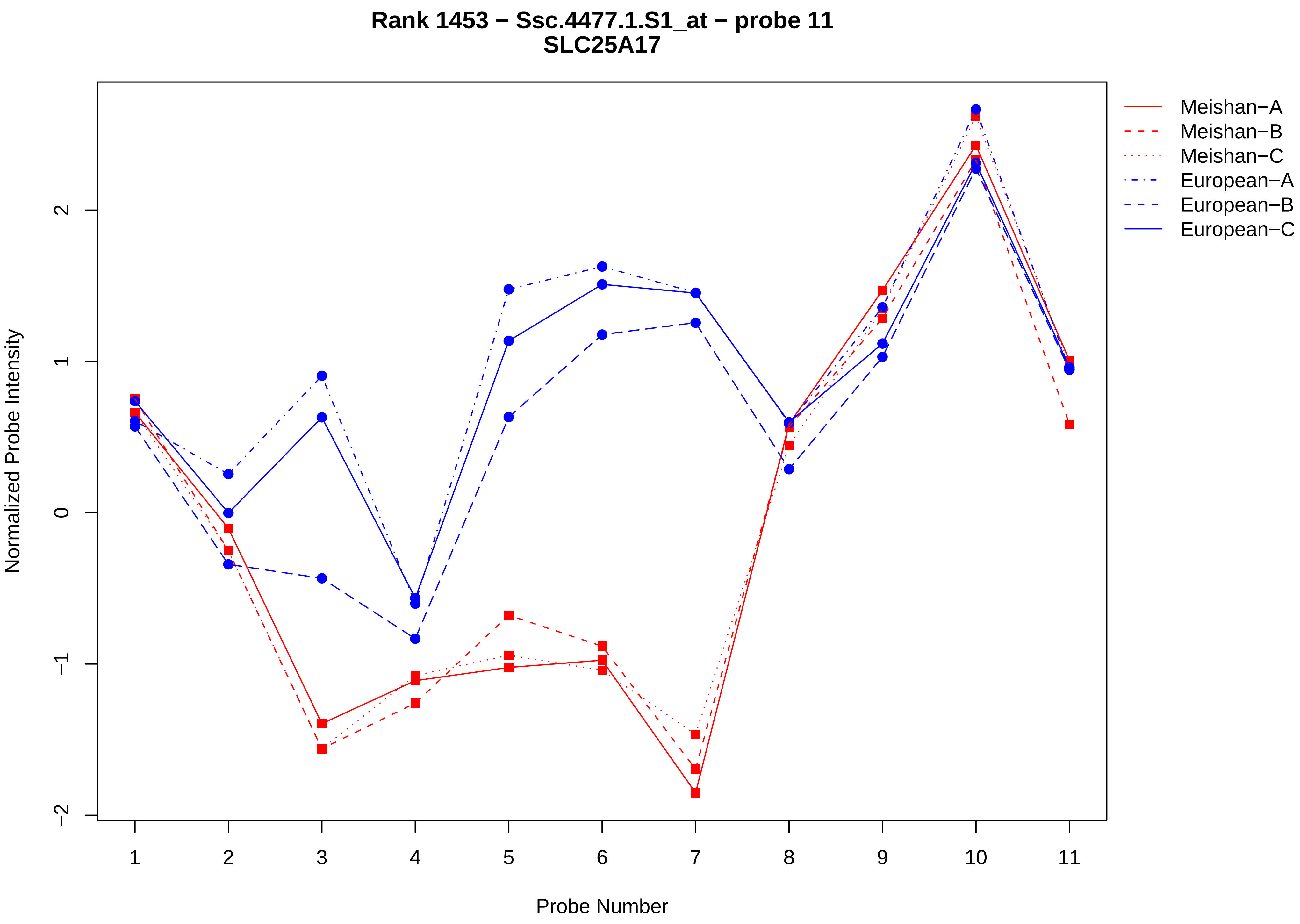
Rank 1453 − Ssc.4477.1.S1_at − probe 11
SLC25A17
Normalized Probe Intensity
Probe Number
−2
−1
0
1
2
1
2
3
4
5
6
7
8
9
10
11
Meishan−A
Meishan−B
Meishan−C
European−A
European−B
European−C

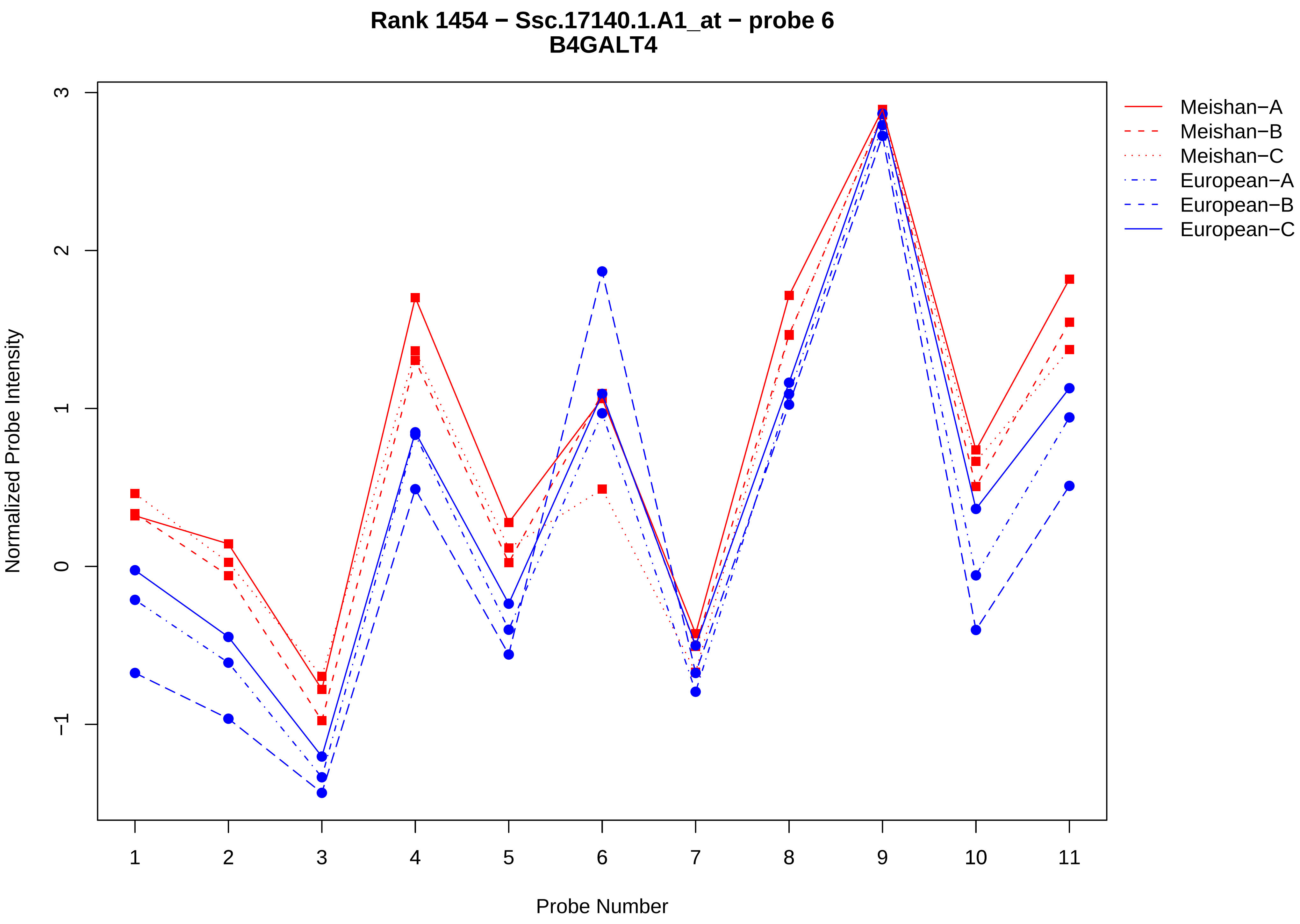

Rank 1454 − Ssc.17140.1.A1_at − probe 6
B4GALT4
Normalized Probe Intensity
Probe Number
Meishan−A
Meishan−B
Meishan−C
European−A
European−B
European−C

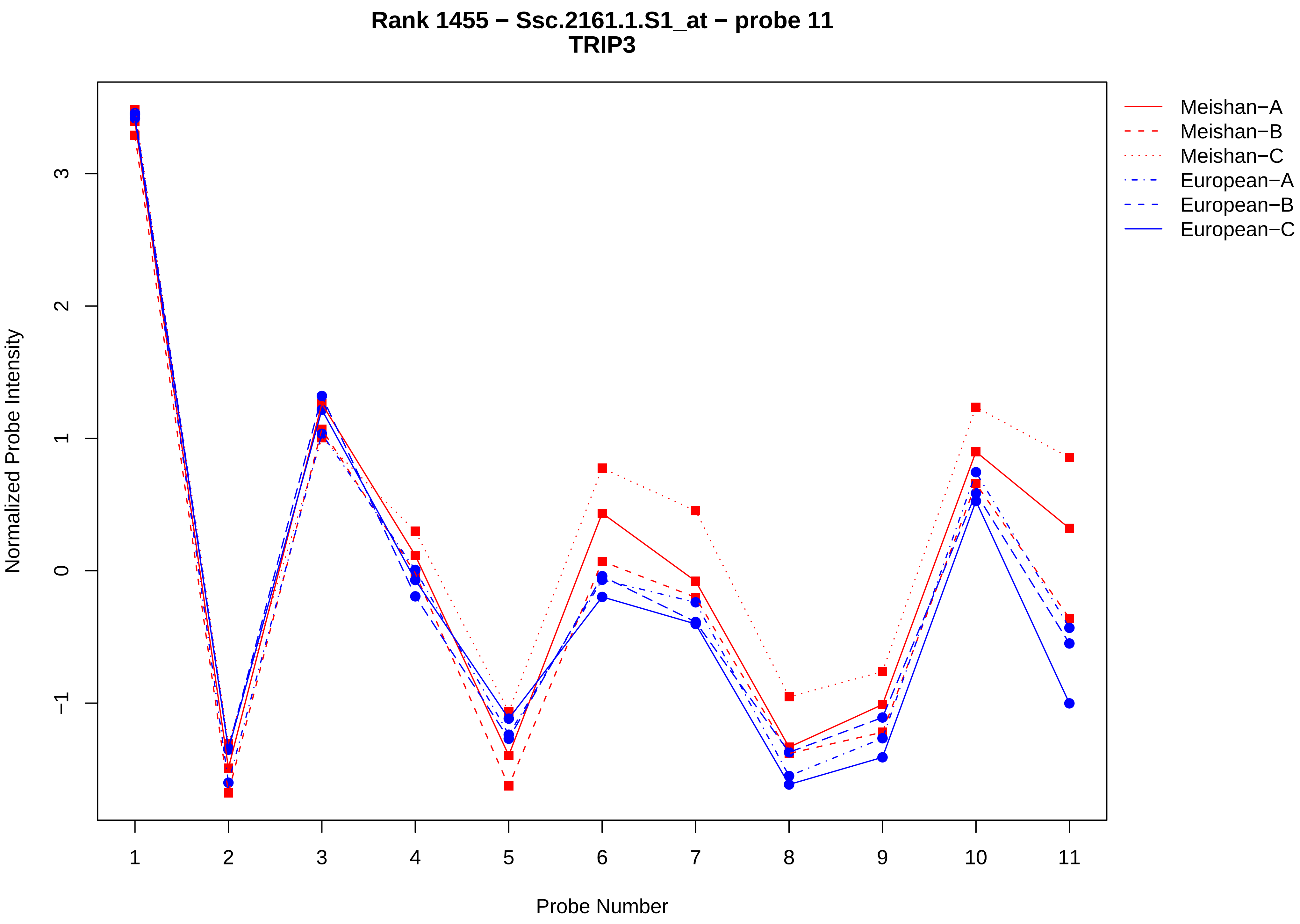

Rank 1455 − Ssc.2161.1.S1_at − probe 11
TRIP3
Normalized Probe Intensity
Probe Number
−1
0
1
2
3
1
2
3
4
5
6
7
8
9
10
11
Meishan−A
Meishan−B
Meishan−C
European−A
European−B
European−C

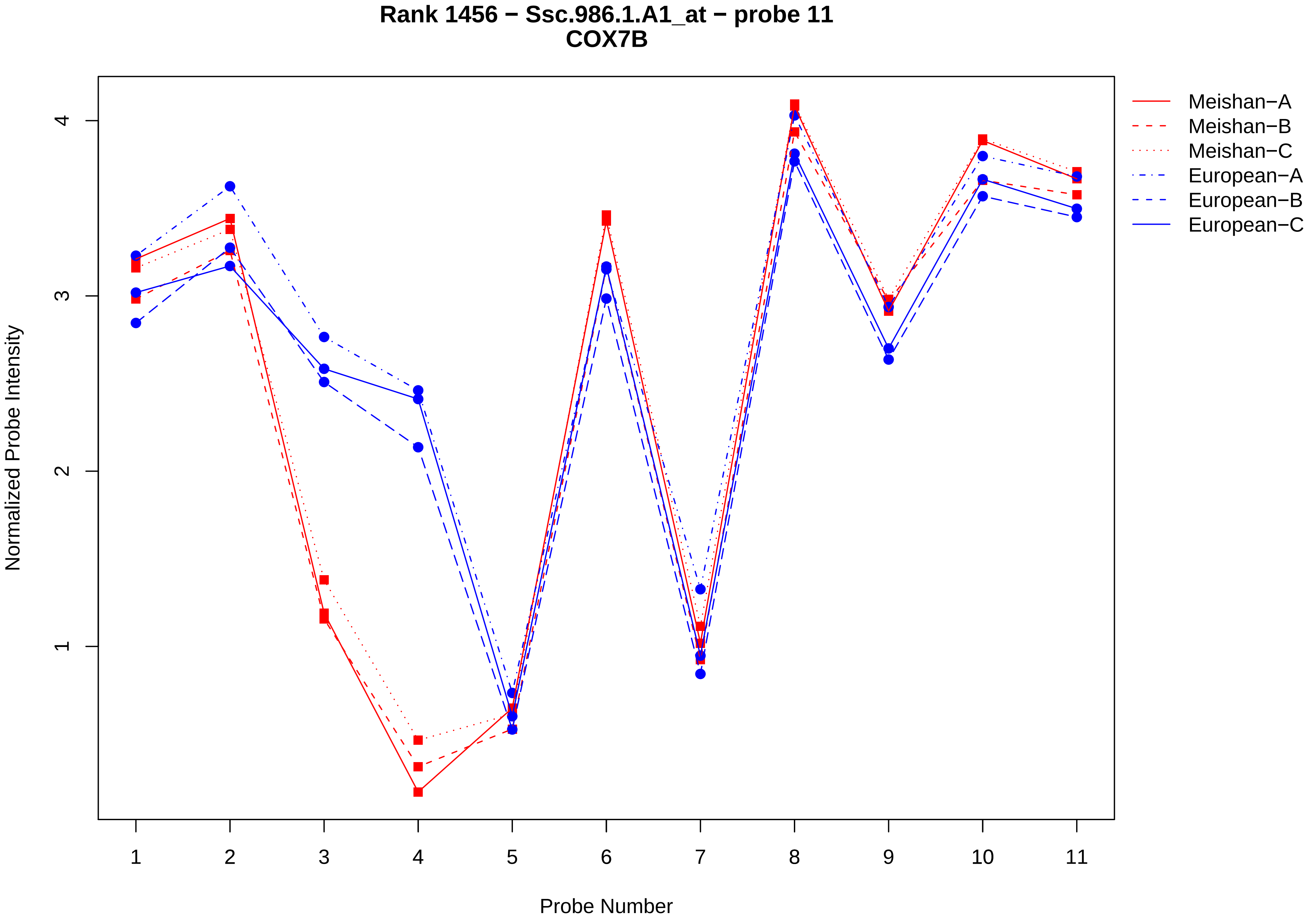

Rank 1456 − Ssc.986.1.A1_at − probe 11
COX7B
Normalized Probe Intensity
Probe Number
1
2
3
4
1
2
3
4
5
6
7
8
9
10
11
Meishan−A
Meishan−B
Meishan−C
European−A
European−B
European−C

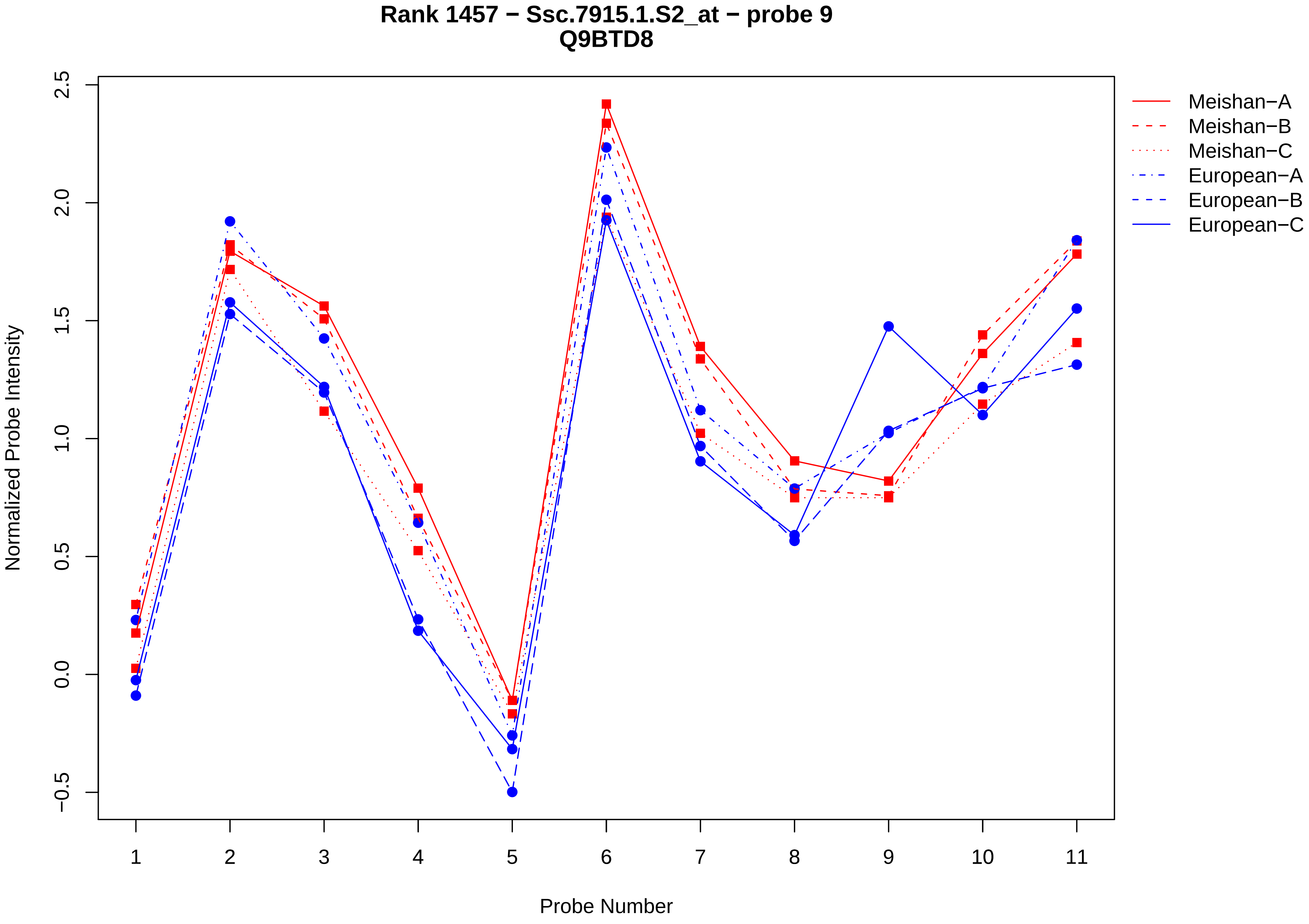

Rank 1457 − Ssc.7915.1.S2_at − probe 9
Q9BTD8
Normalized Probe Intensity
Probe Number
−0.5
0.0
0.5
1.0
1.5
2.0
2.5
1
2
3
4
5
6
7
8
9
10
11
Meishan−A
Meishan−B
Meishan−C
European−A
European−B
European−C

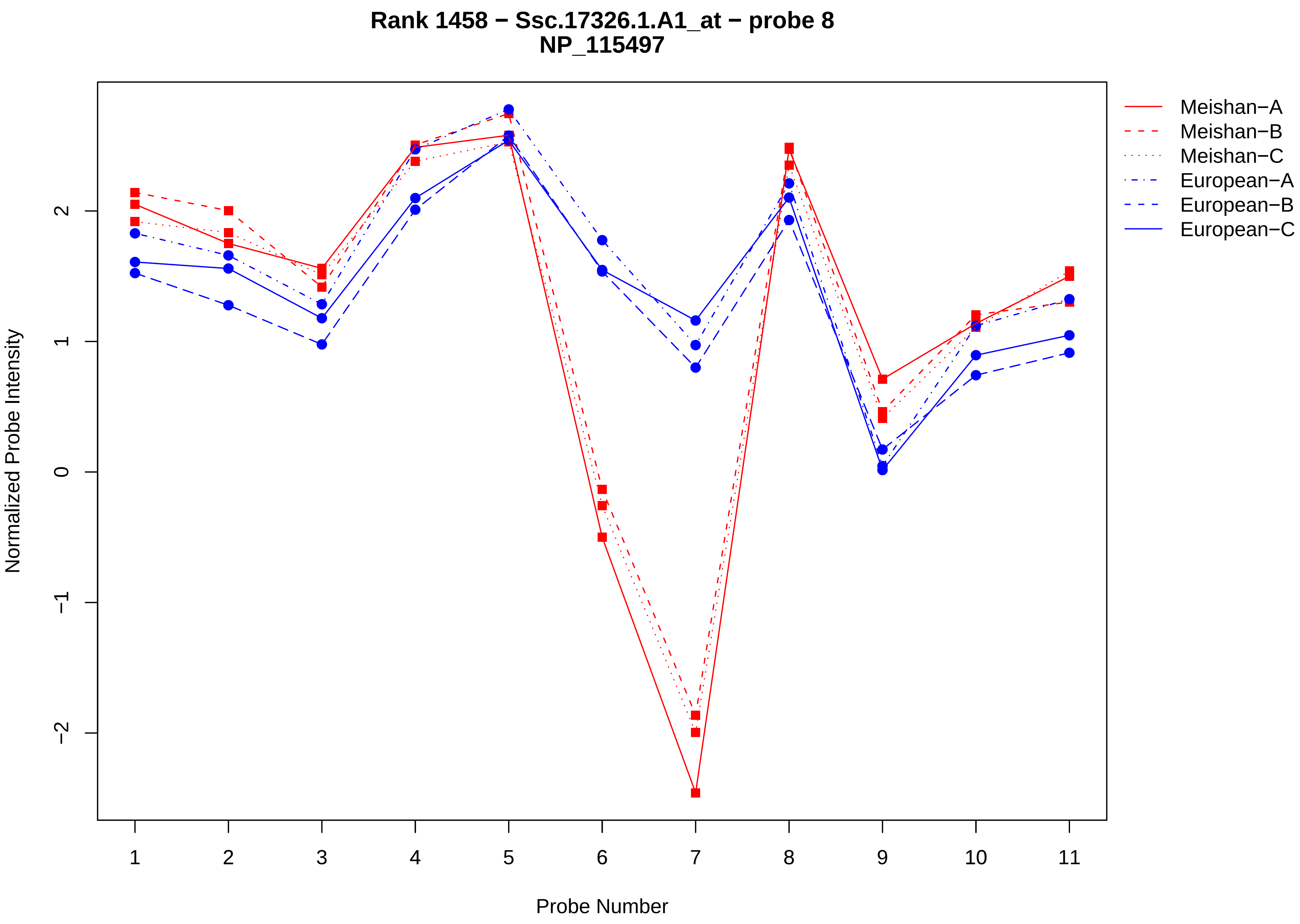
Rank 1458 − Ssc.17326.1.A1_at − probe 8
NP_115497
Normalized Probe Intensity
Probe Number
−2
−1
0
1
2
1
2
3
4
5
6
7
8
9
10
11
Meishan−A
Meishan−B
Meishan−C
European−A
European−B
European−C

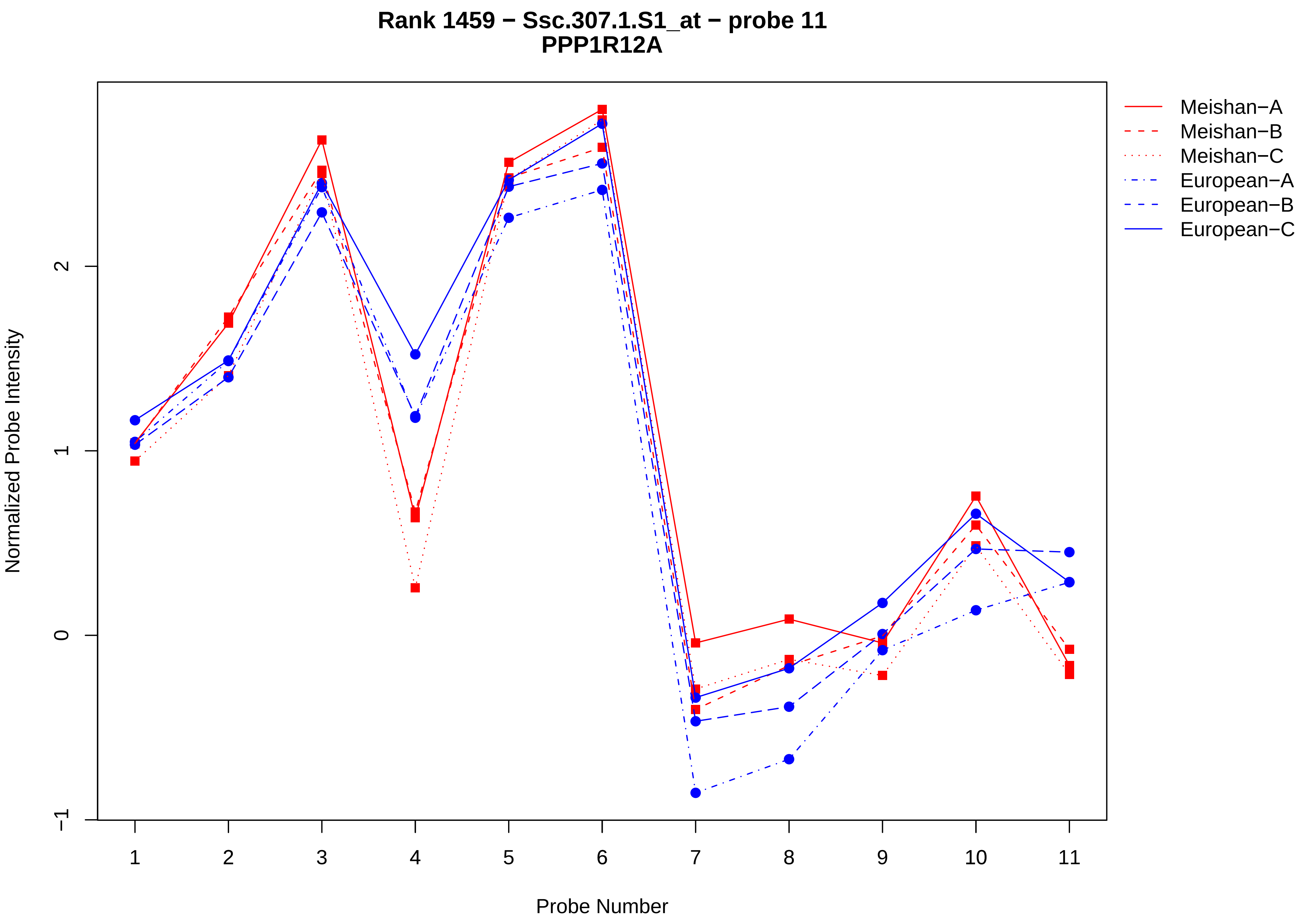

Rank 1459 − Ssc.307.1.S1_at − probe 11
PPP1R12A
Normalized Probe Intensity
Probe Number
−1
0
1
2
1
2
3
4
5
6
7
8
9
10
11
Meishan−A
Meishan−B
Meishan−C
European−A
European−B
European−C

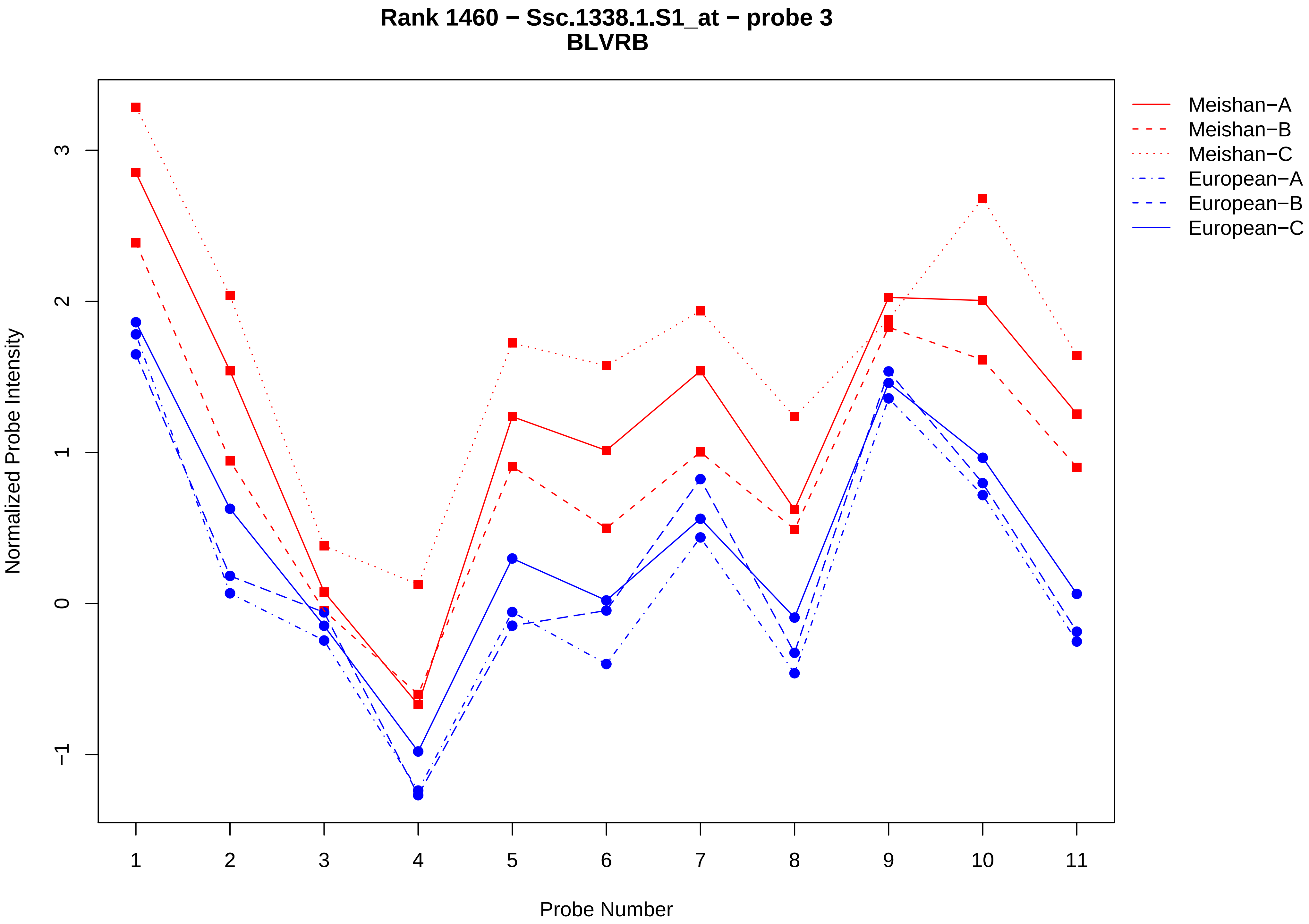

Rank 1460 − Ssc.1338.1.S1_at − probe 3
BLVRB
Normalized Probe Intensity
Probe Number
−1
0
1
2
3
1
2
3
4
5
6
7
8
9
10
11
Meishan−A
Meishan−B
Meishan−C
European−A
European−B
European−C

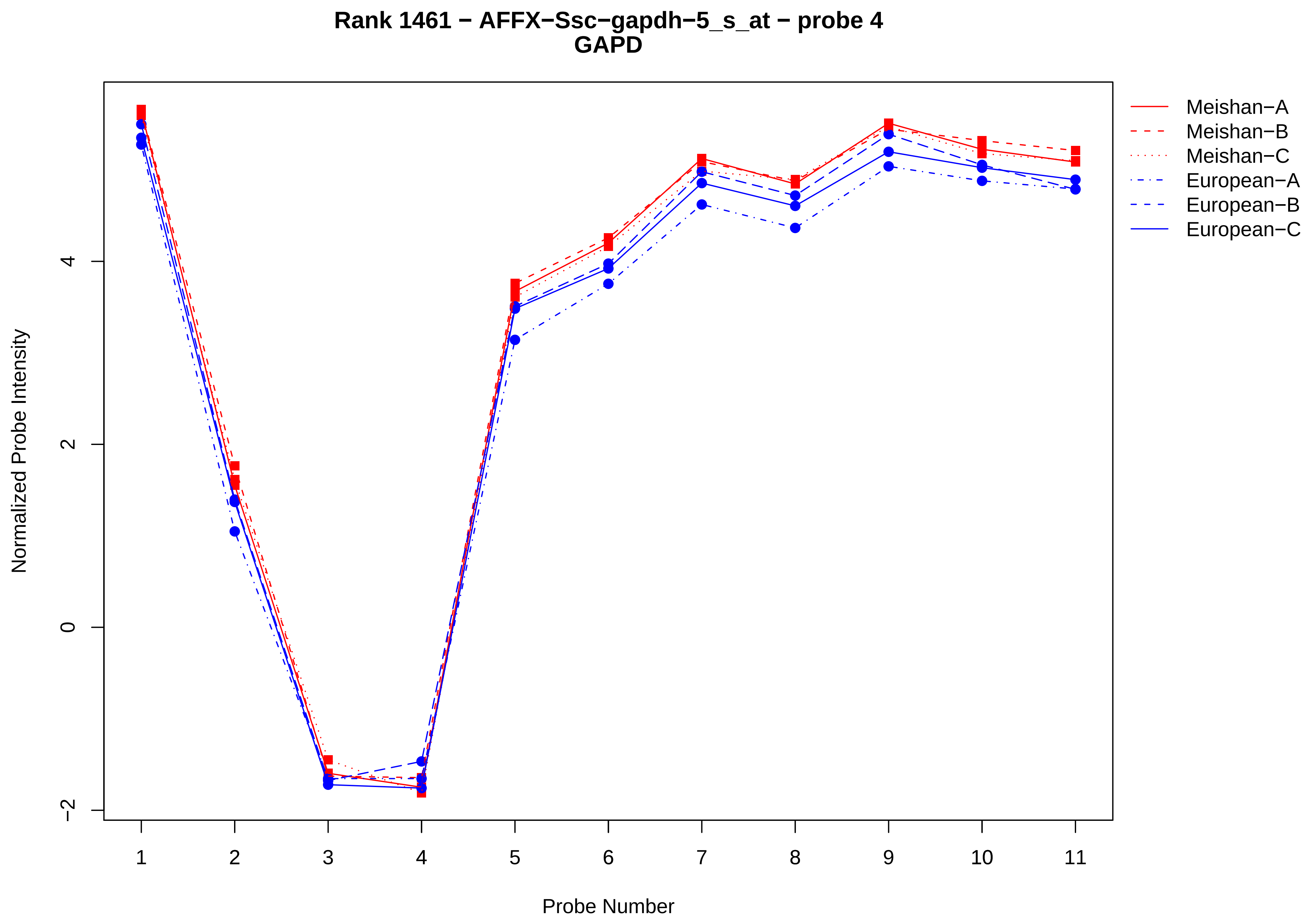

Rank 1461 − AFFX−Ssc−gapdh−5_s_at − probe 4
GAPD
Normalized Probe Intensity
Probe Number
−2
0
2
4
1
2
3
4
5
6
7
8
9
10
11
Meishan−A
Meishan−B
Meishan−C
European−A
European−B
European−C

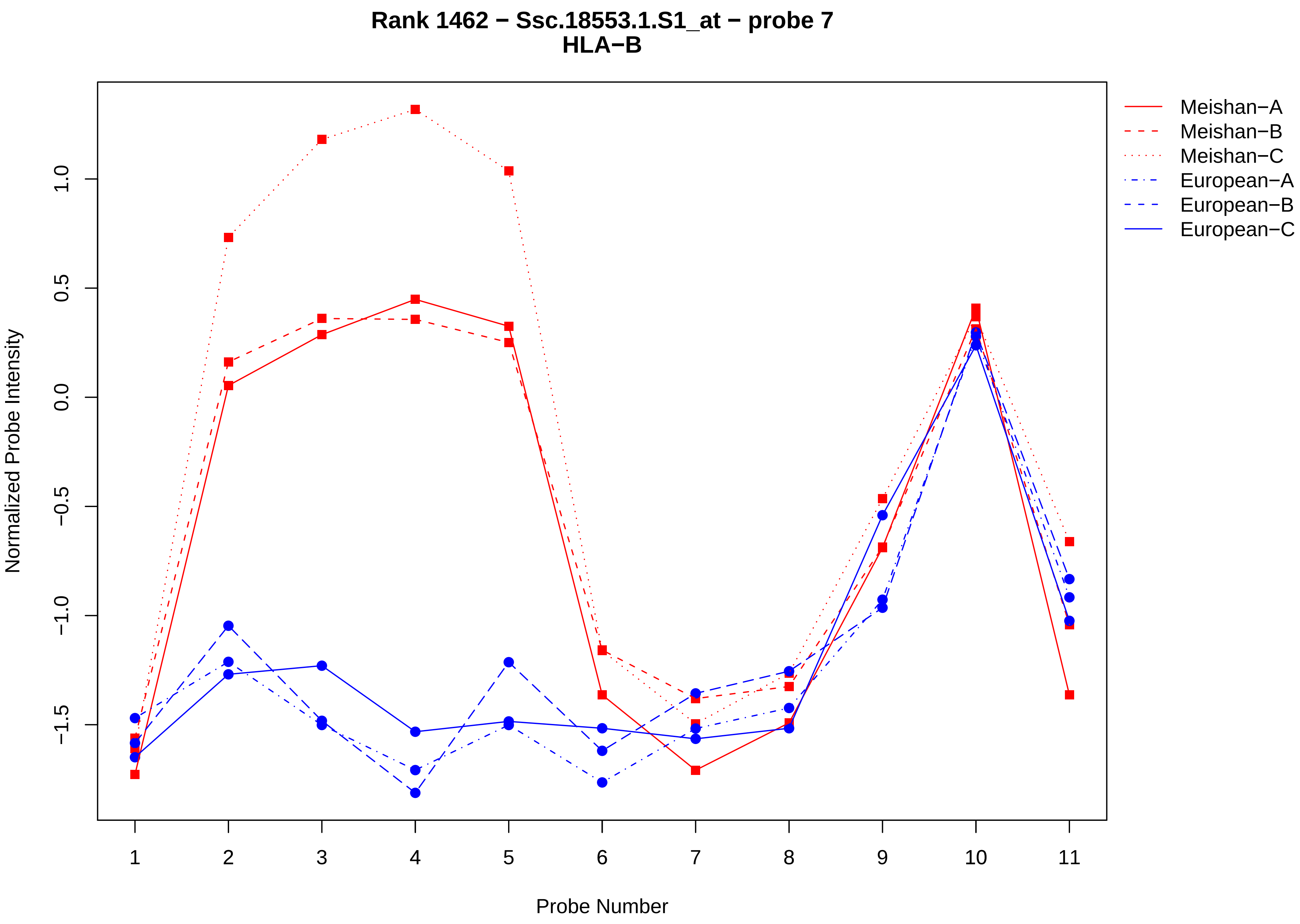

Rank 1462 − Ssc.18553.1.S1_at − probe 7
HLA−B
Normalized Probe Intensity
Probe Number
Meishan−A
Meishan−B
Meishan−C
European−A
European−B
European−C
1.0
0.5
0.0
−0.5
−1.0
−1.5
1
2
3
4
5
6
7
8
9
10
11

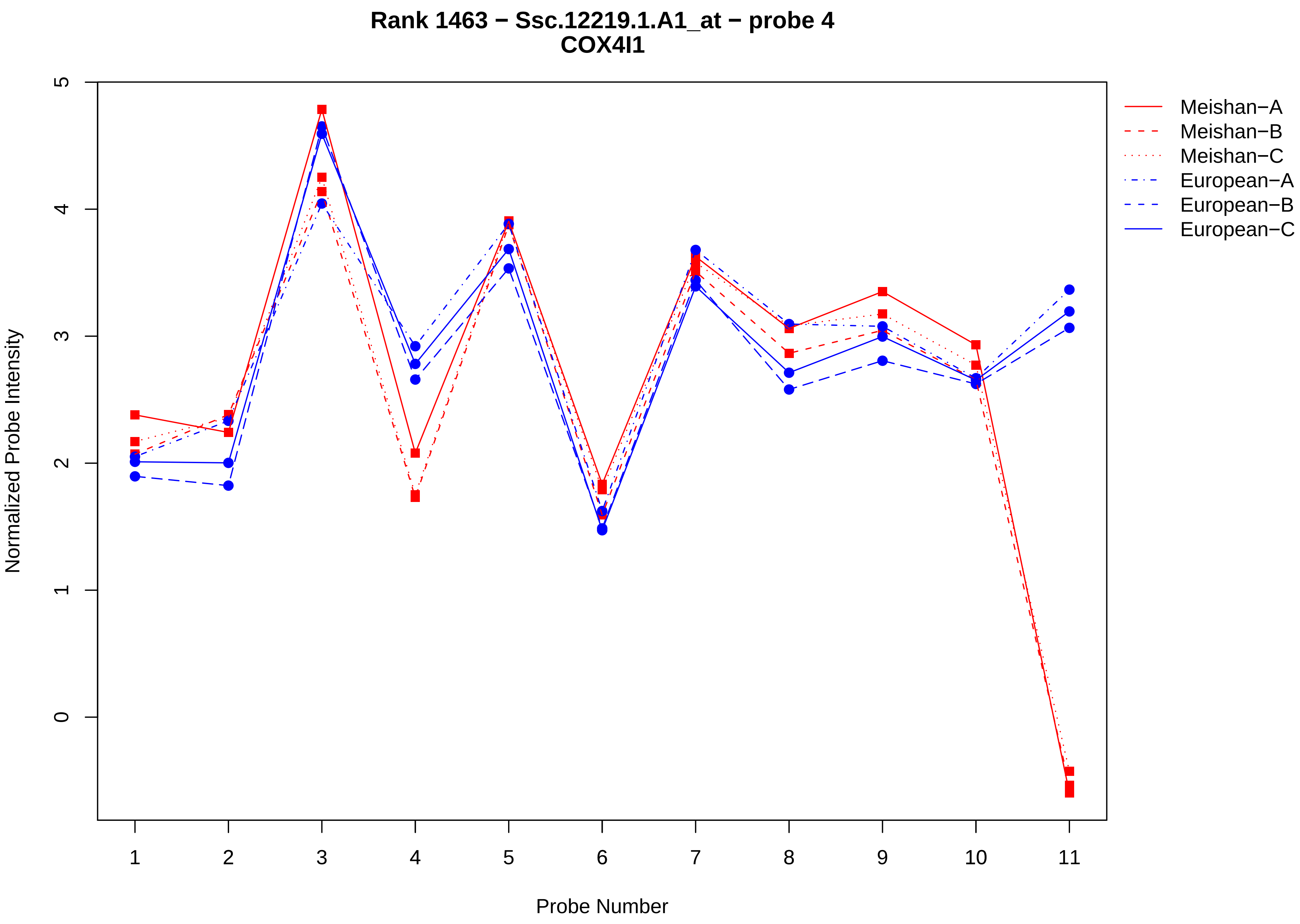
Rank 1463 − Ssc.12219.1.A1_at − probe 4
COX4I1
Normalized Probe Intensity
Probe Number
0
1
2
3
4
5
1
2
3
4
5
6
7
8
9
10
11
Meishan−A
Meishan−B
Meishan−C
European−A
European−B
European−C

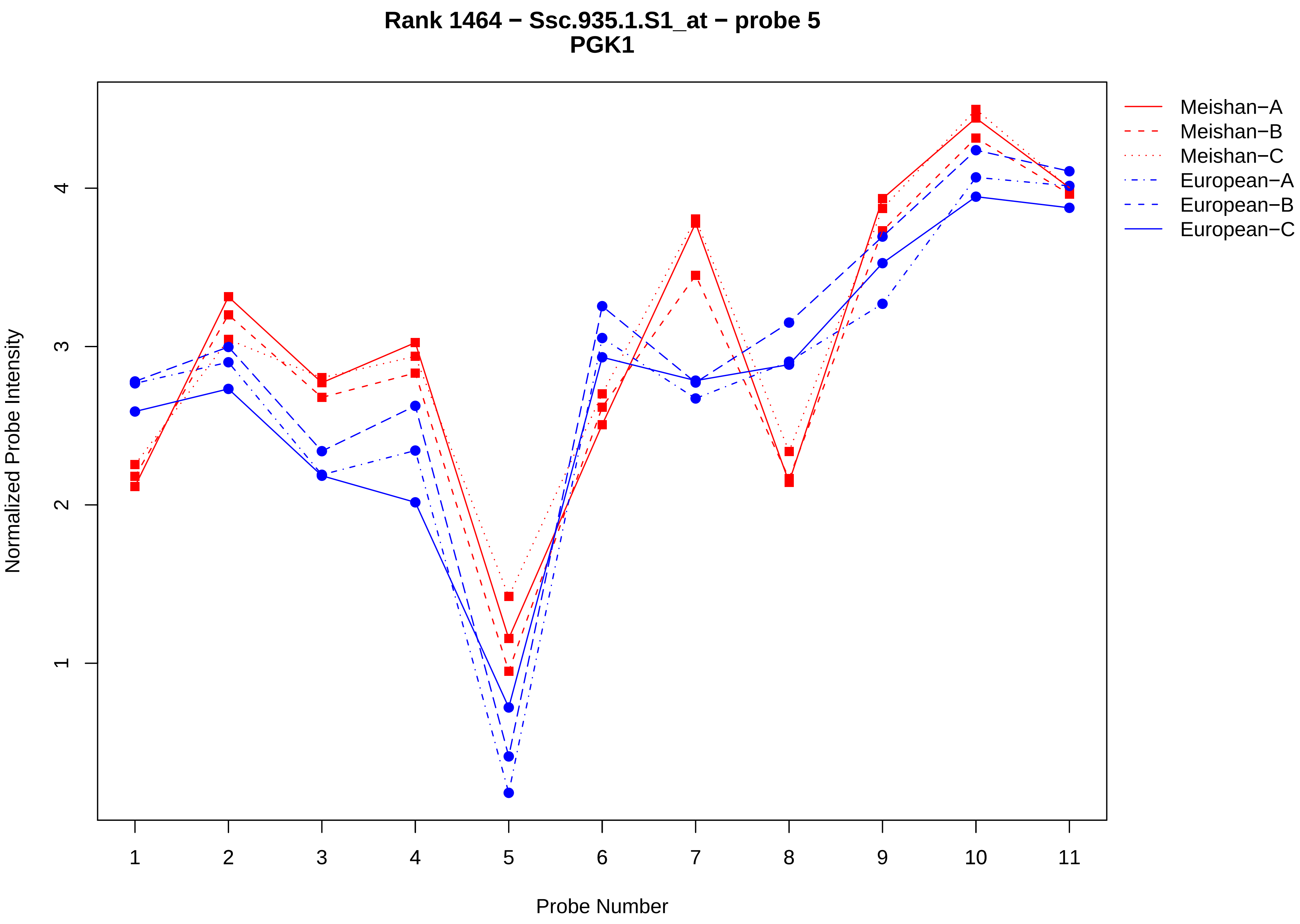
Rank 1464 − Ssc.935.1.S1_at − probe 5
PGK1
Normalized Probe Intensity
Probe Number
1
2
3
4
5
6
7
8
9
10
11
Meishan−A
Meishan−B
Meishan−C
European−A
European−B
European−C

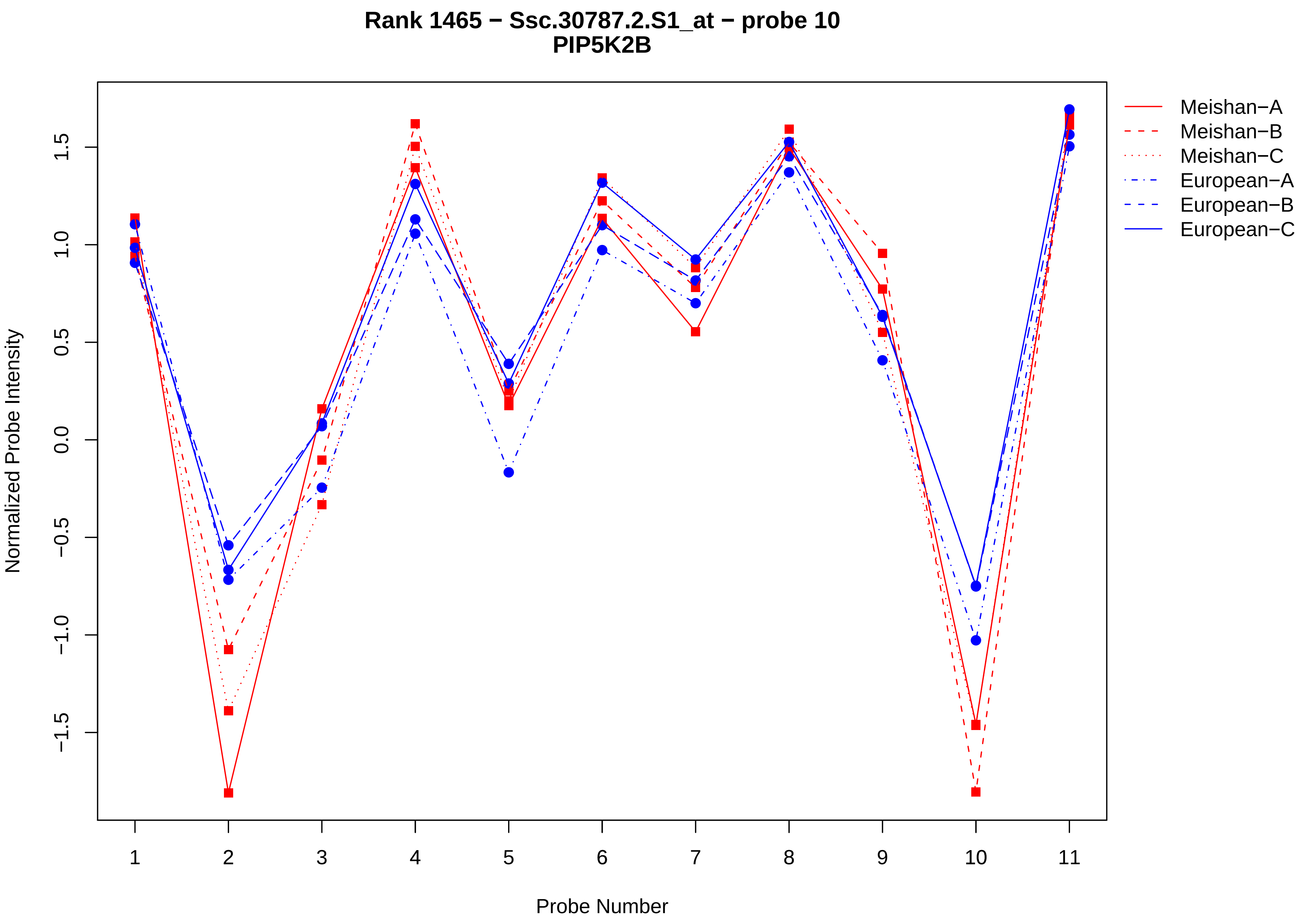

Rank 1465 − Ssc.30787.2.S1_at − probe 10
PIP5K2B
Normalized Probe Intensity
Probe Number
1.5
1.0
0.5
0.0
−0.5
−1.0
−1.5
1
2
3
4
5
6
7
8
9
10
11
Meishan−A
Meishan−B
Meishan−C
European−A
European−B
European−C

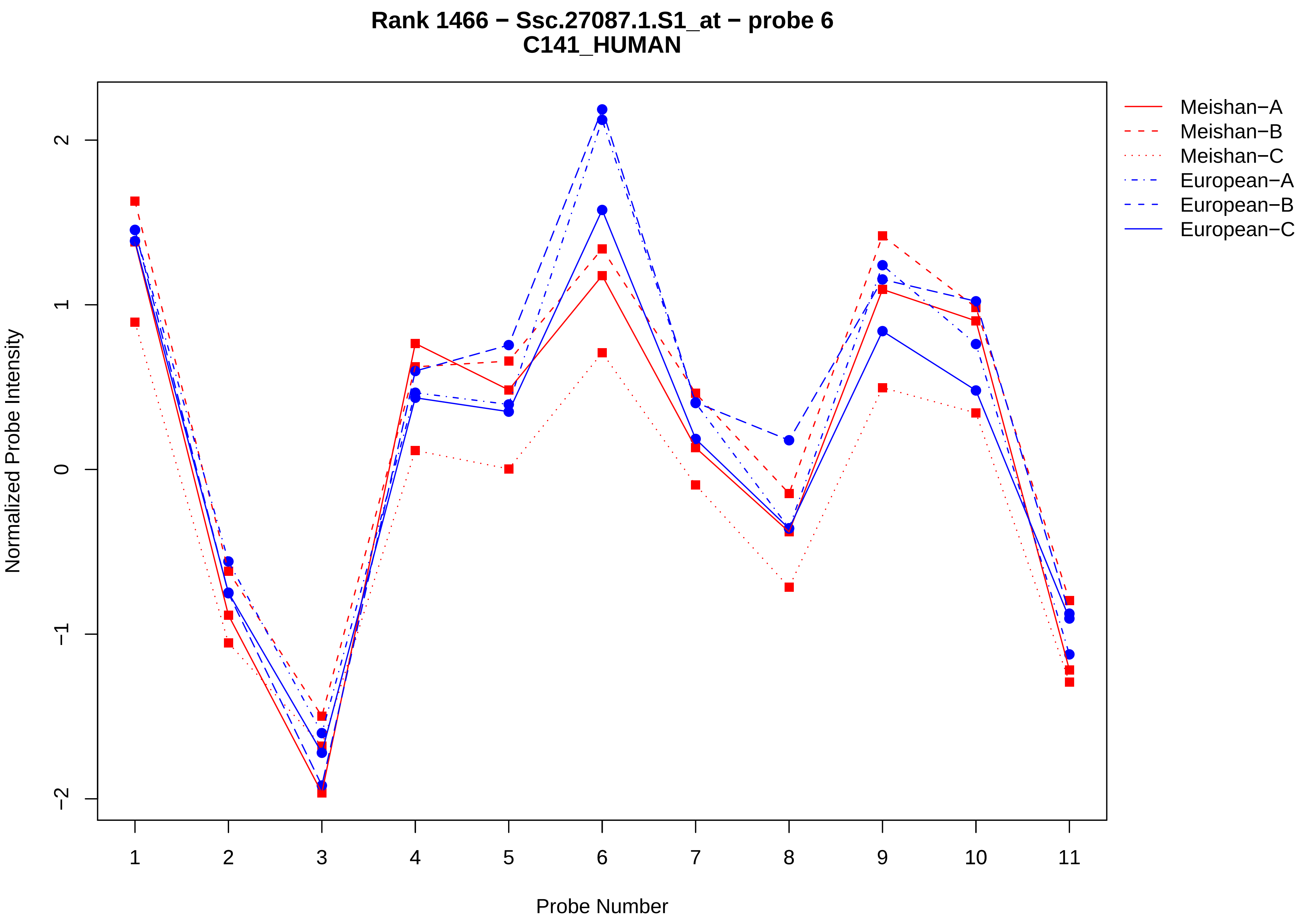

Rank 1466 − Ssc.27087.1.S1_at − probe 6
C141_HUMAN
Normalized Probe Intensity
Probe Number
Meishan−A
Meishan−B
Meishan−C
European−A
European−B
European−C

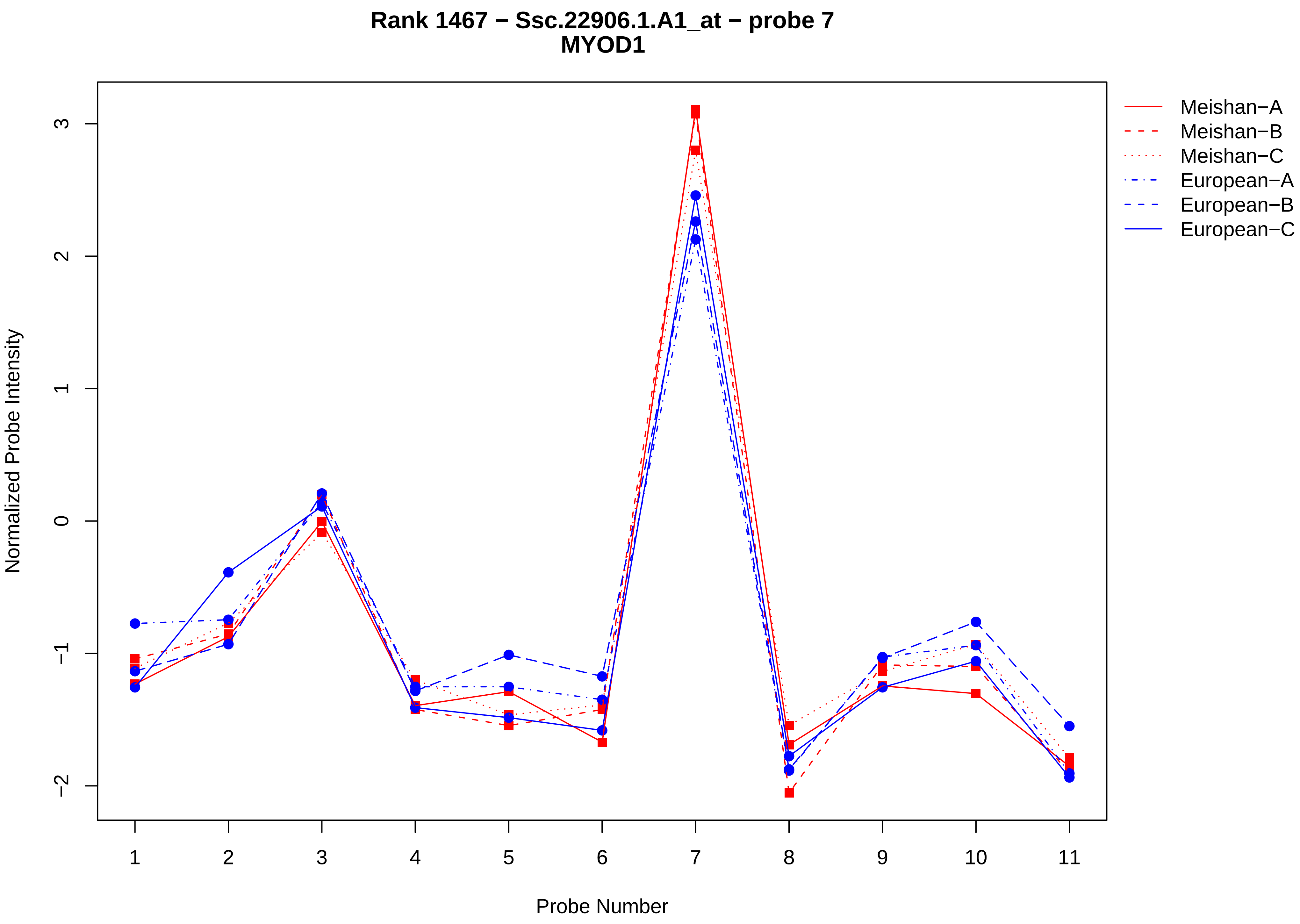

Rank 1467 − Ssc.22906.1.A1_at − probe 7
MYOD1
Normalized Probe Intensity
Probe Number
Meishan−A
Meishan−B
Meishan−C
European−A
European−B
European−C
−2
−1
0
1
2
3
1
2
3
4
5
6
7
8
9
10
11

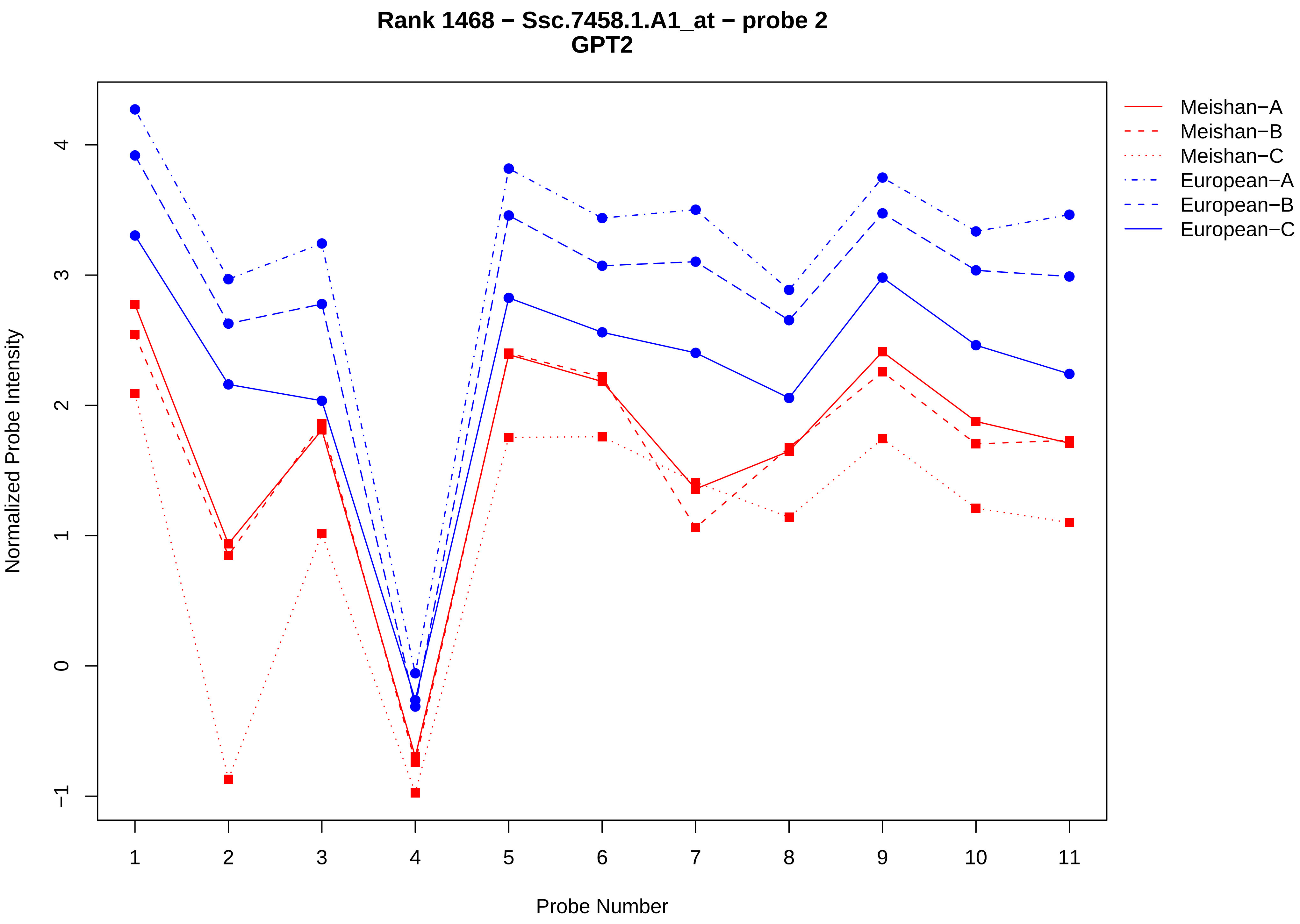

Rank 1468 − Ssc.7458.1.A1_at − probe 2
GPT2
Normalized Probe Intensity
Probe Number
−1
0
1
2
3
4
1
2
3
4
5
6
7
8
9
10
11
Meishan−A
Meishan−B
Meishan−C
European−A
European−B
European−C

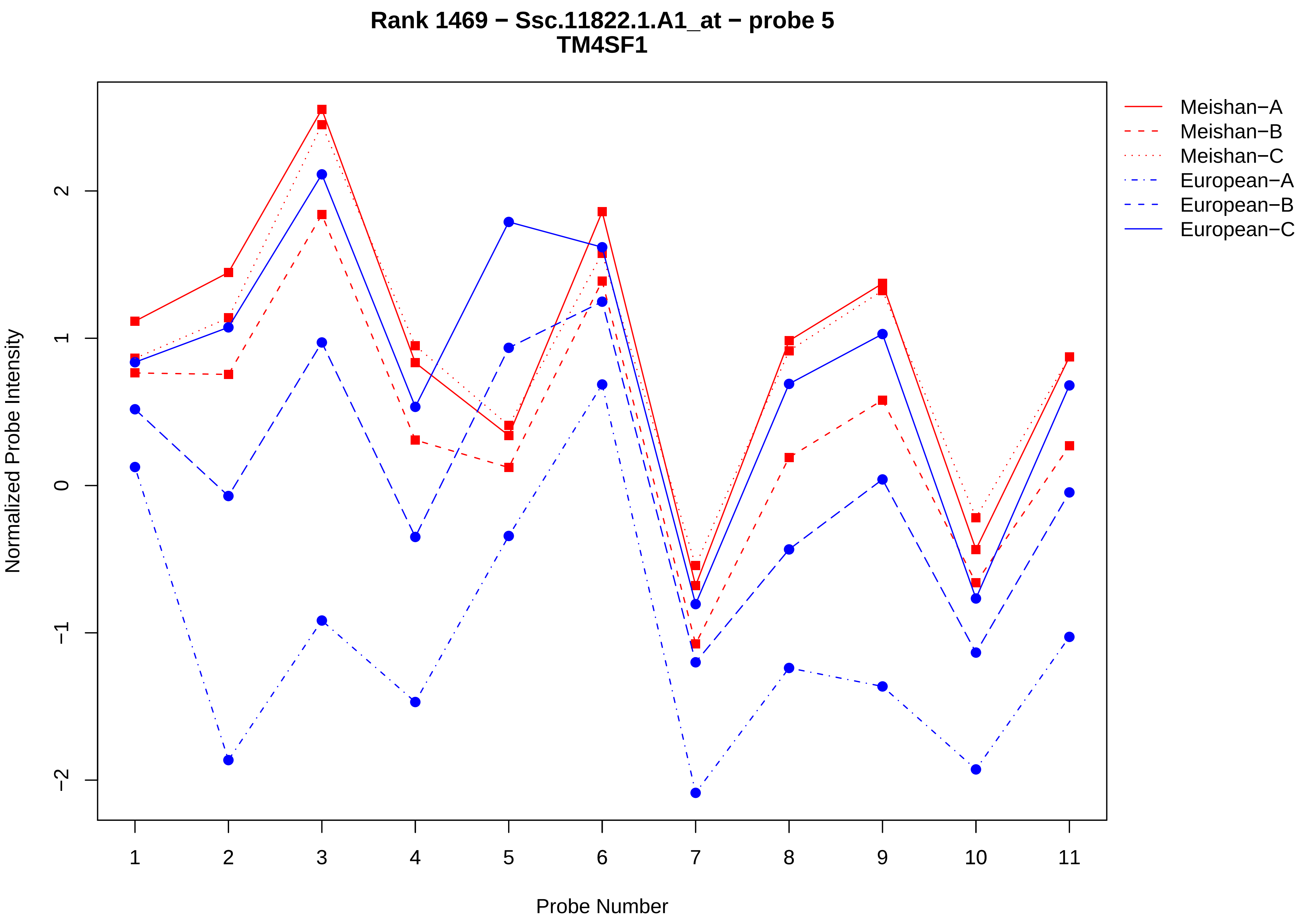

Rank 1469 − Ssc.11822.1.A1_at − probe 5
TM4SF1
Normalized Probe Intensity
Probe Number
−2
−1
0
1
2
1
2
3
4
5
6
7
8
9
10
11
Meishan−A
Meishan−B
Meishan−C
European−A
European−B
European−C

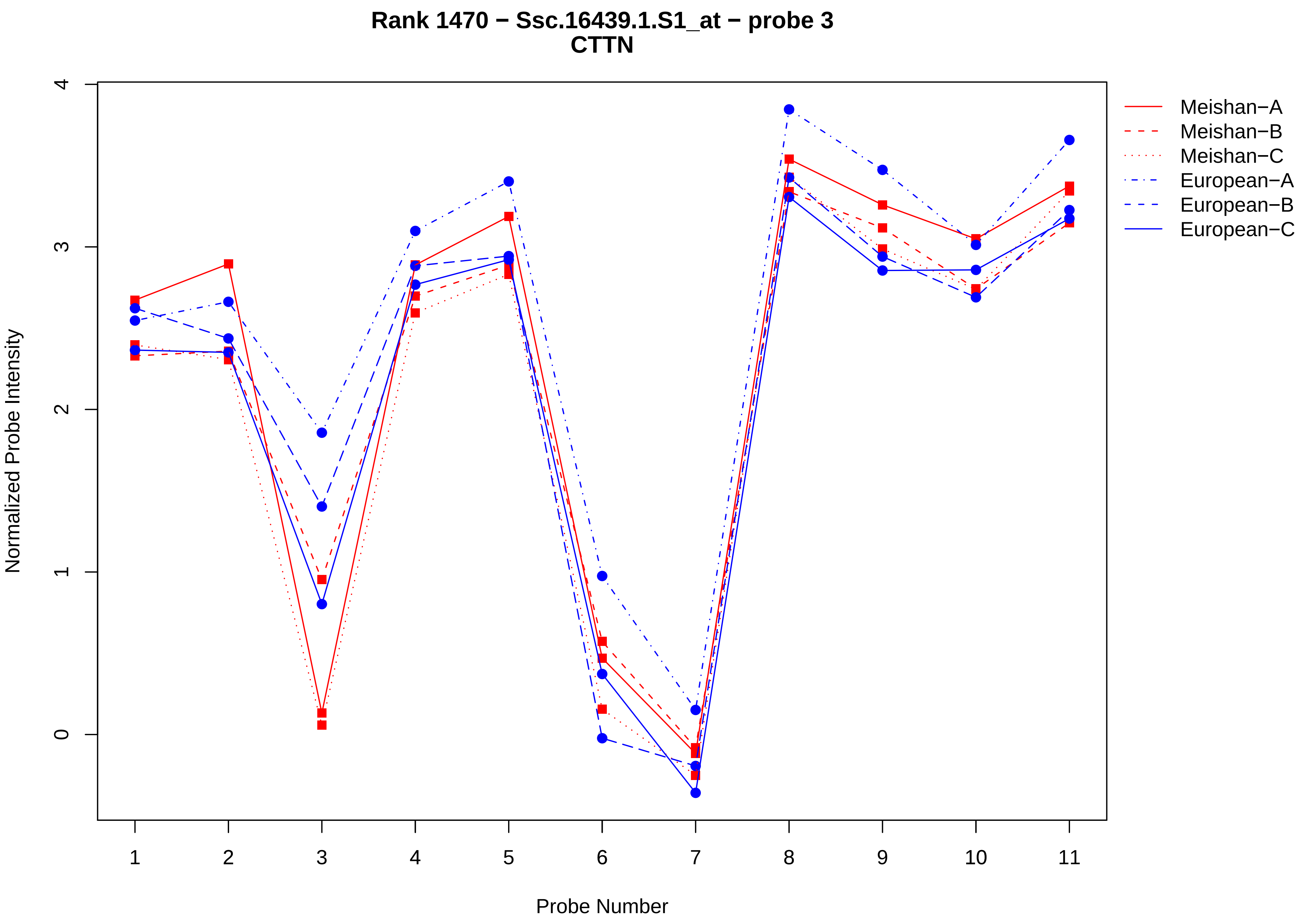

Rank 1470 − Ssc.16439.1.S1_at − probe 3
CTTN
Normalized Probe Intensity
Probe Number
0
1
2
3
4
1
2
3
4
5
6
7
8
9
10
11
Meishan−A
Meishan−B
Meishan−C
European−A
European−B
European−C

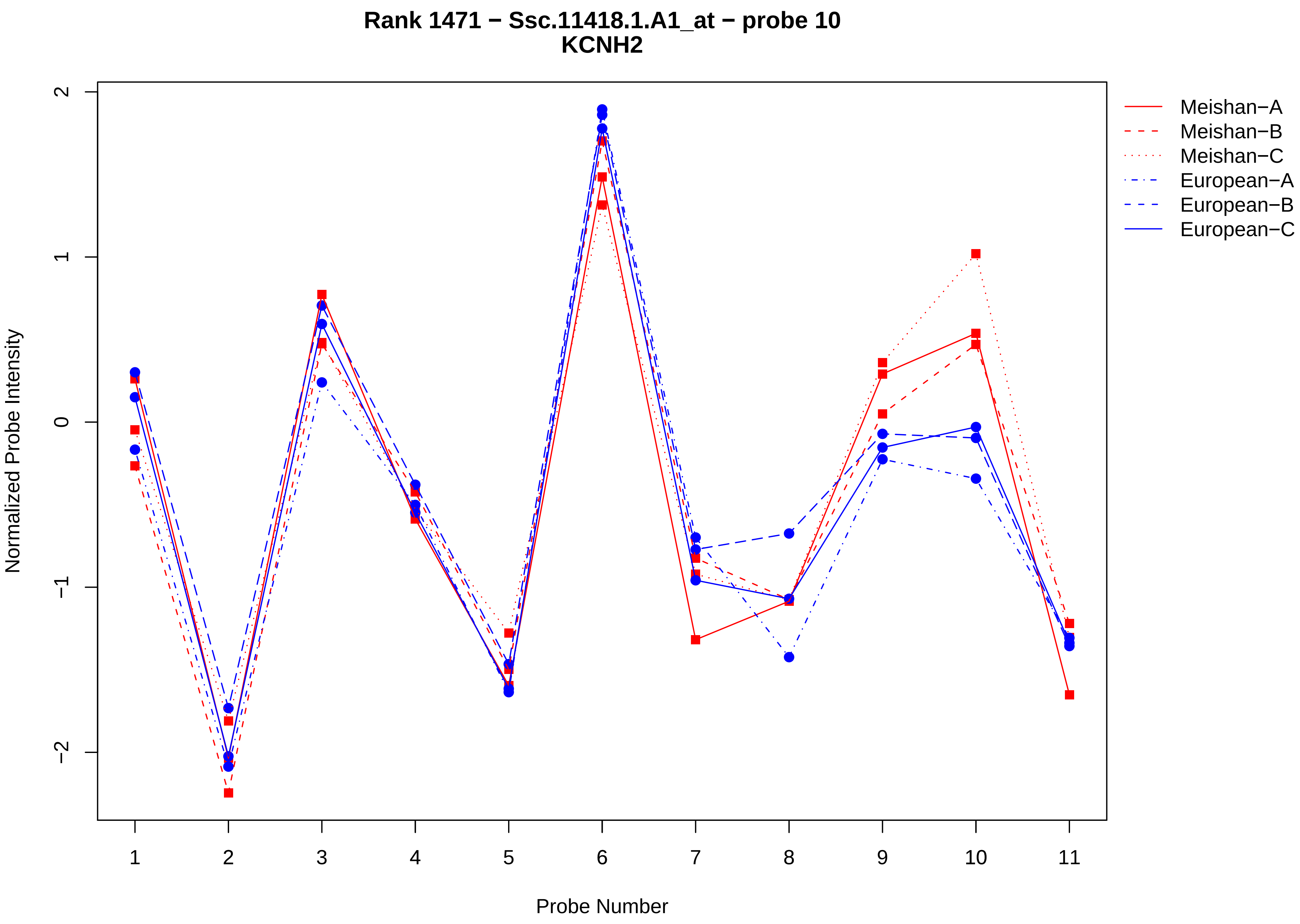

Rank 1471 − Ssc.11418.1.A1_at − probe 10
KCNH2
Normalized Probe Intensity
Probe Number
Meishan−A
Meishan−B
Meishan−C
European−A
European−B
European−C

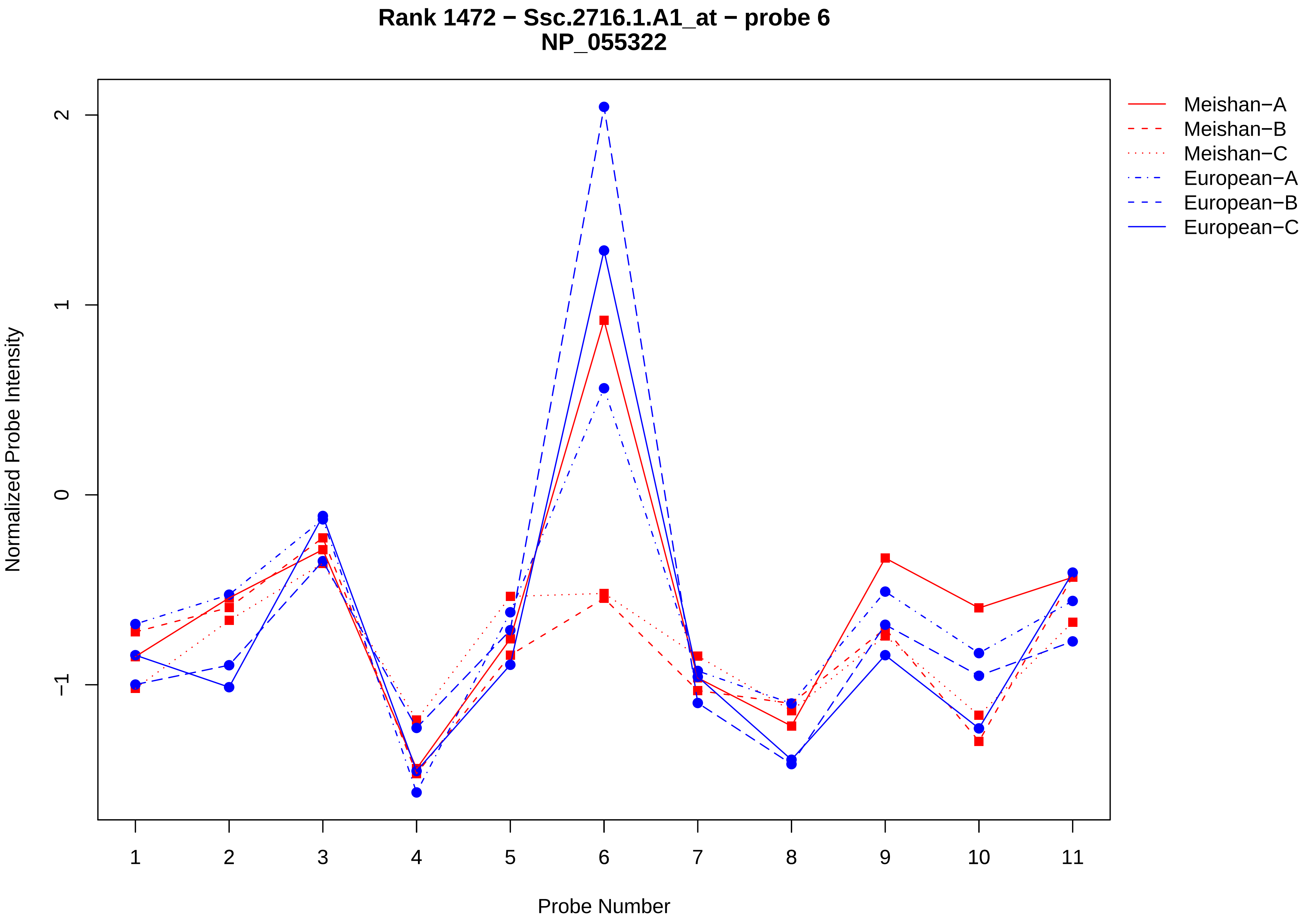
Rank 1472 − Ssc.2716.1.A1_at − probe 6
NP_055322
Normalized Probe Intensity
Probe Number
−1
0
1
2
1
2
3
4
5
6
7
8
9
10
11
Meishan−A
Meishan−B
Meishan−C
European−A
European−B
European−C

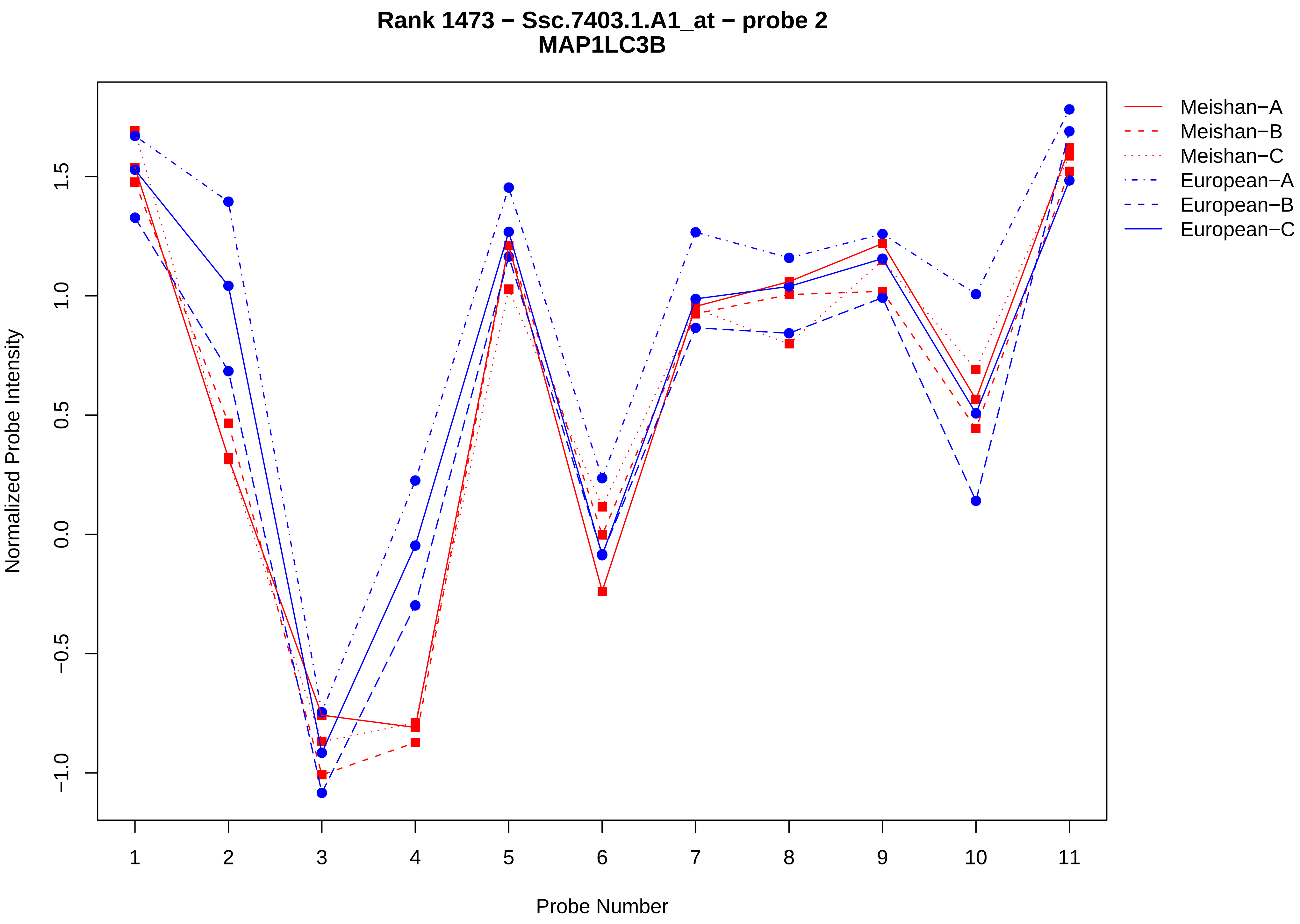

Rank 1473 − Ssc.7403.1.A1_at − probe 2
MAP1LC3B
Normalized Probe Intensity
Probe Number
−1.0
−0.5
0.0
0.5
1.0
1.5
1
2
3
4
5
6
7
8
9
10
11
Meishan−A
Meishan−B
Meishan−C
European−A
European−B
European−C

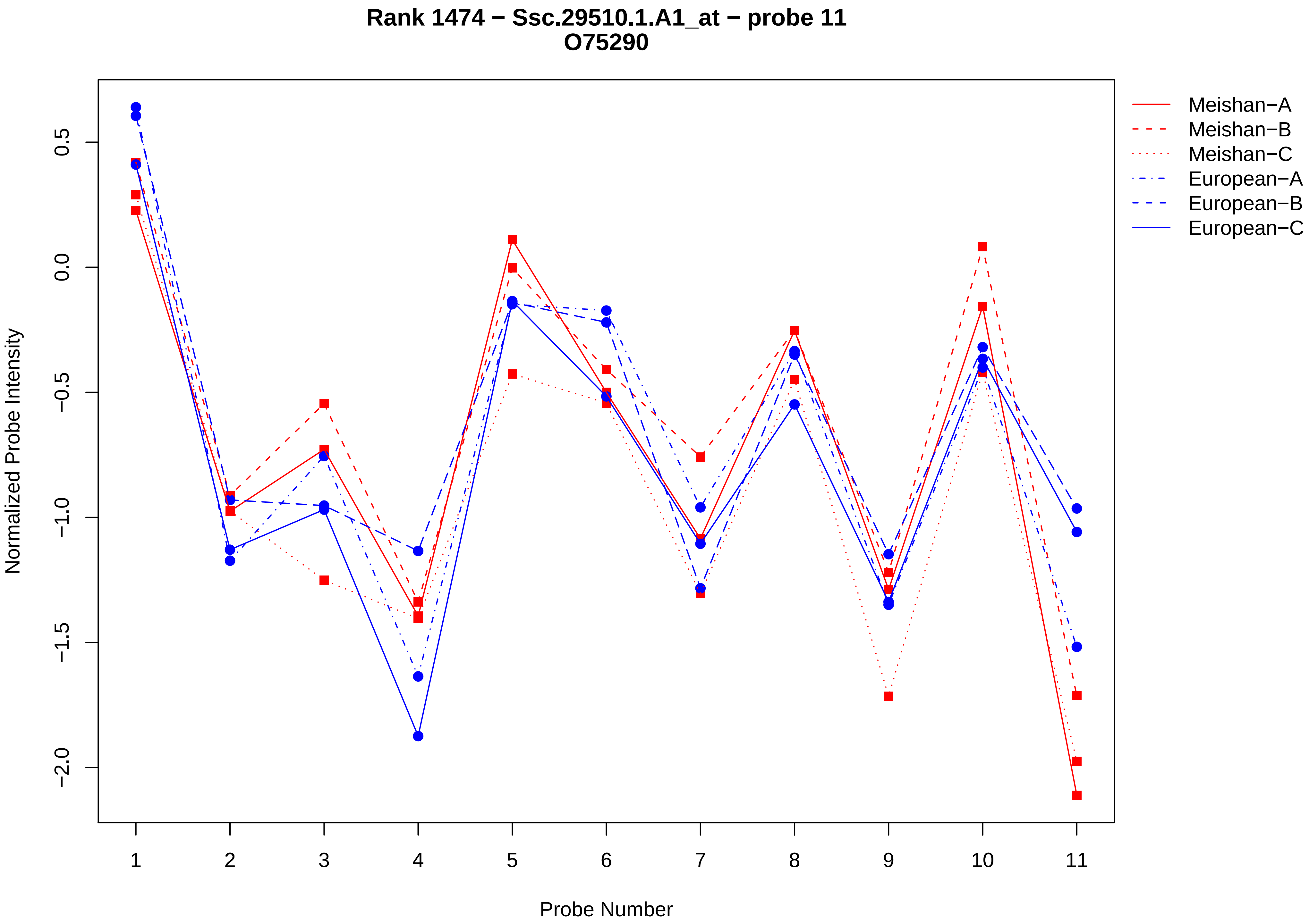

Rank 1474 − Ssc.29510.1.A1_at − probe 11
O75290
Normalized Probe Intensity
Probe Number
Meishan−A
Meishan−B
Meishan−C
European−A
European−B
European−C
0.5
0.0
−0.5
−1.0
−1.5
−2.0
1
2
3
4
5
6
7
8
9
10
11

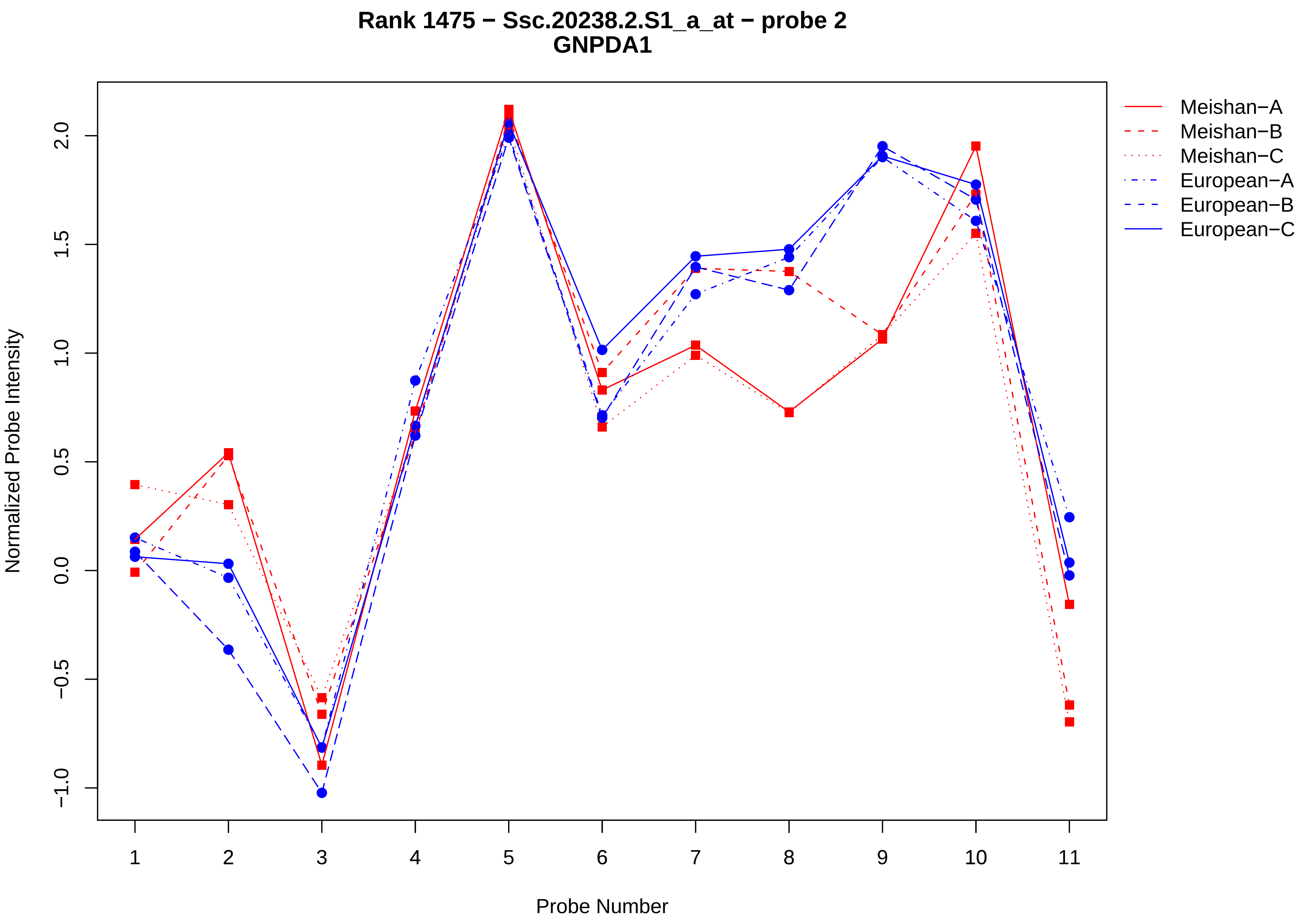

Rank 1475 − Ssc.20238.2.S1_a_at − probe 2
GNPDA1
Normalized Probe Intensity
Probe Number
−1.0
−0.5
0.0
0.5
1.0
1.5
2.0
1
2
3
4
5
6
7
8
9
10
11
Meishan−A
Meishan−B
Meishan−C
European−A
European−B
European−C

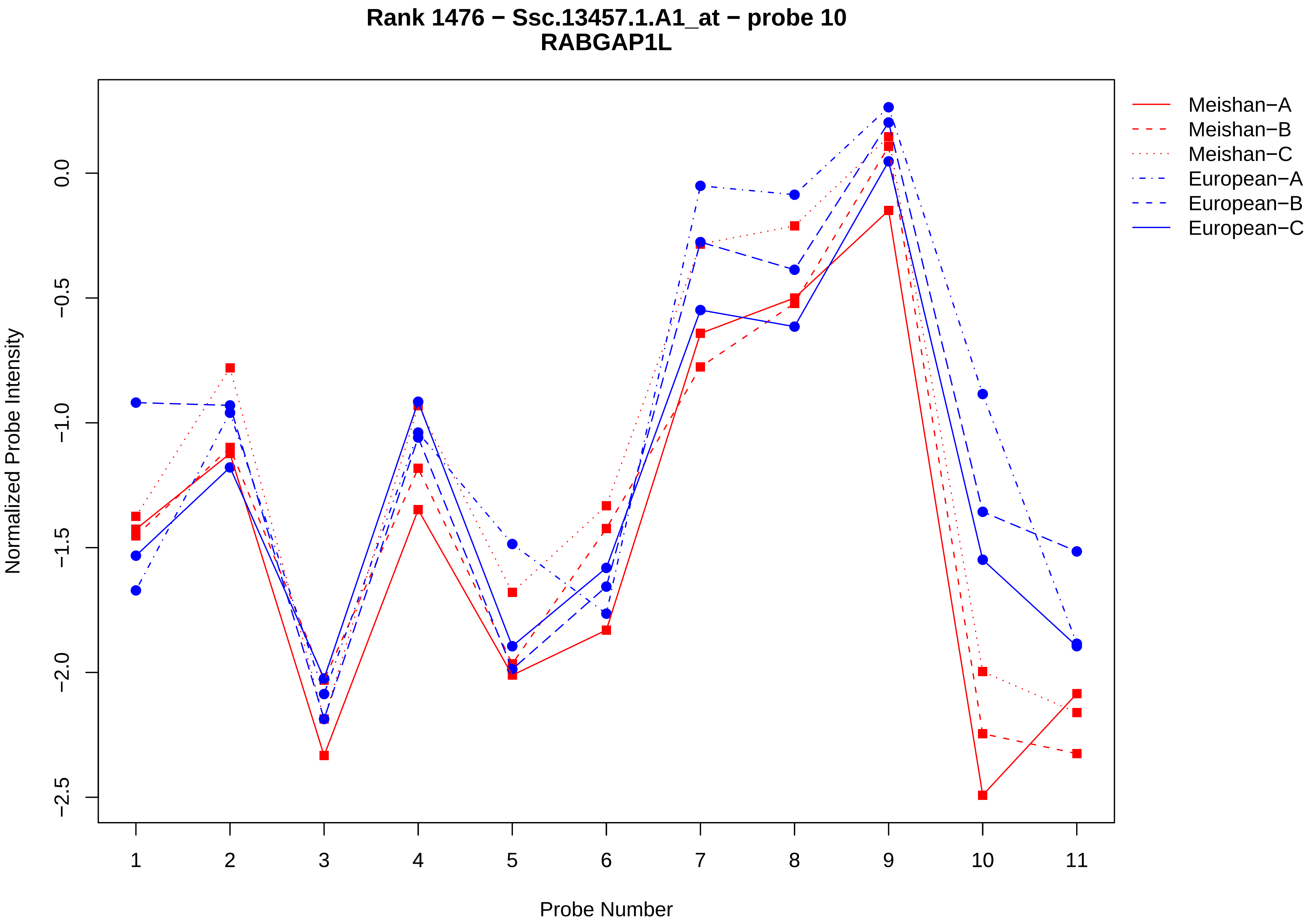

Rank 1476 − Ssc.13457.1.A1_at − probe 10
RABGAP1L
Normalized Probe Intensity
Probe Number
0.0
−0.5
−1.0
−1.5
−2.0
−2.5
1
2
3
4
5
6
7
8
9
10
11
Meishan−A
Meishan−B
Meishan−C
European−A
European−B
European−C

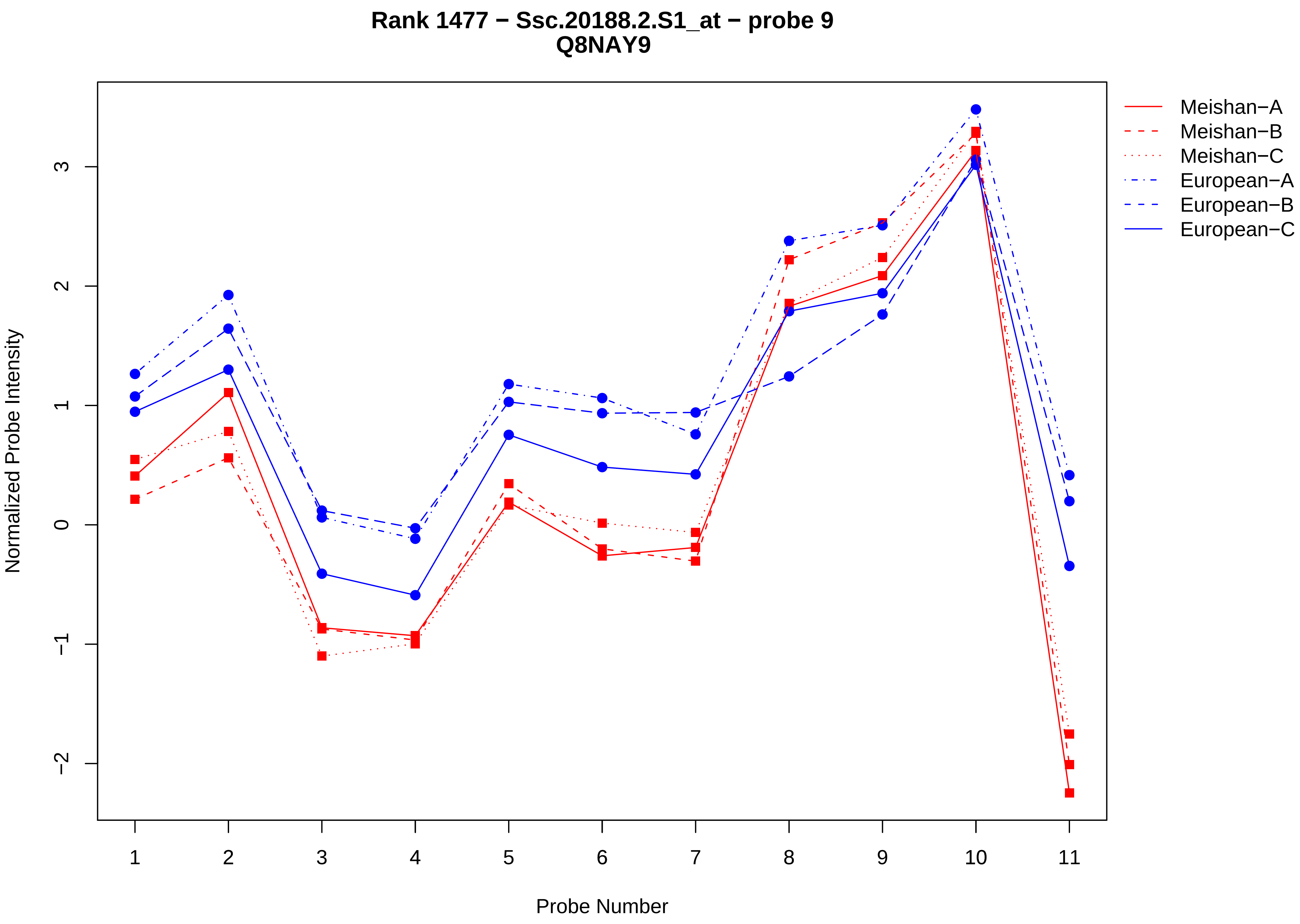

Rank 1477 − Ssc.20188.2.S1_at − probe 9
Q8NAY9
Normalized Probe Intensity
Probe Number
−2
−1
0
1
2
3
1
2
3
4
5
6
7
8
9
10
11
Meishan−A
Meishan−B
Meishan−C
European−A
European−B
European−C

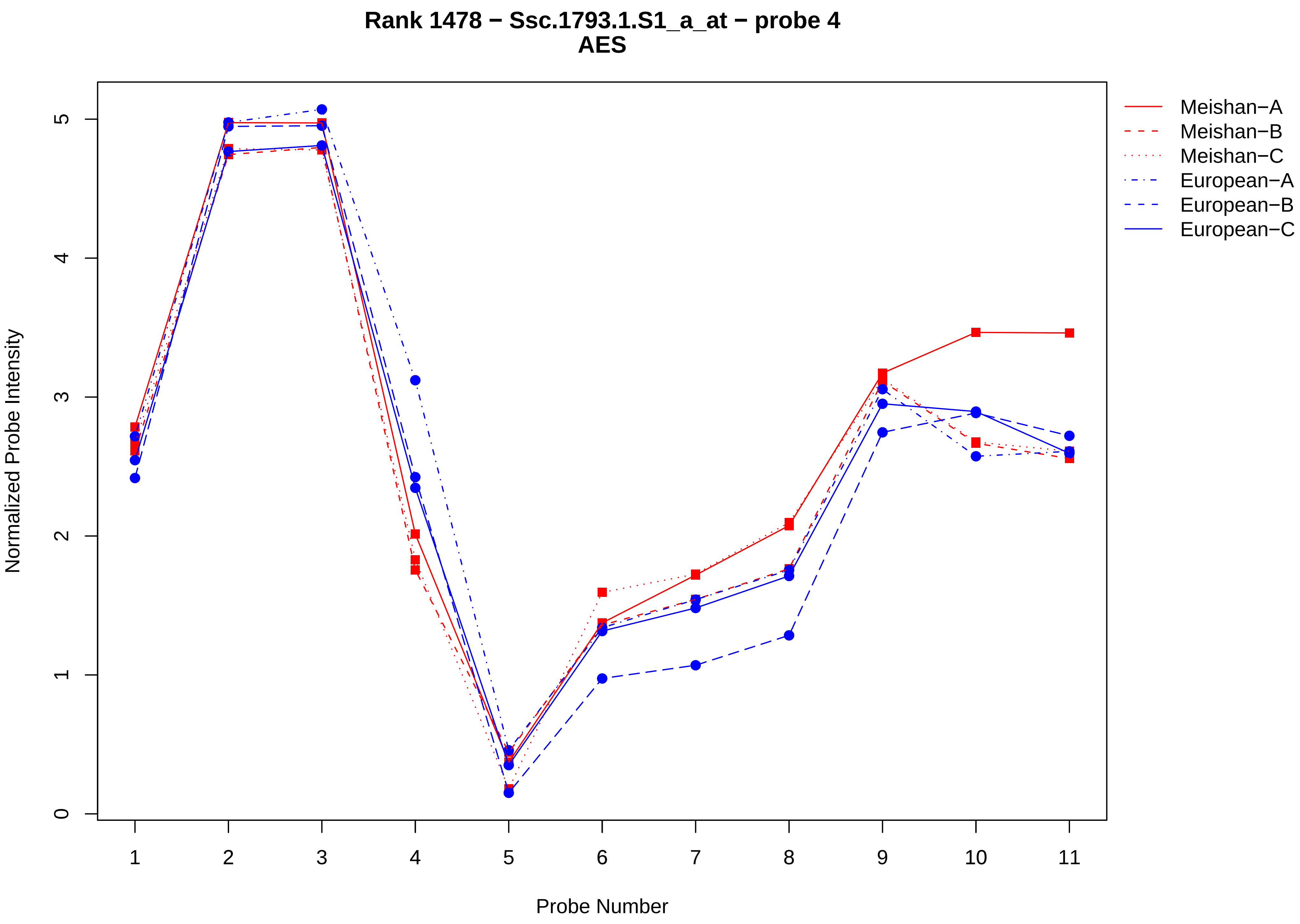

Rank 1478 − Ssc.1793.1.S1_a_at − probe 4
AES
Normalized Probe Intensity
Probe Number
Meishan−A
Meishan−B
Meishan−C
European−A
European−B
European−C

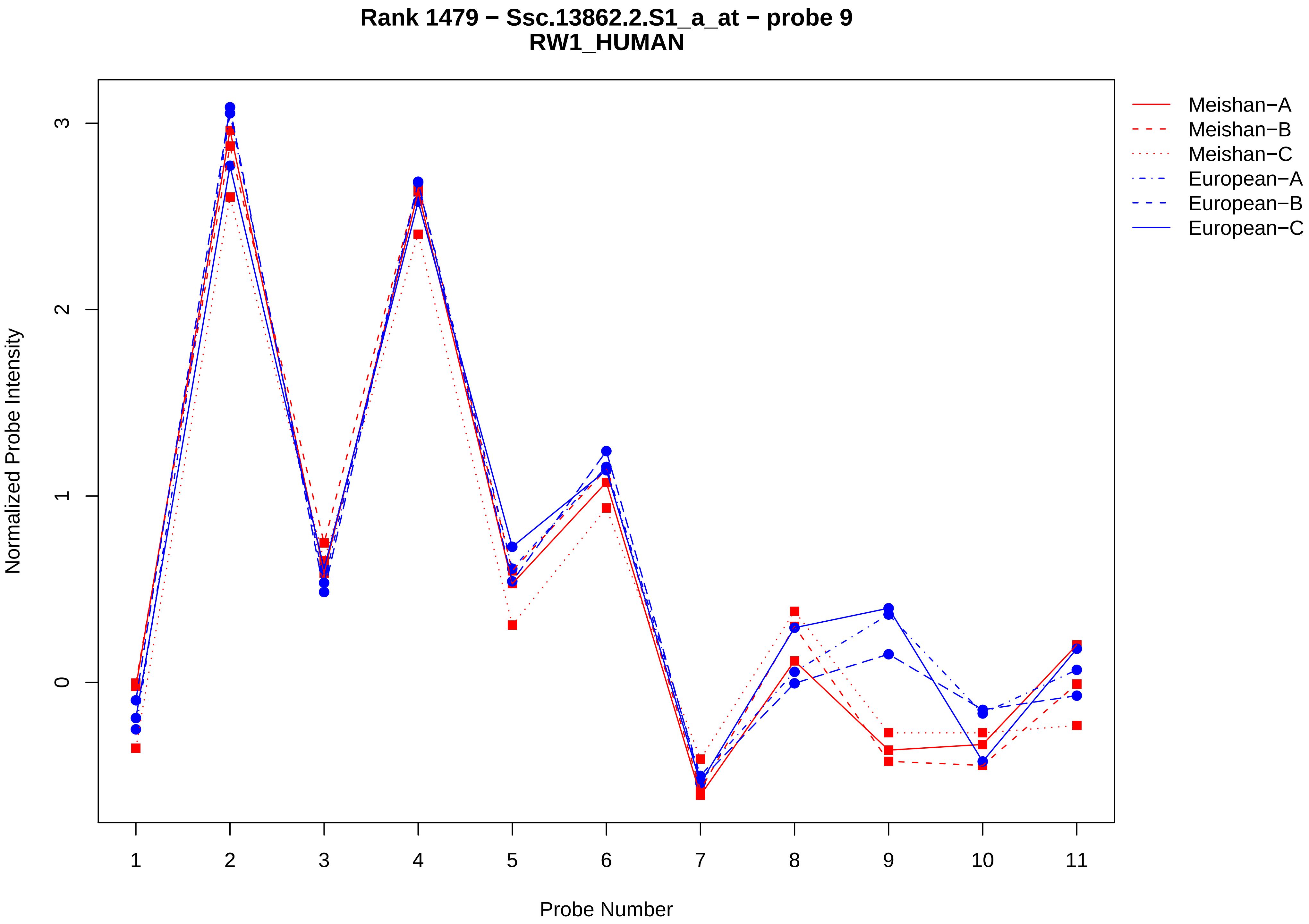

Rank 1479 − Ssc.13862.2.S1_a_at − probe 9
RW1_HUMAN
Normalized Probe Intensity
Probe Number
0
1
2
3
1
2
3
4
5
6
7
8
9
10
11
Meishan−A
Meishan−B
Meishan−C
European−A
European−B
European−C

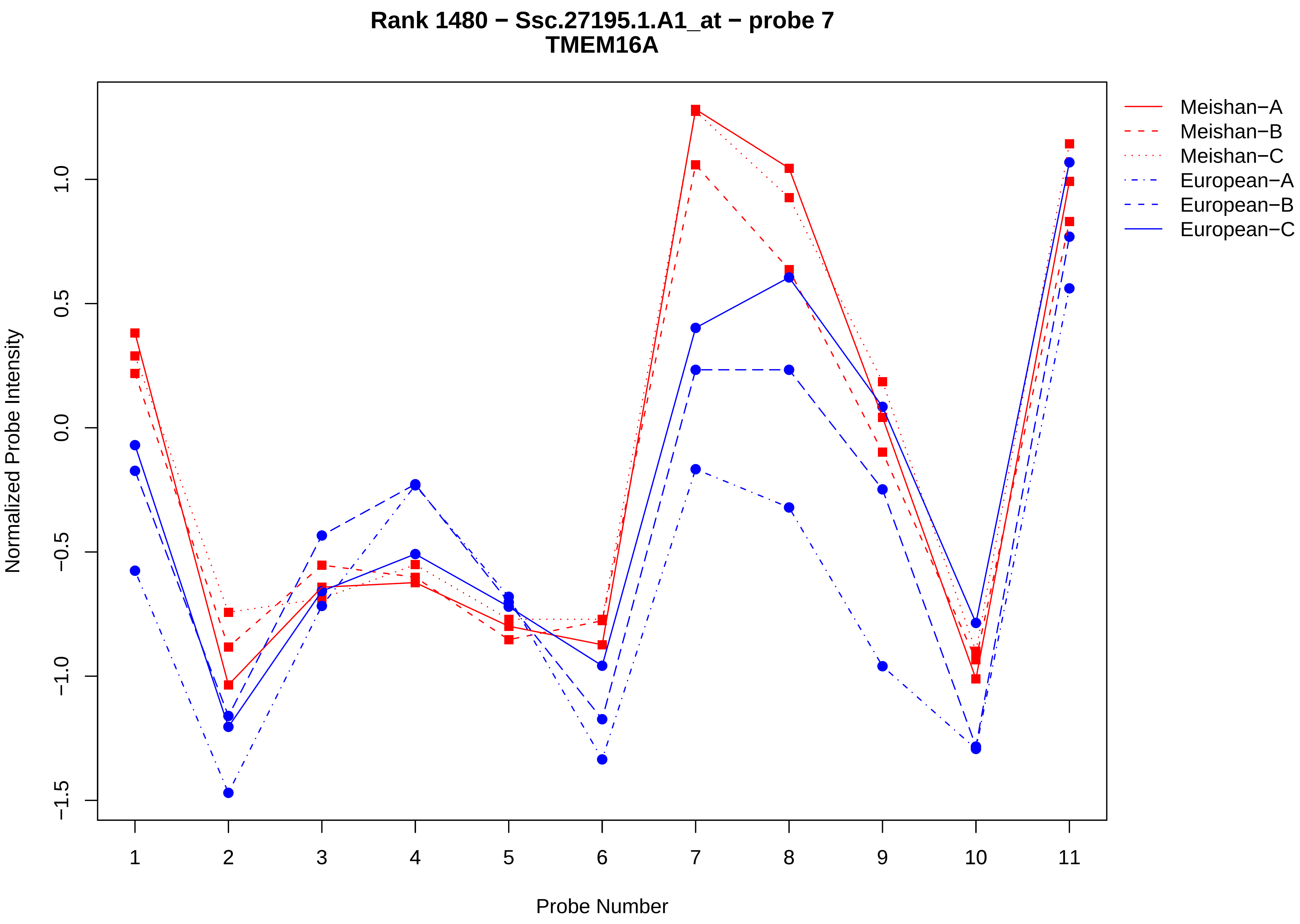

Rank 1480 − Ssc.27195.1.A1_at − probe 7
TMEM16A
Normalized Probe Intensity
Probe Number
−1.5
−1.0
−0.5
0.0
0.5
1.0
1
2
3
4
5
6
7
8
9
10
11
Meishan−A
Meishan−B
Meishan−C
European−A
European−B
European−C

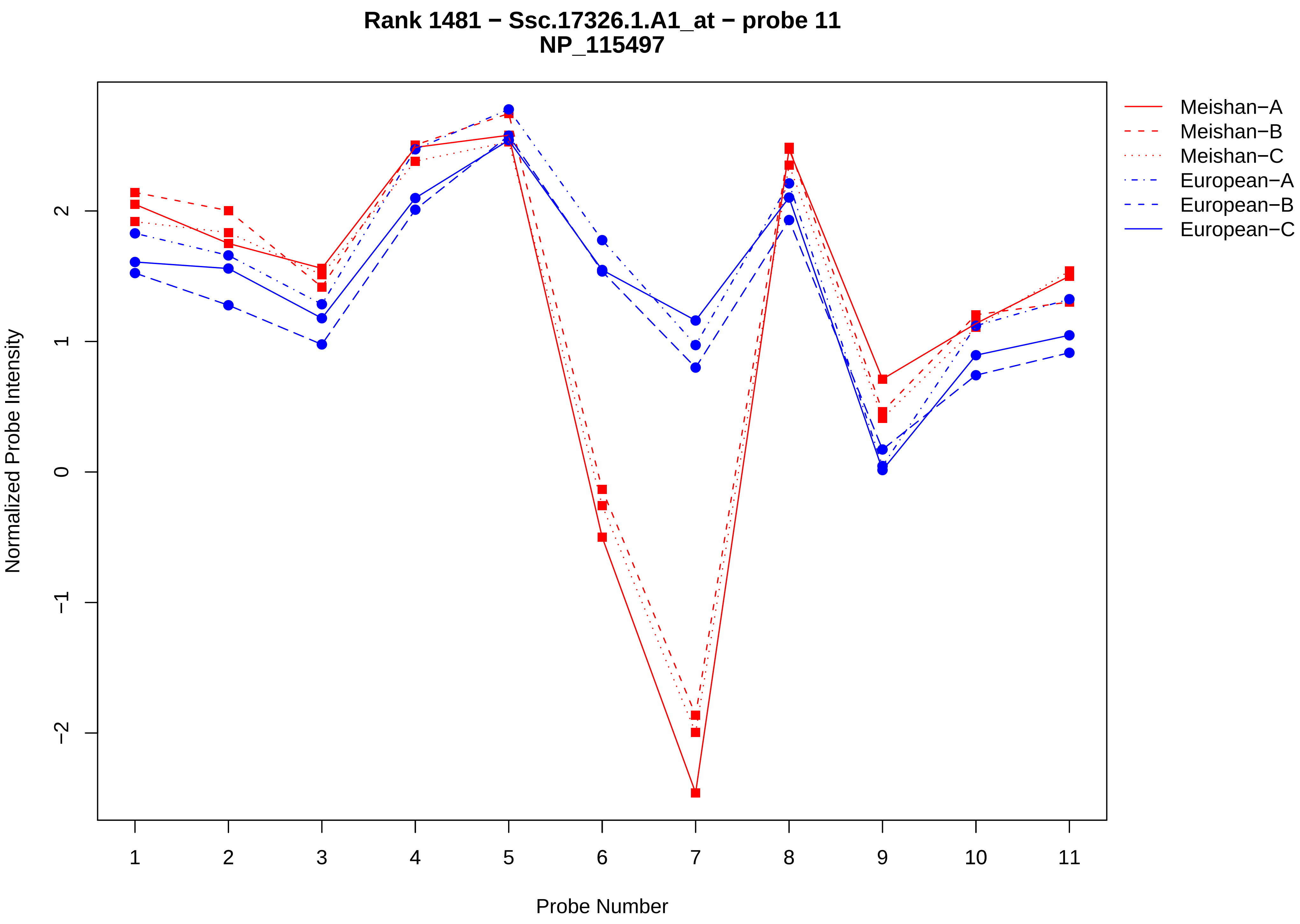
Rank 1481 − Ssc.17326.1.A1_at − probe 11
NP_115497
Normalized Probe Intensity
Probe Number
−2
−1
0
1
2
1
2
3
4
5
6
7
8
9
10
11
Meishan−A
Meishan−B
Meishan−C
European−A
European−B
European−C

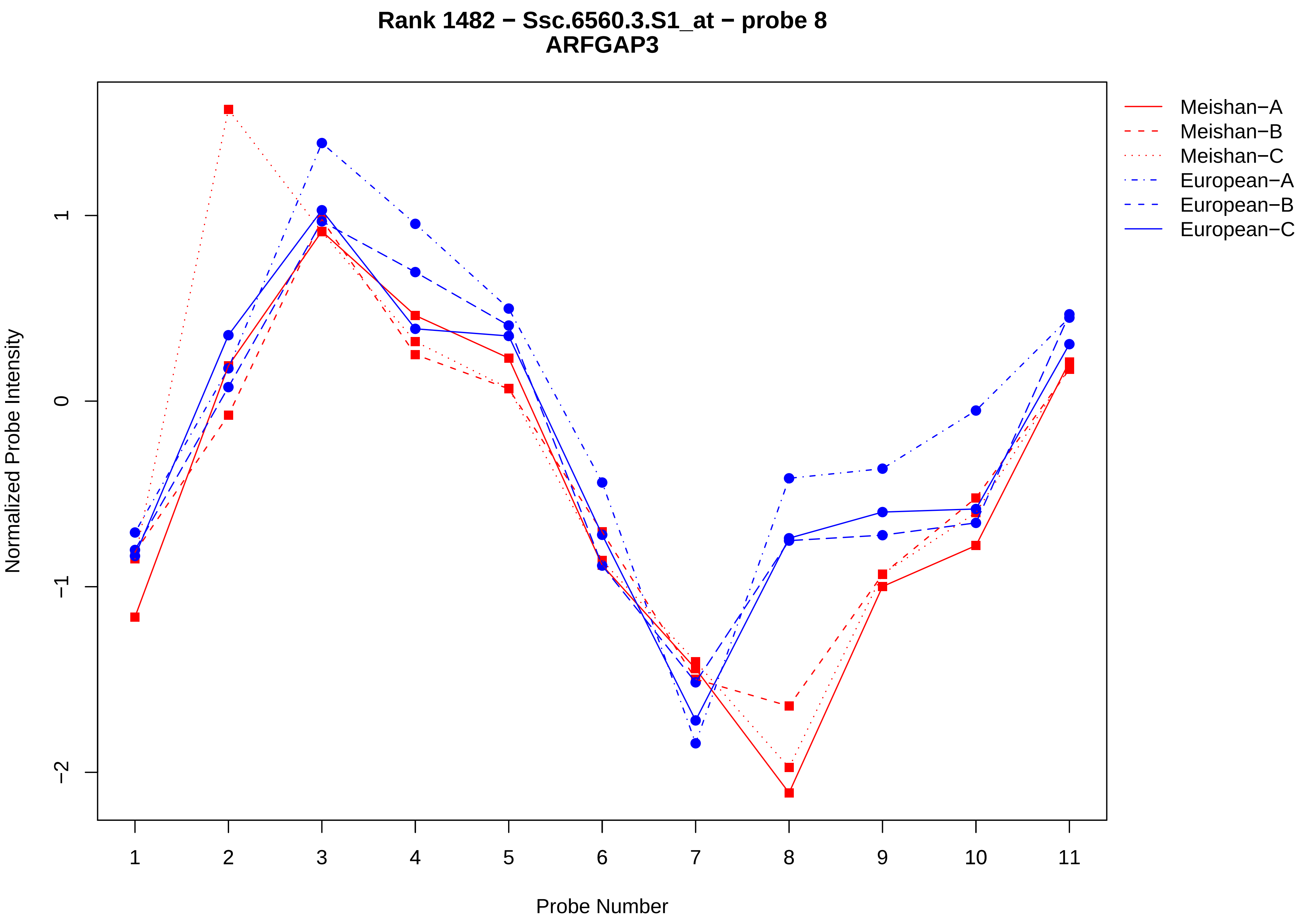

Rank 1482 − Ssc.6560.3.S1_at − probe 8
ARFGAP3
Normalized Probe Intensity
Probe Number
1
0
−1
−2
1
2
3
4
5
6
7
8
9
10
11
Meishan−A
Meishan−B
Meishan−C
European−A
European−B
European−C

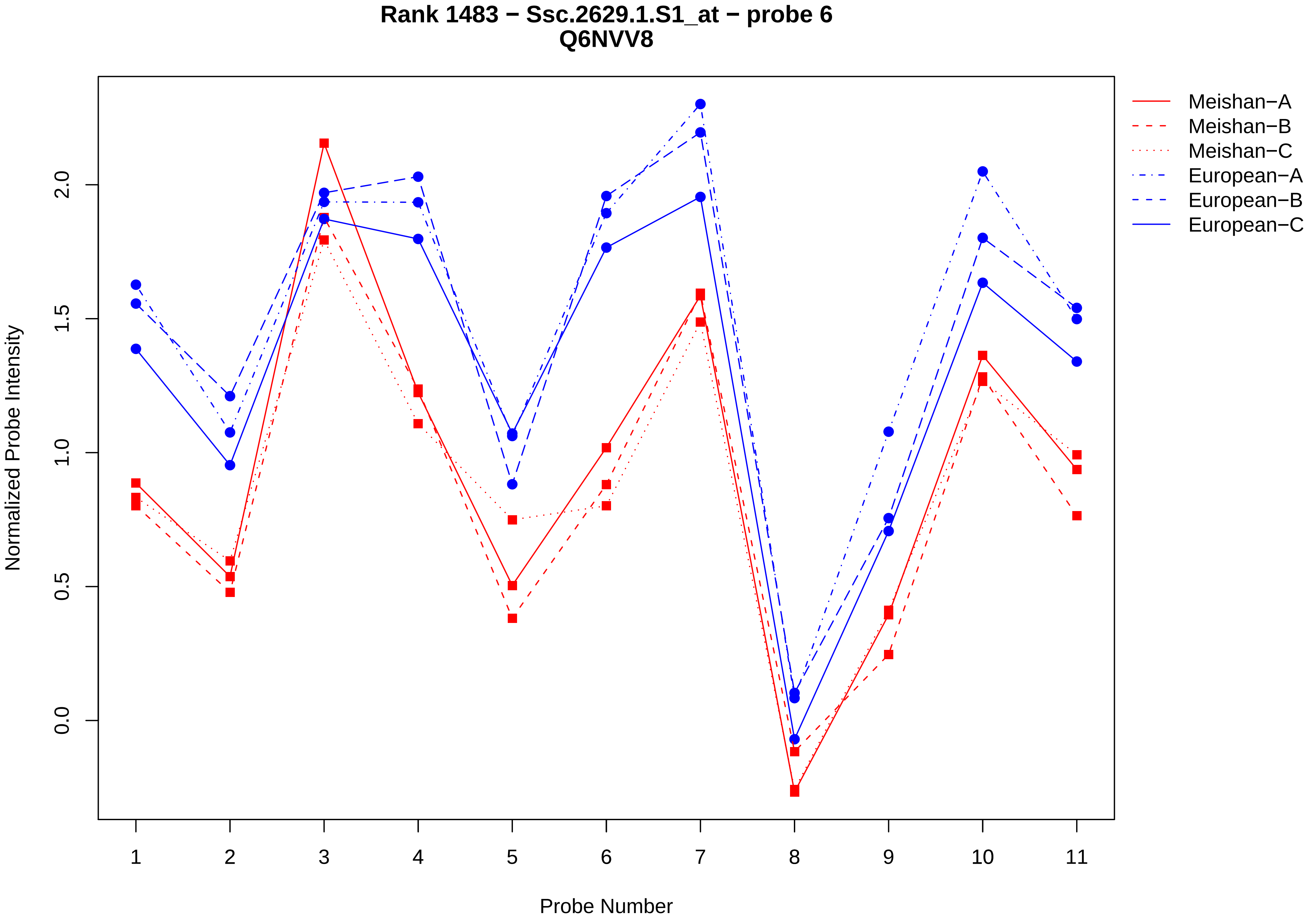

Rank 1483 − Ssc.2629.1.S1_at − probe 6
Q6NVV8
Normalized Probe Intensity
Probe Number
0.0
0.5
1.0
1.5
2.0
1
2
3
4
5
6
7
8
9
10
11
Meishan−A
Meishan−B
Meishan−C
European−A
European−B
European−C

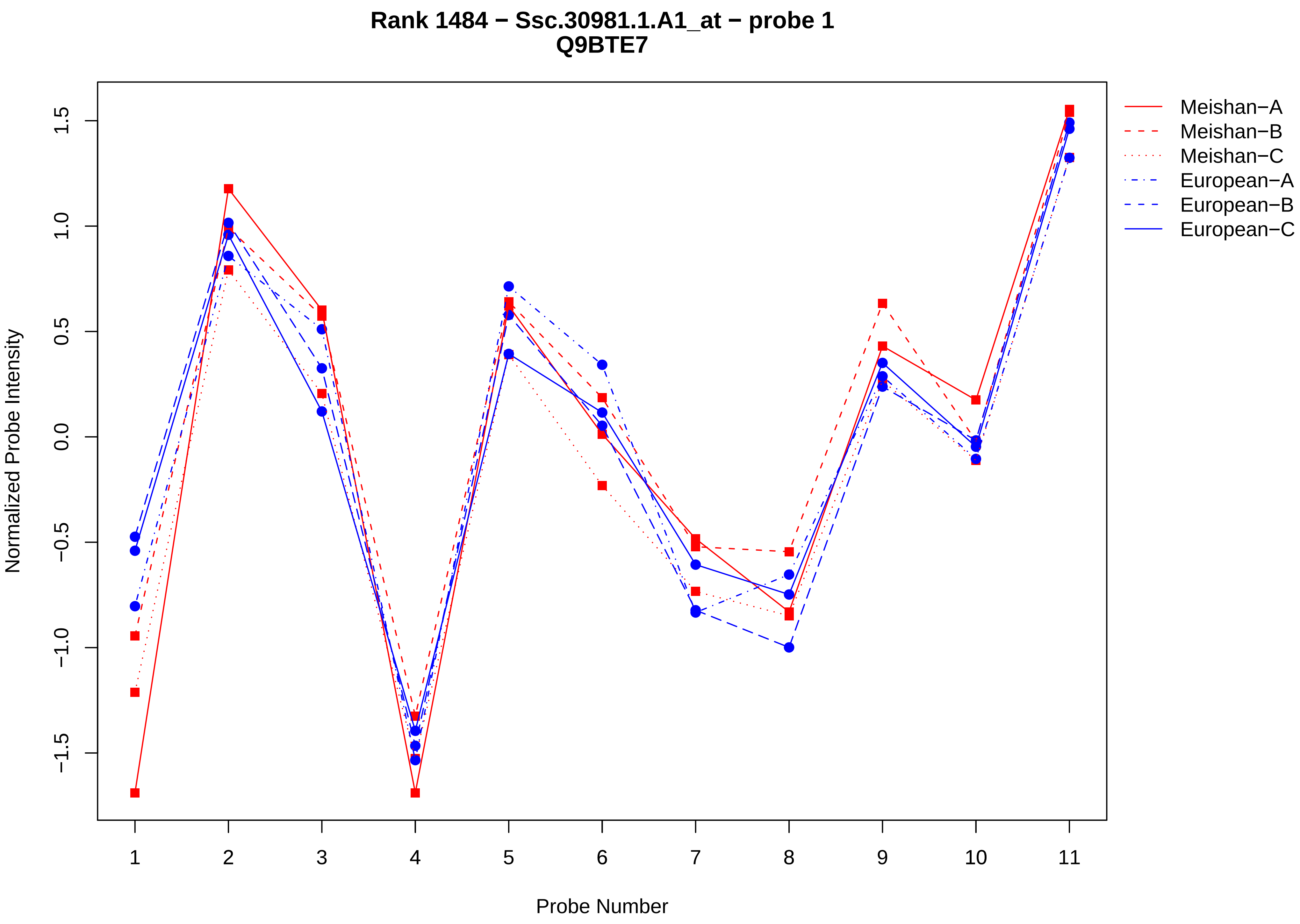

Rank 1484 − Ssc.30981.1.A1_at − probe 1
Q9BTE7
Normalized Probe Intensity
Probe Number
1.5
1.0
0.5
0.0
−0.5
−1.0
−1.5
1
2
3
4
5
6
7
8
9
10
11
Meishan−A
Meishan−B
Meishan−C
European−A
European−B
European−C

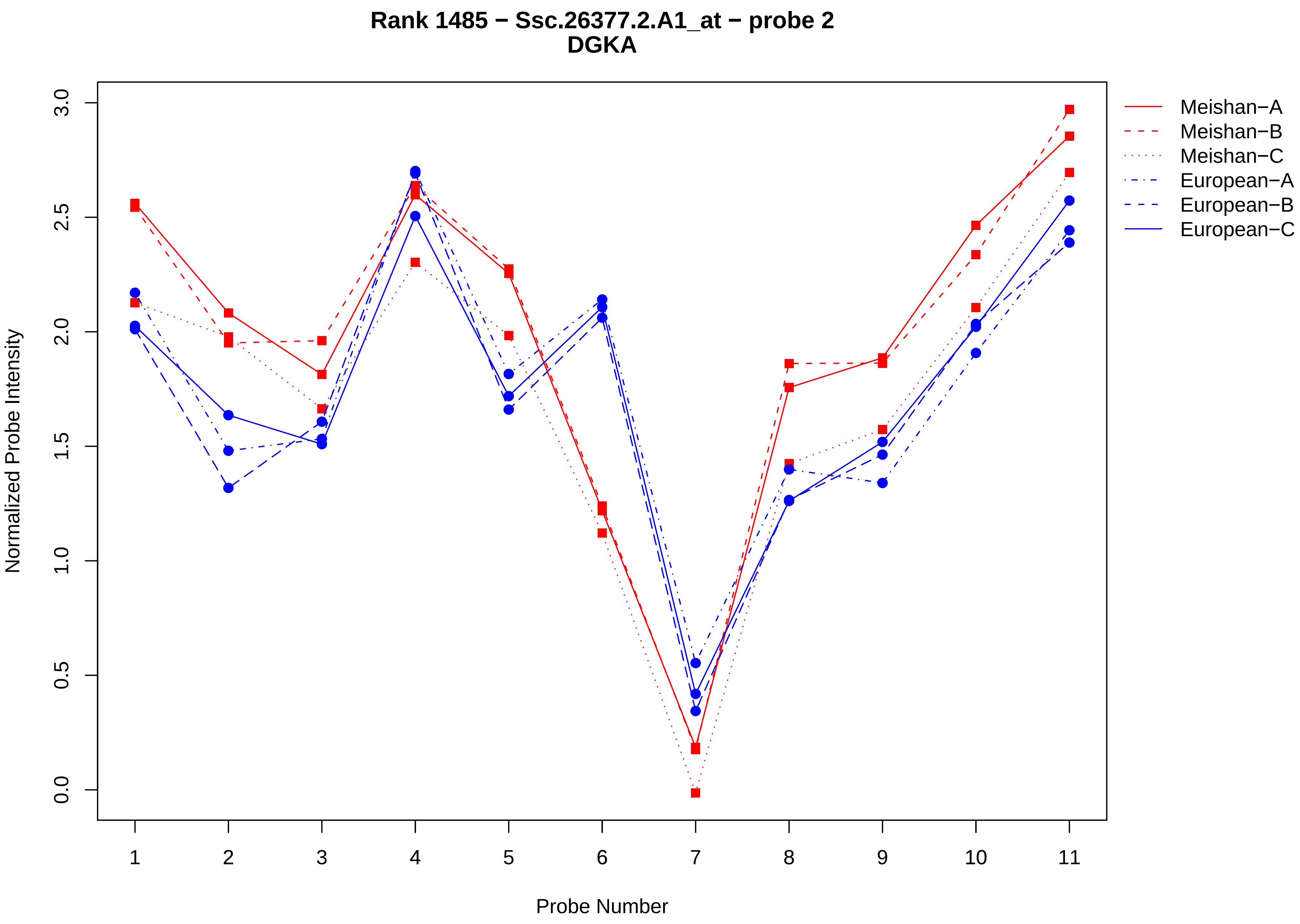
Rank 1485 − Ssc.26377.2.A1_at − probe 2
DGKA
Normalized Probe Intensity
0.0
0.5
1.0
1.5
2.0
2.5
3.0
Probe Number
1
2
3
4
5
6
7
8
9
10
11
Meishan−A
Meishan−B
Meishan−C
European−A
European−B
European−C

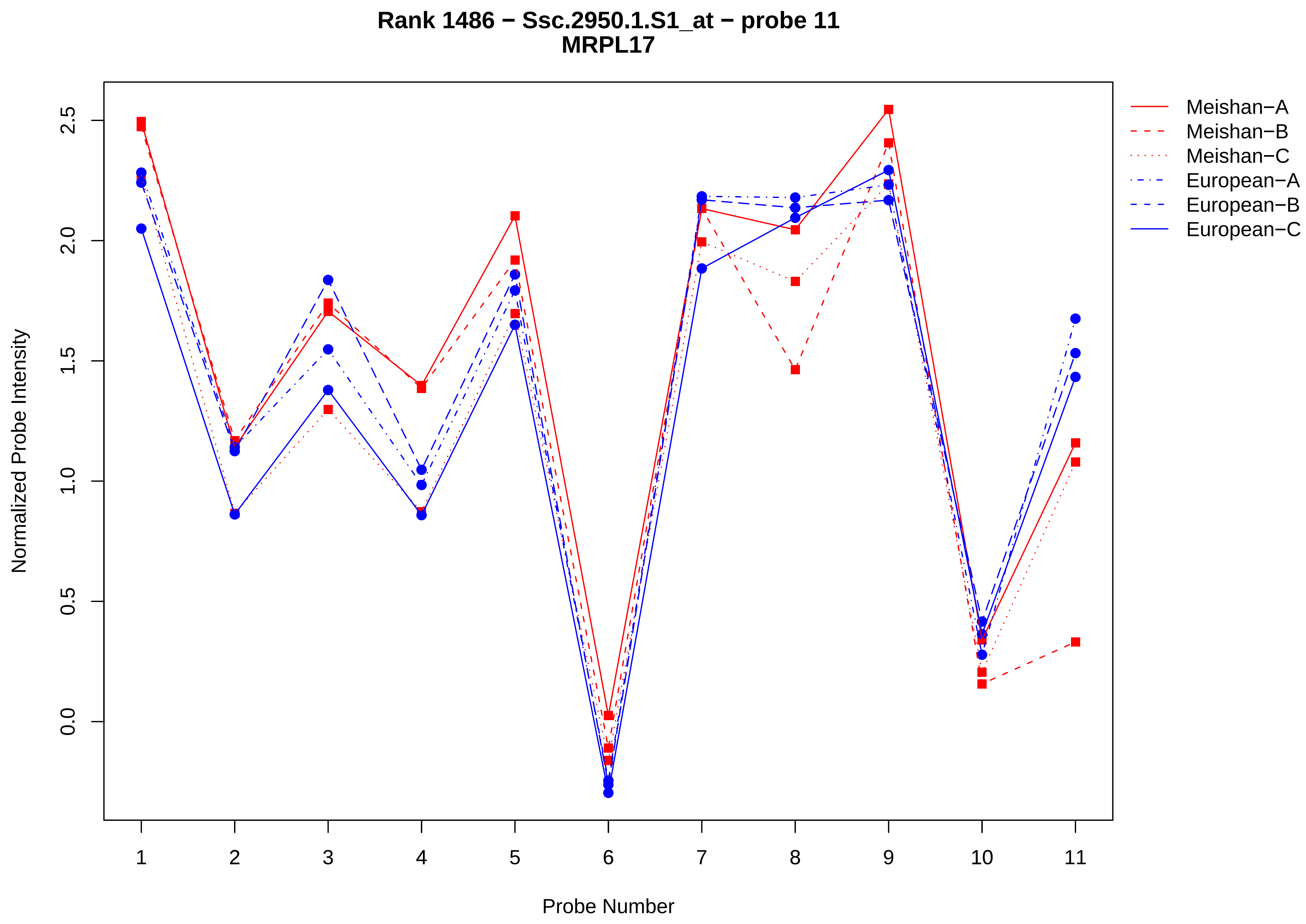

Rank 1486 − Ssc.2950.1.S1_at − probe 11
MRPL17
Normalized Probe Intensity
Probe Number
0.0
0.5
1.0
1.5
2.0
2.5
1
2
3
4
5
6
7
8
9
10
11
Meishan−A
Meishan−B
Meishan−C
European−A
European−B
European−C

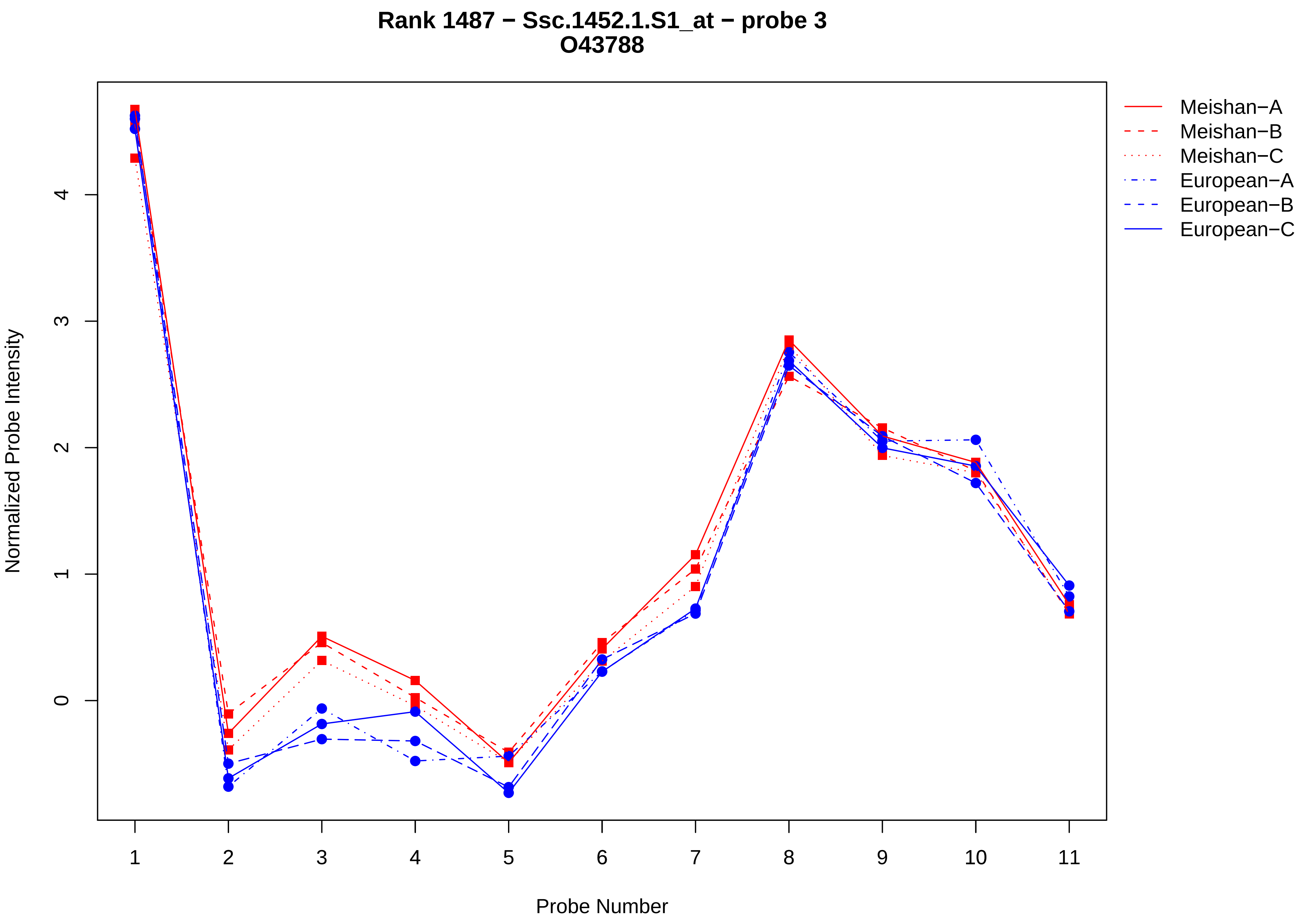

Rank 1487 − Ssc.1452.1.S1_at − probe 3
O43788
Normalized Probe Intensity
Probe Number
0
1
2
3
4
1
2
3
4
5
6
7
8
9
10
11
Meishan−A
Meishan−B
Meishan−C
European−A
European−B
European−C

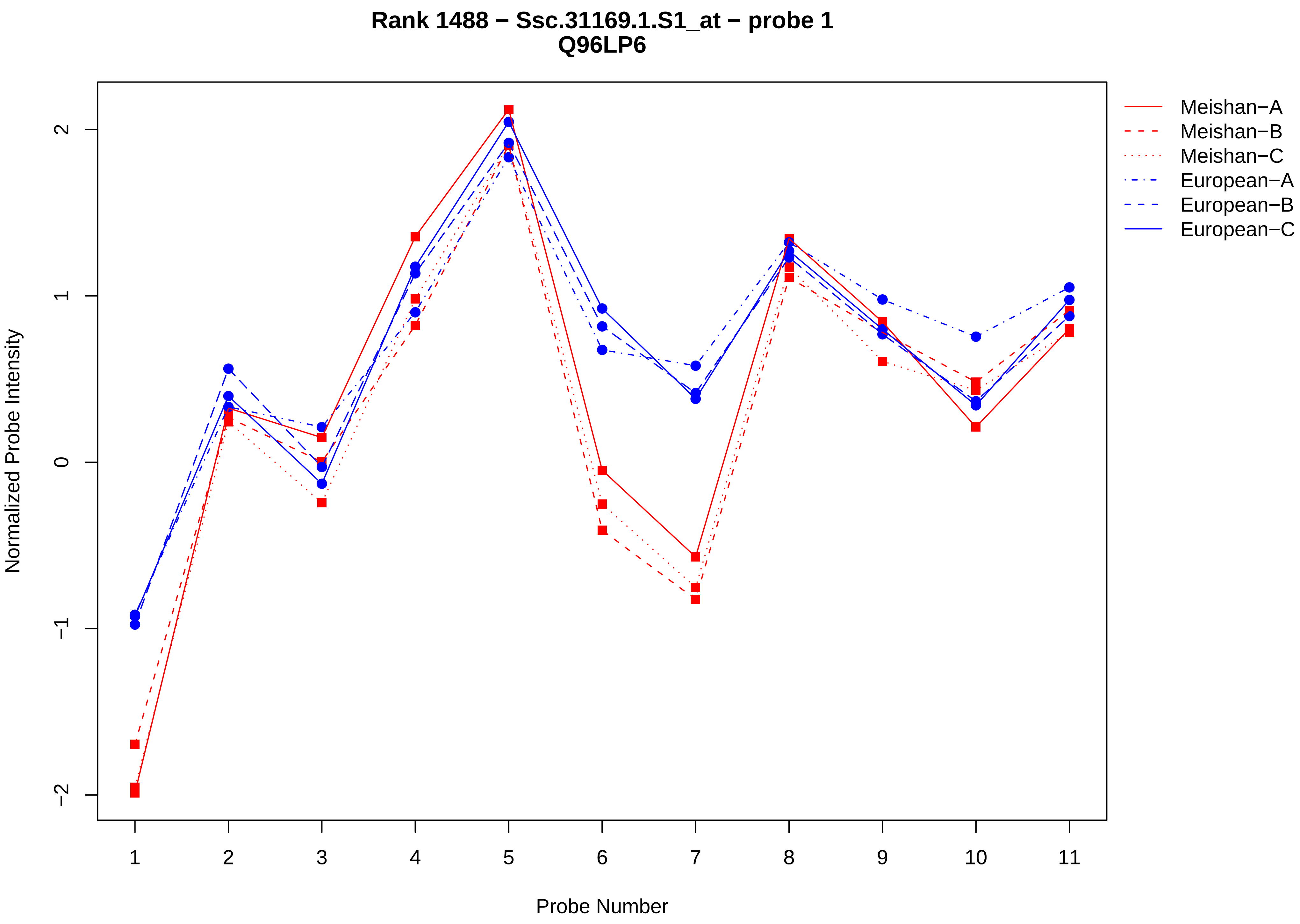

Rank 1488 − Ssc.31169.1.S1_at − probe 1
Q96LP6
Normalized Probe Intensity
Probe Number
−2
−1
0
1
2
1
2
3
4
5
6
7
8
9
10
11
Meishan−A
Meishan−B
Meishan−C
European−A
European−B
European−C

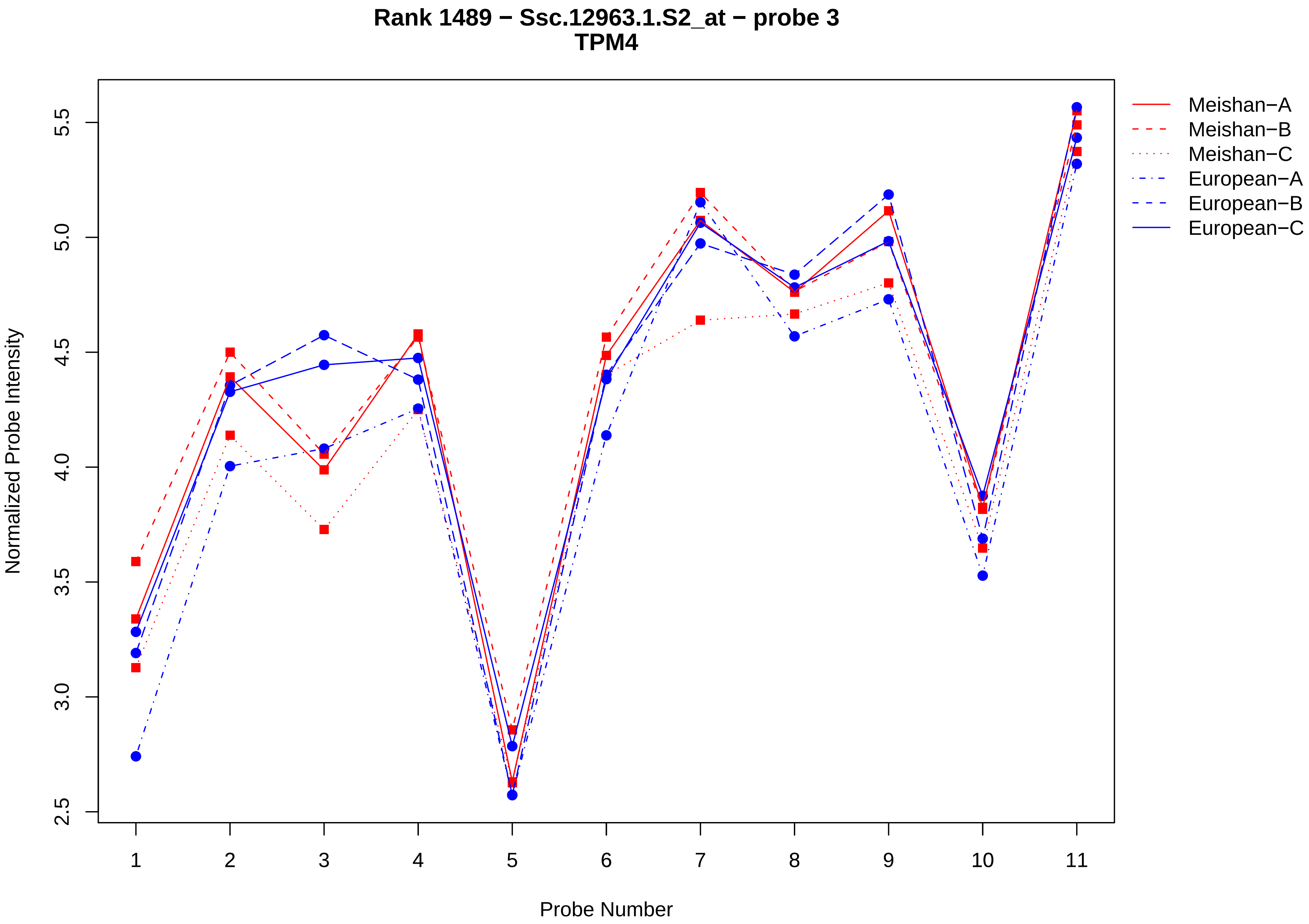

Rank 1489 − Ssc.12963.1.S2_at − probe 3
TPM4
Normalized Probe Intensity
Probe Number
2.5
3.0
3.5
4.0
4.5
5.0
5.5
1
2
3
4
5
6
7
8
9
10
11
Meishan−A
Meishan−B
Meishan−C
European−A
European−B
European−C

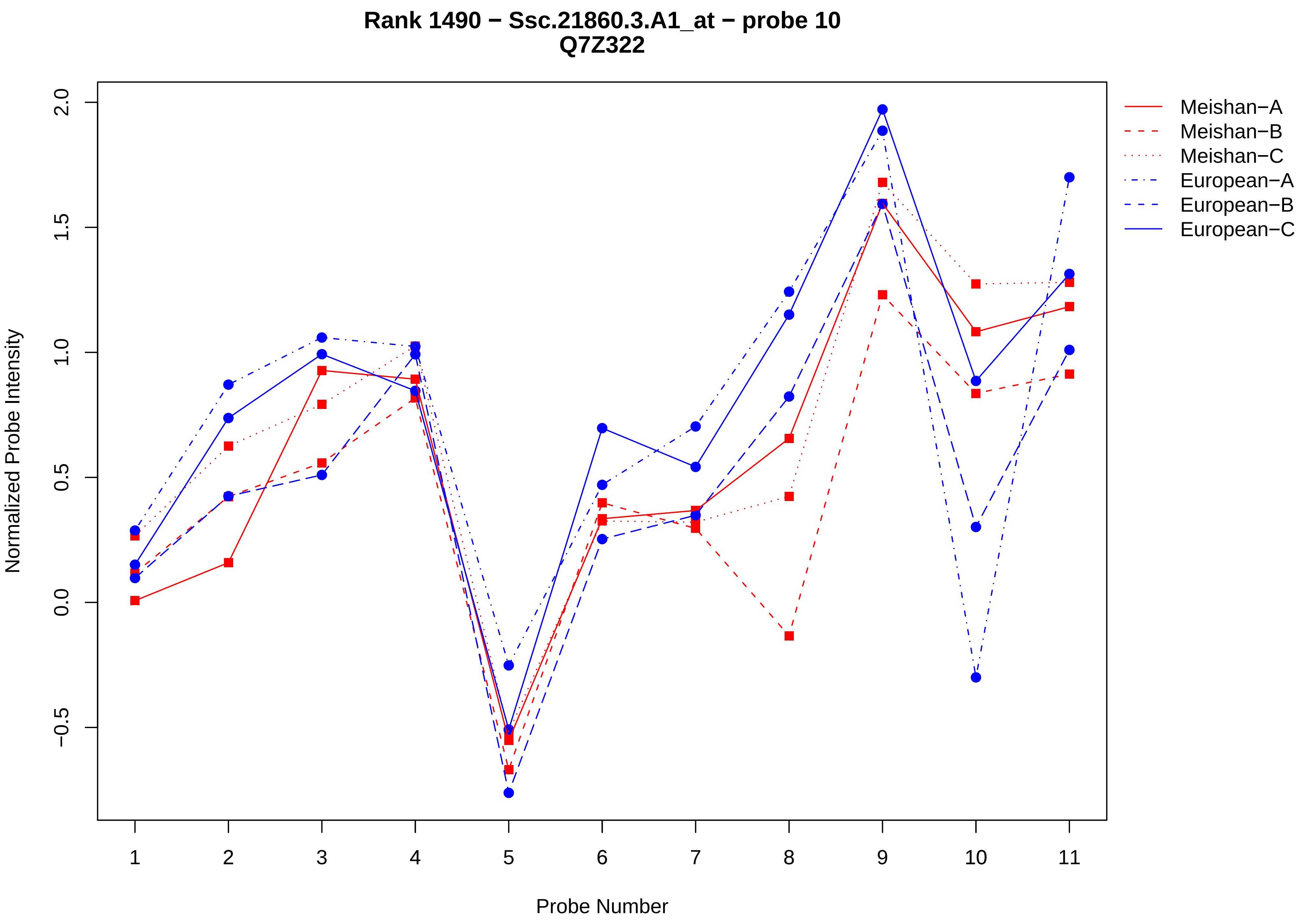

Rank 1490 − Ssc.21860.3.A1_at − probe 10
Q7Z322
Normalized Probe Intensity
Probe Number
Meishan−A
Meishan−B
Meishan−C
European−A
European−B
European−C

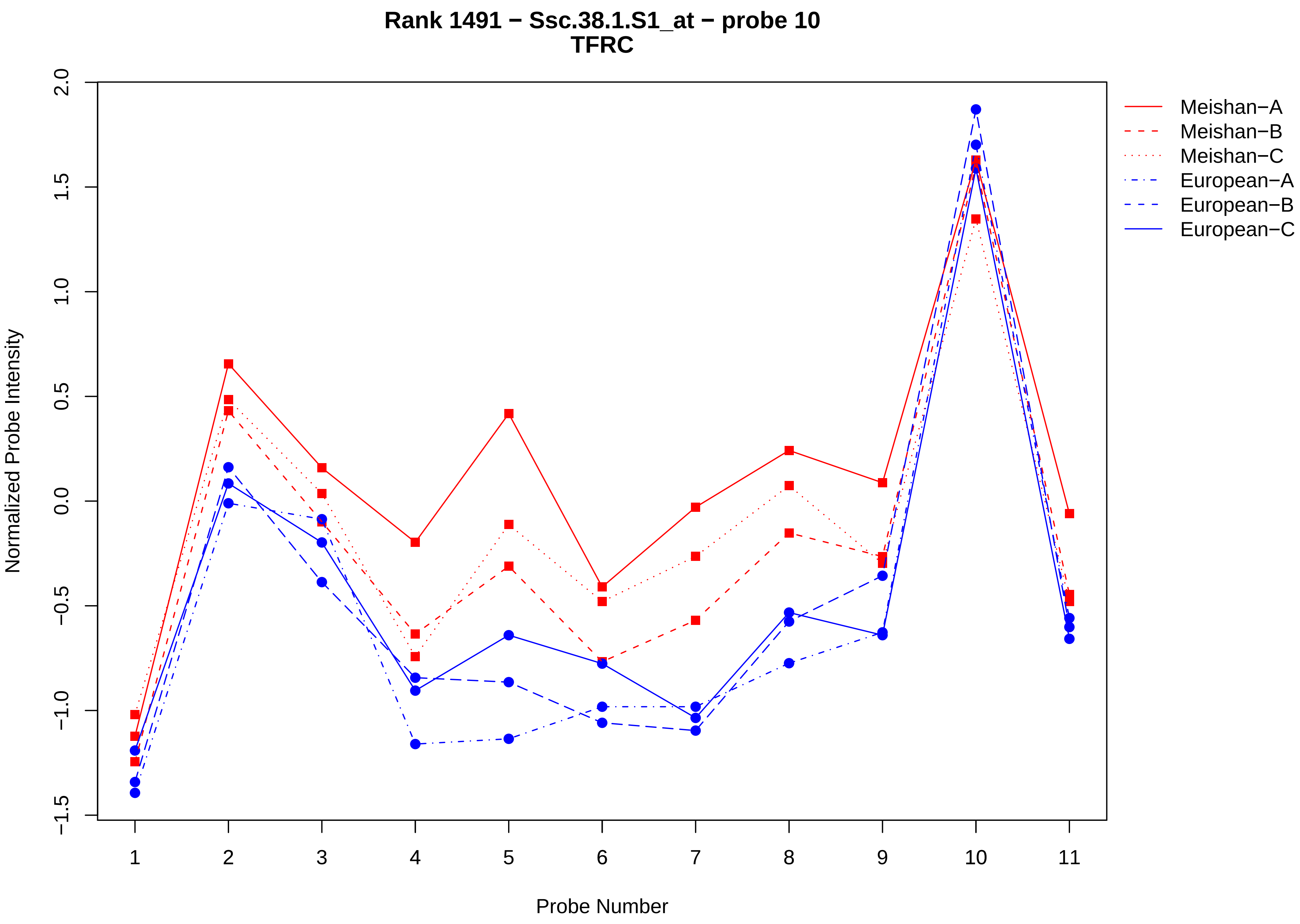
Rank 1491 − Ssc.38.1.S1_at − probe 10
TFRC
Normalized Probe Intensity
Probe Number
Meishan−A
Meishan−B
Meishan−C
European−A
European−B
European−C
−1.5
−1.0
−0.5
0.0
0.5
1.0
1.5
2.0
1
2
3
4
5
6
7
8
9
10
11

Rank 1492 − Ssc.28007.1.A1_at − probe 2
Q7Z6B8

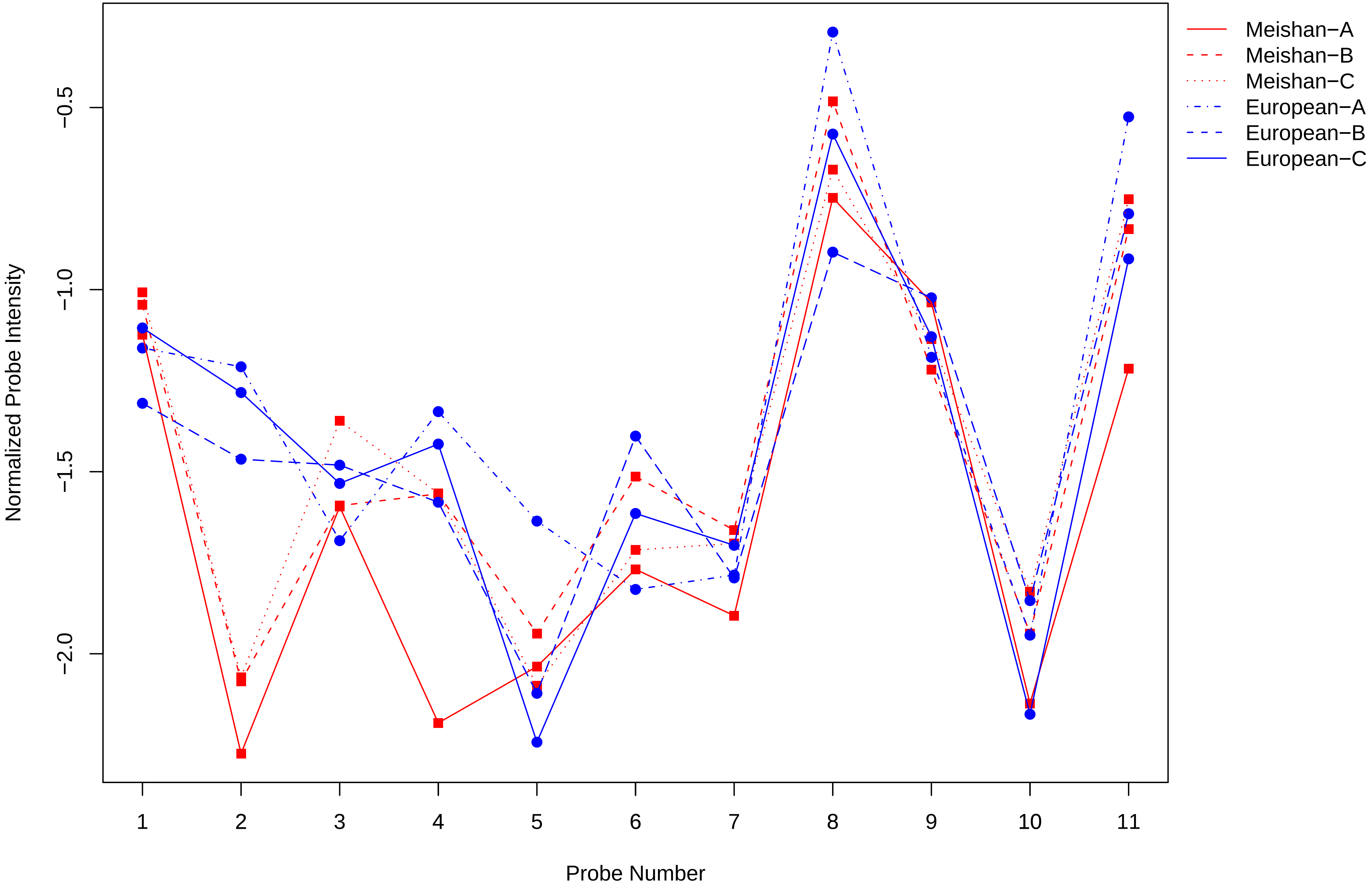

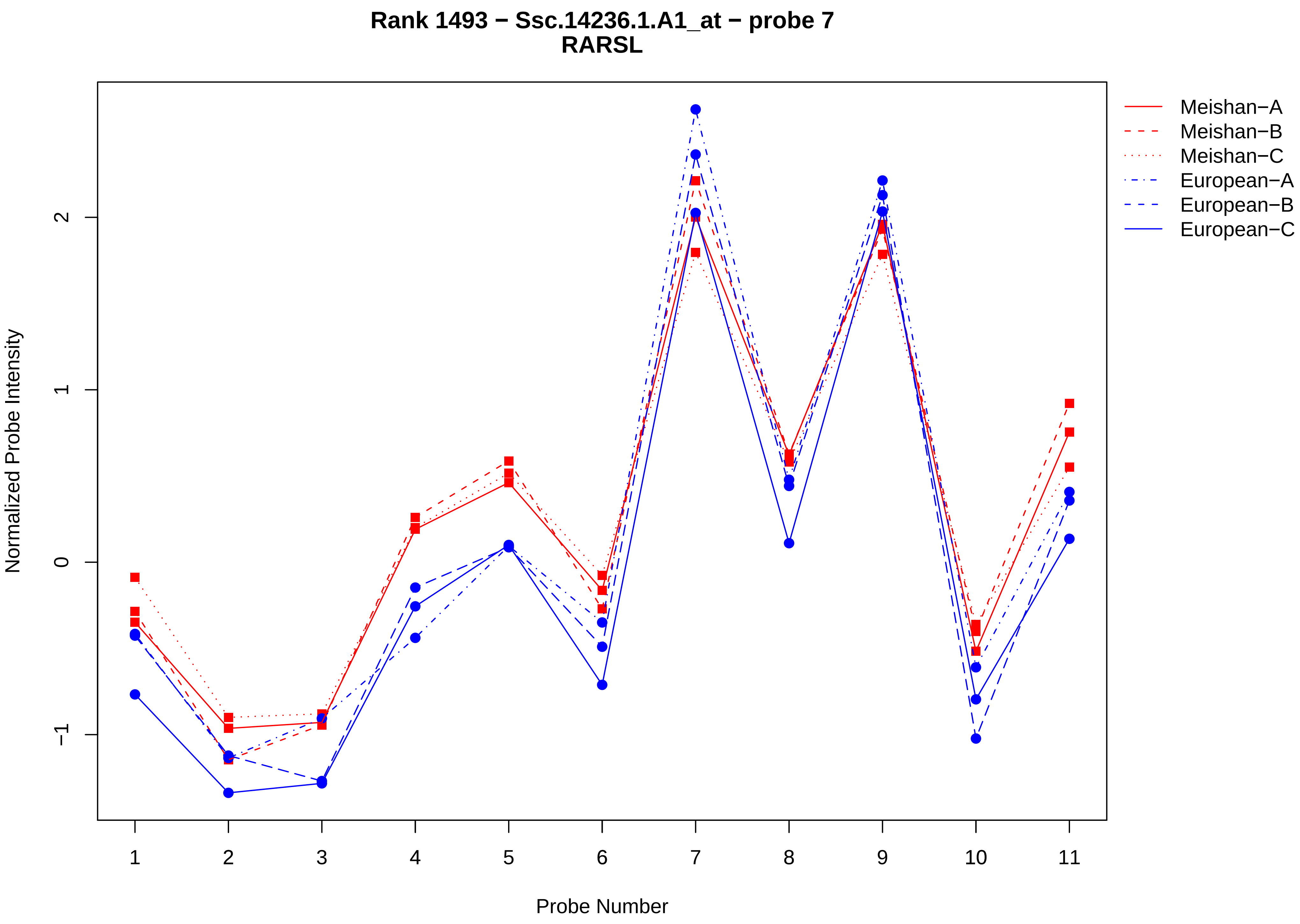

Rank 1493 − Ssc.14236.1.A1_at − probe 7
RARSL
Normalized Probe Intensity
Probe Number
−1
0
1
2
1
2
3
4
5
6
7
8
9
10
11
Meishan−A
Meishan−B
Meishan−C
European−A
European−B
European−C

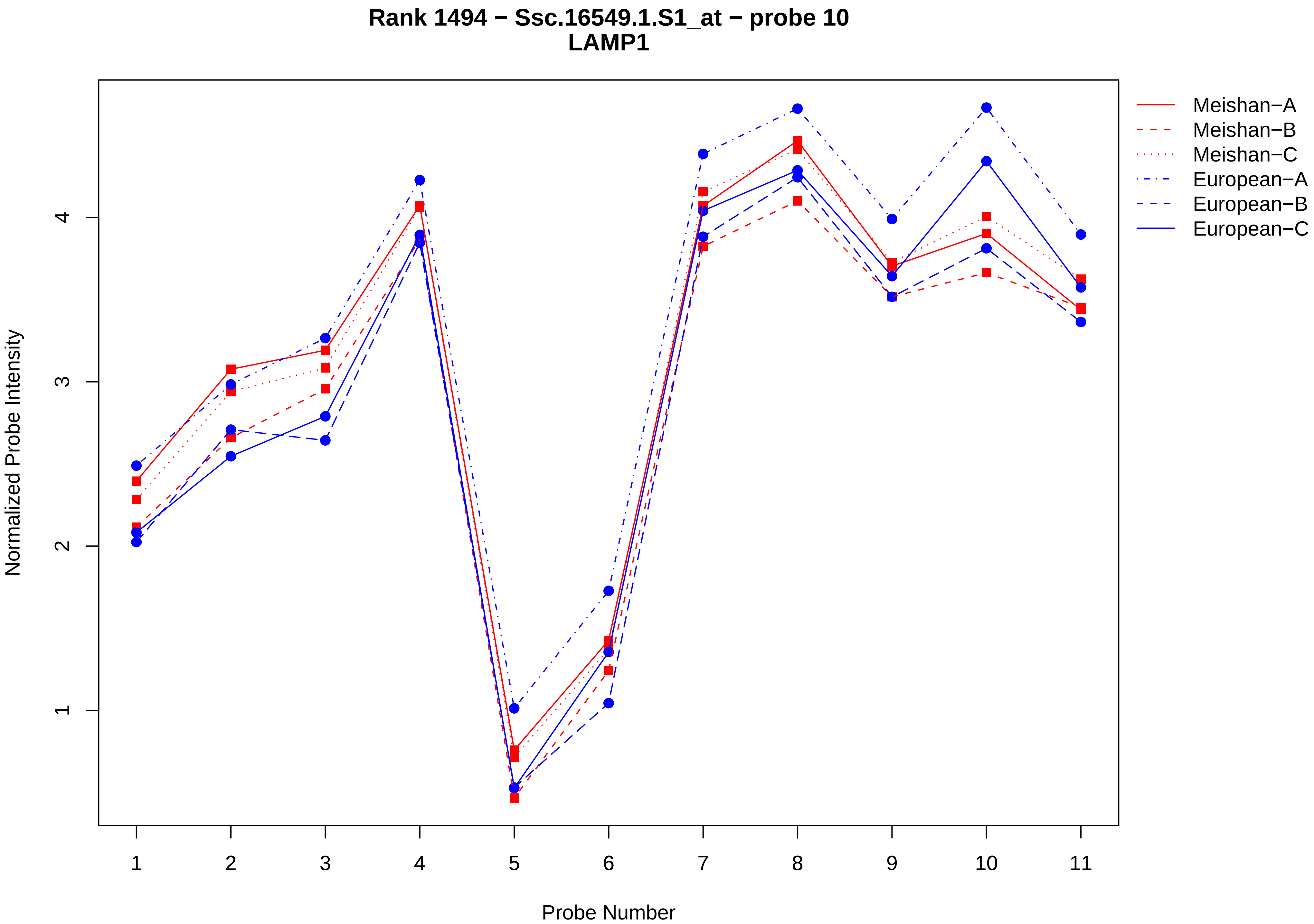

Rank 1494 − Ssc.16549.1.S1_at − probe 10
LAMP1
Normalized Probe Intensity
Probe Number
1
2
3
4
1
2
3
4
5
6
7
8
9
10
11
Meishan−A
Meishan−B
Meishan−C
European−A
European−B
European−C

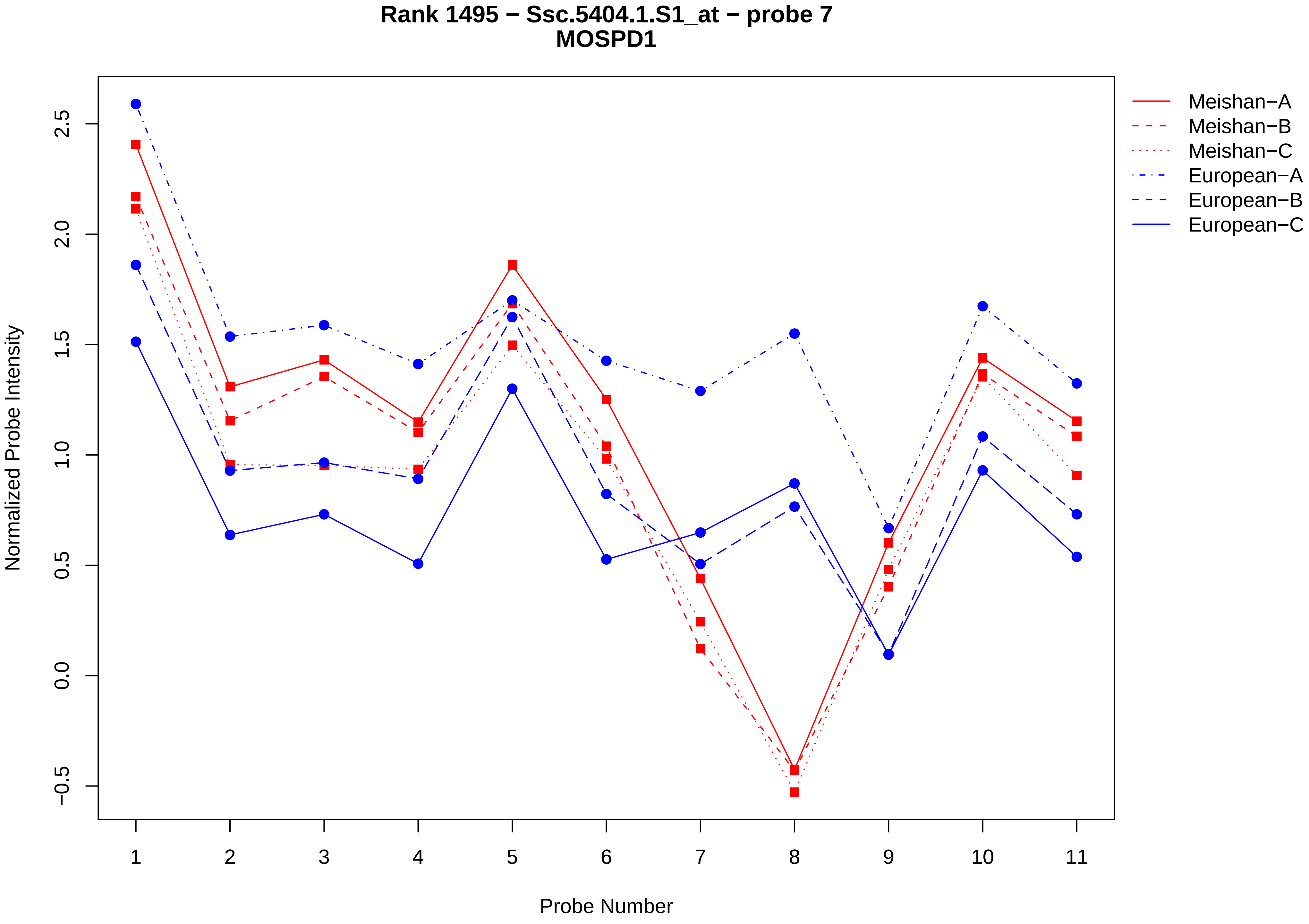

Rank 1495 − Ssc.5404.1.S1_at − probe 7
MOSPD1
Normalized Probe Intensity
Probe Number
−0.5
0.0
0.5
1.0
1.5
2.0
2.5
1
2
3
4
5
6
7
8
9
10
11
Meishan−A
Meishan−B
Meishan−C
European−A
European−B
European−C

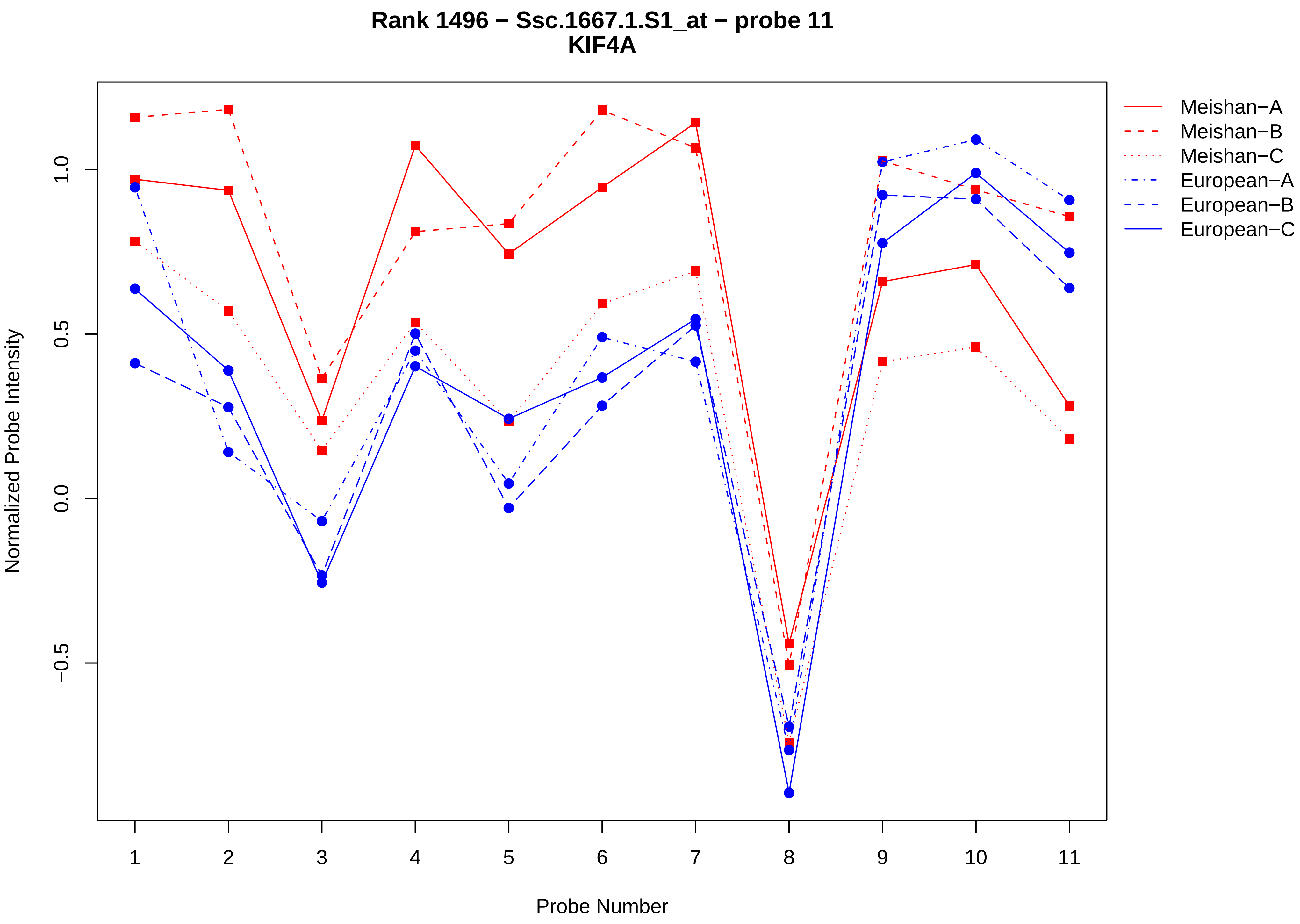

Rank 1496 − Ssc.1667.1.S1_at − probe 11
KIF4A
Normalized Probe Intensity
−0.5
0.0
0.5
1.0
1
2
3
4
5
6
7
8
9
10
11
Probe Number
Meishan−A
Meishan−B
Meishan−C
European−A
European−B
European−C

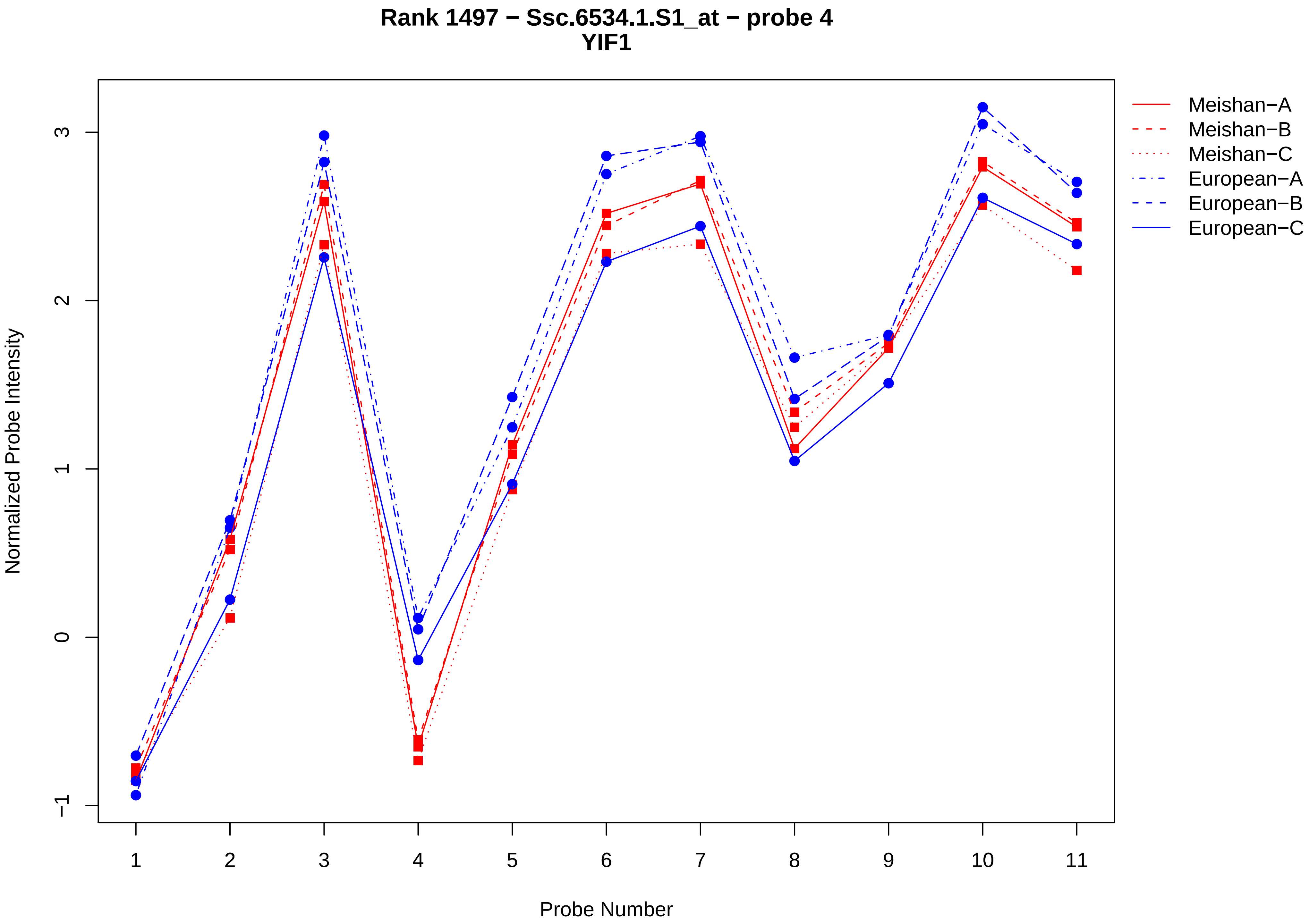

Rank 1497 − Ssc.6534.1.S1_at − probe 4
YIF1
Normalized Probe Intensity
Probe Number
−1
0
1
2
3
1
2
3
4
5
6
7
8
9
10
11
Meishan−A
Meishan−B
Meishan−C
European−A
European−B
European−C

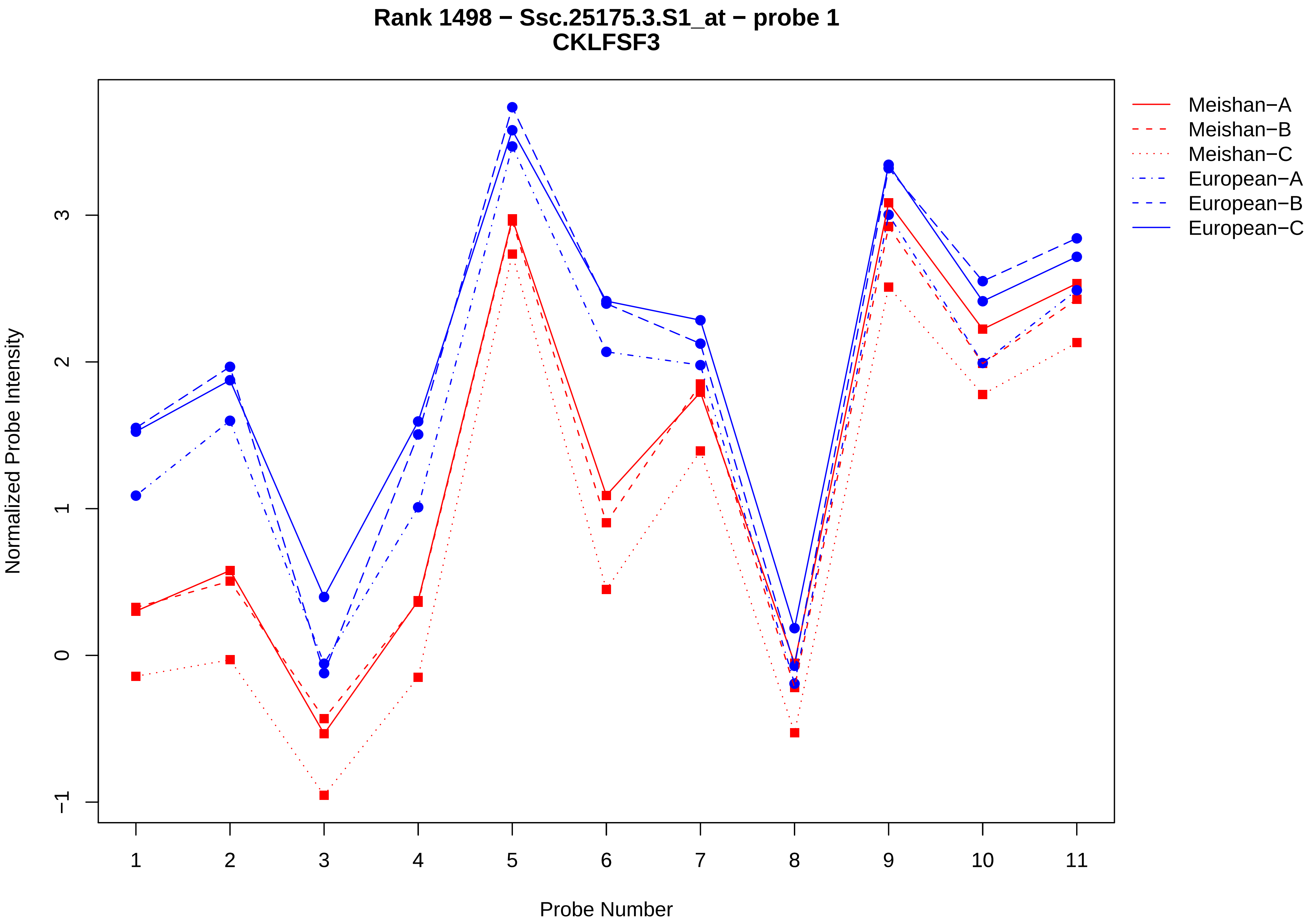

Rank 1498 − Ssc.25175.3.S1_at − probe 1
CKLFSF3
Normalized Probe Intensity
Probe Number
−1
0
1
2
3
1
2
3
4
5
6
7
8
9
10
11
Meishan−A
Meishan−B
Meishan−C
European−A
European−B
European−C

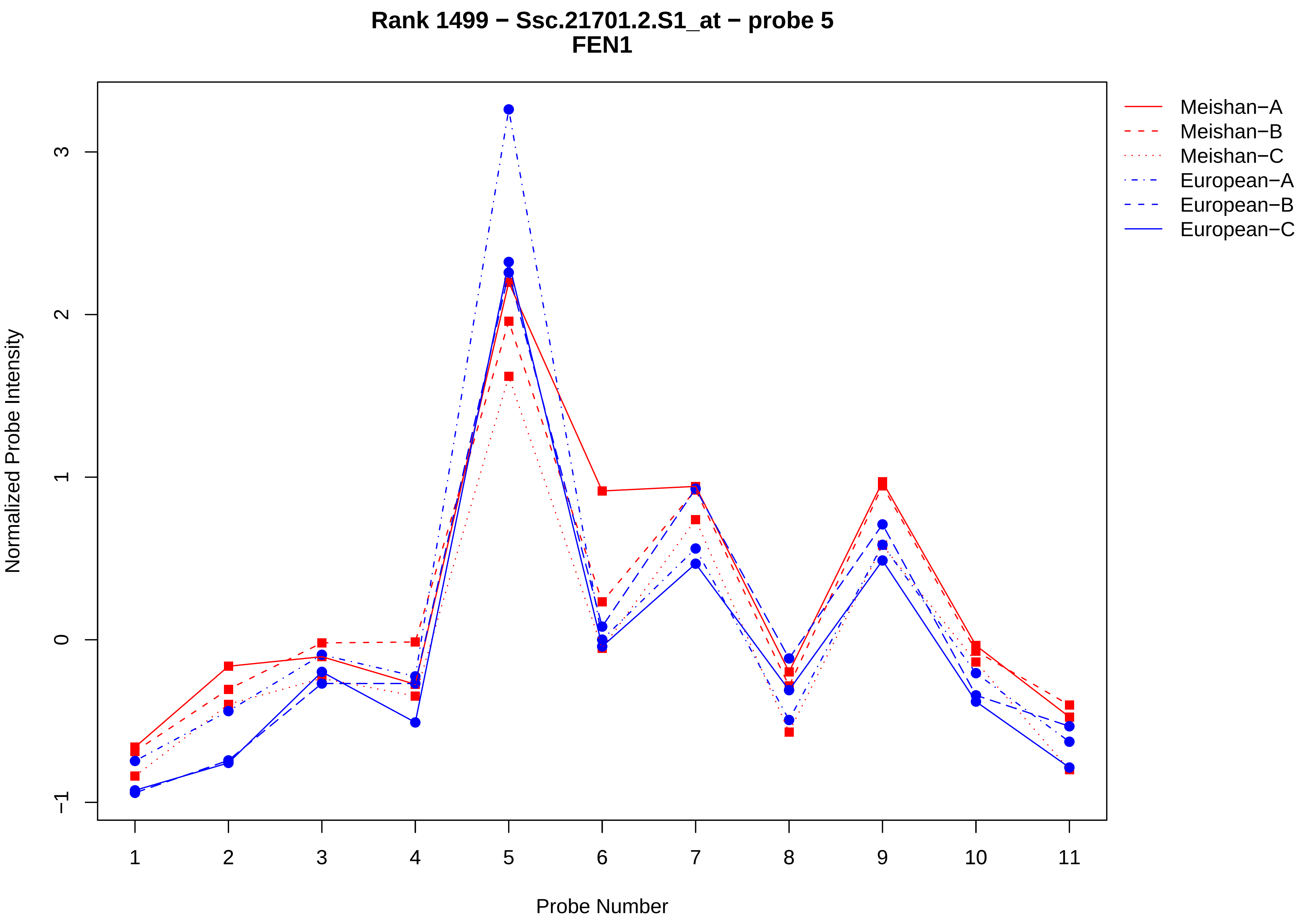
Rank 1499 − Ssc.21701.2.S1_at − probe 5
FEN1
Normalized Probe Intensity
Probe Number
−1
0
1
2
3
1
2
3
4
5
6
7
8
9
10
11
Meishan−A
Meishan−B
Meishan−C
European−A
European−B
European−C

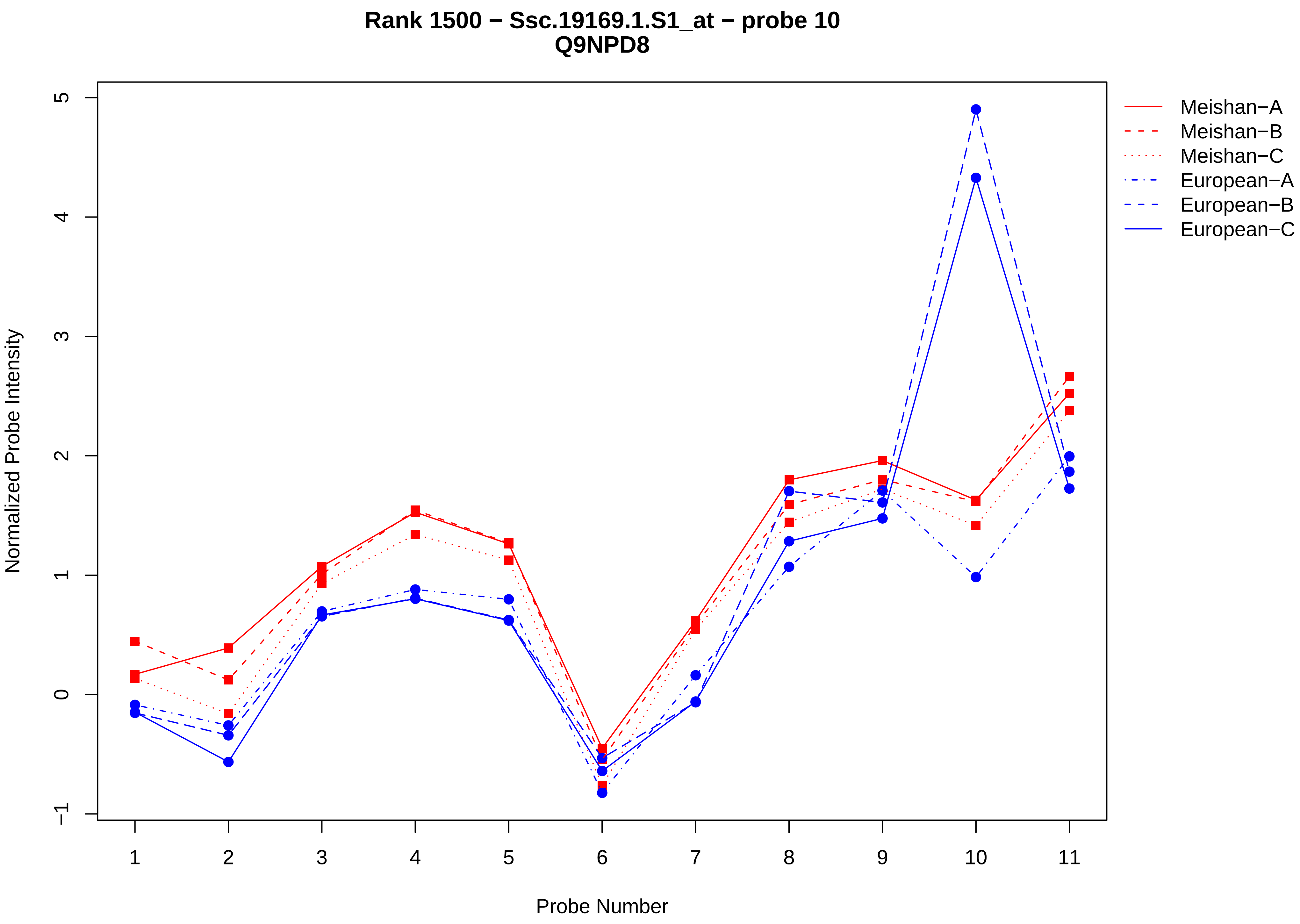

Rank 1500 − Ssc.19169.1.S1_at − probe 10
Q9NPD8
Normalized Probe Intensity
Probe Number
−1
0
1
2
3
4
5
1
2
3
4
5
6
7
8
9
10
11
Meishan−A
Meishan−B
Meishan−C
European−A
European−B
European−C

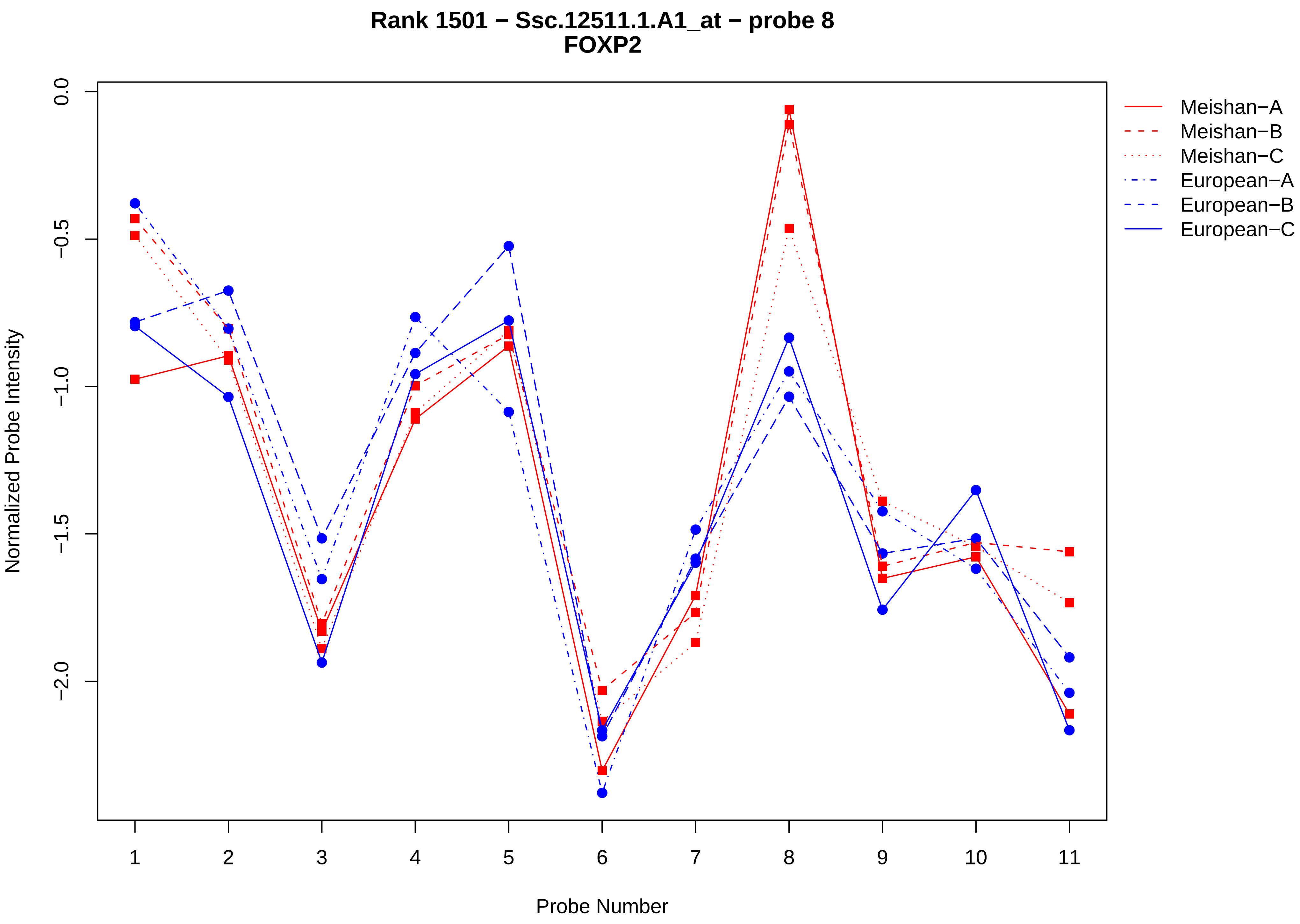
Rank 1501 − Ssc.12511.1.A1_at − probe 8
FOXP2
Normalized Probe Intensity
0.0
−0.5
−1.0
−1.5
−2.0
1
2
3
4
5
6
7
8
9
10
11
Probe Number
Meishan−A
Meishan−B
Meishan−C
European−A
European−B
European−C

Rank 1502 − Ssc.7146.1.A1_at − probe 4
ABCA1

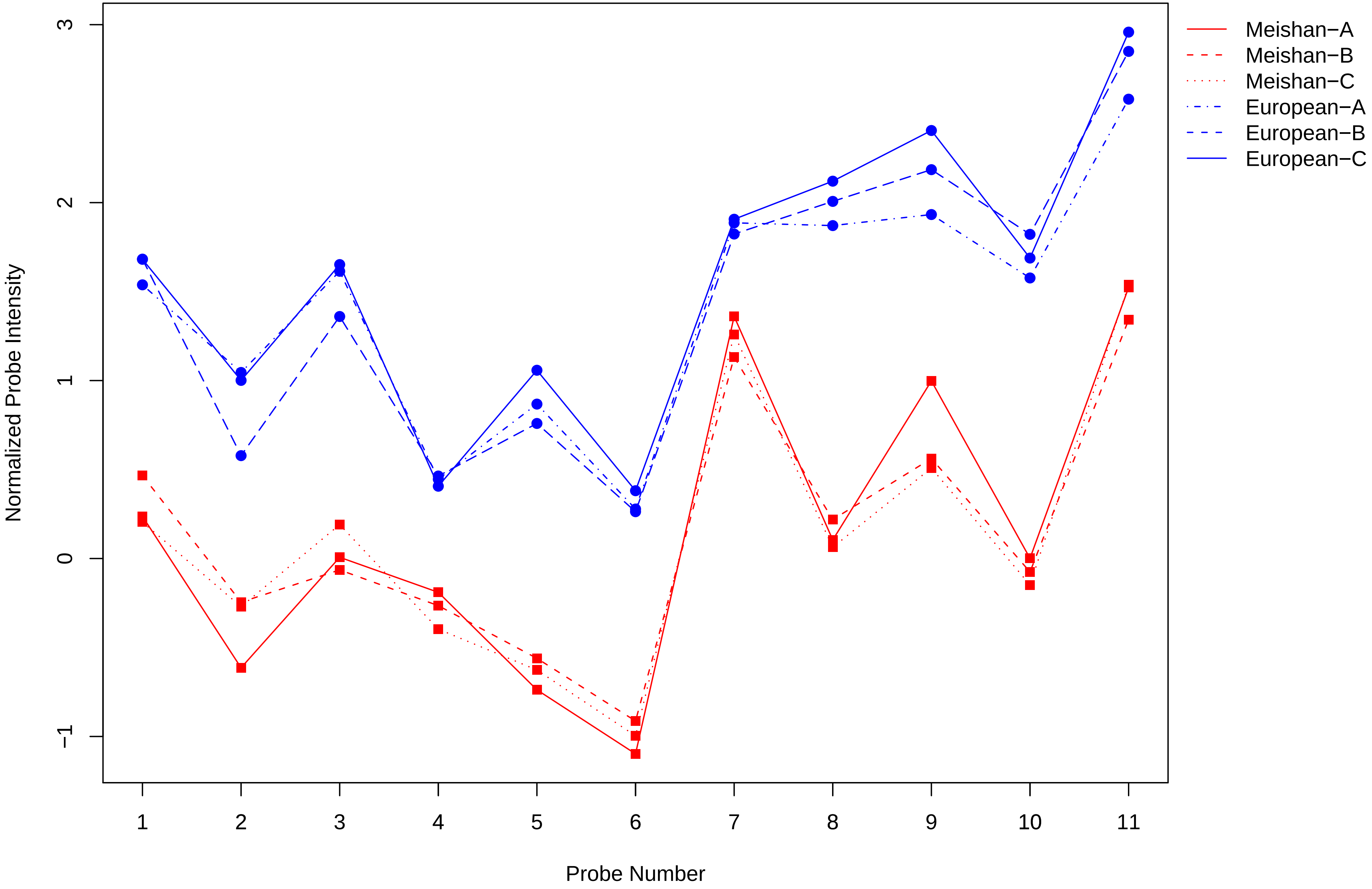

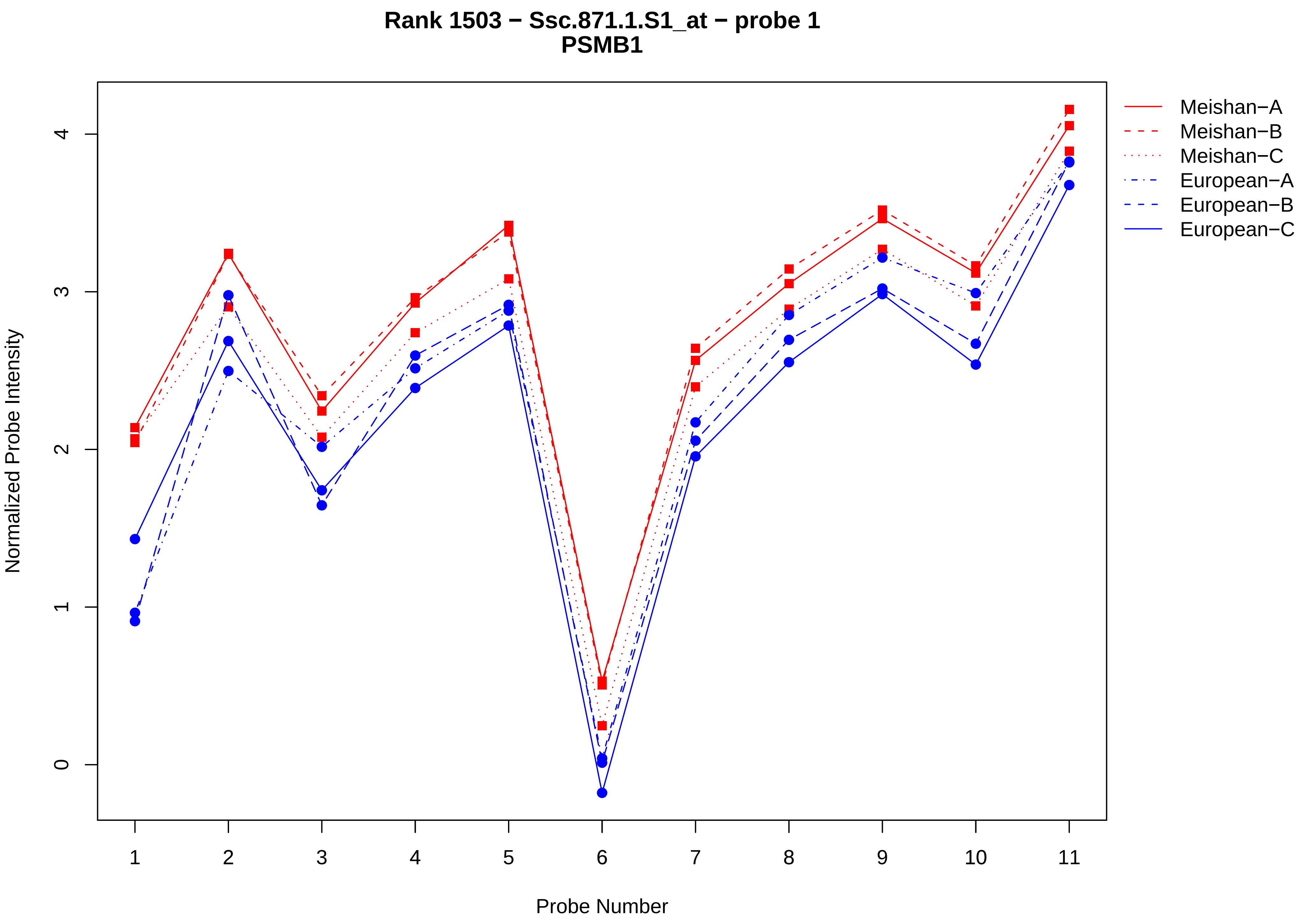

Rank 1503 − Ssc.871.1.S1_at − probe 1
PSMB1
Normalized Probe Intensity
Probe Number
0
1
2
3
4
1
2
3
4
5
6
7
8
9
10
11
Meishan−A
Meishan−B
Meishan−C
European−A
European−B
European−C

Rank 1504 − Ssc.14444.2.S1_at − probe 5
ARG2

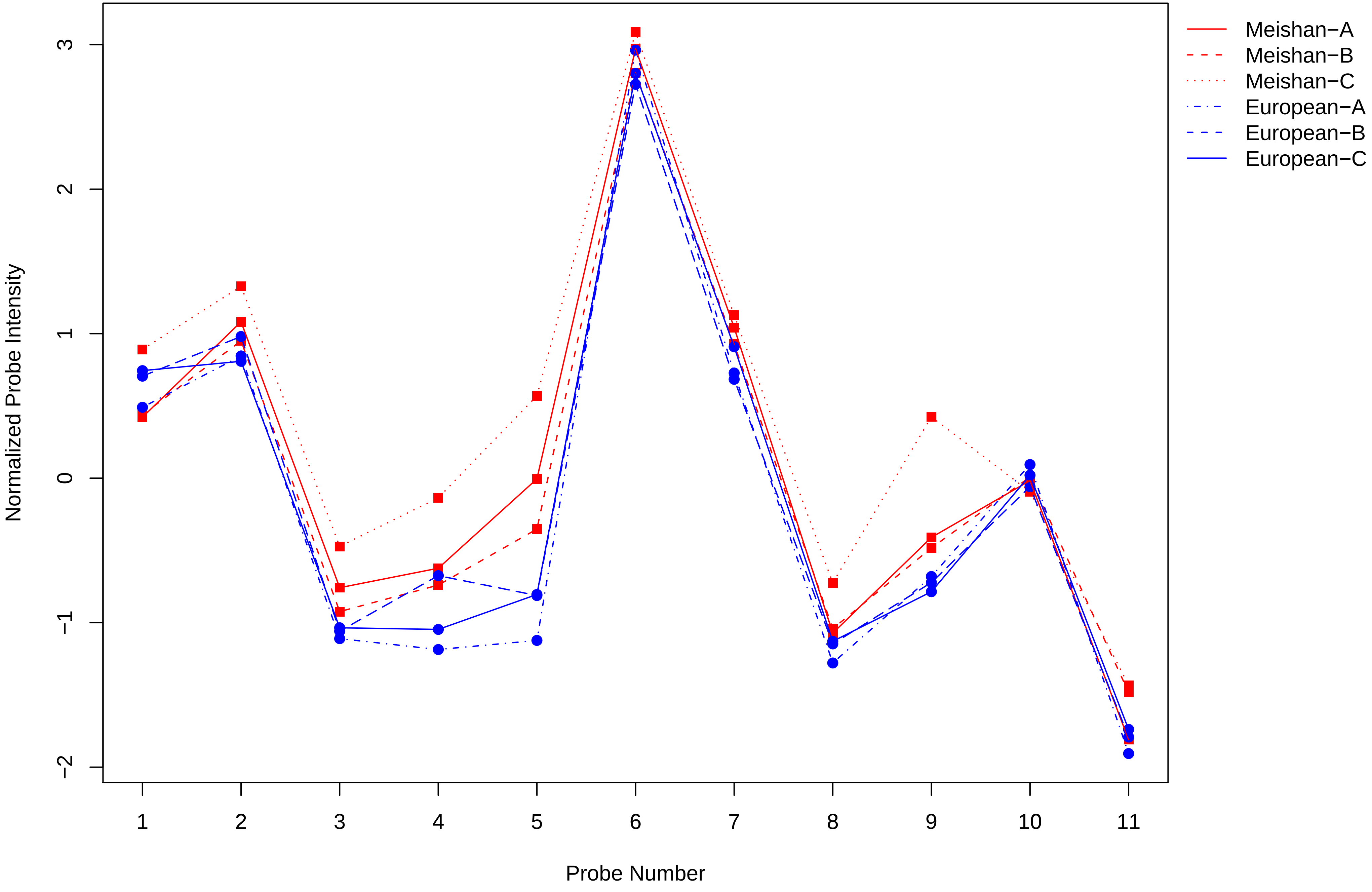

Rank 1505 − Ssc.20770.1.S1_at − probe 10
NSD1

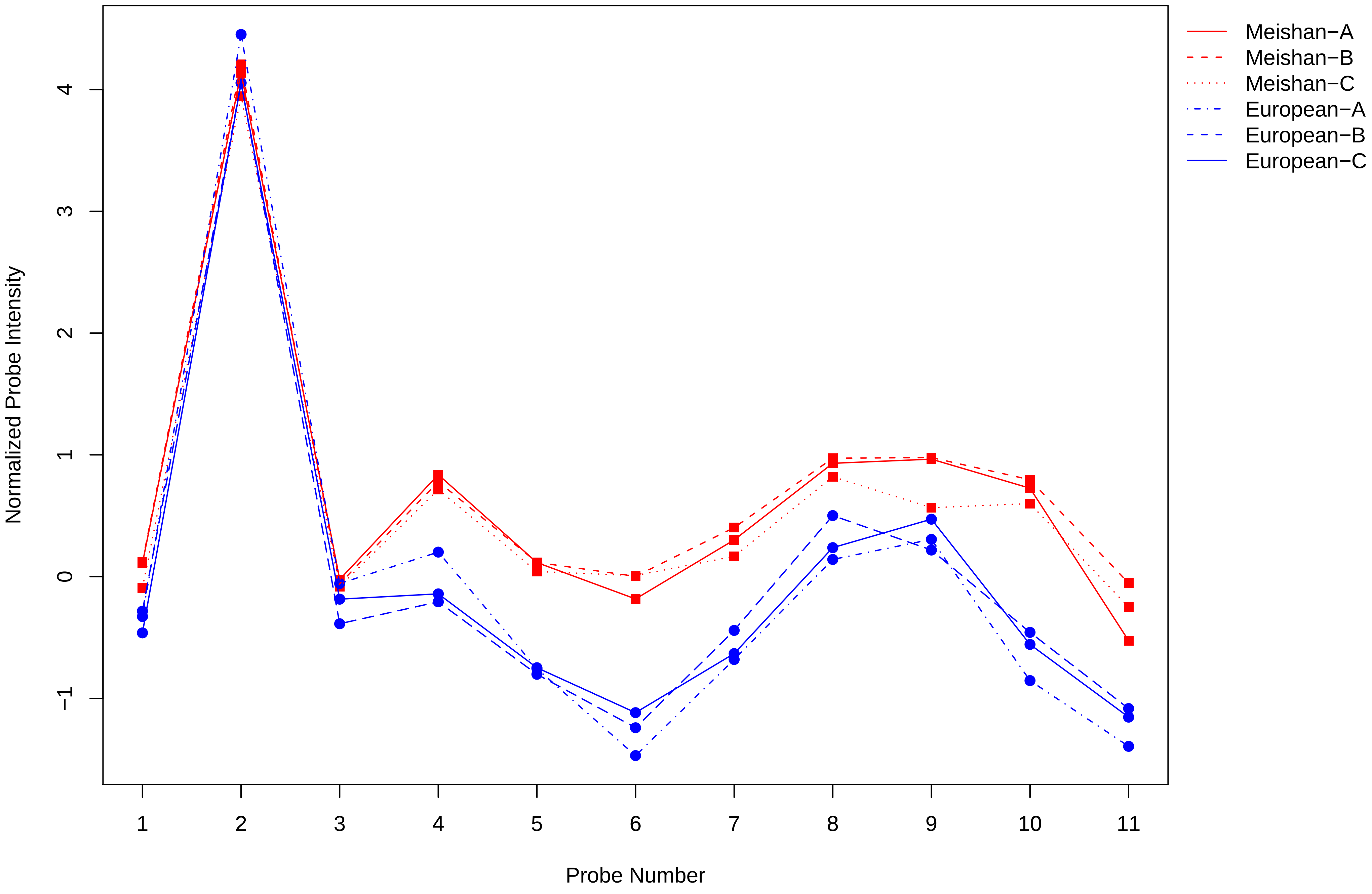

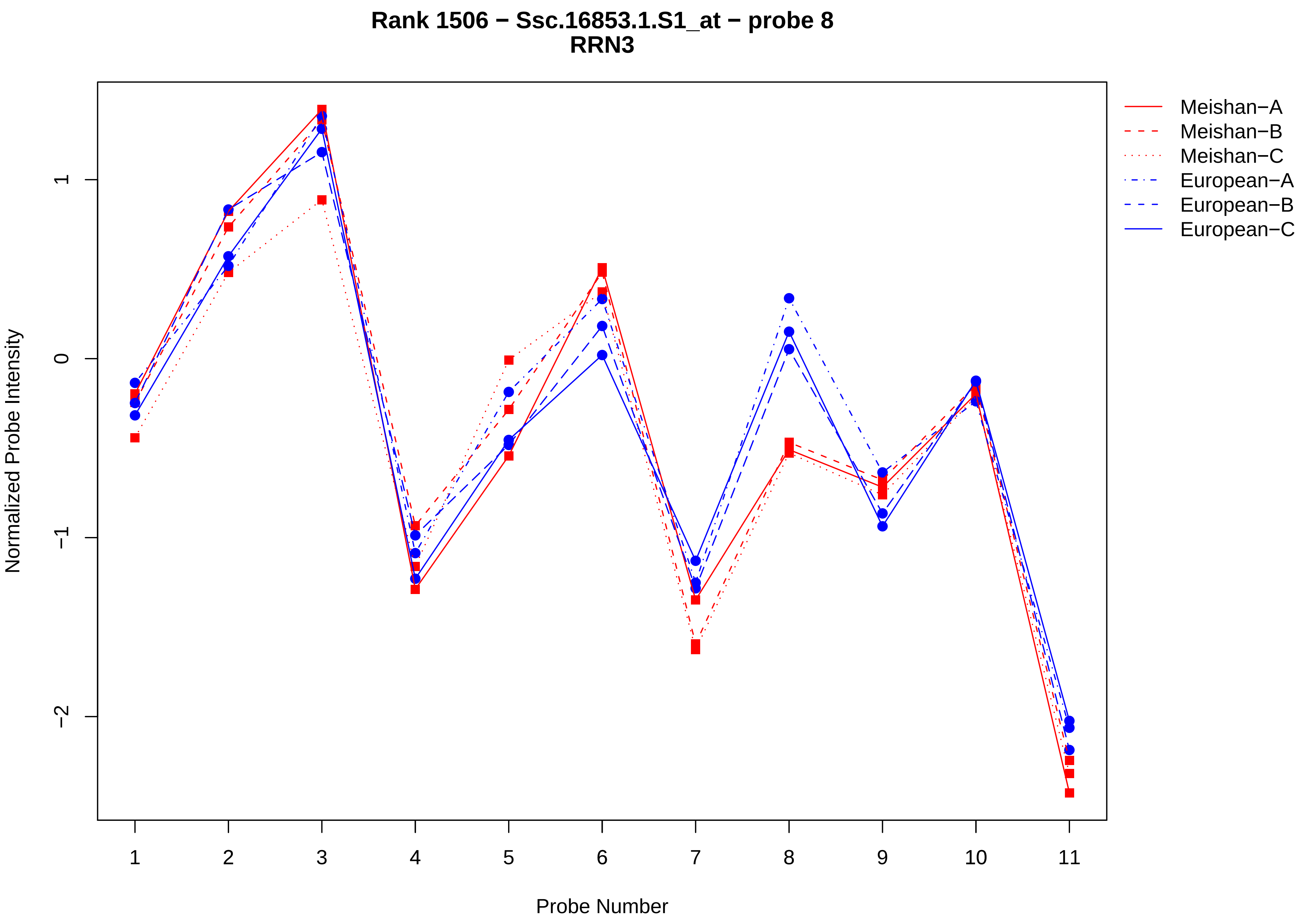

Rank 1506 − Ssc.16853.1.S1_at − probe 8
RRN3
Normalized Probe Intensity
Probe Number
−2
−1
0
1
1
2
3
4
5
6
7
8
9
10
11
Meishan−A
Meishan−B
Meishan−C
European−A
European−B
European−C

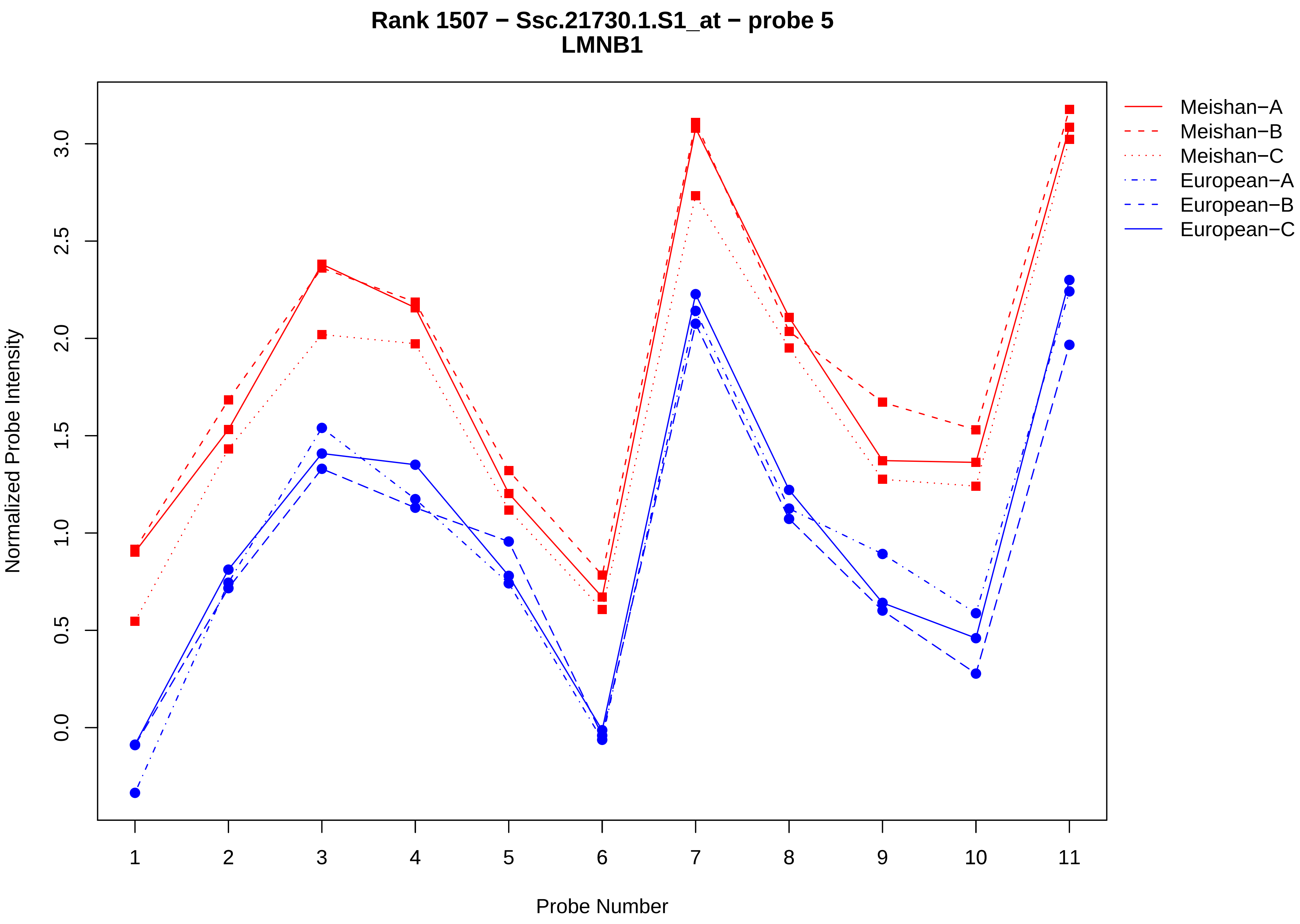

Rank 1507 − Ssc.21730.1.S1_at − probe 5
LMNB1
Normalized Probe Intensity
0.0
0.5
1.0
1.5
2.0
2.5
3.0
Probe Number
1
2
3
4
5
6
7
8
9
10
11
Meishan−A
Meishan−B
Meishan−C
European−A
European−B
European−C

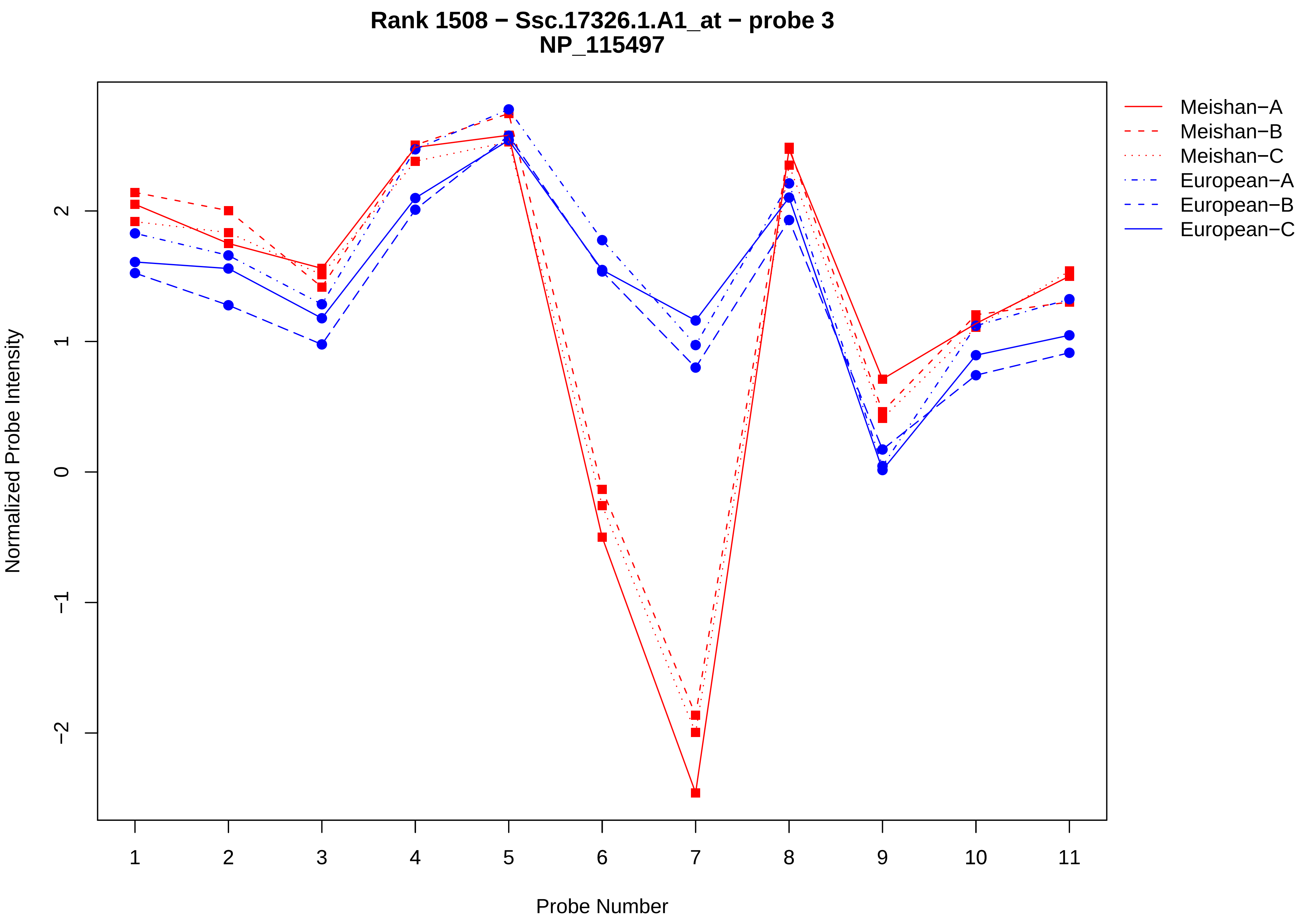

Rank 1508 − Ssc.17326.1.A1_at − probe 3
NP_115497
Normalized Probe Intensity
Probe Number
−2
−1
0
1
2
1
2
3
4
5
6
7
8
9
10
11
Meishan−A
Meishan−B
Meishan−C
European−A
European−B
European−C

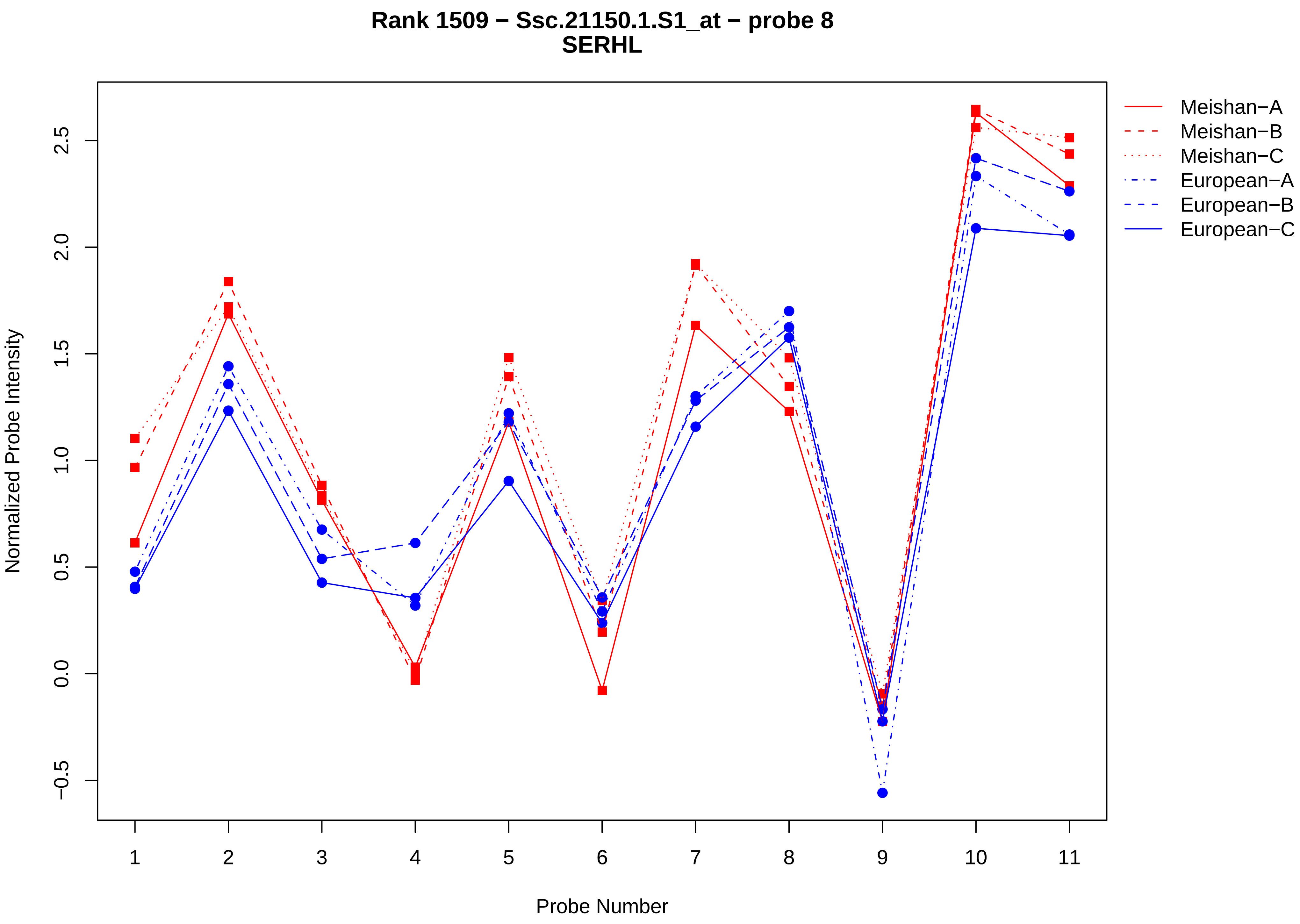

Rank 1509 − Ssc.21150.1.S1_at − probe 8
SERHL
Normalized Probe Intensity
Probe Number
−0.5
0.0
0.5
1.0
1.5
2.0
2.5
1
2
3
4
5
6
7
8
9
10
11
Meishan−A
Meishan−B
Meishan−C
European−A
European−B
European−C

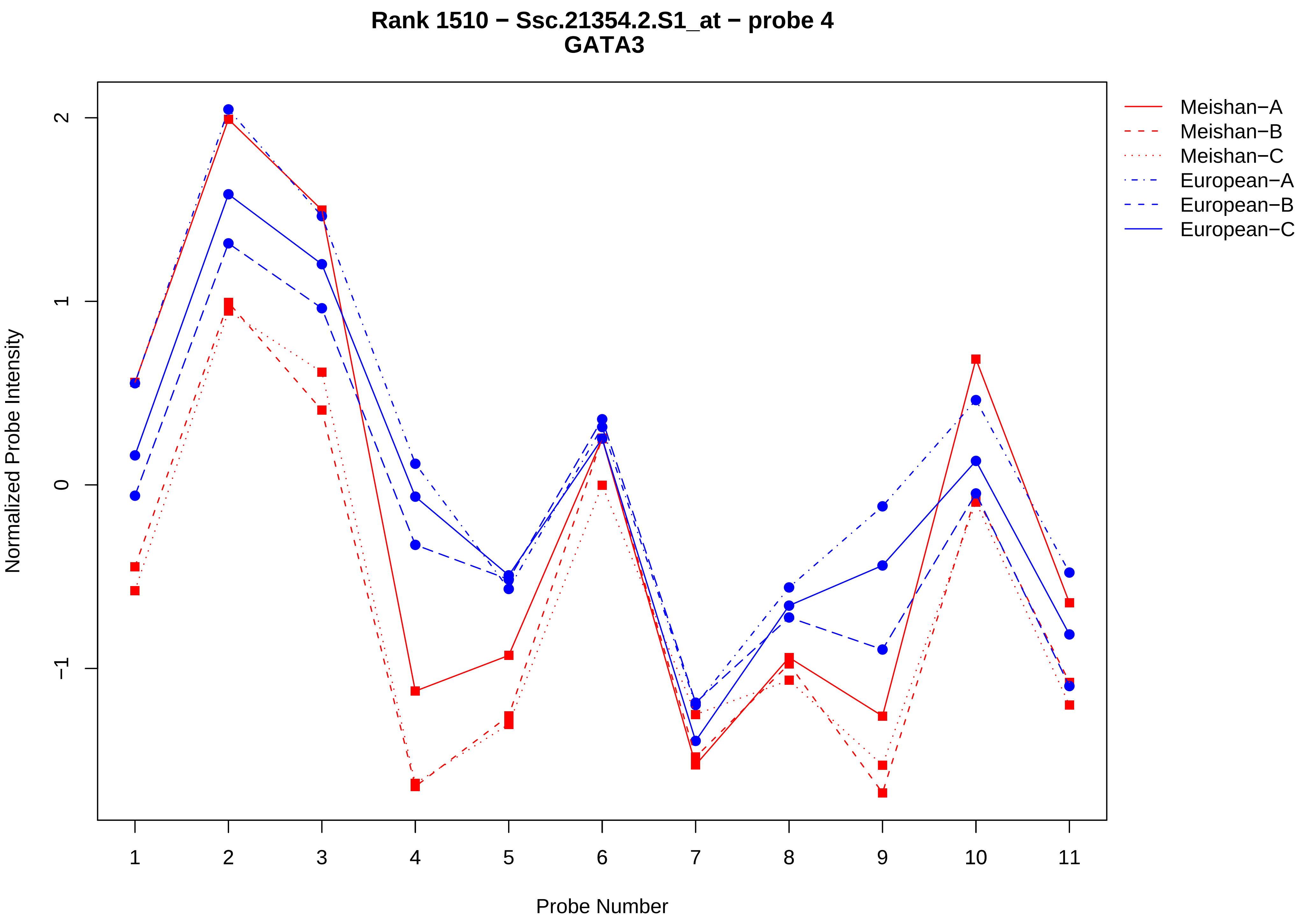

Rank 1510 − Ssc.21354.2.S1_at − probe 4
GATA3
Normalized Probe Intensity
Probe Number
2
1
0
−1
1
2
3
4
5
6
7
8
9
10
11
Meishan−A
Meishan−B
Meishan−C
European−A
European−B
European−C

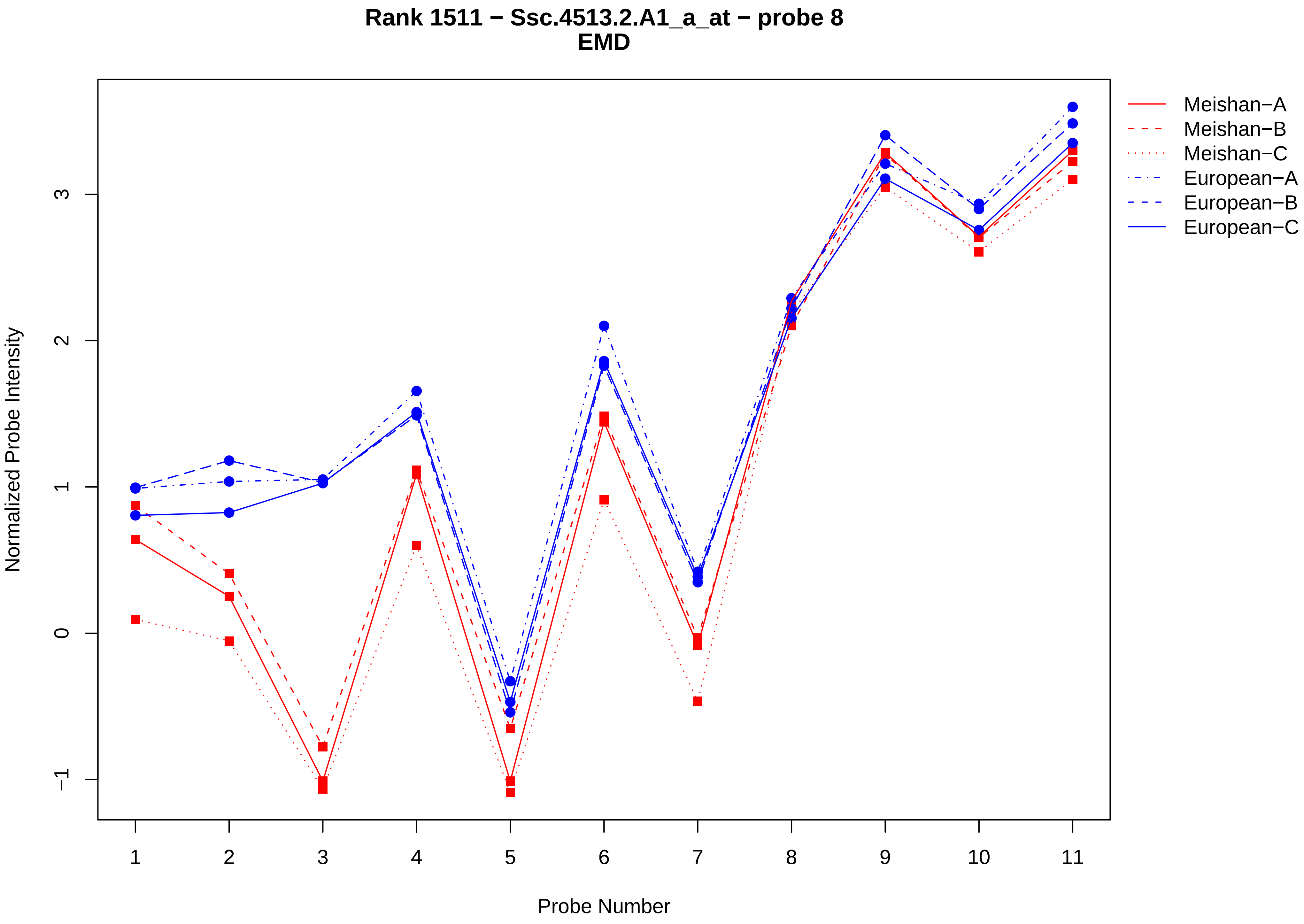
Rank 1511 − Ssc.4513.2.A1_a_at − probe 8
EMD
Normalized Probe Intensity
Probe Number
Meishan−A
Meishan−B
Meishan−C
European−A
European−B
European−C
−1
0
1
2
3
1
2
3
4
5
6
7
8
9
10
11

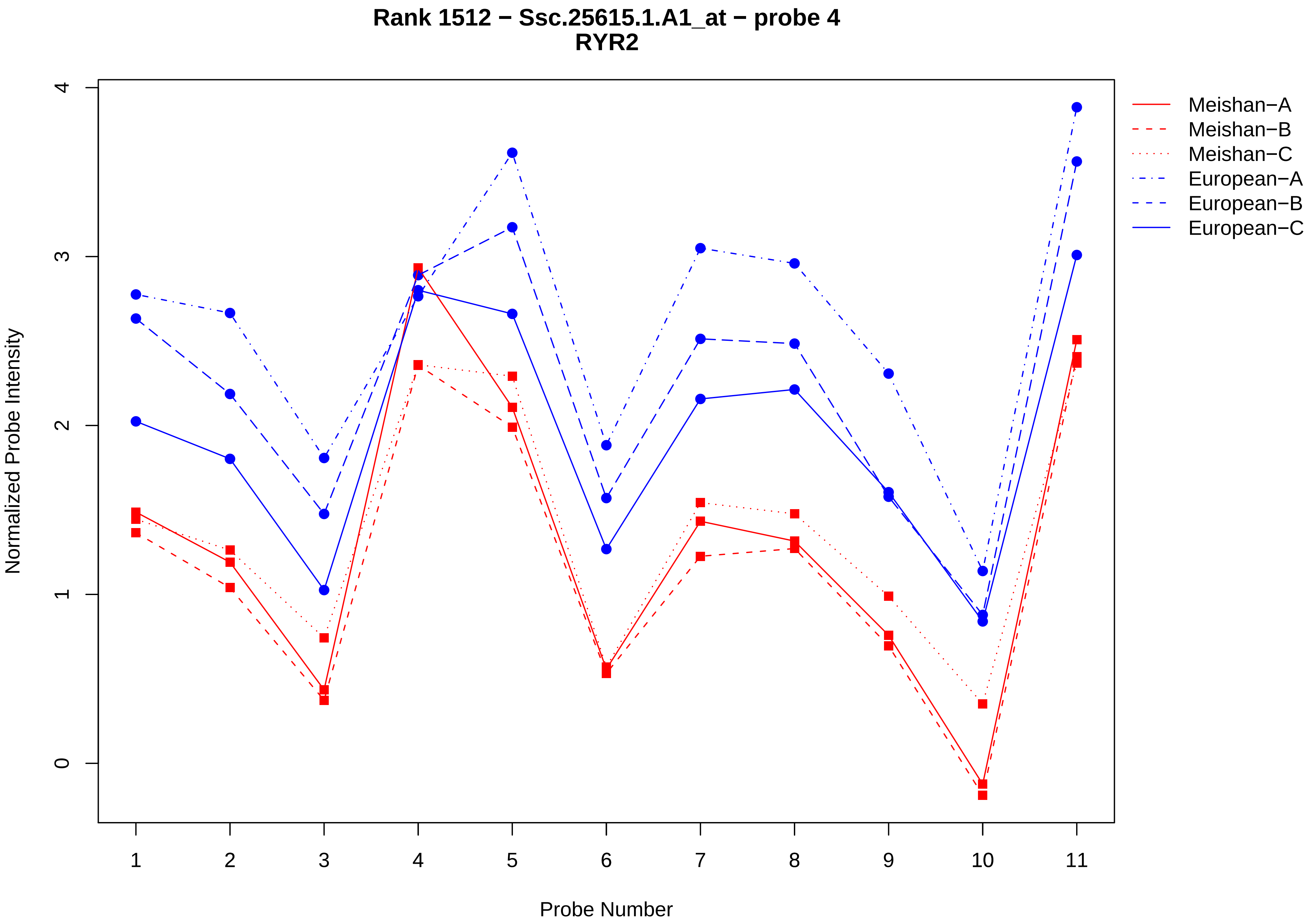

Rank 1512 − Ssc.25615.1.A1_at − probe 4
RYR2
Normalized Probe Intensity
Probe Number
0
1
2
3
4
1
2
3
4
5
6
7
8
9
10
11
Meishan−A
Meishan−B
Meishan−C
European−A
European−B
European−C

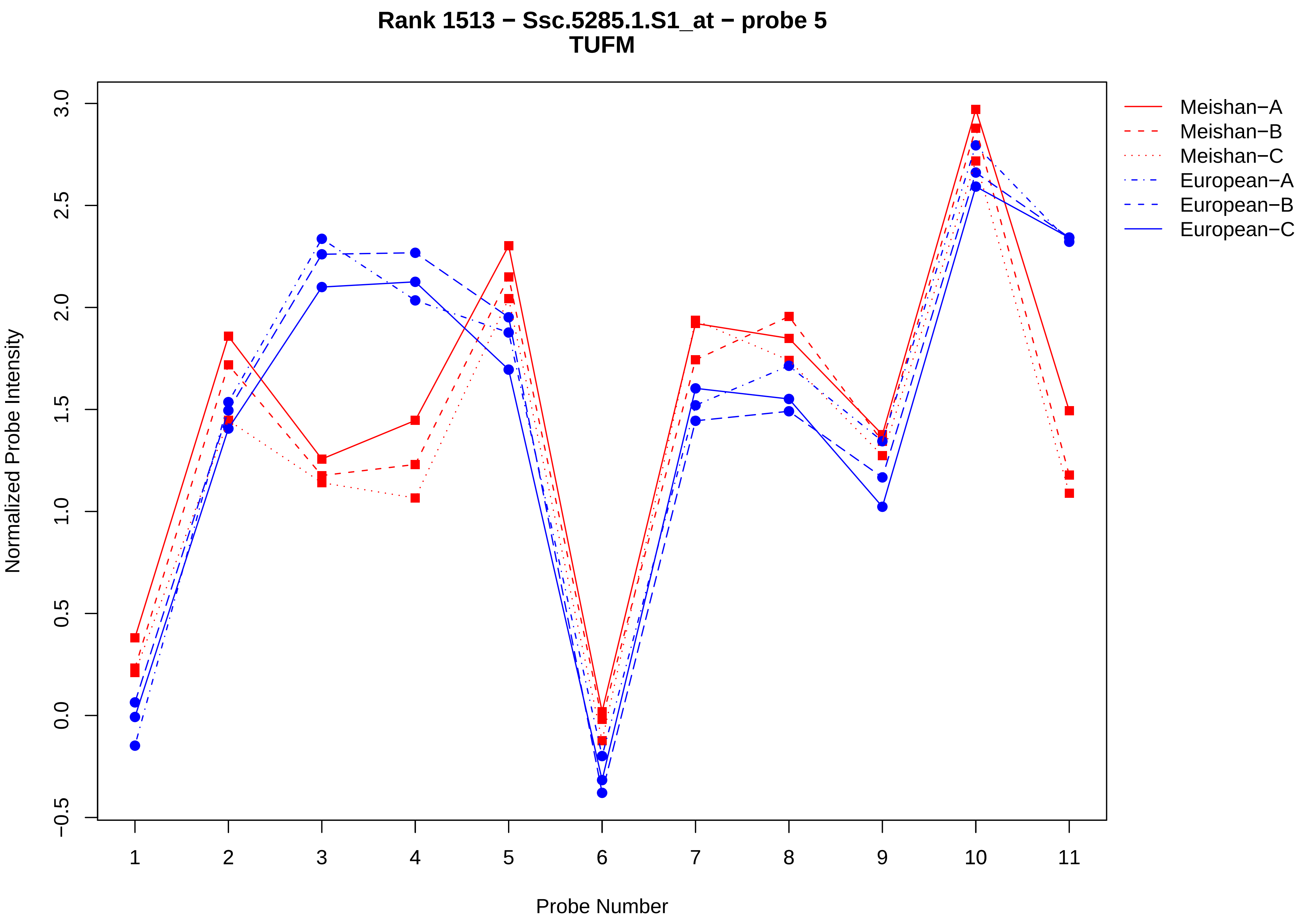

Rank 1513 − Ssc.5285.1.S1_at − probe 5
TUFM
Normalized Probe Intensity
Probe Number
−0.5
0.0
0.5
1.0
1.5
2.0
2.5
3.0
1
2
3
4
5
6
7
8
9
10
11
Meishan−A
Meishan−B
Meishan−C
European−A
European−B
European−C

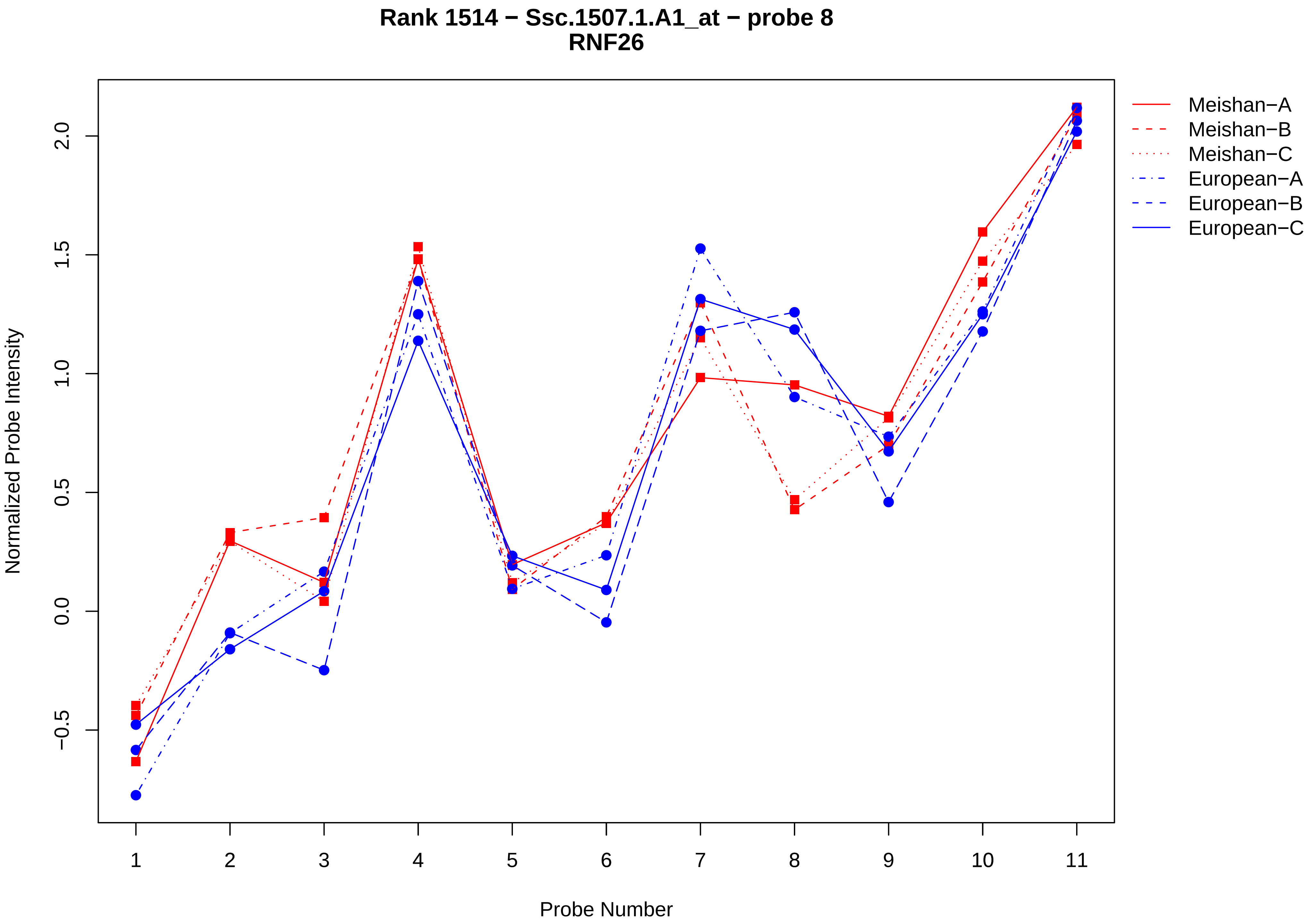

Rank 1514 − Ssc.1507.1.A1_at − probe 8
RNF26
Normalized Probe Intensity
Probe Number
−0.5
0.0
0.5
1.0
1.5
2.0
1
2
3
4
5
6
7
8
9
10
11
Meishan−A
Meishan−B
Meishan−C
European−A
European−B
European−C

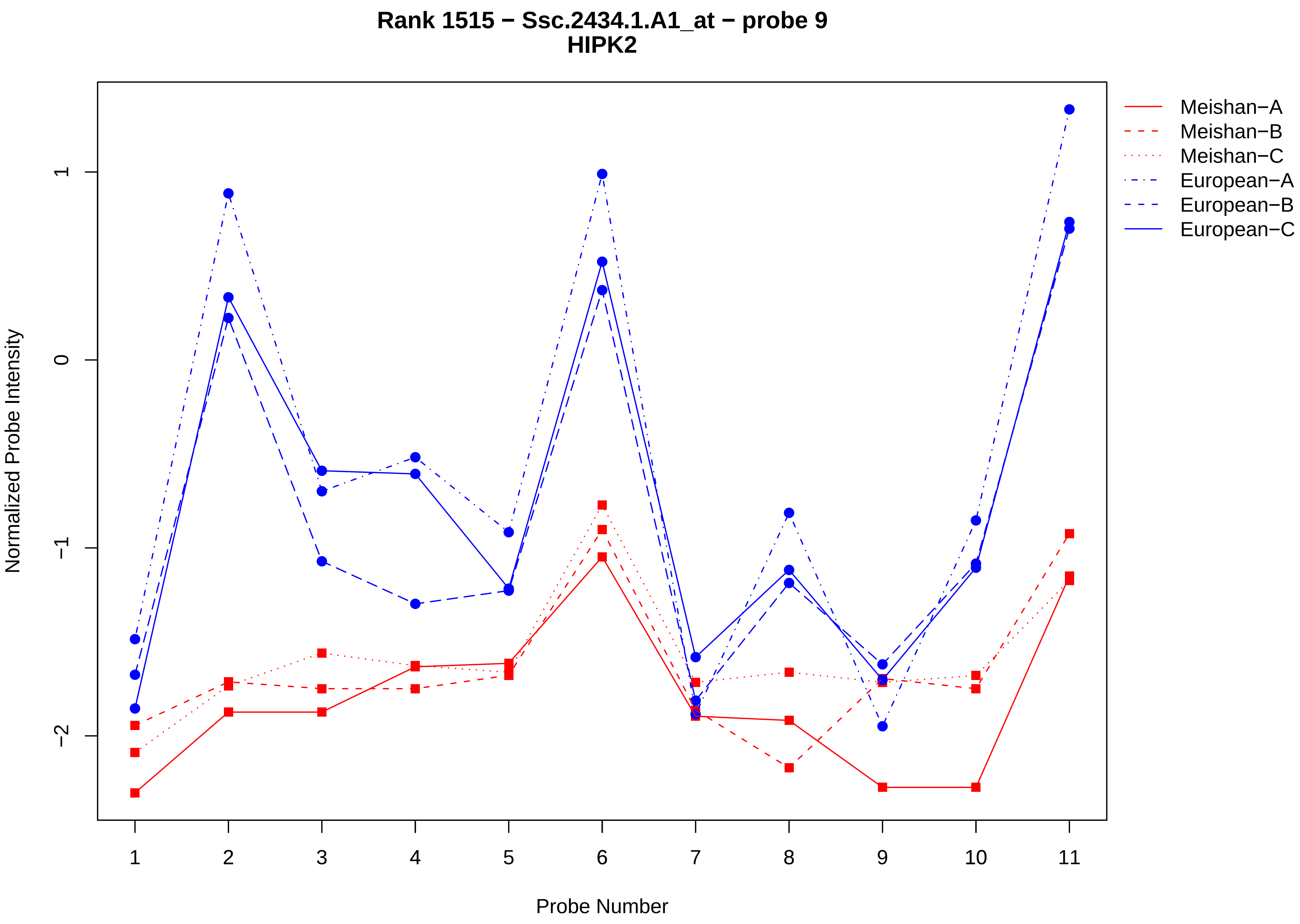

Rank 1515 − Ssc.2434.1.A1_at − probe 9
HIPK2
Normalized Probe Intensity
Probe Number
1
0
−1
−2
1
2
3
4
5
6
7
8
9
10
11
Meishan−A
Meishan−B
Meishan−C
European−A
European−B
European−C

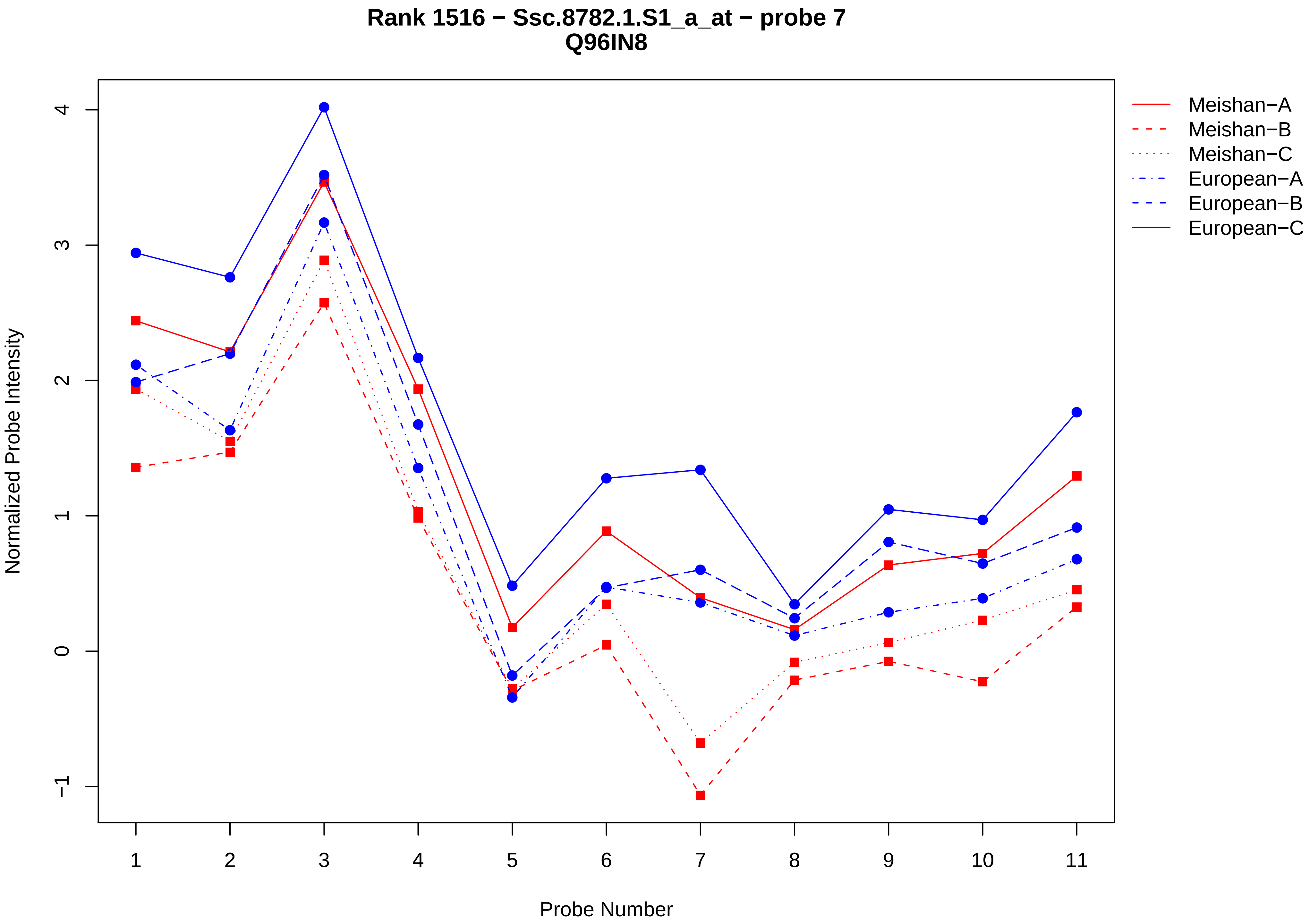

Rank 1516 − Ssc.8782.1.S1_a_at − probe 7
Q96IN8
Normalized Probe Intensity
Probe Number
−1
0
1
2
3
4
1
2
3
4
5
6
7
8
9
10
11
Meishan−A
Meishan−B
Meishan−C
European−A
European−B
European−C

Rank 1517 − Ssc.10303.1.A1_at − probe 6
PDGFD

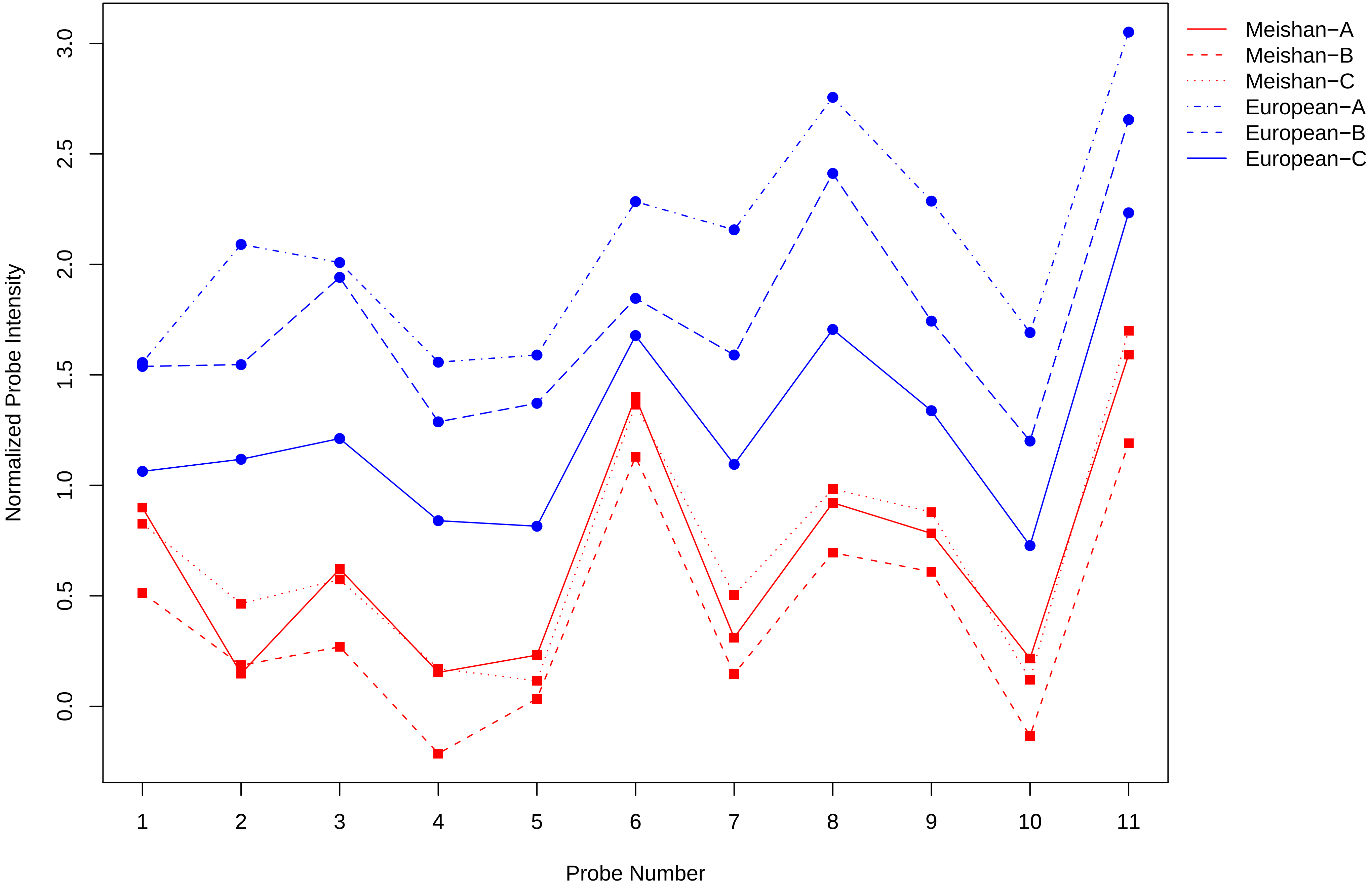

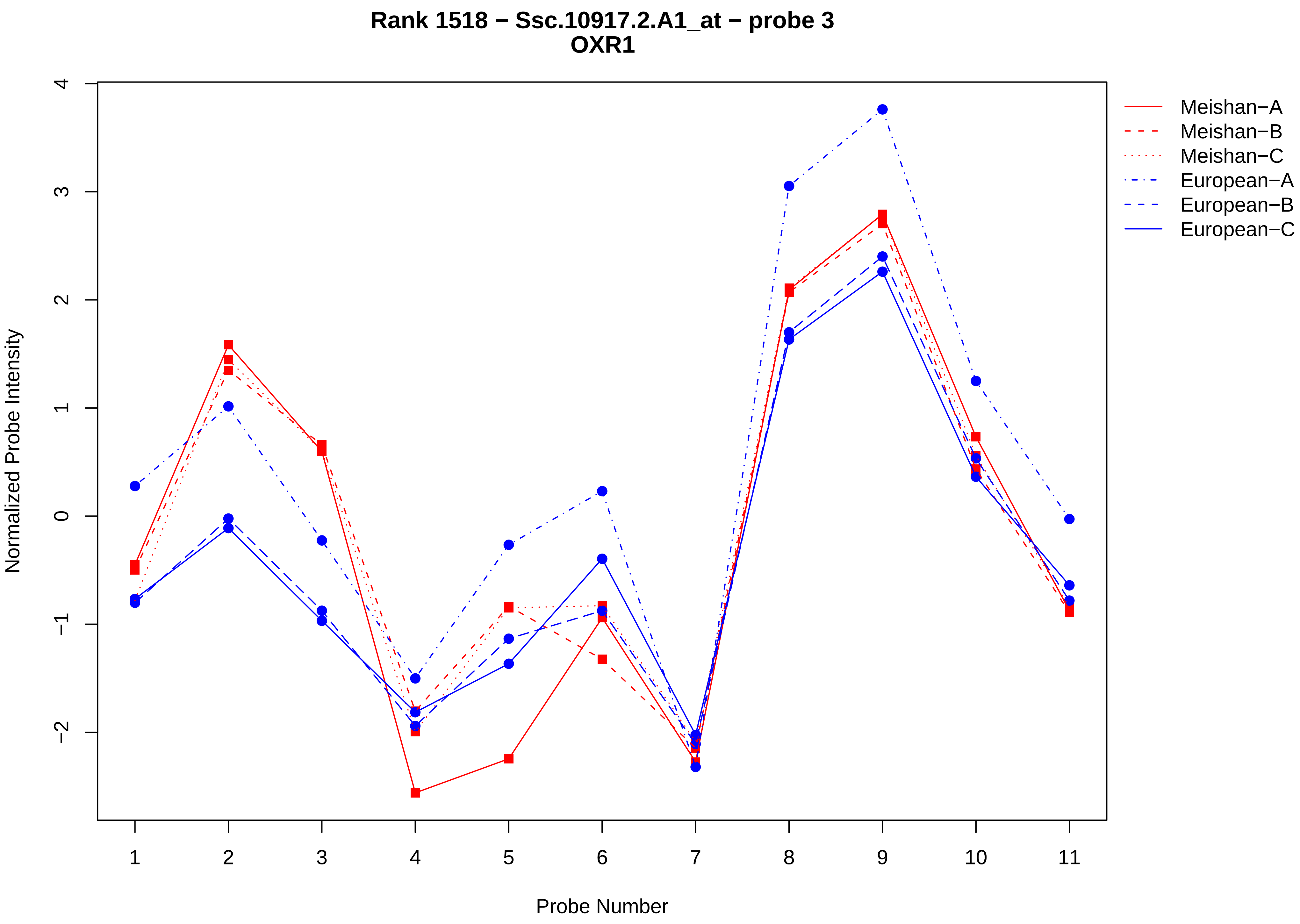

Rank 1518 − Ssc.10917.2.A1_at − probe 3
OXR1
Normalized Probe Intensity
Probe Number
−2
−1
0
1
2
3
4
1
2
3
4
5
6
7
8
9
10
11
Meishan−A
Meishan−B
Meishan−C
European−A
European−B
European−C

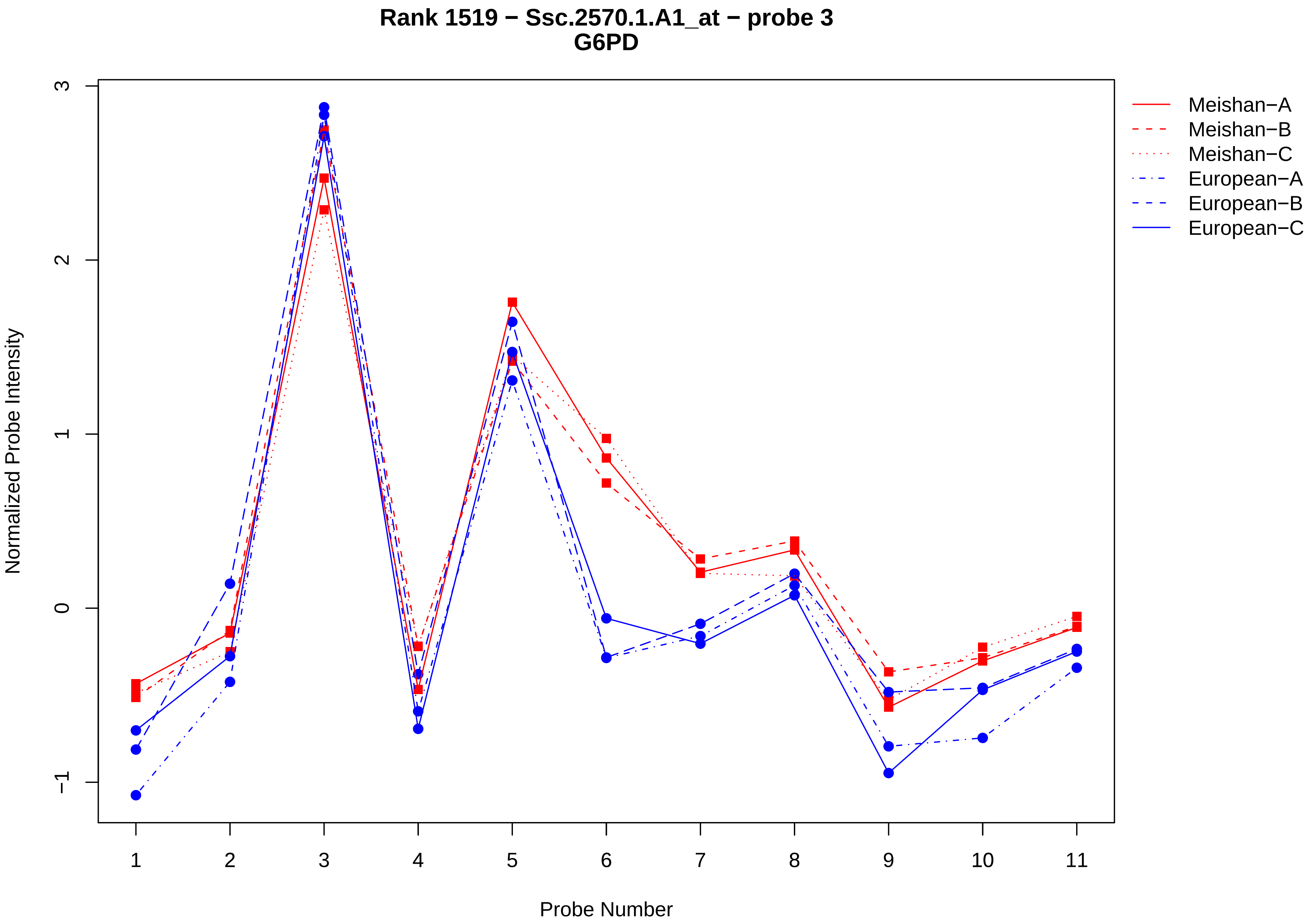

Rank 1519 − Ssc.2570.1.A1_at − probe 3
G6PD
Normalized Probe Intensity
Probe Number
Meishan−A
Meishan−B
Meishan−C
European−A
European−B
European−C
−1
0
1
2
3
1
2
3
4
5
6
7
8
9
10
11

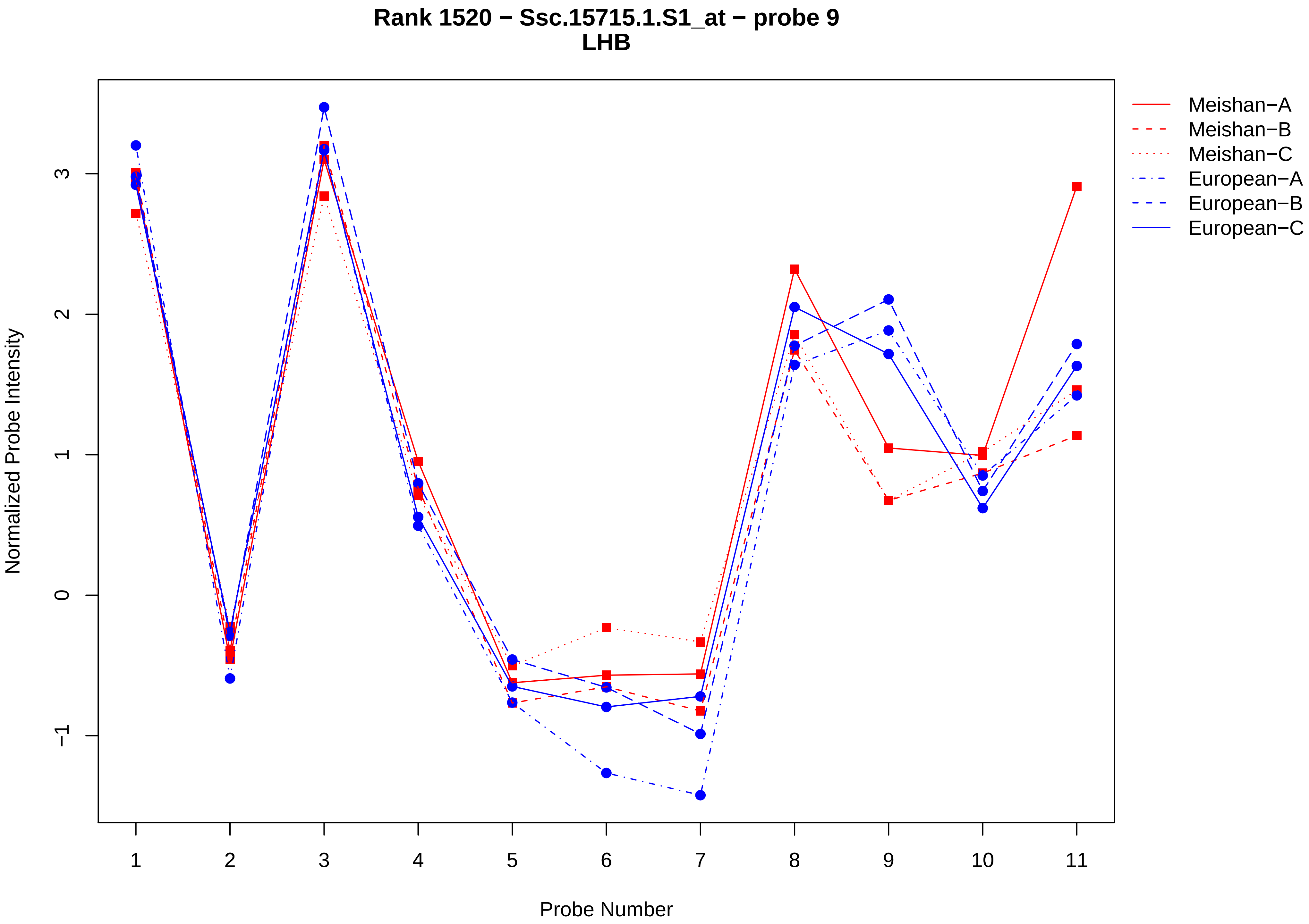
Rank 1520 − Ssc.15715.1.S1_at − probe 9
LHB
Normalized Probe Intensity
Probe Number
−1
0
1
2
3
1
2
3
4
5
6
7
8
9
10
11
Meishan−A
Meishan−B
Meishan−C
European−A
European−B
European−C

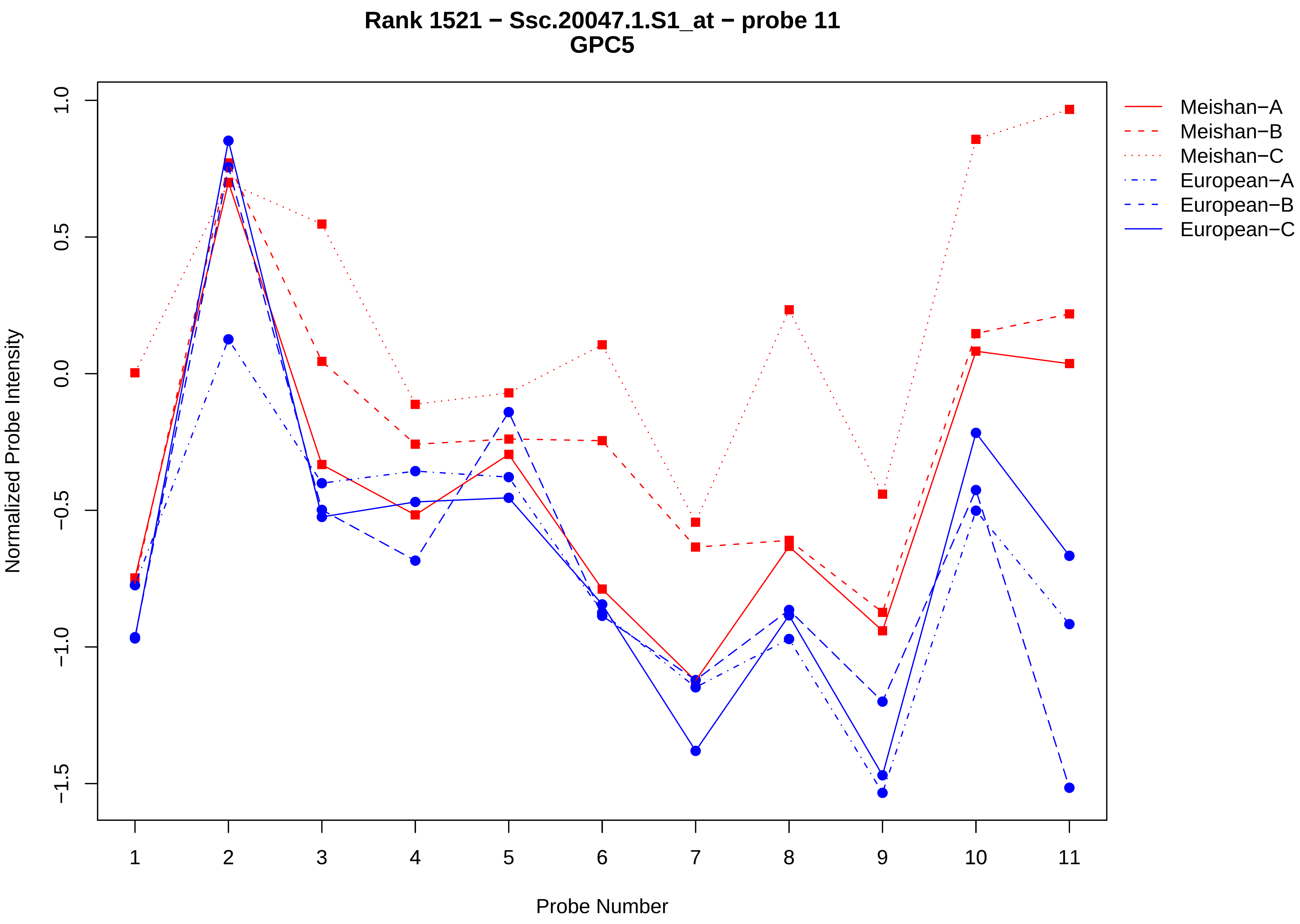

Rank 1521 − Ssc.20047.1.S1_at − probe 11
GPC5
Normalized Probe Intensity
1.0
0.5
0.0
−0.5
−1.0
−1.5
1
2
3
4
5
6
7
8
9
10
11
Probe Number
Meishan−A
Meishan−B
Meishan−C
European−A
European−B
European−C

Rank 1522 − Ssc.791.1.S1_at − probe 1
RPLP2

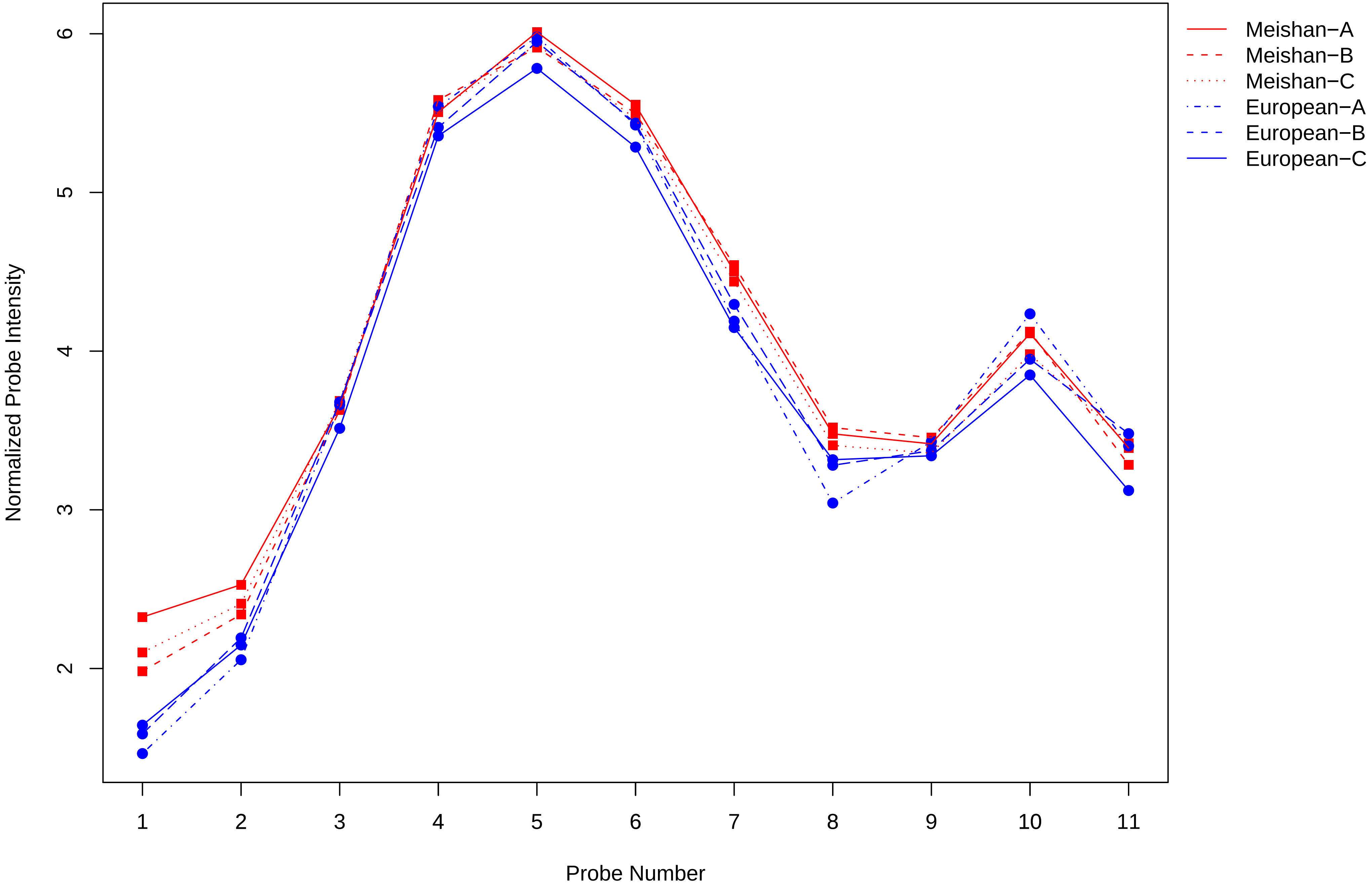

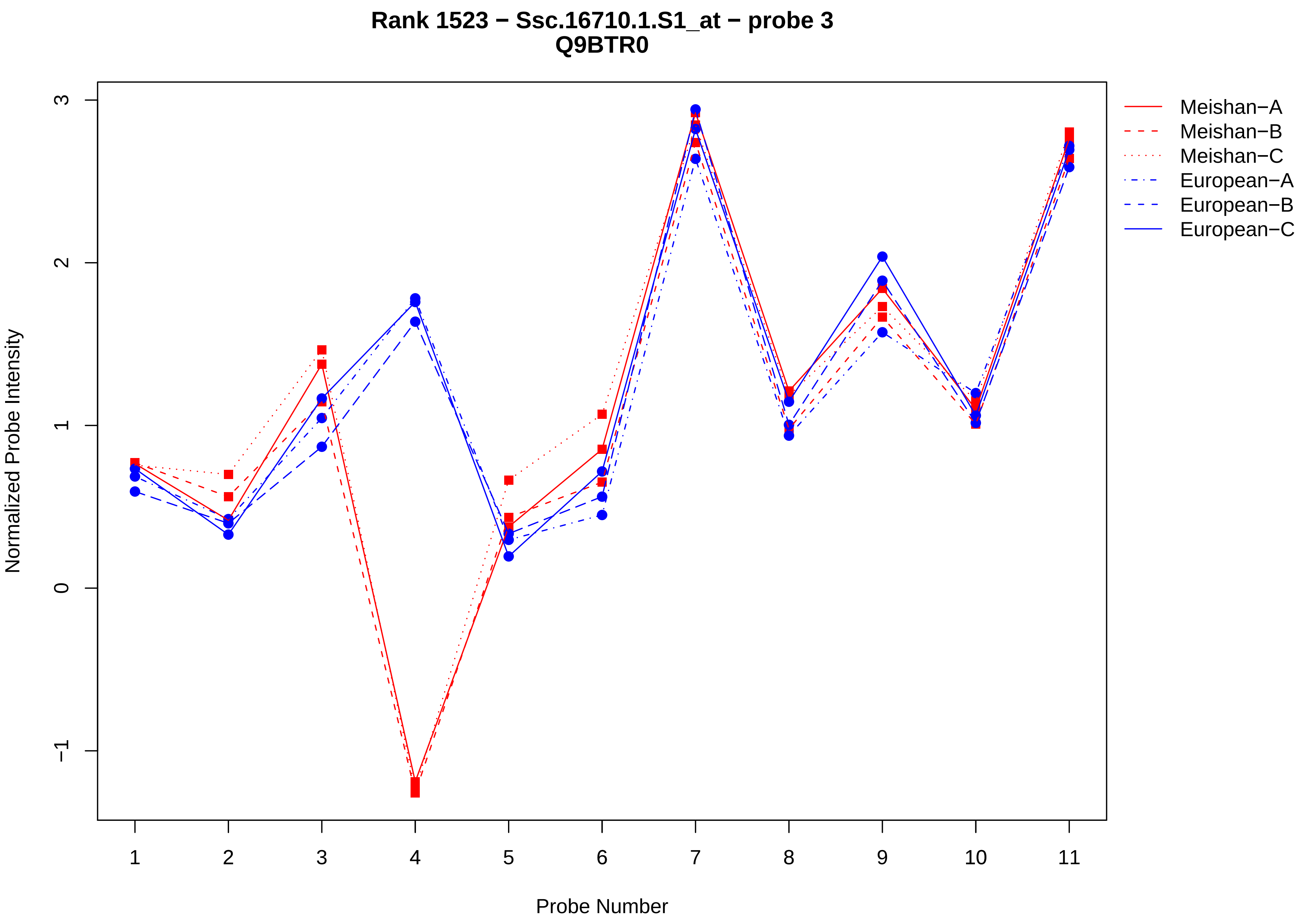

Rank 1523 − Ssc.16710.1.S1_at − probe 3
Q9BTR0
Normalized Probe Intensity
Probe Number
Meishan−A
Meishan−B
Meishan−C
European−A
European−B
European−C
−1
0
1
2
3
1
2
3
4
5
6
7
8
9
10
11

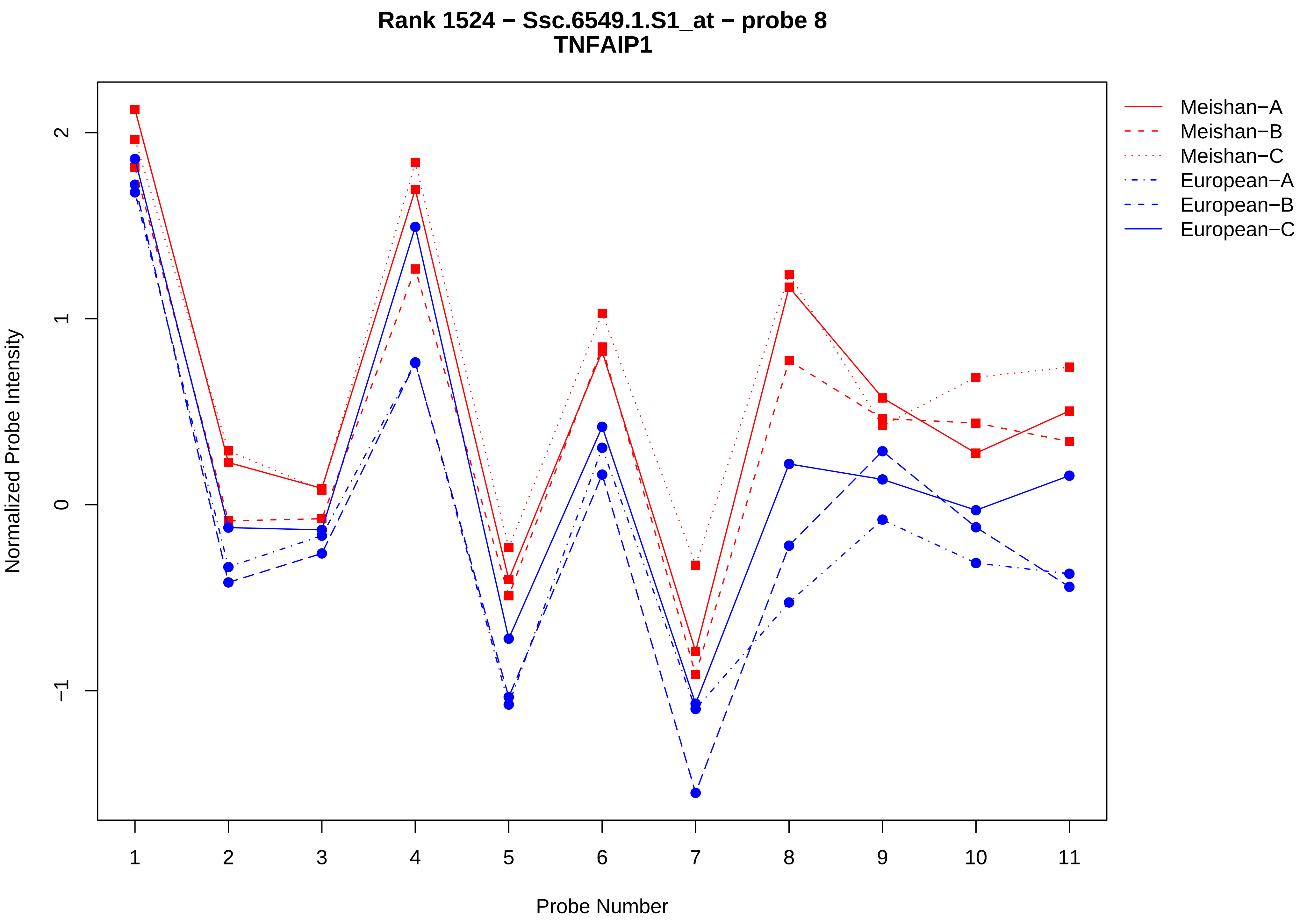

Rank 1524 − Ssc.6549.1.S1_at − probe 8
TNFAIP1
Normalized Probe Intensity
Probe Number
−1
0
1
2
1
2
3
4
5
6
7
8
9
10
11
Meishan−A
Meishan−B
Meishan−C
European−A
European−B
European−C

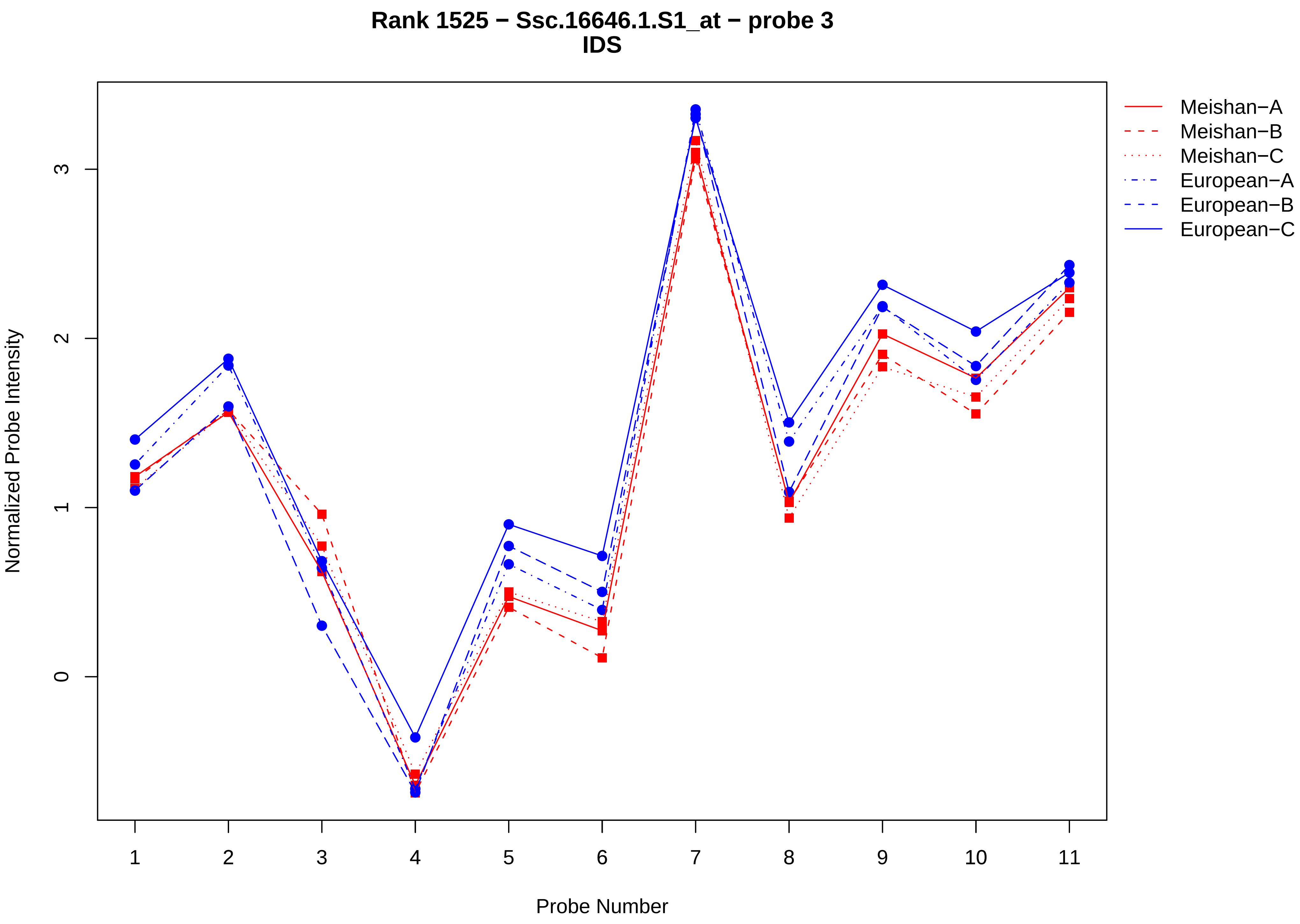

Rank 1525 − Ssc.16646.1.S1_at − probe 3
IDS
Normalized Probe Intensity
Probe Number
0
1
2
3
1
2
3
4
5
6
7
8
9
10
11
Meishan−A
Meishan−B
Meishan−C
European−A
European−B
European−C

Rank 1526 − Ssc.2099.1.S1_at − probe 7
KIF23

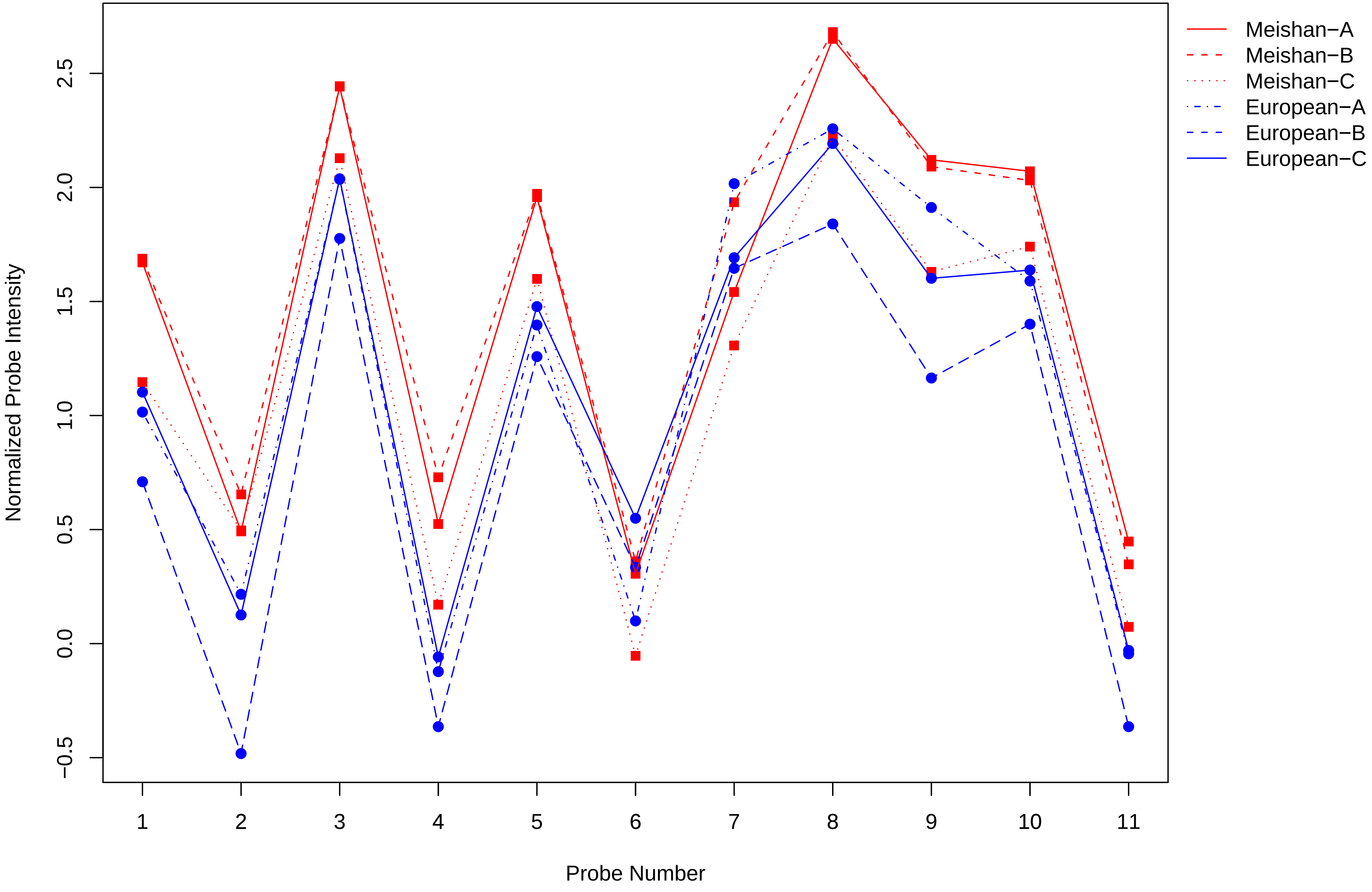

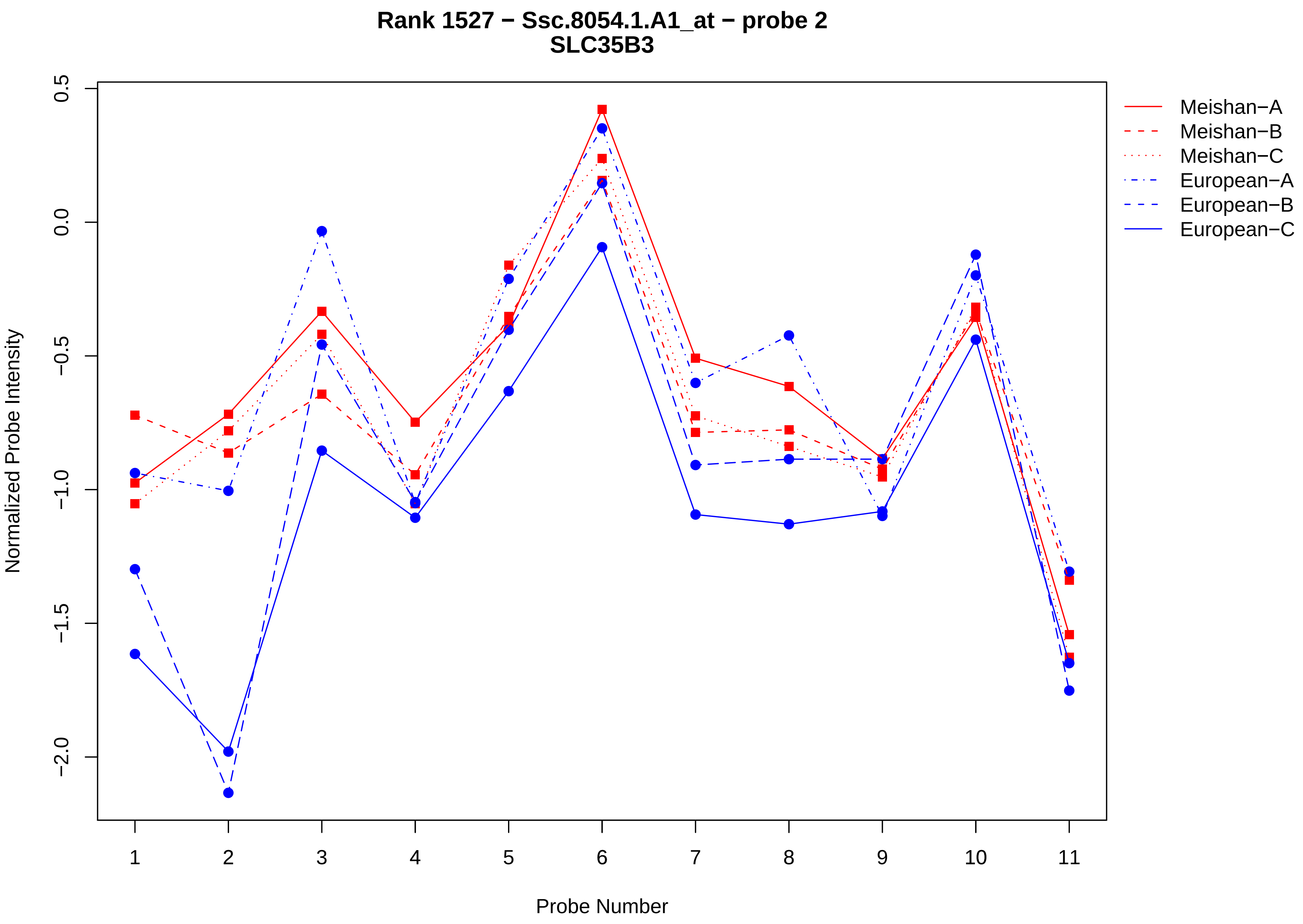

Rank 1527 − Ssc.8054.1.A1_at − probe 2
SLC35B3
Normalized Probe Intensity
Probe Number
0.5
0.0
−0.5
−1.0
−1.5
−2.0
1
2
3
4
5
6
7
8
9
10
11
Meishan−A
Meishan−B
Meishan−C
European−A
European−B
European−C

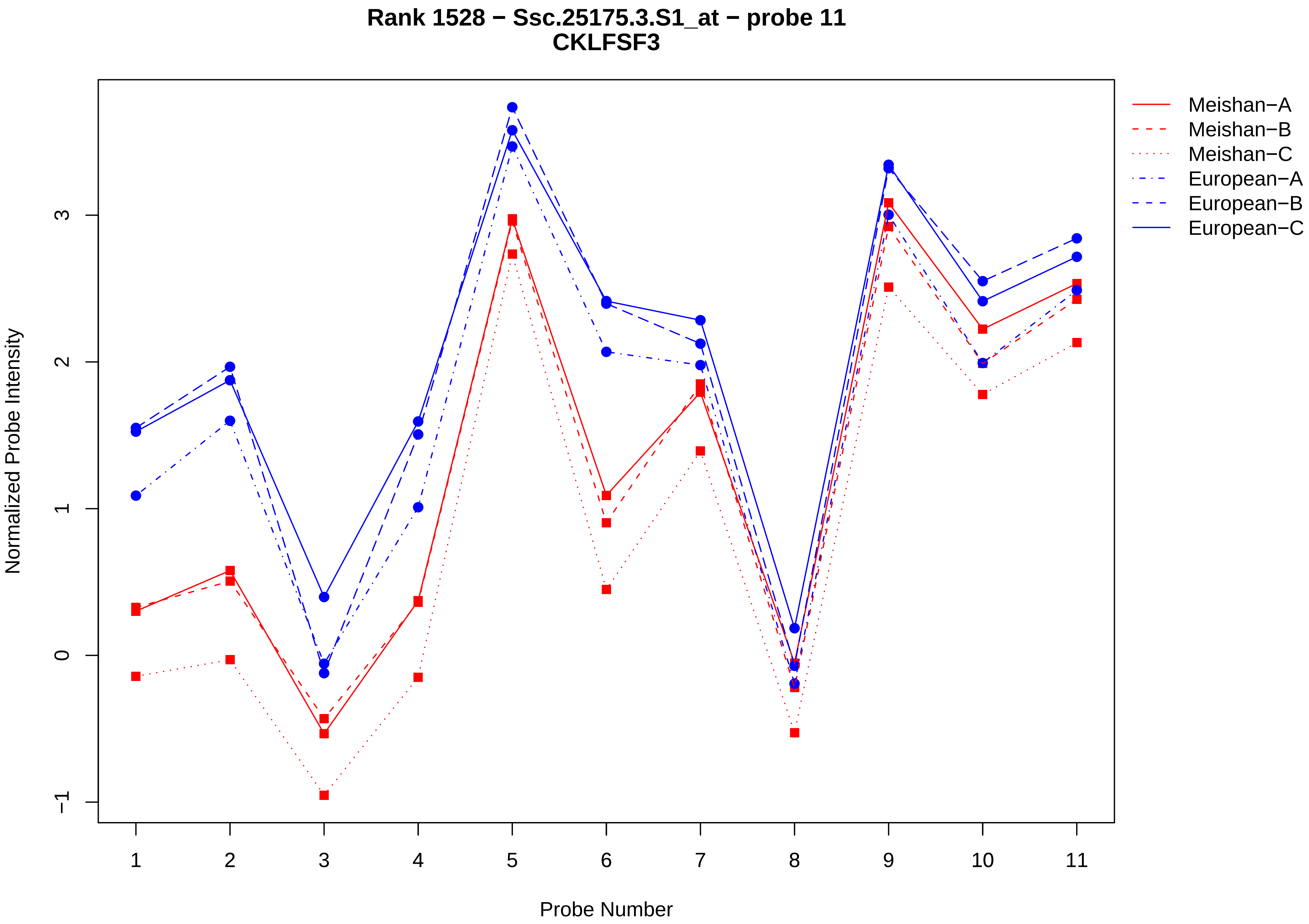

Rank 1528 − Ssc.25175.3.S1_at − probe 11
CKLFSF3
Normalized Probe Intensity
Probe Number
−1
0
1
2
3
1
2
3
4
5
6
7
8
9
10
11
Meishan−A
Meishan−B
Meishan−C
European−A
European−B
European−C

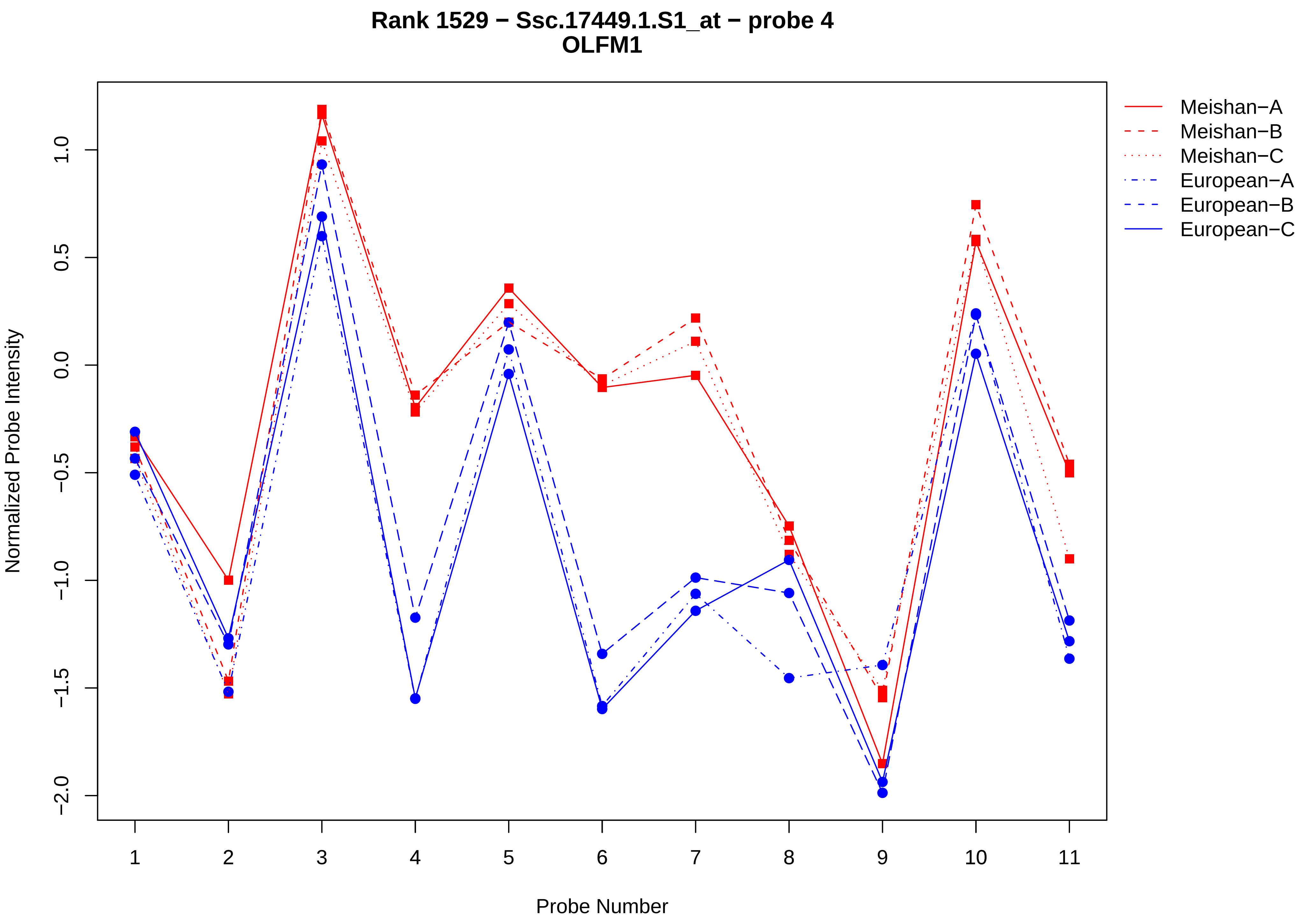

Rank 1529 − Ssc.17449.1.S1_at − probe 4
OLFM1
Normalized Probe Intensity
Probe Number
1.0
0.5
0.0
−0.5
−1.0
−1.5
−2.0
1
2
3
4
5
6
7
8
9
10
11
Meishan−A
Meishan−B
Meishan−C
European−A
European−B
European−C

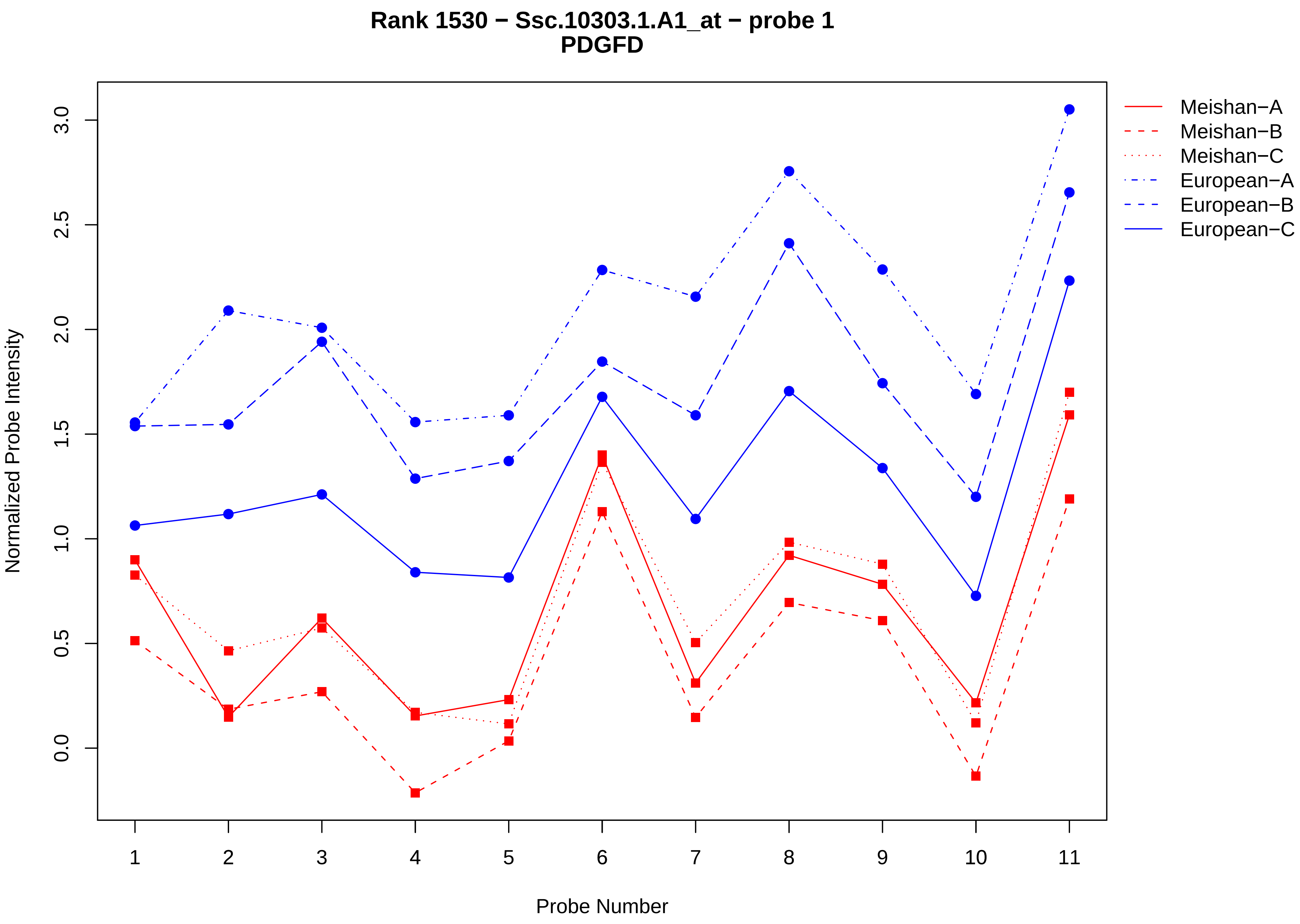

Rank 1530 − Ssc.10303.1.A1_at − probe 1
PDGFD
Normalized Probe Intensity
0.0
0.5
1.0
1.5
2.0
2.5
3.0
1
2
3
4
5
6
7
8
9
10
11
Probe Number
Meishan−A
Meishan−B
Meishan−C
European−A
European−B
European−C

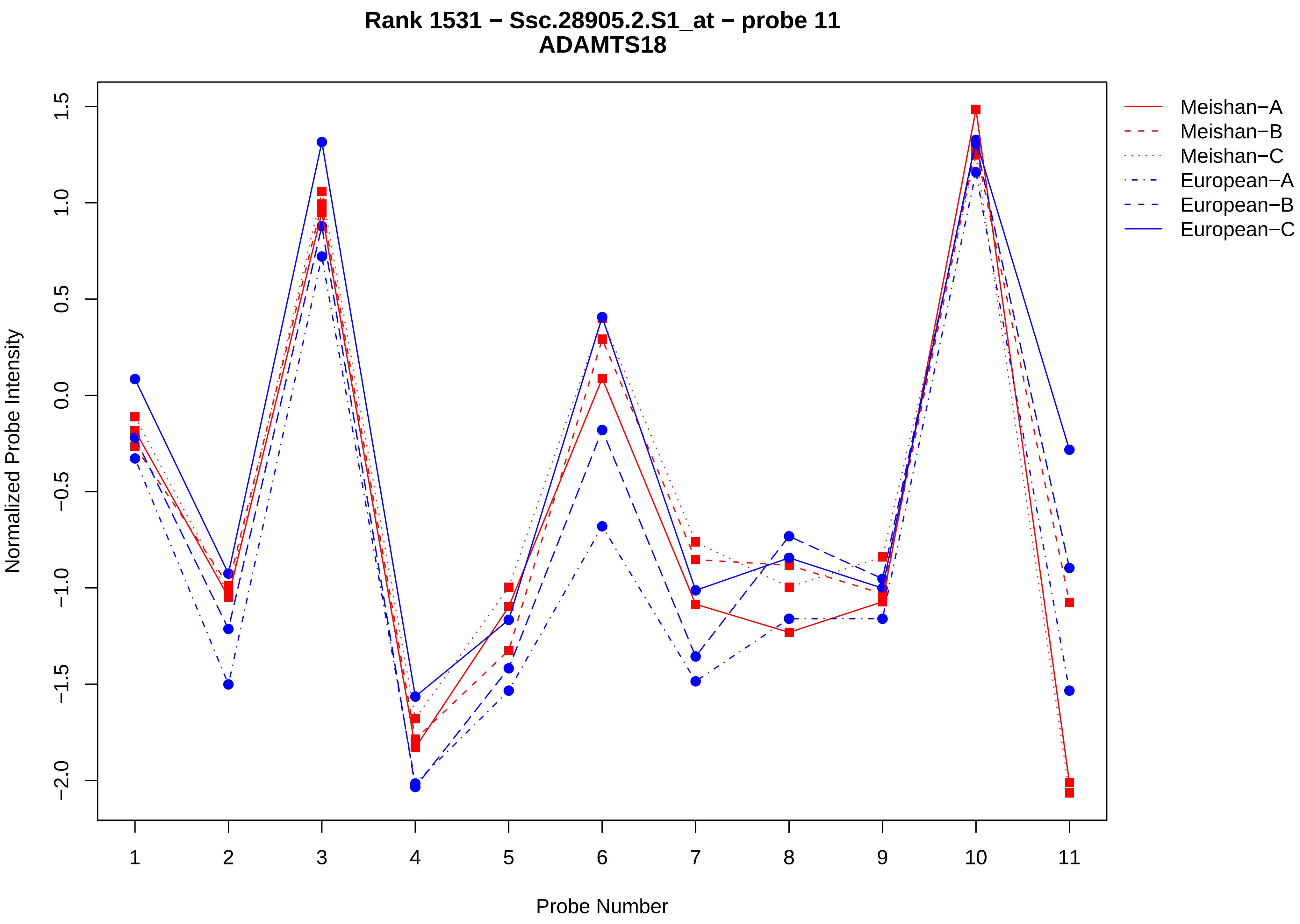

Rank 1531 − Ssc.28905.2.S1_at − probe 11
ADAMTS18
Normalized Probe Intensity
Probe Number
1.5
1.0
0.5
0.0
−0.5
−1.0
−1.5
−2.0
1
2
3
4
5
6
7
8
9
10
11
Meishan−A
Meishan−B
Meishan−C
European−A
European−B
European−C

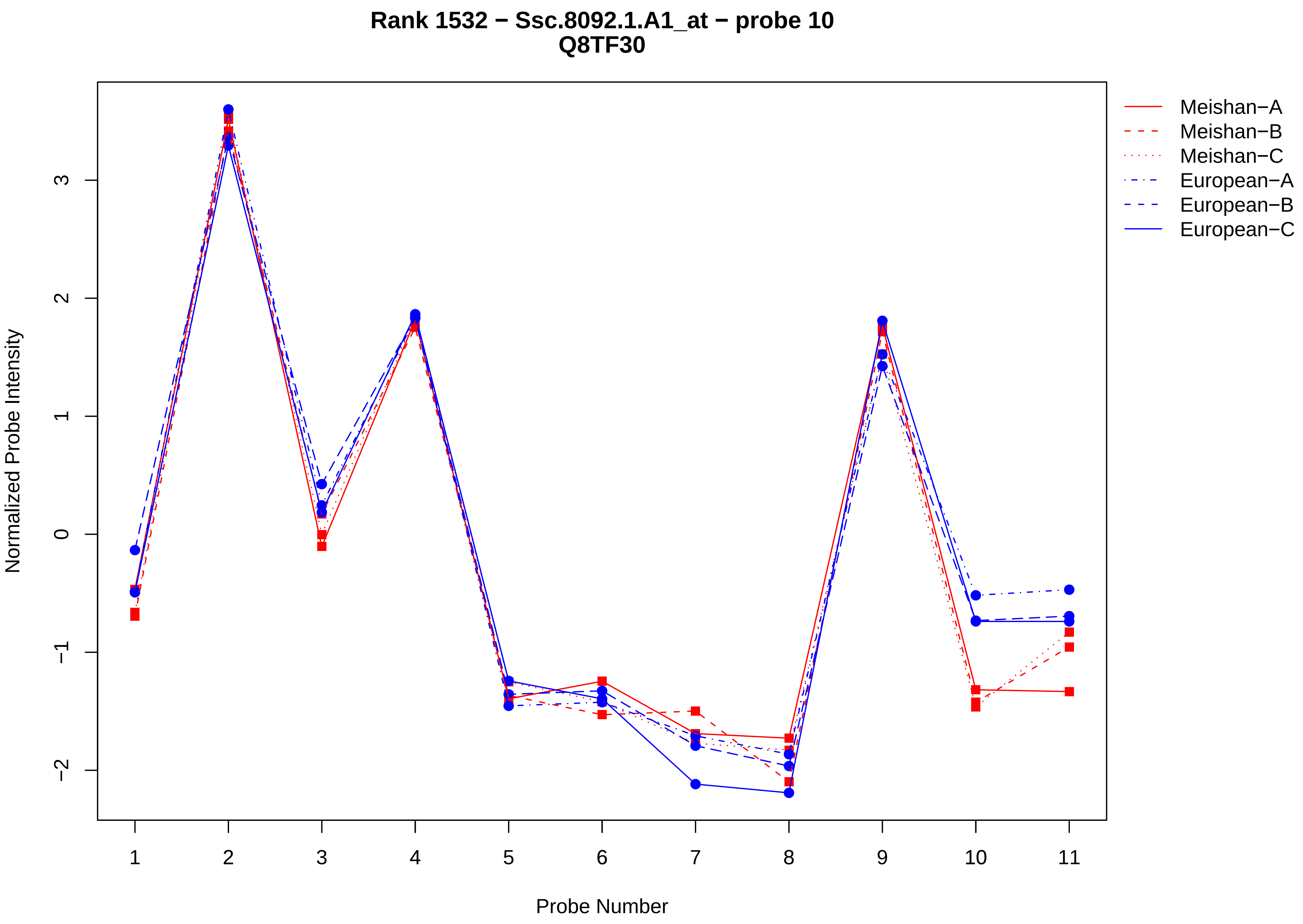
Rank 1532 − Ssc.8092.1.A1_at − probe 10
Q8TF30
Normalized Probe Intensity
Probe Number
−2
−1
0
1
2
3
1
2
3
4
5
6
7
8
9
10
11
Meishan−A
Meishan−B
Meishan−C
European−A
European−B
European−C

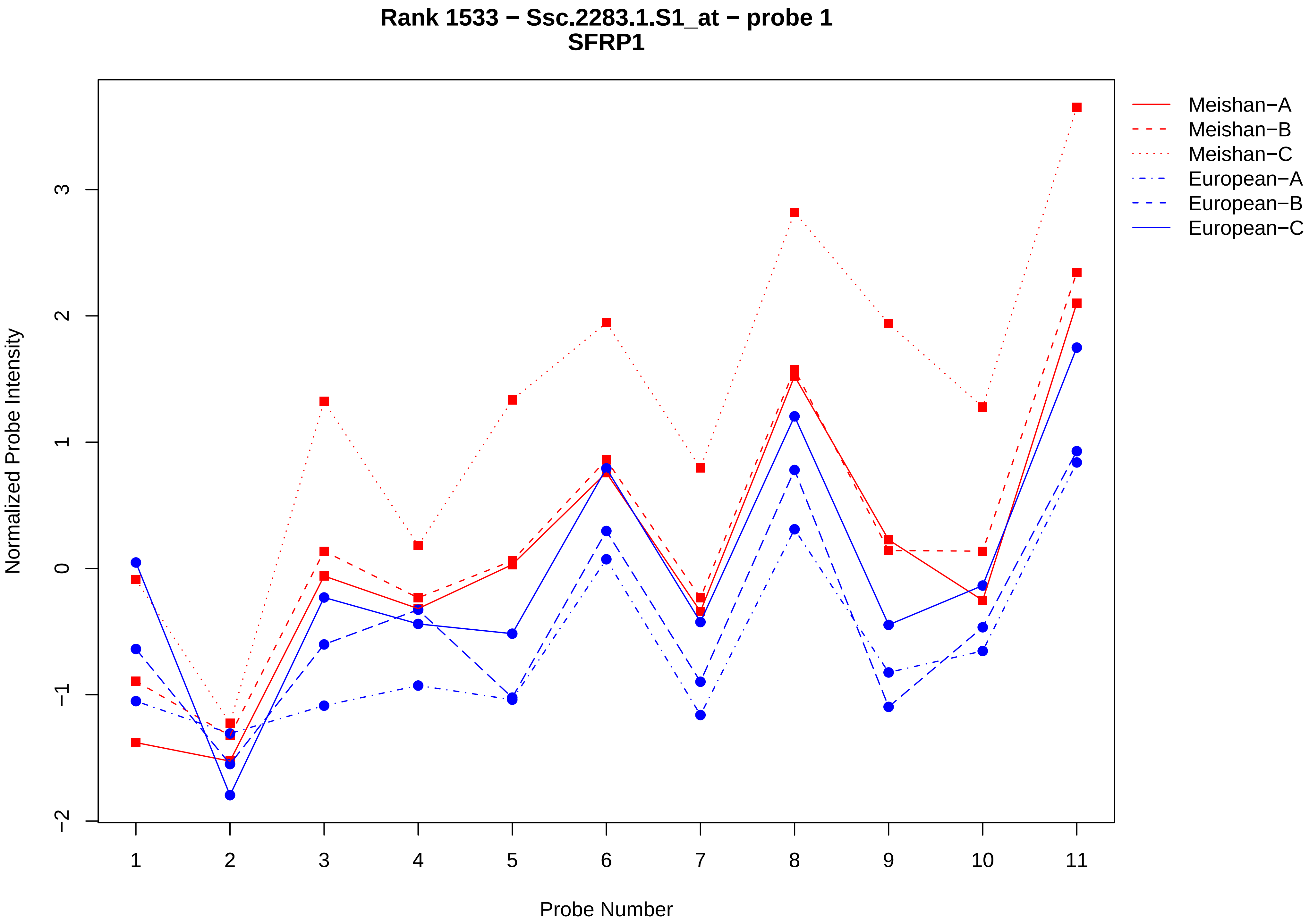

Rank 1533 − Ssc.2283.1.S1_at − probe 1
SFRP1
Normalized Probe Intensity
Probe Number
−2
−1
0
1
2
3
1
2
3
4
5
6
7
8
9
10
11
Meishan−A
Meishan−B
Meishan−C
European−A
European−B
European−C

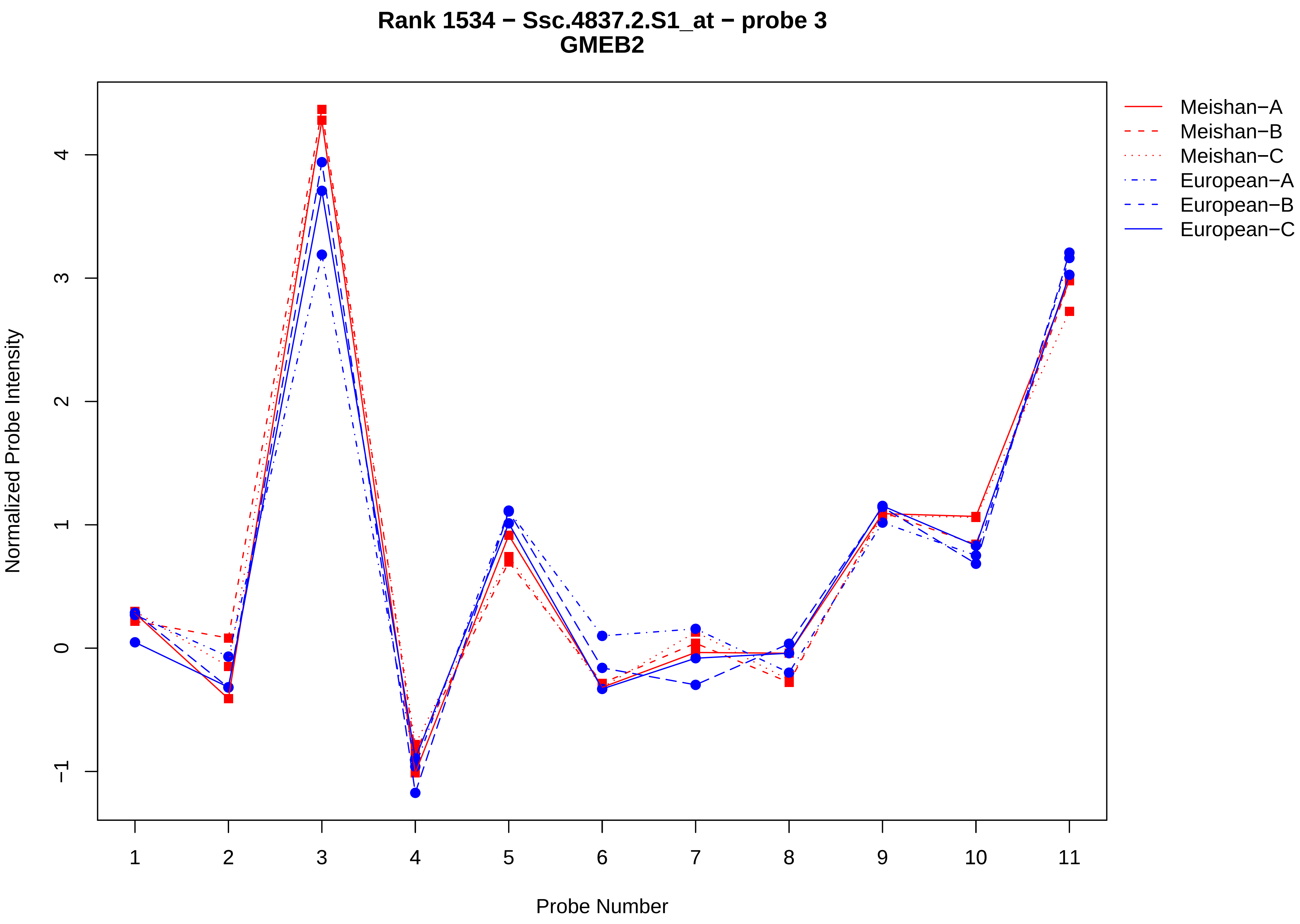

Rank 1534 − Ssc.4837.2.S1_at − probe 3
GMEB2
Meishan−A
Meishan−B
Meishan−C
European−A
European−B
European−C
Normalized Probe Intensity
Probe Number
−1
0
1
2
3
4
1
2
3
4
5
6
7
8
9
10
11

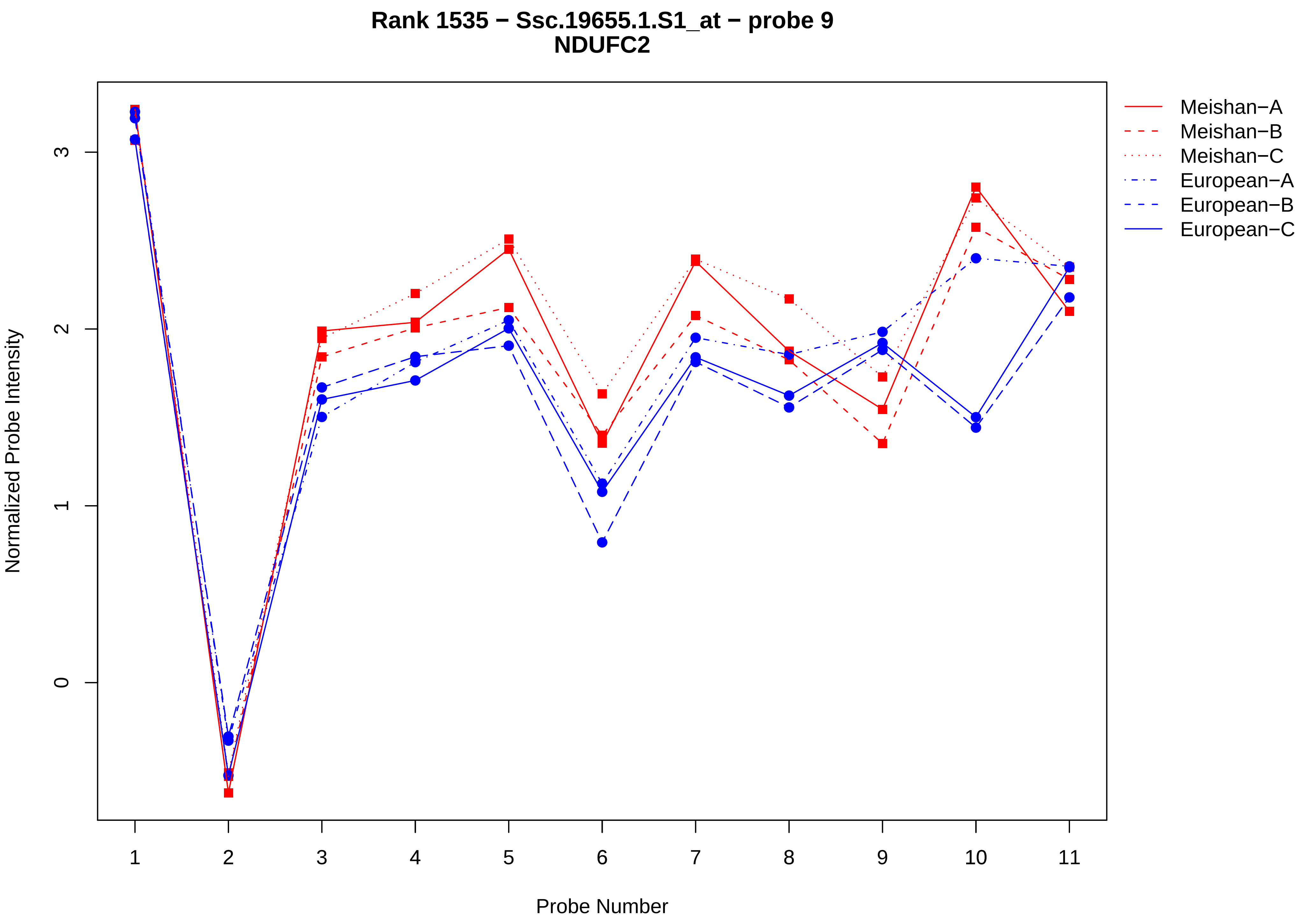

Rank 1535 − Ssc.19655.1.S1_at − probe 9
NDUFC2
Normalized Probe Intensity
Probe Number
0
1
2
3
1
2
3
4
5
6
7
8
9
10
11
Meishan−A
Meishan−B
Meishan−C
European−A
European−B
European−C

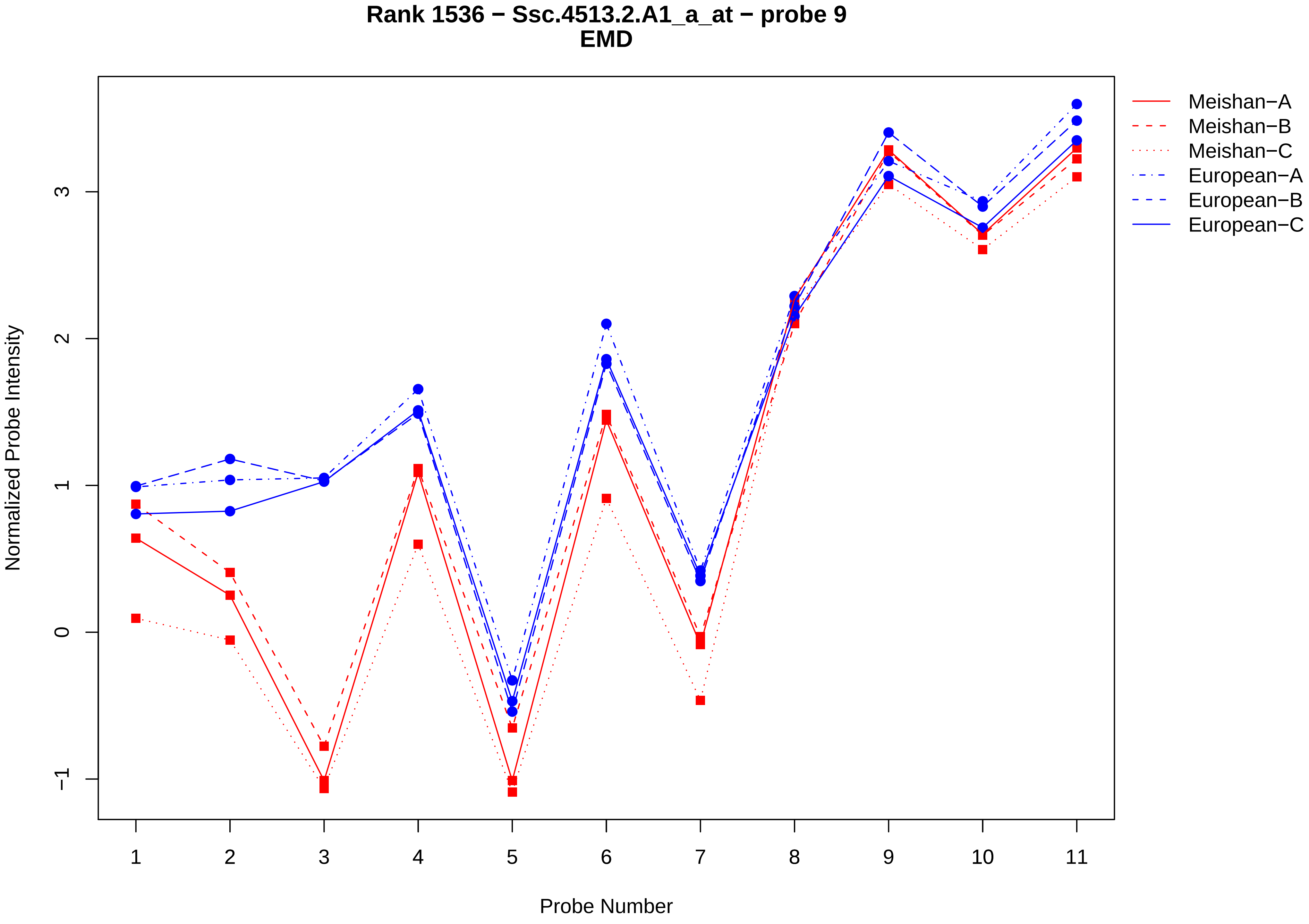

Rank 1536 − Ssc.4513.2.A1_a_at − probe 9
EMD
Normalized Probe Intensity
Probe Number
−1
0
1
2
3
1
2
3
4
5
6
7
8
9
10
11
Meishan−A
Meishan−B
Meishan−C
European−A
European−B
European−C

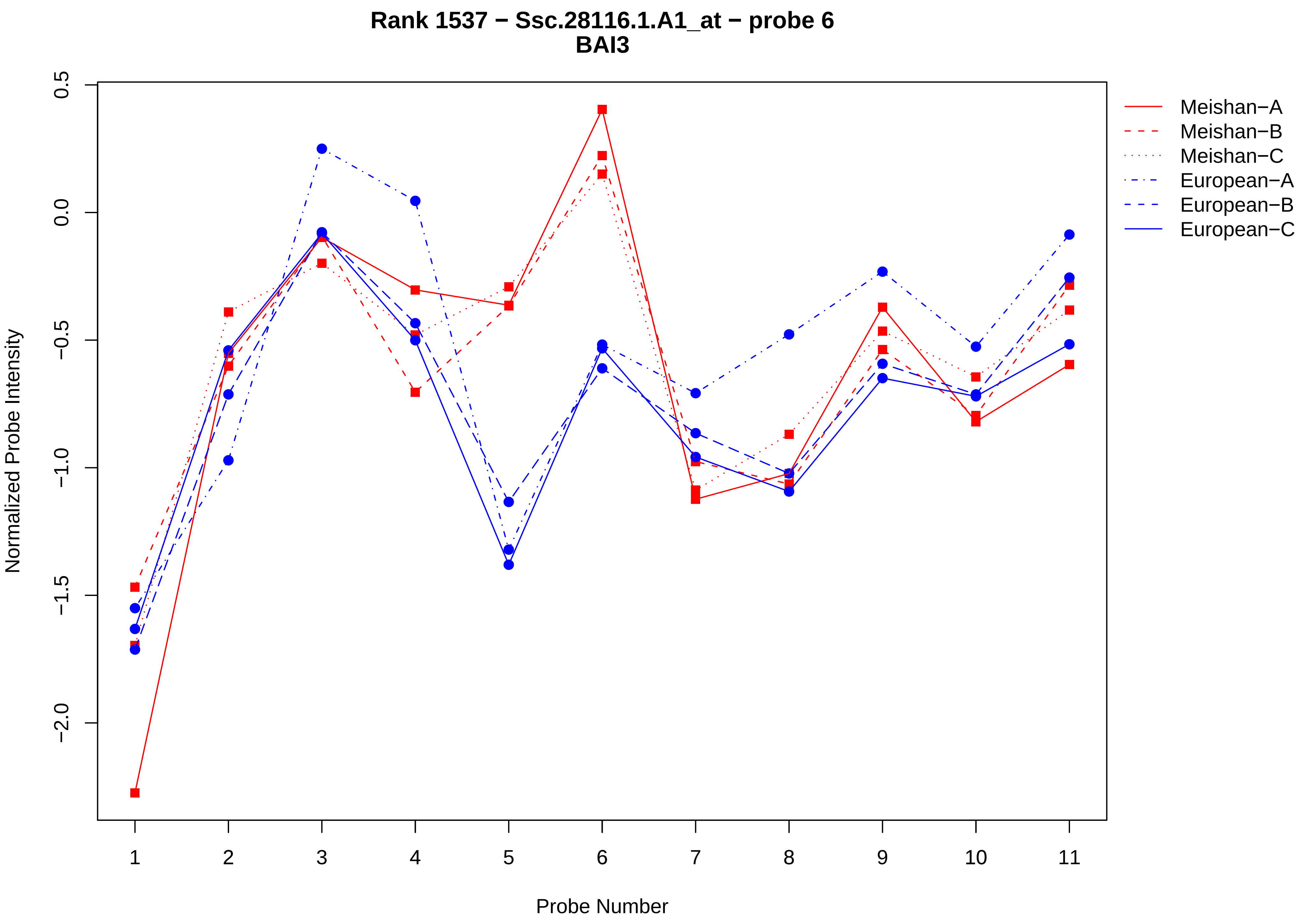

Rank 1537 − Ssc.28116.1.A1_at − probe 6
BAI3
Normalized Probe Intensity
Probe Number
0.5
0.0
−0.5
−1.0
−1.5
−2.0
1
2
3
4
5
6
7
8
9
10
11
Meishan−A
Meishan−B
Meishan−C
European−A
European−B
European−C

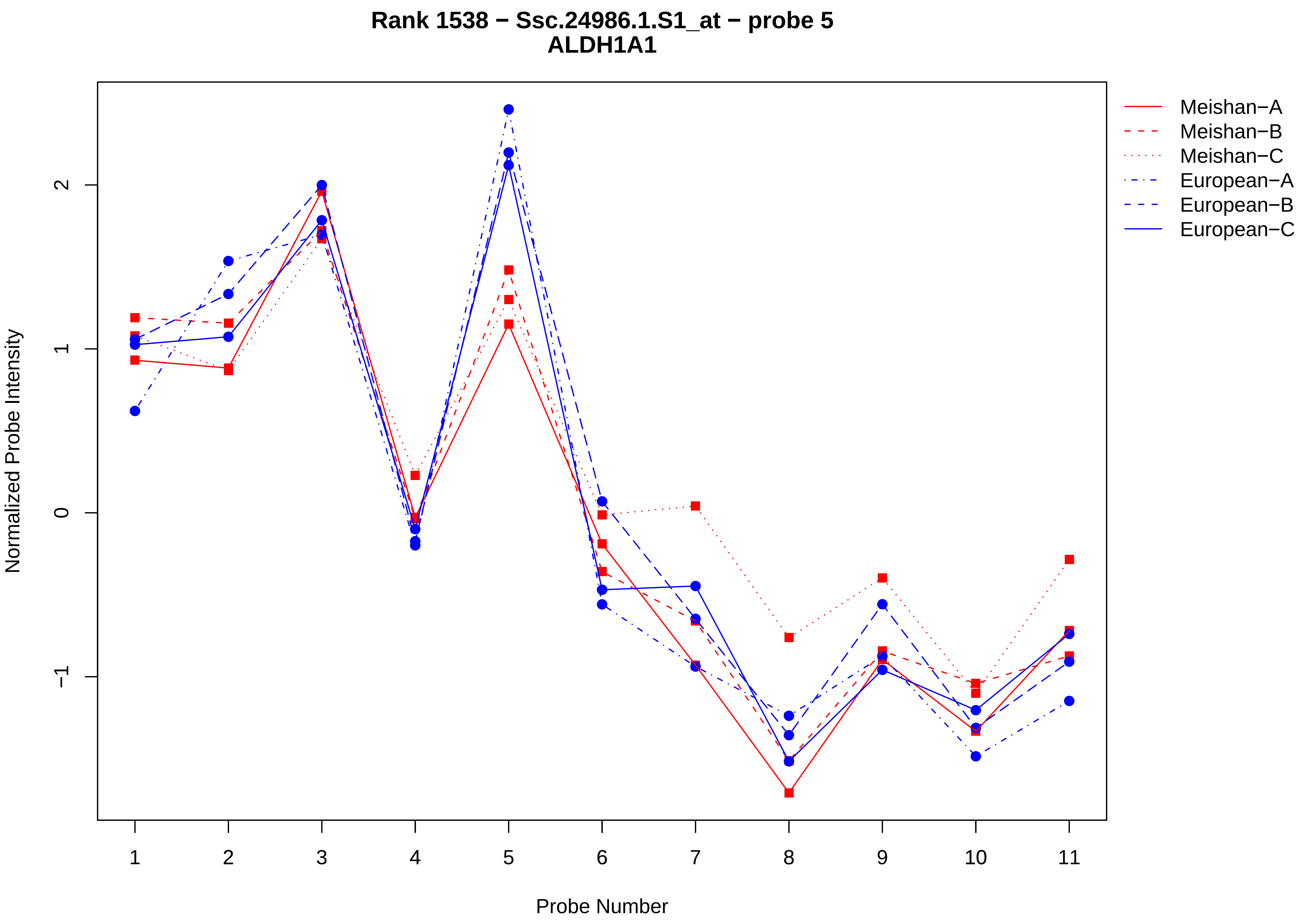

Rank 1538 − Ssc.24986.1.S1_at − probe 5
ALDH1A1
Normalized Probe Intensity
Probe Number
−1
0
1
2
1
2
3
4
5
6
7
8
9
10
11
Meishan−A
Meishan−B
Meishan−C
European−A
European−B
European−C

Rank 1539 − Ssc.8371.1.A1_at − probe 7
KIAA1598

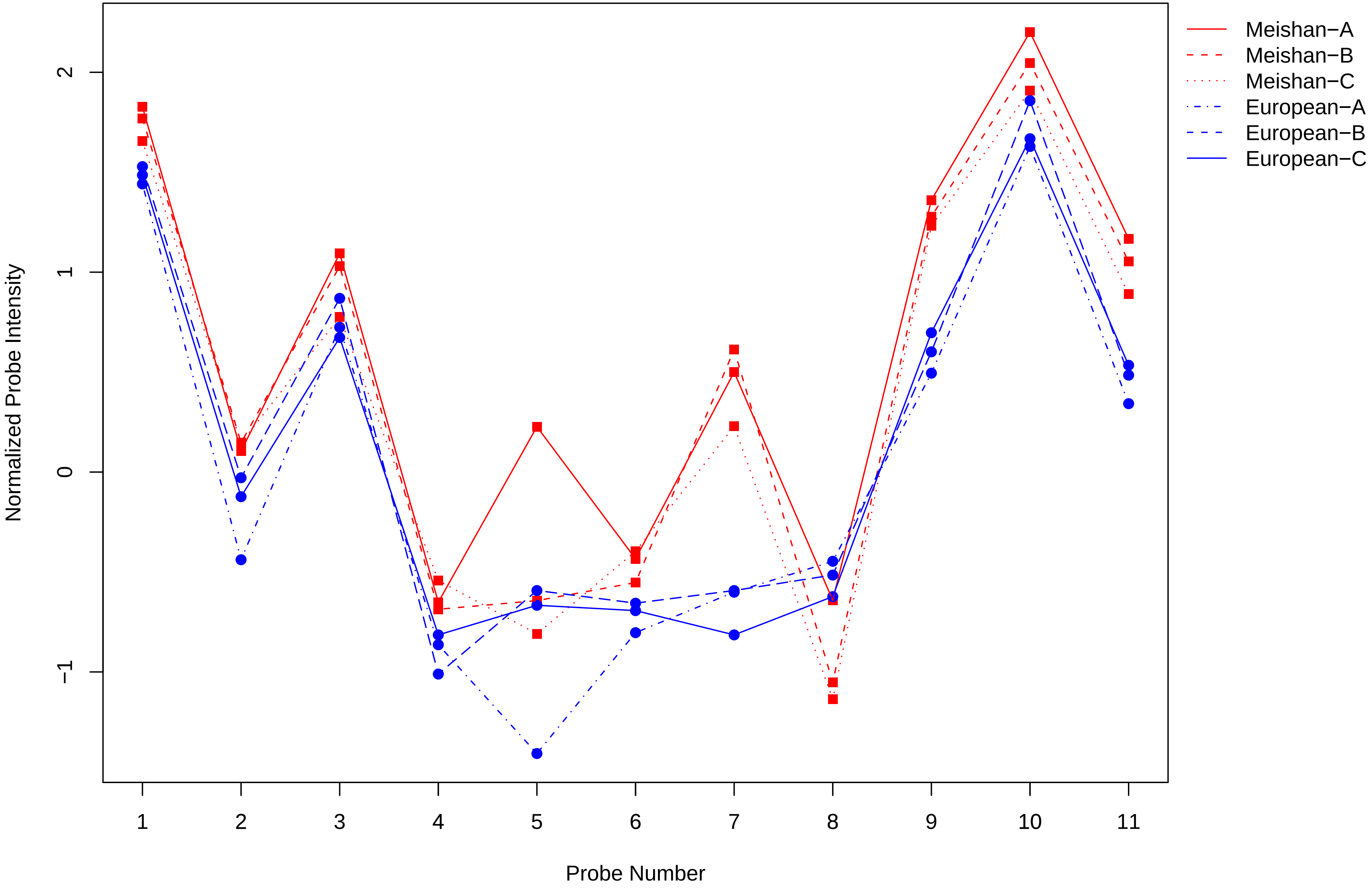

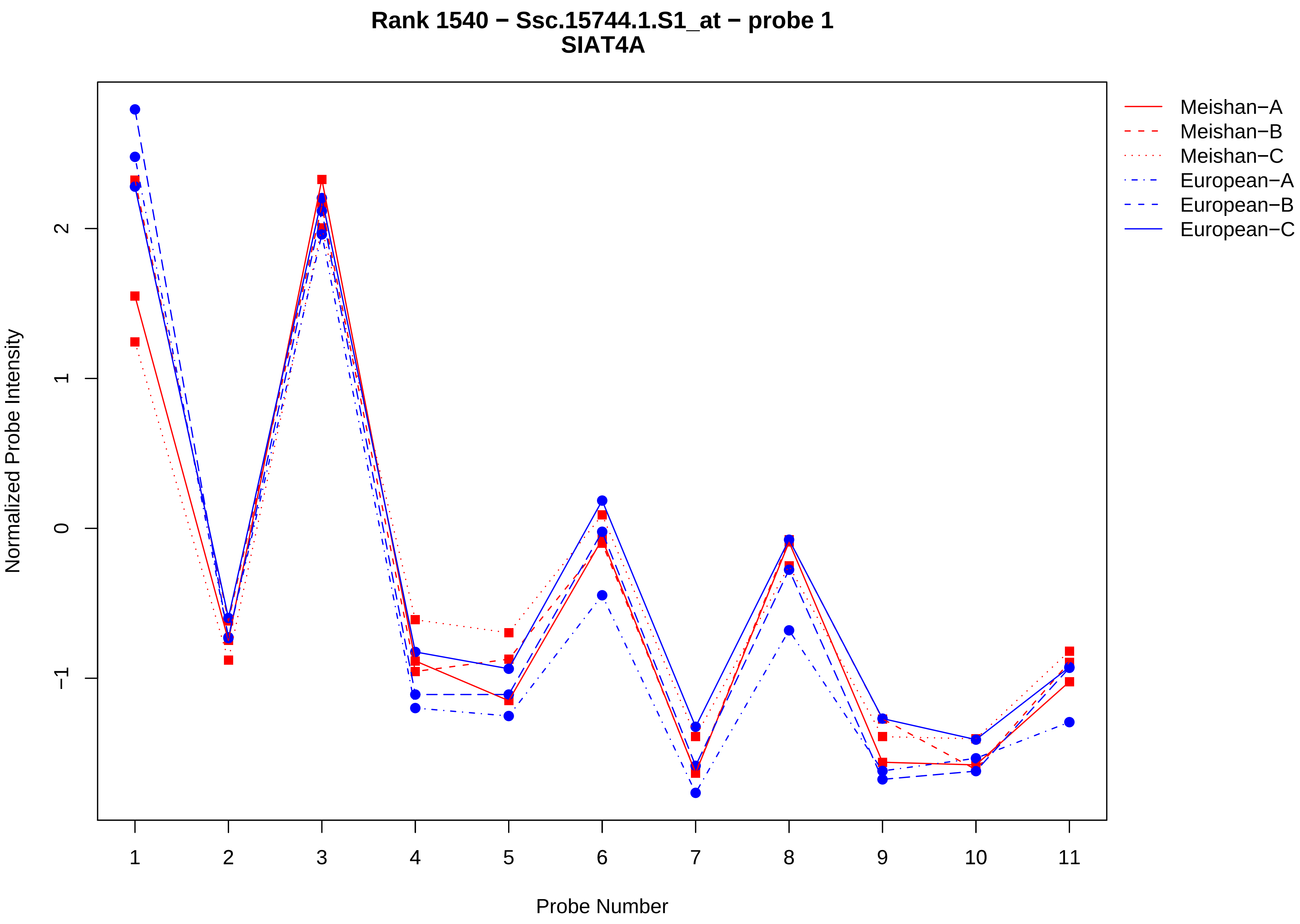
Rank 1540 − Ssc.15744.1.S1_at − probe 1
SIAT4A
Normalized Probe Intensity
Probe Number
−1
0
1
2
1
2
3
4
5
6
7
8
9
10
11
Meishan−A
Meishan−B
Meishan−C
European−A
European−B
European−C

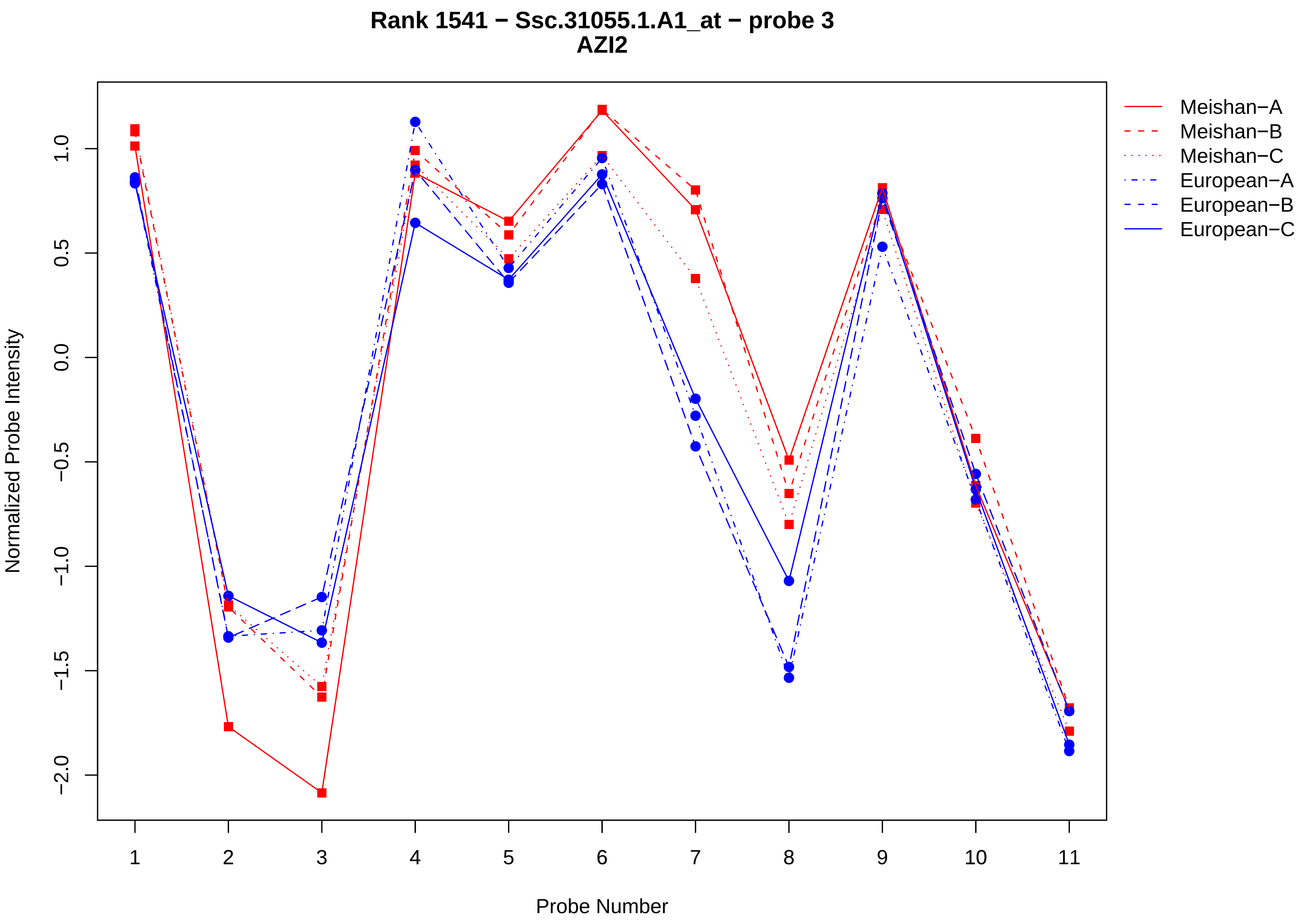

Rank 1541 − Ssc.31055.1.A1_at − probe 3
AZI2
Normalized Probe Intensity
Probe Number
−2.0
−1.5
−1.0
−0.5
0.0
0.5
1.0
1
2
3
4
5
6
7
8
9
10
11
Meishan−A
Meishan−B
Meishan−C
European−A
European−B
European−C

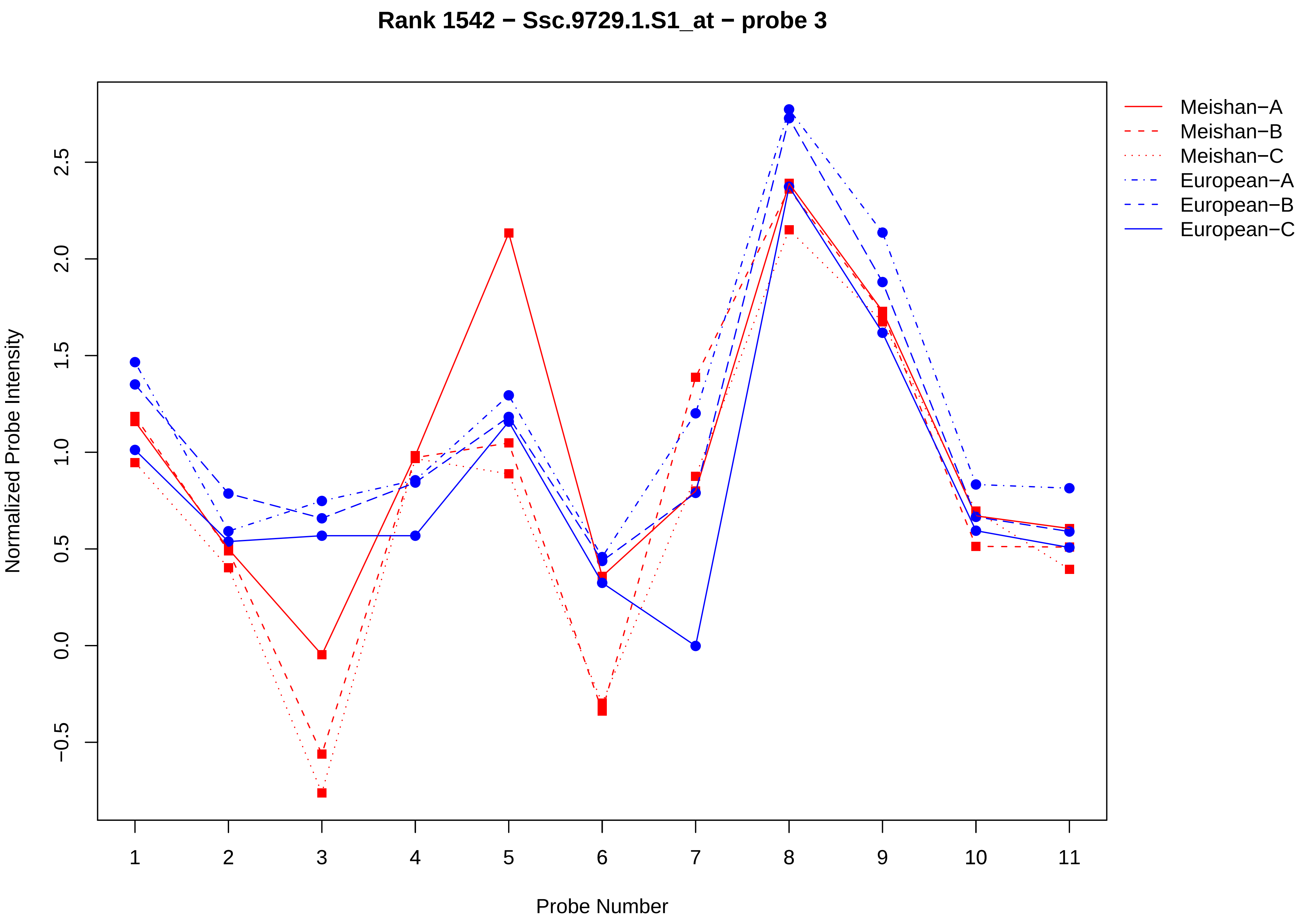
Rank 1542 − Ssc.9729.1.S1_at − probe 3
Normalized Probe Intensity
Probe Number
−0.5
0.0
0.5
1.0
1.5
2.0
2.5
1
2
3
4
5
6
7
8
9
10
11
Meishan−A
Meishan−B
Meishan−C
European−A
European−B
European−C

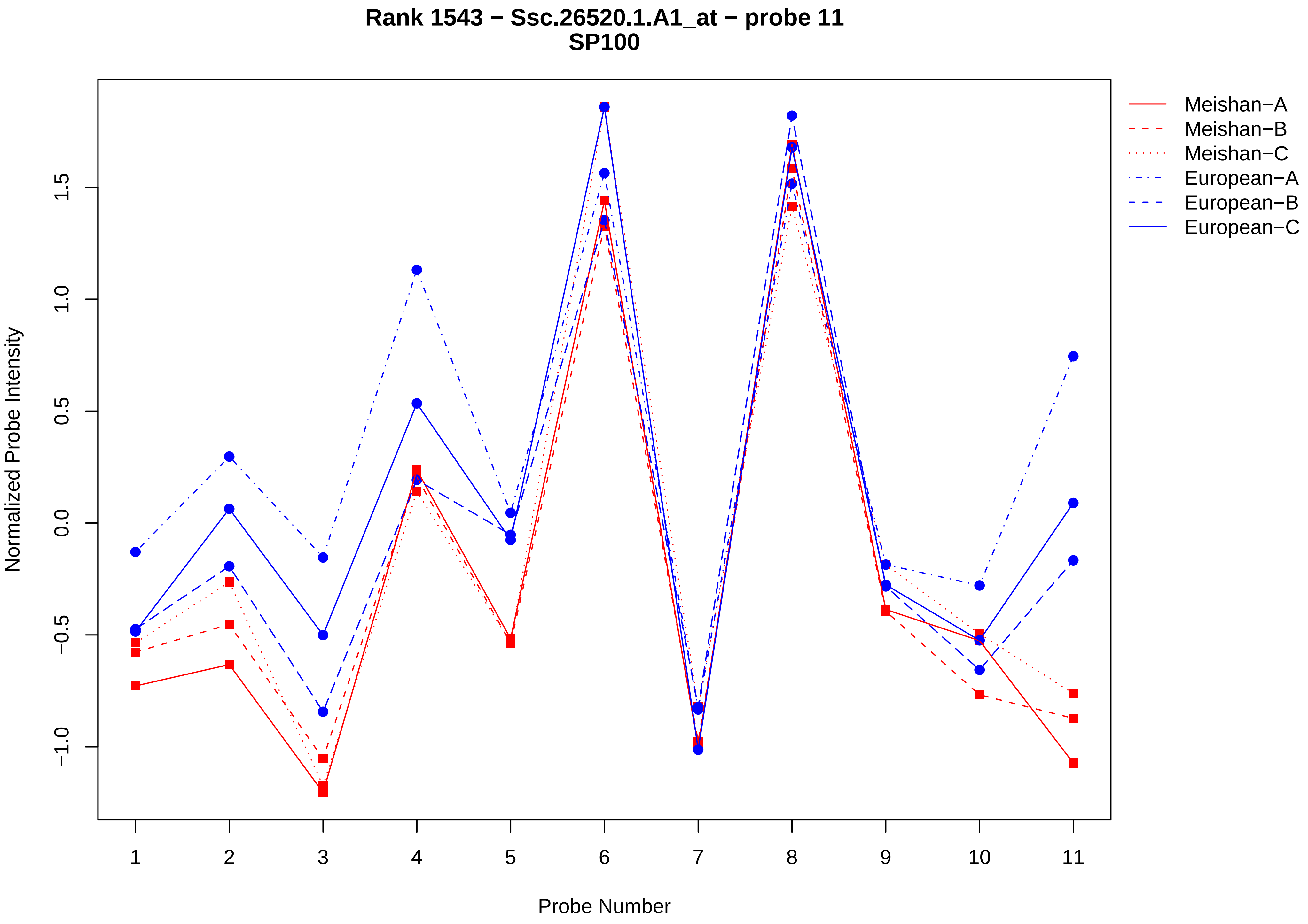

Rank 1543 − Ssc.26520.1.A1_at − probe 11
SP100
Normalized Probe Intensity
Probe Number
−1.0
−0.5
0.0
0.5
1.0
1.5
1
2
3
4
5
6
7
8
9
10
11
Meishan−A
Meishan−B
Meishan−C
European−A
European−B
European−C

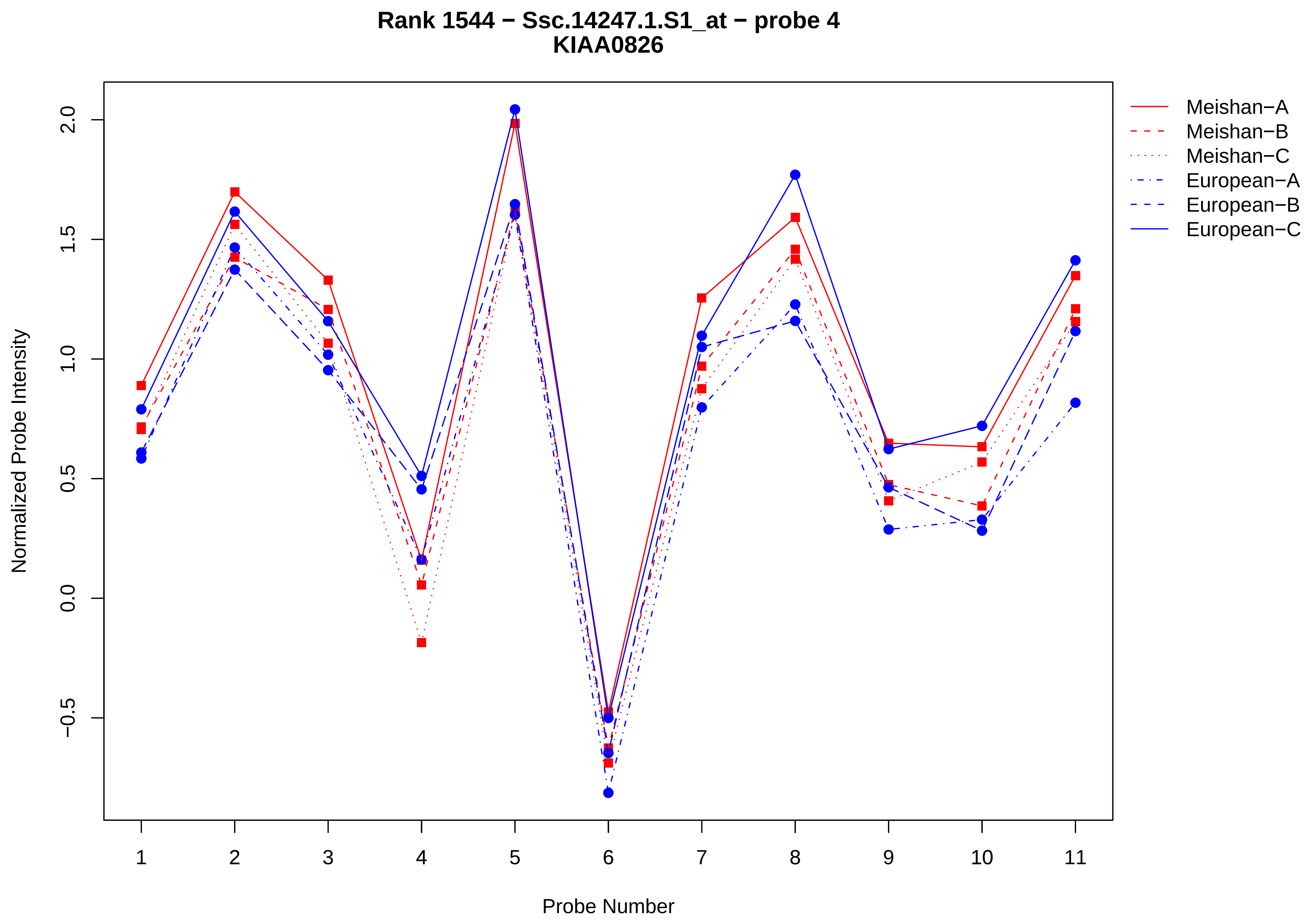

Rank 1544 − Ssc.14247.1.S1_at − probe 4
KIAA0826
Normalized Probe Intensity
−0.5
0.0
0.5
1.0
1.5
2.0
Probe Number
1
2
3
4
5
6
7
8
9
10
11
Meishan−A
Meishan−B
Meishan−C
European−A
European−B
European−C

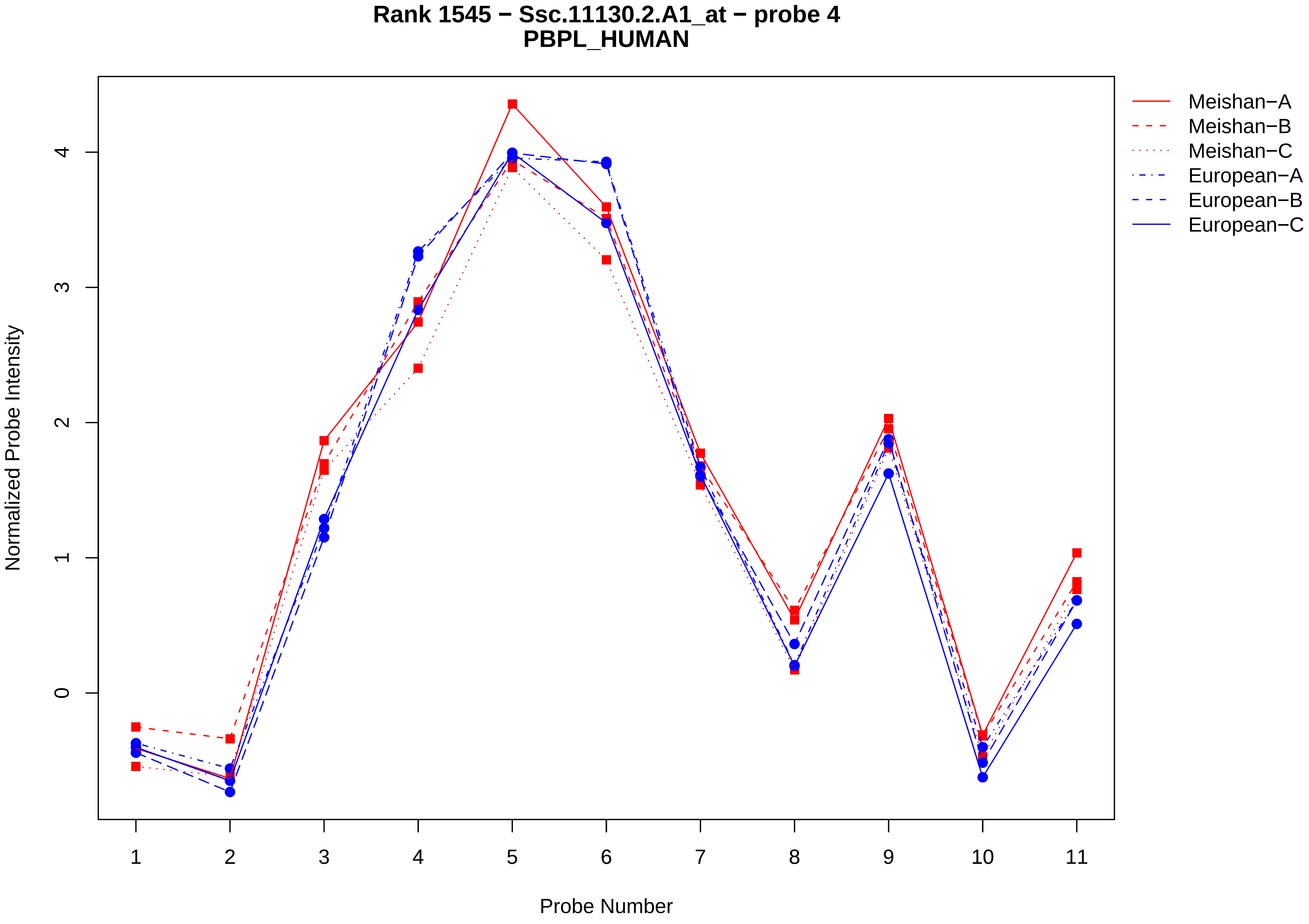

Rank 1545 − Ssc.11130.2.A1_at − probe 4
PBPL_HUMAN
Normalized Probe Intensity
Probe Number
0
1
2
3
4
1
2
3
4
5
6
7
8
9
10
11
Meishan−A
Meishan−B
Meishan−C
European−A
European−B
European−C

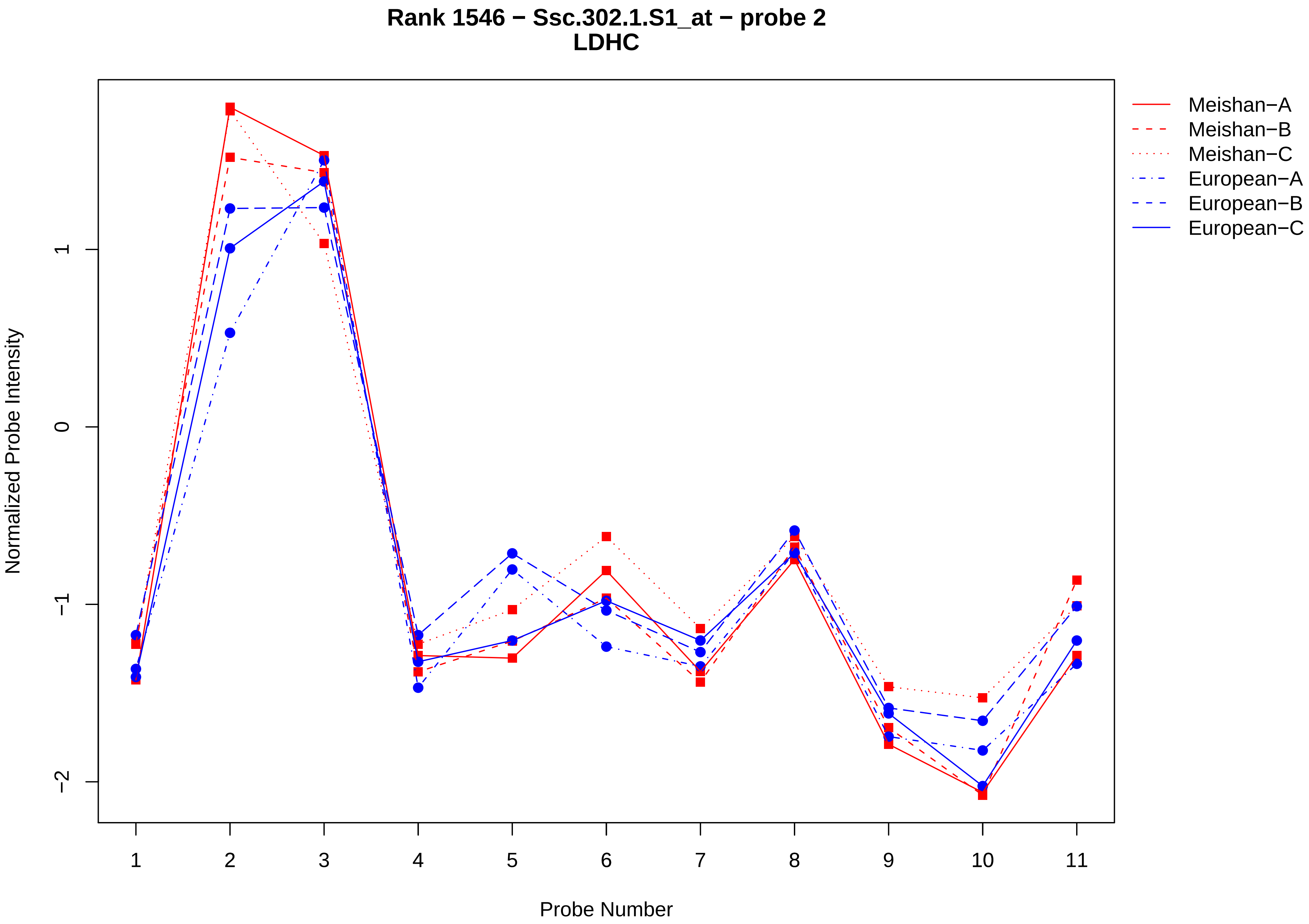

Rank 1546 − Ssc.302.1.S1_at − probe 2
LDHC
Normalized Probe Intensity
Probe Number
−2
−1
0
1
1
2
3
4
5
6
7
8
9
10
11
Meishan−A
Meishan−B
Meishan−C
European−A
European−B
European−C

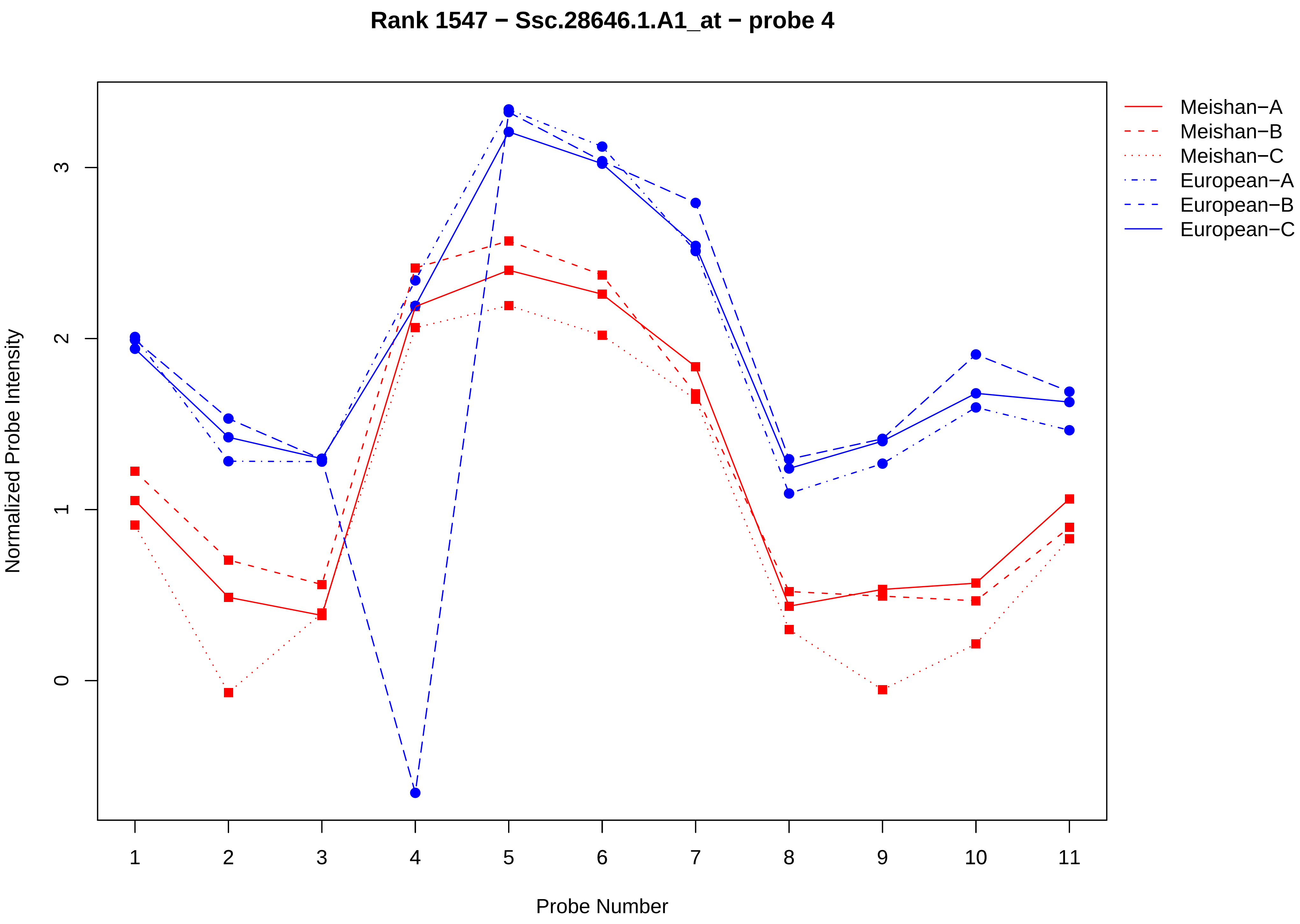

Rank 1547 − Ssc.28646.1.A1_at − probe 4
Normalized Probe Intensity
Probe Number
0
1
2
3
1
2
3
4
5
6
7
8
9
10
11
Meishan−A
Meishan−B
Meishan−C
European−A
European−B
European−C

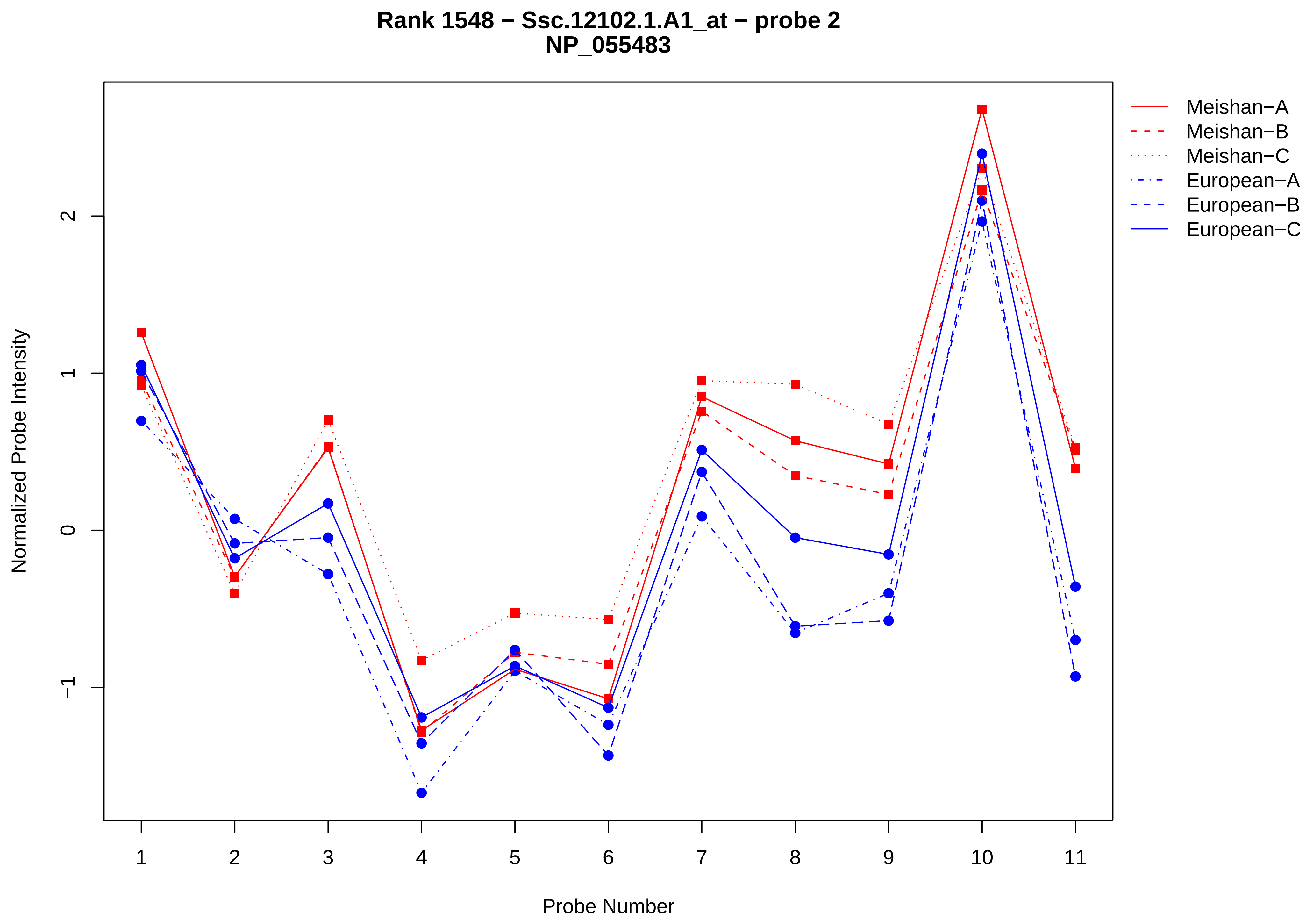

Rank 1548 − Ssc.12102.1.A1_at − probe 2
NP_055483
Normalized Probe Intensity
Probe Number
−1
0
1
2
1
2
3
4
5
6
7
8
9
10
11
Meishan−A
Meishan−B
Meishan−C
European−A
European−B
European−C

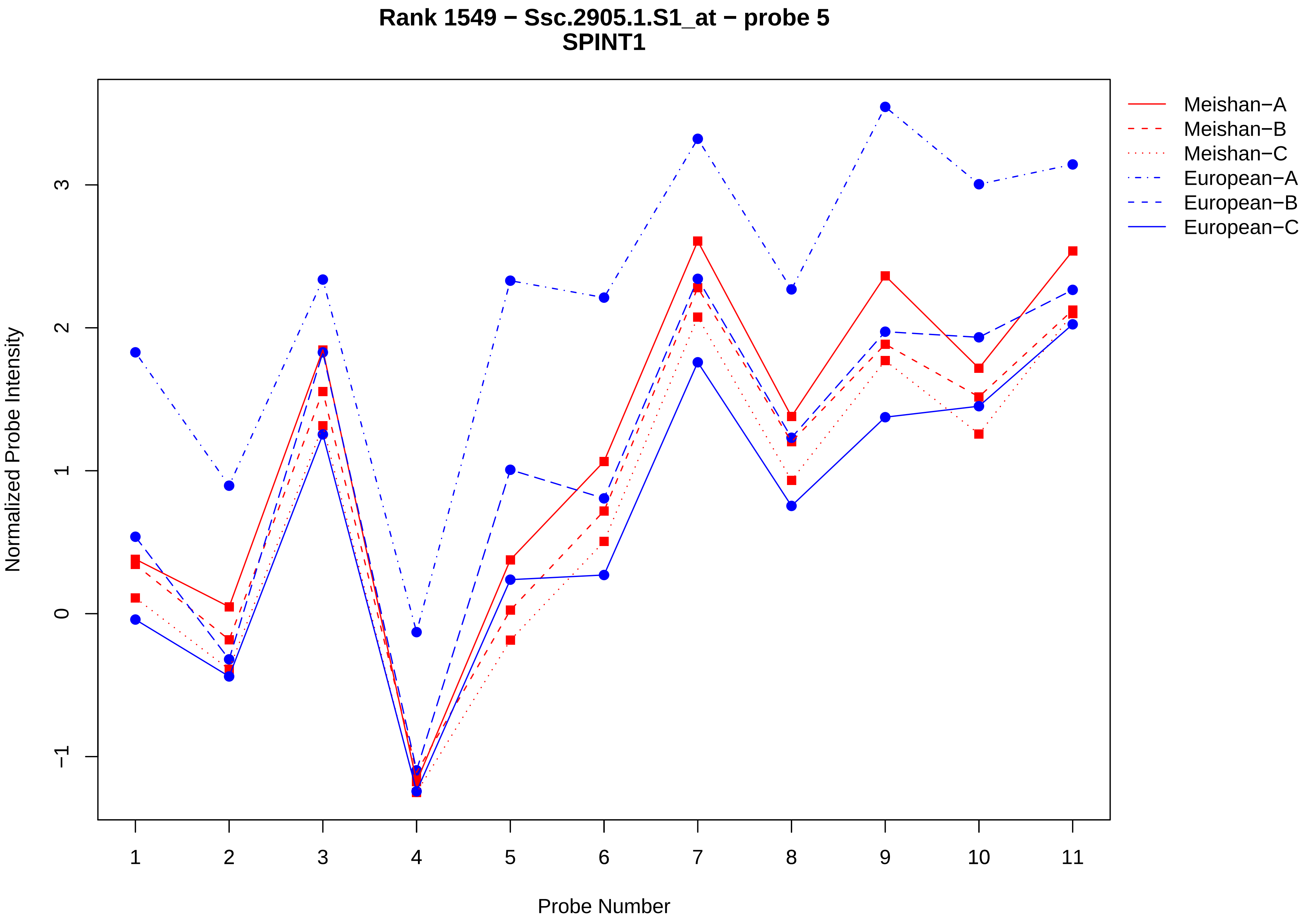

Rank 1549 − Ssc.2905.1.S1_at − probe 5
SPINT1
Normalized Probe Intensity
Probe Number
−1
0
1
2
3
1
2
3
4
5
6
7
8
9
10
11
Meishan−A
Meishan−B
Meishan−C
European−A
European−B
European−C

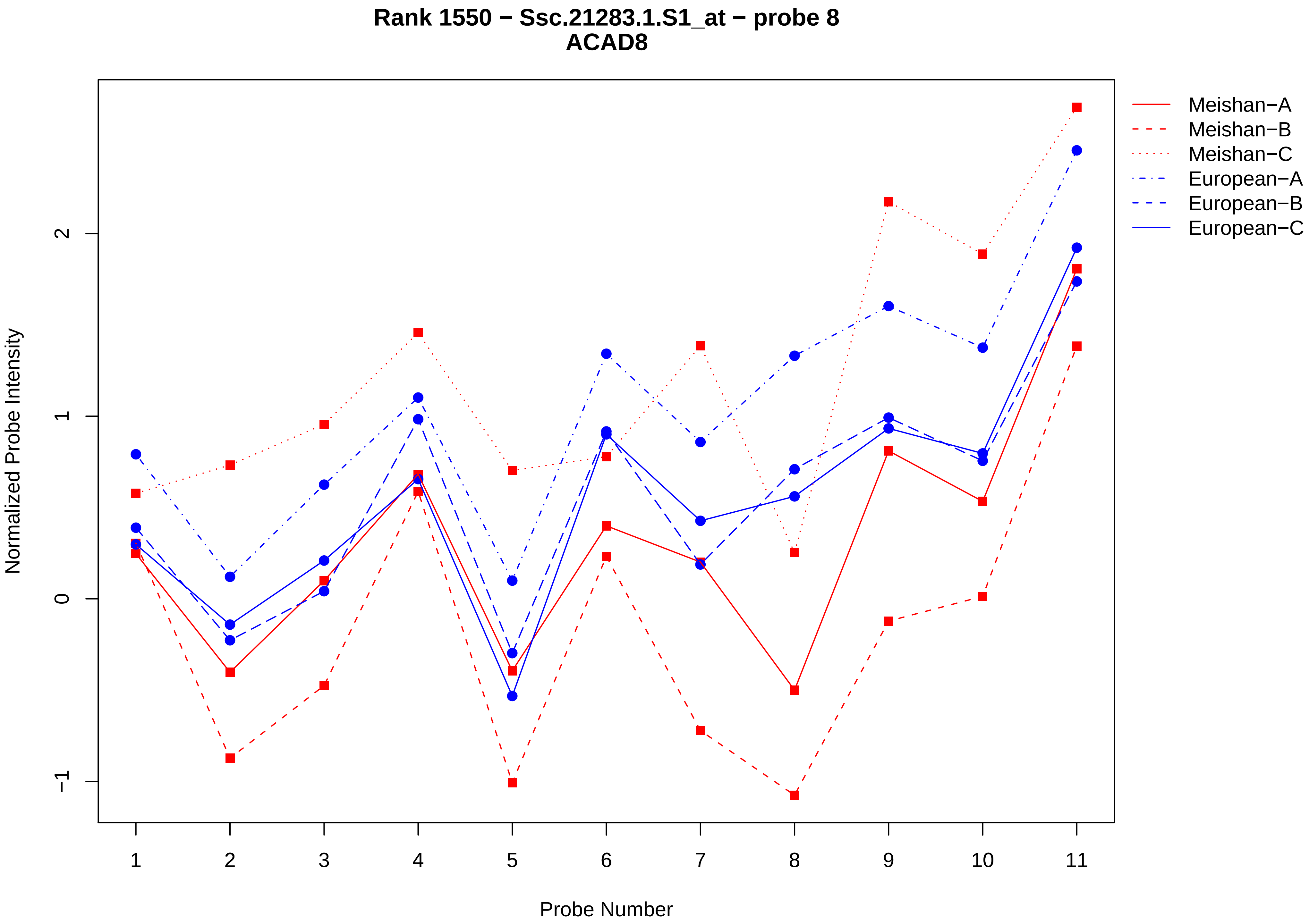

Rank 1550 − Ssc.21283.1.S1_at − probe 8
ACAD8
Normalized Probe Intensity
Probe Number
−1
0
1
2
1
2
3
4
5
6
7
8
9
10
11
Meishan−A
Meishan−B
Meishan−C
European−A
European−B
European−C

Rank 1551 − Ssc.22101.1.S1_at − probe 9
MTHFD1

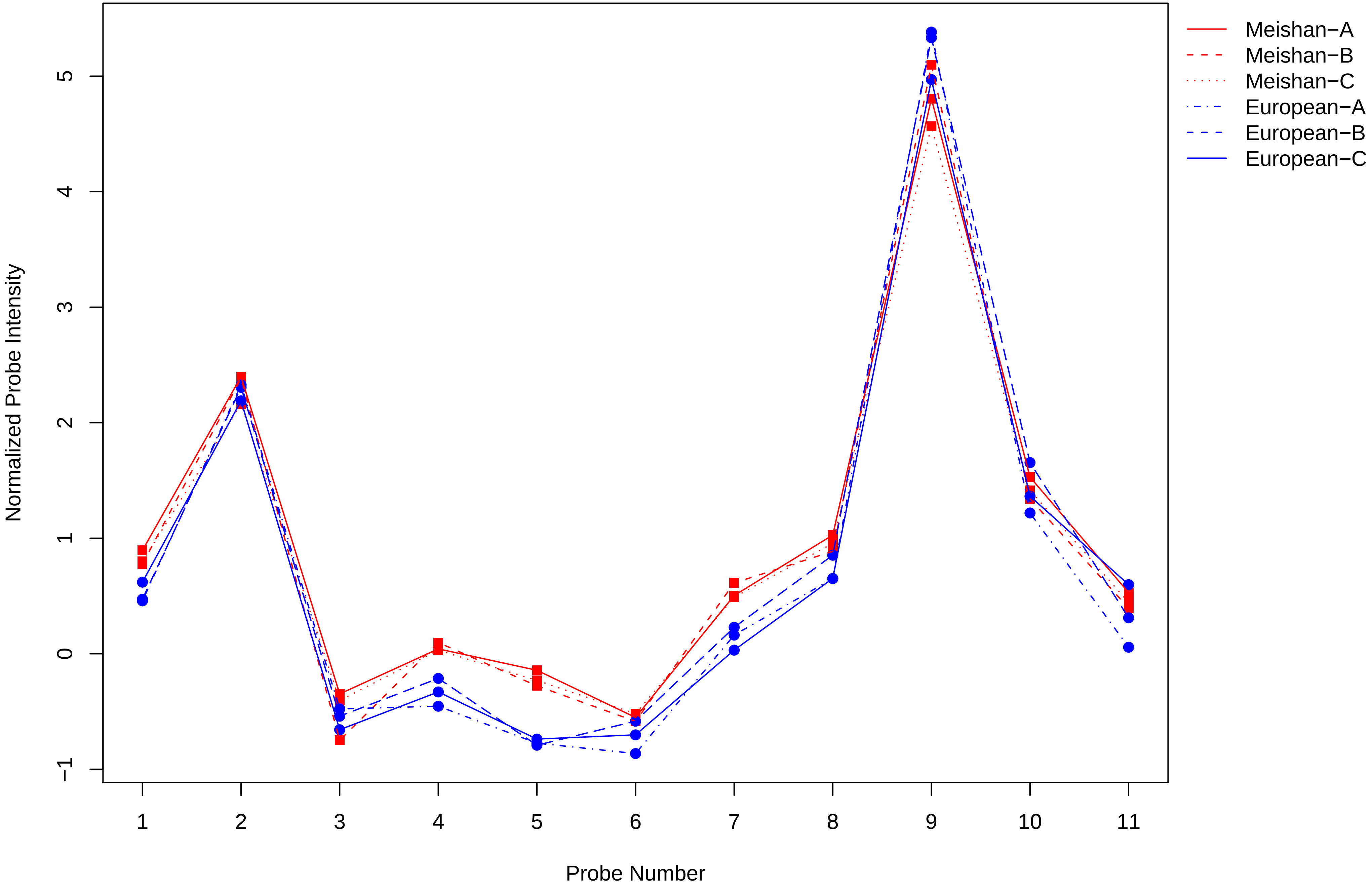

Rank 1552 − Ssc.12996.1.A1_at − probe 9
RBMS3

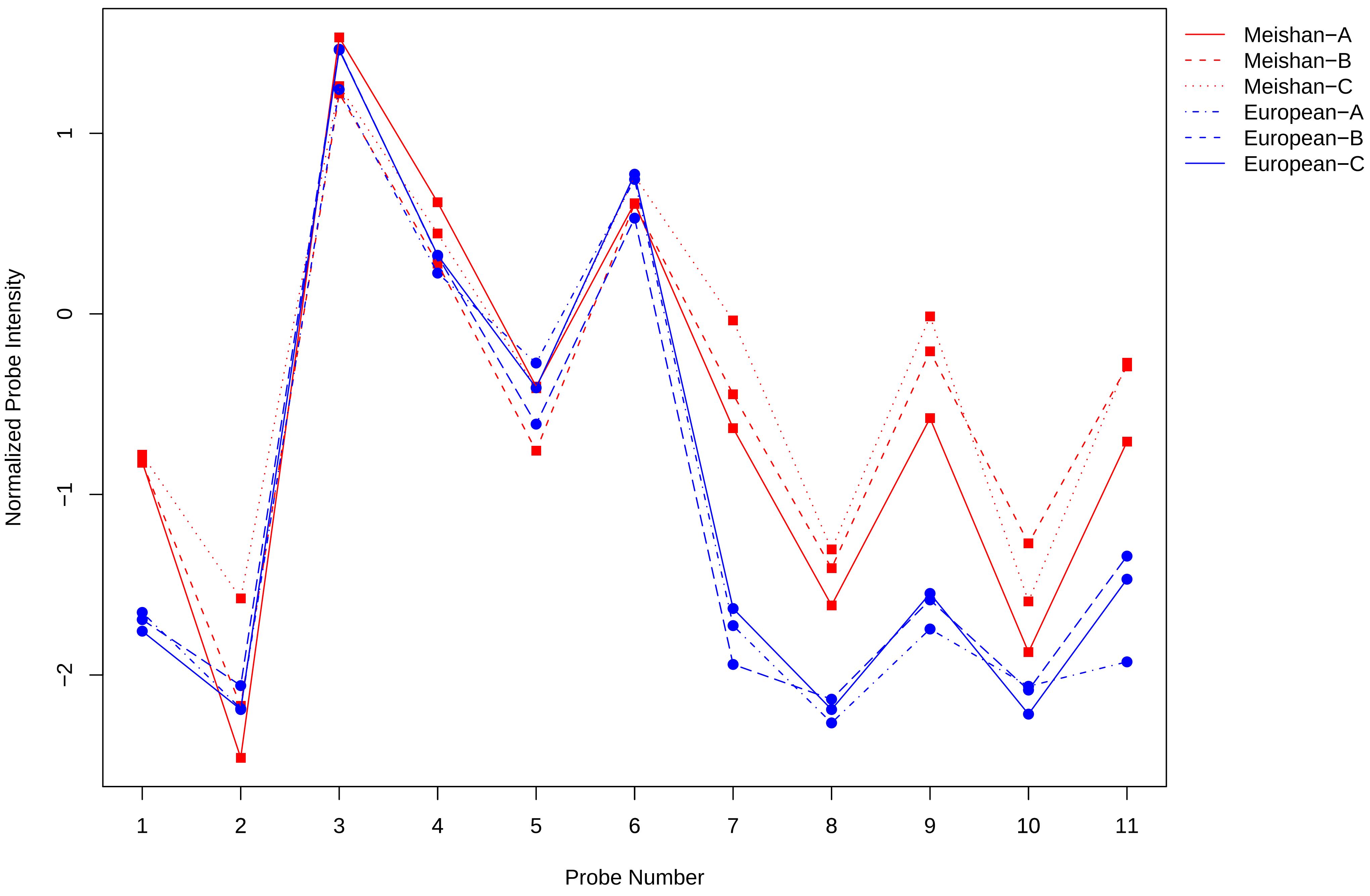

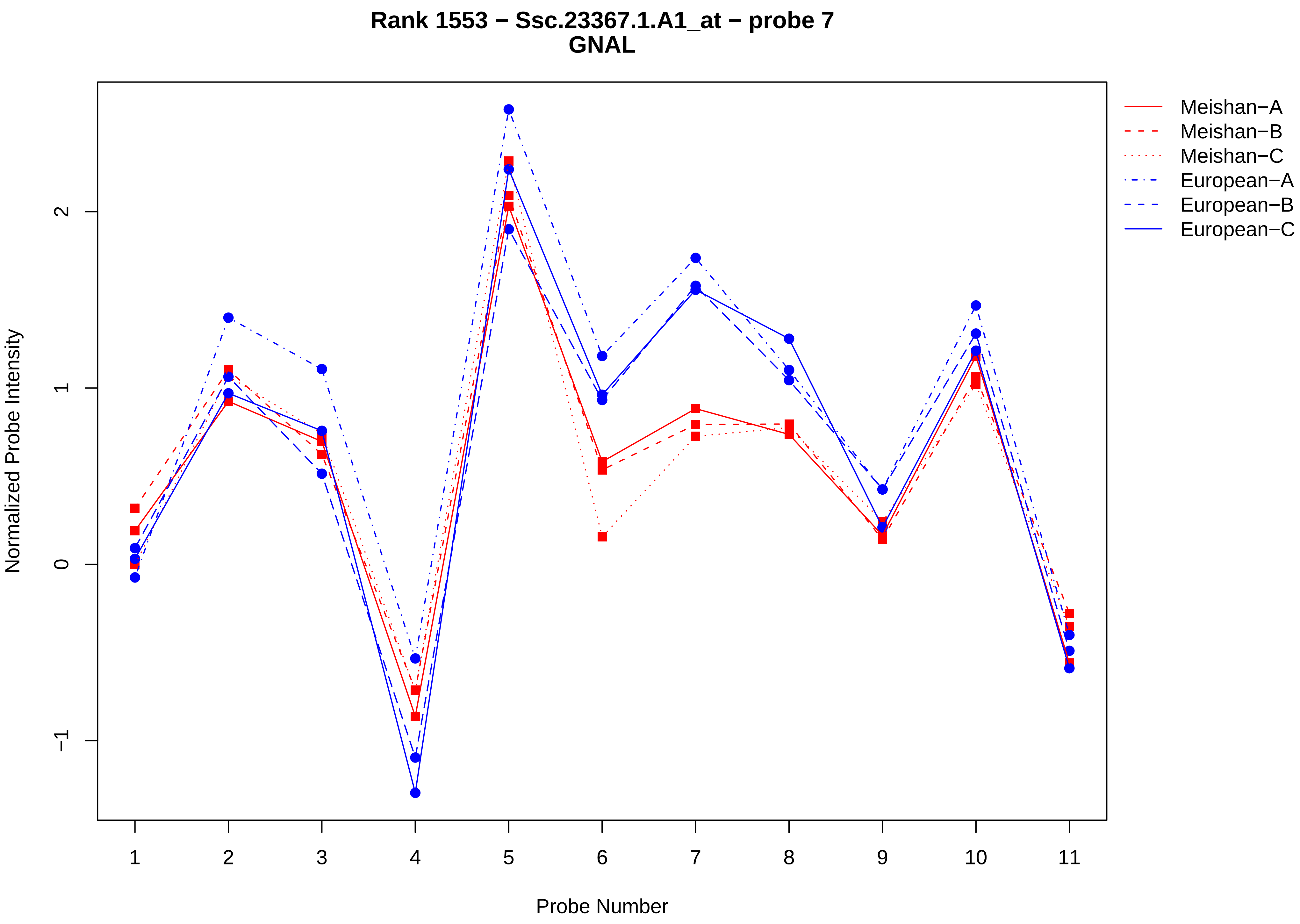

Rank 1553 − Ssc.23367.1.A1_at − probe 7
GNAL
Normalized Probe Intensity
Probe Number
Meishan−A
Meishan−B
Meishan−C
European−A
European−B
European−C
−1
0
1
2
1
2
3
4
5
6
7
8
9
10
11

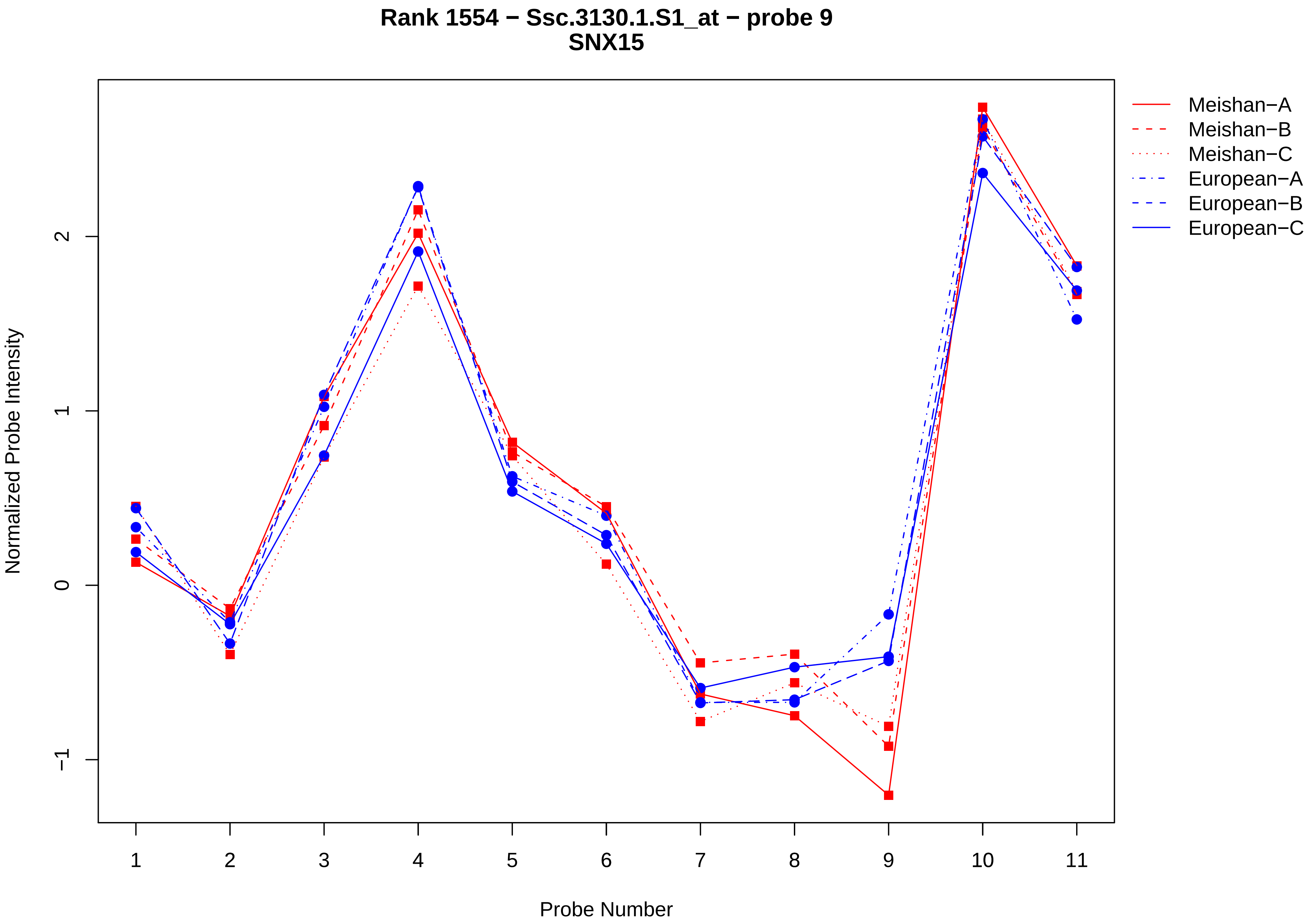

Rank 1554 − Ssc.3130.1.S1_at − probe 9
SNX15
Normalized Probe Intensity
Probe Number
−1
0
1
2
1
2
3
4
5
6
7
8
9
10
11
Meishan−A
Meishan−B
Meishan−C
European−A
European−B
European−C

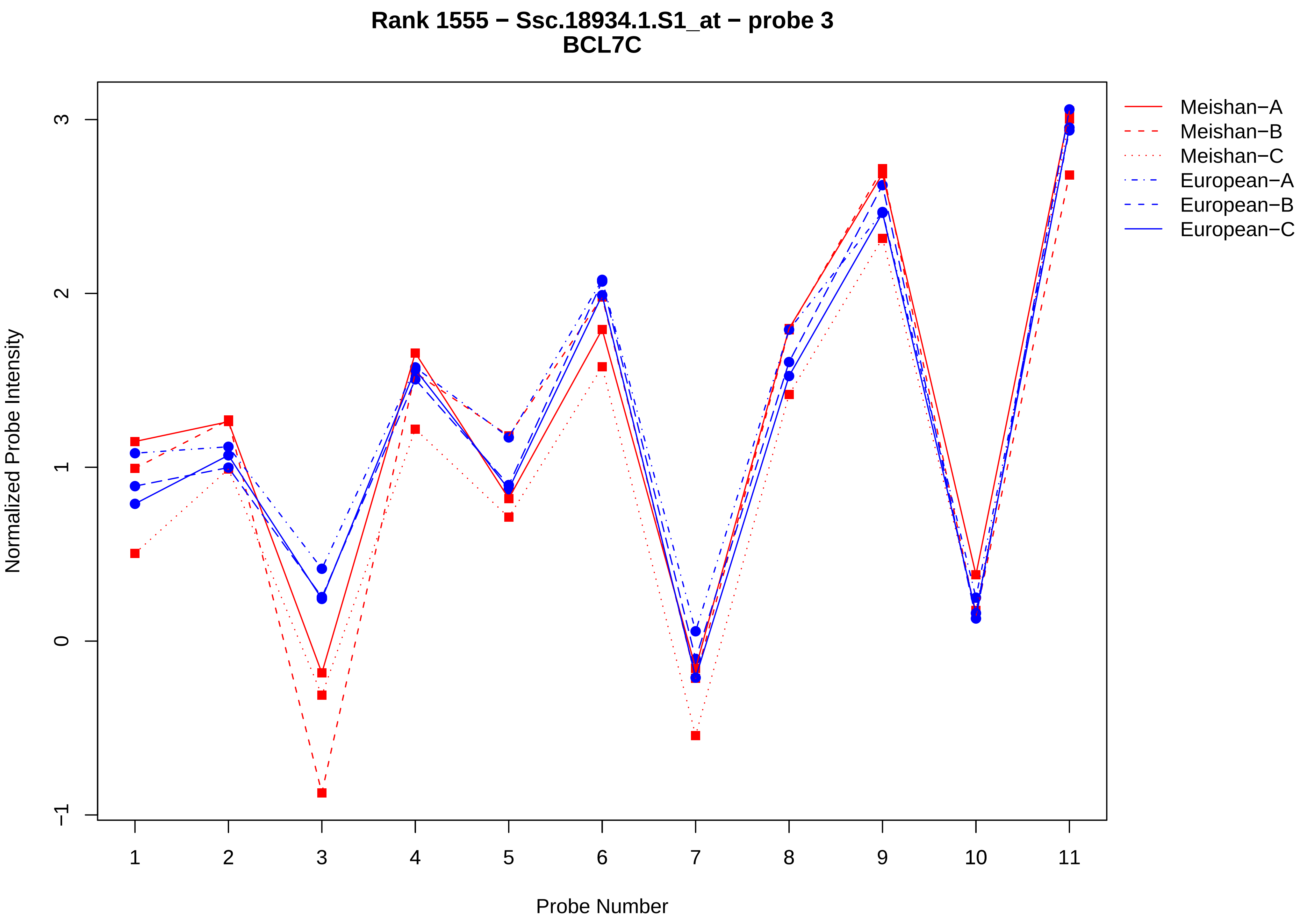

Rank 1555 − Ssc.18934.1.S1_at − probe 3
BCL7C
Normalized Probe Intensity
Probe Number
−1
0
1
2
3
1
2
3
4
5
6
7
8
9
10
11
Meishan−A
Meishan−B
Meishan−C
European−A
European−B
European−C

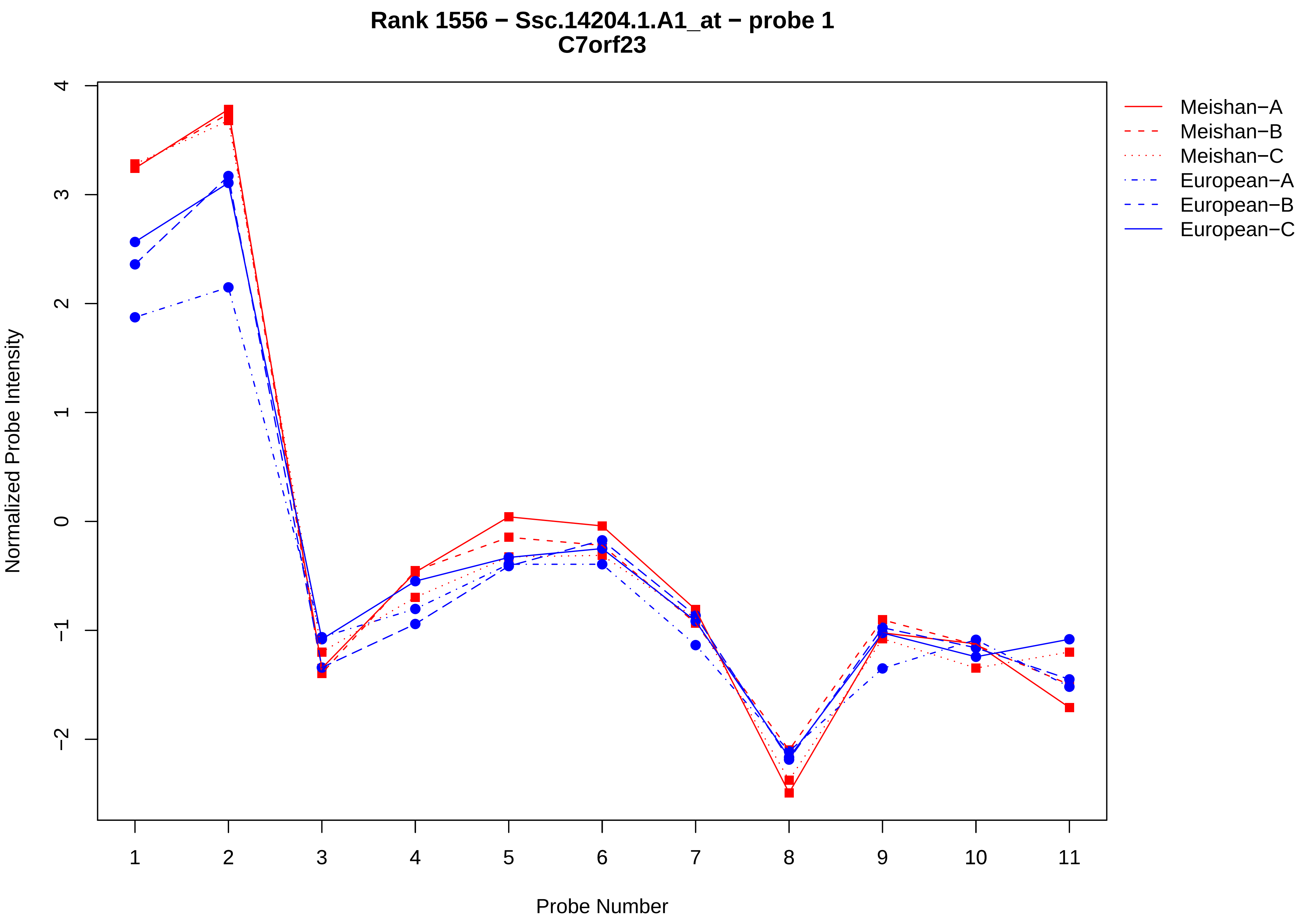
Rank 1556 − Ssc.14204.1.A1_at − probe 1
C7orf23
Normalized Probe Intensity
Probe Number
Meishan−A
Meishan−B
Meishan−C
European−A
European−B
European−C
4
3
2
1
0
−1
−2
1
2
3
4
5
6
7
8
9
10
11

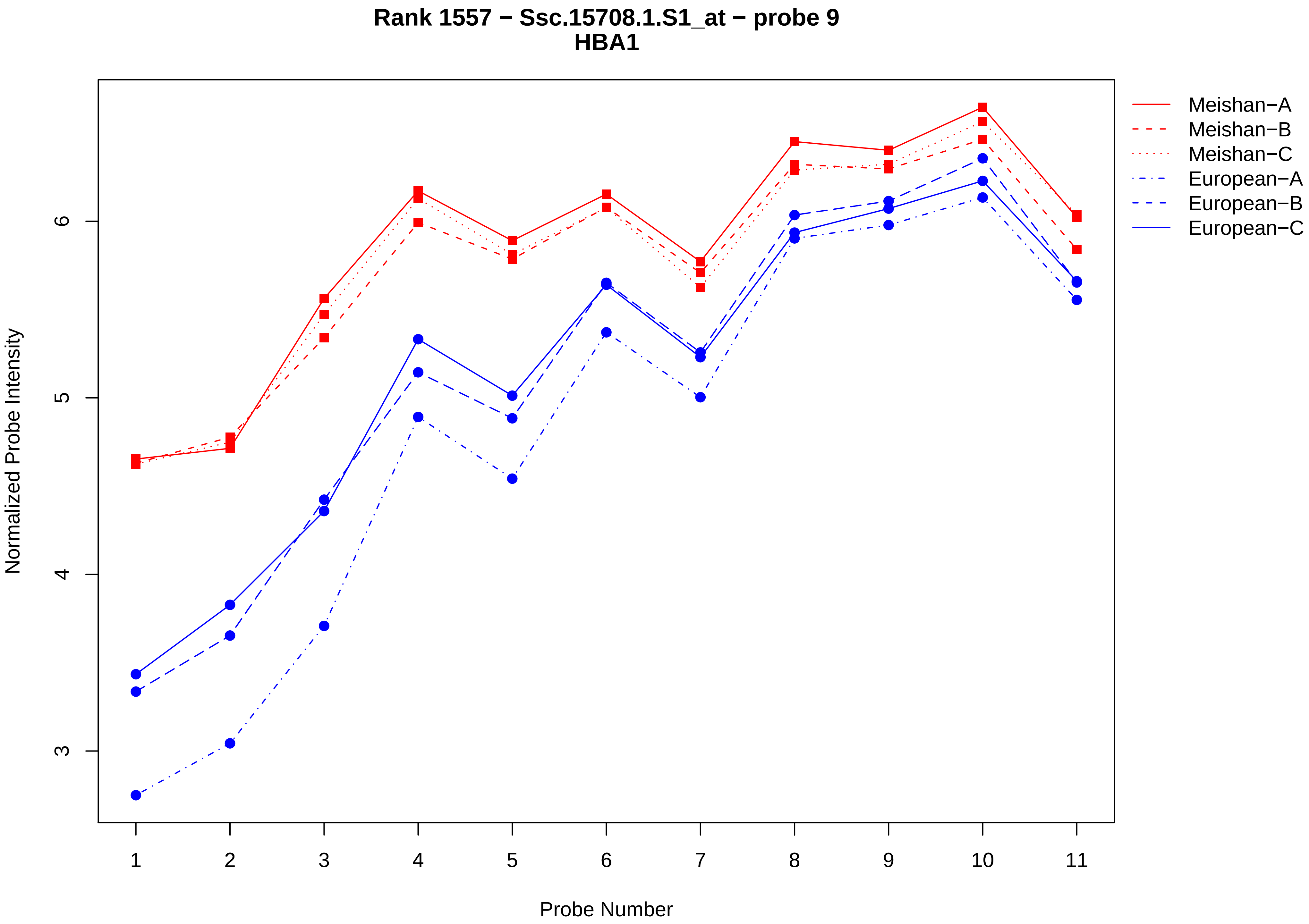

Rank 1557 − Ssc.15708.1.S1_at − probe 9
HBA1
Normalized Probe Intensity
Probe Number
3
4
5
6
1
2
3
4
5
6
7
8
9
10
11
Meishan−A
Meishan−B
Meishan−C
European−A
European−B
European−C

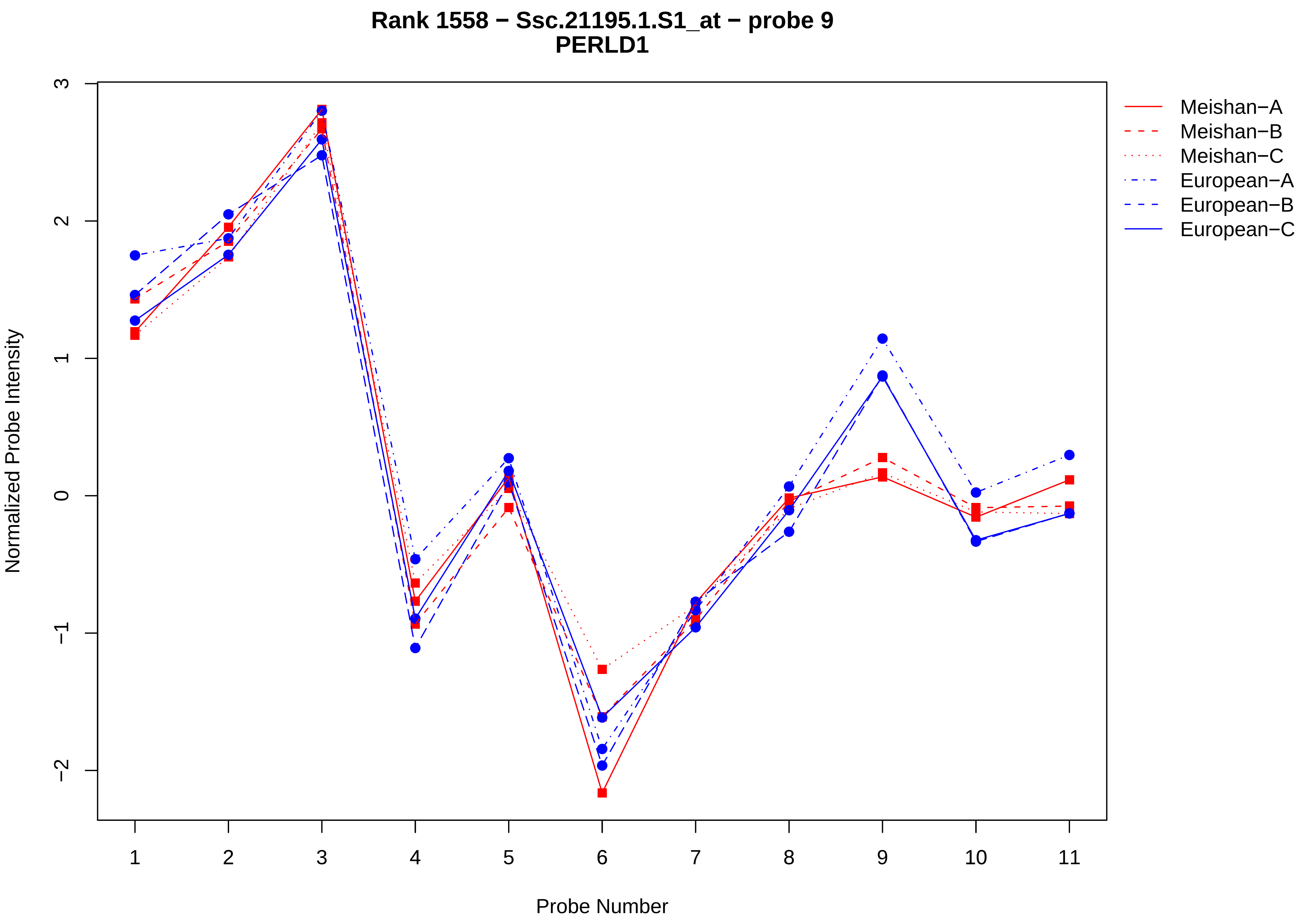
Rank 1558 − Ssc.21195.1.S1_at − probe 9
PERLD1
Normalized Probe Intensity
Probe Number
−2
−1
0
1
2
3
1
2
3
4
5
6
7
8
9
10
11
Meishan−A
Meishan−B
Meishan−C
European−A
European−B
European−C

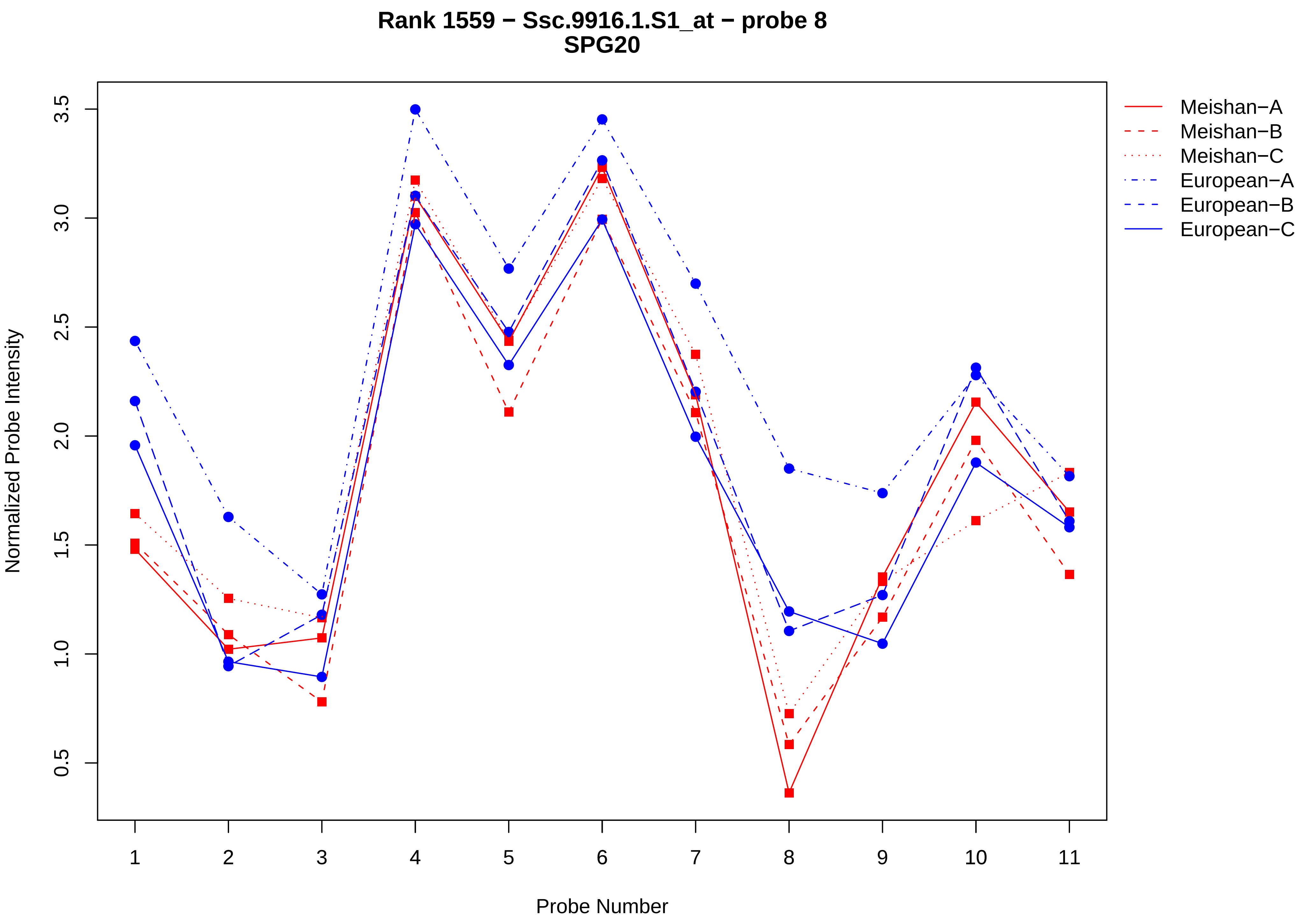

Rank 1559 − Ssc.9916.1.S1_at − probe 8
SPG20
Normalized Probe Intensity
Probe Number
0.5
1.0
1.5
2.0
2.5
3.0
3.5
1
2
3
4
5
6
7
8
9
10
11
Meishan−A
Meishan−B
Meishan−C
European−A
European−B
European−C

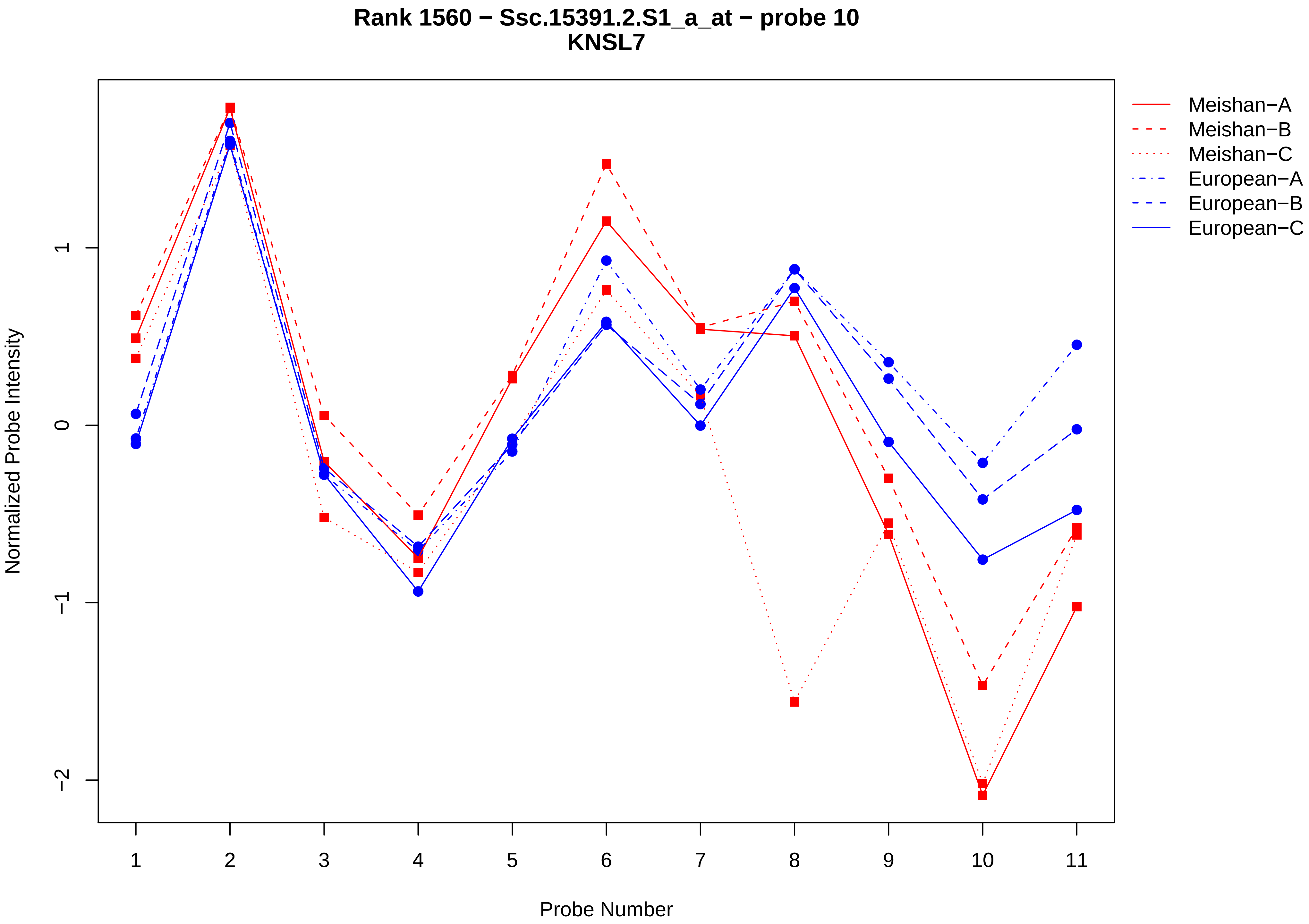

Rank 1560 − Ssc.15391.2.S1_a_at − probe 10
KNSL7
Normalized Probe Intensity
Probe Number
−2
−1
0
1
1
2
3
4
5
6
7
8
9
10
11
Meishan−A
Meishan−B
Meishan−C
European−A
European−B
European−C

Rank 1561 − Ssc.25175.3.S1_at − probe 10
CKLFSF3

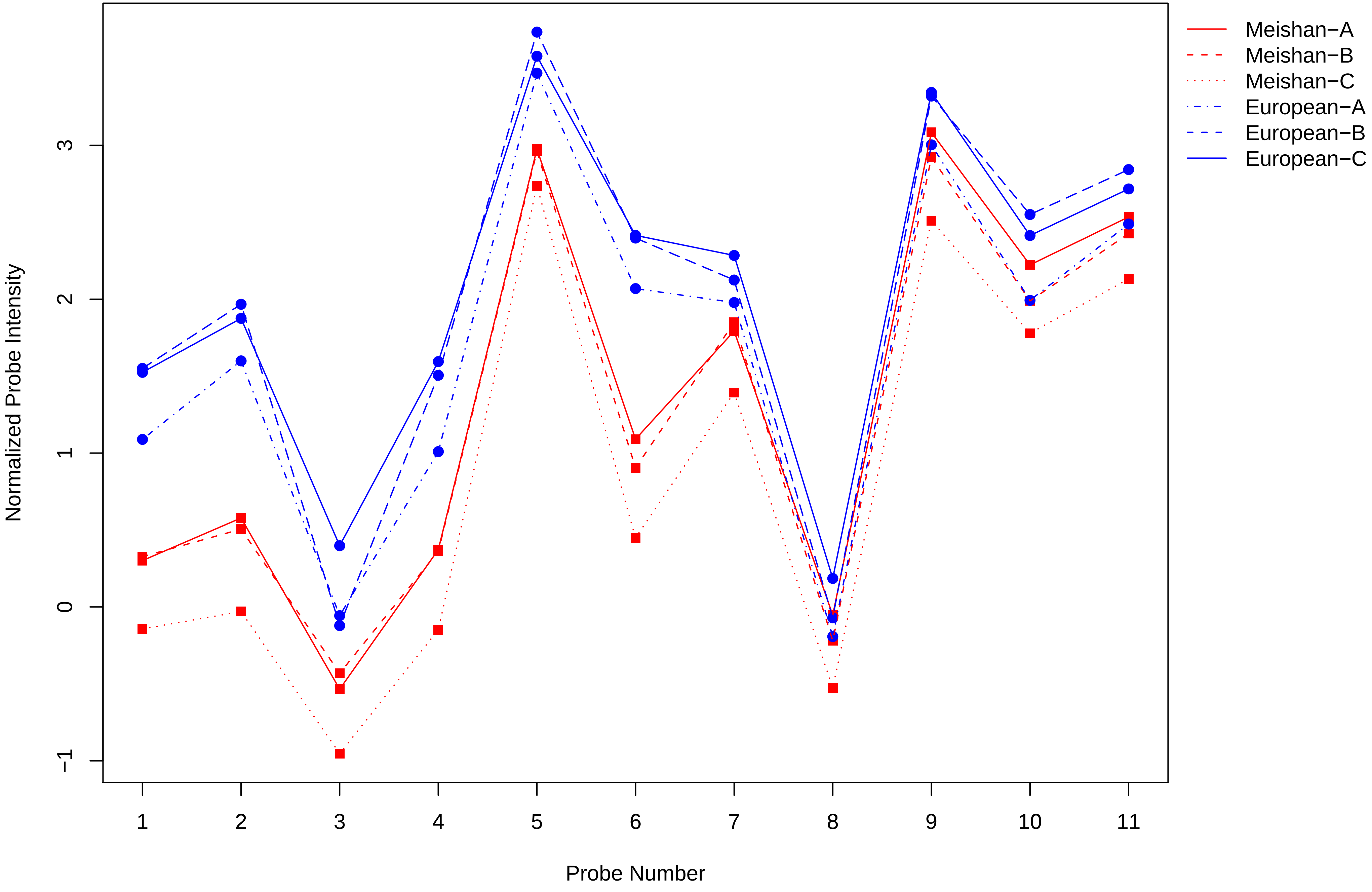

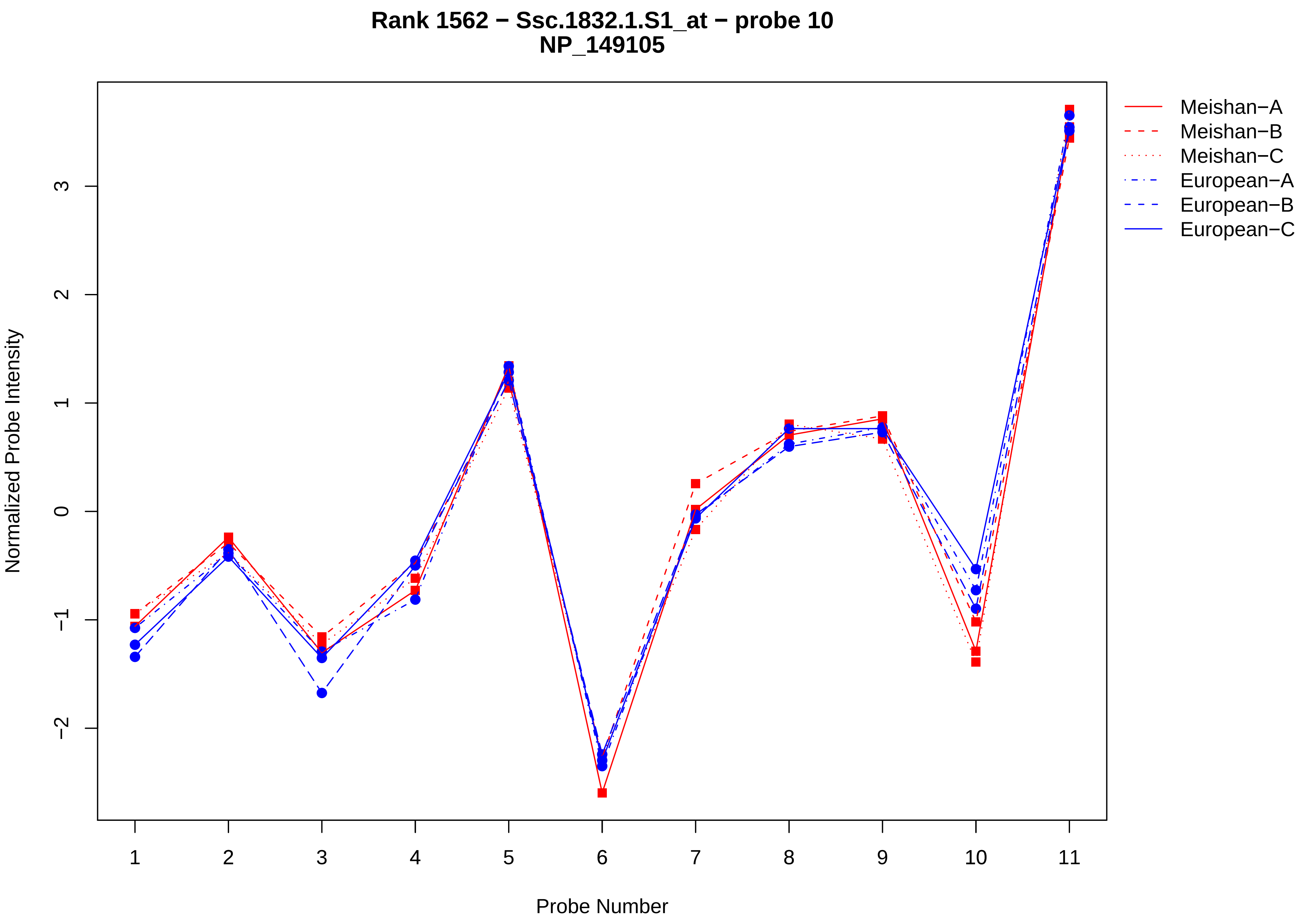
Rank 1562 − Ssc.1832.1.S1_at − probe 10
NP_149105
Normalized Probe Intensity
Probe Number
Meishan−A
Meishan−B
Meishan−C
European−A
European−B
European−C
−2
−1
0
1
2
3
1
2
3
4
5
6
7
8
9
10
11

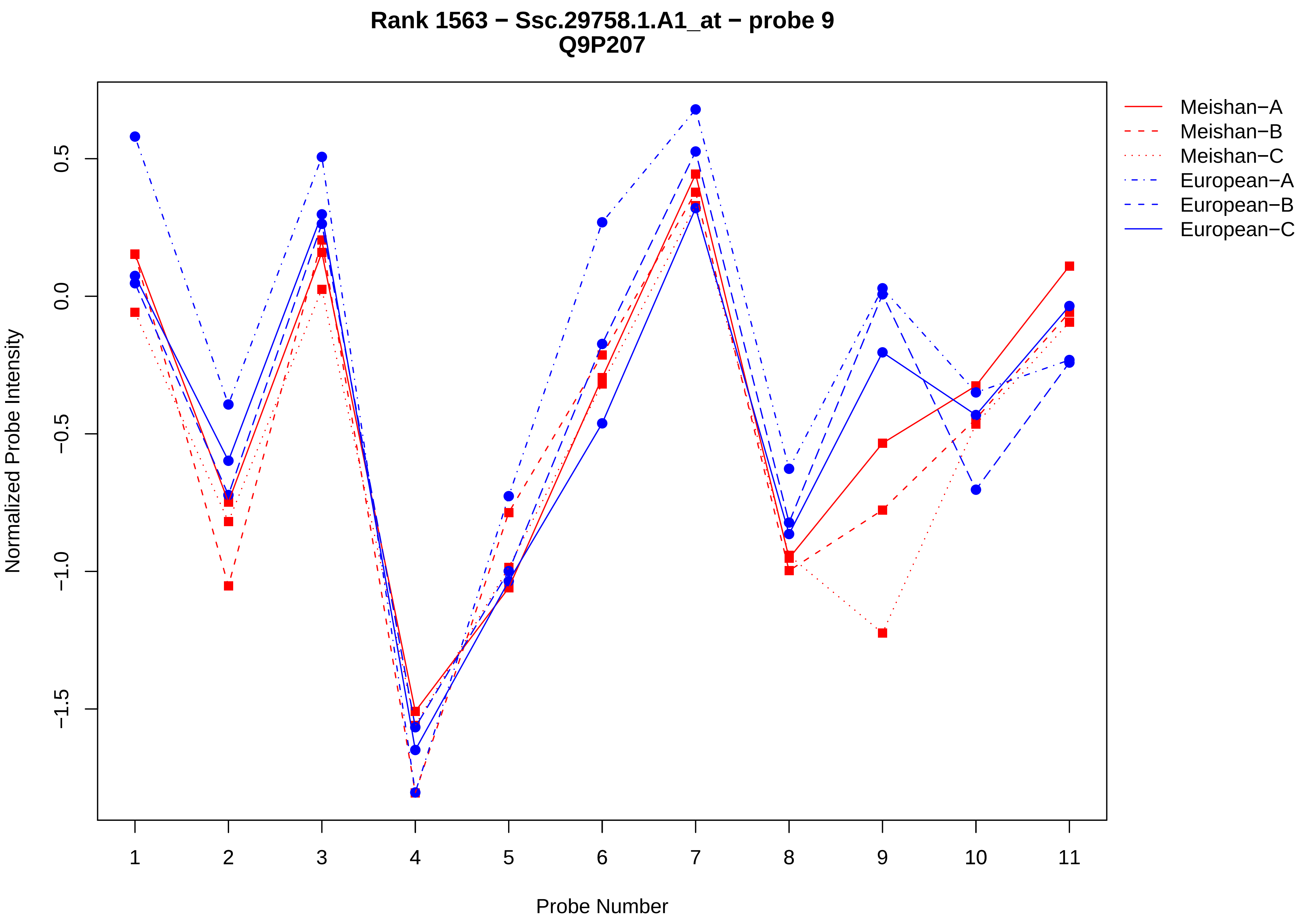

Rank 1563 − Ssc.29758.1.A1_at − probe 9
Q9P207
Normalized Probe Intensity
Probe Number
0.5
0.0
−0.5
−1.0
−1.5
1
2
3
4
5
6
7
8
9
10
11
Meishan−A
Meishan−B
Meishan−C
European−A
European−B
European−C

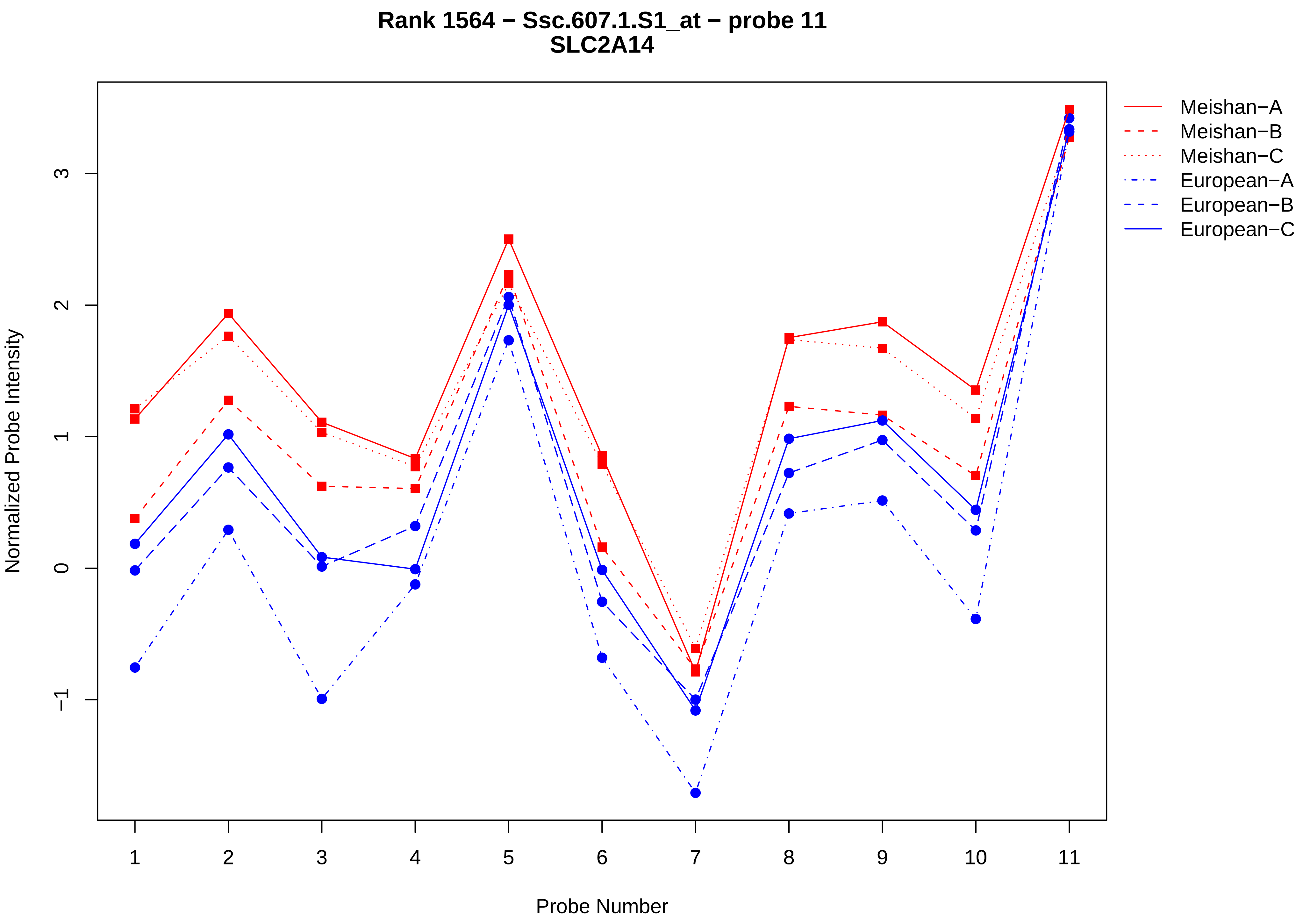
Rank 1564 − Ssc.607.1.S1_at − probe 11
SLC2A14
Normalized Probe Intensity
Probe Number
Meishan−A
Meishan−B
Meishan−C
European−A
European−B
European−C
−1
0
1
2
3
1
2
3
4
5
6
7
8
9
10
11

Rank 1565 − Ssc.9196.1.S1_at − probe 8
Q9NWD8

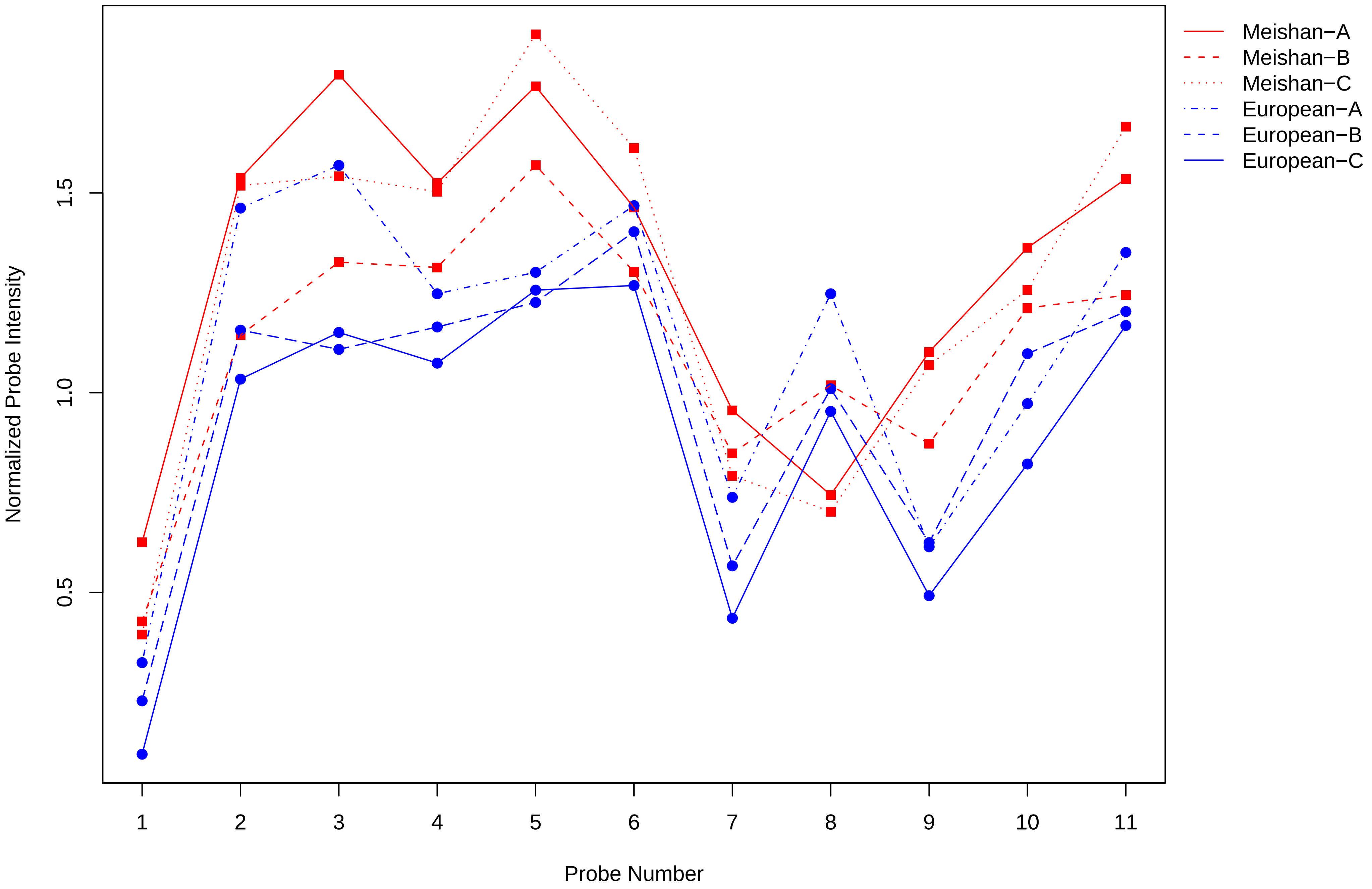

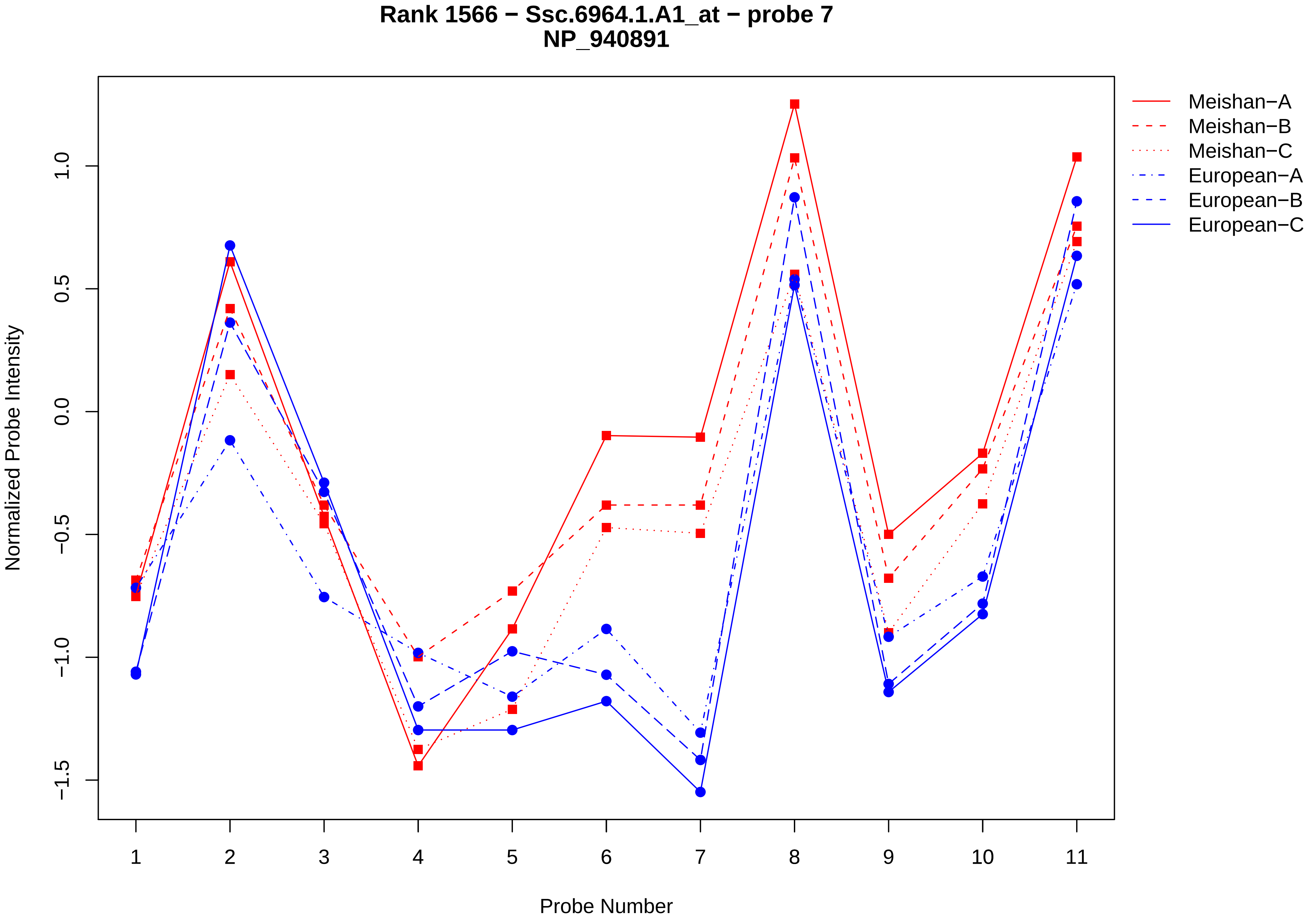
Rank 1566 − Ssc.6964.1.A1_at − probe 7
NP_940891
Normalized Probe Intensity
Probe Number
−1.5
−1.0
−0.5
0.0
0.5
1.0
1
2
3
4
5
6
7
8
9
10
11
Meishan−A
Meishan−B
Meishan−C
European−A
European−B
European−C

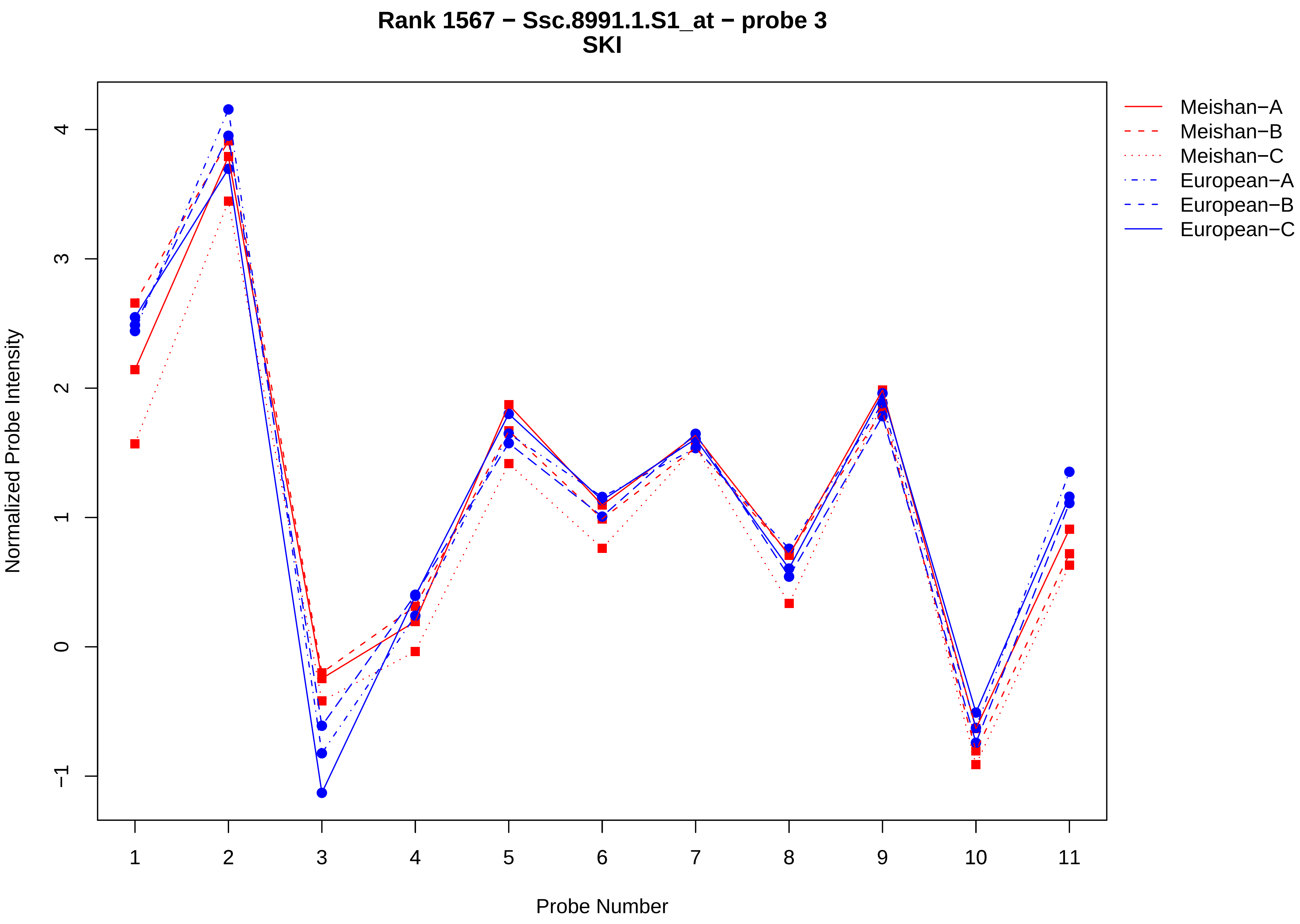

Rank 1567 − Ssc.8991.1.S1_at − probe 3
SKI
Normalized Probe Intensity
Probe Number
−1
0
1
2
3
4
1
2
3
4
5
6
7
8
9
10
11
Meishan−A
Meishan−B
Meishan−C
European−A
European−B
European−C

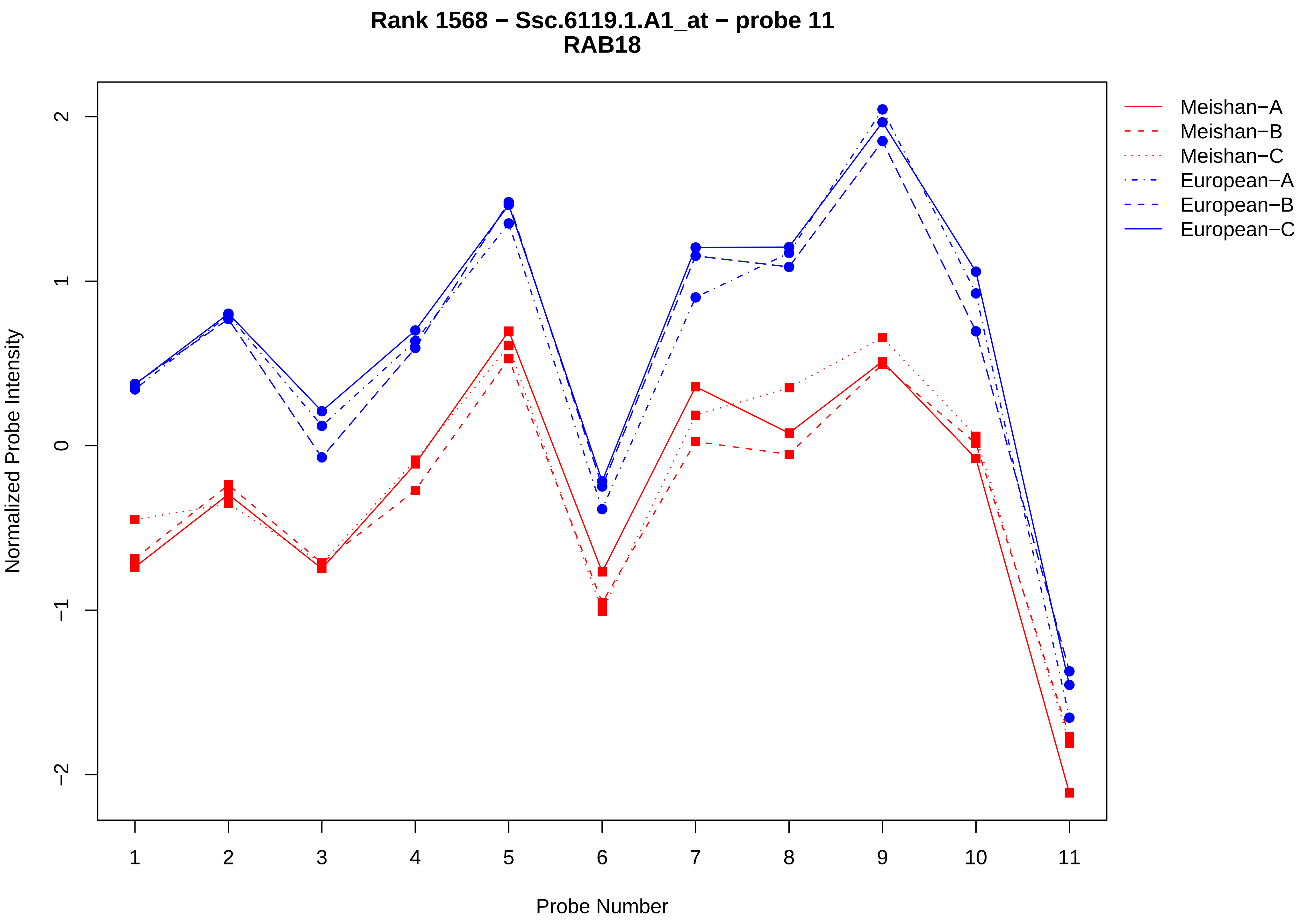

Rank 1568 − Ssc.6119.1.A1_at − probe 11
RAB18
Normalized Probe Intensity
Probe Number
−2
−1
0
1
2
1
2
3
4
5
6
7
8
9
10
11
Meishan−A
Meishan−B
Meishan−C
European−A
European−B
European−C

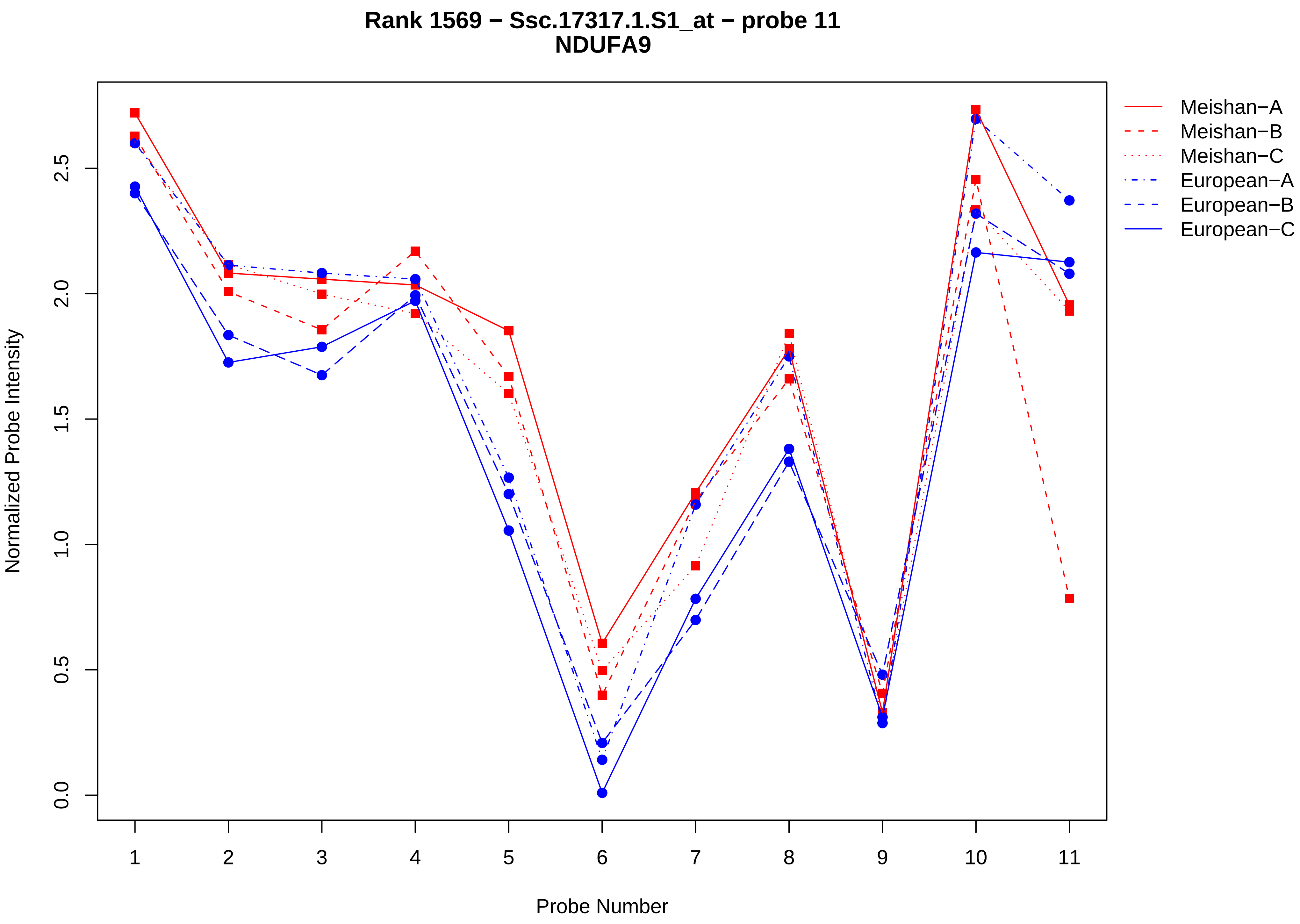

Rank 1569 − Ssc.17317.1.S1_at − probe 11
NDUFA9
Normalized Probe Intensity
Probe Number
0.0
0.5
1.0
1.5
2.0
2.5
1
2
3
4
5
6
7
8
9
10
11
Meishan−A
Meishan−B
Meishan−C
European−A
European−B
European−C

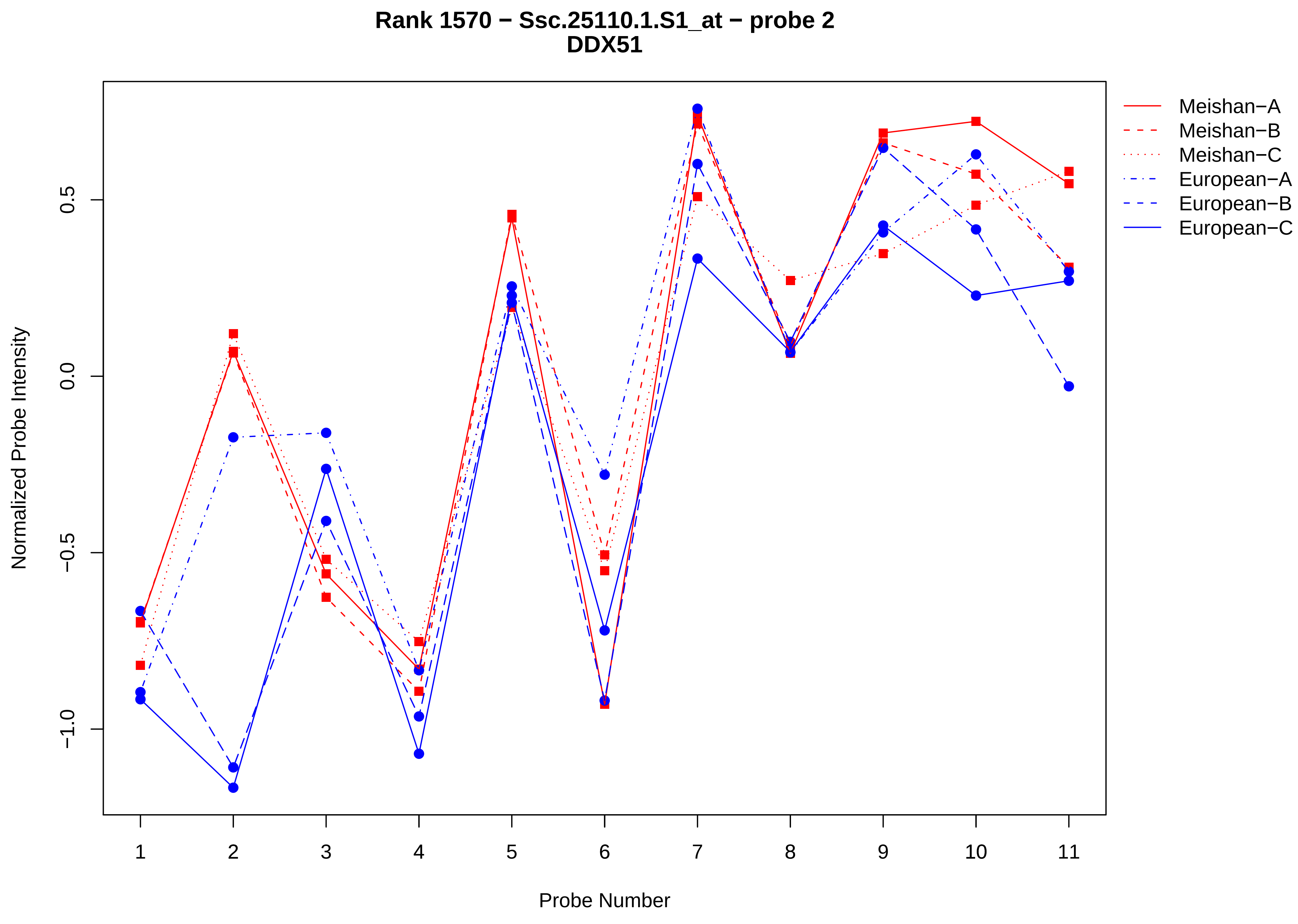
Rank 1570 − Ssc.25110.1.S1_at − probe 2
DDX51
Normalized Probe Intensity
Probe Number
−1.0
−0.5
0.0
0.5
1
2
3
4
5
6
7
8
9
10
11
Meishan−A
Meishan−B
Meishan−C
European−A
European−B
European−C

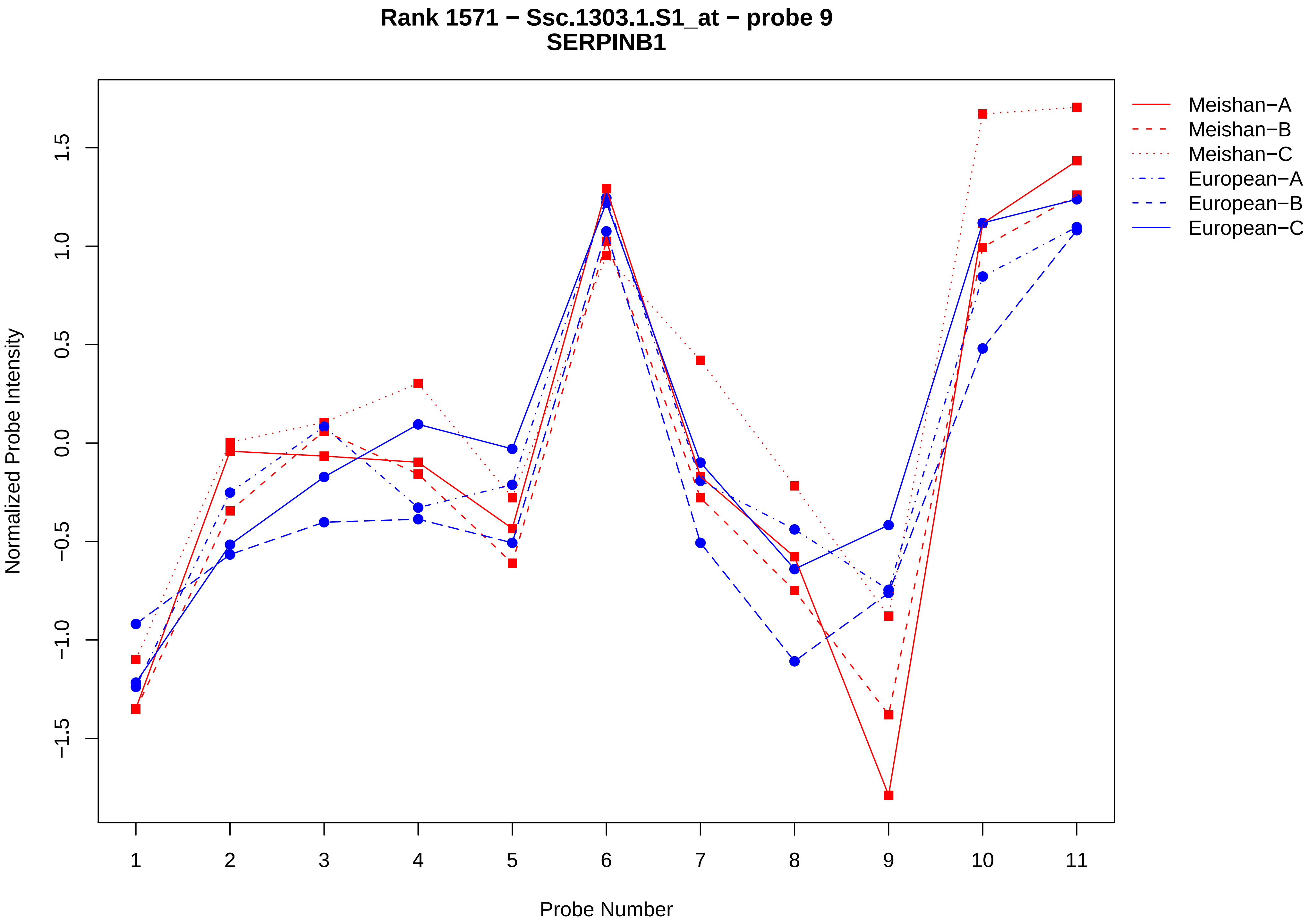

Rank 1571 − Ssc.1303.1.S1_at − probe 9
SERPINB1
Normalized Probe Intensity
Probe Number
Meishan−A
Meishan−B
Meishan−C
European−A
European−B
European−C
1.5
1.0
0.5
0.0
−0.5
−1.0
−1.5
1
2
3
4
5
6
7
8
9
10
11

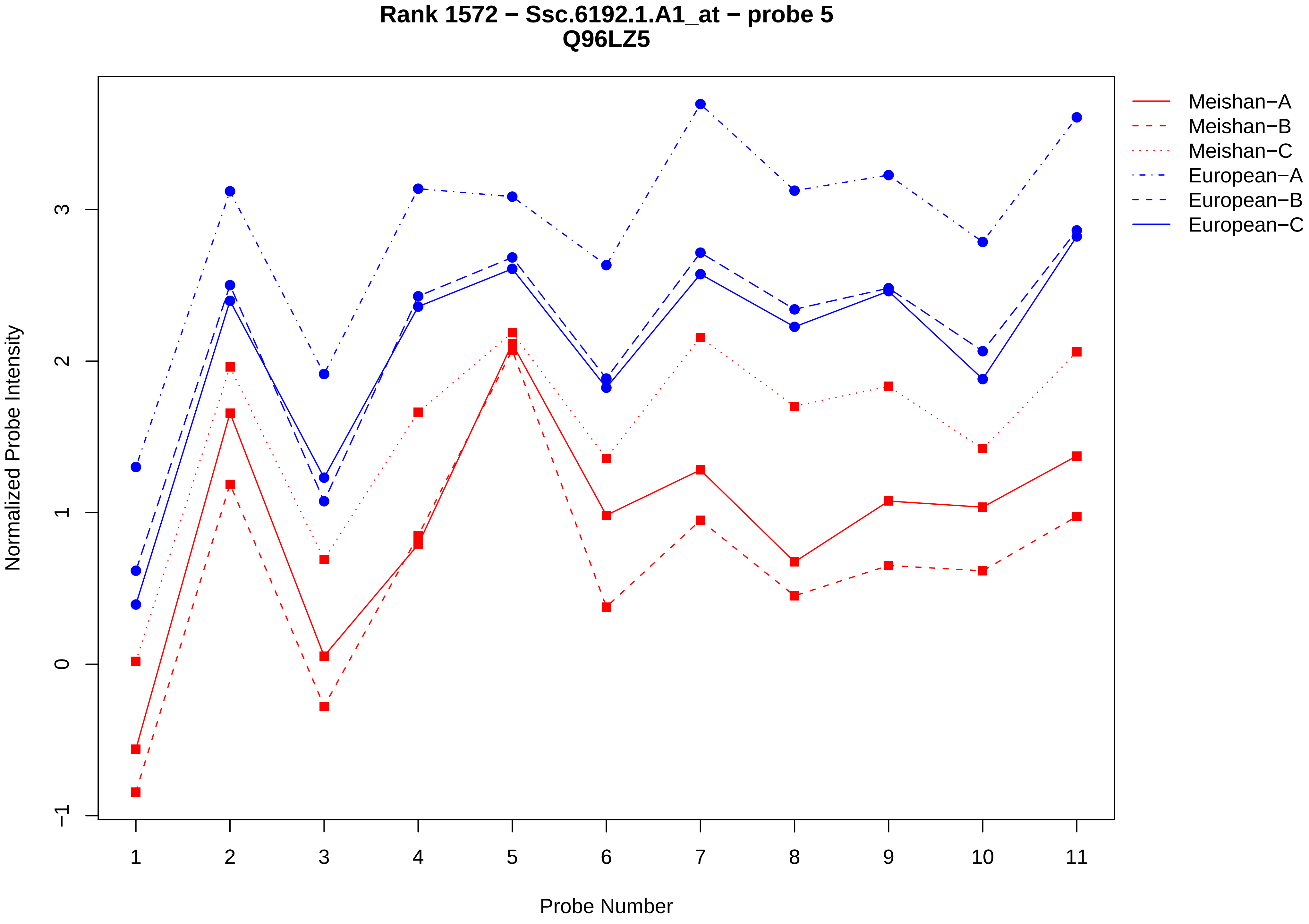

Rank 1572 − Ssc.6192.1.A1_at − probe 5
Q96LZ5
Normalized Probe Intensity
Probe Number
−1
0
1
2
3
1
2
3
4
5
6
7
8
9
10
11
Meishan−A
Meishan−B
Meishan−C
European−A
European−B
European−C

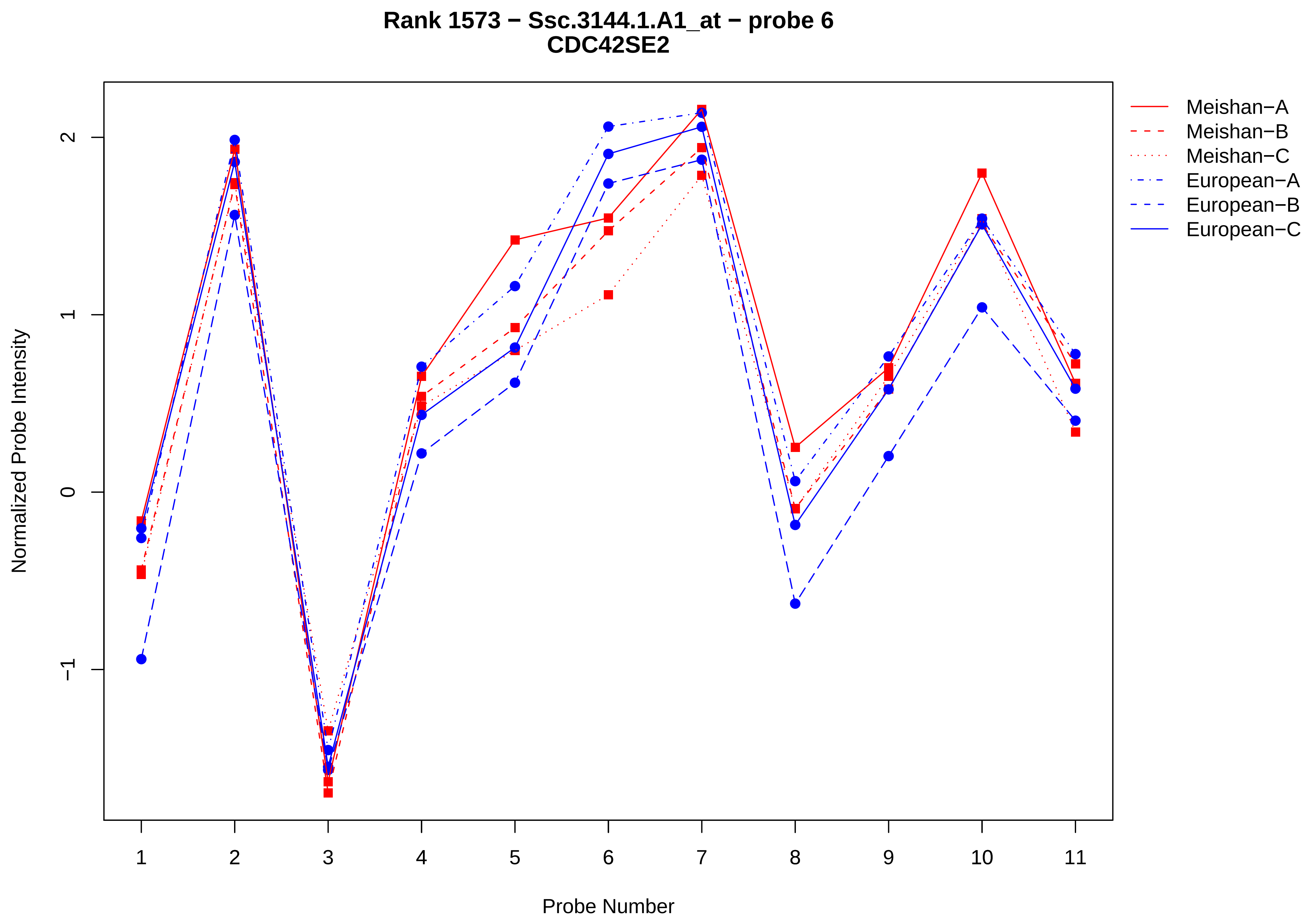

Rank 1573 − Ssc.3144.1.A1_at − probe 6
CDC42SE2
Normalized Probe Intensity
Probe Number
−1
0
1
2
1
2
3
4
5
6
7
8
9
10
11
Meishan−A
Meishan−B
Meishan−C
European−A
European−B
European−C

Rank 1574 − Ssc.11248.1.A1_at − probe 9
MTMR6

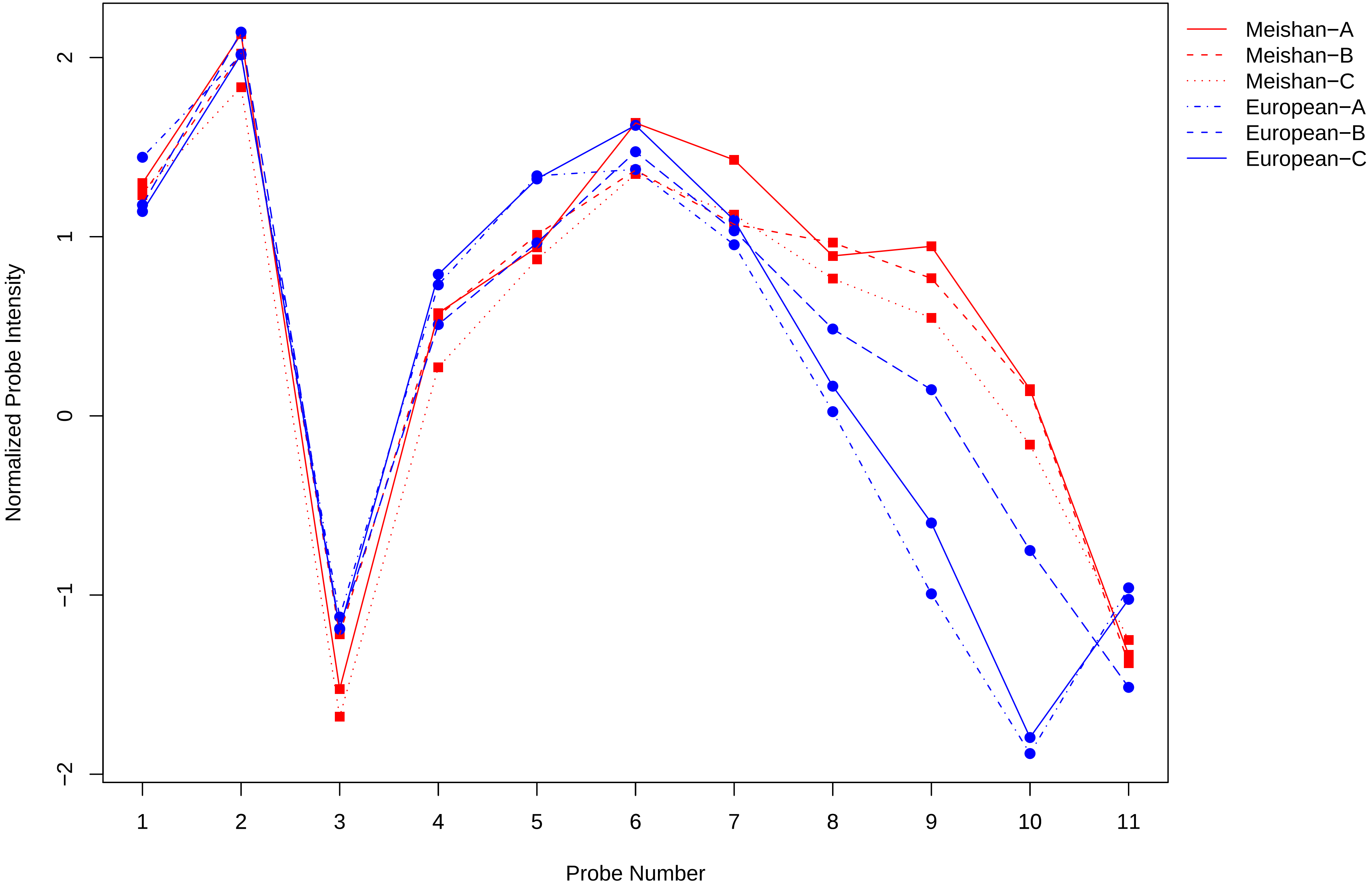

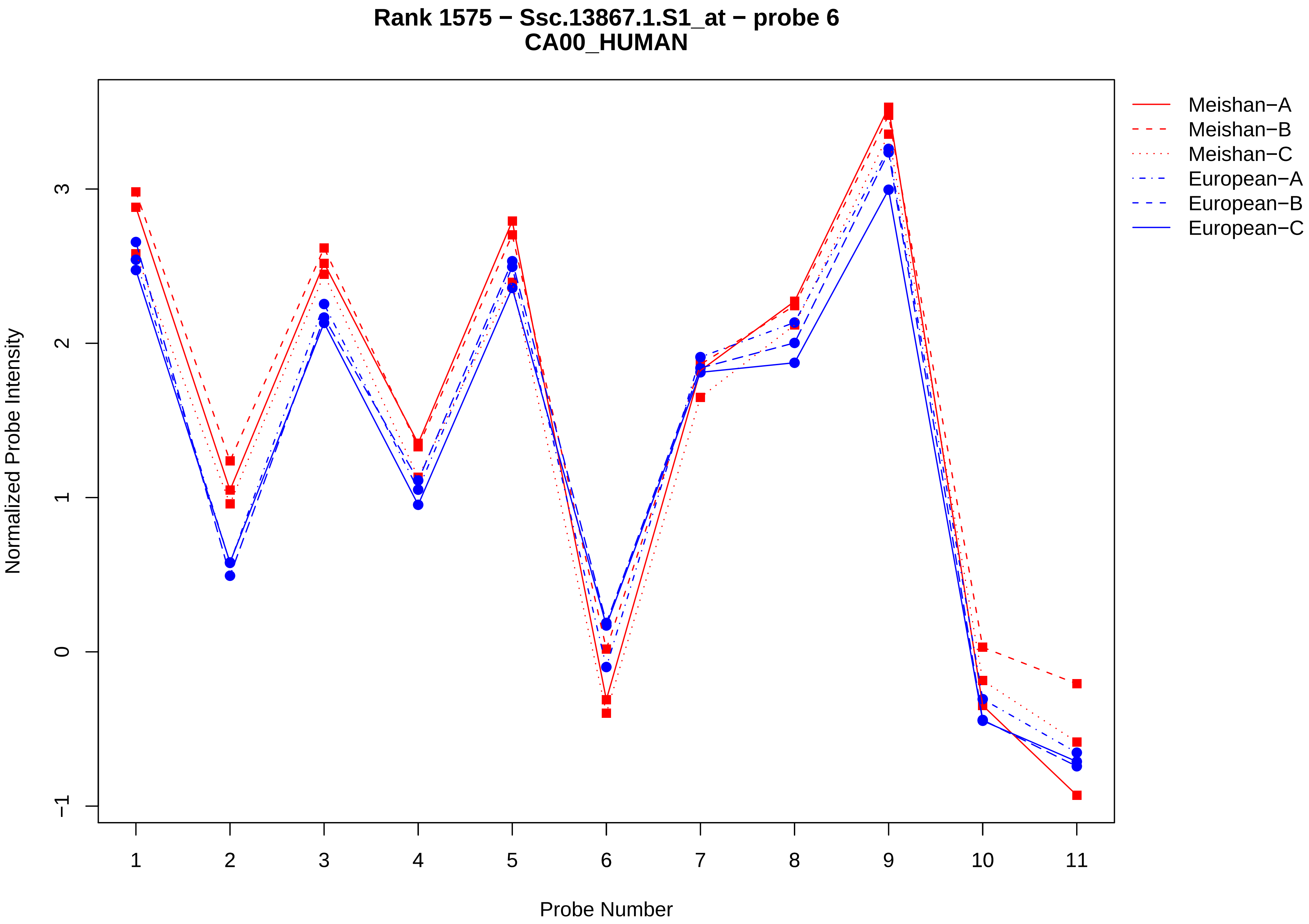
Rank 1575 − Ssc.13867.1.S1_at − probe 6
CA00_HUMAN
Normalized Probe Intensity
Probe Number
−1
0
1
2
3
1
2
3
4
5
6
7
8
9
10
11
Meishan−A
Meishan−B
Meishan−C
European−A
European−B
European−C

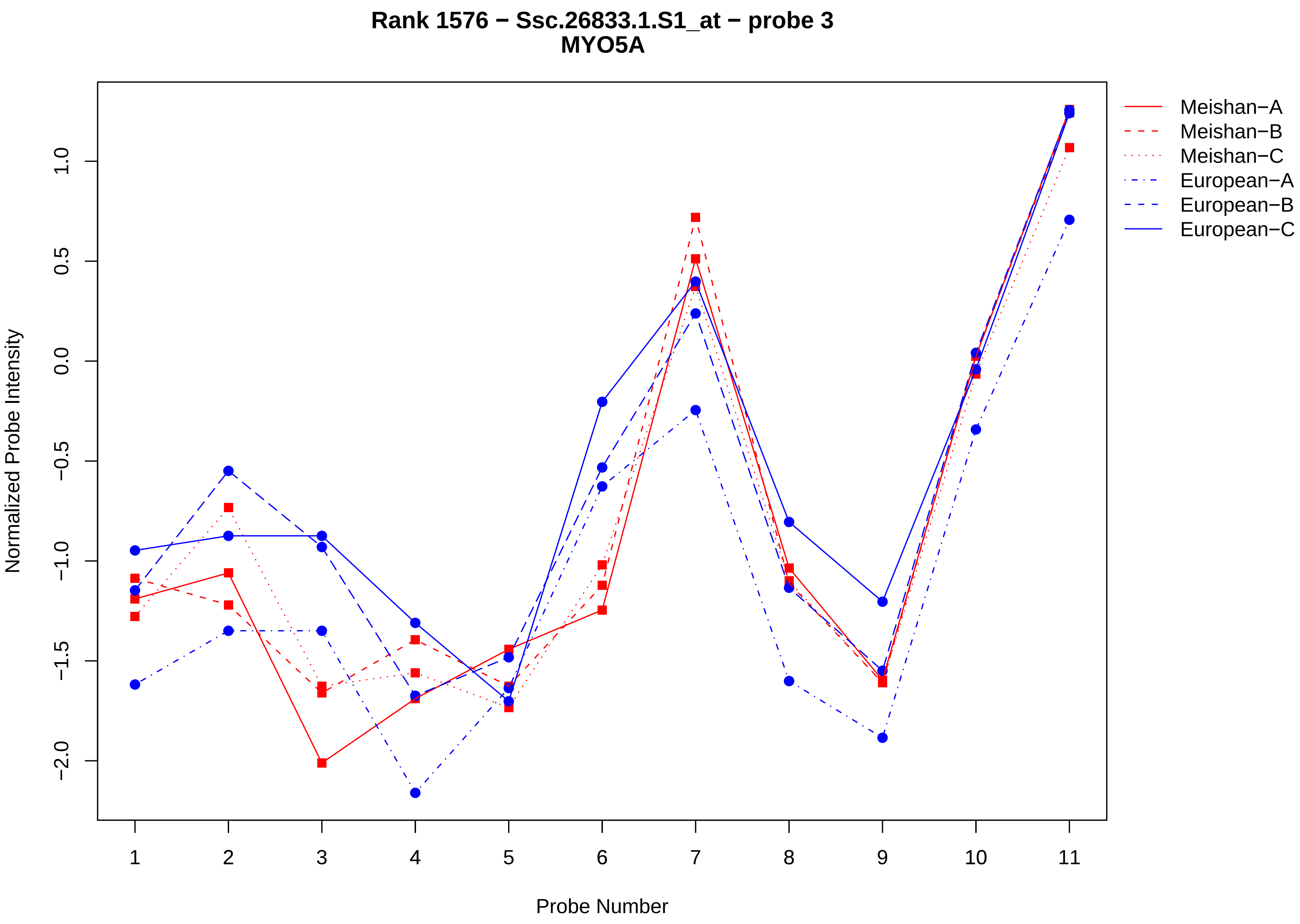

Rank 1576 − Ssc.26833.1.S1_at − probe 3
MYO5A
Normalized Probe Intensity
Probe Number
Meishan−A
Meishan−B
Meishan−C
European−A
European−B
European−C
1.0
0.5
0.0
−0.5
−1.0
−1.5
−2.0
1
2
3
4
5
6
7
8
9
10
11

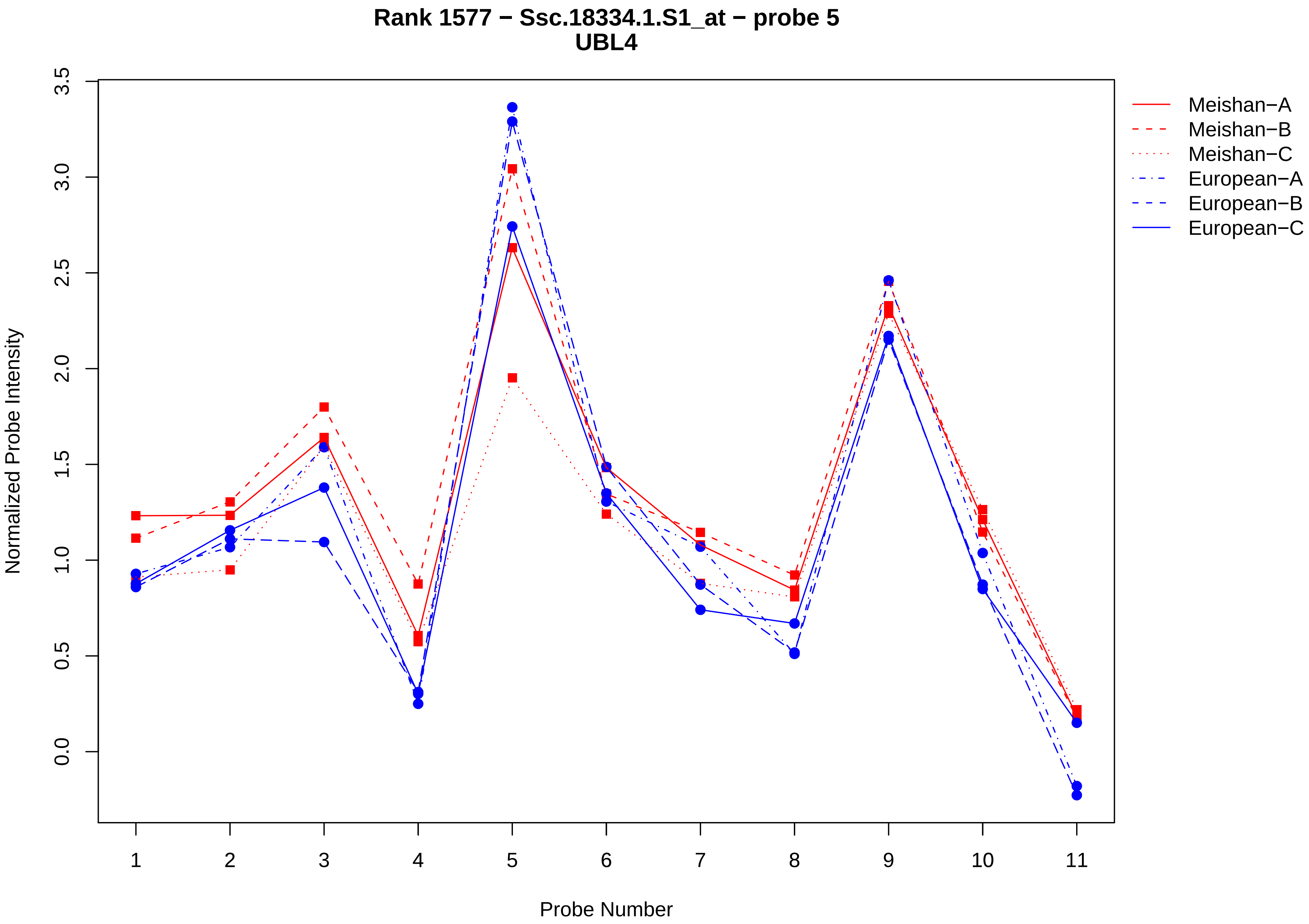

Rank 1577 − Ssc.18334.1.S1_at − probe 5
UBL4
Normalized Probe Intensity
Probe Number
0.0
0.5
1.0
1.5
2.0
2.5
3.0
3.5
1
2
3
4
5
6
7
8
9
10
11
Meishan−A
Meishan−B
Meishan−C
European−A
European−B
European−C

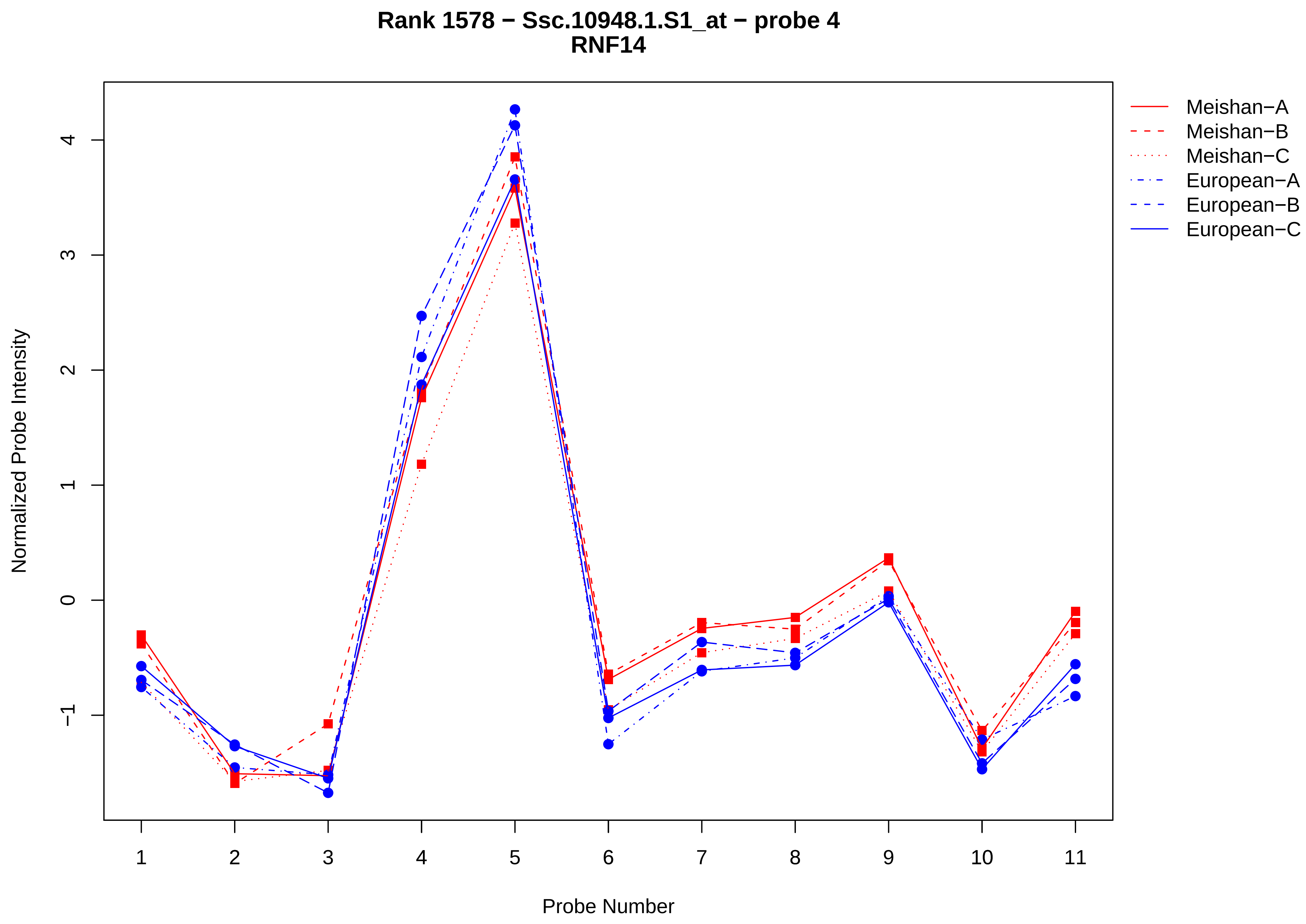
Rank 1578 − Ssc.10948.1.S1_at − probe 4
RNF14
Normalized Probe Intensity
Probe Number
−1
0
1
2
3
4
1
2
3
4
5
6
7
8
9
10
11
Meishan−A
Meishan−B
Meishan−C
European−A
European−B
European−C

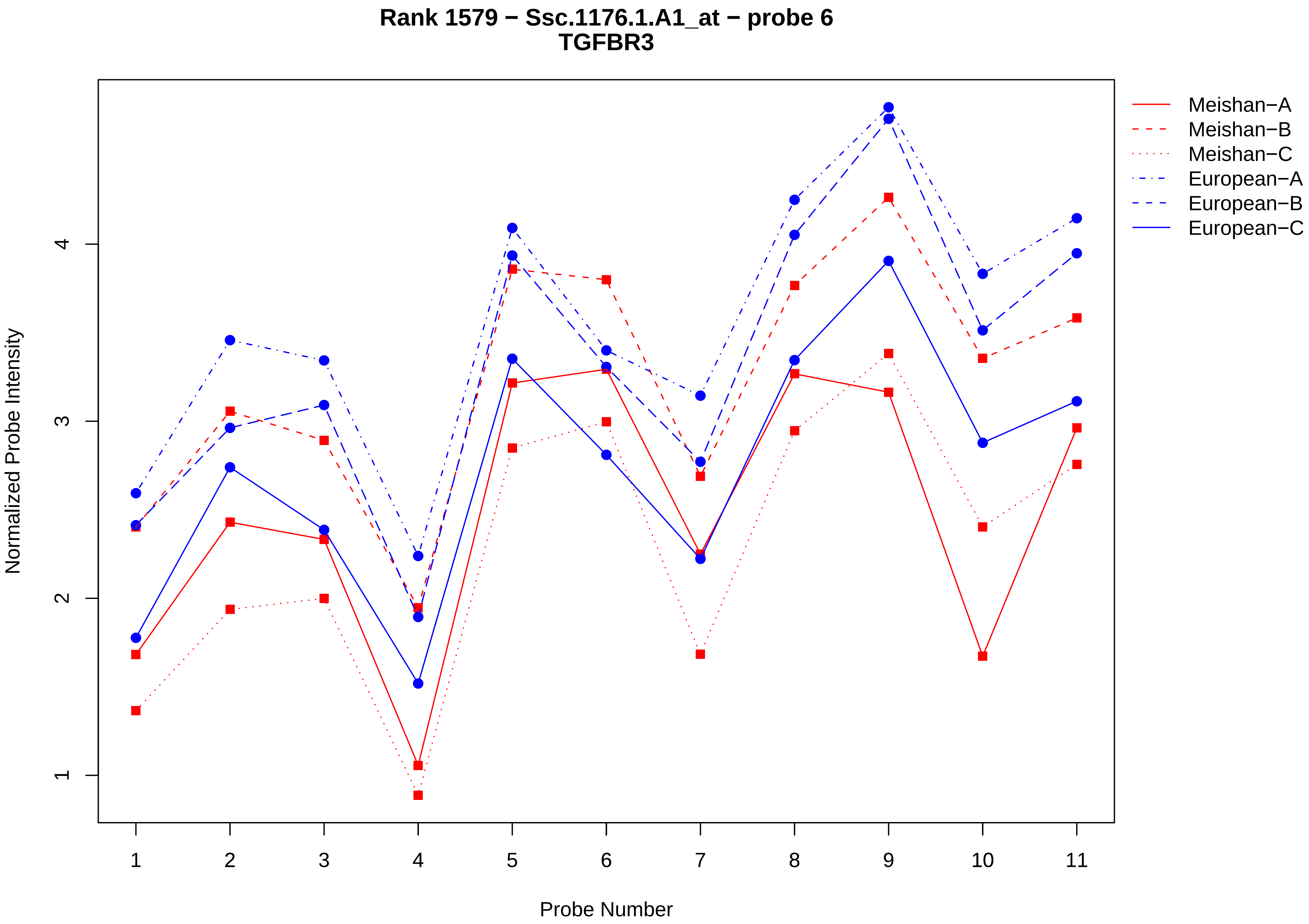

Rank 1579 − Ssc.1176.1.A1_at − probe 6
TGFBR3
Normalized Probe Intensity
1
2
3
4
1
2
3
4
5
6
7
8
9
10
11
Probe Number
Meishan−A
Meishan−B
Meishan−C
European−A
European−B
European−C

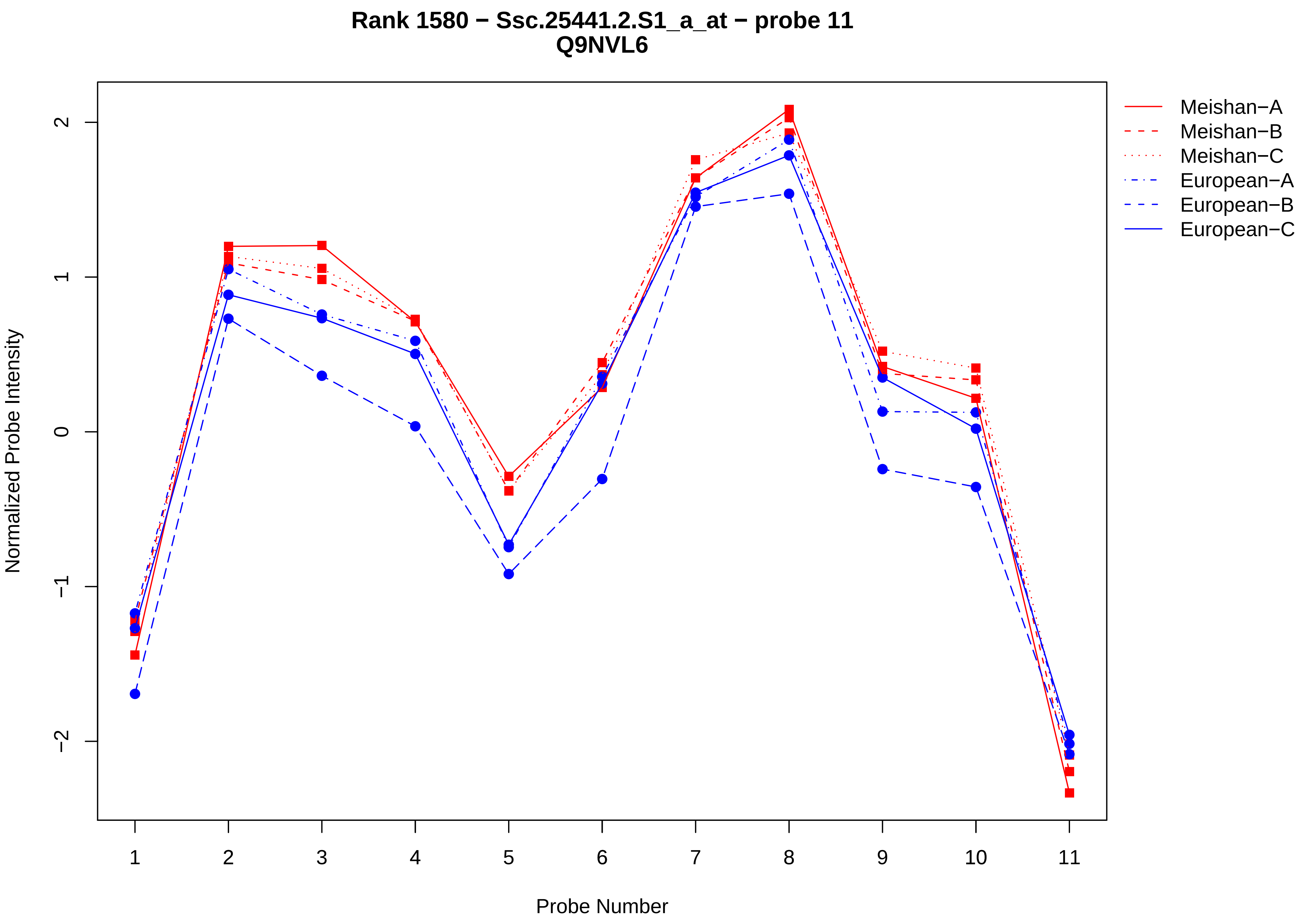
Rank 1580 − Ssc.25441.2.S1_a_at − probe 11
Q9NVL6
Normalized Probe Intensity
Probe Number
Meishan−A
Meishan−B
Meishan−C
European−A
European−B
European−C
−2
−1
0
1
2
1
2
3
4
5
6
7
8
9
10
11

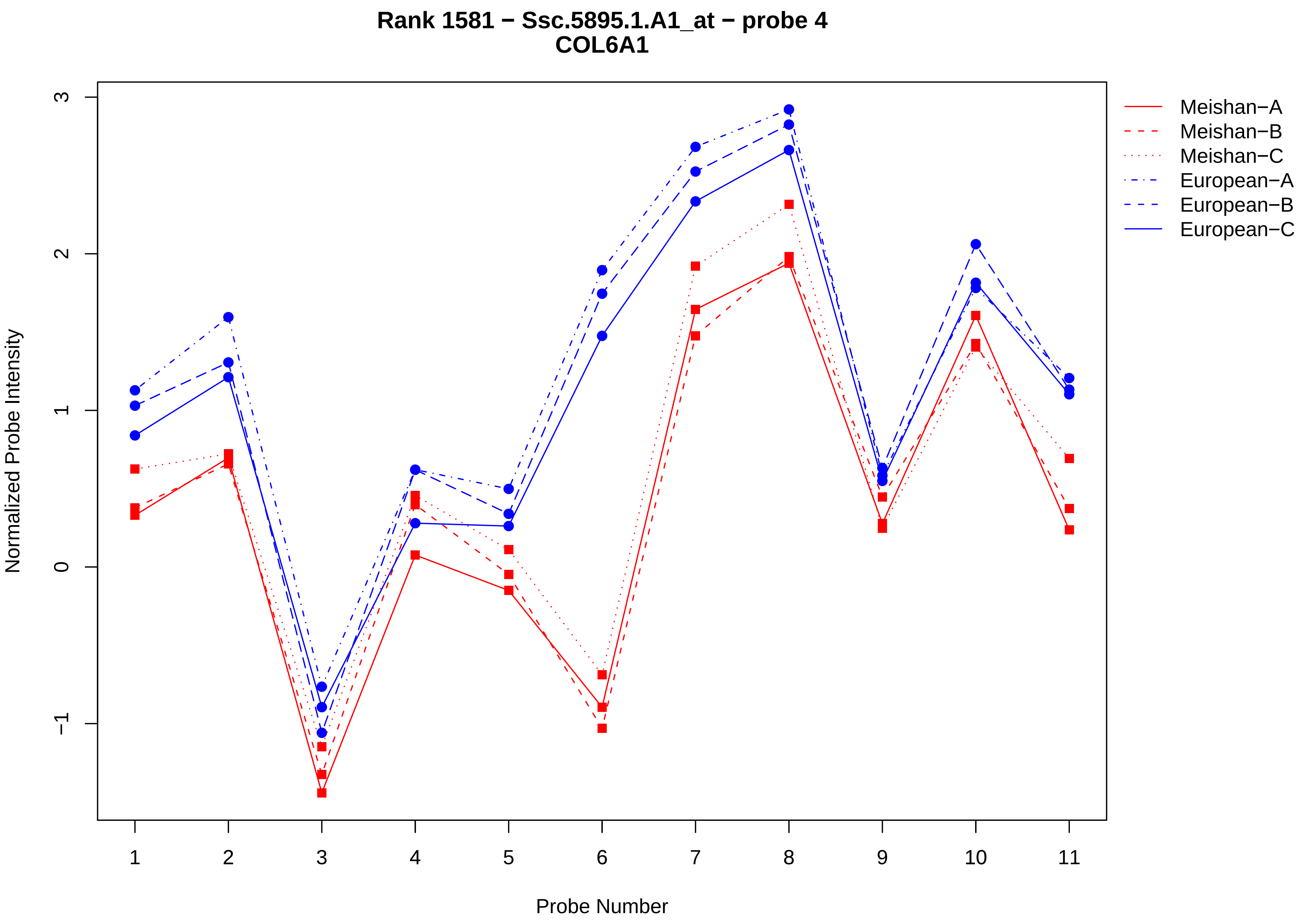
Rank 1581 − Ssc.5895.1.A1_at − probe 4
COL6A1
Normalized Probe Intensity
Probe Number
−1
0
1
2
3
1
2
3
4
5
6
7
8
9
10
11
Meishan−A
Meishan−B
Meishan−C
European−A
European−B
European−C

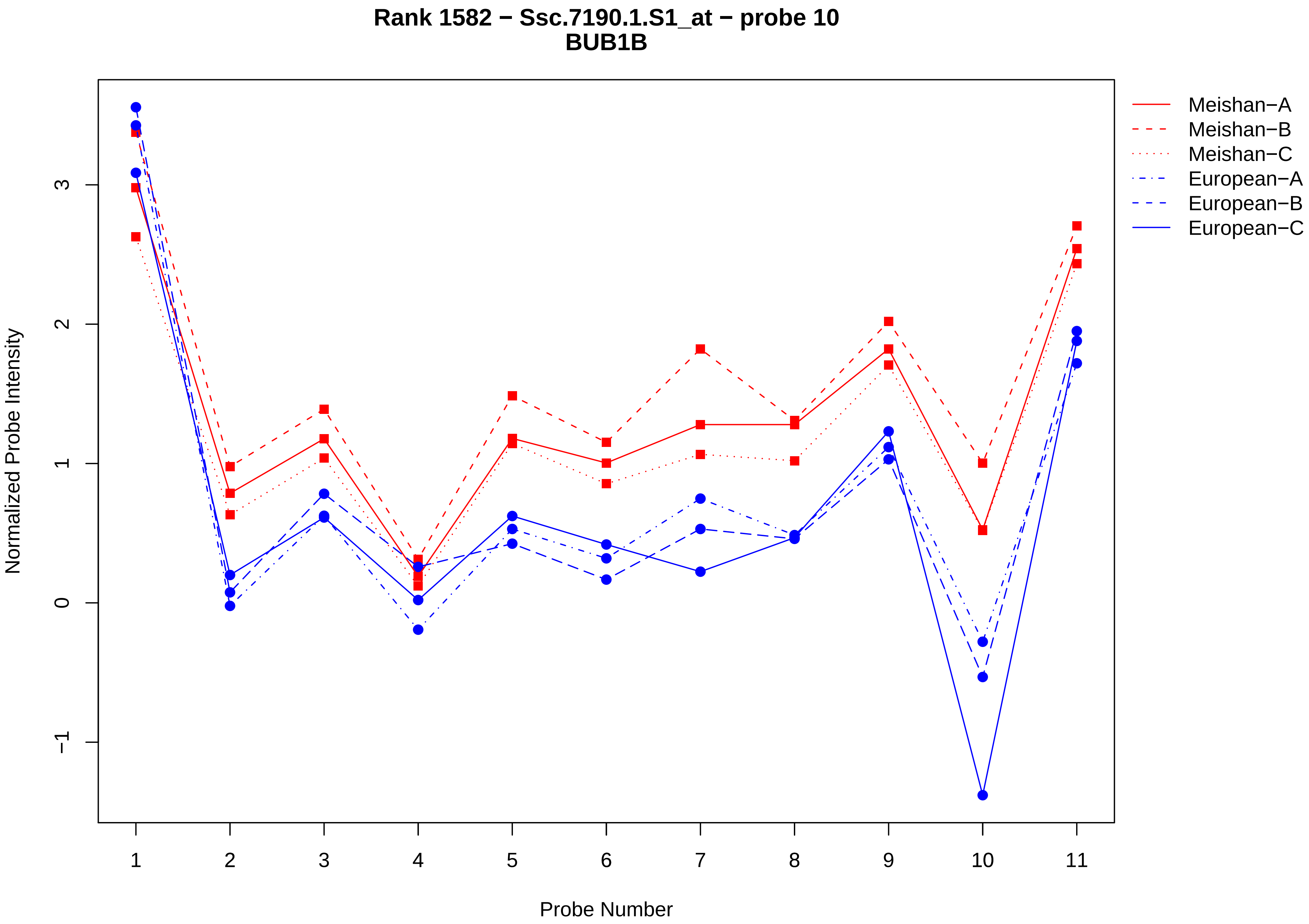
Rank 1582 − Ssc.7190.1.S1_at − probe 10
BUB1B
Normalized Probe Intensity
Probe Number
Meishan−A
Meishan−B
Meishan−C
European−A
European−B
European−C
−1
0
1
2
3
1
2
3
4
5
6
7
8
9
10
11

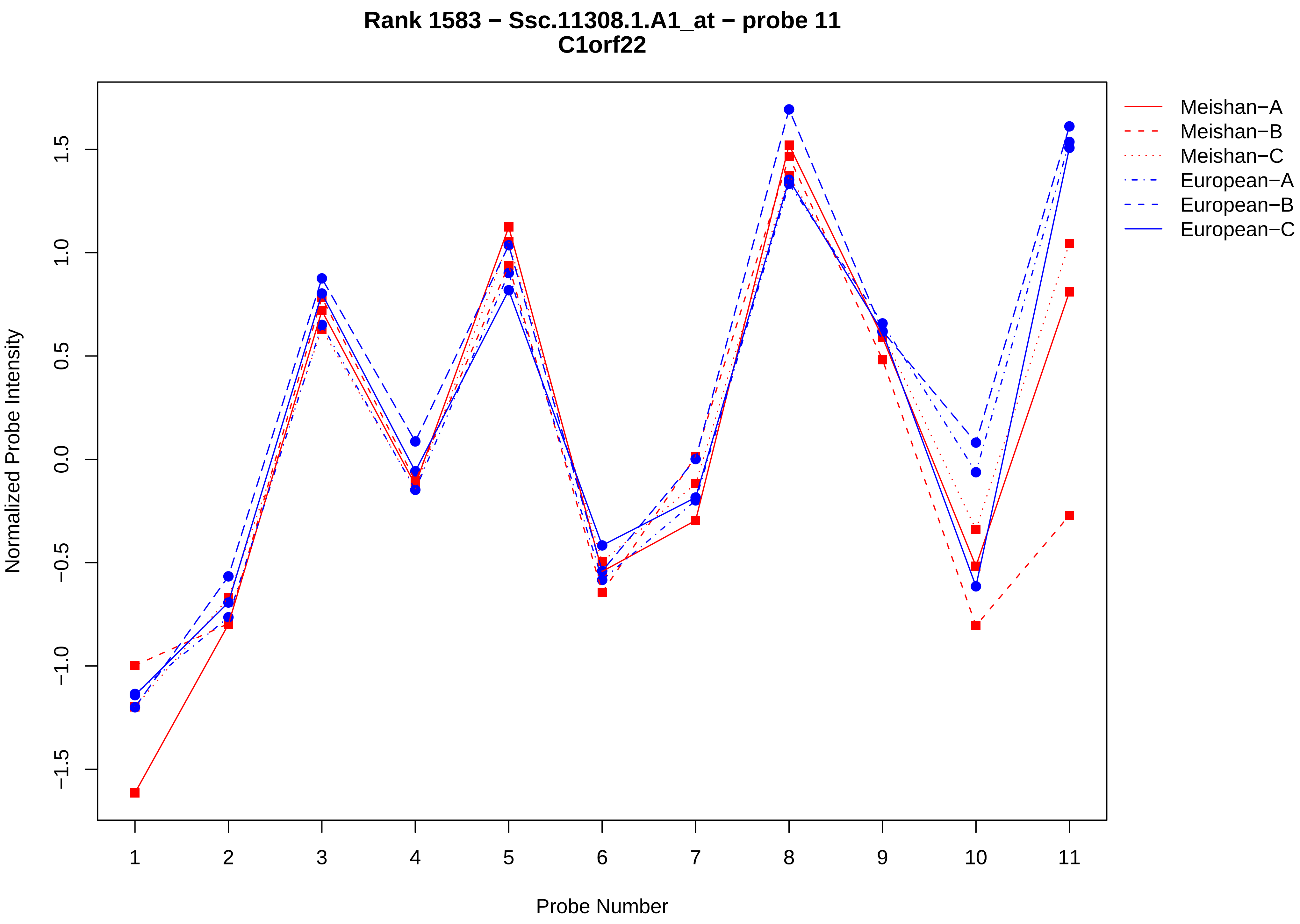

Rank 1583 − Ssc.11308.1.A1_at − probe 11
C1orf22
Normalized Probe Intensity
Probe Number
−1.5
−1.0
−0.5
0.0
0.5
1.0
1.5
1
2
3
4
5
6
7
8
9
10
11
Meishan−A
Meishan−B
Meishan−C
European−A
European−B
European−C

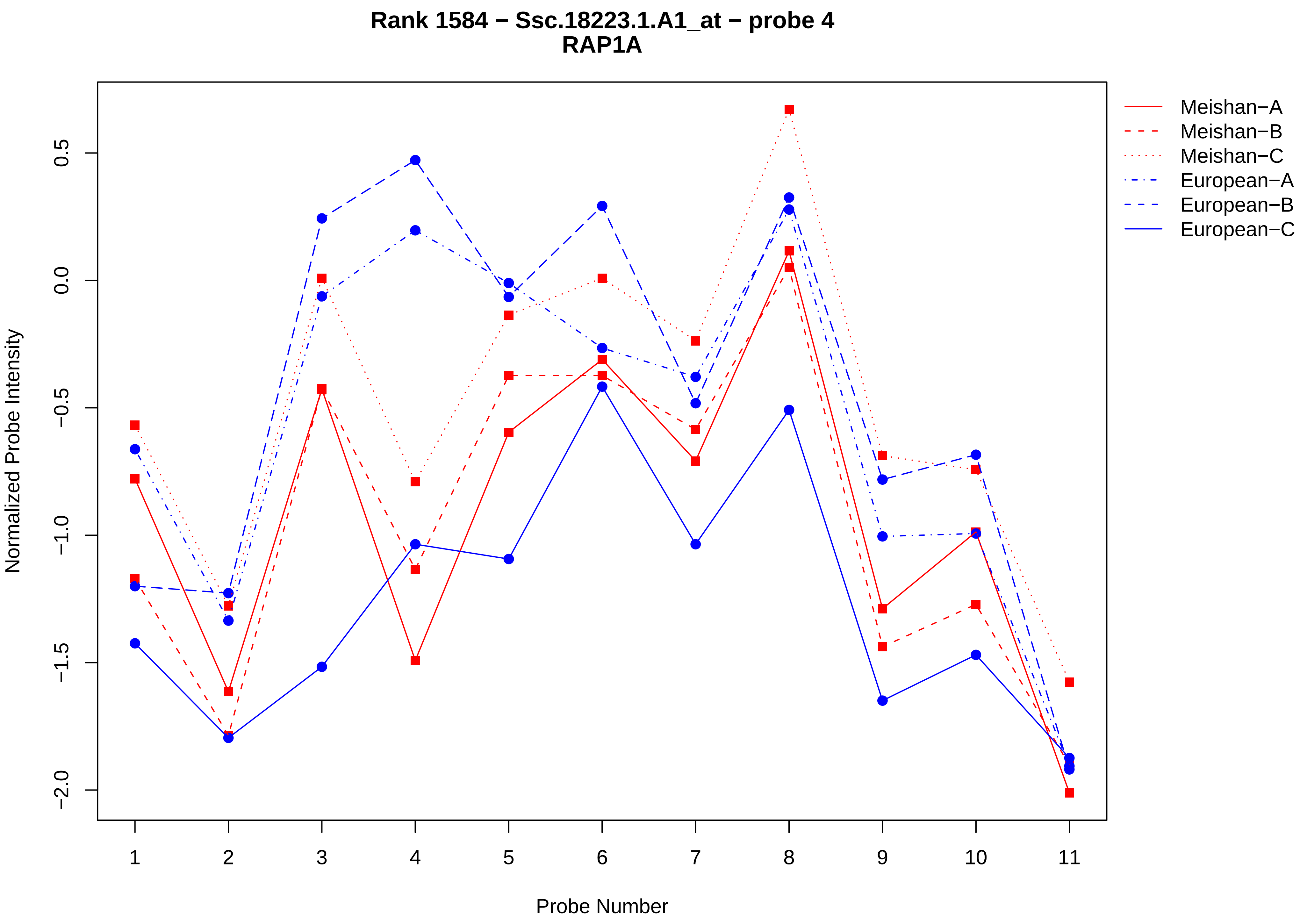
Rank 1584 − Ssc.18223.1.A1_at − probe 4
RAP1A
Normalized Probe Intensity
Probe Number
Meishan−A
Meishan−B
Meishan−C
European−A
European−B
European−C
0.5
0.0
−0.5
−1.0
−1.5
−2.0
1
2
3
4
5
6
7
8
9
10
11

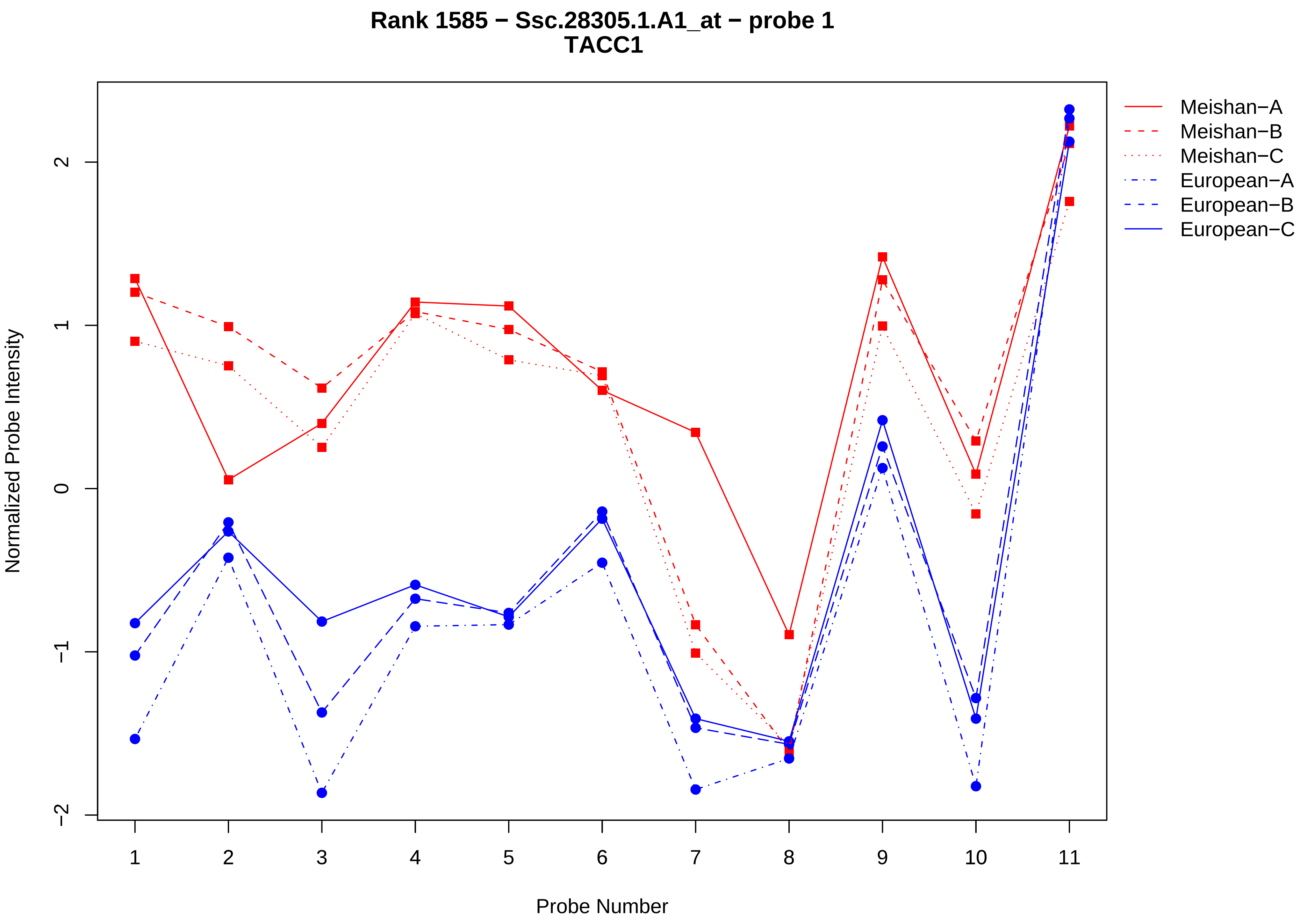
Rank 1585 − Ssc.28305.1.A1_at − probe 1
TACC1
Normalized Probe Intensity
Probe Number
−2
−1
0
1
2
1
2
3
4
5
6
7
8
9
10
11
Meishan−A
Meishan−B
Meishan−C
European−A
European−B
European−C

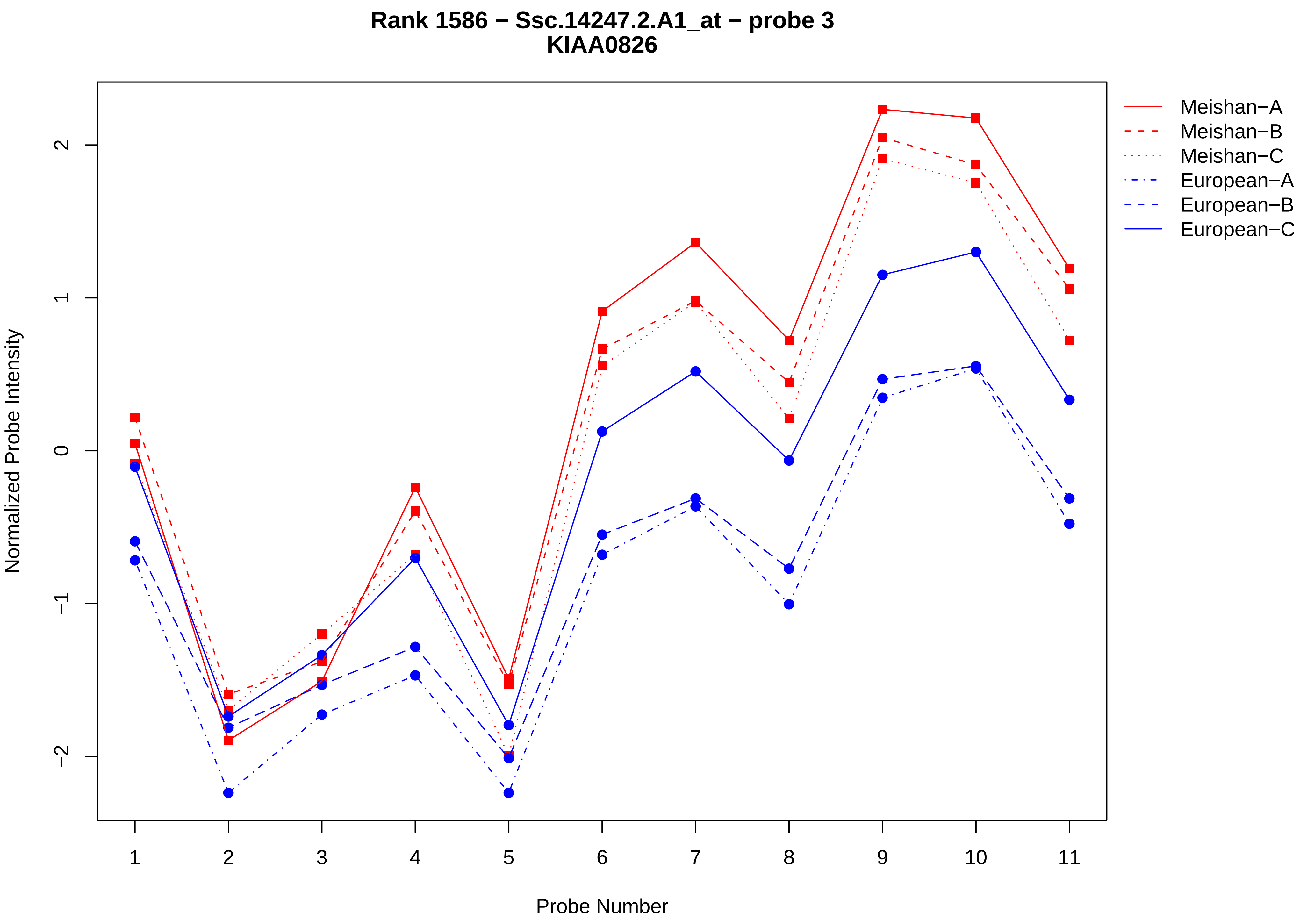

Rank 1586 − Ssc.14247.2.A1_at − probe 3
KIAA0826
Normalized Probe Intensity
Probe Number
Meishan−A
Meishan−B
Meishan−C
European−A
European−B
European−C
−2
−1
0
1
2
1
2
3
4
5
6
7
8
9
10
11

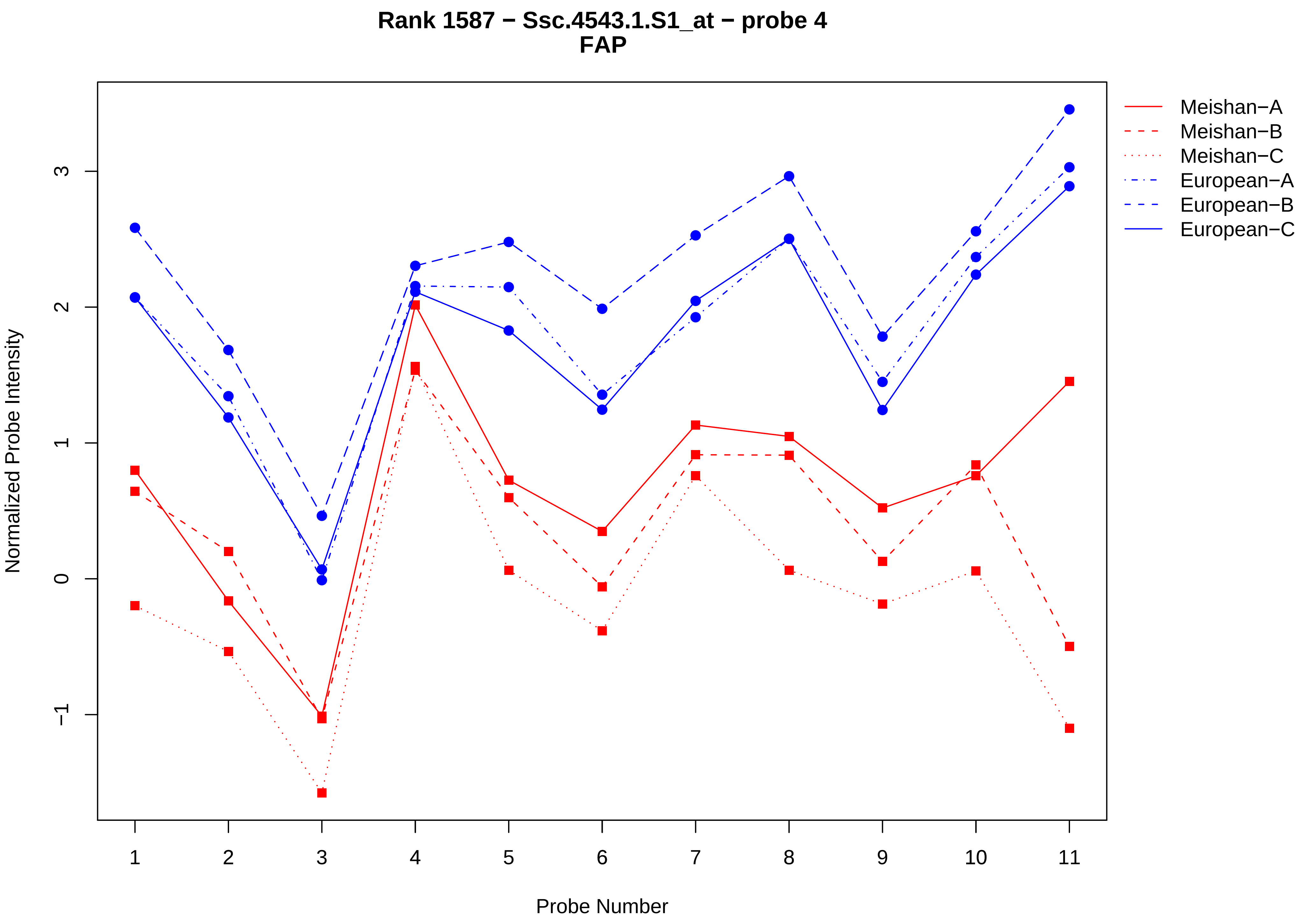

Rank 1587 − Ssc.4543.1.S1_at − probe 4
FAP
Normalized Probe Intensity
Probe Number
−1
0
1
2
3
1
2
3
4
5
6
7
8
9
10
11
Meishan−A
Meishan−B
Meishan−C
European−A
European−B
European−C

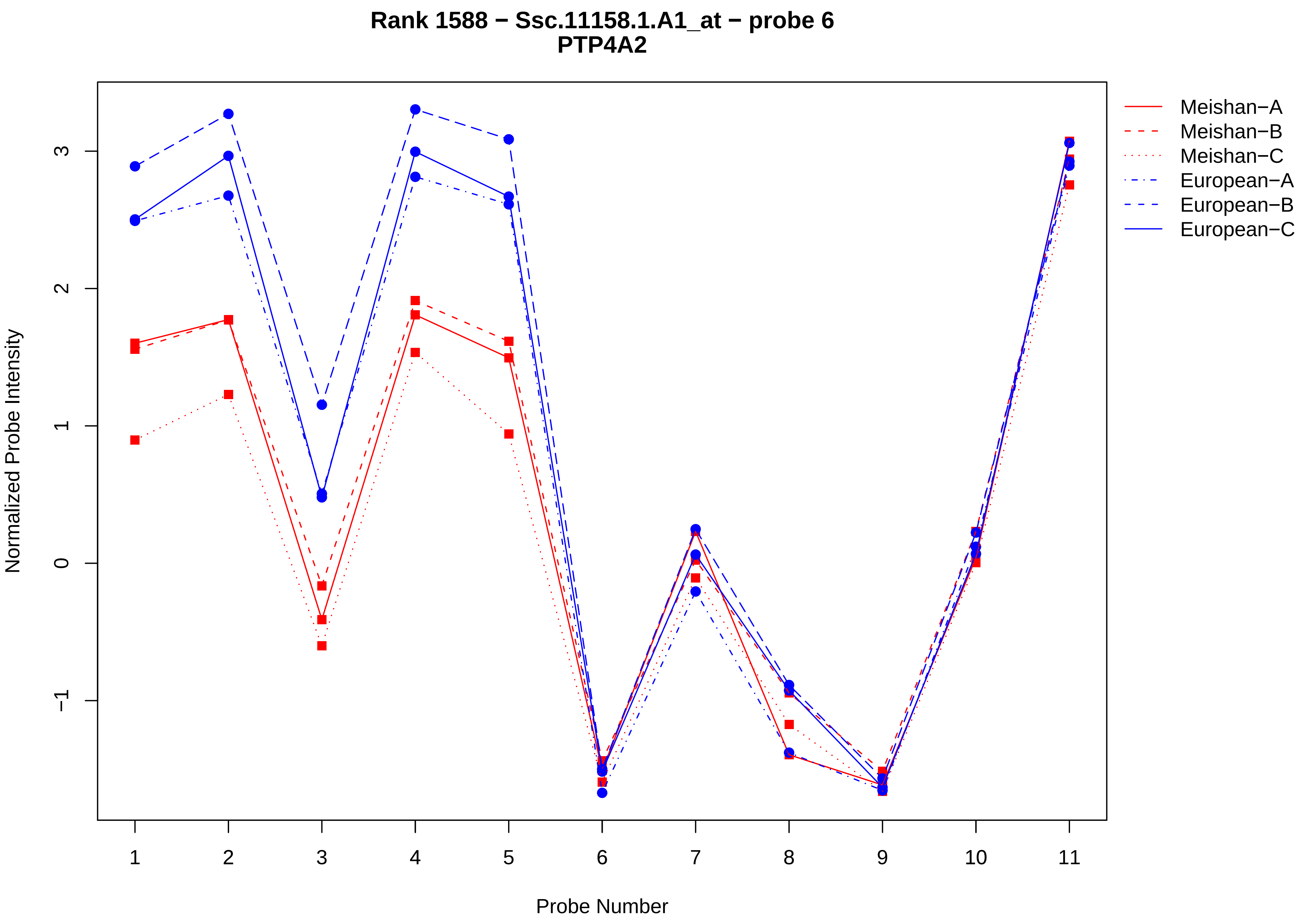
Rank 1588 − Ssc.11158.1.A1_at − probe 6
PTP4A2
Normalized Probe Intensity
Probe Number
−1
0
1
2
3
1
2
3
4
5
6
7
8
9
10
11
Meishan−A
Meishan−B
Meishan−C
European−A
European−B
European−C

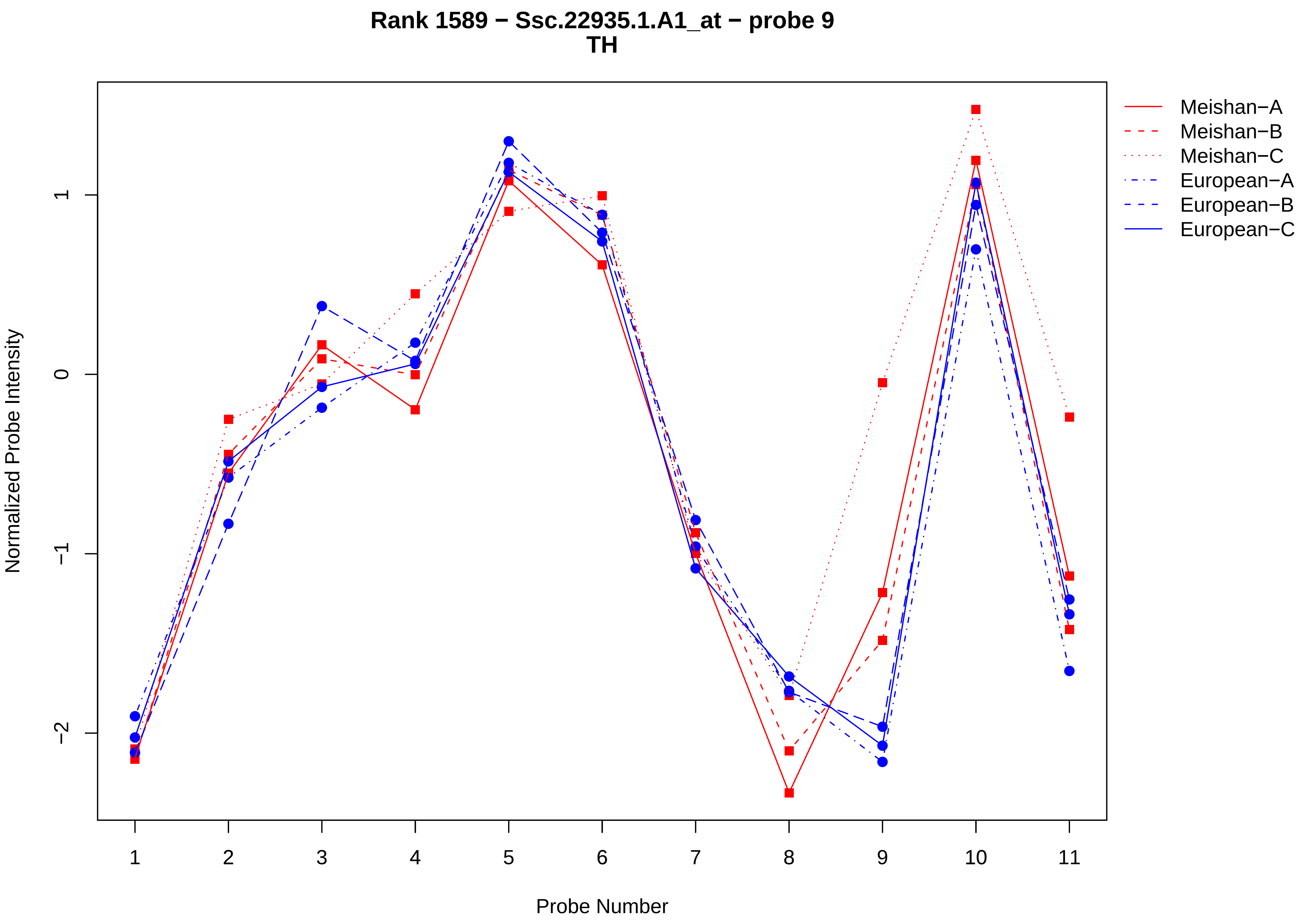

Rank 1589 − Ssc.22935.1.A1_at − probe 9
TH
Normalized Probe Intensity
Probe Number
−2
−1
0
1
1
2
3
4
5
6
7
8
9
10
11
Meishan−A
Meishan−B
Meishan−C
European−A
European−B
European−C

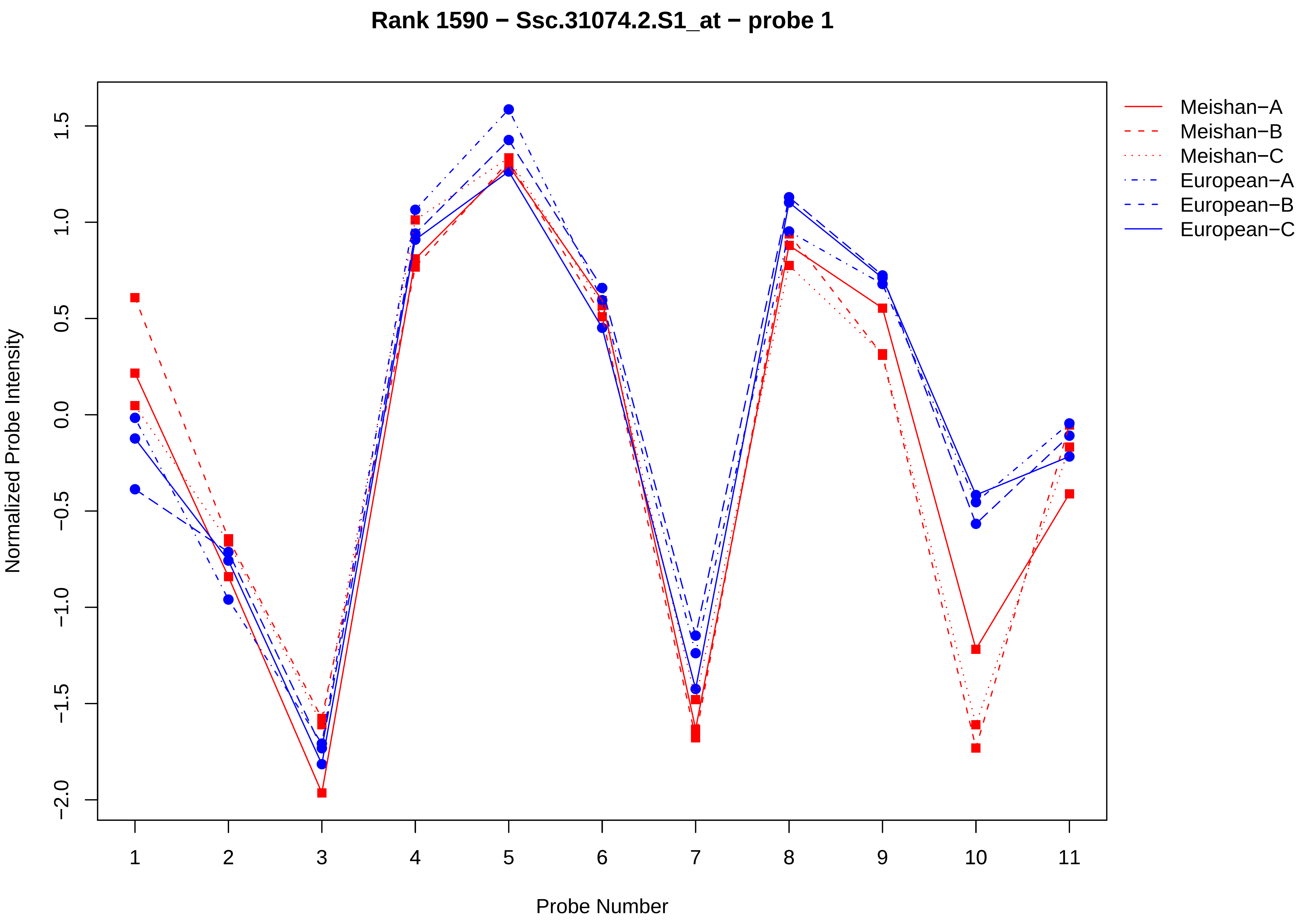

Rank 1590 − Ssc.31074.2.S1_at − probe 1
Normalized Probe Intensity
Probe Number
1.5
1.0
0.5
0.0
−0.5
−1.0
−1.5
−2.0
1
2
3
4
5
6
7
8
9
10
11
Meishan−A
Meishan−B
Meishan−C
European−A
European−B
European−C

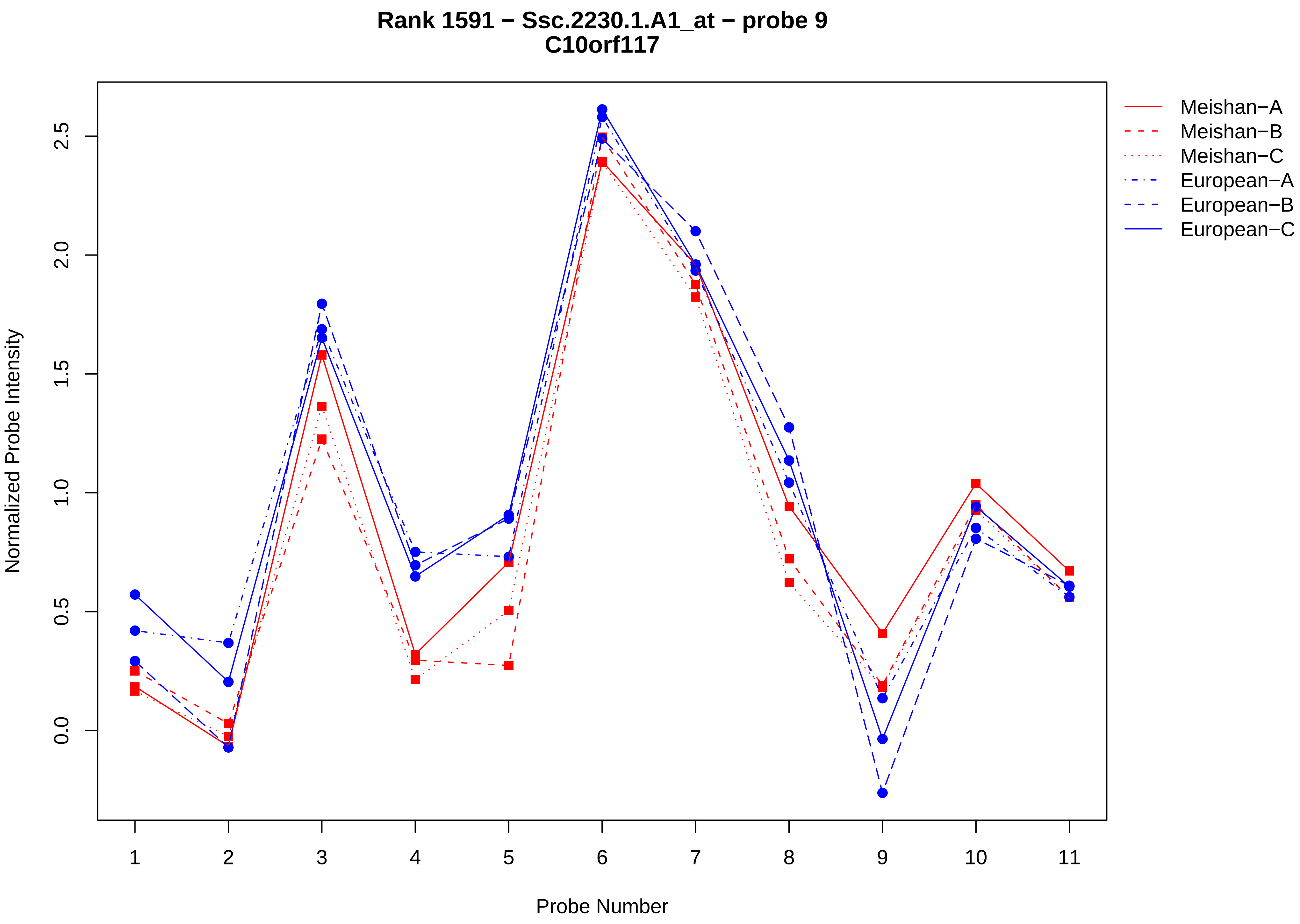

Rank 1591 − Ssc.2230.1.A1_at − probe 9
C10orf117
Normalized Probe Intensity
Probe Number
0.0
0.5
1.0
1.5
2.0
2.5
1
2
3
4
5
6
7
8
9
10
11
Meishan−A
Meishan−B
Meishan−C
European−A
European−B
European−C

Rank 1592 − Ssc.29853.1.A1_at − probe 10
TSC1

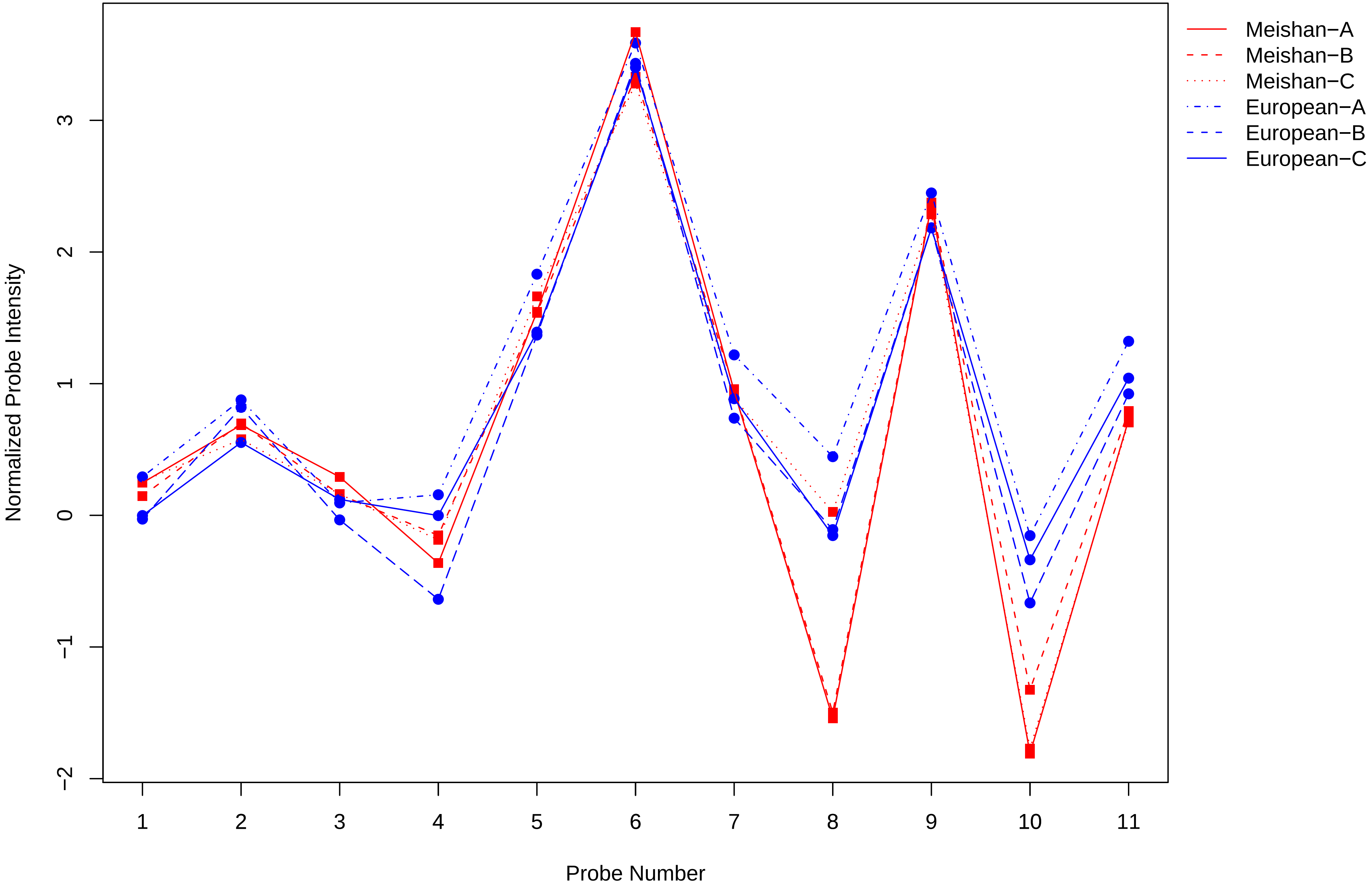

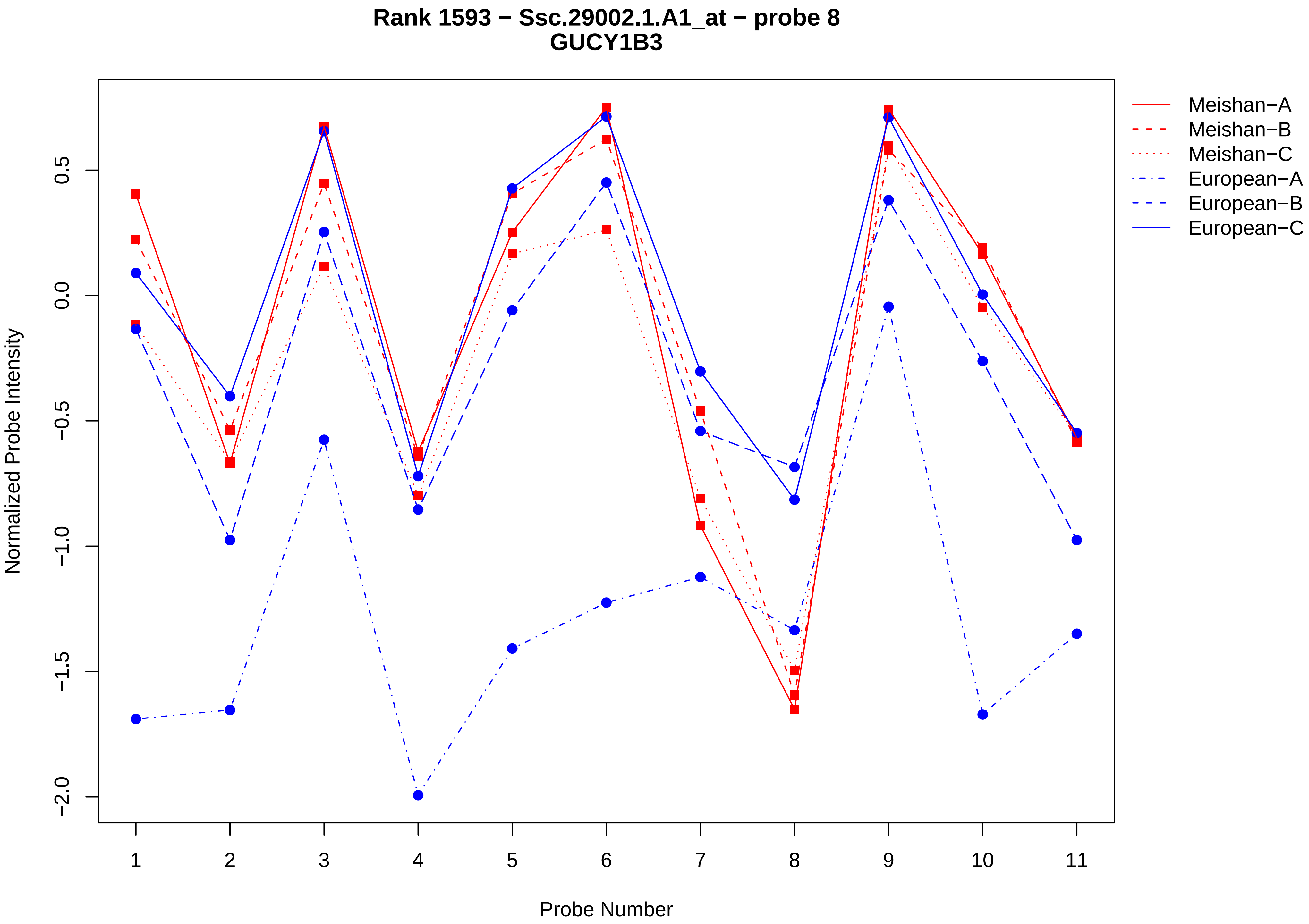

Rank 1593 − Ssc.29002.1.A1_at − probe 8
GUCY1B3
Normalized Probe Intensity
Probe Number
0.5
0.0
−0.5
−1.0
−1.5
−2.0
1
2
3
4
5
6
7
8
9
10
11
Meishan−A
Meishan−B
Meishan−C
European−A
European−B
European−C

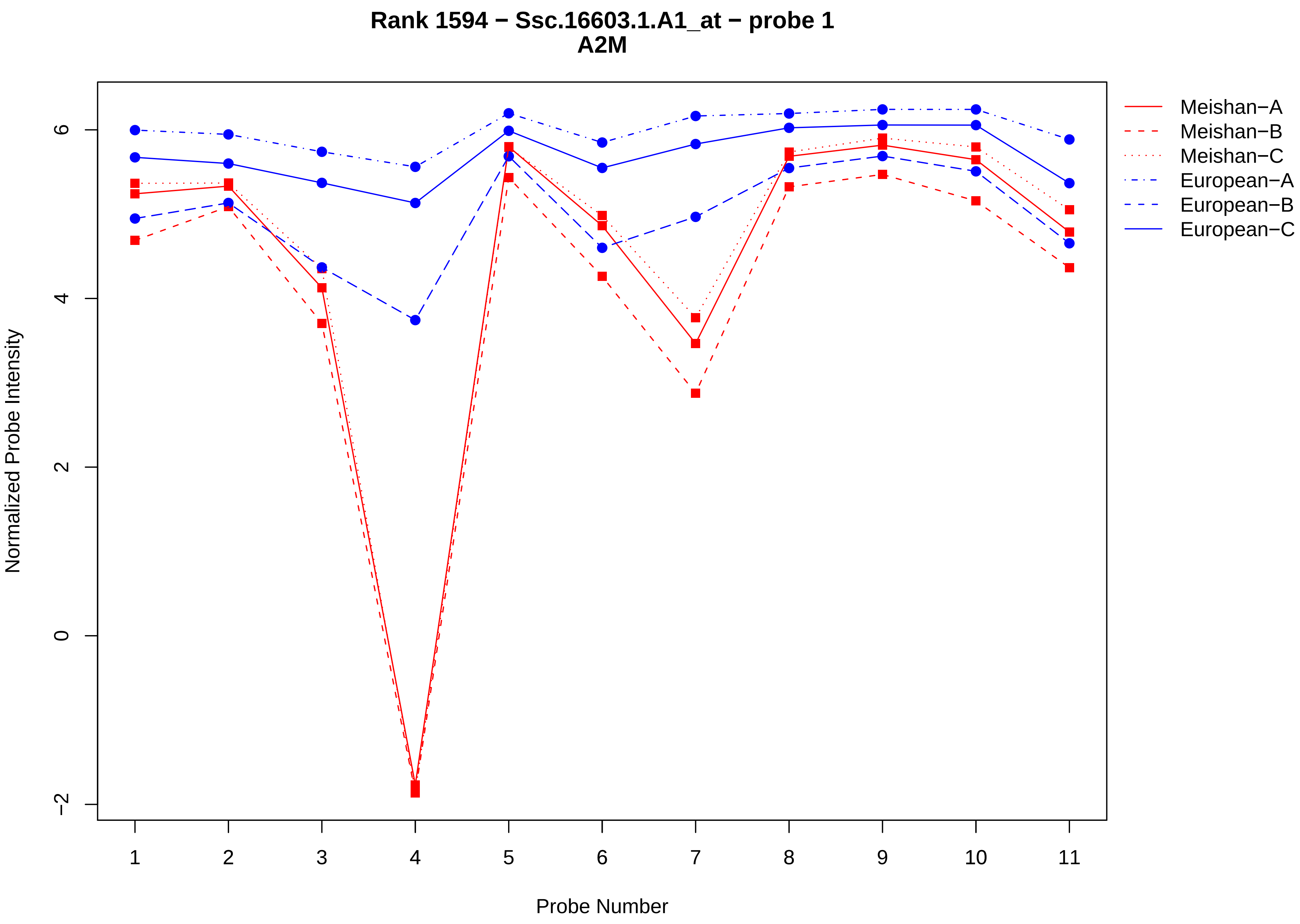

Rank 1594 − Ssc.16603.1.A1_at − probe 1
A2M
Normalized Probe Intensity
Probe Number
−2
0
2
4
6
1
2
3
4
5
6
7
8
9
10
11
Meishan−A
Meishan−B
Meishan−C
European−A
European−B
European−C

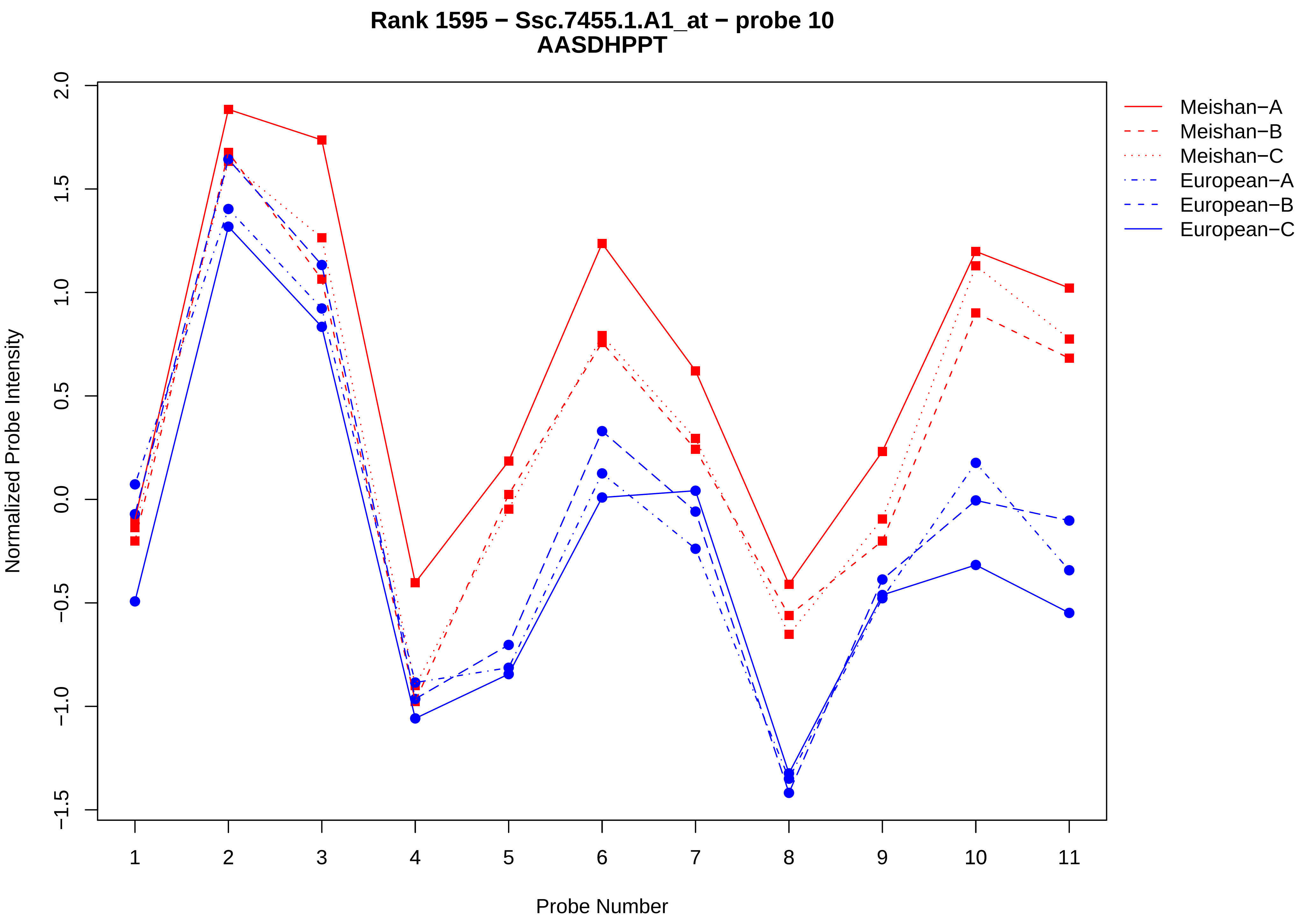

Rank 1595 − Ssc.7455.1.A1_at − probe 10
AASDHPPT
Normalized Probe Intensity
Probe Number
2.0
1.5
1.0
0.5
0.0
−0.5
−1.0
−1.5
1
2
3
4
5
6
7
8
9
10
11
Meishan−A
Meishan−B
Meishan−C
European−A
European−B
European−C

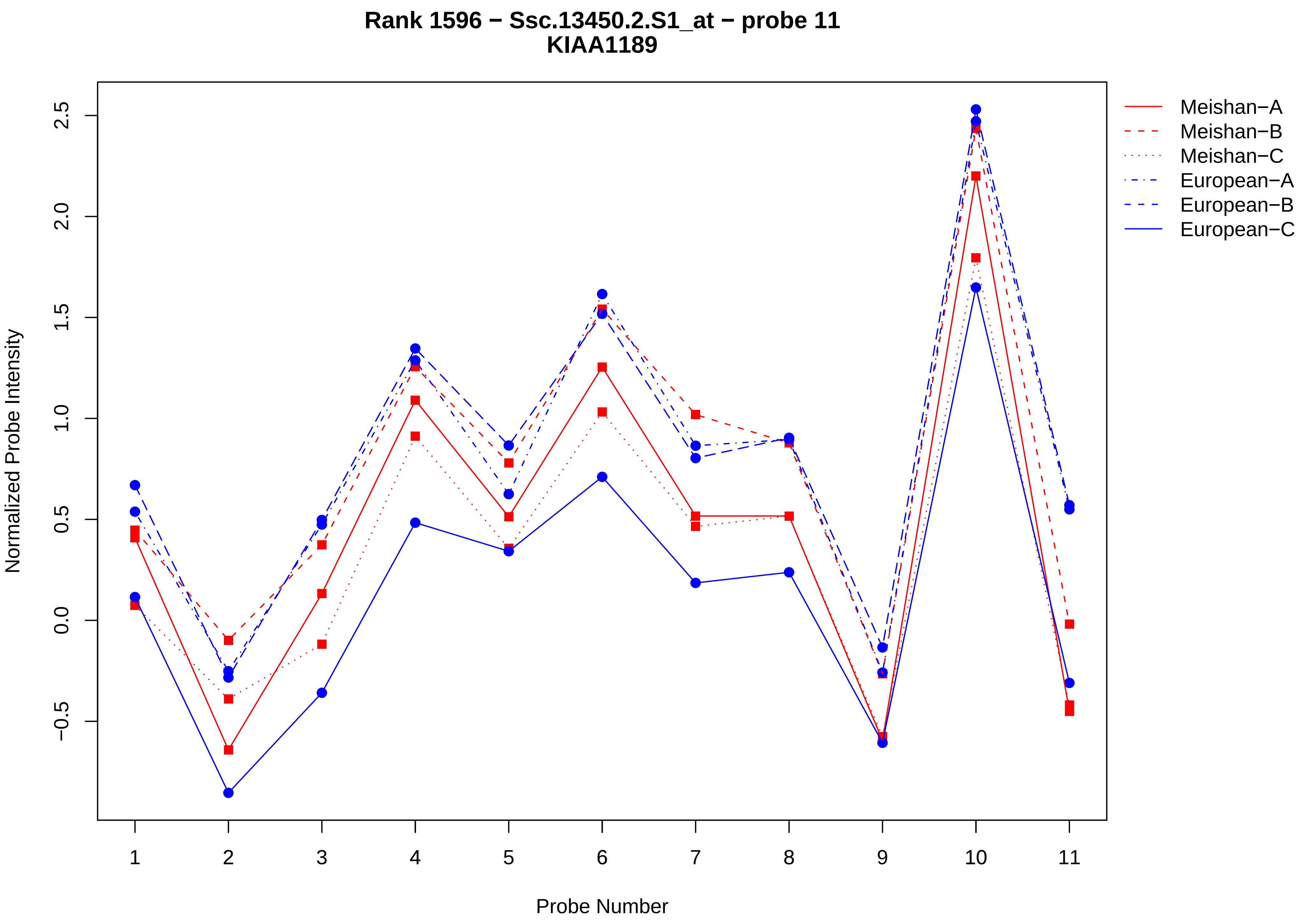

Rank 1596 − Ssc.13450.2.S1_at − probe 11
KIAA1189
Normalized Probe Intensity
Probe Number
−0.5
0.0
0.5
1.0
1.5
2.0
2.5
1
2
3
4
5
6
7
8
9
10
11
Meishan−A
Meishan−B
Meishan−C
European−A
European−B
European−C

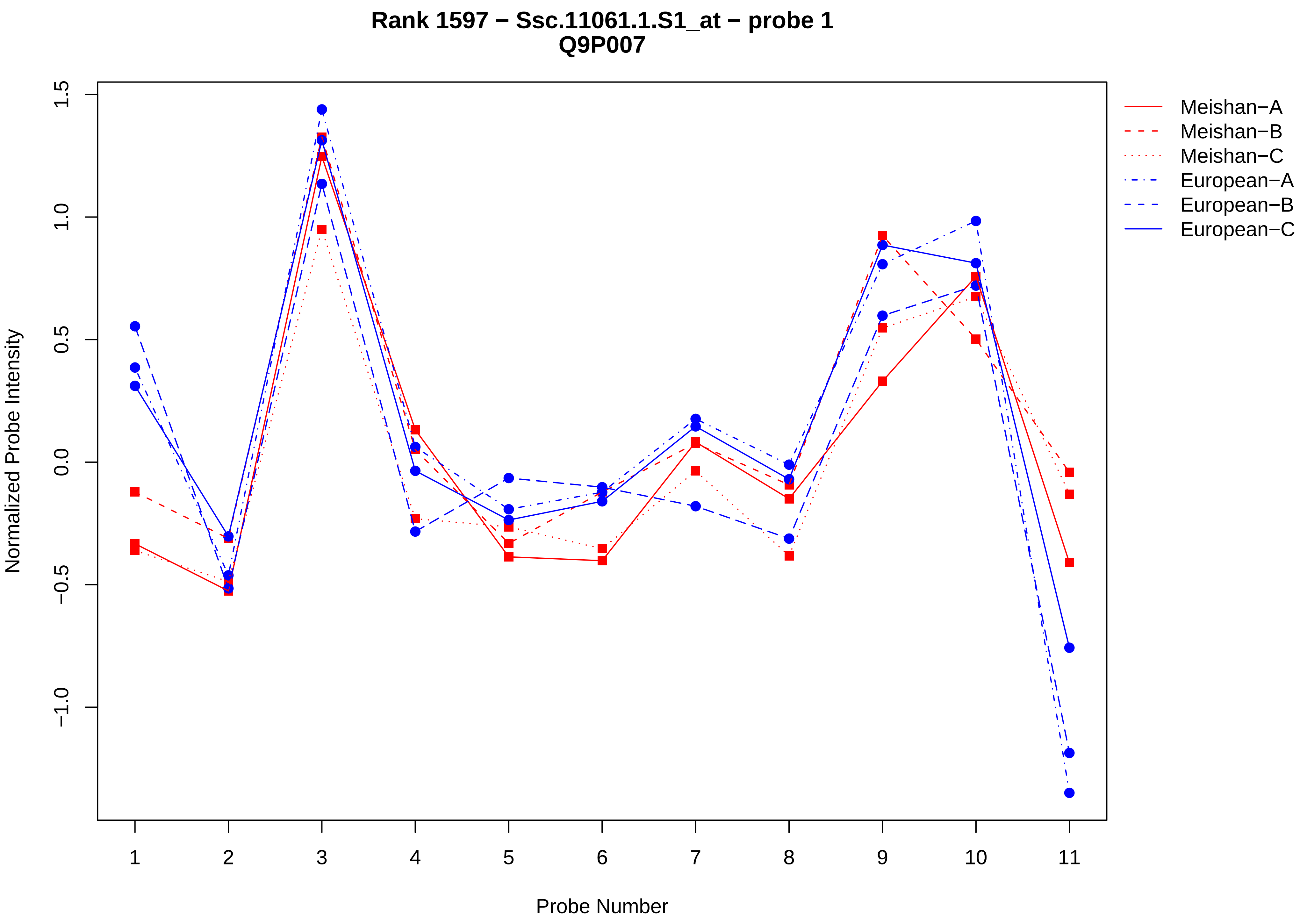

Rank 1597 − Ssc.11061.1.S1_at − probe 1
Q9P007
Normalized Probe Intensity
Probe Number
1.5
1.0
0.5
0.0
−0.5
−1.0
1
2
3
4
5
6
7
8
9
10
11
Meishan−A
Meishan−B
Meishan−C
European−A
European−B
European−C

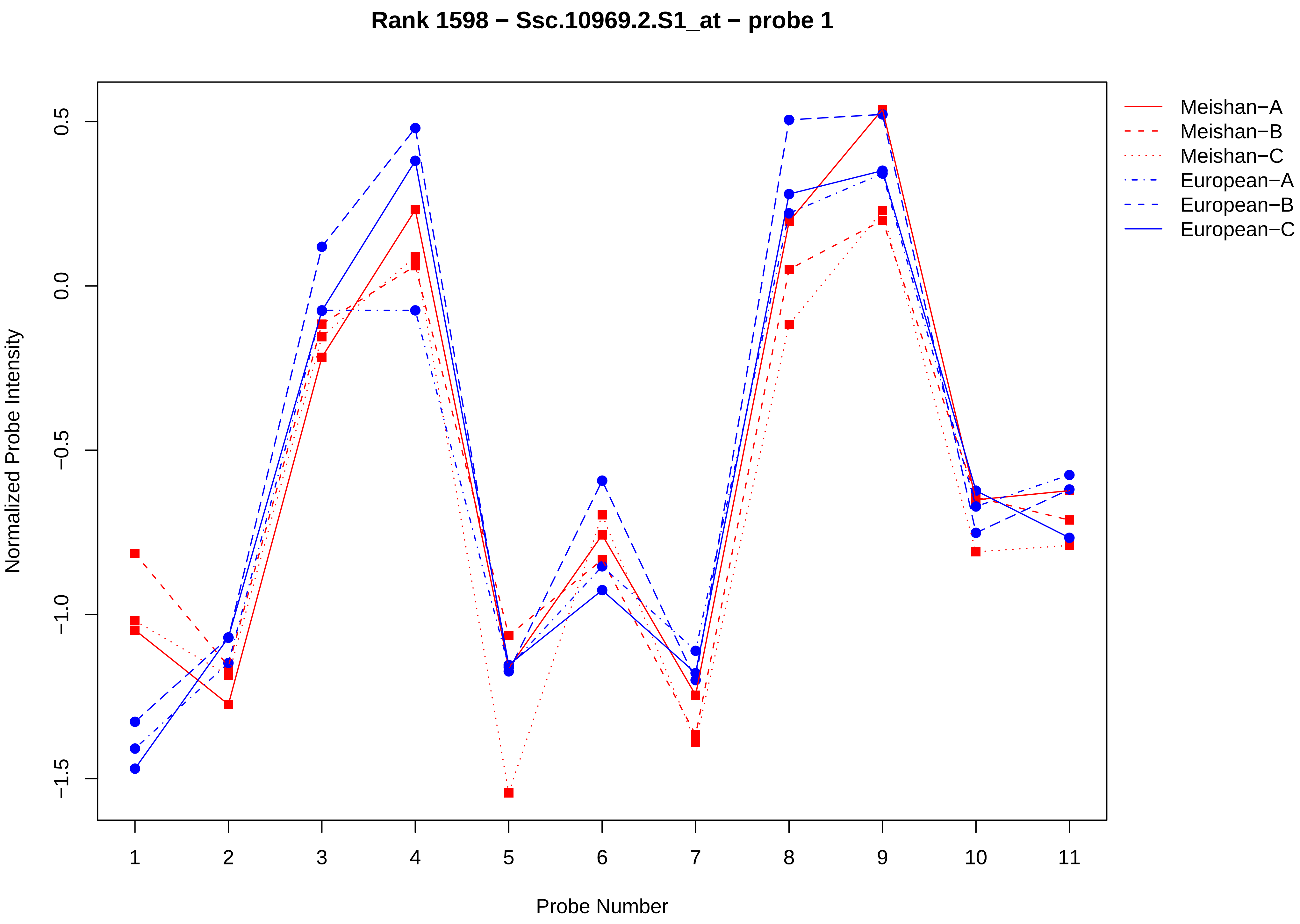

Rank 1598 − Ssc.10969.2.S1_at − probe 1
Normalized Probe Intensity
Probe Number
0.5
0.0
−0.5
−1.0
−1.5
1
2
3
4
5
6
7
8
9
10
11
Meishan−A
Meishan−B
Meishan−C
European−A
European−B
European−C

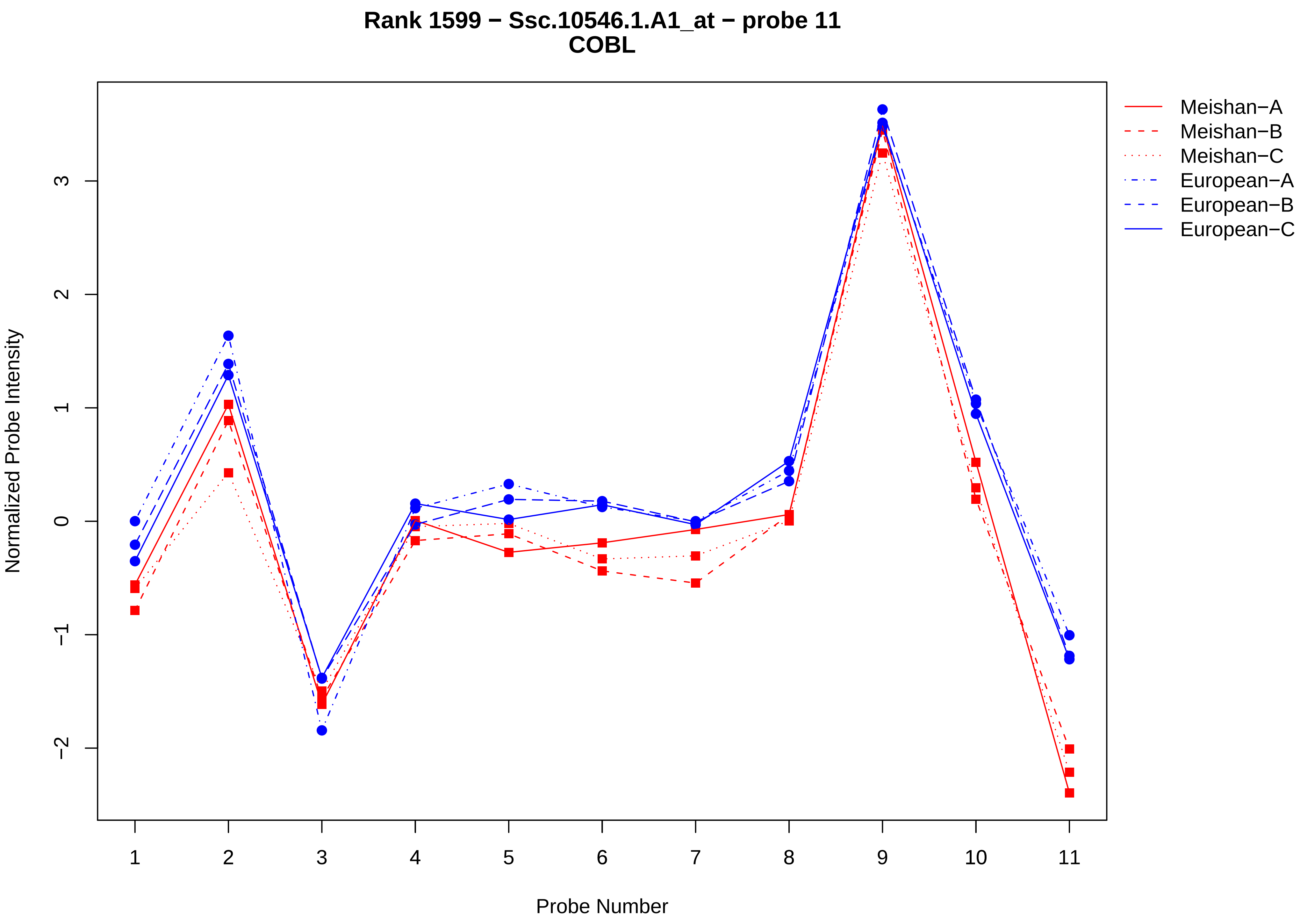

Rank 1599 − Ssc.10546.1.A1_at − probe 11
COBL
Normalized Probe Intensity
Probe Number
Meishan−A
Meishan−B
Meishan−C
European−A
European−B
European−C
−2
−1
0
1
2
3
1
2
3
4
5
6
7
8
9
10
11

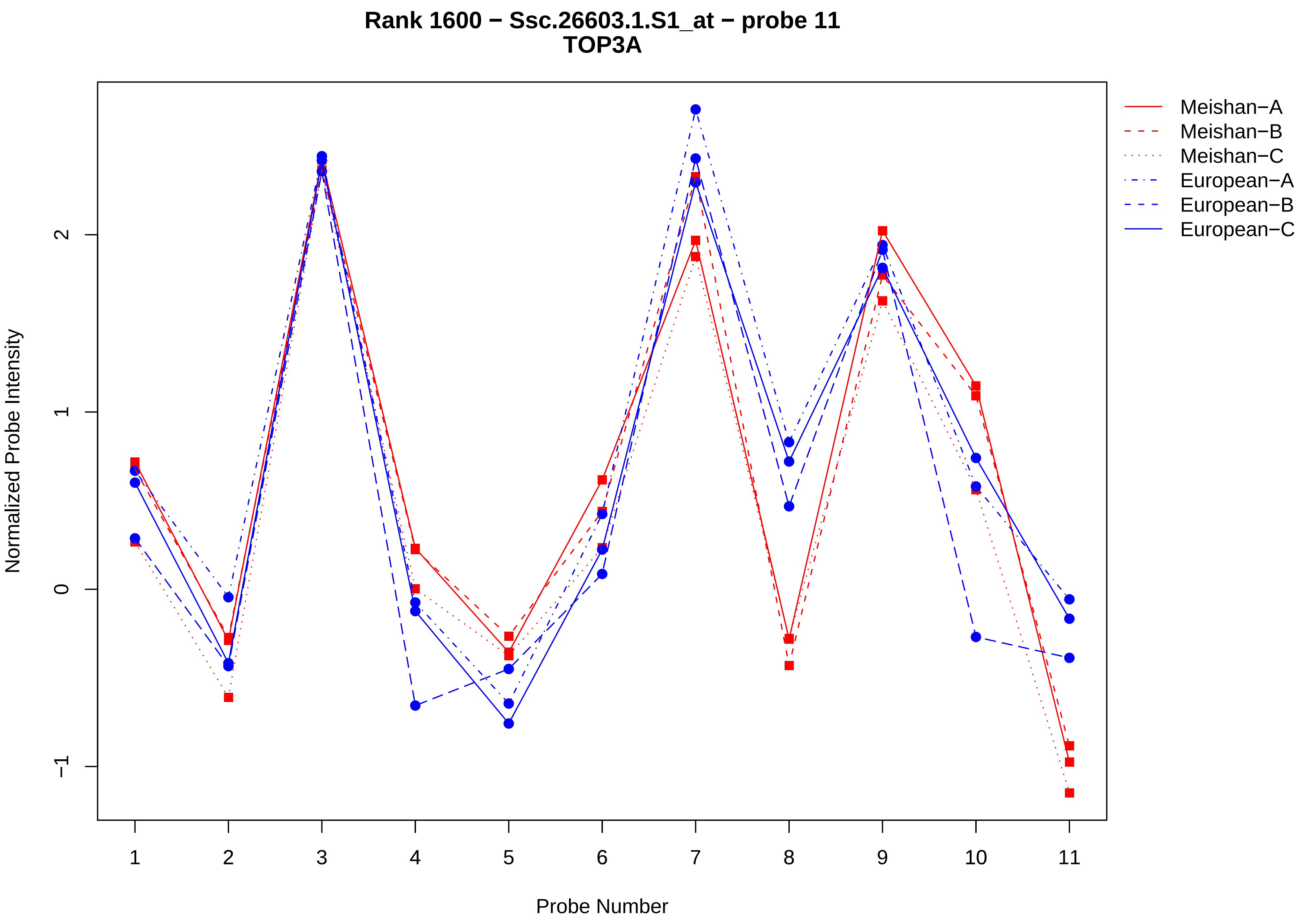

Rank 1600 − Ssc.26603.1.S1_at − probe 11
TOP3A
Normalized Probe Intensity
Probe Number
Meishan−A
Meishan−B
Meishan−C
European−A
European−B
European−C
−1
0
1
2
1
2
3
4
5
6
7
8
9
10
11

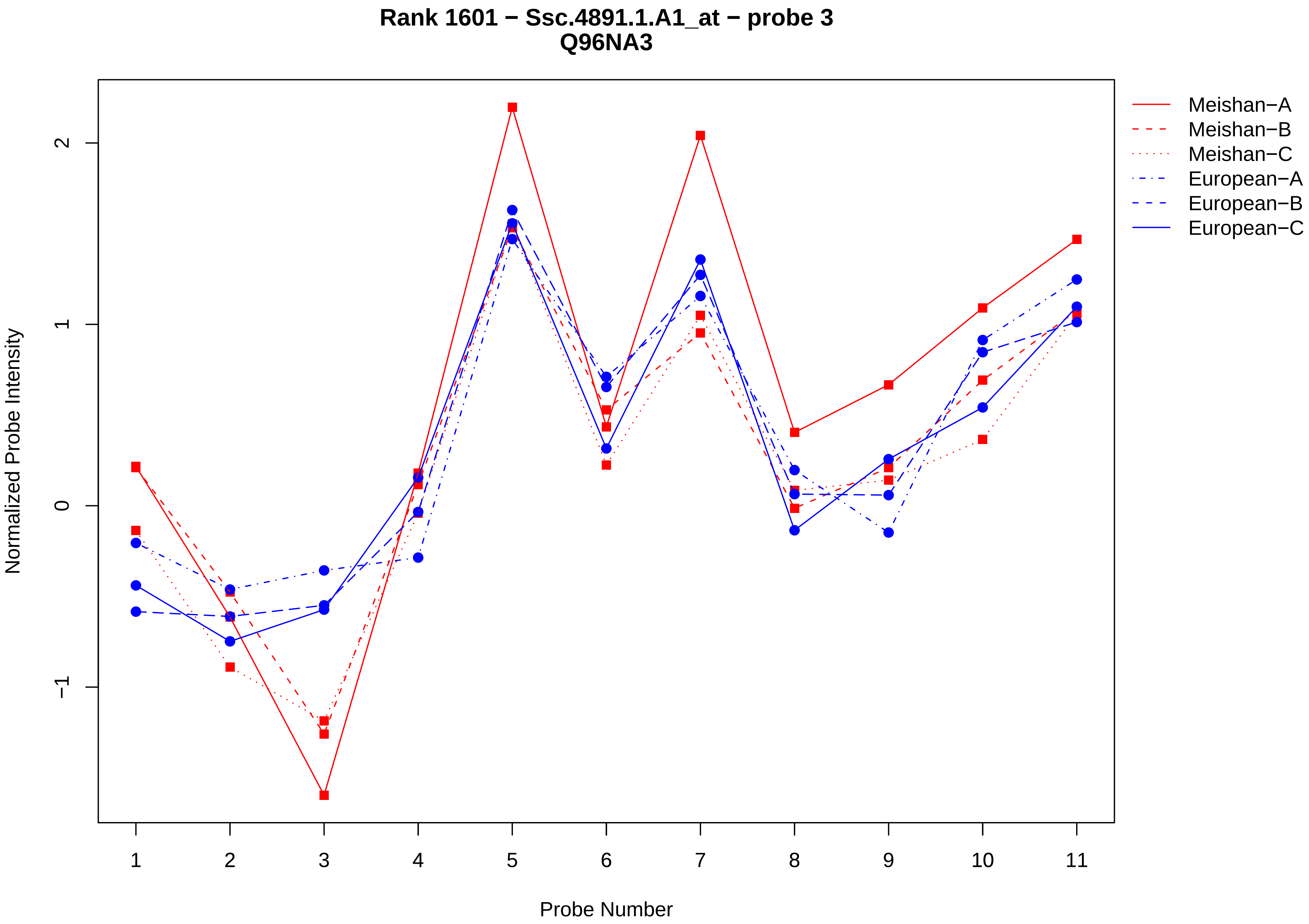
Rank 1601 − Ssc.4891.1.A1_at − probe 3
Q96NA3
Normalized Probe Intensity
Probe Number
−1
0
1
2
1
2
3
4
5
6
7
8
9
10
11
Meishan−A
Meishan−B
Meishan−C
European−A
European−B
European−C

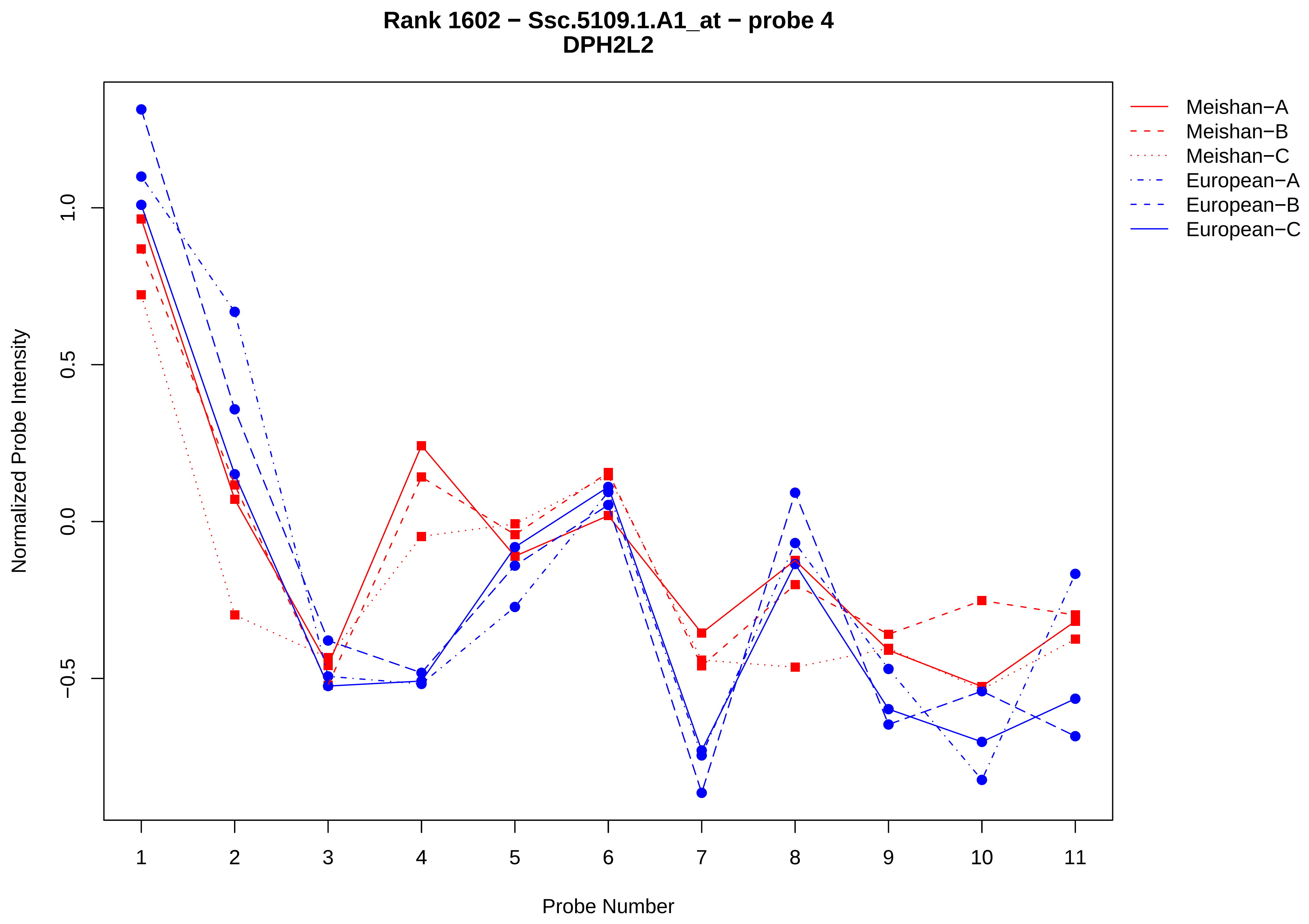

Rank 1602 − Ssc.5109.1.A1_at − probe 4
DPH2L2
Normalized Probe Intensity
−0.5
0.0
0.5
1.0
1
2
3
4
5
6
7
8
9
10
11
Probe Number
Meishan−A
Meishan−B
Meishan−C
European−A
European−B
European−C

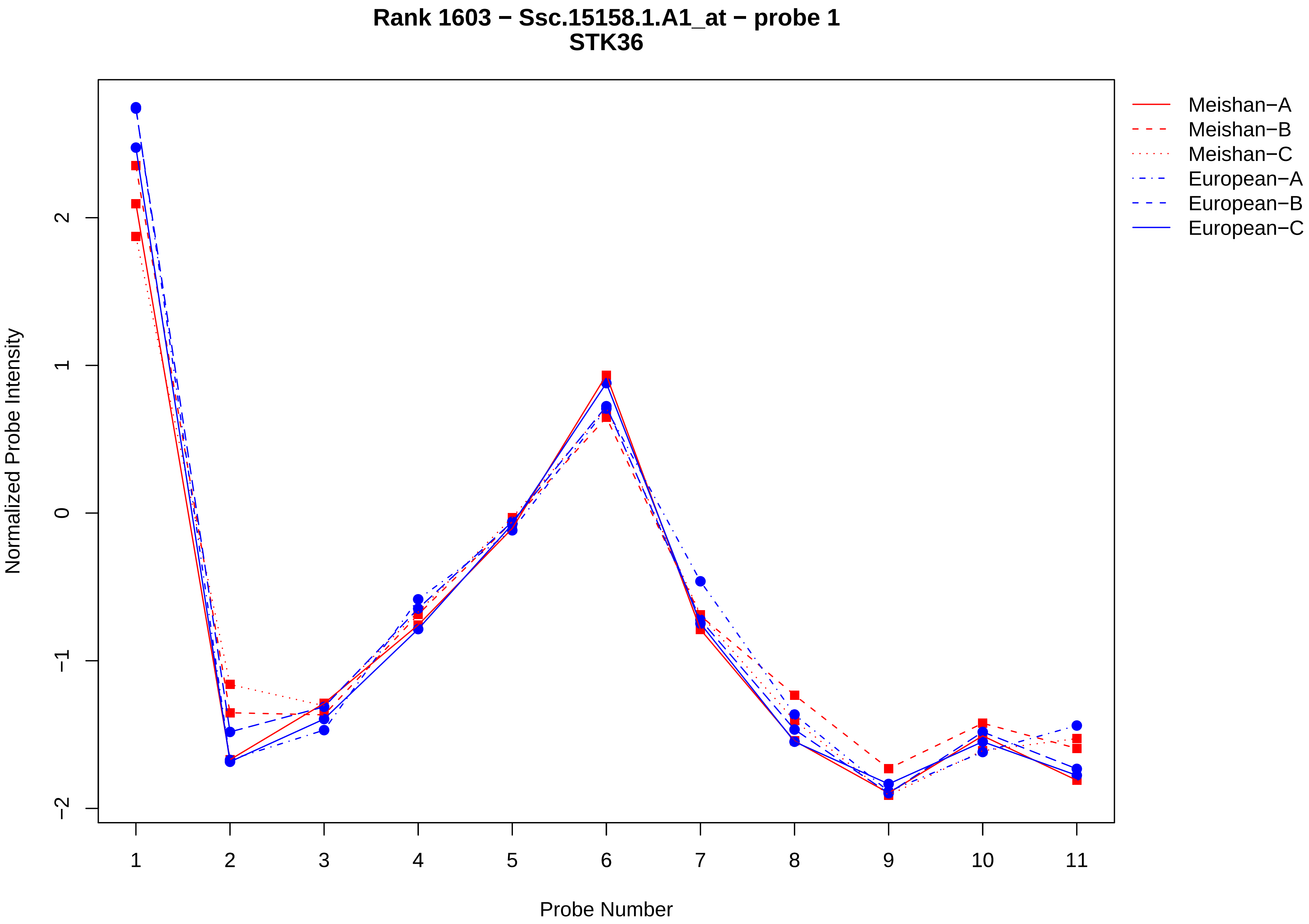

Rank 1603 − Ssc.15158.1.A1_at − probe 1
STK36
Normalized Probe Intensity
Probe Number
−2
−1
0
1
2
1
2
3
4
5
6
7
8
9
10
11
Meishan−A
Meishan−B
Meishan−C
European−A
European−B
European−C

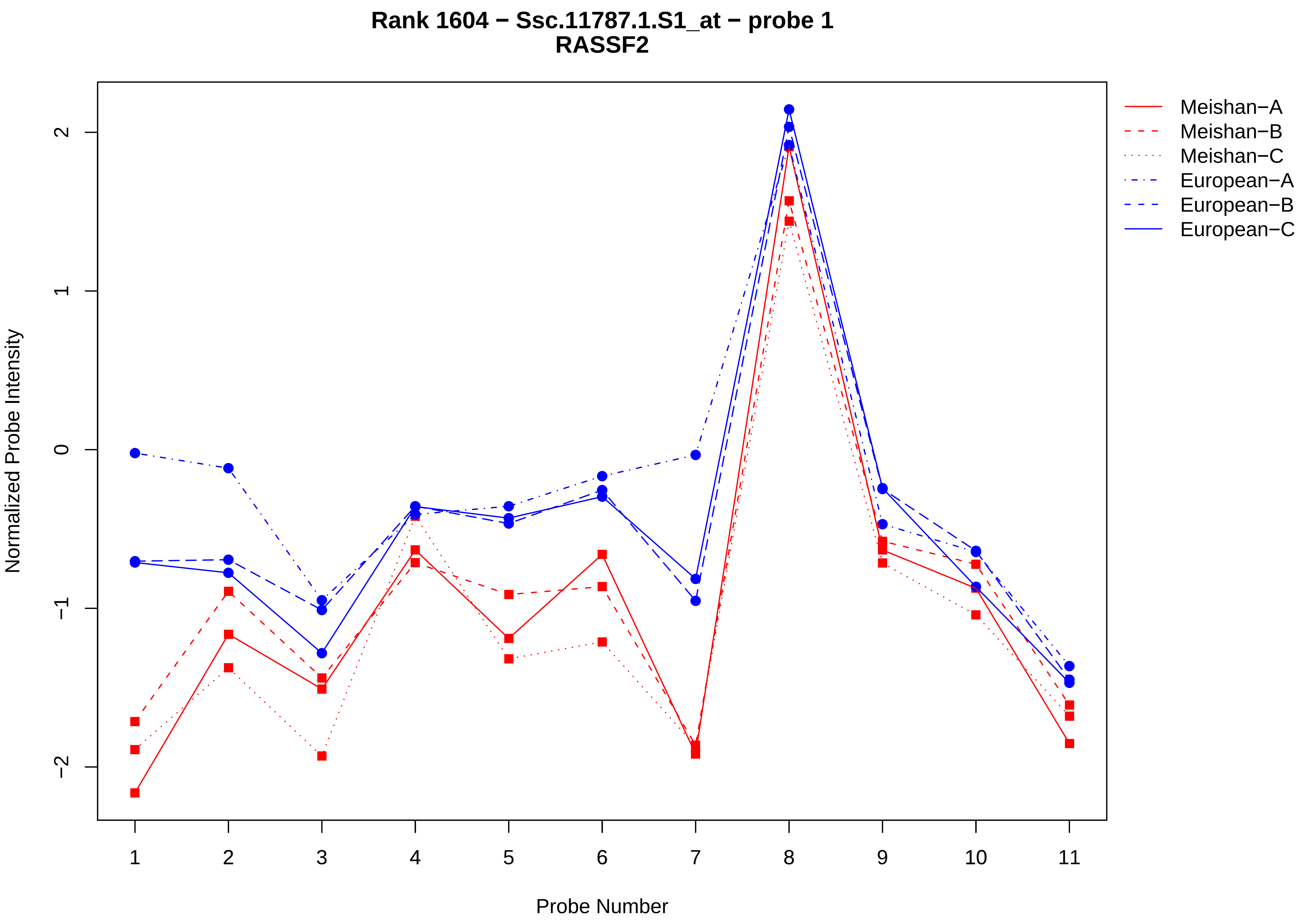

Rank 1604 − Ssc.11787.1.S1_at − probe 1
RASSF2
Normalized Probe Intensity
Probe Number
−2
−1
0
1
2
1
2
3
4
5
6
7
8
9
10
11
Meishan−A
Meishan−B
Meishan−C
European−A
European−B
European−C

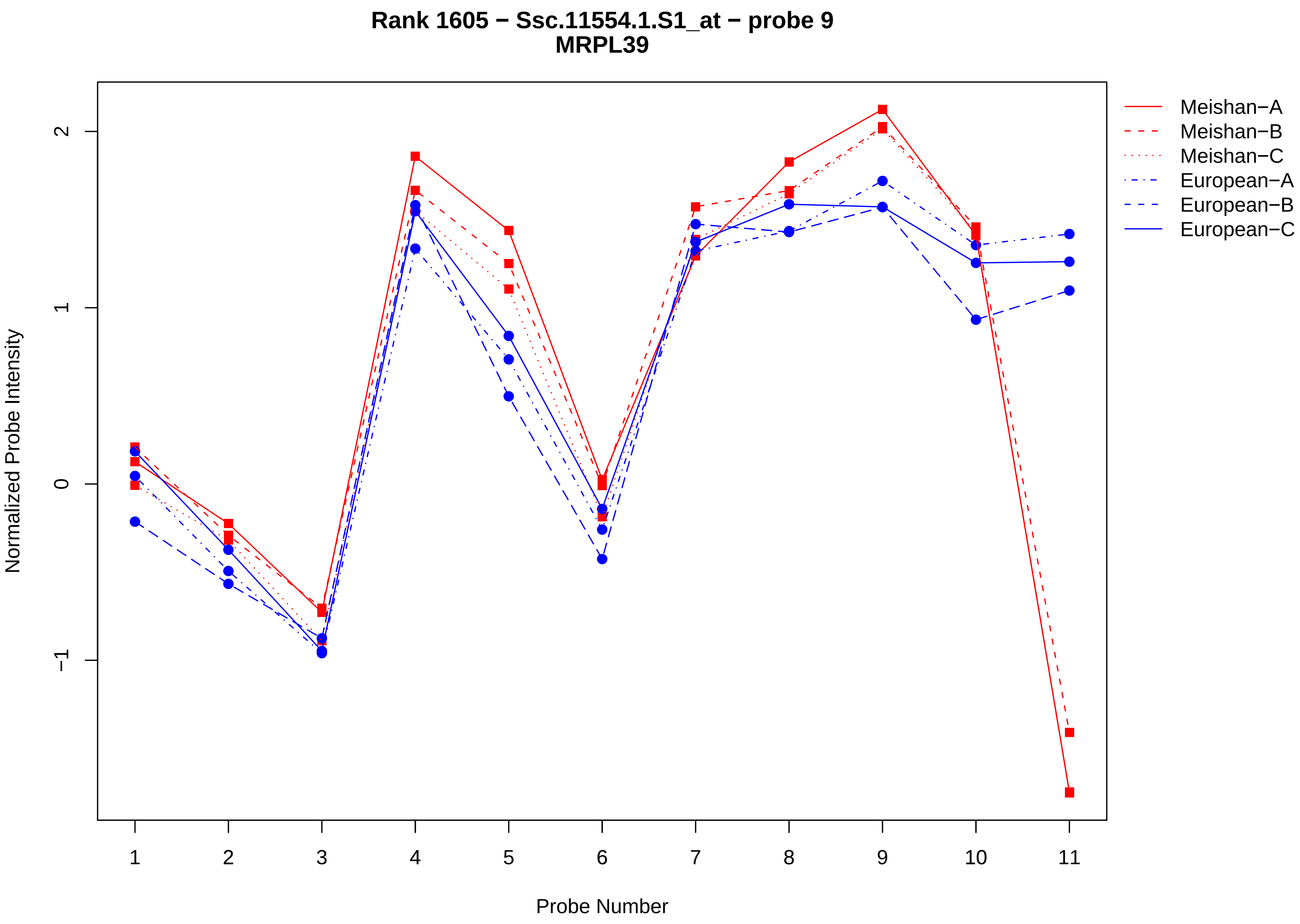

Rank 1605 − Ssc.11554.1.S1_at − probe 9
MRPL39
Normalized Probe Intensity
Probe Number
−1
0
1
2
1
2
3
4
5
6
7
8
9
10
11
Meishan−A
Meishan−B
Meishan−C
European−A
European−B
European−C

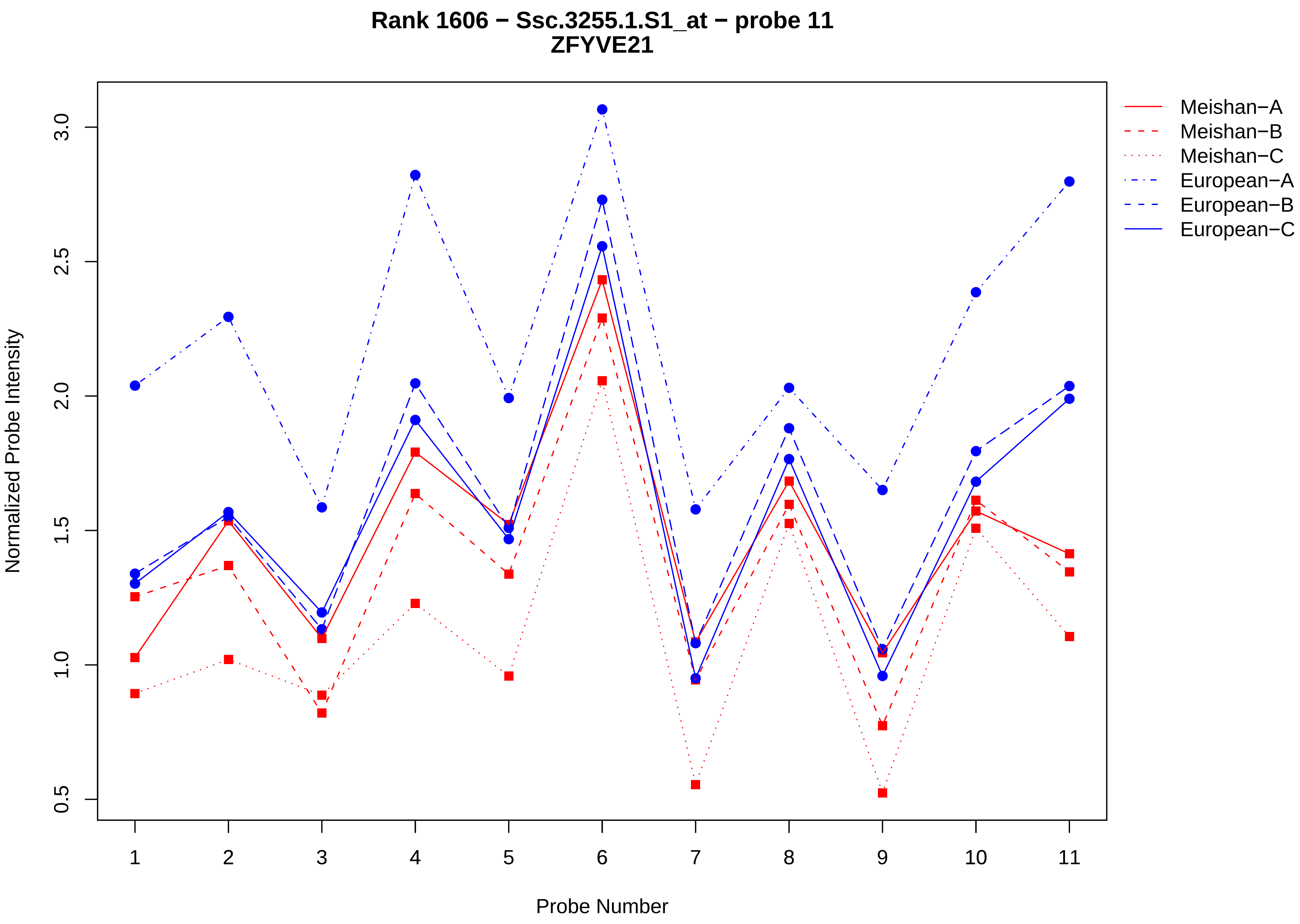

Rank 1606 − Ssc.3255.1.S1_at − probe 11
ZFYVE21
Normalized Probe Intensity
Probe Number
0.5
1.0
1.5
2.0
2.5
3.0
1
2
3
4
5
6
7
8
9
10
11
Meishan−A
Meishan−B
Meishan−C
European−A
European−B
European−C

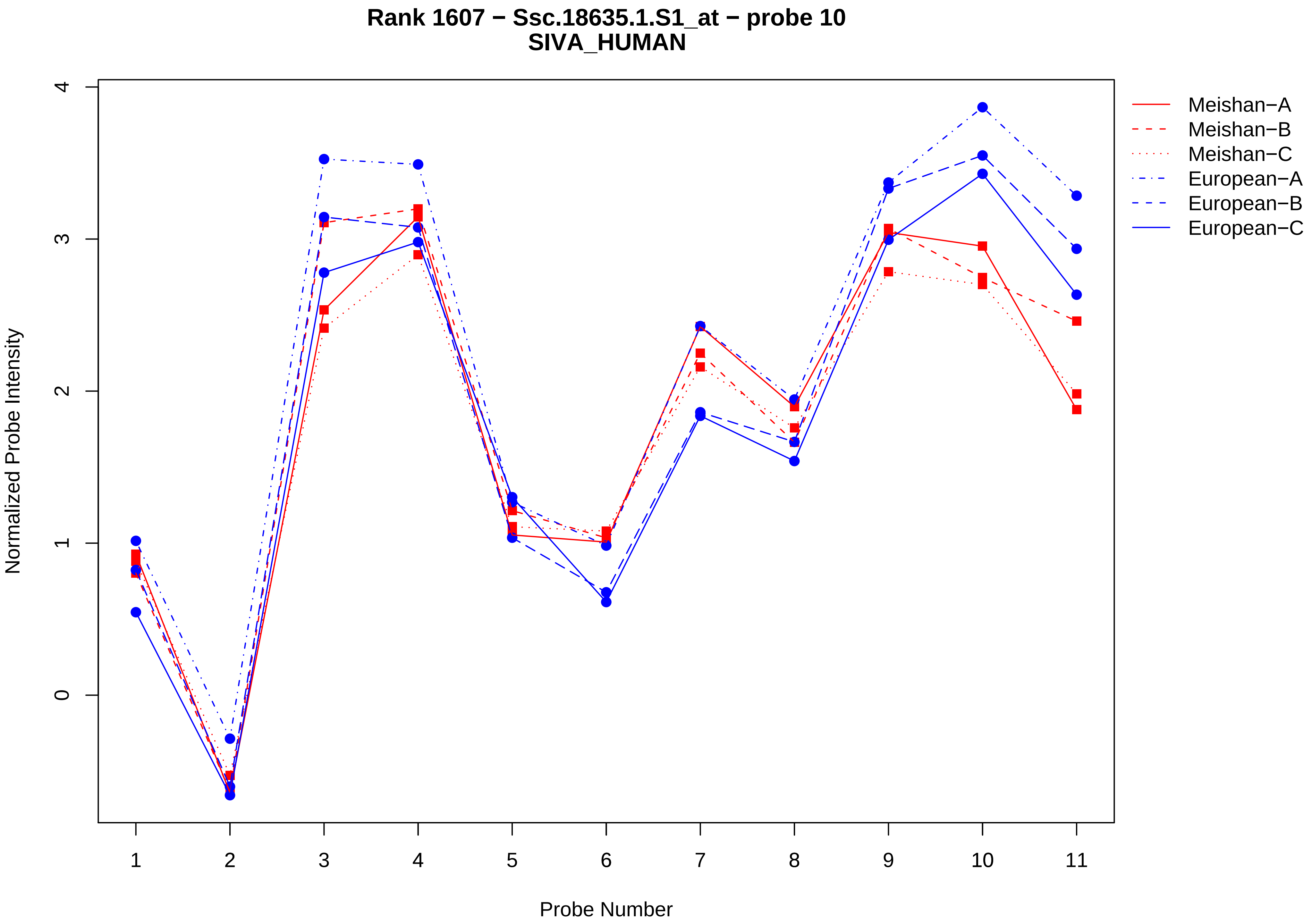

Rank 1607 − Ssc.18635.1.S1_at − probe 10
SIVA_HUMAN
Normalized Probe Intensity
Probe Number
0
1
2
3
4
1
2
3
4
5
6
7
8
9
10
11
Meishan−A
Meishan−B
Meishan−C
European−A
European−B
European−C

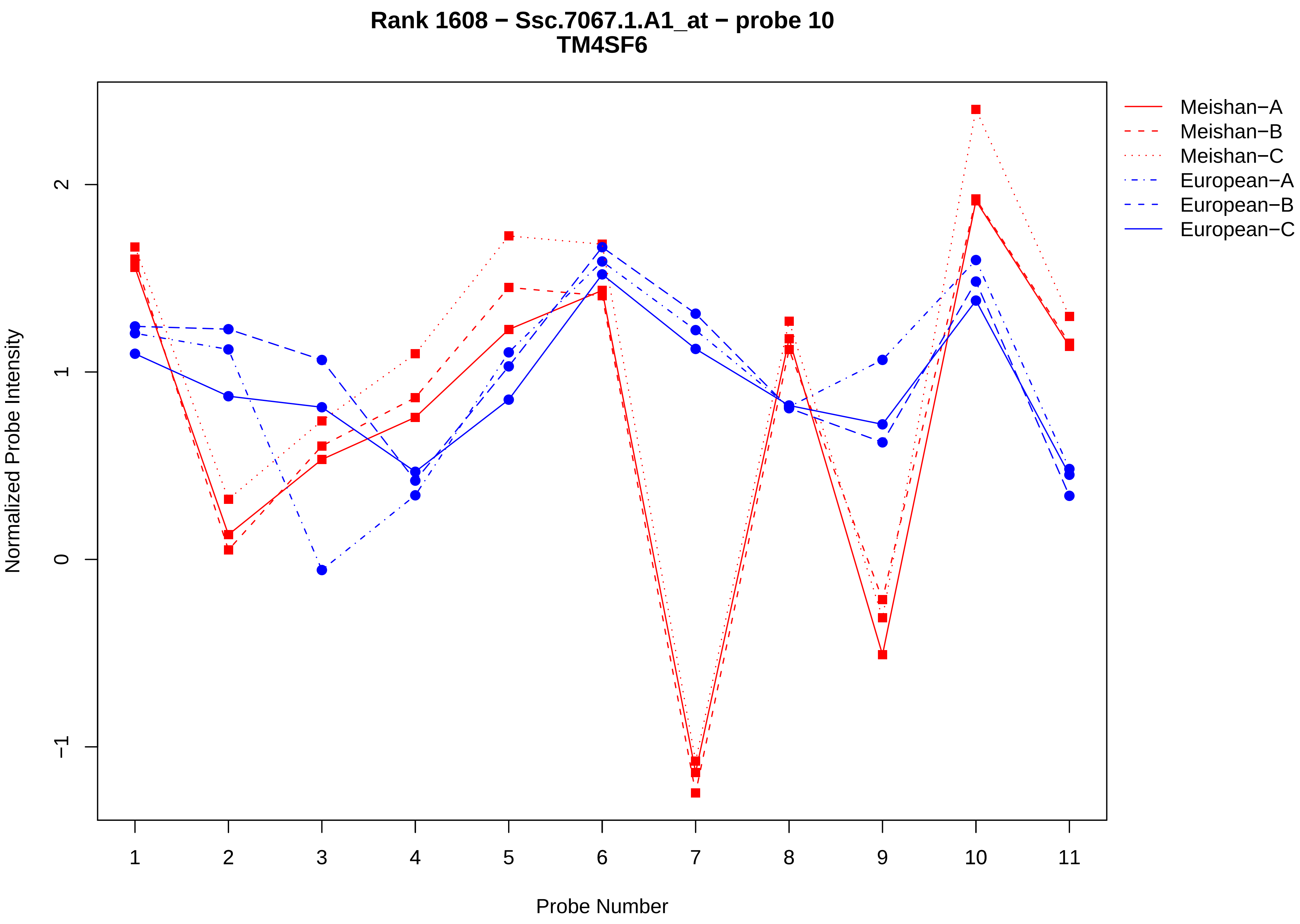

Rank 1608 − Ssc.7067.1.A1_at − probe 10
TM4SF6
Normalized Probe Intensity
Probe Number
Meishan−A
Meishan−B
Meishan−C
European−A
European−B
European−C
−1
0
1
2
1
2
3
4
5
6
7
8
9
10
11

Rank 1609 − Ssc.7450.1.A1_at − probe 7
MRPL35

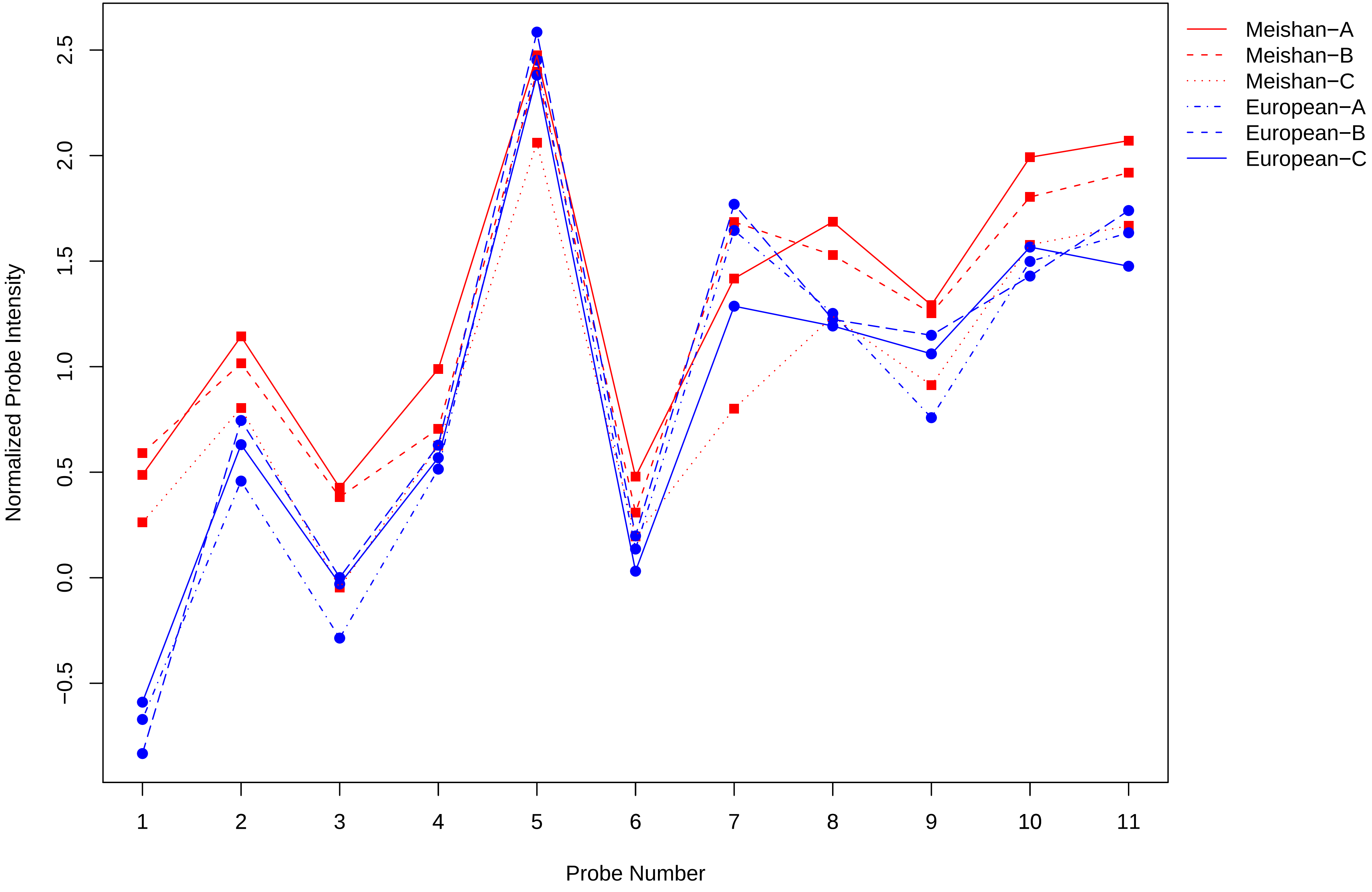

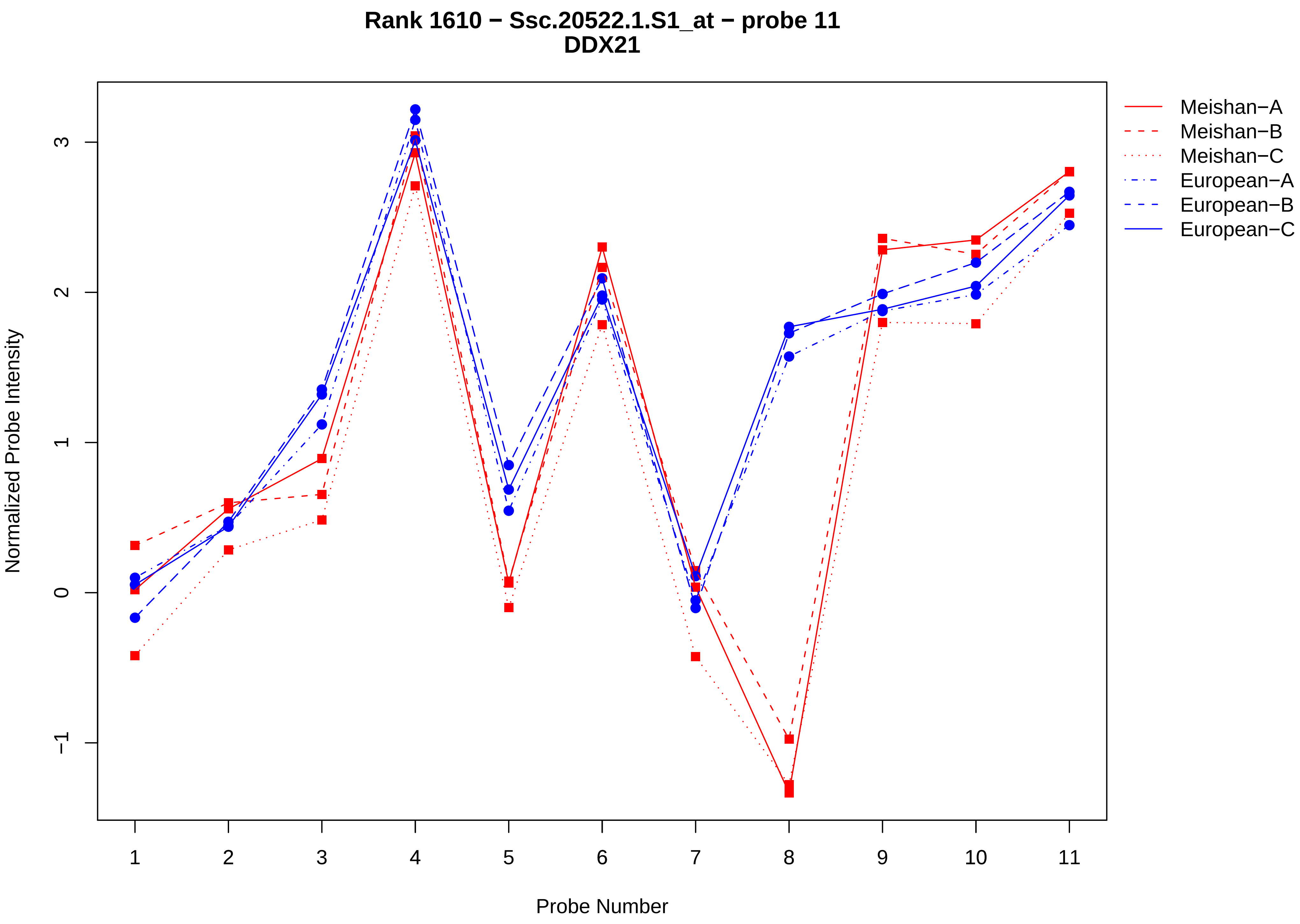

Rank 1610 − Ssc.20522.1.S1_at − probe 11
DDX21
Normalized Probe Intensity
Probe Number
−1
0
1
2
3
1
2
3
4
5
6
7
8
9
10
11
Meishan−A
Meishan−B
Meishan−C
European−A
European−B
European−C

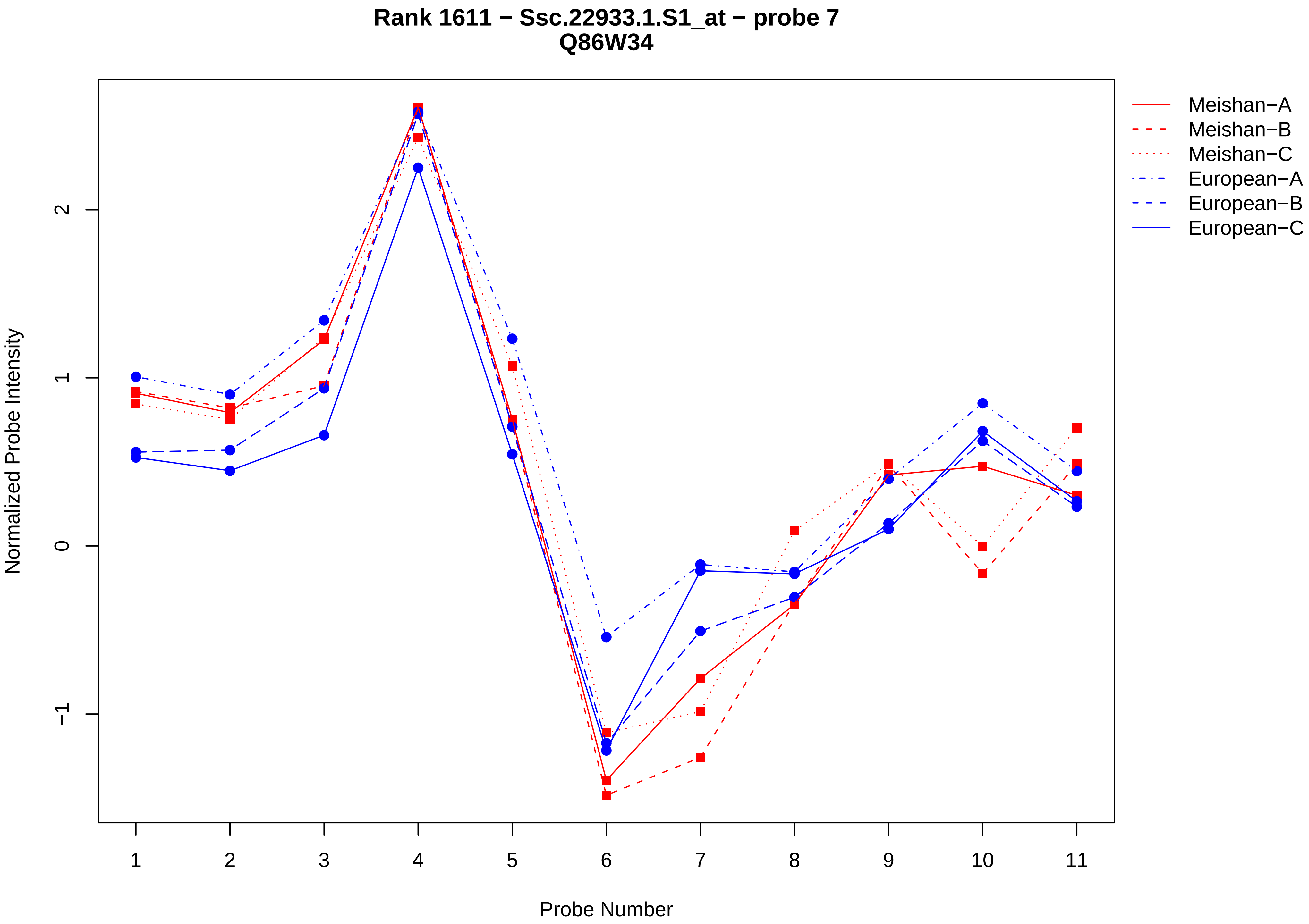

Rank 1611 − Ssc.22933.1.S1_at − probe 7
Q86W34
Normalized Probe Intensity
Probe Number
−1
0
1
2
1
2
3
4
5
6
7
8
9
10
11
Meishan−A
Meishan−B
Meishan−C
European−A
European−B
European−C

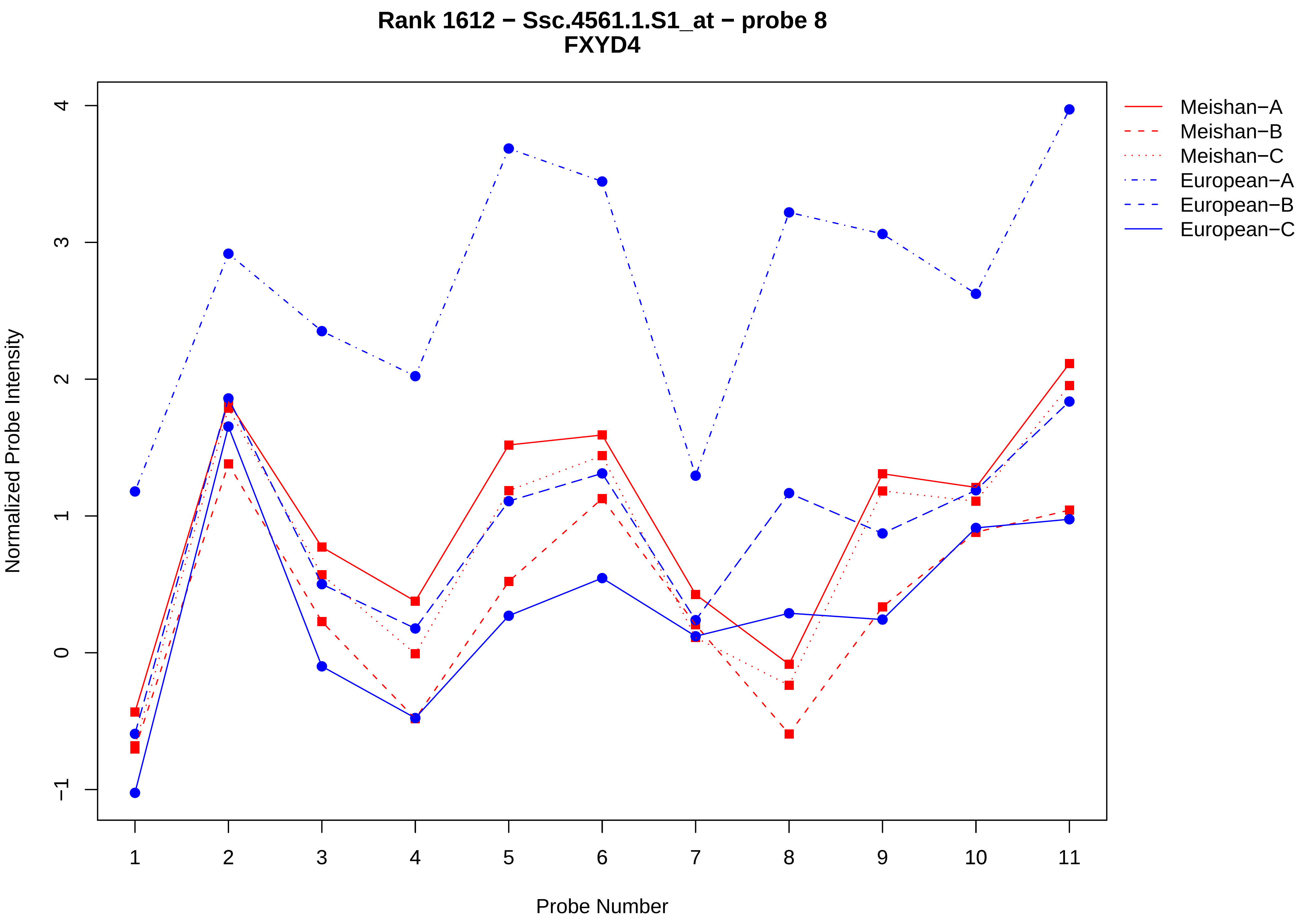

Rank 1612 − Ssc.4561.1.S1_at − probe 8
FXYD4
Normalized Probe Intensity
Probe Number
−1
0
1
2
3
4
1
2
3
4
5
6
7
8
9
10
11
Meishan−A
Meishan−B
Meishan−C
European−A
European−B
European−C

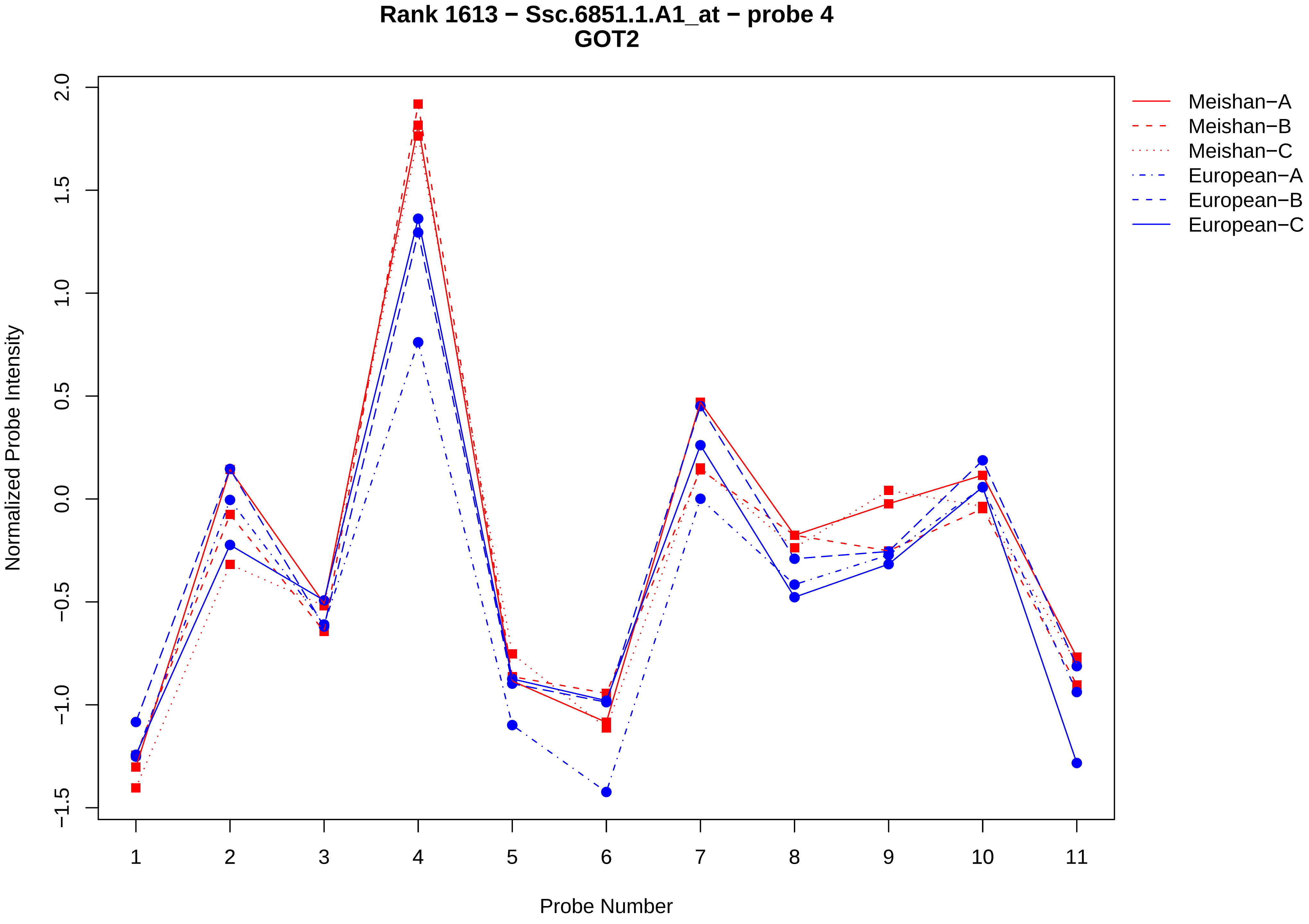

Rank 1613 − Ssc.6851.1.A1_at − probe 4
GOT2
Normalized Probe Intensity
Probe Number
2.0
1.5
1.0
0.5
0.0
−0.5
−1.0
−1.5
1
2
3
4
5
6
7
8
9
10
11
Meishan−A
Meishan−B
Meishan−C
European−A
European−B
European−C

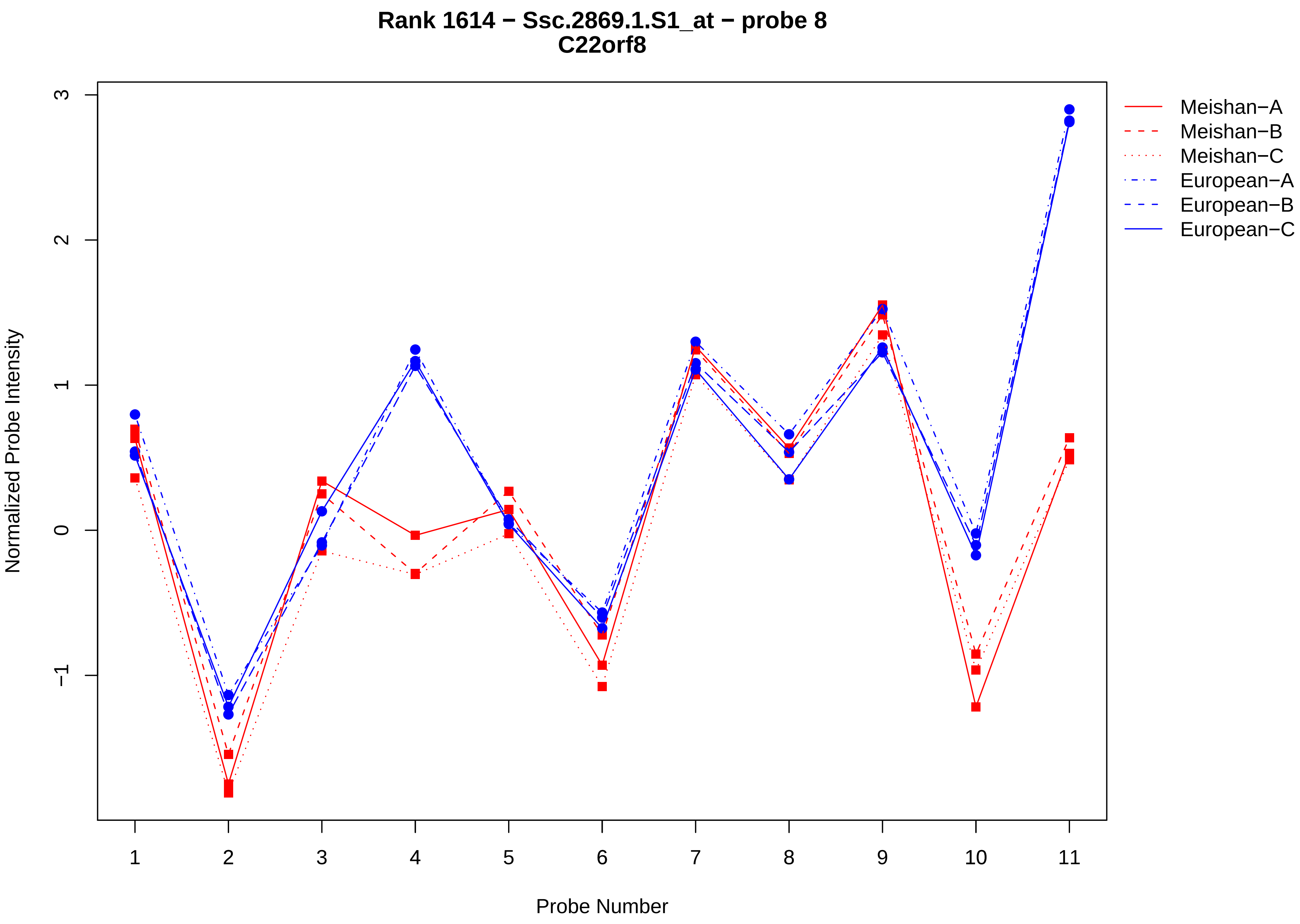

Rank 1614 − Ssc.2869.1.S1_at − probe 8
C22orf8
Normalized Probe Intensity
Probe Number
Meishan−A
Meishan−B
Meishan−C
European−A
European−B
European−C
−1
0
1
2
3
1
2
3
4
5
6
7
8
9
10
11

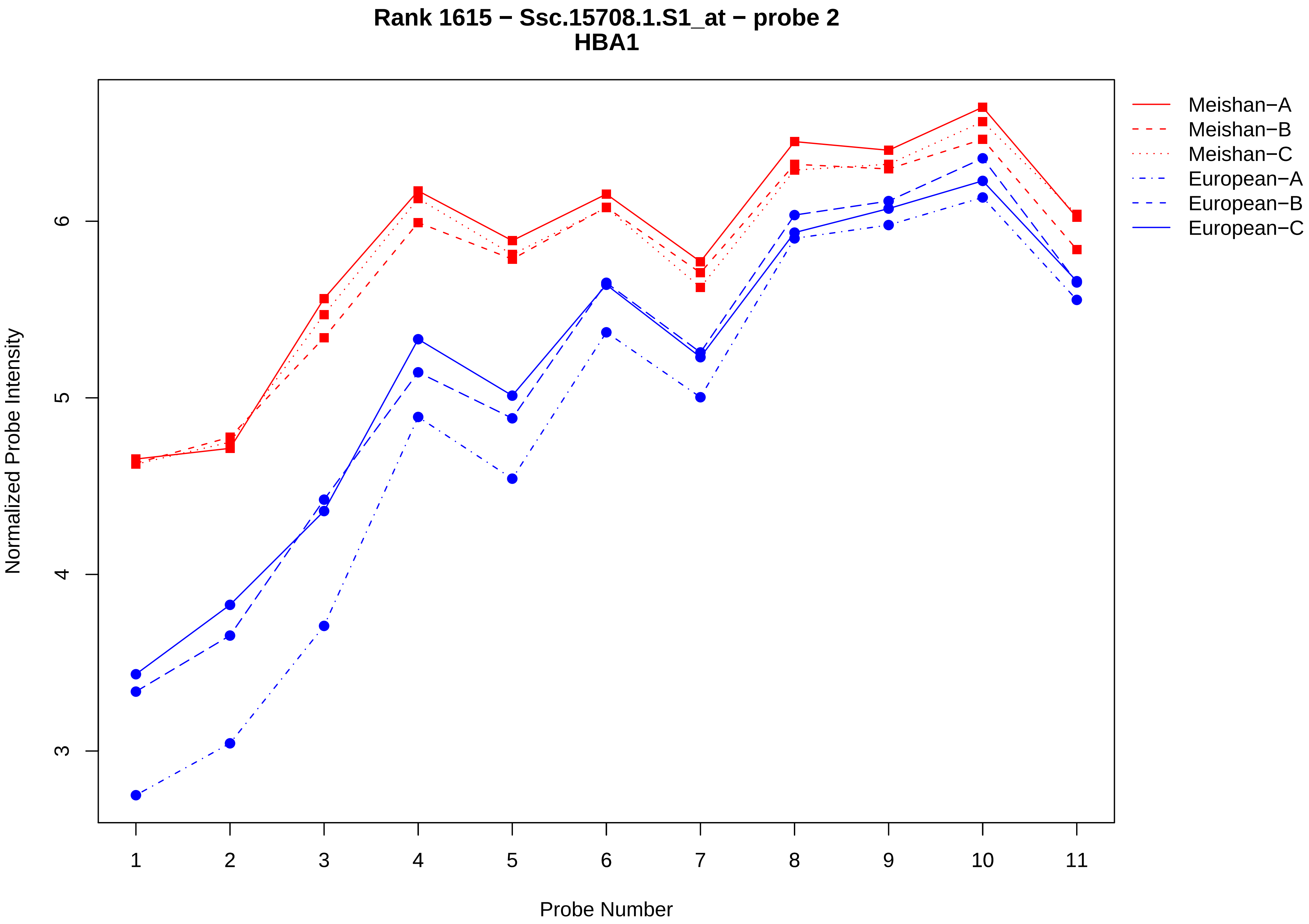
Rank 1615 − Ssc.15708.1.S1_at − probe 2
HBA1
Normalized Probe Intensity
3
4
5
6
Probe Number
1
2
3
4
5
6
7
8
9
10
11
Meishan−A
Meishan−B
Meishan−C
European−A
European−B
European−C

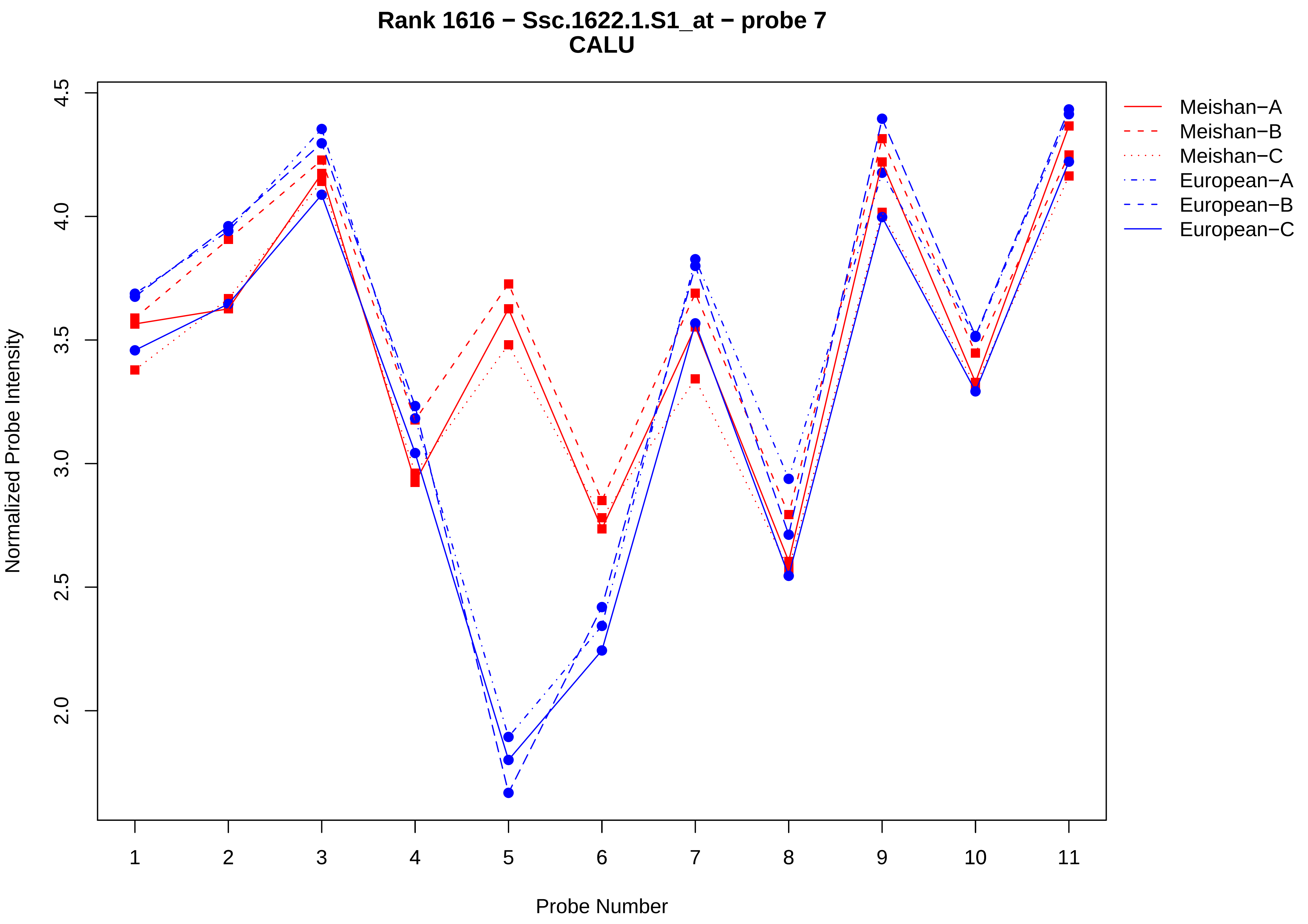

Rank 1616 − Ssc.1622.1.S1_at − probe 7
CALU
Normalized Probe Intensity
Probe Number
2.0
2.5
3.0
3.5
4.0
4.5
1
2
3
4
5
6
7
8
9
10
11
Meishan−A
Meishan−B
Meishan−C
European−A
European−B
European−C

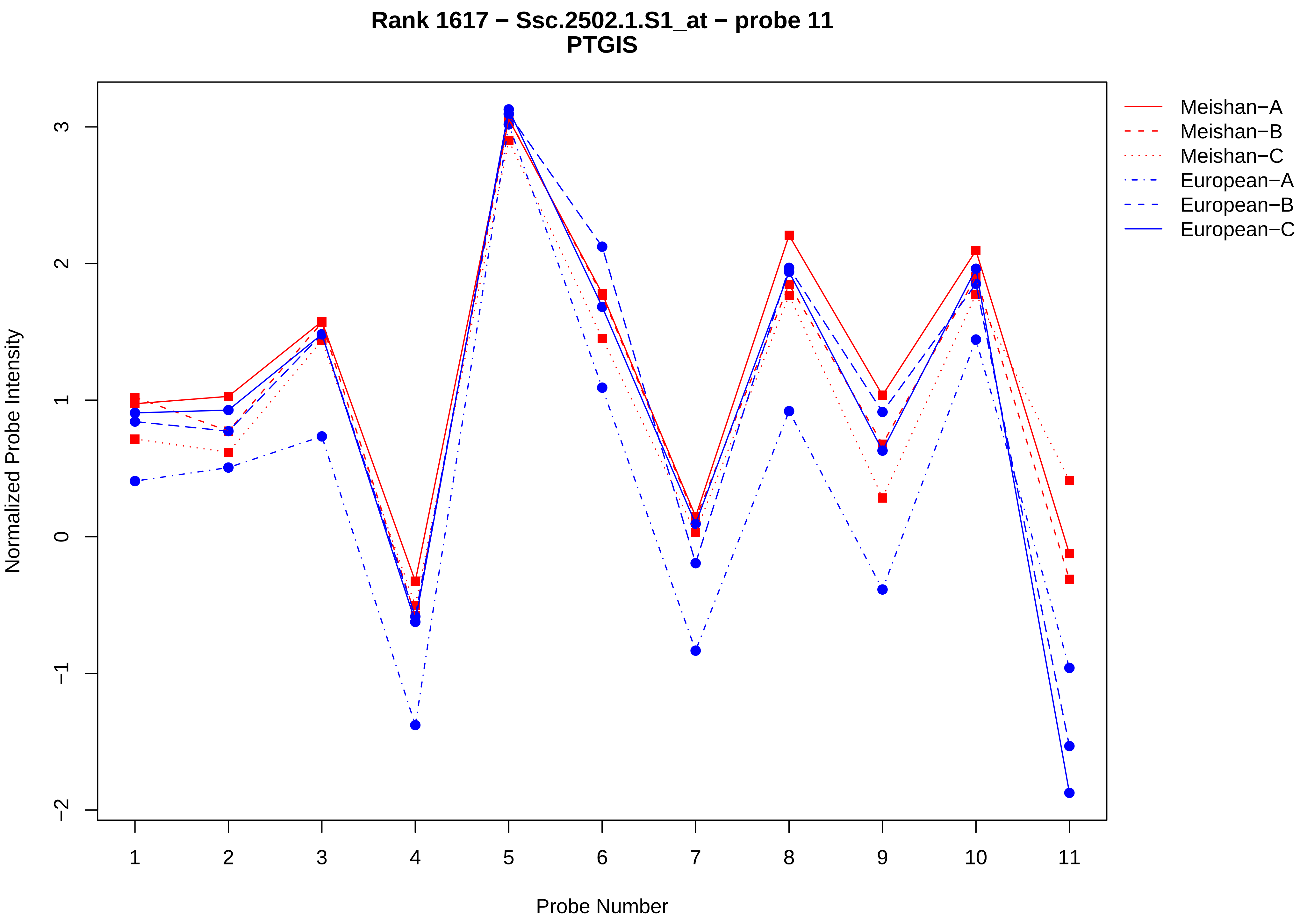
Rank 1617 − Ssc.2502.1.S1_at − probe 11
PTGIS
Normalized Probe Intensity
Probe Number
−2
−1
0
1
2
3
1
2
3
4
5
6
7
8
9
10
11
Meishan−A
Meishan−B
Meishan−C
European−A
European−B
European−C

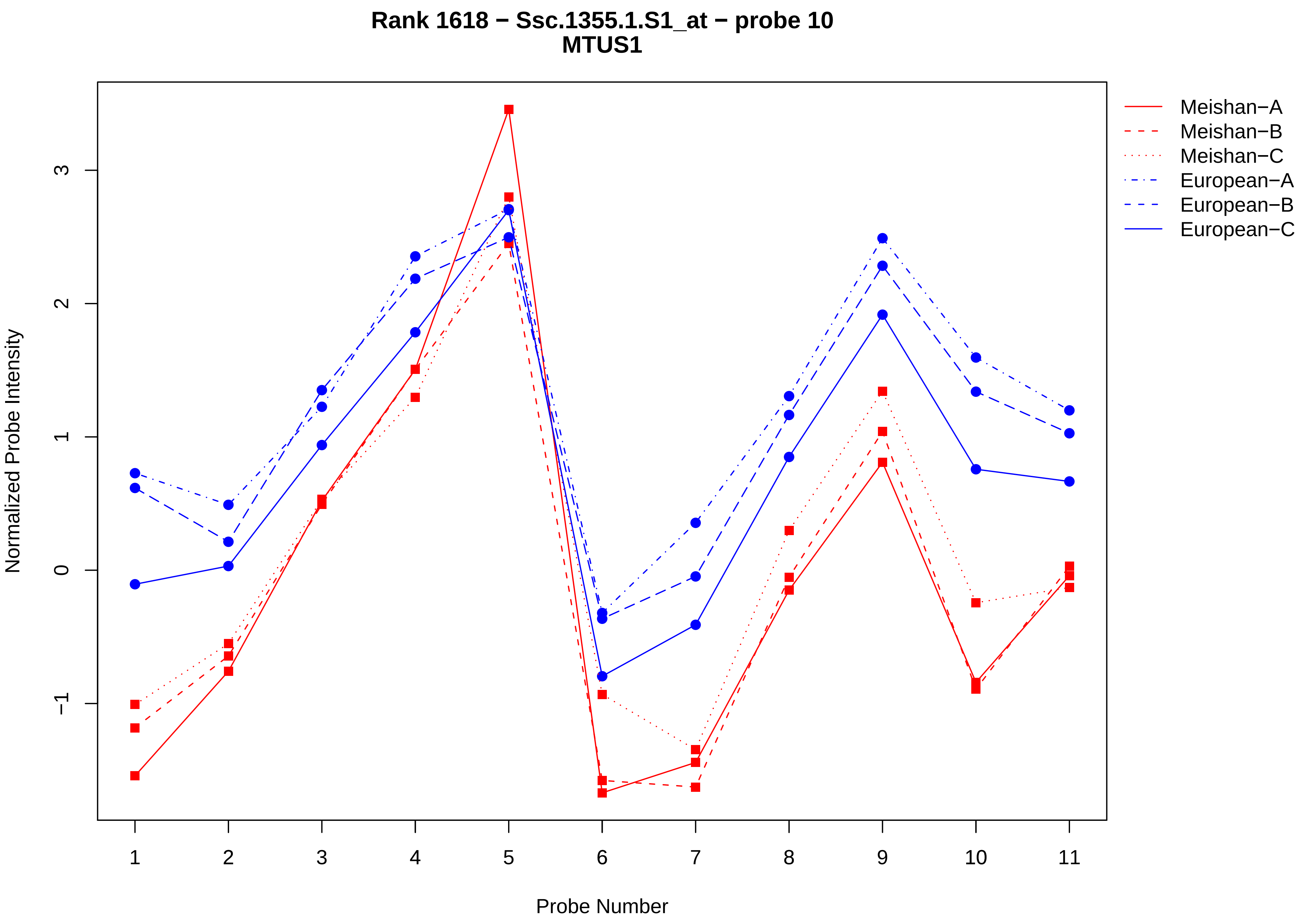

Rank 1618 − Ssc.1355.1.S1_at − probe 10
MTUS1
Normalized Probe Intensity
Probe Number
Meishan−A
Meishan−B
Meishan−C
European−A
European−B
European−C

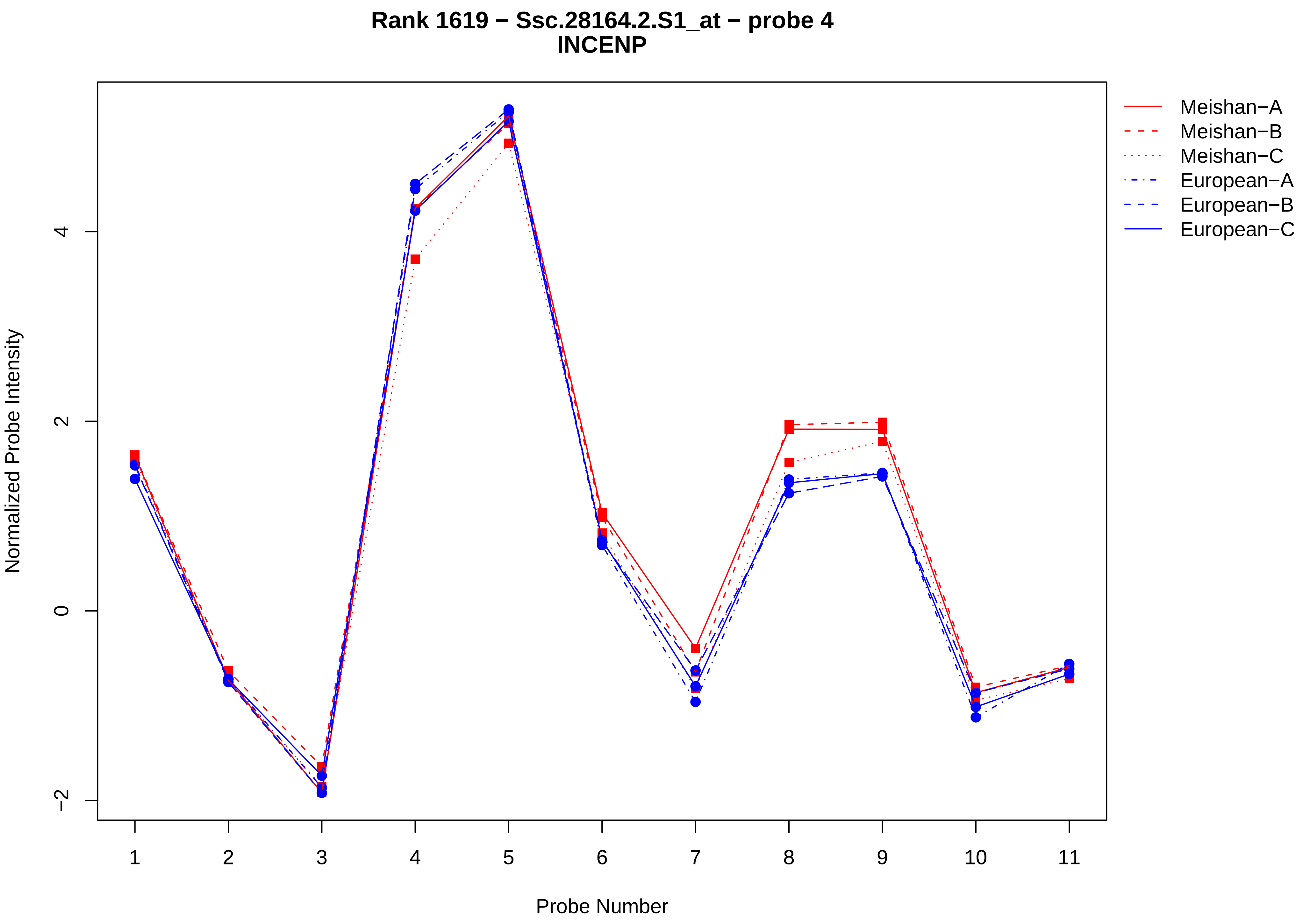

Rank 1619 − Ssc.28164.2.S1_at − probe 4
INCENP
Normalized Probe Intensity
Probe Number
Meishan−A
Meishan−B
Meishan−C
European−A
European−B
European−C
−2
0
2
4
1
2
3
4
5
6
7
8
9
10
11

Rank 1620 − Ssc.7604.2.A1_at − probe 3
C14orf111

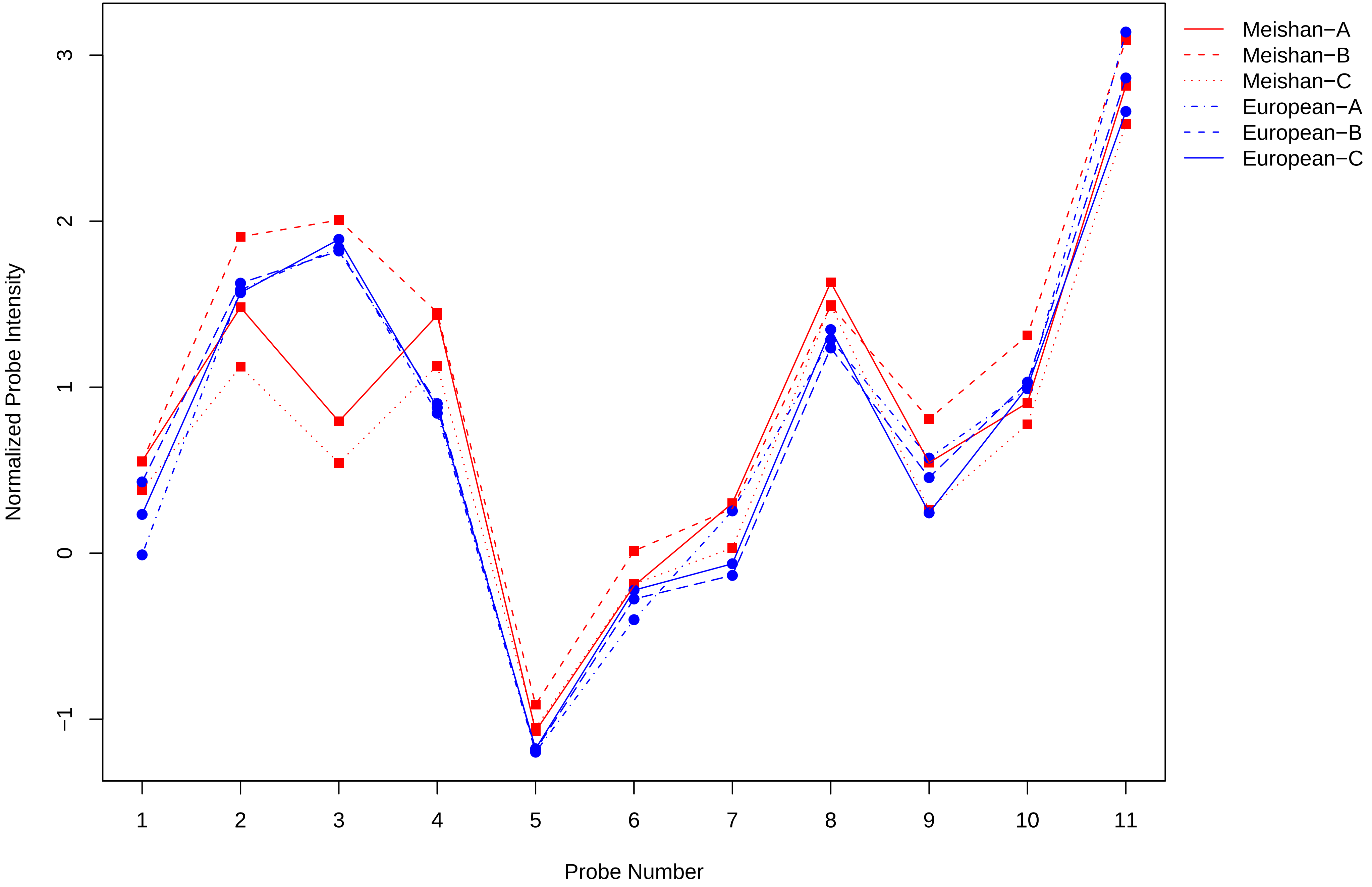

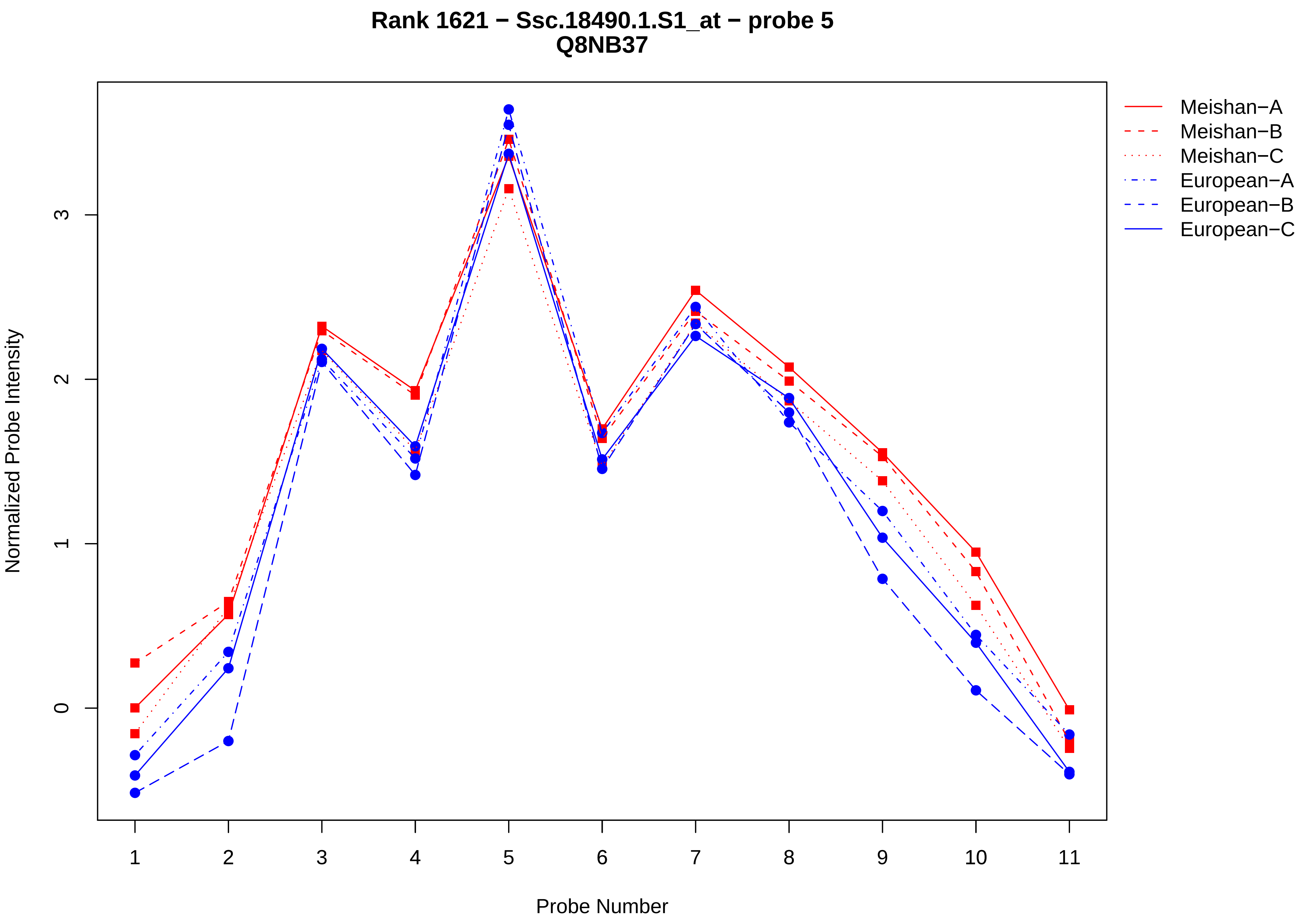

Rank 1621 − Ssc.18490.1.S1_at − probe 5
Q8NB37
Normalized Probe Intensity
Probe Number
0
1
2
3
1
2
3
4
5
6
7
8
9
10
11
Meishan−A
Meishan−B
Meishan−C
European−A
European−B
European−C

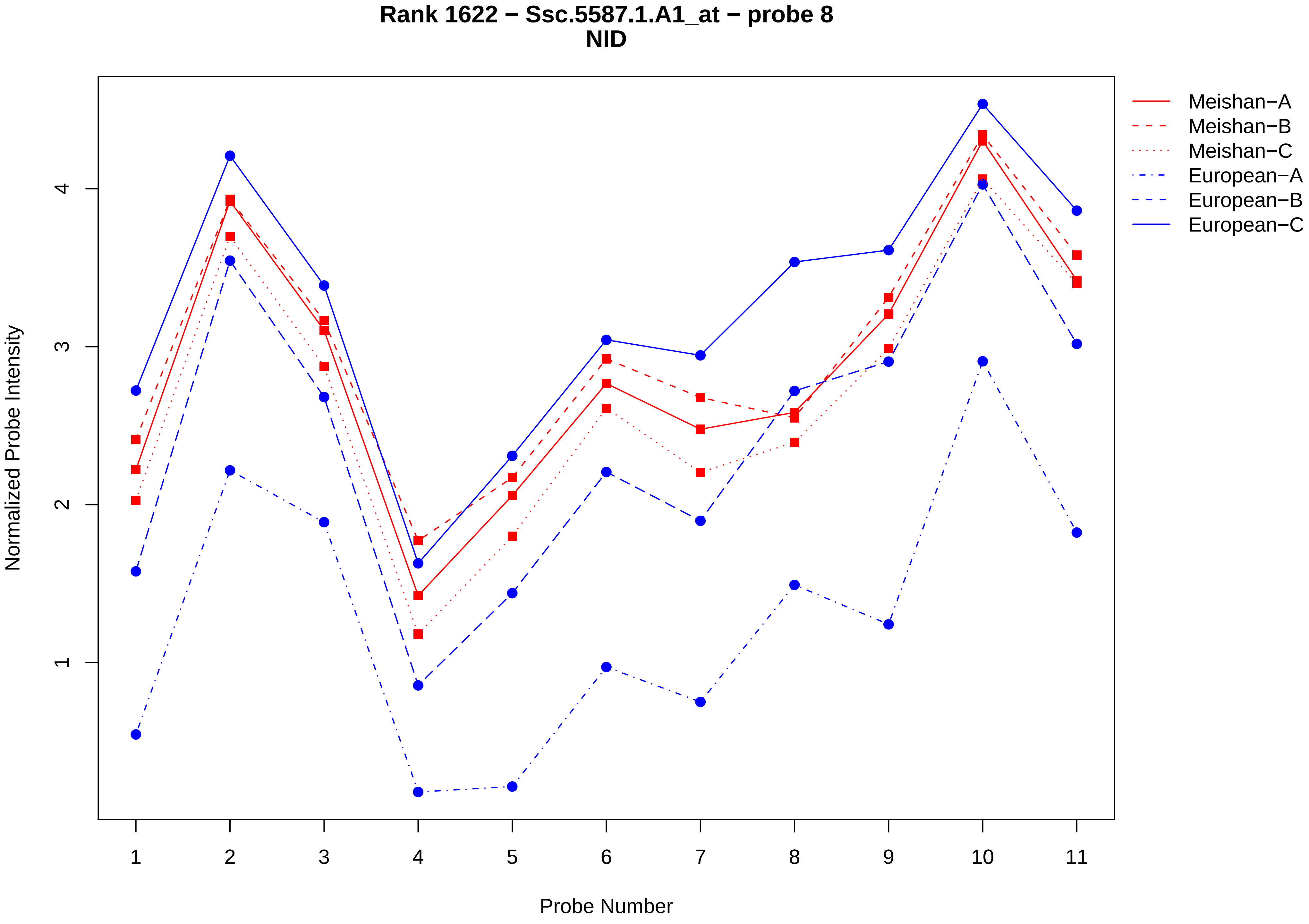

Rank 1622 − Ssc.5587.1.A1_at − probe 8
NID
Normalized Probe Intensity
Probe Number
1
2
3
4
1
2
3
4
5
6
7
8
9
10
11
Meishan−A
Meishan−B
Meishan−C
European−A
European−B
European−C

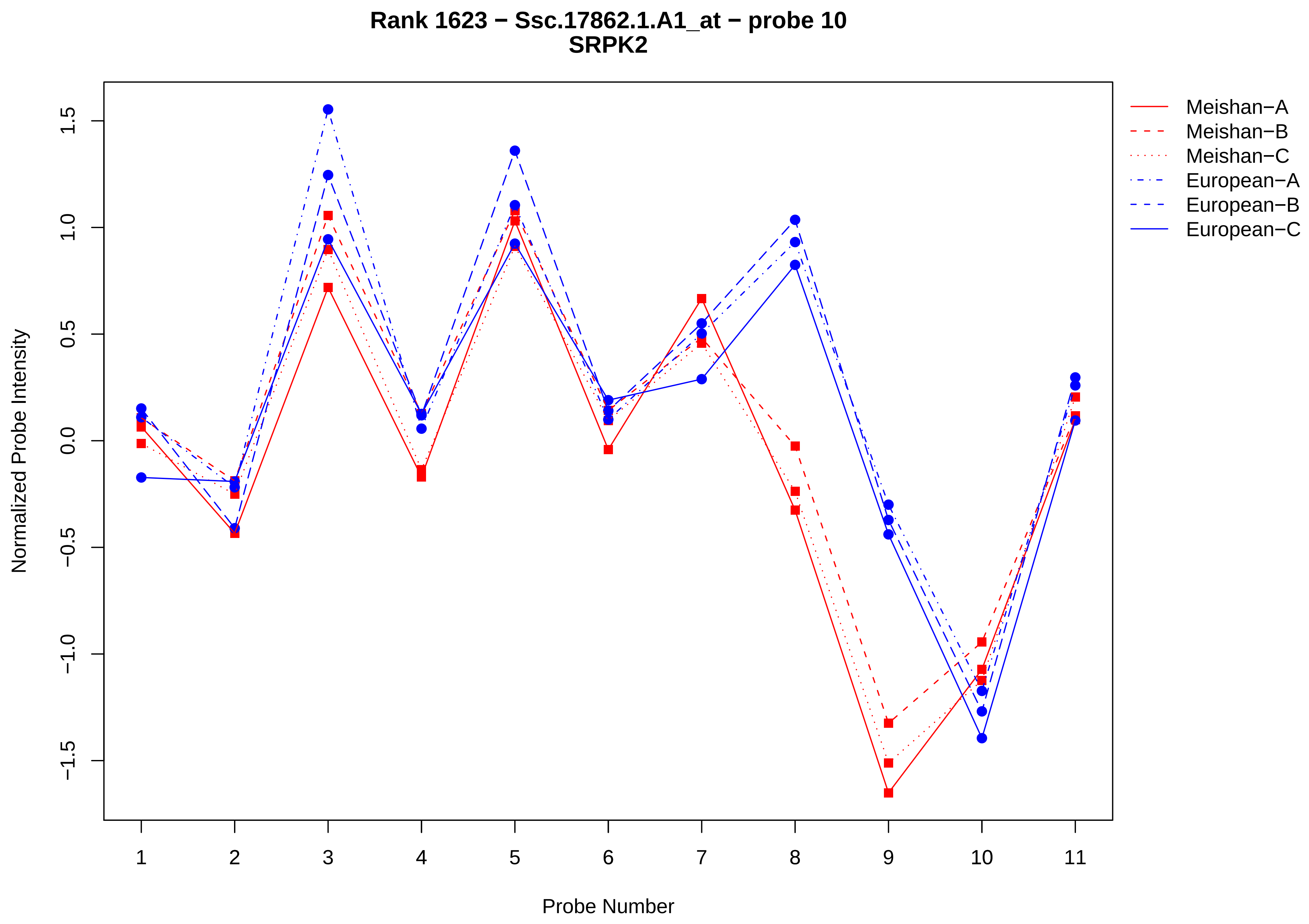
Rank 1623 − Ssc.17862.1.A1_at − probe 10
SRPK2
Normalized Probe Intensity
Probe Number
−1.5
−1.0
−0.5
0.0
0.5
1.0
1.5
1
2
3
4
5
6
7
8
9
10
11
Meishan−A
Meishan−B
Meishan−C
European−A
European−B
European−C

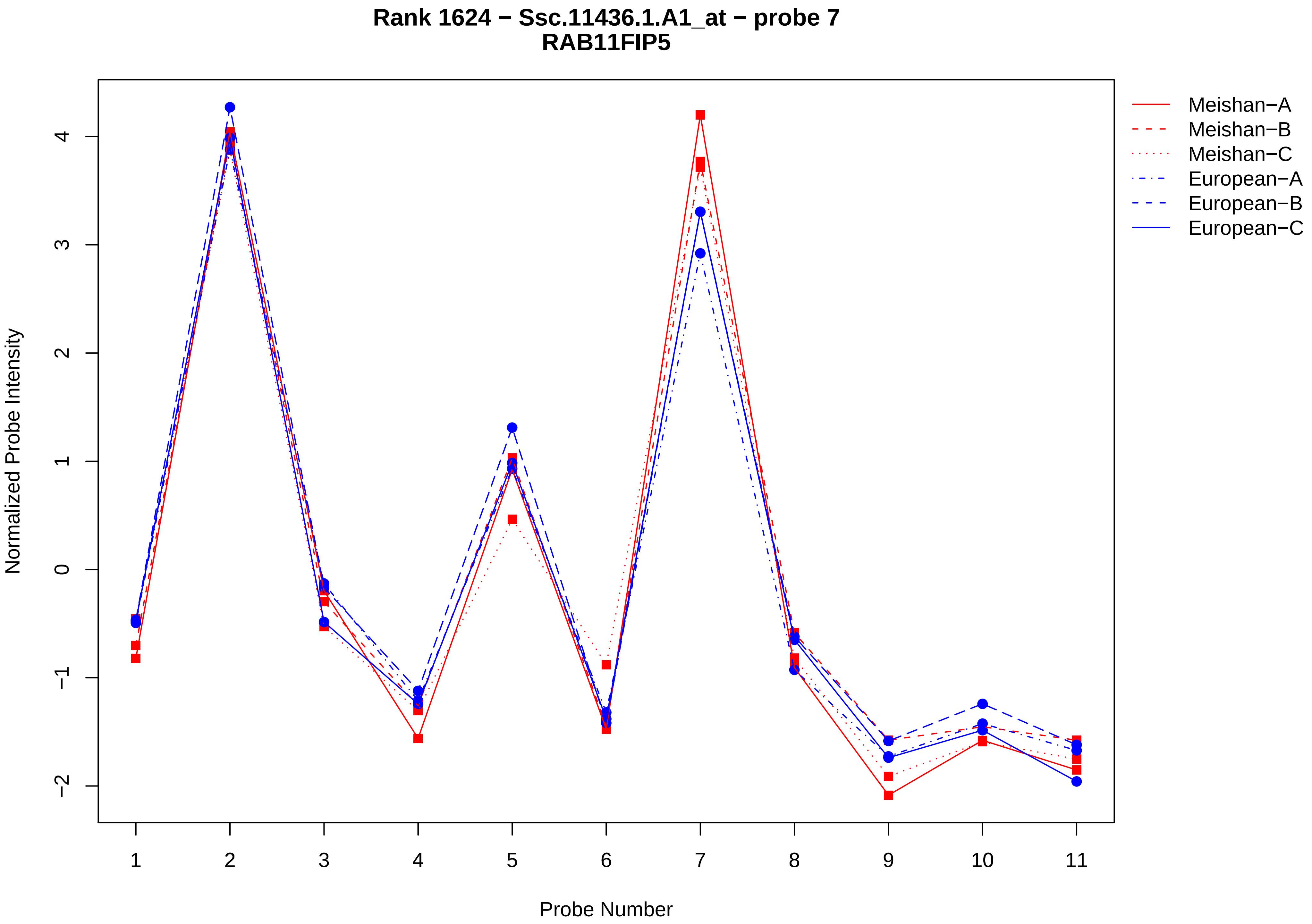

Rank 1624 − Ssc.11436.1.A1_at − probe 7
RAB11FIP5
Normalized Probe Intensity
Probe Number
−2
−1
0
1
2
3
4
1
2
3
4
5
6
7
8
9
10
11
Meishan−A
Meishan−B
Meishan−C
European−A
European−B
European−C

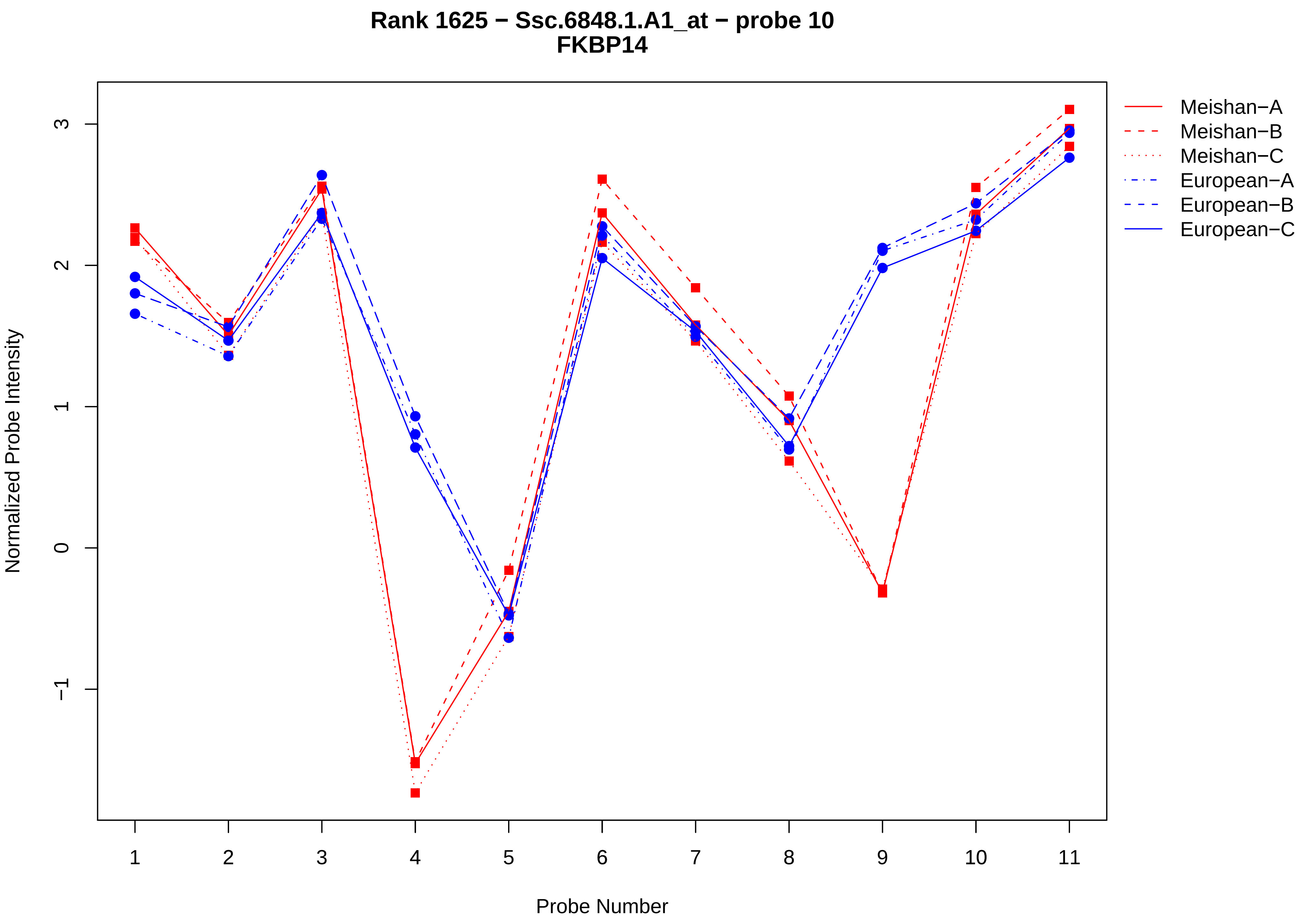

Rank 1625 − Ssc.6848.1.A1_at − probe 10
FKBP14
Normalized Probe Intensity
Probe Number
−1
0
1
2
3
1
2
3
4
5
6
7
8
9
10
11
Meishan−A
Meishan−B
Meishan−C
European−A
European−B
European−C

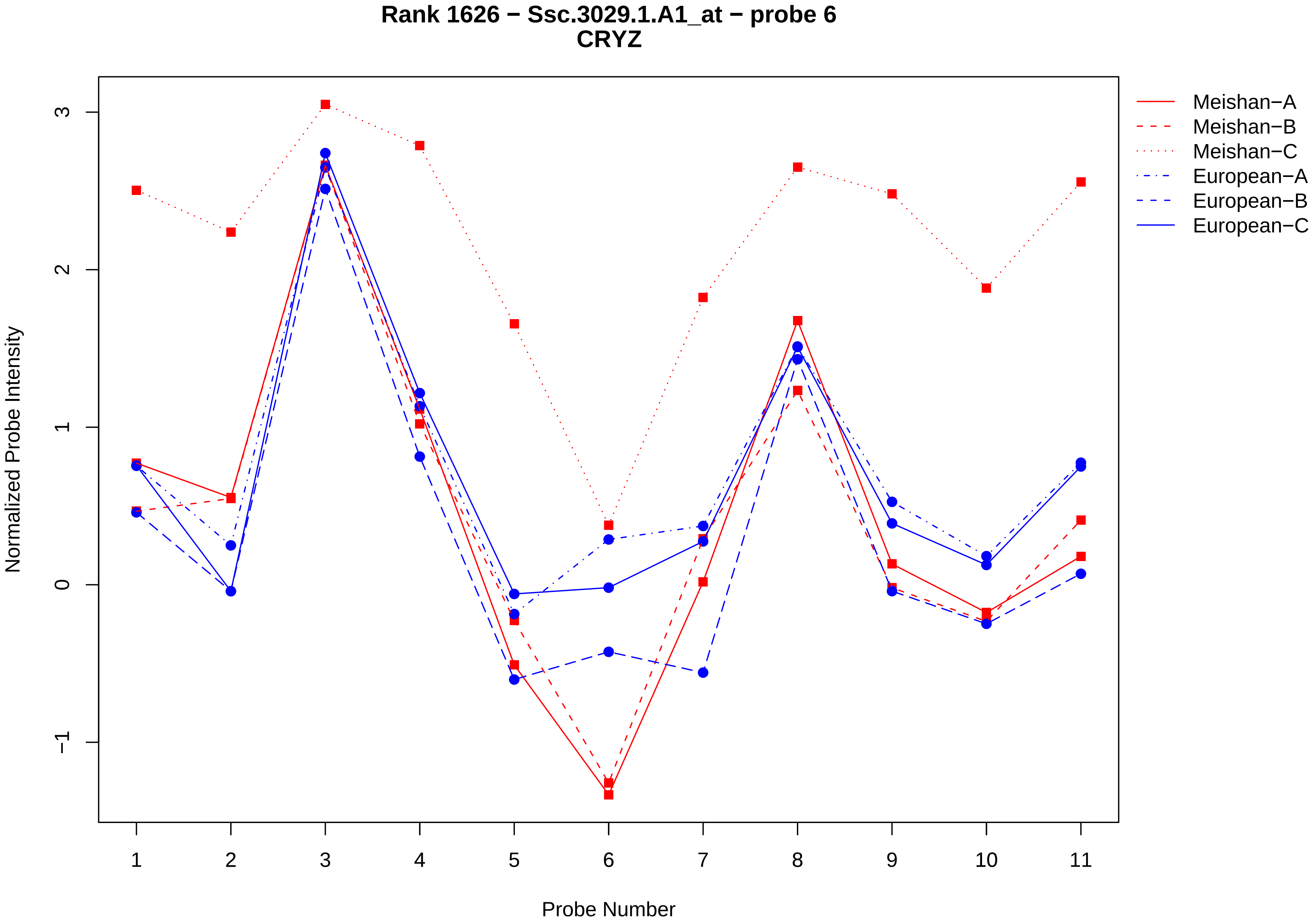

Rank 1626 − Ssc.3029.1.A1_at − probe 6
CRYZ
Normalized Probe Intensity
Probe Number
−1
0
1
2
3
1
2
3
4
5
6
7
8
9
10
11
Meishan−A
Meishan−B
Meishan−C
European−A
European−B
European−C

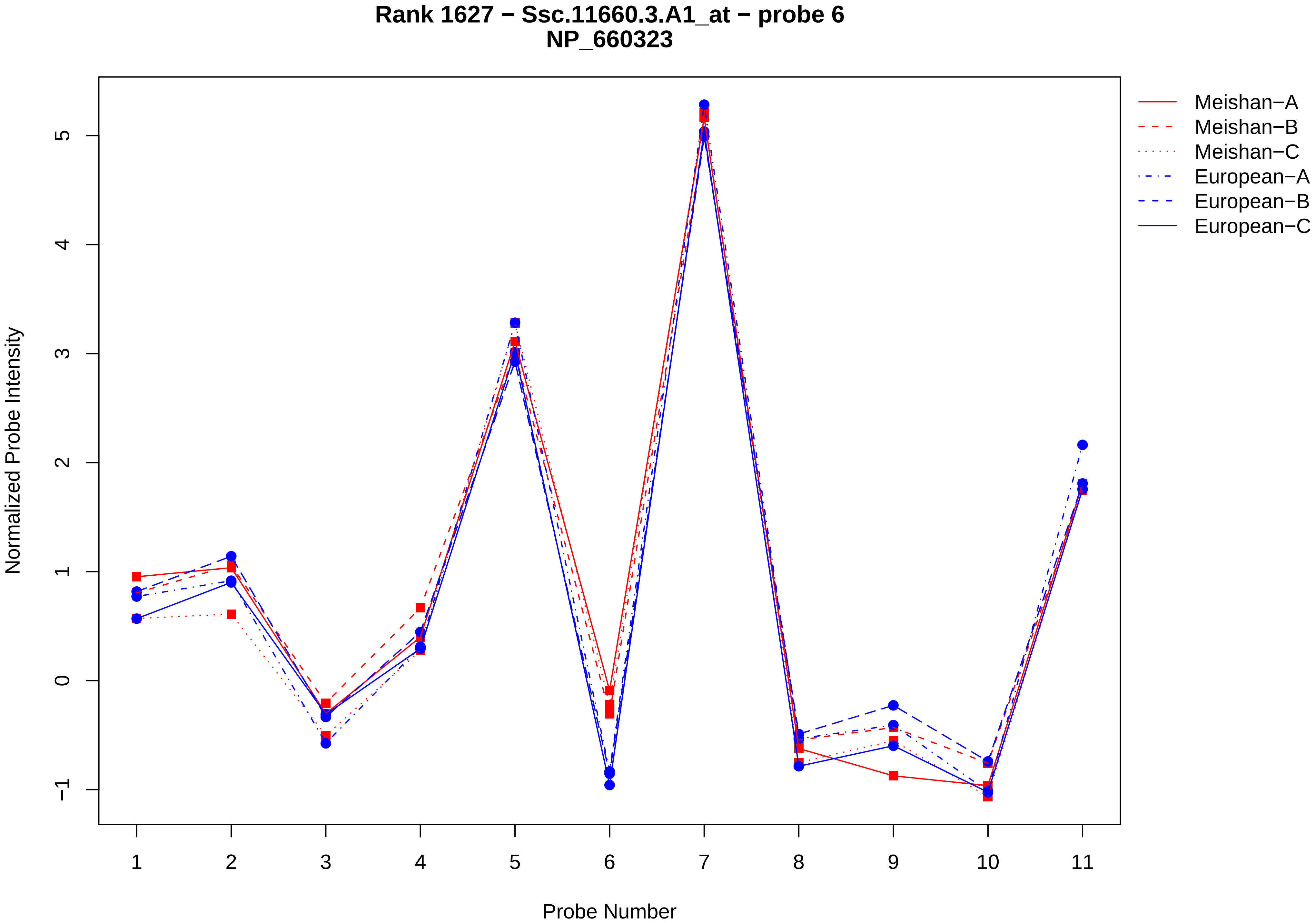
Rank 1627 − Ssc.11660.3.A1_at − probe 6
NP_660323
Normalized Probe Intensity
Probe Number
Meishan−A
Meishan−B
Meishan−C
European−A
European−B
European−C

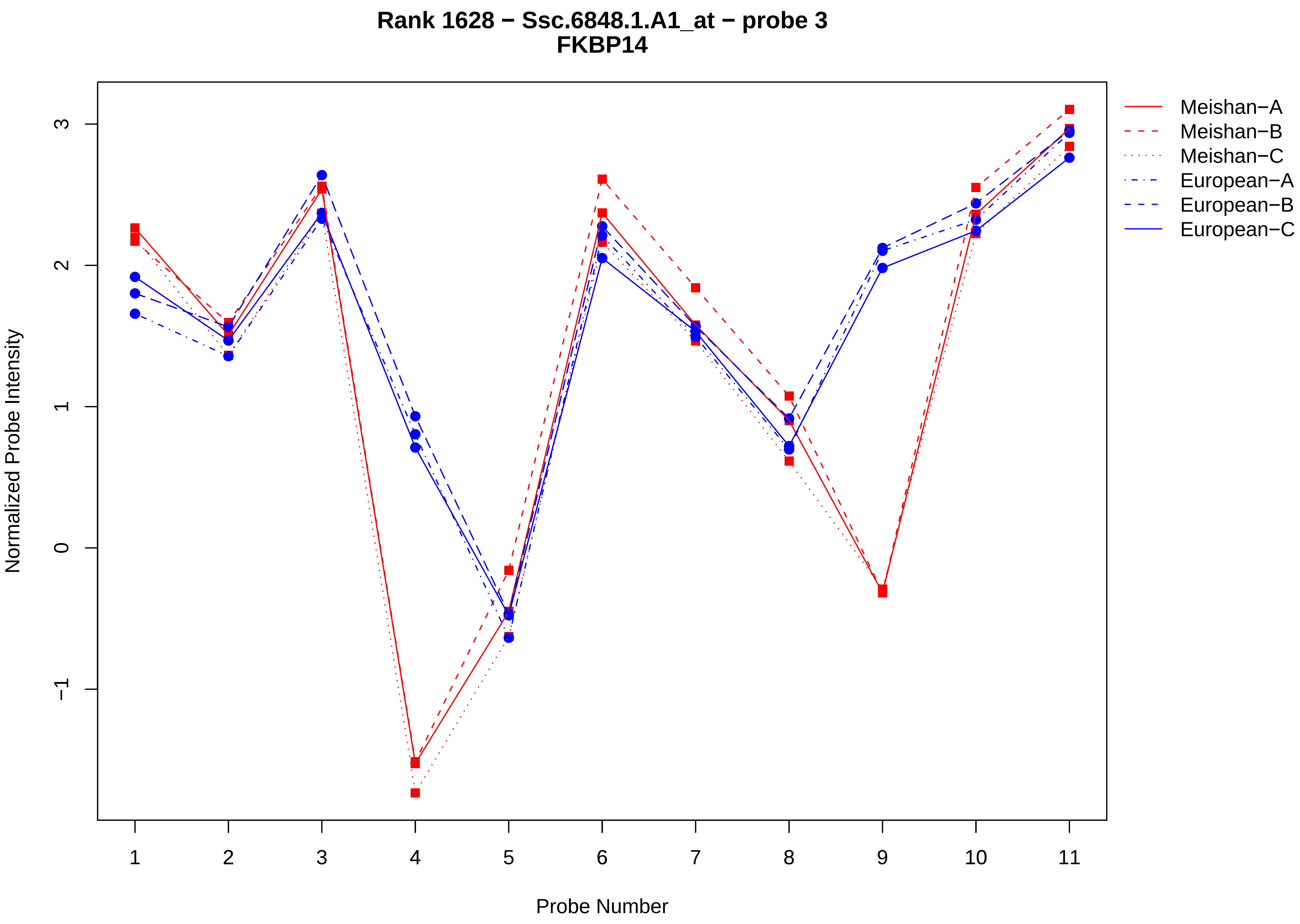

Rank 1628 − Ssc.6848.1.A1_at − probe 3
FKBP14
Normalized Probe Intensity
Probe Number
−1
0
1
2
3
1
2
3
4
5
6
7
8
9
10
11
Meishan−A
Meishan−B
Meishan−C
European−A
European−B
European−C

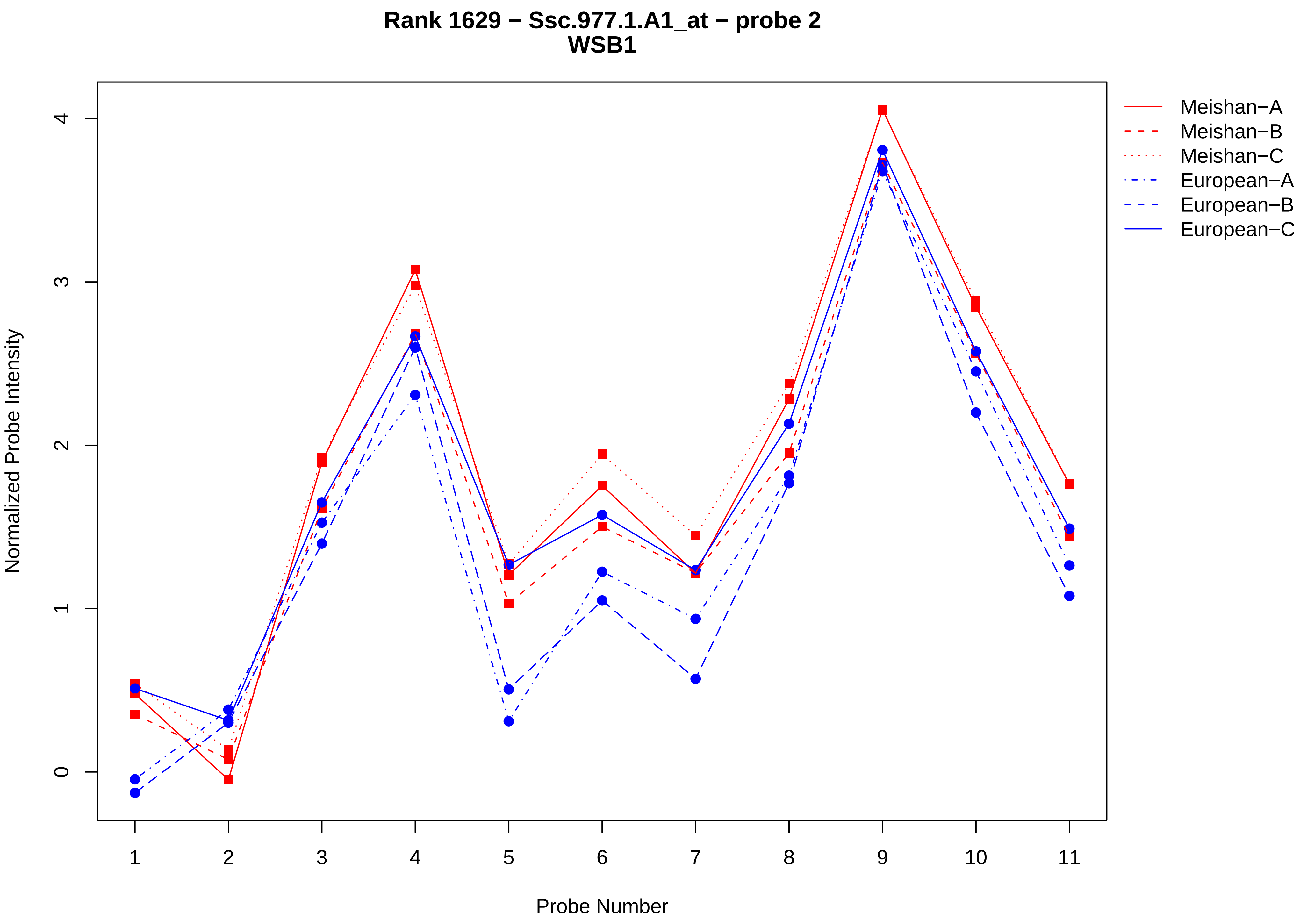

Rank 1629 − Ssc.977.1.A1_at − probe 2
WSB1
Normalized Probe Intensity
Probe Number
0
1
2
3
4
1
2
3
4
5
6
7
8
9
10
11
Meishan−A
Meishan−B
Meishan−C
European−A
European−B
European−C

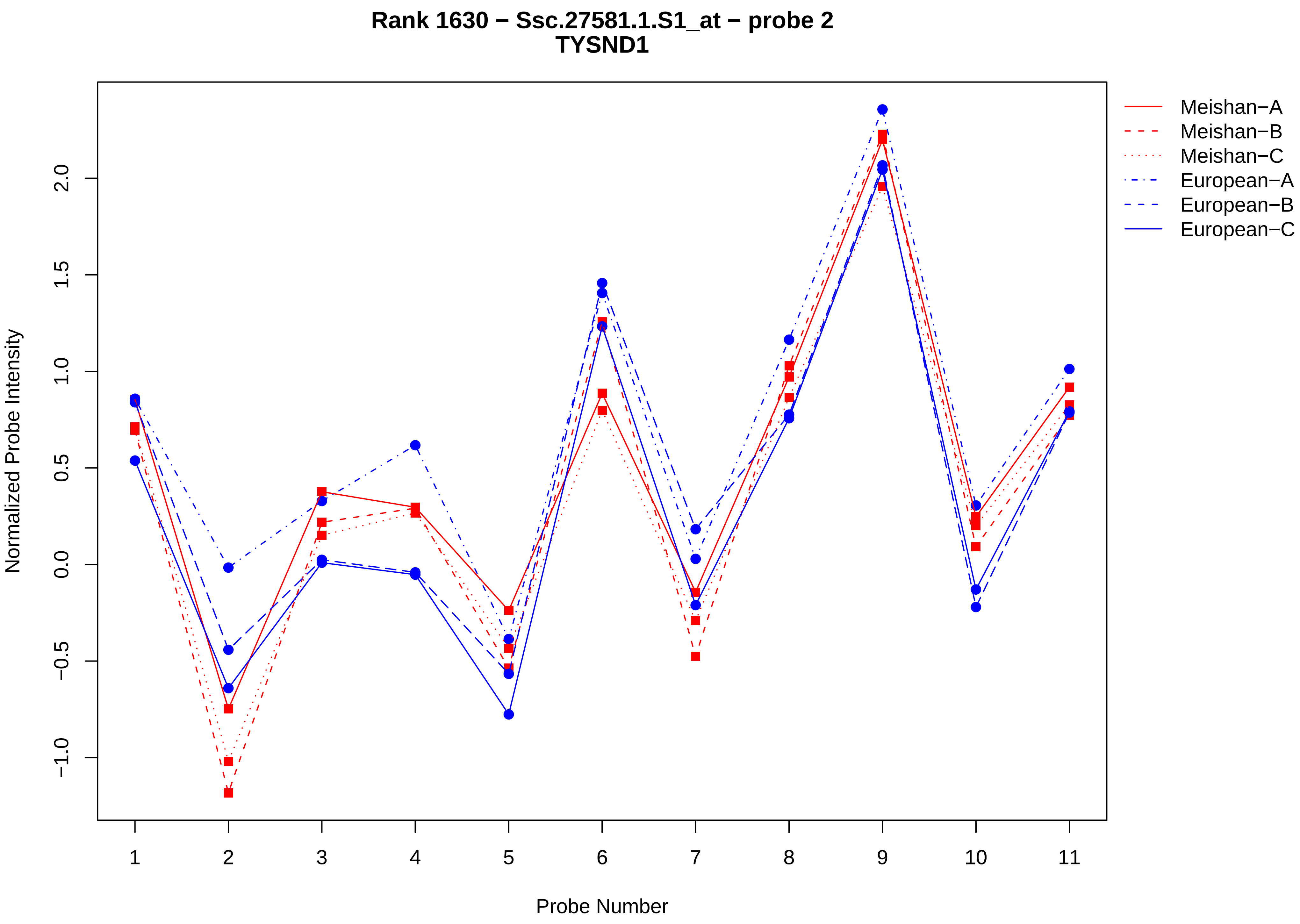

Rank 1630 − Ssc.27581.1.S1_at − probe 2
TYSND1
Normalized Probe Intensity
−1.0
−0.5
0.0
0.5
1.0
1.5
2.0
1
2
3
4
5
6
7
8
9
10
11
Probe Number
Meishan−A
Meishan−B
Meishan−C
European−A
European−B
European−C

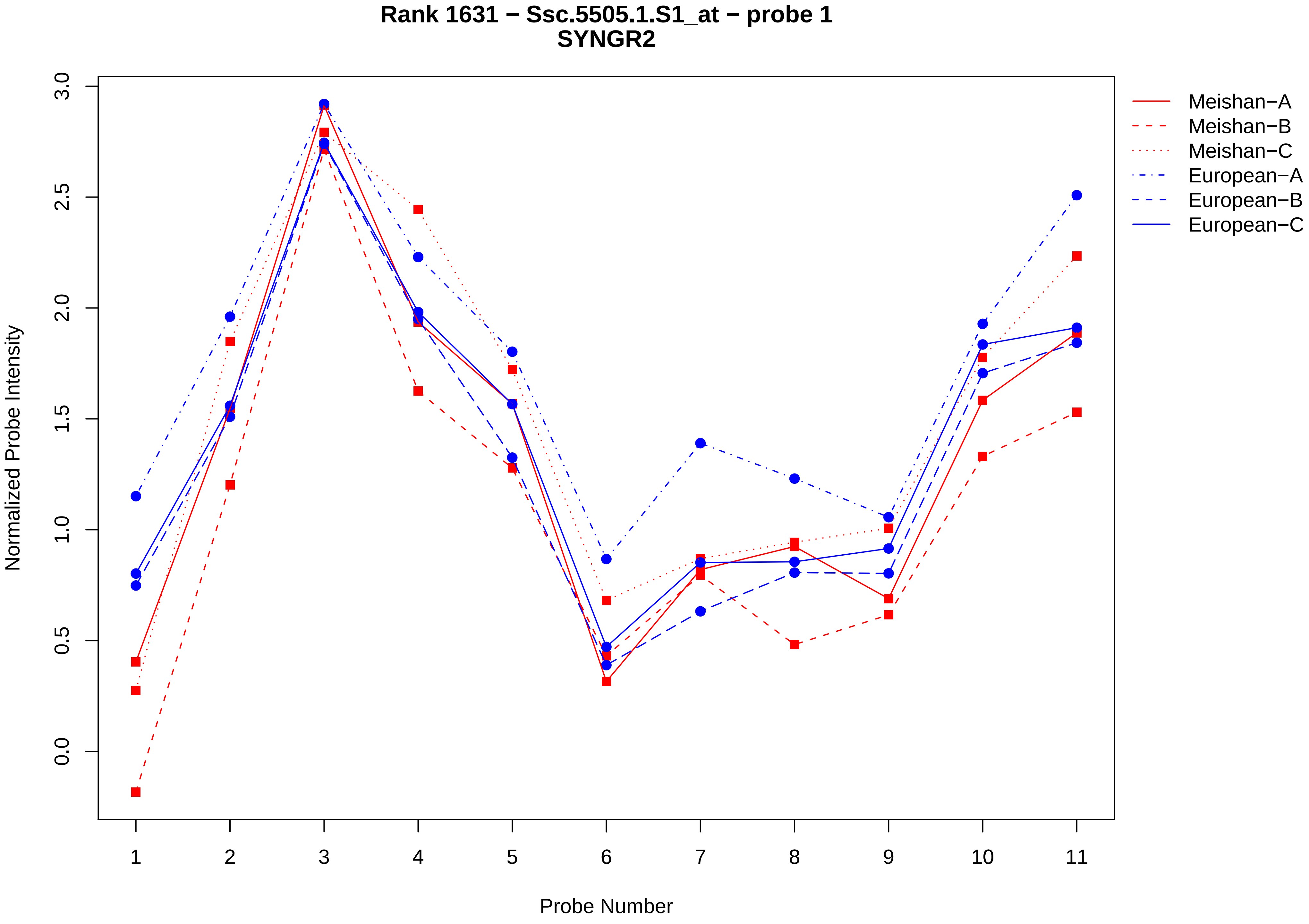

Rank 1631 − Ssc.5505.1.S1_at − probe 1
SYNGR2
Normalized Probe Intensity
Probe Number
0.0
0.5
1.0
1.5
2.0
2.5
3.0
1
2
3
4
5
6
7
8
9
10
11
Meishan−A
Meishan−B
Meishan−C
European−A
European−B
European−C

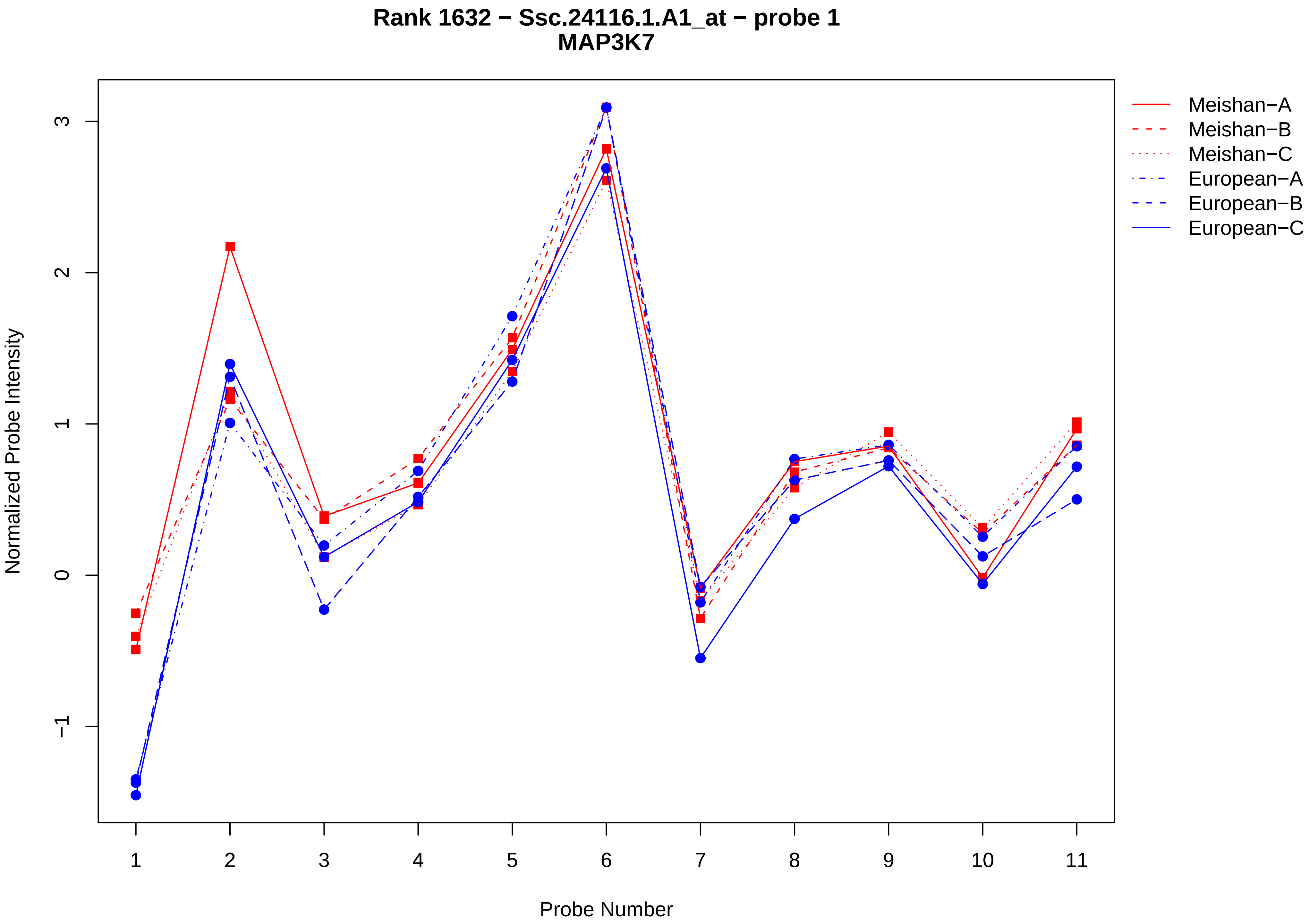

Rank 1632 − Ssc.24116.1.A1_at − probe 1
MAP3K7
Normalized Probe Intensity
Probe Number
−1
0
1
2
3
1
2
3
4
5
6
7
8
9
10
11
Meishan−A
Meishan−B
Meishan−C
European−A
European−B
European−C

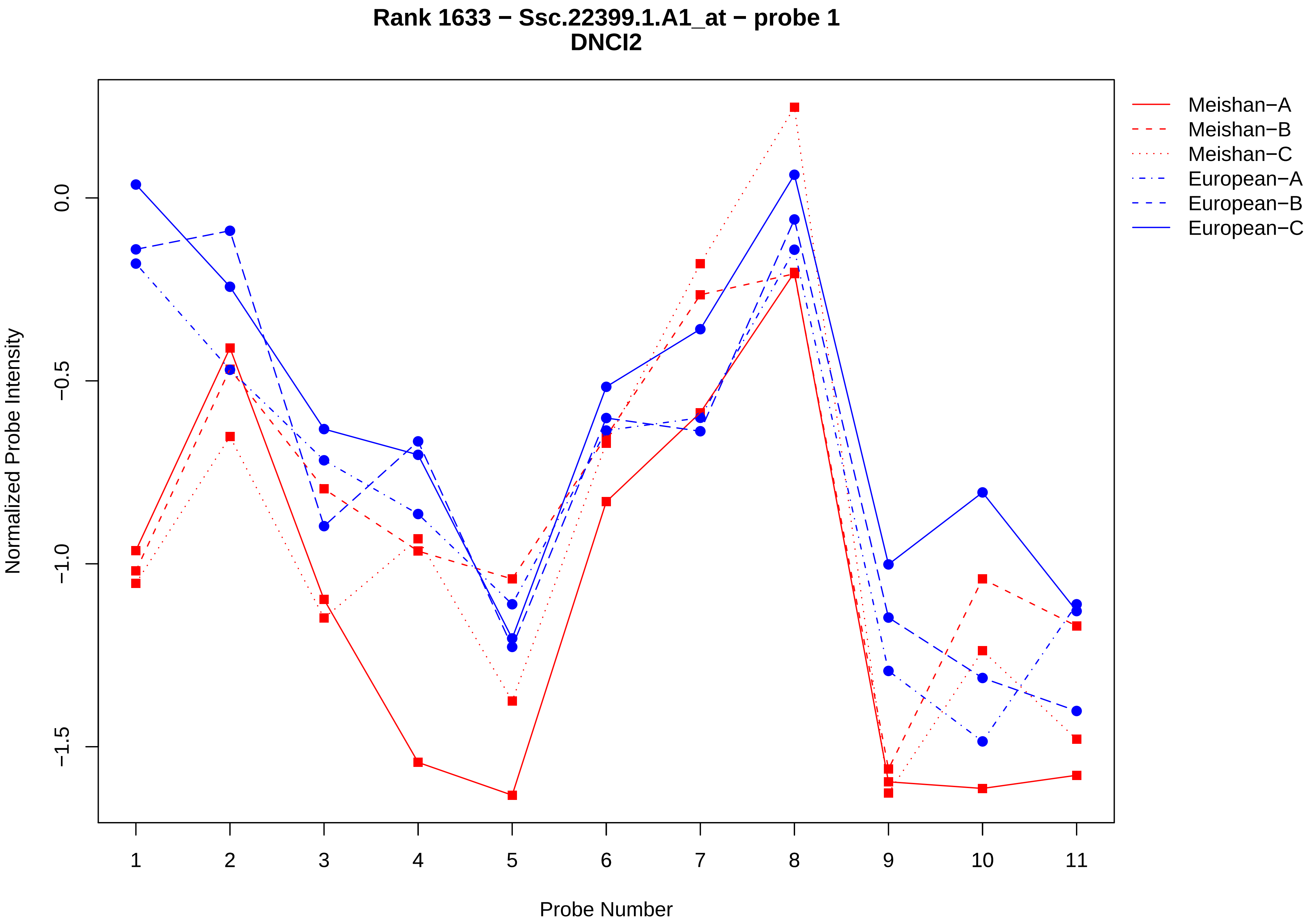

Rank 1633 − Ssc.22399.1.A1_at − probe 1
DNCI2
Normalized Probe Intensity
Probe Number
0.0
−0.5
−1.0
−1.5
1
2
3
4
5
6
7
8
9
10
11
Meishan−A
Meishan−B
Meishan−C
European−A
European−B
European−C

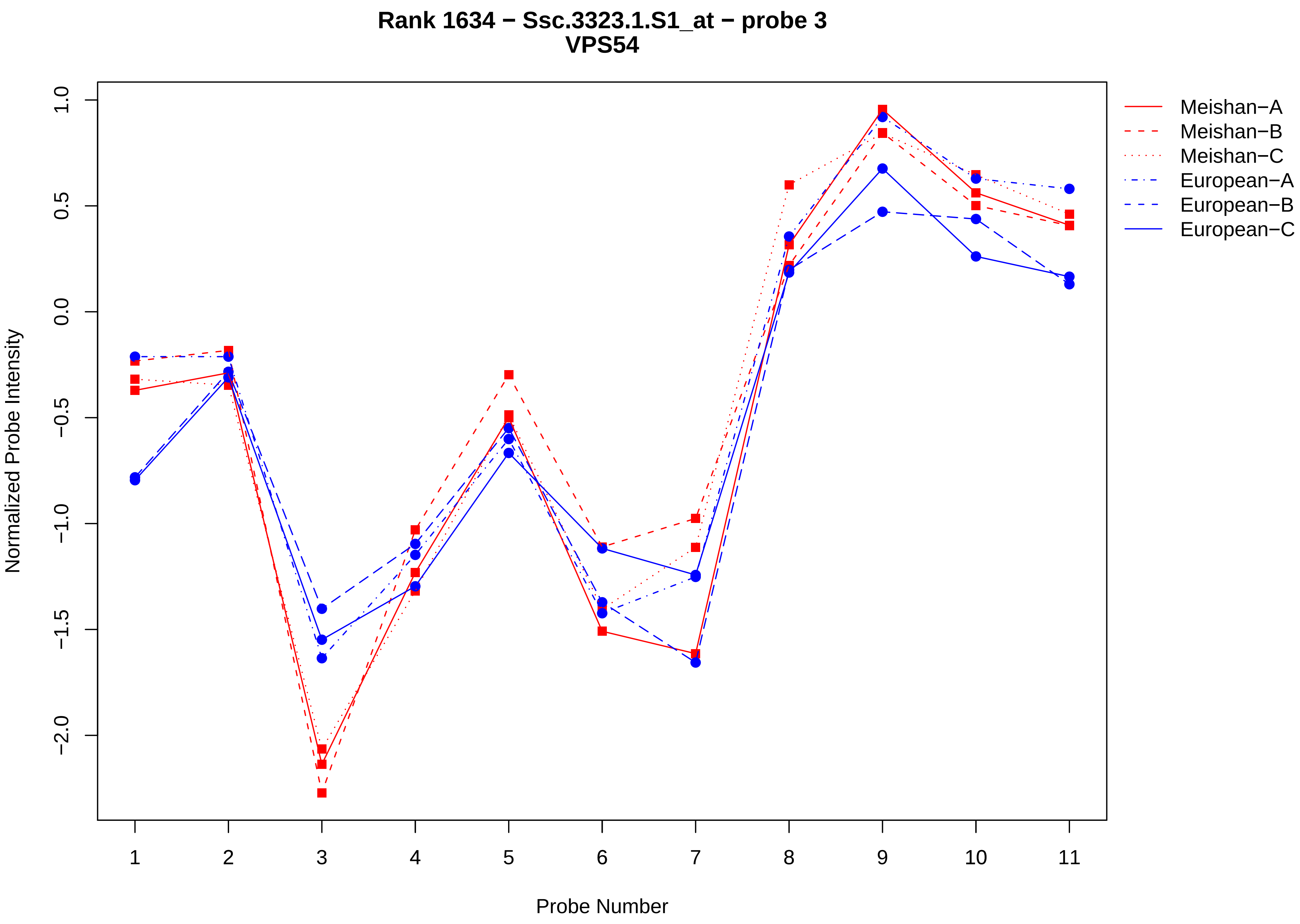

Rank 1634 − Ssc.3323.1.S1_at − probe 3
VPS54
Normalized Probe Intensity
Probe Number
Meishan−A
Meishan−B
Meishan−C
European−A
European−B
European−C

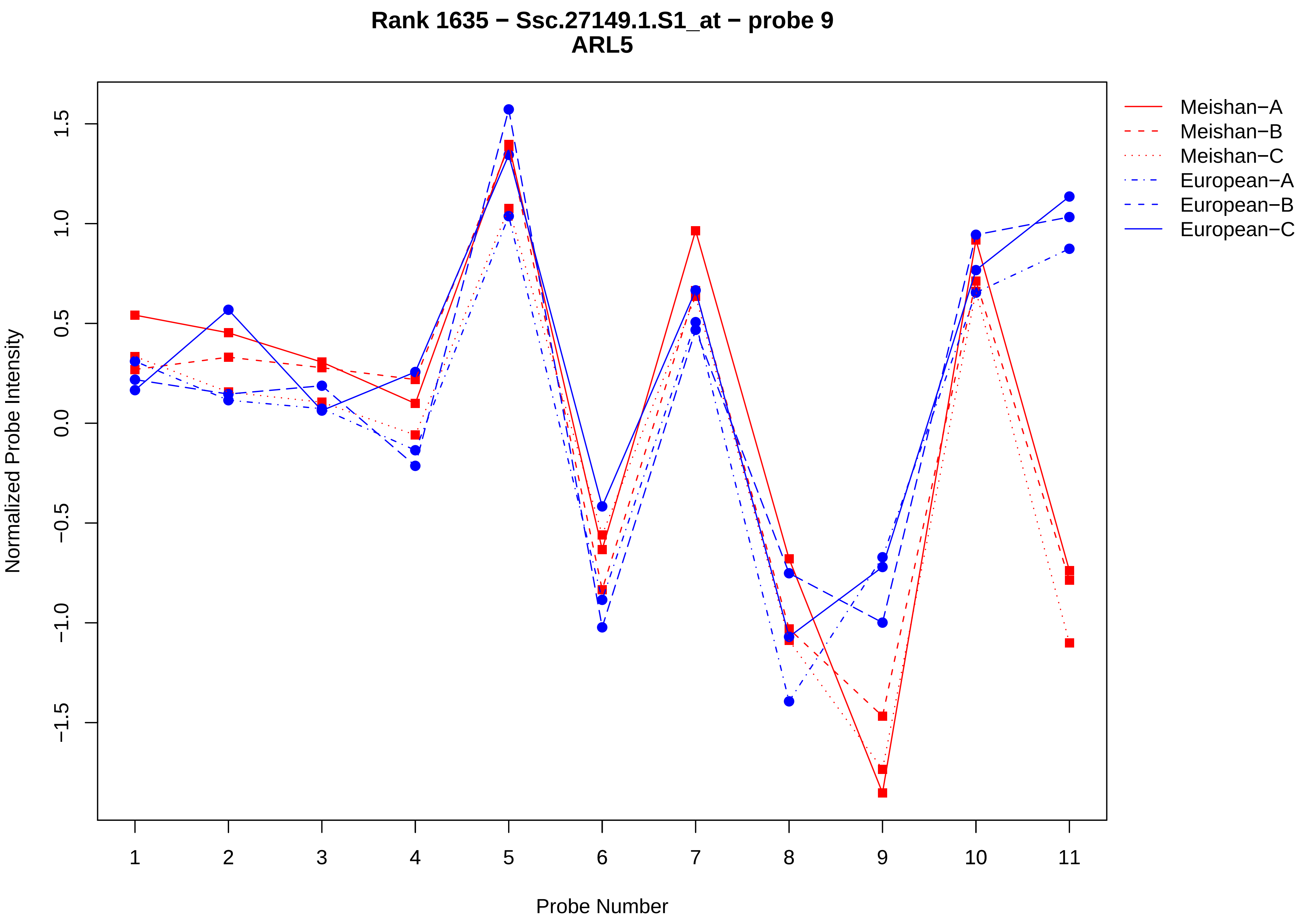

Rank 1635 − Ssc.27149.1.S1_at − probe 9
ARL5
Normalized Probe Intensity
Probe Number
Meishan−A
Meishan−B
Meishan−C
European−A
European−B
European−C

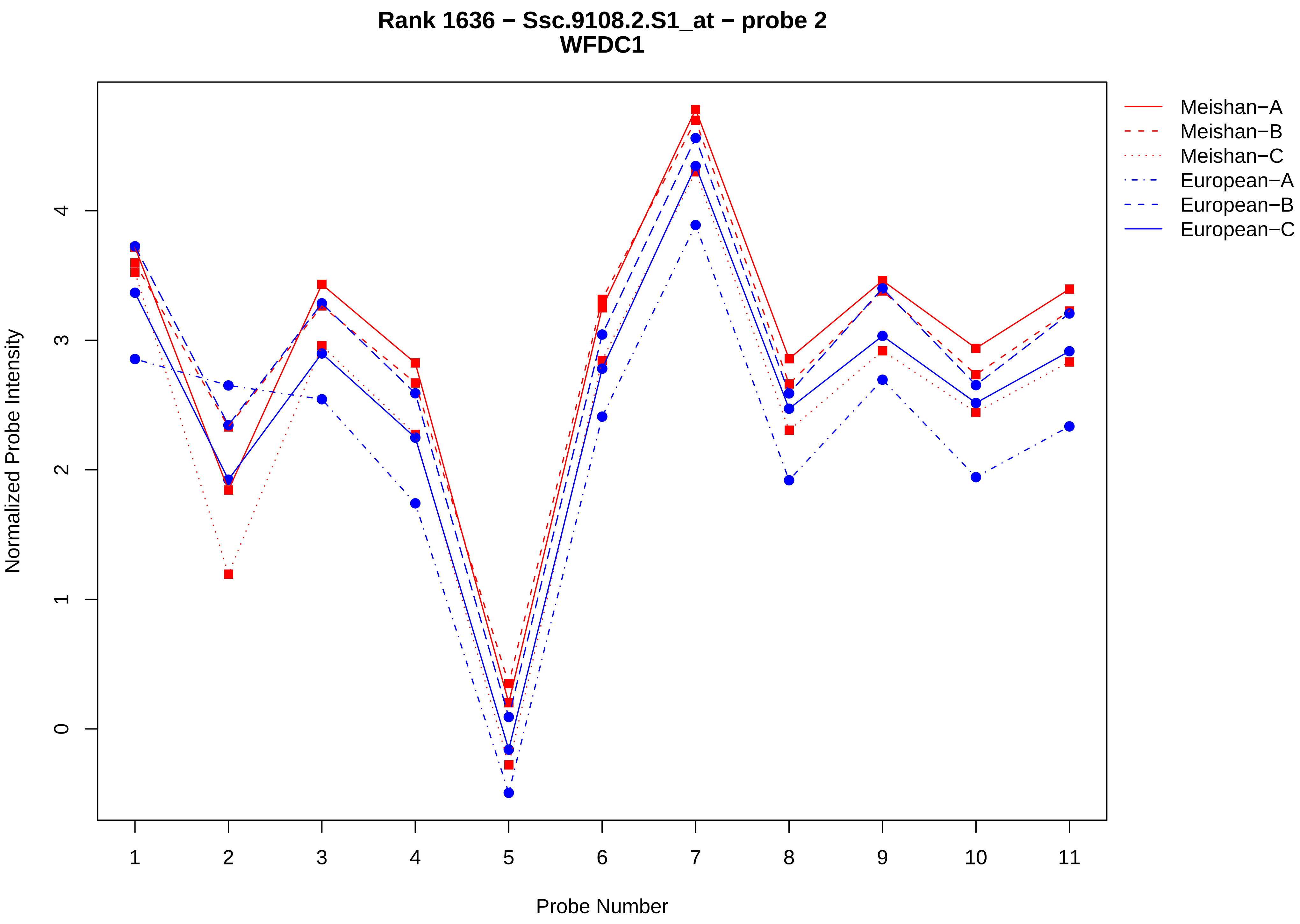

Rank 1636 − Ssc.9108.2.S1_at − probe 2
WFDC1
Normalized Probe Intensity
Probe Number
0
1
2
3
4
1
2
3
4
5
6
7
8
9
10
11
Meishan−A
Meishan−B
Meishan−C
European−A
European−B
European−C

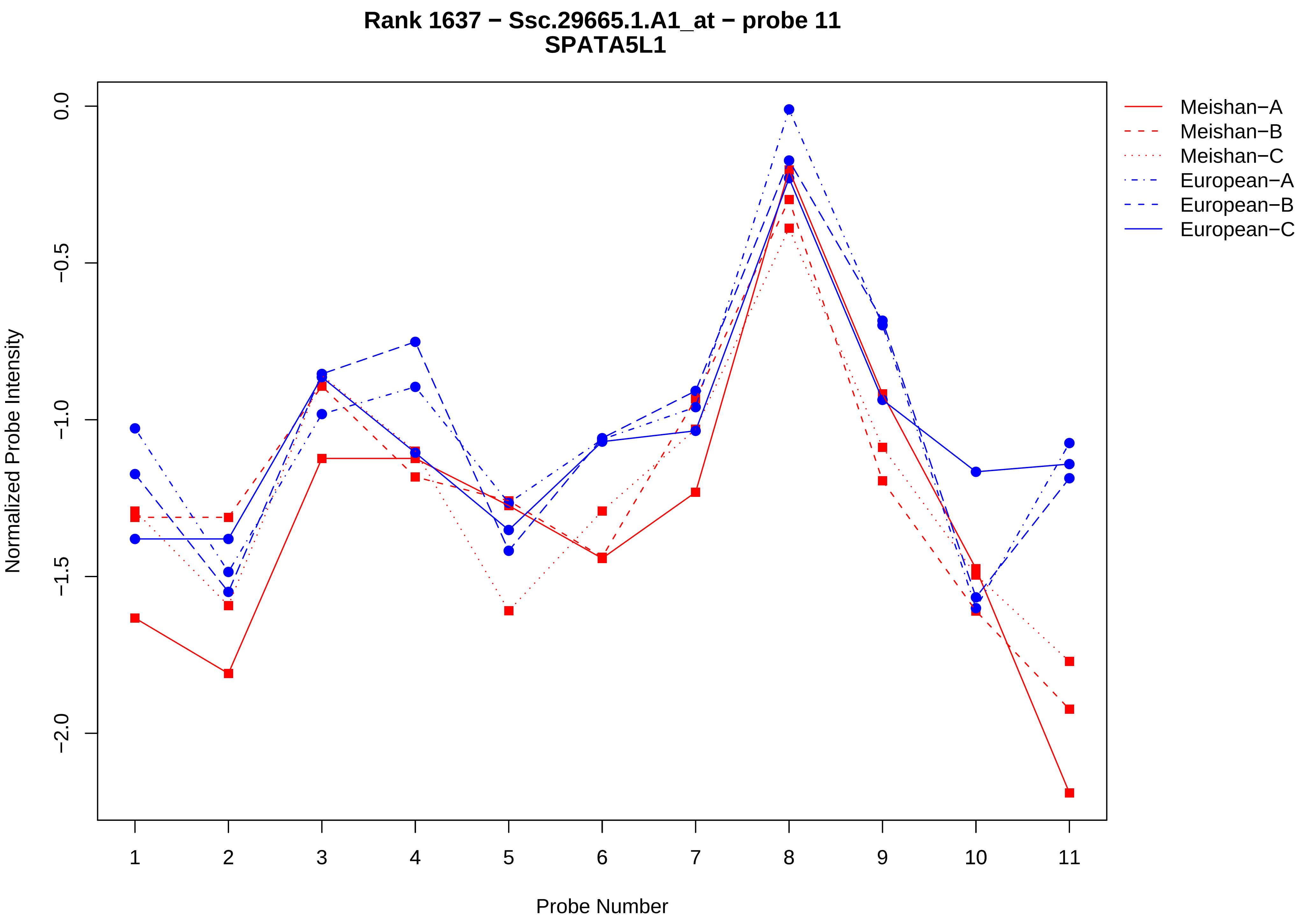

Rank 1637 − Ssc.29665.1.A1_at − probe 11
SPATA5L1
Normalized Probe Intensity
0.0
−0.5
−1.0
−1.5
−2.0
1
2
3
4
5
6
7
8
9
10
11
Probe Number
Meishan−A
Meishan−B
Meishan−C
European−A
European−B
European−C

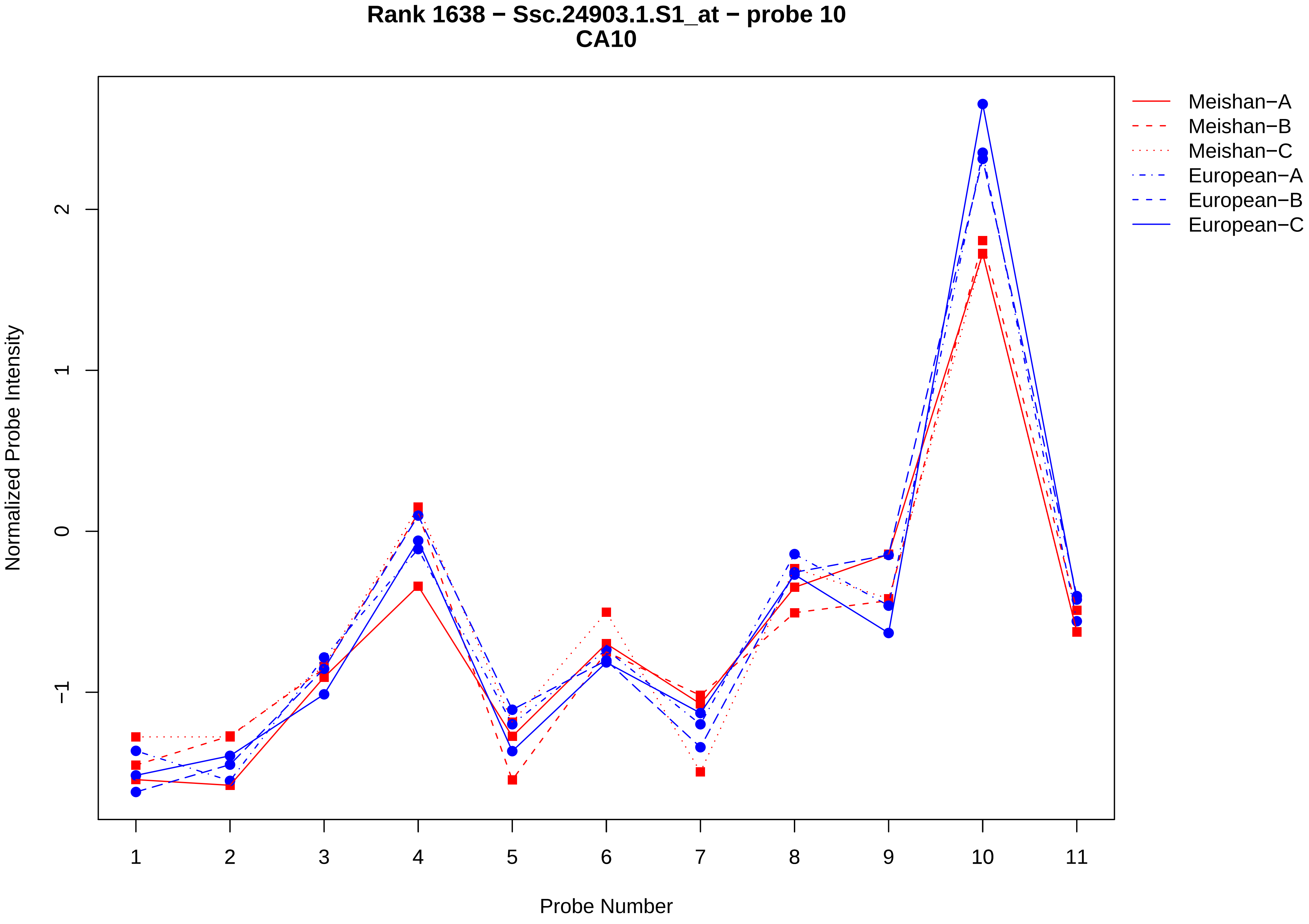

Rank 1638 − Ssc.24903.1.S1_at − probe 10
CA10
Normalized Probe Intensity
Probe Number
Meishan−A
Meishan−B
Meishan−C
European−A
European−B
European−C
−1
0
1
2
1
2
3
4
5
6
7
8
9
10
11

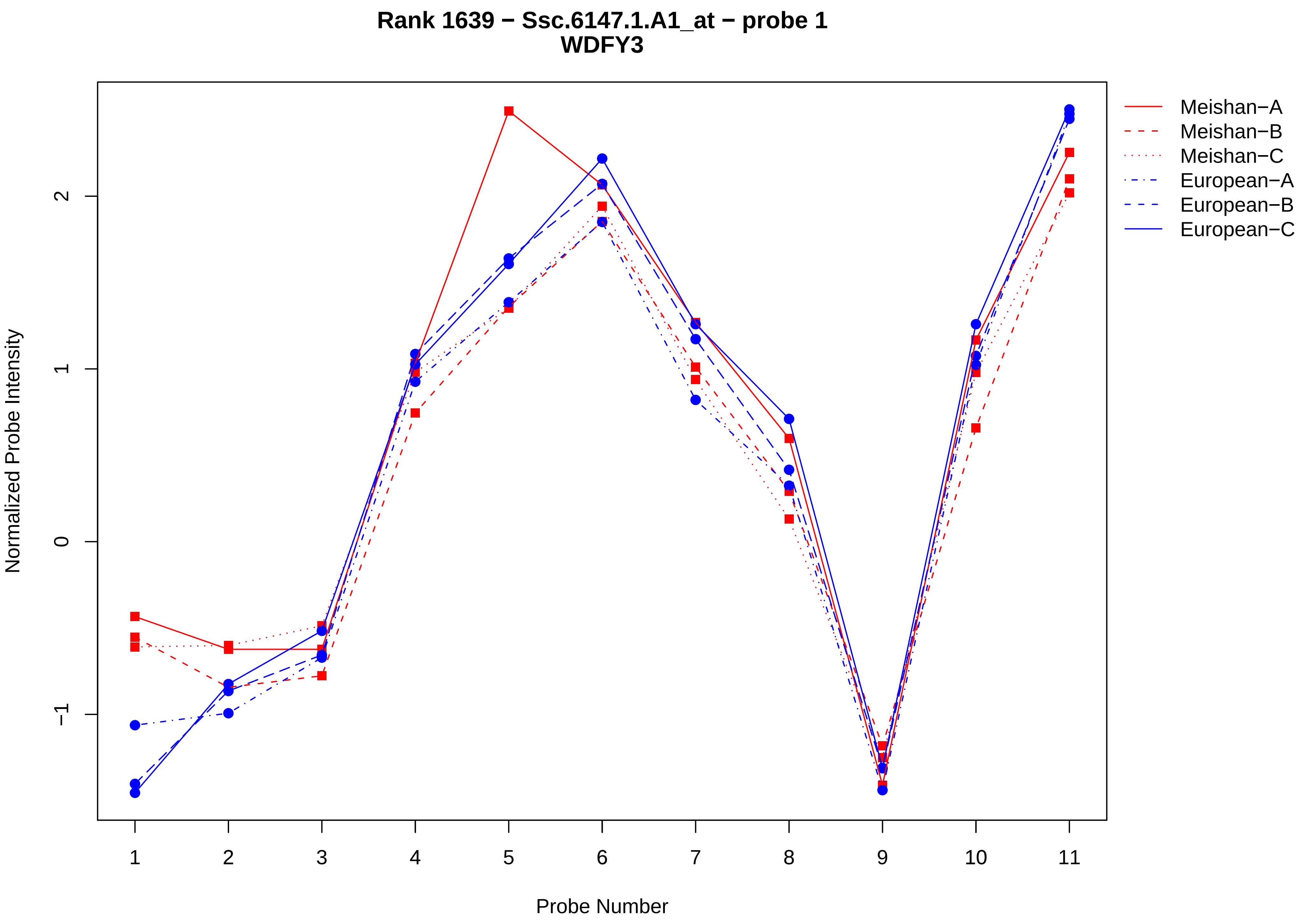
Rank 1639 − Ssc.6147.1.A1_at − probe 1
WDFY3
Normalized Probe Intensity
Probe Number
−1
0
1
2
1
2
3
4
5
6
7
8
9
10
11
Meishan−A
Meishan−B
Meishan−C
European−A
European−B
European−C

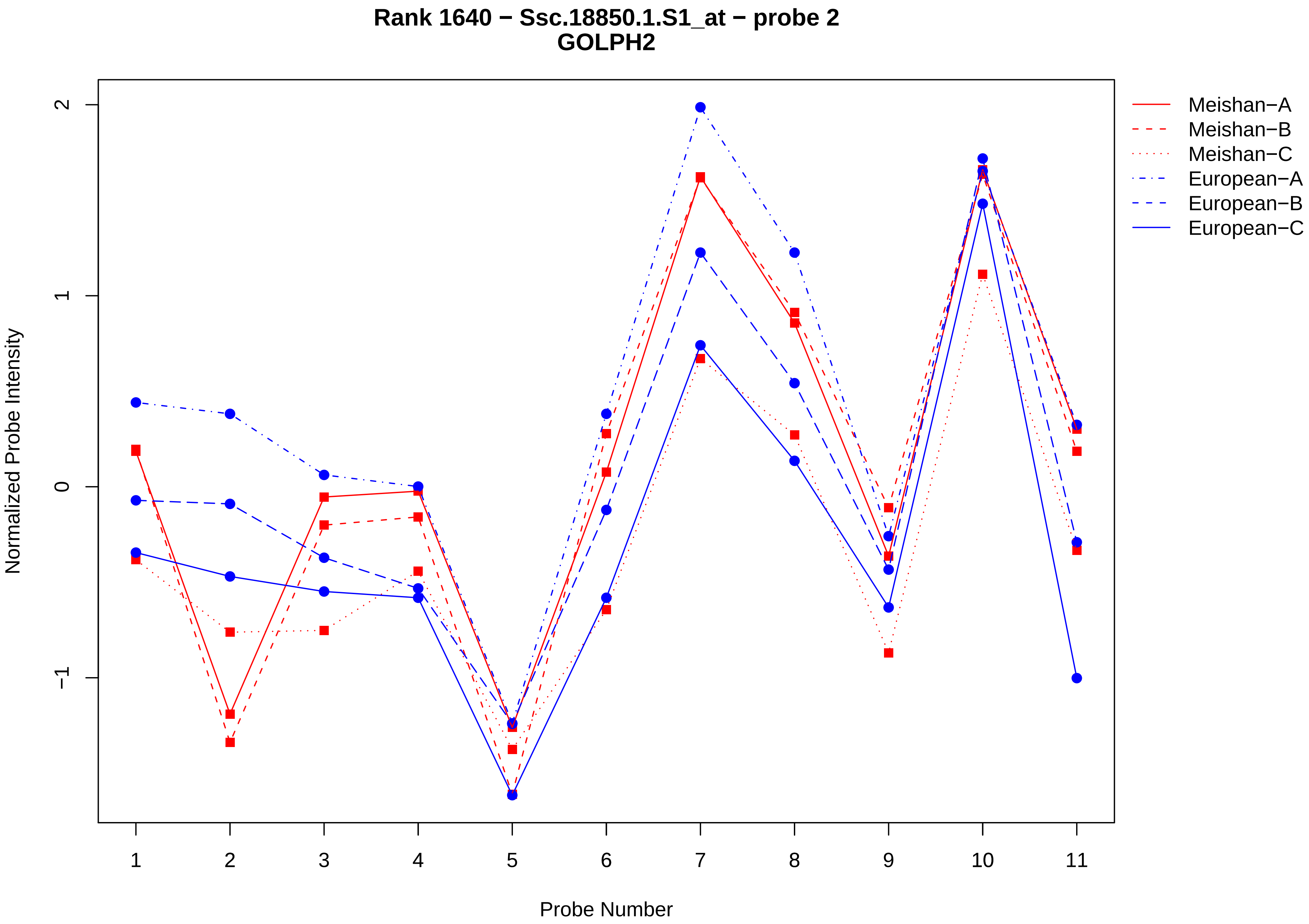

Rank 1640 − Ssc.18850.1.S1_at − probe 2
GOLPH2
Normalized Probe Intensity
Probe Number
−1
0
1
2
1
2
3
4
5
6
7
8
9
10
11
Meishan−A
Meishan−B
Meishan−C
European−A
European−B
European−C

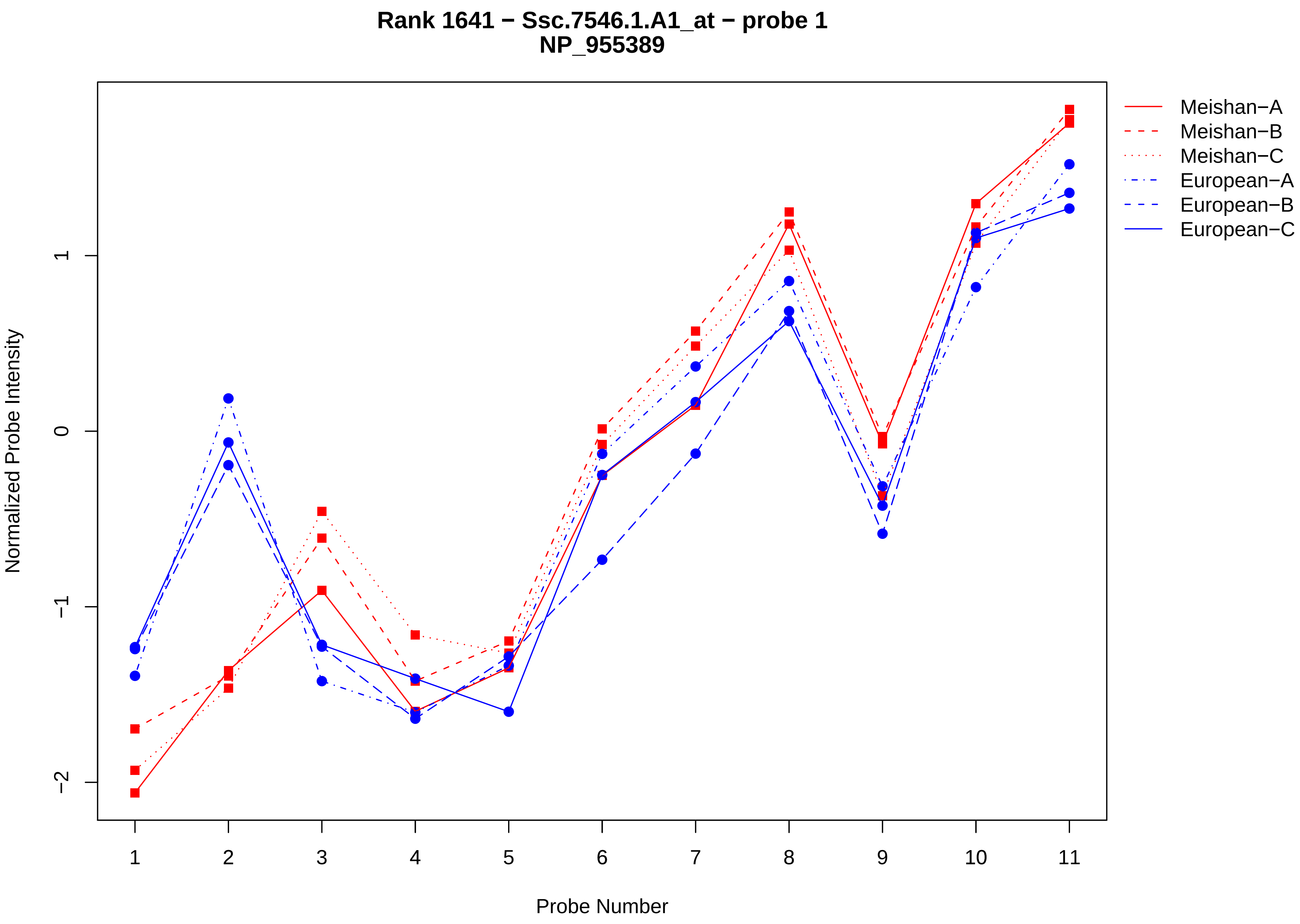

Rank 1641 − Ssc.7546.1.A1_at − probe 1
NP_955389
Normalized Probe Intensity
Probe Number
−2
−1
0
1
1
2
3
4
5
6
7
8
9
10
11
Meishan−A
Meishan−B
Meishan−C
European−A
European−B
European−C

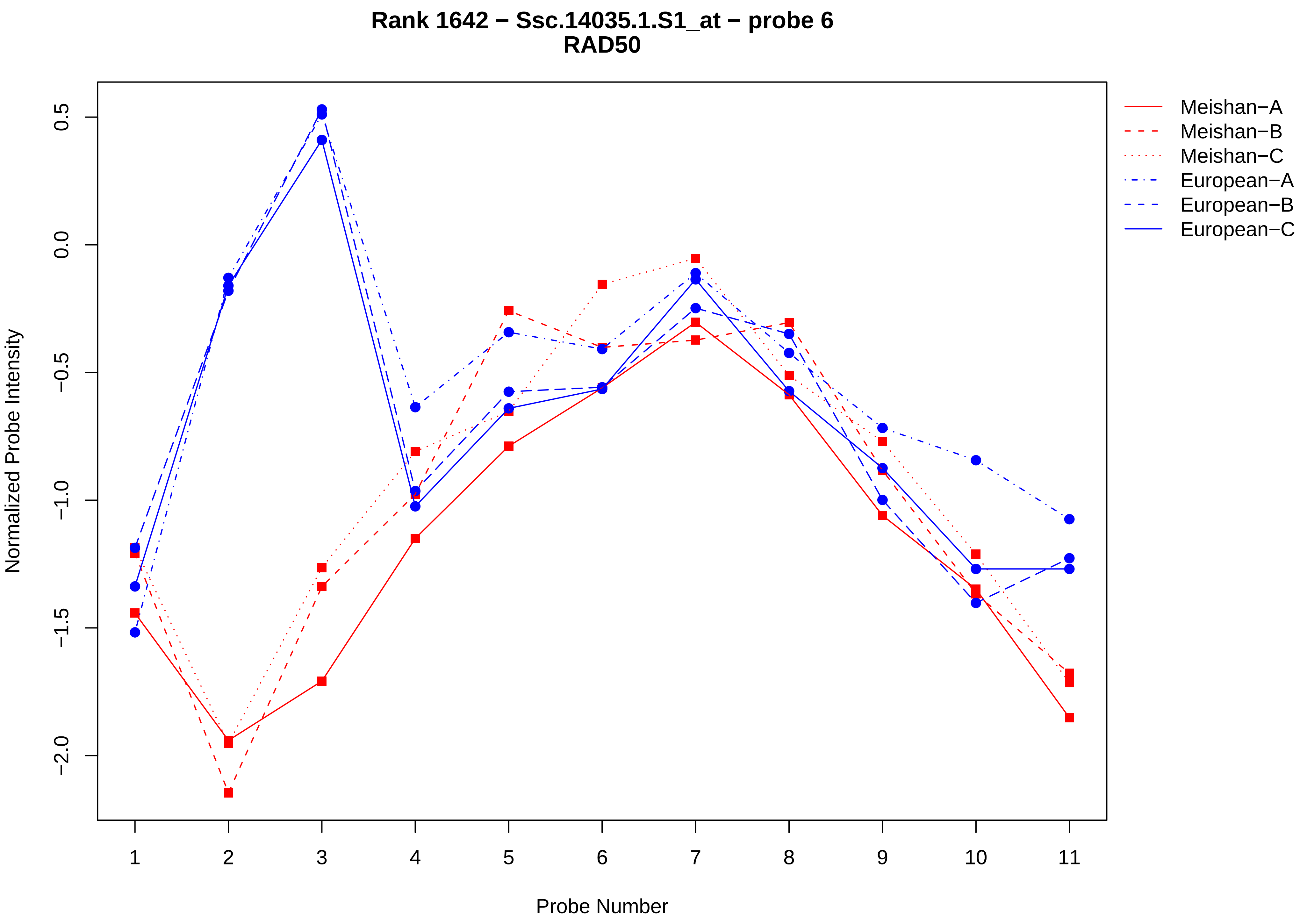
Rank 1642 − Ssc.14035.1.S1_at − probe 6
RAD50
Normalized Probe Intensity
Probe Number
0.5
0.0
−0.5
−1.0
−1.5
−2.0
1
2
3
4
5
6
7
8
9
10
11
Meishan−A
Meishan−B
Meishan−C
European−A
European−B
European−C

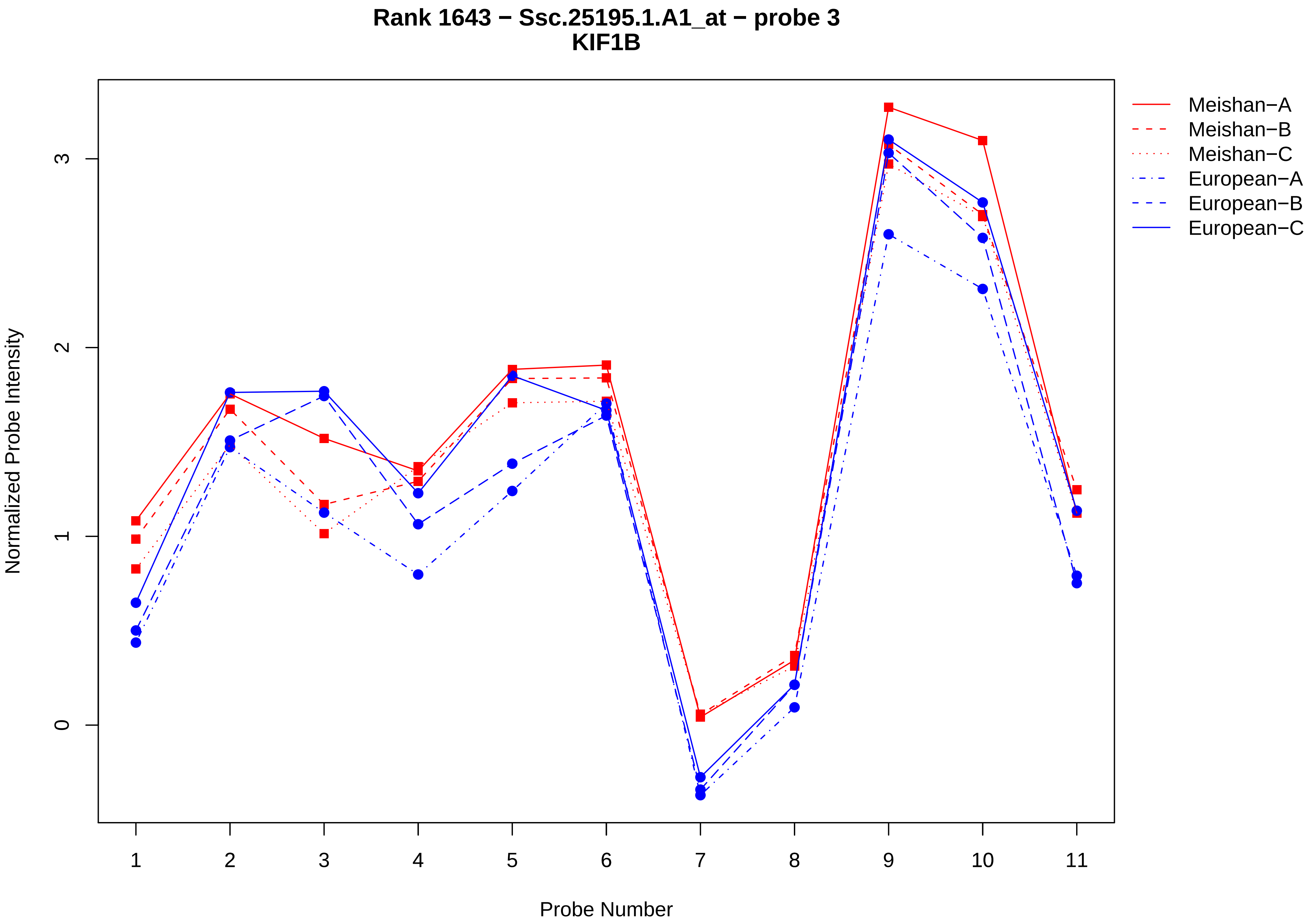

Rank 1643 − Ssc.25195.1.A1_at − probe 3
KIF1B
Normalized Probe Intensity
0
1
2
3
1
2
3
4
5
6
7
8
9
10
11
Probe Number
Meishan−A
Meishan−B
Meishan−C
European−A
European−B
European−C

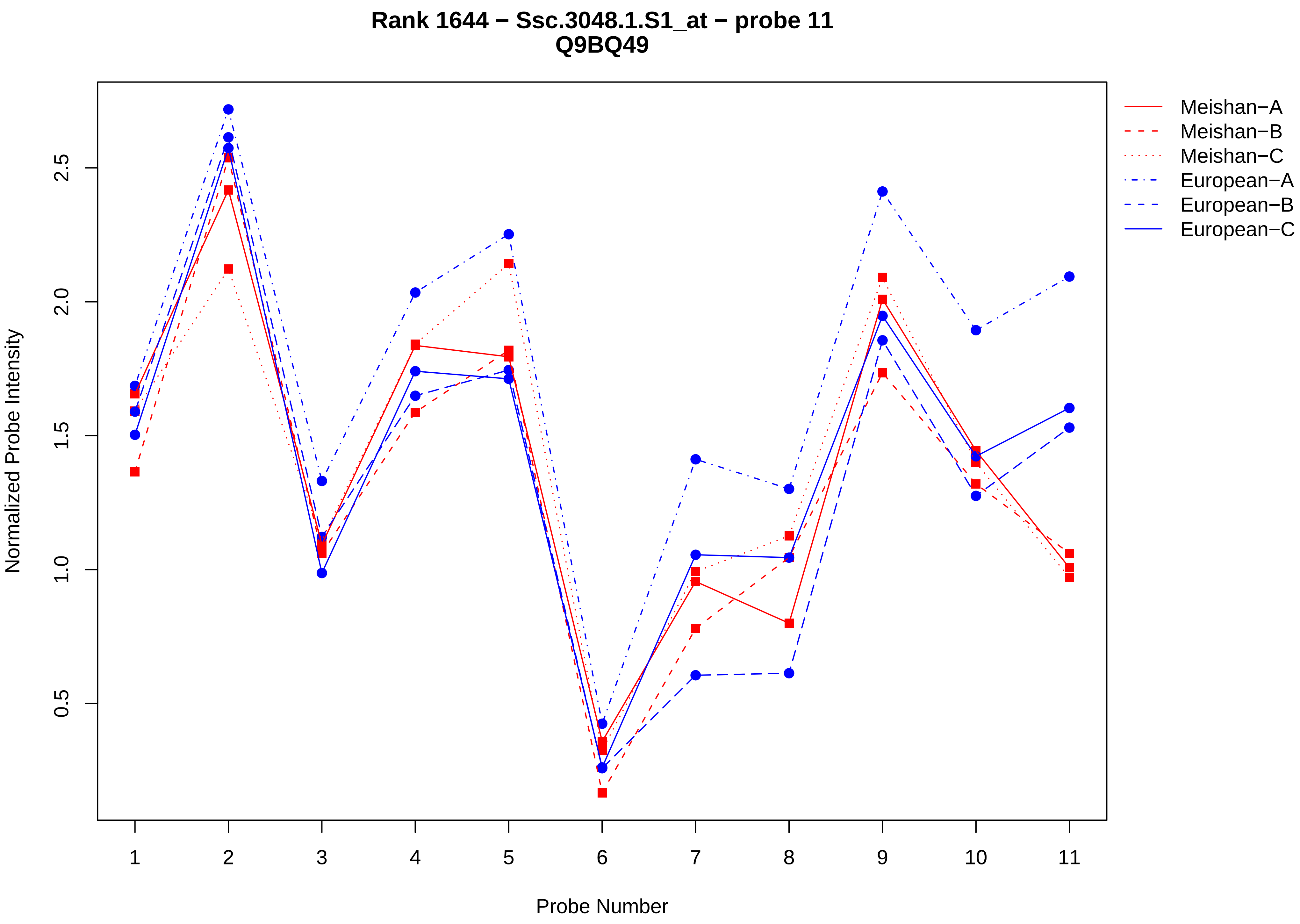

Rank 1644 − Ssc.3048.1.S1_at − probe 11
Q9BQ49
Normalized Probe Intensity
0.5
1.0
1.5
2.0
2.5
Probe Number
1
2
3
4
5
6
7
8
9
10
11
Meishan−A
Meishan−B
Meishan−C
European−A
European−B
European−C

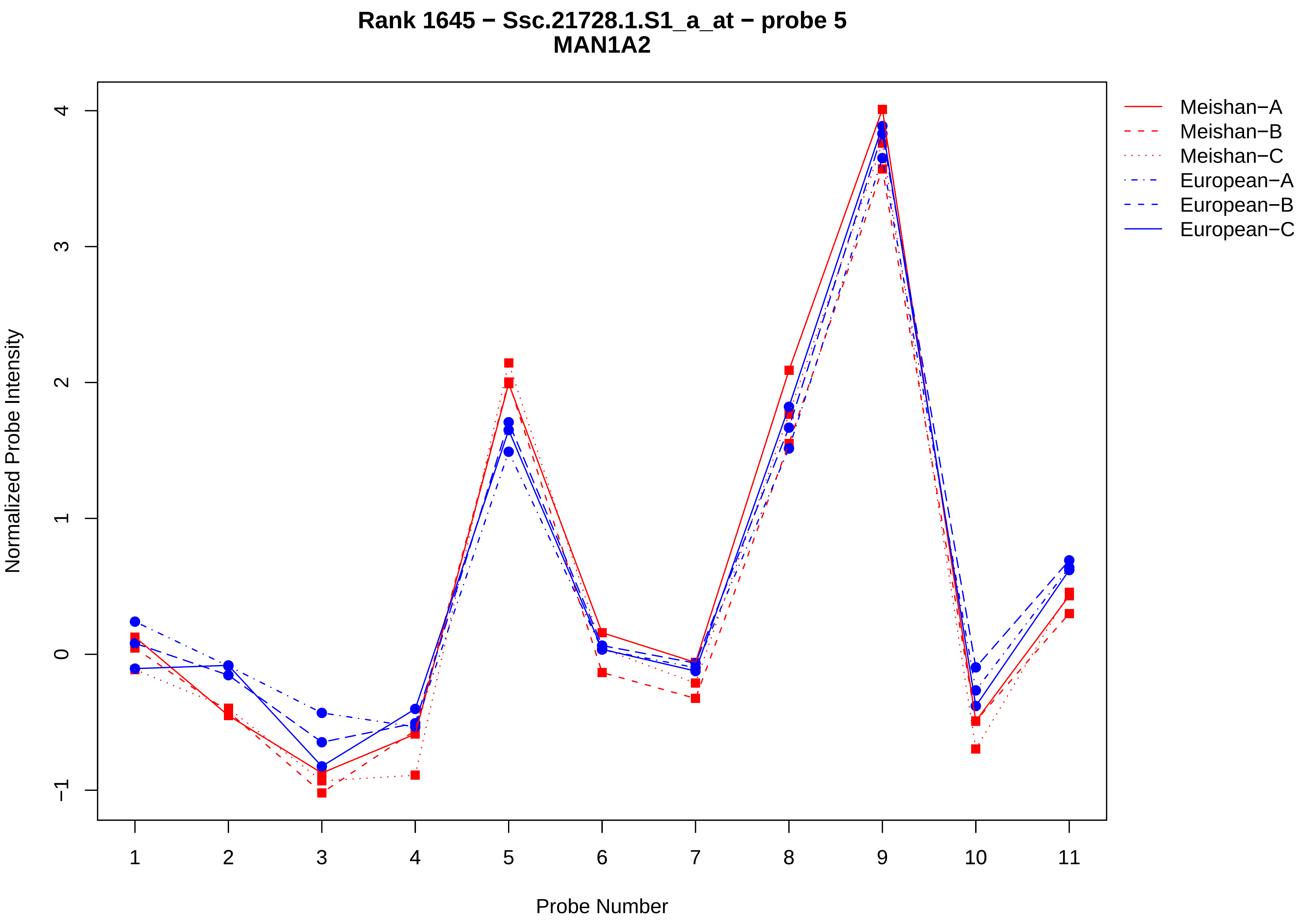
Rank 1645 − Ssc.21728.1.S1_a_at − probe 5
MAN1A2
Normalized Probe Intensity
Probe Number
Meishan−A
Meishan−B
Meishan−C
European−A
European−B
European−C
−1
0
1
2
3
4
1
2
3
4
5
6
7
8
9
10
11

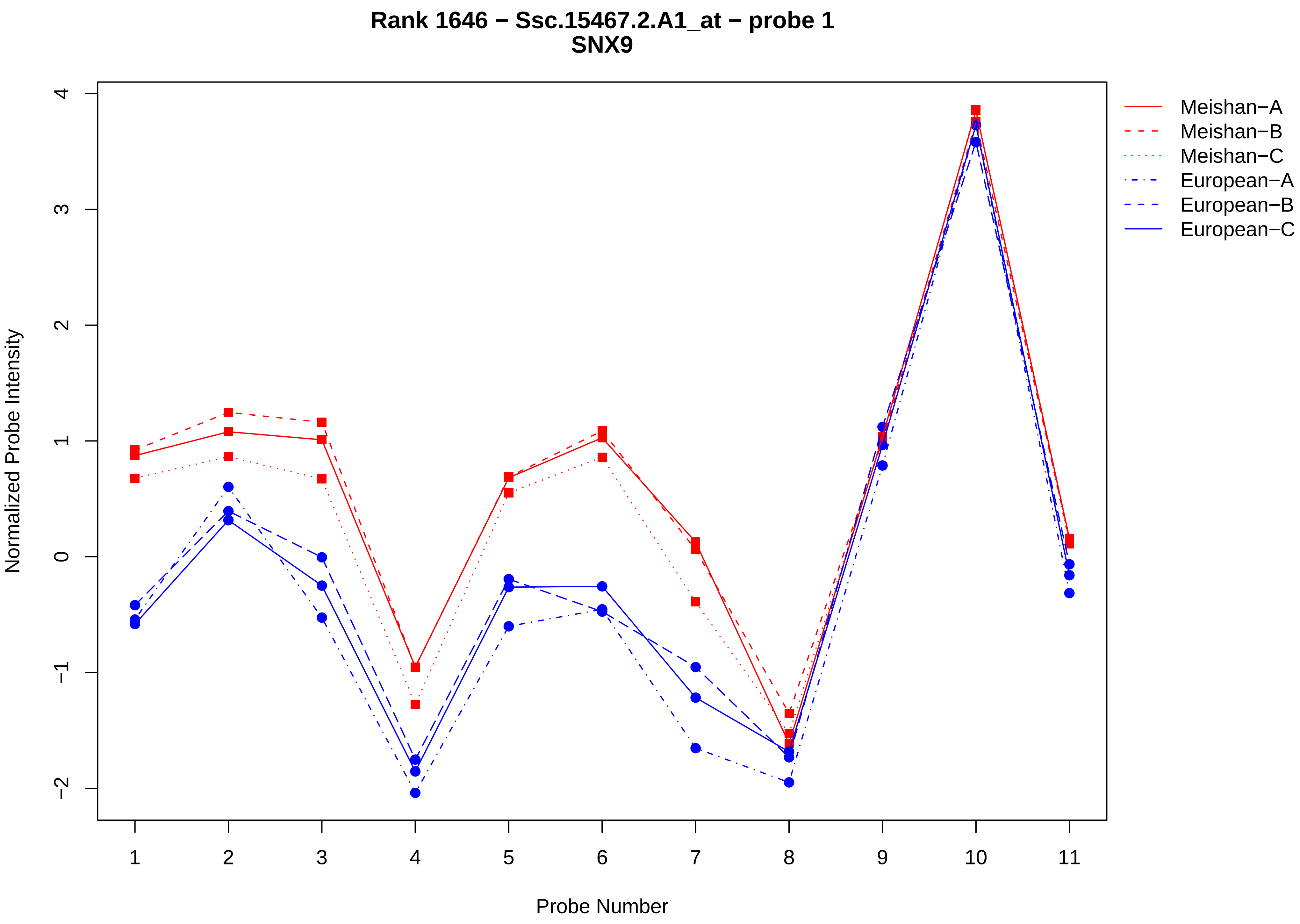

Rank 1646 − Ssc.15467.2.A1_at − probe 1
SNX9
Normalized Probe Intensity
Probe Number
Meishan−A
Meishan−B
Meishan−C
European−A
European−B
European−C

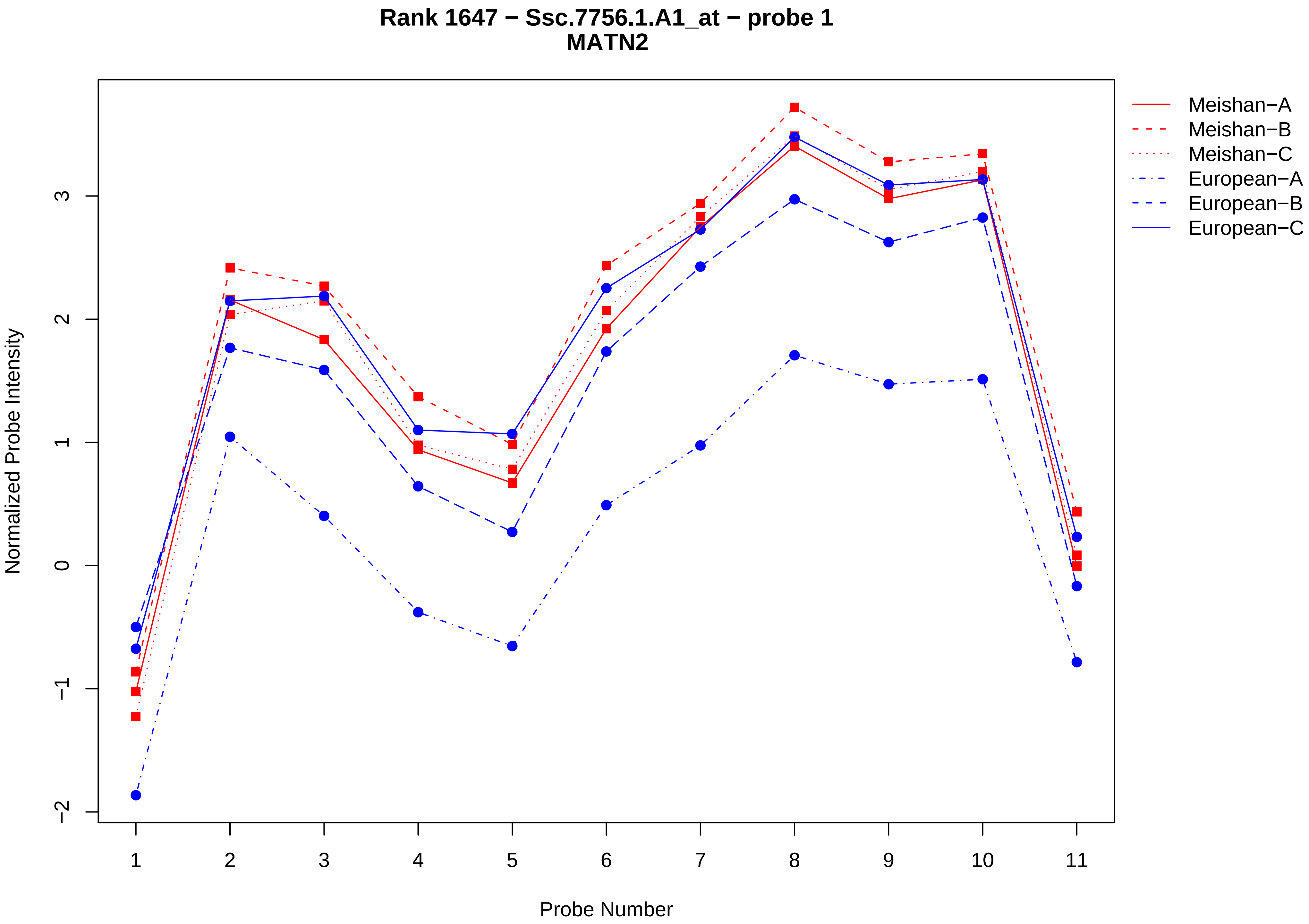

Rank 1647 − Ssc.7756.1.A1_at − probe 1
MATN2
Normalized Probe Intensity
Probe Number
−2
−1
0
1
2
3
1
2
3
4
5
6
7
8
9
10
11
Meishan−A
Meishan−B
Meishan−C
European−A
European−B
European−C

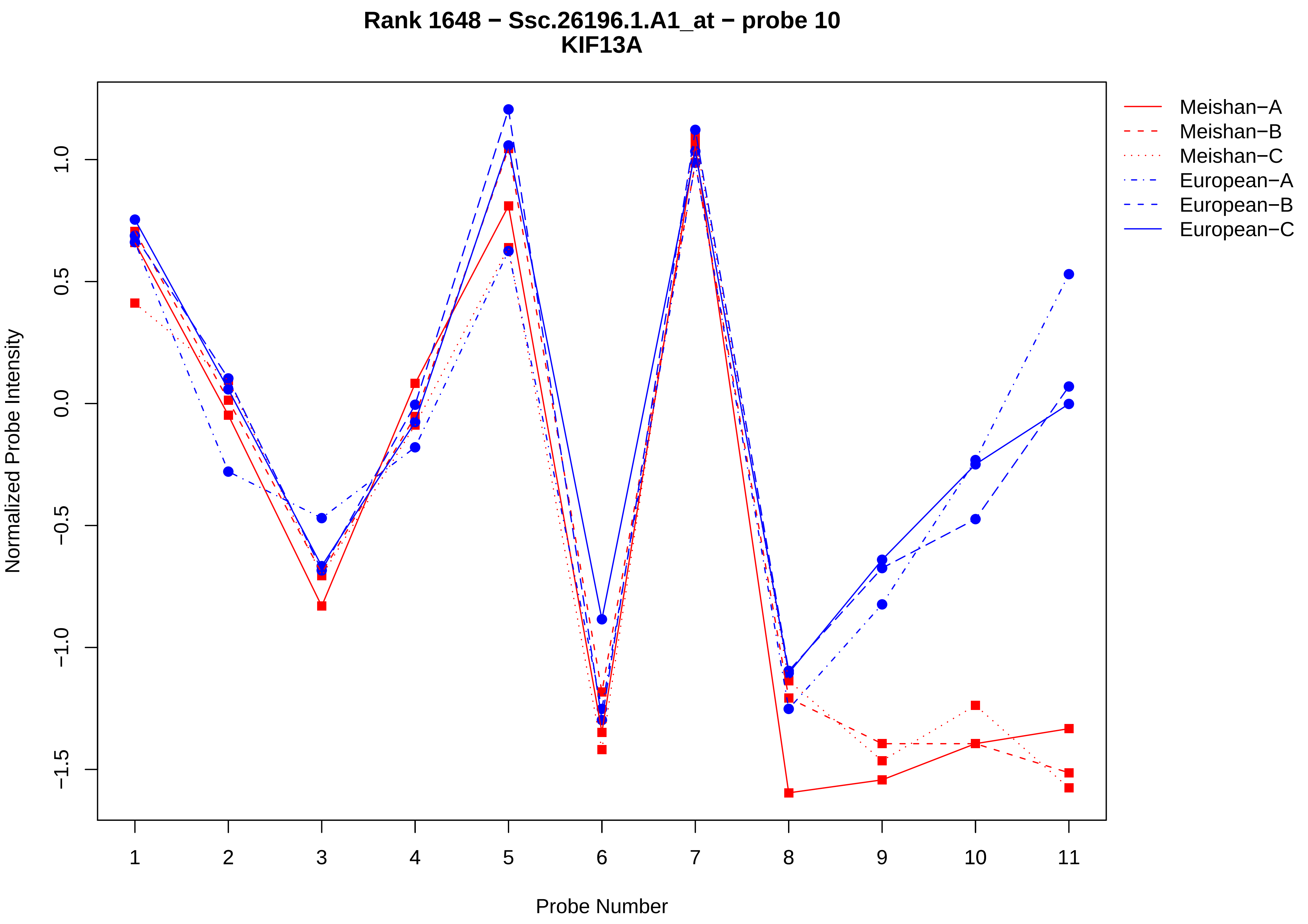

Rank 1648 − Ssc.26196.1.A1_at − probe 10
KIF13A
Normalized Probe Intensity
Probe Number
1.0
0.5
0.0
−0.5
−1.0
−1.5
1
2
3
4
5
6
7
8
9
10
11
Meishan−A
Meishan−B
Meishan−C
European−A
European−B
European−C

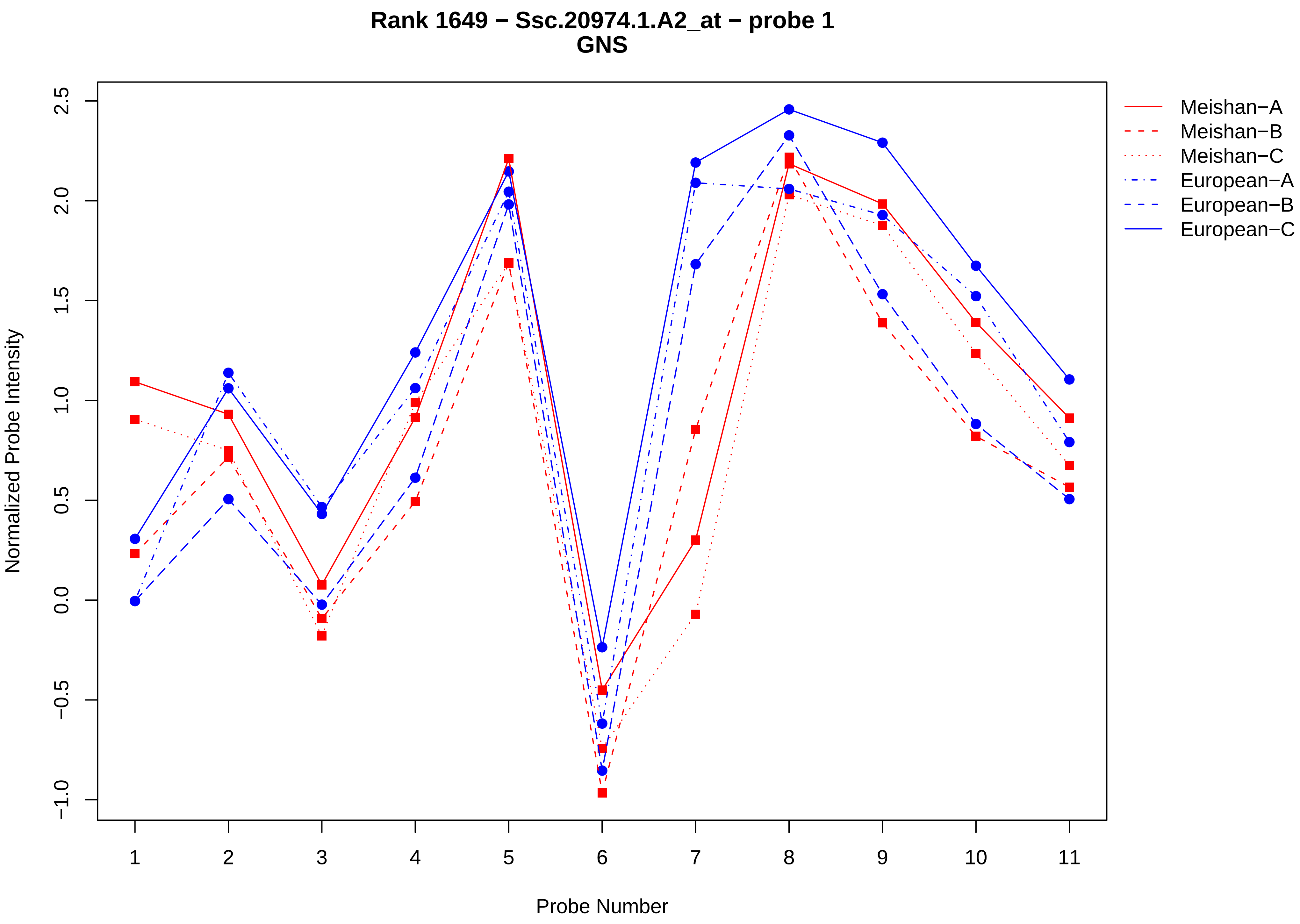

Rank 1649 − Ssc.20974.1.A2_at − probe 1
GNS
Normalized Probe Intensity
Probe Number
Meishan−A
Meishan−B
Meishan−C
European−A
European−B
European−C
−1.0
−0.5
0.0
0.5
1.0
1.5
2.0
2.5
1
2
3
4
5
6
7
8
9
10
11

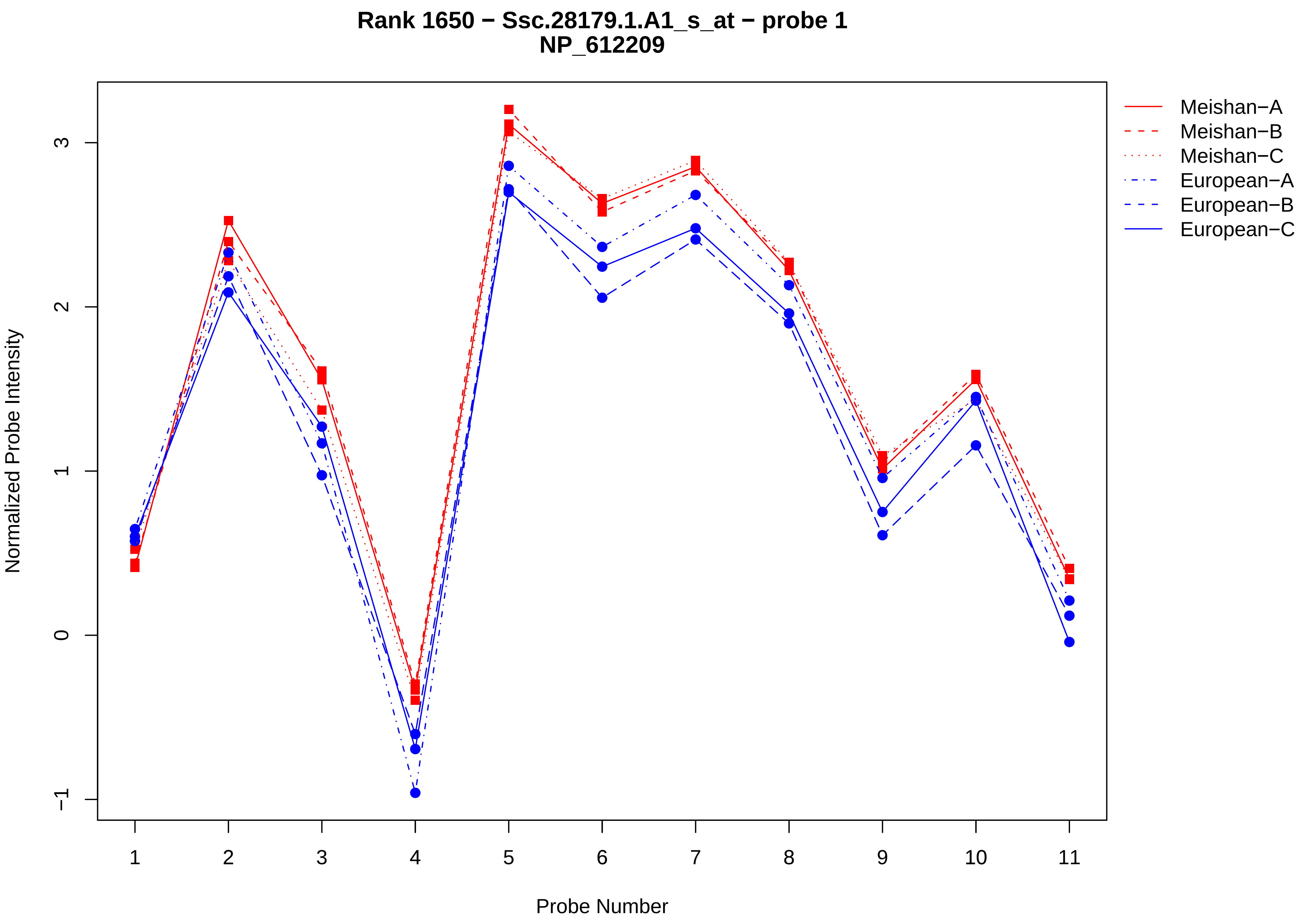

Rank 1650 − Ssc.28179.1.A1_s_at − probe 1
NP_612209
Normalized Probe Intensity
Probe Number
-1
0
1
2
3
1
2
3
4
5
6
7
8
9
10
11
Meishan−A
Meishan−B
Meishan−C
European−A
European−B
European−C

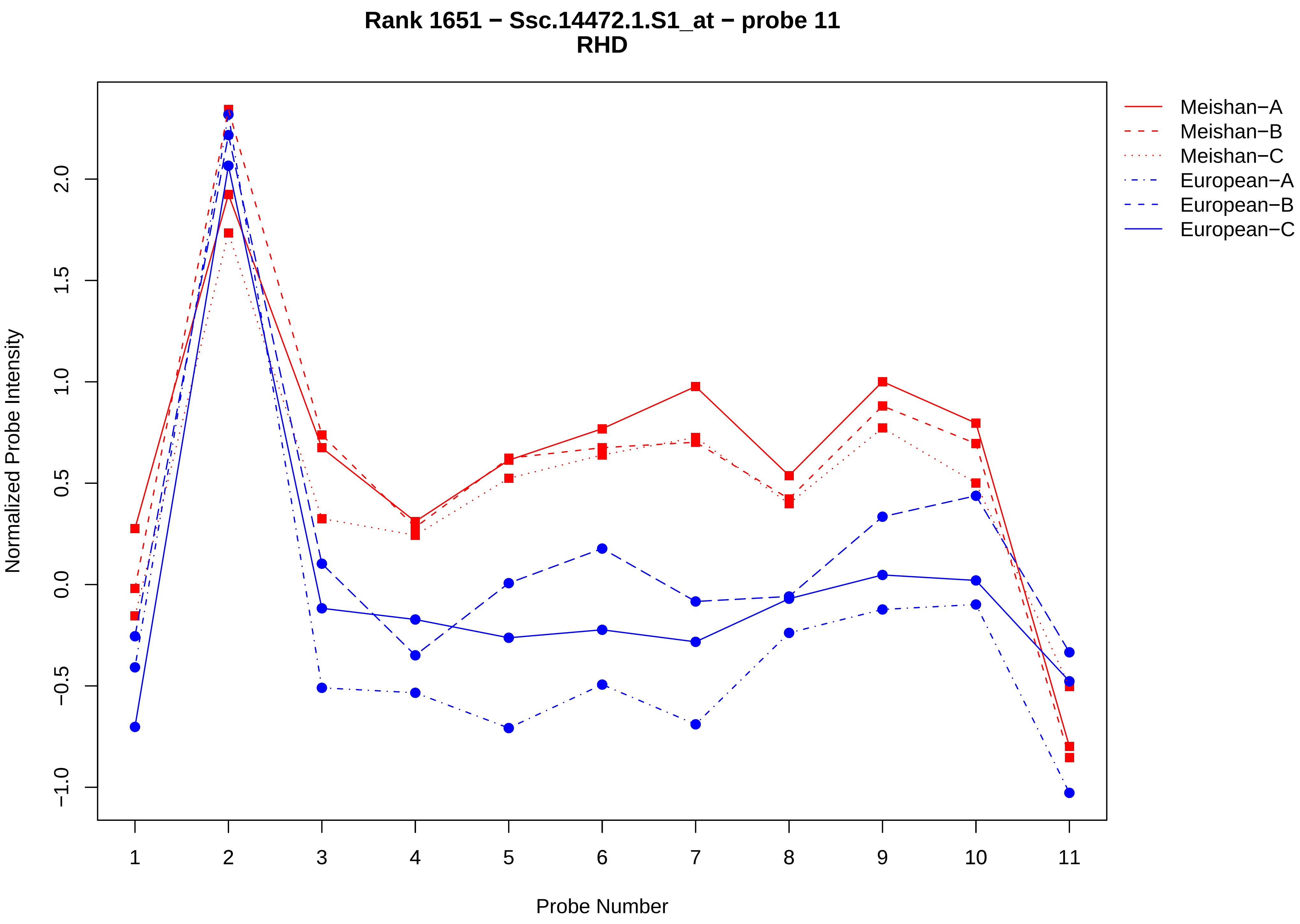
Rank 1651 − Ssc.14472.1.S1_at − probe 11
RHD
Normalized Probe Intensity
Probe Number
Meishan−A
Meishan−B
Meishan−C
European−A
European−B
European−C
−1.0
−0.5
0.0
0.5
1.0
1.5
2.0
1
2
3
4
5
6
7
8
9
10
11

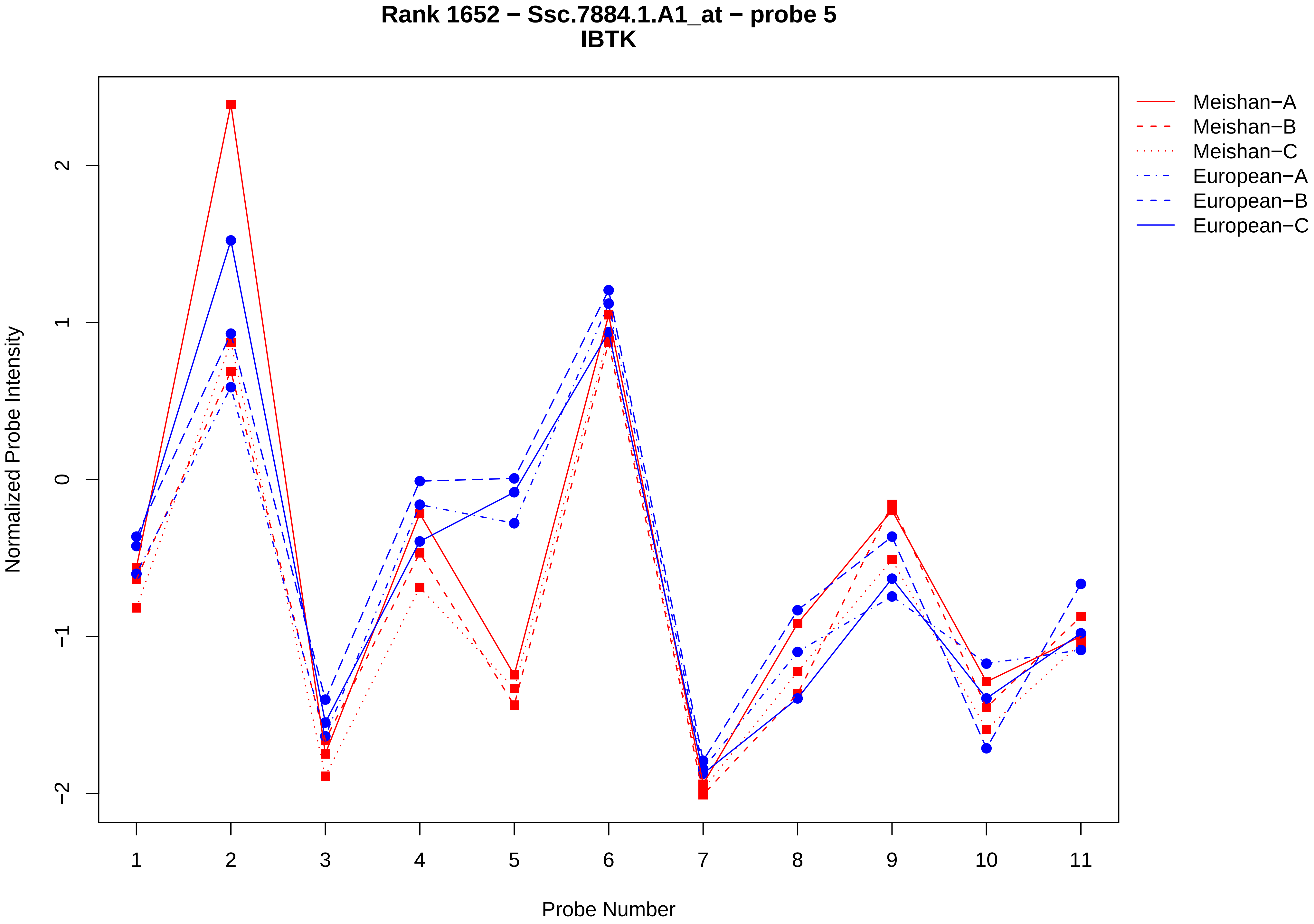
Rank 1652 − Ssc.7884.1.A1_at − probe 5
IBTK
Normalized Probe Intensity
Probe Number
−2
−1
0
1
2
1
2
3
4
5
6
7
8
9
10
11
Meishan−A
Meishan−B
Meishan−C
European−A
European−B
European−C

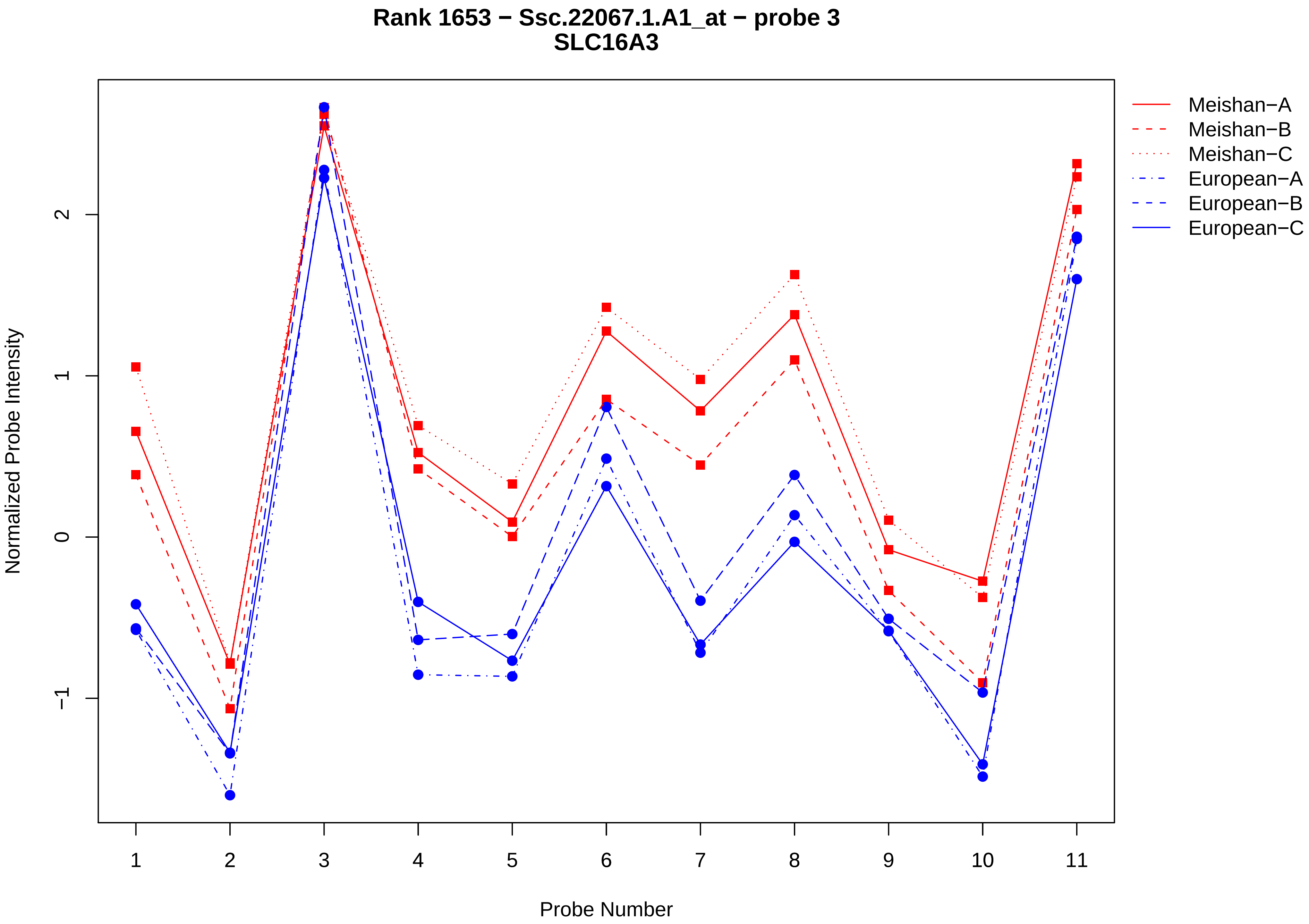

Rank 1653 − Ssc.22067.1.A1_at − probe 3
SLC16A3
Normalized Probe Intensity
Probe Number
−1
0
1
2
1
2
3
4
5
6
7
8
9
10
11
Meishan−A
Meishan−B
Meishan−C
European−A
European−B
European−C

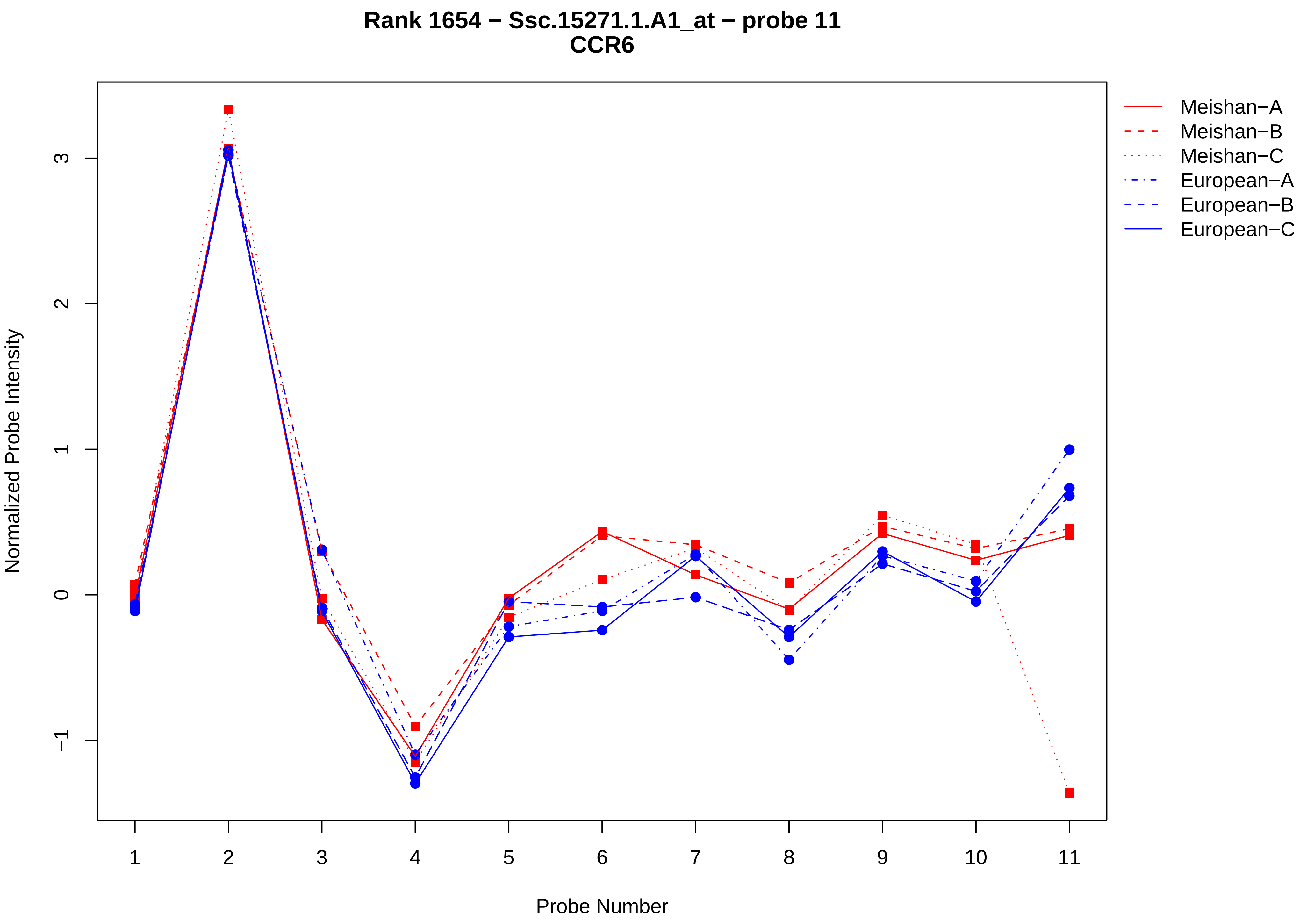

Rank 1654 − Ssc.15271.1.A1_at − probe 11
CCR6
Normalized Probe Intensity
−1
0
1
2
3
Probe Number
1
2
3
4
5
6
7
8
9
10
11
Meishan−A
Meishan−B
Meishan−C
European−A
European−B
European−C

Rank 1655 − Ssc.14502.1.S1_a_at − probe 6
SUCLG1

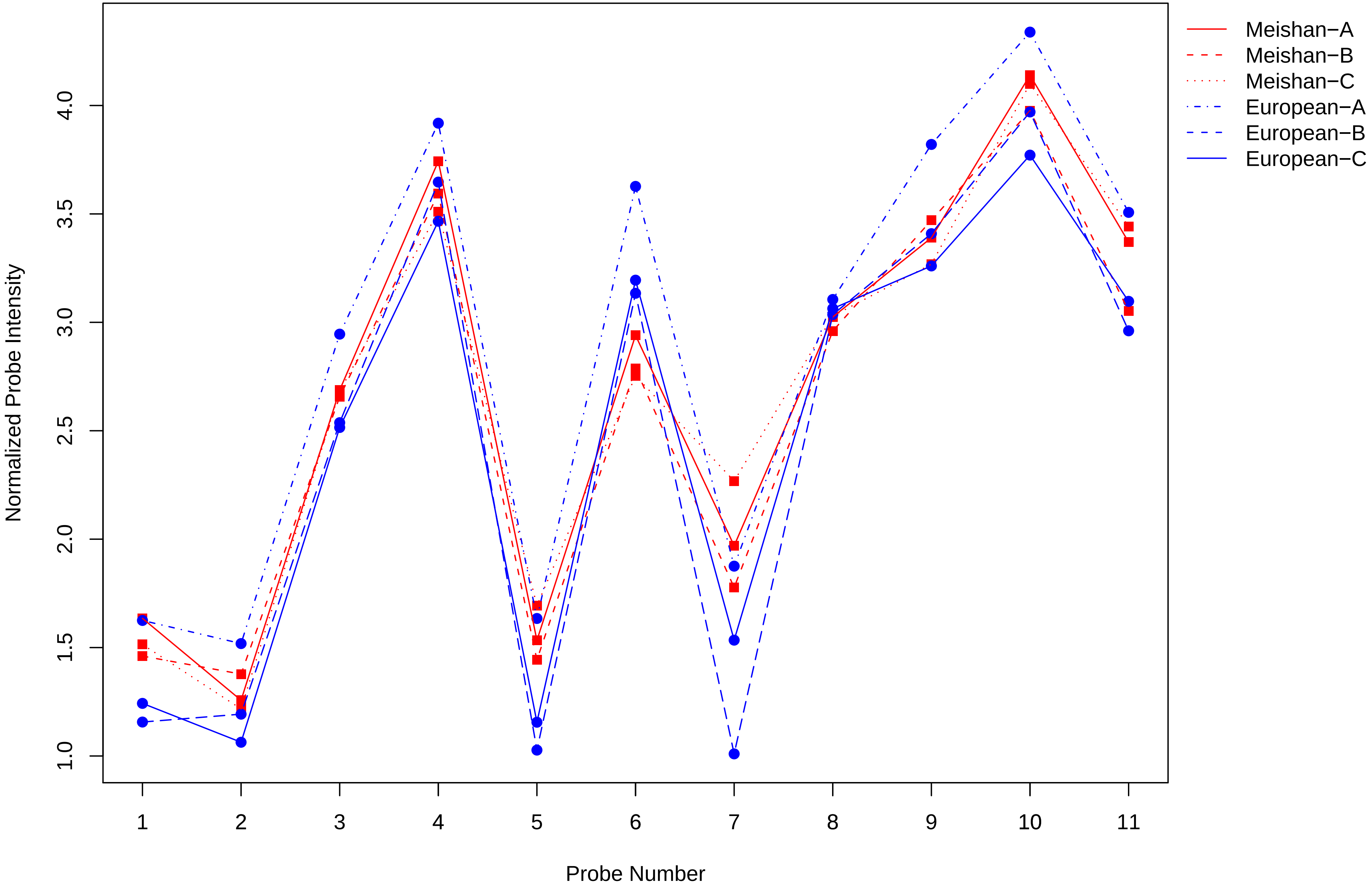

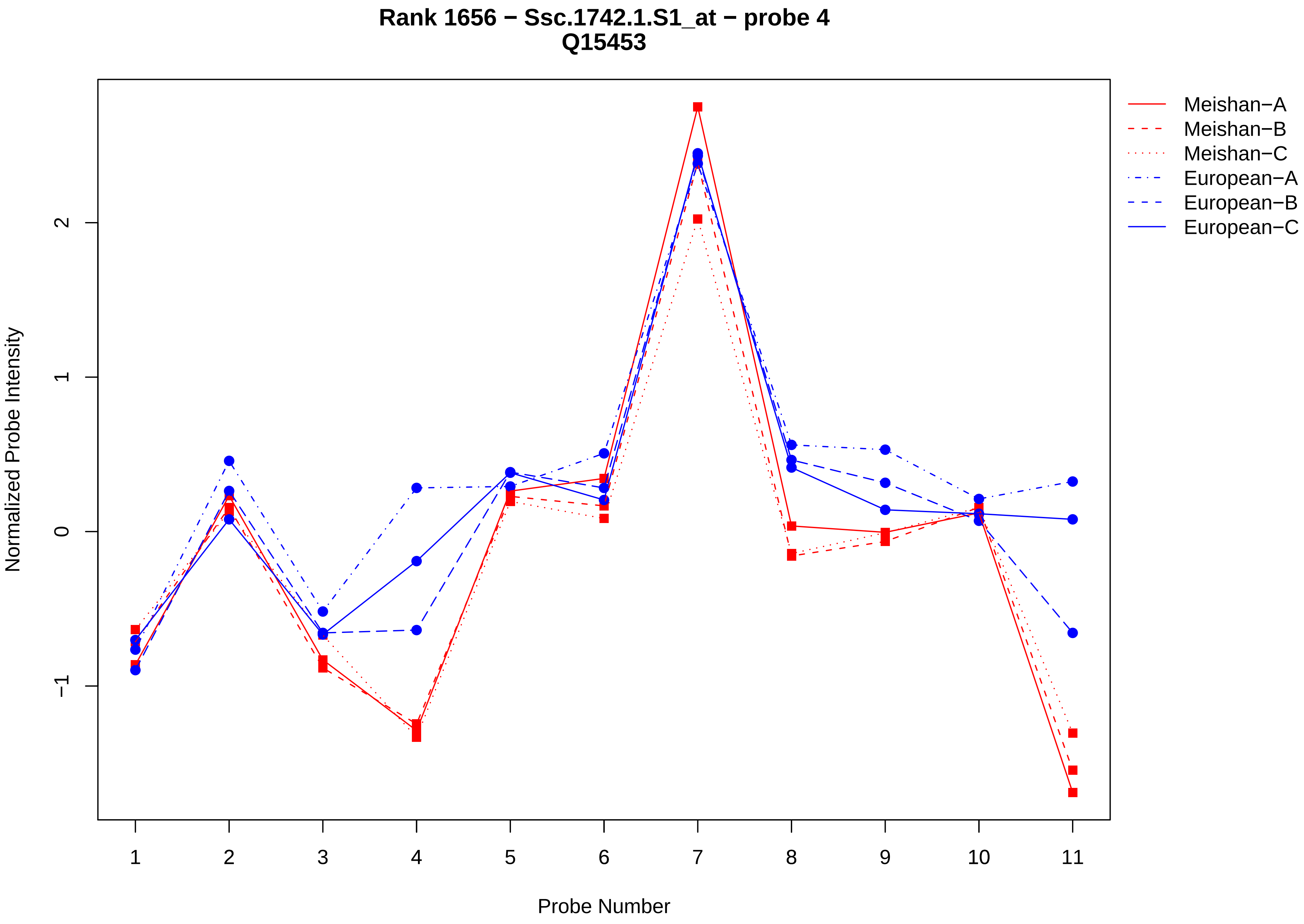

Rank 1656 − Ssc.1742.1.S1_at − probe 4
Q15453
Normalized Probe Intensity
Probe Number
−1
0
1
2
1
2
3
4
5
6
7
8
9
10
11
Meishan−A
Meishan−B
Meishan−C
European−A
European−B
European−C

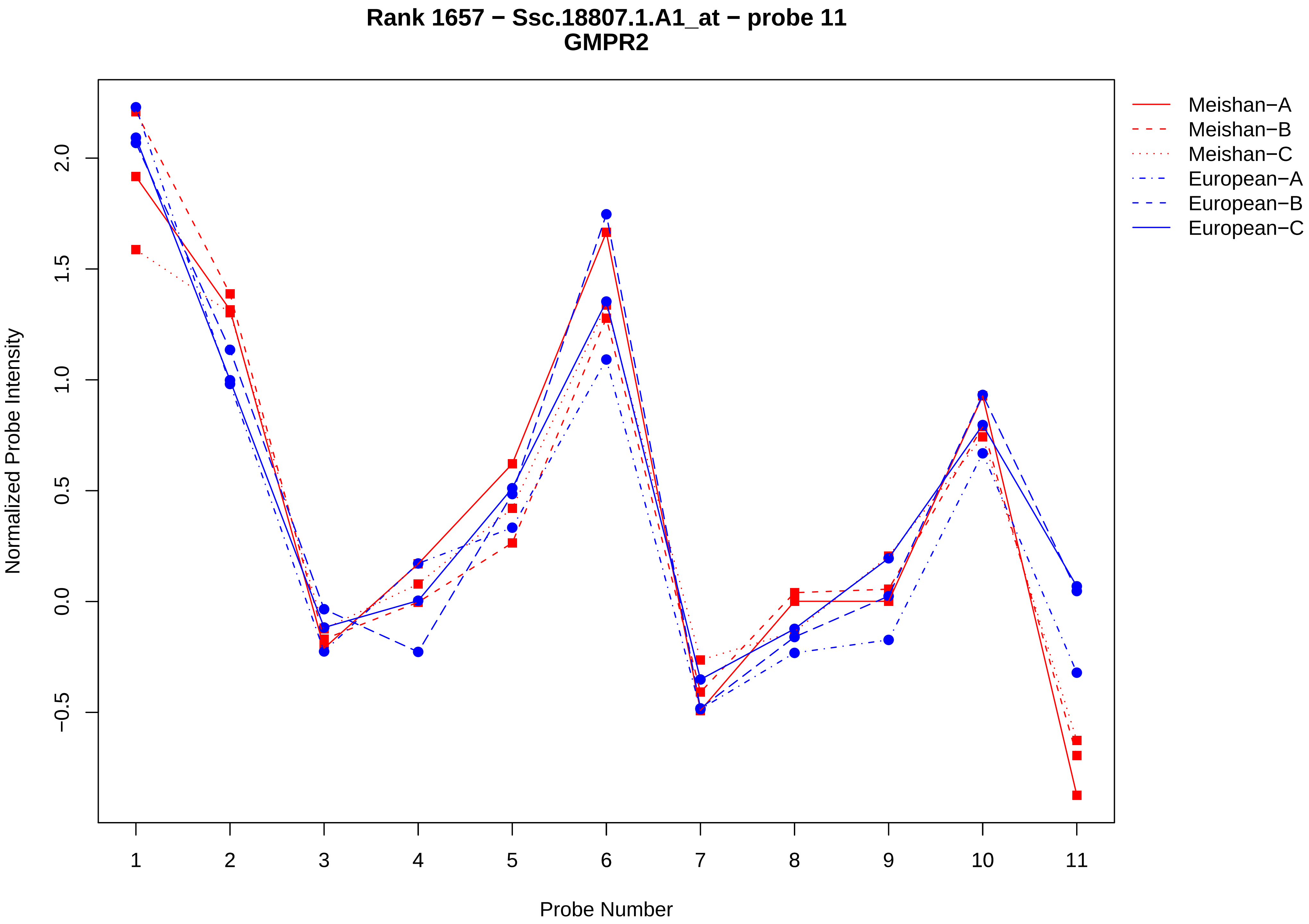

Rank 1657 − Ssc.18807.1.A1_at − probe 11
GMPR2
Normalized Probe Intensity
Probe Number
−0.5
0.0
0.5
1.0
1.5
2.0
1
2
3
4
5
6
7
8
9
10
11
Meishan−A
Meishan−B
Meishan−C
European−A
European−B
European−C

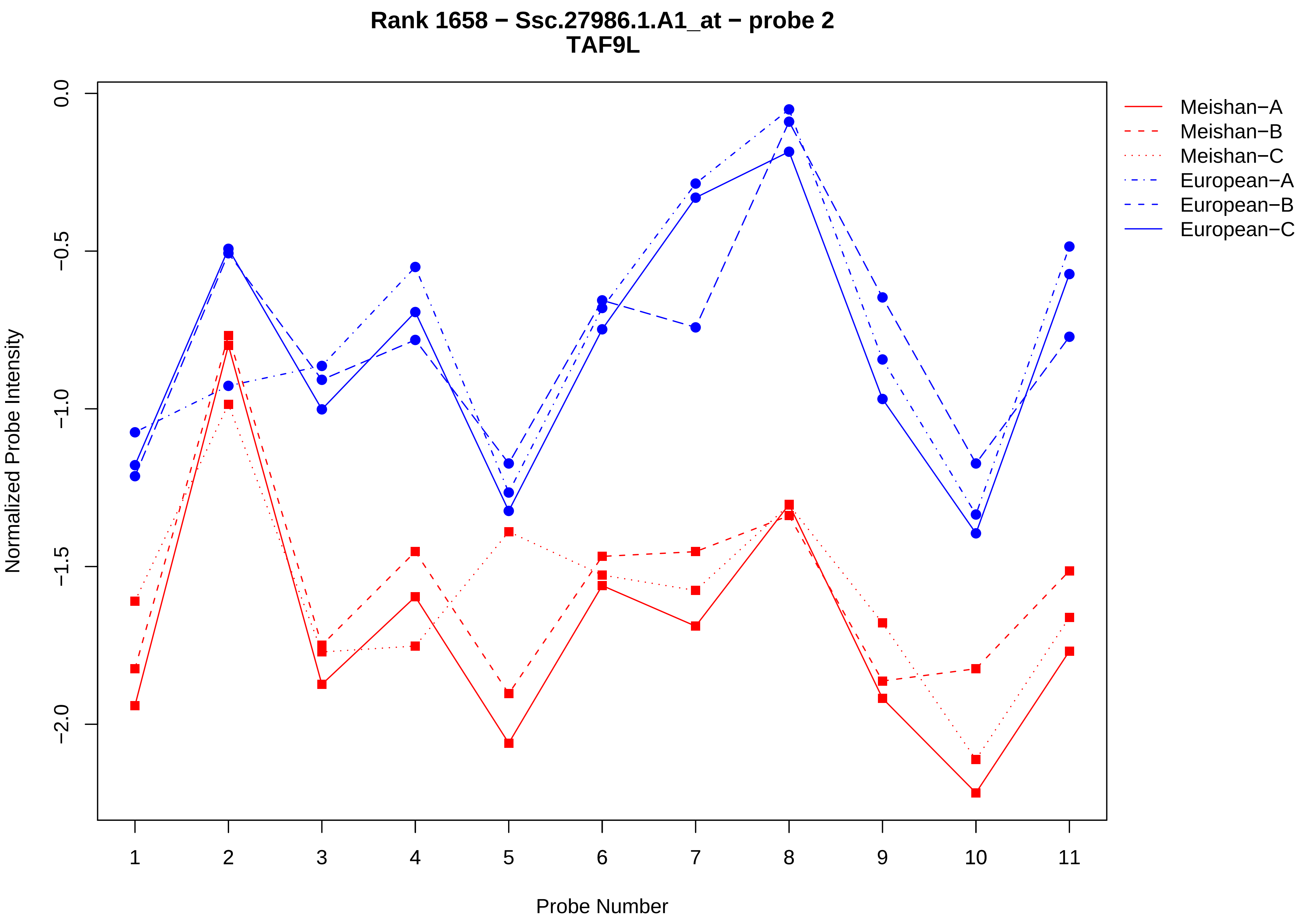

Rank 1658 − Ssc.27986.1.A1_at − probe 2
TAF9L
Normalized Probe Intensity
0.0
−0.5
−1.0
−1.5
−2.0
Probe Number
1
2
3
4
5
6
7
8
9
10
11
Meishan−A
Meishan−B
Meishan−C
European−A
European−B
European−C

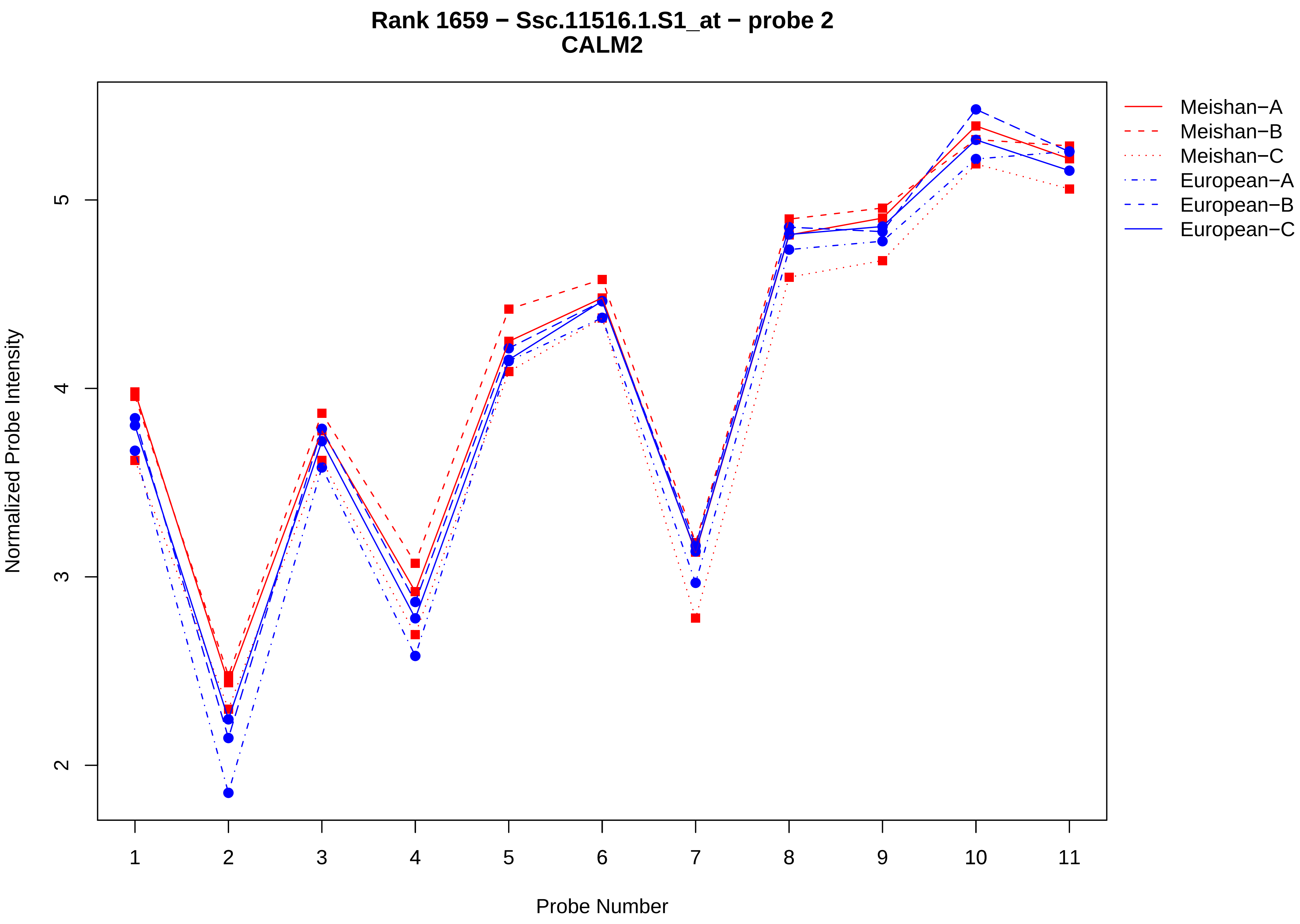
Rank 1659 − Ssc.11516.1.S1_at − probe 2
CALM2
Normalized Probe Intensity
2
3
4
5
1
2
3
4
5
6
7
8
9
10
11
Probe Number
Meishan−A
Meishan−B
Meishan−C
European−A
European−B
European−C

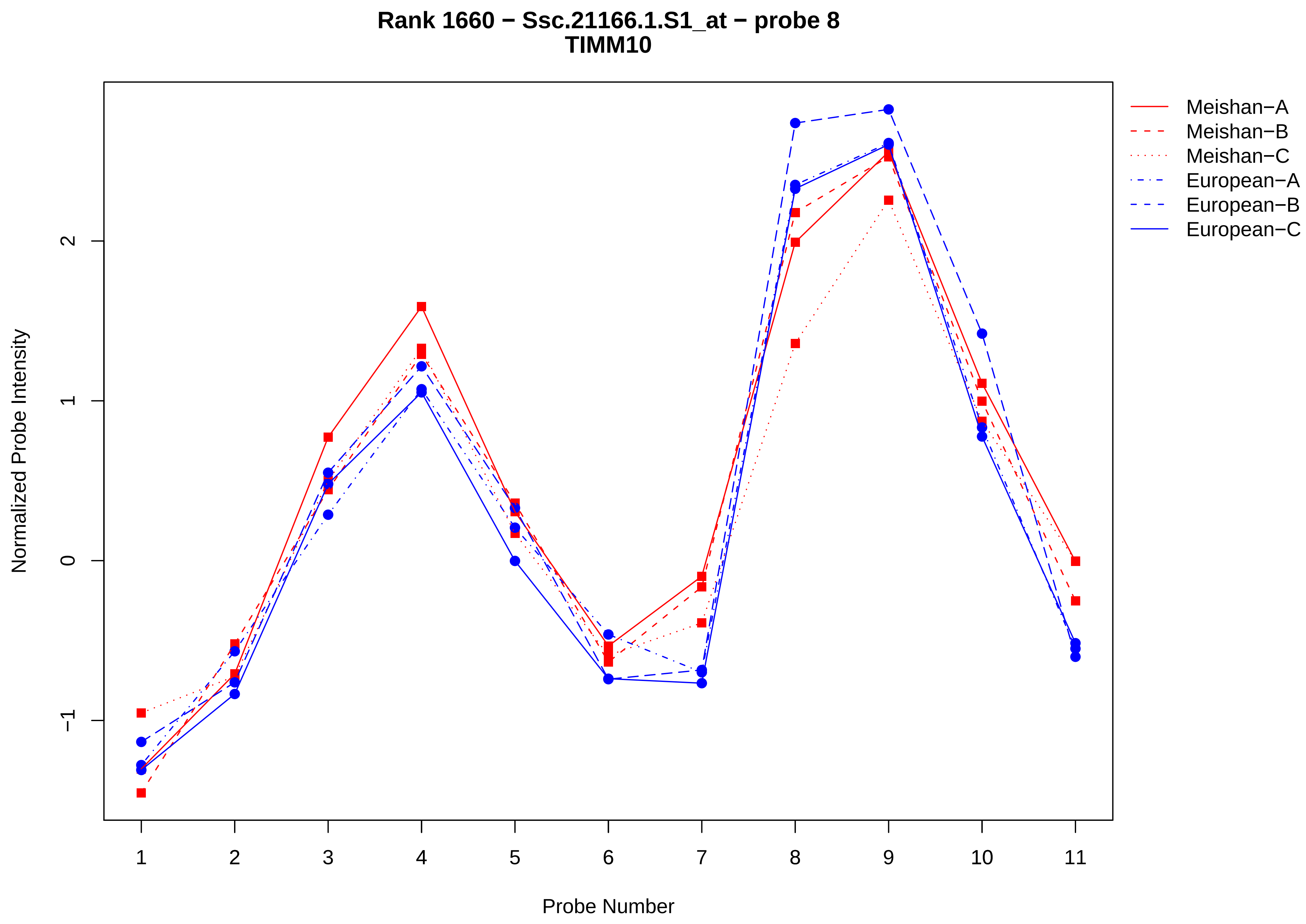

Rank 1660 − Ssc.21166.1.S1_at − probe 8
TIMM10
Normalized Probe Intensity
Probe Number
Meishan−A
Meishan−B
Meishan−C
European−A
European−B
European−C
−1
0
1
2
1
2
3
4
5
6
7
8
9
10
11

Rank 1661 − Ssc.17497.1.S1_at − probe 6
BLOC1S2

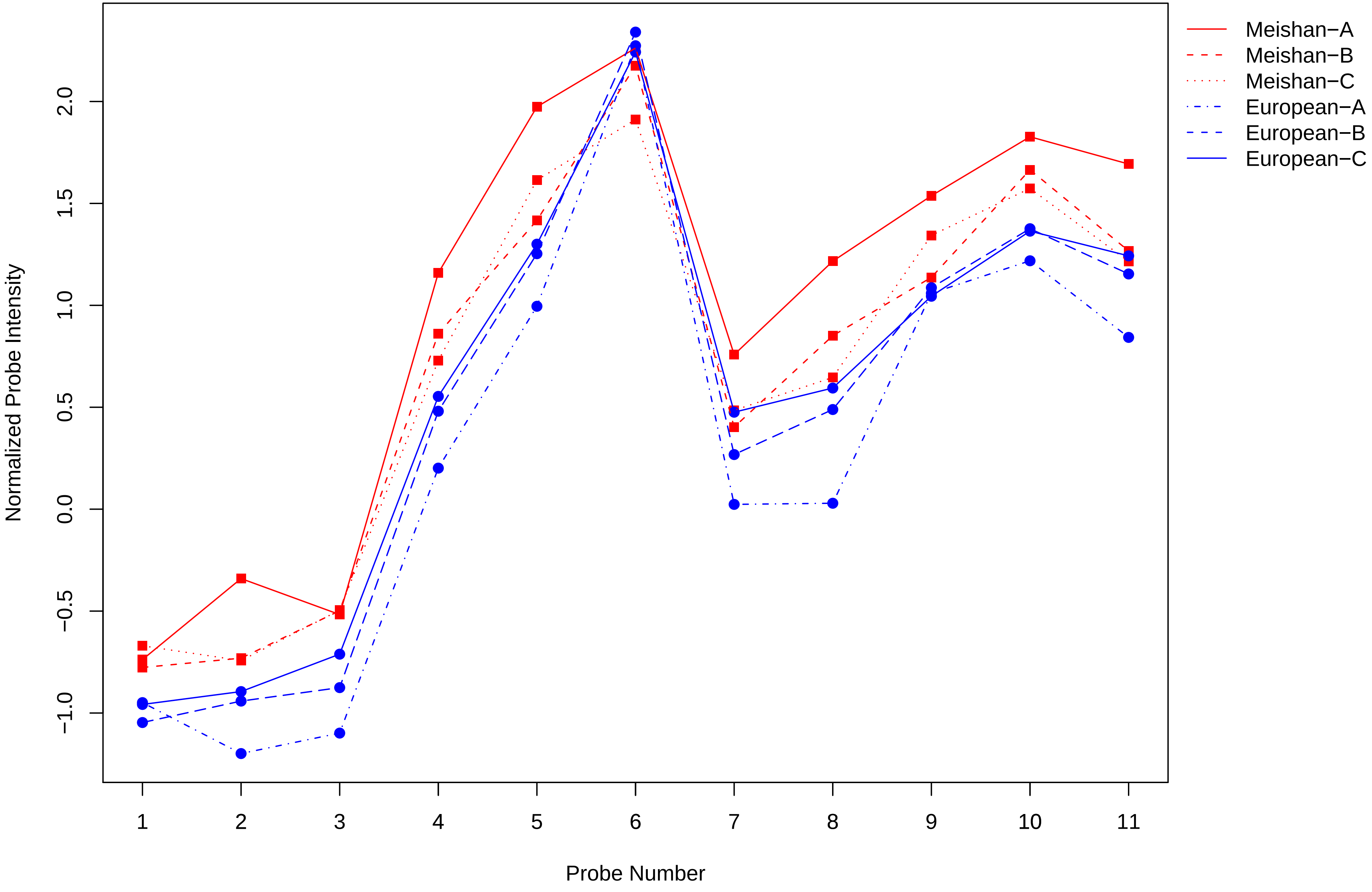

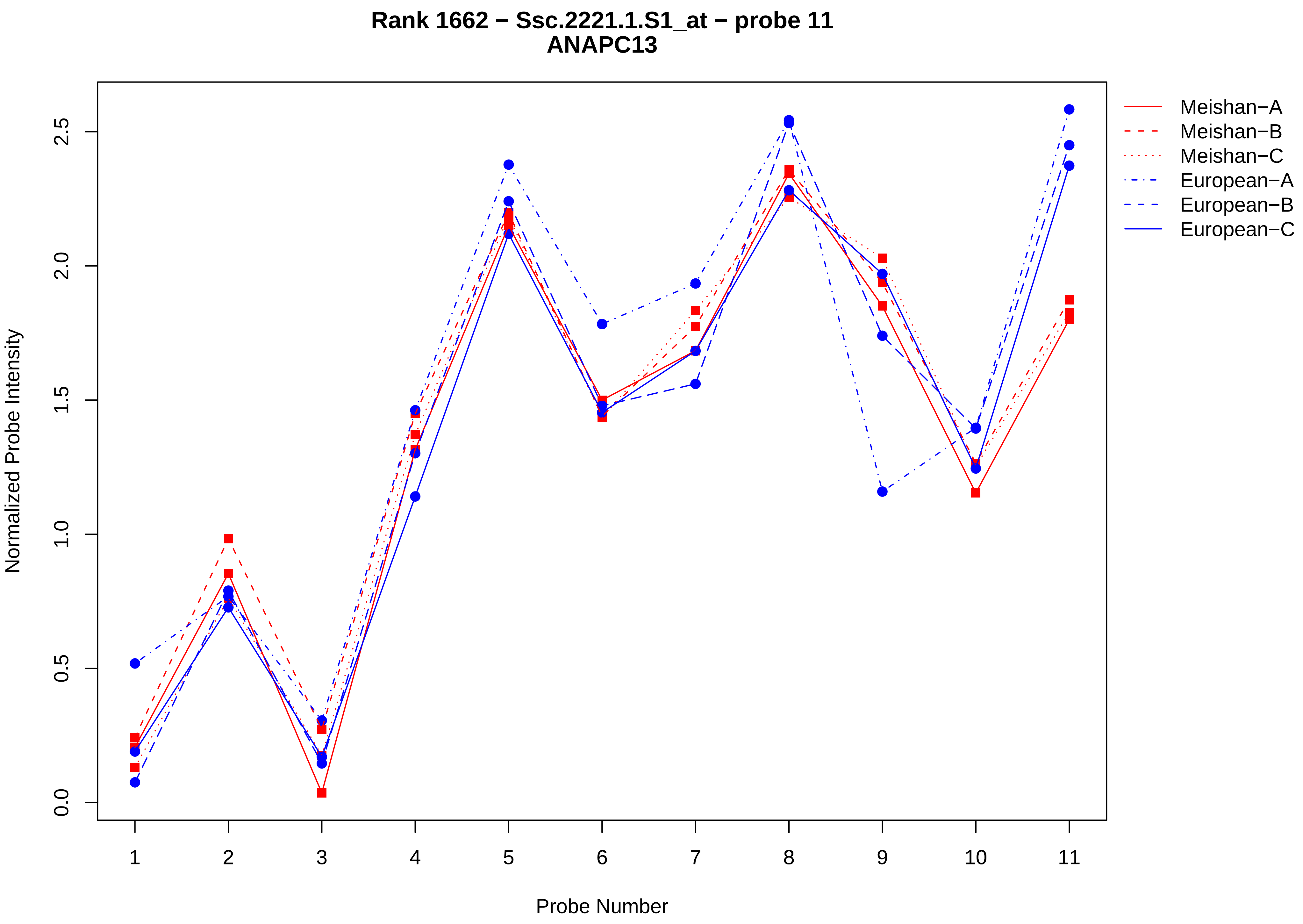
Rank 1662 − Ssc.2221.1.S1_at − probe 11
ANAPC13
Normalized Probe Intensity
Probe Number
0.0
0.5
1.0
1.5
2.0
2.5
1
2
3
4
5
6
7
8
9
10
11
Meishan−A
Meishan−B
Meishan−C
European−A
European−B
European−C

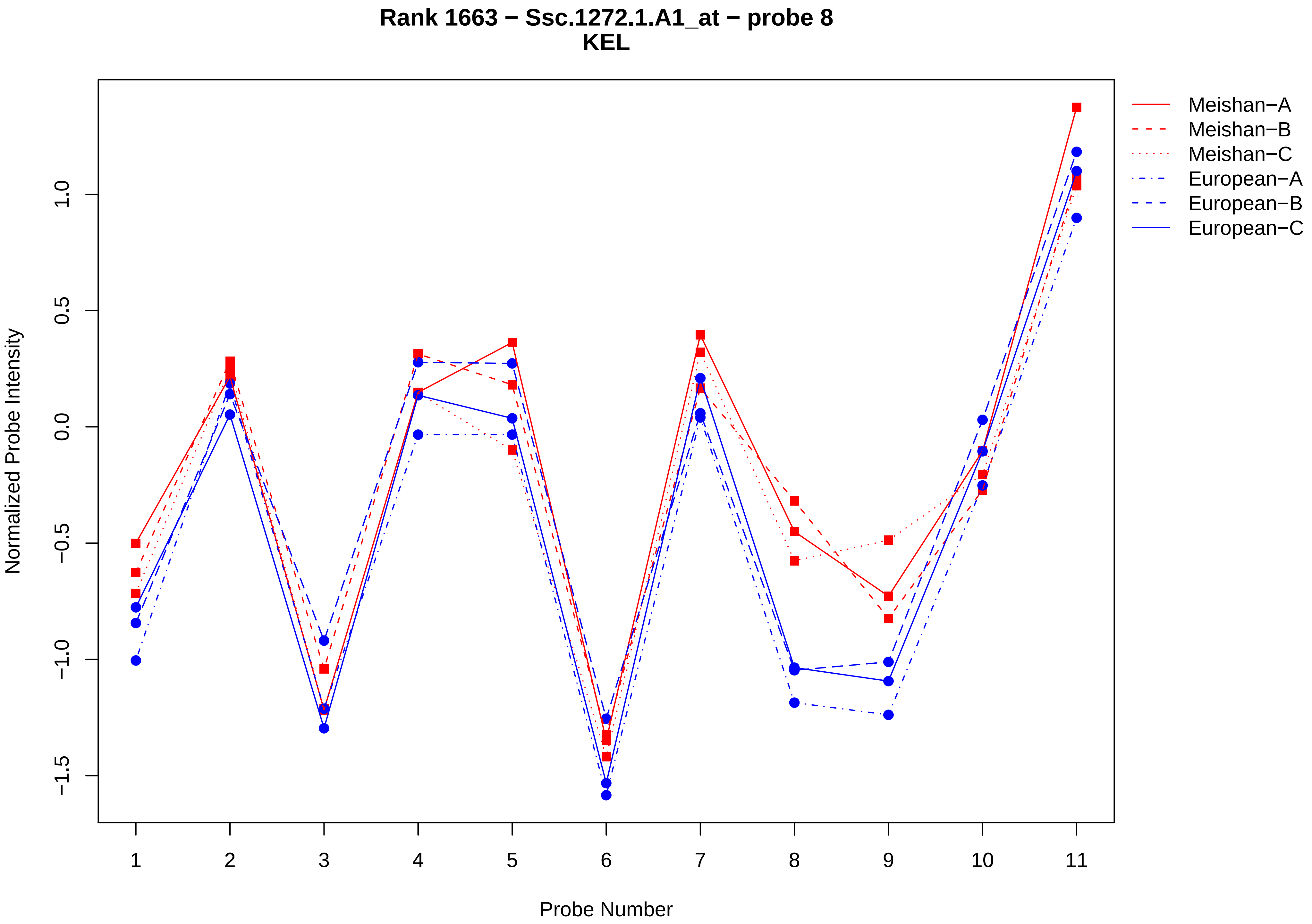

Rank 1663 − Ssc.1272.1.A1_at − probe 8
KEL
Normalized Probe Intensity
Probe Number
1.0
0.5
0.0
−0.5
−1.0
−1.5
1
2
3
4
5
6
7
8
9
10
11
Meishan−A
Meishan−B
Meishan−C
European−A
European−B
European−C

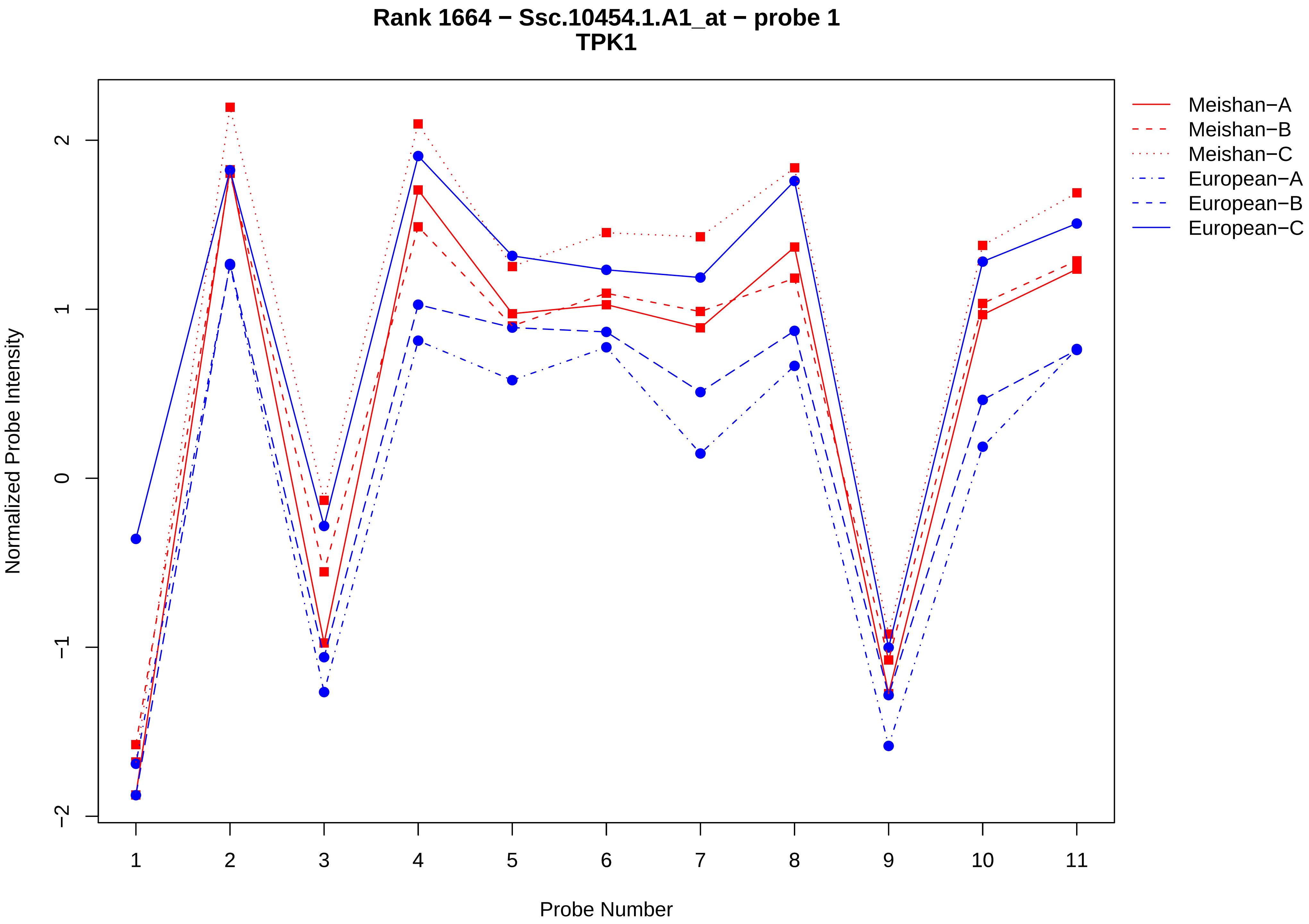
Rank 1664 − Ssc.10454.1.A1_at − probe 1
TPK1
Normalized Probe Intensity
Probe Number
−2
−1
0
1
2
1
2
3
4
5
6
7
8
9
10
11
Meishan−A
Meishan−B
Meishan−C
European−A
European−B
European−C

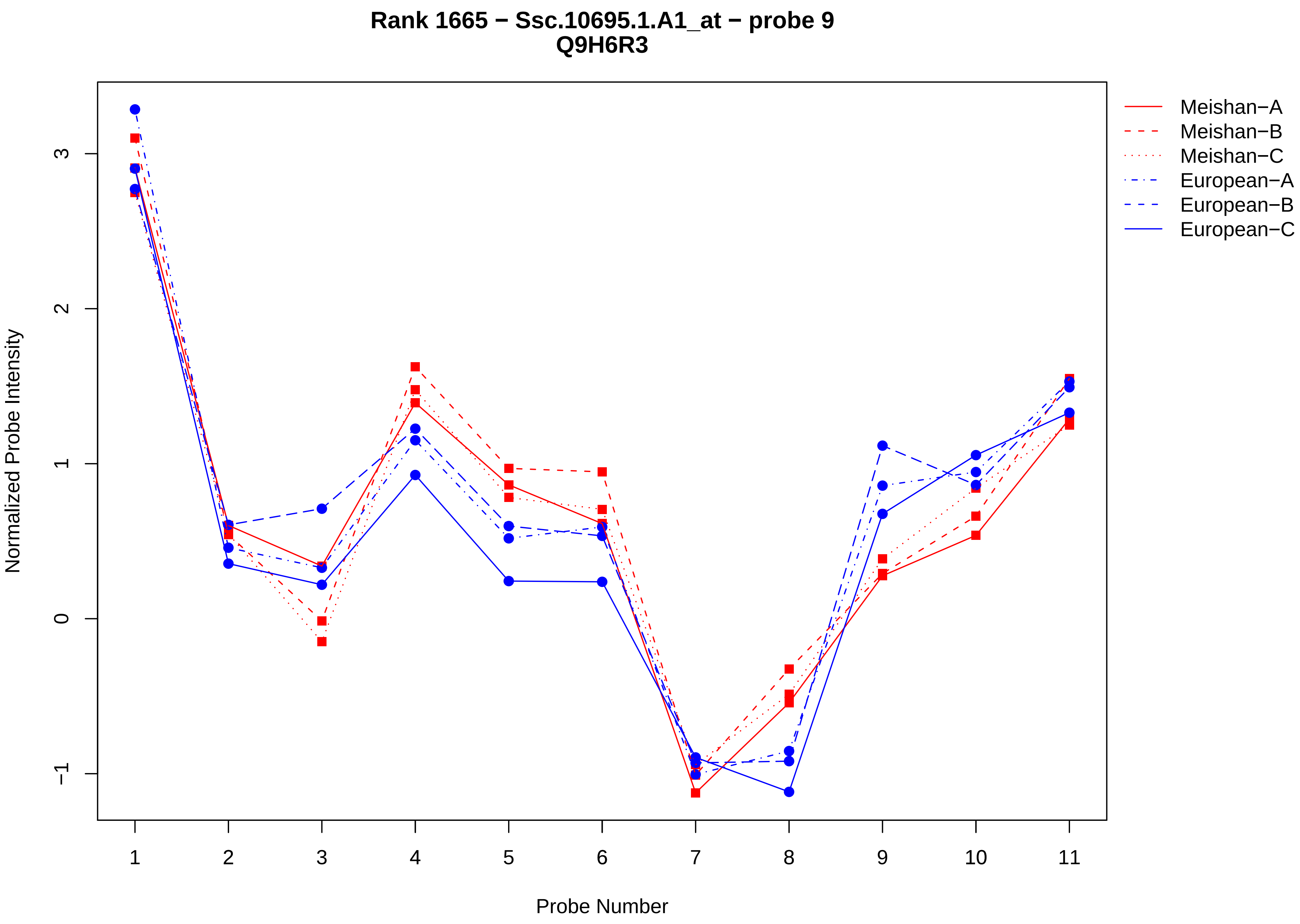
Rank 1665 − Ssc.10695.1.A1_at − probe 9
Q9H6R3
Normalized Probe Intensity
Probe Number
−1
0
1
2
3
1
2
3
4
5
6
7
8
9
10
11
Meishan−A
Meishan−B
Meishan−C
European−A
European−B
European−C

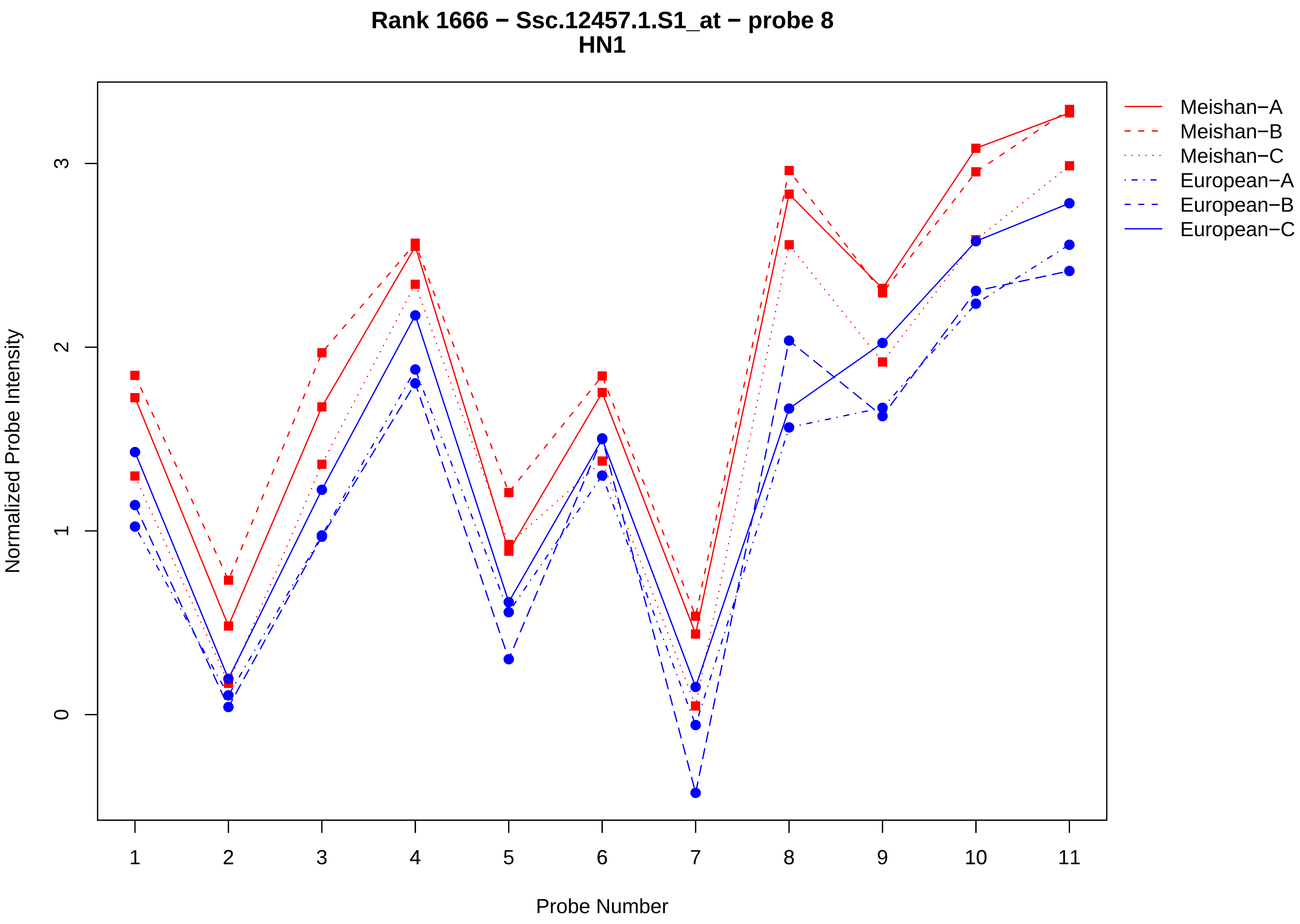

Rank 1666 − Ssc.12457.1.S1_at − probe 8
HN1
Normalized Probe Intensity
Probe Number
0
1
2
3
1
2
3
4
5
6
7
8
9
10
11
Meishan−A
Meishan−B
Meishan−C
European−A
European−B
European−C

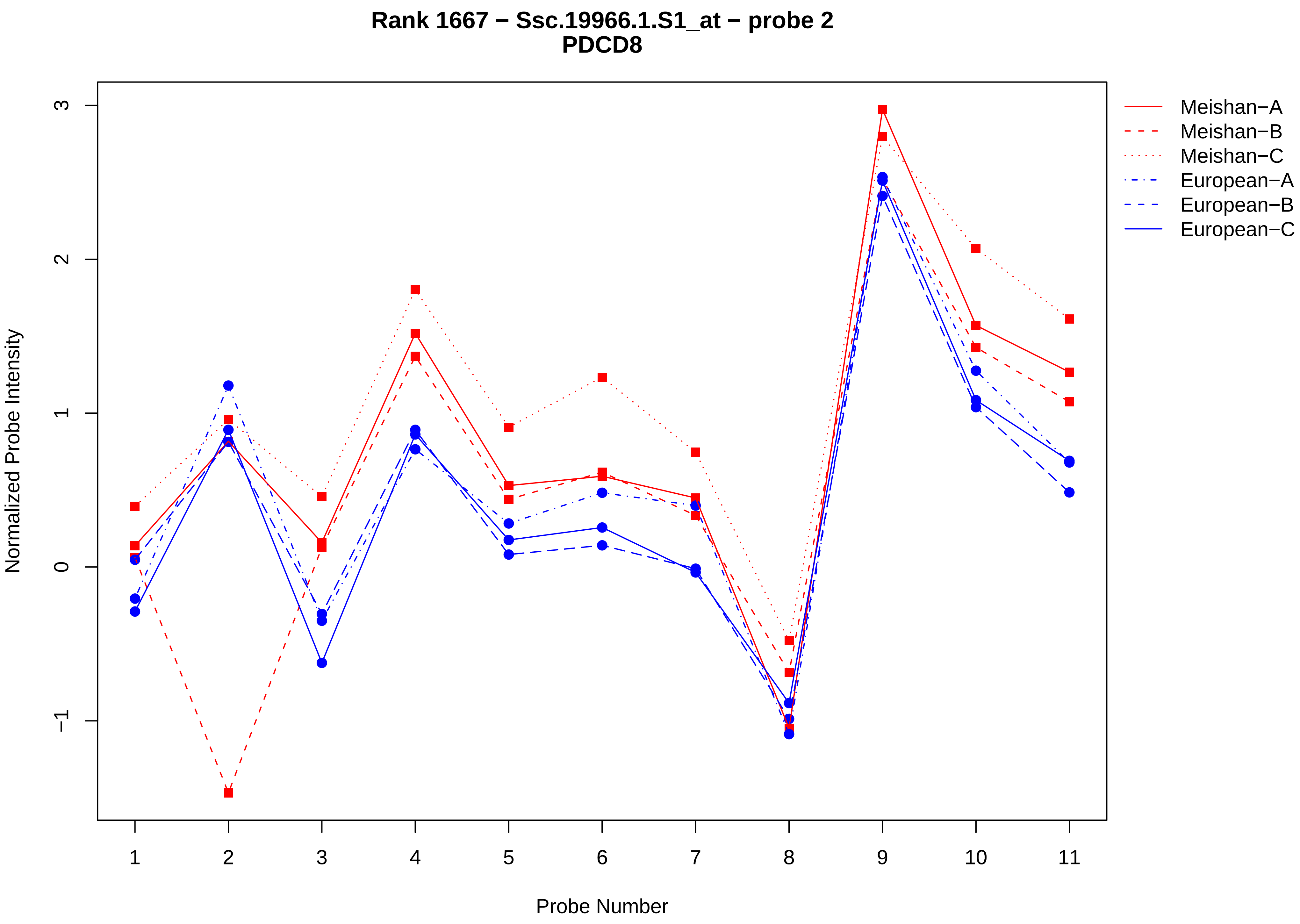

Rank 1667 − Ssc.19966.1.S1_at − probe 2
PDCD8
Normalized Probe Intensity
Probe Number
−1
0
1
2
3
1
2
3
4
5
6
7
8
9
10
11
Meishan−A
Meishan−B
Meishan−C
European−A
European−B
European−C

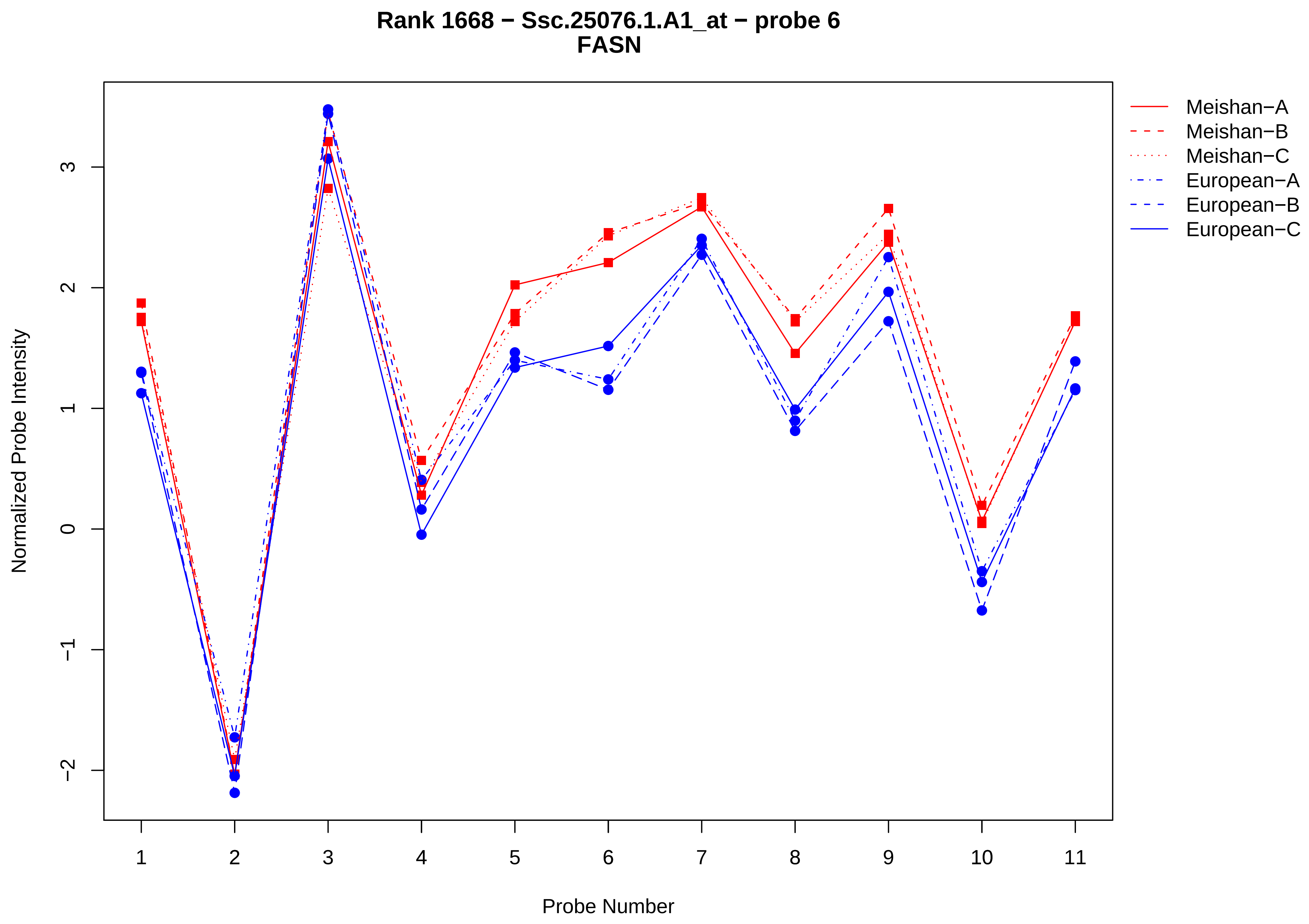

Rank 1668 − Ssc.25076.1.A1_at − probe 6
FASN
Normalized Probe Intensity
−2
−1
0
1
2
3
1
2
3
4
5
6
7
8
9
10
11
Probe Number
Meishan−A
Meishan−B
Meishan−C
European−A
European−B
European−C

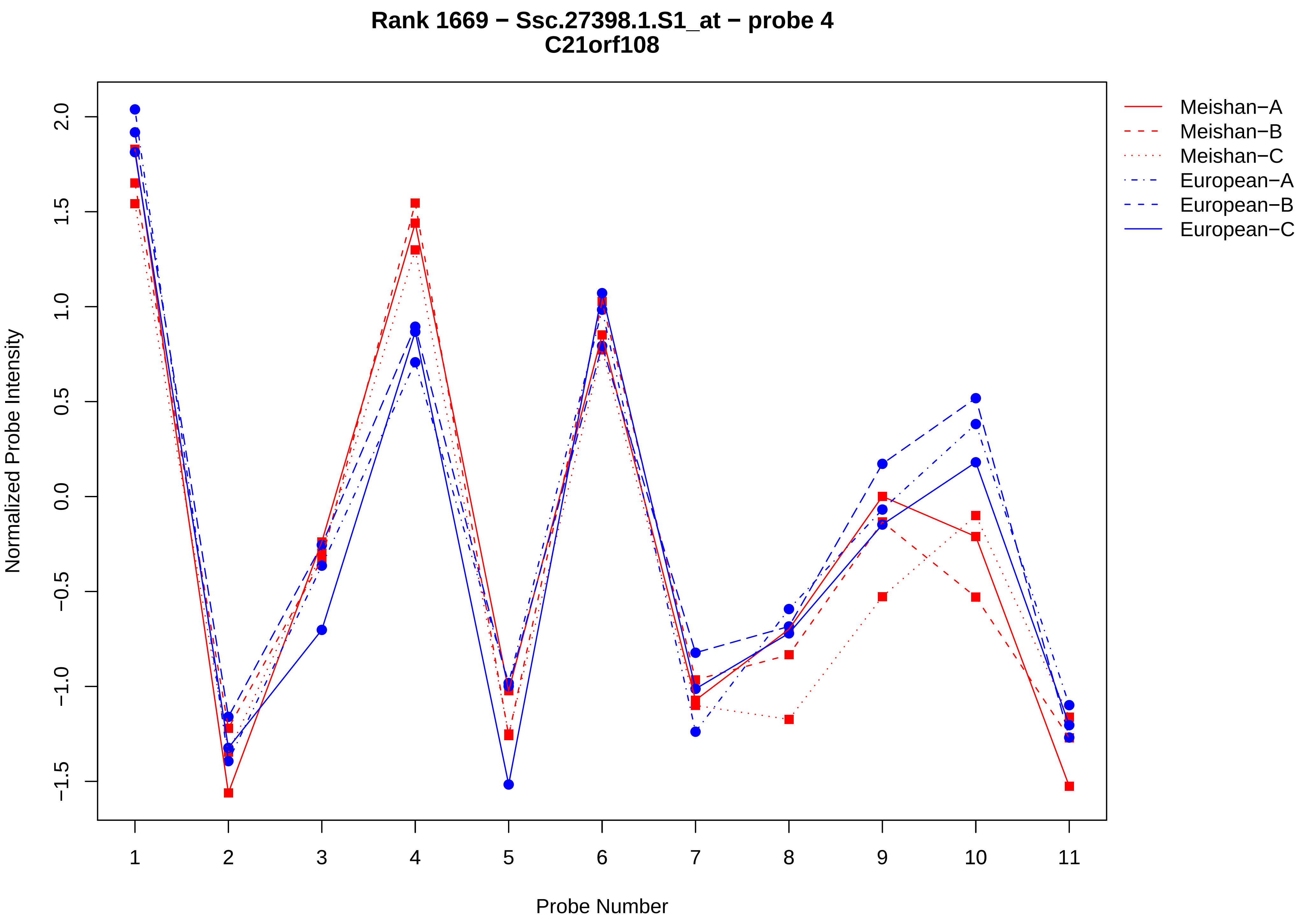
Rank 1669 − Ssc.27398.1.S1_at − probe 4
C21orf108
Normalized Probe Intensity
Probe Number
Meishan−A
Meishan−B
Meishan−C
European−A
European−B
European−C
2.0
1.5
1.0
0.5
0.0
−0.5
−1.0
−1.5
1
2
3
4
5
6
7
8
9
10
11

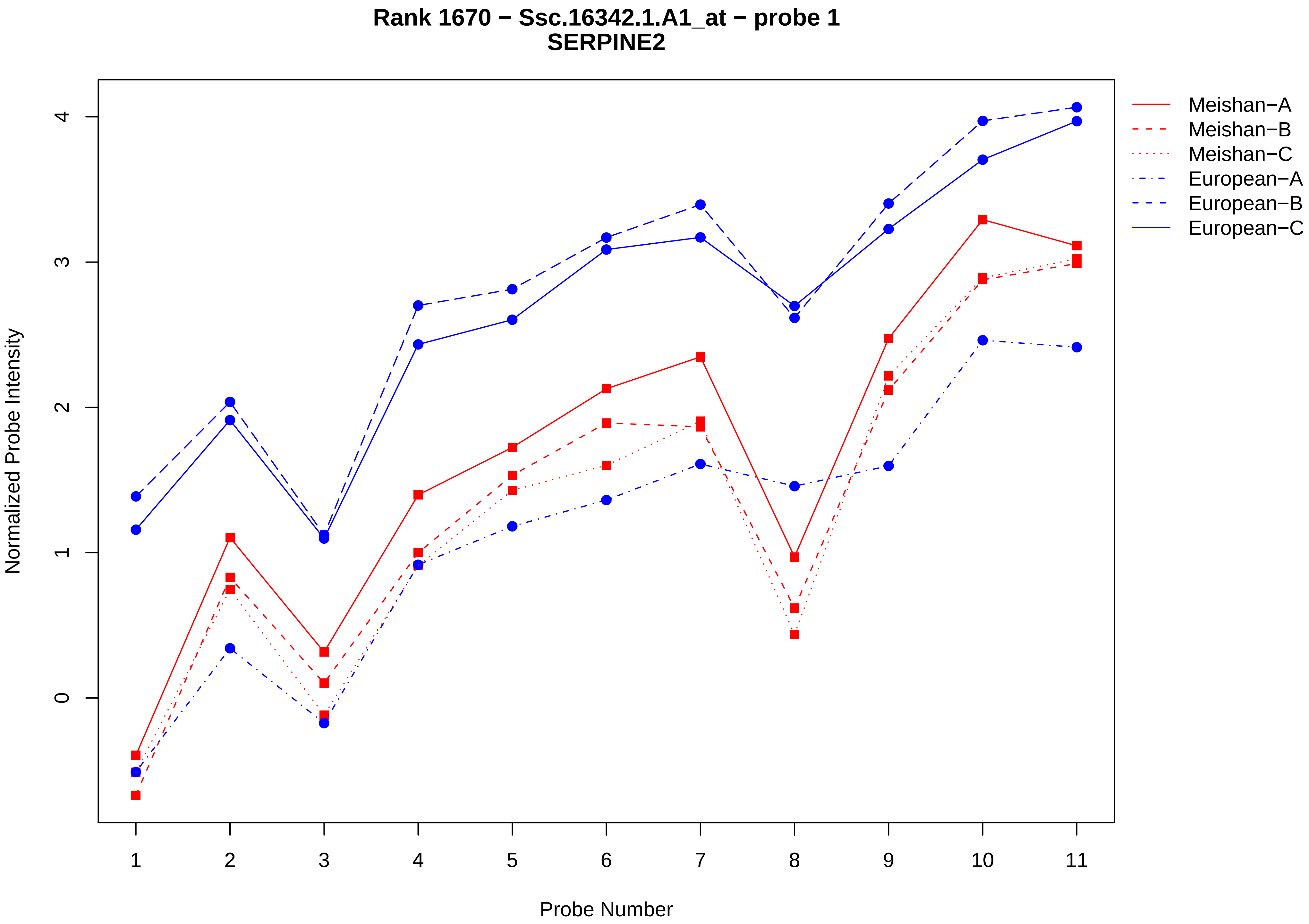

Rank 1670 − Ssc.16342.1.A1_at − probe 1
SERPINE2
Normalized Probe Intensity
Probe Number
0
1
2
3
4
1
2
3
4
5
6
7
8
9
10
11
Meishan−A
Meishan−B
Meishan−C
European−A
European−B
European−C

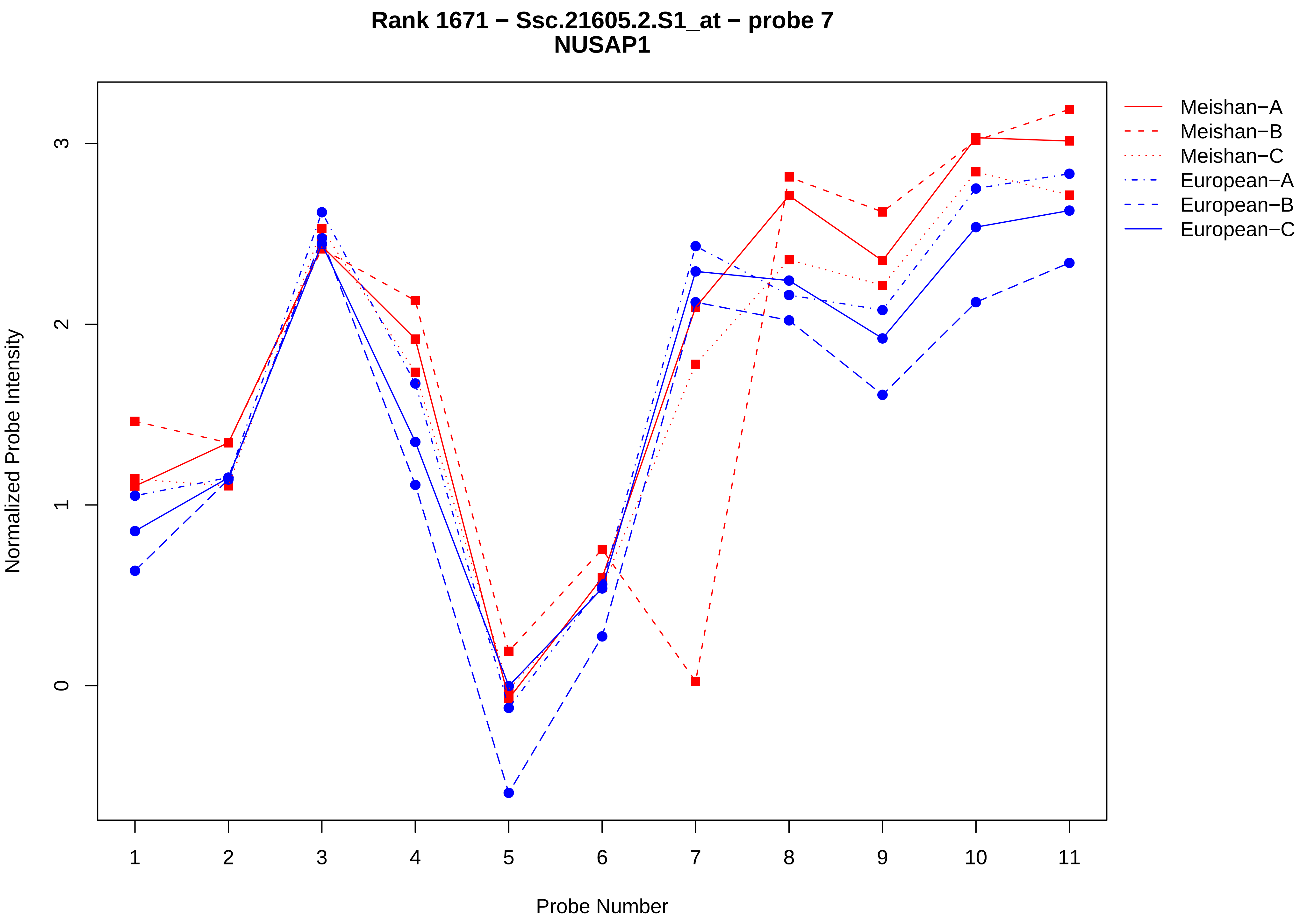
Rank 1671 − Ssc.21605.2.S1_at − probe 7
NUSAP1
Normalized Probe Intensity
0
1
2
3
1
2
3
4
5
6
7
8
9
10
11
Probe Number
Meishan−A
Meishan−B
Meishan−C
European−A
European−B
European−C

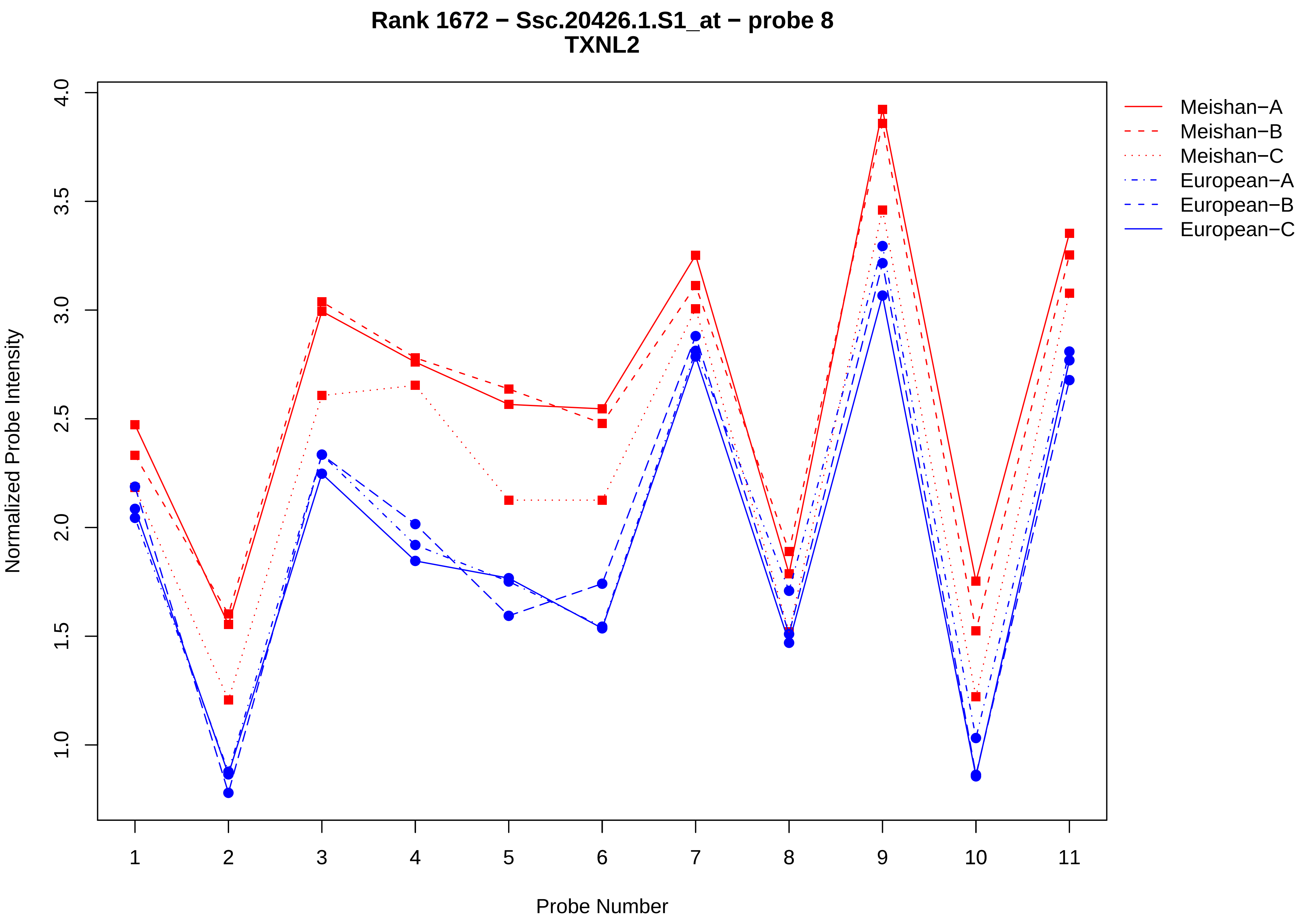

Rank 1672 − Ssc.20426.1.S1_at − probe 8
TXNL2
Normalized Probe Intensity
Probe Number
1.0
1.5
2.0
2.5
3.0
3.5
4.0
1
2
3
4
5
6
7
8
9
10
11
Meishan−A
Meishan−B
Meishan−C
European−A
European−B
European−C

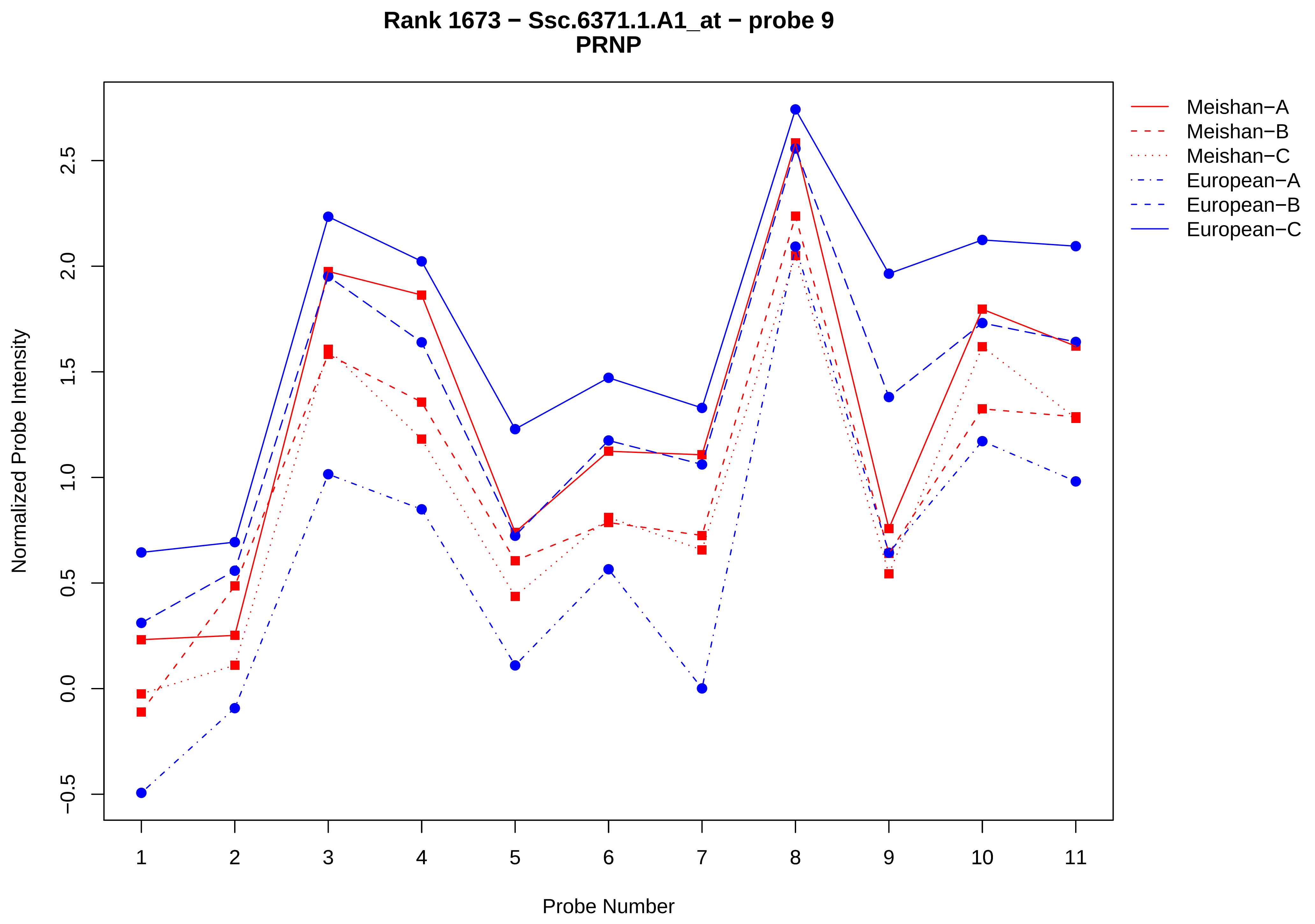

Rank 1673 − Ssc.6371.1.A1_at − probe 9
PRNP
Normalized Probe Intensity
Probe Number
−0.5
0.0
0.5
1.0
1.5
2.0
2.5
1
2
3
4
5
6
7
8
9
10
11
Meishan−A
Meishan−B
Meishan−C
European−A
European−B
European−C

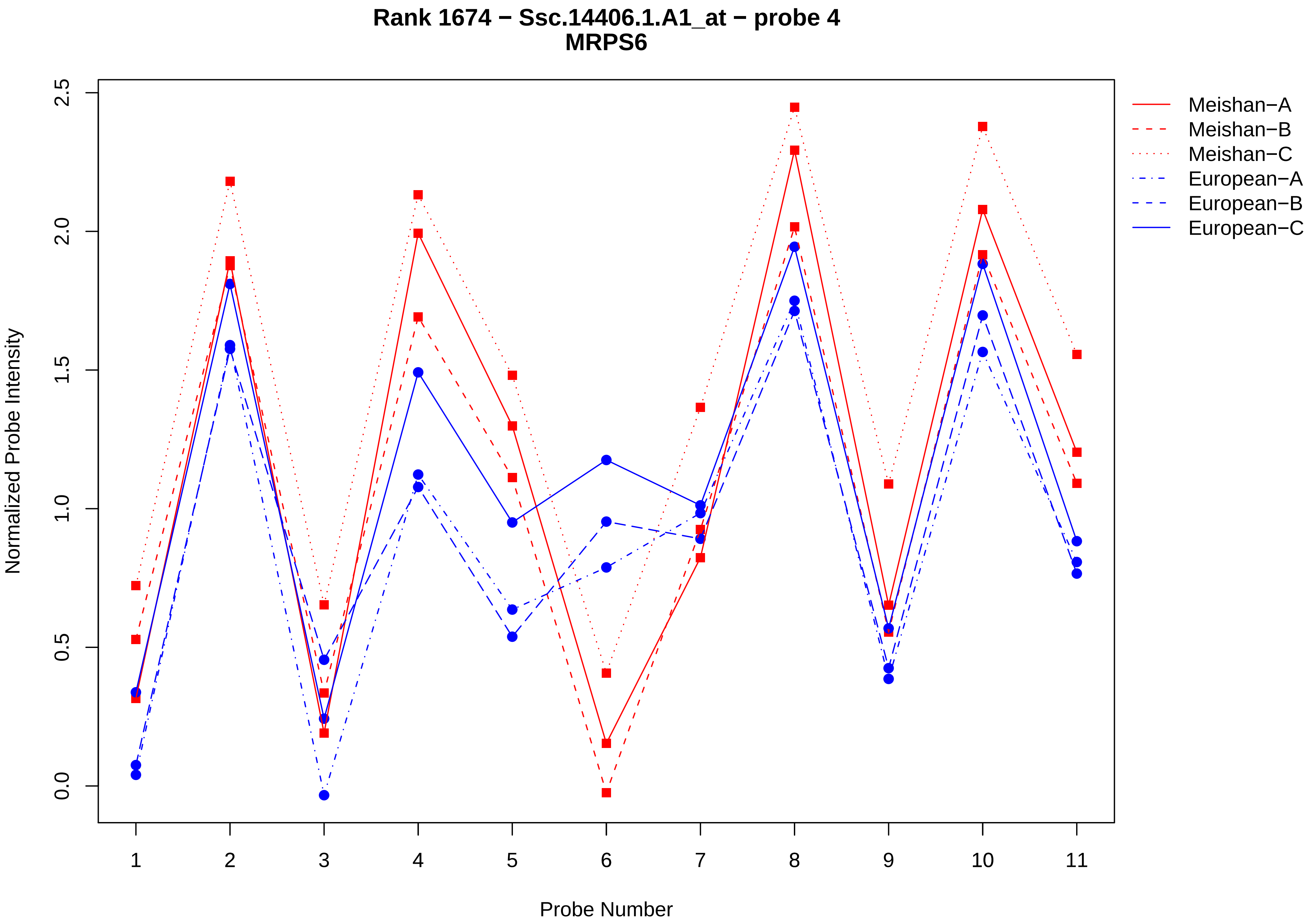
Rank 1674 − Ssc.14406.1.A1_at − probe 4
MRPS6
Normalized Probe Intensity
0.0
0.5
1.0
1.5
2.0
2.5
1
2
3
4
5
6
7
8
9
10
11
Probe Number
Meishan−A
Meishan−B
Meishan−C
European−A
European−B
European−C

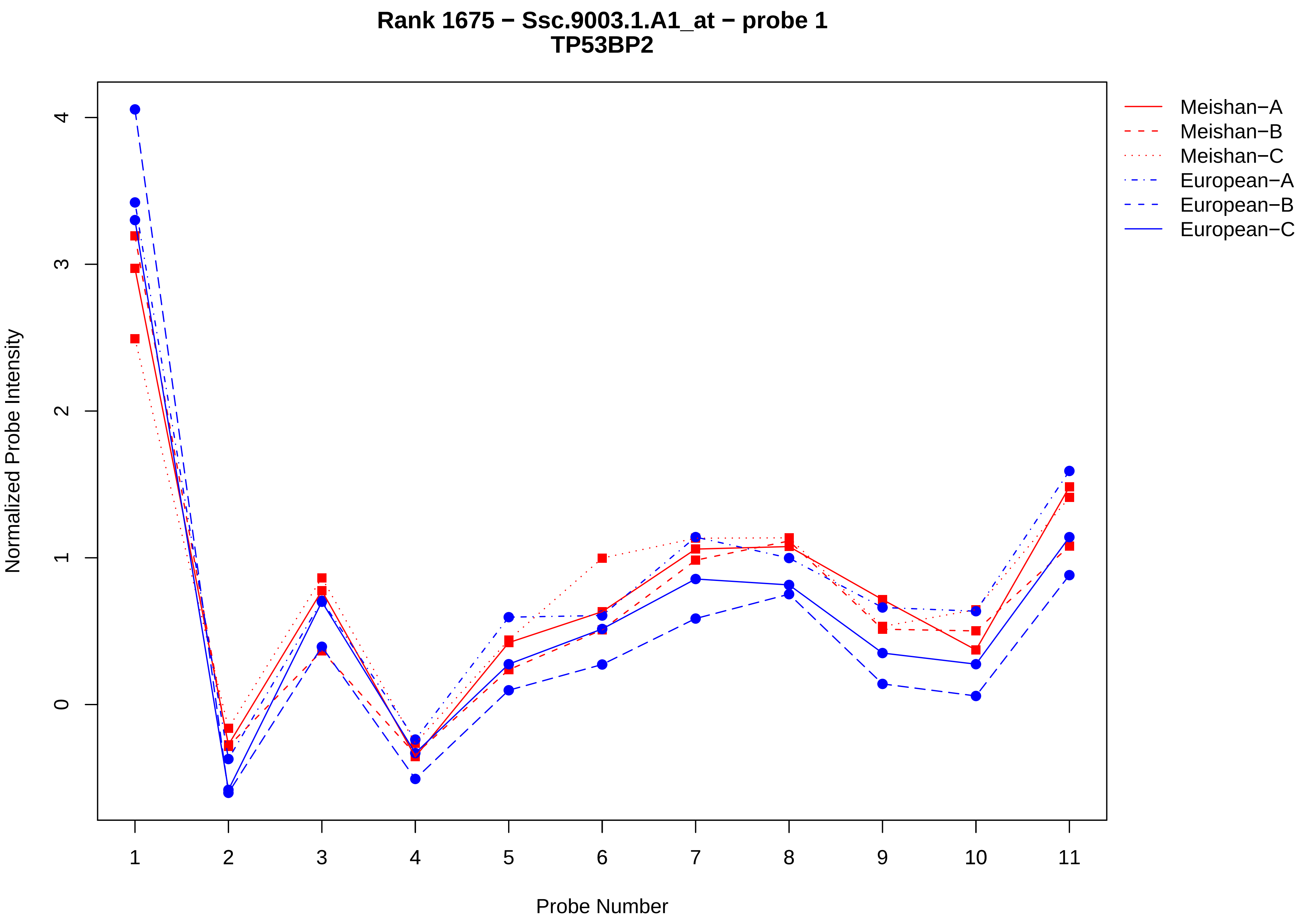

Rank 1675 − Ssc.9003.1.A1_at − probe 1
TP53BP2
Normalized Probe Intensity
Probe Number
0
1
2
3
4
1
2
3
4
5
6
7
8
9
10
11
Meishan−A
Meishan−B
Meishan−C
European−A
European−B
European−C

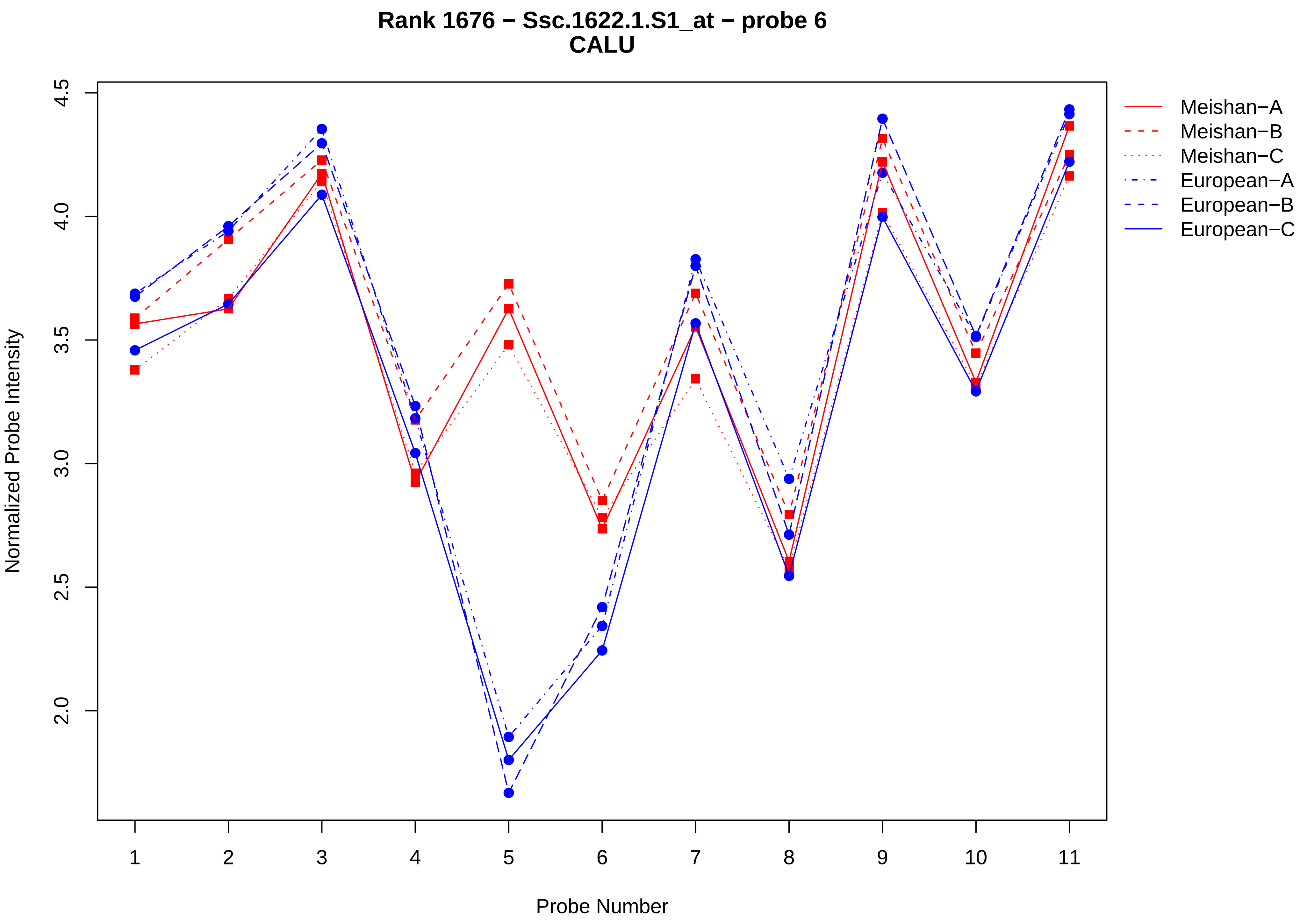

Rank 1676 − Ssc.1622.1.S1_at − probe 6
CALU
Normalized Probe Intensity
Probe Number
Meishan−A
Meishan−B
Meishan−C
European−A
European−B
European−C

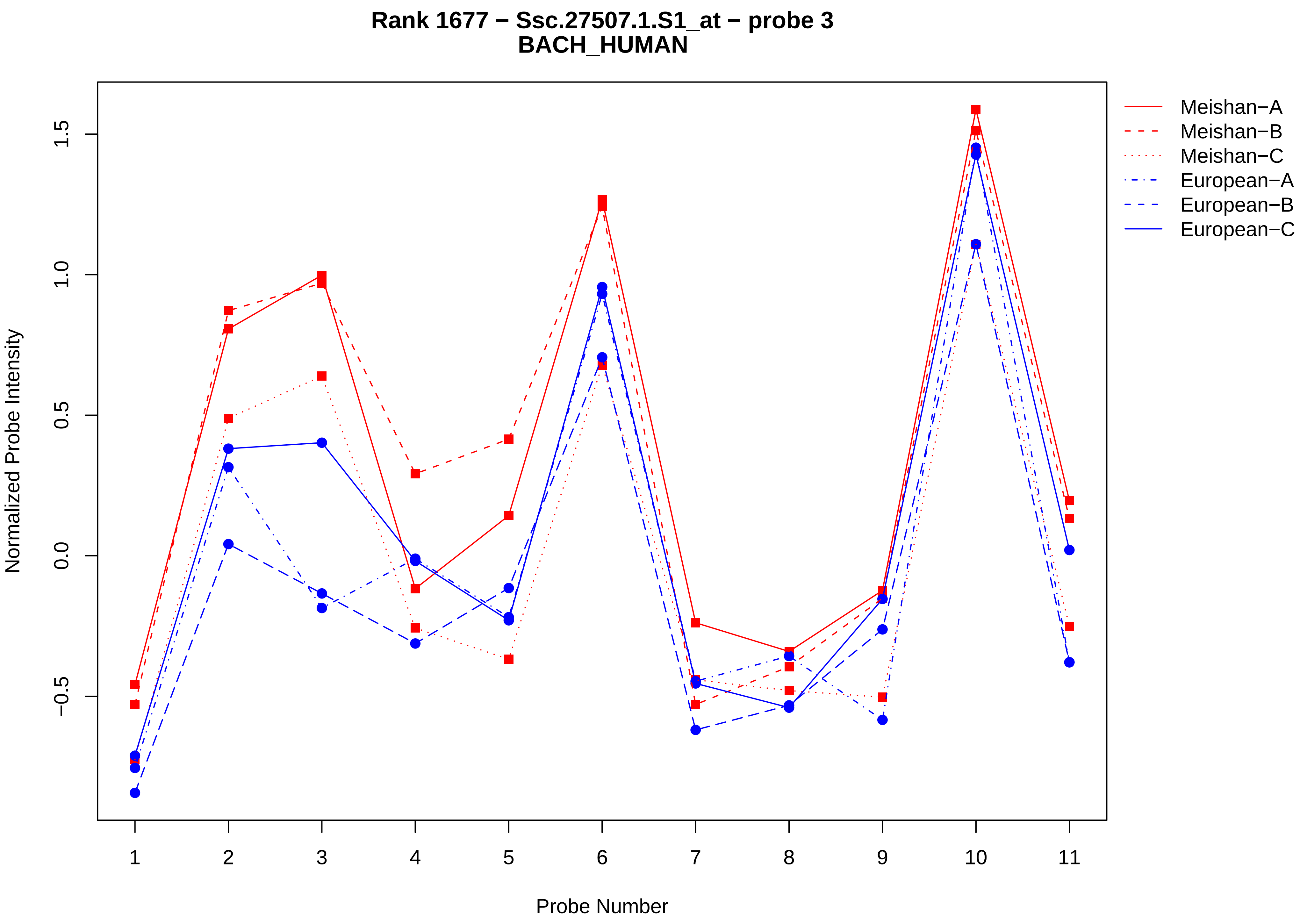

Rank 1677 − Ssc.27507.1.S1_at − probe 3
BACH_HUMAN
Normalized Probe Intensity
Probe Number
−0.5
0.0
0.5
1.0
1.5
1
2
3
4
5
6
7
8
9
10
11
Meishan−A
Meishan−B
Meishan−C
European−A
European−B
European−C

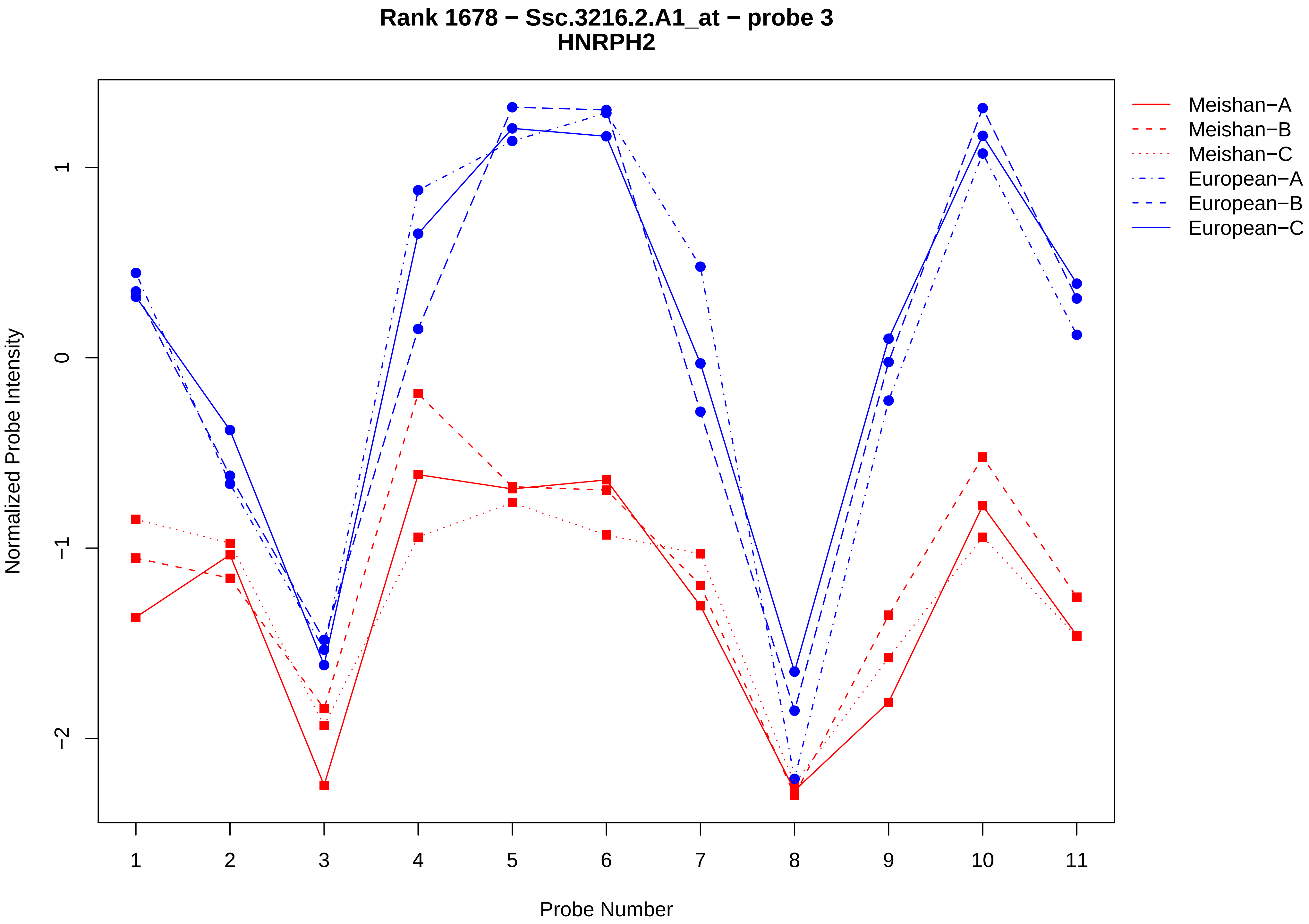

Rank 1678 − Ssc.3216.2.A1_at − probe 3
HNRPH2
Normalized Probe Intensity
Probe Number
−2
−1
0
1
1
2
3
4
5
6
7
8
9
10
11
Meishan−A
Meishan−B
Meishan−C
European−A
European−B
European−C

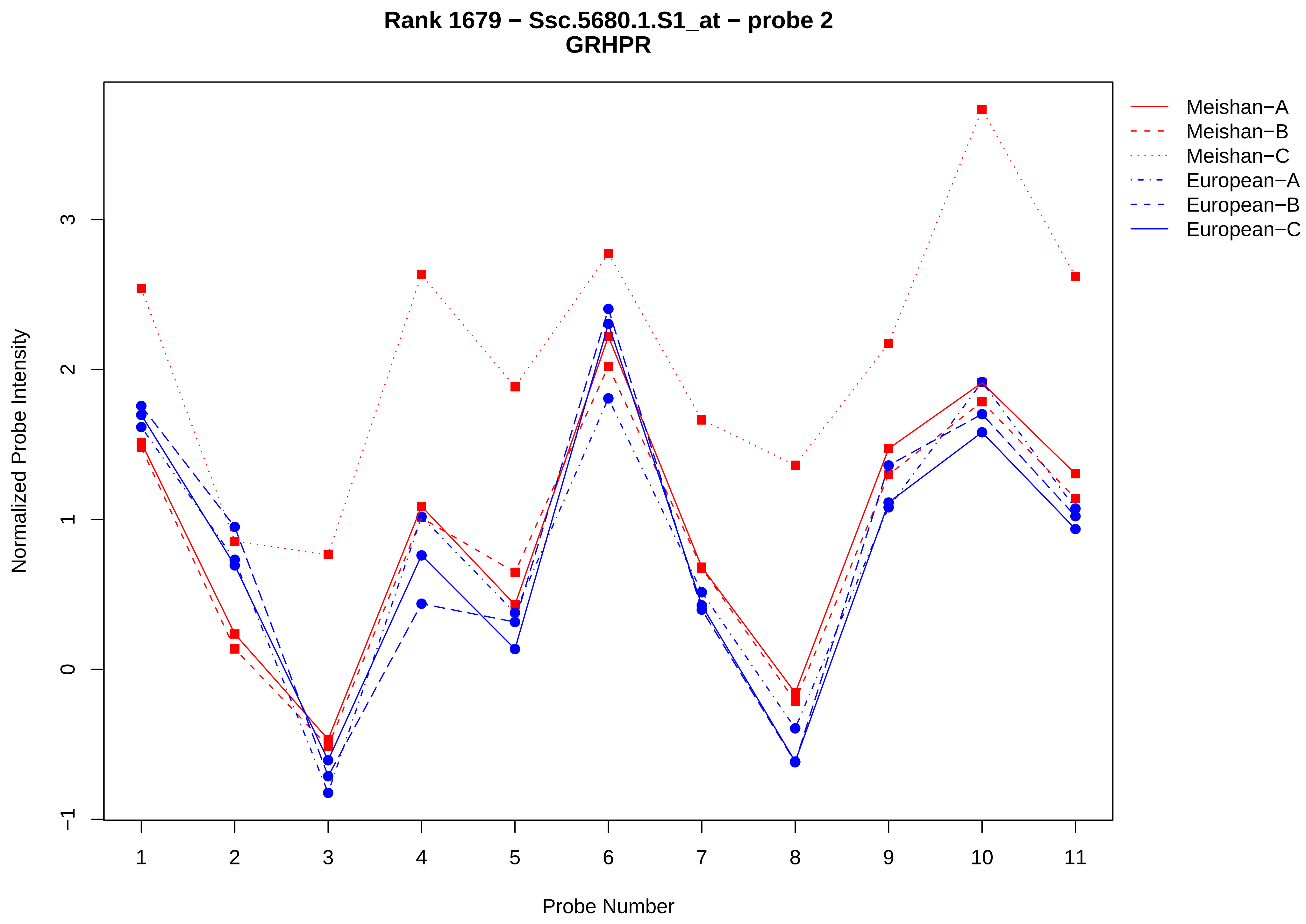
Rank 1679 − Ssc.5680.1.S1_at − probe 2
GRHPR
Normalized Probe Intensity
Probe Number
−1
0
1
2
3
1
2
3
4
5
6
7
8
9
10
11
Meishan−A
Meishan−B
Meishan−C
European−A
European−B
European−C

Rank 1680 − Ssc.12724.1.S1_at − probe 2
ATXN10

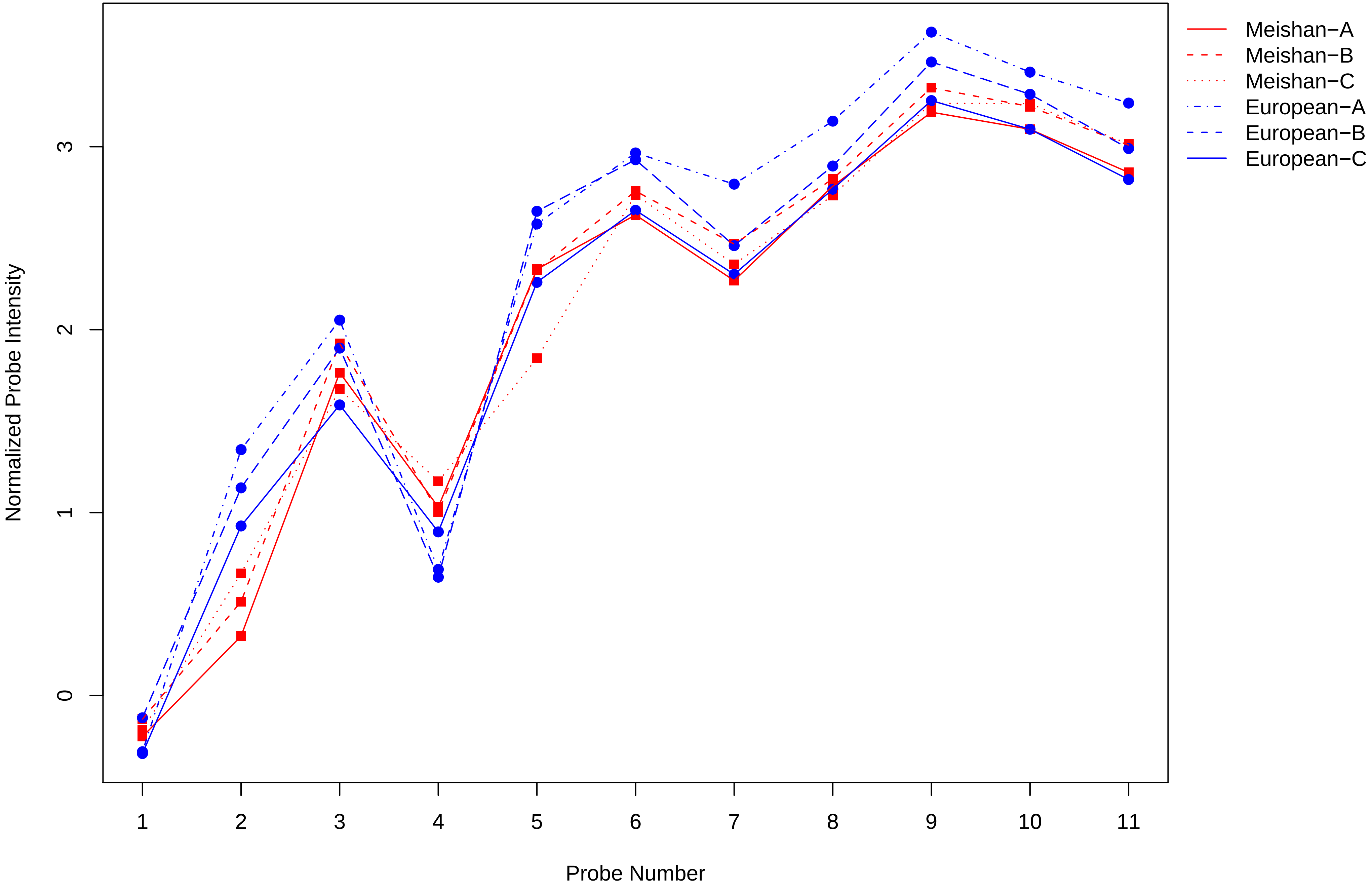

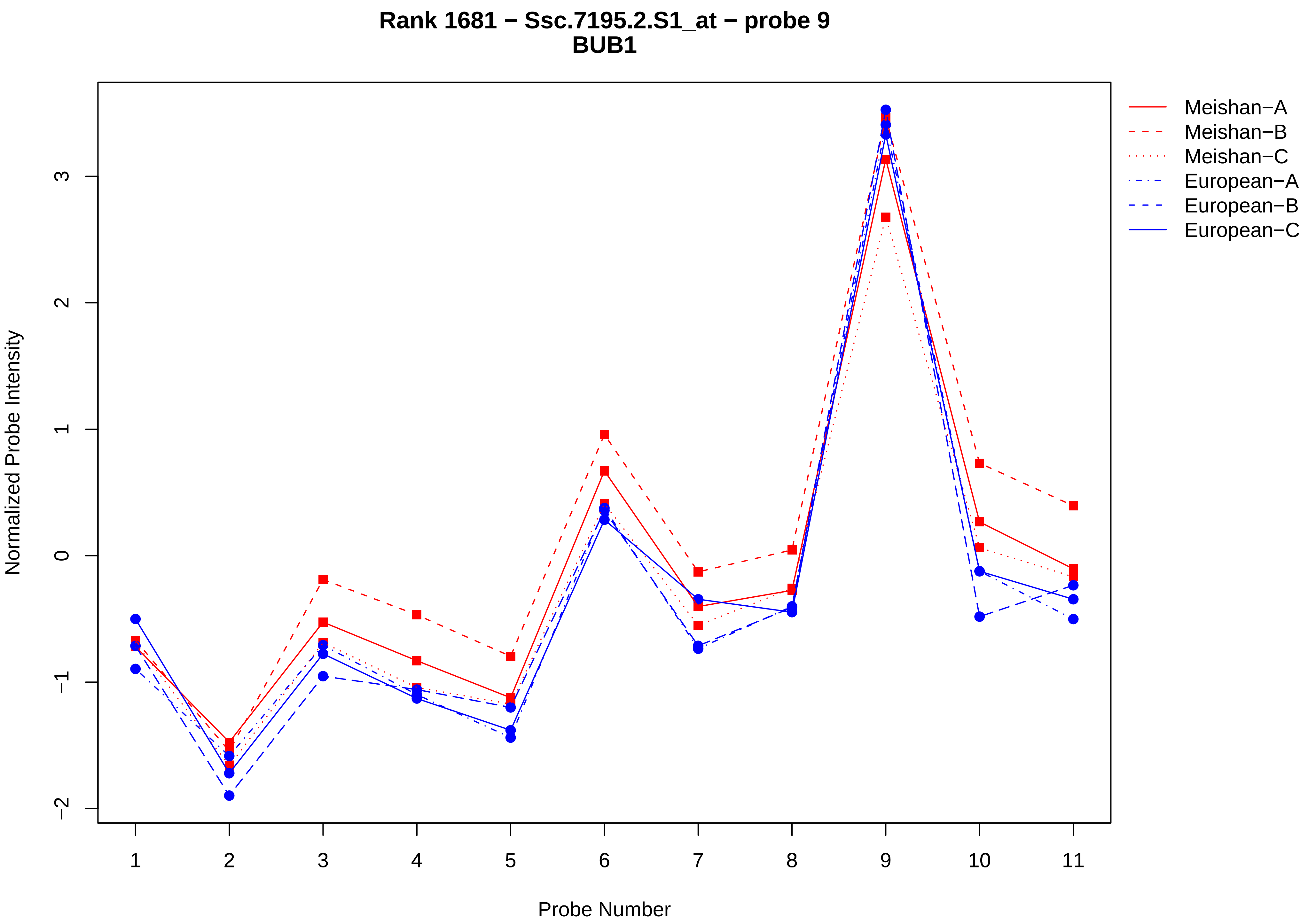

Rank 1681 − Ssc.7195.2.S1_at − probe 9
BUB1
Normalized Probe Intensity
Probe Number
−2
−1
0
1
2
3
1
2
3
4
5
6
7
8
9
10
11
Meishan−A
Meishan−B
Meishan−C
European−A
European−B
European−C

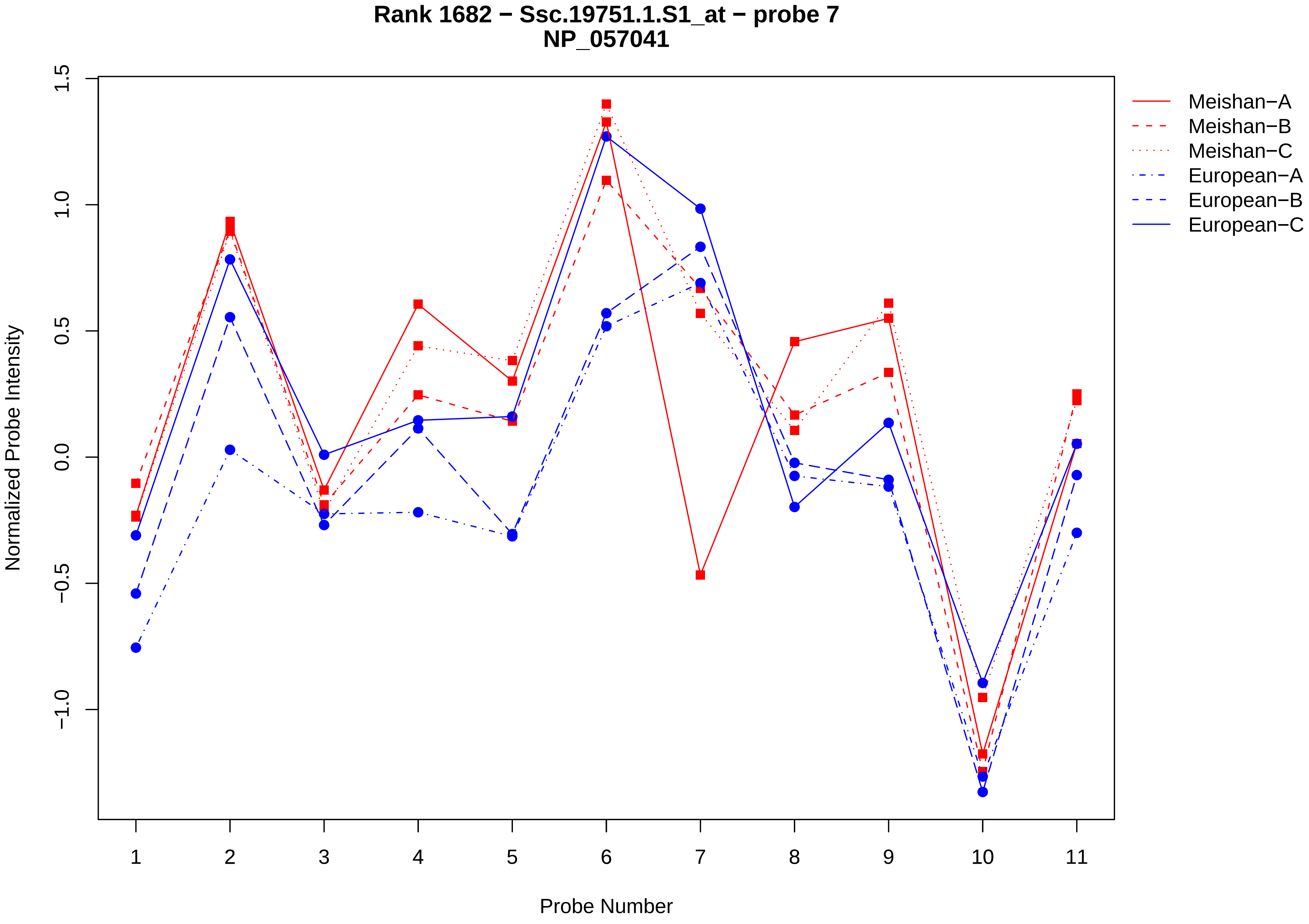

Rank 1682 − Ssc.19751.1.S1_at − probe 7
NP_057041
Normalized Probe Intensity
Probe Number
1.5
1.0
0.5
0.0
−0.5
−1.0
1
2
3
4
5
6
7
8
9
10
11
Meishan−A
Meishan−B
Meishan−C
European−A
European−B
European−C

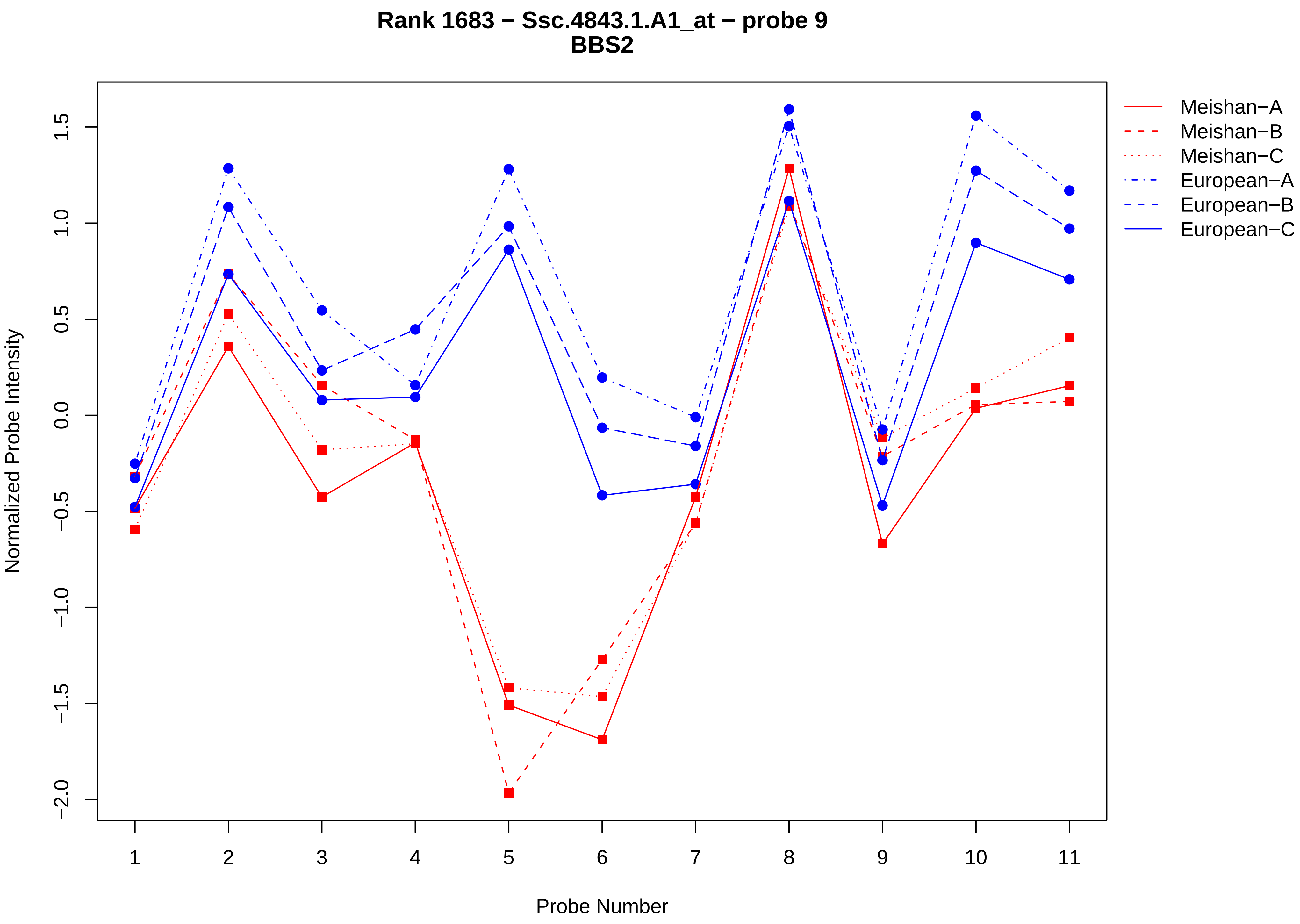

Rank 1683 − Ssc.4843.1.A1_at − probe 9
BBS2
Normalized Probe Intensity
Probe Number
−2.0
−1.5
−1.0
−0.5
0.0
0.5
1.0
1.5
1
2
3
4
5
6
7
8
9
10
11
Meishan−A
Meishan−B
Meishan−C
European−A
European−B
European−C

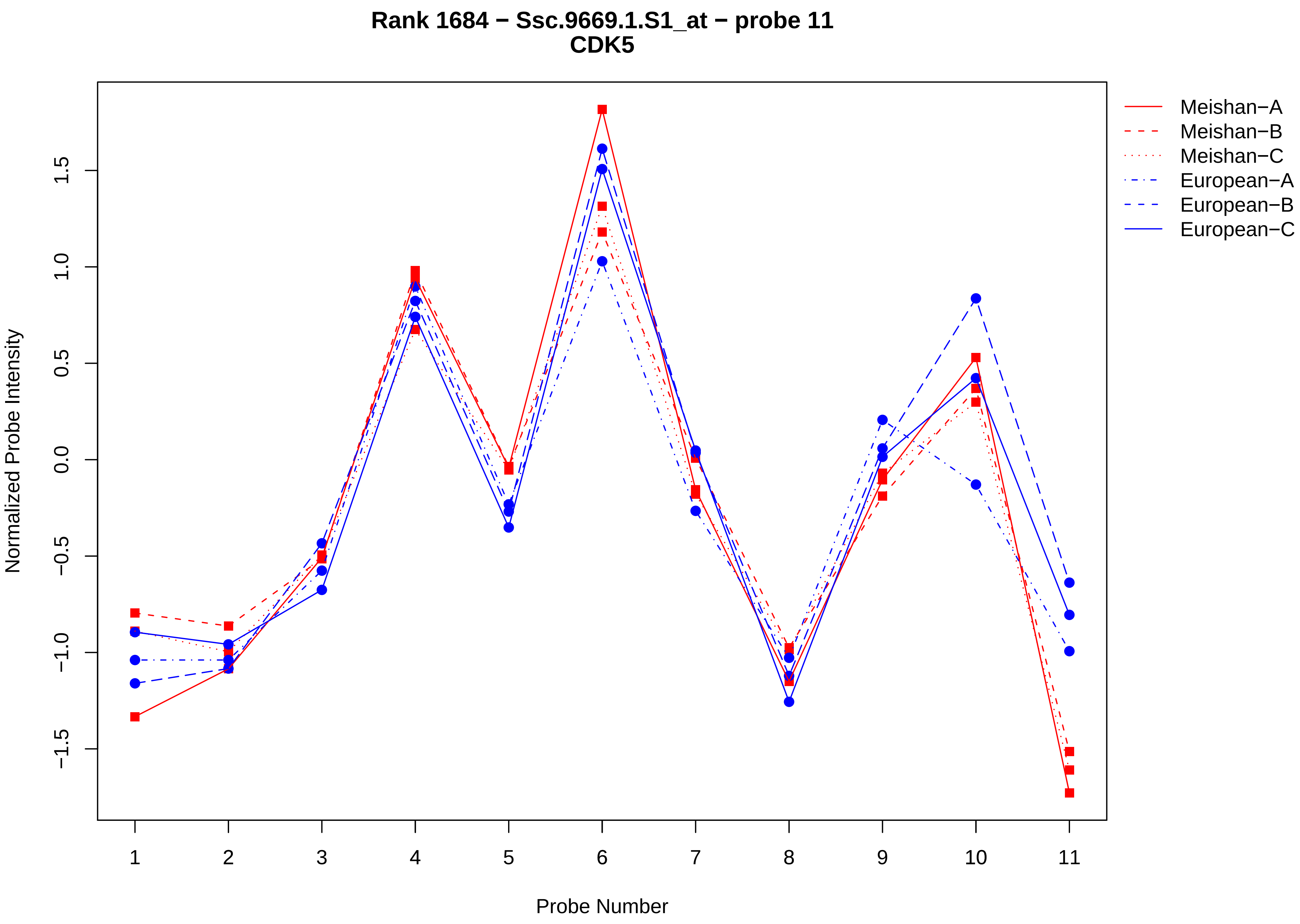

Rank 1684 − Ssc.9669.1.S1_at − probe 11
CDK5
Normalized Probe Intensity
Probe Number
−1.5
−1.0
−0.5
0.0
0.5
1.0
1.5
1
2
3
4
5
6
7
8
9
10
11
Meishan−A
Meishan−B
Meishan−C
European−A
European−B
European−C

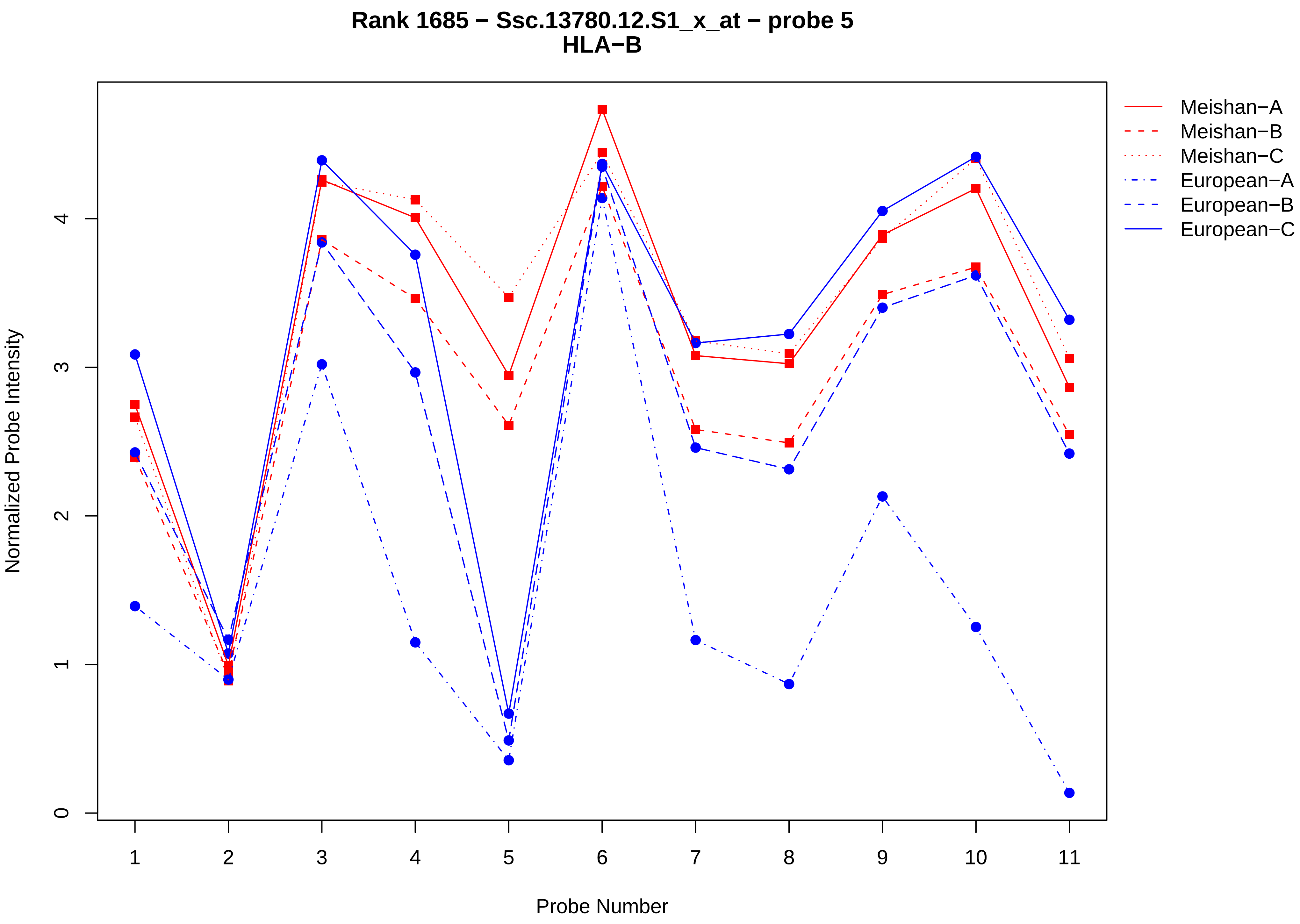
Rank 1685 − Ssc.13780.12.S1_x_at − probe 5
HLA−B
Normalized Probe Intensity
0
1
2
3
4
1
2
3
4
5
6
7
8
9
10
11
Probe Number
Meishan−A
Meishan−B
Meishan−C
European−A
European−B
European−C

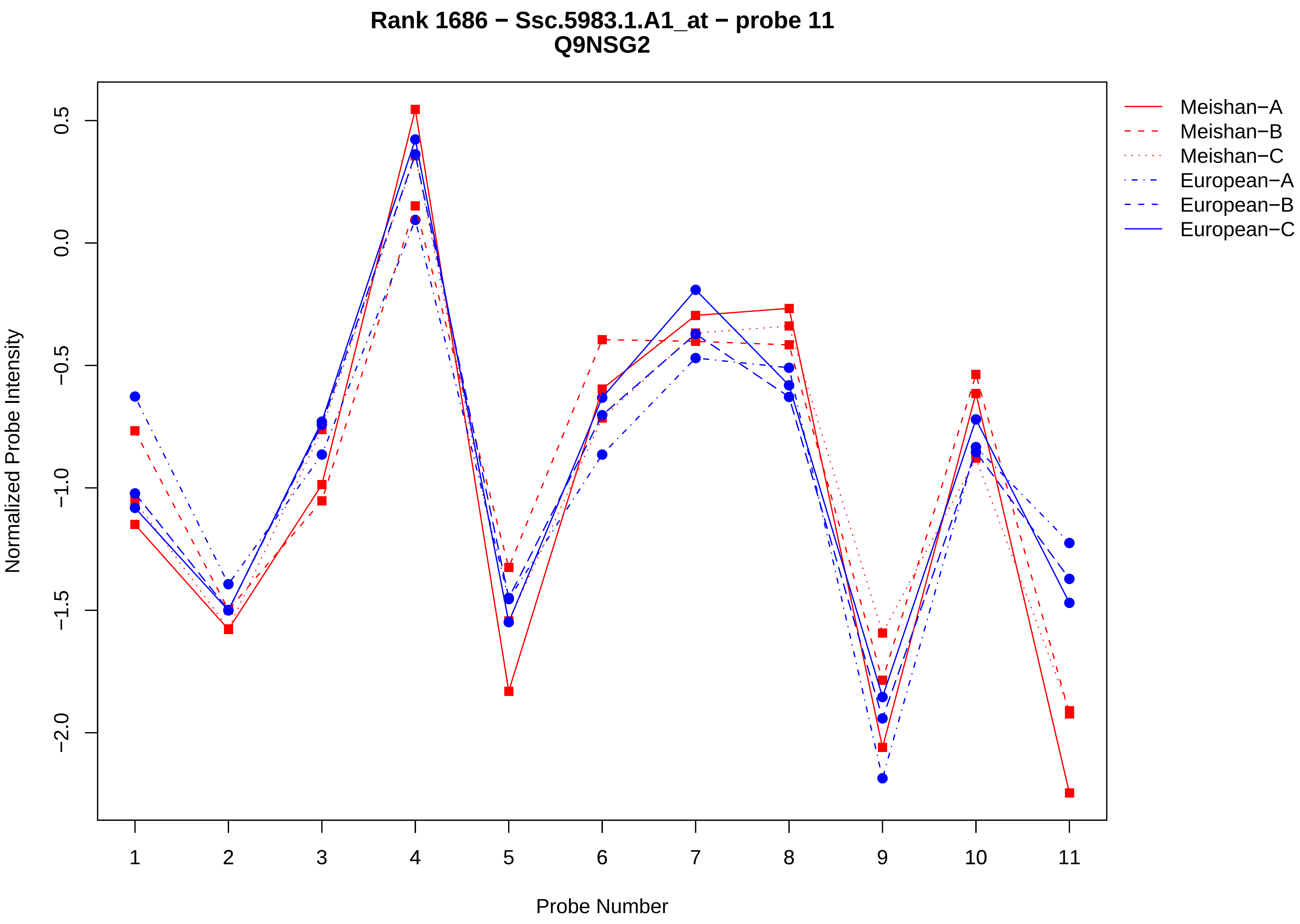

Rank 1686 − Ssc.5983.1.A1_at − probe 11
Q9NSG2
Normalized Probe Intensity
Probe Number
0.5
0.0
−0.5
−1.0
−1.5
−2.0
1
2
3
4
5
6
7
8
9
10
11
Meishan−A
Meishan−B
Meishan−C
European−A
European−B
European−C

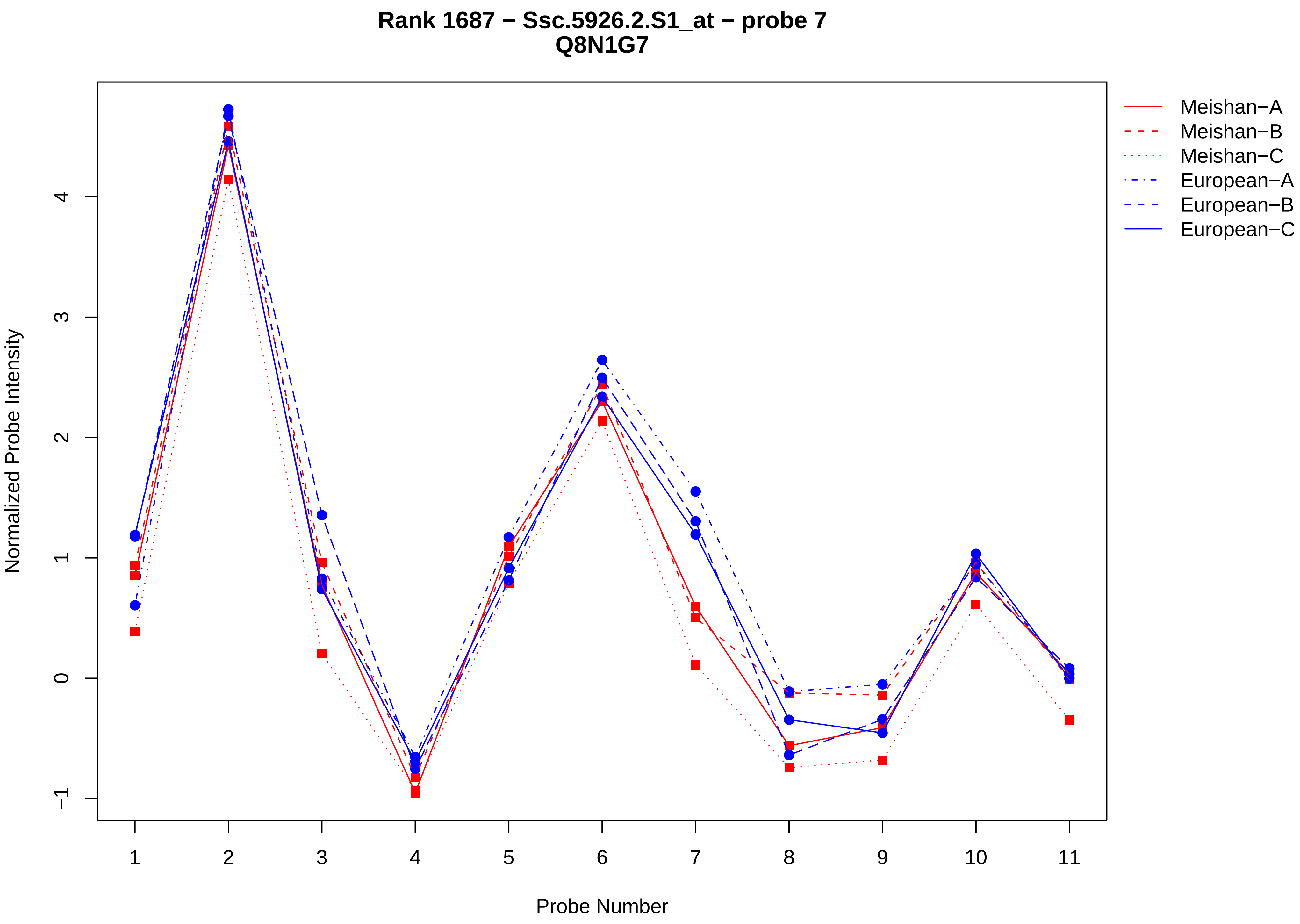

Rank 1687 − Ssc.5926.2.S1_at − probe 7
Q8N1G7
Normalized Probe Intensity
Probe Number
Meishan−A
Meishan−B
Meishan−C
European−A
European−B
European−C
−1
0
1
2
3
4
1
2
3
4
5
6
7
8
9
10
11

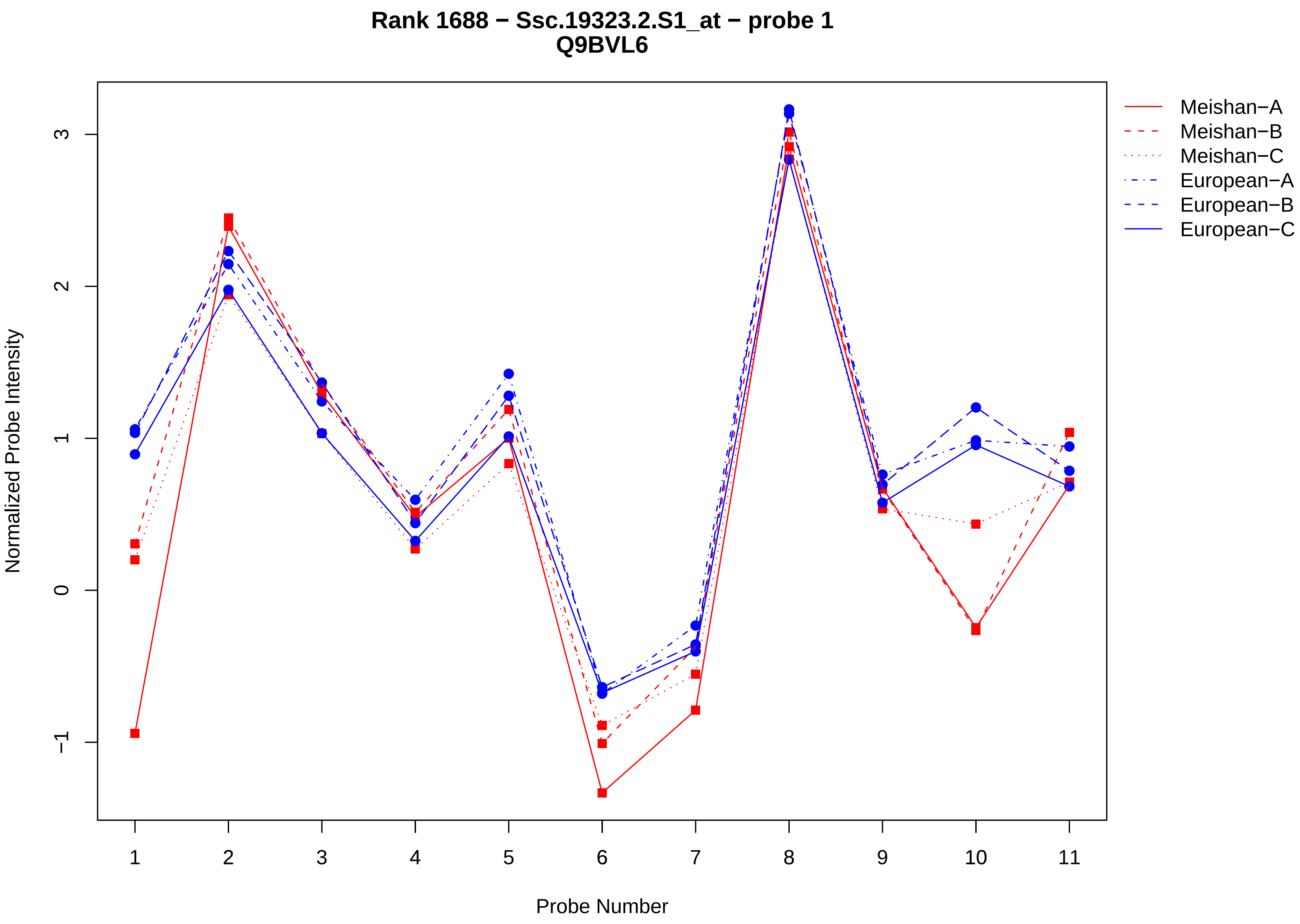

Rank 1688 − Ssc.19323.2.S1_at − probe 1
Q9BVL6
Normalized Probe Intensity
Probe Number
−1
0
1
2
3
1
2
3
4
5
6
7
8
9
10
11
Meishan−A
Meishan−B
Meishan−C
European−A
European−B
European−C

Rank 1689 − Ssc.28333.1.S1_at − probe 9
GEMIN6

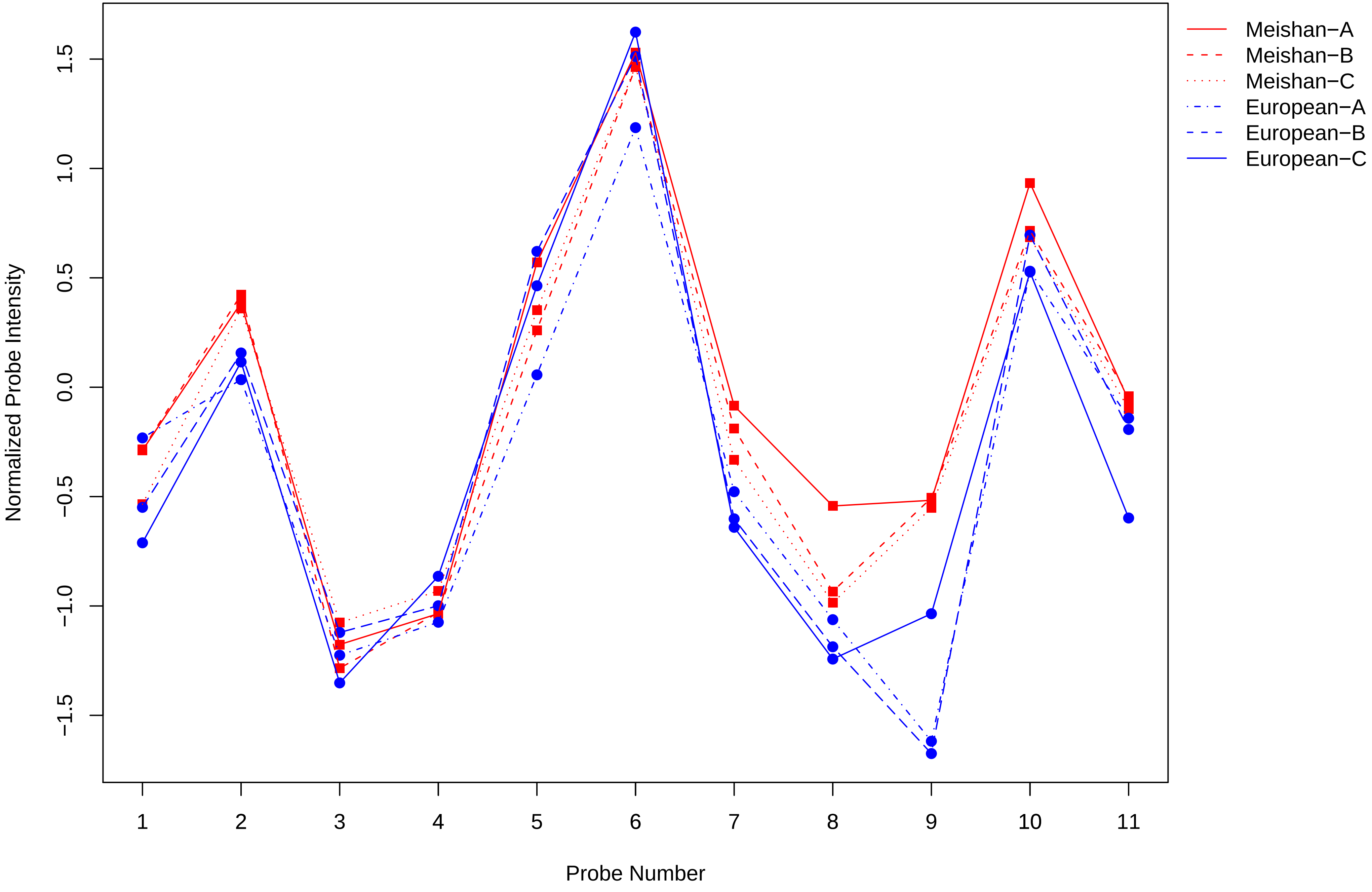

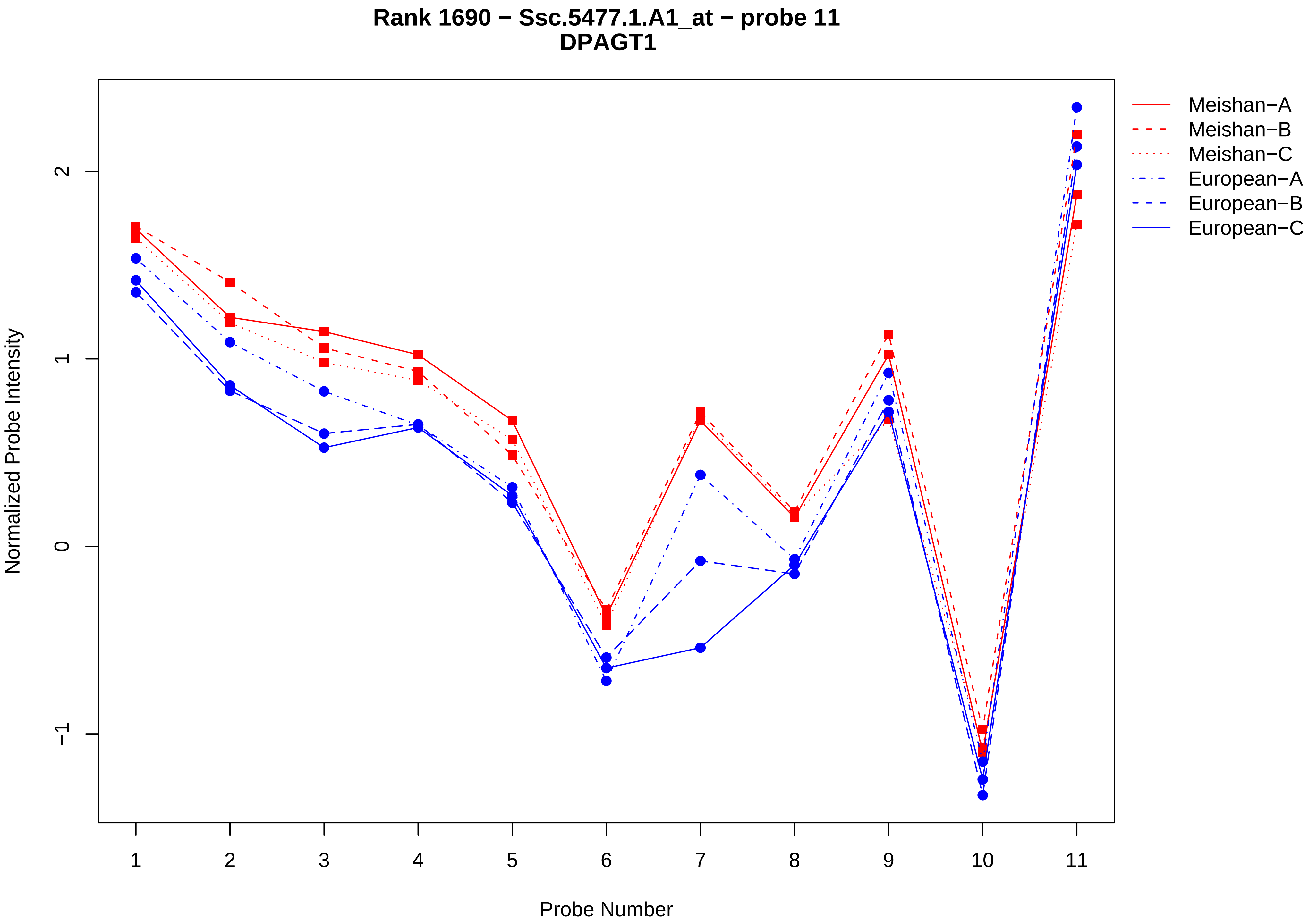

Rank 1690 − Ssc.5477.1.A1_at − probe 11
DPAGT1
Normalized Probe Intensity
Probe Number
−1
0
1
2
1
2
3
4
5
6
7
8
9
10
11
Meishan−A
Meishan−B
Meishan−C
European−A
European−B
European−C

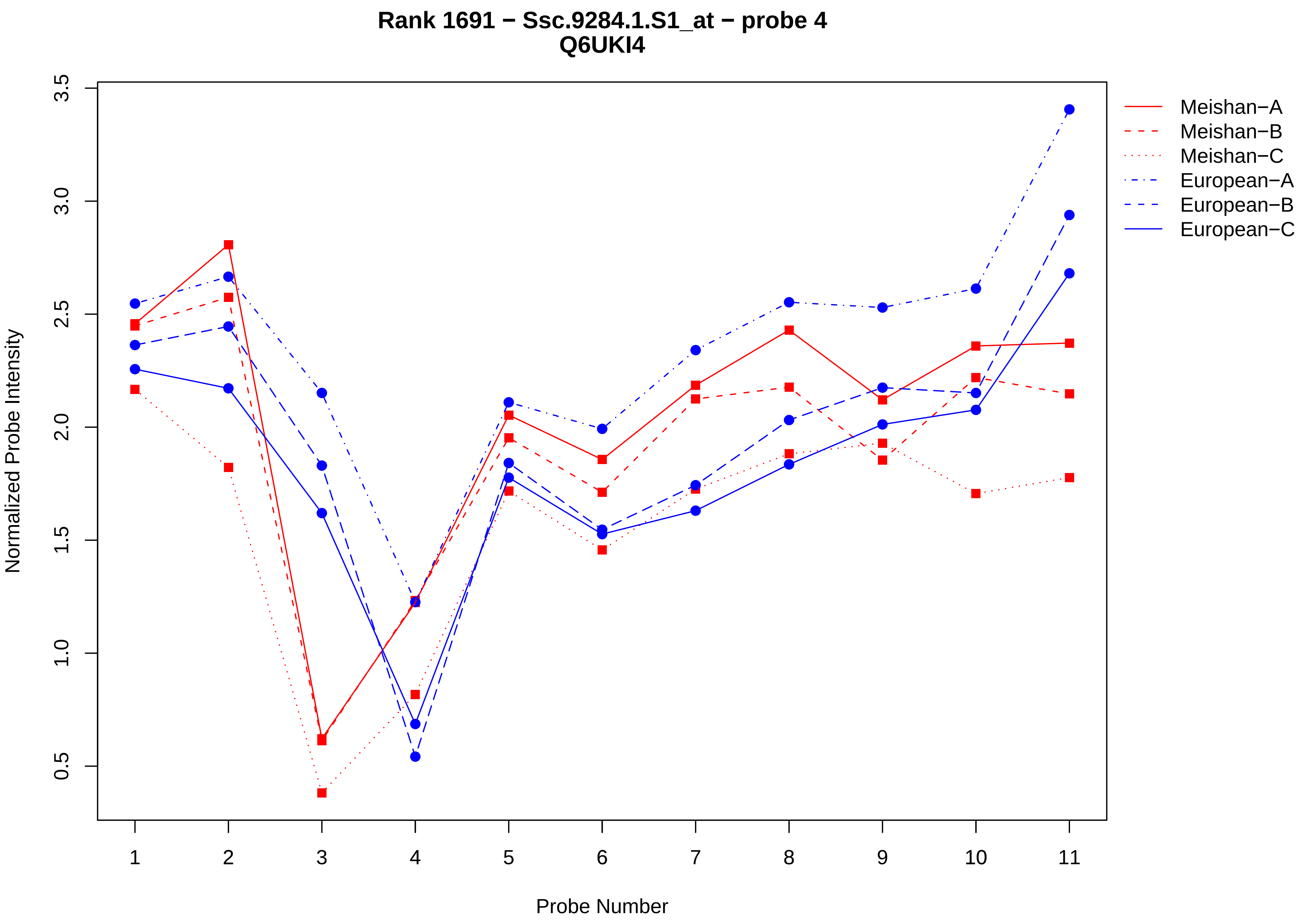
Rank 1691 − Ssc.9284.1.S1_at − probe 4
Q6UKI4
Normalized Probe Intensity
Probe Number
0.5
1.0
1.5
2.0
2.5
3.0
3.5
1
2
3
4
5
6
7
8
9
10
11
Meishan−A
Meishan−B
Meishan−C
European−A
European−B
European−C

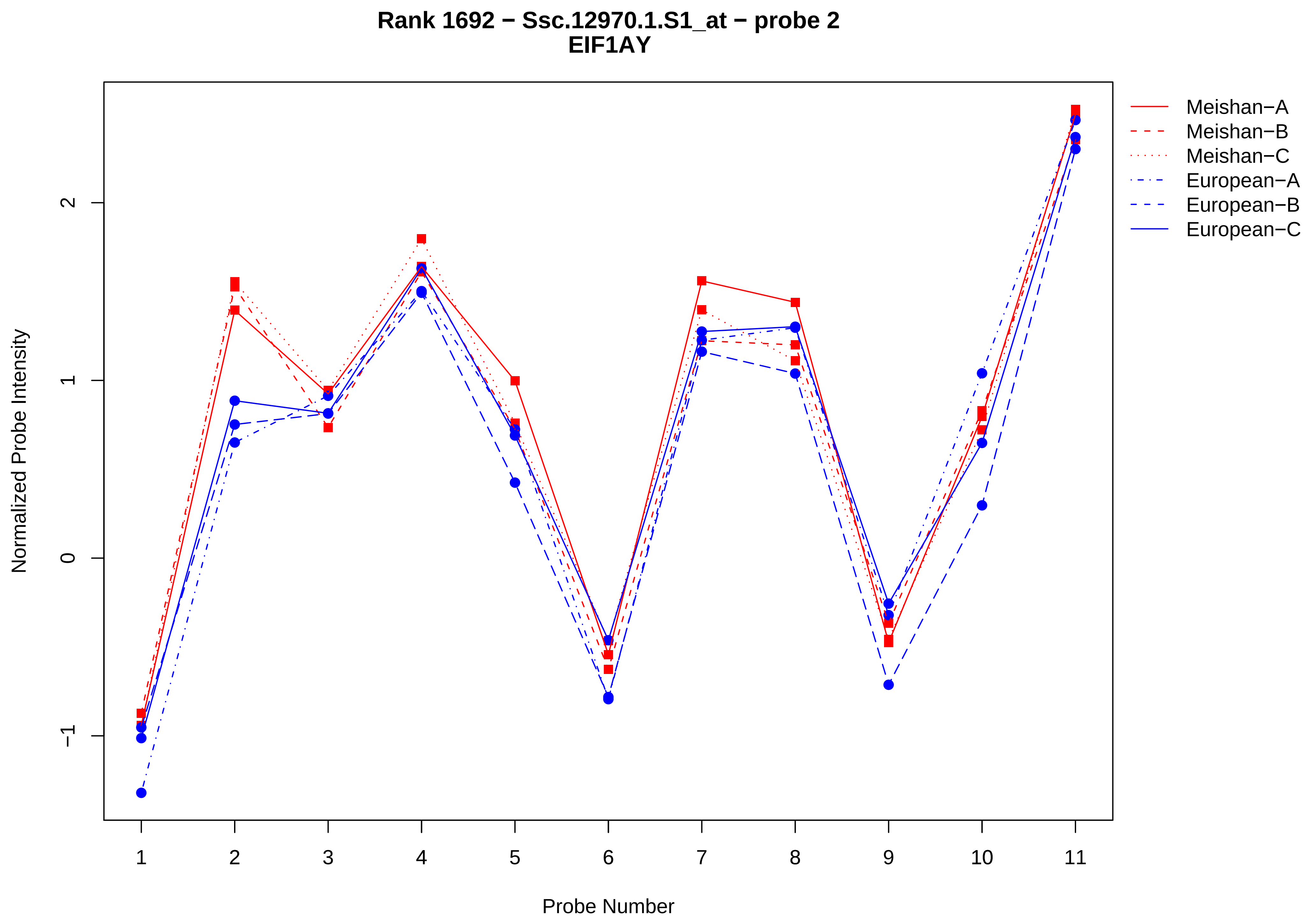

Rank 1692 − Ssc.12970.1.S1_at − probe 2
EIF1AY
Normalized Probe Intensity
Probe Number
−1
0
1
2
1
2
3
4
5
6
7
8
9
10
11
Meishan−A
Meishan−B
Meishan−C
European−A
European−B
European−C

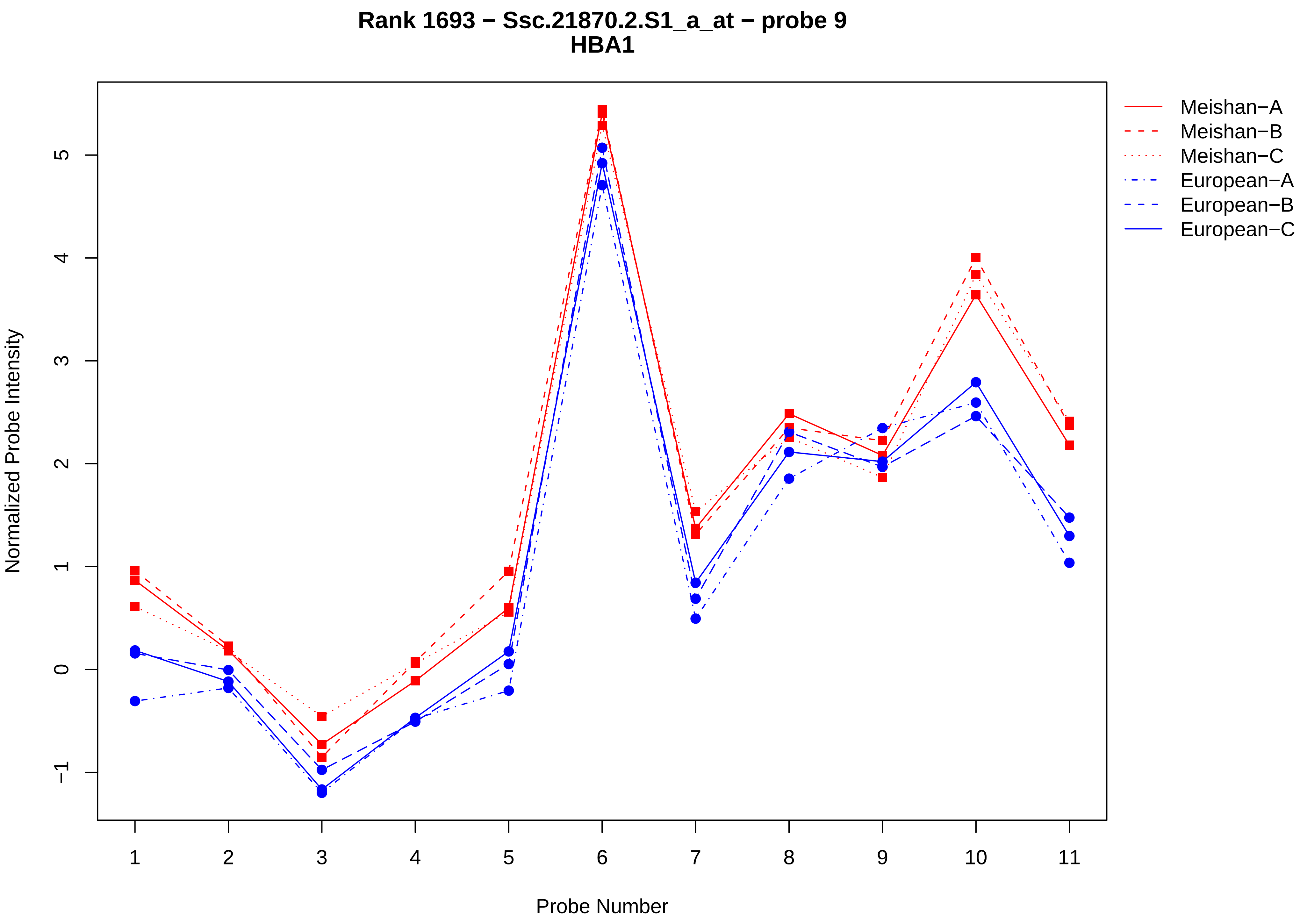

Rank 1693 − Ssc.21870.2.S1_a_at − probe 9
HBA1
Normalized Probe Intensity
−1
0
1
2
3
4
5
1
2
3
4
5
6
7
8
9
10
11
Probe Number
Meishan−A
Meishan−B
Meishan−C
European−A
European−B
European−C

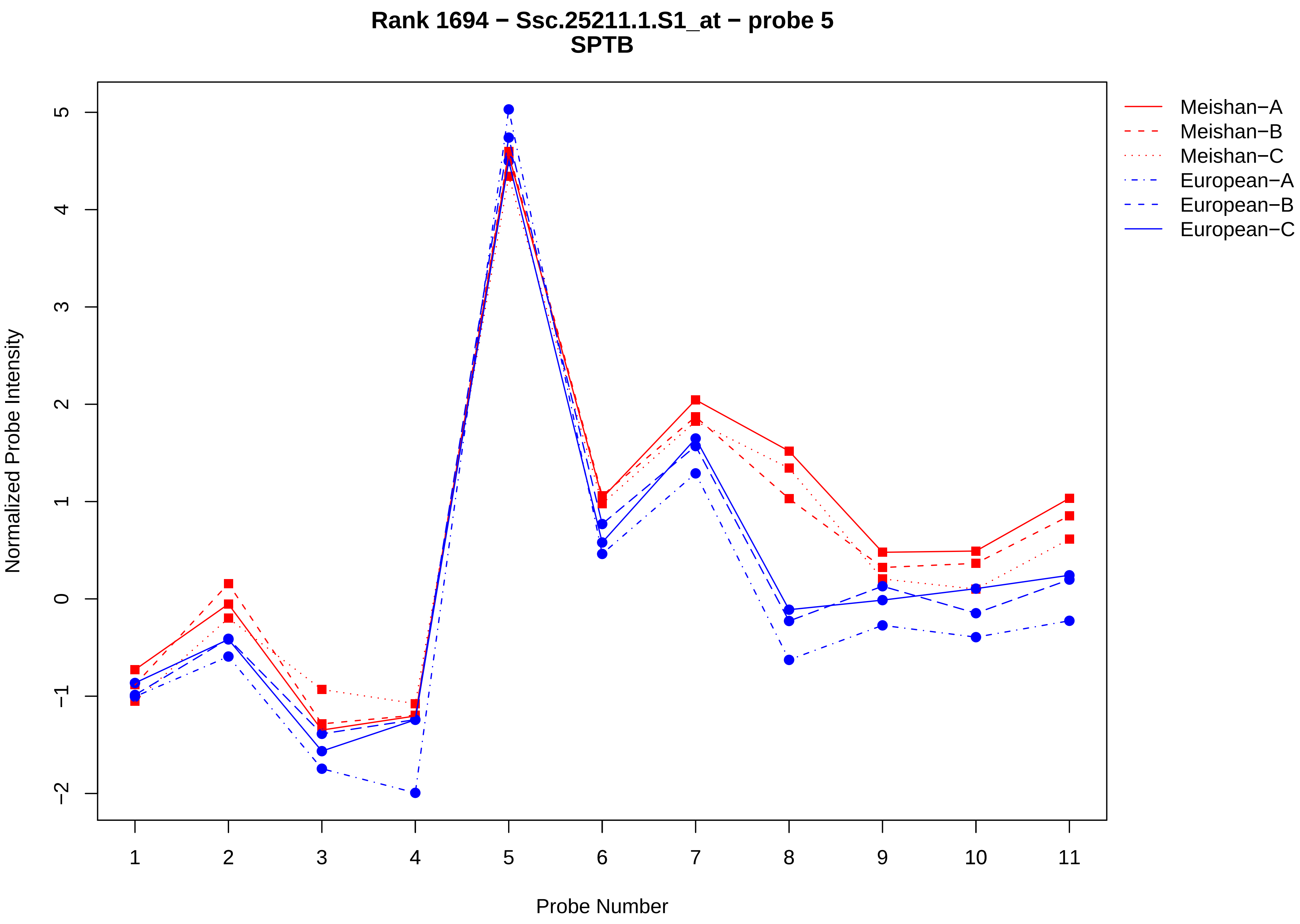

Rank 1694 − Ssc.25211.1.S1_at − probe 5
SPTB
Normalized Probe Intensity
Probe Number
−2
−1
0
1
2
3
4
5
1
2
3
4
5
6
7
8
9
10
11
Meishan−A
Meishan−B
Meishan−C
European−A
European−B
European−C

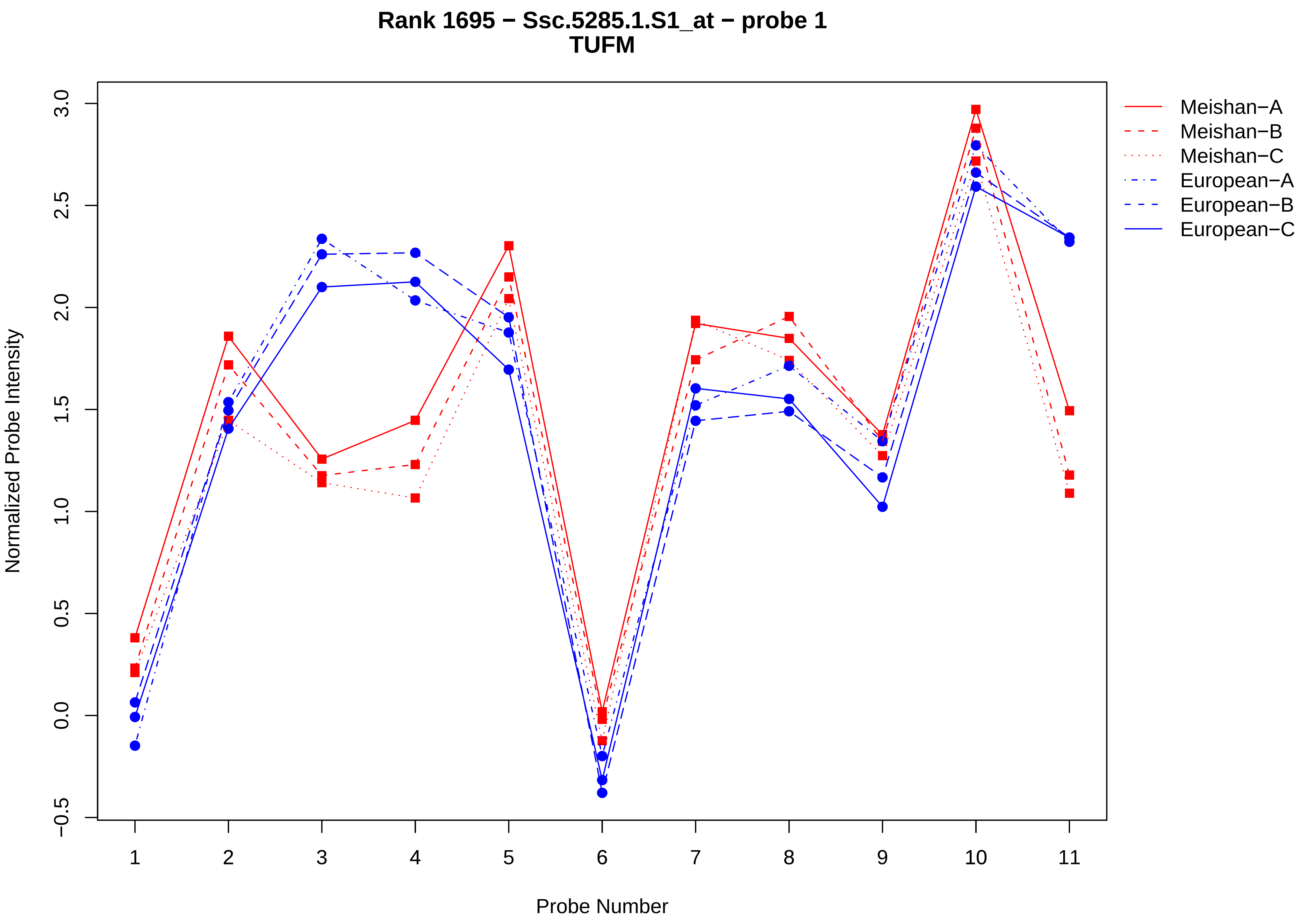

Rank 1695 − Ssc.5285.1.S1_at − probe 1
TUFM
Normalized Probe Intensity
Probe Number
−0.5
0.0
0.5
1.0
1.5
2.0
2.5
3.0
1
2
3
4
5
6
7
8
9
10
11
Meishan−A
Meishan−B
Meishan−C
European−A
European−B
European−C

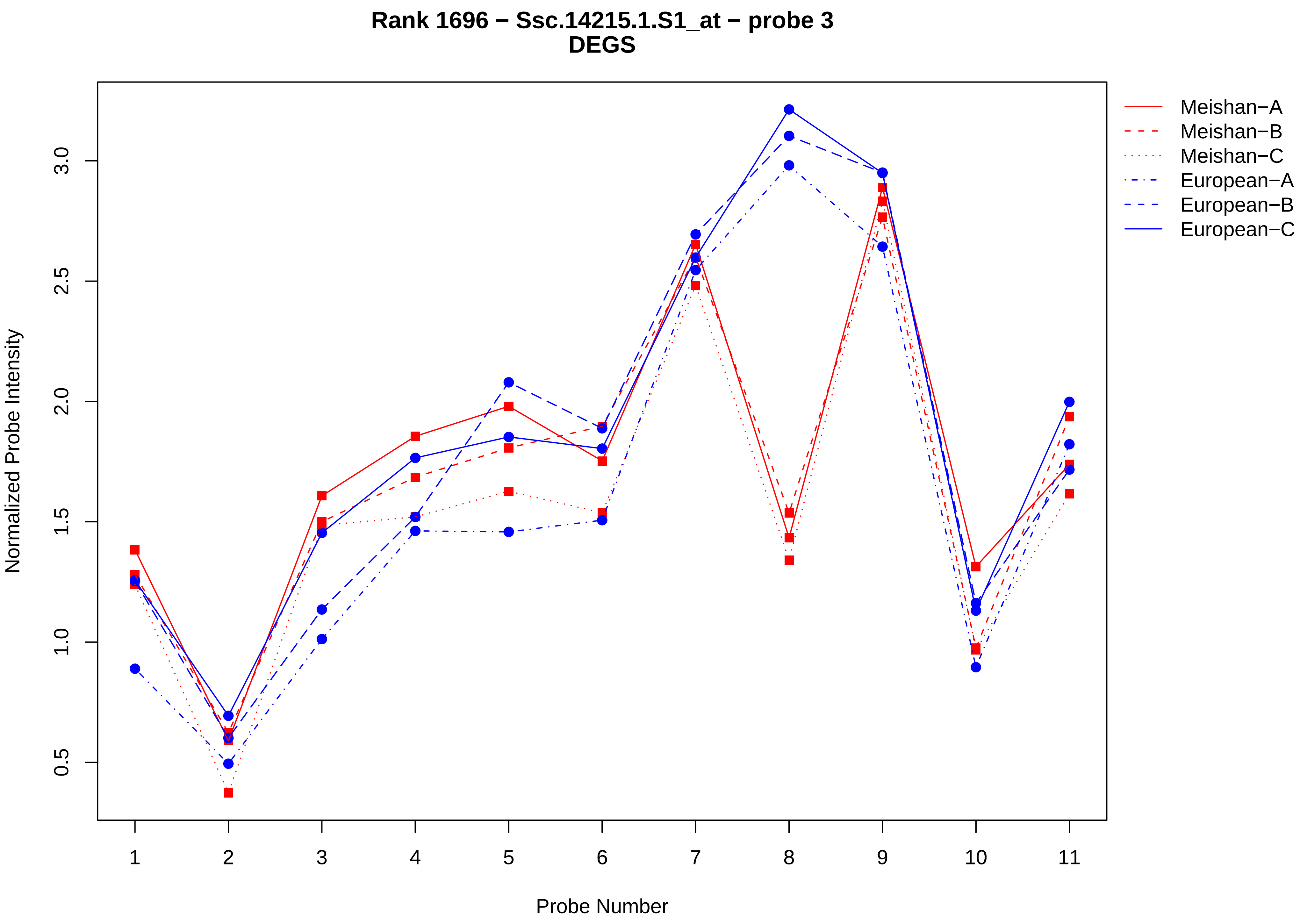

Rank 1696 − Ssc.14215.1.S1_at − probe 3
DEGS
Normalized Probe Intensity
0.5
1.0
1.5
2.0
2.5
3.0
1
2
3
4
5
6
7
8
9
10
11
Probe Number
Meishan−A
Meishan−B
Meishan−C
European−A
European−B
European−C

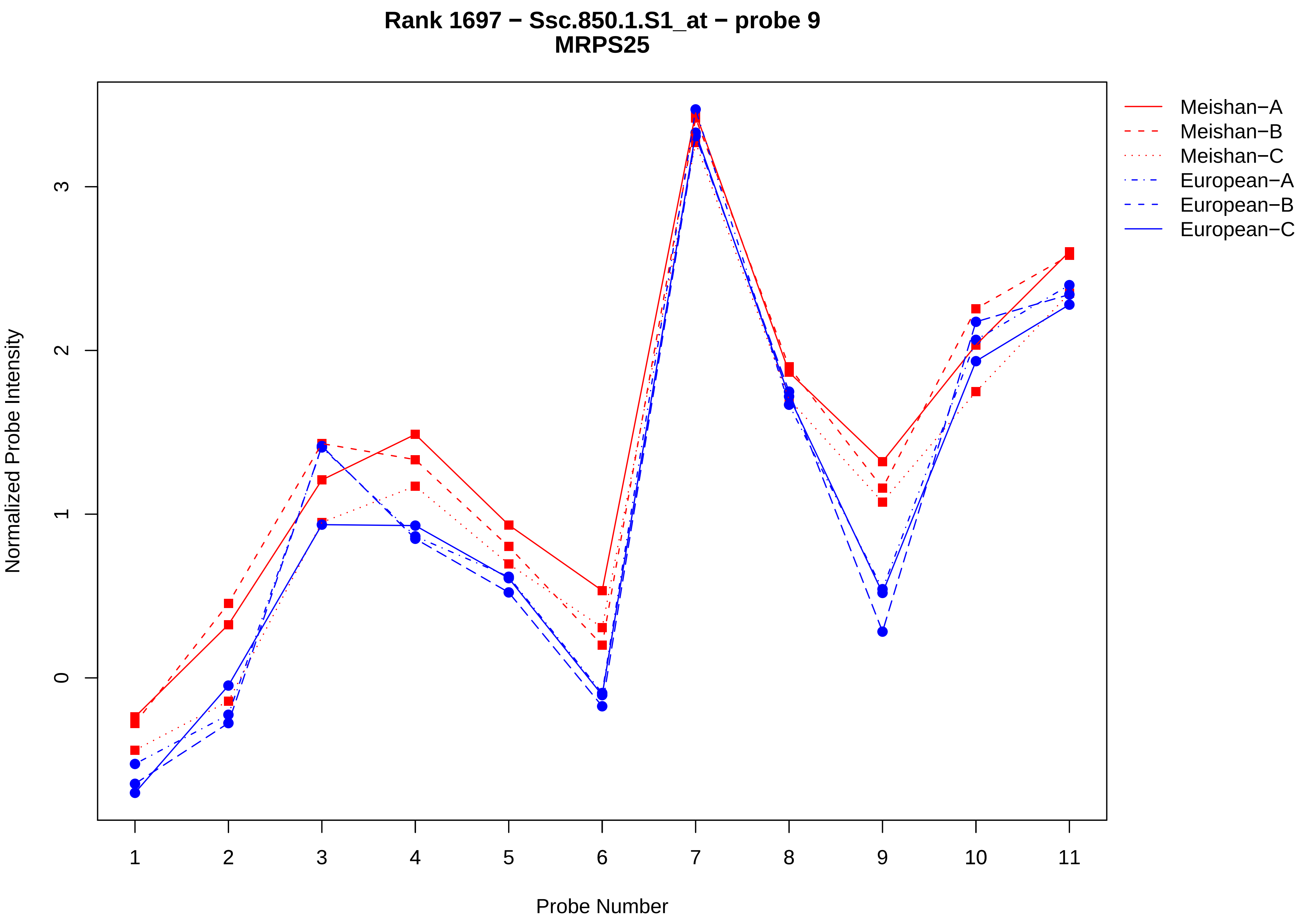

Rank 1697 − Ssc.850.1.S1_at − probe 9
MRPS25
Normalized Probe Intensity
0
1
2
3
1
2
3
4
5
6
7
8
9
10
11
Probe Number
Meishan−A
Meishan−B
Meishan−C
European−A
European−B
European−C

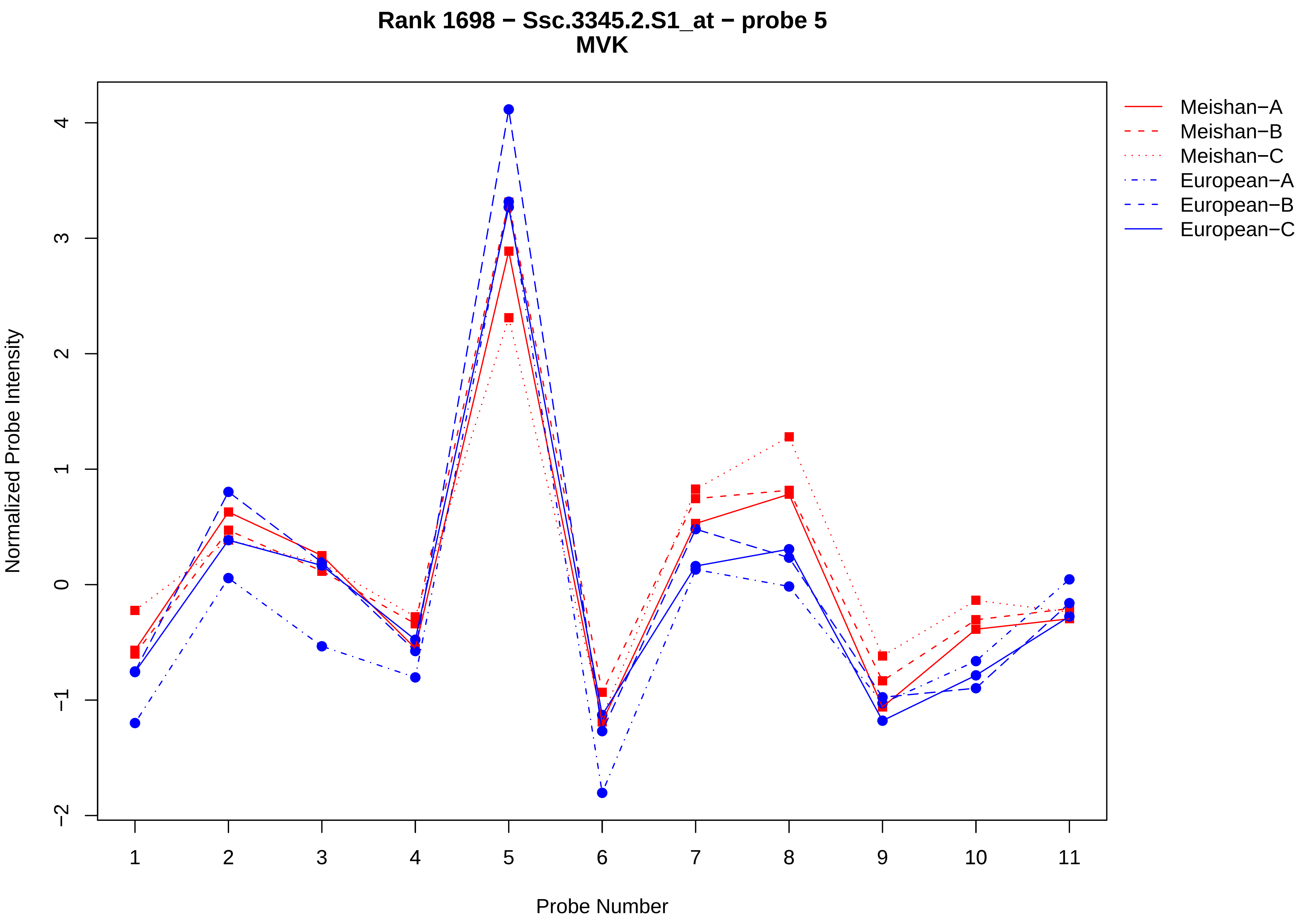

Rank 1698 − Ssc.3345.2.S1_at − probe 5
MVK
Normalized Probe Intensity
Probe Number
Meishan−A
Meishan−B
Meishan−C
European−A
European−B
European−C

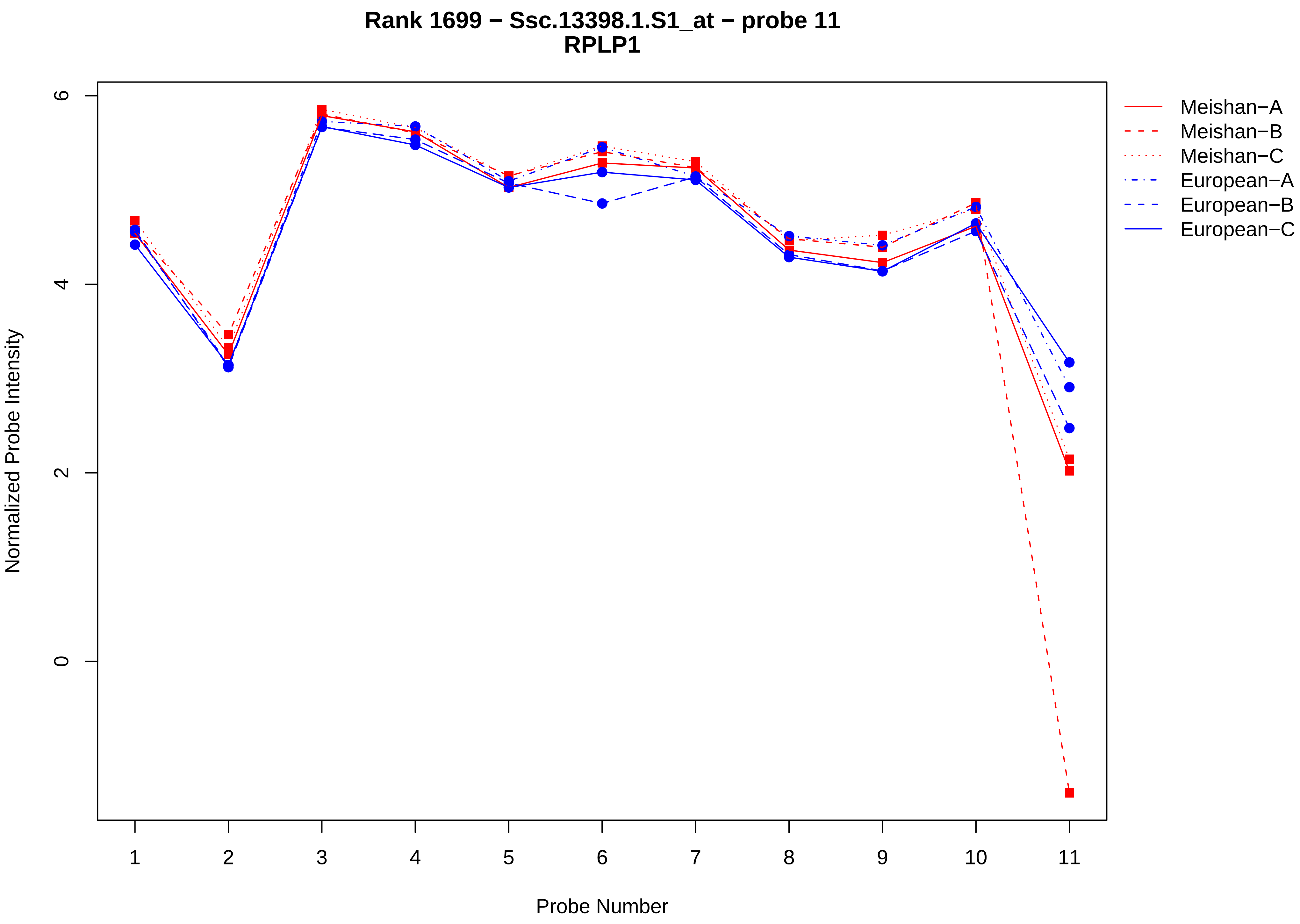

Rank 1699 − Ssc.13398.1.S1_at − probe 11
RPLP1
Normalized Probe Intensity
Probe Number
0
2
4
6
1
2
3
4
5
6
7
8
9
10
11
Meishan−A
Meishan−B
Meishan−C
European−A
European−B
European−C

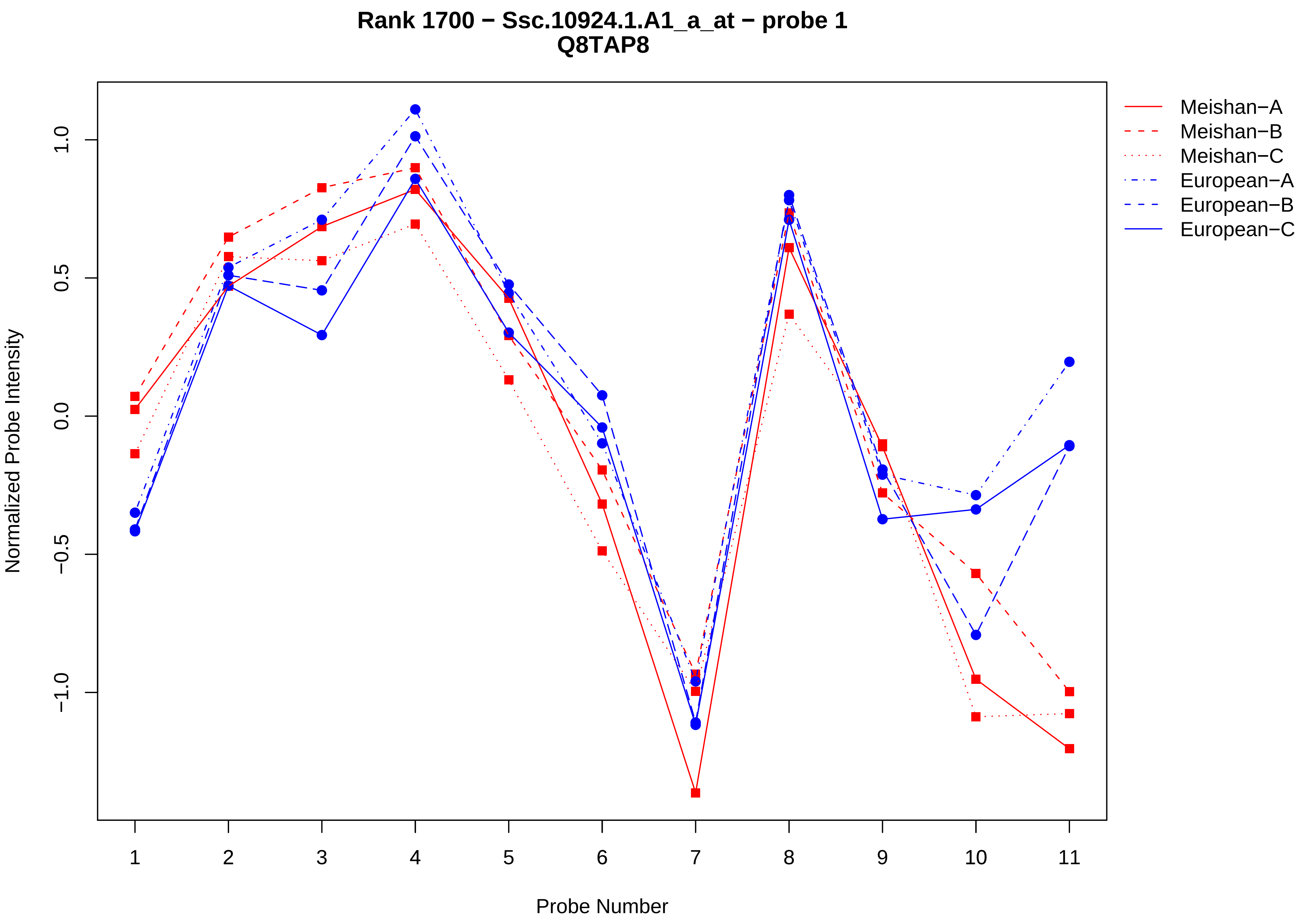

Rank 1700 − Ssc.10924.1.A1_a_at − probe 1
Q8TAP8
Normalized Probe Intensity
Probe Number
1.0
0.5
0.0
−0.5
−1.0
1
2
3
4
5
6
7
8
9
10
11
Meishan−A
Meishan−B
Meishan−C
European−A
European−B
European−C

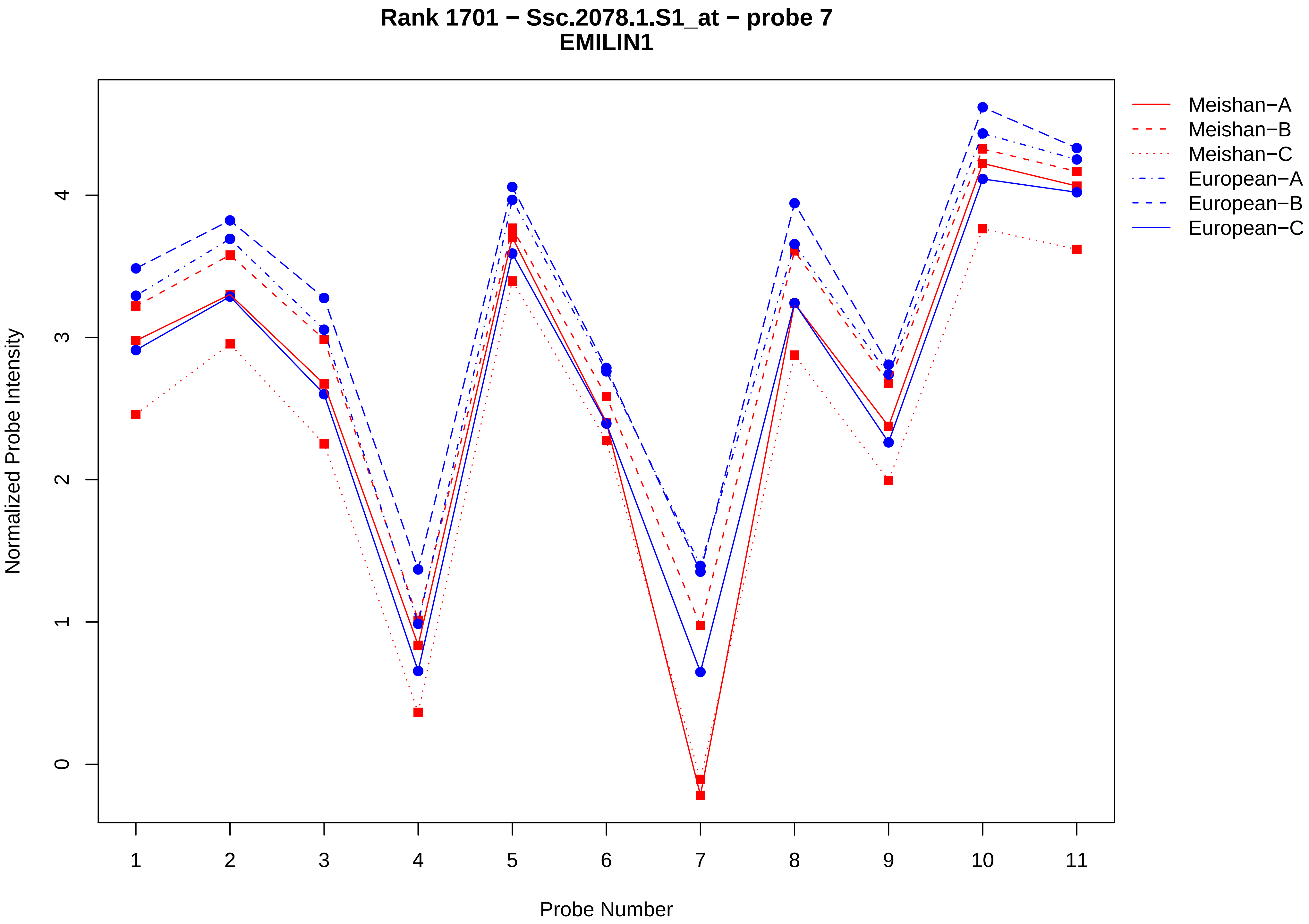
Rank 1701 − Ssc.2078.1.S1_at − probe 7
EMILIN1
Normalized Probe Intensity
Probe Number
0
1
2
3
4
1
2
3
4
5
6
7
8
9
10
11
Meishan−A
Meishan−B
Meishan−C
European−A
European−B
European−C

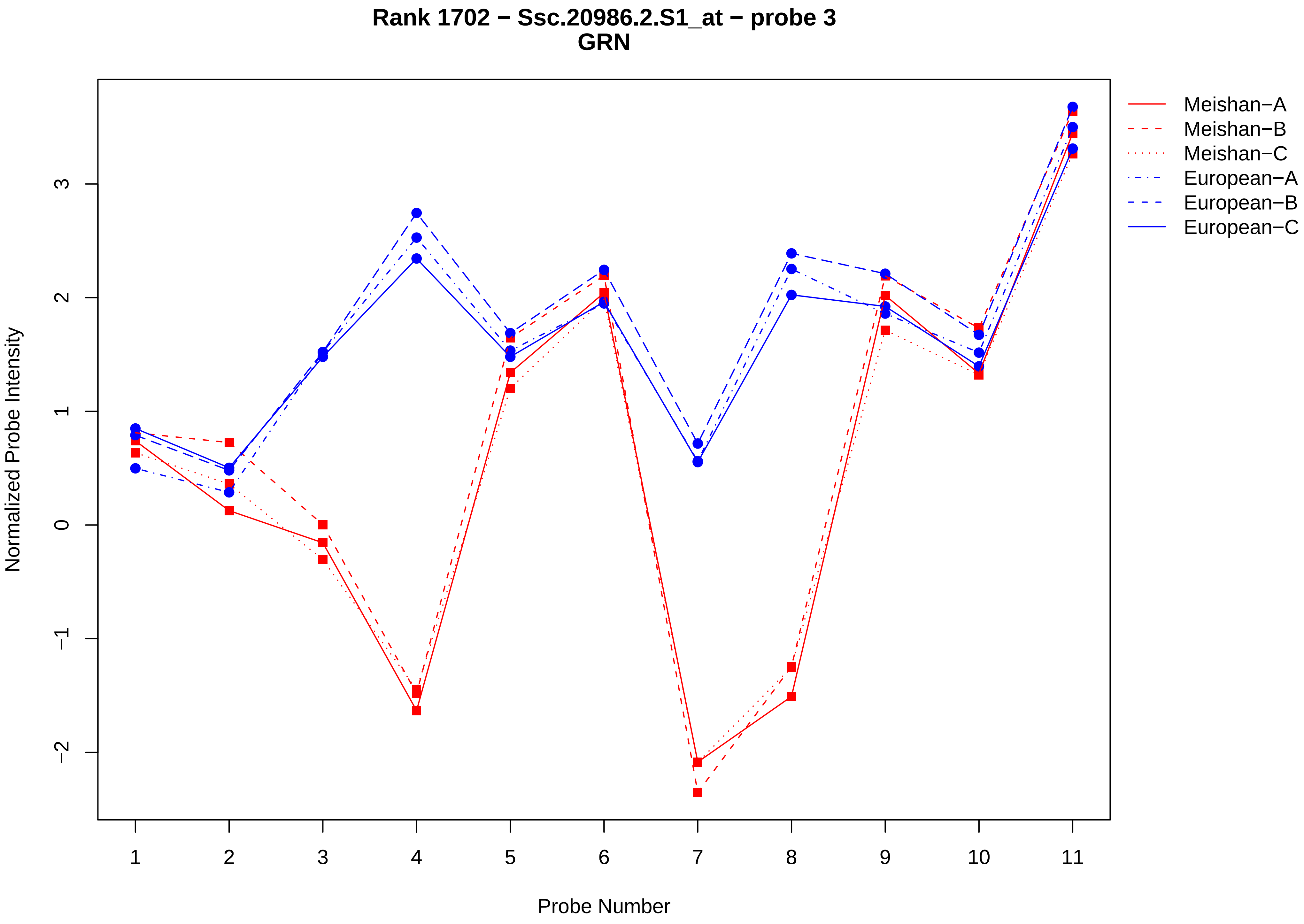

Rank 1702 − Ssc.20986.2.S1_at − probe 3
GRN
Normalized Probe Intensity
Probe Number
−2
−1
0
1
2
3
1
2
3
4
5
6
7
8
9
10
11
Meishan−A
Meishan−B
Meishan−C
European−A
European−B
European−C

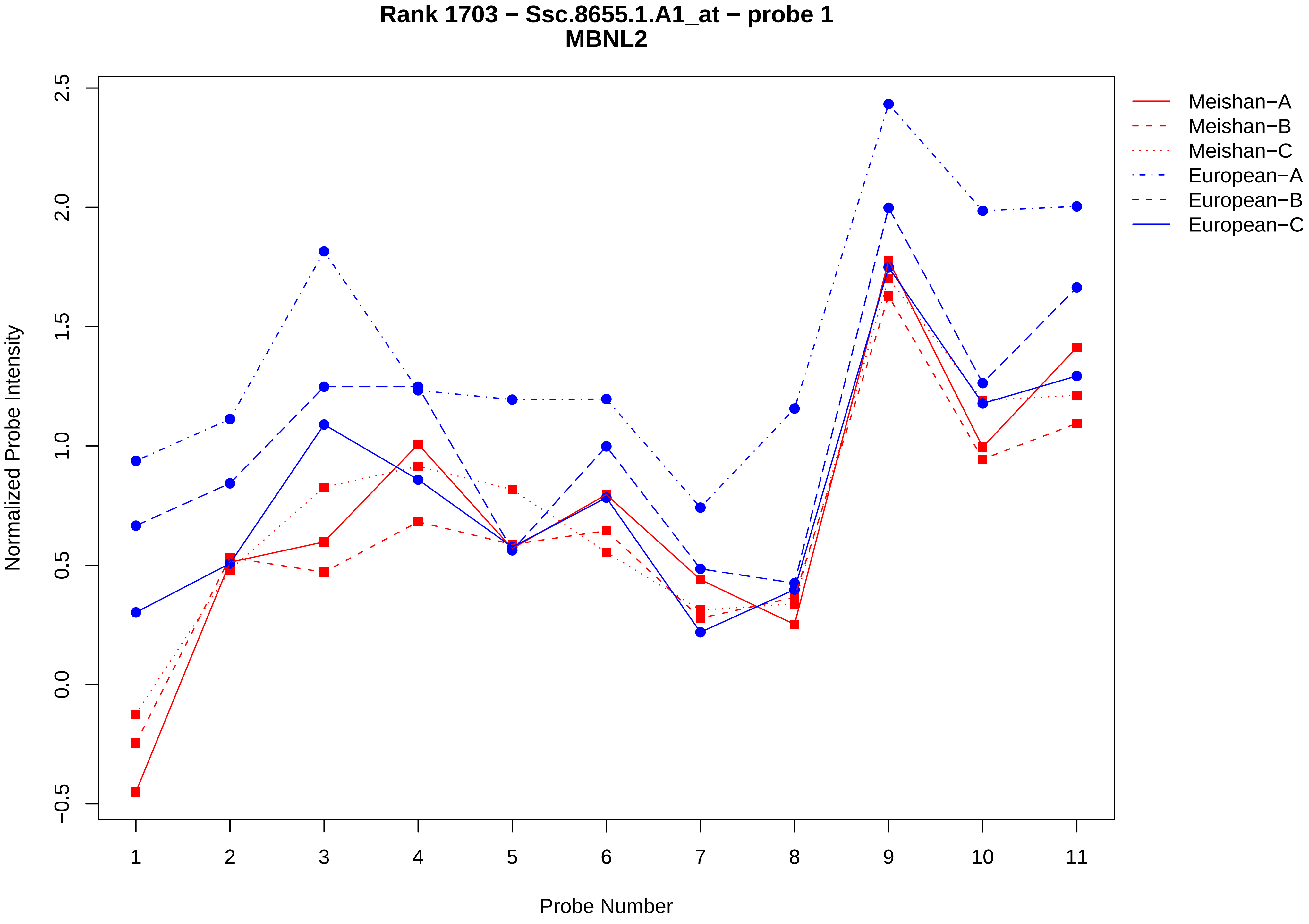

Rank 1703 − Ssc.8655.1.A1_at − probe 1
MBNL2
Normalized Probe Intensity
Probe Number
−0.5
0.0
0.5
1.0
1.5
2.0
2.5
1
2
3
4
5
6
7
8
9
10
11
Meishan−A
Meishan−B
Meishan−C
European−A
European−B
European−C

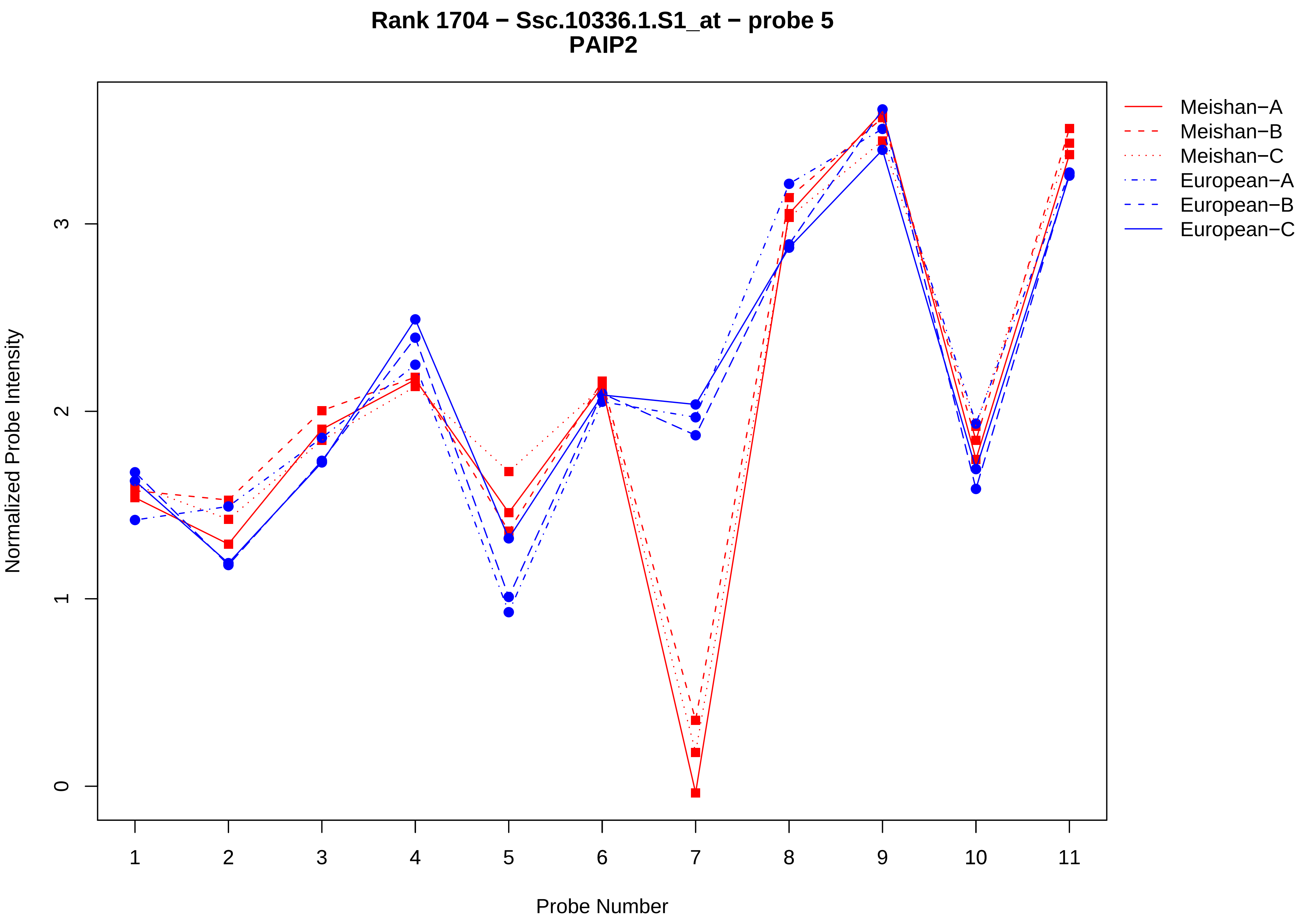

Rank 1704 − Ssc.10336.1.S1_at − probe 5
PAIP2
Normalized Probe Intensity
Probe Number
0
1
2
3
1
2
3
4
5
6
7
8
9
10
11
Meishan−A
Meishan−B
Meishan−C
European−A
European−B
European−C

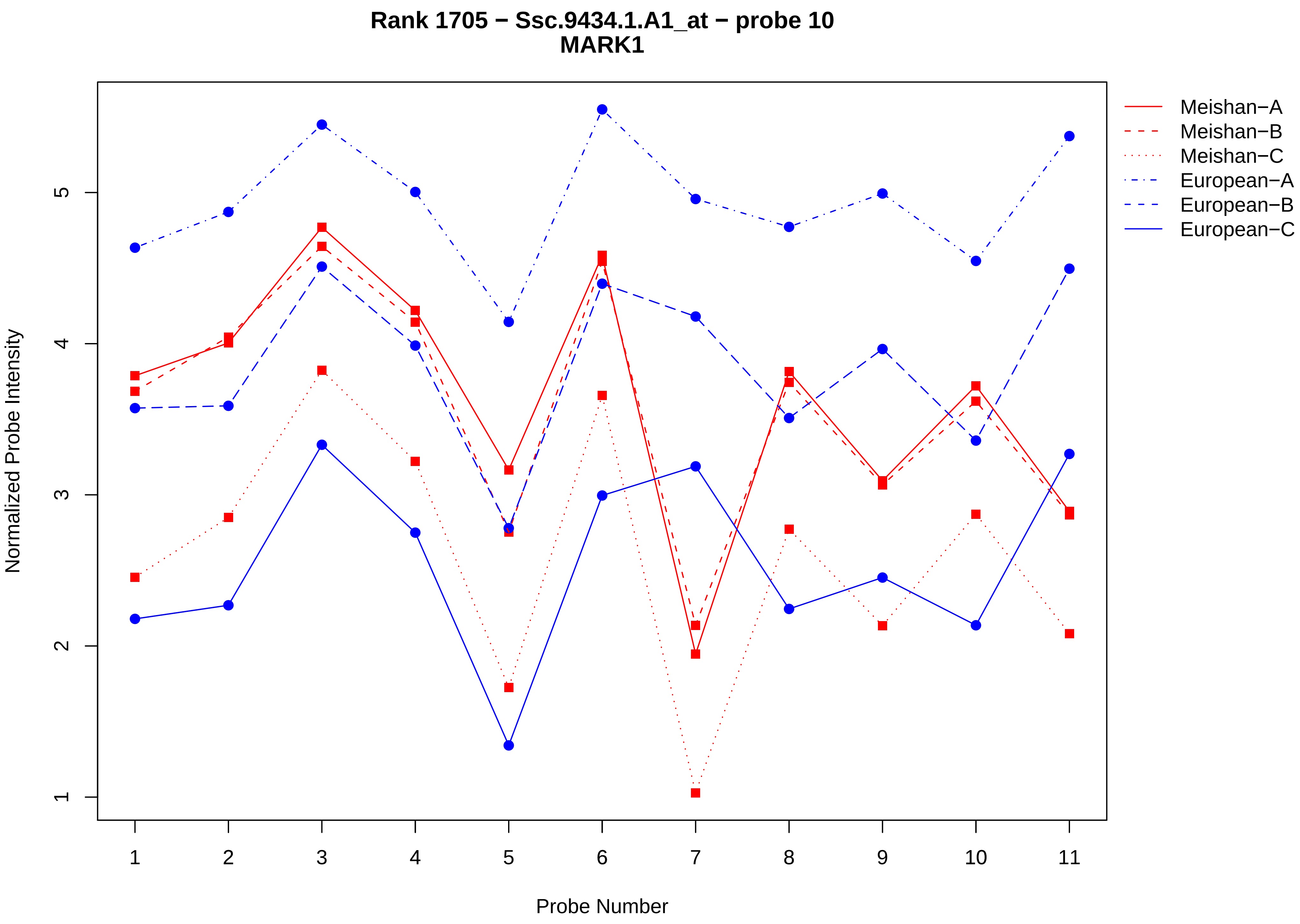

Rank 1705 − Ssc.9434.1.A1_at − probe 10
MARK1
Normalized Probe Intensity
Probe Number
Meishan−A
Meishan−B
Meishan−C
European−A
European−B
European−C
1
2
3
4
5
1
2
3
4
5
6
7
8
9
10
11

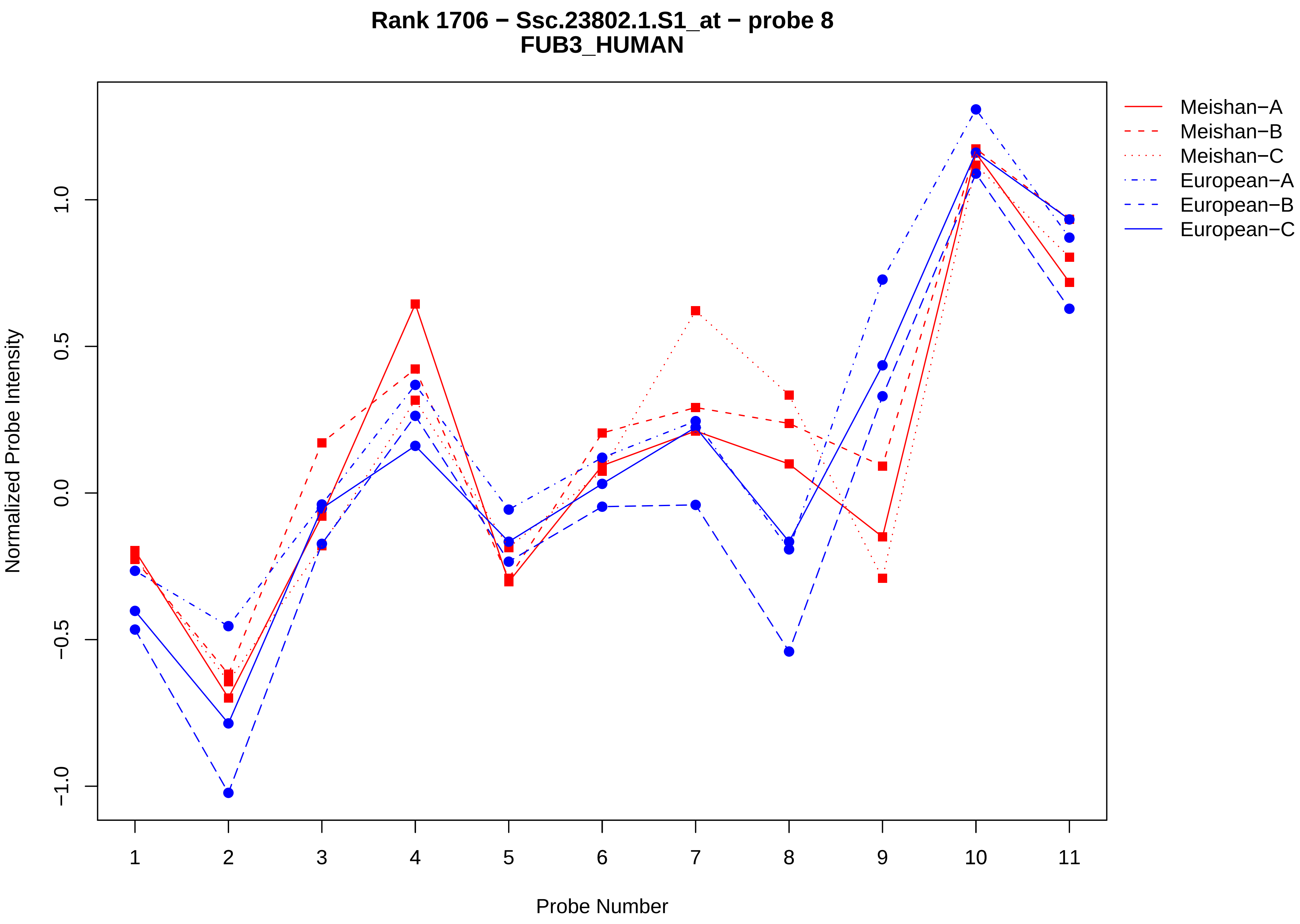

Rank 1706 − Ssc.23802.1.S1_at − probe 8
FUB3_HUMAN
Normalized Probe Intensity
Probe Number
−1.0
−0.5
0.0
0.5
1.0
1
2
3
4
5
6
7
8
9
10
11
Meishan−A
Meishan−B
Meishan−C
European−A
European−B
European−C

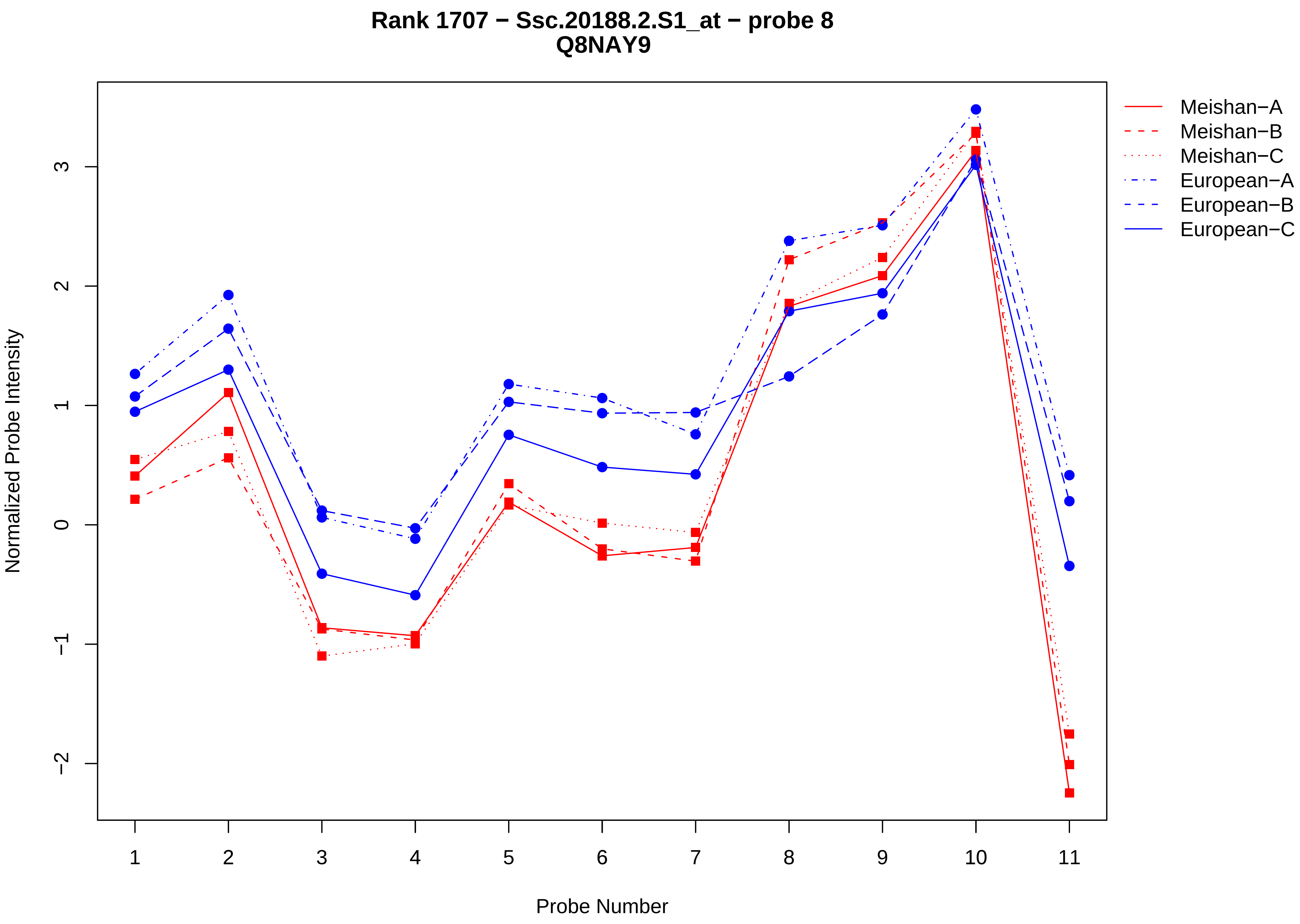

Rank 1707 − Ssc.20188.2.S1_at − probe 8
Q8NAY9
Normalized Probe Intensity
Probe Number
−2
−1
0
1
2
3
1
2
3
4
5
6
7
8
9
10
11
Meishan−A
Meishan−B
Meishan−C
European−A
European−B
European−C

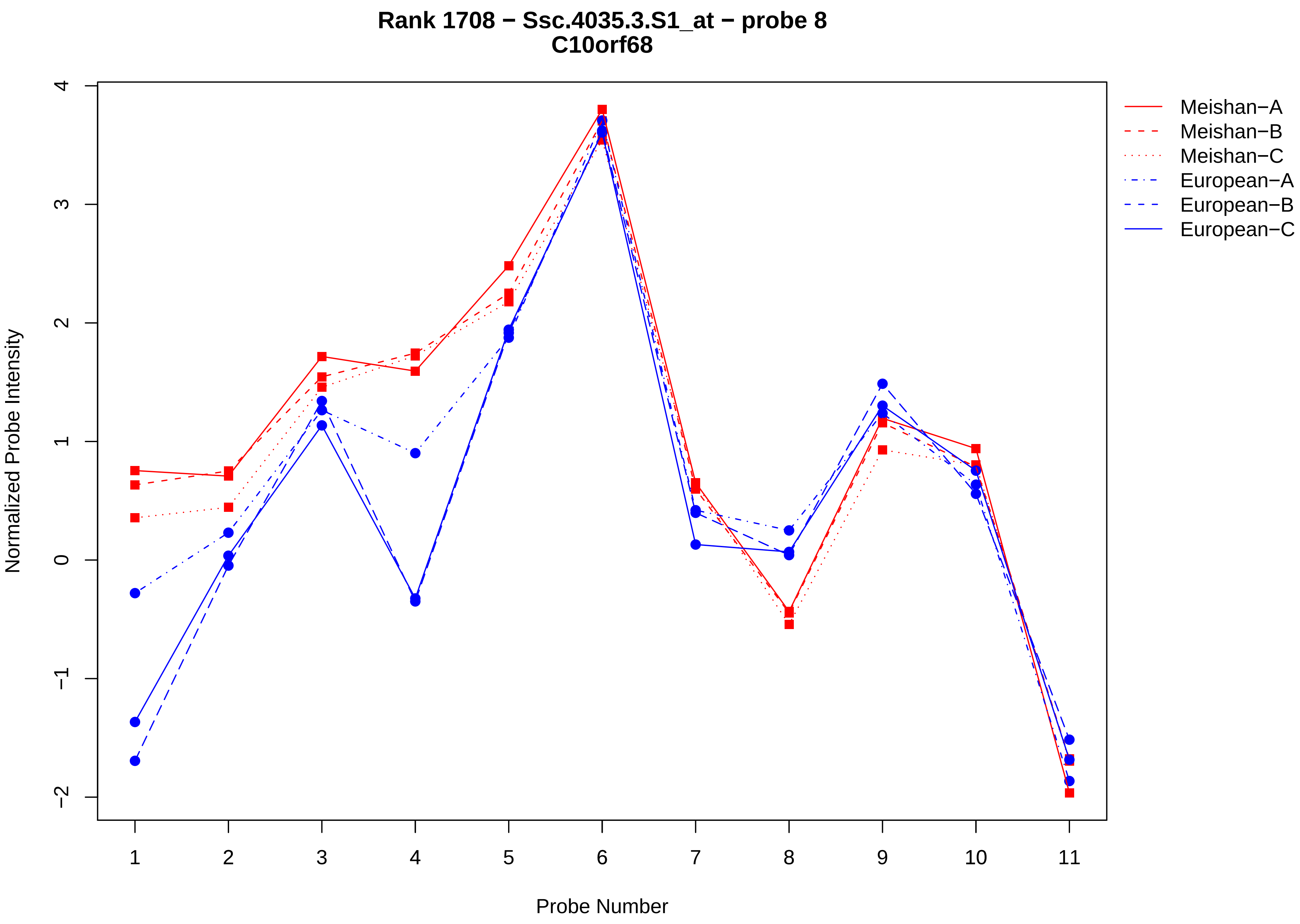

Rank 1708 − Ssc.4035.3.S1_at − probe 8
C10orf68
Normalized Probe Intensity
Probe Number
Meishan−A
Meishan−B
Meishan−C
European−A
European−B
European−C

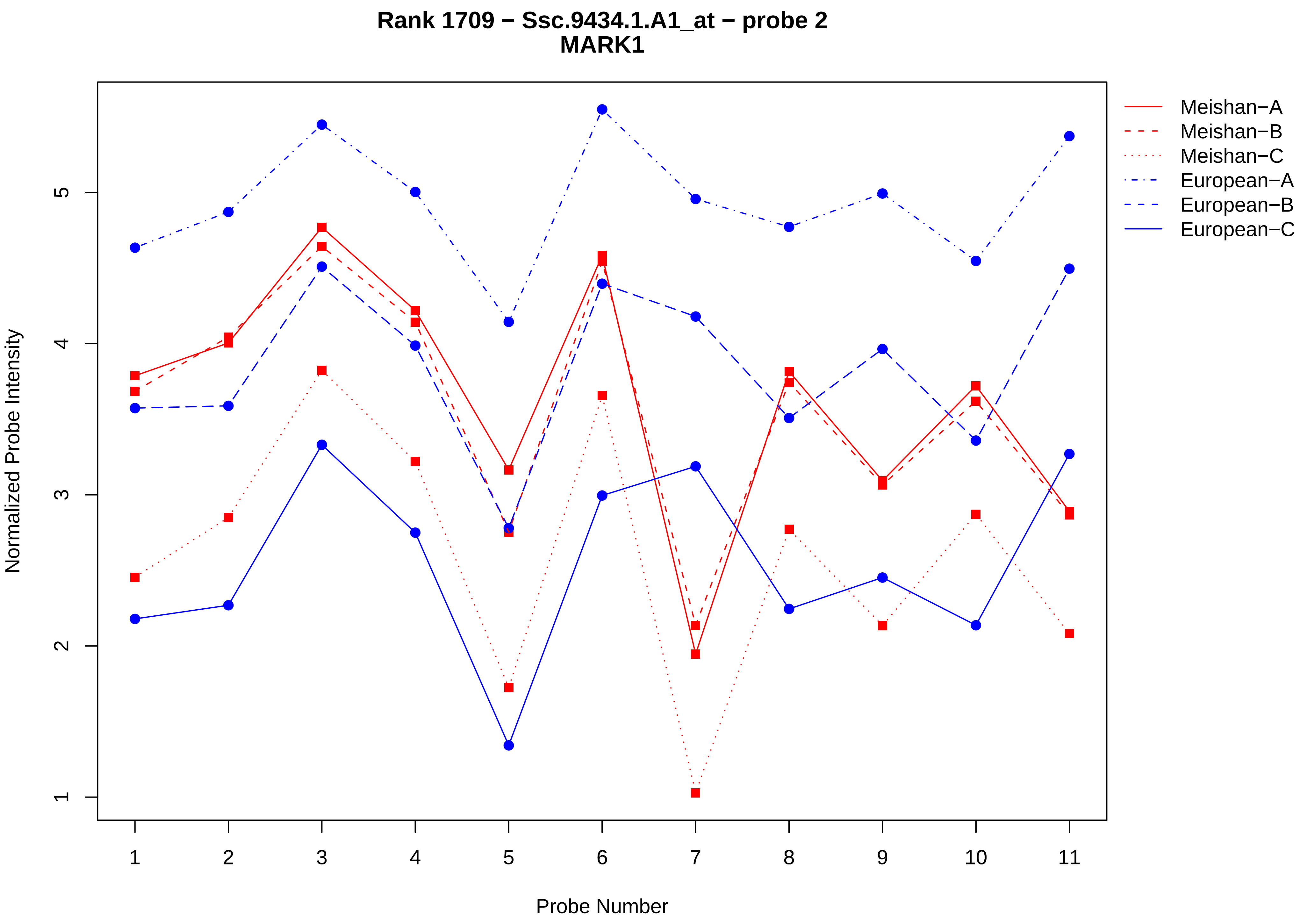

Rank 1709 − Ssc.9434.1.A1_at − probe 2
MARK1
Meishan−A
Meishan−B
Meishan−C
European−A
European−B
European−C
Normalized Probe Intensity
Probe Number
1
2
3
4
5
6
7
8
9
10
11

Rank 1710 − Ssc.13525.1.A1_at − probe 11
SEC24D

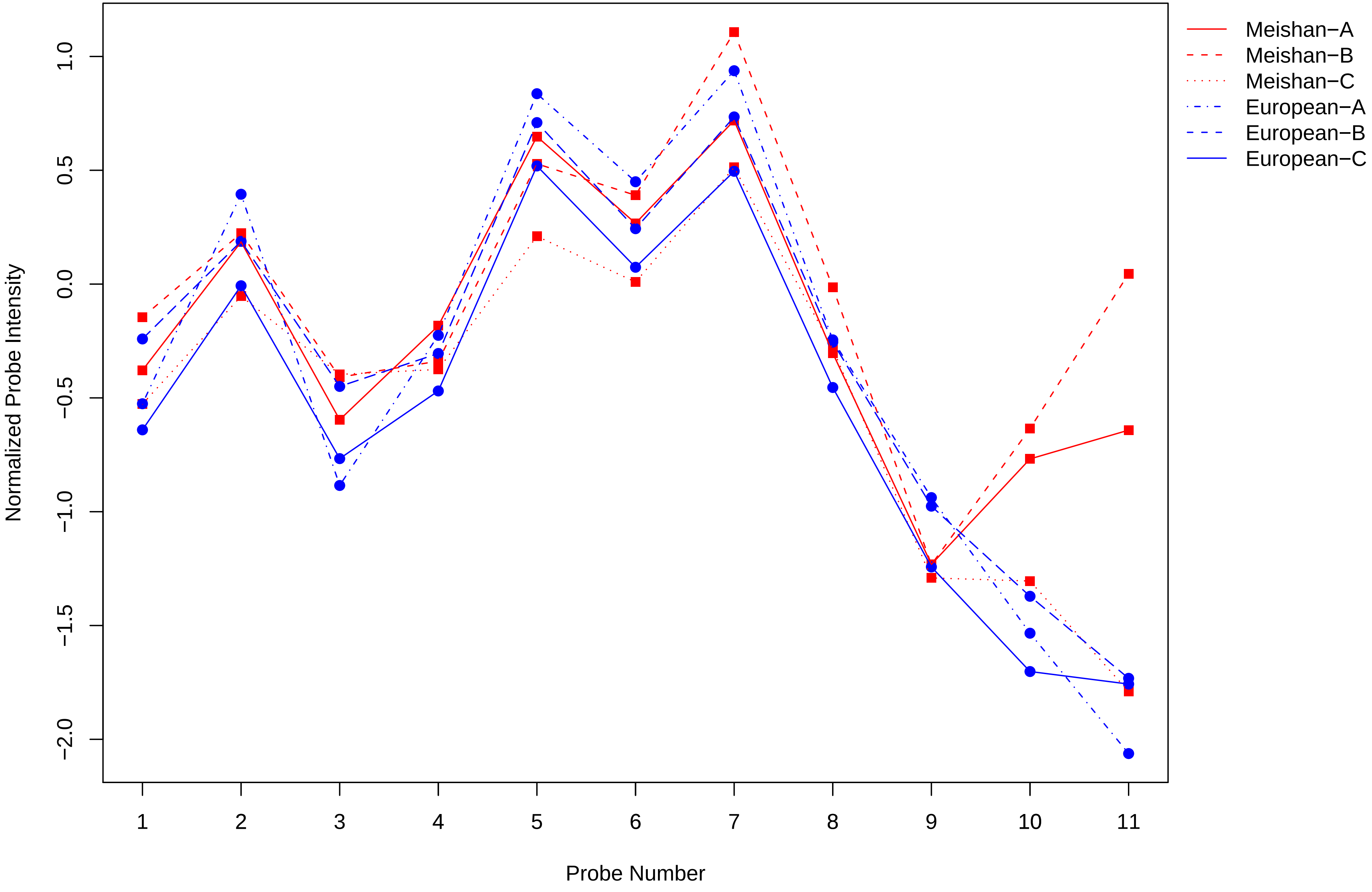

Rank 1711 − Ssc.16710.1.S1_at − probe 6
Q9BTR0

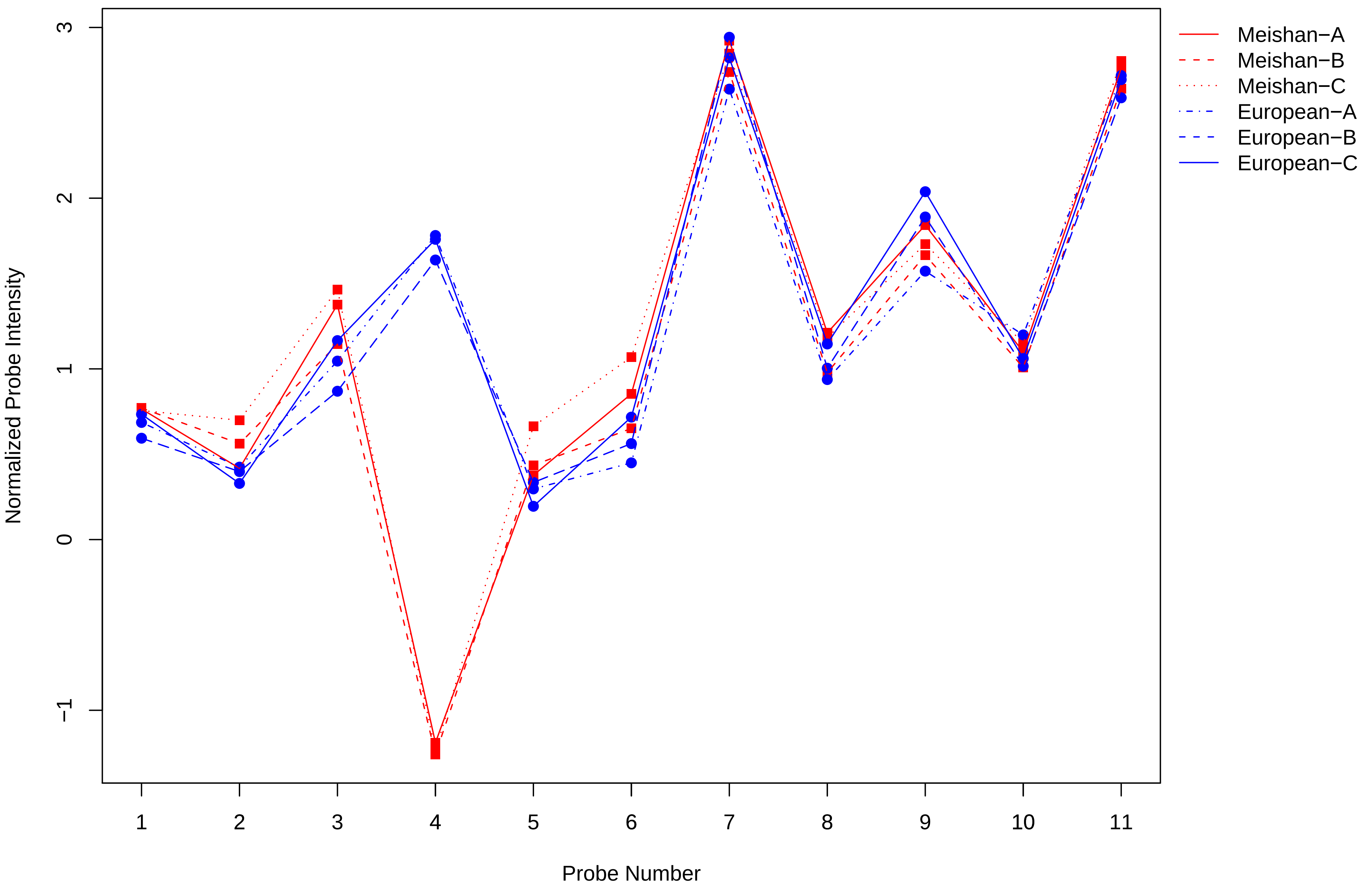

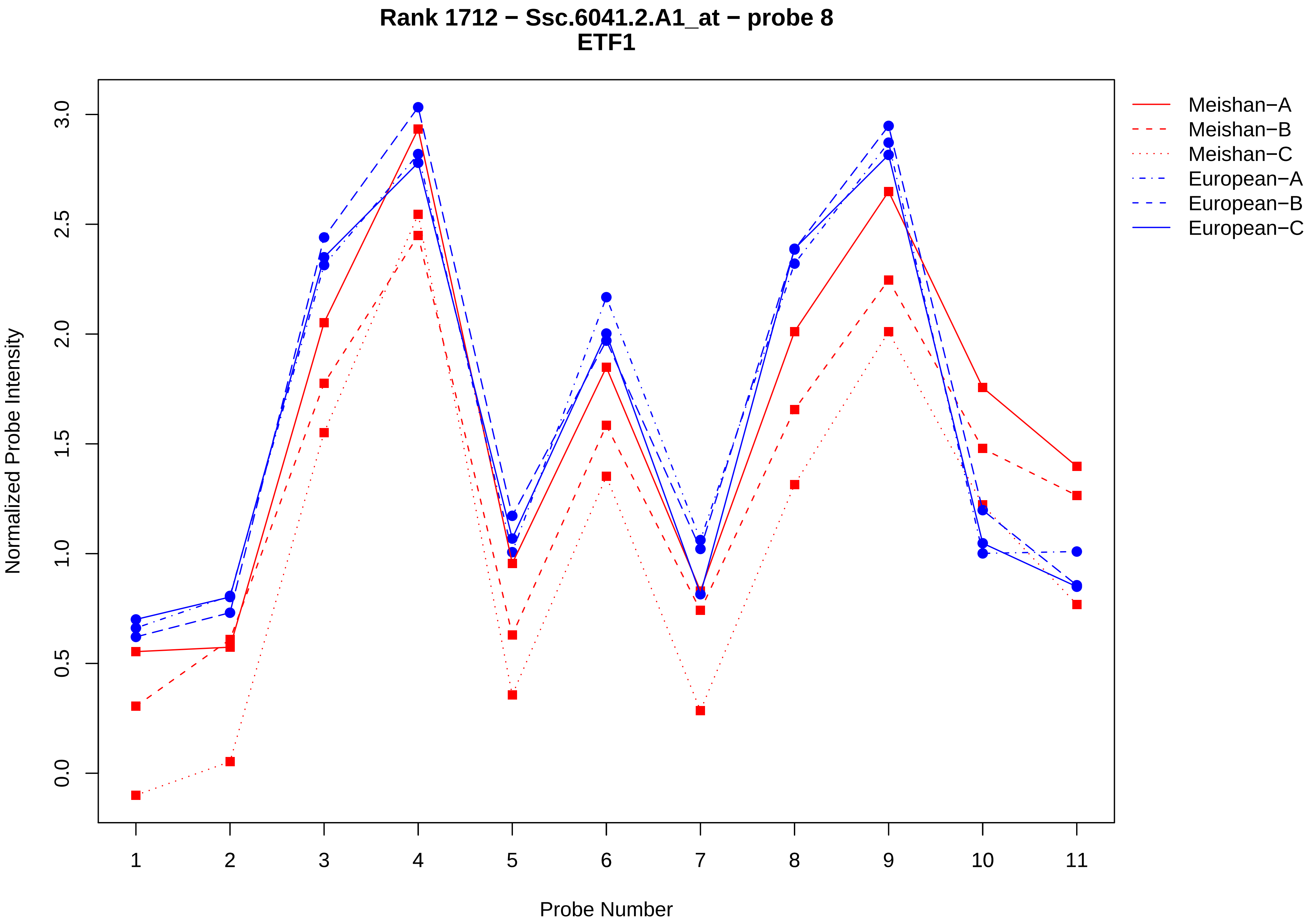

Rank 1712 − Ssc.6041.2.A1_at − probe 8
ETF1
Normalized Probe Intensity
Probe Number
0.0
0.5
1.0
1.5
2.0
2.5
3.0
1
2
3
4
5
6
7
8
9
10
11
Meishan−A
Meishan−B
Meishan−C
European−A
European−B
European−C

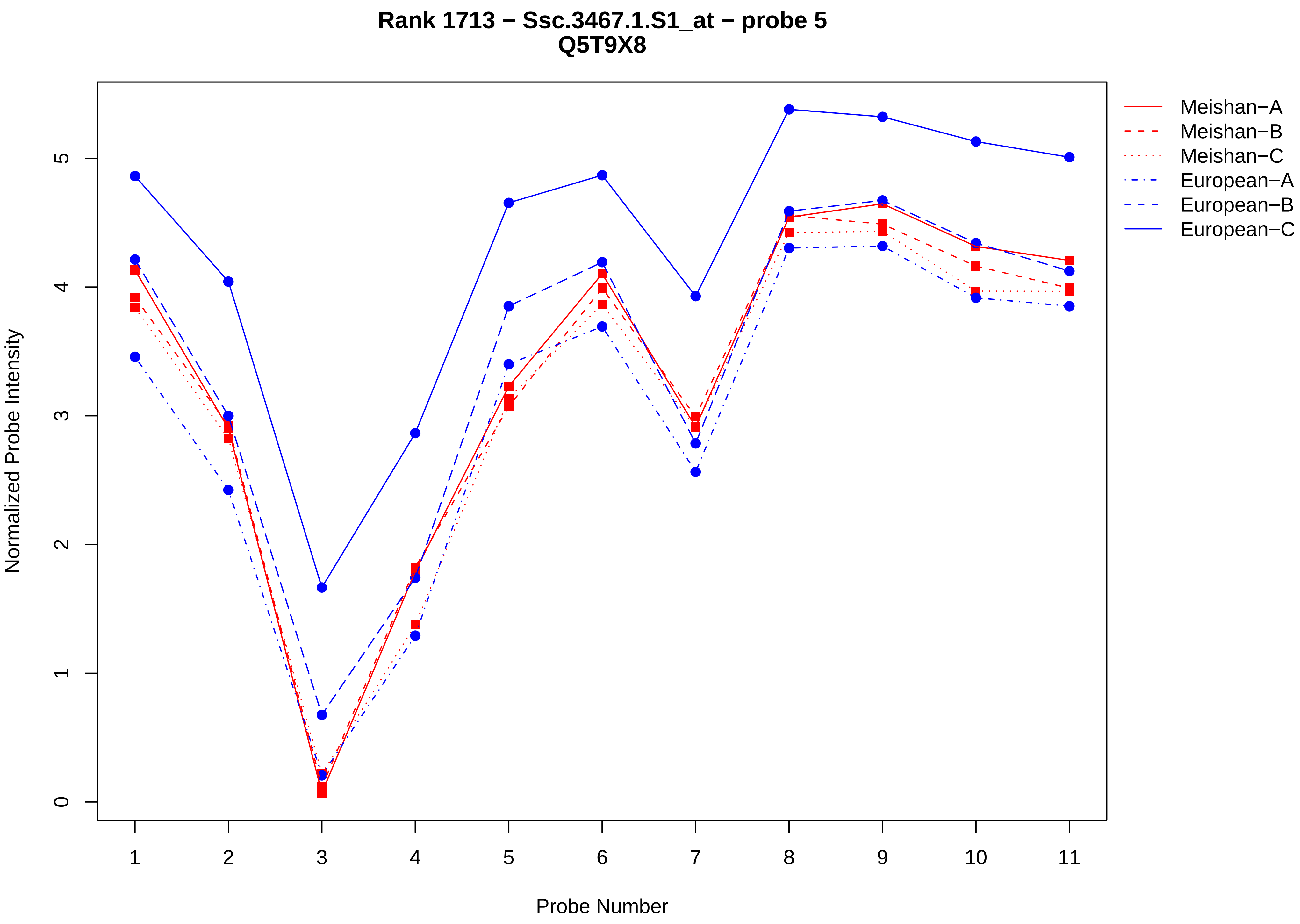

Rank 1713 − Ssc.3467.1.S1_at − probe 5
Q5T9X8
Normalized Probe Intensity
Probe Number
0
1
2
3
4
5
1
2
3
4
5
6
7
8
9
10
11
Meishan−A
Meishan−B
Meishan−C
European−A
European−B
European−C

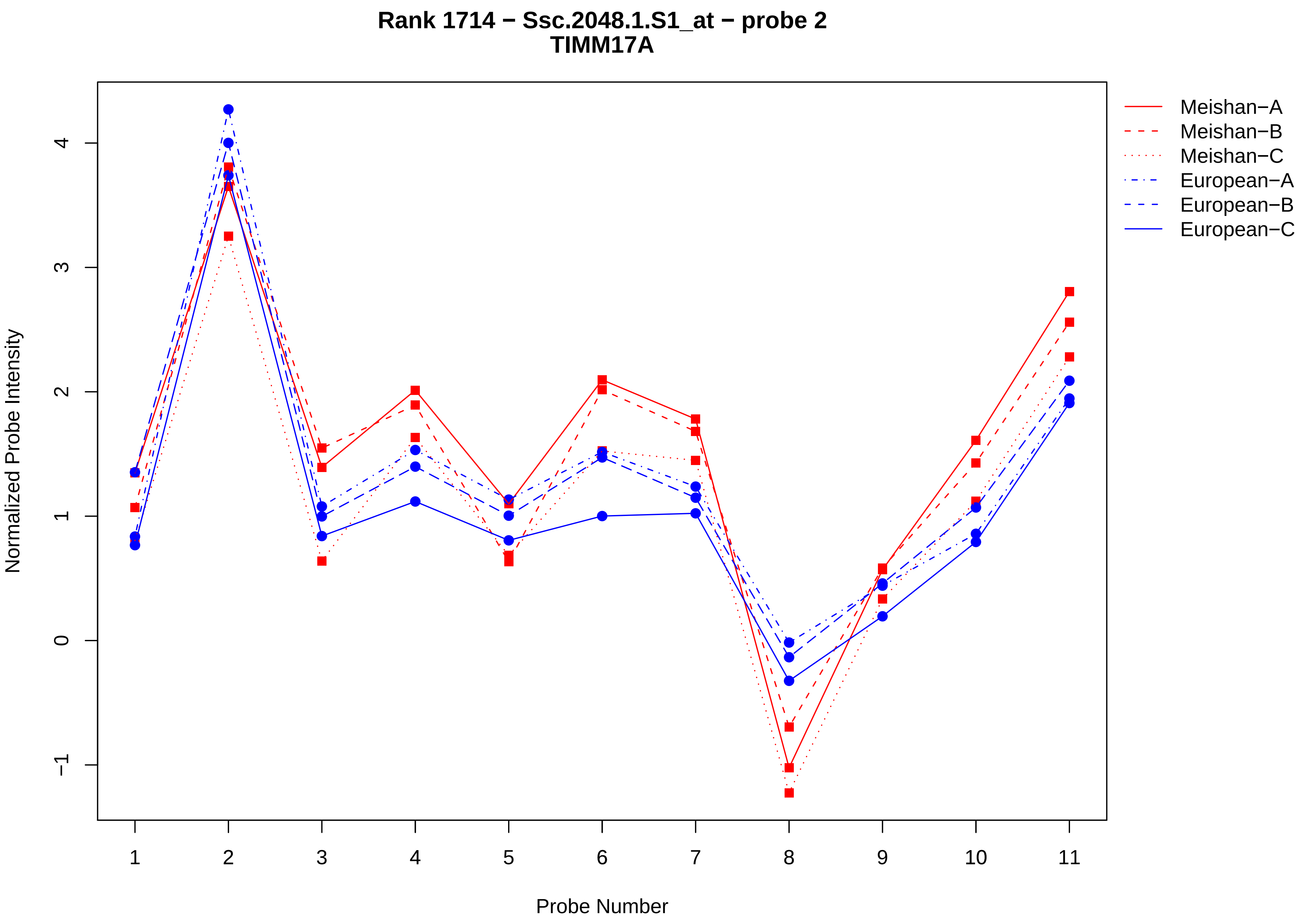

Rank 1714 − Ssc.2048.1.S1_at − probe 2
TIMM17A
Normalized Probe Intensity
Probe Number
−1
0
1
2
3
4
1
2
3
4
5
6
7
8
9
10
11
Meishan−A
Meishan−B
Meishan−C
European−A
European−B
European−C

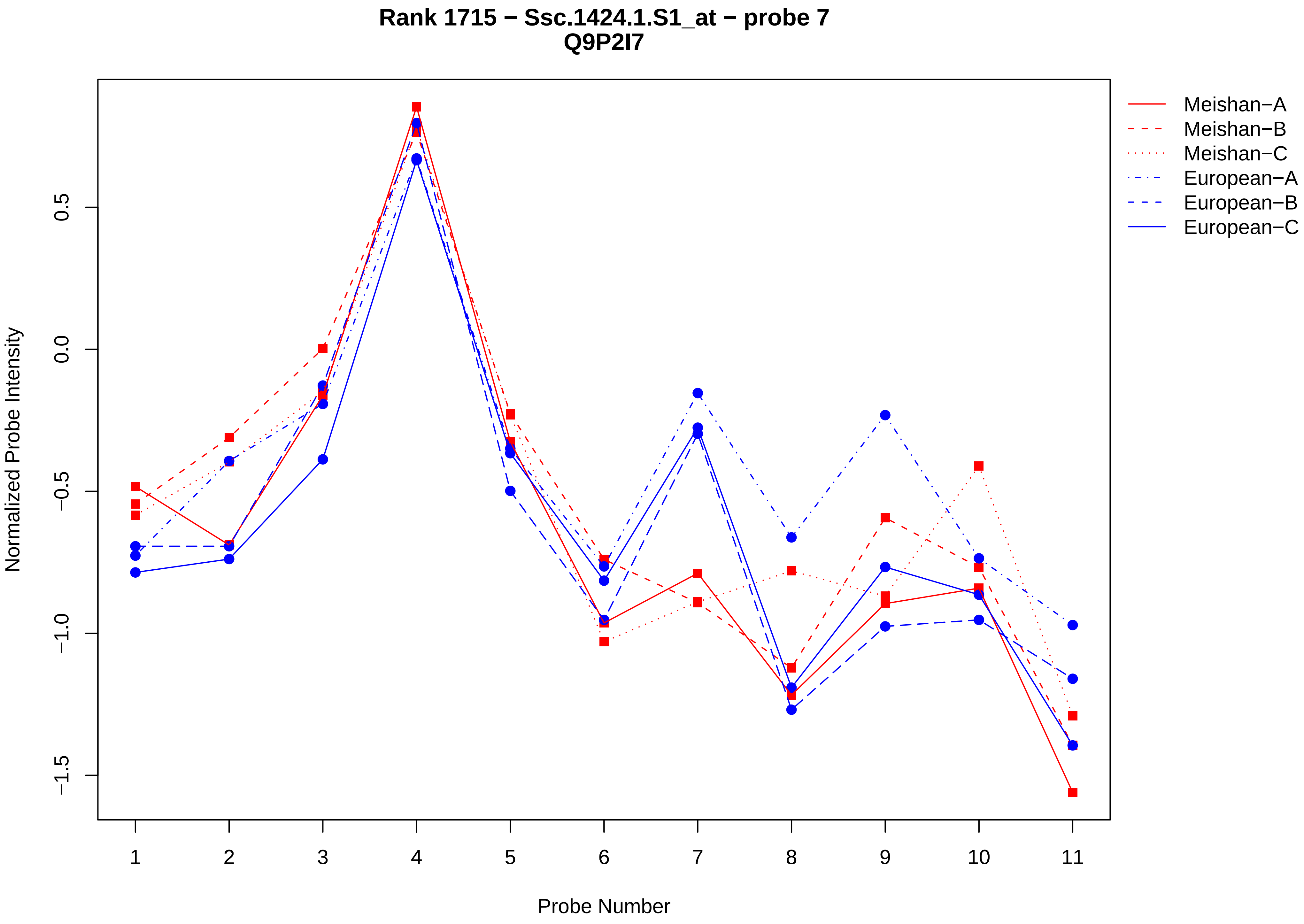

Rank 1715 − Ssc.1424.1.S1_at − probe 7
Q9P2I7
Normalized Probe Intensity
0.5
0.0
−0.5
−1.0
−1.5
1
2
3
4
5
6
7
8
9
10
11
Probe Number
Meishan−A
Meishan−B
Meishan−C
European−A
European−B
European−C

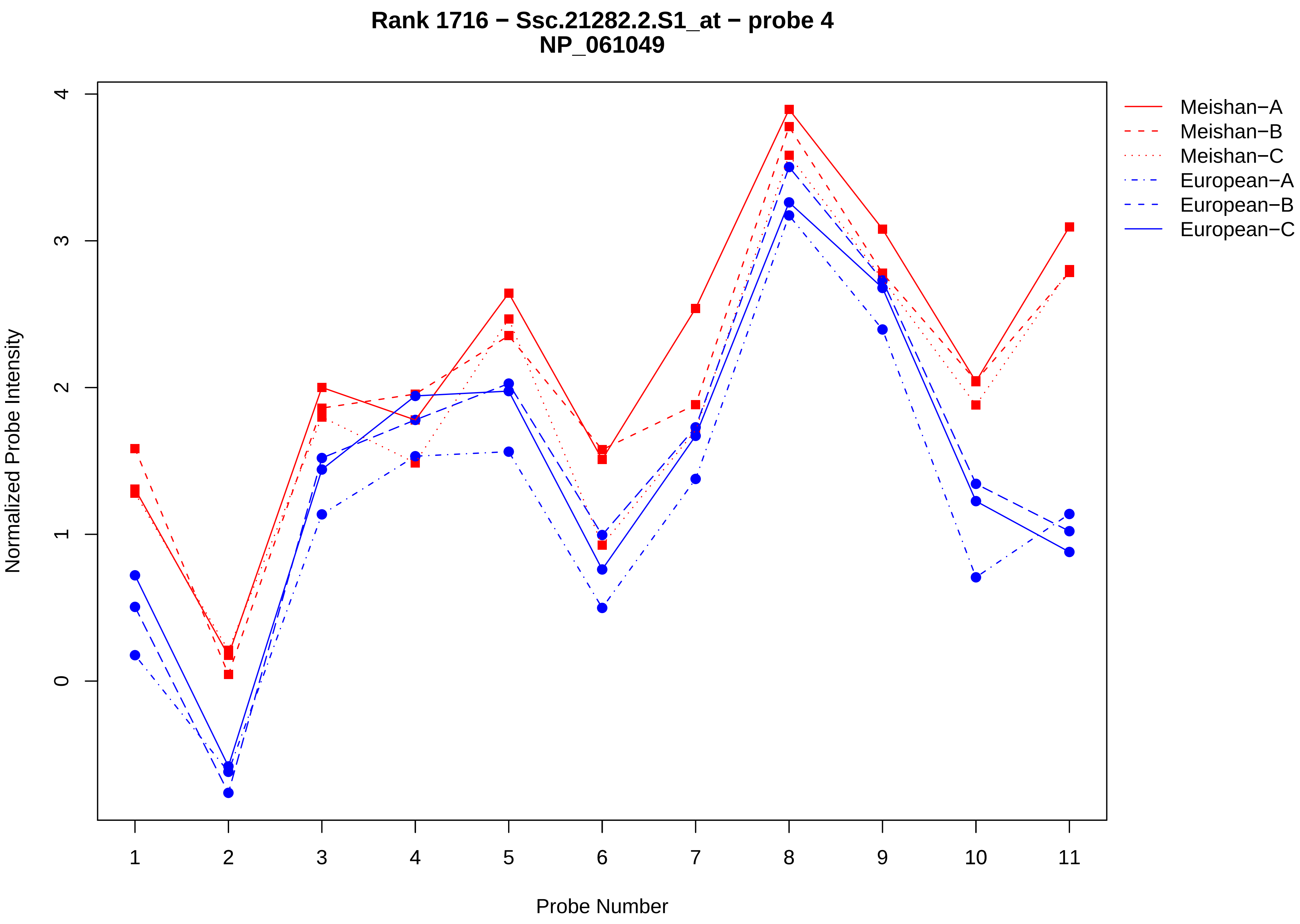

Rank 1716 − Ssc.21282.2.S1_at − probe 4
NP_061049
Normalized Probe Intensity
Probe Number
0
1
2
3
4
1
2
3
4
5
6
7
8
9
10
11
Meishan−A
Meishan−B
Meishan−C
European−A
European−B
European−C

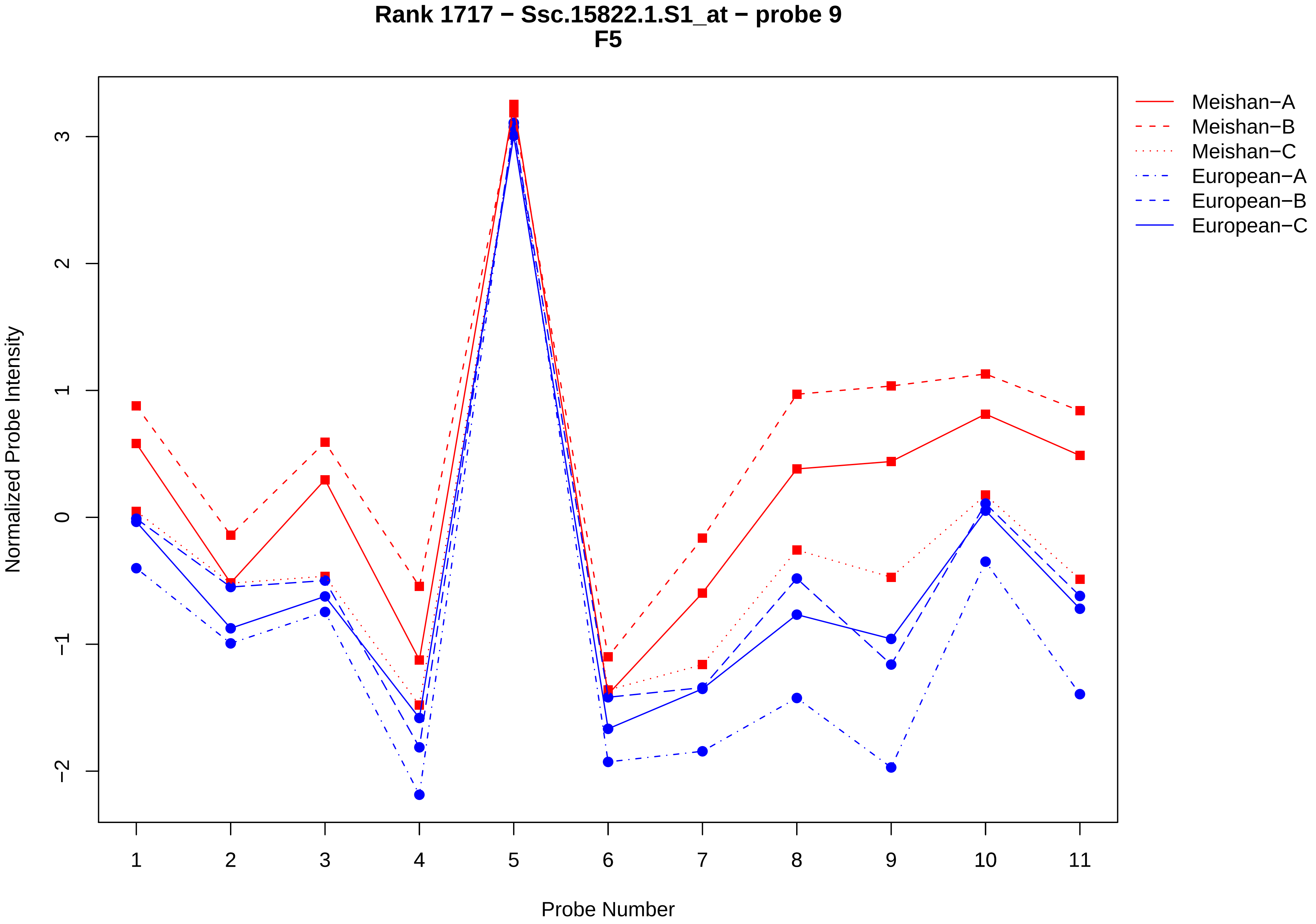

Rank 1717 − Ssc.15822.1.S1_at − probe 9
F5
Normalized Probe Intensity
Probe Number
Meishan−A
Meishan−B
Meishan−C
European−A
European−B
European−C
−2
−1
0
1
2
3
1
2
3
4
5
6
7
8
9
10
11

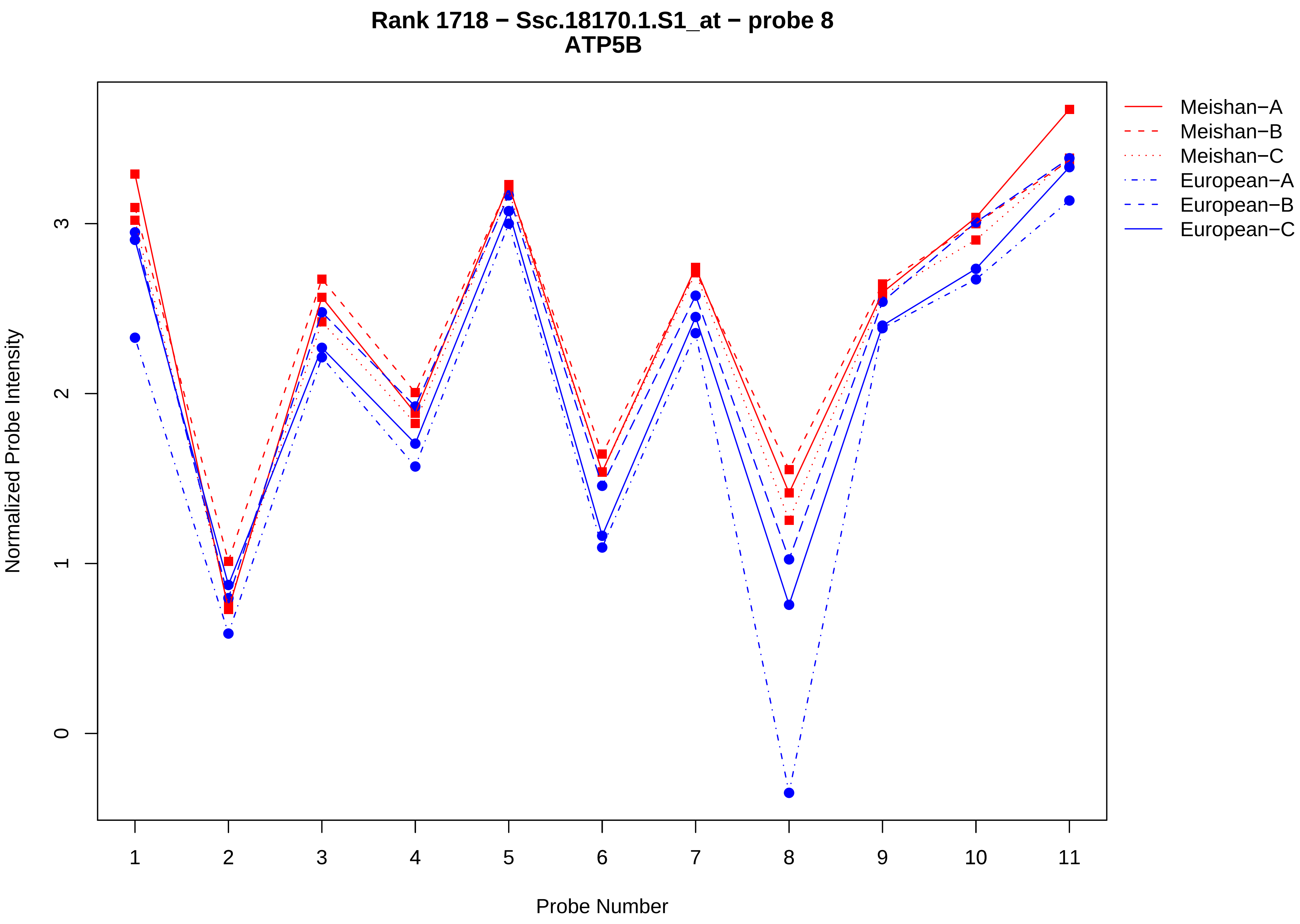
Rank 1718 − Ssc.18170.1.S1_at − probe 8
ATP5B
Normalized Probe Intensity
Probe Number
0
1
2
3
1
2
3
4
5
6
7
8
9
10
11
Meishan−A
Meishan−B
Meishan−C
European−A
European−B
European−C

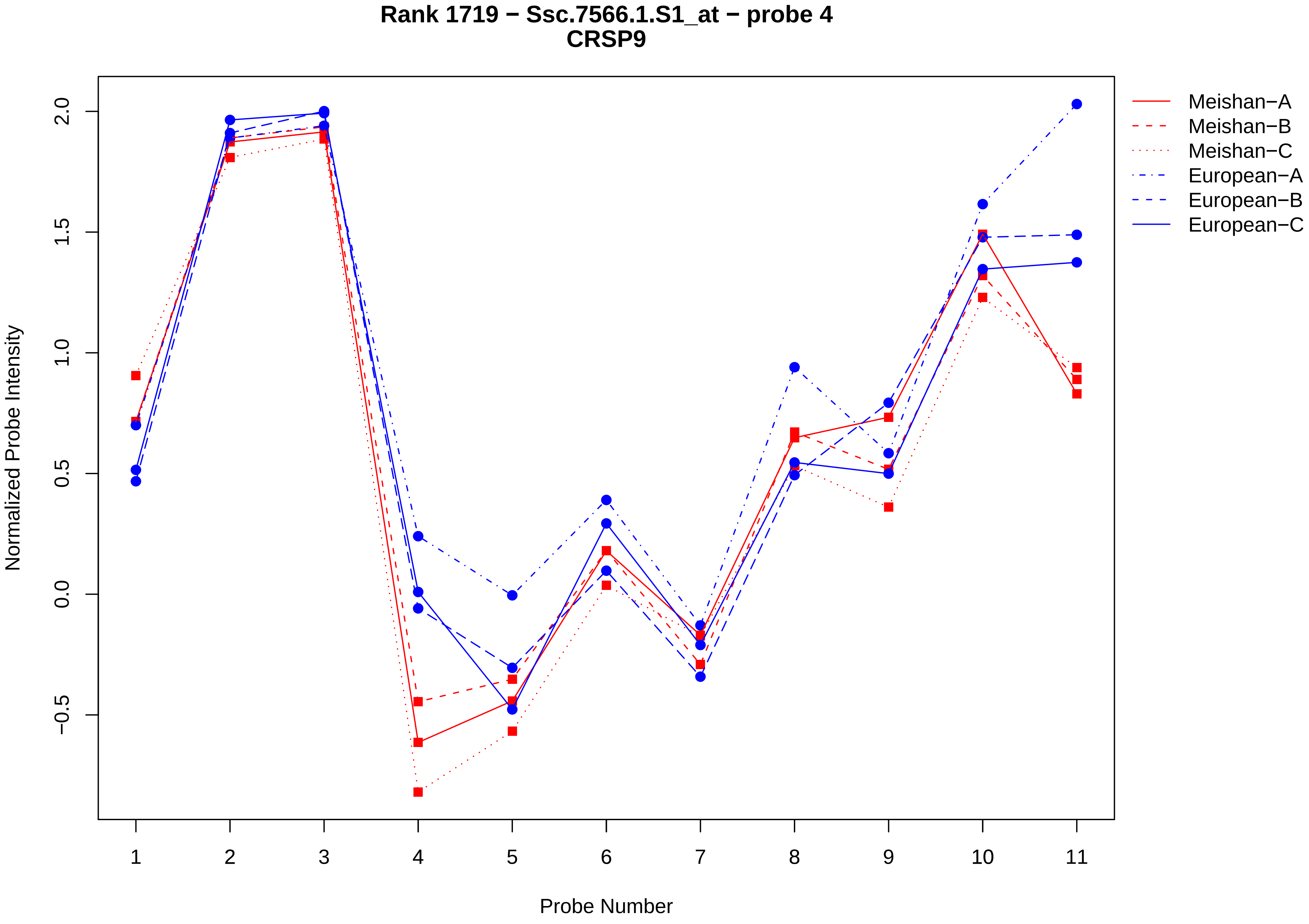

Rank 1719 − Ssc.7566.1.S1_at − probe 4
CRSP9
Normalized Probe Intensity
Probe Number
Meishan−A
Meishan−B
Meishan−C
European−A
European−B
European−C

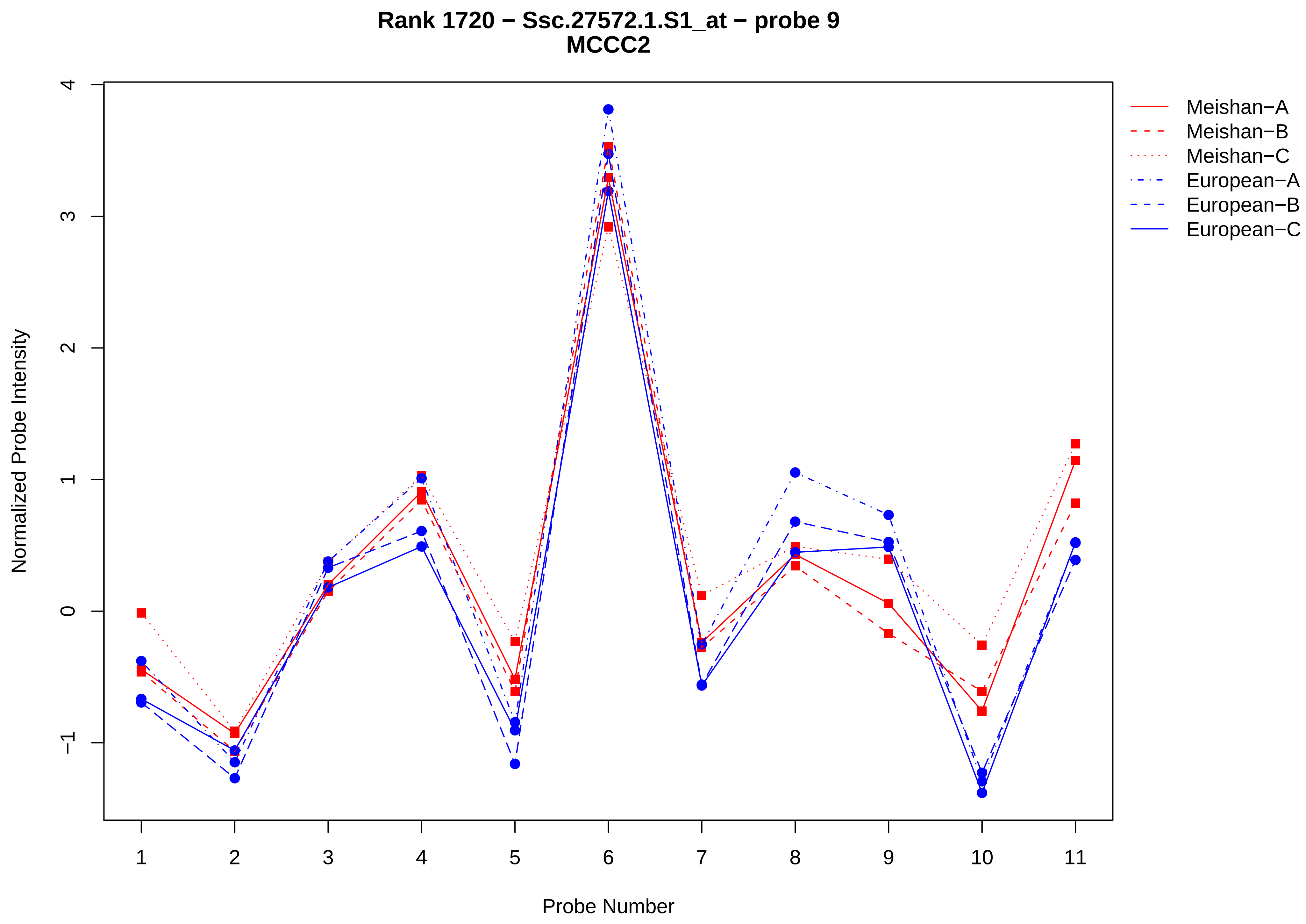

Rank 1720 − Ssc.27572.1.S1_at − probe 9
MCCC2
Normalized Probe Intensity
Probe Number
Meishan−A
Meishan−B
Meishan−C
European−A
European−B
European−C

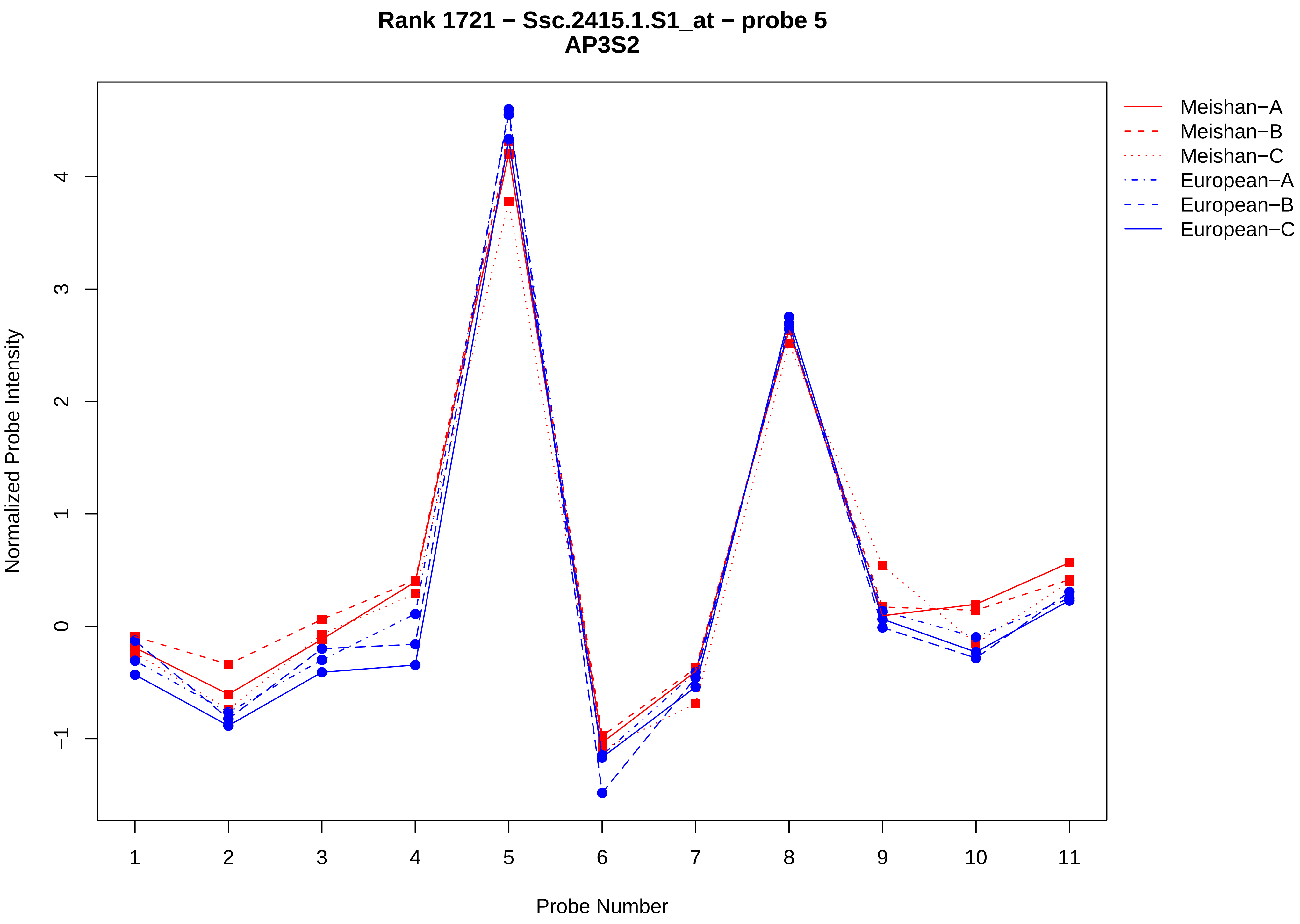

Rank 1721 − Ssc.2415.1.S1_at − probe 5
AP3S2
Normalized Probe Intensity
Probe Number
Meishan−A
Meishan−B
Meishan−C
European−A
European−B
European−C
−1
0
1
2
3
4
1
2
3
4
5
6
7
8
9
10
11

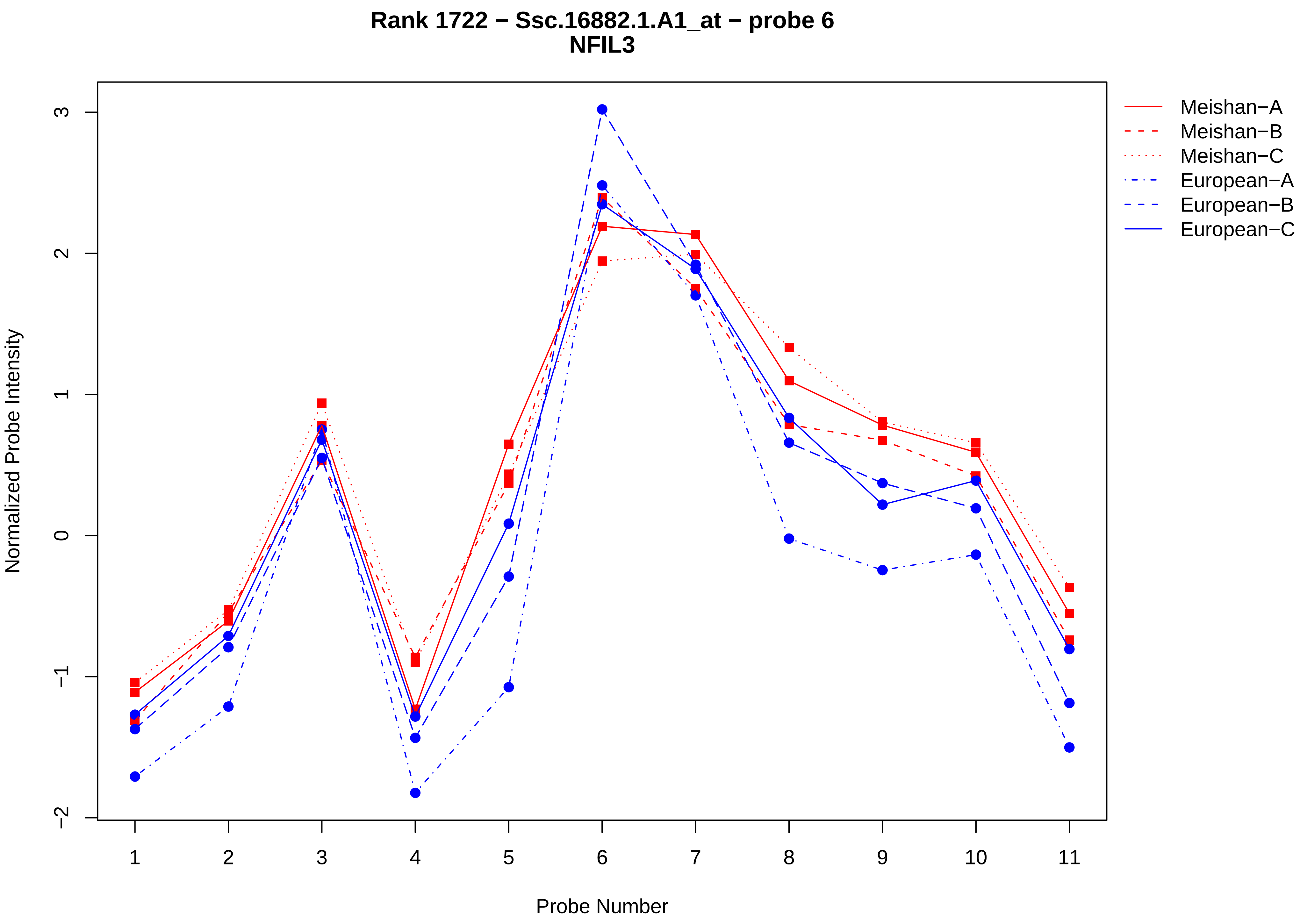

Rank 1722 − Ssc.16882.1.A1_at − probe 6
NFIL3
Normalized Probe Intensity
Probe Number
−2
−1
0
1
2
3
1
2
3
4
5
6
7
8
9
10
11
Meishan−A
Meishan−B
Meishan−C
European−A
European−B
European−C

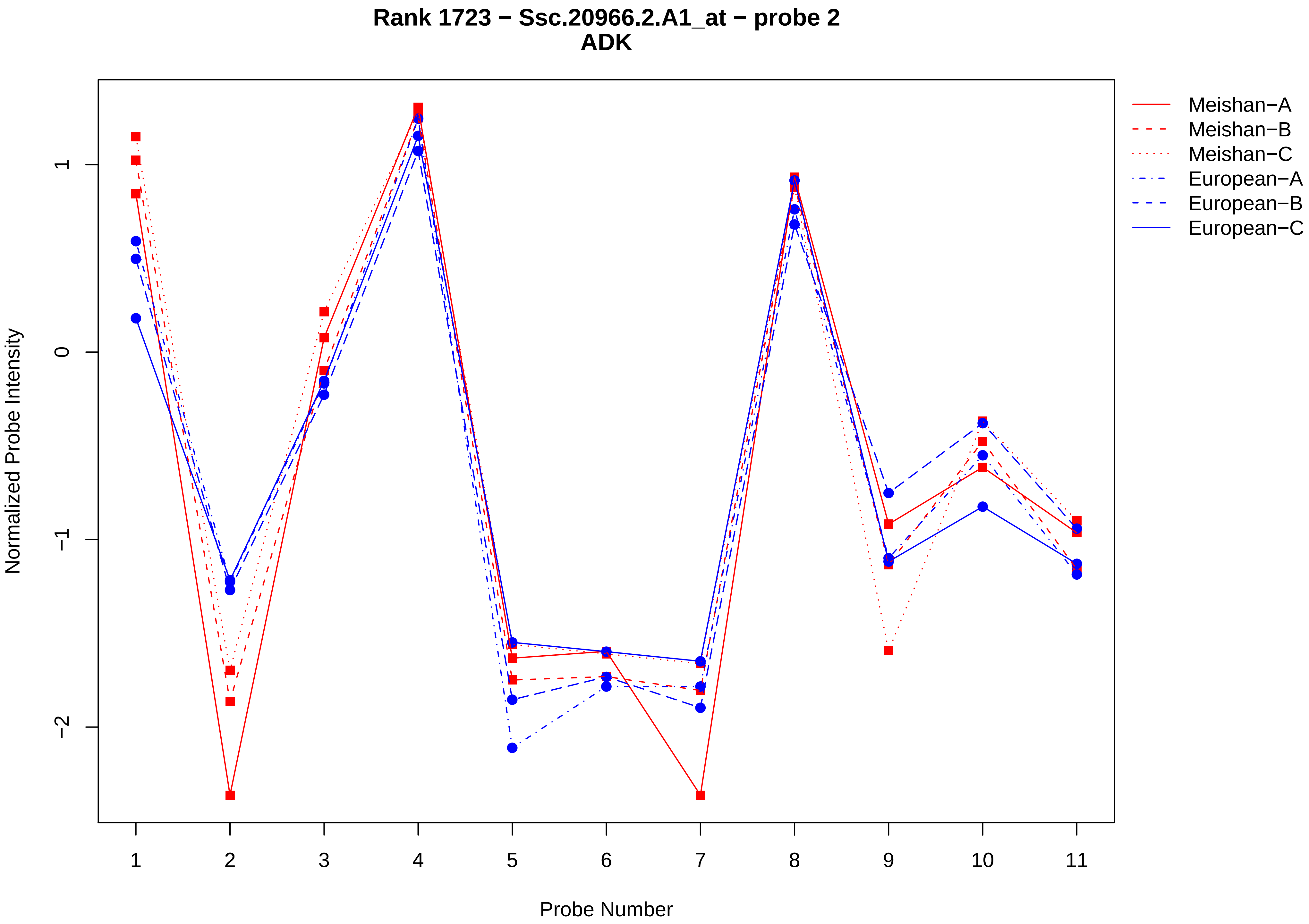

Rank 1723 − Ssc.20966.2.A1_at − probe 2
ADK
Normalized Probe Intensity
Probe Number
−2
−1
0
1
1
2
3
4
5
6
7
8
9
10
11
Meishan−A
Meishan−B
Meishan−C
European−A
European−B
European−C

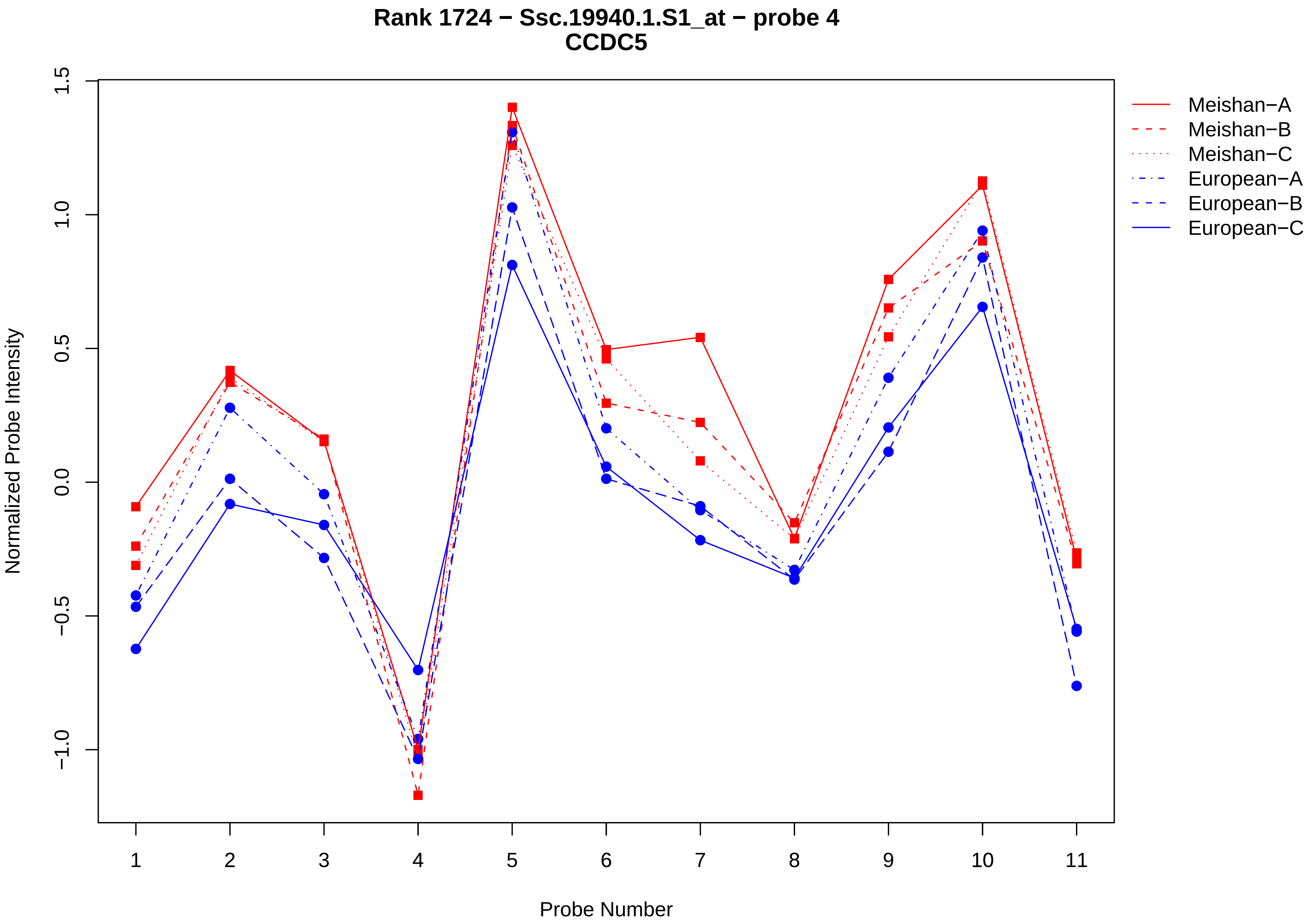

Rank 1724 − Ssc.19940.1.S1_at − probe 4
CCDC5
Normalized Probe Intensity
Probe Number
1.5
1.0
0.5
0.0
−0.5
−1.0
1
2
3
4
5
6
7
8
9
10
11
Meishan−A
Meishan−B
Meishan−C
European−A
European−B
European−C

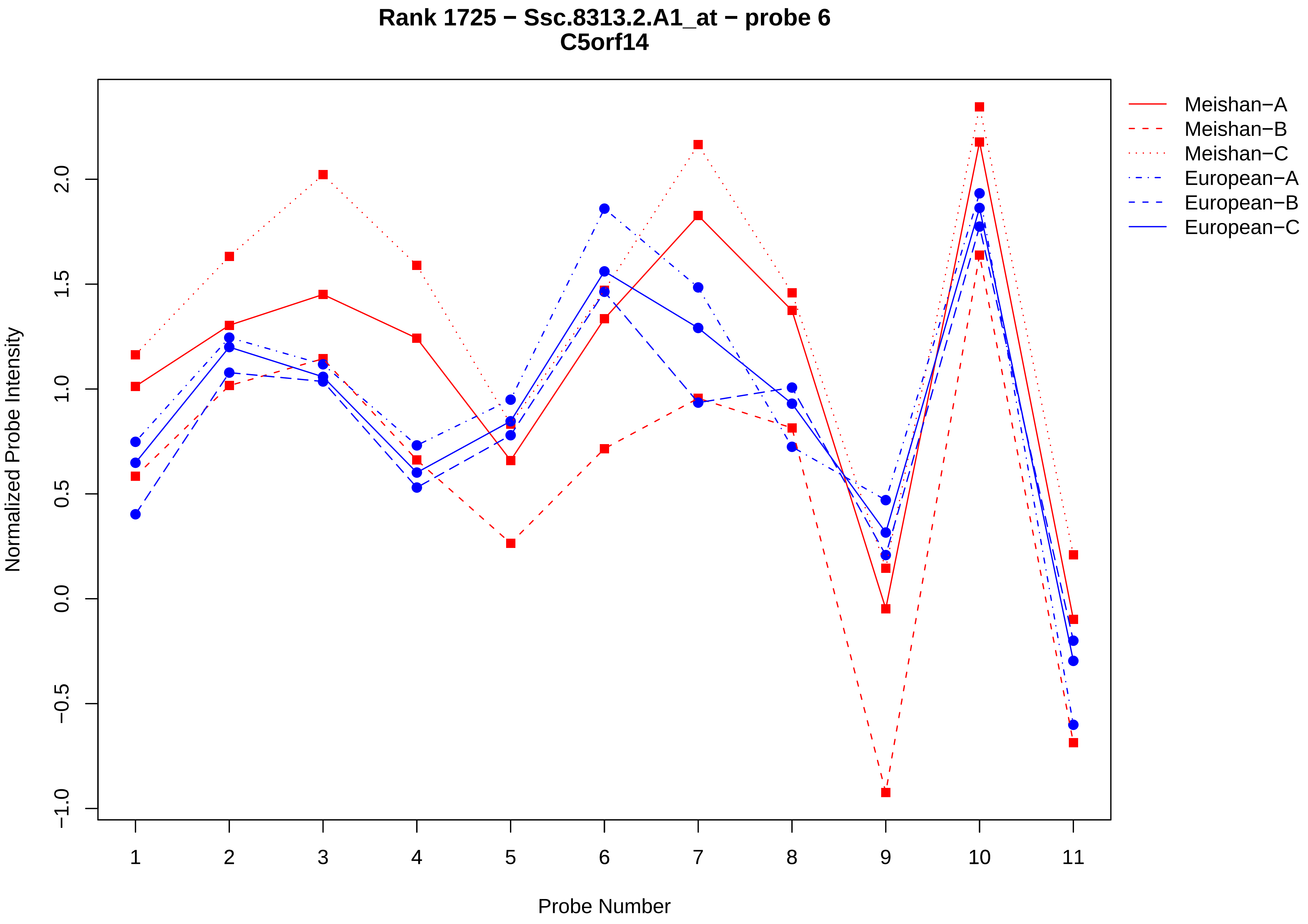

Rank 1725 − Ssc.8313.2.A1_at − probe 6
C5orf14
Normalized Probe Intensity
Probe Number
−1.0
−0.5
0.0
0.5
1.0
1.5
2.0
1
2
3
4
5
6
7
8
9
10
11
Meishan−A
Meishan−B
Meishan−C
European−A
European−B
European−C

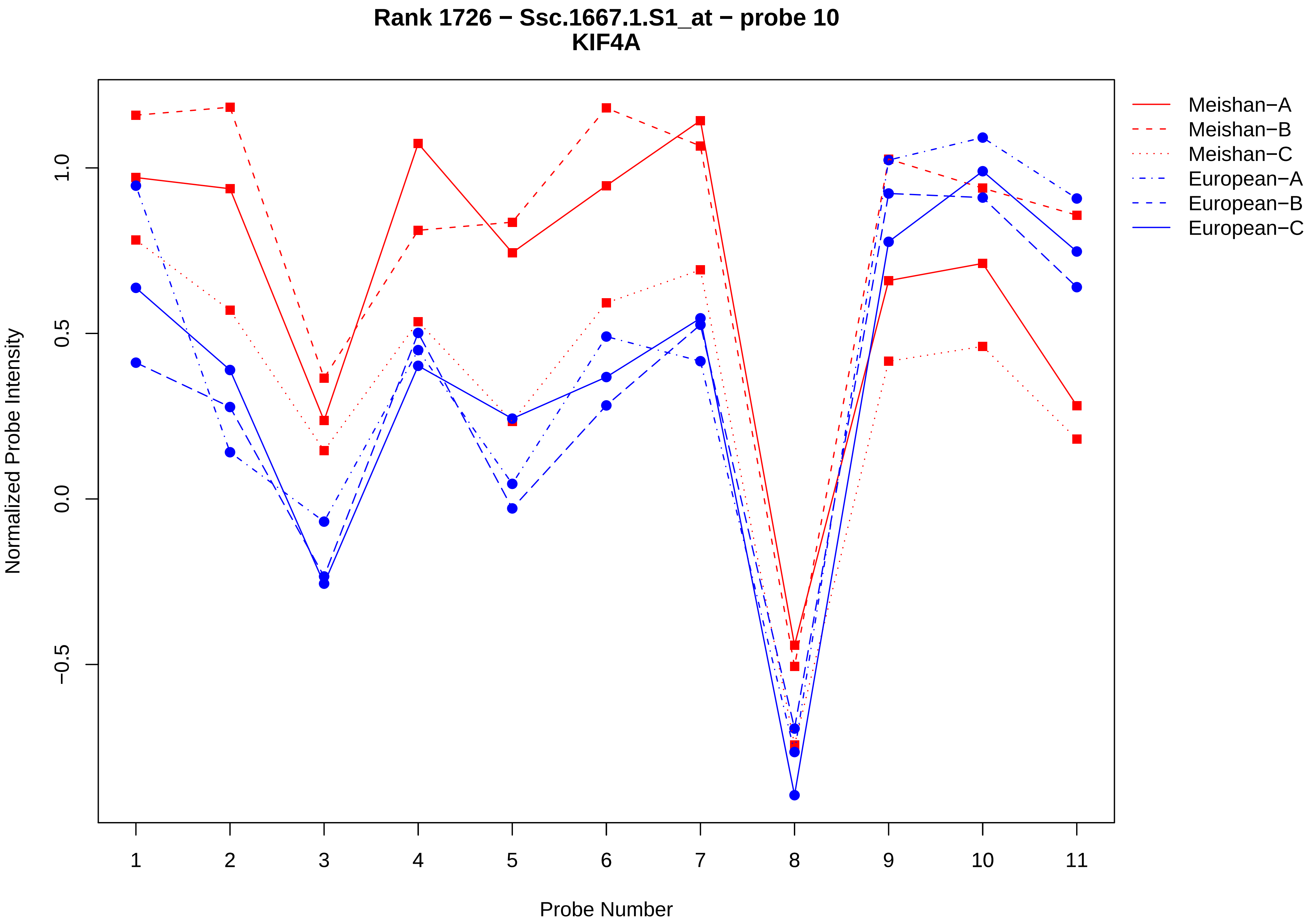

Rank 1726 − Ssc.1667.1.S1_at − probe 10
KIF4A
Normalized Probe Intensity
Probe Number
−0.5
0.0
0.5
1.0
1
2
3
4
5
6
7
8
9
10
11
Meishan−A
Meishan−B
Meishan−C
European−A
European−B
European−C

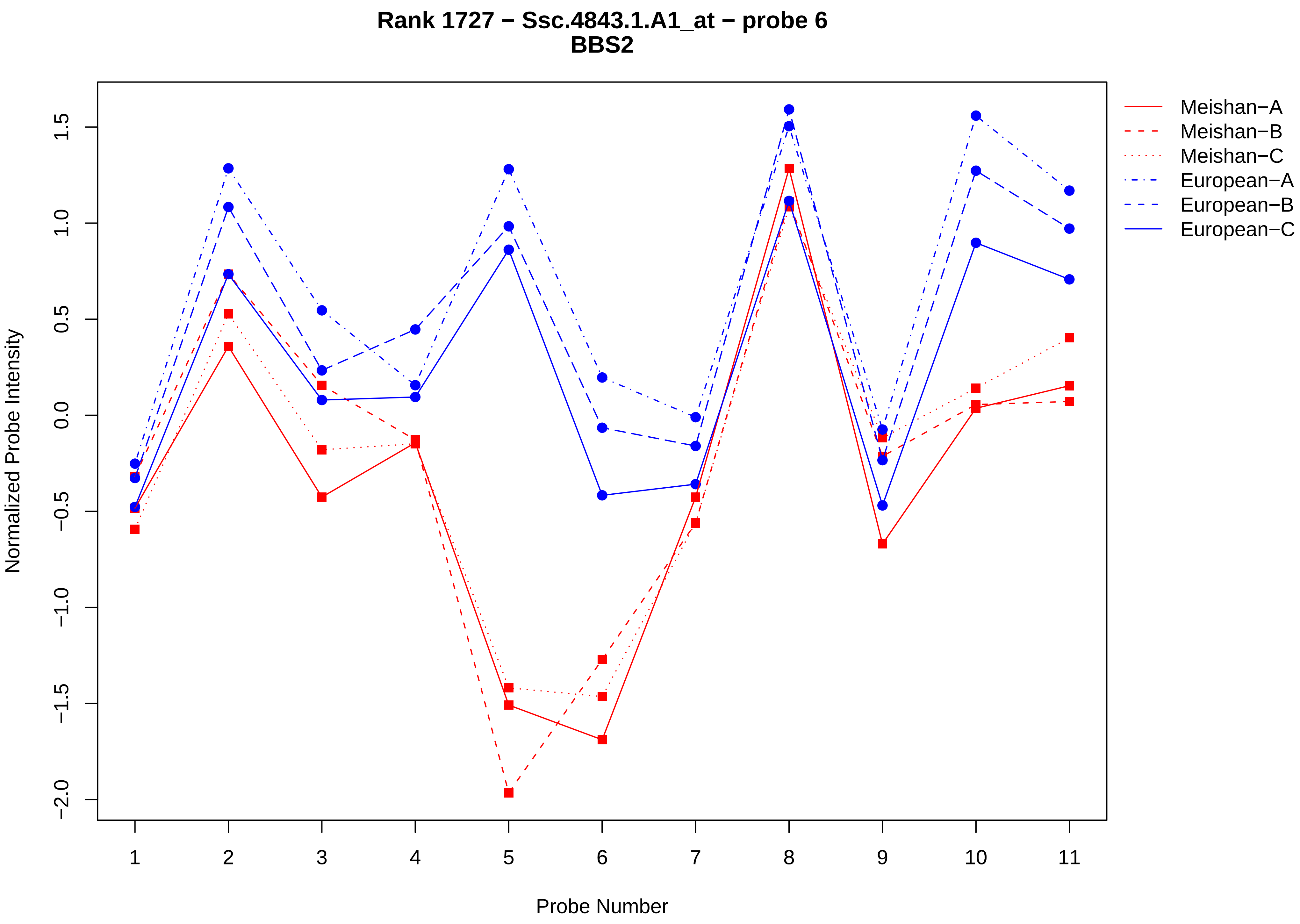

Rank 1727 − Ssc.4843.1.A1_at − probe 6
BBS2
Normalized Probe Intensity
Probe Number
−2.0
−1.5
−1.0
−0.5
0.0
0.5
1.0
1.5
1
2
3
4
5
6
7
8
9
10
11
Meishan−A
Meishan−B
Meishan−C
European−A
European−B
European−C

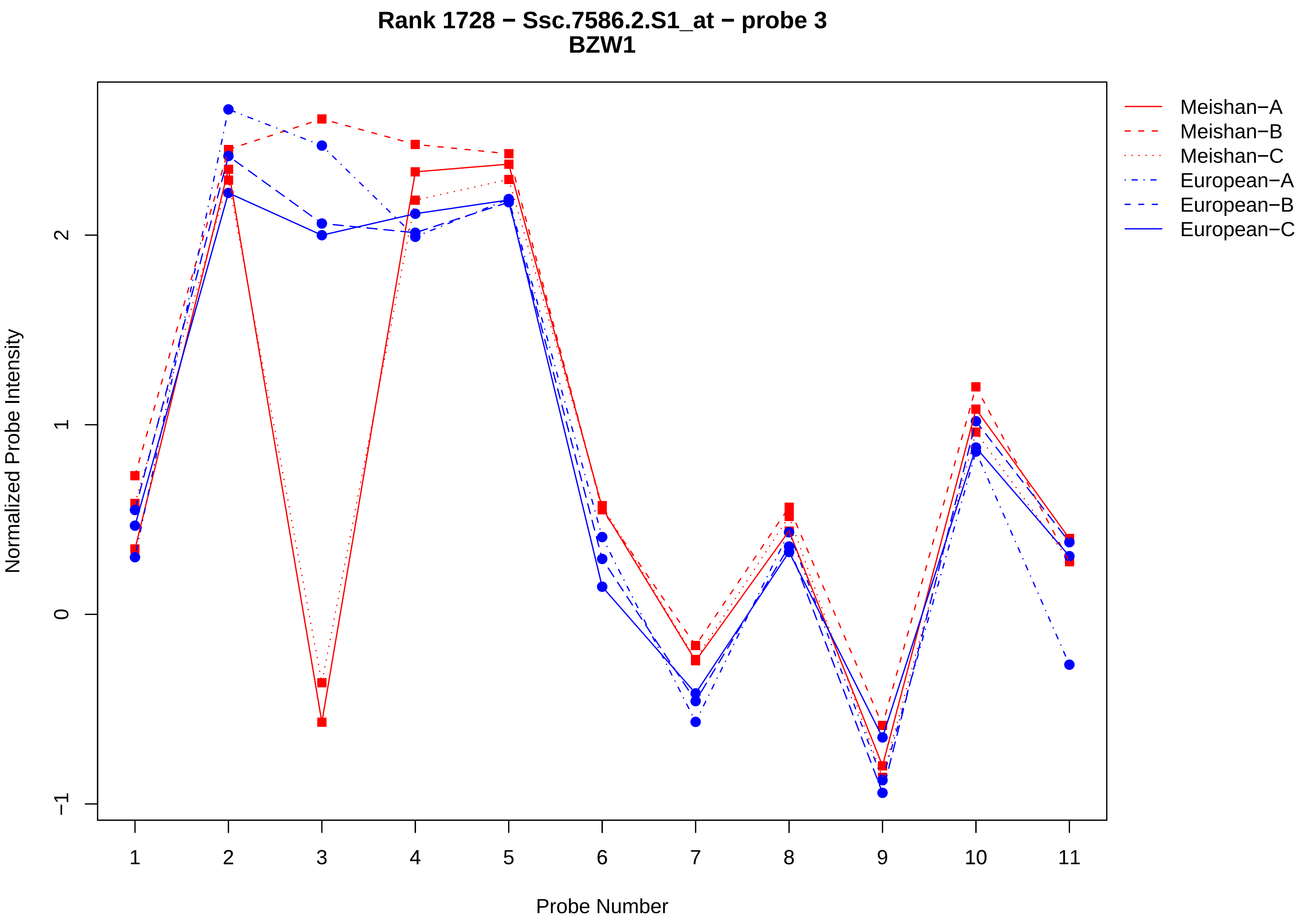

Rank 1728 − Ssc.7586.2.S1_at − probe 3
BZW1
Normalized Probe Intensity
Probe Number
−1
0
1
2
1
2
3
4
5
6
7
8
9
10
11
Meishan−A
Meishan−B
Meishan−C
European−A
European−B
European−C

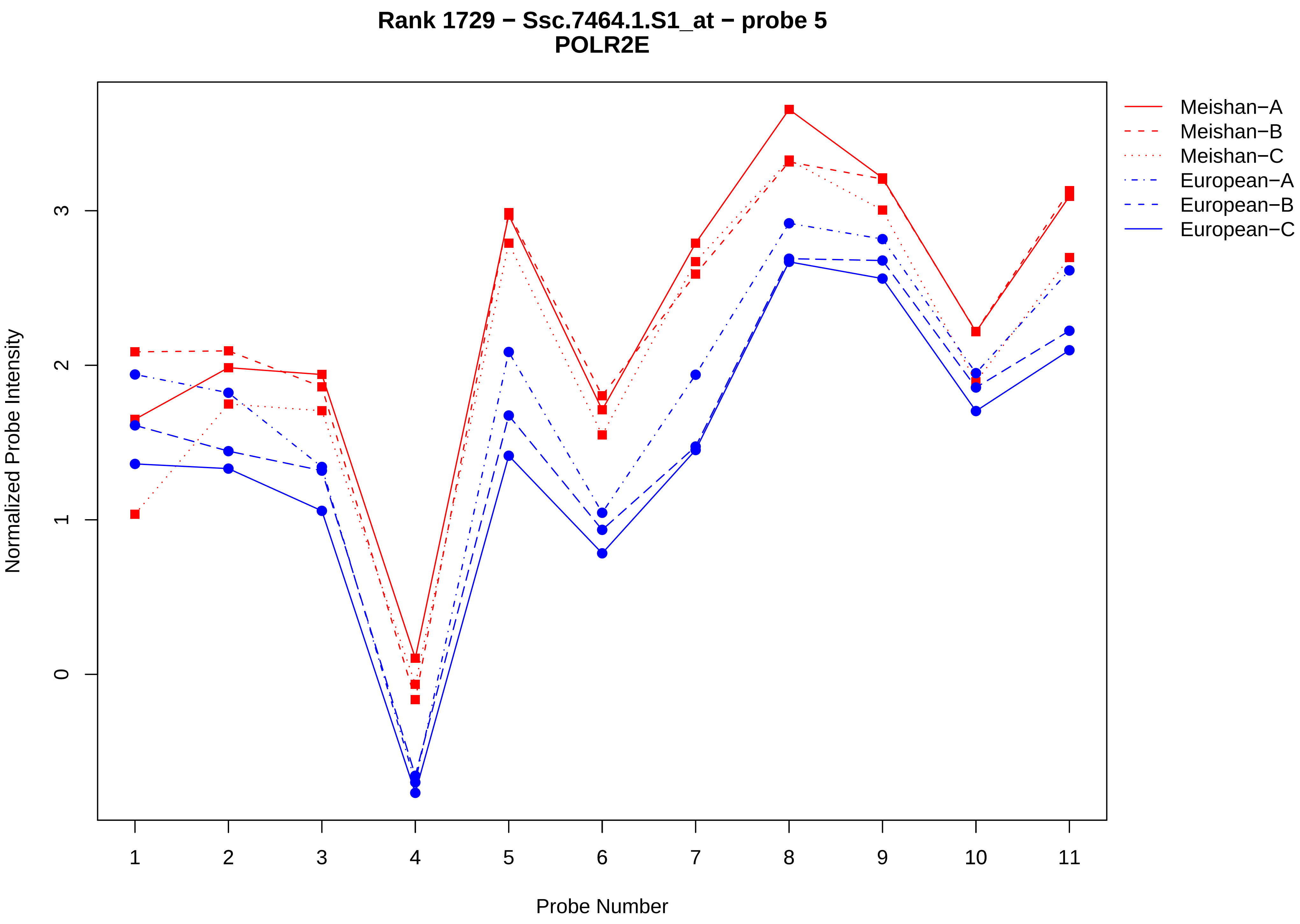

Rank 1729 − Ssc.7464.1.S1_at − probe 5
POLR2E
Normalized Probe Intensity
Probe Number
0
1
2
3
1
2
3
4
5
6
7
8
9
10
11
Meishan−A
Meishan−B
Meishan−C
European−A
European−B
European−C

Rank 1730 − Ssc.986.1.A1_at − probe 1
COX7B

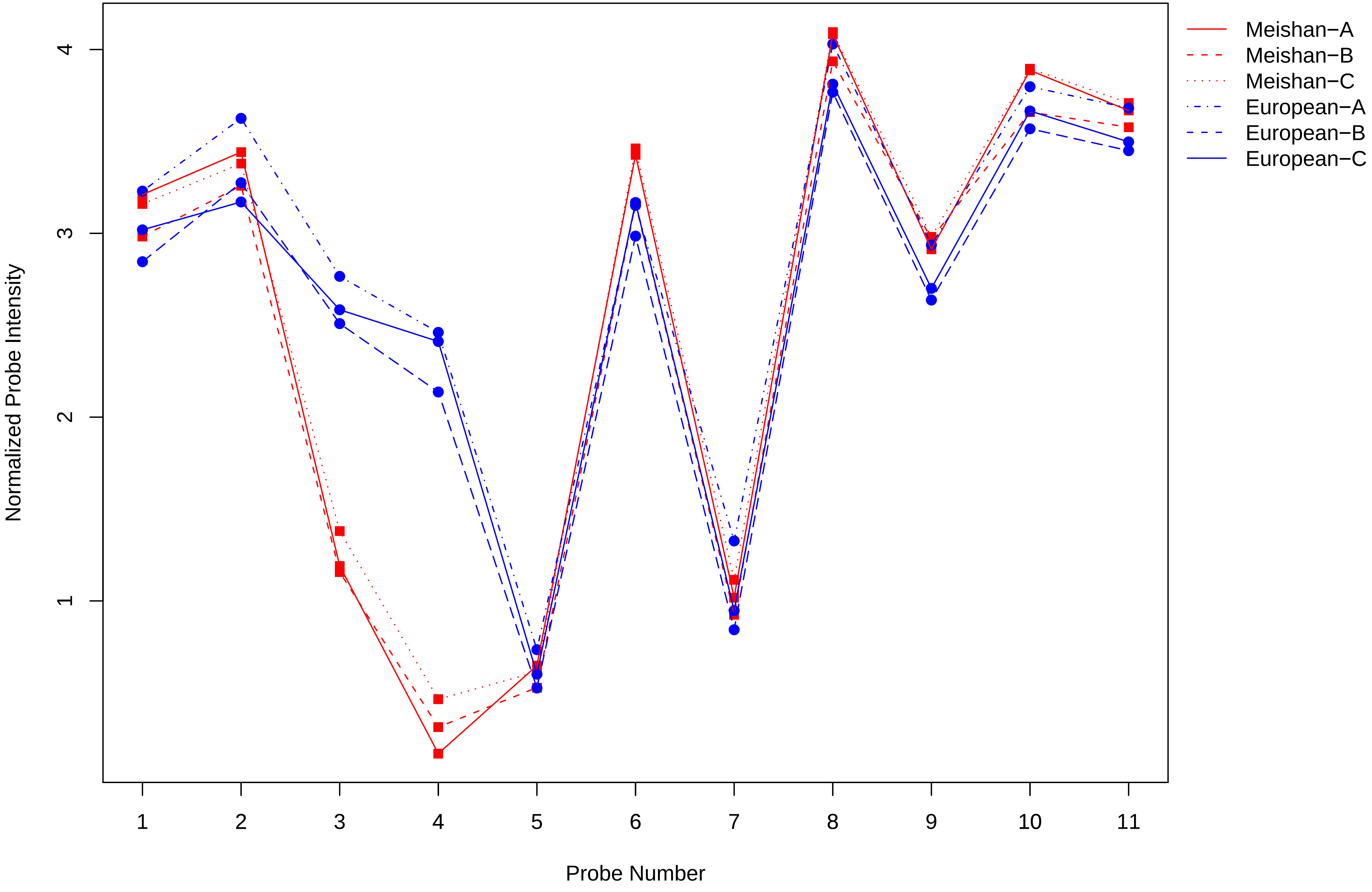

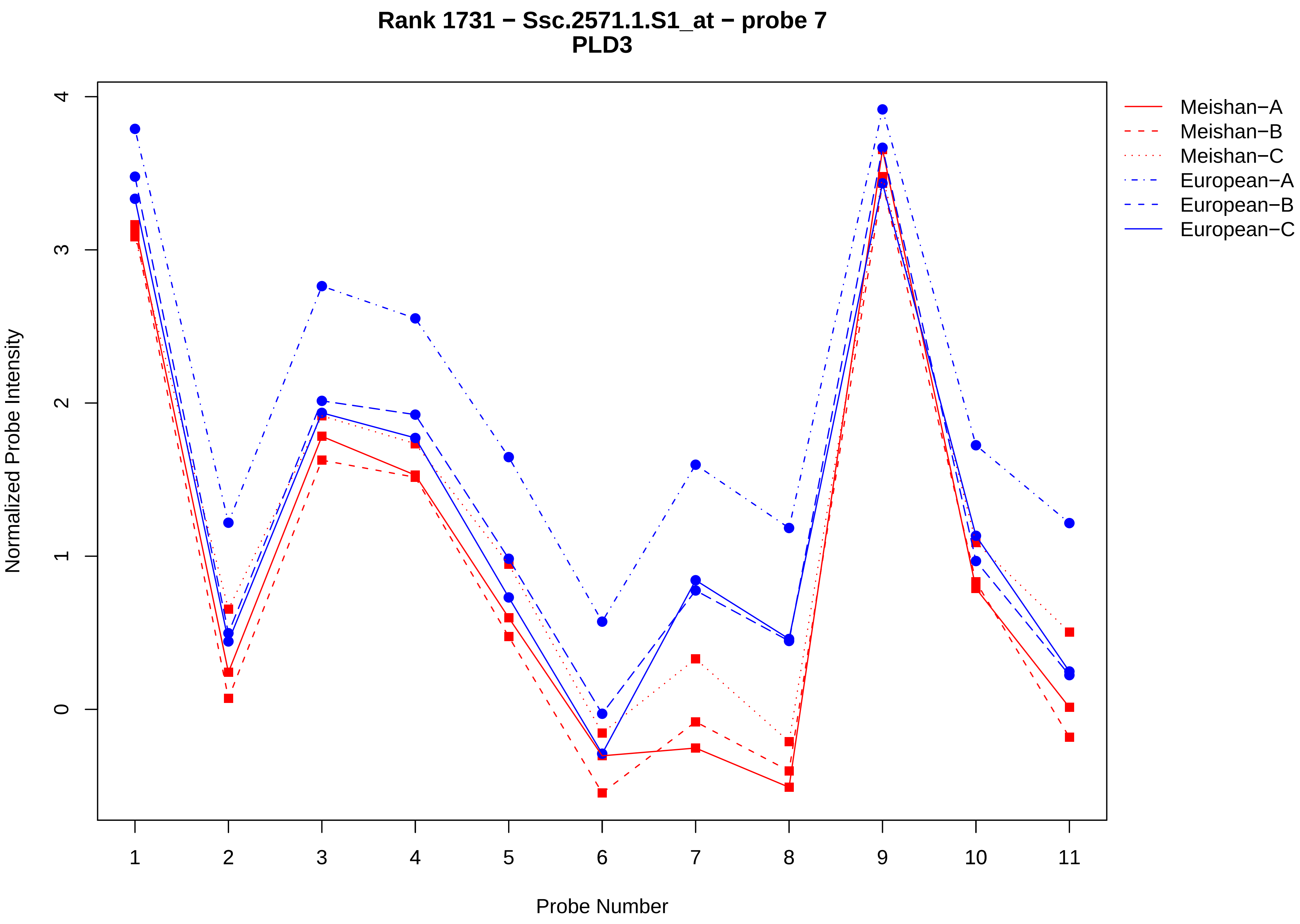

Rank 1731 − Ssc.2571.1.S1_at − probe 7
PLD3
Normalized Probe Intensity
Probe Number
0
1
2
3
4
1
2
3
4
5
6
7
8
9
10
11
Meishan−A
Meishan−B
Meishan−C
European−A
European−B
European−C

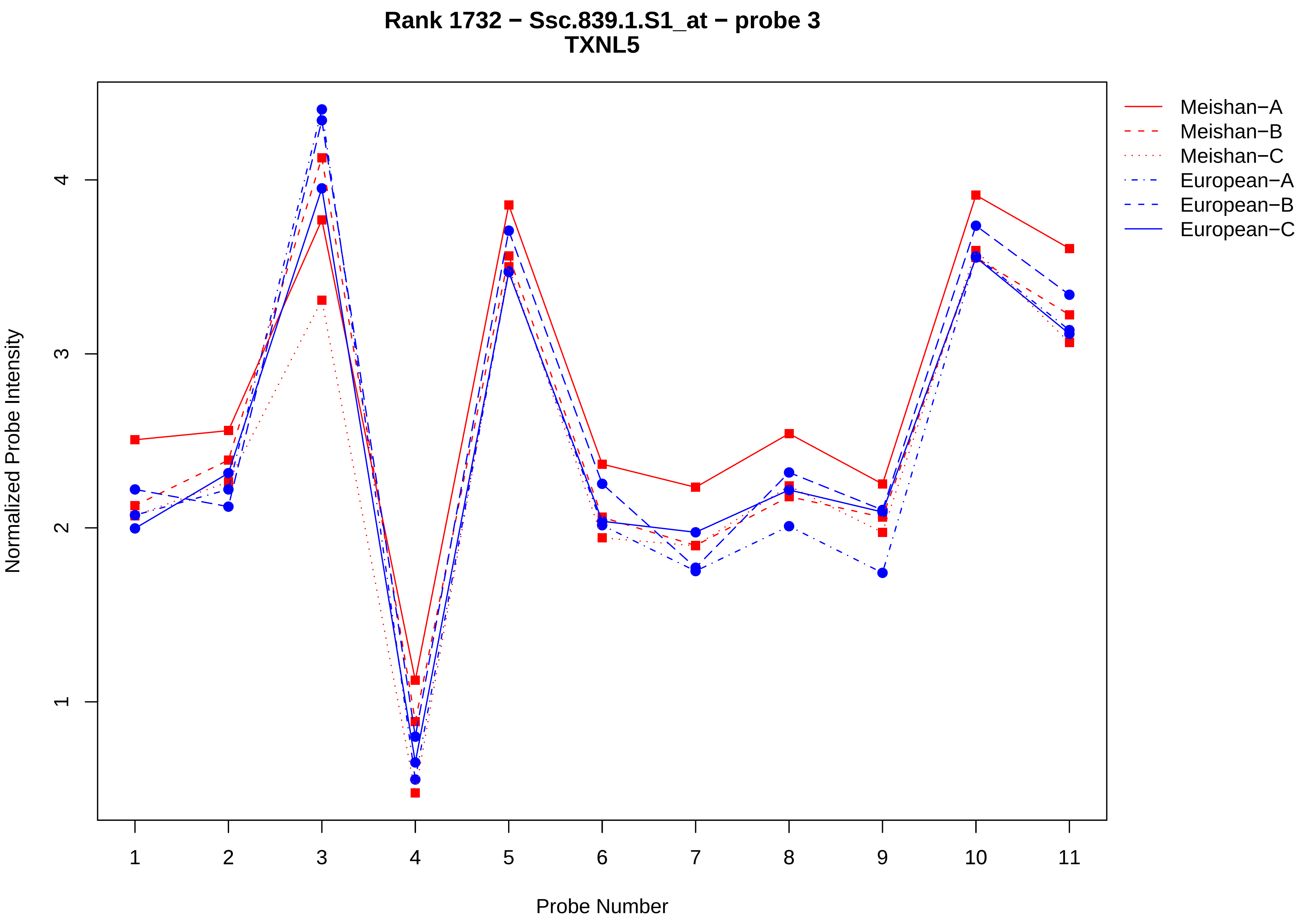
Rank 1732 − Ssc.839.1.S1_at − probe 3
TXNL5
Normalized Probe Intensity
Probe Number
1
2
3
4
5
6
7
8
9
10
11
Meishan−A
Meishan−B
Meishan−C
European−A
European−B
European−C

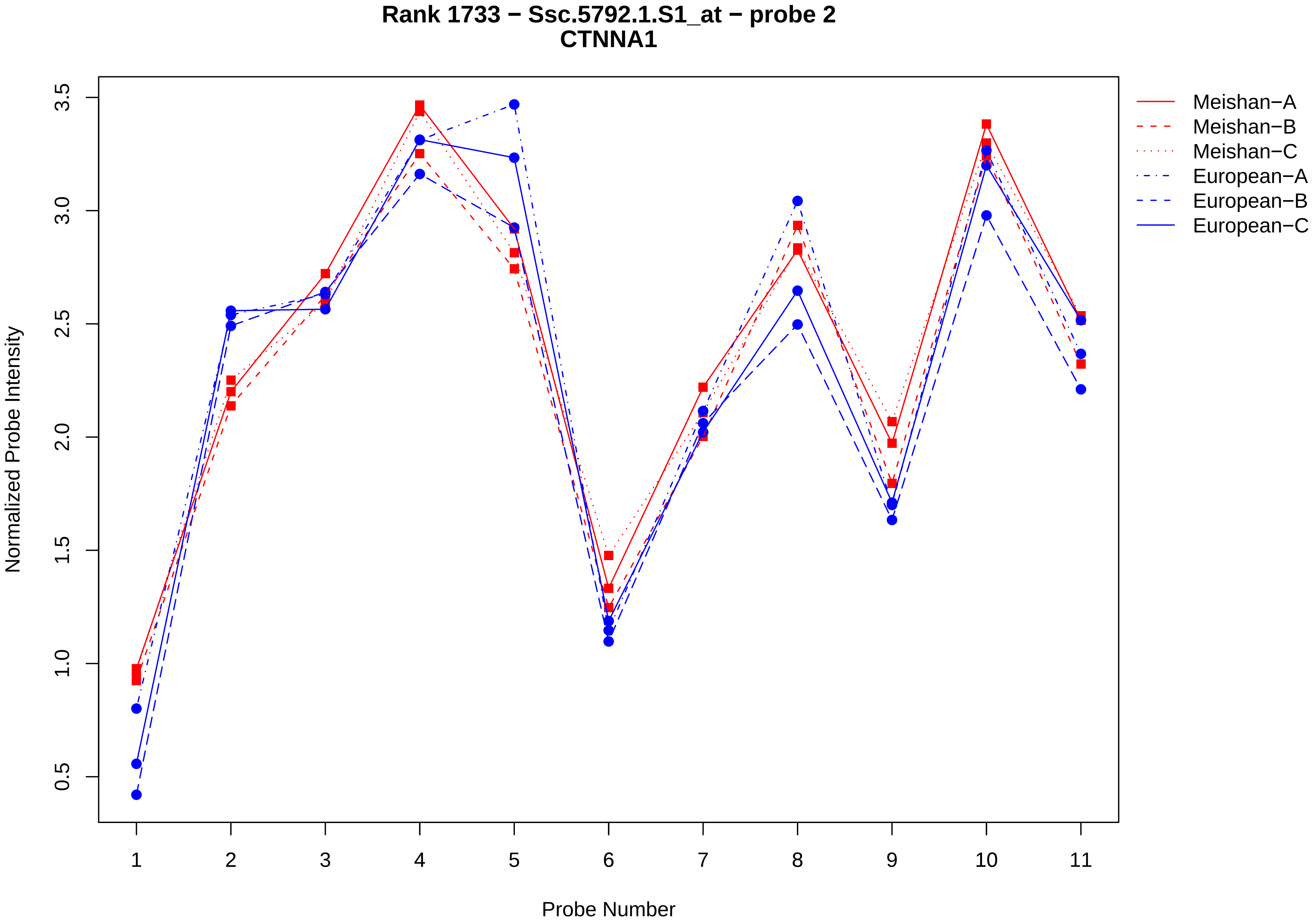

Rank 1733 − Ssc.5792.1.S1_at − probe 2
CTNNA1
Normalized Probe Intensity
Probe Number
Meishan−A
Meishan−B
Meishan−C
European−A
European−B
European−C

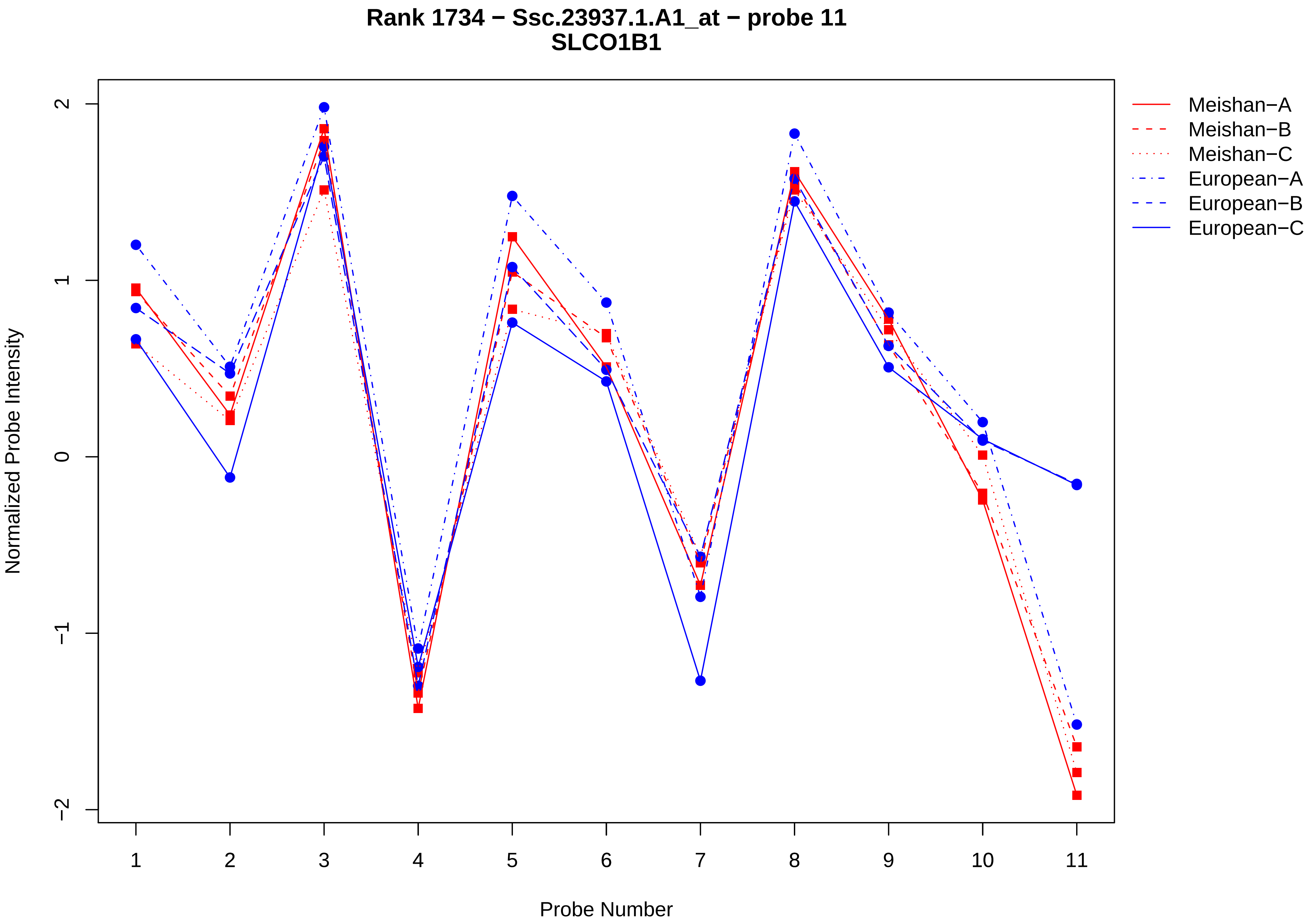

Rank 1734 − Ssc.23937.1.A1_at − probe 11
SLCO1B1
Normalized Probe Intensity
Probe Number
Meishan−A
Meishan−B
Meishan−C
European−A
European−B
European−C

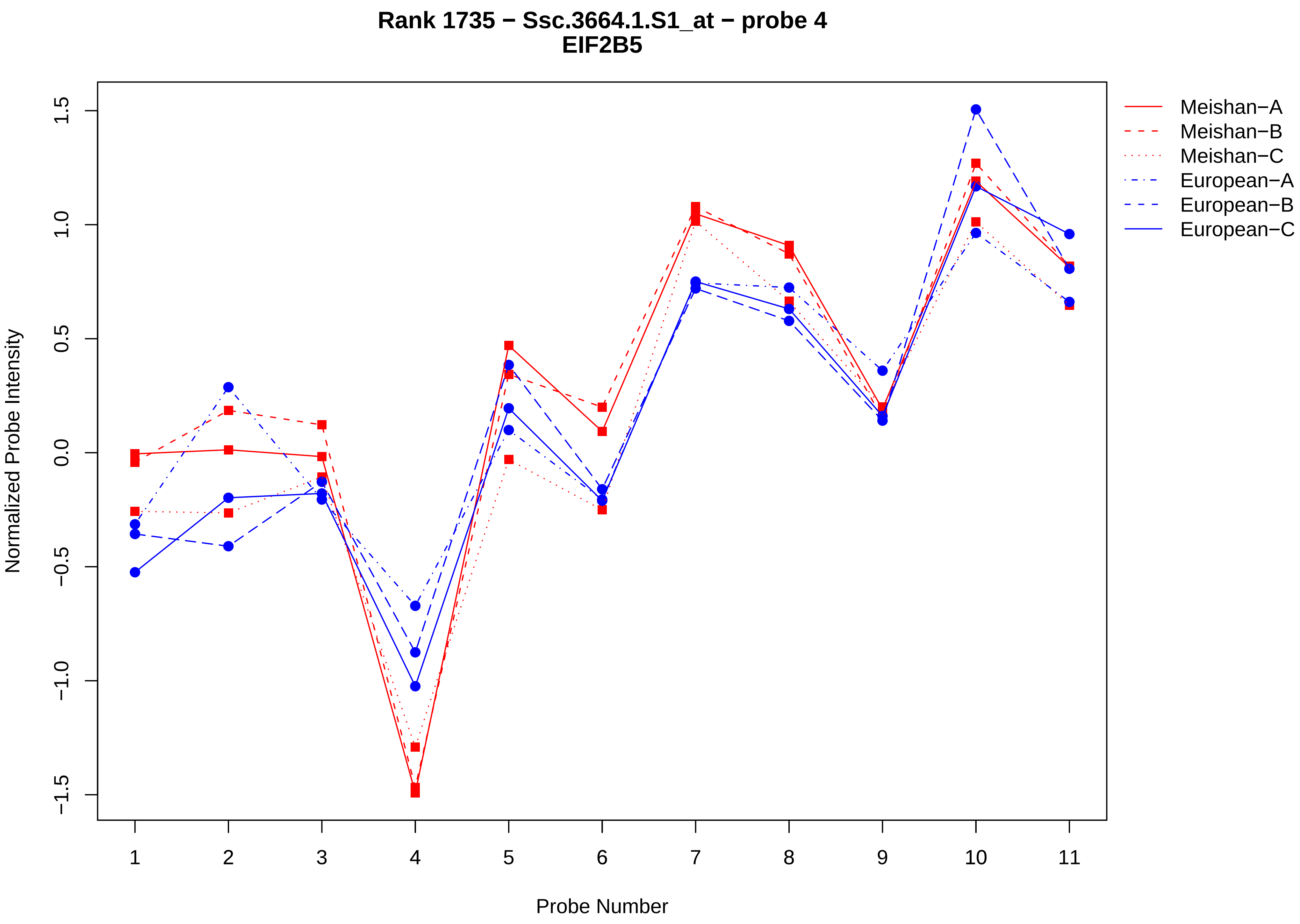

Rank 1735 − Ssc.3664.1.S1_at − probe 4
EIF2B5
Normalized Probe Intensity
Probe Number
1.5
1.0
0.5
0.0
−0.5
−1.0
−1.5
1
2
3
4
5
6
7
8
9
10
11
Meishan−A
Meishan−B
Meishan−C
European−A
European−B
European−C

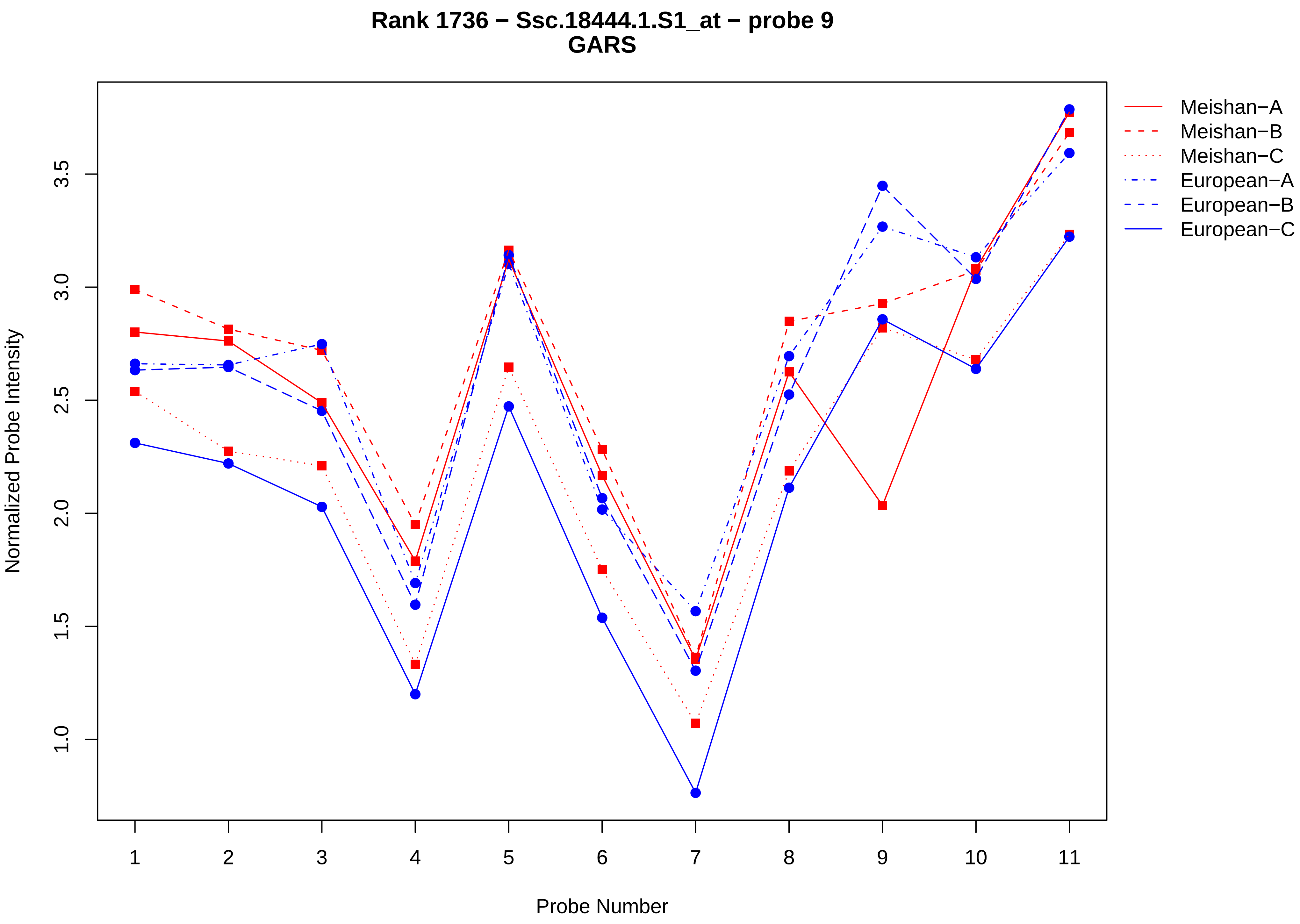

Rank 1736 − Ssc.18444.1.S1_at − probe 9
GARS
Normalized Probe Intensity
1.0
1.5
2.0
2.5
3.0
3.5
1
2
3
4
5
6
7
8
9
10
11
Probe Number
Meishan−A
Meishan−B
Meishan−C
European−A
European−B
European−C

Rank 1737 − Ssc.25441.2.S1_at − probe 4
Q9NVL6

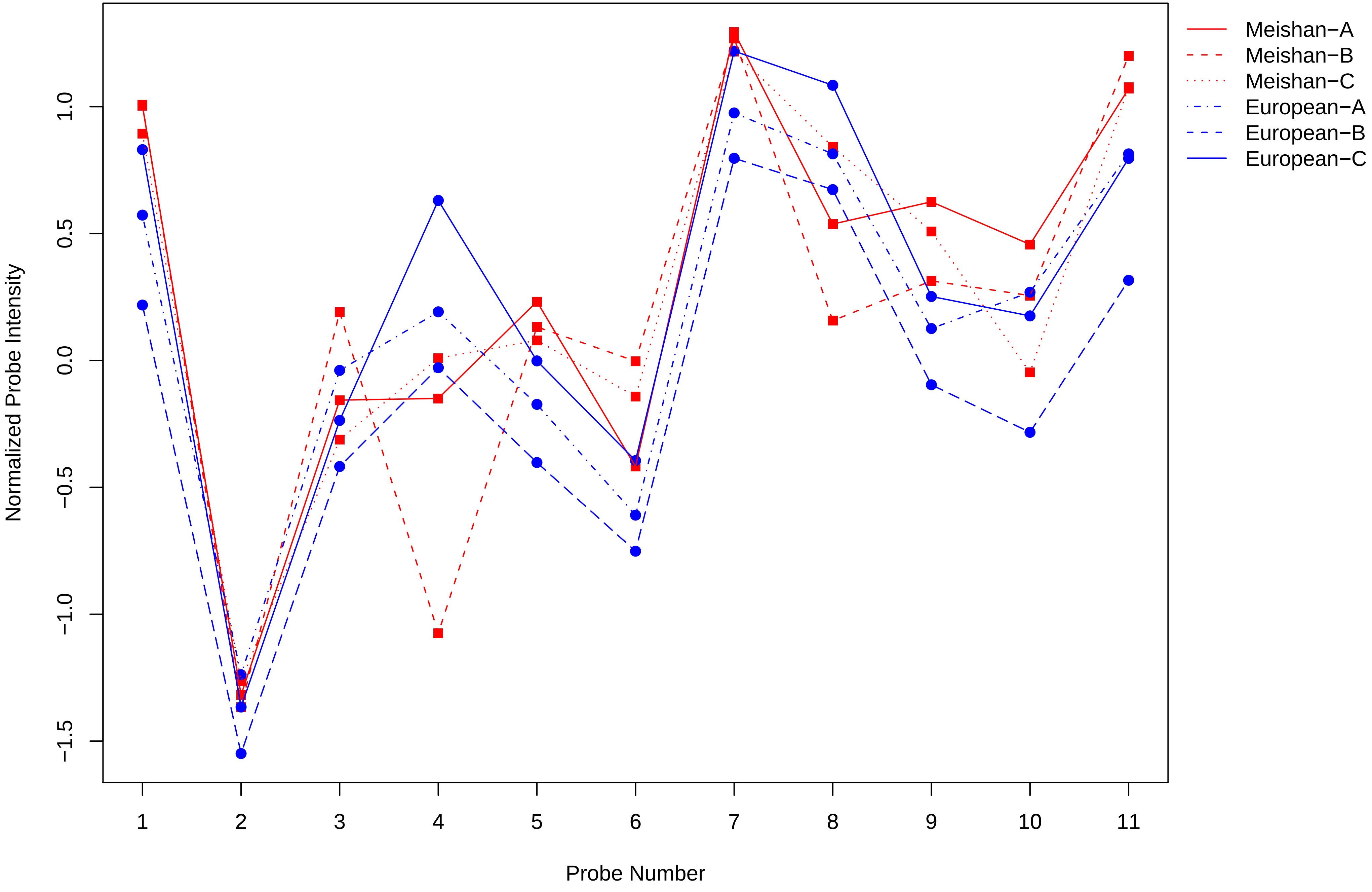

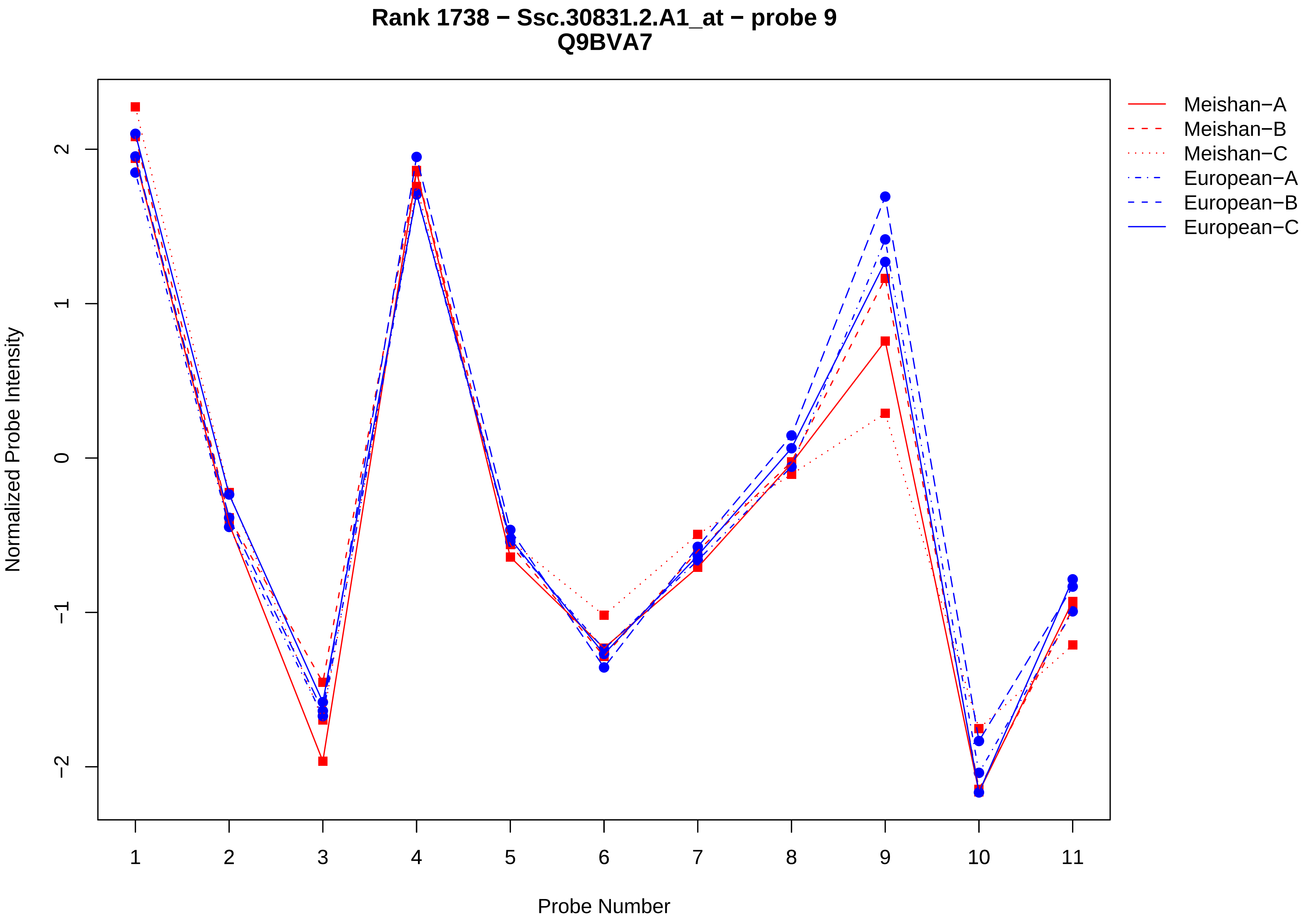

Rank 1738 − Ssc.30831.2.A1_at − probe 9
Q9BVA7
Normalized Probe Intensity
Probe Number
Meishan−A
Meishan−B
Meishan−C
European−A
European−B
European−C
2
1
0
−1
−2
1
2
3
4
5
6
7
8
9
10
11

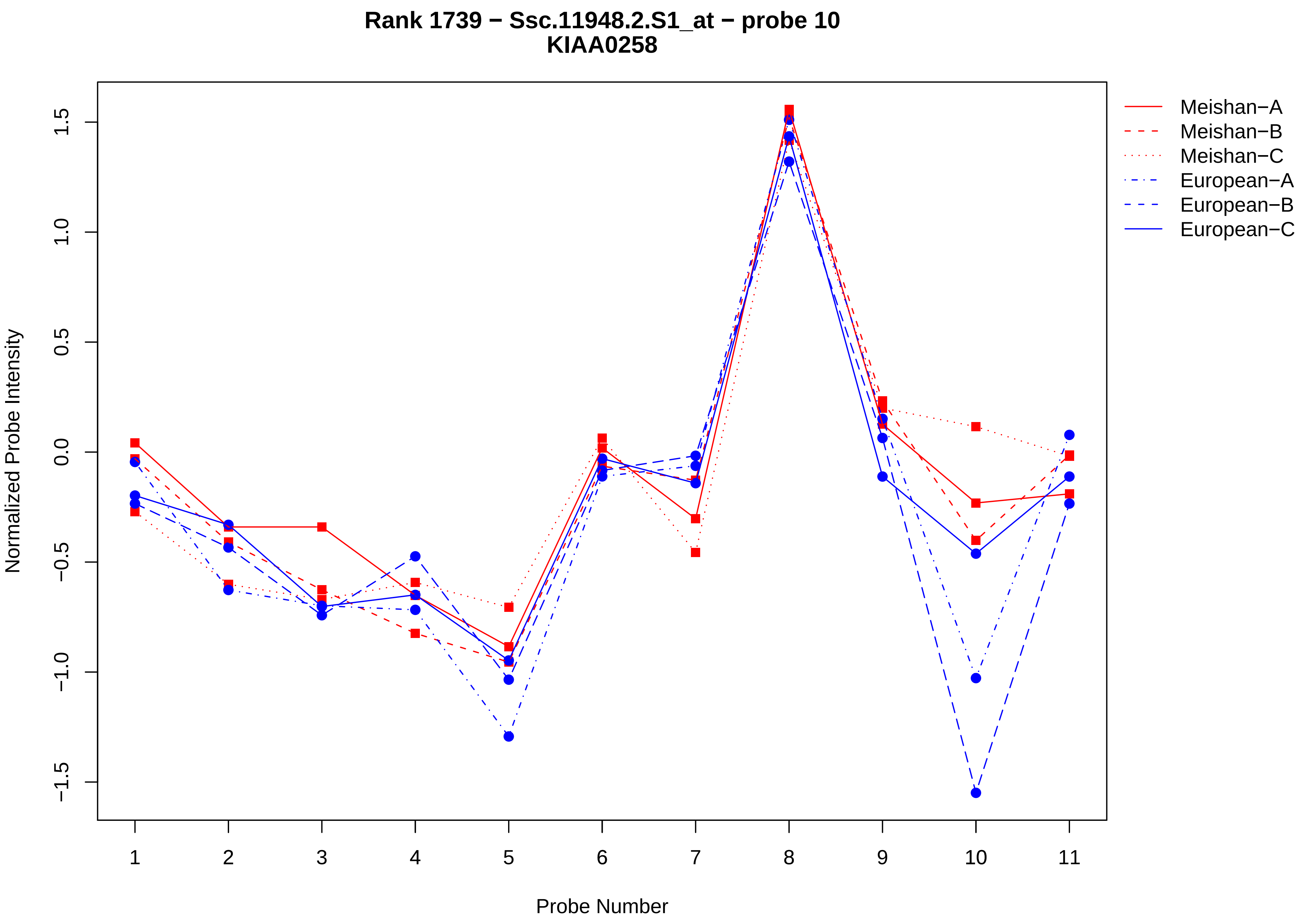

Rank 1739 − Ssc.11948.2.S1_at − probe 10
KIAA0258
Normalized Probe Intensity
Probe Number
1.5
1.0
0.5
0.0
−0.5
−1.0
−1.5
1
2
3
4
5
6
7
8
9
10
11
Meishan−A
Meishan−B
Meishan−C
European−A
European−B
European−C

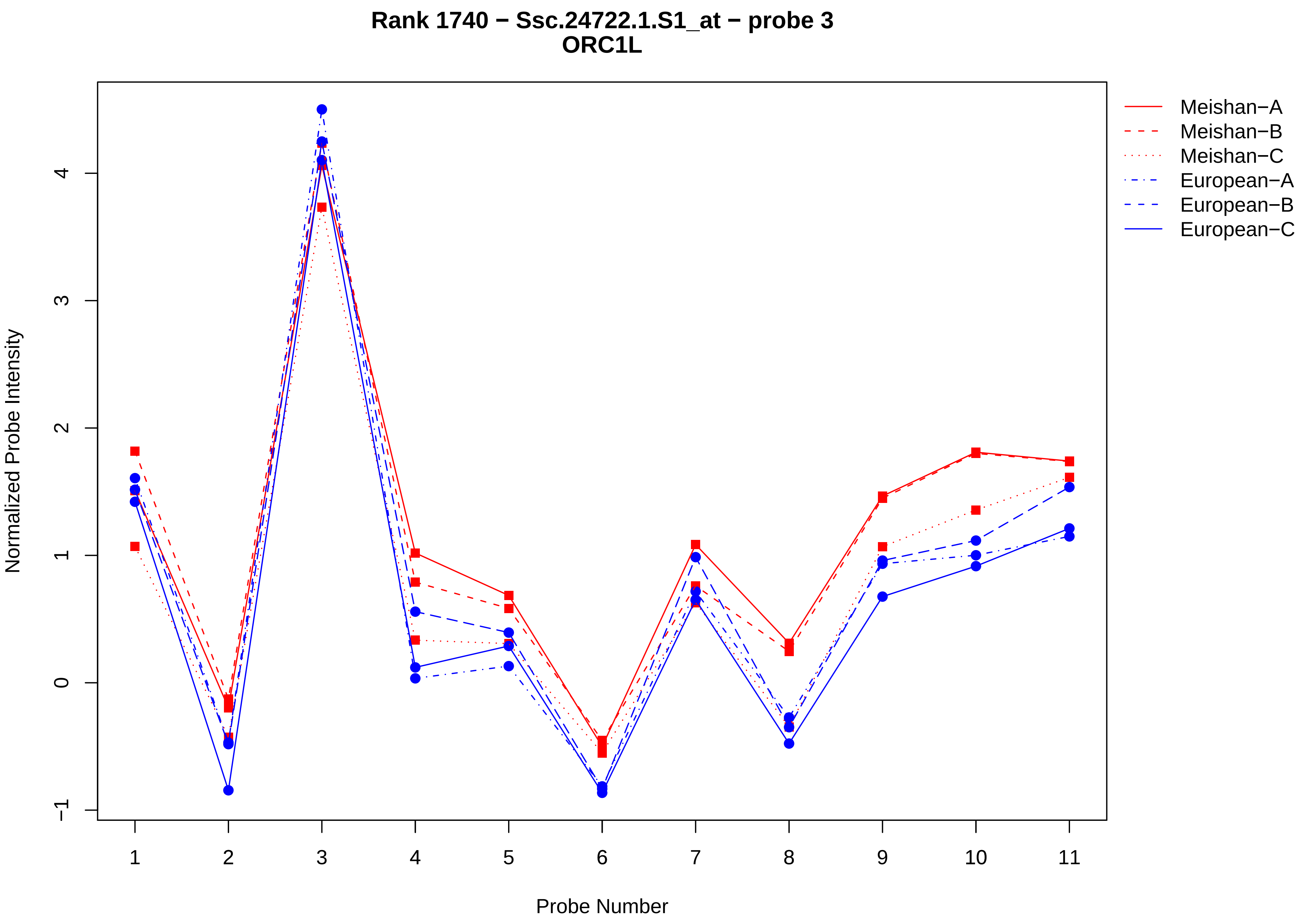

Rank 1740 − Ssc.24722.1.S1_at − probe 3
ORC1L
Normalized Probe Intensity
Probe Number
−1
0
1
2
3
4
1
2
3
4
5
6
7
8
9
10
11
Meishan−A
Meishan−B
Meishan−C
European−A
European−B
European−C

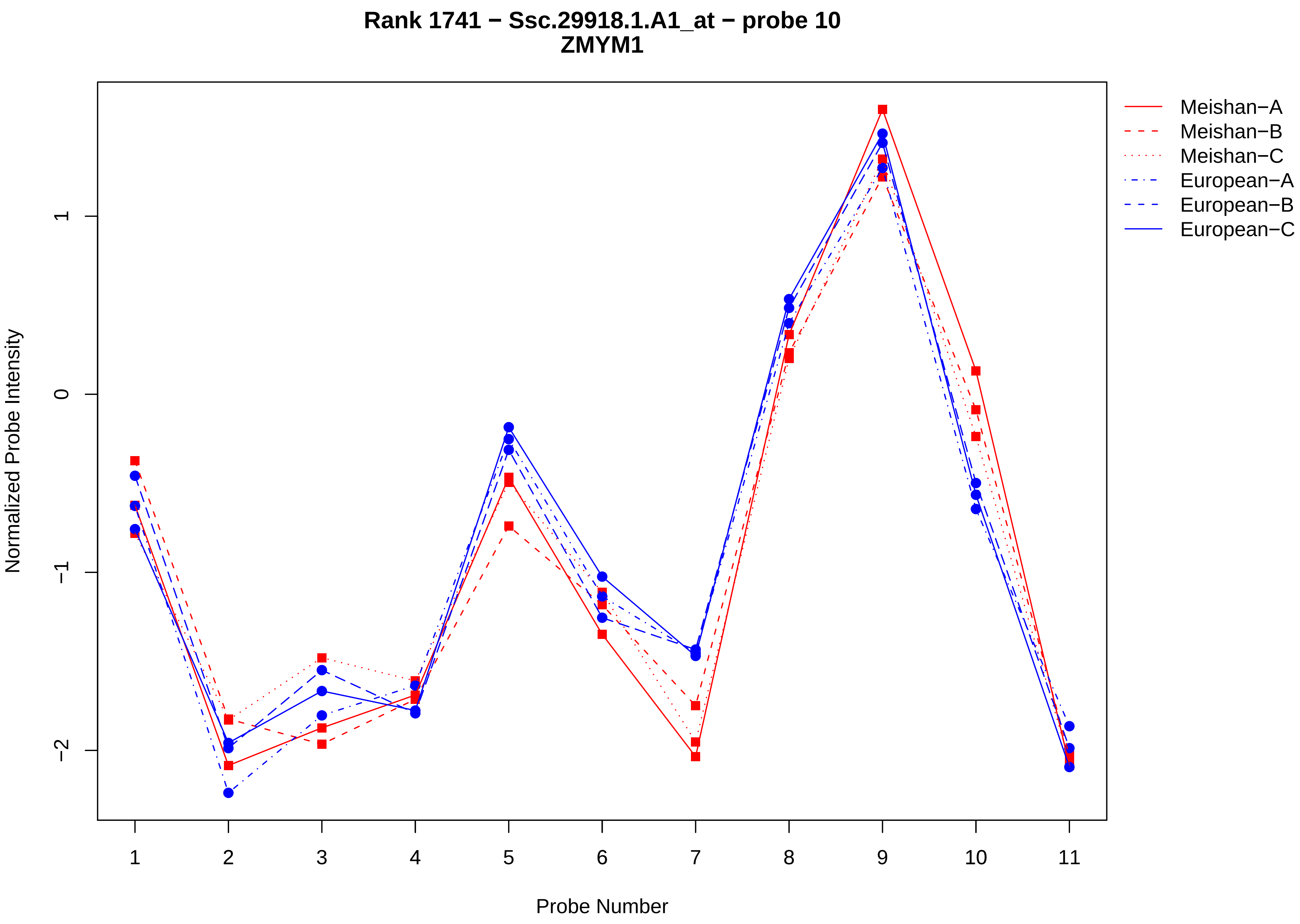
Rank 1741 − Ssc.29918.1.A1_at − probe 10
ZMYM1
Normalized Probe Intensity
Probe Number
−2
−1
0
1
1
2
3
4
5
6
7
8
9
10
11
Meishan−A
Meishan−B
Meishan−C
European−A
European−B
European−C

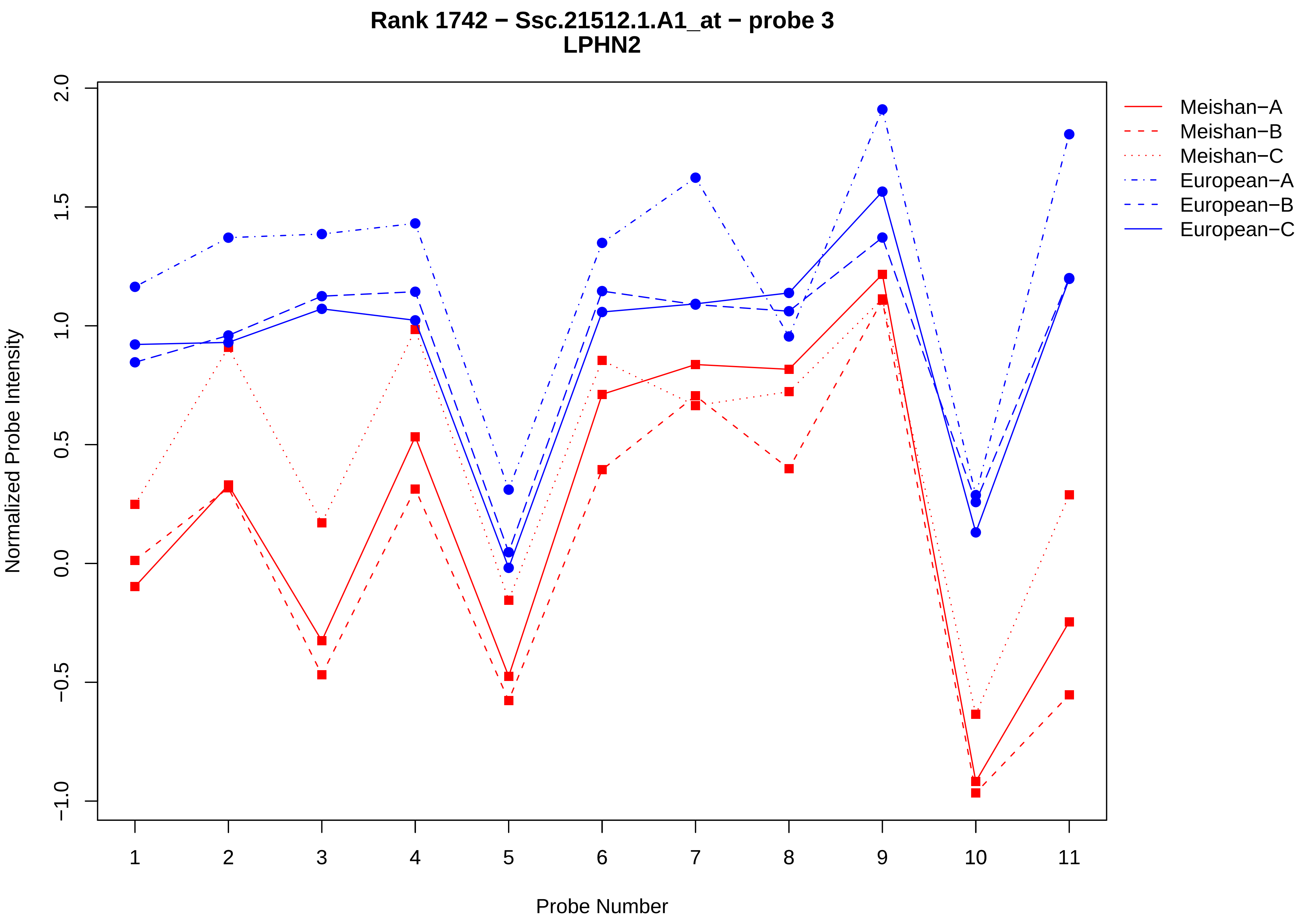

Rank 1742 − Ssc.21512.1.A1_at − probe 3
LPHN2
Normalized Probe Intensity
Probe Number
−1.0
−0.5
0.0
0.5
1.0
1.5
2.0
1
2
3
4
5
6
7
8
9
10
11
Meishan−A
Meishan−B
Meishan−C
European−A
European−B
European−C

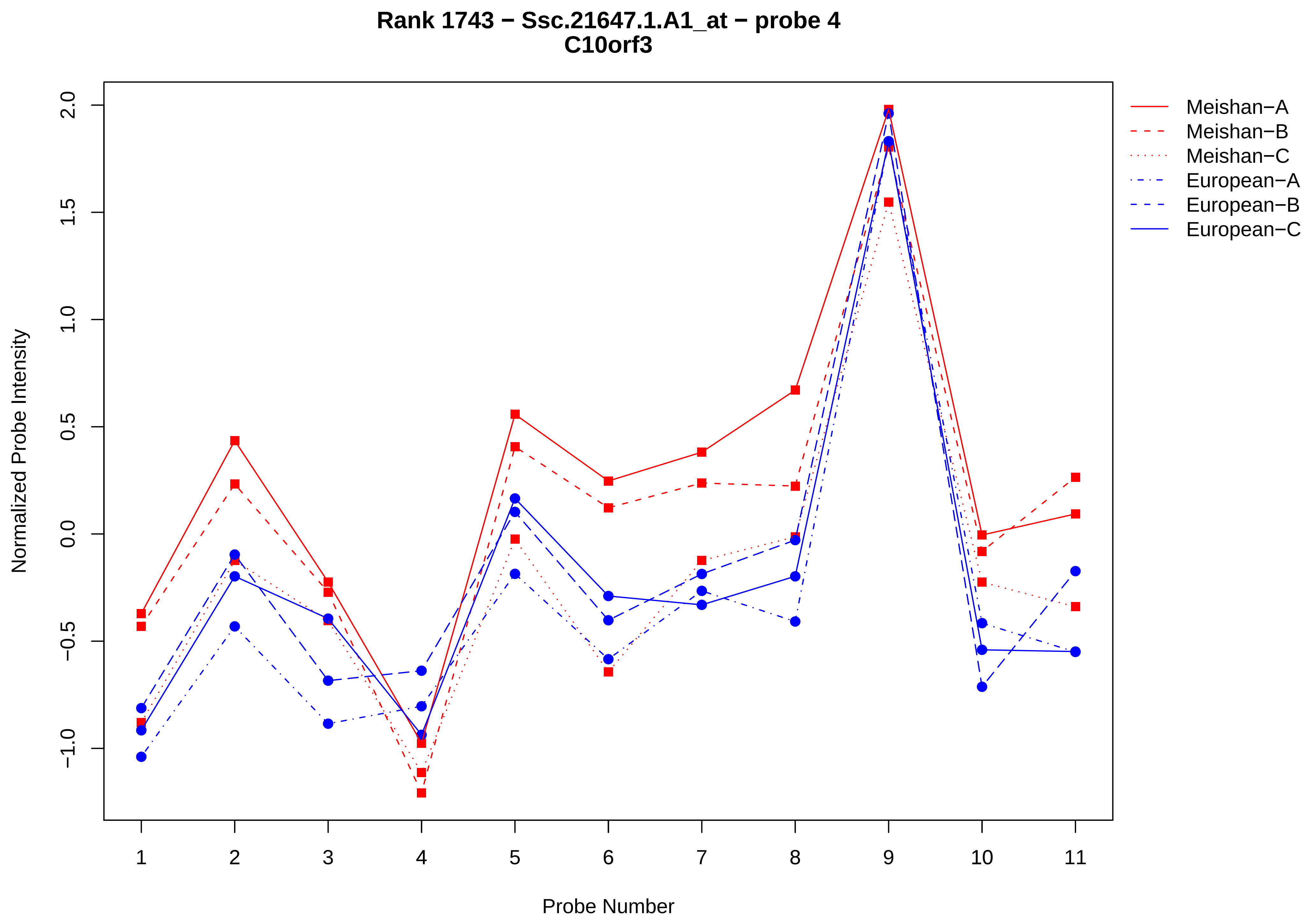

Rank 1743 − Ssc.21647.1.A1_at − probe 4
C10orf3
Normalized Probe Intensity
Probe Number
2.0
1.5
1.0
0.5
0.0
−0.5
−1.0
1
2
3
4
5
6
7
8
9
10
11
Meishan−A
Meishan−B
Meishan−C
European−A
European−B
European−C

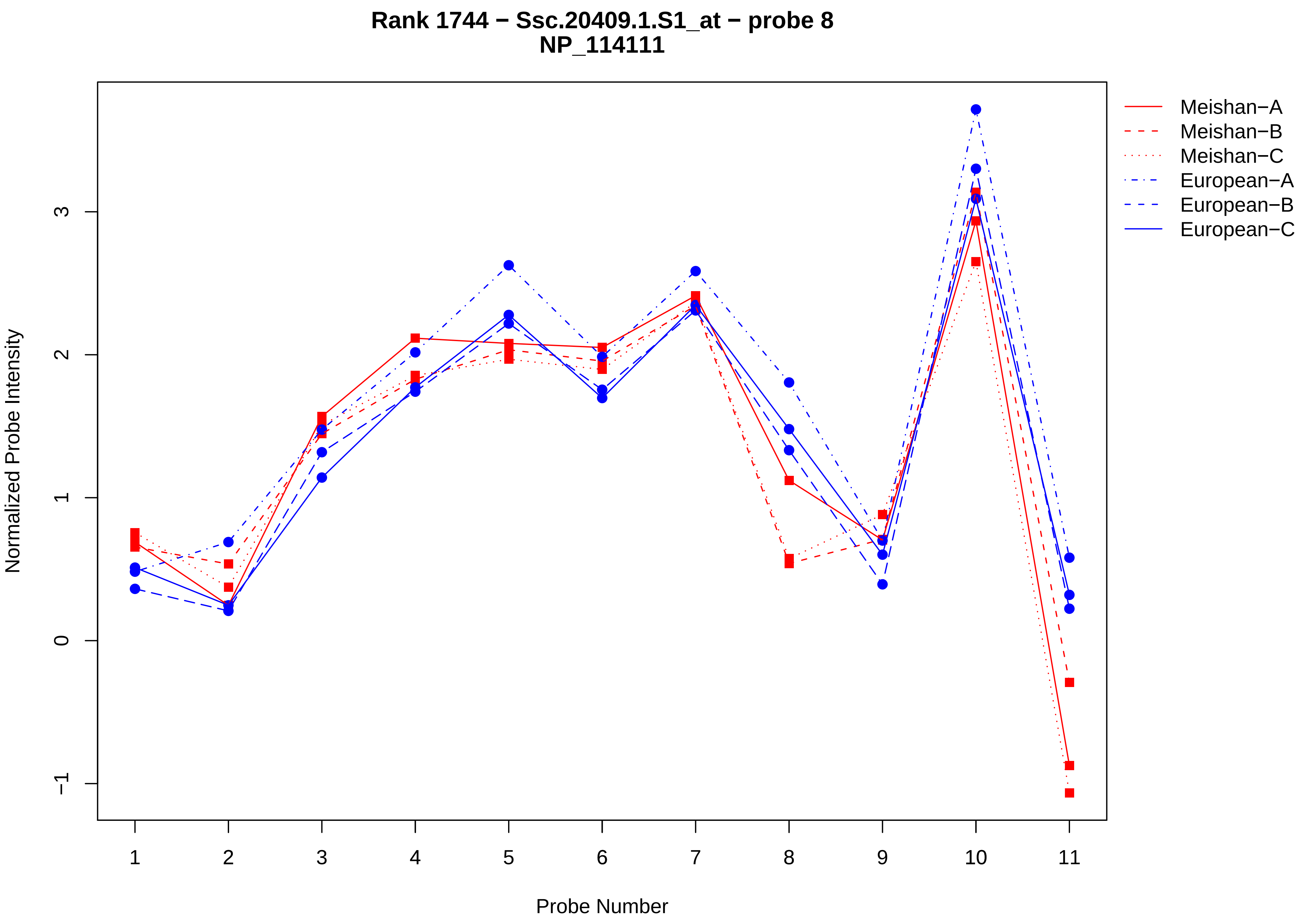

Rank 1744 − Ssc.20409.1.S1_at − probe 8
NP_114111
Normalized Probe Intensity
Probe Number
−1
0
1
2
3
1
2
3
4
5
6
7
8
9
10
11
Meishan−A
Meishan−B
Meishan−C
European−A
European−B
European−C

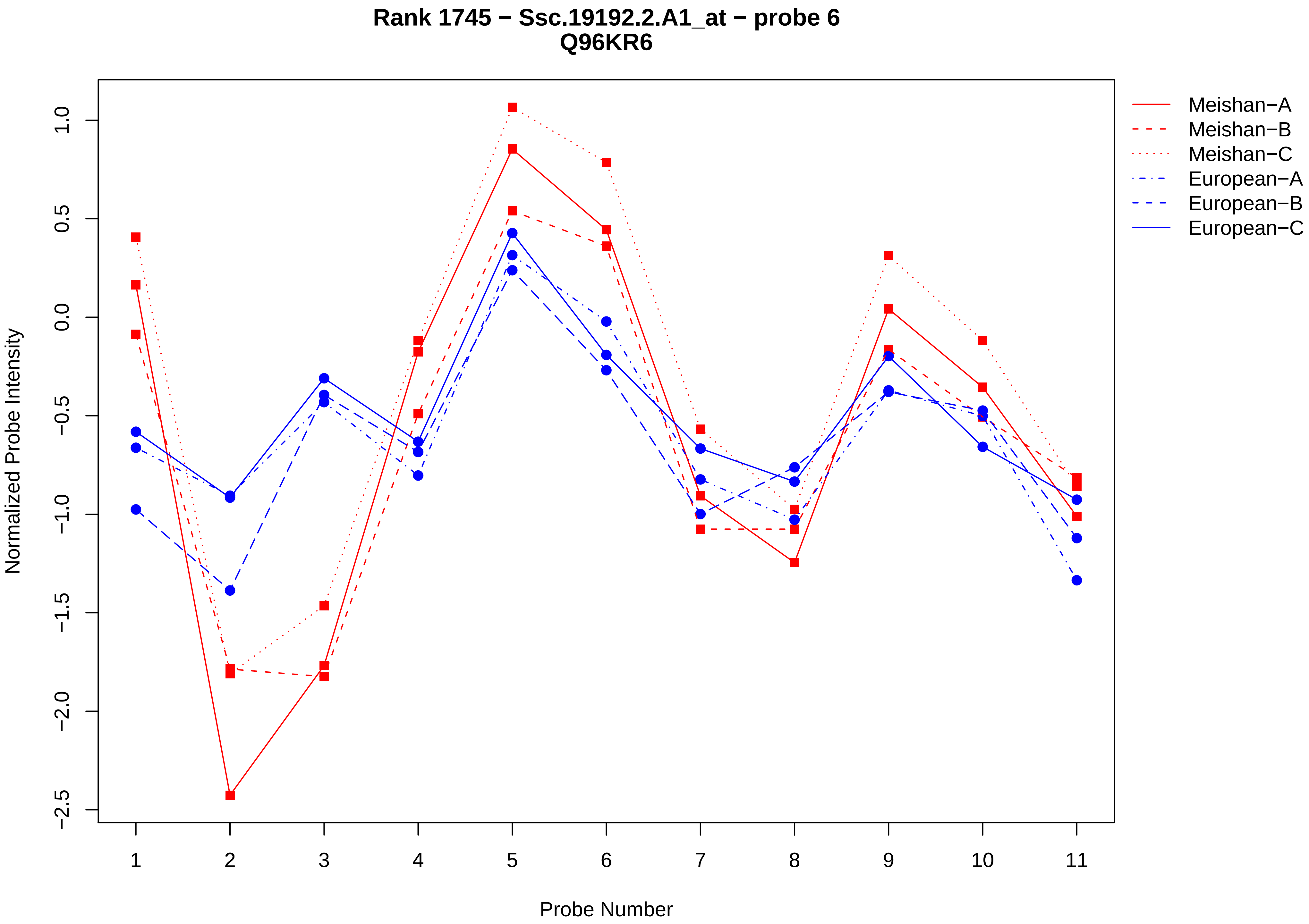

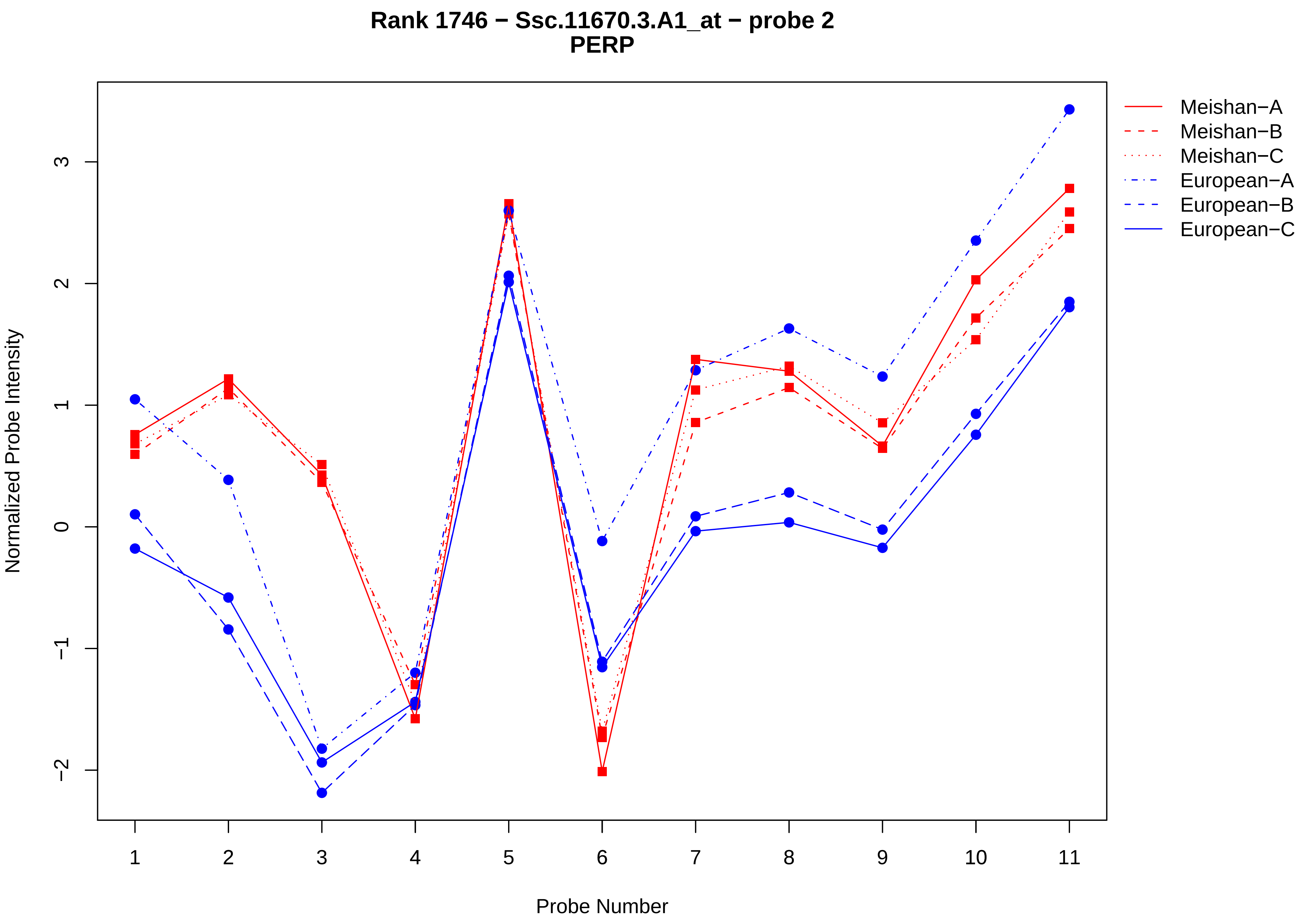

Rank 1746 − Ssc.11670.3.A1_at − probe 2
PERP
Normalized Probe Intensity
Probe Number
−2
−1
0
1
2
3
1
2
3
4
5
6
7
8
9
10
11
Meishan−A
Meishan−B
Meishan−C
European−A
European−B
European−C

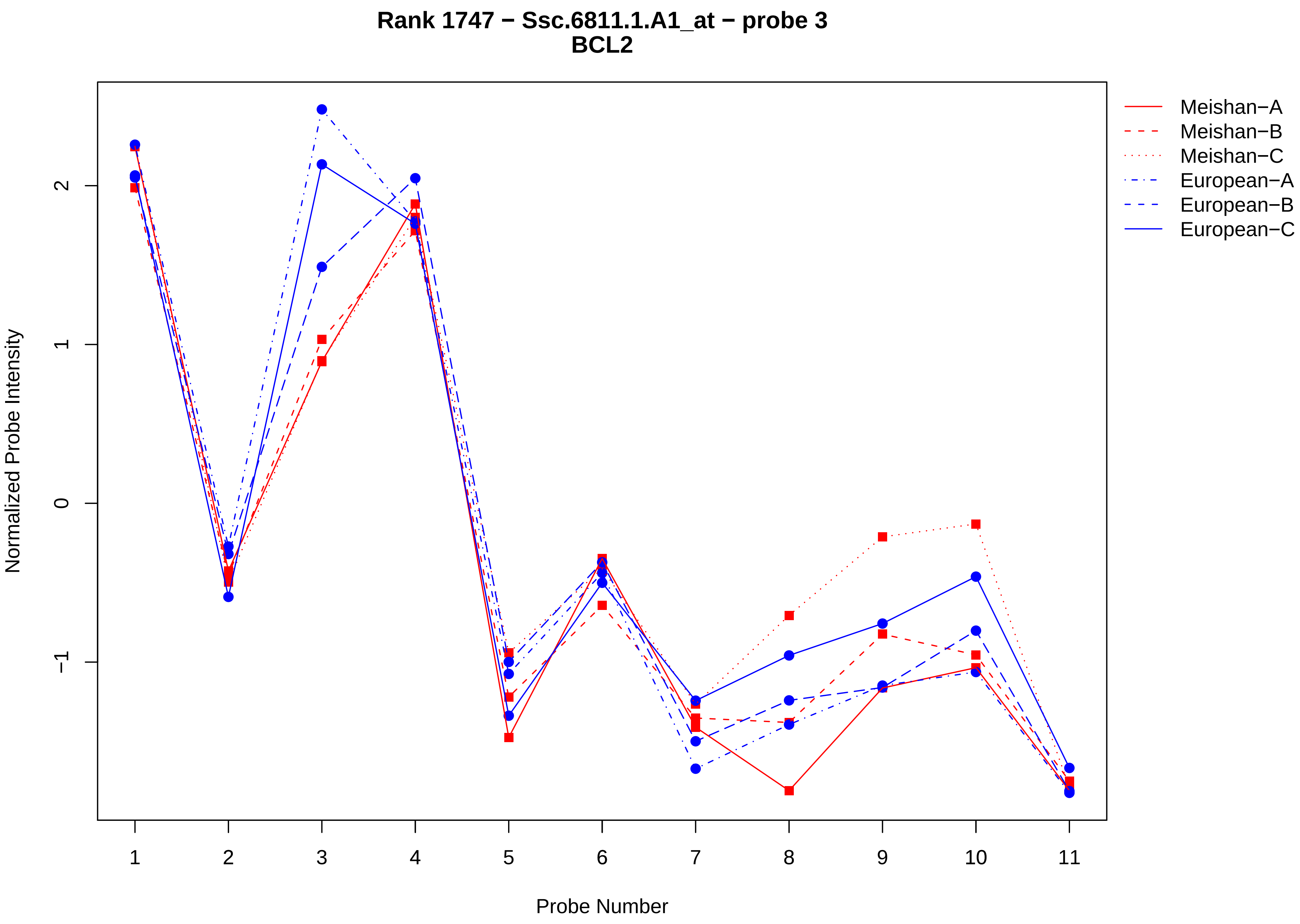
Rank 1747 − Ssc.6811.1.A1_at − probe 3
BCL2
Normalized Probe Intensity
Probe Number
−1
0
1
2
1
2
3
4
5
6
7
8
9
10
11
Meishan−A
Meishan−B
Meishan−C
European−A
European−B
European−C

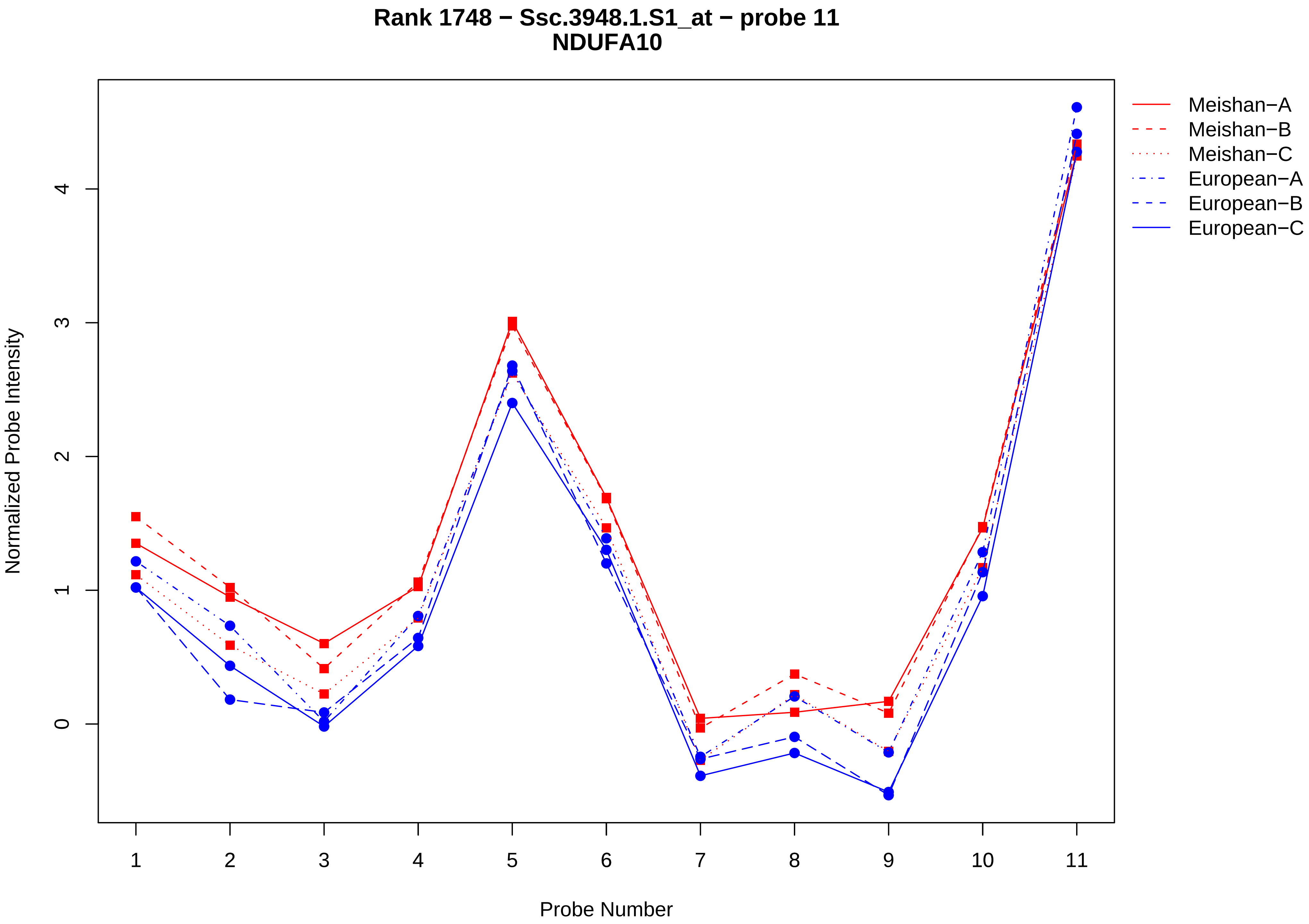

Rank 1748 − Ssc.3948.1.S1_at − probe 11
NDUFA10
Normalized Probe Intensity
Probe Number
0
1
2
3
4
1
2
3
4
5
6
7
8
9
10
11
Meishan−A
Meishan−B
Meishan−C
European−A
European−B
European−C

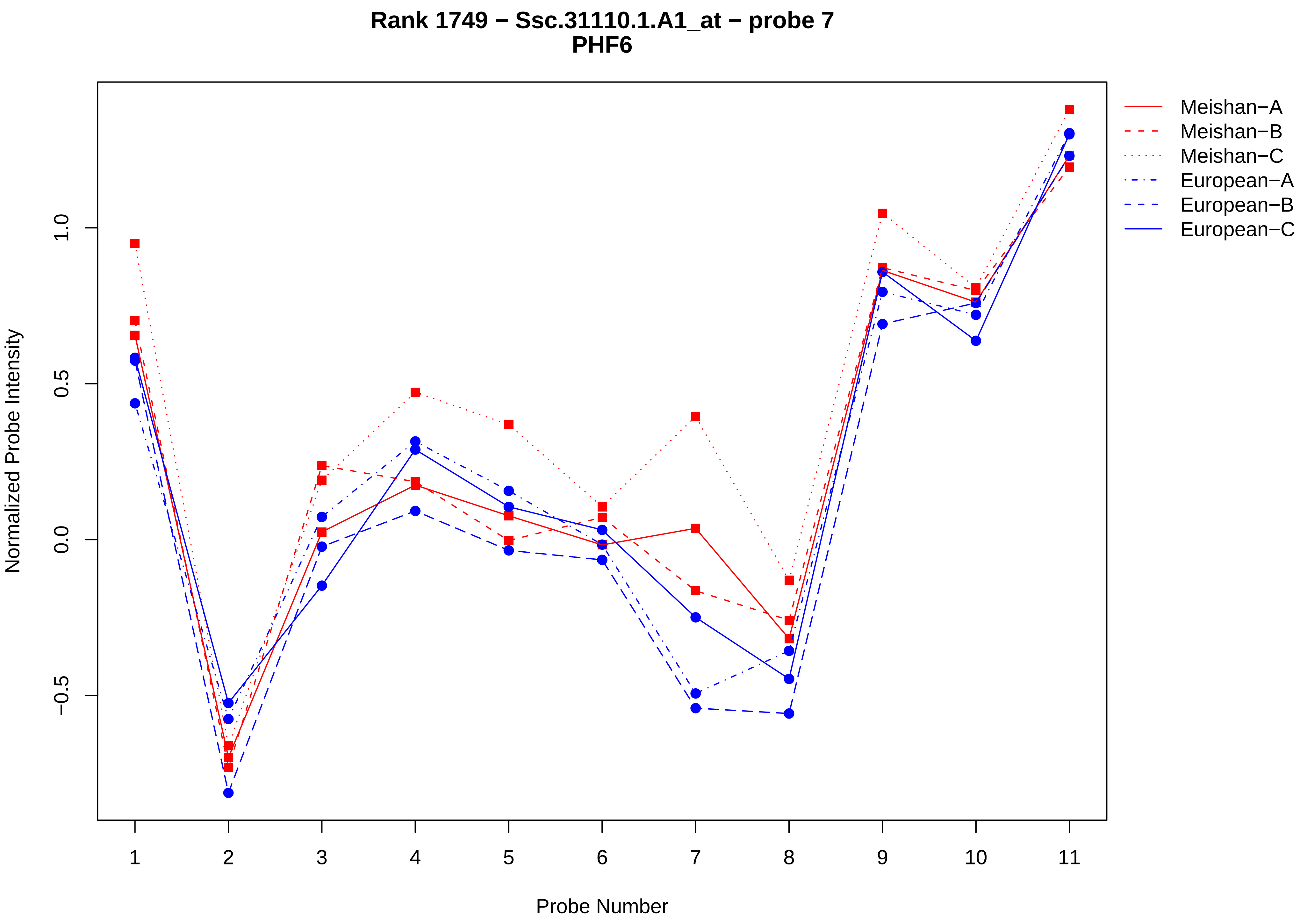

Rank 1749 − Ssc.31110.1.A1_at − probe 7
PHF6
Normalized Probe Intensity
−0.5
0.0
0.5
1.0
1
2
3
4
5
6
7
8
9
10
11
Probe Number
Meishan−A
Meishan−B
Meishan−C
European−A
European−B
European−C

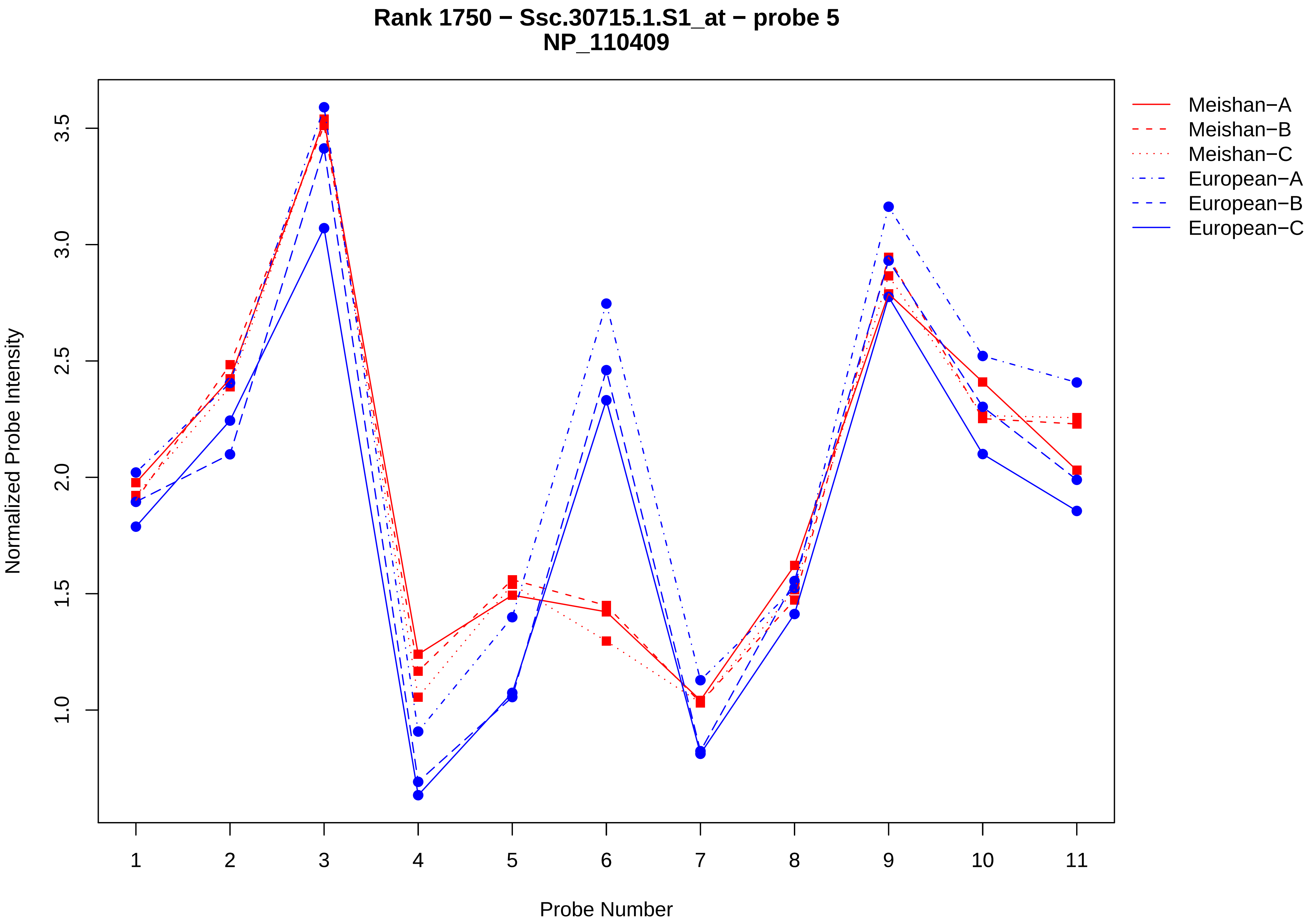

Rank 1750 − Ssc.30715.1.S1_at − probe 5
NP_110409
Normalized Probe Intensity
Probe Number
1.0
1.5
2.0
2.5
3.0
3.5
1
2
3
4
5
6
7
8
9
10
11
Meishan−A
Meishan−B
Meishan−C
European−A
European−B
European−C

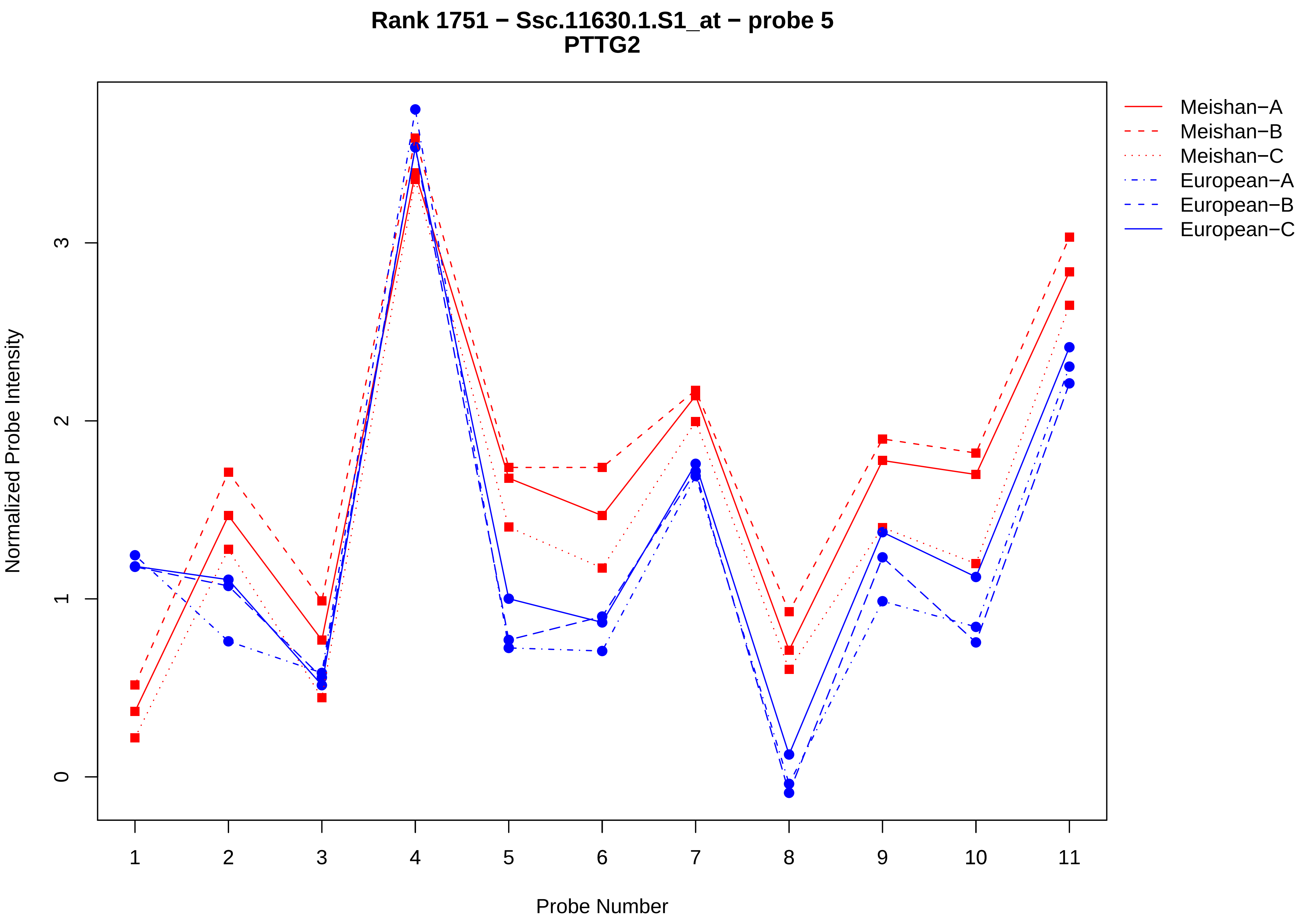
Rank 1751 − Ssc.11630.1.S1_at − probe 5
PTTG2
Normalized Probe Intensity
Probe Number
0
1
2
3
1
2
3
4
5
6
7
8
9
10
11
Meishan−A
Meishan−B
Meishan−C
European−A
European−B
European−C

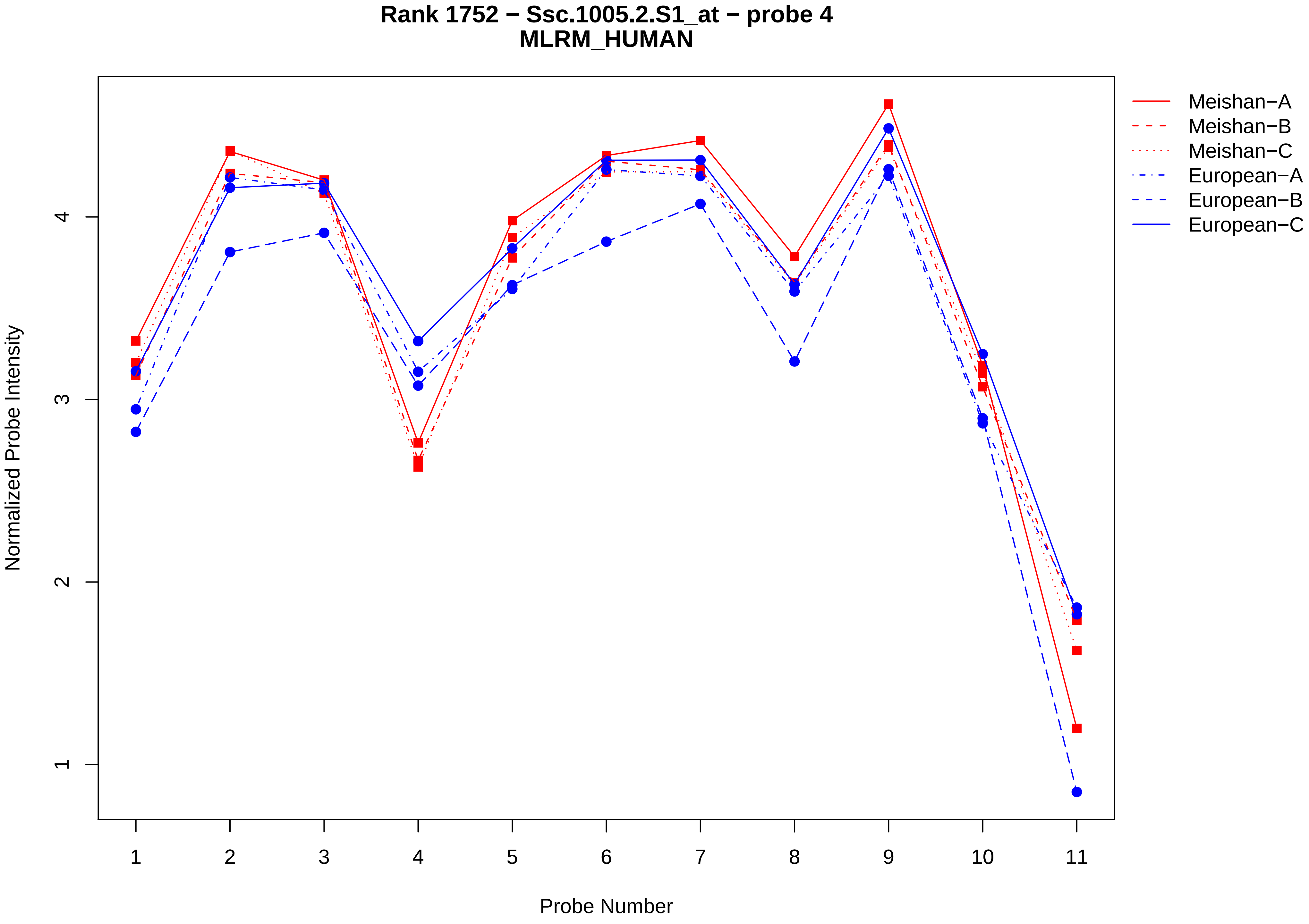

Rank 1752 − Ssc.1005.2.S1_at − probe 4
MLRM_HUMAN
Normalized Probe Intensity
Probe Number
1
2
3
4
1
2
3
4
5
6
7
8
9
10
11
Meishan−A
Meishan−B
Meishan−C
European−A
European−B
European−C

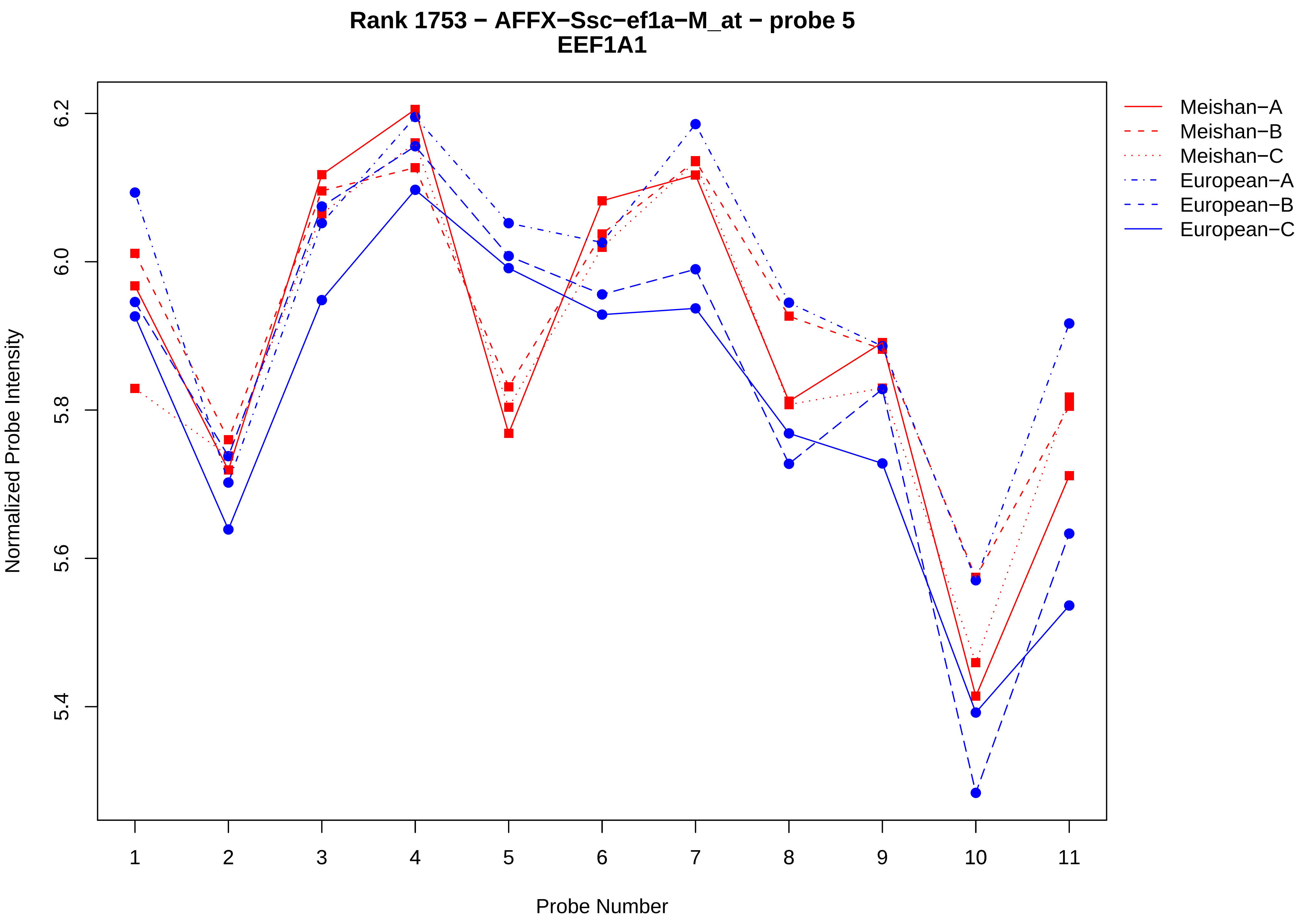

Rank 1753 − AFFX−Ssc−ef1a−M_at − probe 5
EEF1A1
Normalized Probe Intensity
6.2
6.0
5.8
5.6
5.4
Probe Number
1
2
3
4
5
6
7
8
9
10
11
Meishan−A
Meishan−B
Meishan−C
European−A
European−B
European−C

Rank 1754 − Ssc.2571.1.S1_at − probe 8
PLD3

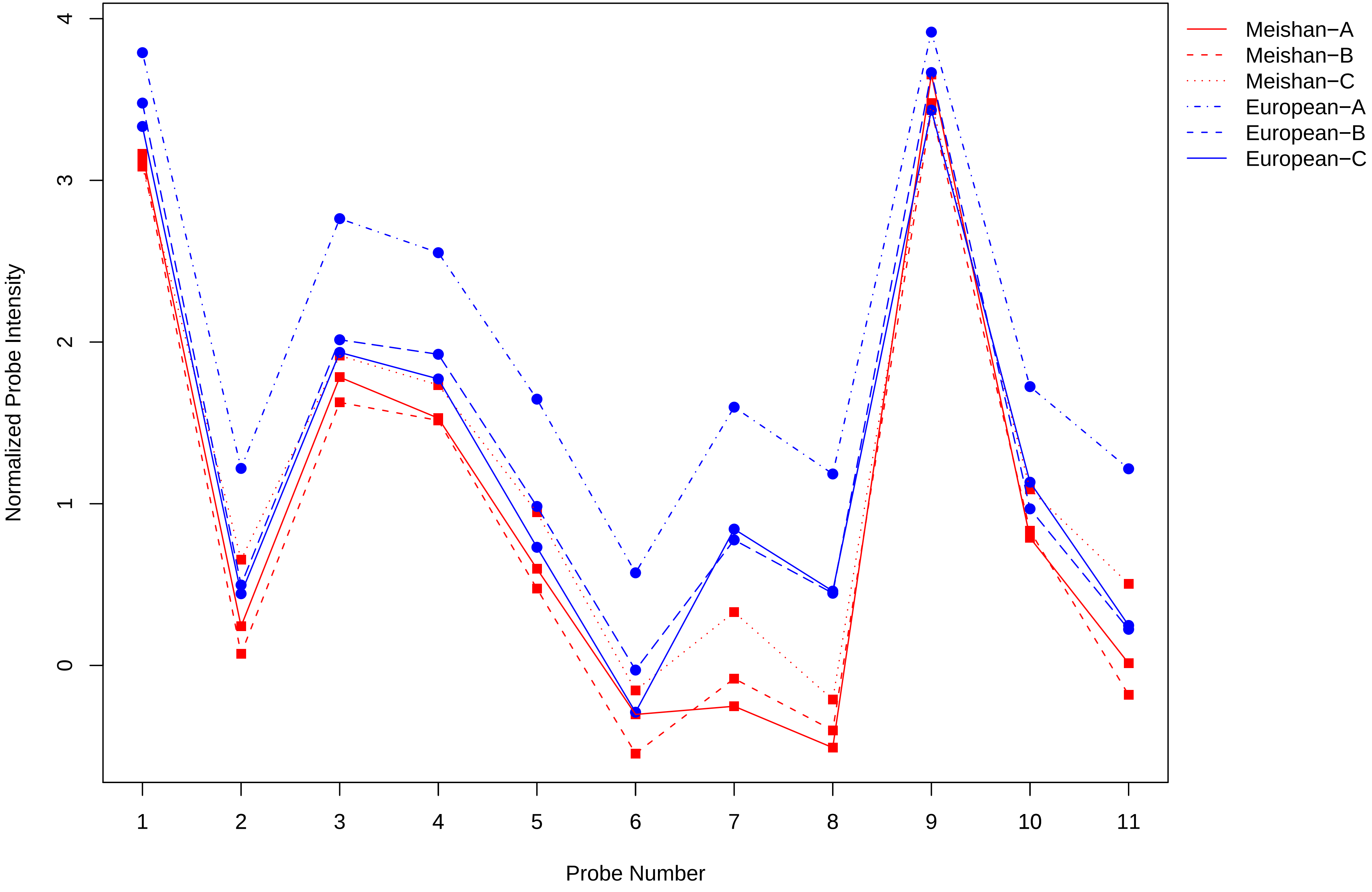

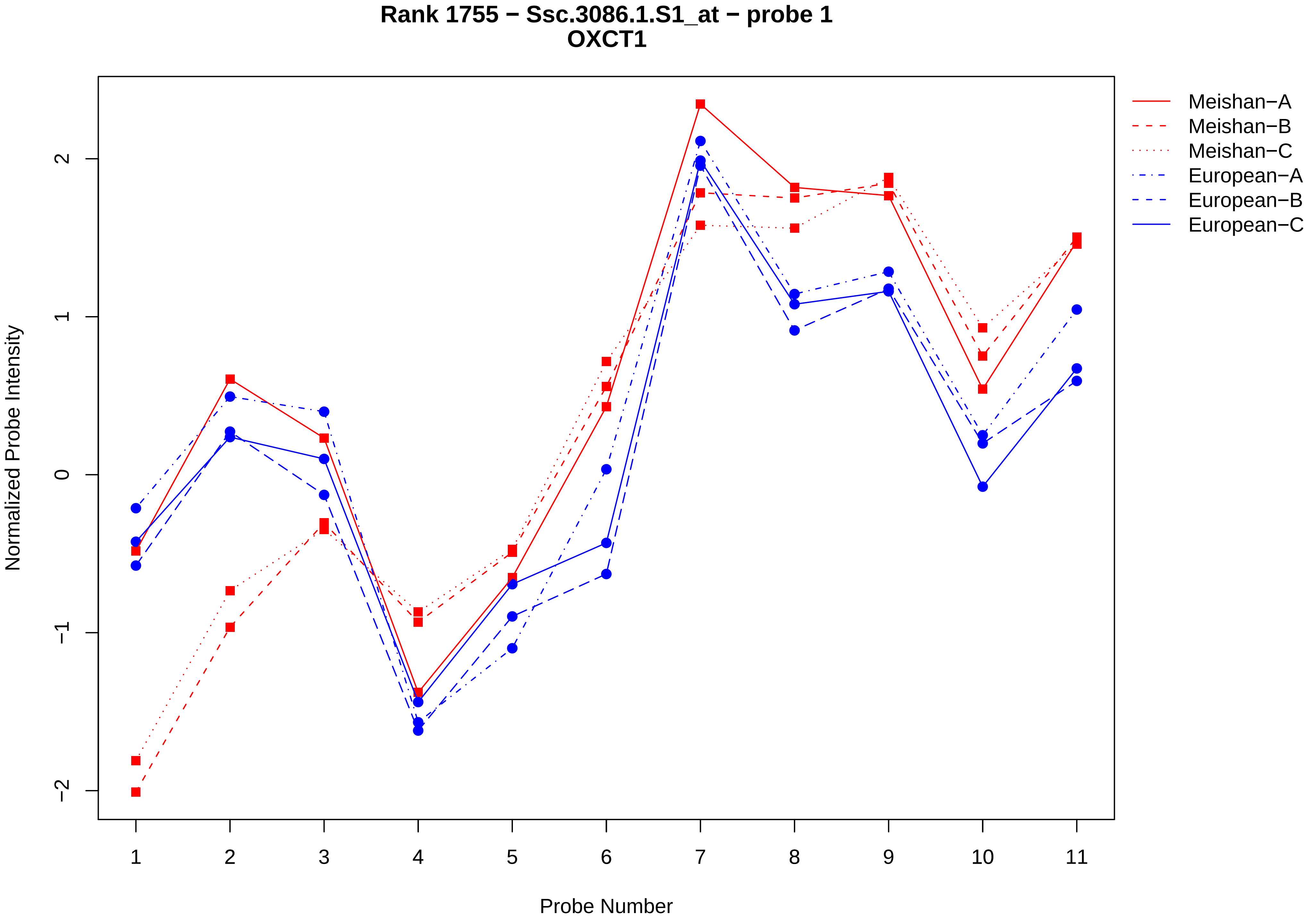

Rank 1755 − Ssc.3086.1.S1_at − probe 1
OXCT1
Normalized Probe Intensity
Probe Number
−2
−1
0
1
2
1
2
3
4
5
6
7
8
9
10
11
Meishan−A
Meishan−B
Meishan−C
European−A
European−B
European−C

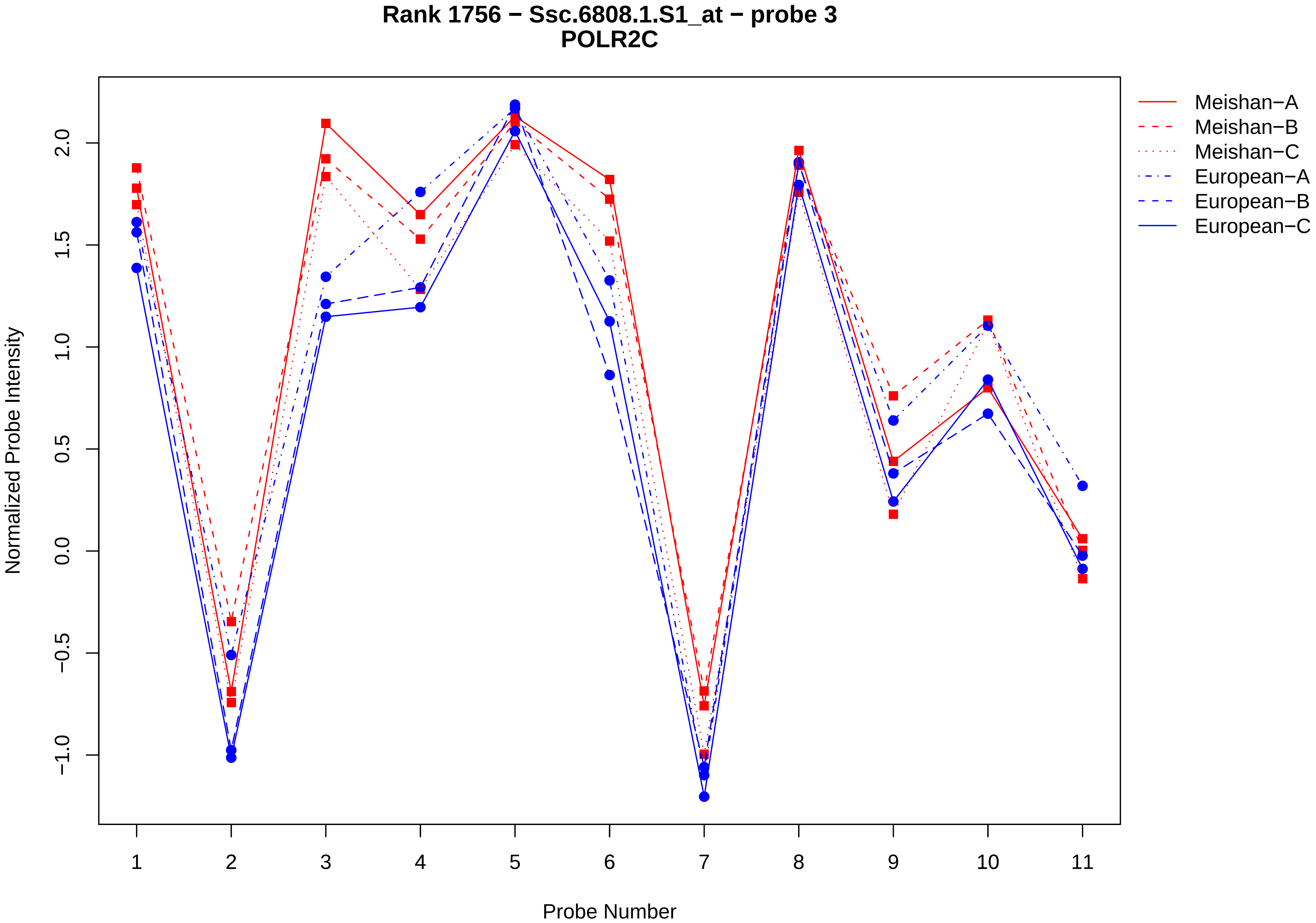

Rank 1756 − Ssc.6808.1.S1_at − probe 3
POLR2C
Normalized Probe Intensity
−1.0
−0.5
0.0
0.5
1.0
1.5
2.0
Probe Number
1
2
3
4
5
6
7
8
9
10
11
Meishan−A
Meishan−B
Meishan−C
European−A
European−B
European−C

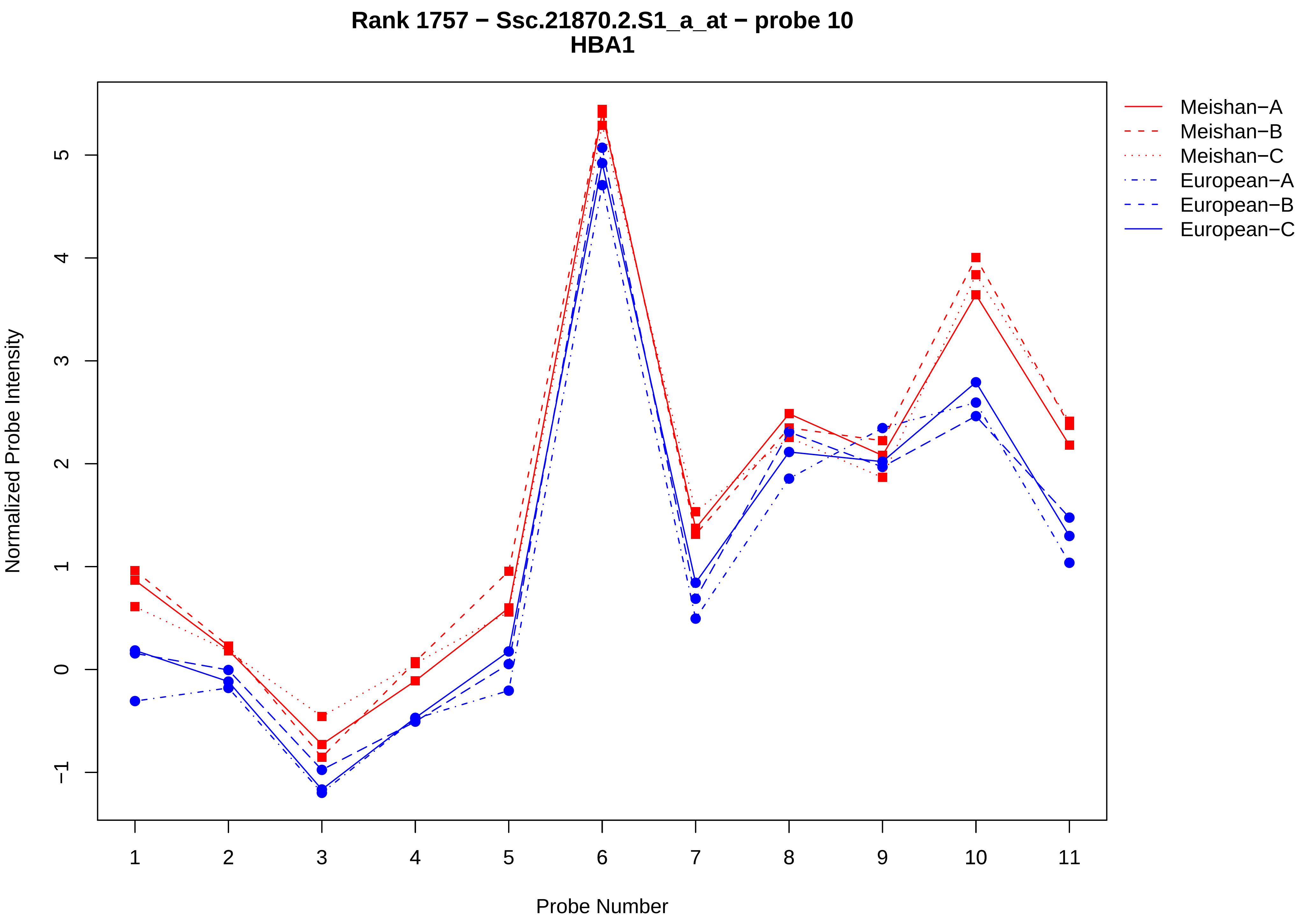

Rank 1757 − Ssc.21870.2.S1_a_at − probe 10
HBA1
Normalized Probe Intensity
Probe Number
Meishan−A
Meishan−B
Meishan−C
European−A
European−B
European−C
−1
0
1
2
3
4
5
1
2
3
4
5
6
7
8
9
10
11

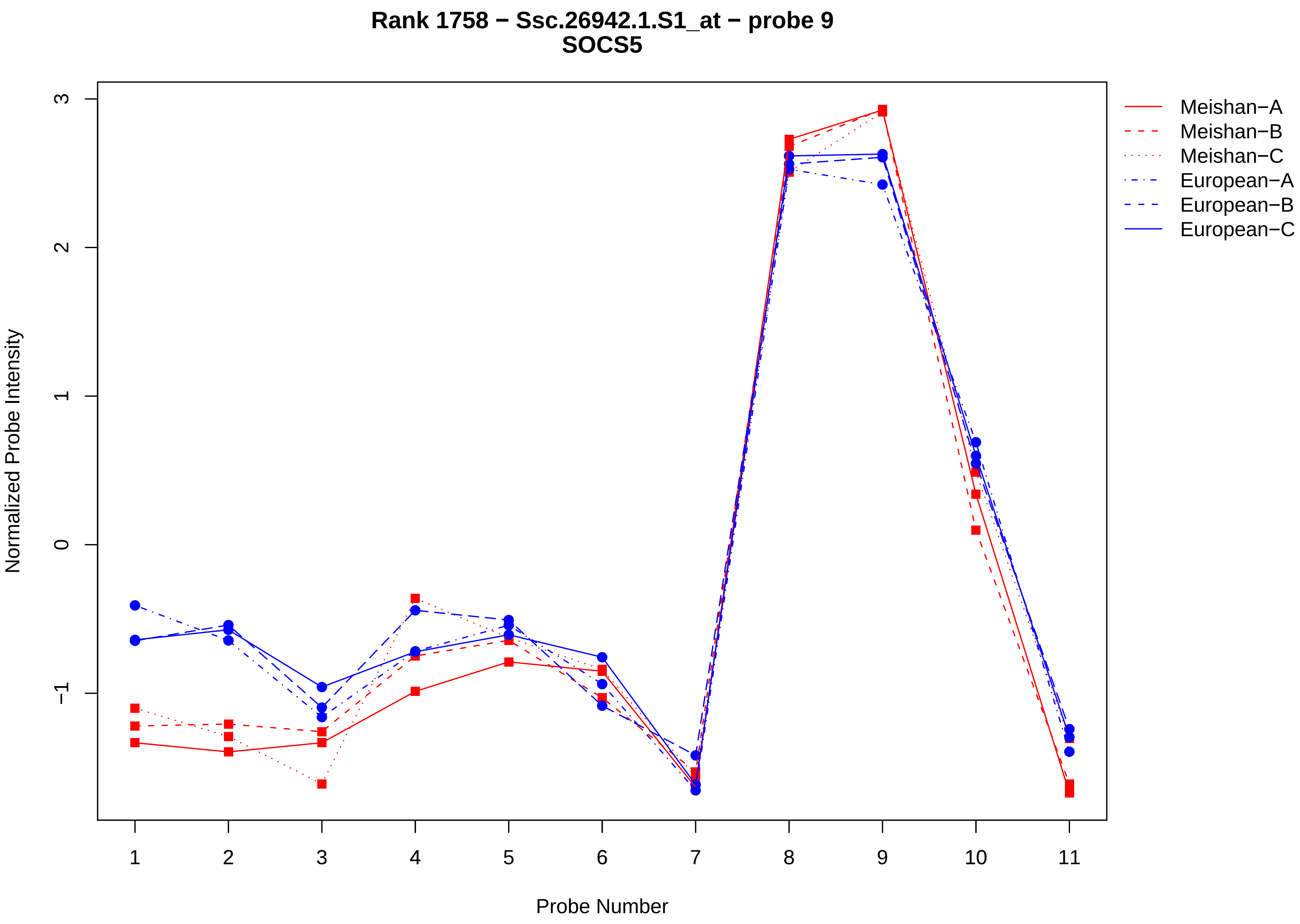
Rank 1758 − Ssc.26942.1.S1_at − probe 9
SOCS5
Normalized Probe Intensity
Probe Number
3
2
1
0
−1
1
2
3
4
5
6
7
8
9
10
11
Meishan−A
Meishan−B
Meishan−C
European−A
European−B
European−C

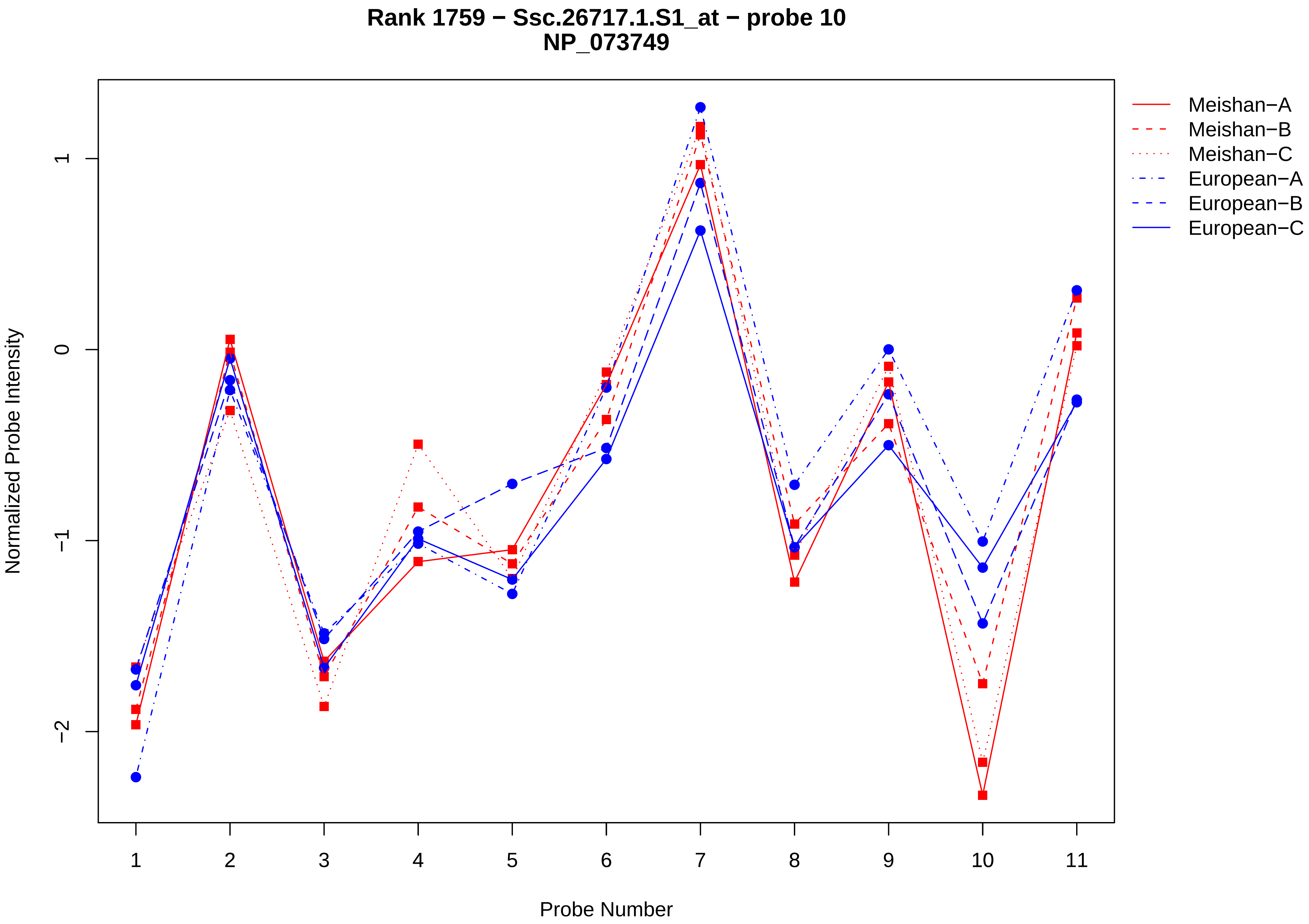

Rank 1759 − Ssc.26717.1.S1_at − probe 10
NP_073749
Normalized Probe Intensity
Probe Number
Meishan−A
Meishan−B
Meishan−C
European−A
European−B
European−C
−2
−1
0
1
1
2
3
4
5
6
7
8
9
10
11

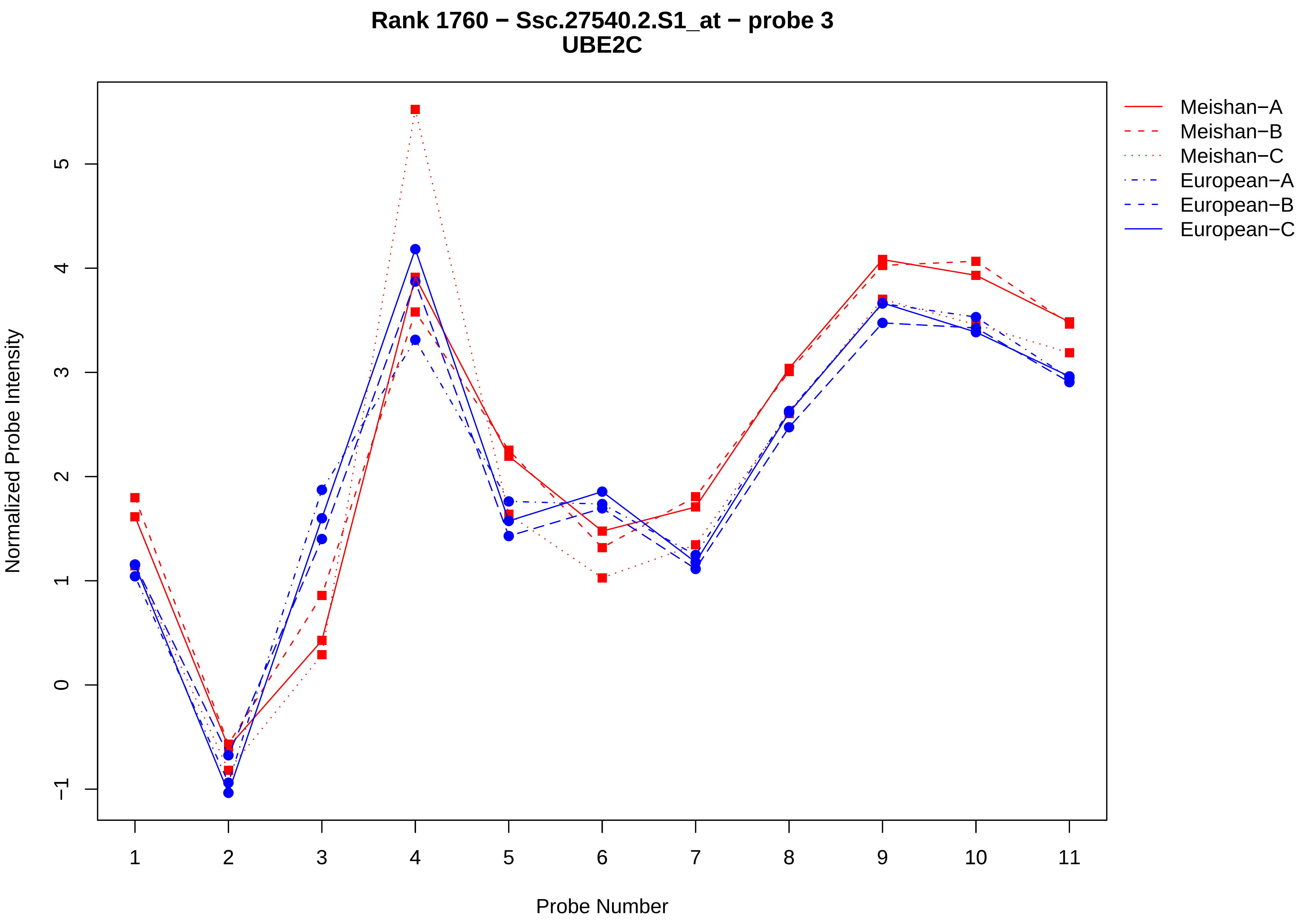

Rank 1760 − Ssc.27540.2.S1_at − probe 3
UBE2C
Normalized Probe Intensity
Probe Number
−1
0
1
2
3
4
5
1
2
3
4
5
6
7
8
9
10
11
Meishan−A
Meishan−B
Meishan−C
European−A
European−B
European−C

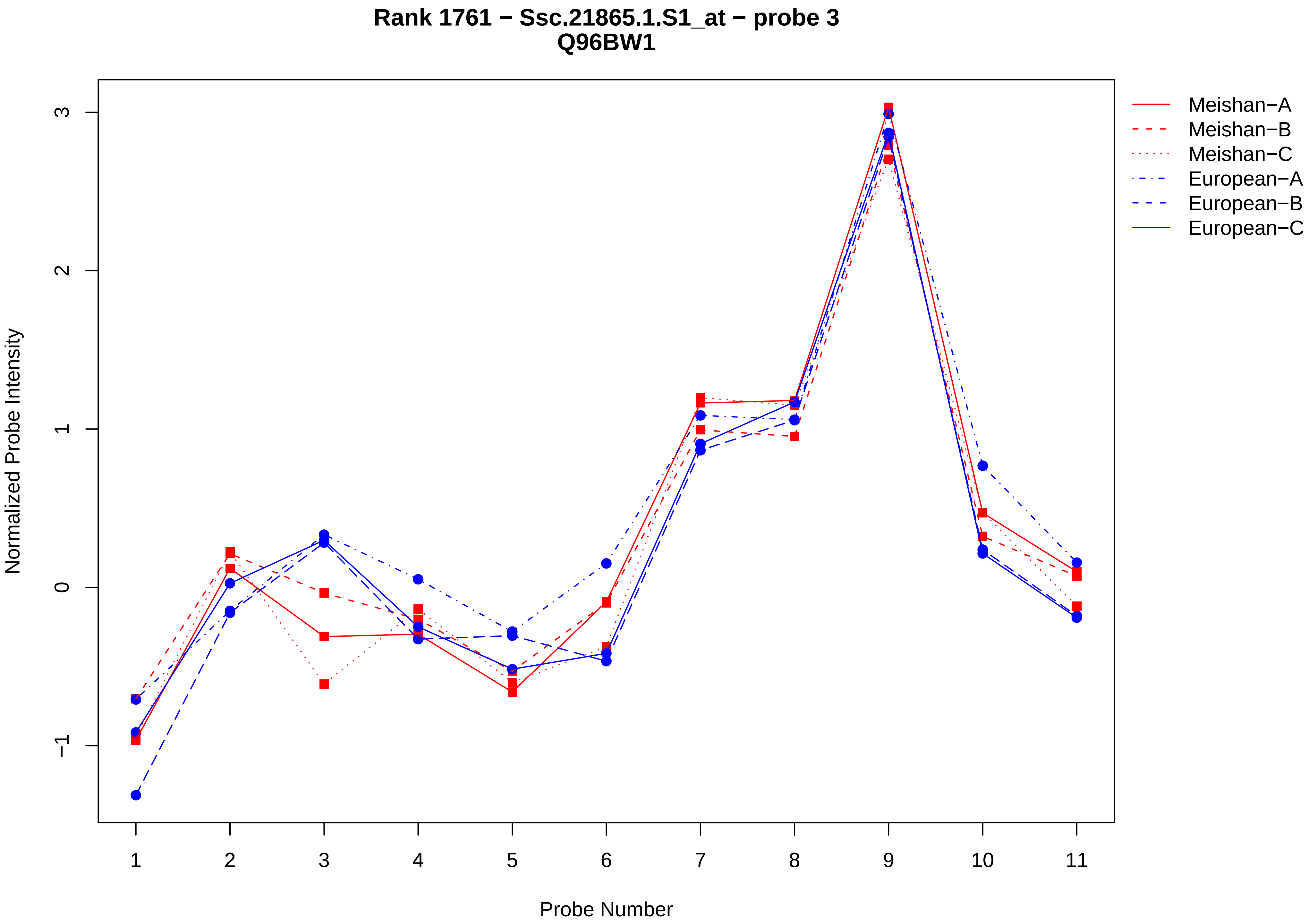

Rank 1761 − Ssc.21865.1.S1_at − probe 3
Q96BW1
Normalized Probe Intensity
Probe Number
−1
0
1
2
3
1
2
3
4
5
6
7
8
9
10
11
Meishan−A
Meishan−B
Meishan−C
European−A
European−B
European−C

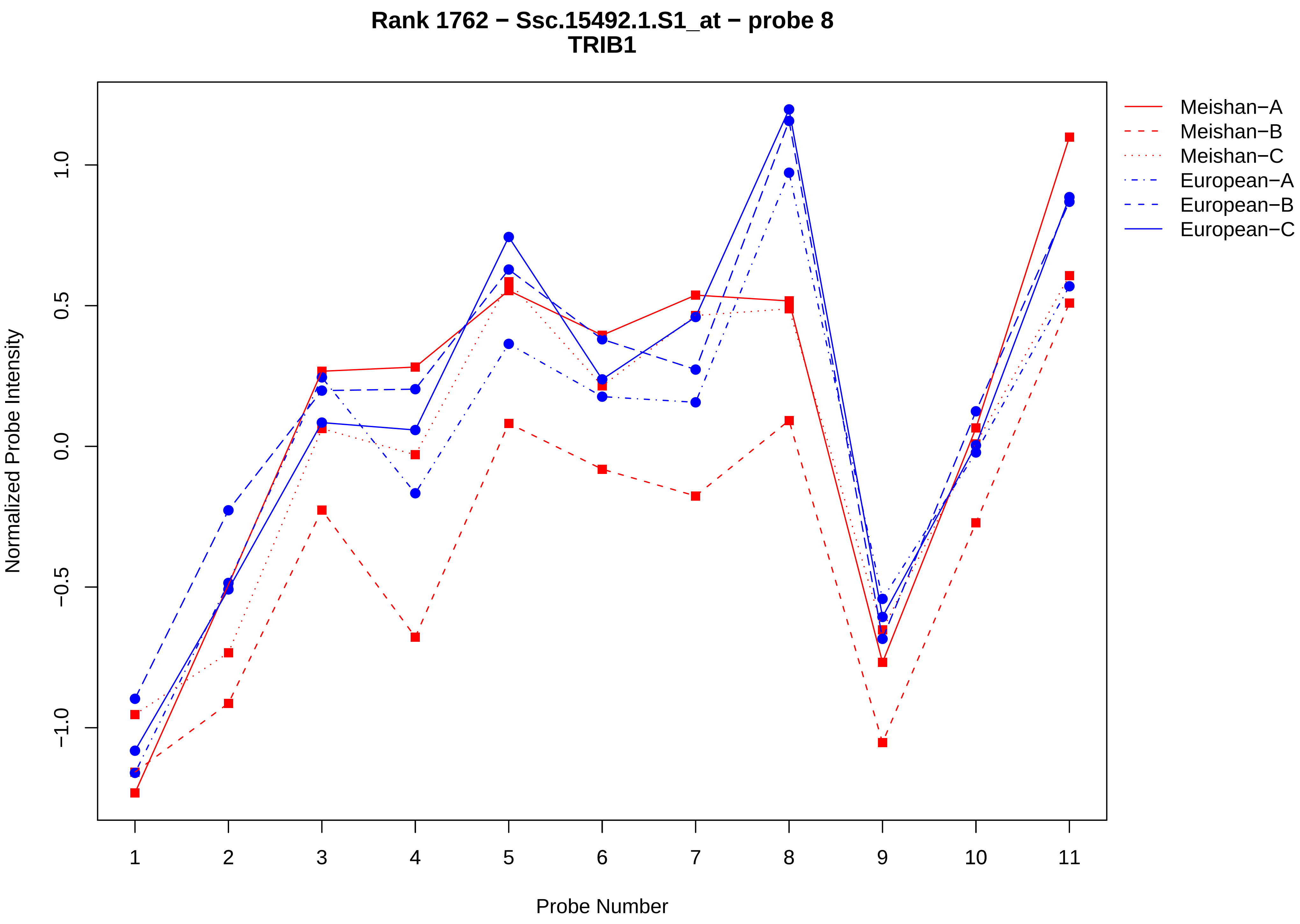

Rank 1762 − Ssc.15492.1.S1_at − probe 8
TRIB1
Normalized Probe Intensity
Probe Number
−1.0
−0.5
0.0
0.5
1.0
1
2
3
4
5
6
7
8
9
10
11
Meishan−A
Meishan−B
Meishan−C
European−A
European−B
European−C

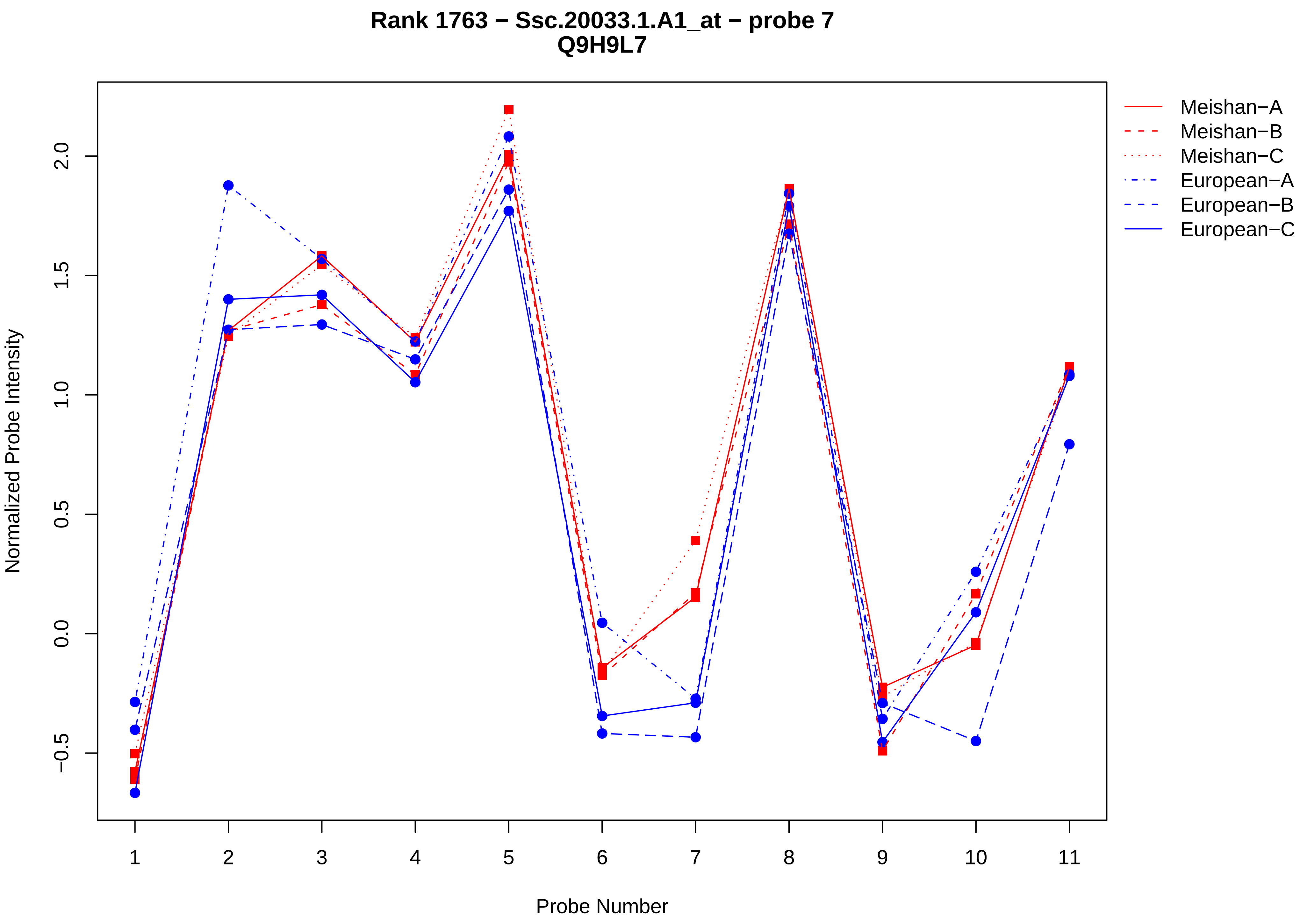

Rank 1763 − Ssc.20033.1.A1_at − probe 7
Q9H9L7
Normalized Probe Intensity
Probe Number
−0.5
0.0
0.5
1.0
1.5
2.0
1
2
3
4
5
6
7
8
9
10
11
Meishan−A
Meishan−B
Meishan−C
European−A
European−B
European−C

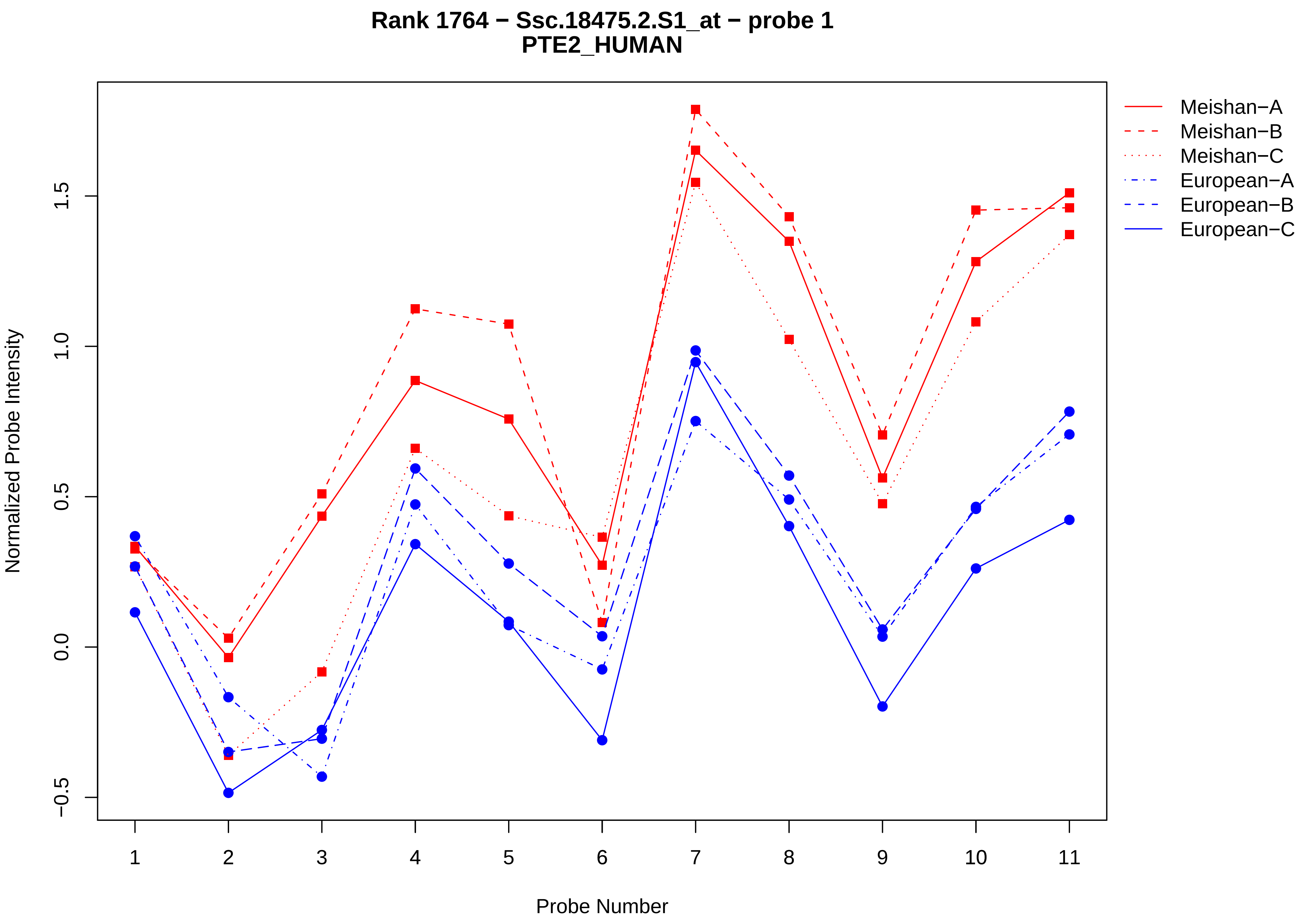

Rank 1764 − Ssc.18475.2.S1_at − probe 1
PTE2_HUMAN
Normalized Probe Intensity
Probe Number
Meishan−A
Meishan−B
Meishan−C
European−A
European−B
European−C
−0.5
0.0
0.5
1.0
1.5
1
2
3
4
5
6
7
8
9
10
11

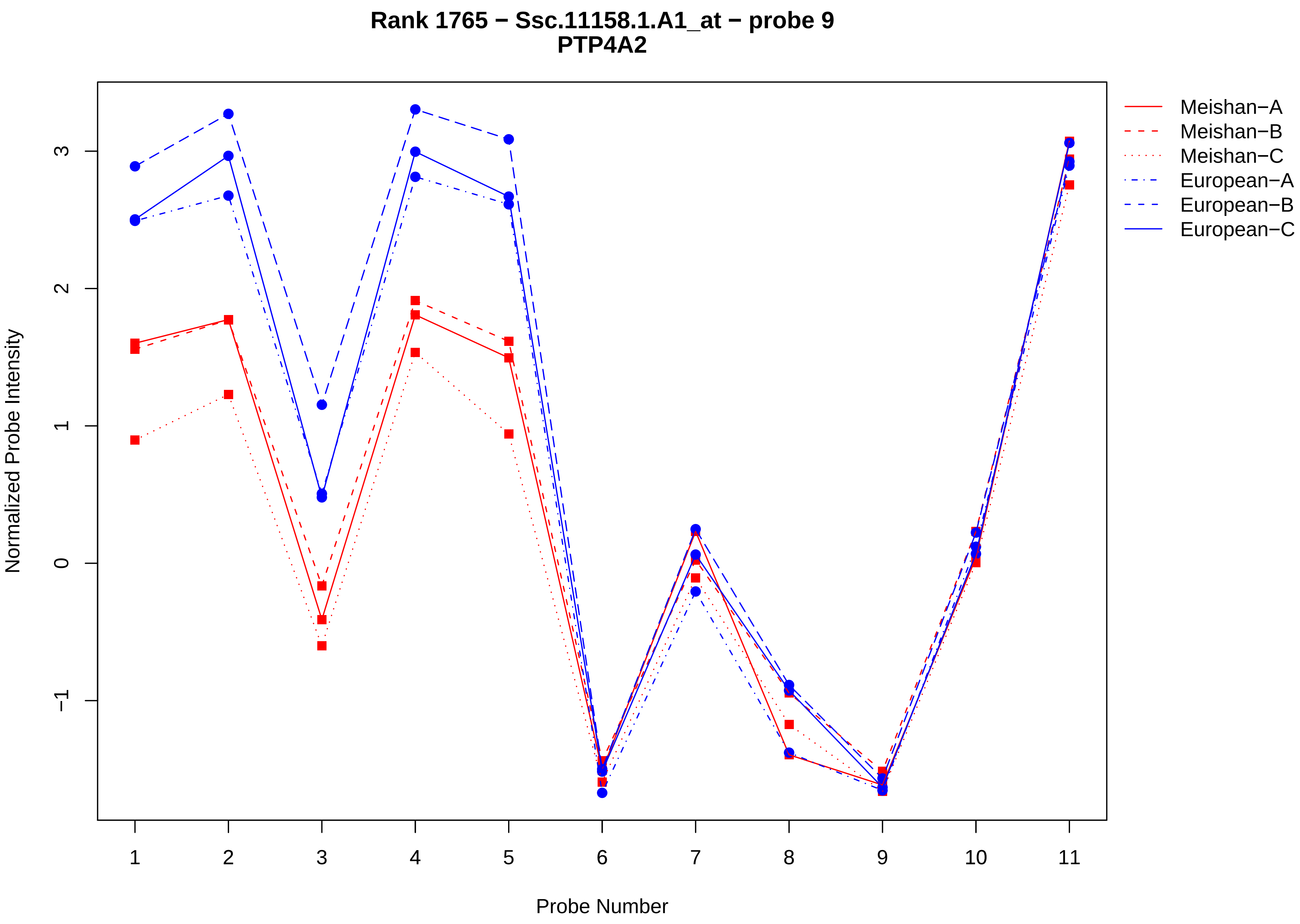

Rank 1765 − Ssc.11158.1.A1_at − probe 9
PTP4A2
Normalized Probe Intensity
Probe Number
−1
0
1
2
3
1
2
3
4
5
6
7
8
9
10
11
Meishan−A
Meishan−B
Meishan−C
European−A
European−B
European−C

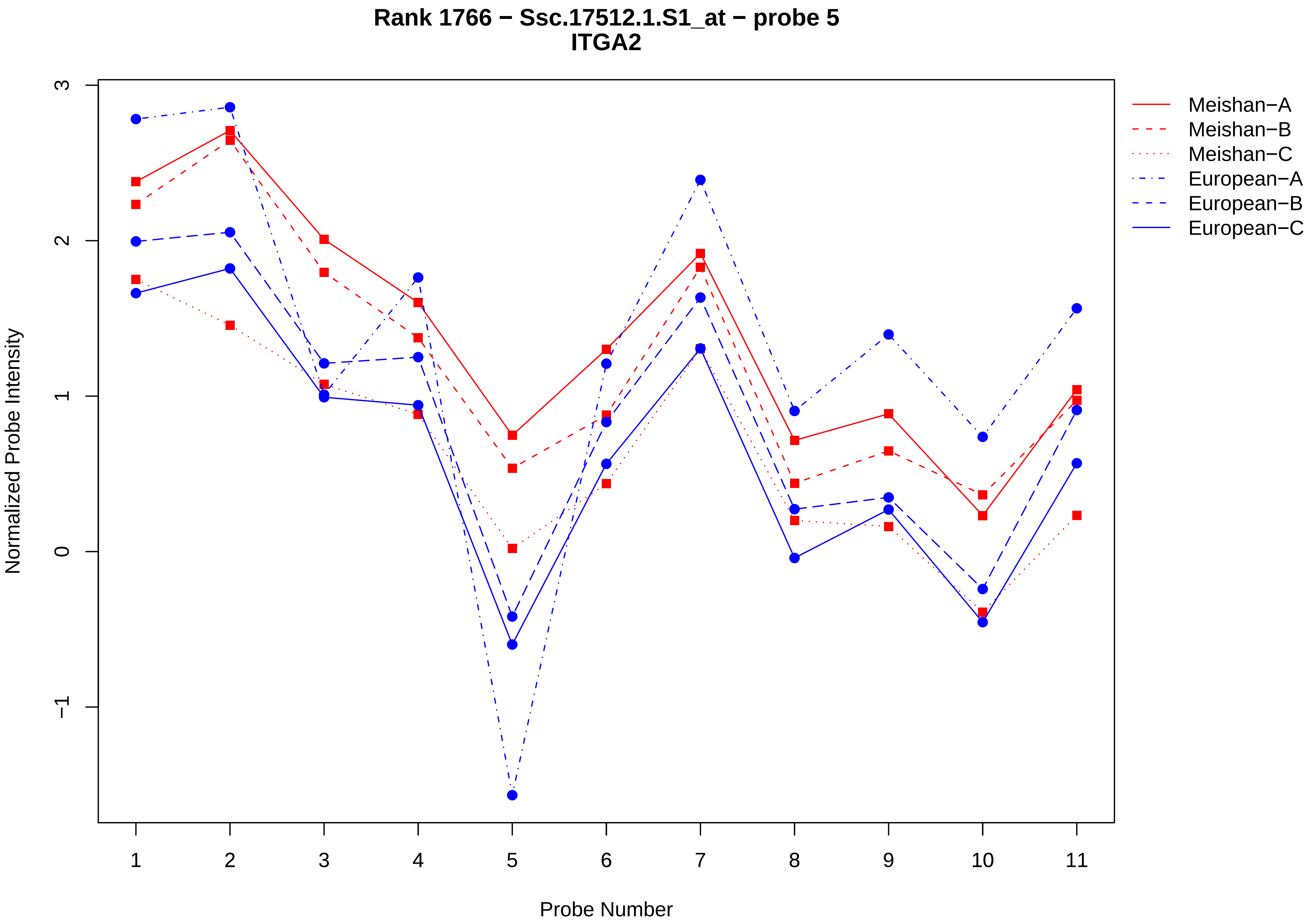
Rank 1766 − Ssc.17512.1.S1_at − probe 5
ITGA2
Normalized Probe Intensity
Probe Number
−1
0
1
2
3
1
2
3
4
5
6
7
8
9
10
11
Meishan−A
Meishan−B
Meishan−C
European−A
European−B
European−C

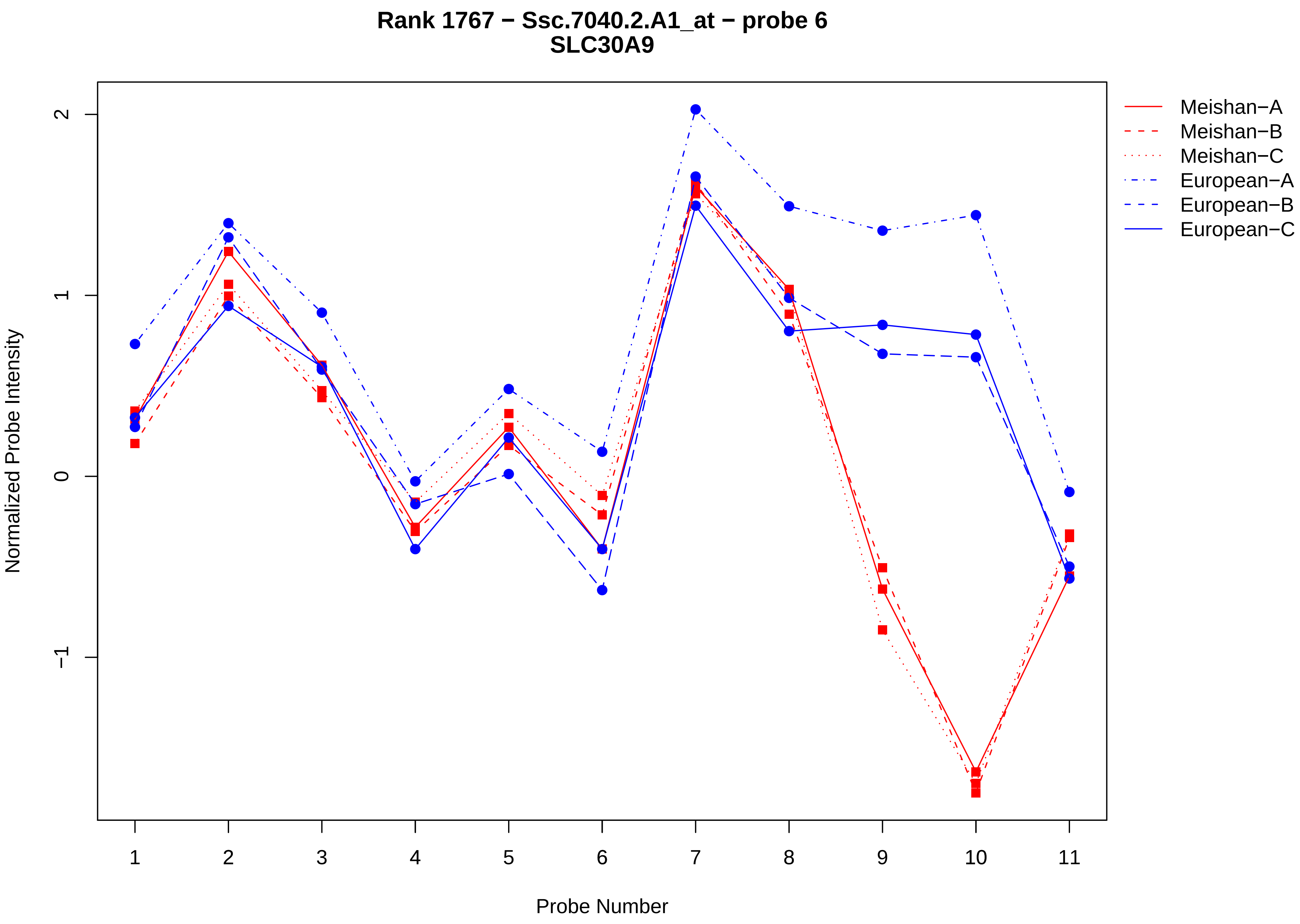

Rank 1767 − Ssc.7040.2.A1_at − probe 6
SLC30A9
Normalized Probe Intensity
Probe Number
−1
0
1
2
1
2
3
4
5
6
7
8
9
10
11
Meishan−A
Meishan−B
Meishan−C
European−A
European−B
European−C

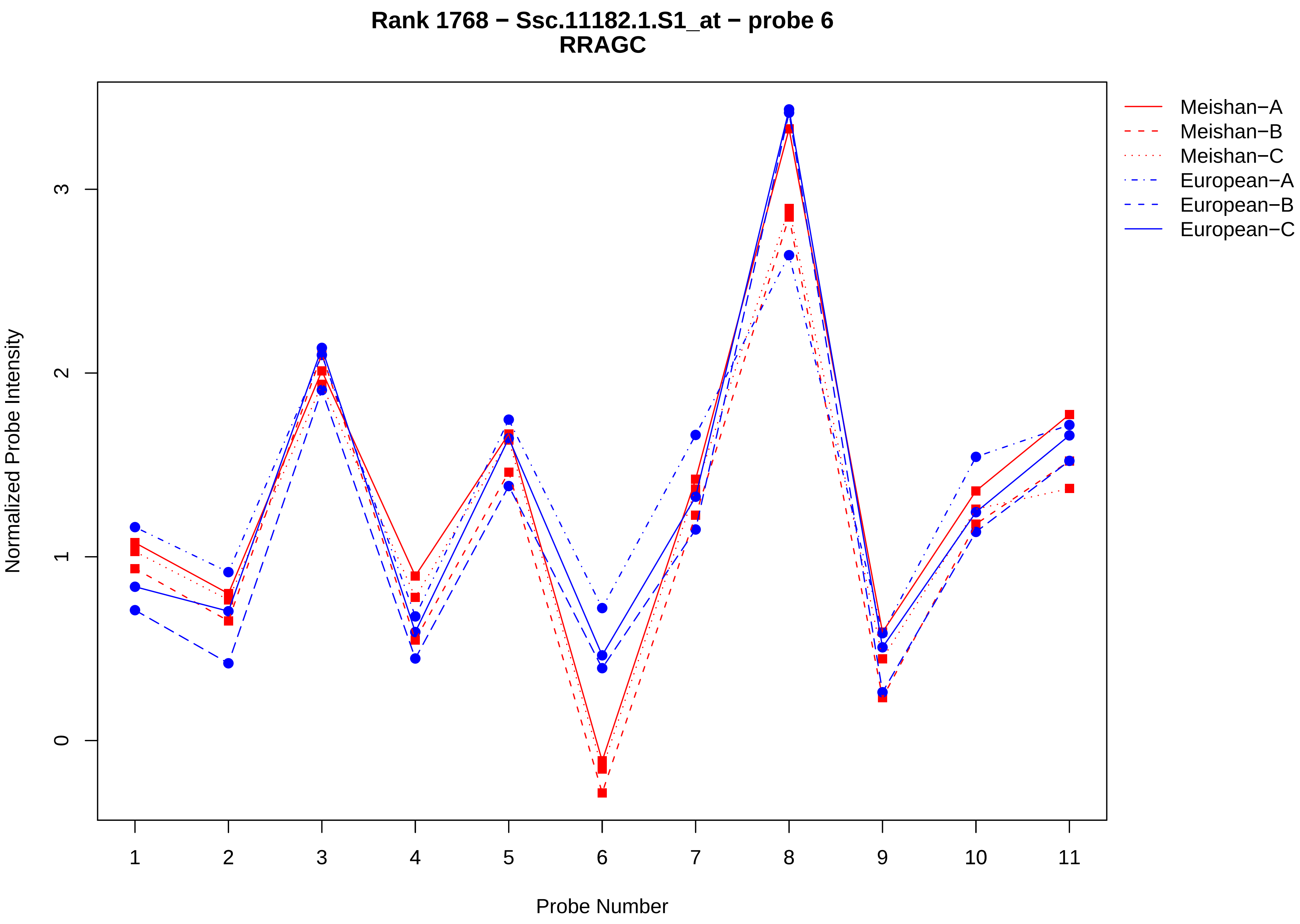

Rank 1768 − Ssc.11182.1.S1_at − probe 6
RRAGC
Normalized Probe Intensity
Probe Number
0
1
2
3
1
2
3
4
5
6
7
8
9
10
11
Meishan−A
Meishan−B
Meishan−C
European−A
European−B
European−C

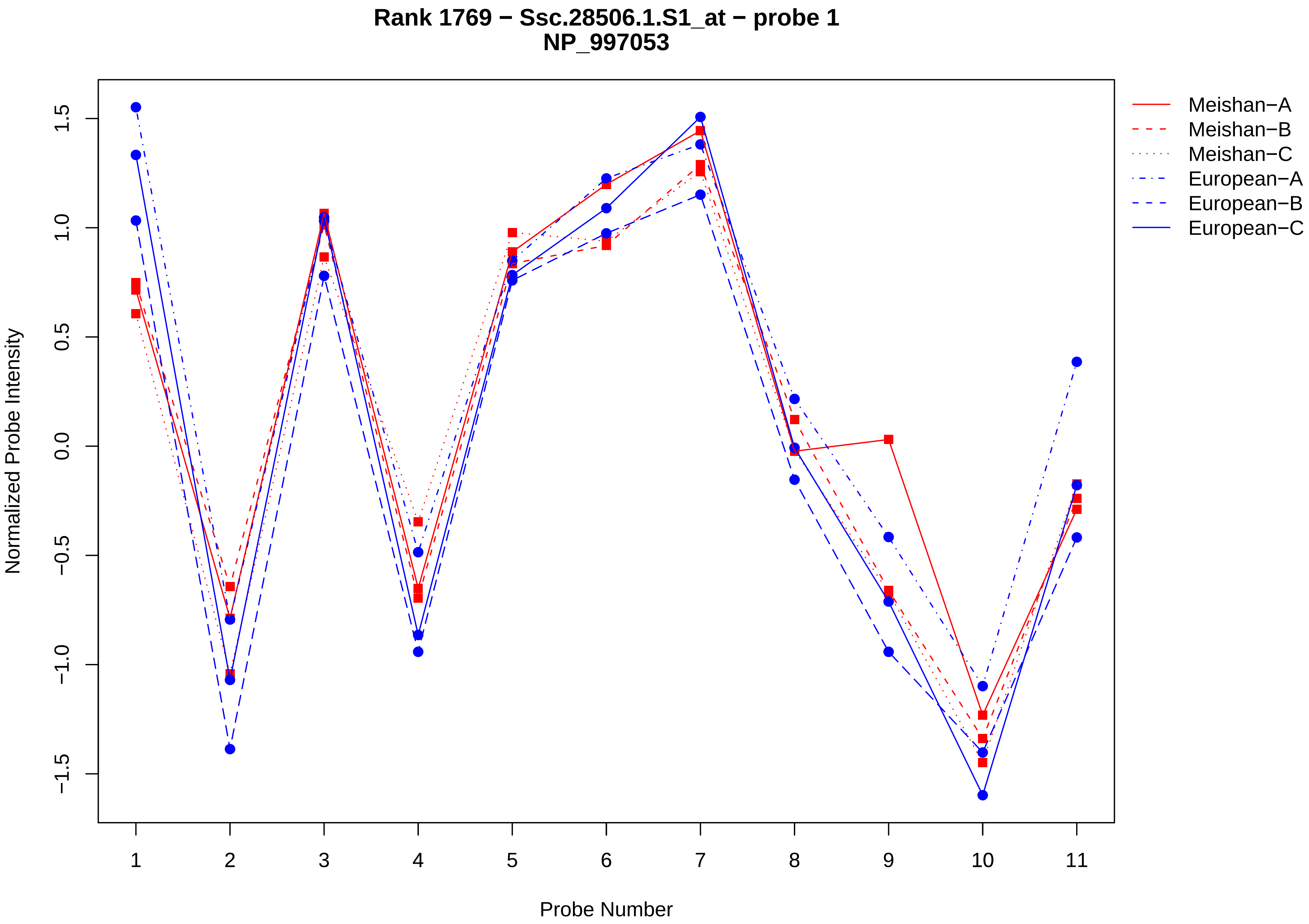

Rank 1769 − Ssc.28506.1.S1_at − probe 1
NP_997053
Normalized Probe Intensity
Probe Number
Meishan−A
Meishan−B
Meishan−C
European−A
European−B
European−C

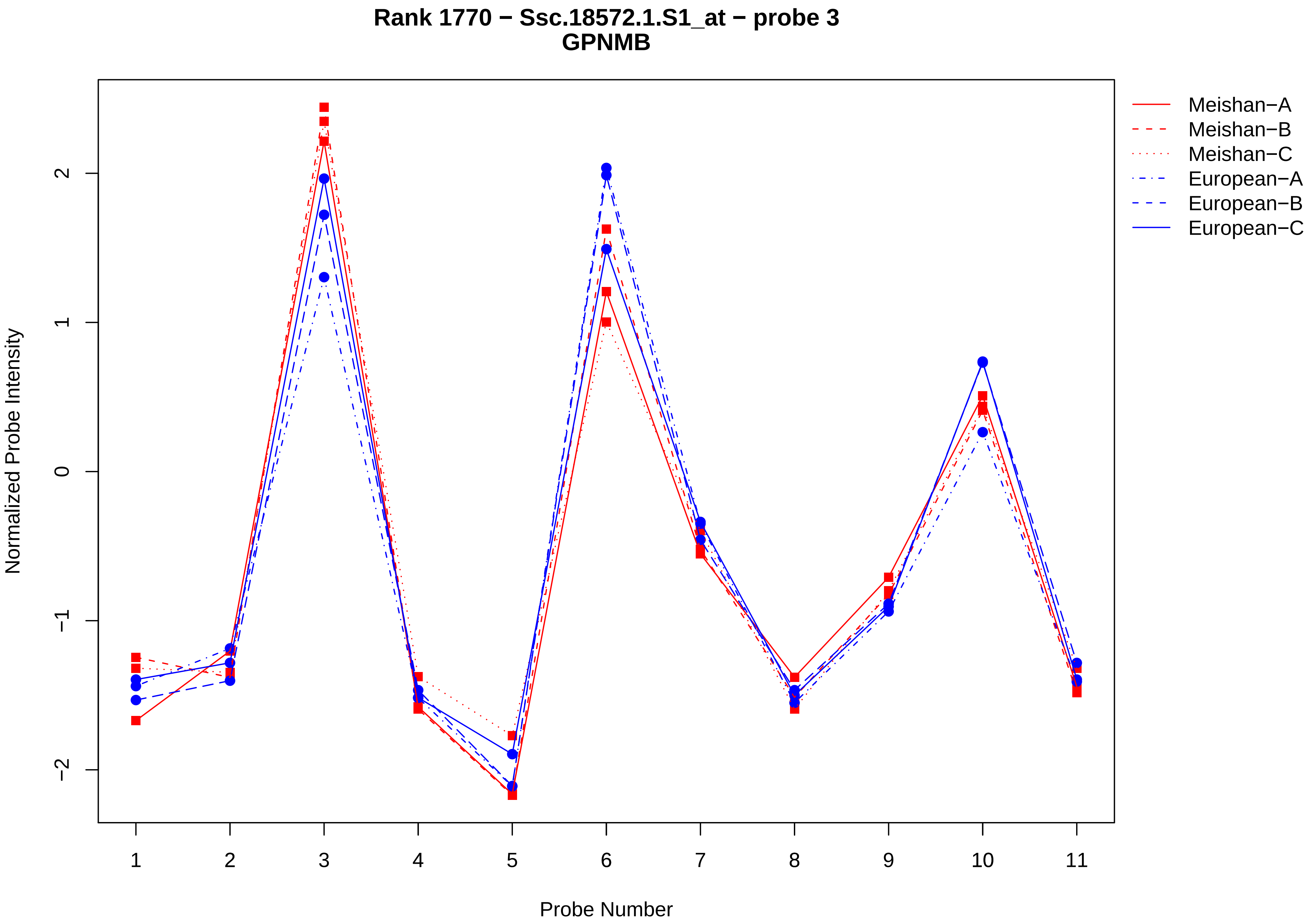
Rank 1770 − Ssc.18572.1.S1_at − probe 3
GPNMB
Normalized Probe Intensity
Probe Number
−2
−1
0
1
2
1
2
3
4
5
6
7
8
9
10
11
Meishan−A
Meishan−B
Meishan−C
European−A
European−B
European−C

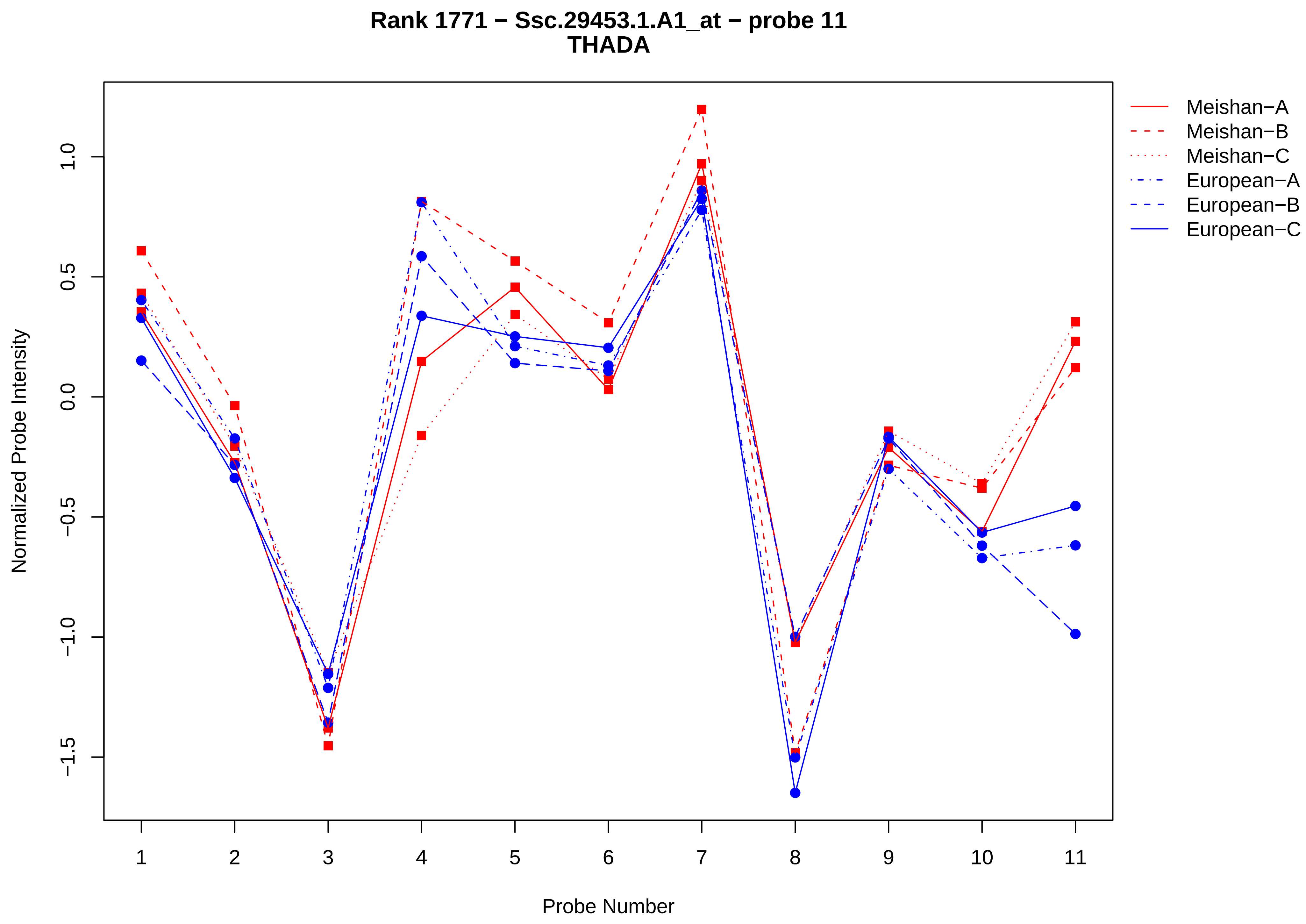

Rank 1771 − Ssc.29453.1.A1_at − probe 11
THADA
Normalized Probe Intensity
Probe Number
−1.5
−1.0
−0.5
0.0
0.5
1.0
1
2
3
4
5
6
7
8
9
10
11
Meishan−A
Meishan−B
Meishan−C
European−A
European−B
European−C

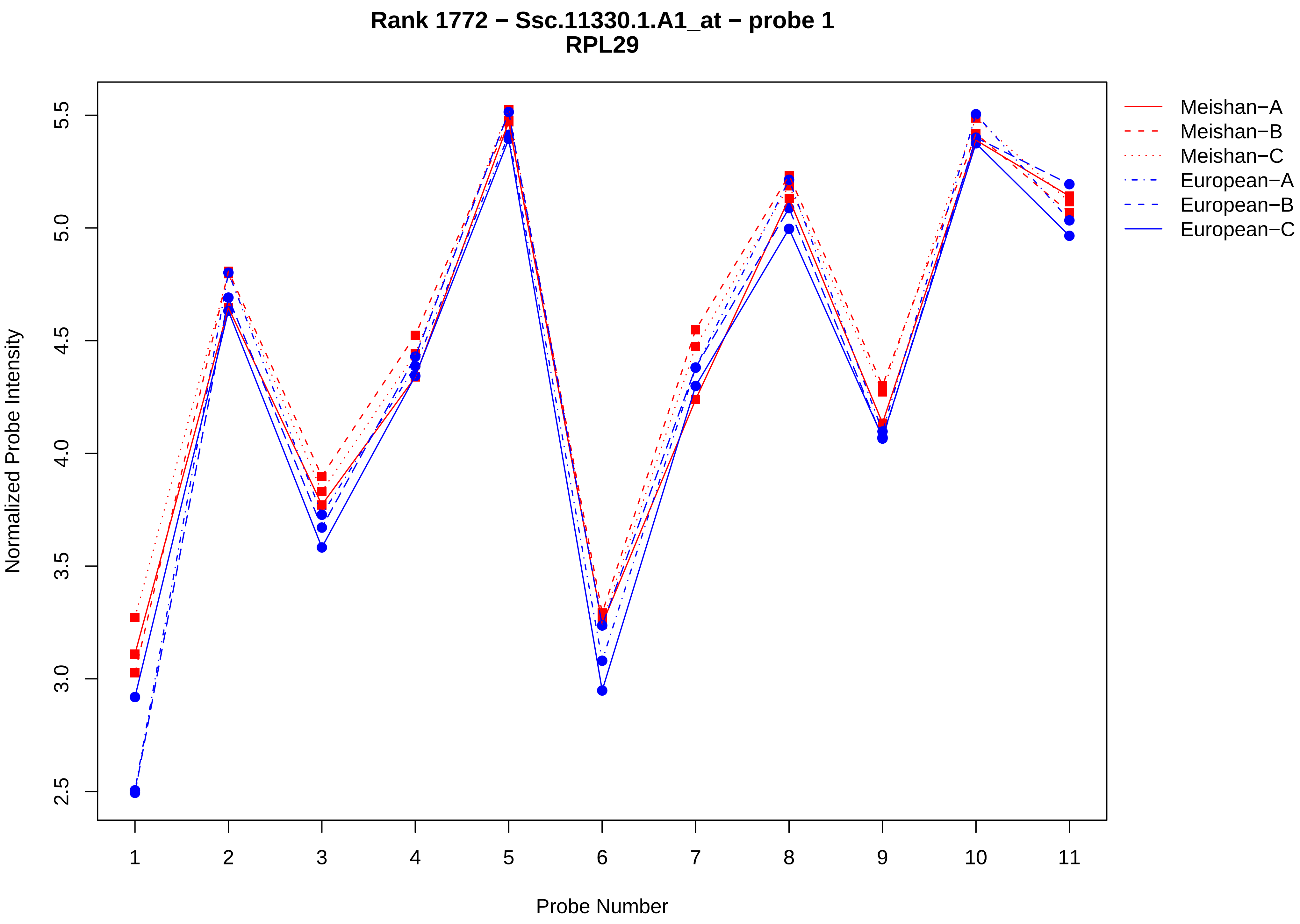
Rank 1772 − Ssc.11330.1.A1_at − probe 1
RPL29
Normalized Probe Intensity
Probe Number
2.5
3.0
3.5
4.0
4.5
5.0
5.5
1
2
3
4
5
6
7
8
9
10
11
Meishan−A
Meishan−B
Meishan−C
European−A
European−B
European−C

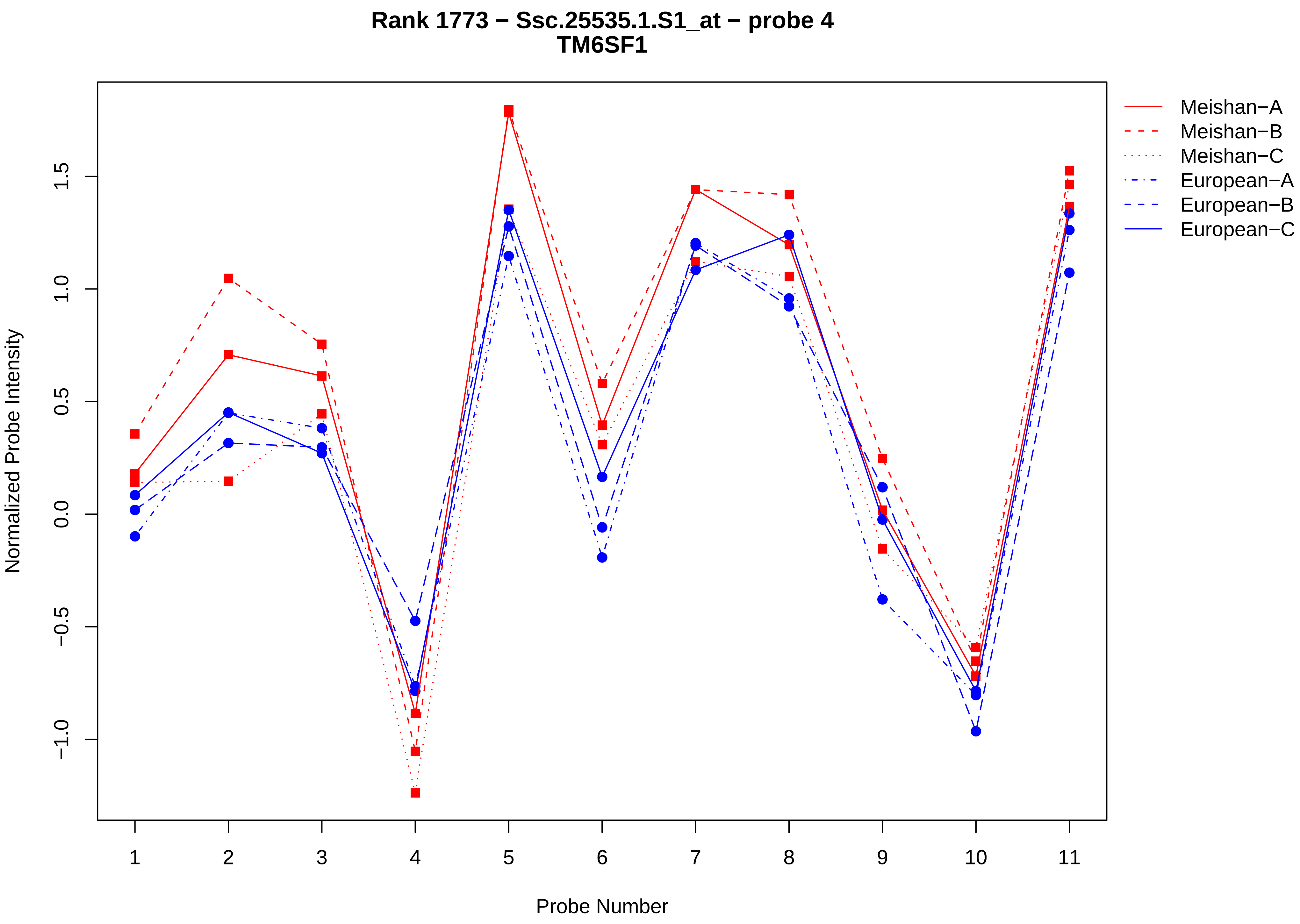

Rank 1773 − Ssc.25535.1.S1_at − probe 4
TM6SF1
Normalized Probe Intensity
Probe Number
Meishan−A
Meishan−B
Meishan−C
European−A
European−B
European−C
−1.0
−0.5
0.0
0.5
1.0
1.5
1
2
3
4
5
6
7
8
9
10
11

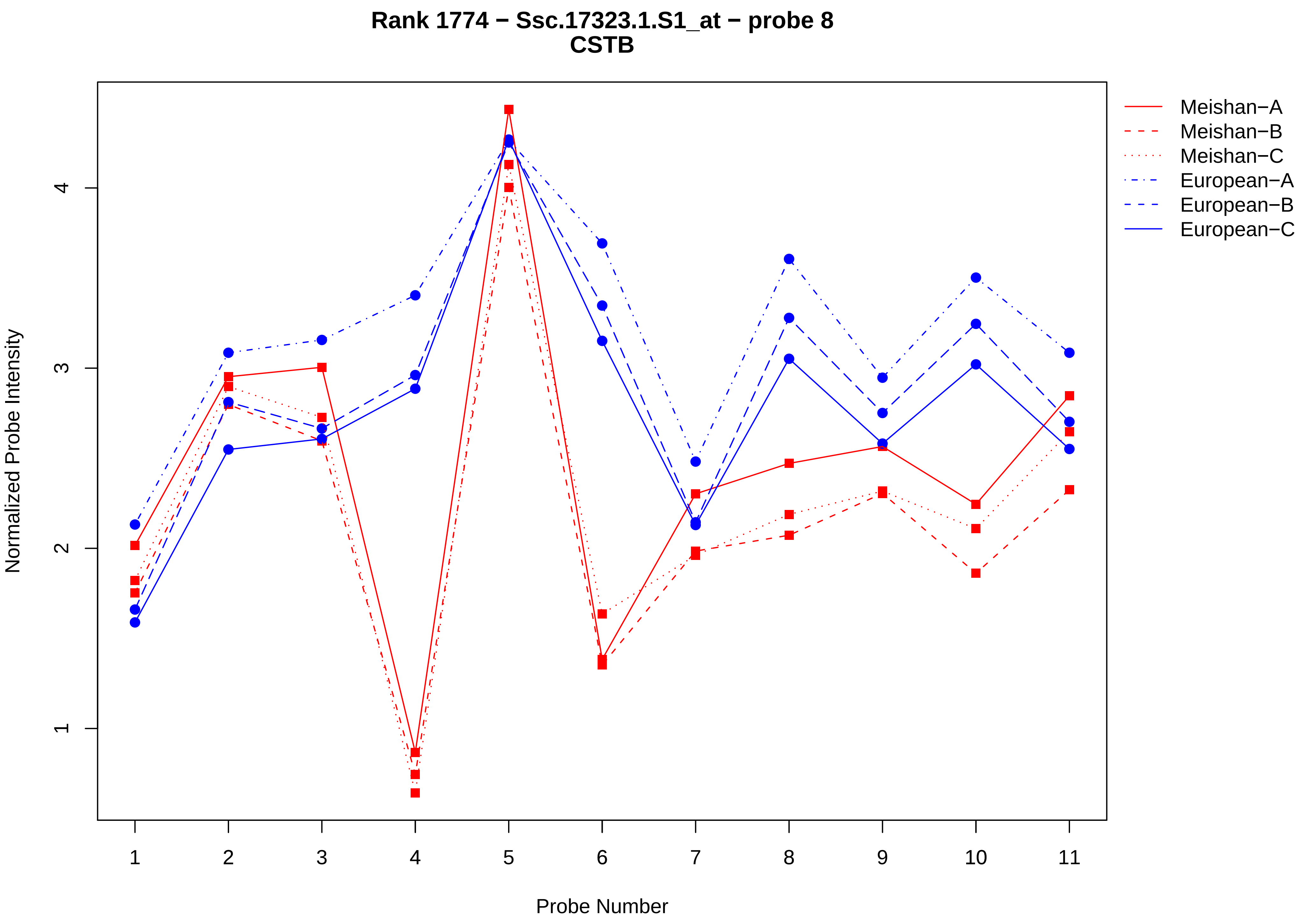

Rank 1774 − Ssc.17323.1.S1_at − probe 8
CSTB
Normalized Probe Intensity
Probe Number
1
2
3
4
1
2
3
4
5
6
7
8
9
10
11
Meishan−A
Meishan−B
Meishan−C
European−A
European−B
European−C

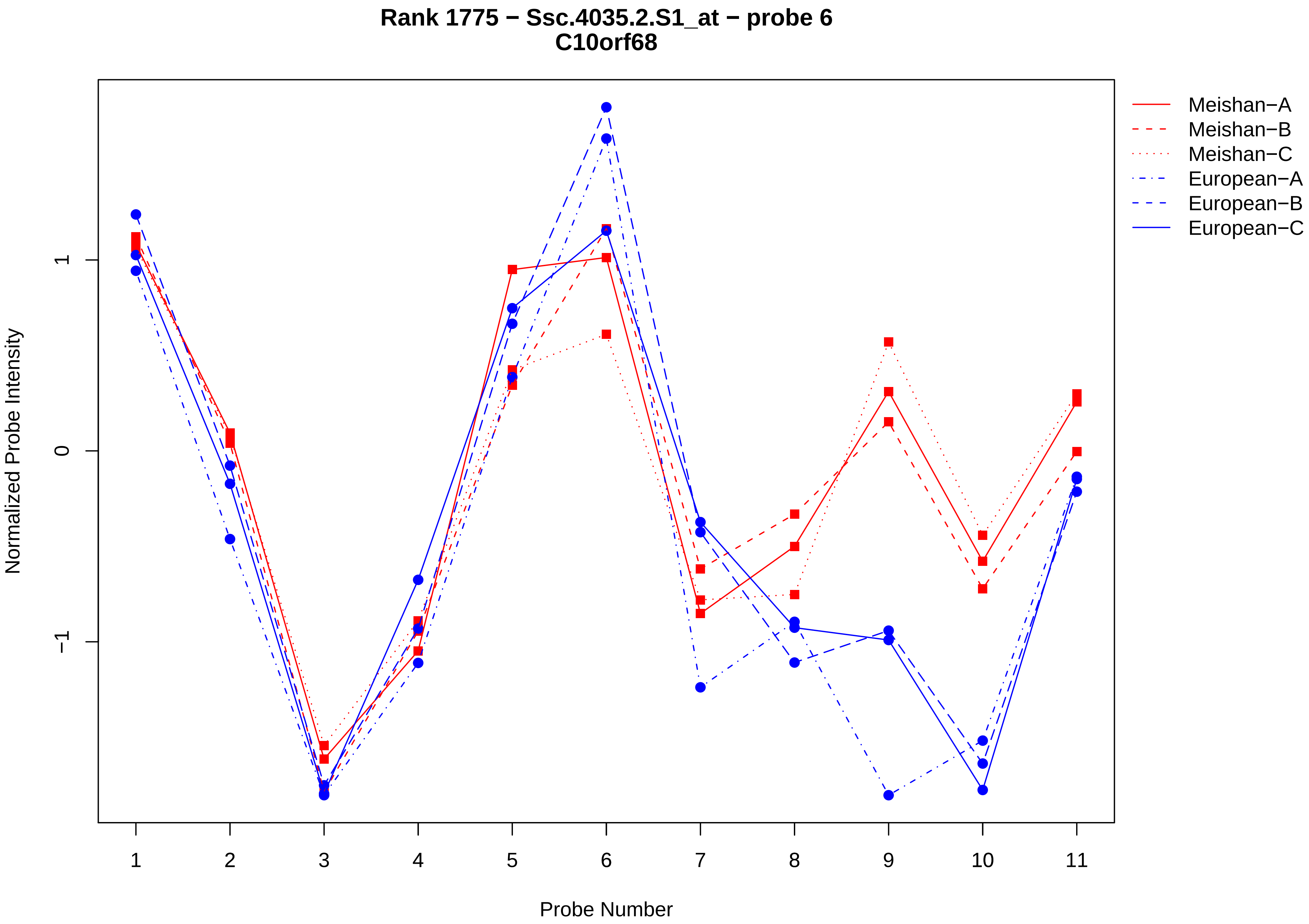

Rank 1775 − Ssc.4035.2.S1_at − probe 6
C10orf68
Normalized Probe Intensity
Probe Number
−1
0
1
1
2
3
4
5
6
7
8
9
10
11
Meishan−A
Meishan−B
Meishan−C
European−A
European−B
European−C

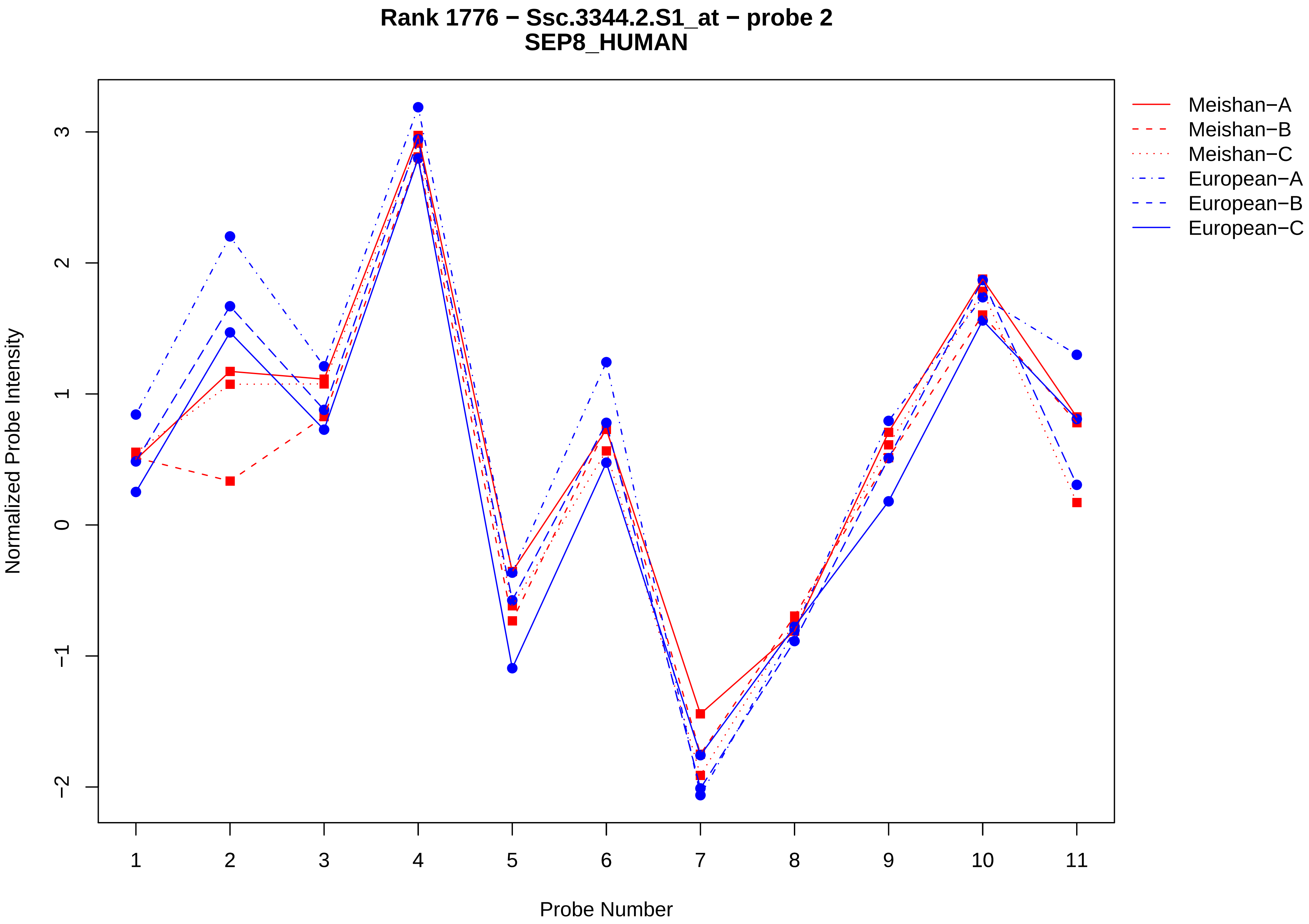

Rank 1776 − Ssc.3344.2.S1_at − probe 2
SEP8_HUMAN
Normalized Probe Intensity
Probe Number
−2
−1
0
1
2
3
1
2
3
4
5
6
7
8
9
10
11
Meishan−A
Meishan−B
Meishan−C
European−A
European−B
European−C

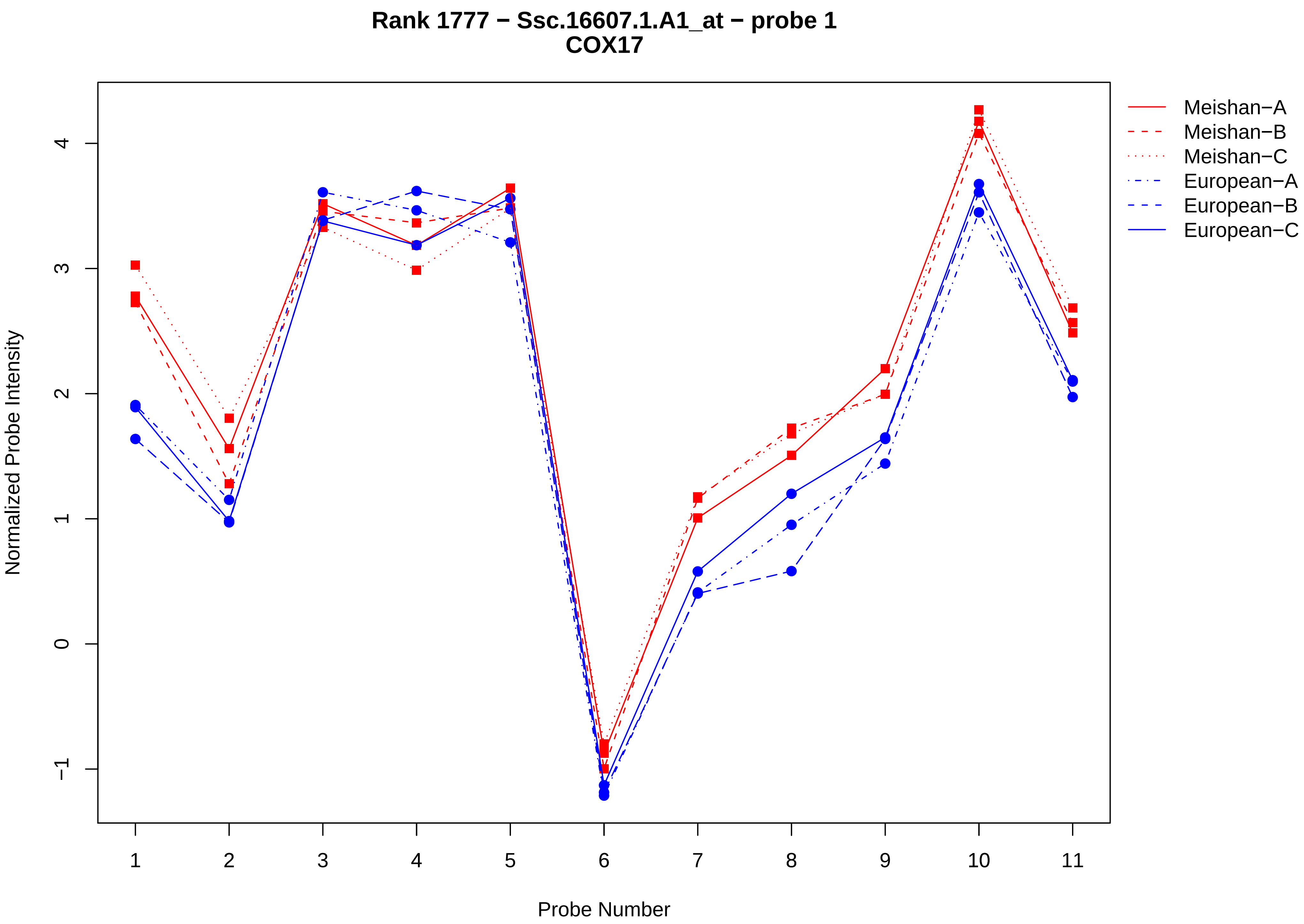
Rank 1777 − Ssc.16607.1.A1_at − probe 1
COX17
Normalized Probe Intensity
Probe Number
Meishan−A
Meishan−B
Meishan−C
European−A
European−B
European−C
−1
0
1
2
3
4
1
2
3
4
5
6
7
8
9
10
11

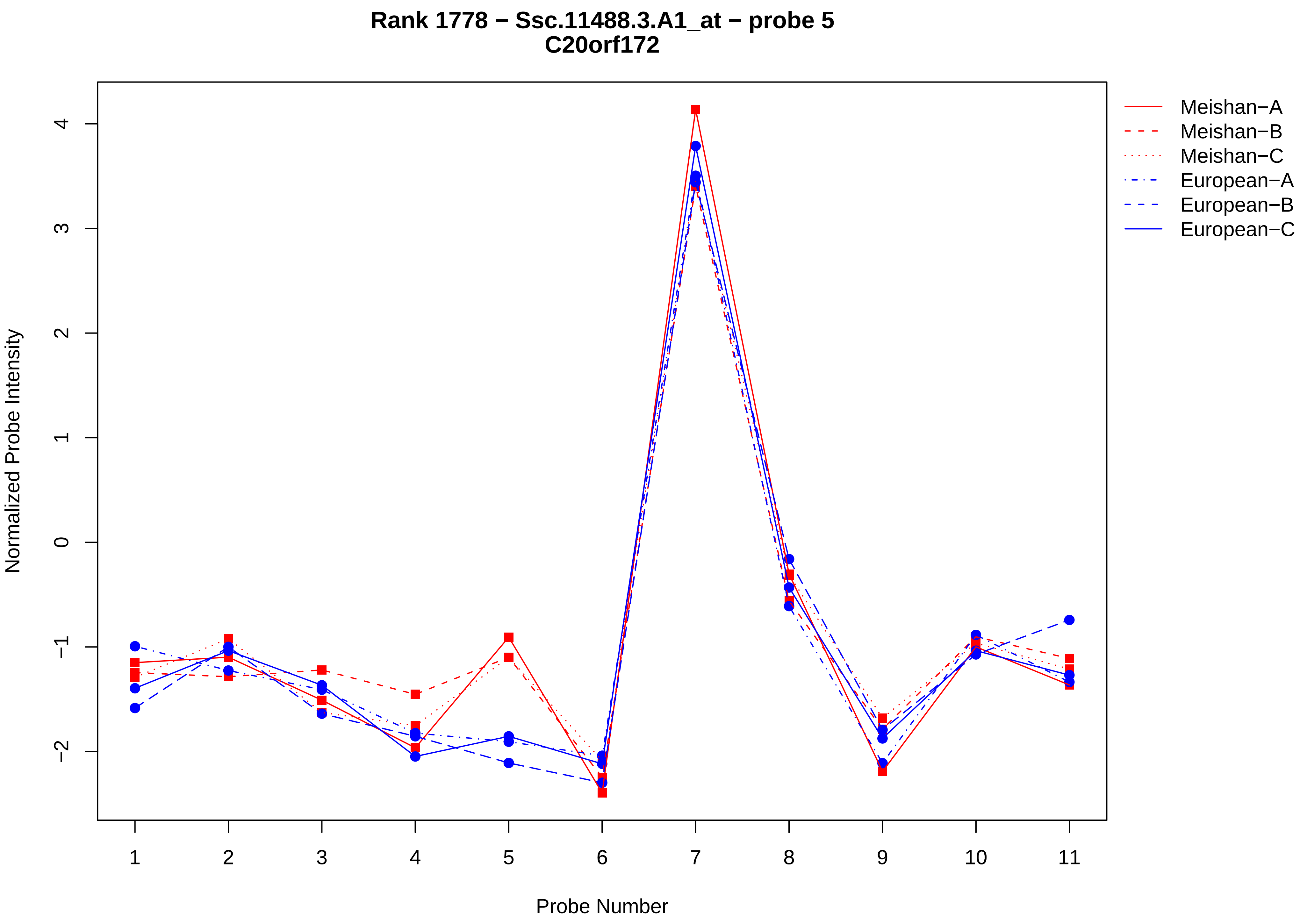
Rank 1778 − Ssc.11488.3.A1_at − probe 5
C20orf172
Normalized Probe Intensity
Probe Number
−2
−1
0
1
2
3
4
1
2
3
4
5
6
7
8
9
10
11
Meishan−A
Meishan−B
Meishan−C
European−A
European−B
European−C

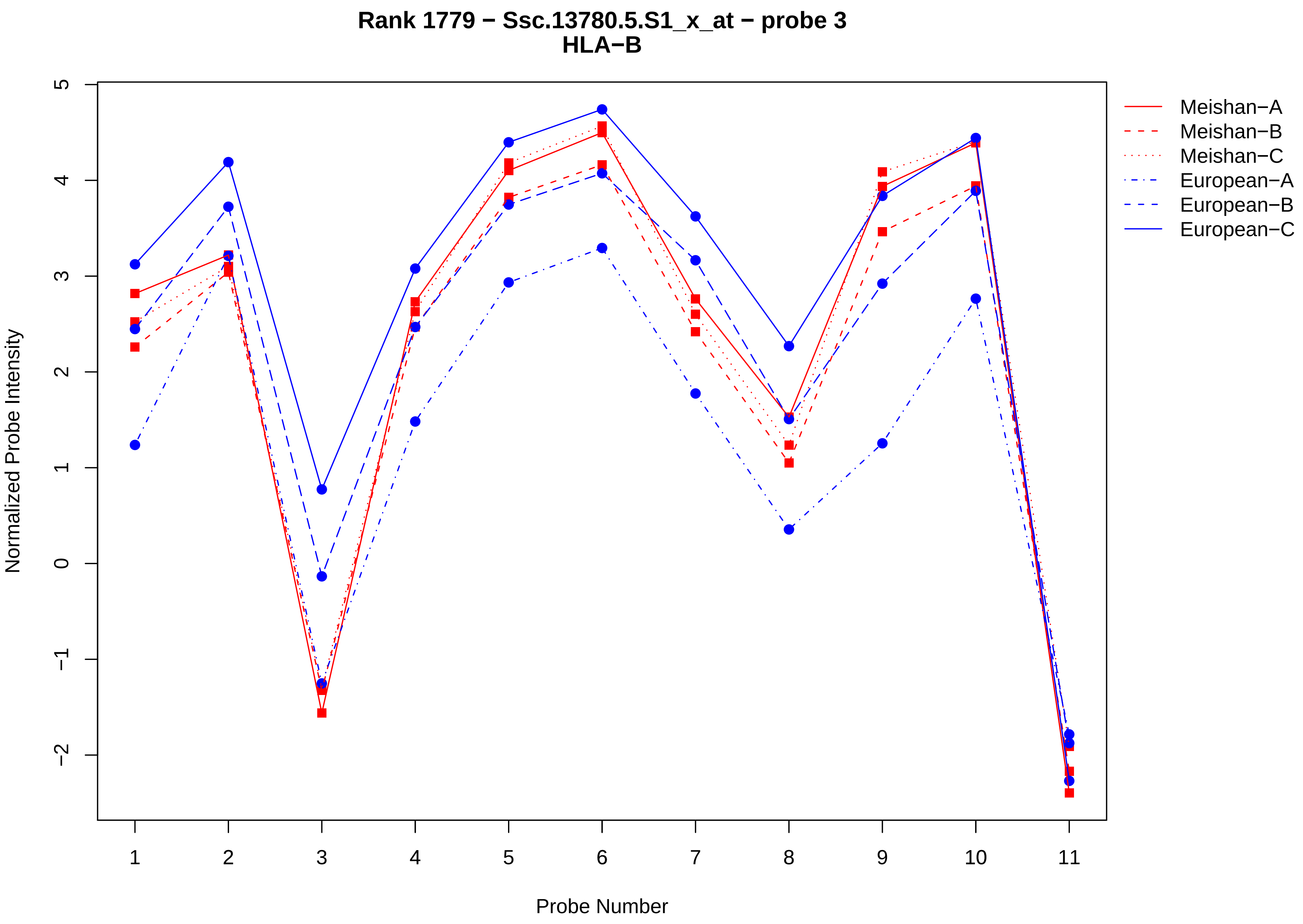

Rank 1779 − Ssc.13780.5.S1_x_at − probe 3
HLA−B
Normalized Probe Intensity
Probe Number
Meishan−A
Meishan−B
Meishan−C
European−A
European−B
European−C

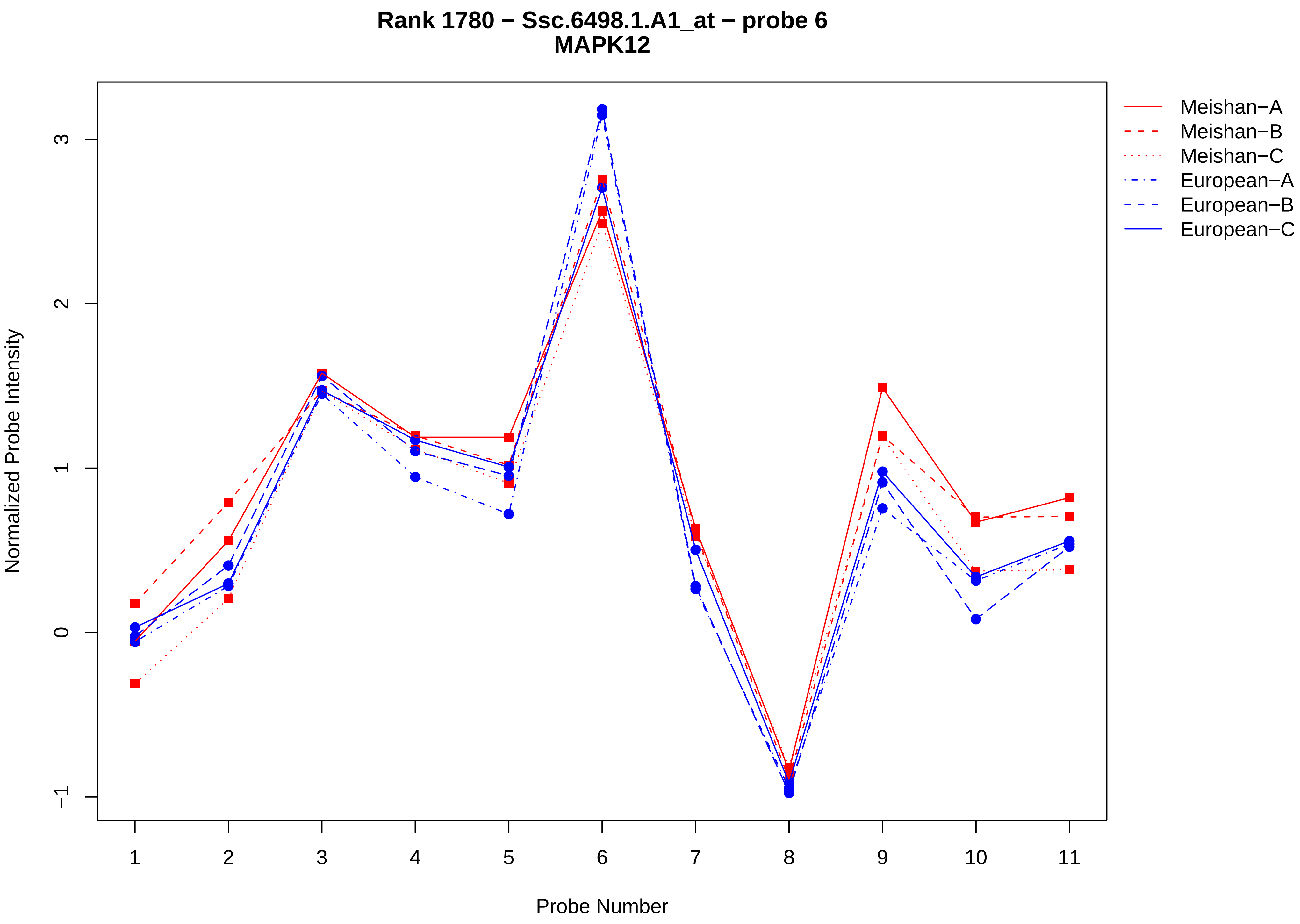

Rank 1780 − Ssc.6498.1.A1_at − probe 6
MAPK12
Normalized Probe Intensity
Probe Number
−1
0
1
2
3
1
2
3
4
5
6
7
8
9
10
11
Meishan−A
Meishan−B
Meishan−C
European−A
European−B
European−C

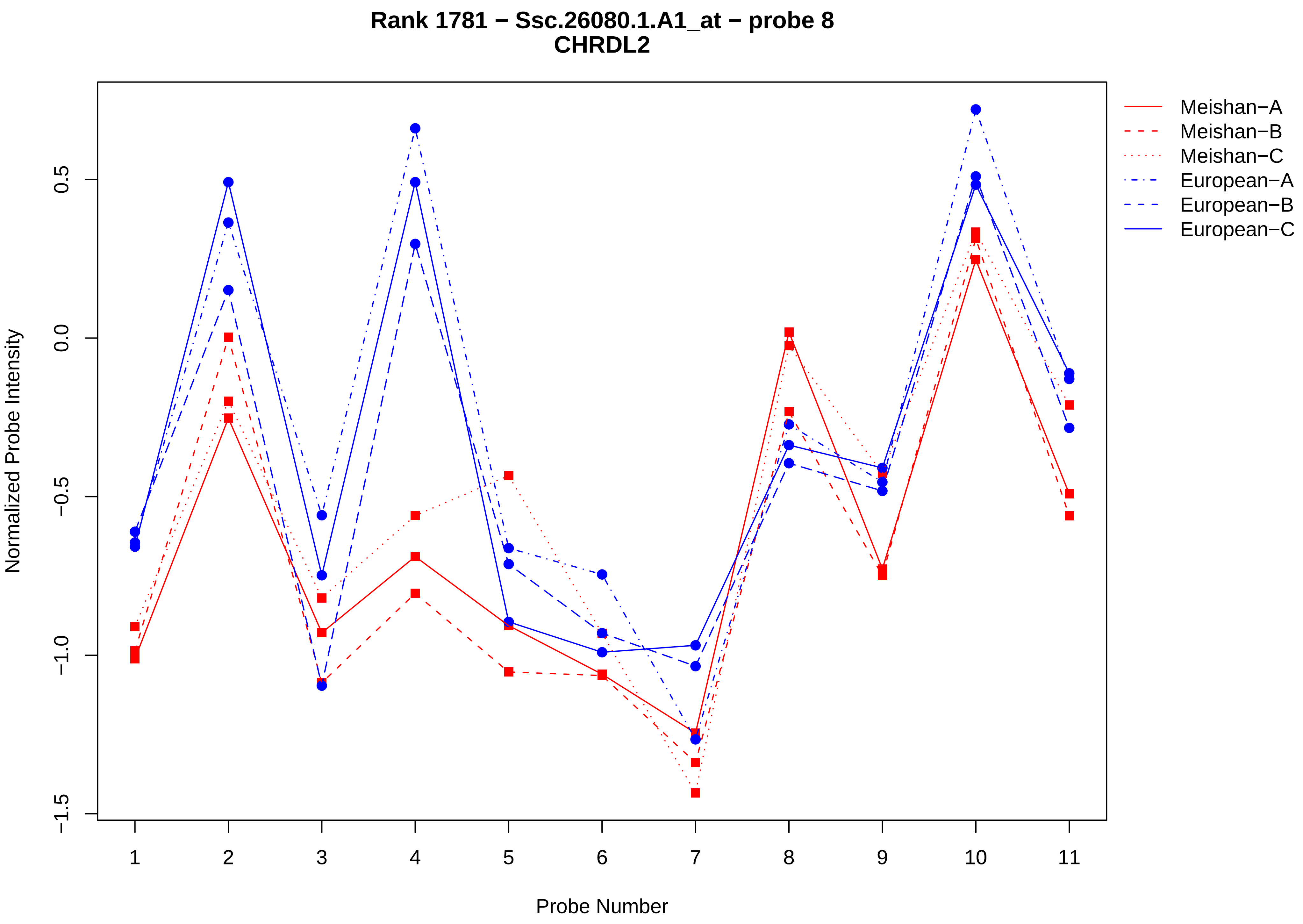
Rank 1781 − Ssc.26080.1.A1_at − probe 8
CHRDL2
Normalized Probe Intensity
Probe Number
−1.5
−1.0
−0.5
0.0
0.5
1
2
3
4
5
6
7
8
9
10
11
Meishan−A
Meishan−B
Meishan−C
European−A
European−B
European−C

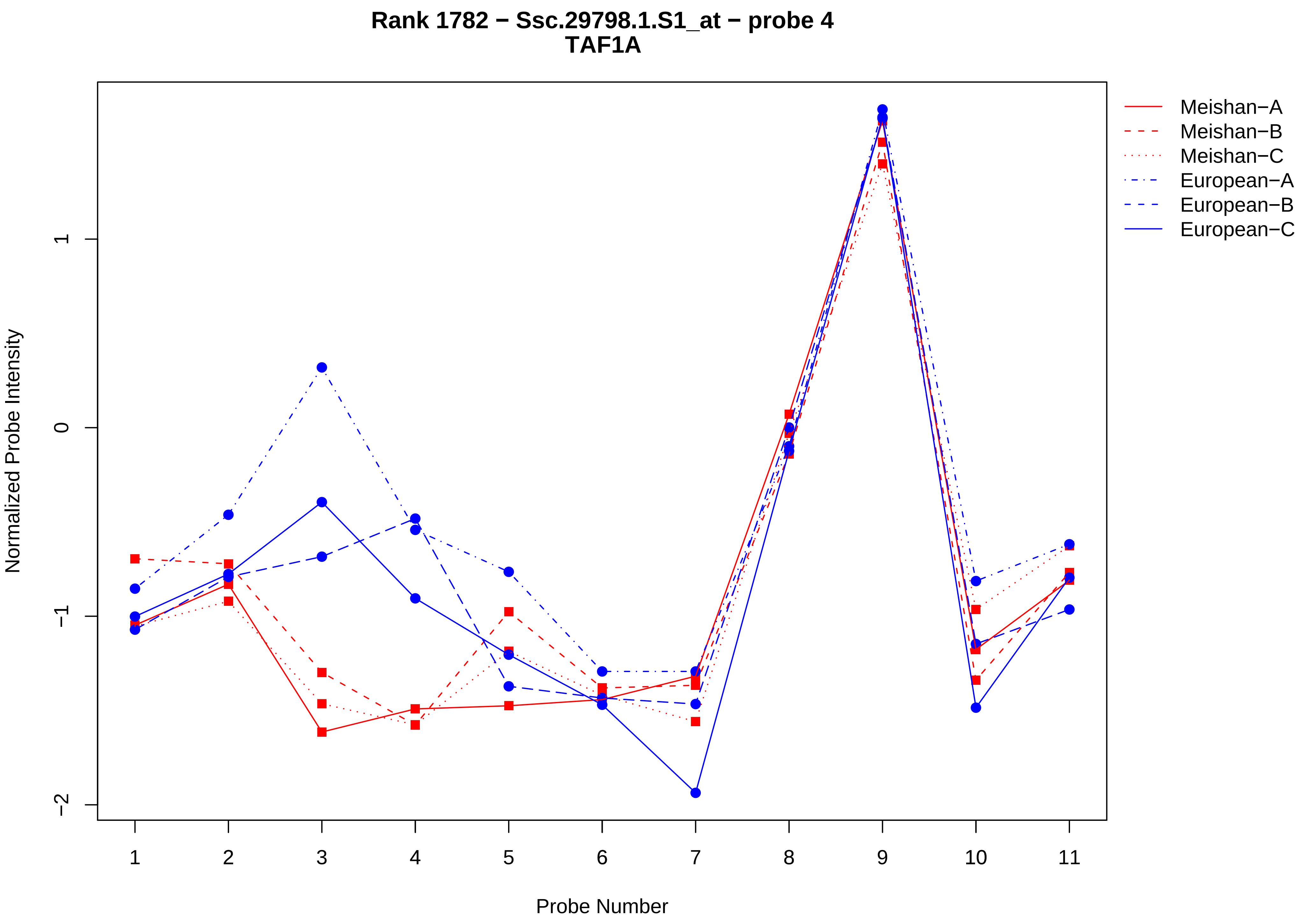

Rank 1782 − Ssc.29798.1.S1_at − probe 4
TAF1A
Normalized Probe Intensity
−2
−1
0
1
1
2
3
4
5
6
7
8
9
10
11
Probe Number
Meishan−A
Meishan−B
Meishan−C
European−A
European−B
European−C

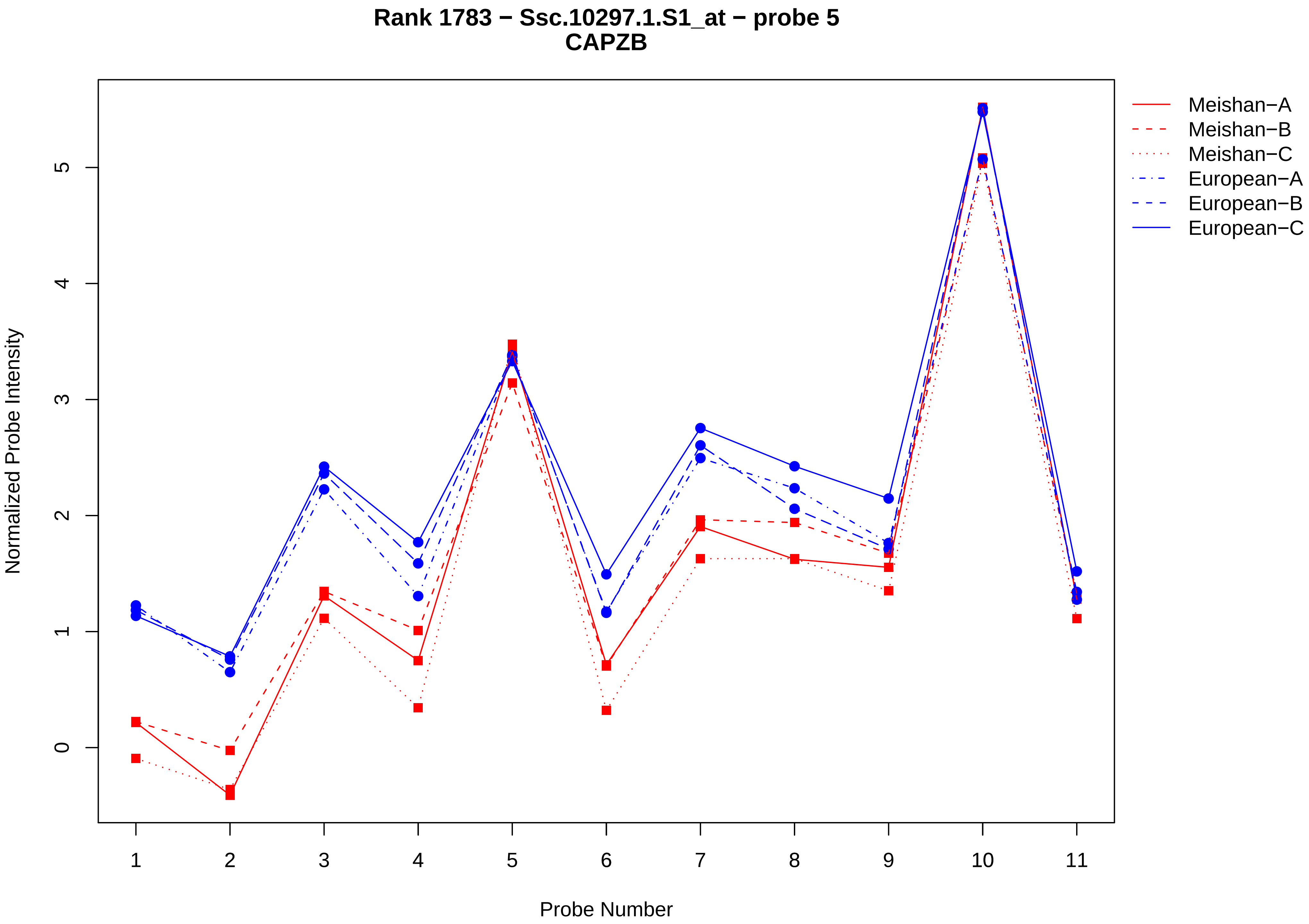

Rank 1783 − Ssc.10297.1.S1_at − probe 5
CAPZB
Normalized Probe Intensity
Probe Number
Meishan−A
Meishan−B
Meishan−C
European−A
European−B
European−C
0
1
2
3
4
5
1
2
3
4
5
6
7
8
9
10
11

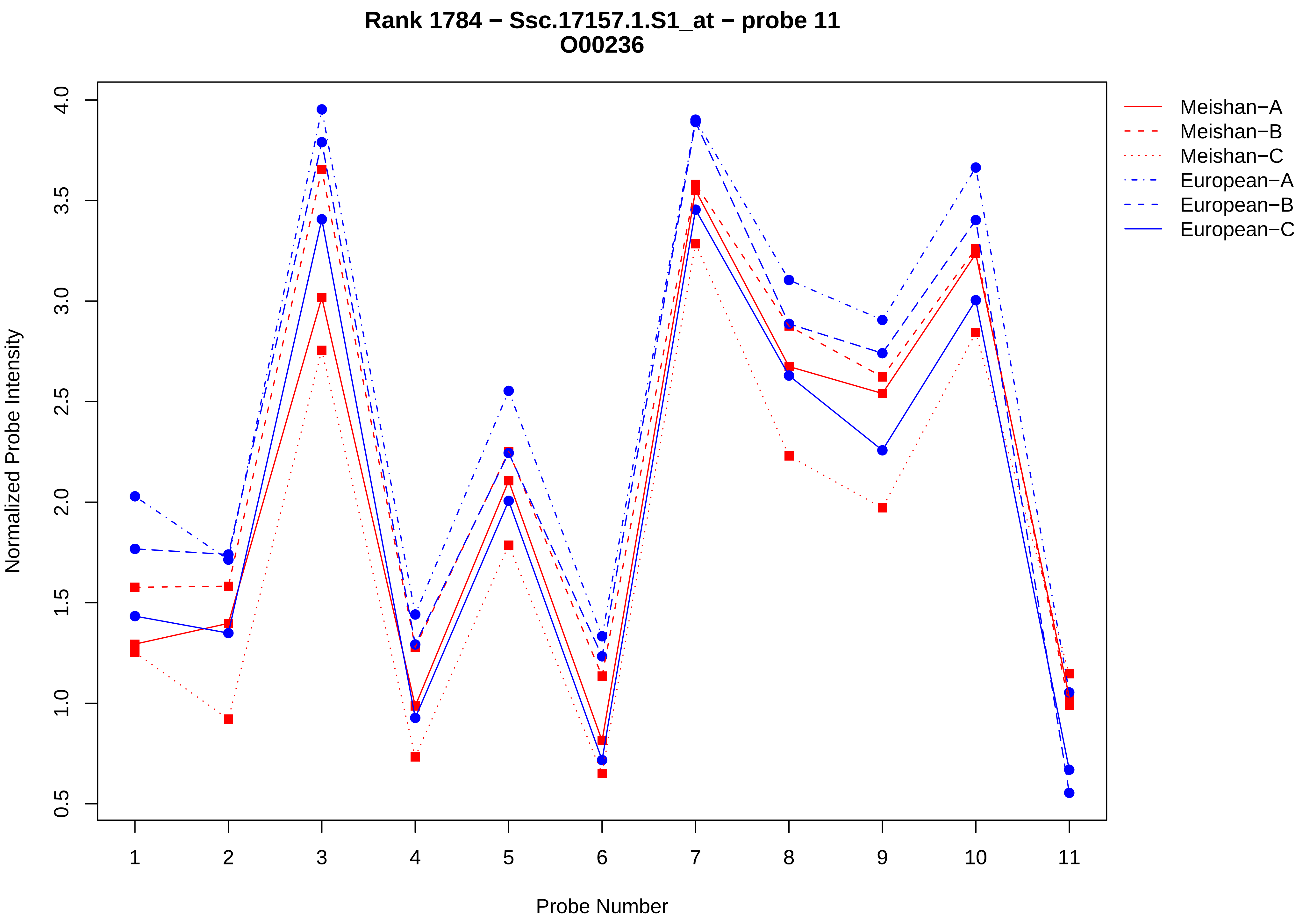
Rank 1784 − Ssc.17157.1.S1_at − probe 11
O00236
Normalized Probe Intensity
0.5
1.0
1.5
2.0
2.5
3.0
3.5
4.0
1
2
3
4
5
6
7
8
9
10
11
Probe Number
Meishan−A
Meishan−B
Meishan−C
European−A
European−B
European−C

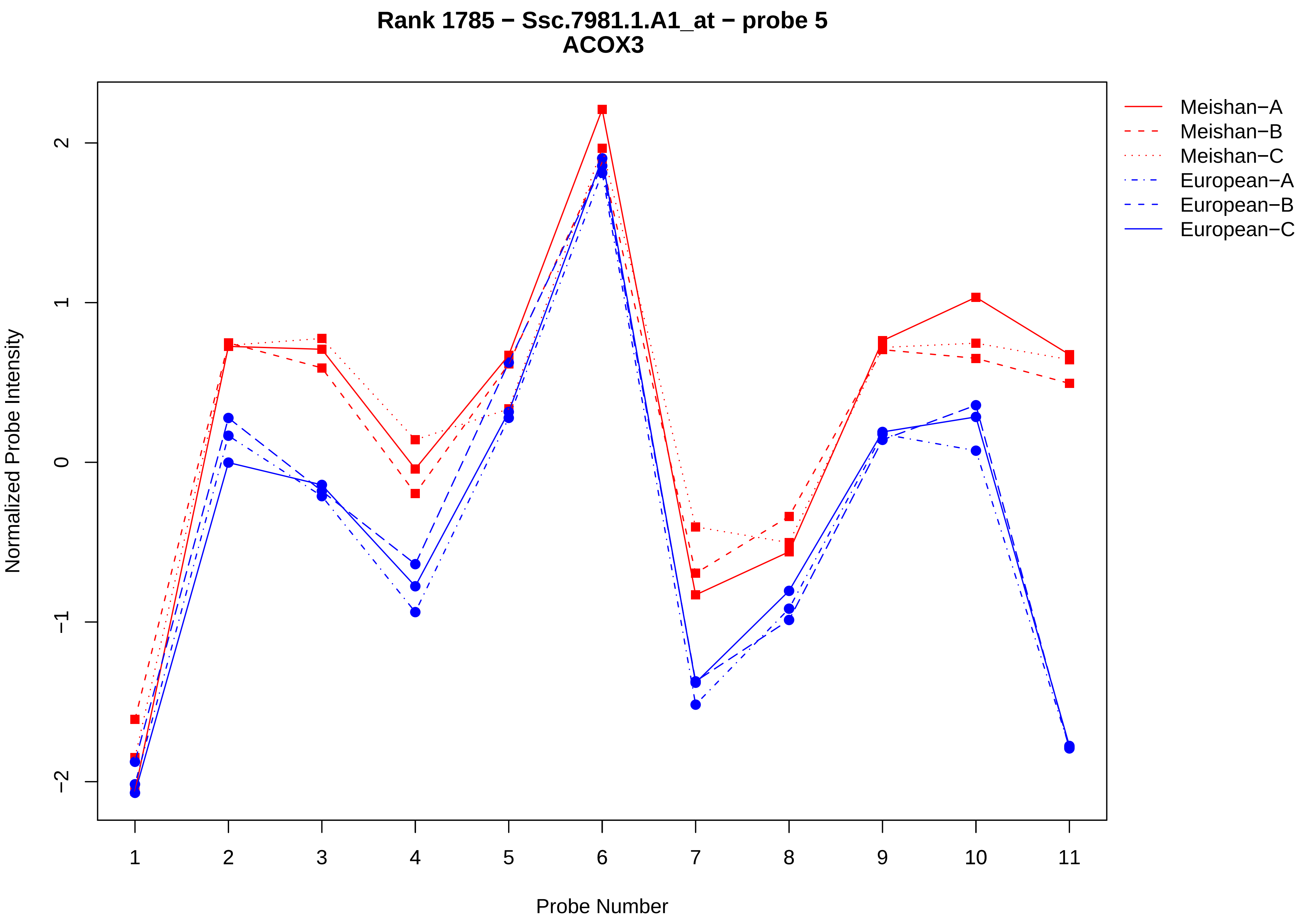
Rank 1785 − Ssc.7981.1.A1_at − probe 5
ACOX3
Normalized Probe Intensity
Probe Number
Meishan−A
Meishan−B
Meishan−C
European−A
European−B
European−C
−2
−1
0
1
2
1
2
3
4
5
6
7
8
9
10
11

Rank 1786 − Ssc.13661.1.A1_at − probe 9
Q96BU1

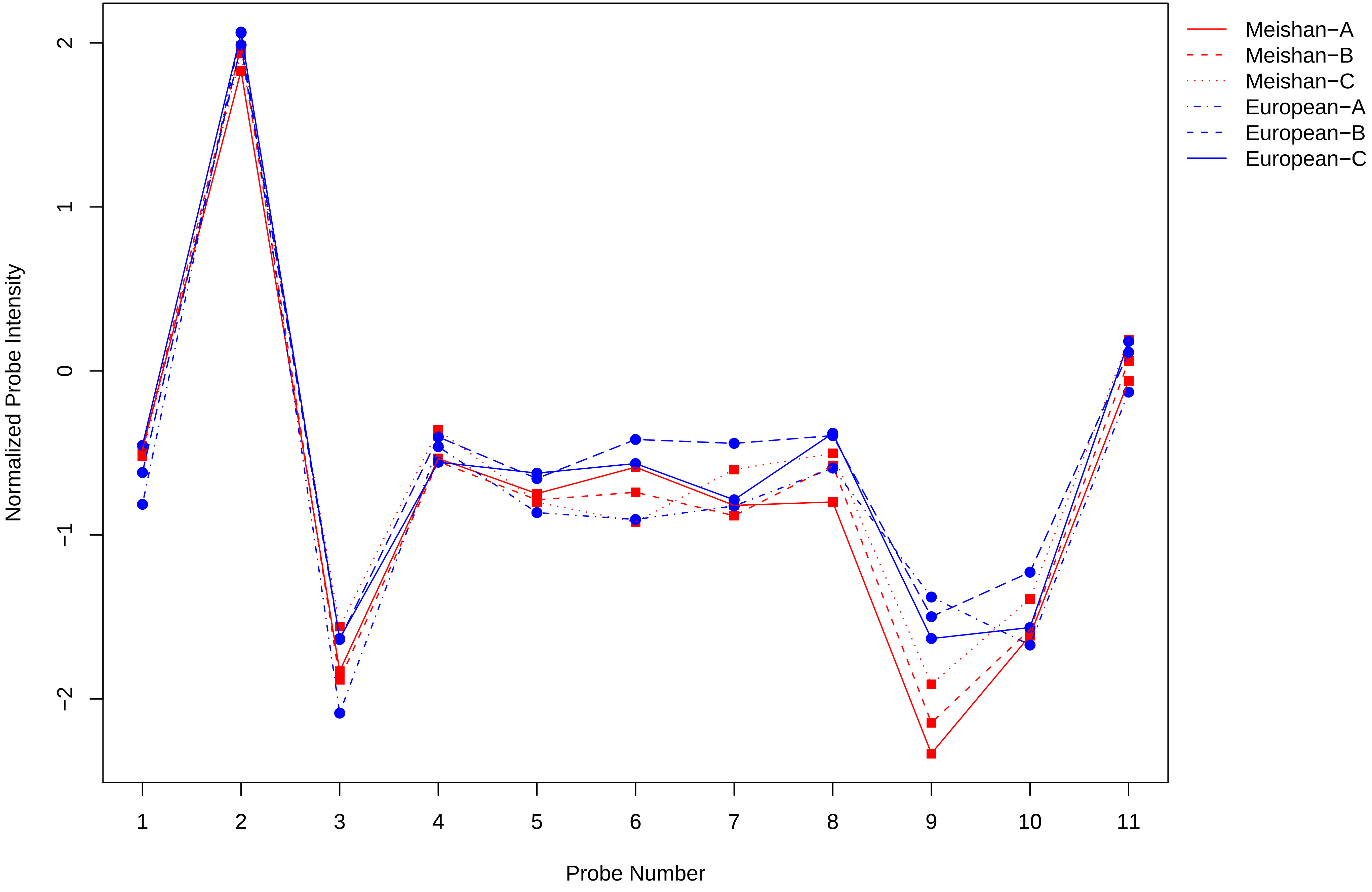

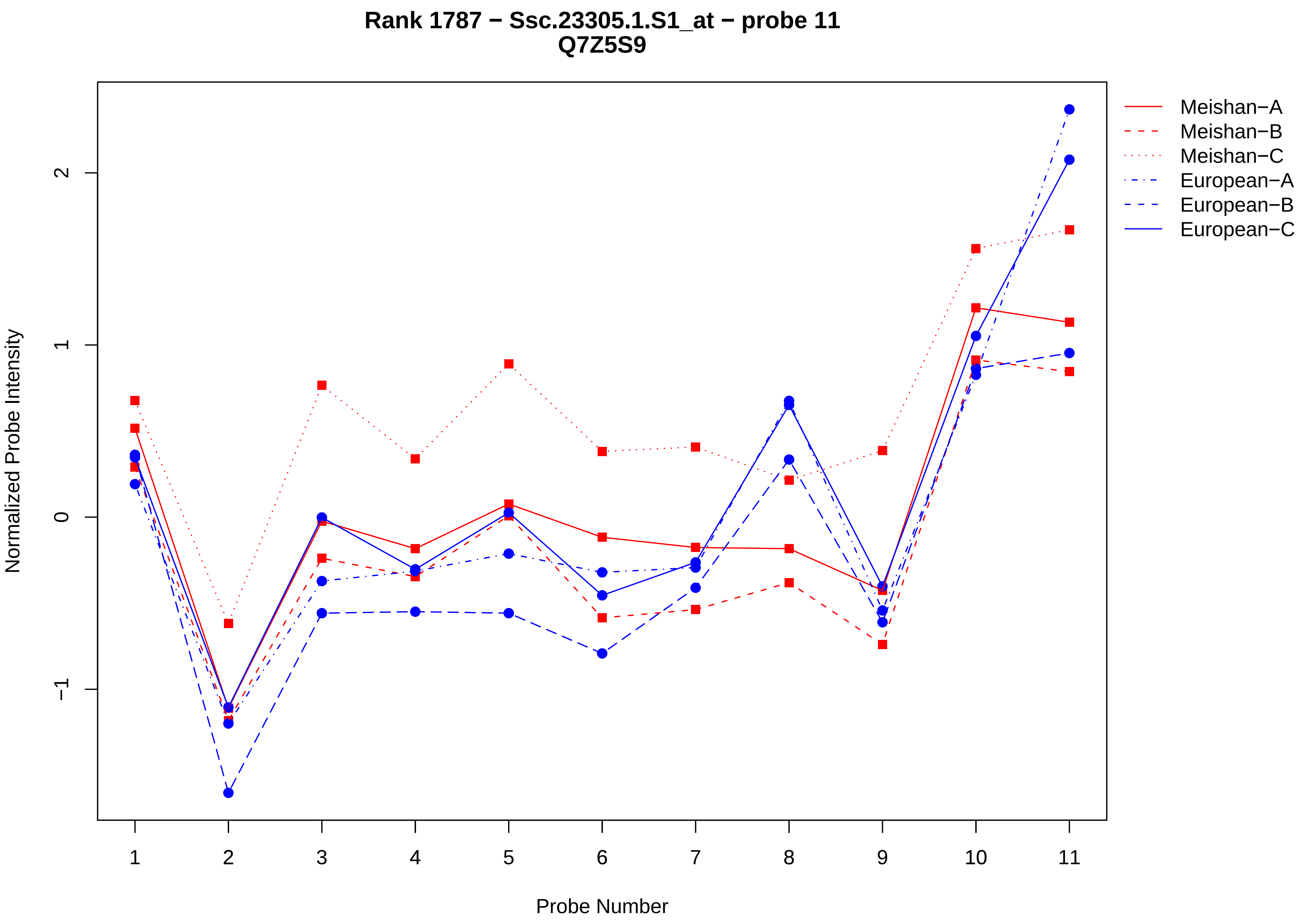

Rank 1787 − Ssc.23305.1.S1_at − probe 11
Q7Z5S9
Normalized Probe Intensity
−1
0
1
2
1
2
3
4
5
6
7
8
9
10
11
Probe Number
Meishan−A
Meishan−B
Meishan−C
European−A
European−B
European−C

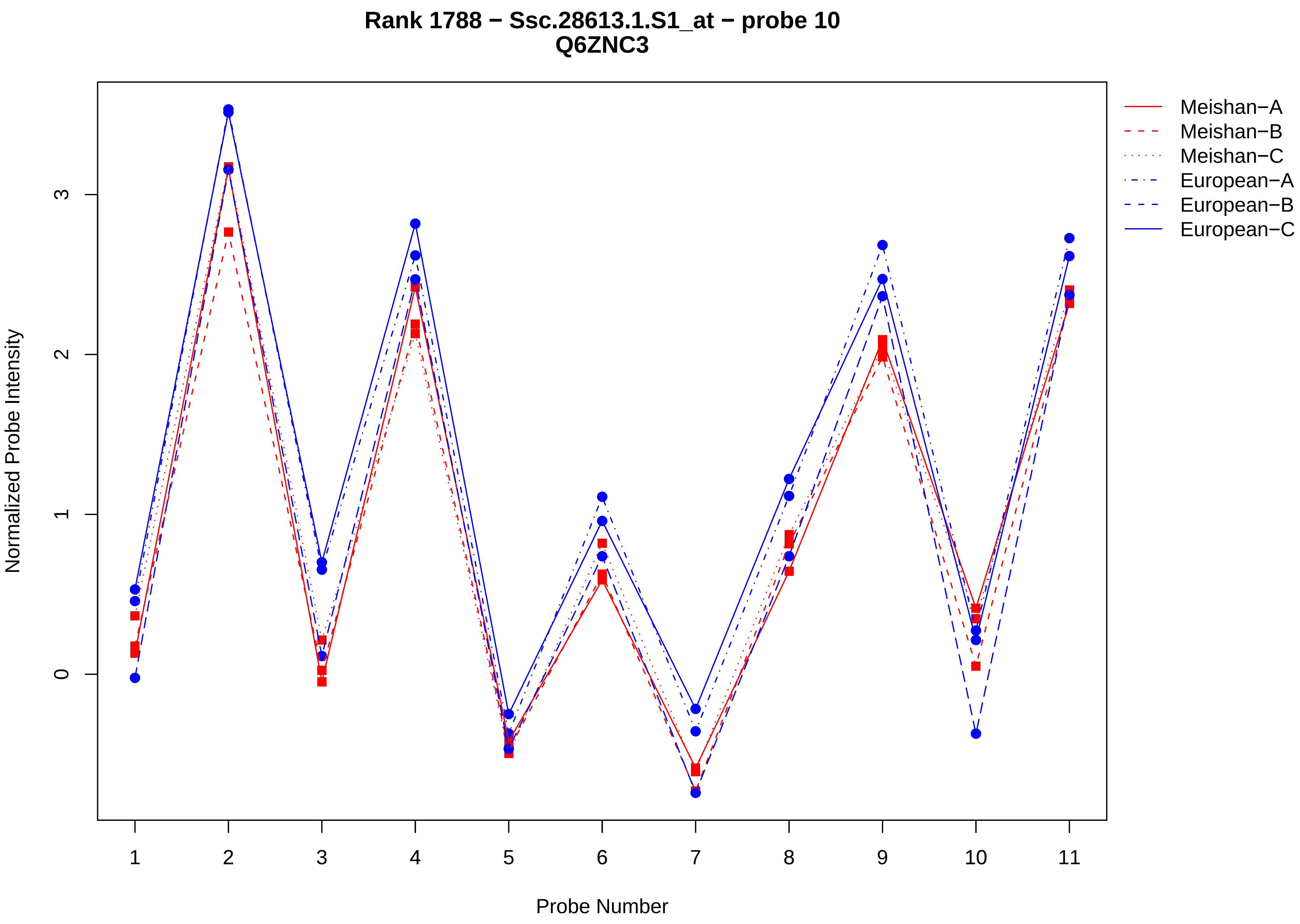

Rank 1788 − Ssc.28613.1.S1_at − probe 10
Q6ZNC3
Normalized Probe Intensity
0
1
2
3
1
2
3
4
5
6
7
8
9
10
11
Probe Number
Meishan−A
Meishan−B
Meishan−C
European−A
European−B
European−C

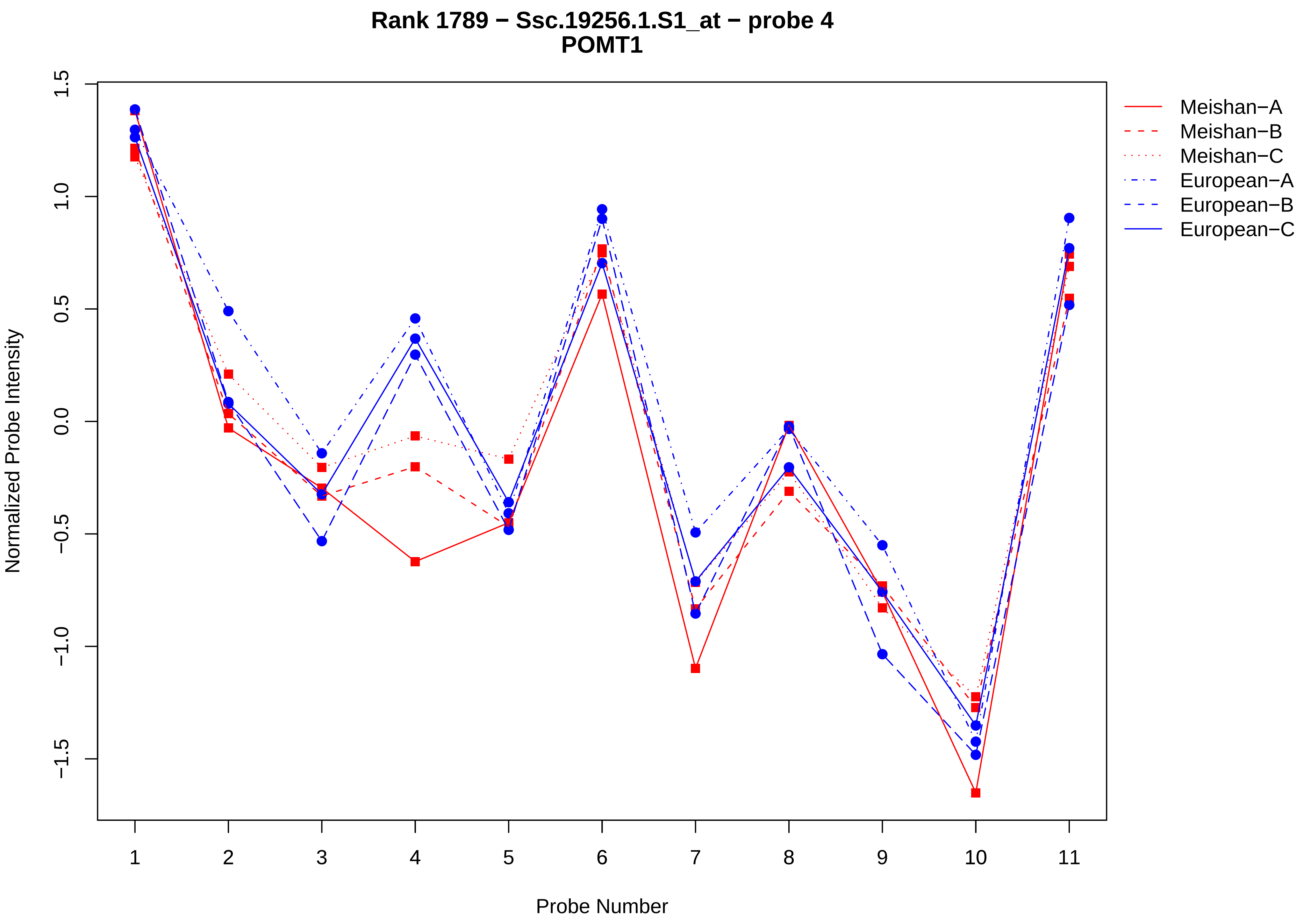

Rank 1789 − Ssc.19256.1.S1_at − probe 4
POMT1
Normalized Probe Intensity
Probe Number
1.5
1.0
0.5
0.0
−0.5
−1.0
−1.5
1
2
3
4
5
6
7
8
9
10
11
Meishan−A
Meishan−B
Meishan−C
European−A
European−B
European−C

Rank 1790 − Ssc.26449.3.S1_a_at − probe 11
Q9P0N5

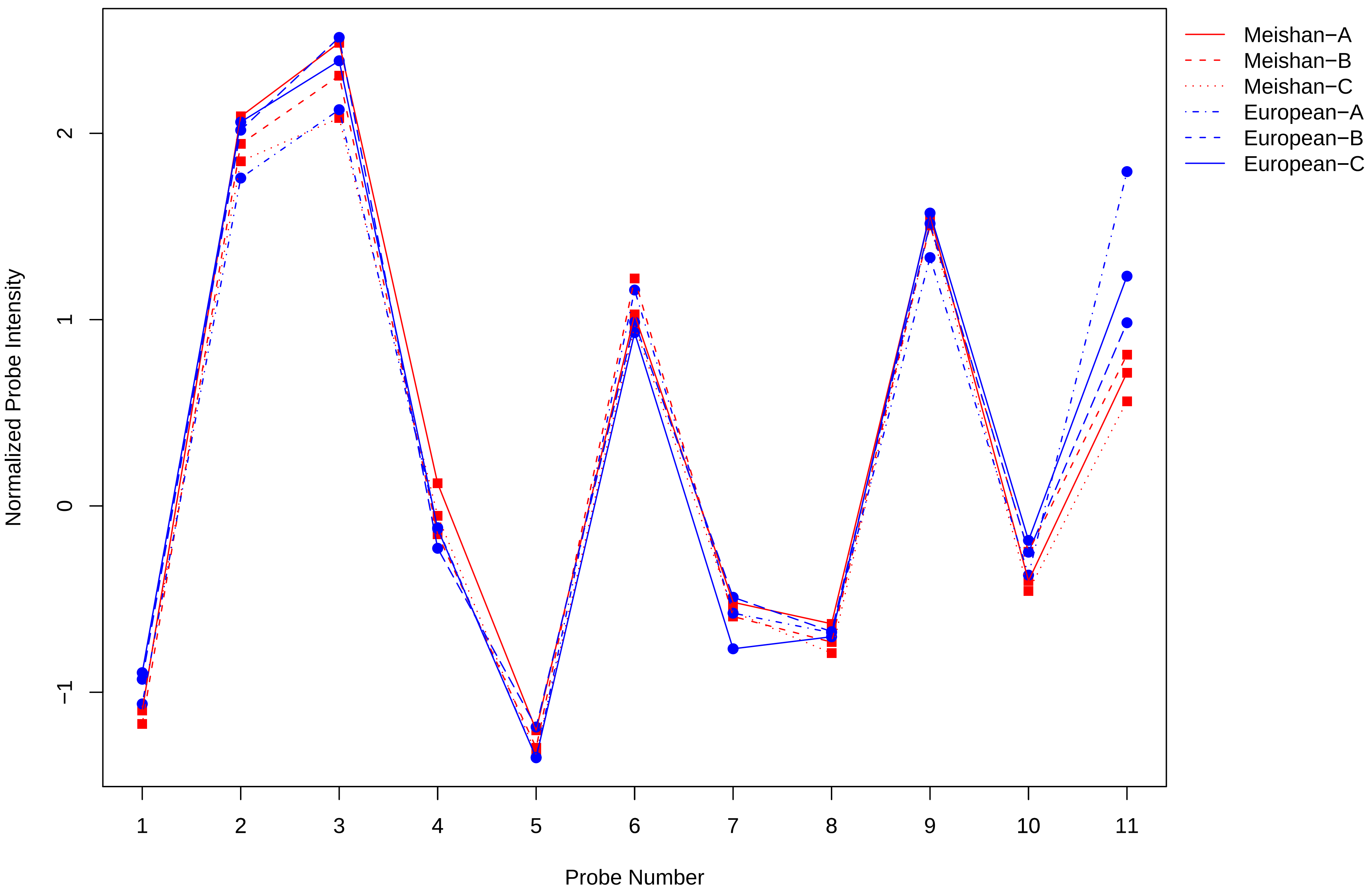

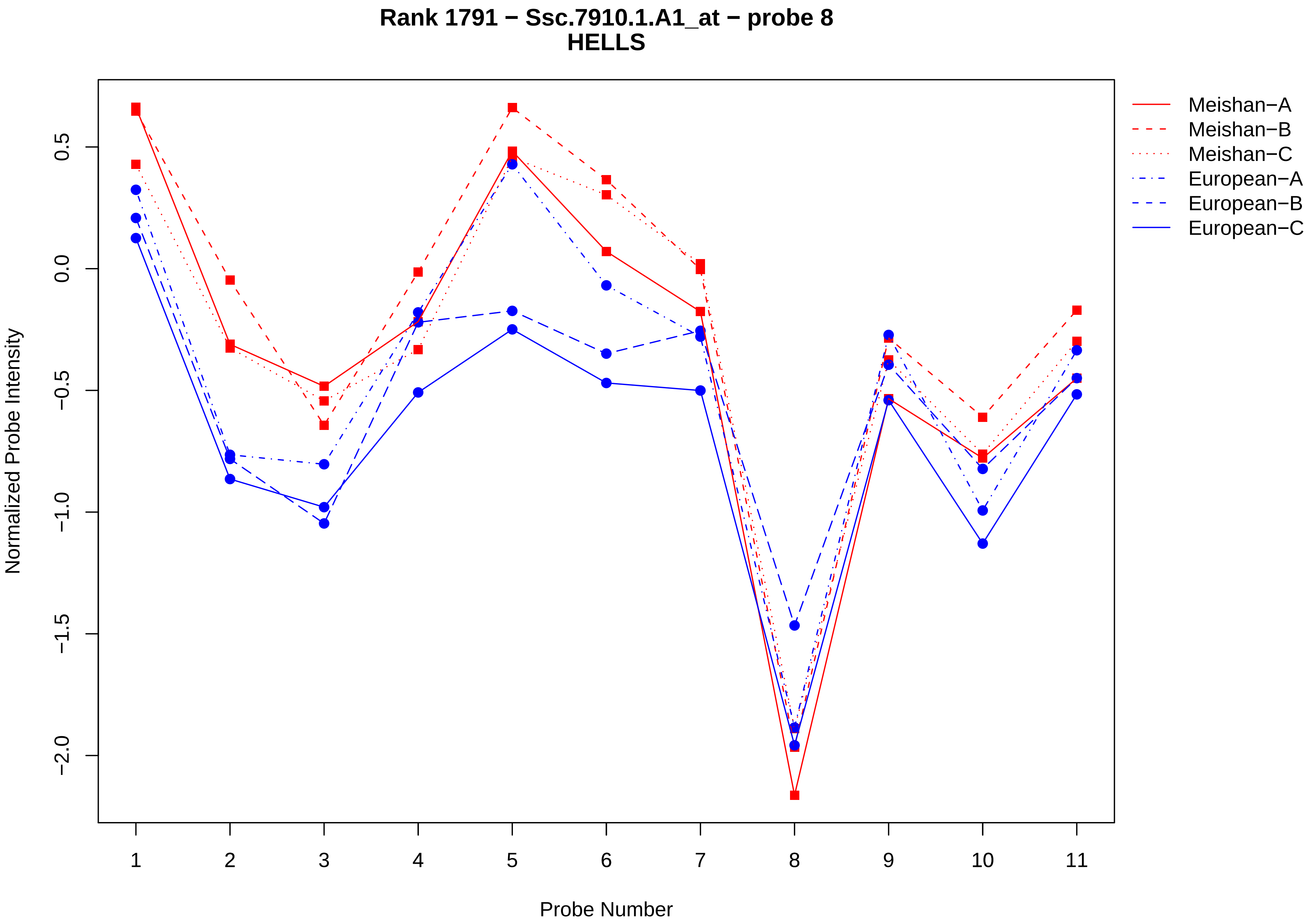

Rank 1791 − Ssc.7910.1.A1_at − probe 8
HELLS
Normalized Probe Intensity
Probe Number
0.5
0.0
−0.5
−1.0
−1.5
−2.0
1
2
3
4
5
6
7
8
9
10
11
Meishan−A
Meishan−B
Meishan−C
European−A
European−B
European−C

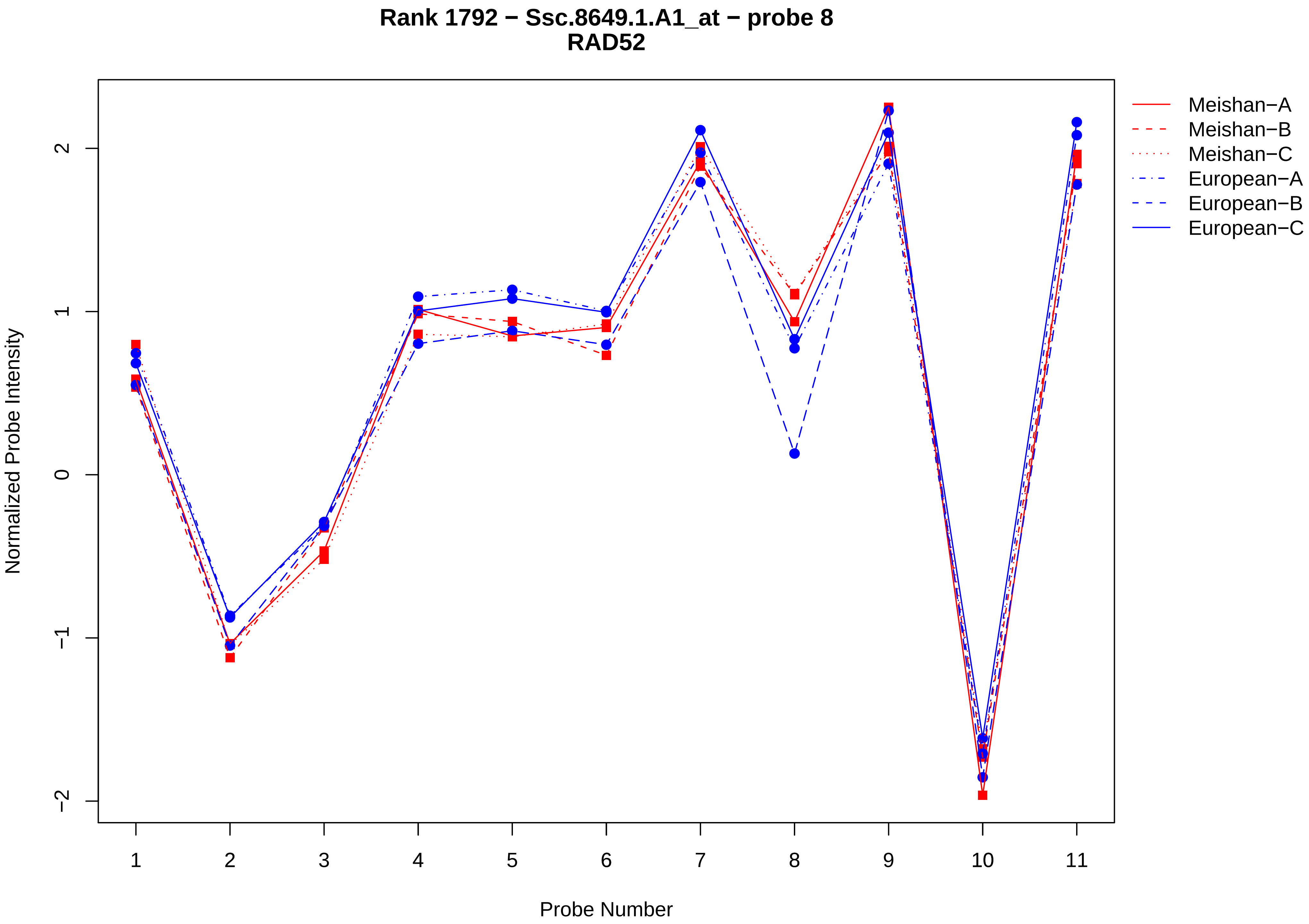

Rank 1792 − Ssc.8649.1.A1_at − probe 8
RAD52
Normalized Probe Intensity
Probe Number
−2
−1
0
1
2
1
2
3
4
5
6
7
8
9
10
11
Meishan−A
Meishan−B
Meishan−C
European−A
European−B
European−C

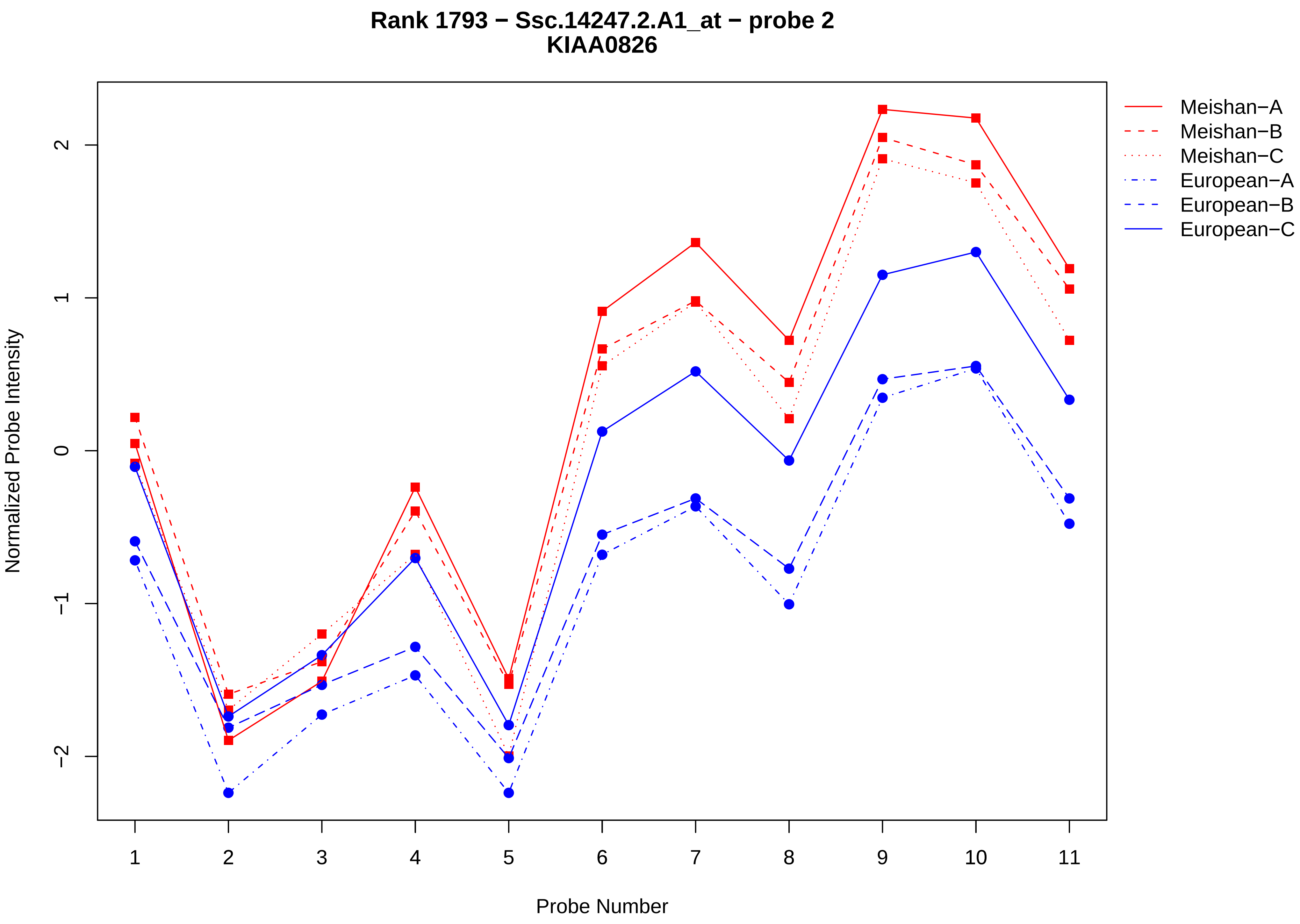

Rank 1793 − Ssc.14247.2.A1_at − probe 2
KIAA0826
Normalized Probe Intensity
Probe Number
−2
−1
0
1
2
1
2
3
4
5
6
7
8
9
10
11
Meishan−A
Meishan−B
Meishan−C
European−A
European−B
European−C

Rank 1794 − Ssc.26850.1.S1_at − probe 5
NP_065161

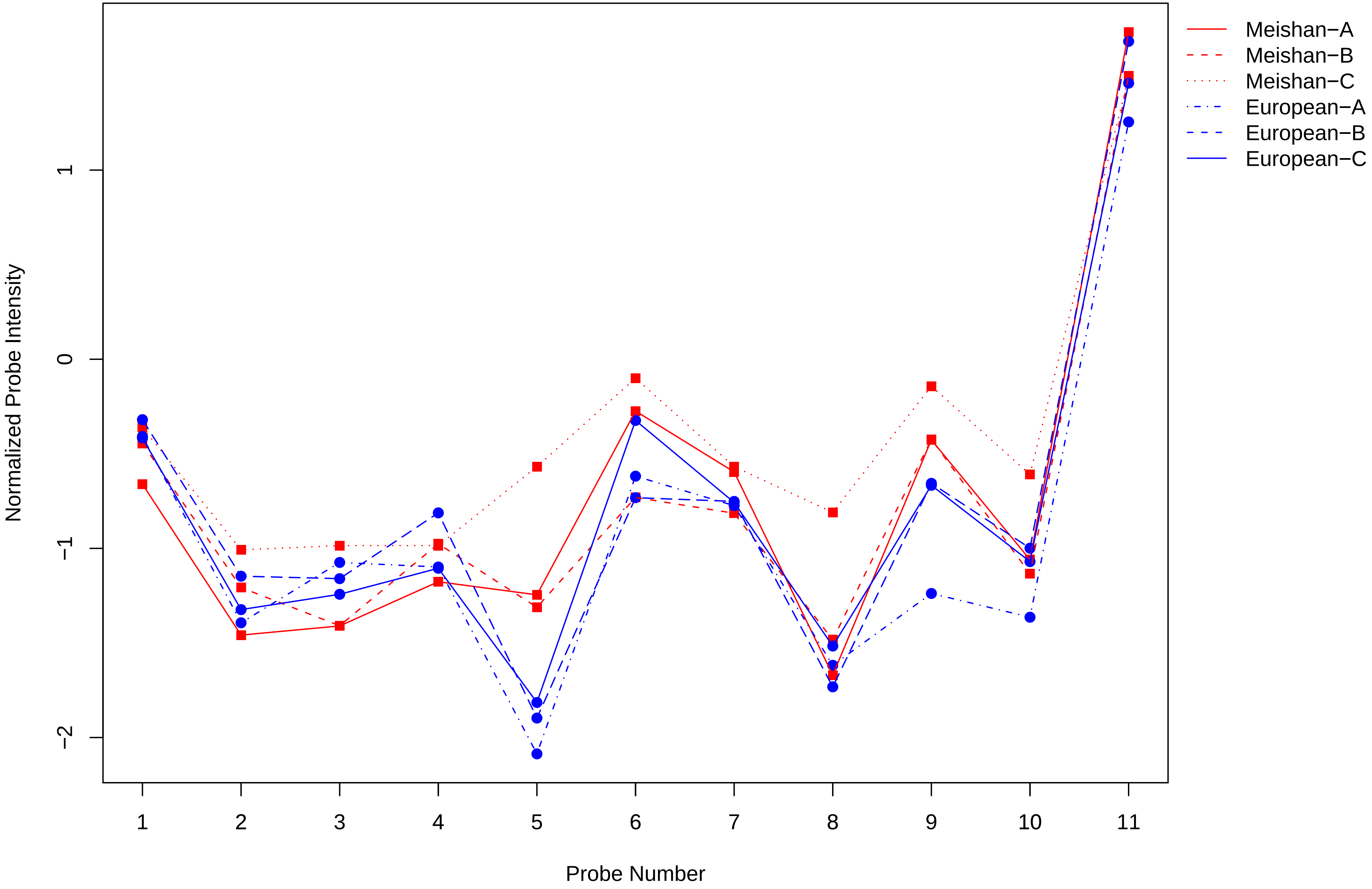

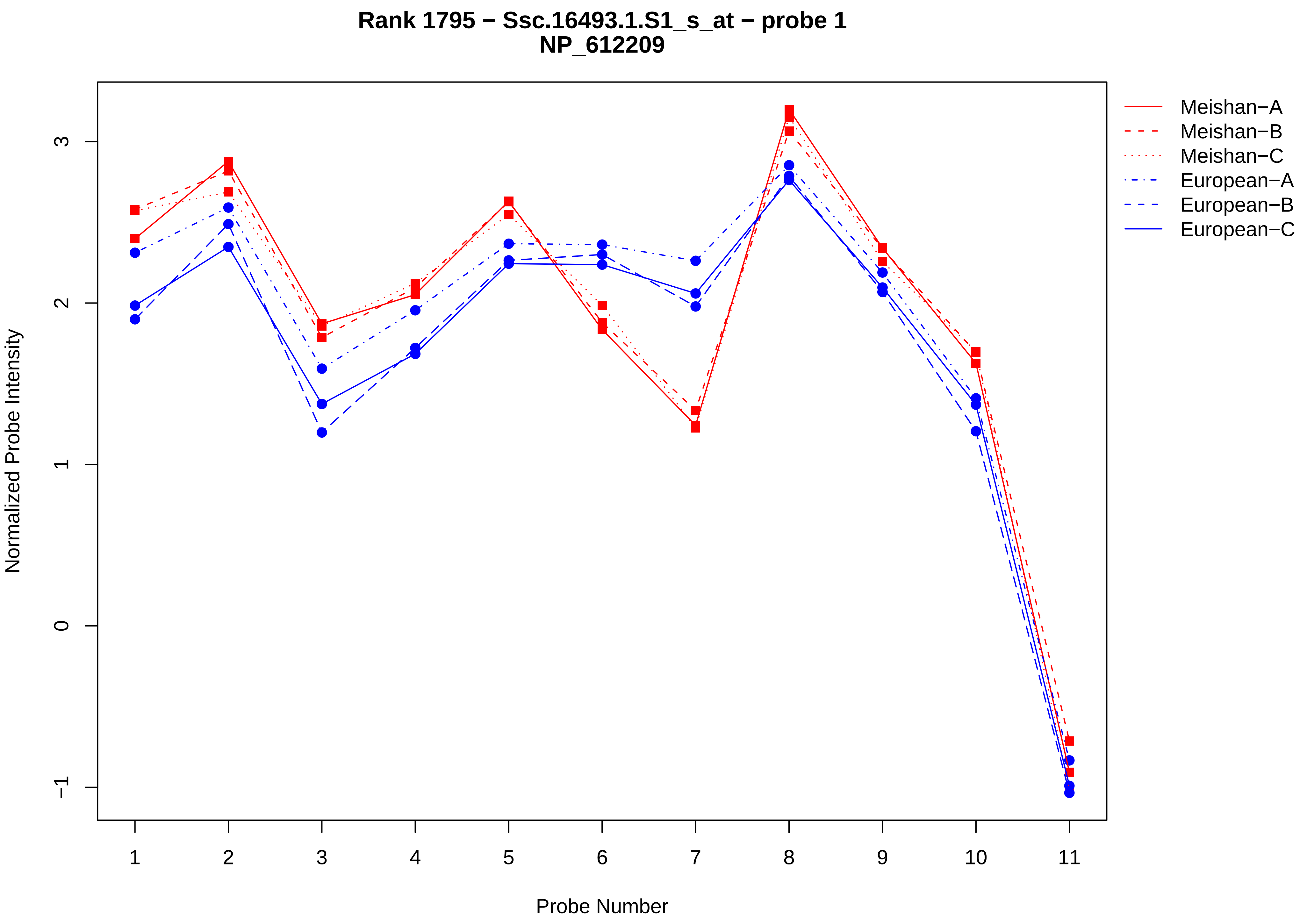

Rank 1795 − Ssc.16493.1.S1_s_at − probe 1
NP_612209
Normalized Probe Intensity
Probe Number
−1
0
1
2
3
1
2
3
4
5
6
7
8
9
10
11
Meishan−A
Meishan−B
Meishan−C
European−A
European−B
European−C

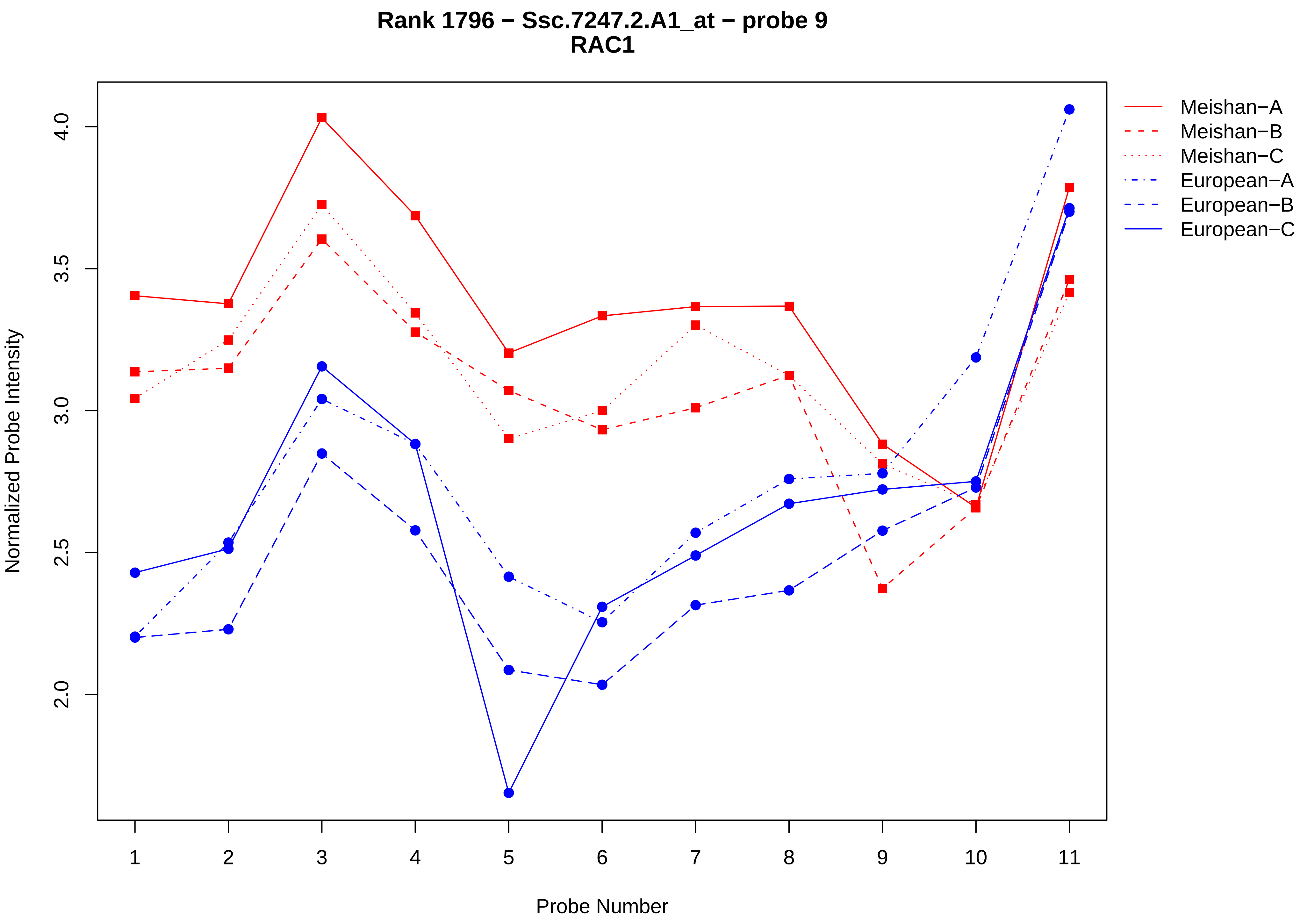

Rank 1796 − Ssc.7247.2.A1_at − probe 9
RAC1
Normalized Probe Intensity
2.0
2.5
3.0
3.5
4.0
1
2
3
4
5
6
7
8
9
10
11
Probe Number
Meishan−A
Meishan−B
Meishan−C
European−A
European−B
European−C

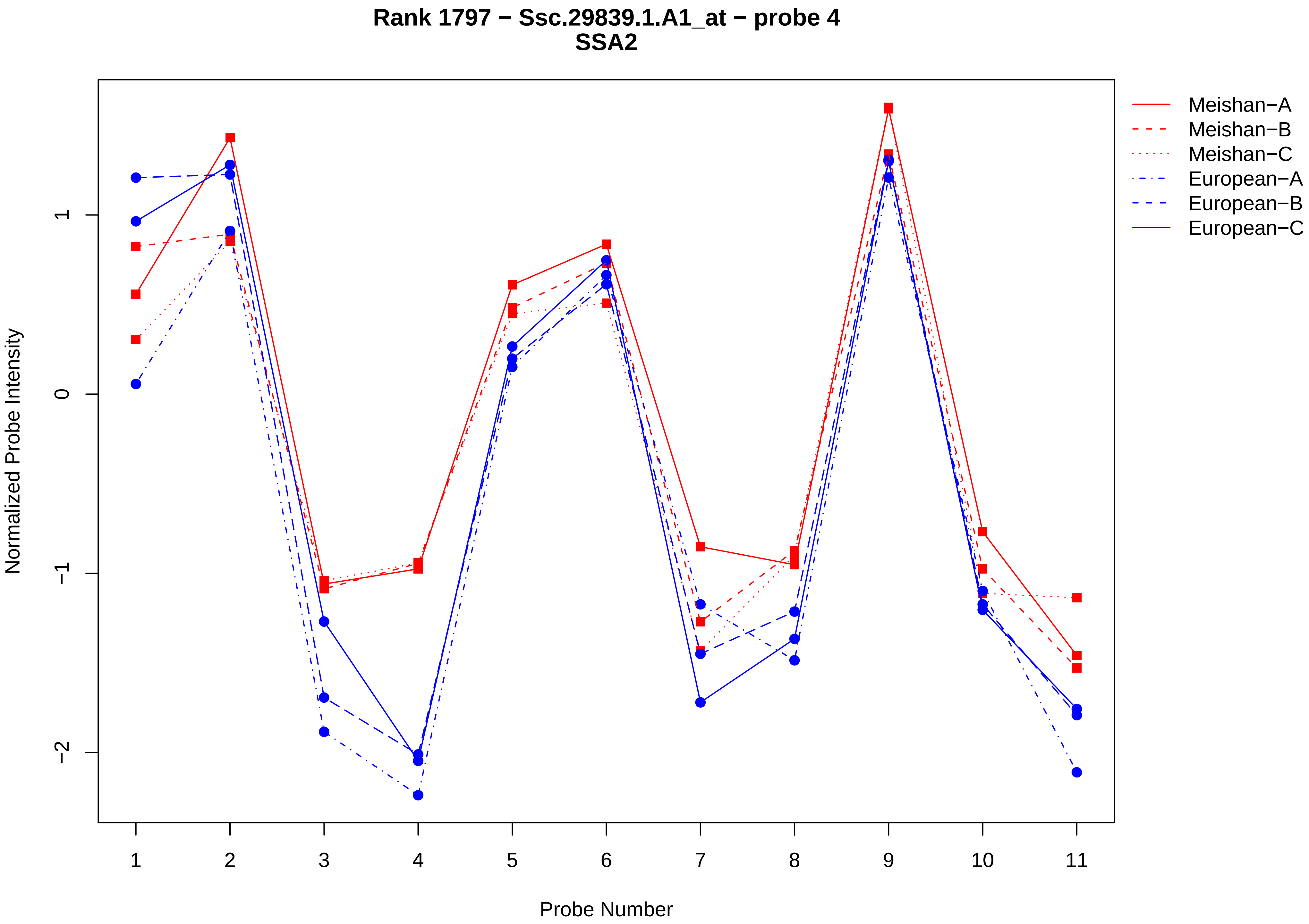

Rank 1797 − Ssc.29839.1.A1_at − probe 4
SSA2
Normalized Probe Intensity
Probe Number
1
2
3
4
5
6
7
8
9
10
11
−2
−1
0
1
Meishan−A
Meishan−B
Meishan−C
European−A
European−B
European−C

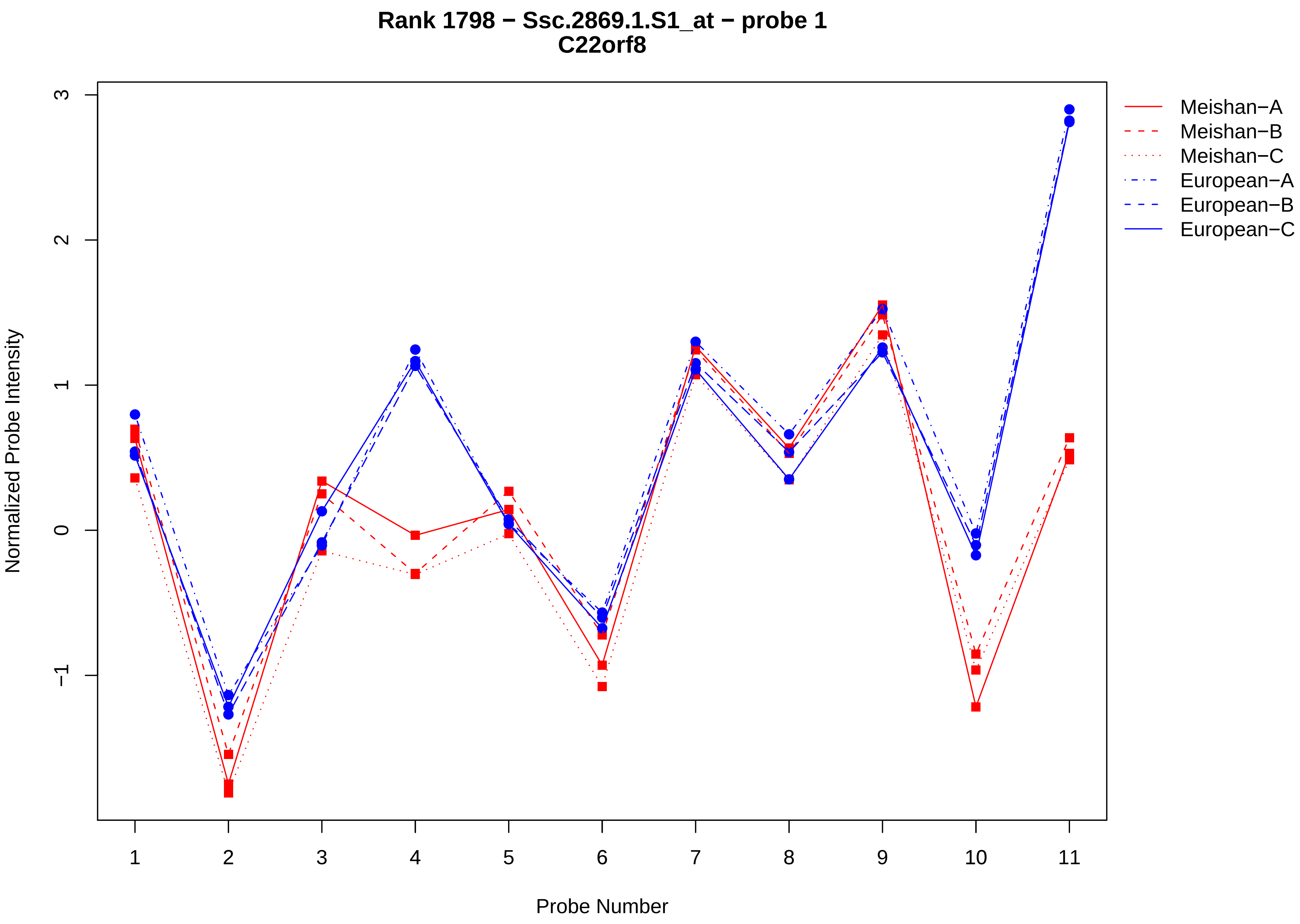
Rank 1798 − Ssc.2869.1.S1_at − probe 1
C22orf8
Normalized Probe Intensity
Probe Number
Meishan−A
Meishan−B
Meishan−C
European−A
European−B
European−C
−1
0
1
2
3
1
2
3
4
5
6
7
8
9
10
11

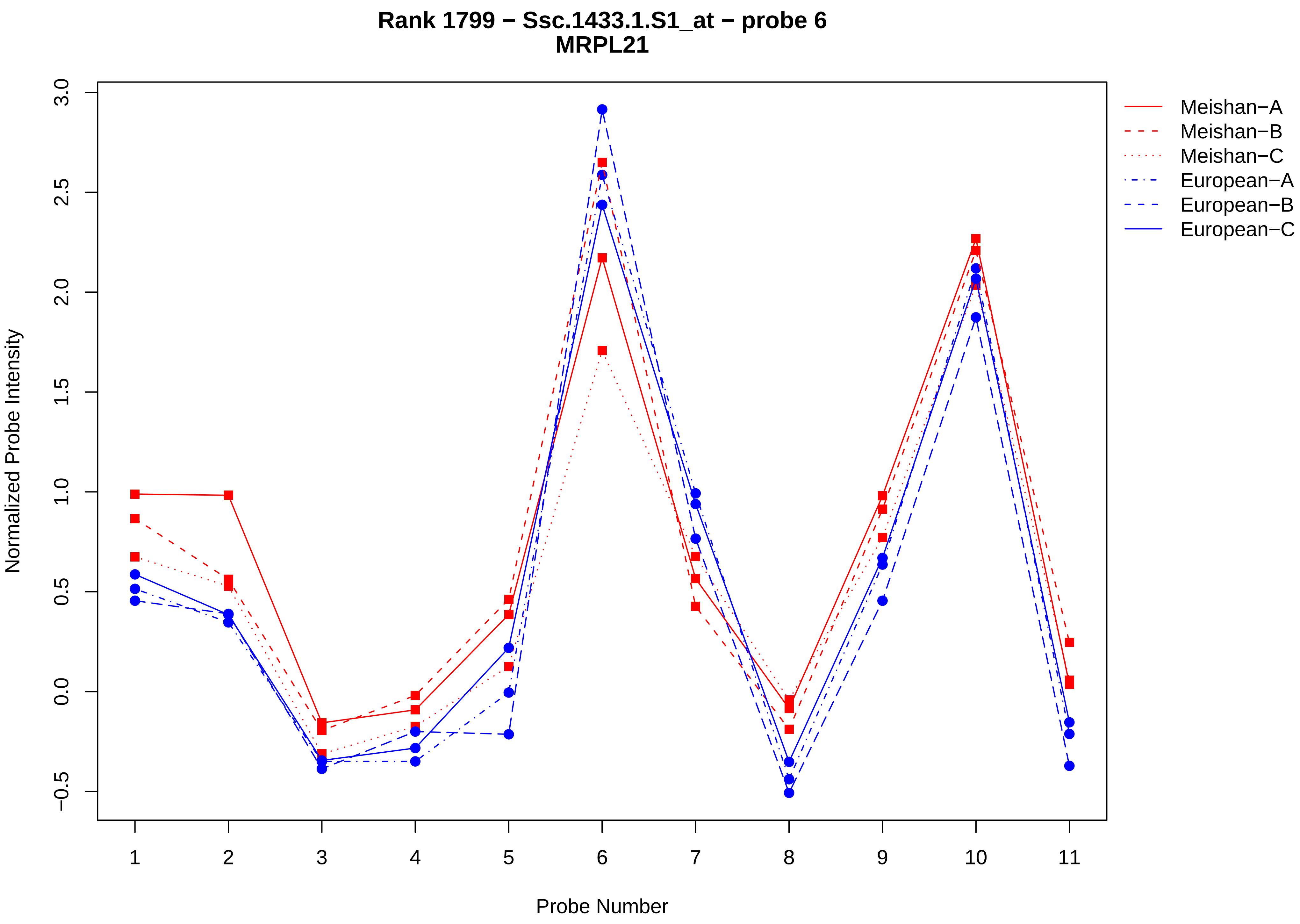

Rank 1799 − Ssc.1433.1.S1_at − probe 6
MRPL21
Normalized Probe Intensity
Probe Number
Meishan−A
Meishan−B
Meishan−C
European−A
European−B
European−C

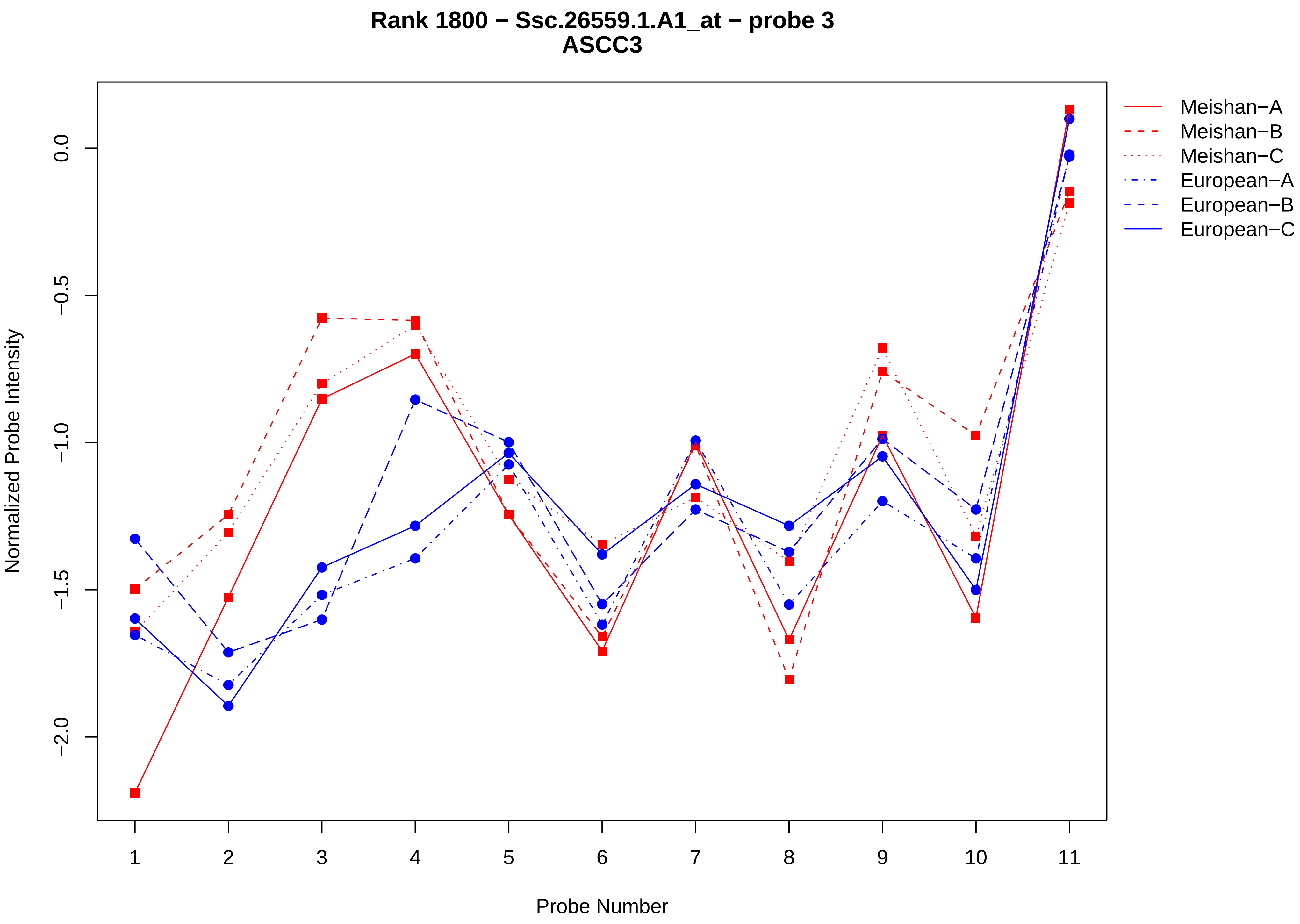

Rank 1800 − Ssc.26559.1.A1_at − probe 3
ASCC3
Normalized Probe Intensity
Probe Number
0.0
−0.5
−1.0
−1.5
−2.0
1
2
3
4
5
6
7
8
9
10
11
Meishan−A
Meishan−B
Meishan−C
European−A
European−B
European−C

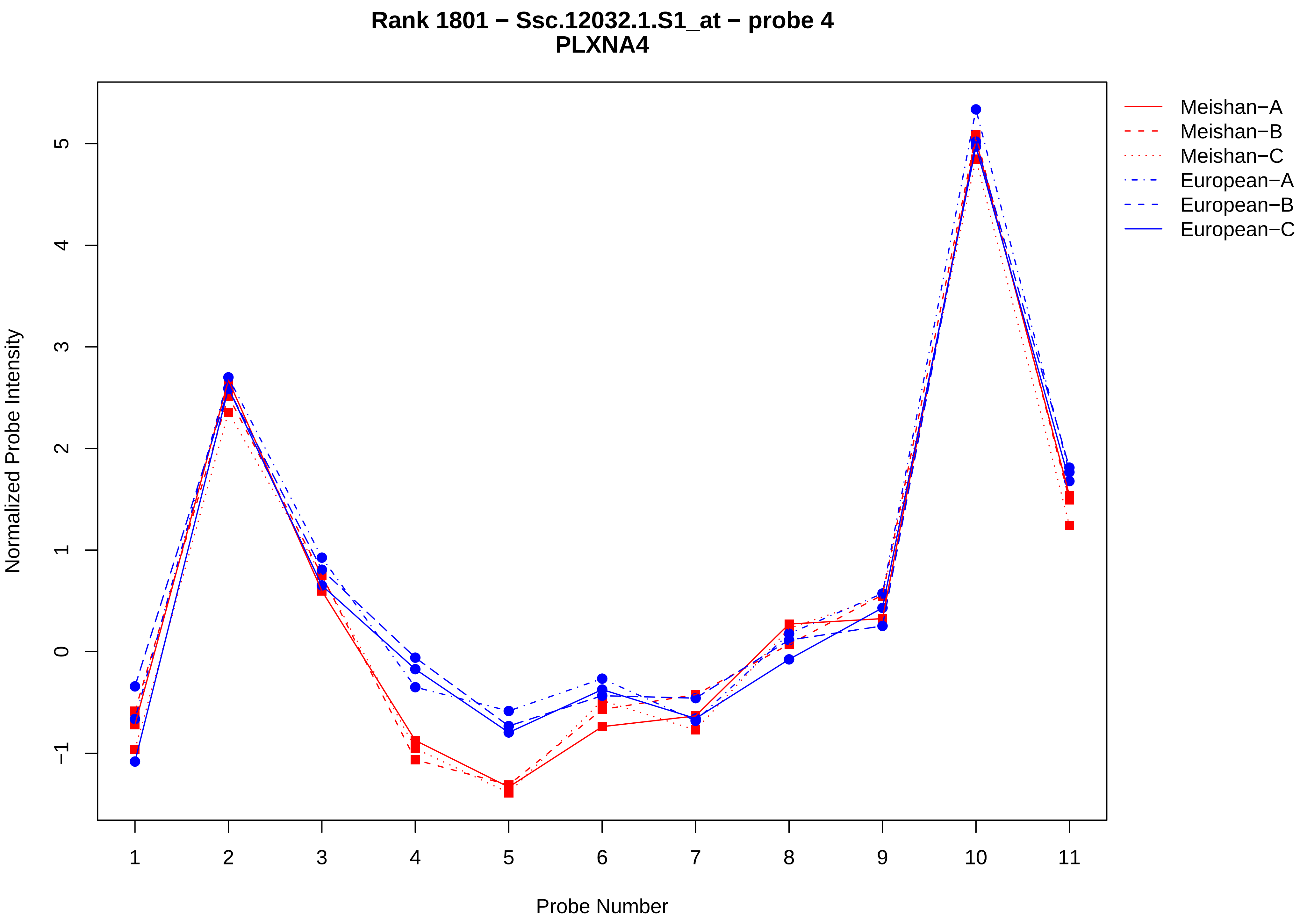

Rank 1801 − Ssc.12032.1.S1_at − probe 4
PLXNA4
Normalized Probe Intensity
Probe Number
−1
0
1
2
3
4
5
1
2
3
4
5
6
7
8
9
10
11
Meishan−A
Meishan−B
Meishan−C
European−A
European−B
European−C

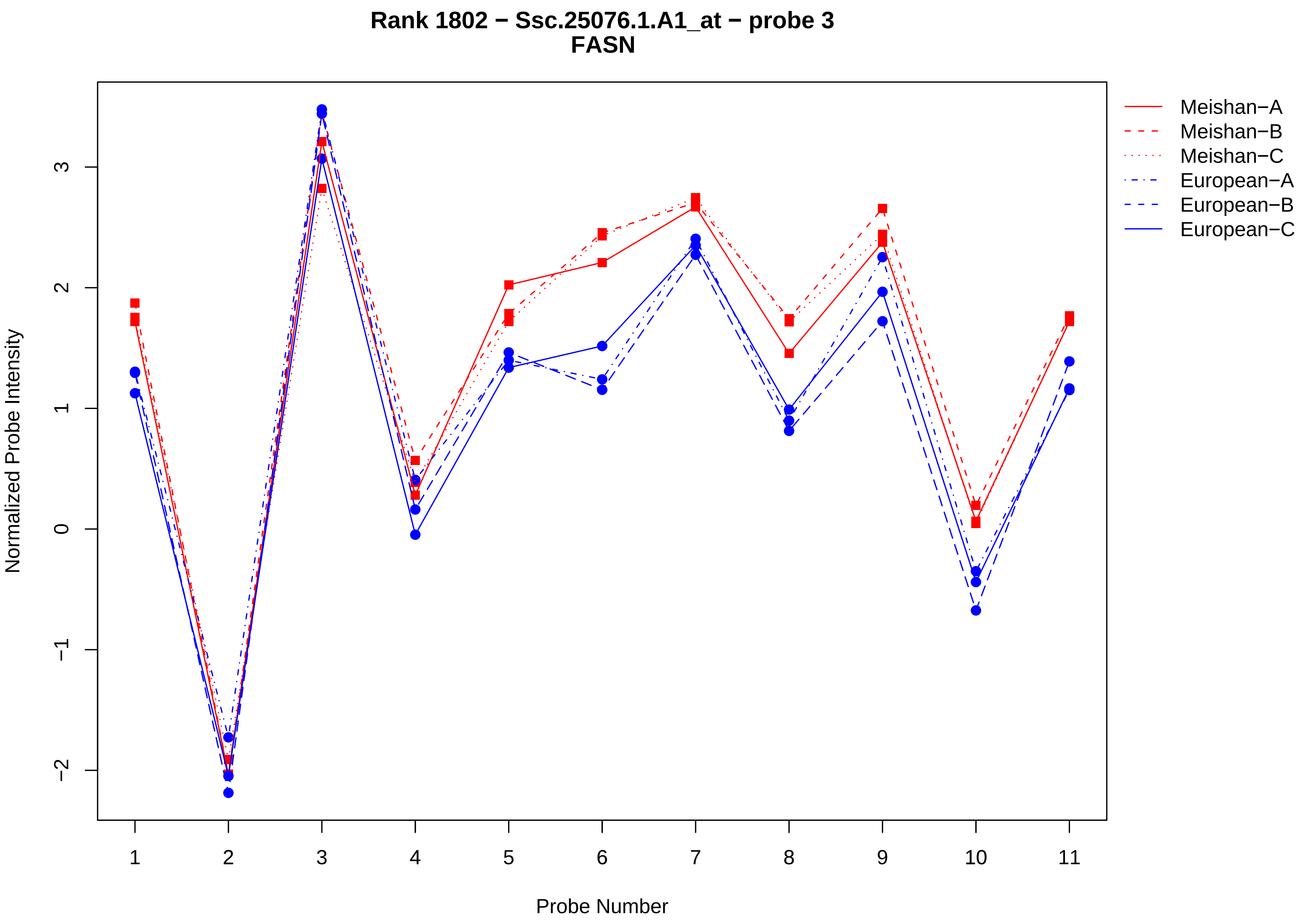

Rank 1802 − Ssc.25076.1.A1_at − probe 3
FASN
Normalized Probe Intensity
Probe Number
−2
−1
0
1
2
3
1
2
3
4
5
6
7
8
9
10
11
Meishan−A
Meishan−B
Meishan−C
European−A
European−B
European−C

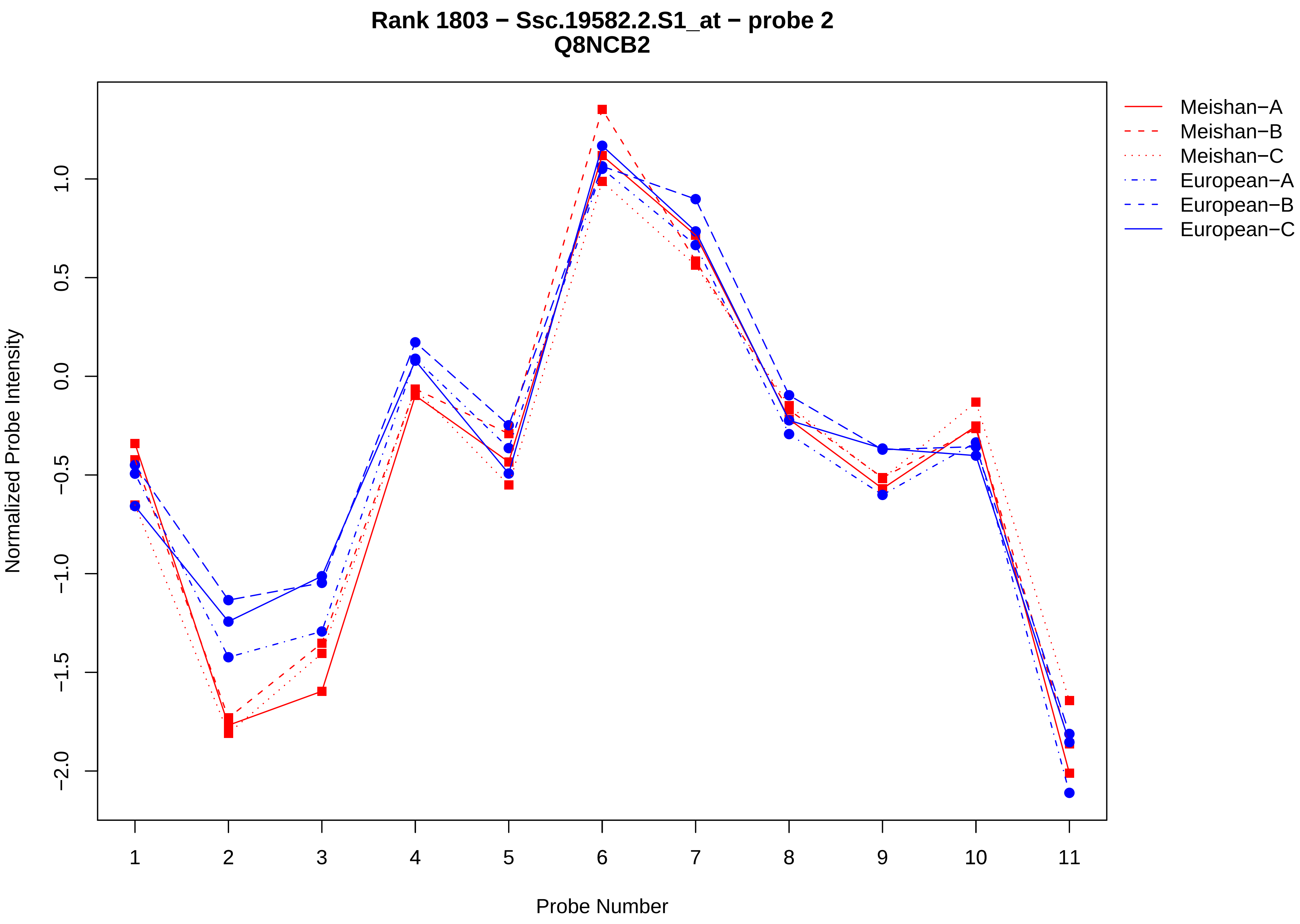

Rank 1803 − Ssc.19582.2.S1_at − probe 2
Q8NCB2
Normalized Probe Intensity
Probe Number
1.0
0.5
0.0
−0.5
−1.0
−1.5
−2.0
1
2
3
4
5
6
7
8
9
10
11
Meishan−A
Meishan−B
Meishan−C
European−A
European−B
European−C

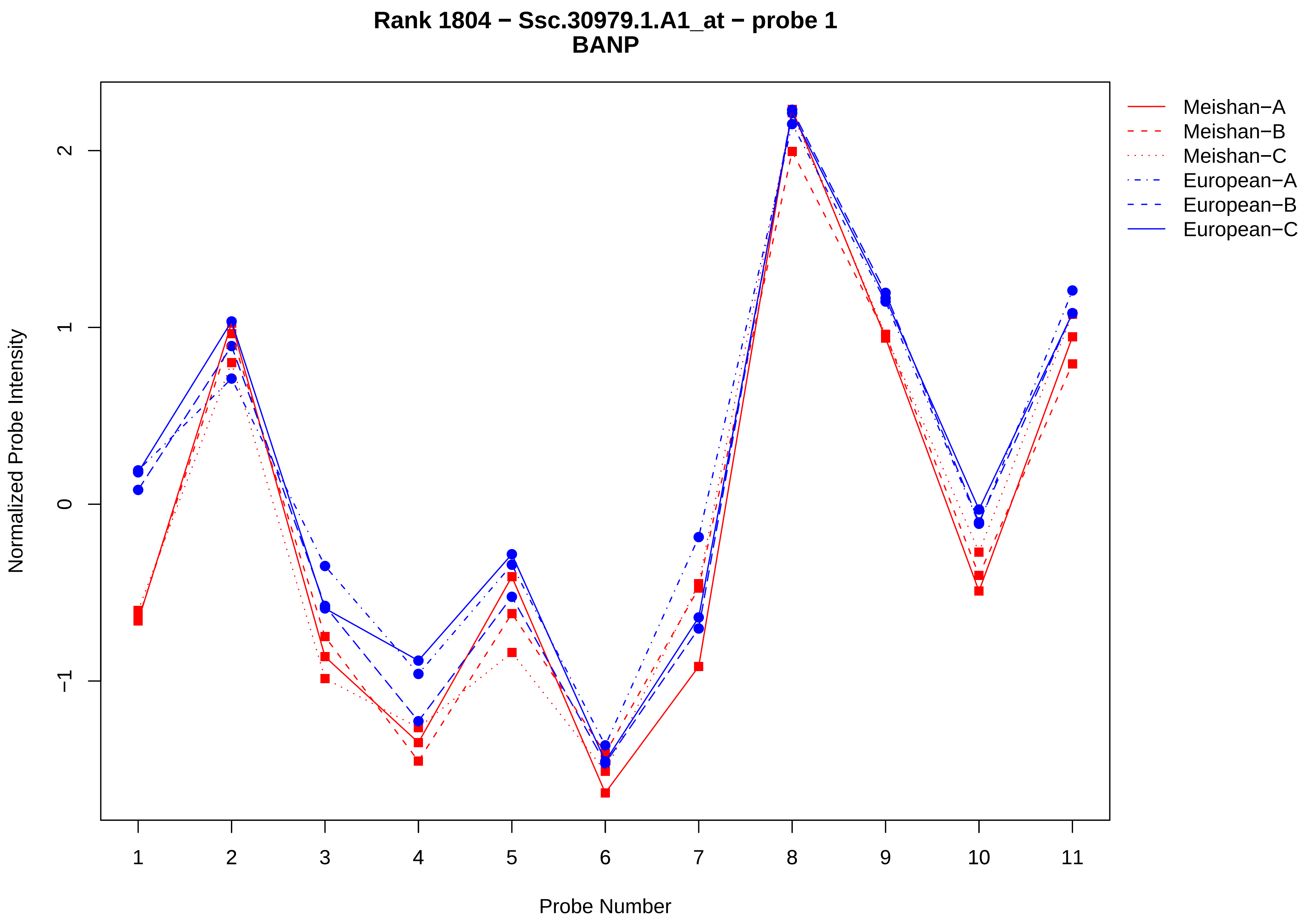

Rank 1804 − Ssc.30979.1.A1_at − probe 1
BANP
Normalized Probe Intensity
Probe Number
Meishan−A
Meishan−B
Meishan−C
European−A
European−B
European−C

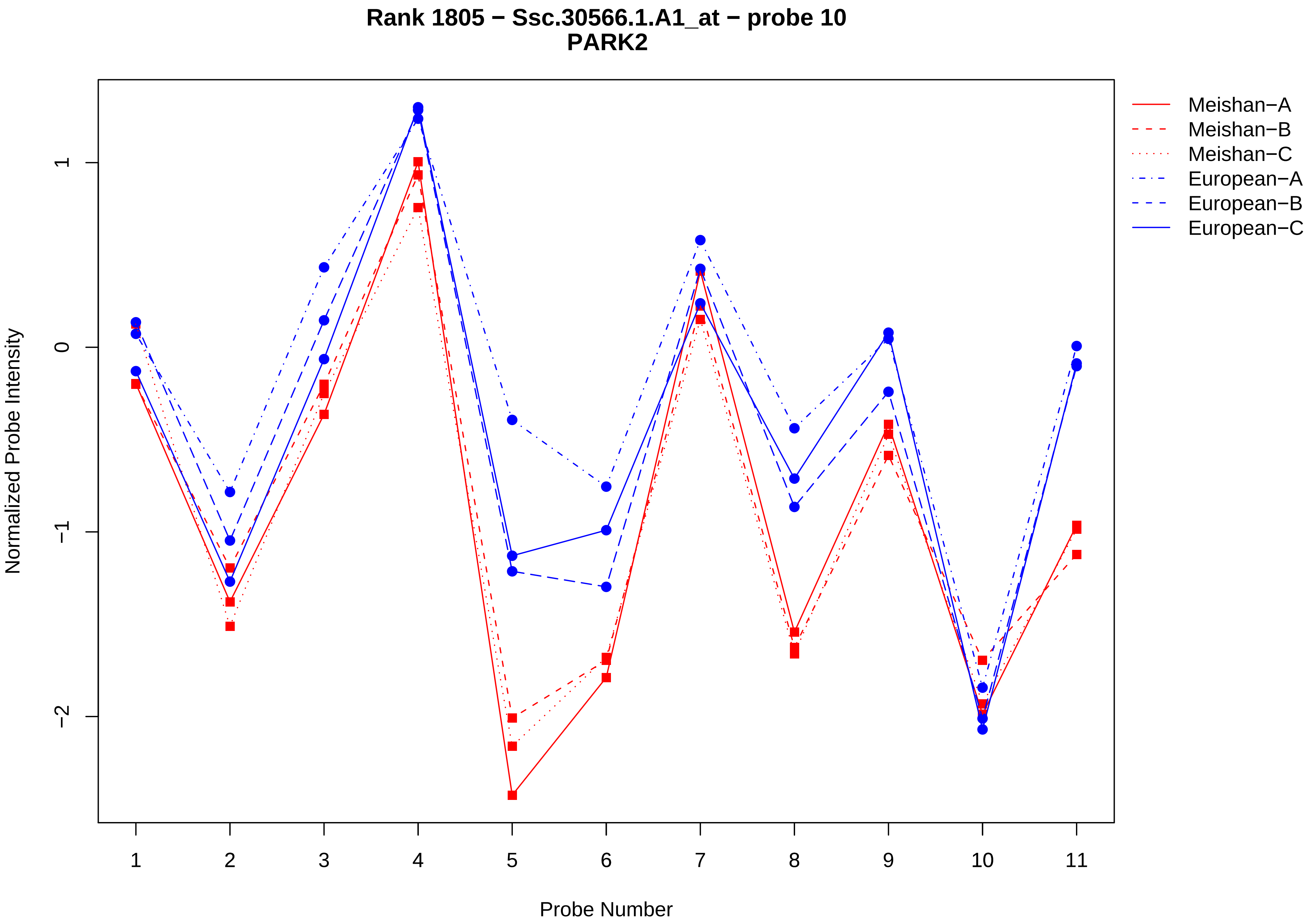

Rank 1805 − Ssc.30566.1.A1_at − probe 10
PARK2
Normalized Probe Intensity
Probe Number
1
0
−1
−2
1
2
3
4
5
6
7
8
9
10
11
Meishan−A
Meishan−B
Meishan−C
European−A
European−B
European−C

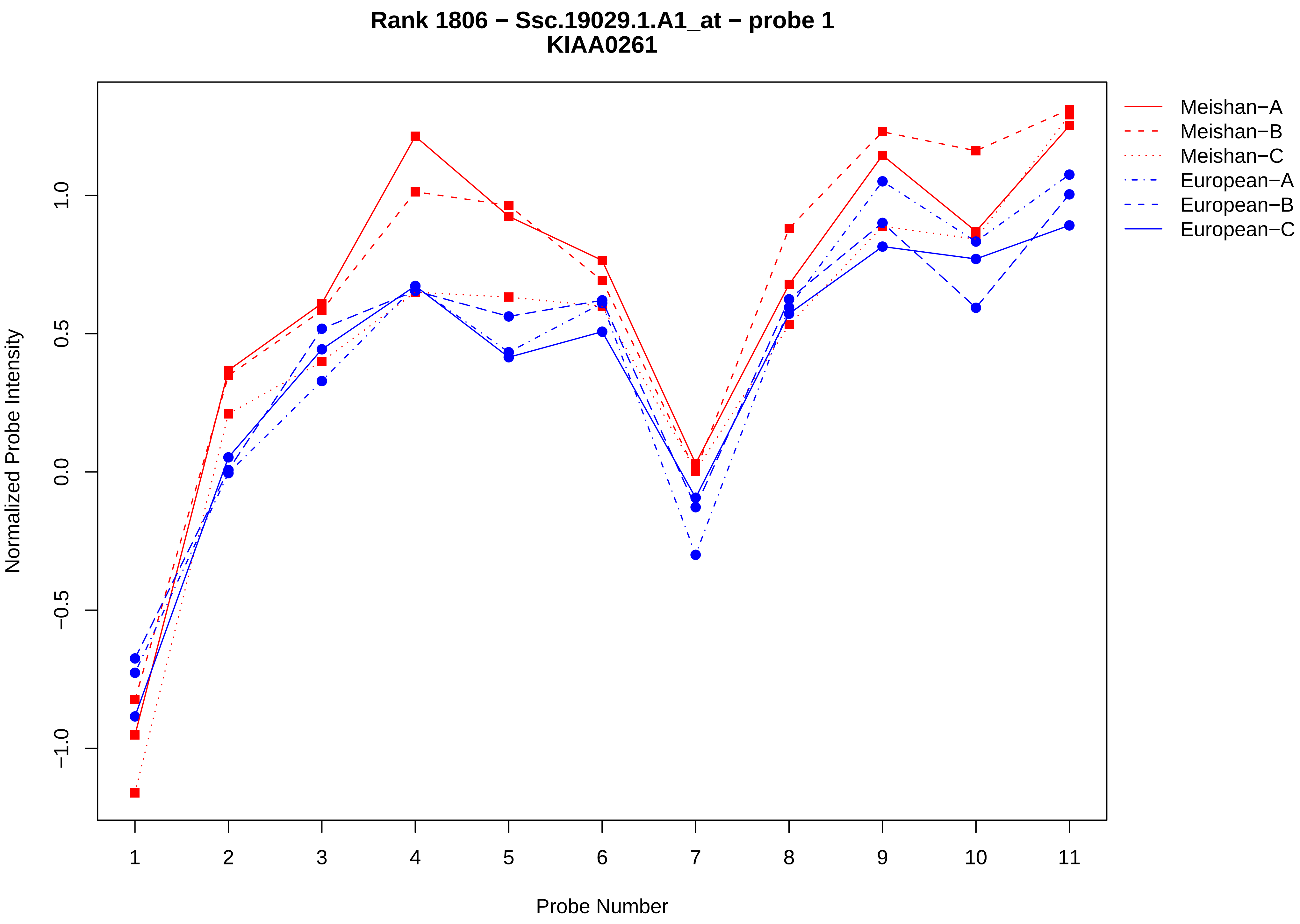
Rank 1806 − Ssc.19029.1.A1_at − probe 1
KIAA0261
Normalized Probe Intensity
−1.0
−0.5
0.0
0.5
1.0
Probe Number
1
2
3
4
5
6
7
8
9
10
11
Meishan−A
Meishan−B
Meishan−C
European−A
European−B
European−C

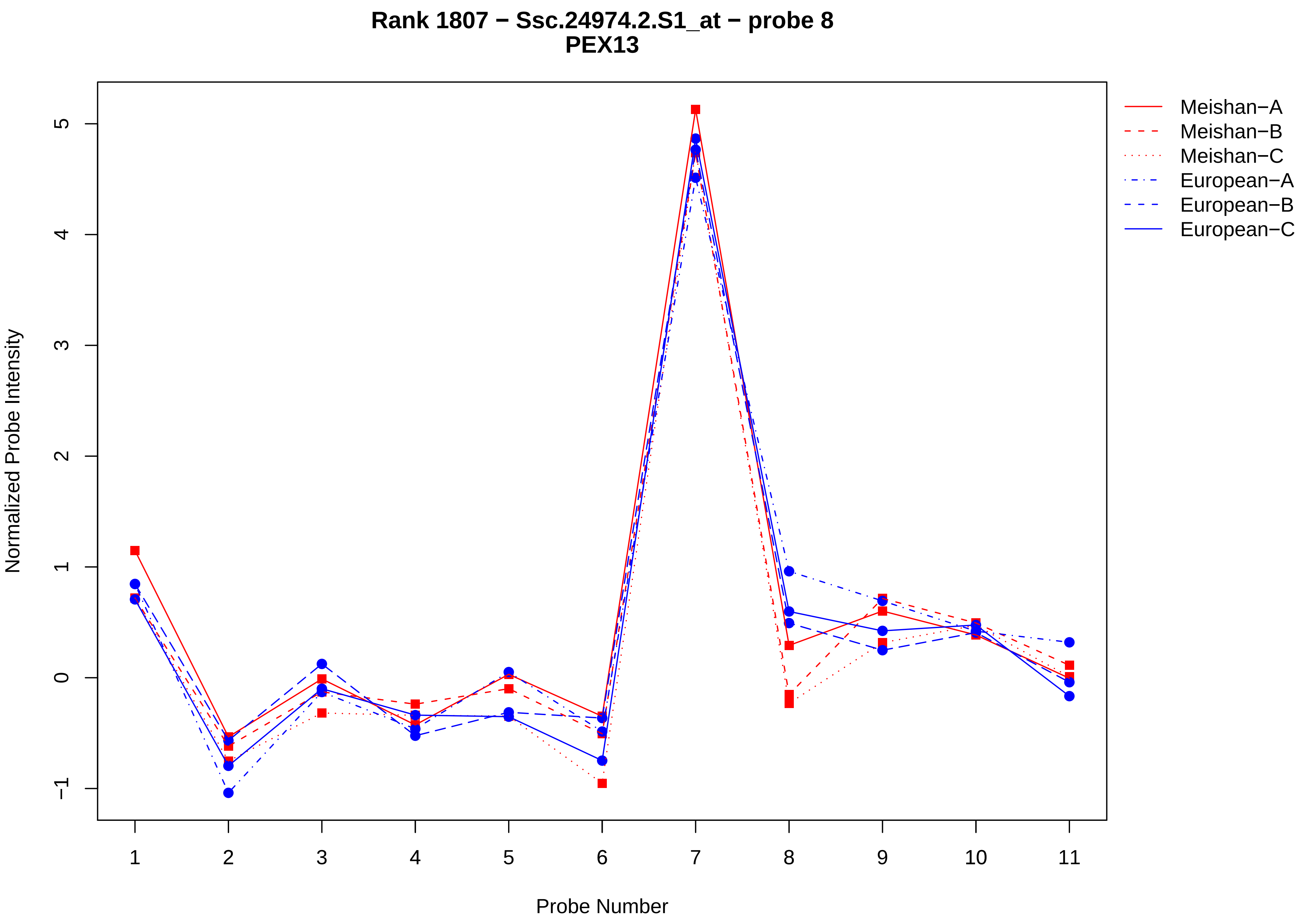

Rank 1807 − Ssc.24974.2.S1_at − probe 8
PEX13
Normalized Probe Intensity
Probe Number
−1
0
1
2
3
4
5
1
2
3
4
5
6
7
8
9
10
11
Meishan−A
Meishan−B
Meishan−C
European−A
European−B
European−C

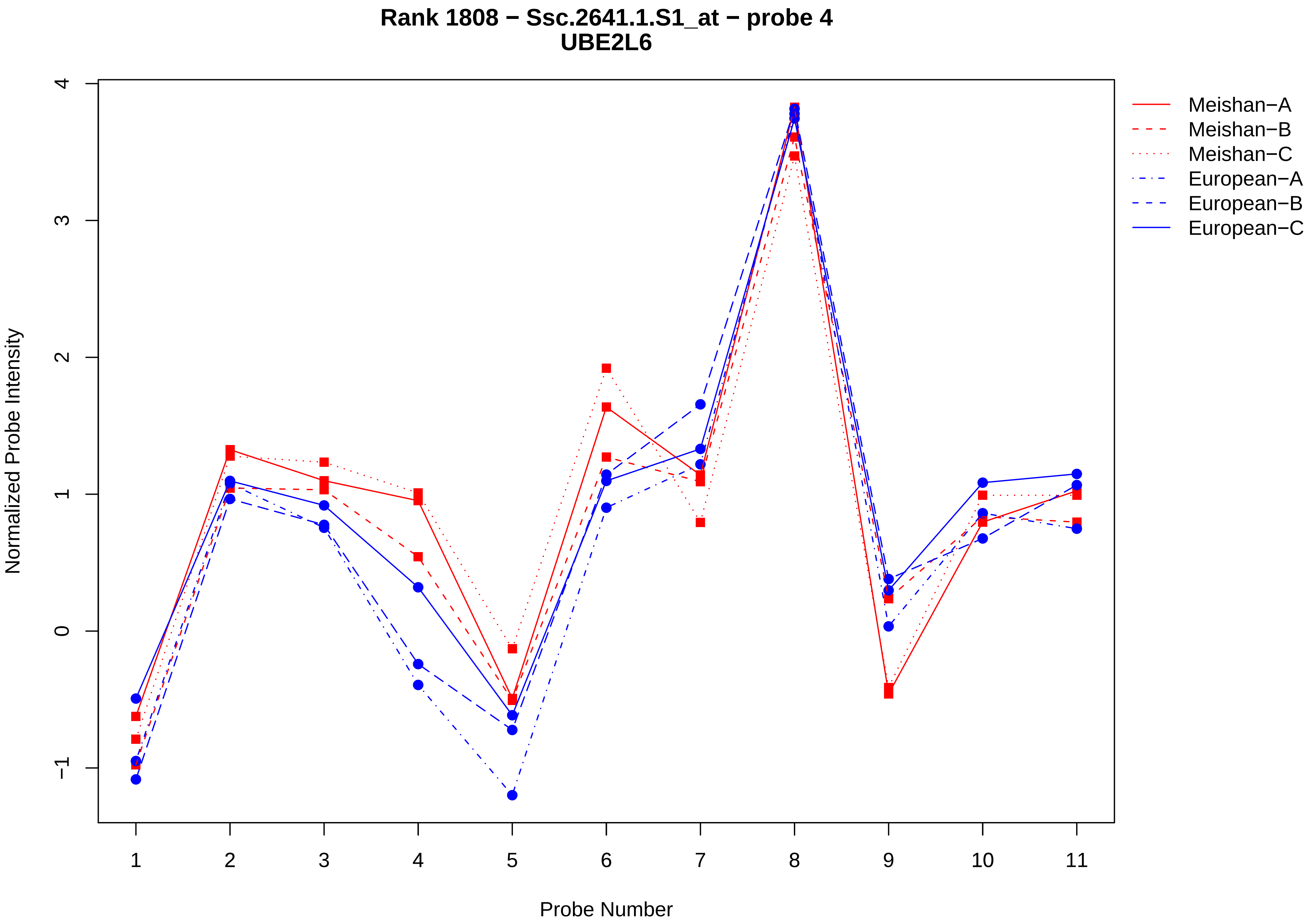

Rank 1808 − Ssc.2641.1.S1_at − probe 4
UBE2L6
Normalized Probe Intensity
Probe Number
−1
0
1
2
3
4
1
2
3
4
5
6
7
8
9
10
11
Meishan−A
Meishan−B
Meishan−C
European−A
European−B
European−C

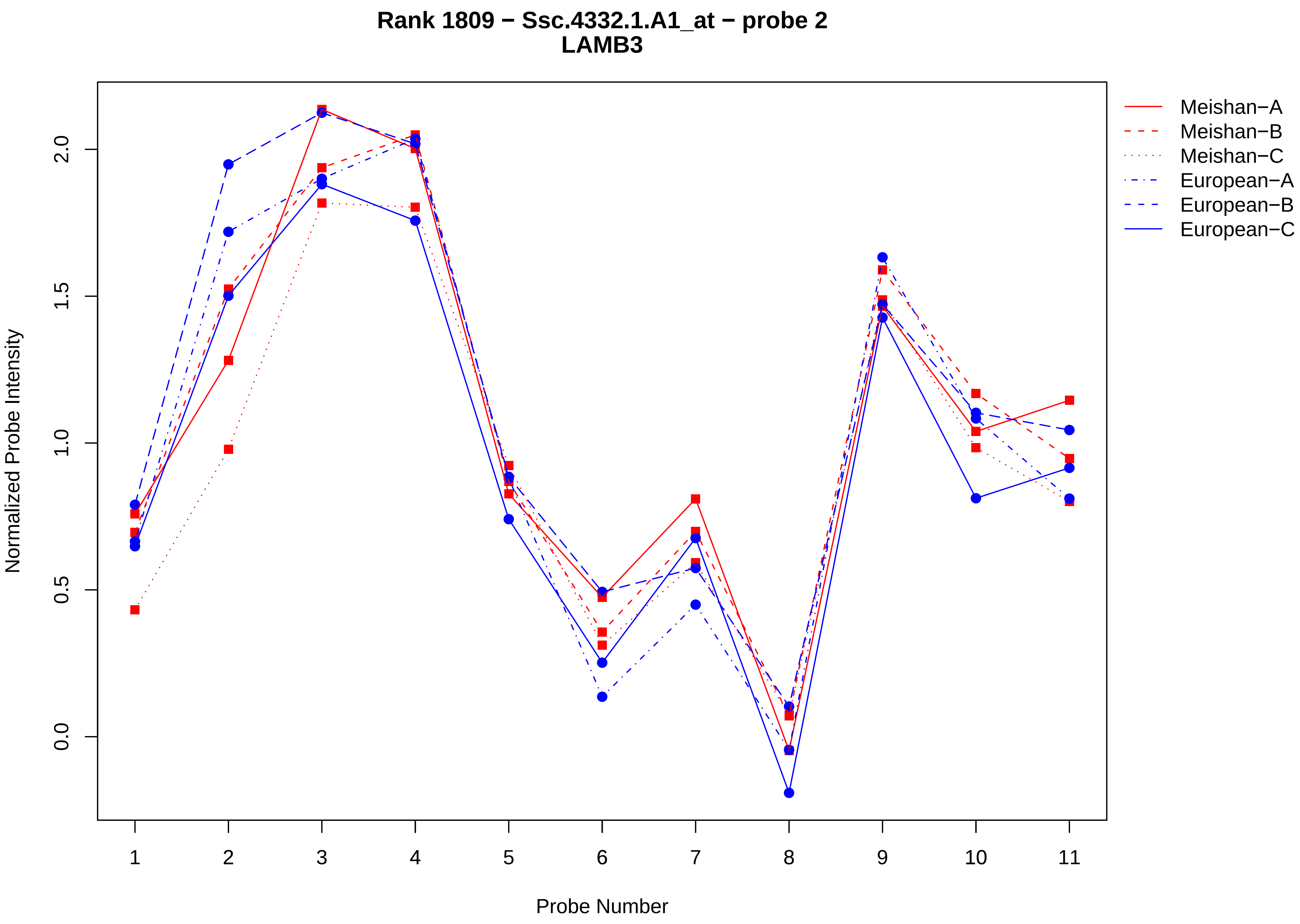

Rank 1809 − Ssc.4332.1.A1_at − probe 2
LAMB3
Normalized Probe Intensity
Probe Number
0.0
0.5
1.0
1.5
2.0
1
2
3
4
5
6
7
8
9
10
11
Meishan−A
Meishan−B
Meishan−C
European−A
European−B
European−C

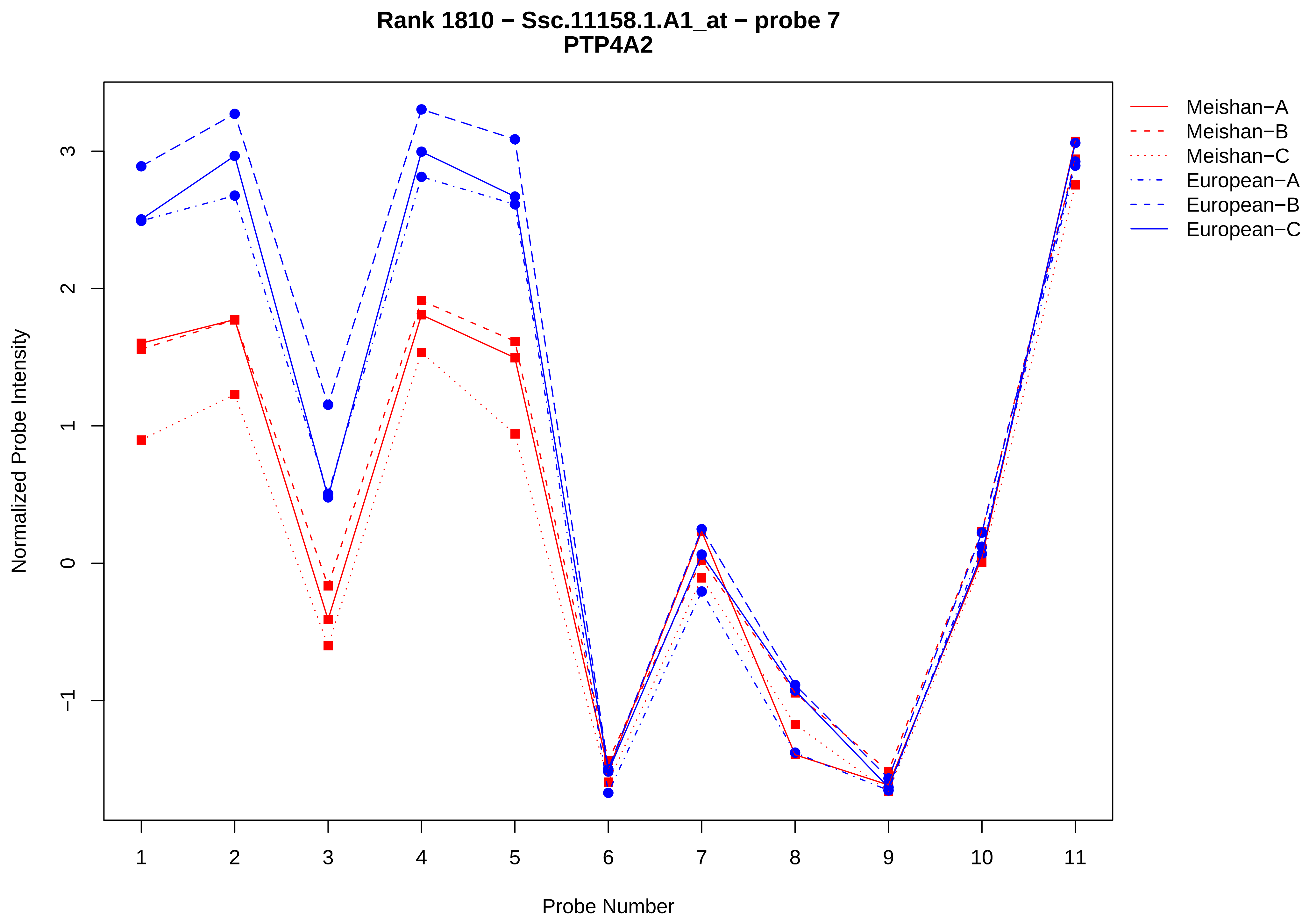

Rank 1810 − Ssc.11158.1.A1_at − probe 7
PTP4A2
Normalized Probe Intensity
Probe Number
−1
0
1
2
3
1
2
3
4
5
6
7
8
9
10
11
Meishan−A
Meishan−B
Meishan−C
European−A
European−B
European−C

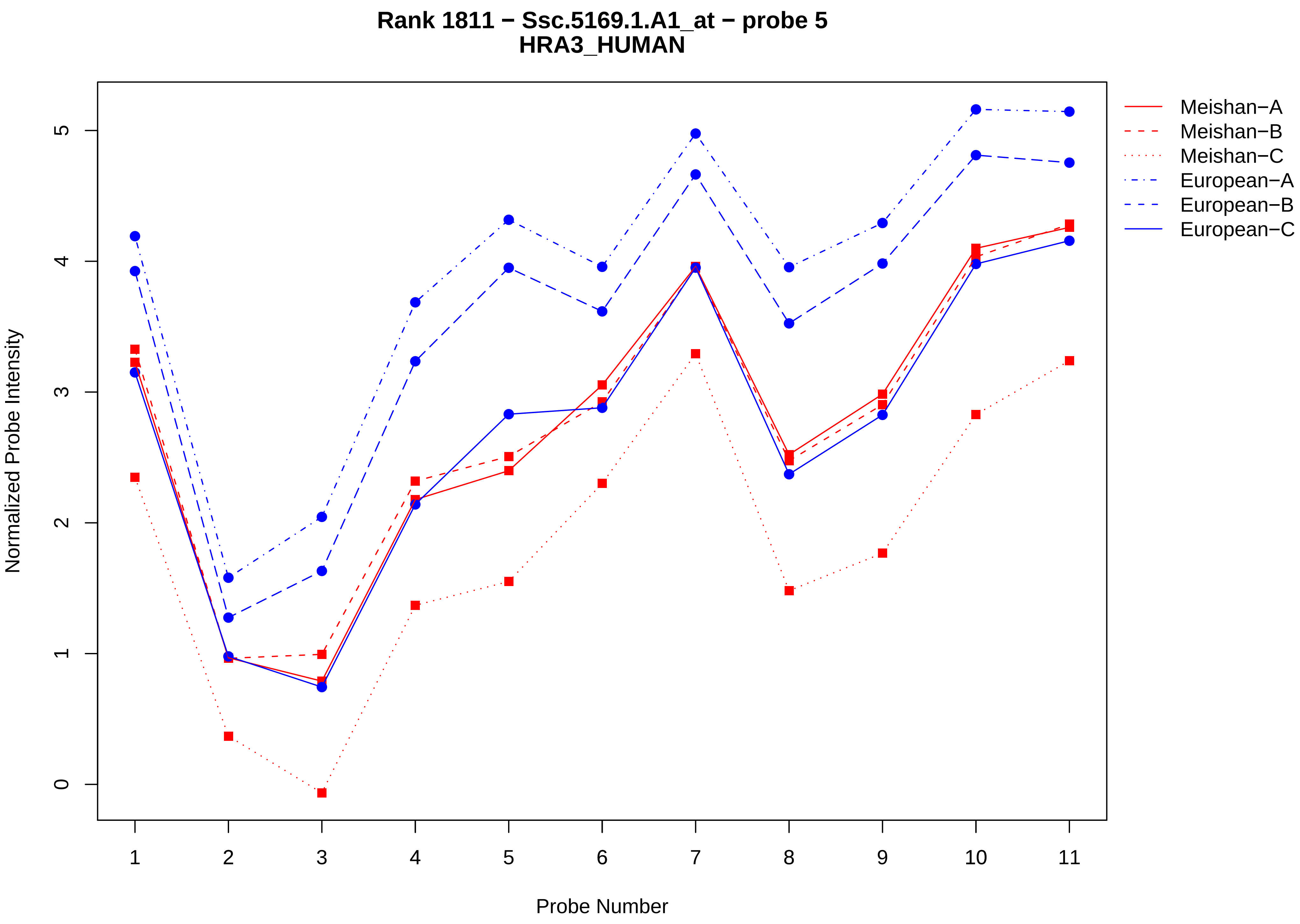
Rank 1811 − Ssc.5169.1.A1_at − probe 5
HRA3_HUMAN
Normalized Probe Intensity
Probe Number
0
1
2
3
4
5
1
2
3
4
5
6
7
8
9
10
11
Meishan−A
Meishan−B
Meishan−C
European−A
European−B
European−C

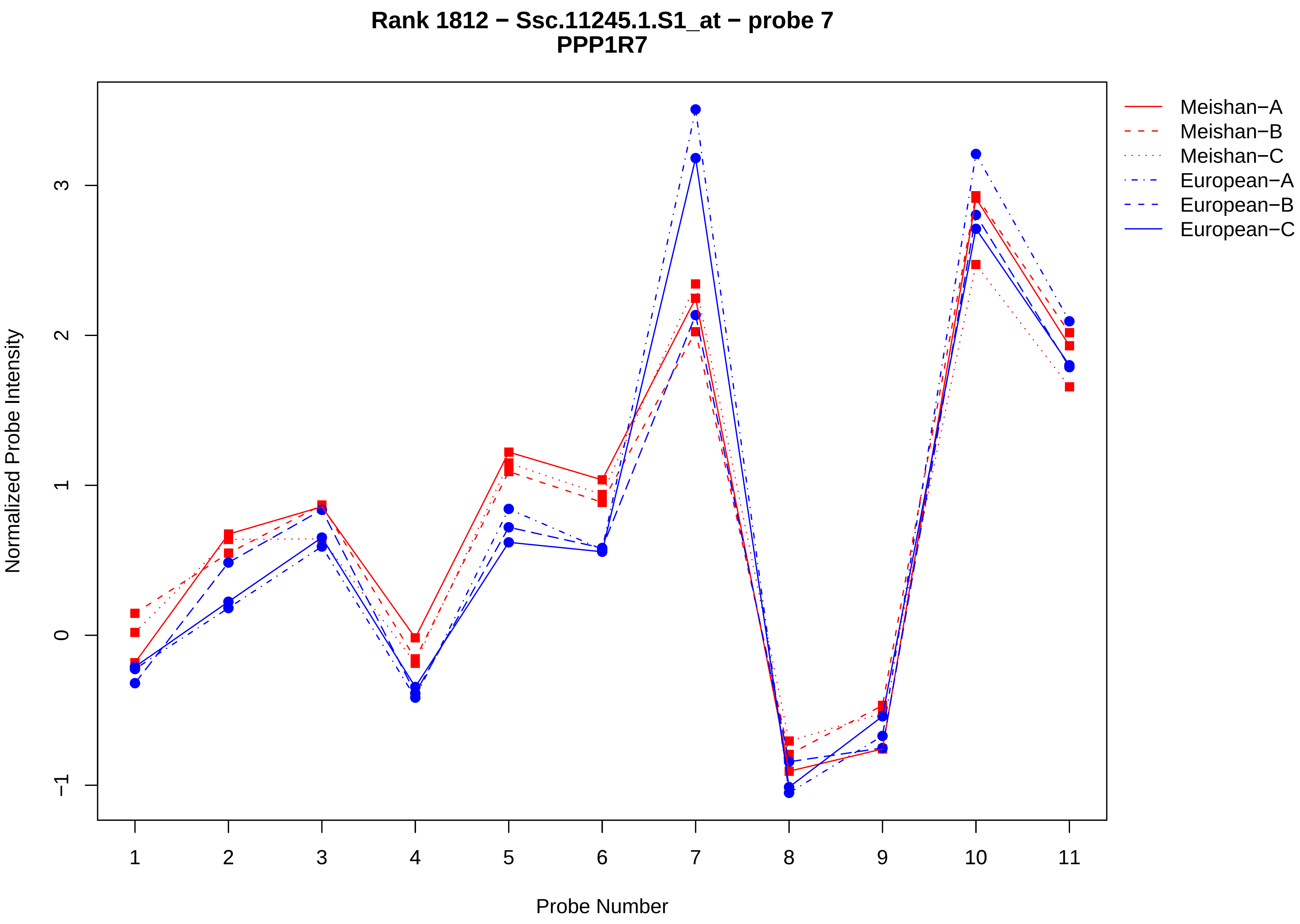

Rank 1812 − Ssc.11245.1.S1_at − probe 7
PPP1R7
Normalized Probe Intensity
Probe Number
Meishan−A
Meishan−B
Meishan−C
European−A
European−B
European−C
−1
0
1
2
3
1
2
3
4
5
6
7
8
9
10
11

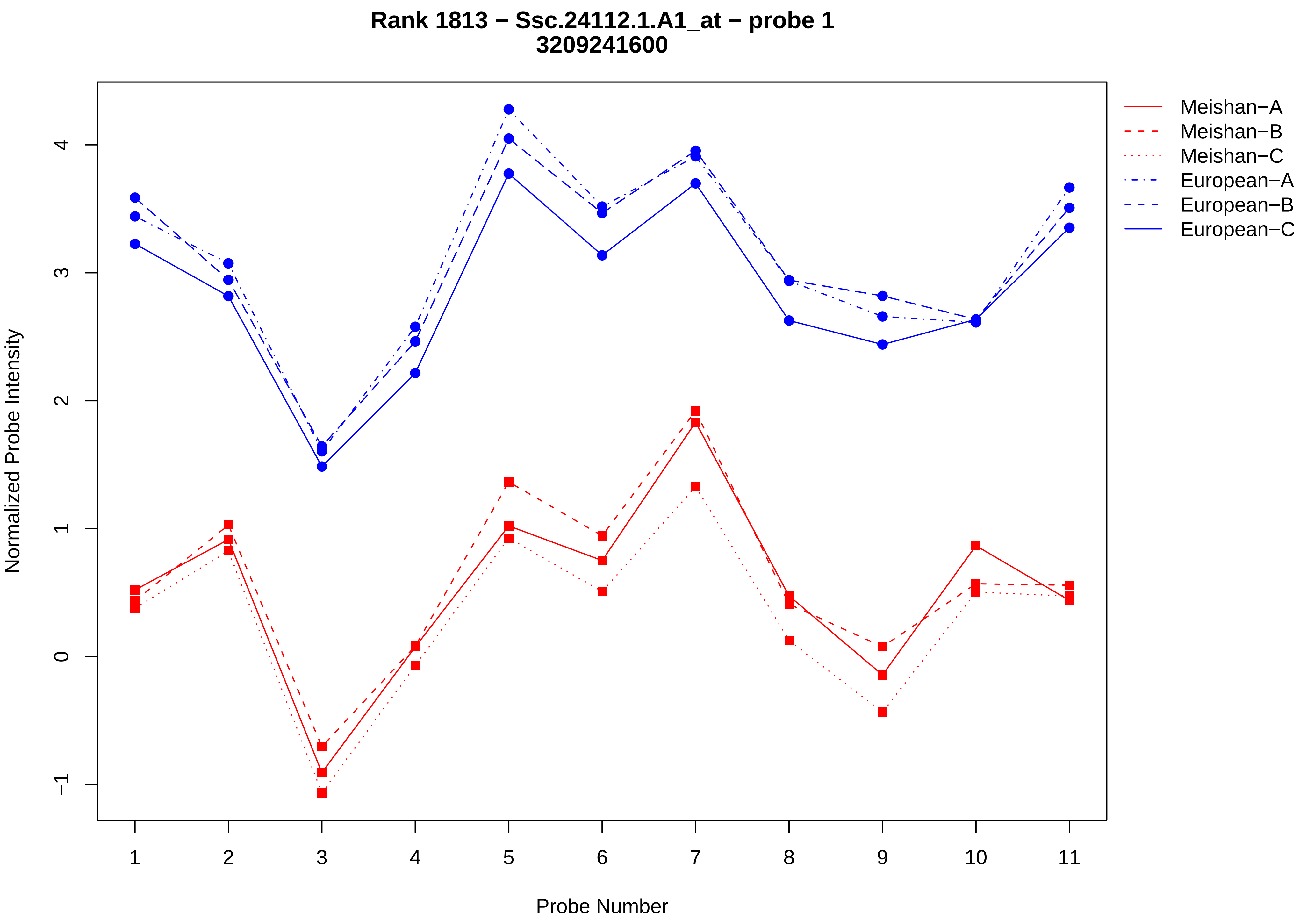

Rank 1813 − Ssc.24112.1.A1_at − probe 1
3209241600
Normalized Probe Intensity
Probe Number
Meishan−A
Meishan−B
Meishan−C
European−A
European−B
European−C
−1
0
1
2
3
4
1
2
3
4
5
6
7
8
9
10
11

Rank 1814 − Ssc.24415.1.A1_at − probe 4
NP_115914

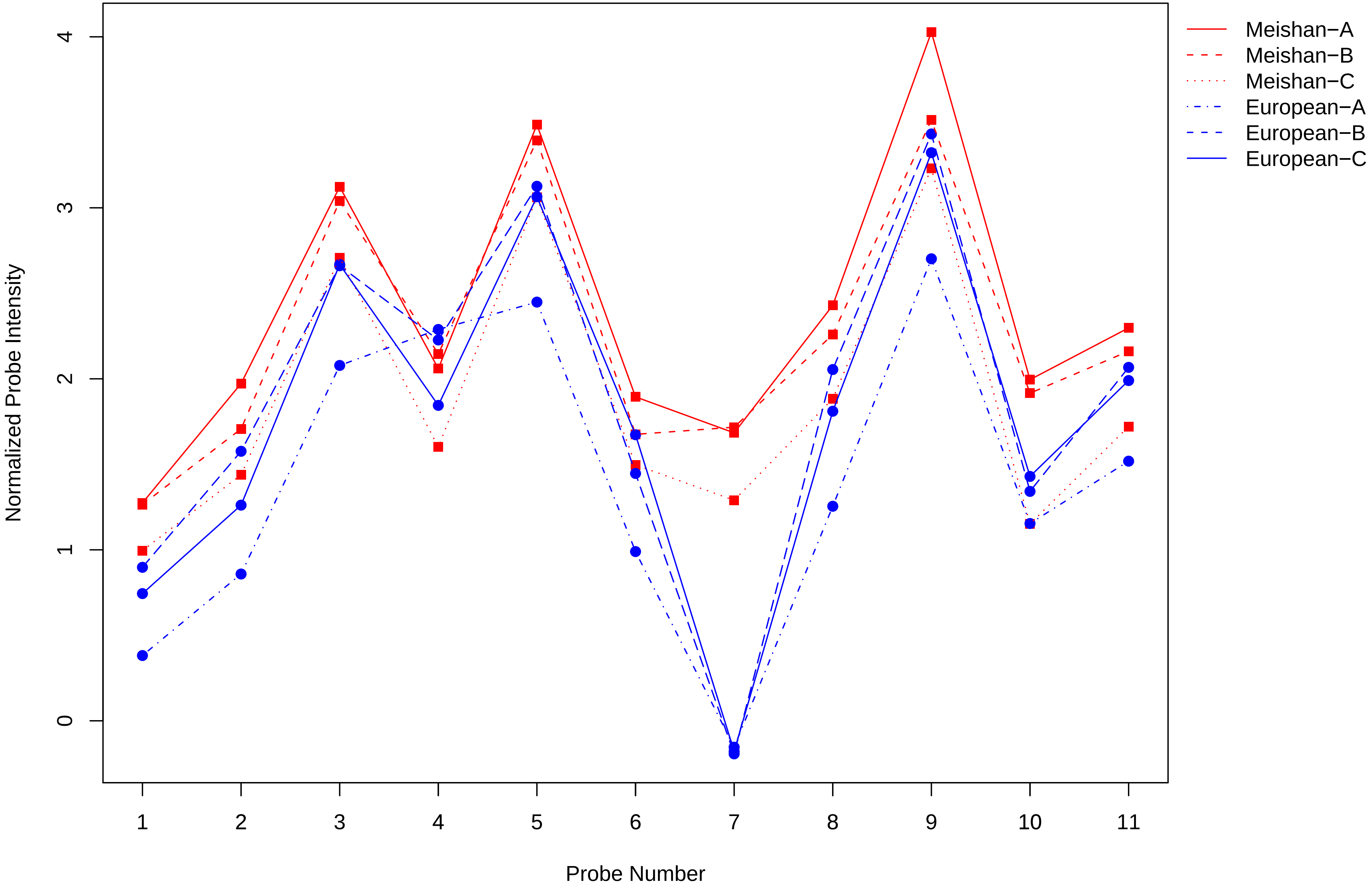

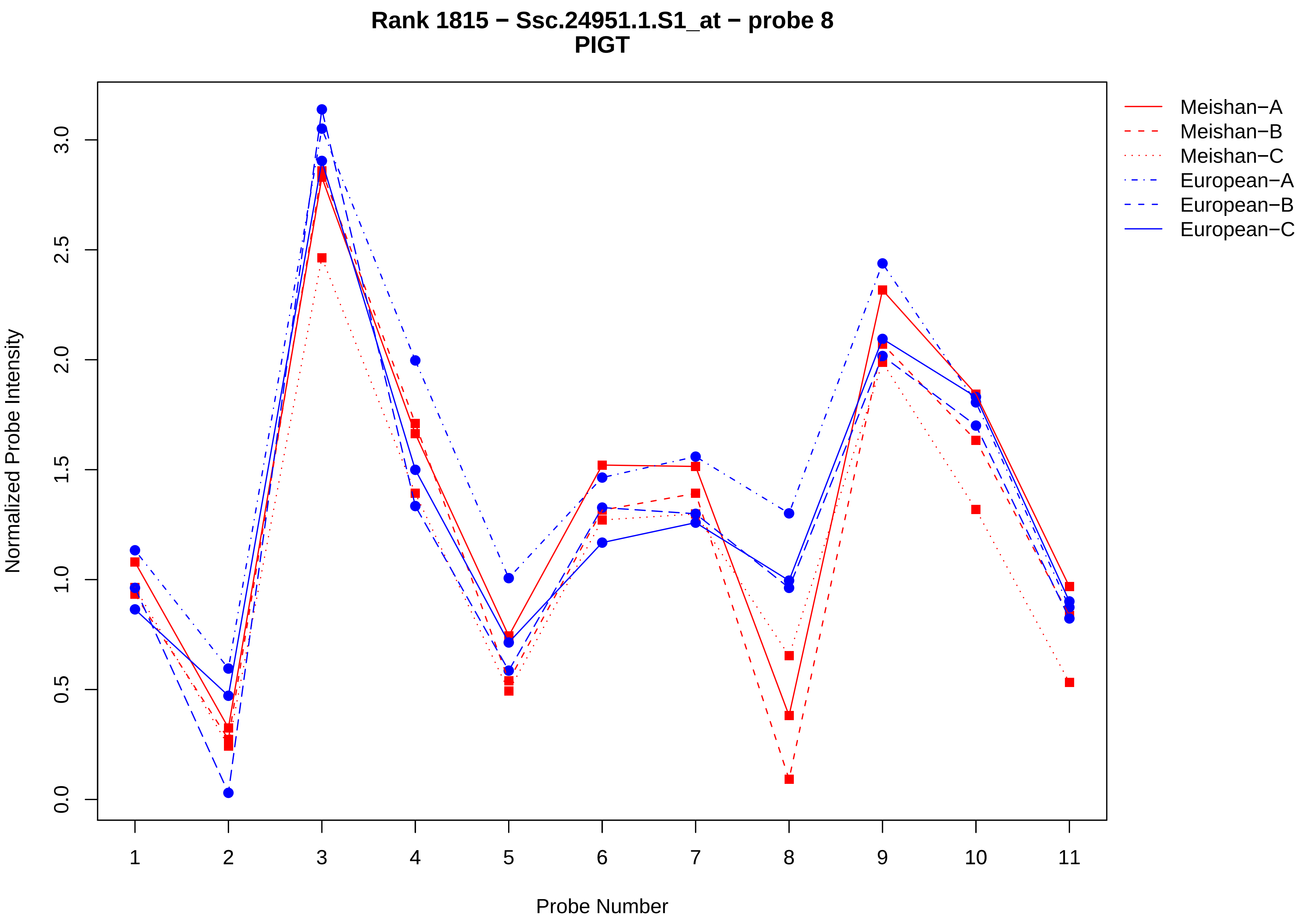

Rank 1815 − Ssc.24951.1.S1_at − probe 8
PIGT
Normalized Probe Intensity
0.0
0.5
1.0
1.5
2.0
2.5
3.0
1
2
3
4
5
6
7
8
9
10
11
Probe Number
Meishan−A
Meishan−B
Meishan−C
European−A
European−B
European−C

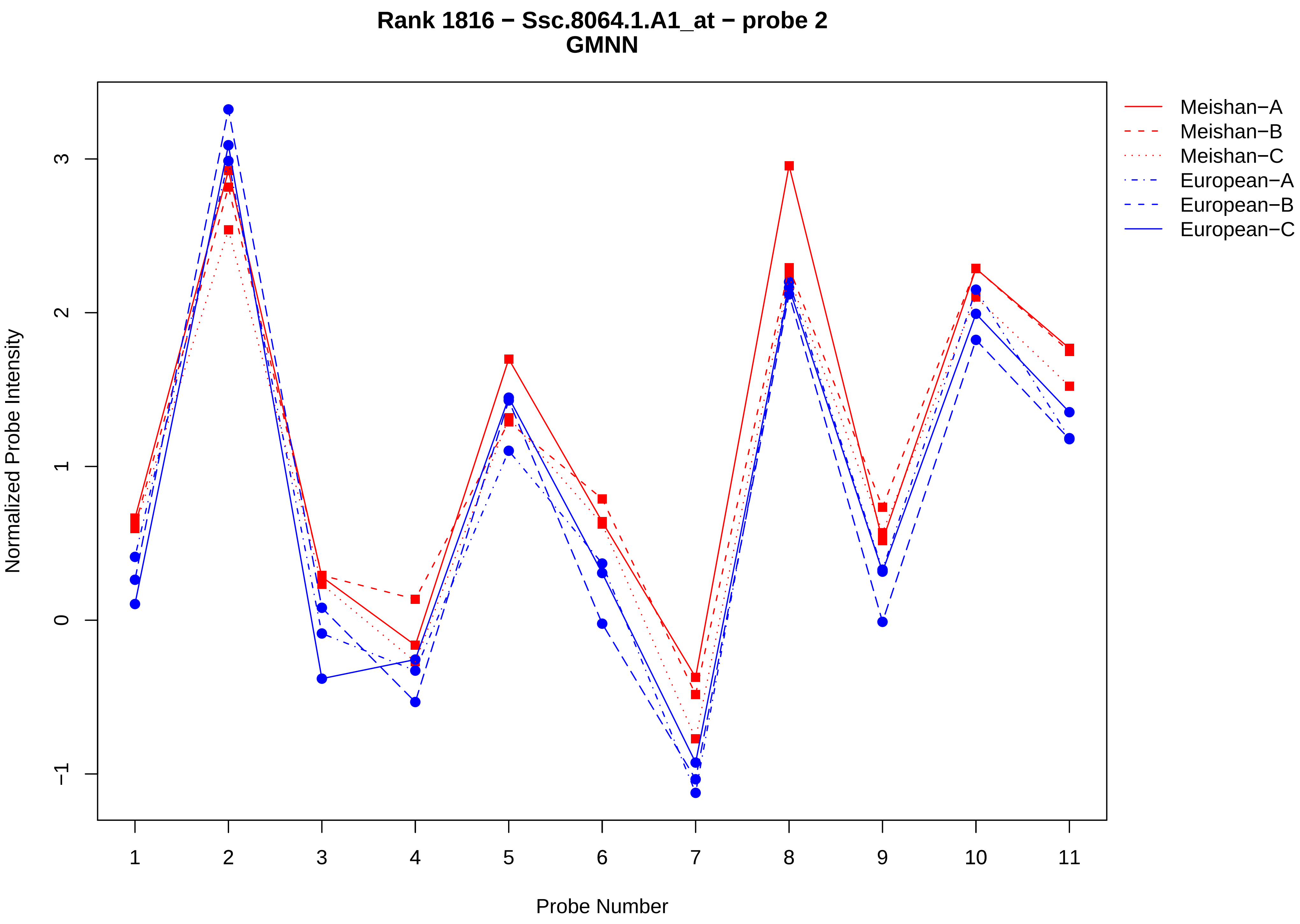

Rank 1816 − Ssc.8064.1.A1_at − probe 2
GMNN
Normalized Probe Intensity
Probe Number
−1
0
1
2
3
1
2
3
4
5
6
7
8
9
10
11
Meishan−A
Meishan−B
Meishan−C
European−A
European−B
European−C

Rank 1817 − Ssc.10796.1.A1_at − probe 4
CNTNAP5

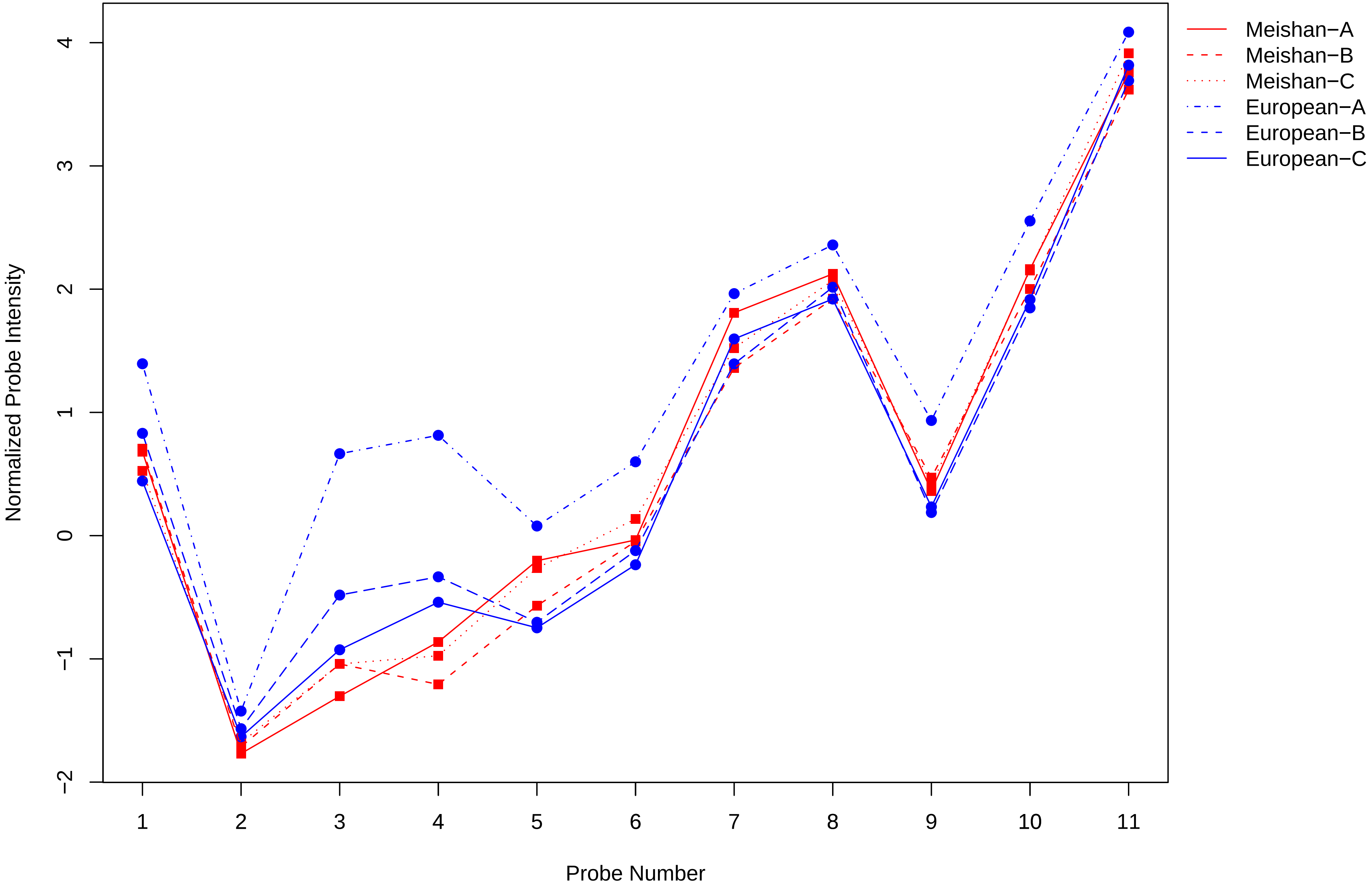

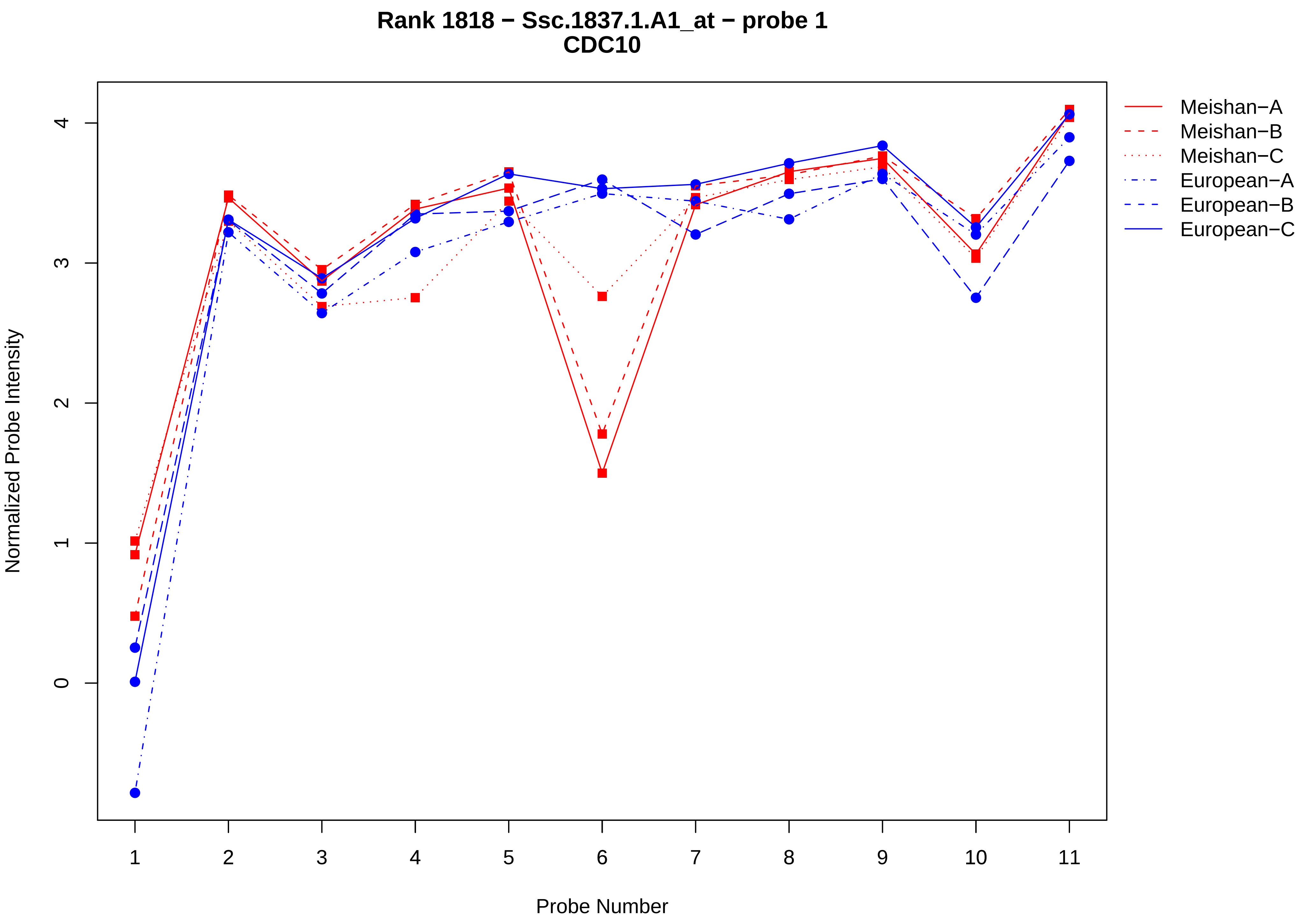
Rank 1818 − Ssc.1837.1.A1_at − probe 1
CDC10
Normalized Probe Intensity
Probe Number
0
1
2
3
4
1
2
3
4
5
6
7
8
9
10
11
Meishan−A
Meishan−B
Meishan−C
European−A
European−B
European−C

Rank 1819 − Ssc.30826.1.A1_at − probe 5
MMP16

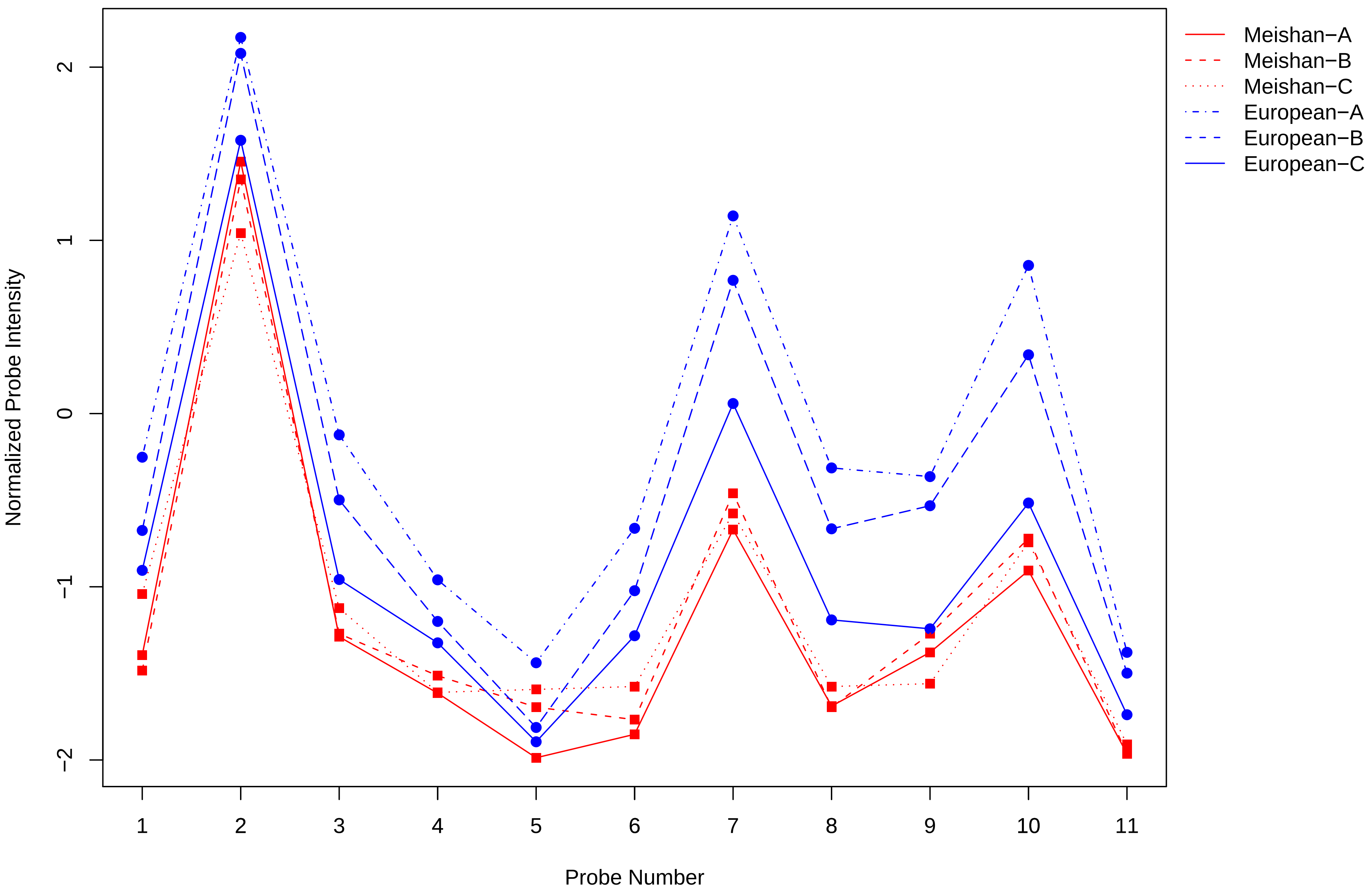

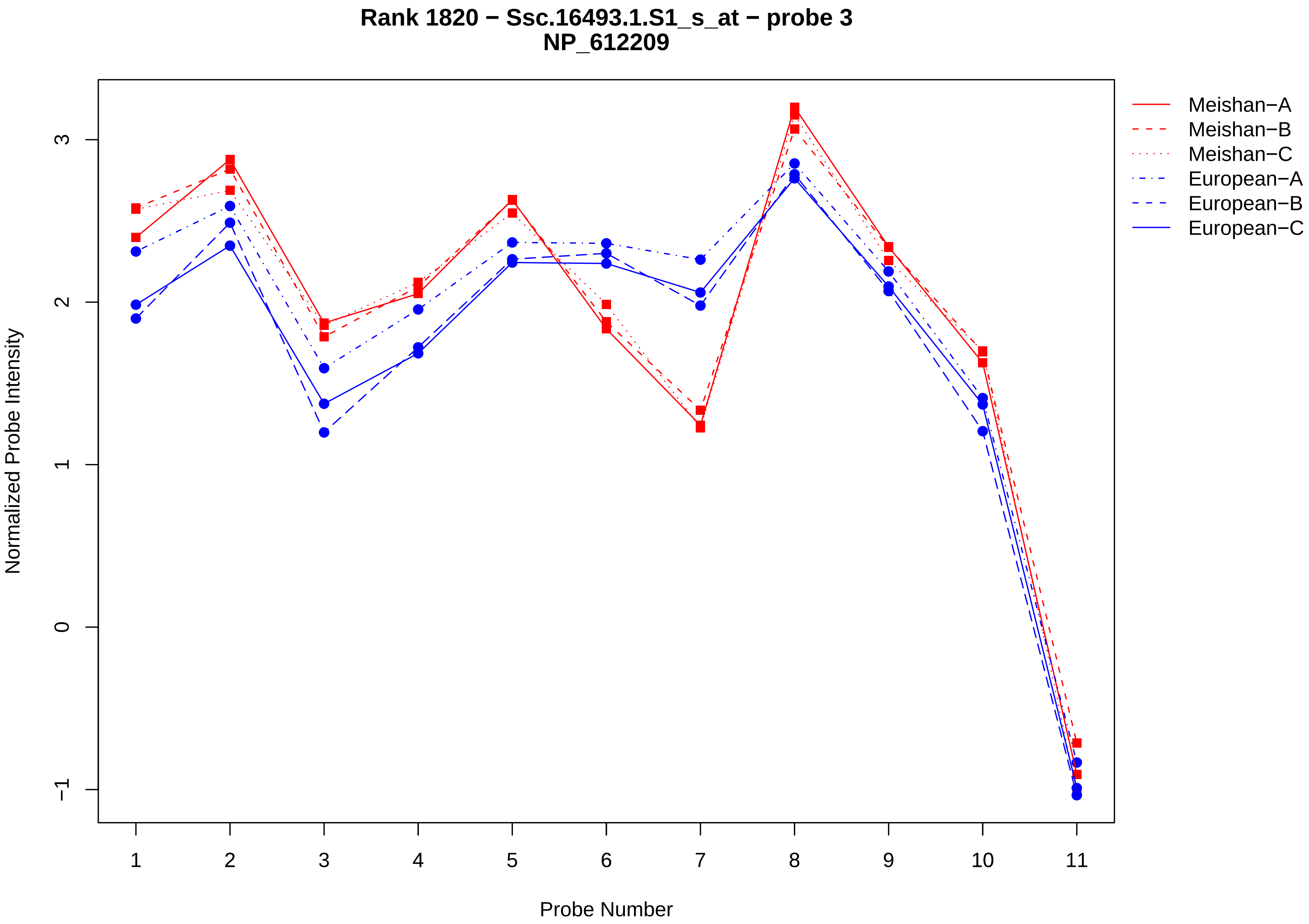
Rank 1820 − Ssc.16493.1.S1_s_at − probe 3
NP_612209
Normalized Probe Intensity
Probe Number
−1
0
1
2
3
1
2
3
4
5
6
7
8
9
10
11
Meishan−A
Meishan−B
Meishan−C
European−A
European−B
European−C

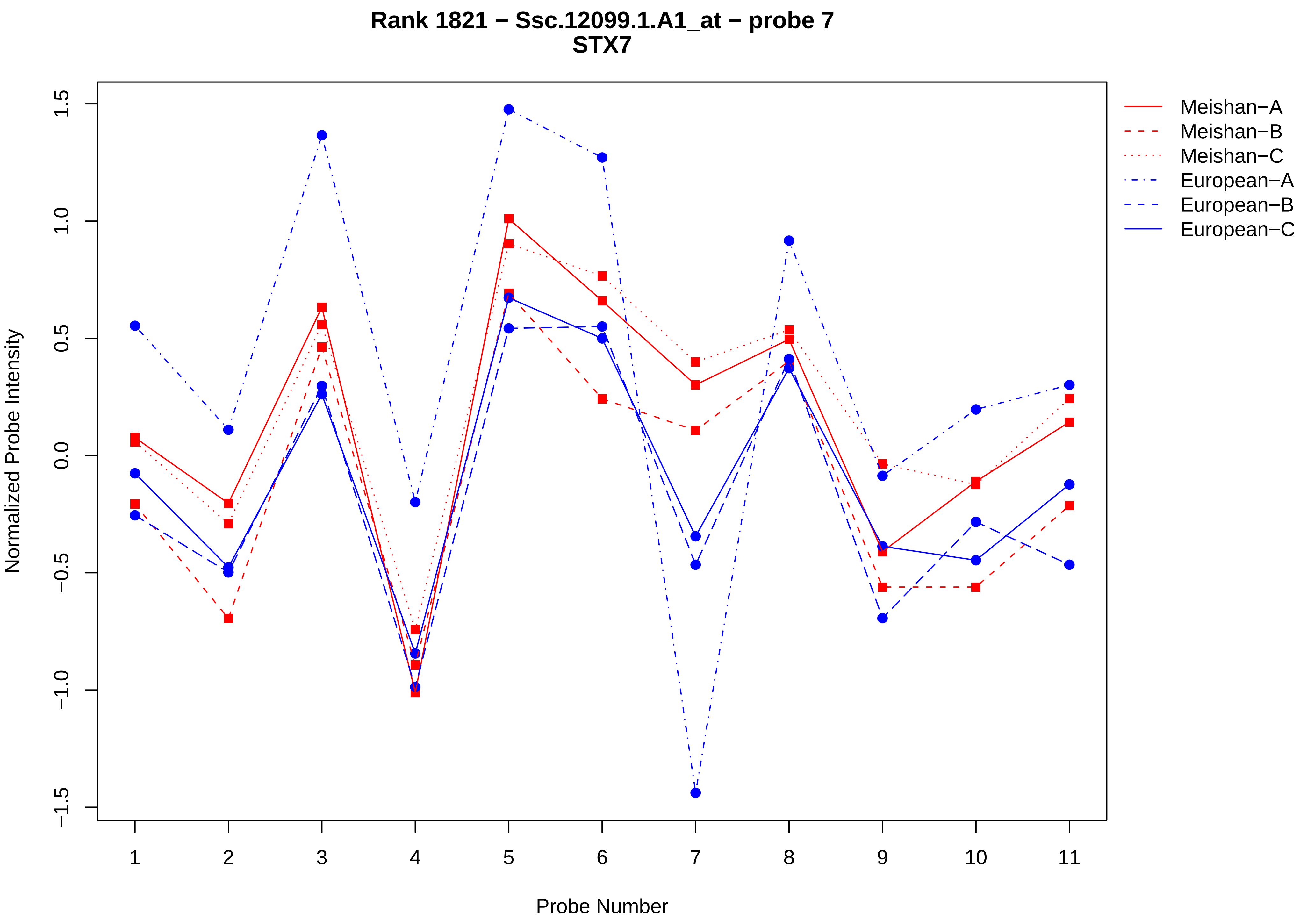

Rank 1821 − Ssc.12099.1.A1_at − probe 7
STX7
Normalized Probe Intensity
Probe Number
Meishan−A
Meishan−B
Meishan−C
European−A
European−B
European−C

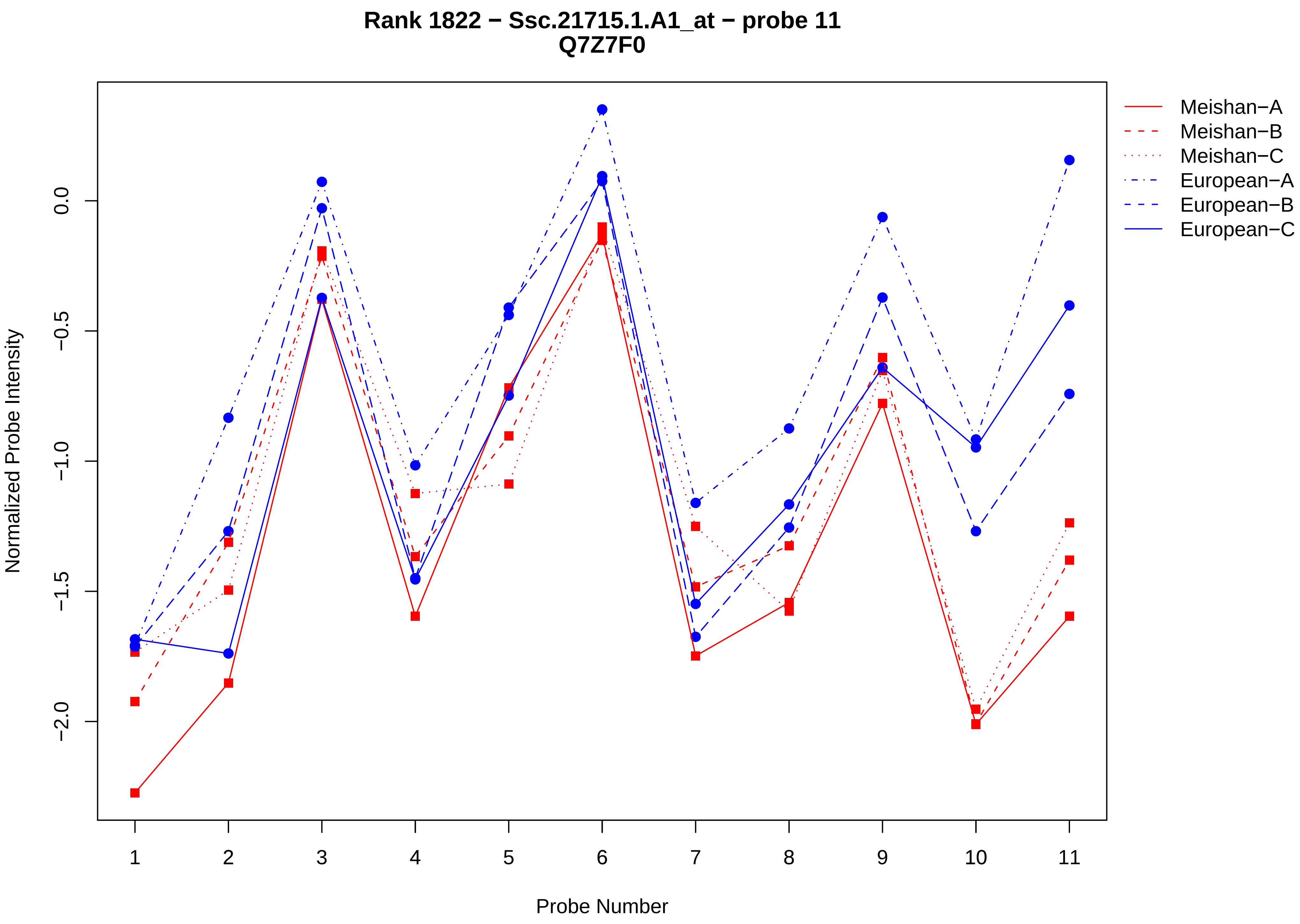

Rank 1822 − Ssc.21715.1.A1_at − probe 11
Q7Z7F0
Normalized Probe Intensity
Probe Number
0.0
−0.5
−1.0
−1.5
−2.0
1
2
3
4
5
6
7
8
9
10
11
Meishan−A
Meishan−B
Meishan−C
European−A
European−B
European−C

Rank 1823 − Ssc.23516.1.S1_at − probe 3
SATB1

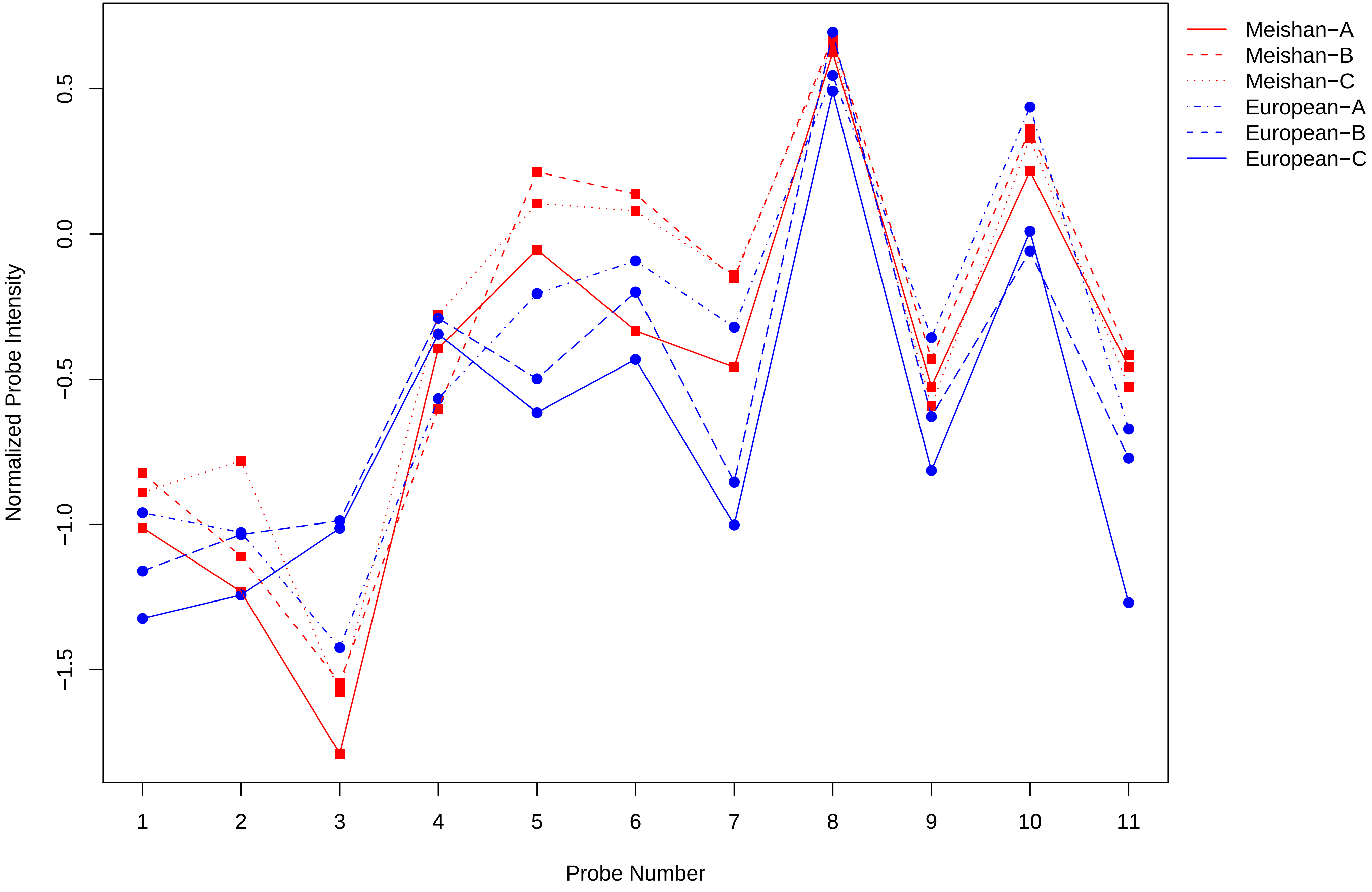

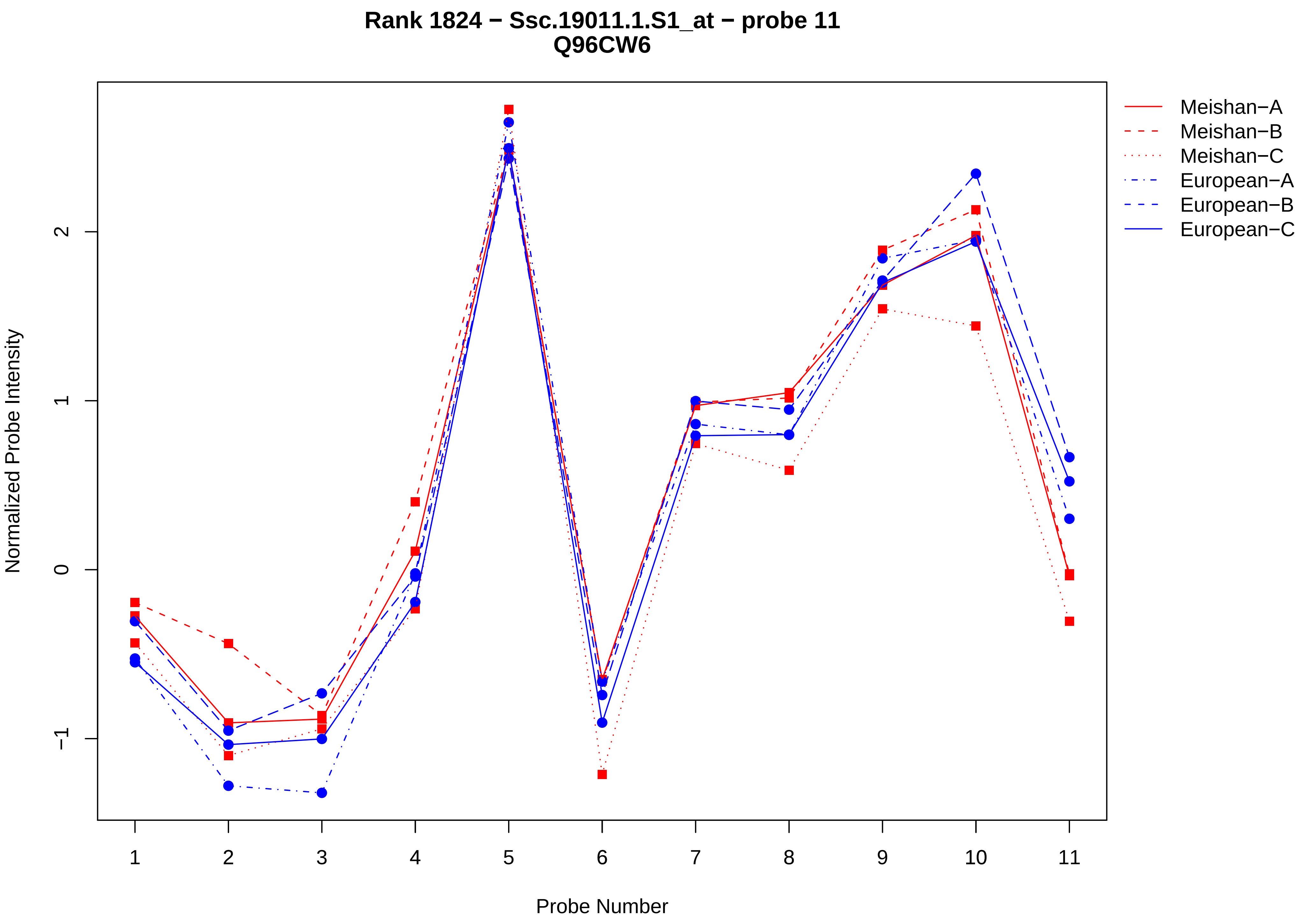

Rank 1824 − Ssc.19011.1.S1_at − probe 11
Q96CW6
Normalized Probe Intensity
Probe Number
−1
0
1
2
1
2
3
4
5
6
7
8
9
10
11
Meishan−A
Meishan−B
Meishan−C
European−A
European−B
European−C

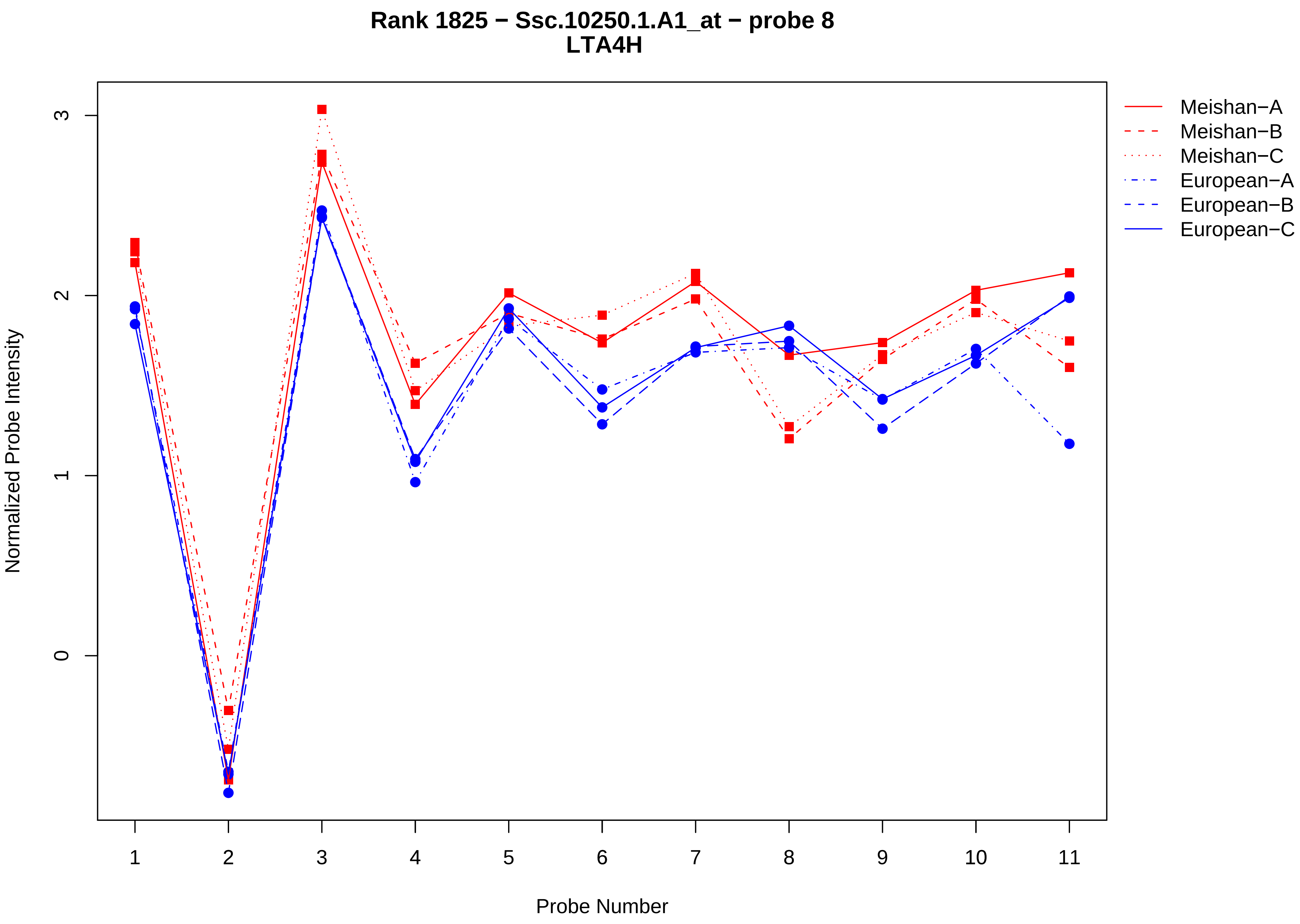

Rank 1825 − Ssc.10250.1.A1_at − probe 8
LTA4H
Normalized Probe Intensity
0
1
2
3
1
2
3
4
5
6
7
8
9
10
11
Probe Number
Meishan−A
Meishan−B
Meishan−C
European−A
European−B
European−C

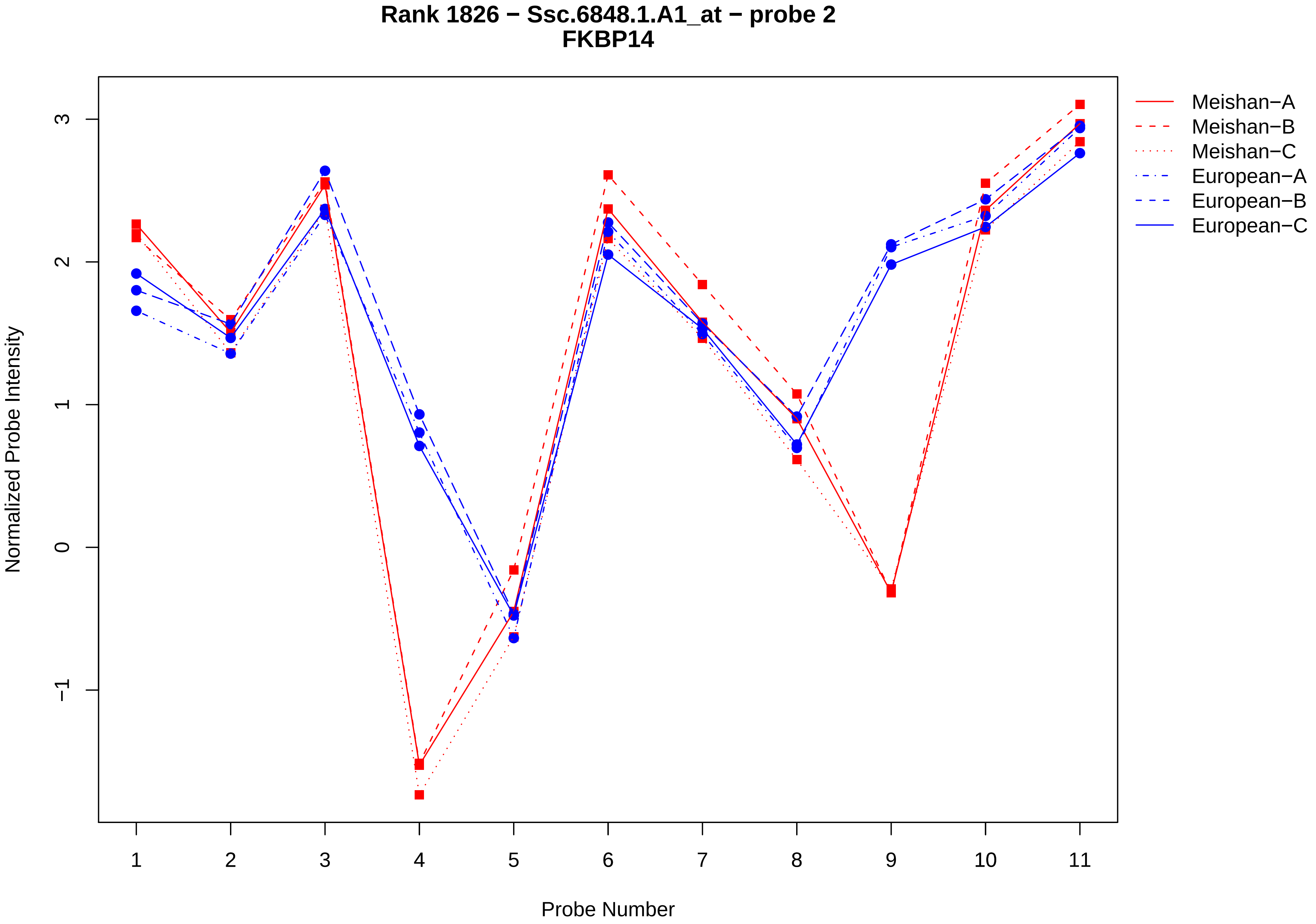

Rank 1826 − Ssc.6848.1.A1_at − probe 2
FKBP14
Normalized Probe Intensity
Probe Number
Meishan−A
Meishan−B
Meishan−C
European−A
European−B
European−C
−1
0
1
2
3
1
2
3
4
5
6
7
8
9
10
11

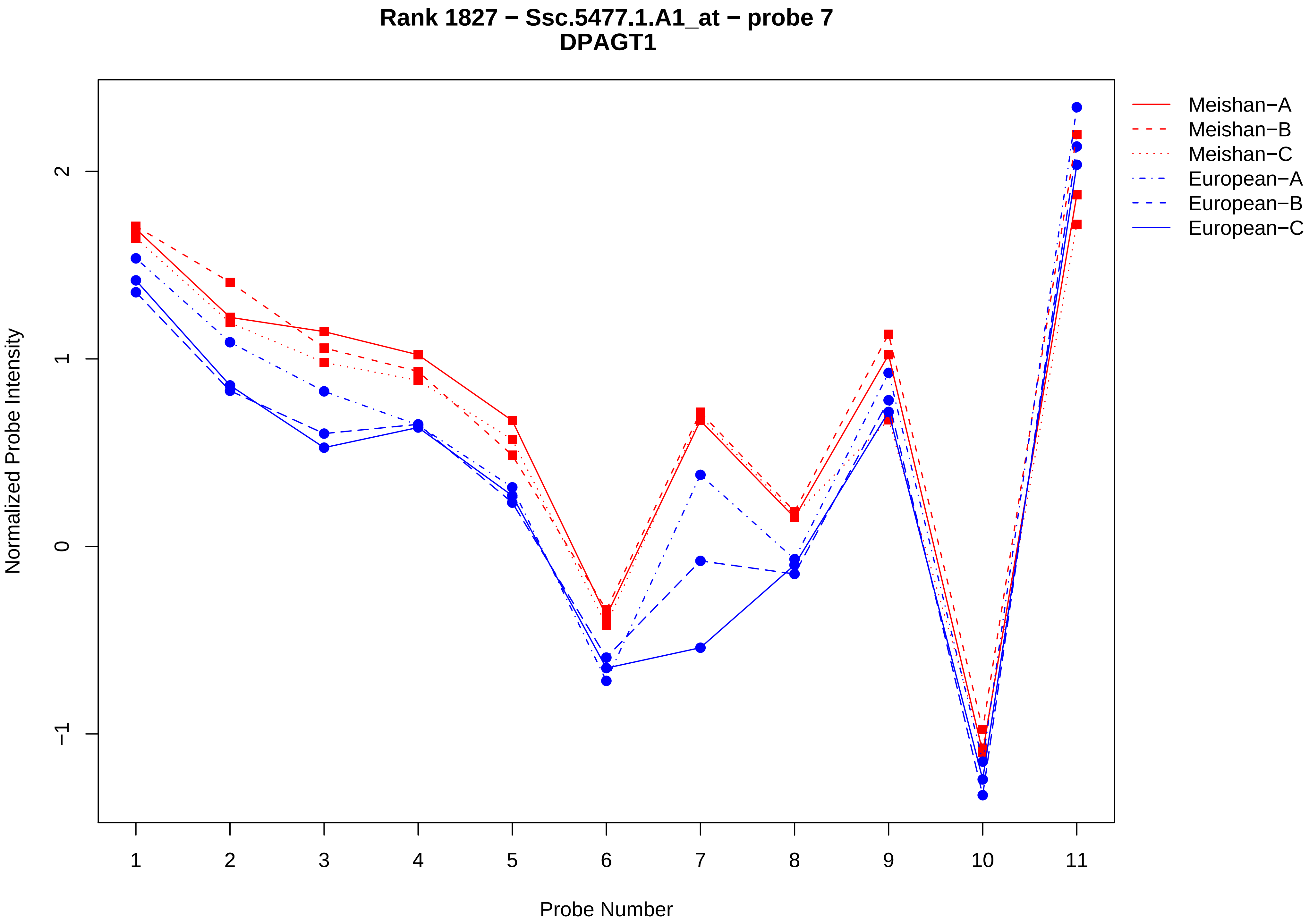
Rank 1827 − Ssc.5477.1.A1_at − probe 7
DPAGT1
Normalized Probe Intensity
Probe Number
Meishan−A
Meishan−B
Meishan−C
European−A
European−B
European−C
−1
0
1
2
1
2
3
4
5
6
7
8
9
10
11

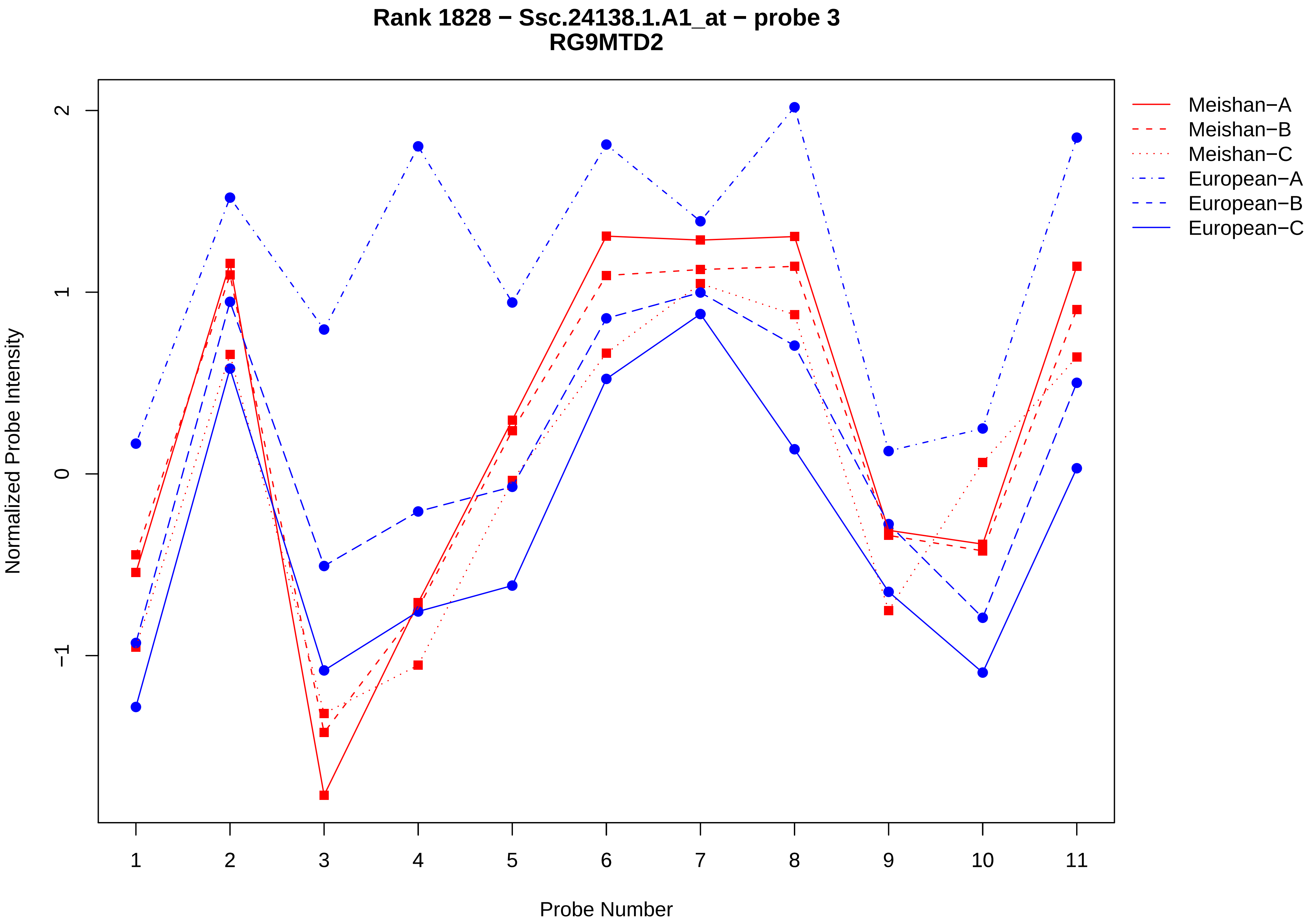
Rank 1828 − Ssc.24138.1.A1_at − probe 3
RG9MTD2
Normalized Probe Intensity
Probe Number
−1
0
1
2
1
2
3
4
5
6
7
8
9
10
11
Meishan−A
Meishan−B
Meishan−C
European−A
European−B
European−C

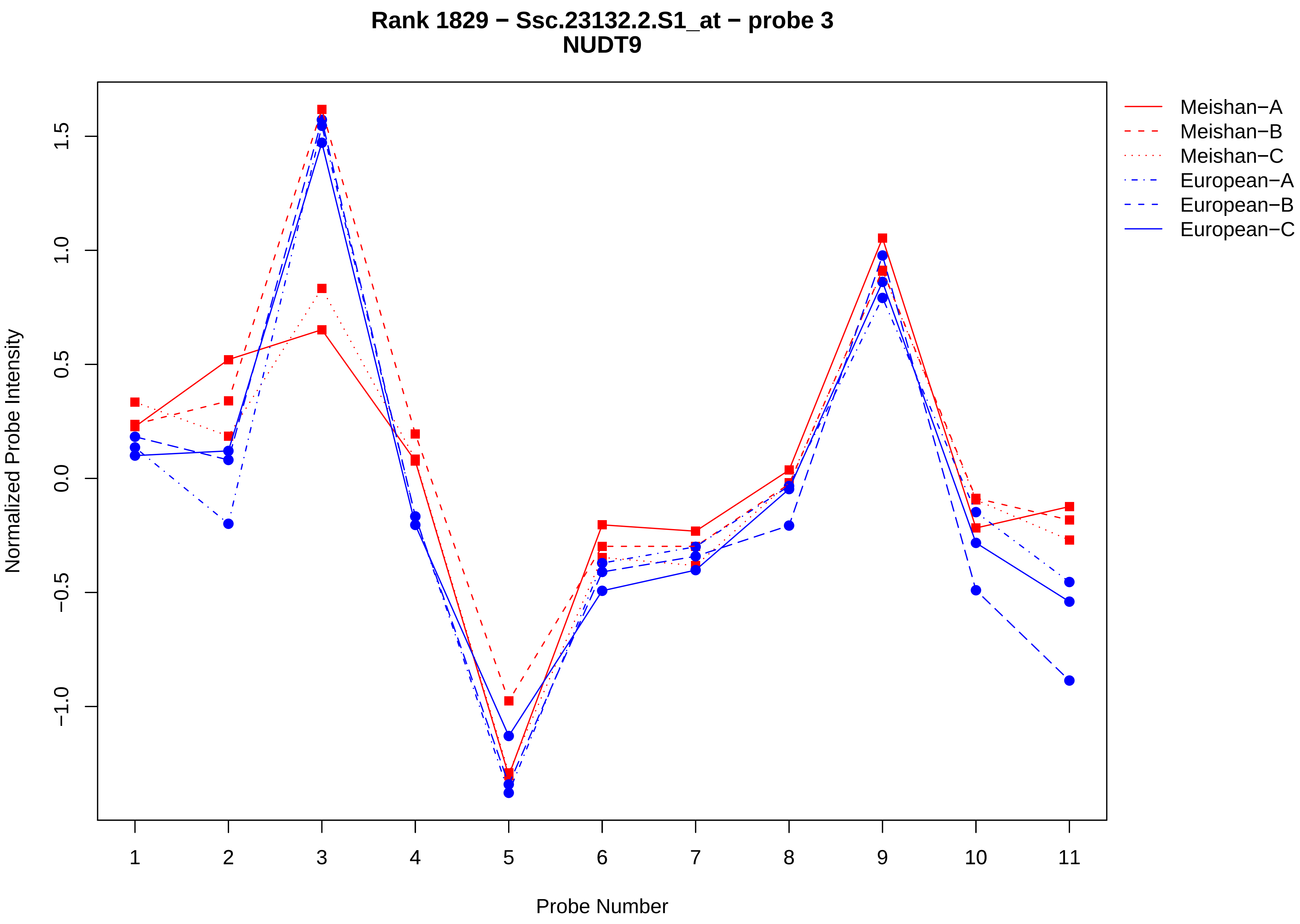

Rank 1829 − Ssc.23132.2.S1_at − probe 3
NUDT9
Normalized Probe Intensity
−1.0
−0.5
0.0
0.5
1.0
1.5
1
2
3
4
5
6
7
8
9
10
11
Probe Number
Meishan−A
Meishan−B
Meishan−C
European−A
European−B
European−C

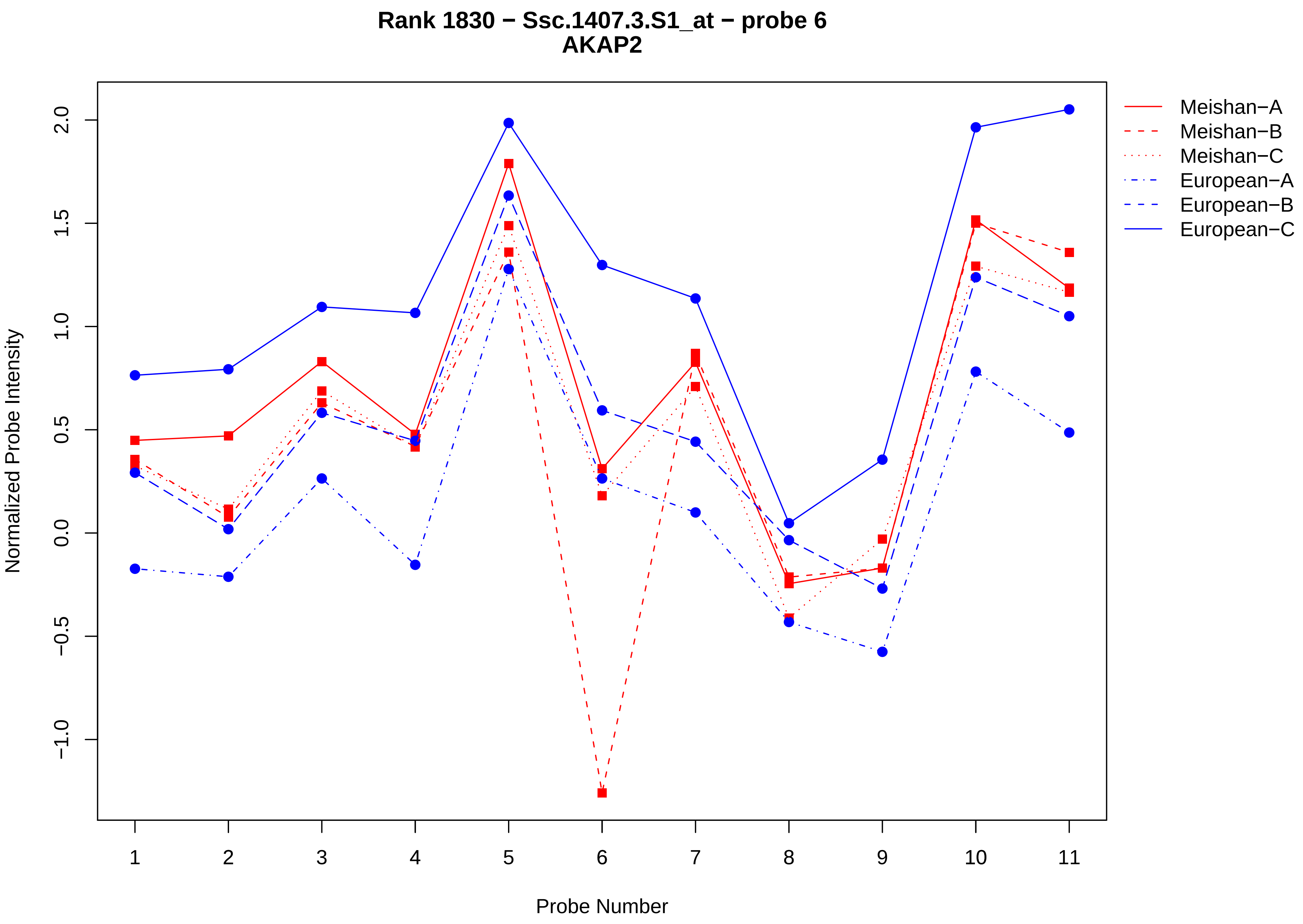

Rank 1830 − Ssc.1407.3.S1_at − probe 6
AKAP2
Normalized Probe Intensity
Probe Number
Meishan−A
Meishan−B
Meishan−C
European−A
European−B
European−C

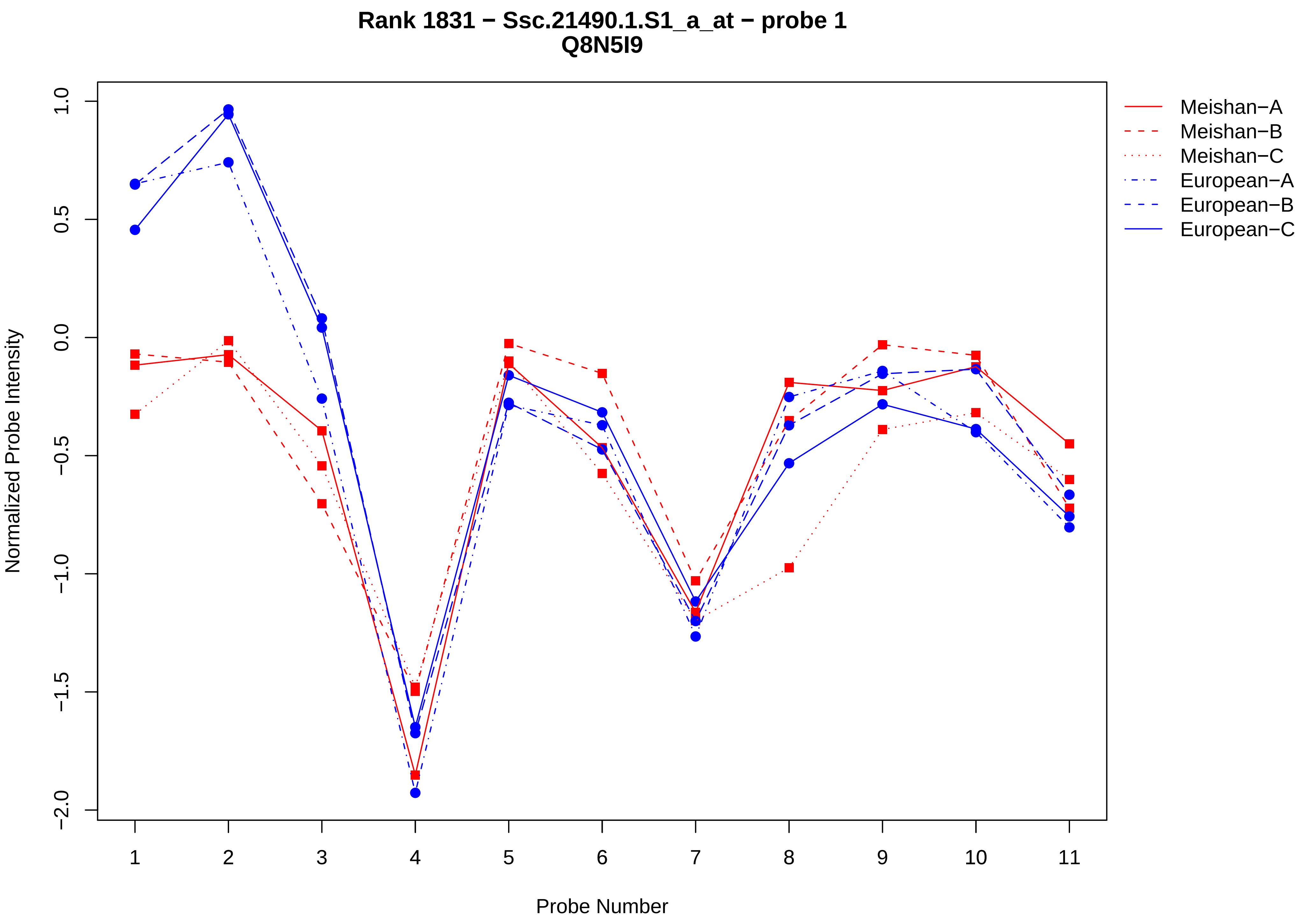

Rank 1831 − Ssc.21490.1.S1_a_at − probe 1
Q8N5I9
Normalized Probe Intensity
Probe Number
1.0
0.5
0.0
−0.5
−1.0
−1.5
−2.0
1
2
3
4
5
6
7
8
9
10
11
Meishan−A
Meishan−B
Meishan−C
European−A
European−B
European−C

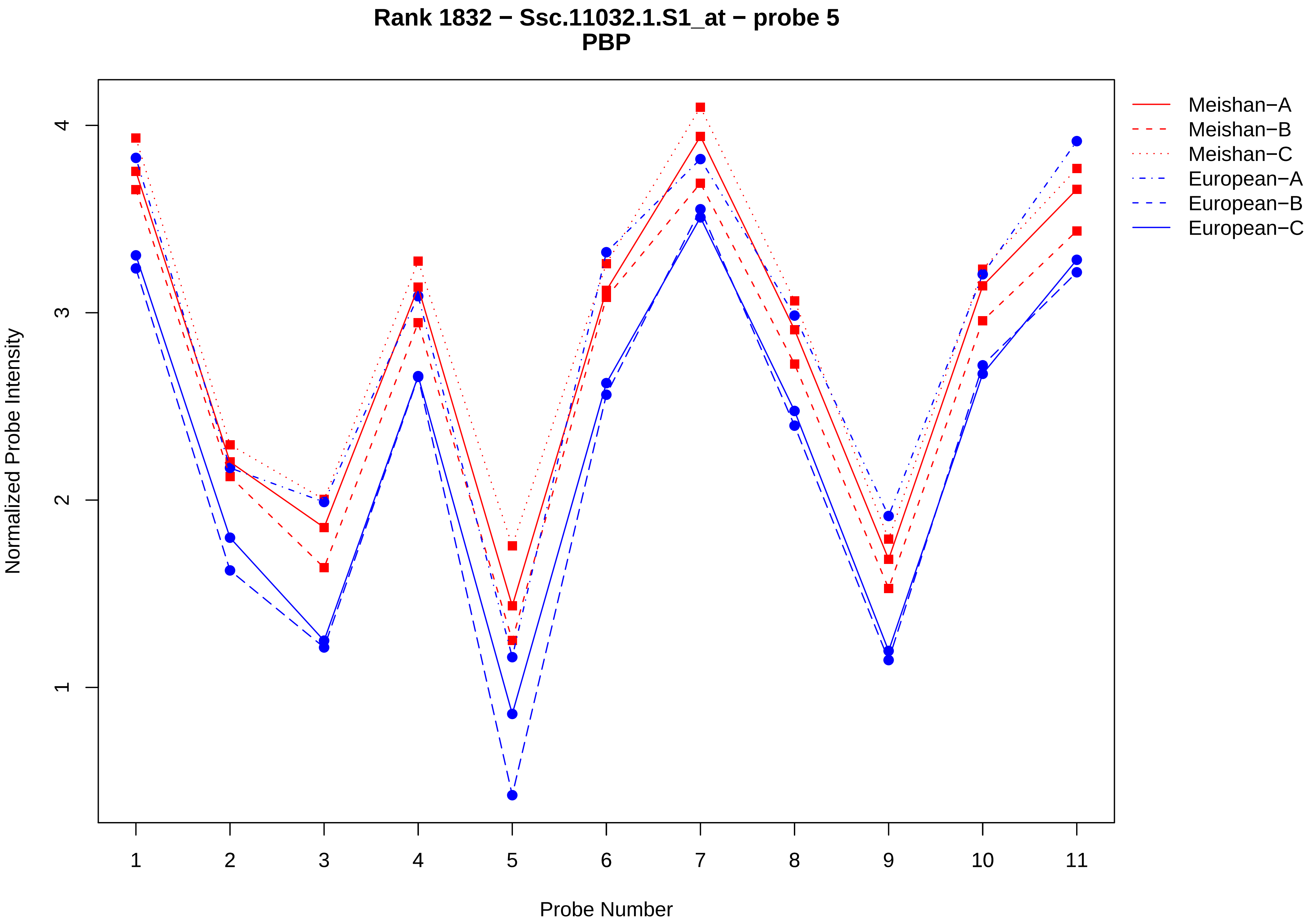

Rank 1832 − Ssc.11032.1.S1_at − probe 5
PBP
Normalized Probe Intensity
Probe Number
1
2
3
4
1
2
3
4
5
6
7
8
9
10
11
Meishan−A
Meishan−B
Meishan−C
European−A
European−B
European−C

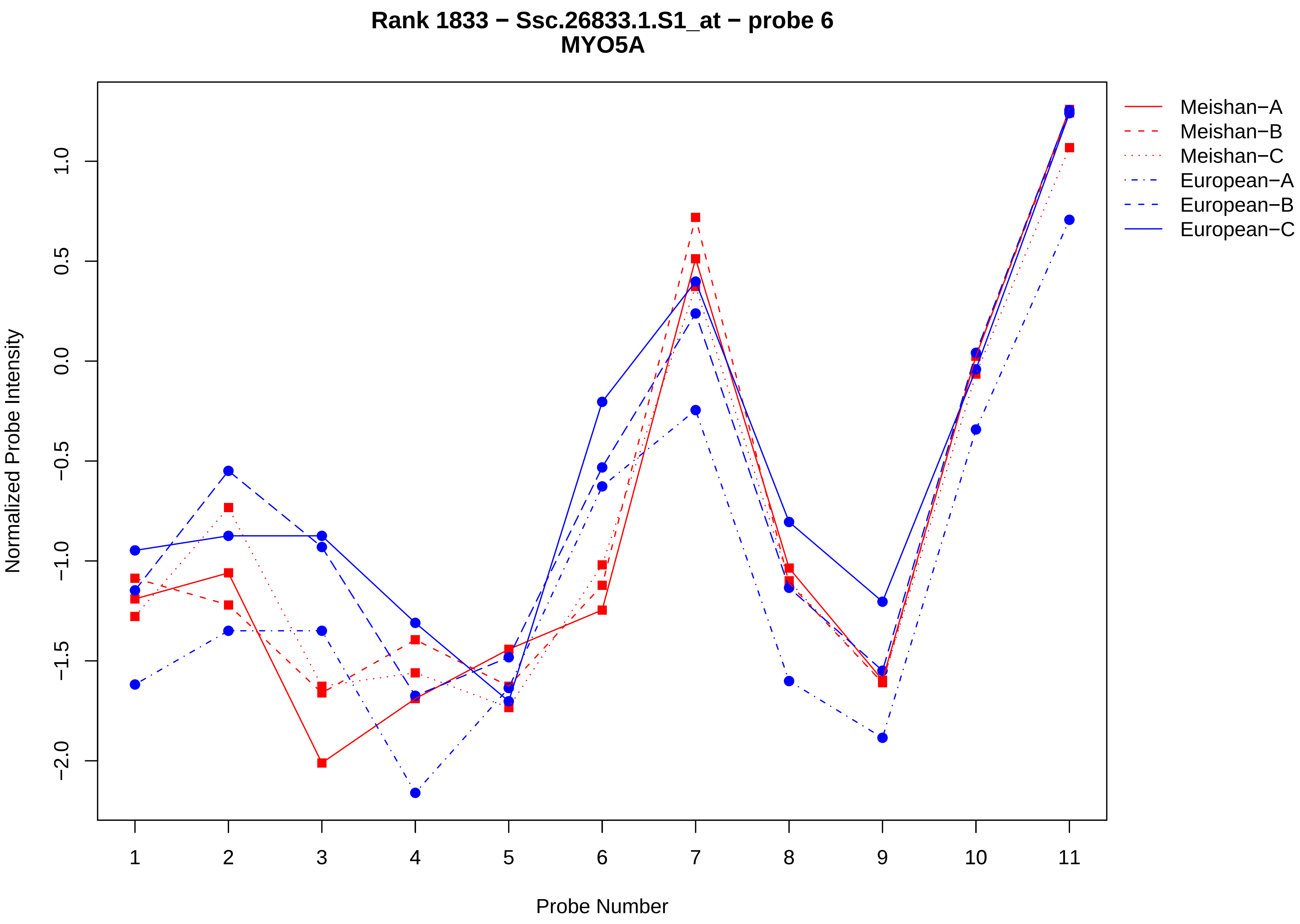

Rank 1833 − Ssc.26833.1.S1_at − probe 6
MYO5A
Normalized Probe Intensity
Probe Number
Meishan−A
Meishan−B
Meishan−C
European−A
European−B
European−C
−2.0
−1.5
−1.0
−0.5
0.0
0.5
1.0
1
2
3
4
5
6
7
8
9
10
11

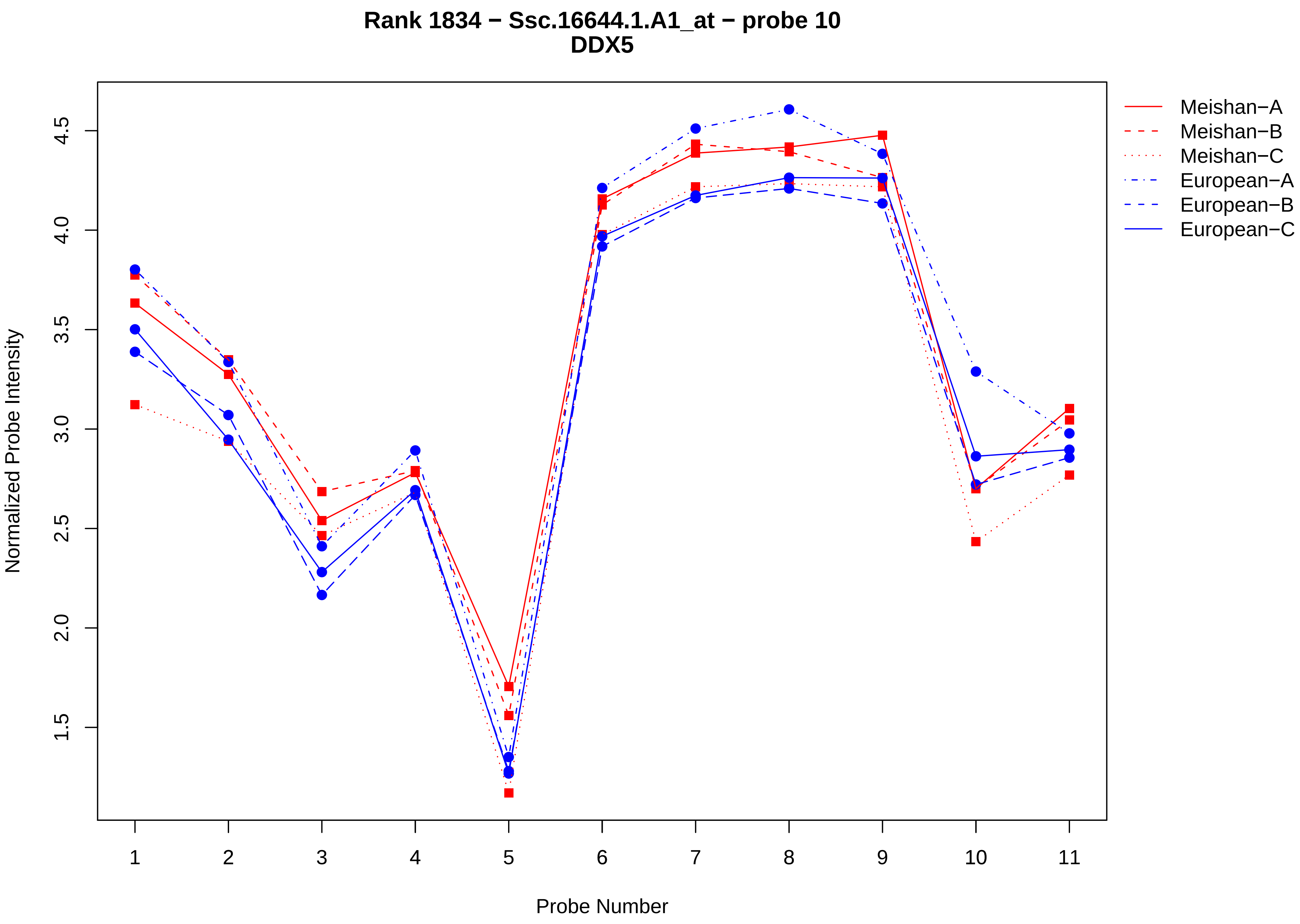

Rank 1834 − Ssc.16644.1.A1_at − probe 10
DDX5
Normalized Probe Intensity
Probe Number
1.5
2.0
2.5
3.0
3.5
4.0
4.5
1
2
3
4
5
6
7
8
9
10
11
Meishan−A
Meishan−B
Meishan−C
European−A
European−B
European−C

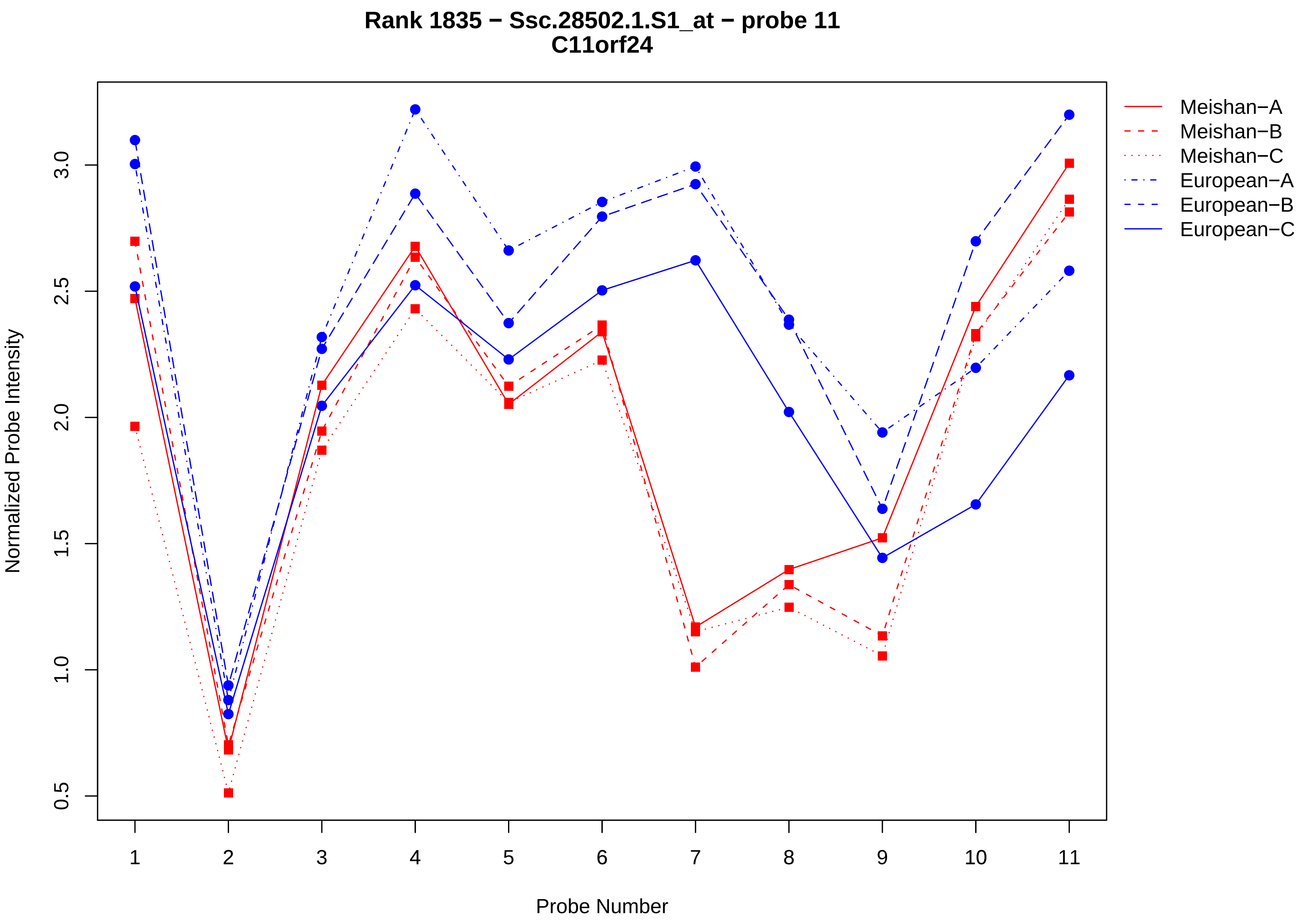

Rank 1835 − Ssc.28502.1.S1_at − probe 11
C11orf24
Normalized Probe Intensity
Probe Number
0.5
1.0
1.5
2.0
2.5
3.0
1
2
3
4
5
6
7
8
9
10
11
Meishan−A
Meishan−B
Meishan−C
European−A
European−B
European−C

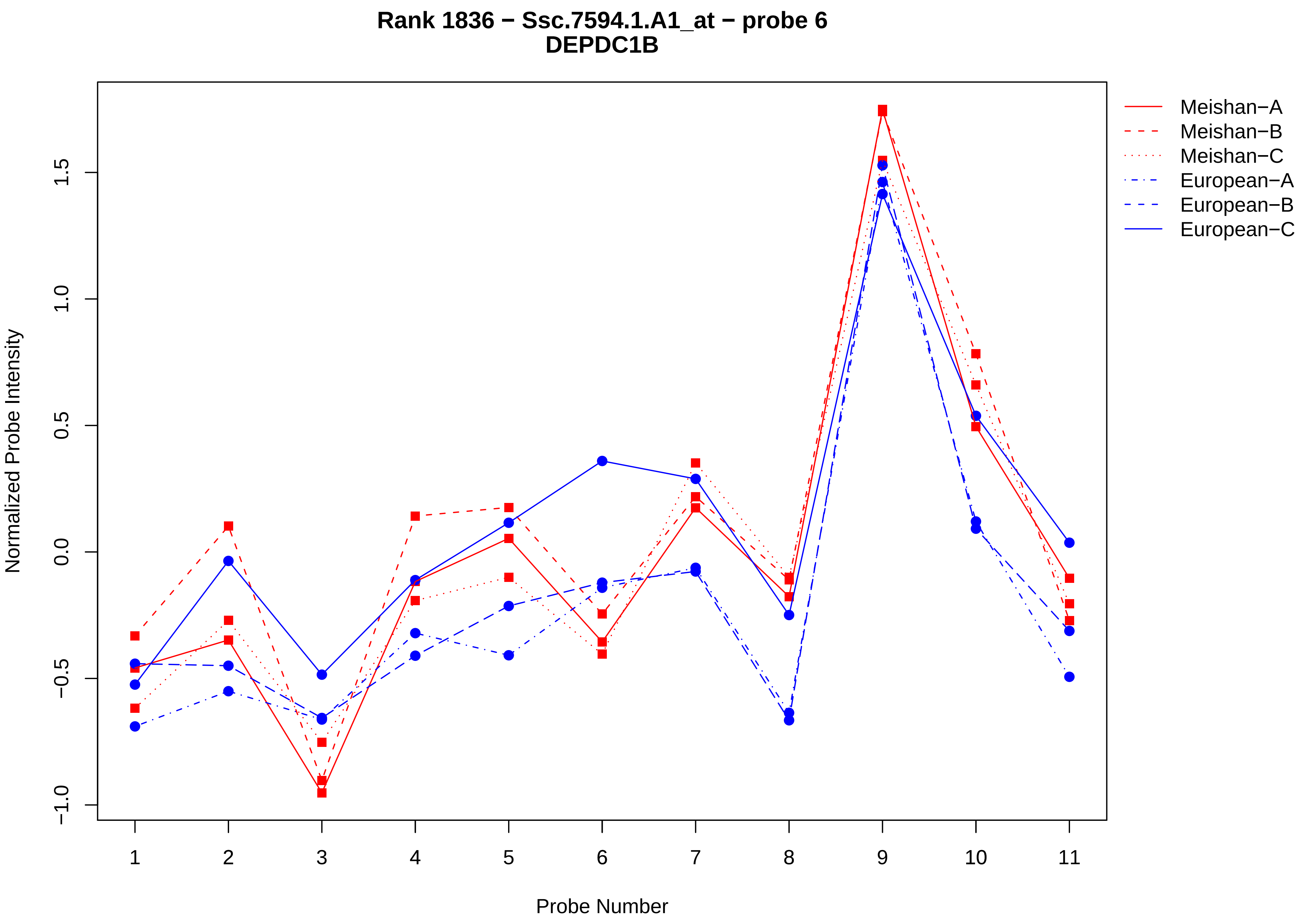

Rank 1836 − Ssc.7594.1.A1_at − probe 6
DEPDC1B
Normalized Probe Intensity
Probe Number
−1.0
−0.5
0.0
0.5
1.0
1.5
1
2
3
4
5
6
7
8
9
10
11
Meishan−A
Meishan−B
Meishan−C
European−A
European−B
European−C

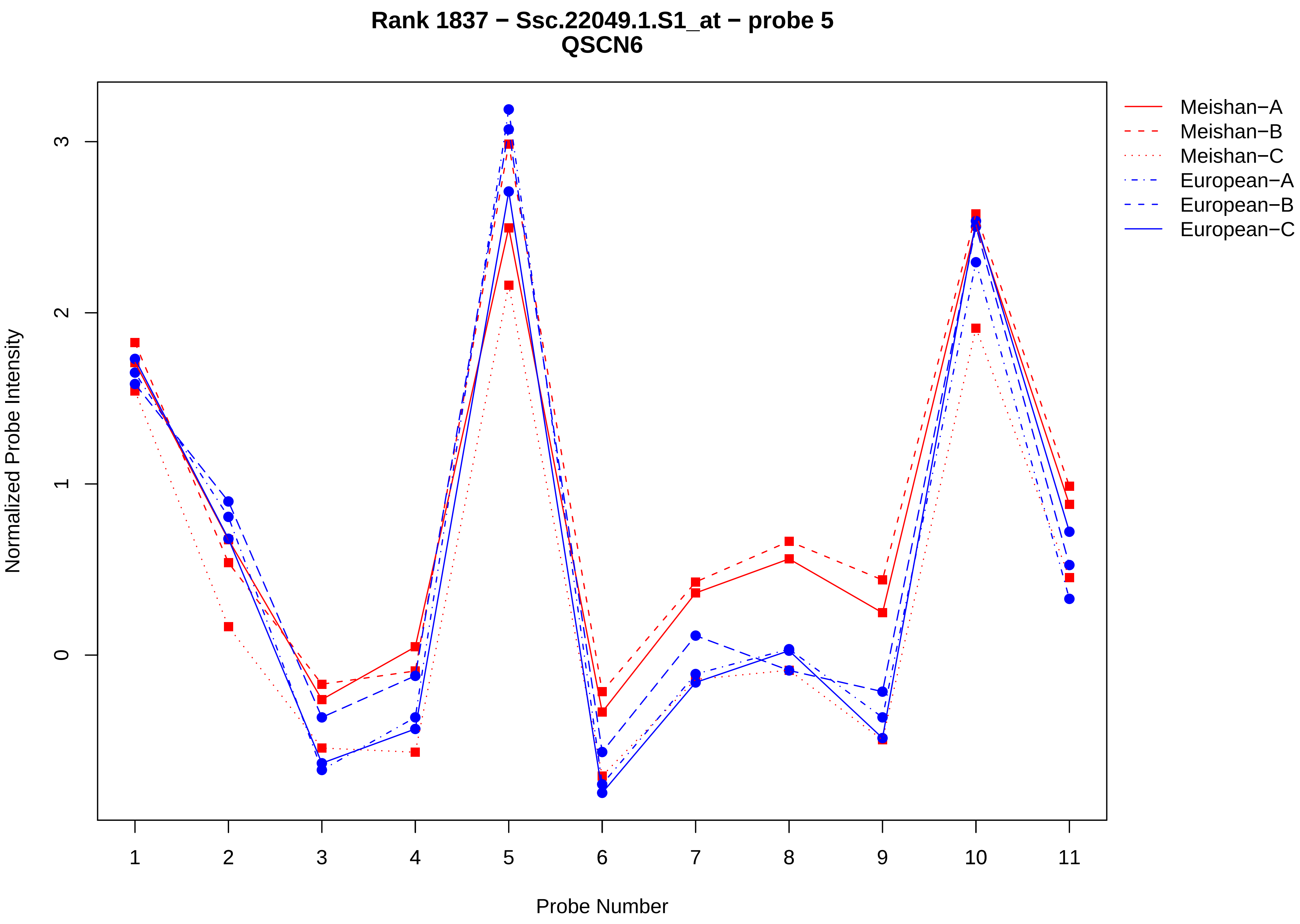
Rank 1837 − Ssc.22049.1.S1_at − probe 5
QSCN6
Normalized Probe Intensity
Probe Number
0
1
2
3
1
2
3
4
5
6
7
8
9
10
11
Meishan−A
Meishan−B
Meishan−C
European−A
European−B
European−C

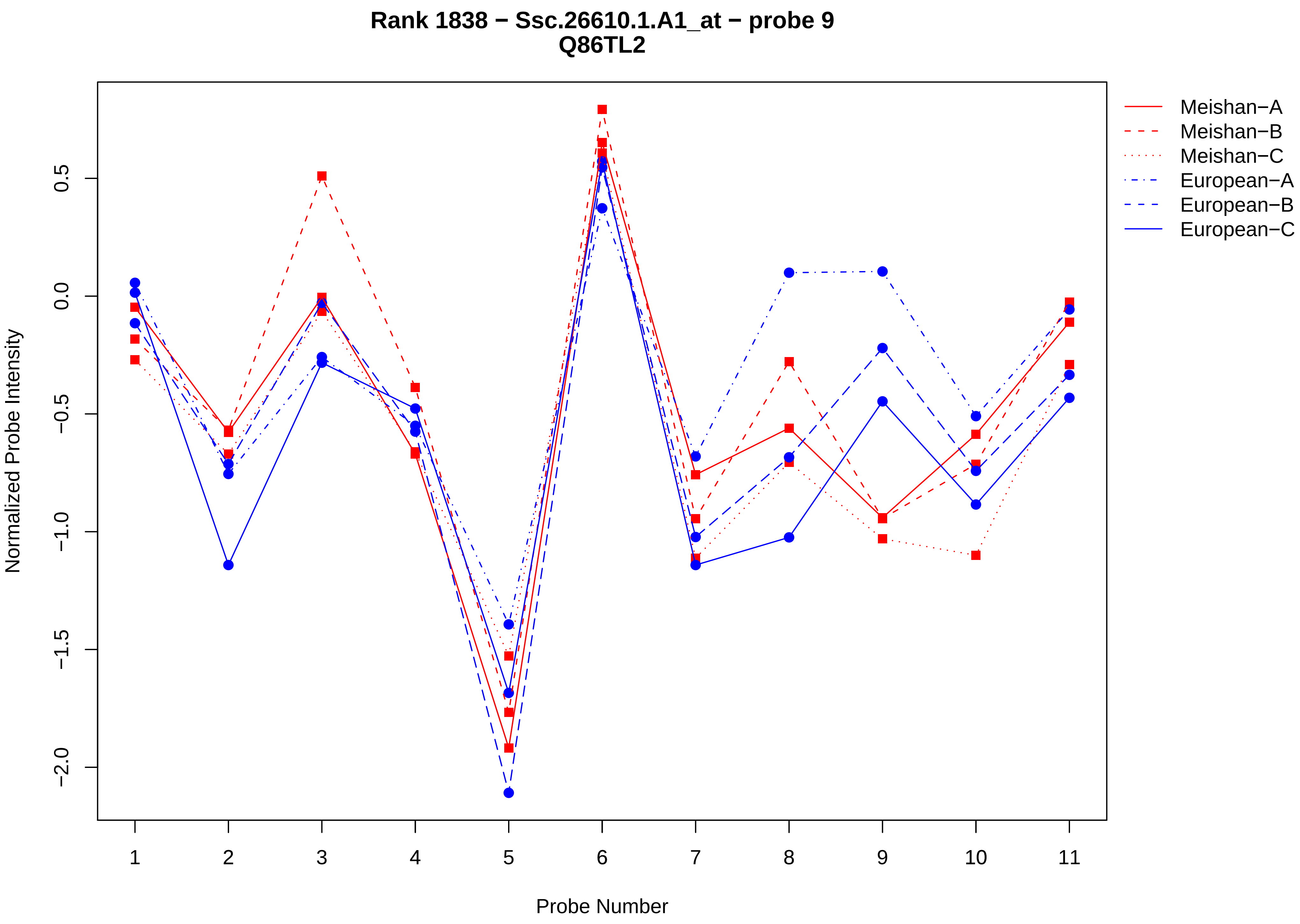

Rank 1838 − Ssc.26610.1.A1_at − probe 9
Q86TL2
Normalized Probe Intensity
Probe Number
0.5
0.0
−0.5
−1.0
−1.5
−2.0
1
2
3
4
5
6
7
8
9
10
11
Meishan−A
Meishan−B
Meishan−C
European−A
European−B
European−C

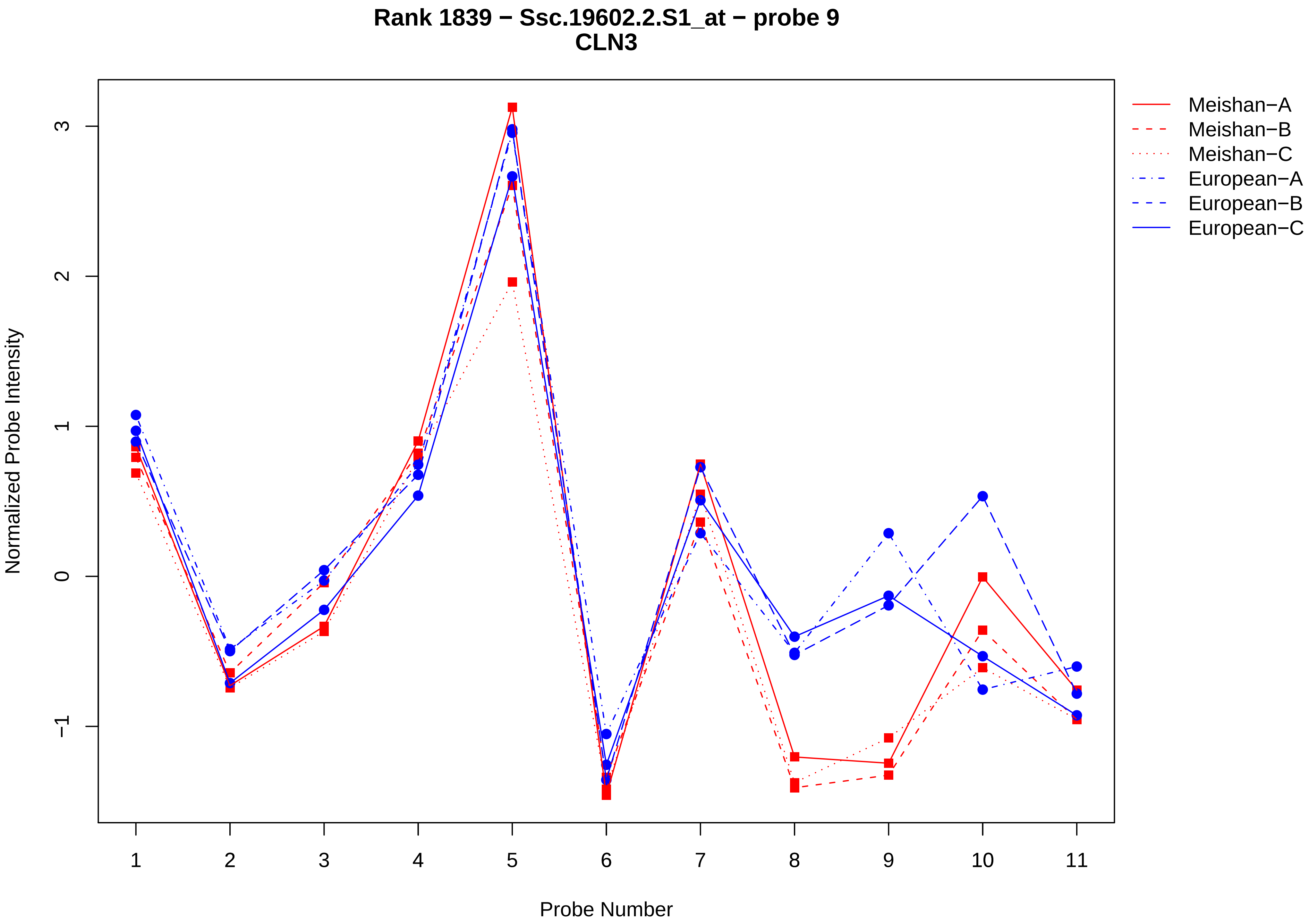

Rank 1839 − Ssc.19602.2.S1_at − probe 9
CLN3
Normalized Probe Intensity
Probe Number
−1
0
1
2
3
1
2
3
4
5
6
7
8
9
10
11
Meishan−A
Meishan−B
Meishan−C
European−A
European−B
European−C

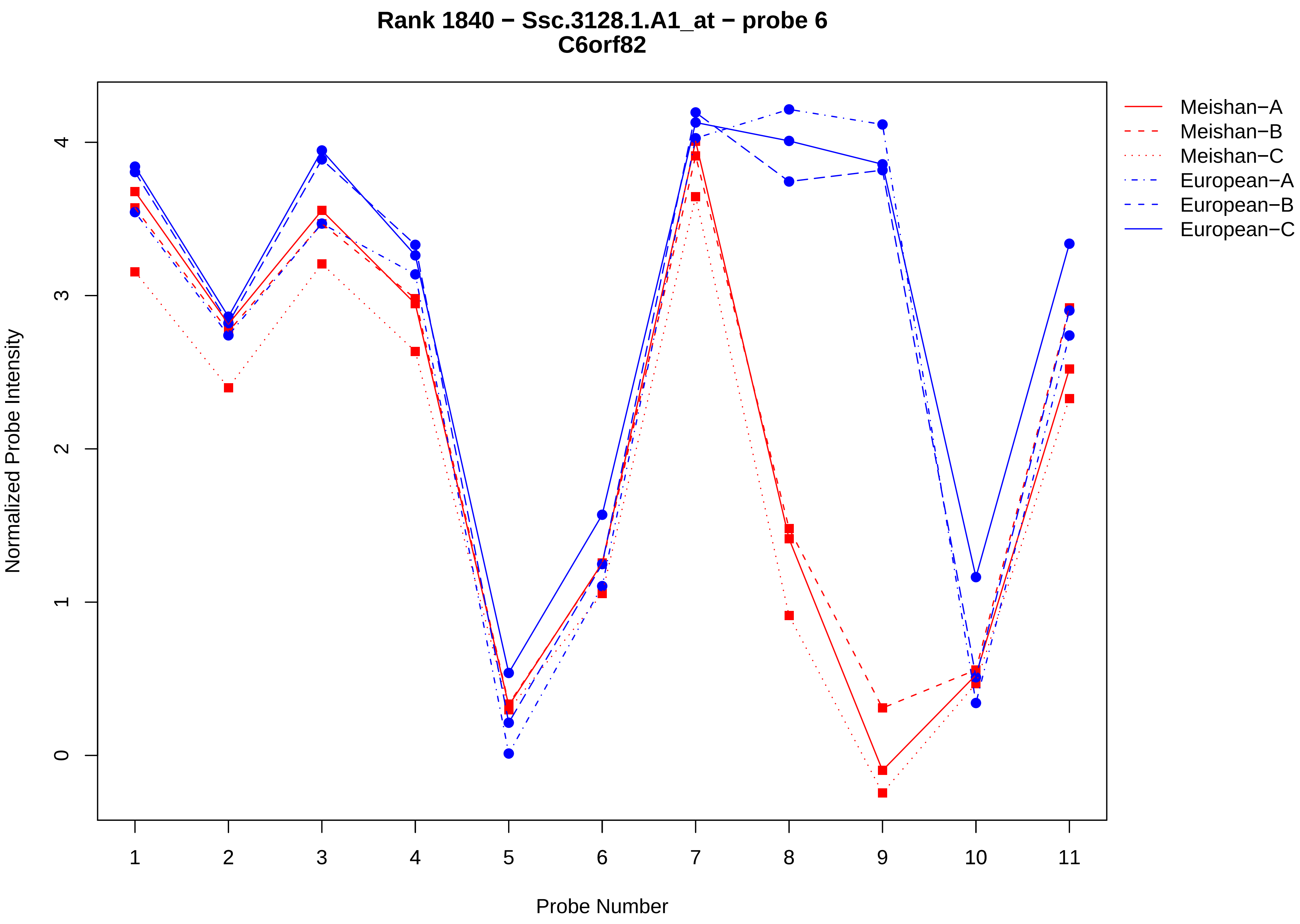

Rank 1840 − Ssc.3128.1.A1_at − probe 6
C6orf82
Normalized Probe Intensity
Probe Number
0
1
2
3
4
1
2
3
4
5
6
7
8
9
10
11
Meishan−A
Meishan−B
Meishan−C
European−A
European−B
European−C

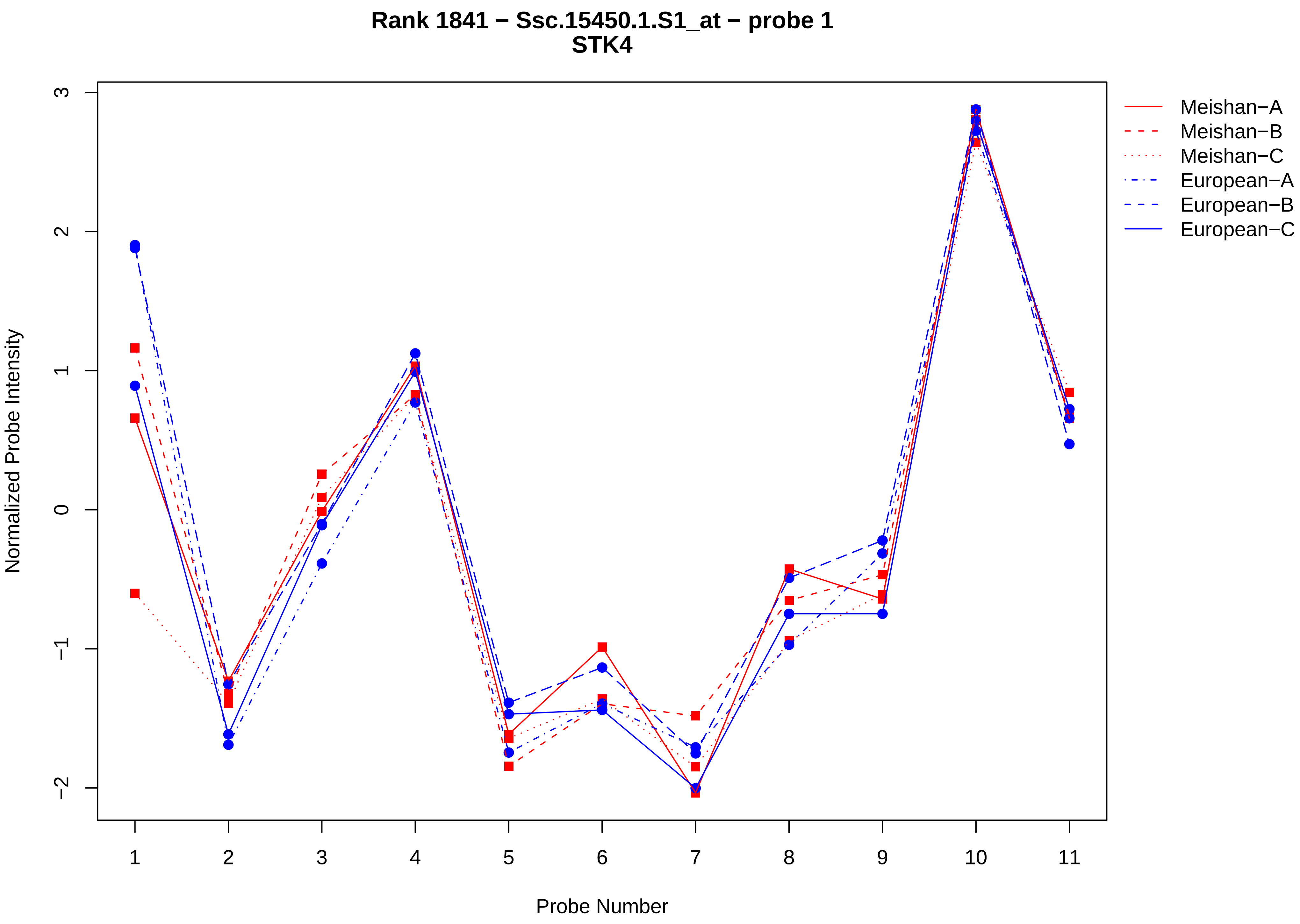

Rank 1841 − Ssc.15450.1.S1_at − probe 1
STK4
Normalized Probe Intensity
Probe Number
Meishan−A
Meishan−B
Meishan−C
European−A
European−B
European−C

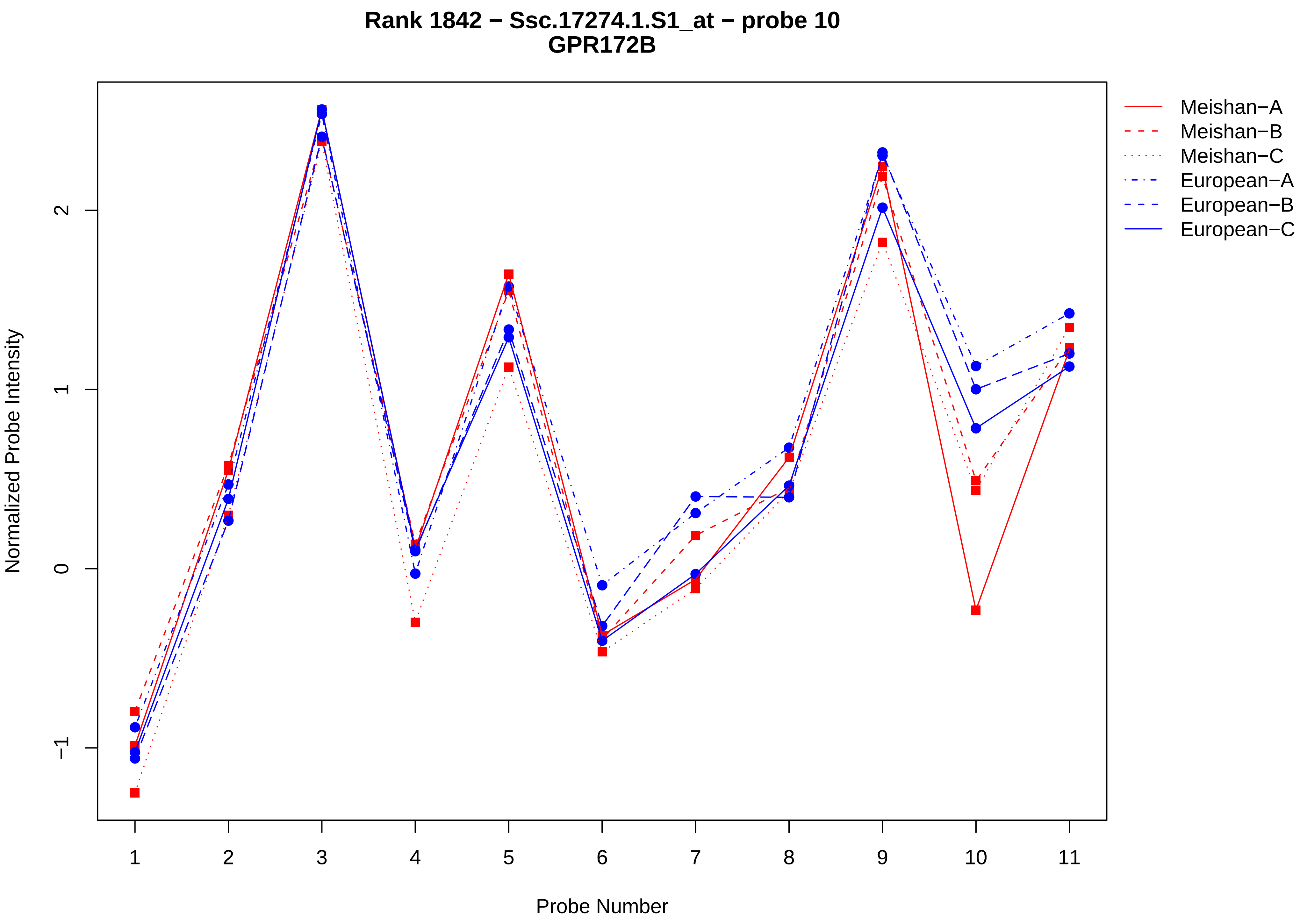
Rank 1842 − Ssc.17274.1.S1_at − probe 10
GPR172B
Normalized Probe Intensity
Probe Number
−1
0
1
2
1
2
3
4
5
6
7
8
9
10
11
Meishan−A
Meishan−B
Meishan−C
European−A
European−B
European−C

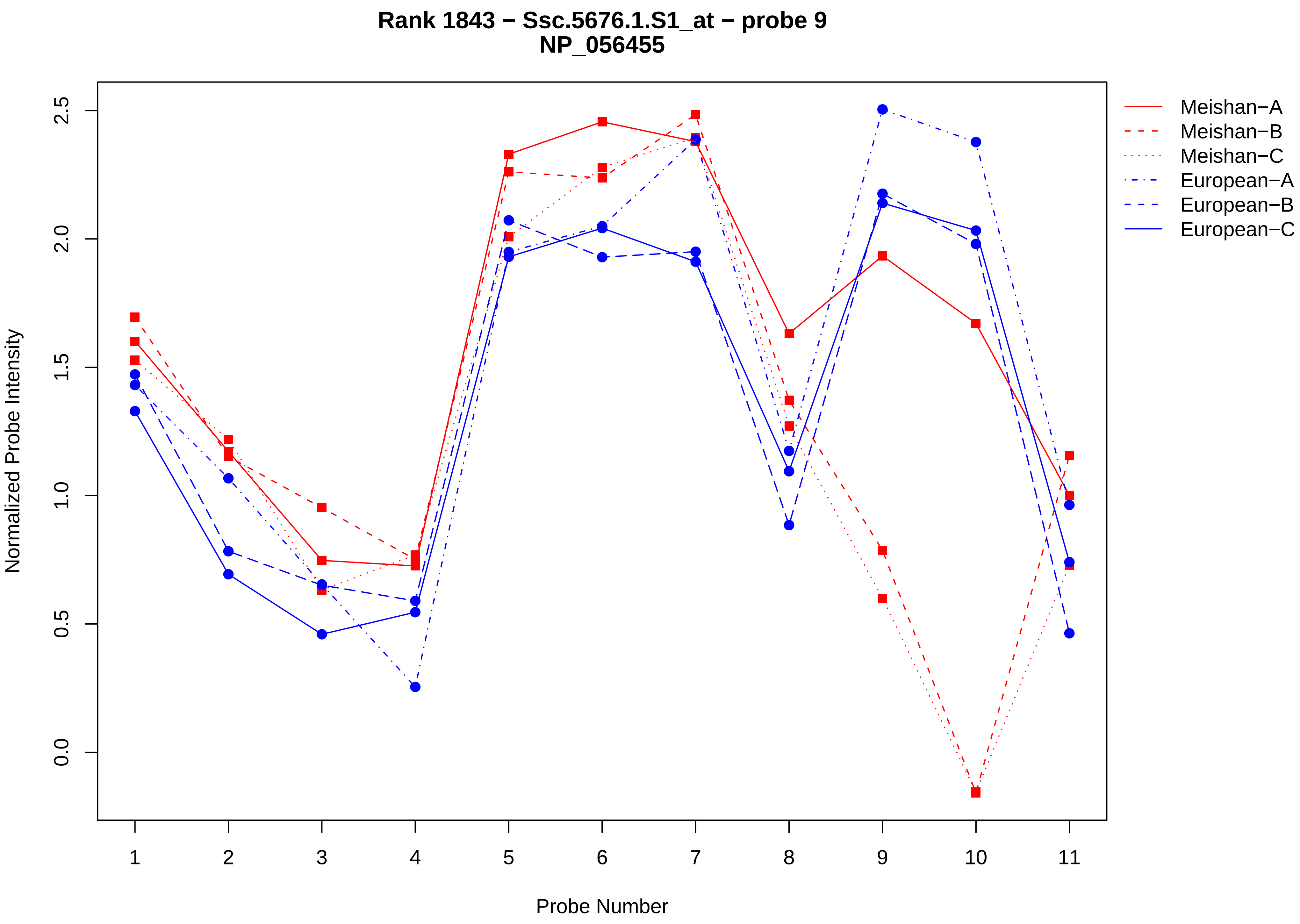
Rank 1843 − Ssc.5676.1.S1_at − probe 9
NP_056455
Normalized Probe Intensity
0.0
0.5
1.0
1.5
2.0
2.5
Probe Number
1
2
3
4
5
6
7
8
9
10
11
Meishan−A
Meishan−B
Meishan−C
European−A
European−B
European−C

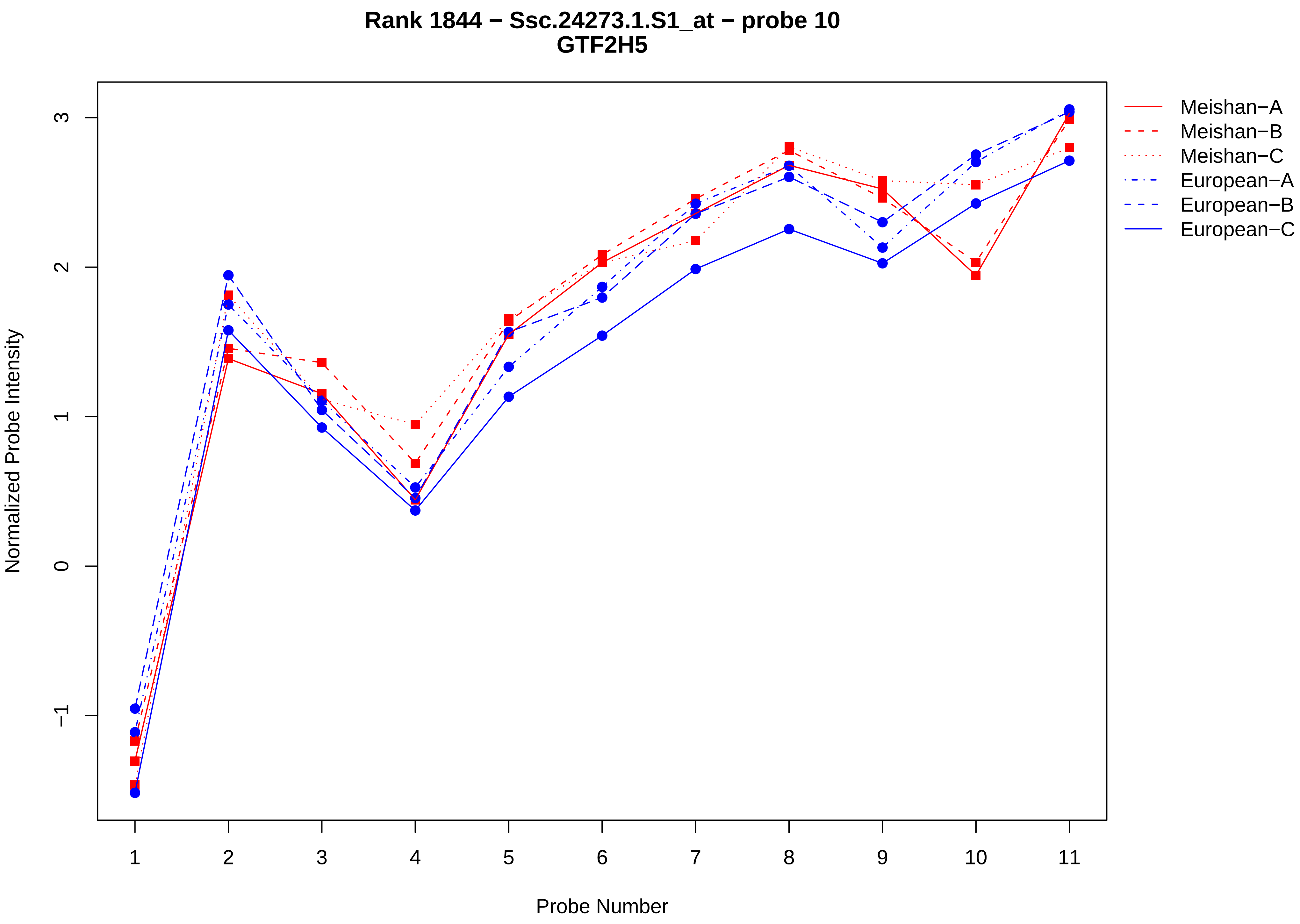

Rank 1844 − Ssc.24273.1.S1_at − probe 10
GTF2H5
Normalized Probe Intensity
Probe Number
Meishan−A
Meishan−B
Meishan−C
European−A
European−B
European−C
−1
0
1
2
3
1
2
3
4
5
6
7
8
9
10
11

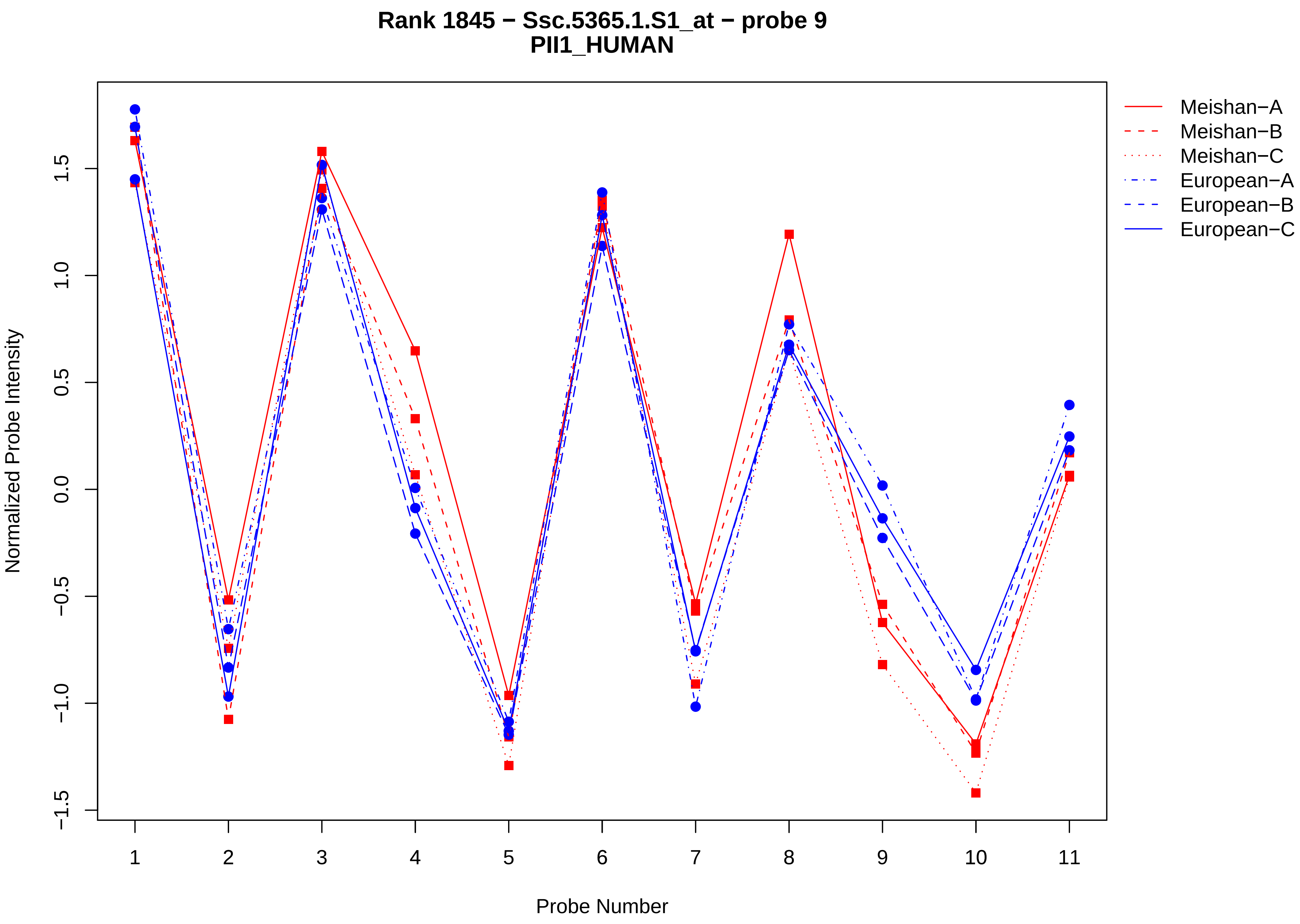
Rank 1845 − Ssc.5365.1.S1_at − probe 9
PII1_HUMAN
Normalized Probe Intensity
Probe Number
−1.5
−1.0
−0.5
0.0
0.5
1.0
1.5
1
2
3
4
5
6
7
8
9
10
11
Meishan−A
Meishan−B
Meishan−C
European−A
European−B
European−C

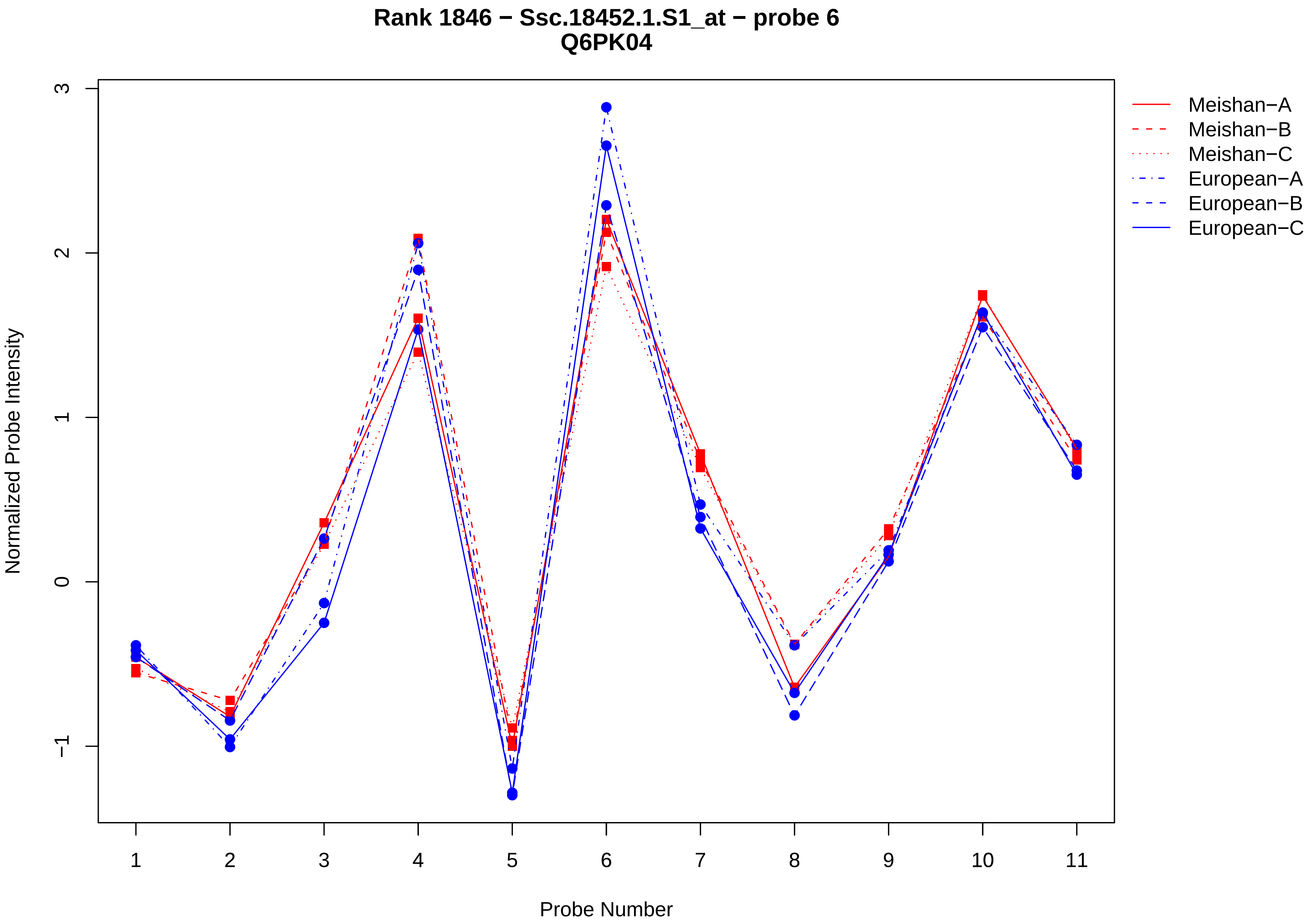

Rank 1846 − Ssc.18452.1.S1_at − probe 6
Q6PK04
Normalized Probe Intensity
Probe Number
−1
0
1
2
3
1
2
3
4
5
6
7
8
9
10
11
Meishan−A
Meishan−B
Meishan−C
European−A
European−B
European−C

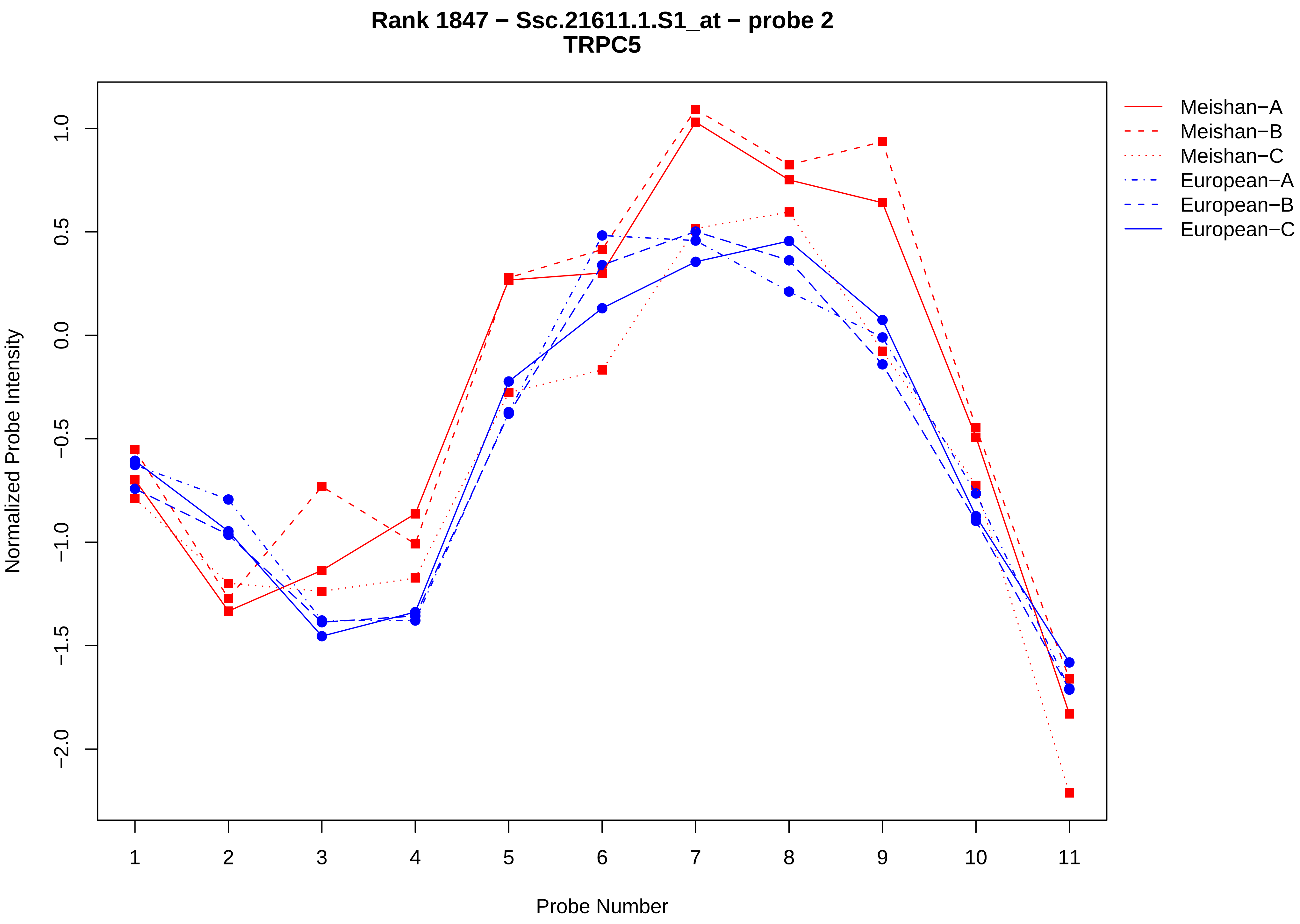

Rank 1847 − Ssc.21611.1.S1_at − probe 2
TRPC5
Normalized Probe Intensity
Probe Number
1.0
0.5
0.0
−0.5
−1.0
−1.5
−2.0
1
2
3
4
5
6
7
8
9
10
11
Meishan−A
Meishan−B
Meishan−C
European−A
European−B
European−C

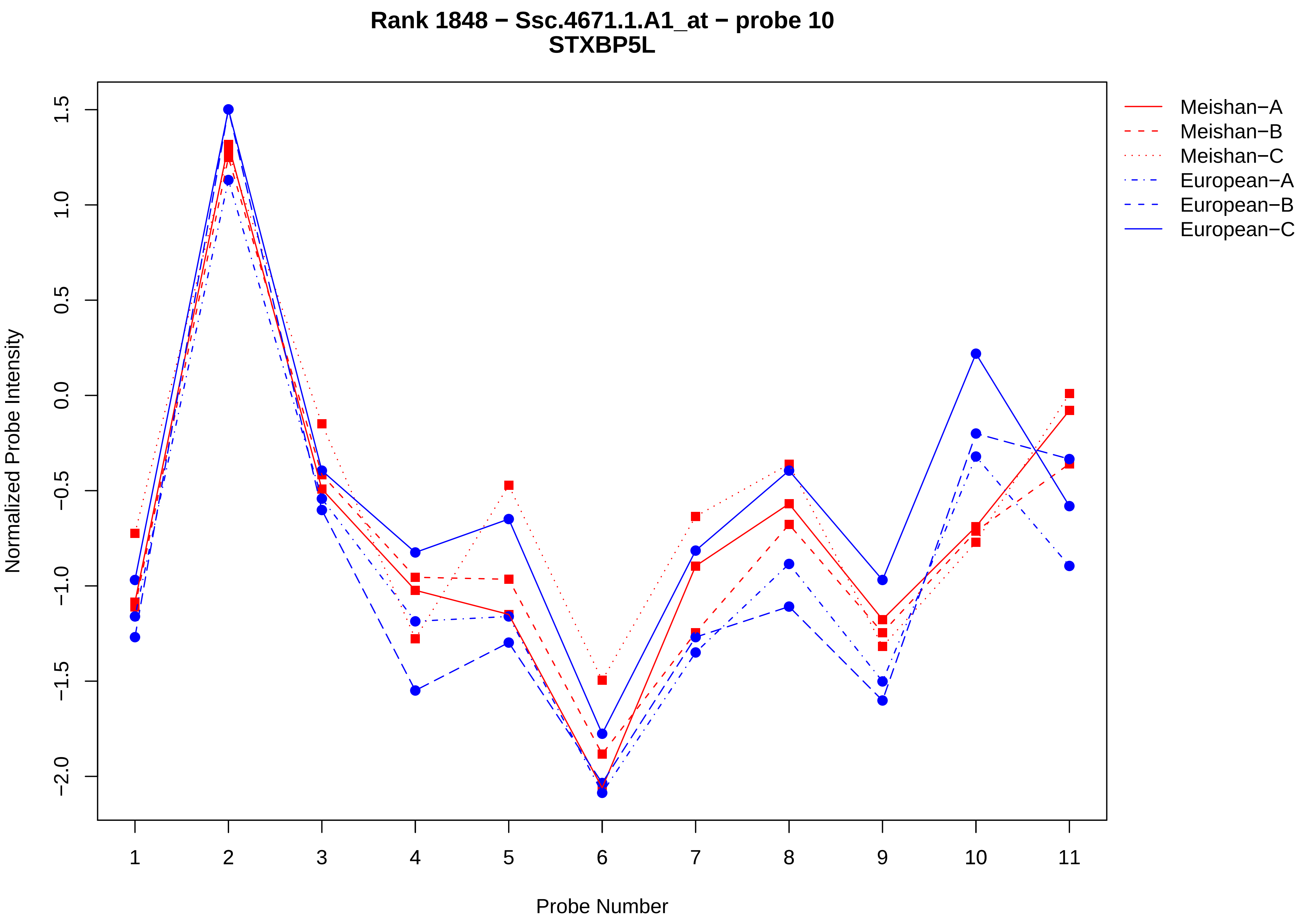

Rank 1848 − Ssc.4671.1.A1_at − probe 10
STXBP5L
Normalized Probe Intensity
Probe Number
1.5
1.0
0.5
0.0
−0.5
−1.0
−1.5
−2.0
1
2
3
4
5
6
7
8
9
10
11
Meishan−A
Meishan−B
Meishan−C
European−A
European−B
European−C

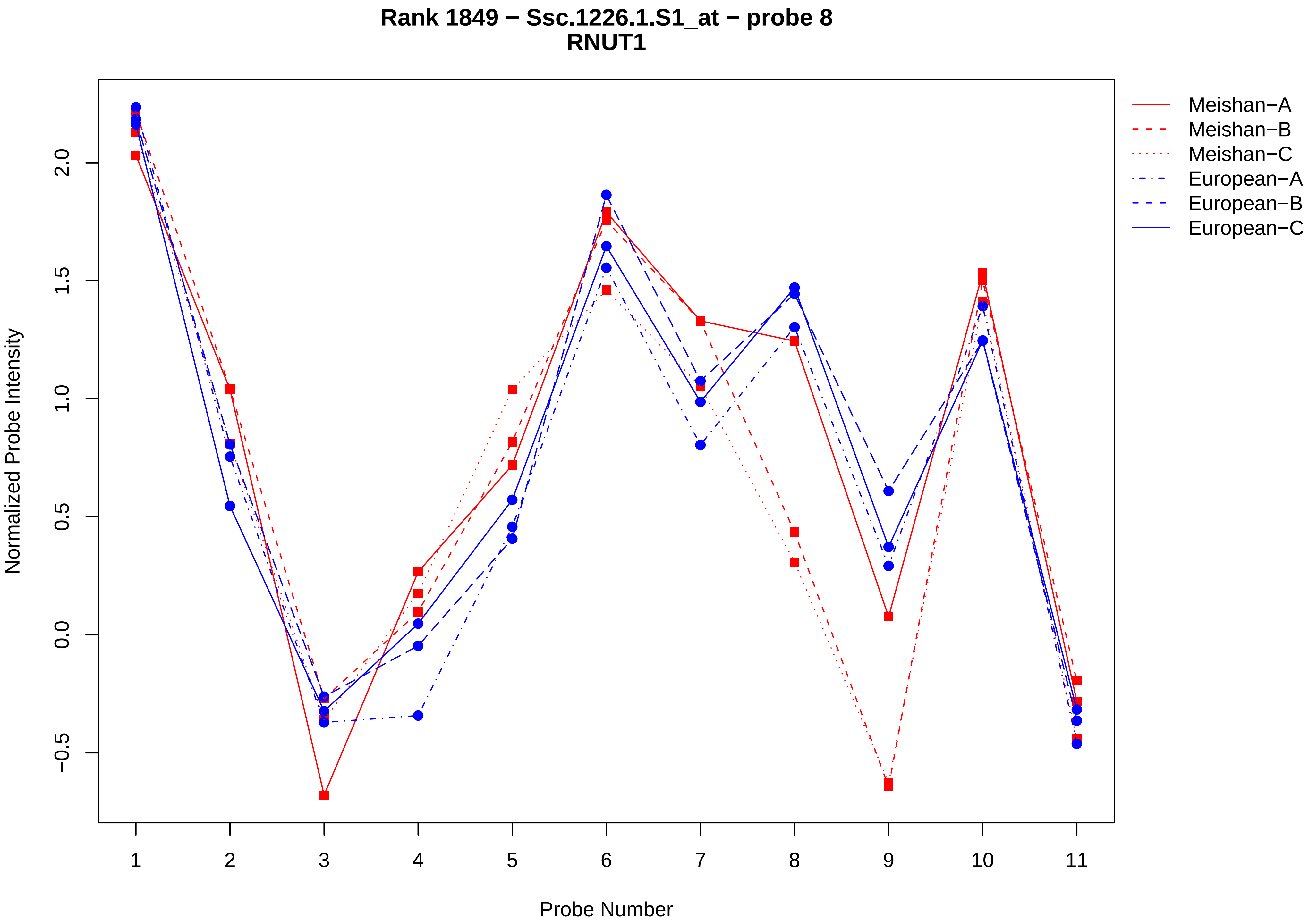

Rank 1849 − Ssc.1226.1.S1_at − probe 8
RNUT1
Normalized Probe Intensity
Probe Number
−0.5
0.0
0.5
1.0
1.5
2.0
1
2
3
4
5
6
7
8
9
10
11
Meishan−A
Meishan−B
Meishan−C
European−A
European−B
European−C

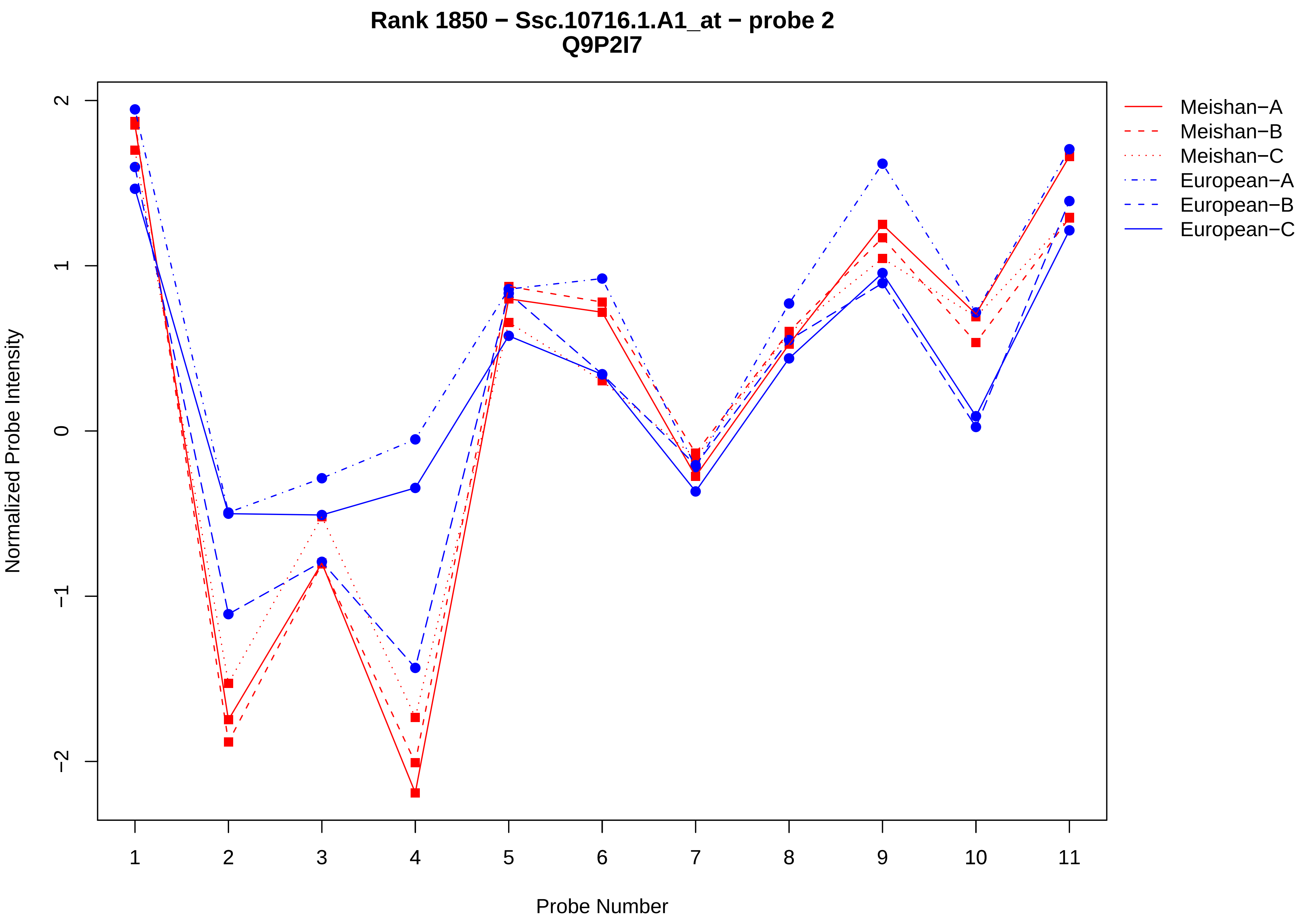

Rank 1850 − Ssc.10716.1.A1_at − probe 2
Q9P2I7
Normalized Probe Intensity
Probe Number
Meishan−A
Meishan−B
Meishan−C
European−A
European−B
European−C

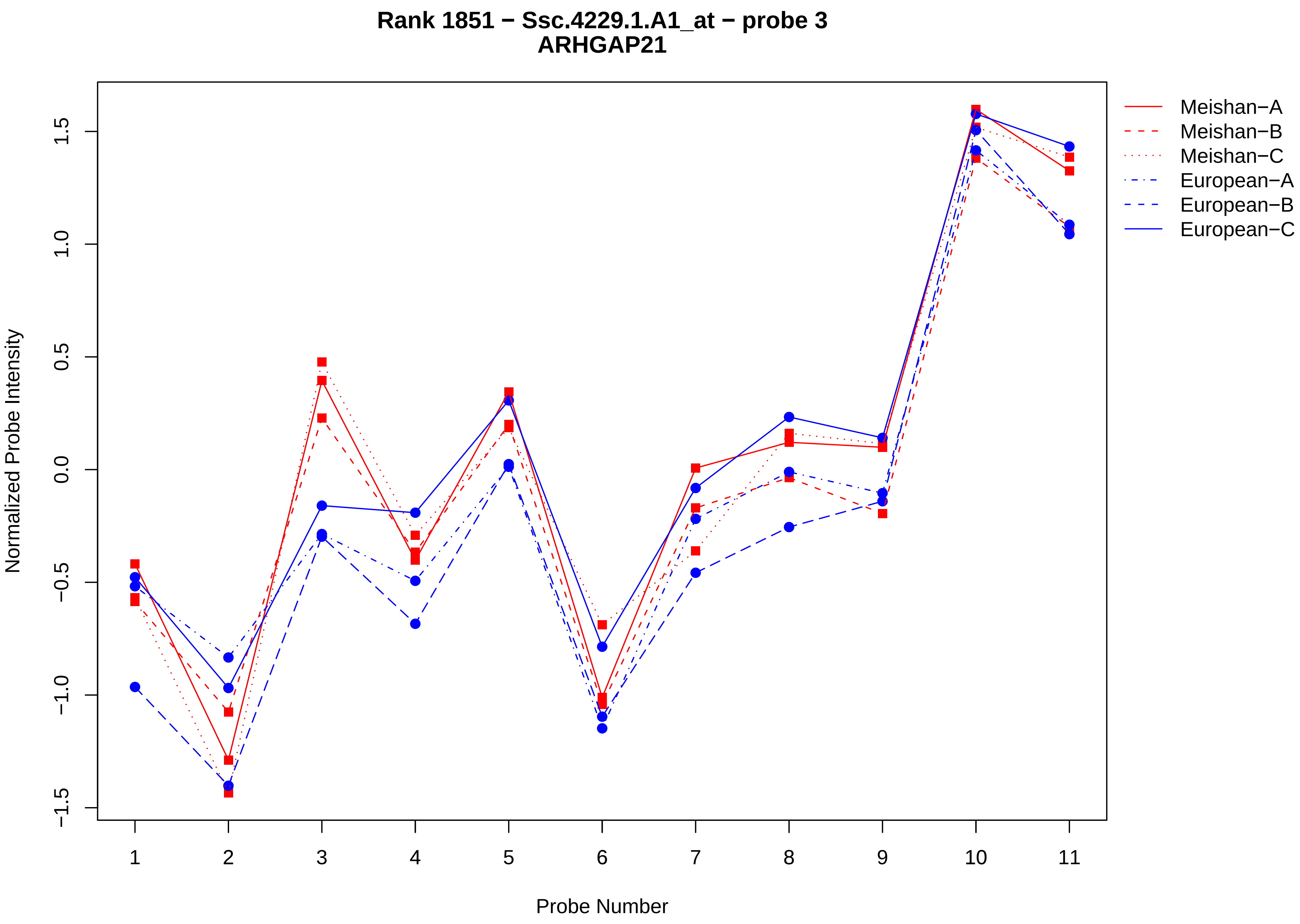

Rank 1851 − Ssc.4229.1.A1_at − probe 3
ARHGAP21
Normalized Probe Intensity
Probe Number
−1.5
−1.0
−0.5
0.0
0.5
1.0
1.5
1
2
3
4
5
6
7
8
9
10
11
Meishan−A
Meishan−B
Meishan−C
European−A
European−B
European−C

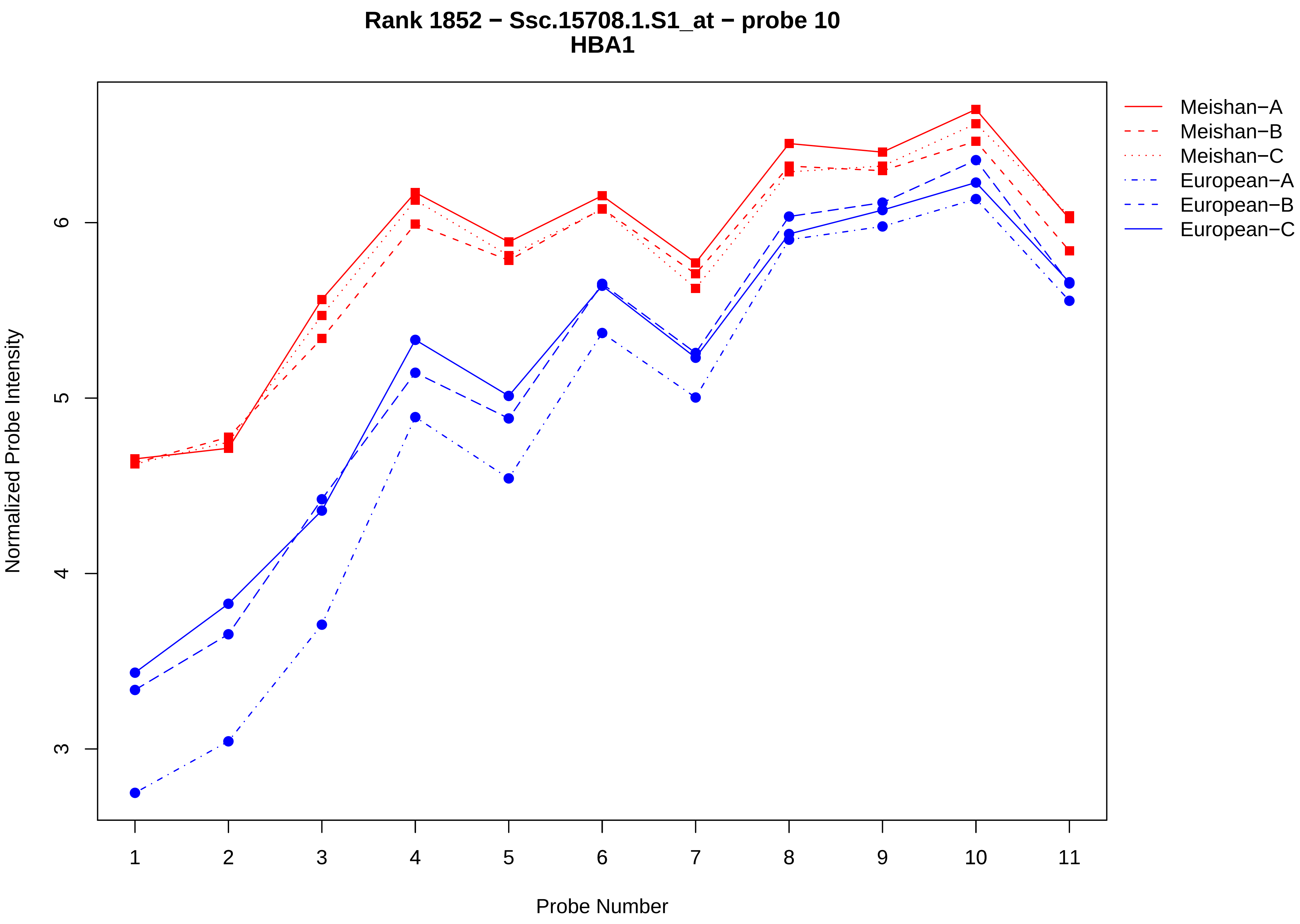

Rank 1852 − Ssc.15708.1.S1_at − probe 10
HBA1
Normalized Probe Intensity
Probe Number
3
4
5
6
1
2
3
4
5
6
7
8
9
10
11
Meishan−A
Meishan−B
Meishan−C
European−A
European−B
European−C

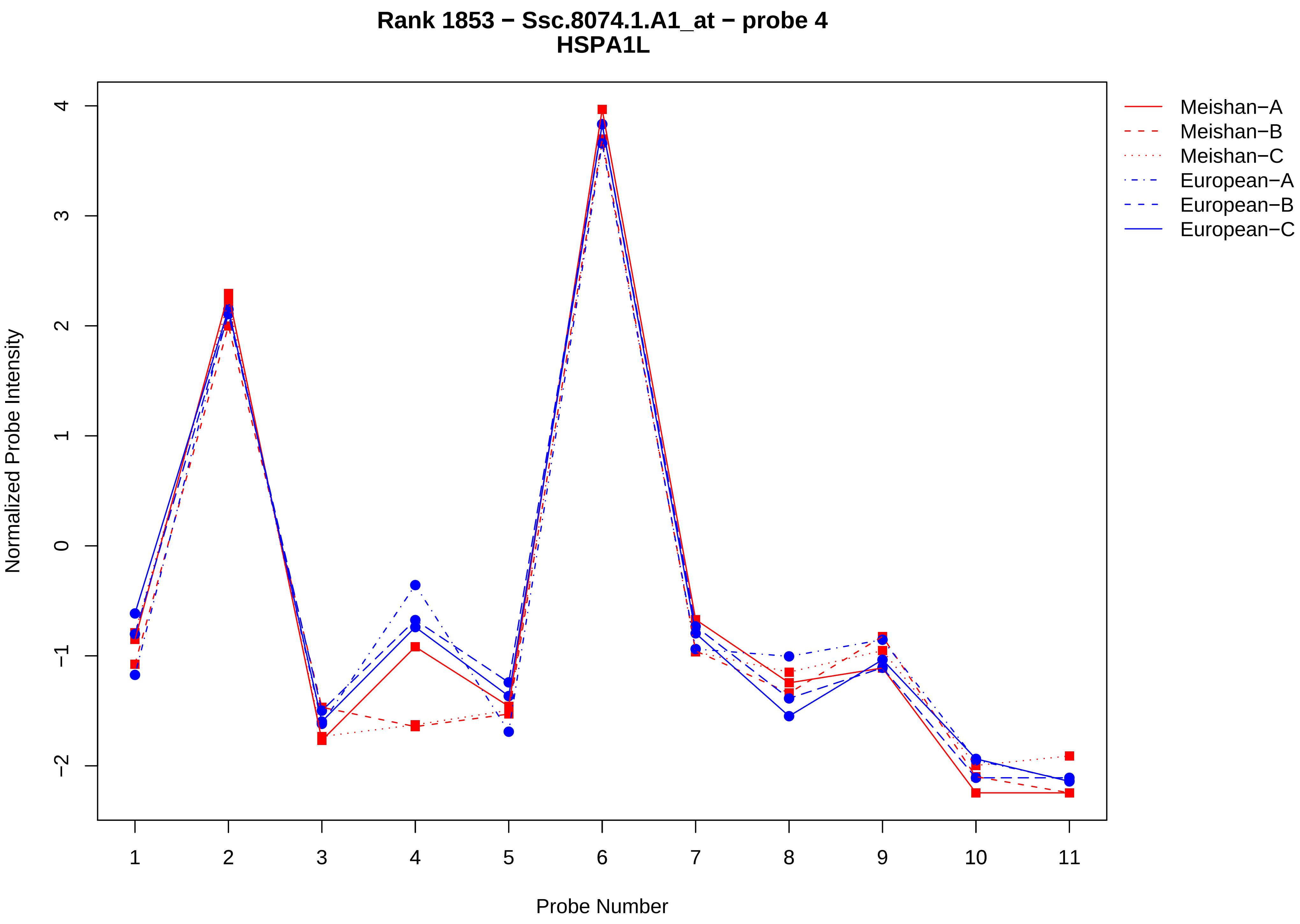

Rank 1853 − Ssc.8074.1.A1_at − probe 4
HSPA1L
Normalized Probe Intensity
Probe Number
4
3
2
1
0
−1
−2
1
2
3
4
5
6
7
8
9
10
11
Meishan−A
Meishan−B
Meishan−C
European−A
European−B
European−C

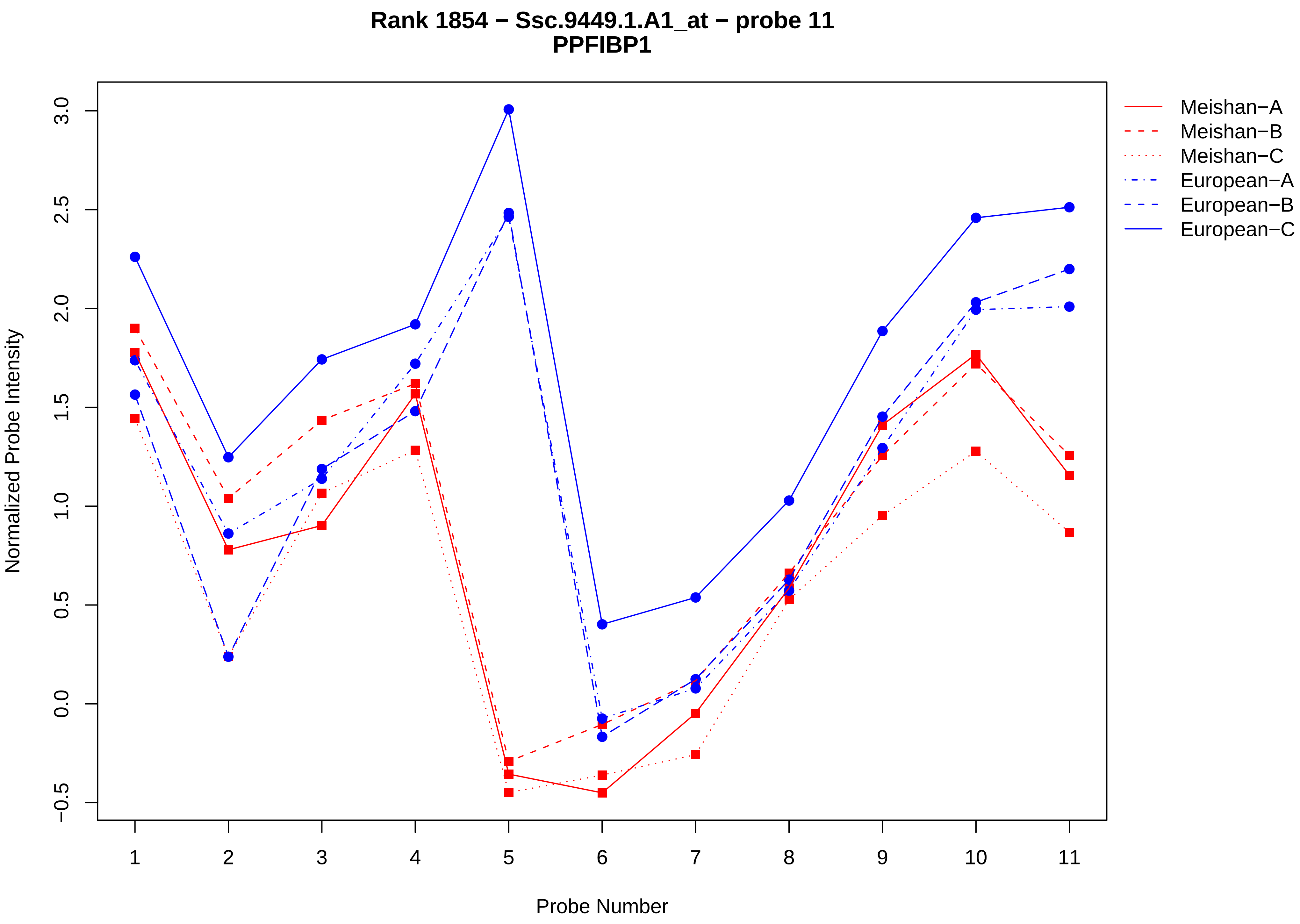

Rank 1854 − Ssc.9449.1.A1_at − probe 11
PPFIBP1
Normalized Probe Intensity
Probe Number
−0.5
0.0
0.5
1.0
1.5
2.0
2.5
3.0
1
2
3
4
5
6
7
8
9
10
11
Meishan−A
Meishan−B
Meishan−C
European−A
European−B
European−C

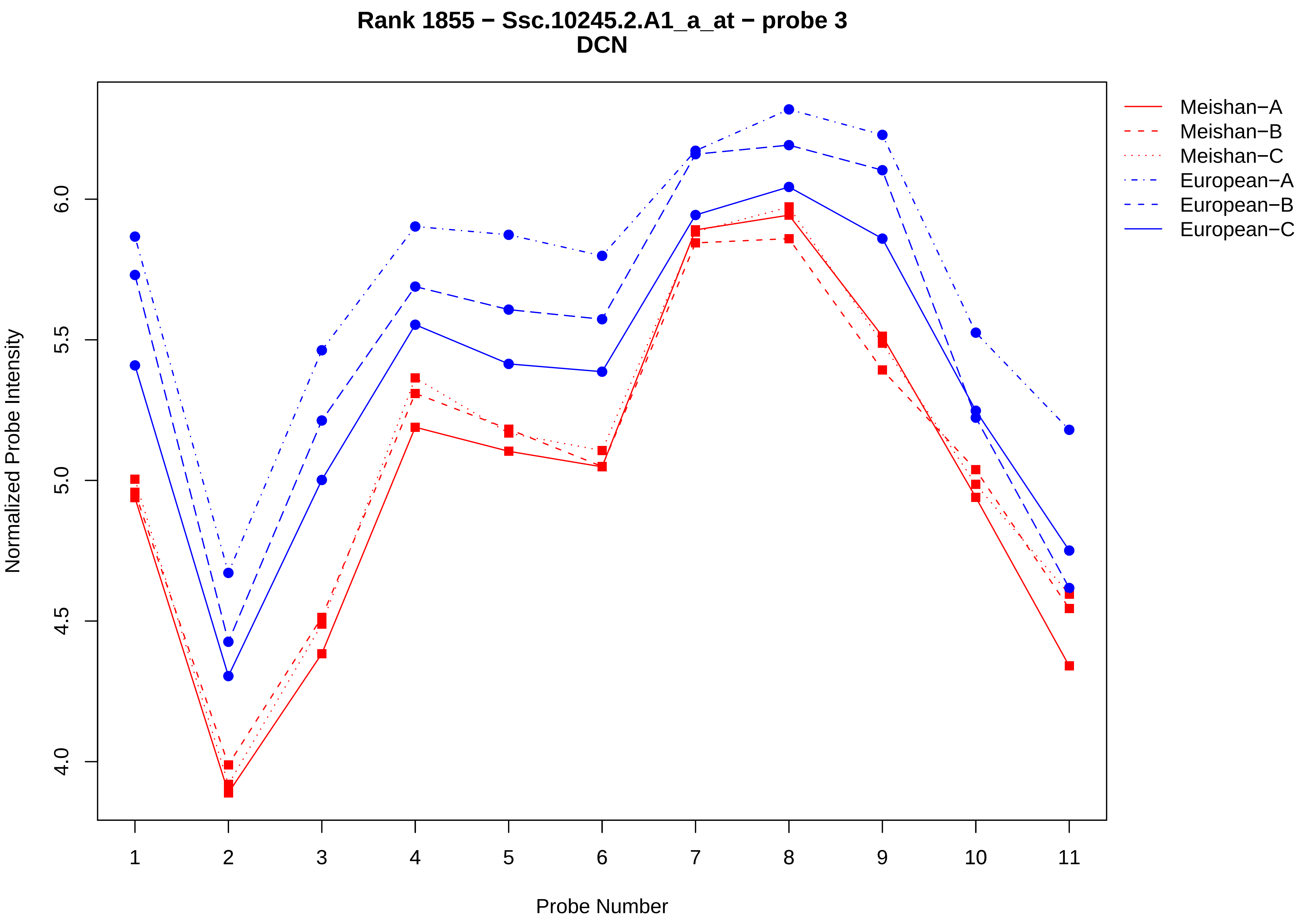

Rank 1855 − Ssc.10245.2.A1_a_at − probe 3
DCN
Normalized Probe Intensity
4.0
4.5
5.0
5.5
6.0
Probe Number
1
2
3
4
5
6
7
8
9
10
11
Meishan−A
Meishan−B
Meishan−C
European−A
European−B
European−C

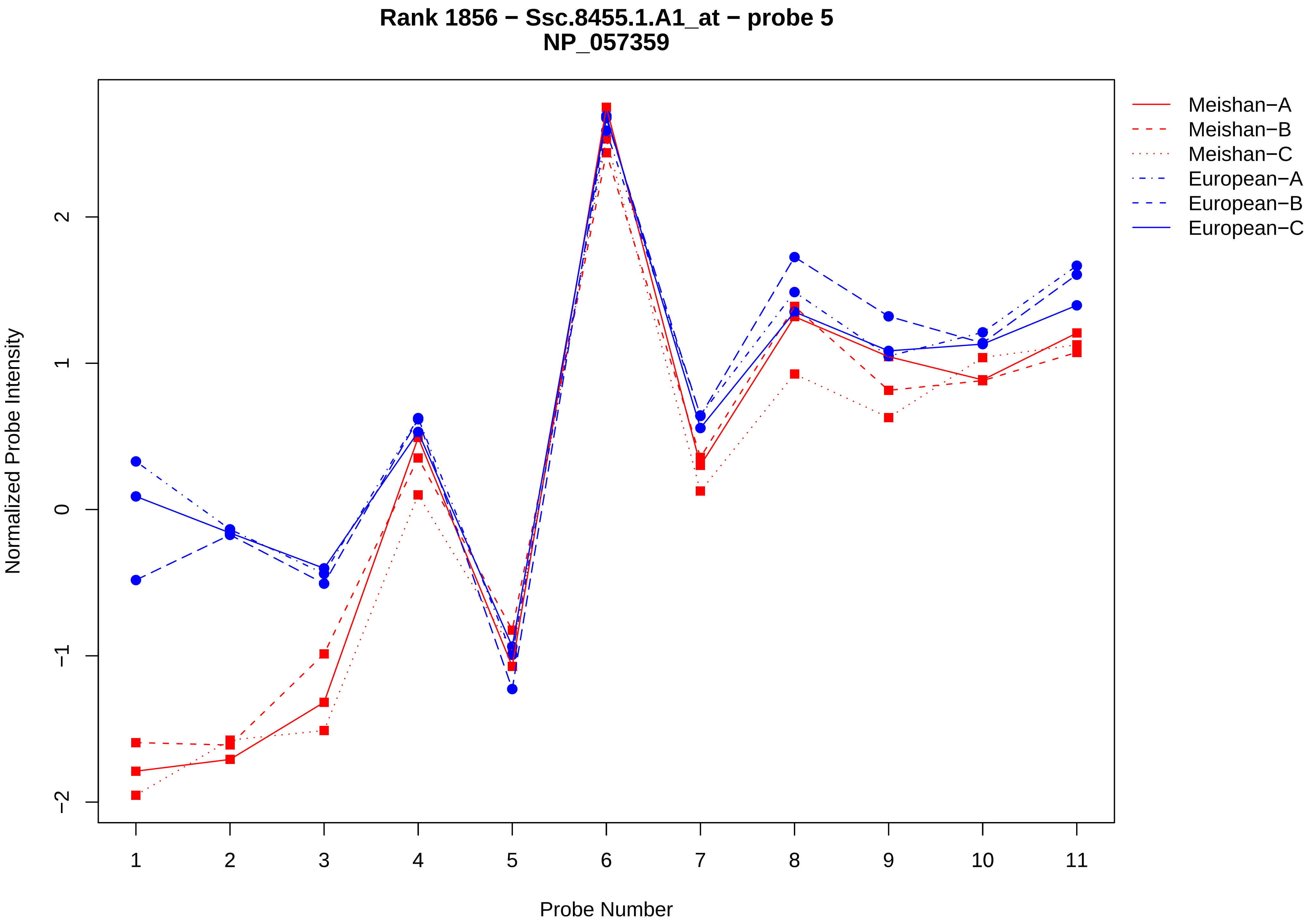

Rank 1856 − Ssc.8455.1.A1_at − probe 5
NP_057359
Normalized Probe Intensity
Probe Number
−2
−1
0
1
2
1
2
3
4
5
6
7
8
9
10
11
Meishan−A
Meishan−B
Meishan−C
European−A
European−B
European−C

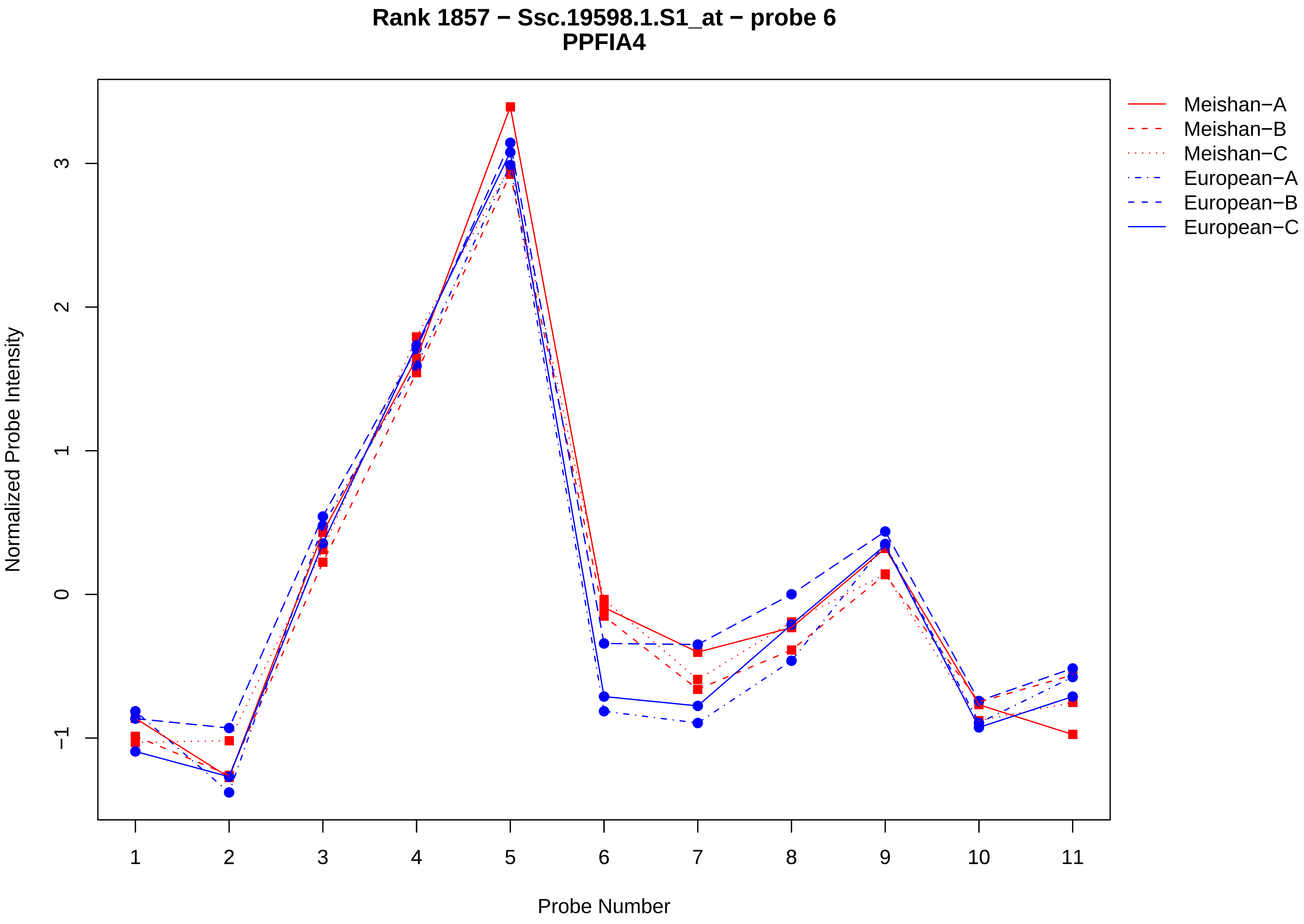

Rank 1857 − Ssc.19598.1.S1_at − probe 6
PPFIA4
Normalized Probe Intensity
Probe Number
−1
0
1
2
3
1
2
3
4
5
6
7
8
9
10
11
Meishan−A
Meishan−B
Meishan−C
European−A
European−B
European−C

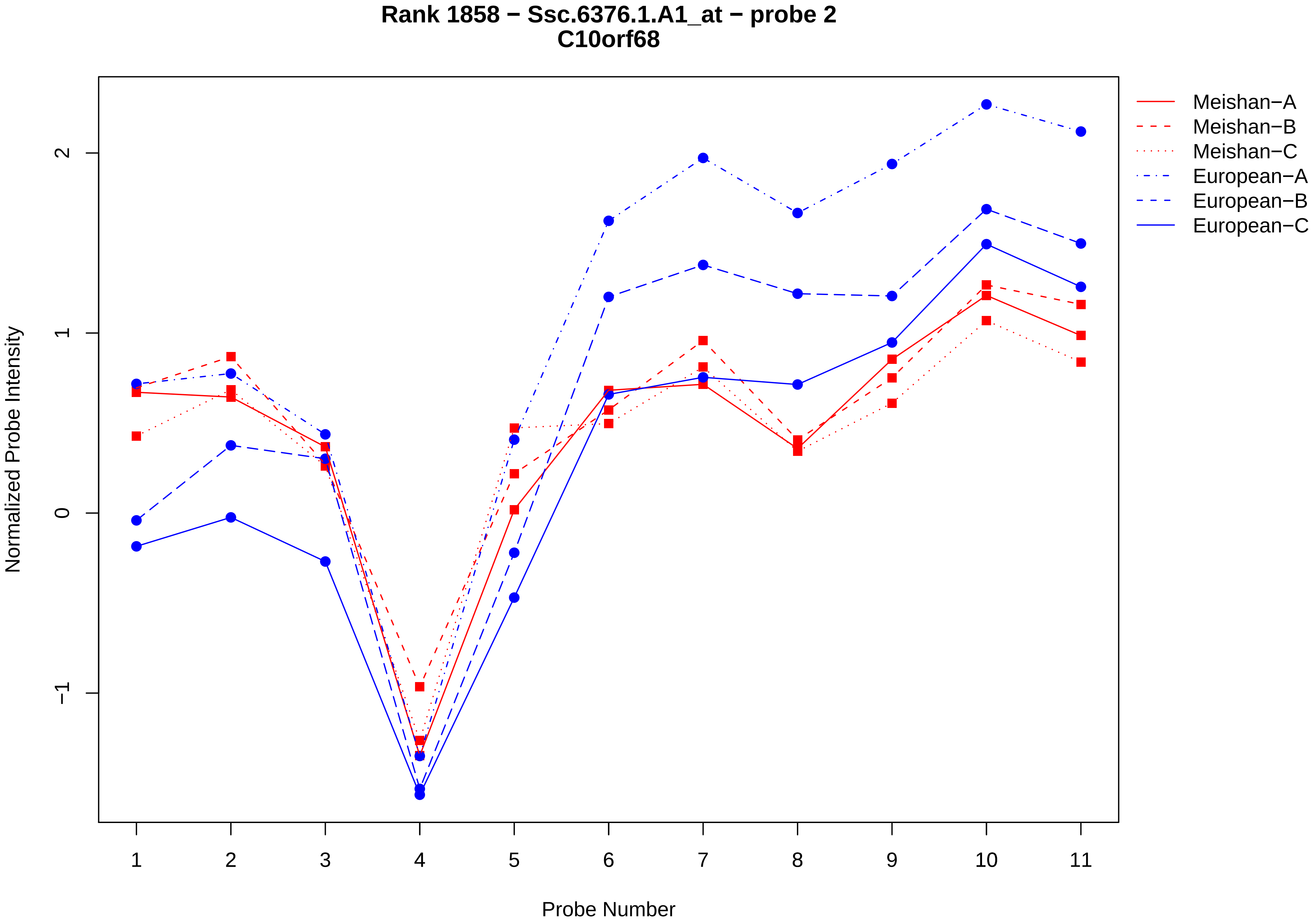

Rank 1858 − Ssc.6376.1.A1_at − probe 2
C10orf68
Normalized Probe Intensity
Probe Number
−1
0
1
2
1
2
3
4
5
6
7
8
9
10
11
Meishan−A
Meishan−B
Meishan−C
European−A
European−B
European−C

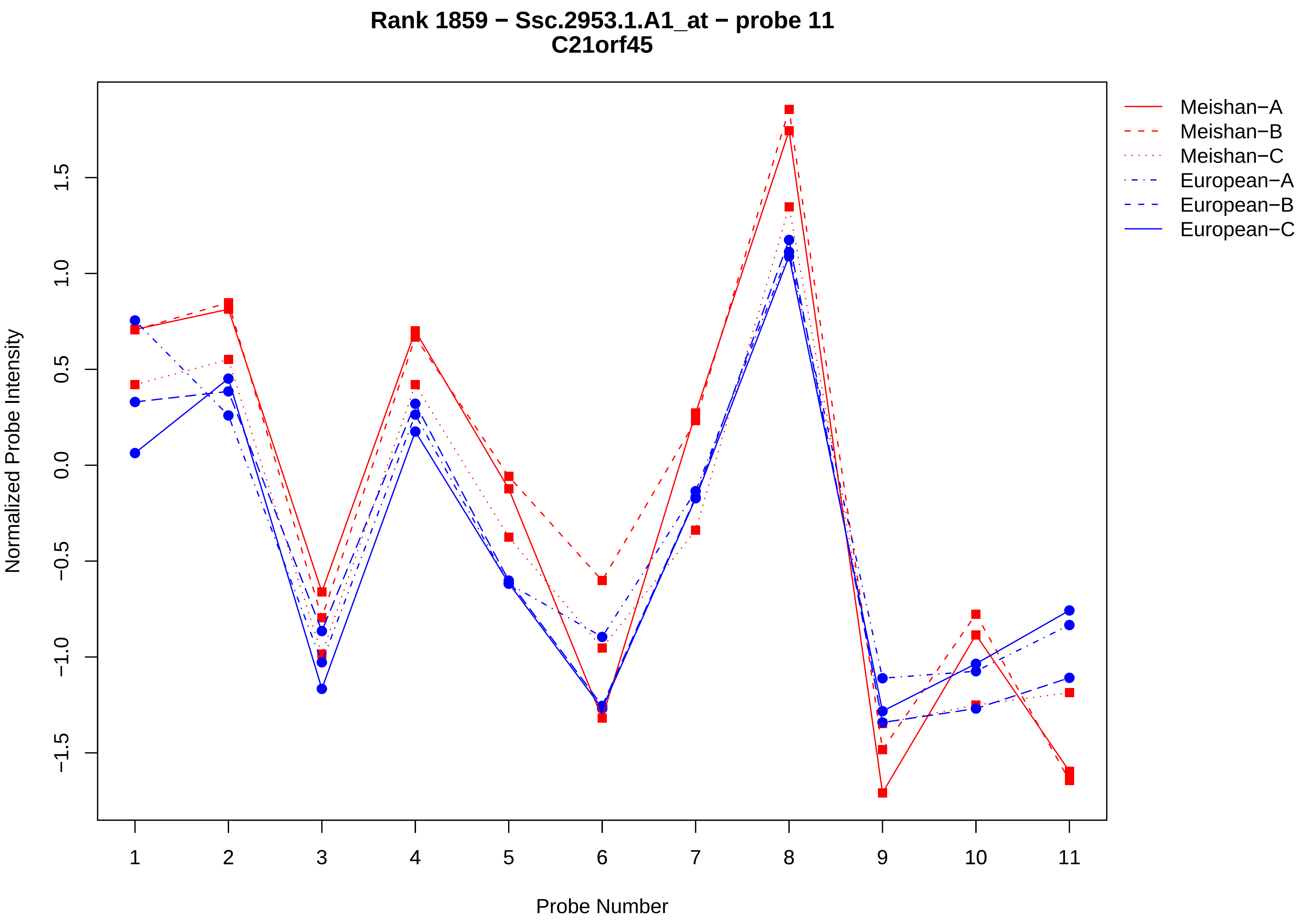

Rank 1859 − Ssc.2953.1.A1_at − probe 11
C21orf45
Normalized Probe Intensity
Probe Number
−1.5
−1.0
−0.5
0.0
0.5
1.0
1.5
1
2
3
4
5
6
7
8
9
10
11
Meishan−A
Meishan−B
Meishan−C
European−A
European−B
European−C

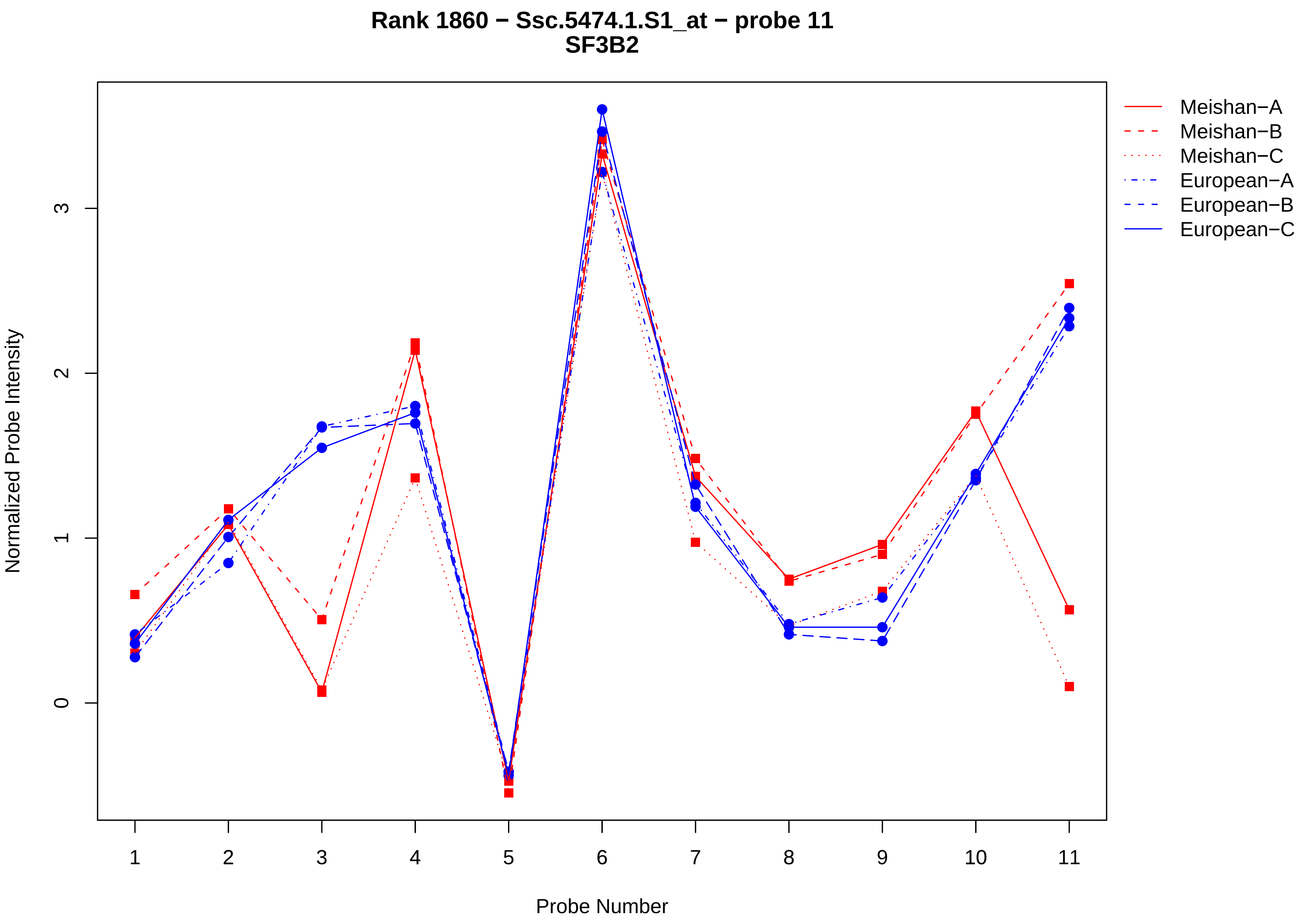

Rank 1860 − Ssc.5474.1.S1_at − probe 11
SF3B2
Meishan−A
Meishan−B
Meishan−C
European−A
European−B
European−C
Normalized Probe Intensity
Probe Number
0
1
2
3
1
2
3
4
5
6
7
8
9
10
11

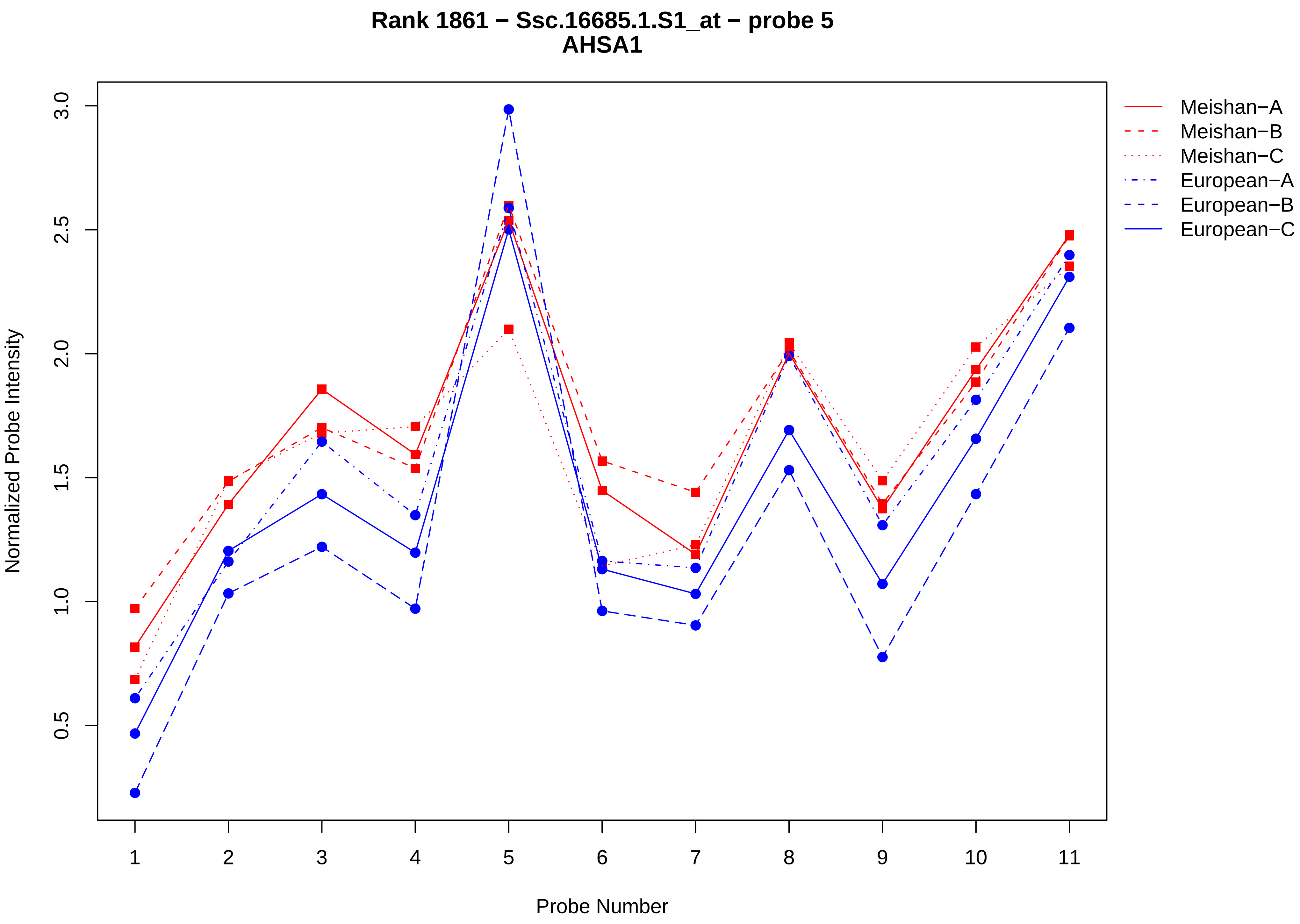

Rank 1861 − Ssc.16685.1.S1_at − probe 5
AHSA1
Normalized Probe Intensity
Probe Number
Meishan−A
Meishan−B
Meishan−C
European−A
European−B
European−C

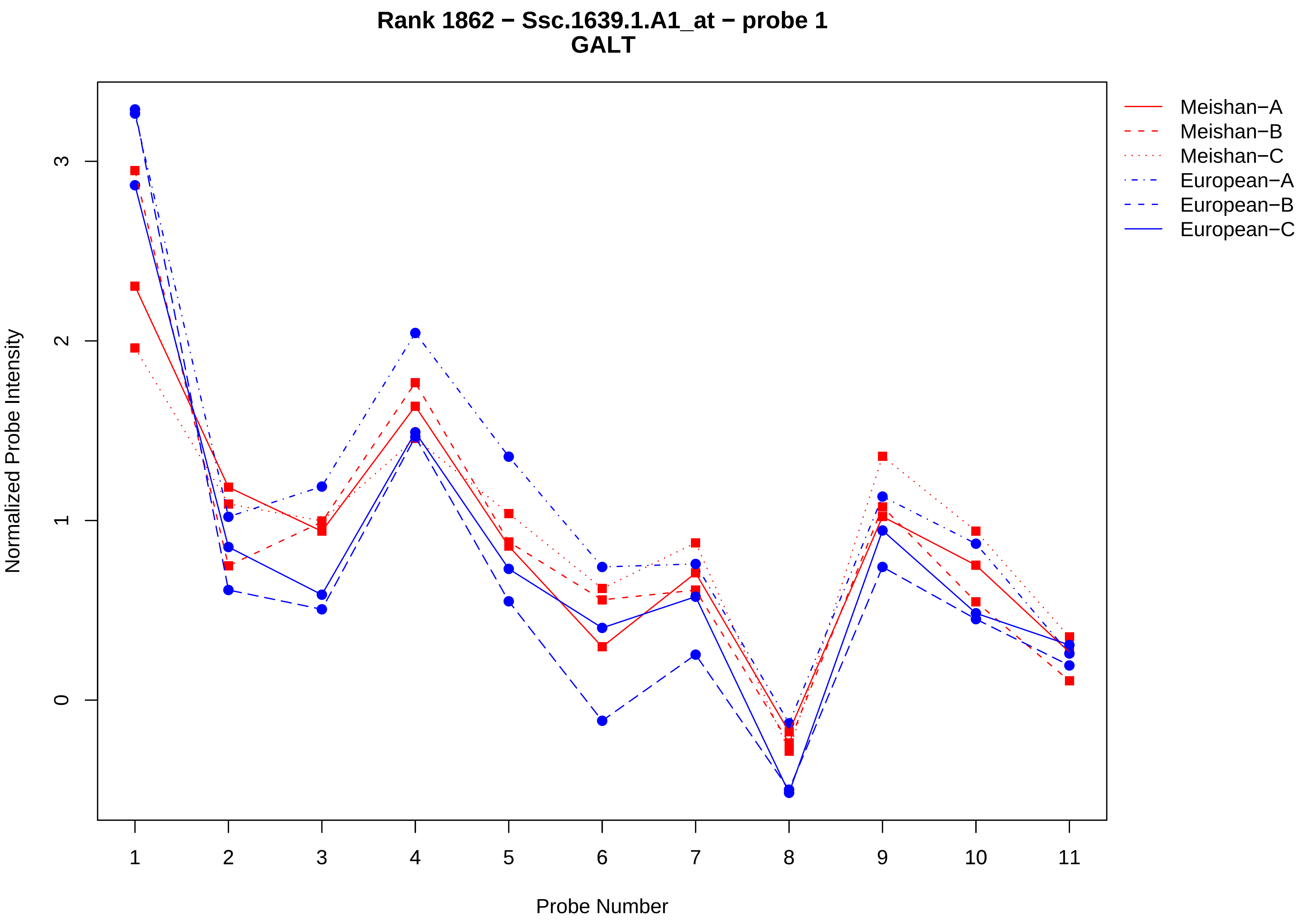

Rank 1862 − Ssc.1639.1.A1_at − probe 1
GALT
Normalized Probe Intensity
Probe Number
0
1
2
3
1
2
3
4
5
6
7
8
9
10
11
Meishan−A
Meishan−B
Meishan−C
European−A
European−B
European−C

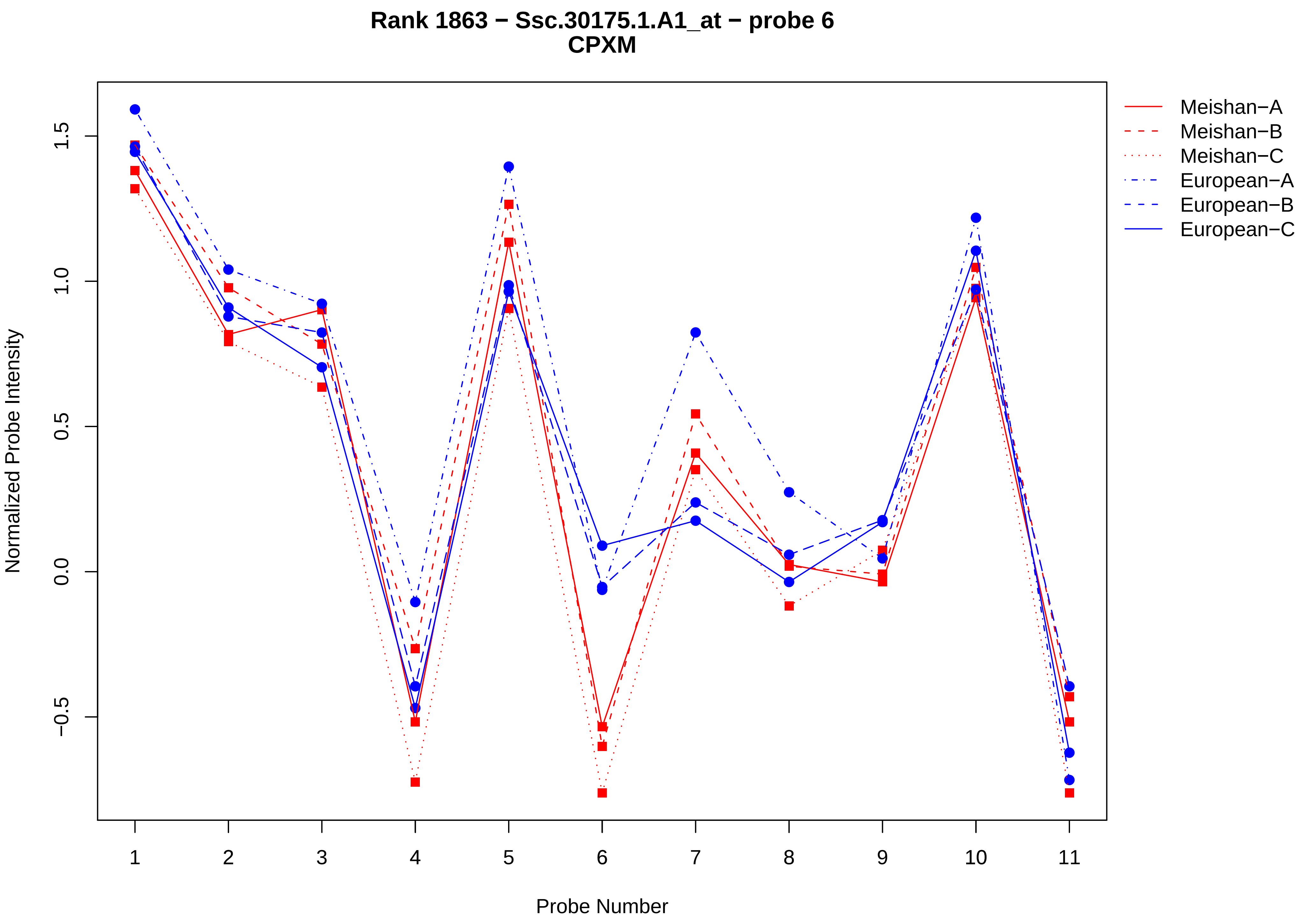

Rank 1863 − Ssc.30175.1.A1_at − probe 6
CPXM
Normalized Probe Intensity
Probe Number
−0.5
0.0
0.5
1.0
1.5
1
2
3
4
5
6
7
8
9
10
11
Meishan−A
Meishan−B
Meishan−C
European−A
European−B
European−C

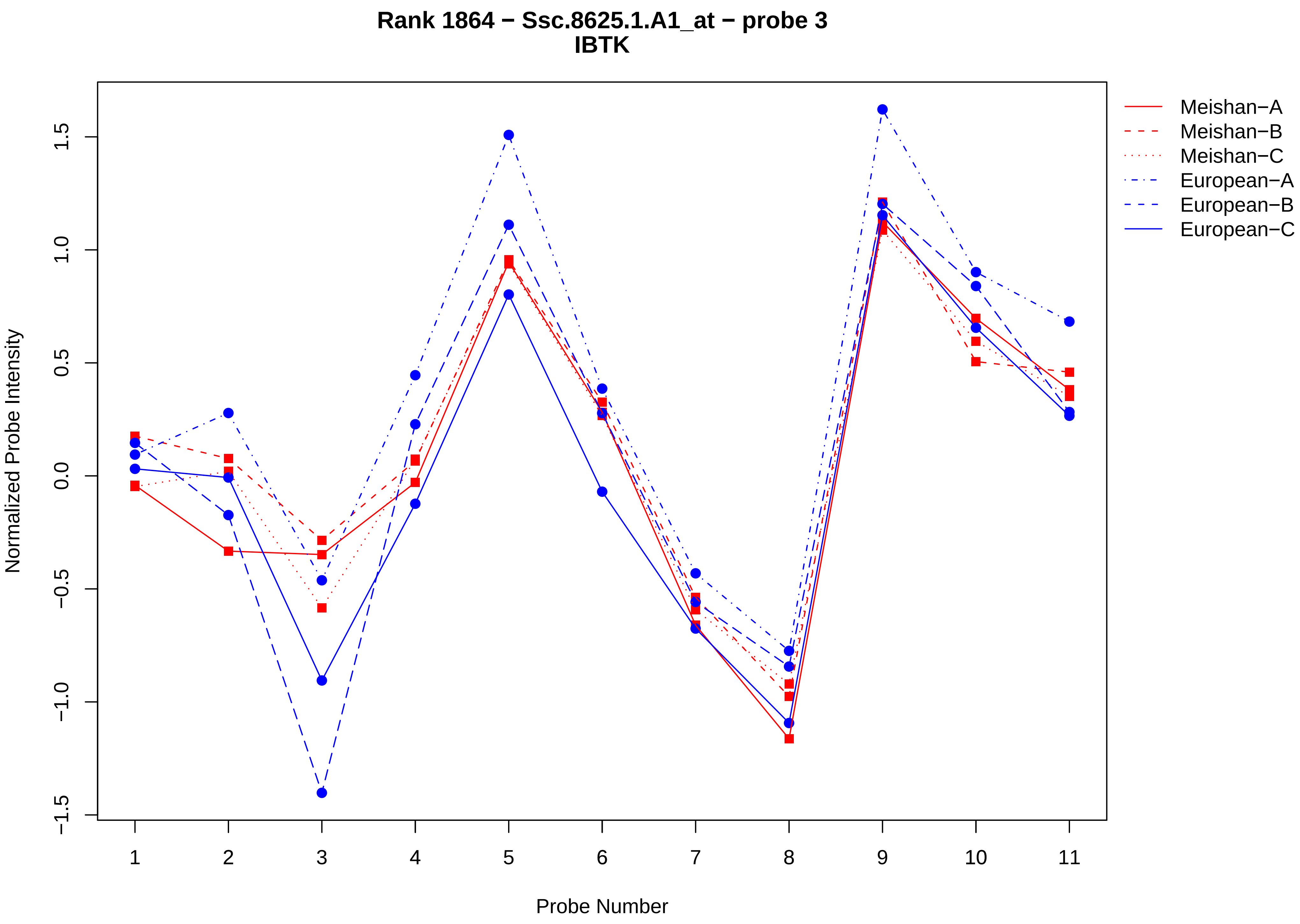

Rank 1864 − Ssc.8625.1.A1_at − probe 3
IBTK
Normalized Probe Intensity
Probe Number
−1.5
−1.0
−0.5
0.0
0.5
1.0
1.5
1
2
3
4
5
6
7
8
9
10
11
Meishan−A
Meishan−B
Meishan−C
European−A
European−B
European−C

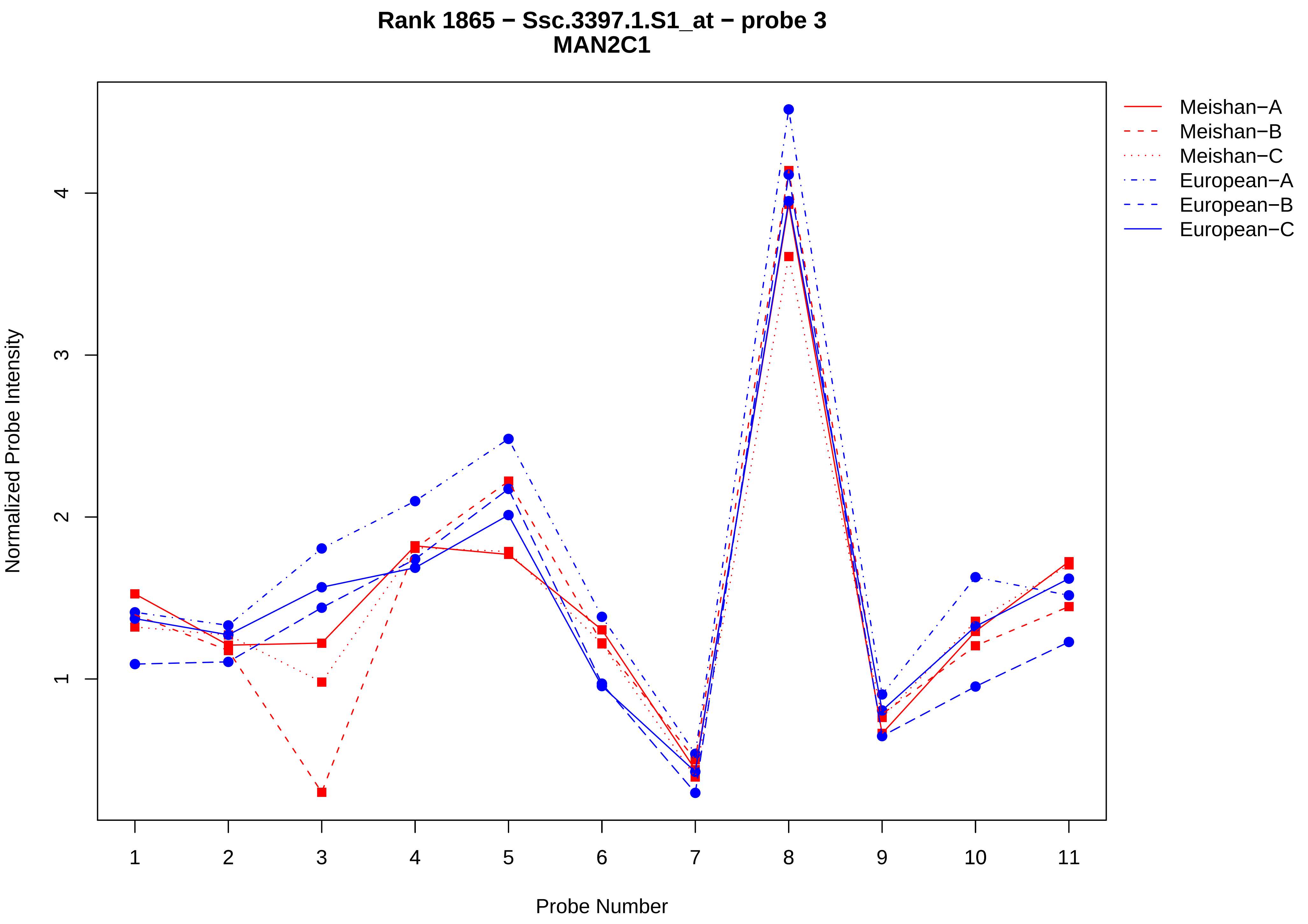

Rank 1865 − Ssc.3397.1.S1_at − probe 3
MAN2C1
Normalized Probe Intensity
Probe Number
1
2
3
4
1
2
3
4
5
6
7
8
9
10
11
Meishan−A
Meishan−B
Meishan−C
European−A
European−B
European−C

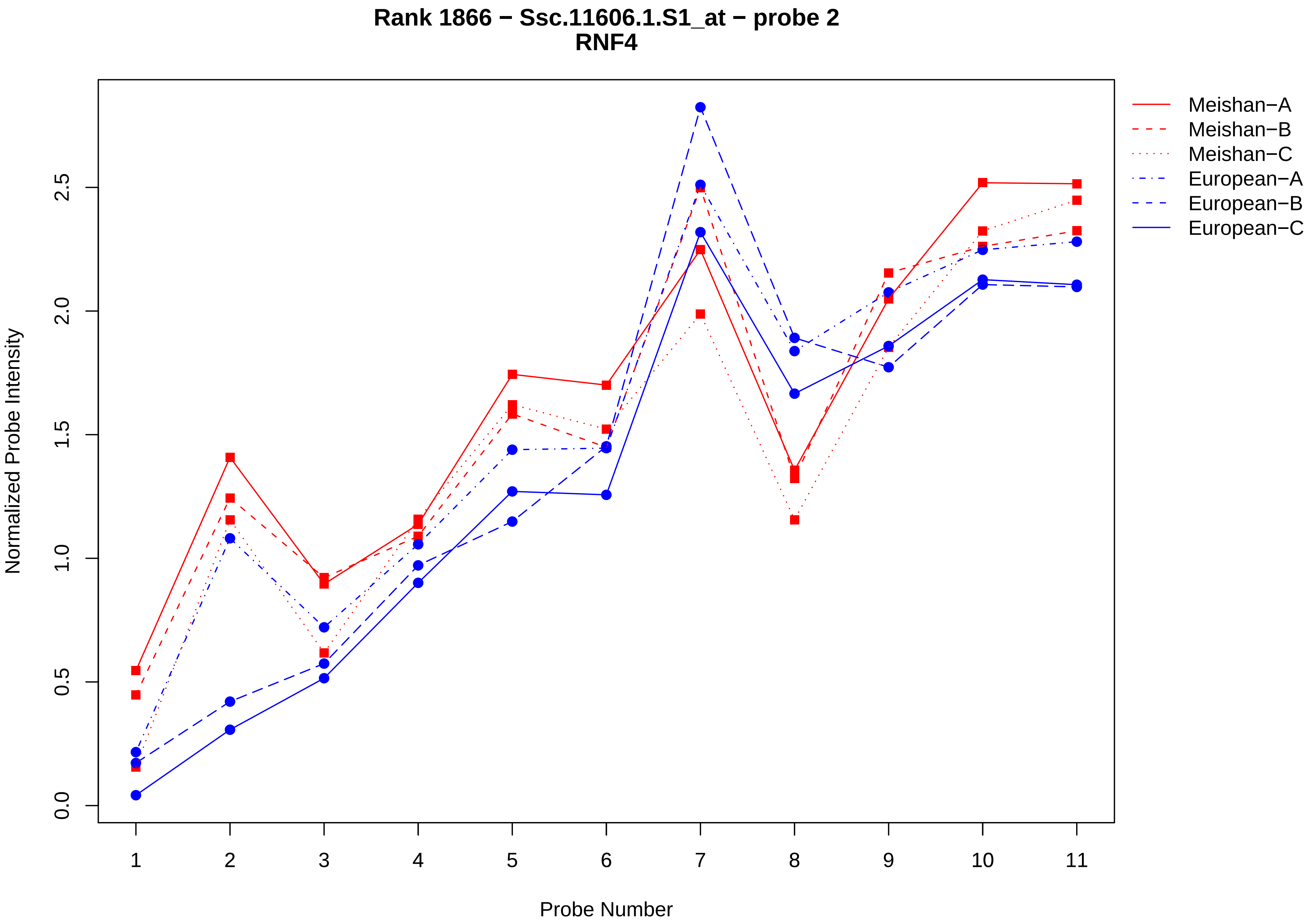

Rank 1866 − Ssc.11606.1.S1_at − probe 2
RNF4
Normalized Probe Intensity
Probe Number
0.0
0.5
1.0
1.5
2.0
2.5
1
2
3
4
5
6
7
8
9
10
11
Meishan−A
Meishan−B
Meishan−C
European−A
European−B
European−C

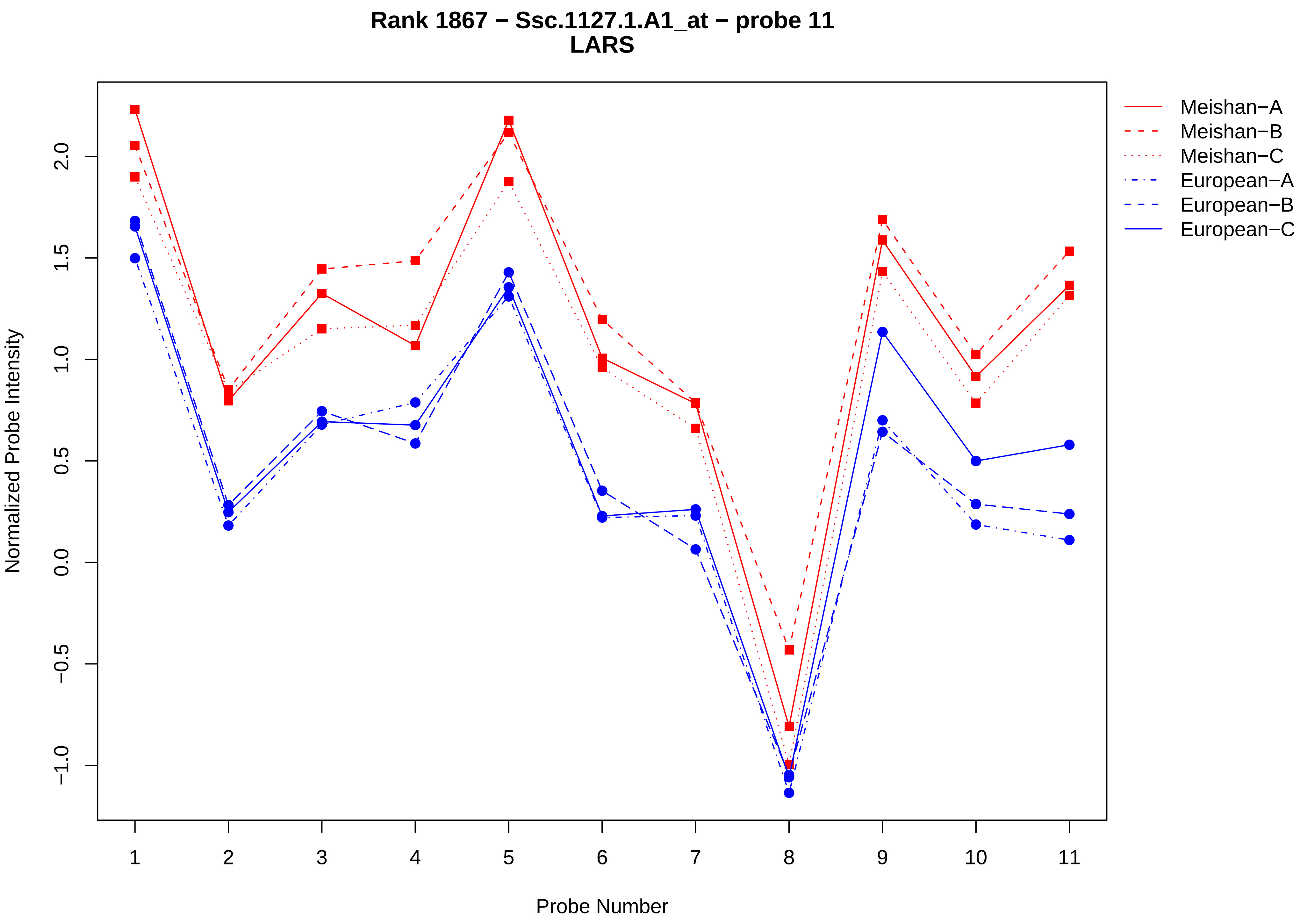
Rank 1867 − Ssc.1127.1.A1_at − probe 11
LARS
Normalized Probe Intensity
Probe Number
−1.0
−0.5
0.0
0.5
1.0
1.5
2.0
1
2
3
4
5
6
7
8
9
10
11
Meishan−A
Meishan−B
Meishan−C
European−A
European−B
European−C

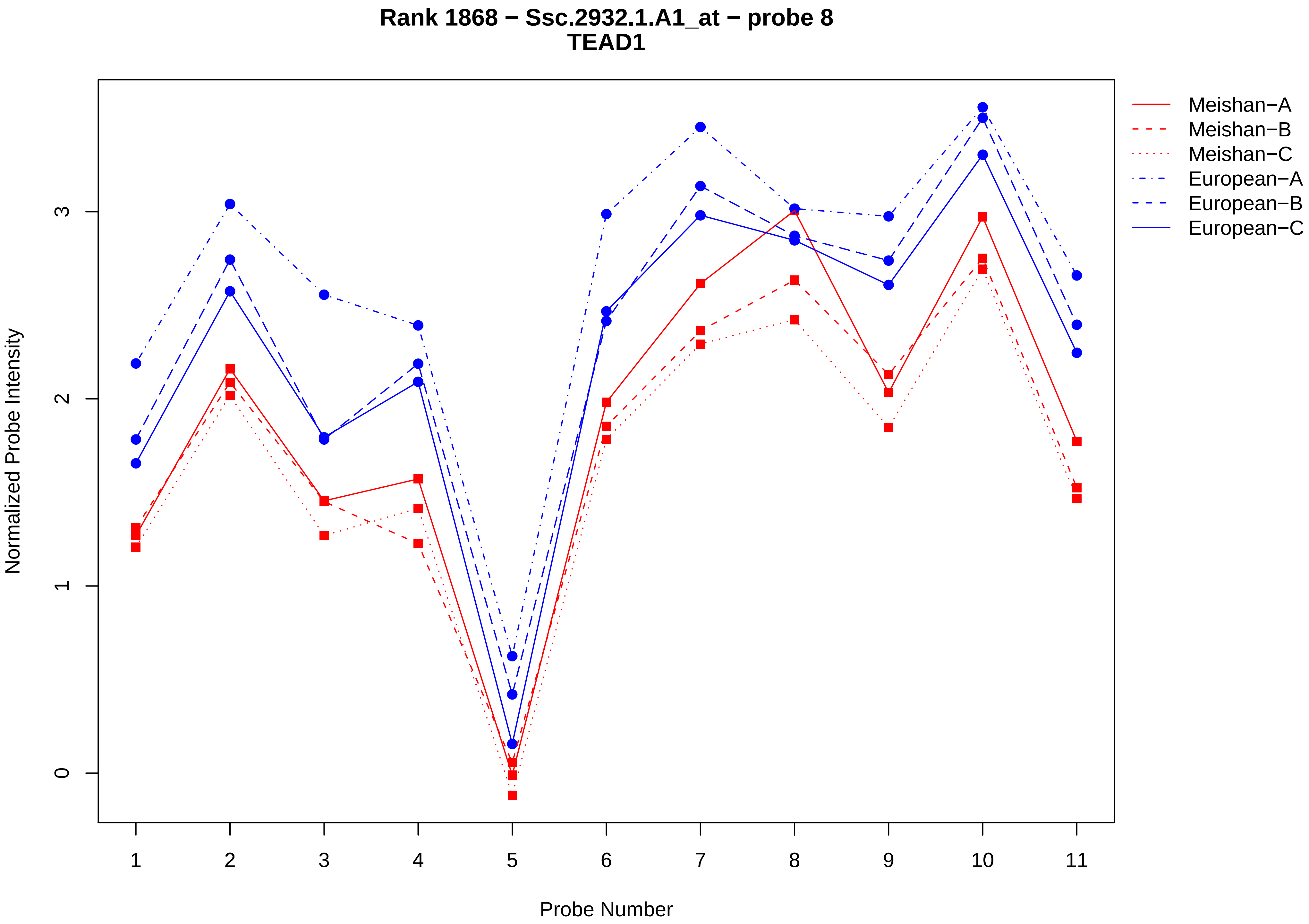

Rank 1868 − Ssc.2932.1.A1_at − probe 8
TEAD1
Normalized Probe Intensity
Probe Number
0
1
2
3
1
2
3
4
5
6
7
8
9
10
11
Meishan−A
Meishan−B
Meishan−C
European−A
European−B
European−C

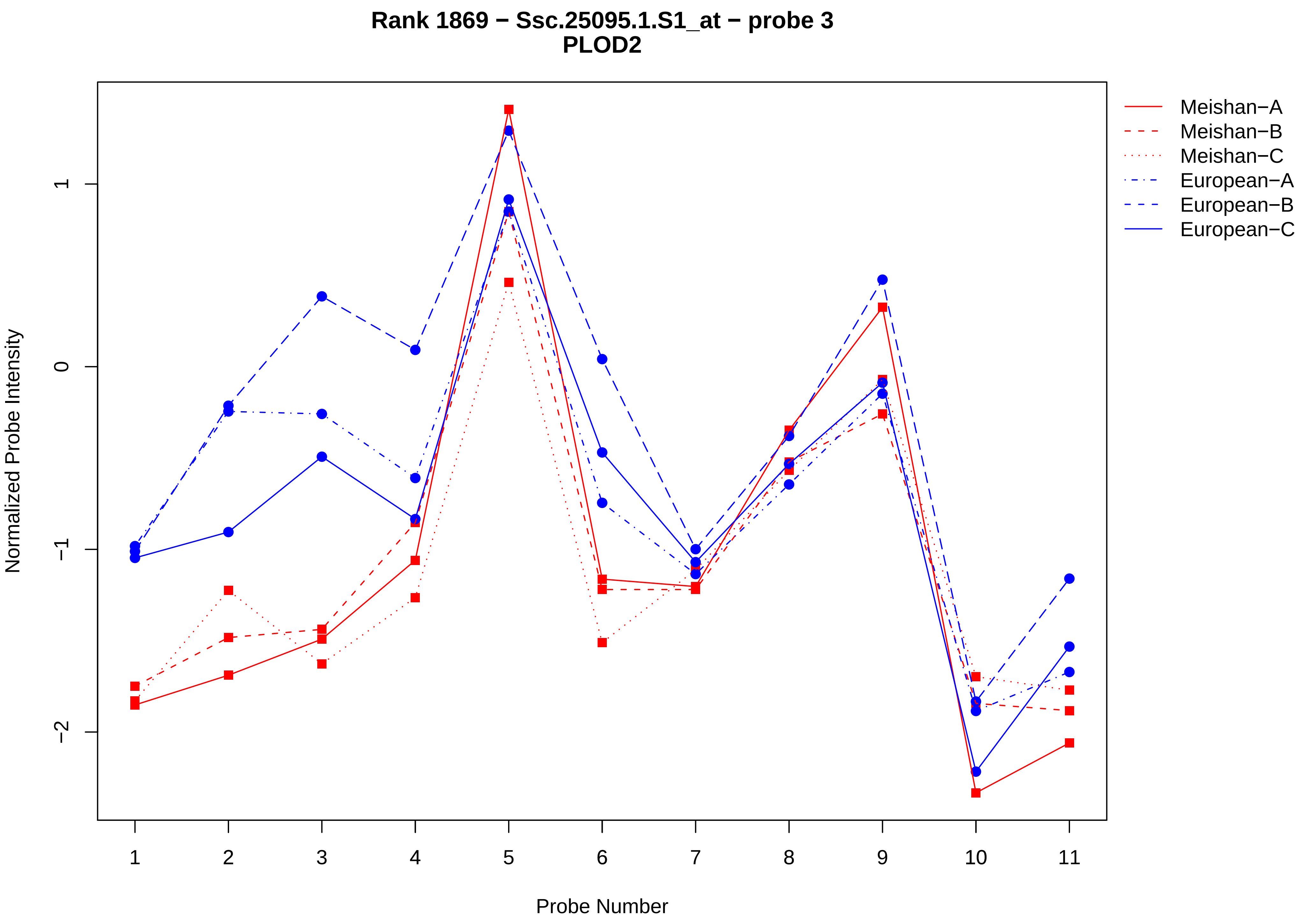

Rank 1869 − Ssc.25095.1.S1_at − probe 3
PLOD2
Normalized Probe Intensity
Probe Number
1
0
−1
−2
1
2
3
4
5
6
7
8
9
10
11
Meishan−A
Meishan−B
Meishan−C
European−A
European−B
European−C

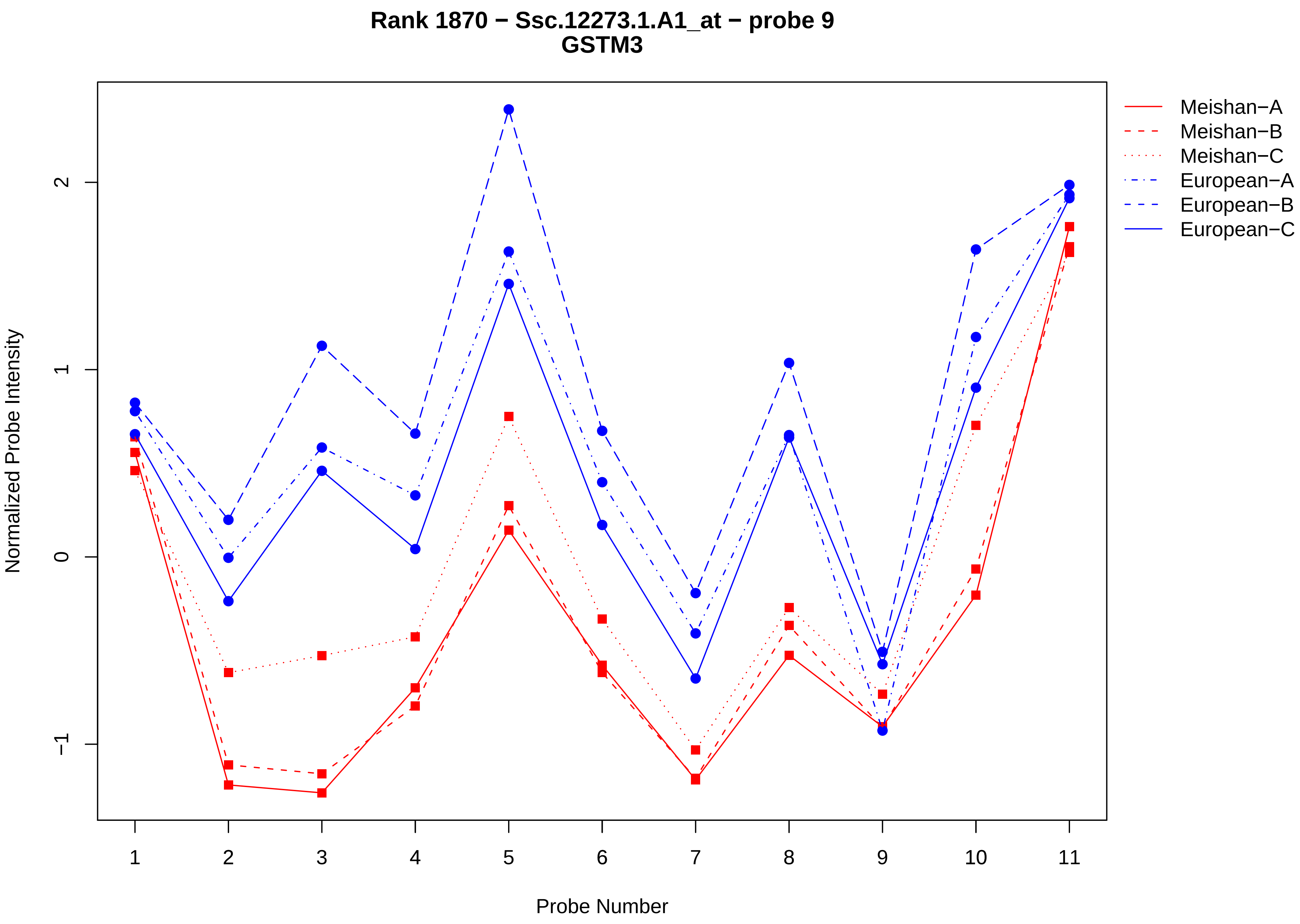
Rank 1870 − Ssc.12273.1.A1_at − probe 9
GSTM3
Normalized Probe Intensity
Probe Number
Meishan−A
Meishan−B
Meishan−C
European−A
European−B
European−C
−1
0
1
2
1
2
3
4
5
6
7
8
9
10
11

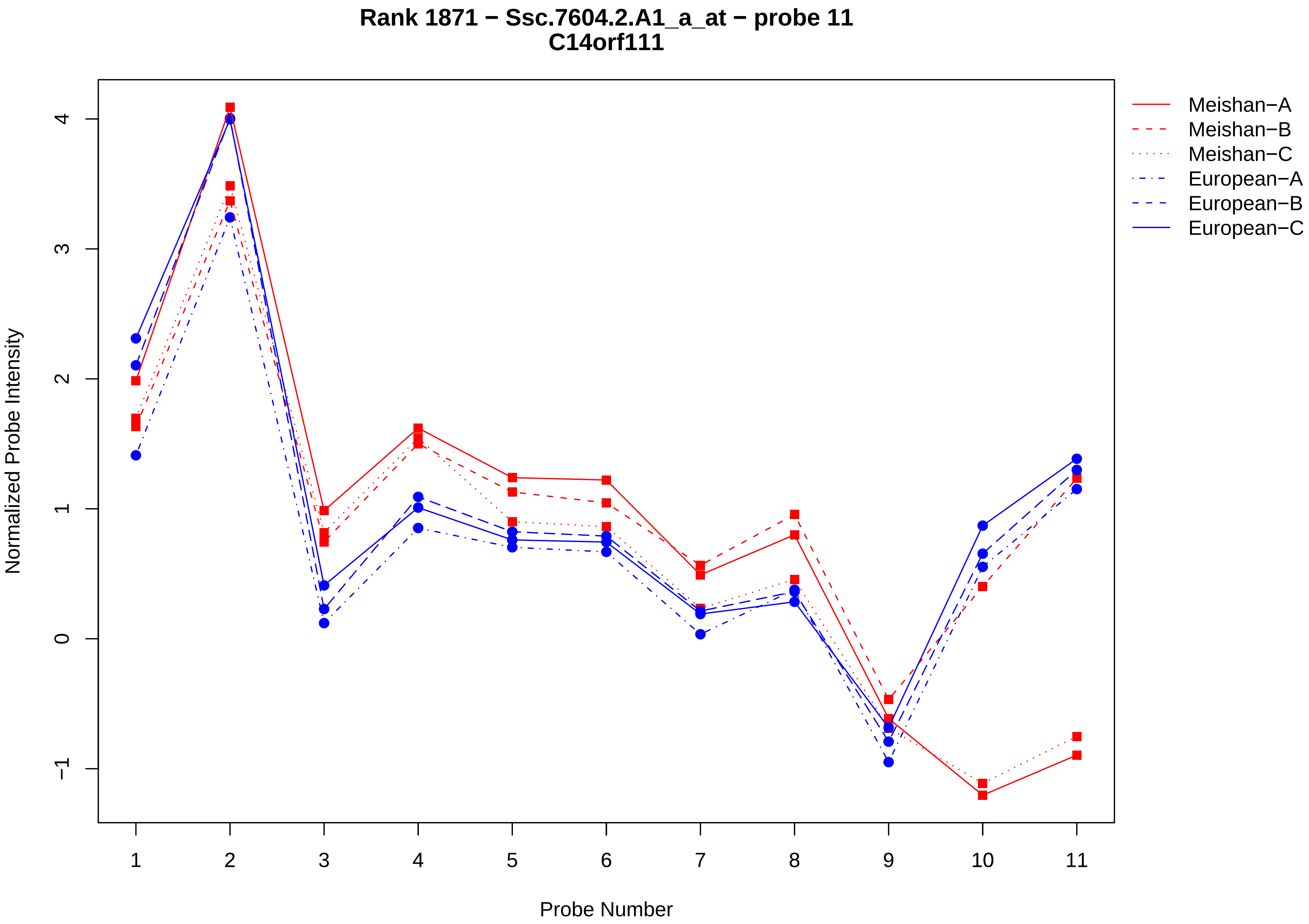

Rank 1871 − Ssc.7604.2.A1_a_at − probe 11
C14orf111
Normalized Probe Intensity
Probe Number
Meishan−A
Meishan−B
Meishan−C
European−A
European−B
European−C
−1
0
1
2
3
4
1
2
3
4
5
6
7
8
9
10
11

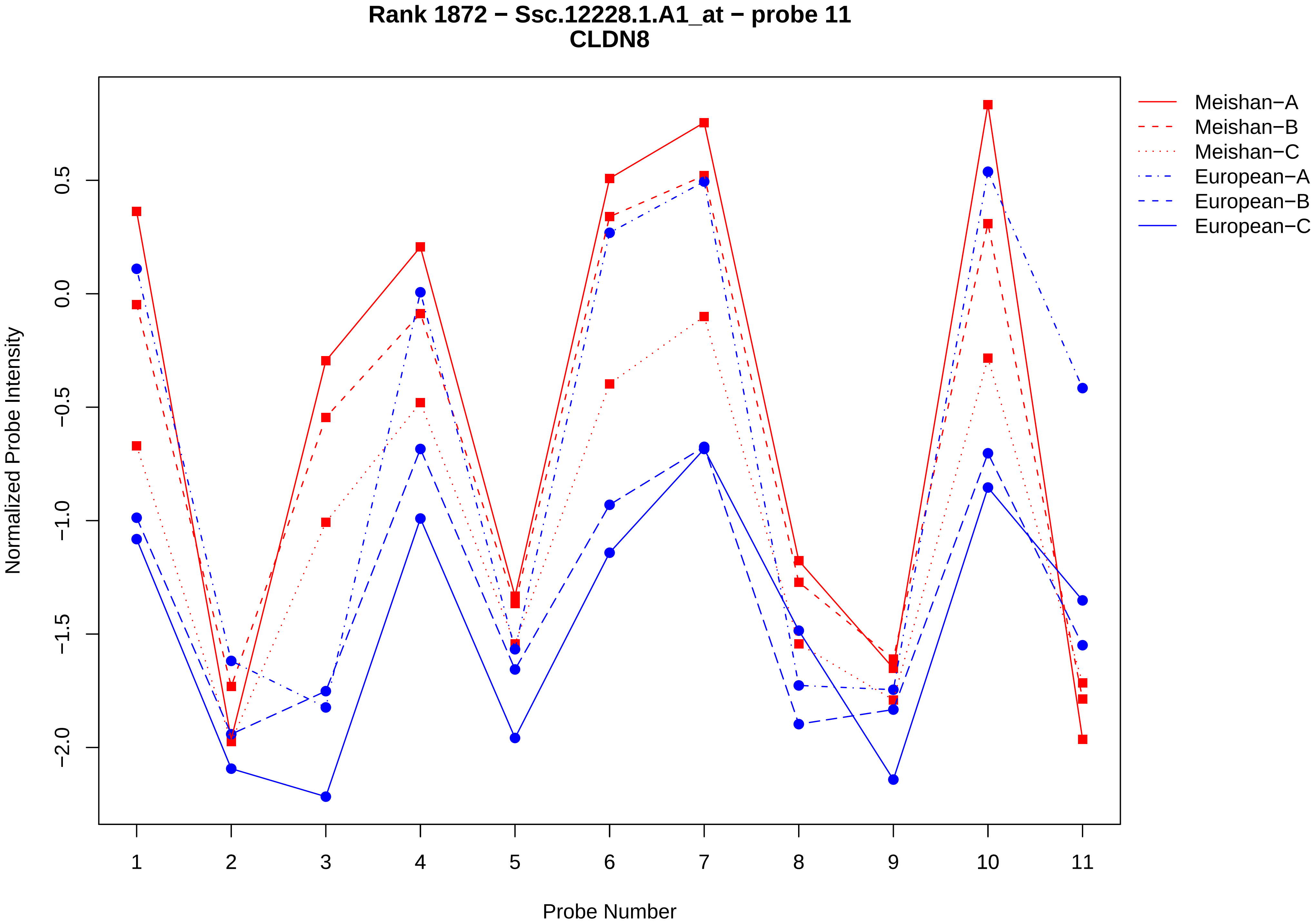

Rank 1872 − Ssc.12228.1.A1_at − probe 11
CLDN8
Normalized Probe Intensity
Probe Number
0.5
0.0
−0.5
−1.0
−1.5
−2.0
1
2
3
4
5
6
7
8
9
10
11
Meishan−A
Meishan−B
Meishan−C
European−A
European−B
European−C

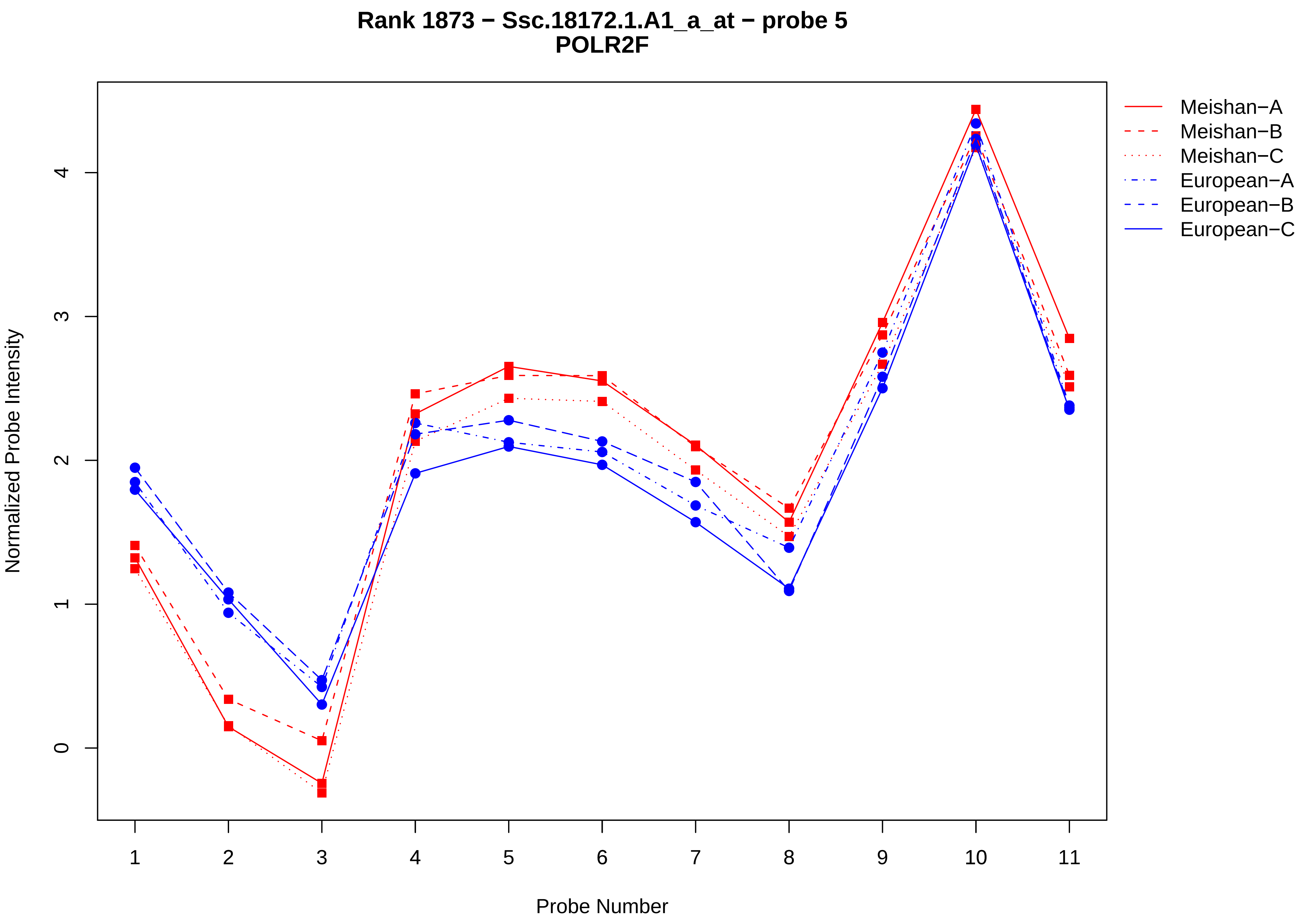
Rank 1873 − Ssc.18172.1.A1_a_at − probe 5
POLR2F
Normalized Probe Intensity
Probe Number
0
1
2
3
4
1
2
3
4
5
6
7
8
9
10
11
Meishan−A
Meishan−B
Meishan−C
European−A
European−B
European−C

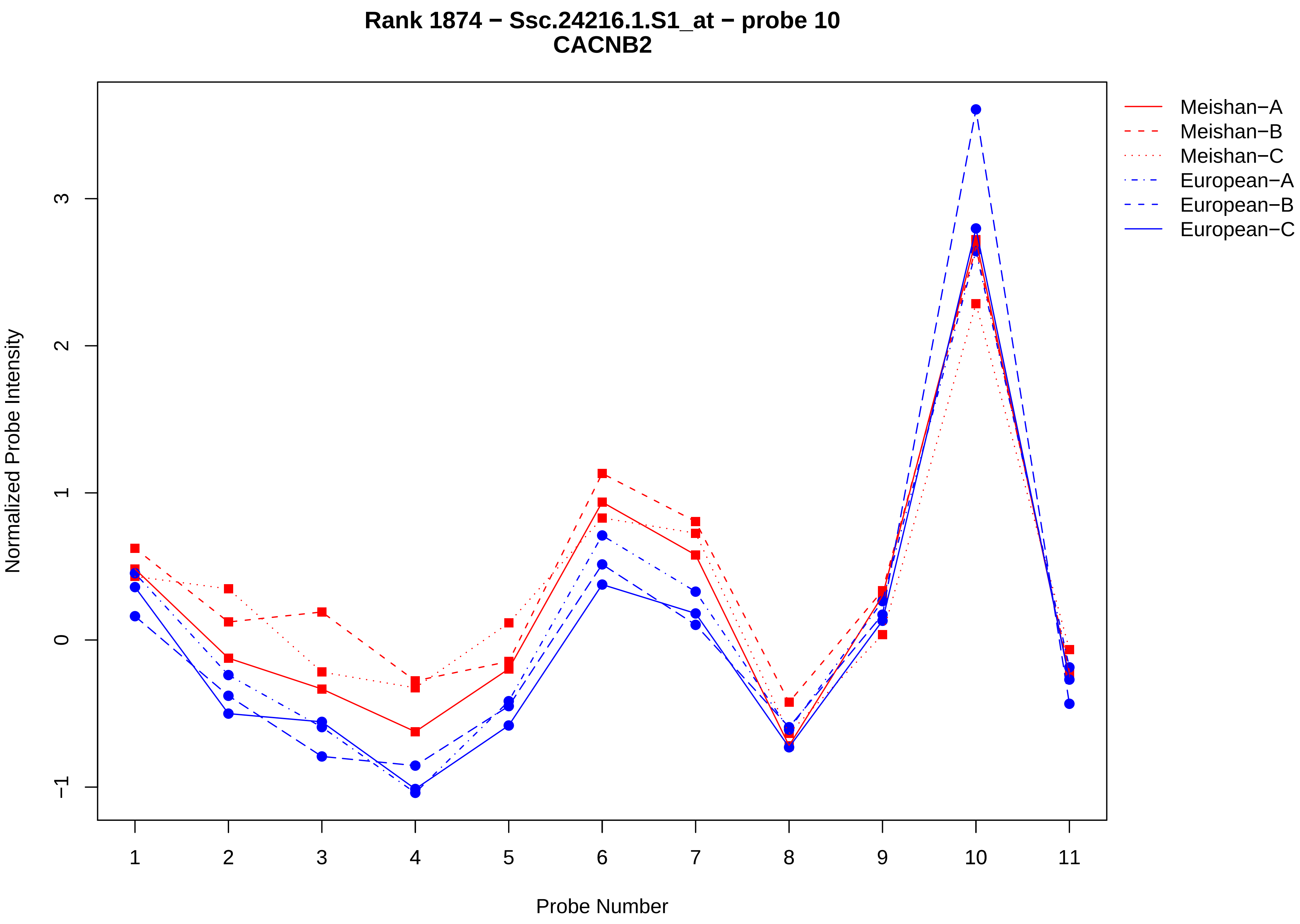

Rank 1874 − Ssc.24216.1.S1_at − probe 10
CACNB2
Normalized Probe Intensity
Probe Number
−1
0
1
2
3
1
2
3
4
5
6
7
8
9
10
11
Meishan−A
Meishan−B
Meishan−C
European−A
European−B
European−C

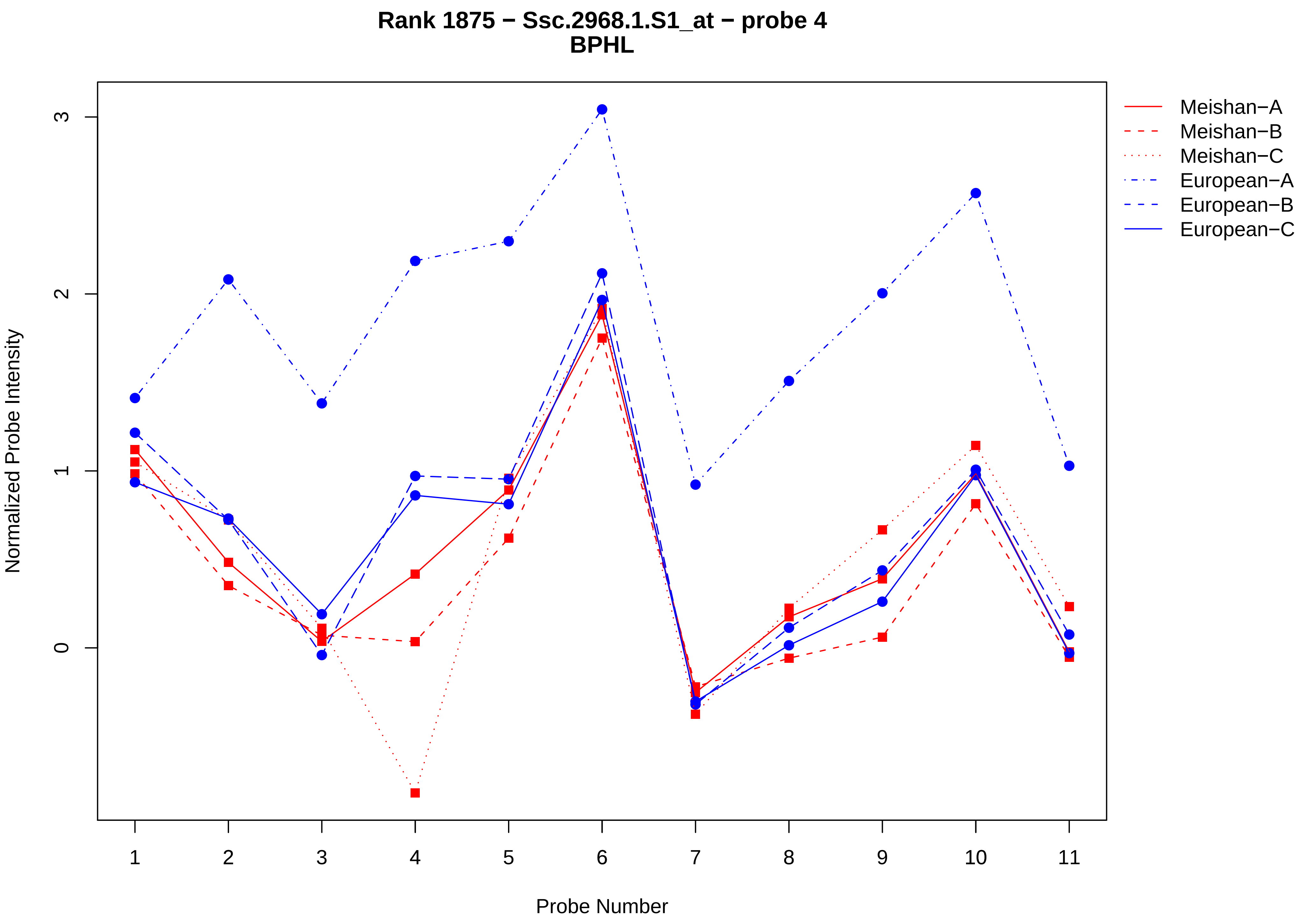

Rank 1875 − Ssc.2968.1.S1_at − probe 4
BPHL
Normalized Probe Intensity
0
1
2
3
1
2
3
4
5
6
7
8
9
10
11
Probe Number
Meishan−A
Meishan−B
Meishan−C
European−A
European−B
European−C

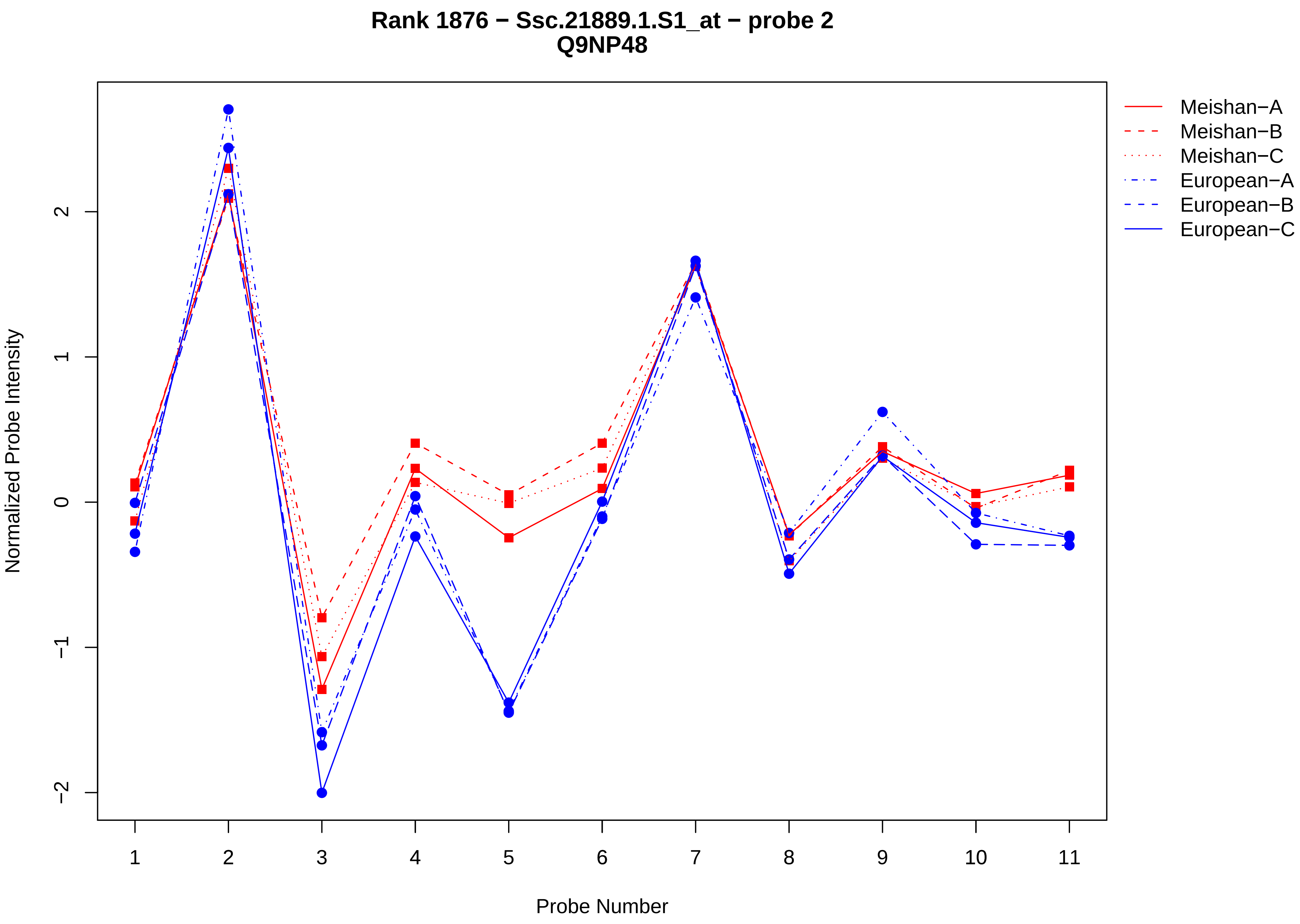

Rank 1876 − Ssc.21889.1.S1_at − probe 2
Q9NP48
Normalized Probe Intensity
Probe Number
Meishan−A
Meishan−B
Meishan−C
European−A
European−B
European−C
−2
−1
0
1
2
1
2
3
4
5
6
7
8
9
10
11

Rank 1877 − Ssc.8779.1.A1_at − probe 3
Q6UXV4

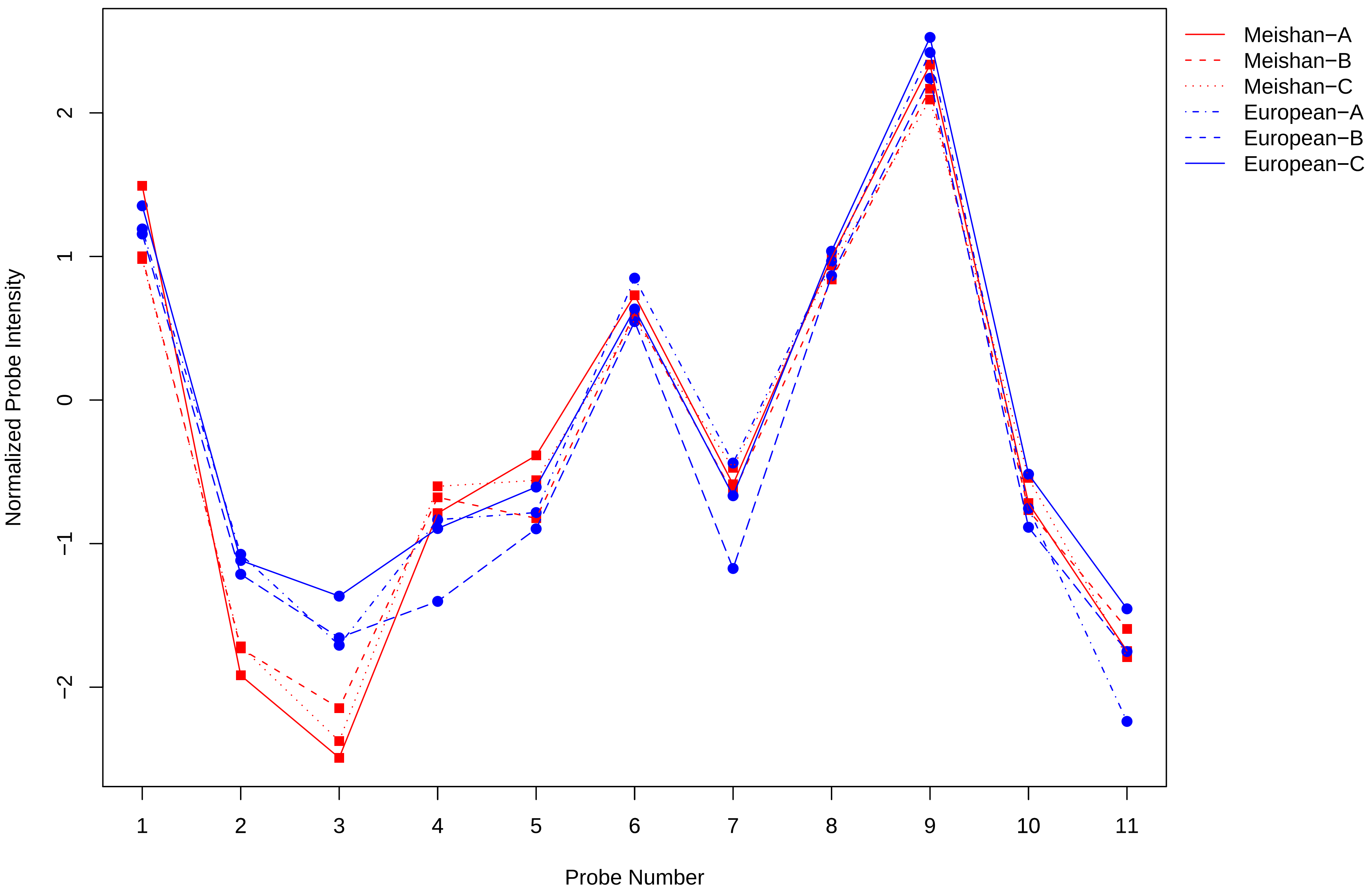

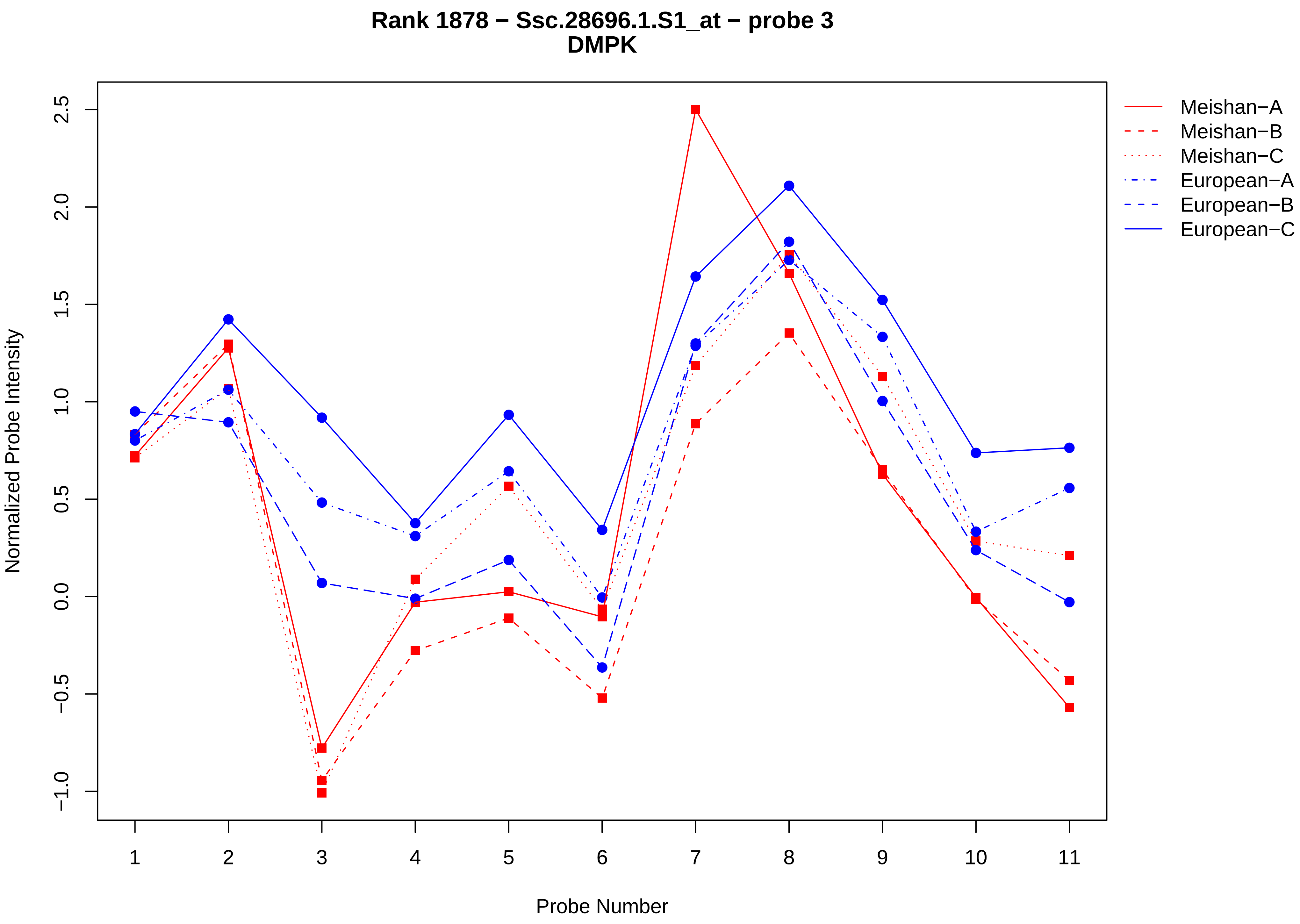

Rank 1878 − Ssc.28696.1.S1_at − probe 3
DMPK
Normalized Probe Intensity
Probe Number
Meishan−A
Meishan−B
Meishan−C
European−A
European−B
European−C

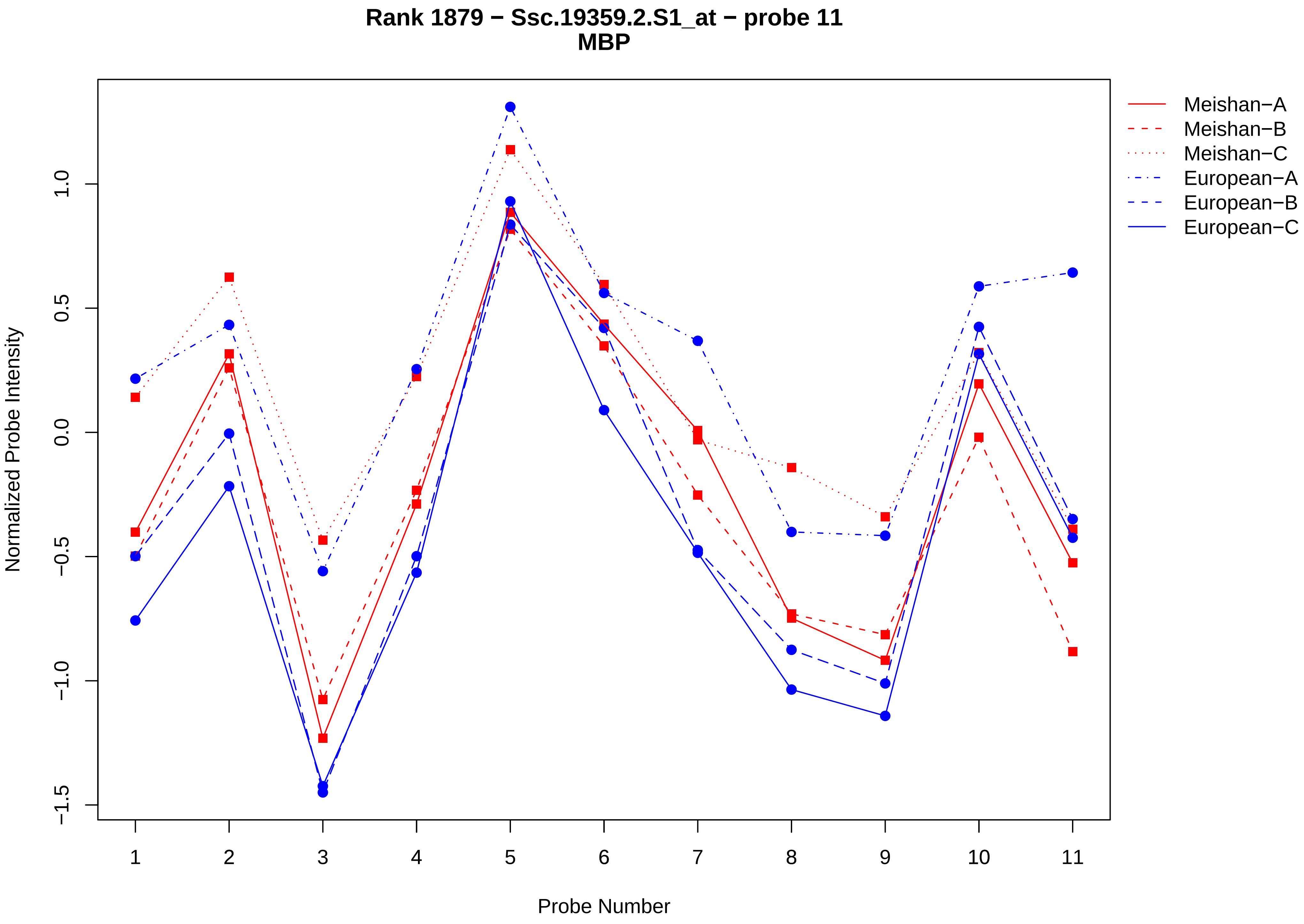
Rank 1879 − Ssc.19359.2.S1_at − probe 11
MBP
Normalized Probe Intensity
Probe Number
−1.5
−1.0
−0.5
0.0
0.5
1.0
1
2
3
4
5
6
7
8
9
10
11
Meishan−A
Meishan−B
Meishan−C
European−A
European−B
European−C

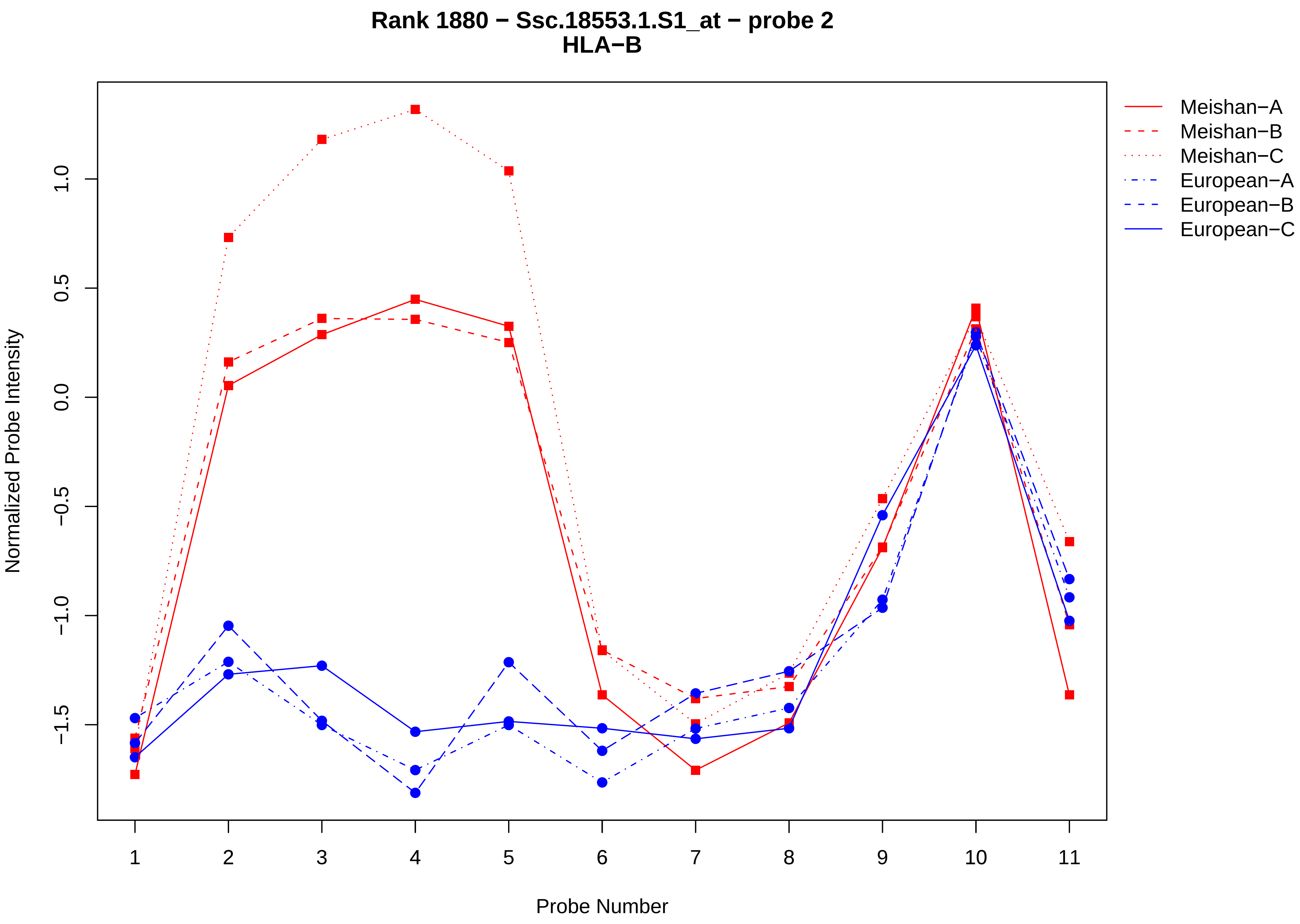
Rank 1880 − Ssc.18553.1.S1_at − probe 2
HLA−B
Normalized Probe Intensity
Probe Number
1.0
0.5
0.0
−0.5
−1.0
−1.5
1
2
3
4
5
6
7
8
9
10
11
Meishan−A
Meishan−B
Meishan−C
European−A
European−B
European−C

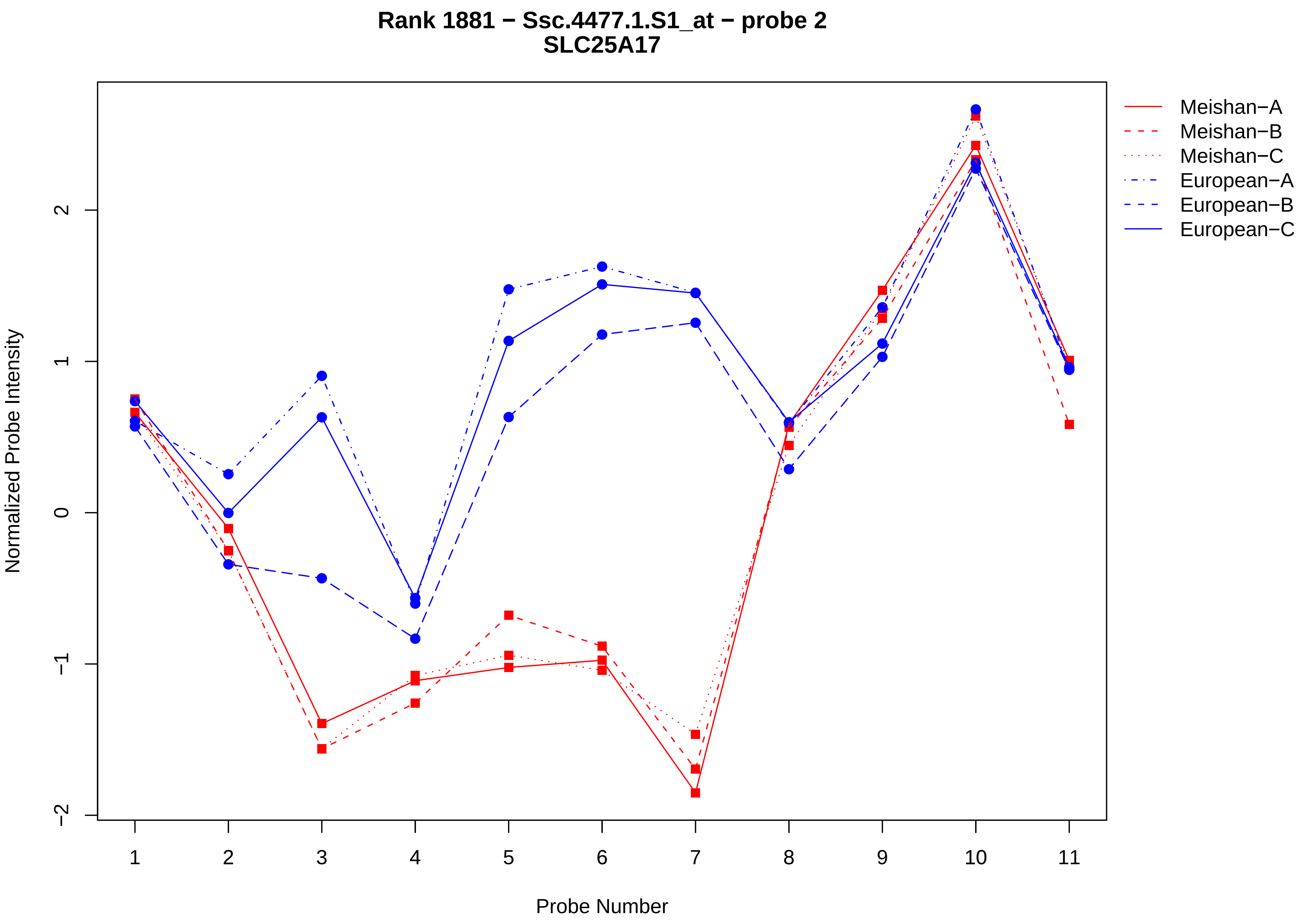

Rank 1881 − Ssc.4477.1.S1_at − probe 2
SLC25A17
Normalized Probe Intensity
Probe Number
−2
−1
0
1
2
1
2
3
4
5
6
7
8
9
10
11
Meishan−A
Meishan−B
Meishan−C
European−A
European−B
European−C

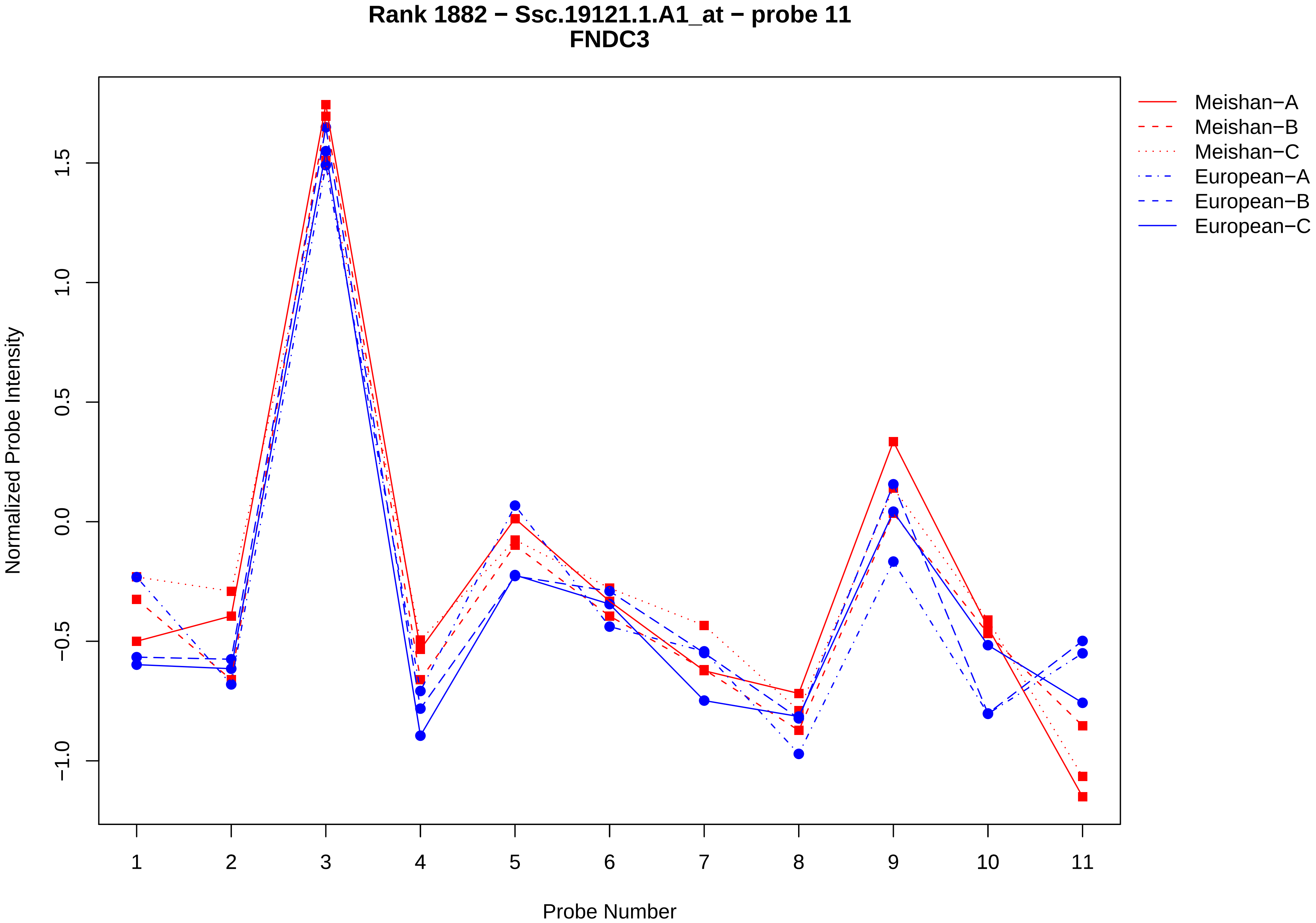
Rank 1882 − Ssc.19121.1.A1_at − probe 11
FNDC3
Normalized Probe Intensity
Probe Number
−1.0
−0.5
0.0
0.5
1.0
1.5
1
2
3
4
5
6
7
8
9
10
11
Meishan−A
Meishan−B
Meishan−C
European−A
European−B
European−C

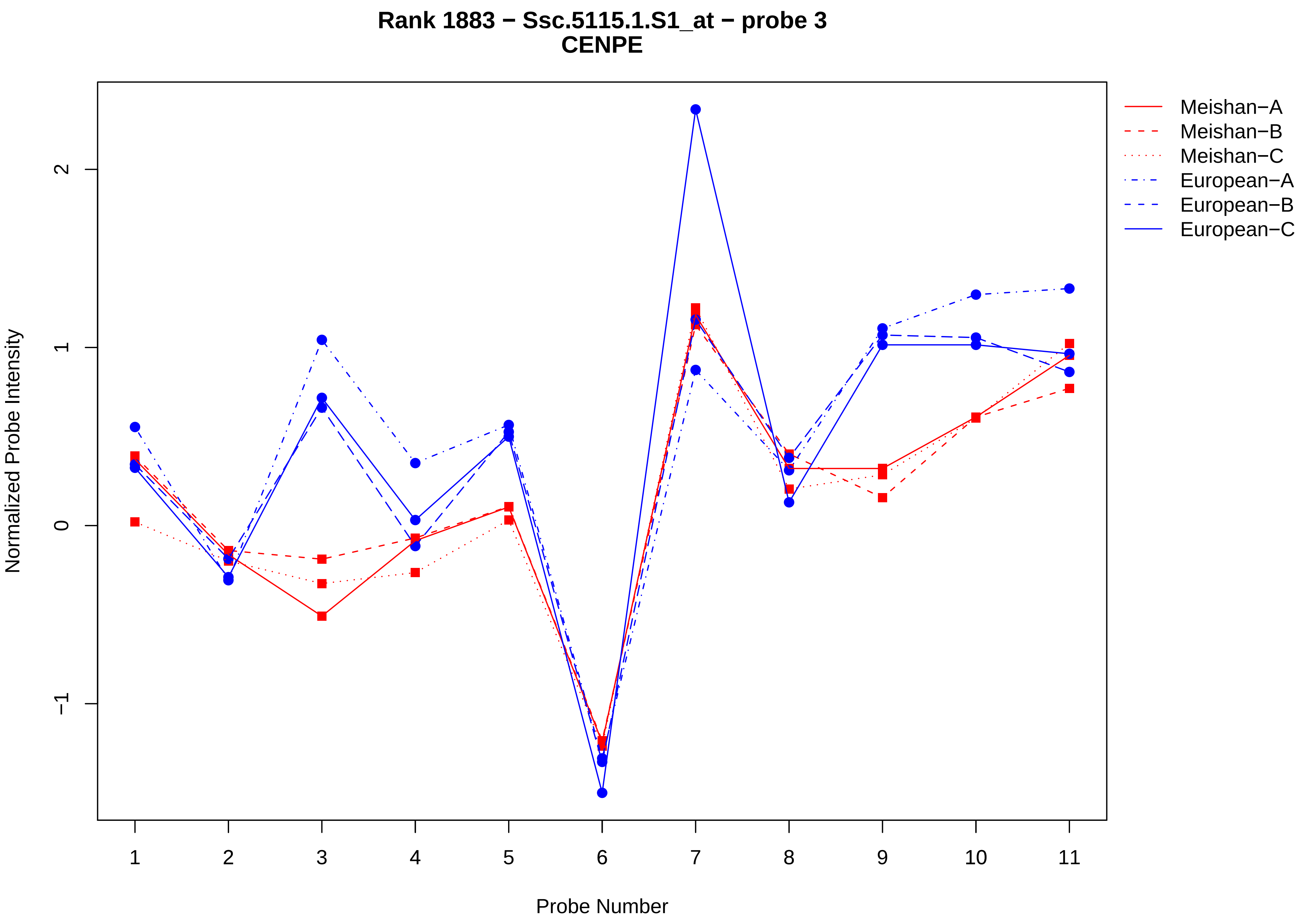

Rank 1883 − Ssc.5115.1.S1_at − probe 3
CENPE
Normalized Probe Intensity
−1
0
1
2
1
2
3
4
5
6
7
8
9
10
11
Probe Number
Meishan−A
Meishan−B
Meishan−C
European−A
European−B
European−C

Rank 1884 − Ssc.1595.1.S1_at − probe 1
PHEMX

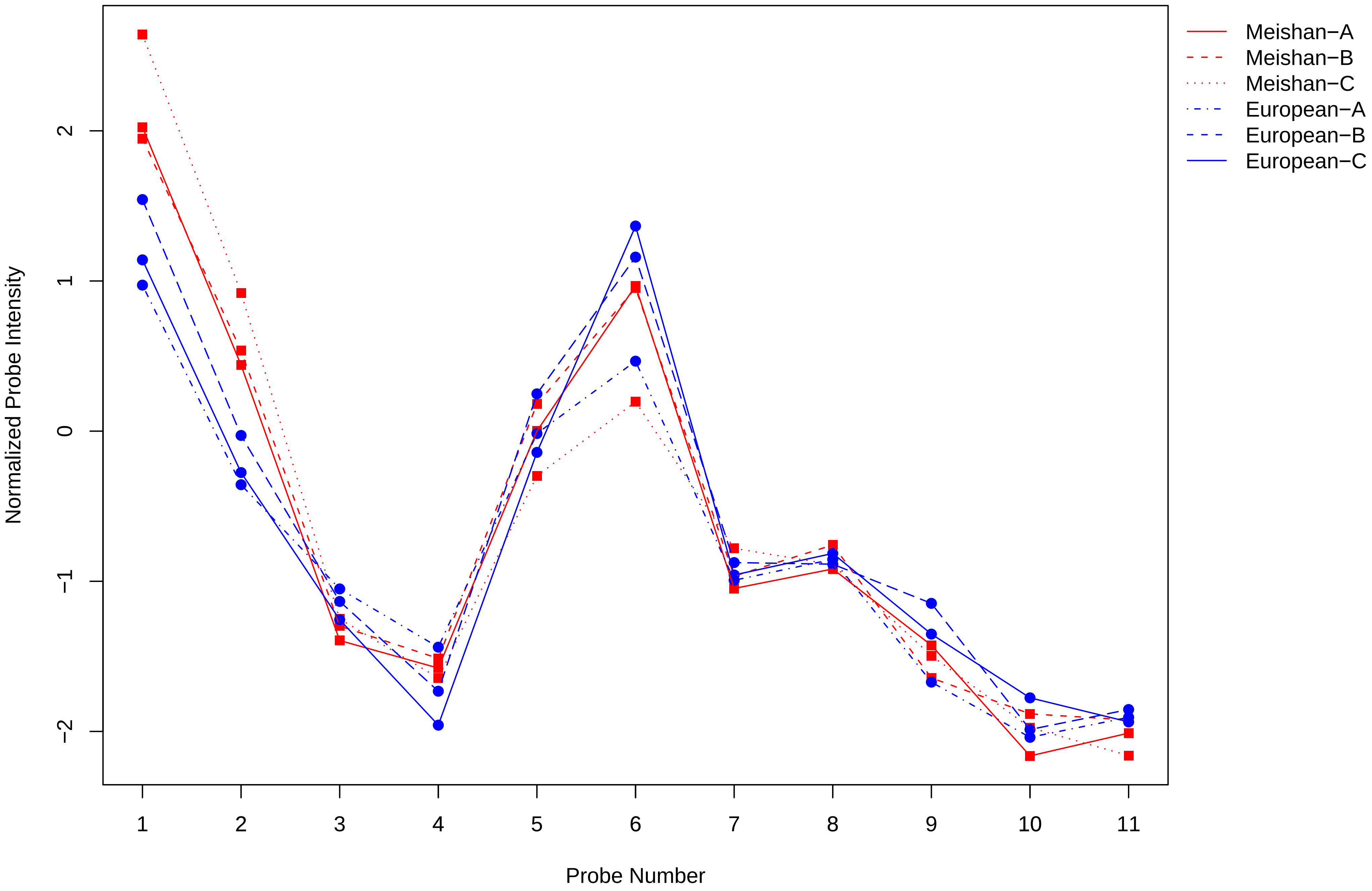

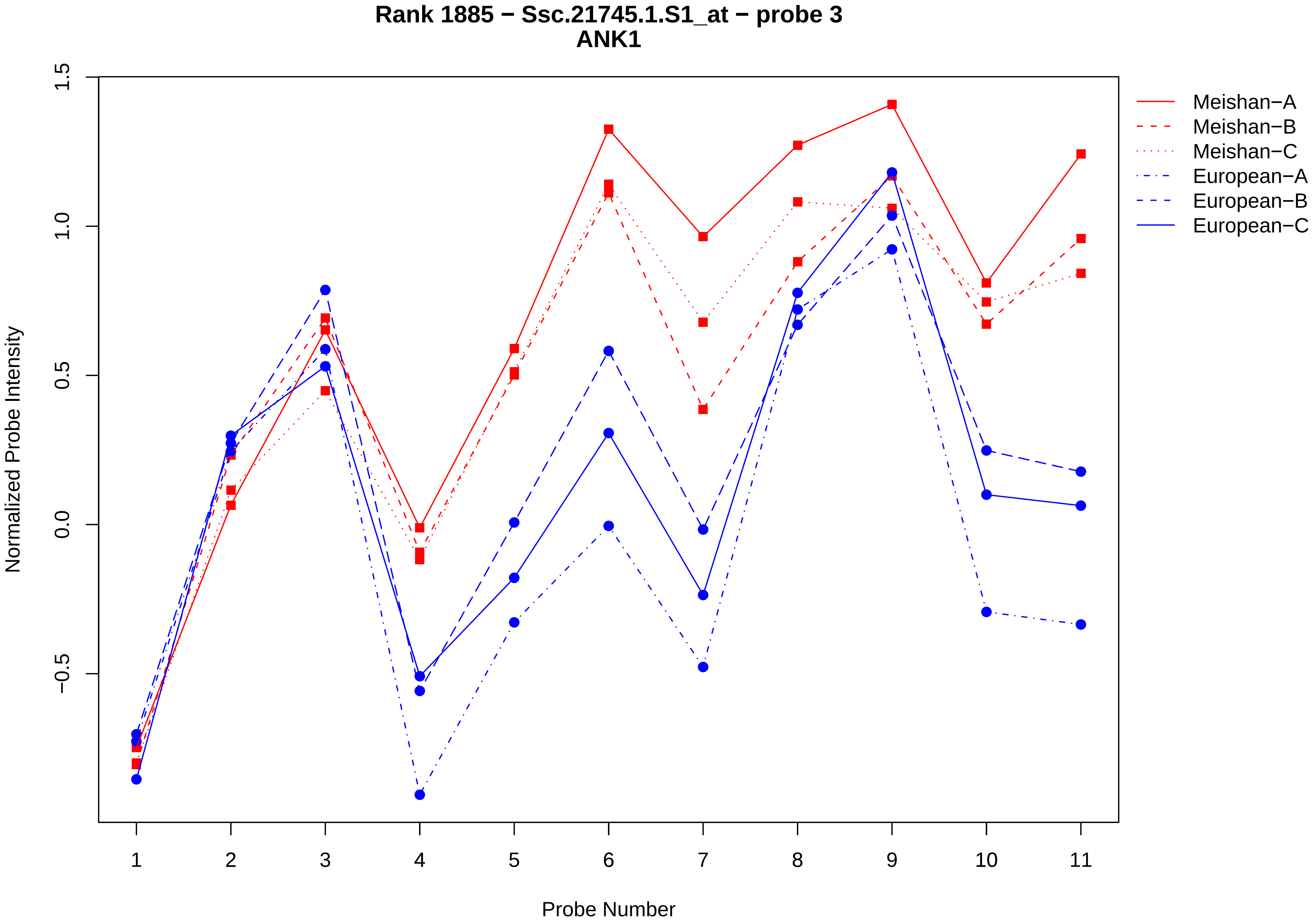

Rank 1885 − Ssc.21745.1.S1_at − probe 3
ANK1
Normalized Probe Intensity
Probe Number
Meishan−A
Meishan−B
Meishan−C
European−A
European−B
European−C
−0.5
0.0
0.5
1.0
1.5
1
2
3
4
5
6
7
8
9
10
11

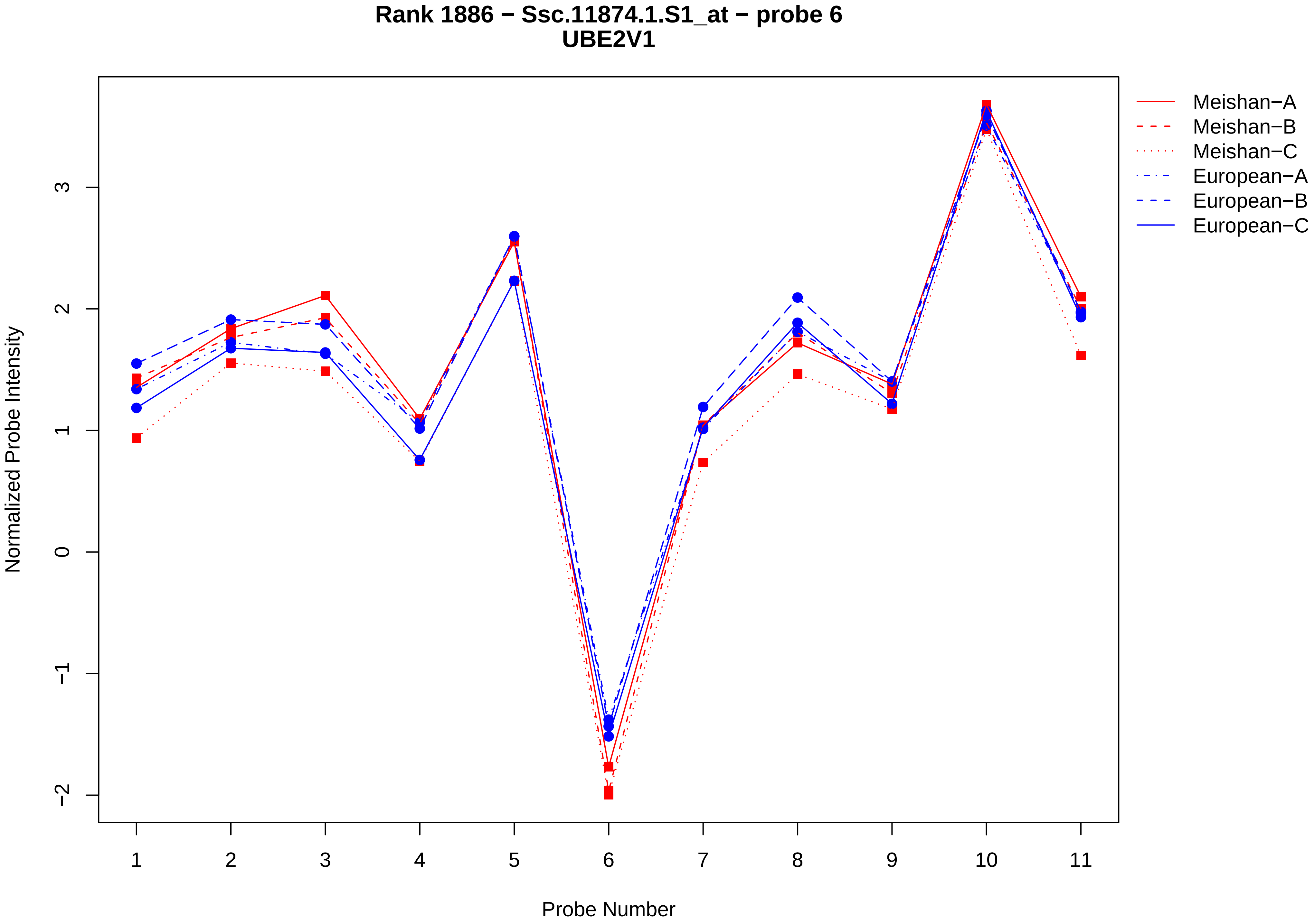

Rank 1886 − Ssc.11874.1.S1_at − probe 6
UBE2V1
Normalized Probe Intensity
Probe Number
−2
−1
0
1
2
3
1
2
3
4
5
6
7
8
9
10
11
Meishan−A
Meishan−B
Meishan−C
European−A
European−B
European−C

Rank 1887 − Ssc.1790.2.S1_at − probe 2
NUP50

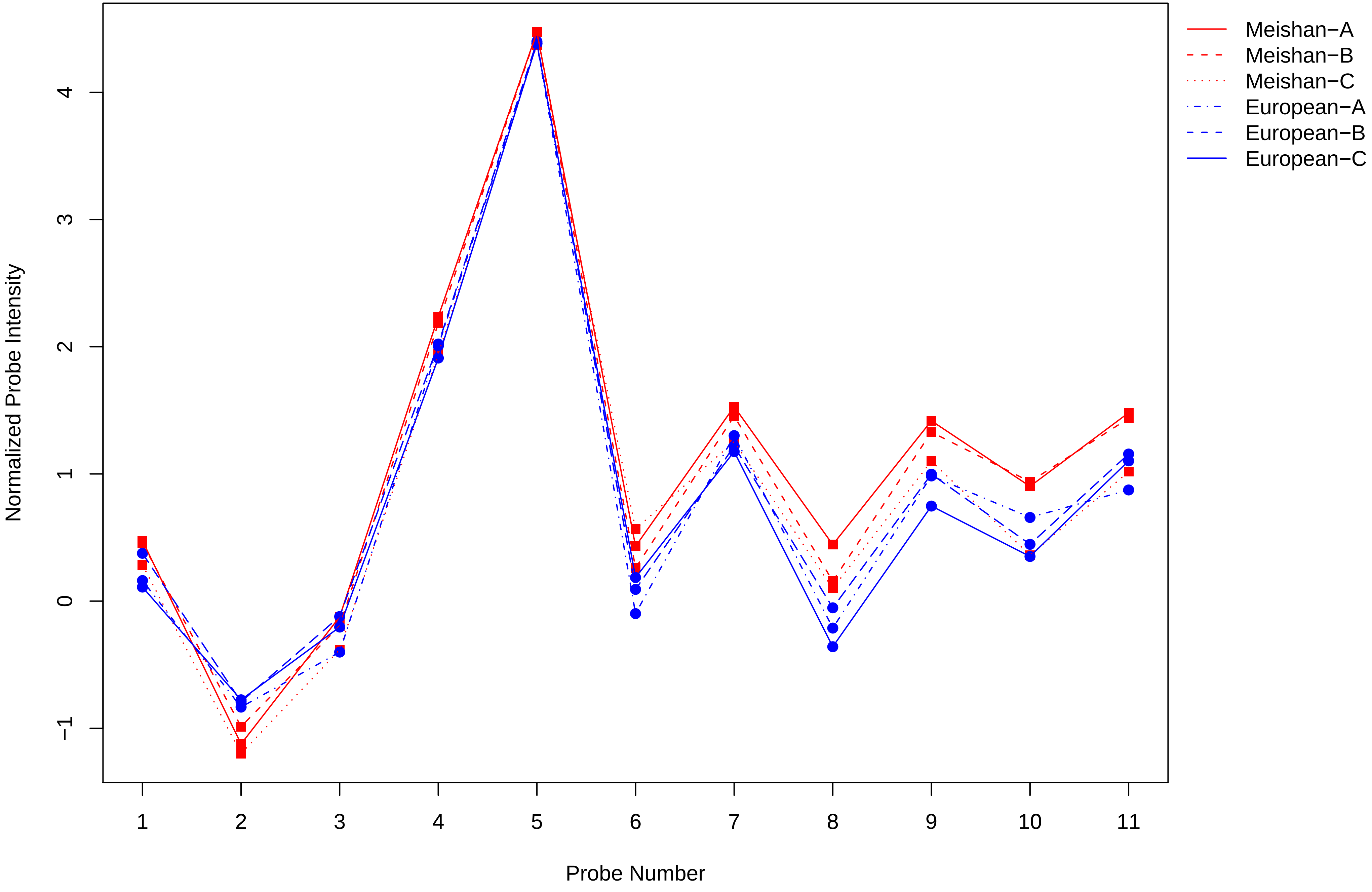

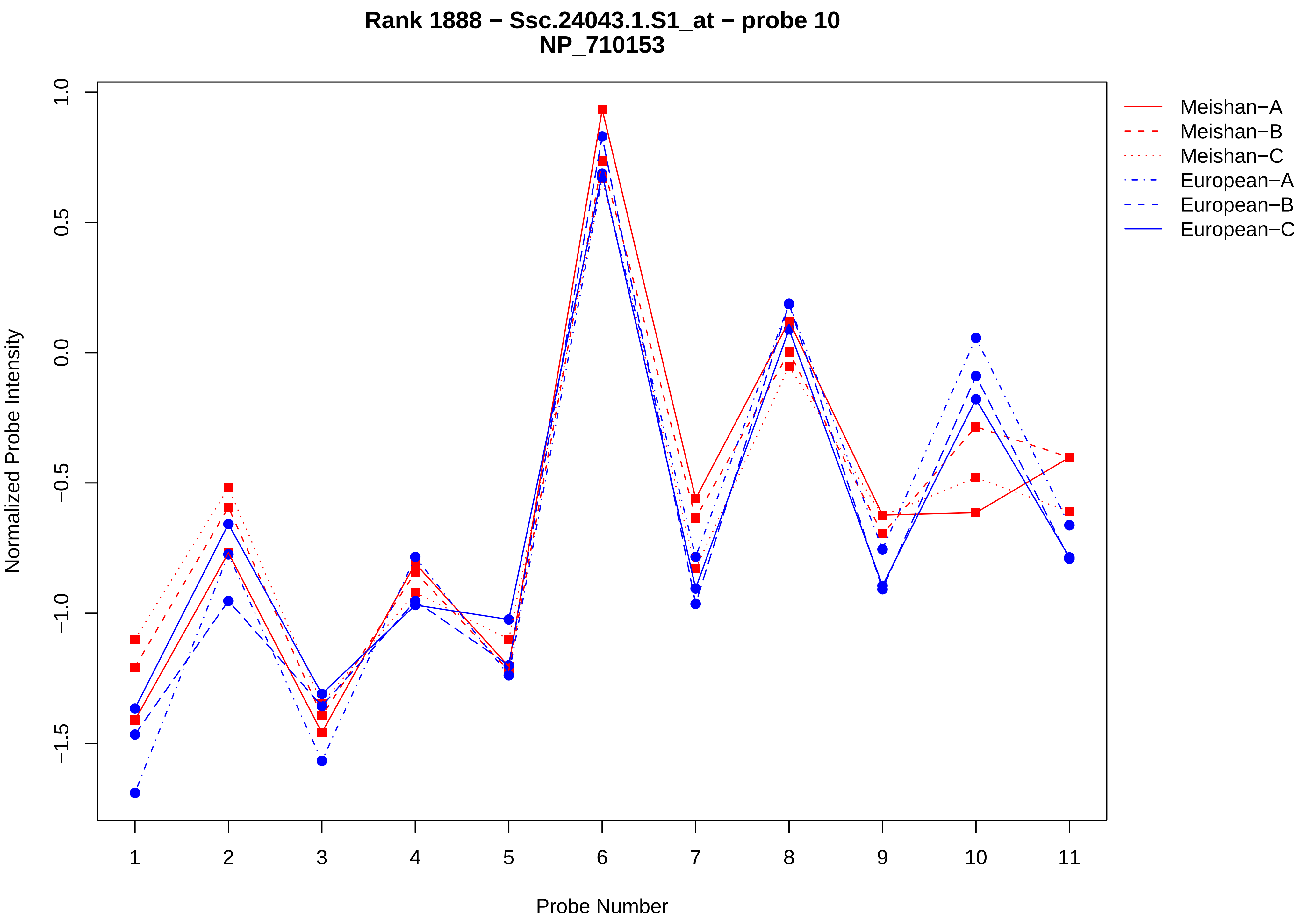

Rank 1888 − Ssc.24043.1.S1_at − probe 10
NP_710153
Normalized Probe Intensity
Probe Number
1.0
0.5
0.0
−0.5
−1.0
−1.5
1
2
3
4
5
6
7
8
9
10
11
Meishan−A
Meishan−B
Meishan−C
European−A
European−B
European−C

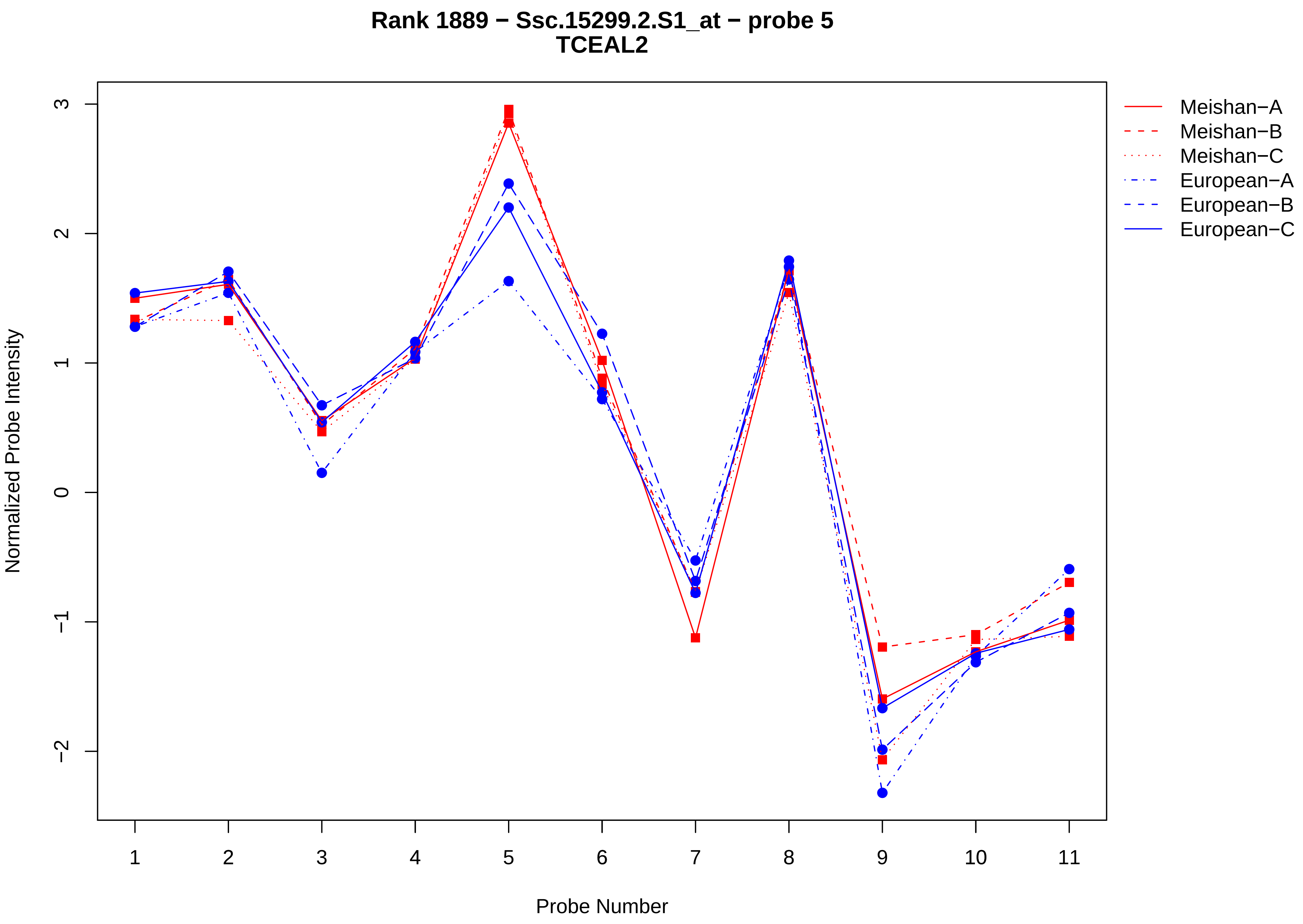

Rank 1889 − Ssc.15299.2.S1_at − probe 5
TCEAL2
Normalized Probe Intensity
Probe Number
Meishan−A
Meishan−B
Meishan−C
European−A
European−B
European−C

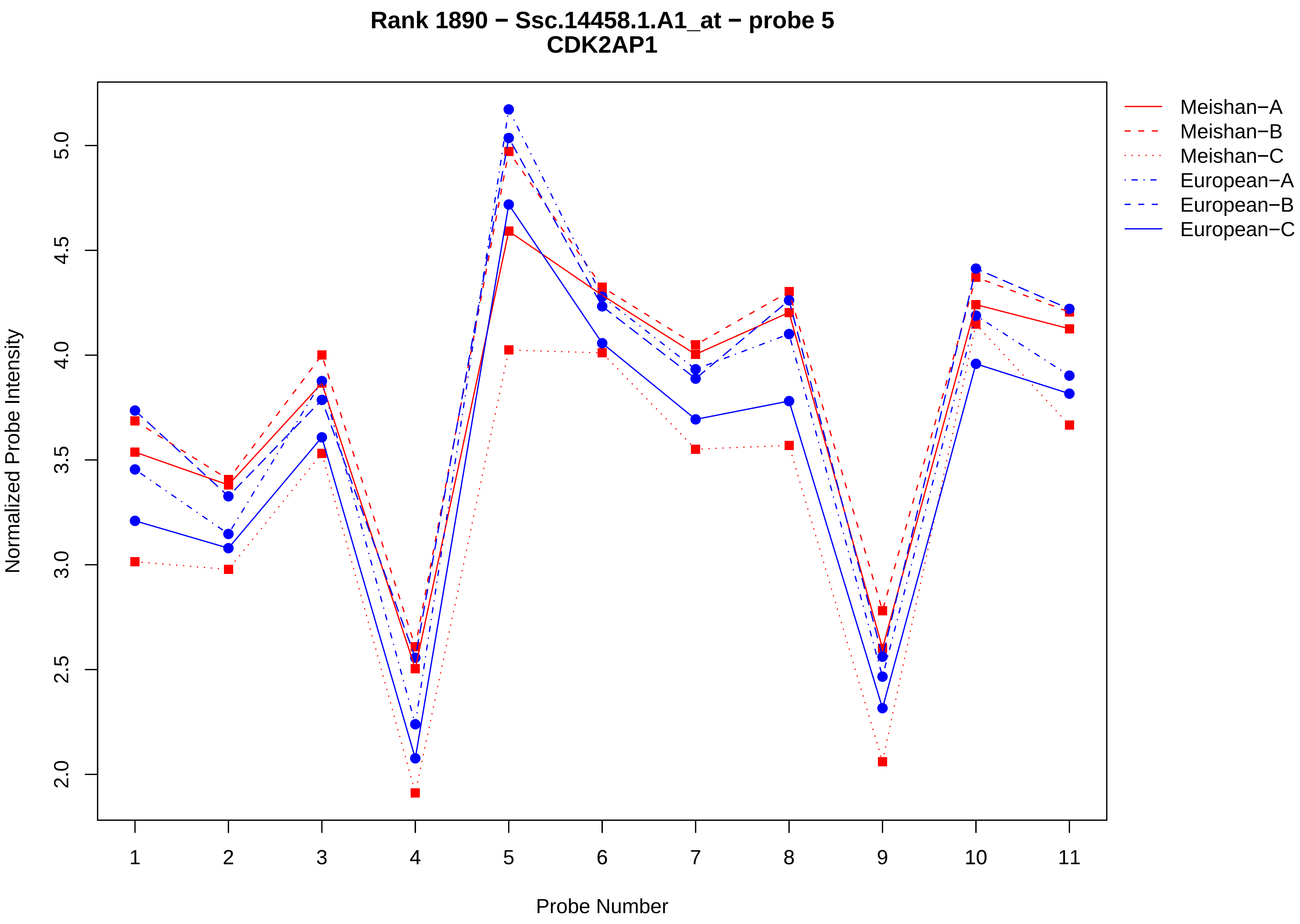

Rank 1890 − Ssc.14458.1.A1_at − probe 5
CDK2AP1
Normalized Probe Intensity
Probe Number
2.0
2.5
3.0
3.5
4.0
4.5
5.0
1
2
3
4
5
6
7
8
9
10
11
Meishan−A
Meishan−B
Meishan−C
European−A
European−B
European−C

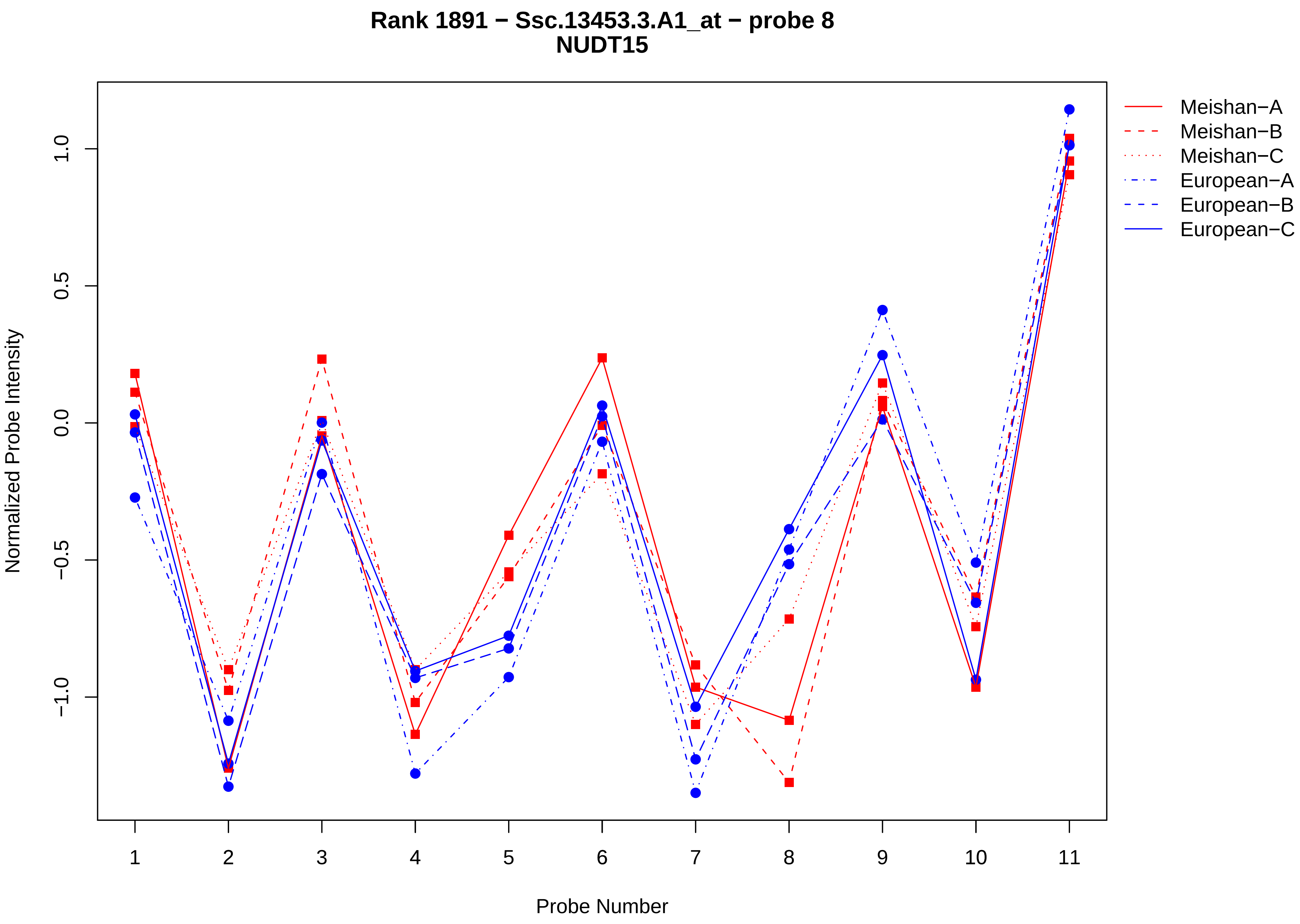
Rank 1891 − Ssc.13453.3.A1_at − probe 8
NUDT15
Normalized Probe Intensity
Probe Number
1.0
0.5
0.0
−0.5
−1.0
1
2
3
4
5
6
7
8
9
10
11
Meishan−A
Meishan−B
Meishan−C
European−A
European−B
European−C

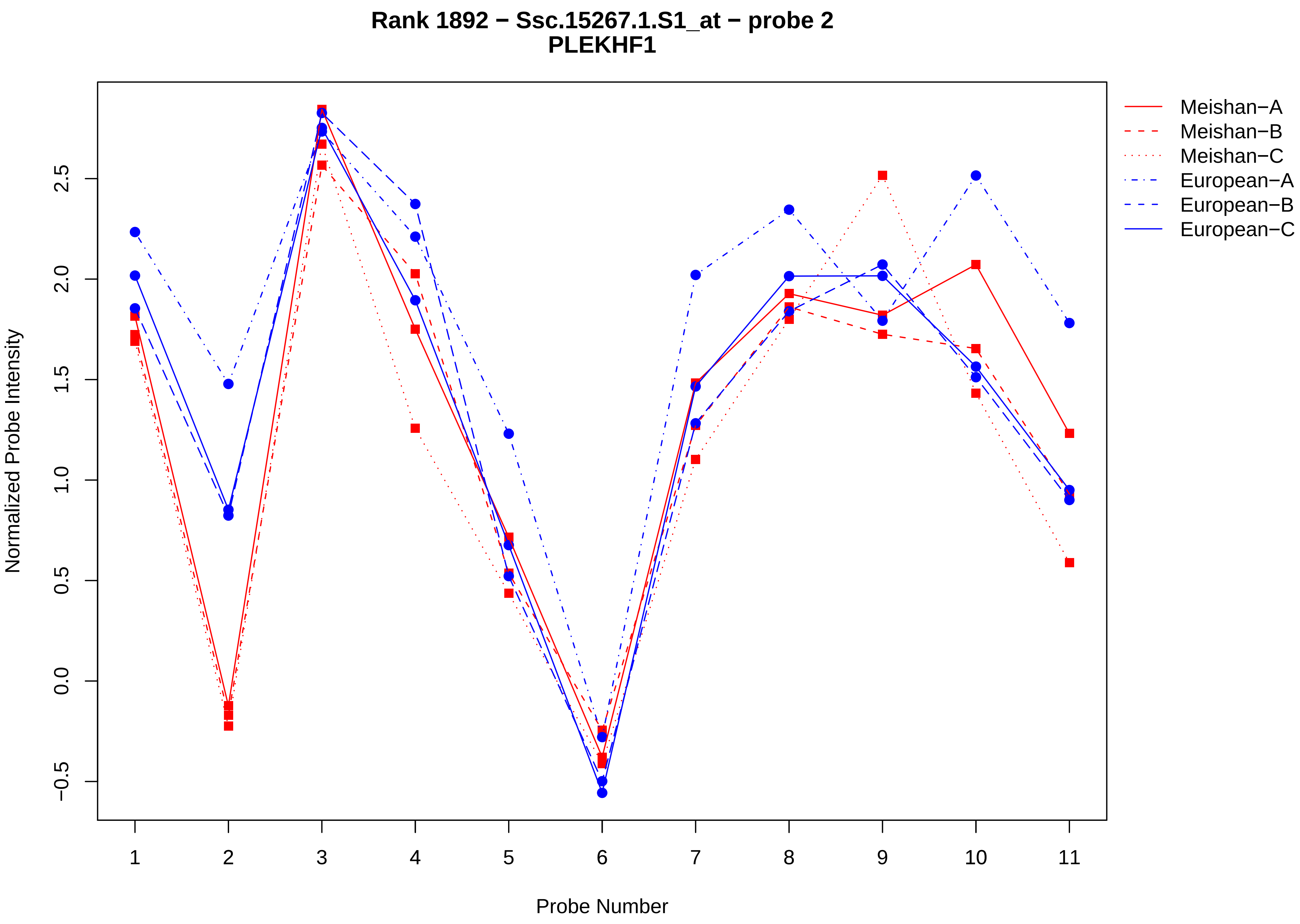

Rank 1892 − Ssc.15267.1.S1_at − probe 2
PLEKHF1
Normalized Probe Intensity
Probe Number
−0.5
0.0
0.5
1.0
1.5
2.0
2.5
1
2
3
4
5
6
7
8
9
10
11
Meishan−A
Meishan−B
Meishan−C
European−A
European−B
European−C

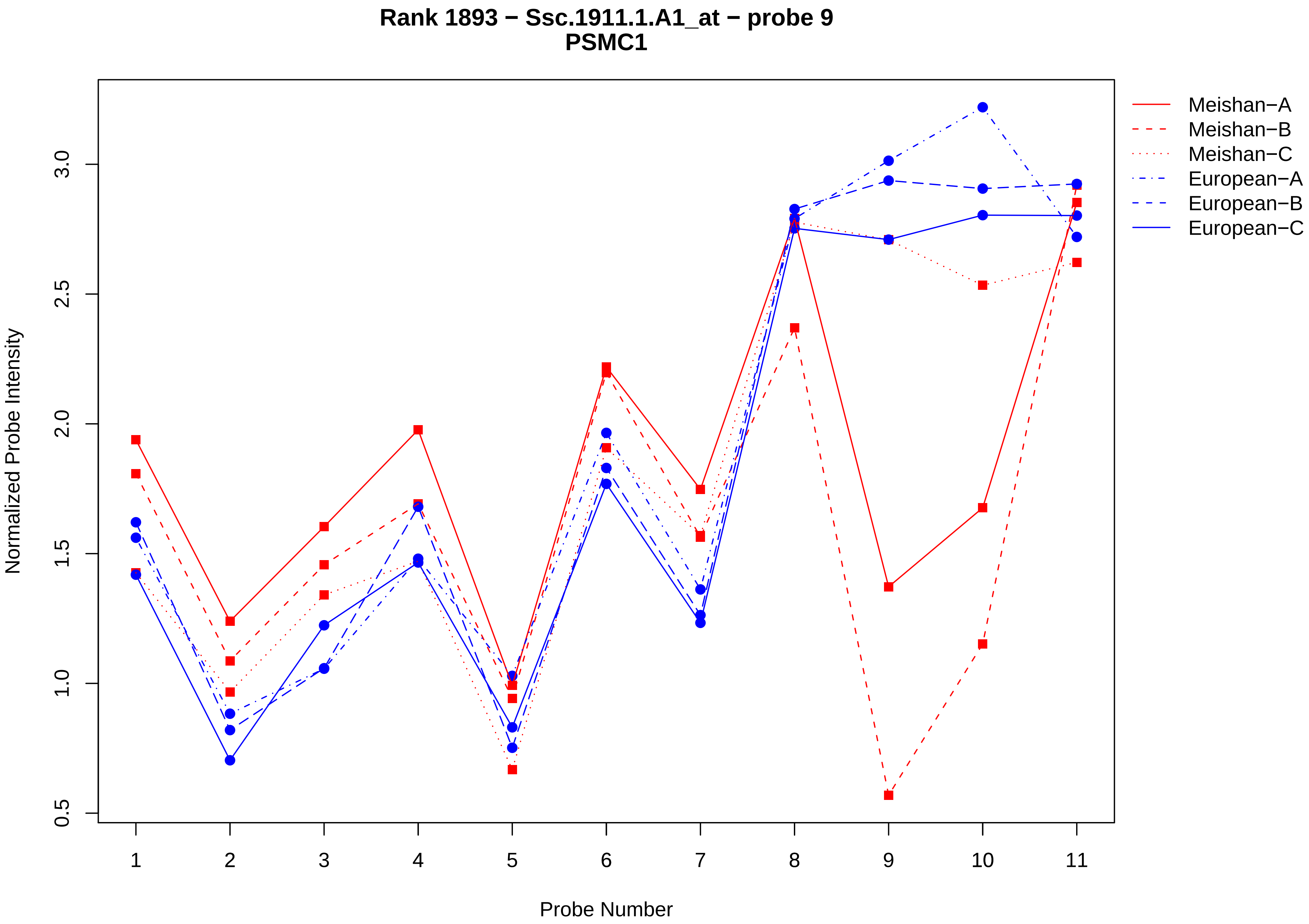

Rank 1893 − Ssc.1911.1.A1_at − probe 9
PSMC1
Normalized Probe Intensity
0.5
1.0
1.5
2.0
2.5
3.0
Probe Number
1
2
3
4
5
6
7
8
9
10
11
Meishan−A
Meishan−B
Meishan−C
European−A
European−B
European−C

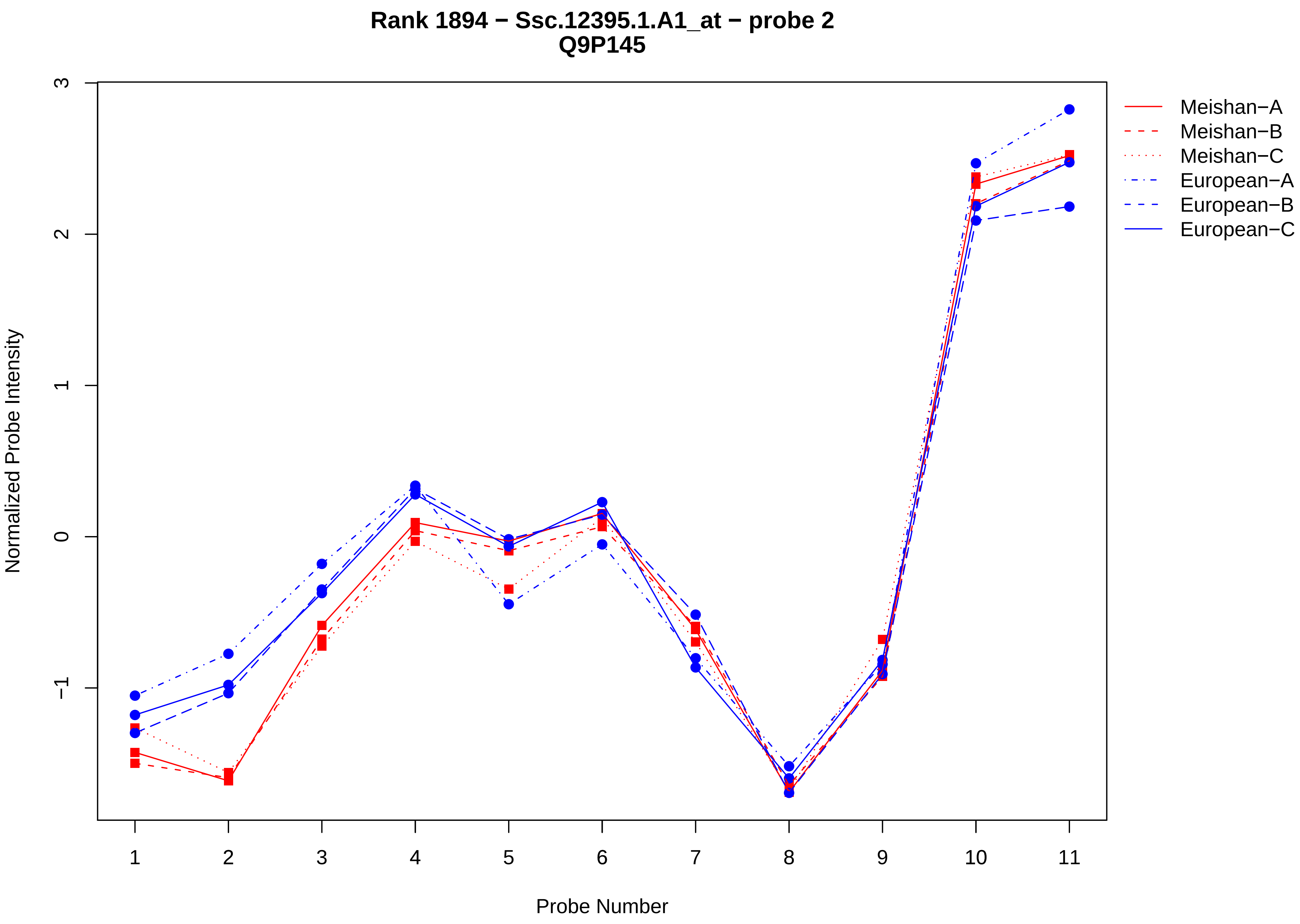

Rank 1894 − Ssc.12395.1.A1_at − probe 2
Q9P145
Normalized Probe Intensity
Probe Number
Meishan−A
Meishan−B
Meishan−C
European−A
European−B
European−C

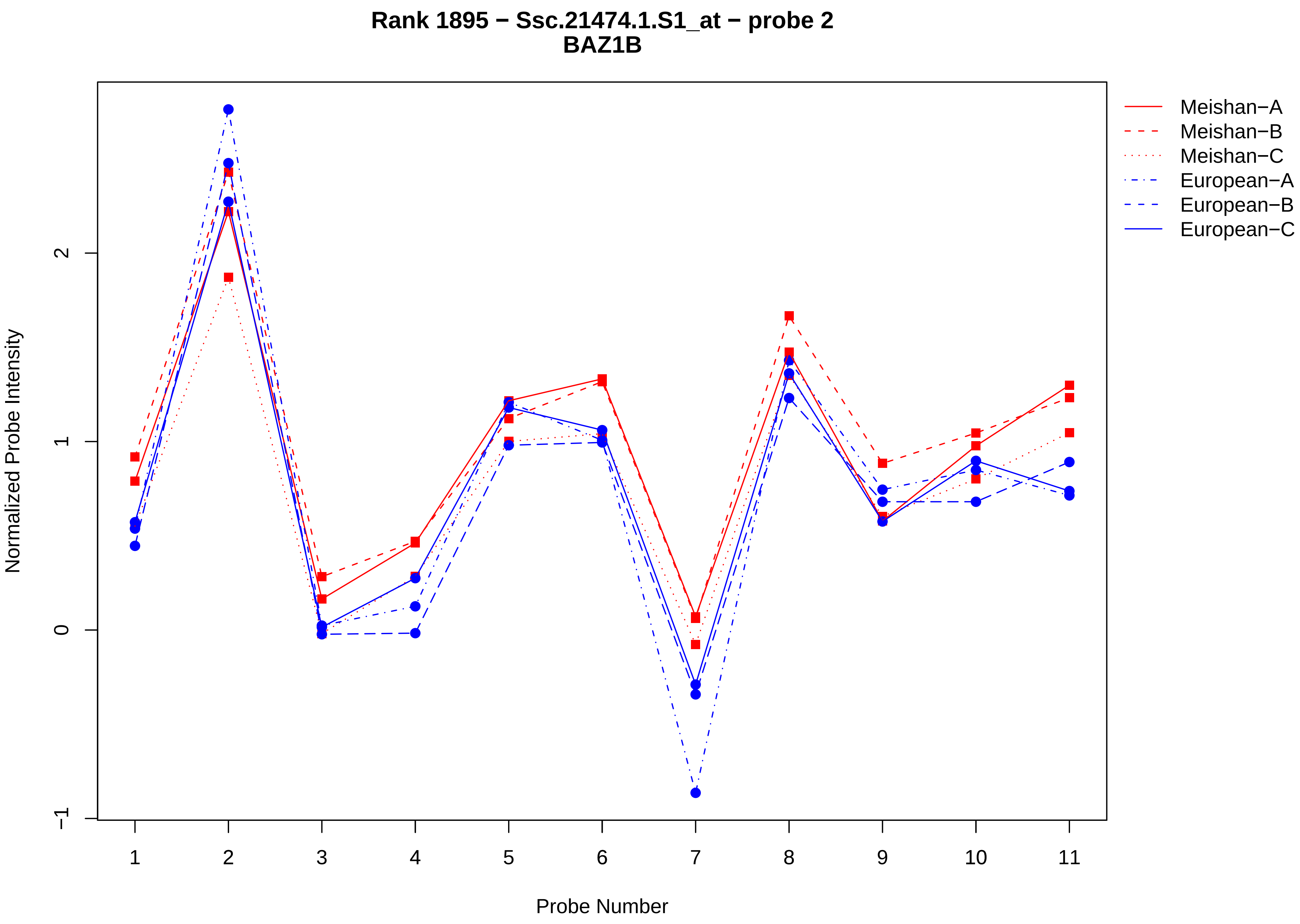

Rank 1895 − Ssc.21474.1.S1_at − probe 2
BAZ1B
Normalized Probe Intensity
Probe Number
−1
0
1
2
1
2
3
4
5
6
7
8
9
10
11
Meishan−A
Meishan−B
Meishan−C
European−A
European−B
European−C

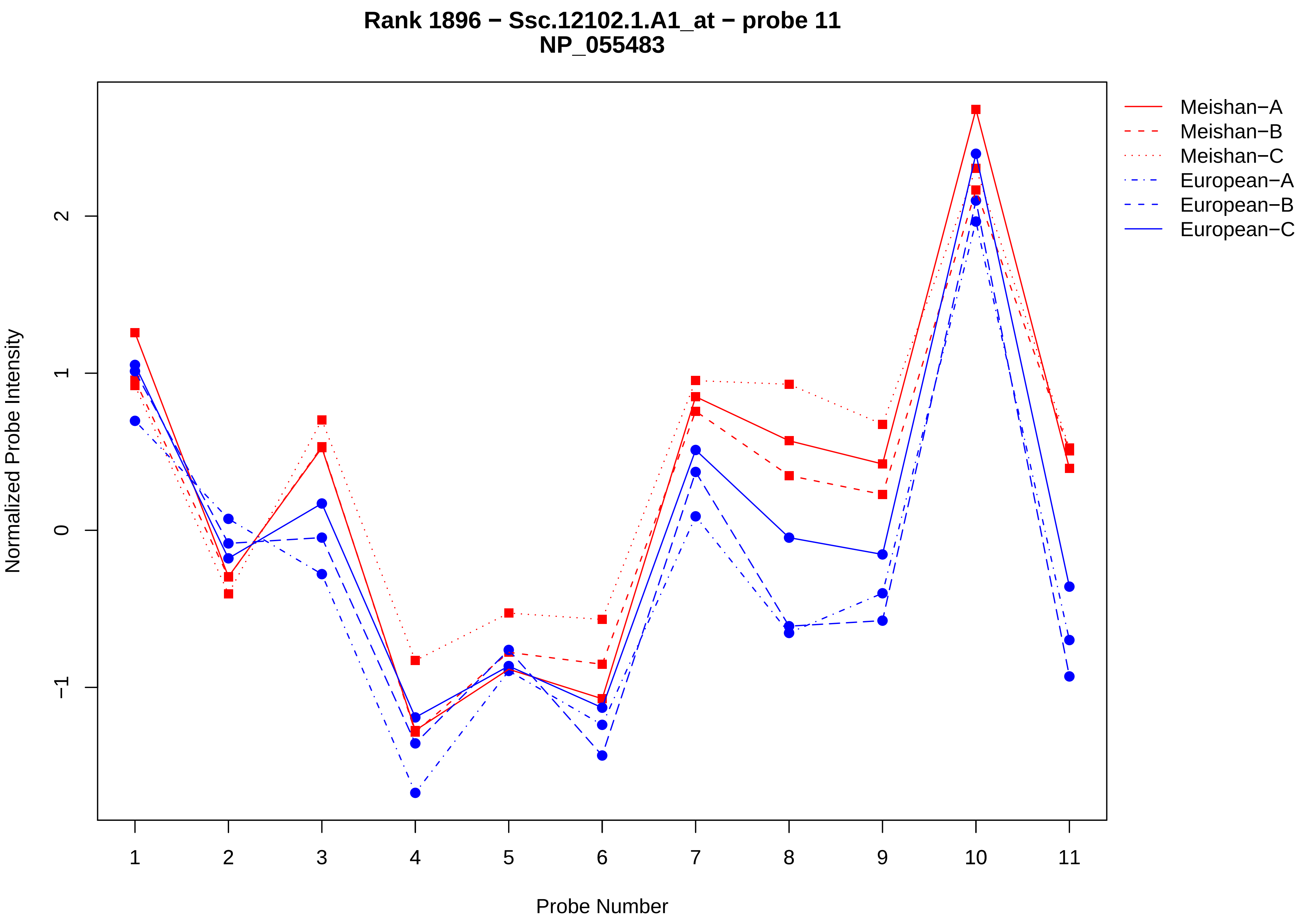
Rank 1896 − Ssc.12102.1.A1_at − probe 11
NP_055483
Normalized Probe Intensity
Probe Number
−1
0
1
2
1
2
3
4
5
6
7
8
9
10
11
Meishan−A
Meishan−B
Meishan−C
European−A
European−B
European−C

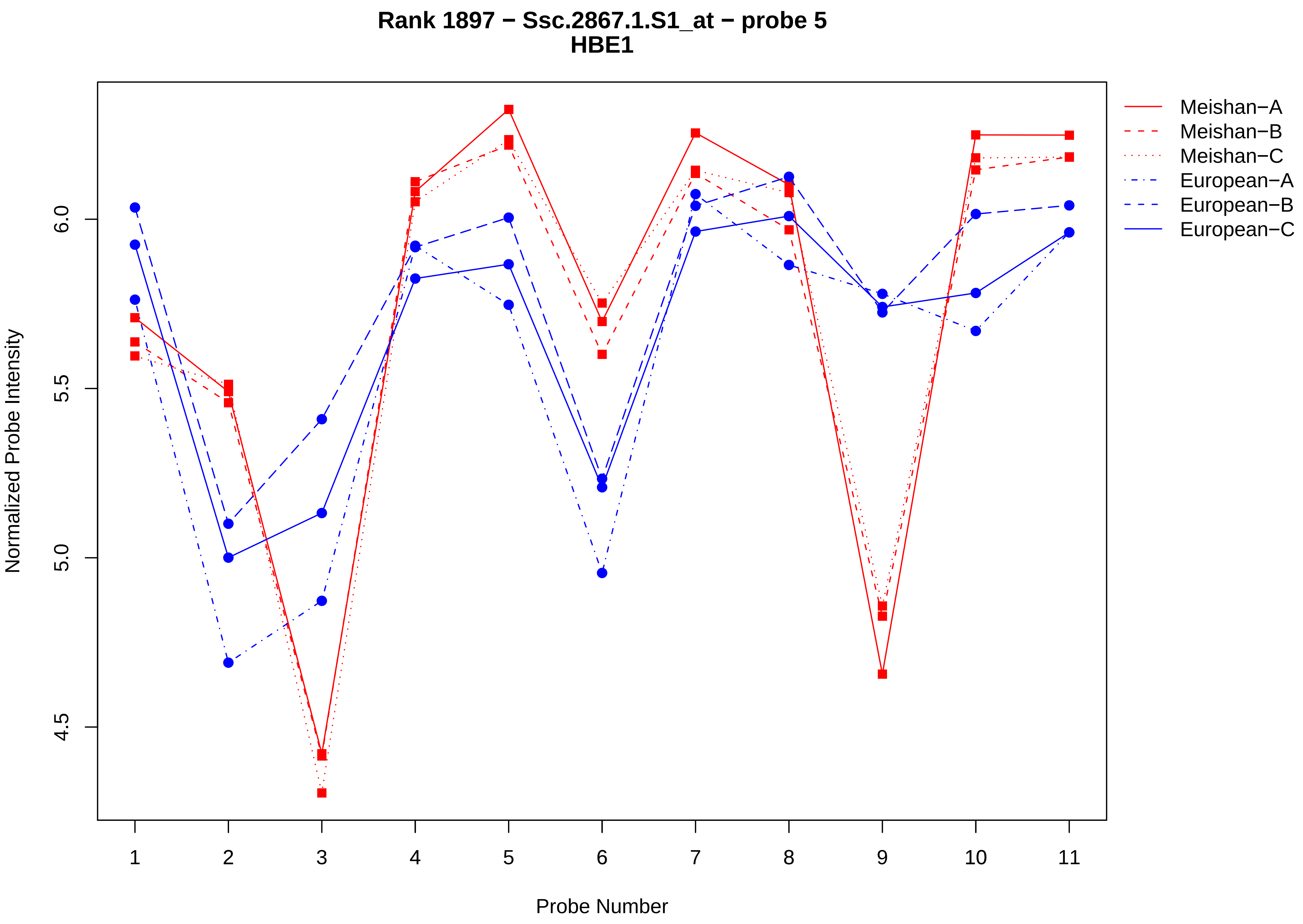

Rank 1897 − Ssc.2867.1.S1_at − probe 5
HBE1
Normalized Probe Intensity
Probe Number
4.5
5.0
5.5
6.0
1
2
3
4
5
6
7
8
9
10
11
Meishan−A
Meishan−B
Meishan−C
European−A
European−B
European−C

Rank 1898 − Ssc.24556.2.S1_a_at − probe 8
SLC35F5

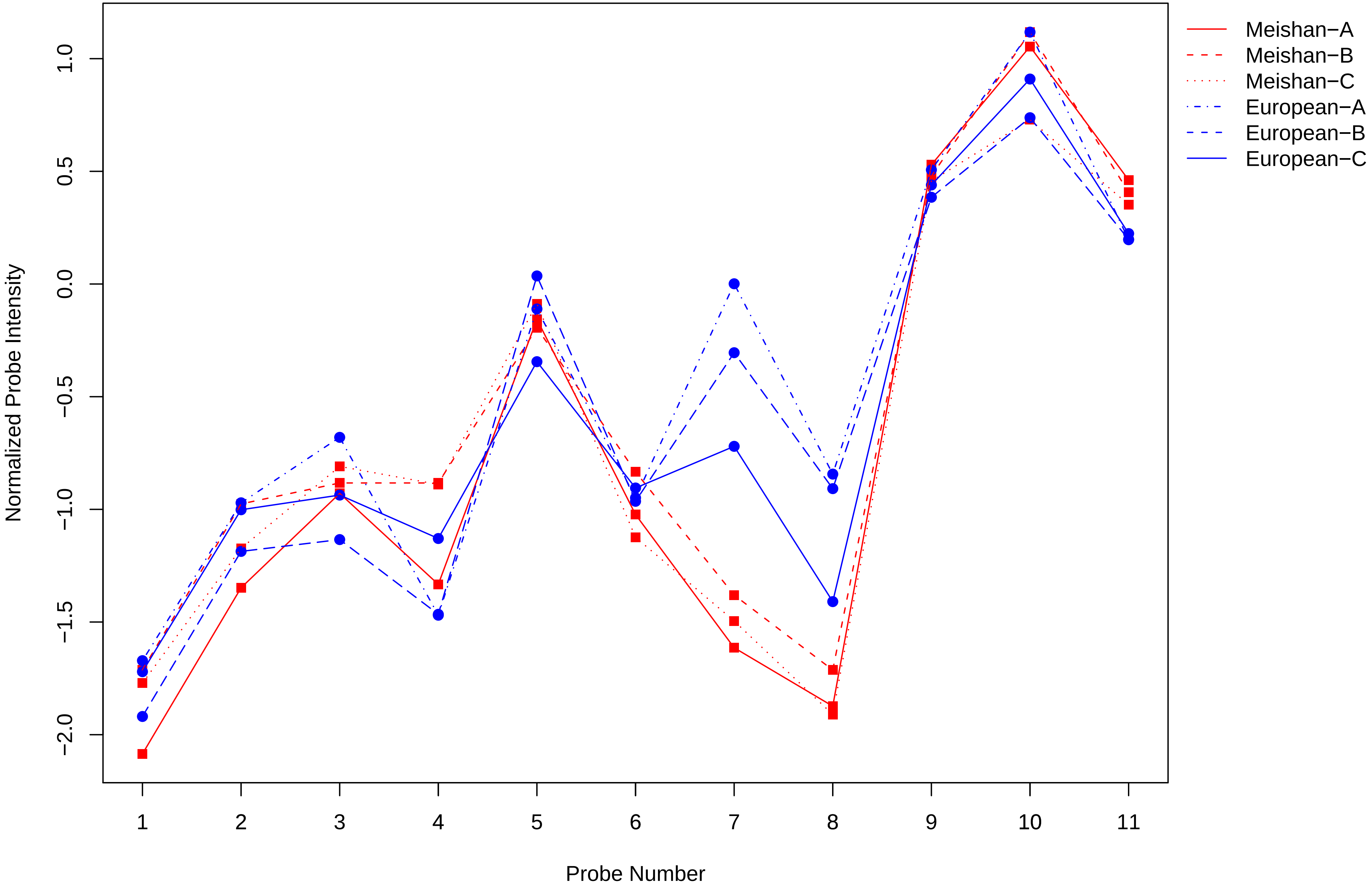

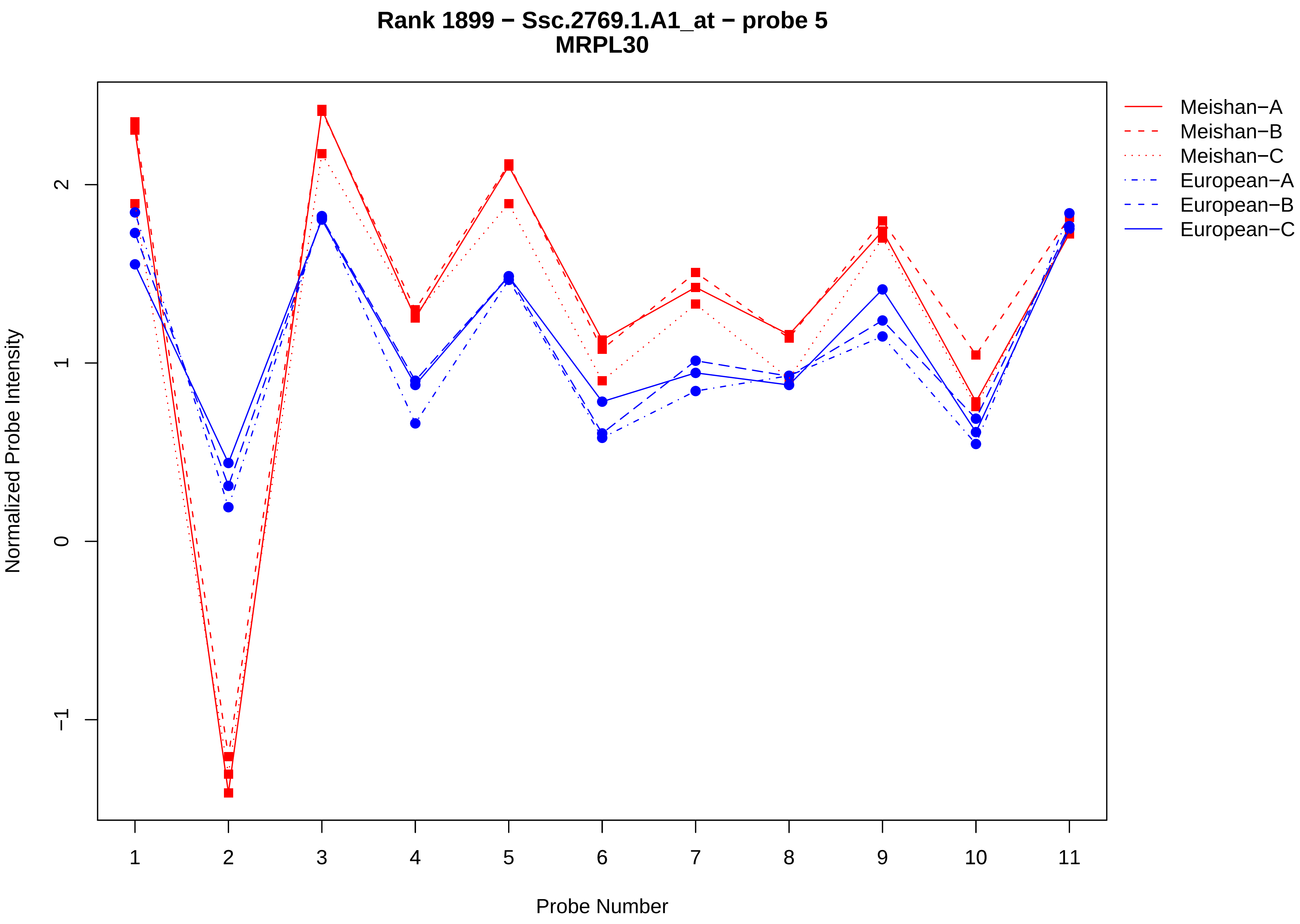

Rank 1899 − Ssc.2769.1.A1_at − probe 5
MRPL30
Normalized Probe Intensity
Probe Number
−1
0
1
2
1
2
3
4
5
6
7
8
9
10
11
Meishan−A
Meishan−B
Meishan−C
European−A
European−B
European−C

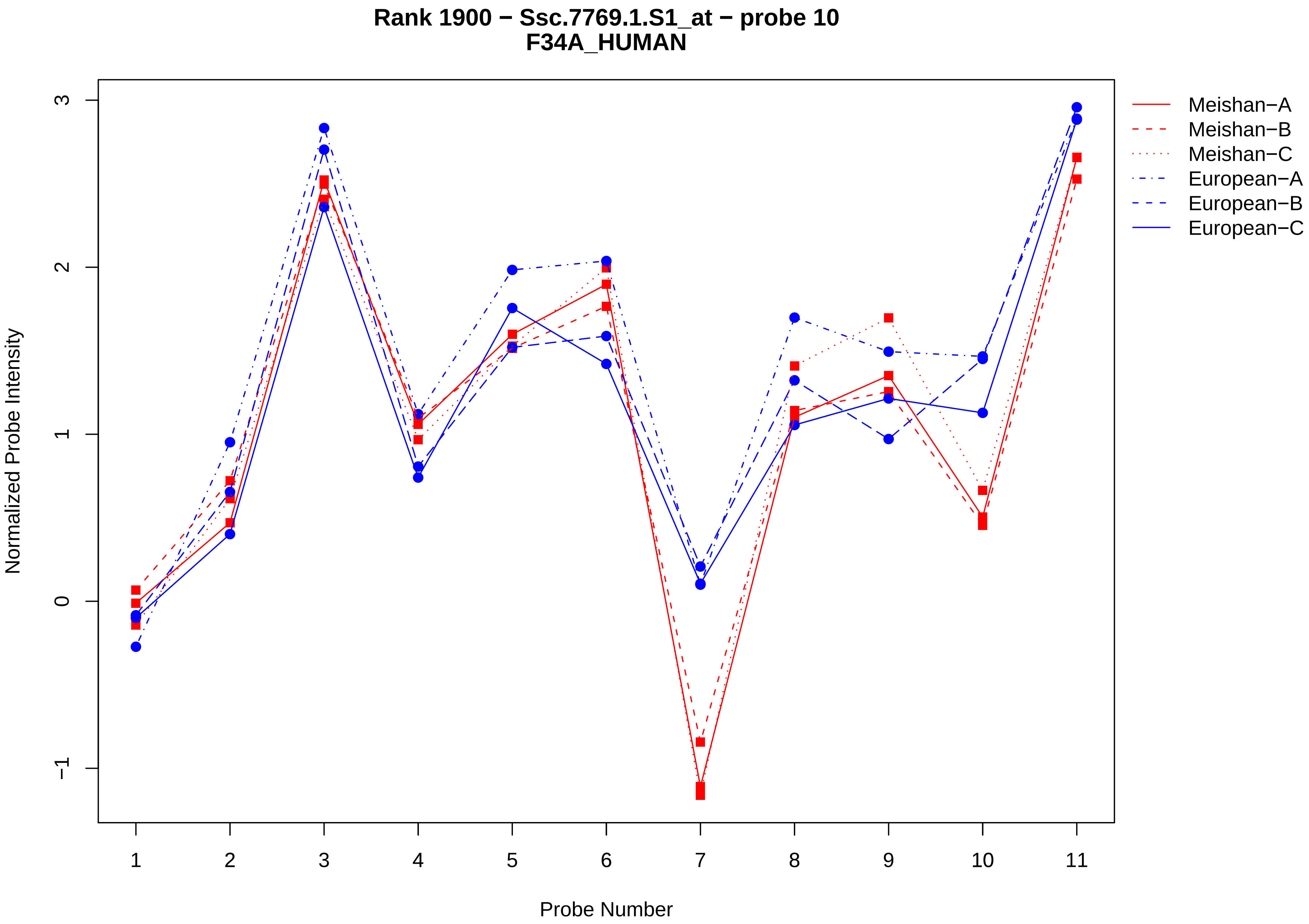

Rank 1900 − Ssc.7769.1.S1_at − probe 10
F34A_HUMAN
Normalized Probe Intensity
Probe Number
−1
0
1
2
3
1
2
3
4
5
6
7
8
9
10
11
Meishan−A
Meishan−B
Meishan−C
European−A
European−B
European−C

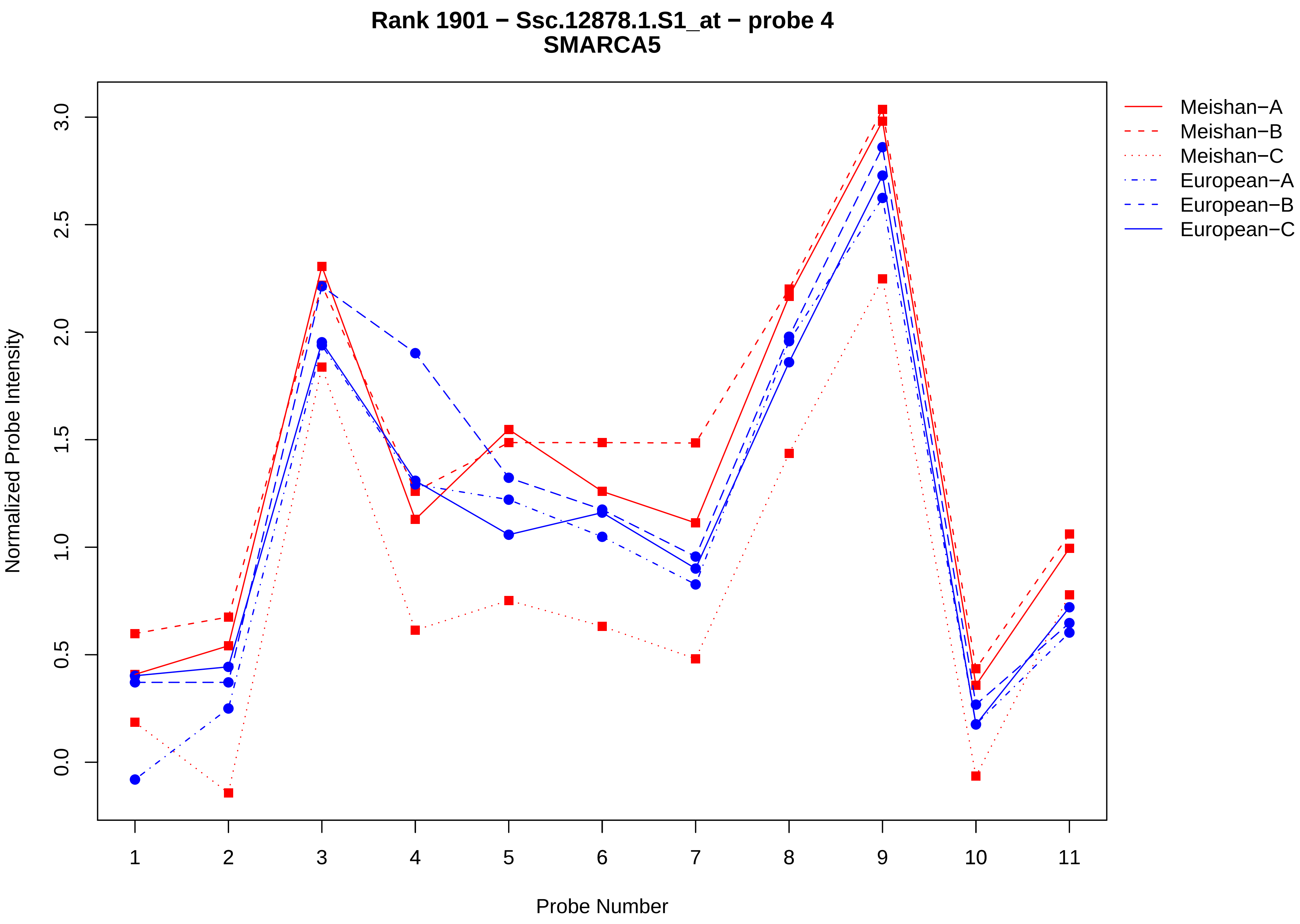
Rank 1901 − Ssc.12878.1.S1_at − probe 4
SMARCA5
Normalized Probe Intensity
Probe Number
0.0
0.5
1.0
1.5
2.0
2.5
3.0
1
2
3
4
5
6
7
8
9
10
11
Meishan−A
Meishan−B
Meishan−C
European−A
European−B
European−C

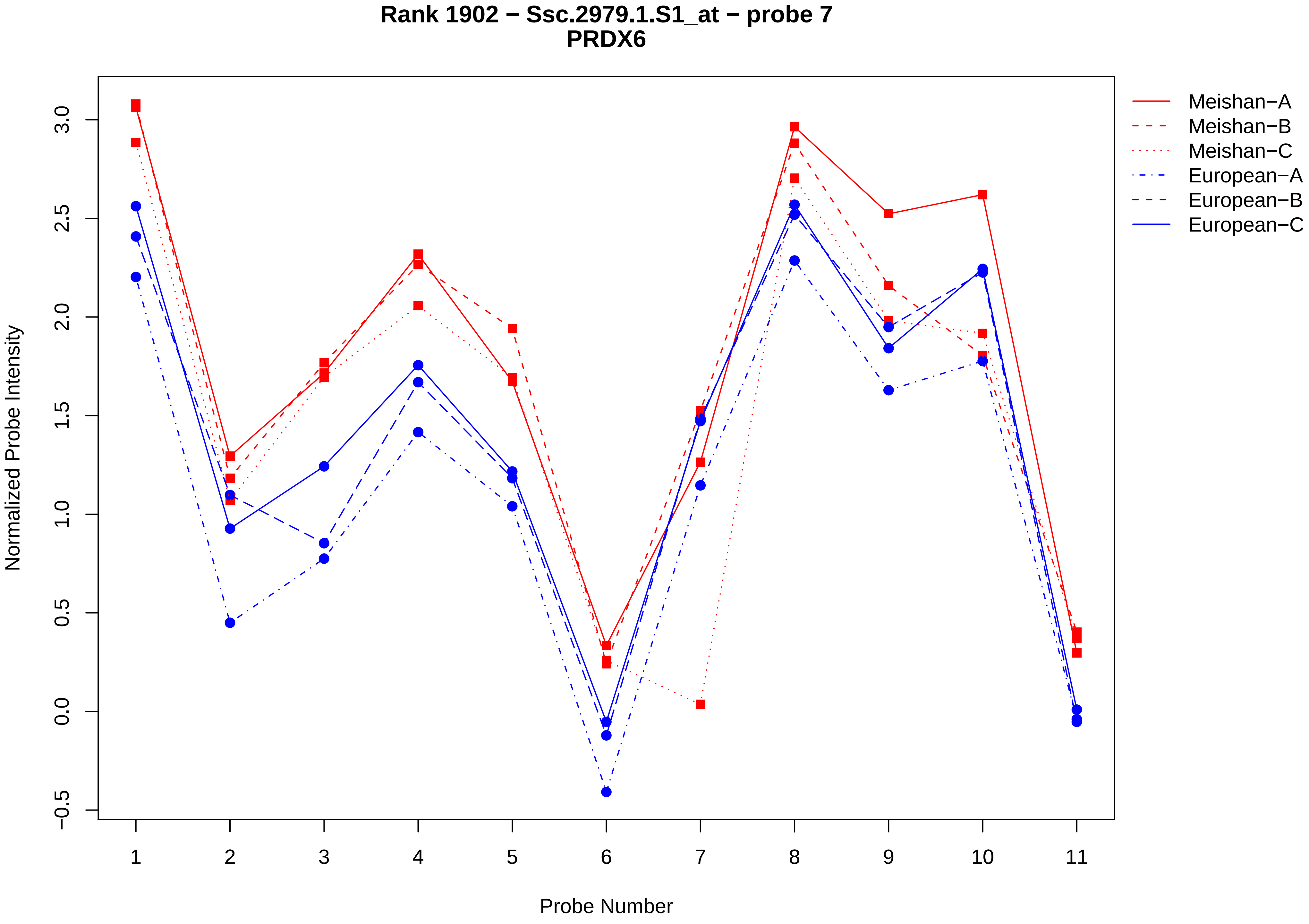

Rank 1902 − Ssc.2979.1.S1_at − probe 7
PRDX6
Normalized Probe Intensity
Probe Number
−0.5
0.0
0.5
1.0
1.5
2.0
2.5
3.0
1
2
3
4
5
6
7
8
9
10
11
Meishan−A
Meishan−B
Meishan−C
European−A
European−B
European−C

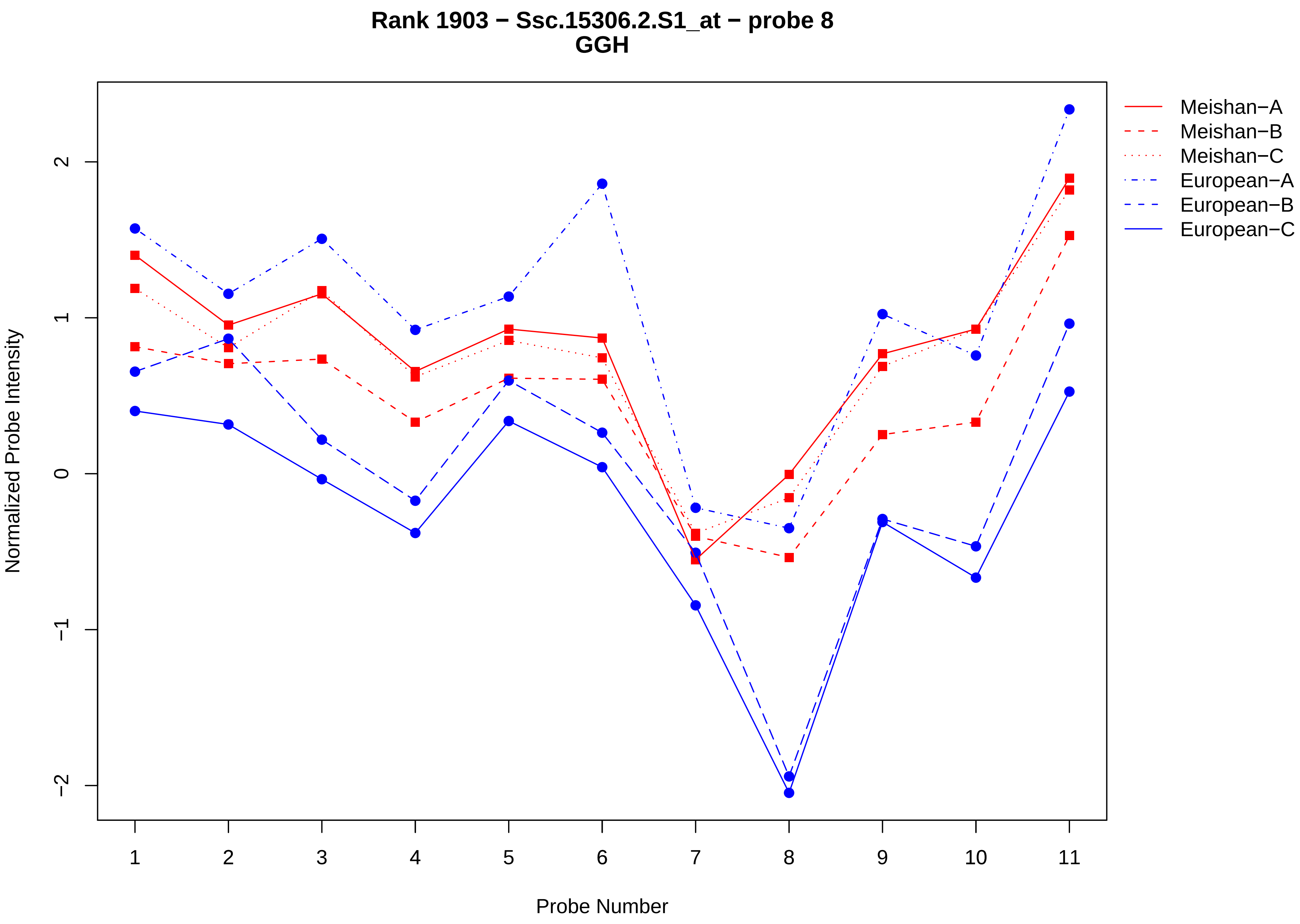

Rank 1903 − Ssc.15306.2.S1_at − probe 8
GGH
Normalized Probe Intensity
Probe Number
−2
−1
0
1
2
1
2
3
4
5
6
7
8
9
10
11
Meishan−A
Meishan−B
Meishan−C
European−A
European−B
European−C

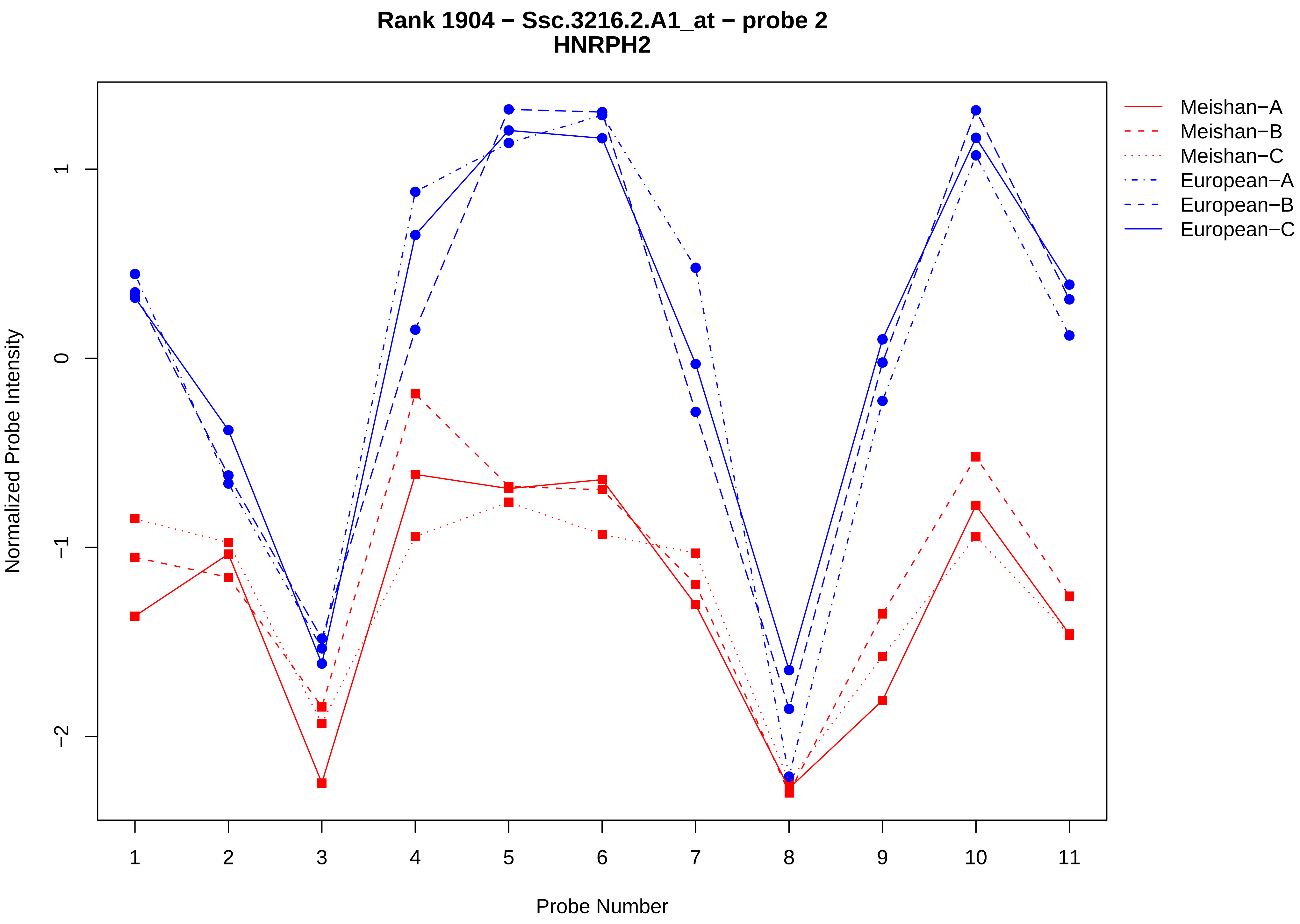

Rank 1904 − Ssc.3216.2.A1_at − probe 2
HNRPH2
Normalized Probe Intensity
Probe Number
1
0
−1
−2
1
2
3
4
5
6
7
8
9
10
11
Meishan−A
Meishan−B
Meishan−C
European−A
European−B
European−C

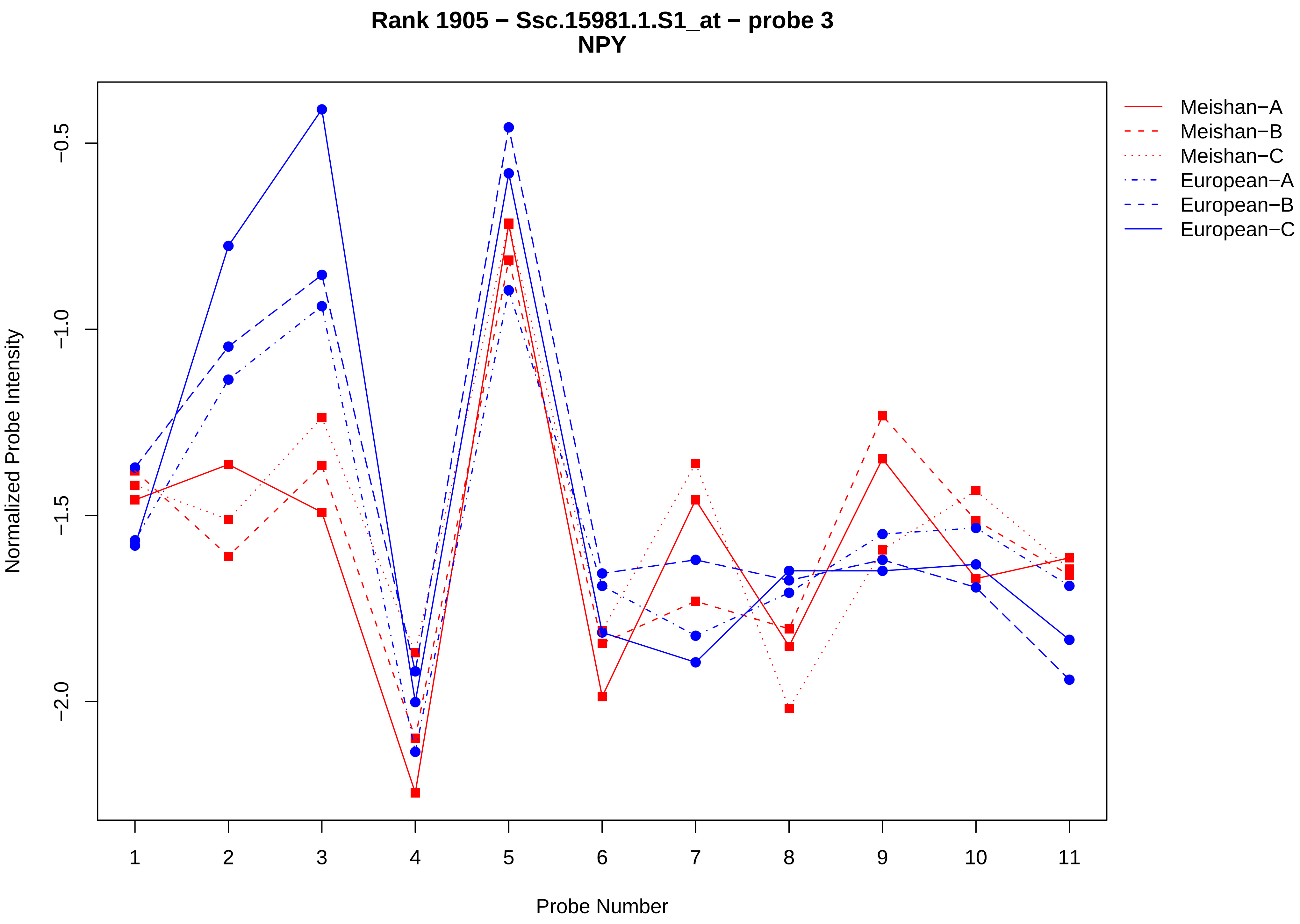

Rank 1905 − Ssc.15981.1.S1_at − probe 3
NPY
Normalized Probe Intensity
−0.5
−1.0
−1.5
−2.0
1
2
3
4
5
6
7
8
9
10
11
Probe Number
Meishan−A
Meishan−B
Meishan−C
European−A
European−B
European−C

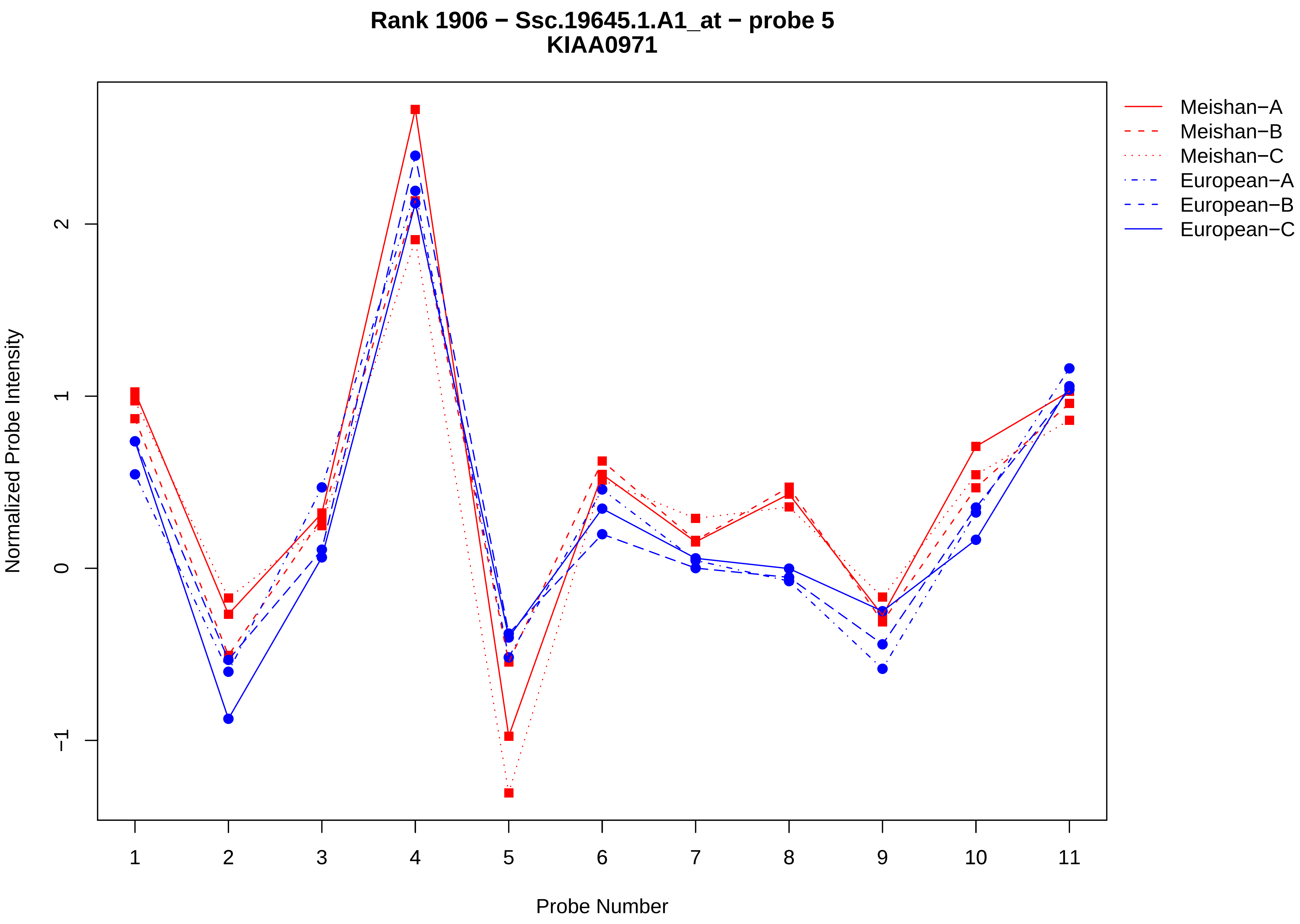

Rank 1906 − Ssc.19645.1.A1_at − probe 5
KIAA0971
Normalized Probe Intensity
Probe Number
−1
0
1
2
1
2
3
4
5
6
7
8
9
10
11
Meishan−A
Meishan−B
Meishan−C
European−A
European−B
European−C

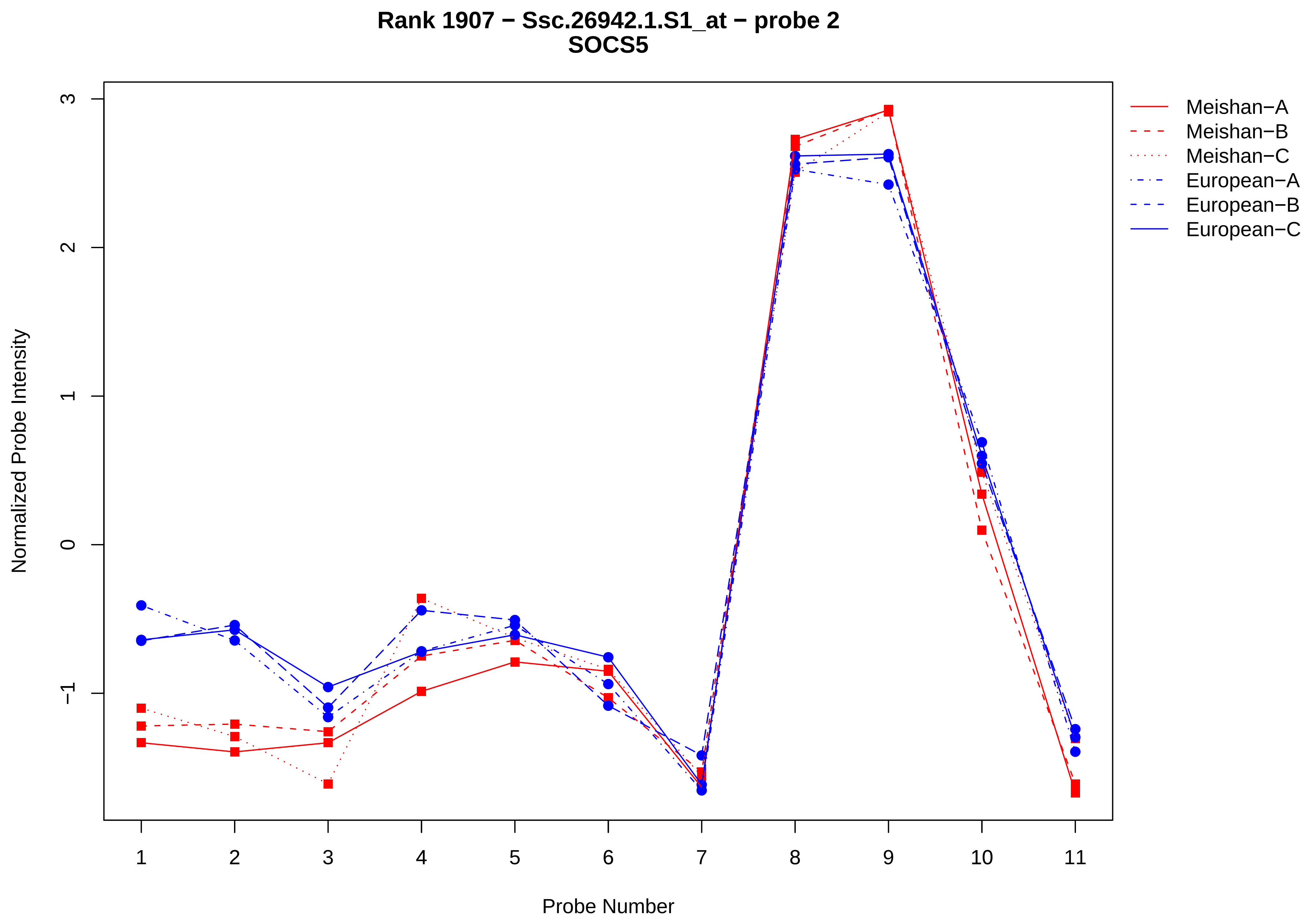

Rank 1907 − Ssc.26942.1.S1_at − probe 2
SOCS5
Normalized Probe Intensity
Probe Number
Meishan−A
Meishan−B
Meishan−C
European−A
European−B
European−C

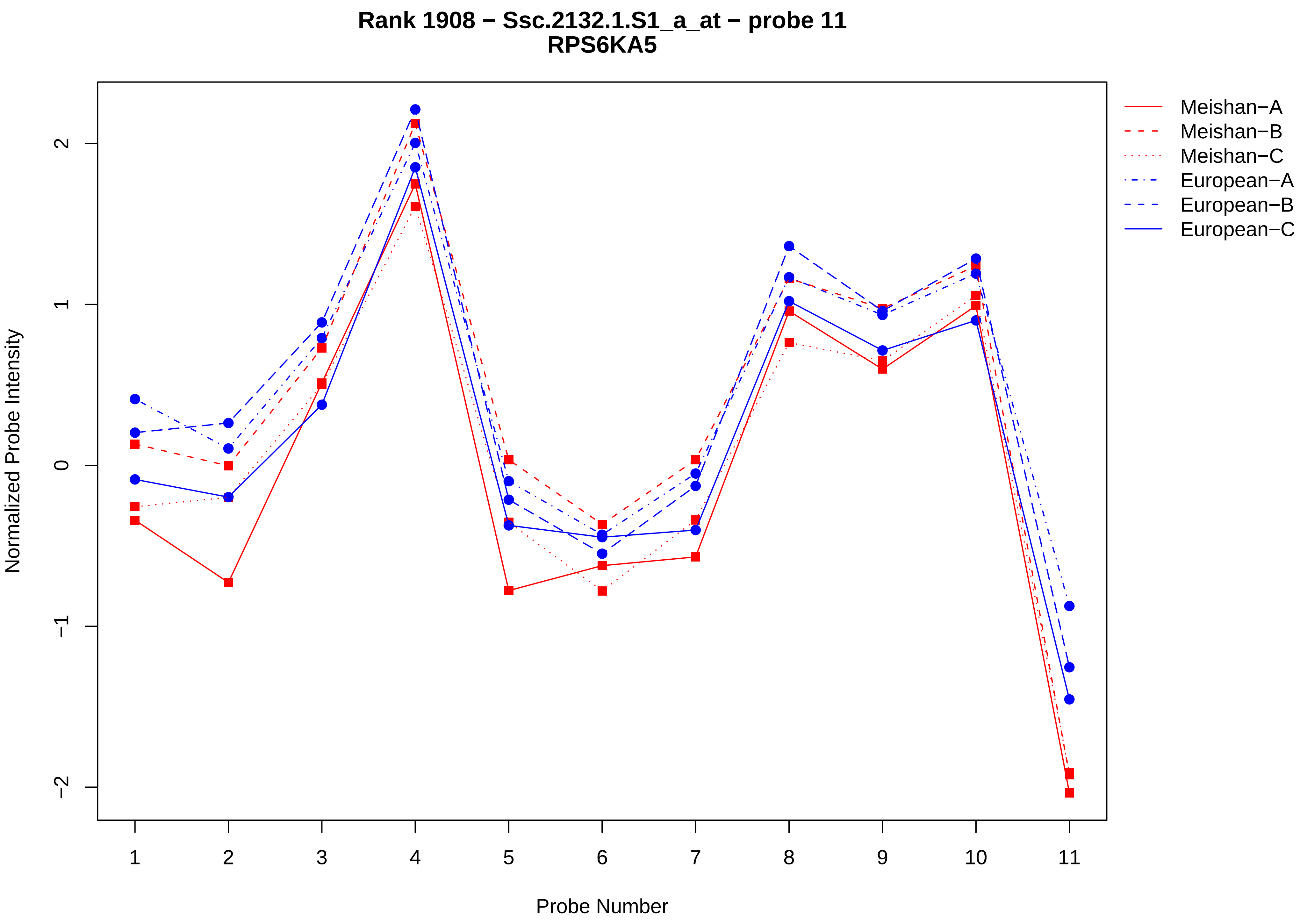

Rank 1908 − Ssc.2132.1.S1_a_at − probe 11
RPS6KA5
Normalized Probe Intensity
Probe Number
Meishan−A
Meishan−B
Meishan−C
European−A
European−B
European−C

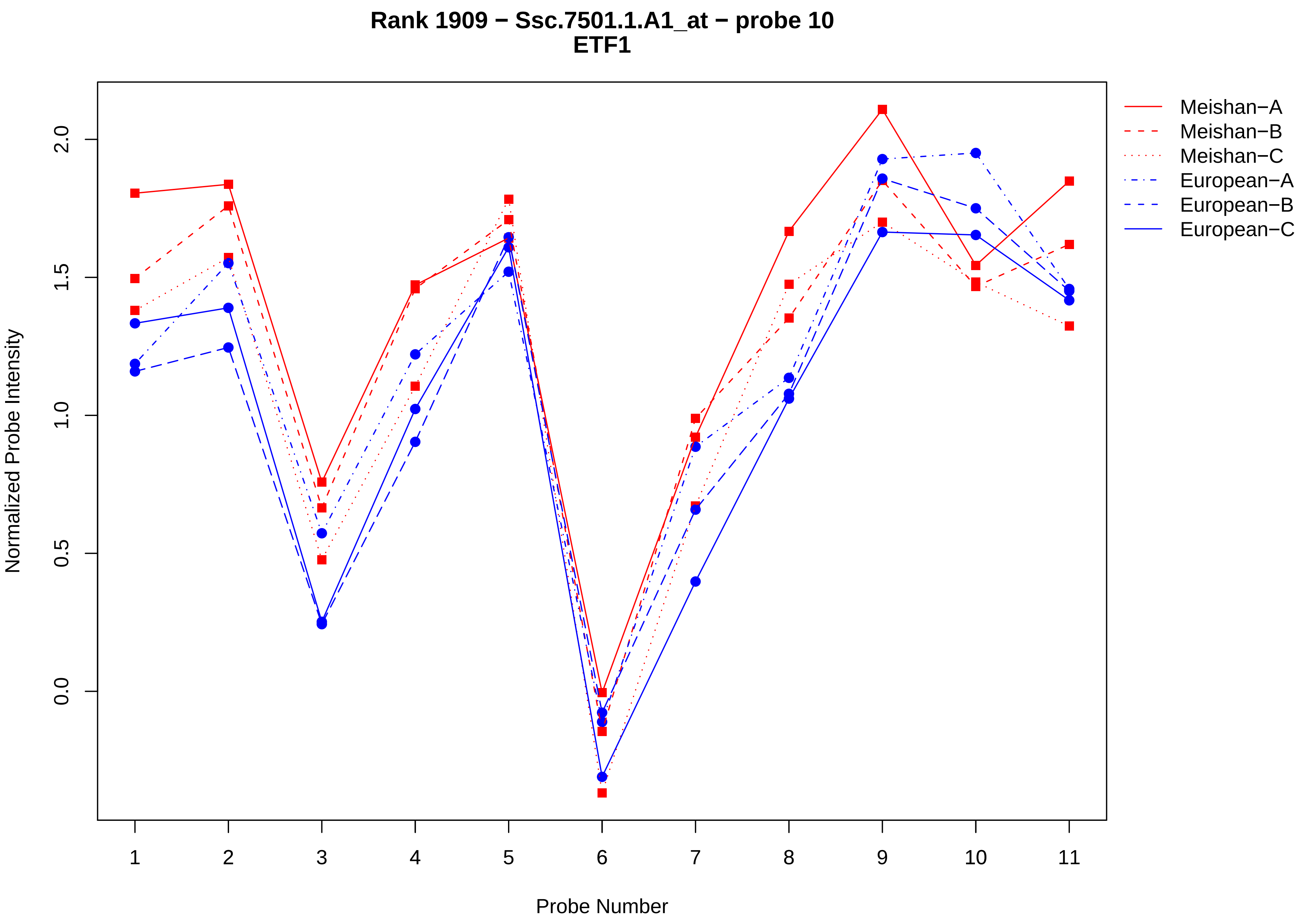

Rank 1909 − Ssc.7501.1.A1_at − probe 10
ETF1
Normalized Probe Intensity
Probe Number
0.0
0.5
1.0
1.5
2.0
1
2
3
4
5
6
7
8
9
10
11
Meishan−A
Meishan−B
Meishan−C
European−A
European−B
European−C

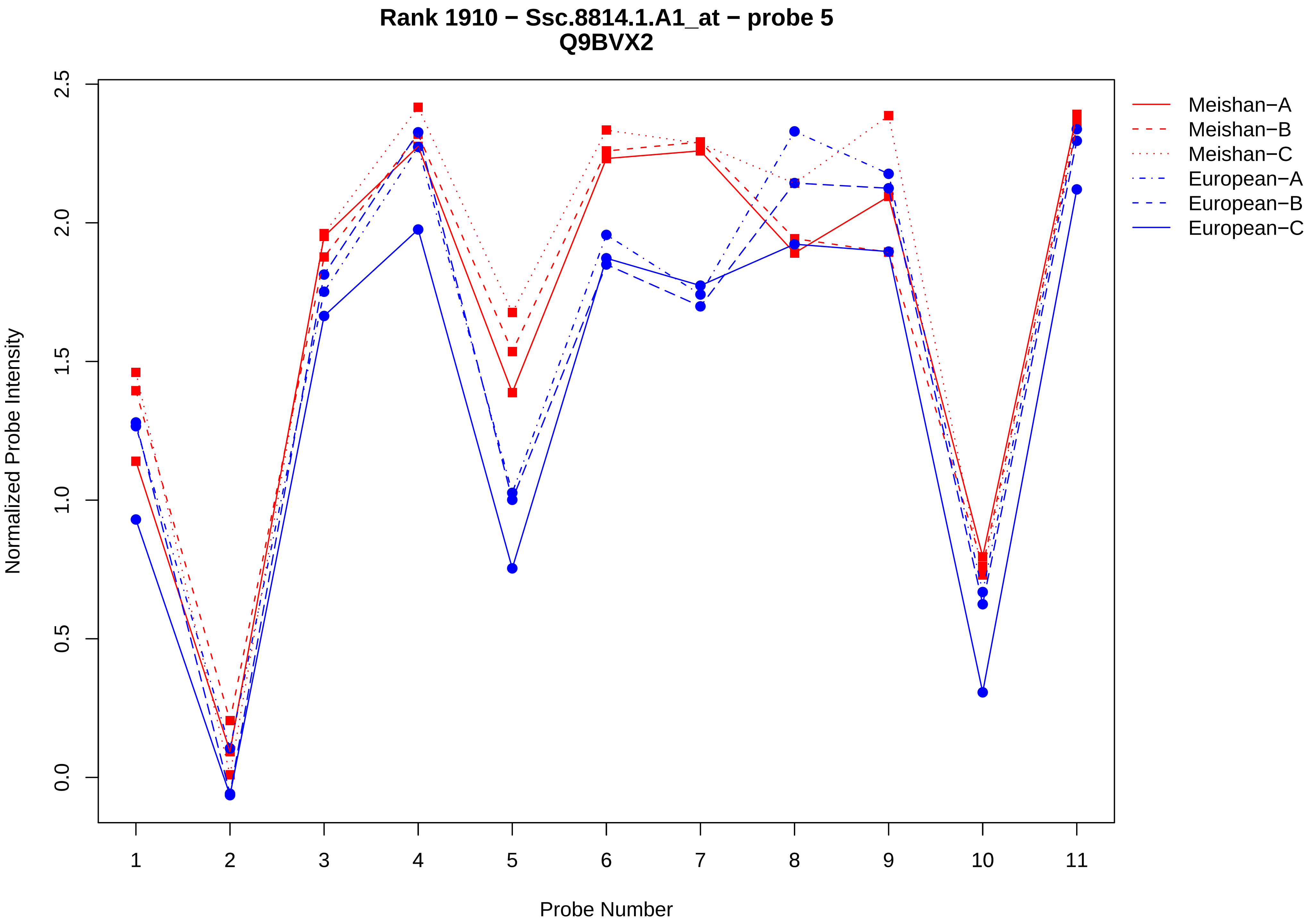

Rank 1910 − Ssc.8814.1.A1_at − probe 5
Q9BVX2
Normalized Probe Intensity
Probe Number
0.0
0.5
1.0
1.5
2.0
2.5
1
2
3
4
5
6
7
8
9
10
11
Meishan−A
Meishan−B
Meishan−C
European−A
European−B
European−C

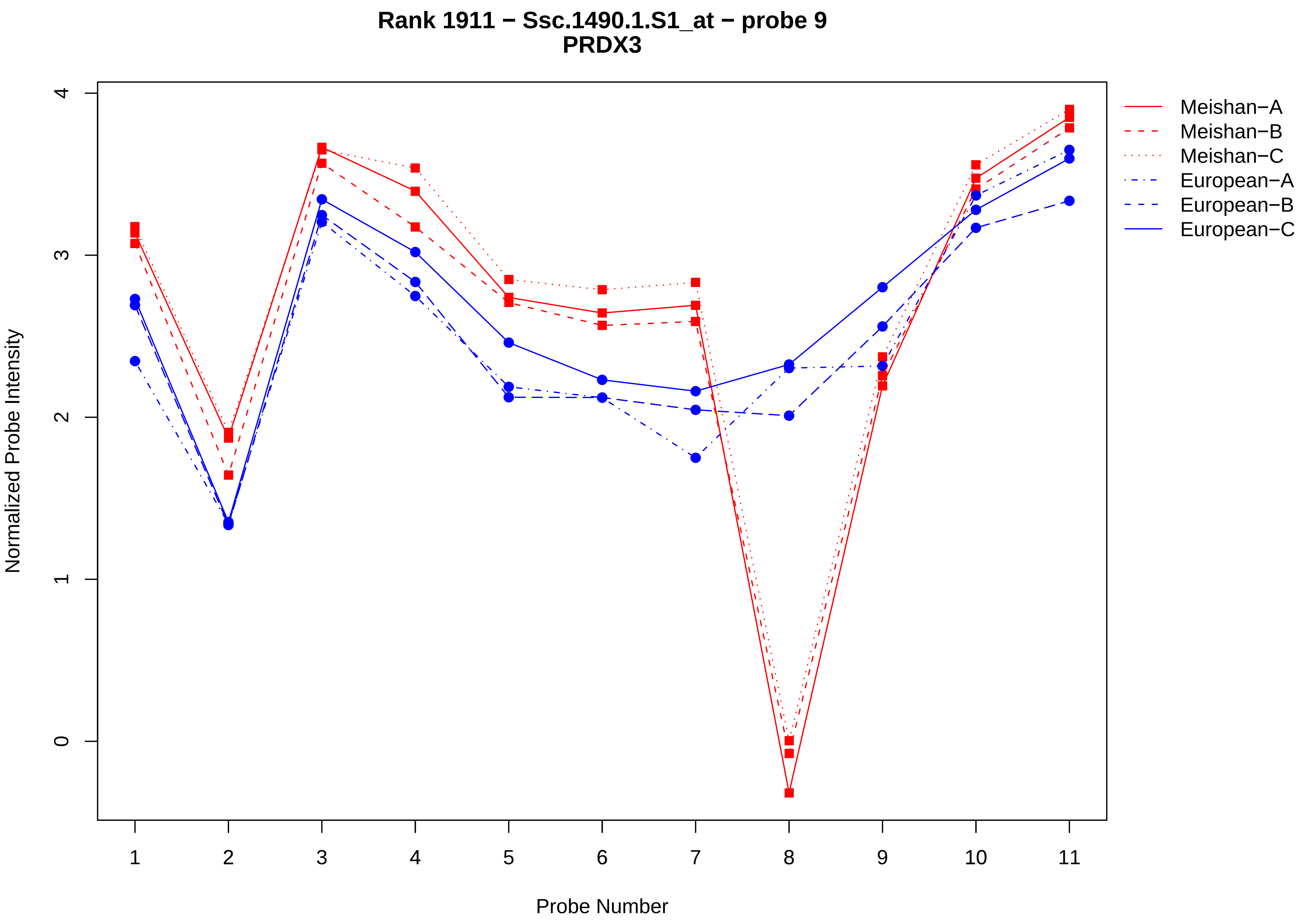
Rank 1911 − Ssc.1490.1.S1_at − probe 9
PRDX3
Normalized Probe Intensity
Probe Number
0
1
2
3
4
1
2
3
4
5
6
7
8
9
10
11
Meishan−A
Meishan−B
Meishan−C
European−A
European−B
European−C

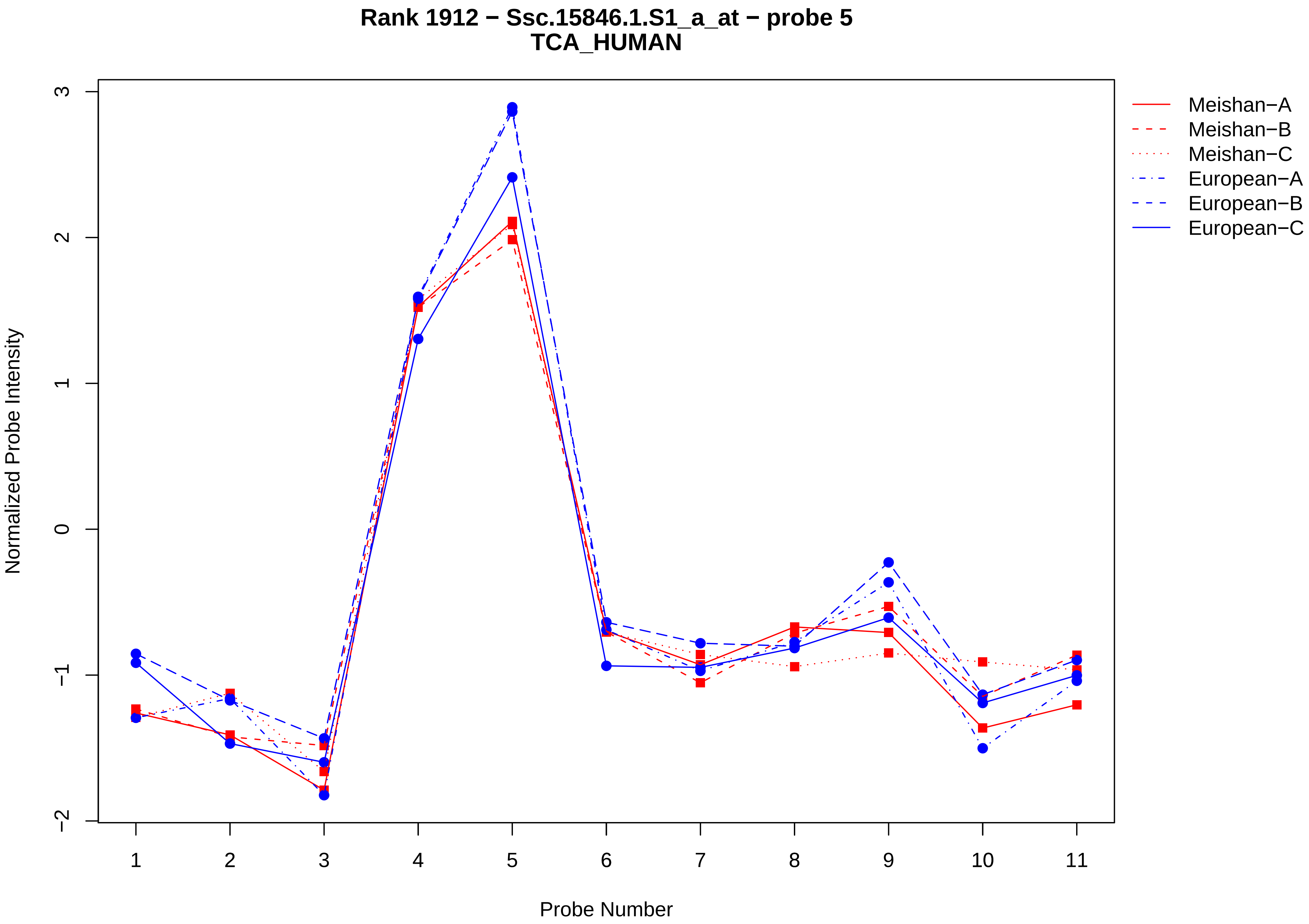

Rank 1912 − Ssc.15846.1.S1_a_at − probe 5
TCA_HUMAN
Normalized Probe Intensity
Probe Number
−2
−1
0
1
2
3
1
2
3
4
5
6
7
8
9
10
11
Meishan−A
Meishan−B
Meishan−C
European−A
European−B
European−C

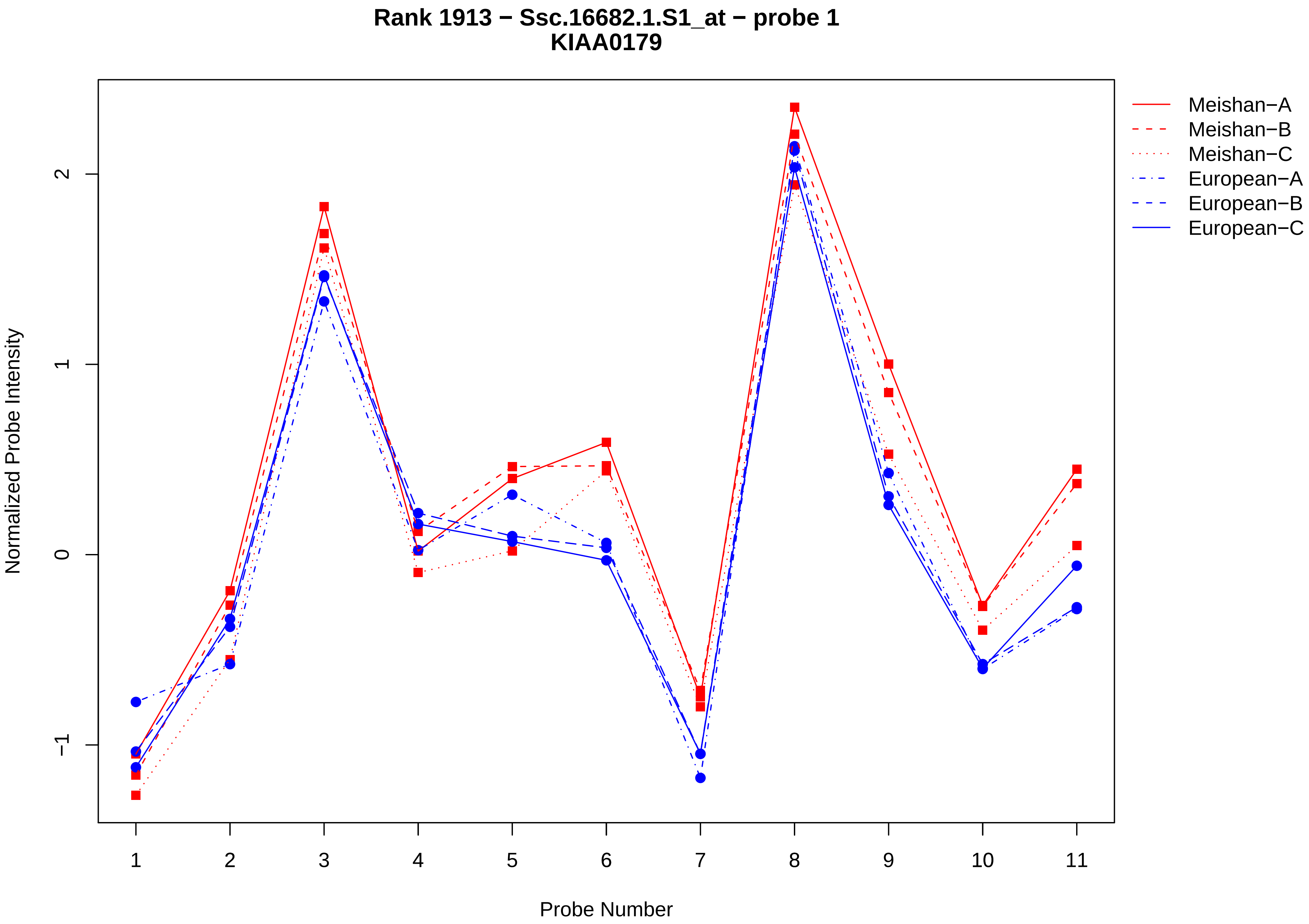
Rank 1913 − Ssc.16682.1.S1_at − probe 1
KIAA0179
Normalized Probe Intensity
Probe Number
−1
0
1
2
1
2
3
4
5
6
7
8
9
10
11
Meishan−A
Meishan−B
Meishan−C
European−A
European−B
European−C

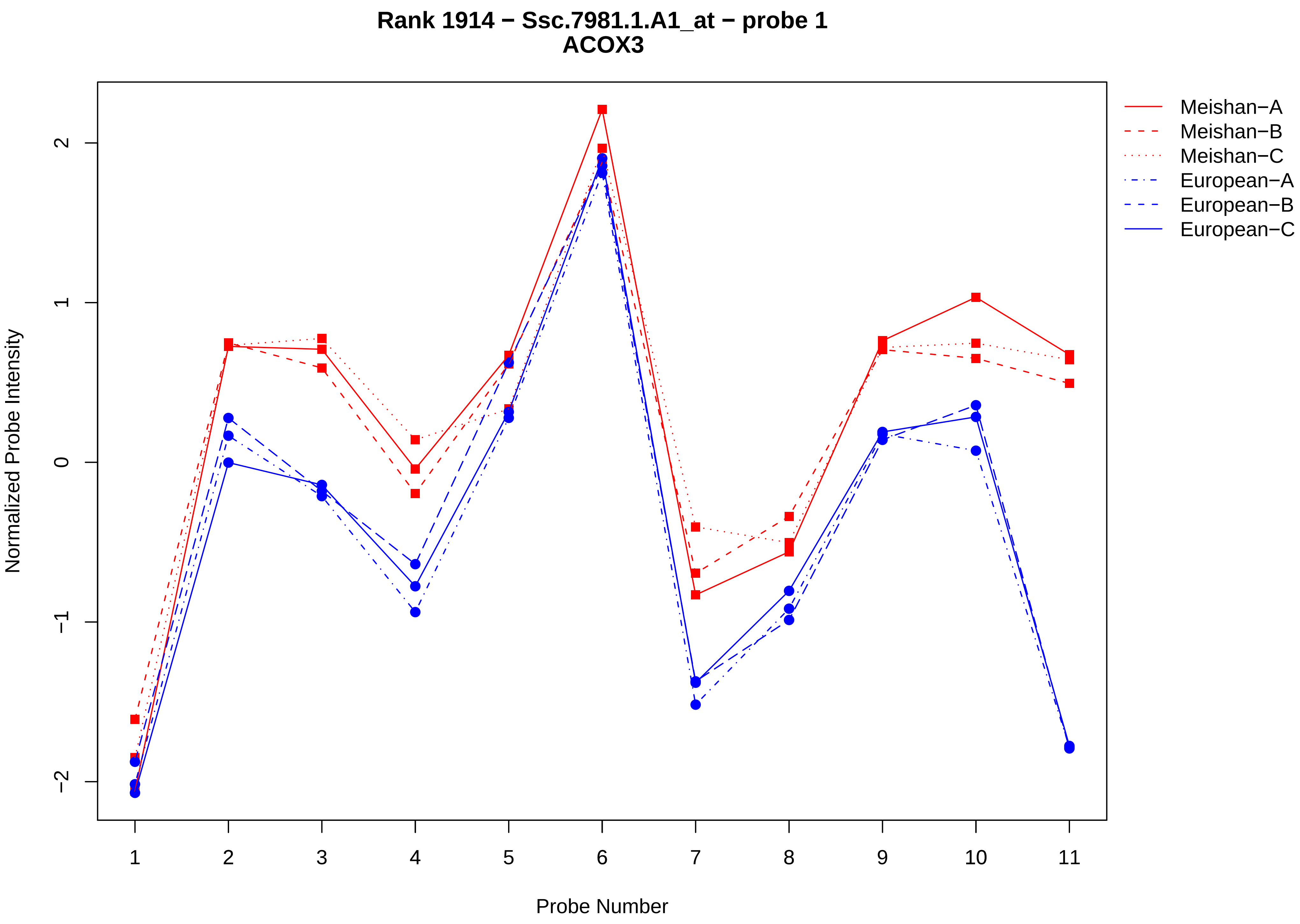

Rank 1914 − Ssc.7981.1.A1_at − probe 1
ACOX3
Normalized Probe Intensity
Probe Number
Meishan−A
Meishan−B
Meishan−C
European−A
European−B
European−C

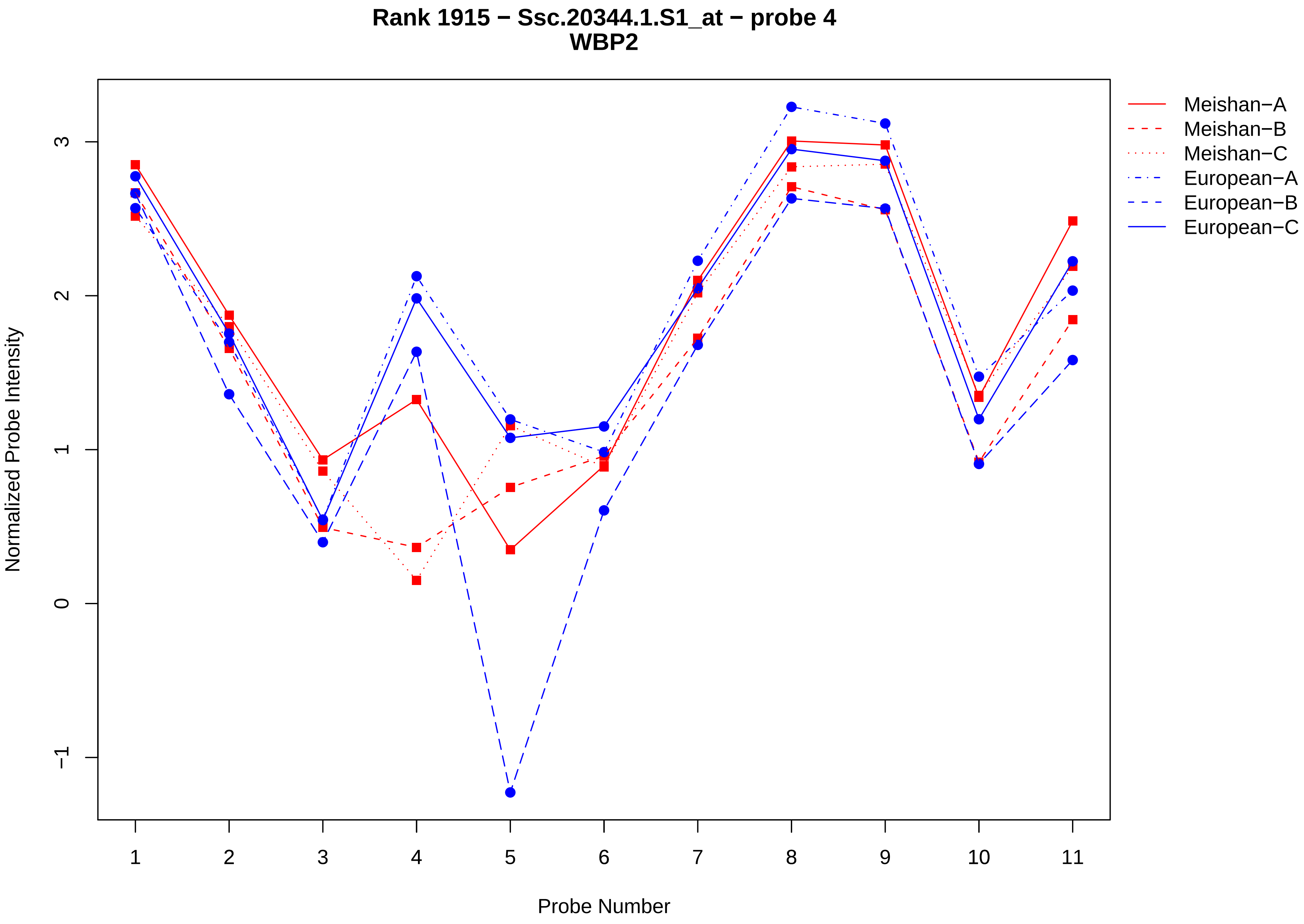

Rank 1915 − Ssc.20344.1.S1_at − probe 4
WBP2
Normalized Probe Intensity
Probe Number
−1
0
1
2
3
1
2
3
4
5
6
7
8
9
10
11
Meishan−A
Meishan−B
Meishan−C
European−A
European−B
European−C

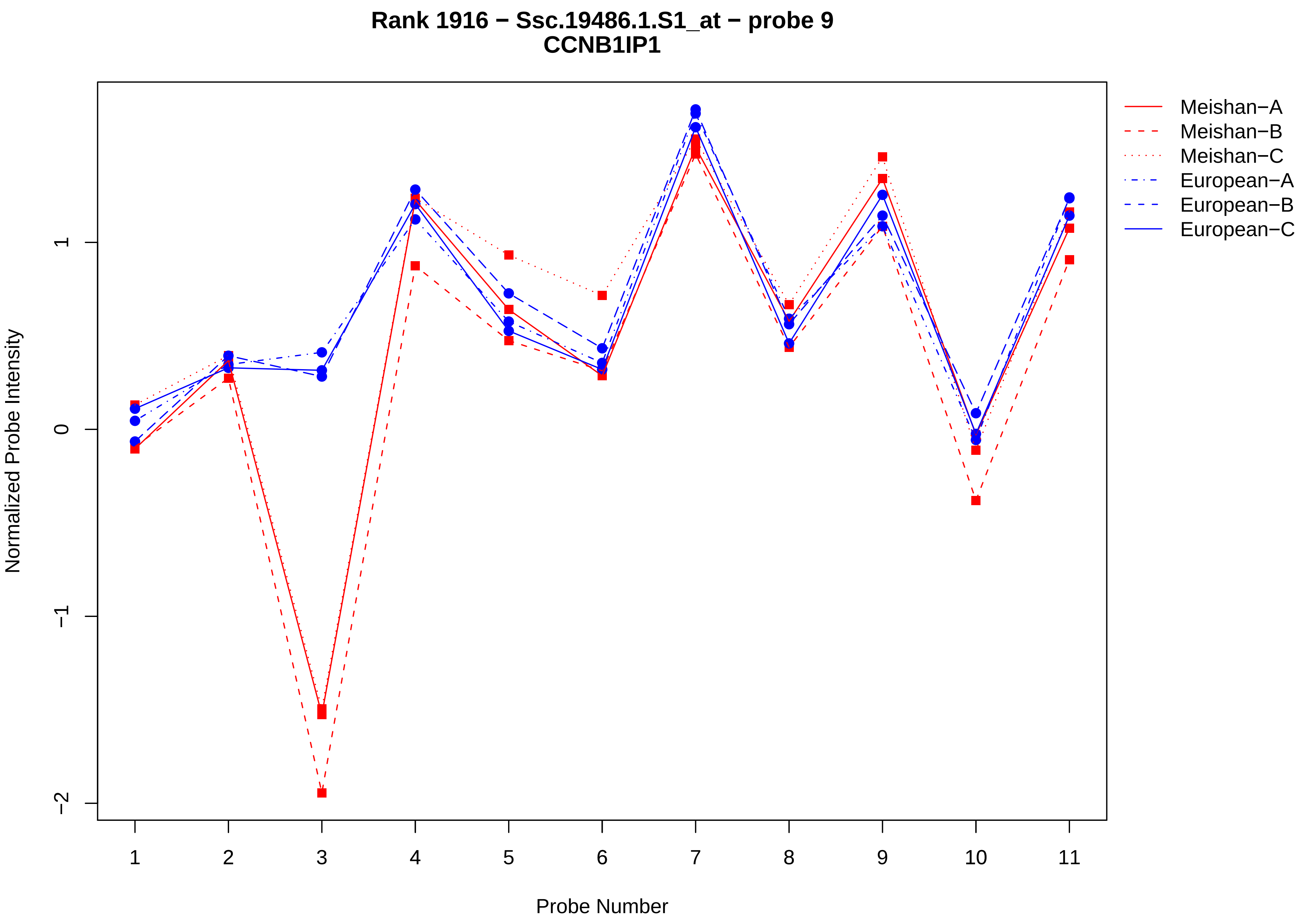

Rank 1916 − Ssc.19486.1.S1_at − probe 9
CCNB1IP1
Normalized Probe Intensity
Probe Number
−2
−1
0
1
1
2
3
4
5
6
7
8
9
10
11
Meishan−A
Meishan−B
Meishan−C
European−A
European−B
European−C

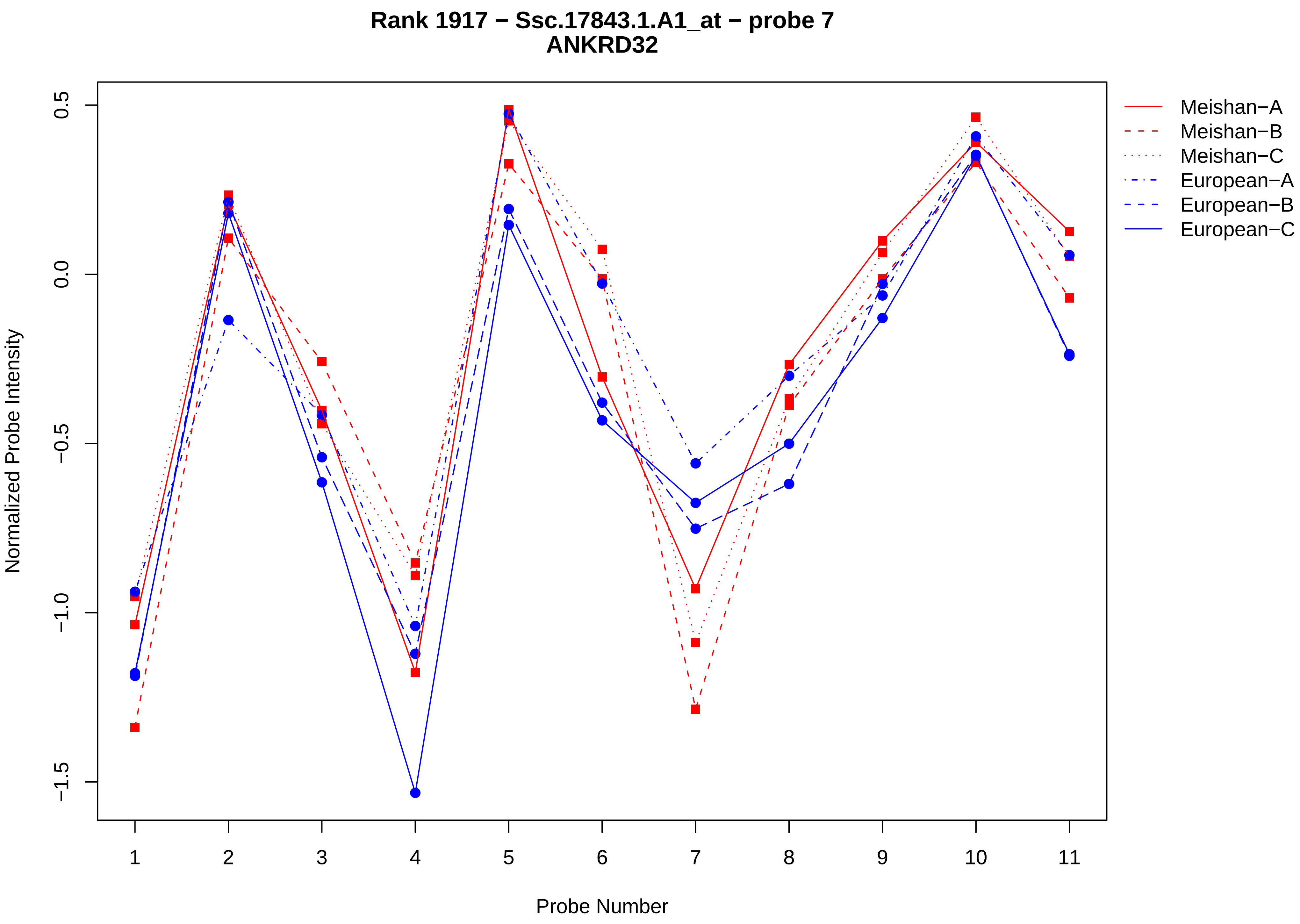

Rank 1917 − Ssc.17843.1.A1_at − probe 7
ANKRD32
Normalized Probe Intensity
Probe Number
0.5
0.0
−0.5
−1.0
−1.5
1
2
3
4
5
6
7
8
9
10
11
Meishan−A
Meishan−B
Meishan−C
European−A
European−B
European−C

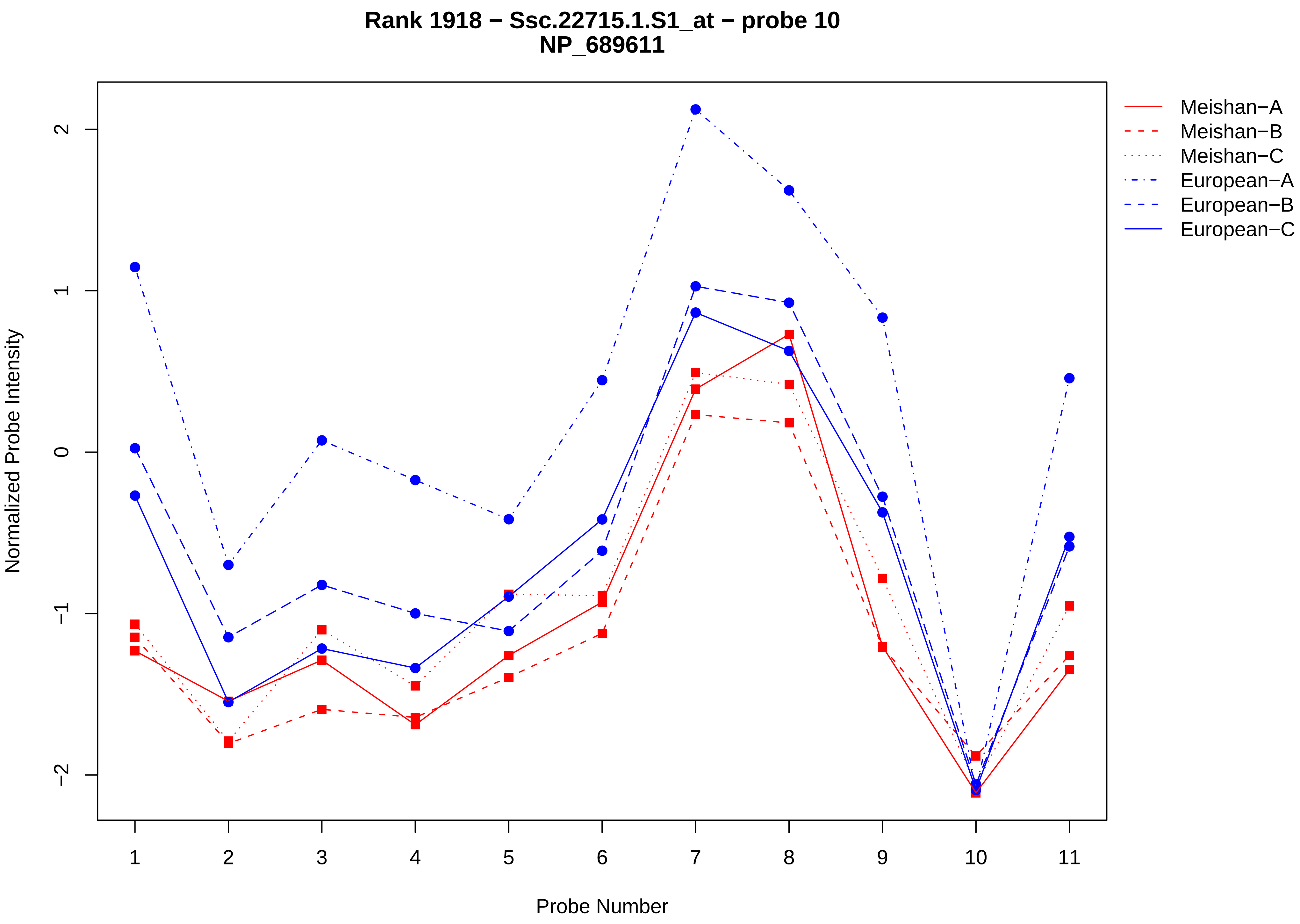

Rank 1918 − Ssc.22715.1.S1_at − probe 10
NP_689611
Normalized Probe Intensity
Probe Number
Meishan−A
Meishan−B
Meishan−C
European−A
European−B
European−C

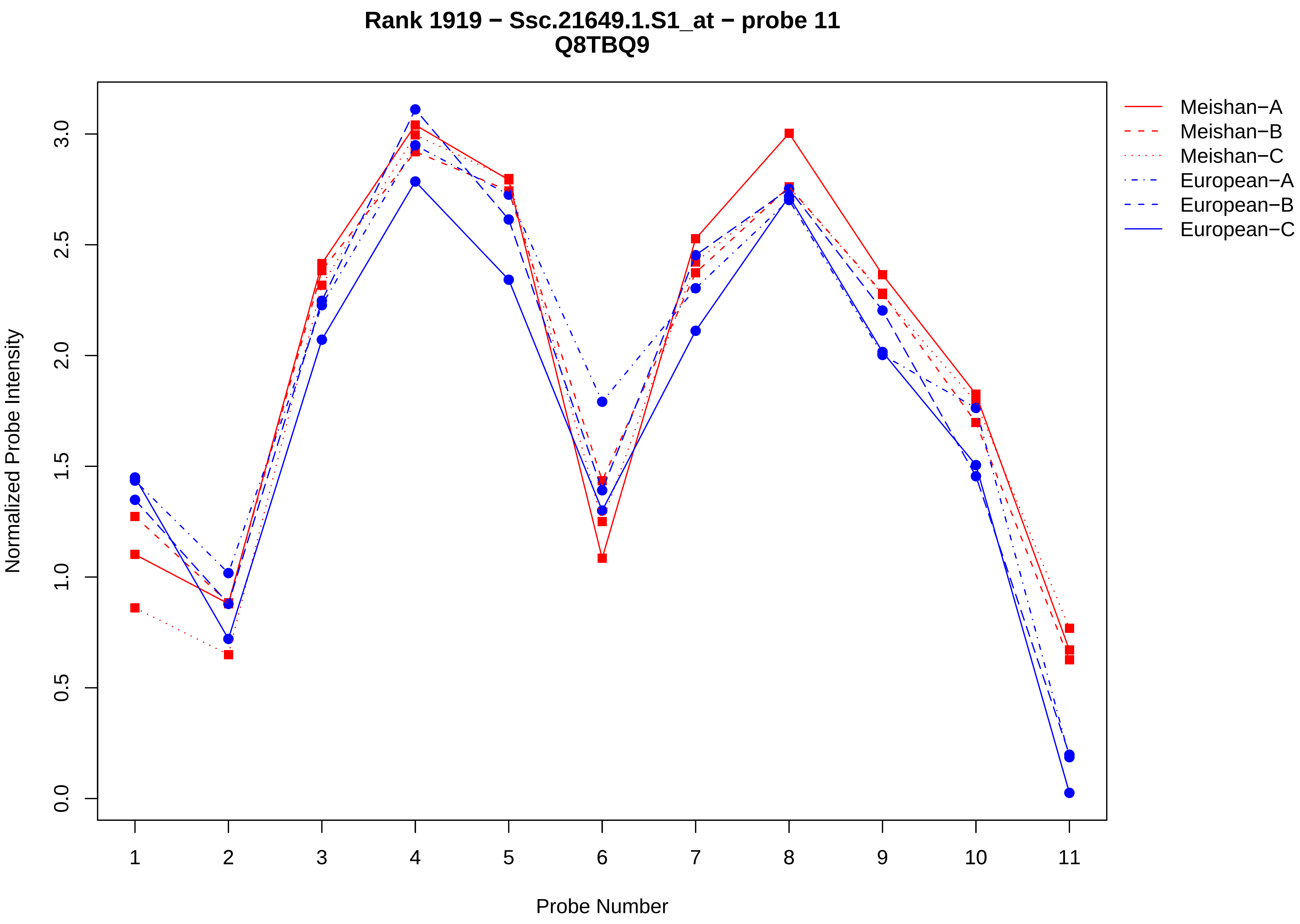

Rank 1919 − Ssc.21649.1.S1_at − probe 11
Q8TBQ9
Normalized Probe Intensity
Probe Number
0.0
0.5
1.0
1.5
2.0
2.5
3.0
1
2
3
4
5
6
7
8
9
10
11
Meishan−A
Meishan−B
Meishan−C
European−A
European−B
European−C

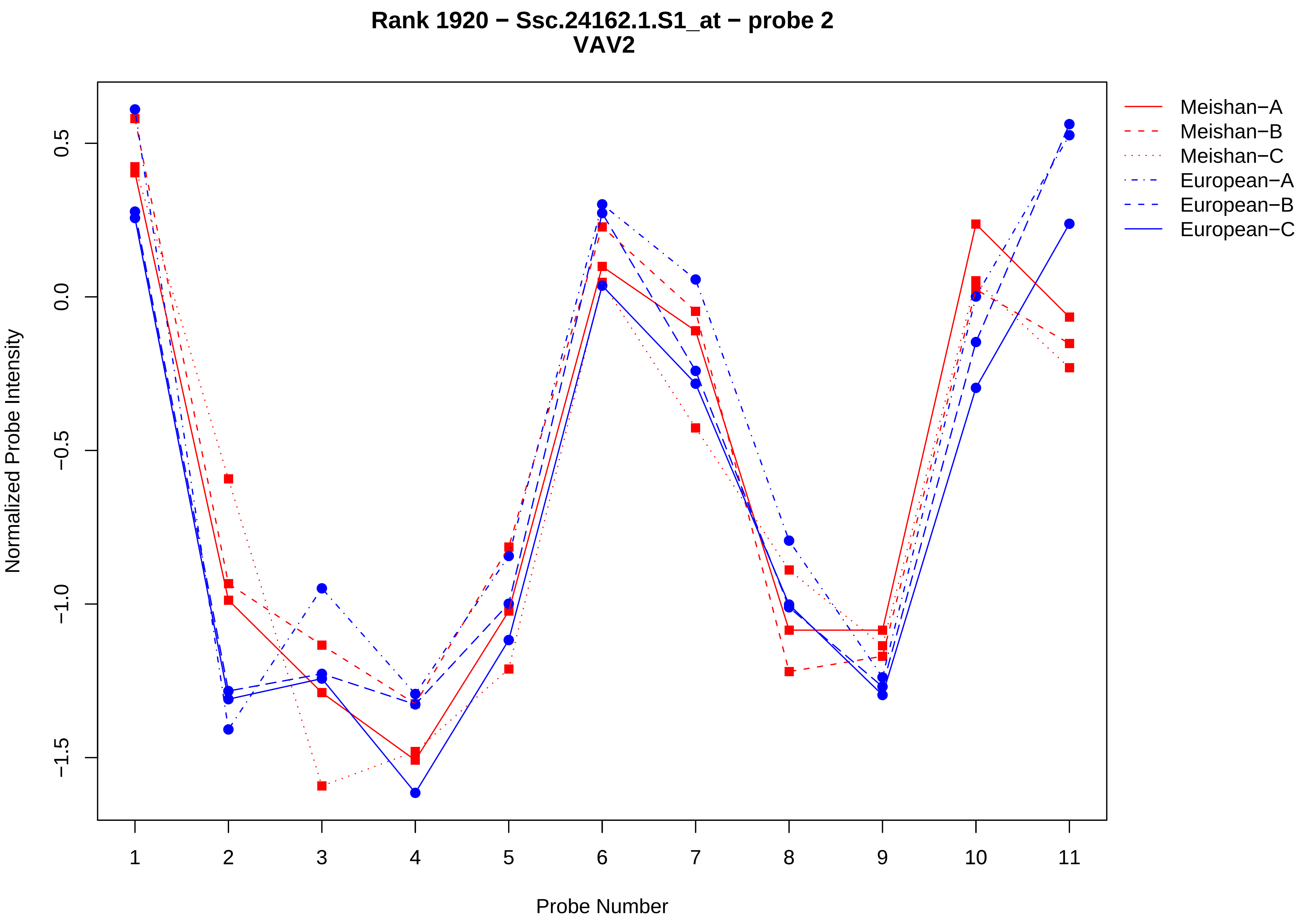

Rank 1920 − Ssc.24162.1.S1_at − probe 2
VAV2
Normalized Probe Intensity
Probe Number
0.5
0.0
−0.5
−1.0
−1.5
1
2
3
4
5
6
7
8
9
10
11
Meishan−A
Meishan−B
Meishan−C
European−A
European−B
European−C

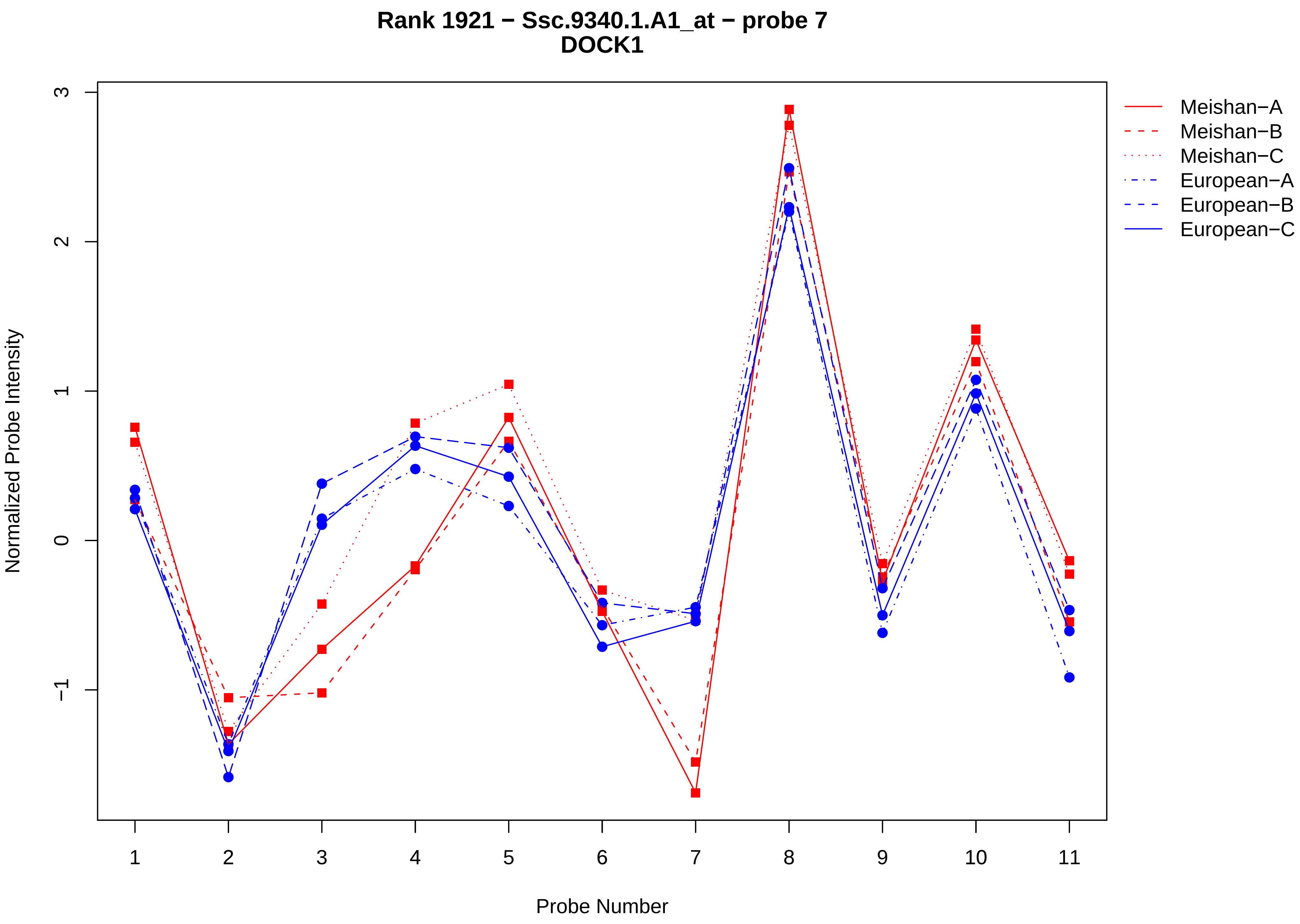

Rank 1921 − Ssc.9340.1.A1_at − probe 7
DOCK1
Normalized Probe Intensity
Probe Number
Meishan−A
Meishan−B
Meishan−C
European−A
European−B
European−C

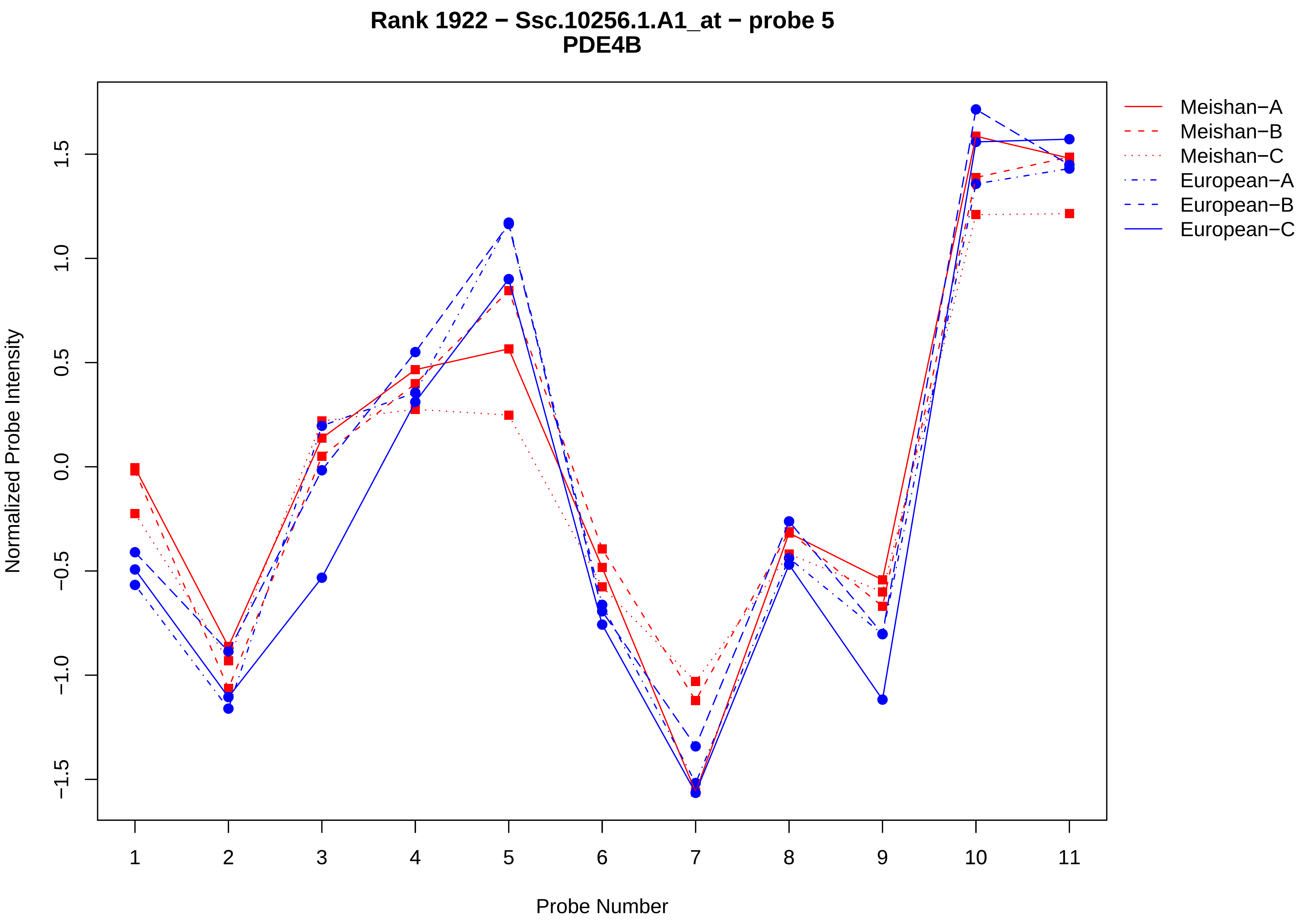

Rank 1922 − Ssc.10256.1.A1_at − probe 5
PDE4B
Normalized Probe Intensity
Probe Number
1.5
1.0
0.5
0.0
−0.5
−1.0
−1.5
1
2
3
4
5
6
7
8
9
10
11
Meishan−A
Meishan−B
Meishan−C
European−A
European−B
European−C

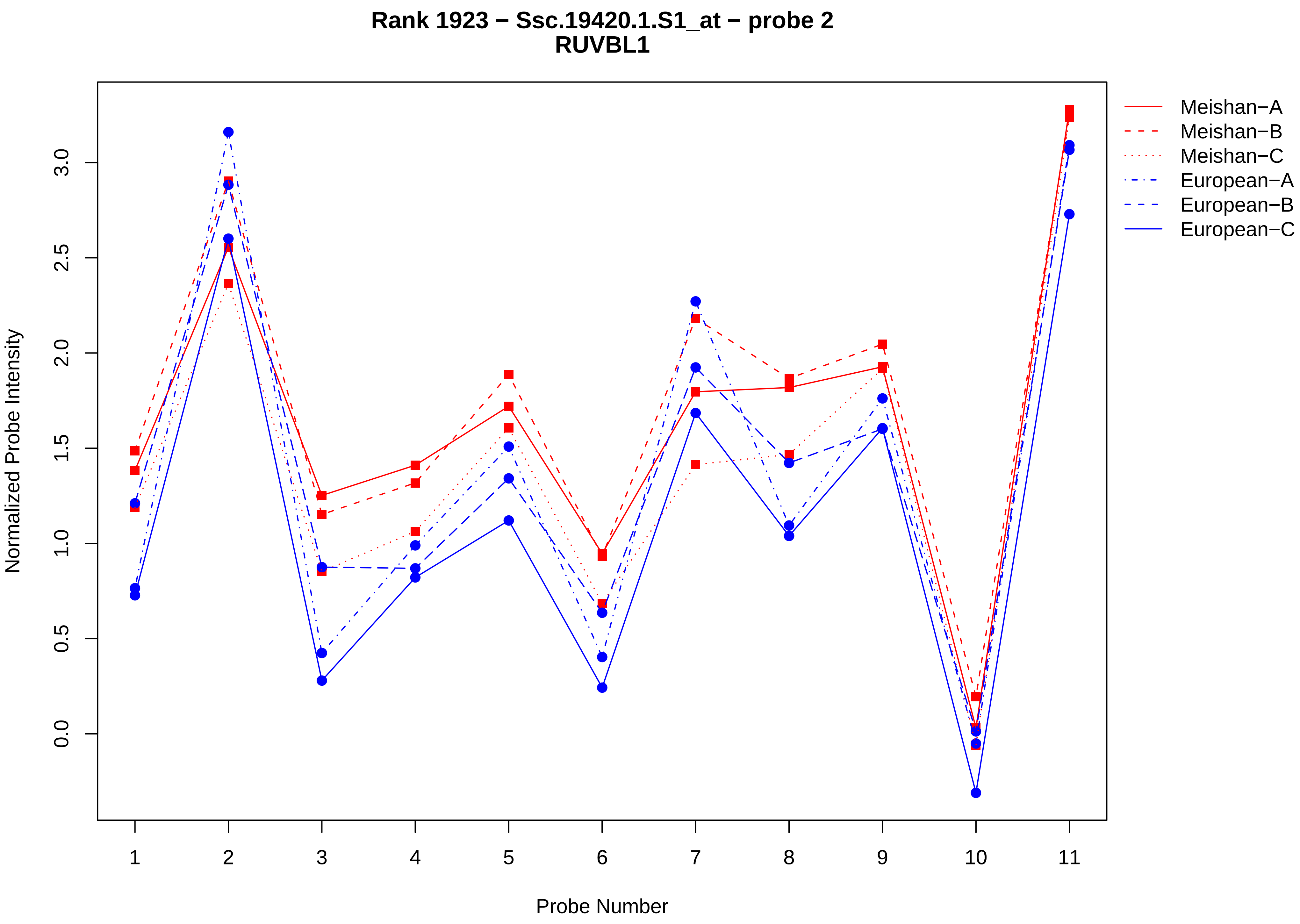

Rank 1923 − Ssc.19420.1.S1_at − probe 2
RUVBL1
Normalized Probe Intensity
Probe Number
0.0
0.5
1.0
1.5
2.0
2.5
3.0
1
2
3
4
5
6
7
8
9
10
11
Meishan−A
Meishan−B
Meishan−C
European−A
European−B
European−C

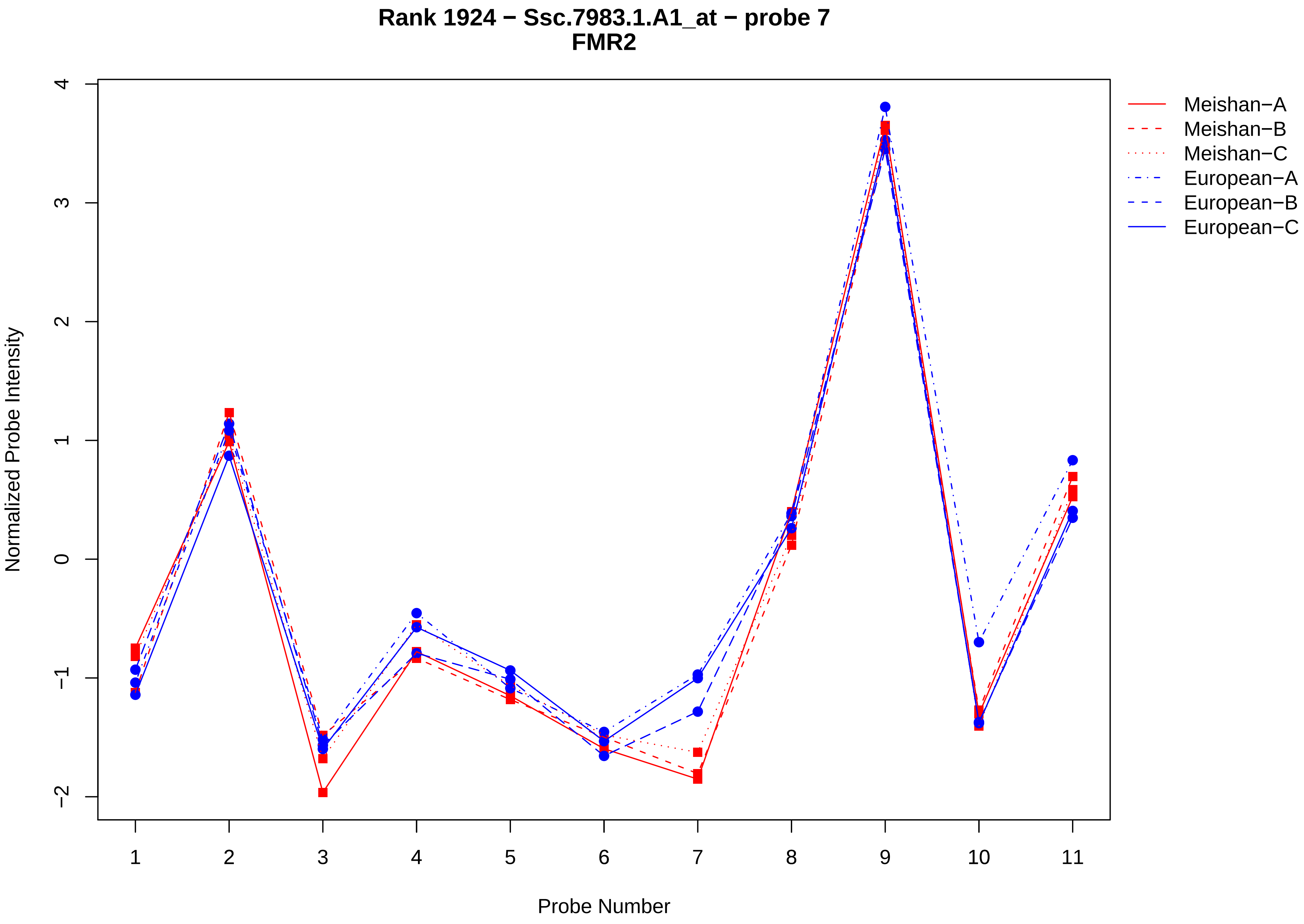

Rank 1924 − Ssc.7983.1.A1_at − probe 7
FMR2
Normalized Probe Intensity
Probe Number
−2
−1
0
1
2
3
4
1
2
3
4
5
6
7
8
9
10
11
Meishan−A
Meishan−B
Meishan−C
European−A
European−B
European−C

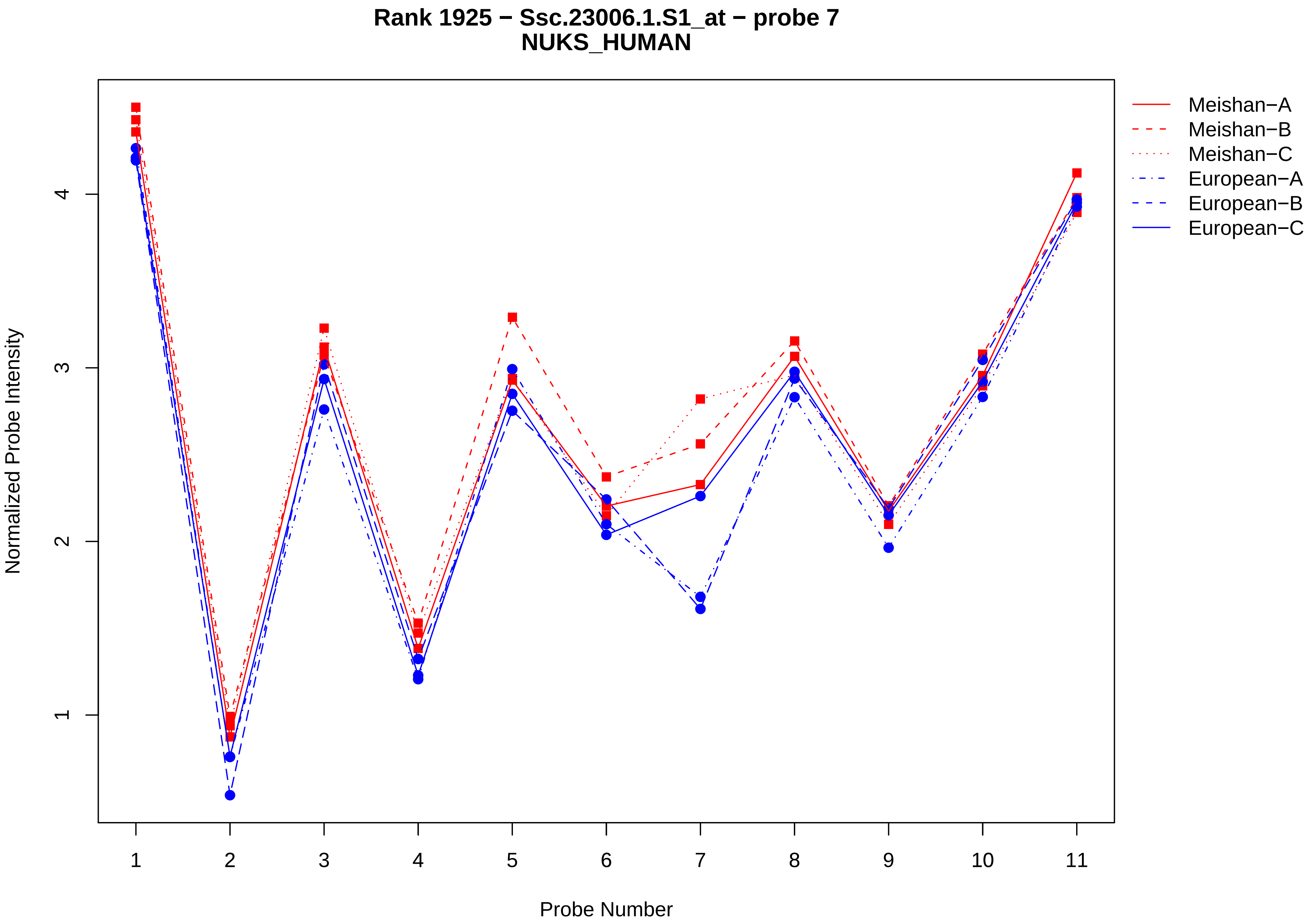

Rank 1925 − Ssc.23006.1.S1_at − probe 7
NUKS_HUMAN
Normalized Probe Intensity
Probe Number
1
2
3
4
1
2
3
4
5
6
7
8
9
10
11
Meishan−A
Meishan−B
Meishan−C
European−A
European−B
European−C

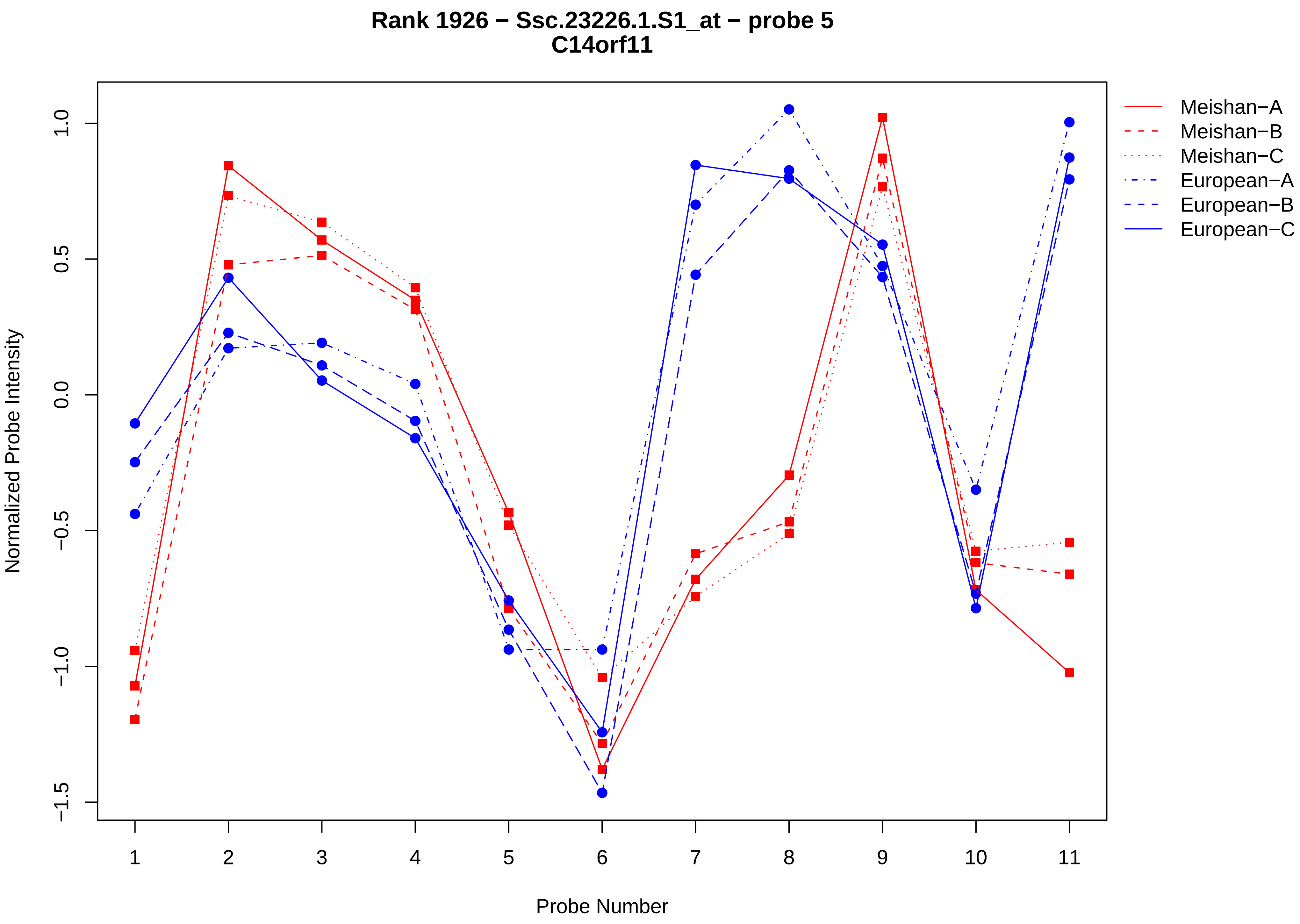
Rank 1926 − Ssc.23226.1.S1_at − probe 5
C14orf11
Normalized Probe Intensity
Probe Number
1.0
0.5
0.0
−0.5
−1.0
−1.5
1
2
3
4
5
6
7
8
9
10
11
Meishan−A
Meishan−B
Meishan−C
European−A
European−B
European−C

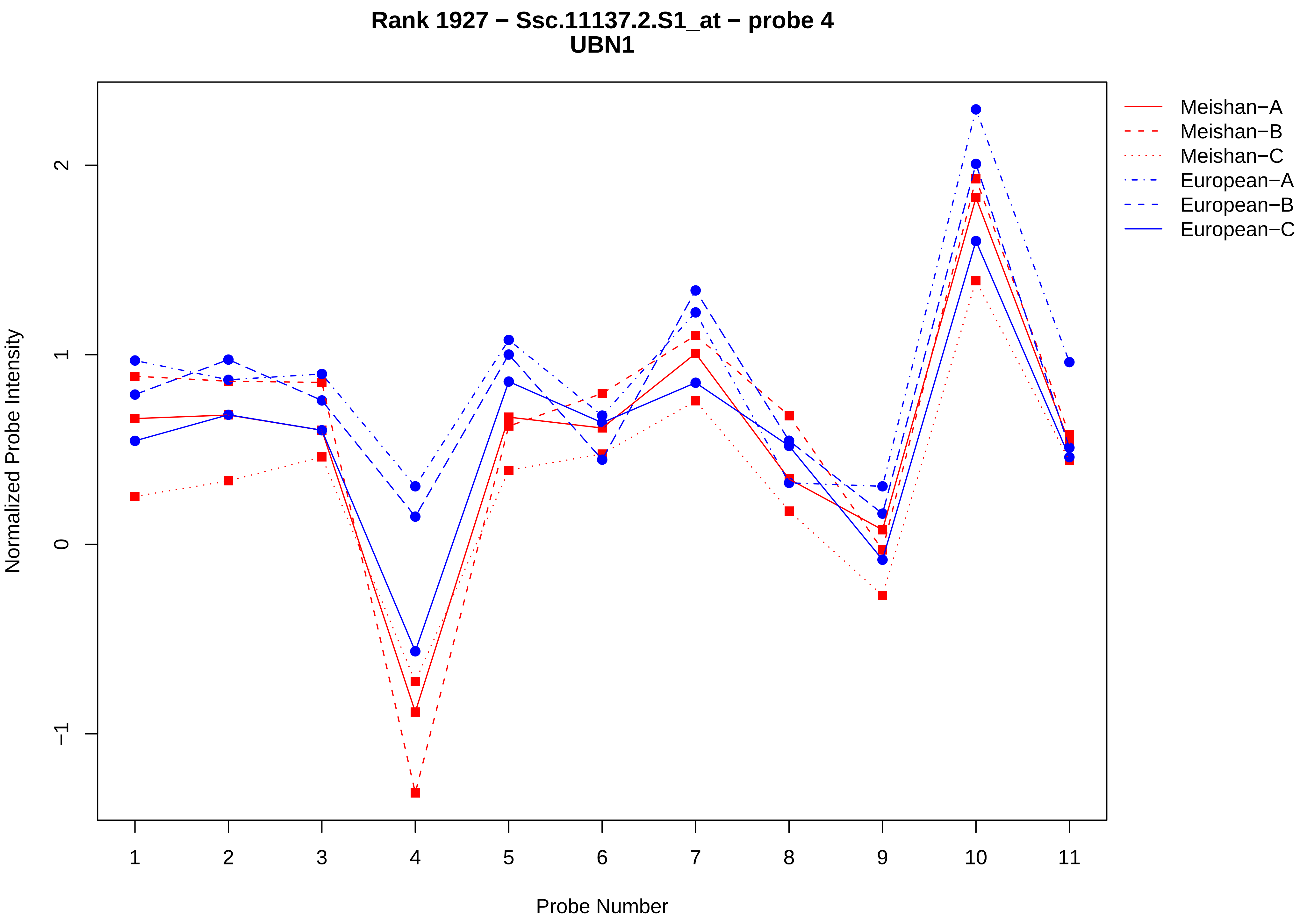
Rank 1927 − Ssc.11137.2.S1_at − probe 4
UBN1
Normalized Probe Intensity
Probe Number
−1
0
1
2
1
2
3
4
5
6
7
8
9
10
11
Meishan−A
Meishan−B
Meishan−C
European−A
European−B
European−C

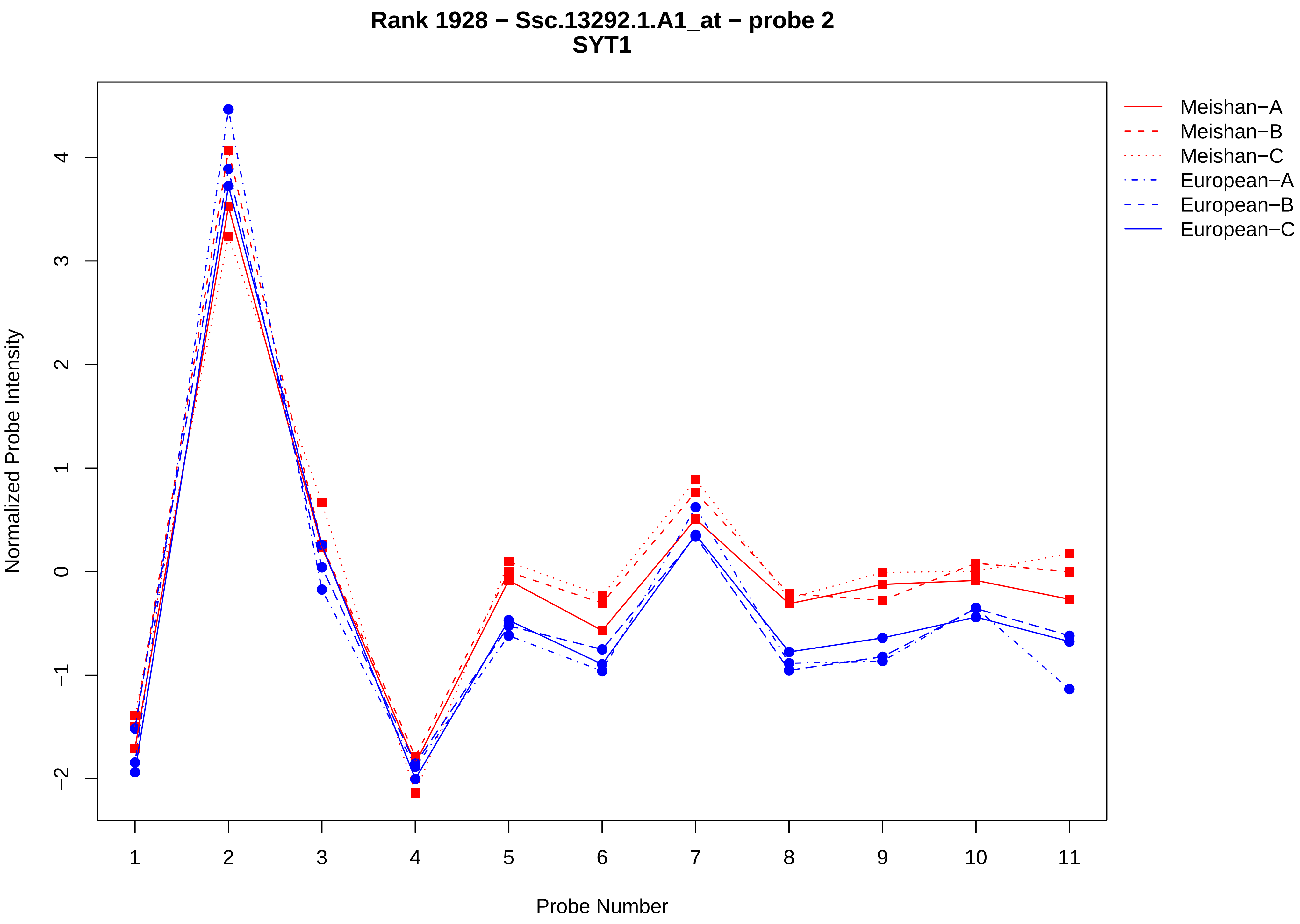

Rank 1928 − Ssc.13292.1.A1_at − probe 2
SYT1
Normalized Probe Intensity
Probe Number
Meishan−A
Meishan−B
Meishan−C
European−A
European−B
European−C

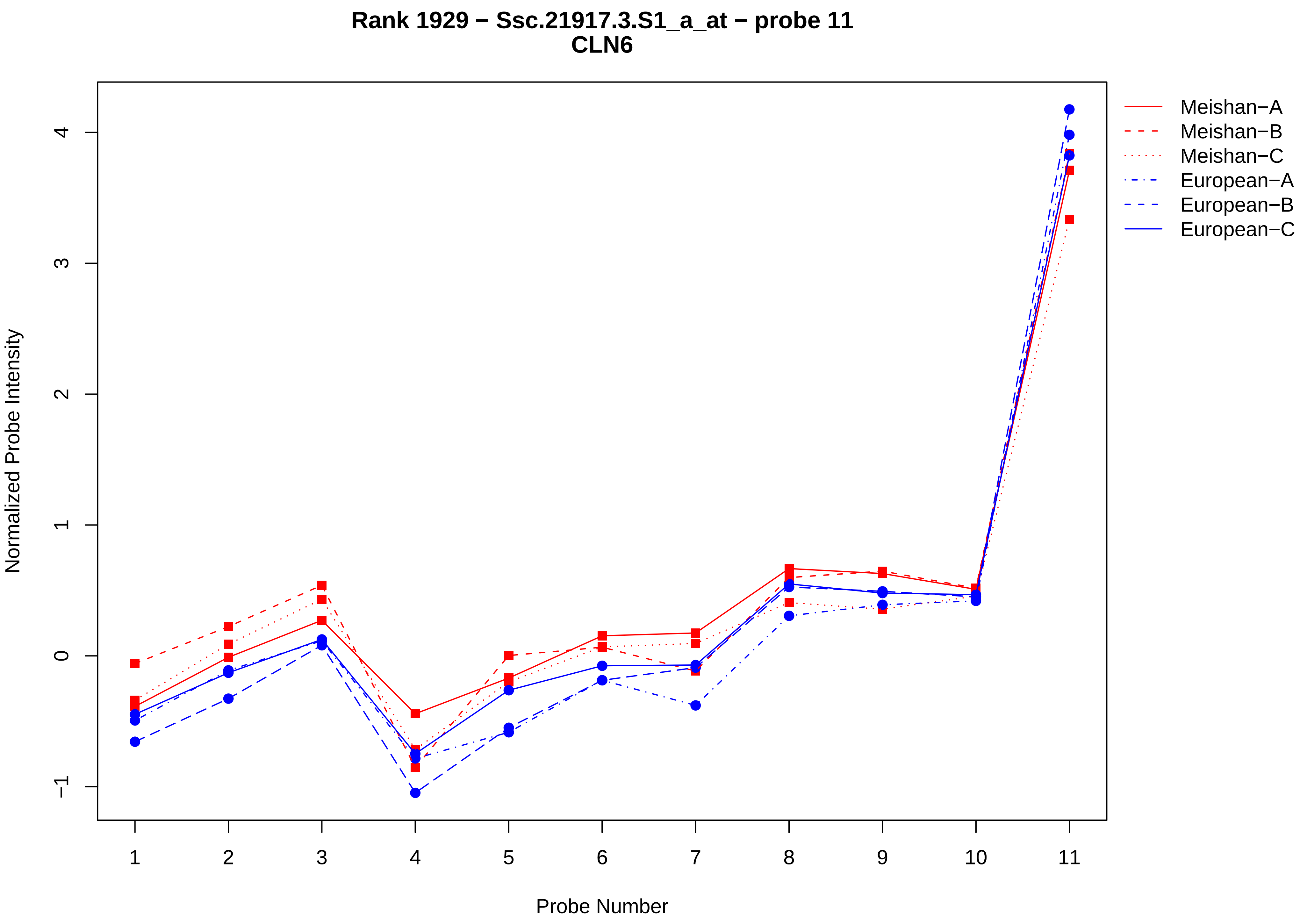

Rank 1929 − Ssc.21917.3.S1_a_at − probe 11
CLN6
Normalized Probe Intensity
Probe Number
−1
0
1
2
3
4
1
2
3
4
5
6
7
8
9
10
11
Meishan−A
Meishan−B
Meishan−C
European−A
European−B
European−C

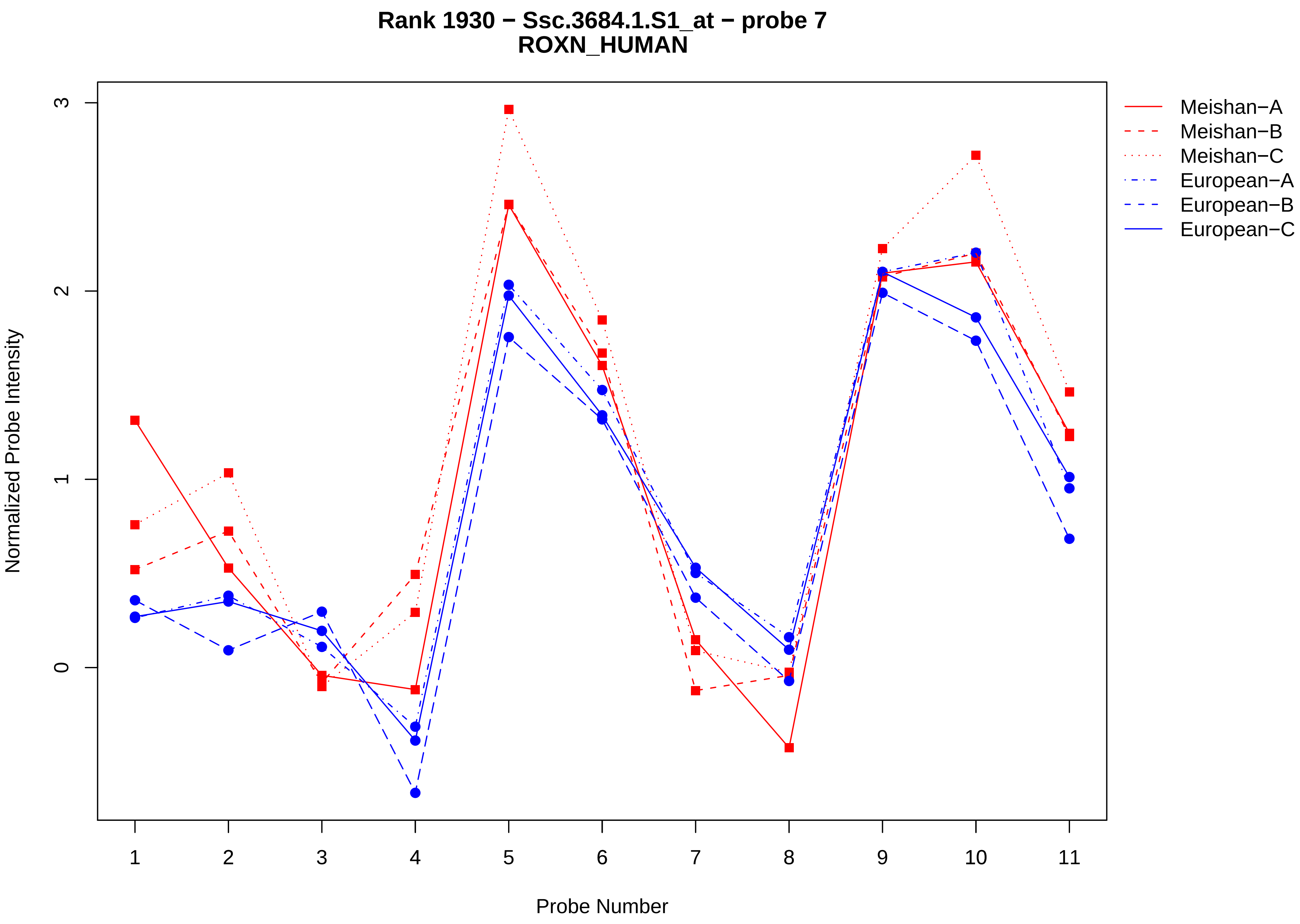

Rank 1930 − Ssc.3684.1.S1_at − probe 7
ROXN_HUMAN
Normalized Probe Intensity
Probe Number
0
1
2
3
1
2
3
4
5
6
7
8
9
10
11
Meishan−A
Meishan−B
Meishan−C
European−A
European−B
European−C

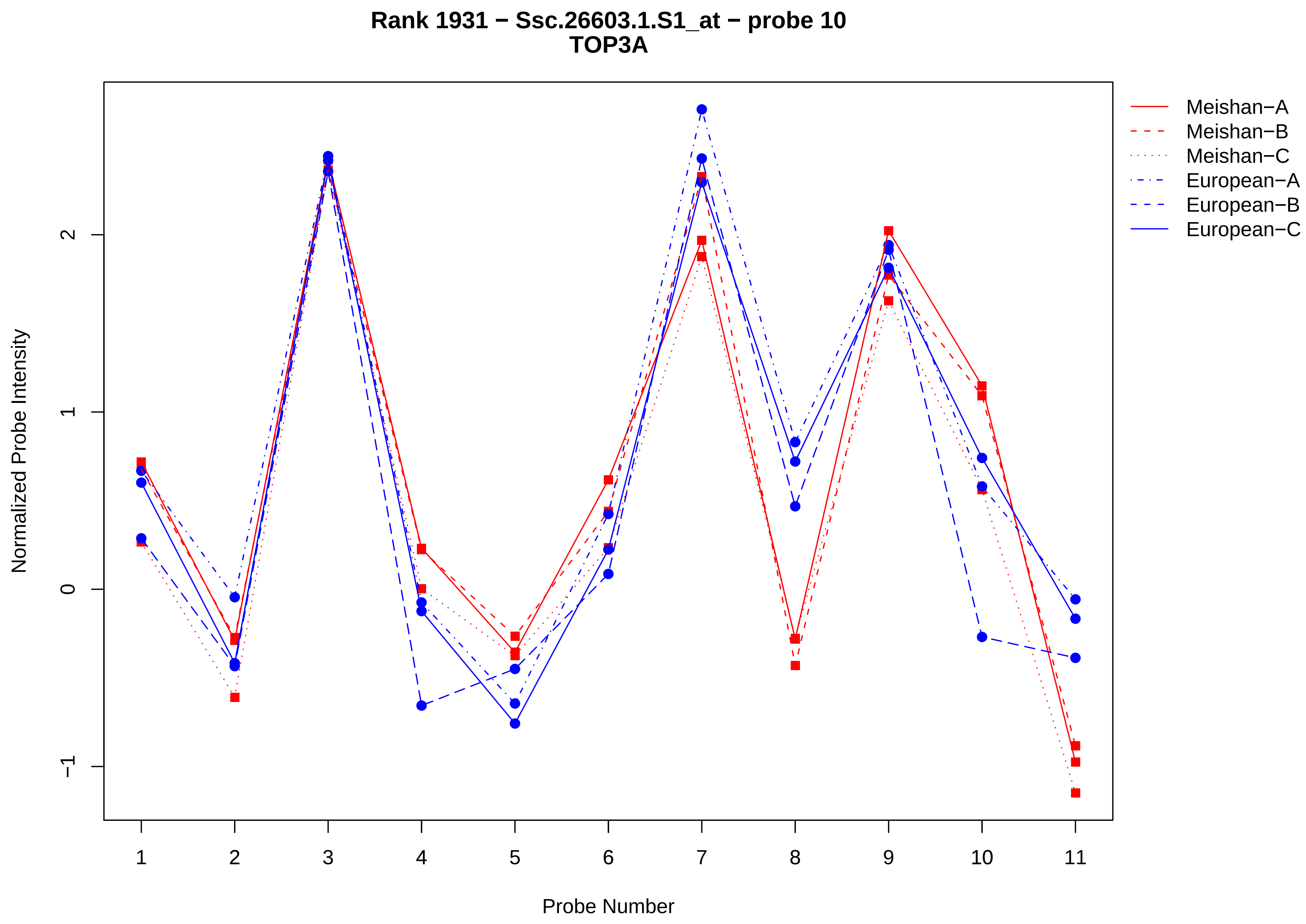

Rank 1931 − Ssc.26603.1.S1_at − probe 10
TOP3A
Normalized Probe Intensity
Probe Number
−1
0
1
2
1
2
3
4
5
6
7
8
9
10
11
Meishan−A
Meishan−B
Meishan−C
European−A
European−B
European−C

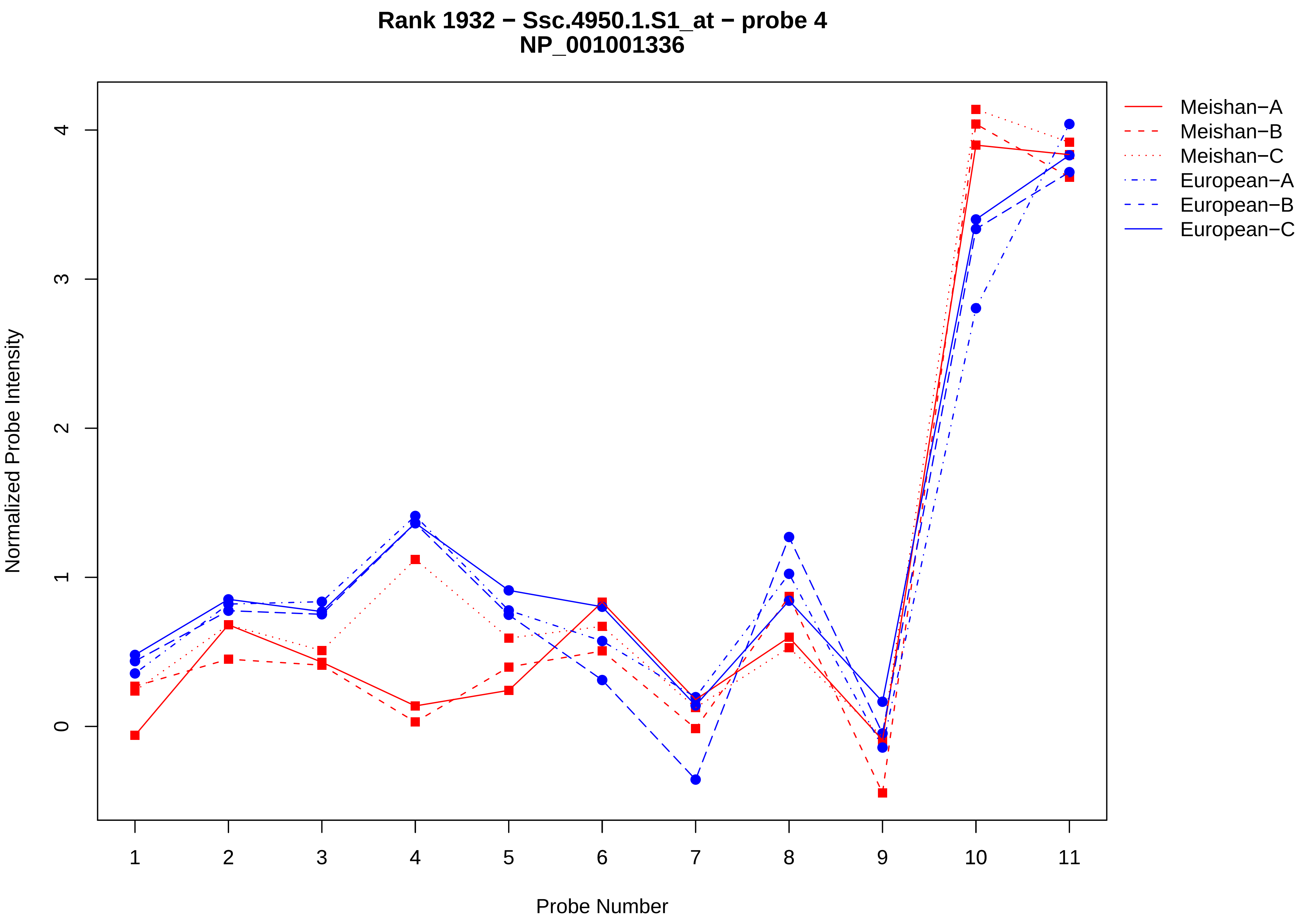

Rank 1932 − Ssc.4950.1.S1_at − probe 4
NP_001001336
Normalized Probe Intensity
Probe Number
0
1
2
3
4
1
2
3
4
5
6
7
8
9
10
11
Meishan−A
Meishan−B
Meishan−C
European−A
European−B
European−C

Rank 1933 − Ssc.25064.1.S1_at − probe 2
GRID2

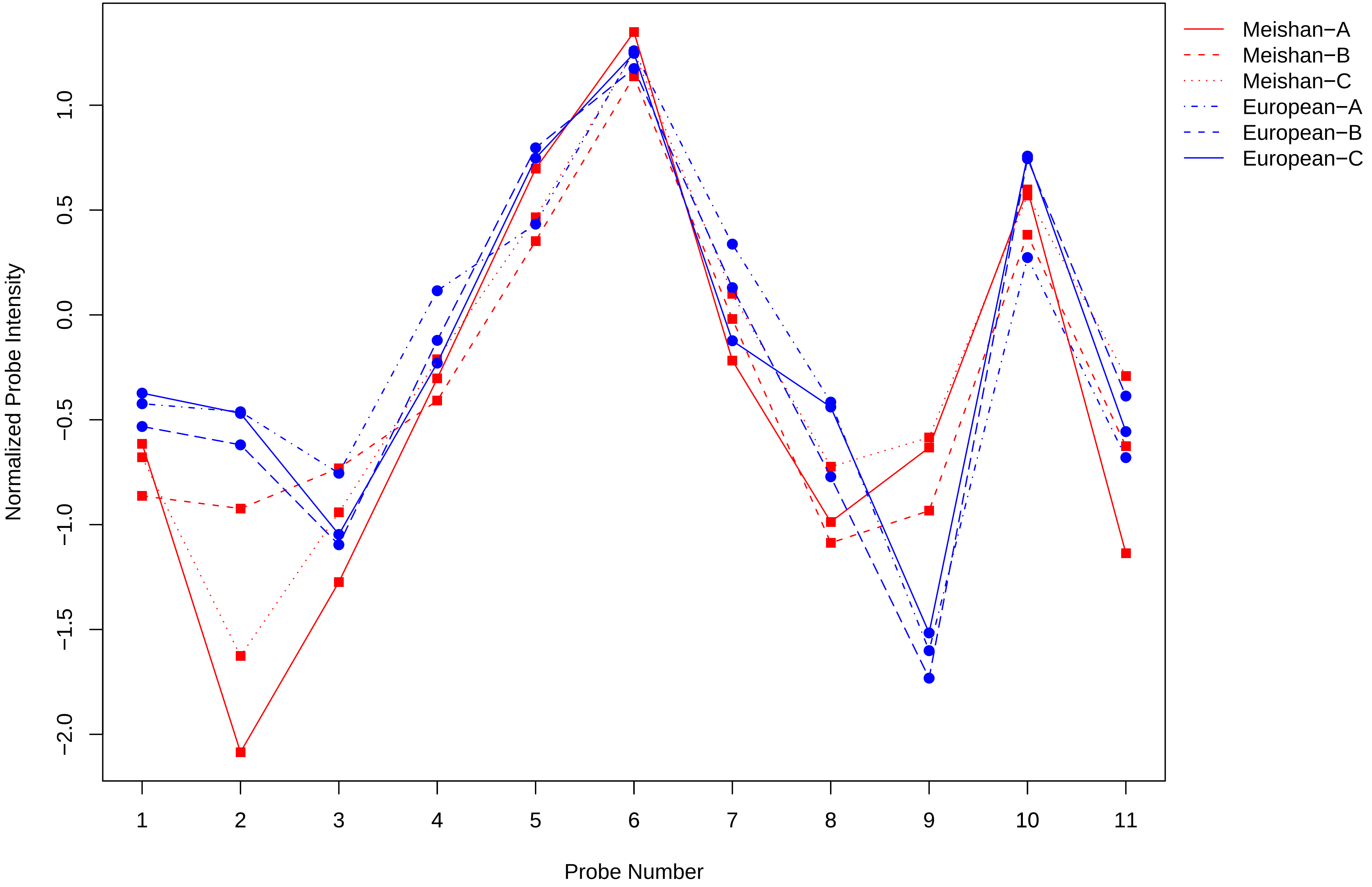

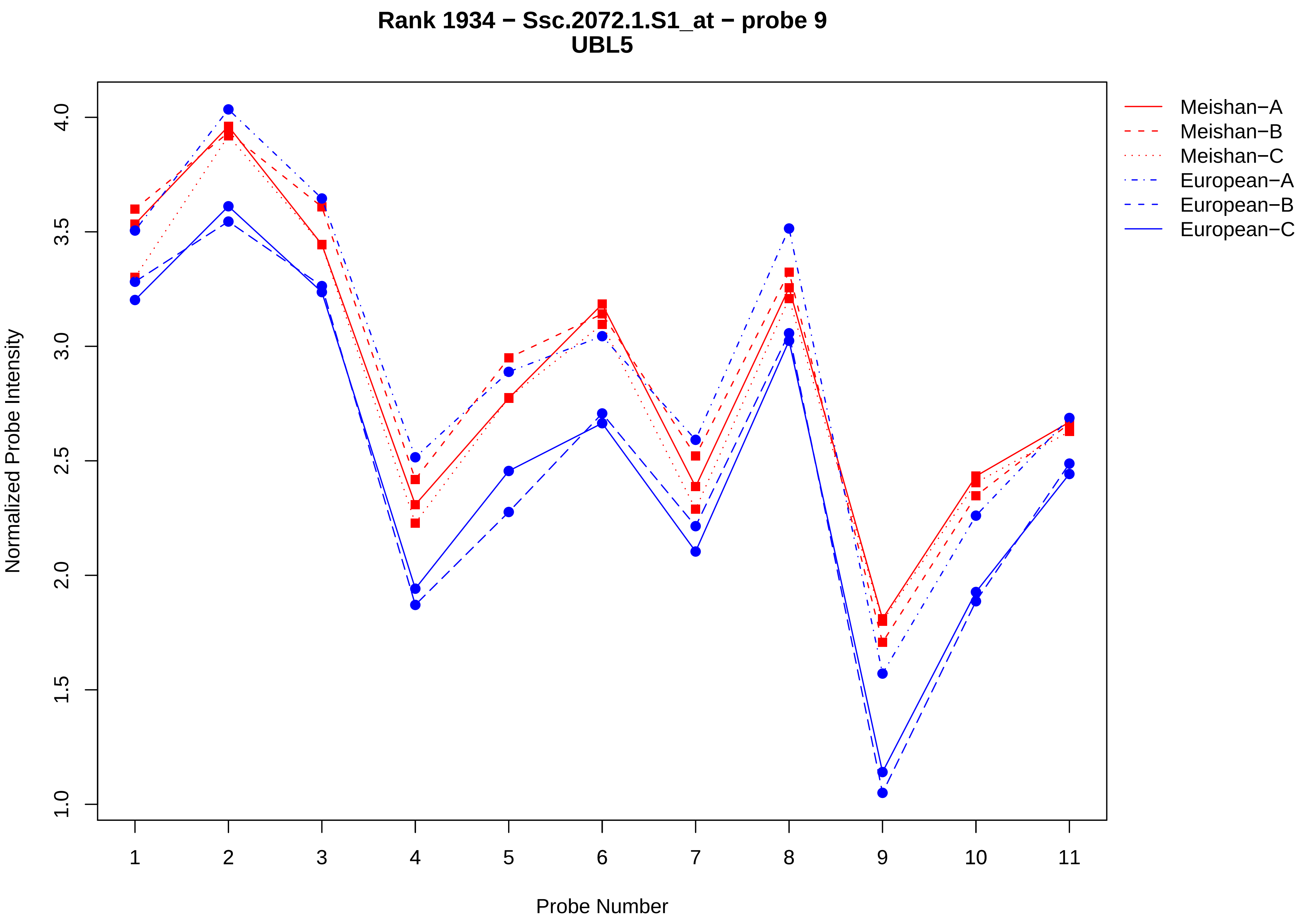

Rank 1934 − Ssc.2072.1.S1_at − probe 9
UBL5
Normalized Probe Intensity
1.0
1.5
2.0
2.5
3.0
3.5
4.0
1
2
3
4
5
6
7
8
9
10
11
Probe Number
Meishan−A
Meishan−B
Meishan−C
European−A
European−B
European−C

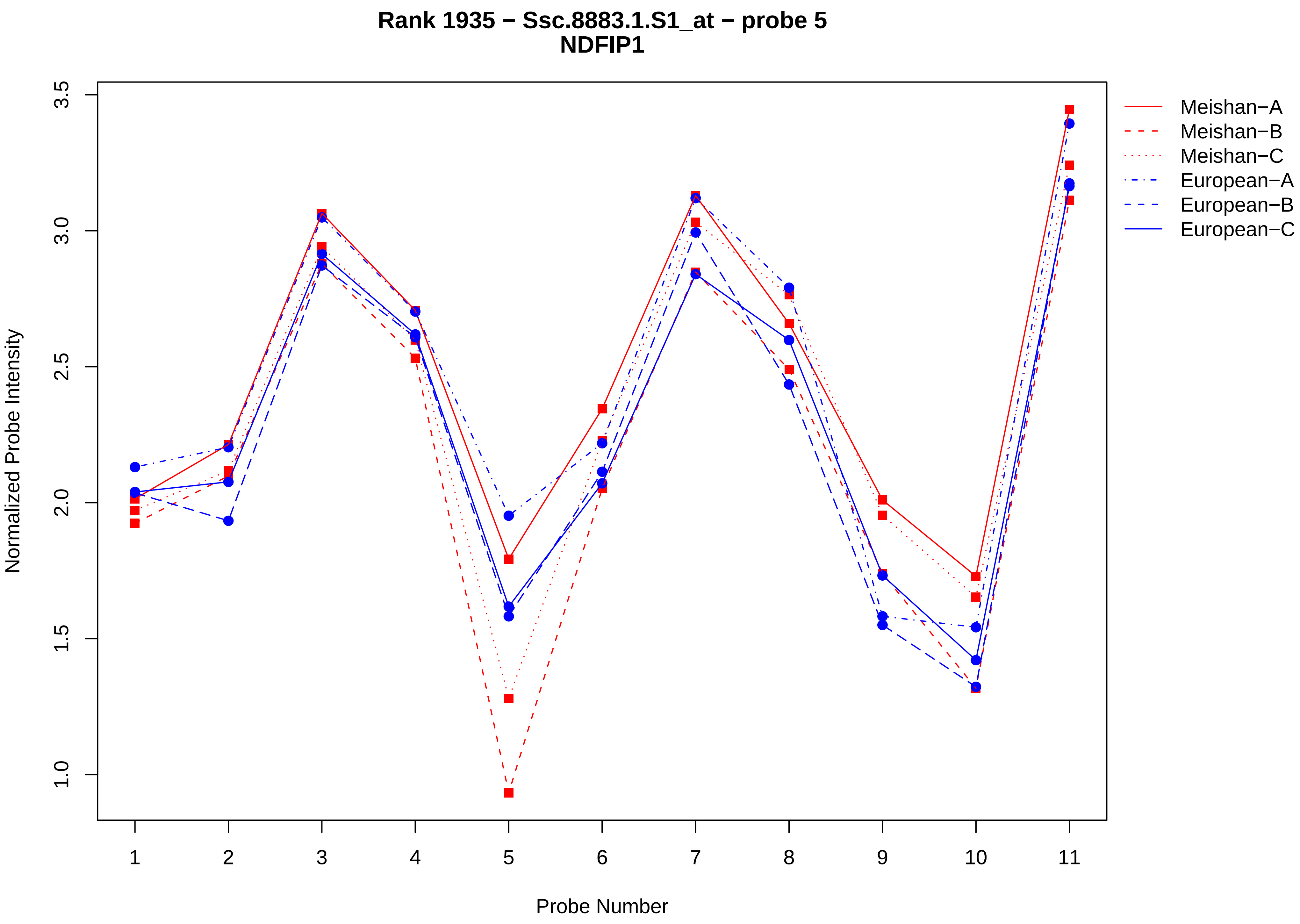

Rank 1935 − Ssc.8883.1.S1_at − probe 5
NDFIP1
Normalized Probe Intensity
Probe Number
1.0
1.5
2.0
2.5
3.0
3.5
1
2
3
4
5
6
7
8
9
10
11
Meishan−A
Meishan−B
Meishan−C
European−A
European−B
European−C

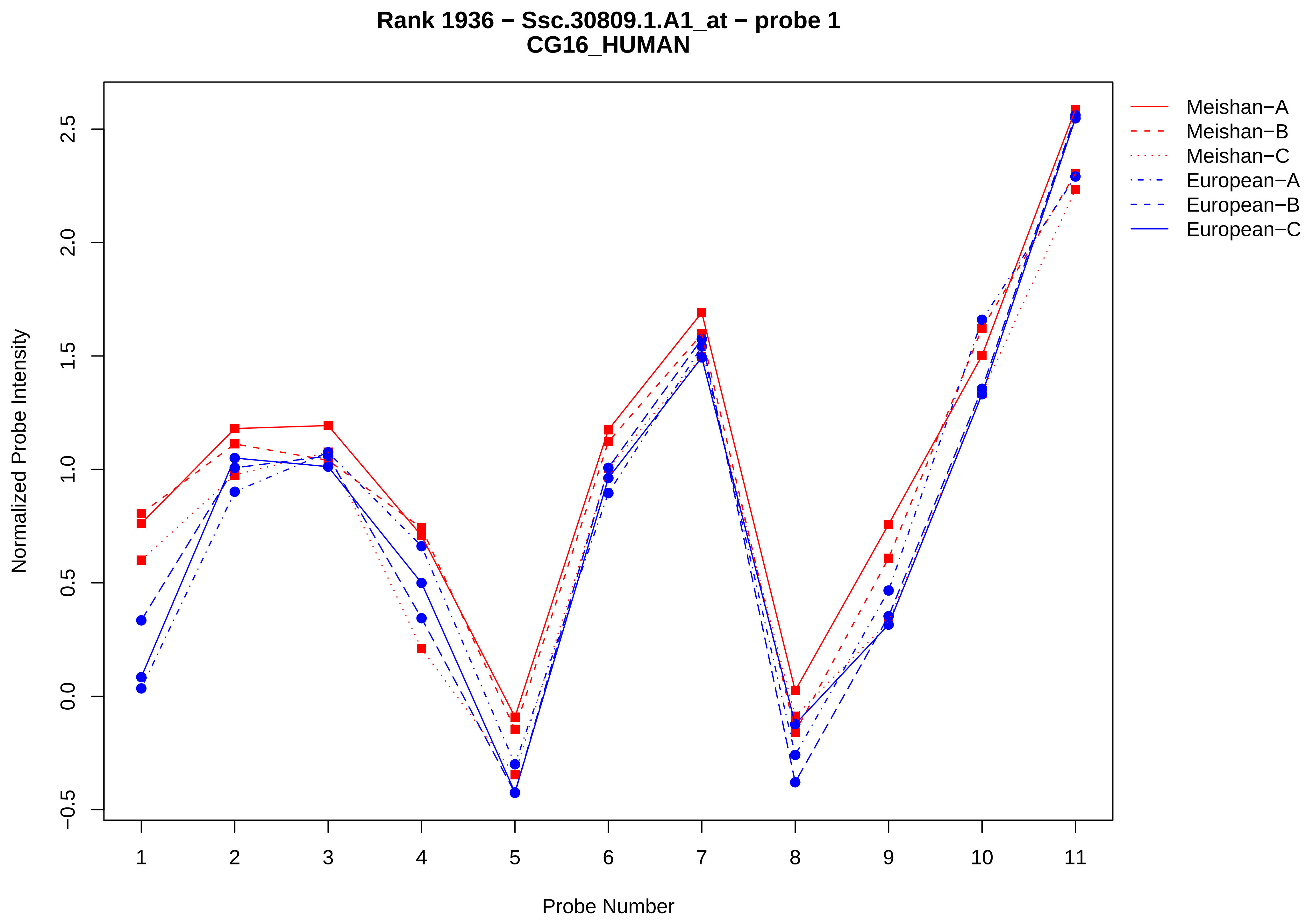

Rank 1936 − Ssc.30809.1.A1_at − probe 1
CG16_HUMAN
Normalized Probe Intensity
Probe Number
−0.5
0.0
0.5
1.0
1.5
2.0
2.5
1
2
3
4
5
6
7
8
9
10
11
Meishan−A
Meishan−B
Meishan−C
European−A
European−B
European−C

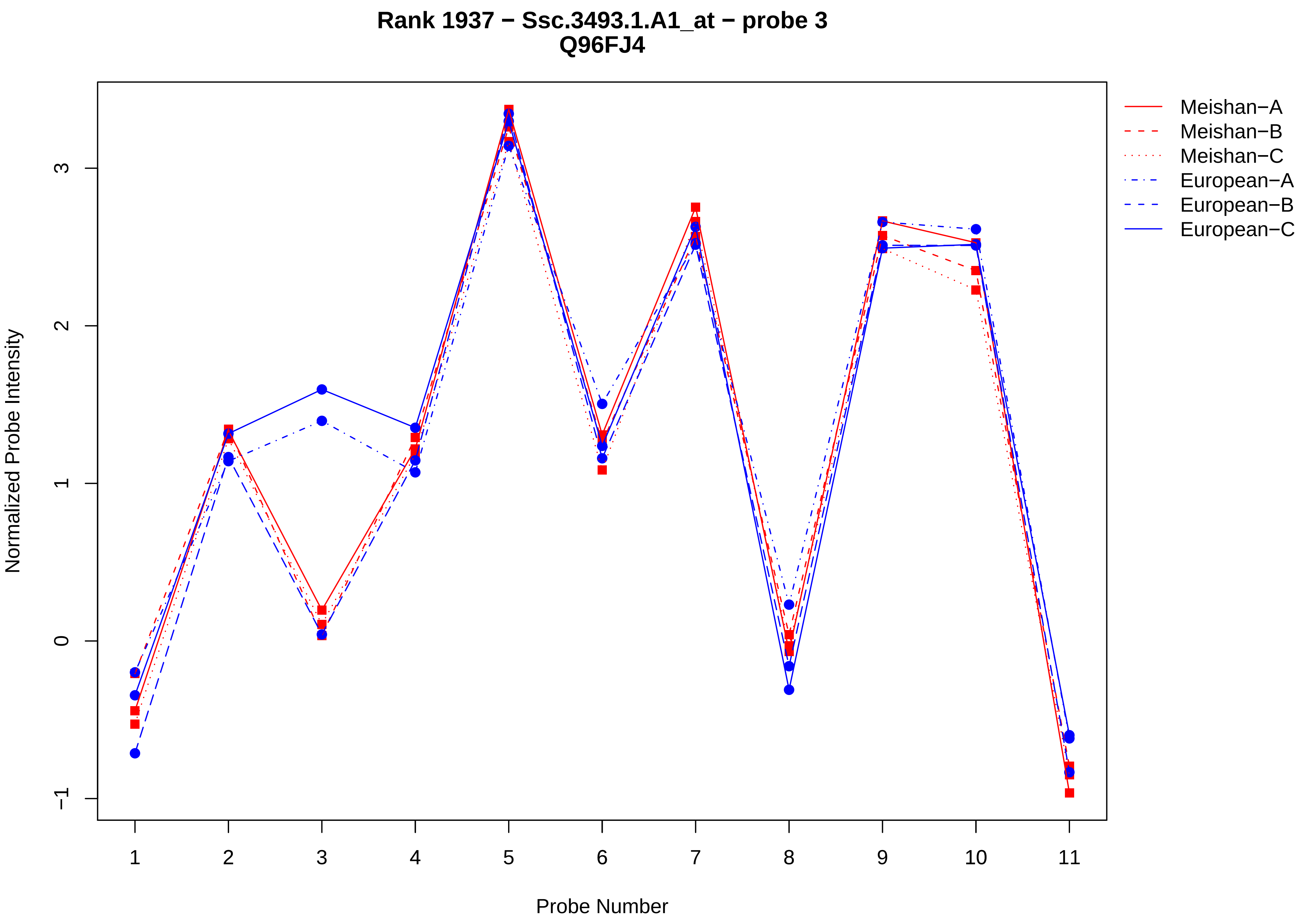

Rank 1937 − Ssc.3493.1.A1_at − probe 3
Q96FJ4
Normalized Probe Intensity
Probe Number
Meishan−A
Meishan−B
Meishan−C
European−A
European−B
European−C

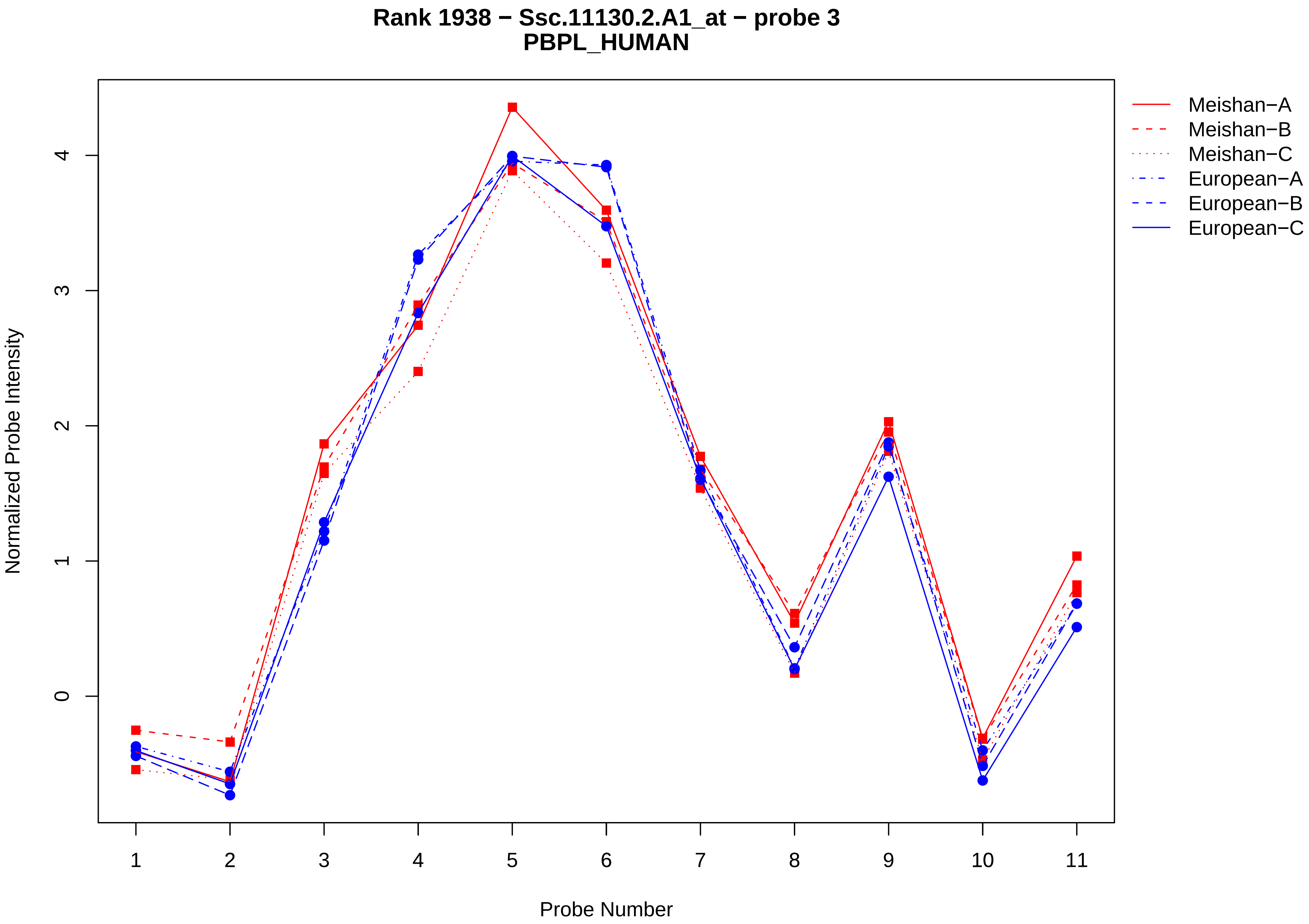

Rank 1938 − Ssc.11130.2.A1_at − probe 3
PBPL_HUMAN
Normalized Probe Intensity
Probe Number
0
1
2
3
4
1
2
3
4
5
6
7
8
9
10
11
Meishan−A
Meishan−B
Meishan−C
European−A
European−B
European−C

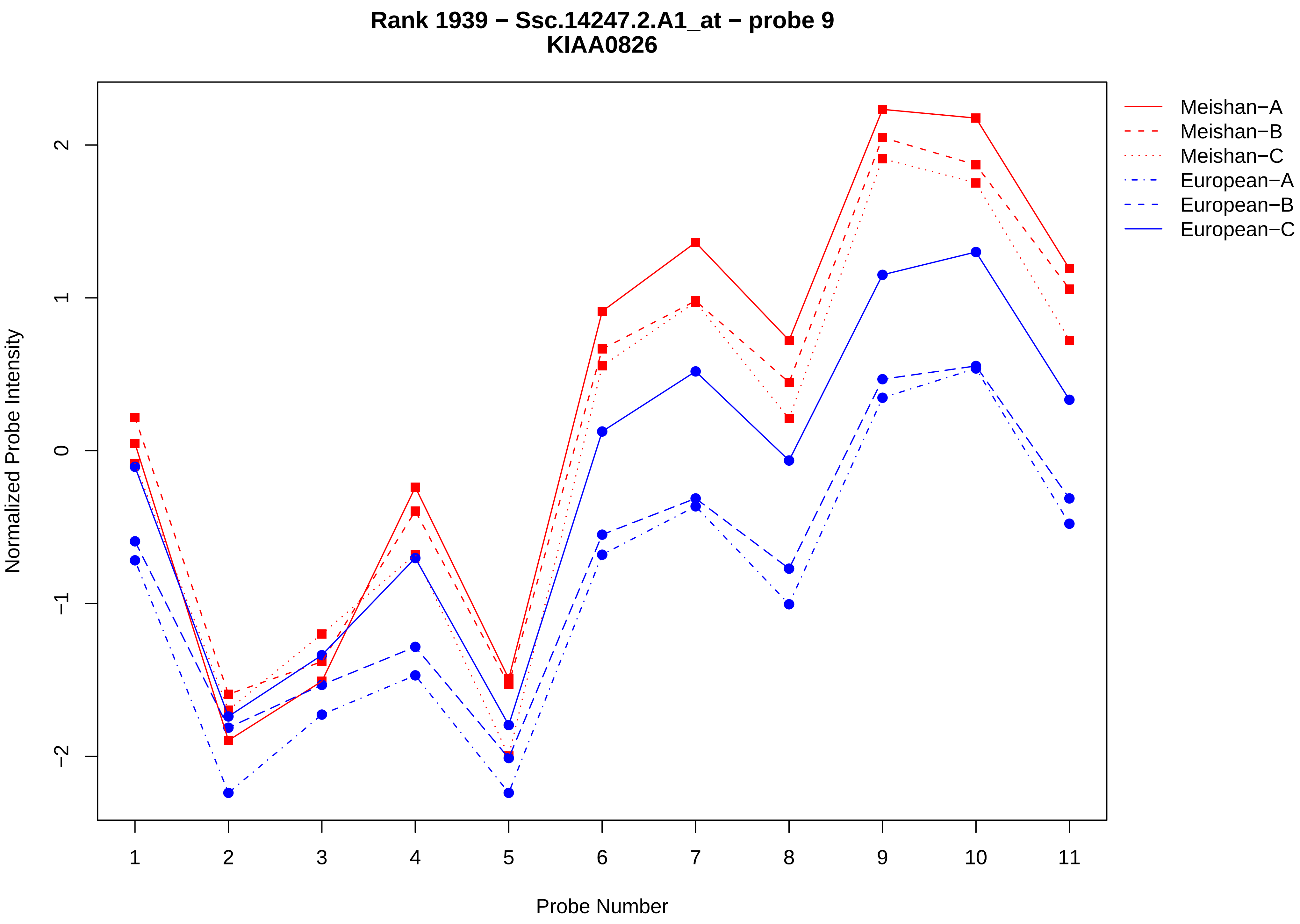

Rank 1939 − Ssc.14247.2.A1_at − probe 9
KIAA0826
Normalized Probe Intensity
Probe Number
−2
−1
0
1
2
1
2
3
4
5
6
7
8
9
10
11
Meishan−A
Meishan−B
Meishan−C
European−A
European−B
European−C

Rank 1940 − Ssc.2138.1.S1_at − probe 8
CDC42BPA

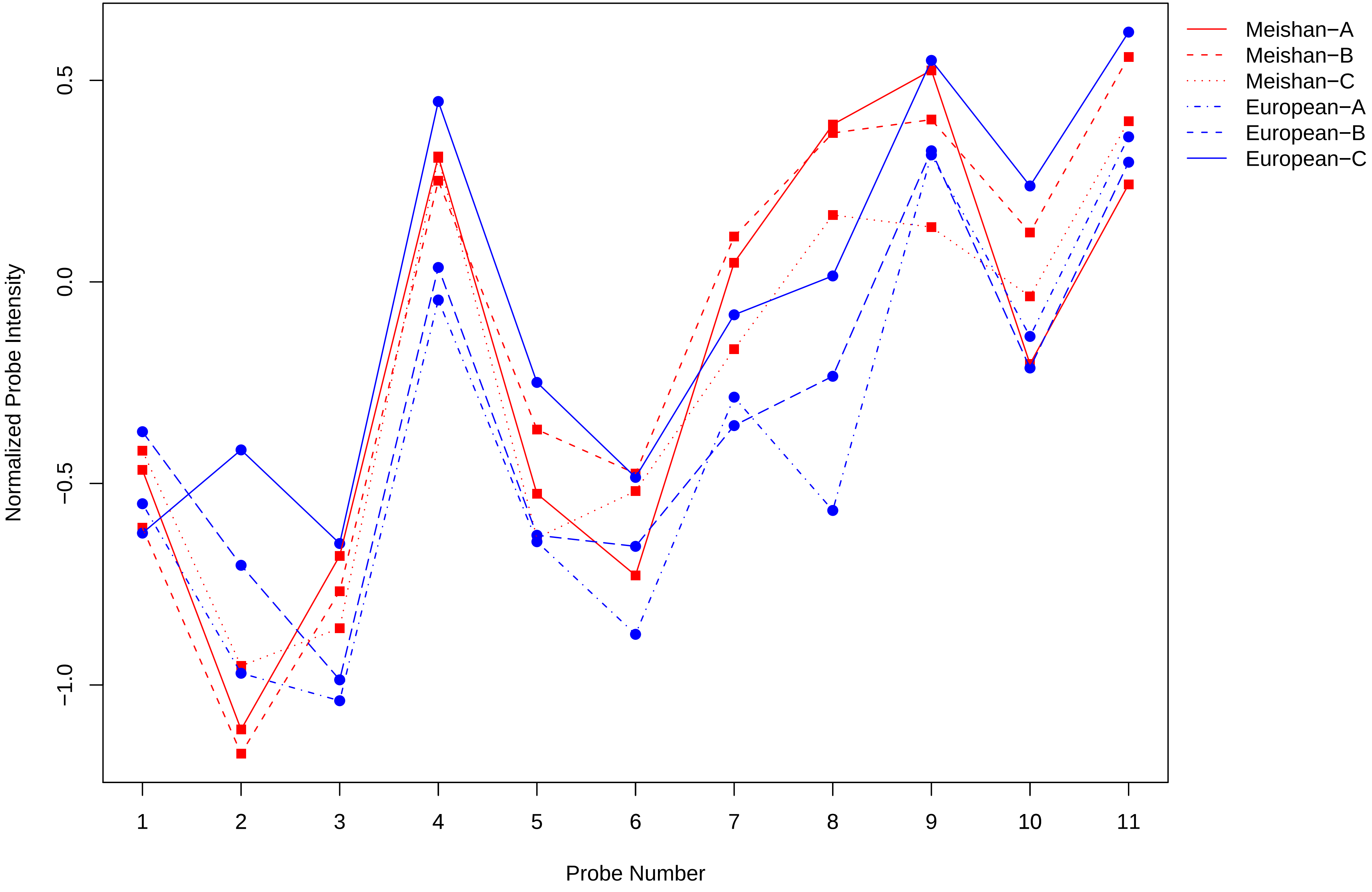

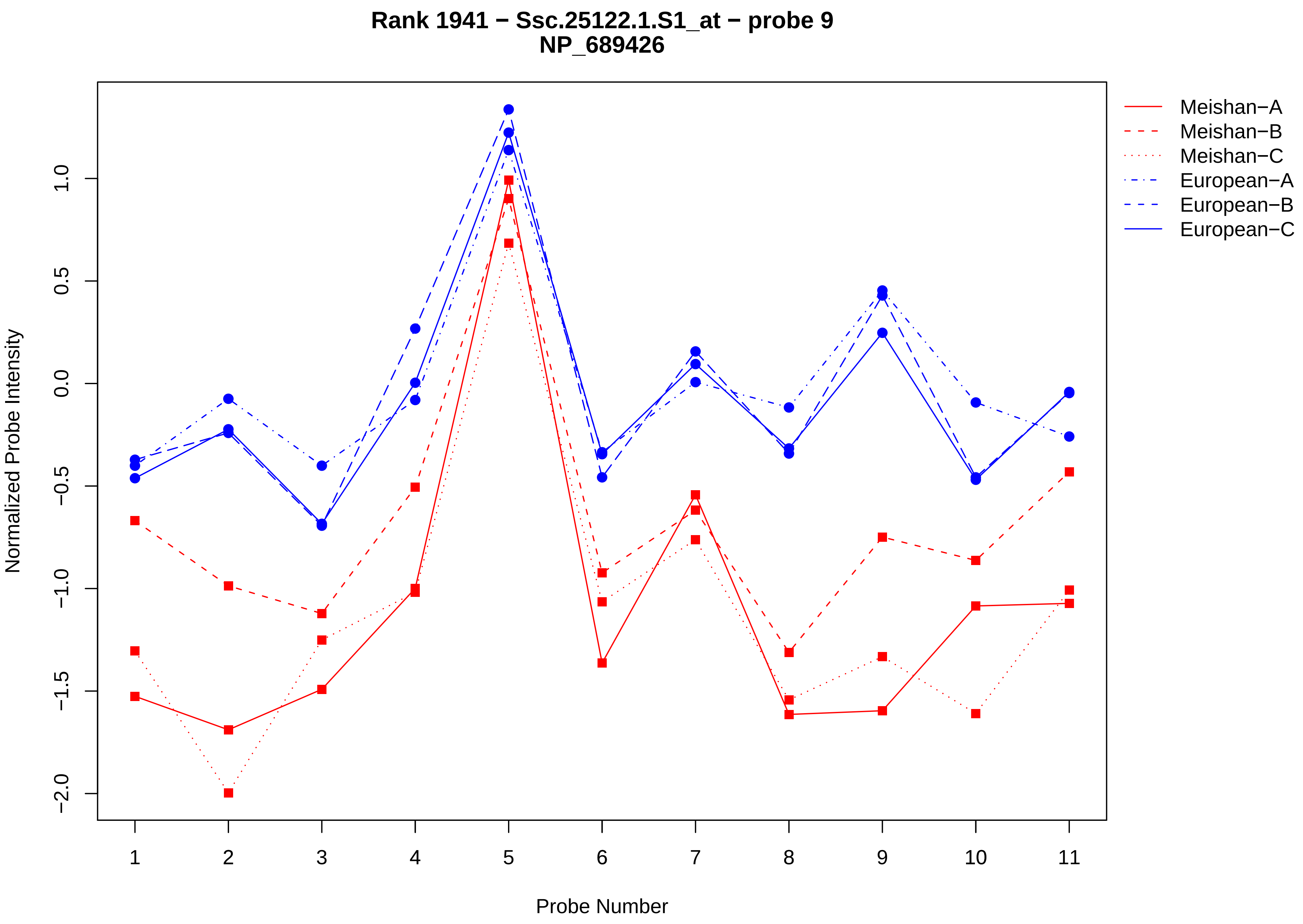
Rank 1941 − Ssc.25122.1.S1_at − probe 9
NP_689426
Normalized Probe Intensity
Probe Number
Meishan−A
Meishan−B
Meishan−C
European−A
European−B
European−C
−2.0
−1.5
−1.0
−0.5
0.0
0.5
1.0
1
2
3
4
5
6
7
8
9
10
11

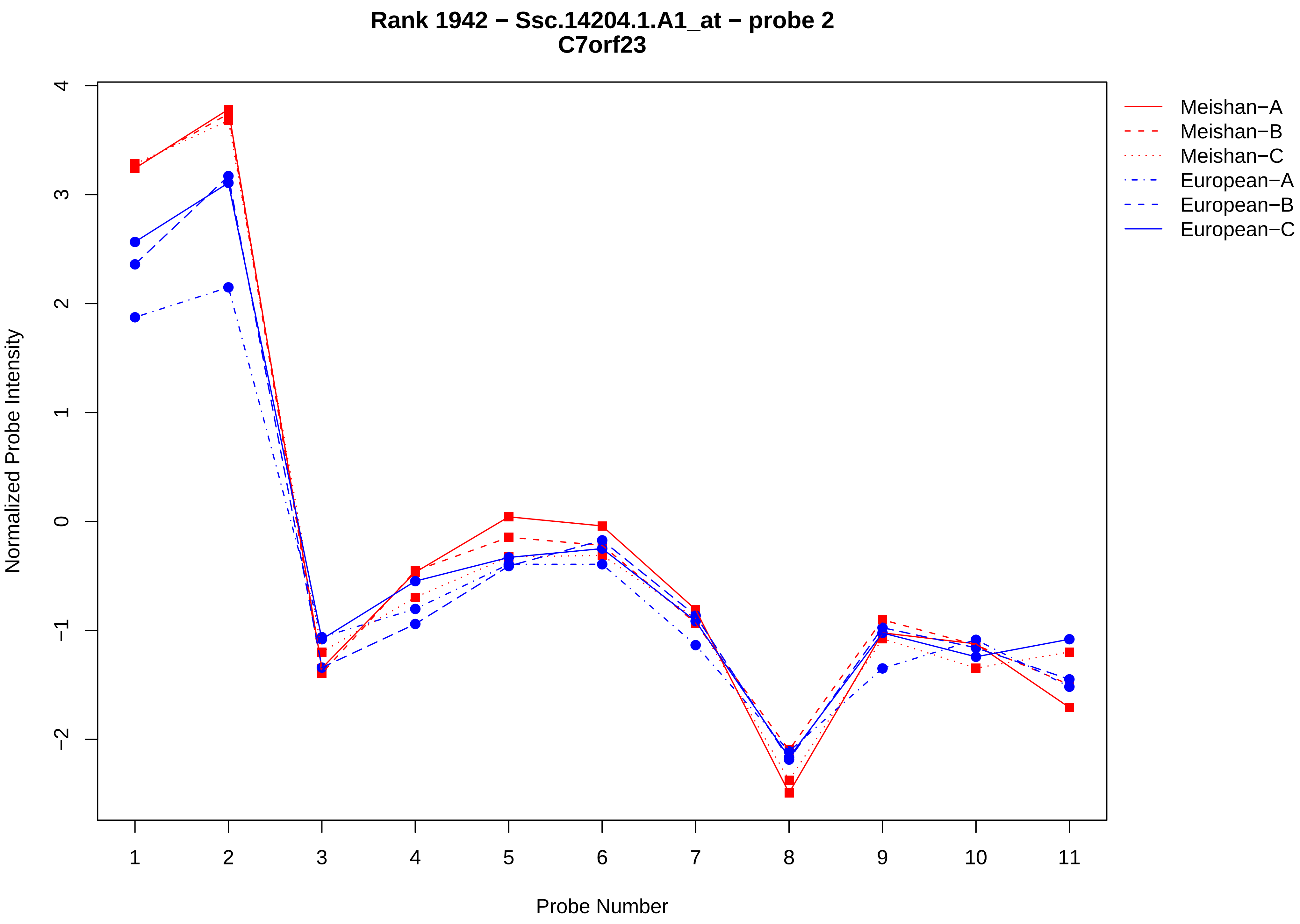

Rank 1942 − Ssc.14204.1.A1_at − probe 2
C7orf23
Normalized Probe Intensity
Probe Number
4
3
2
1
0
−1
−2
1
2
3
4
5
6
7
8
9
10
11
Meishan−A
Meishan−B
Meishan−C
European−A
European−B
European−C

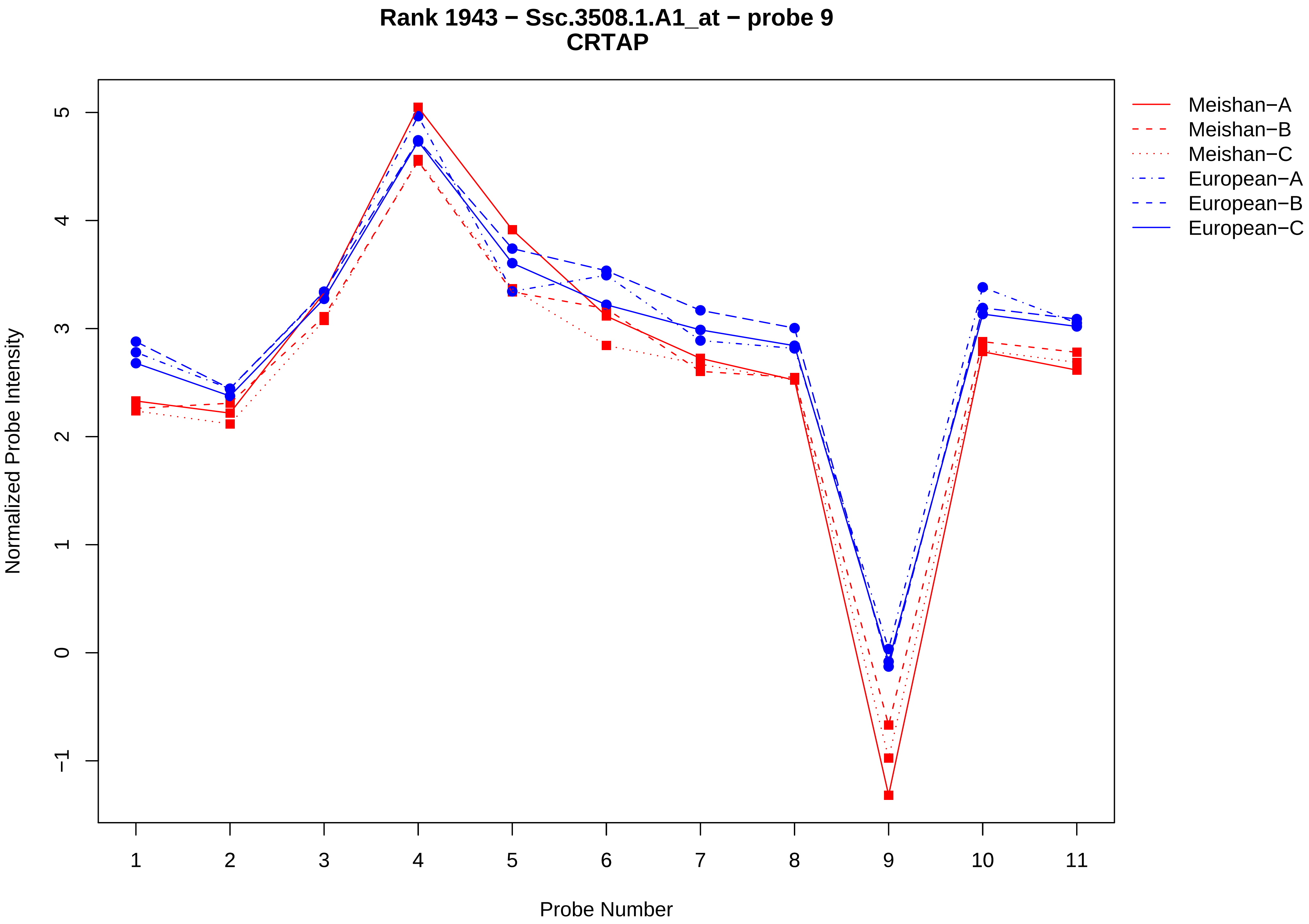

Rank 1943 − Ssc.3508.1.A1_at − probe 9
CRTAP
Normalized Probe Intensity
Probe Number
−1
0
1
2
3
4
5
1
2
3
4
5
6
7
8
9
10
11
Meishan−A
Meishan−B
Meishan−C
European−A
European−B
European−C

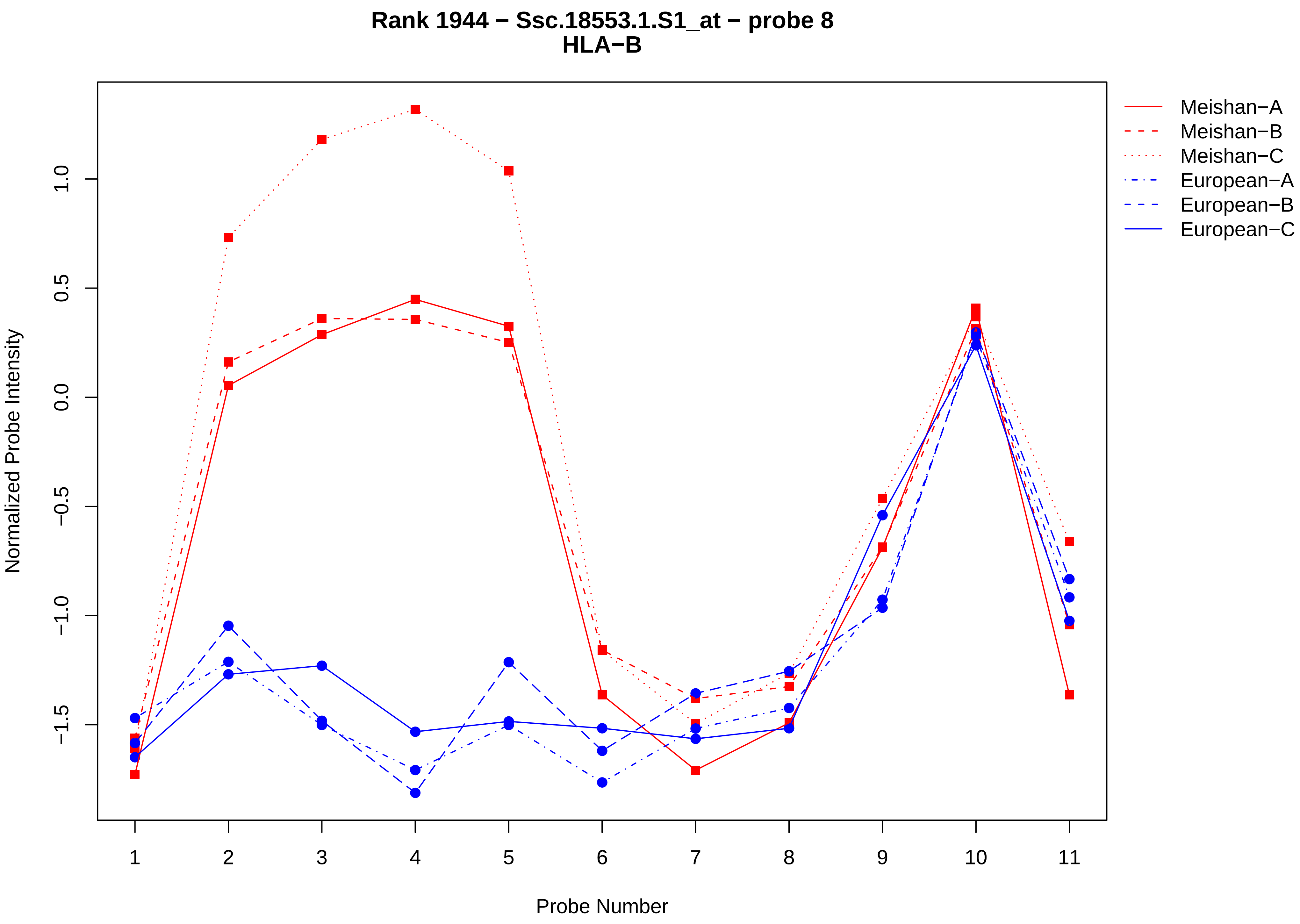

Rank 1944 − Ssc.18553.1.S1_at − probe 8
HLA−B
Normalized Probe Intensity
Probe Number
−1.5
−1.0
−0.5
0.0
0.5
1.0
1
2
3
4
5
6
7
8
9
10
11
Meishan−A
Meishan−B
Meishan−C
European−A
European−B
European−C

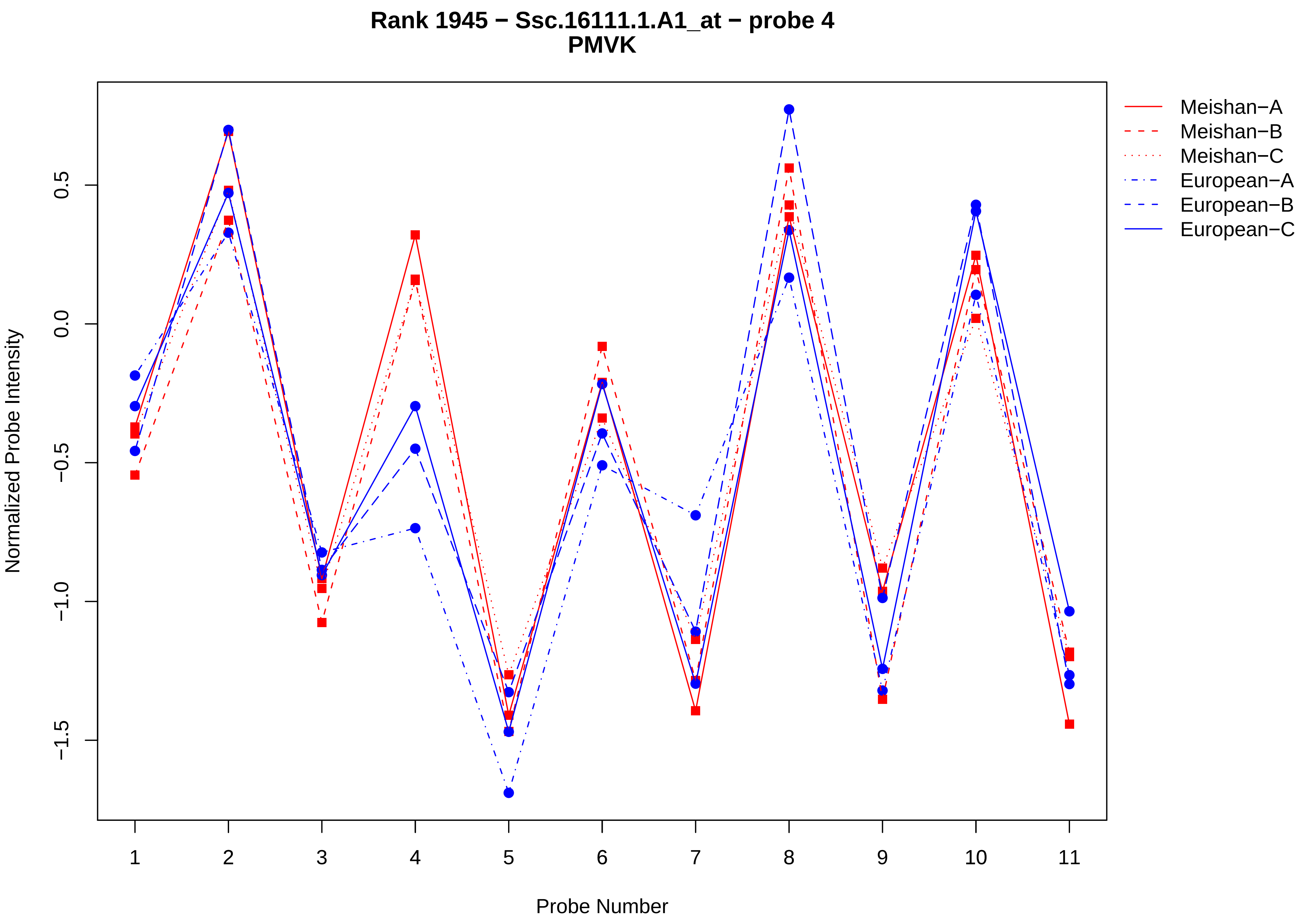
Rank 1945 − Ssc.16111.1.A1_at − probe 4
PMVK
Normalized Probe Intensity
Probe Number
0.5
0.0
−0.5
−1.0
−1.5
1
2
3
4
5
6
7
8
9
10
11
Meishan−A
Meishan−B
Meishan−C
European−A
European−B
European−C

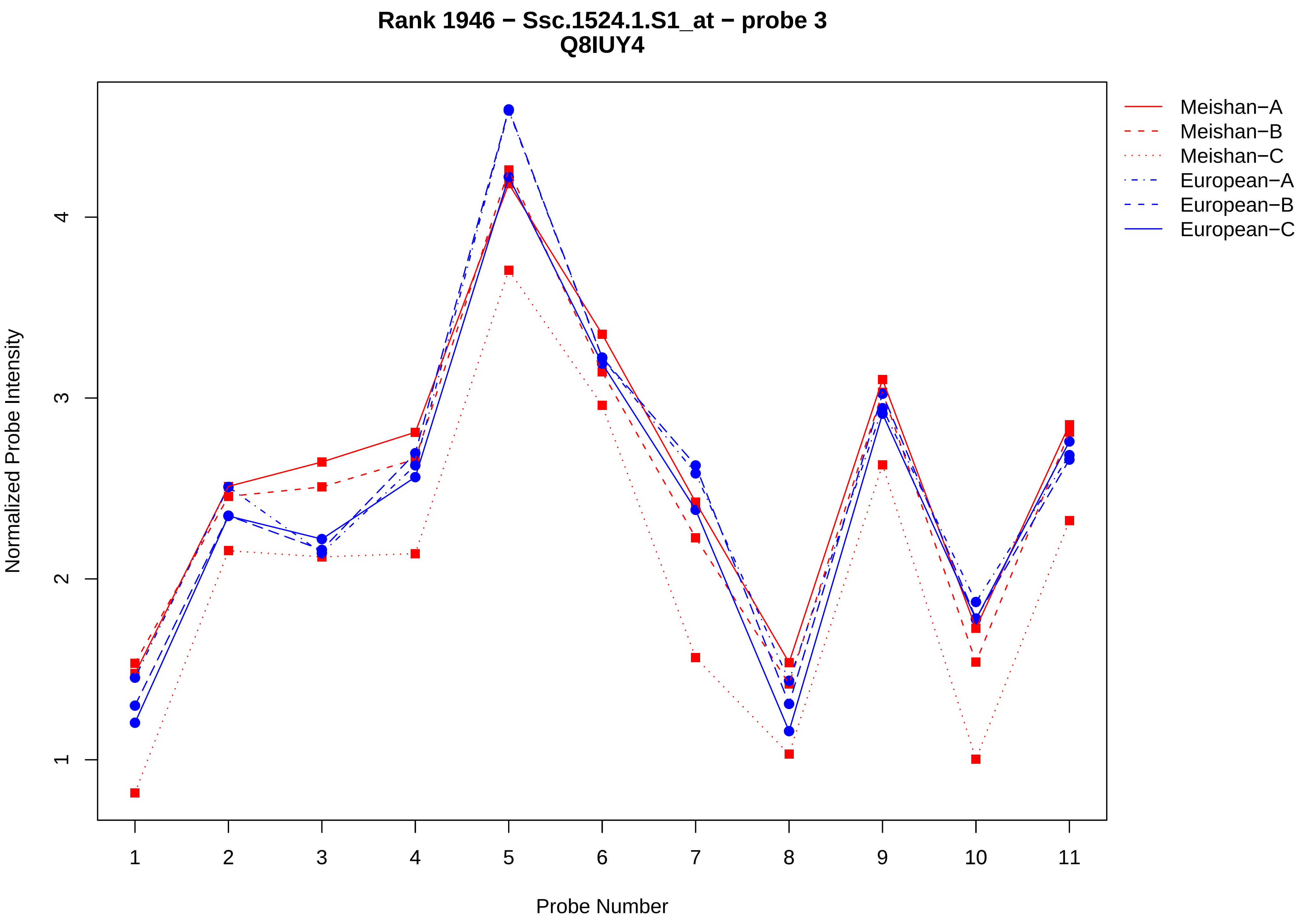
Rank 1946 − Ssc.1524.1.S1_at − probe 3
Q8IUY4
Normalized Probe Intensity
Probe Number
1
2
3
4
1
2
3
4
5
6
7
8
9
10
11
Meishan−A
Meishan−B
Meishan−C
European−A
European−B
European−C

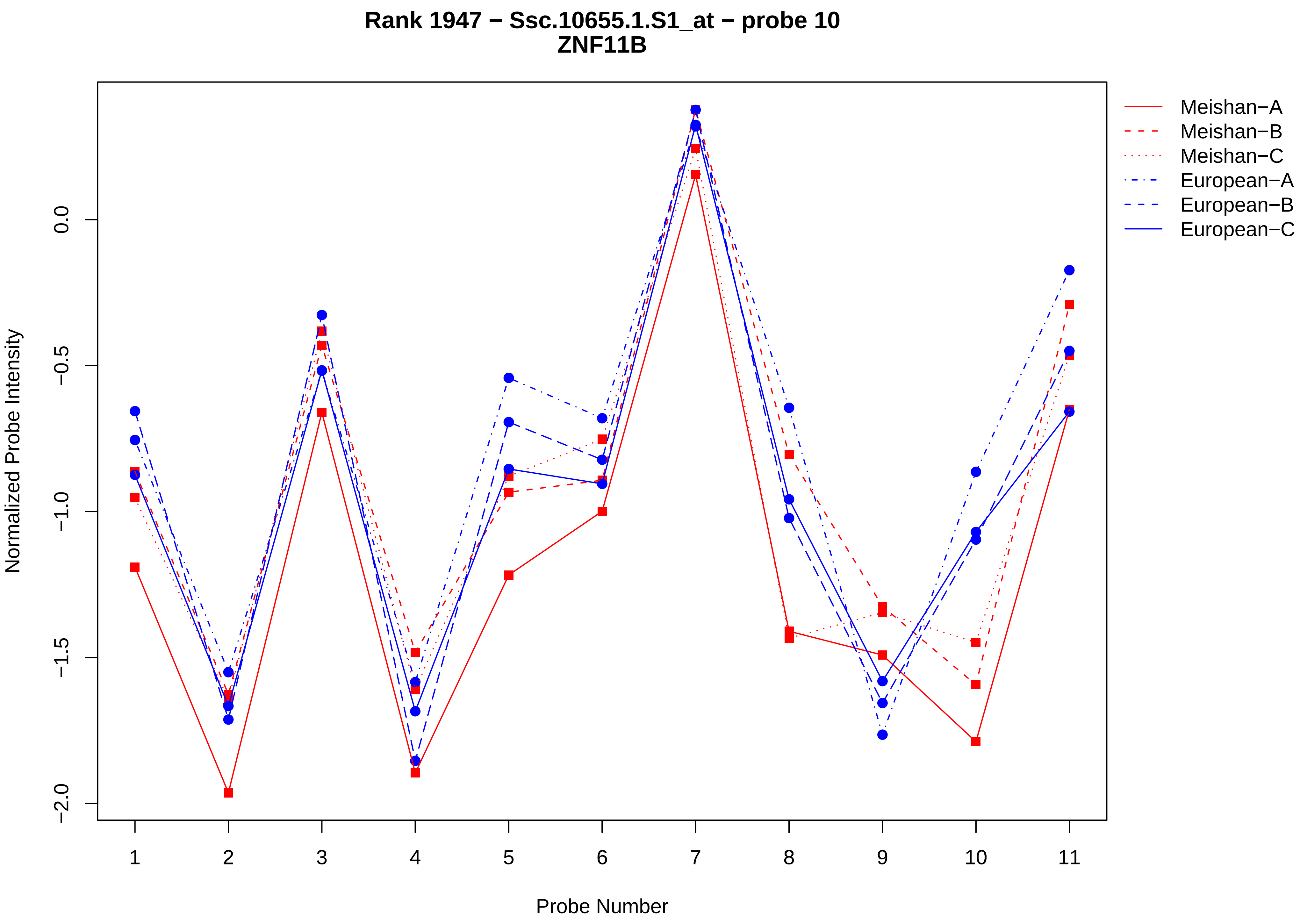

Rank 1947 − Ssc.10655.1.S1_at − probe 10
ZNF11B
Normalized Probe Intensity
Probe Number
0.0
−0.5
−1.0
−1.5
−2.0
1
2
3
4
5
6
7
8
9
10
11
Meishan−A
Meishan−B
Meishan−C
European−A
European−B
European−C

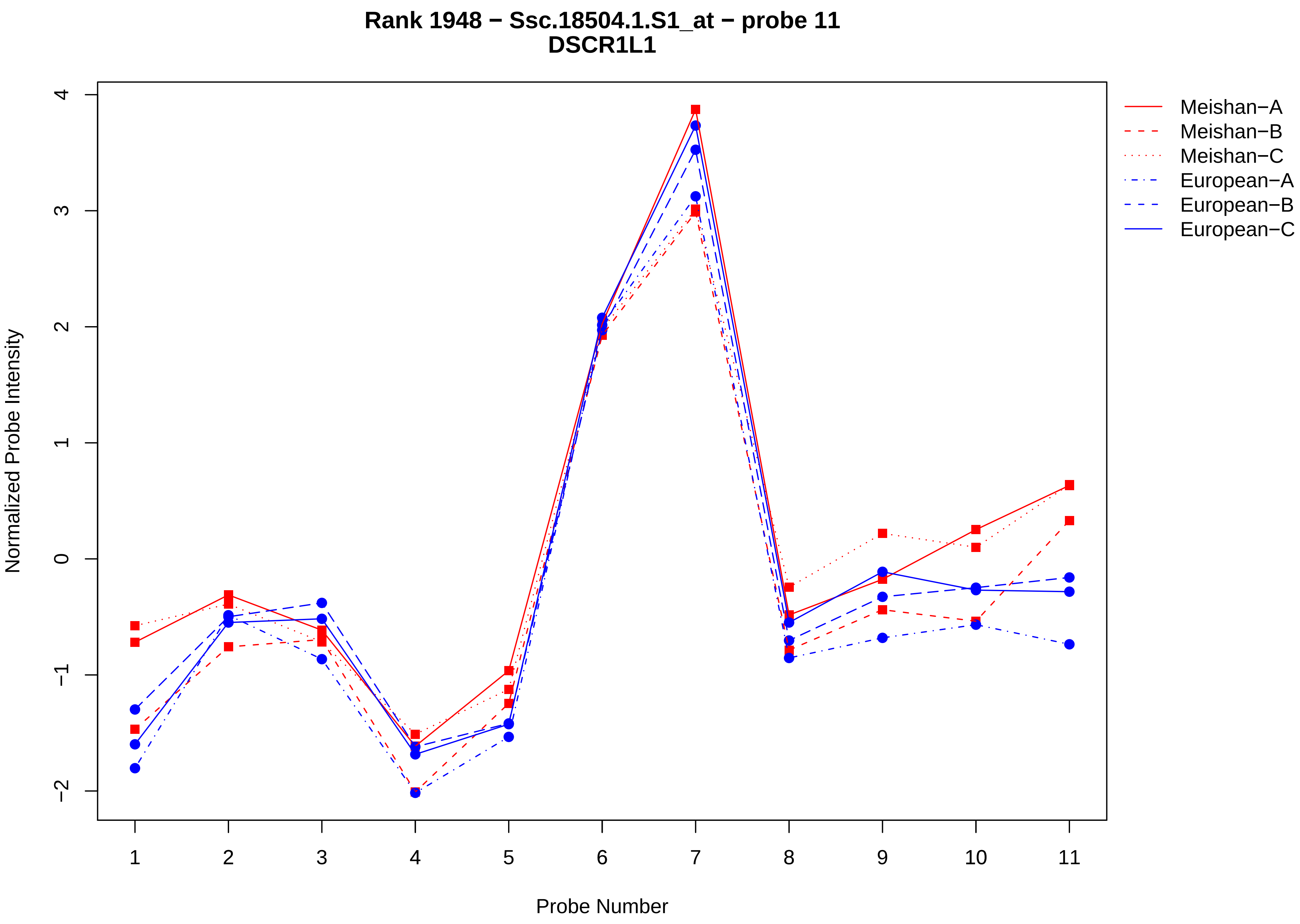

Rank 1948 − Ssc.18504.1.S1_at − probe 11
DSCR1L1
Normalized Probe Intensity
Probe Number
−2
−1
0
1
2
3
4
1
2
3
4
5
6
7
8
9
10
11
Meishan−A
Meishan−B
Meishan−C
European−A
European−B
European−C

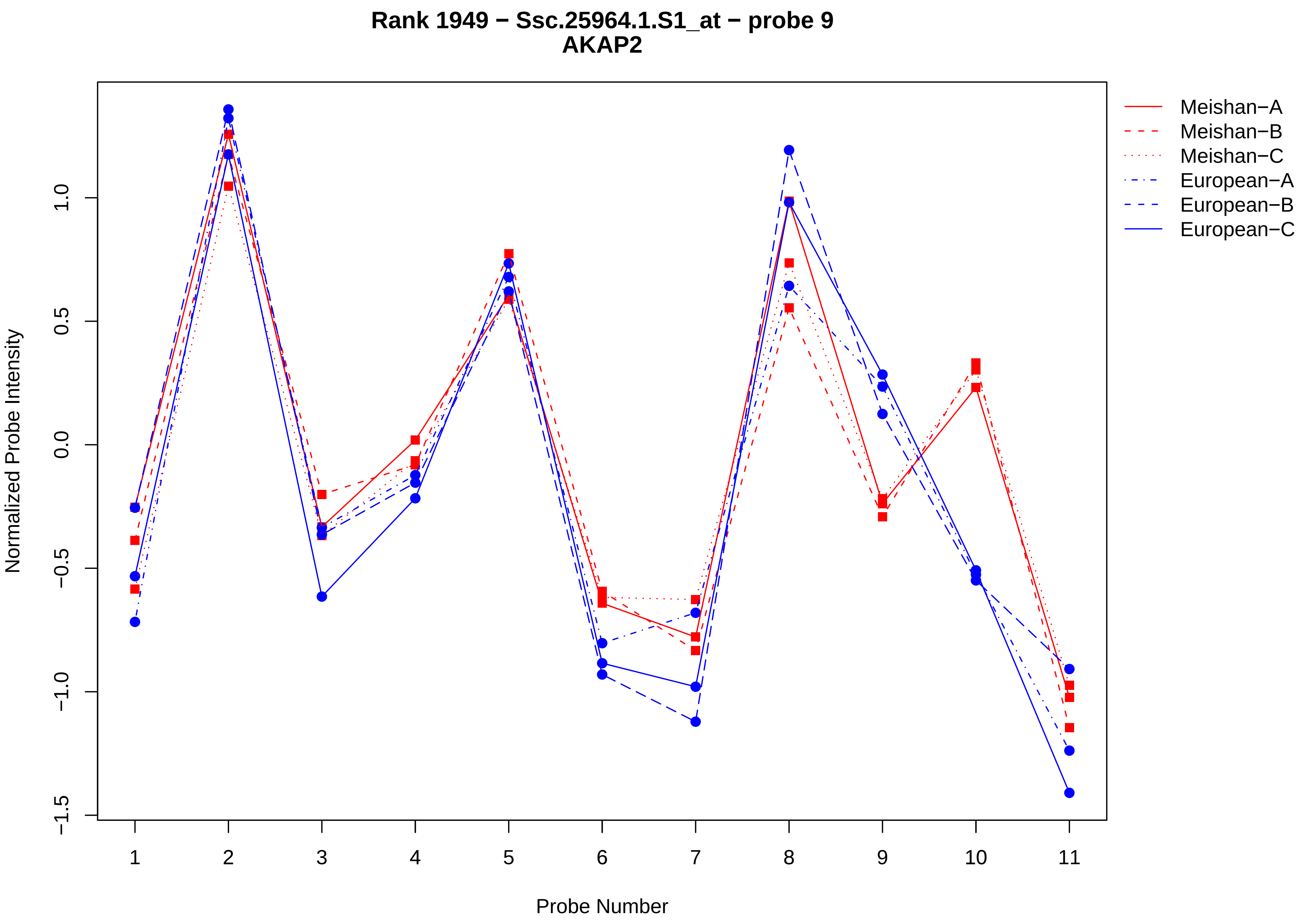

Rank 1949 − Ssc.25964.1.S1_at − probe 9
AKAP2
Normalized Probe Intensity
Probe Number
−1.5
−1.0
−0.5
0.0
0.5
1.0
1
2
3
4
5
6
7
8
9
10
11
Meishan−A
Meishan−B
Meishan−C
European−A
European−B
European−C

Rank 1950 − Ssc.5780.2.S1_at − probe 3
PCNX

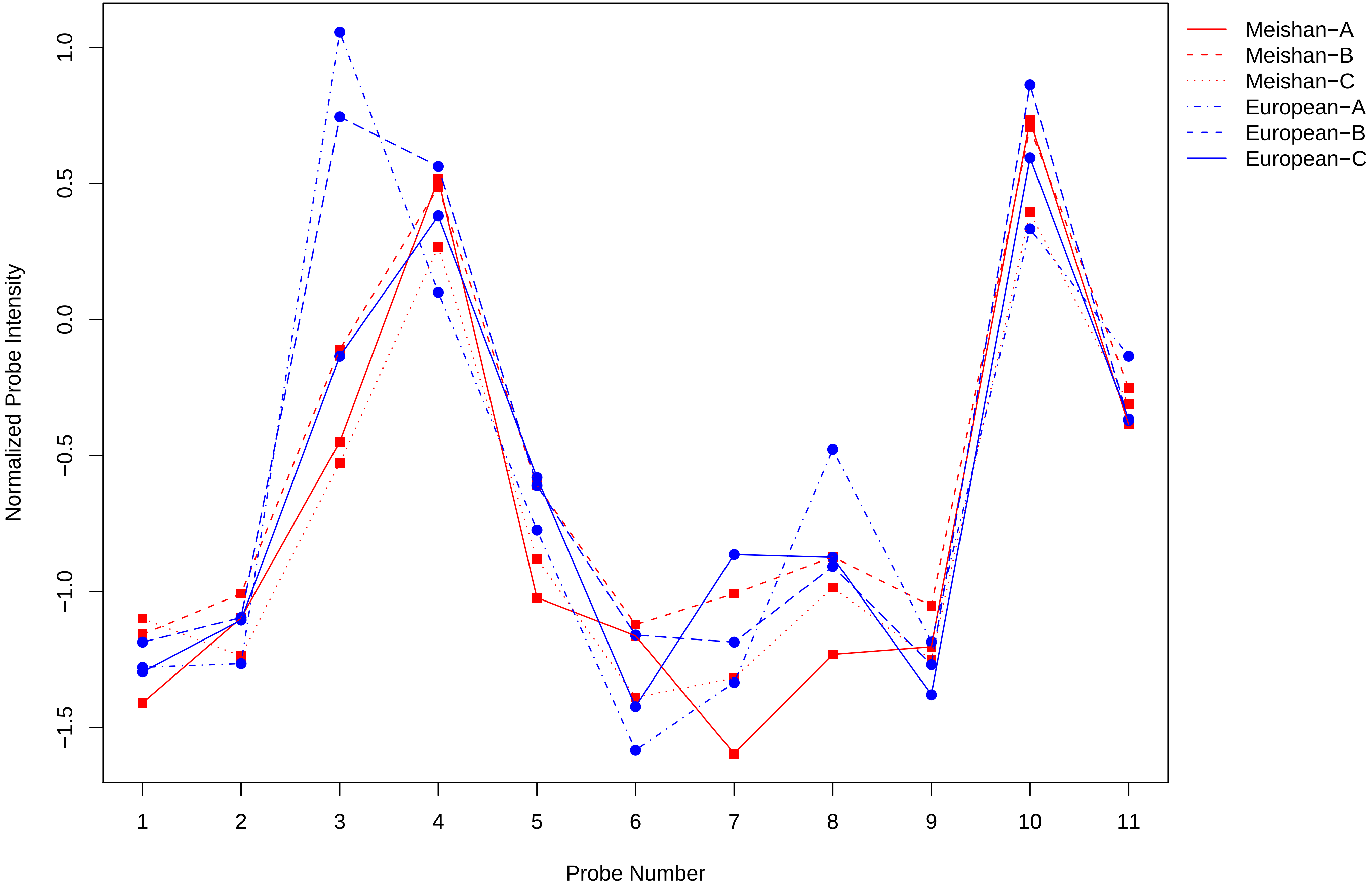

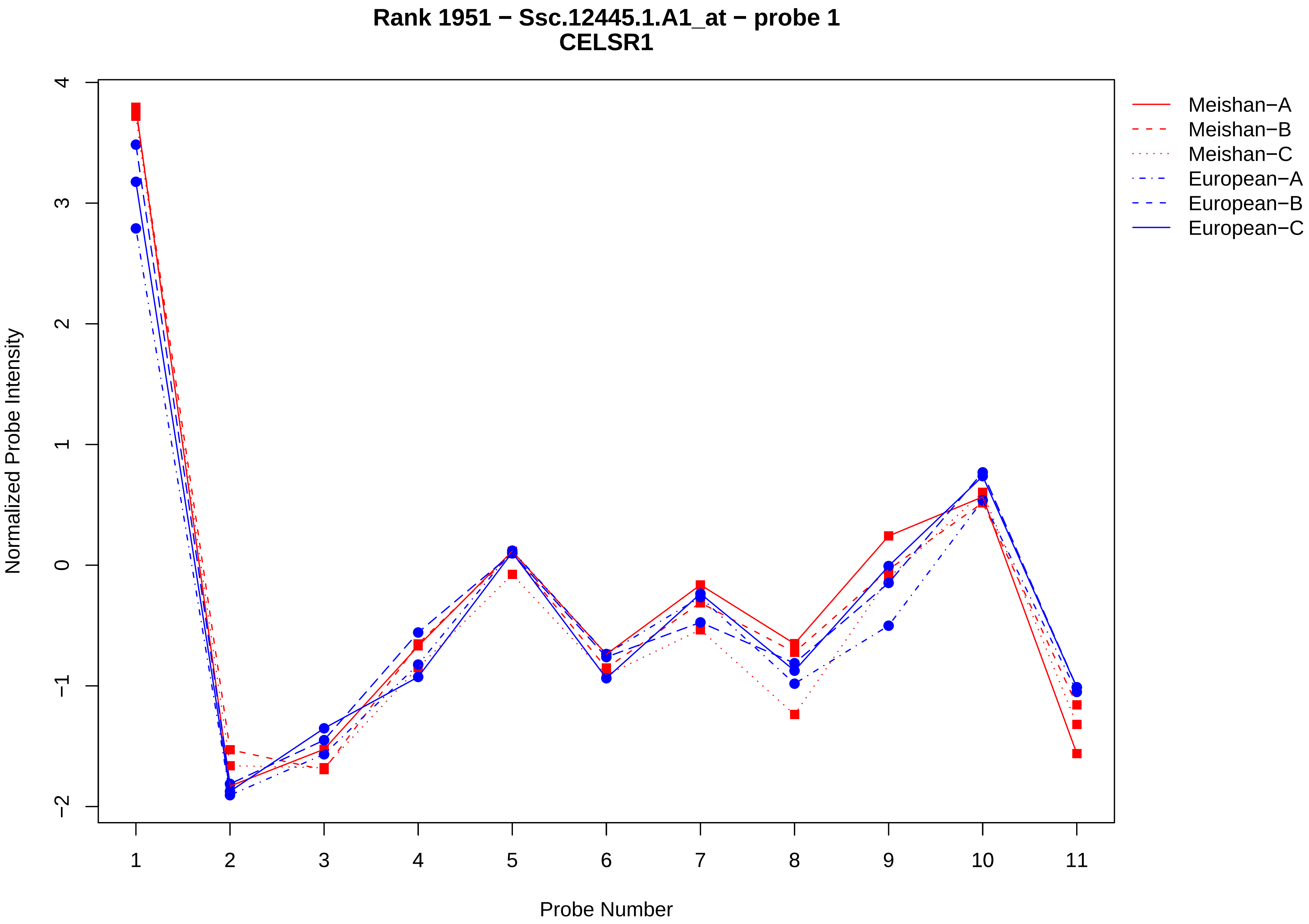

Rank 1951 − Ssc.12445.1.A1_at − probe 1
CELSR1
Normalized Probe Intensity
Probe Number
4
3
2
1
0
−1
−2
1
2
3
4
5
6
7
8
9
10
11
Meishan−A
Meishan−B
Meishan−C
European−A
European−B
European−C

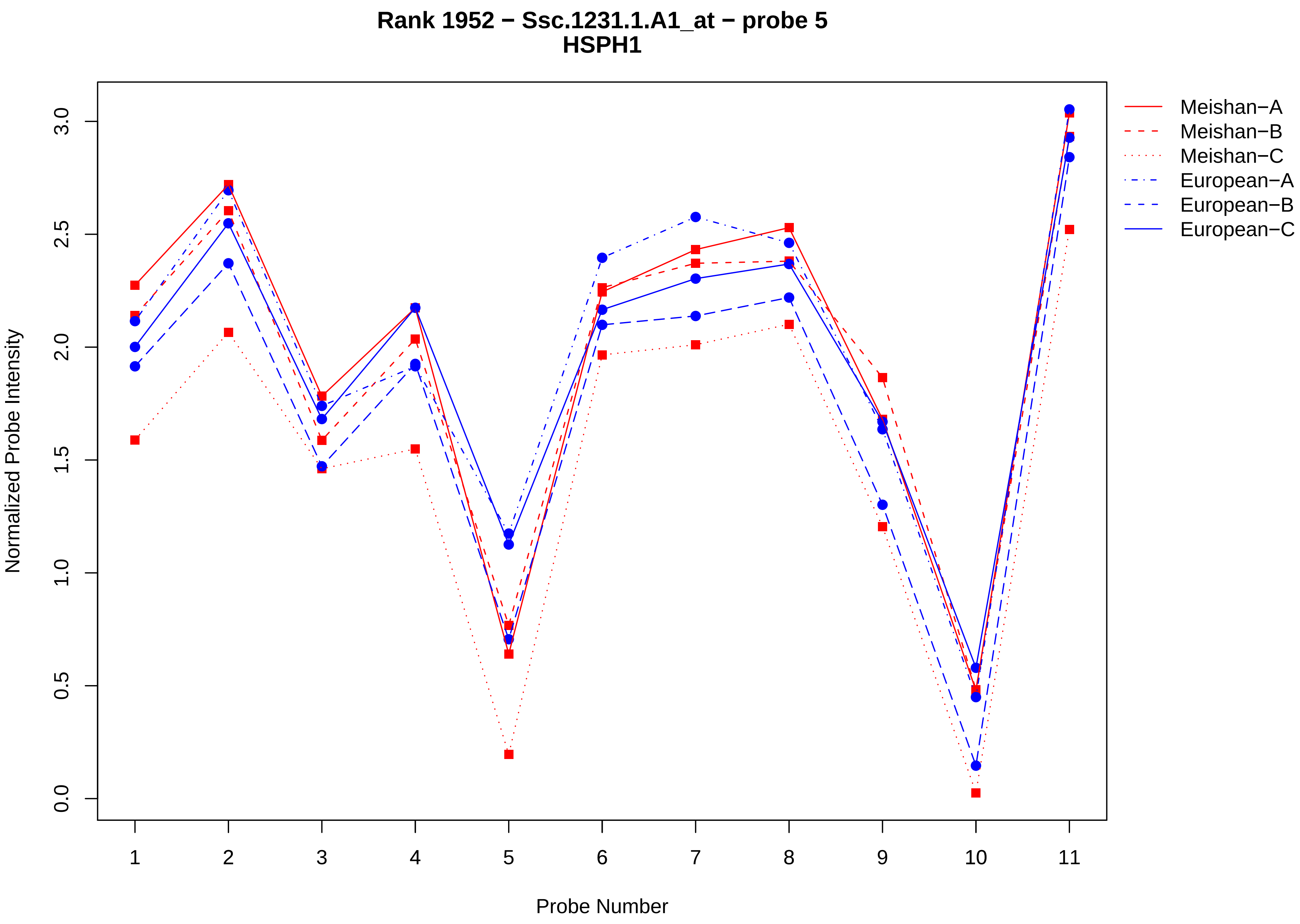
Rank 1952 − Ssc.1231.1.A1_at − probe 5
HSPH1
Normalized Probe Intensity
Probe Number
0.0
0.5
1.0
1.5
2.0
2.5
3.0
1
2
3
4
5
6
7
8
9
10
11
Meishan−A
Meishan−B
Meishan−C
European−A
European−B
European−C

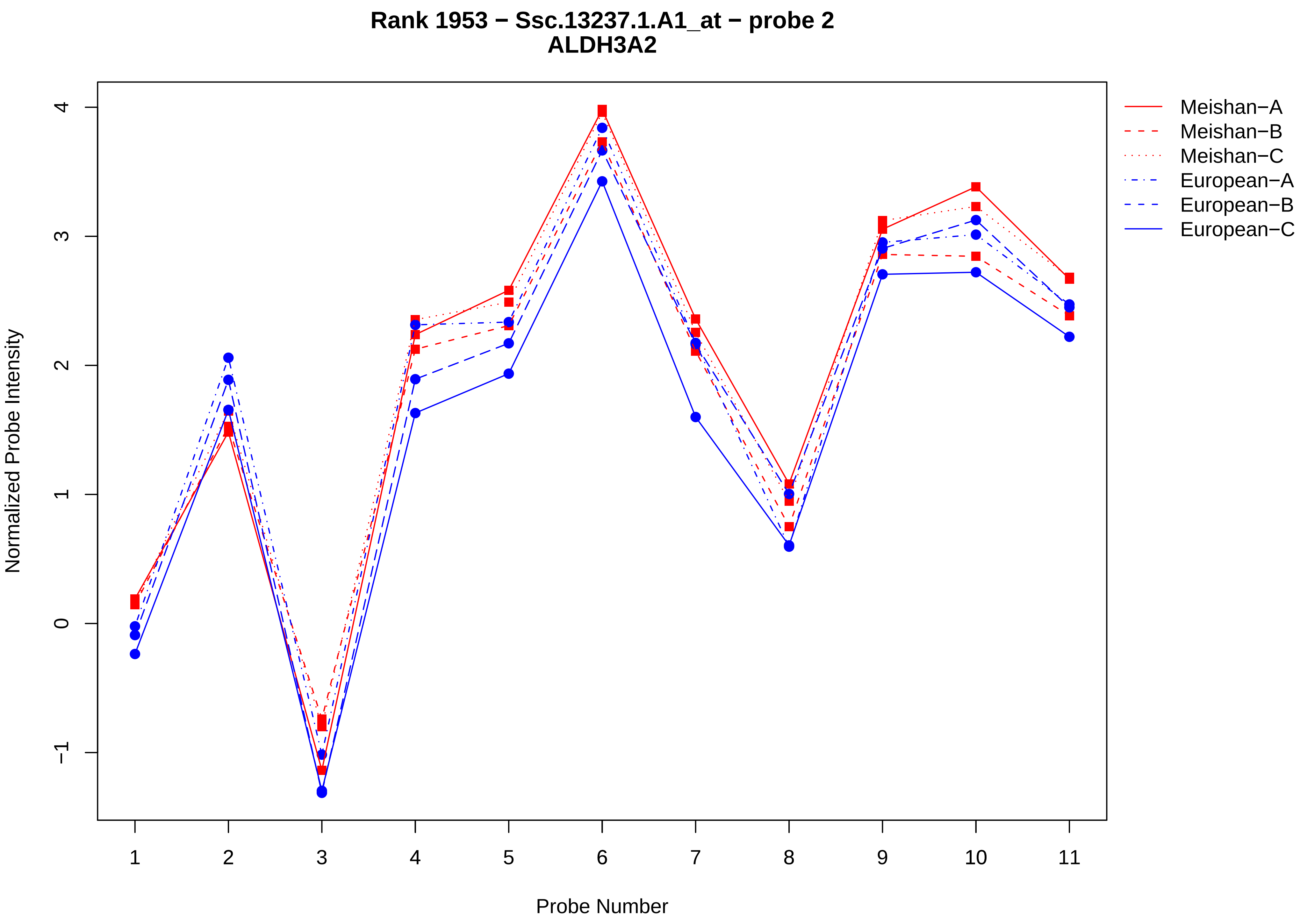

Rank 1953 − Ssc.13237.1.A1_at − probe 2
ALDH3A2
Normalized Probe Intensity
Probe Number
−1
0
1
2
3
4
1
2
3
4
5
6
7
8
9
10
11
Meishan−A
Meishan−B
Meishan−C
European−A
European−B
European−C

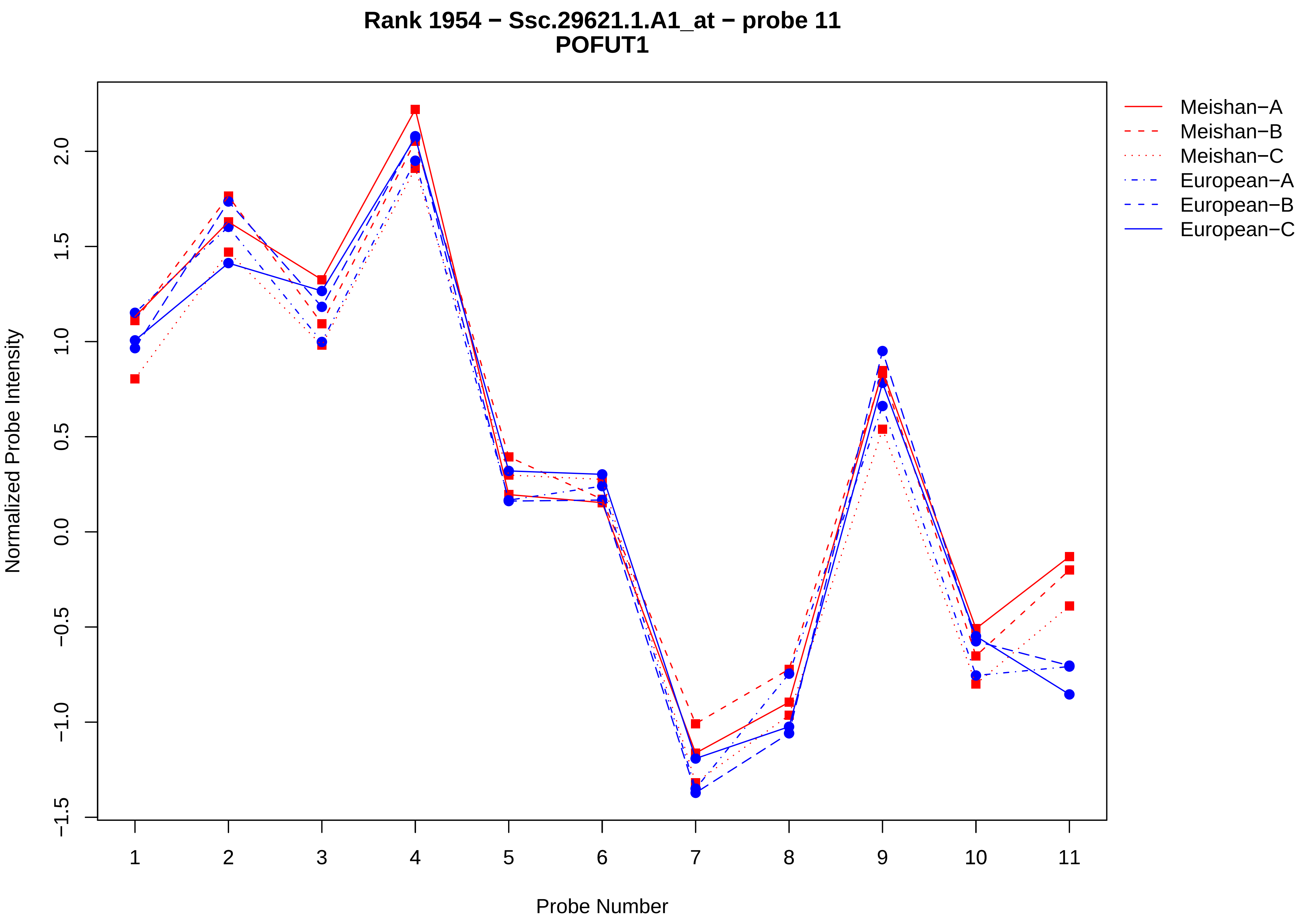

Rank 1954 − Ssc.29621.1.A1_at − probe 11
POFUT1
Normalized Probe Intensity
Probe Number
2.0
1.5
1.0
0.5
0.0
−0.5
−1.0
−1.5
1
2
3
4
5
6
7
8
9
10
11
Meishan−A
Meishan−B
Meishan−C
European−A
European−B
European−C

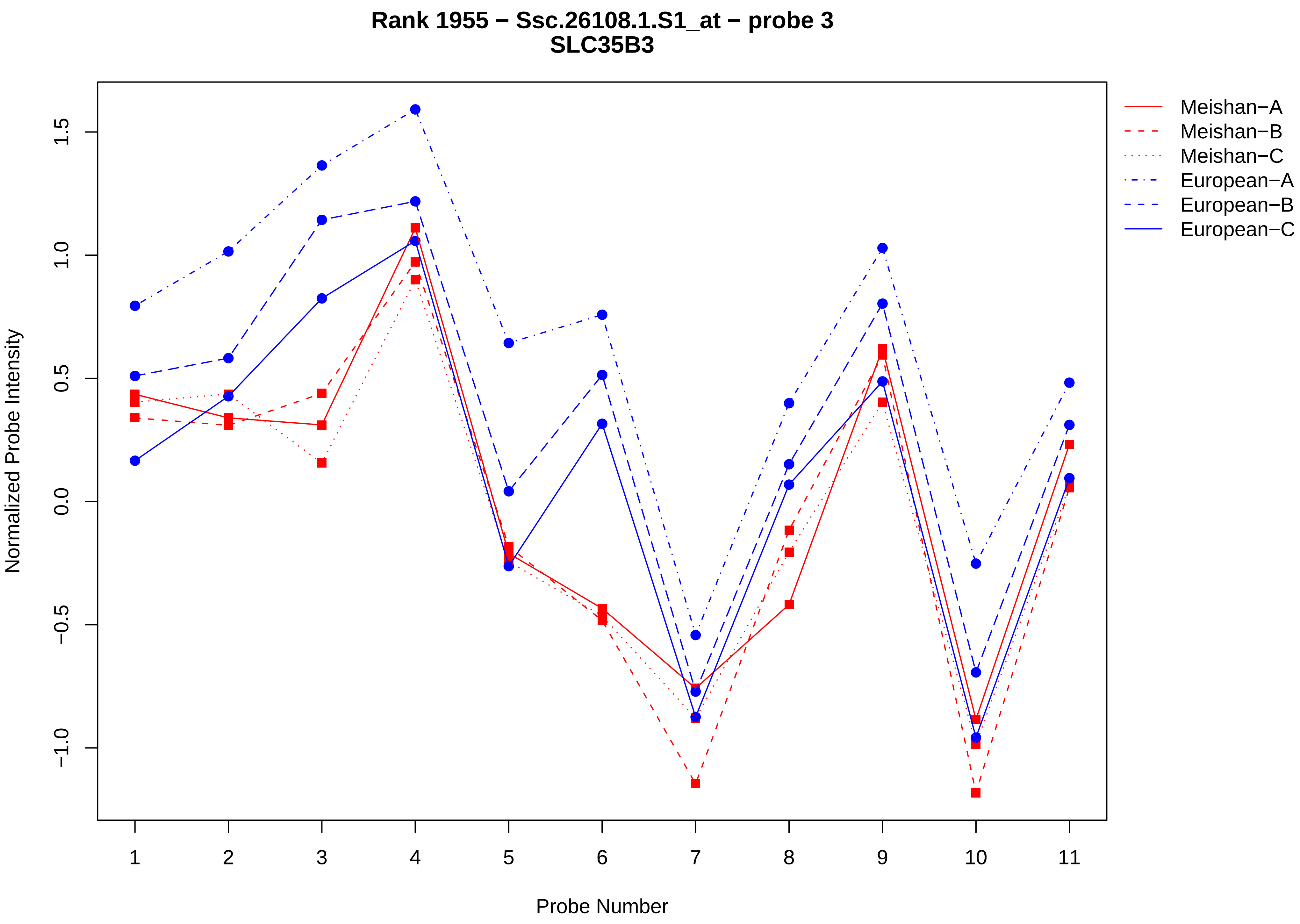

Rank 1955 − Ssc.26108.1.S1_at − probe 3
SLC35B3
Normalized Probe Intensity
Probe Number
1.5
1.0
0.5
0.0
−0.5
−1.0
1
2
3
4
5
6
7
8
9
10
11
Meishan−A
Meishan−B
Meishan−C
European−A
European−B
European−C

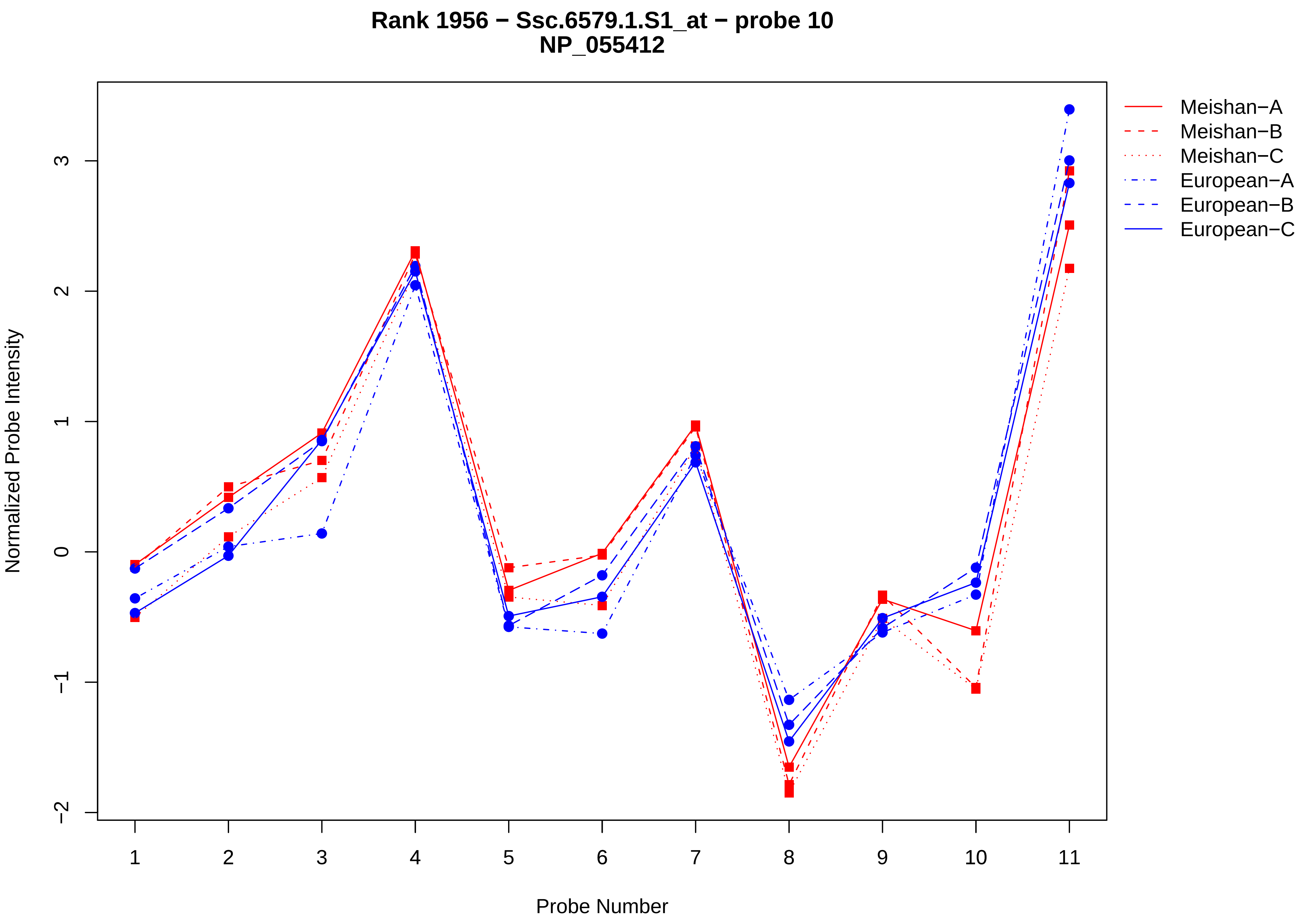

Rank 1956 − Ssc.6579.1.S1_at − probe 10
NP_055412
Normalized Probe Intensity
Probe Number
−2
−1
0
1
2
3
1
2
3
4
5
6
7
8
9
10
11
Meishan−A
Meishan−B
Meishan−C
European−A
European−B
European−C

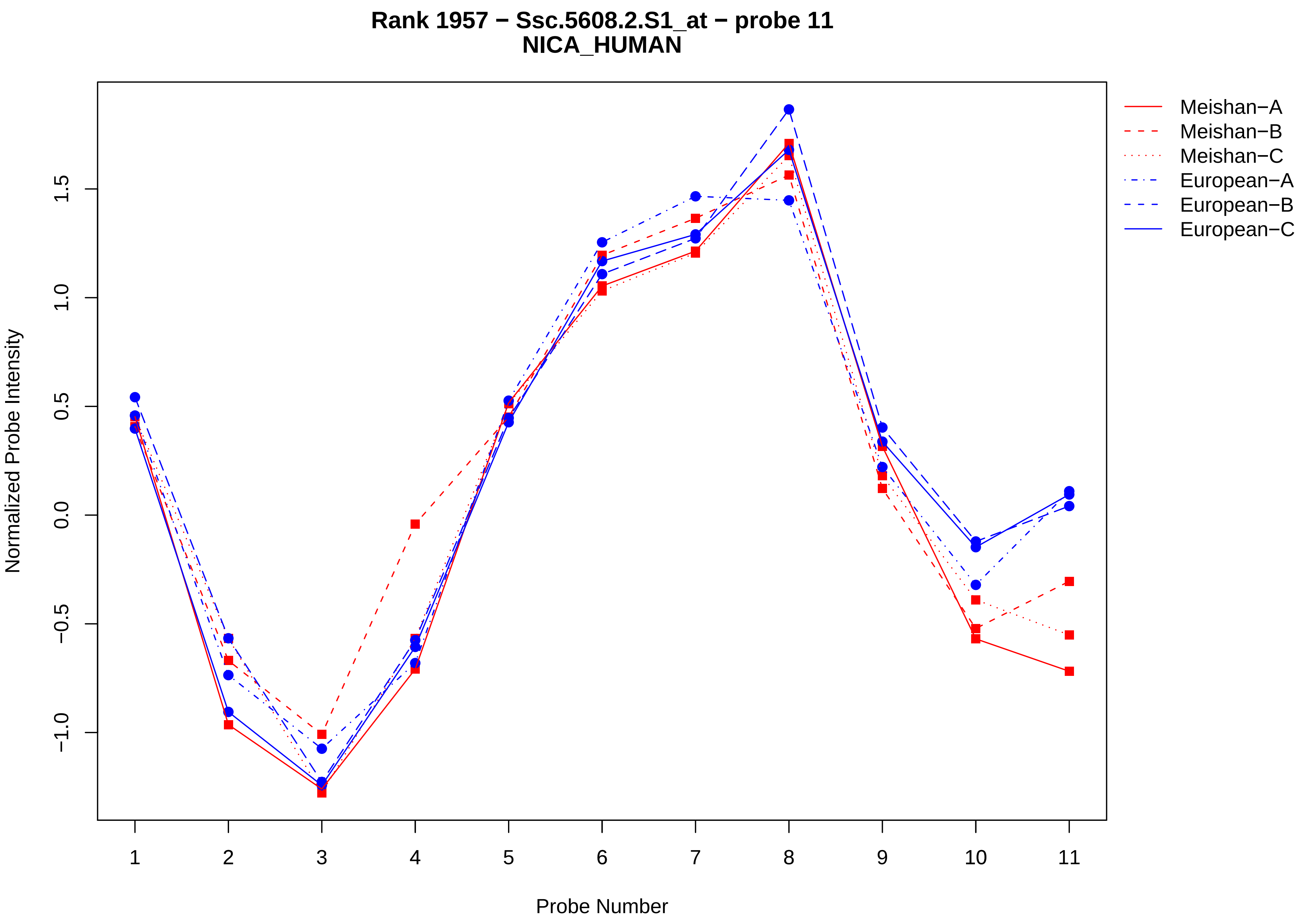

Rank 1957 − Ssc.5608.2.S1_at − probe 11
NICA_HUMAN
Normalized Probe Intensity
Probe Number
−1.0
−0.5
0.0
0.5
1.0
1.5
1
2
3
4
5
6
7
8
9
10
11
Meishan−A
Meishan−B
Meishan−C
European−A
European−B
European−C

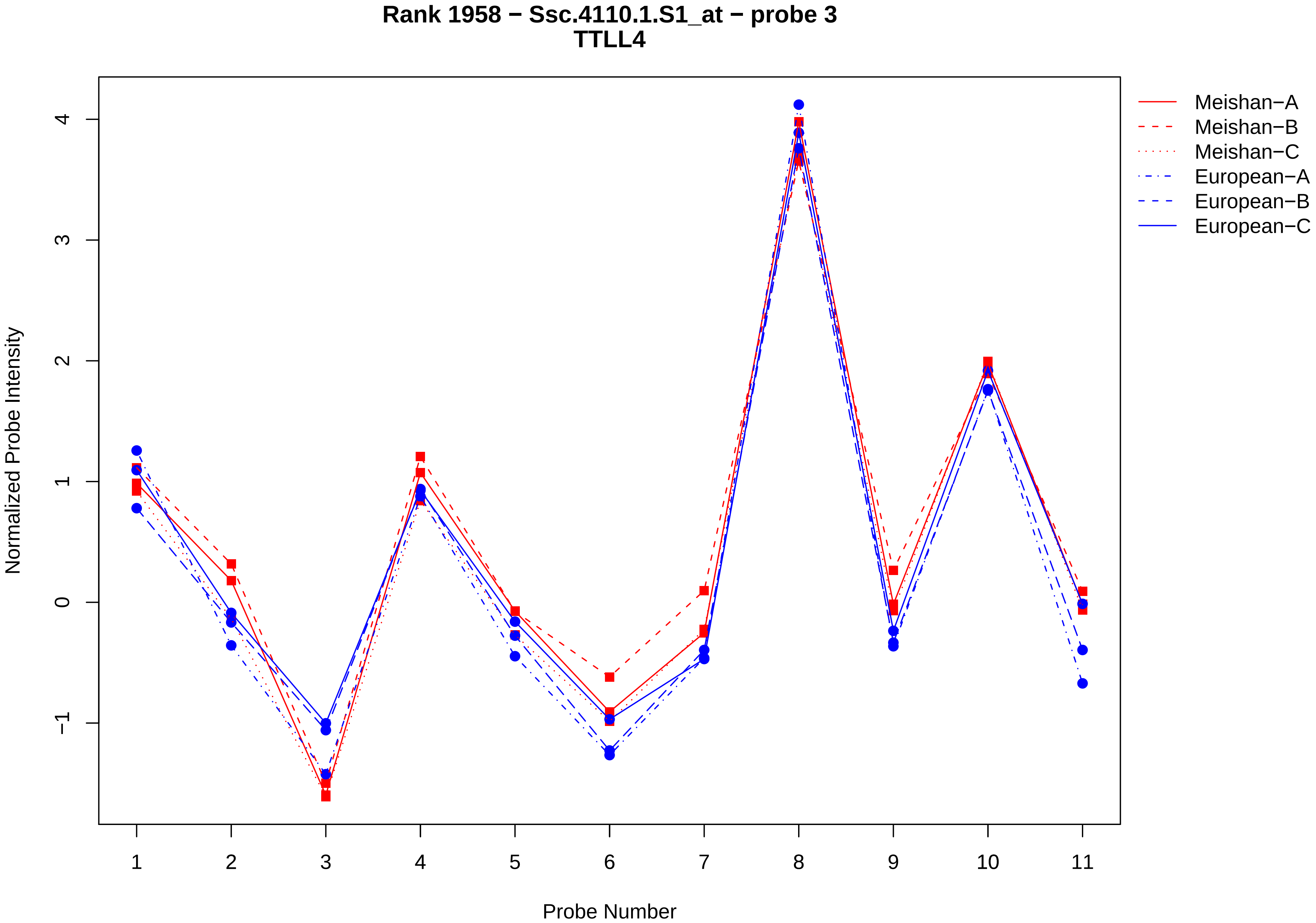

Rank 1958 − Ssc.4110.1.S1_at − probe 3
TTLL4
Normalized Probe Intensity
Probe Number
−1
0
1
2
3
4
1
2
3
4
5
6
7
8
9
10
11
Meishan−A
Meishan−B
Meishan−C
European−A
European−B
European−C

Rank 1959 − Ssc.4843.1.A1_at − probe 1
BBS2

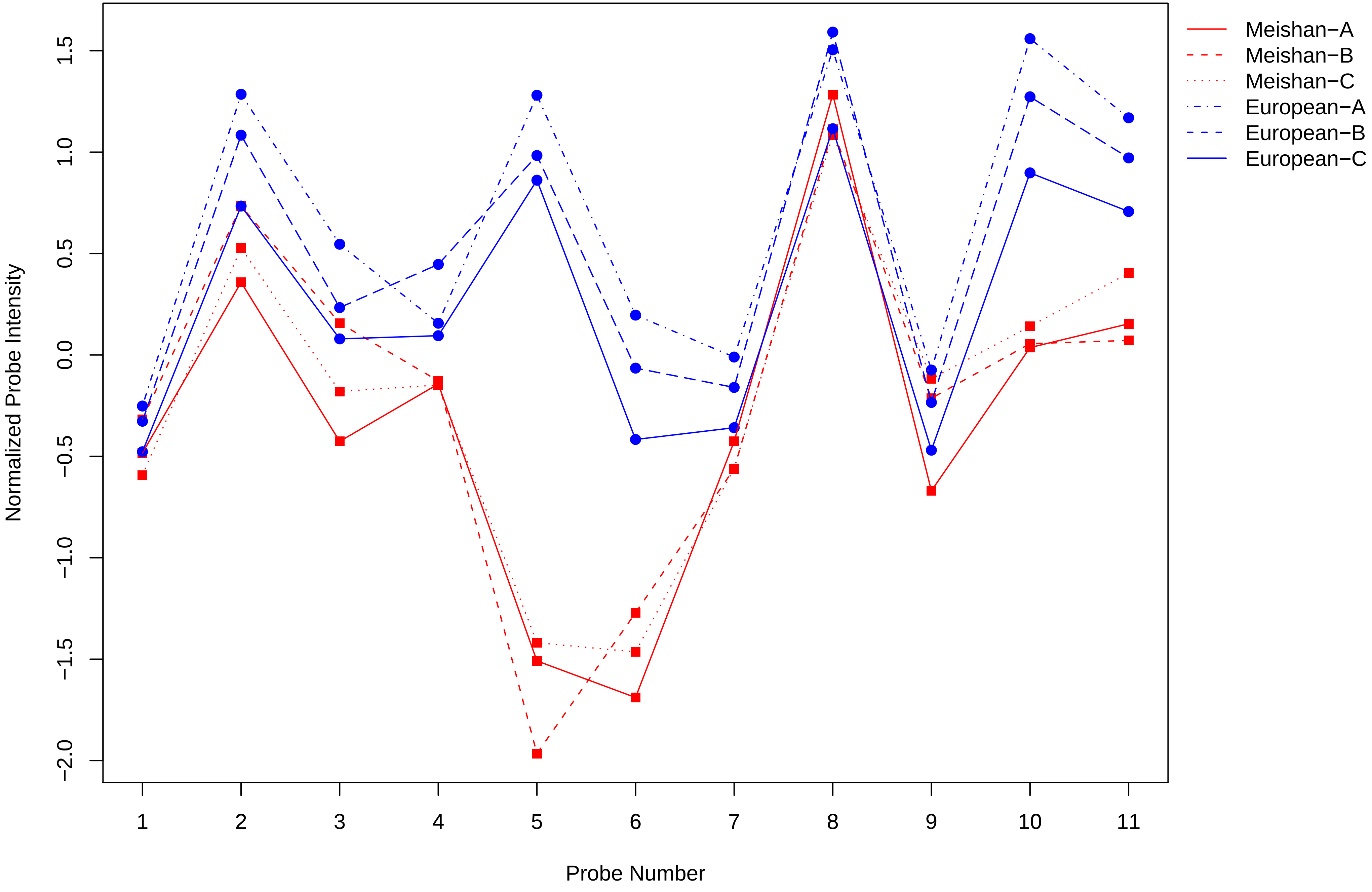

Rank 1960 − Ssc.3079.1.S1_at − probe 11
PHLDA3

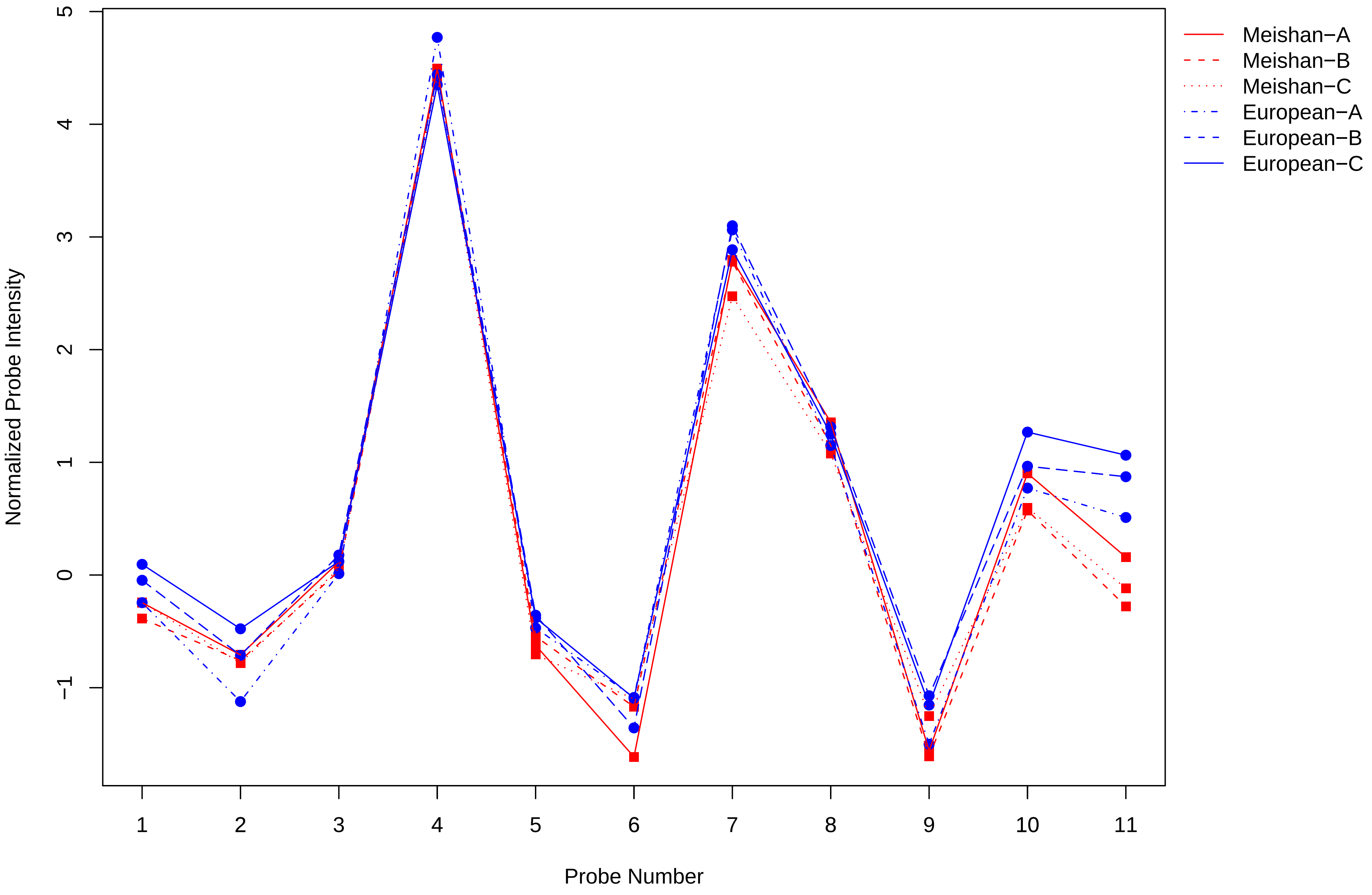

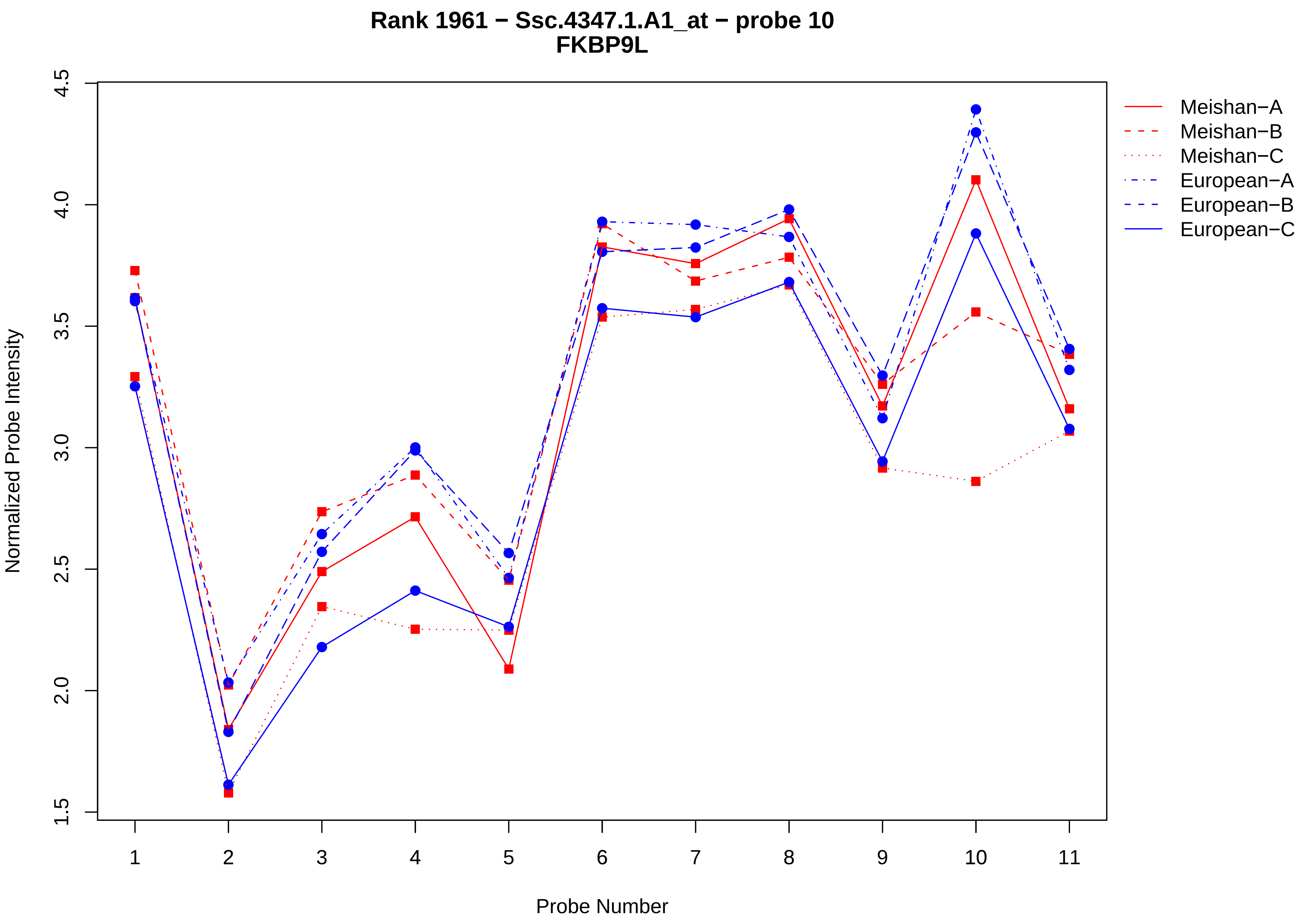
Rank 1961 − Ssc.4347.1.A1_at − probe 10
FKBP9L
Normalized Probe Intensity
Probe Number
1.5
2.0
2.5
3.0
3.5
4.0
4.5
1
2
3
4
5
6
7
8
9
10
11
Meishan−A
Meishan−B
Meishan−C
European−A
European−B
European−C

Rank 1962 − Ssc.12809.4.S1_a_at − probe 8
NP_060177

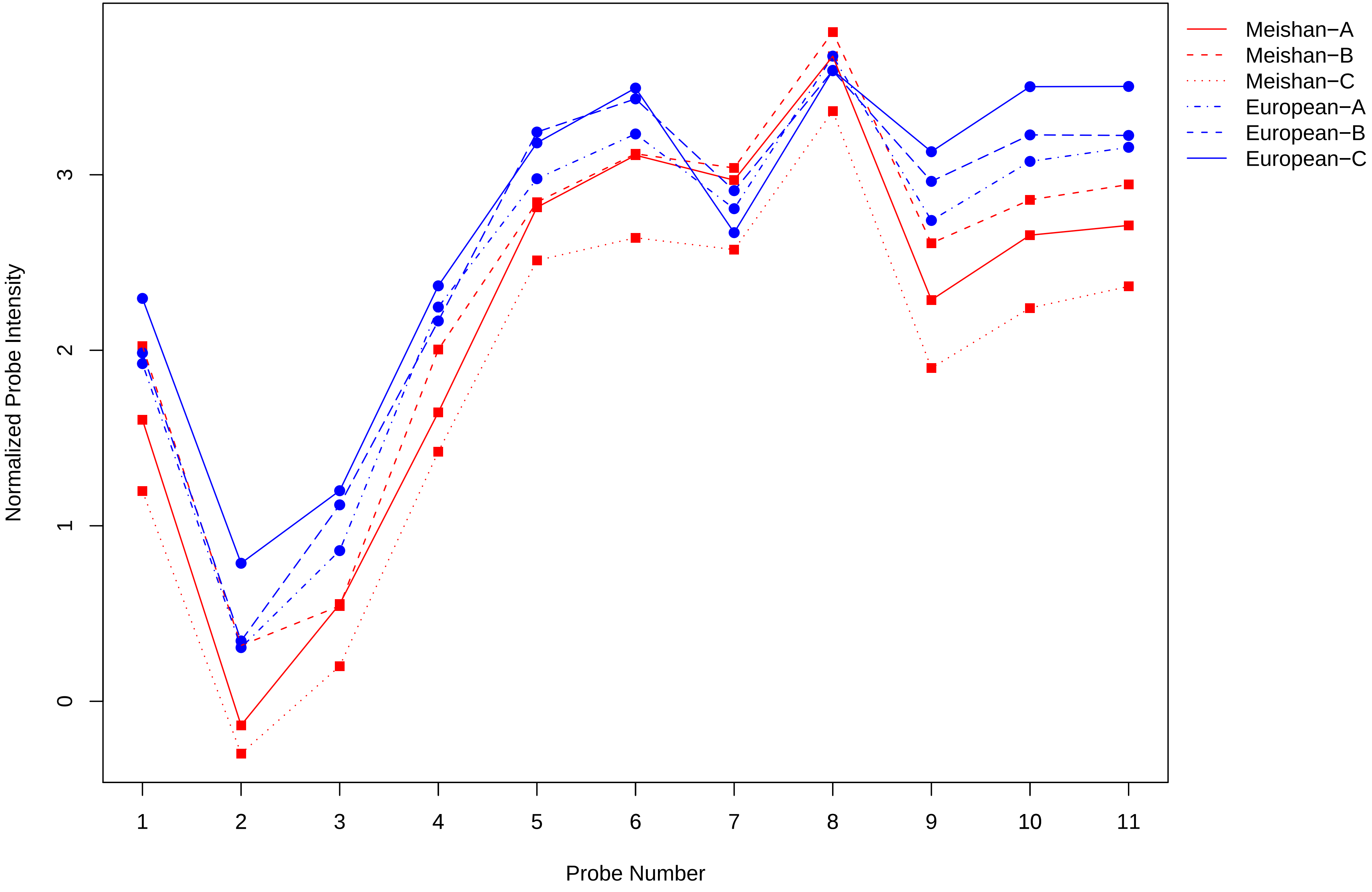

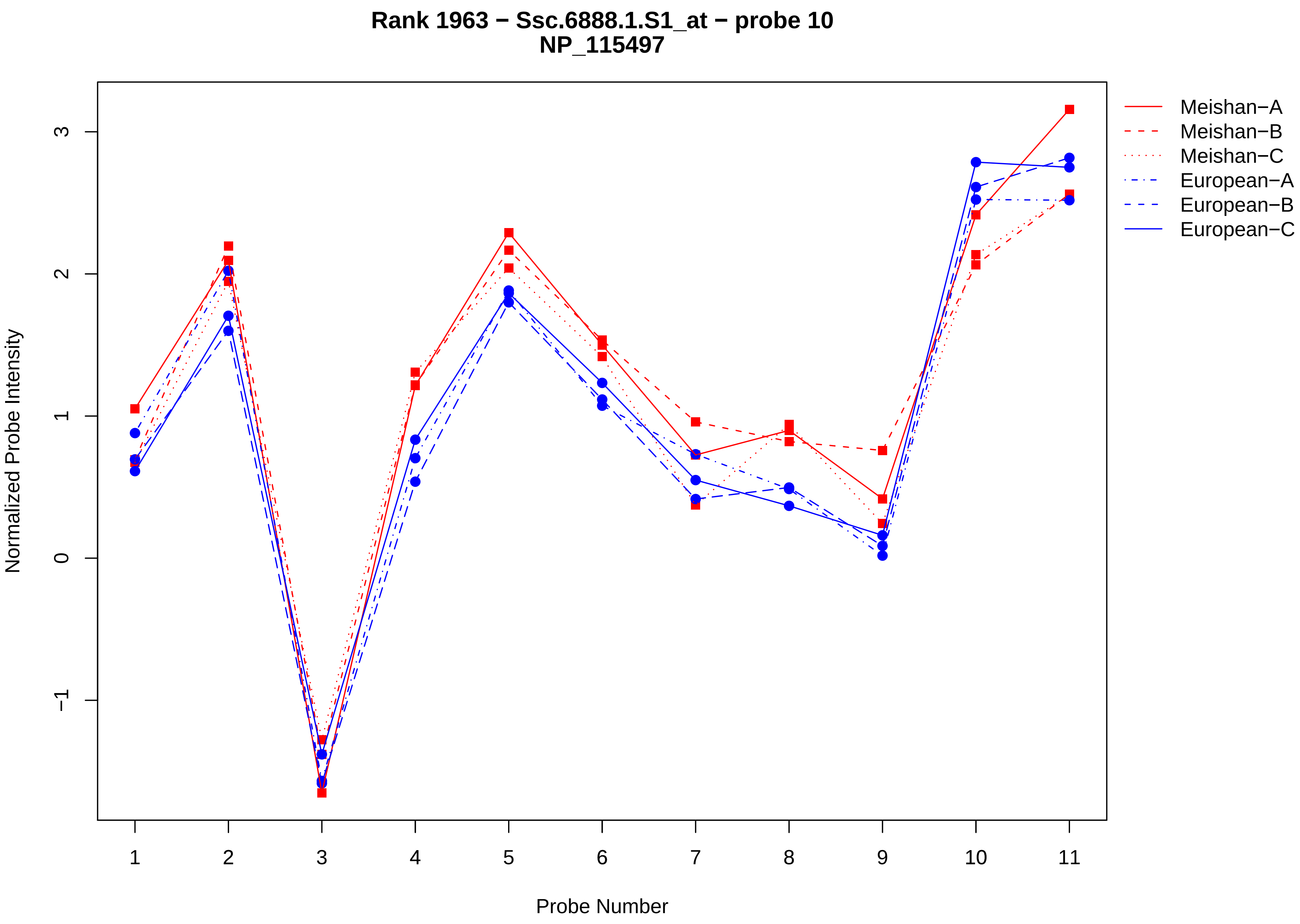

Rank 1963 − Ssc.6888.1.S1_at − probe 10
NP_115497
Normalized Probe Intensity
Probe Number
−1
0
1
2
3
1
2
3
4
5
6
7
8
9
10
11
Meishan−A
Meishan−B
Meishan−C
European−A
European−B
European−C

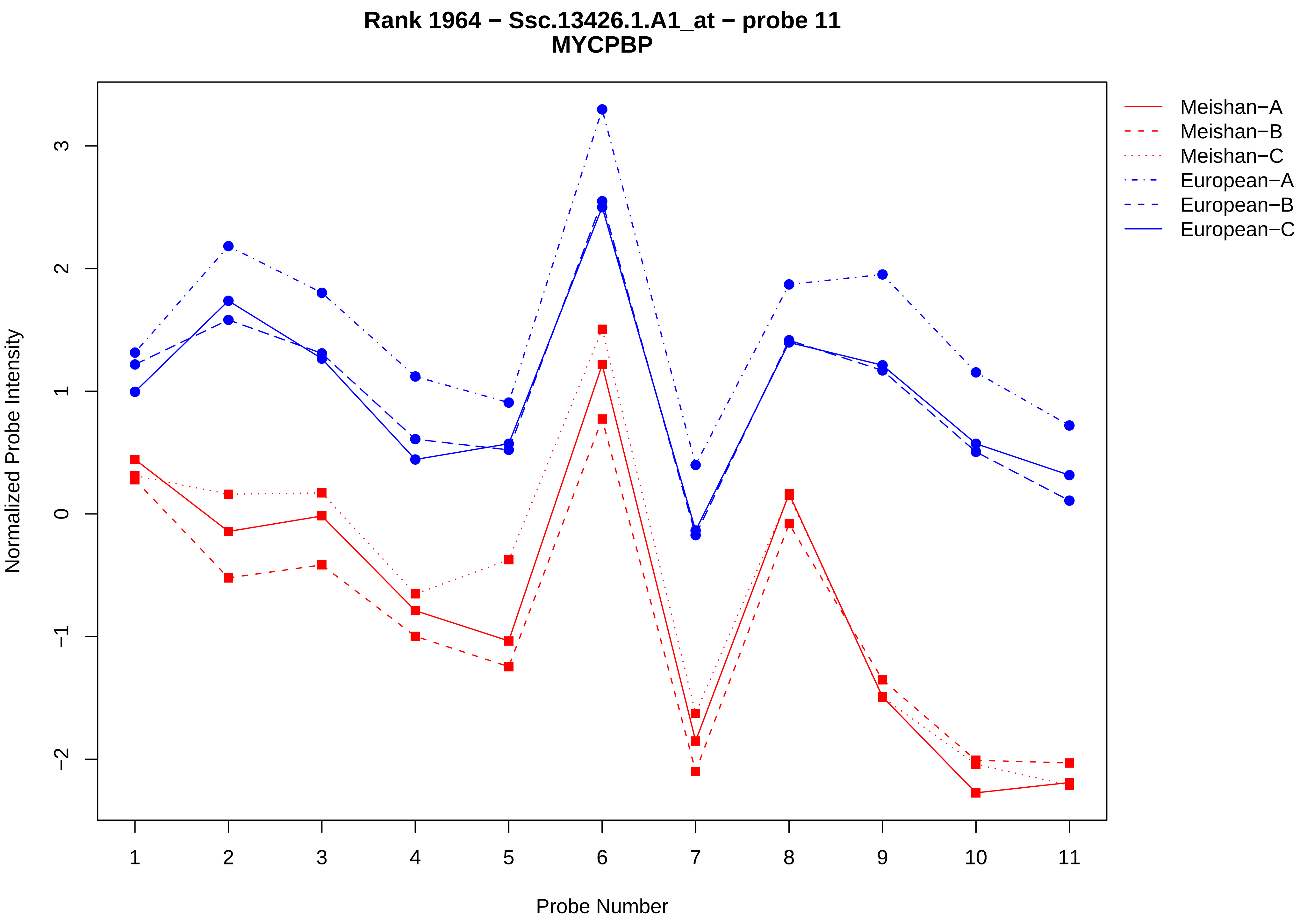

Rank 1964 − Ssc.13426.1.A1_at − probe 11
MYCPBP
Normalized Probe Intensity
Probe Number
Meishan−A
Meishan−B
Meishan−C
European−A
European−B
European−C
3
2
1
0
−1
−2
1
2
3
4
5
6
7
8
9
10
11

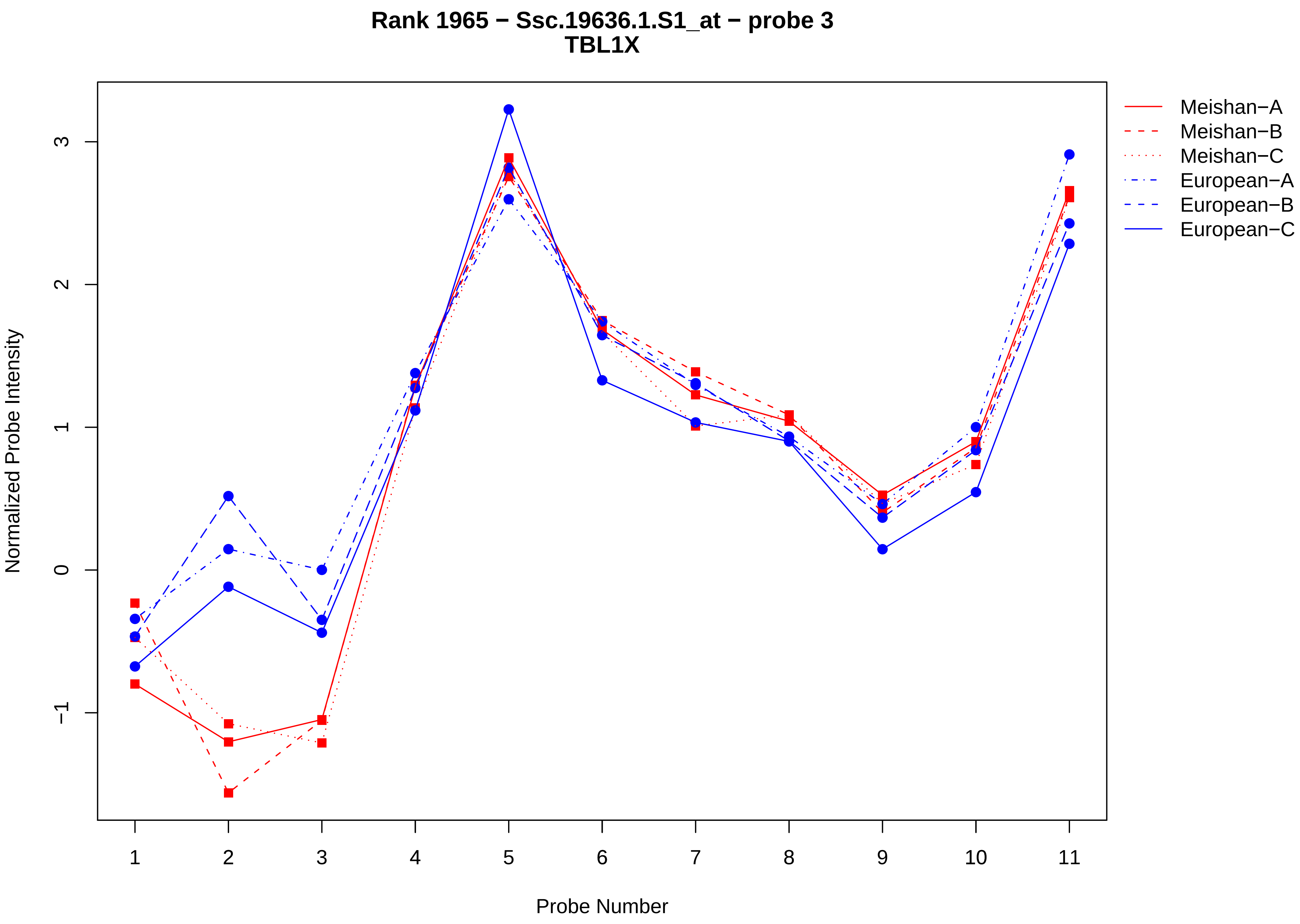
Rank 1965 − Ssc.19636.1.S1_at − probe 3
TBL1X
Normalized Probe Intensity
Probe Number
Meishan−A
Meishan−B
Meishan−C
European−A
European−B
European−C
−1
0
1
2
3
1
2
3
4
5
6
7
8
9
10
11

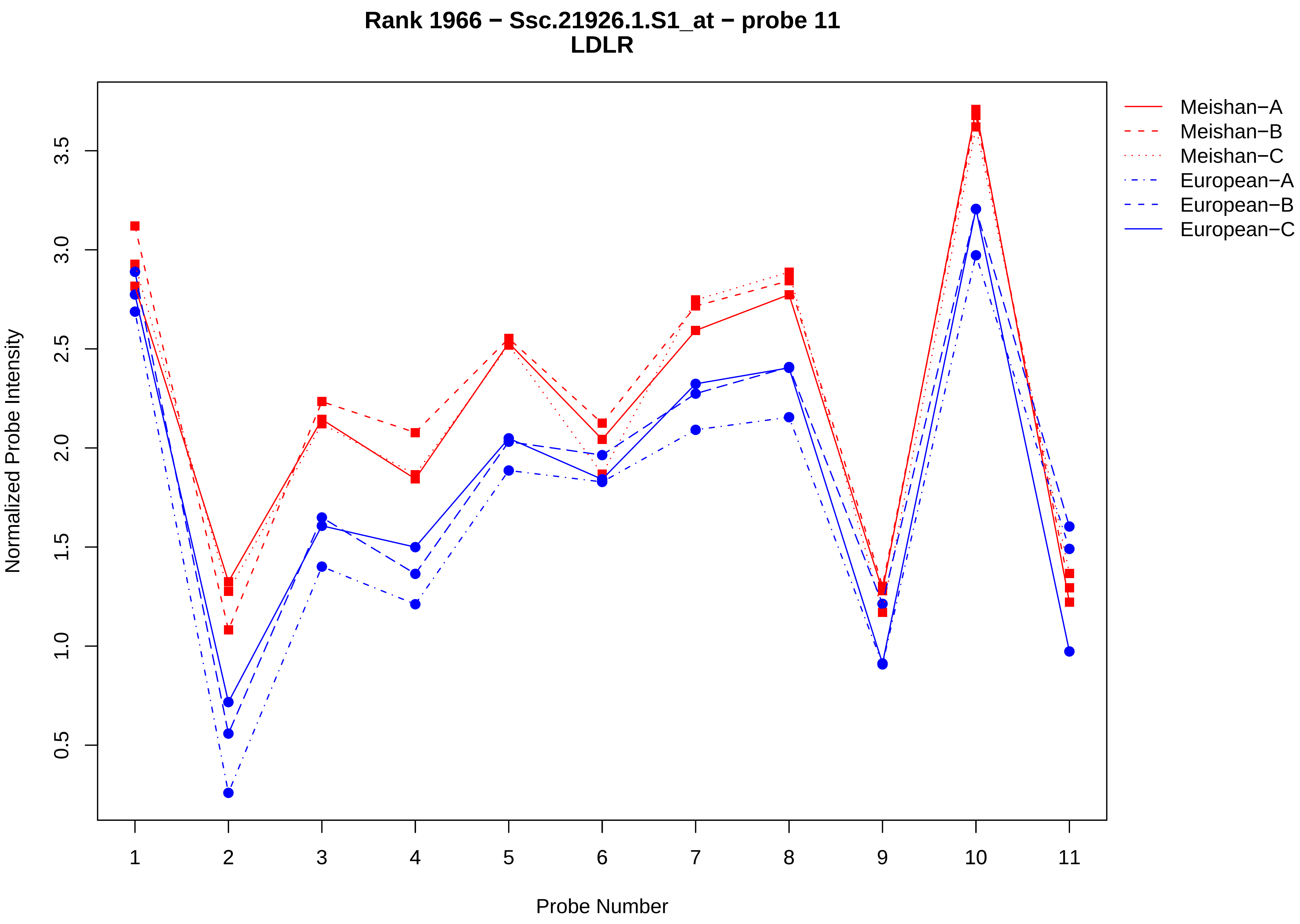

Rank 1966 − Ssc.21926.1.S1_at − probe 11
LDLR
Normalized Probe Intensity
Probe Number
0.5
1.0
1.5
2.0
2.5
3.0
3.5
1
2
3
4
5
6
7
8
9
10
11
Meishan−A
Meishan−B
Meishan−C
European−A
European−B
European−C

Rank 1967 − Ssc.29668.1.A1_at − probe 5
ZNF184

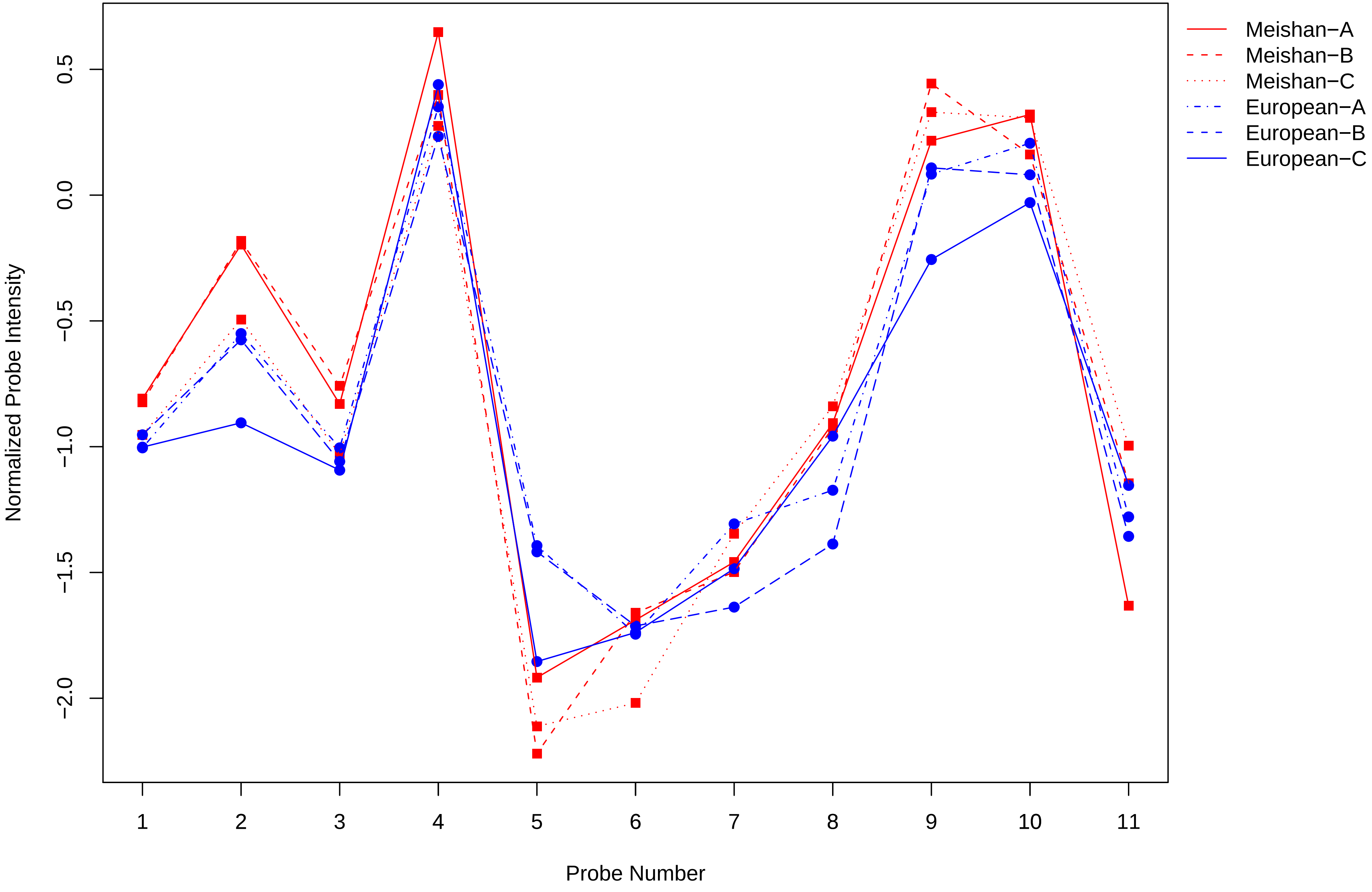

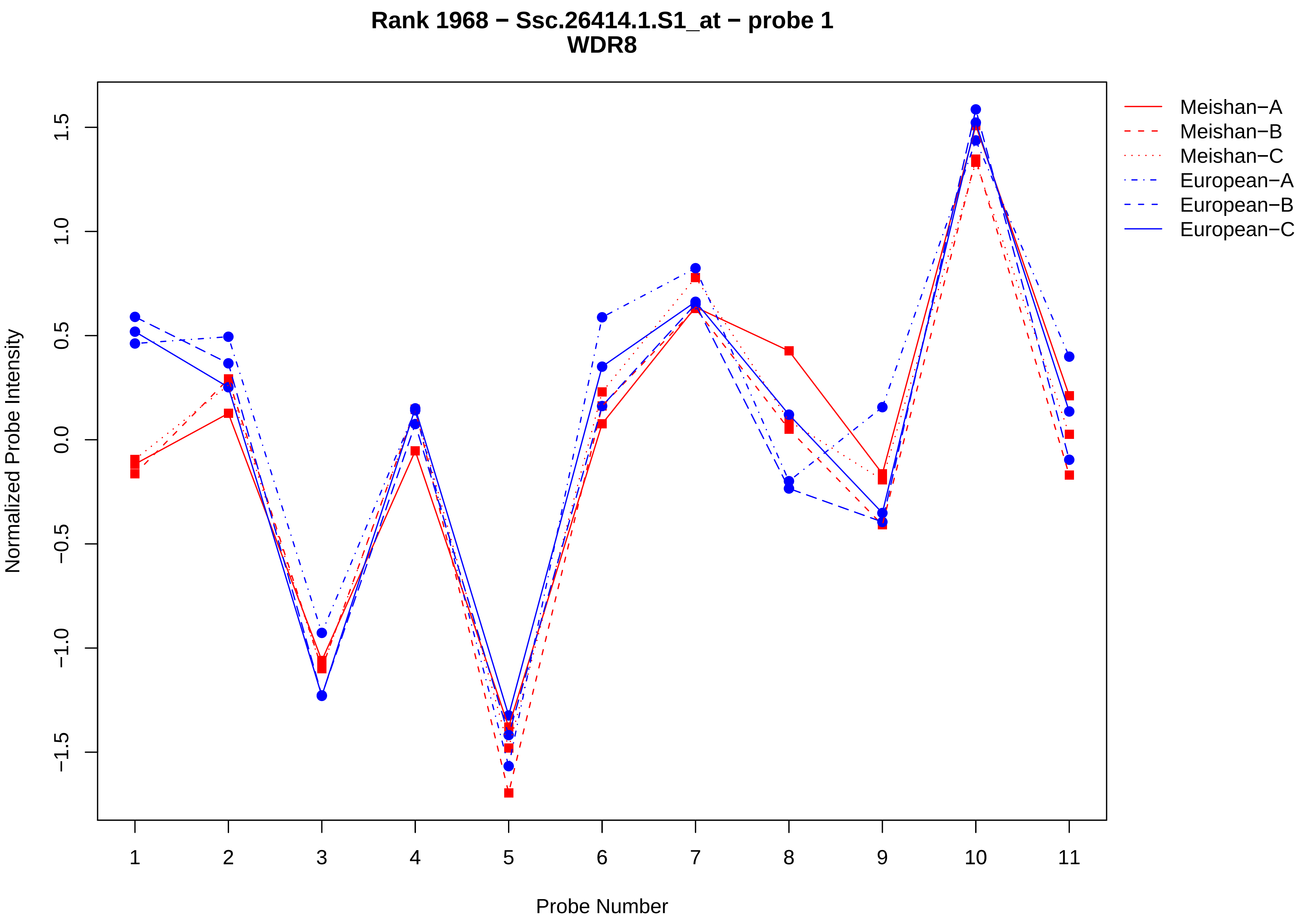

Rank 1968 − Ssc.26414.1.S1_at − probe 1
WDR8
Normalized Probe Intensity
Probe Number
1.5
1.0
0.5
0.0
−0.5
−1.0
−1.5
1
2
3
4
5
6
7
8
9
10
11
Meishan−A
Meishan−B
Meishan−C
European−A
European−B
European−C

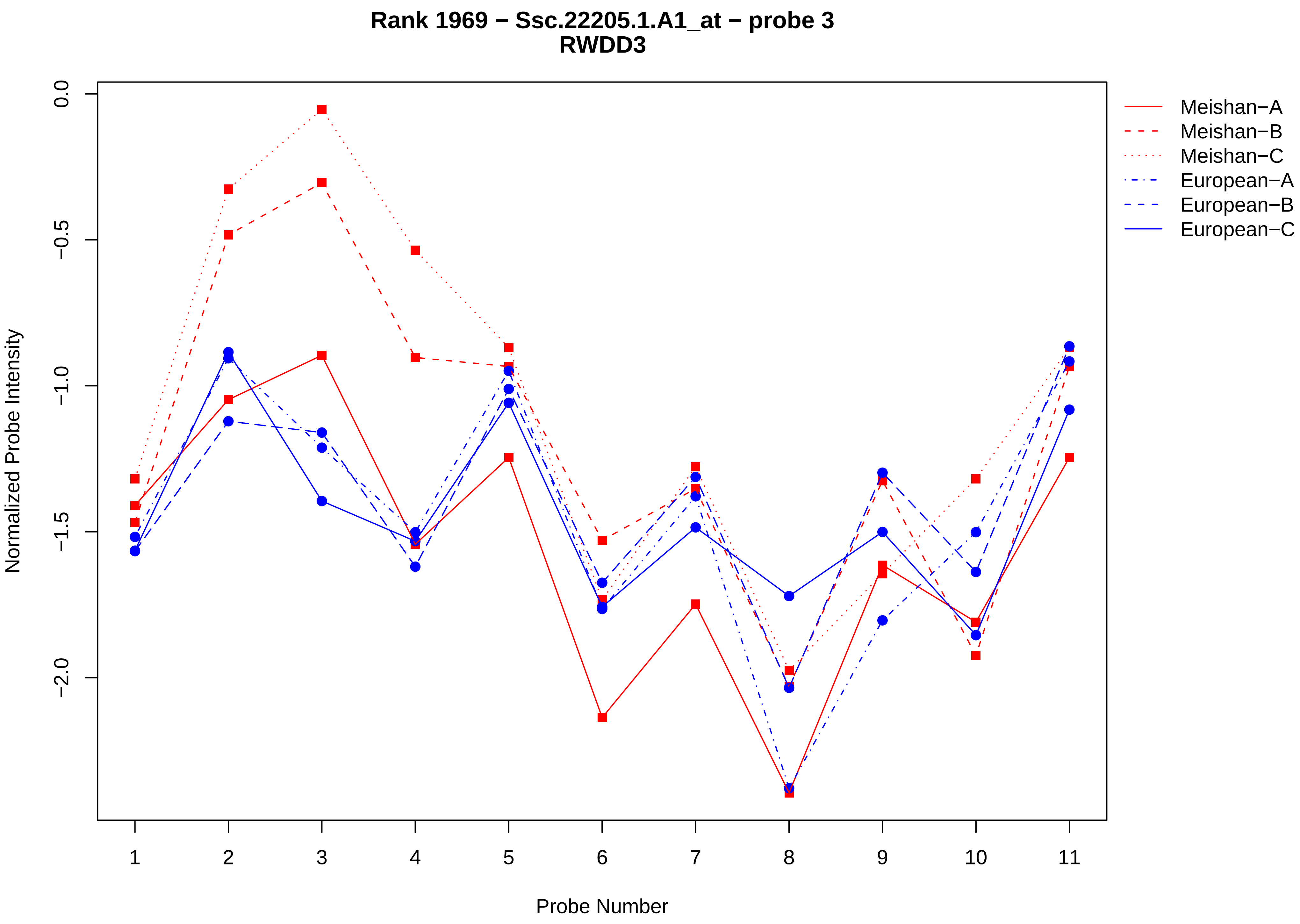

Rank 1969 − Ssc.22205.1.A1_at − probe 3
RWDD3
Normalized Probe Intensity
0.0
−0.5
−1.0
−1.5
−2.0
Probe Number
1
2
3
4
5
6
7
8
9
10
11
Meishan−A
Meishan−B
Meishan−C
European−A
European−B
European−C

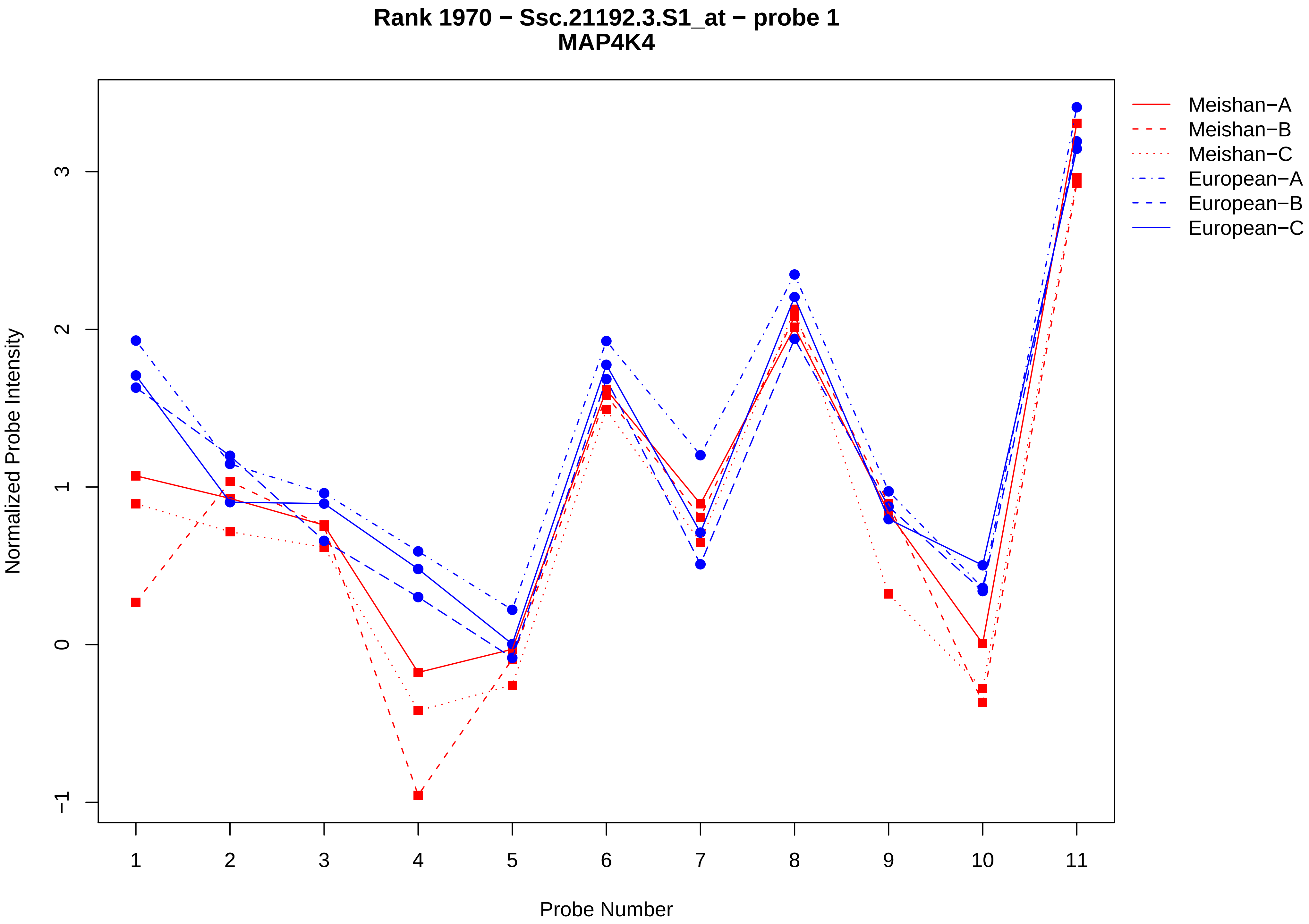

Rank 1970 − Ssc.21192.3.S1_at − probe 1
MAP4K4
Normalized Probe Intensity
Probe Number
−1
0
1
2
3
1
2
3
4
5
6
7
8
9
10
11
Meishan−A
Meishan−B
Meishan−C
European−A
European−B
European−C

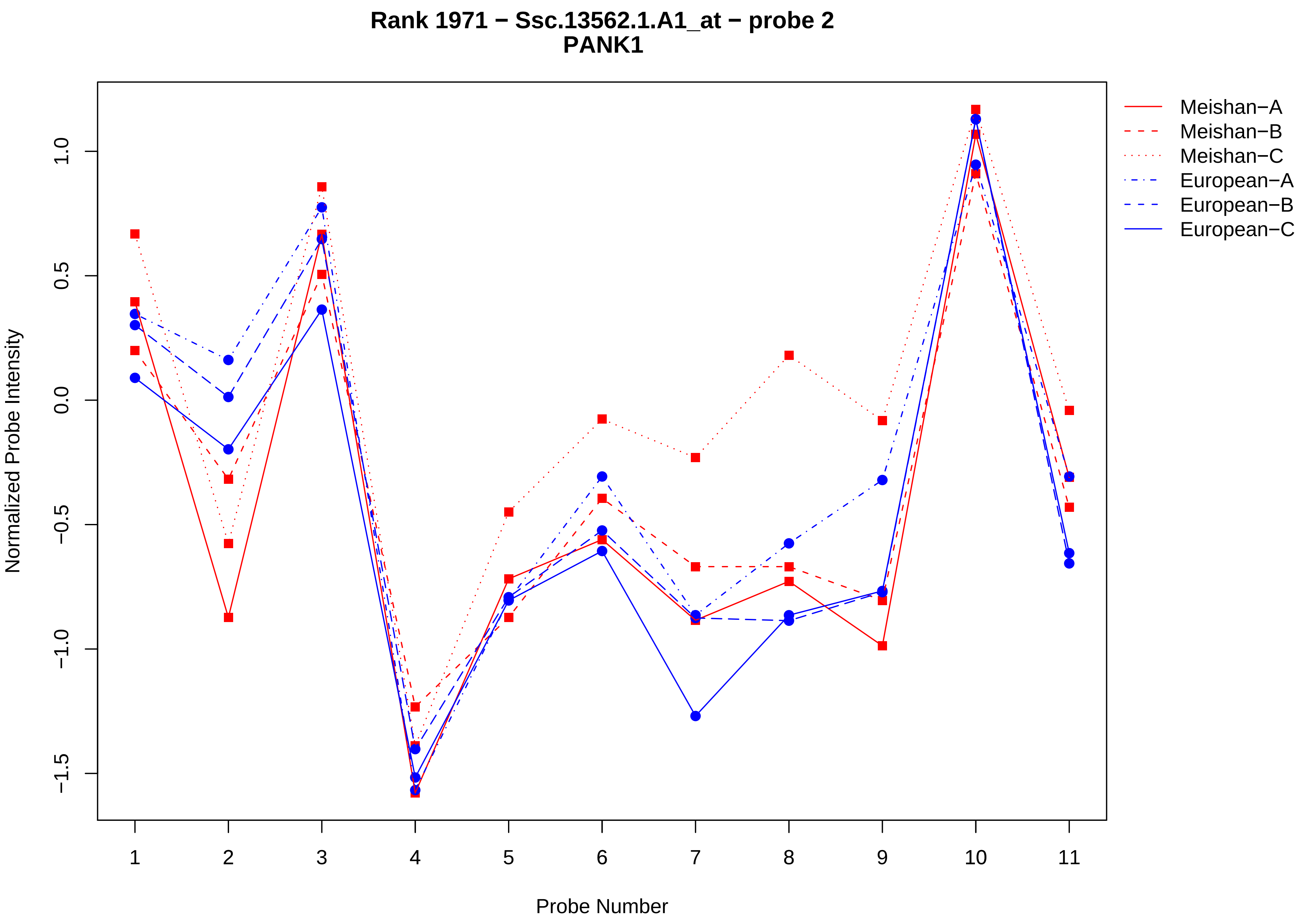

Rank 1971 − Ssc.13562.1.A1_at − probe 2
PANK1
Normalized Probe Intensity
Probe Number
Meishan−A
Meishan−B
Meishan−C
European−A
European−B
European−C
−1.5
−1.0
−0.5
0.0
0.5
1.0
1
2
3
4
5
6
7
8
9
10
11

Rank 1972 − Ssc.23128.1.S1_at − probe 7
Q6UWP3

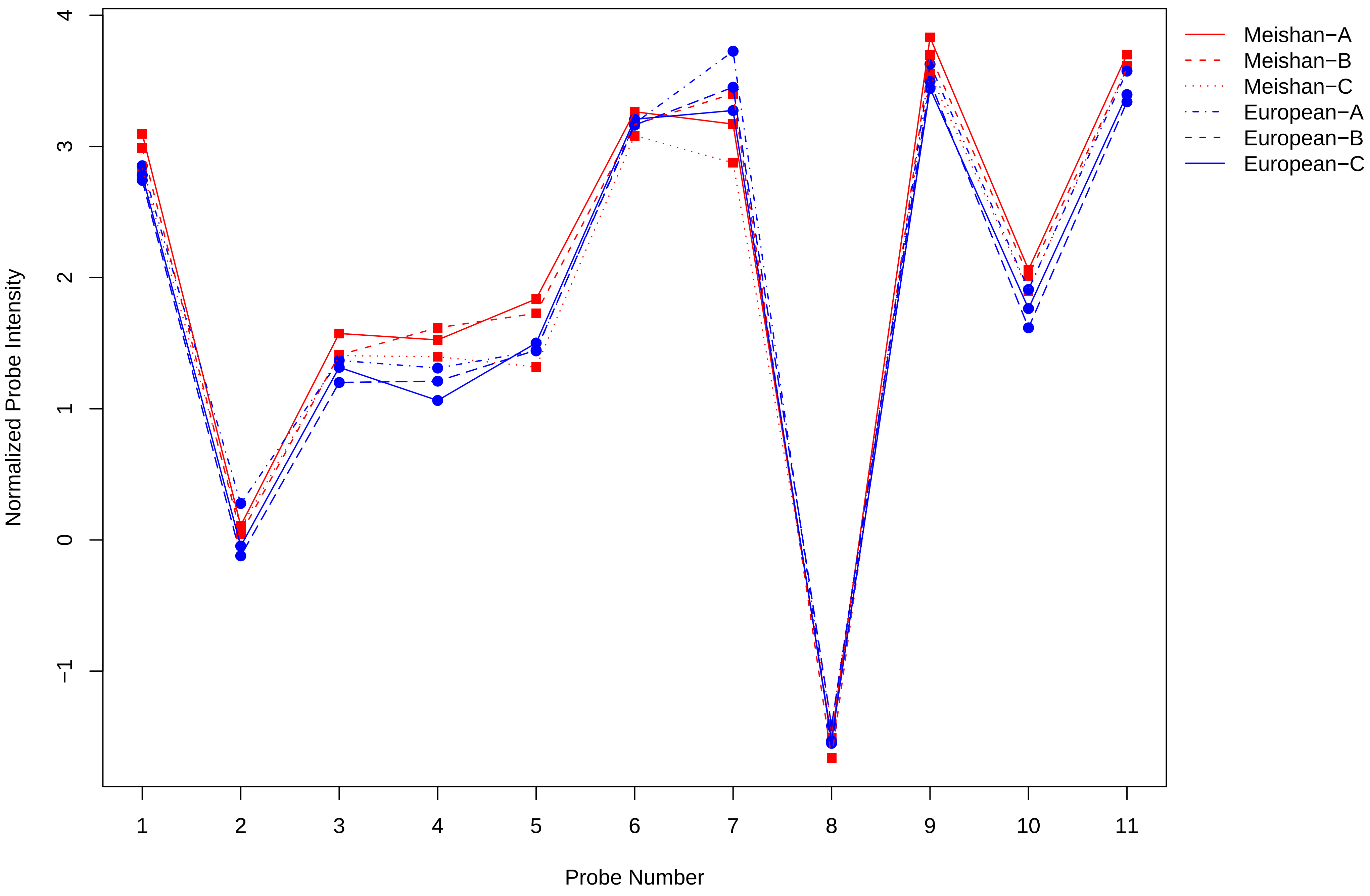

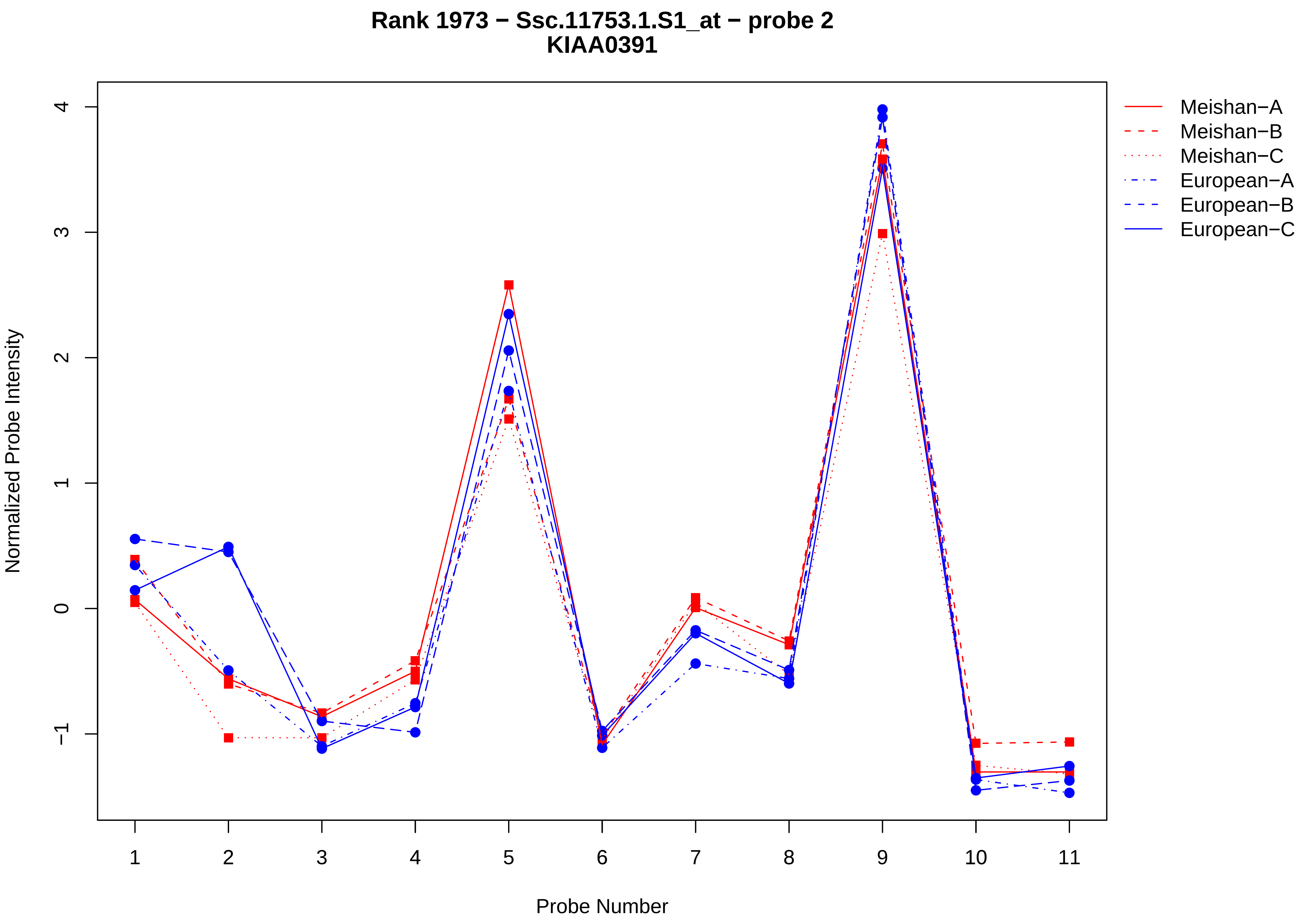
Rank 1973 − Ssc.11753.1.S1_at − probe 2
KIAA0391
Normalized Probe Intensity
Probe Number
−1
0
1
2
3
4
1
2
3
4
5
6
7
8
9
10
11
Meishan−A
Meishan−B
Meishan−C
European−A
European−B
European−C

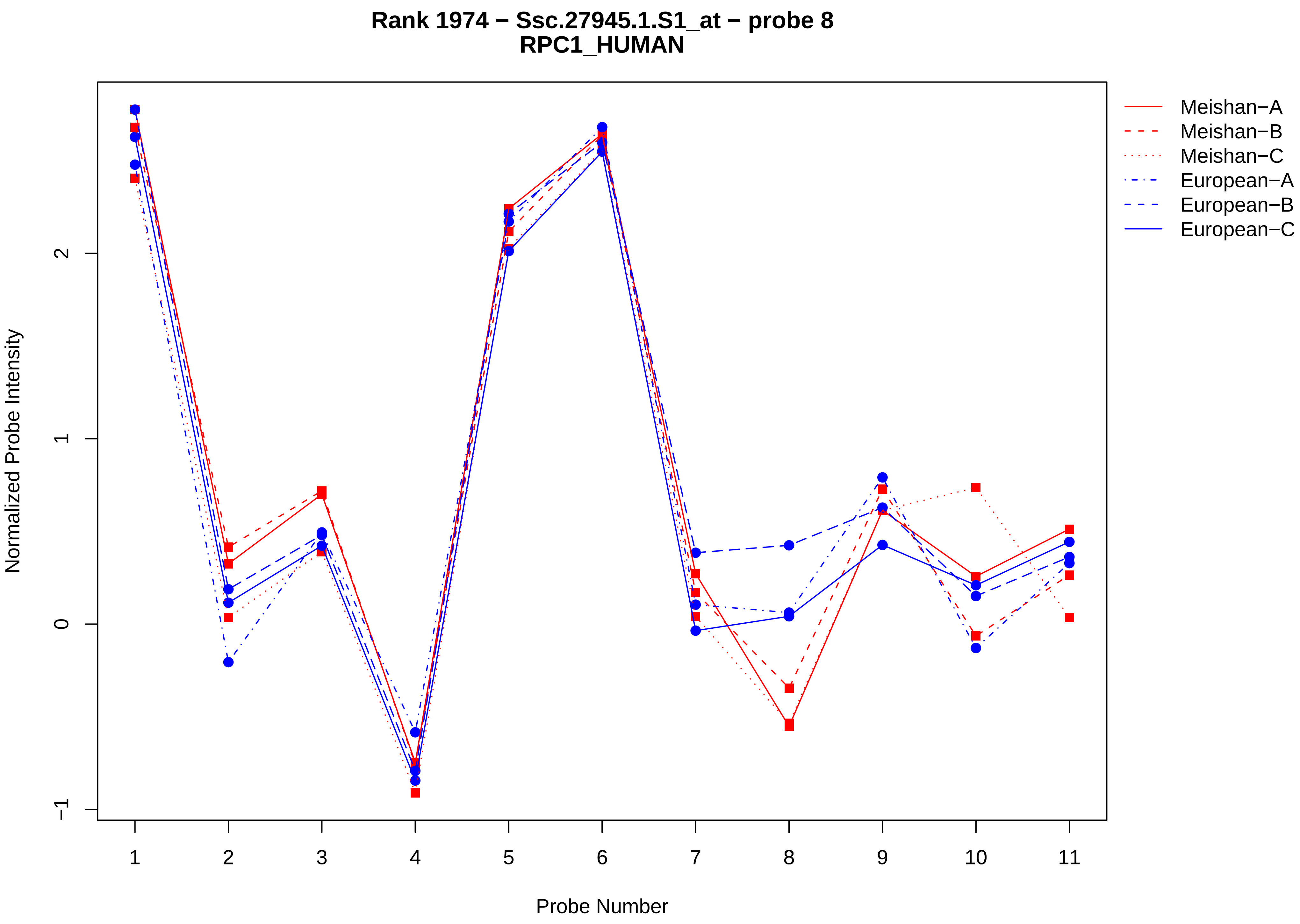
Rank 1974 − Ssc.27945.1.S1_at − probe 8
RPC1_HUMAN
Normalized Probe Intensity
Probe Number
−1
0
1
2
1
2
3
4
5
6
7
8
9
10
11
Meishan−A
Meishan−B
Meishan−C
European−A
European−B
European−C

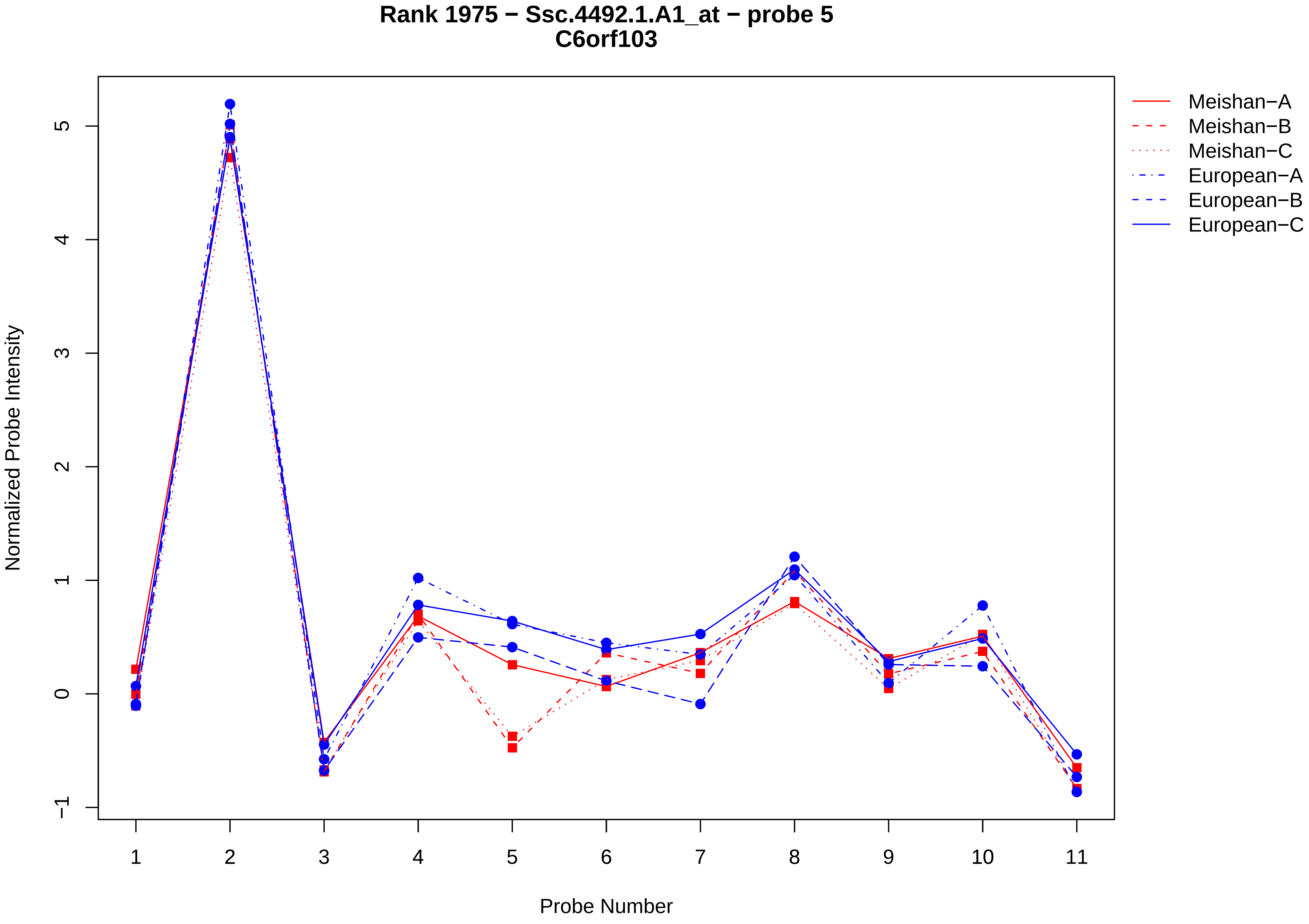

Rank 1975 − Ssc.4492.1.A1_at − probe 5
C6orf103
Normalized Probe Intensity
Probe Number
Meishan−A
Meishan−B
Meishan−C
European−A
European−B
European−C
−1
0
1
2
3
4
5
1
2
3
4
5
6
7
8
9
10
11

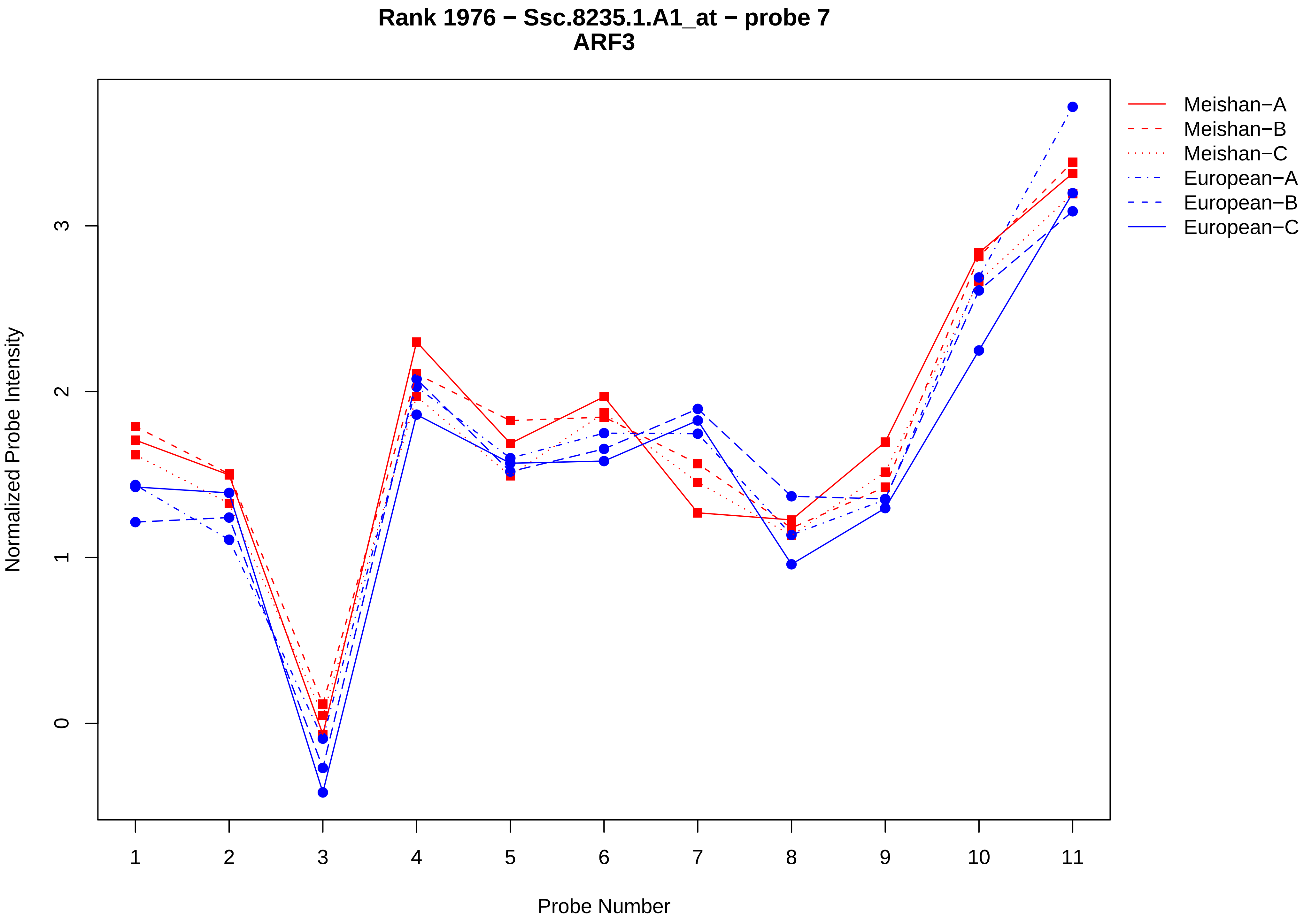

Rank 1976 − Ssc.8235.1.A1_at − probe 7
ARF3
Normalized Probe Intensity
Probe Number
0
1
2
3
1
2
3
4
5
6
7
8
9
10
11
Meishan−A
Meishan−B
Meishan−C
European−A
European−B
European−C

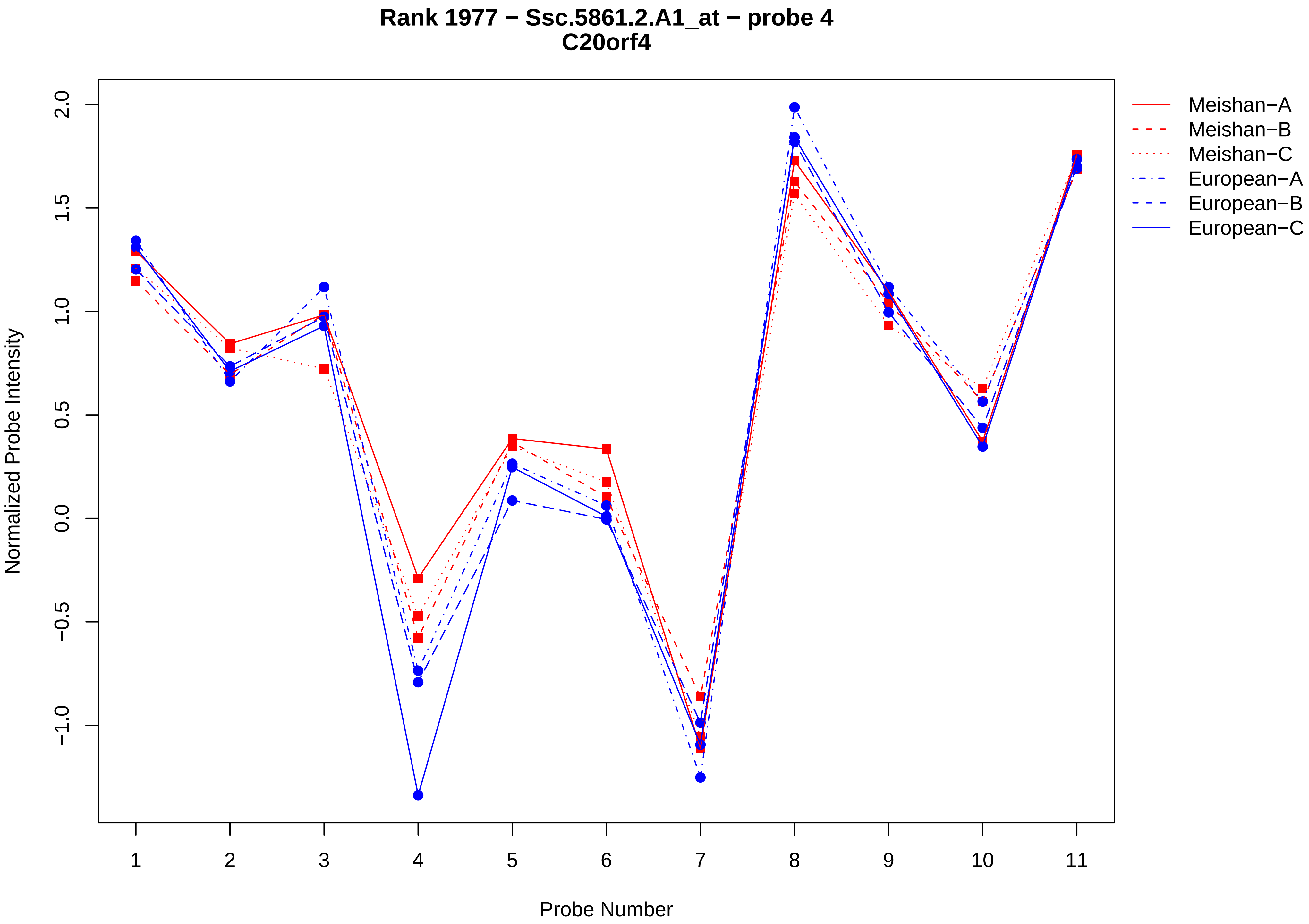

Rank 1977 − Ssc.5861.2.A1_at − probe 4
C20orf4
Normalized Probe Intensity
Probe Number
−1.0
−0.5
0.0
0.5
1.0
1.5
2.0
1
2
3
4
5
6
7
8
9
10
11
Meishan−A
Meishan−B
Meishan−C
European−A
European−B
European−C

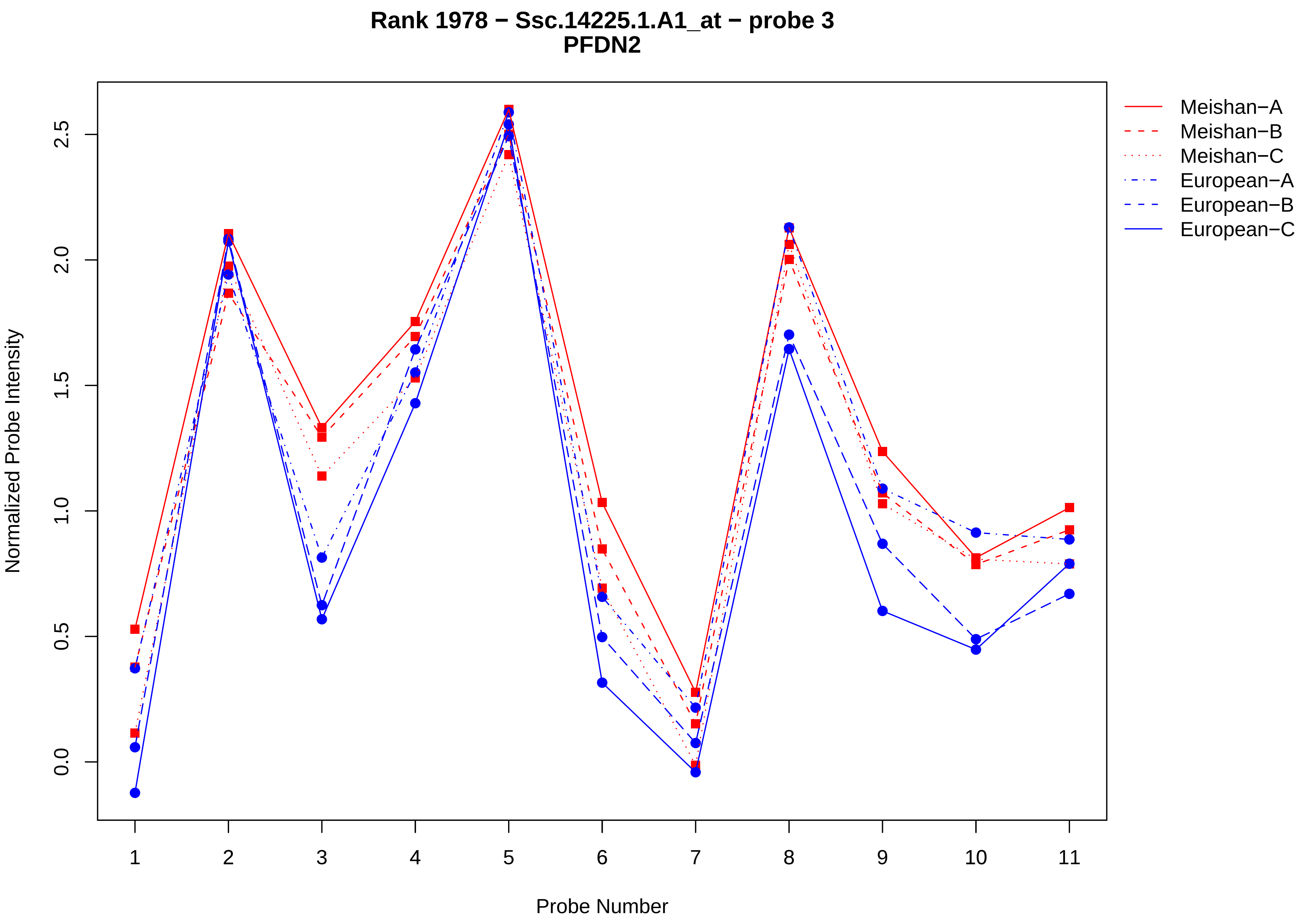

Rank 1978 − Ssc.14225.1.A1_at − probe 3
PFDN2
Normalized Probe Intensity
Probe Number
Meishan−A
Meishan−B
Meishan−C
European−A
European−B
European−C
0.0
0.5
1.0
1.5
2.0
2.5
1
2
3
4
5
6
7
8
9
10
11

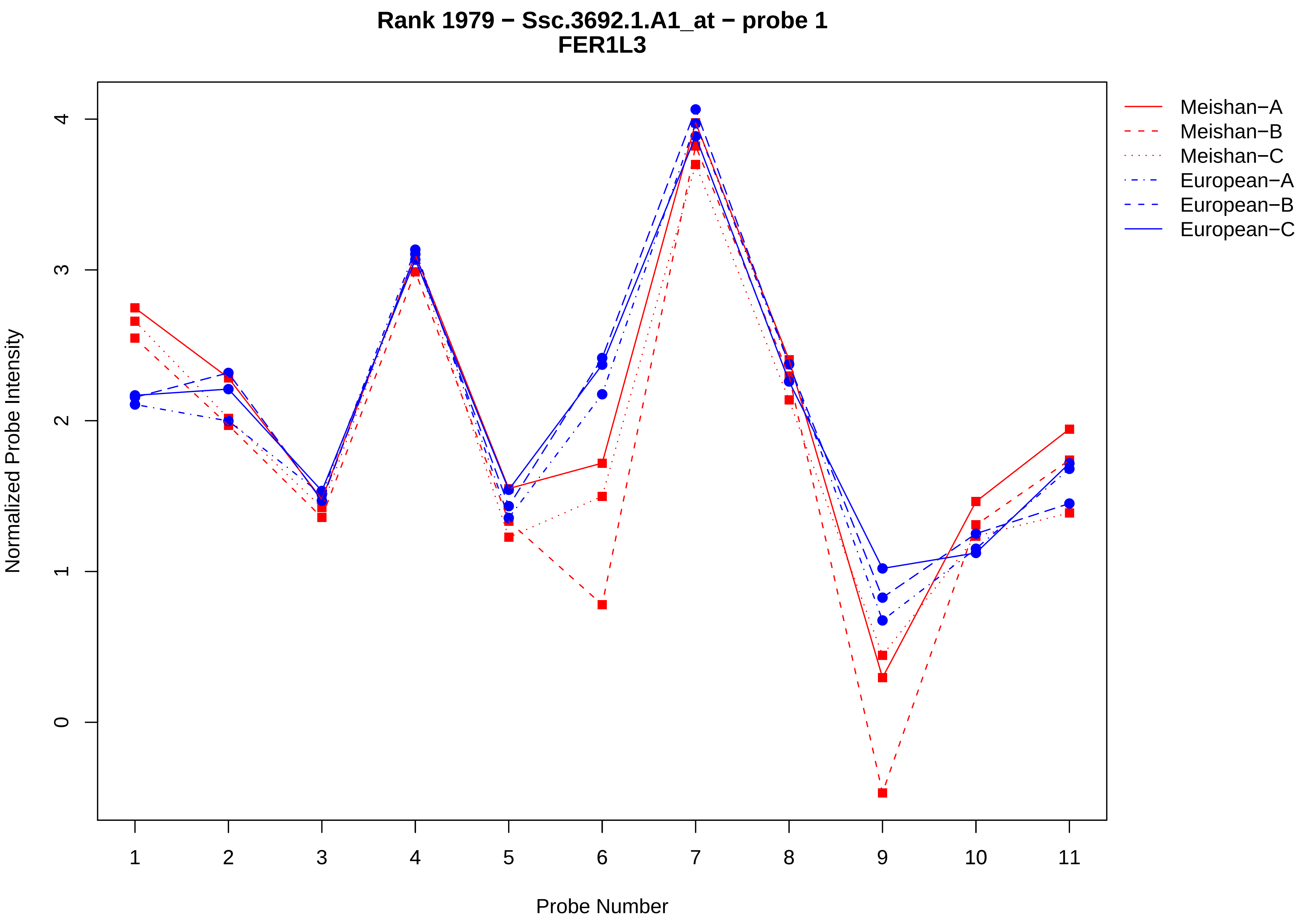

Rank 1979 − Ssc.3692.1.A1_at − probe 1
FER1L3
Normalized Probe Intensity
Probe Number
0
1
2
3
4
1
2
3
4
5
6
7
8
9
10
11
Meishan−A
Meishan−B
Meishan−C
European−A
European−B
European−C

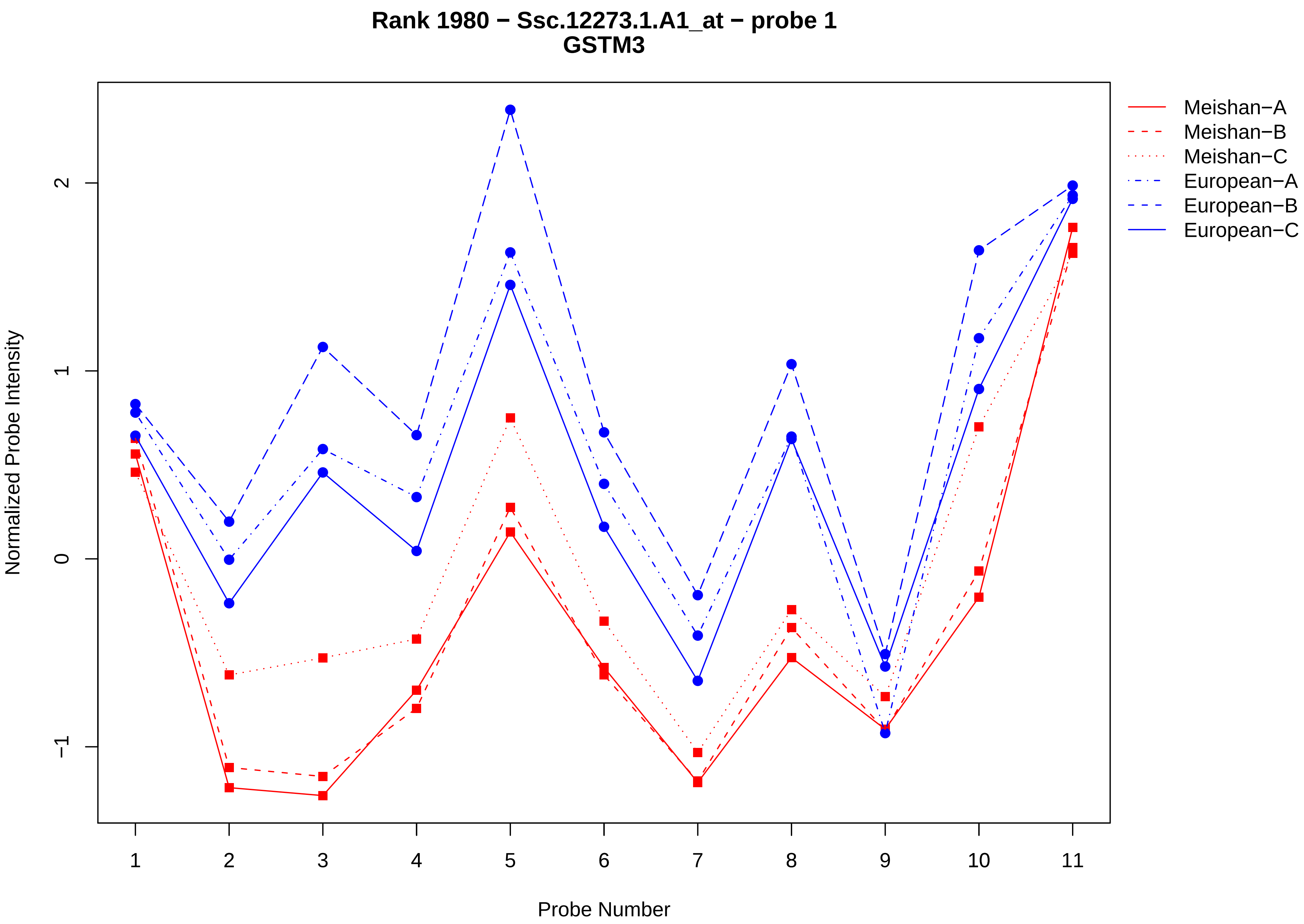

Rank 1980 − Ssc.12273.1.A1_at − probe 1
GSTM3
Normalized Probe Intensity
Probe Number
−1
0
1
2
1
2
3
4
5
6
7
8
9
10
11
Meishan−A
Meishan−B
Meishan−C
European−A
European−B
European−C

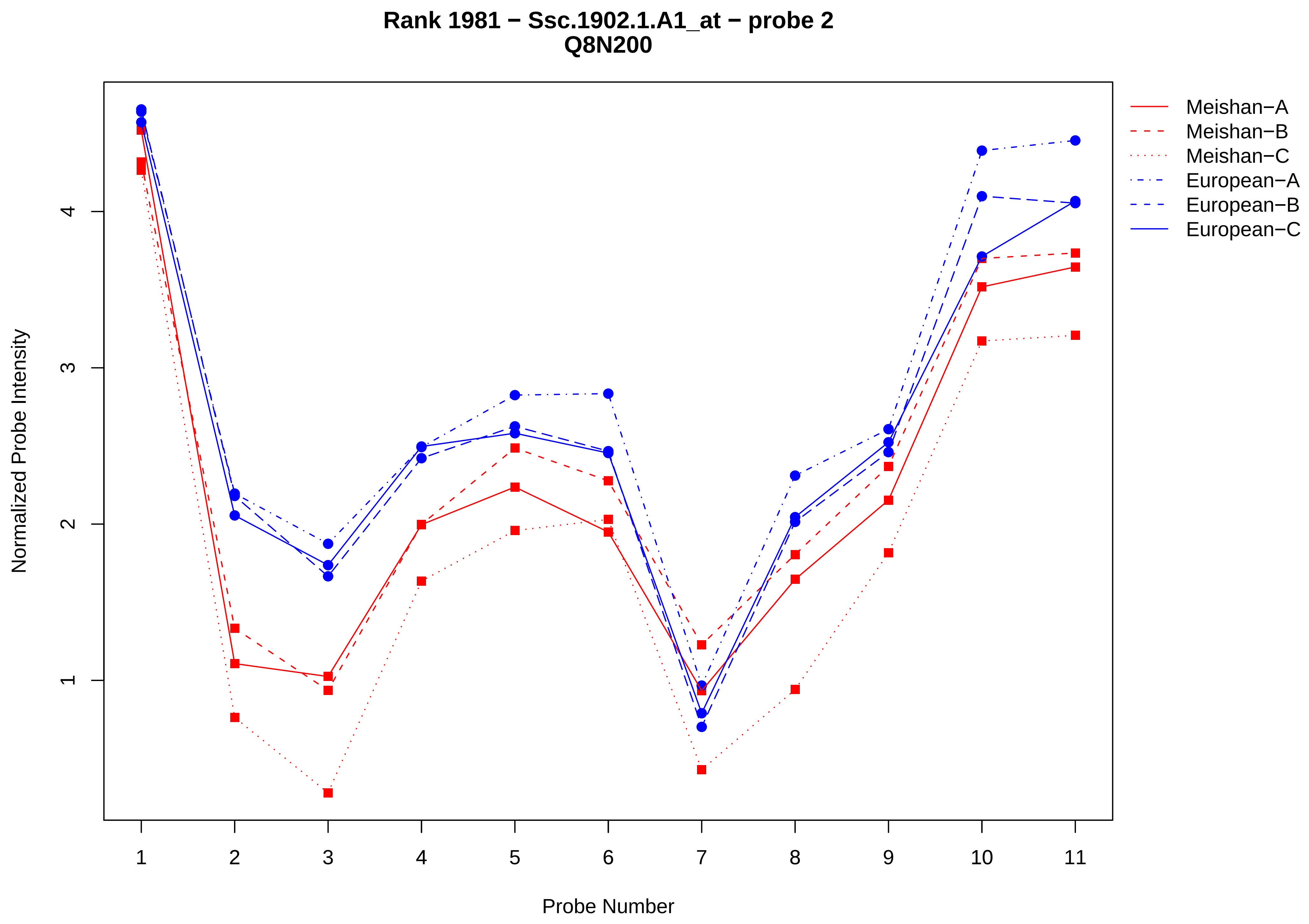

Rank 1981 − Ssc.1902.1.A1_at − probe 2
Q8N200
Normalized Probe Intensity
Probe Number
1
2
3
4
1
2
3
4
5
6
7
8
9
10
11
Meishan−A
Meishan−B
Meishan−C
European−A
European−B
European−C

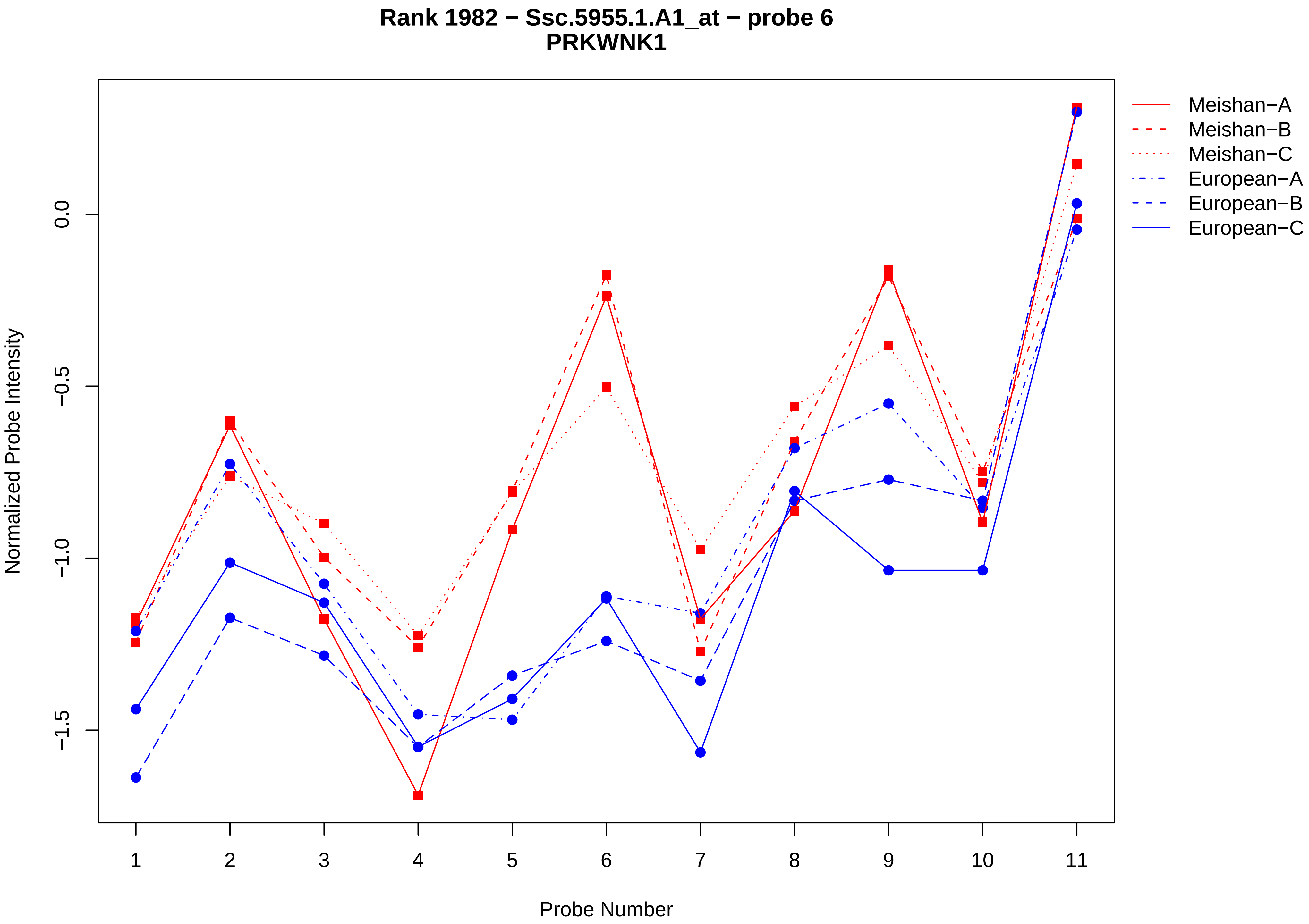

Rank 1982 − Ssc.5955.1.A1_at − probe 6
PRKWNK1
Normalized Probe Intensity
Probe Number
0.0
−0.5
−1.0
−1.5
1
2
3
4
5
6
7
8
9
10
11
Meishan−A
Meishan−B
Meishan−C
European−A
European−B
European−C

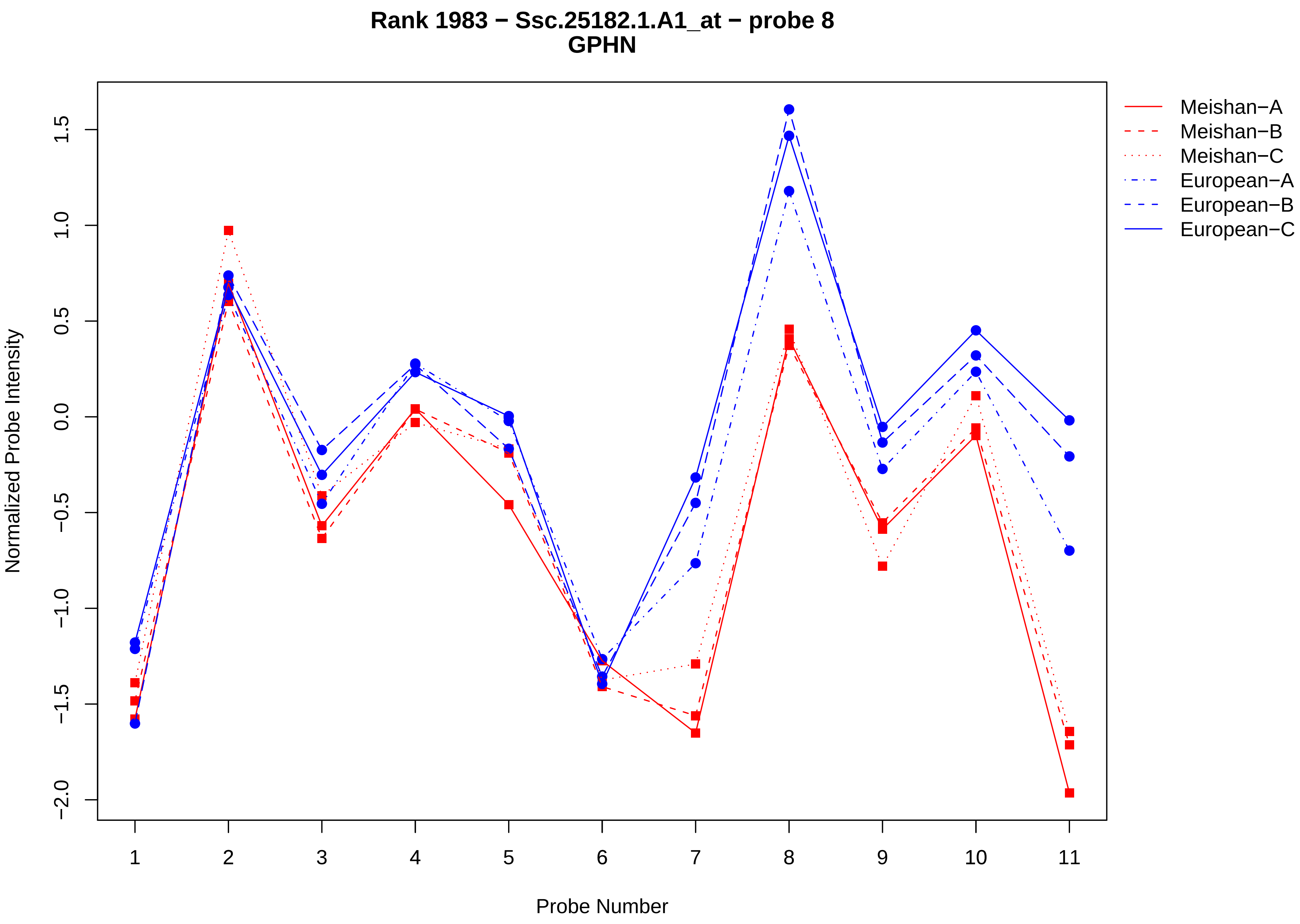
Rank 1983 − Ssc.25182.1.A1_at − probe 8
GPHN
Normalized Probe Intensity
Probe Number
1.5
1.0
0.5
0.0
−0.5
−1.0
−1.5
−2.0
1
2
3
4
5
6
7
8
9
10
11
Meishan−A
Meishan−B
Meishan−C
European−A
European−B
European−C

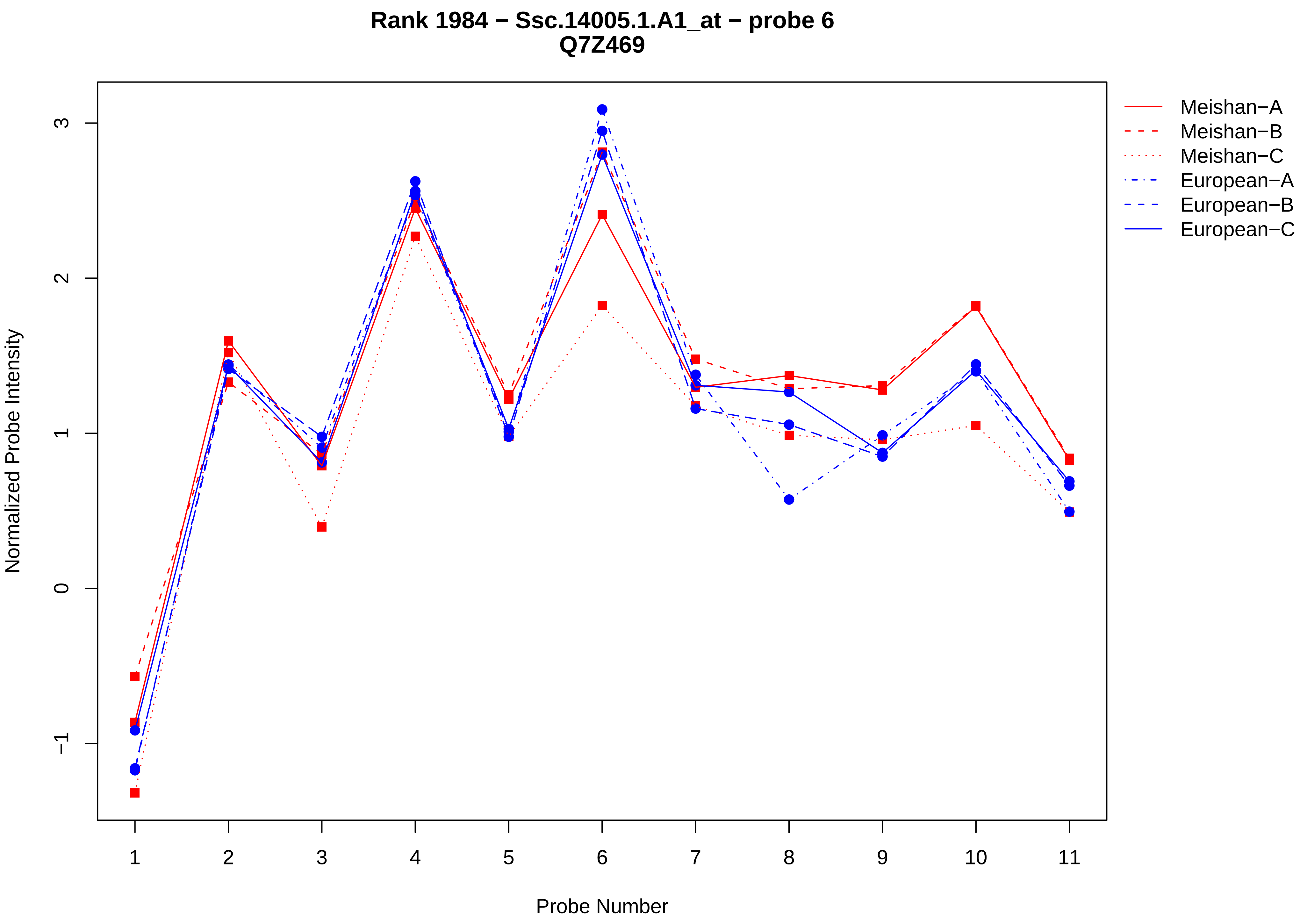

Rank 1984 − Ssc.14005.1.A1_at − probe 6
Q7Z469
Normalized Probe Intensity
Probe Number
−1
0
1
2
3
1
2
3
4
5
6
7
8
9
10
11
Meishan−A
Meishan−B
Meishan−C
European−A
European−B
European−C

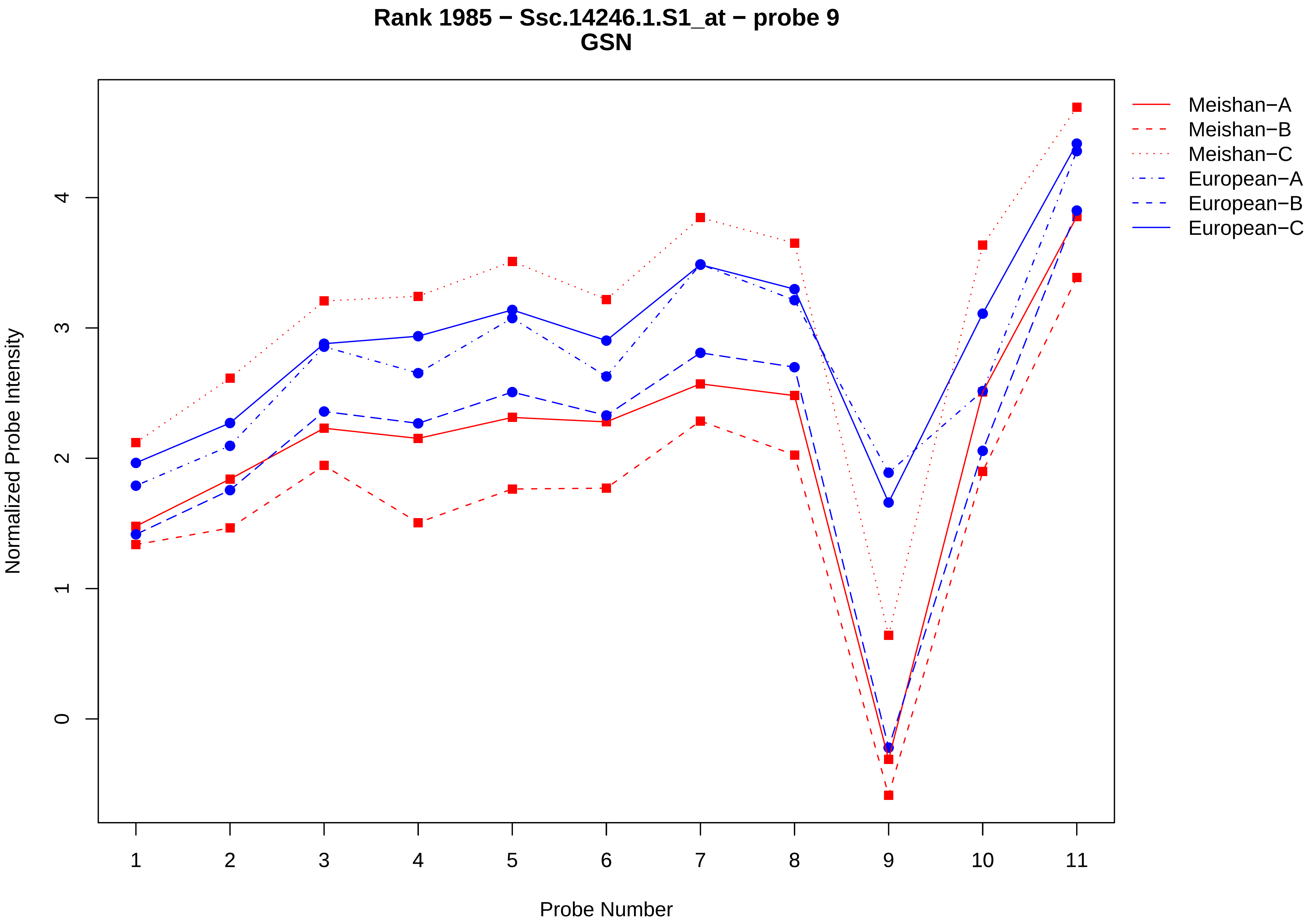
Rank 1985 − Ssc.14246.1.S1_at − probe 9
GSN
Normalized Probe Intensity
Probe Number
0
1
2
3
4
1
2
3
4
5
6
7
8
9
10
11
Meishan−A
Meishan−B
Meishan−C
European−A
European−B
European−C

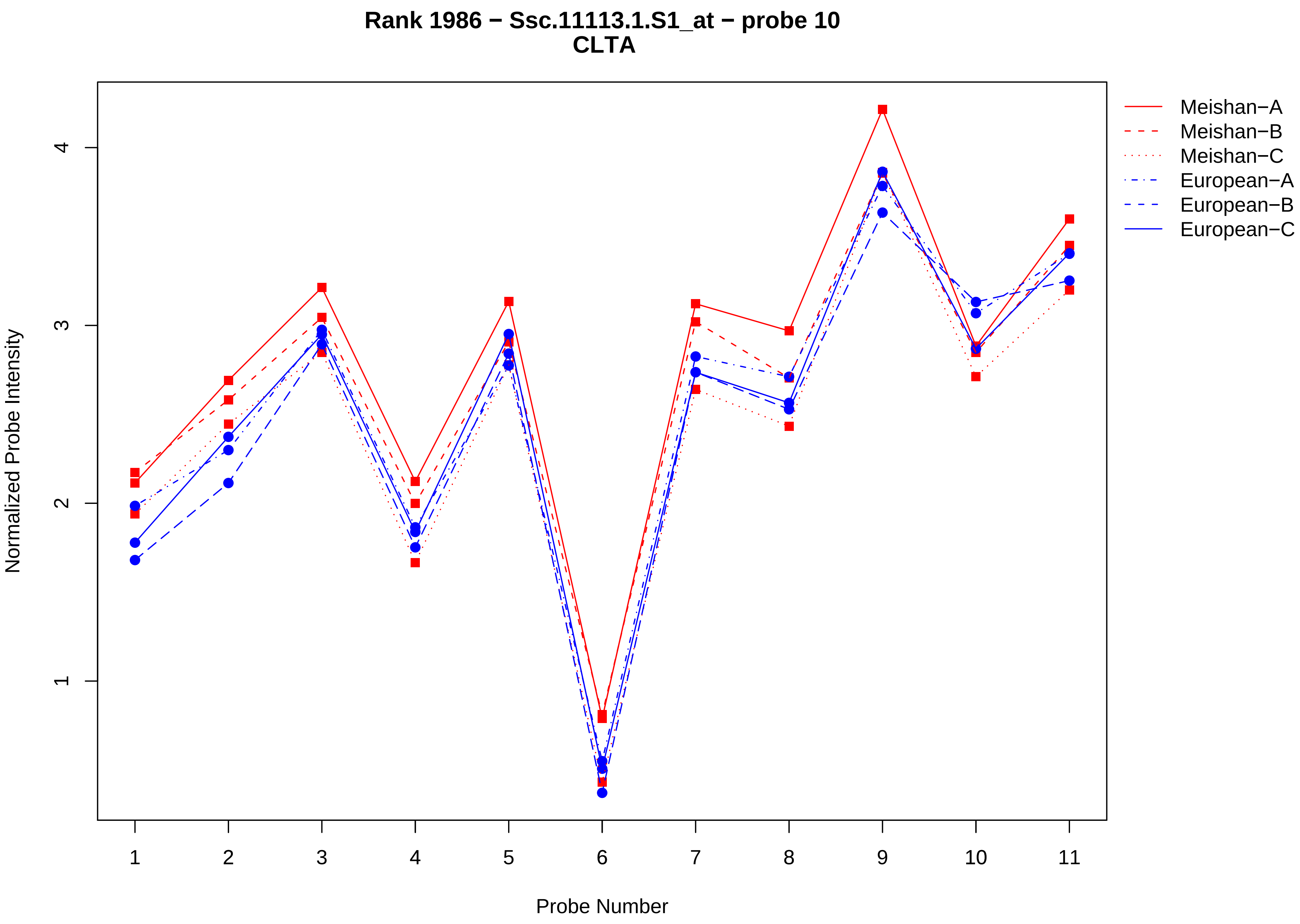
Rank 1986 − Ssc.11113.1.S1_at − probe 10
CLTA
Normalized Probe Intensity
Probe Number
1
2
3
4
5
6
7
8
9
10
11
Meishan−A
Meishan−B
Meishan−C
European−A
European−B
European−C

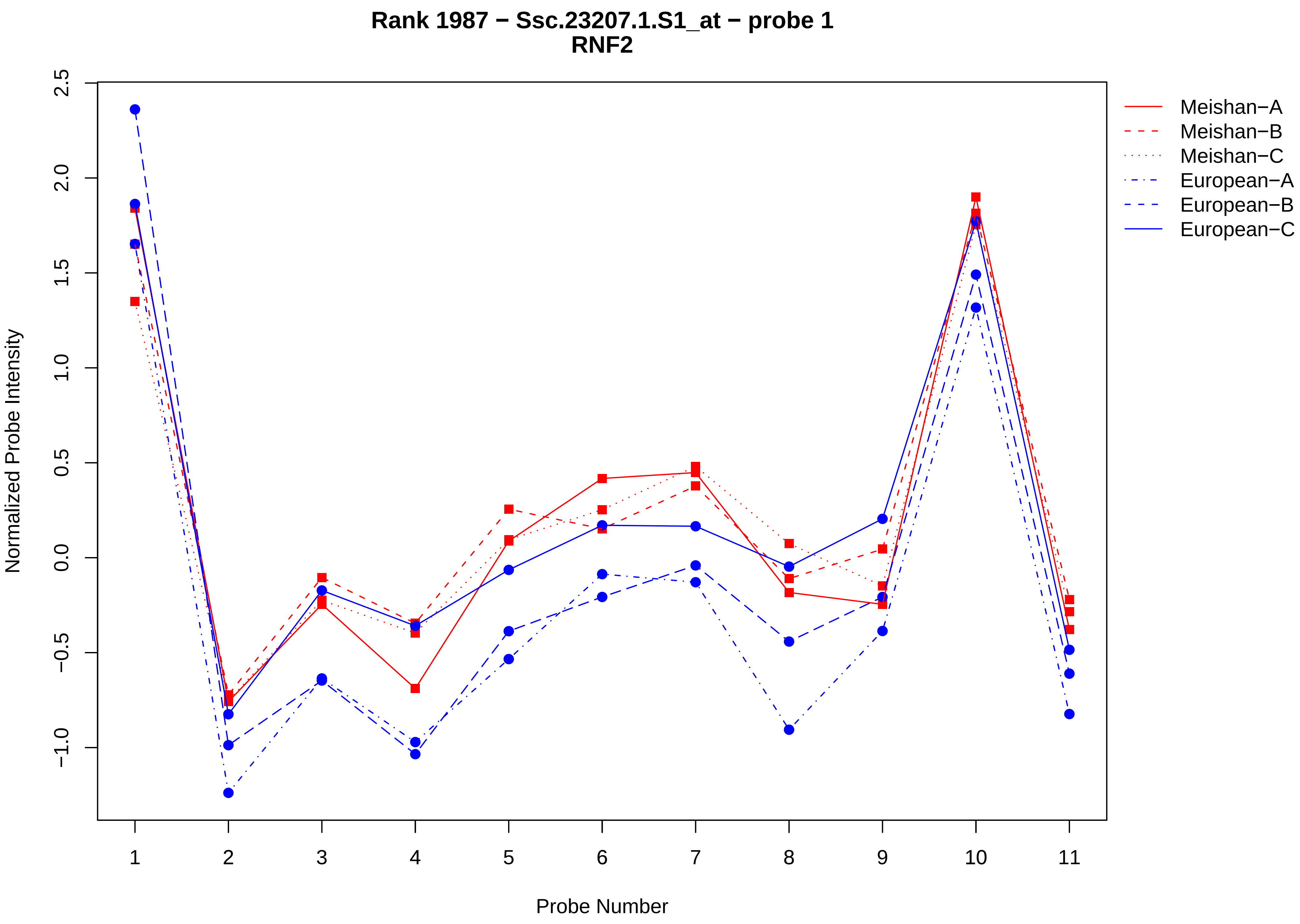
Rank 1987 − Ssc.23207.1.S1_at − probe 1
RNF2
Normalized Probe Intensity
Probe Number
−1.0
−0.5
0.0
0.5
1.0
1.5
2.0
2.5
1
2
3
4
5
6
7
8
9
10
11
Meishan−A
Meishan−B
Meishan−C
European−A
European−B
European−C

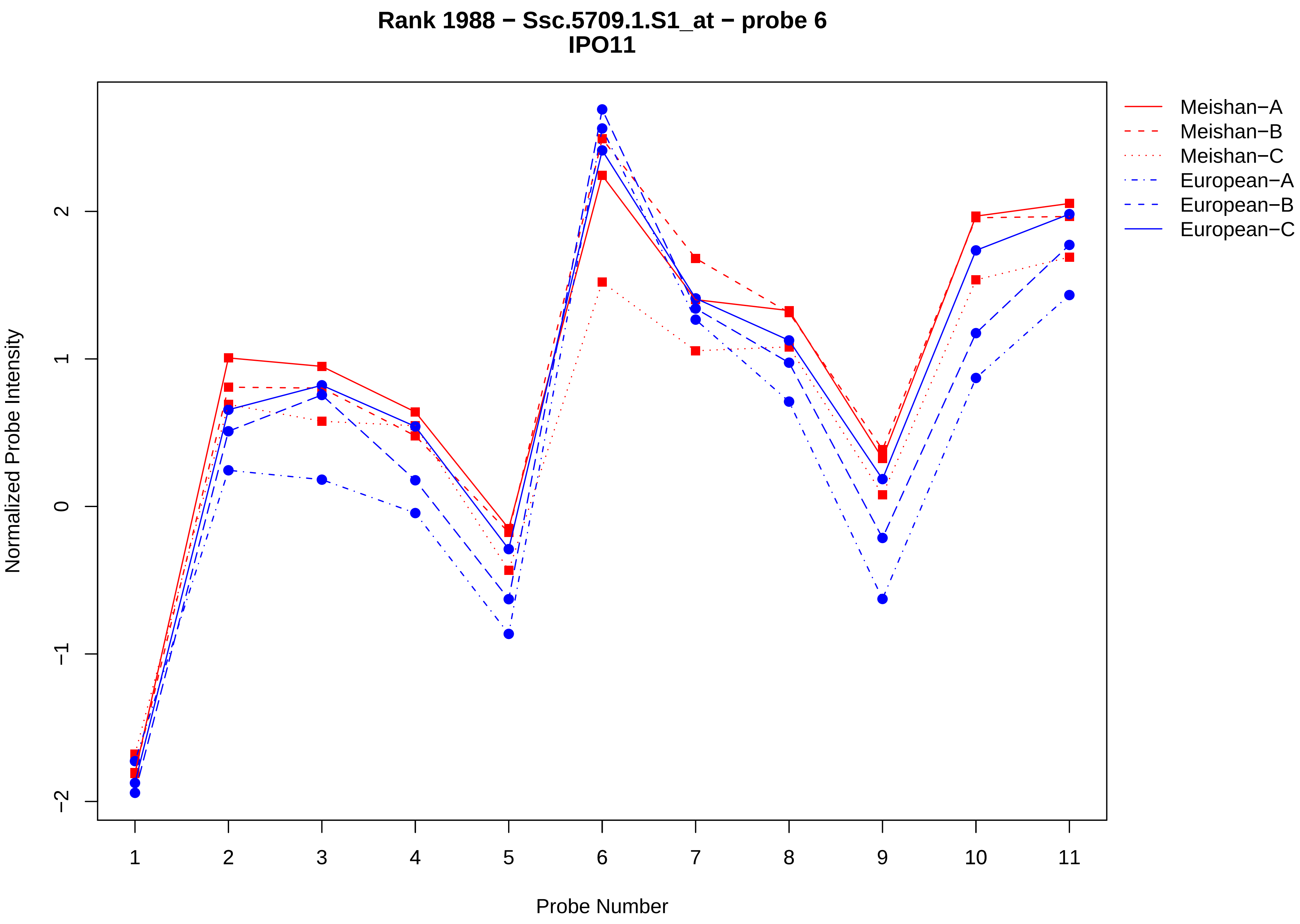

Rank 1988 − Ssc.5709.1.S1_at − probe 6
IPO11
Normalized Probe Intensity
Probe Number
−2
−1
0
1
2
1
2
3
4
5
6
7
8
9
10
11
Meishan−A
Meishan−B
Meishan−C
European−A
European−B
European−C

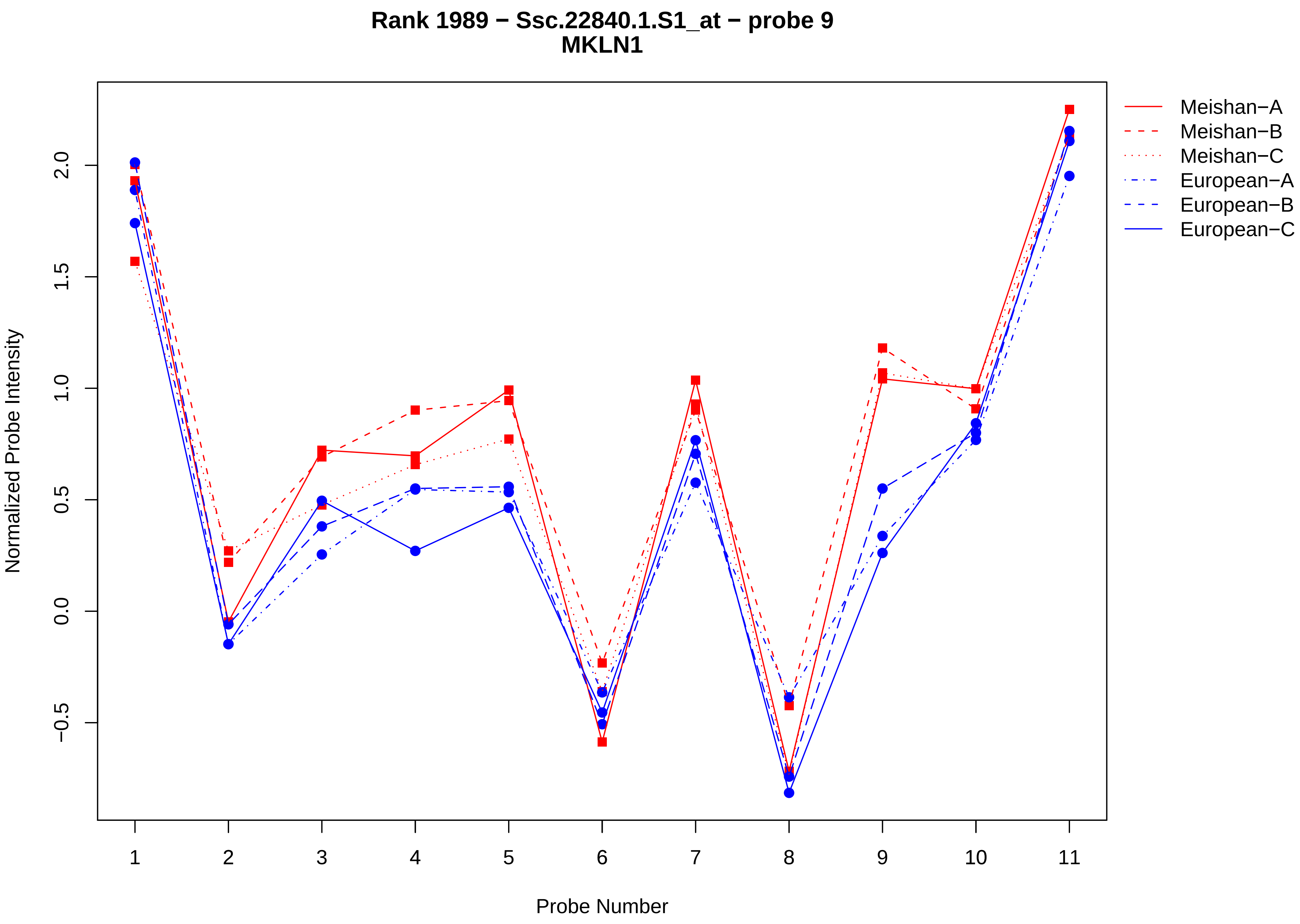
Rank 1989 − Ssc.22840.1.S1_at − probe 9
MKLN1
Normalized Probe Intensity
Probe Number
−0.5
0.0
0.5
1.0
1.5
2.0
1
2
3
4
5
6
7
8
9
10
11
Meishan−A
Meishan−B
Meishan−C
European−A
European−B
European−C

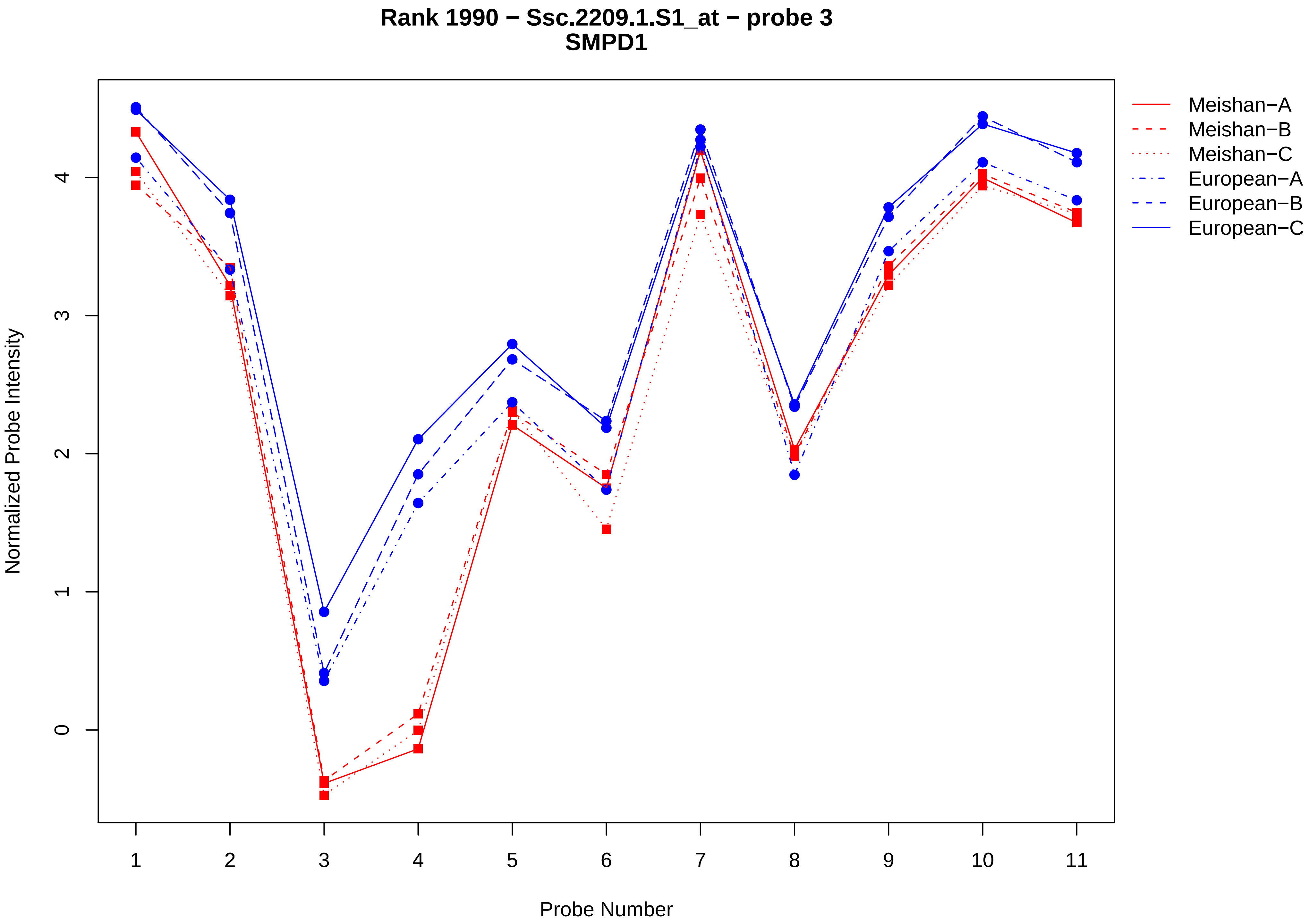

Rank 1990 − Ssc.2209.1.S1_at − probe 3
SMPD1
Normalized Probe Intensity
Probe Number
0
1
2
3
4
1 2 3 4 5 6 7 8 9 10 11
Meishan−A
Meishan−B
Meishan−C
European−A
European−B
European−C

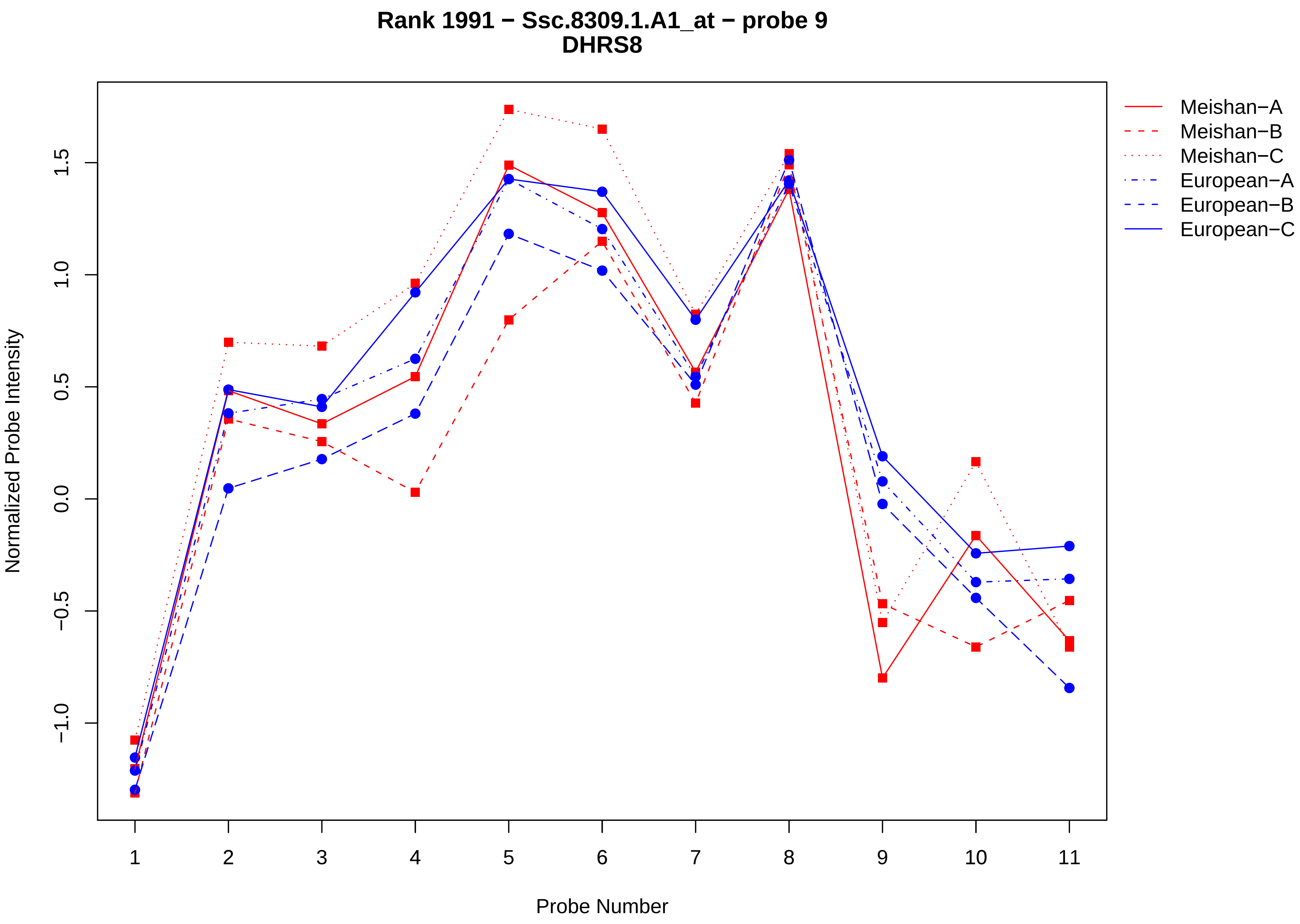

Rank 1991 − Ssc.8309.1.A1_at − probe 9
DHRS8
Normalized Probe Intensity
Probe Number
−1.0
−0.5
0.0
0.5
1.0
1.5
1
2
3
4
5
6
7
8
9
10
11
Meishan−A
Meishan−B
Meishan−C
European−A
European−B
European−C

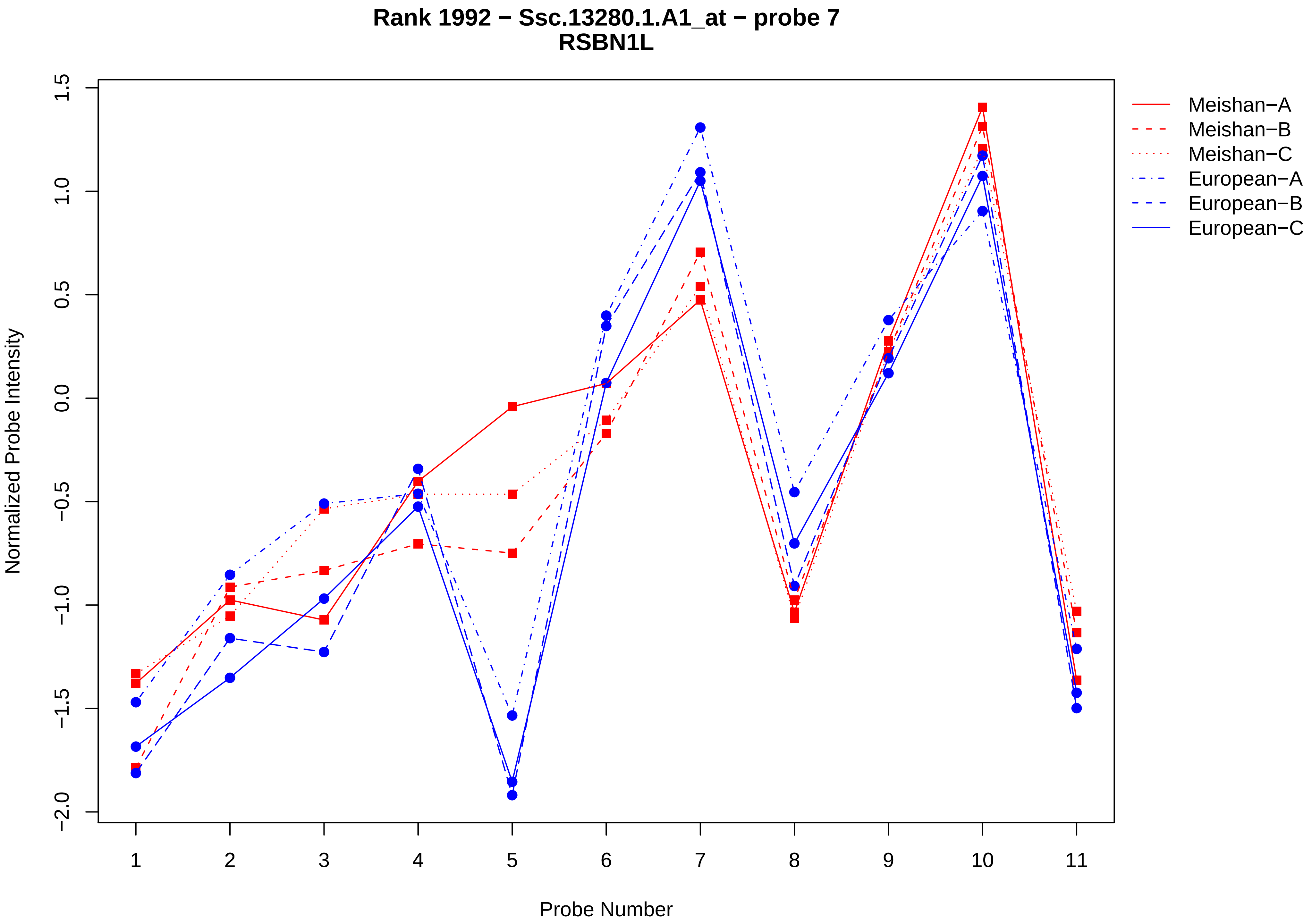

Rank 1992 − Ssc.13280.1.A1_at − probe 7
RSBN1L
Normalized Probe Intensity
Probe Number
1.5
1.0
0.5
0.0
−0.5
−1.0
−1.5
−2.0
1
2
3
4
5
6
7
8
9
10
11
Meishan−A
Meishan−B
Meishan−C
European−A
European−B
European−C

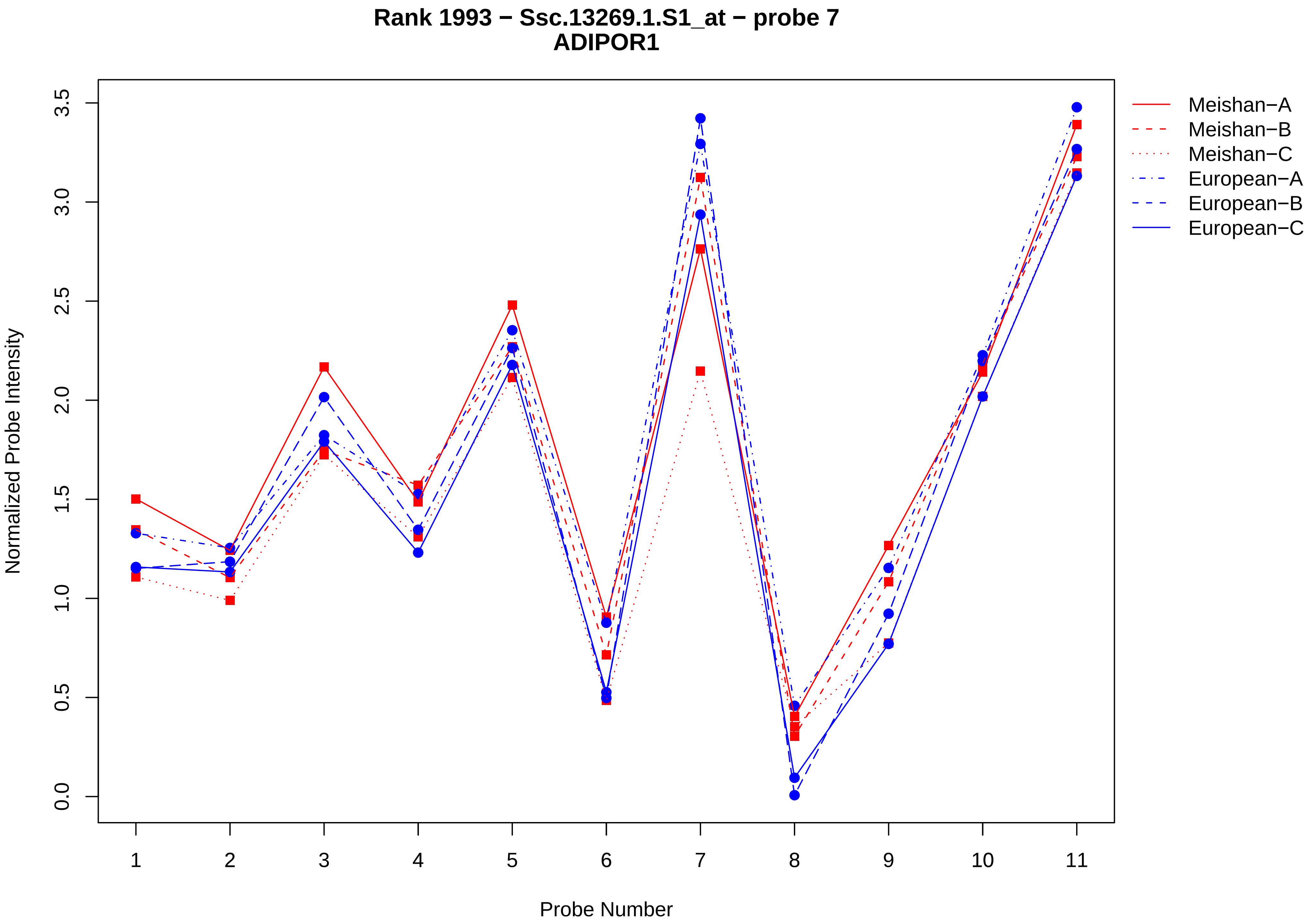
Rank 1993 − Ssc.13269.1.S1_at − probe 7
ADIPOR1
Normalized Probe Intensity
0.0
0.5
1.0
1.5
2.0
2.5
3.0
3.5
1
2
3
4
5
6
7
8
9
10
11
Probe Number
Meishan−A
Meishan−B
Meishan−C
European−A
European−B
European−C

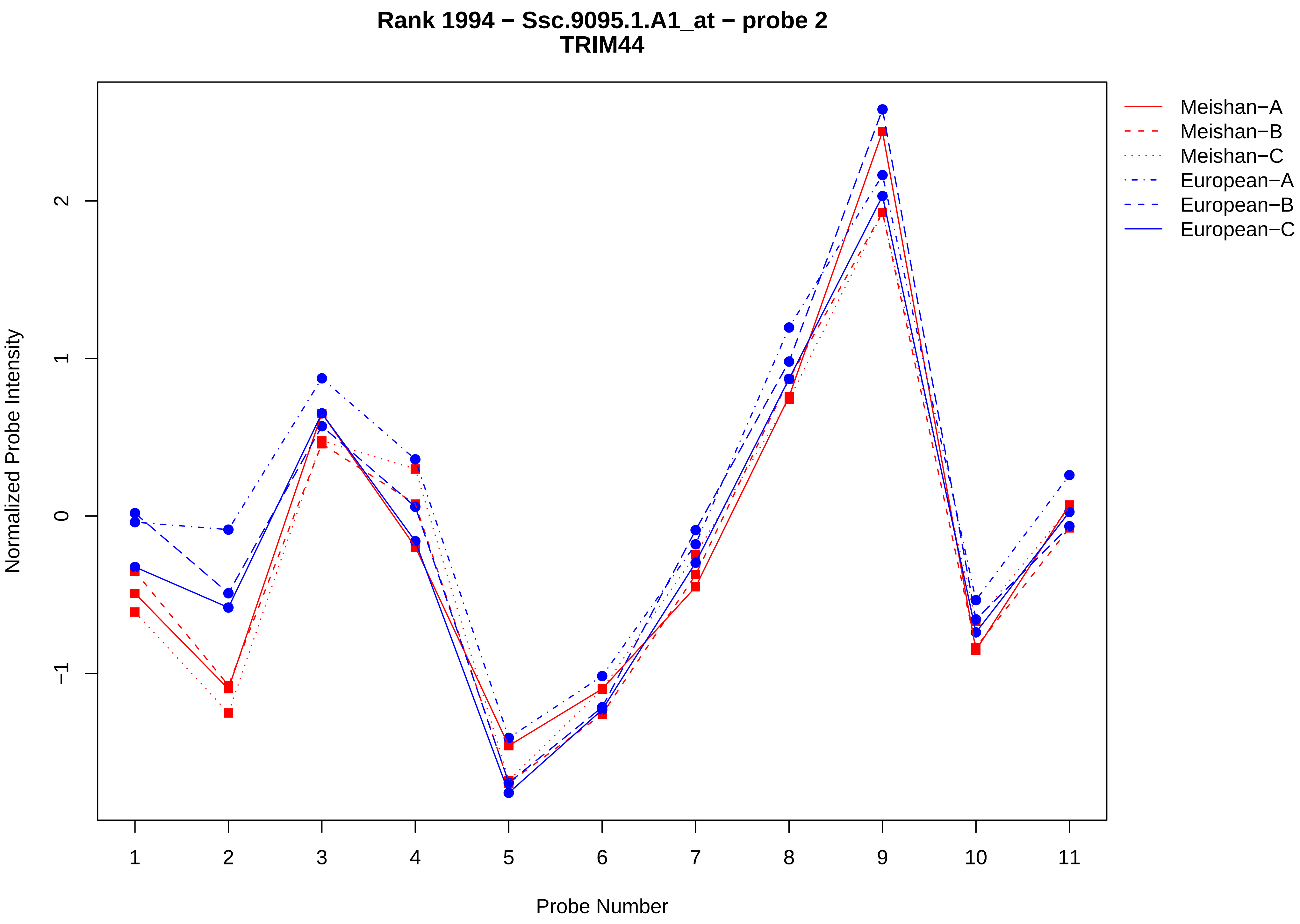

Rank 1994 − Ssc.9095.1.A1_at − probe 2
TRIM44
Normalized Probe Intensity
Probe Number
−1
0
1
2
1
2
3
4
5
6
7
8
9
10
11
Meishan−A
Meishan−B
Meishan−C
European−A
European−B
European−C

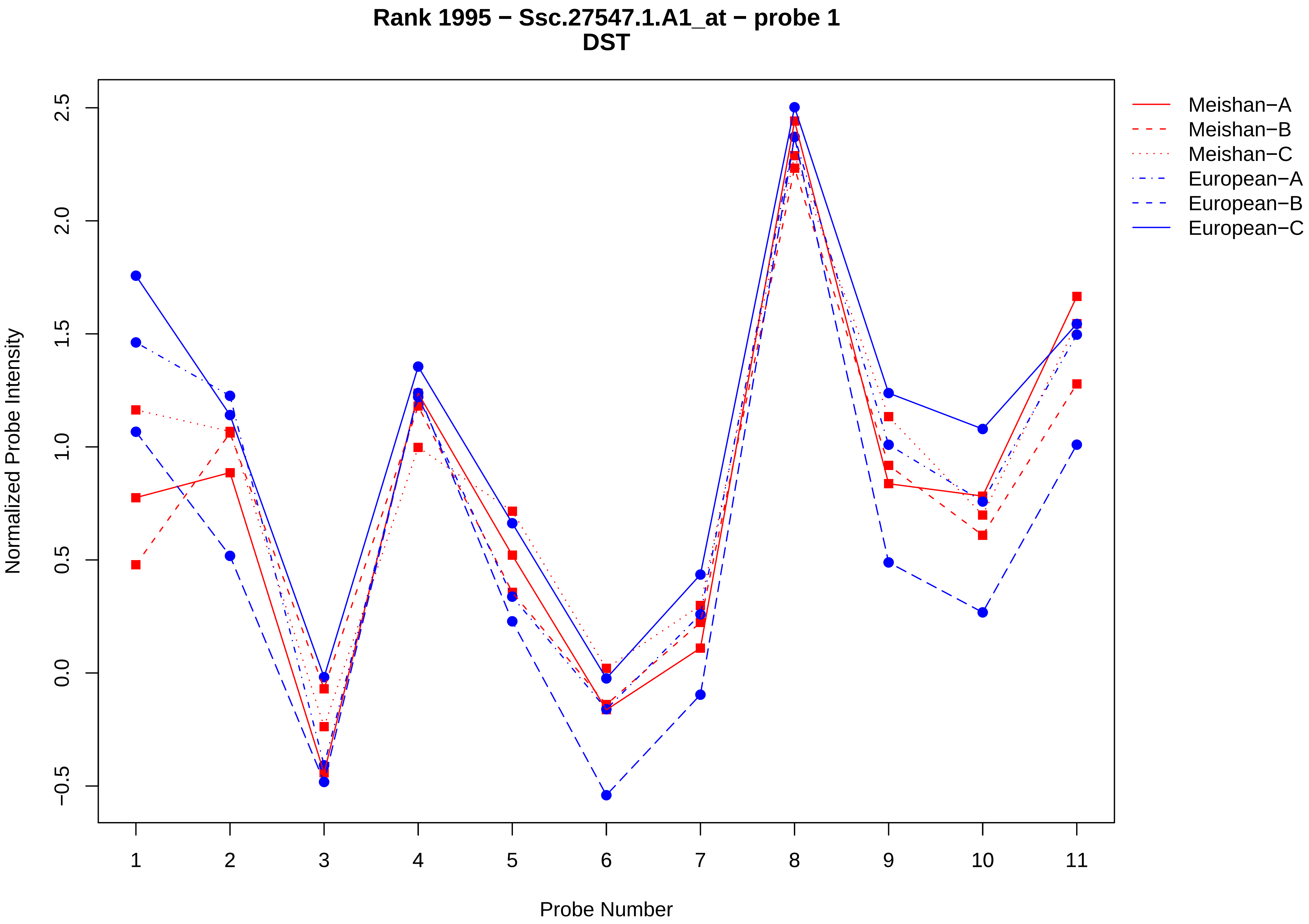
Rank 1995 − Ssc.27547.1.A1_at − probe 1
DST
Normalized Probe Intensity
Probe Number
−0.5
0.0
0.5
1.0
1.5
2.0
2.5
1
2
3
4
5
6
7
8
9
10
11
Meishan−A
Meishan−B
Meishan−C
European−A
European−B
European−C

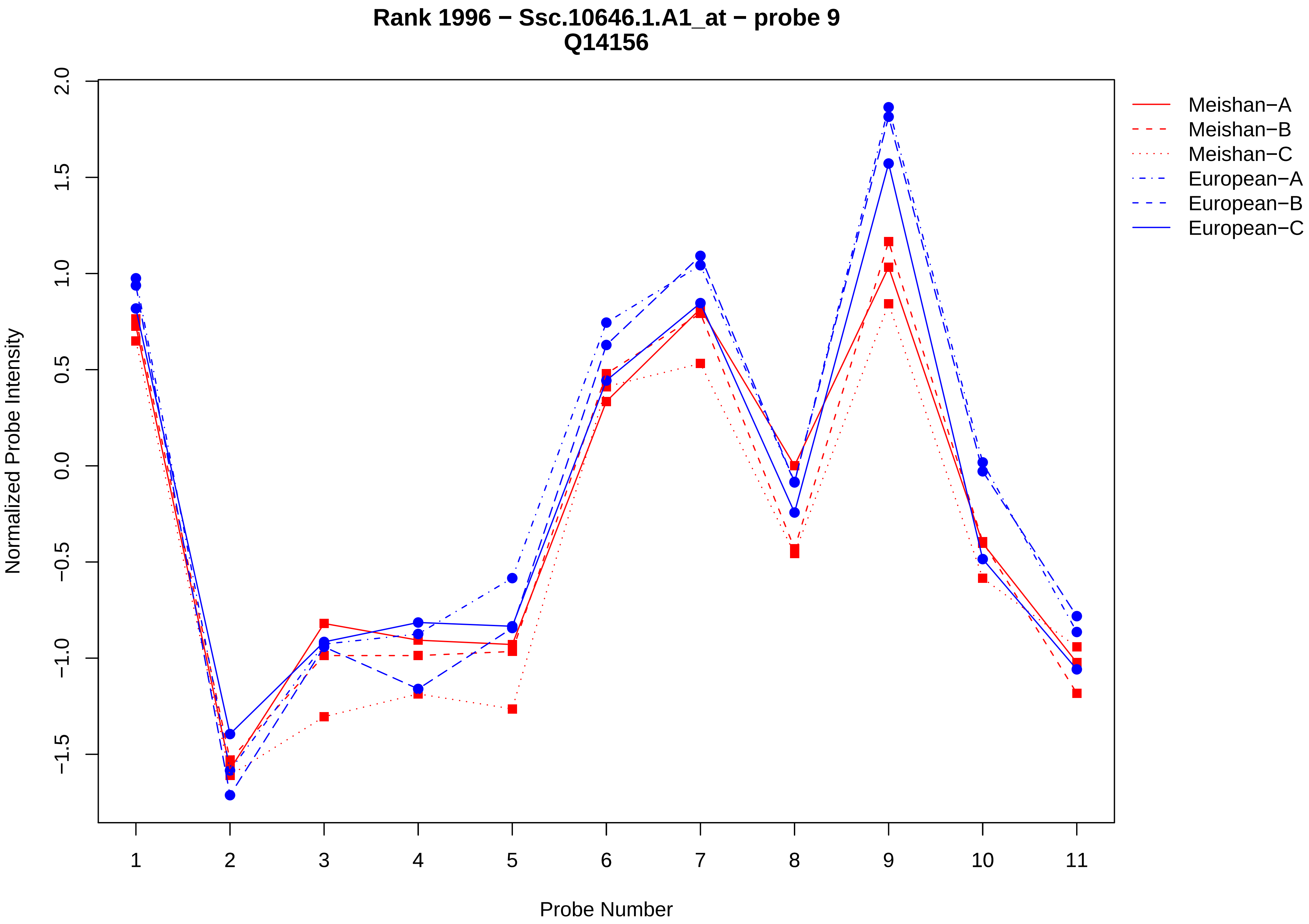

Rank 1996 − Ssc.10646.1.A1_at − probe 9
Q14156
Normalized Probe Intensity
Probe Number
Meishan−A
Meishan−B
Meishan−C
European−A
European−B
European−C
2.0
1.5
1.0
0.5
0.0
−0.5
−1.0
−1.5
1
2
3
4
5
6
7
8
9
10
11

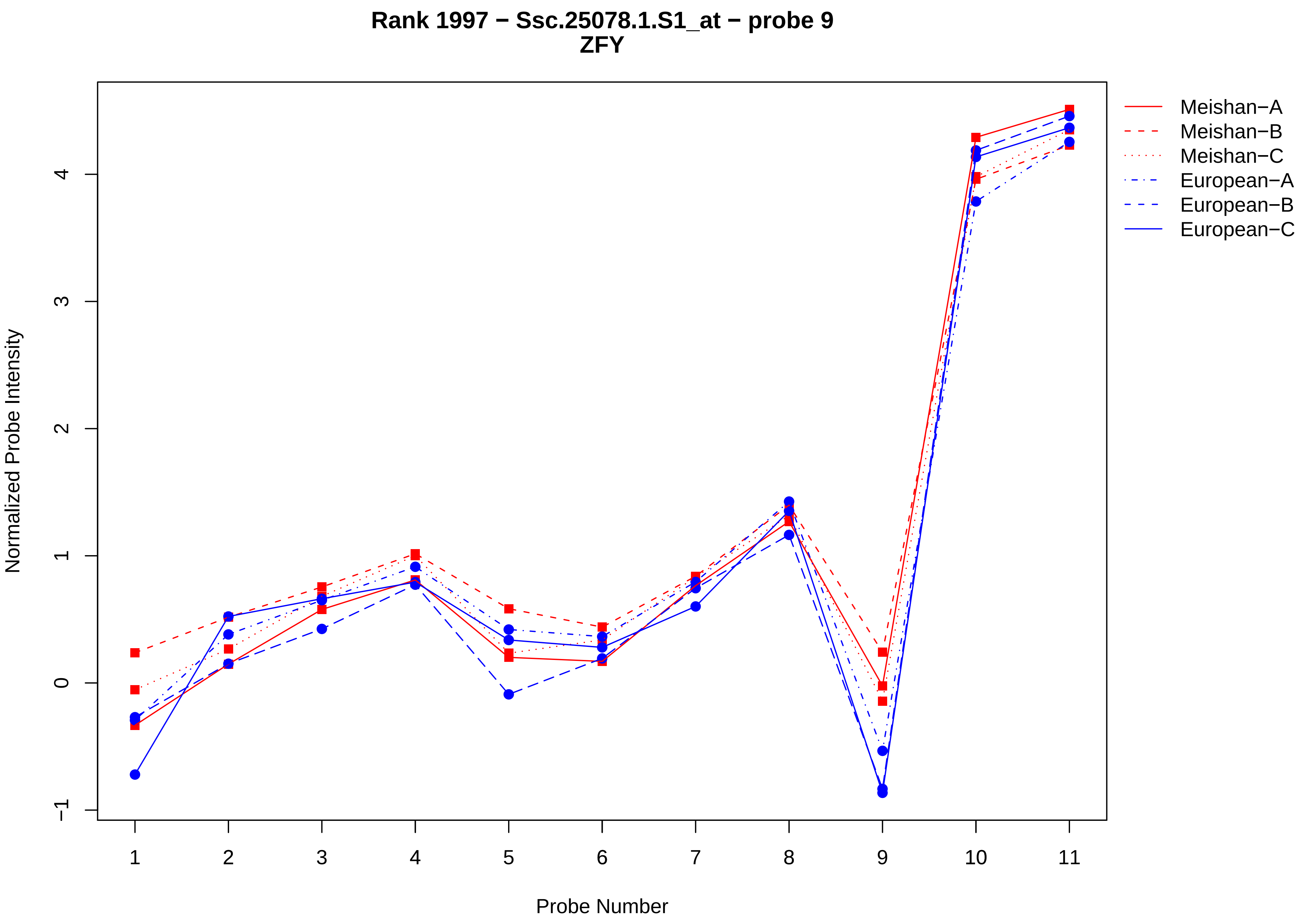
Rank 1997 − Ssc.25078.1.S1_at − probe 9
ZFY
Normalized Probe Intensity
Probe Number
−1
0
1
2
3
4
1
2
3
4
5
6
7
8
9
10
11
Meishan−A
Meishan−B
Meishan−C
European−A
European−B
European−C

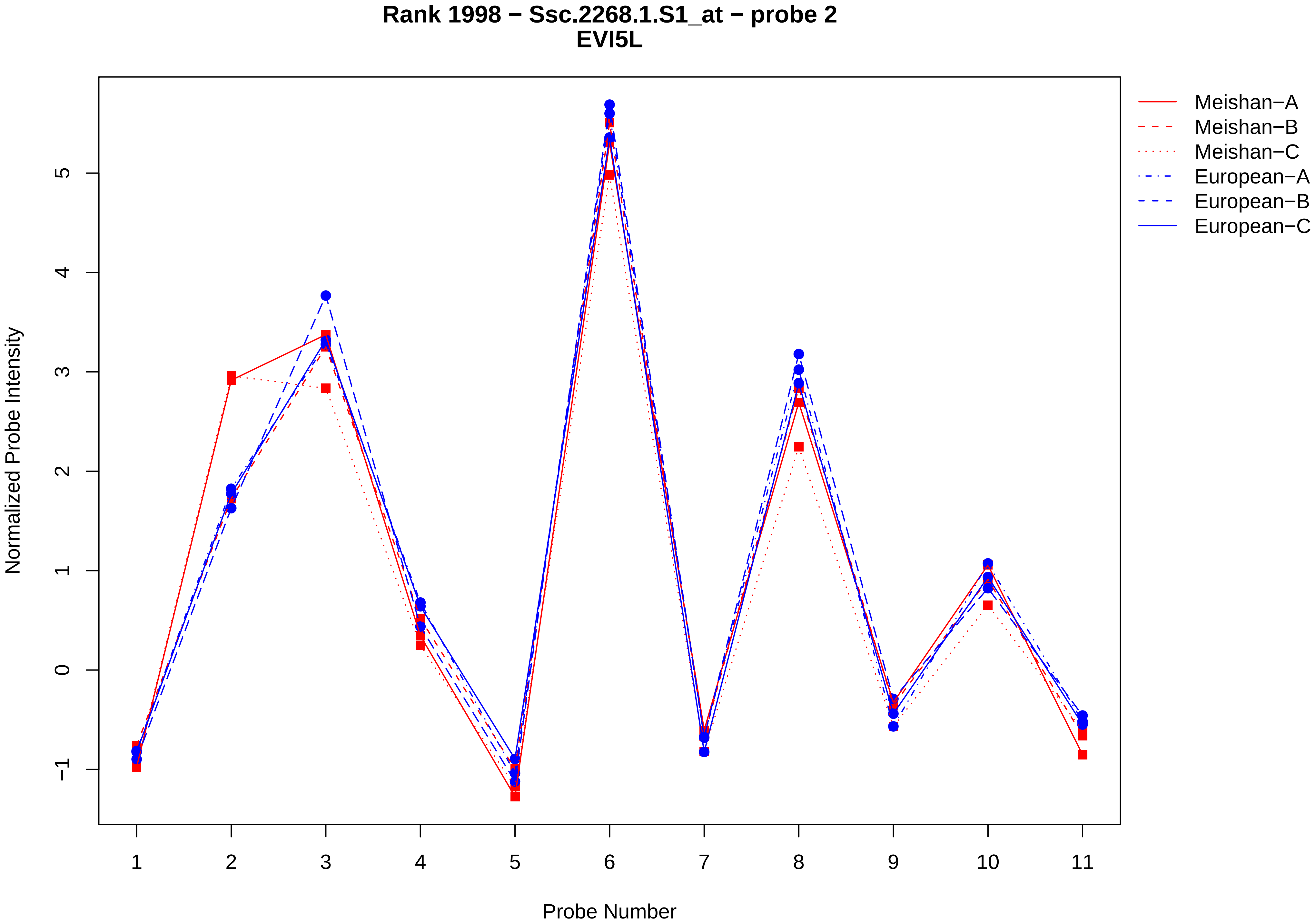

Rank 1998 − Ssc.2268.1.S1_at − probe 2
EVI5L
Normalized Probe Intensity
Probe Number
−1
0
1
2
3
4
5
1
2
3
4
5
6
7
8
9
10
11
Meishan−A
Meishan−B
Meishan−C
European−A
European−B
European−C

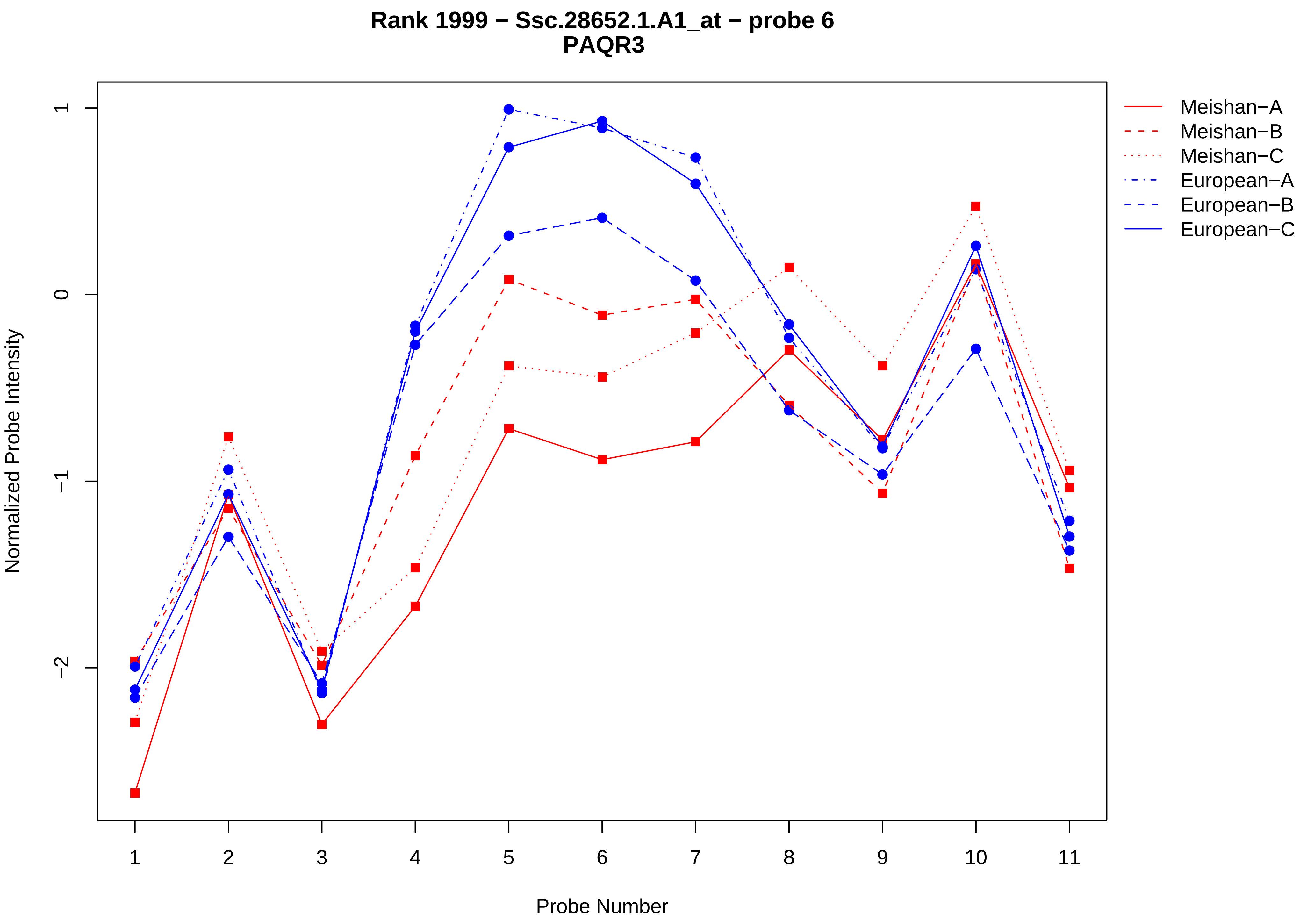

Rank 1999 − Ssc.28652.1.A1_at − probe 6
PAQR3
Normalized Probe Intensity
Probe Number
1
0
−1
−2
1
2
3
4
5
6
7
8
9
10
11
Meishan−A
Meishan−B
Meishan−C
European−A
European−B
European−C

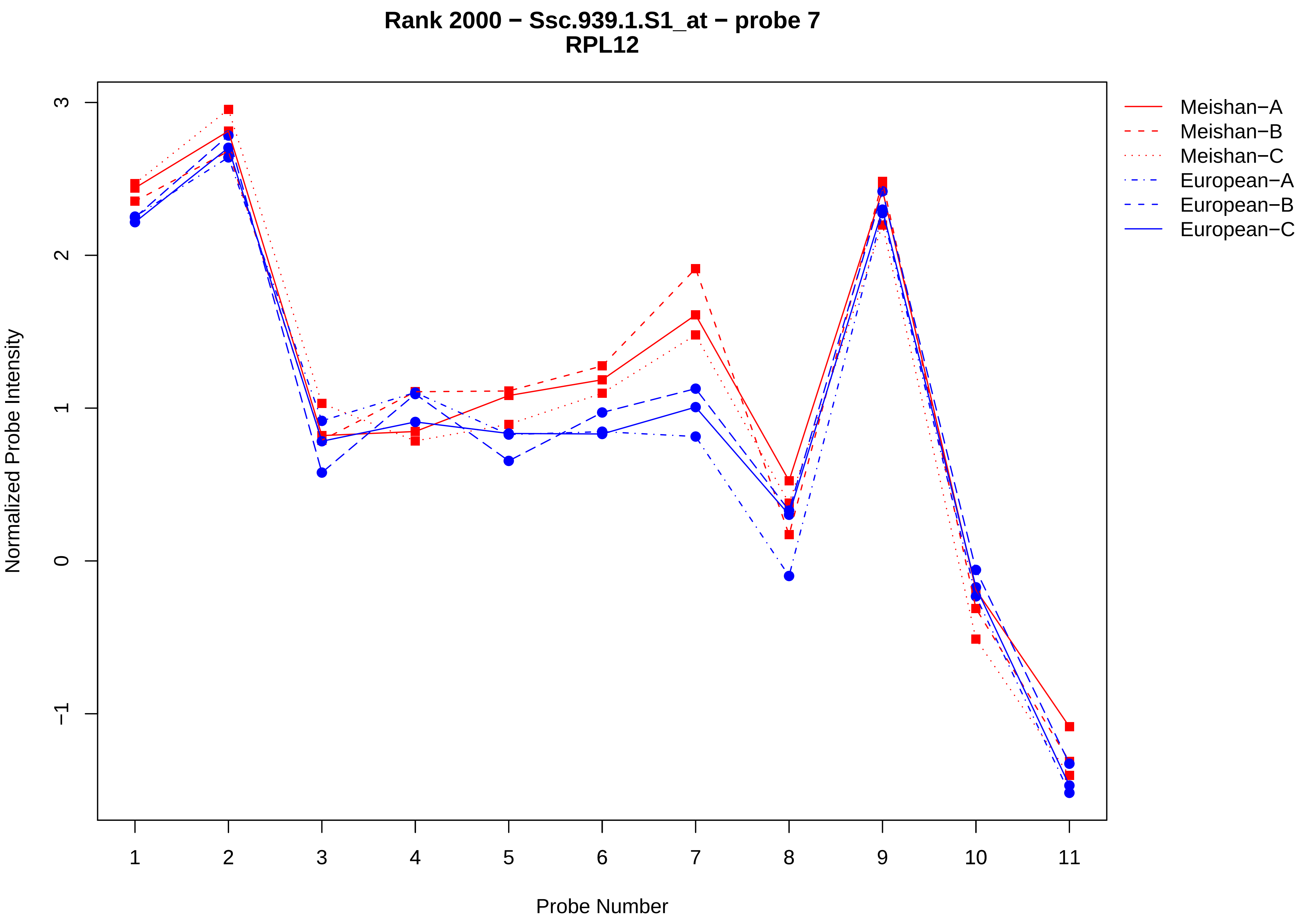

Rank 2000 − Ssc.939.1.S1_at − probe 7
RPL12
Normalized Probe Intensity
Probe Number
Meishan−A
Meishan−B
Meishan−C
European−A
European−B
European−C

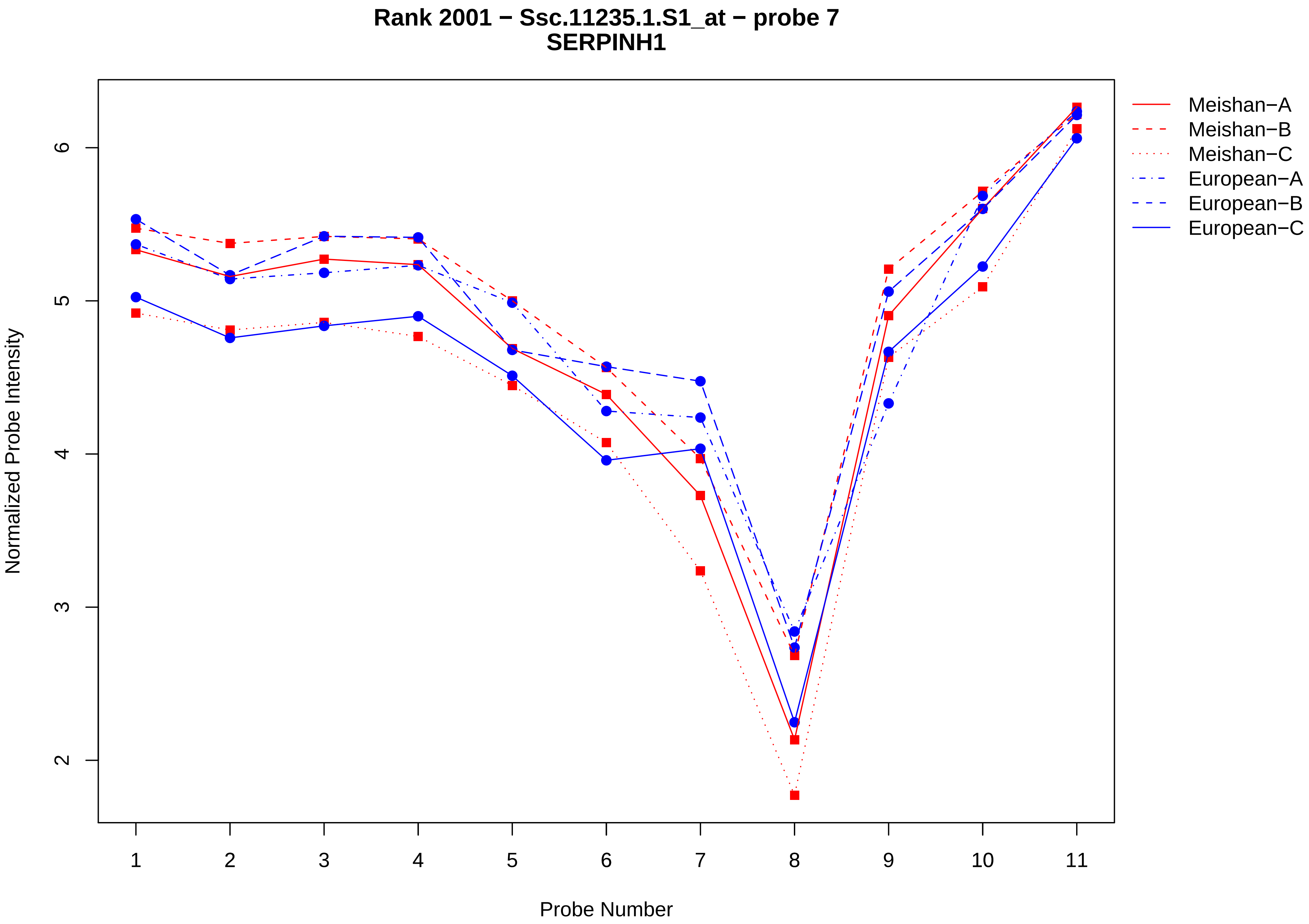
Rank 2001 − Ssc.11235.1.S1_at − probe 7
SERPINH1
Normalized Probe Intensity
Probe Number
Meishan−A
Meishan−B
Meishan−C
European−A
European−B
European−C

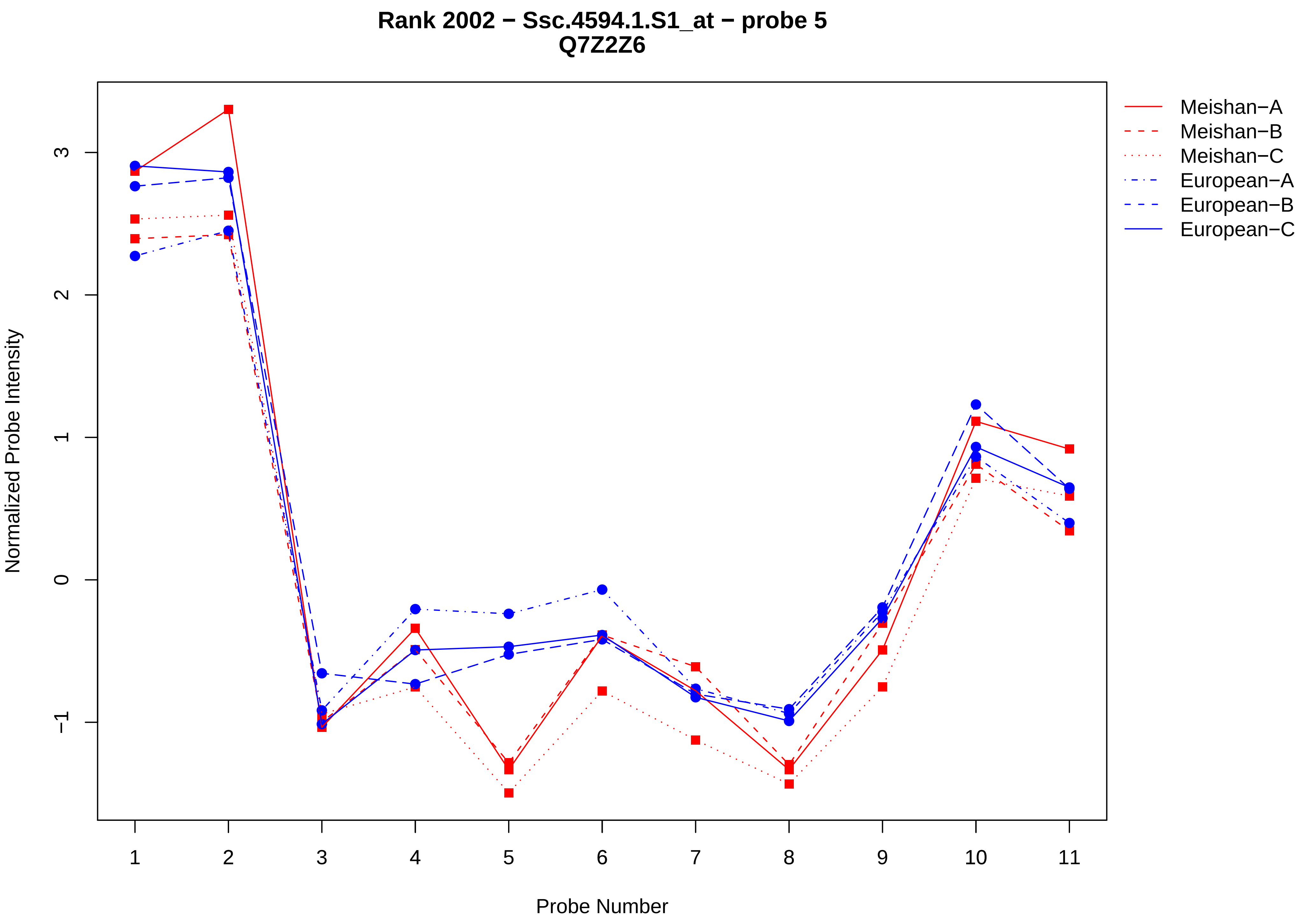

Rank 2002 − Ssc.4594.1.S1_at − probe 5
Q7Z2Z6
Normalized Probe Intensity
Probe Number
Meishan−A
Meishan−B
Meishan−C
European−A
European−B
European−C

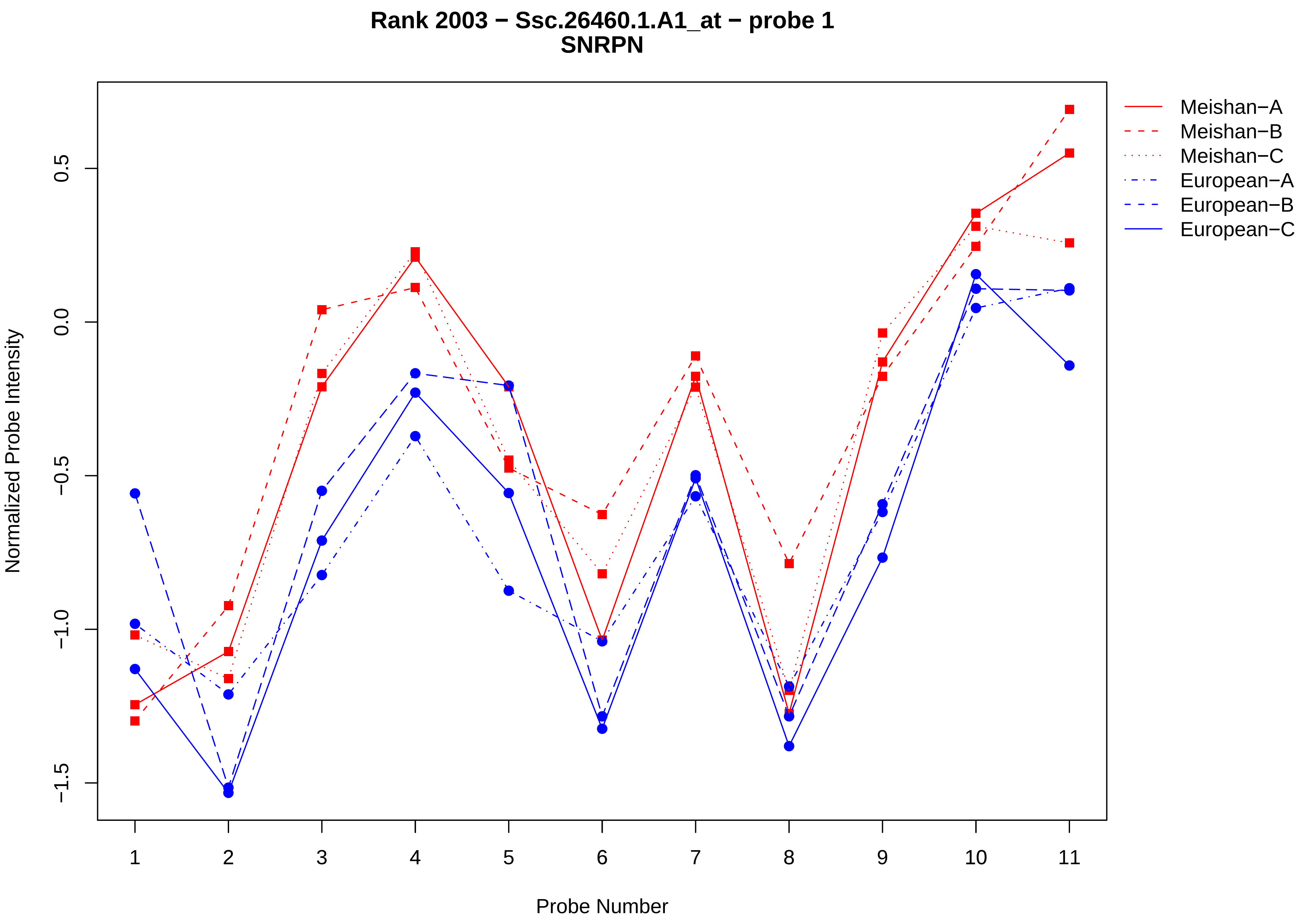
Rank 2003 − Ssc.26460.1.A1_at − probe 1
SNRPN
Normalized Probe Intensity
Probe Number
0.5
0.0
−0.5
−1.0
−1.5
1
2
3
4
5
6
7
8
9
10
11
Meishan−A
Meishan−B
Meishan−C
European−A
European−B
European−C

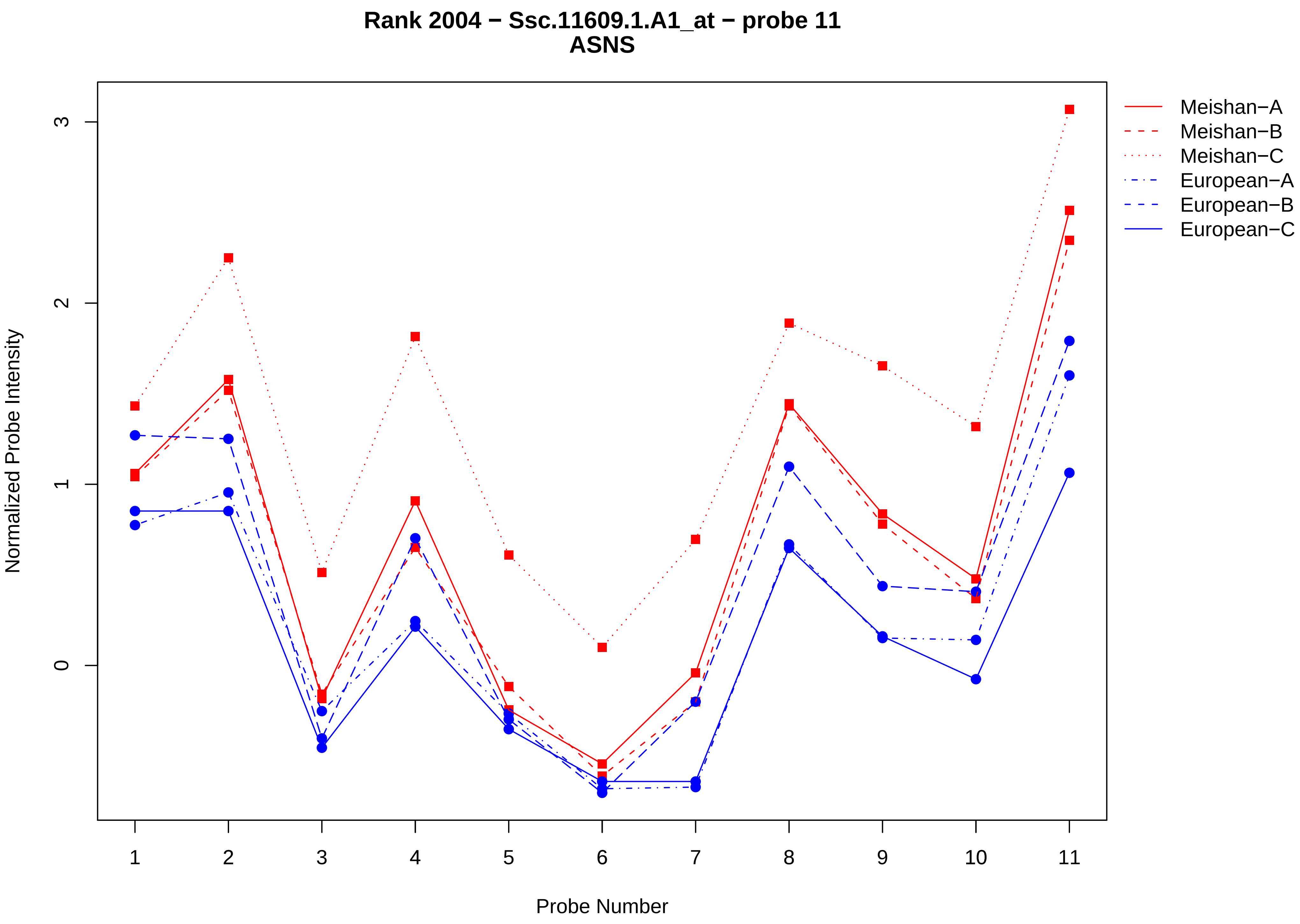

Rank 2004 − Ssc.11609.1.A1_at − probe 11
ASNS
Normalized Probe Intensity
Probe Number
0
1
2
3
1
2
3
4
5
6
7
8
9
10
11
Meishan−A
Meishan−B
Meishan−C
European−A
European−B
European−C

Rank 2005 − Ssc.7144.1.S1_at − probe 5
PDE1A

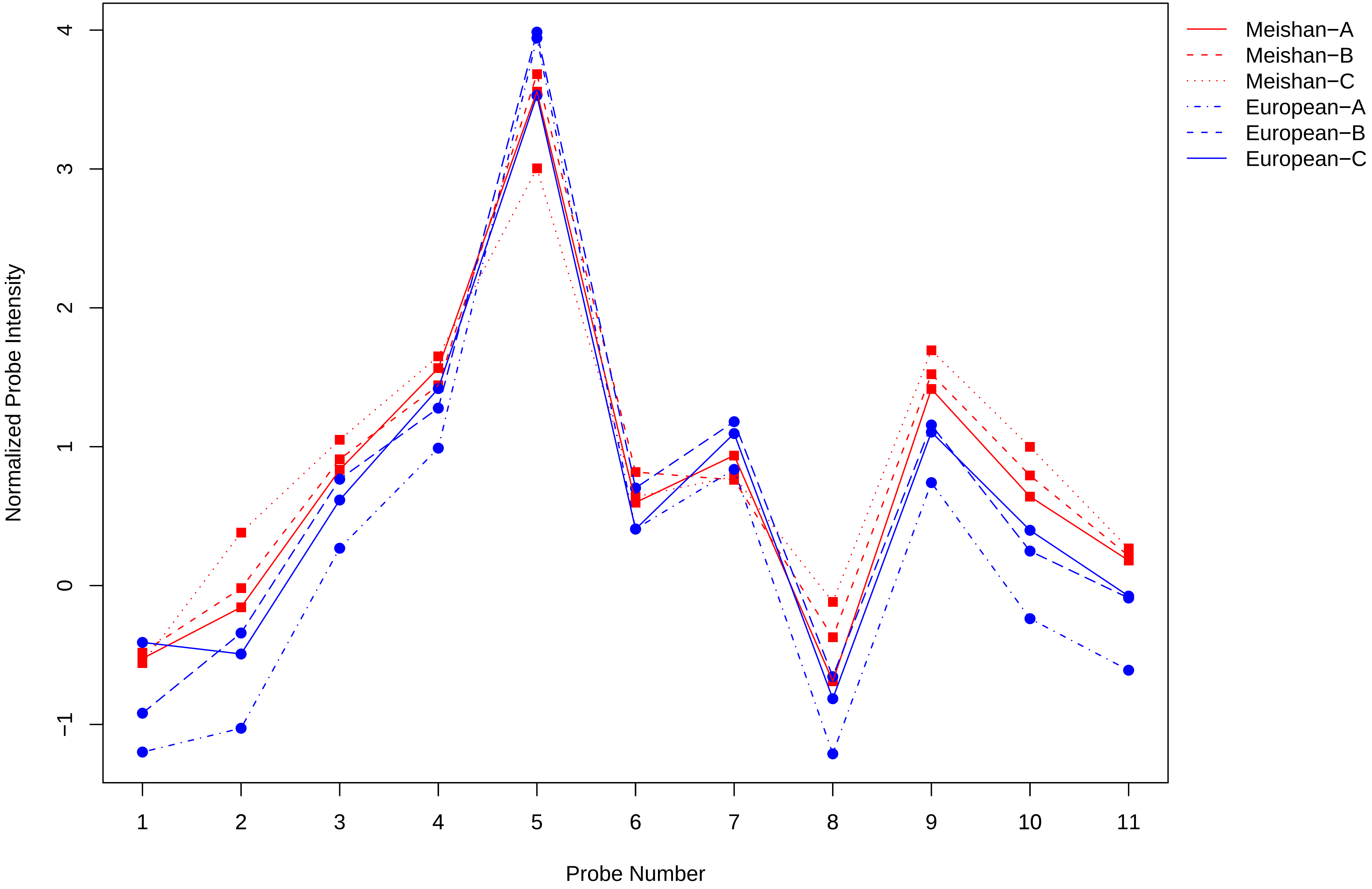

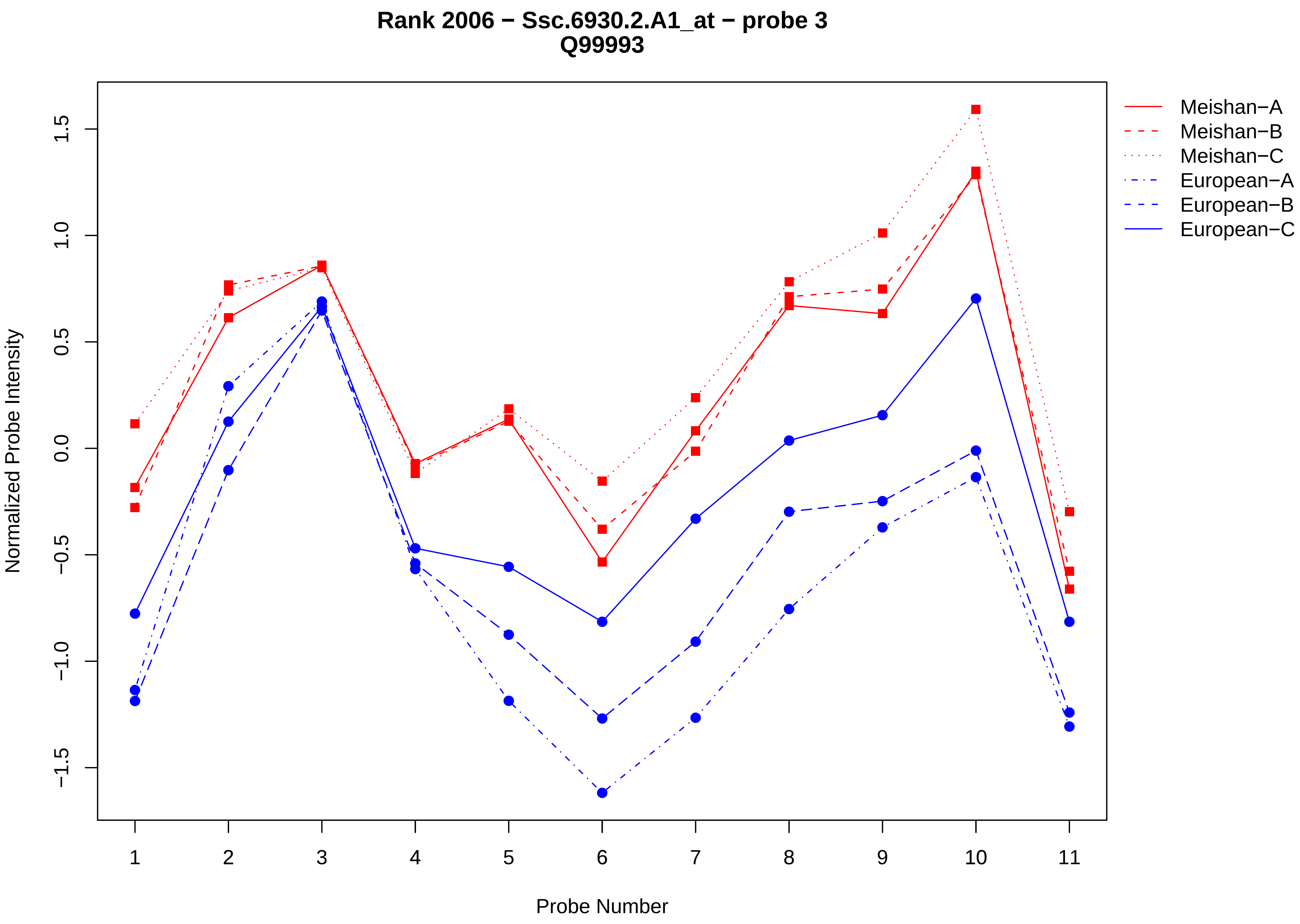
Rank 2006 − Ssc.6930.2.A1_at − probe 3
Q99993
Normalized Probe Intensity
Probe Number
Meishan−A
Meishan−B
Meishan−C
European−A
European−B
European−C
−1.5
−1.0
−0.5
0.0
0.5
1.0
1.5
1
2
3
4
5
6
7
8
9
10
11

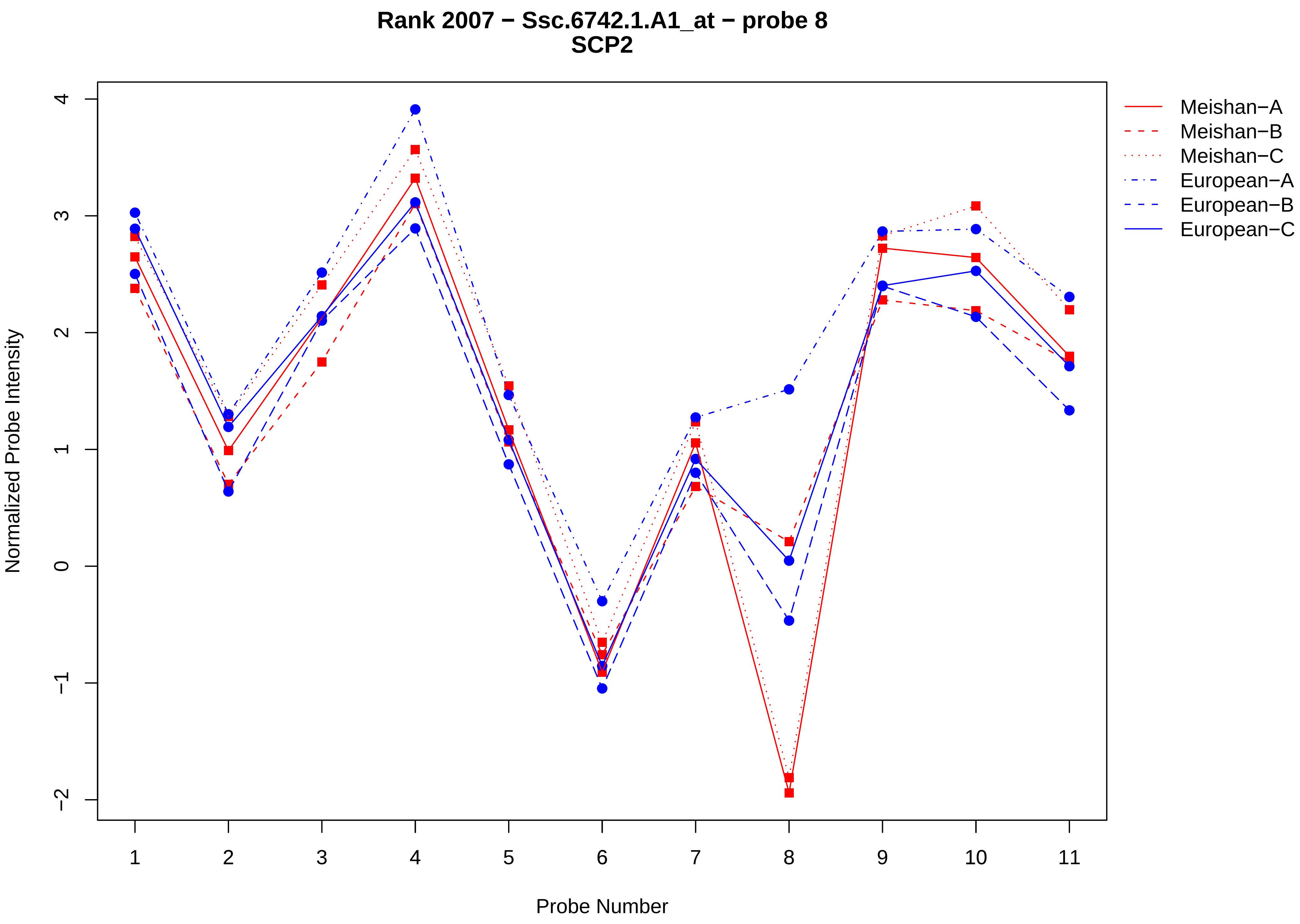

Rank 2007 − Ssc.6742.1.A1_at − probe 8
SCP2
Normalized Probe Intensity
Probe Number
Meishan−A
Meishan−B
Meishan−C
European−A
European−B
European−C

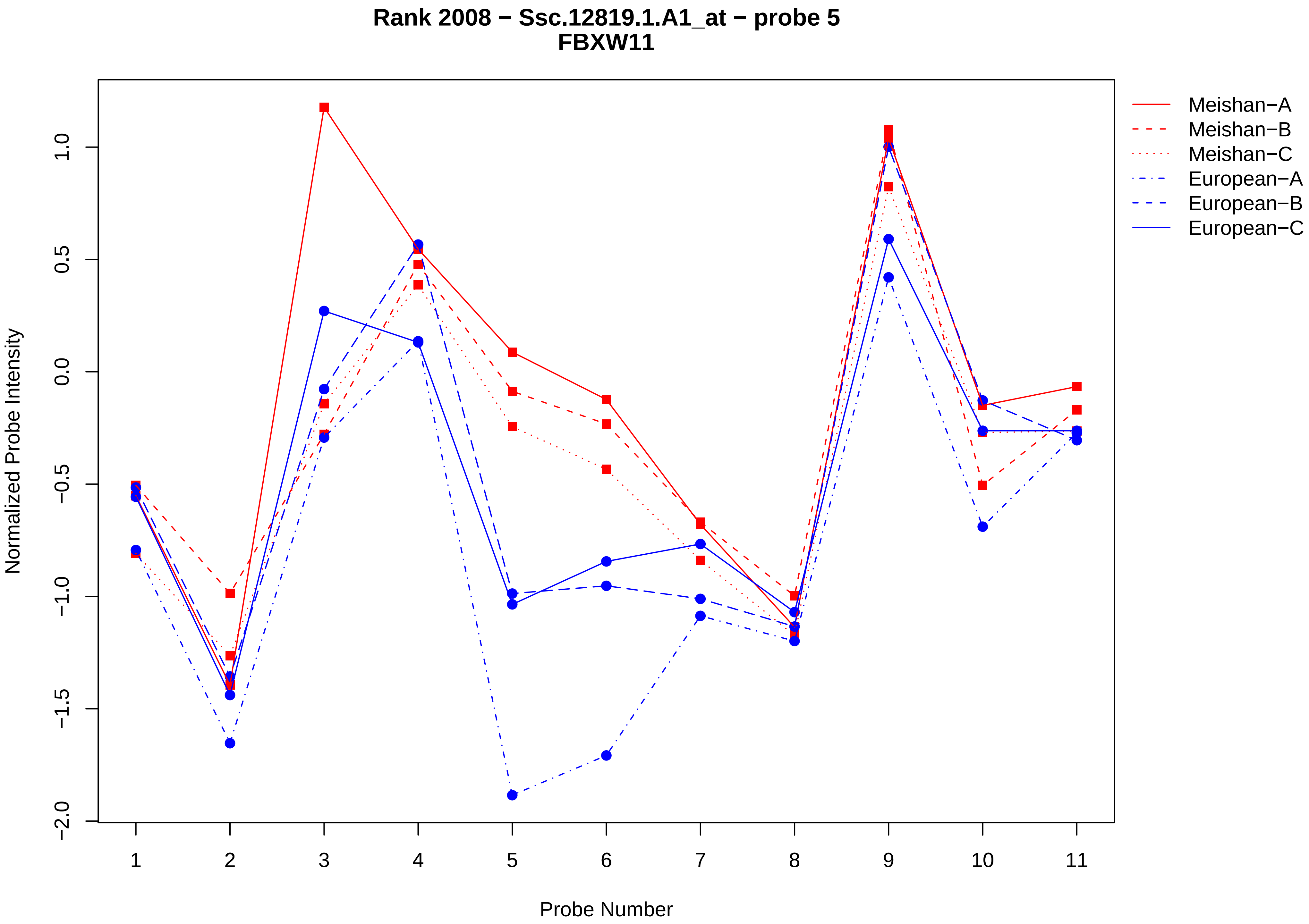

Rank 2008 − Ssc.12819.1.A1_at − probe 5
FBXW11
Normalized Probe Intensity
Probe Number
Meishan−A
Meishan−B
Meishan−C
European−A
European−B
European−C
1.0
0.5
0.0
−0.5
−1.0
−1.5
−2.0
1
2
3
4
5
6
7
8
9
10
11

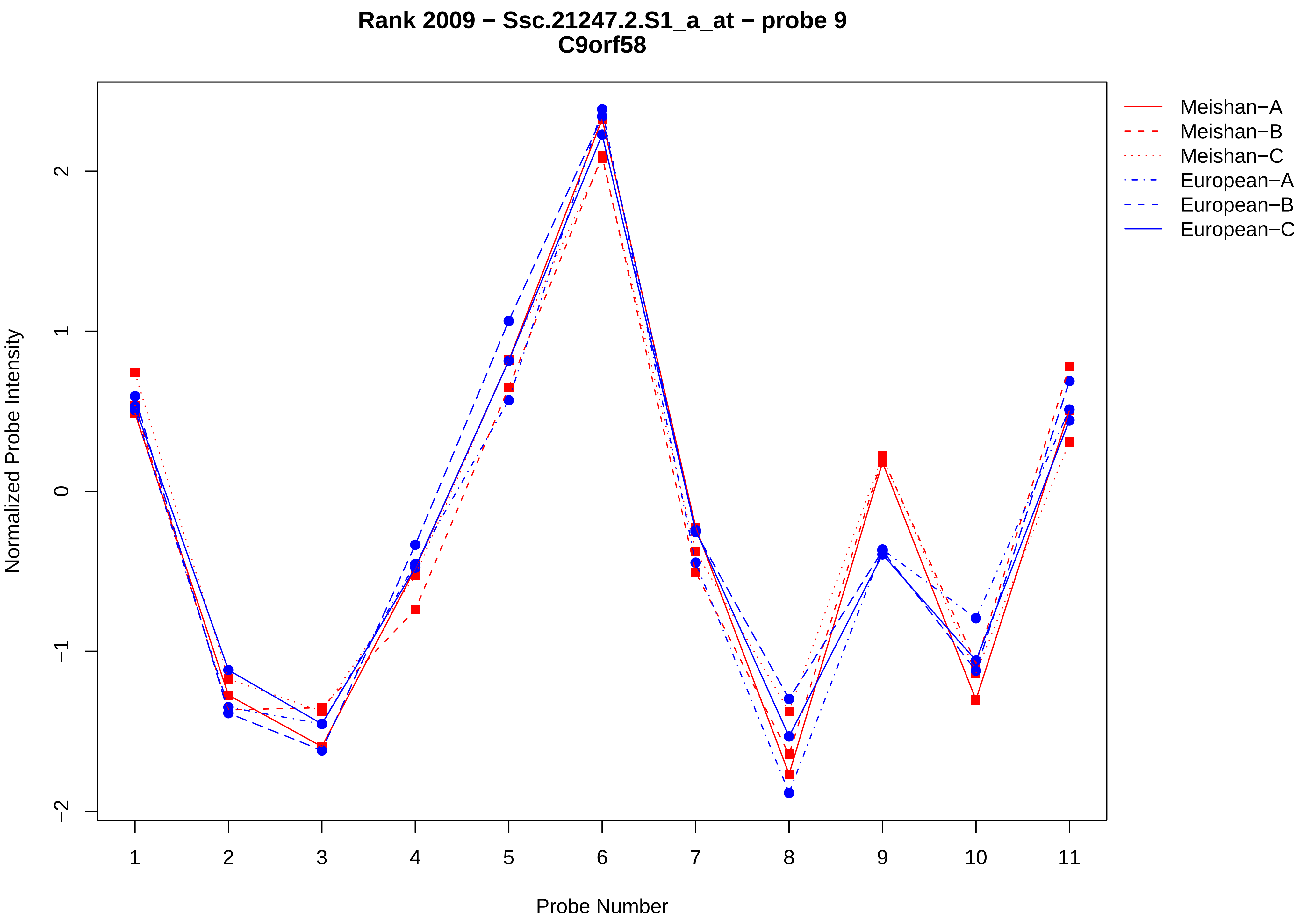

Rank 2009 − Ssc.21247.2.S1_a_at − probe 9
C9orf58
Normalized Probe Intensity
Probe Number
−2
−1
0
1
2
1
2
3
4
5
6
7
8
9
10
11
Meishan−A
Meishan−B
Meishan−C
European−A
European−B
European−C

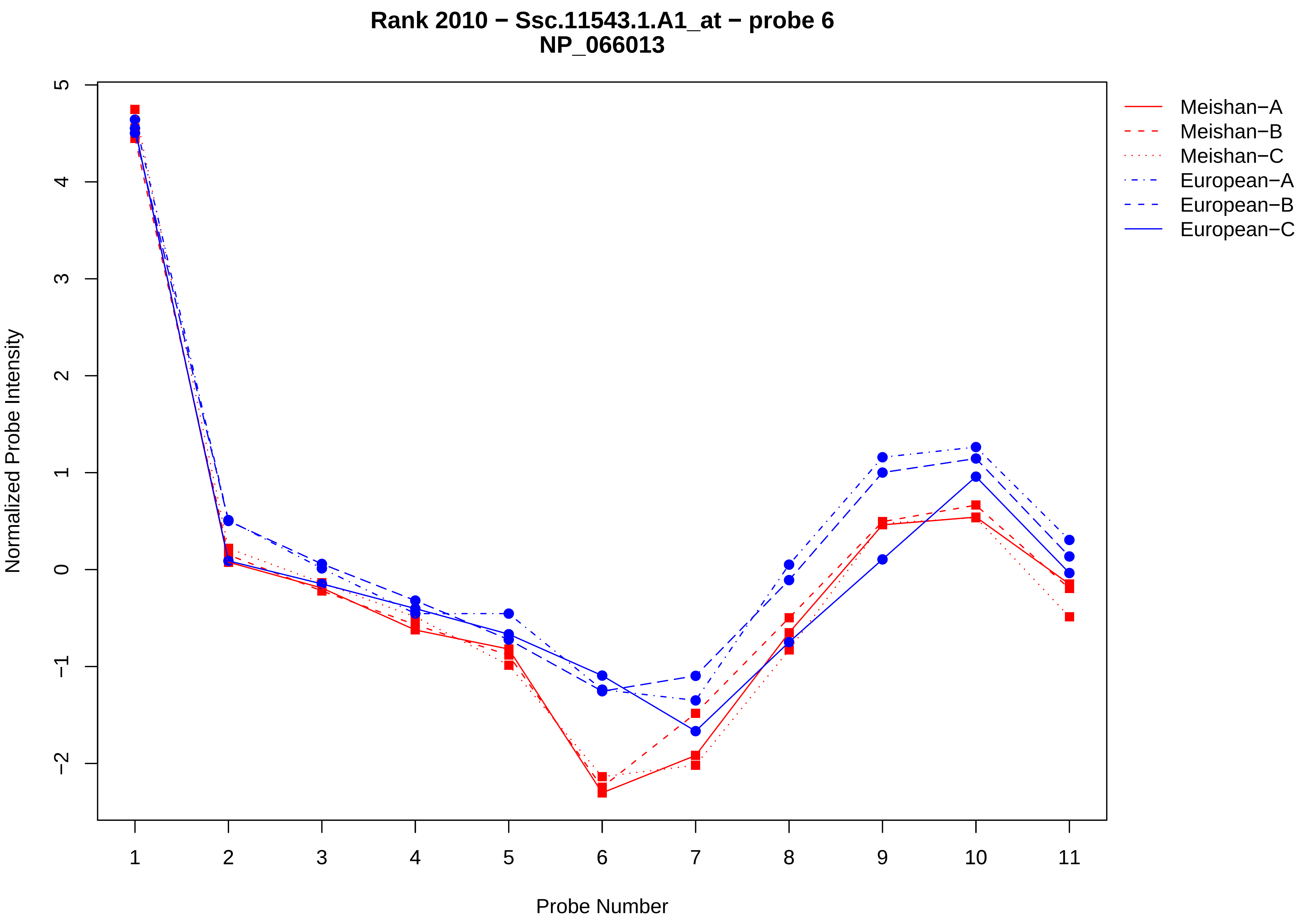

Rank 2010 − Ssc.11543.1.A1_at − probe 6
NP_066013
Normalized Probe Intensity
Probe Number
Meishan−A
Meishan−B
Meishan−C
European−A
European−B
European−C

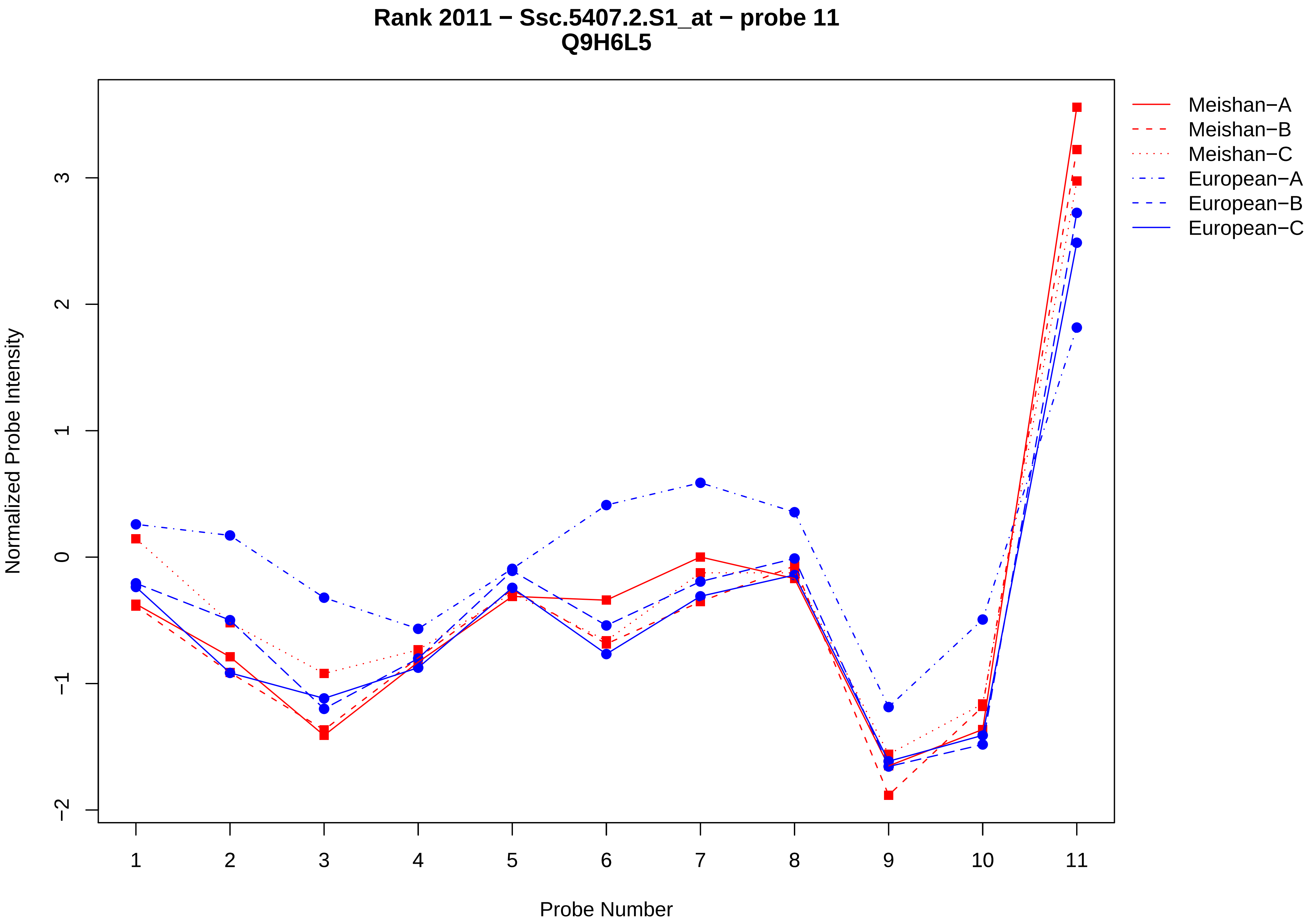

Rank 2011 − Ssc.5407.2.S1_at − probe 11
Q9H6L5
Normalized Probe Intensity
Probe Number
Meishan−A
Meishan−B
Meishan−C
European−A
European−B
European−C
−2
−1
0
1
2
3
1
2
3
4
5
6
7
8
9
10
11

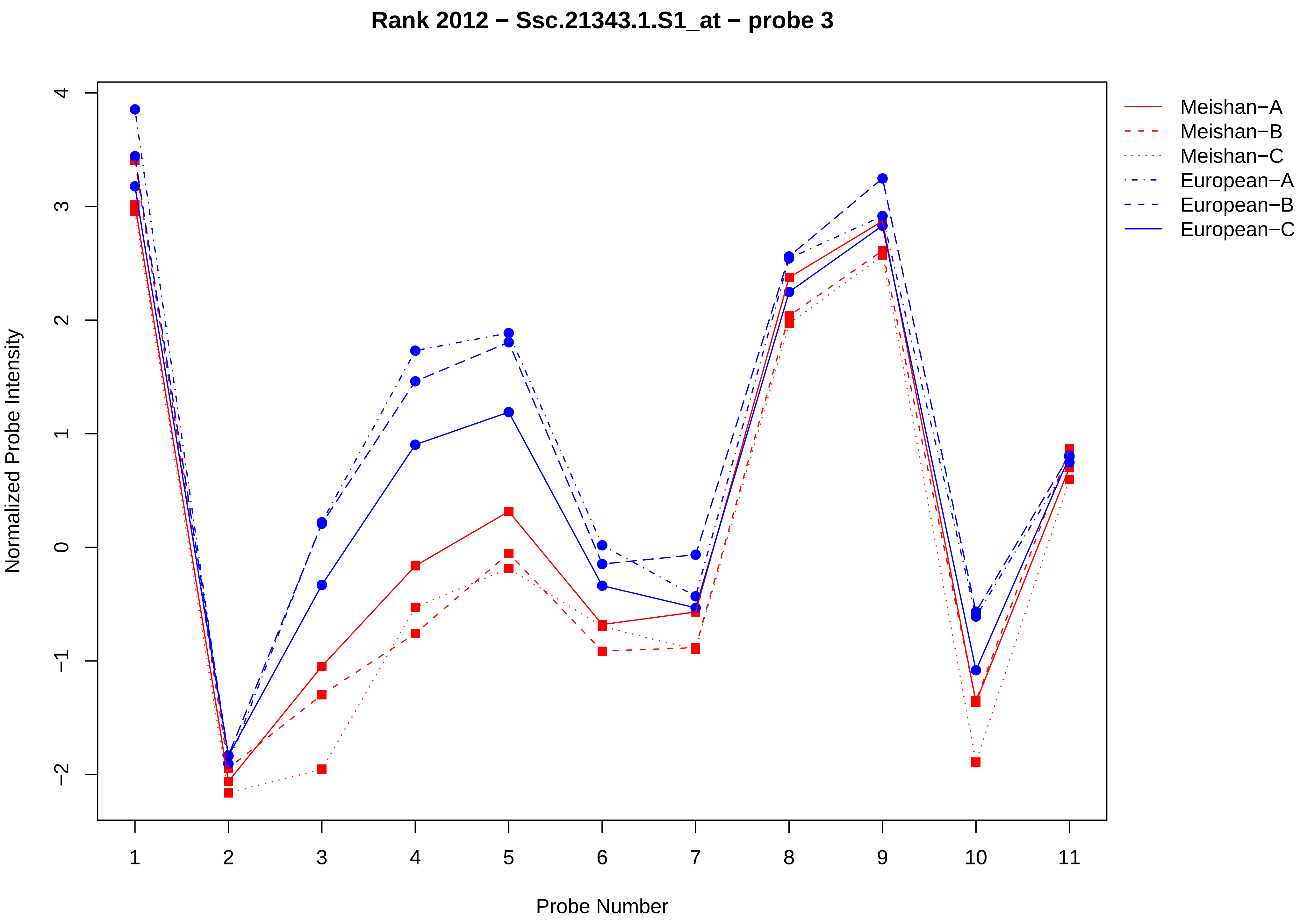
Rank 2012 − Ssc.21343.1.S1_at − probe 3
Normalized Probe Intensity
Probe Number
Meishan−A
Meishan−B
Meishan−C
European−A
European−B
European−C

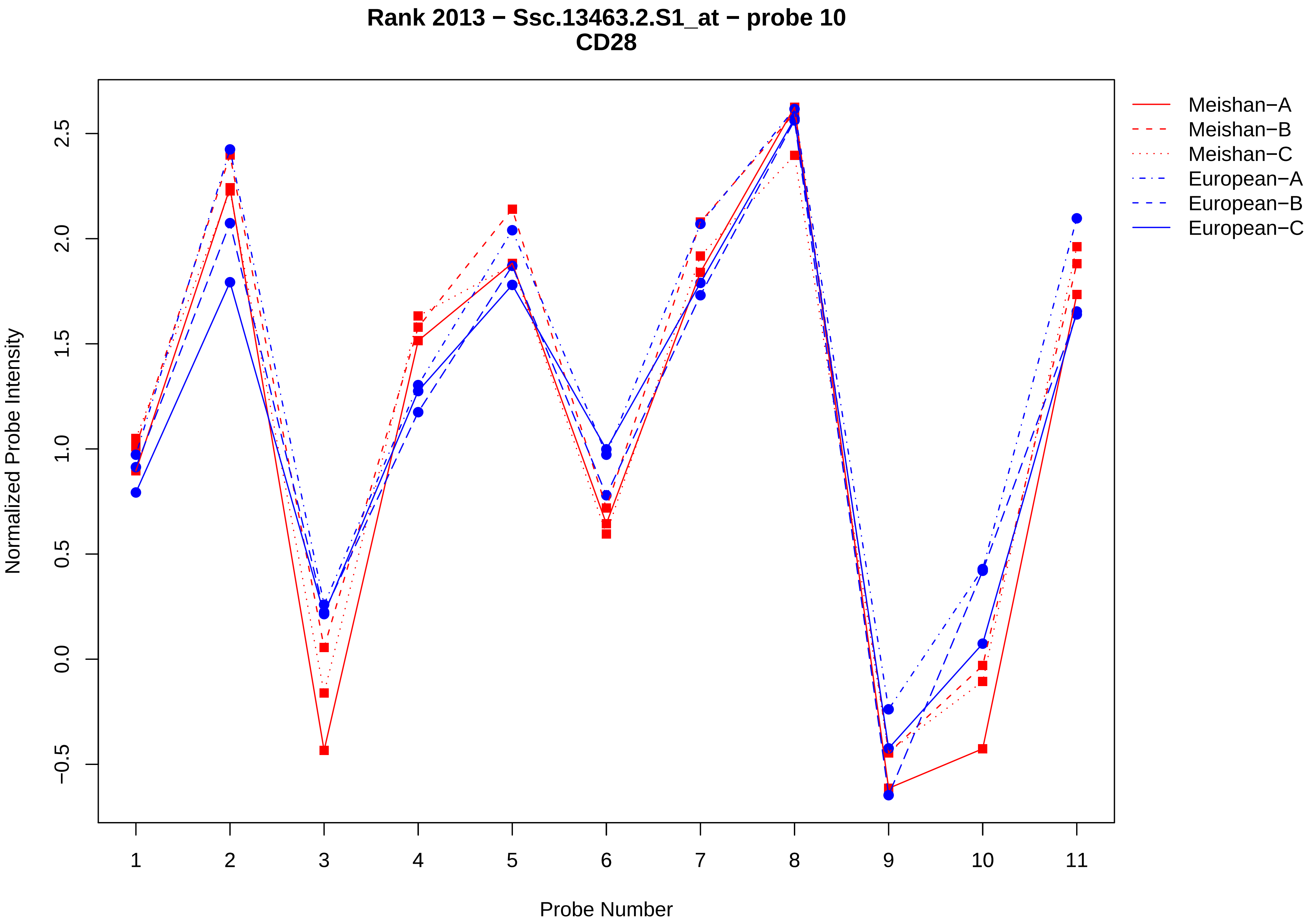
Rank 2013 − Ssc.13463.2.S1_at − probe 10
CD28
Normalized Probe Intensity
Probe Number
−0.5
0.0
0.5
1.0
1.5
2.0
2.5
1
2
3
4
5
6
7
8
9
10
11
Meishan−A
Meishan−B
Meishan−C
European−A
European−B
European−C

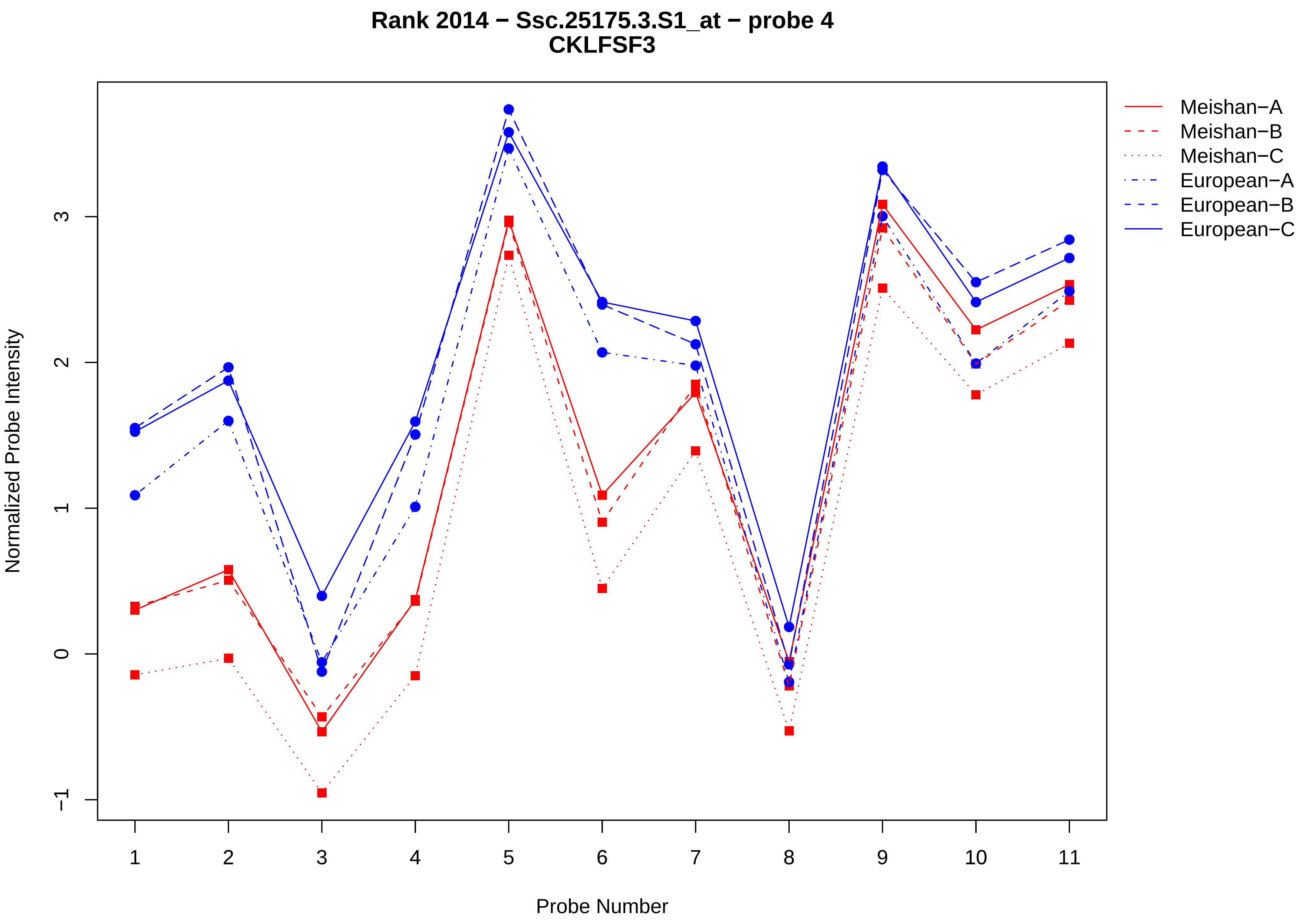

Rank 2014 − Ssc.25175.3.S1_at − probe 4
CKLFSF3
Normalized Probe Intensity
Probe Number
−1
0
1
2
3
1
2
3
4
5
6
7
8
9
10
11
Meishan−A
Meishan−B
Meishan−C
European−A
European−B
European−C

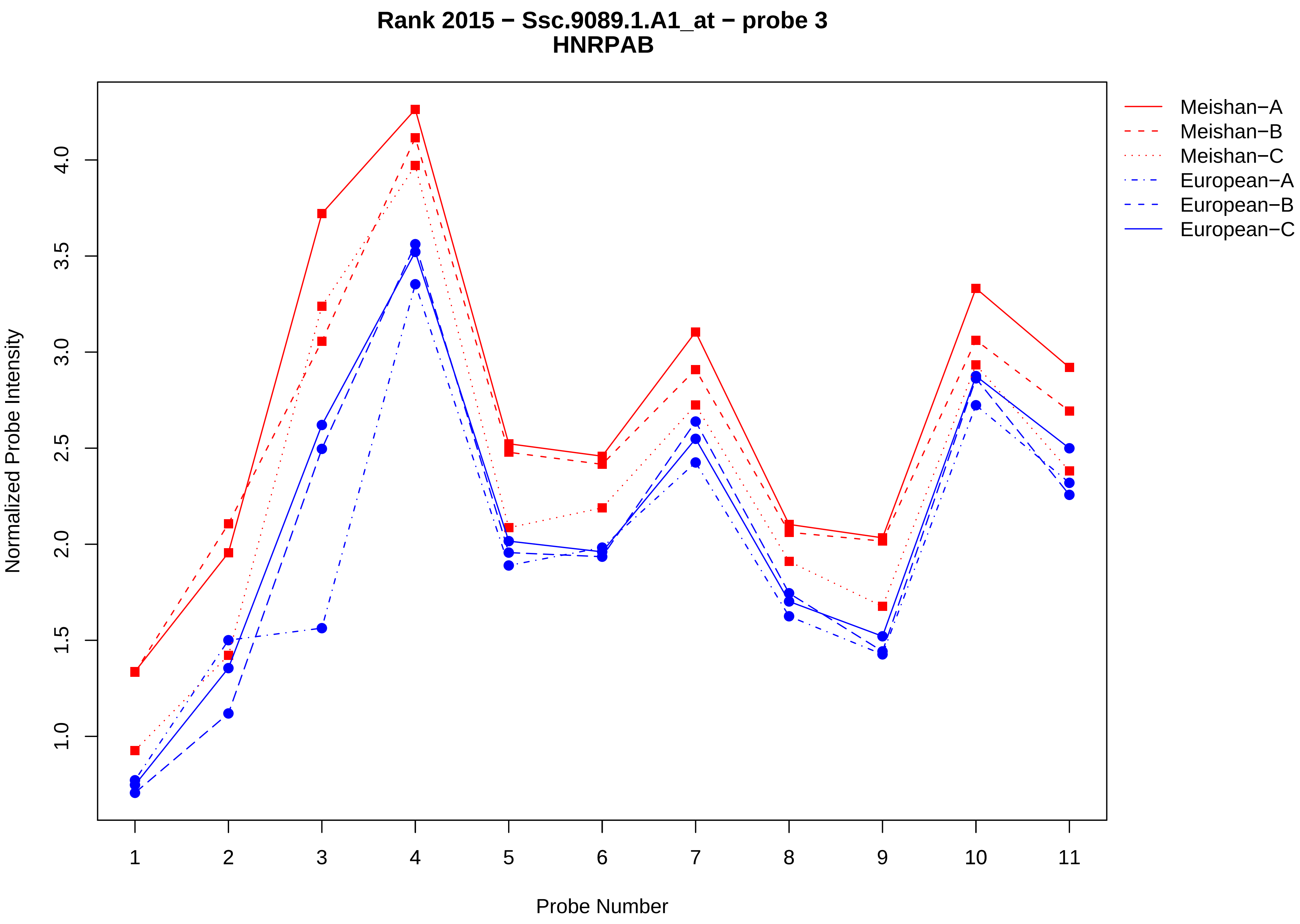
Rank 2015 − Ssc.9089.1.A1_at − probe 3
HNRPAB
Normalized Probe Intensity
1.0
1.5
2.0
2.5
3.0
3.5
4.0
Probe Number
1
2
3
4
5
6
7
8
9
10
11
Meishan−A
Meishan−B
Meishan−C
European−A
European−B
European−C

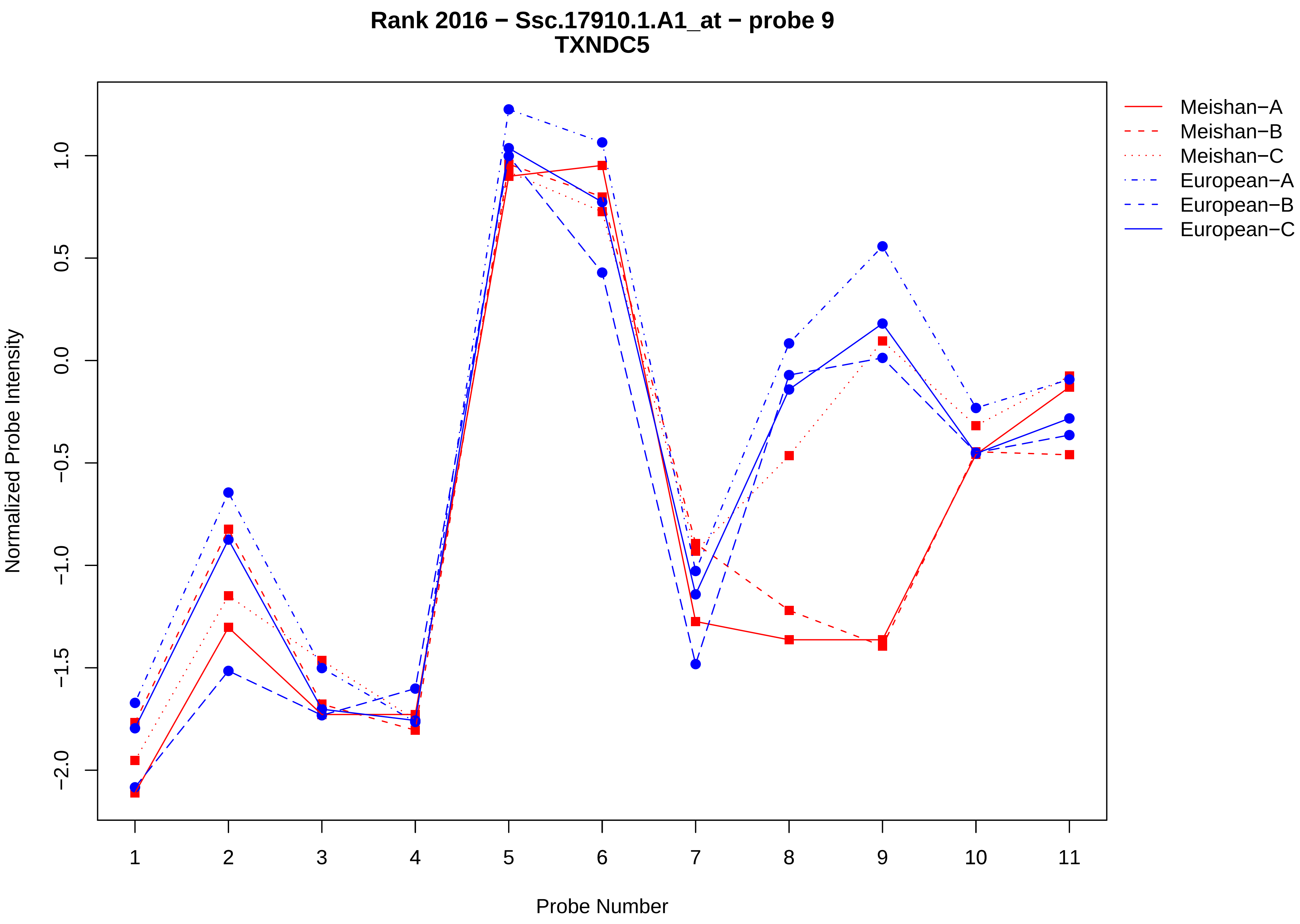

Rank 2016 − Ssc.17910.1.A1_at − probe 9
TXNDC5
Normalized Probe Intensity
−2.0
−1.5
−1.0
−0.5
0.0
0.5
1.0
1
2
3
4
5
6
7
8
9
10
11
Probe Number
Meishan−A
Meishan−B
Meishan−C
European−A
European−B
European−C

Rank 2017 − Ssc.29318.1.A1_at − probe 4
MTMR1

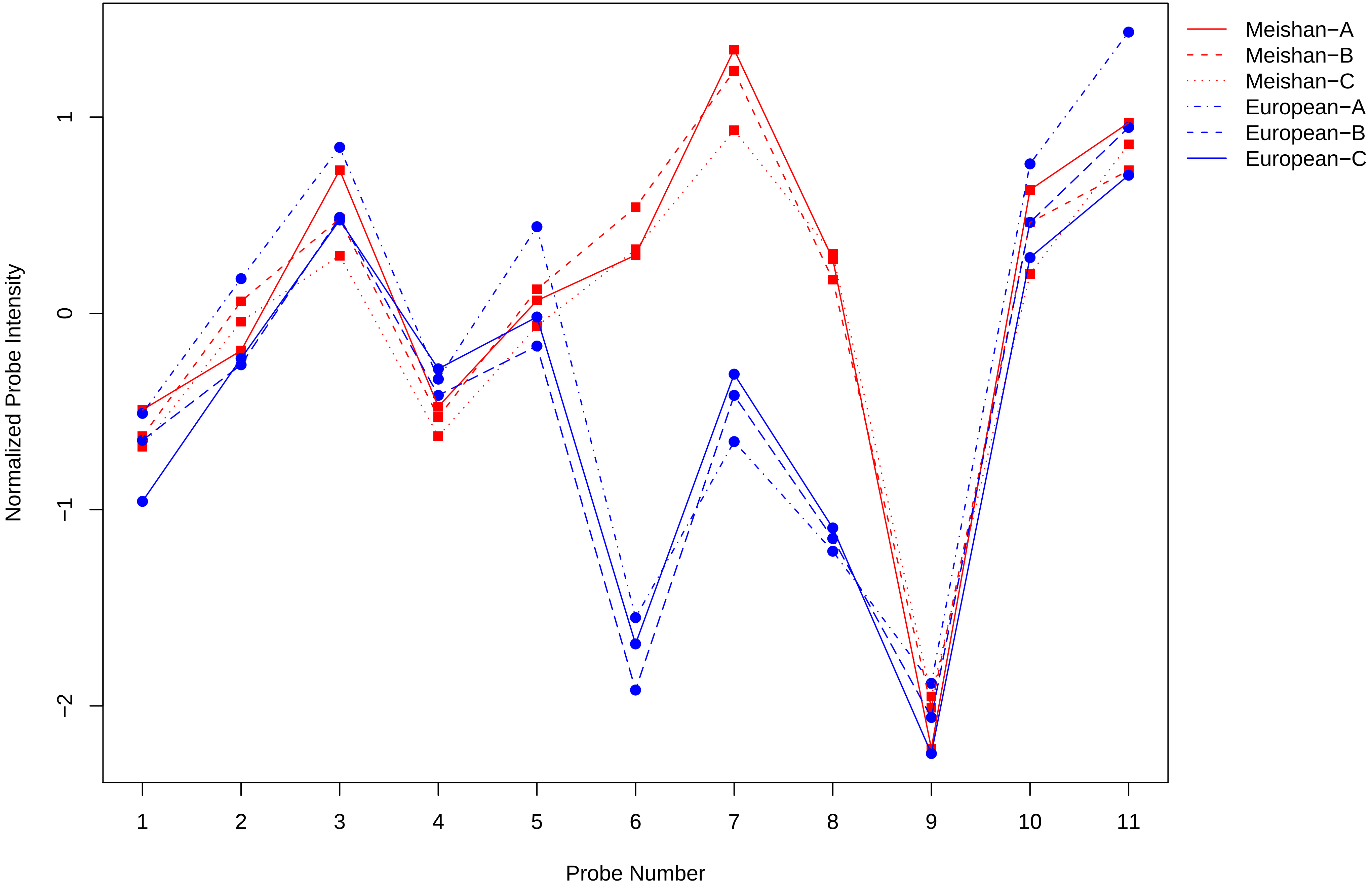

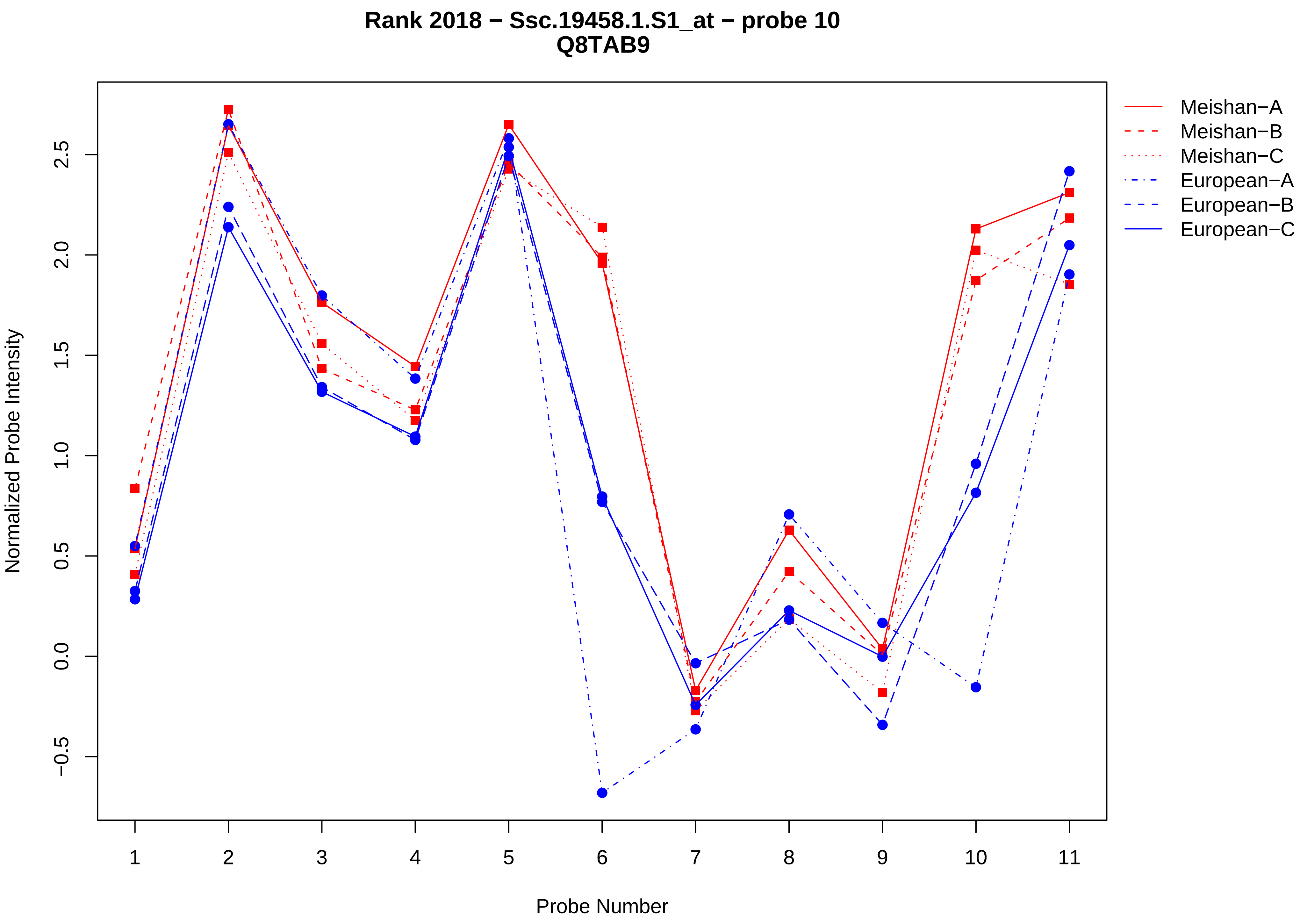

Rank 2018 − Ssc.19458.1.S1_at − probe 10
Q8TAB9
Normalized Probe Intensity
Probe Number
−0.5
0.0
0.5
1.0
1.5
2.0
2.5
1
2
3
4
5
6
7
8
9
10
11
Meishan−A
Meishan−B
Meishan−C
European−A
European−B
European−C

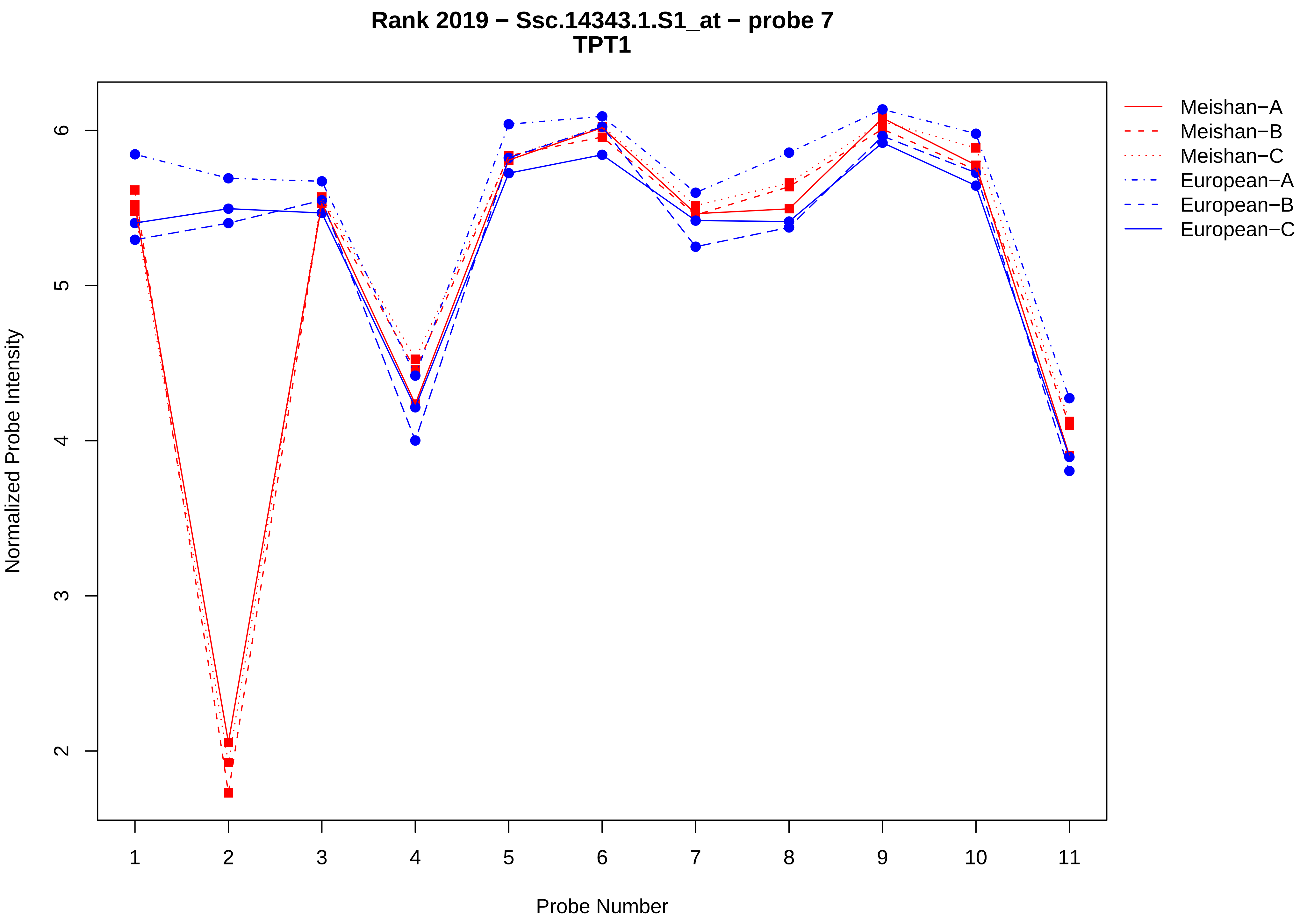

Rank 2019 − Ssc.14343.1.S1_at − probe 7
TPT1
Normalized Probe Intensity
Probe Number
Meishan−A
Meishan−B
Meishan−C
European−A
European−B
European−C

Rank 2020 − Ssc.31196.1.S1_at − probe 11
3208550400

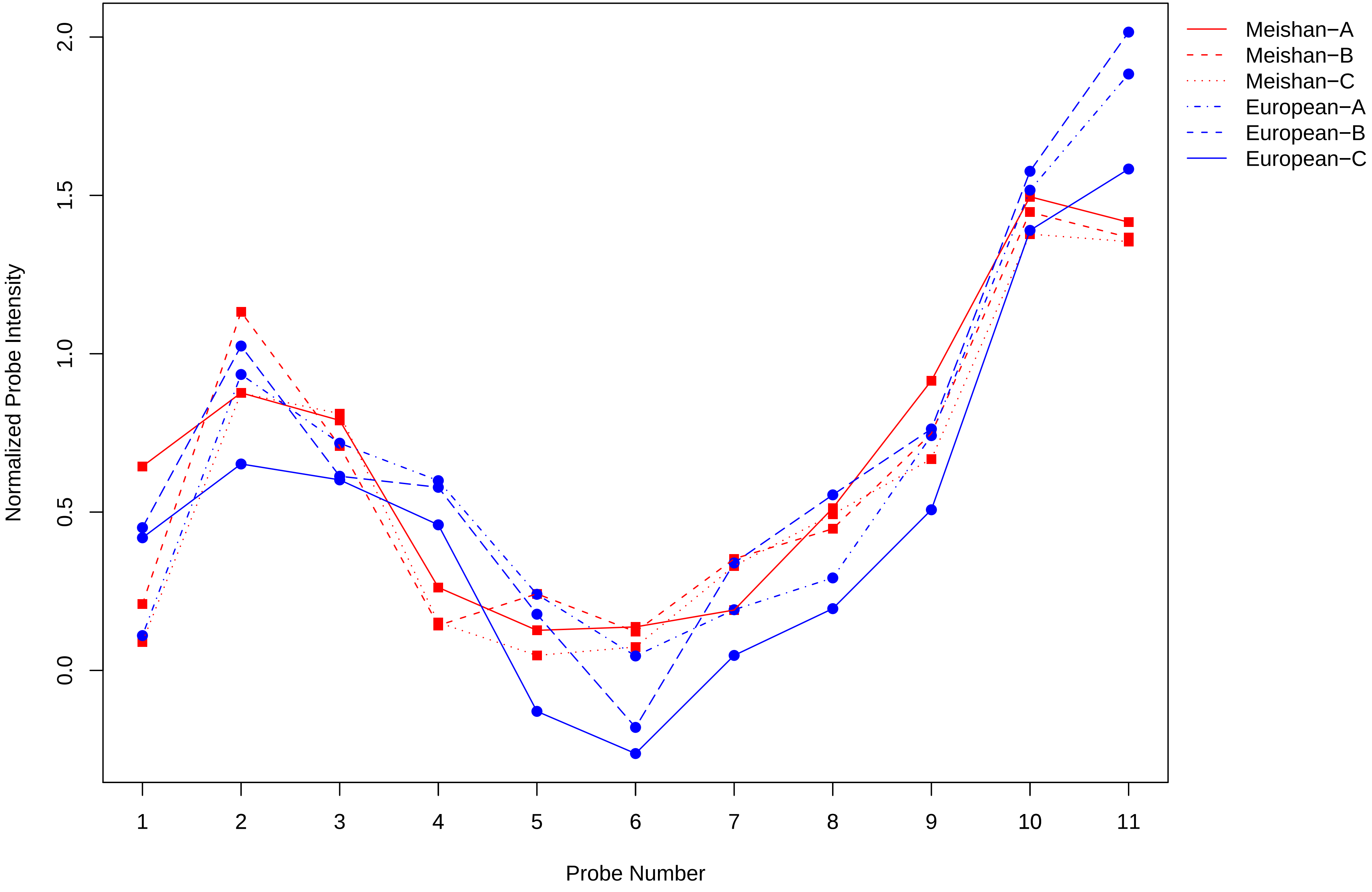

Rank 2021 − Ssc.10373.1.S1_at − probe 10
PREX_HUMAN

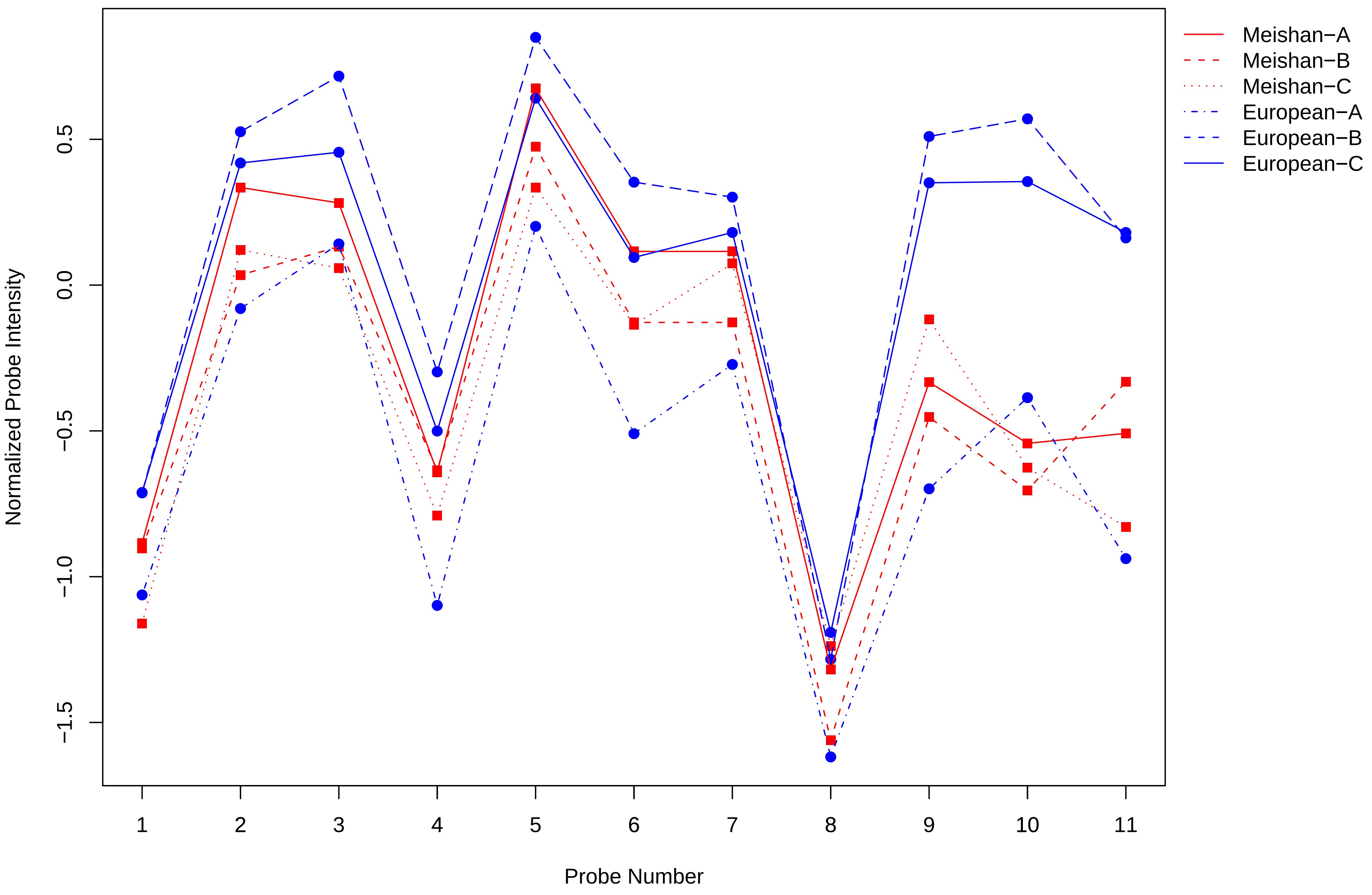

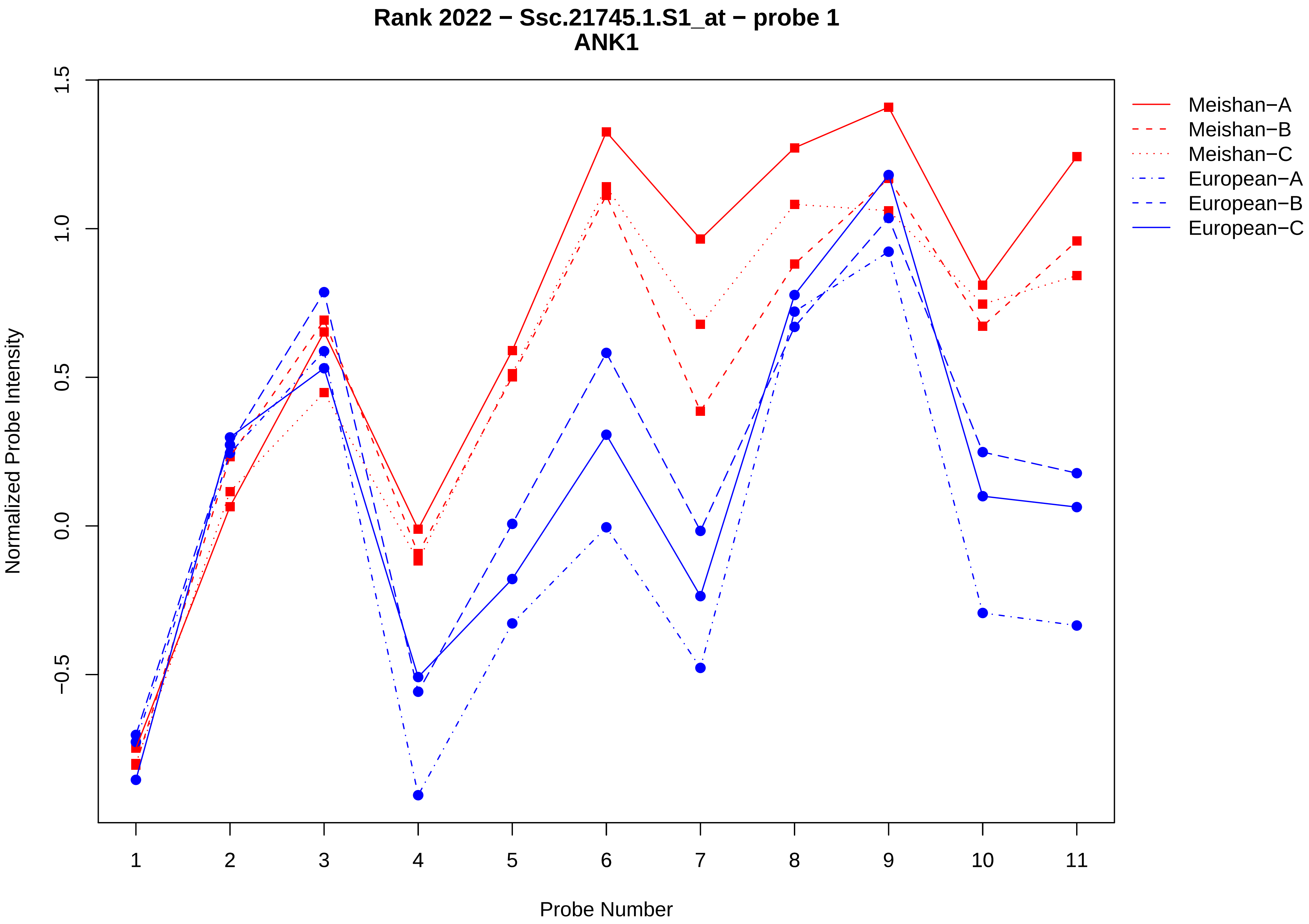

Rank 2022 − Ssc.21745.1.S1_at − probe 1
ANK1
Normalized Probe Intensity
Probe Number
−0.5
0.0
0.5
1.0
1.5
1
2
3
4
5
6
7
8
9
10
11
Meishan−A
Meishan−B
Meishan−C
European−A
European−B
European−C

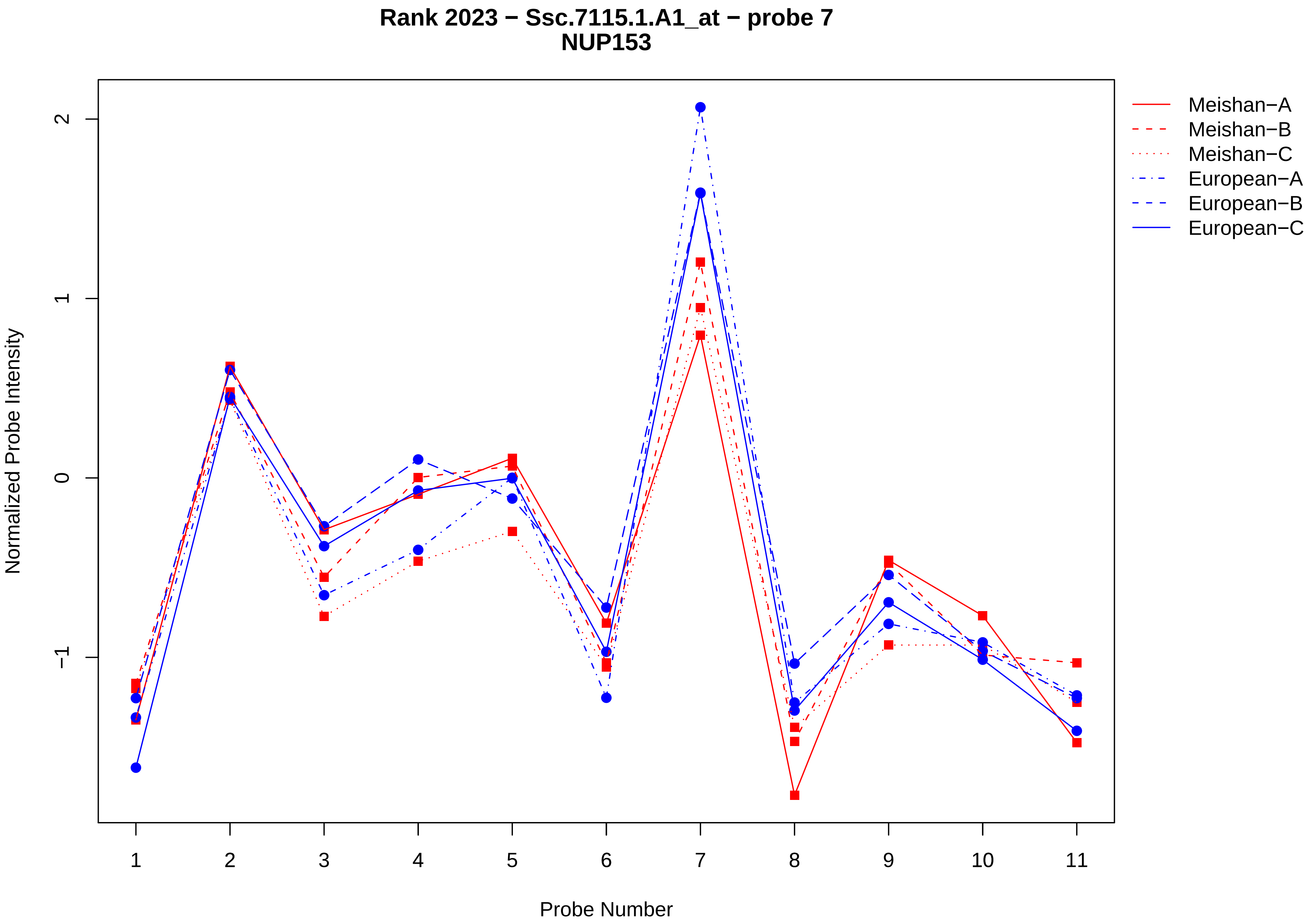

Rank 2023 − Ssc.7115.1.A1_at − probe 7
NUP153
Normalized Probe Intensity
Probe Number
Meishan−A
Meishan−B
Meishan−C
European−A
European−B
European−C
−1
0
1
2
1
2
3
4
5
6
7
8
9
10
11

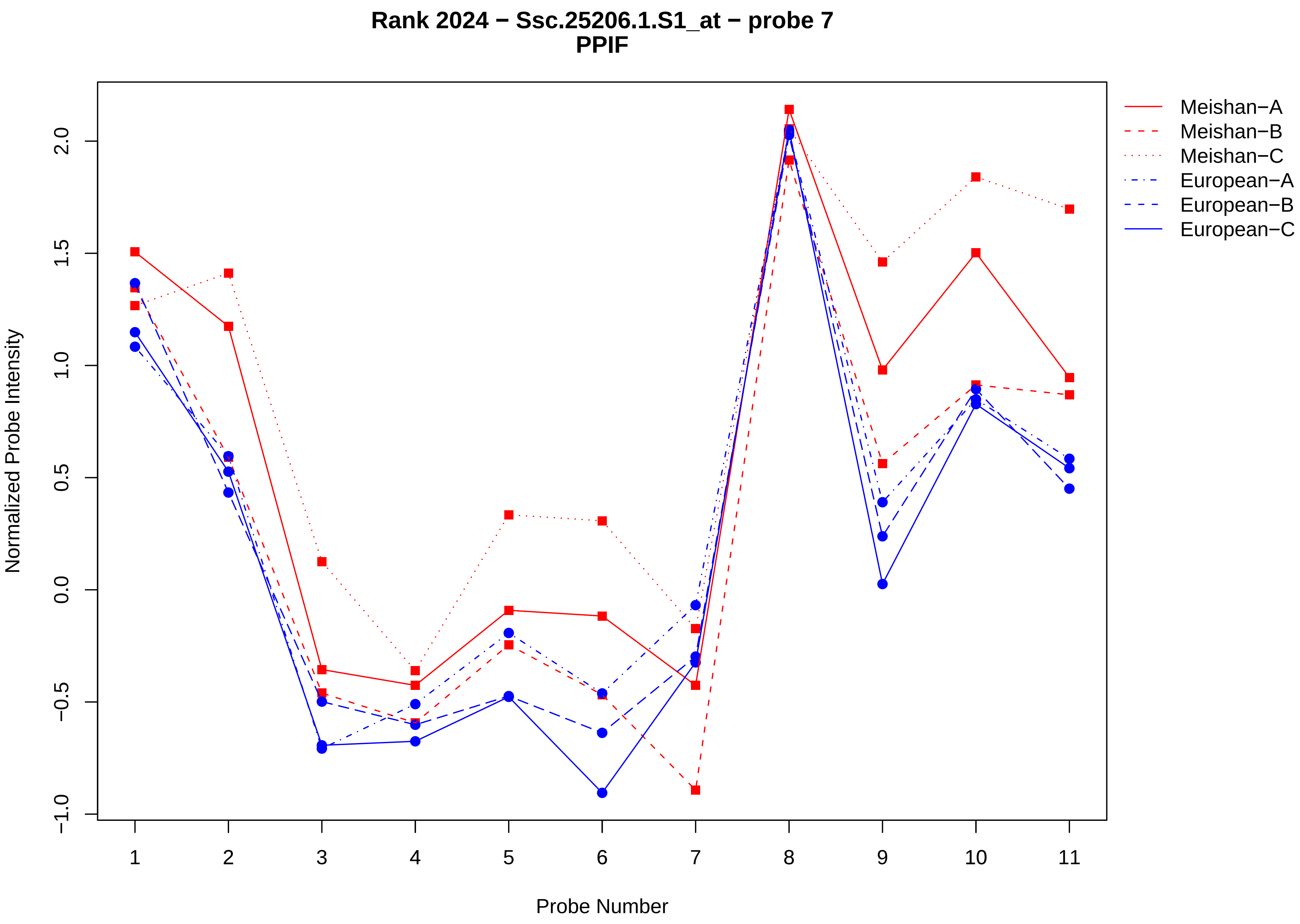
Rank 2024 − Ssc.25206.1.S1_at − probe 7
PPIF
Normalized Probe Intensity
Probe Number
Meishan−A
Meishan−B
Meishan−C
European−A
European−B
European−C
−1.0
−0.5
0.0
0.5
1.0
1.5
2.0
1
2
3
4
5
6
7
8
9
10
11

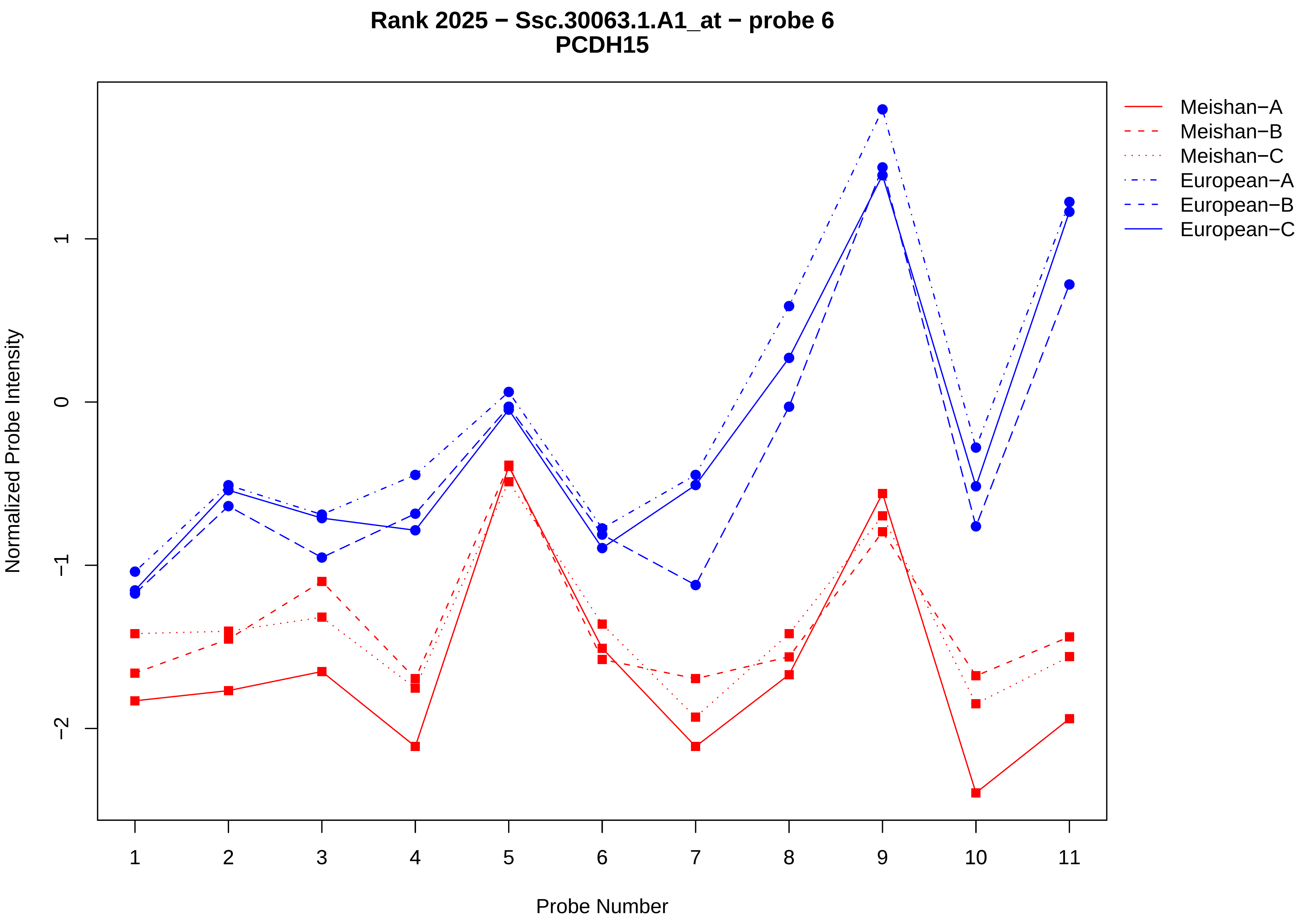

Rank 2025 − Ssc.30063.1.A1_at − probe 6
PCDH15
Normalized Probe Intensity
Probe Number
−2
−1
0
1
1
2
3
4
5
6
7
8
9
10
11
Meishan−A
Meishan−B
Meishan−C
European−A
European−B
European−C

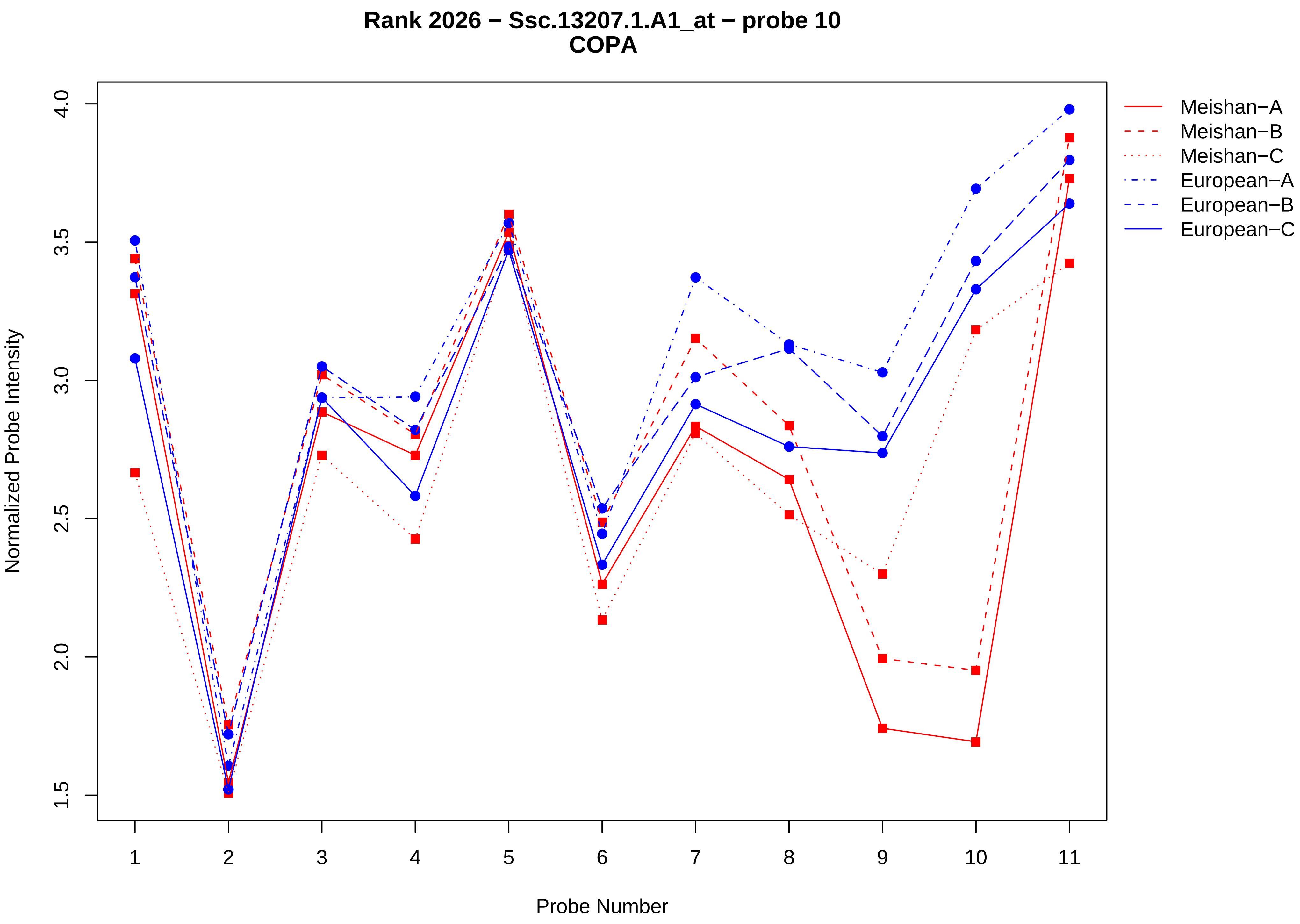
Rank 2026 − Ssc.13207.1.A1_at − probe 10
COPA
Normalized Probe Intensity
Probe Number
1.5
2.0
2.5
3.0
3.5
4.0
1
2
3
4
5
6
7
8
9
10
11
Meishan−A
Meishan−B
Meishan−C
European−A
European−B
European−C

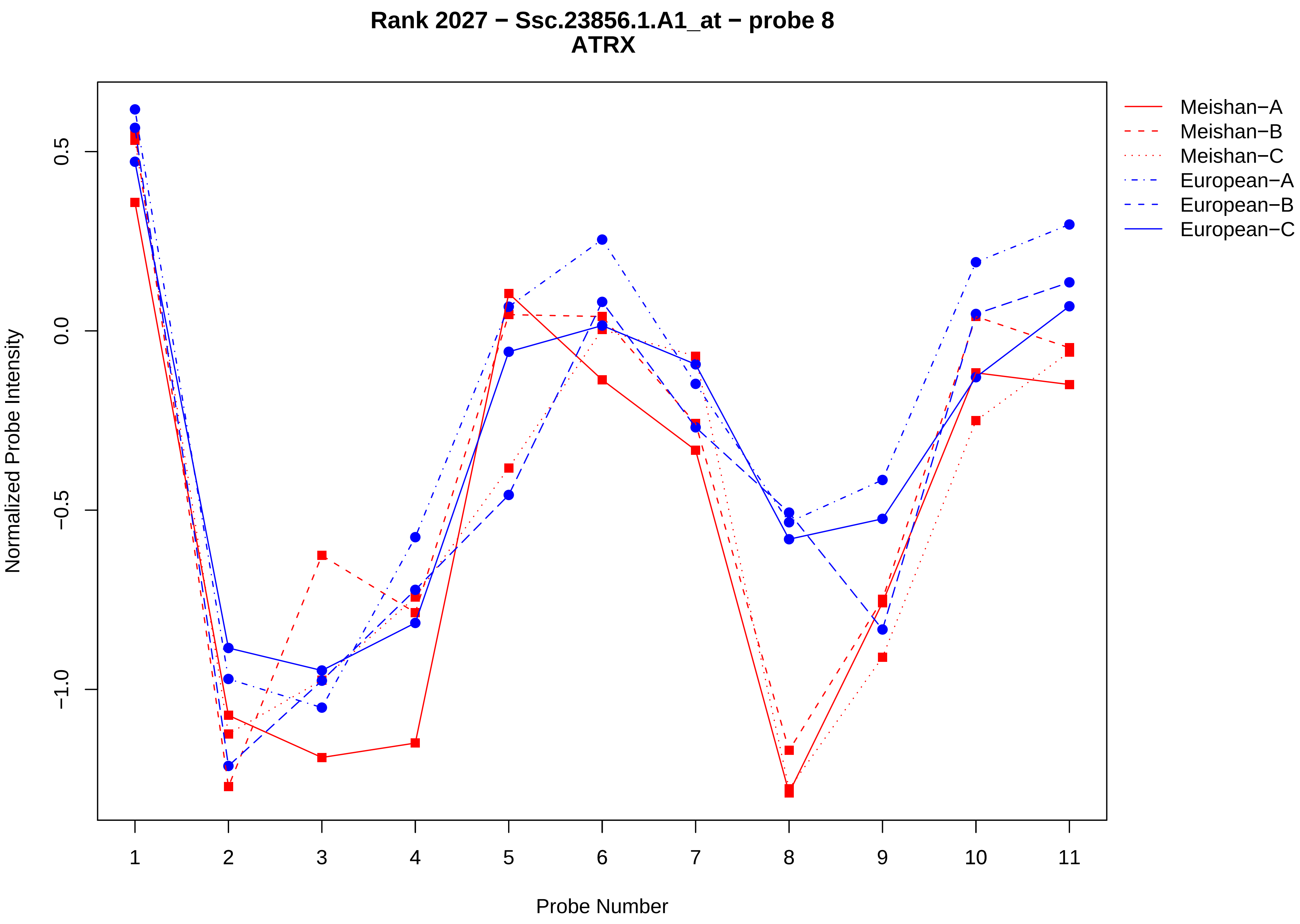

Rank 2027 − Ssc.23856.1.A1_at − probe 8
ATRX
Normalized Probe Intensity
0.5
0.0
−0.5
−1.0
1
2
3
4
5
6
7
8
9
10
11
Probe Number
Meishan−A
Meishan−B
Meishan−C
European−A
European−B
European−C

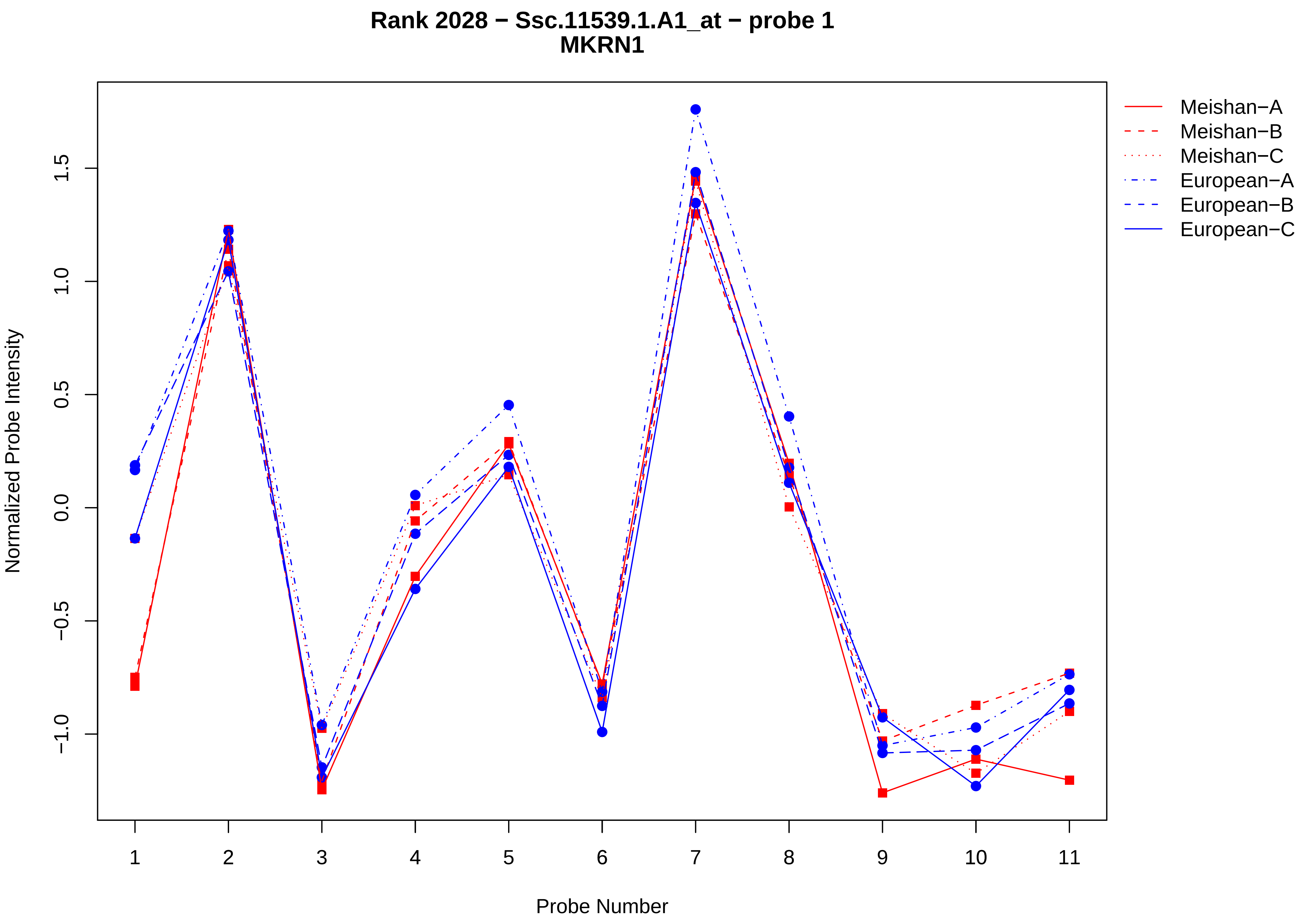

Rank 2028 − Ssc.11539.1.A1_at − probe 1
MKRN1
Normalized Probe Intensity
Probe Number
Meishan−A
Meishan−B
Meishan−C
European−A
European−B
European−C
−1.0
−0.5
0.0
0.5
1.0
1.5
1
2
3
4
5
6
7
8
9
10
11

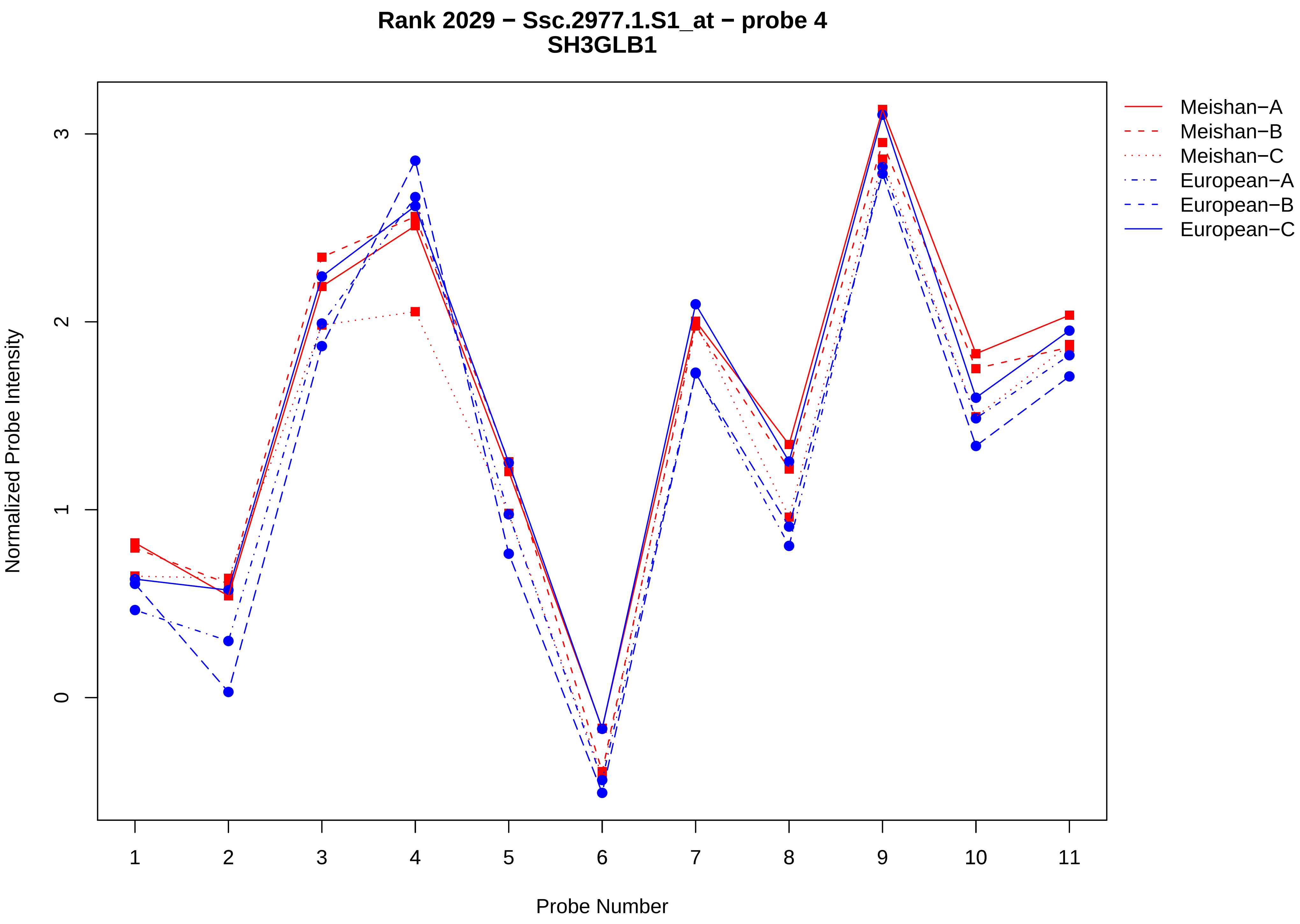
Rank 2029 − Ssc.2977.1.S1_at − probe 4
SH3GLB1
Normalized Probe Intensity
Probe Number
0
1
2
3
1
2
3
4
5
6
7
8
9
10
11
Meishan−A
Meishan−B
Meishan−C
European−A
European−B
European−C

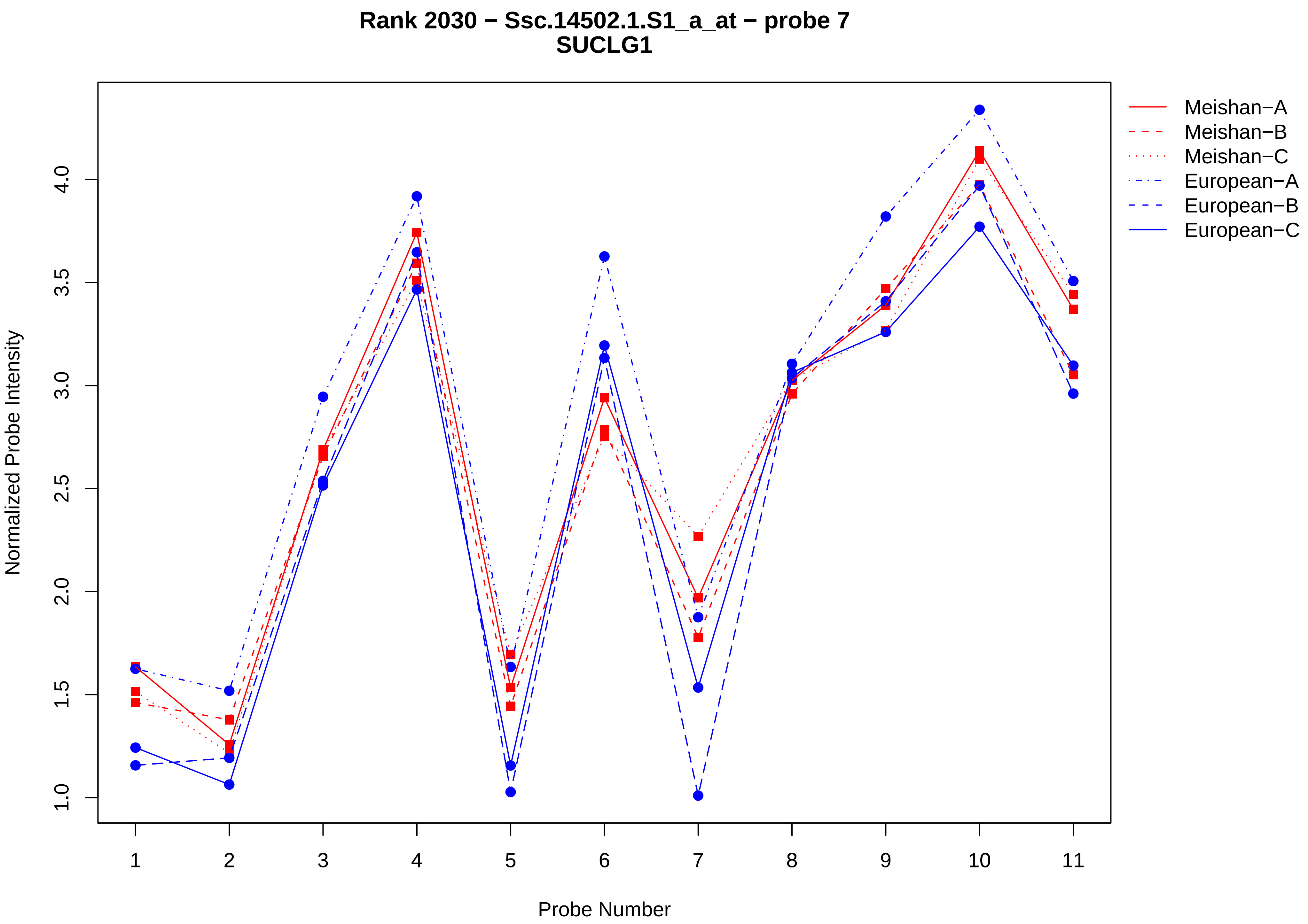
Rank 2030 − Ssc.14502.1.S1_a_at − probe 7
SUCLG1
Normalized Probe Intensity
Probe Number
1.0
1.5
2.0
2.5
3.0
3.5
4.0
1
2
3
4
5
6
7
8
9
10
11
Meishan−A
Meishan−B
Meishan−C
European−A
European−B
European−C

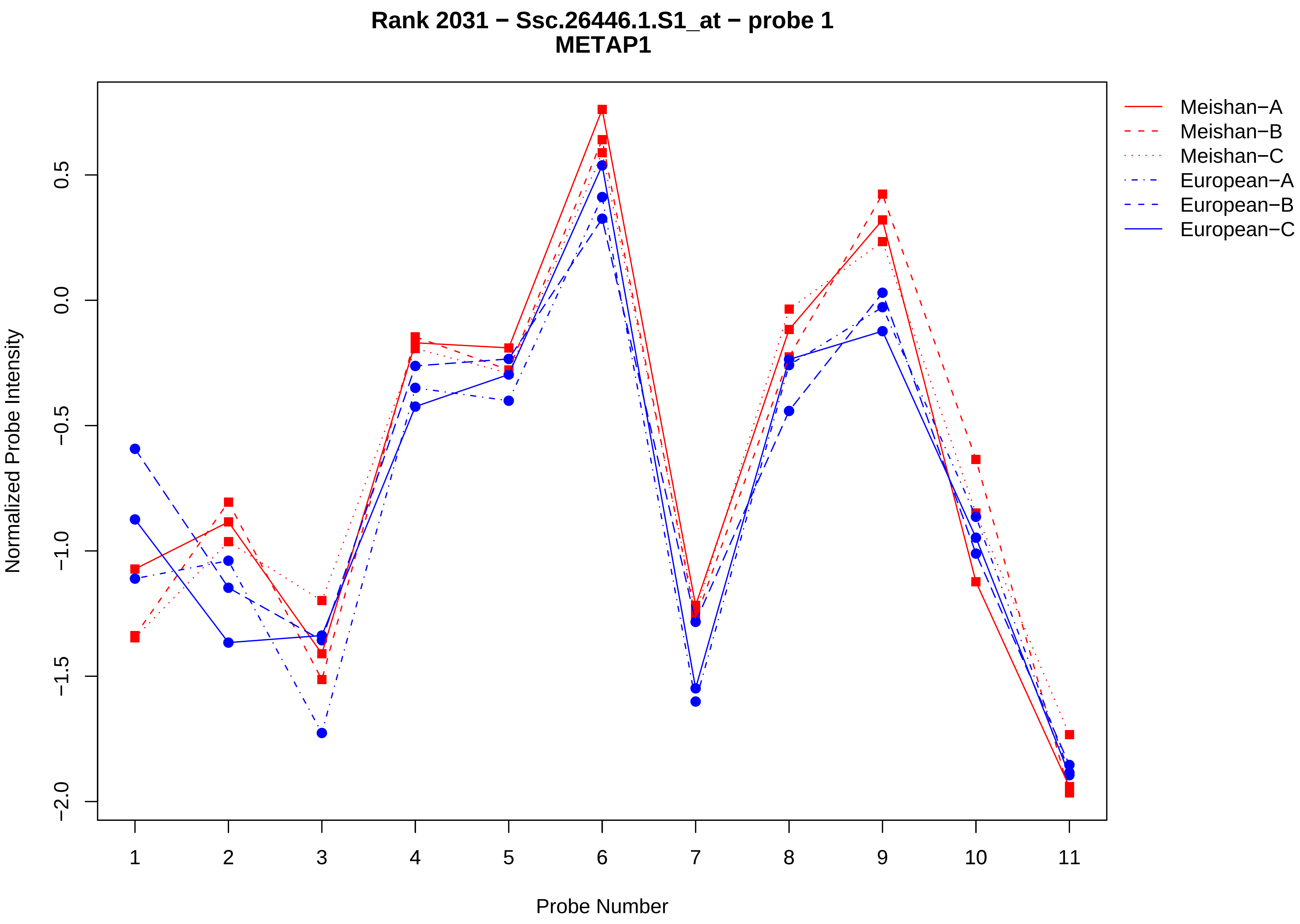
Rank 2031 − Ssc.26446.1.S1_at − probe 1
METAP1
Normalized Probe Intensity
Probe Number
0.5
0.0
−0.5
−1.0
−1.5
−2.0
1
2
3
4
5
6
7
8
9
10
11
Meishan−A
Meishan−B
Meishan−C
European−A
European−B
European−C

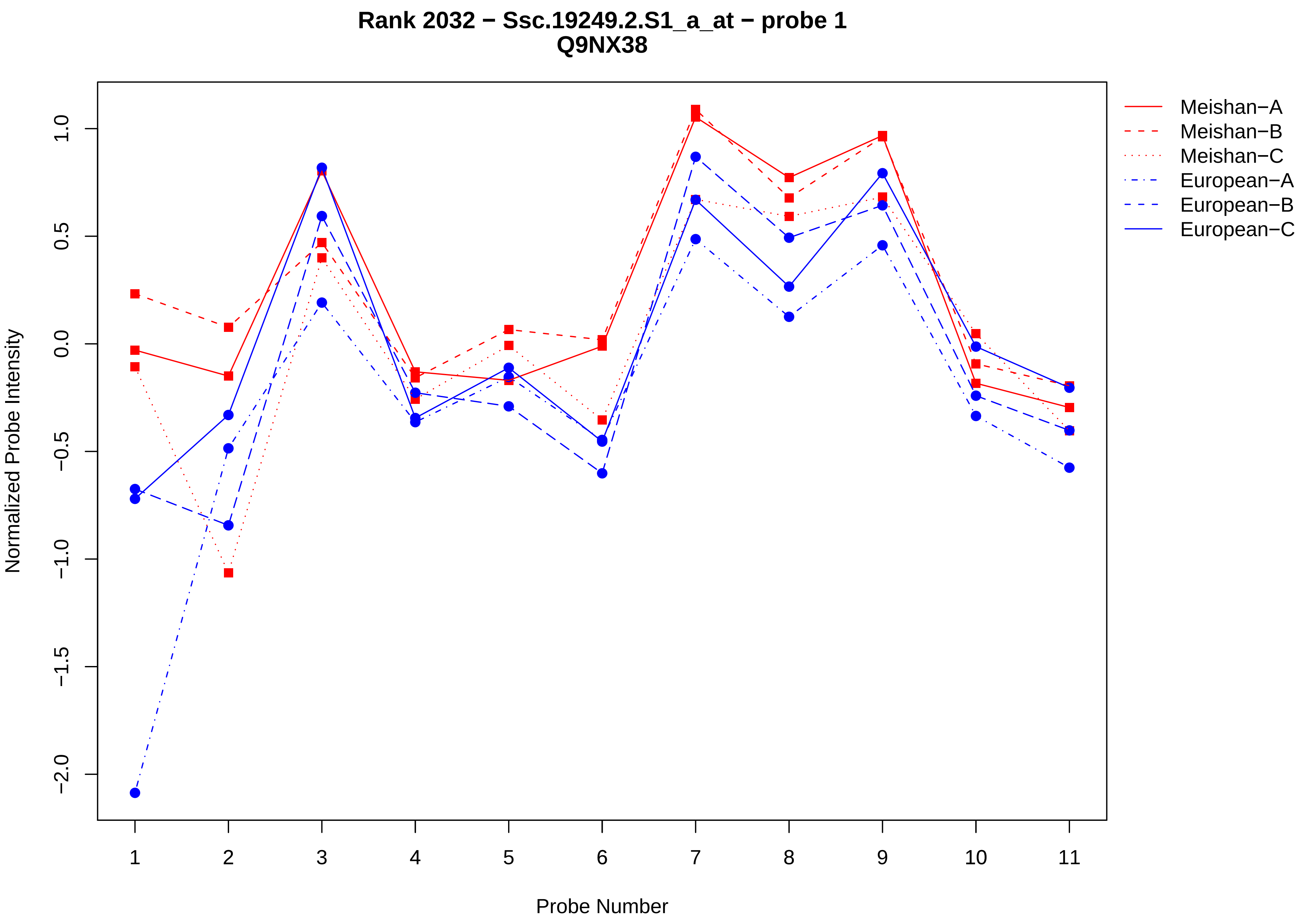
Rank 2032 − Ssc.19249.2.S1_a_at − probe 1
Q9NX38
Normalized Probe Intensity
Probe Number
Meishan−A
Meishan−B
Meishan−C
European−A
European−B
European−C
1.0
0.5
0.0
−0.5
−1.0
−1.5
−2.0
1
2
3
4
5
6
7
8
9
10
11

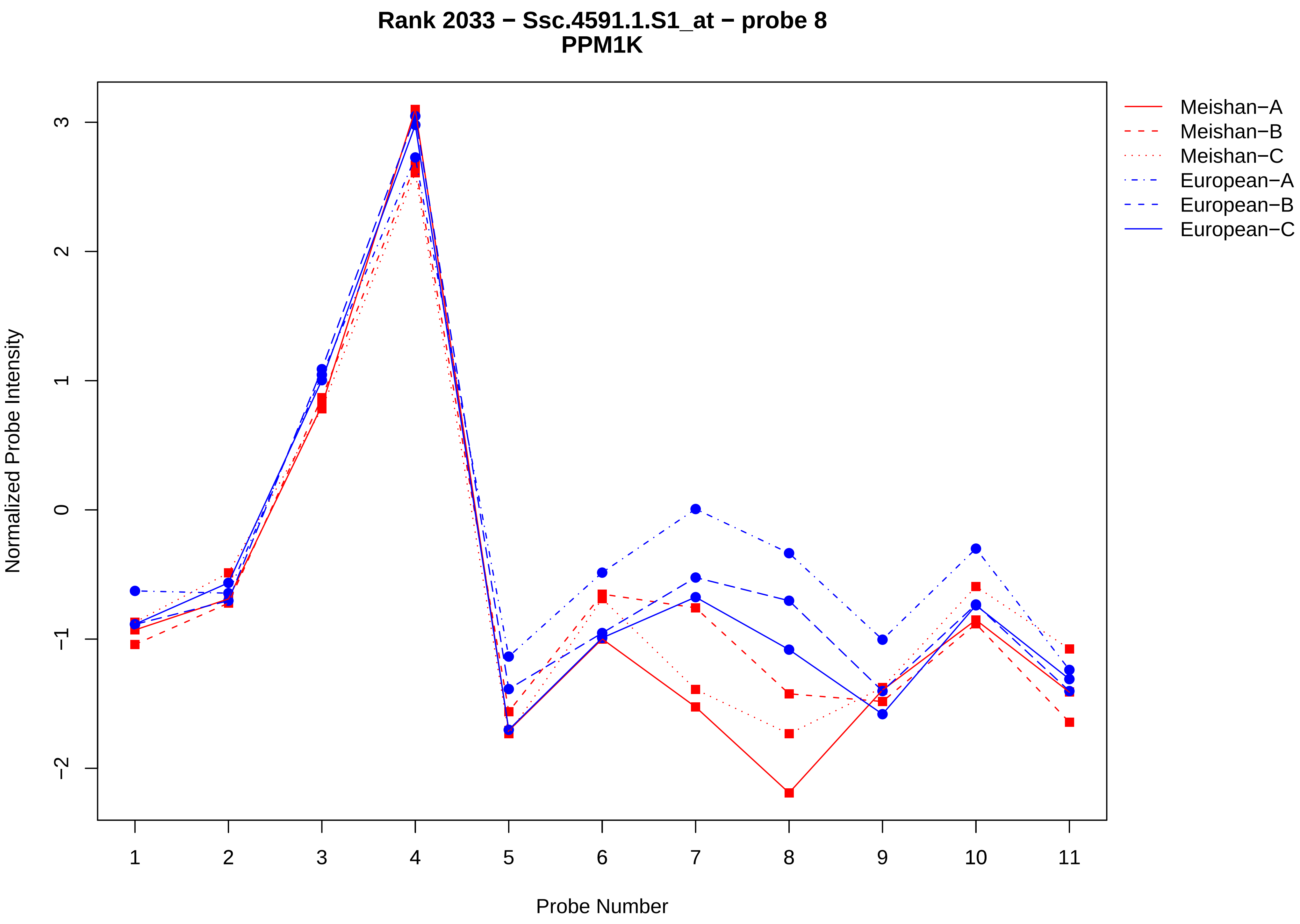
Rank 2033 − Ssc.4591.1.S1_at − probe 8
PPM1K
Normalized Probe Intensity
Probe Number
Meishan−A
Meishan−B
Meishan−C
European−A
European−B
European−C
3
2
1
0
−1
−2
1
2
3
4
5
6
7
8
9
10
11

Rank 2034 − Ssc.17987.1.A1_at − probe 5
HBA1

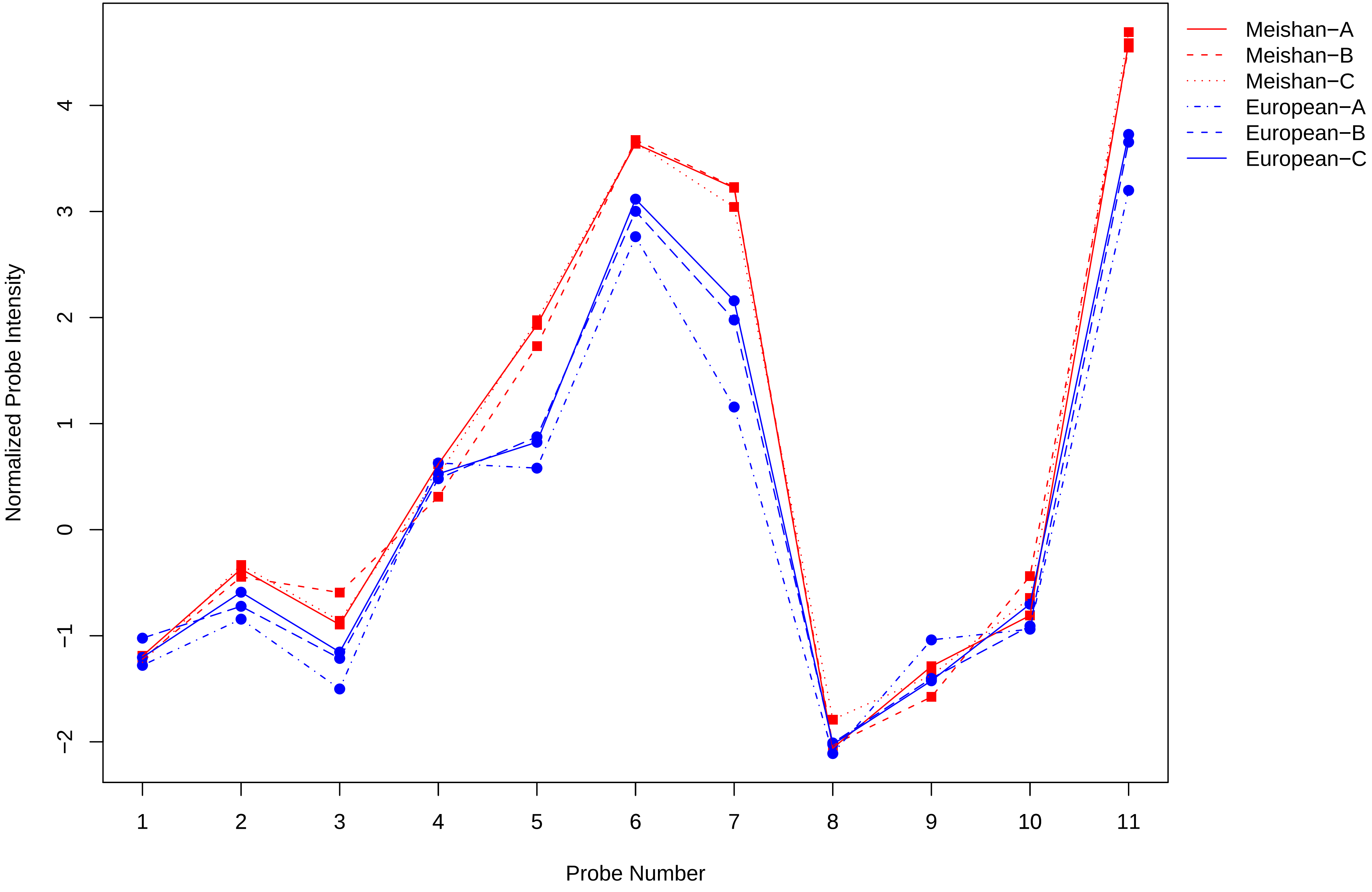

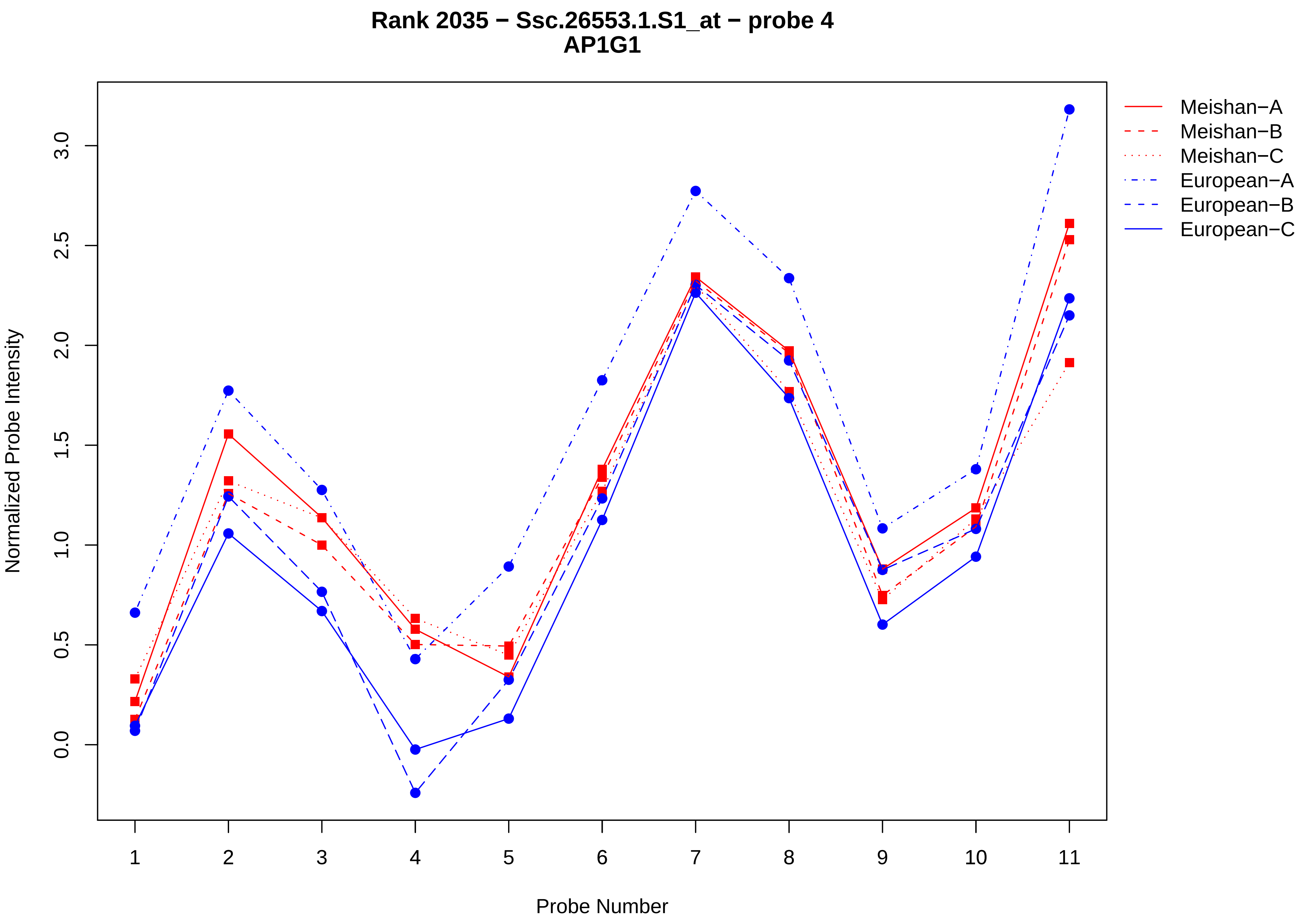

Rank 2035 − Ssc.26553.1.S1_at − probe 4
AP1G1
Normalized Probe Intensity
Probe Number
0.0
0.5
1.0
1.5
2.0
2.5
3.0
1
2
3
4
5
6
7
8
9
10
11
Meishan−A
Meishan−B
Meishan−C
European−A
European−B
European−C

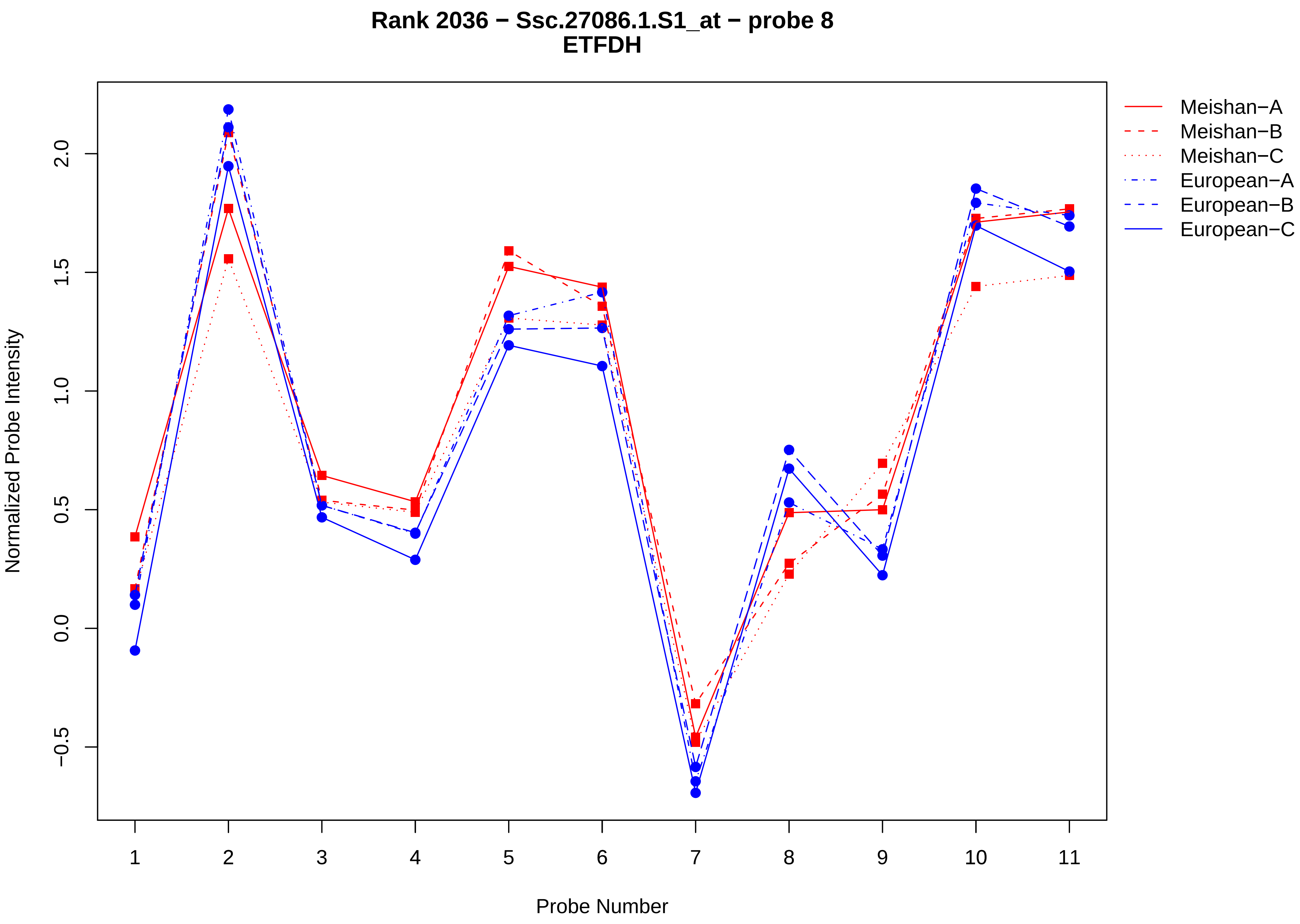
Rank 2036 − Ssc.27086.1.S1_at − probe 8
ETFDH
Normalized Probe Intensity
Probe Number
Meishan−A
Meishan−B
Meishan−C
European−A
European−B
European−C
−0.5
0.0
0.5
1.0
1.5
2.0
1
2
3
4
5
6
7
8
9
10
11

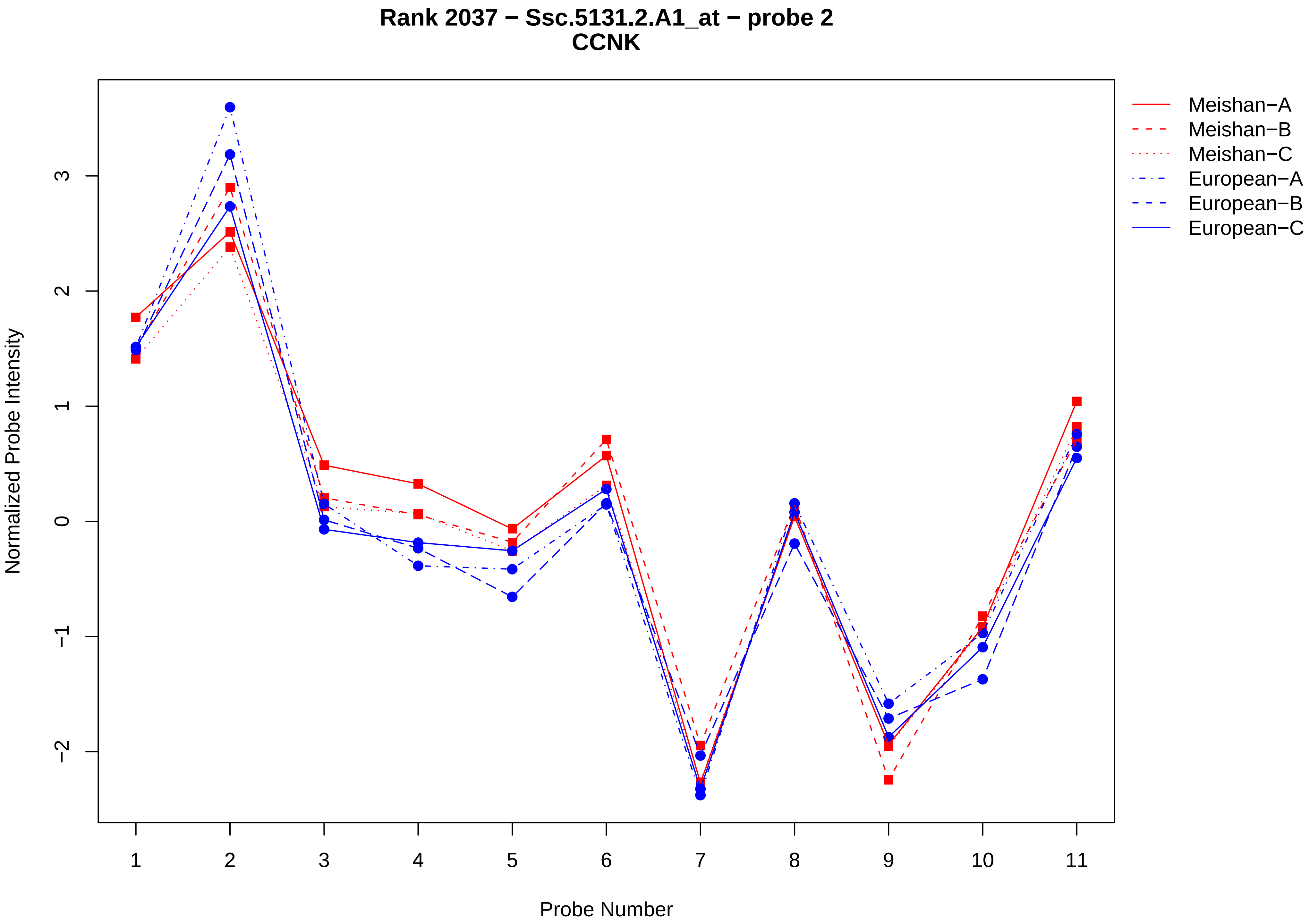

Rank 2037 − Ssc.5131.2.A1_at − probe 2
CCNK
Normalized Probe Intensity
Probe Number
−2
−1
0
1
2
3
1
2
3
4
5
6
7
8
9
10
11
Meishan−A
Meishan−B
Meishan−C
European−A
European−B
European−C

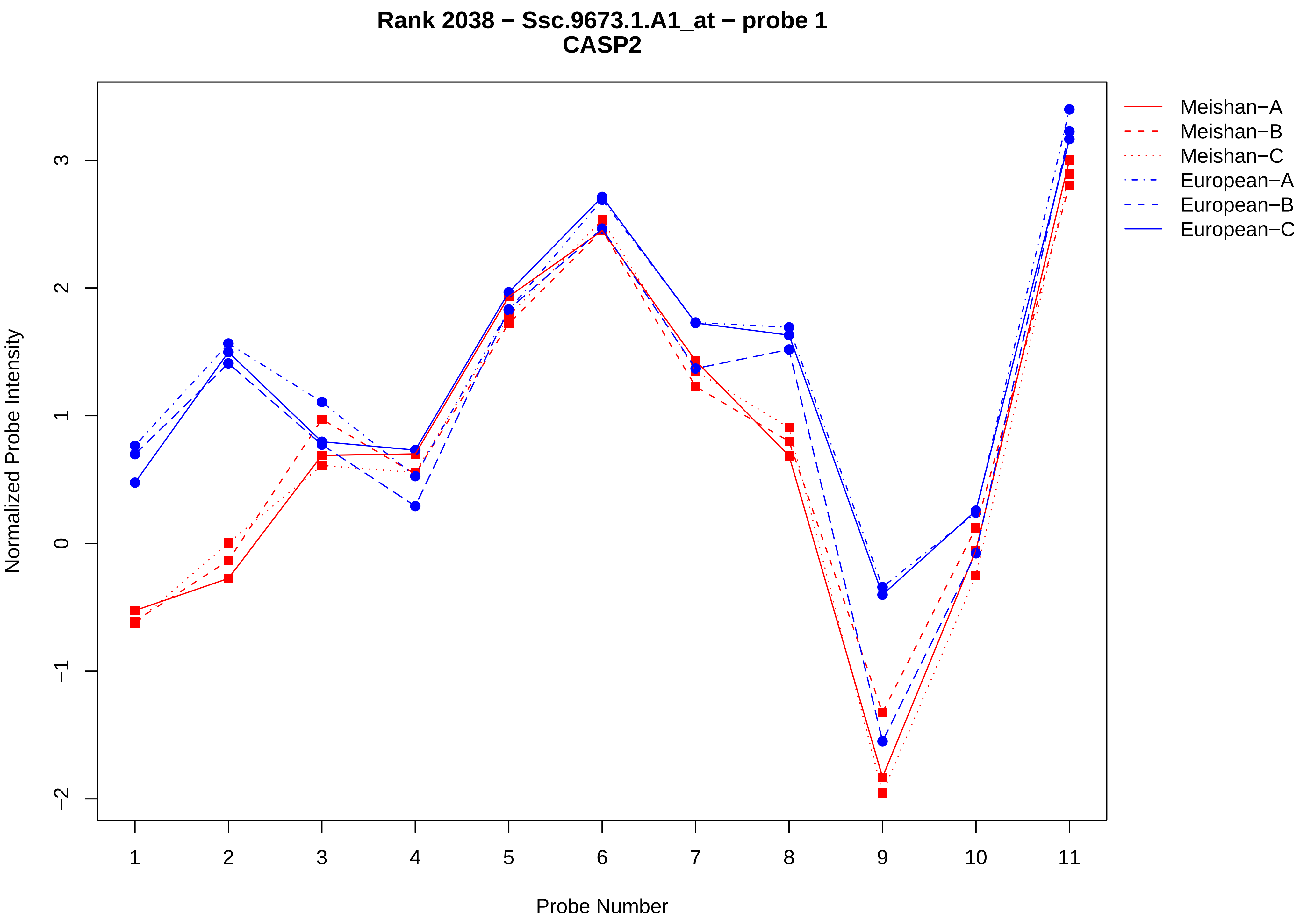

Rank 2038 − Ssc.9673.1.A1_at − probe 1
CASP2
Normalized Probe Intensity
Probe Number
−2
−1
0
1
2
3
1
2
3
4
5
6
7
8
9
10
11
Meishan−A
Meishan−B
Meishan−C
European−A
European−B
European−C

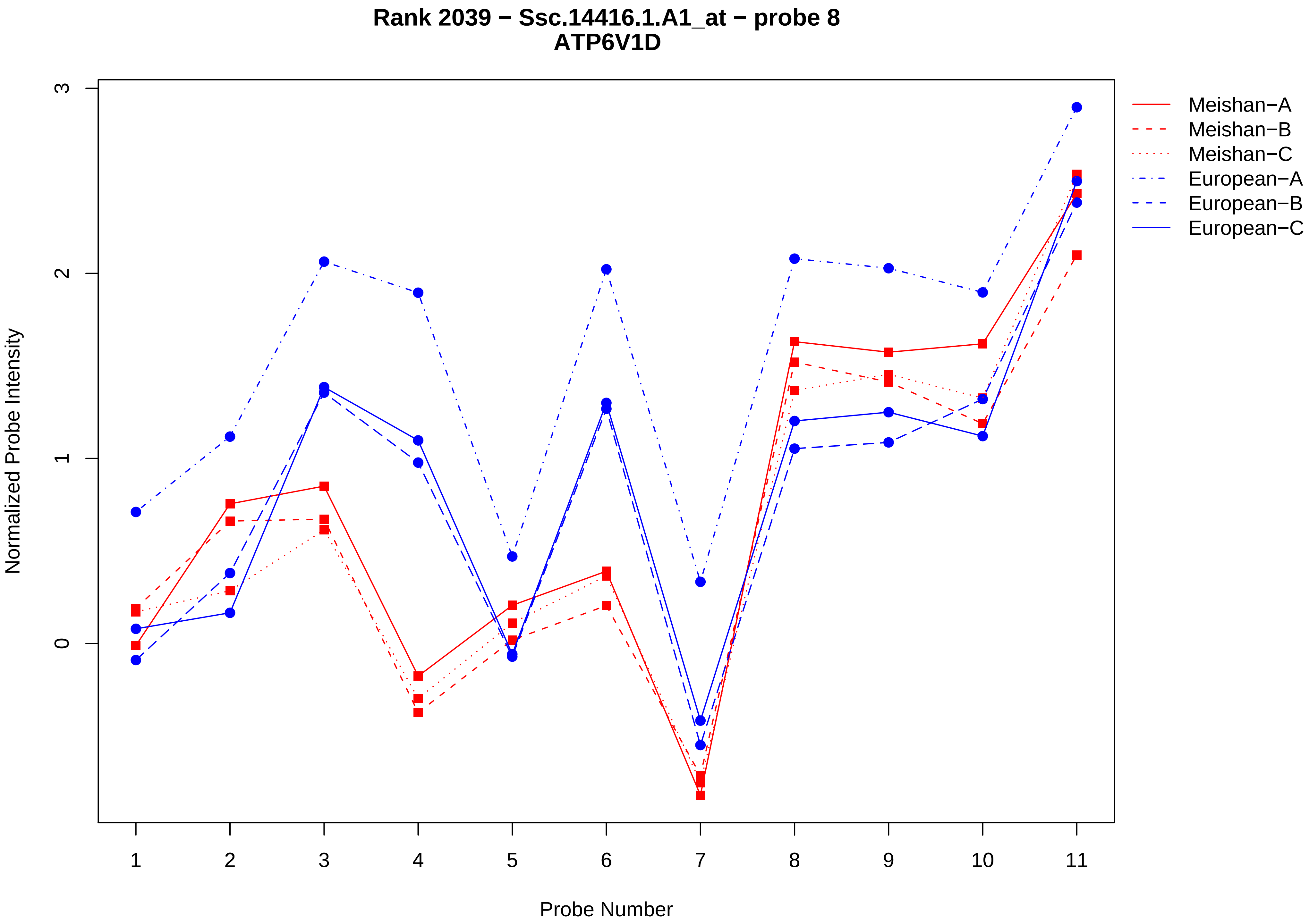

Rank 2039 − Ssc.14416.1.A1_at − probe 8
ATP6V1D
Normalized Probe Intensity
Probe Number
Meishan−A
Meishan−B
Meishan−C
European−A
European−B
European−C
0
1
2
3
1
2
3
4
5
6
7
8
9
10
11

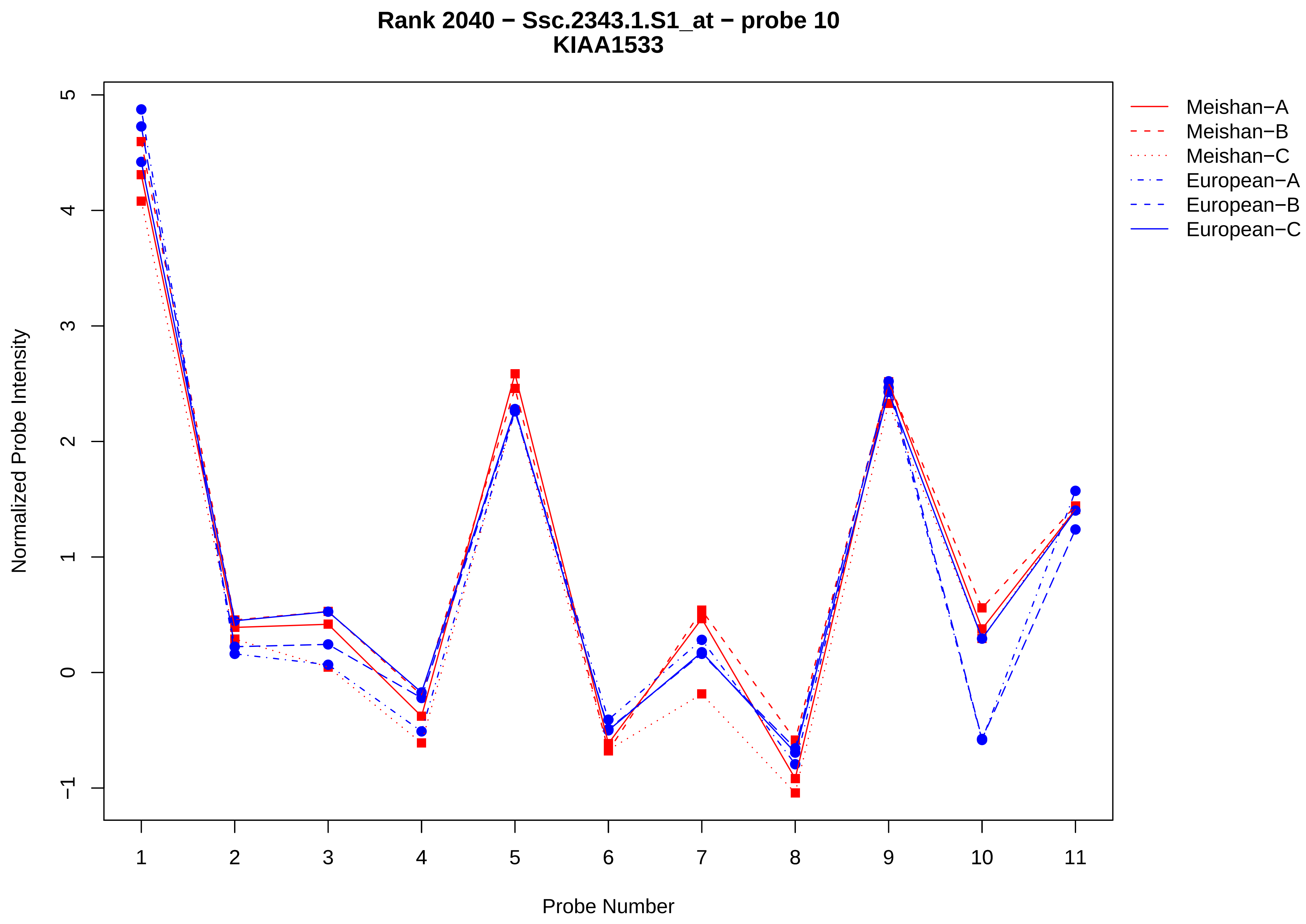

Rank 2040 − Ssc.2343.1.S1_at − probe 10
KIAA1533
Normalized Probe Intensity
Probe Number
−1
0
1
2
3
4
5
1
2
3
4
5
6
7
8
9
10
11
Meishan−A
Meishan−B
Meishan−C
European−A
European−B
European−C

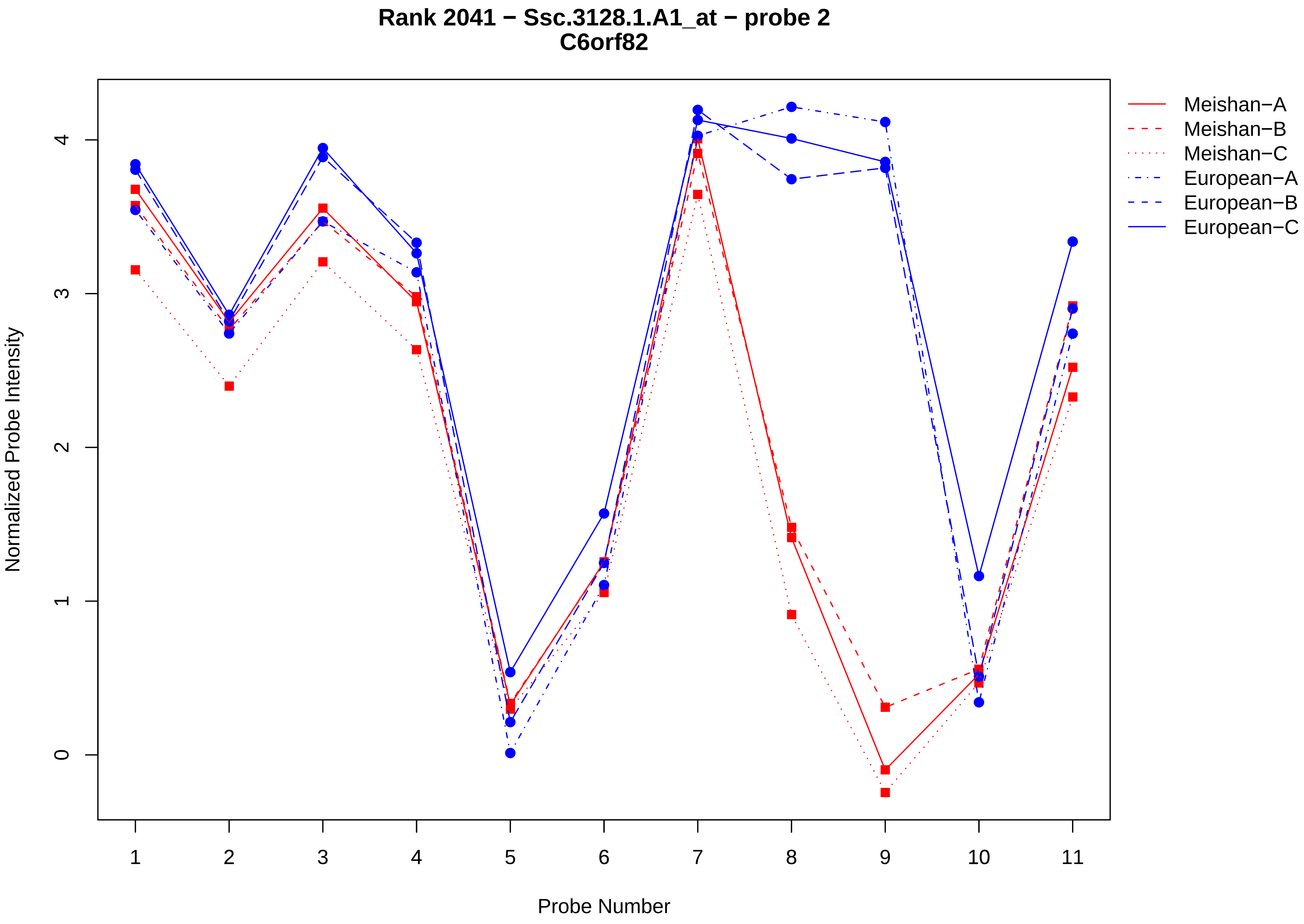
Rank 2041 − Ssc.3128.1.A1_at − probe 2
C6orf82
Normalized Probe Intensity
Probe Number
0
1
2
3
4
1
2
3
4
5
6
7
8
9
10
11
Meishan−A
Meishan−B
Meishan−C
European−A
European−B
European−C

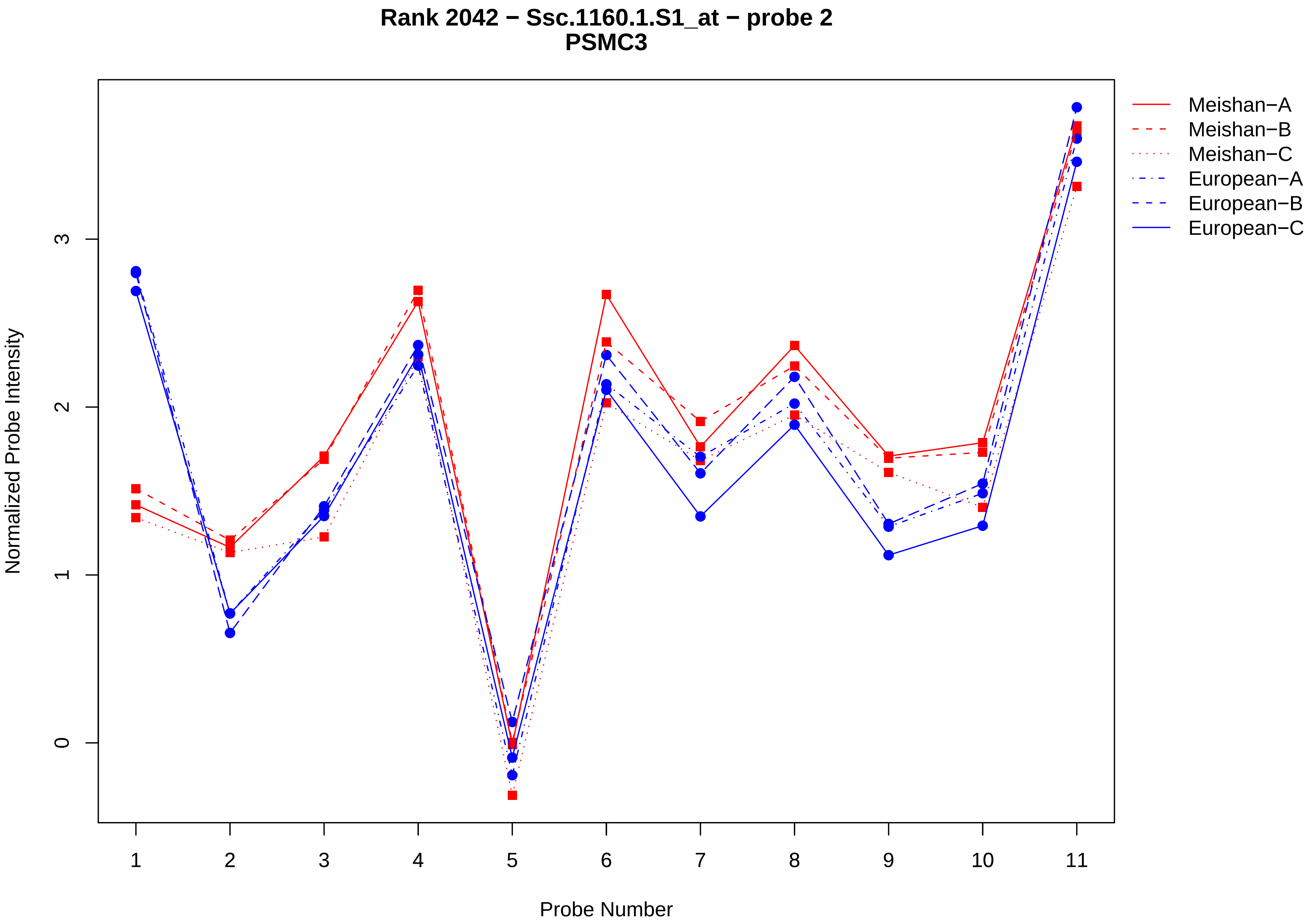

Rank 2042 − Ssc.1160.1.S1_at − probe 2
PSMC3
Normalized Probe Intensity
Probe Number
0
1
2
3
1
2
3
4
5
6
7
8
9
10
11
Meishan−A
Meishan−B
Meishan−C
European−A
European−B
European−C

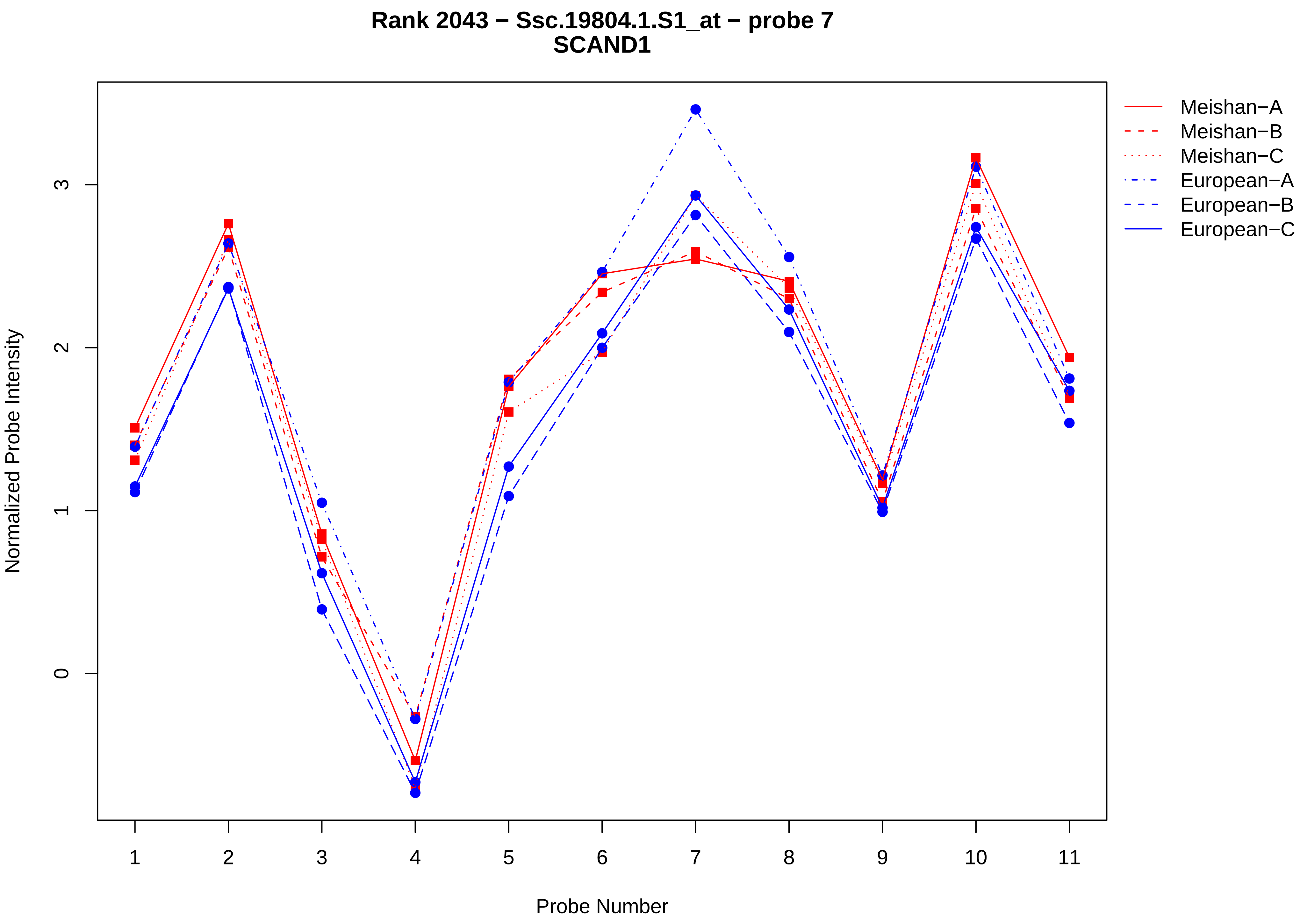

Rank 2043 − Ssc.19804.1.S1_at − probe 7
SCAND1
Normalized Probe Intensity
Probe Number
0
1
2
3
1
2
3
4
5
6
7
8
9
10
11
Meishan−A
Meishan−B
Meishan−C
European−A
European−B
European−C

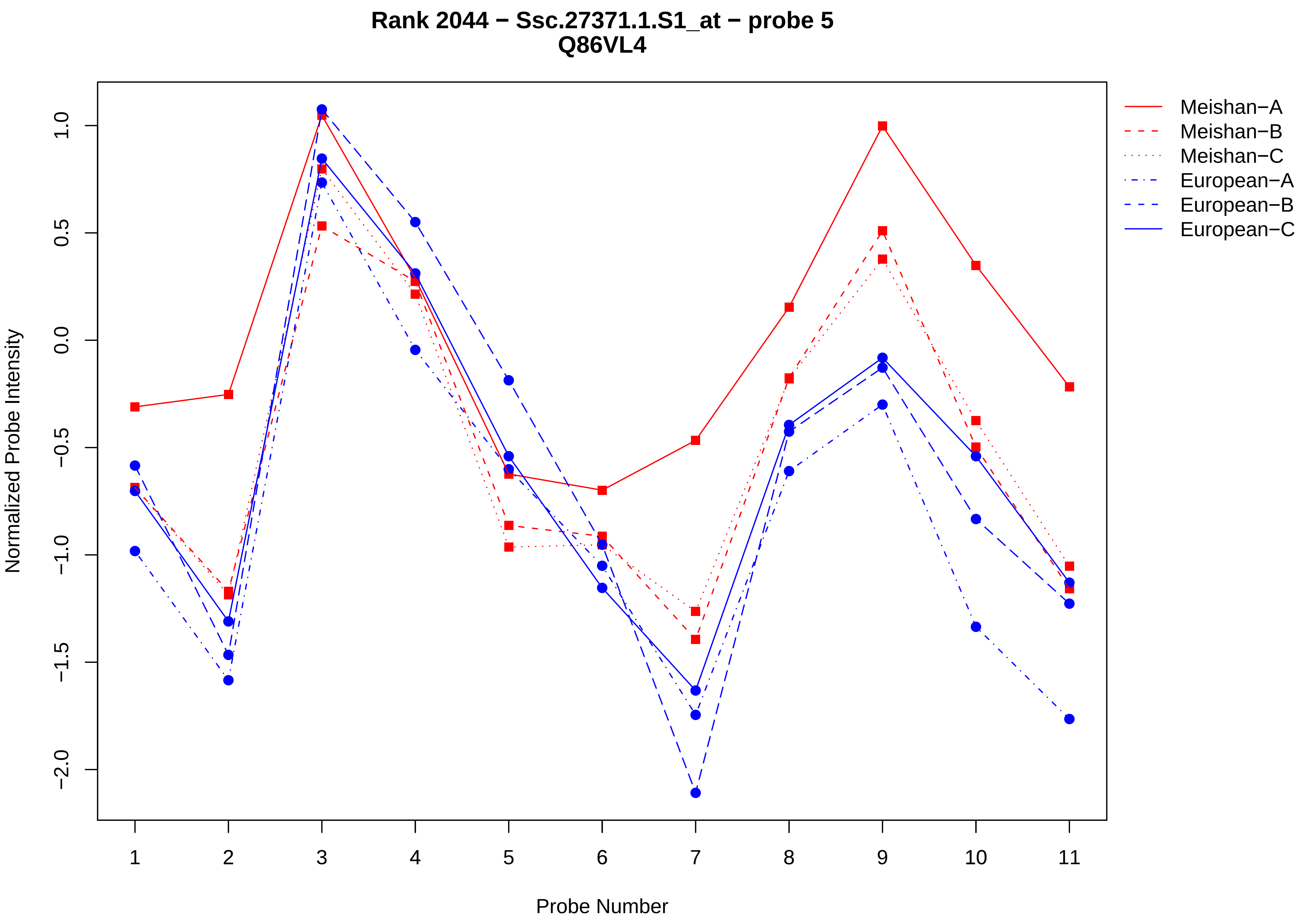

Rank 2044 − Ssc.27371.1.S1_at − probe 5
Q86VL4
Normalized Probe Intensity
Probe Number
Meishan−A
Meishan−B
Meishan−C
European−A
European−B
European−C
1.0
0.5
0.0
−0.5
−1.0
−1.5
−2.0
1
2
3
4
5
6
7
8
9
10
11

Rank 2045 − Ssc.6097.1.S1_at − probe 8
TXNRD1

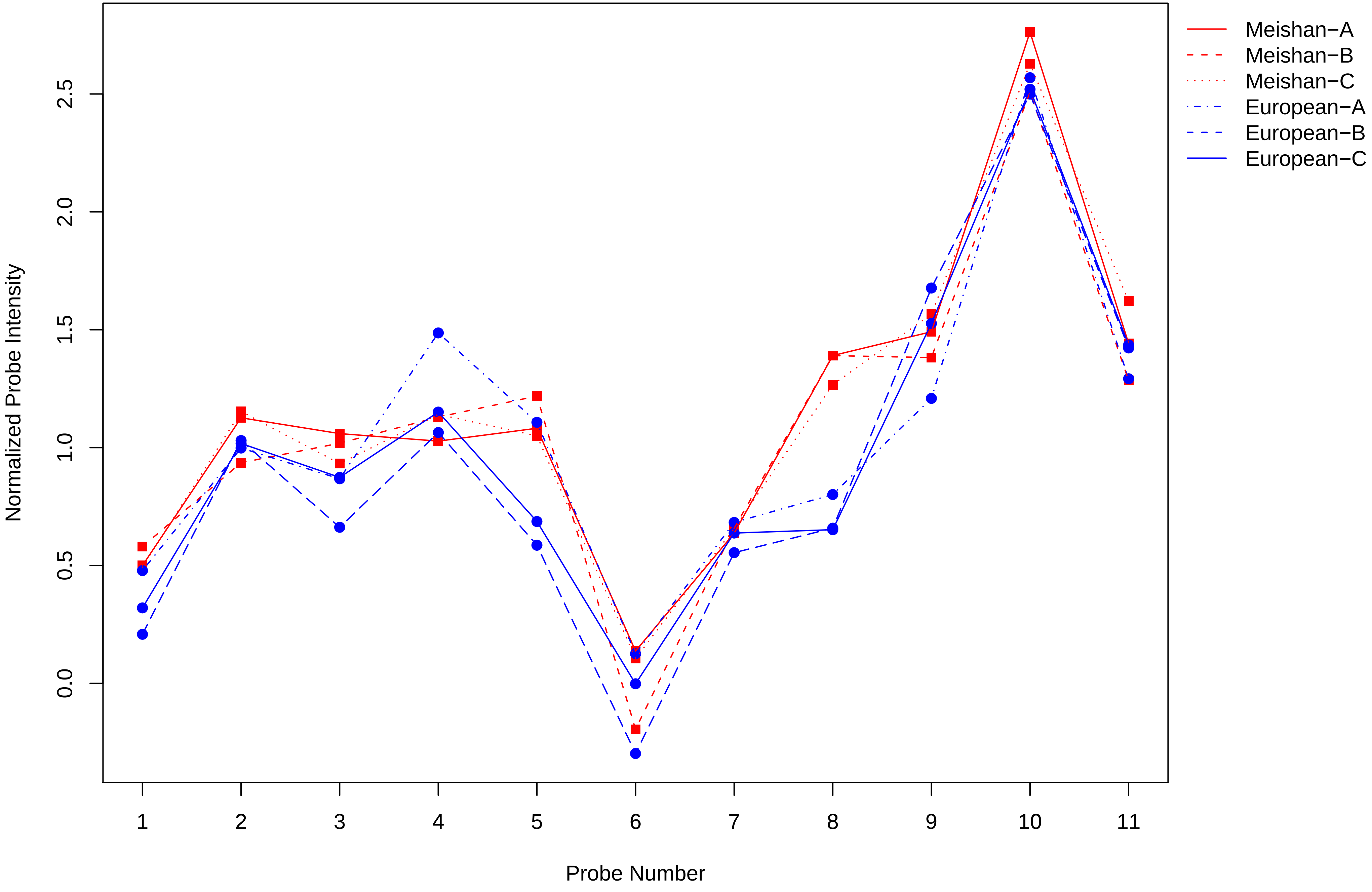

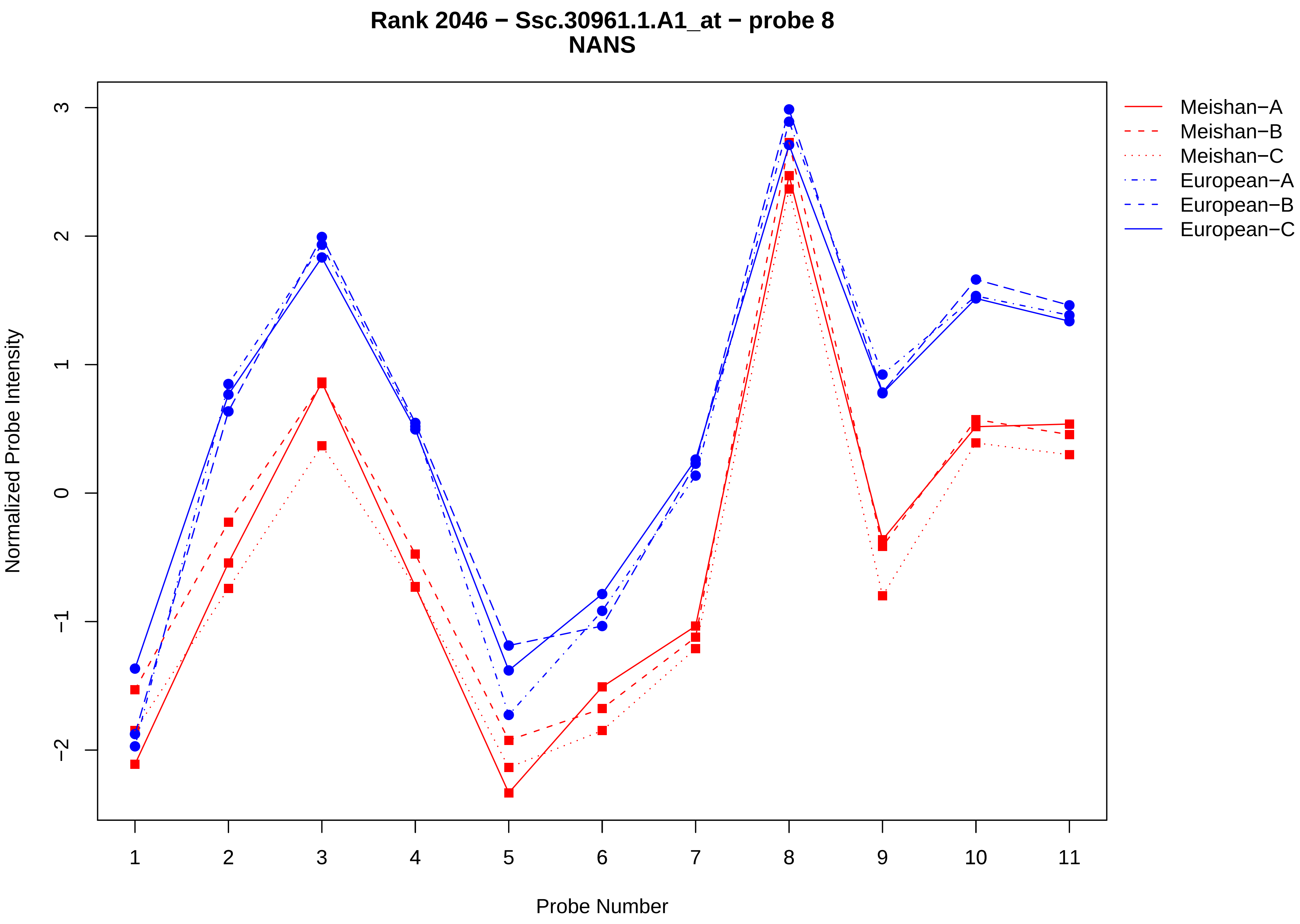

Rank 2046 − Ssc.30961.1.A1_at − probe 8
NANS
Normalized Probe Intensity
Probe Number
−2
−1
0
1
2
3
1
2
3
4
5
6
7
8
9
10
11
Meishan−A
Meishan−B
Meishan−C
European−A
European−B
European−C

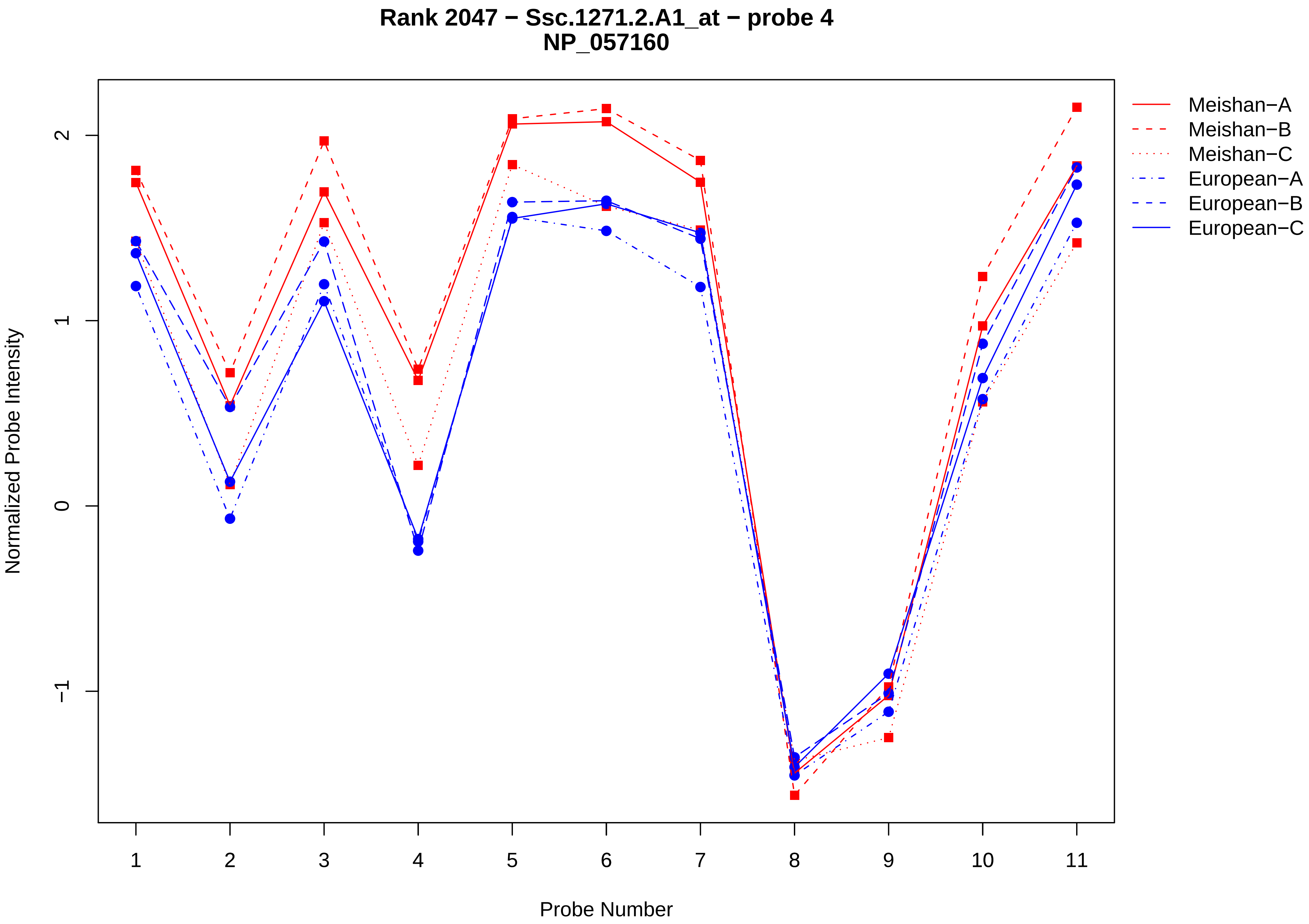

Rank 2047 − Ssc.1271.2.A1_at − probe 4
NP_057160
Normalized Probe Intensity
Probe Number
−1
0
1
2
1
2
3
4
5
6
7
8
9
10
11
Meishan−A
Meishan−B
Meishan−C
European−A
European−B
European−C

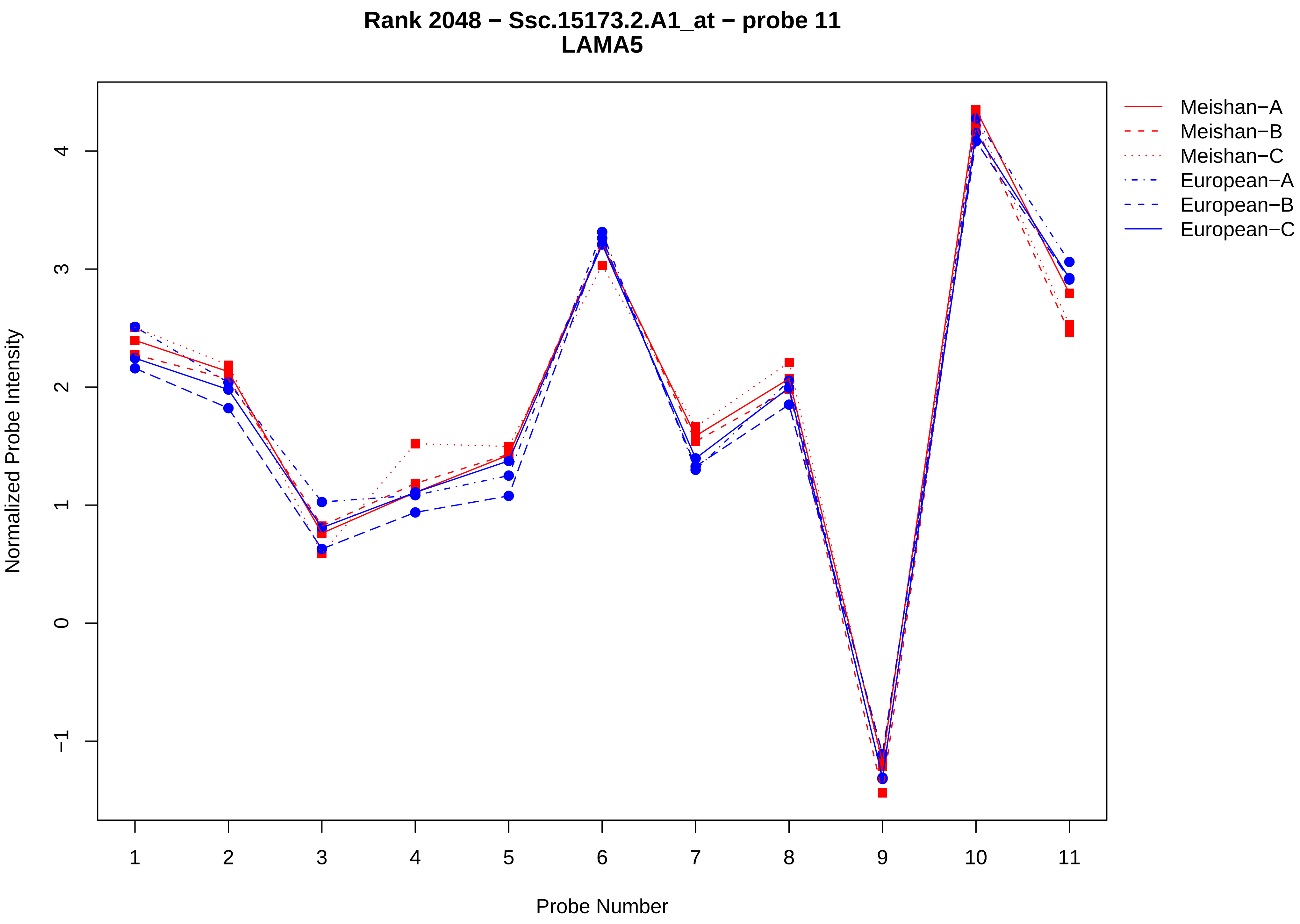

Rank 2048 − Ssc.15173.2.A1_at − probe 11
LAMA5
Normalized Probe Intensity
Probe Number
−1
0
1
2
3
4
1
2
3
4
5
6
7
8
9
10
11
Meishan−A
Meishan−B
Meishan−C
European−A
European−B
European−C

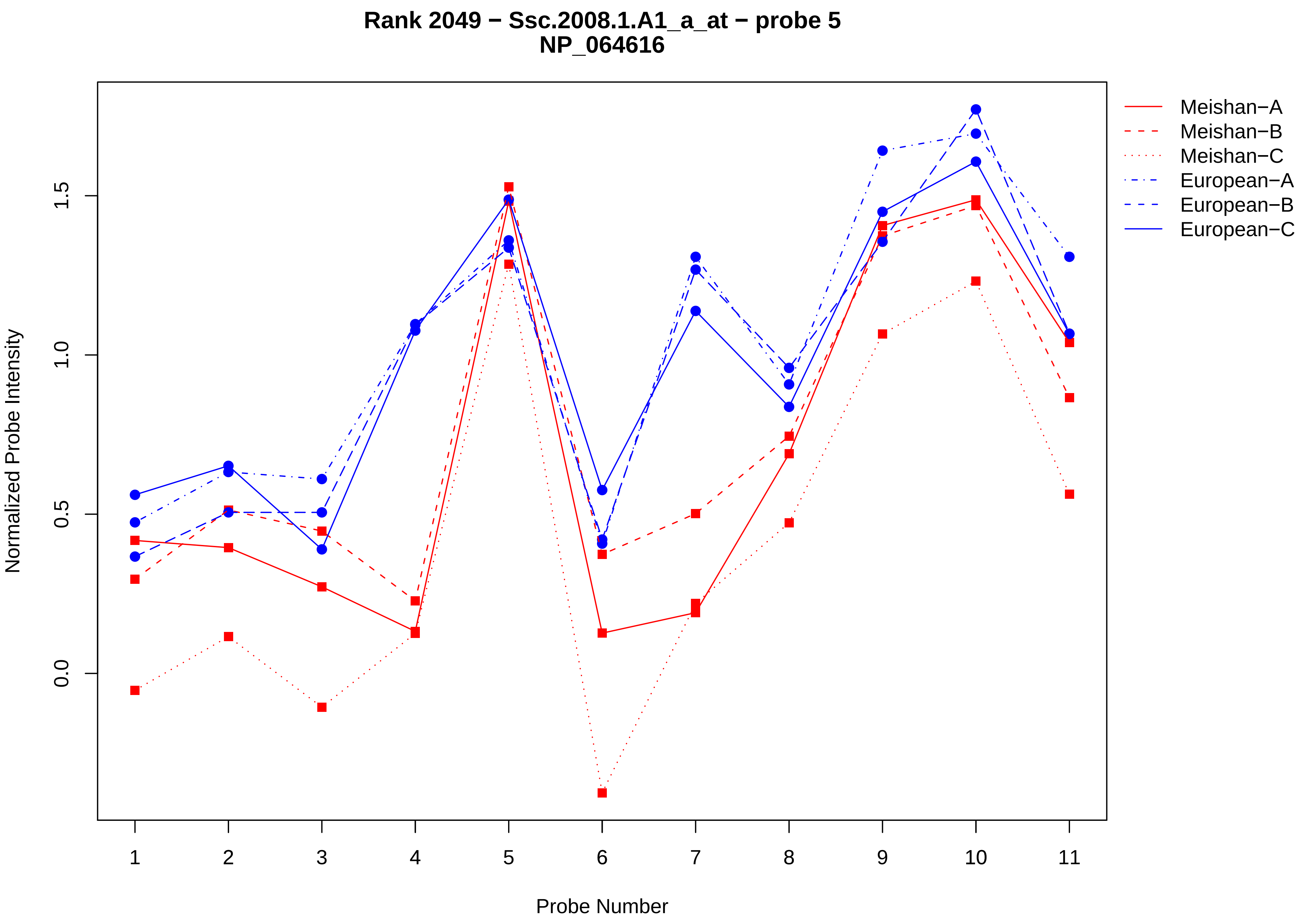
Rank 2049 − Ssc.2008.1.A1_a_at − probe 5
NP_064616
Normalized Probe Intensity
0.0
0.5
1.0
1.5
1
2
3
4
5
6
7
8
9
10
11
Probe Number
Meishan−A
Meishan−B
Meishan−C
European−A
European−B
European−C

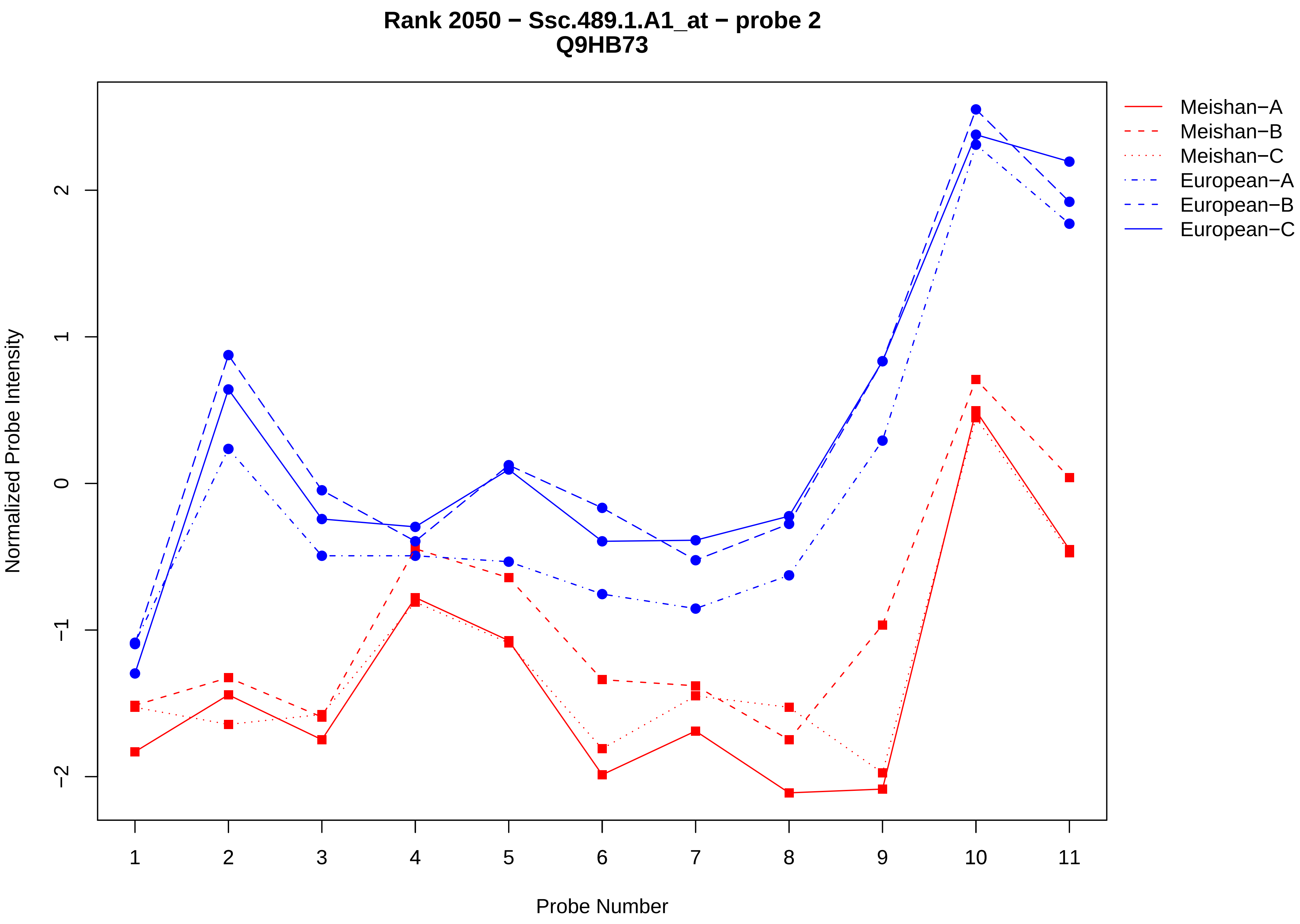

Rank 2050 − Ssc.489.1.A1_at − probe 2
Q9HB73
Normalized Probe Intensity
Probe Number
−2
−1
0
1
2
1
2
3
4
5
6
7
8
9
10
11
Meishan−A
Meishan−B
Meishan−C
European−A
European−B
European−C

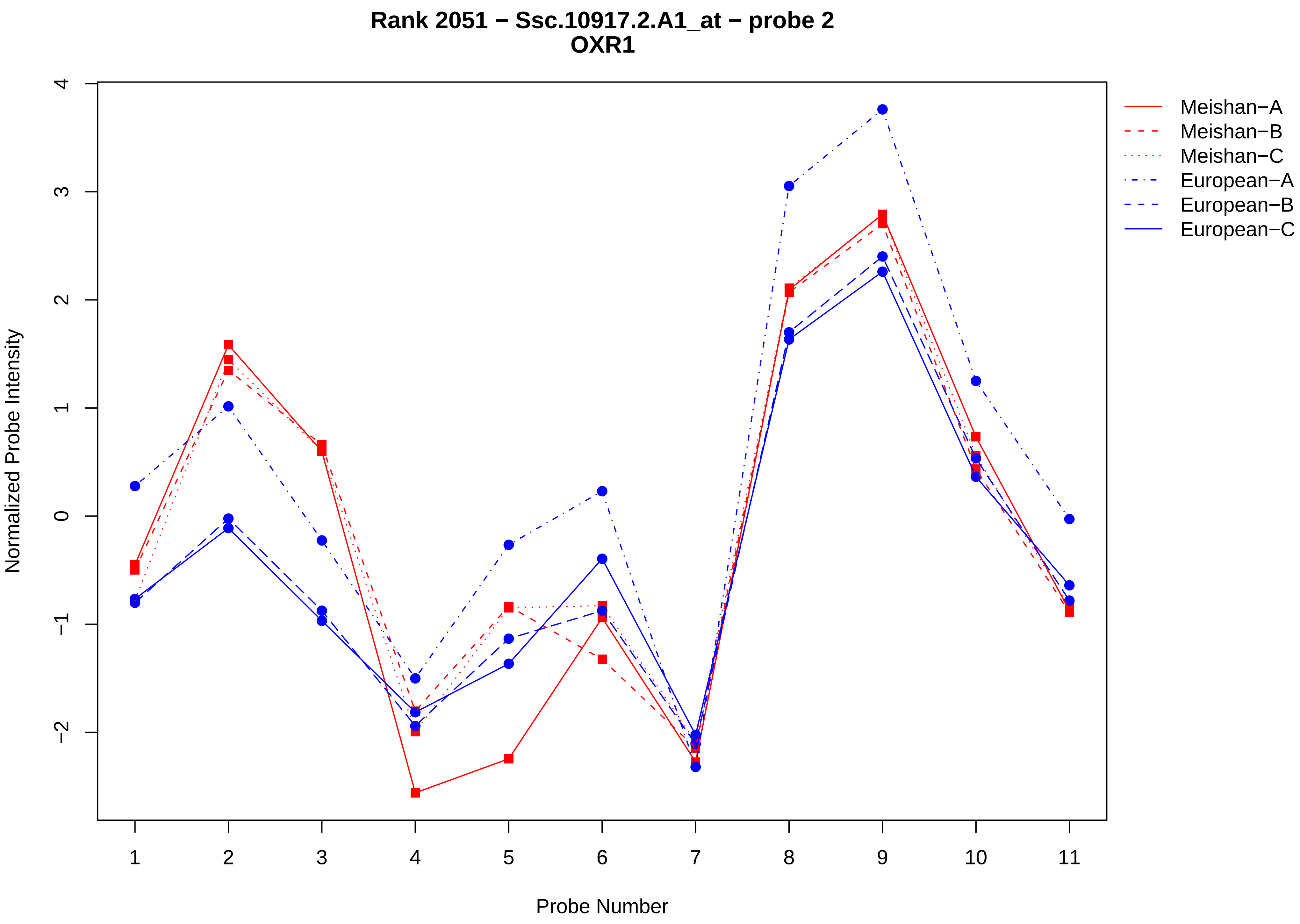

Rank 2051 − Ssc.10917.2.A1_at − probe 2
OXR1
Normalized Probe Intensity
Probe Number
4
3
2
1
0
−1
−2
1
2
3
4
5
6
7
8
9
10
11
Meishan−A
Meishan−B
Meishan−C
European−A
European−B
European−C

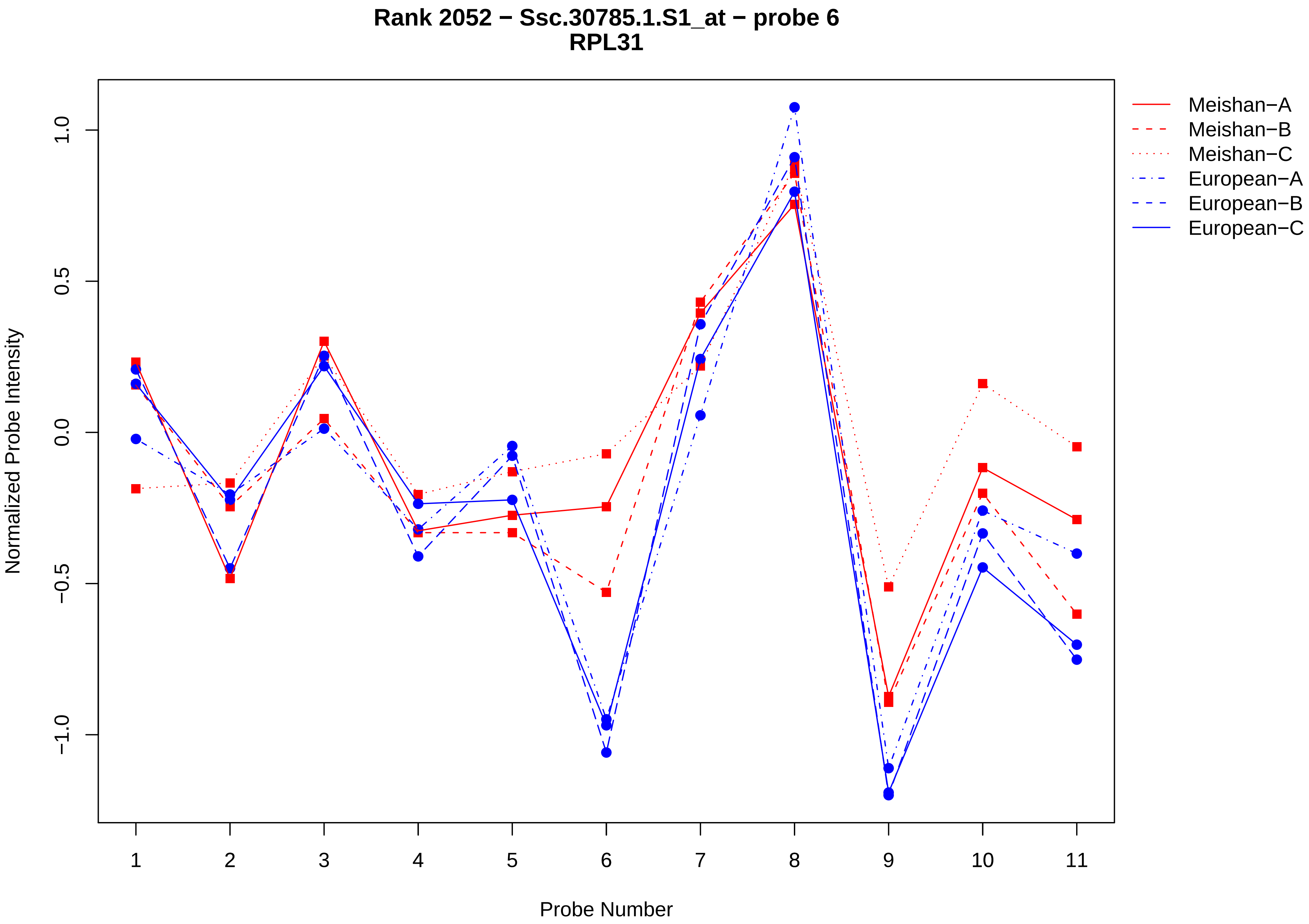

Rank 2052 − Ssc.30785.1.S1_at − probe 6
RPL31
Normalized Probe Intensity
Probe Number
1.0
0.5
0.0
−0.5
−1.0
1
2
3
4
5
6
7
8
9
10
11
Meishan−A
Meishan−B
Meishan−C
European−A
European−B
European−C

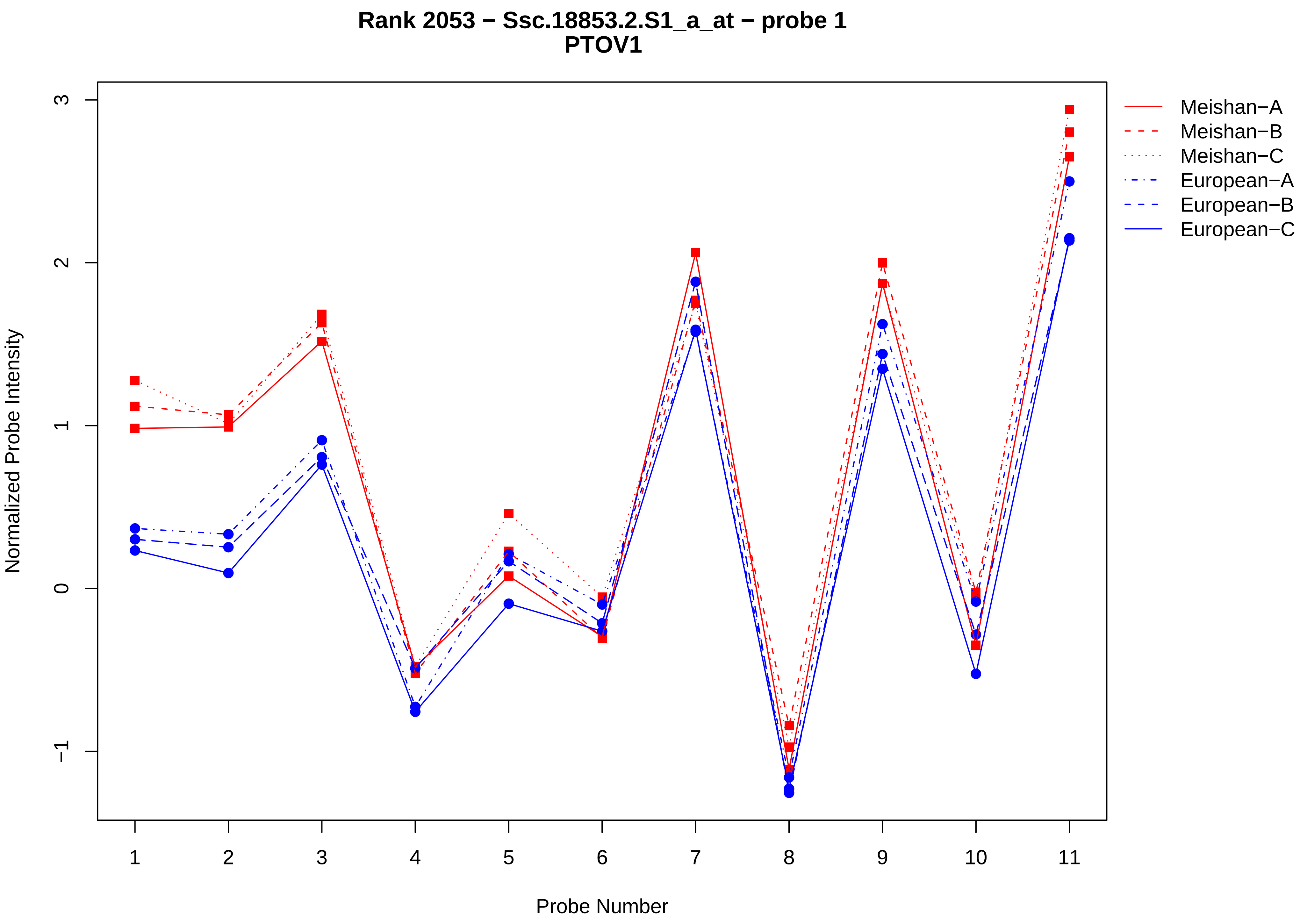
Rank 2053 − Ssc.18853.2.S1_a_at − probe 1
PTOV1
Normalized Probe Intensity
Probe Number
Meishan−A
Meishan−B
Meishan−C
European−A
European−B
European−C
−1
0
1
2
3
1
2
3
4
5
6
7
8
9
10
11

Rank 2054 − Ssc.7981.1.A1_at − probe 6
ACOX3

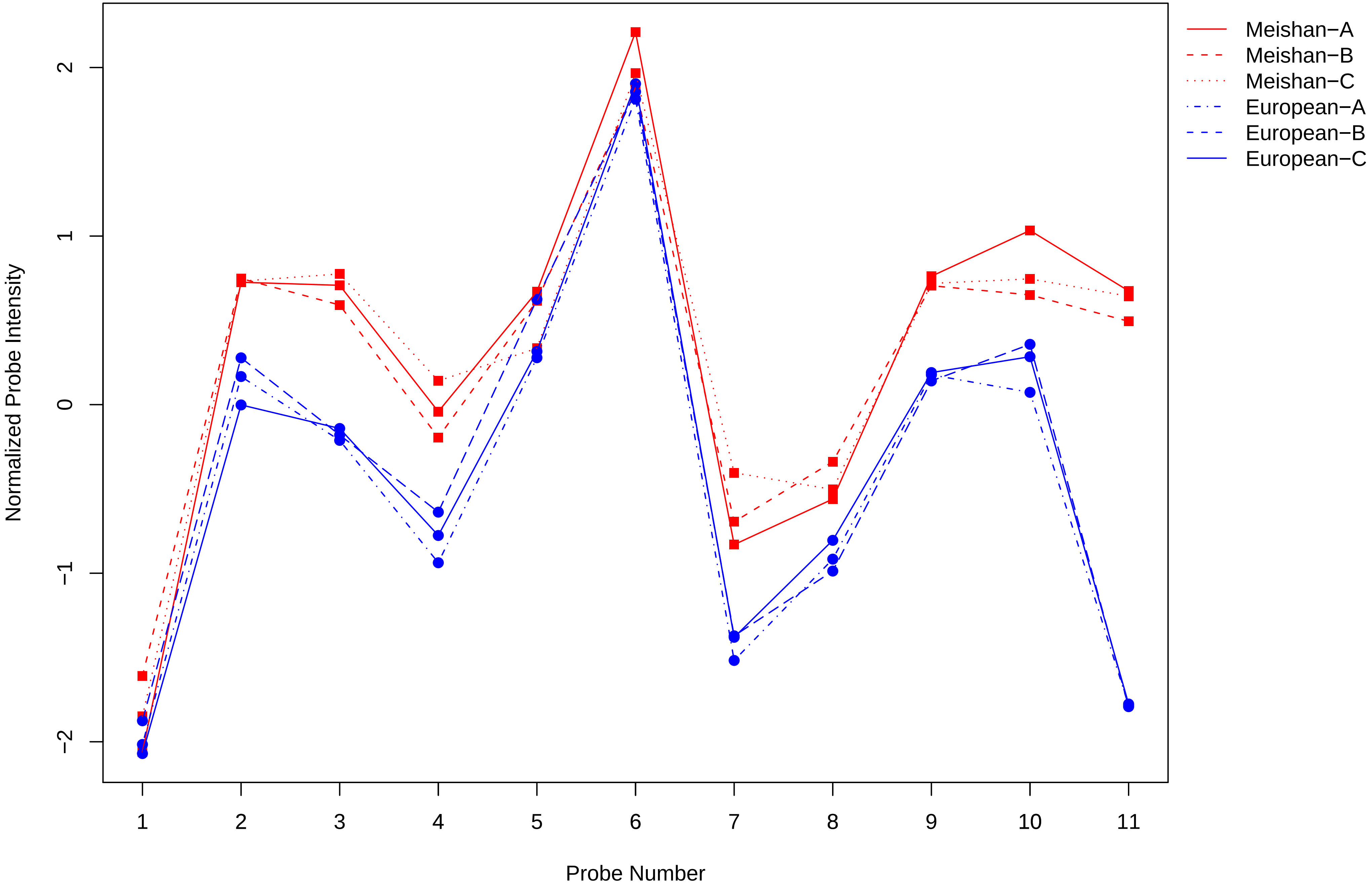

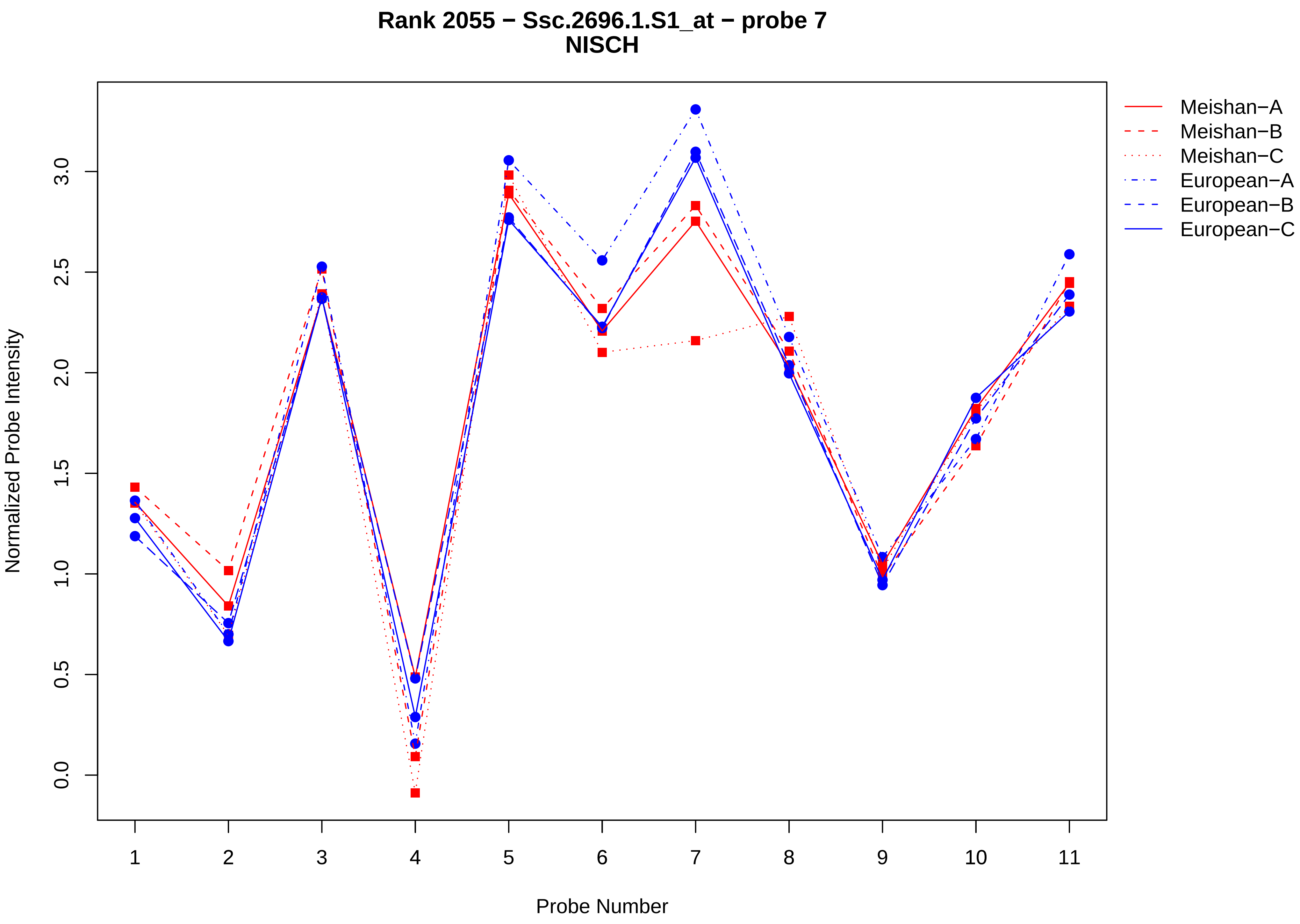

Rank 2055 − Ssc.2696.1.S1_at − probe 7
NISCH
Normalized Probe Intensity
Probe Number
0.0
0.5
1.0
1.5
2.0
2.5
3.0
1
2
3
4
5
6
7
8
9
10
11
Meishan−A
Meishan−B
Meishan−C
European−A
European−B
European−C

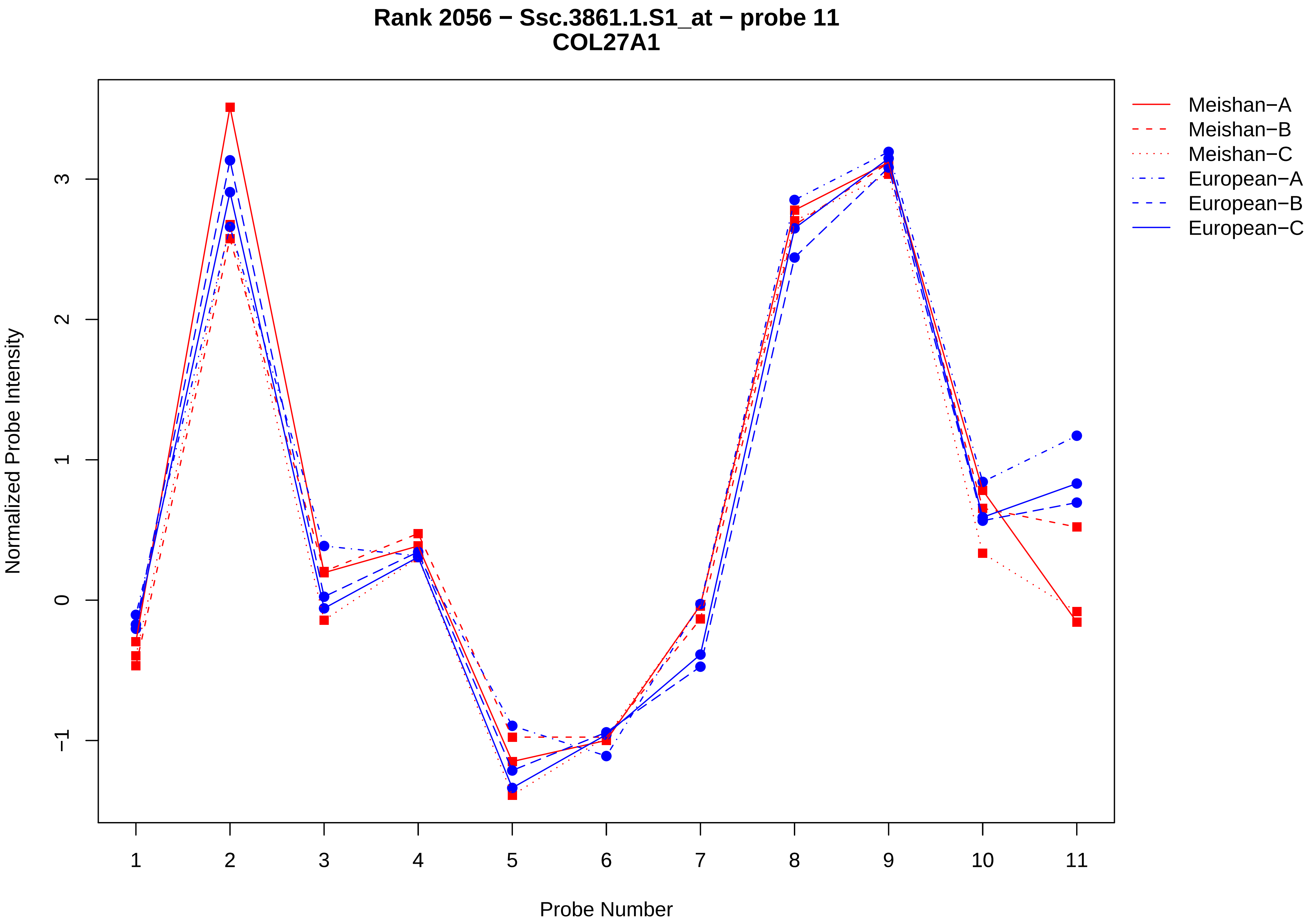

Rank 2056 − Ssc.3861.1.S1_at − probe 11
COL27A1
Normalized Probe Intensity
Probe Number
−1
0
1
2
3
1
2
3
4
5
6
7
8
9
10
11
Meishan−A
Meishan−B
Meishan−C
European−A
European−B
European−C

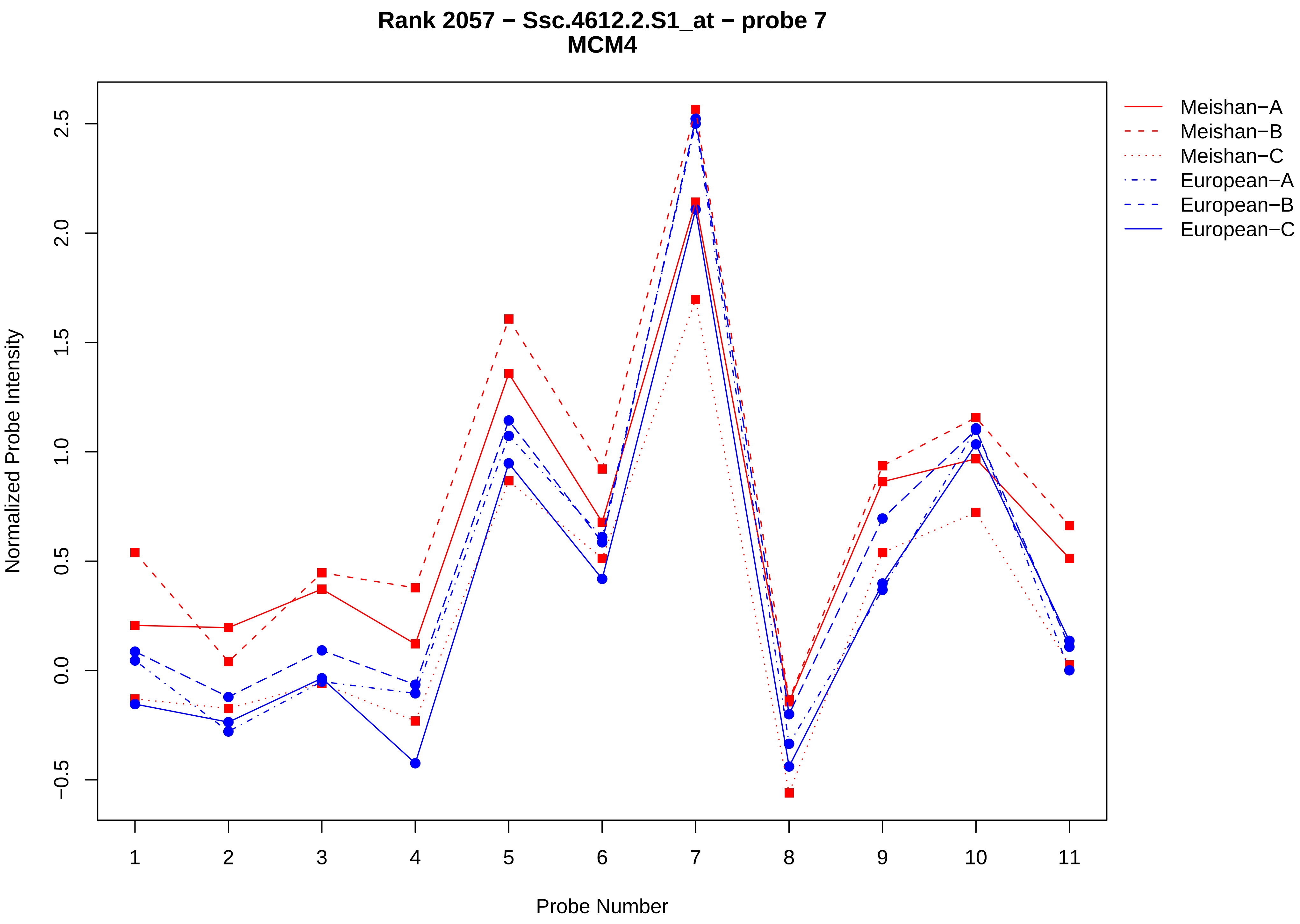

Rank 2057 − Ssc.4612.2.S1_at − probe 7
MCM4
Normalized Probe Intensity
Probe Number
Meishan−A
Meishan−B
Meishan−C
European−A
European−B
European−C

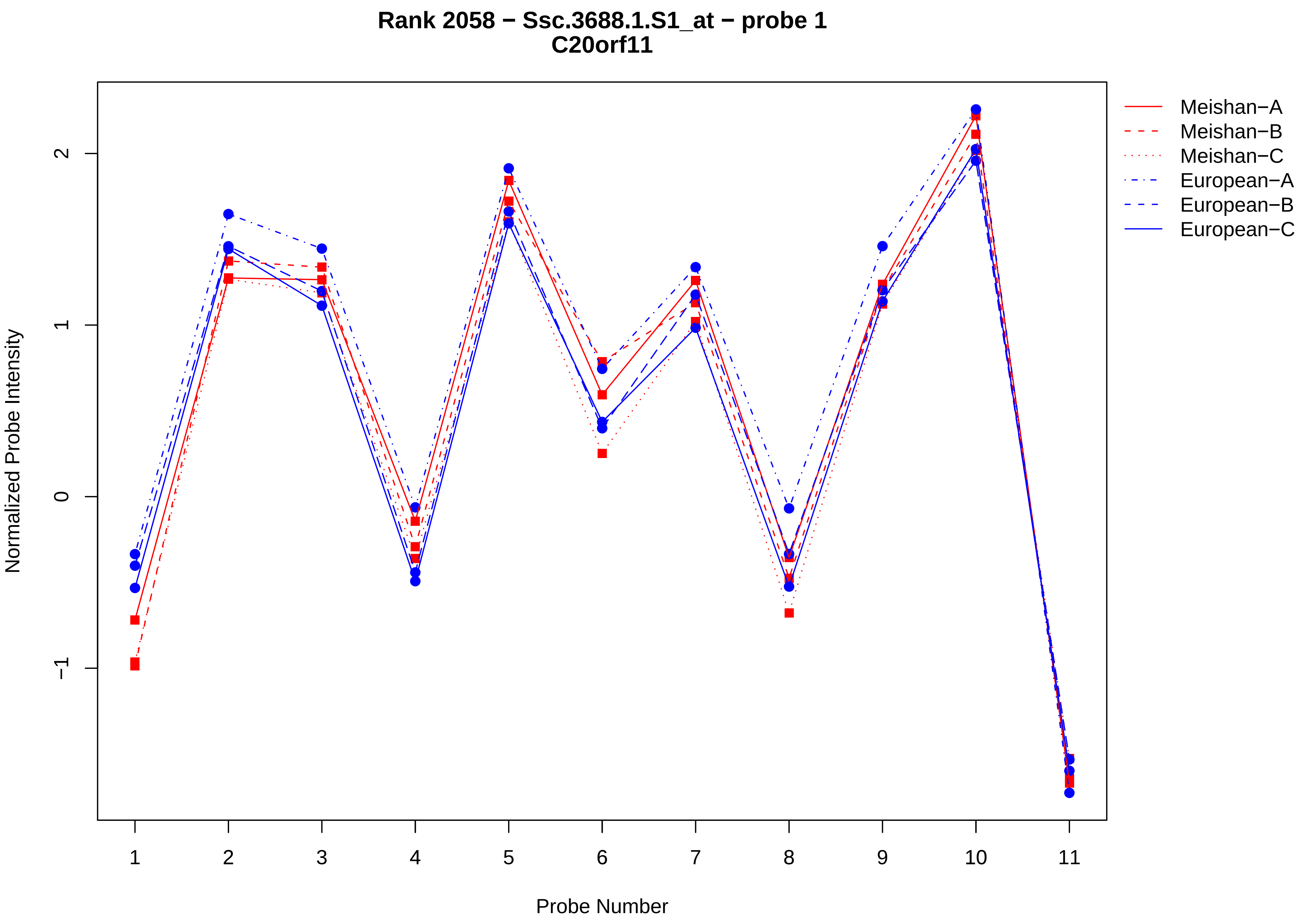

Rank 2058 − Ssc.3688.1.S1_at − probe 1
C20orf11
Normalized Probe Intensity
Probe Number
−1
0
1
2
1
2
3
4
5
6
7
8
9
10
11
Meishan−A
Meishan−B
Meishan−C
European−A
European−B
European−C

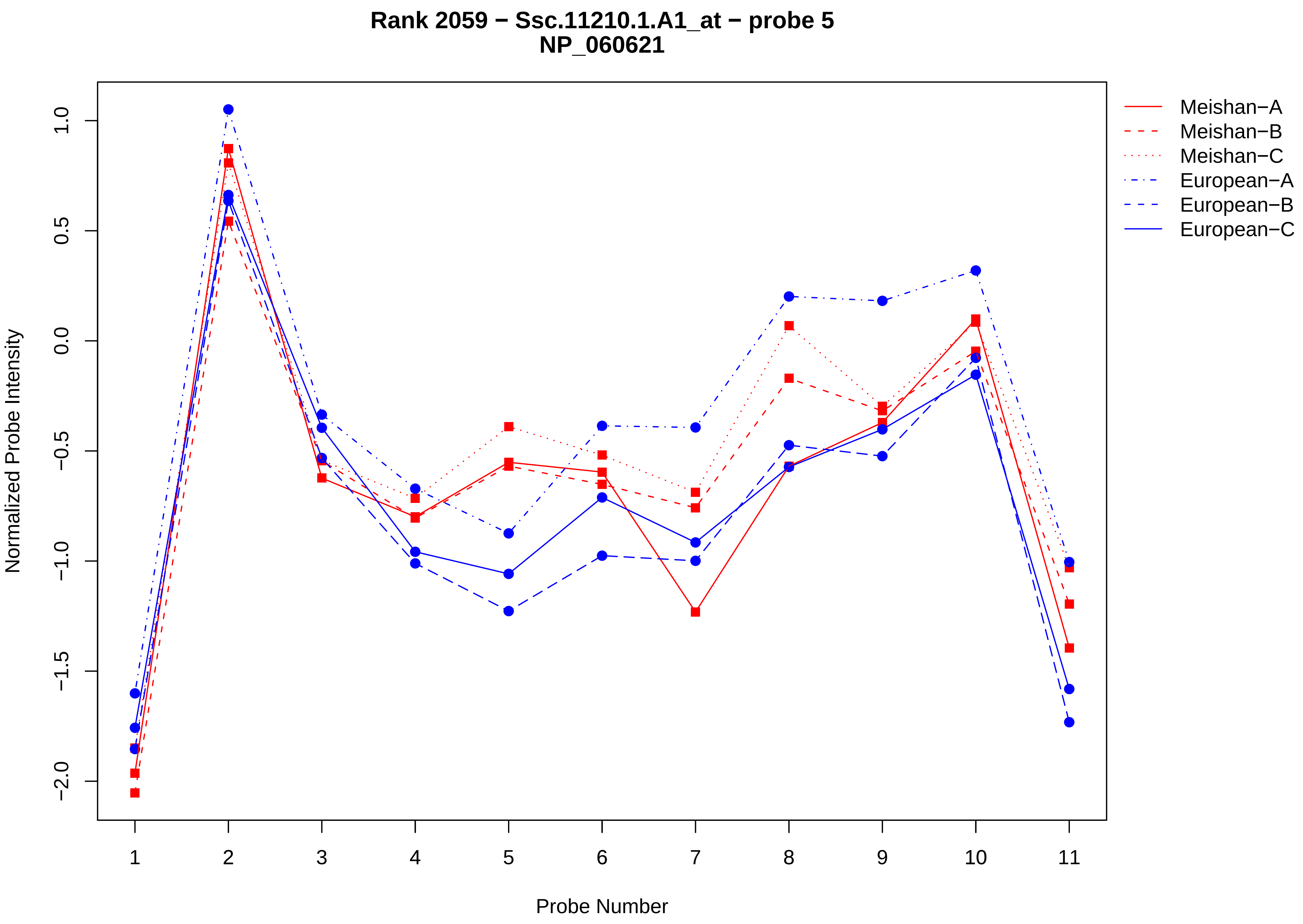

Rank 2059 − Ssc.11210.1.A1_at − probe 5
NP_060621
Normalized Probe Intensity
Probe Number
Meishan−A
Meishan−B
Meishan−C
European−A
European−B
European−C
1.0
0.5
0.0
−0.5
−1.0
−1.5
−2.0
1
2
3
4
5
6
7
8
9
10
11

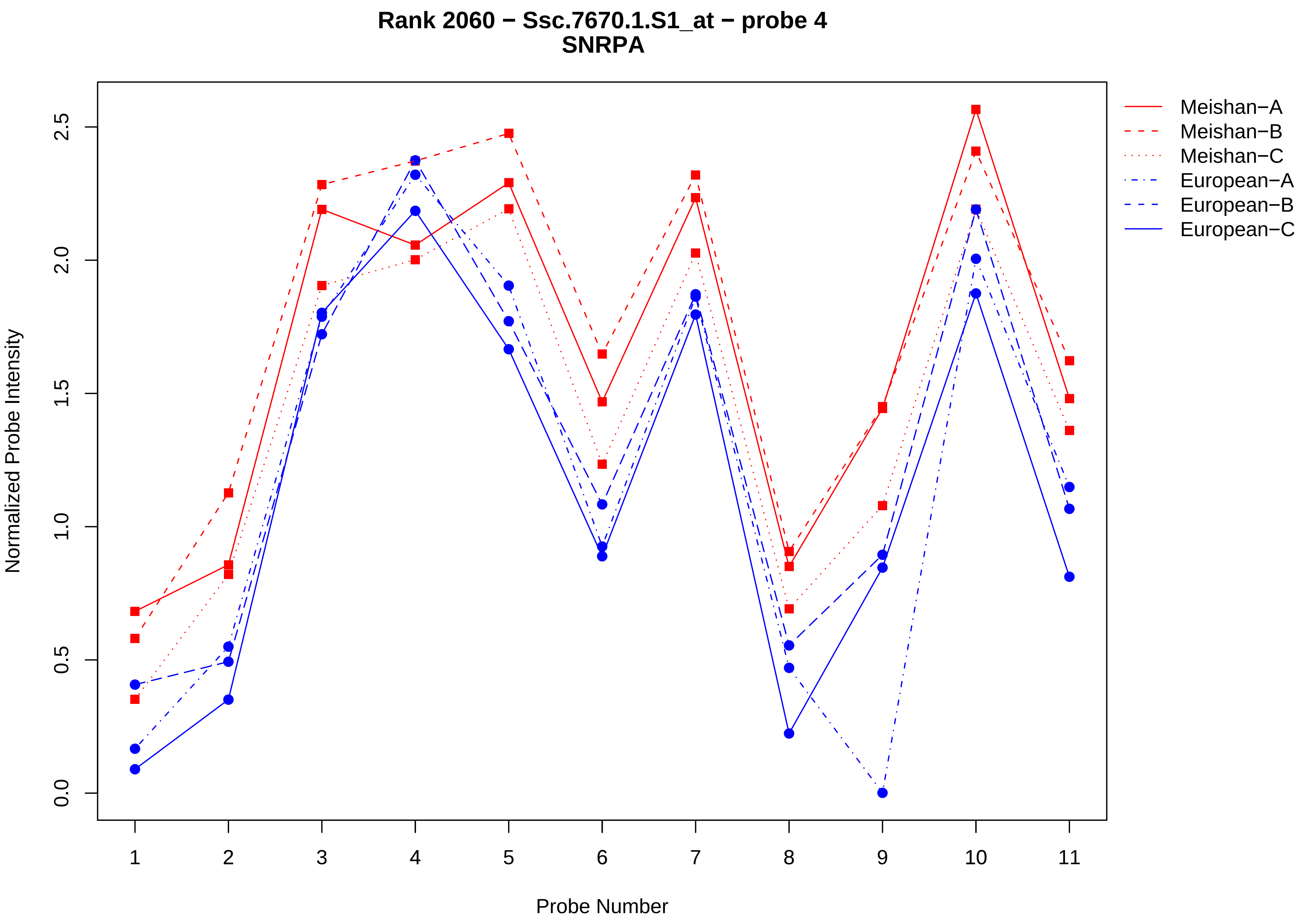

Rank 2060 − Ssc.7670.1.S1_at − probe 4
SNRPA
Normalized Probe Intensity
Probe Number
0.0
0.5
1.0
1.5
2.0
2.5
1
2
3
4
5
6
7
8
9
10
11
Meishan−A
Meishan−B
Meishan−C
European−A
European−B
European−C

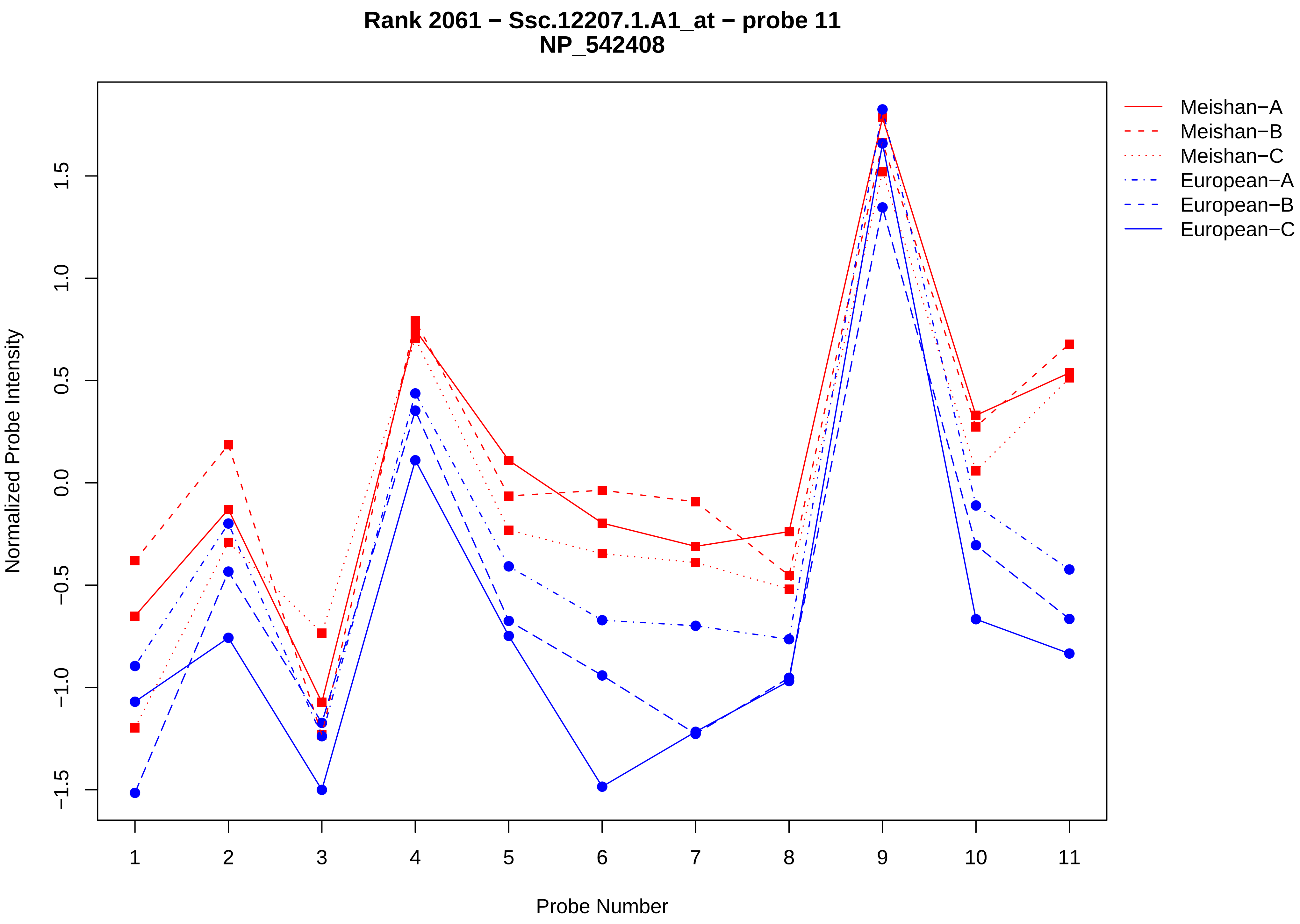

Rank 2061 − Ssc.12207.1.A1_at − probe 11
NP_542408
Normalized Probe Intensity
Probe Number
−1.5
−1.0
−0.5
0.0
0.5
1.0
1.5
1
2
3
4
5
6
7
8
9
10
11
Meishan−A
Meishan−B
Meishan−C
European−A
European−B
European−C

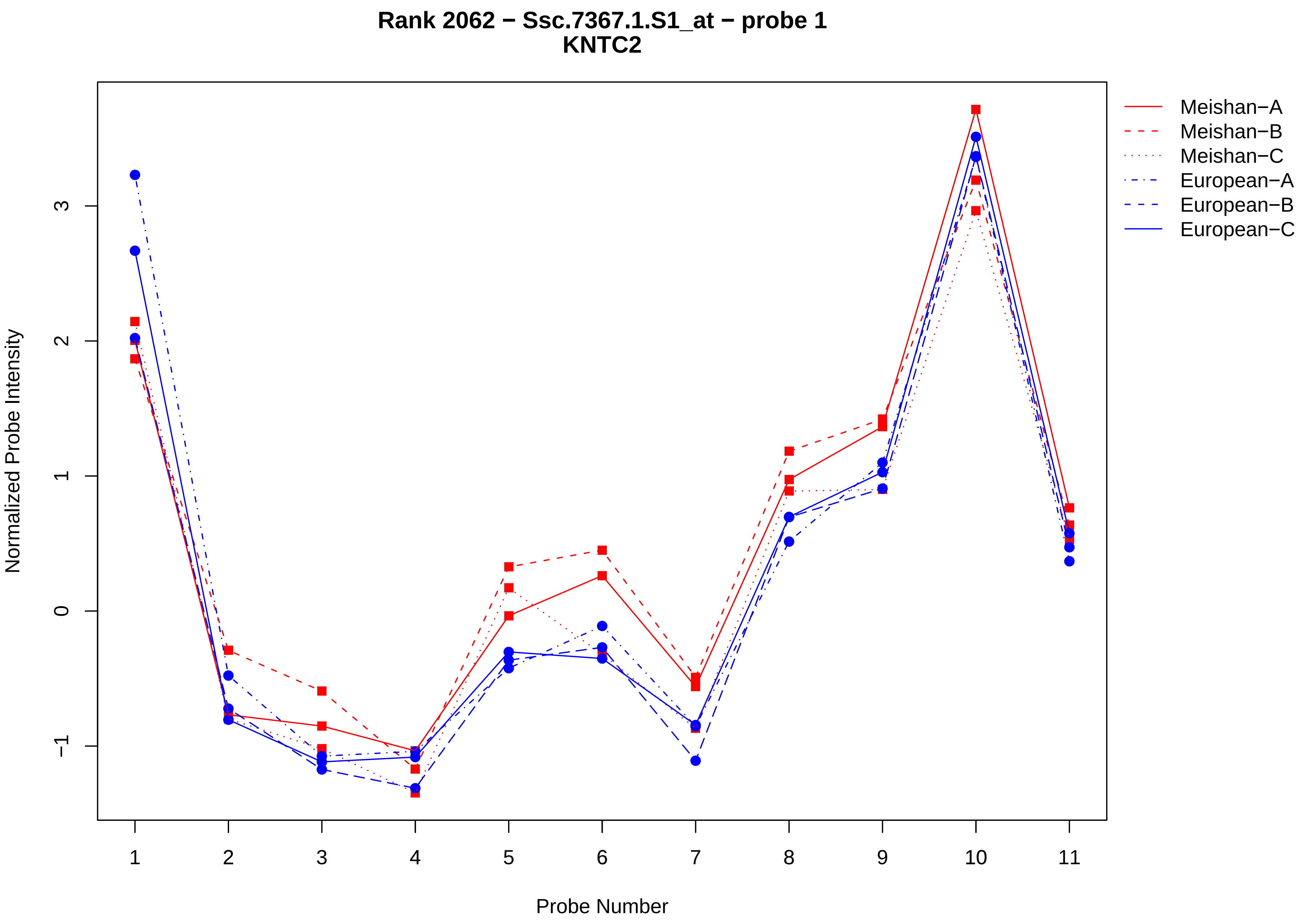

Rank 2062 − Ssc.7367.1.S1_at − probe 1
KNTC2
Normalized Probe Intensity
Probe Number
−1
0
1
2
3
1
2
3
4
5
6
7
8
9
10
11
Meishan−A
Meishan−B
Meishan−C
European−A
European−B
European−C

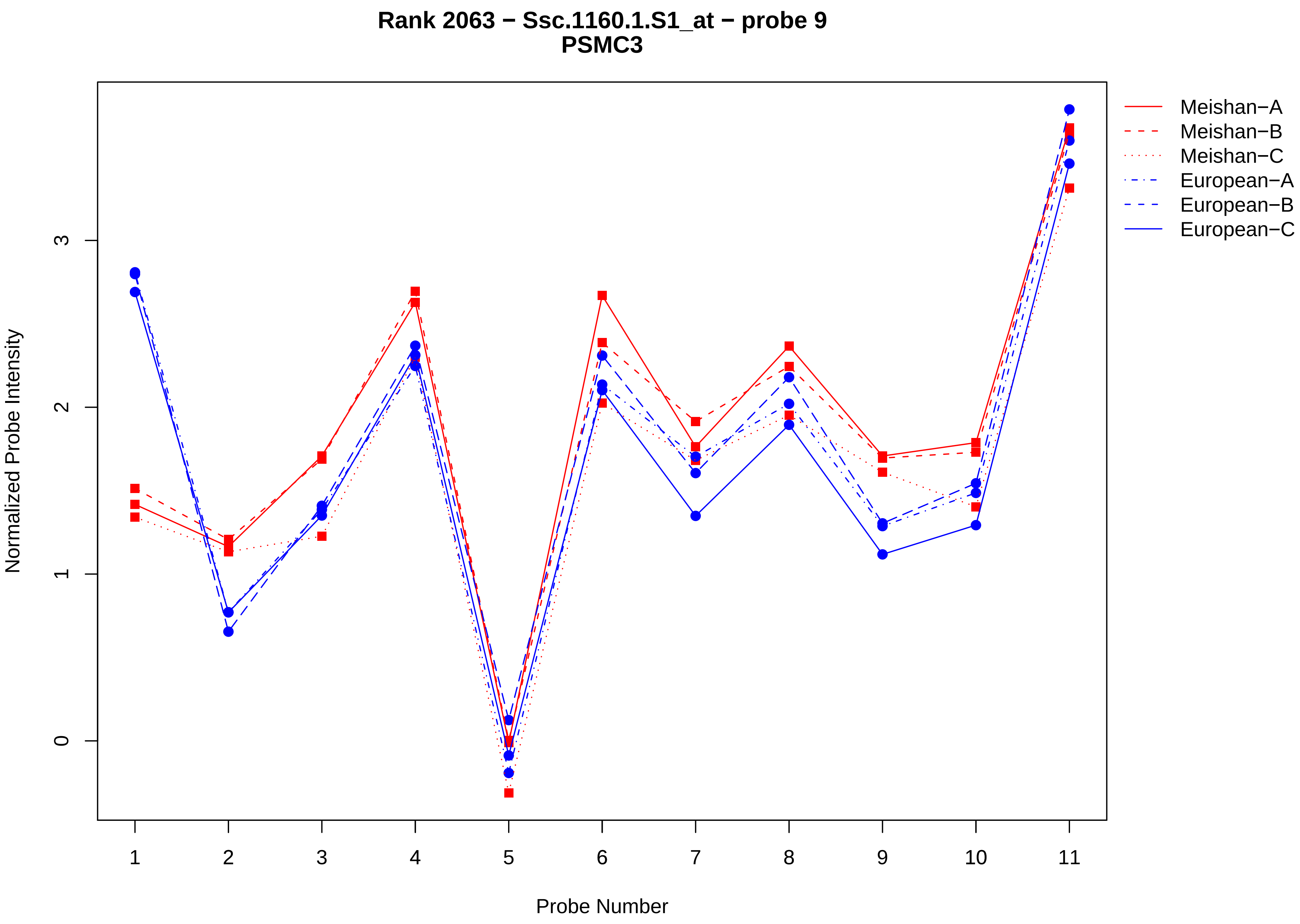

Rank 2063 − Ssc.1160.1.S1_at − probe 9
PSMC3
Normalized Probe Intensity
0
1
2
3
1
2
3
4
5
6
7
8
9
10
11
Probe Number
Meishan−A
Meishan−B
Meishan−C
European−A
European−B
European−C

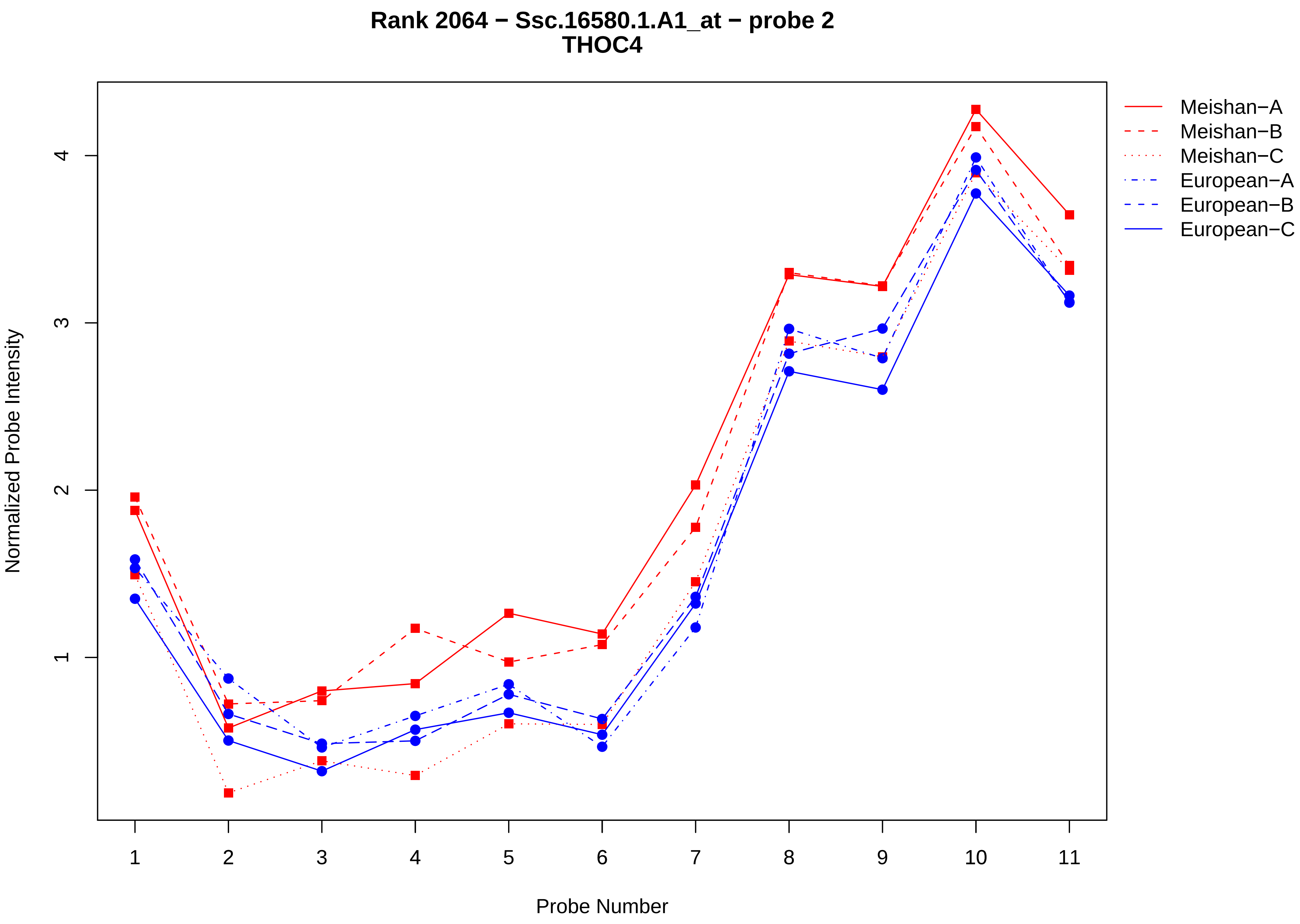

Rank 2064 − Ssc.16580.1.A1_at − probe 2
THOC4
Normalized Probe Intensity
Probe Number
Meishan−A
Meishan−B
Meishan−C
European−A
European−B
European−C

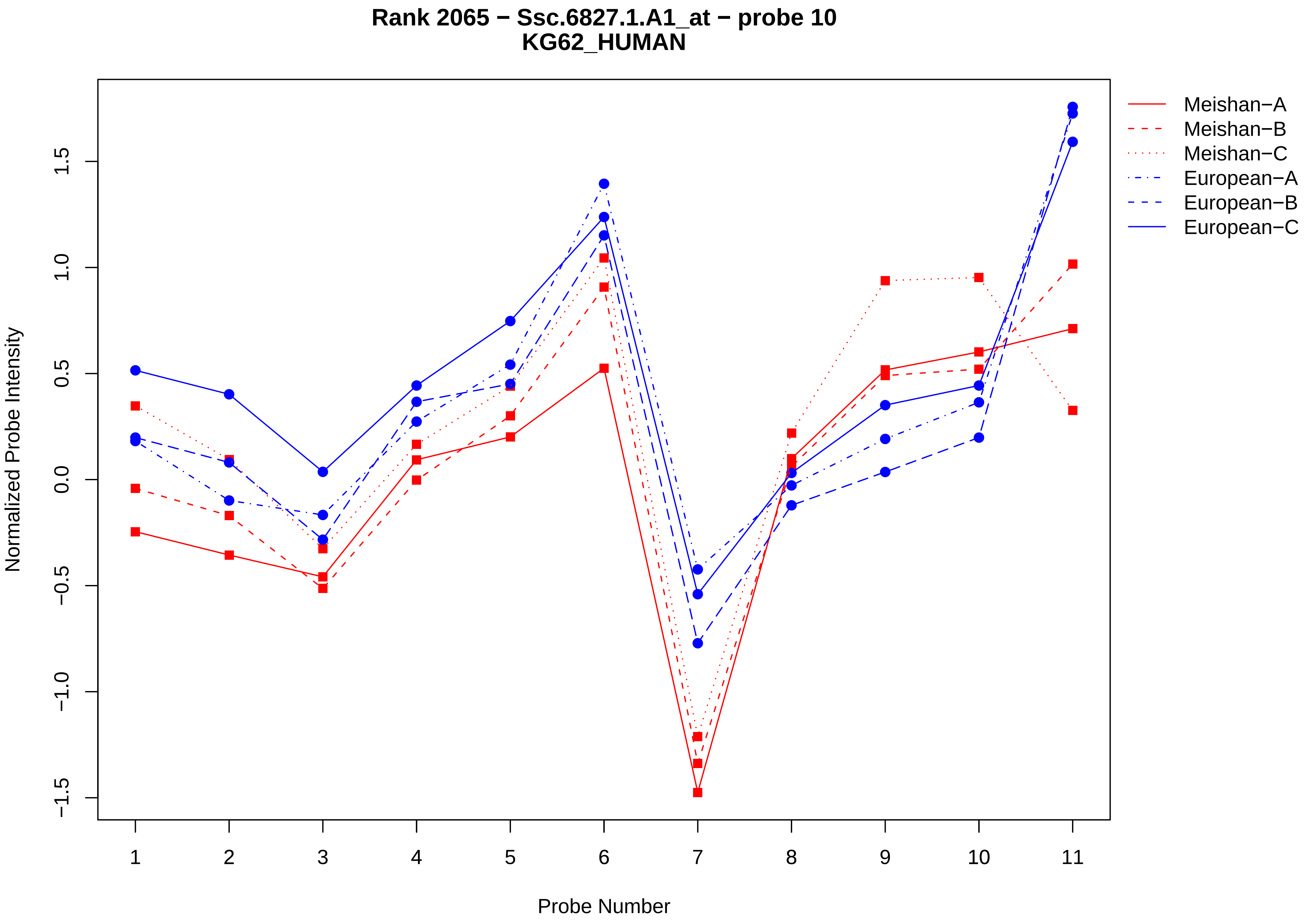

Rank 2065 − Ssc.6827.1.A1_at − probe 10
KG62_HUMAN
Normalized Probe Intensity
Probe Number
Meishan−A
Meishan−B
Meishan−C
European−A
European−B
European−C
−1.5
−1.0
−0.5
0.0
0.5
1.0
1.5
1
2
3
4
5
6
7
8
9
10
11

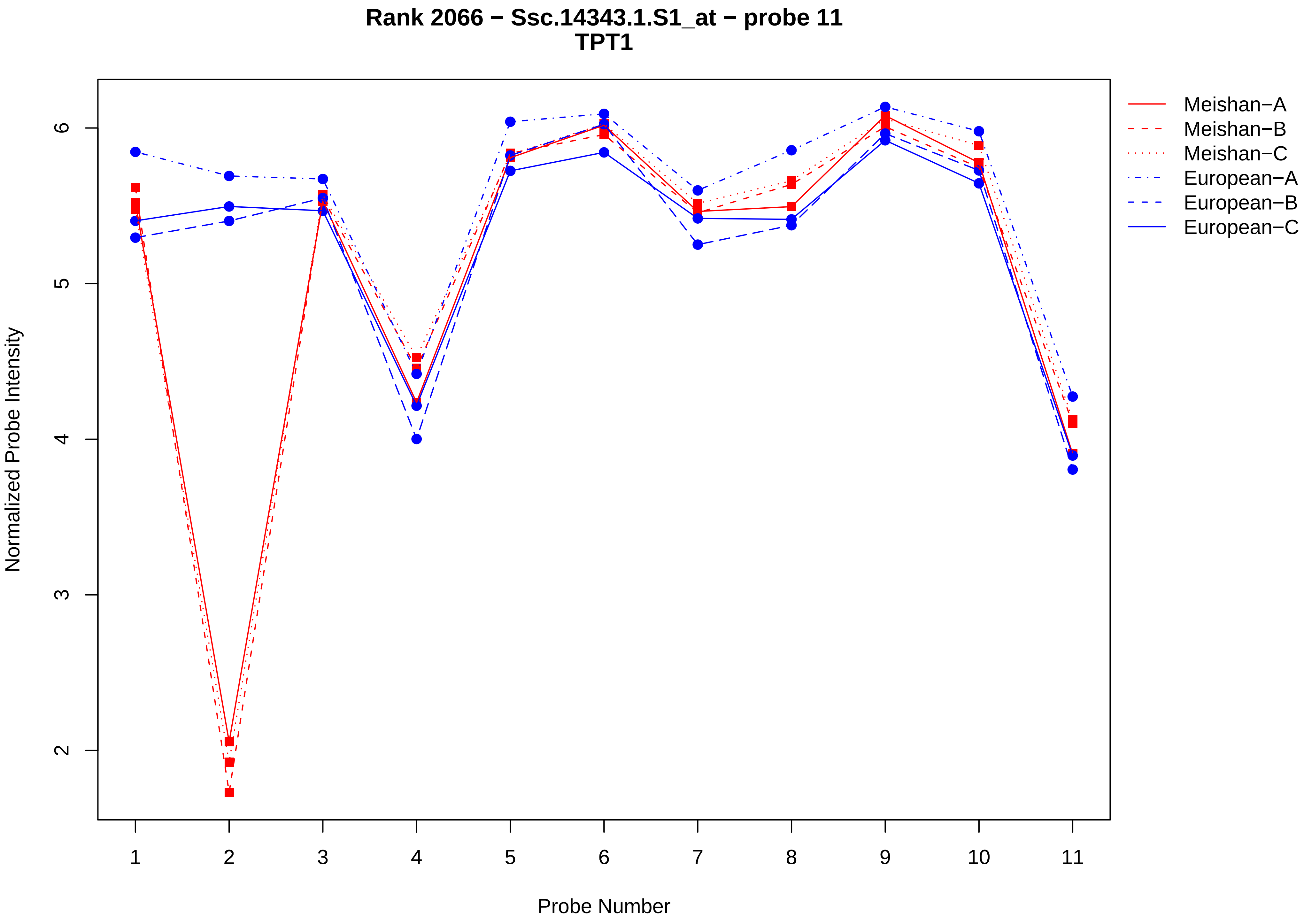

Rank 2066 − Ssc.14343.1.S1_at − probe 11
TPT1
Normalized Probe Intensity
Probe Number
Meishan−A
Meishan−B
Meishan−C
European−A
European−B
European−C
2
3
4
5
6
1
2
3
4
5
6
7
8
9
10
11

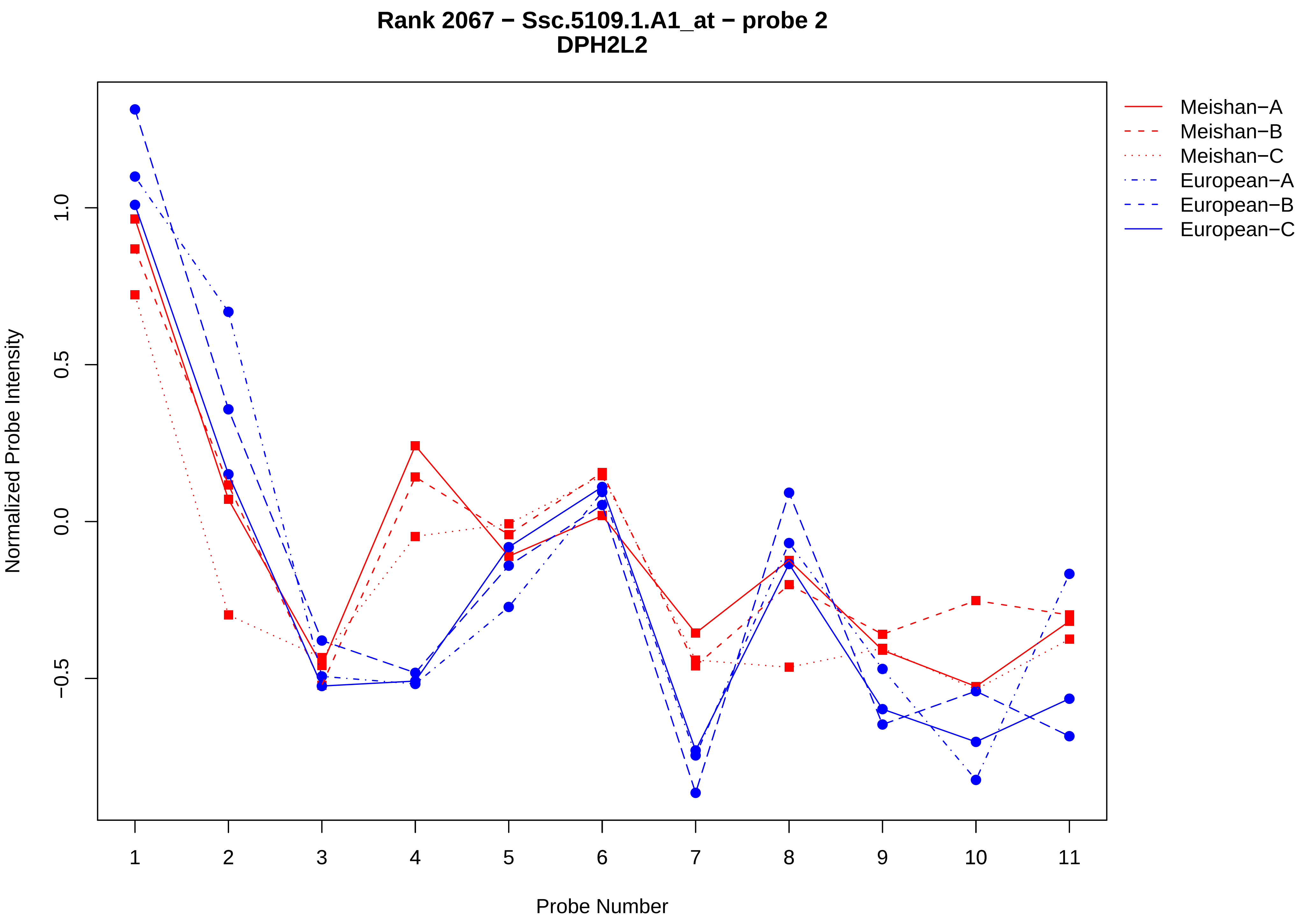

Rank 2067 − Ssc.5109.1.A1_at − probe 2
DPH2L2
Normalized Probe Intensity
Probe Number
1.0
0.5
0.0
−0.5
1
2
3
4
5
6
7
8
9
10
11
Meishan−A
Meishan−B
Meishan−C
European−A
European−B
European−C

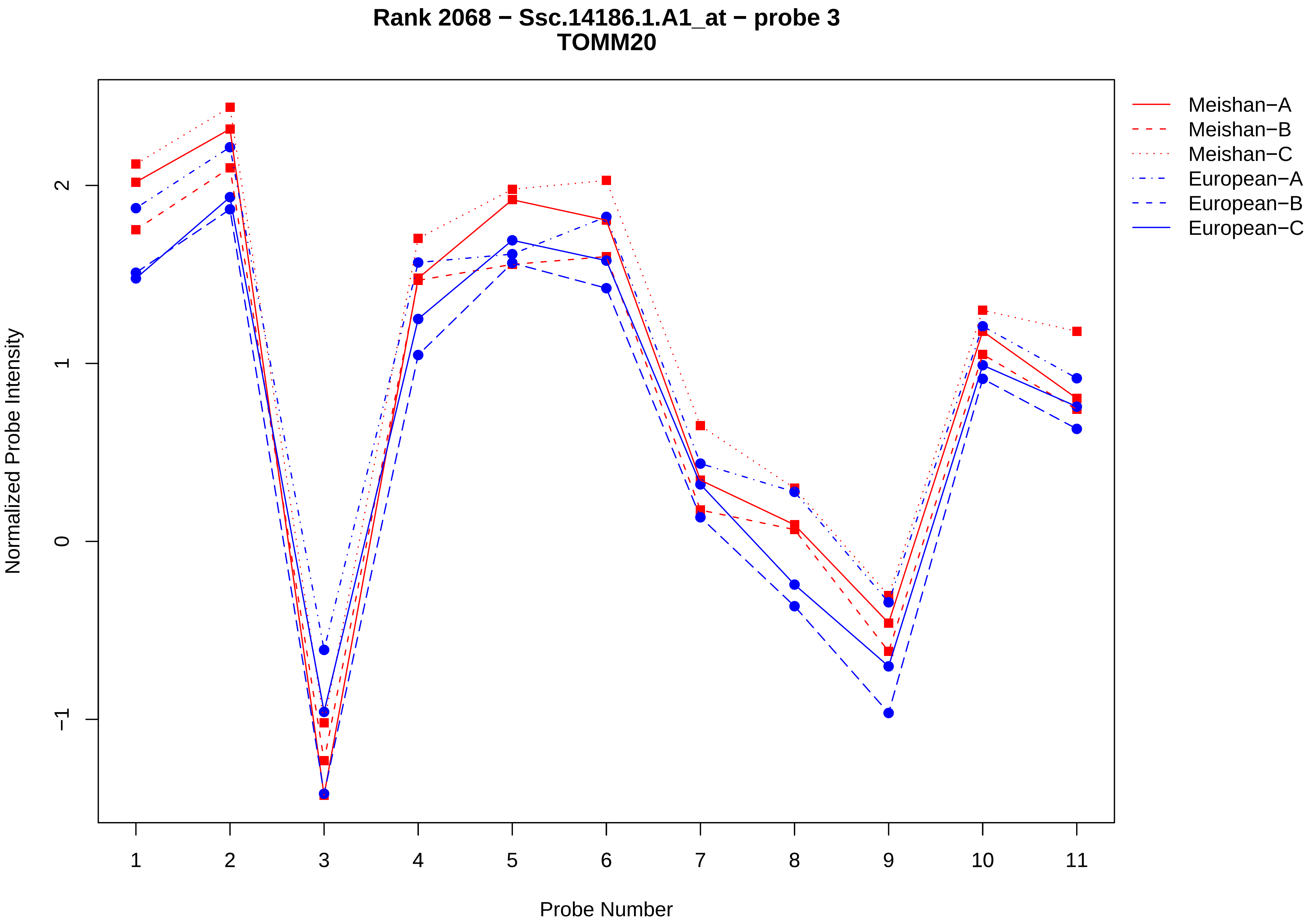

Rank 2068 − Ssc.14186.1.A1_at − probe 3
TOMM20
Normalized Probe Intensity
Probe Number
−1
0
1
2
1
2
3
4
5
6
7
8
9
10
11
Meishan−A
Meishan−B
Meishan−C
European−A
European−B
European−C

Rank 2069 − Ssc.1250.1.A1_at − probe 10
TCF12

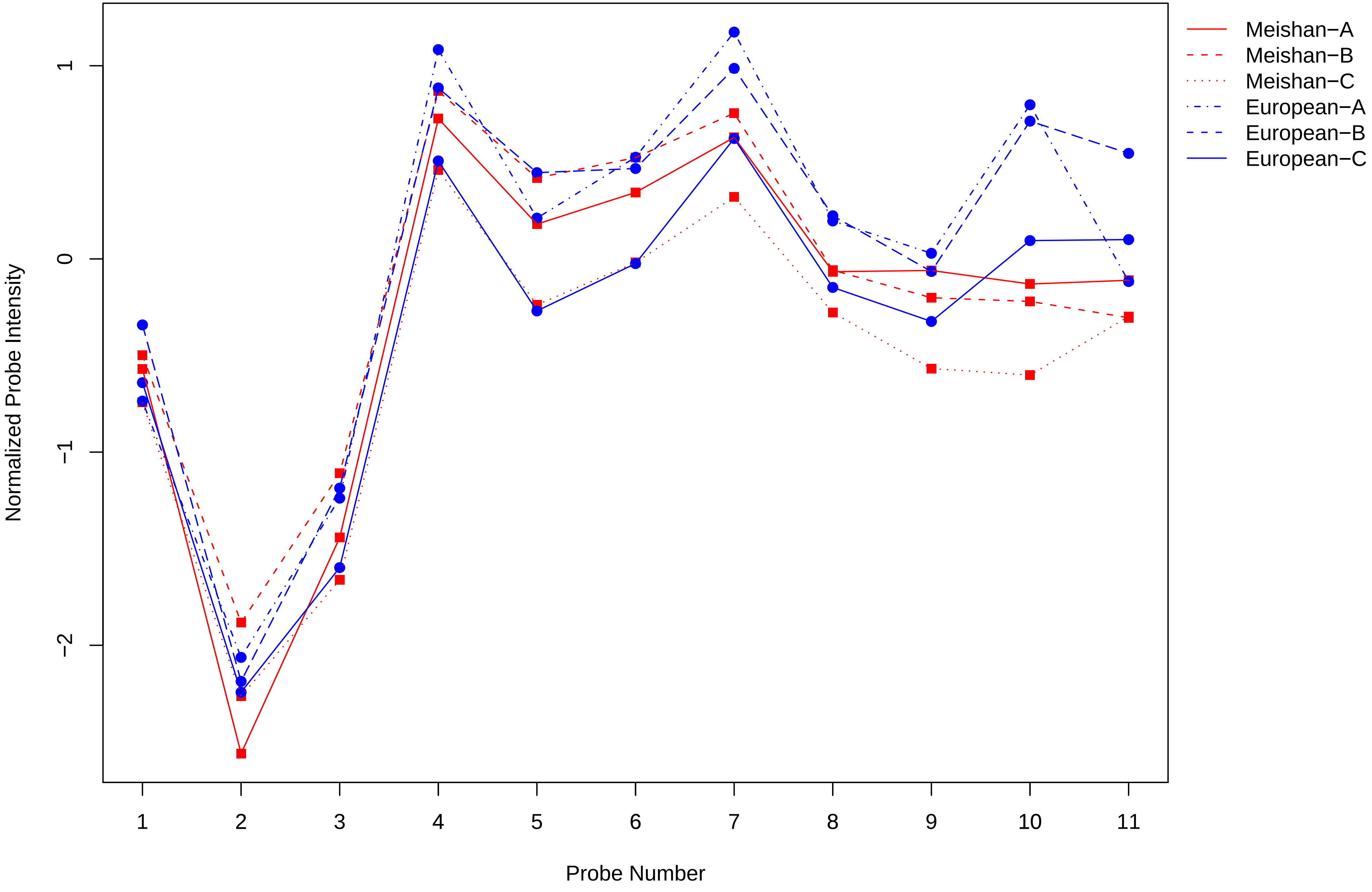

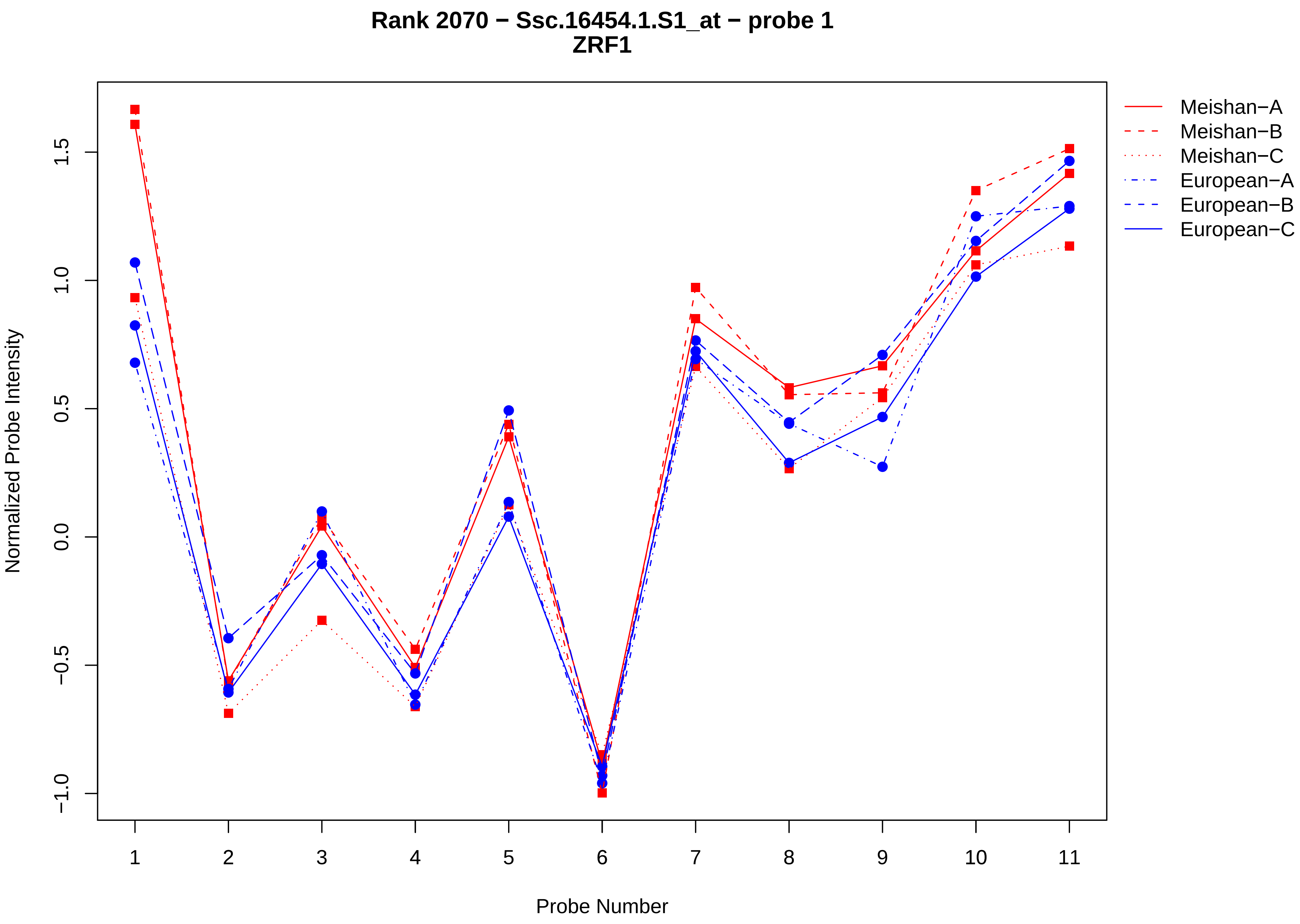

Rank 2070 − Ssc.16454.1.S1_at − probe 1
ZRF1
Normalized Probe Intensity
Probe Number
−1.0
−0.5
0.0
0.5
1.0
1.5
1
2
3
4
5
6
7
8
9
10
11
Meishan−A
Meishan−B
Meishan−C
European−A
European−B
European−C

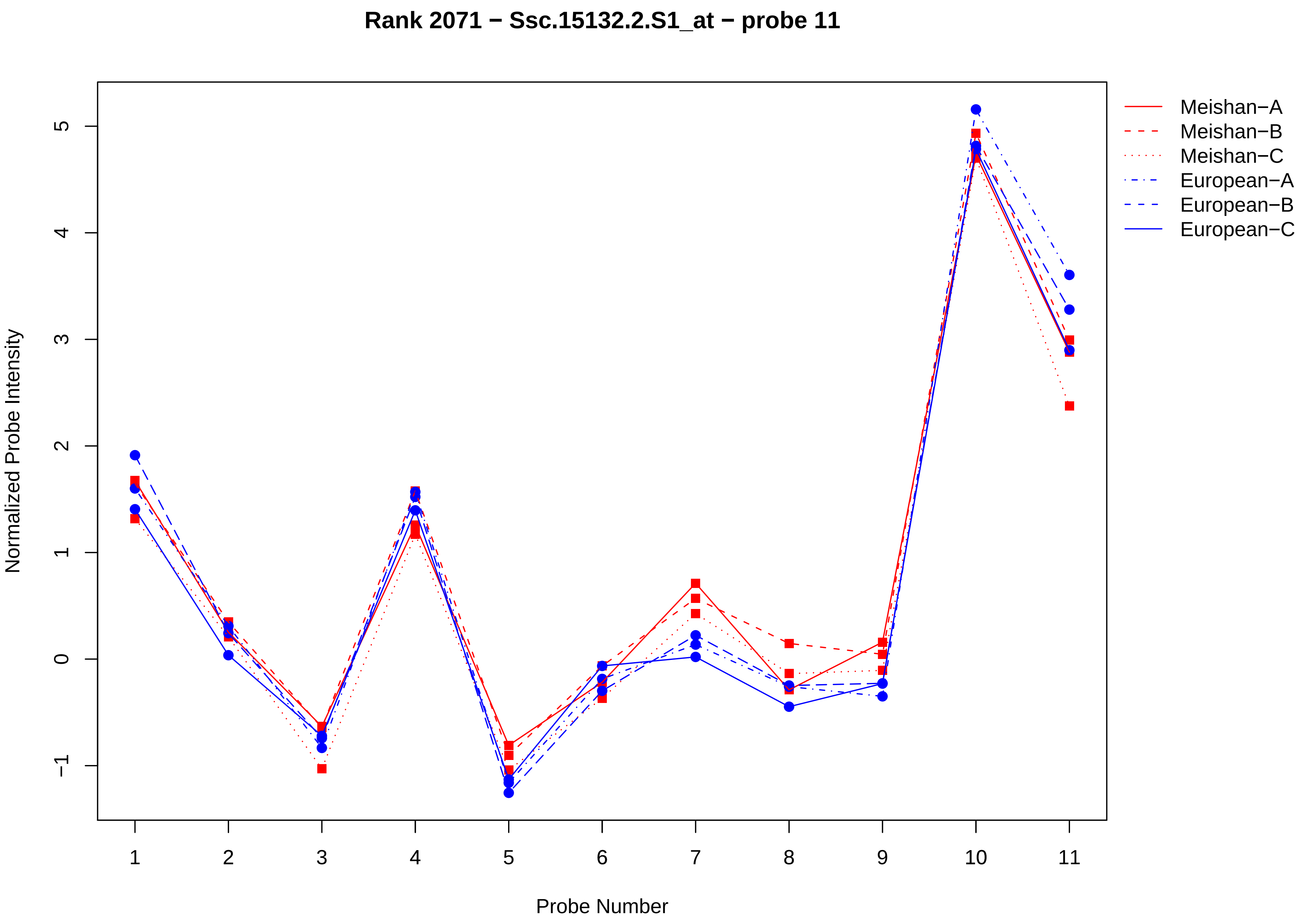

Rank 2071 − Ssc.15132.2.S1_at − probe 11
Normalized Probe Intensity
Probe Number
Meishan−A
Meishan−B
Meishan−C
European−A
European−B
European−C
−1
0
1
2
3
4
5
1
2
3
4
5
6
7
8
9
10
11

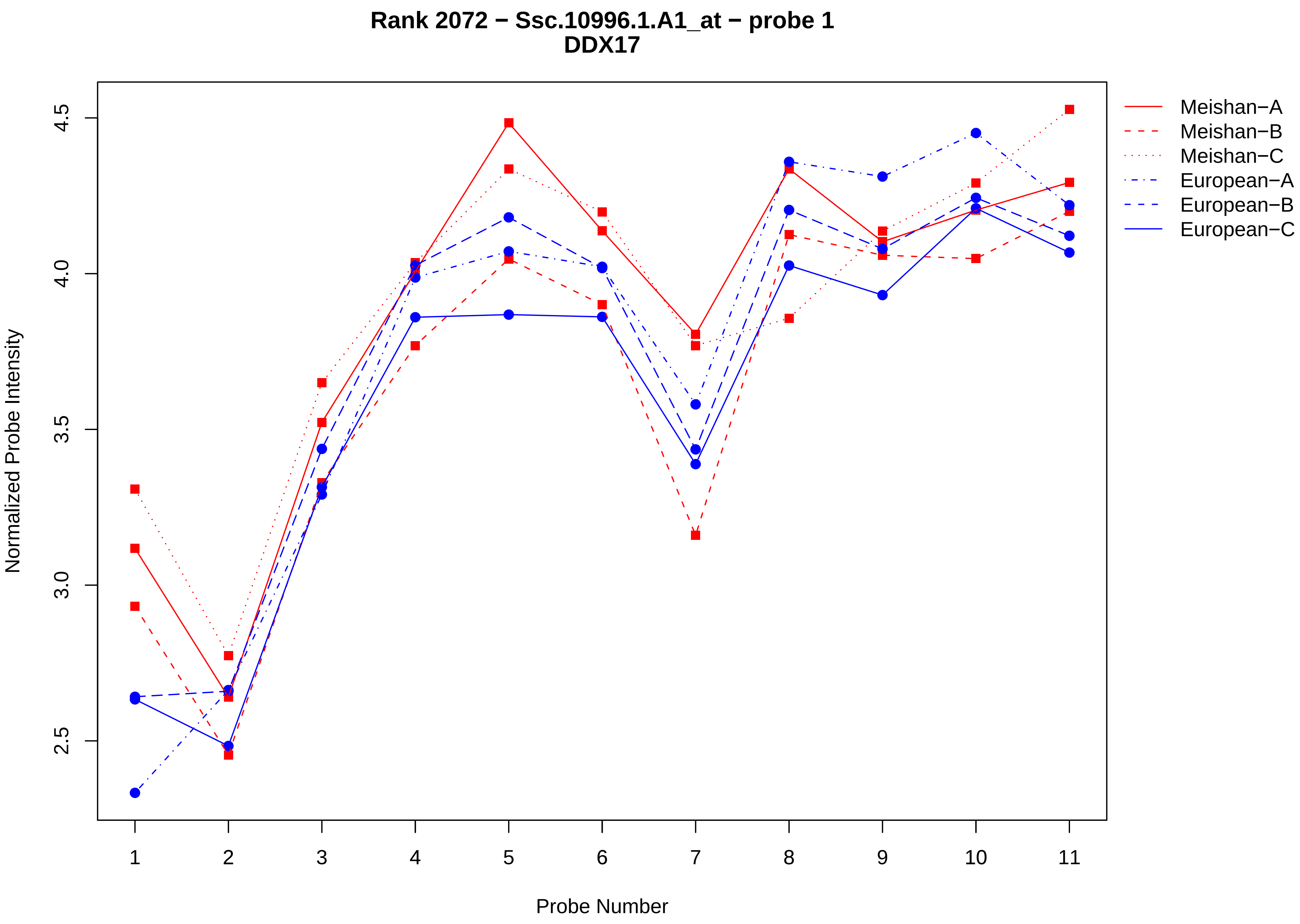

Rank 2072 − Ssc.10996.1.A1_at − probe 1
DDX17
Normalized Probe Intensity
Probe Number
Meishan−A
Meishan−B
Meishan−C
European−A
European−B
European−C

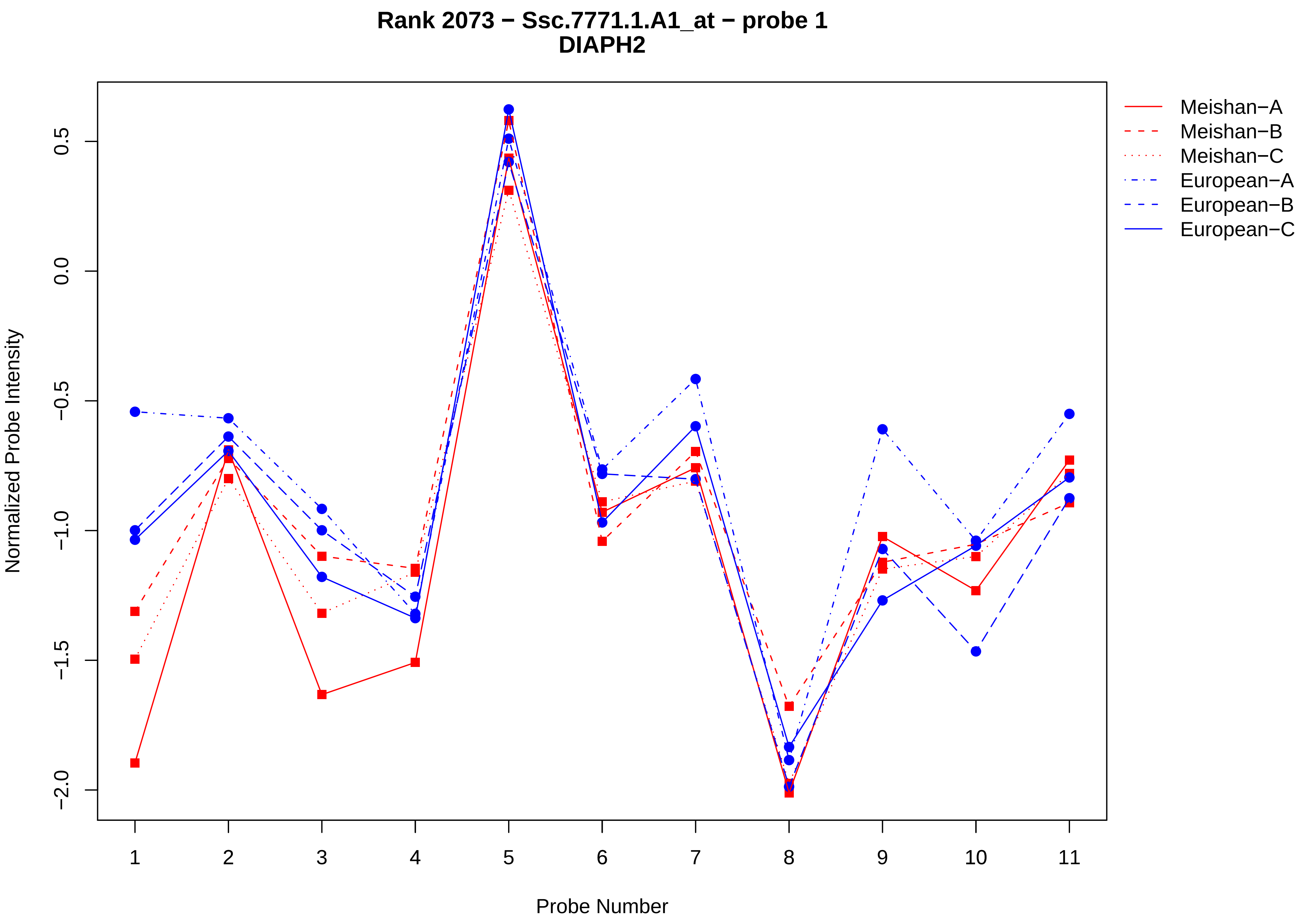

Rank 2073 − Ssc.7771.1.A1_at − probe 1
DIAPH2
Normalized Probe Intensity
Probe Number
0.5
0.0
−0.5
−1.0
−1.5
−2.0
1
2
3
4
5
6
7
8
9
10
11
Meishan−A
Meishan−B
Meishan−C
European−A
European−B
European−C

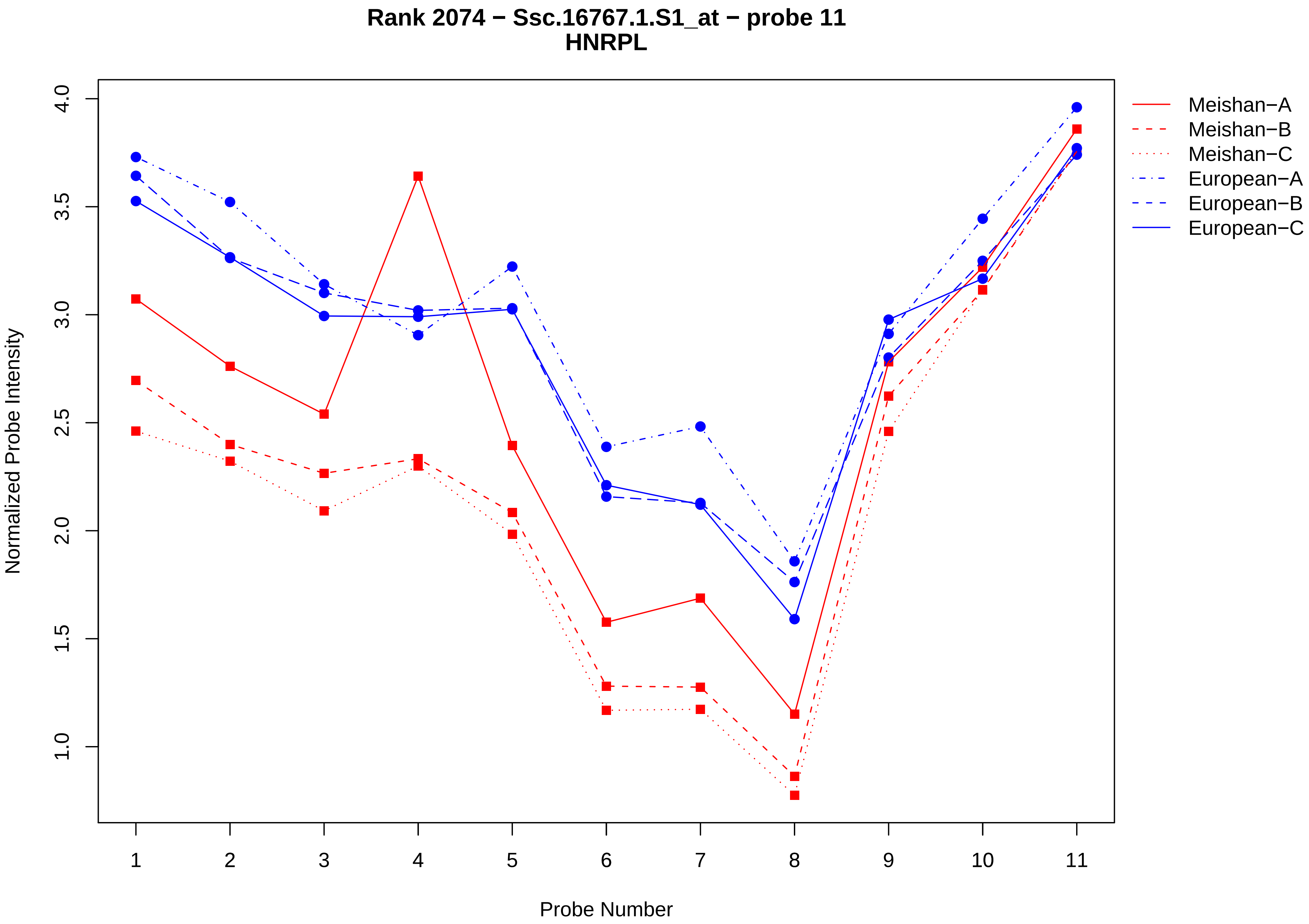

Rank 2074 − Ssc.16767.1.S1_at − probe 11
HNRPL
Normalized Probe Intensity
Probe Number
1.0
1.5
2.0
2.5
3.0
3.5
4.0
1
2
3
4
5
6
7
8
9
10
11
Meishan−A
Meishan−B
Meishan−C
European−A
European−B
European−C

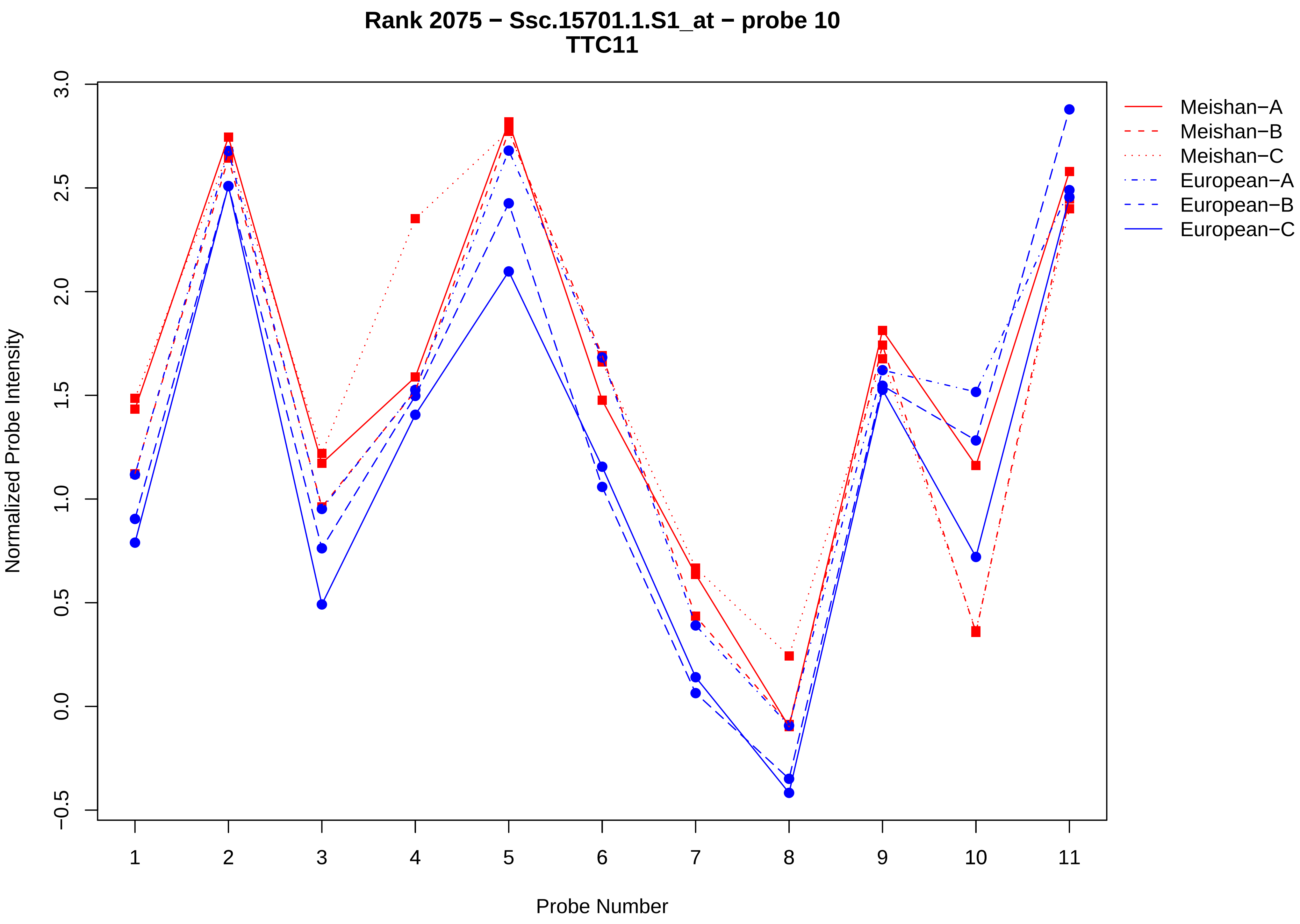

Rank 2075 − Ssc.15701.1.S1_at − probe 10
TTC11
Normalized Probe Intensity
Probe Number
−0.5
0.0
0.5
1.0
1.5
2.0
2.5
3.0
1
2
3
4
5
6
7
8
9
10
11
Meishan−A
Meishan−B
Meishan−C
European−A
European−B
European−C

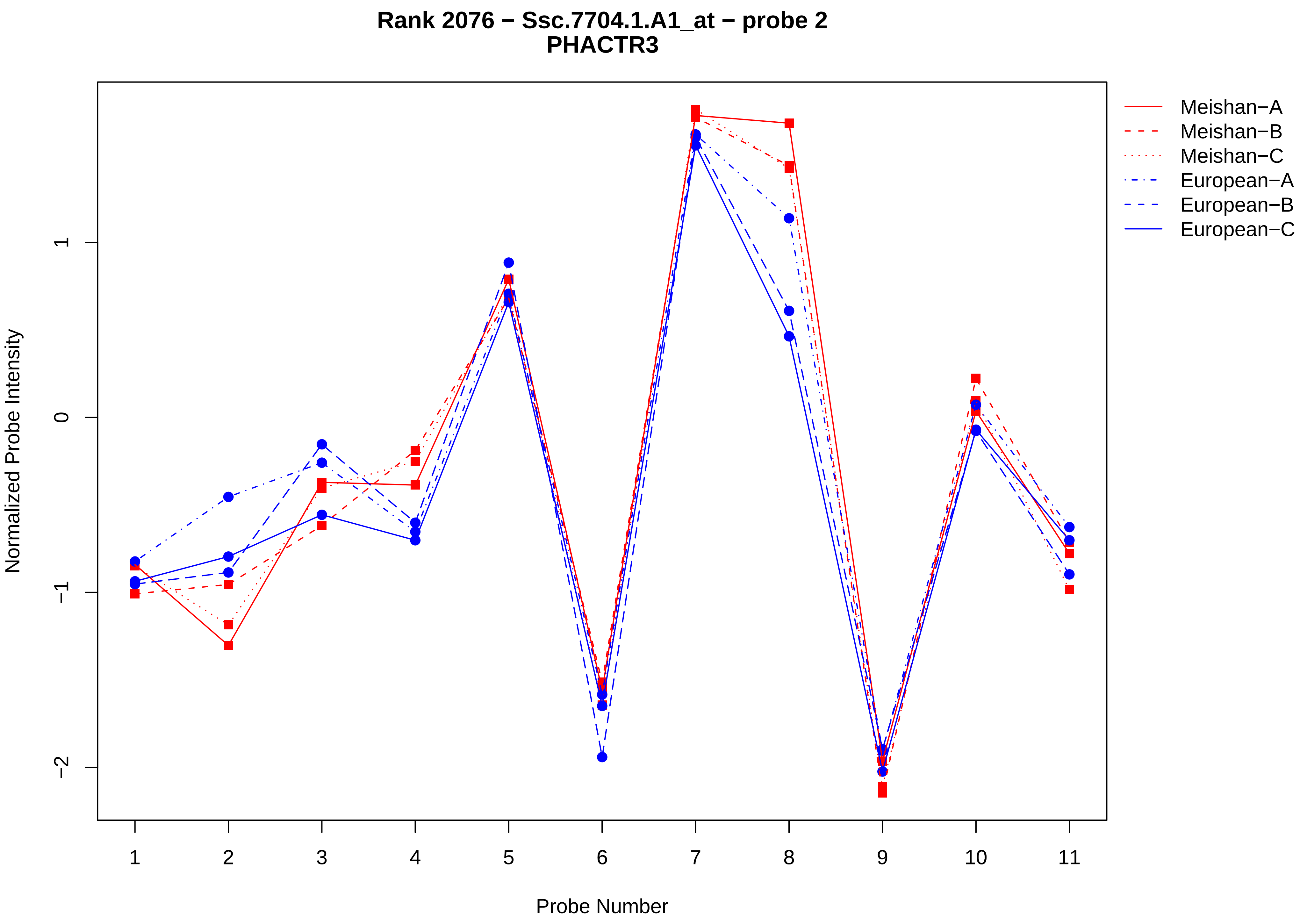

Rank 2076 − Ssc.7704.1.A1_at − probe 2
PHACTR3
Normalized Probe Intensity
Probe Number
Meishan−A
Meishan−B
Meishan−C
European−A
European−B
European−C
1
0
−1
−2
1
2
3
4
5
6
7
8
9
10
11

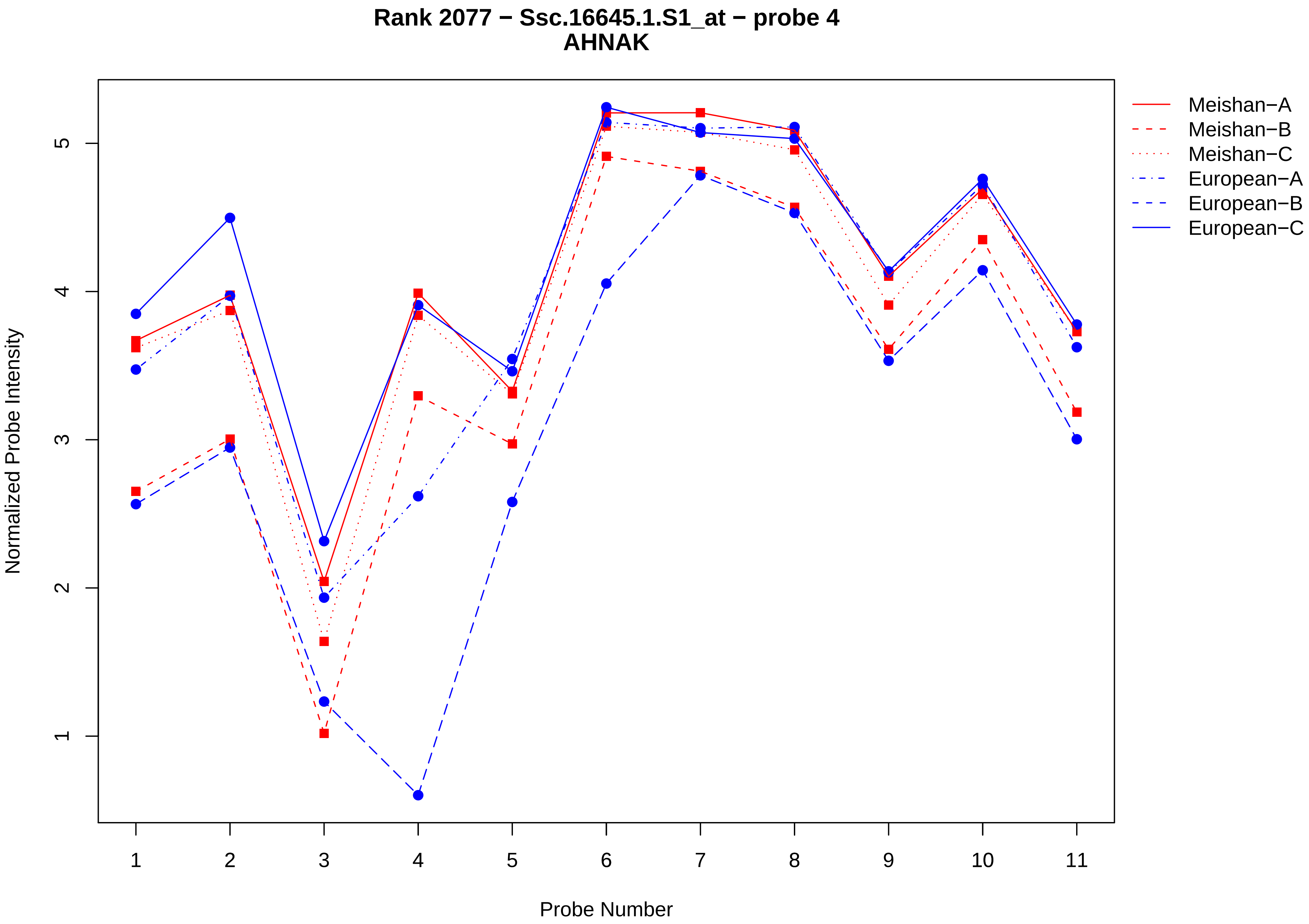

Rank 2077 − Ssc.16645.1.S1_at − probe 4
AHNAK
Normalized Probe Intensity
Probe Number
1
2
3
4
5
1
2
3
4
5
6
7
8
9
10
11
Meishan−A
Meishan−B
Meishan−C
European−A
European−B
European−C

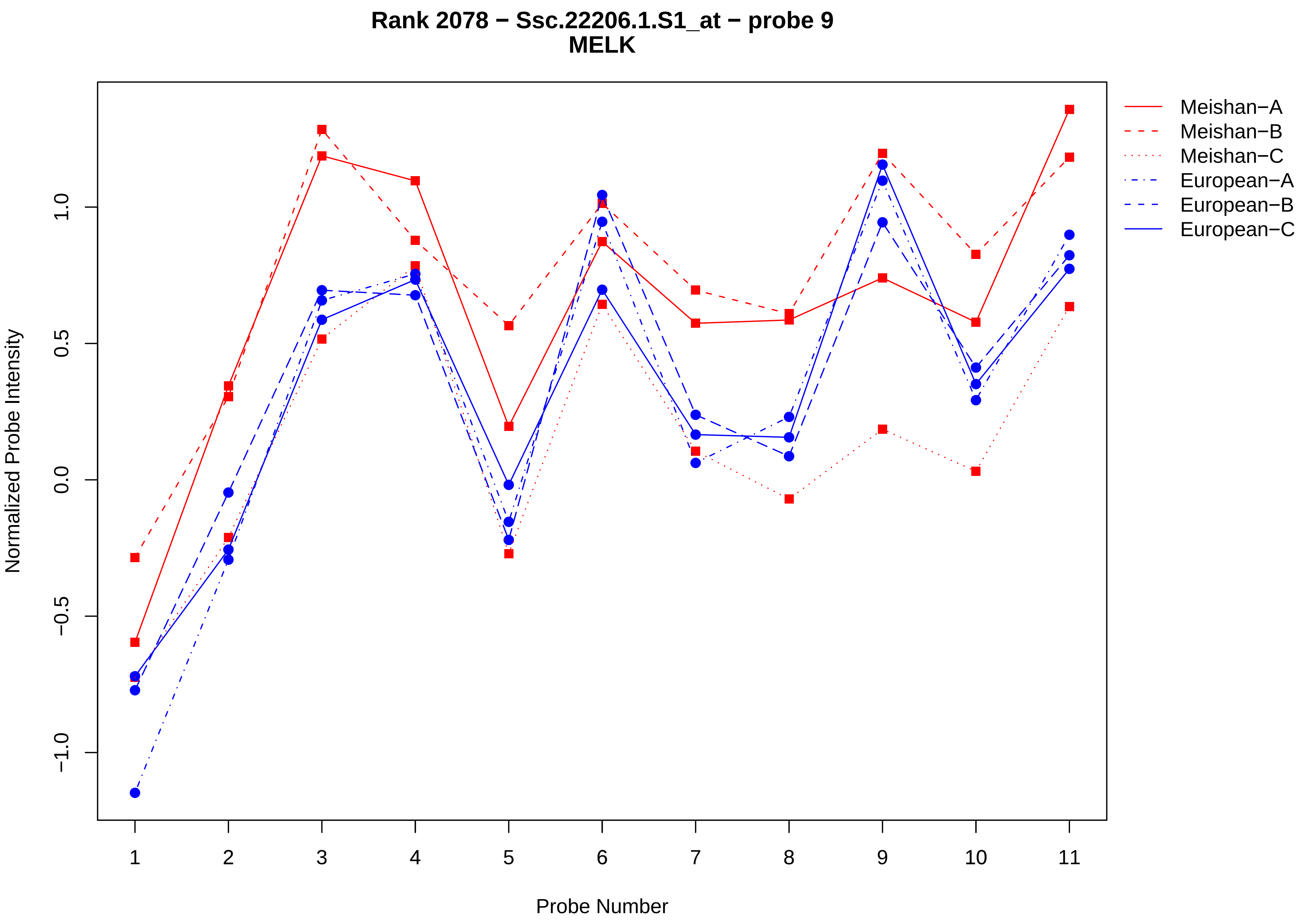

Rank 2078 − Ssc.22206.1.S1_at − probe 9
MELK
Normalized Probe Intensity
Probe Number
Meishan−A
Meishan−B
Meishan−C
European−A
European−B
European−C
−1.0
−0.5
0.0
0.5
1.0
1
2
3
4
5
6
7
8
9
10
11

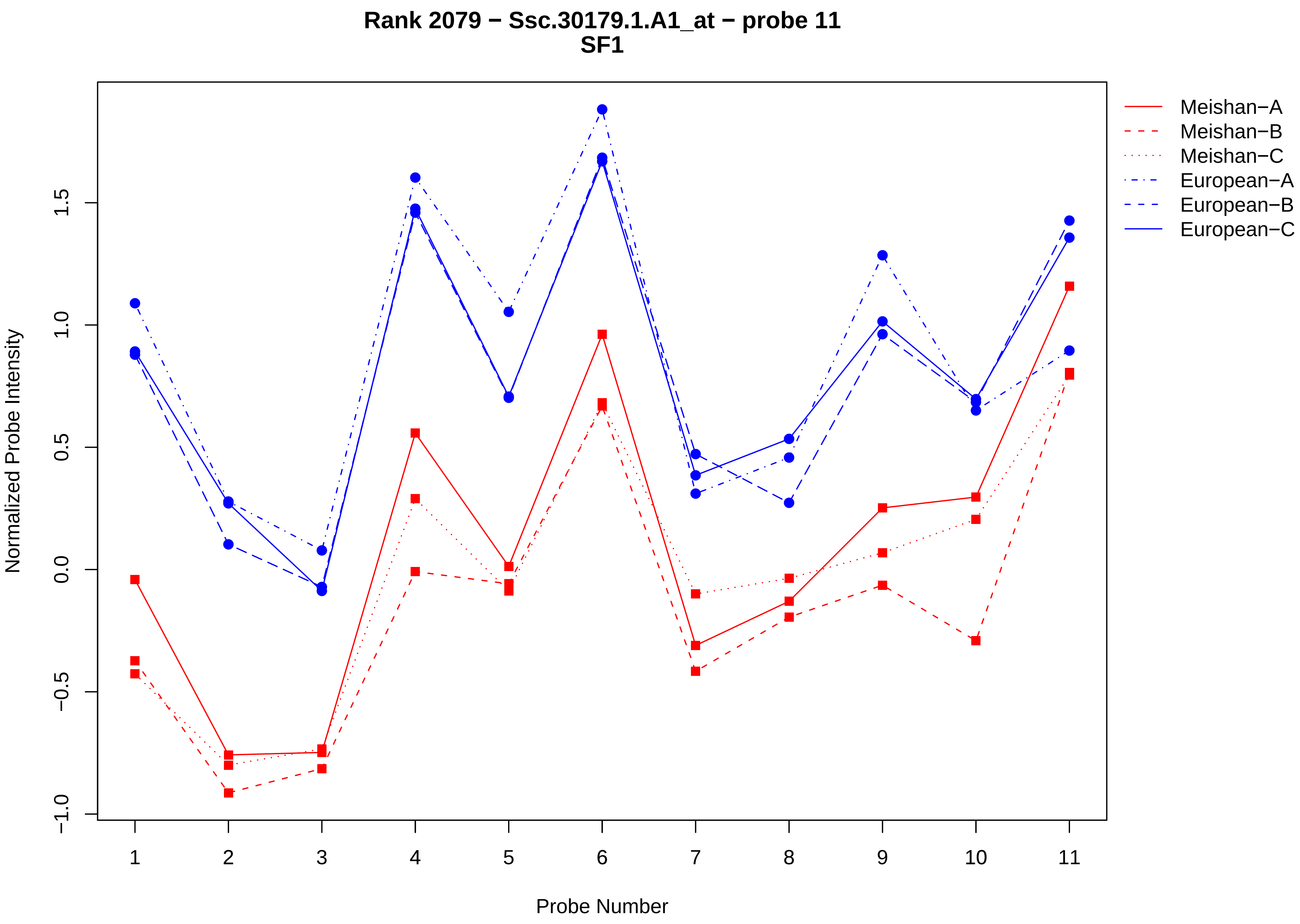

Rank 2079 − Ssc.30179.1.A1_at − probe 11
SF1
Normalized Probe Intensity
Probe Number
−1.0
−0.5
0.0
0.5
1.0
1.5
1
2
3
4
5
6
7
8
9
10
11
Meishan−A
Meishan−B
Meishan−C
European−A
European−B
European−C

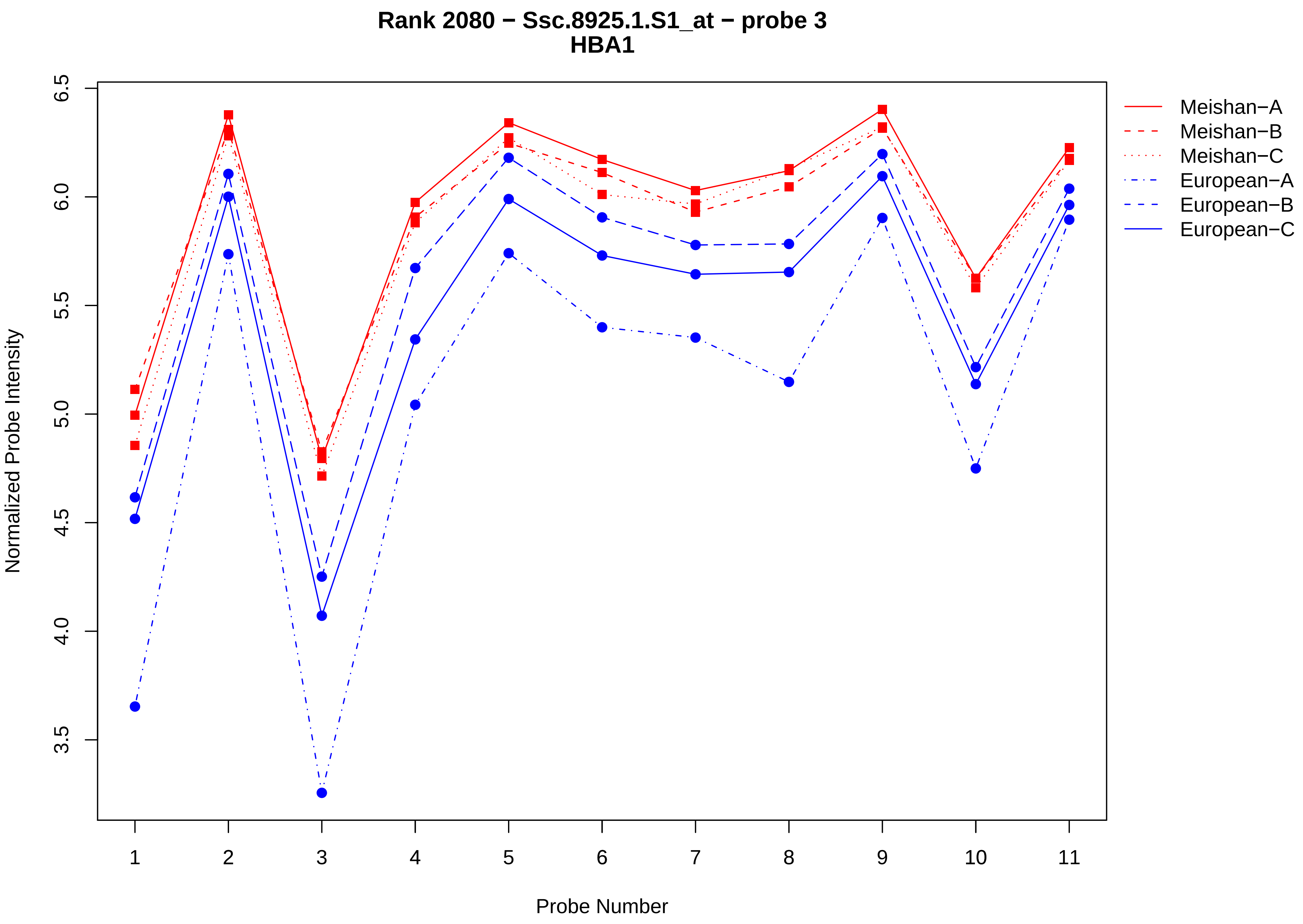

Rank 2080 − Ssc.8925.1.S1_at − probe 3
HBA1
Normalized Probe Intensity
Probe Number
3.5
4.0
4.5
5.0
5.5
6.0
6.5
1
2
3
4
5
6
7
8
9
10
11
Meishan−A
Meishan−B
Meishan−C
European−A
European−B
European−C

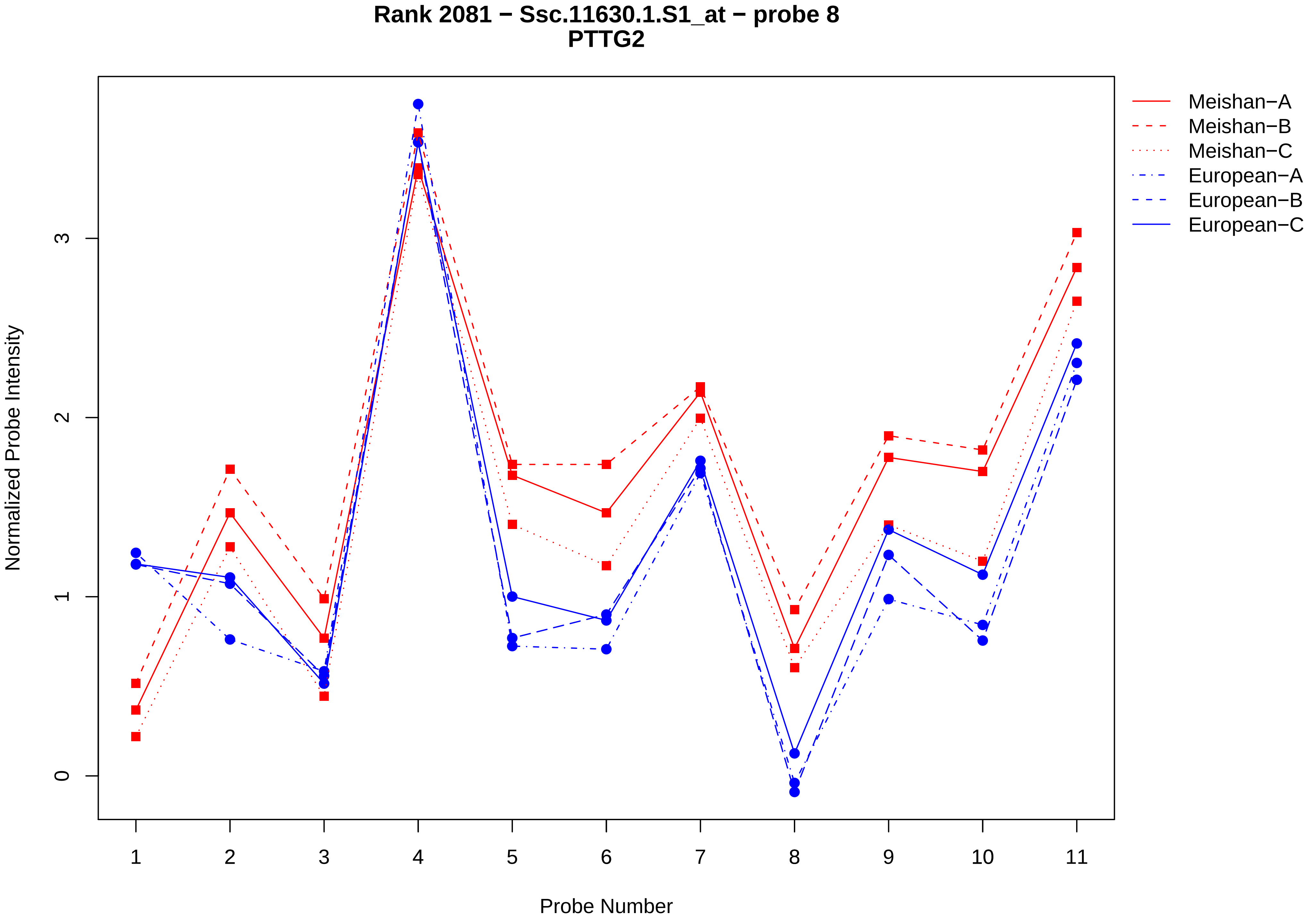
Rank 2081 − Ssc.11630.1.S1_at − probe 8
PTTG2
Normalized Probe Intensity
Probe Number
0
1
2
3
1
2
3
4
5
6
7
8
9
10
11
Meishan−A
Meishan−B
Meishan−C
European−A
European−B
European−C

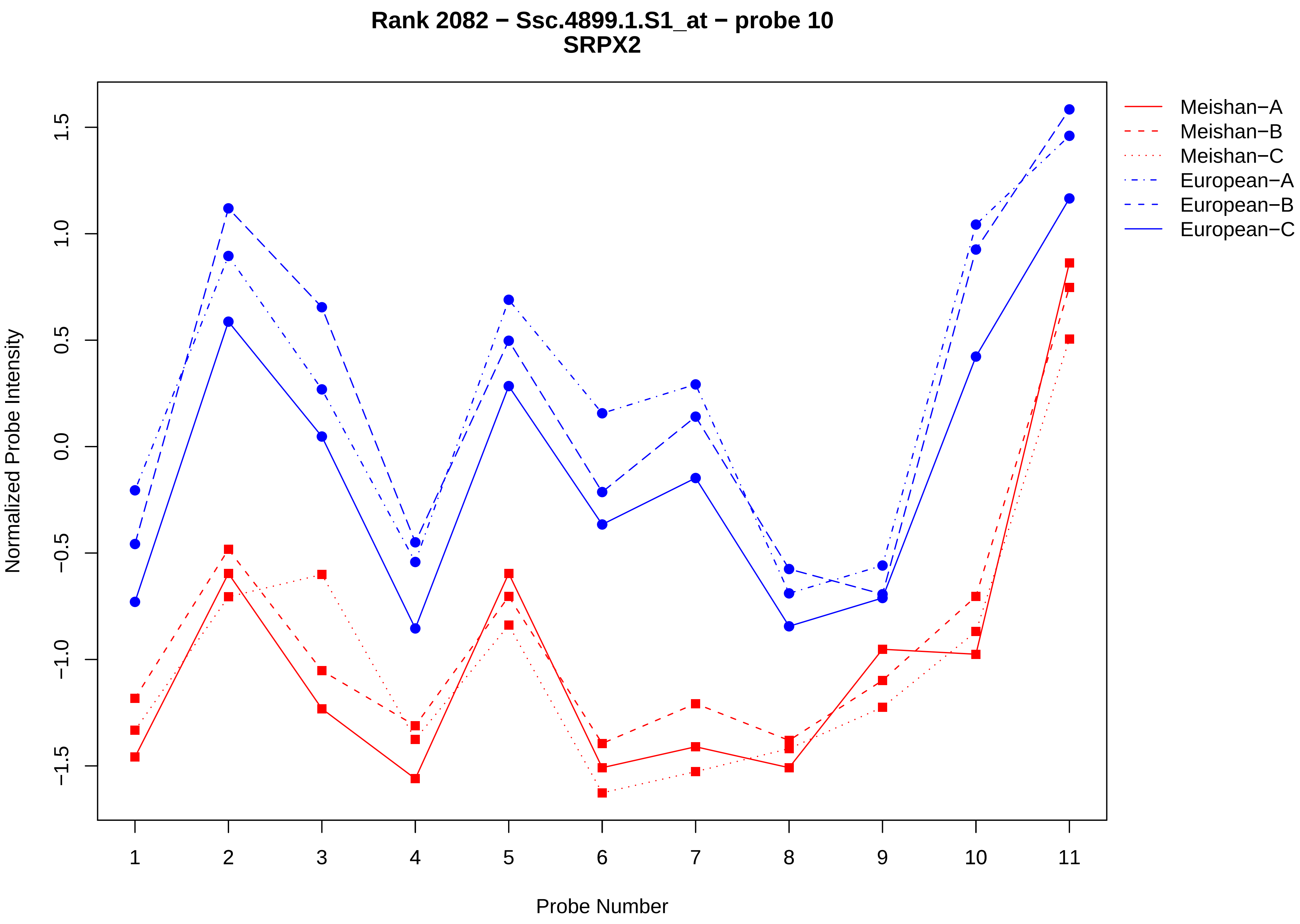

Rank 2082 − Ssc.4899.1.S1_at − probe 10
SRPX2
Normalized Probe Intensity
Probe Number
Meishan−A
Meishan−B
Meishan−C
European−A
European−B
European−C
1.5
1.0
0.5
0.0
−0.5
−1.0
−1.5
1
2
3
4
5
6
7
8
9
10
11

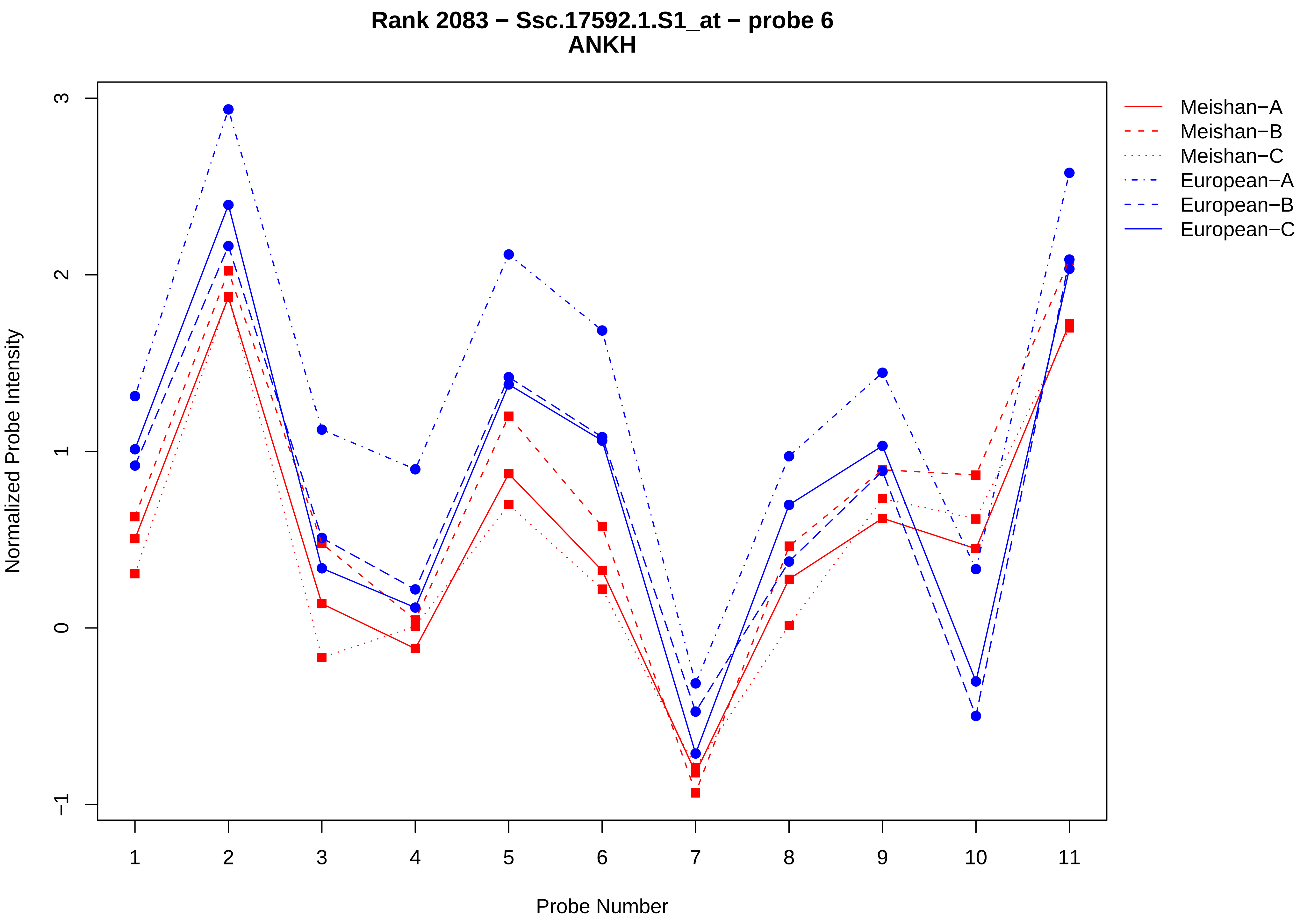

Rank 2083 − Ssc.17592.1.S1_at − probe 6
ANKH
Normalized Probe Intensity
Probe Number
−1
0
1
2
3
1
2
3
4
5
6
7
8
9
10
11
Meishan−A
Meishan−B
Meishan−C
European−A
European−B
European−C

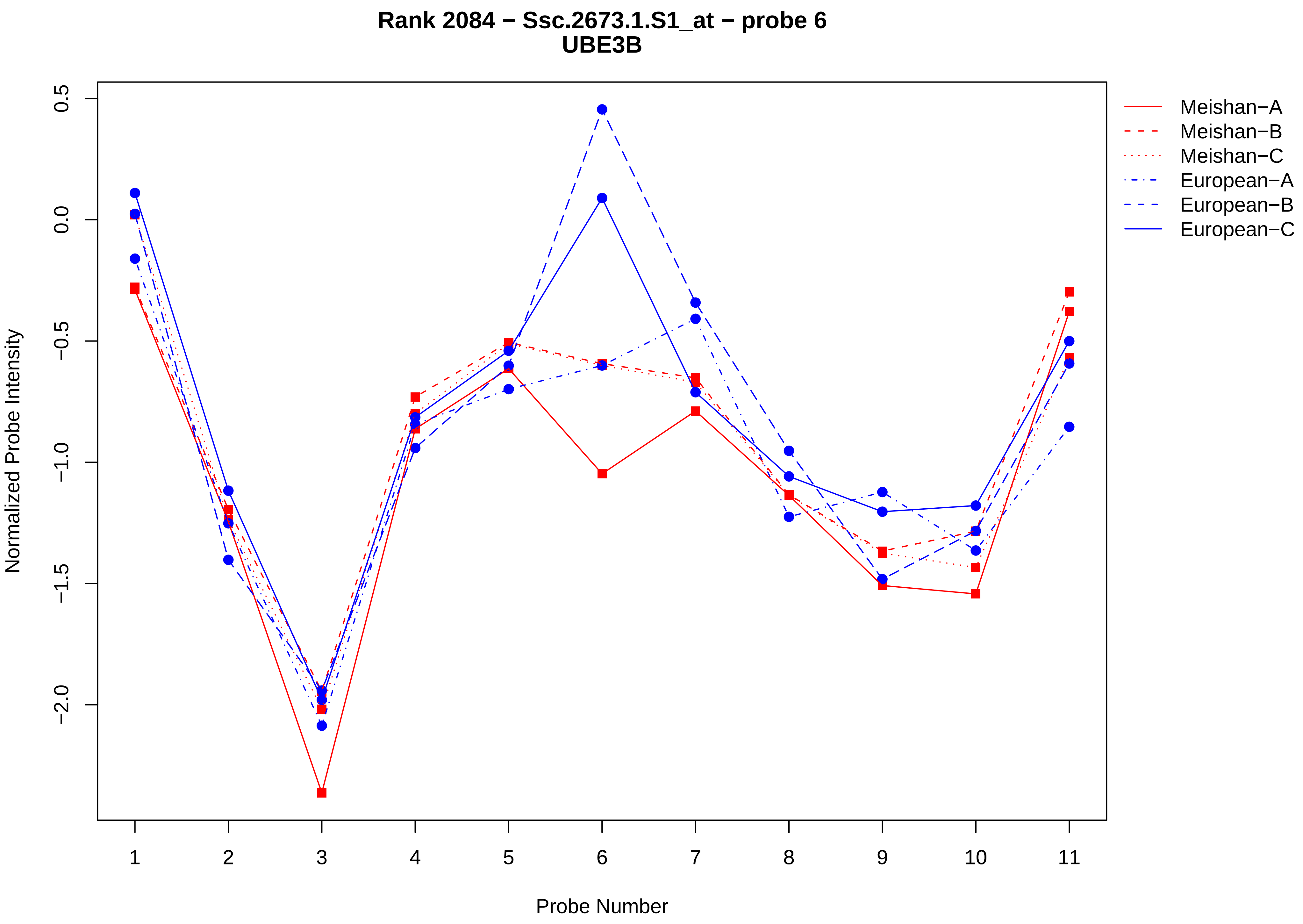
Rank 2084 − Ssc.2673.1.S1_at − probe 6
UBE3B
Normalized Probe Intensity
Probe Number
0.5
0.0
−0.5
−1.0
−1.5
−2.0
1
2
3
4
5
6
7
8
9
10
11
Meishan−A
Meishan−B
Meishan−C
European−A
European−B
European−C

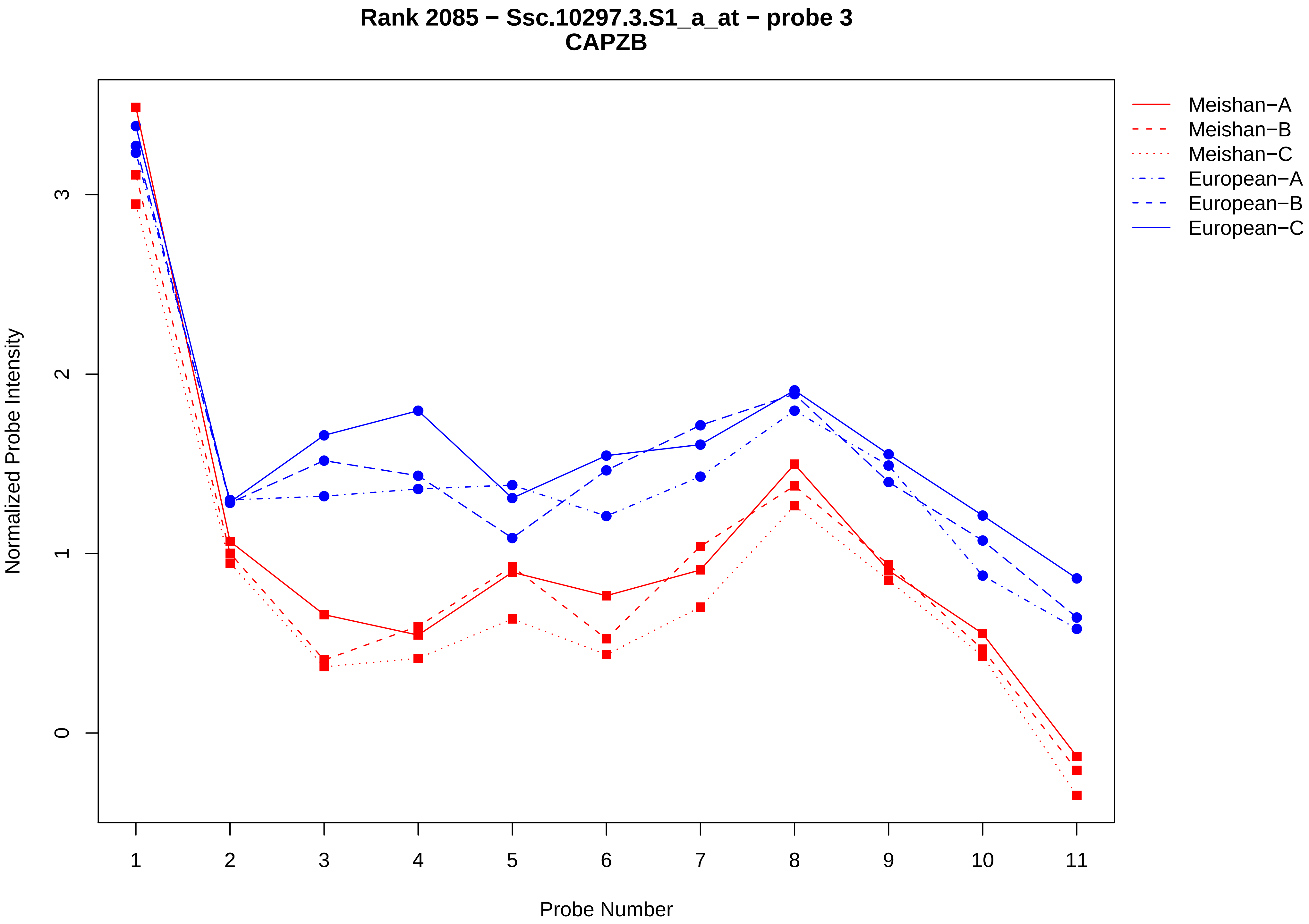

Rank 2085 − Ssc.10297.3.S1_a_at − probe 3
CAPZB
Normalized Probe Intensity
Probe Number
0
1
2
3
1
2
3
4
5
6
7
8
9
10
11
Meishan−A
Meishan−B
Meishan−C
European−A
European−B
European−C

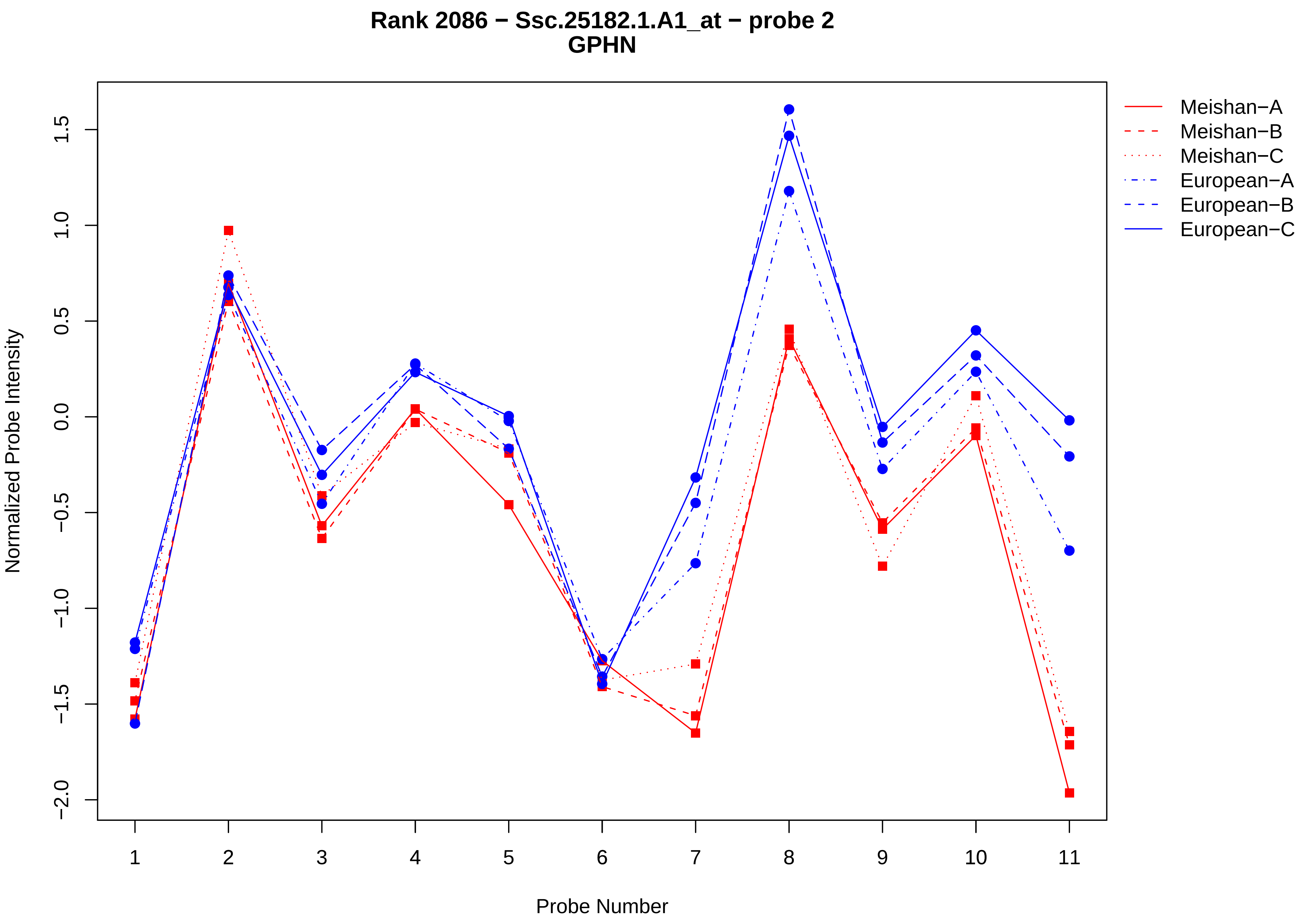

Rank 2086 − Ssc.25182.1.A1_at − probe 2
GPHN
Normalized Probe Intensity
Probe Number
Meishan−A
Meishan−B
Meishan−C
European−A
European−B
European−C
1.5
1.0
0.5
0.0
−0.5
−1.0
−1.5
−2.0
1
2
3
4
5
6
7
8
9
10
11

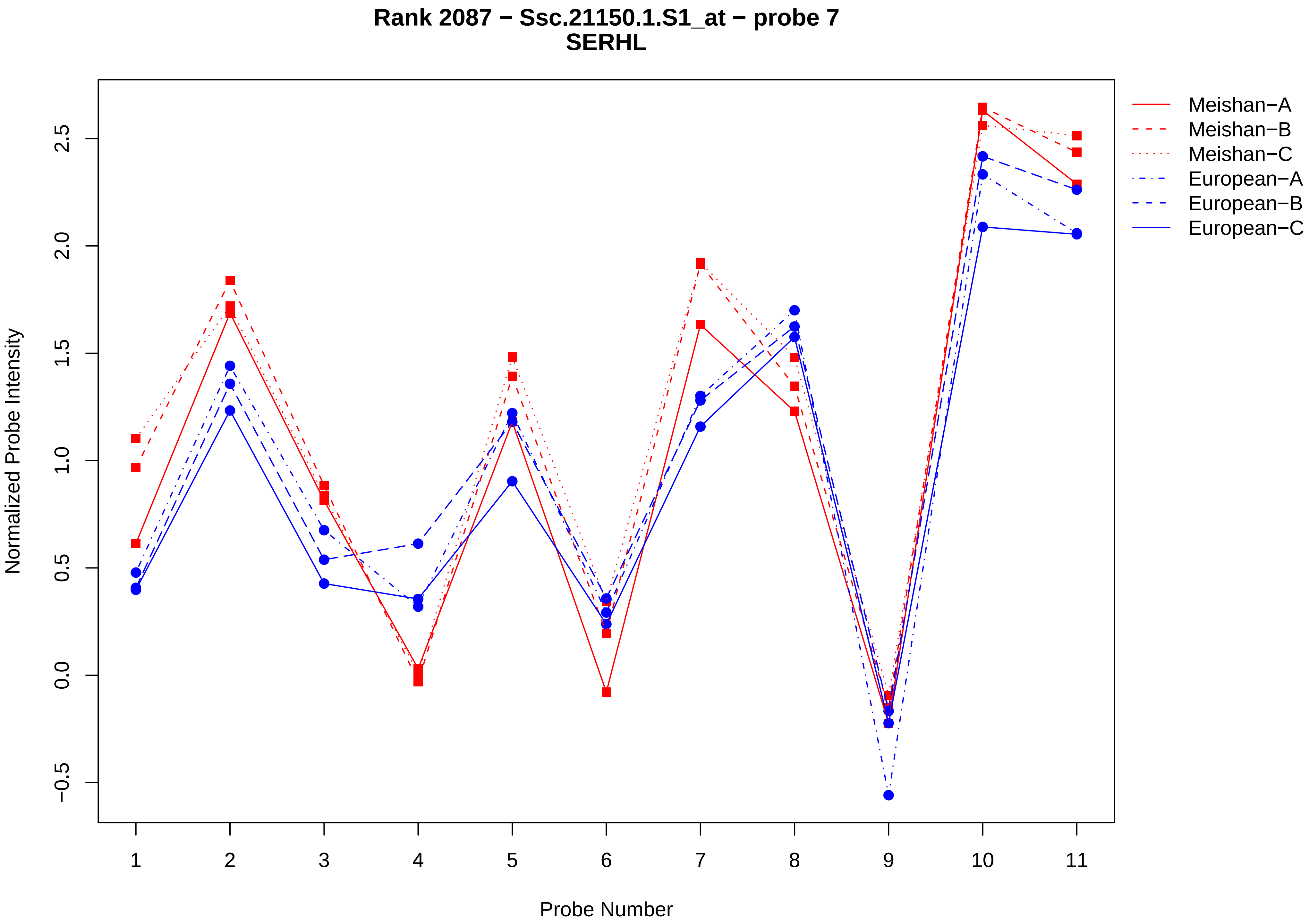

Rank 2087 − Ssc.21150.1.S1_at − probe 7
SERHL
Normalized Probe Intensity
Probe Number
−0.5
0.0
0.5
1.0
1.5
2.0
2.5
1
2
3
4
5
6
7
8
9
10
11
Meishan−A
Meishan−B
Meishan−C
European−A
European−B
European−C

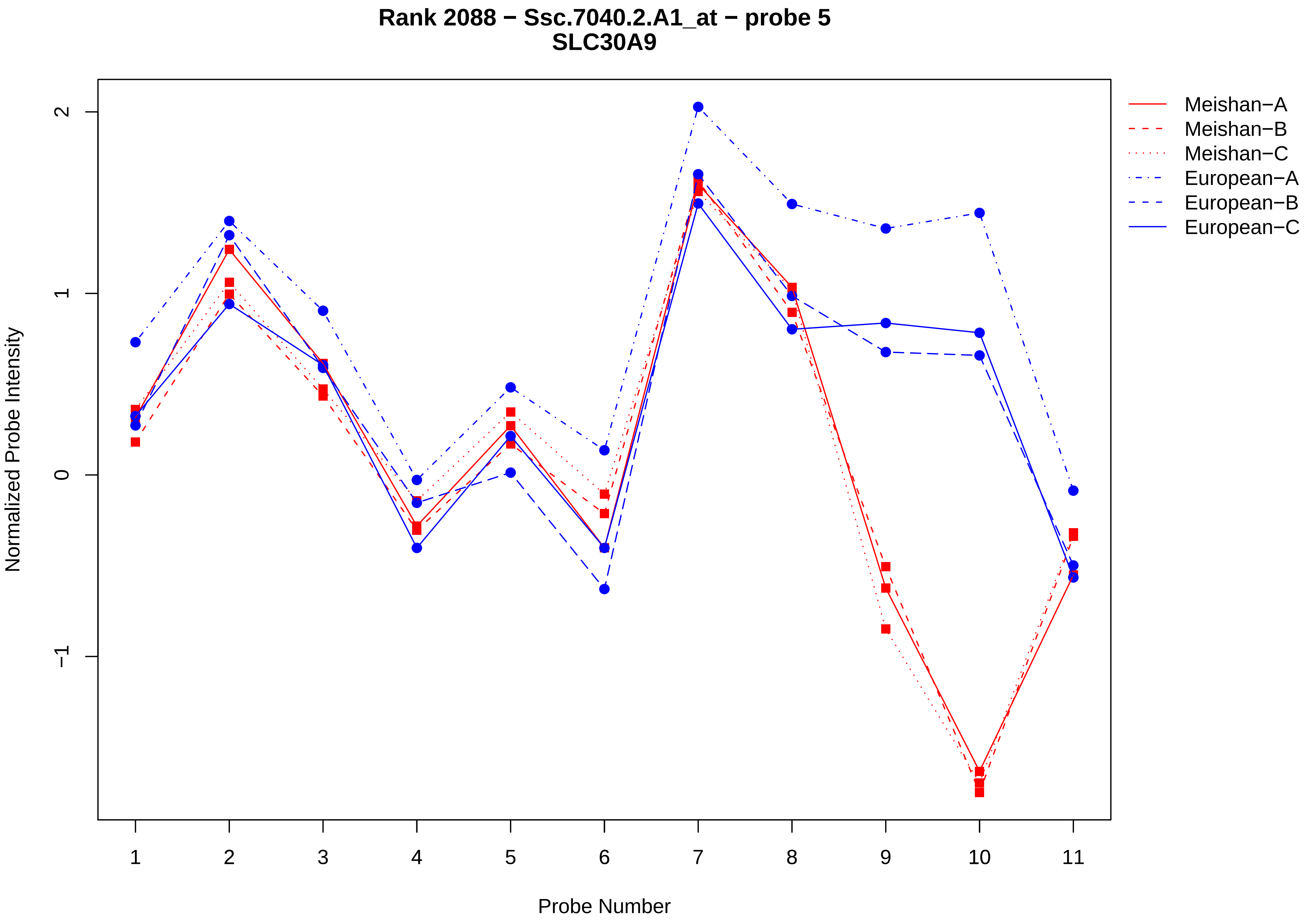
Rank 2088 − Ssc.7040.2.A1_at − probe 5
SLC30A9
Normalized Probe Intensity
Probe Number
Meishan−A
Meishan−B
Meishan−C
European−A
European−B
European−C
−1
0
1
2
1
2
3
4
5
6
7
8
9
10
11

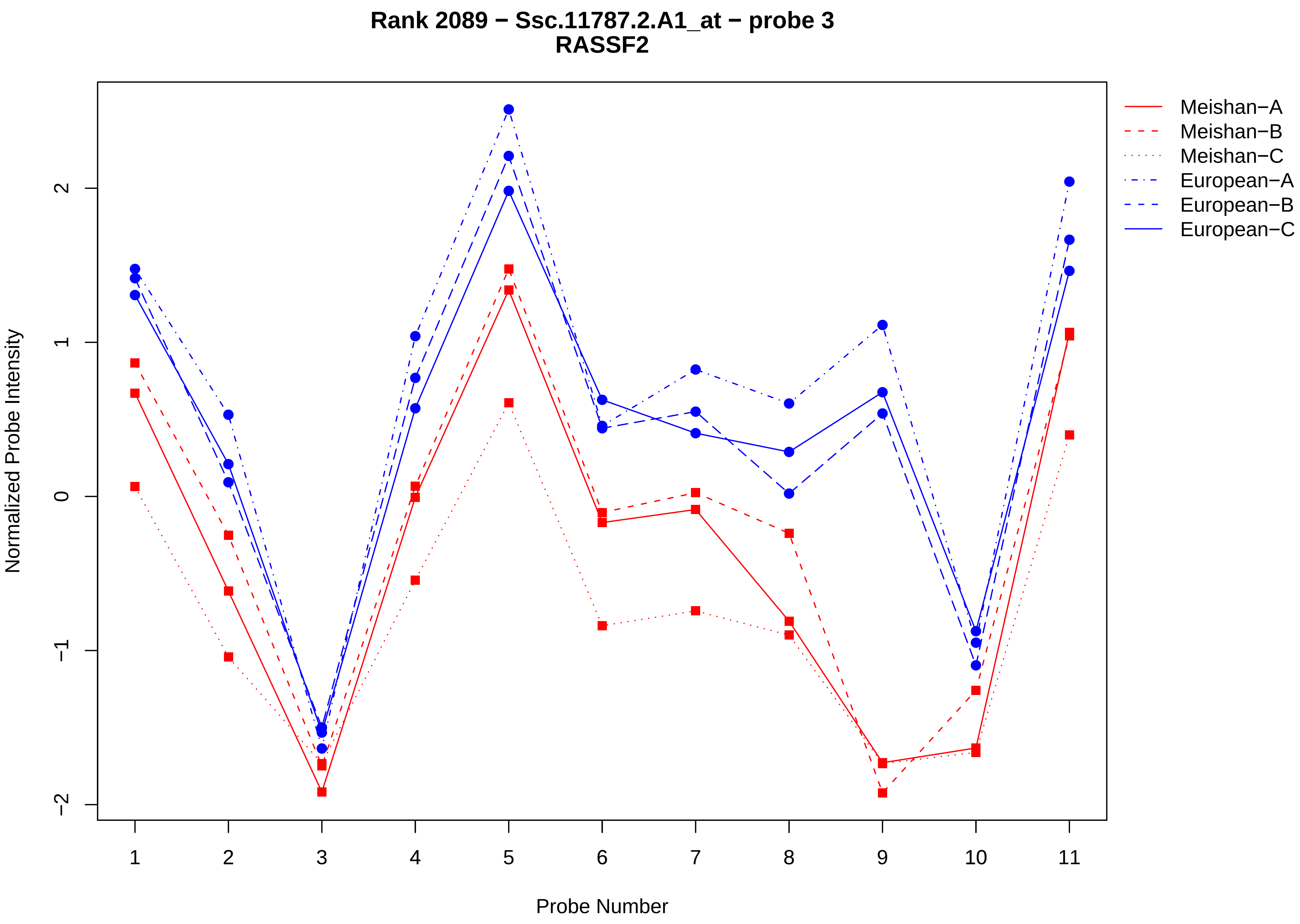

Rank 2089 − Ssc.11787.2.A1_at − probe 3
RASSF2
Normalized Probe Intensity
Probe Number
−2
−1
0
1
2
1
2
3
4
5
6
7
8
9
10
11
Meishan−A
Meishan−B
Meishan−C
European−A
European−B
European−C

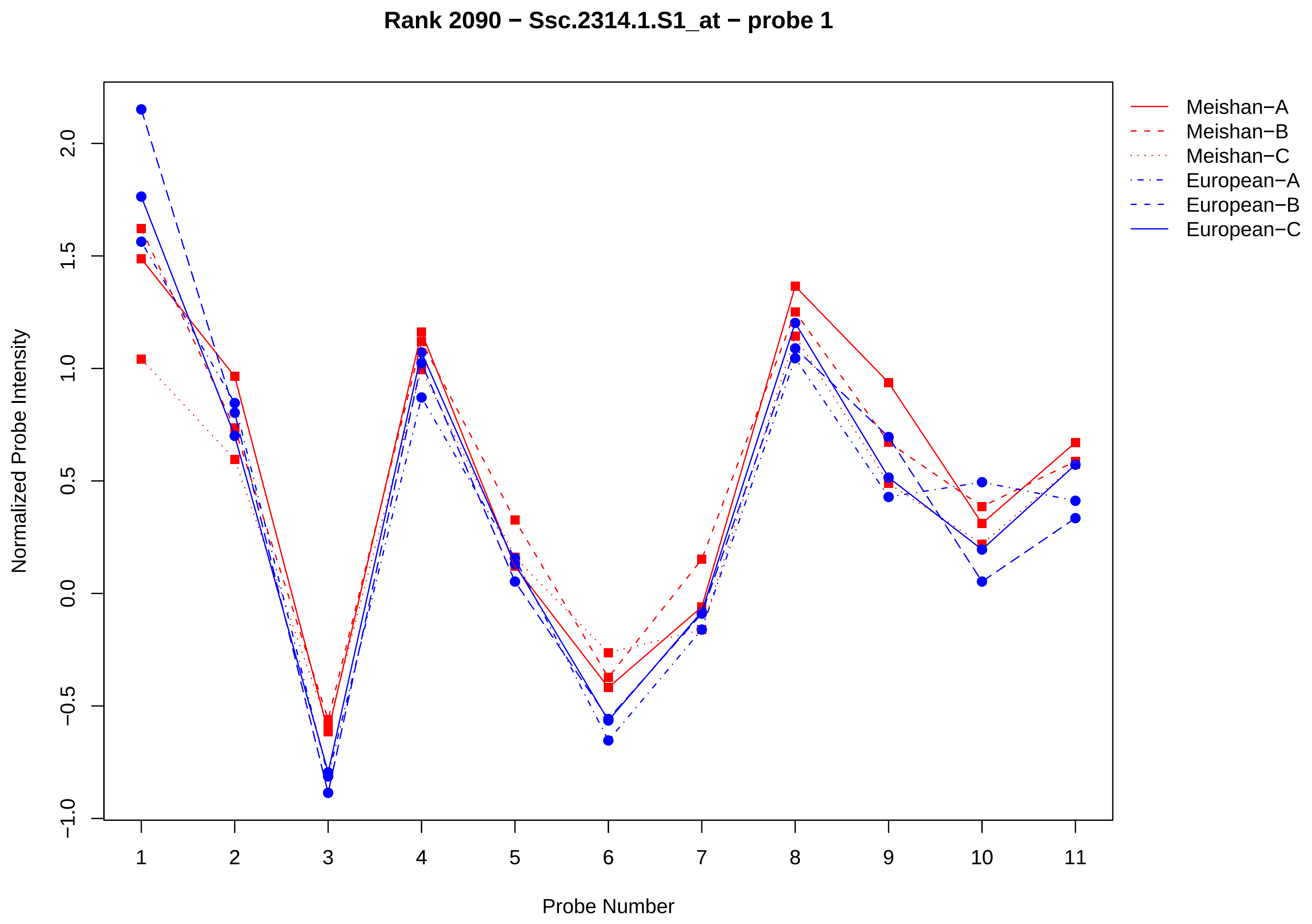

Rank 2090 − Ssc.2314.1.S1_at − probe 1
Normalized Probe Intensity
Probe Number
−1.0
−0.5
0.0
0.5
1.0
1.5
2.0
1
2
3
4
5
6
7
8
9
10
11
Meishan−A
Meishan−B
Meishan−C
European−A
European−B
European−C

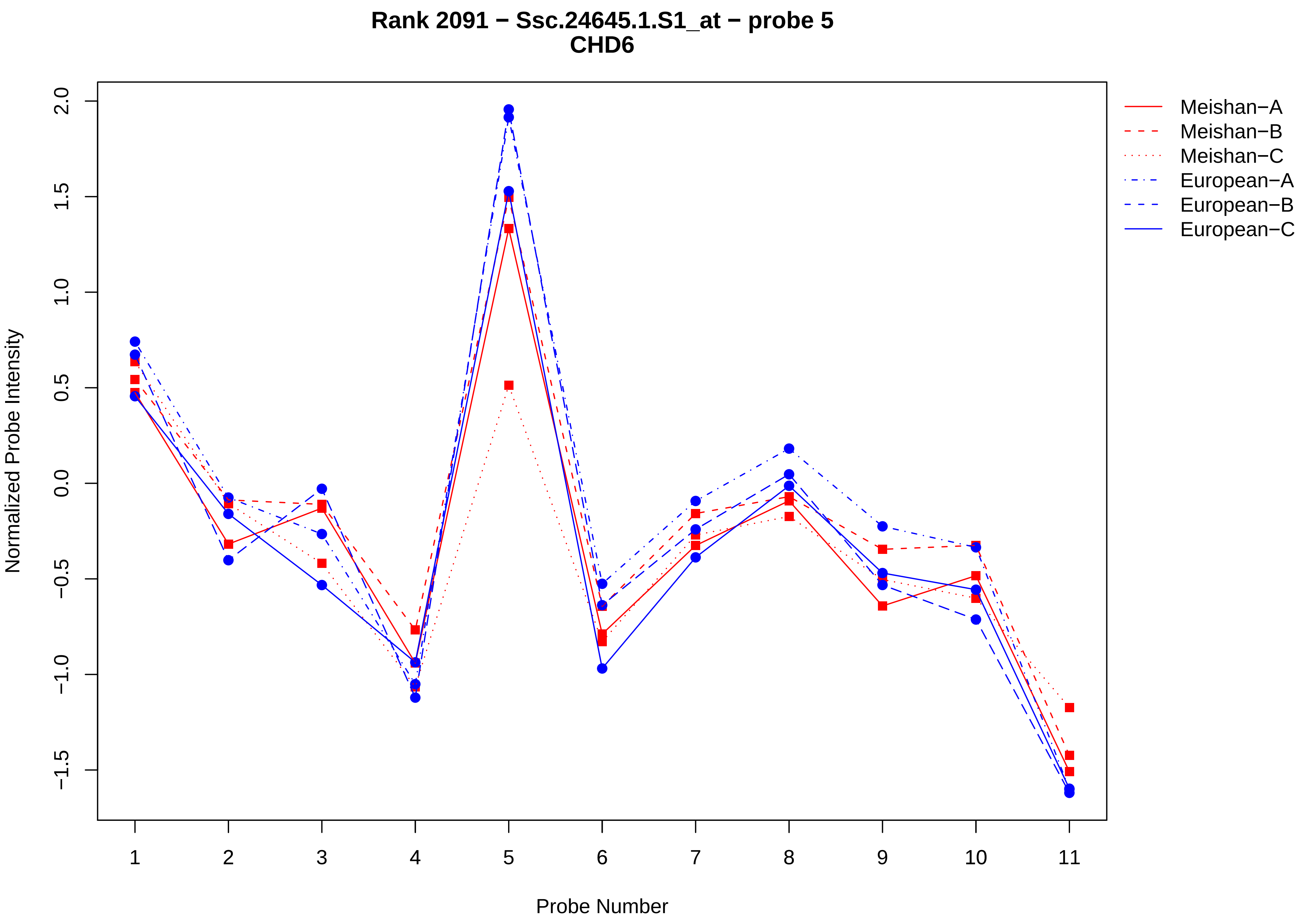
Rank 2091 − Ssc.24645.1.S1_at − probe 5
CHD6
Normalized Probe Intensity
Probe Number
2.0
1.5
1.0
0.5
0.0
−0.5
−1.0
−1.5
1
2
3
4
5
6
7
8
9
10
11
Meishan−A
Meishan−B
Meishan−C
European−A
European−B
European−C

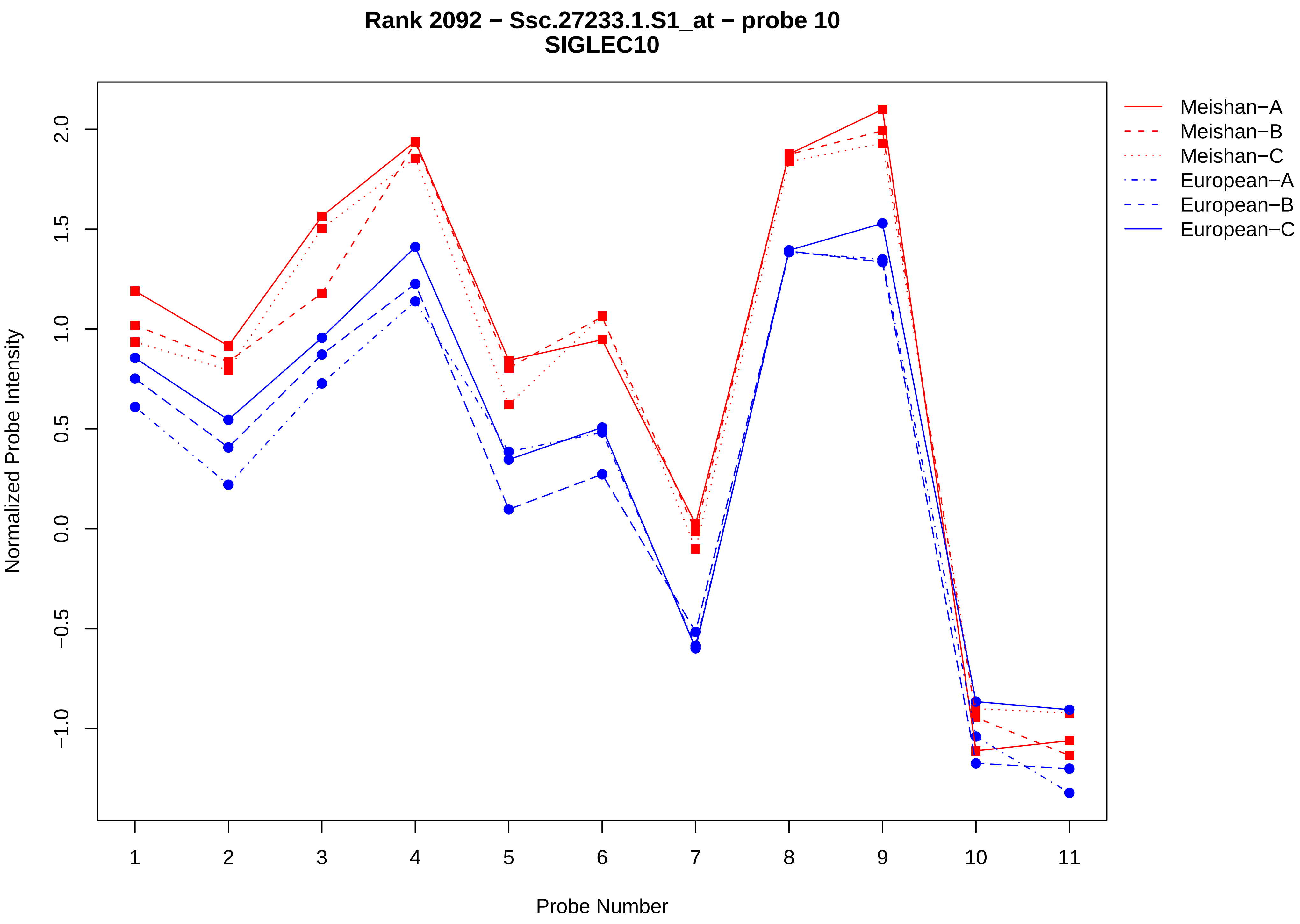
Rank 2092 − Ssc.27233.1.S1_at − probe 10
SIGLEC10
Normalized Probe Intensity
Probe Number
Meishan−A
Meishan−B
Meishan−C
European−A
European−B
European−C
2.0
1.5
1.0
0.5
0.0
−0.5
−1.0
1
2
3
4
5
6
7
8
9
10
11

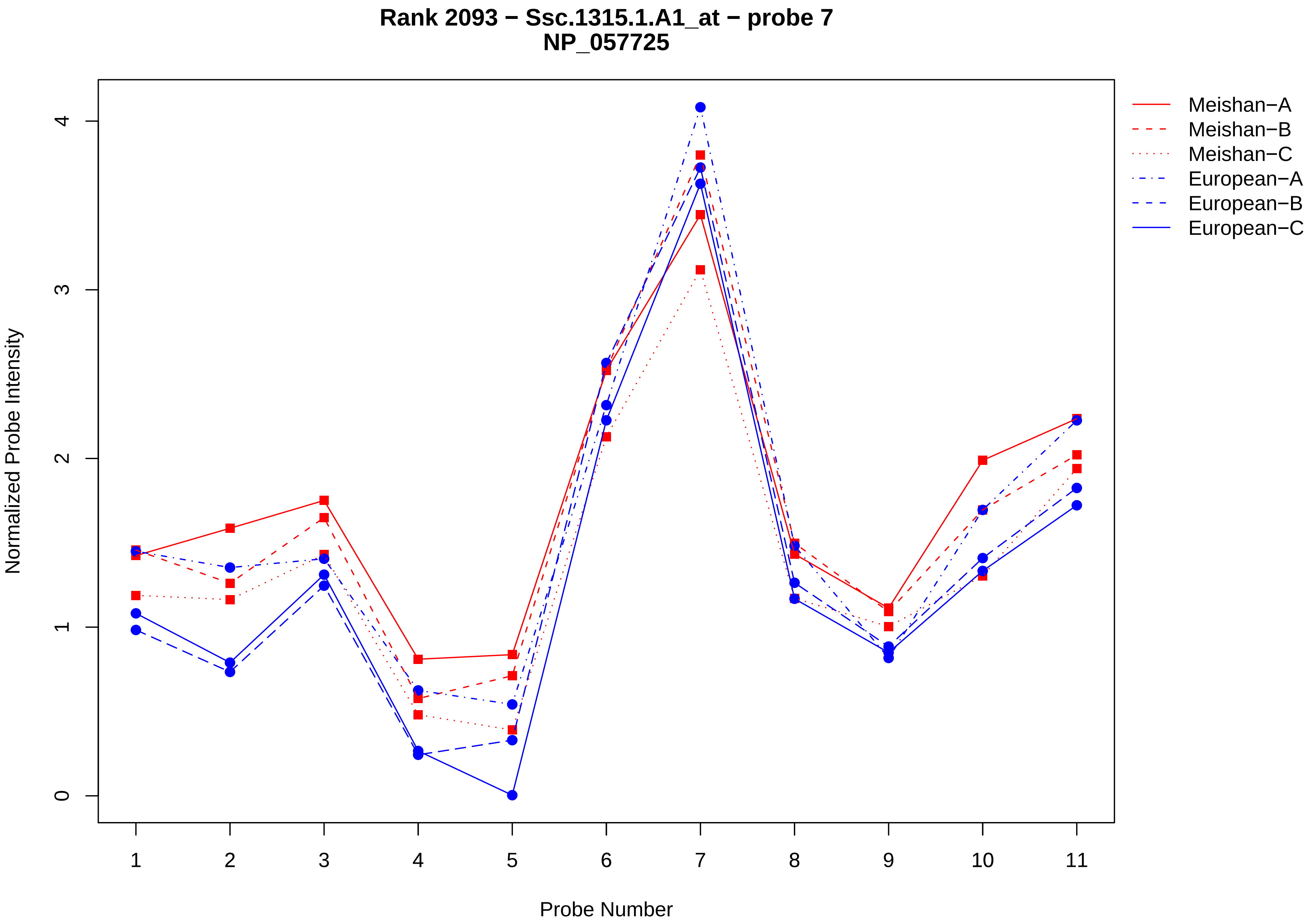

Rank 2093 − Ssc.1315.1.A1_at − probe 7
NP_057725
Normalized Probe Intensity
0
1
2
3
4
1
2
3
4
5
6
7
8
9
10
11
Probe Number
Meishan−A
Meishan−B
Meishan−C
European−A
European−B
European−C

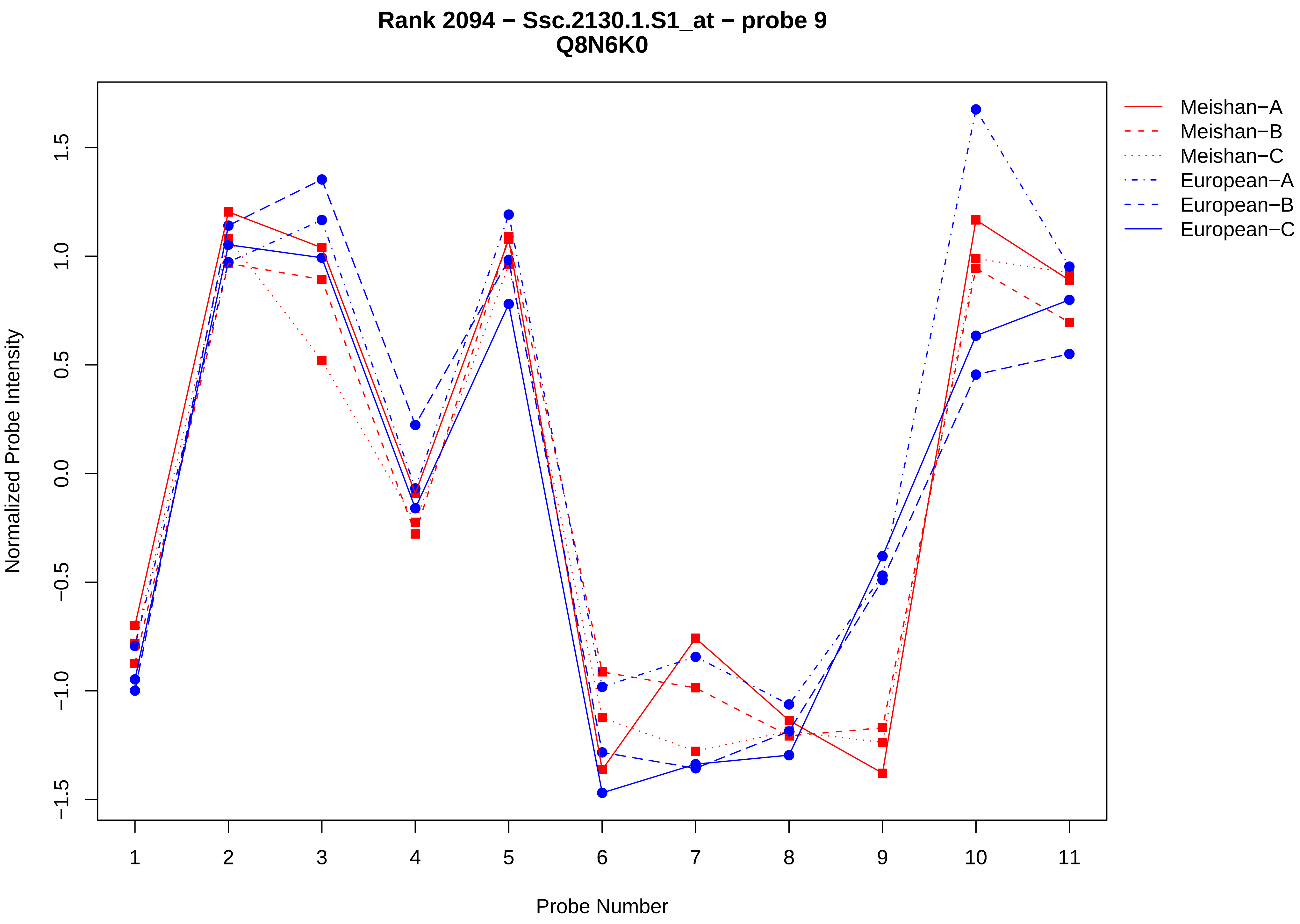

Rank 2094 − Ssc.2130.1.S1_at − probe 9
Q8N6K0
Normalized Probe Intensity
Probe Number
−1.5
−1.0
−0.5
0.0
0.5
1.0
1.5
1
2
3
4
5
6
7
8
9
10
11
Meishan−A
Meishan−B
Meishan−C
European−A
European−B
European−C

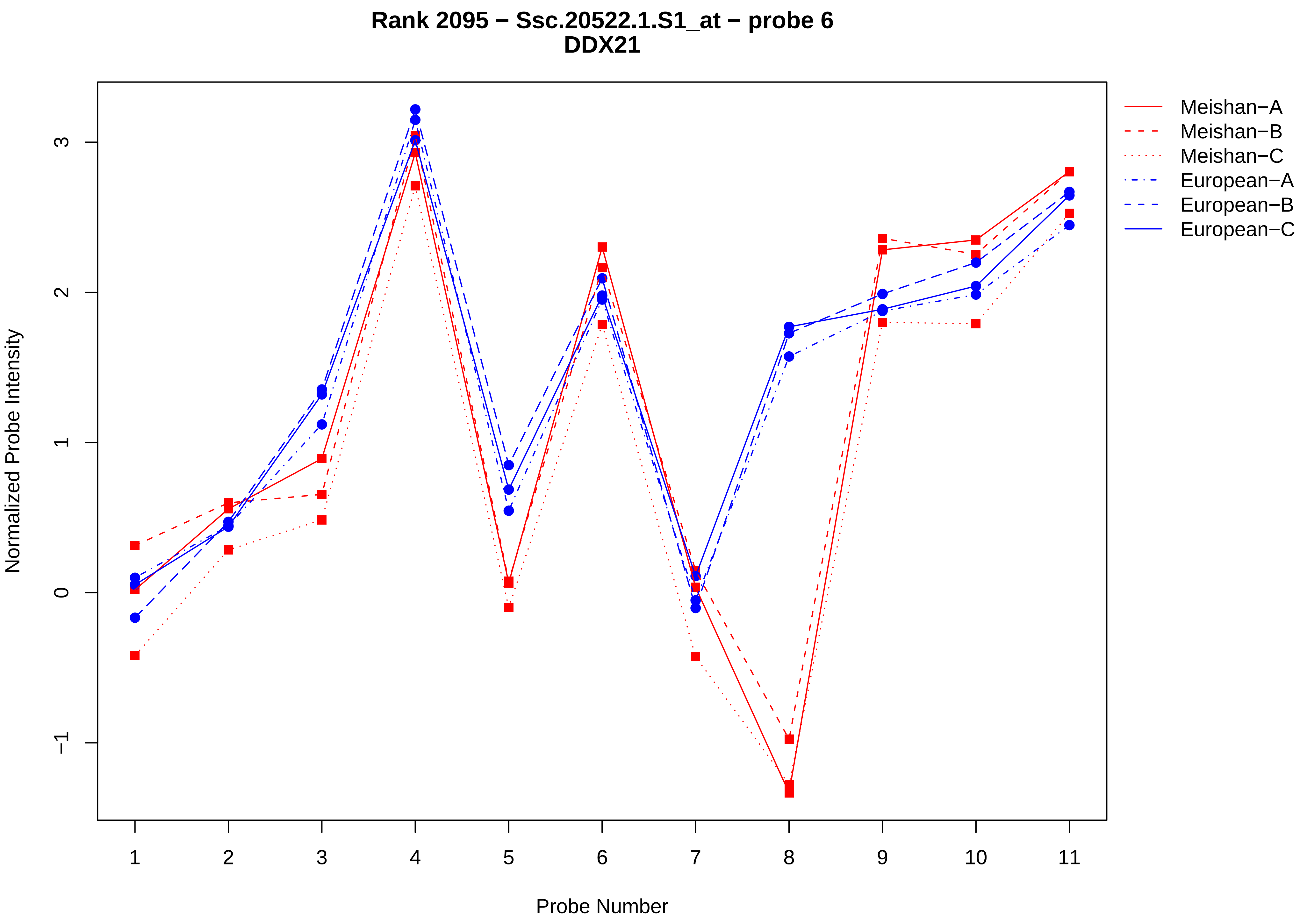

Rank 2095 − Ssc.20522.1.S1_at − probe 6
DDX21
Normalized Probe Intensity
Probe Number
−1
0
1
2
3
1
2
3
4
5
6
7
8
9
10
11
Meishan−A
Meishan−B
Meishan−C
European−A
European−B
European−C

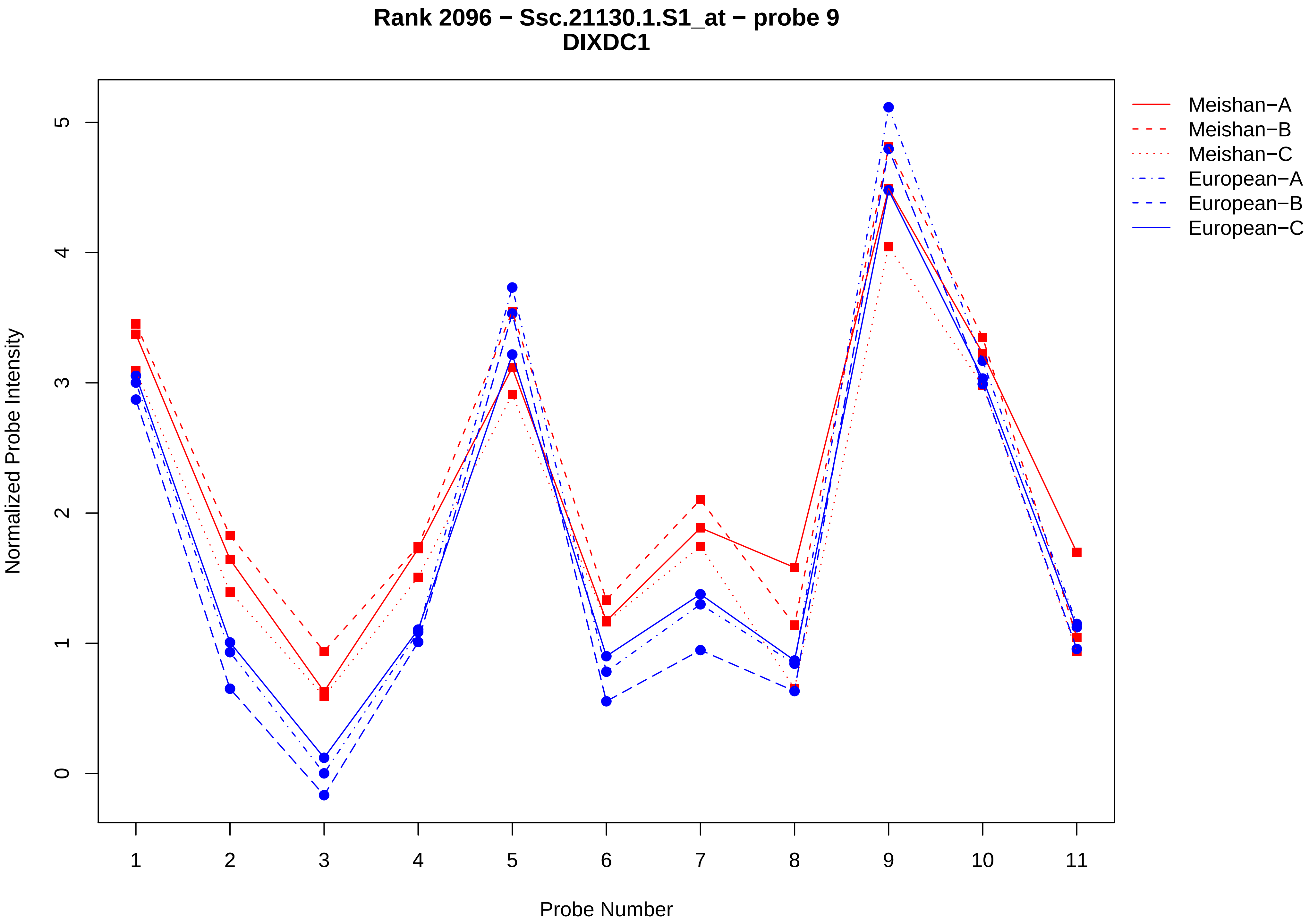

Rank 2096 − Ssc.21130.1.S1_at − probe 9
DIXDC1
Normalized Probe Intensity
Probe Number
0
1
2
3
4
5
1
2
3
4
5
6
7
8
9
10
11
Meishan−A
Meishan−B
Meishan−C
European−A
European−B
European−C

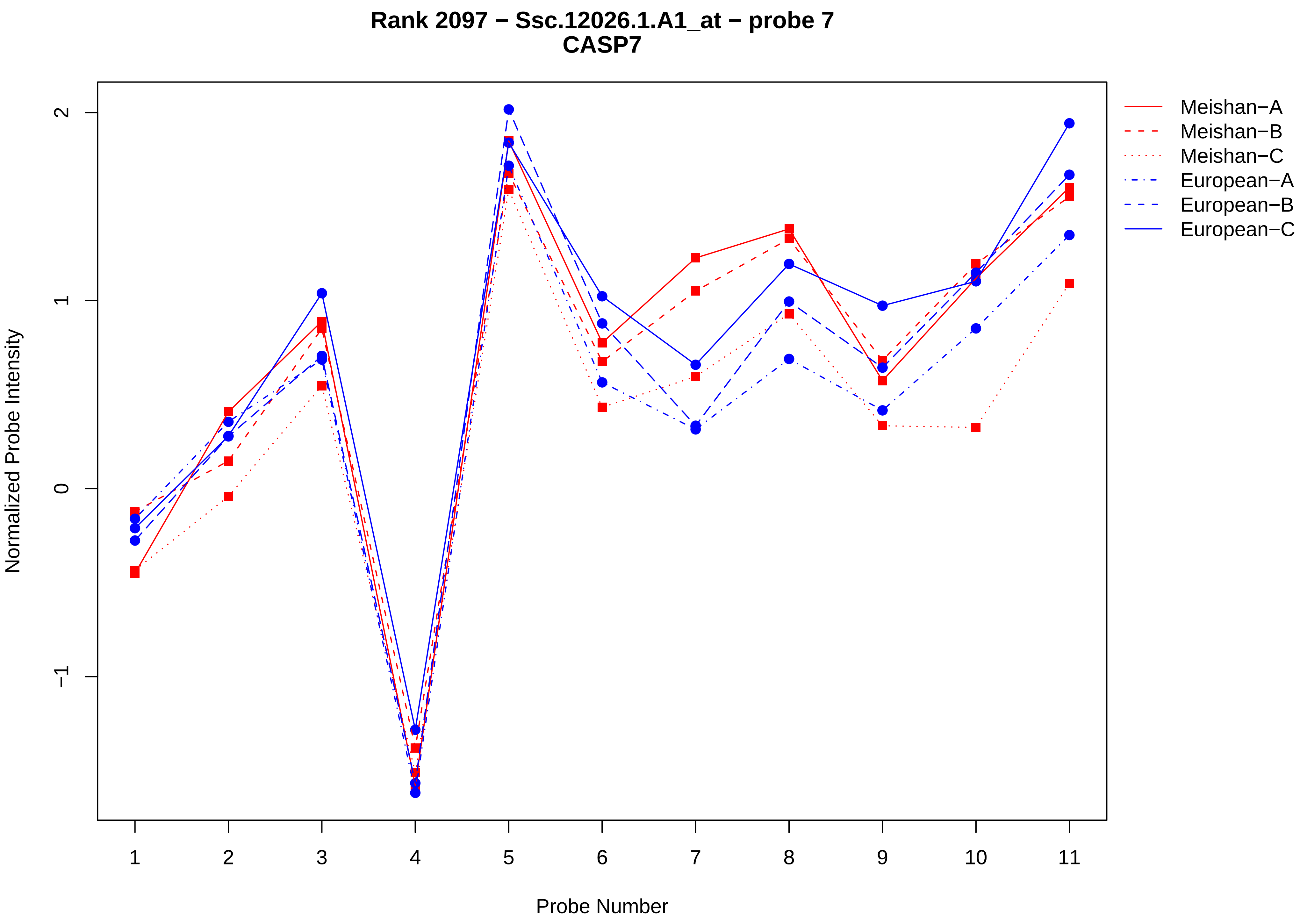
Rank 2097 − Ssc.12026.1.A1_at − probe 7
CASP7
Normalized Probe Intensity
Probe Number
Meishan−A
Meishan−B
Meishan−C
European−A
European−B
European−C

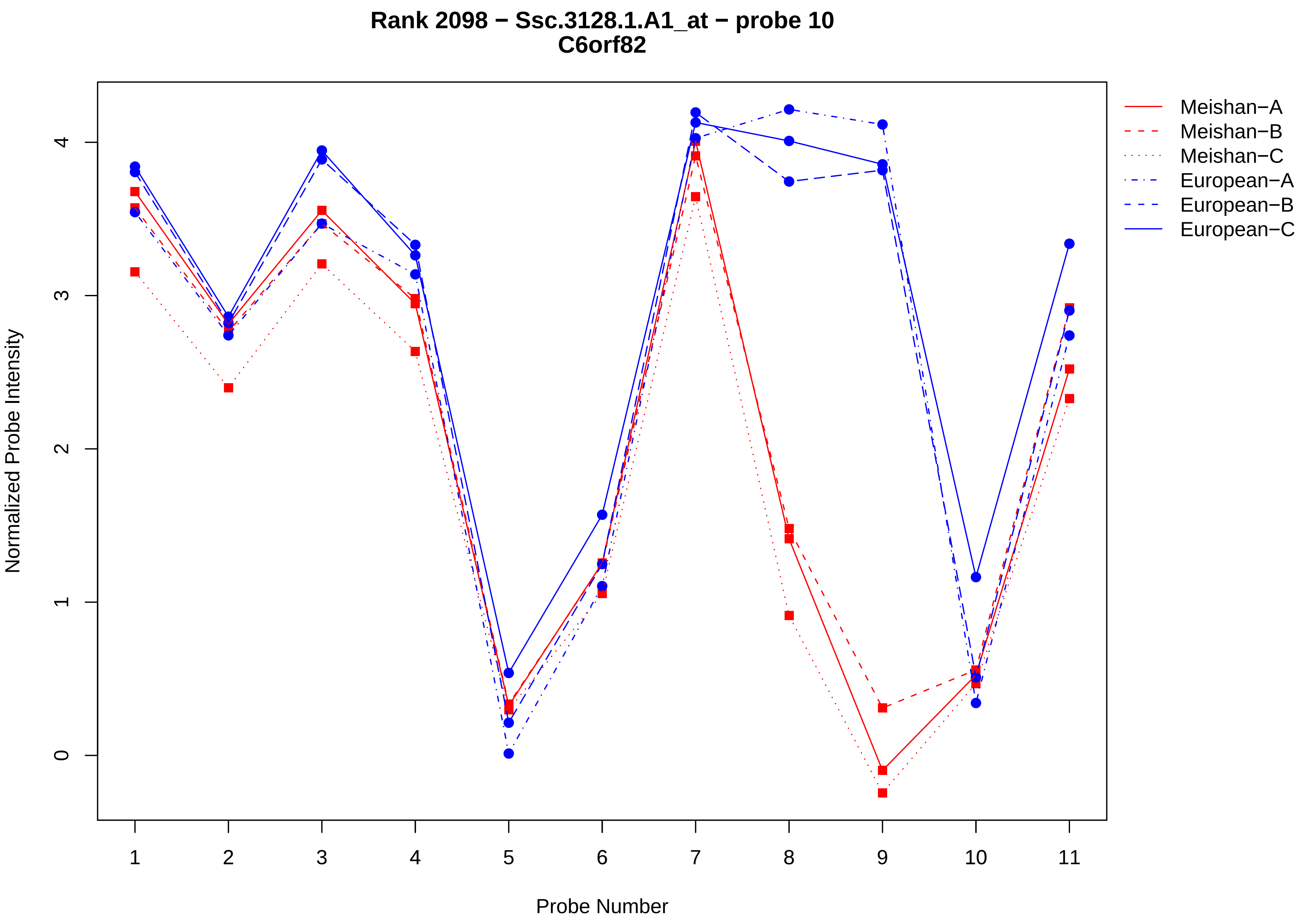

Rank 2098 − Ssc.3128.1.A1_at − probe 10
C6orf82
Normalized Probe Intensity
Probe Number
0
1
2
3
4
1
2
3
4
5
6
7
8
9
10
11
Meishan−A
Meishan−B
Meishan−C
European−A
European−B
European−C

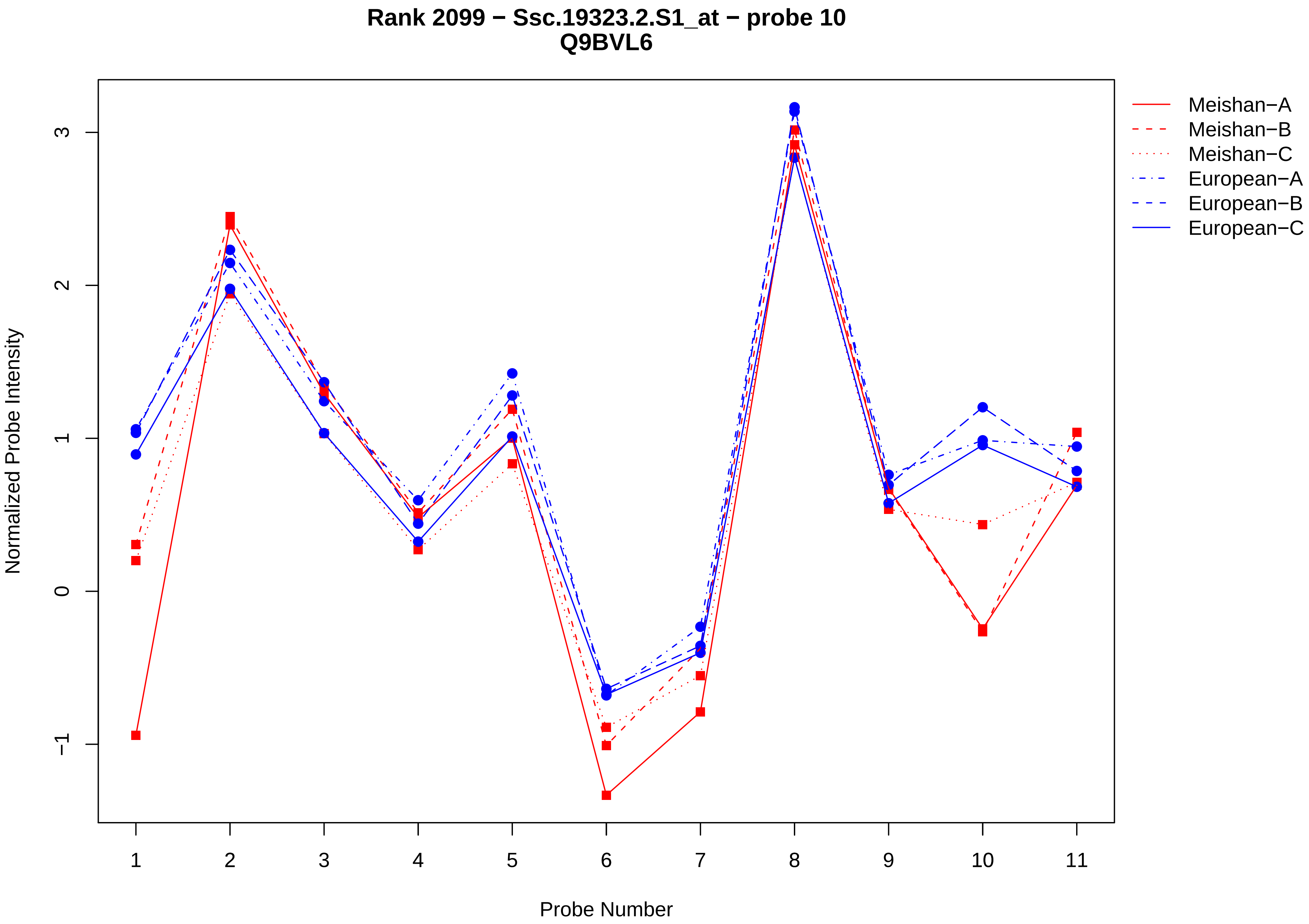

Rank 2099 − Ssc.19323.2.S1_at − probe 10
Q9BVL6
Normalized Probe Intensity
Probe Number
Meishan−A
Meishan−B
Meishan−C
European−A
European−B
European−C
−1
0
1
2
3
1
2
3
4
5
6
7
8
9
10
11

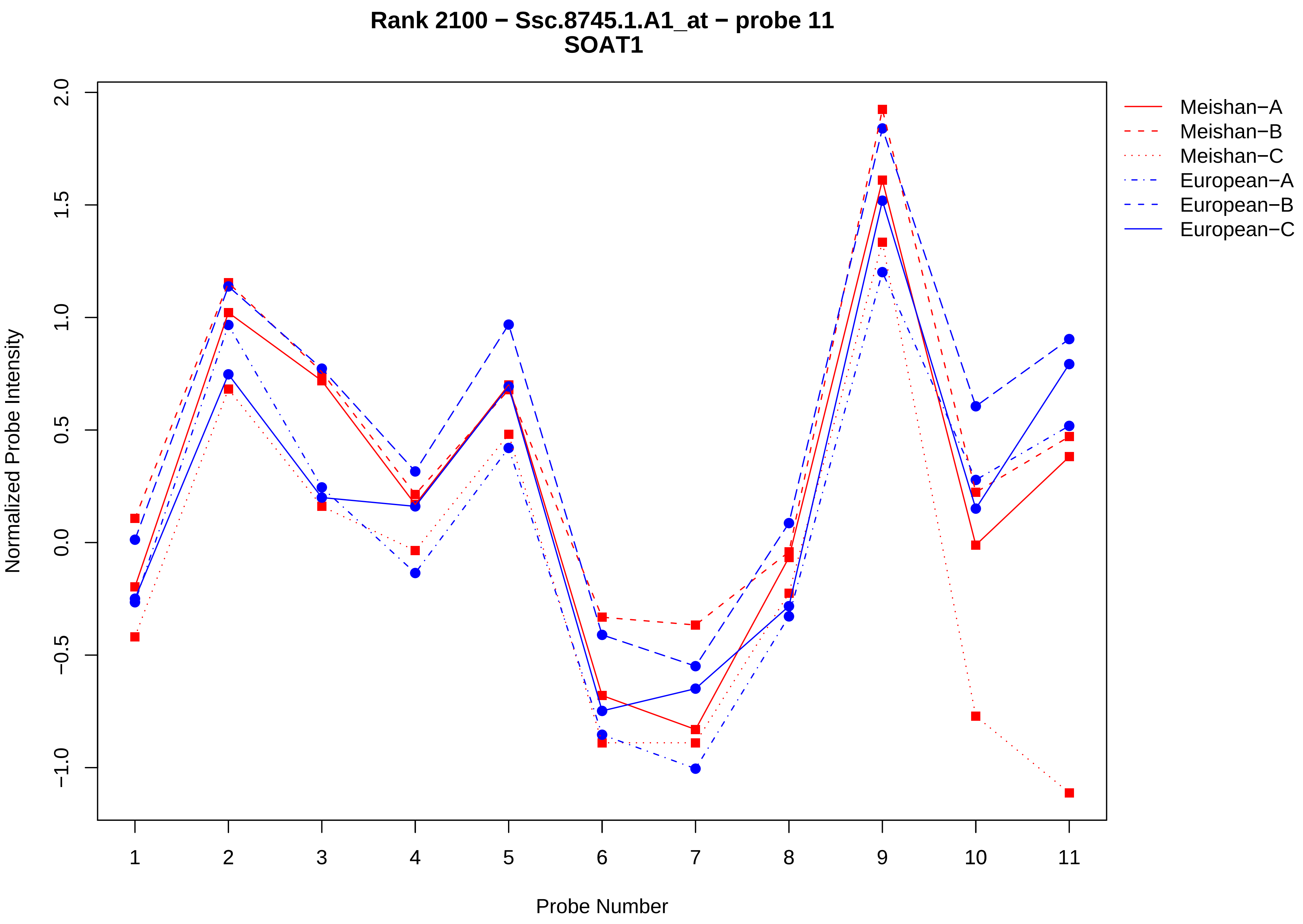

Rank 2100 − Ssc.8745.1.A1_at − probe 11
SOAT1
Normalized Probe Intensity
Probe Number
−1.0
−0.5
0.0
0.5
1.0
1.5
2.0
1
2
3
4
5
6
7
8
9
10
11
Meishan−A
Meishan−B
Meishan−C
European−A
European−B
European−C

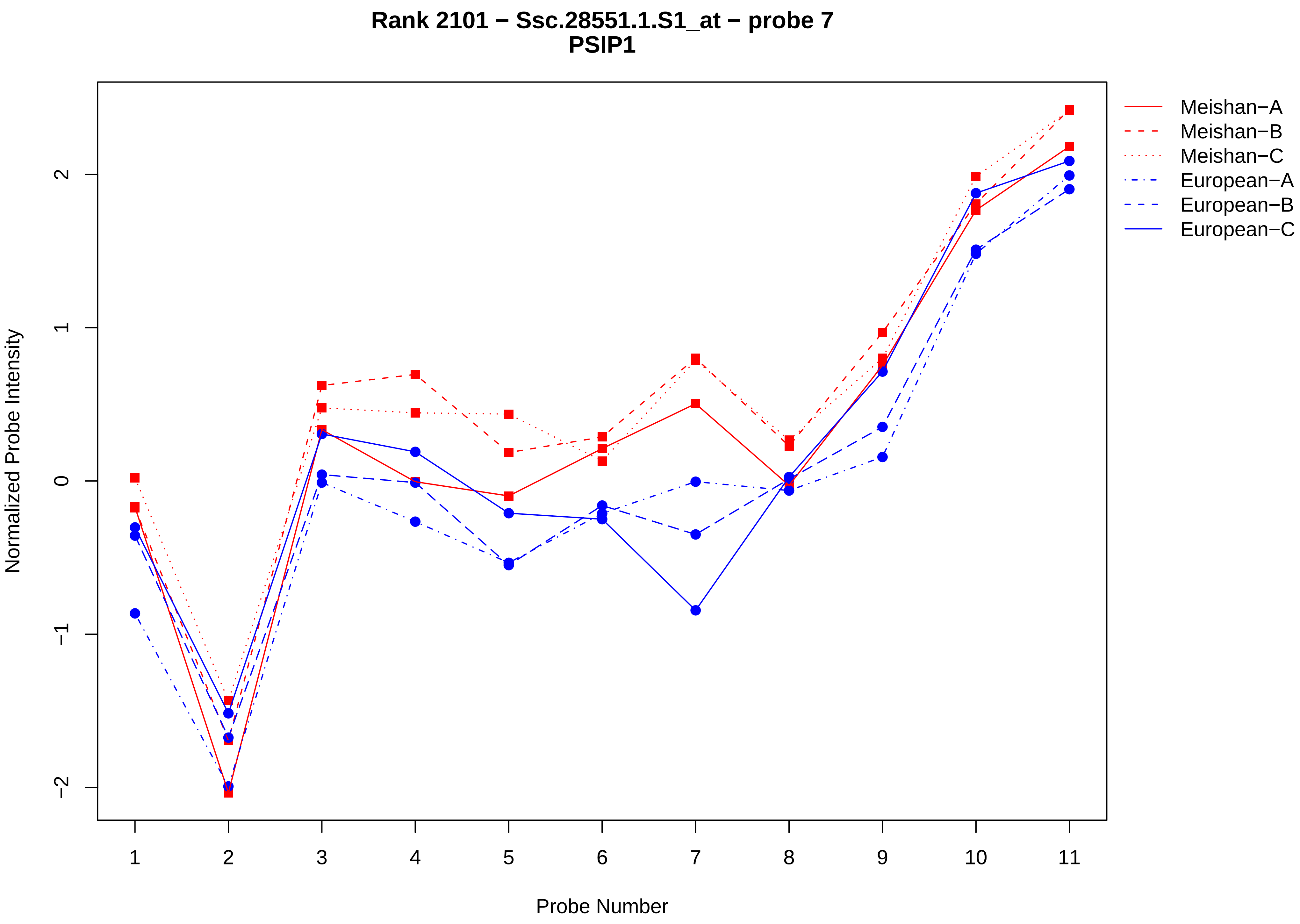

Rank 2101 − Ssc.28551.1.S1_at − probe 7
PSIP1
Normalized Probe Intensity
Probe Number
−2
−1
0
1
2
1
2
3
4
5
6
7
8
9
10
11
Meishan−A
Meishan−B
Meishan−C
European−A
European−B
European−C

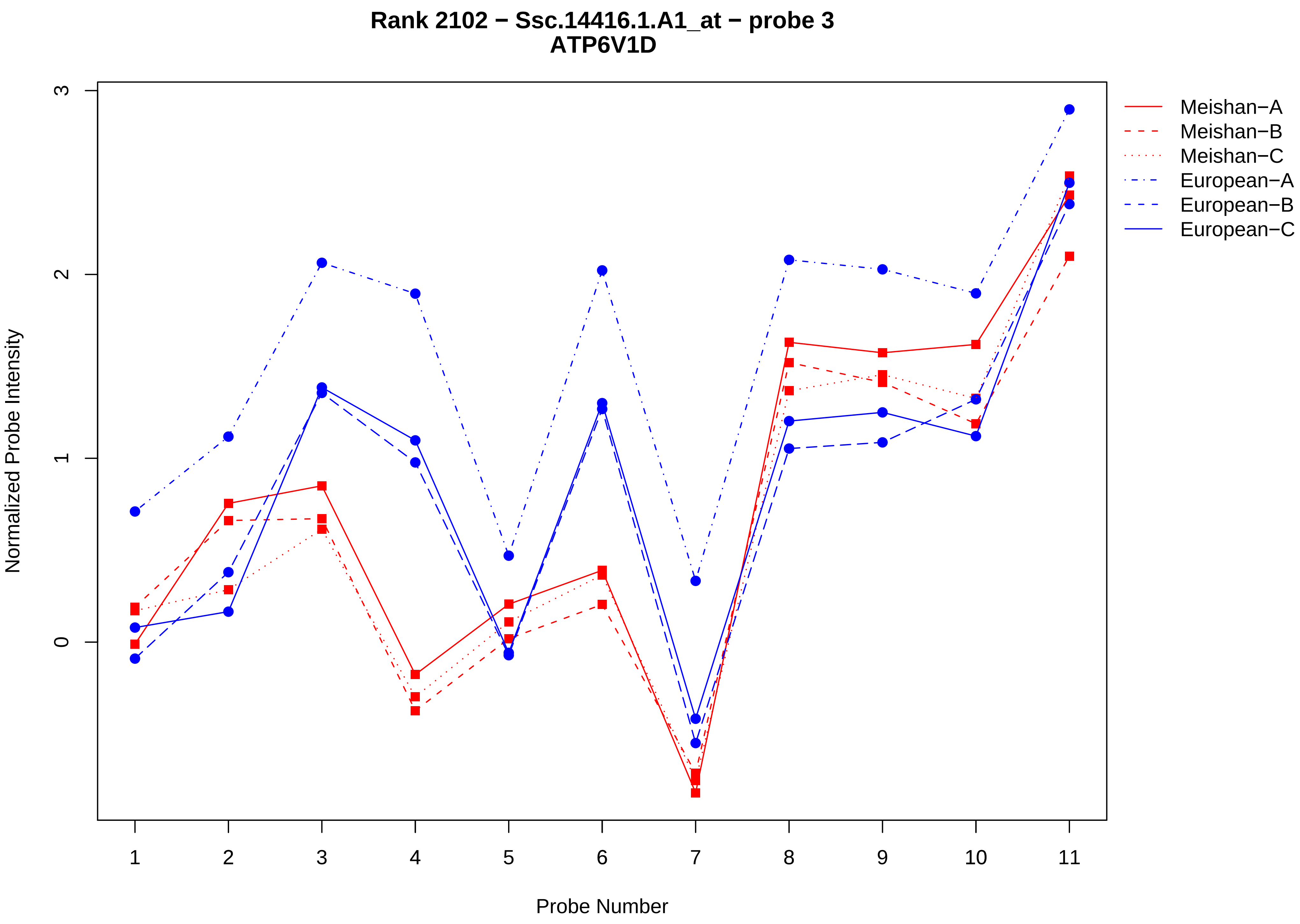
Rank 2102 − Ssc.14416.1.A1_at − probe 3
ATP6V1D
Normalized Probe Intensity
Probe Number
0
1
2
3
1
2
3
4
5
6
7
8
9
10
11
Meishan−A
Meishan−B
Meishan−C
European−A
European−B
European−C

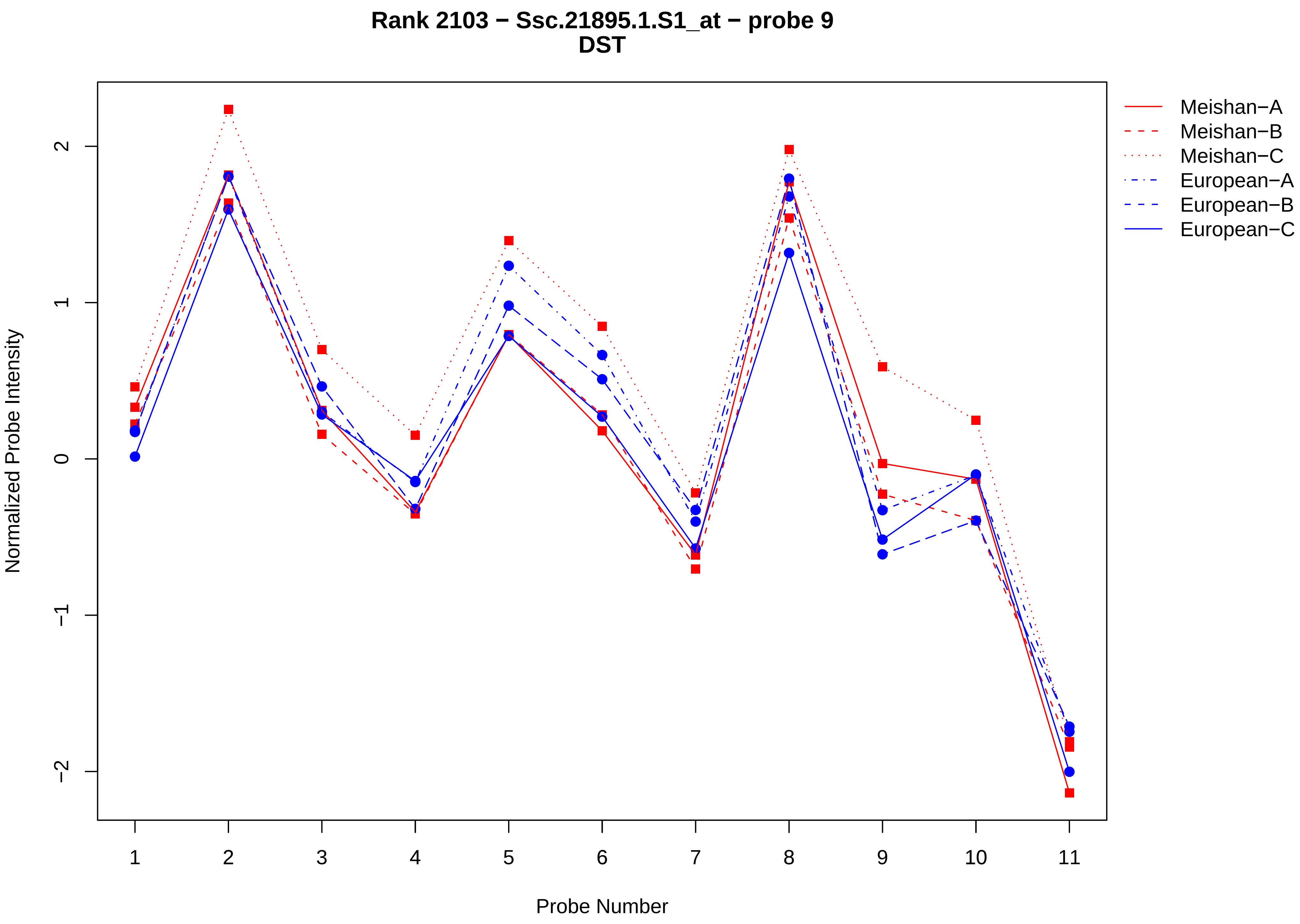

Rank 2103 − Ssc.21895.1.S1_at − probe 9
DST
Normalized Probe Intensity
Probe Number
−2
−1
0
1
2
1
2
3
4
5
6
7
8
9
10
11
Meishan−A
Meishan−B
Meishan−C
European−A
European−B
European−C

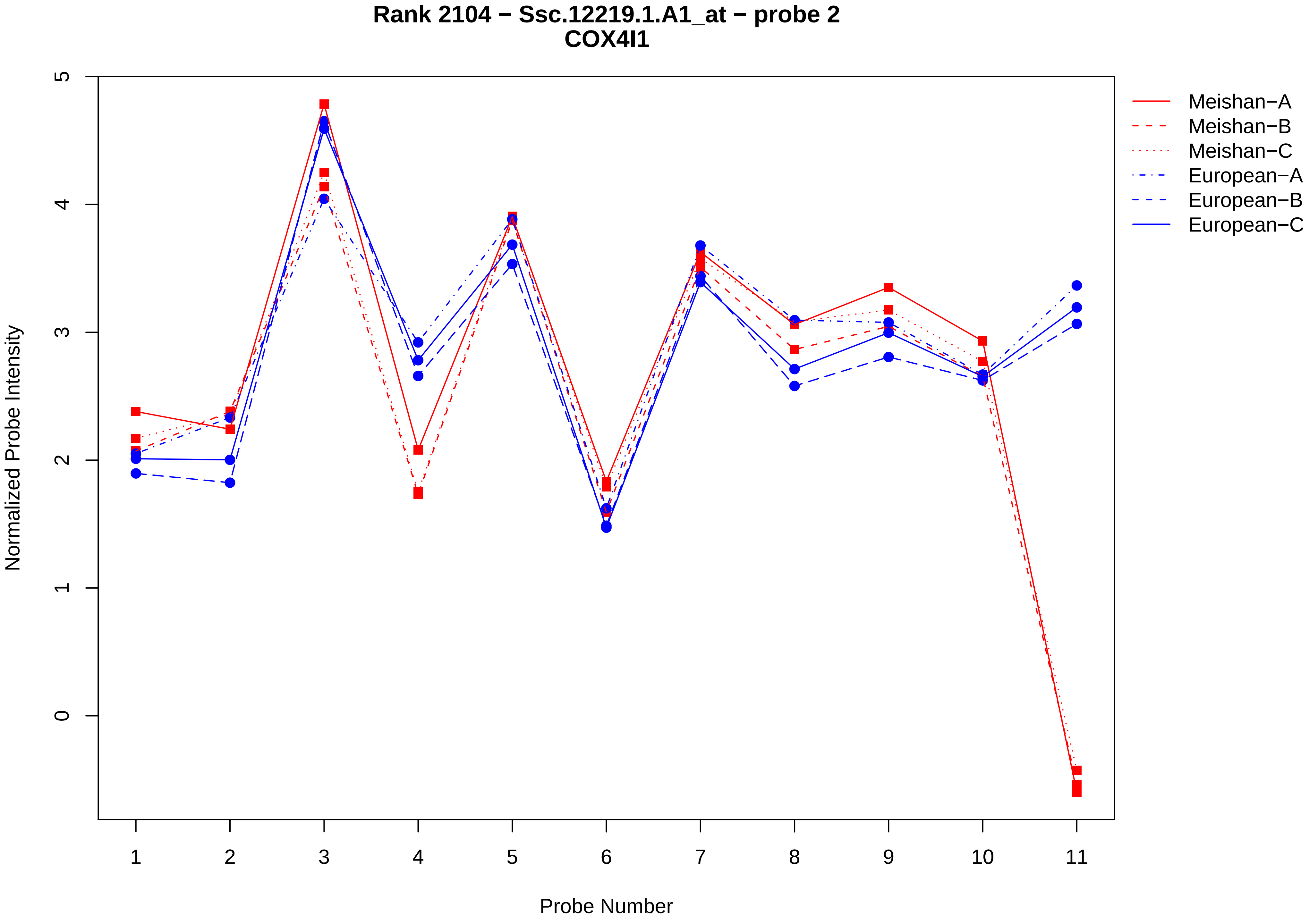

Rank 2104 − Ssc.12219.1.A1_at − probe 2
COX4I1
Normalized Probe Intensity
Probe Number
0
1
2
3
4
5
1
2
3
4
5
6
7
8
9
10
11
Meishan−A
Meishan−B
Meishan−C
European−A
European−B
European−C

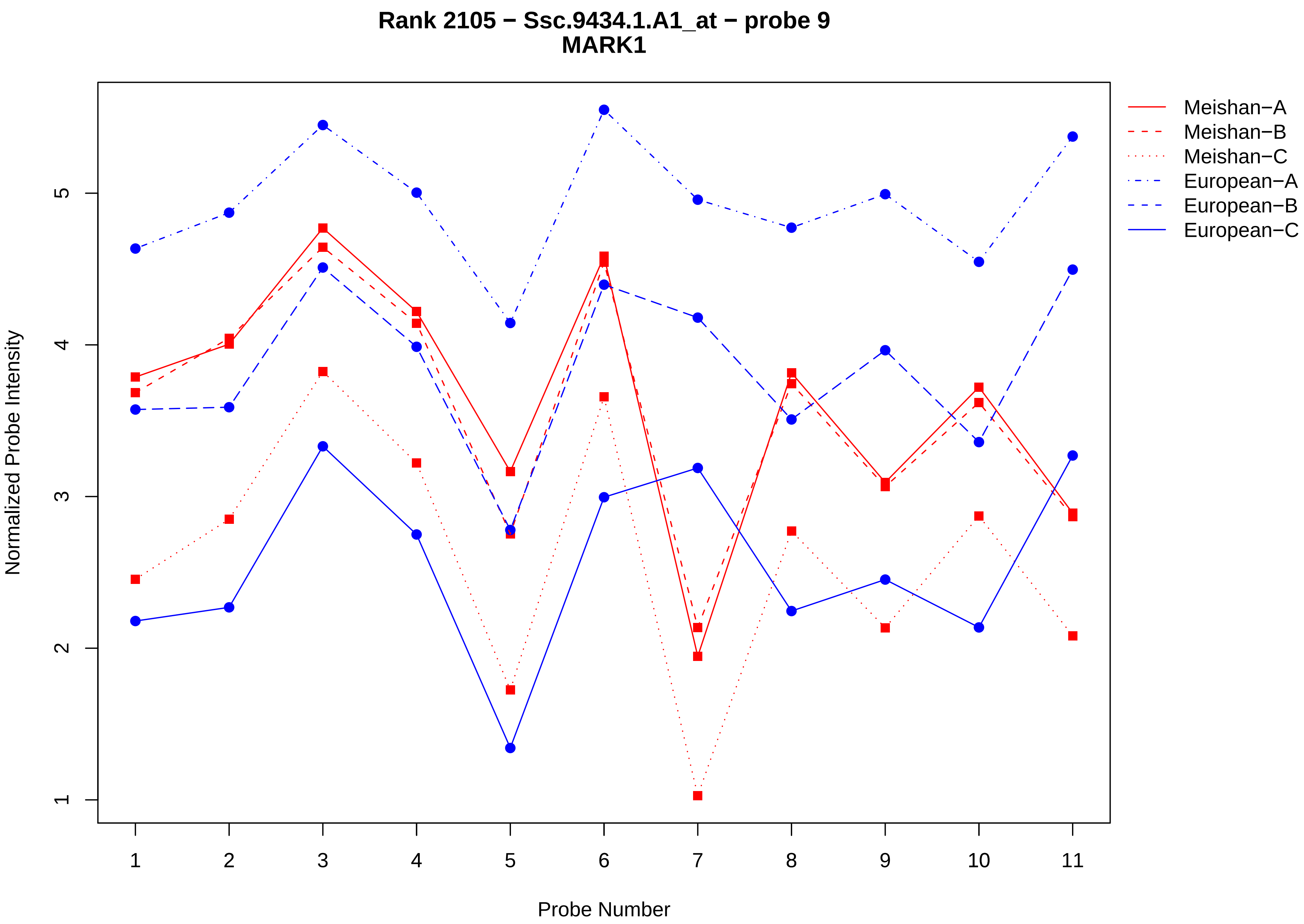

Rank 2105 − Ssc.9434.1.A1_at − probe 9
MARK1
Normalized Probe Intensity
Probe Number
1
2
3
4
5
1
2
3
4
5
6
7
8
9
10
11
Meishan−A
Meishan−B
Meishan−C
European−A
European−B
European−C

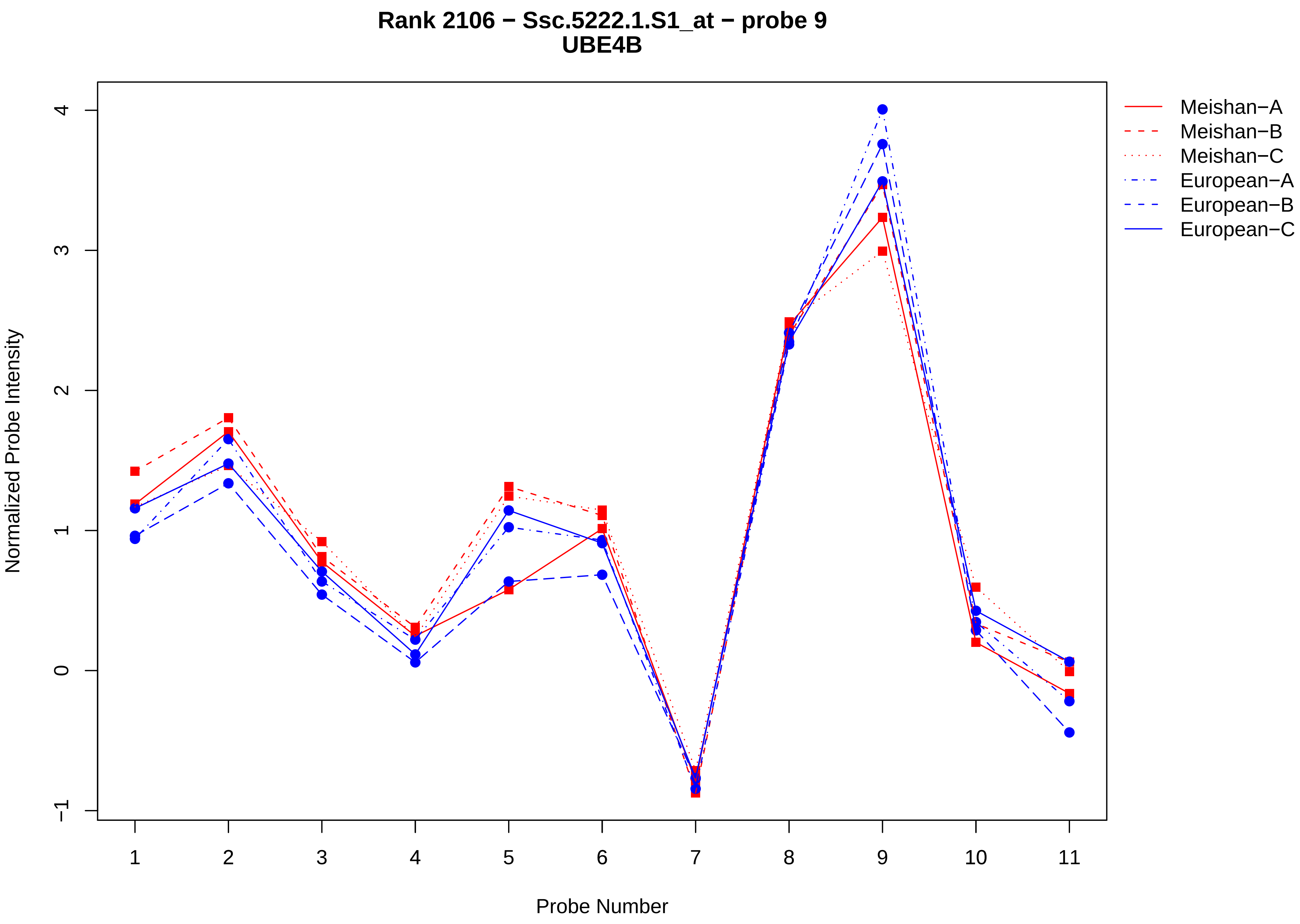

Rank 2106 − Ssc.5222.1.S1_at − probe 9
UBE4B
Normalized Probe Intensity
Probe Number
Meishan−A
Meishan−B
Meishan−C
European−A
European−B
European−C
−1
0
1
2
3
4
1
2
3
4
5
6
7
8
9
10
11

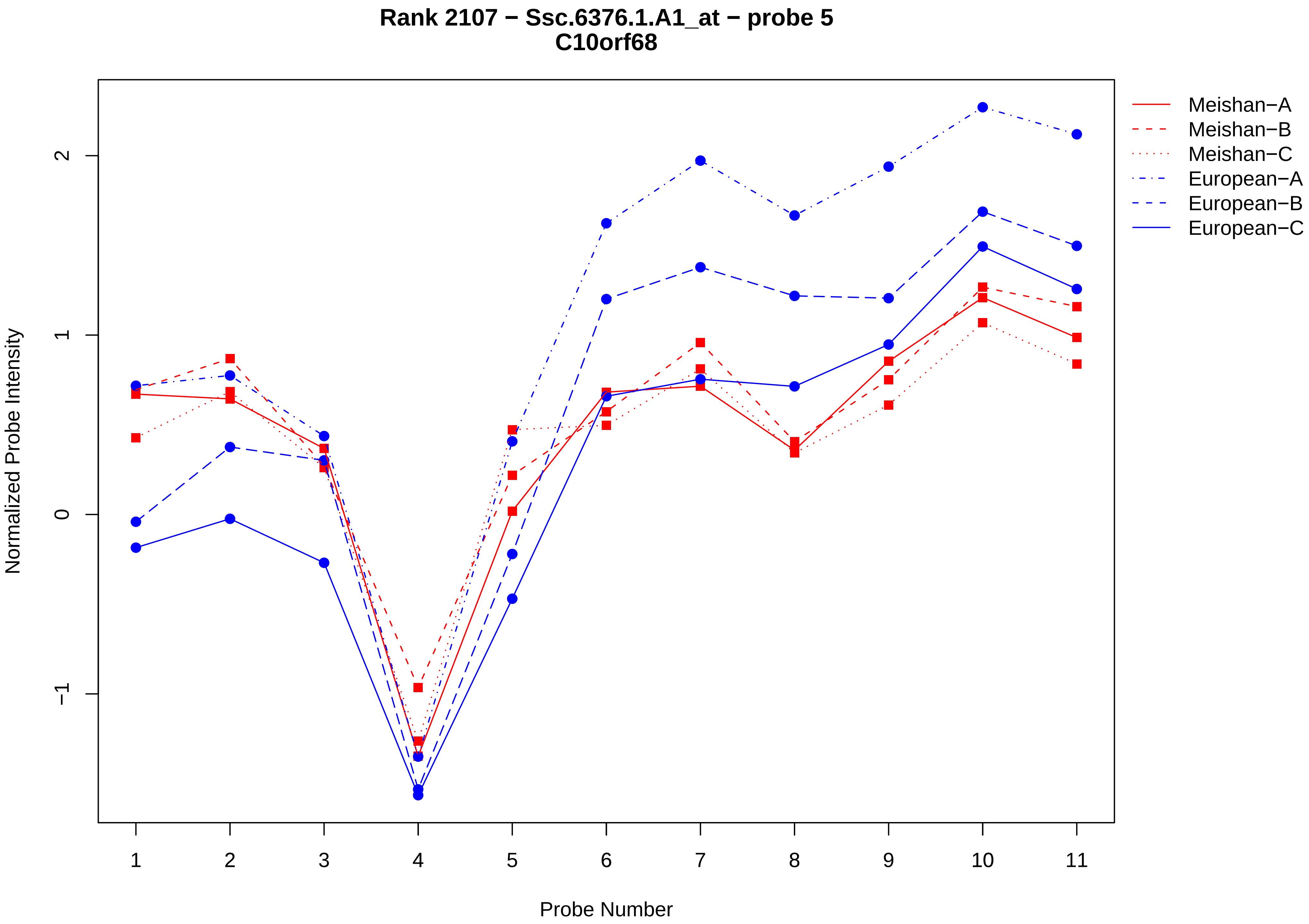

Rank 2107 − Ssc.6376.1.A1_at − probe 5
C10orf68
Normalized Probe Intensity
Probe Number
−1
0
1
2
1
2
3
4
5
6
7
8
9
10
11
Meishan−A
Meishan−B
Meishan−C
European−A
European−B
European−C

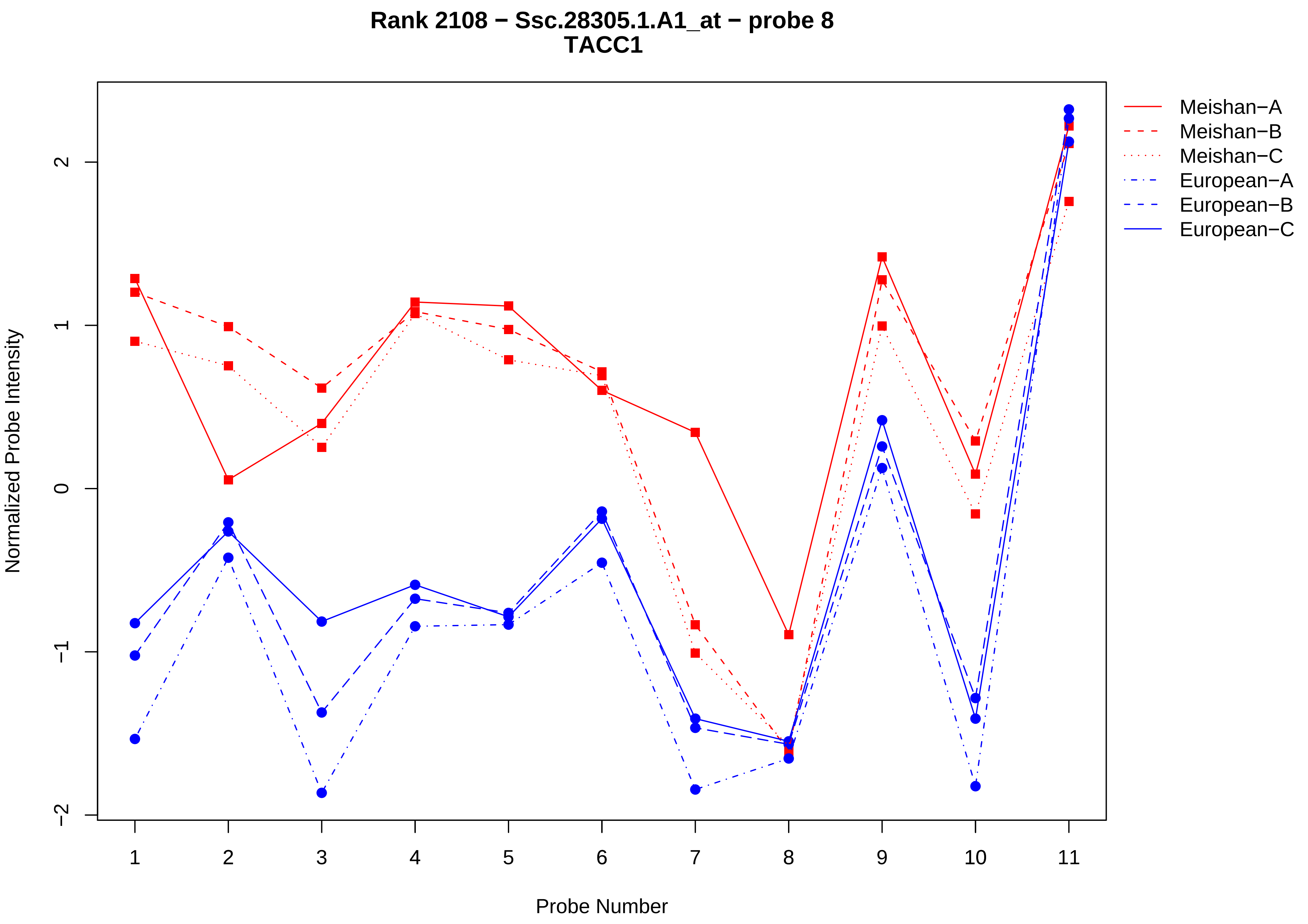

Rank 2108 − Ssc.28305.1.A1_at − probe 8
TACC1
Normalized Probe Intensity
Probe Number
−2
−1
0
1
2
1
2
3
4
5
6
7
8
9
10
11
Meishan−A
Meishan−B
Meishan−C
European−A
European−B
European−C

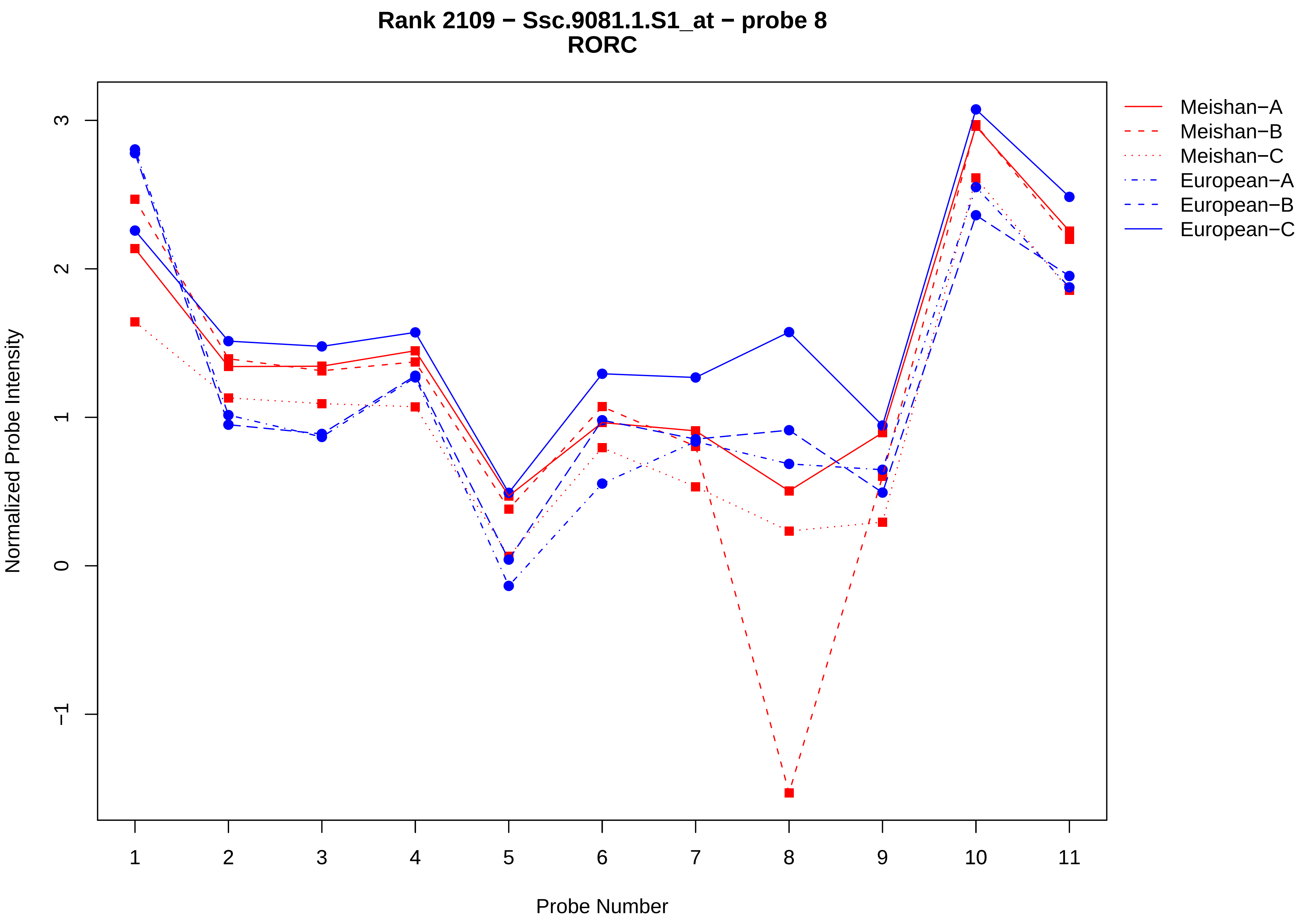

Rank 2109 − Ssc.9081.1.S1_at − probe 8
RORC
Normalized Probe Intensity
Probe Number
Meishan−A
Meishan−B
Meishan−C
European−A
European−B
European−C

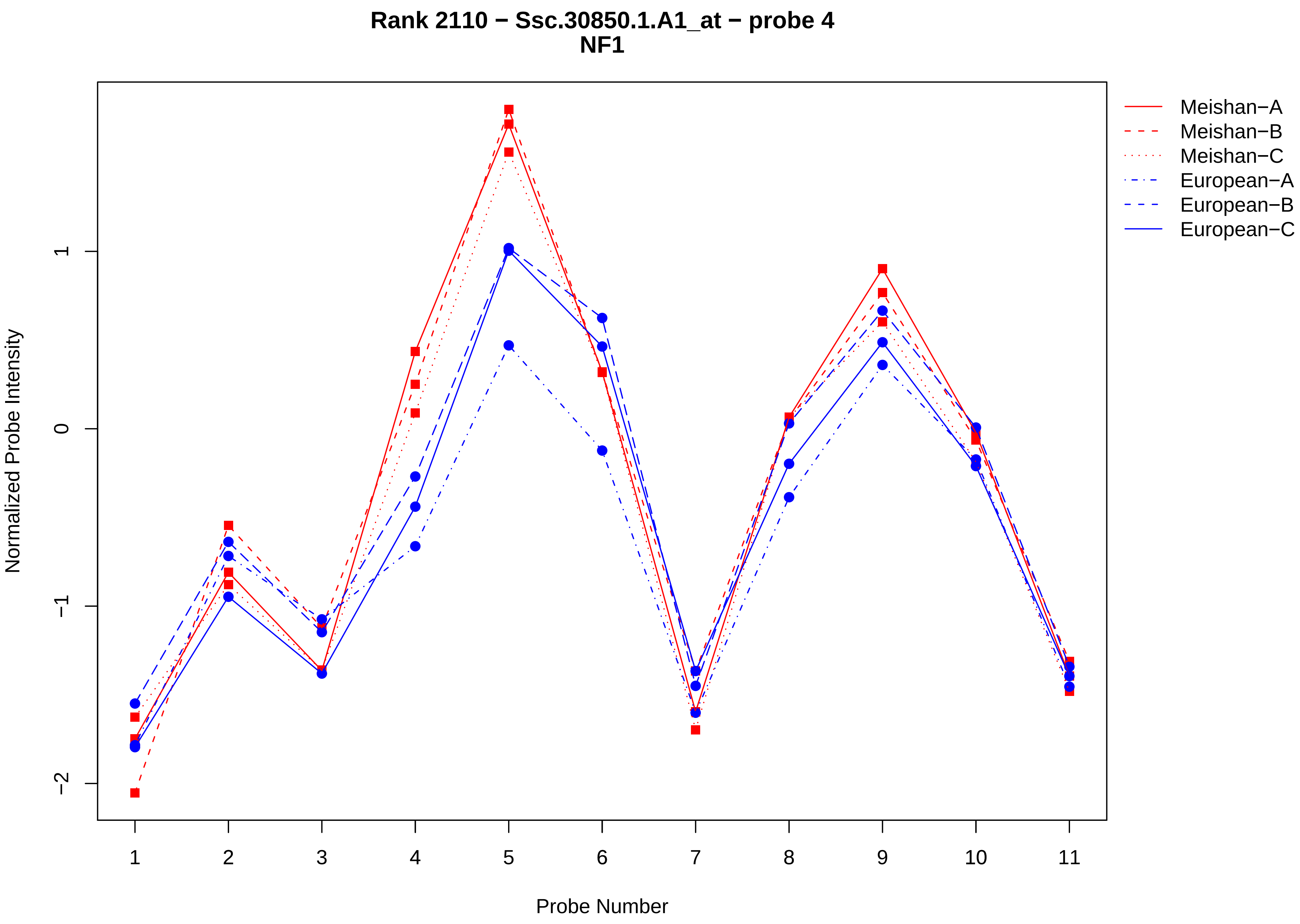

Rank 2110 − Ssc.30850.1.A1_at − probe 4
NF1
Normalized Probe Intensity
Probe Number
−2
−1
0
1
1
2
3
4
5
6
7
8
9
10
11
Meishan−A
Meishan−B
Meishan−C
European−A
European−B
European−C

Rank 2111 − Ssc.17449.1.S1_at − probe 7
OLFM1

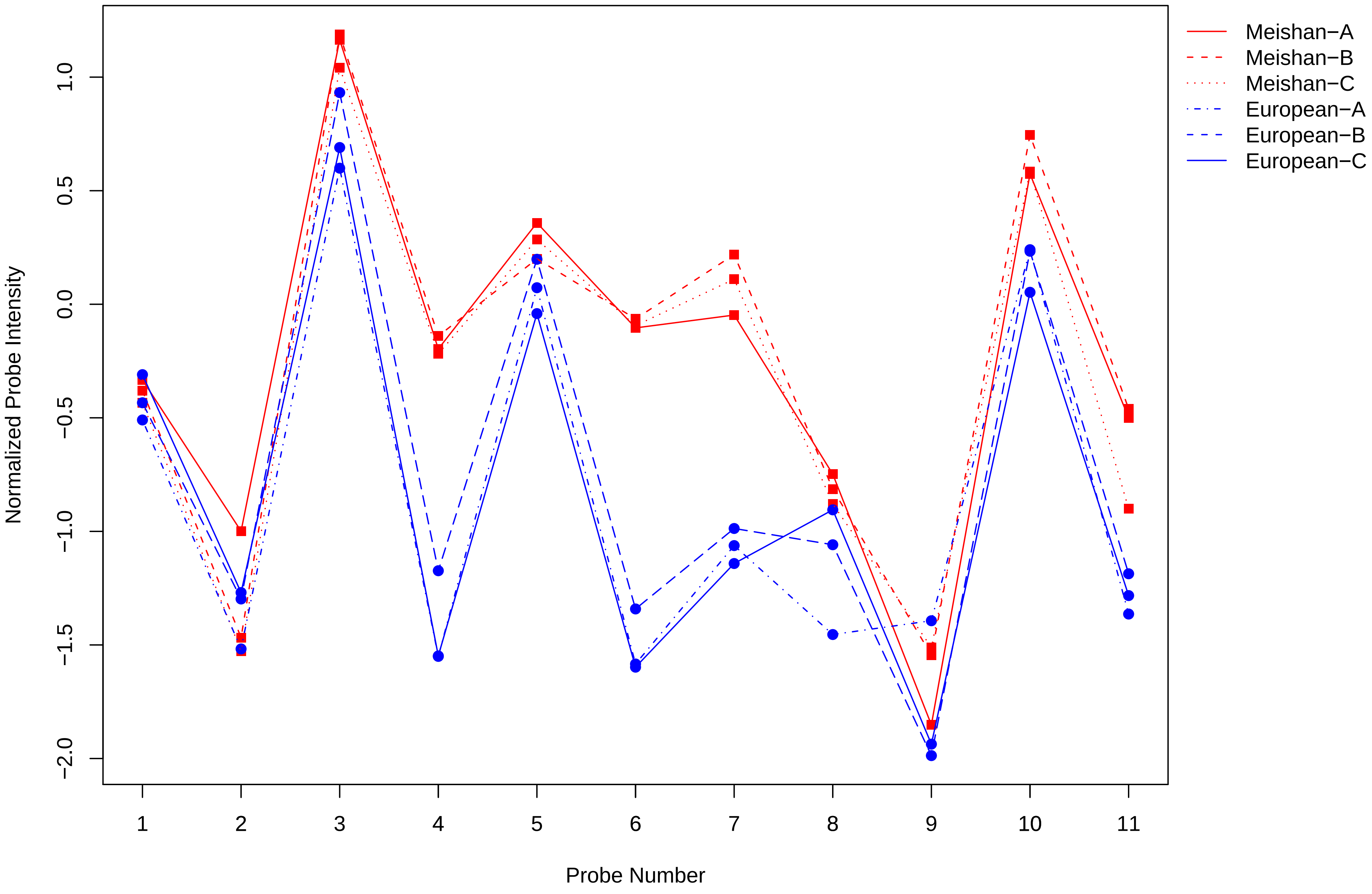

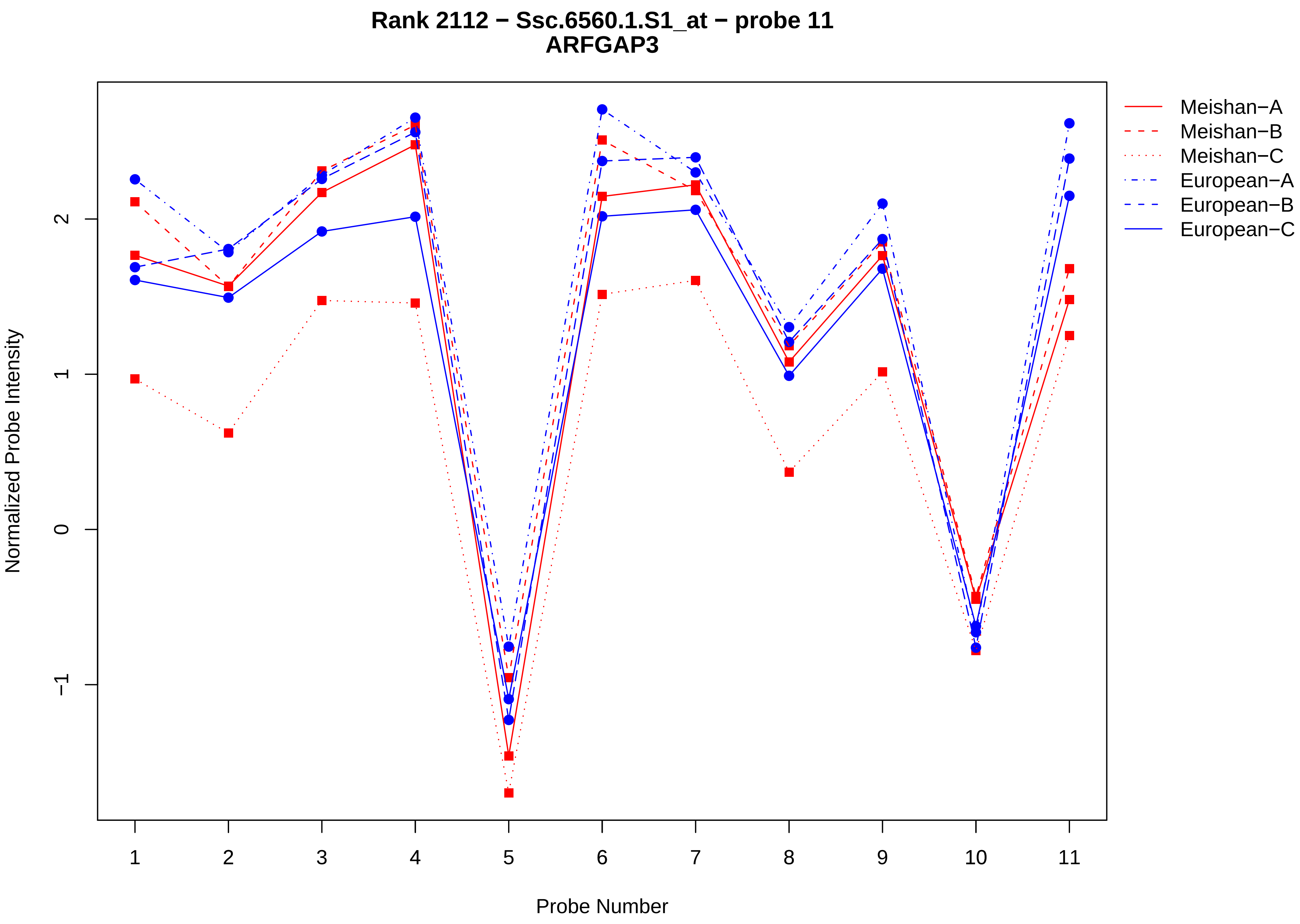

Rank 2112 − Ssc.6560.1.S1_at − probe 11
ARFGAP3
Normalized Probe Intensity
Probe Number
−1
0
1
2
1
2
3
4
5
6
7
8
9
10
11
Meishan−A
Meishan−B
Meishan−C
European−A
European−B
European−C

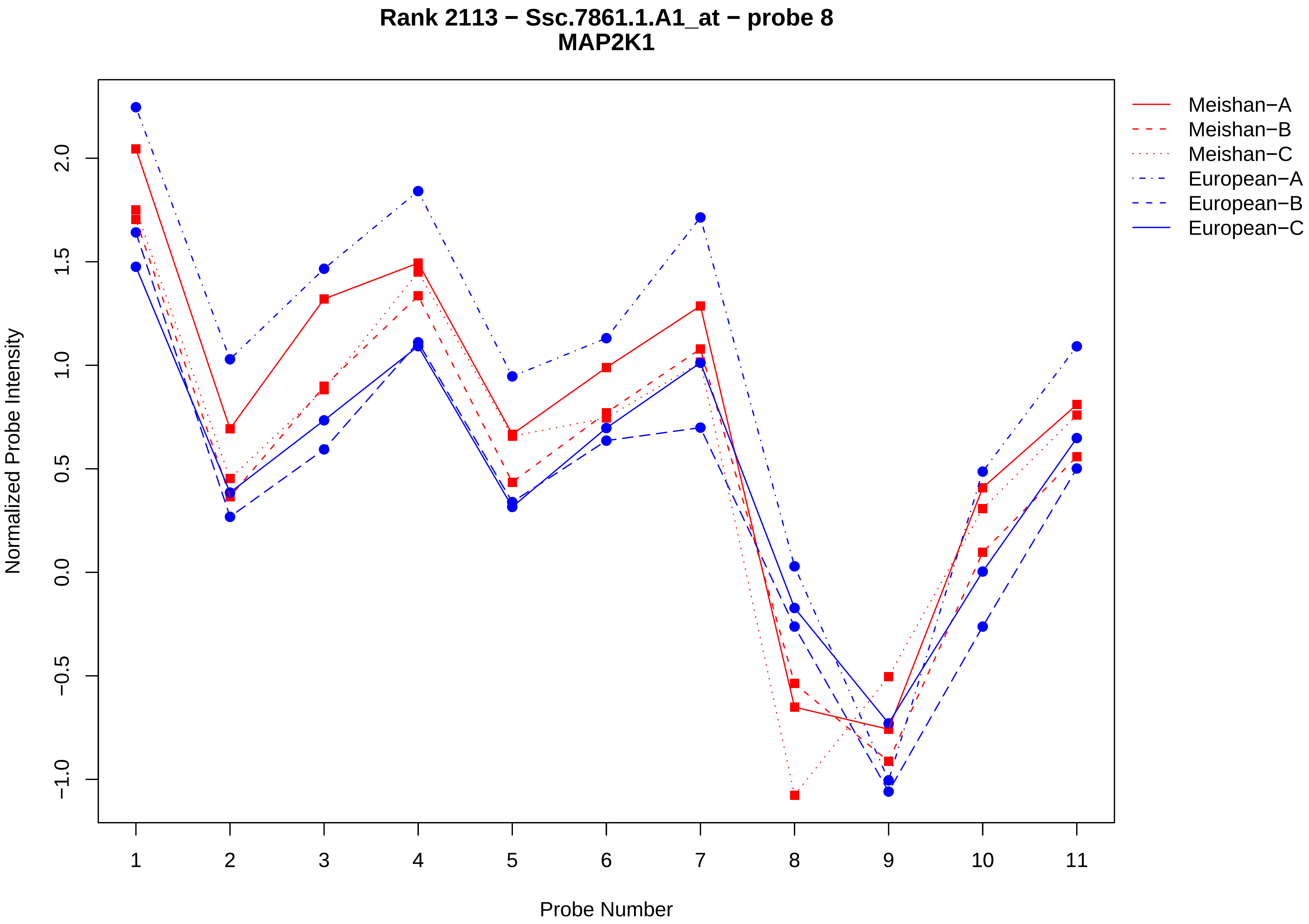
Rank 2113 − Ssc.7861.1.A1_at − probe 8
MAP2K1
Normalized Probe Intensity
Probe Number
−1.0
−0.5
0.0
0.5
1.0
1.5
2.0
1
2
3
4
5
6
7
8
9
10
11
Meishan−A
Meishan−B
Meishan−C
European−A
European−B
European−C

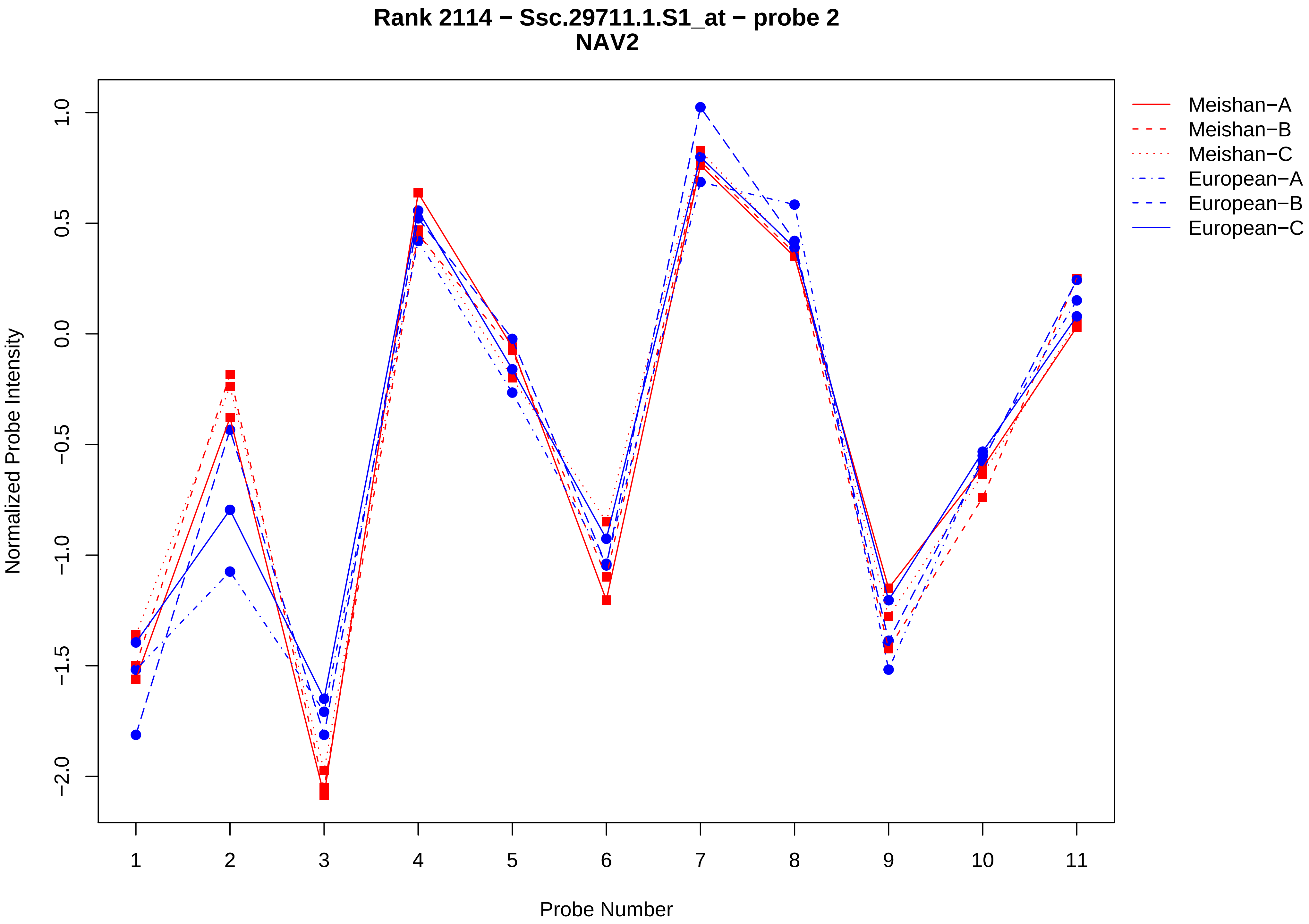

Rank 2114 − Ssc.29711.1.S1_at − probe 2
NAV2
Normalized Probe Intensity
Probe Number
1.0
0.5
0.0
−0.5
−1.0
−1.5
−2.0
1
2
3
4
5
6
7
8
9
10
11
Meishan−A
Meishan−B
Meishan−C
European−A
European−B
European−C

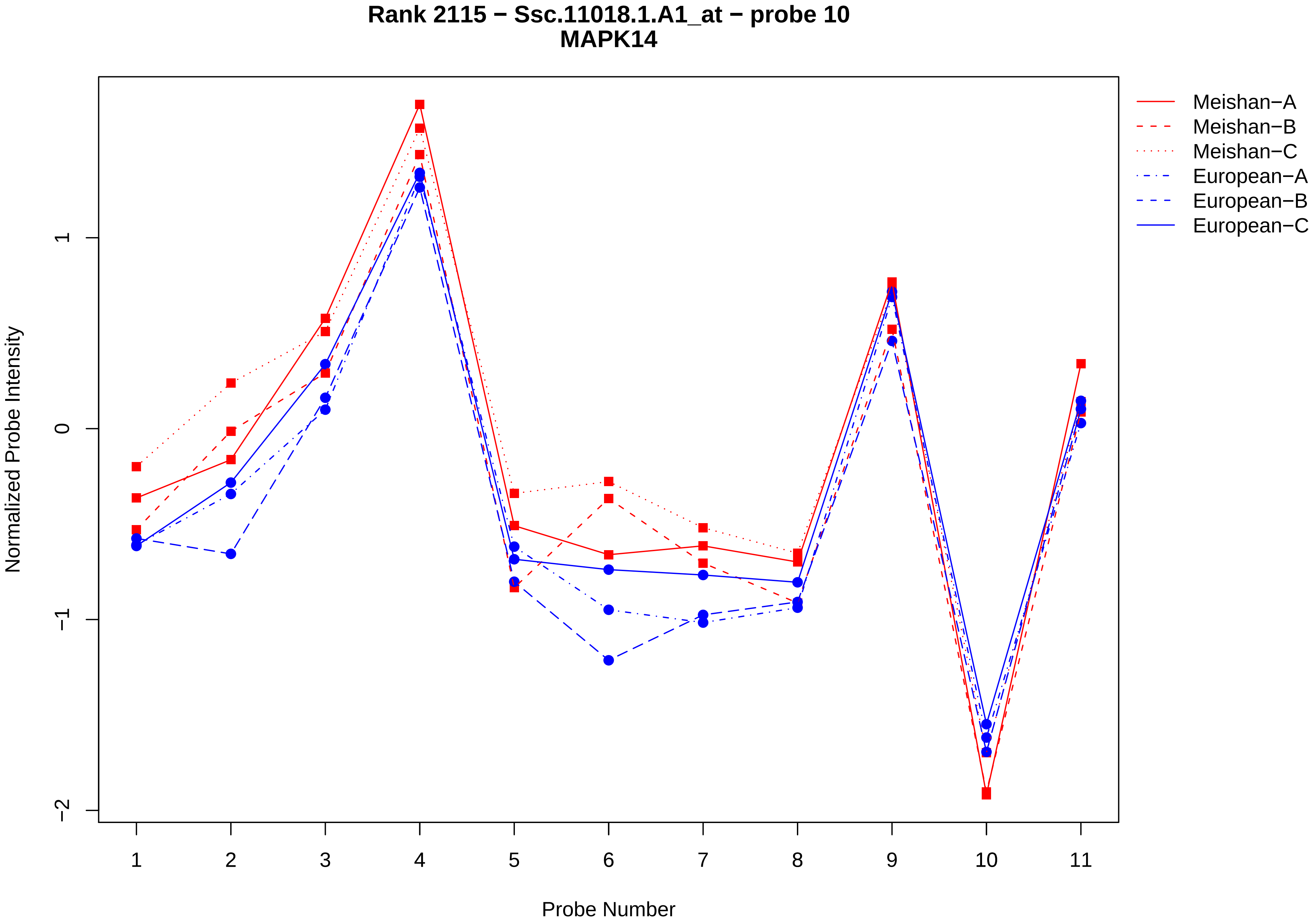

Rank 2115 − Ssc.11018.1.A1_at − probe 10
MAPK14
Normalized Probe Intensity
Probe Number
−2
−1
0
1
1
2
3
4
5
6
7
8
9
10
11
Meishan−A
Meishan−B
Meishan−C
European−A
European−B
European−C

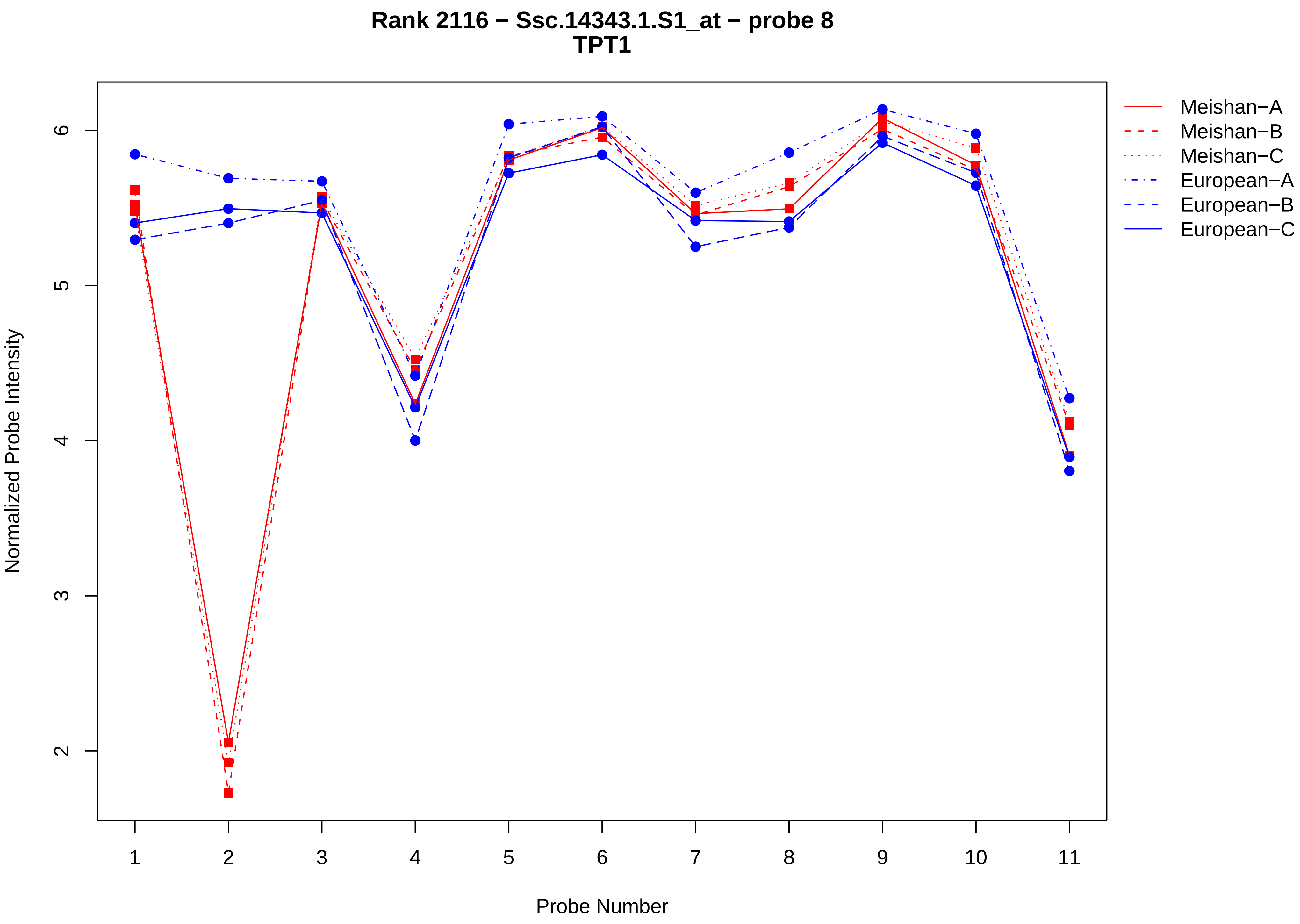

Rank 2116 − Ssc.14343.1.S1_at − probe 8
TPT1
Normalized Probe Intensity
Probe Number
Meishan−A
Meishan−B
Meishan−C
European−A
European−B
European−C
1
2
3
4
5
6
7
8
9
10
11
2
3
4
5
6

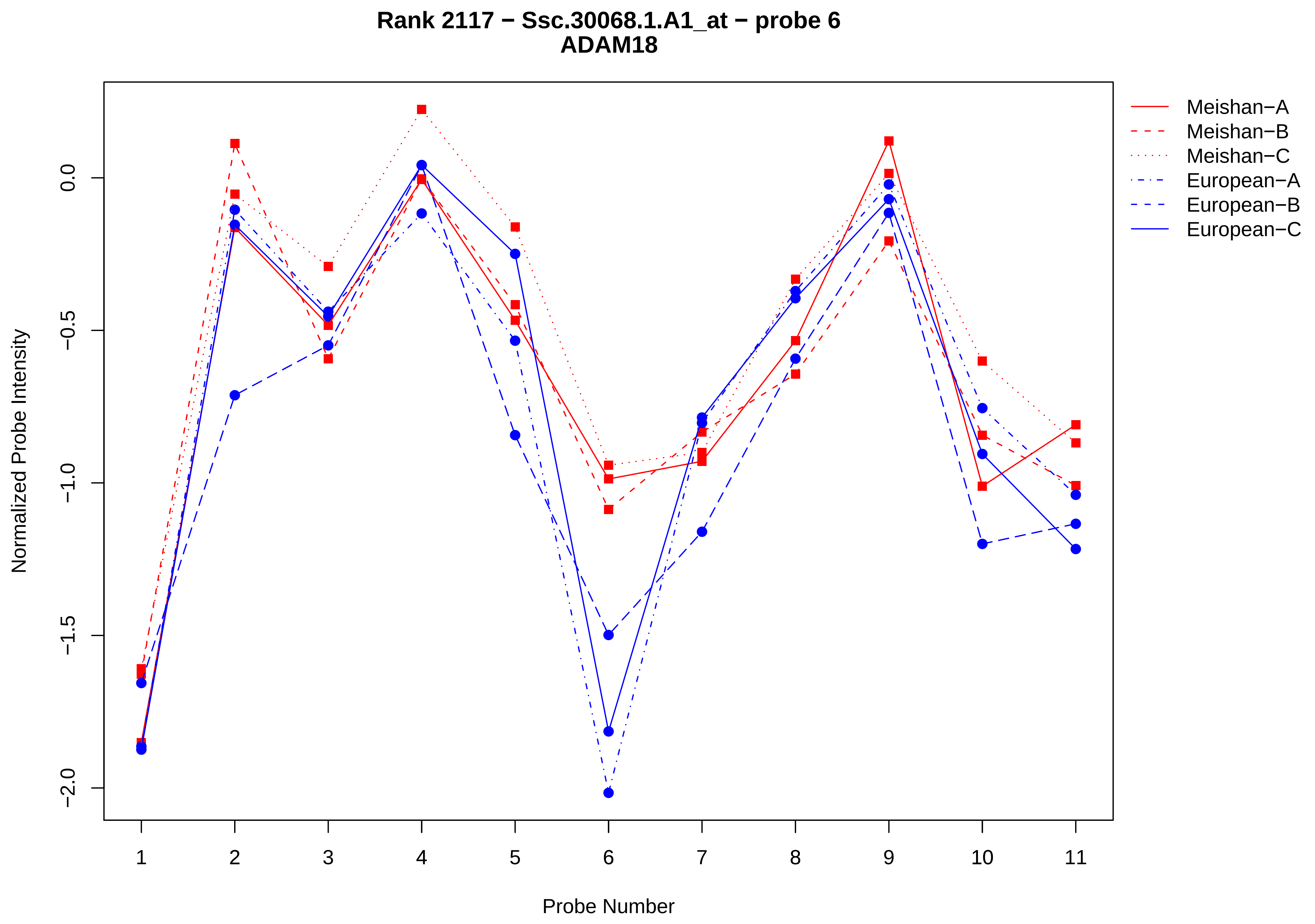

Rank 2117 − Ssc.30068.1.A1_at − probe 6
ADAM18
Normalized Probe Intensity
Probe Number
0.0
−0.5
−1.0
−1.5
−2.0
1
2
3
4
5
6
7
8
9
10
11
Meishan−A
Meishan−B
Meishan−C
European−A
European−B
European−C

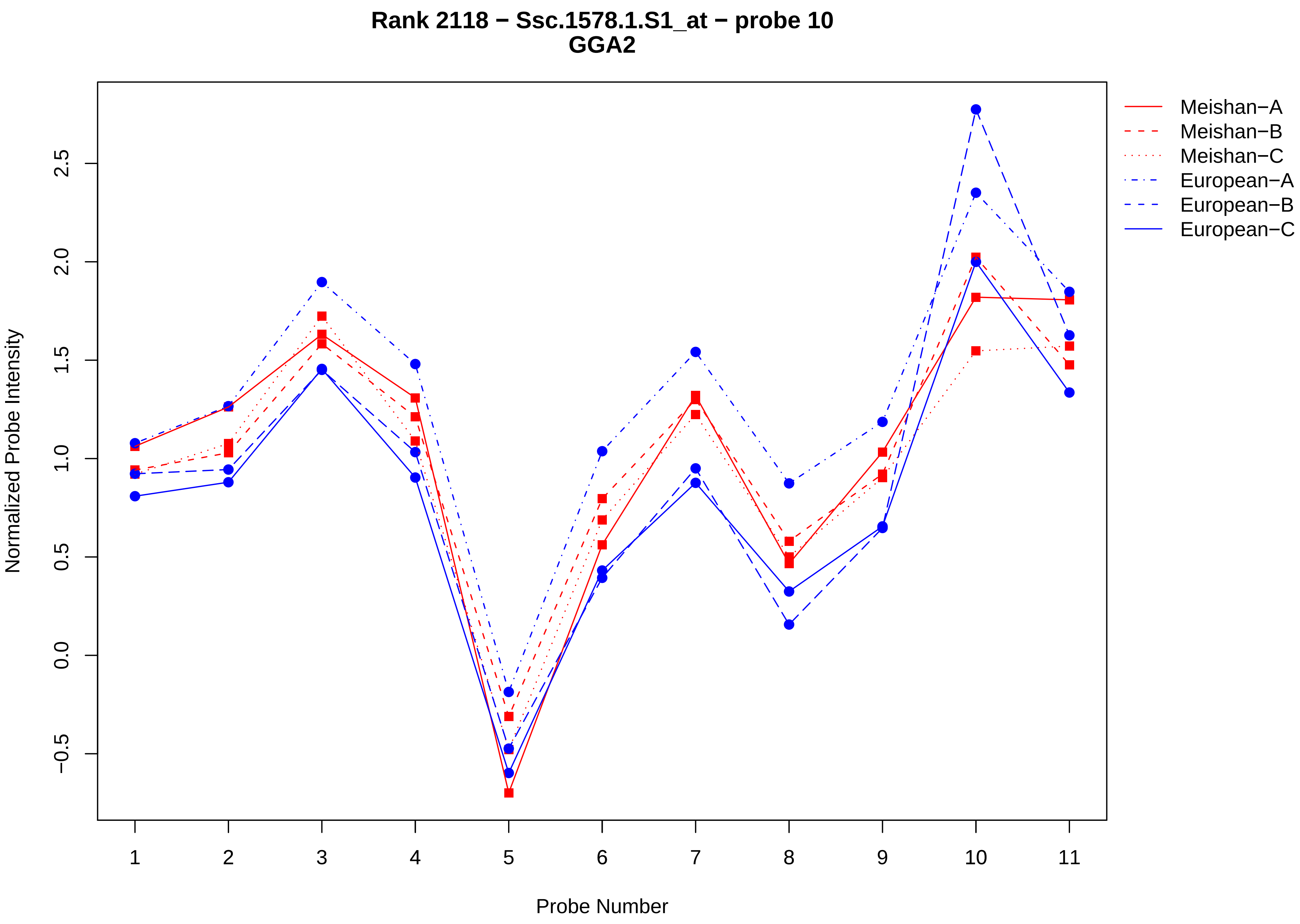

Rank 2118 − Ssc.1578.1.S1_at − probe 10
GGA2
Normalized Probe Intensity
Probe Number
Meishan−A
Meishan−B
Meishan−C
European−A
European−B
European−C

Rank 2119 − Ssc.18489.1.S1_at − probe 7
MAP3K3

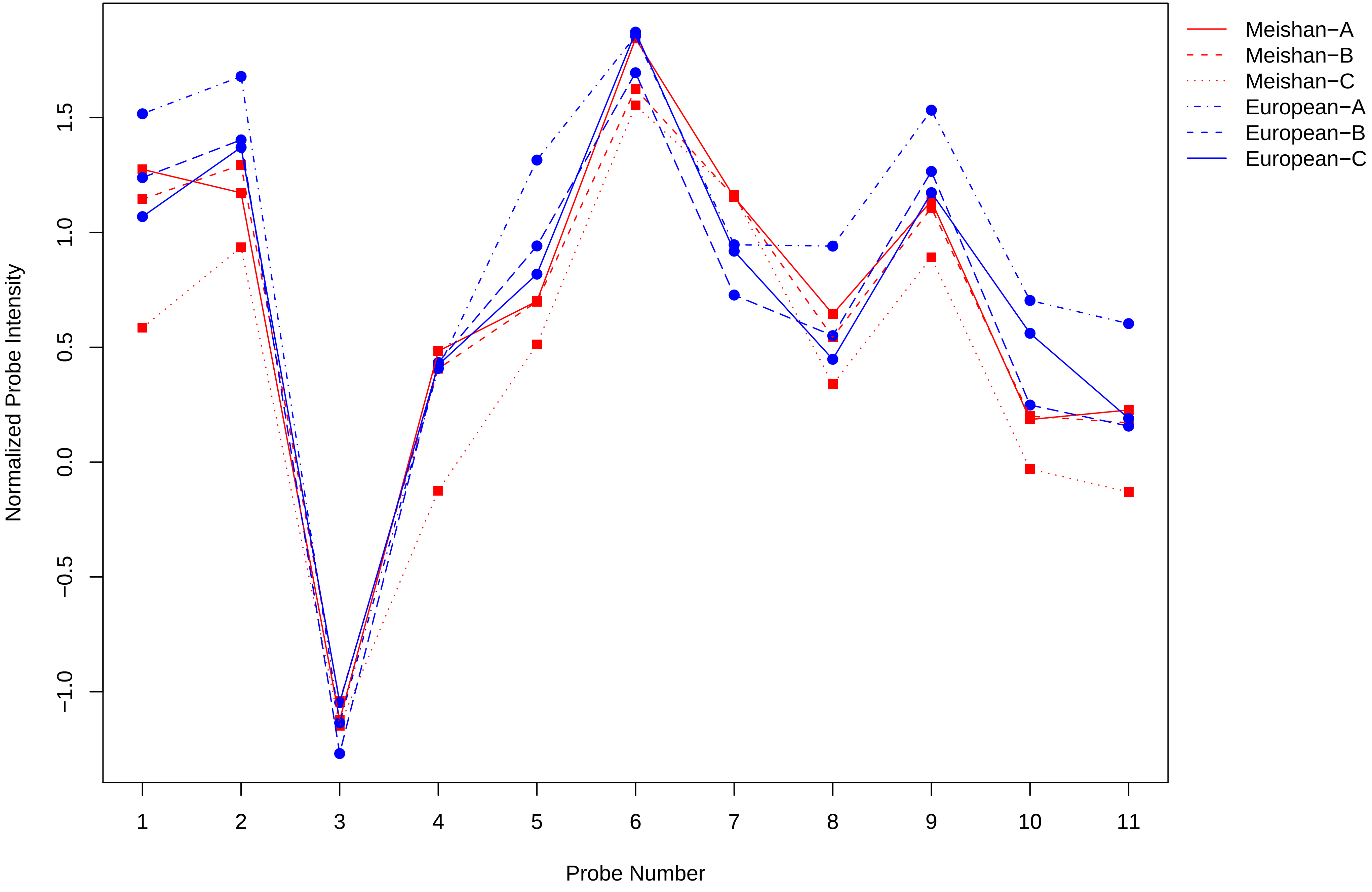

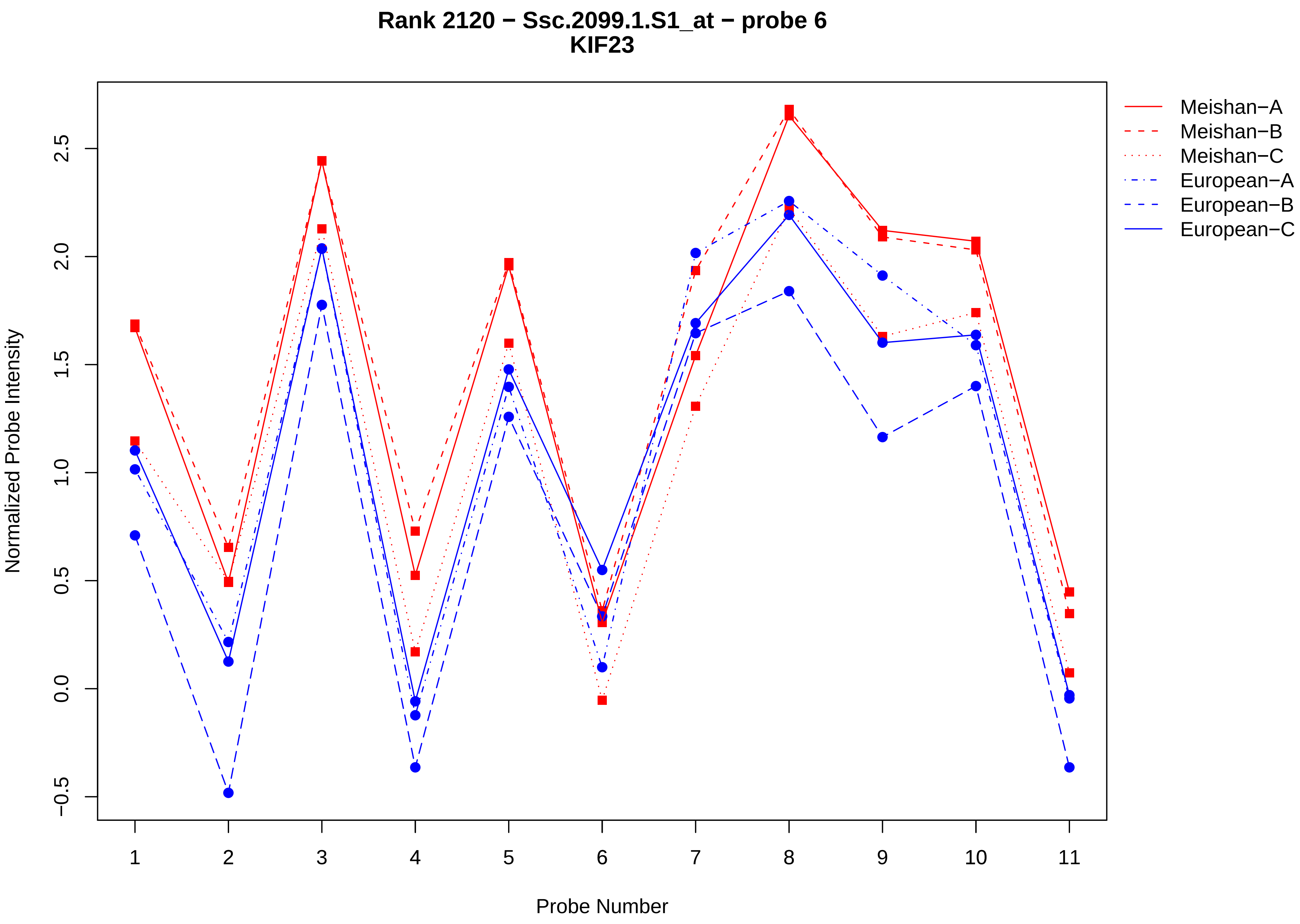

Rank 2120 − Ssc.2099.1.S1_at − probe 6
KIF23
Normalized Probe Intensity
Probe Number
−0.5
0.0
0.5
1.0
1.5
2.0
2.5
1
2
3
4
5
6
7
8
9
10
11
Meishan−A
Meishan−B
Meishan−C
European−A
European−B
European−C

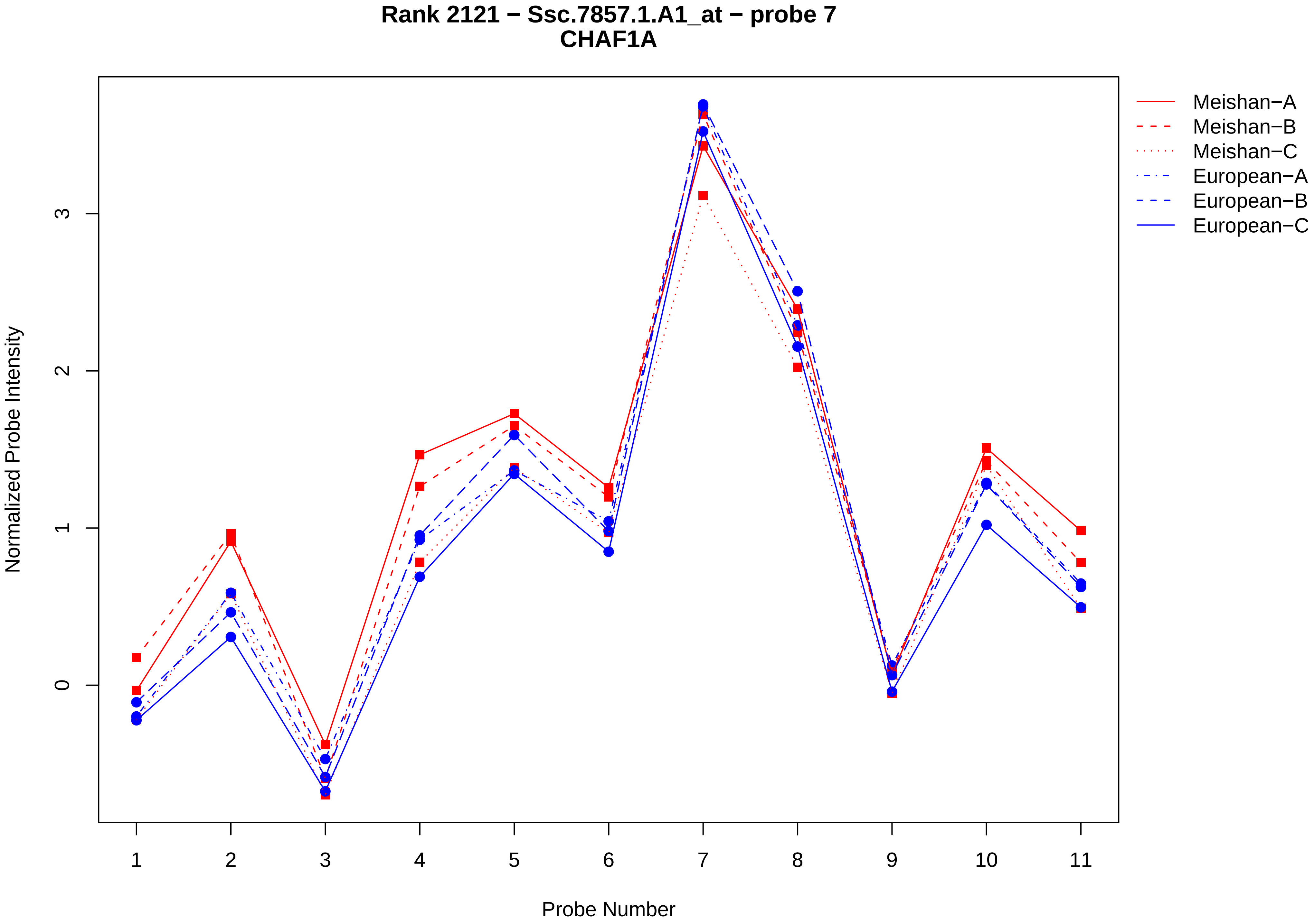

Rank 2121 − Ssc.7857.1.A1_at − probe 7
CHAF1A
Normalized Probe Intensity
Probe Number
0
1
2
3
1
2
3
4
5
6
7
8
9
10
11
Meishan−A
Meishan−B
Meishan−C
European−A
European−B
European−C

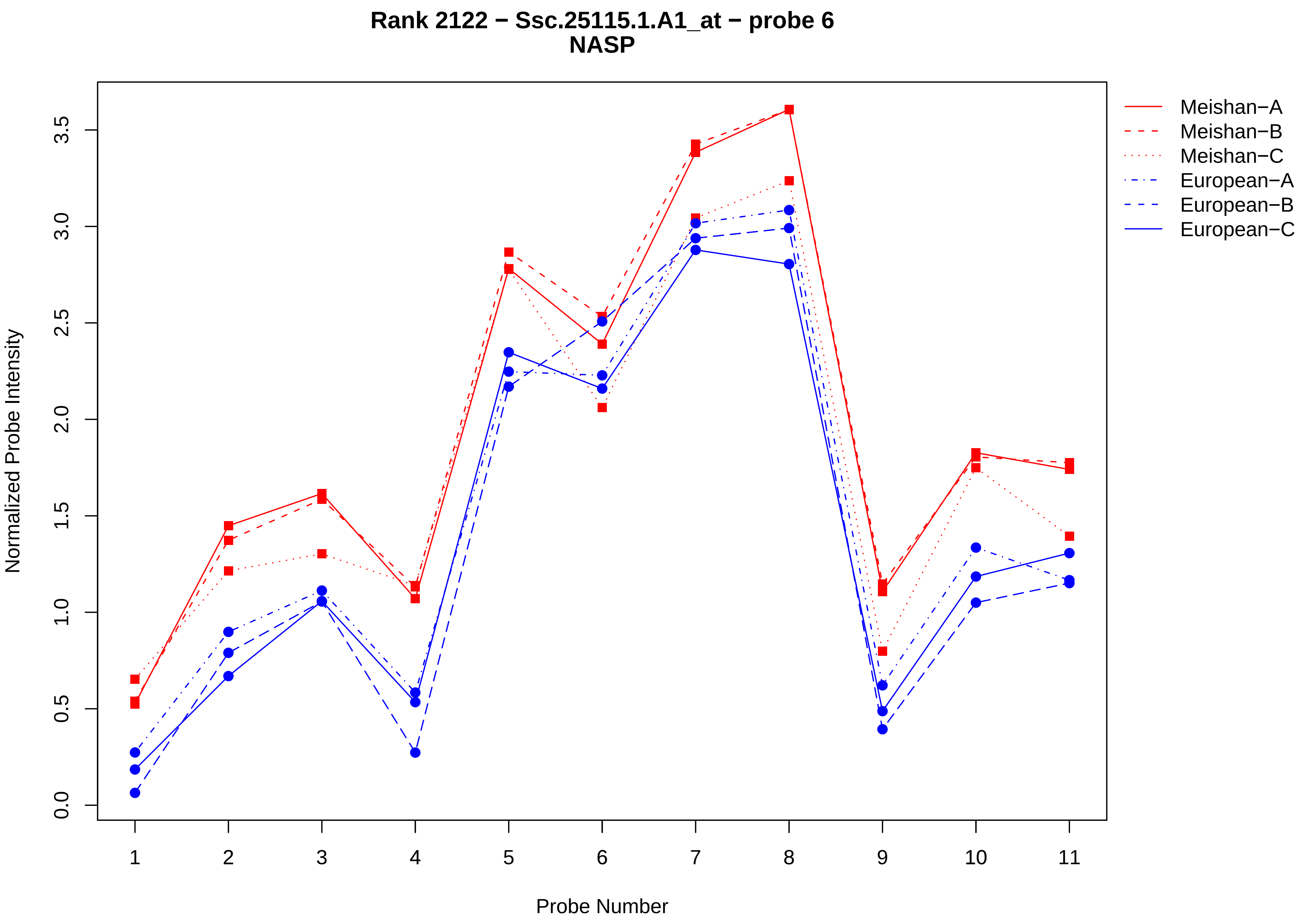
Rank 2122 − Ssc.25115.1.A1_at − probe 6
NASP
Normalized Probe Intensity
Probe Number
0.0
0.5
1.0
1.5
2.0
2.5
3.0
3.5
1
2
3
4
5
6
7
8
9
10
11
Meishan−A
Meishan−B
Meishan−C
European−A
European−B
European−C

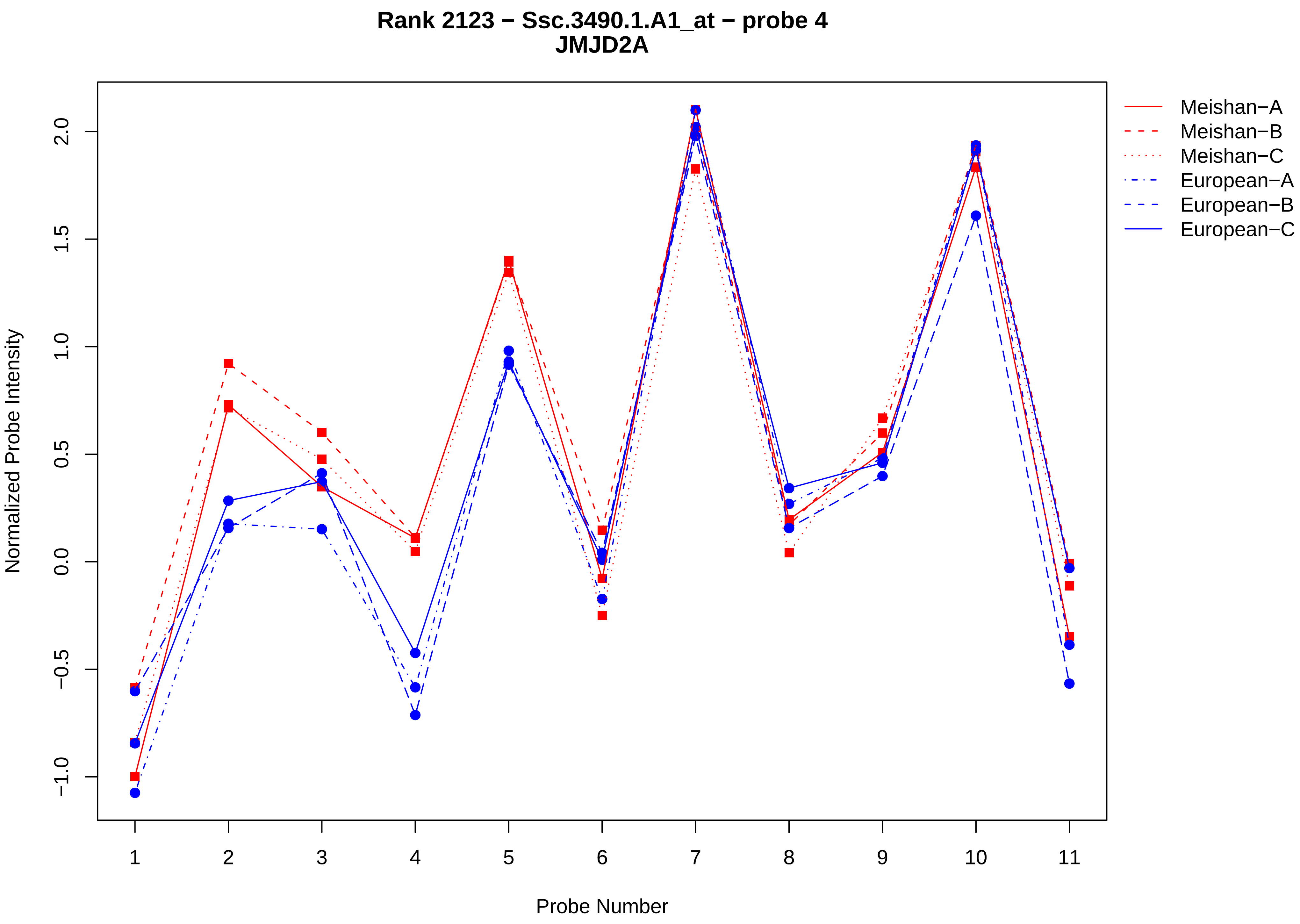

Rank 2123 − Ssc.3490.1.A1_at − probe 4
JMJD2A
Normalized Probe Intensity
Probe Number
−1.0
−0.5
0.0
0.5
1.0
1.5
2.0
1
2
3
4
5
6
7
8
9
10
11
Meishan−A
Meishan−B
Meishan−C
European−A
European−B
European−C

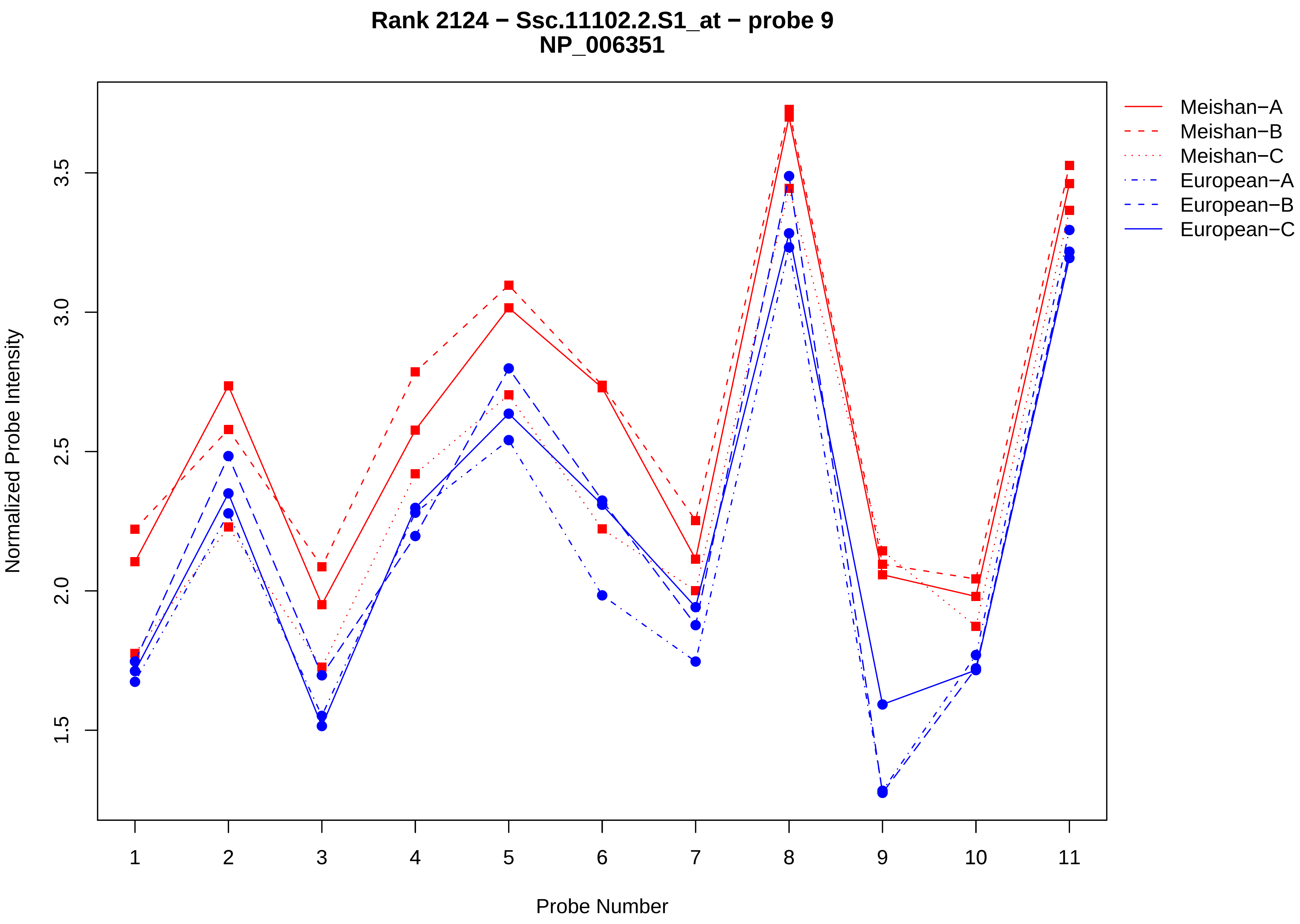

Rank 2124 − Ssc.11102.2.S1_at − probe 9
NP_006351
Normalized Probe Intensity
1.5
2.0
2.5
3.0
3.5
1
2
3
4
5
6
7
8
9
10
11
Probe Number
Meishan−A
Meishan−B
Meishan−C
European−A
European−B
European−C

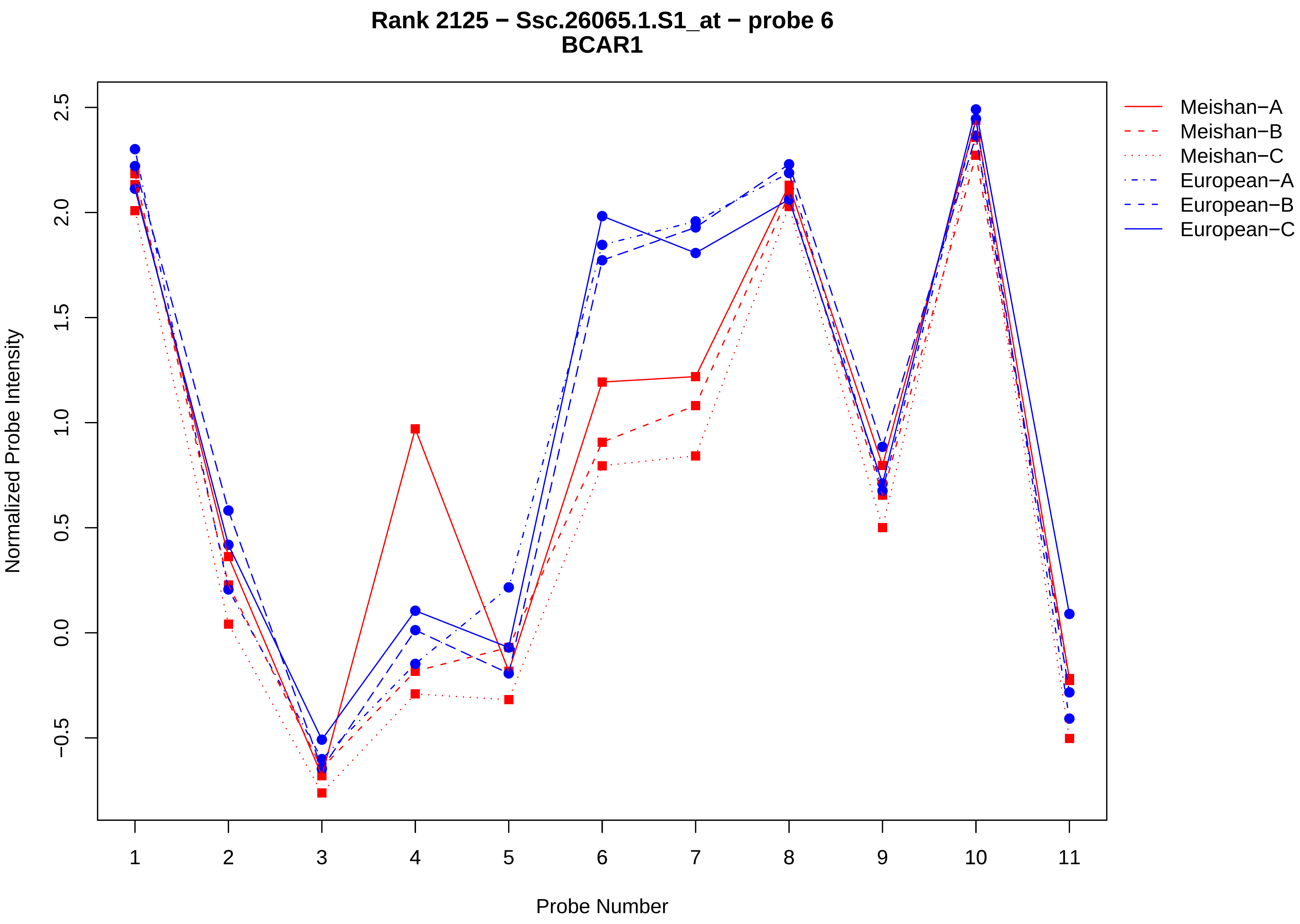
Rank 2125 − Ssc.26065.1.S1_at − probe 6
BCAR1
Normalized Probe Intensity
Probe Number
−0.5
0.0
0.5
1.0
1.5
2.0
2.5
1
2
3
4
5
6
7
8
9
10
11
Meishan−A
Meishan−B
Meishan−C
European−A
European−B
European−C

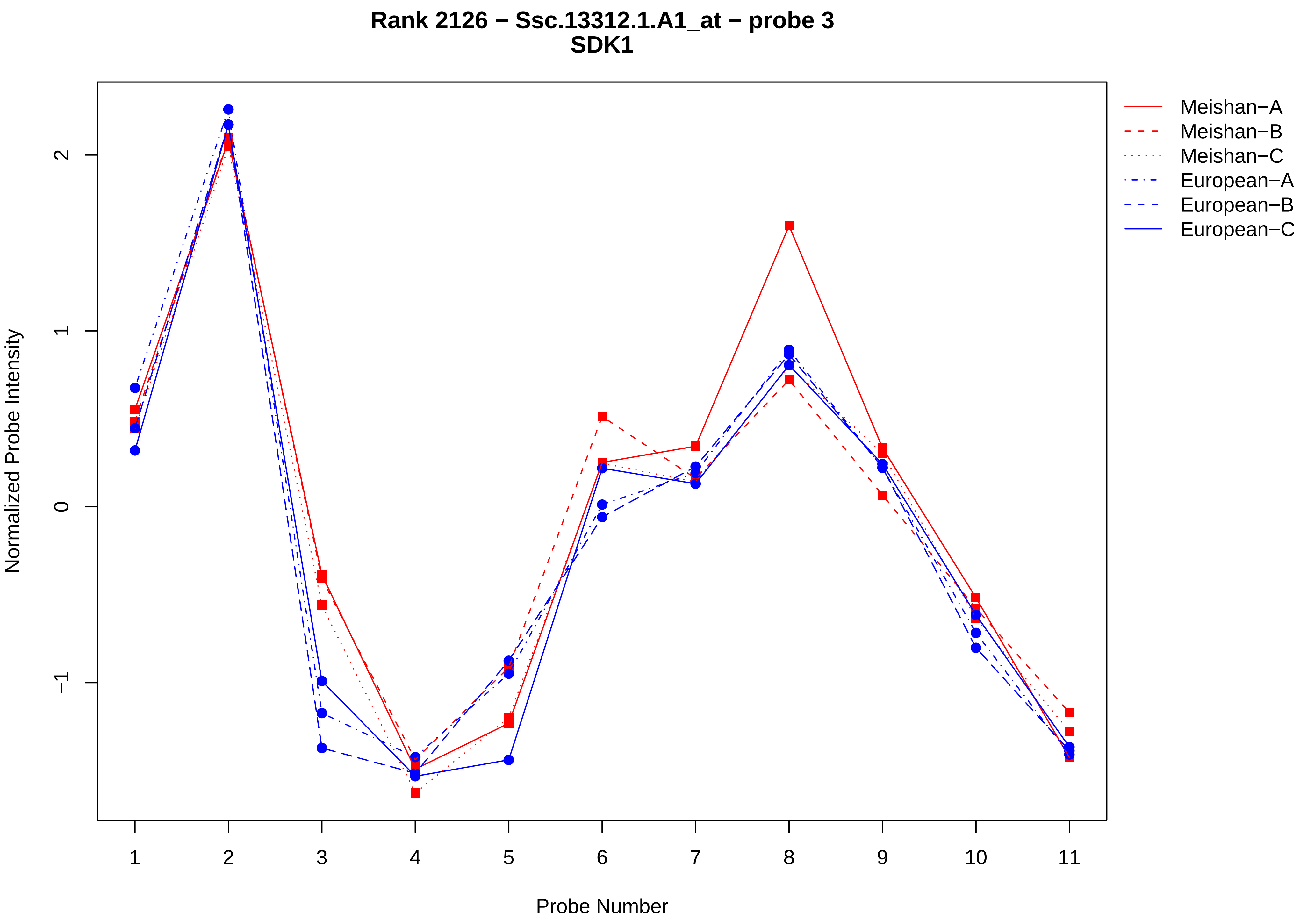
Rank 2126 − Ssc.13312.1.A1_at − probe 3
SDK1
Normalized Probe Intensity
Probe Number
Meishan−A
Meishan−B
Meishan−C
European−A
European−B
European−C
2
1
0
−1
1
2
3
4
5
6
7
8
9
10
11

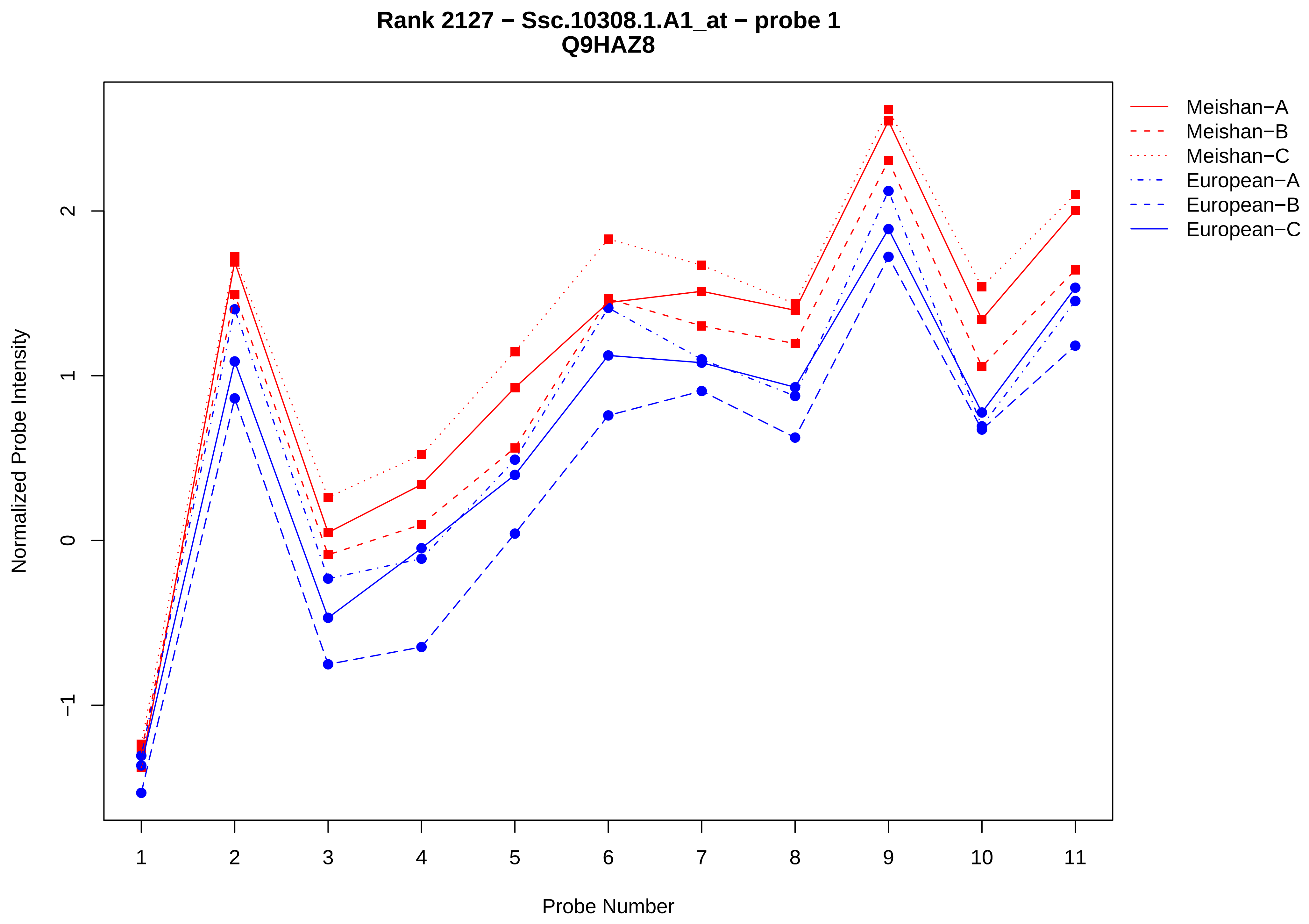
Rank 2127 − Ssc.10308.1.A1_at − probe 1
Q9HAZ8
Normalized Probe Intensity
Probe Number
−1
0
1
2
1
2
3
4
5
6
7
8
9
10
11
Meishan−A
Meishan−B
Meishan−C
European−A
European−B
European−C

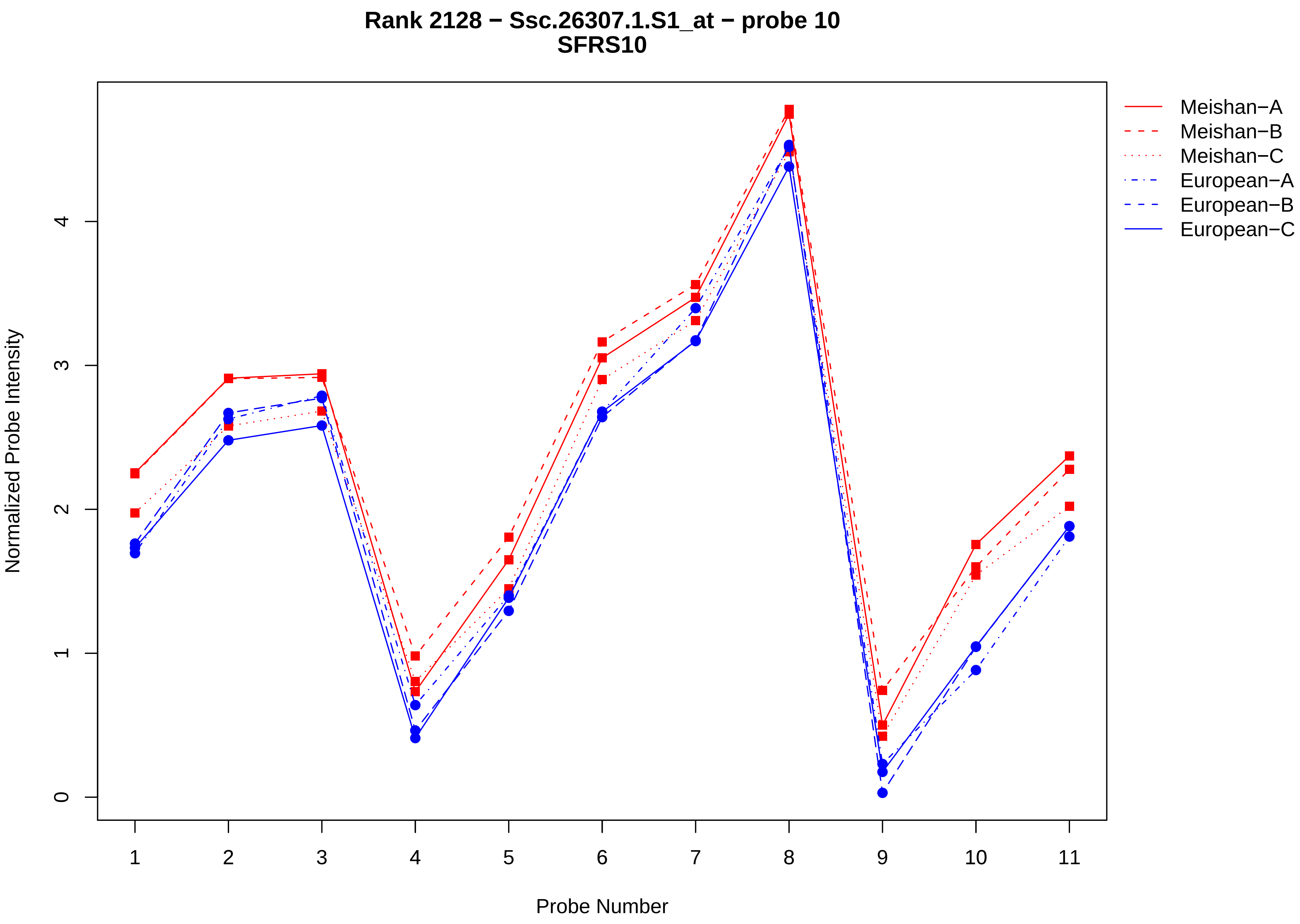

Rank 2128 − Ssc.26307.1.S1_at − probe 10
SFRS10
Normalized Probe Intensity
Probe Number
0
1
2
3
4
1
2
3
4
5
6
7
8
9
10
11
Meishan−A
Meishan−B
Meishan−C
European−A
European−B
European−C

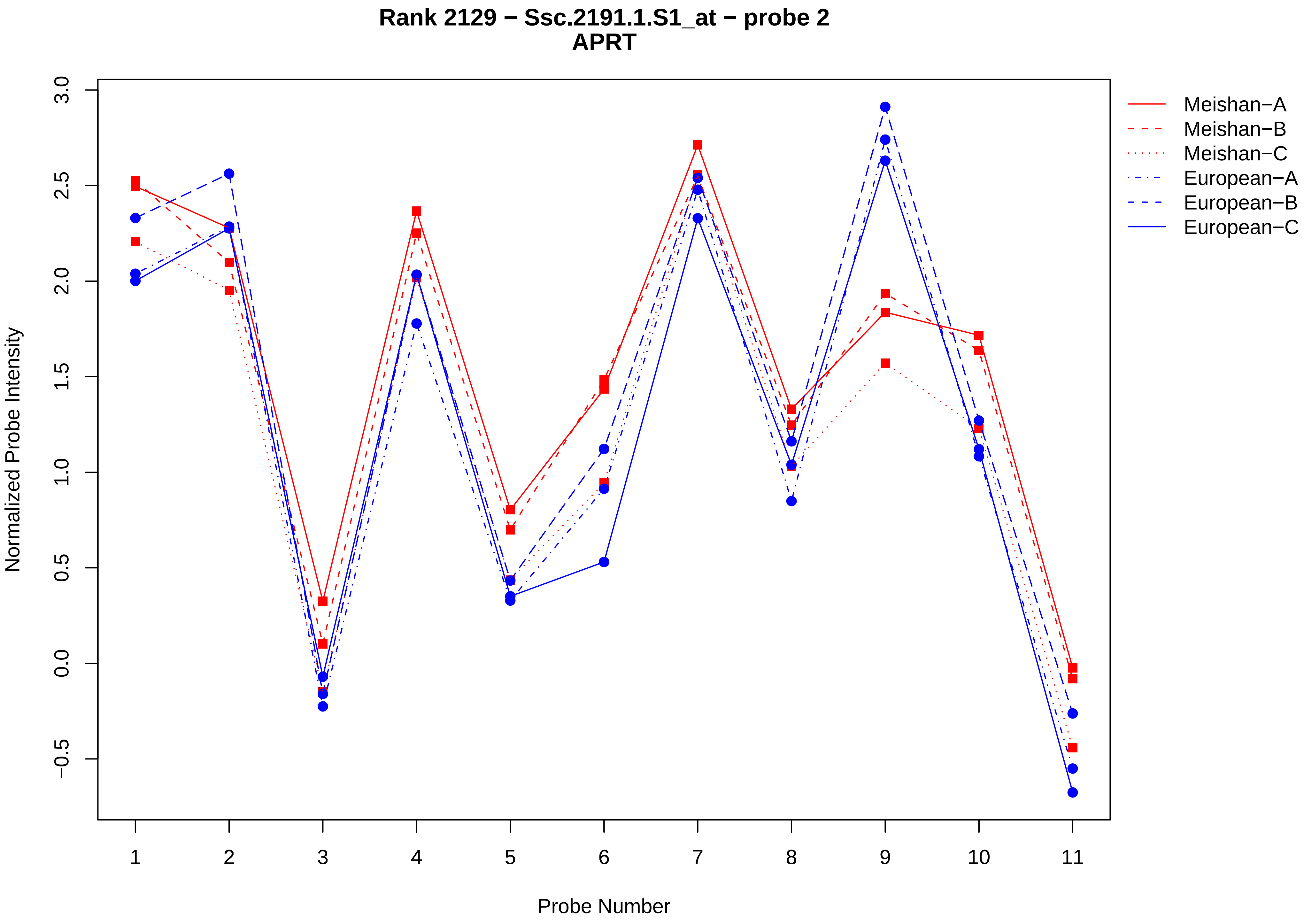

Rank 2129 − Ssc.2191.1.S1_at − probe 2
APRT
Normalized Probe Intensity
Probe Number
−0.5
0.0
0.5
1.0
1.5
2.0
2.5
3.0
1
2
3
4
5
6
7
8
9
10
11
Meishan−A
Meishan−B
Meishan−C
European−A
European−B
European−C

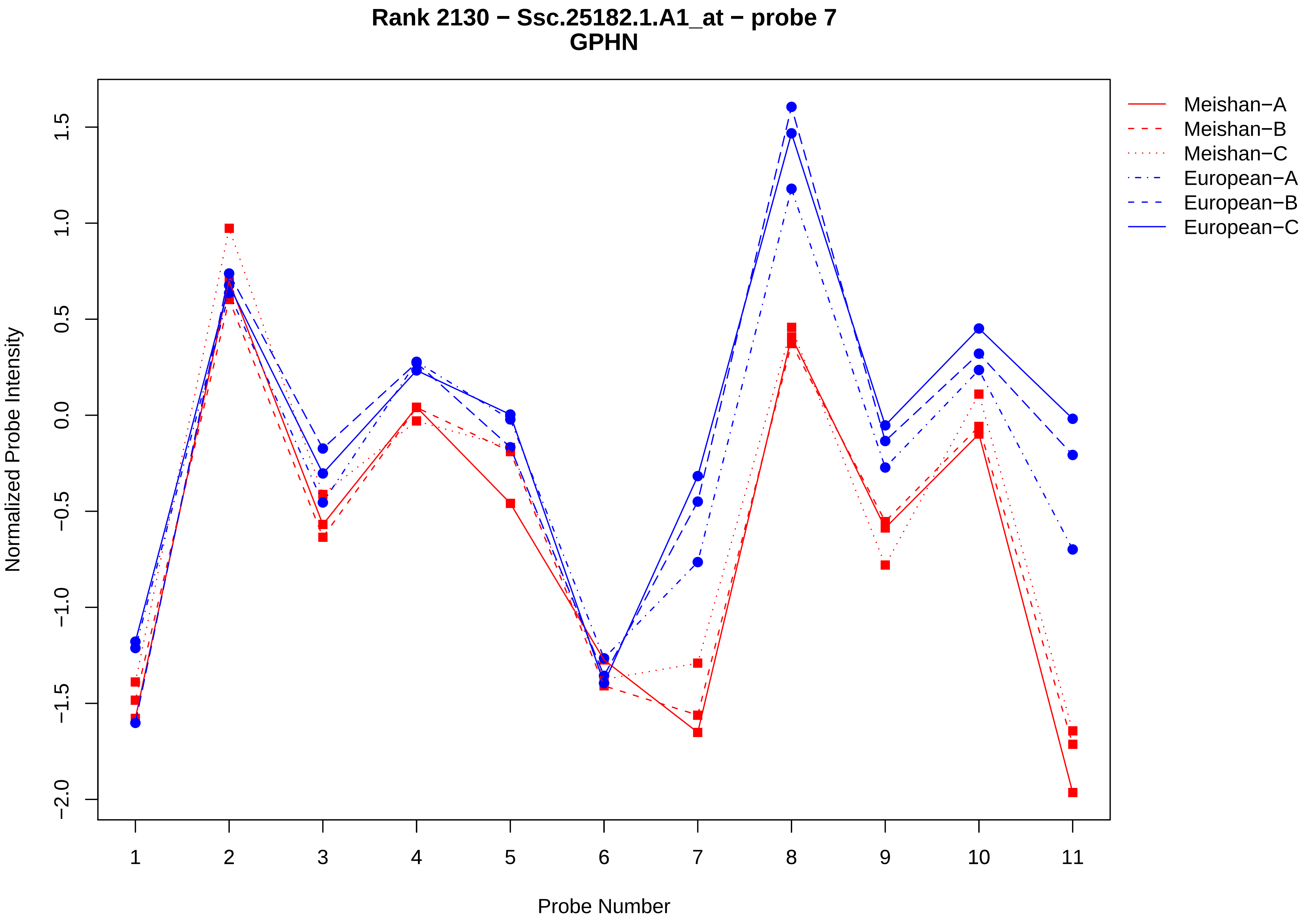

Rank 2130 − Ssc.25182.1.A1_at − probe 7
GPHN
Normalized Probe Intensity
Probe Number
−2.0
−1.5
−1.0
−0.5
0.0
0.5
1.0
1.5
1
2
3
4
5
6
7
8
9
10
11
Meishan−A
Meishan−B
Meishan−C
European−A
European−B
European−C

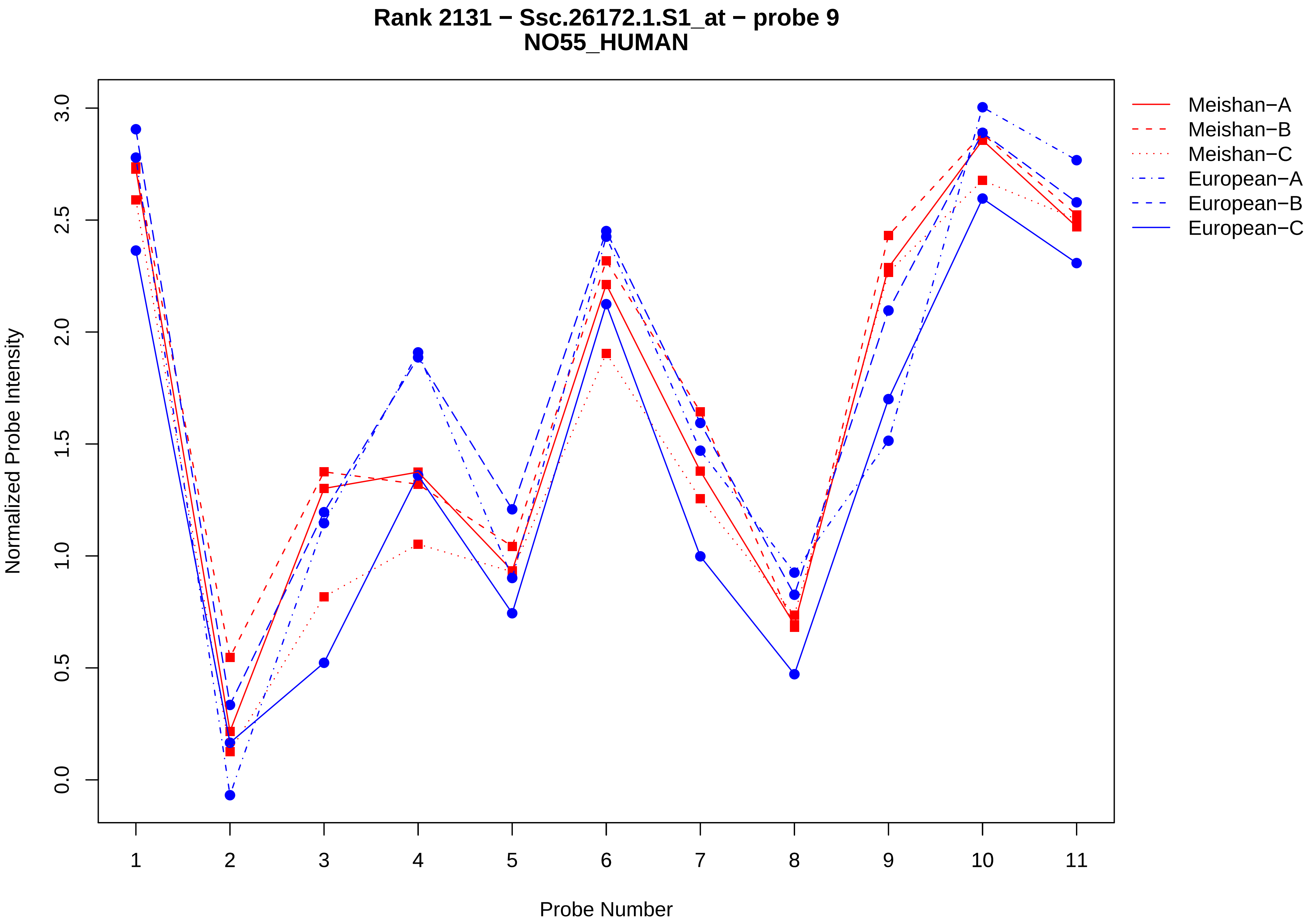

Rank 2131 − Ssc.26172.1.S1_at − probe 9
NO55_HUMAN
Normalized Probe Intensity
0.0
0.5
1.0
1.5
2.0
2.5
3.0
1
2
3
4
5
6
7
8
9
10
11
Probe Number
Meishan−A
Meishan−B
Meishan−C
European−A
European−B
European−C

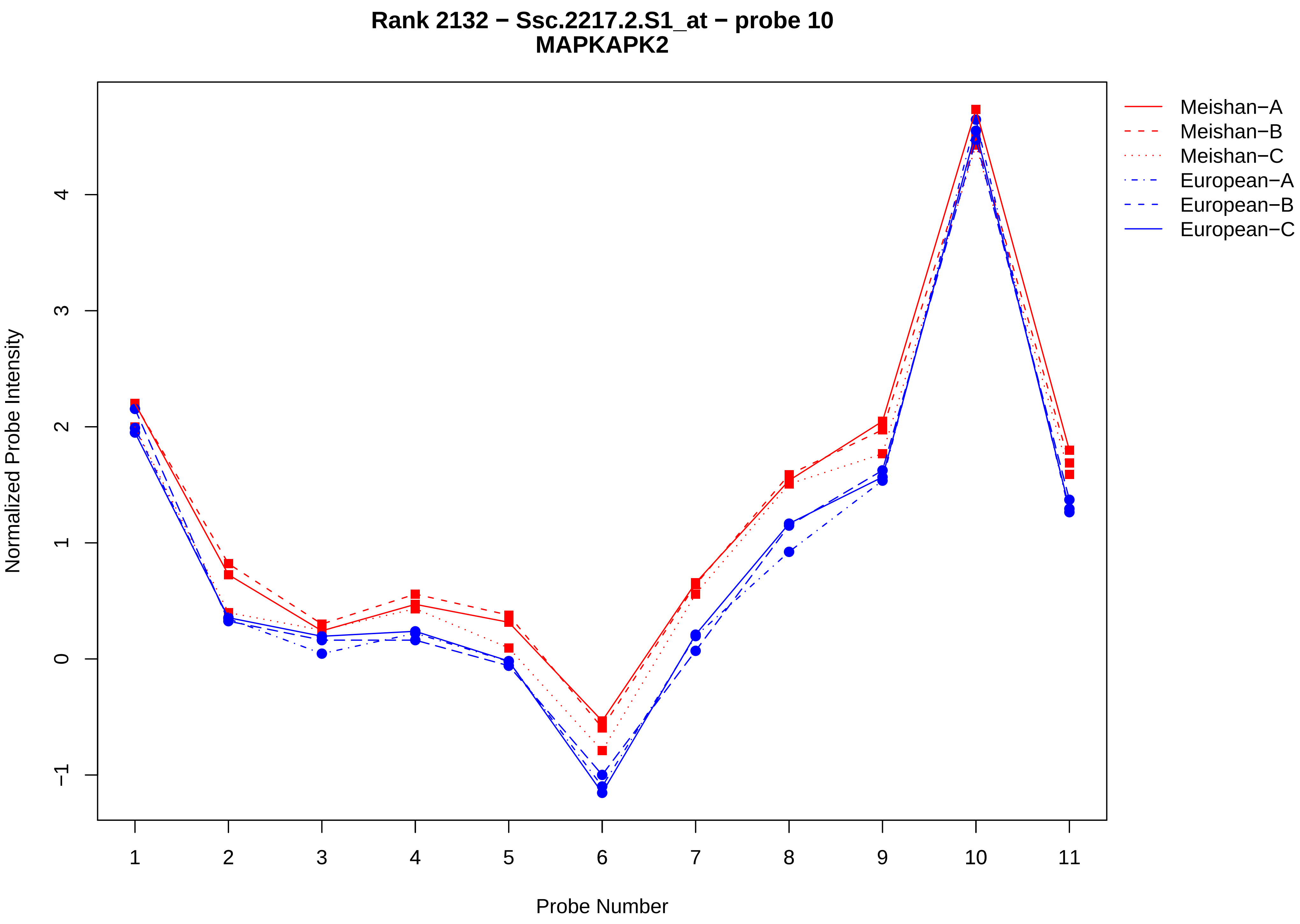

Rank 2132 − Ssc.2217.2.S1_at − probe 10
MAPKAPK2
Normalized Probe Intensity
Probe Number
−1
0
1
2
3
4
1
2
3
4
5
6
7
8
9
10
11
Meishan−A
Meishan−B
Meishan−C
European−A
European−B
European−C

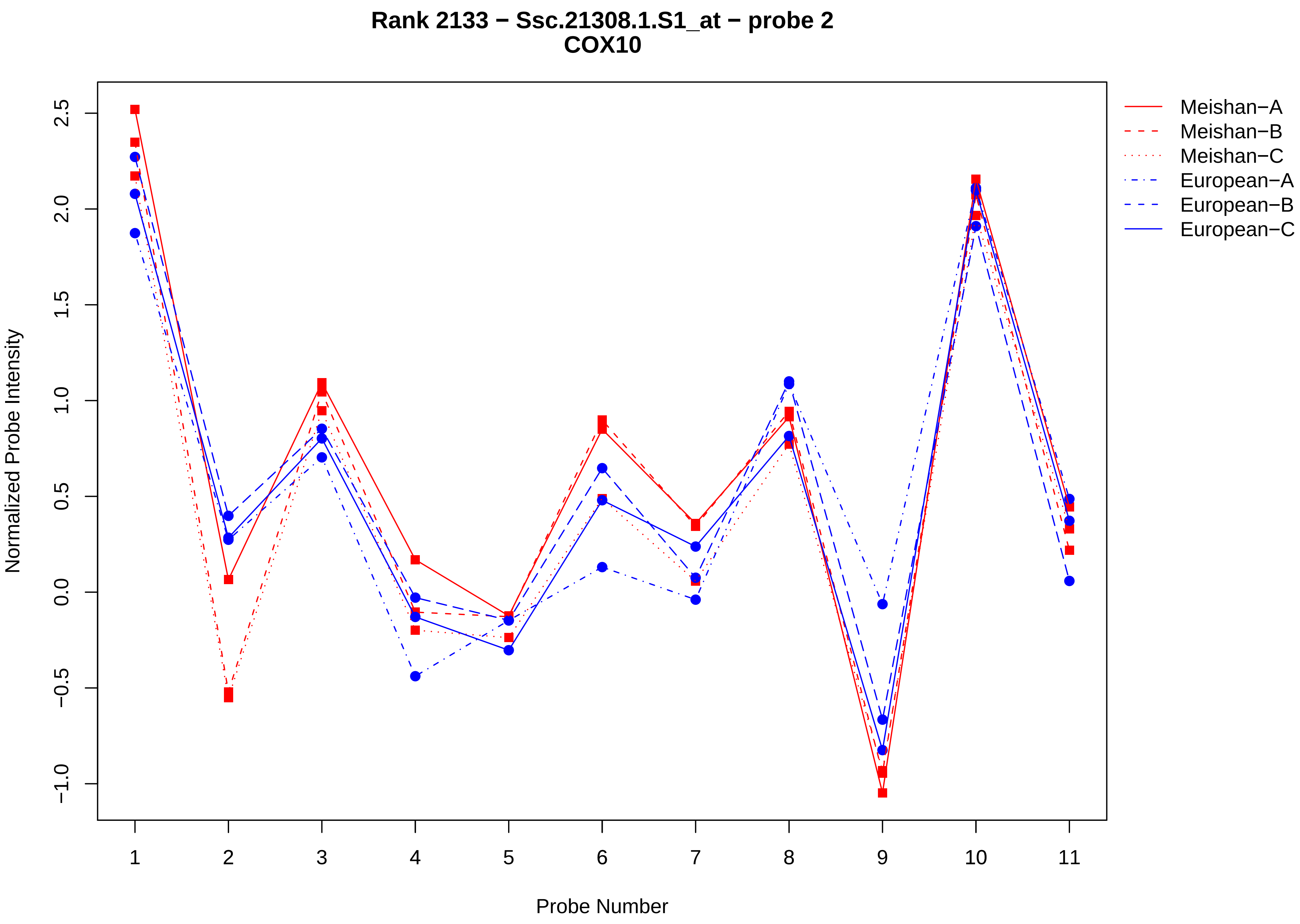
Rank 2133 − Ssc.21308.1.S1_at − probe 2
COX10
Normalized Probe Intensity
Probe Number
Meishan−A
Meishan−B
Meishan−C
European−A
European−B
European−C

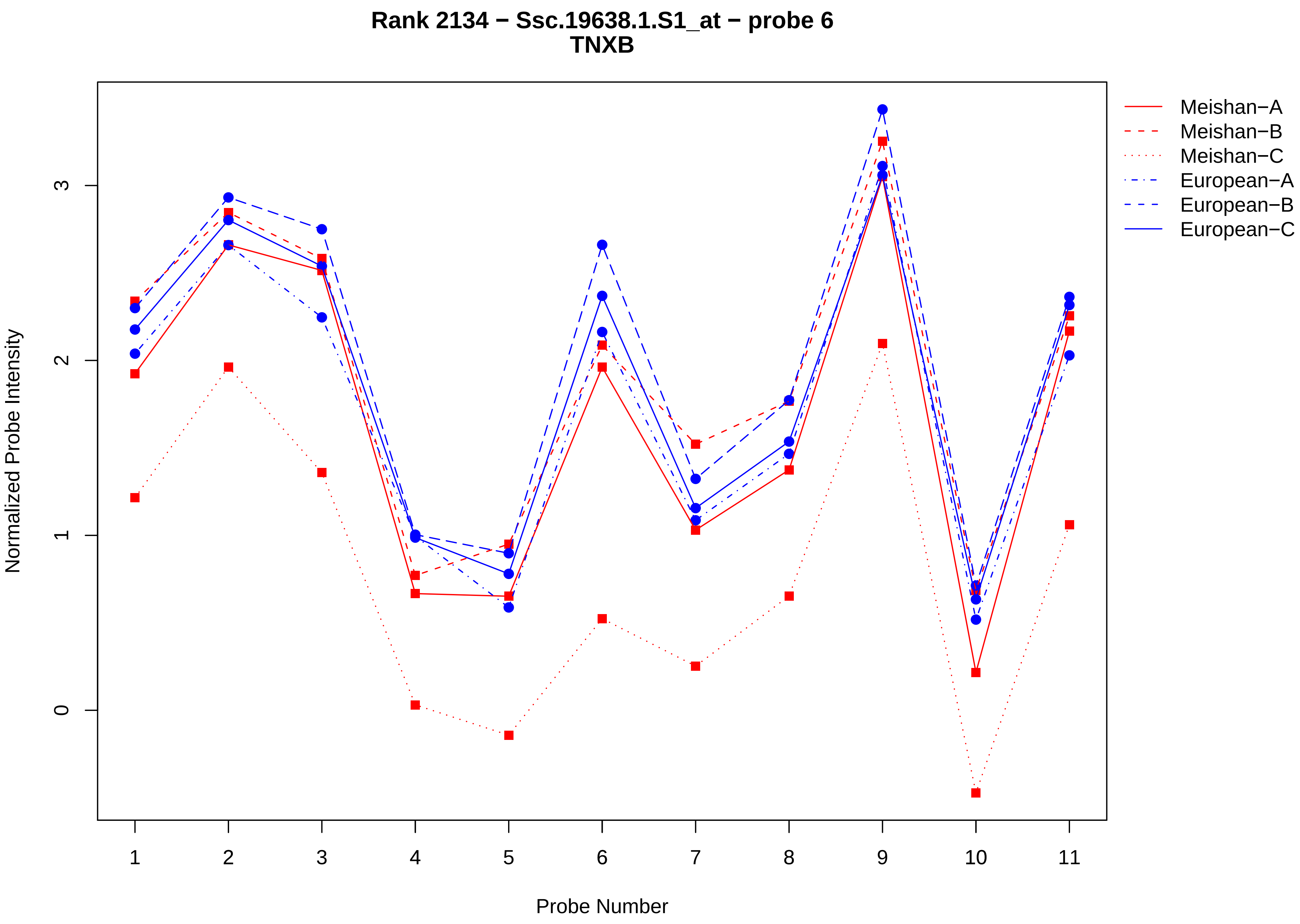

Rank 2134 − Ssc.19638.1.S1_at − probe 6
TNXB
Normalized Probe Intensity
Probe Number
0
1
2
3
1
2
3
4
5
6
7
8
9
10
11
Meishan−A
Meishan−B
Meishan−C
European−A
European−B
European−C

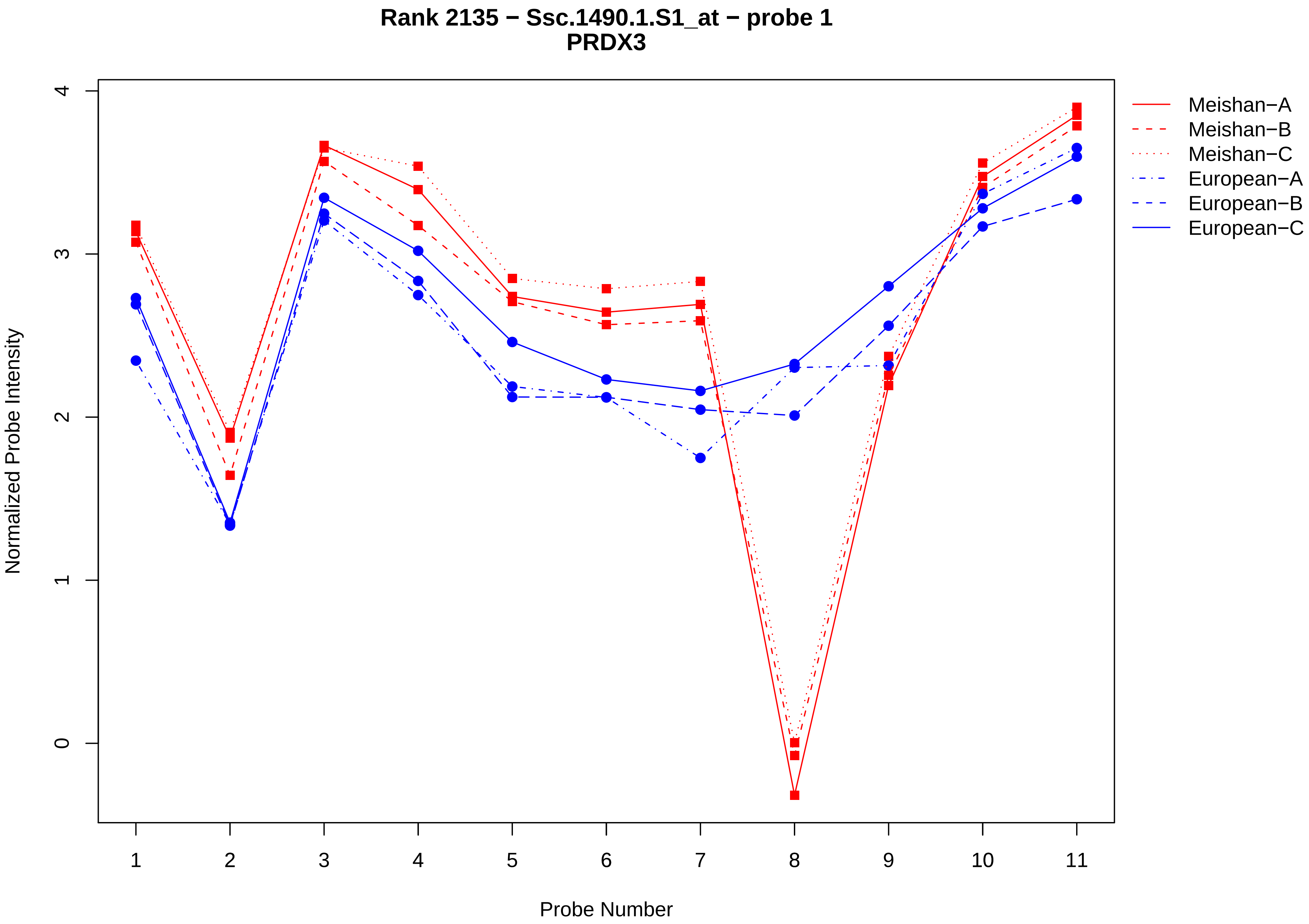

Rank 2135 − Ssc.1490.1.S1_at − probe 1
PRDX3
Normalized Probe Intensity
Probe Number
0
1
2
3
4
1
2
3
4
5
6
7
8
9
10
11
Meishan−A
Meishan−B
Meishan−C
European−A
European−B
European−C

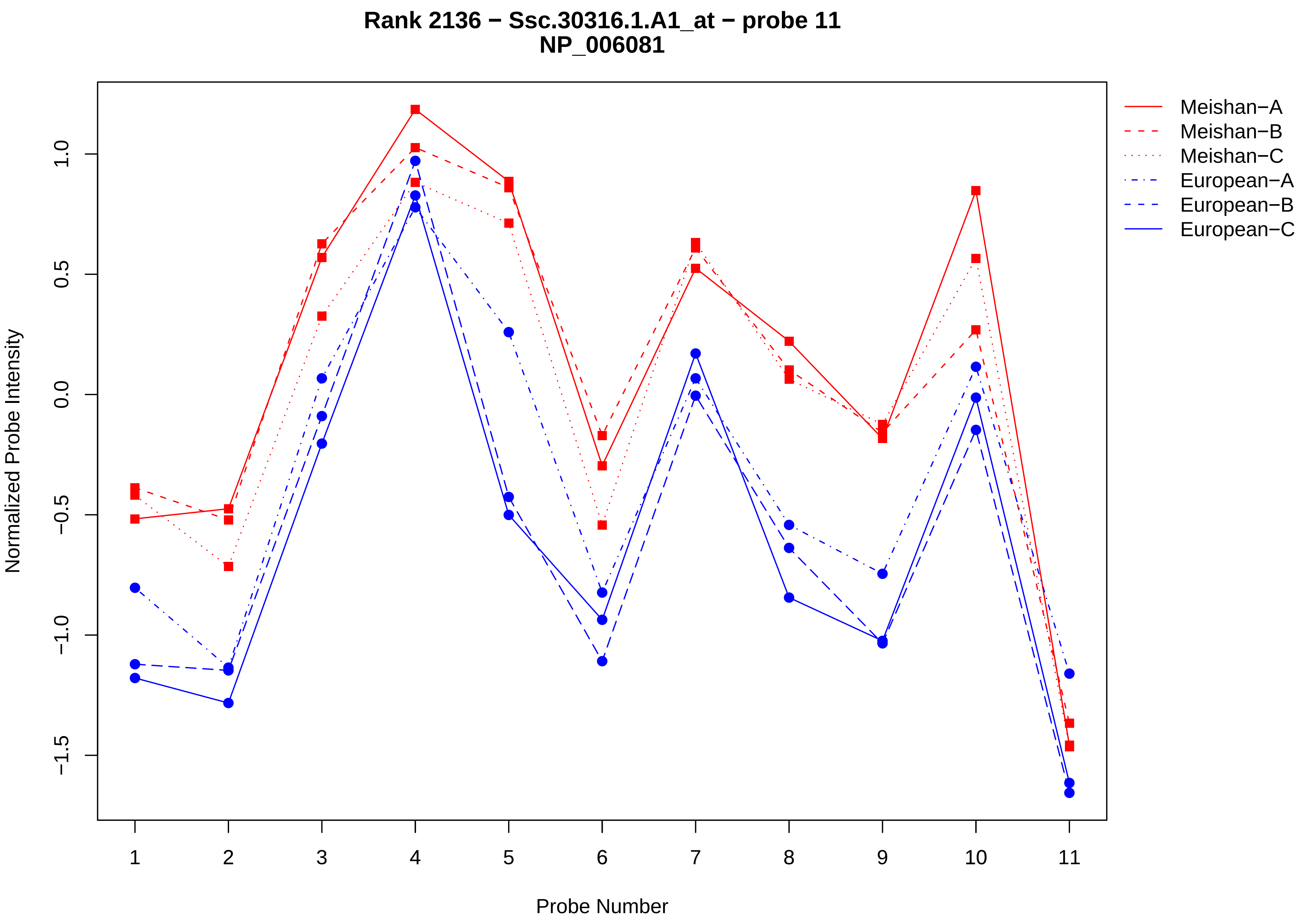

Rank 2136 − Ssc.30316.1.A1_at − probe 11
NP_006081
Normalized Probe Intensity
Probe Number
Meishan−A
Meishan−B
Meishan−C
European−A
European−B
European−C

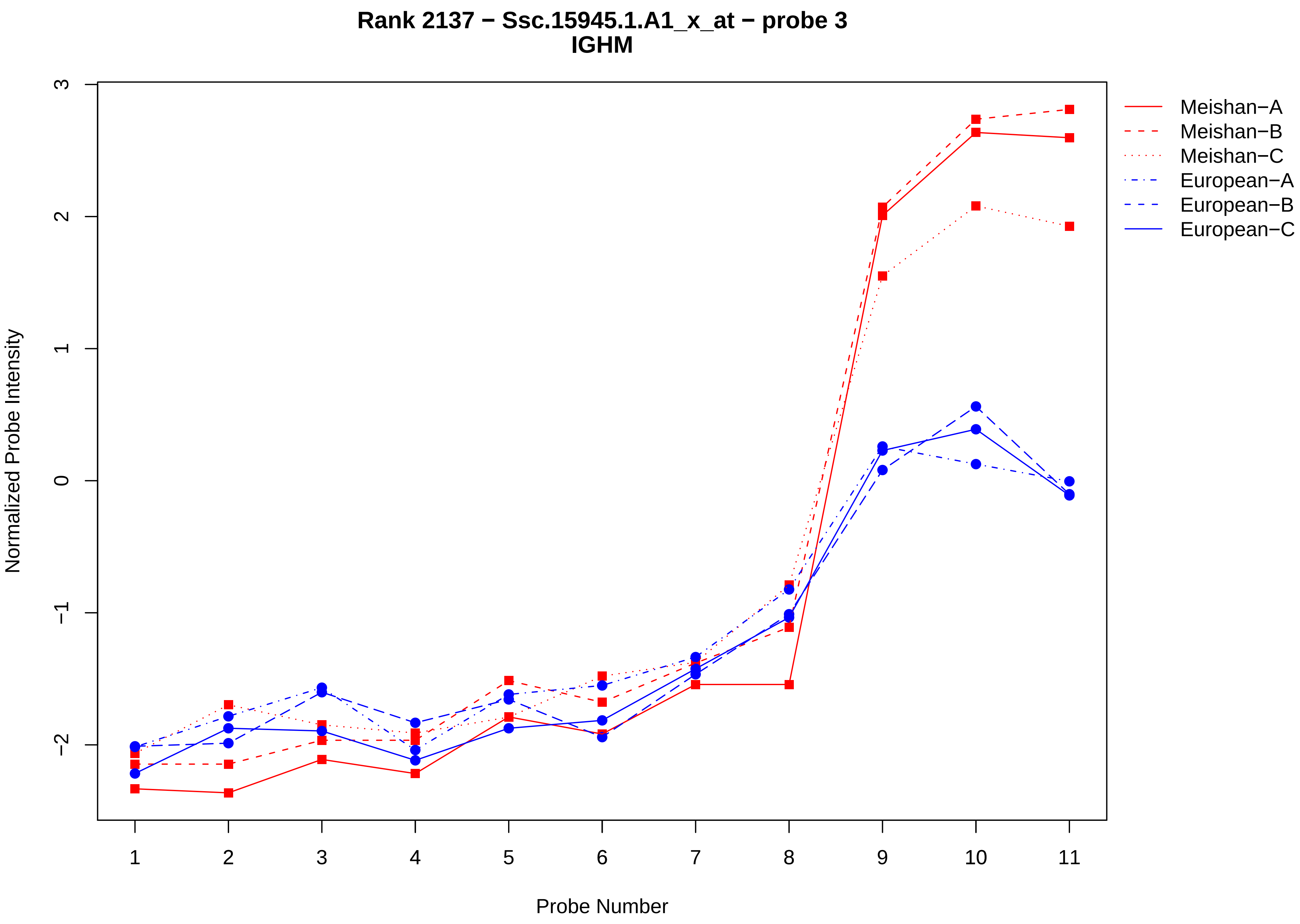

Rank 2137 − Ssc.15945.1.A1_x_at − probe 3
IGHM
Normalized Probe Intensity
Probe Number
Meishan−A
Meishan−B
Meishan−C
European−A
European−B
European−C

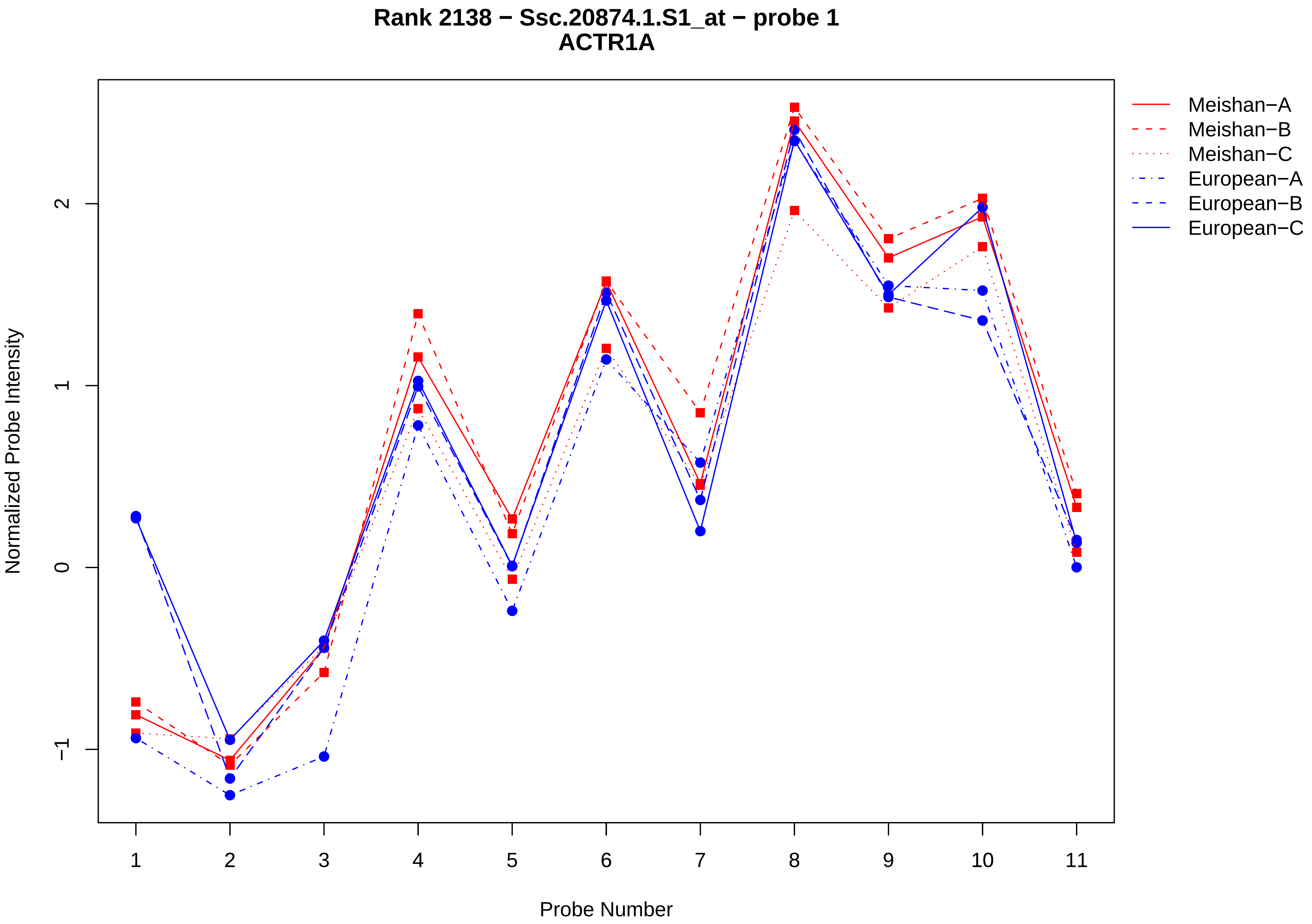

Rank 2138 − Ssc.20874.1.S1_at − probe 1
ACTR1A
Normalized Probe Intensity
Probe Number
−1
0
1
2
1
2
3
4
5
6
7
8
9
10
11
Meishan−A
Meishan−B
Meishan−C
European−A
European−B
European−C

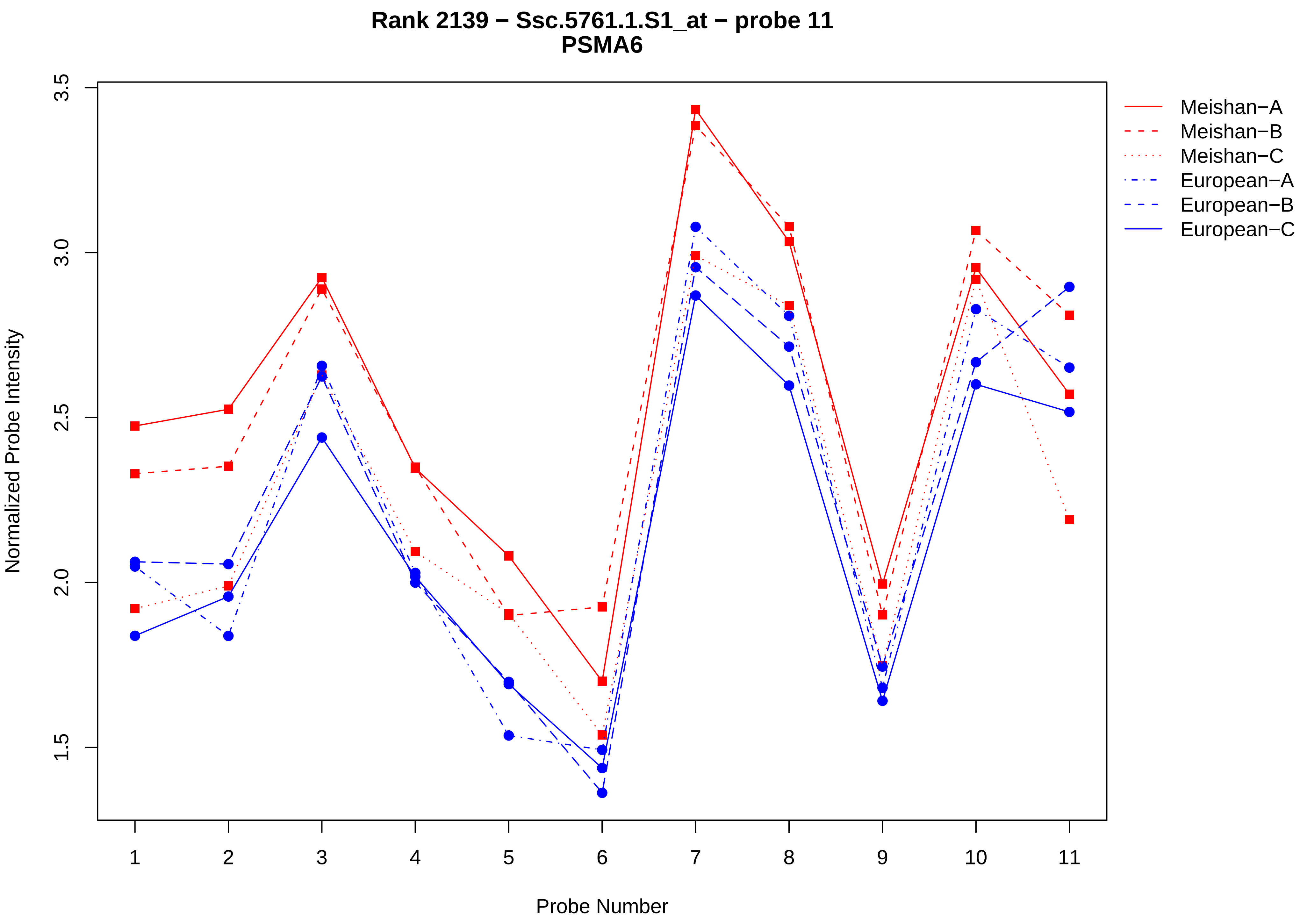

Rank 2139 − Ssc.5761.1.S1_at − probe 11
PSMA6
Normalized Probe Intensity
Probe Number
1.5
2.0
2.5
3.0
3.5
1
2
3
4
5
6
7
8
9
10
11
Meishan−A
Meishan−B
Meishan−C
European−A
European−B
European−C

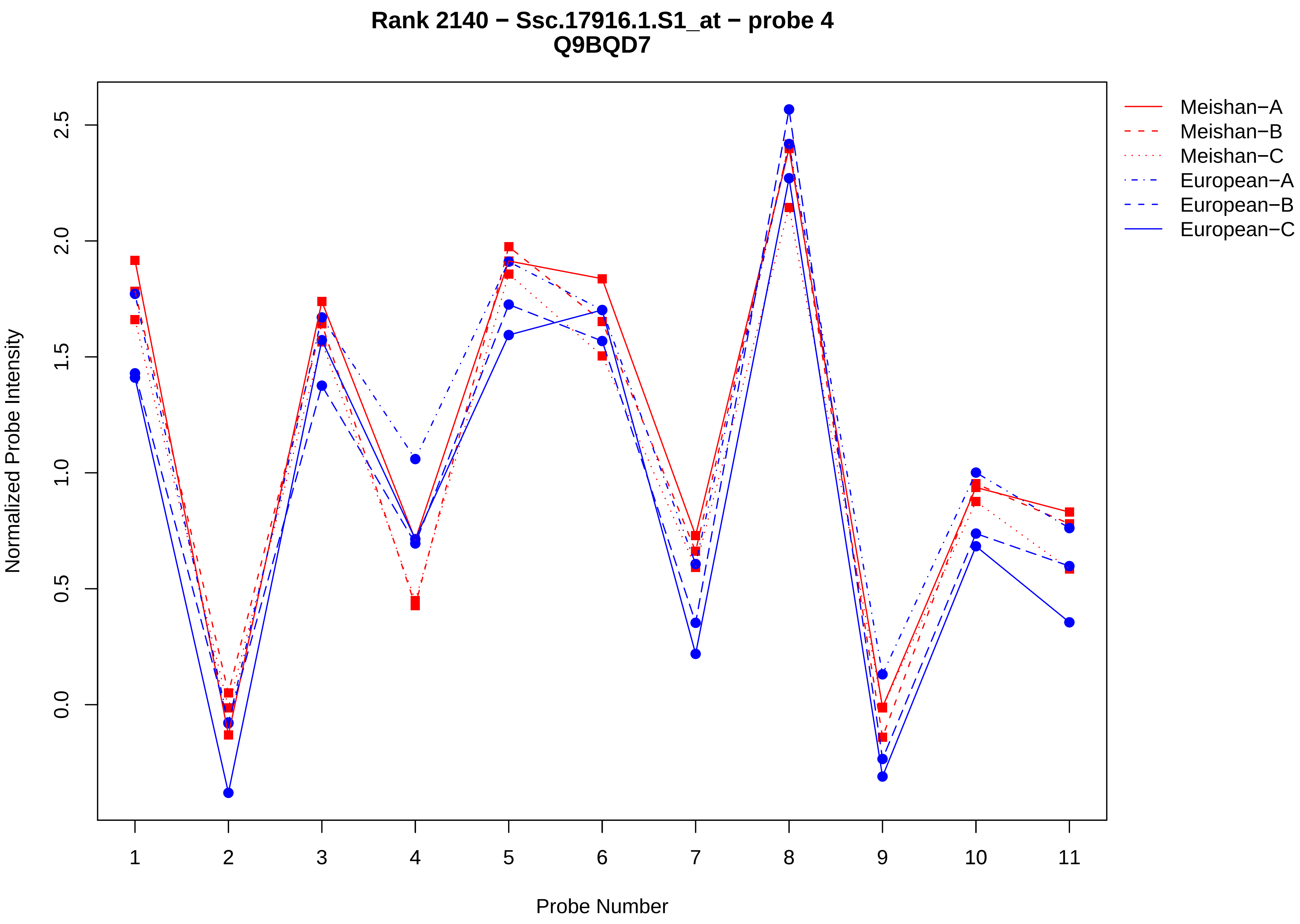

Rank 2140 − Ssc.17916.1.S1_at − probe 4
Q9BQD7
Normalized Probe Intensity
0.0
0.5
1.0
1.5
2.0
2.5
1
2
3
4
5
6
7
8
9
10
11
Probe Number
Meishan−A
Meishan−B
Meishan−C
European−A
European−B
European−C

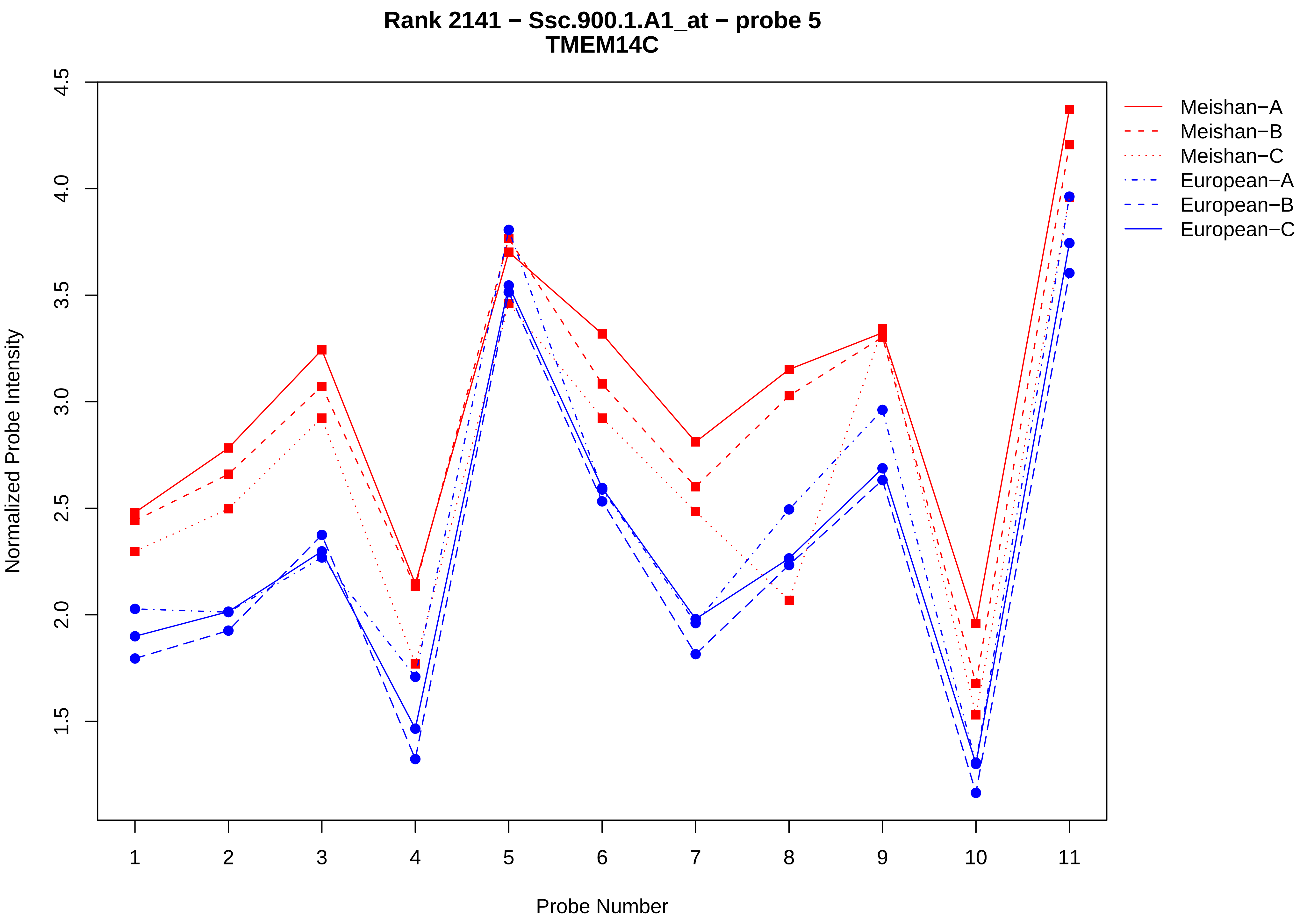
Rank 2141 − Ssc.900.1.A1_at − probe 5
TMEM14C
Normalized Probe Intensity
Probe Number
1.5
2.0
2.5
3.0
3.5
4.0
4.5
1
2
3
4
5
6
7
8
9
10
11
Meishan−A
Meishan−B
Meishan−C
European−A
European−B
European−C

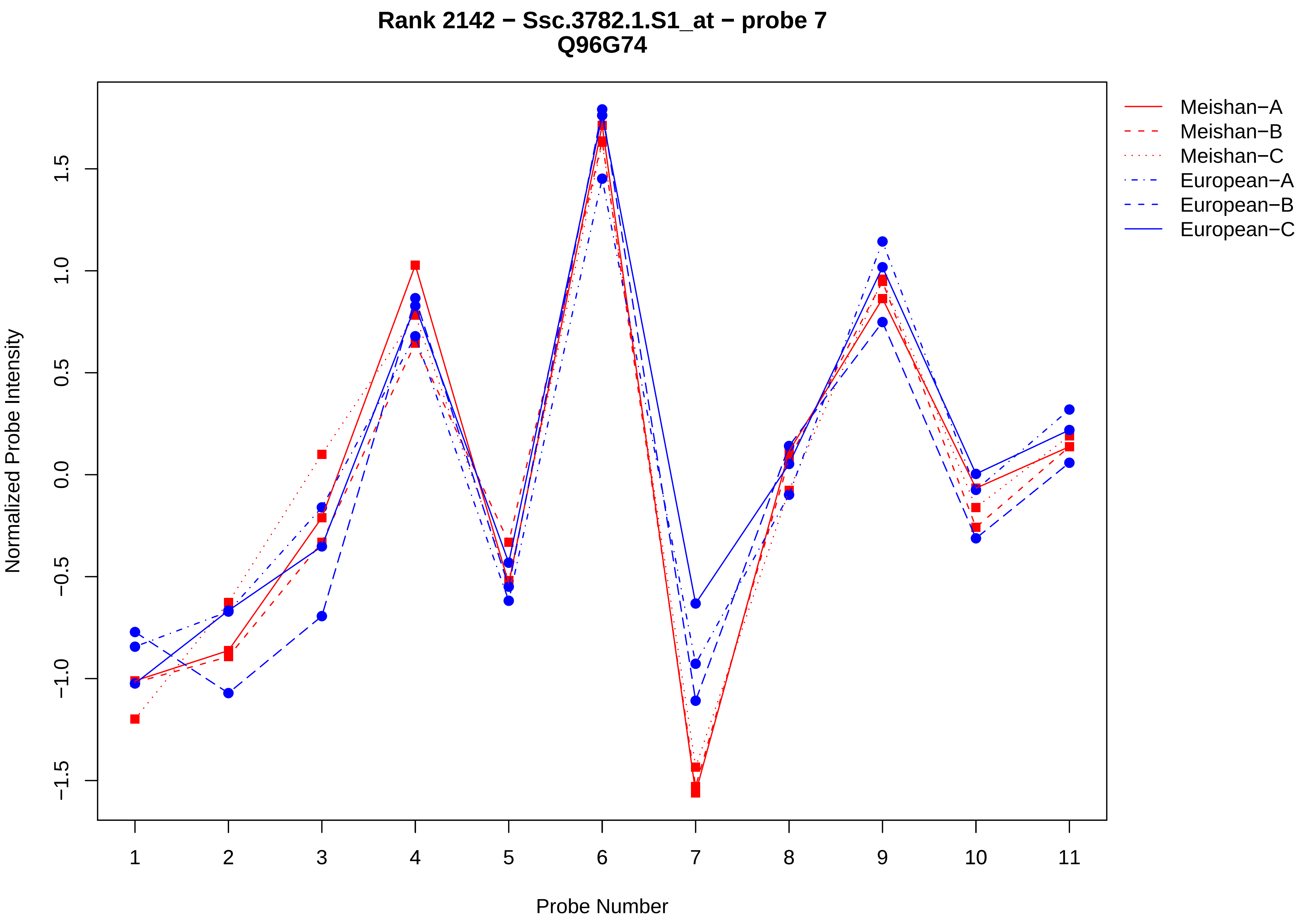

Rank 2142 − Ssc.3782.1.S1_at − probe 7
Q96G74
Normalized Probe Intensity
Probe Number
−1.5
−1.0
−0.5
0.0
0.5
1.0
1.5
1
2
3
4
5
6
7
8
9
10
11
Meishan−A
Meishan−B
Meishan−C
European−A
European−B
European−C

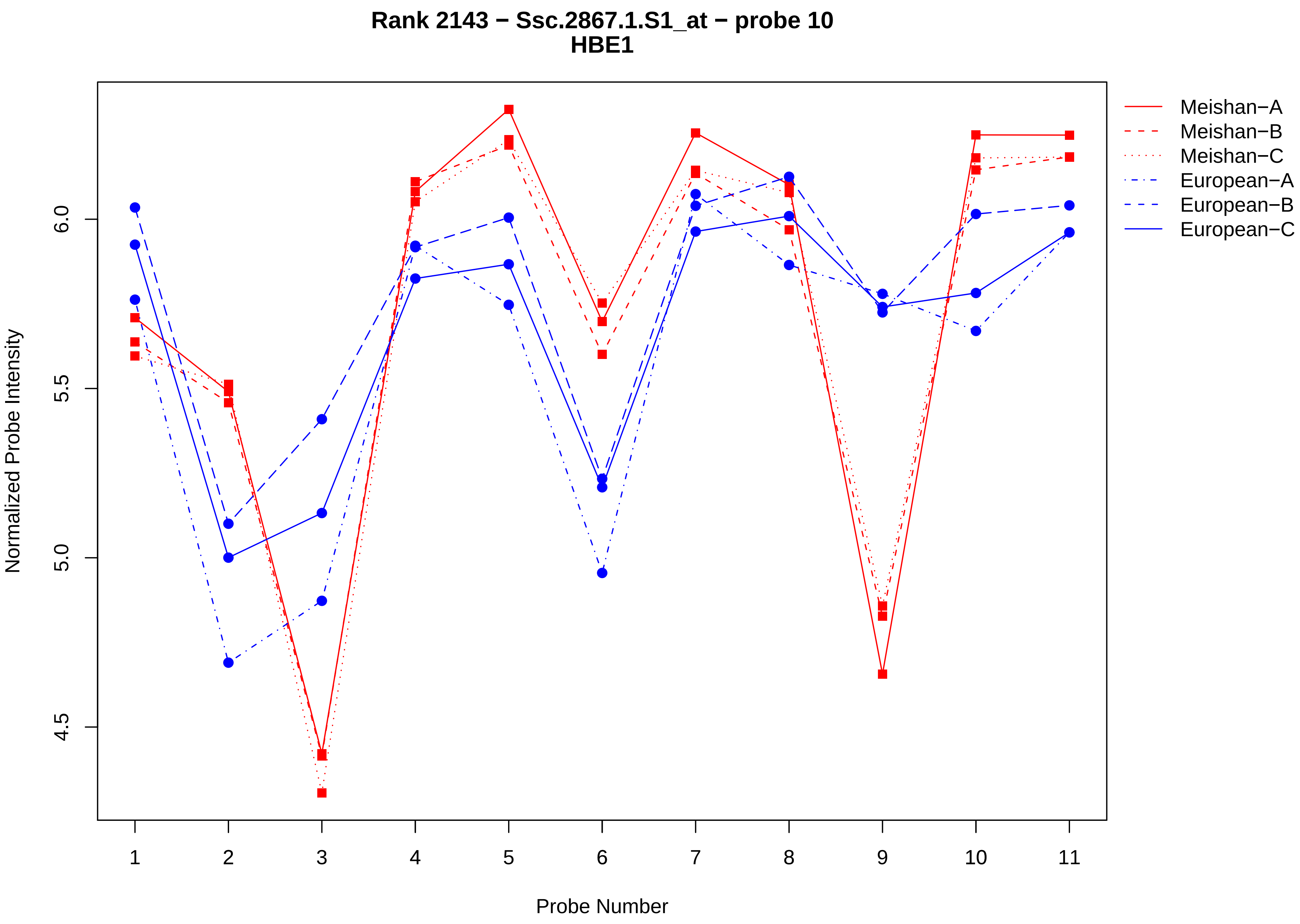
Rank 2143 − Ssc.2867.1.S1_at − probe 10
HBE1
Normalized Probe Intensity
4.5
5.0
5.5
6.0
1
2
3
4
5
6
7
8
9
10
11
Probe Number
Meishan−A
Meishan−B
Meishan−C
European−A
European−B
European−C

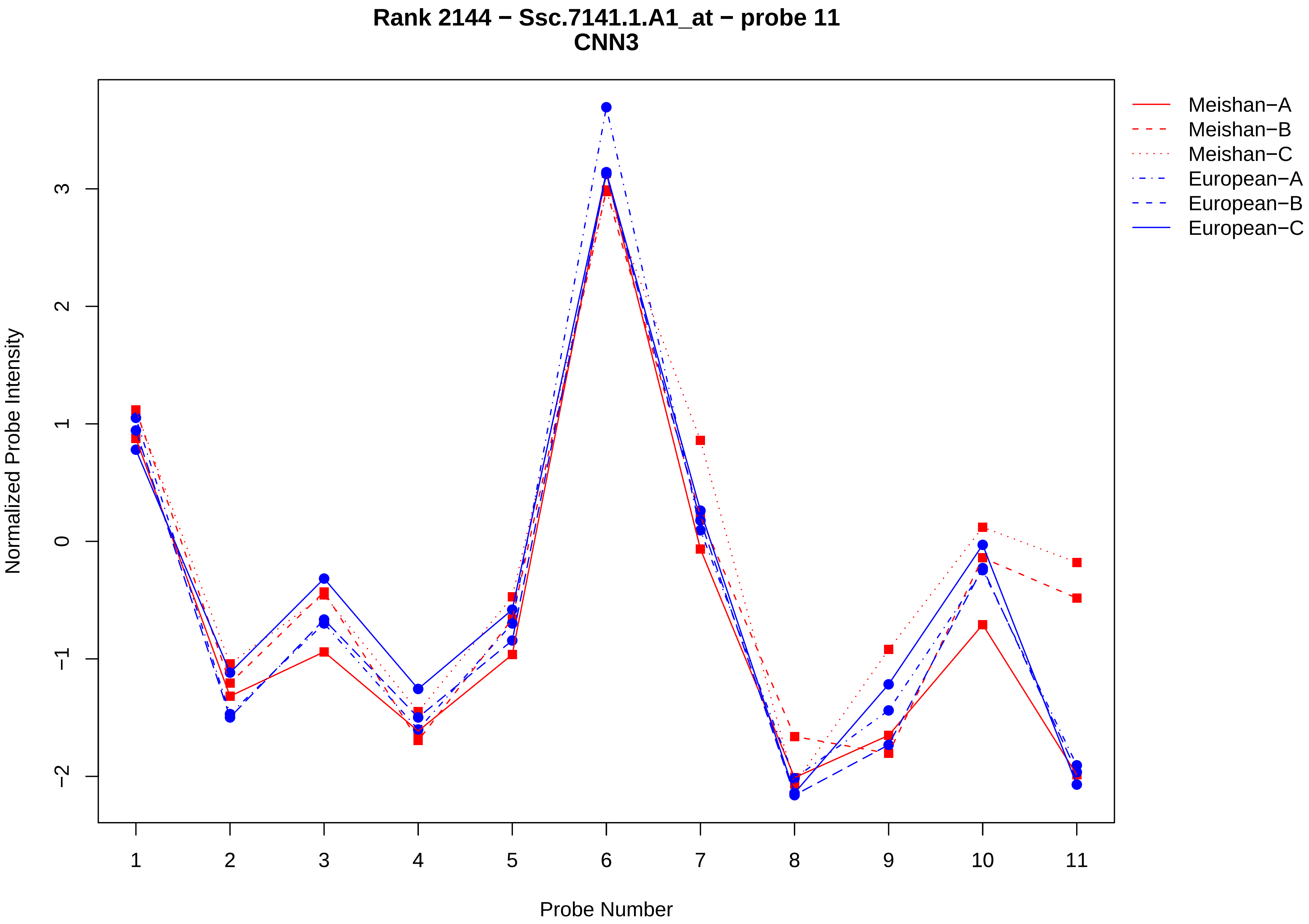

Rank 2144 − Ssc.7141.1.A1_at − probe 11
CNN3
Normalized Probe Intensity
Probe Number
Meishan−A
Meishan−B
Meishan−C
European−A
European−B
European−C

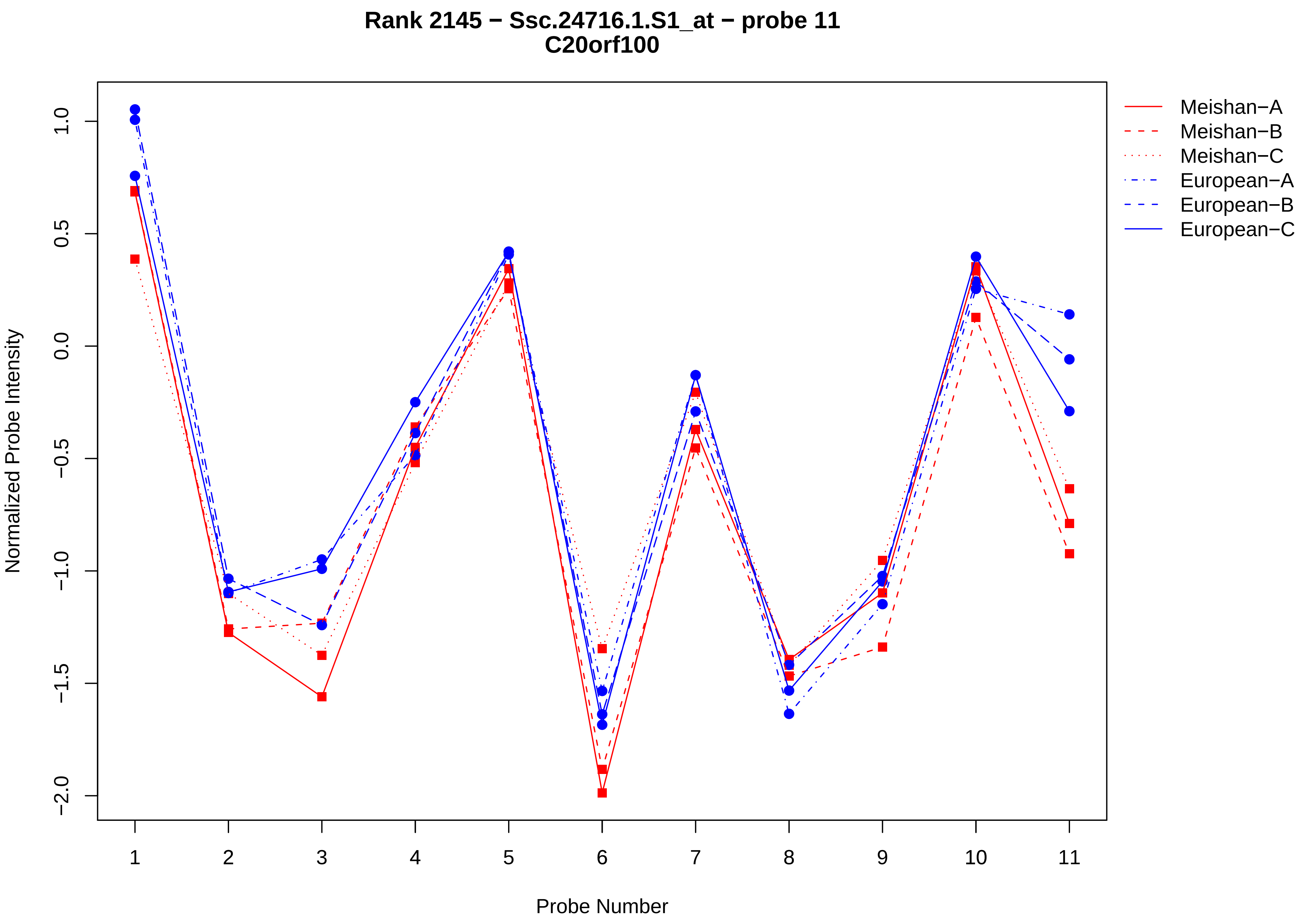

Rank 2145 − Ssc.24716.1.S1_at − probe 11
C20orf100
Normalized Probe Intensity
Probe Number
Meishan−A
Meishan−B
Meishan−C
European−A
European−B
European−C
1.0
0.5
0.0
−0.5
−1.0
−1.5
−2.0
1
2
3
4
5
6
7
8
9
10
11

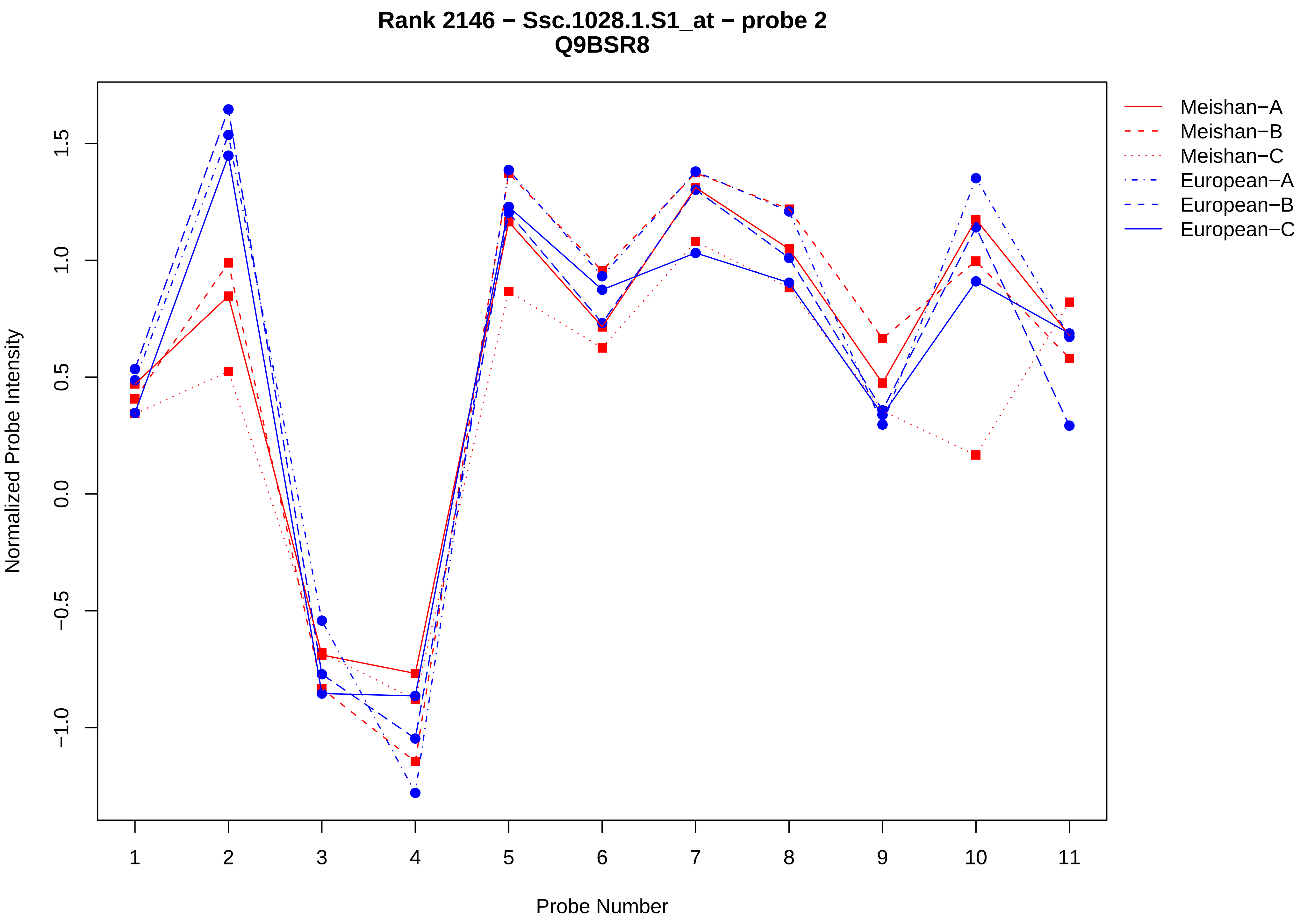
Rank 2146 − Ssc.1028.1.S1_at − probe 2
Q9BSR8
Normalized Probe Intensity
Probe Number
−1.0
−0.5
0.0
0.5
1.0
1.5
1
2
3
4
5
6
7
8
9
10
11
Meishan−A
Meishan−B
Meishan−C
European−A
European−B
European−C

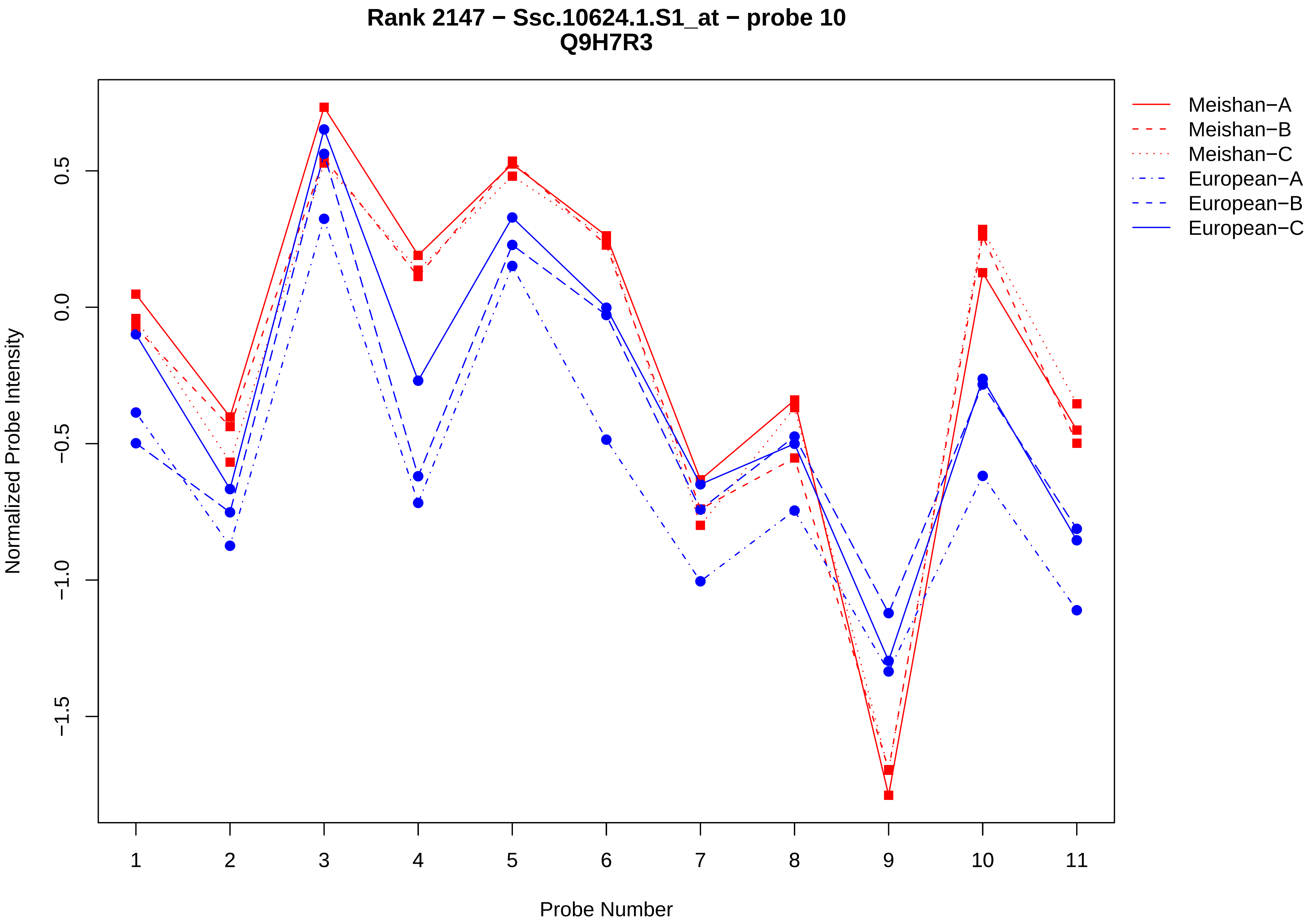

Rank 2147 − Ssc.10624.1.S1_at − probe 10
Q9H7R3
Normalized Probe Intensity
Probe Number
0.5
0.0
−0.5
−1.0
−1.5
1
2
3
4
5
6
7
8
9
10
11
Meishan−A
Meishan−B
Meishan−C
European−A
European−B
European−C

Rank 2148 − Ssc.28425.1.A1_at − probe 1
CHES1

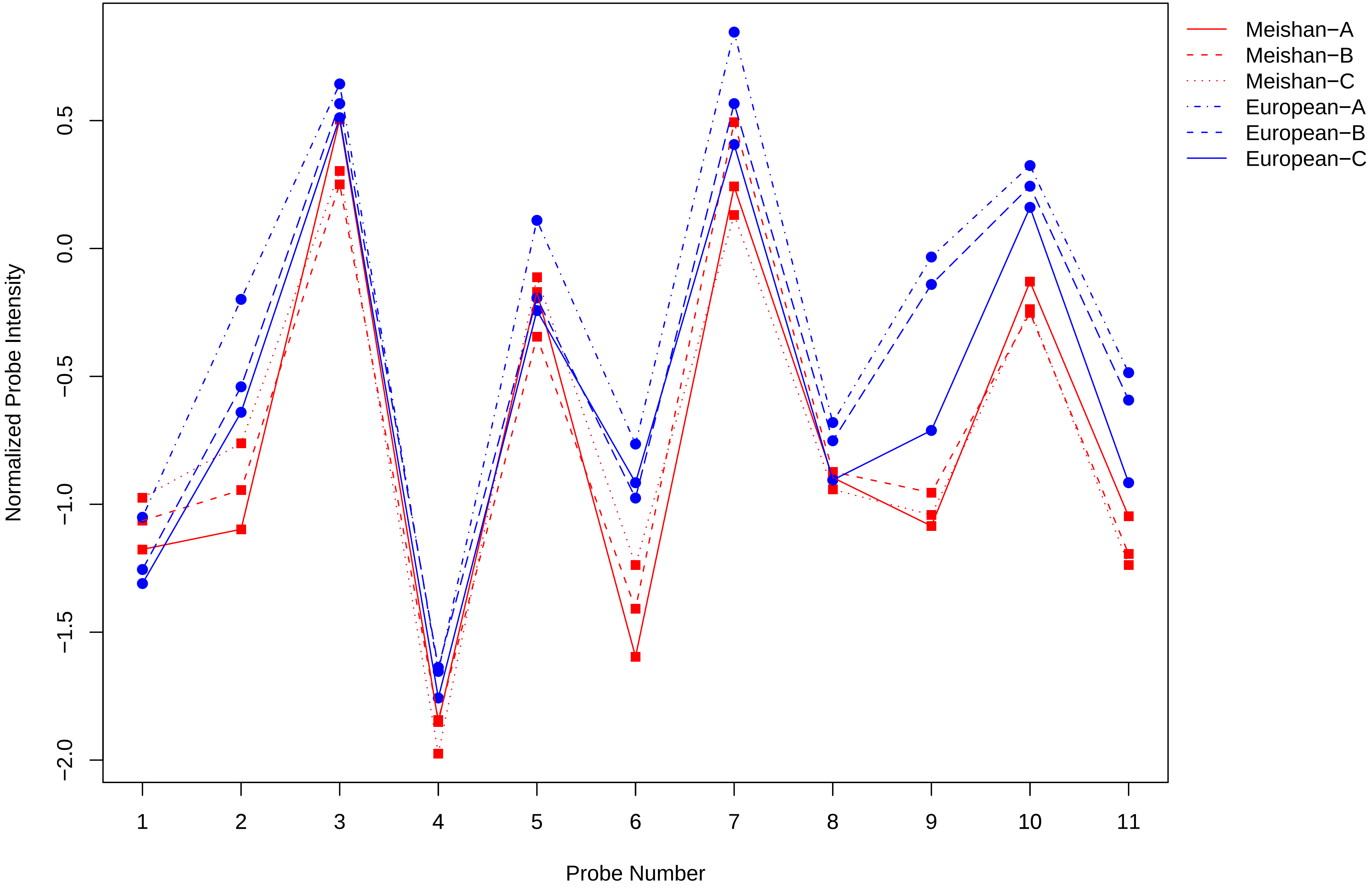

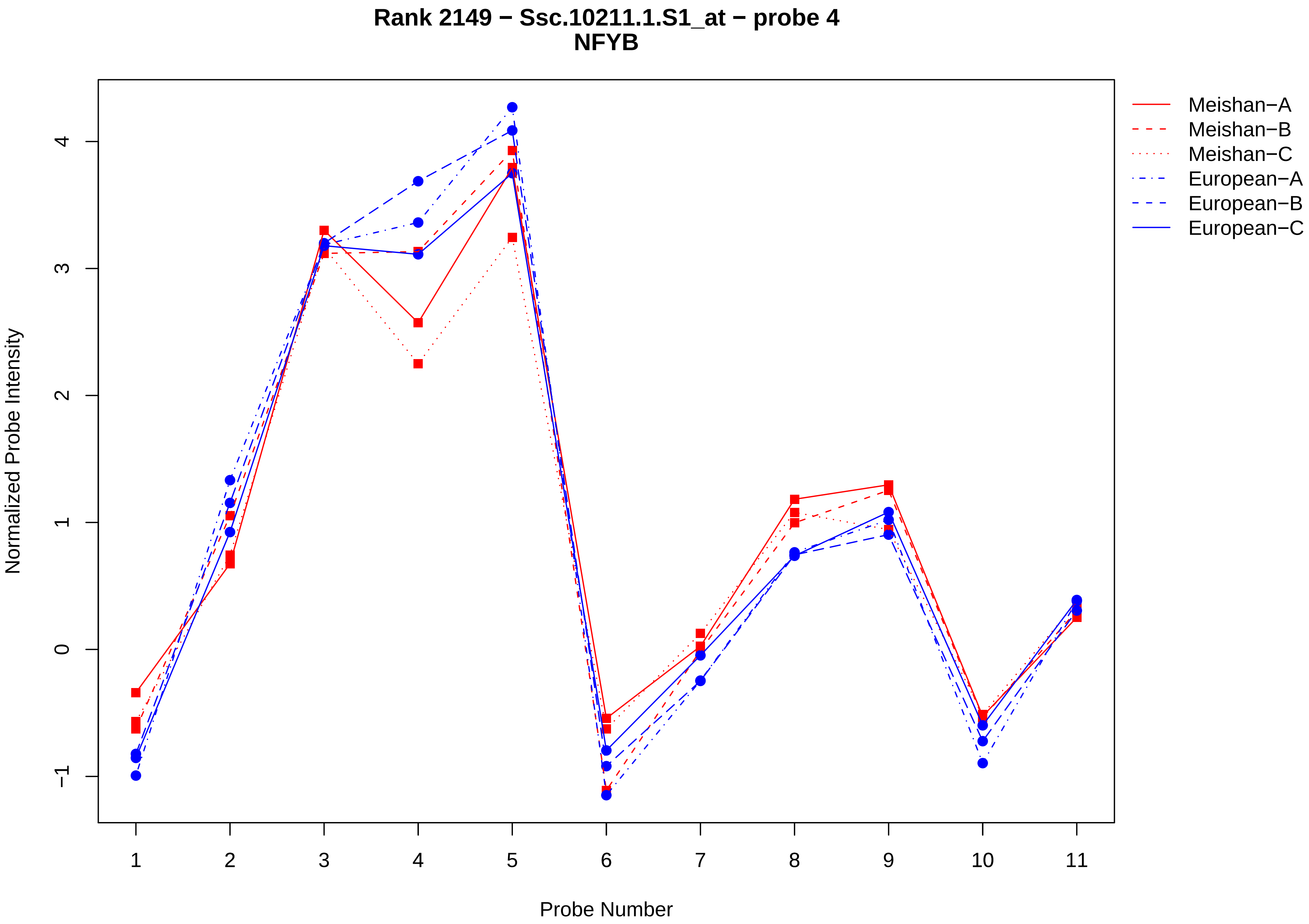
Rank 2149 − Ssc.10211.1.S1_at − probe 4
NFYB
Normalized Probe Intensity
Probe Number
−1
0
1
2
3
4
1
2
3
4
5
6
7
8
9
10
11
Meishan−A
Meishan−B
Meishan−C
European−A
European−B
European−C

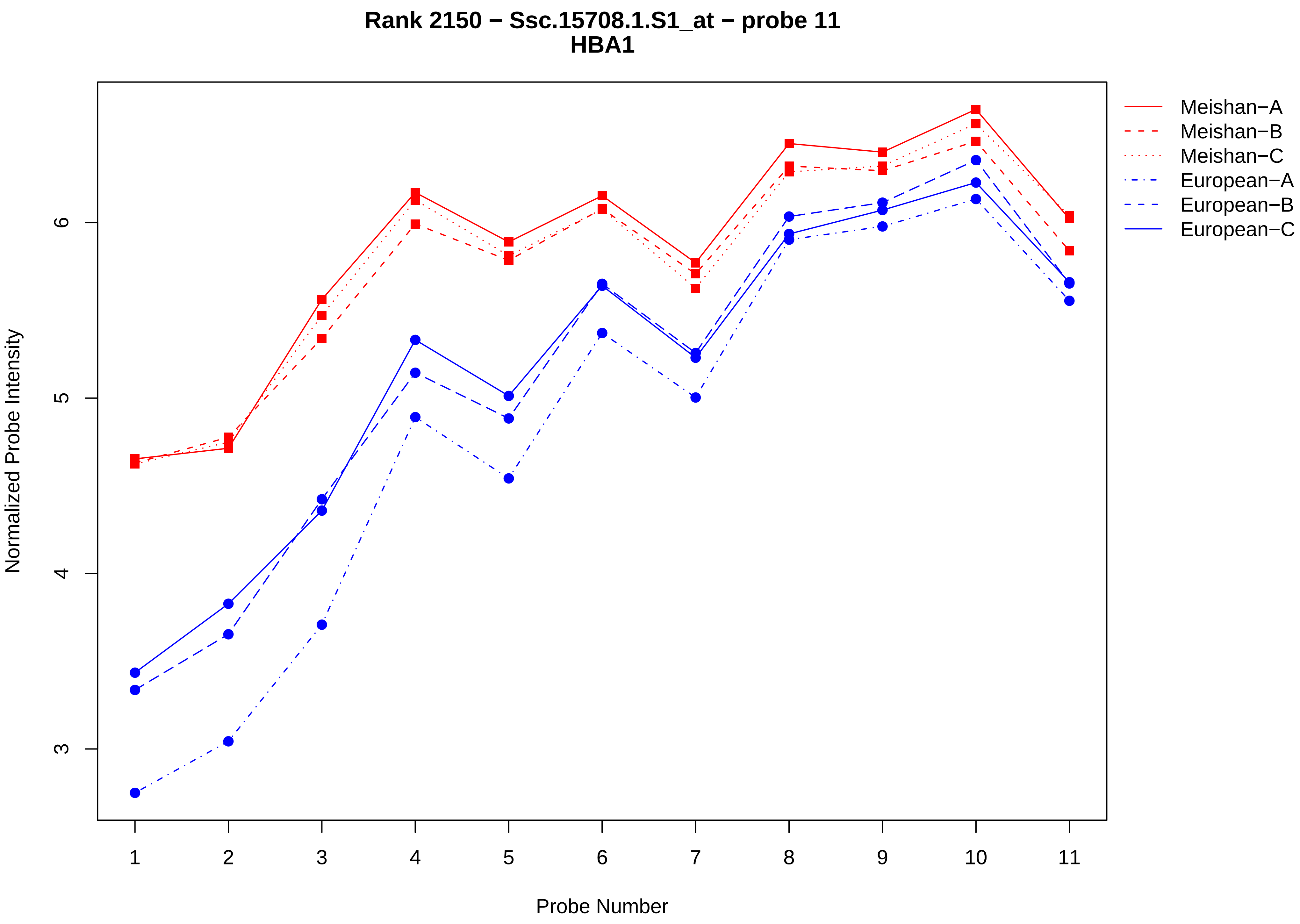

Rank 2150 − Ssc.15708.1.S1_at − probe 11
HBA1
Normalized Probe Intensity
Probe Number
3
4
5
6
1
2
3
4
5
6
7
8
9
10
11
Meishan−A
Meishan−B
Meishan−C
European−A
European−B
European−C

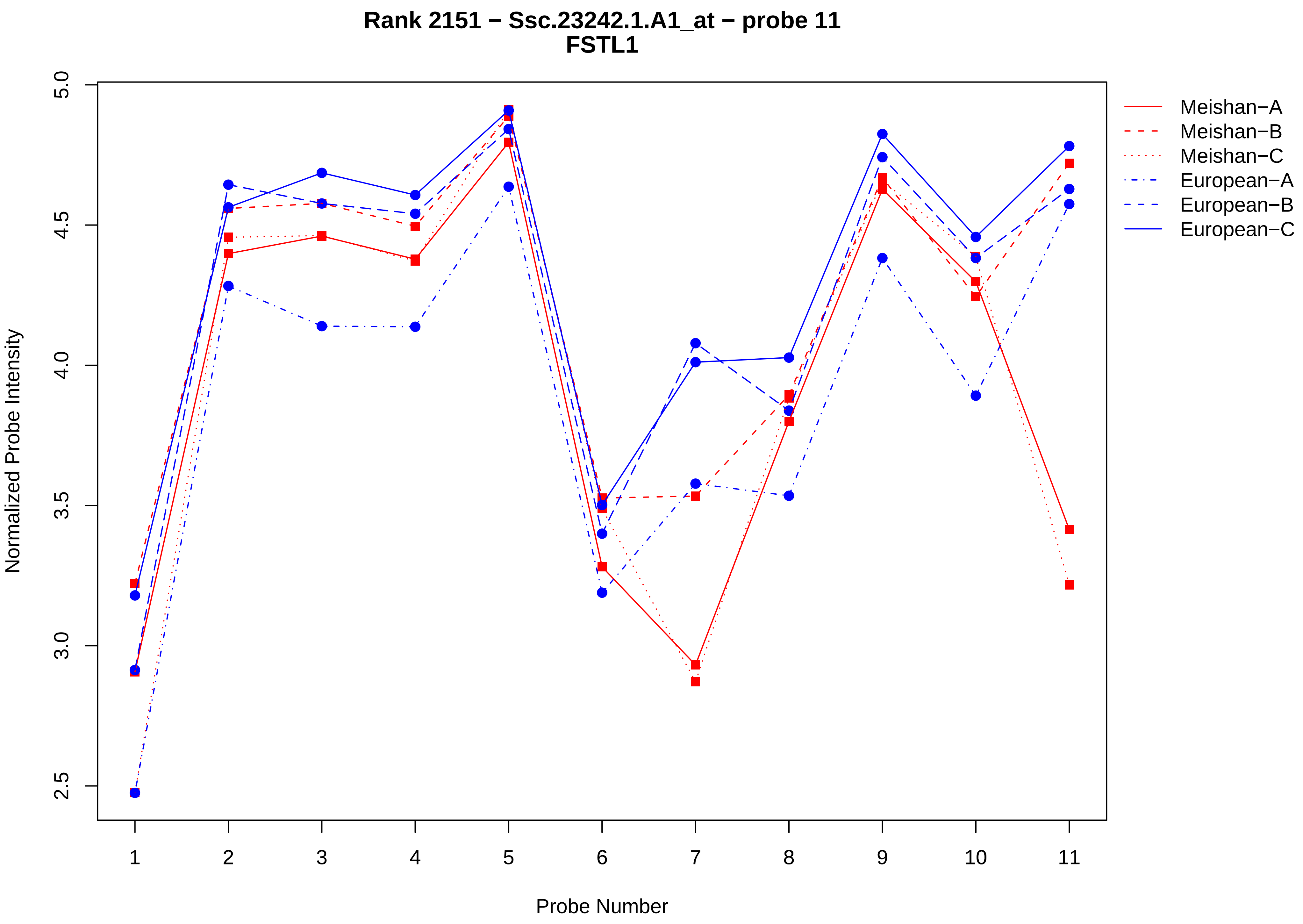

Rank 2151 − Ssc.23242.1.A1_at − probe 11
FSTL1
Normalized Probe Intensity
Probe Number
2.5
3.0
3.5
4.0
4.5
5.0
1
2
3
4
5
6
7
8
9
10
11
Meishan−A
Meishan−B
Meishan−C
European−A
European−B
European−C

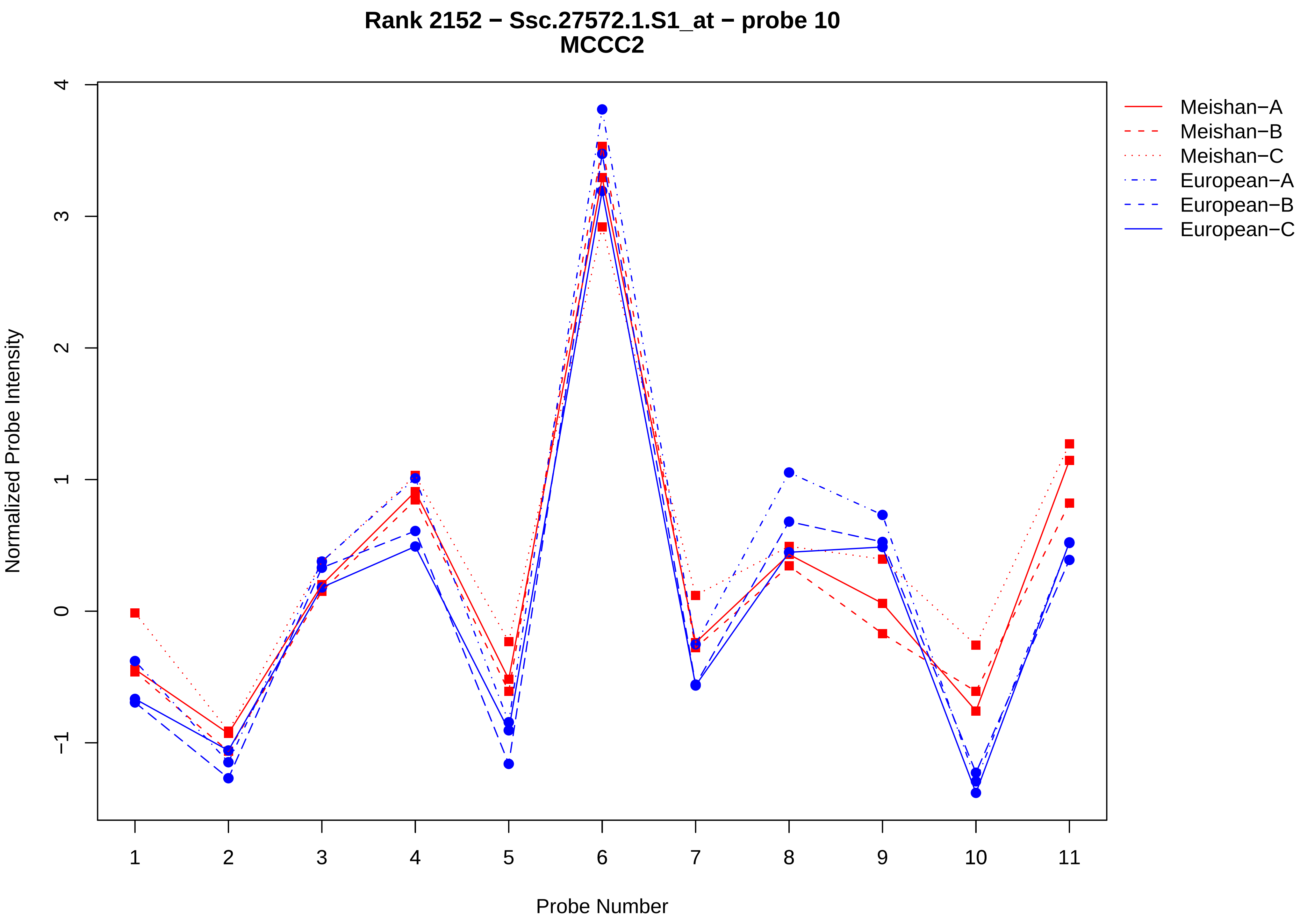

Rank 2152 − Ssc.27572.1.S1_at − probe 10
MCCC2
Normalized Probe Intensity
Probe Number
Meishan−A
Meishan−B
Meishan−C
European−A
European−B
European−C

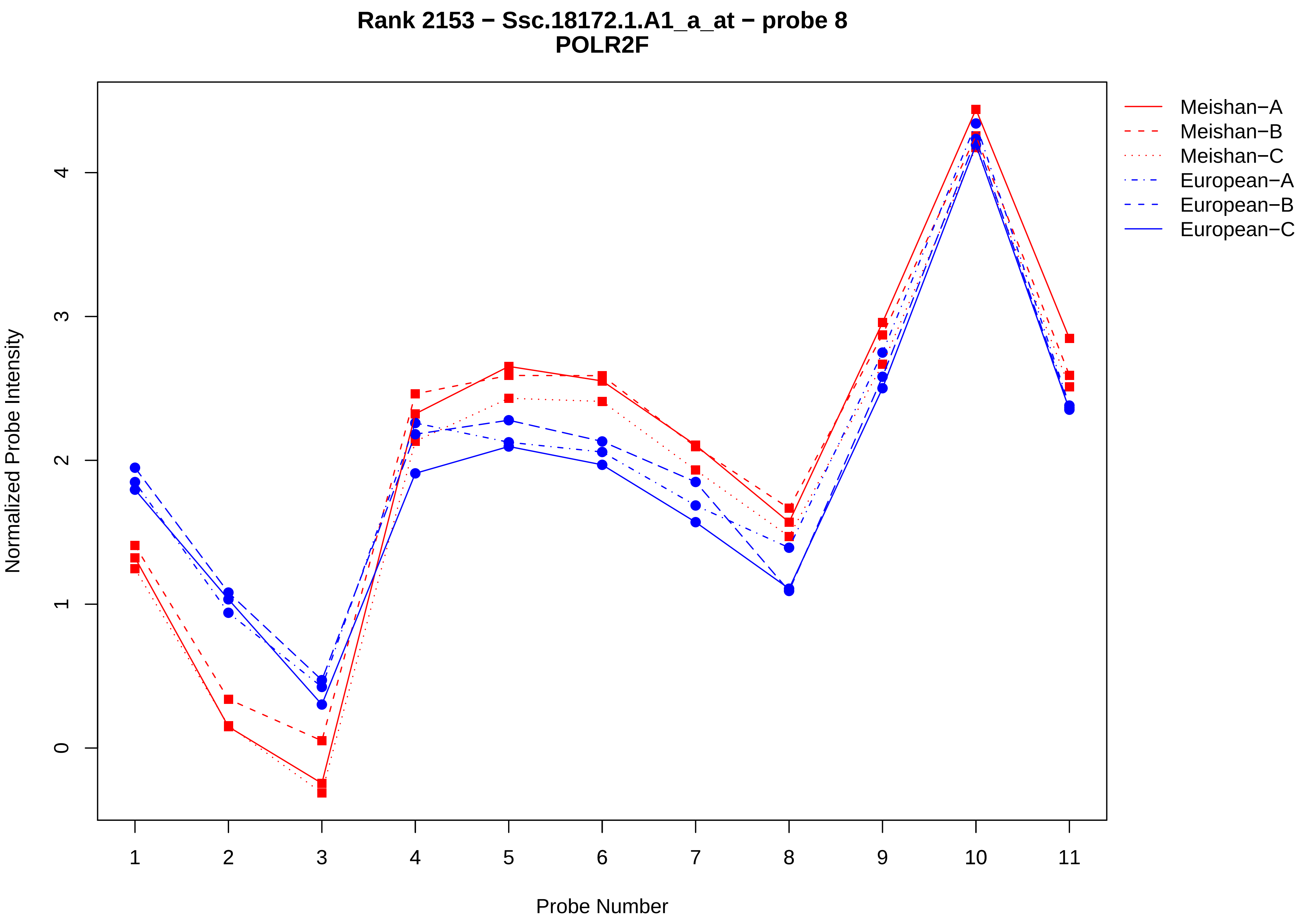

Rank 2153 − Ssc.18172.1.A1_a_at − probe 8
POLR2F
Normalized Probe Intensity
0
1
2
3
4
1
2
3
4
5
6
7
8
9
10
11
Probe Number
Meishan−A
Meishan−B
Meishan−C
European−A
European−B
European−C

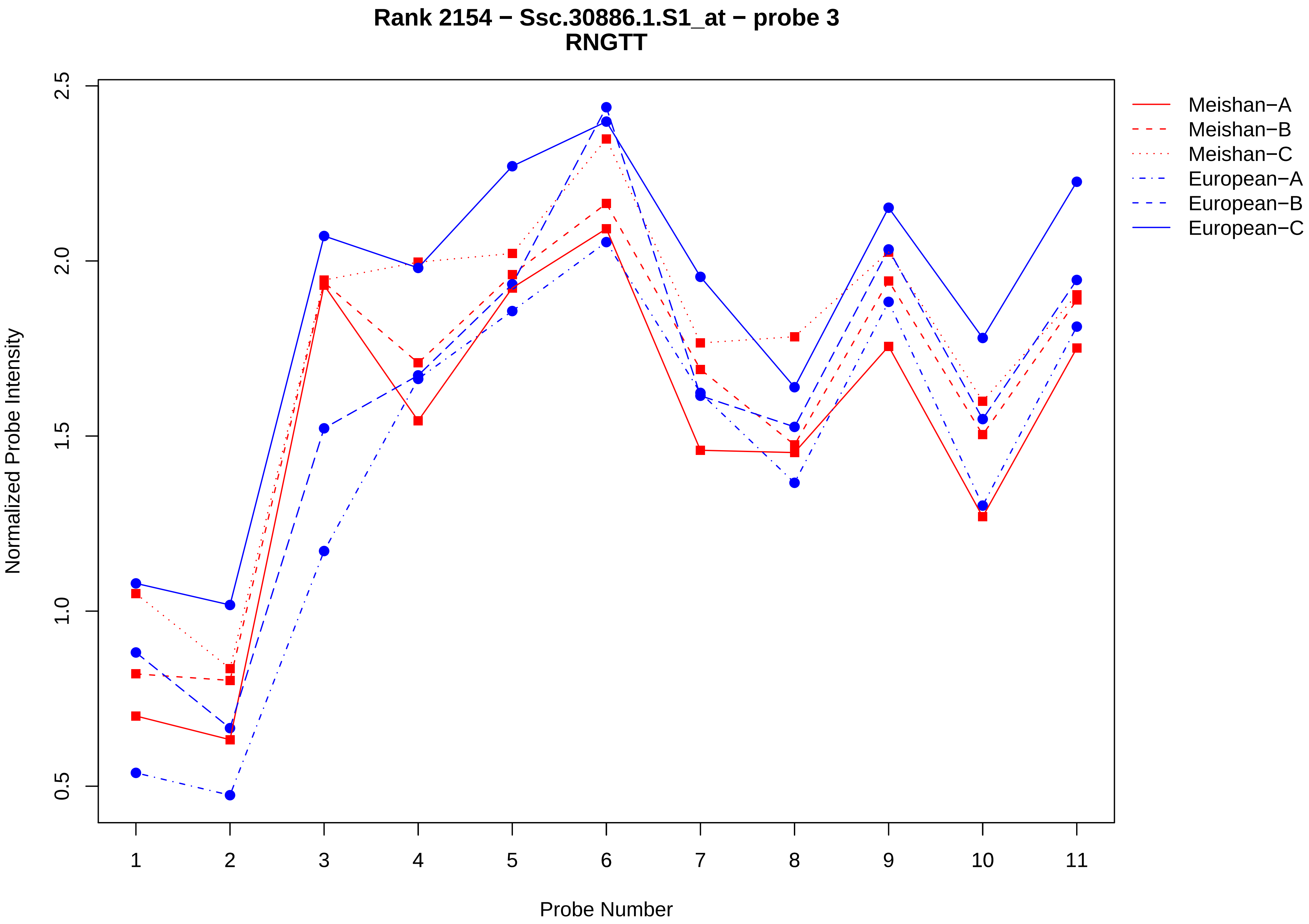

Rank 2154 − Ssc.30886.1.S1_at − probe 3
RNGTT
Normalized Probe Intensity
Probe Number
0.5
1.0
1.5
2.0
2.5
1
2
3
4
5
6
7
8
9
10
11
Meishan−A
Meishan−B
Meishan−C
European−A
European−B
European−C

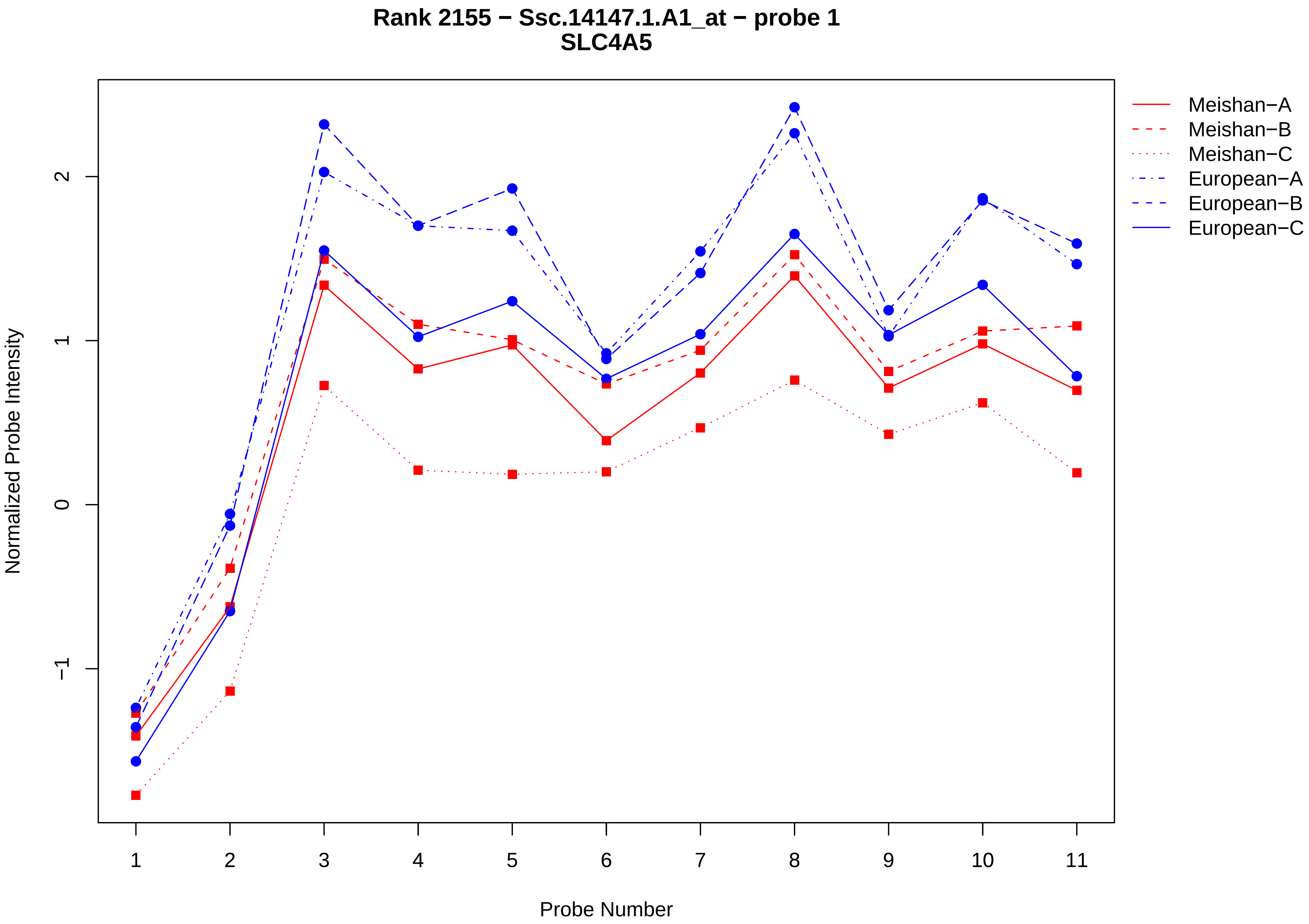

Rank 2155 − Ssc.14147.1.A1_at − probe 1
SLC4A5
Normalized Probe Intensity
Probe Number
−1
0
1
2
1
2
3
4
5
6
7
8
9
10
11
Meishan−A
Meishan−B
Meishan−C
European−A
European−B
European−C

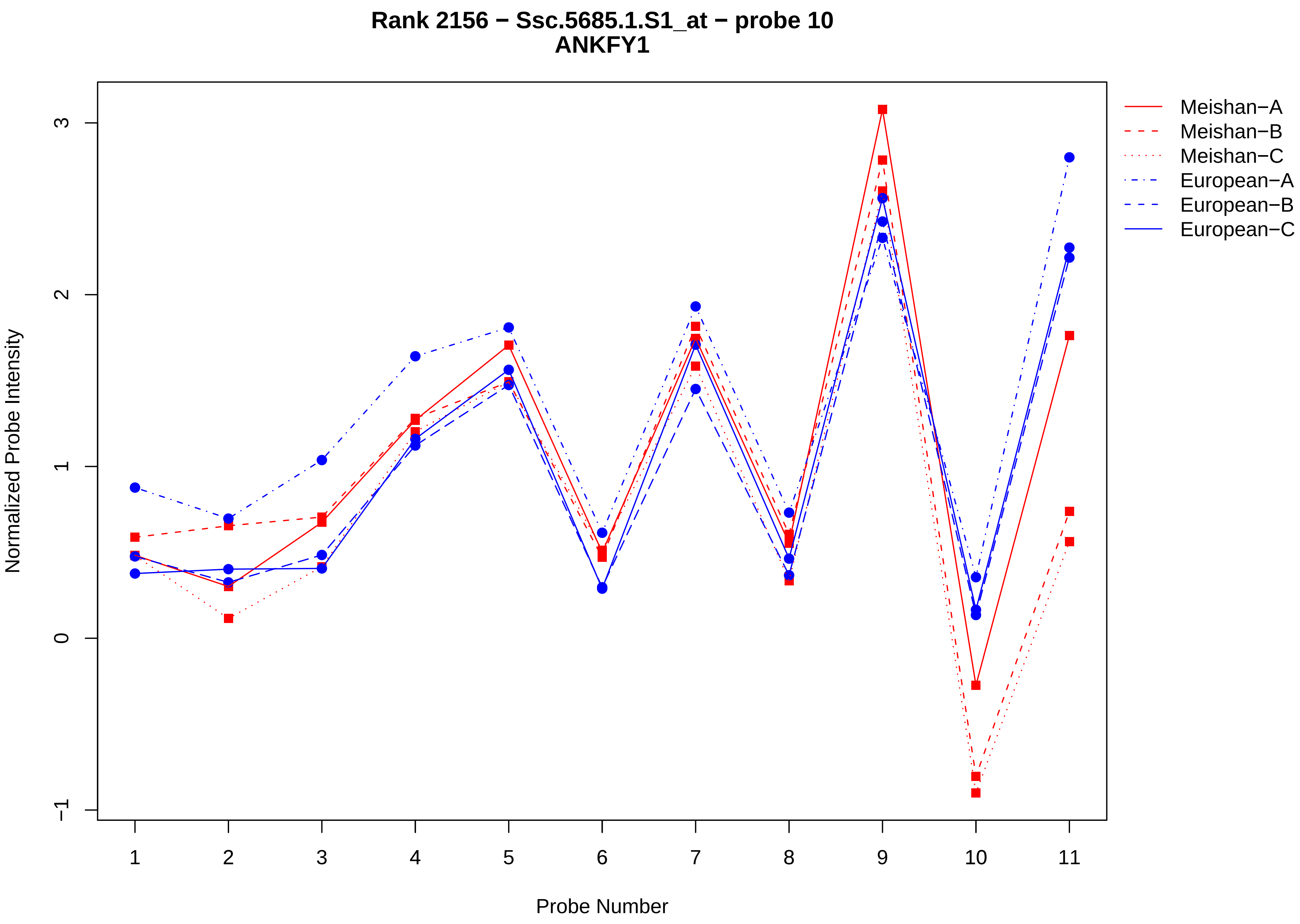

Rank 2156 − Ssc.5685.1.S1_at − probe 10
ANKFY1
Normalized Probe Intensity
Probe Number
−1
0
1
2
3
1
2
3
4
5
6
7
8
9
10
11
Meishan−A
Meishan−B
Meishan−C
European−A
European−B
European−C

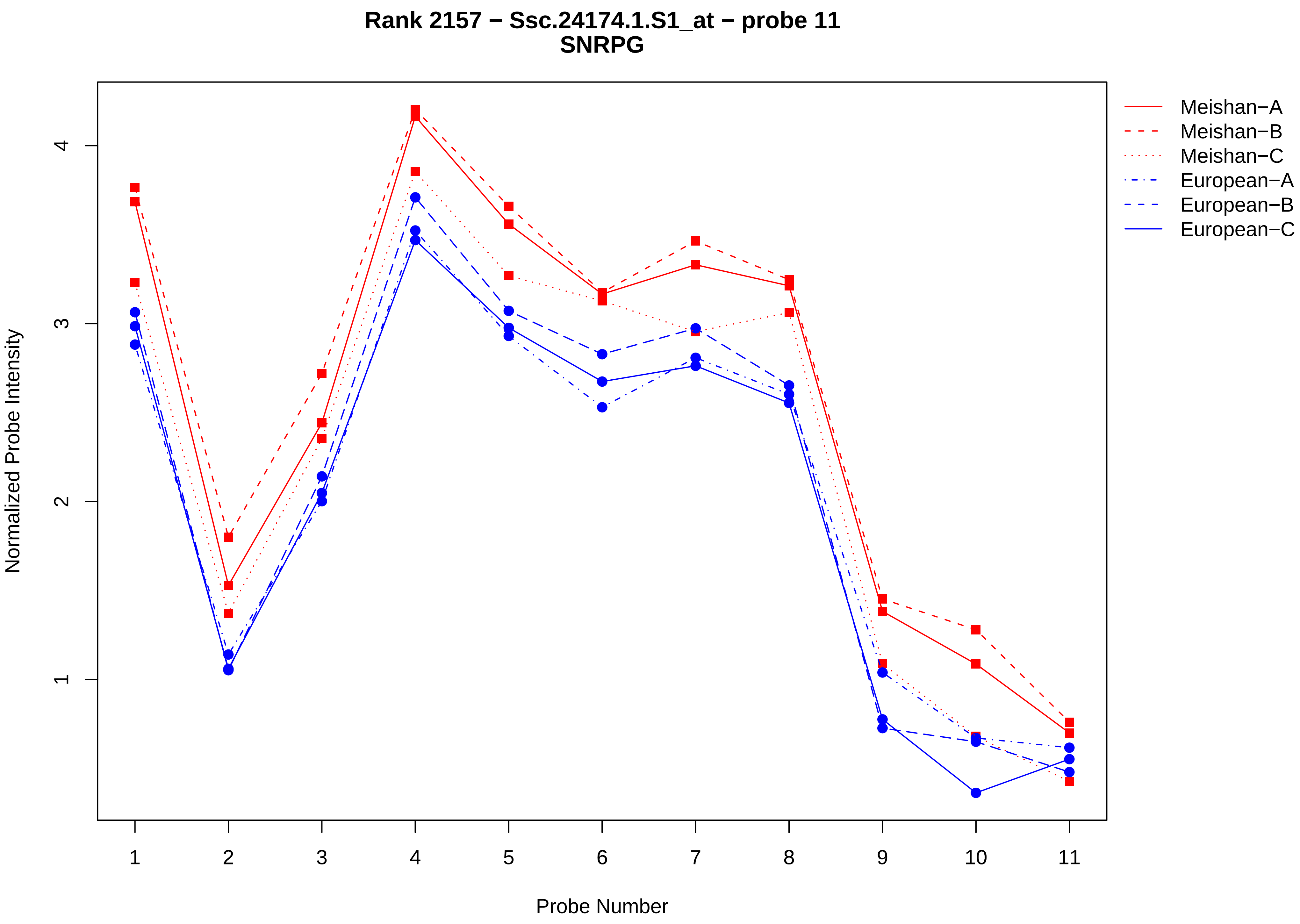

Rank 2157 − Ssc.24174.1.S1_at − probe 11
SNRPG
Normalized Probe Intensity
1
2
3
4
Probe Number
1
2
3
4
5
6
7
8
9
10
11
Meishan−A
Meishan−B
Meishan−C
European−A
European−B
European−C

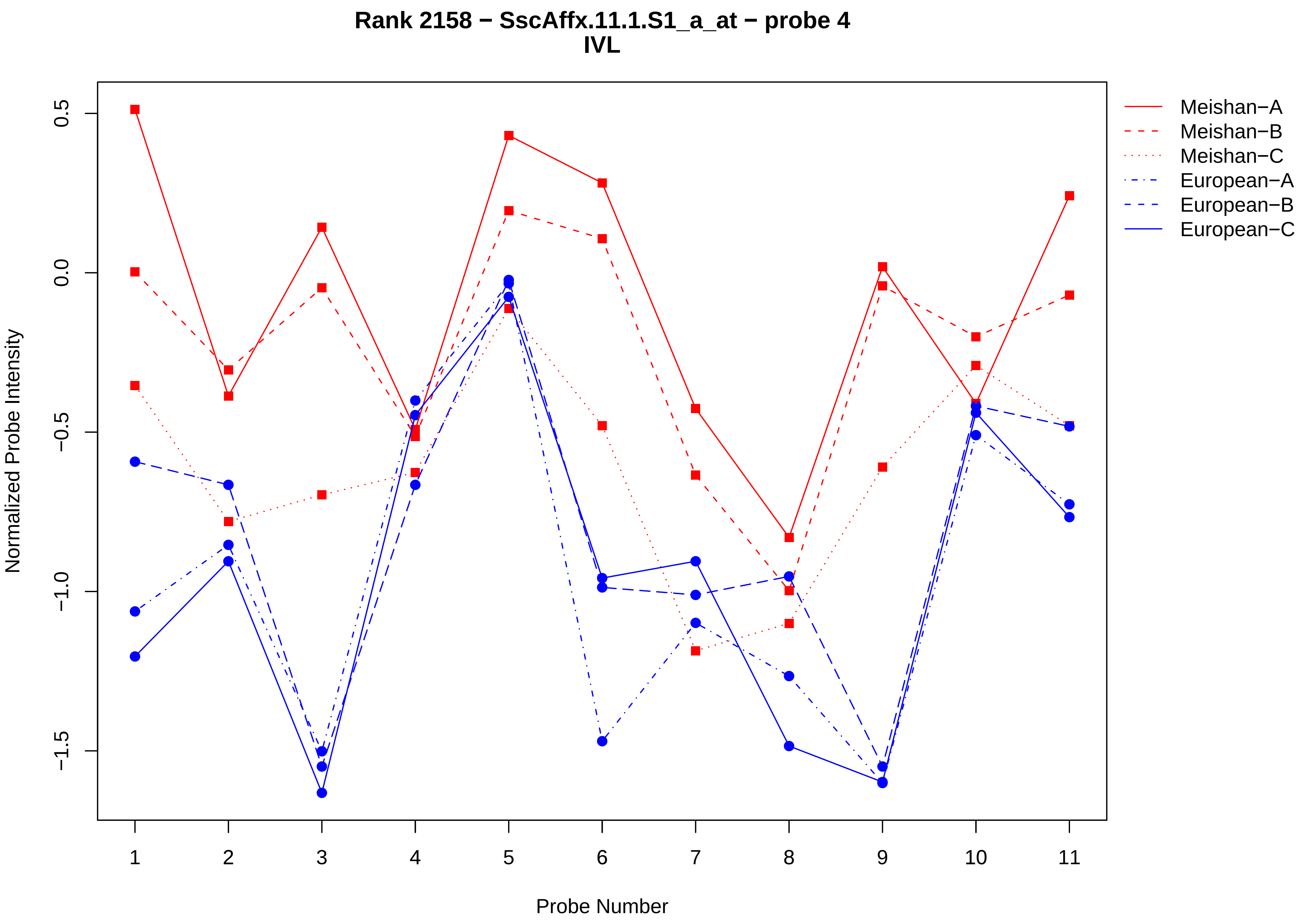

Rank 2158 − SscAffx.11.1.S1_a_at − probe 4
IVL
Normalized Probe Intensity
Probe Number
0.5
0.0
−0.5
−1.0
−1.5
1
2
3
4
5
6
7
8
9
10
11
Meishan−A
Meishan−B
Meishan−C
European−A
European−B
European−C

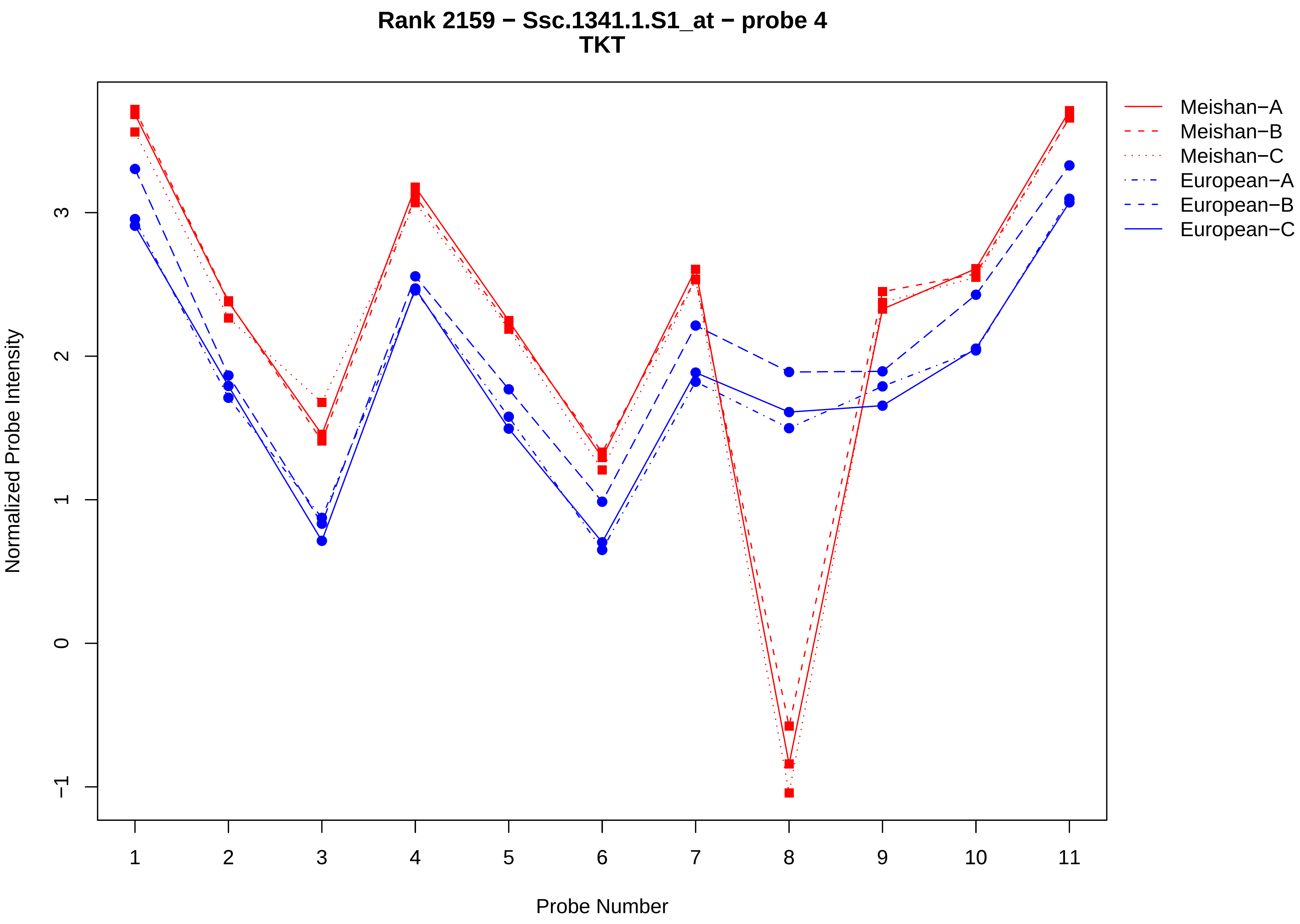
Rank 2159 − Ssc.1341.1.S1_at − probe 4
TKT
Normalized Probe Intensity
Probe Number
−1
0
1
2
3
1
2
3
4
5
6
7
8
9
10
11
Meishan−A
Meishan−B
Meishan−C
European−A
European−B
European−C

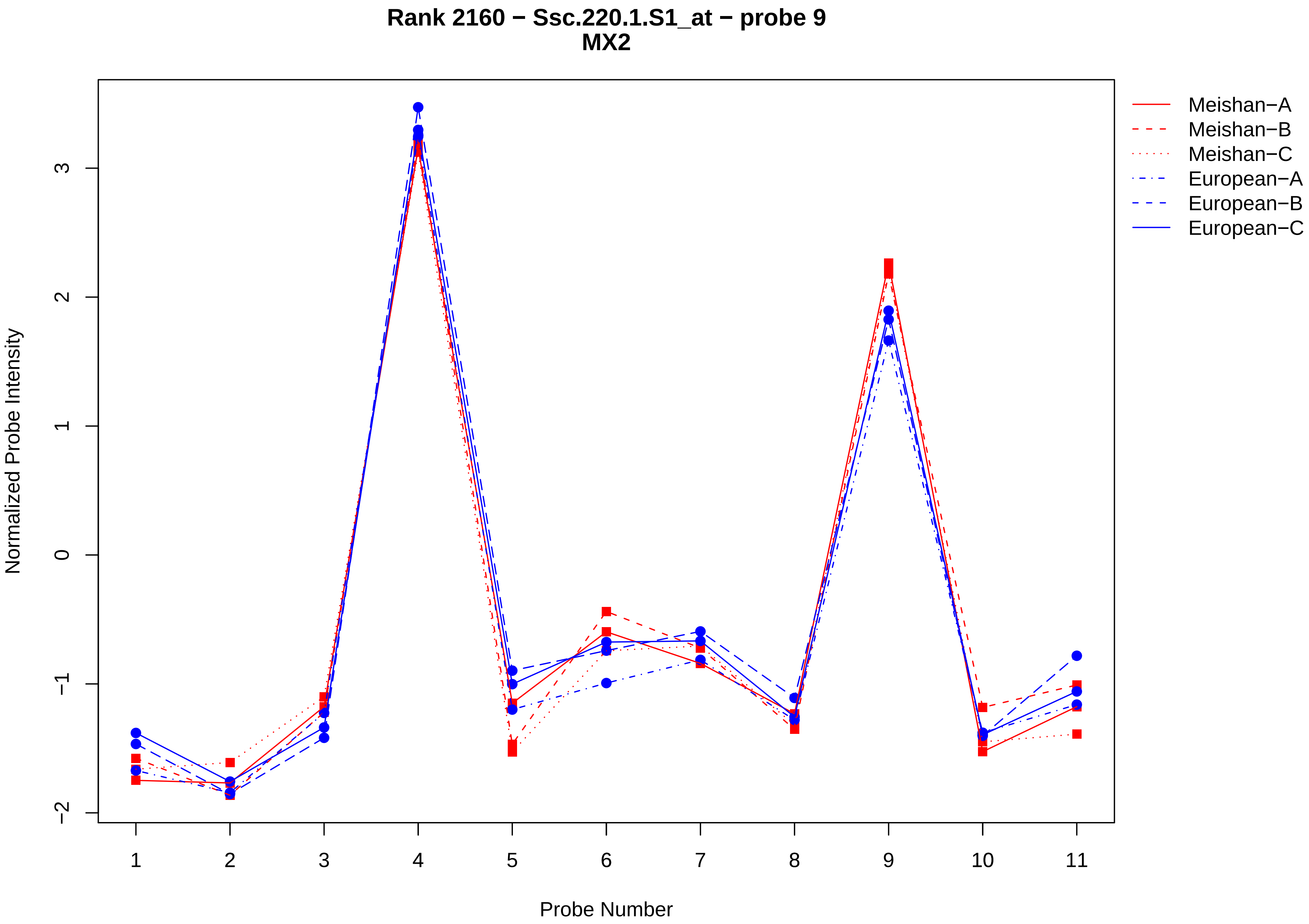

Rank 2160 − Ssc.220.1.S1_at − probe 9
MX2
Normalized Probe Intensity
Probe Number
−2
−1
0
1
2
3
1
2
3
4
5
6
7
8
9
10
11
Meishan−A
Meishan−B
Meishan−C
European−A
European−B
European−C

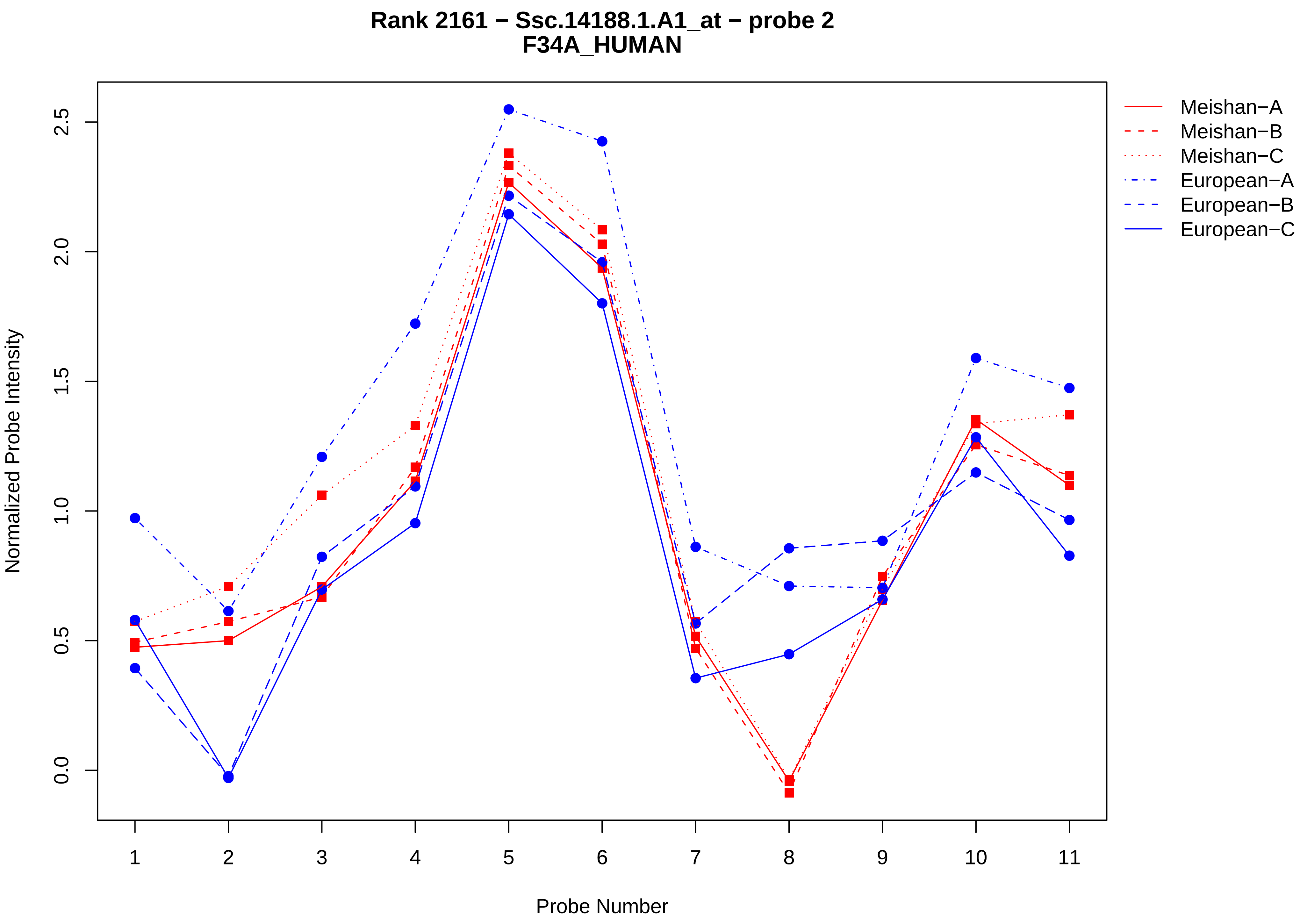
Rank 2161 − Ssc.14188.1.A1_at − probe 2
F34A_HUMAN
Normalized Probe Intensity
0.0
0.5
1.0
1.5
2.0
2.5
1
2
3
4
5
6
7
8
9
10
11
Probe Number
Meishan−A
Meishan−B
Meishan−C
European−A
European−B
European−C

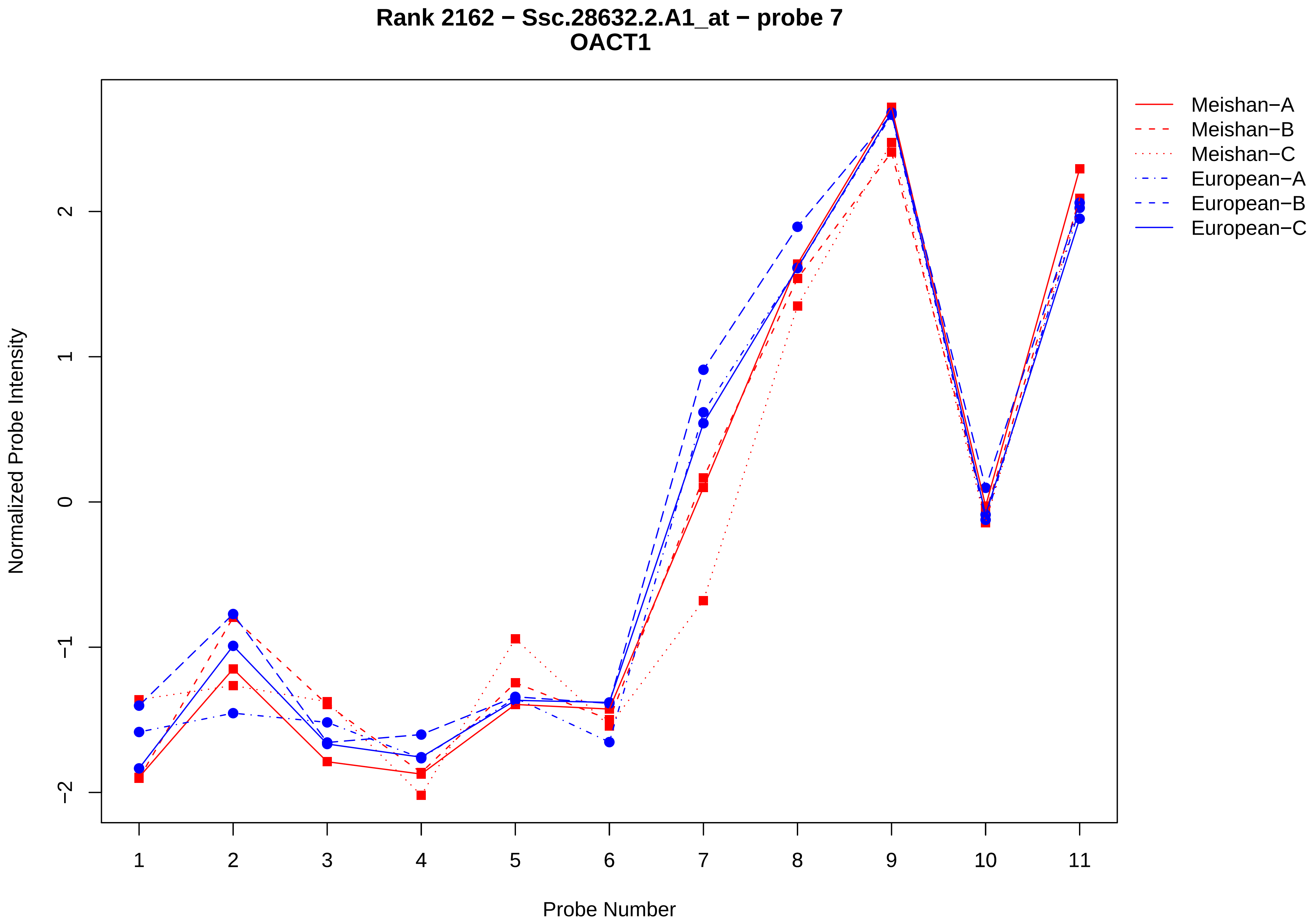

Rank 2162 − Ssc.28632.2.A1_at − probe 7
OACT1
Normalized Probe Intensity
Probe Number
−2
−1
0
1
2
1
2
3
4
5
6
7
8
9
10
11
Meishan−A
Meishan−B
Meishan−C
European−A
European−B
European−C

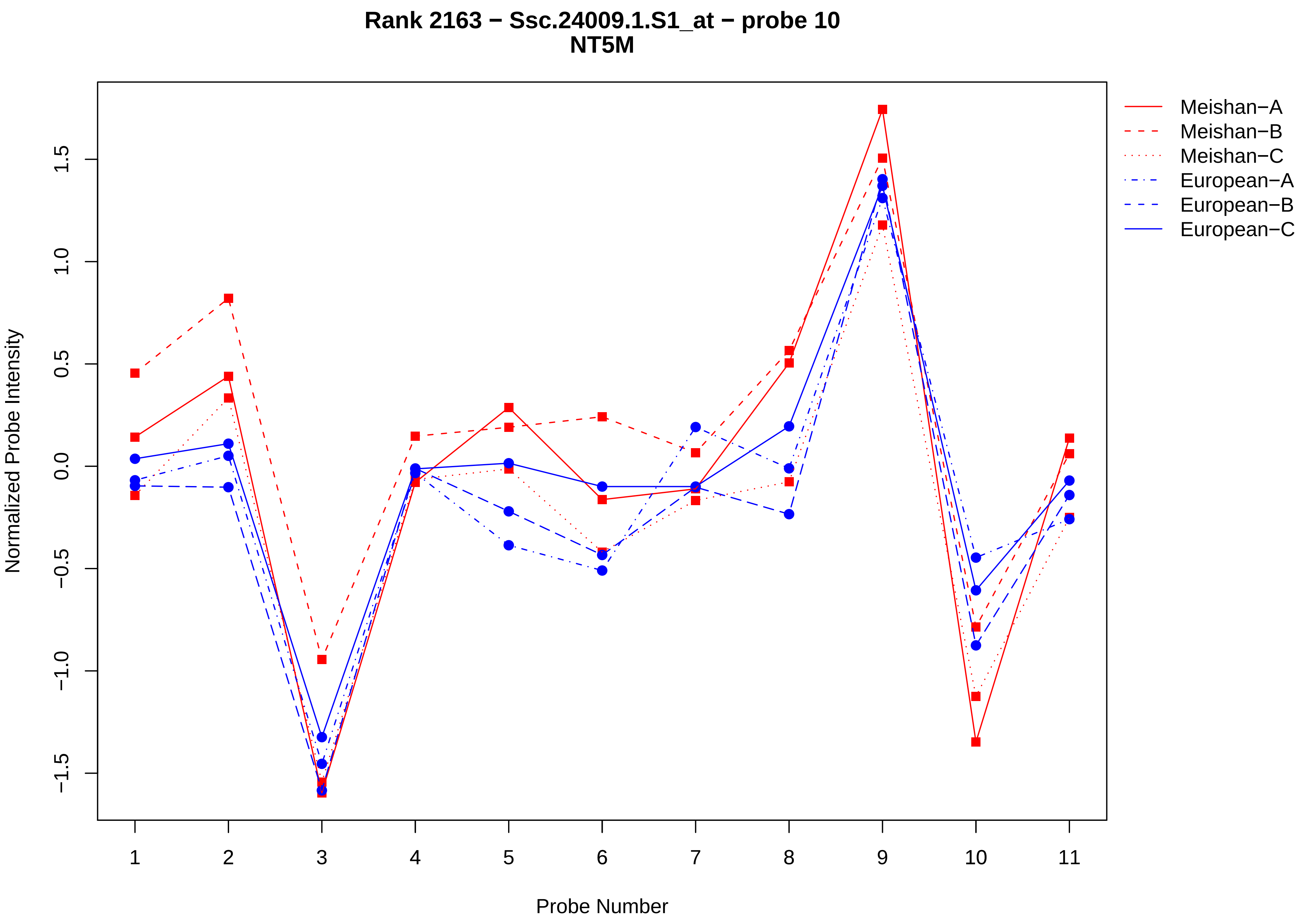

Rank 2163 − Ssc.24009.1.S1_at − probe 10
NT5M
Normalized Probe Intensity
Probe Number
1.5
1.0
0.5
0.0
−0.5
−1.0
−1.5
1
2
3
4
5
6
7
8
9
10
11
Meishan−A
Meishan−B
Meishan−C
European−A
European−B
European−C

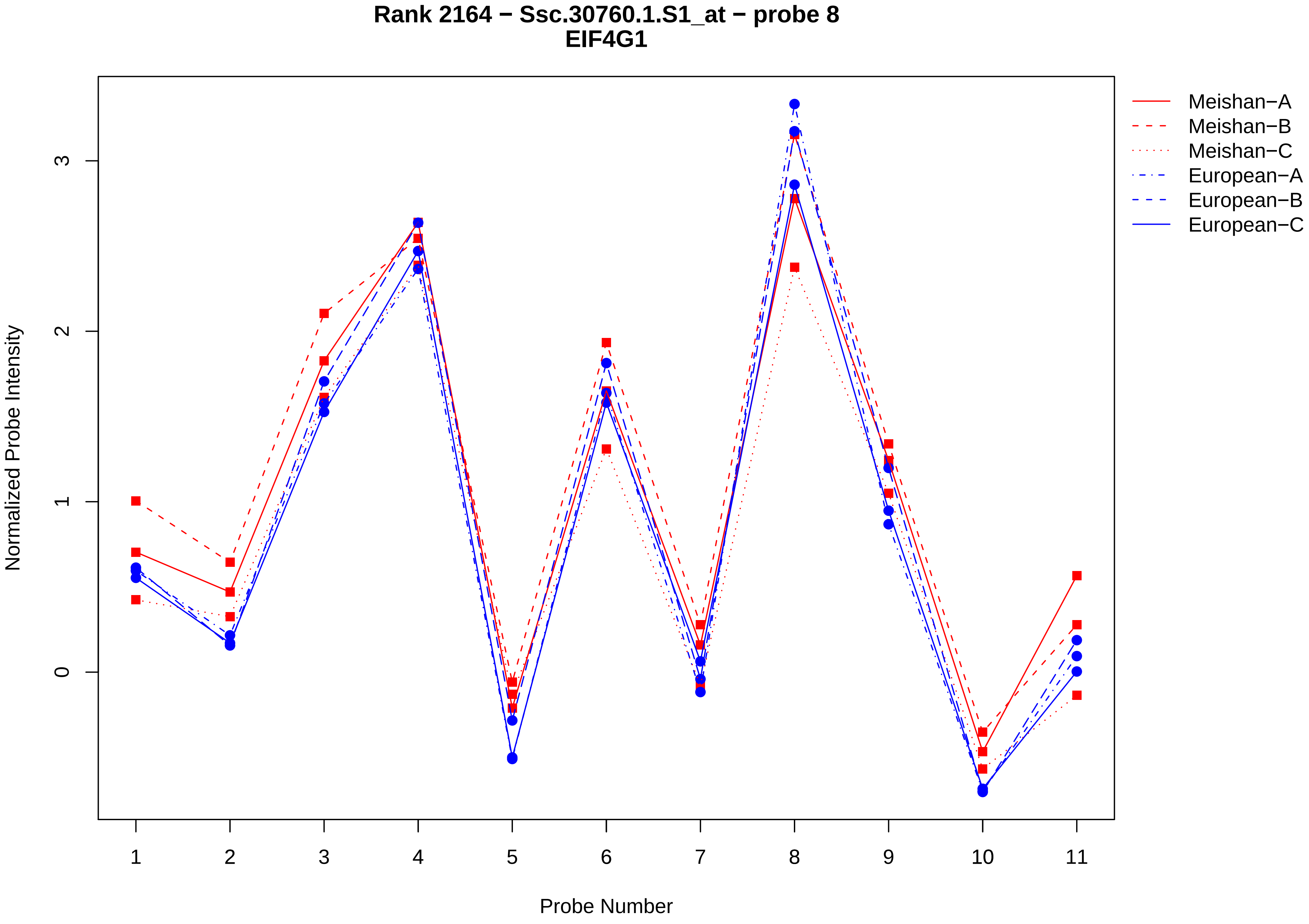
Rank 2164 − Ssc.30760.1.S1_at − probe 8
EIF4G1
Normalized Probe Intensity
Probe Number
0
1
2
3
1
2
3
4
5
6
7
8
9
10
11
Meishan−A
Meishan−B
Meishan−C
European−A
European−B
European−C

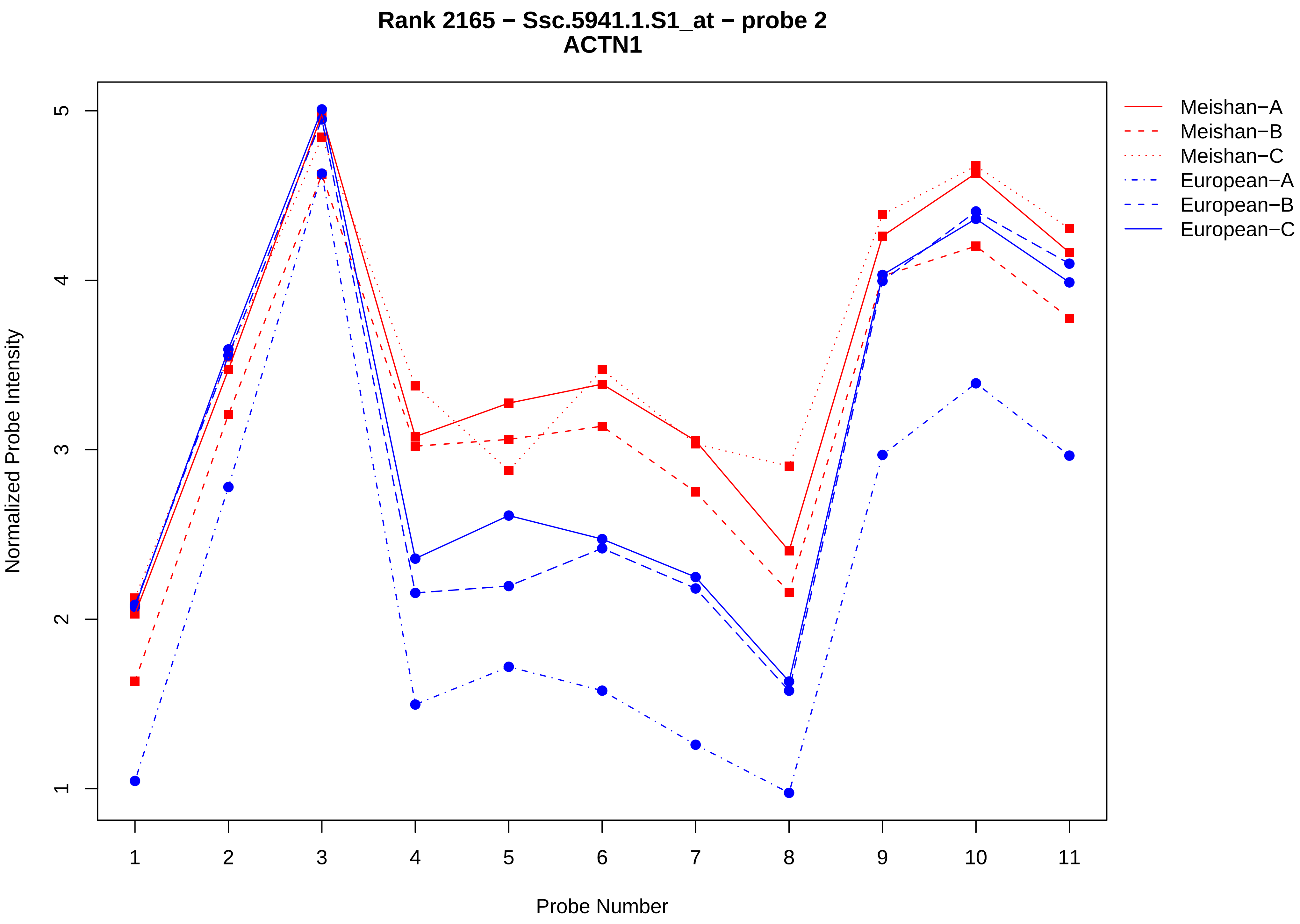
Rank 2165 − Ssc.5941.1.S1_at − probe 2
ACTN1
Normalized Probe Intensity
Probe Number
1
2
3
4
5
1
2
3
4
5
6
7
8
9
10
11
Meishan−A
Meishan−B
Meishan−C
European−A
European−B
European−C

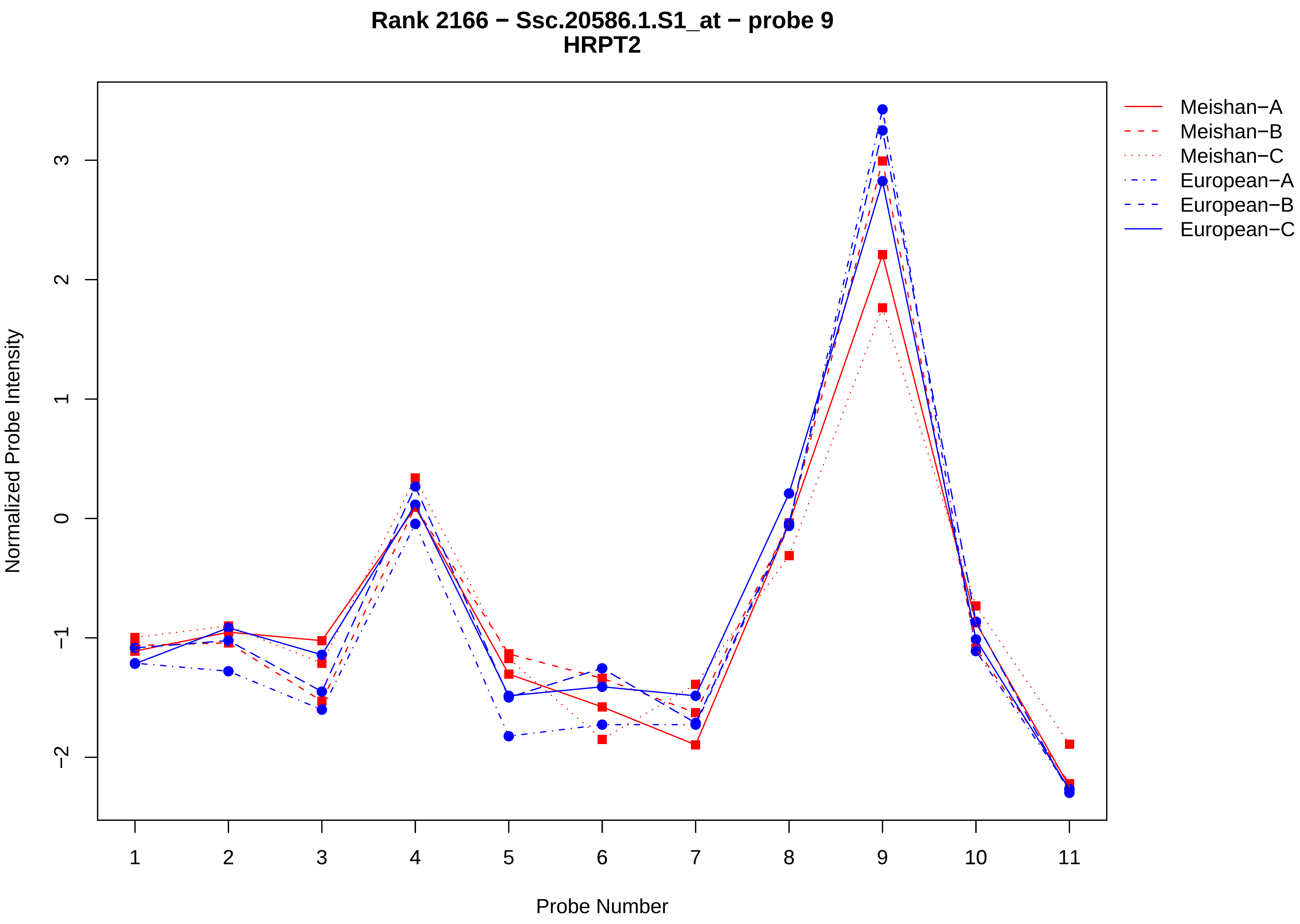

Rank 2166 − Ssc.20586.1.S1_at − probe 9
HRPT2
Normalized Probe Intensity
Probe Number
−2
−1
0
1
2
3
1
2
3
4
5
6
7
8
9
10
11
Meishan−A
Meishan−B
Meishan−C
European−A
European−B
European−C

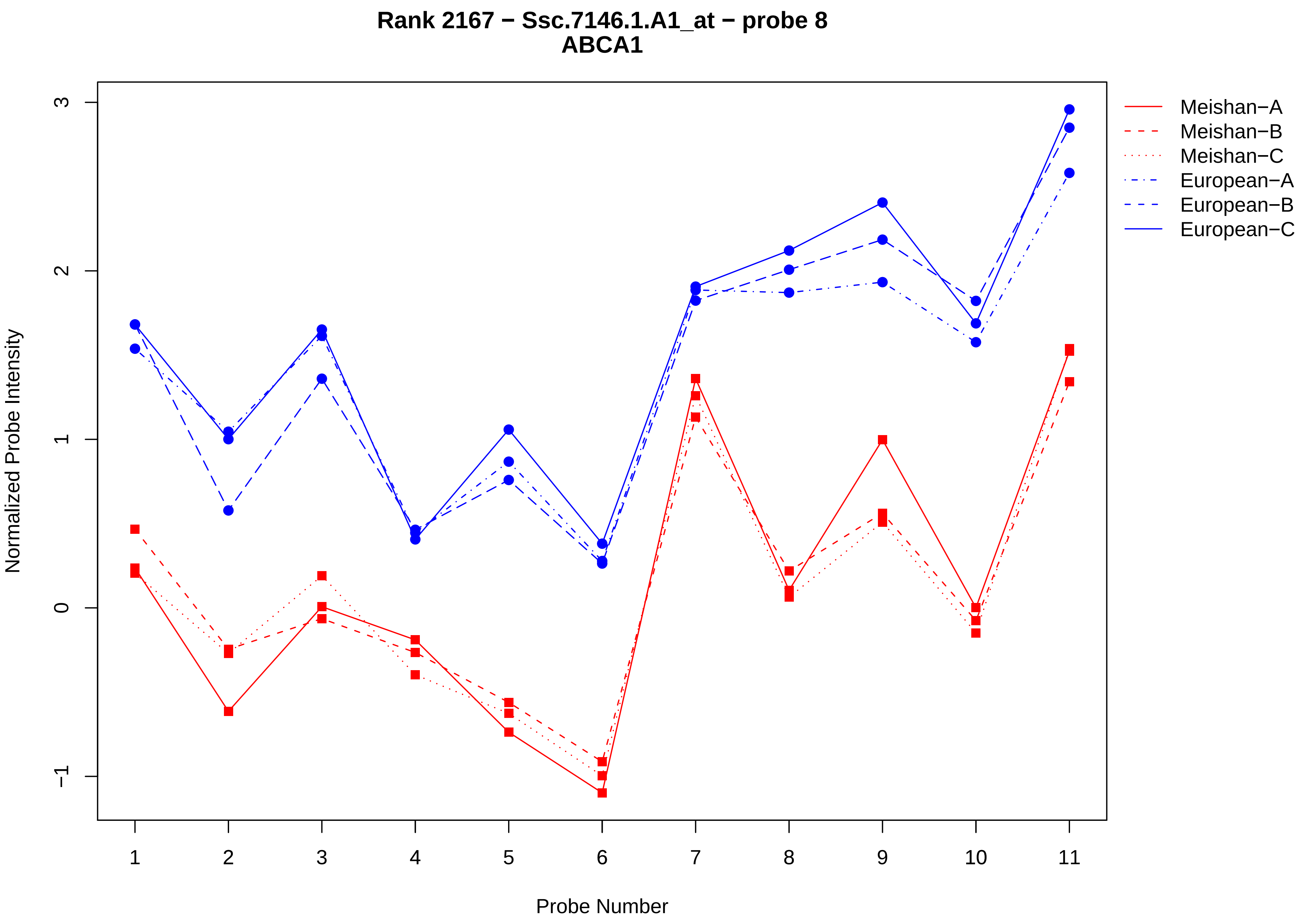

Rank 2167 − Ssc.7146.1.A1_at − probe 8
ABCA1
Normalized Probe Intensity
Probe Number
−1
0
1
2
3
1
2
3
4
5
6
7
8
9
10
11
Meishan−A
Meishan−B
Meishan−C
European−A
European−B
European−C

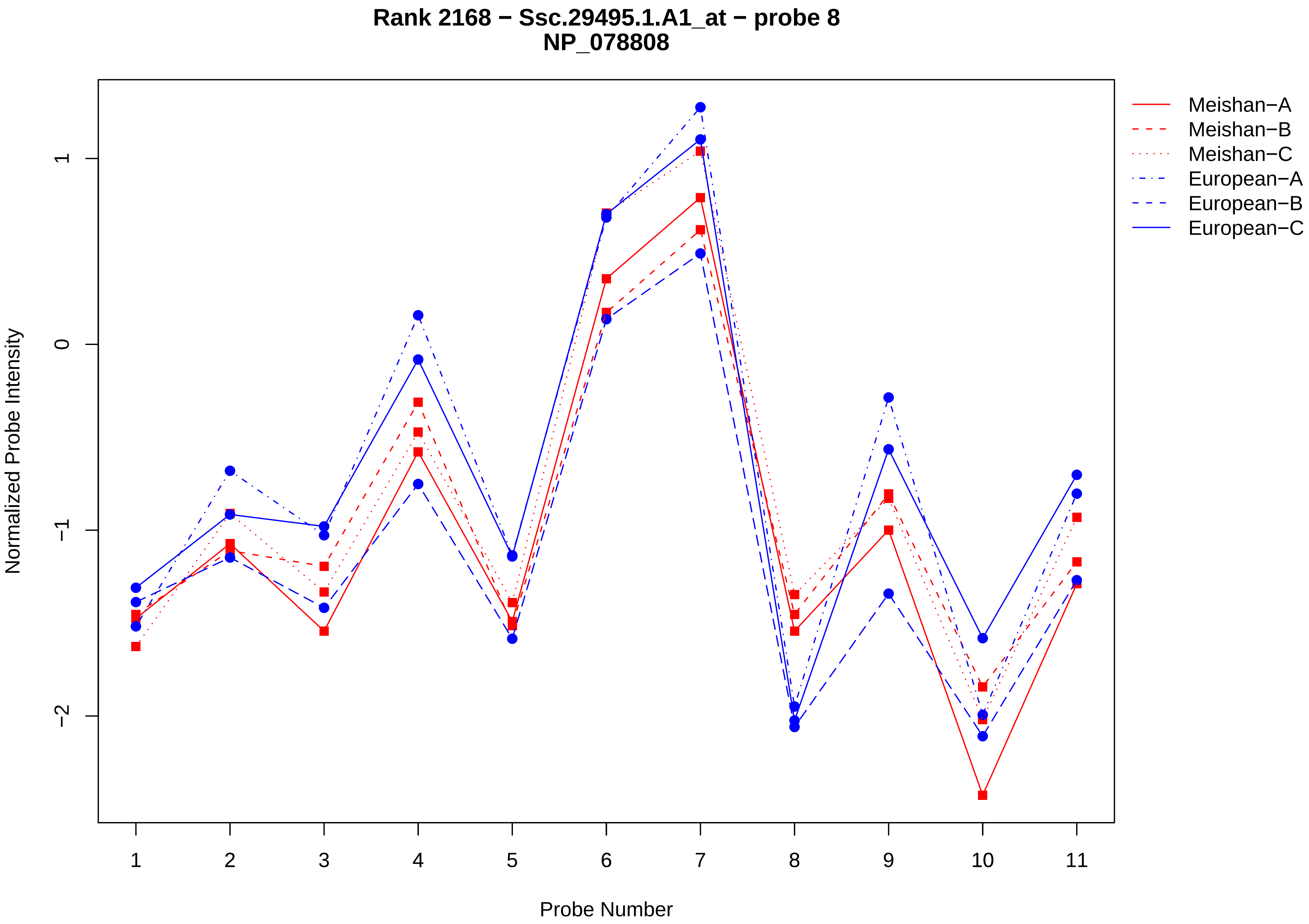

Rank 2168 − Ssc.29495.1.A1_at − probe 8
NP_078808
Normalized Probe Intensity
Probe Number
1
0
−1
−2
1
2
3
4
5
6
7
8
9
10
11
Meishan−A
Meishan−B
Meishan−C
European−A
European−B
European−C

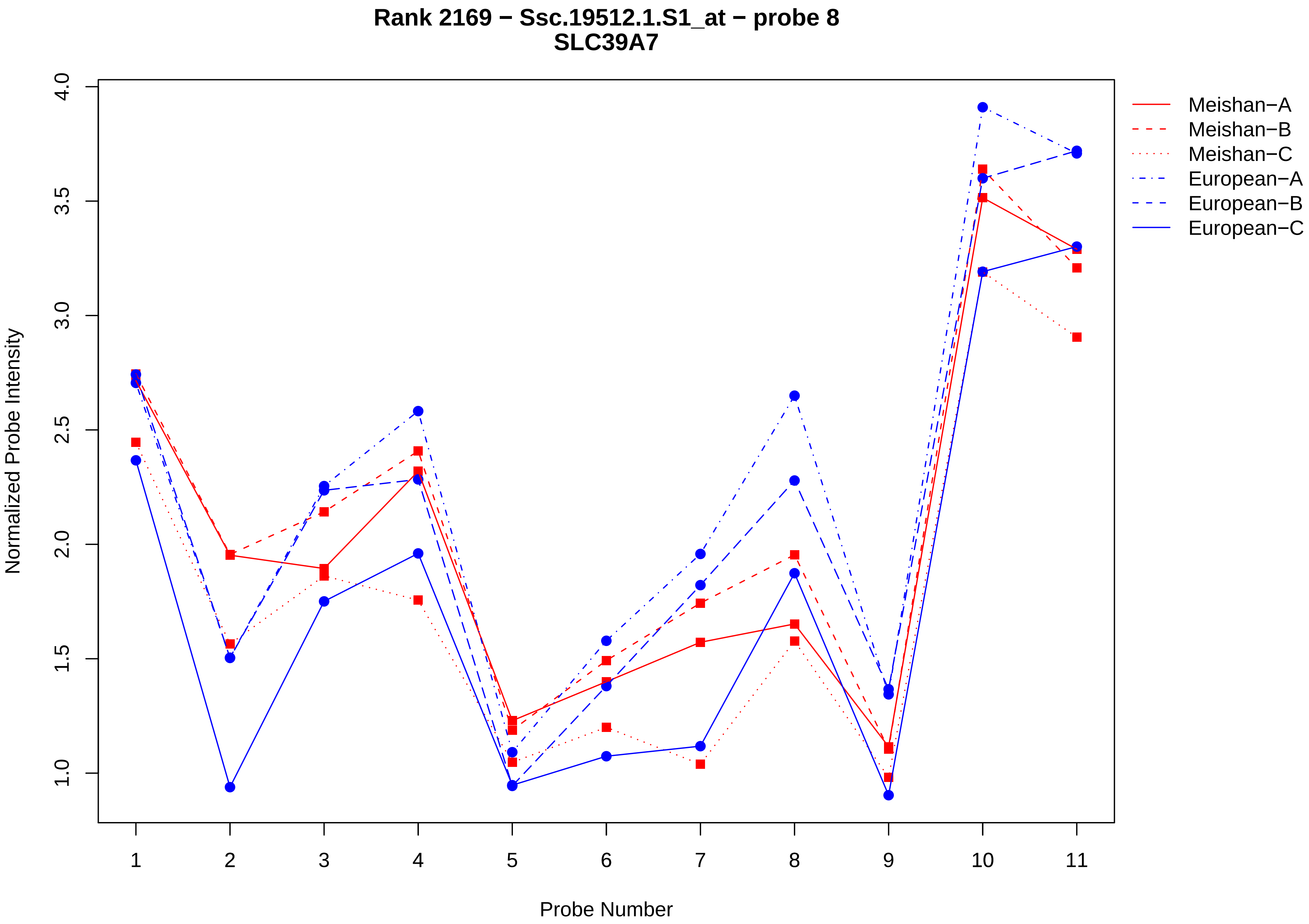

Rank 2169 − Ssc.19512.1.S1_at − probe 8
SLC39A7
Normalized Probe Intensity
Probe Number
1.0
1.5
2.0
2.5
3.0
3.5
4.0
1
2
3
4
5
6
7
8
9
10
11
Meishan−A
Meishan−B
Meishan−C
European−A
European−B
European−C

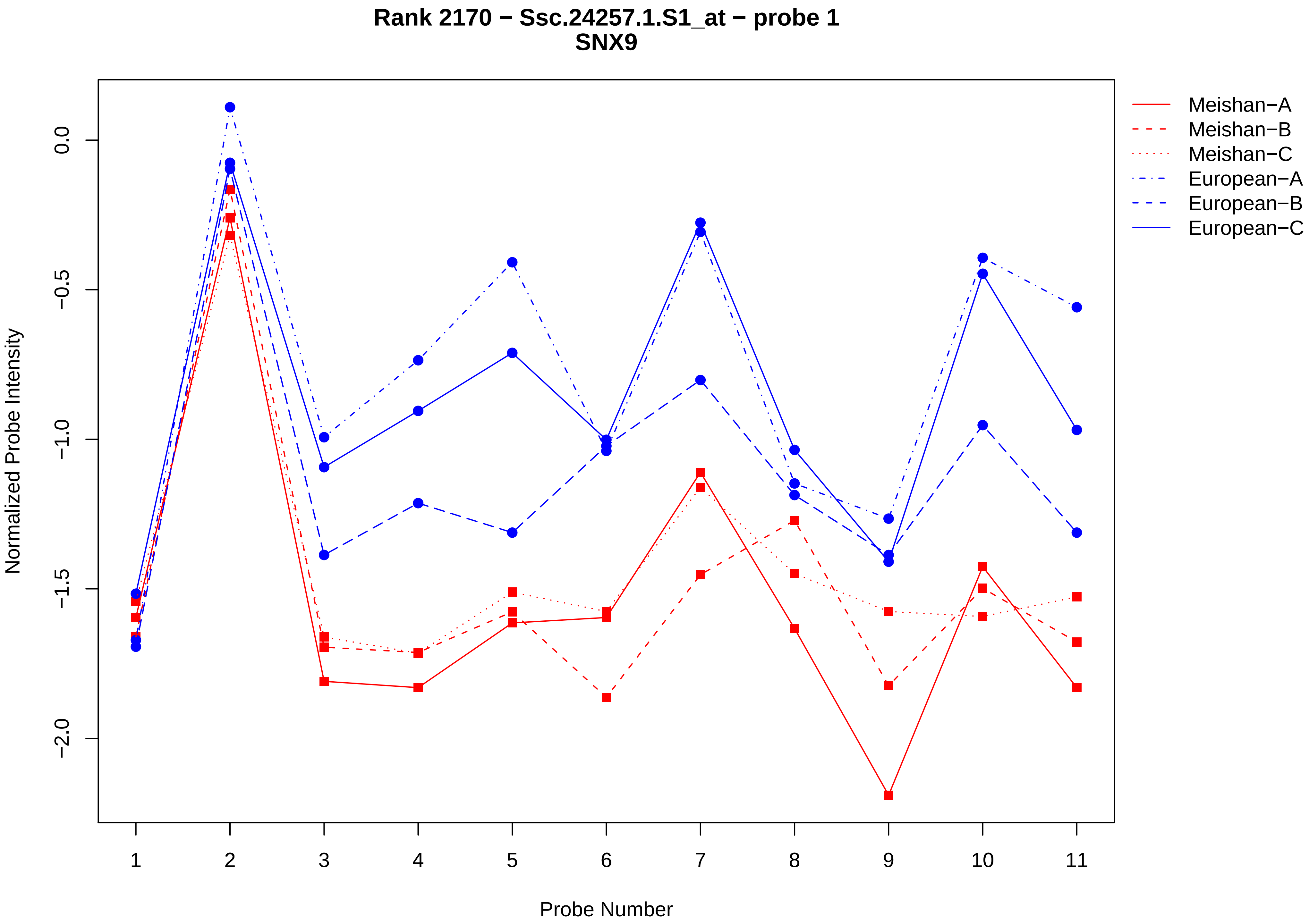

Rank 2170 − Ssc.24257.1.S1_at − probe 1
SNX9
Normalized Probe Intensity
Probe Number
0.0
−0.5
−1.0
−1.5
−2.0
1
2
3
4
5
6
7
8
9
10
11
Meishan−A
Meishan−B
Meishan−C
European−A
European−B
European−C

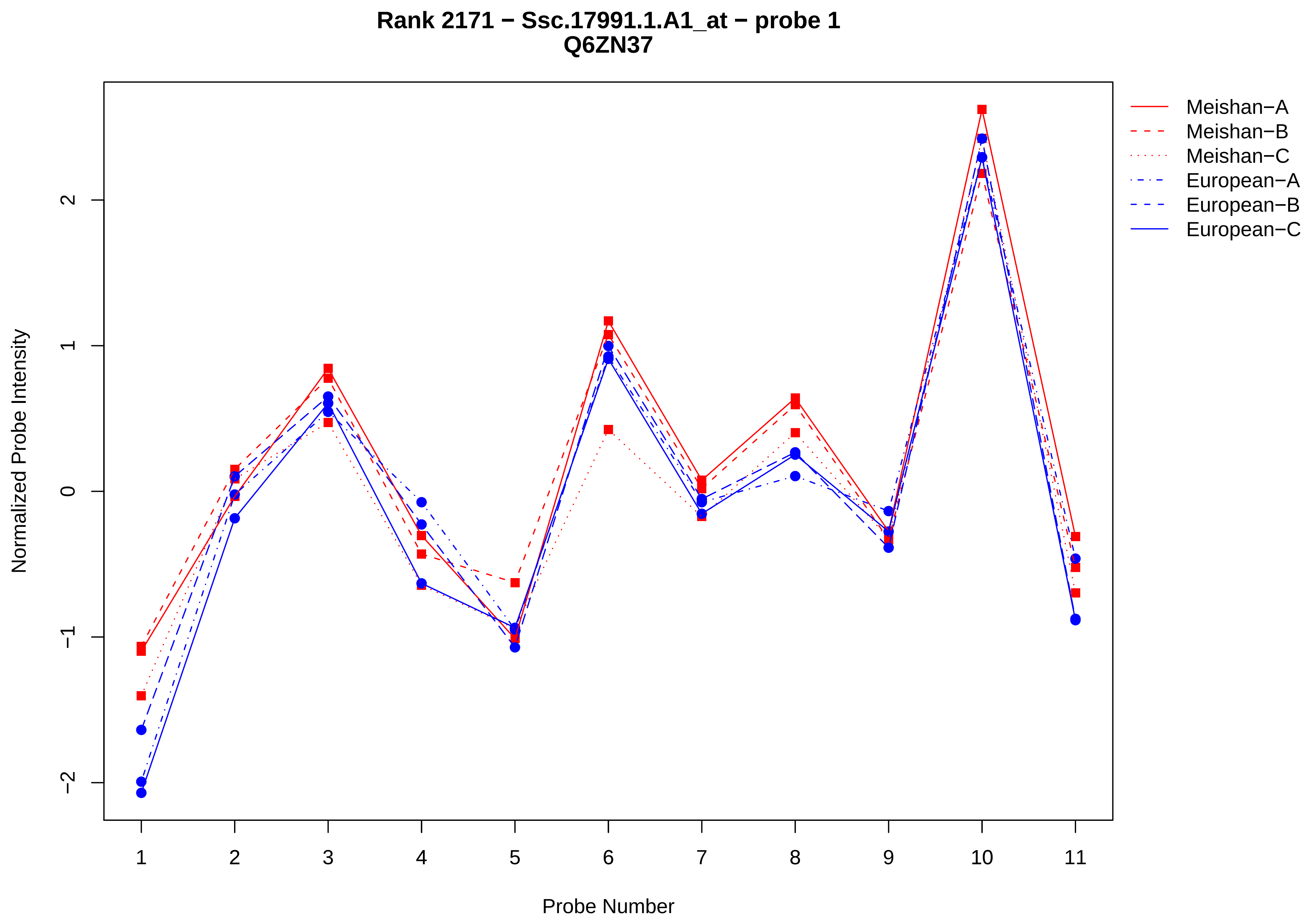

Rank 2171 − Ssc.17991.1.A1_at − probe 1
Q6ZN37
Normalized Probe Intensity
Probe Number
−2
−1
0
1
2
1
2
3
4
5
6
7
8
9
10
11
Meishan−A
Meishan−B
Meishan−C
European−A
European−B
European−C

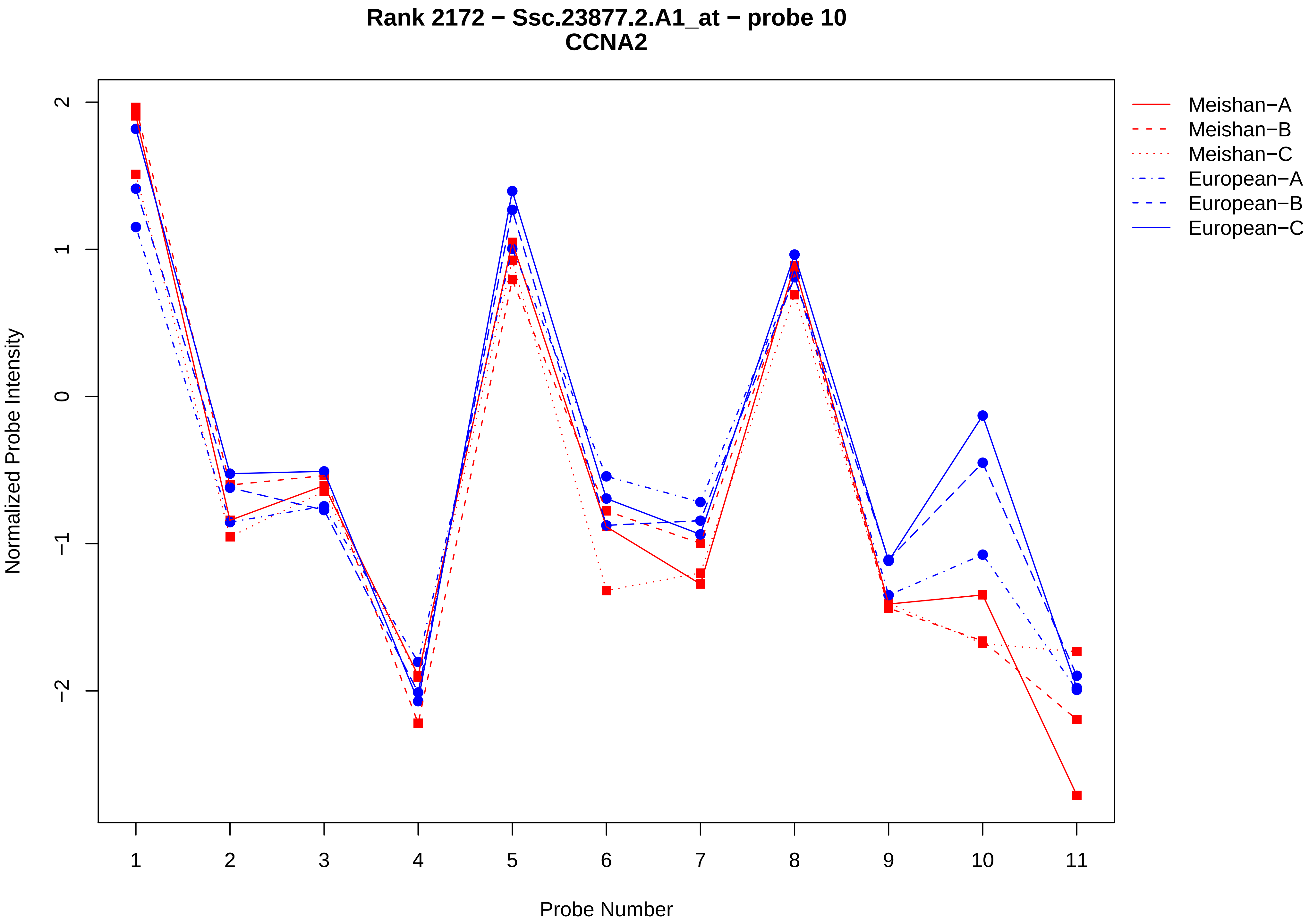
Rank 2172 − Ssc.23877.2.A1_at − probe 10
CCNA2
Normalized Probe Intensity
Probe Number
Meishan−A
Meishan−B
Meishan−C
European−A
European−B
European−C
2
1
0
−1
−2
1
2
3
4
5
6
7
8
9
10
11

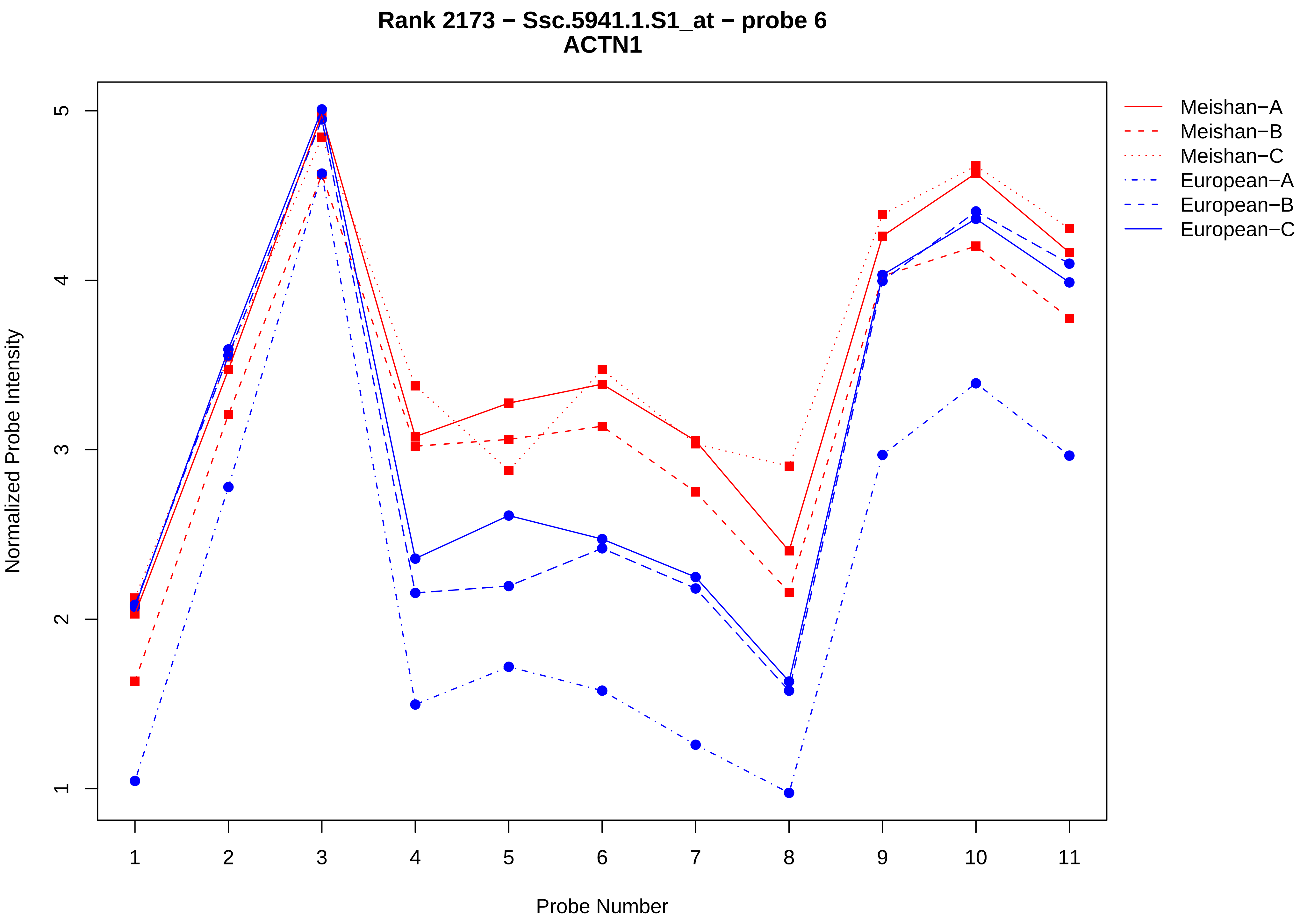

Rank 2173 − Ssc.5941.1.S1_at − probe 6
ACTN1
Normalized Probe Intensity
Probe Number
1
2
3
4
5
1
2
3
4
5
6
7
8
9
10
11
Meishan−A
Meishan−B
Meishan−C
European−A
European−B
European−C

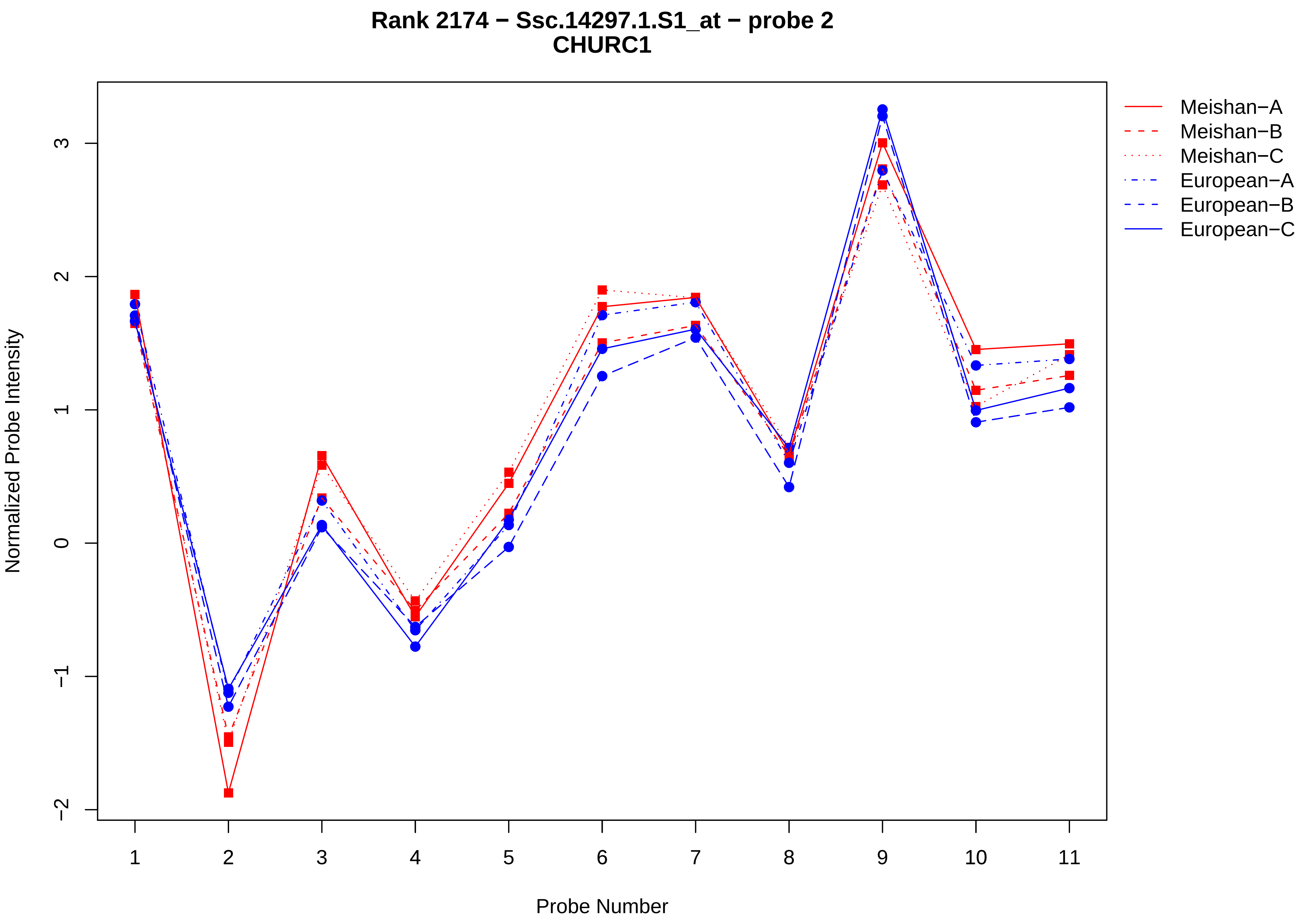

Rank 2174 − Ssc.14297.1.S1_at − probe 2
CHURC1
Normalized Probe Intensity
Probe Number
−2
−1
0
1
2
3
1
2
3
4
5
6
7
8
9
10
11
Meishan−A
Meishan−B
Meishan−C
European−A
European−B
European−C

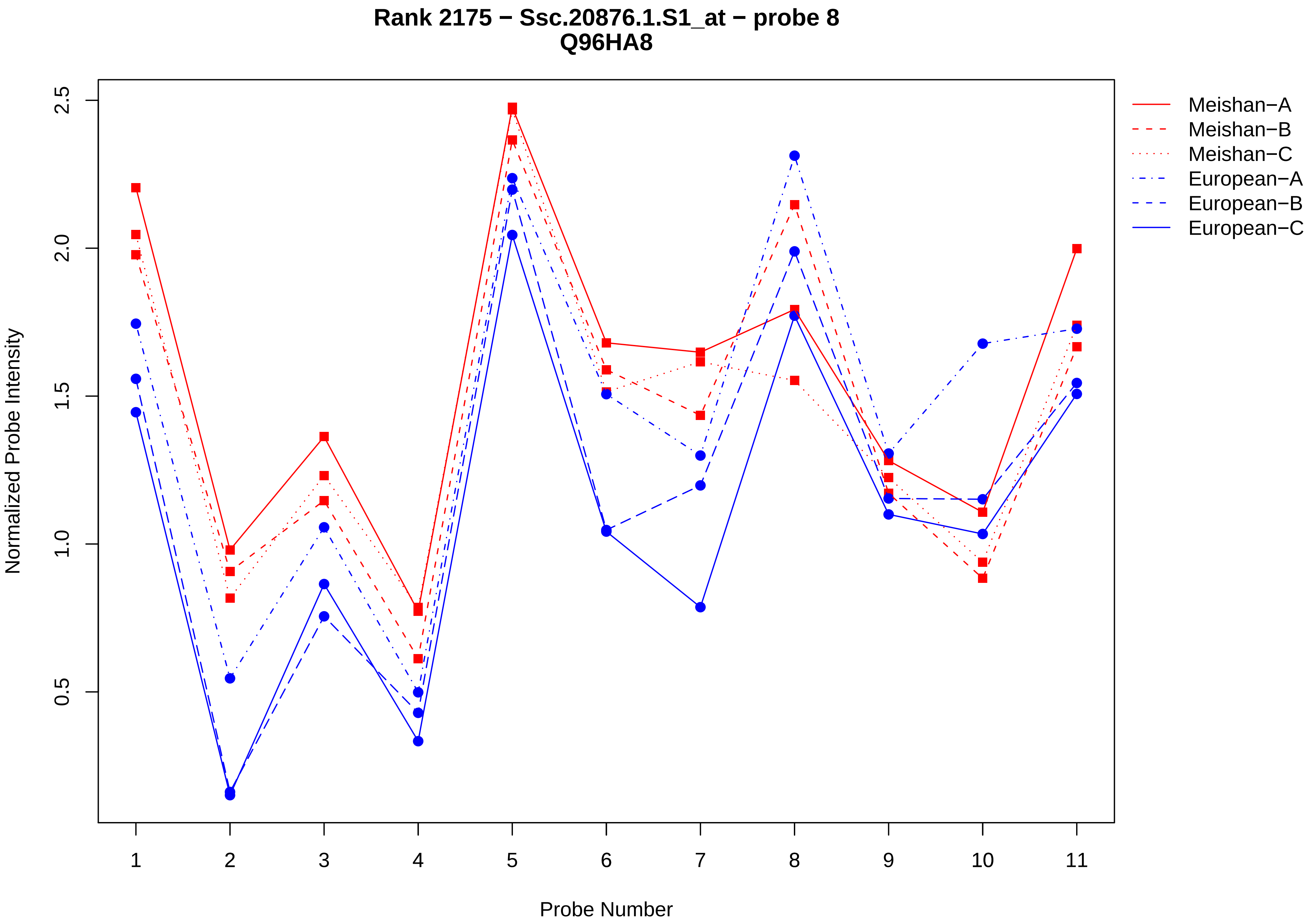

Rank 2175 − Ssc.20876.1.S1_at − probe 8
Q96HA8
Normalized Probe Intensity
0.5
1.0
1.5
2.0
2.5
1
2
3
4
5
6
7
8
9
10
11
Probe Number
Meishan−A
Meishan−B
Meishan−C
European−A
European−B
European−C

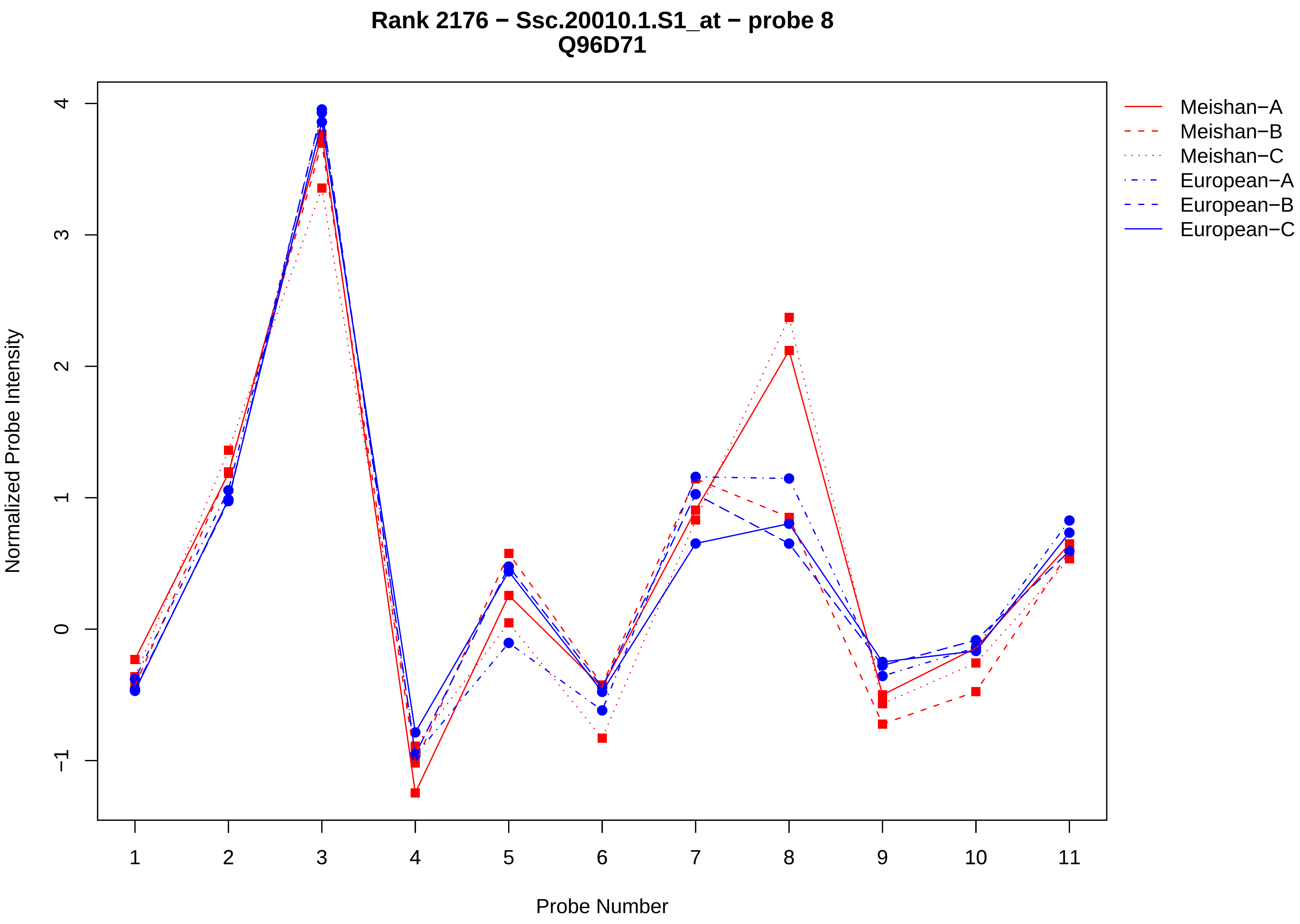
Rank 2176 − Ssc.20010.1.S1_at − probe 8
Q96D71
Normalized Probe Intensity
Probe Number
Meishan−A
Meishan−B
Meishan−C
European−A
European−B
European−C
−1
0
1
2
3
4
1
2
3
4
5
6
7
8
9
10
11

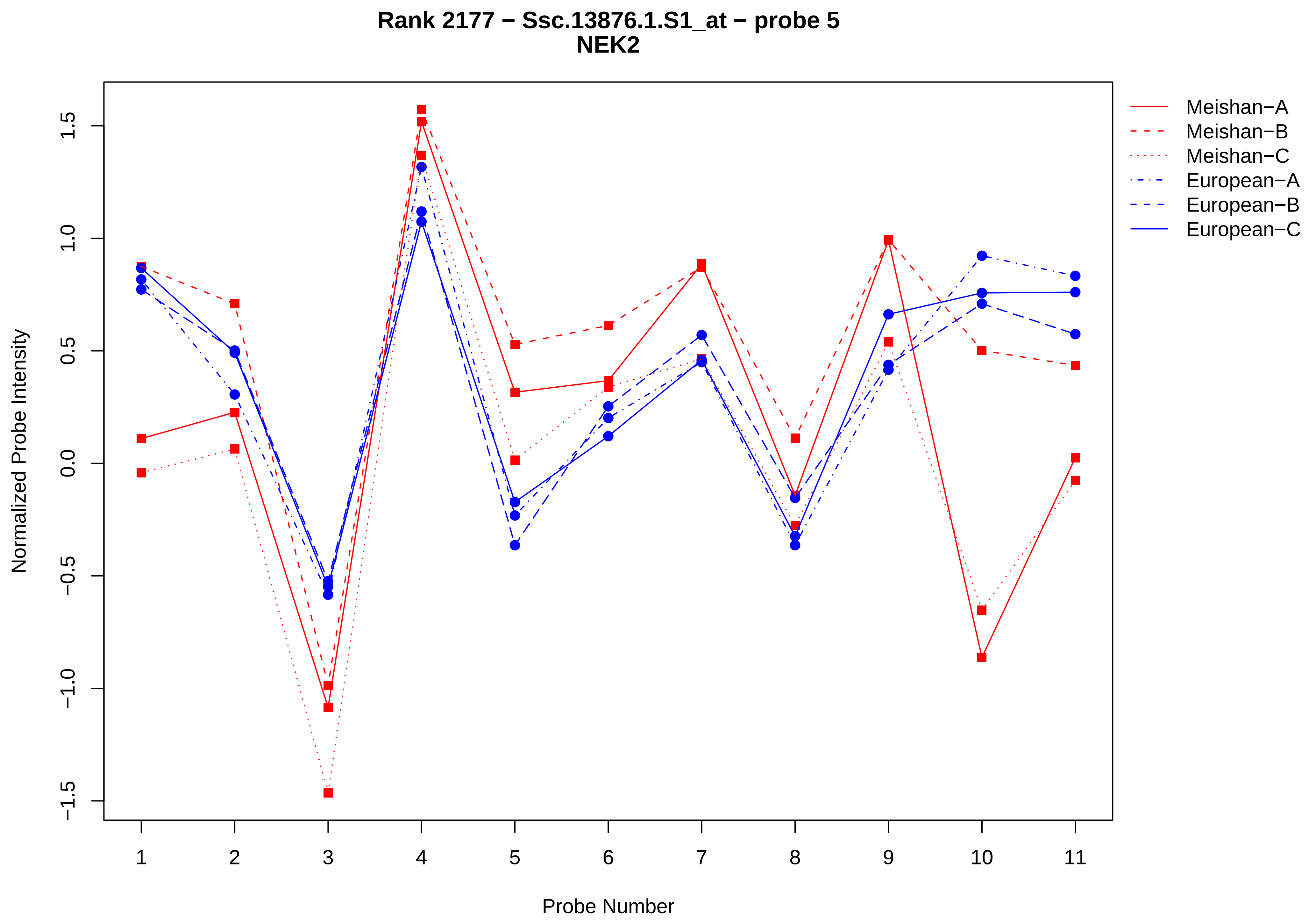

Rank 2177 − Ssc.13876.1.S1_at − probe 5
NEK2
Normalized Probe Intensity
Probe Number
1.5
1.0
0.5
0.0
−0.5
−1.0
−1.5
1
2
3
4
5
6
7
8
9
10
11
Meishan−A
Meishan−B
Meishan−C
European−A
European−B
European−C

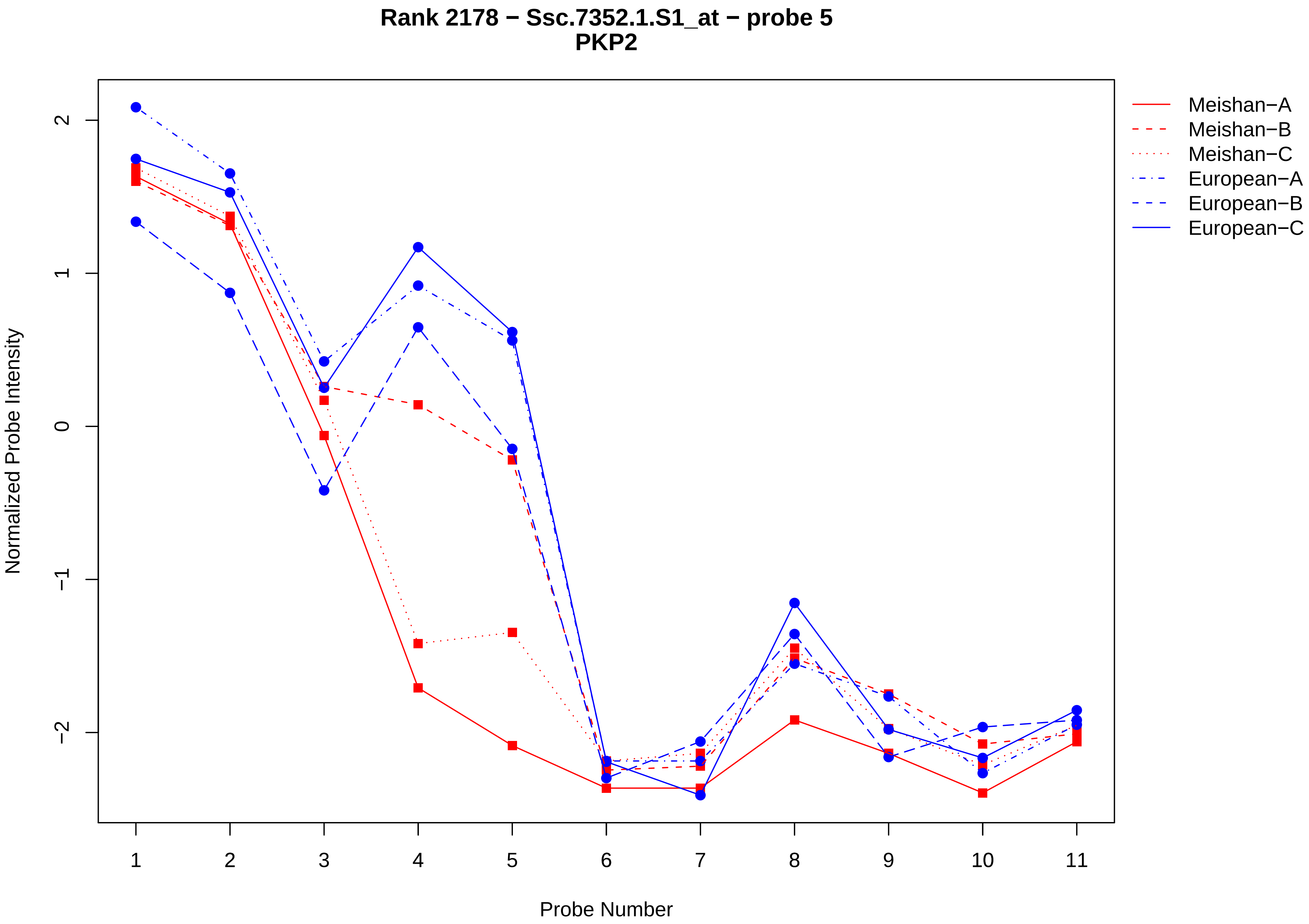

Rank 2178 − Ssc.7352.1.S1_at − probe 5
PKP2
Normalized Probe Intensity
Probe Number
Meishan−A
Meishan−B
Meishan−C
European−A
European−B
European−C
−2
−1
0
1
2
1
2
3
4
5
6
7
8
9
10
11

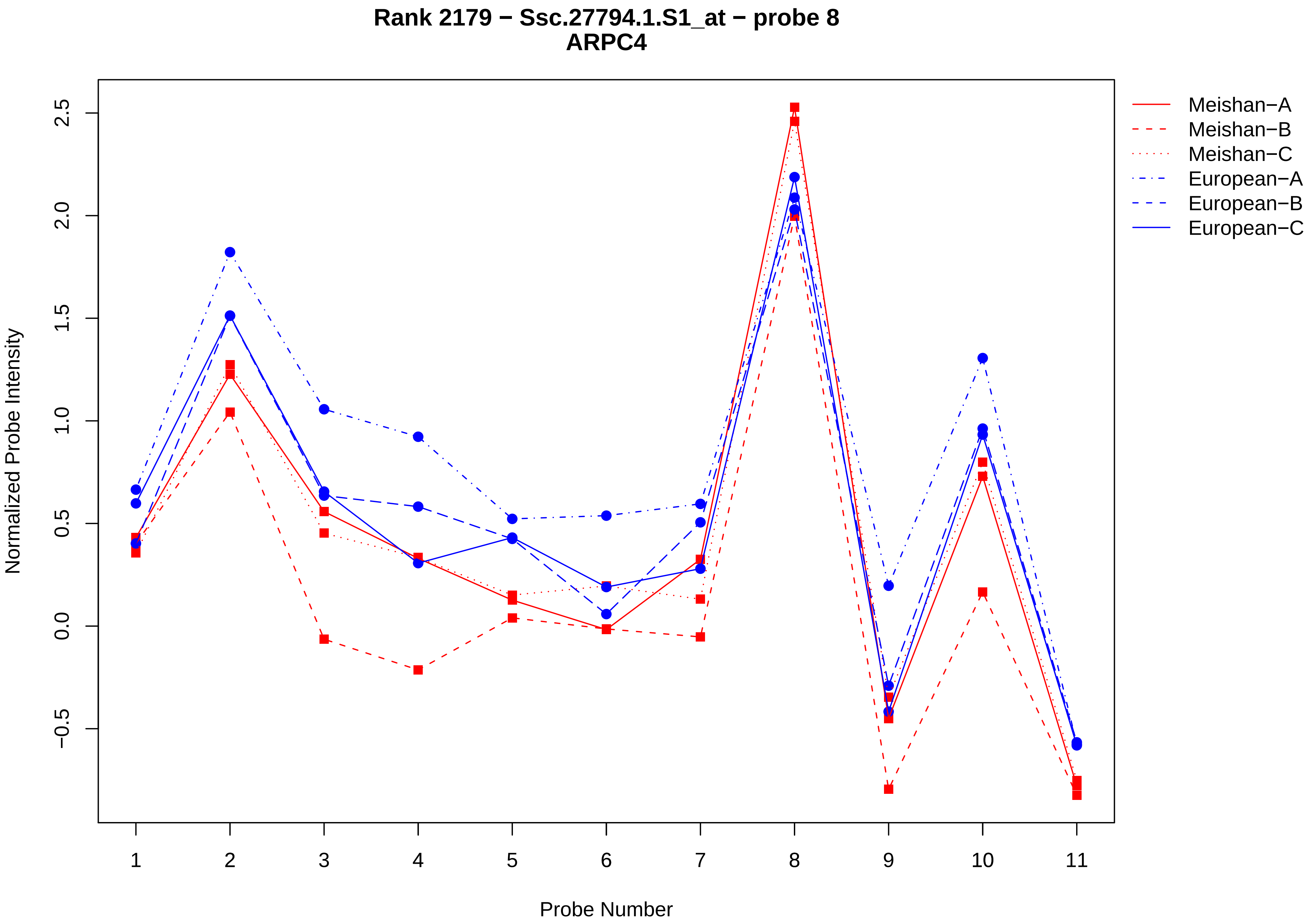

Rank 2179 − Ssc.27794.1.S1_at − probe 8
ARPC4
Normalized Probe Intensity
Probe Number
Meishan−A
Meishan−B
Meishan−C
European−A
European−B
European−C
−0.5
0.0
0.5
1.0
1.5
2.0
2.5
1
2
3
4
5
6
7
8
9
10
11

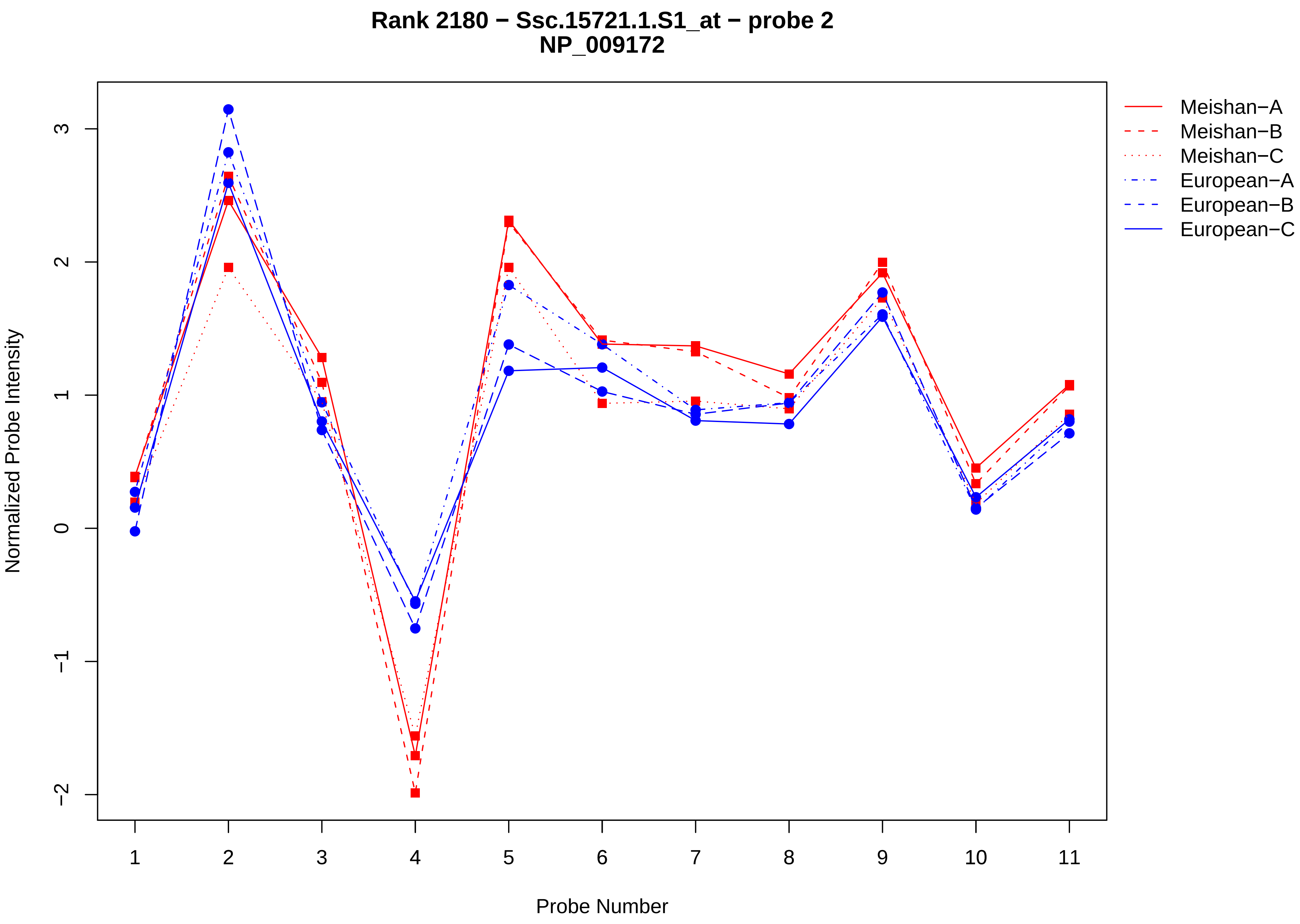

Rank 2180 − Ssc.15721.1.S1_at − probe 2
NP_009172
Normalized Probe Intensity
Probe Number
−2
−1
0
1
2
3
1
2
3
4
5
6
7
8
9
10
11
Meishan−A
Meishan−B
Meishan−C
European−A
European−B
European−C

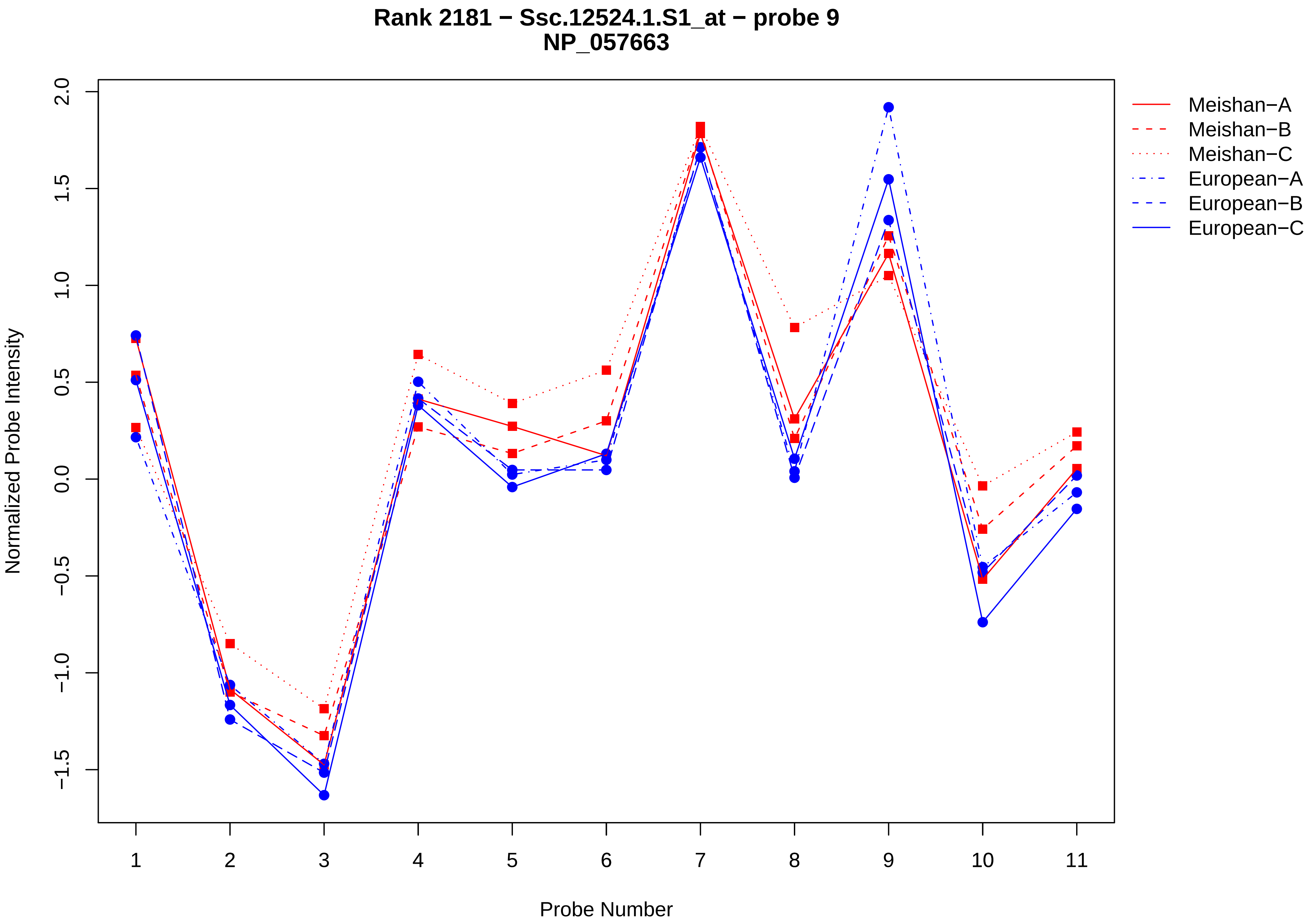

Rank 2181 − Ssc.12524.1.S1_at − probe 9
NP_057663
Normalized Probe Intensity
Probe Number
Meishan−A
Meishan−B
Meishan−C
European−A
European−B
European−C

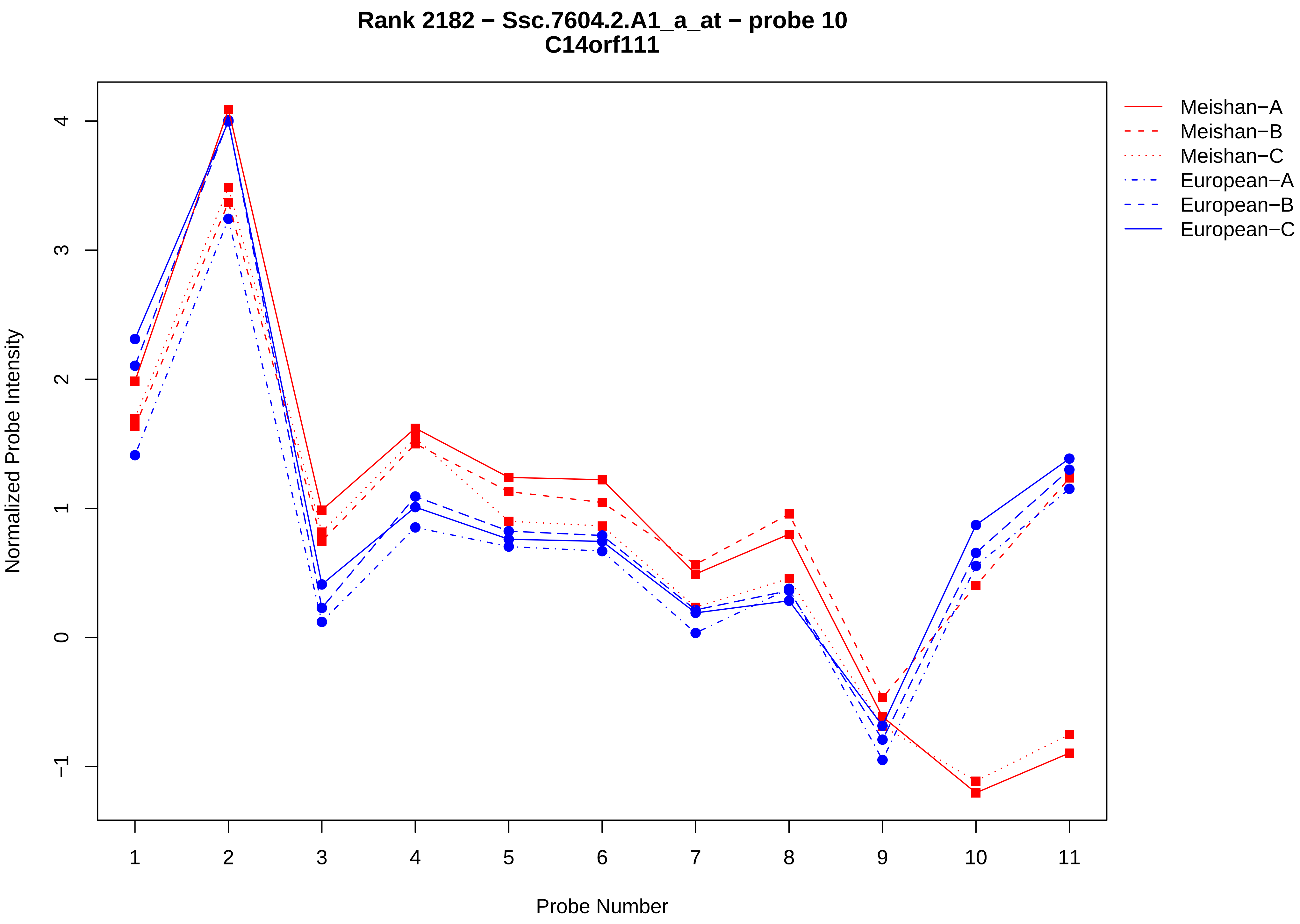

Rank 2182 − Ssc.7604.2.A1_a_at − probe 10
C14orf111
Normalized Probe Intensity
Probe Number
Meishan−A
Meishan−B
Meishan−C
European−A
European−B
European−C

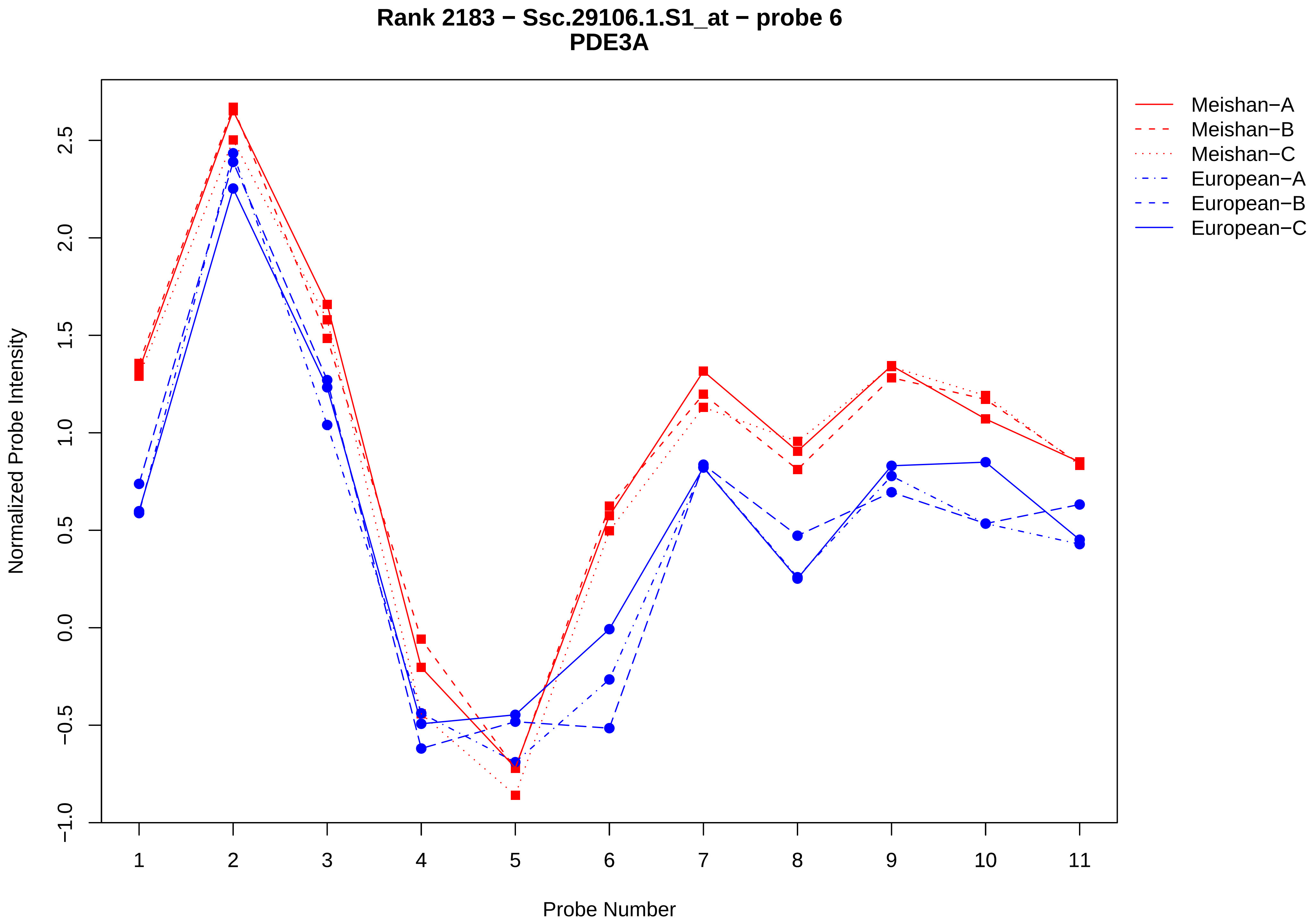

Rank 2183 − Ssc.29106.1.S1_at − probe 6
PDE3A
Normalized Probe Intensity
Probe Number
−1.0
−0.5
0.0
0.5
1.0
1.5
2.0
2.5
1
2
3
4
5
6
7
8
9
10
11
Meishan−A
Meishan−B
Meishan−C
European−A
European−B
European−C

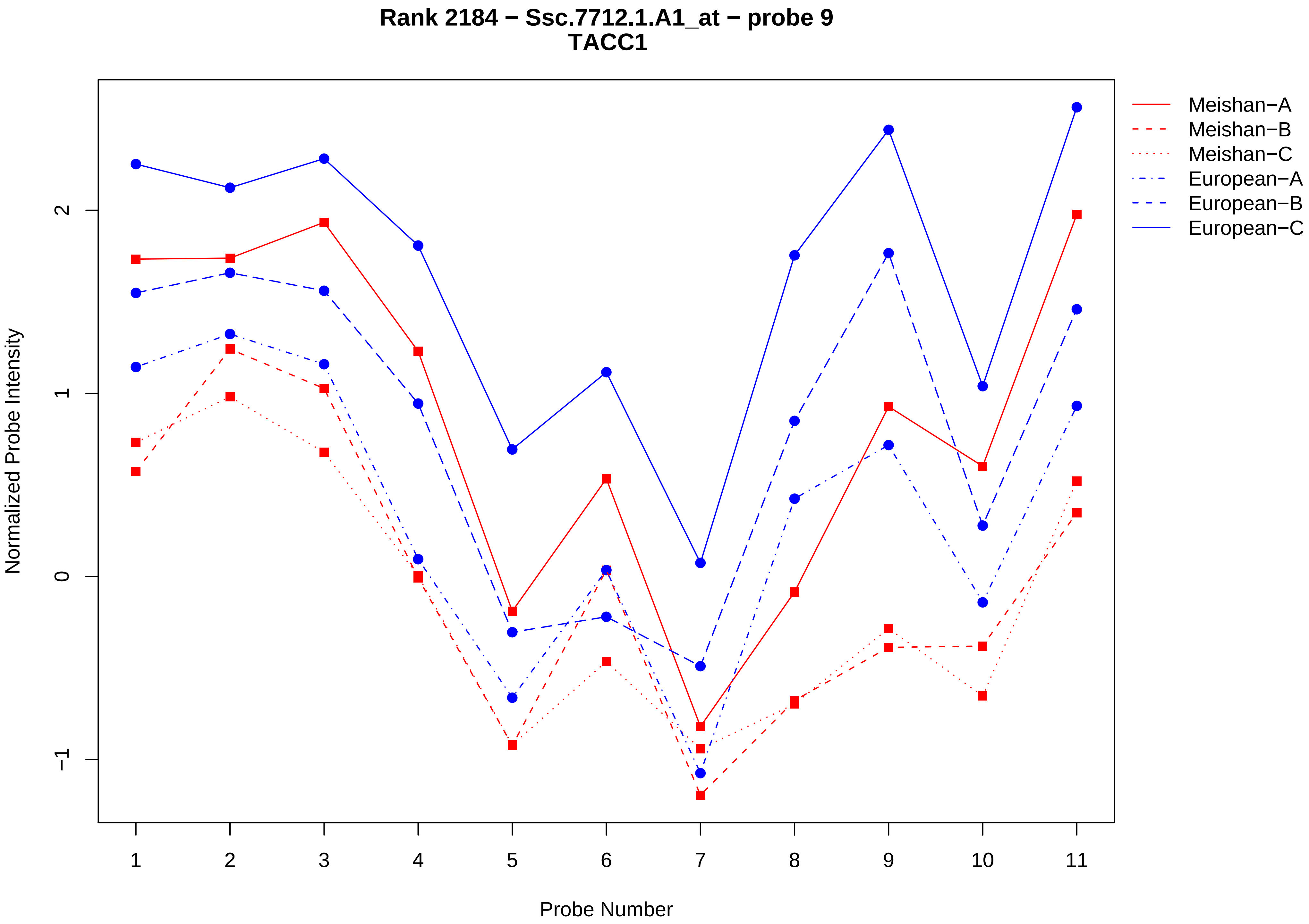

Rank 2184 − Ssc.7712.1.A1_at − probe 9
TACC1
Normalized Probe Intensity
Probe Number
−1
0
1
2
1
2
3
4
5
6
7
8
9
10
11
Meishan−A
Meishan−B
Meishan−C
European−A
European−B
European−C

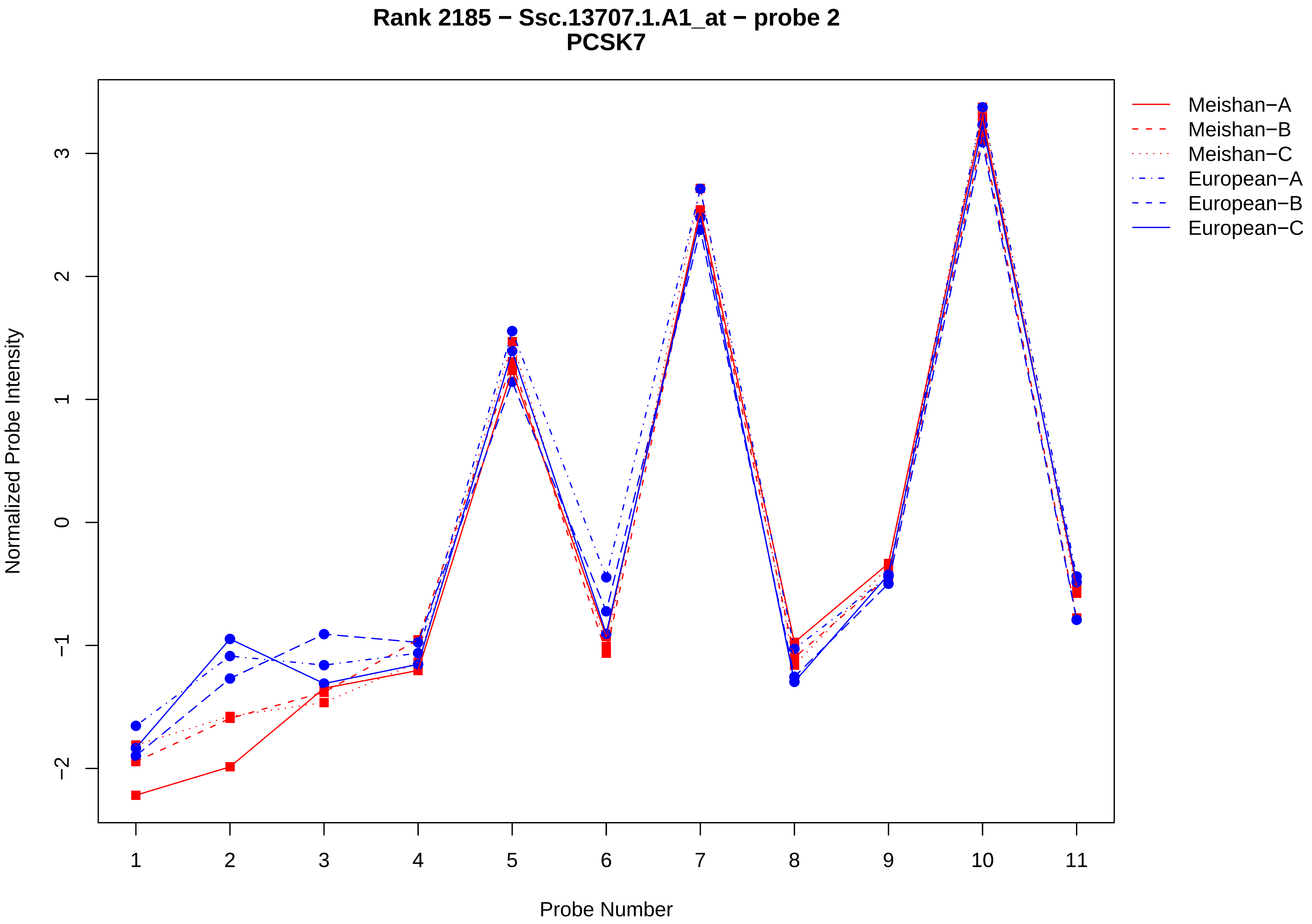

Rank 2185 − Ssc.13707.1.A1_at − probe 2
PCSK7
Normalized Probe Intensity
−2
−1
0
1
2
3
1
2
3
4
5
6
7
8
9
10
11
Probe Number
Meishan−A
Meishan−B
Meishan−C
European−A
European−B
European−C

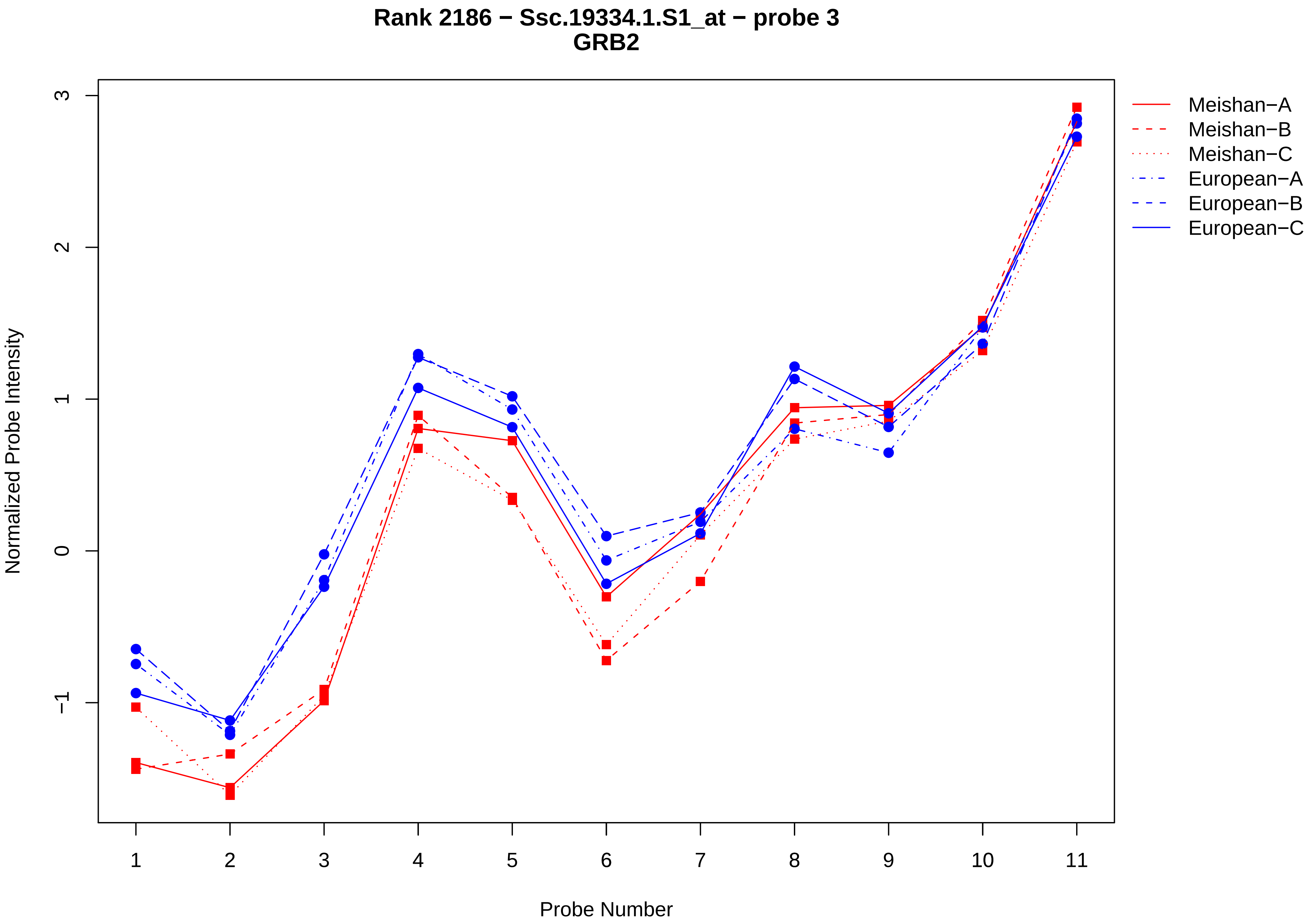

Rank 2186 − Ssc.19334.1.S1_at − probe 3
GRB2
Normalized Probe Intensity
Probe Number
−1
0
1
2
3
1
2
3
4
5
6
7
8
9
10
11
Meishan−A
Meishan−B
Meishan−C
European−A
European−B
European−C

Rank 2187 − Ssc.1354.1.S1_at − probe 6
ERAF

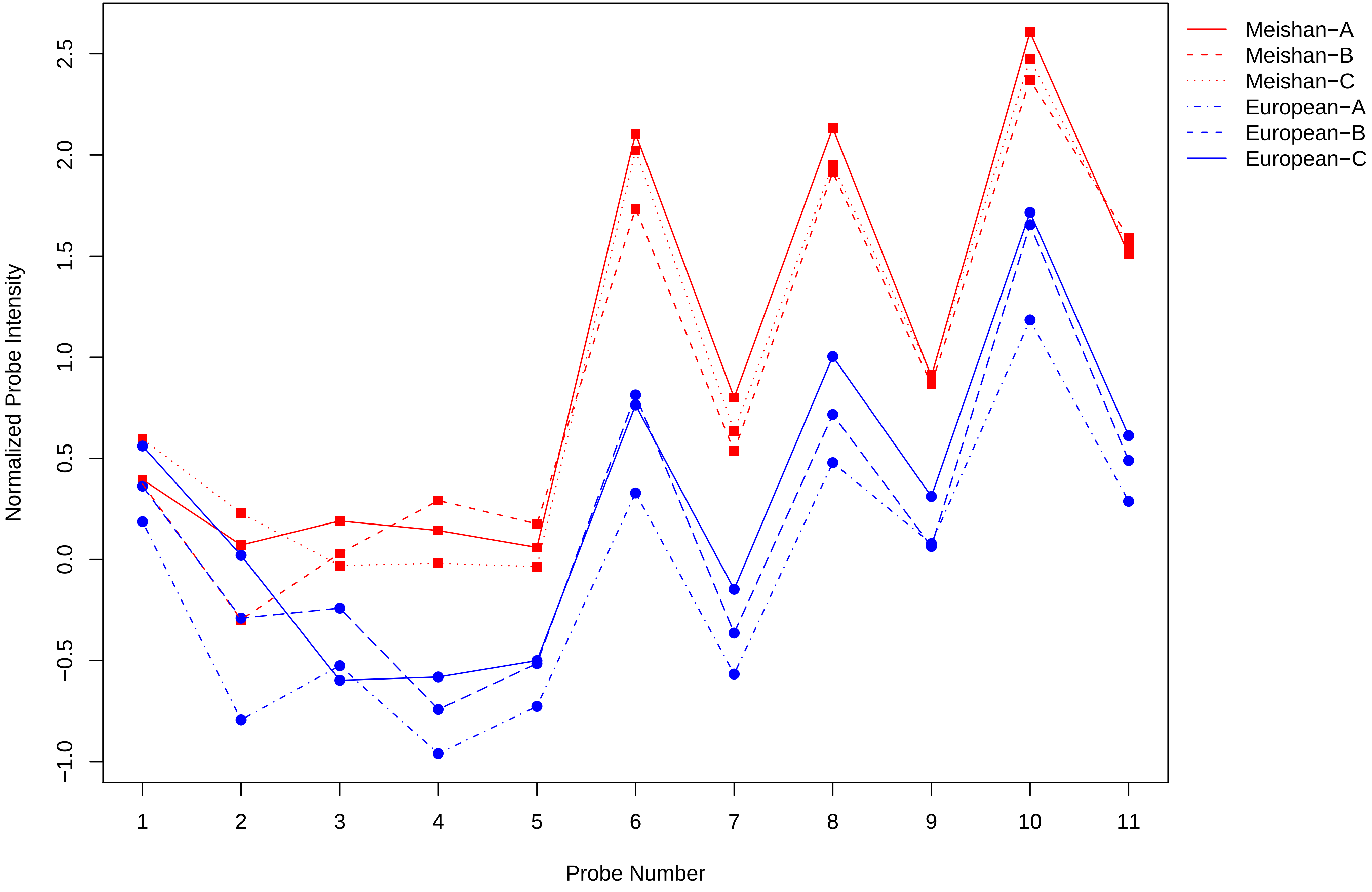

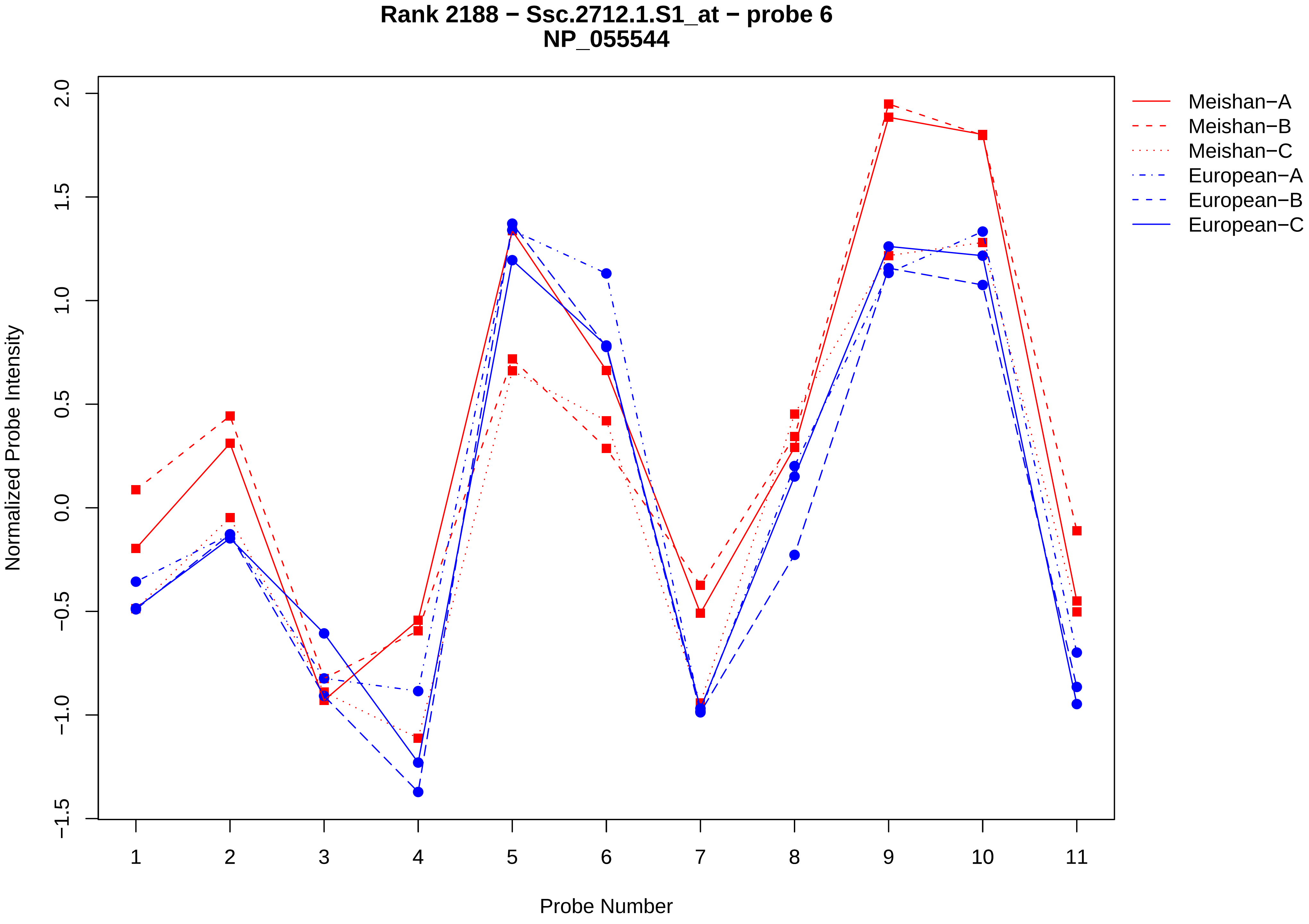
Rank 2188 − Ssc.2712.1.S1_at − probe 6
NP_055544
Normalized Probe Intensity
Probe Number
2.0
1.5
1.0
0.5
0.0
−0.5
−1.0
−1.5
1
2
3
4
5
6
7
8
9
10
11
Meishan−A
Meishan−B
Meishan−C
European−A
European−B
European−C

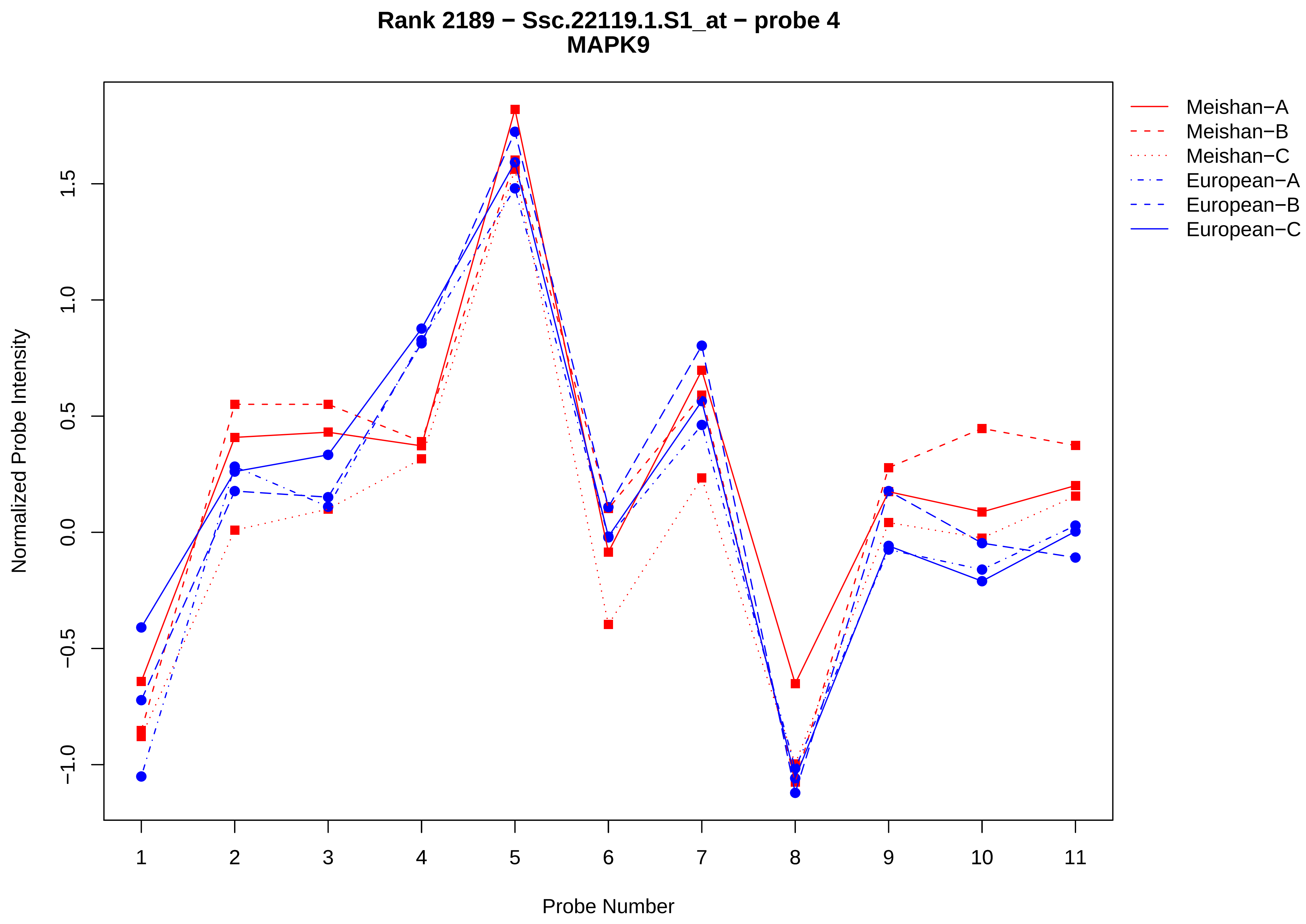
Rank 2189 − Ssc.22119.1.S1_at − probe 4
MAPK9
Normalized Probe Intensity
Probe Number
−1.0
−0.5
0.0
0.5
1.0
1.5
1
2
3
4
5
6
7
8
9
10
11
Meishan−A
Meishan−B
Meishan−C
European−A
European−B
European−C

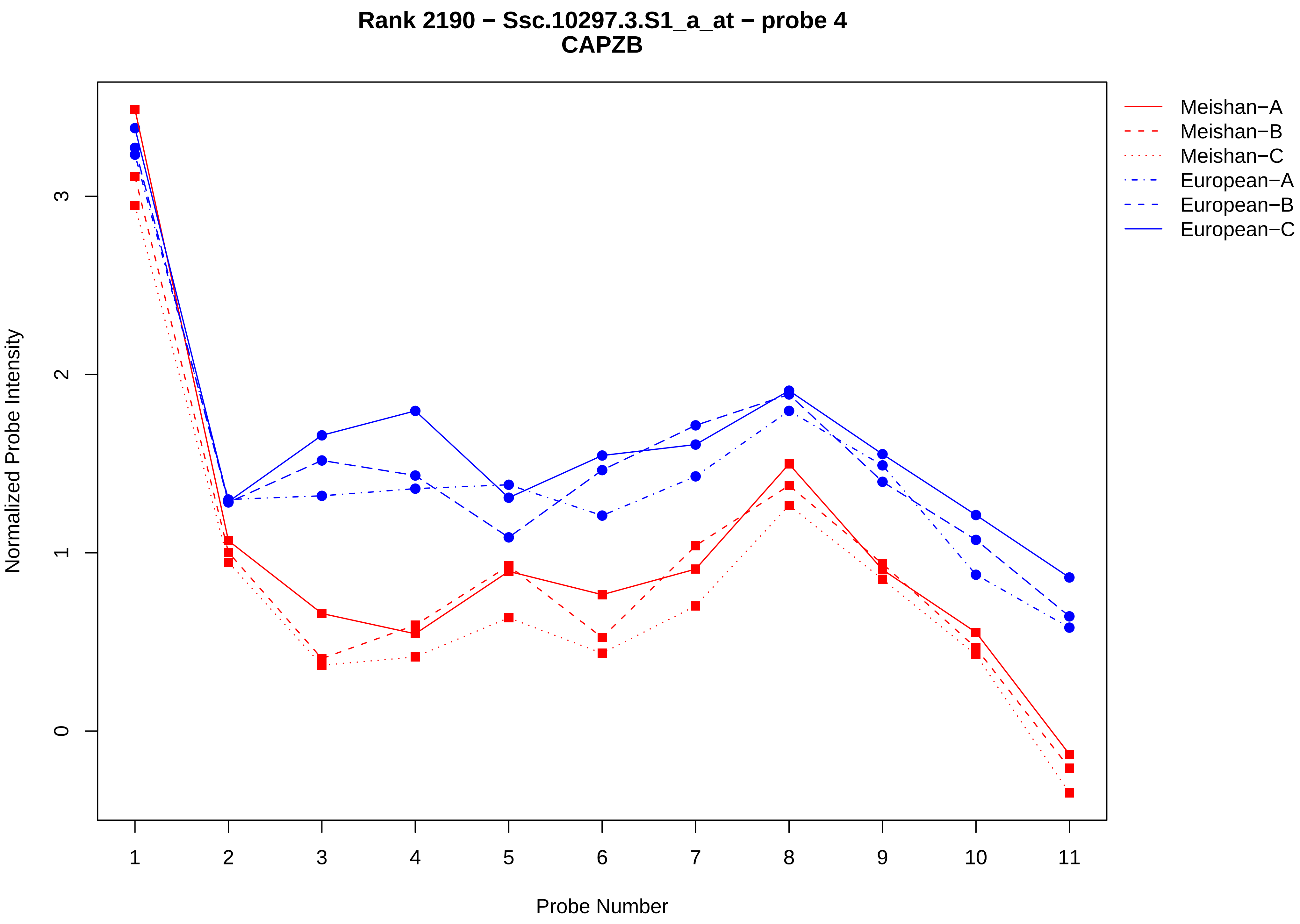

Rank 2190 − Ssc.10297.3.S1_a_at − probe 4
CAPZB
Normalized Probe Intensity
Probe Number
0
1
2
3
1
2
3
4
5
6
7
8
9
10
11
Meishan−A
Meishan−B
Meishan−C
European−A
European−B
European−C

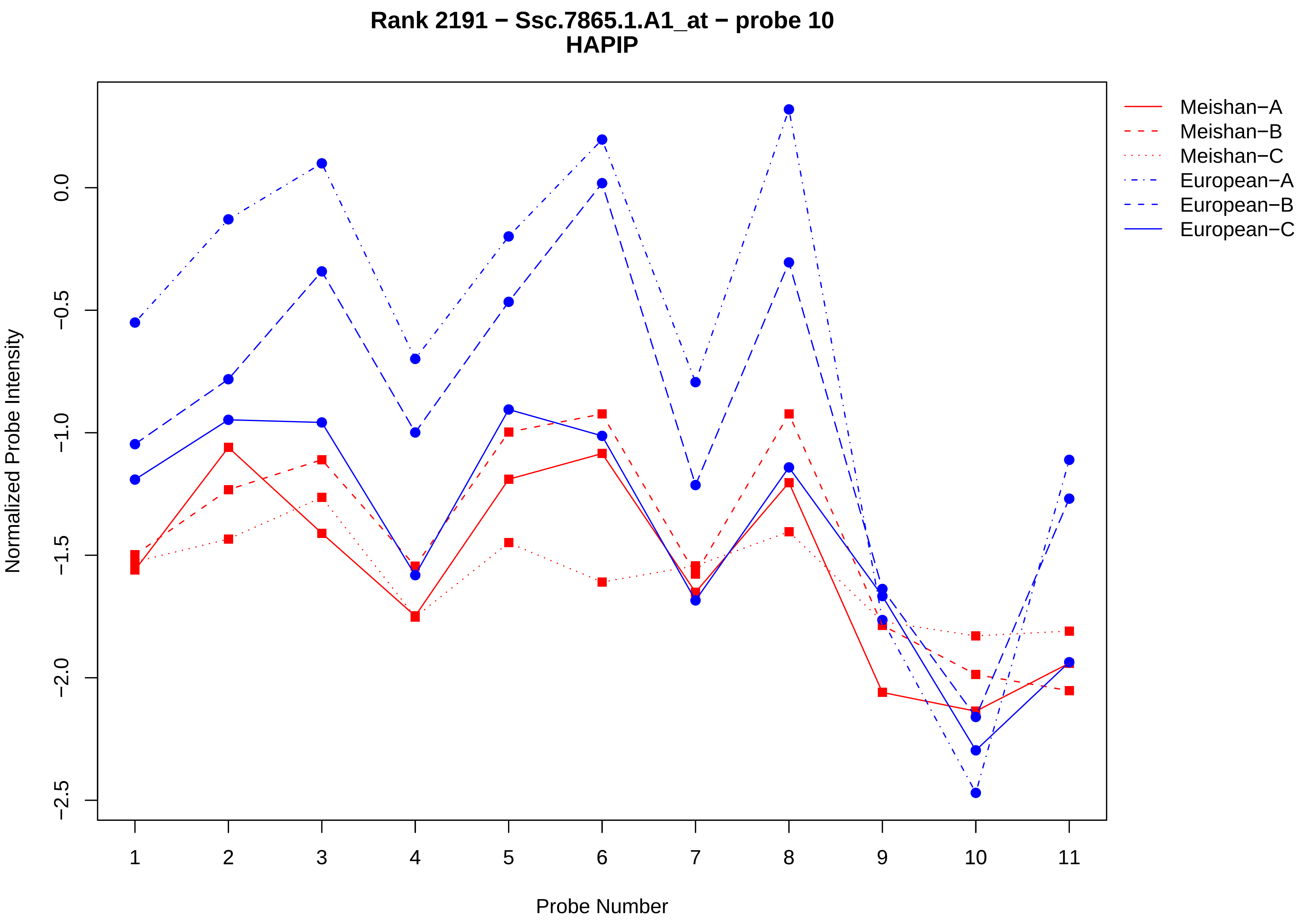

Rank 2191 − Ssc.7865.1.A1_at − probe 10
HAPIP
Normalized Probe Intensity
Probe Number
0.0
−0.5
−1.0
−1.5
−2.0
−2.5
1
2
3
4
5
6
7
8
9
10
11
Meishan−A
Meishan−B
Meishan−C
European−A
European−B
European−C

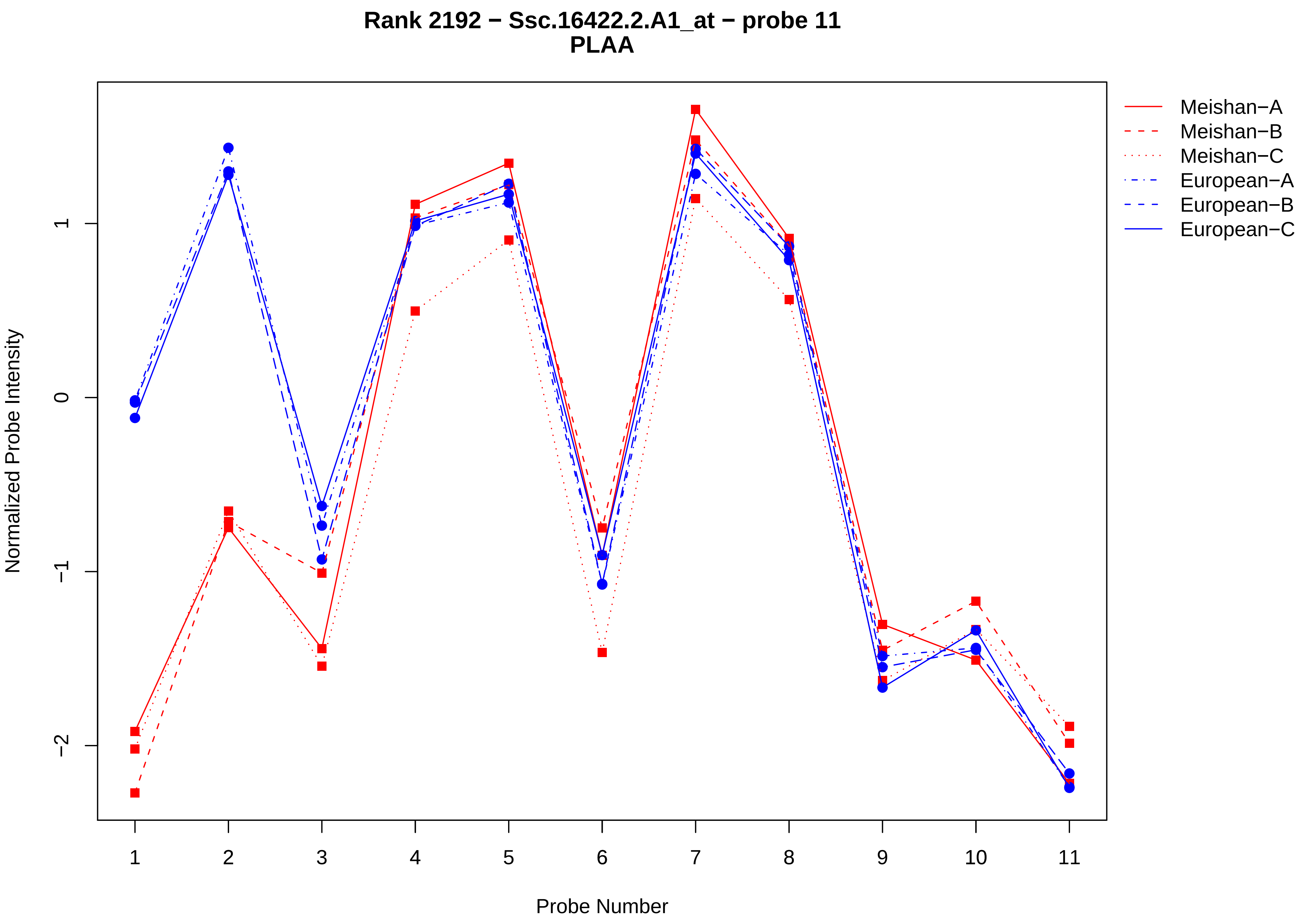

Rank 2192 − Ssc.16422.2.A1_at − probe 11
PLAA
Normalized Probe Intensity
Probe Number
−2
−1
0
1
1
2
3
4
5
6
7
8
9
10
11
Meishan−A
Meishan−B
Meishan−C
European−A
European−B
European−C

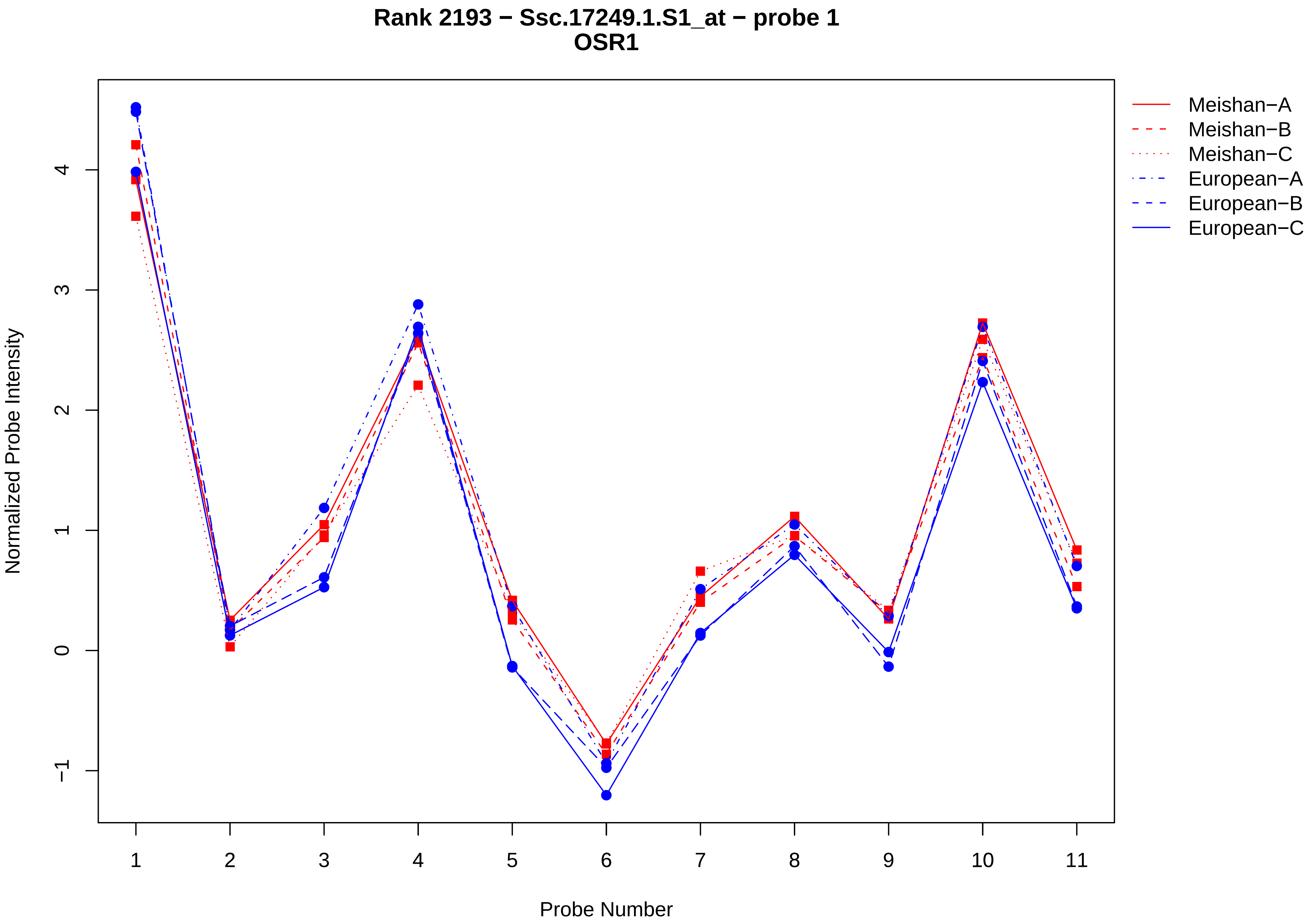

Rank 2193 − Ssc.17249.1.S1_at − probe 1
OSR1
Normalized Probe Intensity
Probe Number
Meishan−A
Meishan−B
Meishan−C
European−A
European−B
European−C

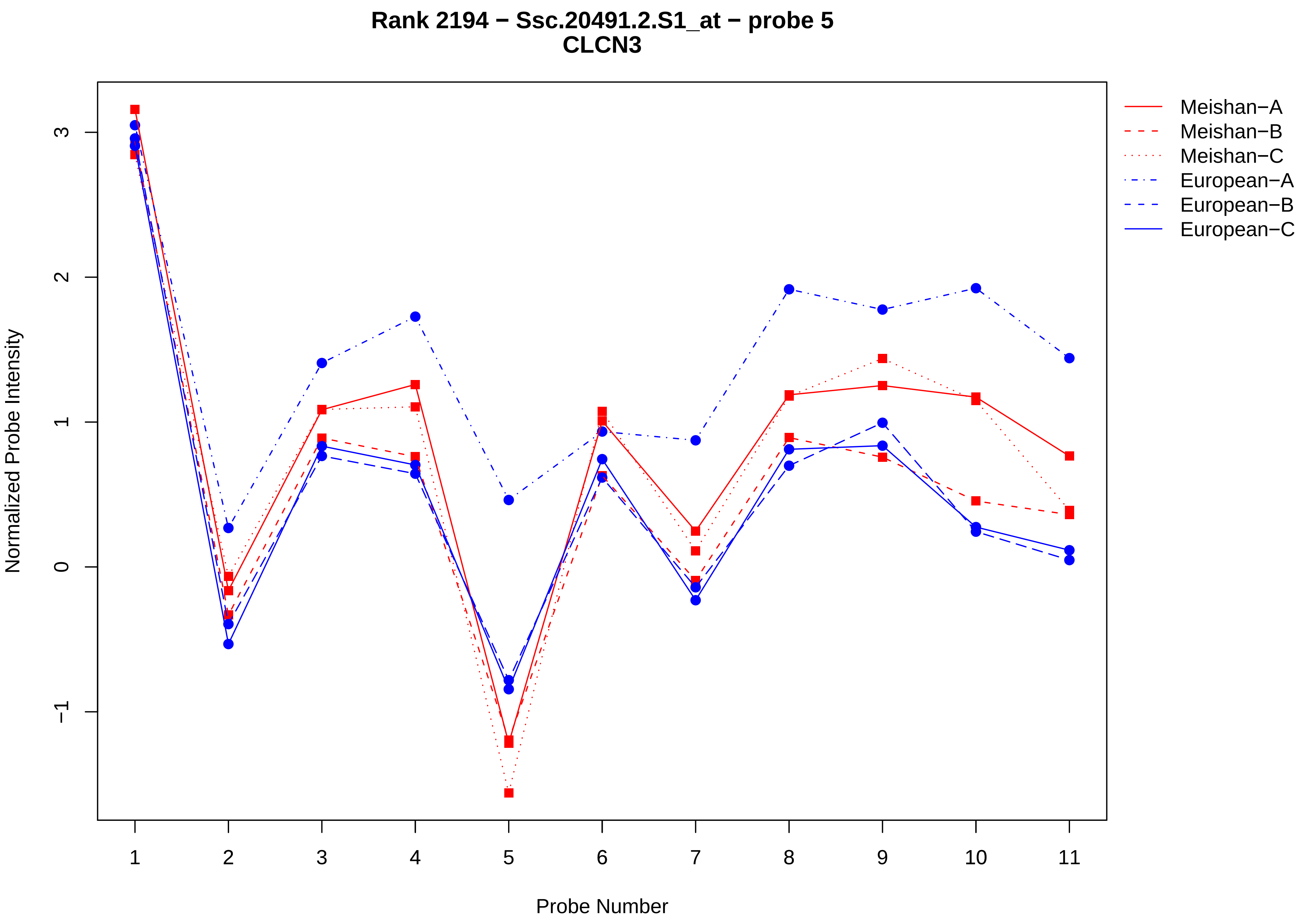

Rank 2194 − Ssc.20491.2.S1_at − probe 5
CLCN3
Normalized Probe Intensity
Probe Number
Meishan−A
Meishan−B
Meishan−C
European−A
European−B
European−C

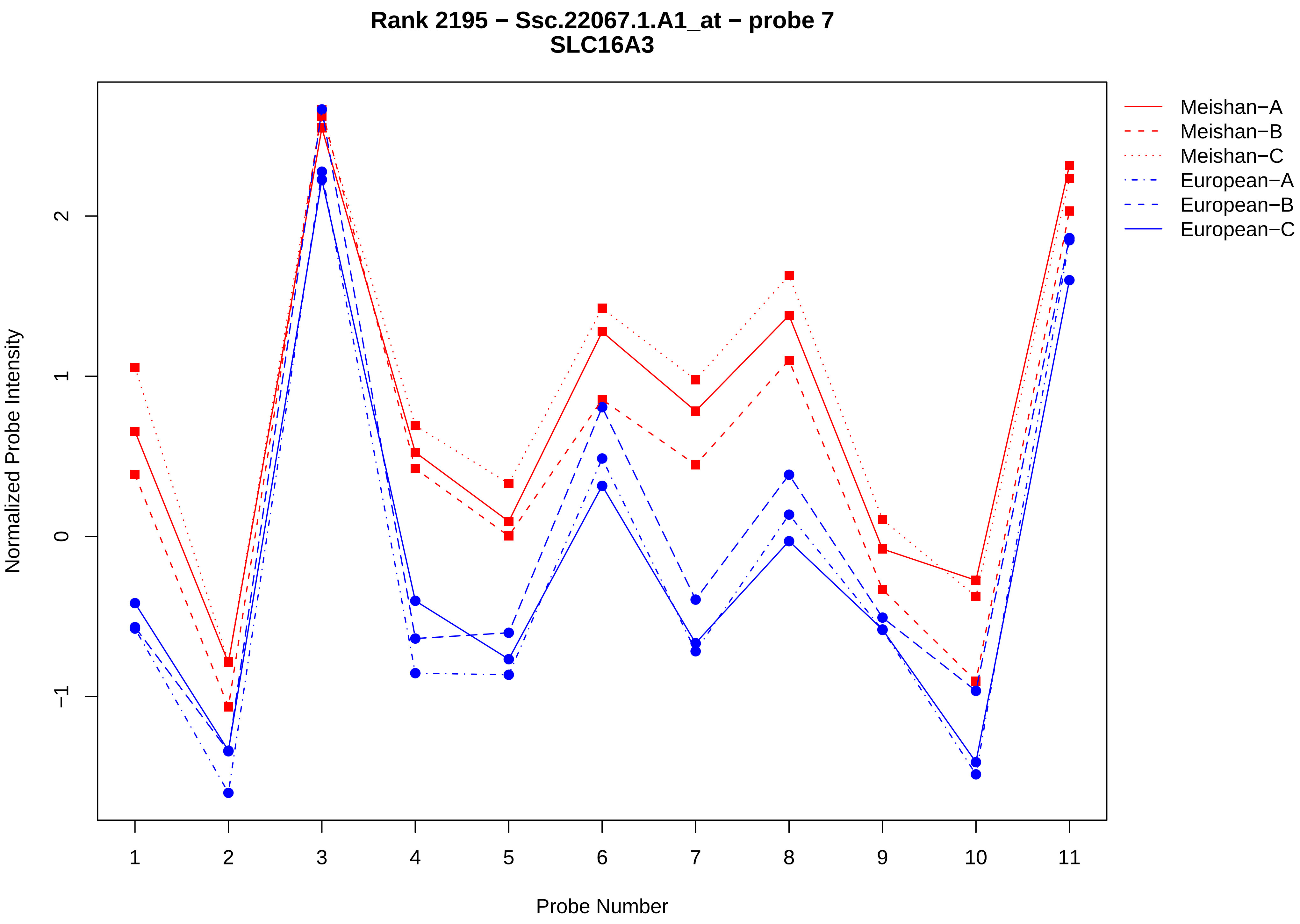

Rank 2195 − Ssc.22067.1.A1_at − probe 7
SLC16A3
Normalized Probe Intensity
Probe Number
Meishan−A
Meishan−B
Meishan−C
European−A
European−B
European−C
−1
0
1
2
1
2
3
4
5
6
7
8
9
10
11

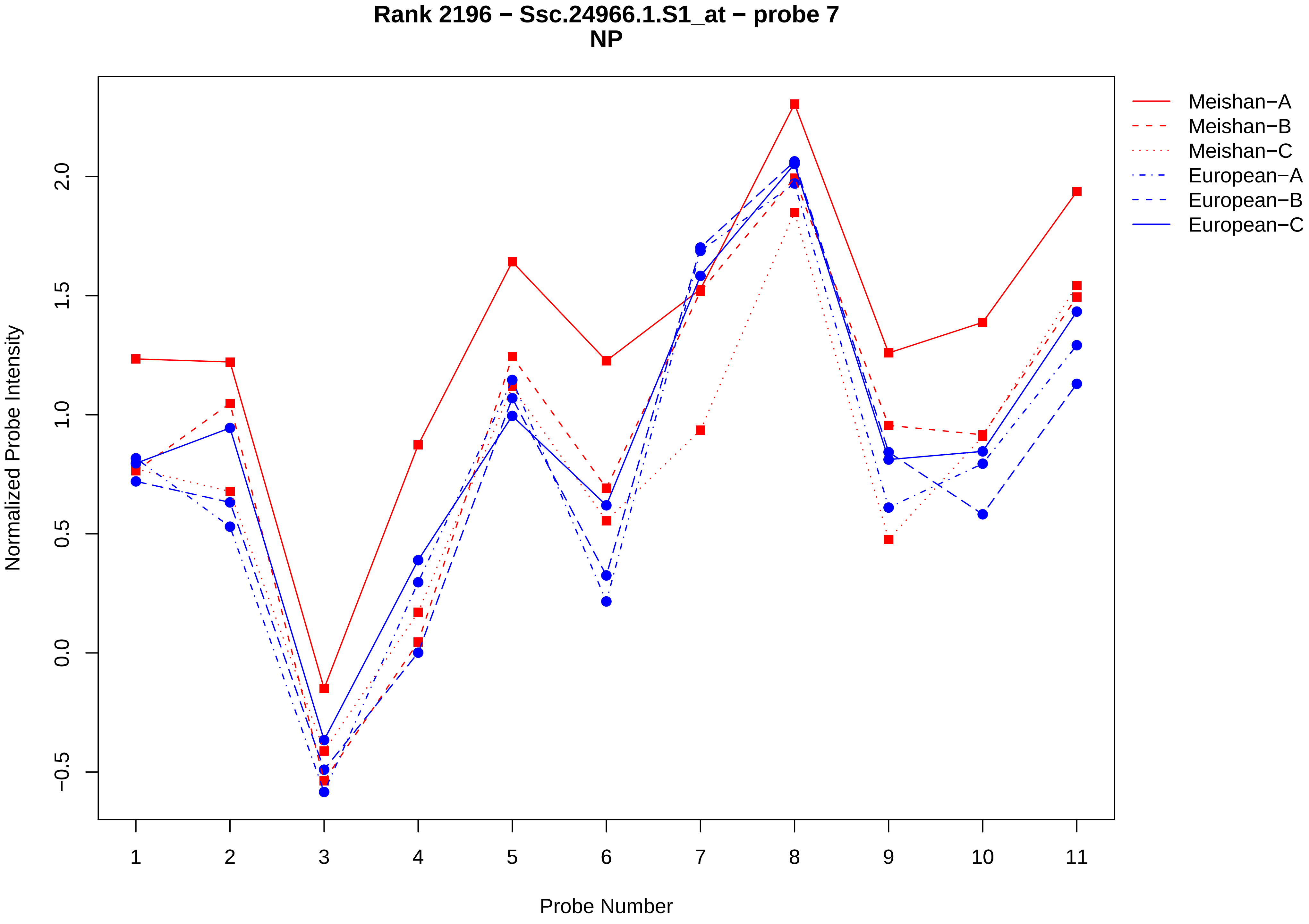

Rank 2196 − Ssc.24966.1.S1_at − probe 7
NP
Normalized Probe Intensity
−0.5
0.0
0.5
1.0
1.5
2.0
1
2
3
4
5
6
7
8
9
10
11
Probe Number
Meishan−A
Meishan−B
Meishan−C
European−A
European−B
European−C

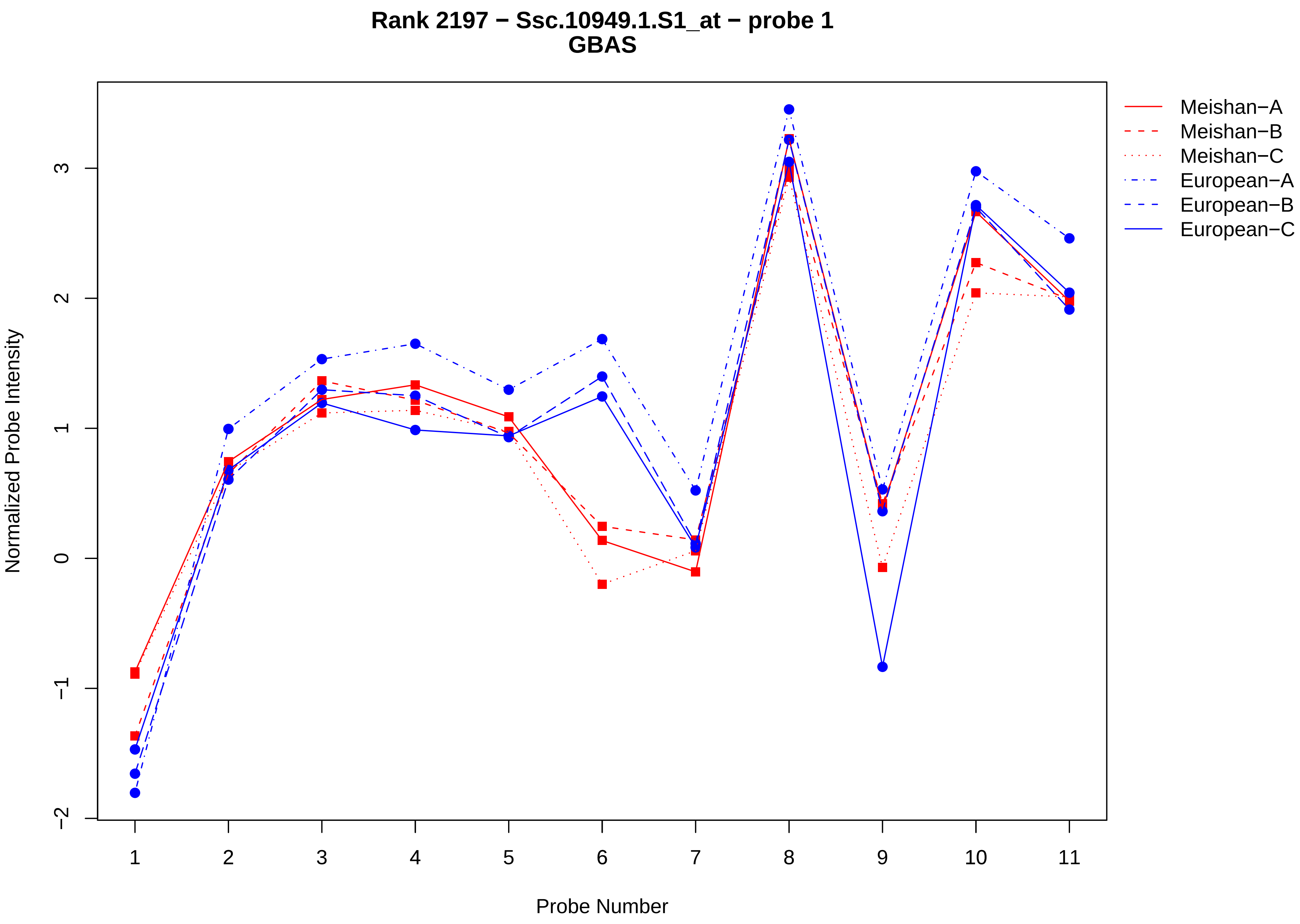

Rank 2197 − Ssc.10949.1.S1_at − probe 1
GBAS
Normalized Probe Intensity
Probe Number
−2
−1
0
1
2
3
1
2
3
4
5
6
7
8
9
10
11
Meishan−A
Meishan−B
Meishan−C
European−A
European−B
European−C

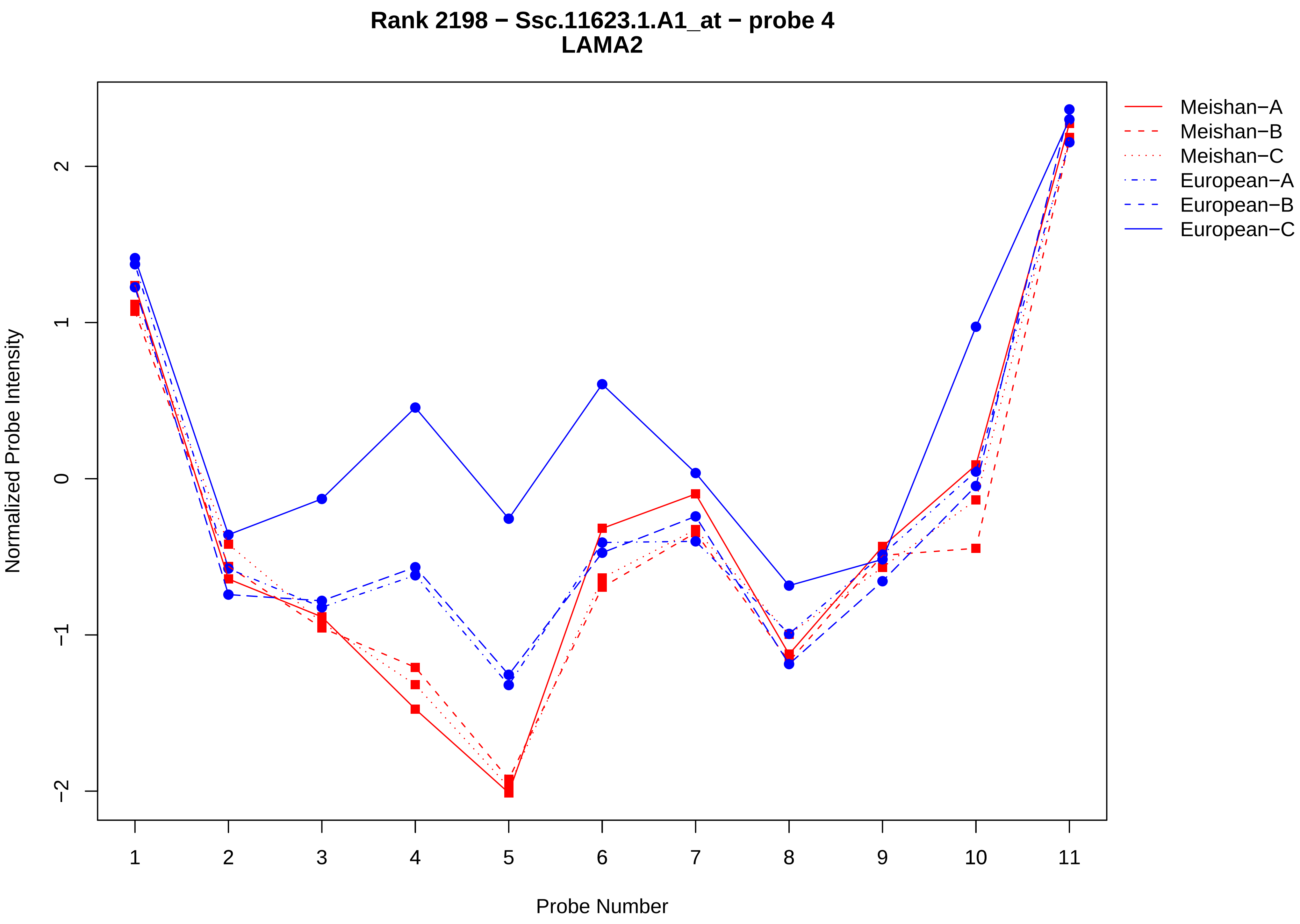
Rank 2198 − Ssc.11623.1.A1_at − probe 4
LAMA2
Meishan−A
Meishan−B
Meishan−C
European−A
European−B
European−C
Normalized Probe Intensity
Probe Number
−2
−1
0
1
2
1
2
3
4
5
6
7
8
9
10
11

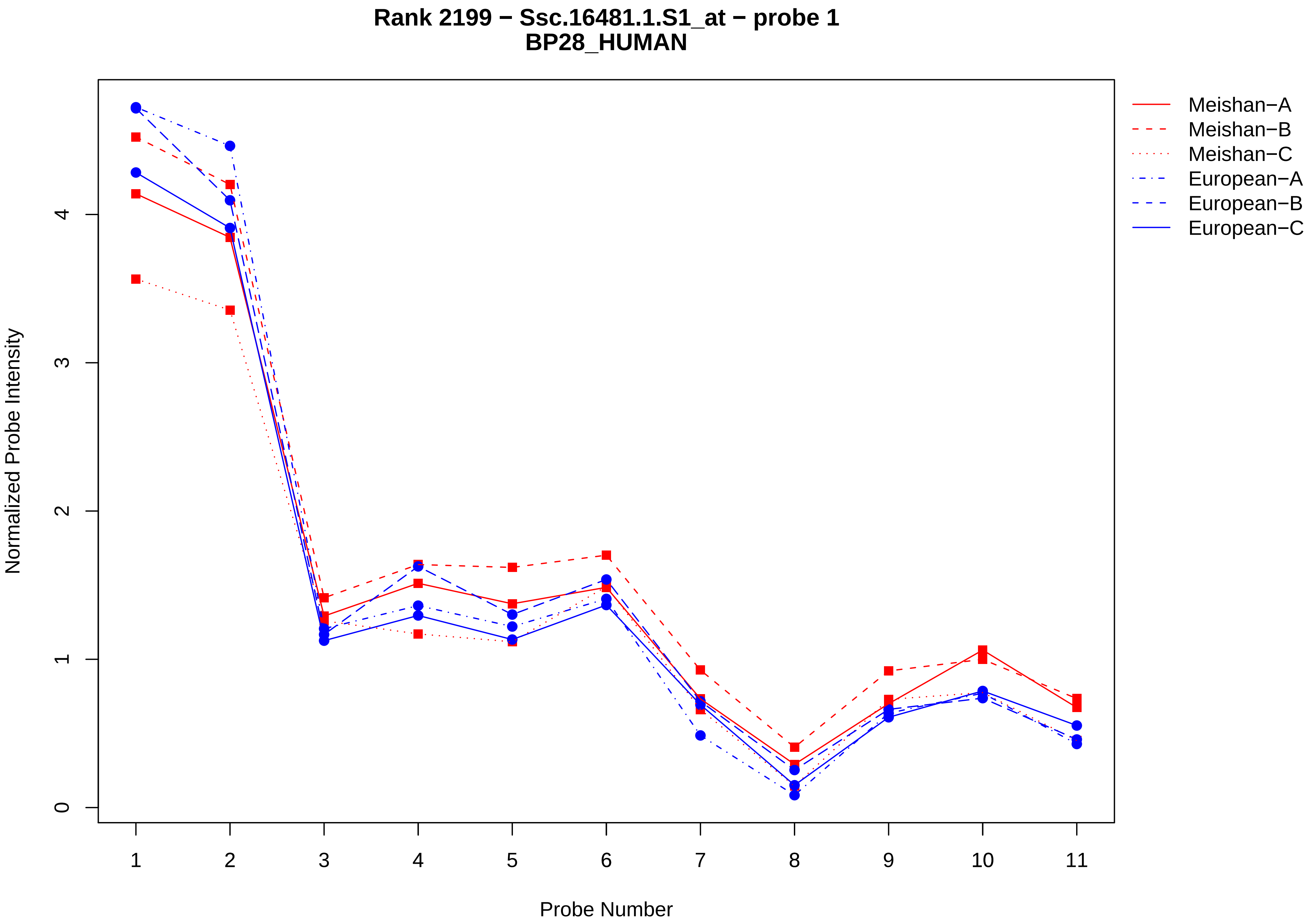

Rank 2199 − Ssc.16481.1.S1_at − probe 1
BP28_HUMAN
Normalized Probe Intensity
Probe Number
0
1
2
3
4
1
2
3
4
5
6
7
8
9
10
11
Meishan−A
Meishan−B
Meishan−C
European−A
European−B
European−C

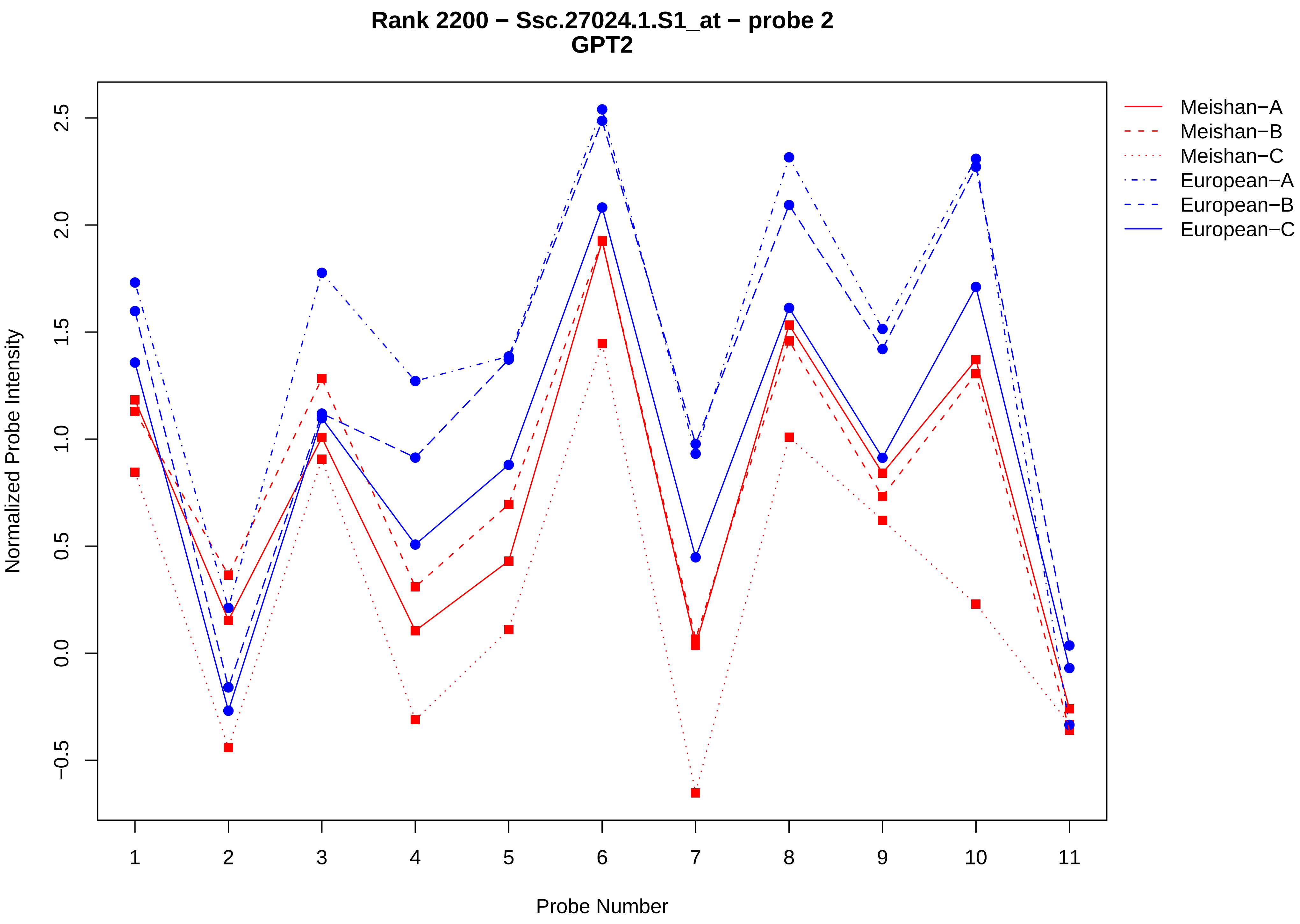

Rank 2200 − Ssc.27024.1.S1_at − probe 2
GPT2
Normalized Probe Intensity
Probe Number
−0.5
0.0
0.5
1.0
1.5
2.0
2.5
1
2
3
4
5
6
7
8
9
10
11
Meishan−A
Meishan−B
Meishan−C
European−A
European−B
European−C

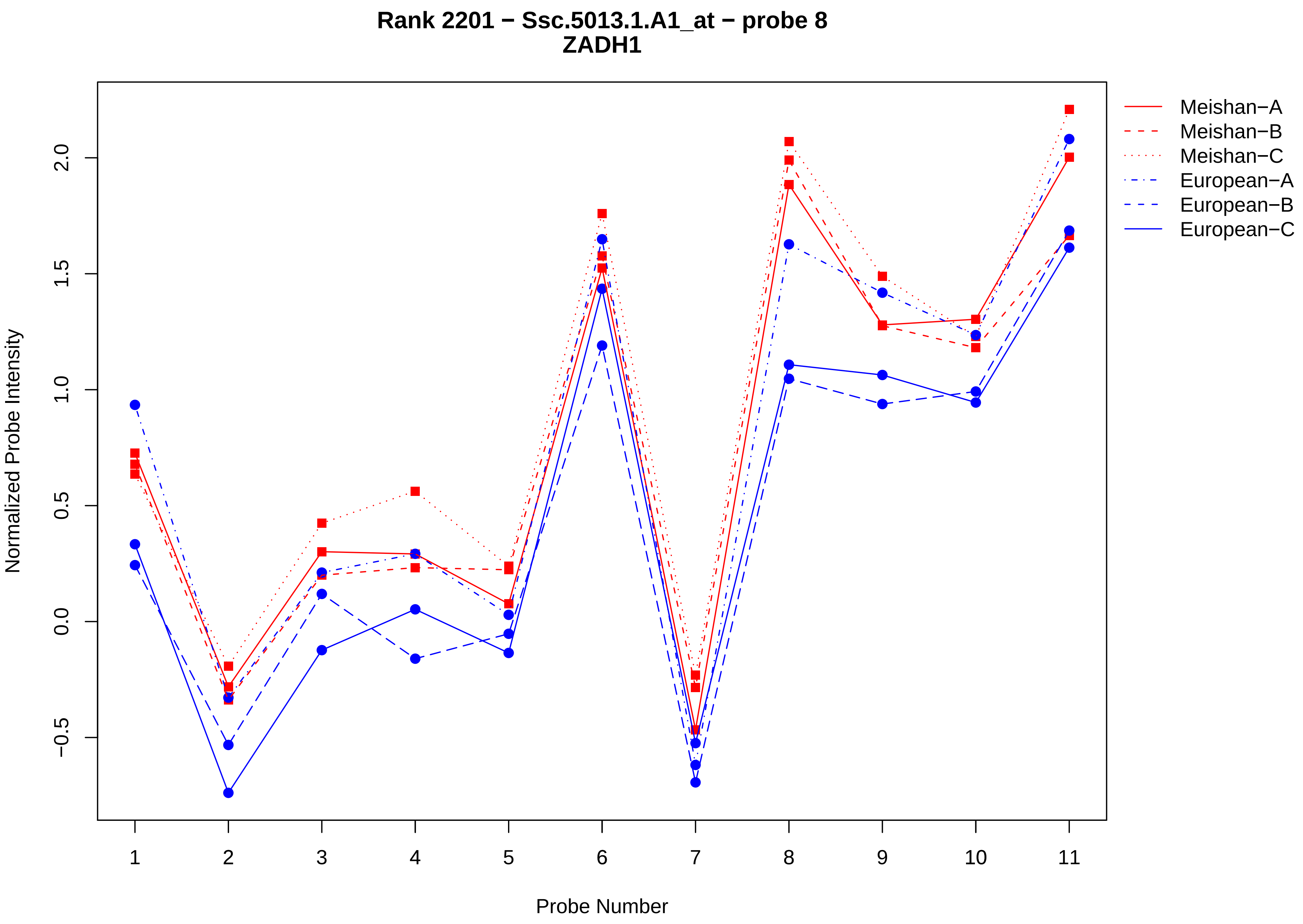

Rank 2201 − Ssc.5013.1.A1_at − probe 8
ZADH1
Normalized Probe Intensity
−0.5
0.0
0.5
1.0
1.5
2.0
1
2
3
4
5
6
7
8
9
10
11
Probe Number
Meishan−A
Meishan−B
Meishan−C
European−A
European−B
European−C

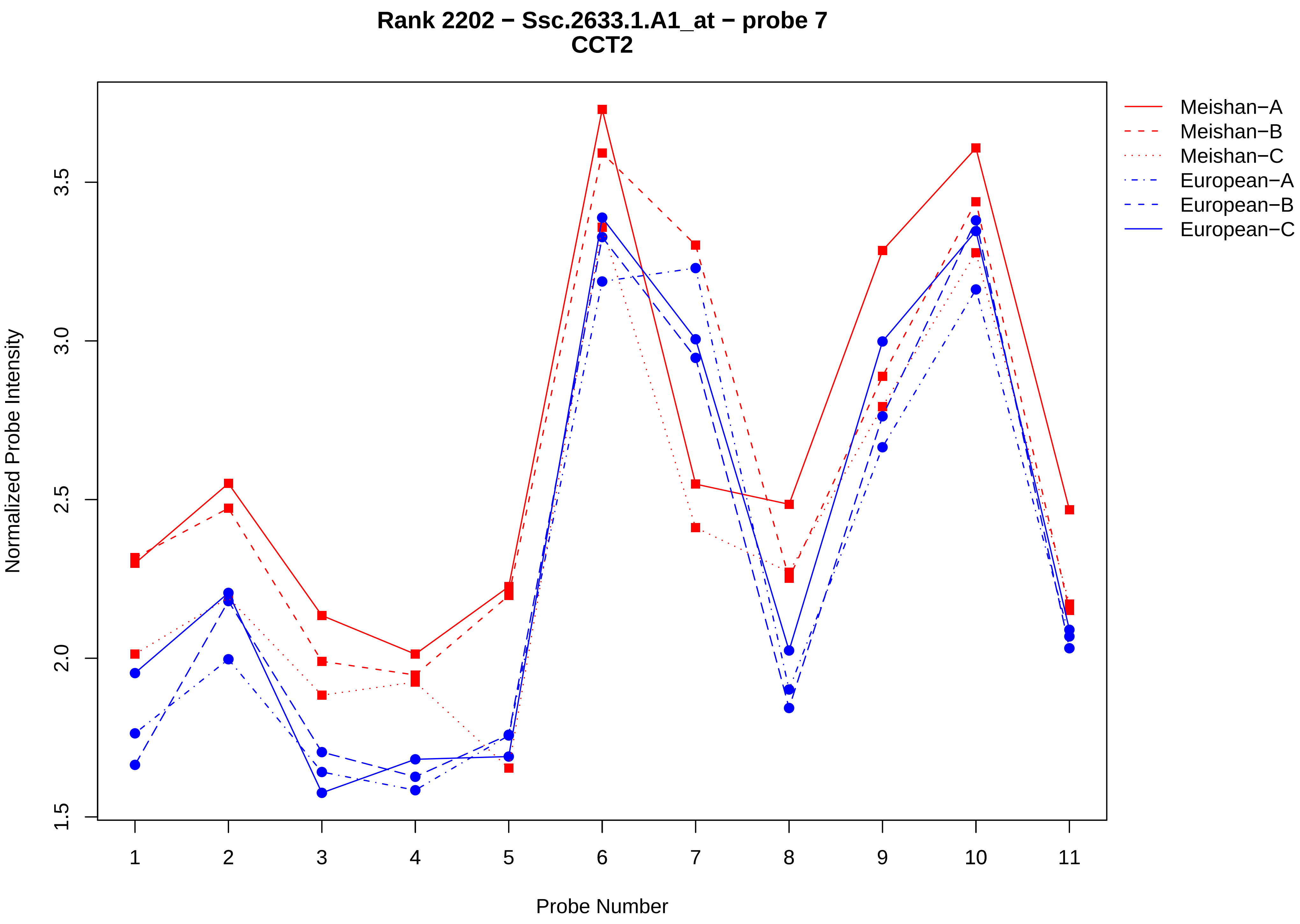

Rank 2202 − Ssc.2633.1.A1_at − probe 7
CCT2
Normalized Probe Intensity
Probe Number
1.5
2.0
2.5
3.0
3.5
1
2
3
4
5
6
7
8
9
10
11
Meishan−A
Meishan−B
Meishan−C
European−A
European−B
European−C

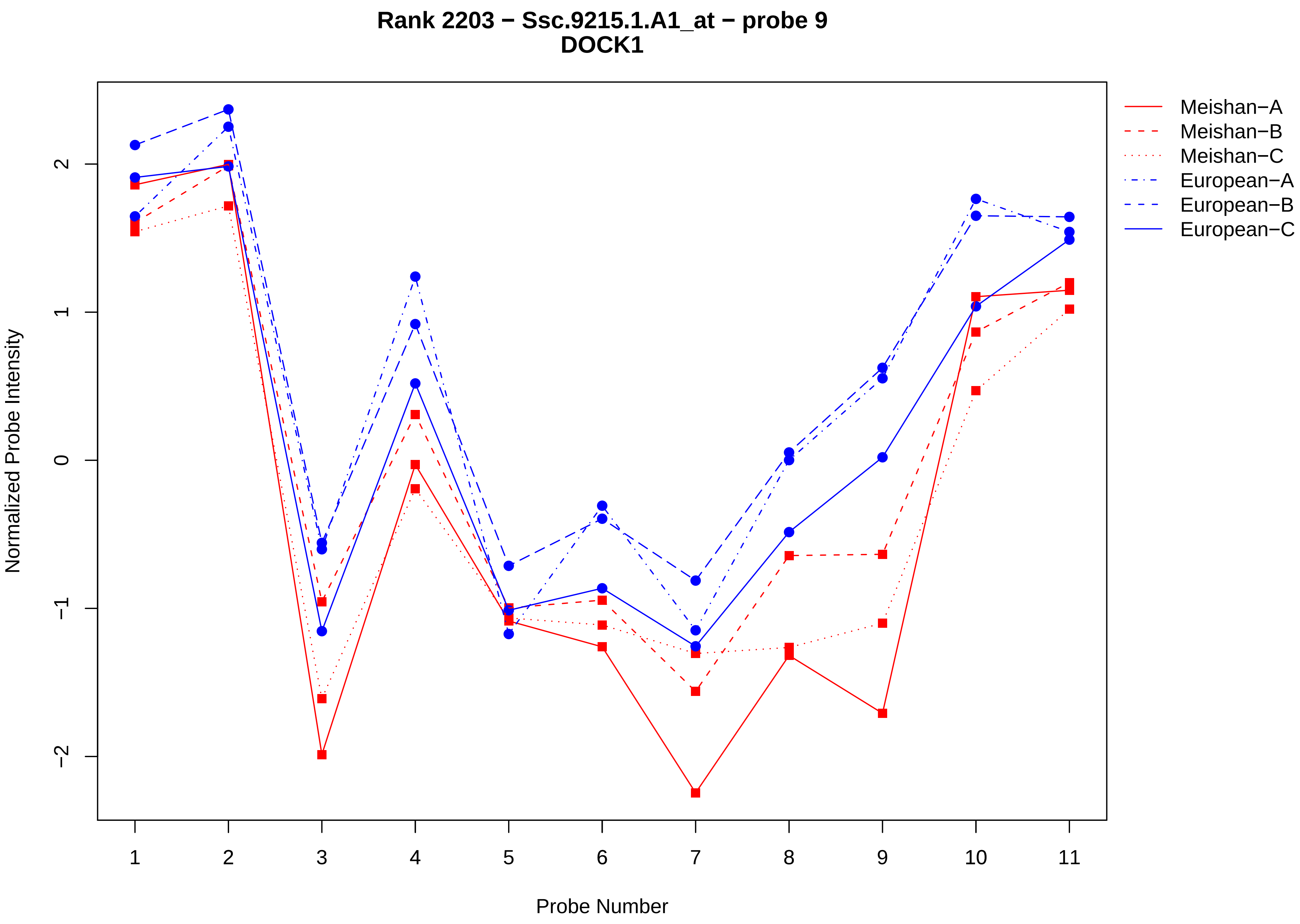

Rank 2203 − Ssc.9215.1.A1_at − probe 9
DOCK1
Normalized Probe Intensity
Probe Number
−2
−1
0
1
2
1
2
3
4
5
6
7
8
9
10
11
Meishan−A
Meishan−B
Meishan−C
European−A
European−B
European−C

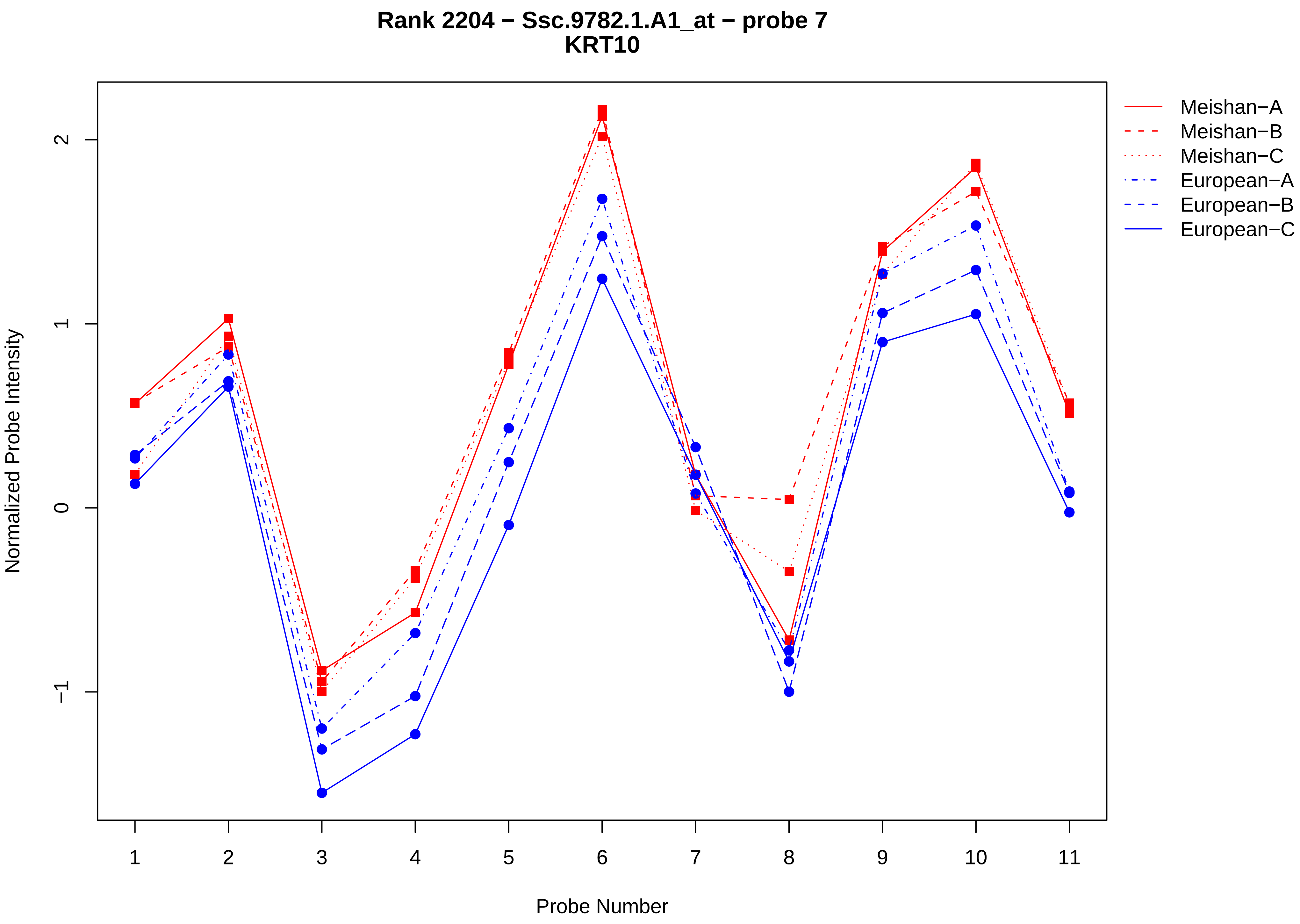

Rank 2204 − Ssc.9782.1.A1_at − probe 7
KRT10
Normalized Probe Intensity
Probe Number
−1
0
1
2
1
2
3
4
5
6
7
8
9
10
11
Meishan−A
Meishan−B
Meishan−C
European−A
European−B
European−C

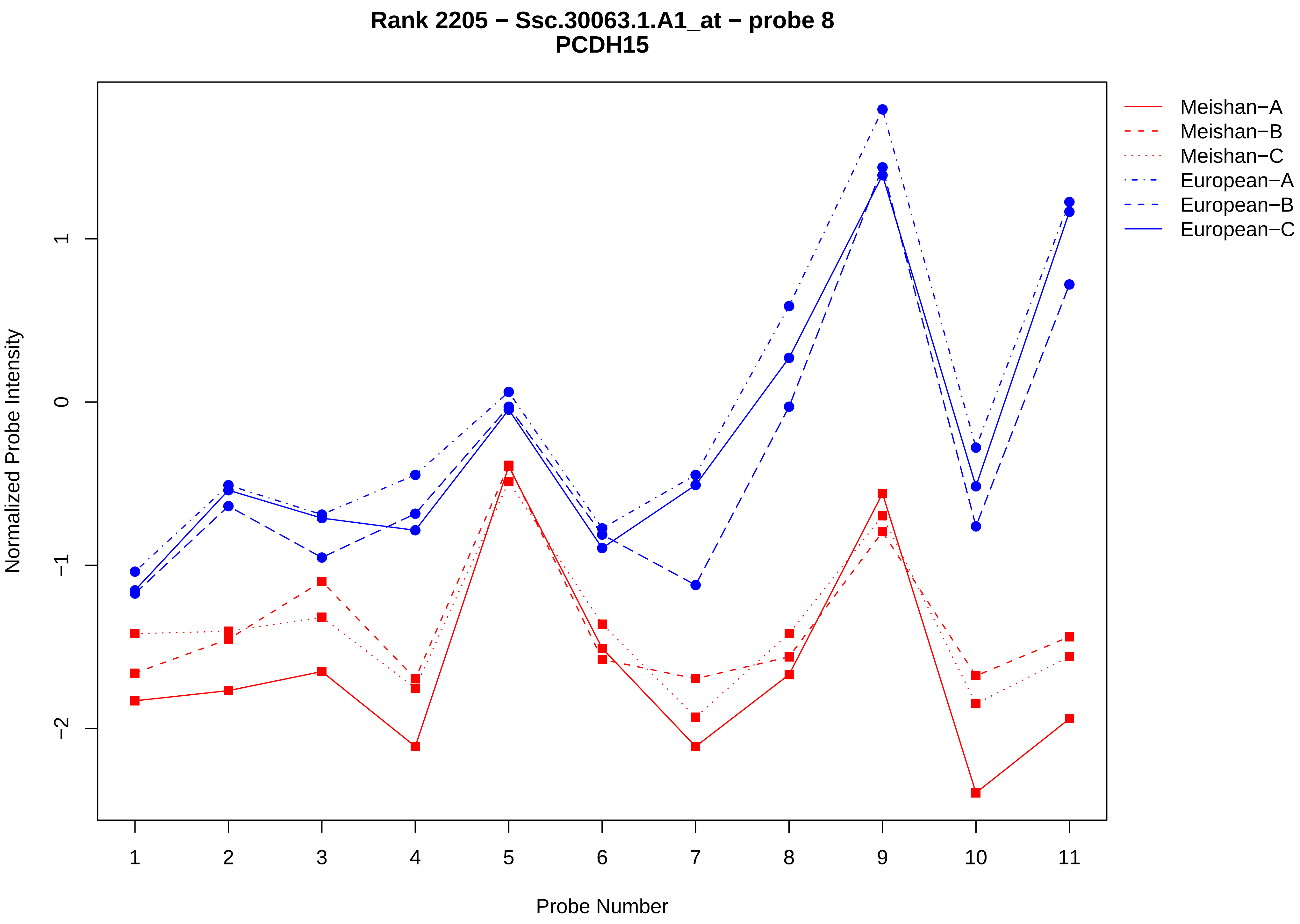
Rank 2205 − Ssc.30063.1.A1_at − probe 8
PCDH15
Normalized Probe Intensity
Probe Number
Meishan−A
Meishan−B
Meishan−C
European−A
European−B
European−C
−2
−1
0
1
1
2
3
4
5
6
7
8
9
10
11

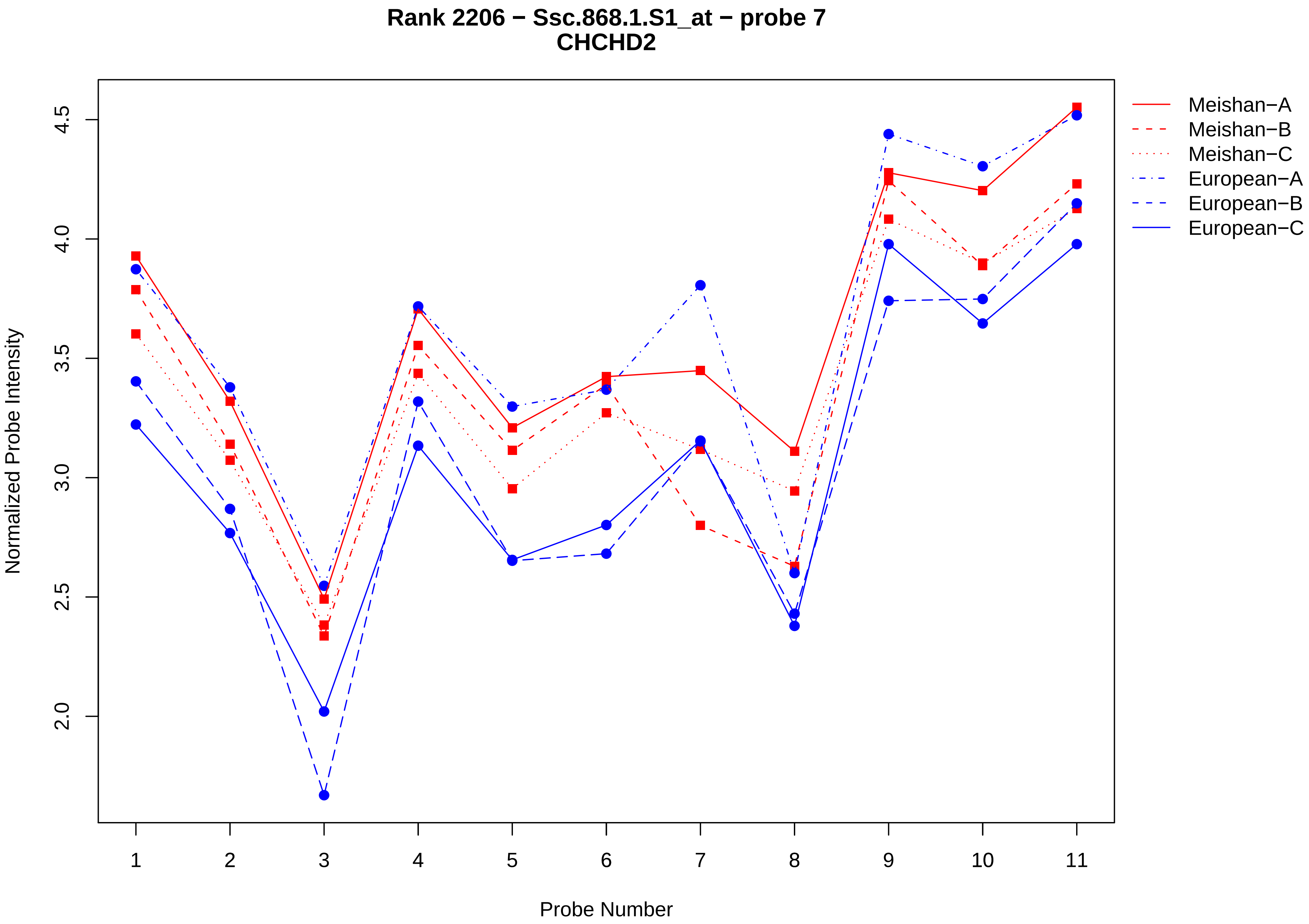

Rank 2206 − Ssc.868.1.S1_at − probe 7
CHCHD2
Normalized Probe Intensity
Probe Number
2.0
2.5
3.0
3.5
4.0
4.5
1
2
3
4
5
6
7
8
9
10
11
Meishan−A
Meishan−B
Meishan−C
European−A
European−B
European−C

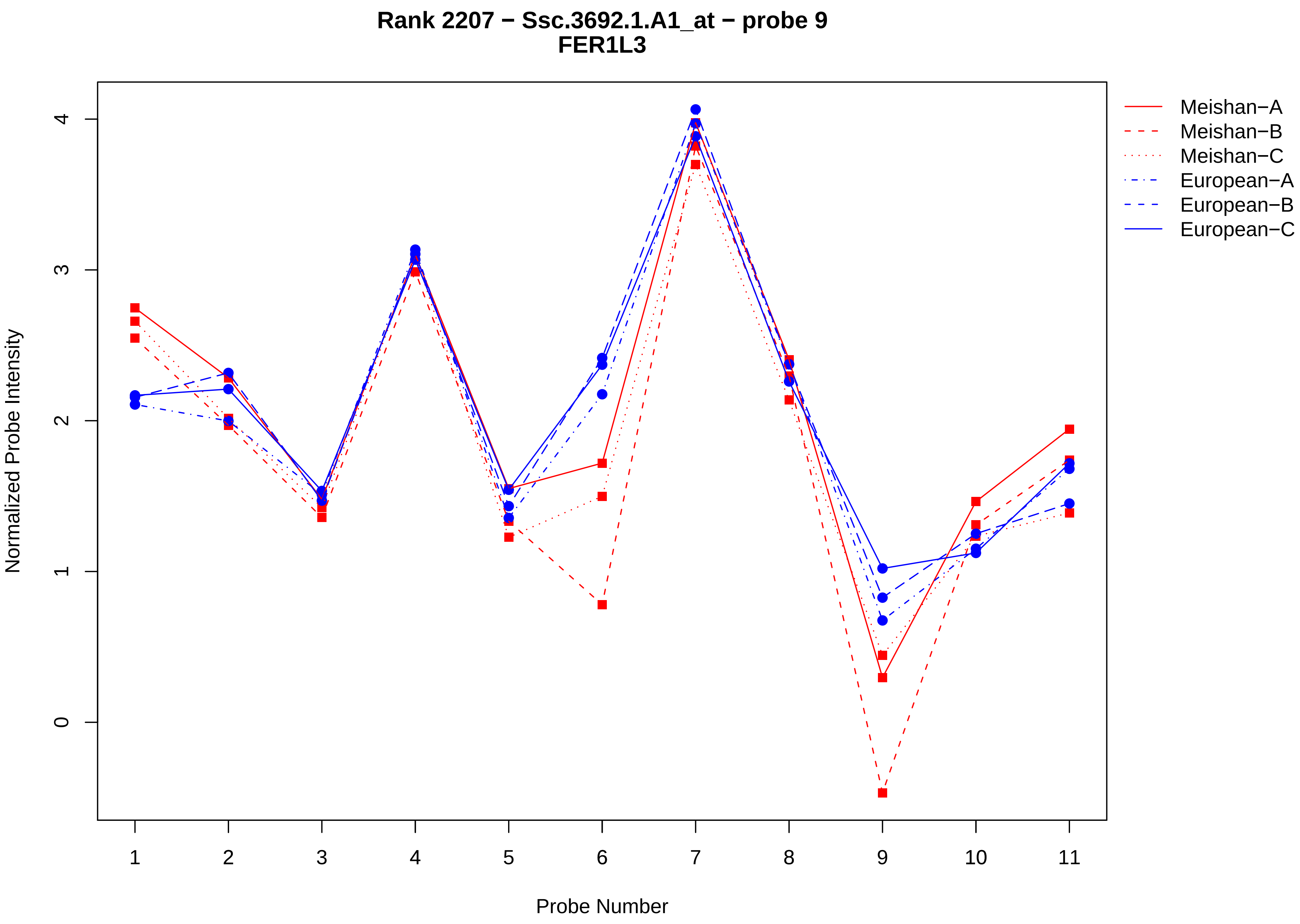

Rank 2207 − Ssc.3692.1.A1_at − probe 9
FER1L3
Normalized Probe Intensity
Probe Number
0
1
2
3
4
1
2
3
4
5
6
7
8
9
10
11
Meishan−A
Meishan−B
Meishan−C
European−A
European−B
European−C

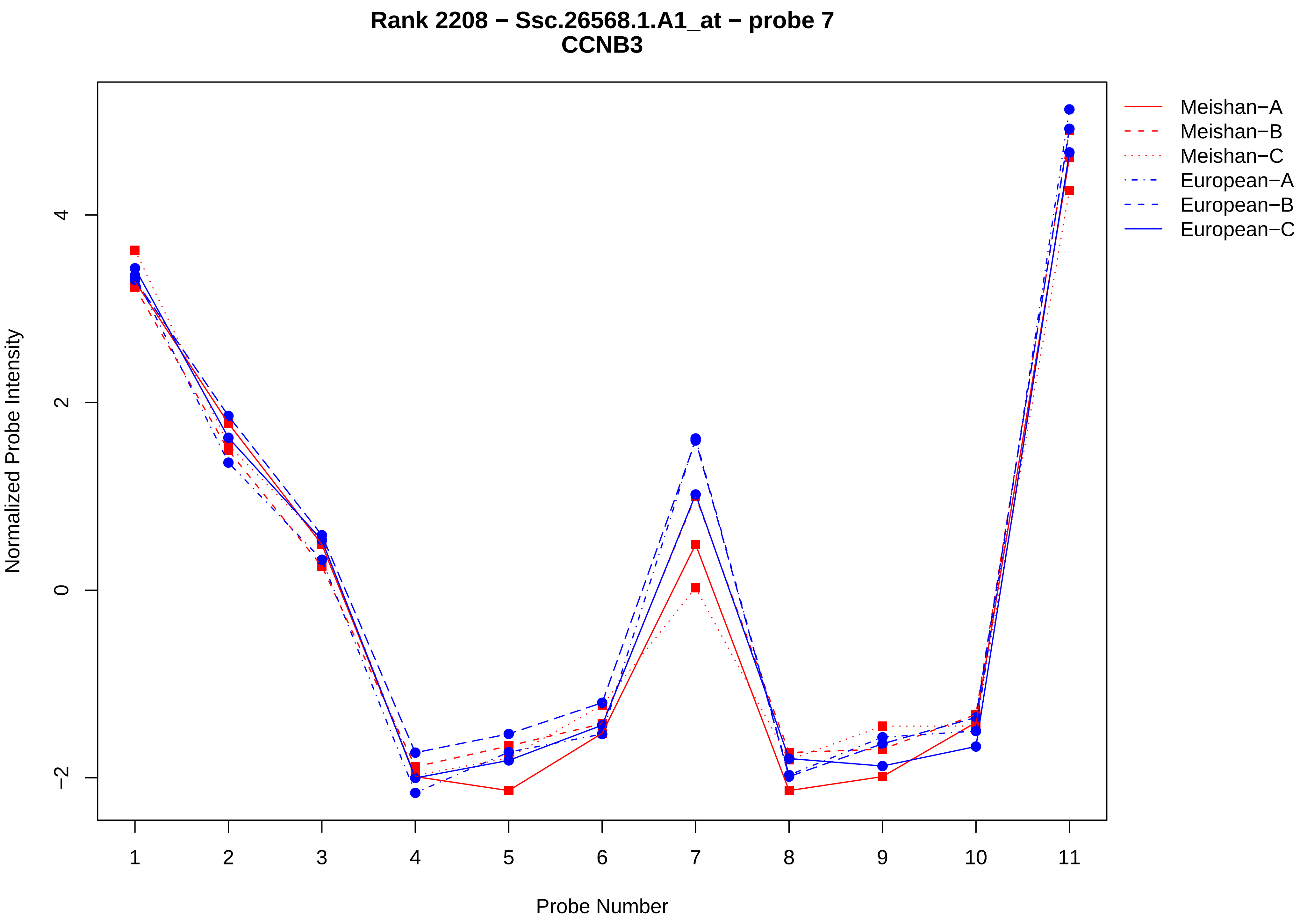

Rank 2208 − Ssc.26568.1.A1_at − probe 7
CCNB3
Normalized Probe Intensity
Probe Number
−2
0
2
4
1
2
3
4
5
6
7
8
9
10
11
Meishan−A
Meishan−B
Meishan−C
European−A
European−B
European−C

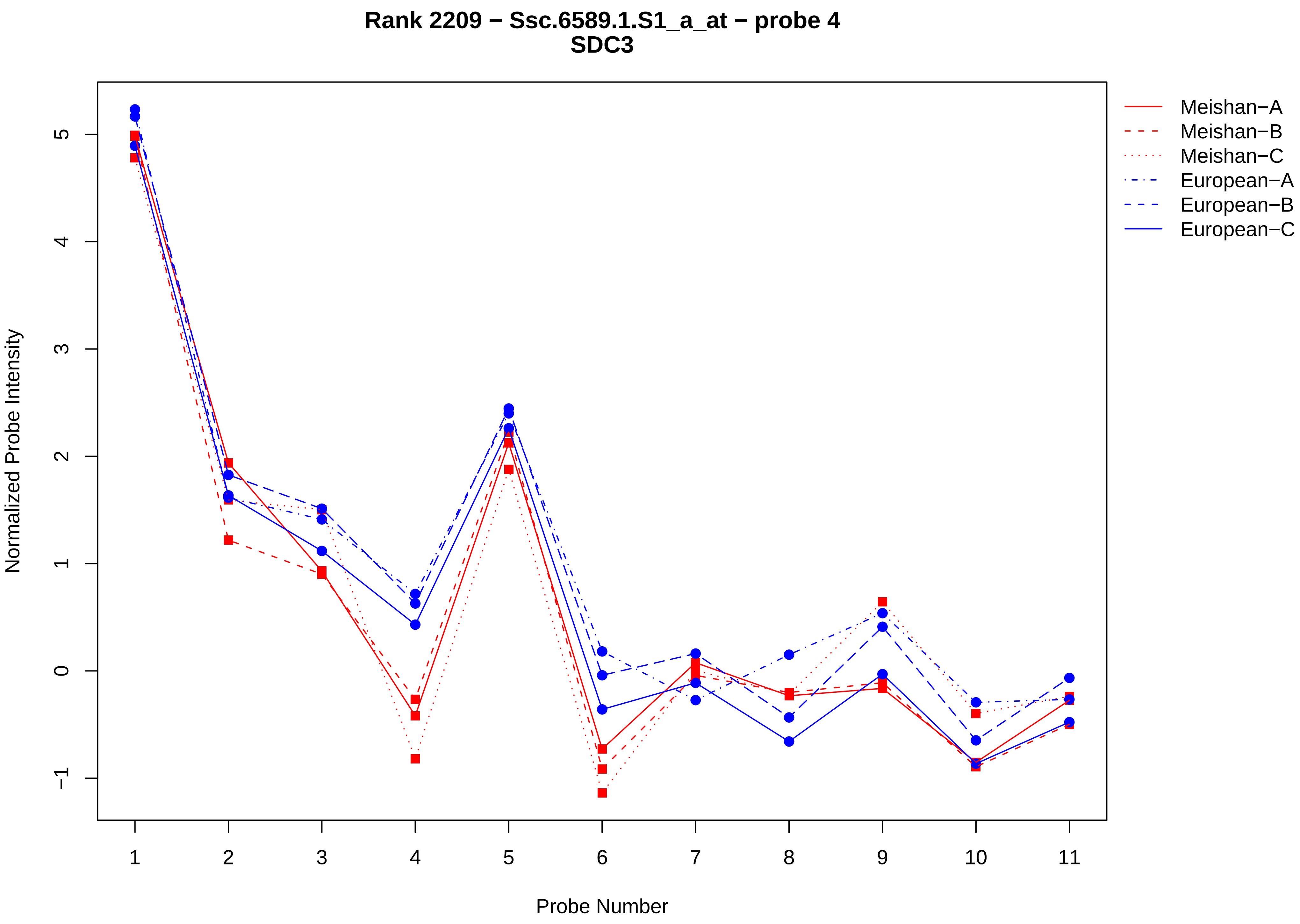

Rank 2209 − Ssc.6589.1.S1_a_at − probe 4
SDC3
Normalized Probe Intensity
Probe Number
Meishan−A
Meishan−B
Meishan−C
European−A
European−B
European−C

Rank 2210 − Ssc.21893.1.S1_at − probe 4
Q96GB2

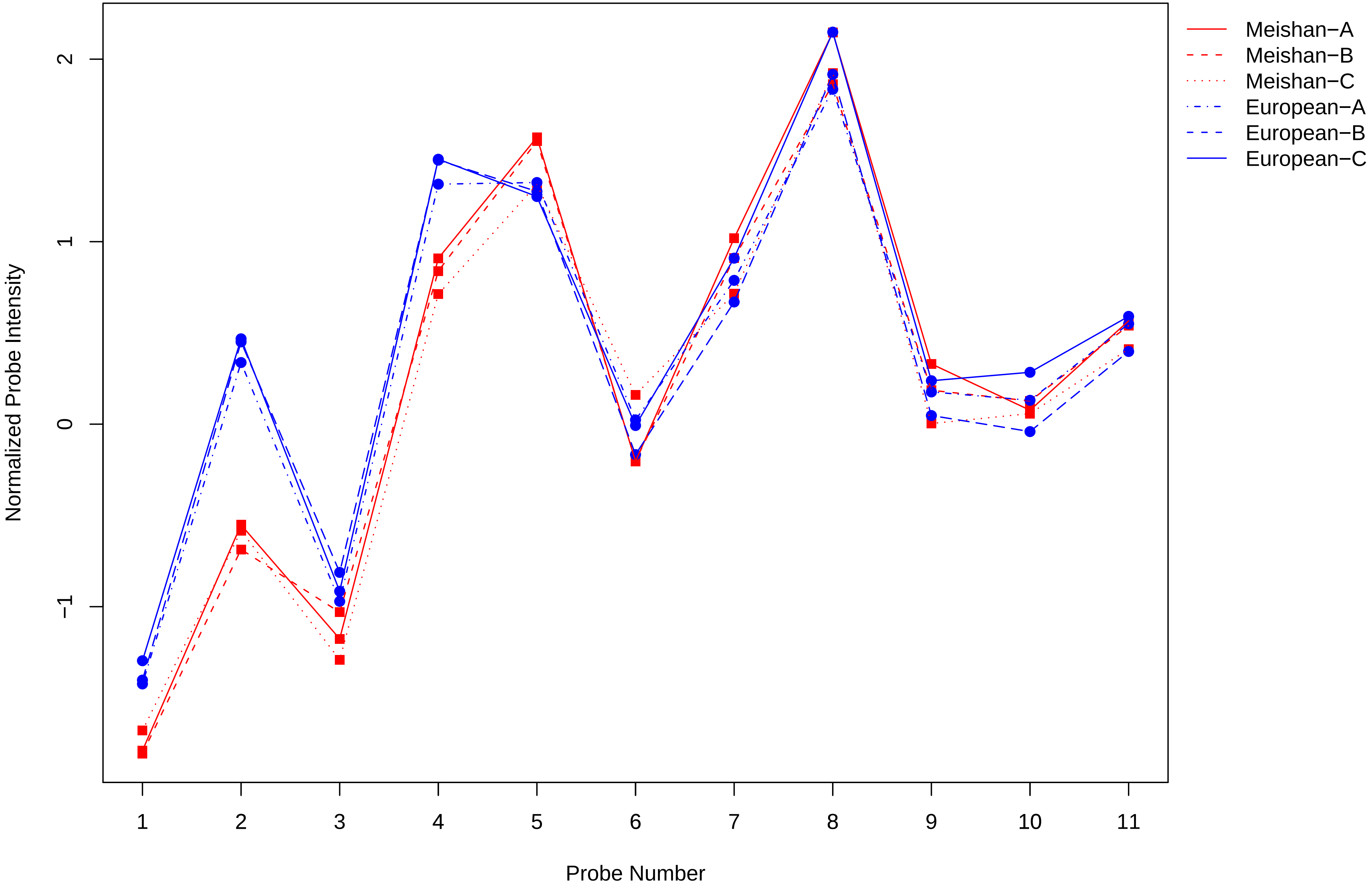

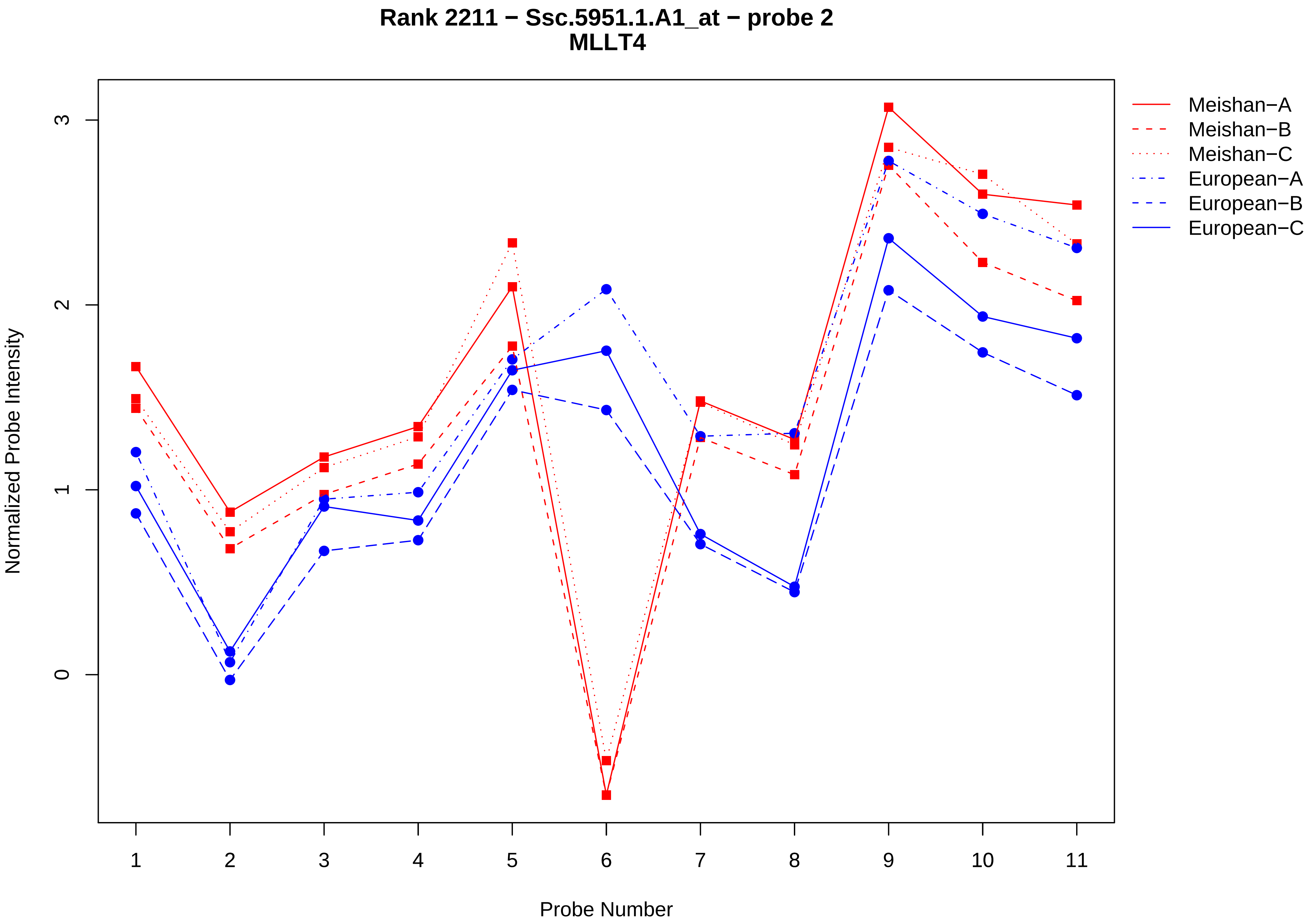

Rank 2211 − Ssc.5951.1.A1_at − probe 2
MLLT4
Normalized Probe Intensity
Probe Number
0
1
2
3
1
2
3
4
5
6
7
8
9
10
11
Meishan−A
Meishan−B
Meishan−C
European−A
European−B
European−C

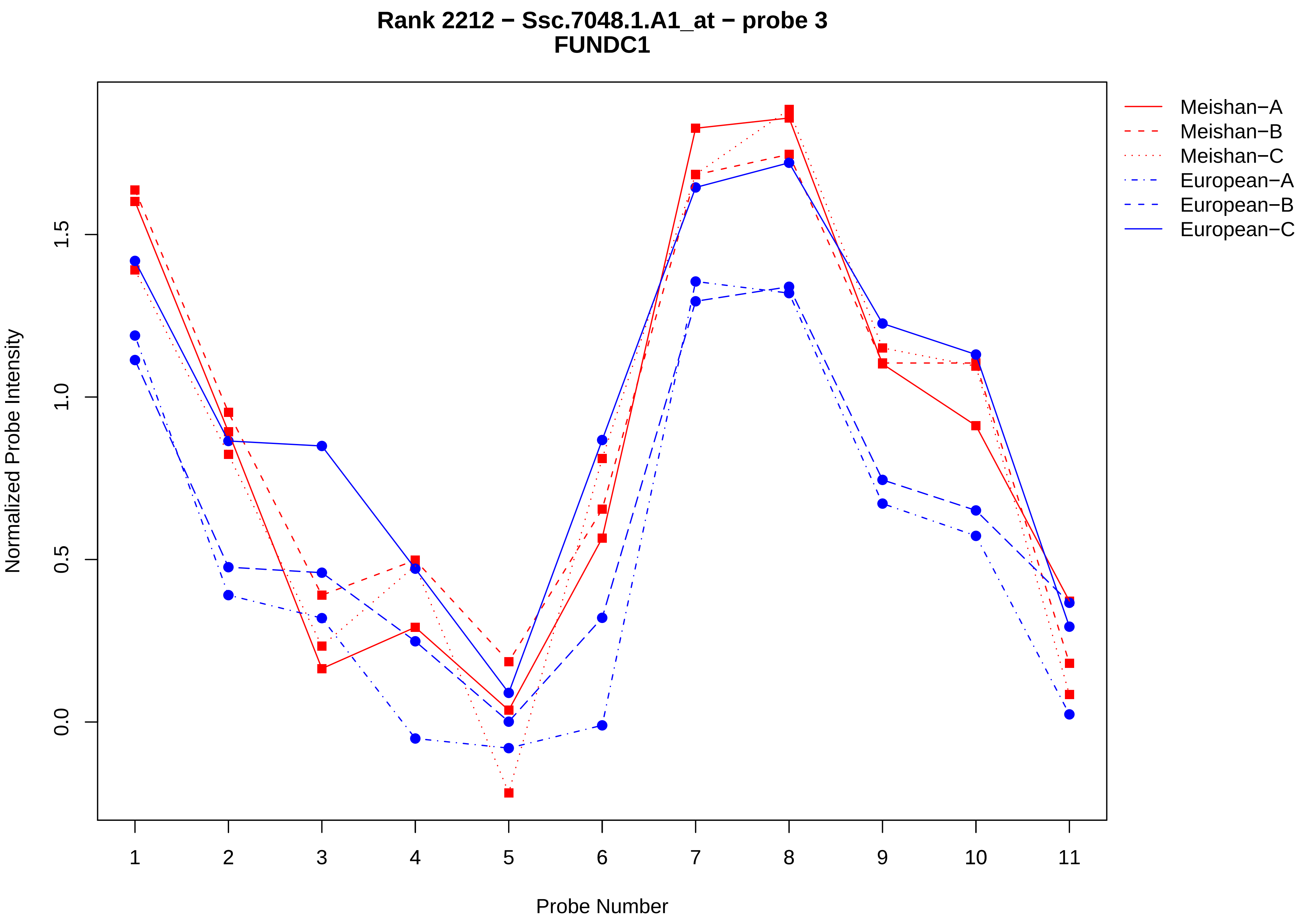

Rank 2212 − Ssc.7048.1.A1_at − probe 3
FUNDC1
Normalized Probe Intensity
0.0
0.5
1.0
1.5
Probe Number
1
2
3
4
5
6
7
8
9
10
11
Meishan−A
Meishan−B
Meishan−C
European−A
European−B
European−C

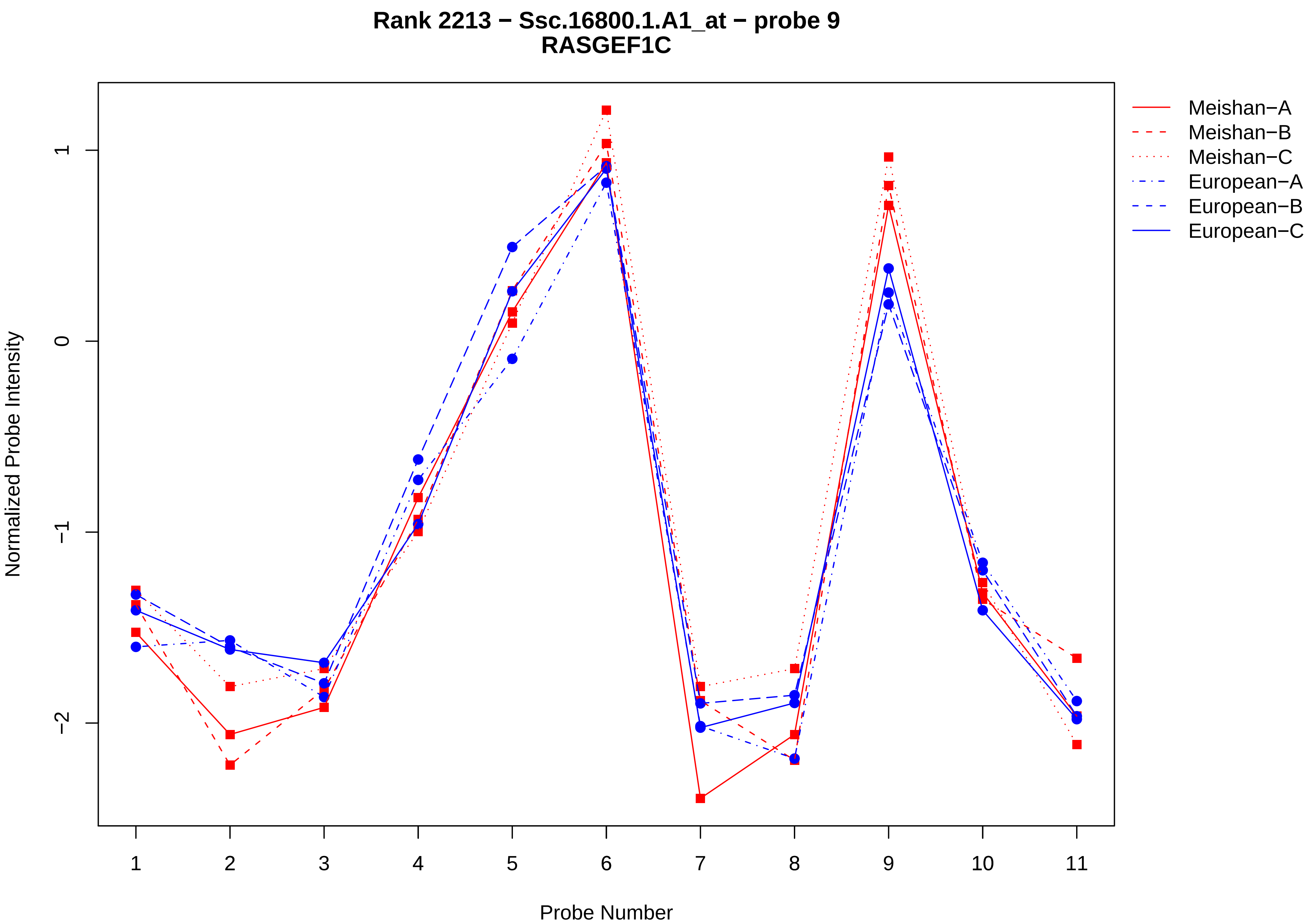

Rank 2213 − Ssc.16800.1.A1_at − probe 9
RASGEF1C
Normalized Probe Intensity
Probe Number
−2
−1
0
1
1
2
3
4
5
6
7
8
9
10
11
Meishan−A
Meishan−B
Meishan−C
European−A
European−B
European−C

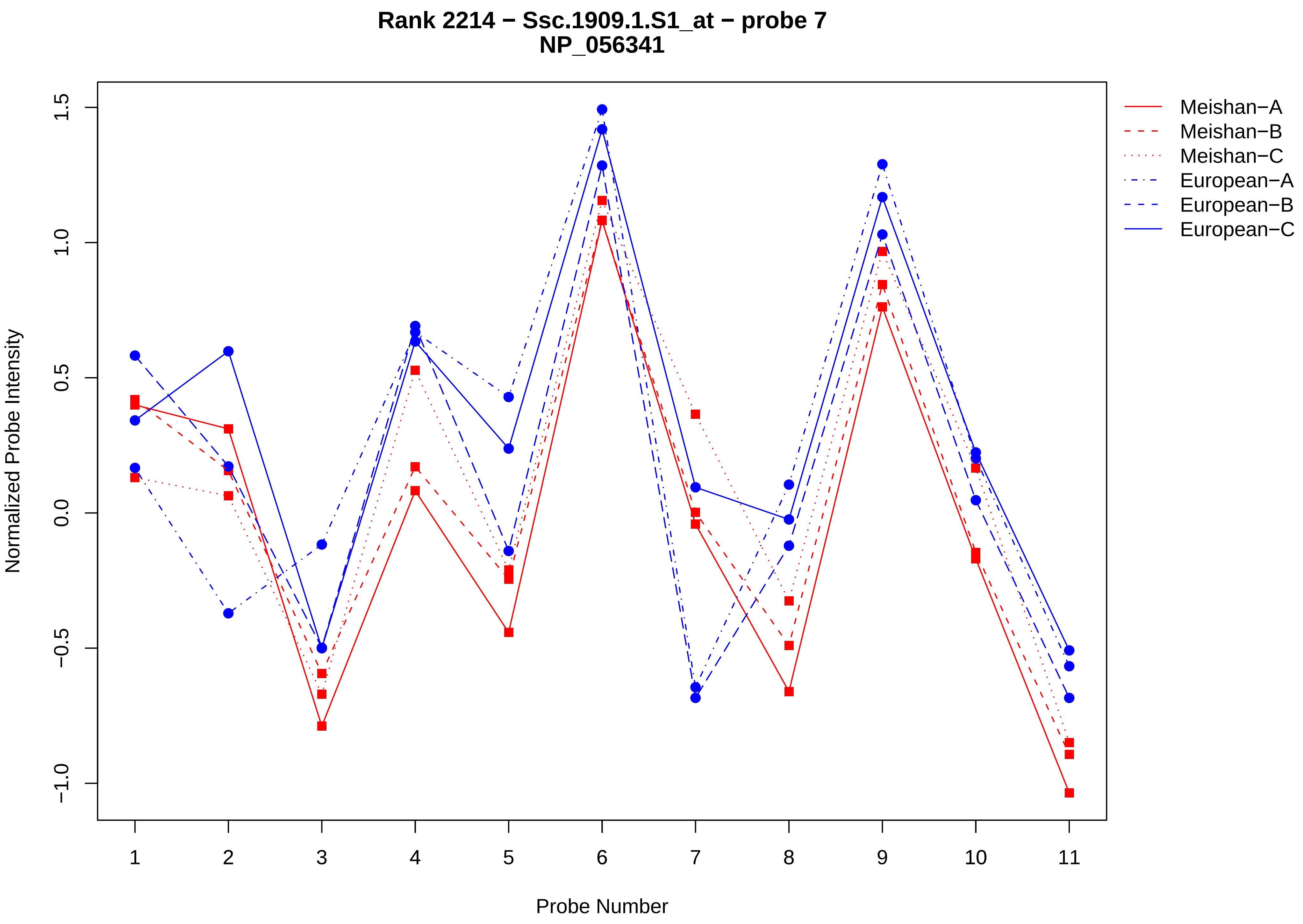
Rank 2214 − Ssc.1909.1.S1_at − probe 7
NP_056341
Normalized Probe Intensity
Probe Number
Meishan−A
Meishan−B
Meishan−C
European−A
European−B
European−C
1.5
1.0
0.5
0.0
−0.5
−1.0
1
2
3
4
5
6
7
8
9
10
11

Rank 2215 − Ssc.898.1.S1_at − probe 6
Q9Y3E2

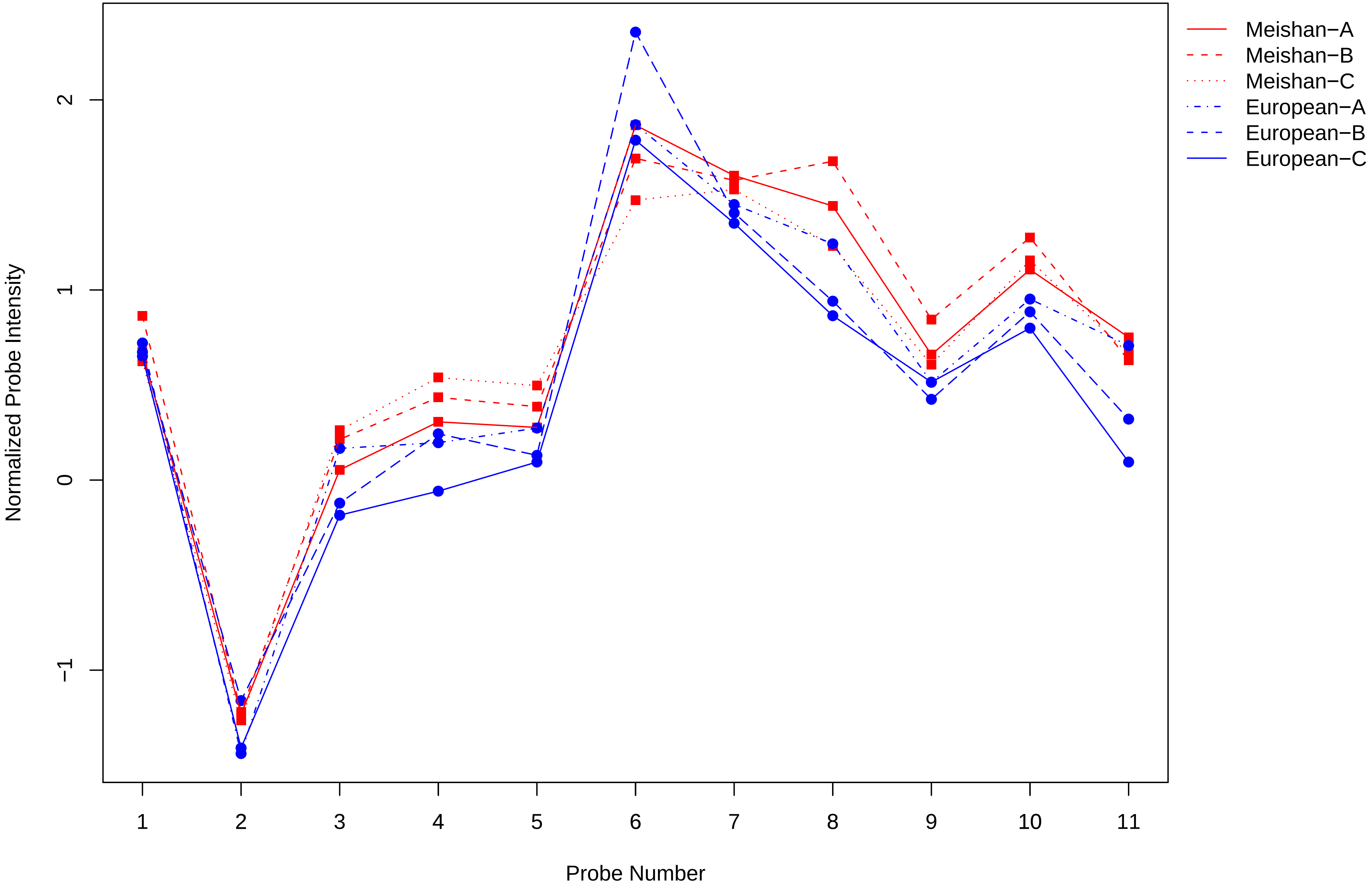

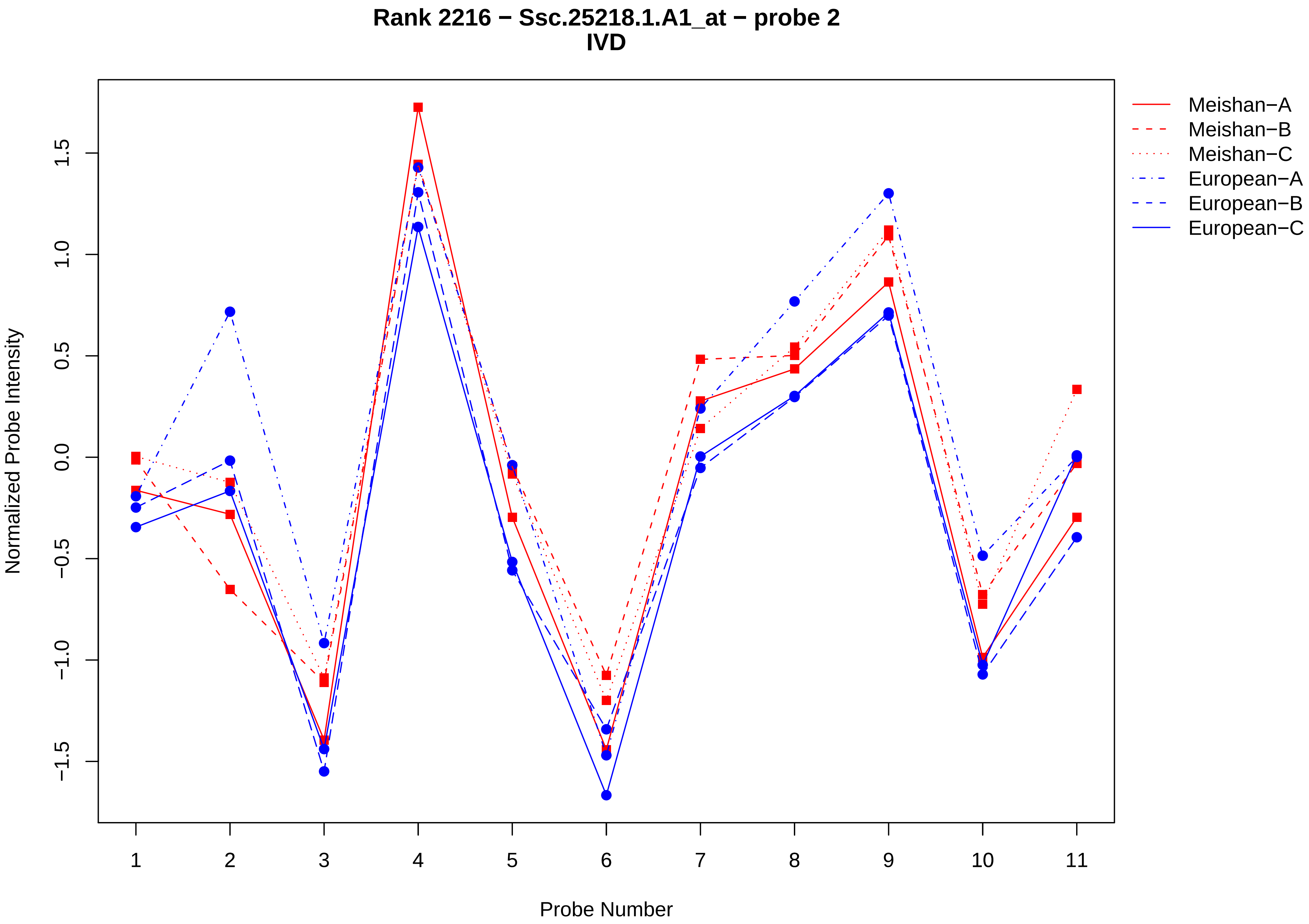

Rank 2216 − Ssc.25218.1.A1_at − probe 2
IVD
Normalized Probe Intensity
Probe Number
−1.5
−1.0
−0.5
0.0
0.5
1.0
1.5
1
2
3
4
5
6
7
8
9
10
11
Meishan−A
Meishan−B
Meishan−C
European−A
European−B
European−C

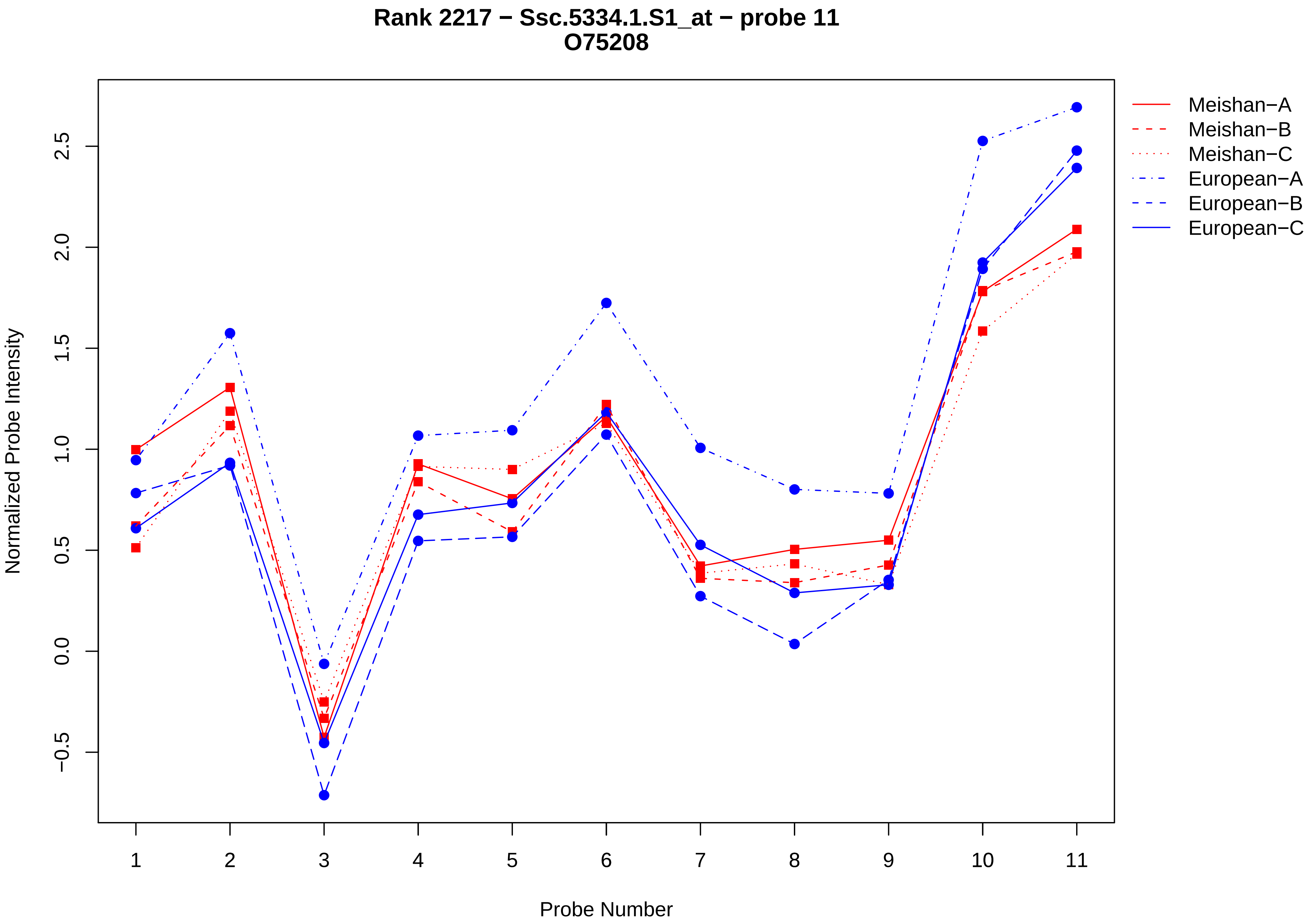

Rank 2217 − Ssc.5334.1.S1_at − probe 11
O75208
Normalized Probe Intensity
Probe Number
Meishan−A
Meishan−B
Meishan−C
European−A
European−B
European−C

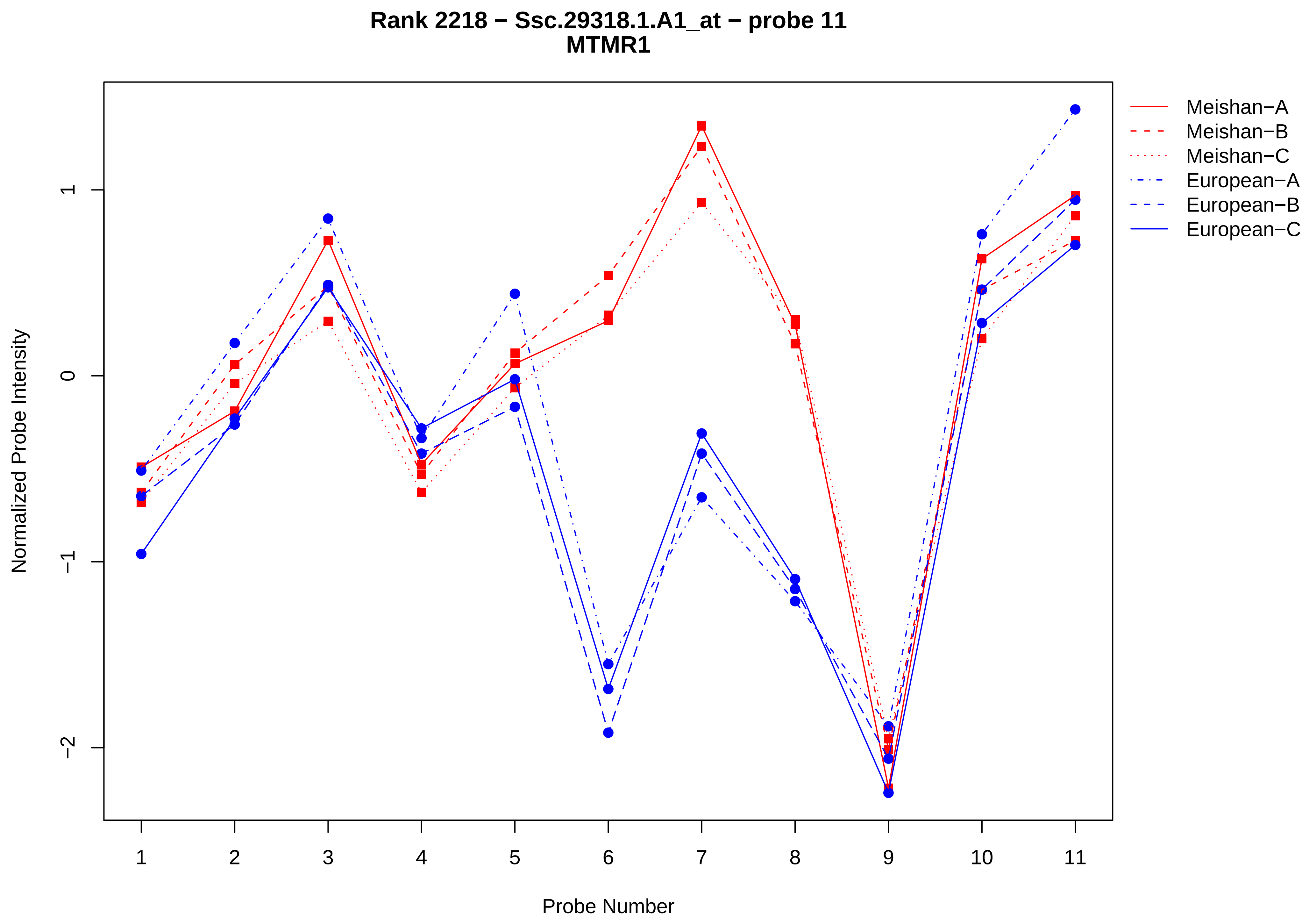

Rank 2218 − Ssc.29318.1.A1_at − probe 11
MTMR1
Normalized Probe Intensity
Probe Number
1
0
−1
−2
1
2
3
4
5
6
7
8
9
10
11
Meishan−A
Meishan−B
Meishan−C
European−A
European−B
European−C

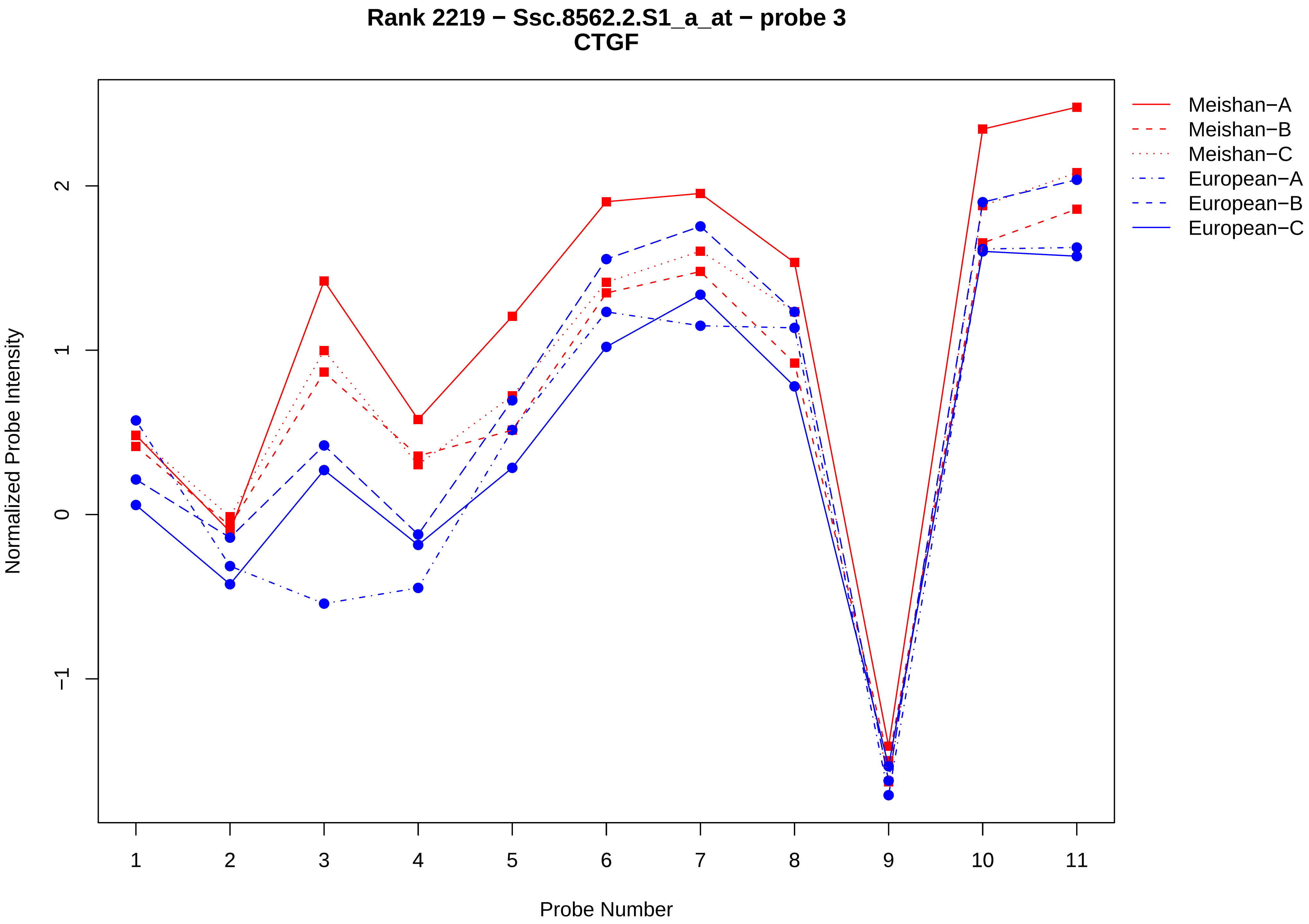

Rank 2219 − Ssc.8562.2.S1_a_at − probe 3
CTGF
Normalized Probe Intensity
Probe Number
Meishan−A
Meishan−B
Meishan−C
European−A
European−B
European−C
−1
0
1
2
1
2
3
4
5
6
7
8
9
10
11

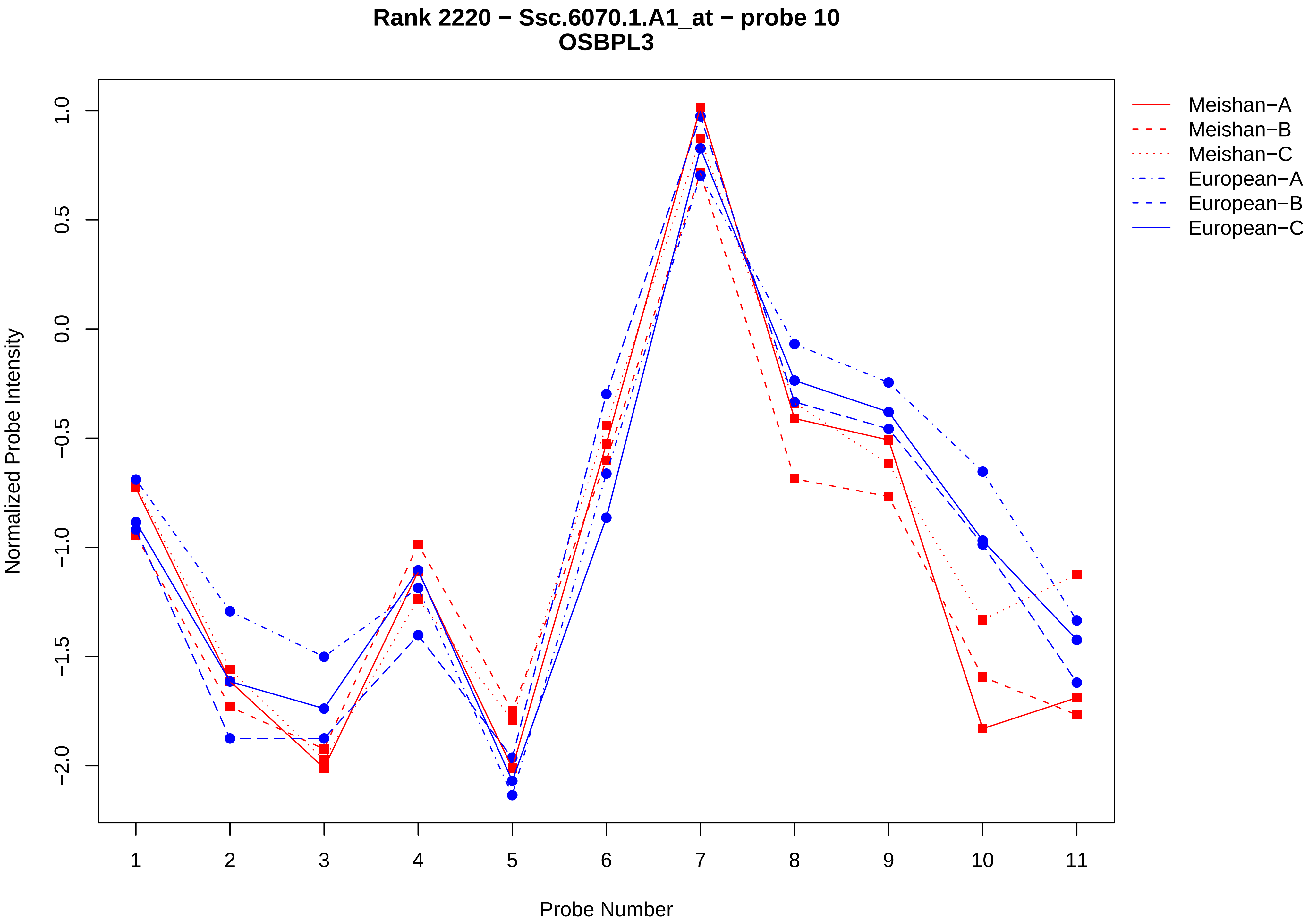

Rank 2220 − Ssc.6070.1.A1_at − probe 10
OSBPL3
Normalized Probe Intensity
Probe Number
1.0
0.5
0.0
−0.5
−1.0
−1.5
−2.0
1
2
3
4
5
6
7
8
9
10
11
Meishan−A
Meishan−B
Meishan−C
European−A
European−B
European−C

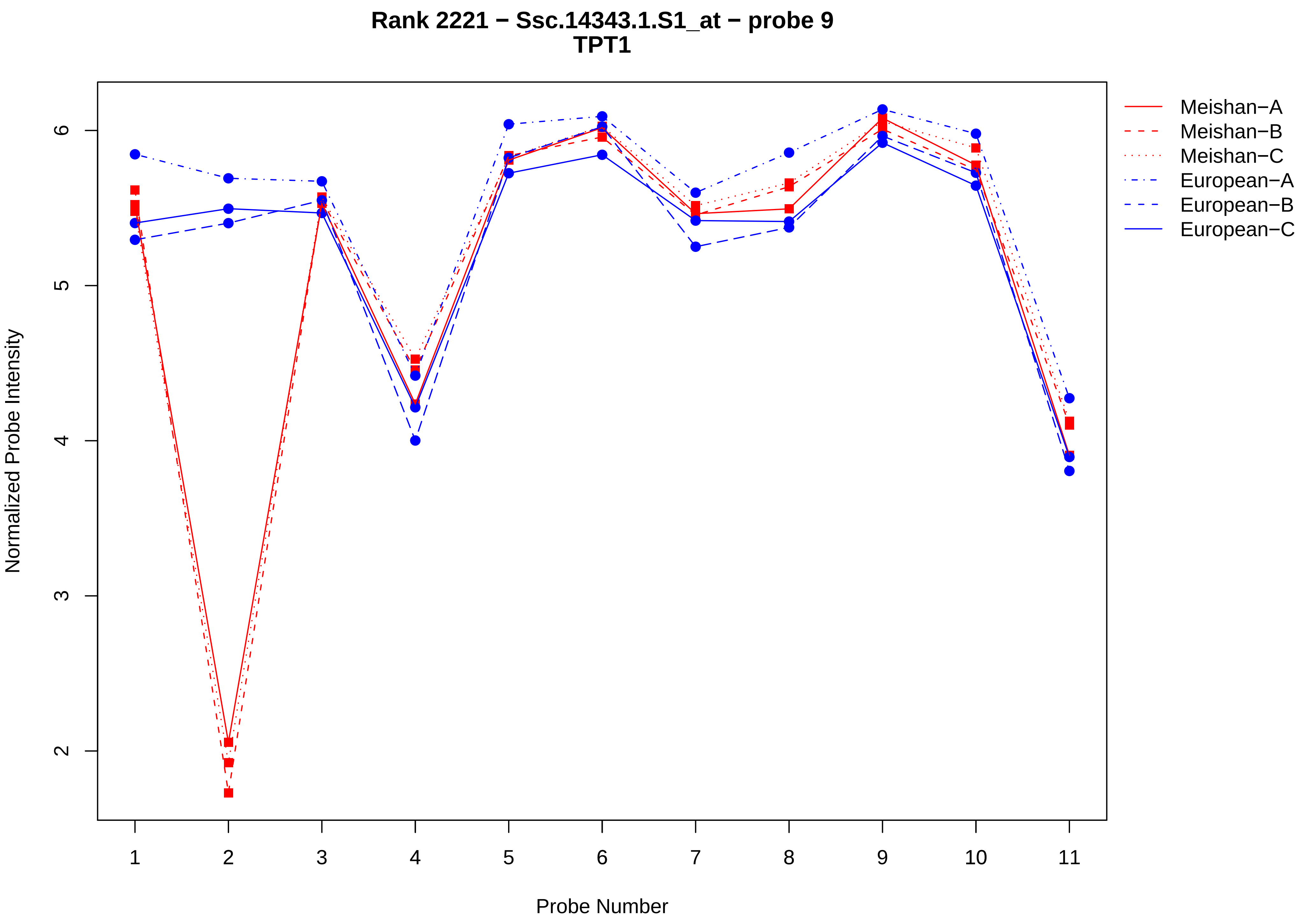
Rank 2221 − Ssc.14343.1.S1_at − probe 9
TPT1
Normalized Probe Intensity
2
3
4
5
6
1
2
3
4
5
6
7
8
9
10
11
Probe Number
Meishan−A
Meishan−B
Meishan−C
European−A
European−B
European−C

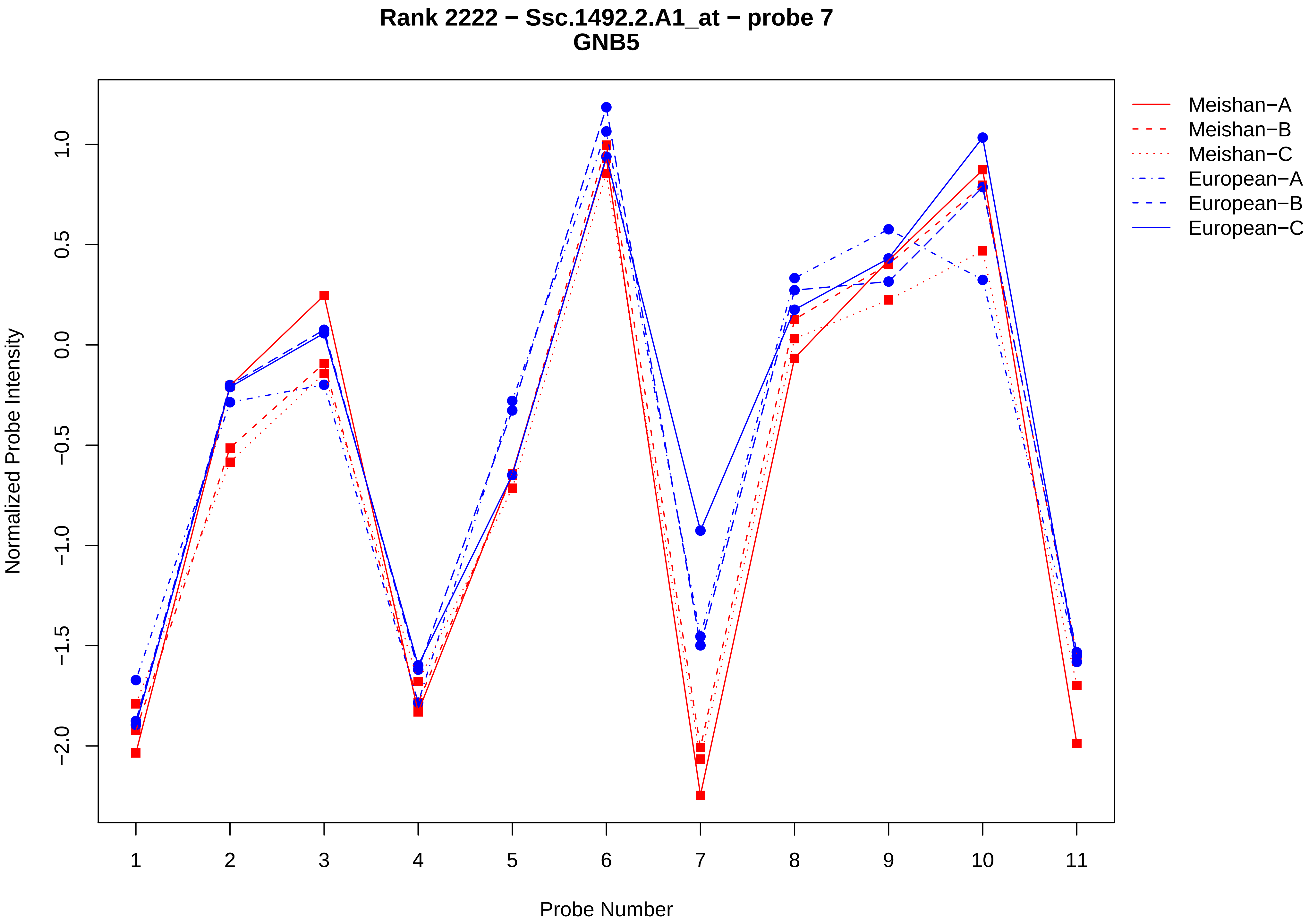

Rank 2222 − Ssc.1492.2.A1_at − probe 7
GNB5
Normalized Probe Intensity
Probe Number
Meishan−A
Meishan−B
Meishan−C
European−A
European−B
European−C

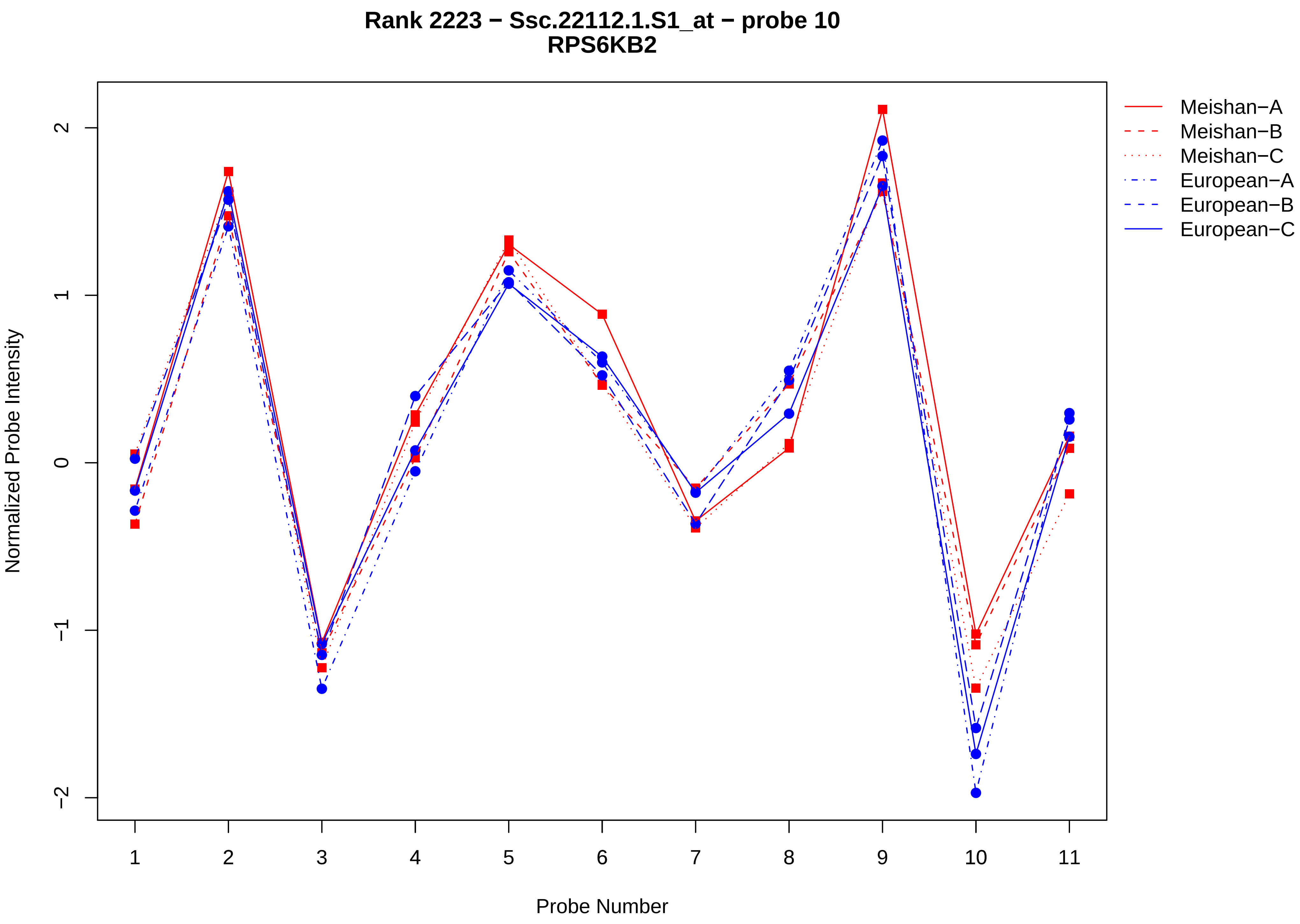

Rank 2223 − Ssc.22112.1.S1_at − probe 10
RPS6KB2
Normalized Probe Intensity
Probe Number
−2
−1
0
1
2
1
2
3
4
5
6
7
8
9
10
11
Meishan−A
Meishan−B
Meishan−C
European−A
European−B
European−C

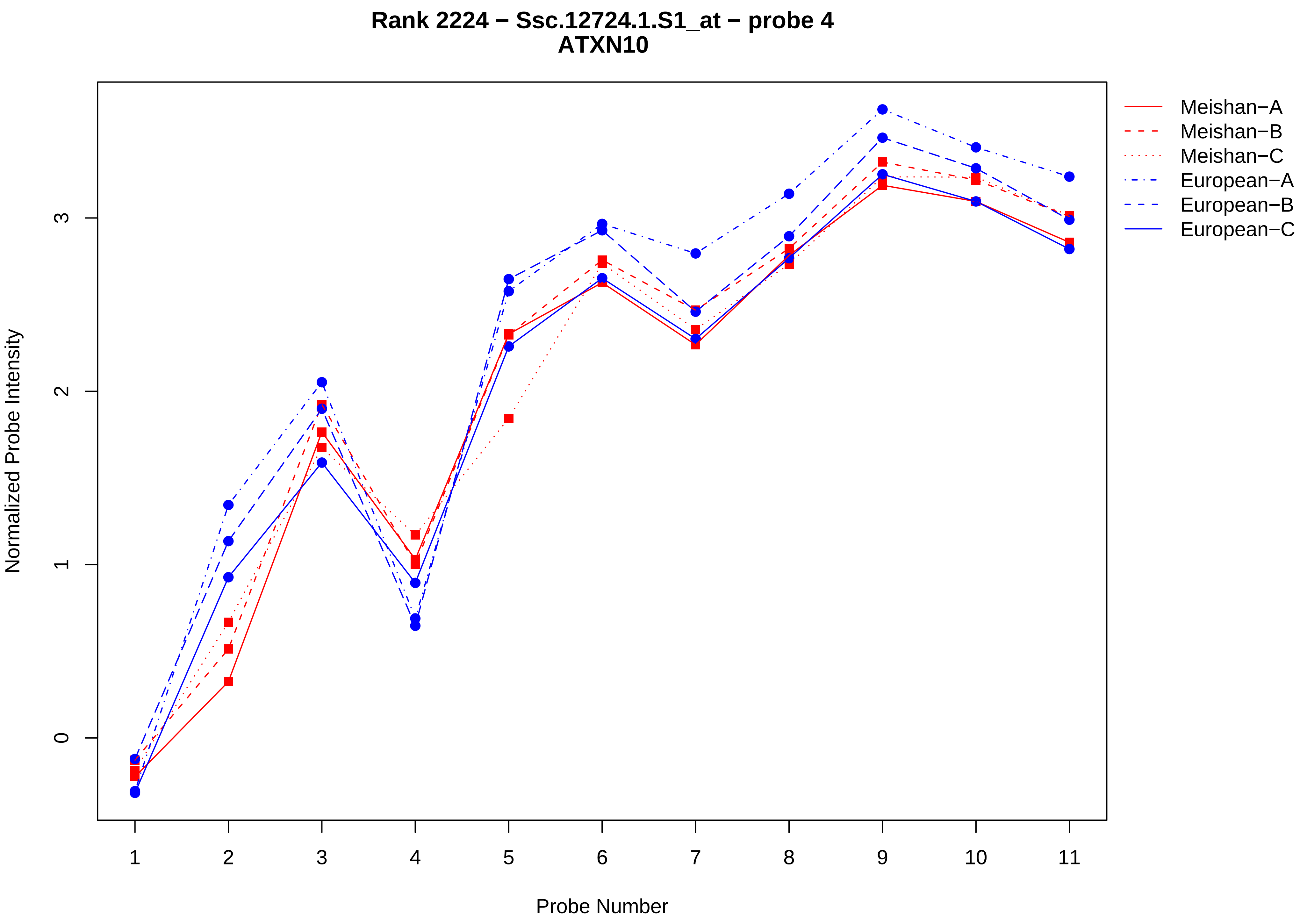

Rank 2224 − Ssc.12724.1.S1_at − probe 4
ATXN10
Normalized Probe Intensity
Probe Number
0
1
2
3
1
2
3
4
5
6
7
8
9
10
11
Meishan−A
Meishan−B
Meishan−C
European−A
European−B
European−C

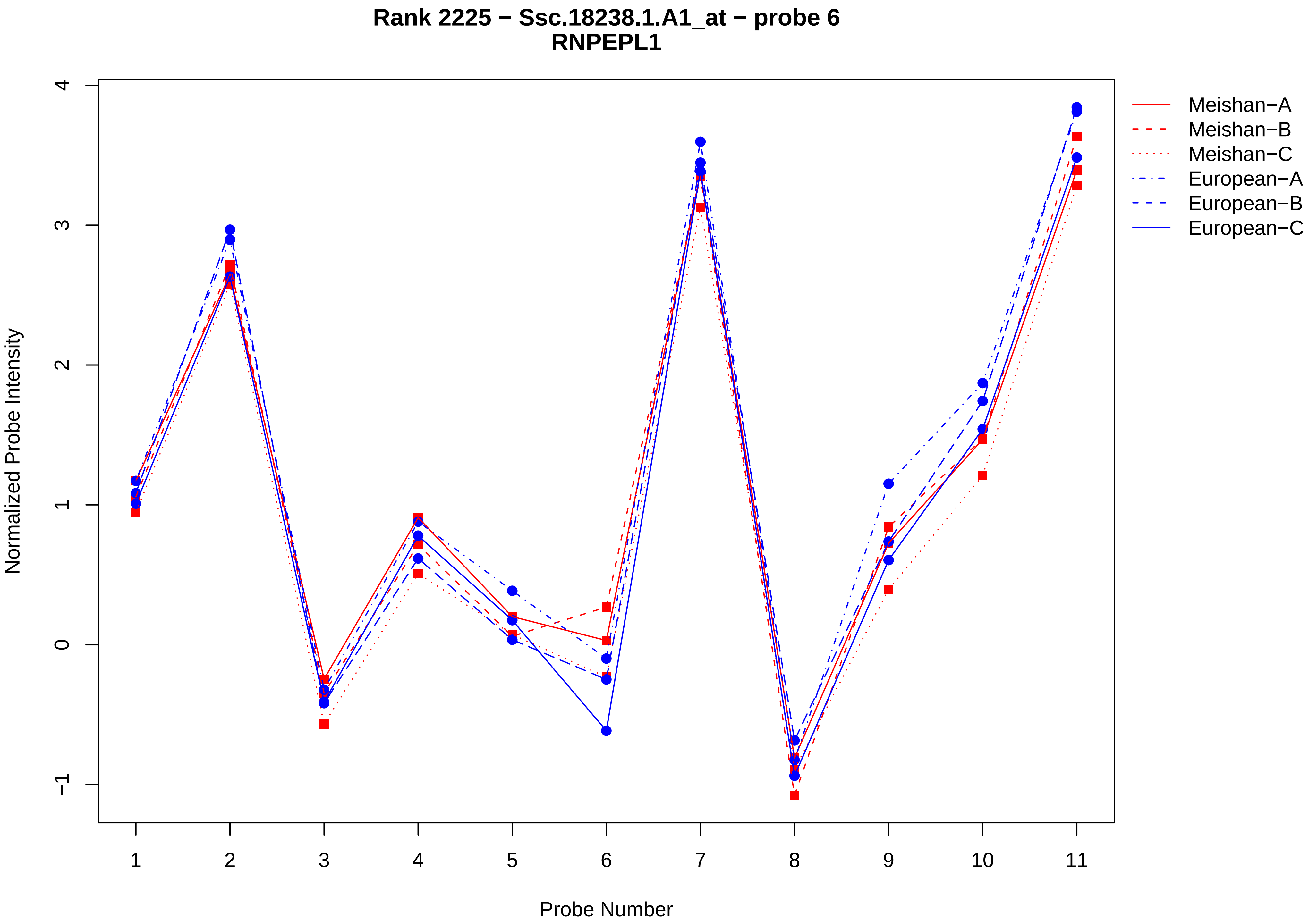

Rank 2225 − Ssc.18238.1.A1_at − probe 6
RNPEPL1
Normalized Probe Intensity
Probe Number
−1
0
1
2
3
4
1
2
3
4
5
6
7
8
9
10
11
Meishan−A
Meishan−B
Meishan−C
European−A
European−B
European−C

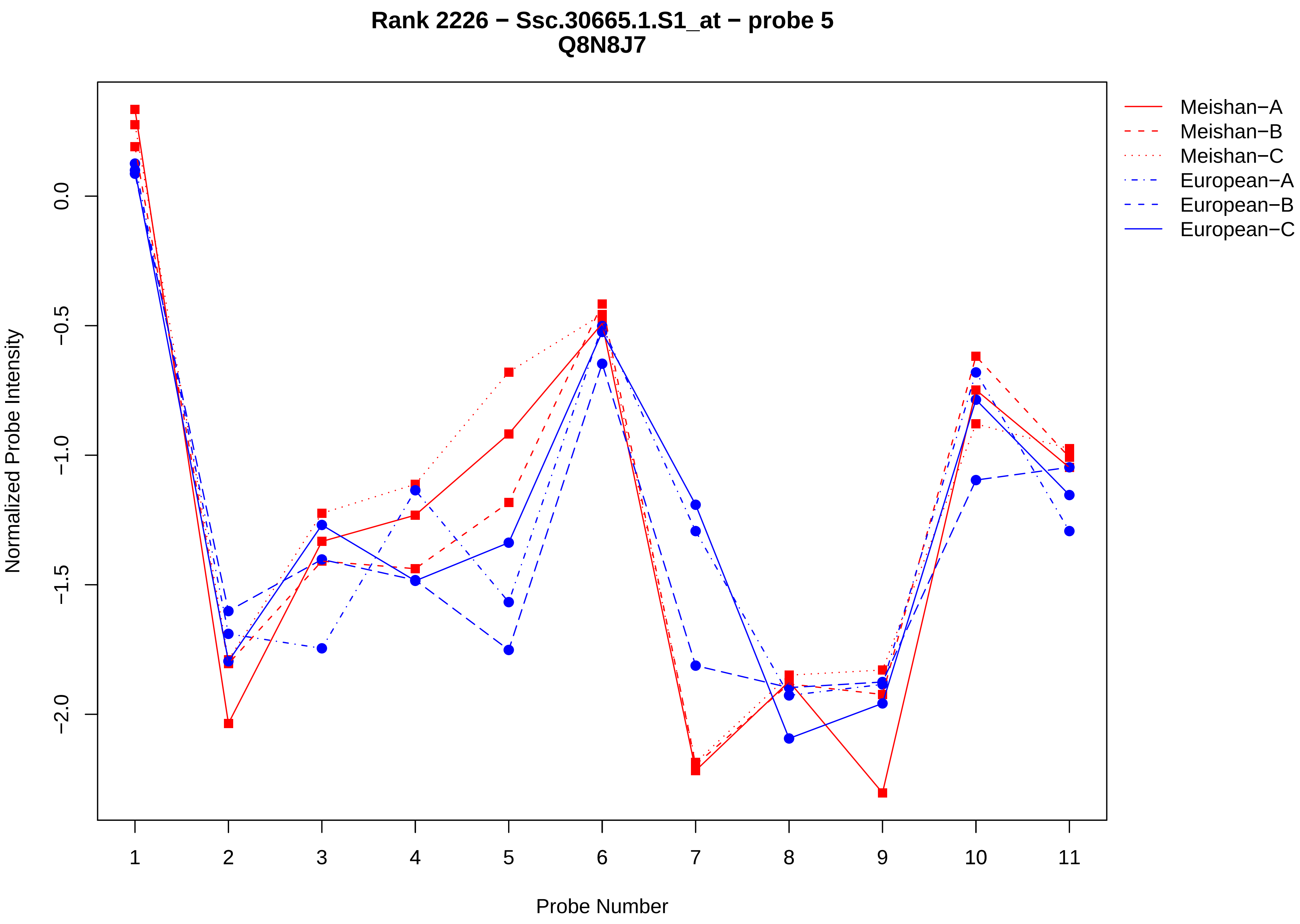
Rank 2226 − Ssc.30665.1.S1_at − probe 5
Q8N8J7
Normalized Probe Intensity
Probe Number
0.0
−0.5
−1.0
−1.5
−2.0
1
2
3
4
5
6
7
8
9
10
11
Meishan−A
Meishan−B
Meishan−C
European−A
European−B
European−C

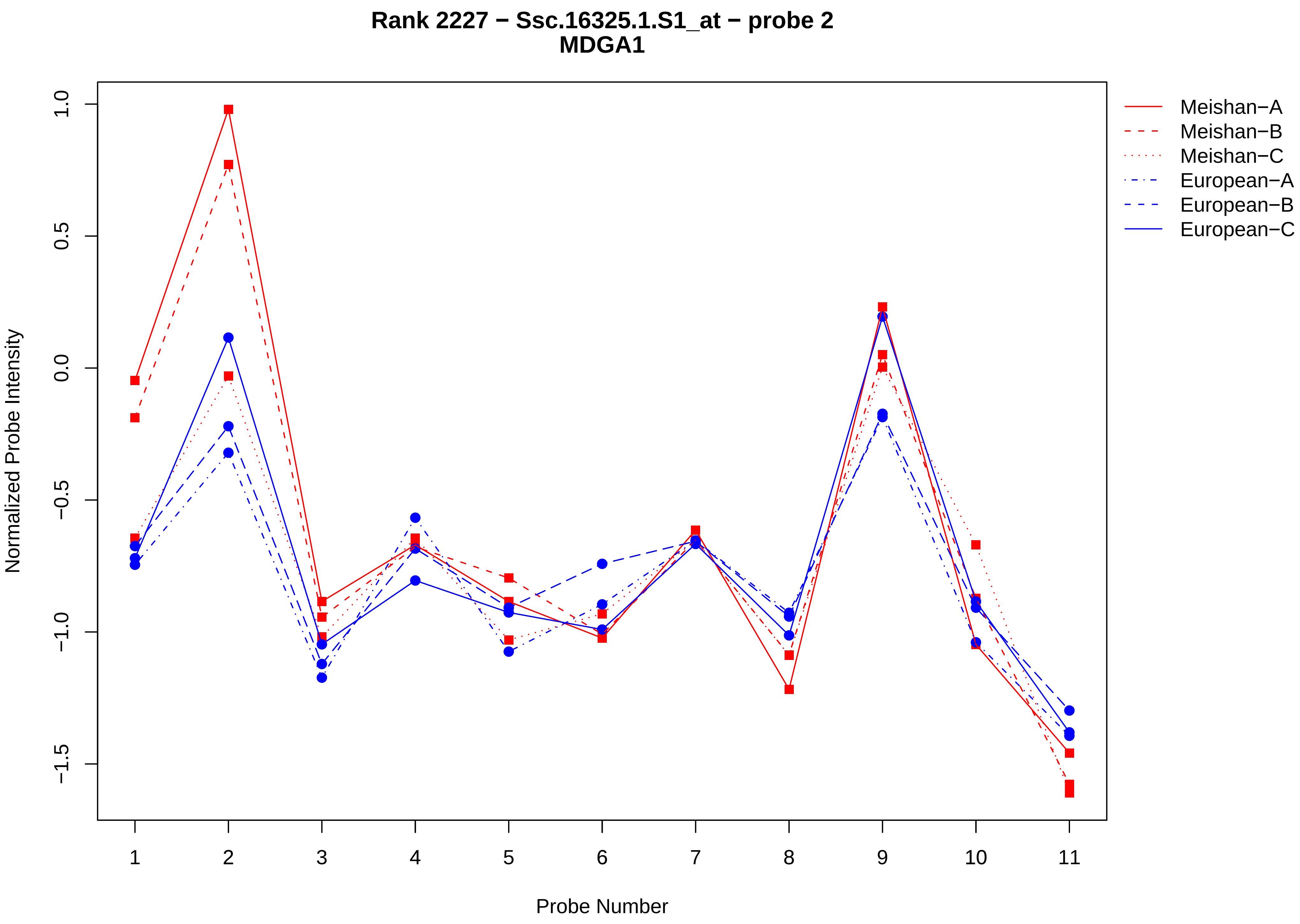

Rank 2227 − Ssc.16325.1.S1_at − probe 2
MDGA1
Normalized Probe Intensity
Probe Number
1.0
0.5
0.0
−0.5
−1.0
−1.5
1
2
3
4
5
6
7
8
9
10
11
Meishan−A
Meishan−B
Meishan−C
European−A
European−B
European−C

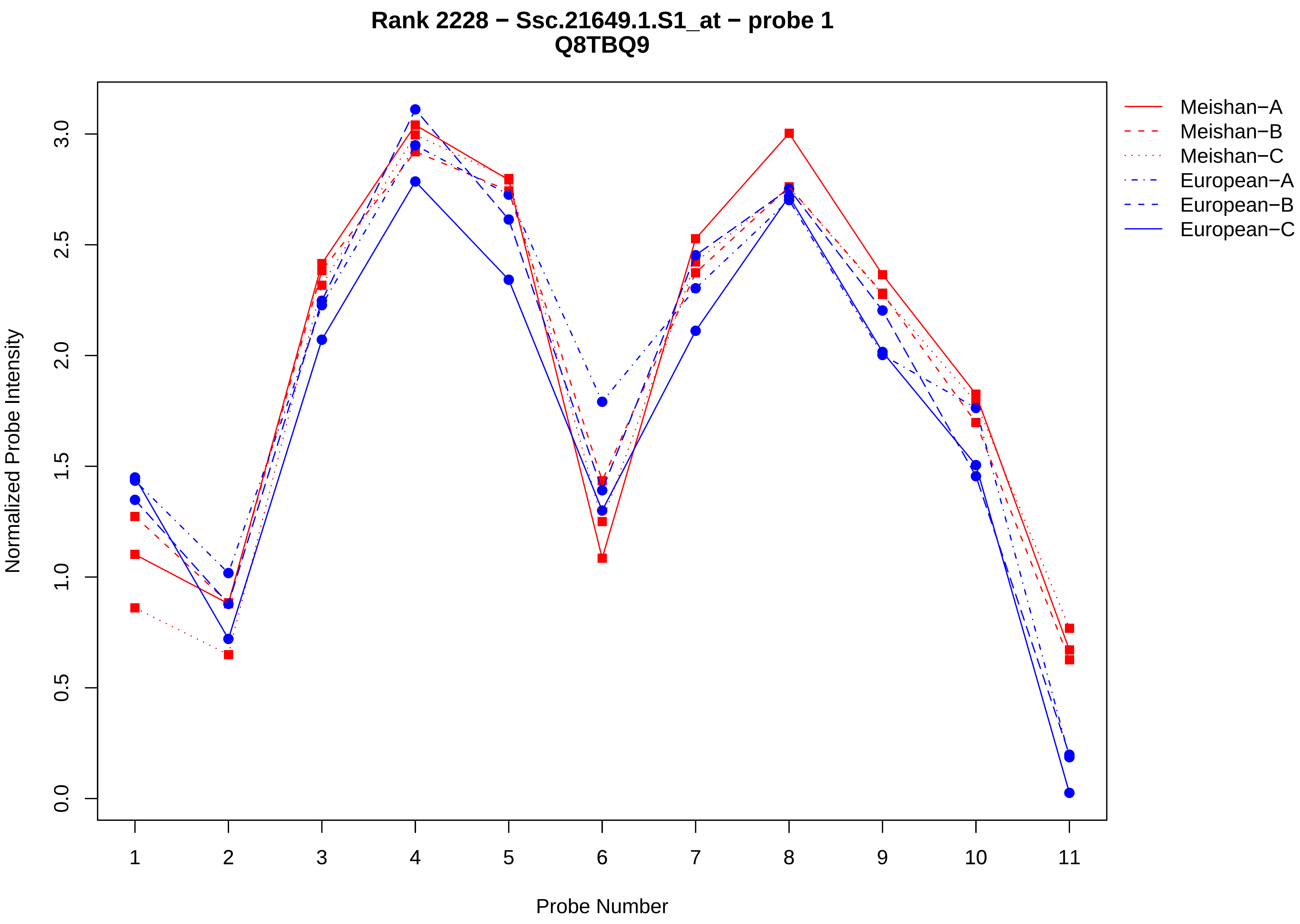

Rank 2228 − Ssc.21649.1.S1_at − probe 1
Q8TBQ9
Normalized Probe Intensity
Probe Number
0.0
0.5
1.0
1.5
2.0
2.5
3.0
1
2
3
4
5
6
7
8
9
10
11
Meishan−A
Meishan−B
Meishan−C
European−A
European−B
European−C

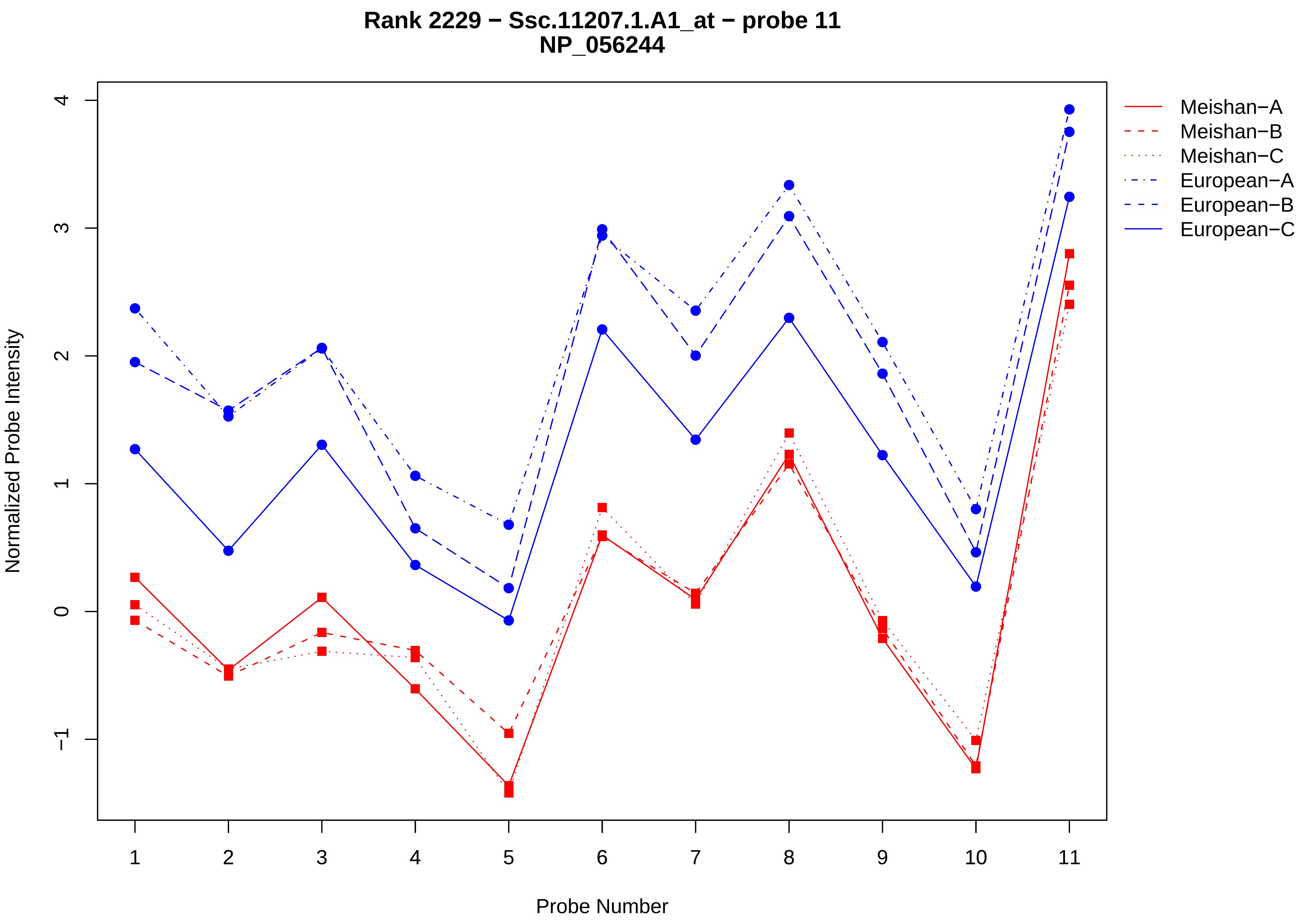

Rank 2229 − Ssc.11207.1.A1_at − probe 11
NP_056244
Normalized Probe Intensity
Probe Number
−1
0
1
2
3
4
1
2
3
4
5
6
7
8
9
10
11
Meishan−A
Meishan−B
Meishan−C
European−A
European−B
European−C

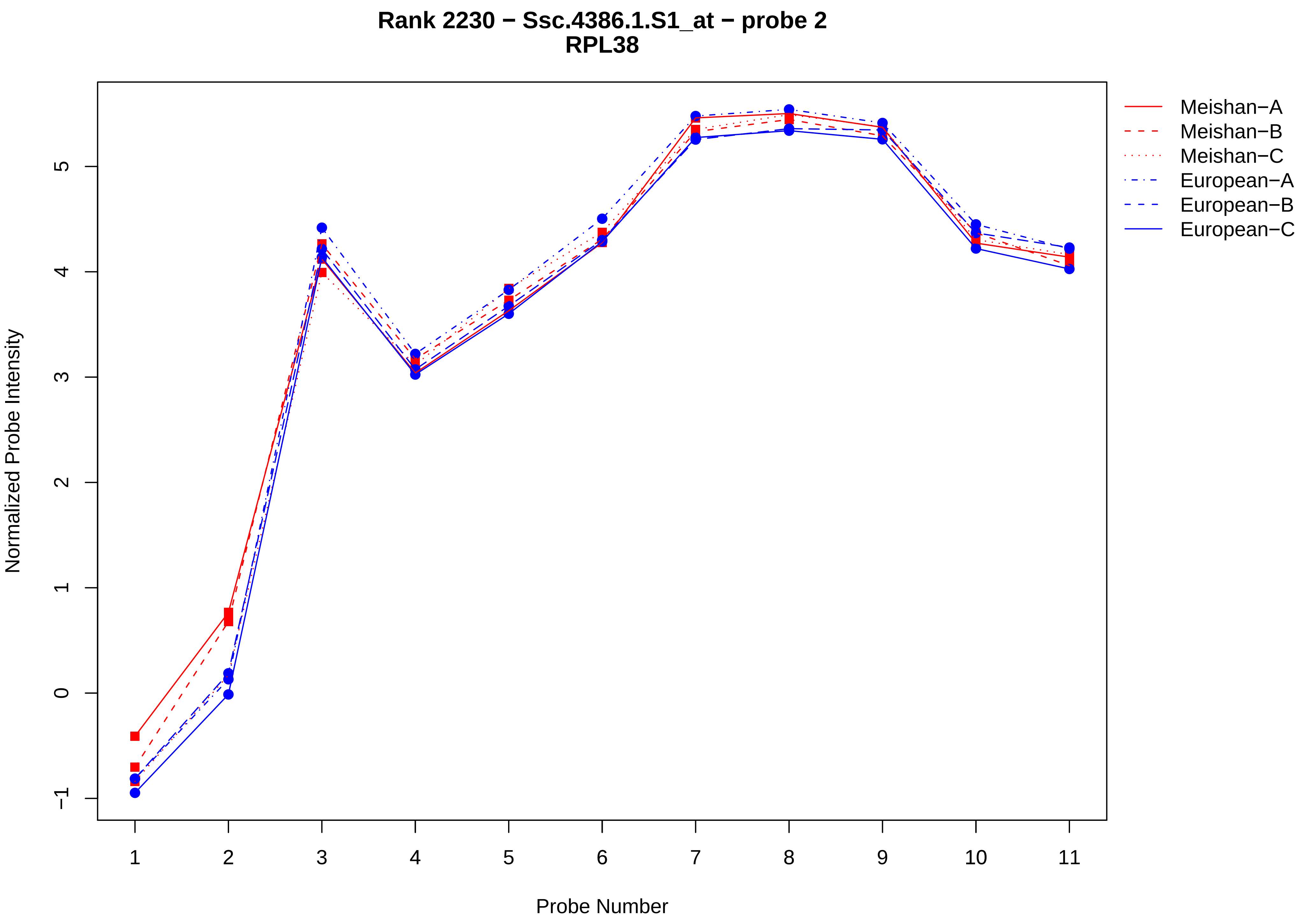

Rank 2230 − Ssc.4386.1.S1_at − probe 2
RPL38
Normalized Probe Intensity
Probe Number
−1
0
1
2
3
4
5
1
2
3
4
5
6
7
8
9
10
11
Meishan−A
Meishan−B
Meishan−C
European−A
European−B
European−C

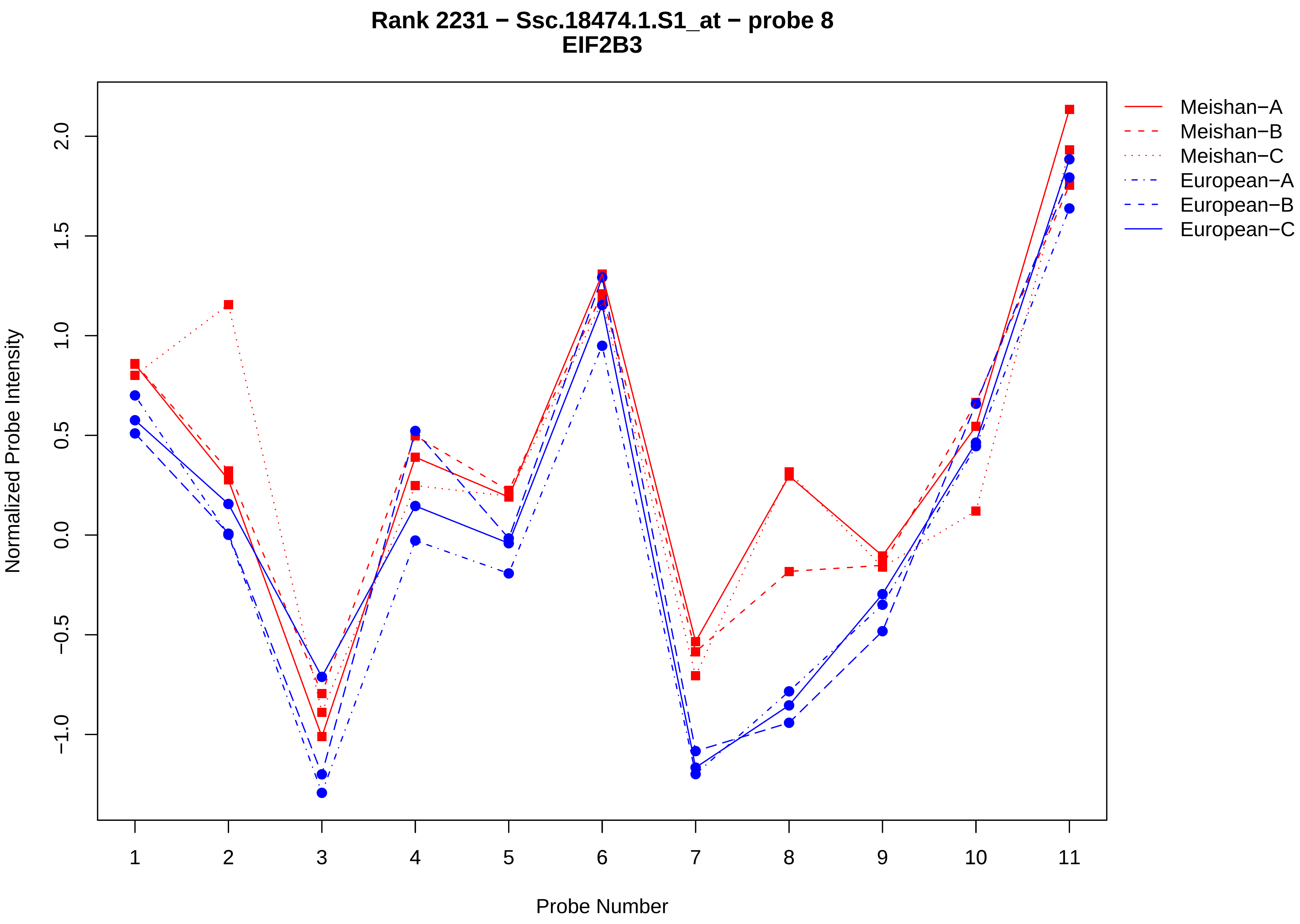

Rank 2231 − Ssc.18474.1.S1_at − probe 8
EIF2B3
Normalized Probe Intensity
Probe Number
Meishan−A
Meishan−B
Meishan−C
European−A
European−B
European−C

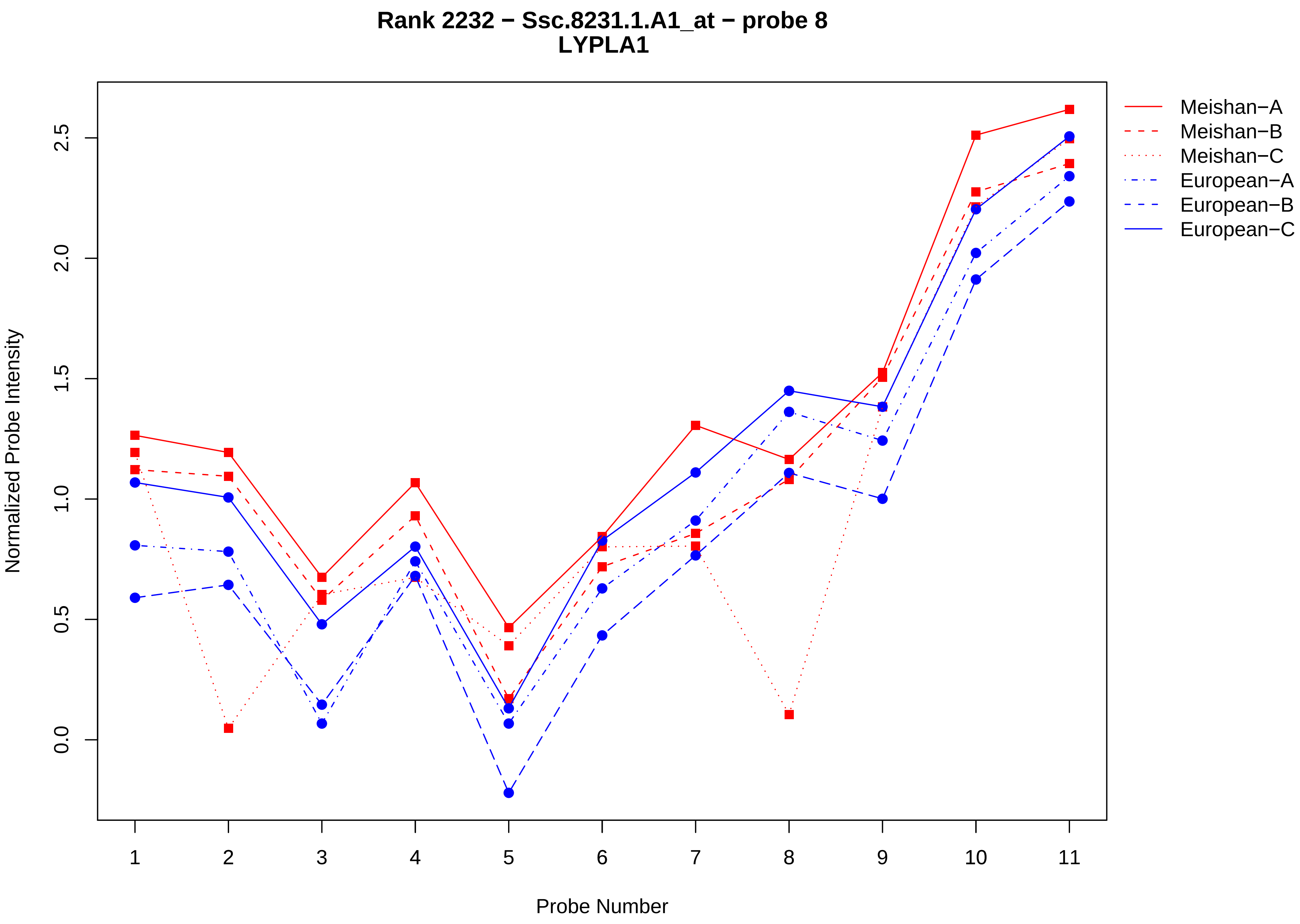

Rank 2232 − Ssc.8231.1.A1_at − probe 8
LYPLA1
Normalized Probe Intensity
Probe Number
0.0
0.5
1.0
1.5
2.0
2.5
1
2
3
4
5
6
7
8
9
10
11
Meishan−A
Meishan−B
Meishan−C
European−A
European−B
European−C

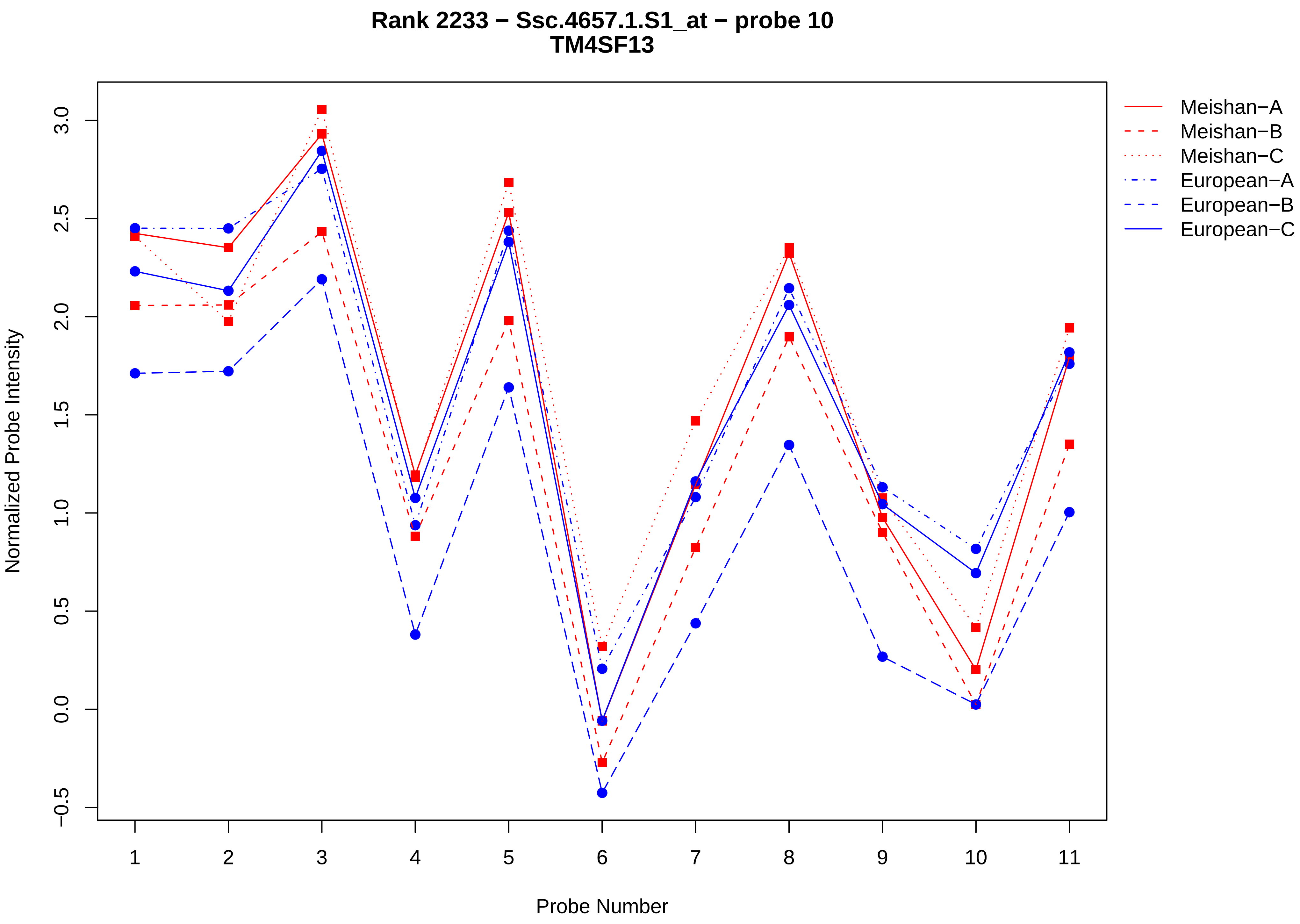

Rank 2233 − Ssc.4657.1.S1_at − probe 10
TM4SF13
Normalized Probe Intensity
Probe Number
−0.5
0.0
0.5
1.0
1.5
2.0
2.5
3.0
1
2
3
4
5
6
7
8
9
10
11
Meishan−A
Meishan−B
Meishan−C
European−A
European−B
European−C

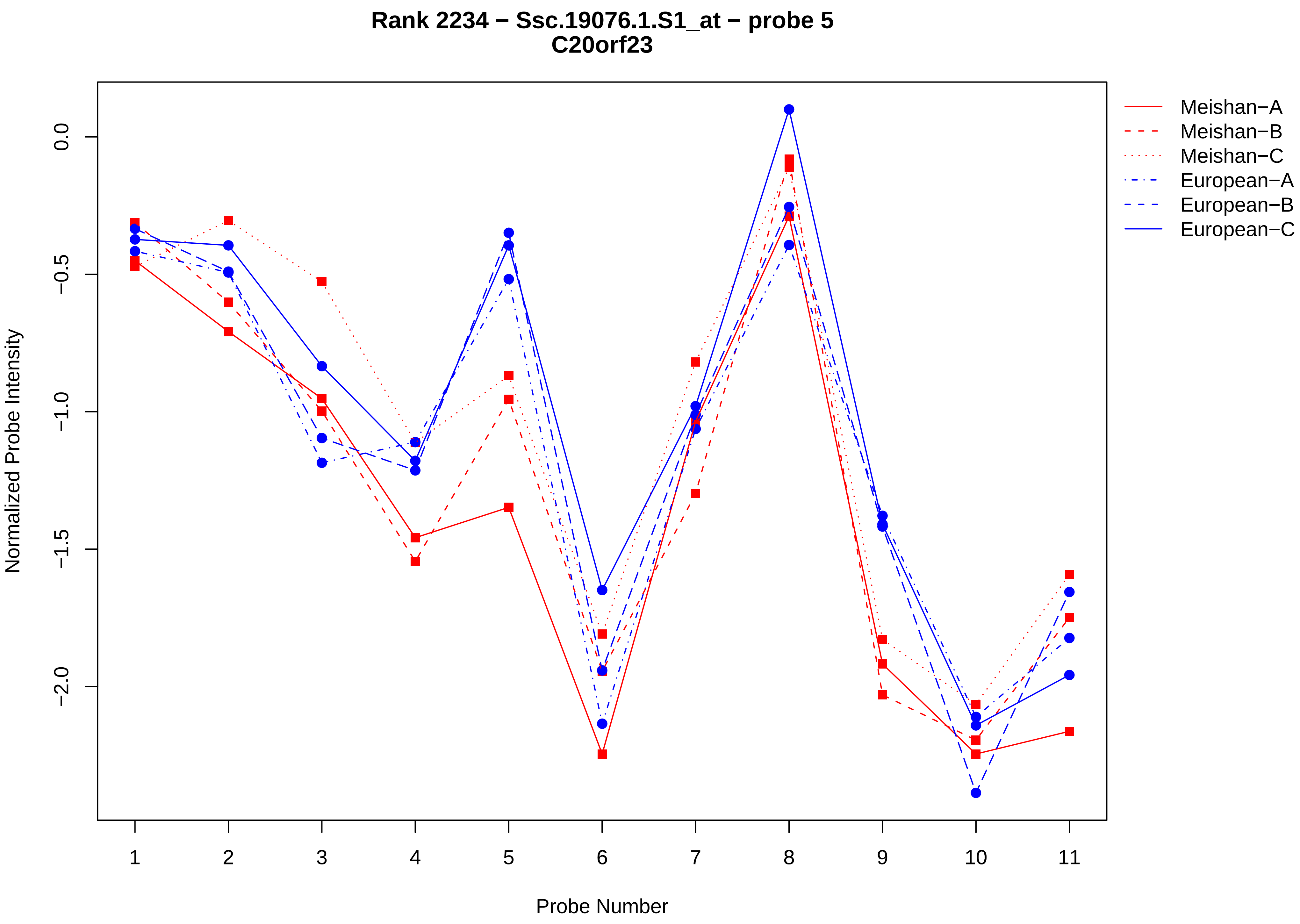

Rank 2234 − Ssc.19076.1.S1_at − probe 5
C20orf23
Normalized Probe Intensity
0.0
−0.5
−1.0
−1.5
−2.0
1
2
3
4
5
6
7
8
9
10
11
Probe Number
Meishan−A
Meishan−B
Meishan−C
European−A
European−B
European−C

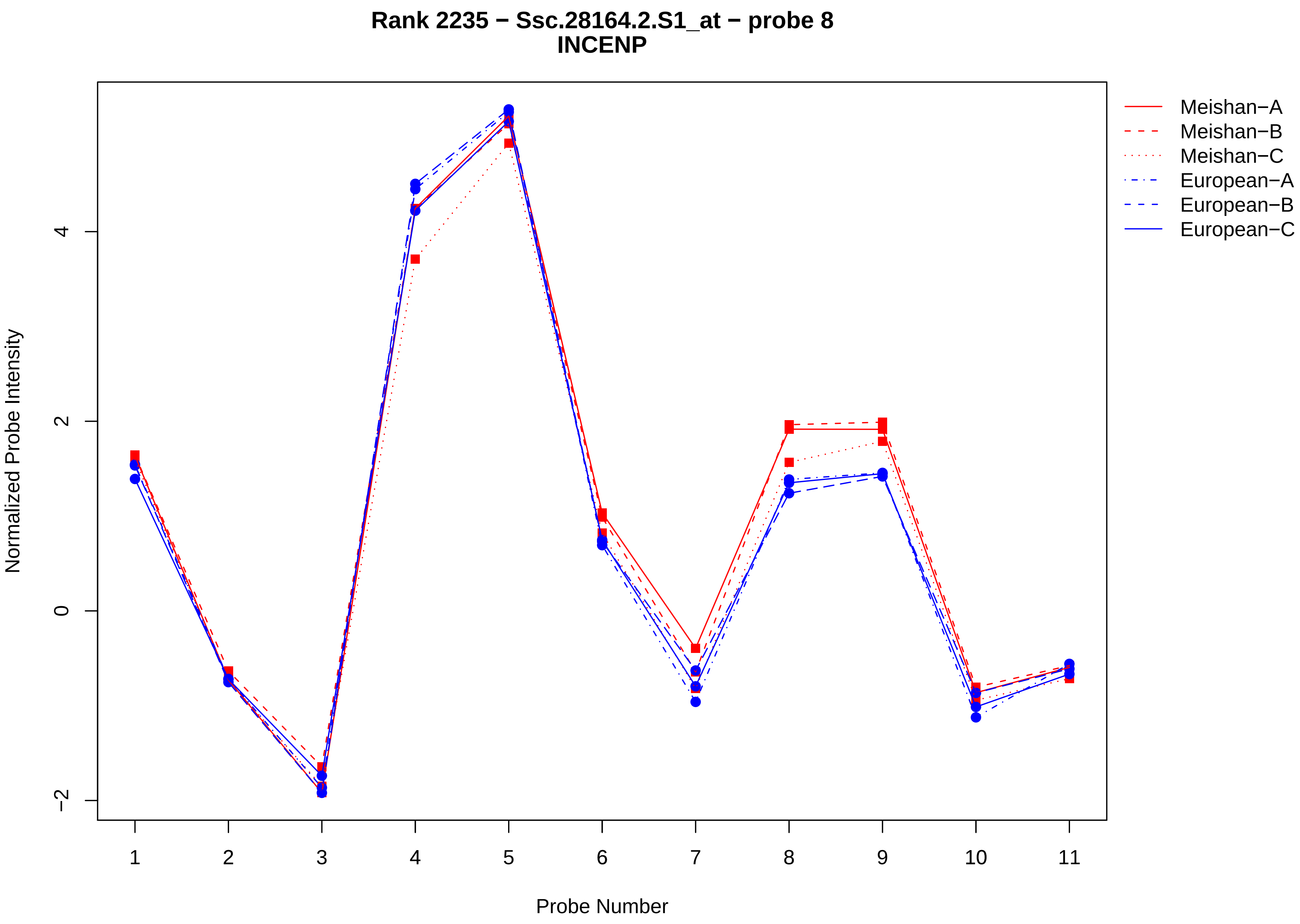

Rank 2235 − Ssc.28164.2.S1_at − probe 8
INCENP
Normalized Probe Intensity
Probe Number
−2
0
2
4
1
2
3
4
5
6
7
8
9
10
11
Meishan−A
Meishan−B
Meishan−C
European−A
European−B
European−C

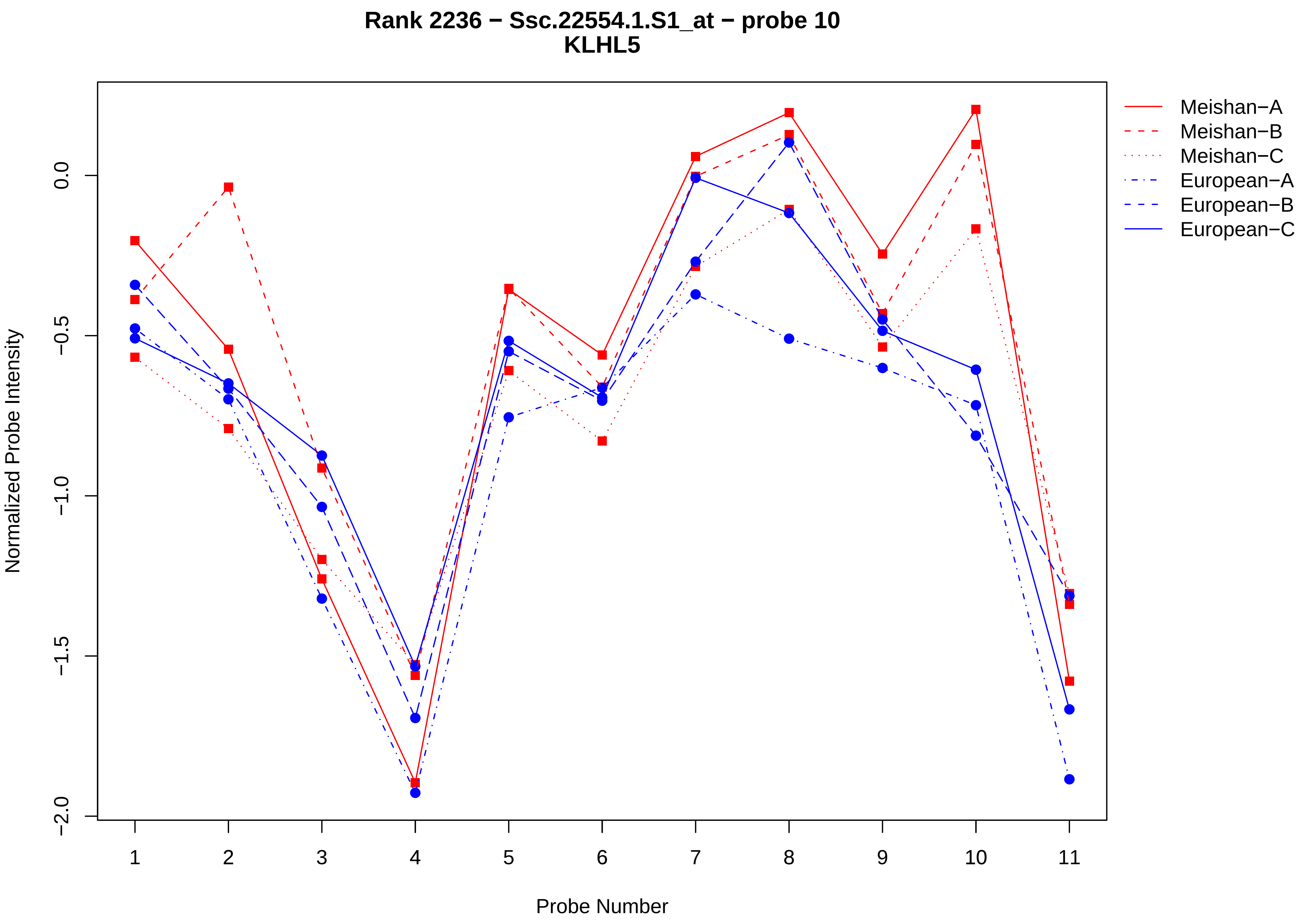

Rank 2236 − Ssc.22554.1.S1_at − probe 10
KLHL5
Normalized Probe Intensity
Probe Number
0.0
−0.5
−1.0
−1.5
−2.0
1
2
3
4
5
6
7
8
9
10
11
Meishan−A
Meishan−B
Meishan−C
European−A
European−B
European−C

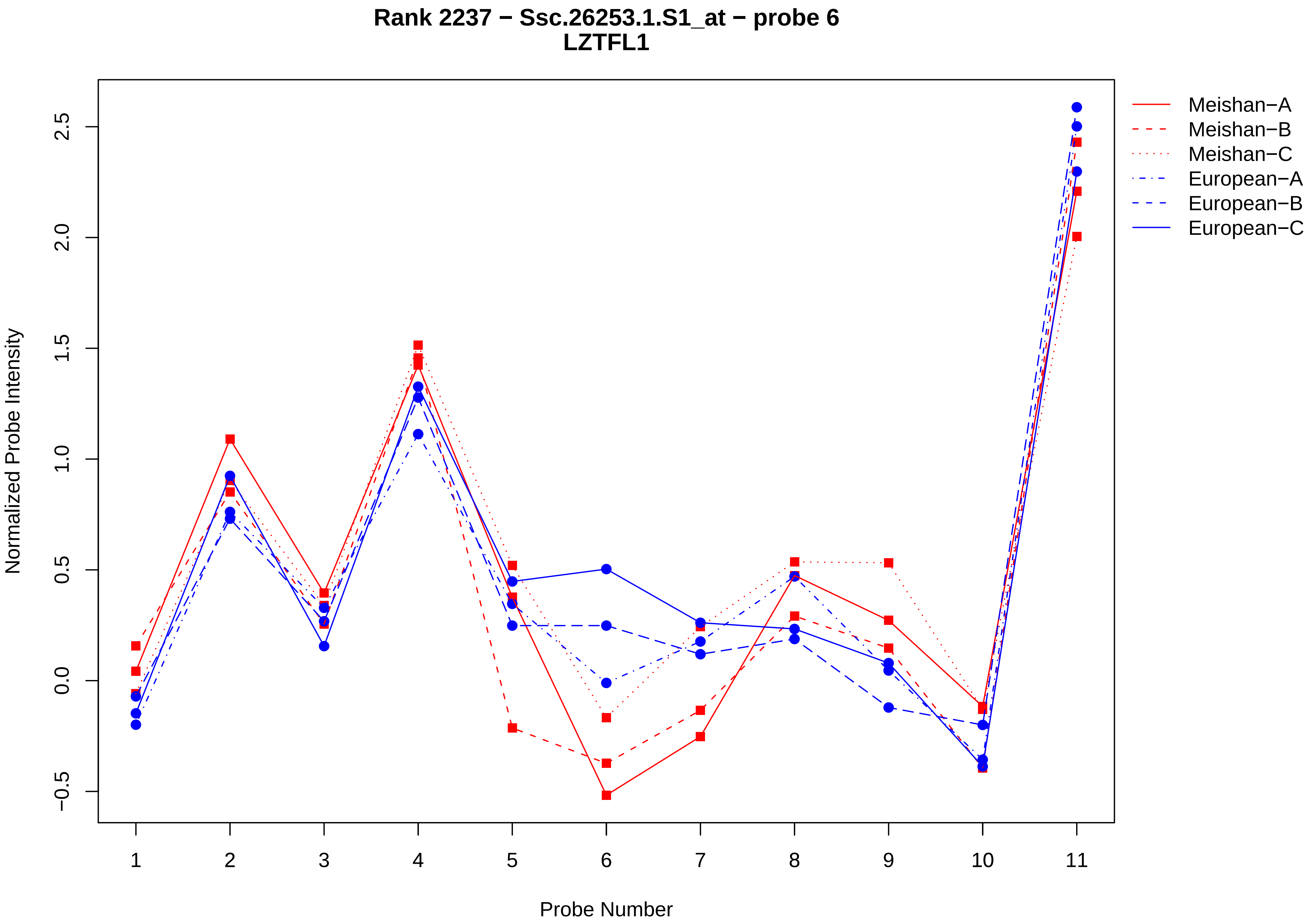
Rank 2237 − Ssc.26253.1.S1_at − probe 6
LZTFL1
Normalized Probe Intensity
Probe Number
Meishan−A
Meishan−B
Meishan−C
European−A
European−B
European−C
−0.5
0.0
0.5
1.0
1.5
2.0
2.5
1
2
3
4
5
6
7
8
9
10
11

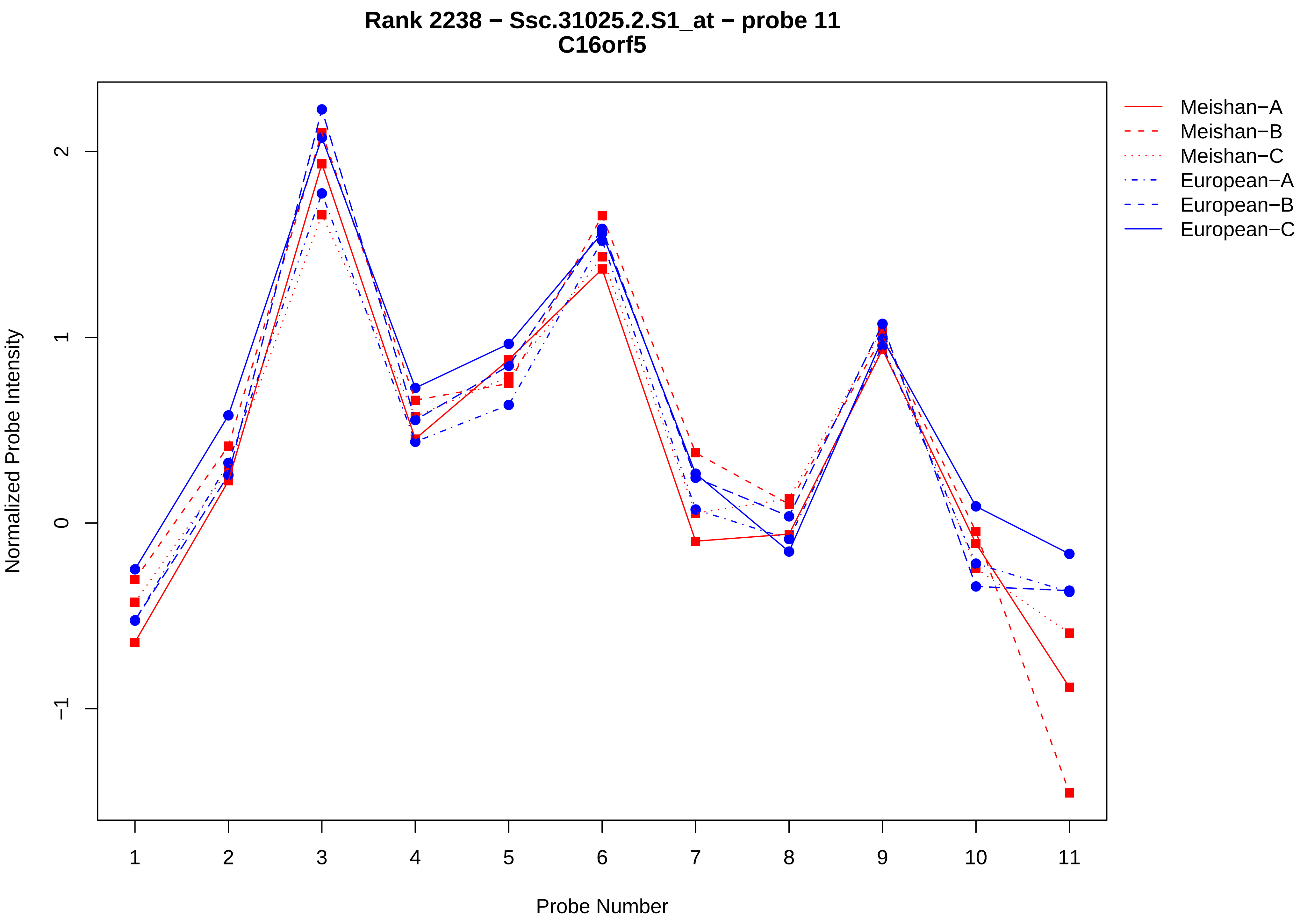
Rank 2238 − Ssc.31025.2.S1_at − probe 11
C16orf5
Normalized Probe Intensity
Probe Number
−1
0
1
2
1
2
3
4
5
6
7
8
9
10
11
Meishan−A
Meishan−B
Meishan−C
European−A
European−B
European−C

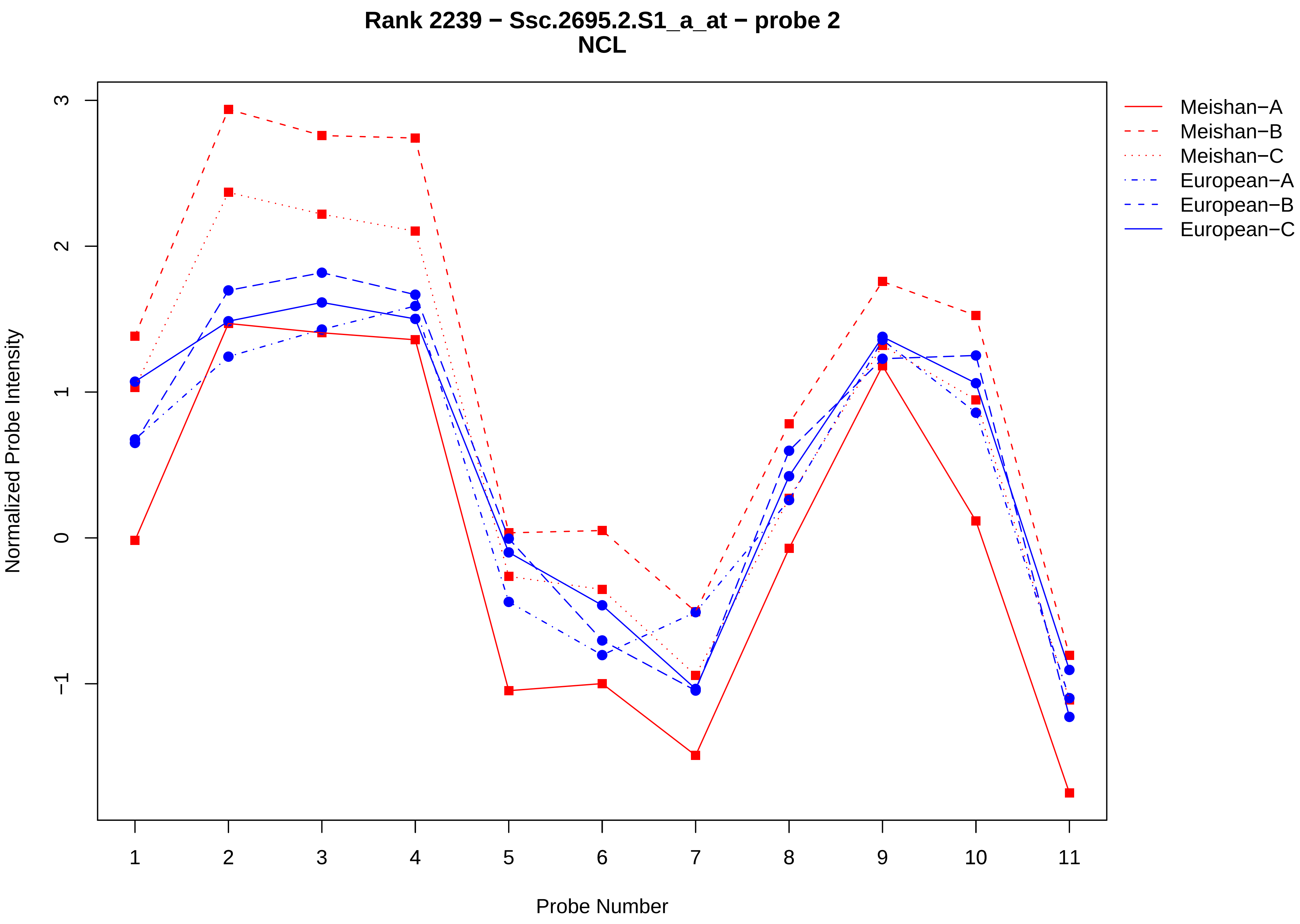
Rank 2239 − Ssc.2695.2.S1_a_at − probe 2
NCL
Normalized Probe Intensity
−1
0
1
2
3
Probe Number
1
2
3
4
5
6
7
8
9
10
11
Meishan−A
Meishan−B
Meishan−C
European−A
European−B
European−C

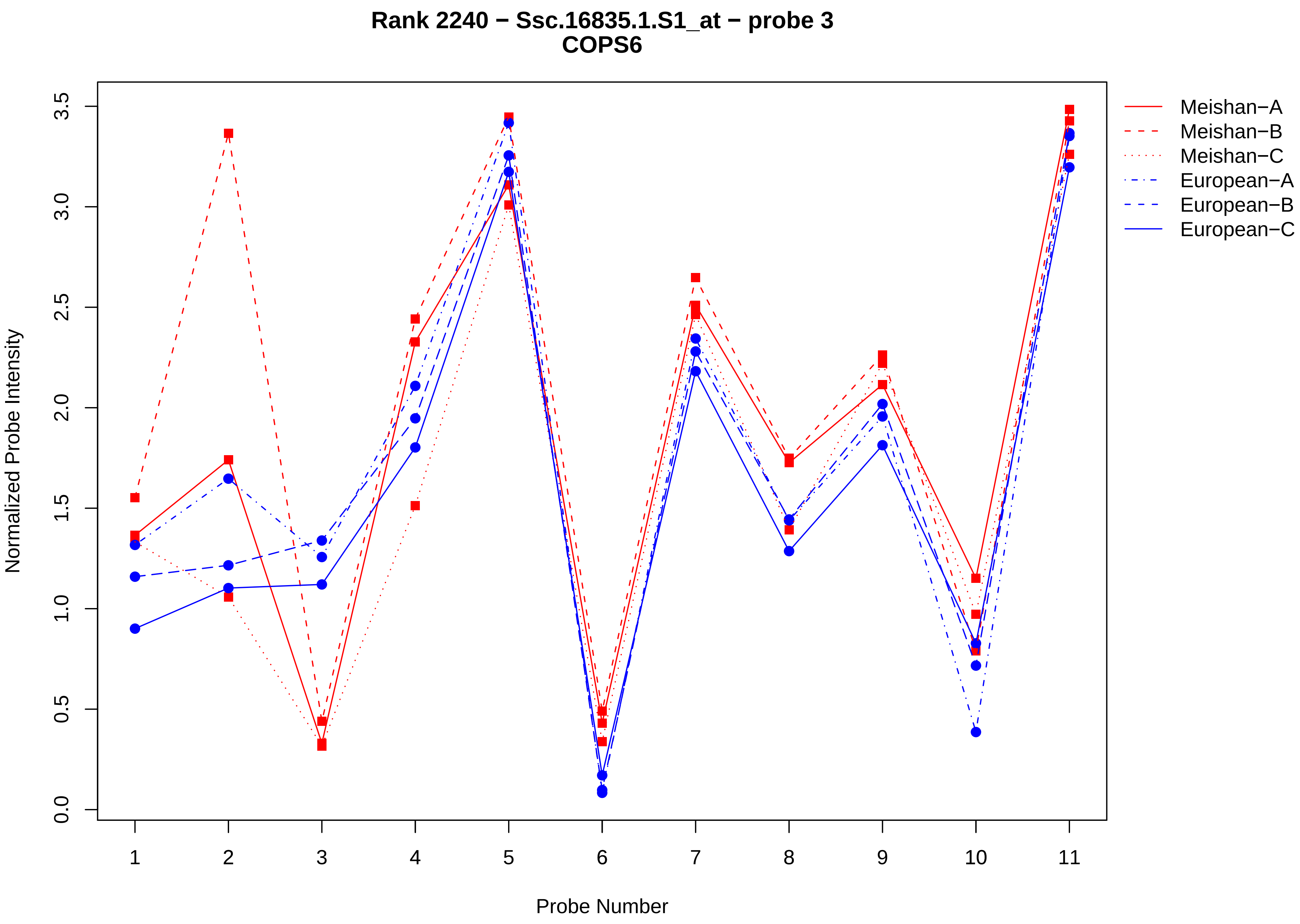

Rank 2240 − Ssc.16835.1.S1_at − probe 3
COPS6
Normalized Probe Intensity
Probe Number
0.0
0.5
1.0
1.5
2.0
2.5
3.0
3.5
1
2
3
4
5
6
7
8
9
10
11
Meishan−A
Meishan−B
Meishan−C
European−A
European−B
European−C

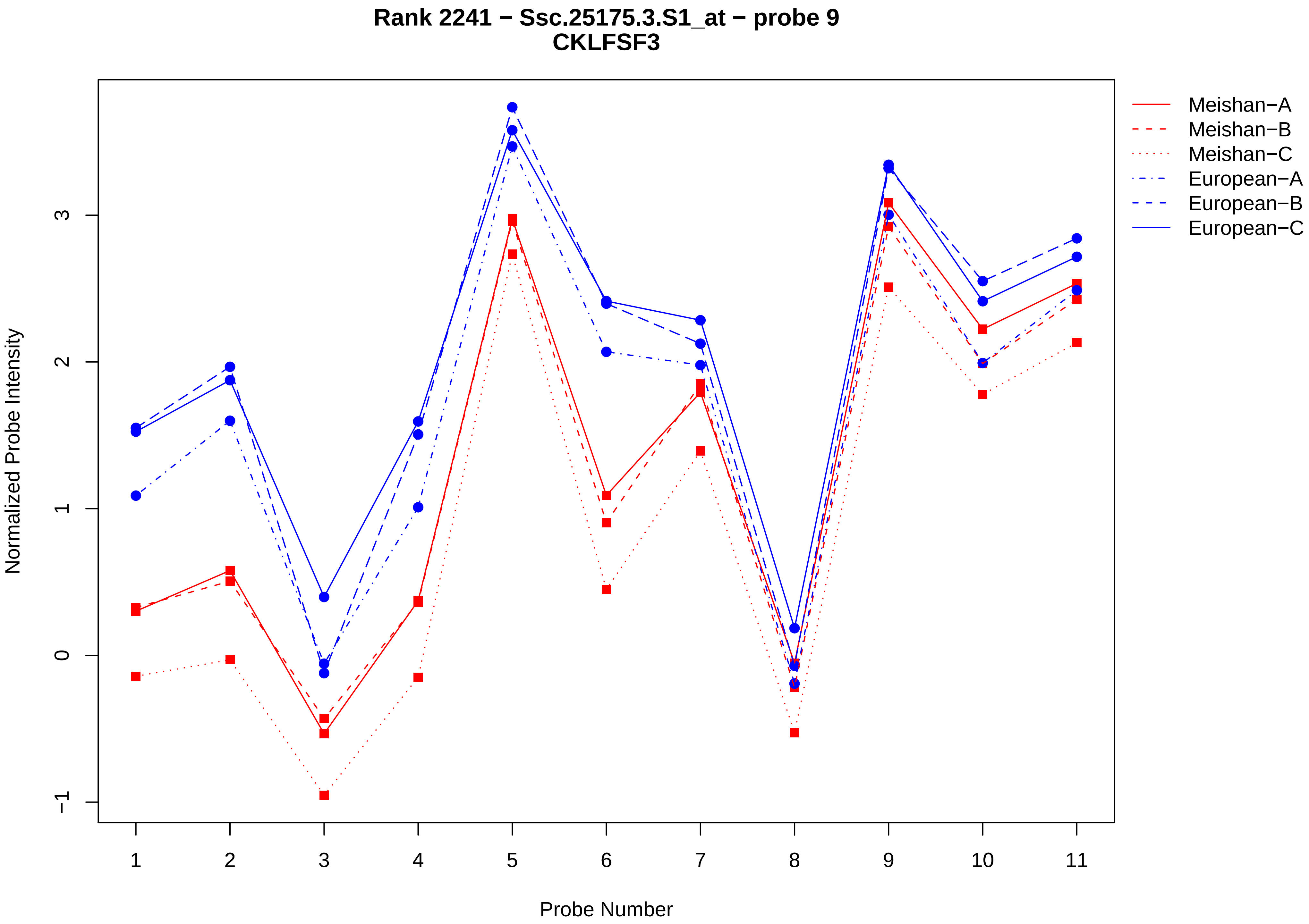

Rank 2241 − Ssc.25175.3.S1_at − probe 9
CKLFSF3
Normalized Probe Intensity
Probe Number
Meishan−A
Meishan−B
Meishan−C
European−A
European−B
European−C
−1
0
1
2
3
1
2
3
4
5
6
7
8
9
10
11

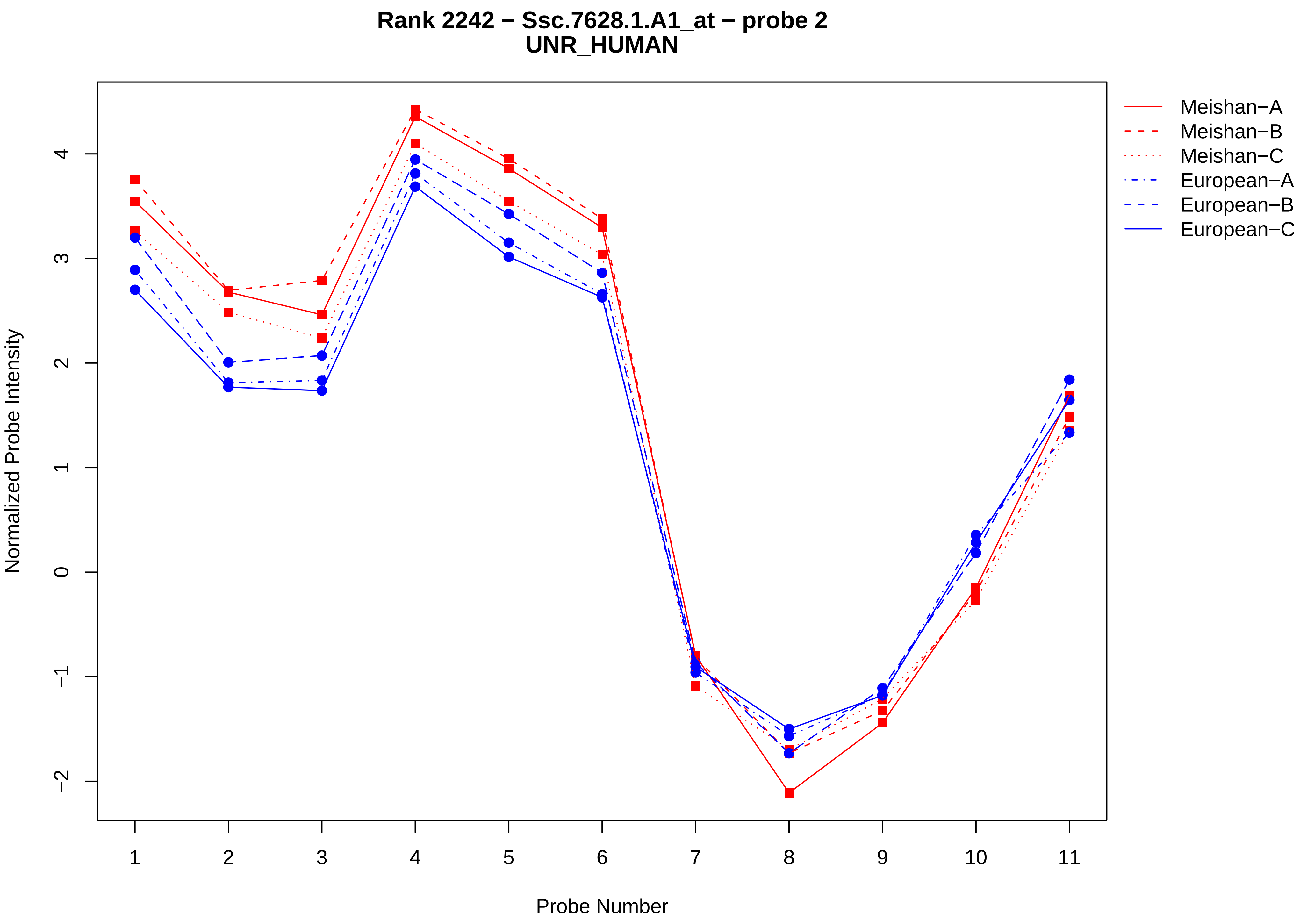

Rank 2242 − Ssc.7628.1.A1_at − probe 2
UNR_HUMAN
Normalized Probe Intensity
Probe Number
Meishan−A
Meishan−B
Meishan−C
European−A
European−B
European−C

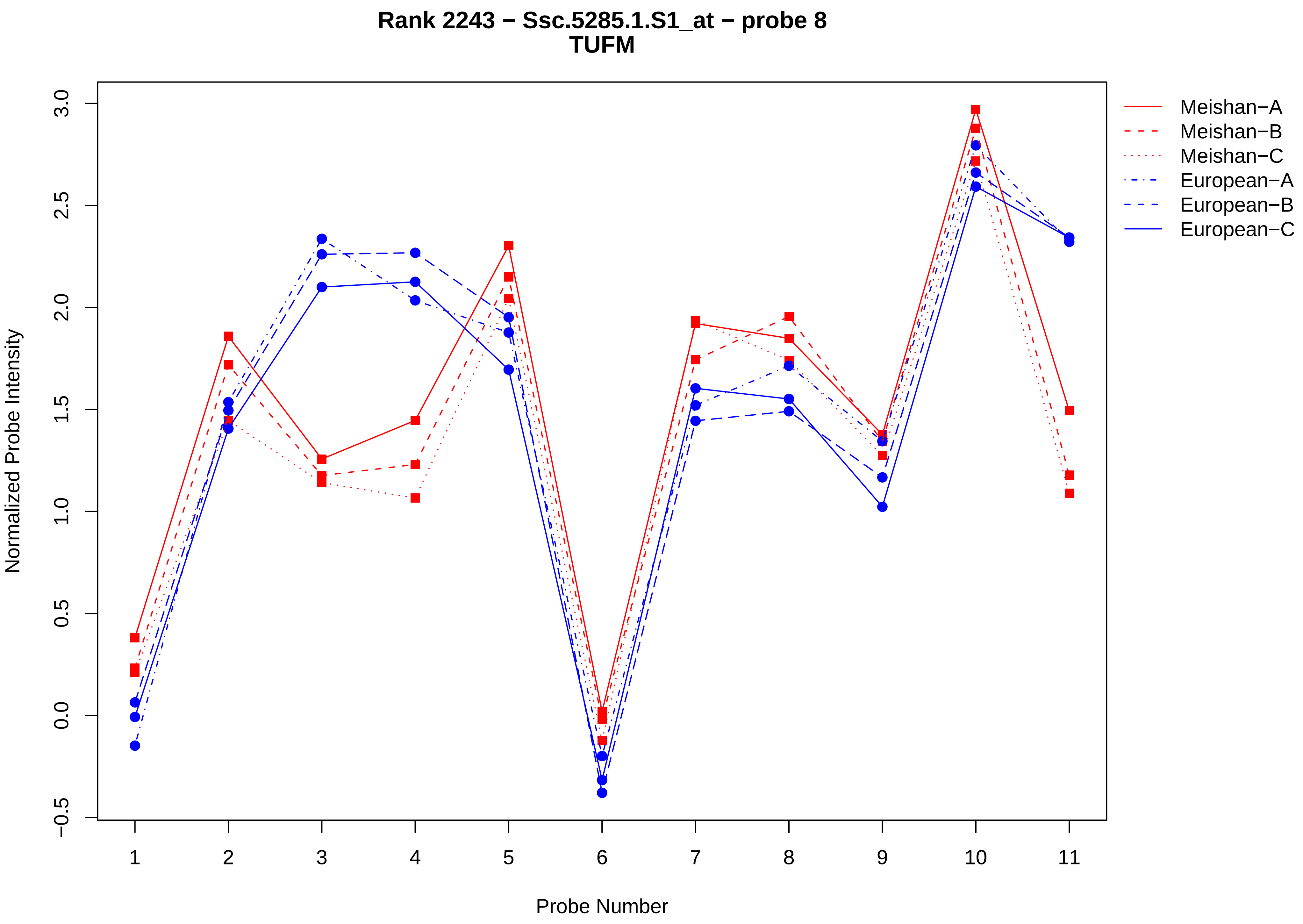

Rank 2243 − Ssc.5285.1.S1_at − probe 8
TUFM
Normalized Probe Intensity
Probe Number
−0.5
0.0
0.5
1.0
1.5
2.0
2.5
3.0
1
2
3
4
5
6
7
8
9
10
11
Meishan−A
Meishan−B
Meishan−C
European−A
European−B
European−C

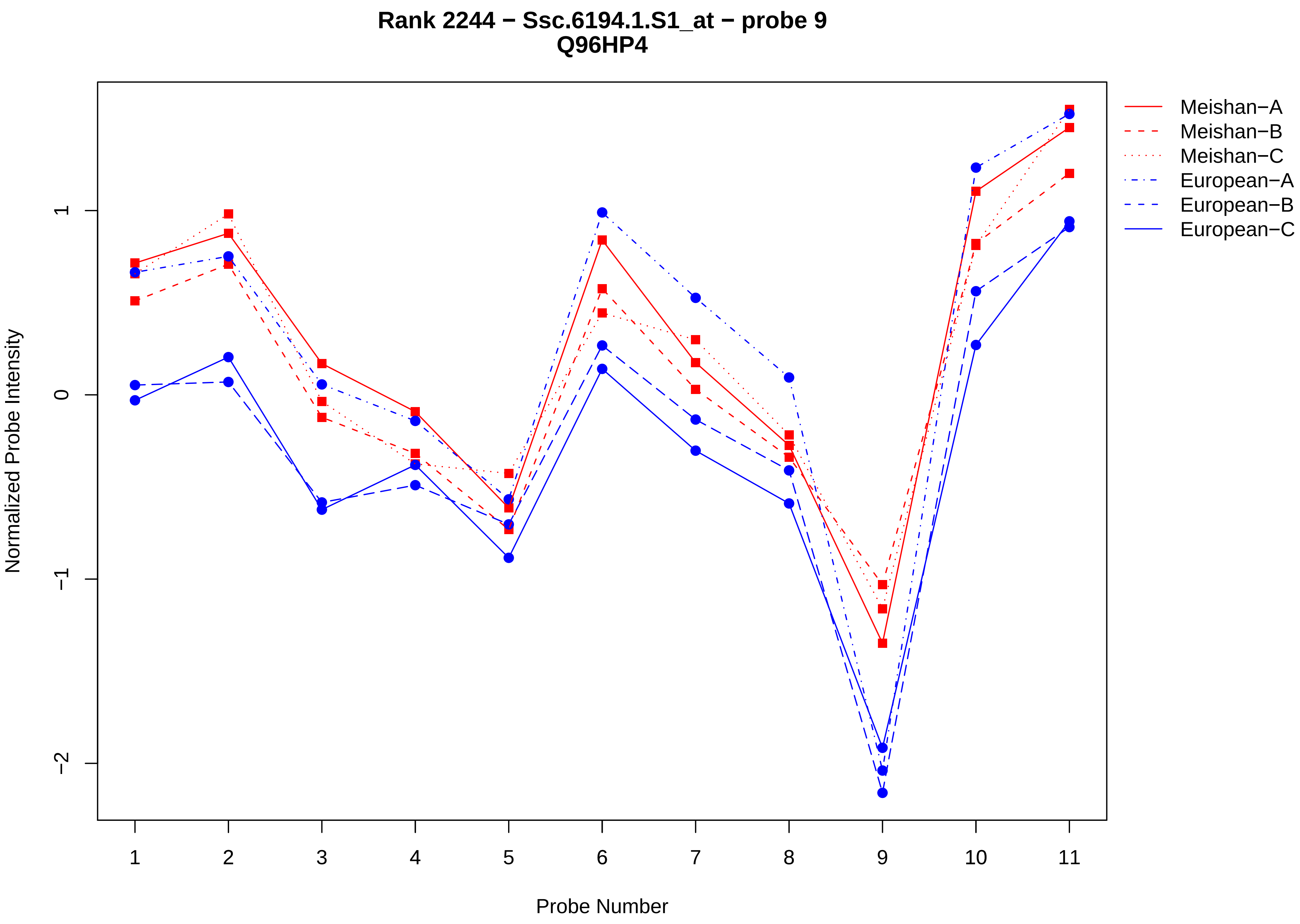

Rank 2244 − Ssc.6194.1.S1_at − probe 9
Q96HP4
Normalized Probe Intensity
Probe Number
Meishan−A
Meishan−B
Meishan−C
European−A
European−B
European−C
−2
−1
0
1
1
2
3
4
5
6
7
8
9
10
11

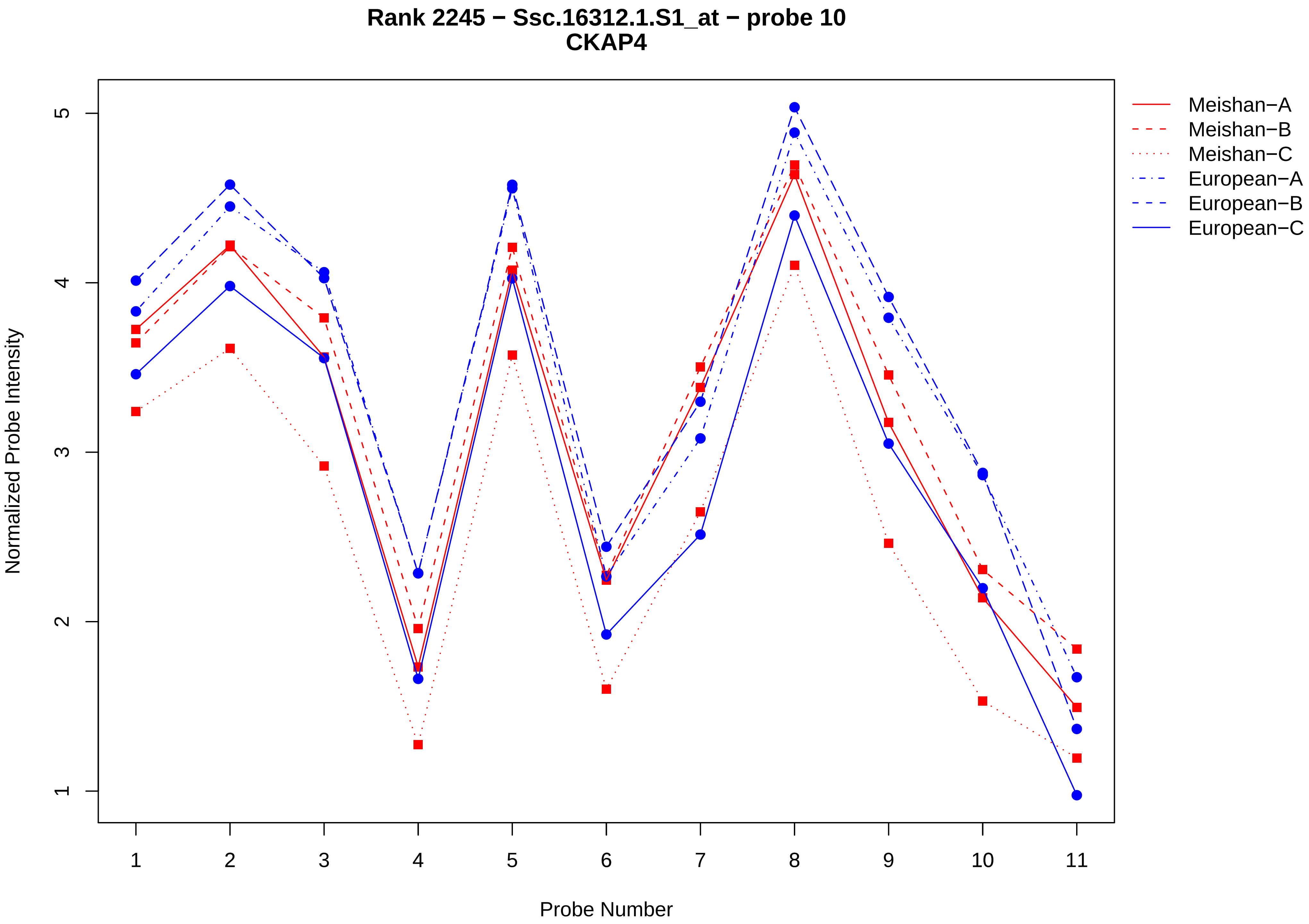
Rank 2245 − Ssc.16312.1.S1_at − probe 10
CKAP4
Normalized Probe Intensity
Probe Number
1
2
3
4
5
1
2
3
4
5
6
7
8
9
10
11
Meishan−A
Meishan−B
Meishan−C
European−A
European−B
European−C

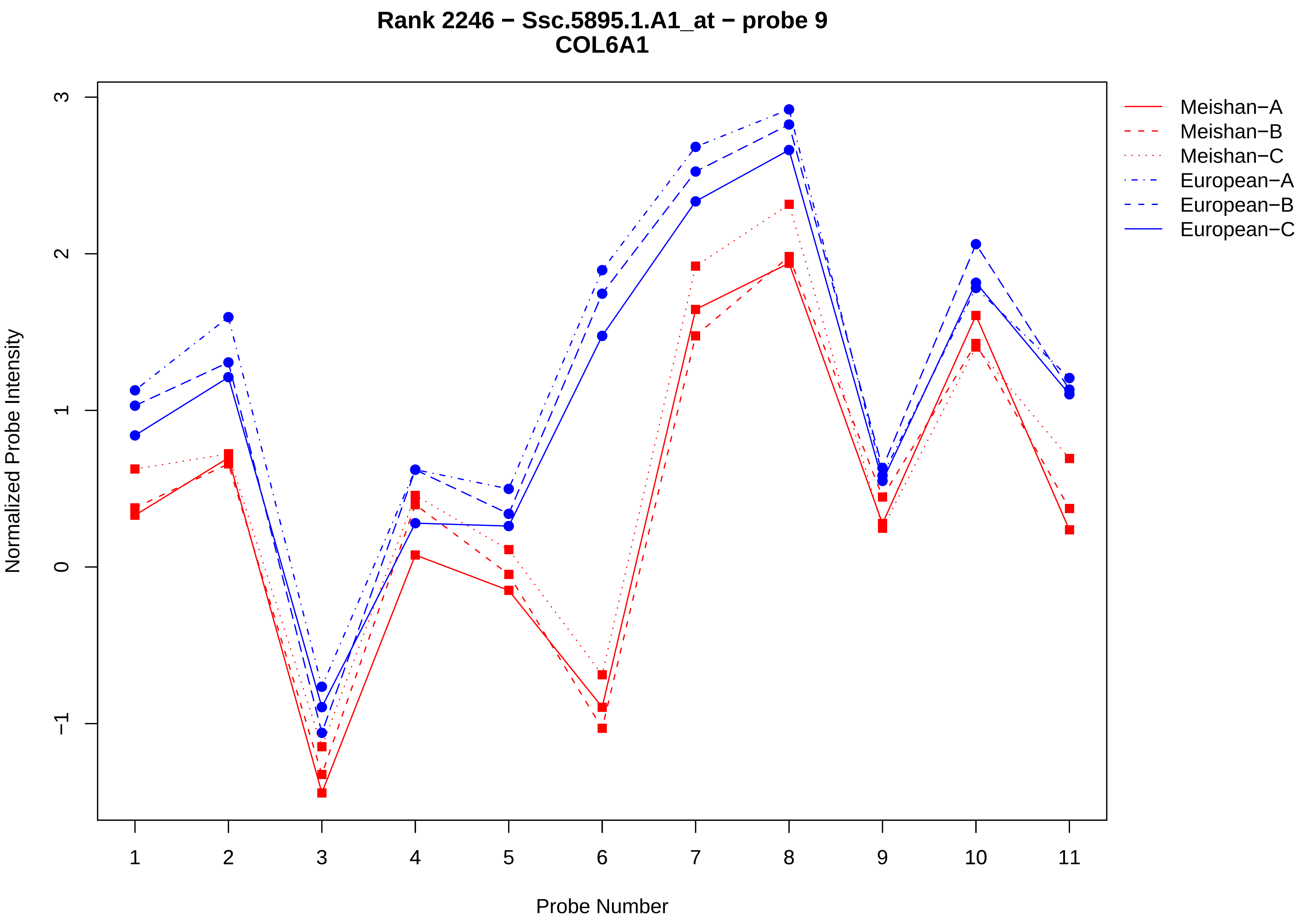

Rank 2246 − Ssc.5895.1.A1_at − probe 9
COL6A1
Normalized Probe Intensity
Probe Number
Meishan−A
Meishan−B
Meishan−C
European−A
European−B
European−C
−1
0
1
2
3
1
2
3
4
5
6
7
8
9
10
11

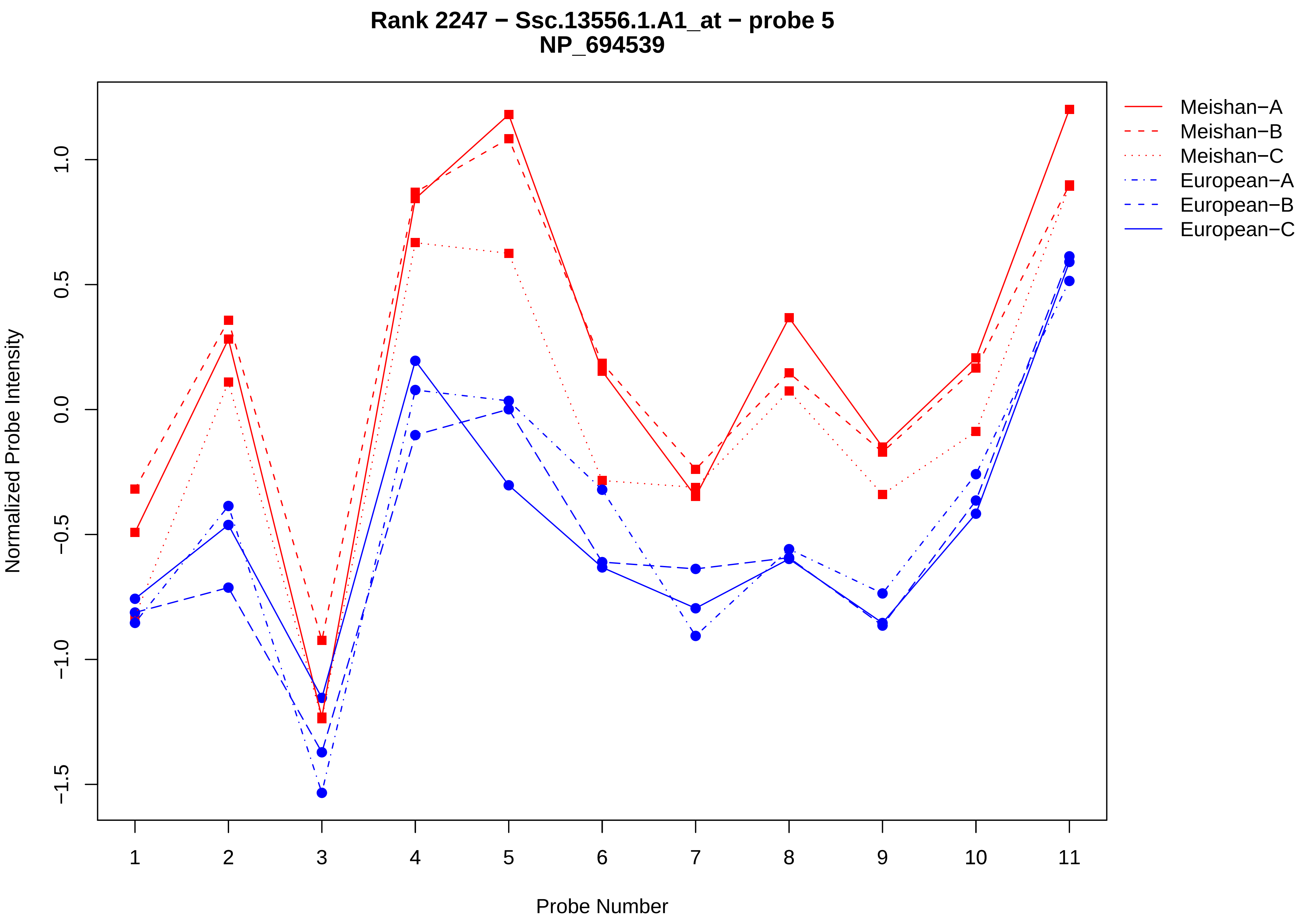

Rank 2247 − Ssc.13556.1.A1_at − probe 5
NP_694539
Normalized Probe Intensity
Probe Number
1.0
0.5
0.0
−0.5
−1.0
−1.5
1
2
3
4
5
6
7
8
9
10
11
Meishan−A
Meishan−B
Meishan−C
European−A
European−B
European−C

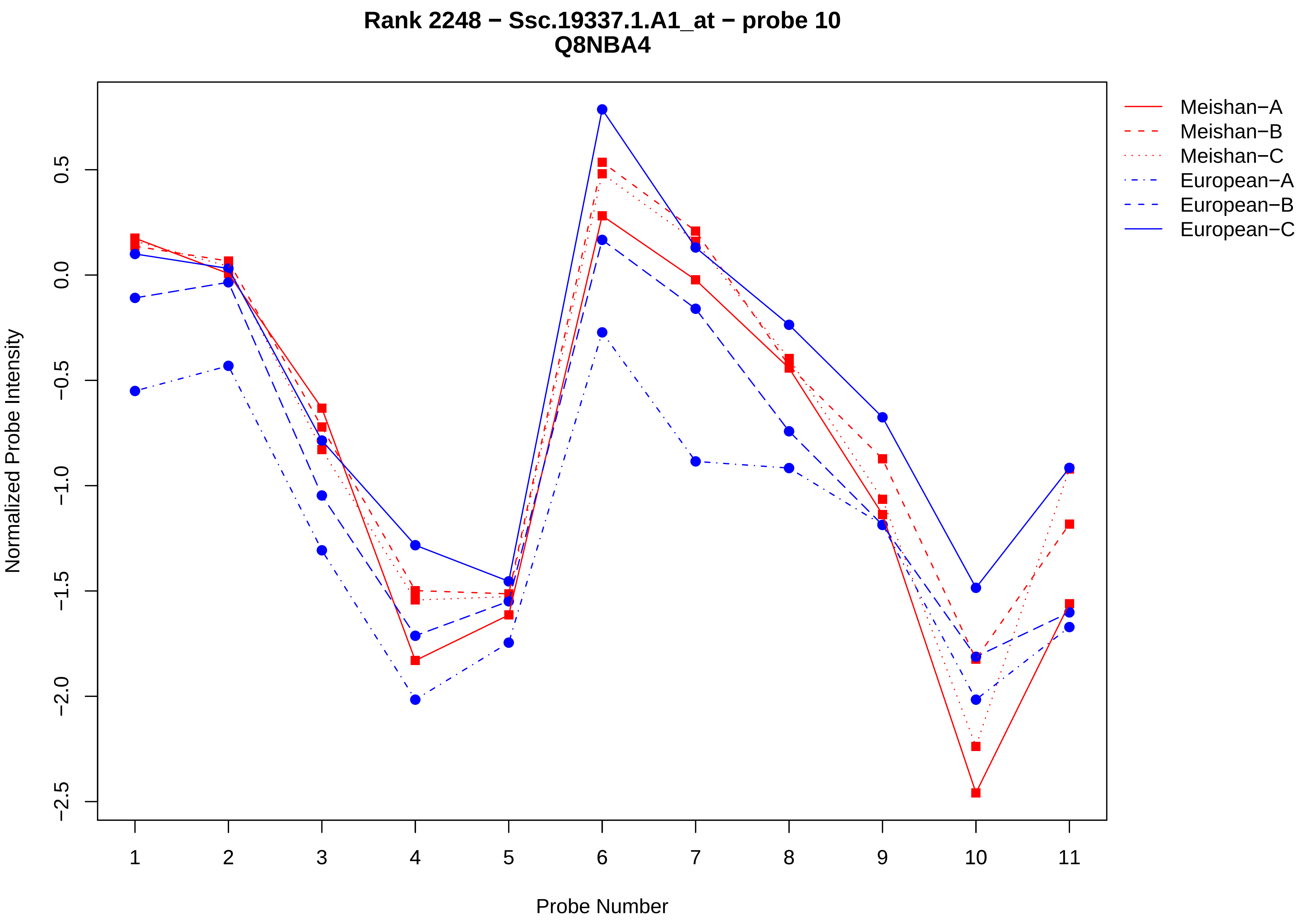

Rank 2248 − Ssc.19337.1.A1_at − probe 10
Q8NBA4
Normalized Probe Intensity
Probe Number
0.5
0.0
−0.5
−1.0
−1.5
−2.0
−2.5
1
2
3
4
5
6
7
8
9
10
11
Meishan−A
Meishan−B
Meishan−C
European−A
European−B
European−C

Rank 2249 − Ssc.9306.1.A1_at − probe 10
Q5SWH9

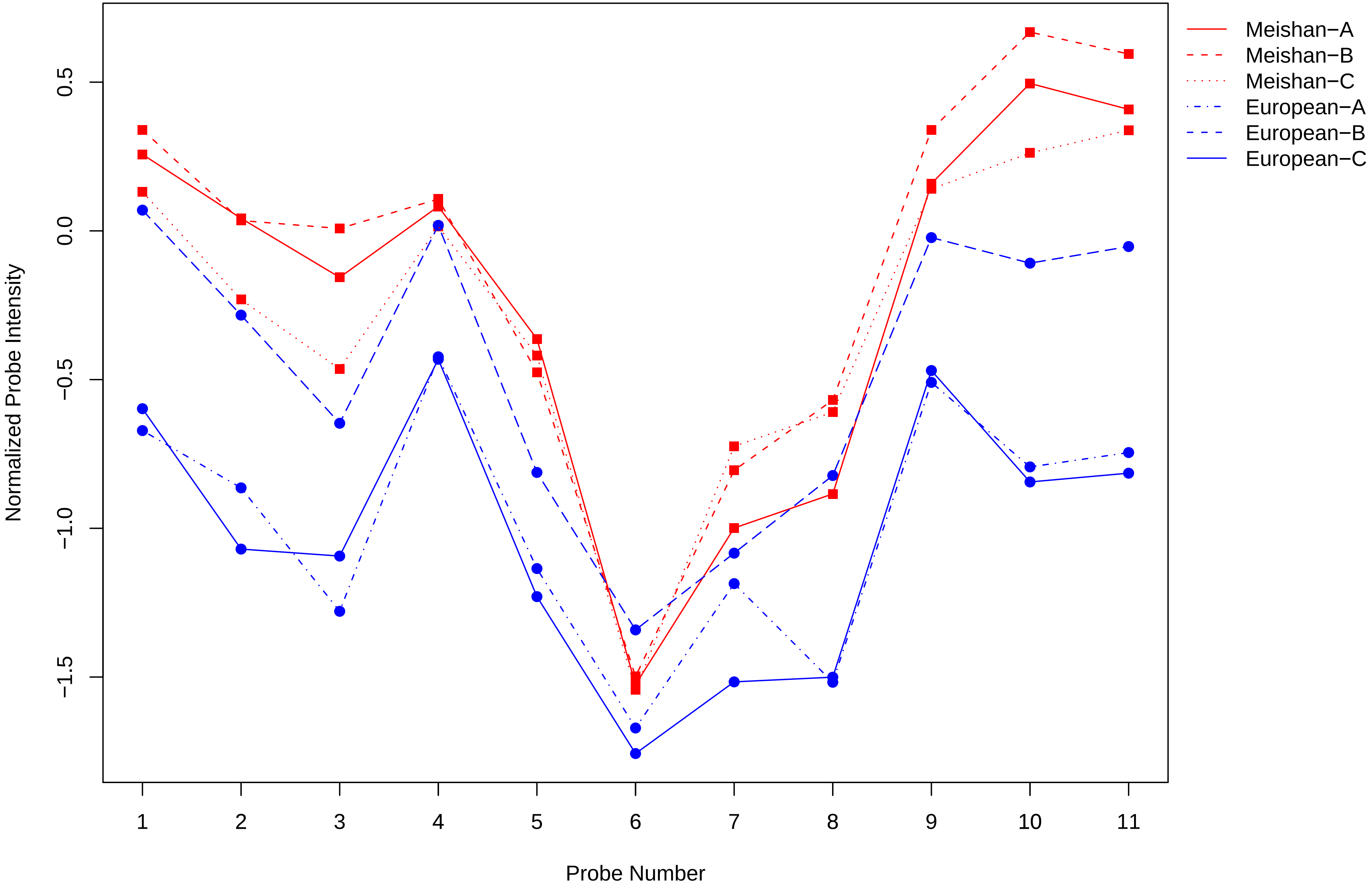

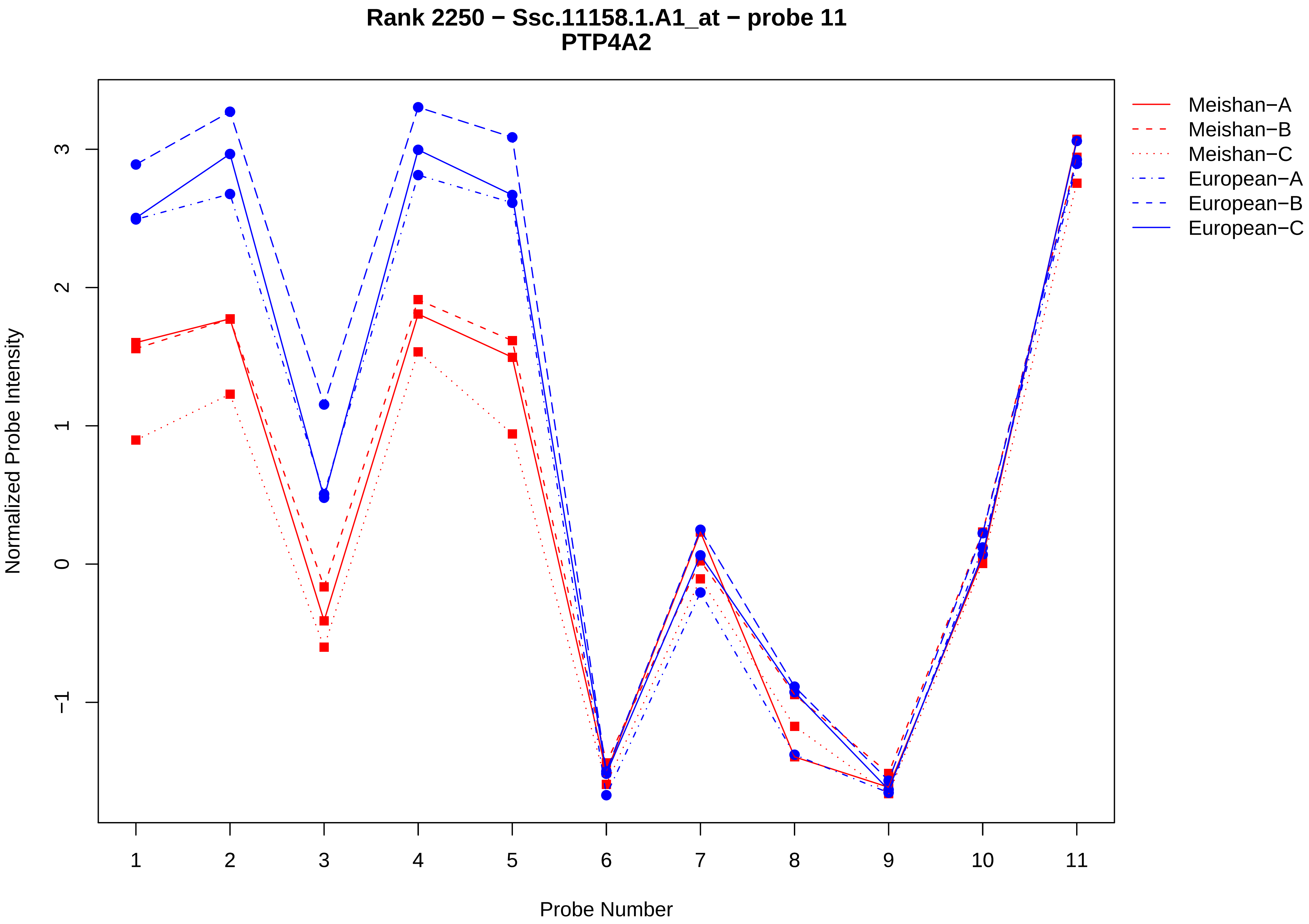

Rank 2250 − Ssc.11158.1.A1_at − probe 11
PTP4A2
Normalized Probe Intensity
Probe Number
−1
0
1
2
3
1
2
3
4
5
6
7
8
9
10
11
Meishan−A
Meishan−B
Meishan−C
European−A
European−B
European−C

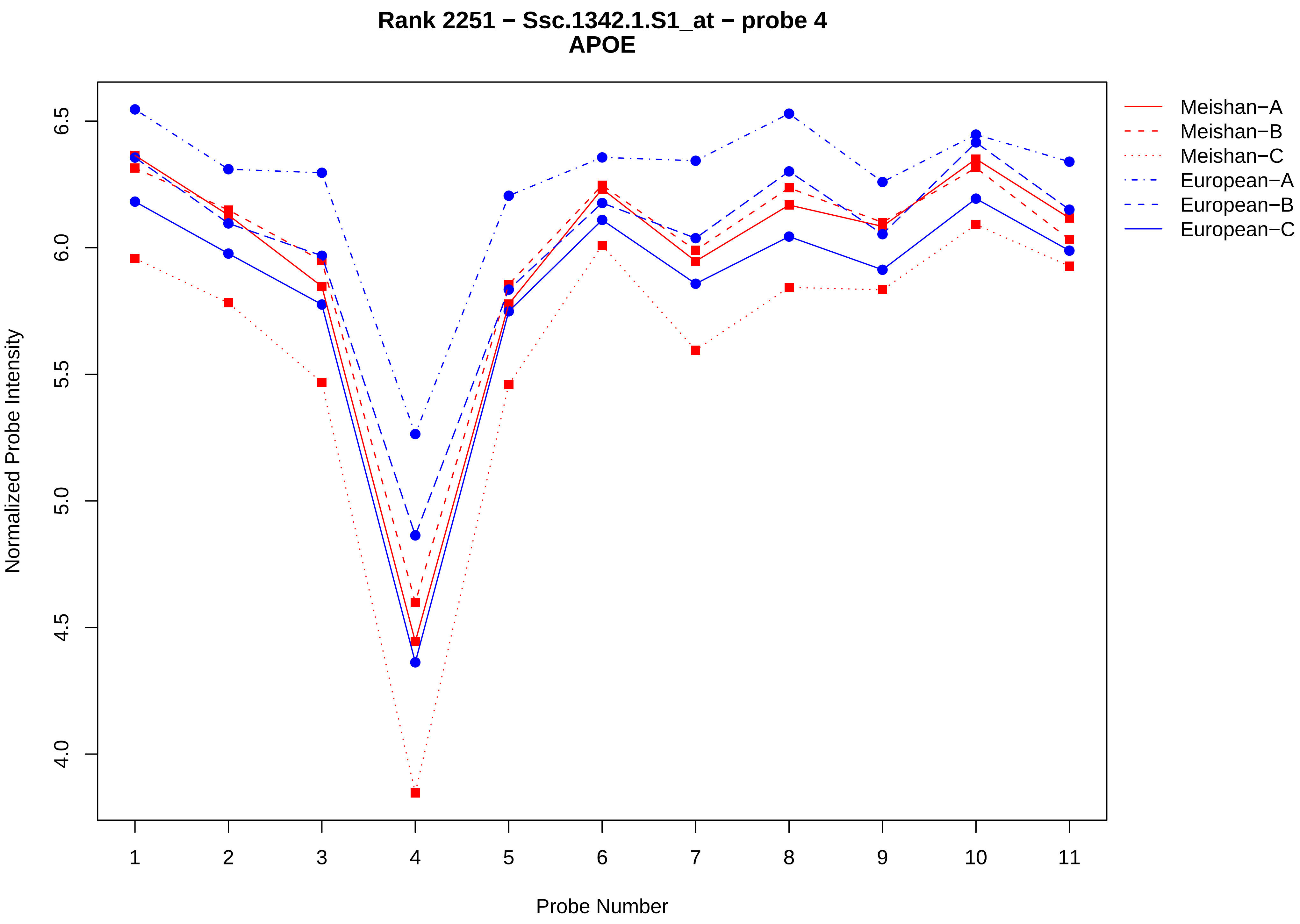

Rank 2251 − Ssc.1342.1.S1_at − probe 4
APOE
Normalized Probe Intensity
Probe Number
4.0
4.5
5.0
5.5
6.0
6.5
1
2
3
4
5
6
7
8
9
10
11
Meishan−A
Meishan−B
Meishan−C
European−A
European−B
European−C

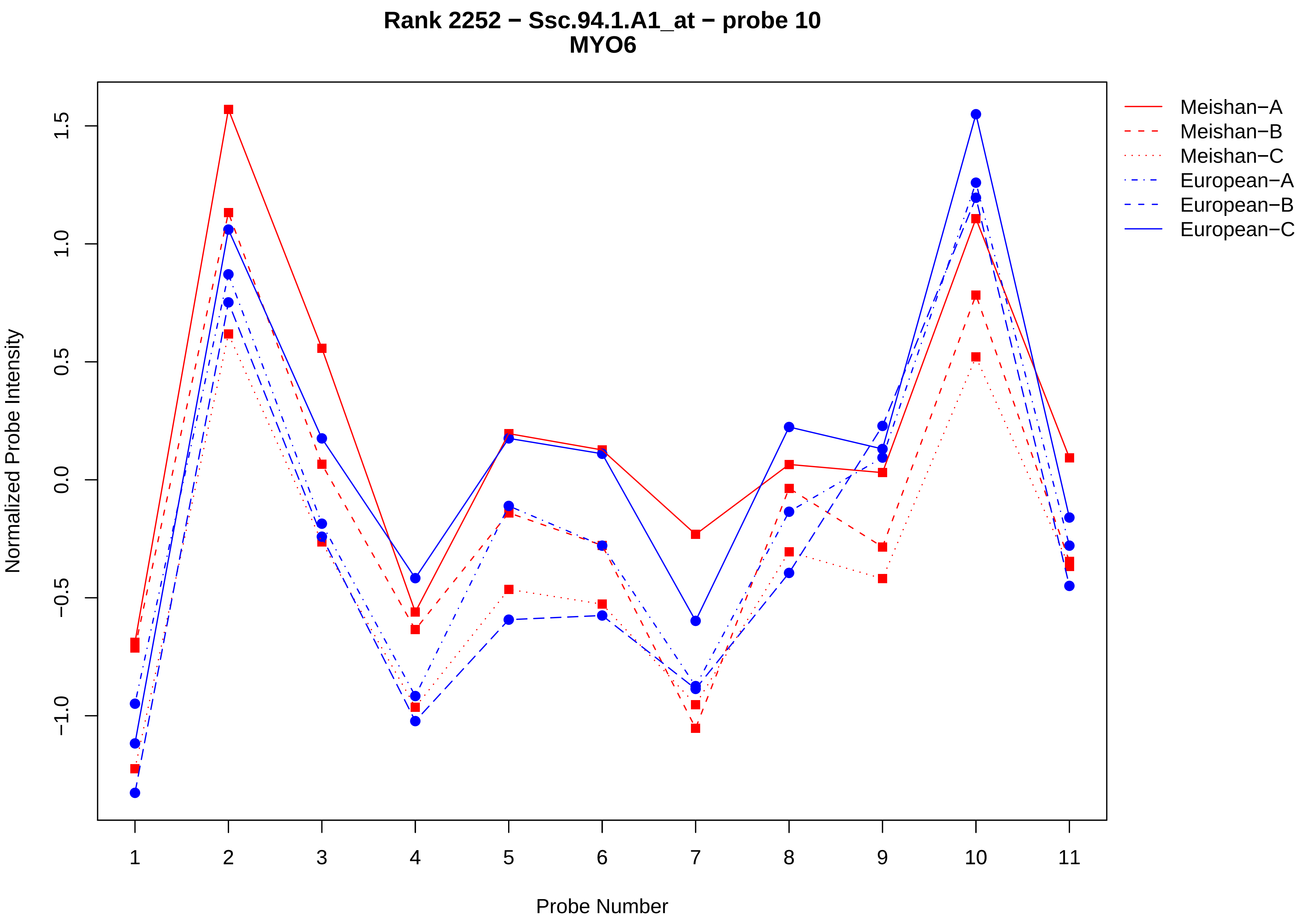

Rank 2252 − Ssc.94.1.A1_at − probe 10
MYO6
Normalized Probe Intensity
Probe Number
−1.0
−0.5
0.0
0.5
1.0
1.5
1
2
3
4
5
6
7
8
9
10
11
Meishan−A
Meishan−B
Meishan−C
European−A
European−B
European−C

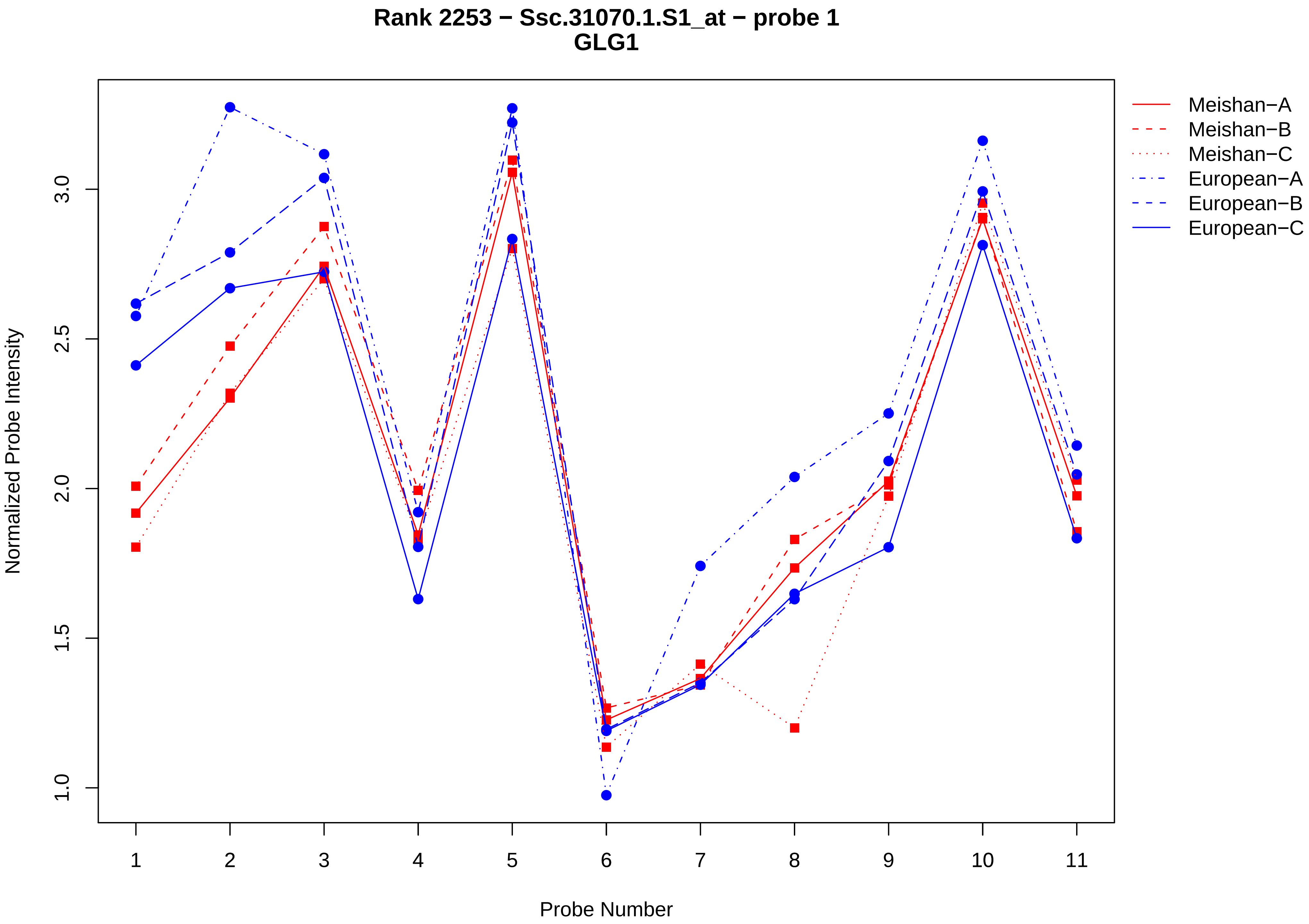

Rank 2253 − Ssc.31070.1.S1_at − probe 1
GLG1
Normalized Probe Intensity
Probe Number
Meishan−A
Meishan−B
Meishan−C
European−A
European−B
European−C
1.0
1.5
2.0
2.5
3.0
1
2
3
4
5
6
7
8
9
10
11

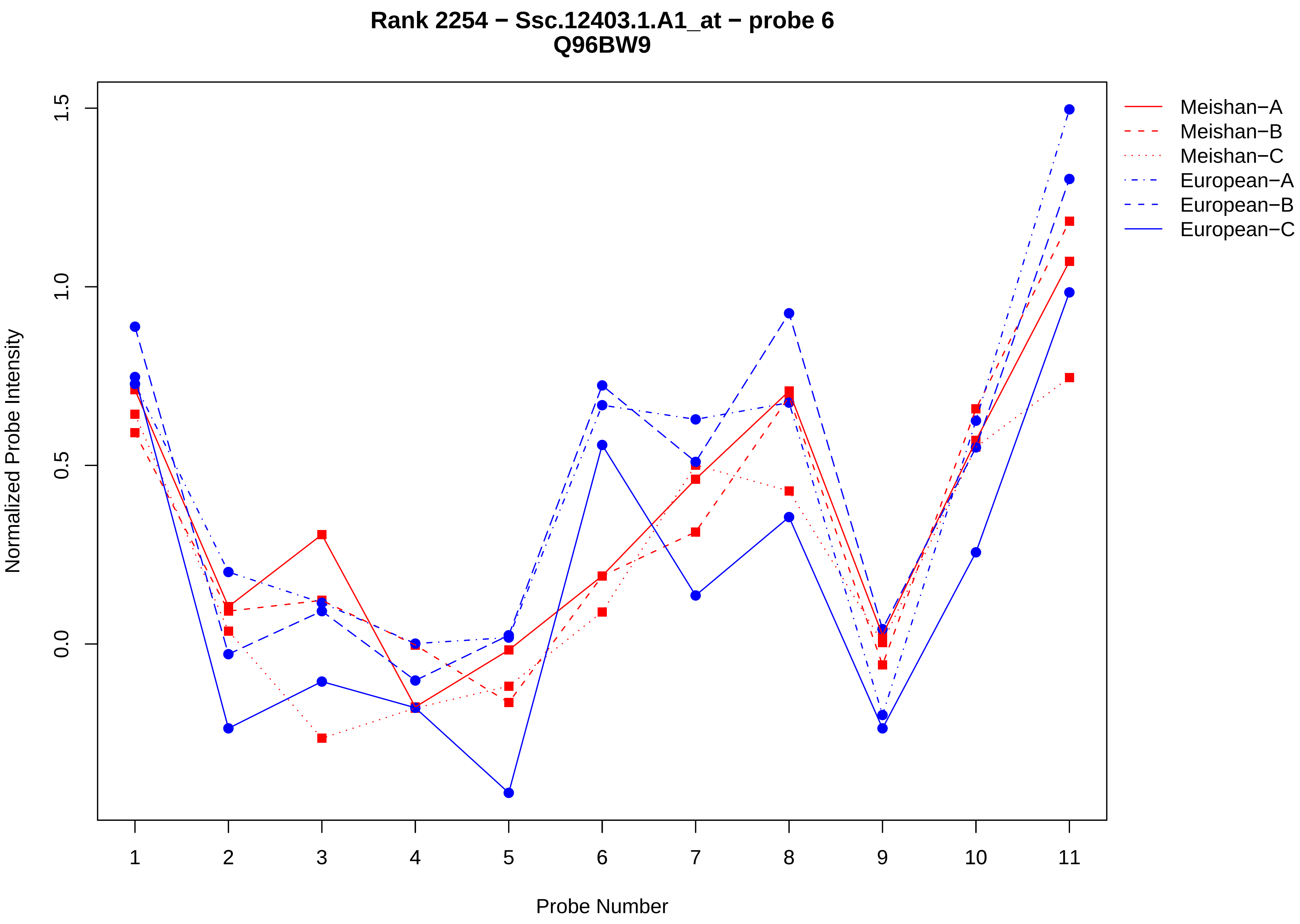

Rank 2254 − Ssc.12403.1.A1_at − probe 6
Q96BW9
Normalized Probe Intensity
Probe Number
Meishan−A
Meishan−B
Meishan−C
European−A
European−B
European−C
0.0
0.5
1.0
1.5
1
2
3
4
5
6
7
8
9
10
11

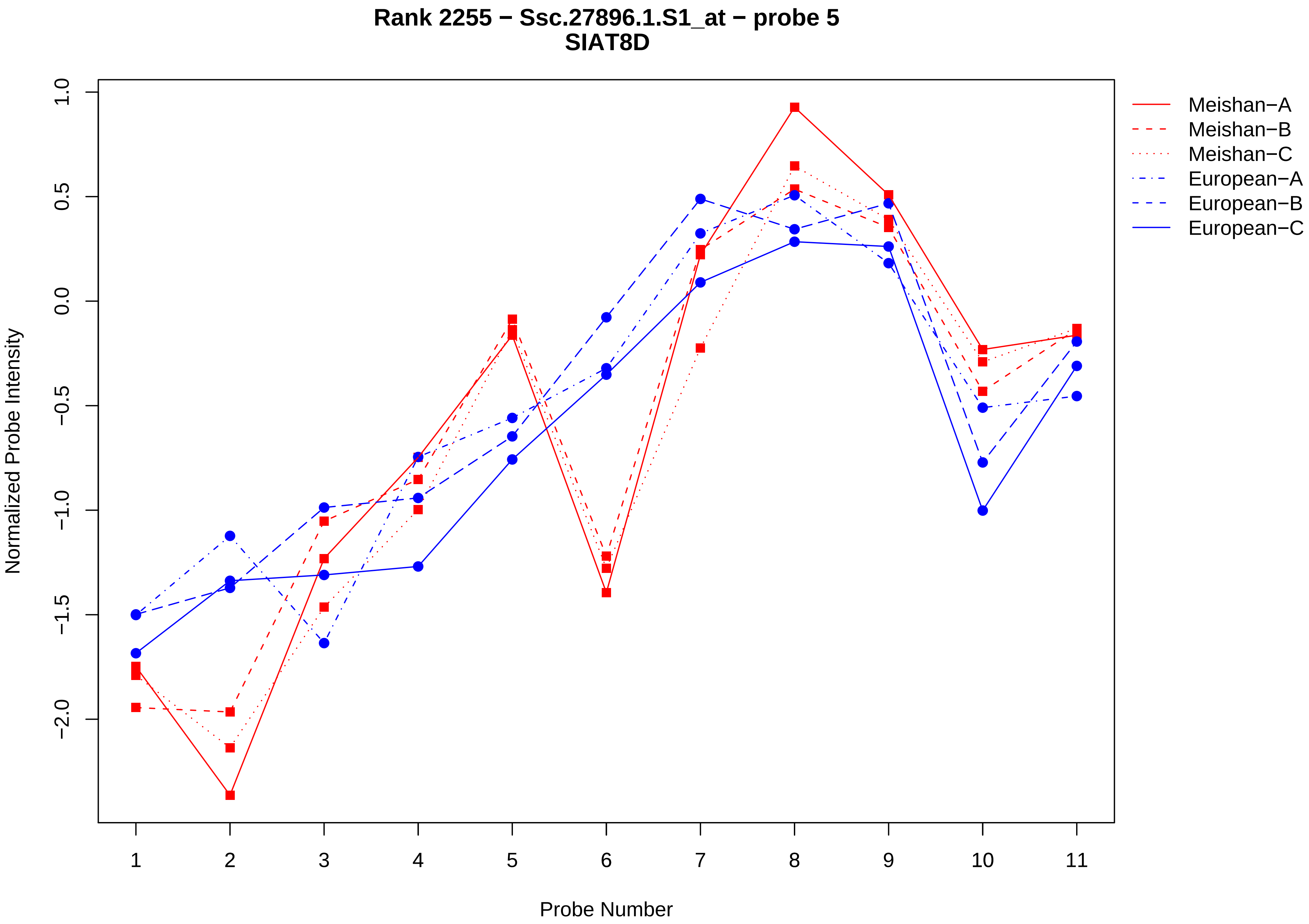

Rank 2255 − Ssc.27896.1.S1_at − probe 5
SIAT8D
Normalized Probe Intensity
Probe Number
1.0
0.5
0.0
−0.5
−1.0
−1.5
−2.0
1
2
3
4
5
6
7
8
9
10
11
Meishan−A
Meishan−B
Meishan−C
European−A
European−B
European−C

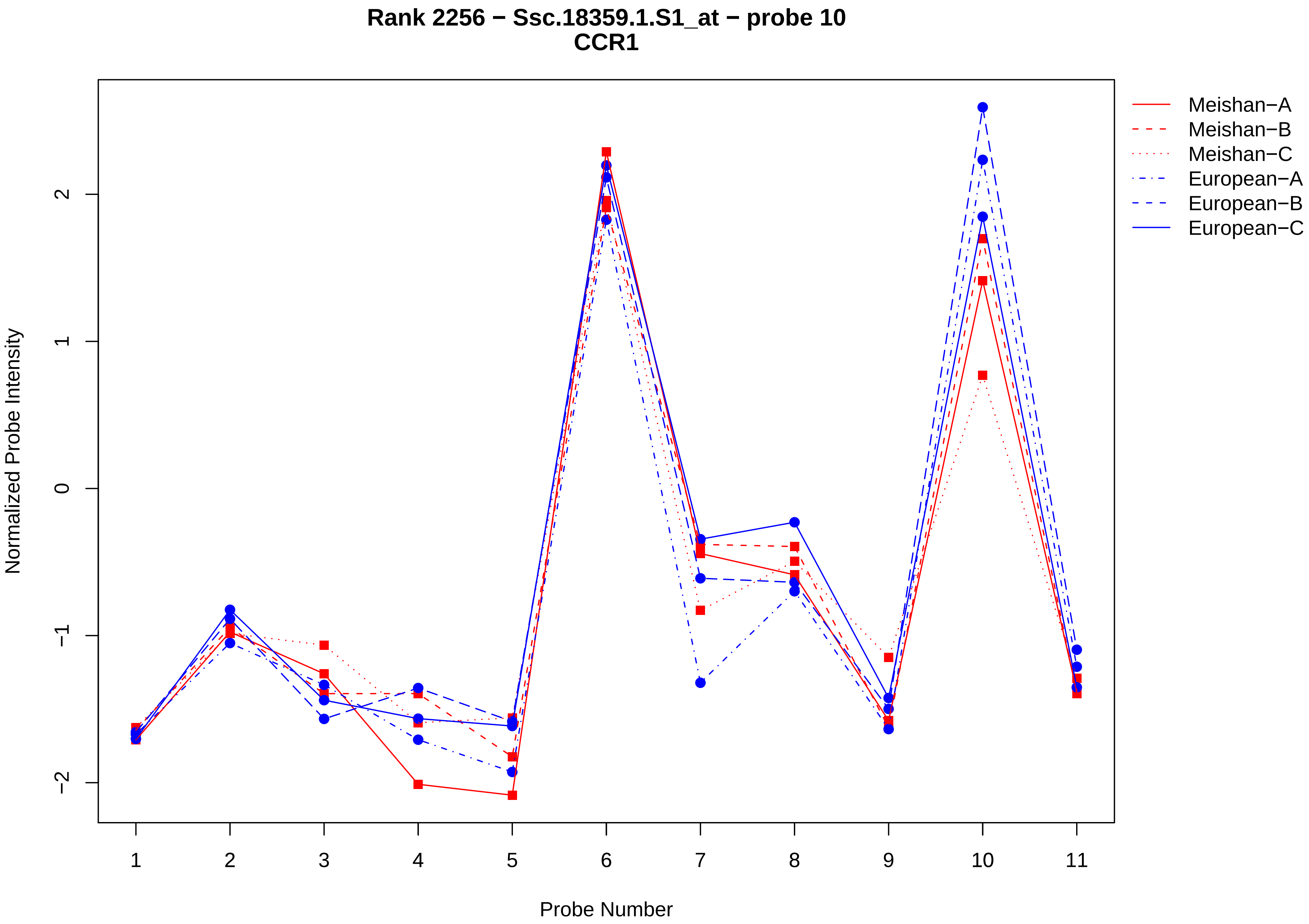
Rank 2256 − Ssc.18359.1.S1_at − probe 10
CCR1
Normalized Probe Intensity
Probe Number
−2
−1
0
1
2
1
2
3
4
5
6
7
8
9
10
11
Meishan−A
Meishan−B
Meishan−C
European−A
European−B
European−C

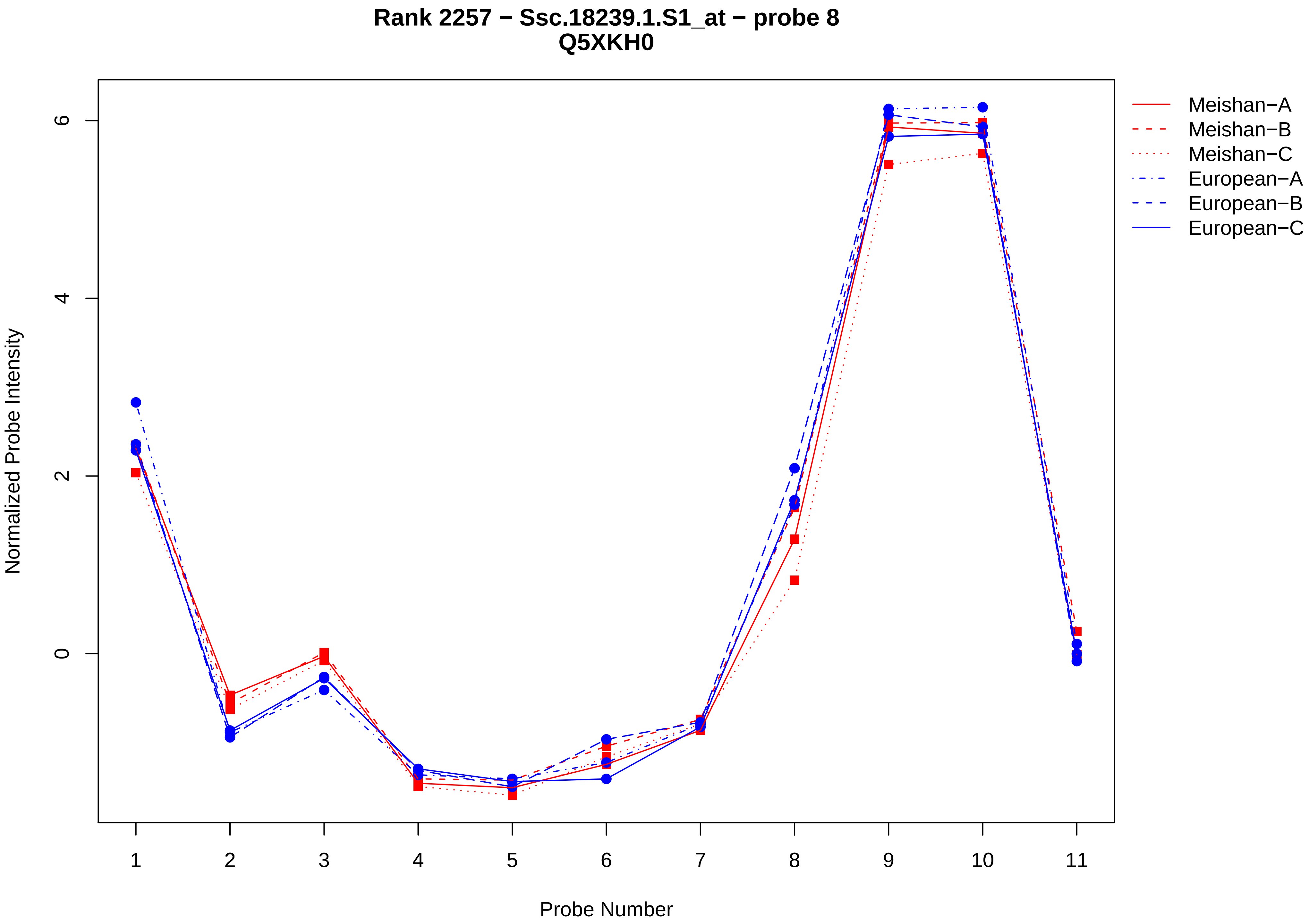

Rank 2257 − Ssc.18239.1.S1_at − probe 8
Q5XKH0
Normalized Probe Intensity
Probe Number
0
2
4
6
1
2
3
4
5
6
7
8
9
10
11
Meishan−A
Meishan−B
Meishan−C
European−A
European−B
European−C

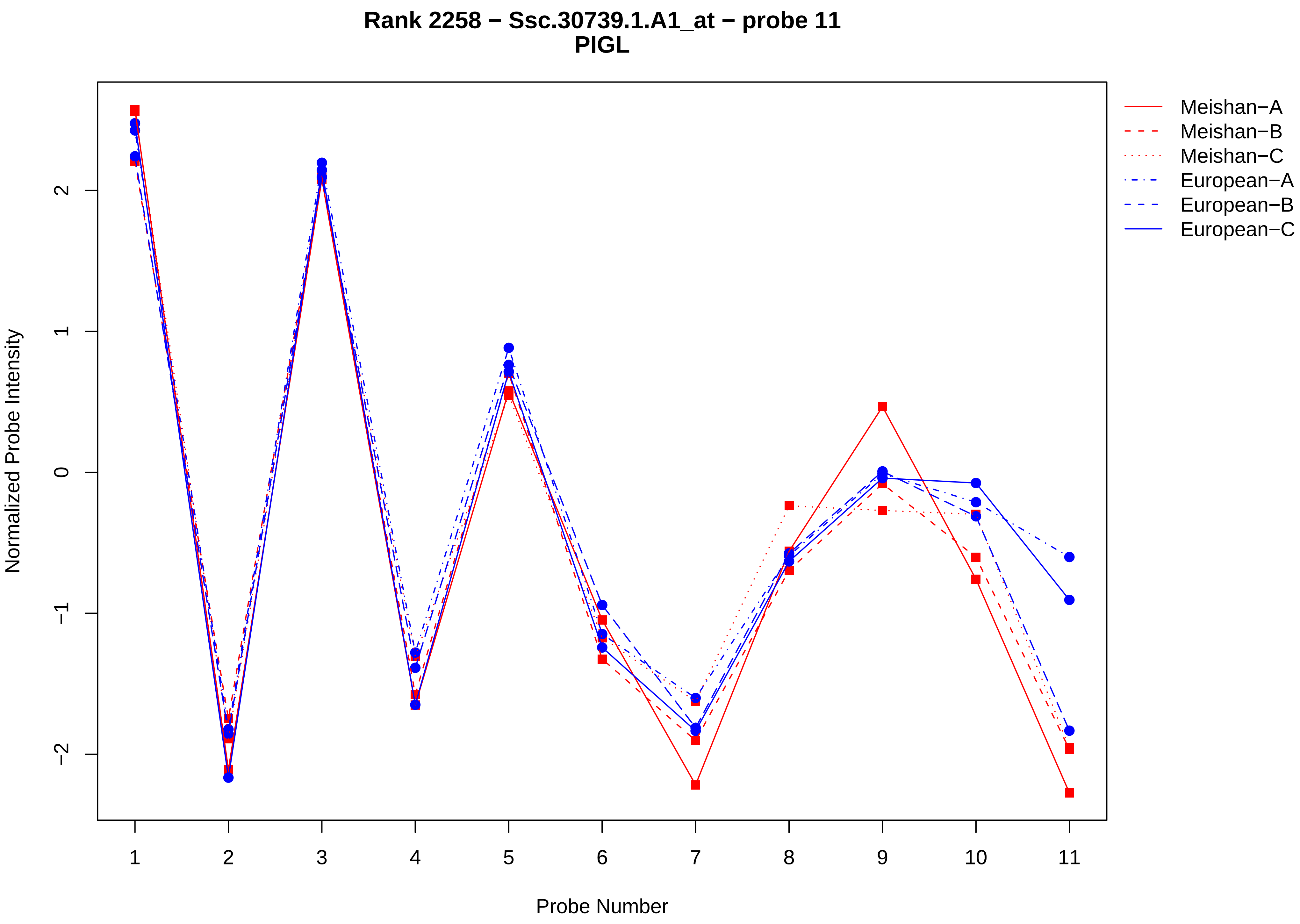
Rank 2258 − Ssc.30739.1.A1_at − probe 11
PIGL
Normalized Probe Intensity
Probe Number
−2
−1
0
1
2
1
2
3
4
5
6
7
8
9
10
11
Meishan−A
Meishan−B
Meishan−C
European−A
European−B
European−C

Rank 2259 − Ssc.31057.1.A1_s_at − probe 3
HSPA9B

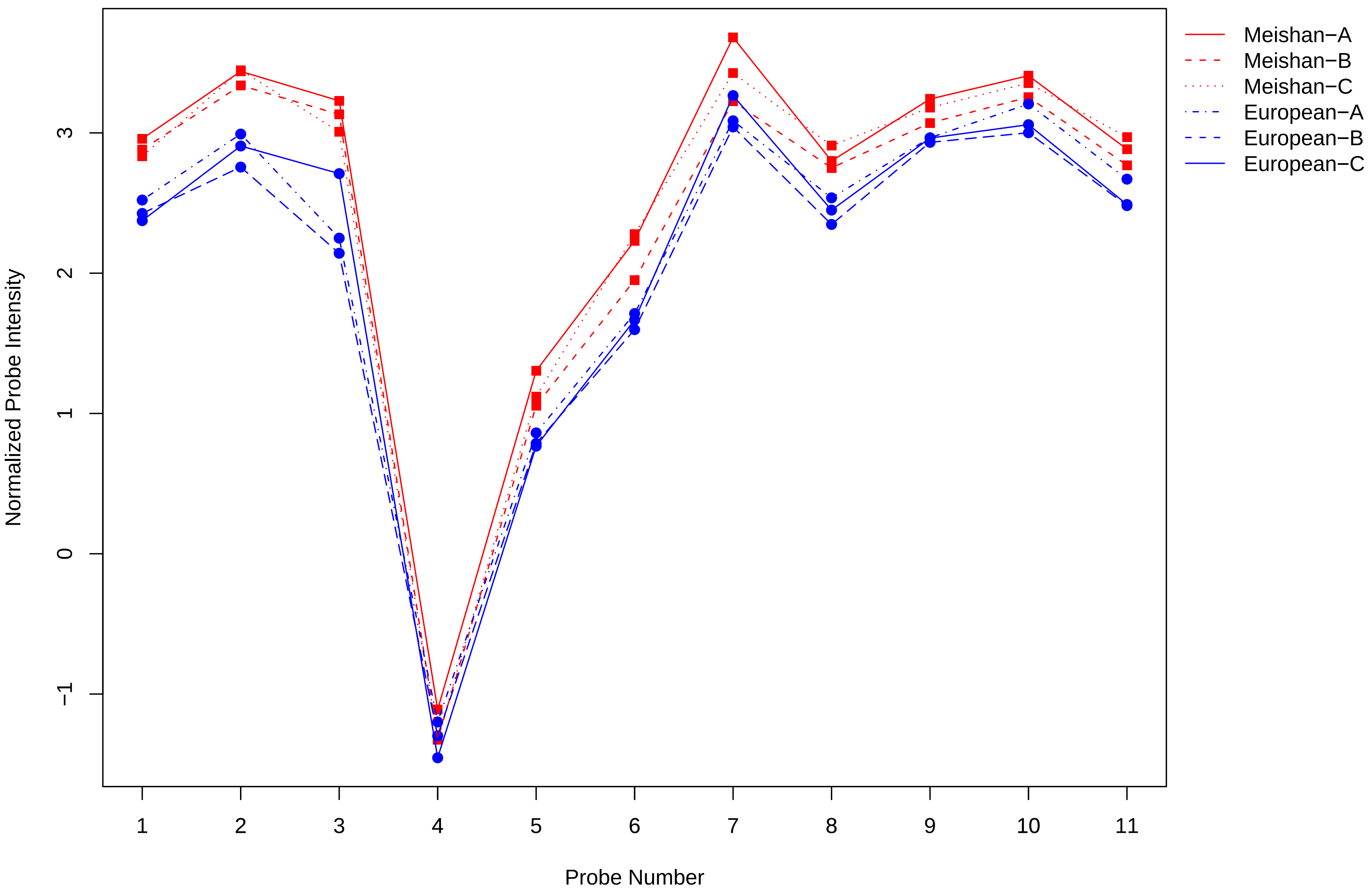

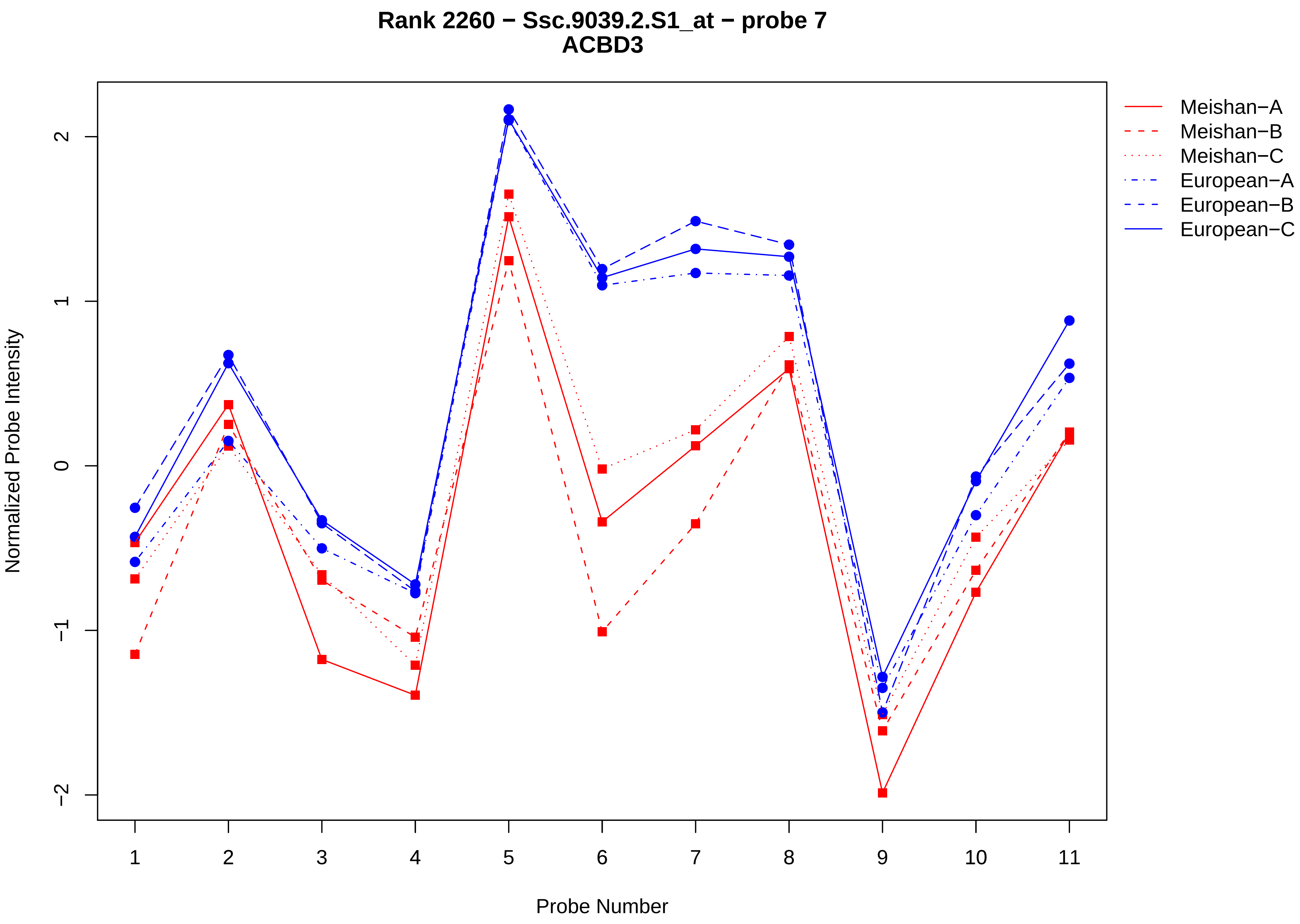

Rank 2260 − Ssc.9039.2.S1_at − probe 7
ACBD3
Normalized Probe Intensity
Probe Number
−2
−1
0
1
2
1
2
3
4
5
6
7
8
9
10
11
Meishan−A
Meishan−B
Meishan−C
European−A
European−B
European−C

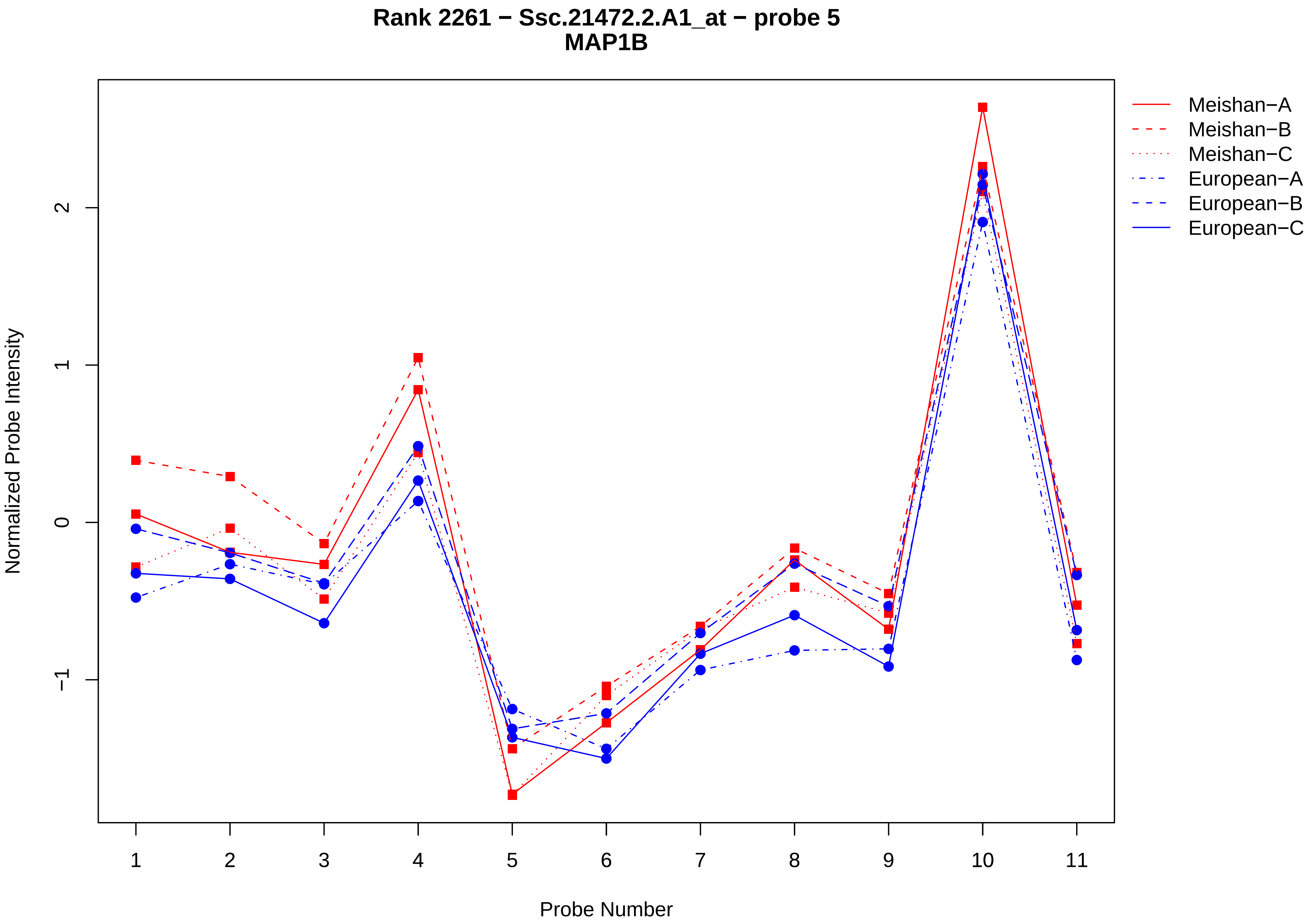

Rank 2261 − Ssc.21472.2.A1_at − probe 5
MAP1B
Normalized Probe Intensity
Probe Number
−1
0
1
2
1
2
3
4
5
6
7
8
9
10
11
Meishan−A
Meishan−B
Meishan−C
European−A
European−B
European−C

Rank 2262 − Ssc.20029.1.S1_at − probe 2
SNI1_HUMAN

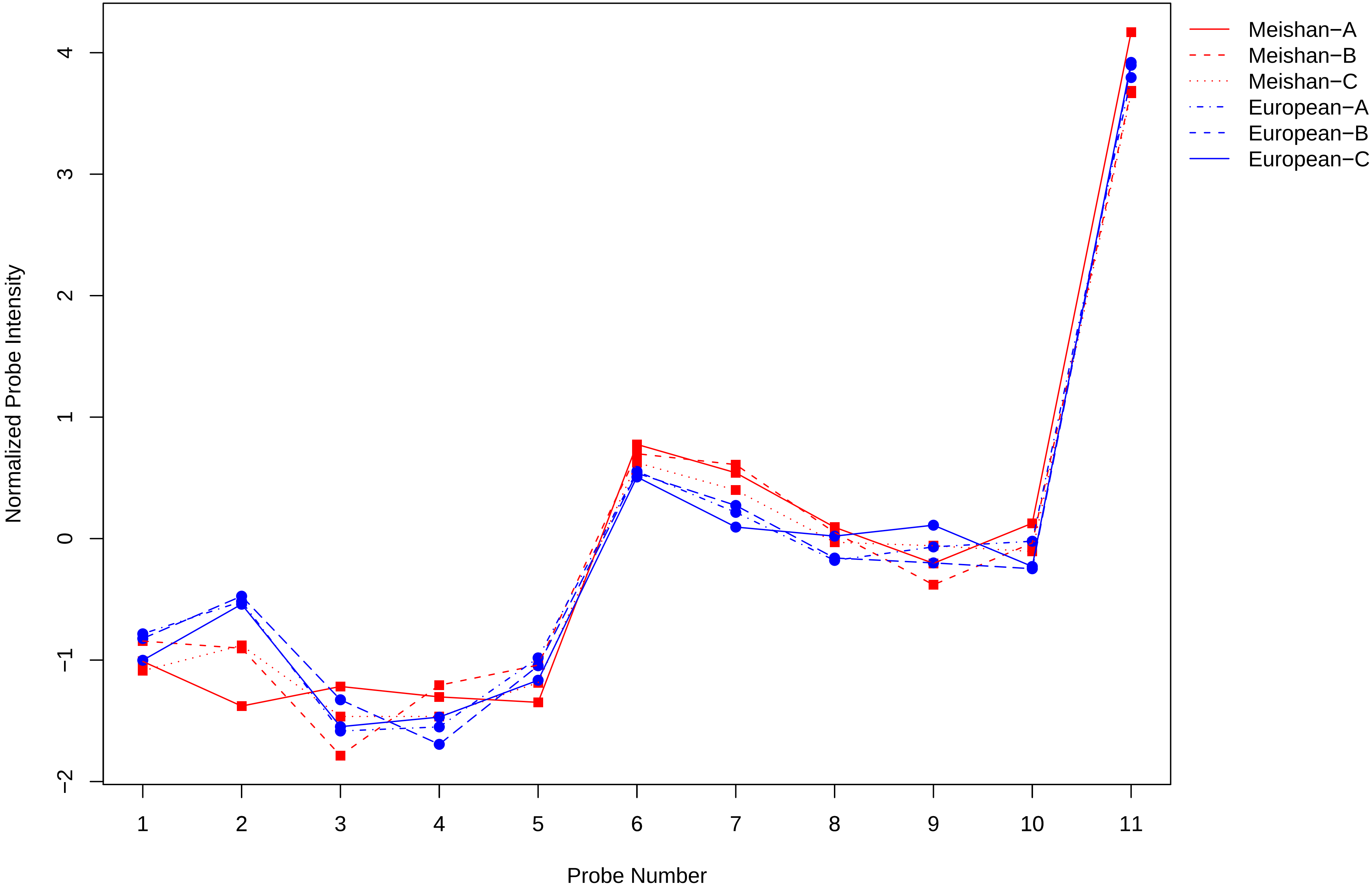

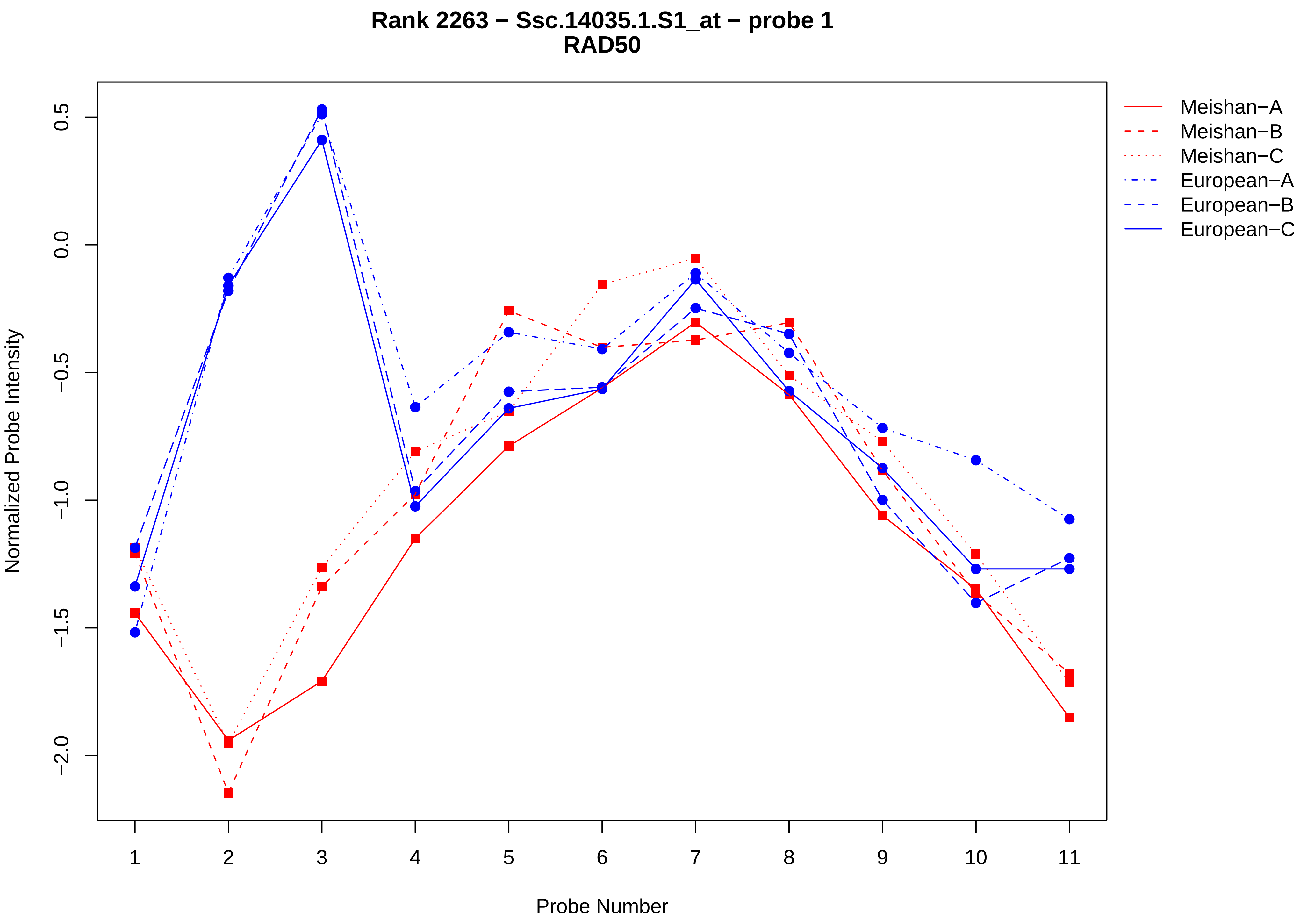
Rank 2263 − Ssc.14035.1.S1_at − probe 1
RAD50
Normalized Probe Intensity
Probe Number
Meishan−A
Meishan−B
Meishan−C
European−A
European−B
European−C

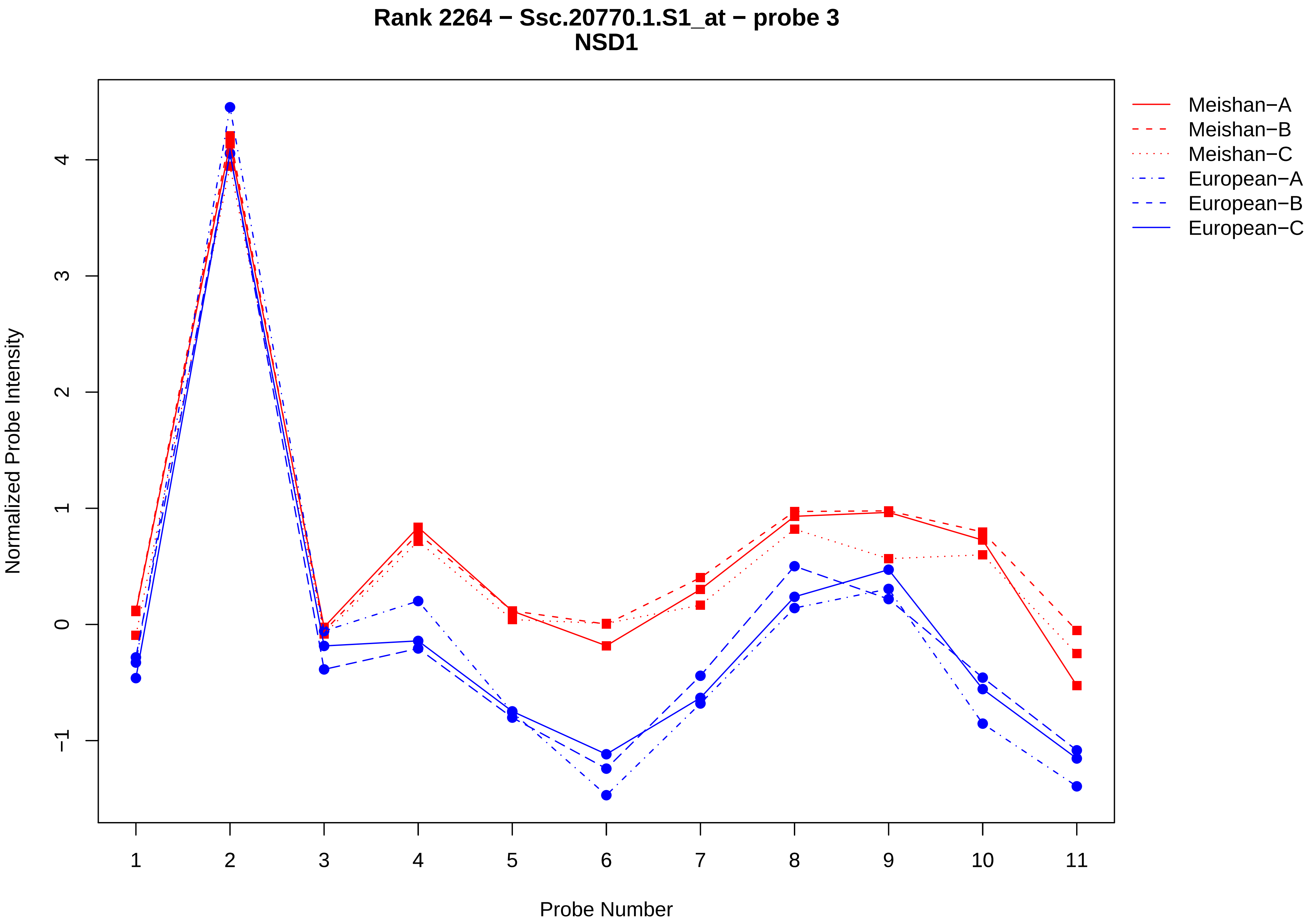
Rank 2264 − Ssc.20770.1.S1_at − probe 3
NSD1
Normalized Probe Intensity
Probe Number
−1
0
1
2
3
4
1
2
3
4
5
6
7
8
9
10
11
Meishan−A
Meishan−B
Meishan−C
European−A
European−B
European−C

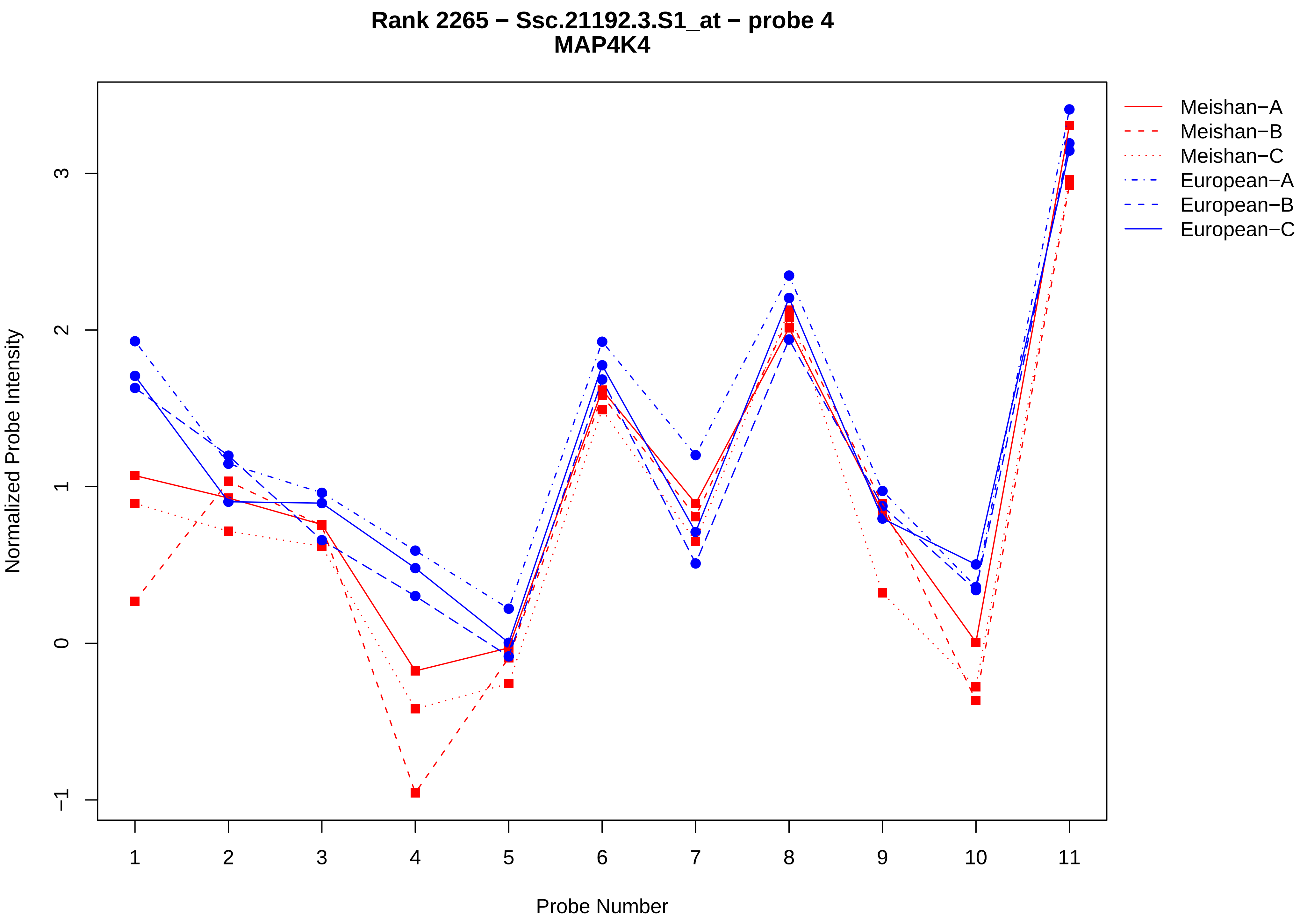

Rank 2265 − Ssc.21192.3.S1_at − probe 4
MAP4K4
Meishan−A
Meishan−B
Meishan−C
European−A
European−B
European−C
Normalized Probe Intensity
Probe Number
−1
0
1
2
3
1
2
3
4
5
6
7
8
9
10
11

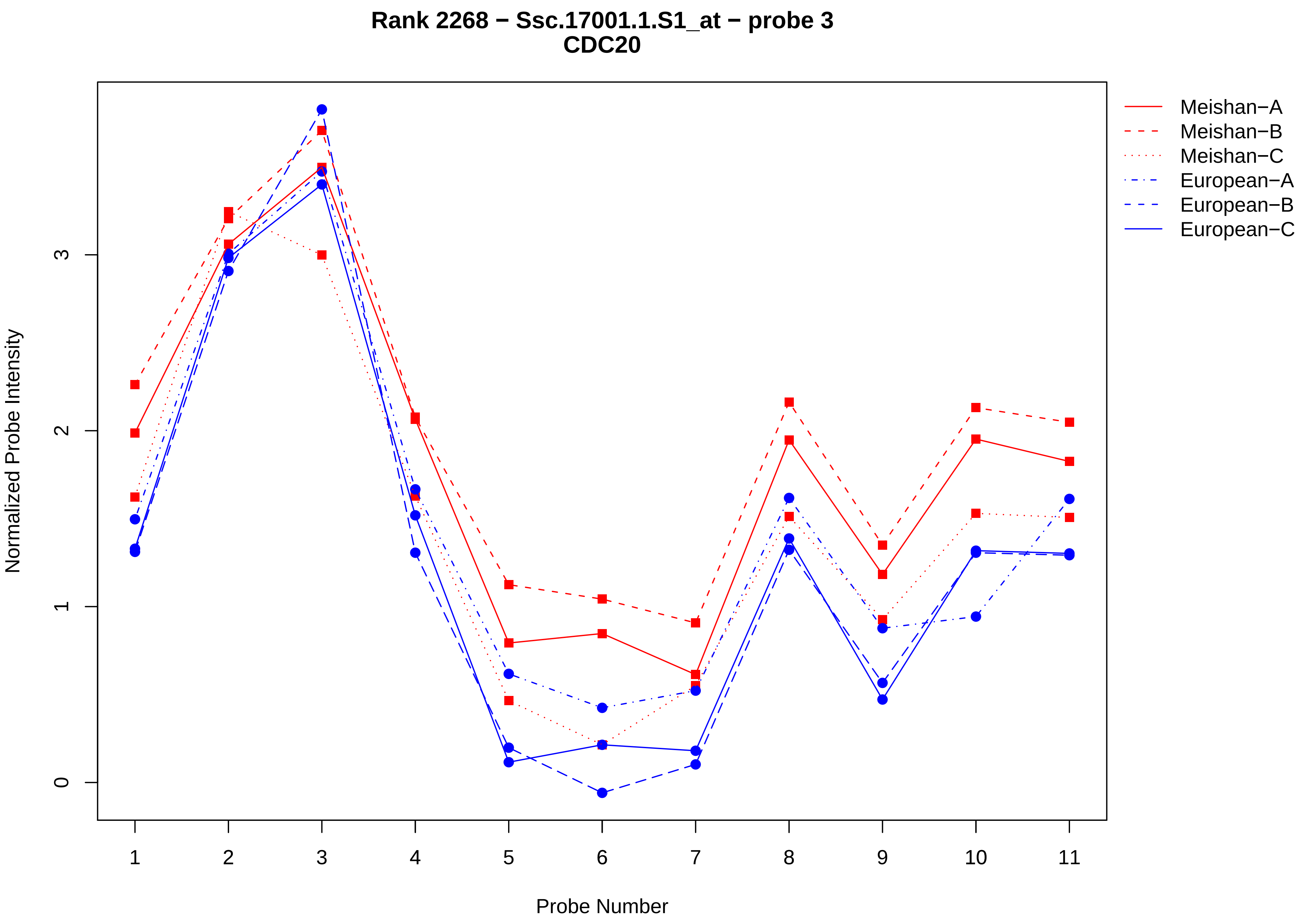
Rank 2268 − Ssc.17001.1.S1_at − probe 3
CDC20
Normalized Probe Intensity
Probe Number
0
1
2
3
1
2
3
4
5
6
7
8
9
10
11
Meishan−A
Meishan−B
Meishan−C
European−A
European−B
European−C

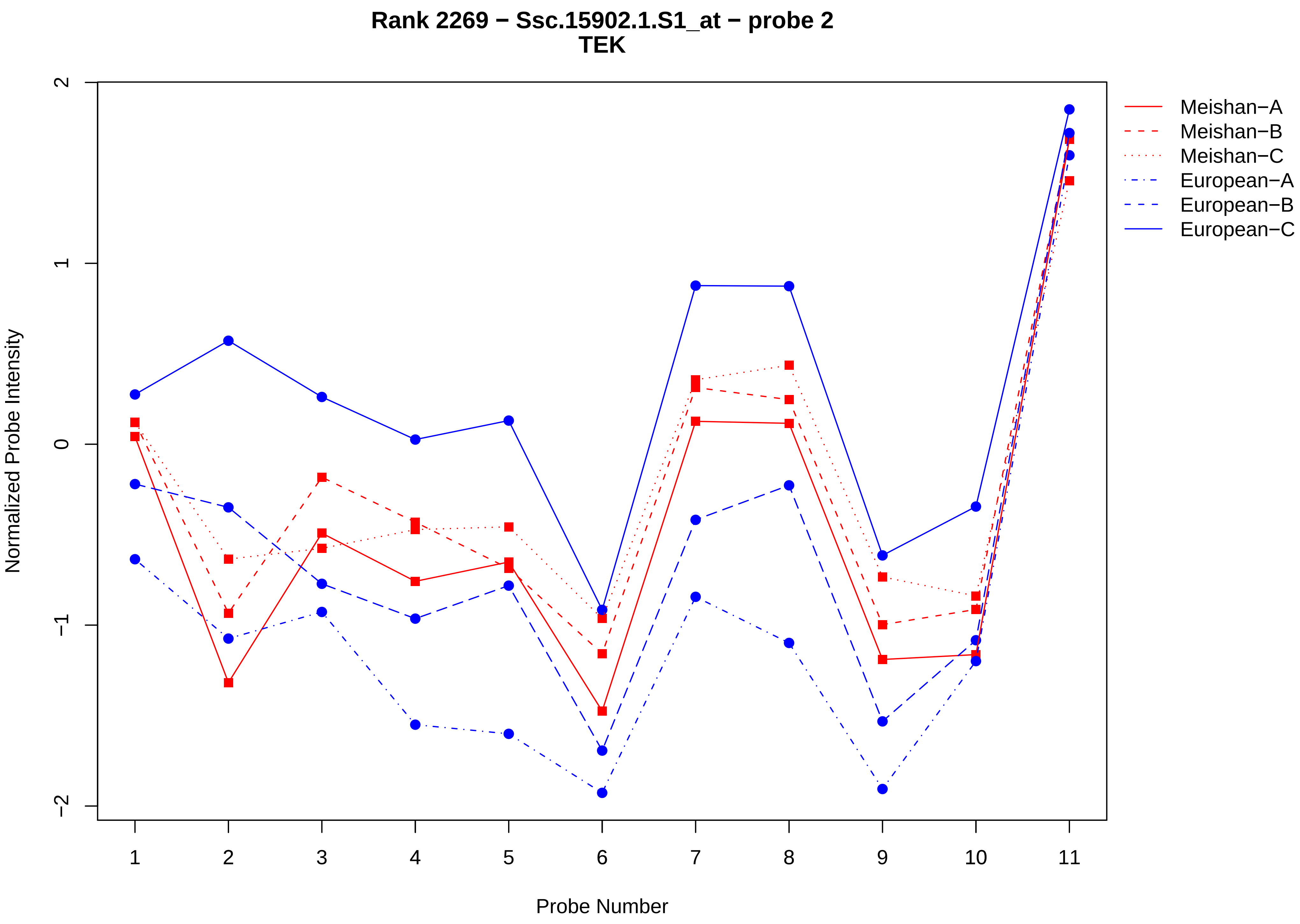
Rank 2269 − Ssc.15902.1.S1_at − probe 2
TEK
Normalized Probe Intensity
Probe Number
2
1
0
−1
−2
1
2
3
4
5
6
7
8
9
10
11
Meishan−A
Meishan−B
Meishan−C
European−A
European−B
European−C

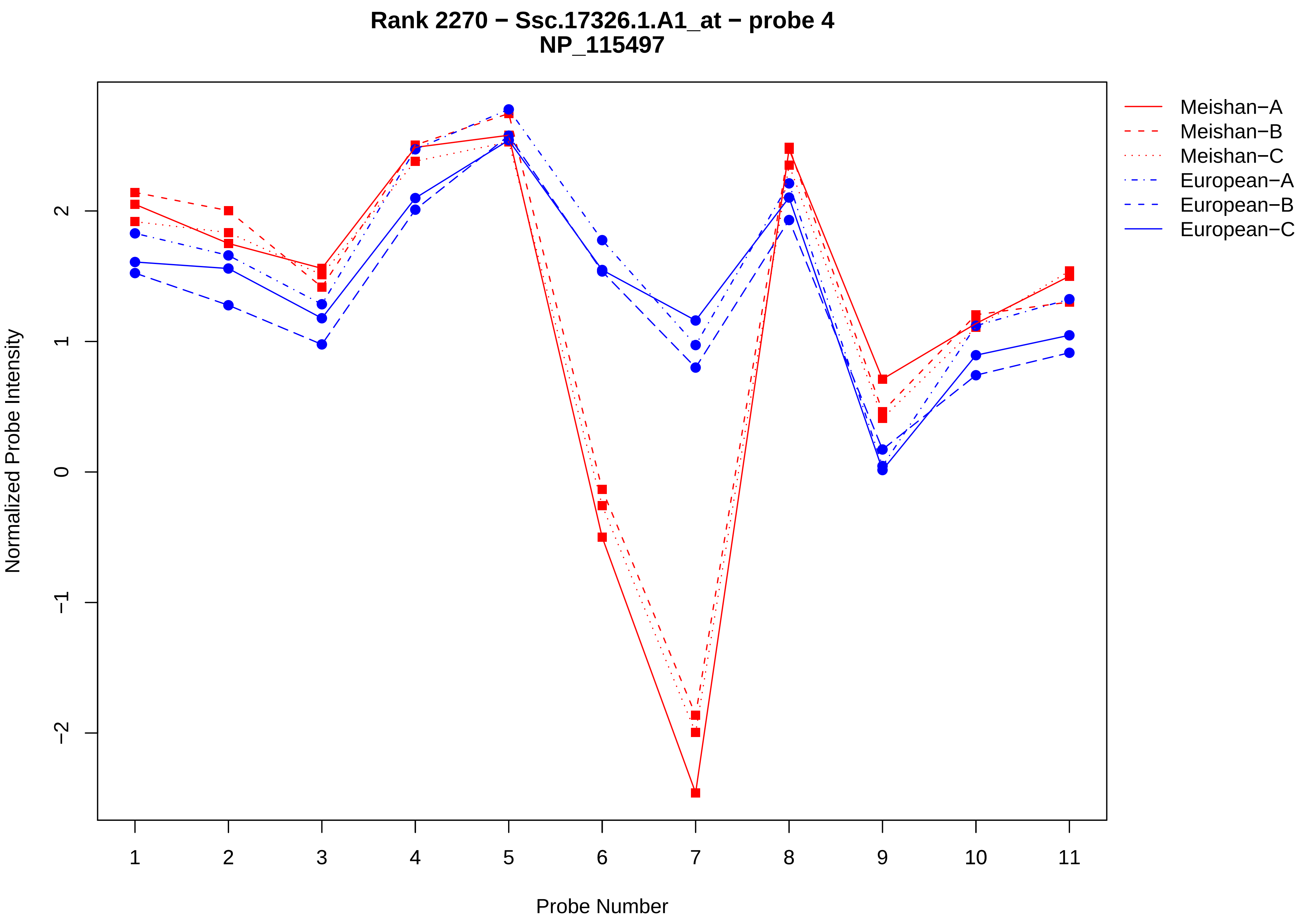

Rank 2270 − Ssc.17326.1.A1_at − probe 4
NP_115497
Normalized Probe Intensity
Probe Number
−2
−1
0
1
2
1
2
3
4
5
6
7
8
9
10
11
Meishan−A
Meishan−B
Meishan−C
European−A
European−B
European−C

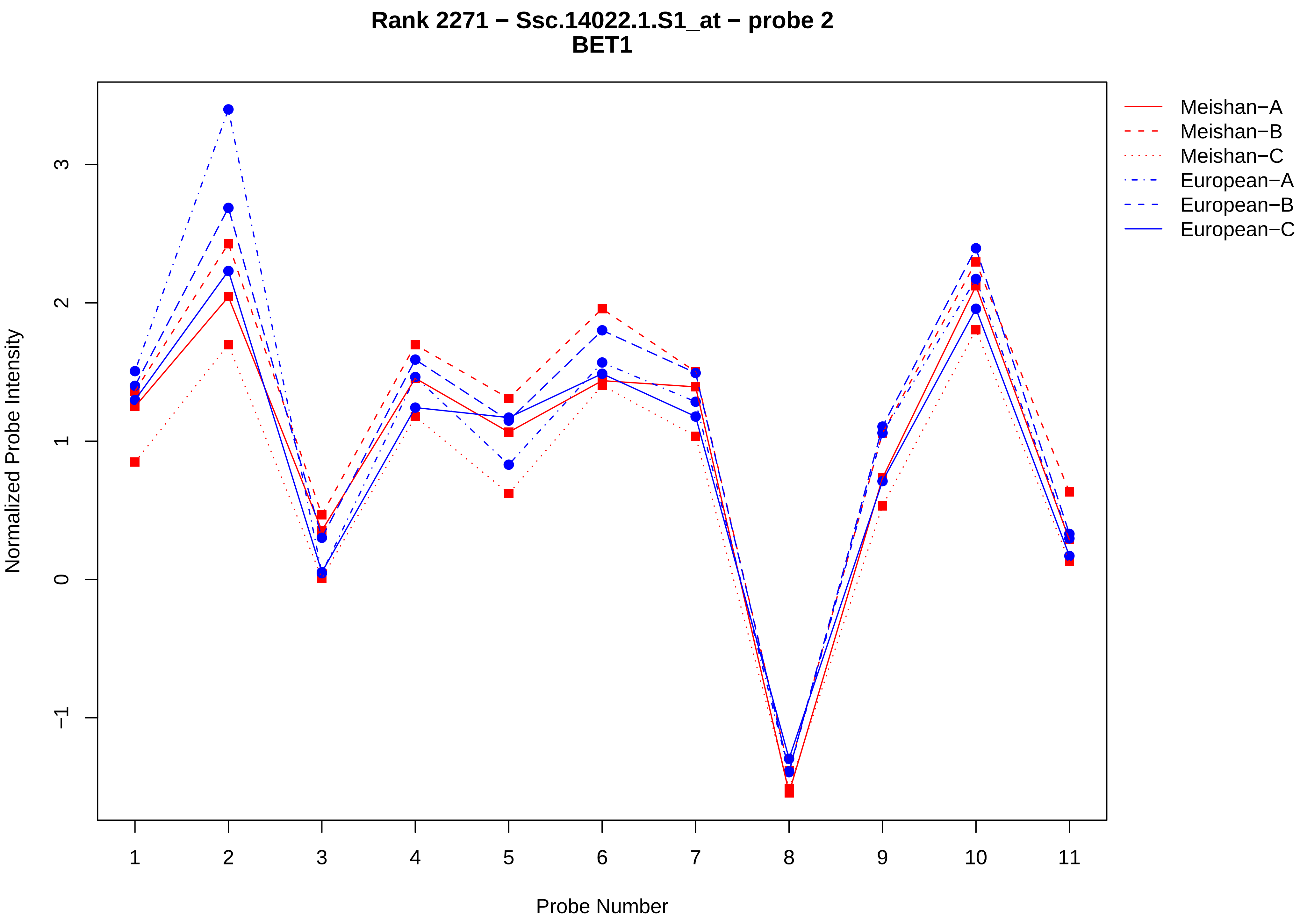

Rank 2271 − Ssc.14022.1.S1_at − probe 2
BET1
Normalized Probe Intensity
Probe Number
Meishan−A
Meishan−B
Meishan−C
European−A
European−B
European−C

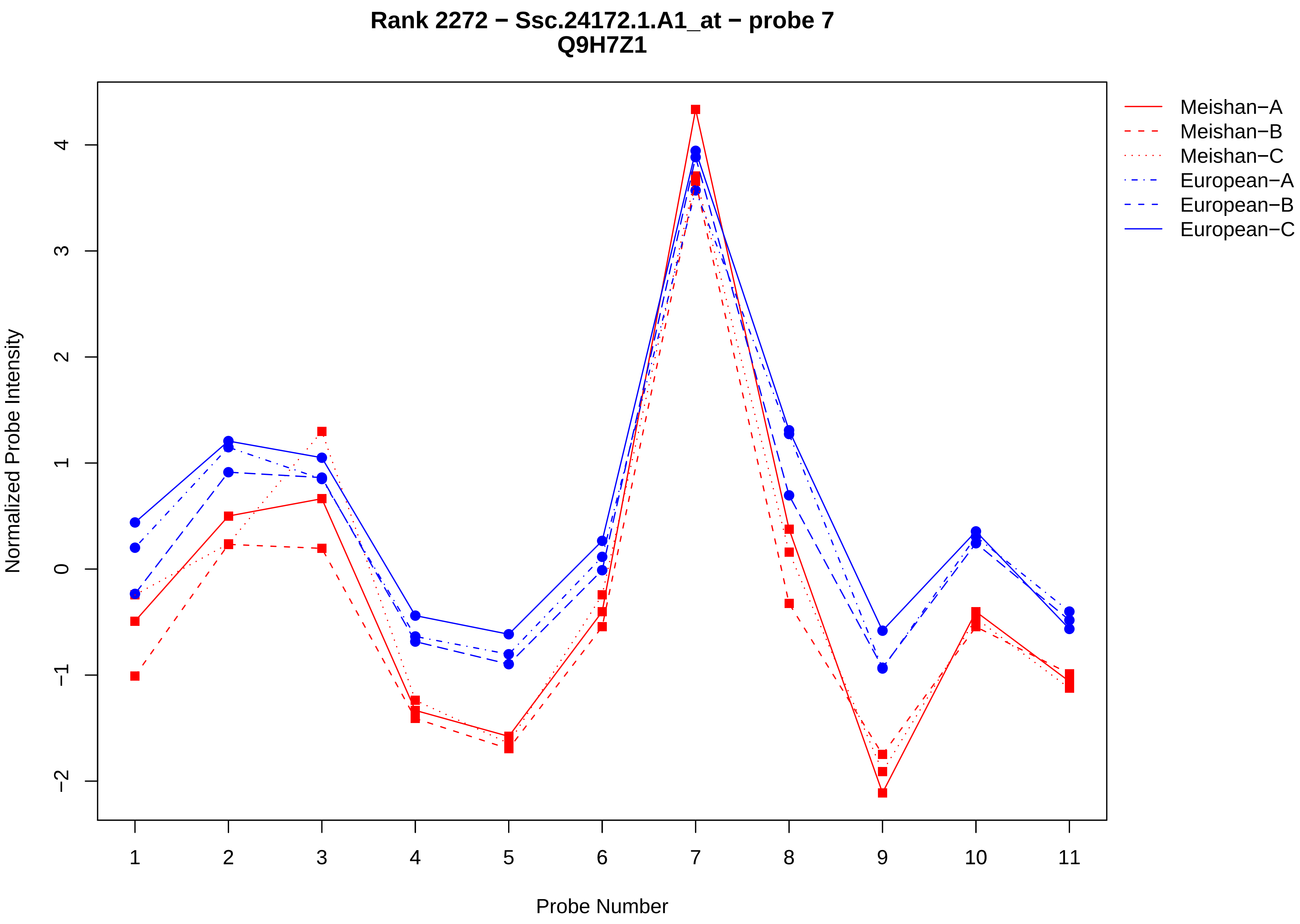

Rank 2272 − Ssc.24172.1.A1_at − probe 7
Q9H7Z1
Normalized Probe Intensity
Probe Number
−2
−1
0
1
2
3
4
1
2
3
4
5
6
7
8
9
10
11
Meishan−A
Meishan−B
Meishan−C
European−A
European−B
European−C

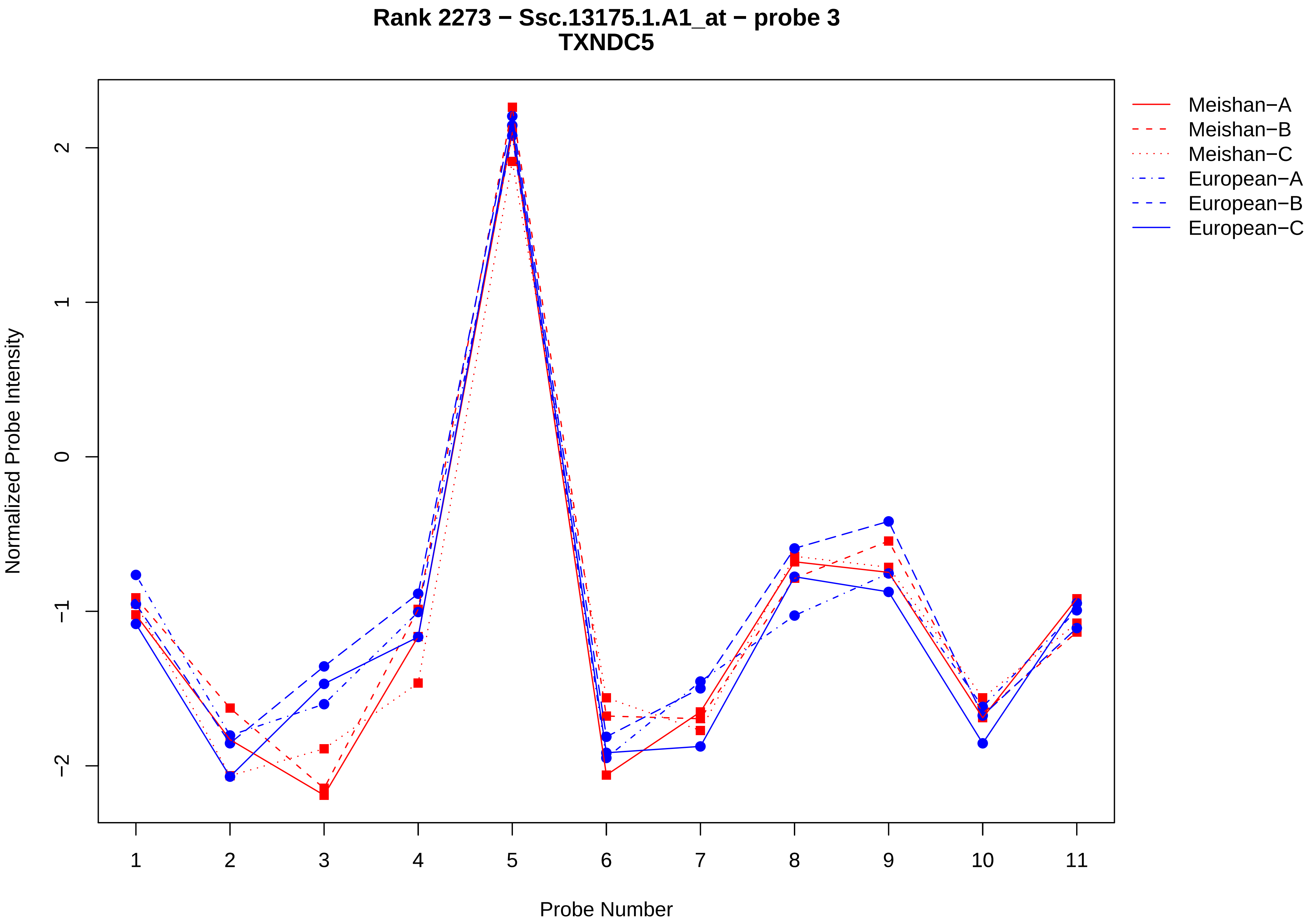

Rank 2273 − Ssc.13175.1.A1_at − probe 3
TXNDC5
Normalized Probe Intensity
Probe Number
−2
−1
0
1
2
1
2
3
4
5
6
7
8
9
10
11
Meishan−A
Meishan−B
Meishan−C
European−A
European−B
European−C

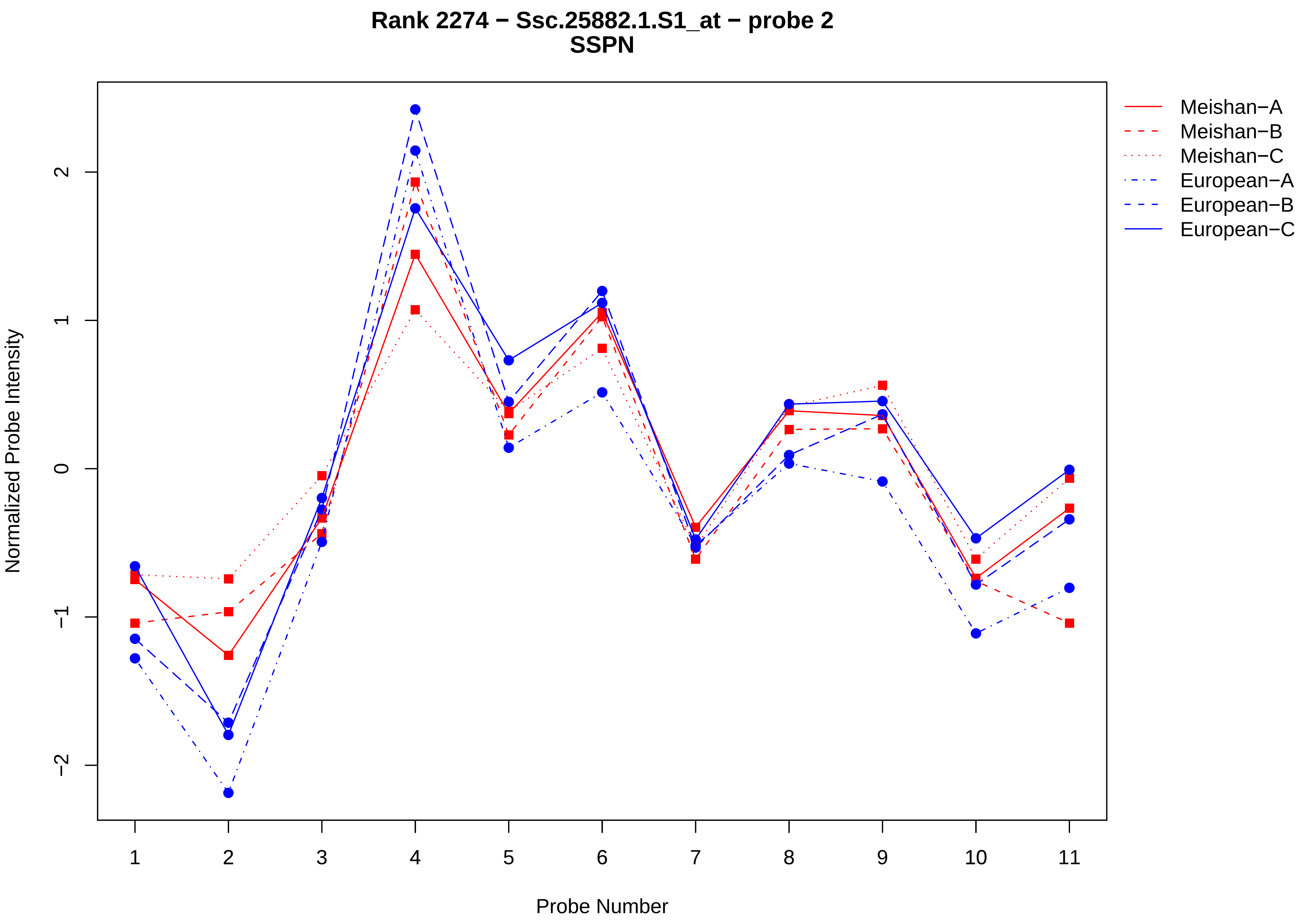

Rank 2274 − Ssc.25882.1.S1_at − probe 2
SSPN
Normalized Probe Intensity
Probe Number
−2
−1
0
1
2
1
2
3
4
5
6
7
8
9
10
11
Meishan−A
Meishan−B
Meishan−C
European−A
European−B
European−C

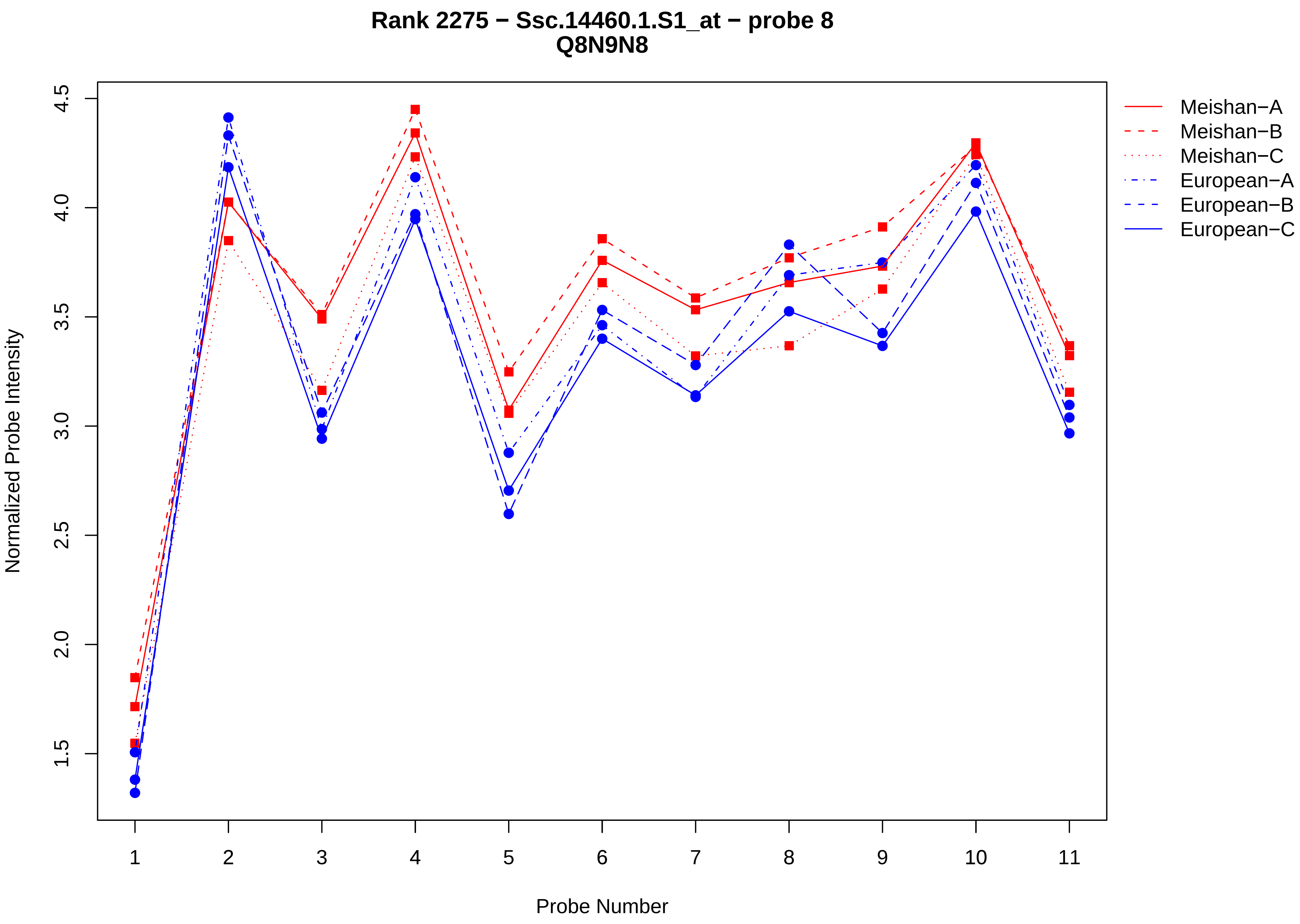
Rank 2275 − Ssc.14460.1.S1_at − probe 8
Q8N9N8
Normalized Probe Intensity
Probe Number
1.5
2.0
2.5
3.0
3.5
4.0
4.5
1
2
3
4
5
6
7
8
9
10
11
Meishan−A
Meishan−B
Meishan−C
European−A
European−B
European−C

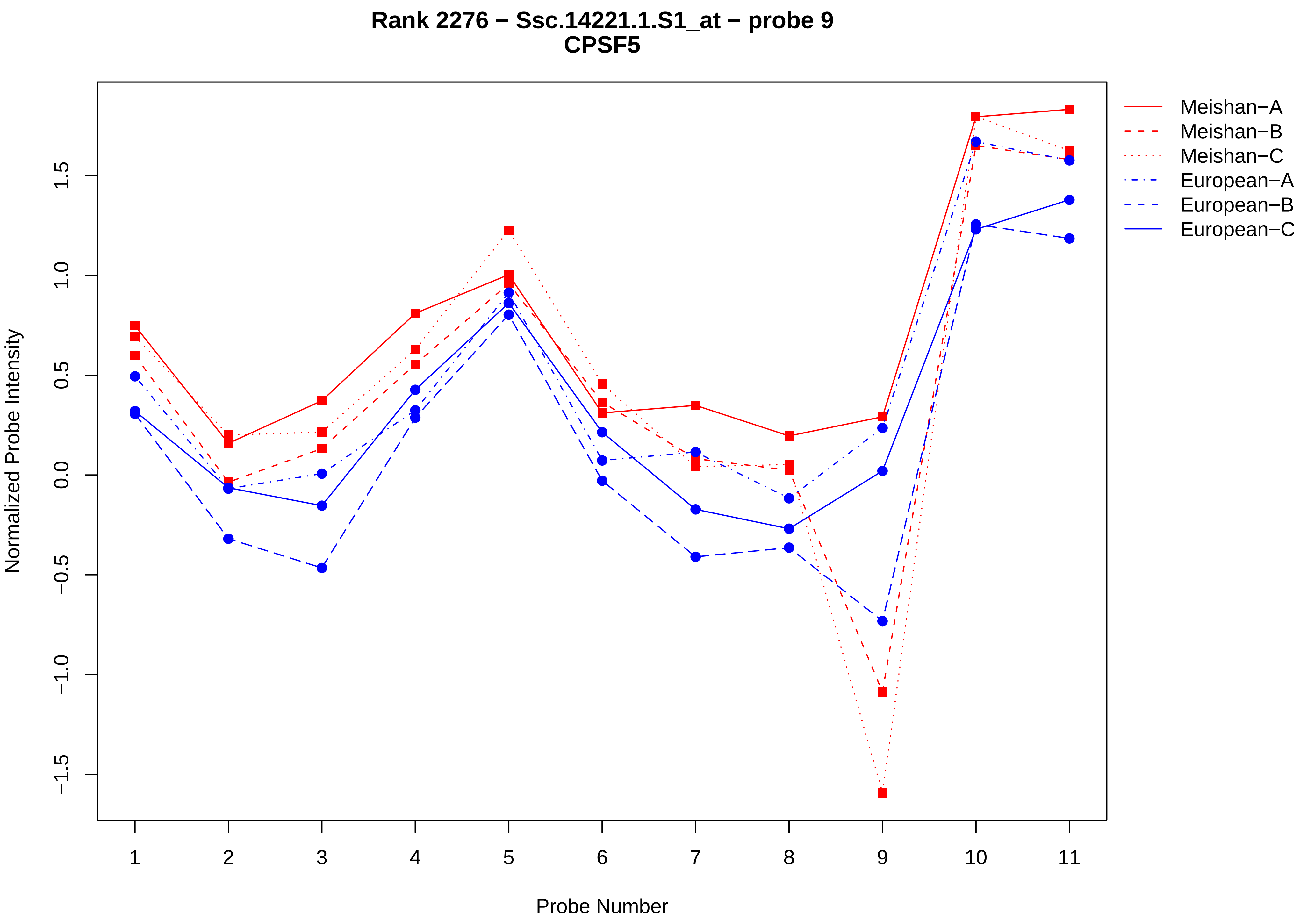

Rank 2276 − Ssc.14221.1.S1_at − probe 9
CPSF5
Normalized Probe Intensity
Probe Number
Meishan−A
Meishan−B
Meishan−C
European−A
European−B
European−C
−1.5
−1.0
−0.5
0.0
0.5
1.0
1.5
1
2
3
4
5
6
7
8
9
10
11

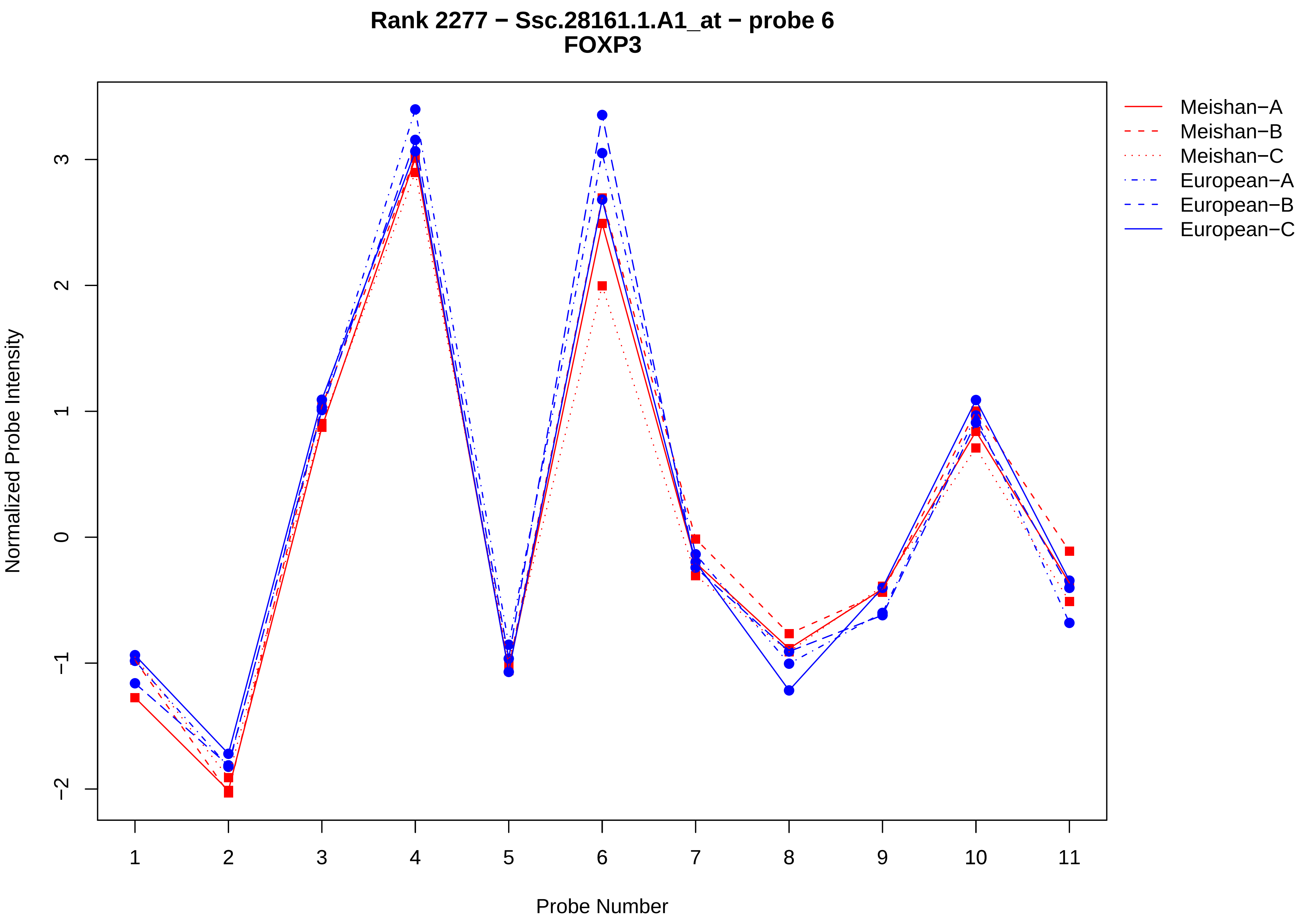
Rank 2277 − Ssc.28161.1.A1_at − probe 6
FOXP3
Normalized Probe Intensity
Probe Number
Meishan−A
Meishan−B
Meishan−C
European−A
European−B
European−C
−2
−1
0
1
2
3
1
2
3
4
5
6
7
8
9
10
11

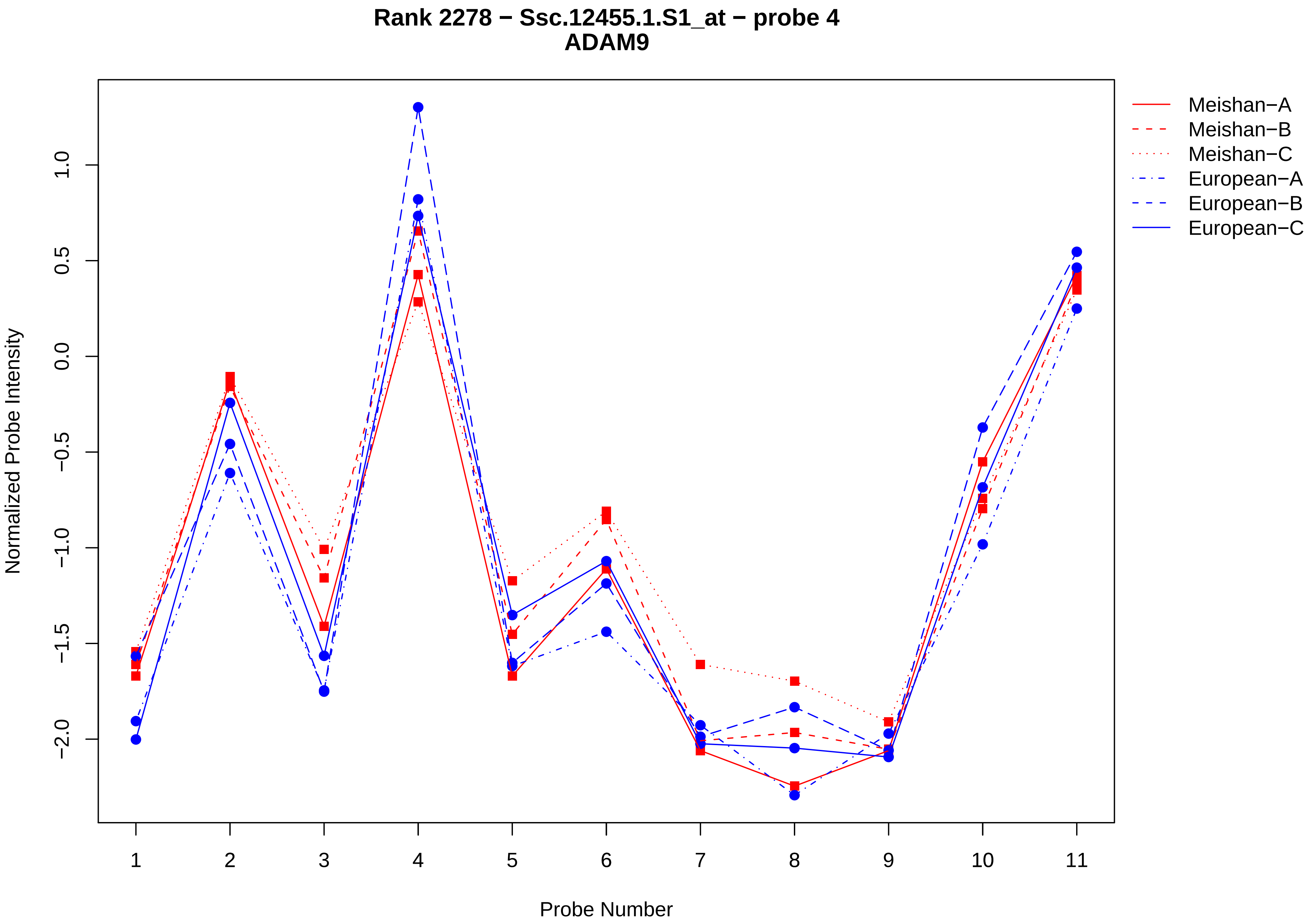

Rank 2278 − Ssc.12455.1.S1_at − probe 4
ADAM9
Normalized Probe Intensity
Probe Number
Meishan−A
Meishan−B
Meishan−C
European−A
European−B
European−C

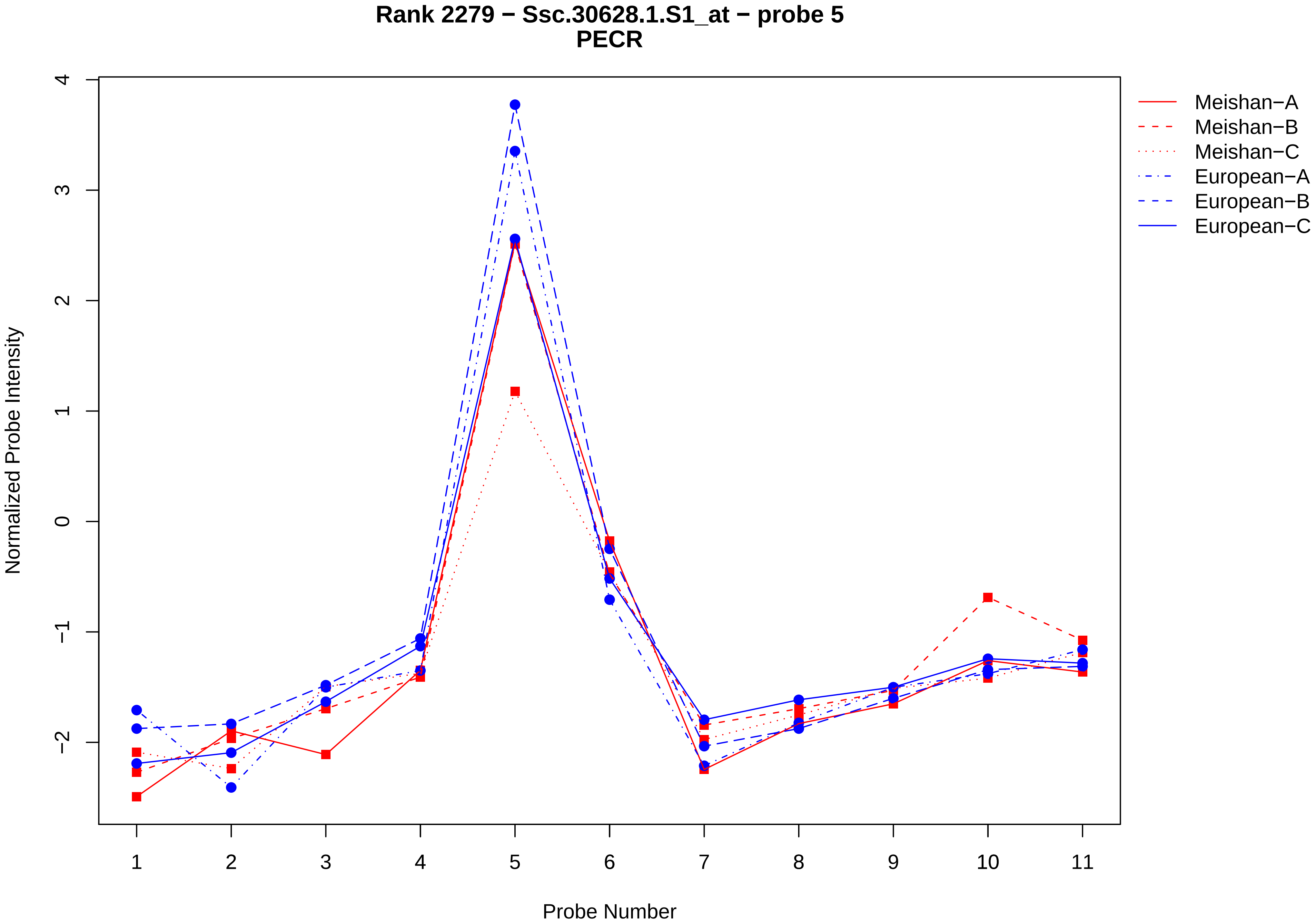

Rank 2279 − Ssc.30628.1.S1_at − probe 5
PECR
Normalized Probe Intensity
Probe Number
Meishan−A
Meishan−B
Meishan−C
European−A
European−B
European−C

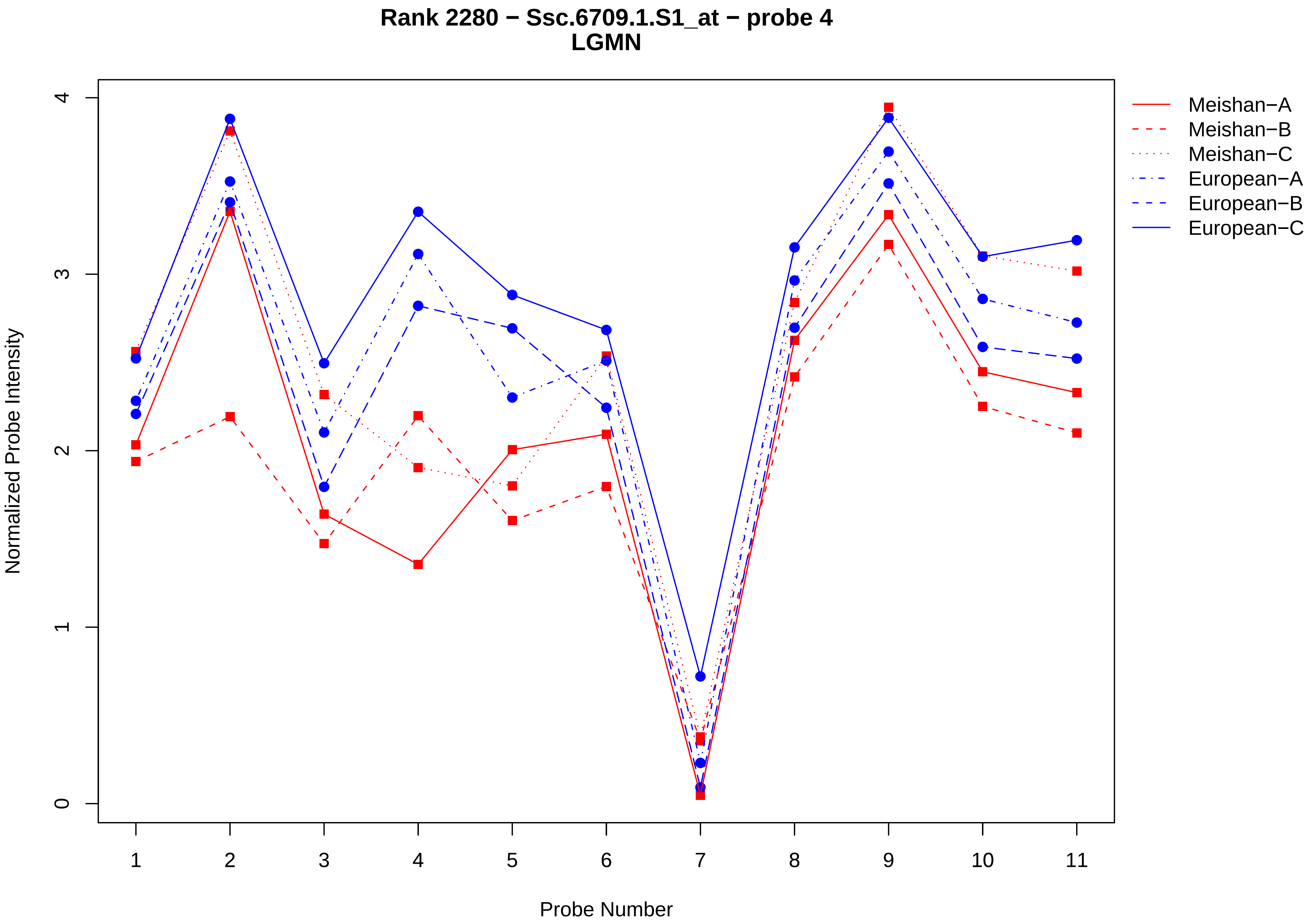

Rank 2280 − Ssc.6709.1.S1_at − probe 4
LGMN
Normalized Probe Intensity
Probe Number
0
1
2
3
4
1
2
3
4
5
6
7
8
9
10
11
Meishan−A
Meishan−B
Meishan−C
European−A
European−B
European−C

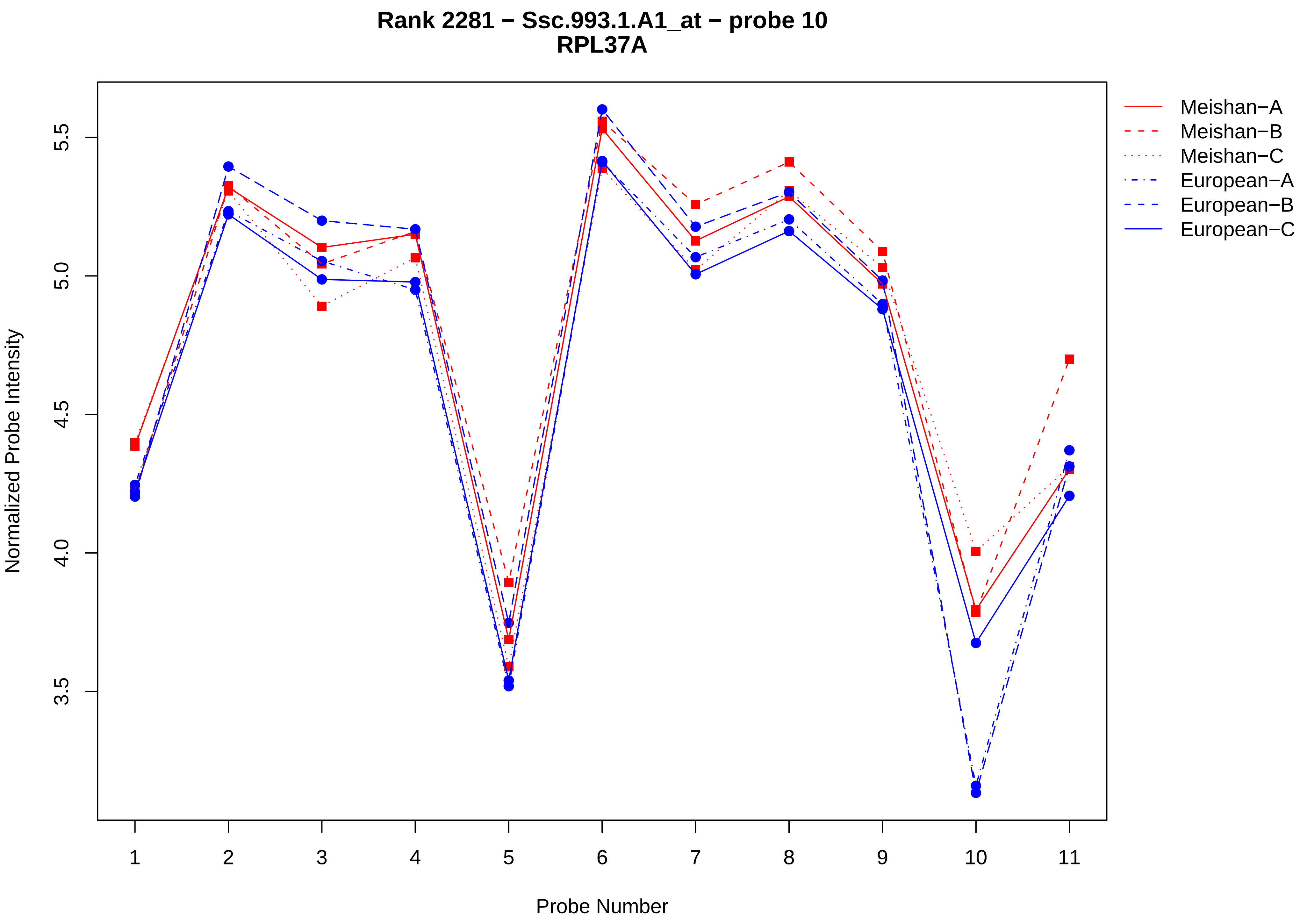

Rank 2281 − Ssc.993.1.A1_at − probe 10
RPL37A
Normalized Probe Intensity
Probe Number
3.5
4.0
4.5
5.0
5.5
1
2
3
4
5
6
7
8
9
10
11
Meishan−A
Meishan−B
Meishan−C
European−A
European−B
European−C

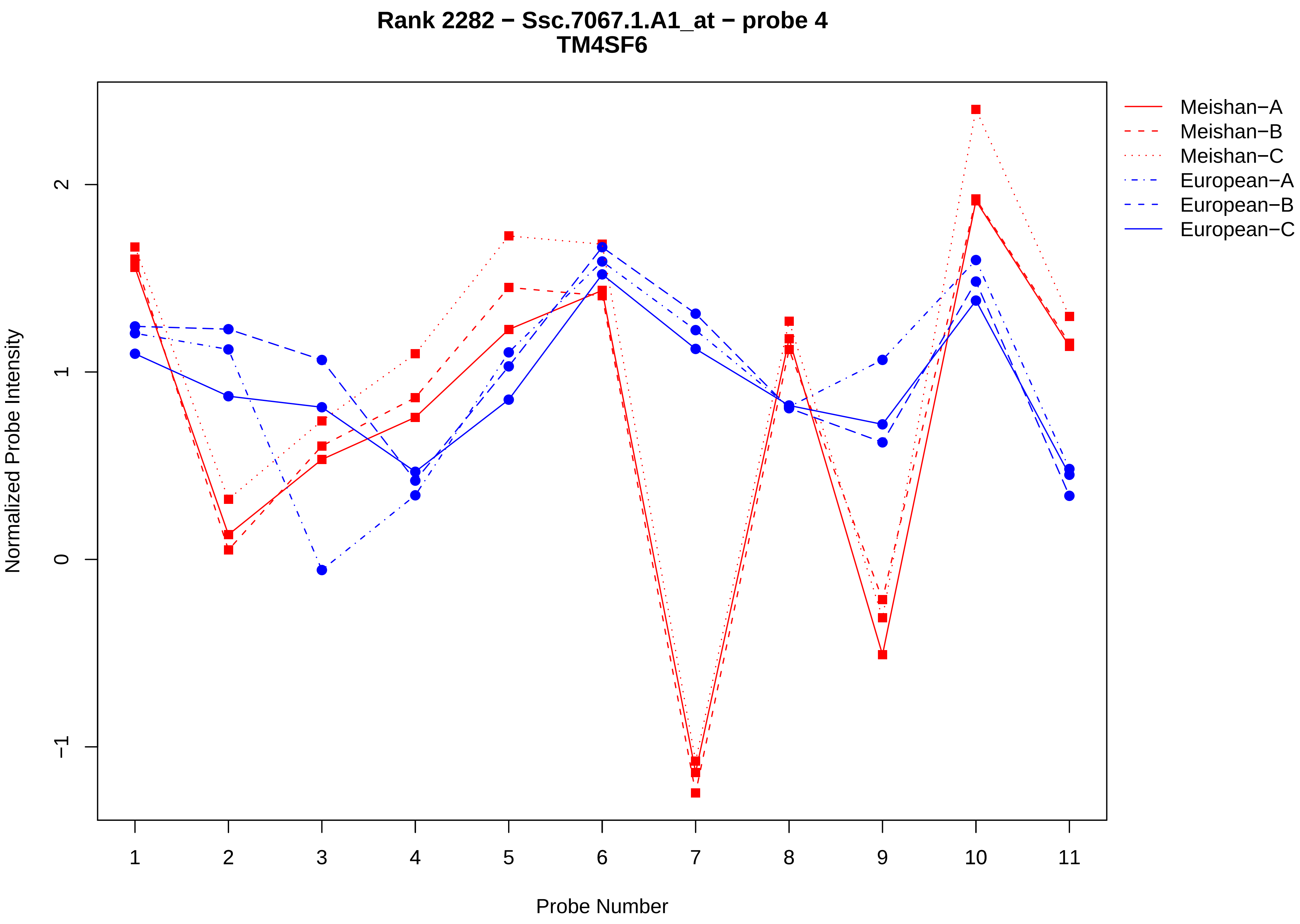
Rank 2282 − Ssc.7067.1.A1_at − probe 4
TM4SF6
Normalized Probe Intensity
Probe Number
−1
0
1
2
1
2
3
4
5
6
7
8
9
10
11
Meishan−A
Meishan−B
Meishan−C
European−A
European−B
European−C

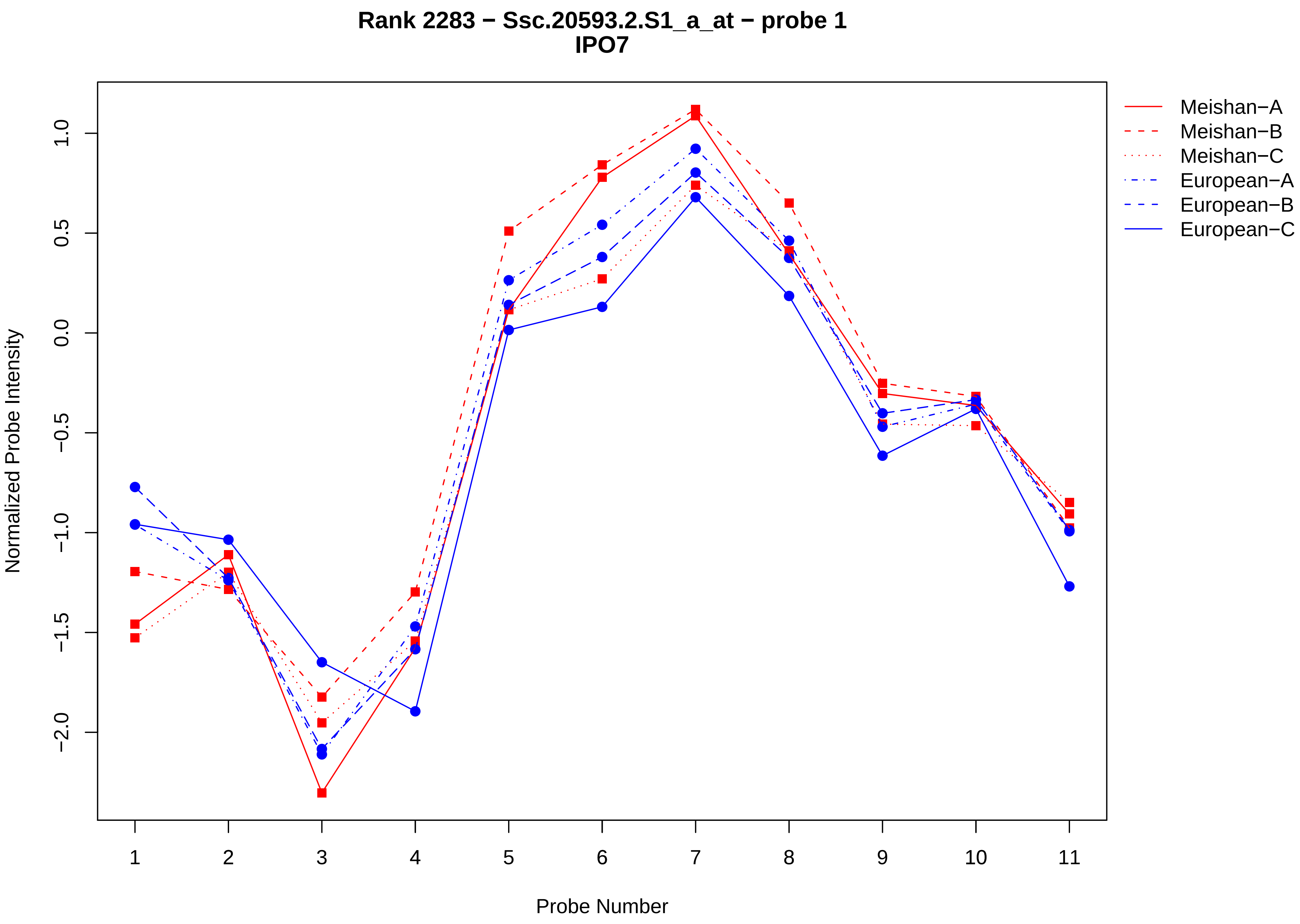

Rank 2283 − Ssc.20593.2.S1_a_at − probe 1
IPO7
Normalized Probe Intensity
−2.0
−1.5
−1.0
−0.5
0.0
0.5
1.0
Probe Number
1
2
3
4
5
6
7
8
9
10
11
Meishan−A
Meishan−B
Meishan−C
European−A
European−B
European−C

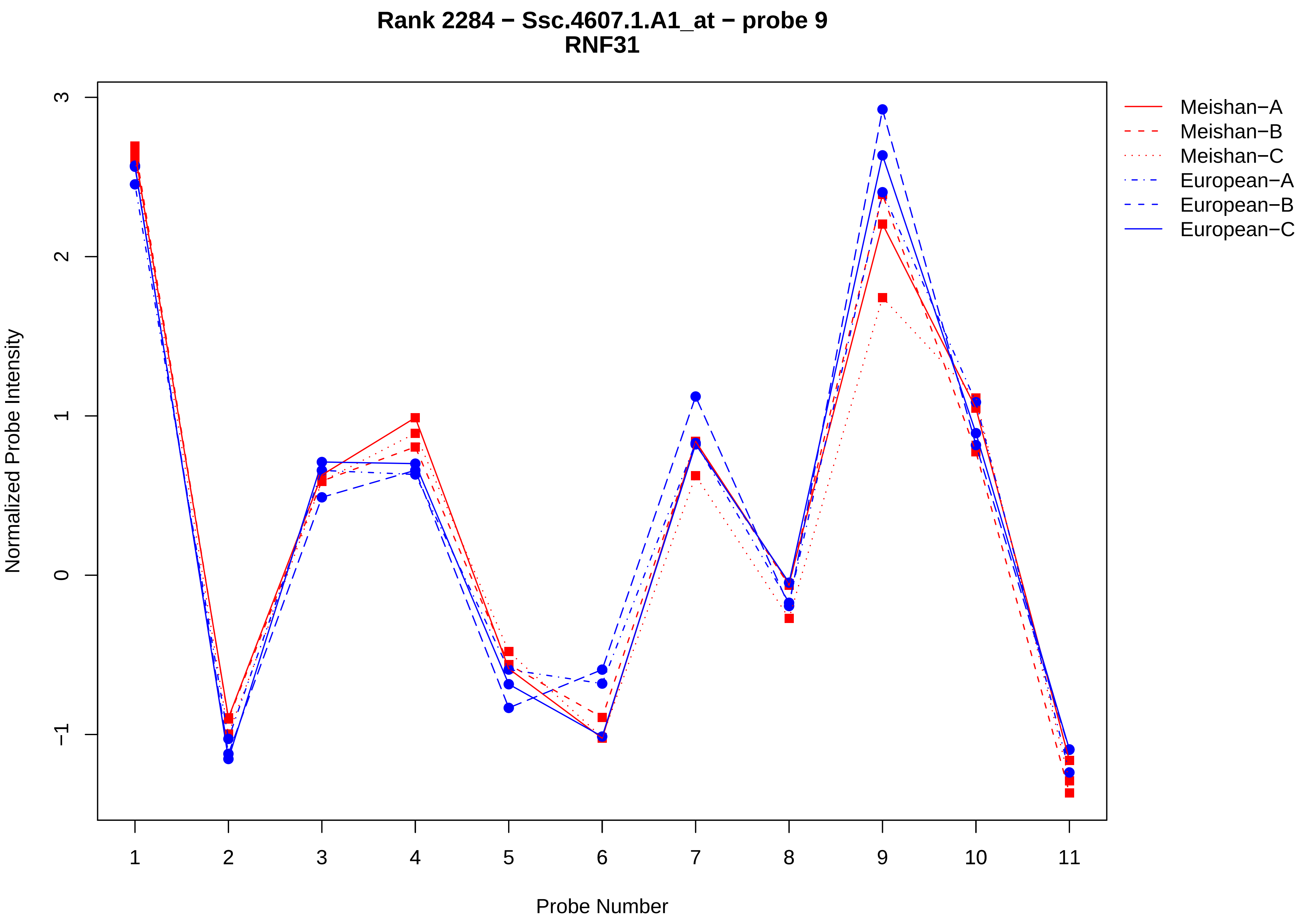

Rank 2284 − Ssc.4607.1.A1_at − probe 9
RNF31
Normalized Probe Intensity
Probe Number
Meishan−A
Meishan−B
Meishan−C
European−A
European−B
European−C
−1
0
1
2
3
1
2
3
4
5
6
7
8
9
10
11

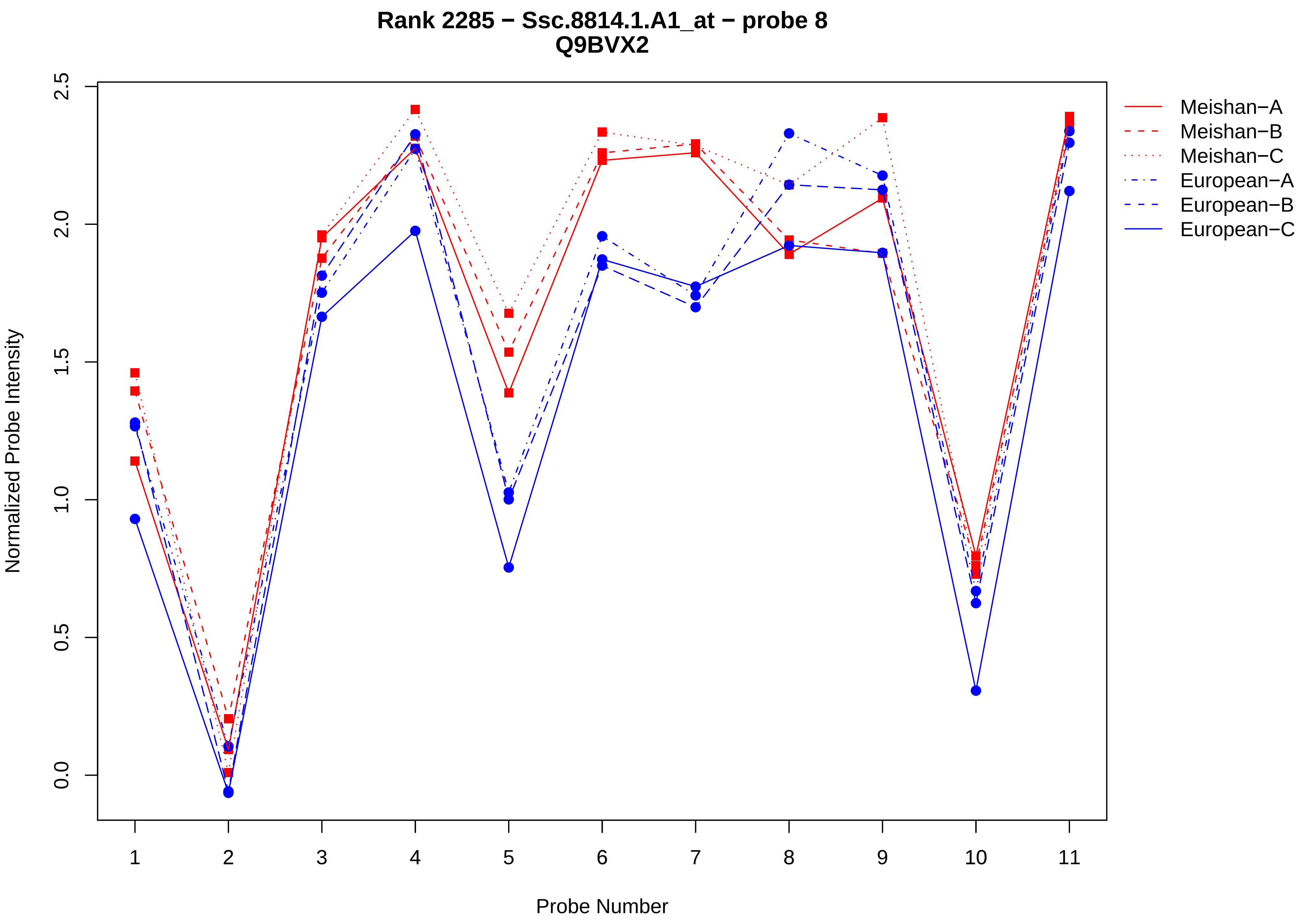

Rank 2285 − Ssc.8814.1.A1_at − probe 8
Q9BVX2
Normalized Probe Intensity
0.0
0.5
1.0
1.5
2.0
2.5
1
2
3
4
5
6
7
8
9
10
11
Probe Number
Meishan−A
Meishan−B
Meishan−C
European−A
European−B
European−C

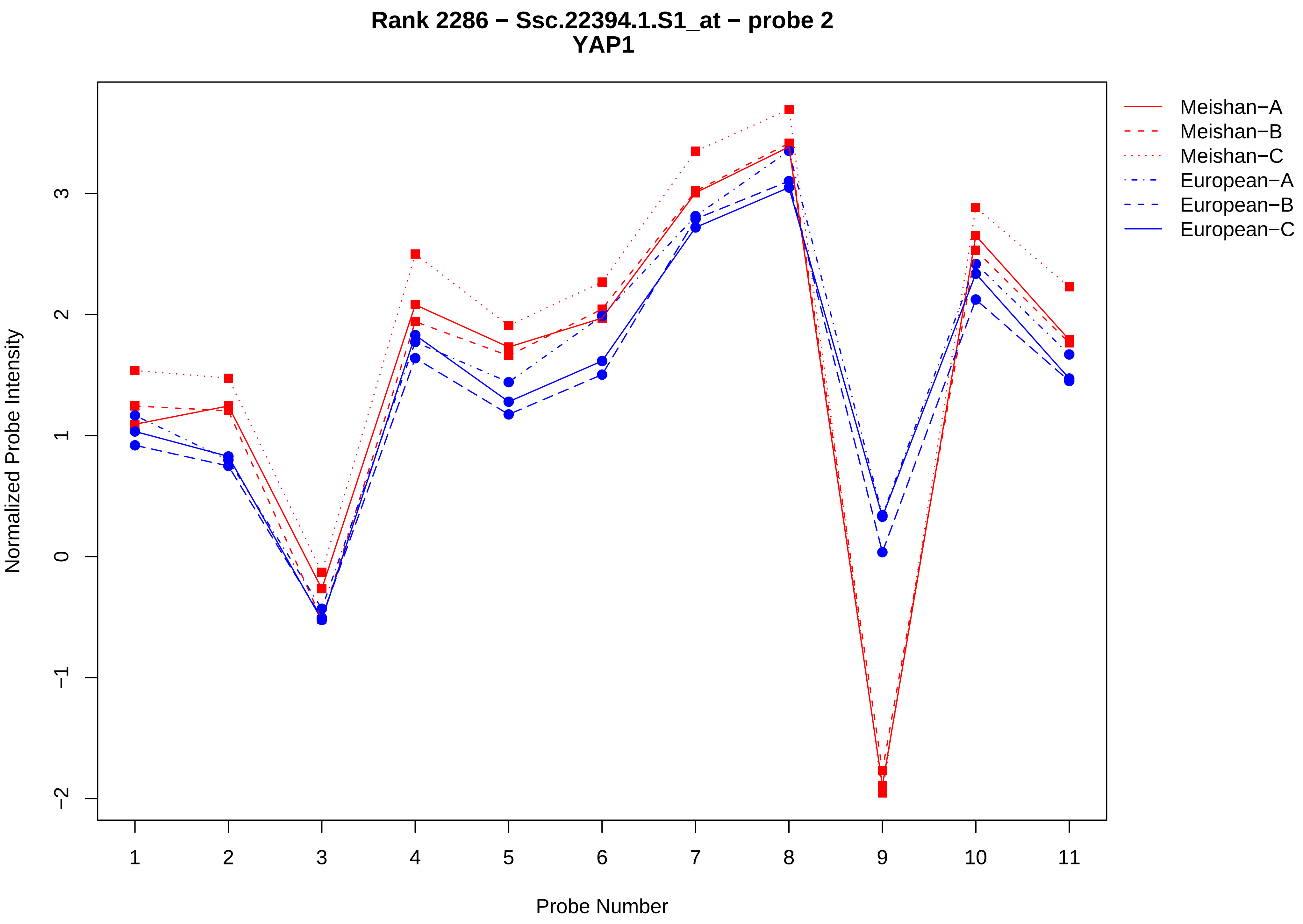

Rank 2286 − Ssc.22394.1.S1_at − probe 2
YAP1
Normalized Probe Intensity
Probe Number
−2
−1
0
1
2
3
1
2
3
4
5
6
7
8
9
10
11
Meishan−A
Meishan−B
Meishan−C
European−A
European−B
European−C

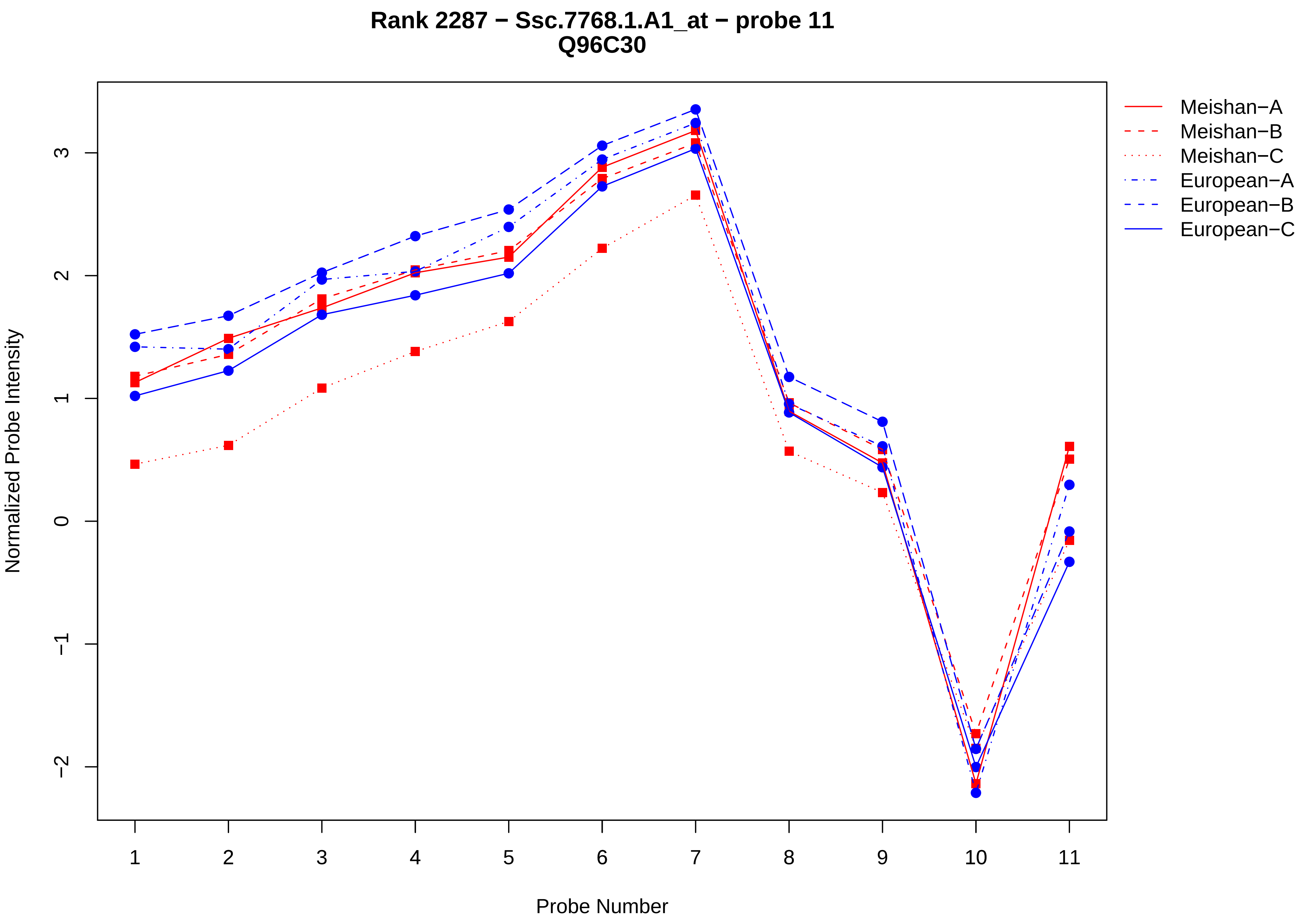

Rank 2287 − Ssc.7768.1.A1_at − probe 11
Q96C30
Normalized Probe Intensity
Probe Number
Meishan−A
Meishan−B
Meishan−C
European−A
European−B
European−C
−2
−1
0
1
2
3
1
2
3
4
5
6
7
8
9
10
11

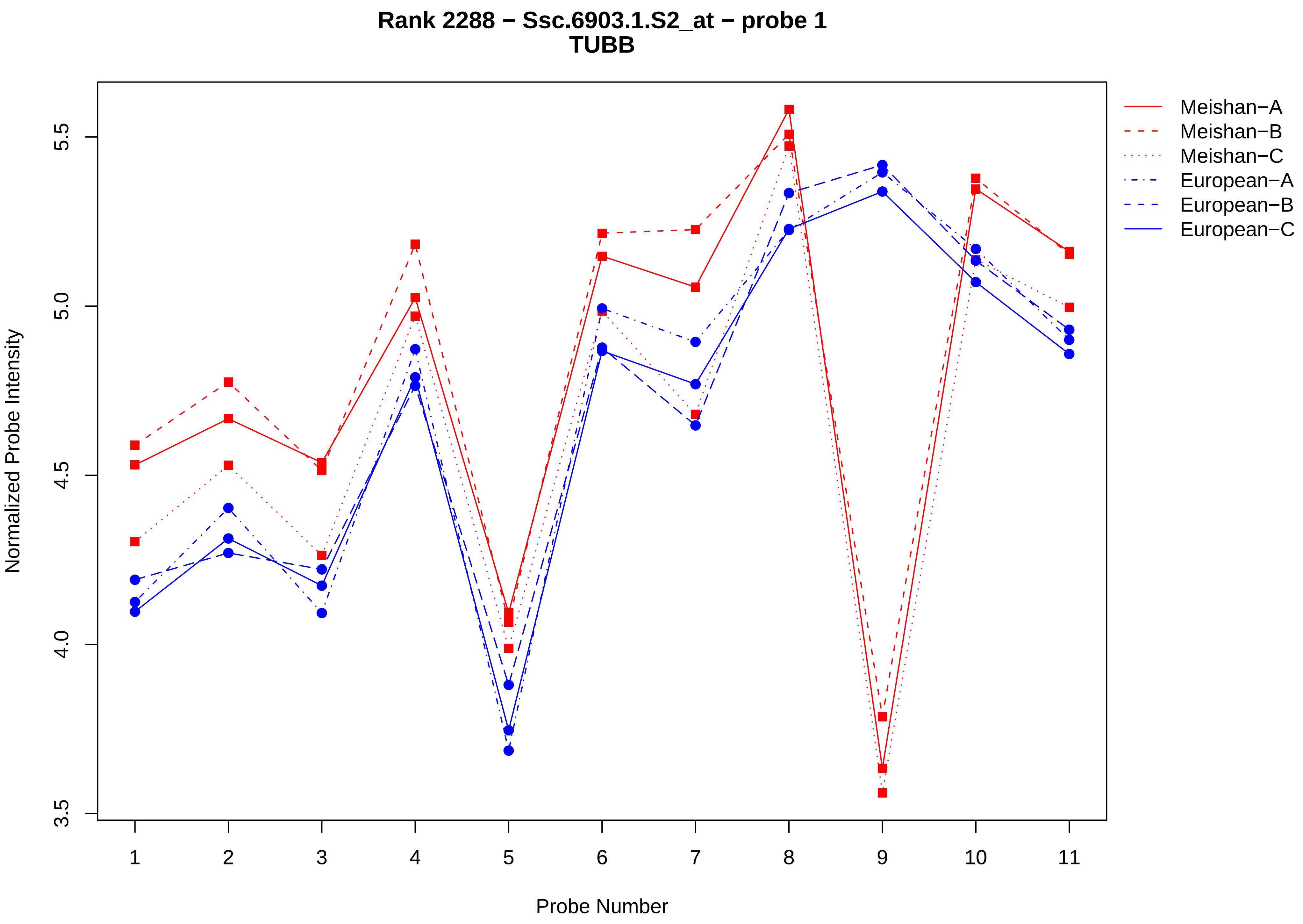
Rank 2288 − Ssc.6903.1.S2_at − probe 1
TUBB
Normalized Probe Intensity
Probe Number
3.5
4.0
4.5
5.0
5.5
1
2
3
4
5
6
7
8
9
10
11
Meishan−A
Meishan−B
Meishan−C
European−A
European−B
European−C

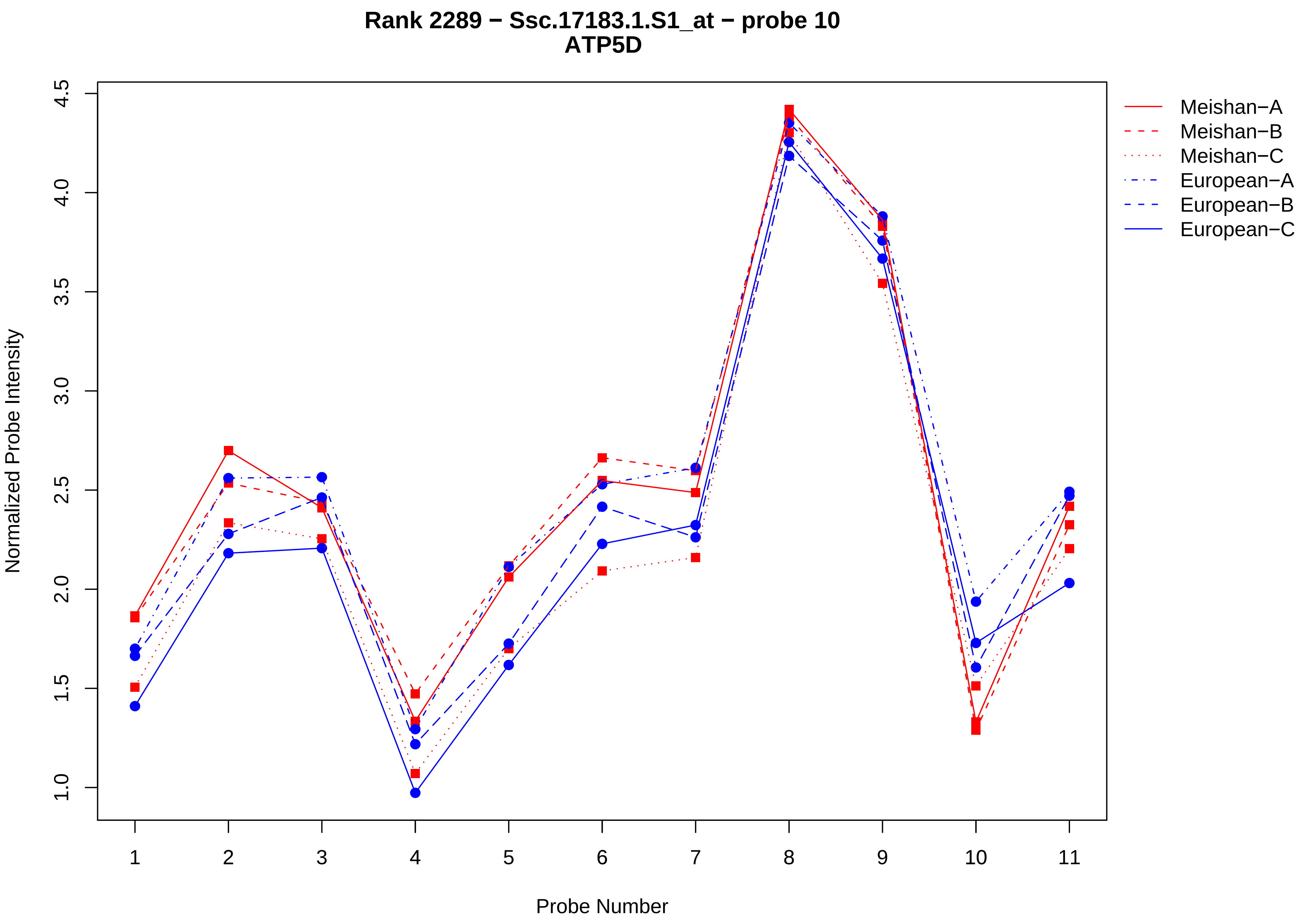
Rank 2289 − Ssc.17183.1.S1_at − probe 10
ATP5D
Normalized Probe Intensity
Probe Number
1.0
1.5
2.0
2.5
3.0
3.5
4.0
4.5
1
2
3
4
5
6
7
8
9
10
11
Meishan−A
Meishan−B
Meishan−C
European−A
European−B
European−C

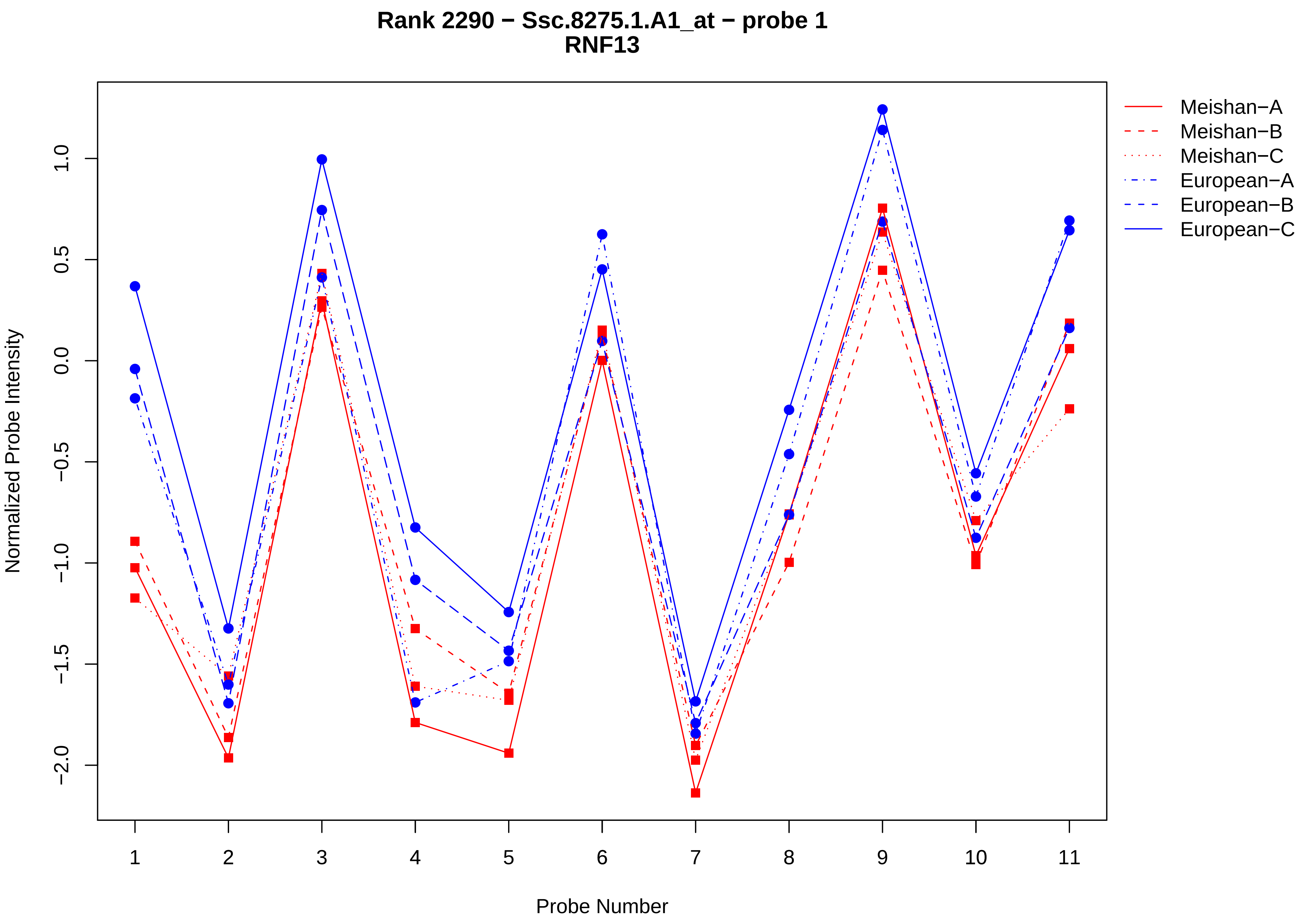

Rank 2290 − Ssc.8275.1.A1_at − probe 1
RNF13
Normalized Probe Intensity
Probe Number
1.0
0.5
0.0
−0.5
−1.0
−1.5
−2.0
1
2
3
4
5
6
7
8
9
10
11
Meishan−A
Meishan−B
Meishan−C
European−A
European−B
European−C

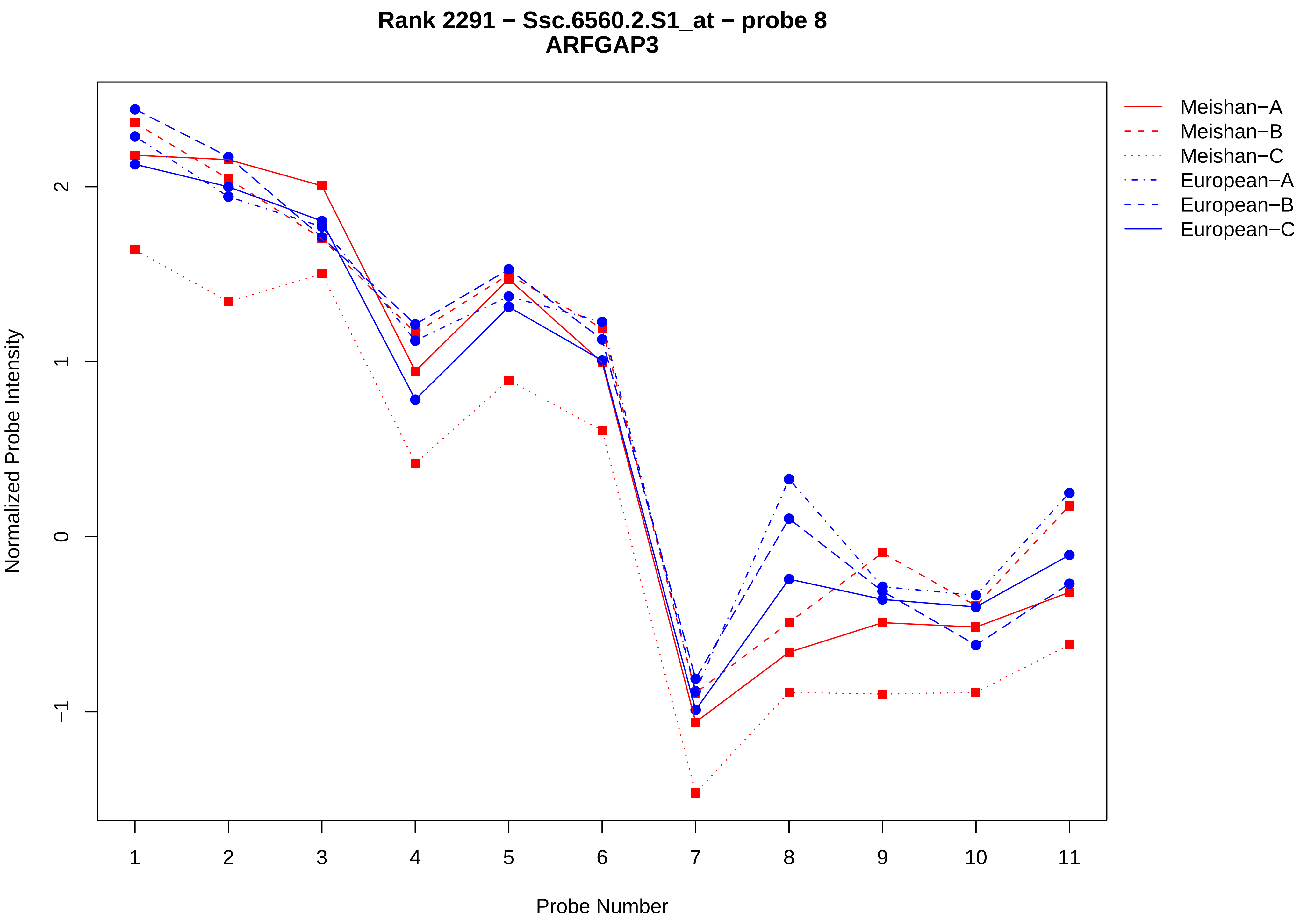

Rank 2291 − Ssc.6560.2.S1_at − probe 8
ARFGAP3
Normalized Probe Intensity
Probe Number
−1
0
1
2
1
2
3
4
5
6
7
8
9
10
11
Meishan−A
Meishan−B
Meishan−C
European−A
European−B
European−C

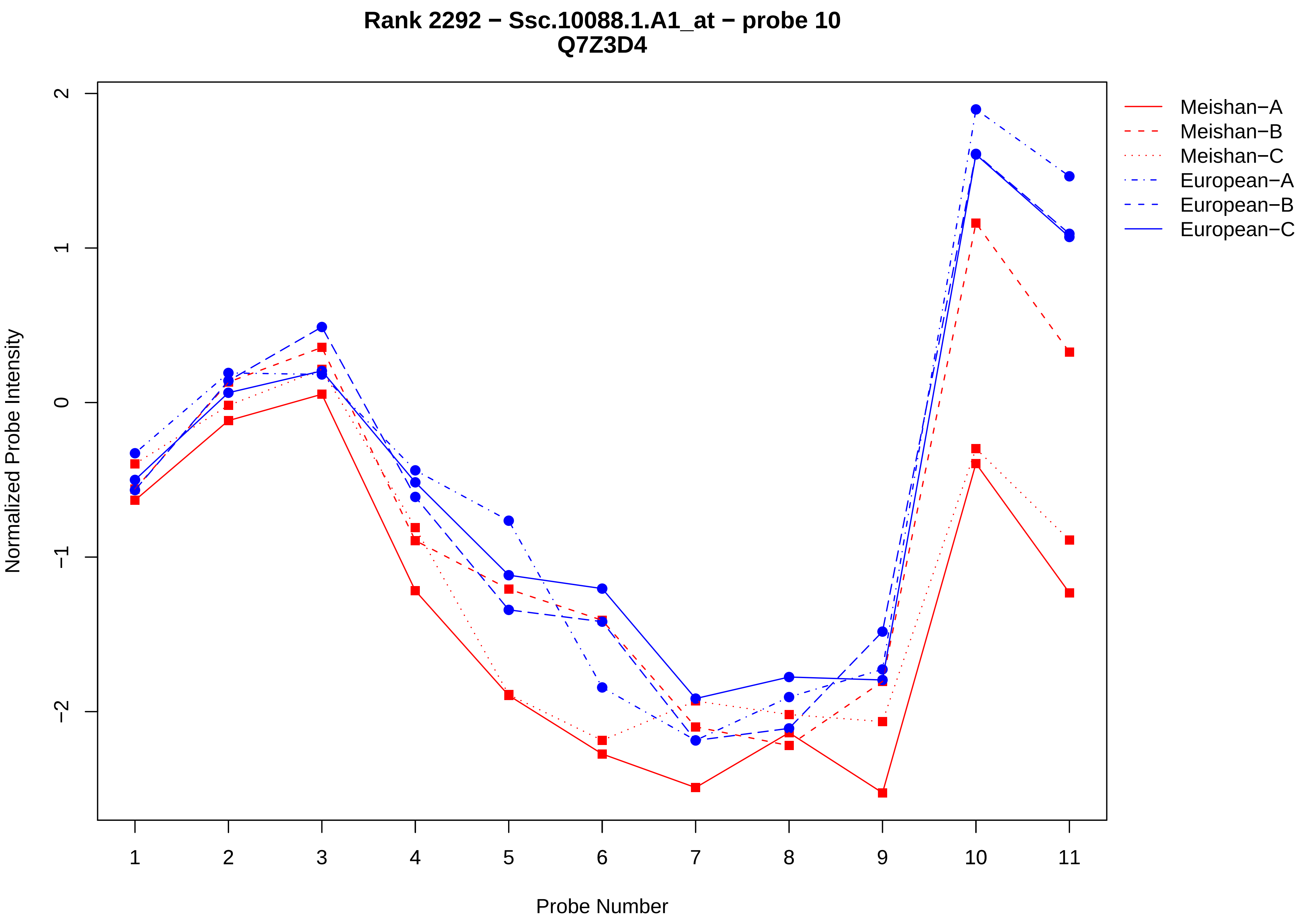

Rank 2292 − Ssc.10088.1.A1_at − probe 10
Q7Z3D4
Normalized Probe Intensity
Probe Number
Meishan−A
Meishan−B
Meishan−C
European−A
European−B
European−C
−2
−1
0
1
2
1
2
3
4
5
6
7
8
9
10
11

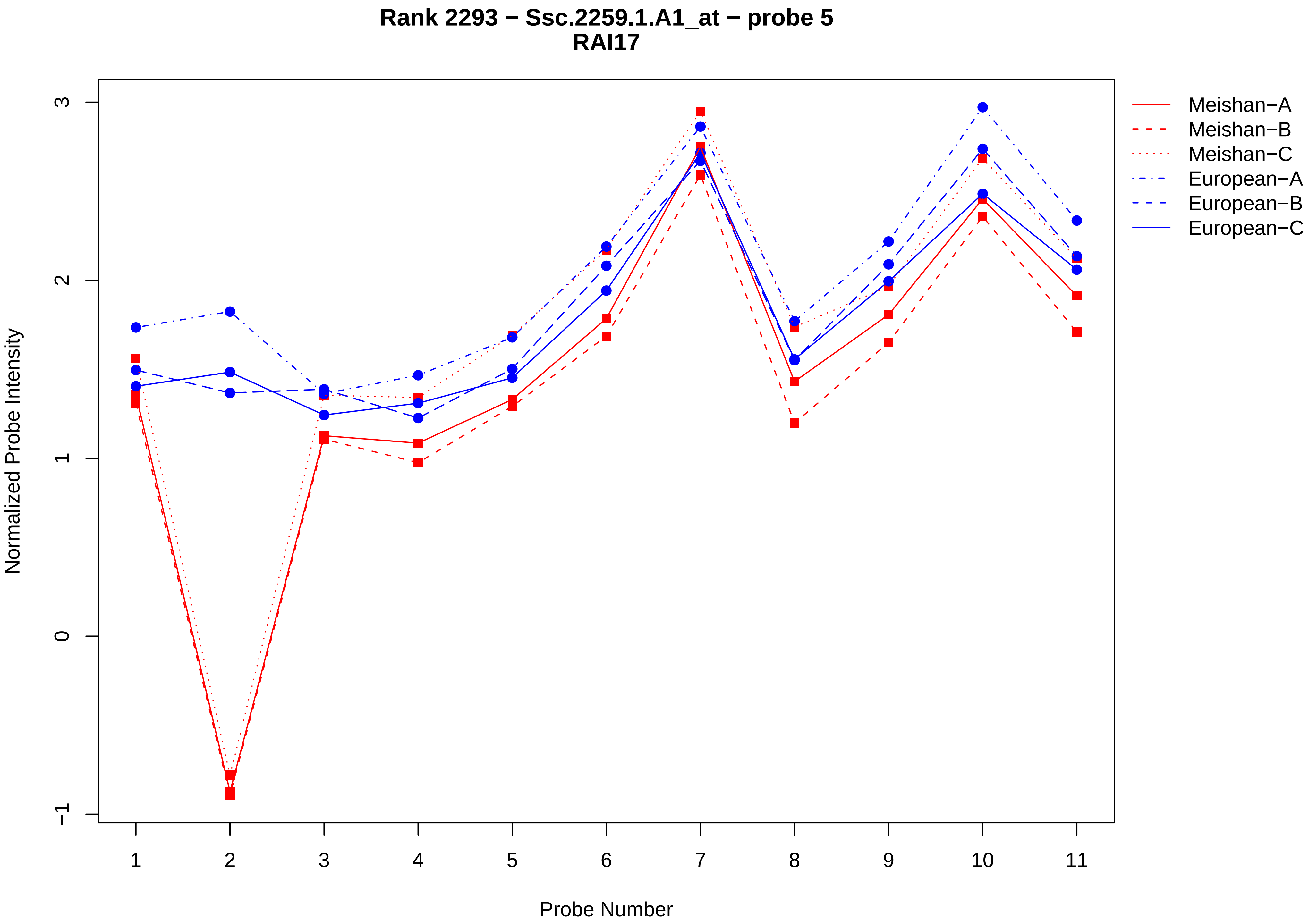

Rank 2293 − Ssc.2259.1.A1_at − probe 5
RAI17
Normalized Probe Intensity
Probe Number
−1
0
1
2
3
1
2
3
4
5
6
7
8
9
10
11
Meishan−A
Meishan−B
Meishan−C
European−A
European−B
European−C

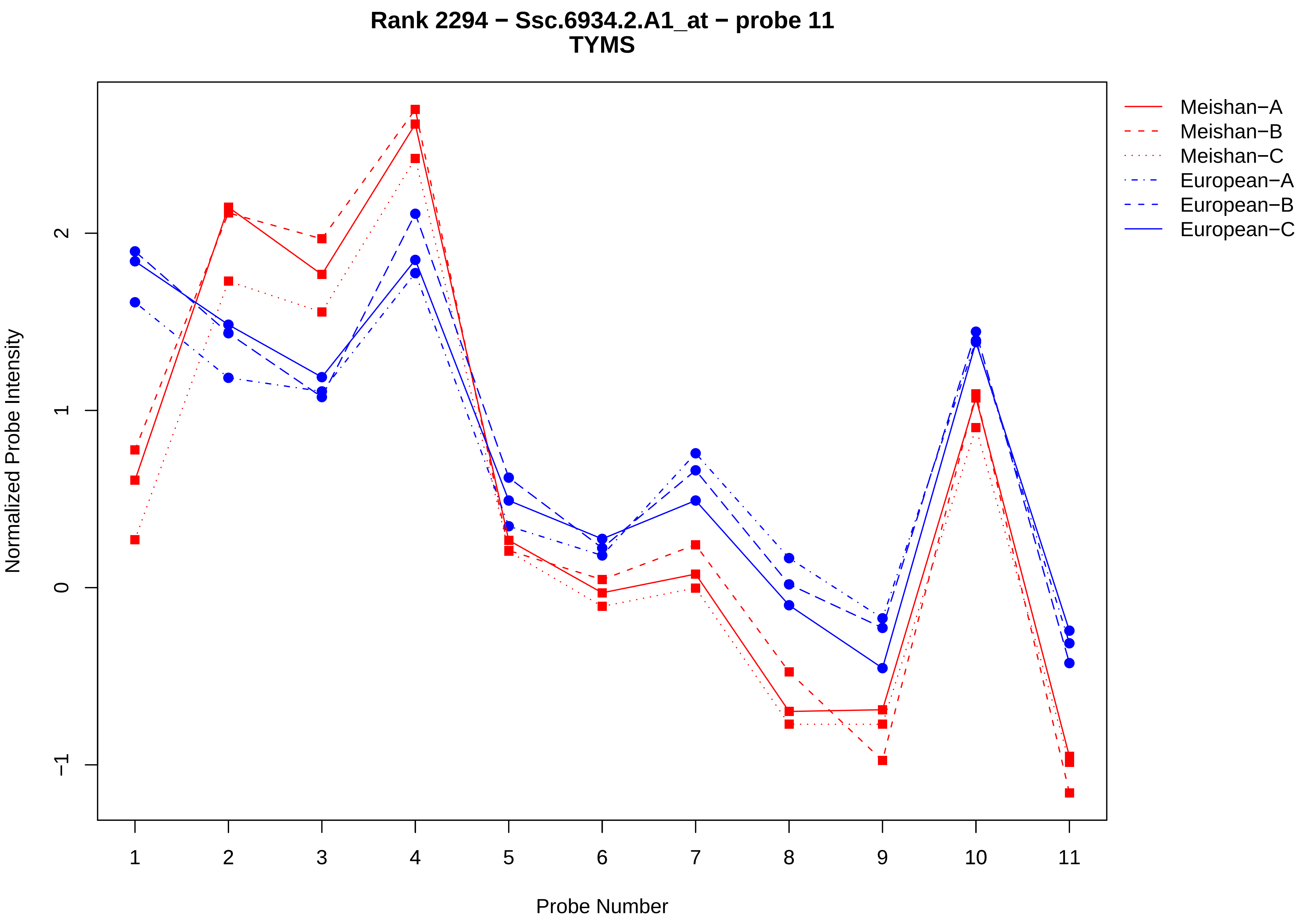

Rank 2294 − Ssc.6934.2.A1_at − probe 11
TYMS
Normalized Probe Intensity
Probe Number
−1
0
1
2
1
2
3
4
5
6
7
8
9
10
11
Meishan−A
Meishan−B
Meishan−C
European−A
European−B
European−C

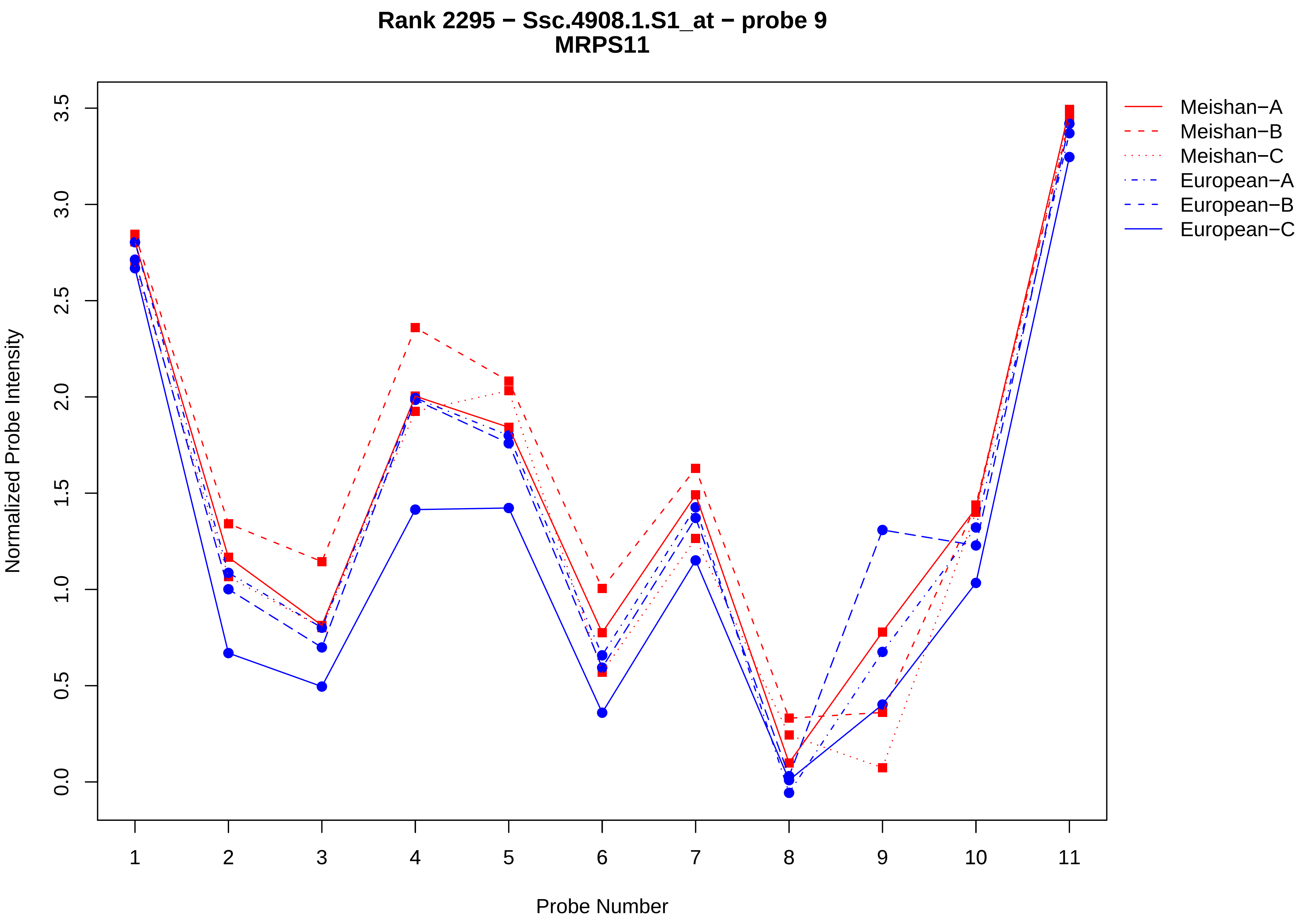
Rank 2295 − Ssc.4908.1.S1_at − probe 9
MRPS11
Normalized Probe Intensity
0.0
0.5
1.0
1.5
2.0
2.5
3.0
3.5
Probe Number
1
2
3
4
5
6
7
8
9
10
11
Meishan−A
Meishan−B
Meishan−C
European−A
European−B
European−C

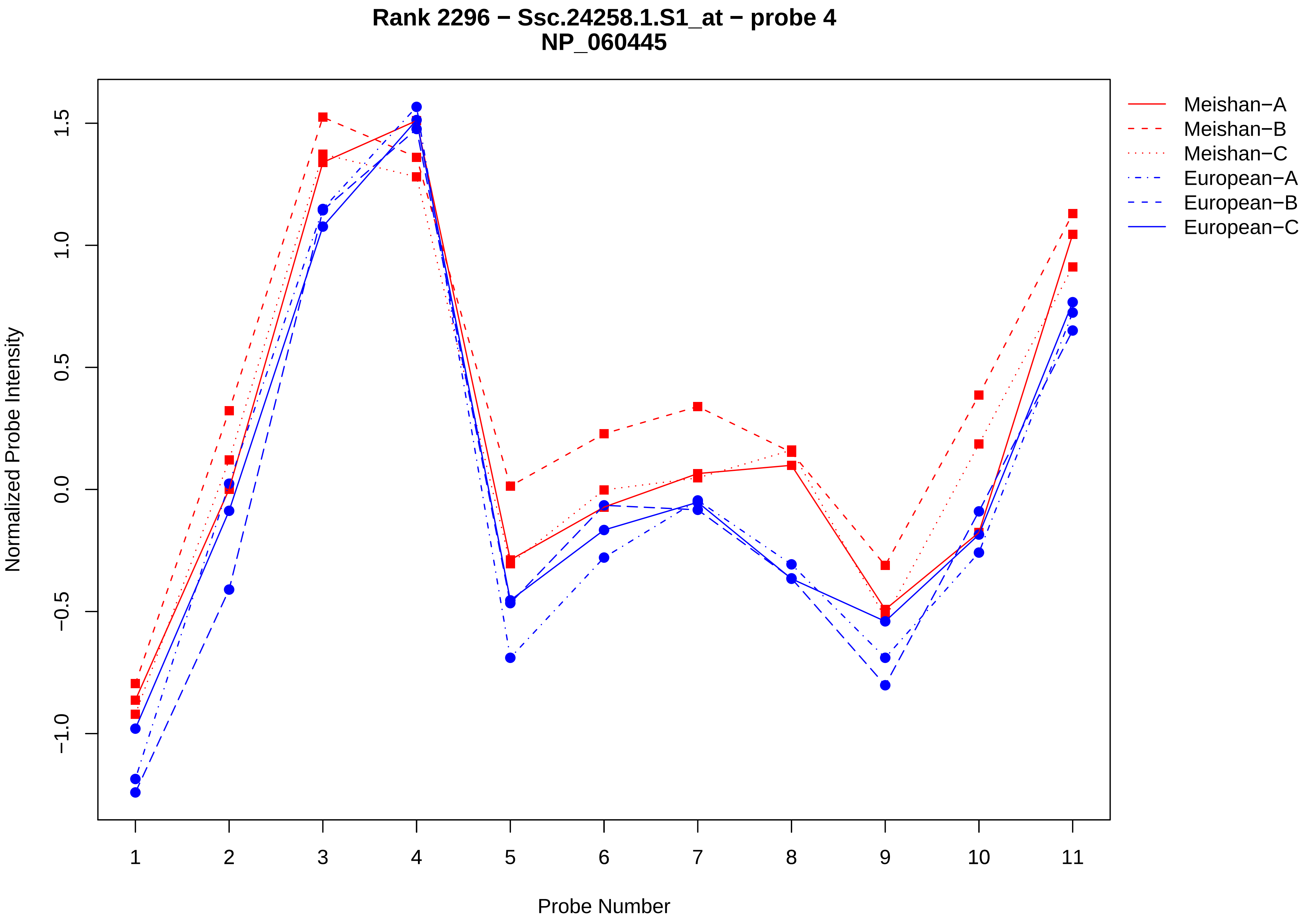

Rank 2296 − Ssc.24258.1.S1_at − probe 4
NP_060445
Normalized Probe Intensity
Probe Number
−1.0
−0.5
0.0
0.5
1.0
1.5
1
2
3
4
5
6
7
8
9
10
11
Meishan−A
Meishan−B
Meishan−C
European−A
European−B
European−C

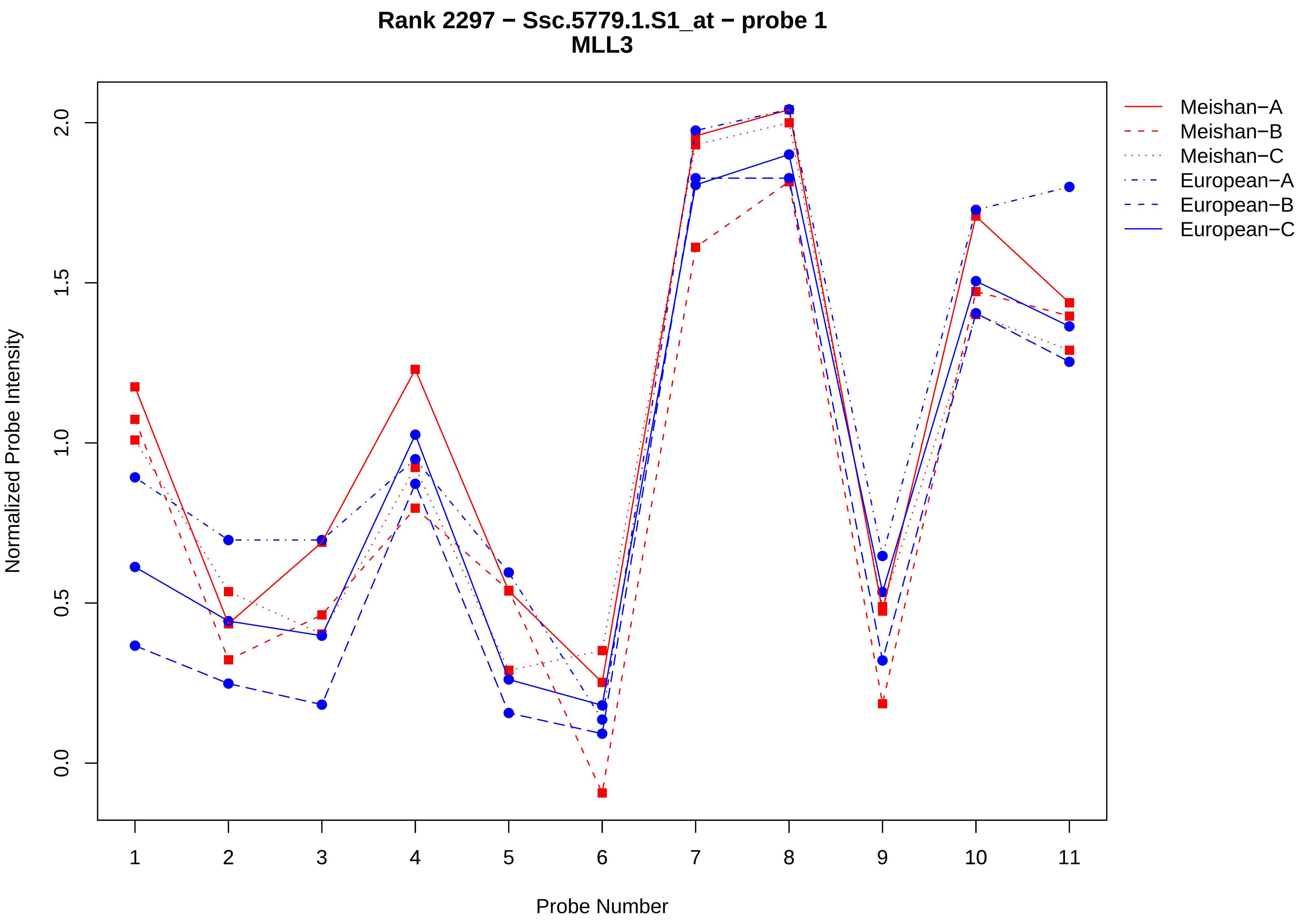

Rank 2297 − Ssc.5779.1.S1_at − probe 1
MLL3
Normalized Probe Intensity
0.0
0.5
1.0
1.5
2.0
Probe Number
1
2
3
4
5
6
7
8
9
10
11
Meishan−A
Meishan−B
Meishan−C
European−A
European−B
European−C

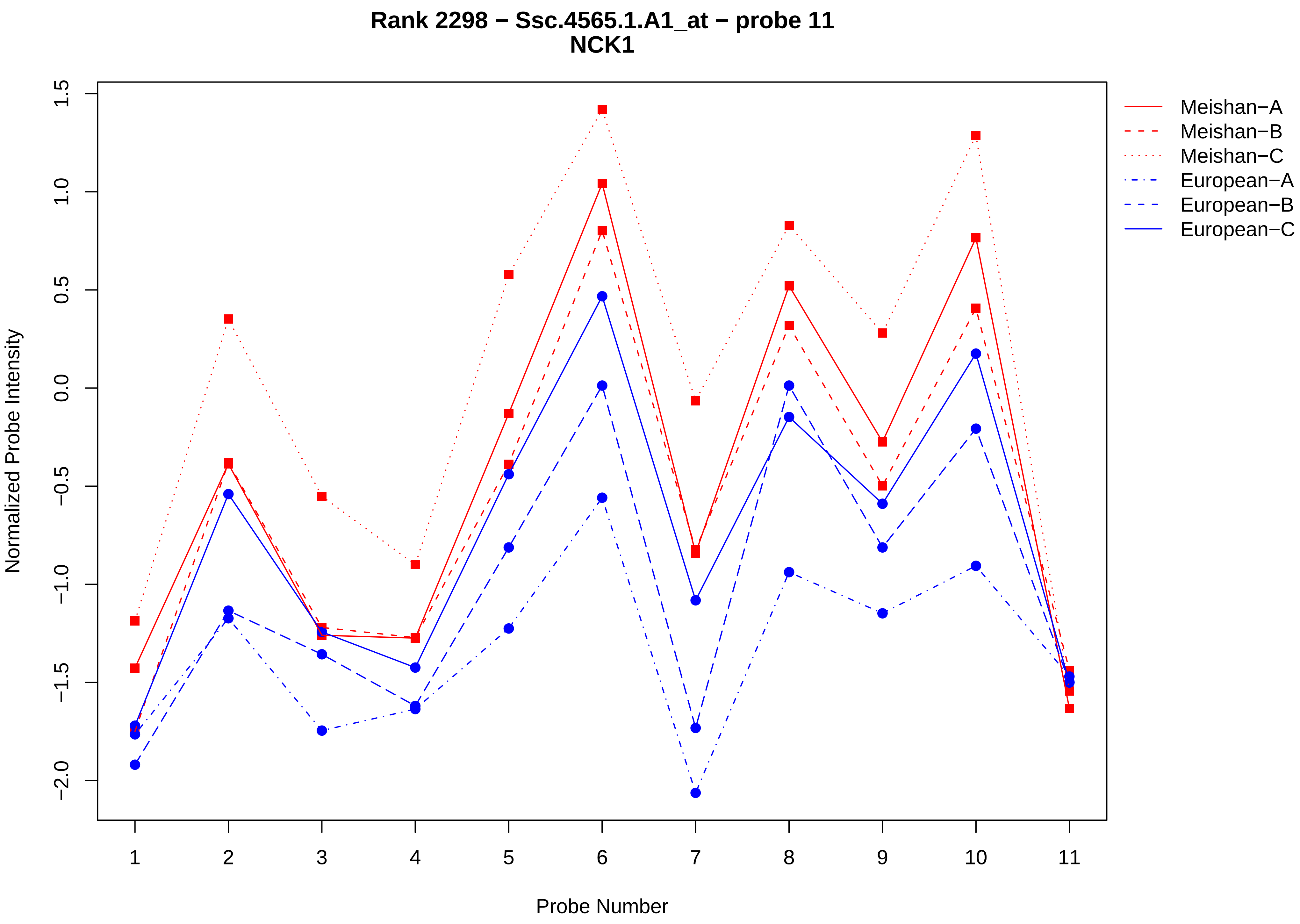

Rank 2298 − Ssc.4565.1.A1_at − probe 11
NCK1
Normalized Probe Intensity
Probe Number
1.5
1.0
0.5
0.0
−0.5
−1.0
−1.5
−2.0
1
2
3
4
5
6
7
8
9
10
11
Meishan−A
Meishan−B
Meishan−C
European−A
European−B
European−C

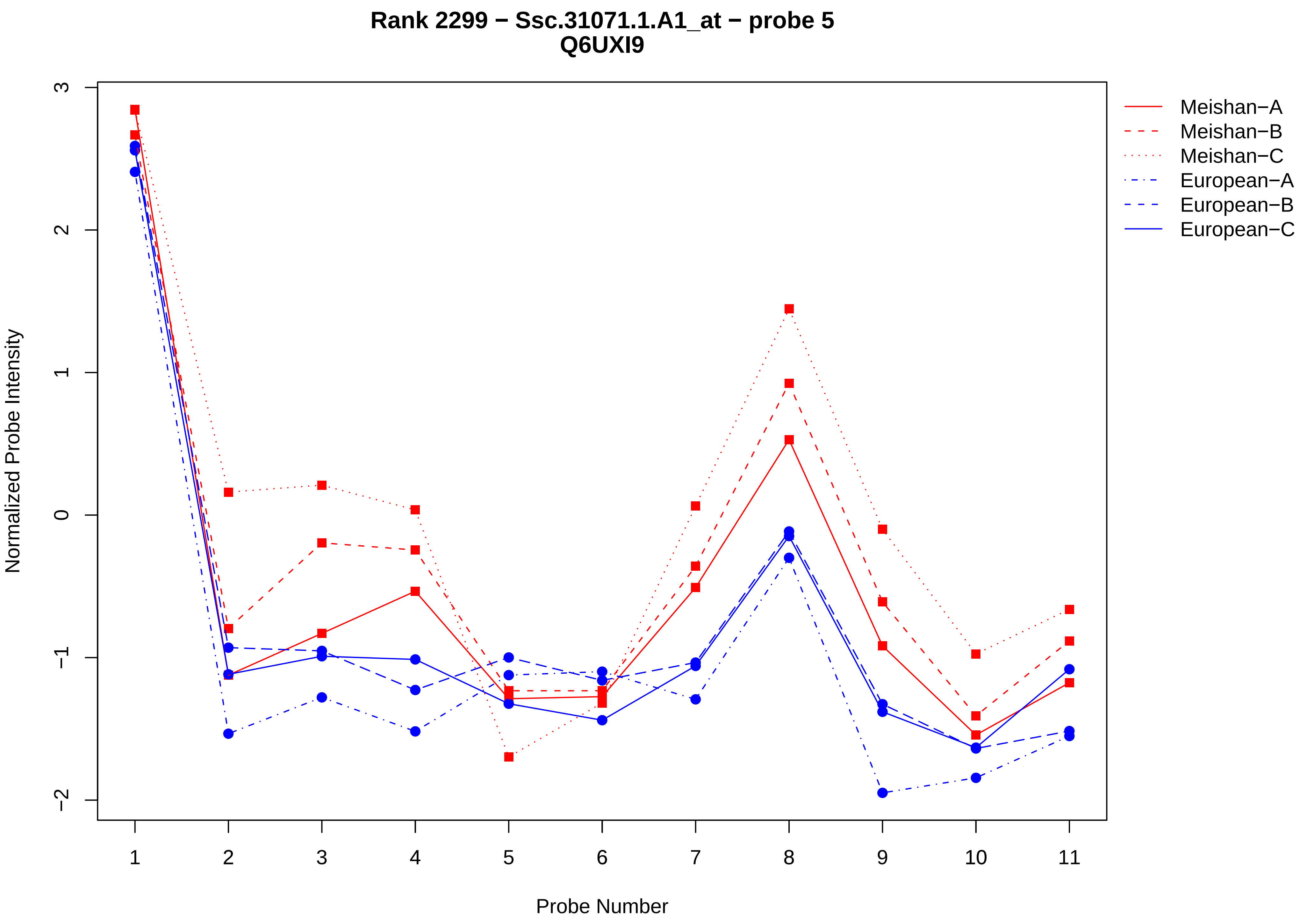

Rank 2299 − Ssc.31071.1.A1_at − probe 5
Q6UXI9
Normalized Probe Intensity
Probe Number
Meishan−A
Meishan−B
Meishan−C
European−A
European−B
European−C

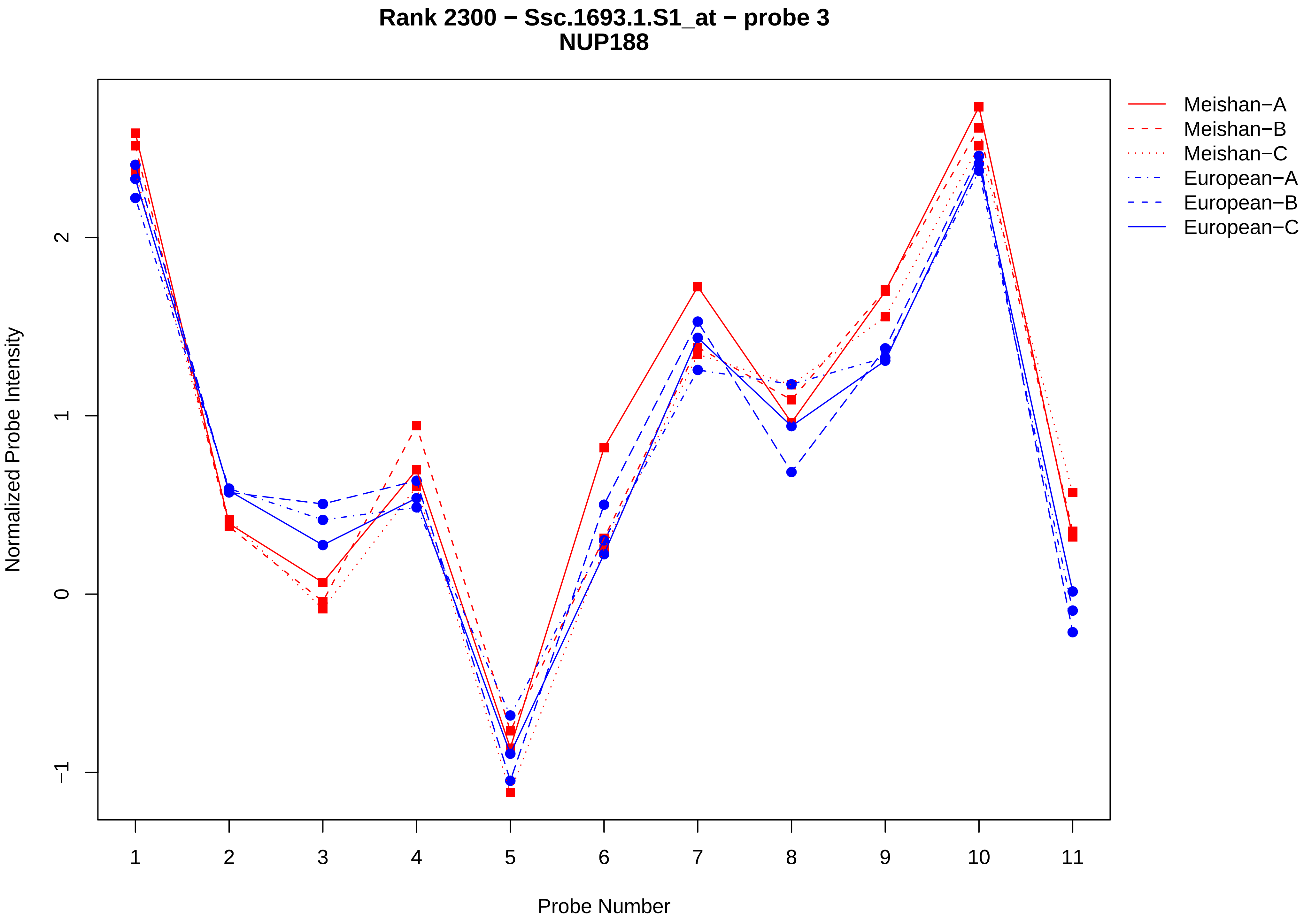

Rank 2300 − Ssc.1693.1.S1_at − probe 3
NUP188
Normalized Probe Intensity
Probe Number
−1
0
1
2
1
2
3
4
5
6
7
8
9
10
11
Meishan−A
Meishan−B
Meishan−C
European−A
European−B
European−C

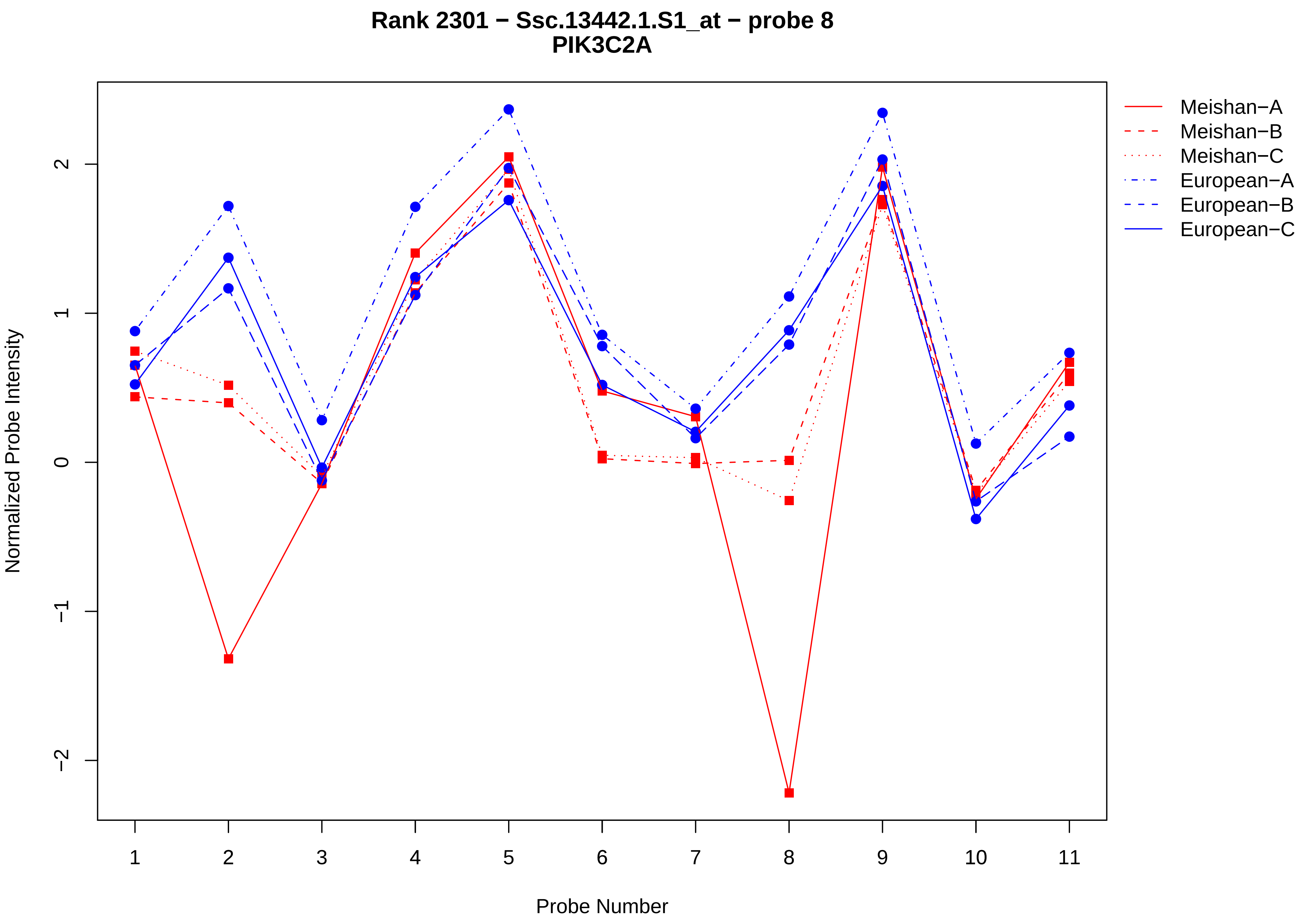
Rank 2301 − Ssc.13442.1.S1_at − probe 8
PIK3C2A
Normalized Probe Intensity
Probe Number
−2
−1
0
1
2
1
2
3
4
5
6
7
8
9
10
11
Meishan−A
Meishan−B
Meishan−C
European−A
European−B
European−C

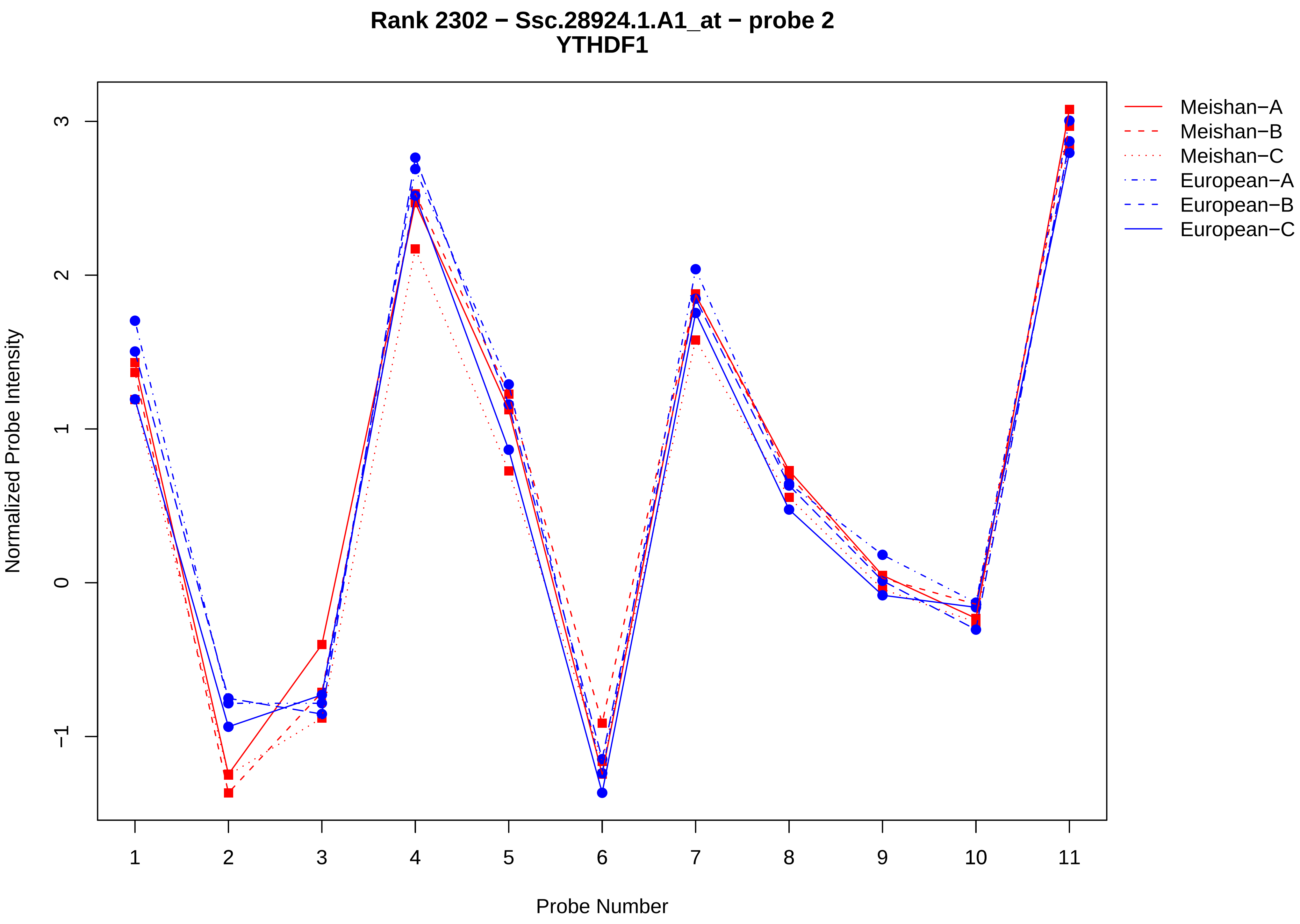

Rank 2302 − Ssc.28924.1.A1_at − probe 2
YTHDF1
Normalized Probe Intensity
Probe Number
Meishan−A
Meishan−B
Meishan−C
European−A
European−B
European−C
−1
0
1
2
3
1
2
3
4
5
6
7
8
9
10
11

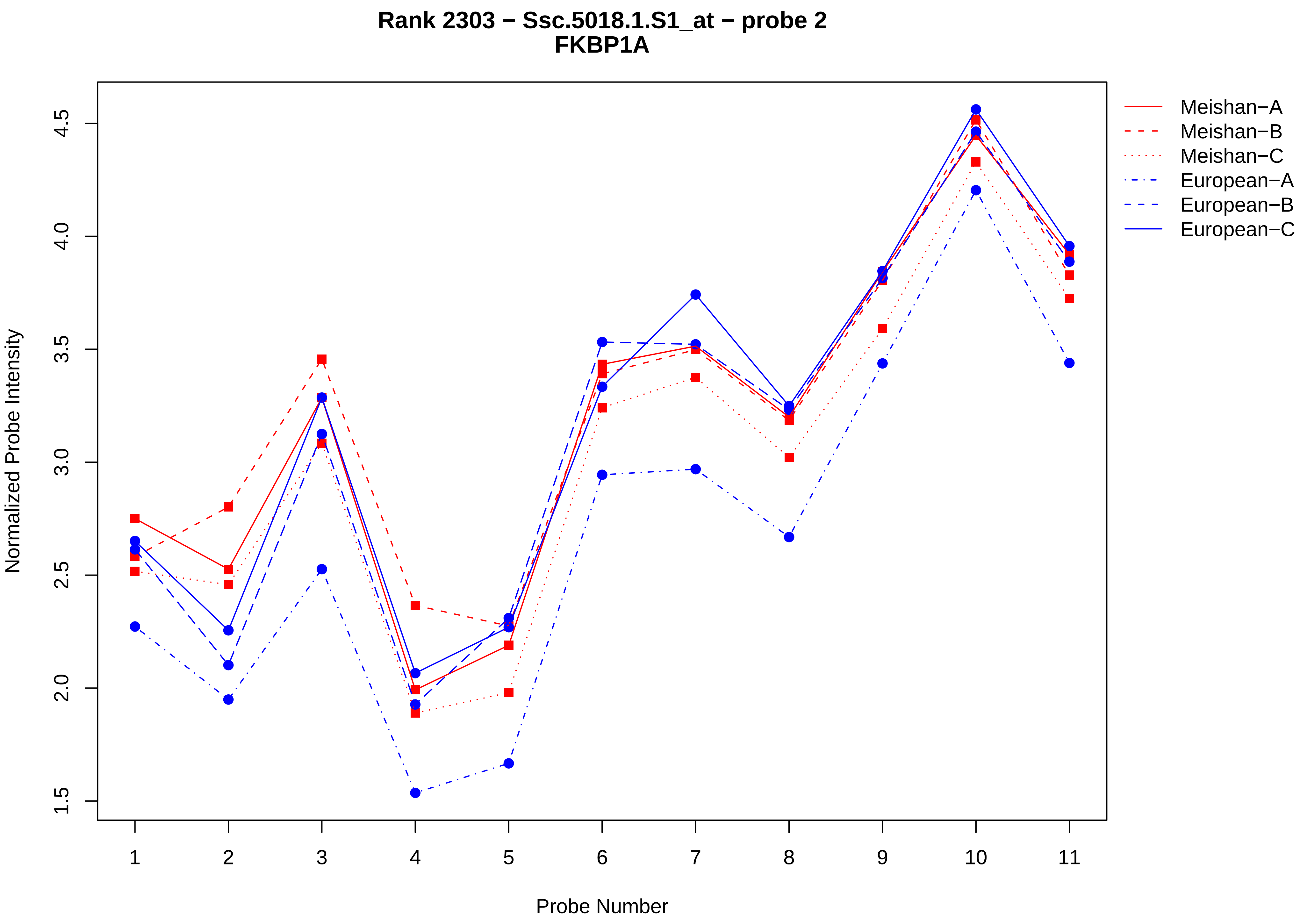

Rank 2303 − Ssc.5018.1.S1_at − probe 2
FKBP1A
Normalized Probe Intensity
Probe Number
1.5
2.0
2.5
3.0
3.5
4.0
4.5
1
2
3
4
5
6
7
8
9
10
11
Meishan−A
Meishan−B
Meishan−C
European−A
European−B
European−C

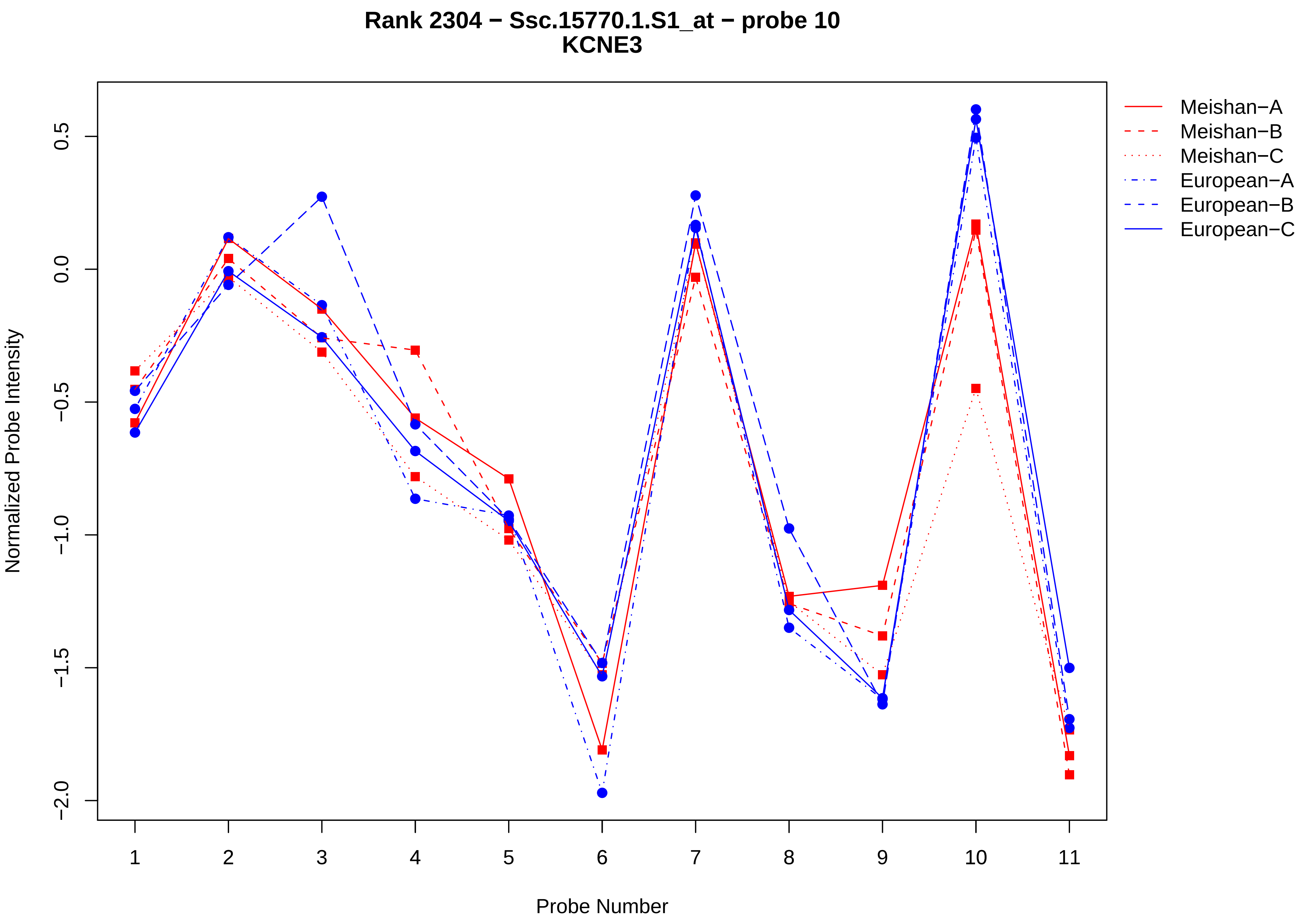
Rank 2304 − Ssc.15770.1.S1_at − probe 10
KCNE3
Normalized Probe Intensity
0.5
0.0
−0.5
−1.0
−1.5
−2.0
1
2
3
4
5
6
7
8
9
10
11
Probe Number
Meishan−A
Meishan−B
Meishan−C
European−A
European−B
European−C

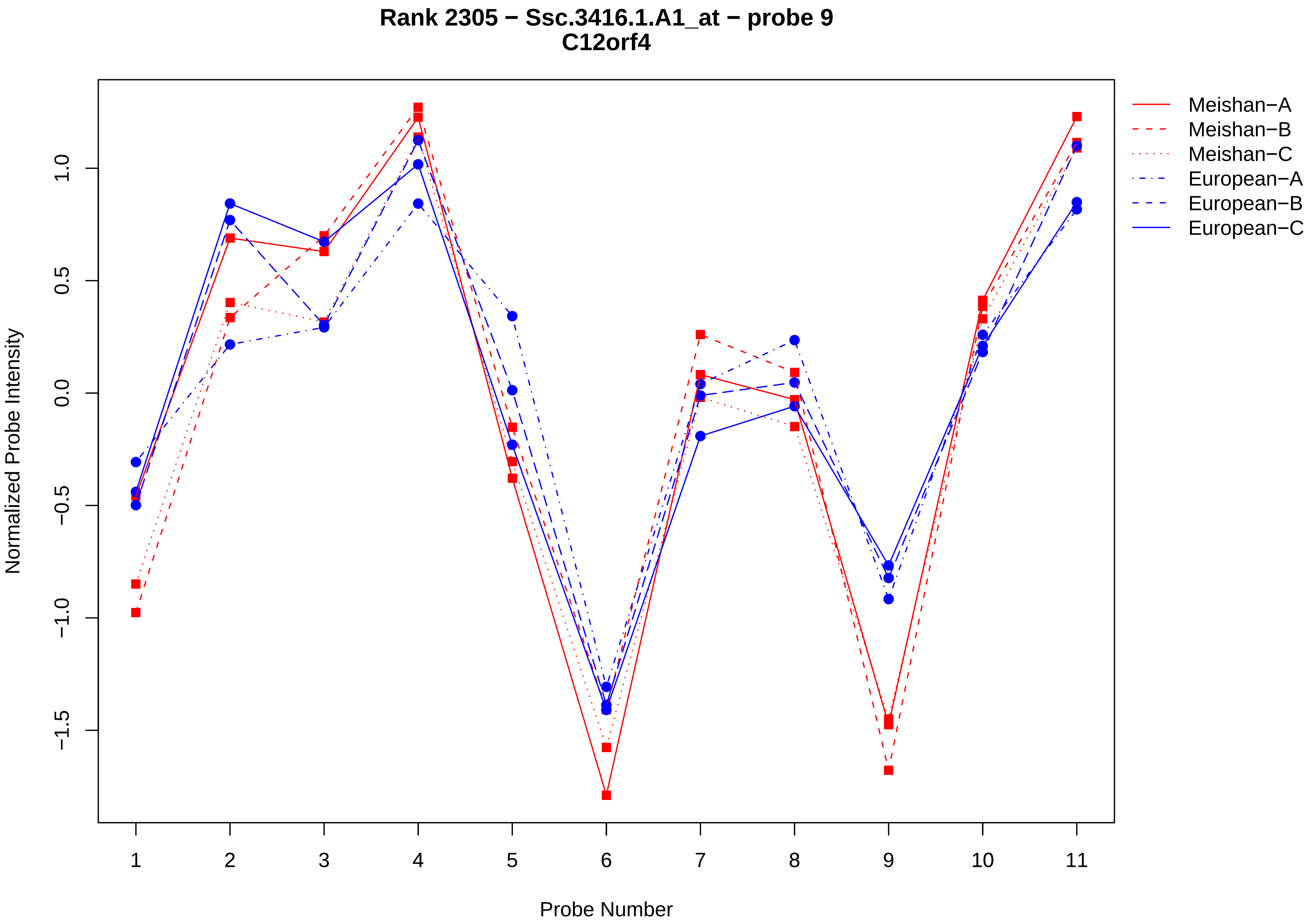

Rank 2305 − Ssc.3416.1.A1_at − probe 9
C12orf4
Normalized Probe Intensity
−1.5
−1.0
−0.5
0.0
0.5
1.0
Probe Number
1
2
3
4
5
6
7
8
9
10
11
Meishan−A
Meishan−B
Meishan−C
European−A
European−B
European−C

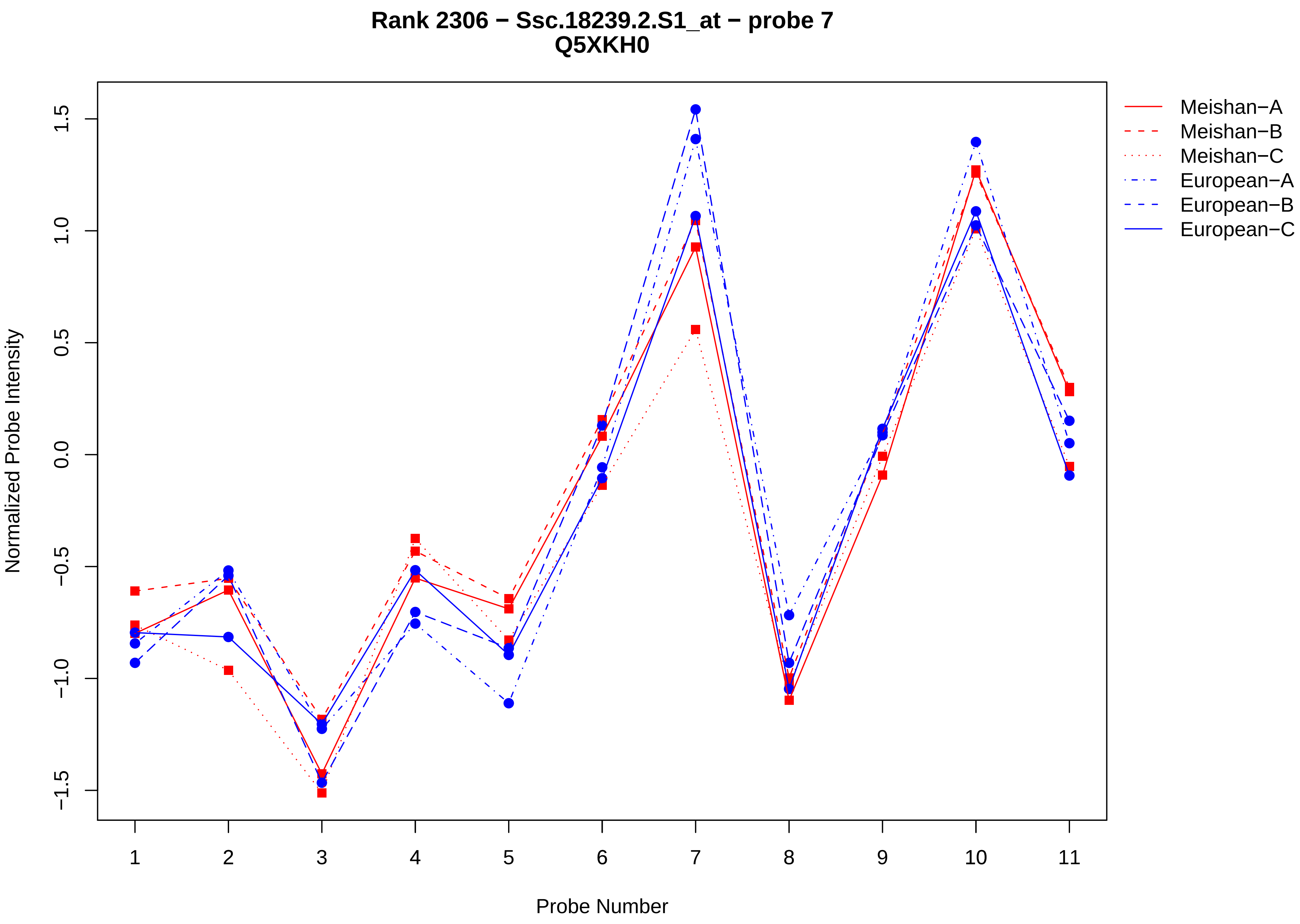

Rank 2306 − Ssc.18239.2.S1_at − probe 7
Q5XKH0
Normalized Probe Intensity
Probe Number
Meishan−A
Meishan−B
Meishan−C
European−A
European−B
European−C
1.5
1.0
0.5
0.0
−0.5
−1.0
−1.5
1
2
3
4
5
6
7
8
9
10
11

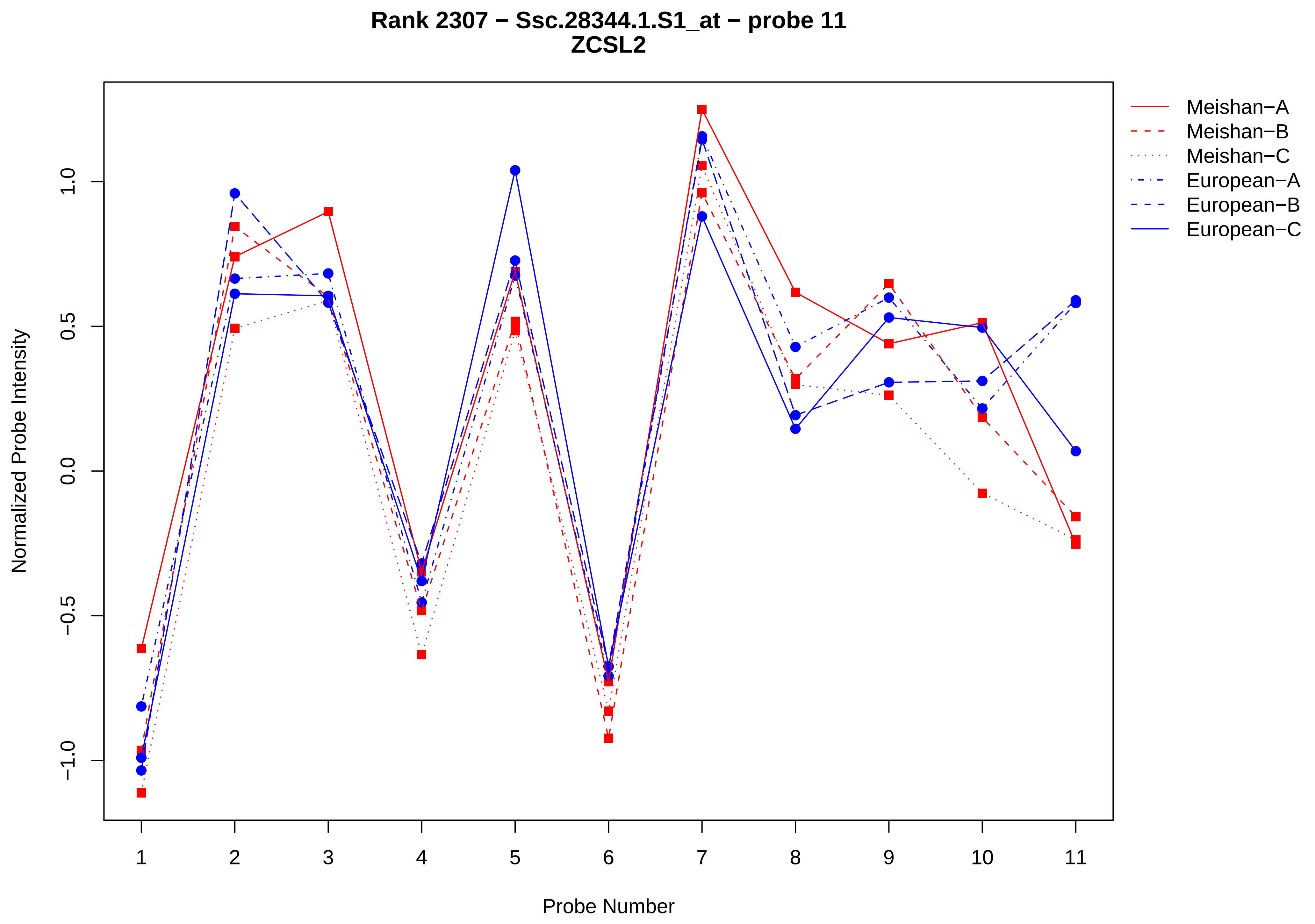

Rank 2307 − Ssc.28344.1.S1_at − probe 11
ZCSL2
Normalized Probe Intensity
−1.0
−0.5
0.0
0.5
1.0
1
2
3
4
5
6
7
8
9
10
11
Probe Number
Meishan−A
Meishan−B
Meishan−C
European−A
European−B
European−C

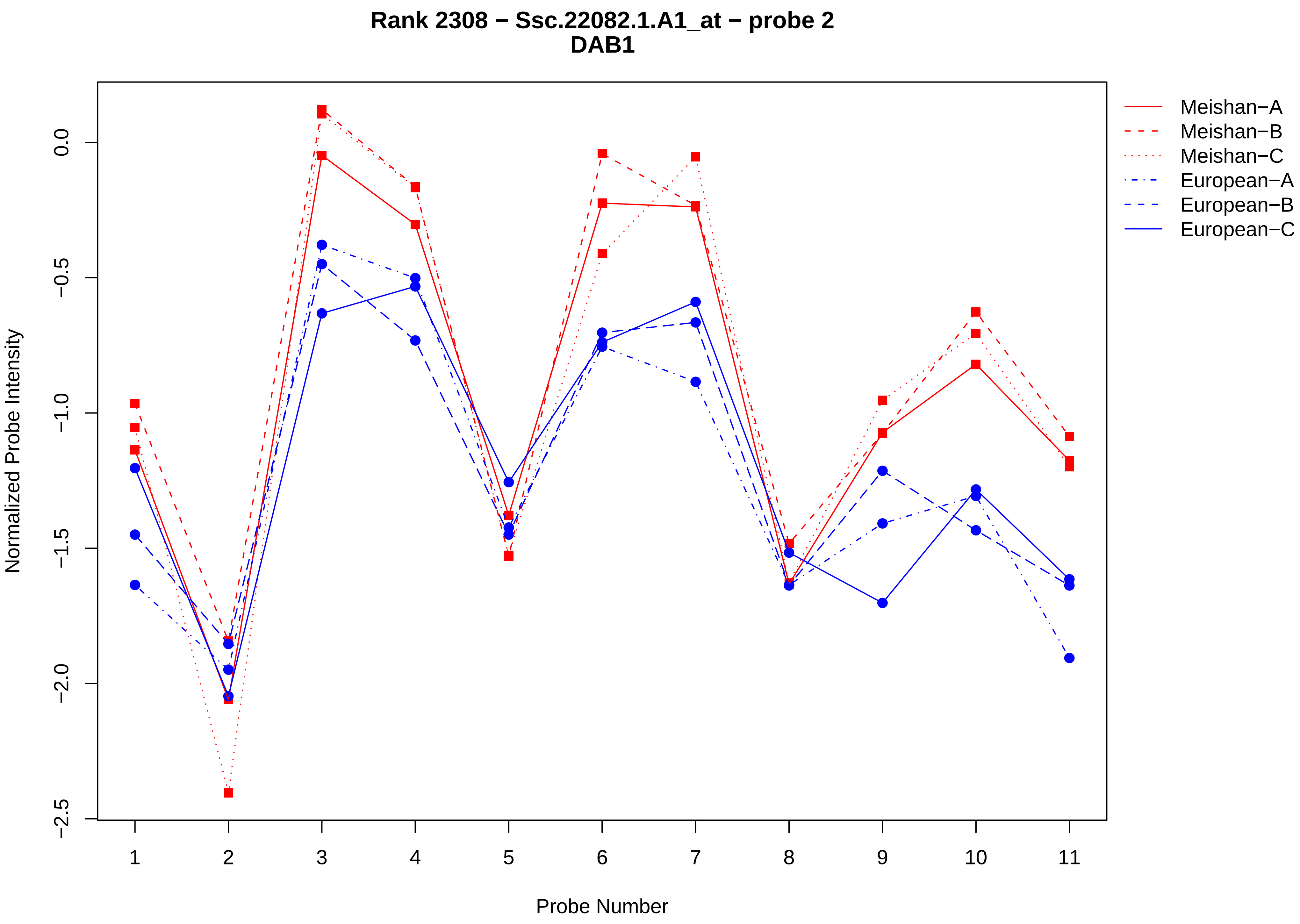

Rank 2308 − Ssc.22082.1.A1_at − probe 2
DAB1
Normalized Probe Intensity
Probe Number
0.0
−0.5
−1.0
−1.5
−2.0
−2.5
1
2
3
4
5
6
7
8
9
10
11
Meishan−A
Meishan−B
Meishan−C
European−A
European−B
European−C

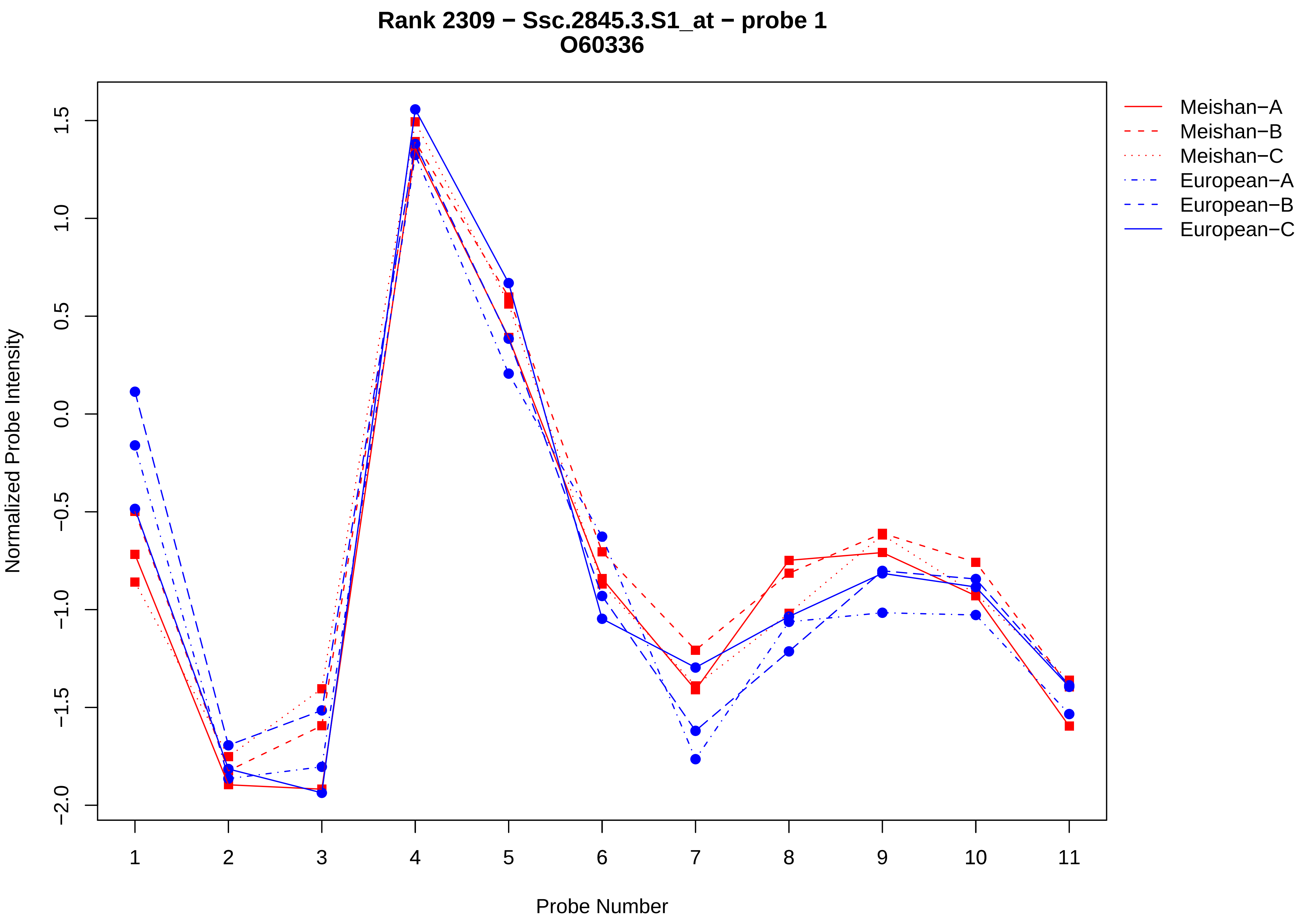

Rank 2309 − Ssc.2845.3.S1_at − probe 1
O60336
Normalized Probe Intensity
Probe Number
1.5
1.0
0.5
0.0
−0.5
−1.0
−1.5
−2.0
1
2
3
4
5
6
7
8
9
10
11
Meishan−A
Meishan−B
Meishan−C
European−A
European−B
European−C

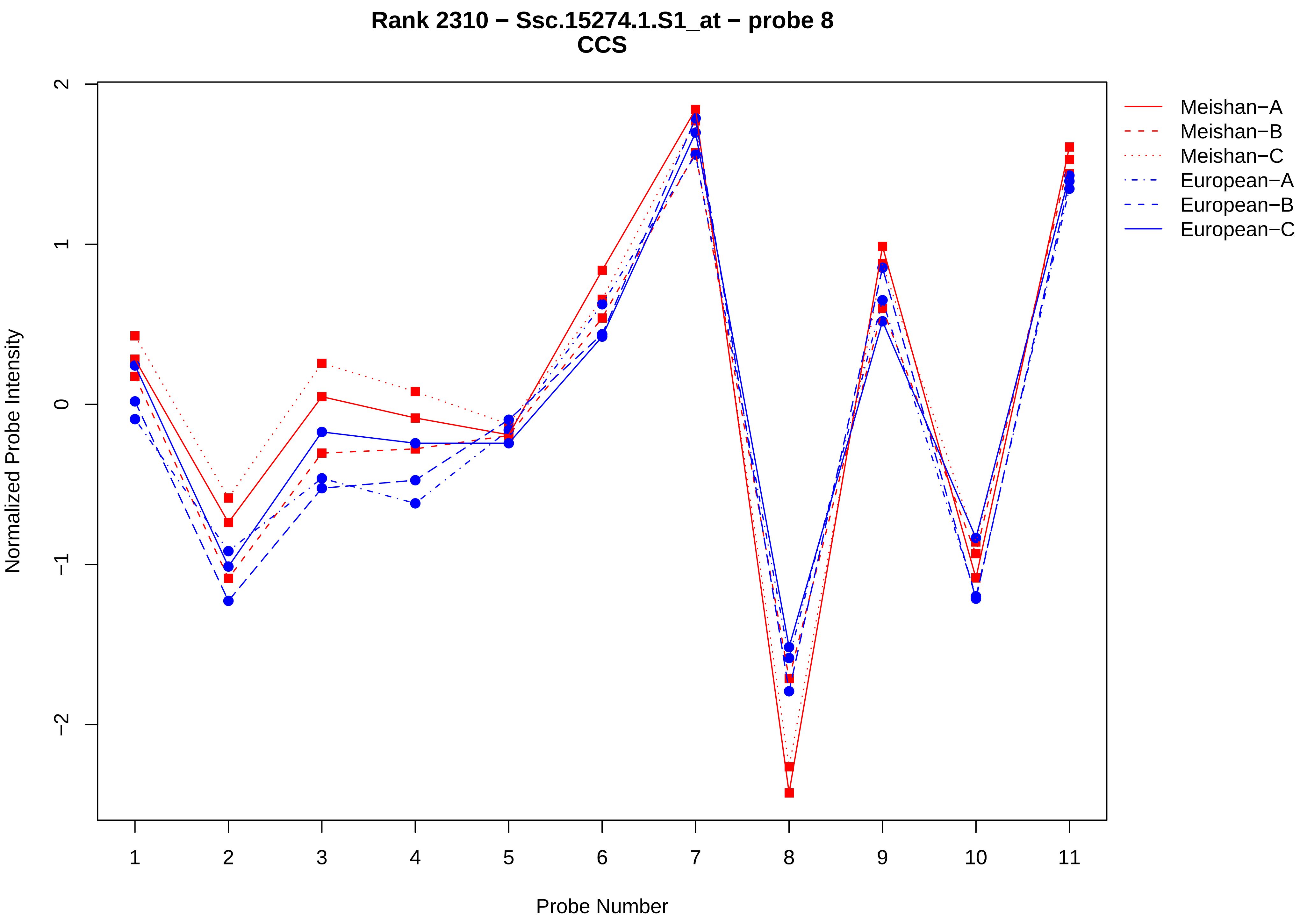

Rank 2310 − Ssc.15274.1.S1_at − probe 8
CCS
Normalized Probe Intensity
Probe Number
−2
−1
0
1
2
1
2
3
4
5
6
7
8
9
10
11
Meishan−A
Meishan−B
Meishan−C
European−A
European−B
European−C

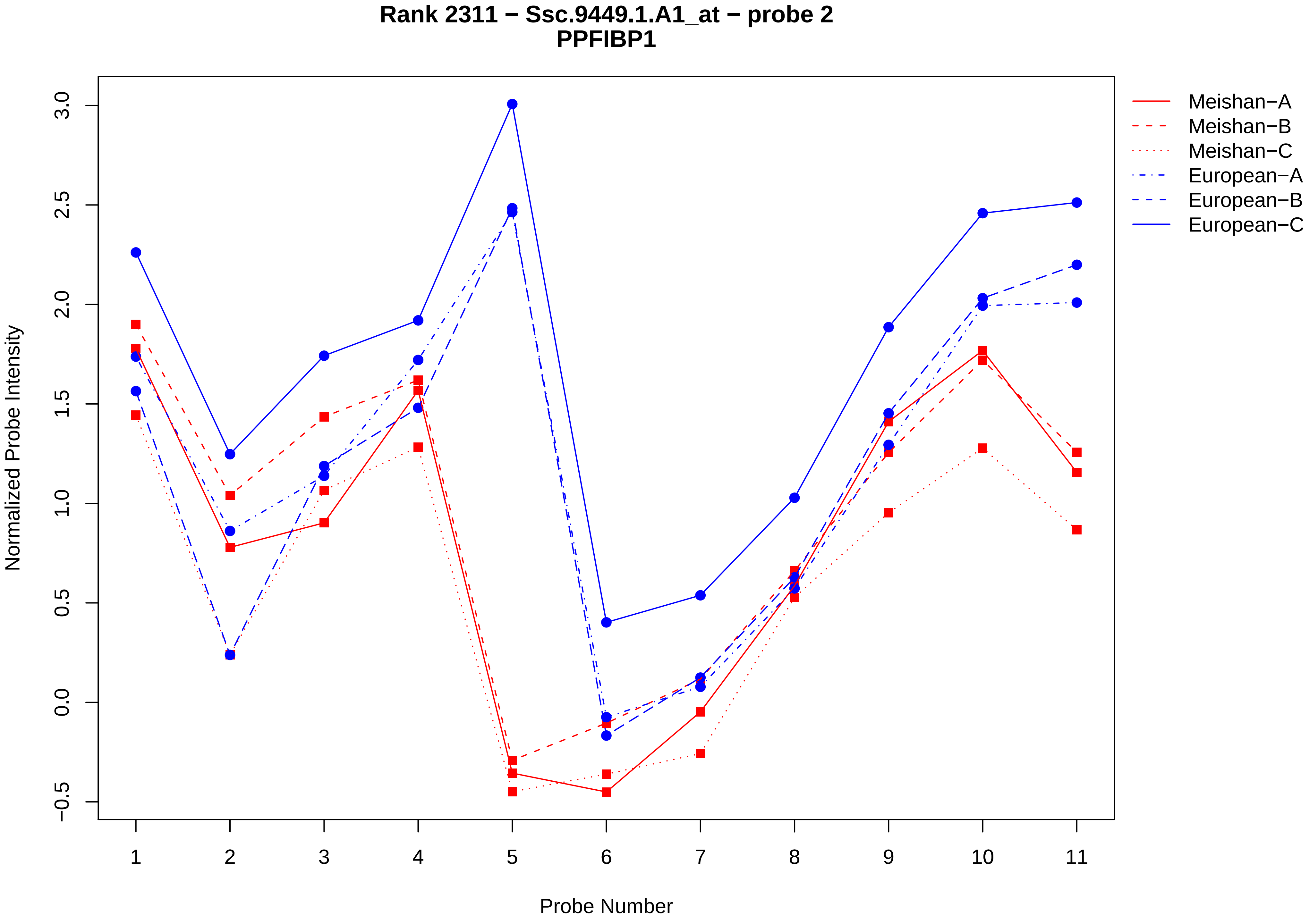

Rank 2311 − Ssc.9449.1.A1_at − probe 2
PPFIBP1
Normalized Probe Intensity
Probe Number
−0.5
0.0
0.5
1.0
1.5
2.0
2.5
3.0
1
2
3
4
5
6
7
8
9
10
11
Meishan−A
Meishan−B
Meishan−C
European−A
European−B
European−C

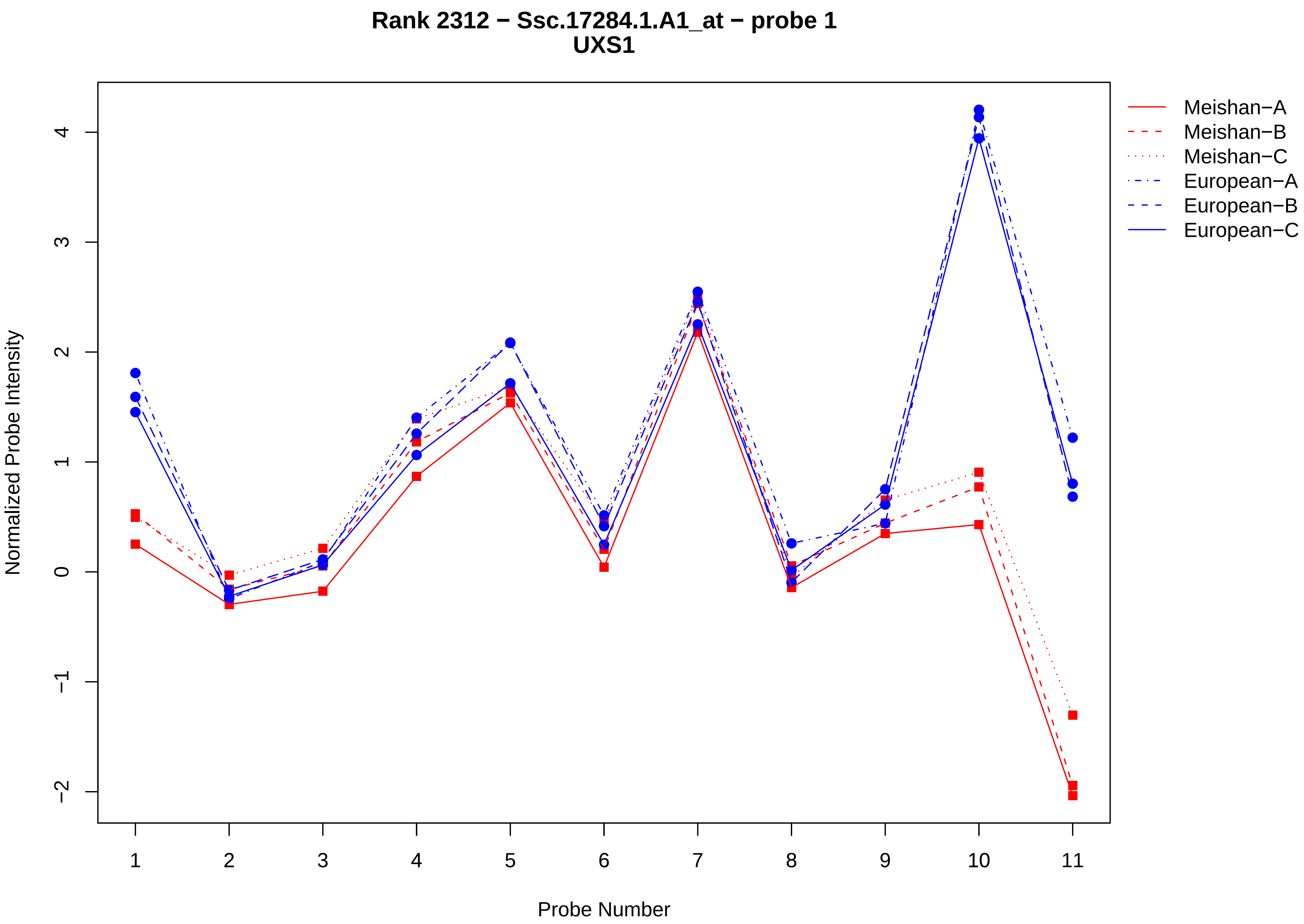

Rank 2312 − Ssc.17284.1.A1_at − probe 1
UXS1
Normalized Probe Intensity
Probe Number
Meishan−A
Meishan−B
Meishan−C
European−A
European−B
European−C

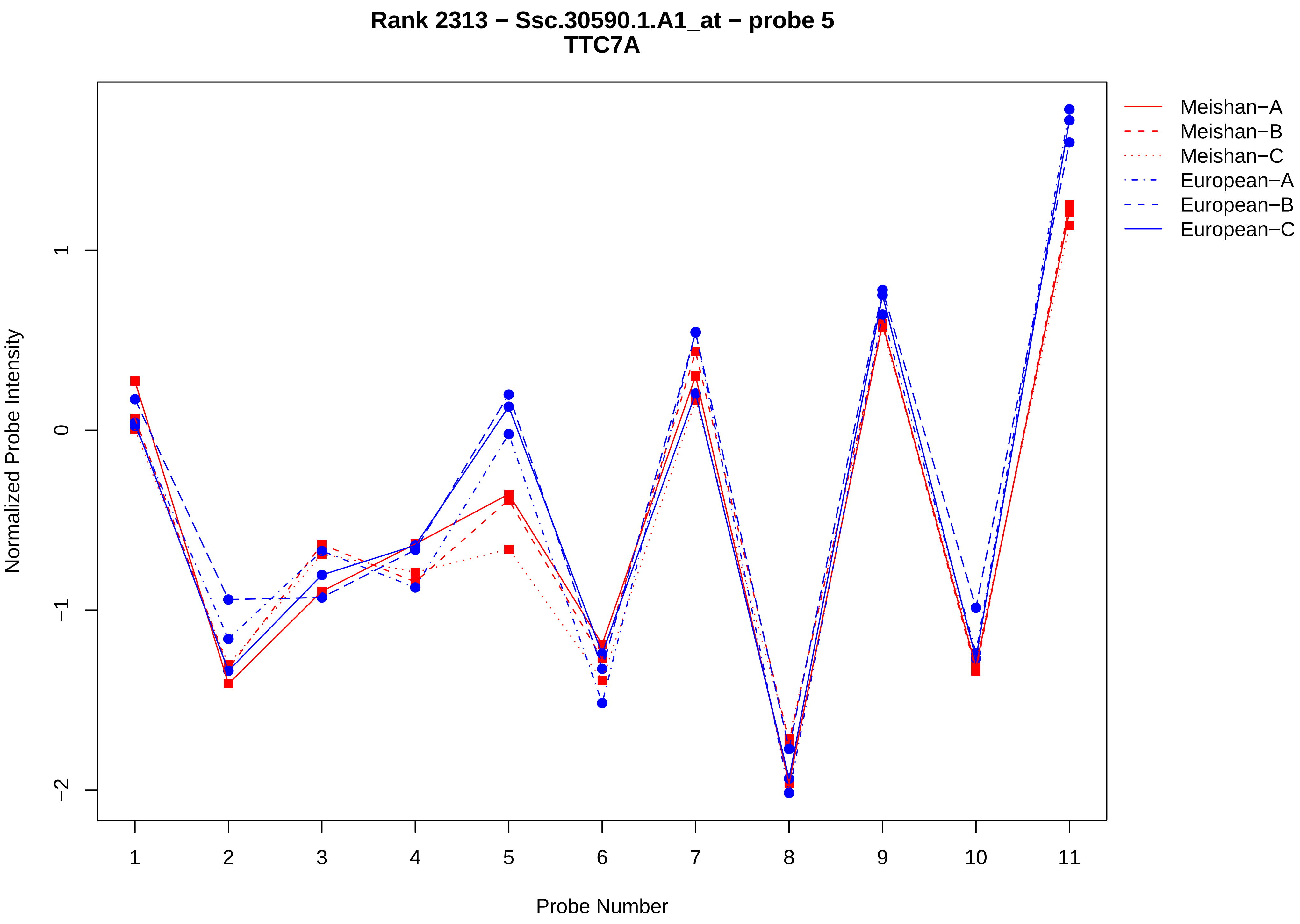

Rank 2313 − Ssc.30590.1.A1_at − probe 5
TTC7A
Normalized Probe Intensity
Probe Number
−2
−1
0
1
1
2
3
4
5
6
7
8
9
10
11
Meishan−A
Meishan−B
Meishan−C
European−A
European−B
European−C

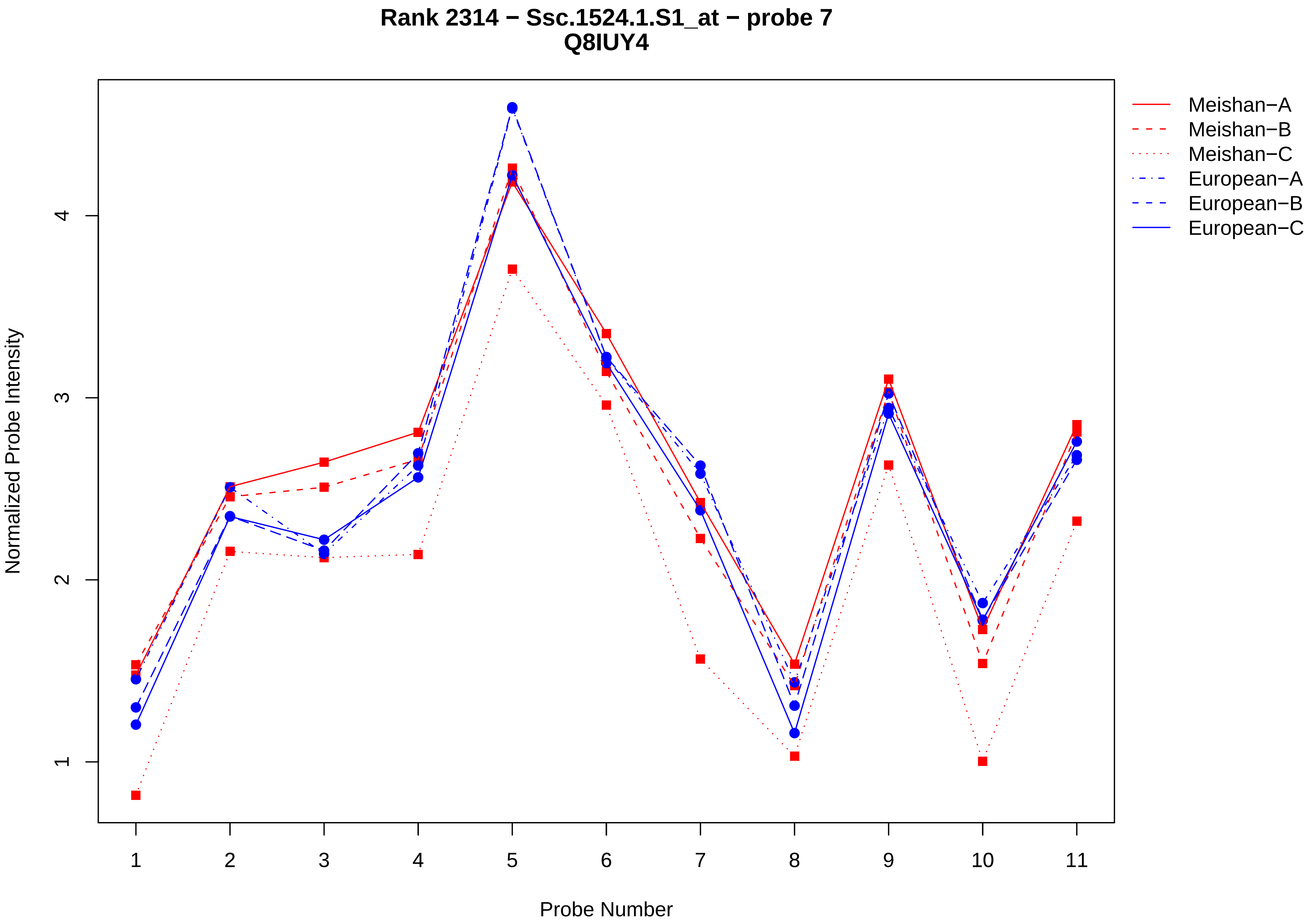

Rank 2314 − Ssc.1524.1.S1_at − probe 7
Q8IUY4
Normalized Probe Intensity
Probe Number
1
2
3
4
1
2
3
4
5
6
7
8
9
10
11
Meishan−A
Meishan−B
Meishan−C
European−A
European−B
European−C

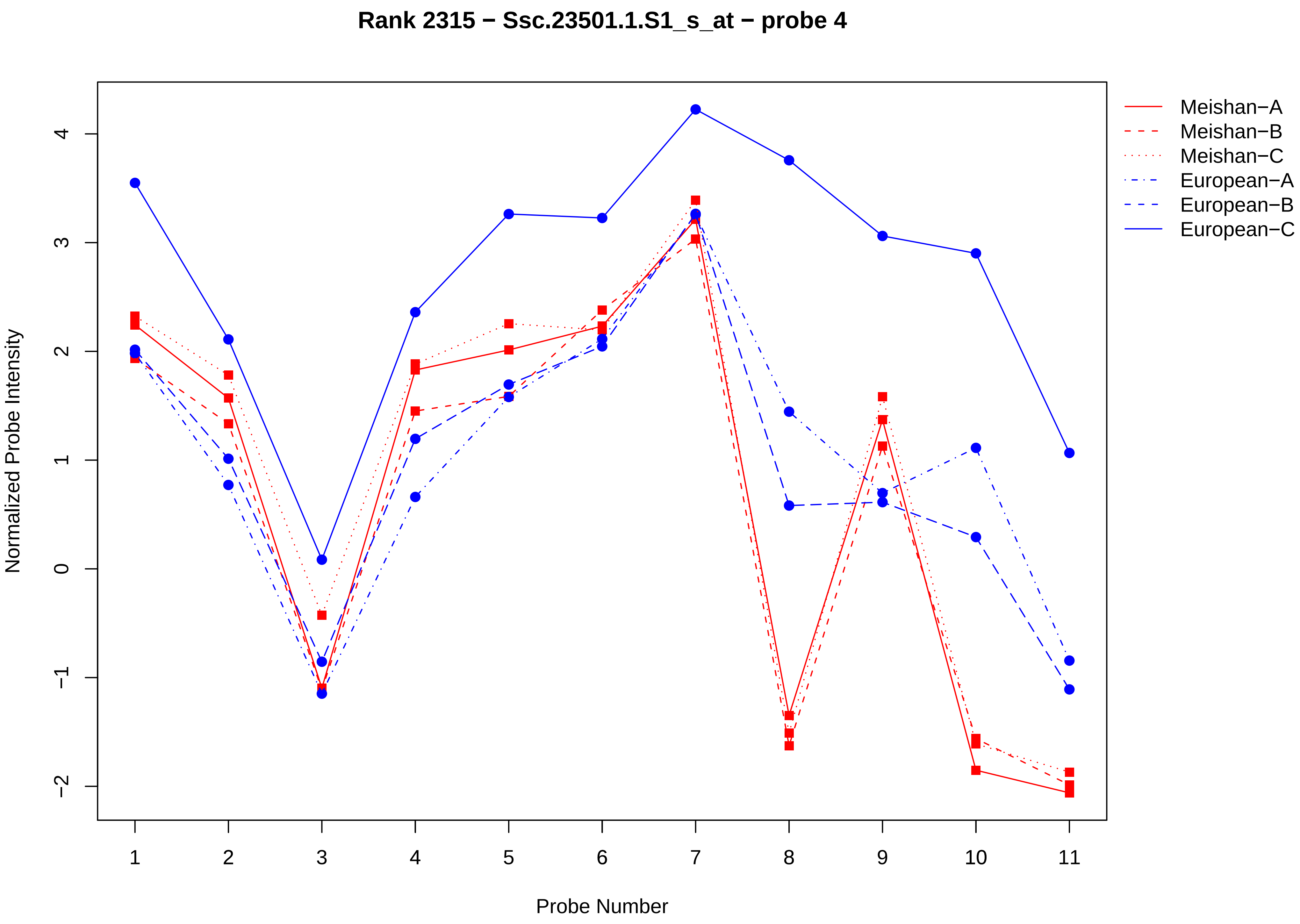
Rank 2315 − Ssc.23501.1.S1_s_at − probe 4
Normalized Probe Intensity
Probe Number
−2
−1
0
1
2
3
4
1
2
3
4
5
6
7
8
9
10
11
Meishan−A
Meishan−B
Meishan−C
European−A
European−B
European−C

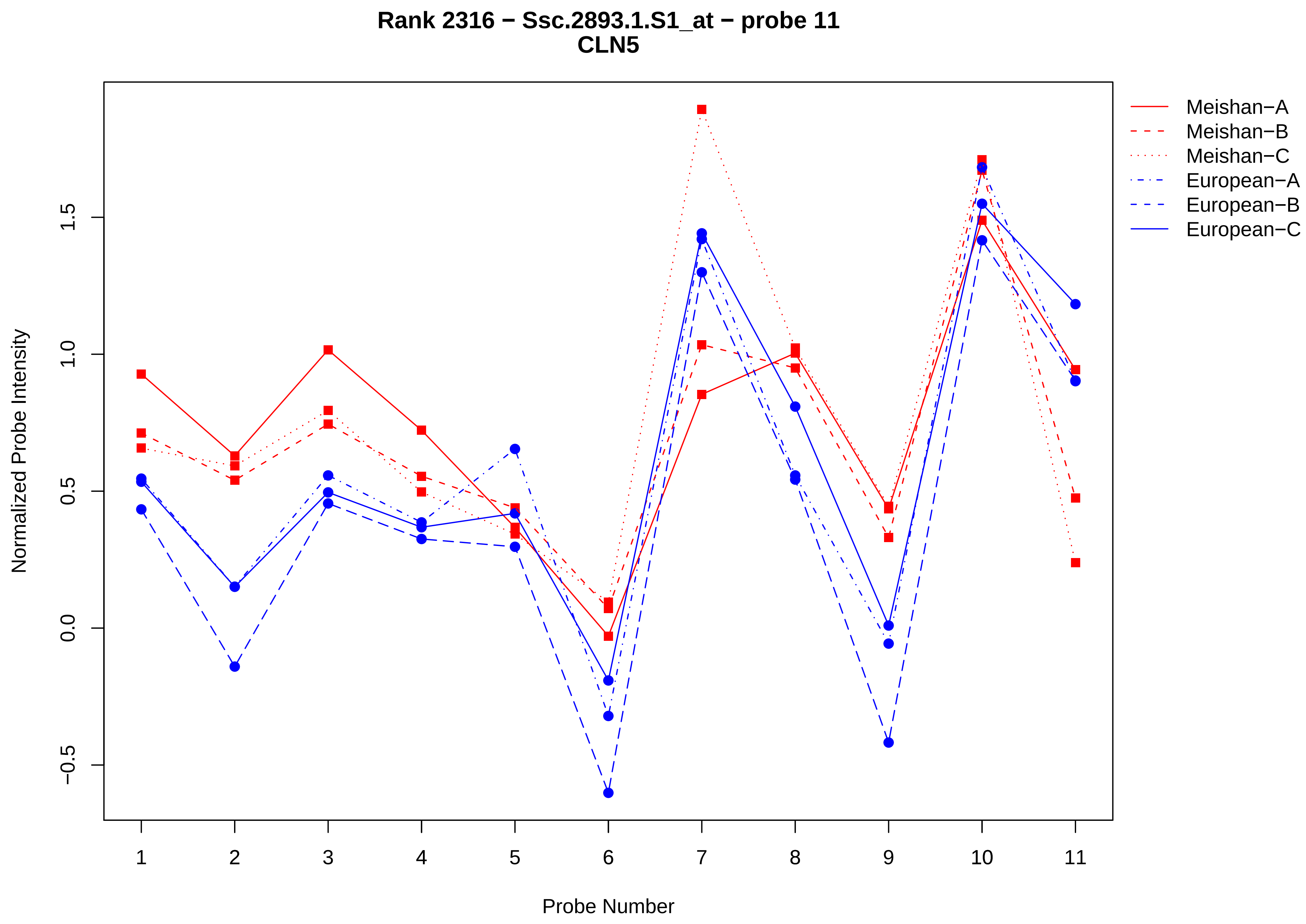

Rank 2316 − Ssc.2893.1.S1_at − probe 11
CLN5
Normalized Probe Intensity
Probe Number
Meishan−A
Meishan−B
Meishan−C
European−A
European−B
European−C

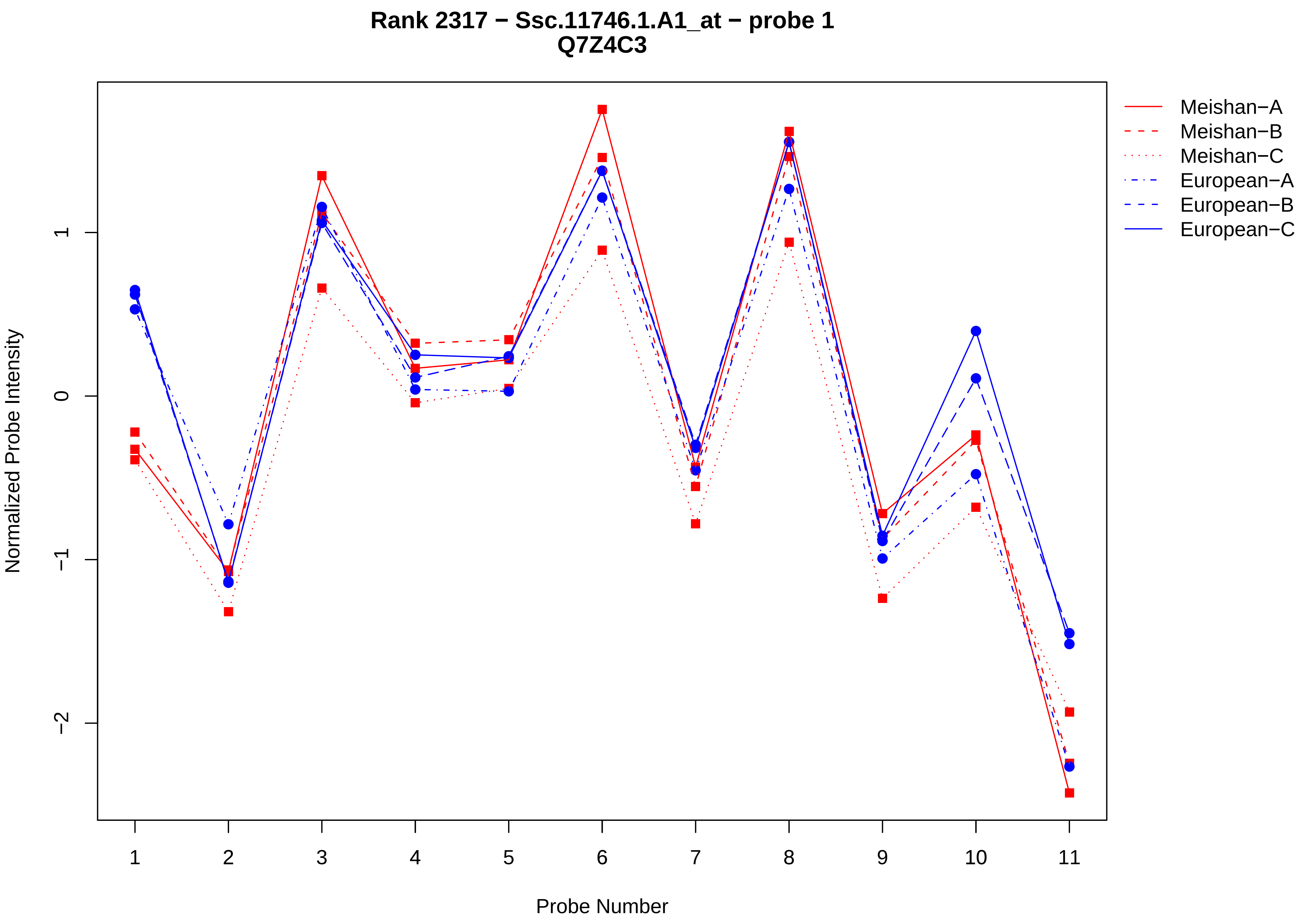

Rank 2317 − Ssc.11746.1.A1_at − probe 1
Q7Z4C3
Normalized Probe Intensity
Probe Number
Meishan−A
Meishan−B
Meishan−C
European−A
European−B
European−C
1
2
3
4
5
6
7
8
9
10
11
−2
−1
0
1

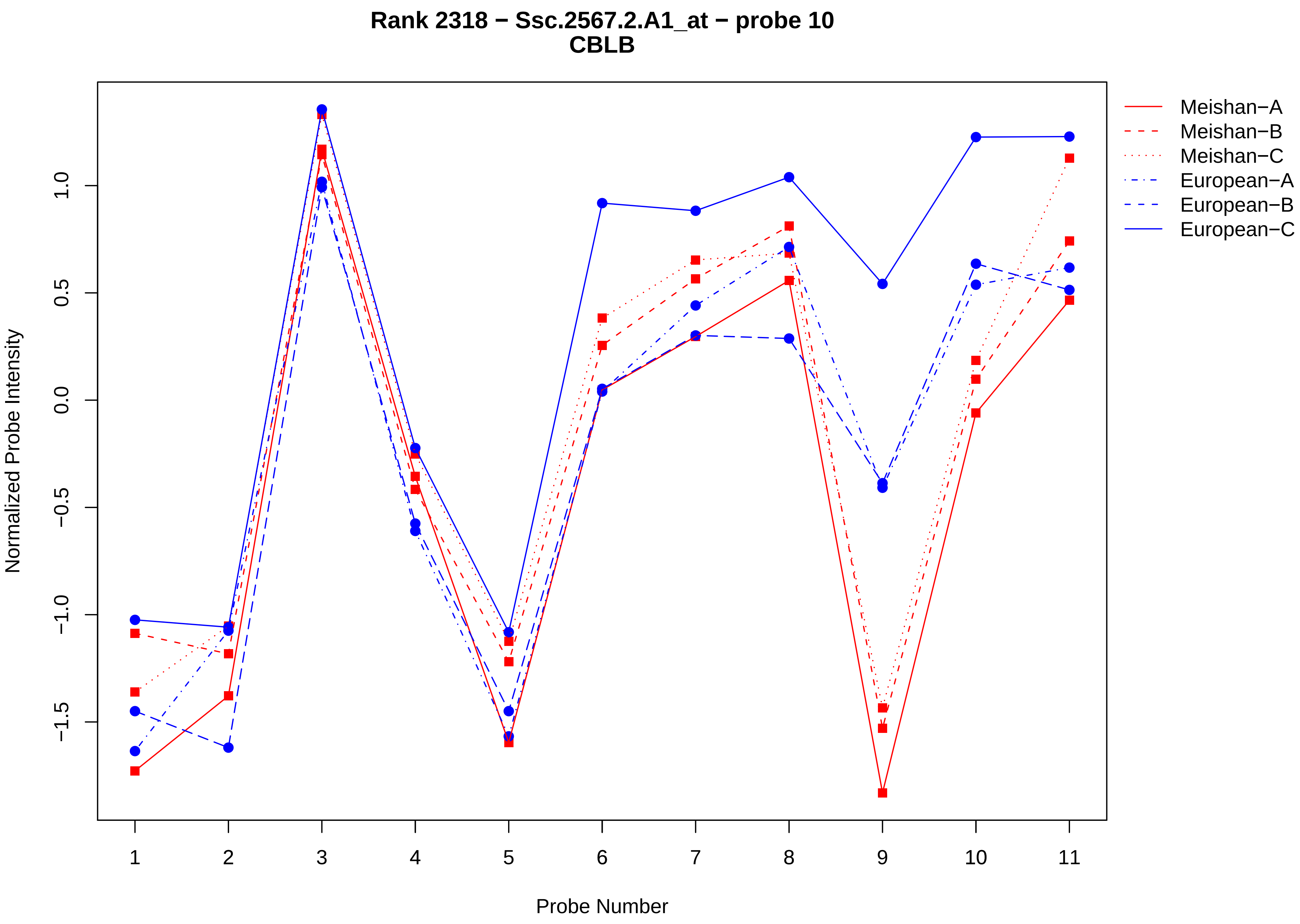

Rank 2318 − Ssc.2567.2.A1_at − probe 10
CBLB
Normalized Probe Intensity
Probe Number
Meishan−A
Meishan−B
Meishan−C
European−A
European−B
European−C

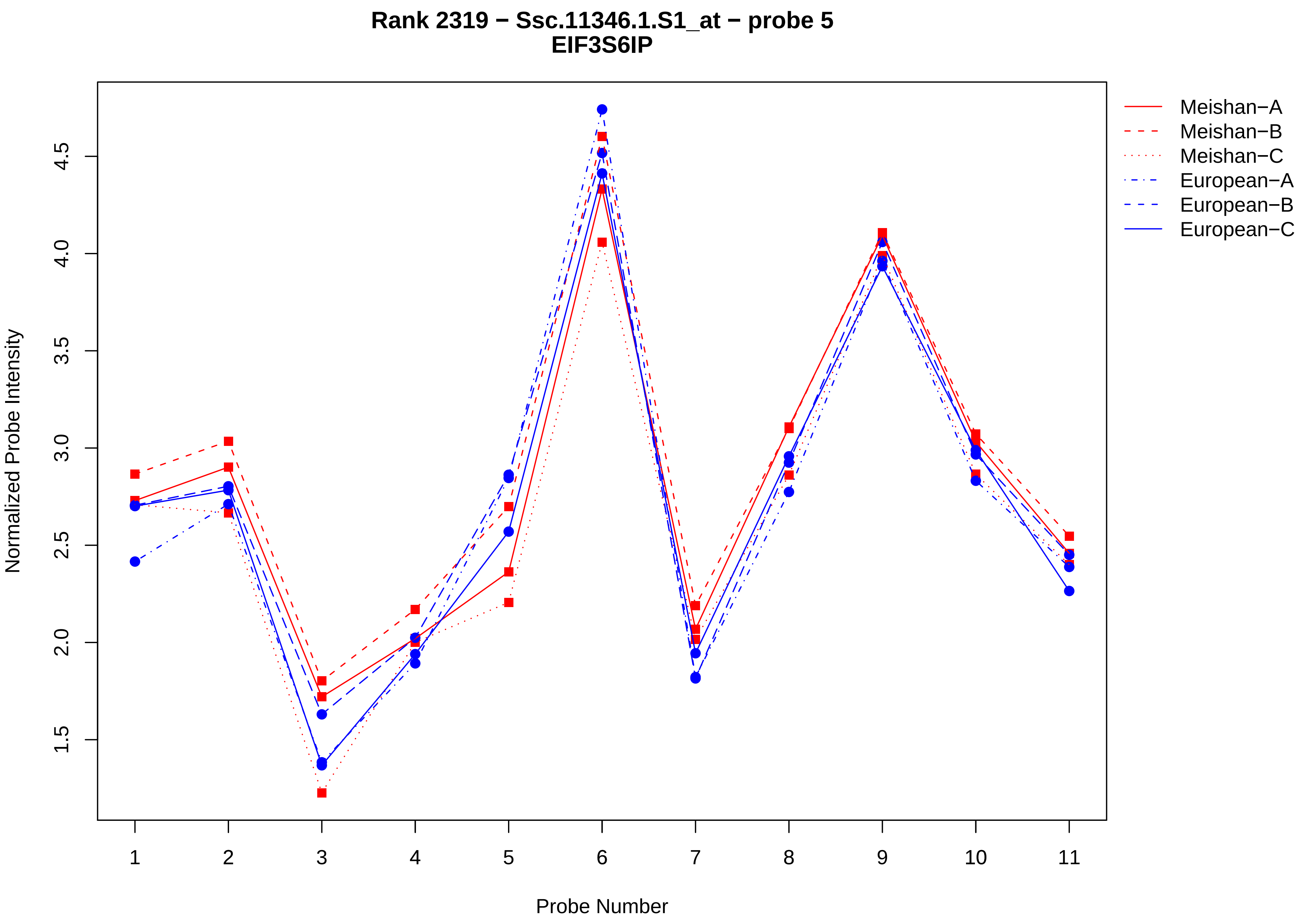

Rank 2319 − Ssc.11346.1.S1_at − probe 5
EIF3S6IP
Normalized Probe Intensity
Probe Number
1.5
2.0
2.5
3.0
3.5
4.0
4.5
1
2
3
4
5
6
7
8
9
10
11
Meishan−A
Meishan−B
Meishan−C
European−A
European−B
European−C

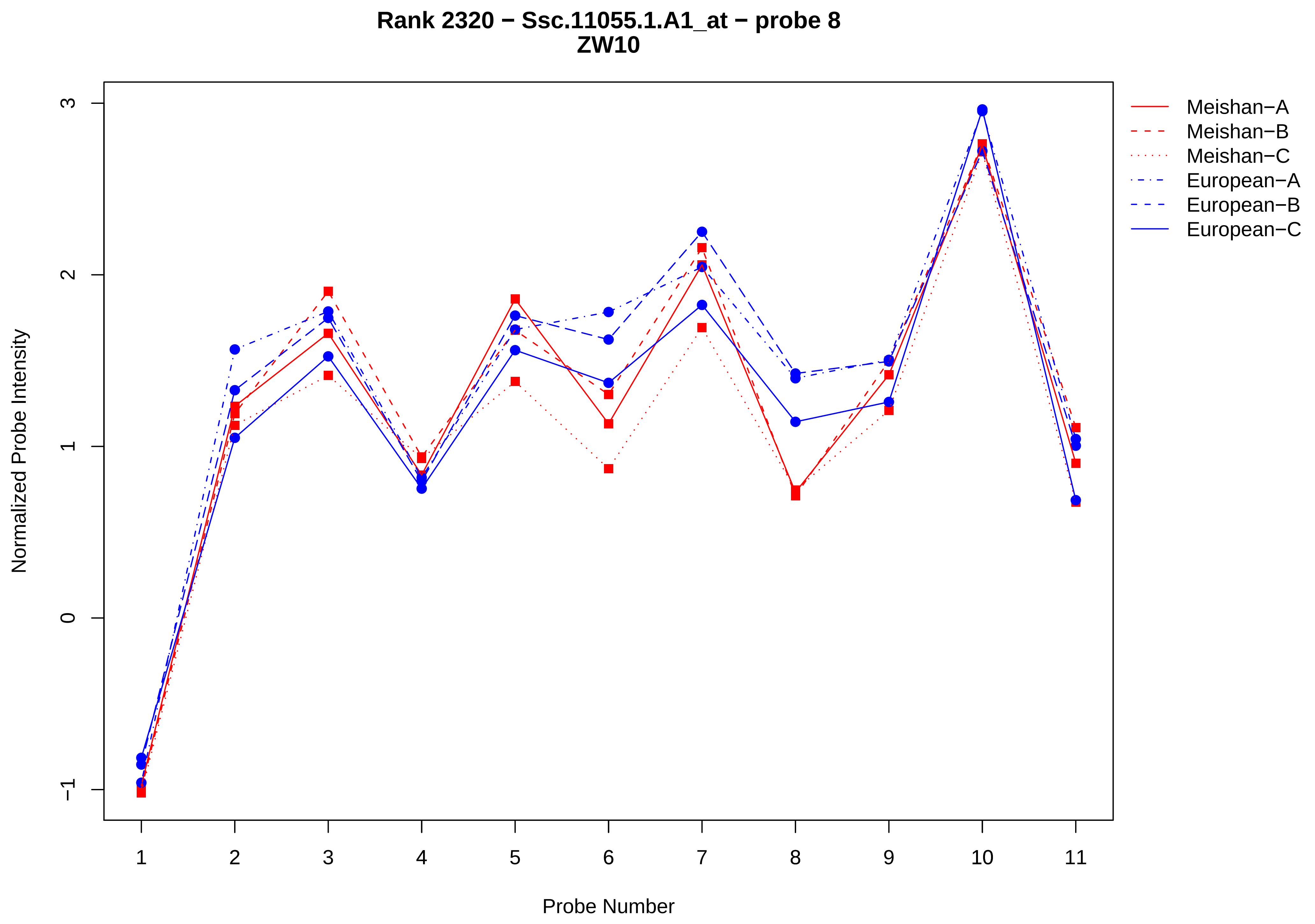

Rank 2320 − Ssc.11055.1.A1_at − probe 8
ZW10
Normalized Probe Intensity
Probe Number
Meishan−A
Meishan−B
Meishan−C
European−A
European−B
European−C

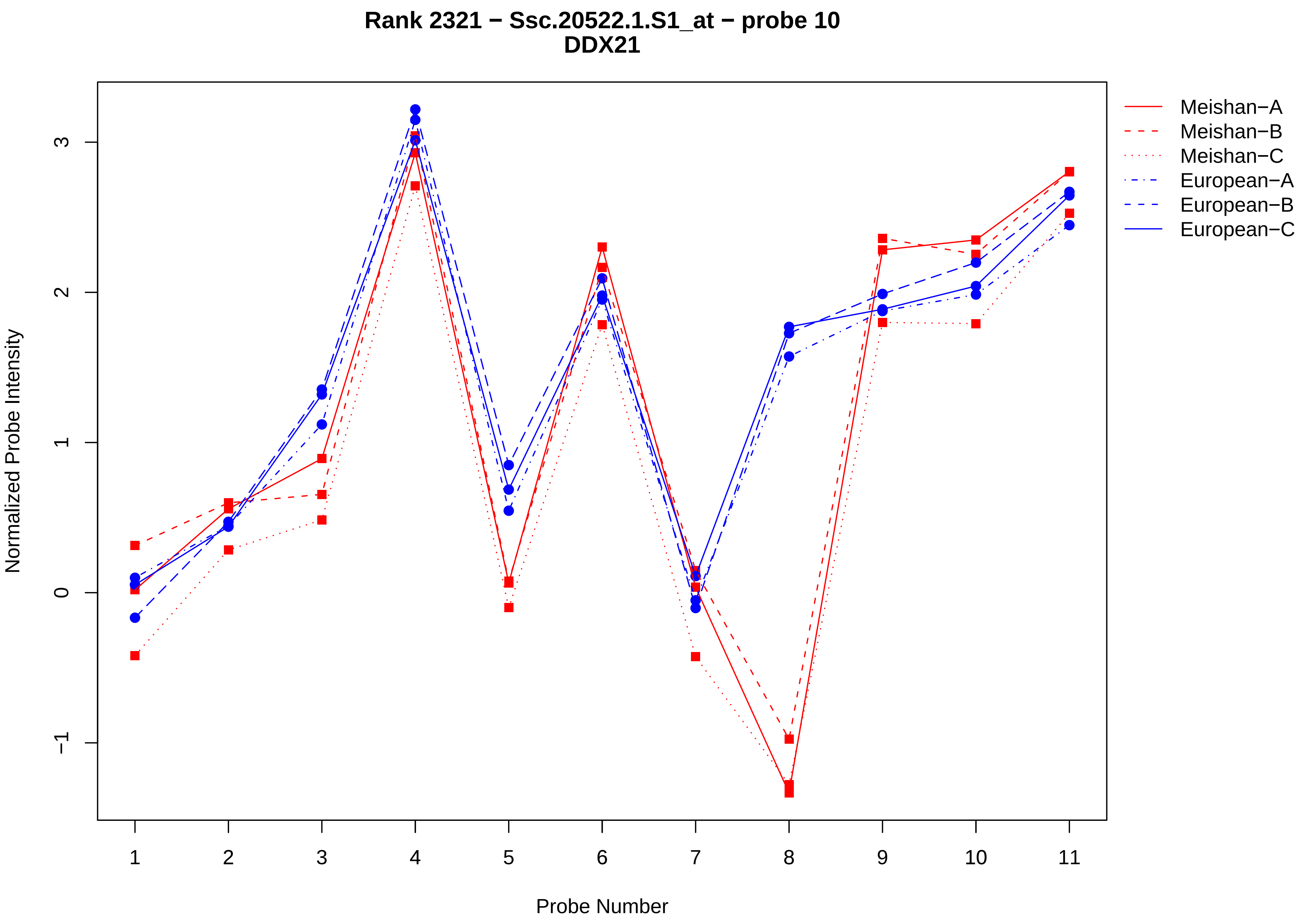

Rank 2321 − Ssc.20522.1.S1_at − probe 10
DDX21
Normalized Probe Intensity
Probe Number
−1
0
1
2
3
1
2
3
4
5
6
7
8
9
10
11
Meishan−A
Meishan−B
Meishan−C
European−A
European−B
European−C

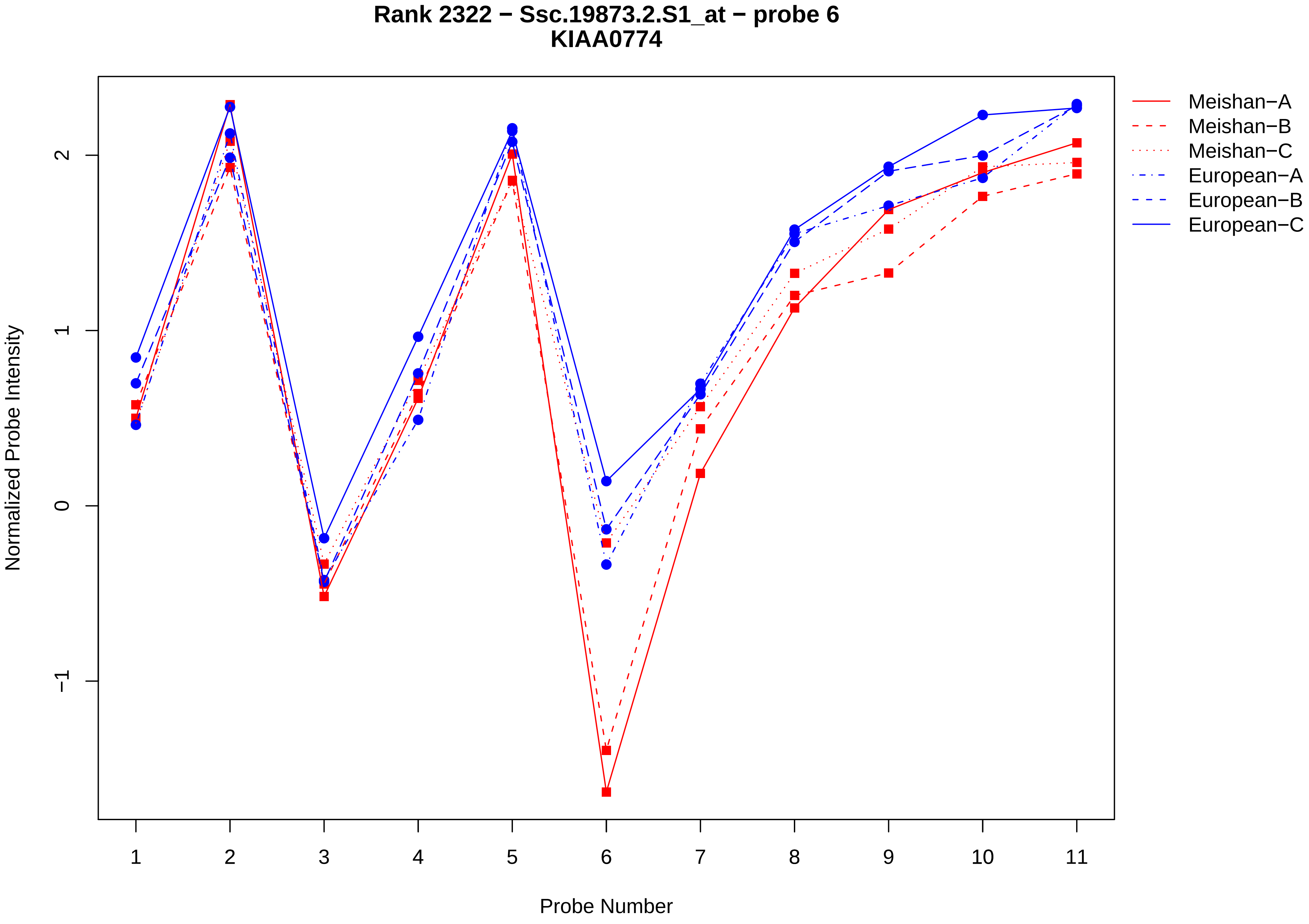

Rank 2322 − Ssc.19873.2.S1_at − probe 6
KIAA0774
Normalized Probe Intensity
Probe Number
−1
0
1
2
1
2
3
4
5
6
7
8
9
10
11
Meishan−A
Meishan−B
Meishan−C
European−A
European−B
European−C

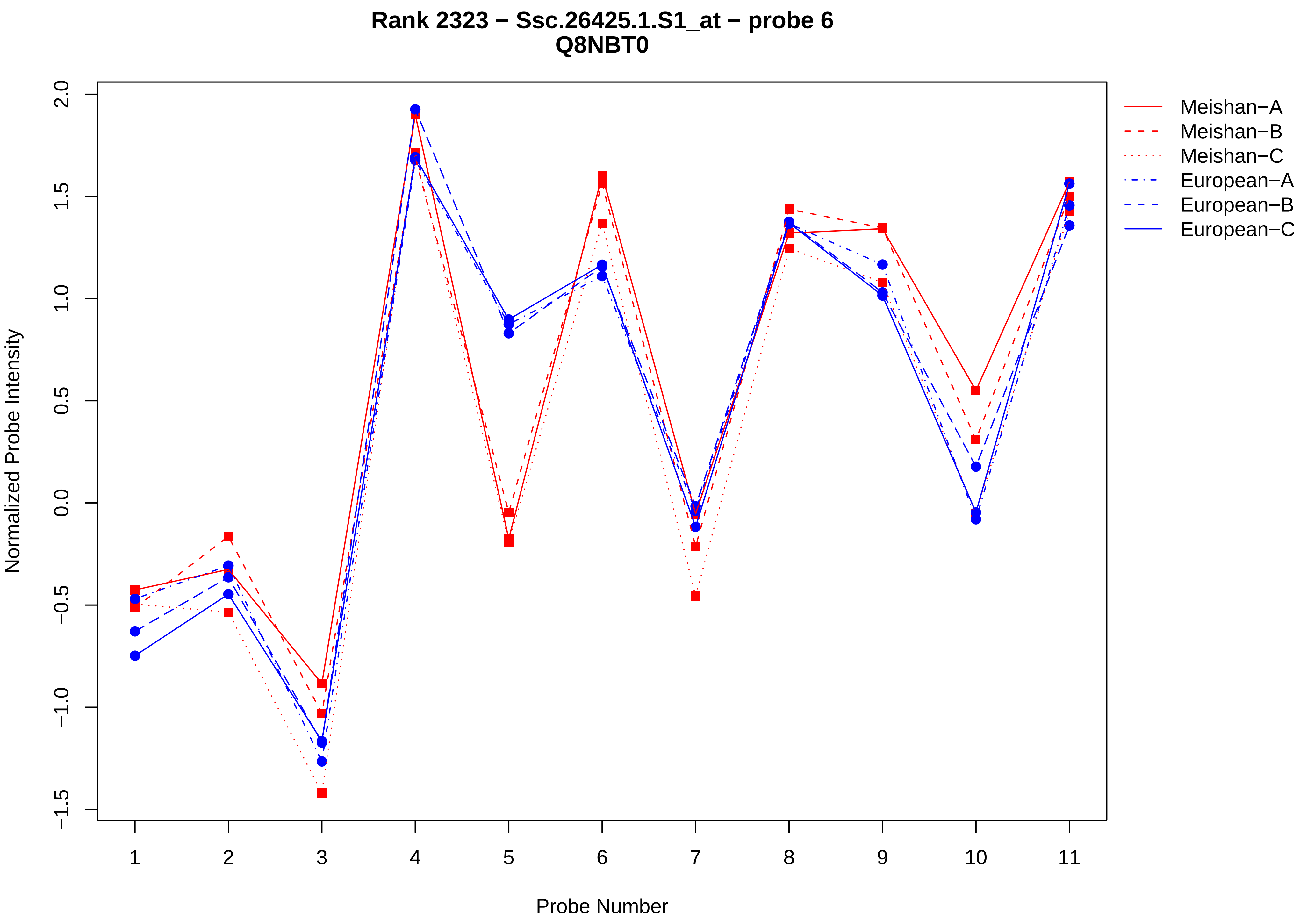
Rank 2323 − Ssc.26425.1.S1_at − probe 6
Q8NBT0
Normalized Probe Intensity
Probe Number
2.0
1.5
1.0
0.5
0.0
−0.5
−1.0
−1.5
1
2
3
4
5
6
7
8
9
10
11
Meishan−A
Meishan−B
Meishan−C
European−A
European−B
European−C

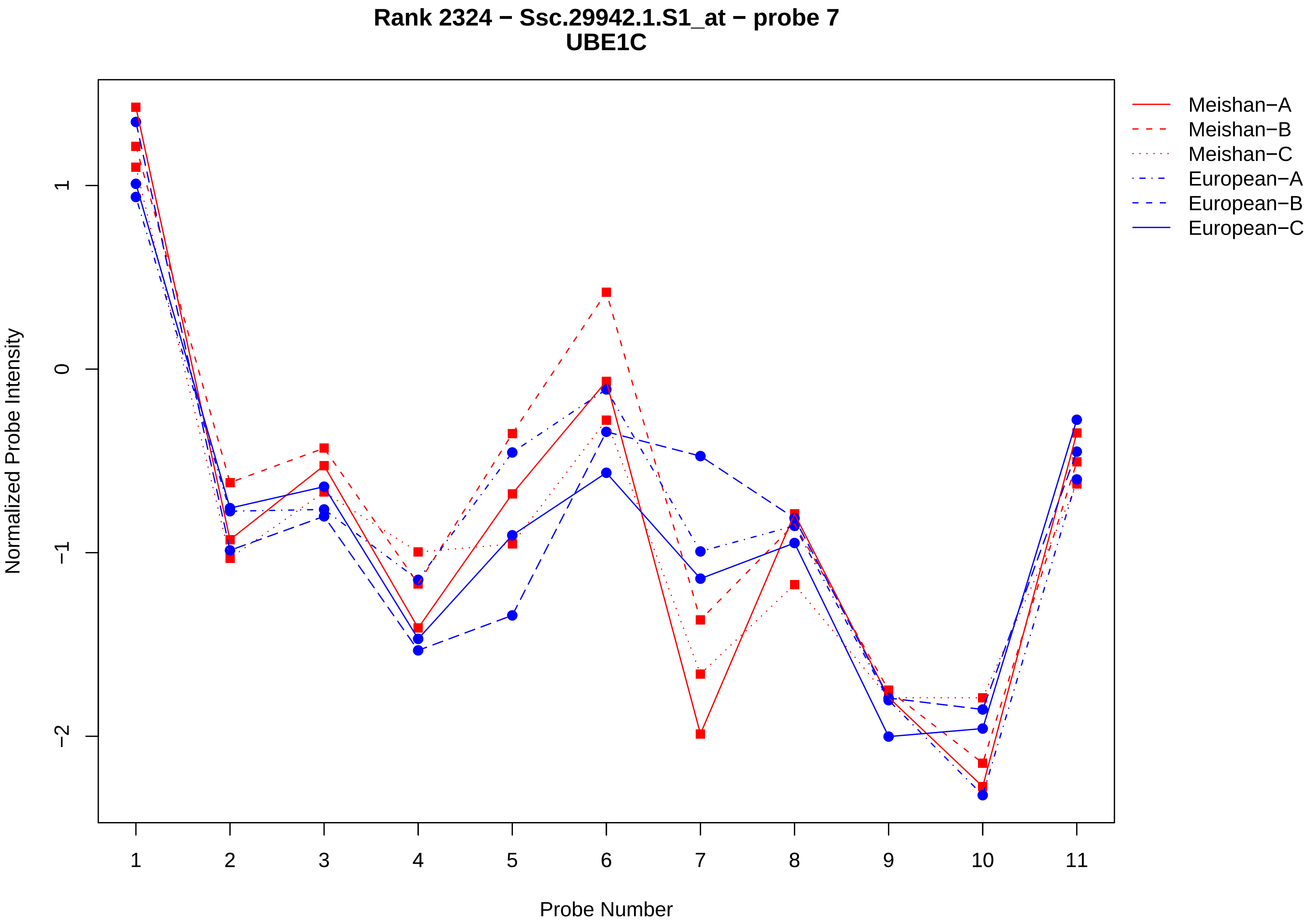

Rank 2324 − Ssc.29942.1.S1_at − probe 7
UBE1C
Normalized Probe Intensity
Probe Number
1
0
−1
−2
1
2
3
4
5
6
7
8
9
10
11
Meishan−A
Meishan−B
Meishan−C
European−A
European−B
European−C

Rank 2325 − Ssc.17577.1.S1_at − probe 1
SHOC2

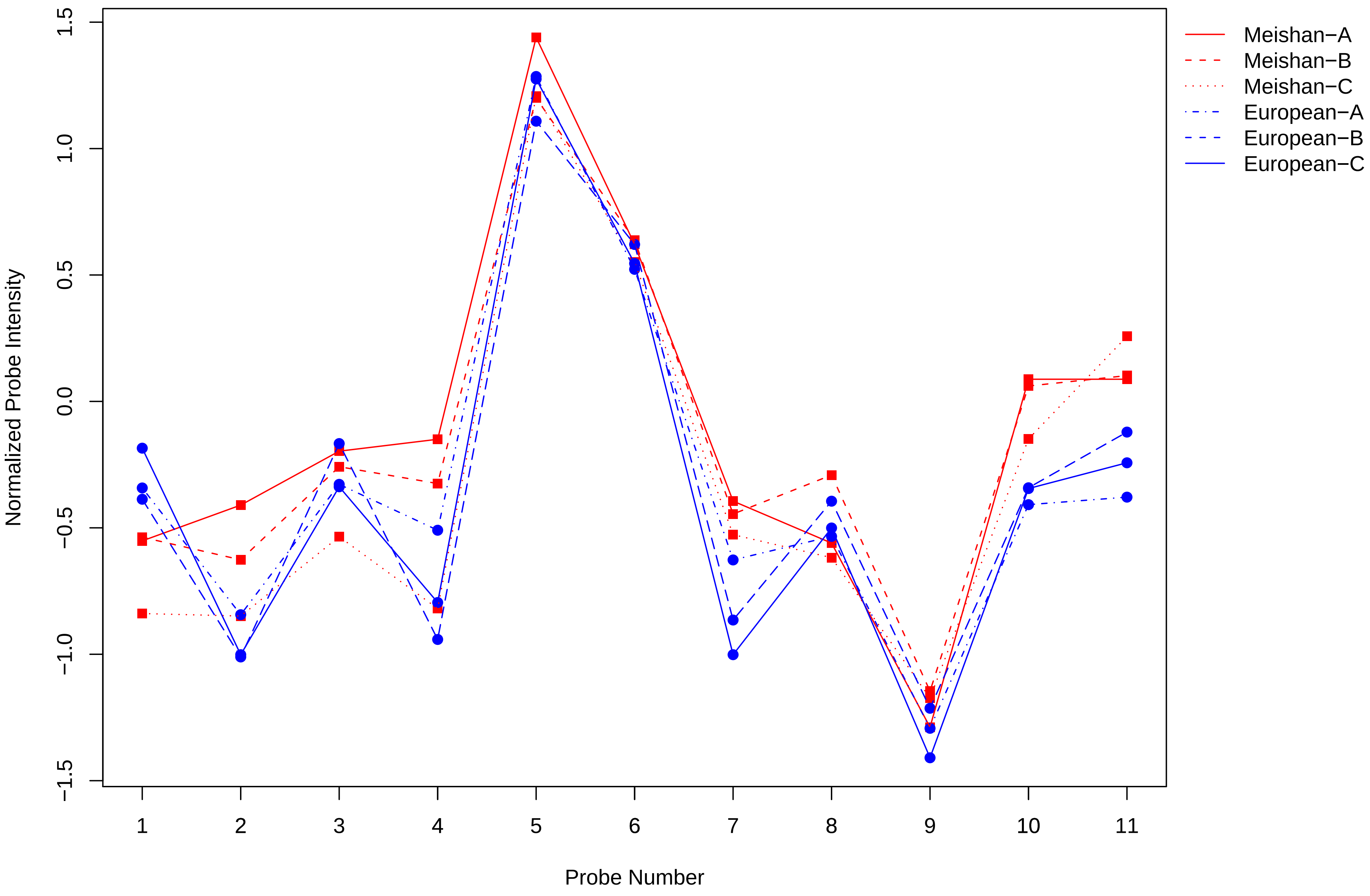

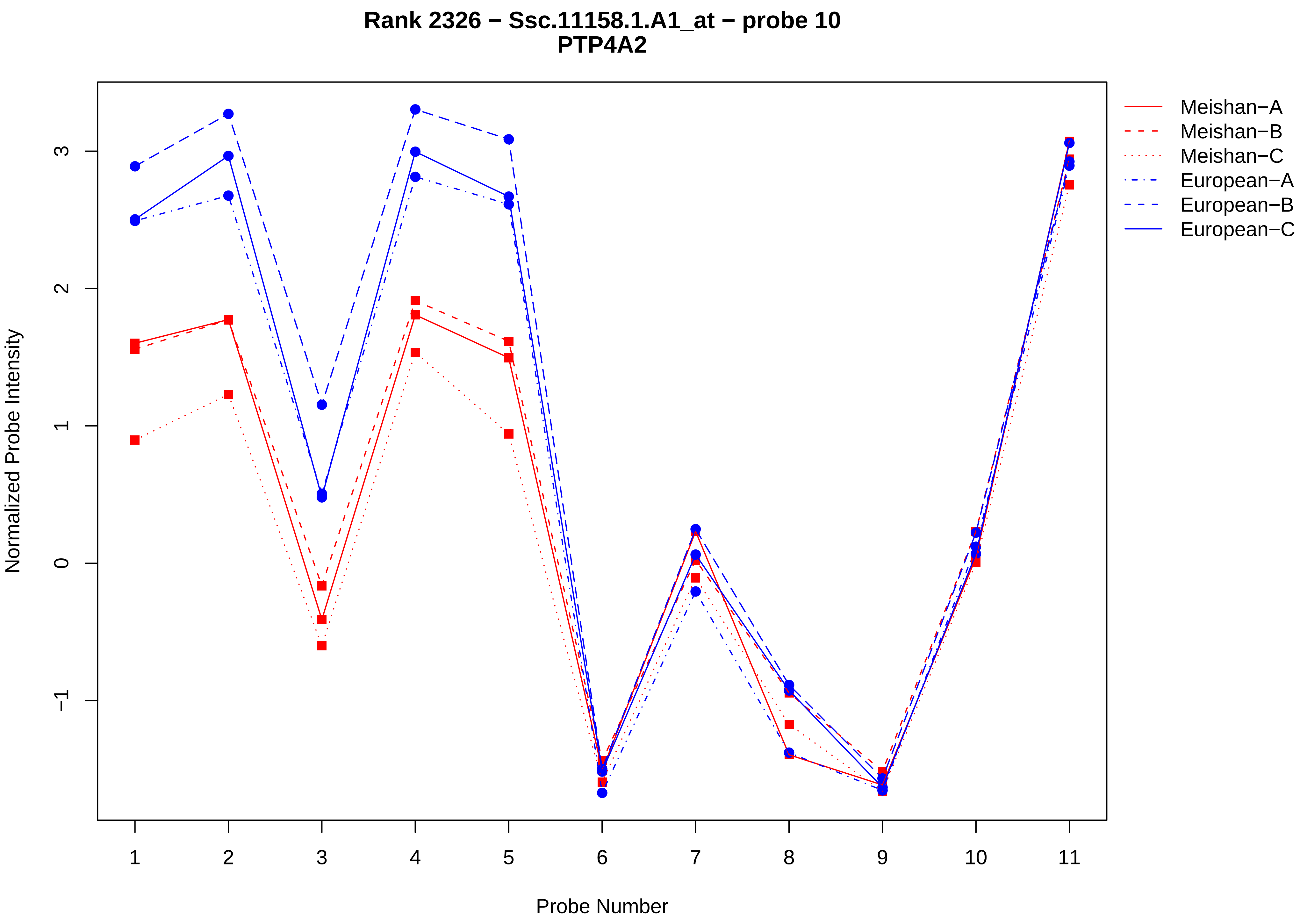
Rank 2326 − Ssc.11158.1.A1_at − probe 10
PTP4A2
Normalized Probe Intensity
Probe Number
−1
0
1
2
3
1
2
3
4
5
6
7
8
9
10
11
Meishan−A
Meishan−B
Meishan−C
European−A
European−B
European−C

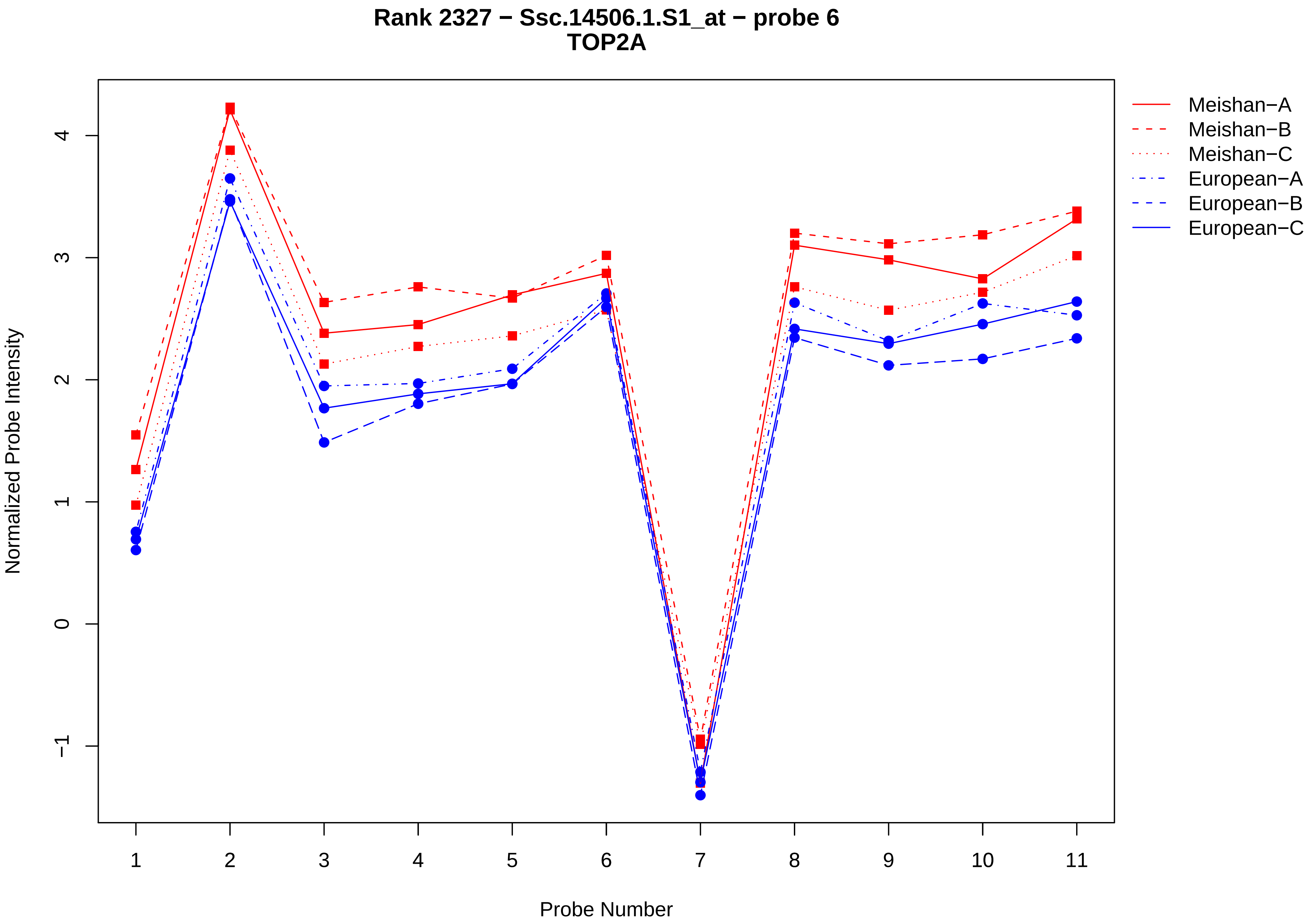

Rank 2327 − Ssc.14506.1.S1_at − probe 6
TOP2A
Normalized Probe Intensity
Probe Number
−1
0
1
2
3
4
1
2
3
4
5
6
7
8
9
10
11
Meishan−A
Meishan−B
Meishan−C
European−A
European−B
European−C

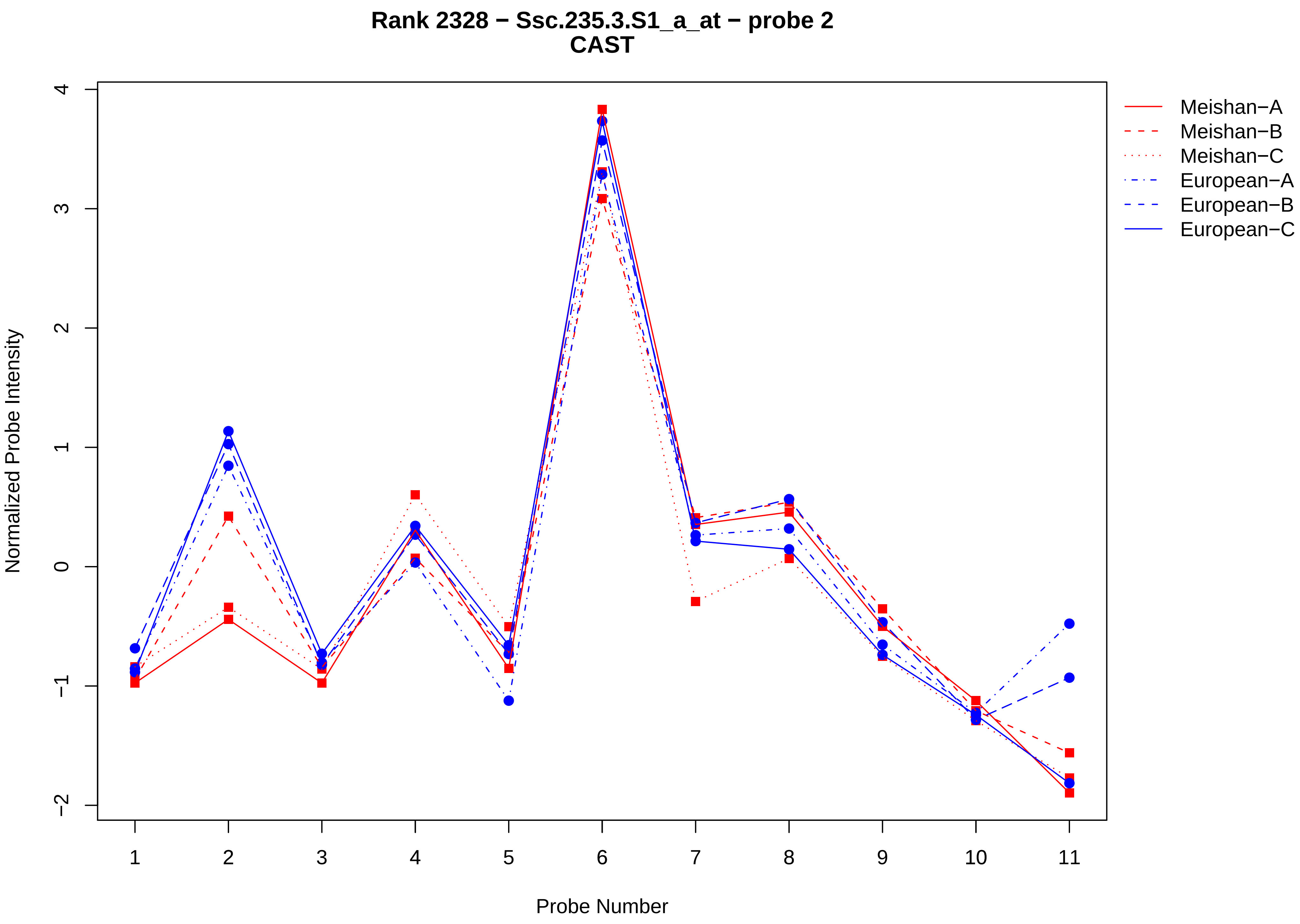

Rank 2328 − Ssc.235.3.S1_a_at − probe 2
CAST
Normalized Probe Intensity
Probe Number
Meishan−A
Meishan−B
Meishan−C
European−A
European−B
European−C
4
3
2
1
0
−1
−2
1
2
3
4
5
6
7
8
9
10
11

Rank 2329 − Ssc.11096.1.S1_at − probe 8
OXCT1

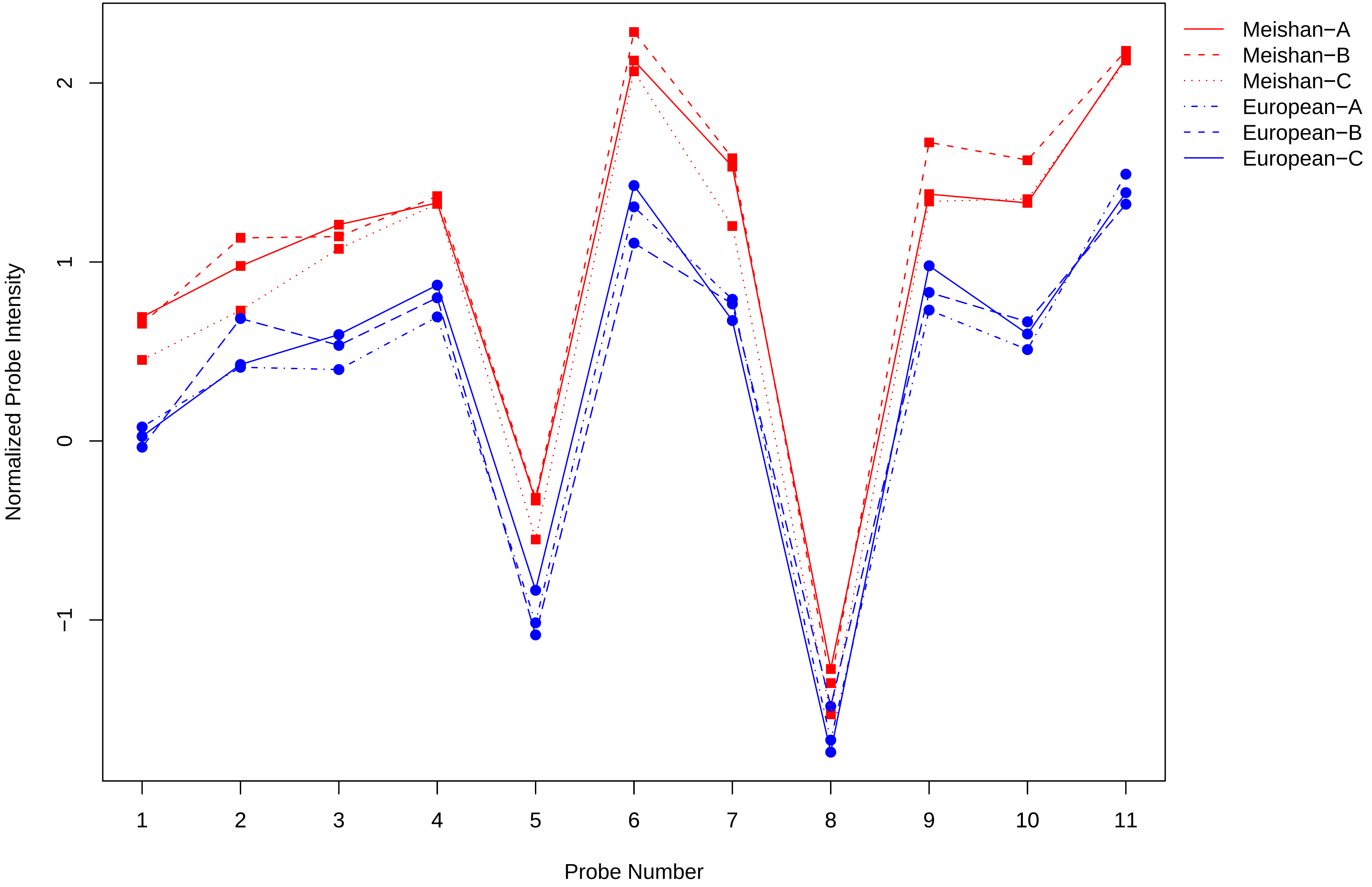

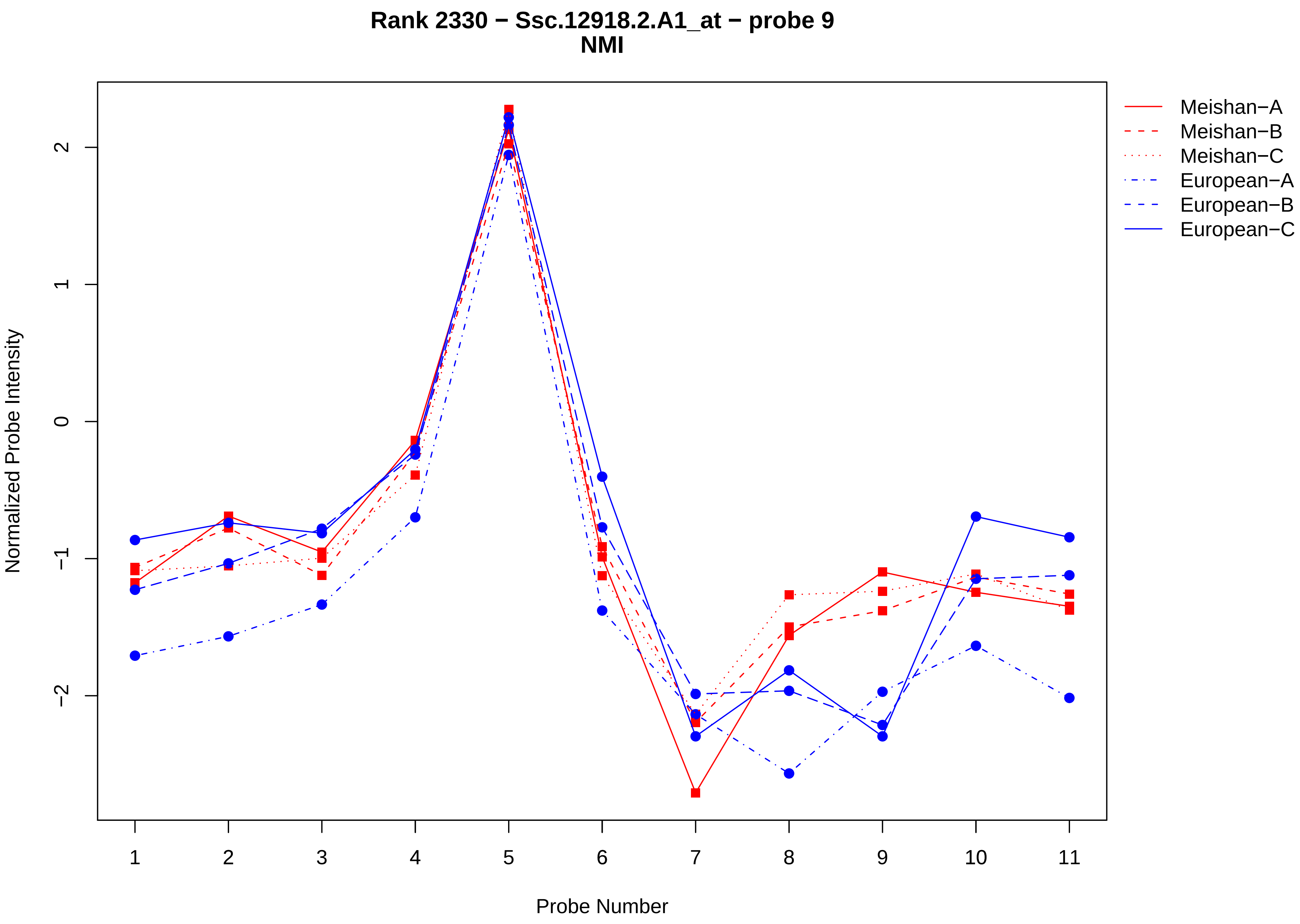

Rank 2330 − Ssc.12918.2.A1_at − probe 9
NMI
Normalized Probe Intensity
Probe Number
Meishan−A
Meishan−B
Meishan−C
European−A
European−B
European−C
−2
−1
0
1
2
1
2
3
4
5
6
7
8
9
10
11

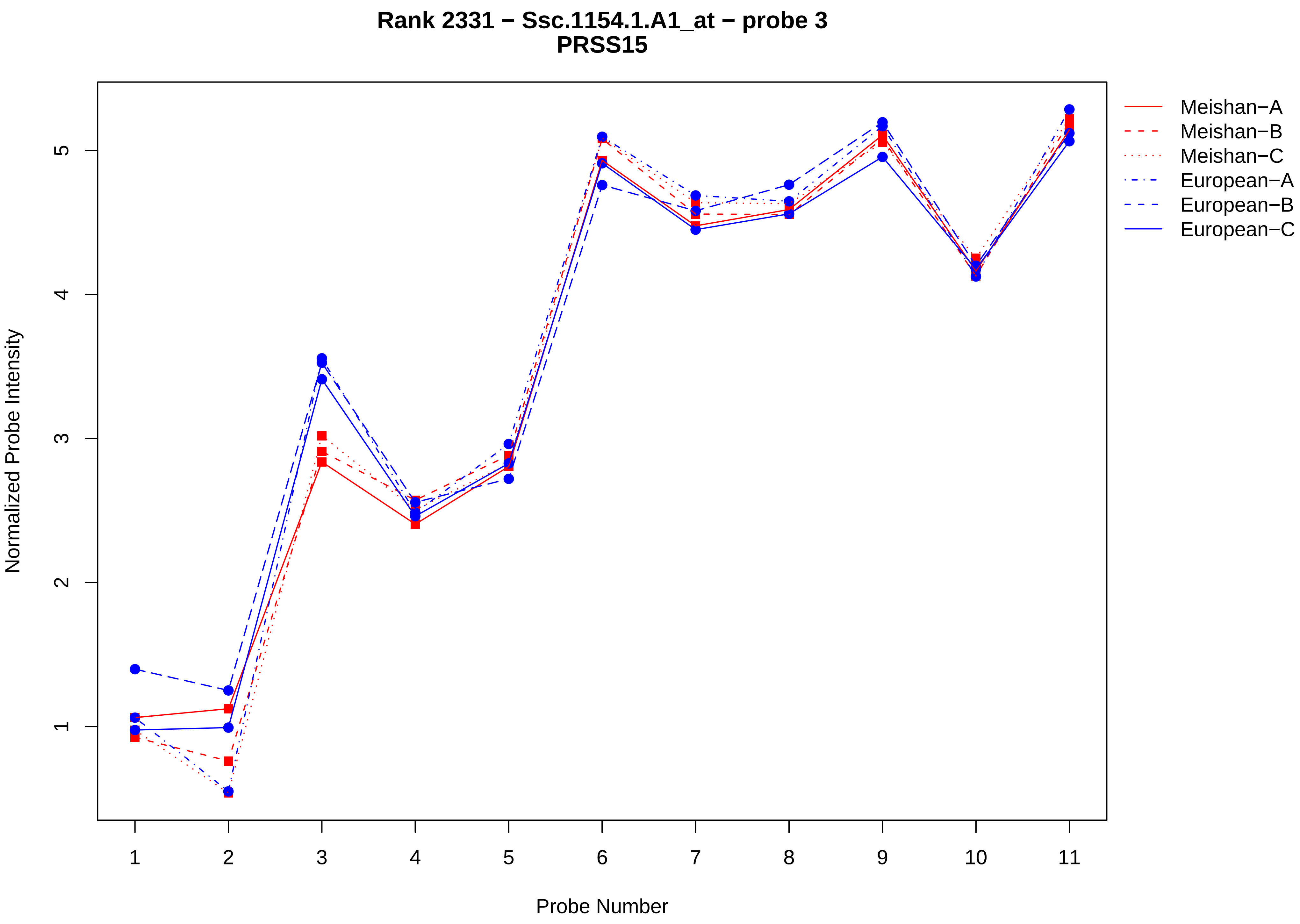

Rank 2331 − Ssc.1154.1.A1_at − probe 3
PRSS15
Normalized Probe Intensity
Probe Number
1
2
3
4
5
1
2
3
4
5
6
7
8
9
10
11
Meishan−A
Meishan−B
Meishan−C
European−A
European−B
European−C

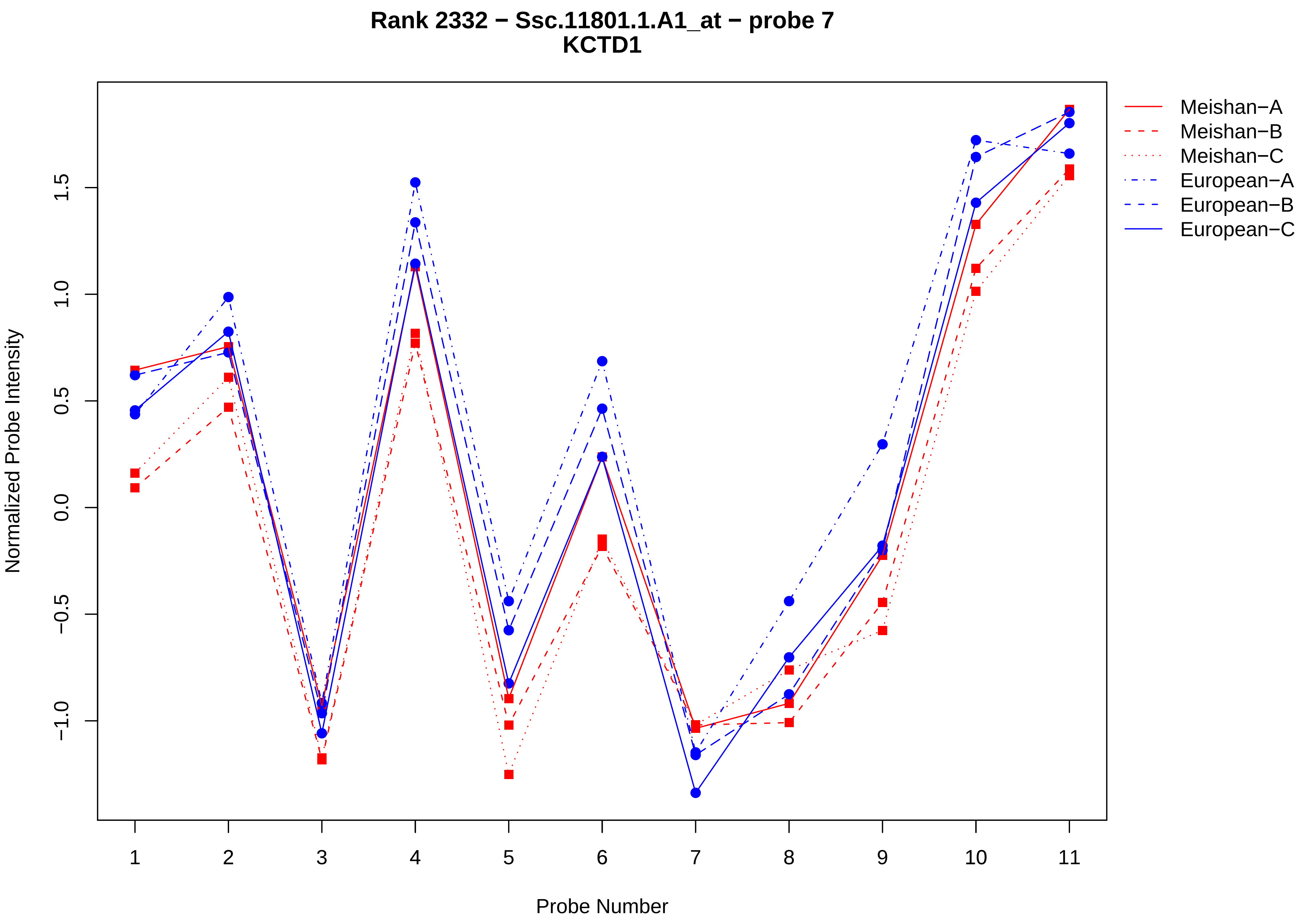

Rank 2332 − Ssc.11801.1.A1_at − probe 7
KCTD1
Normalized Probe Intensity
Probe Number
1.5
1.0
0.5
0.0
−0.5
−1.0
1
2
3
4
5
6
7
8
9
10
11
Meishan−A
Meishan−B
Meishan−C
European−A
European−B
European−C

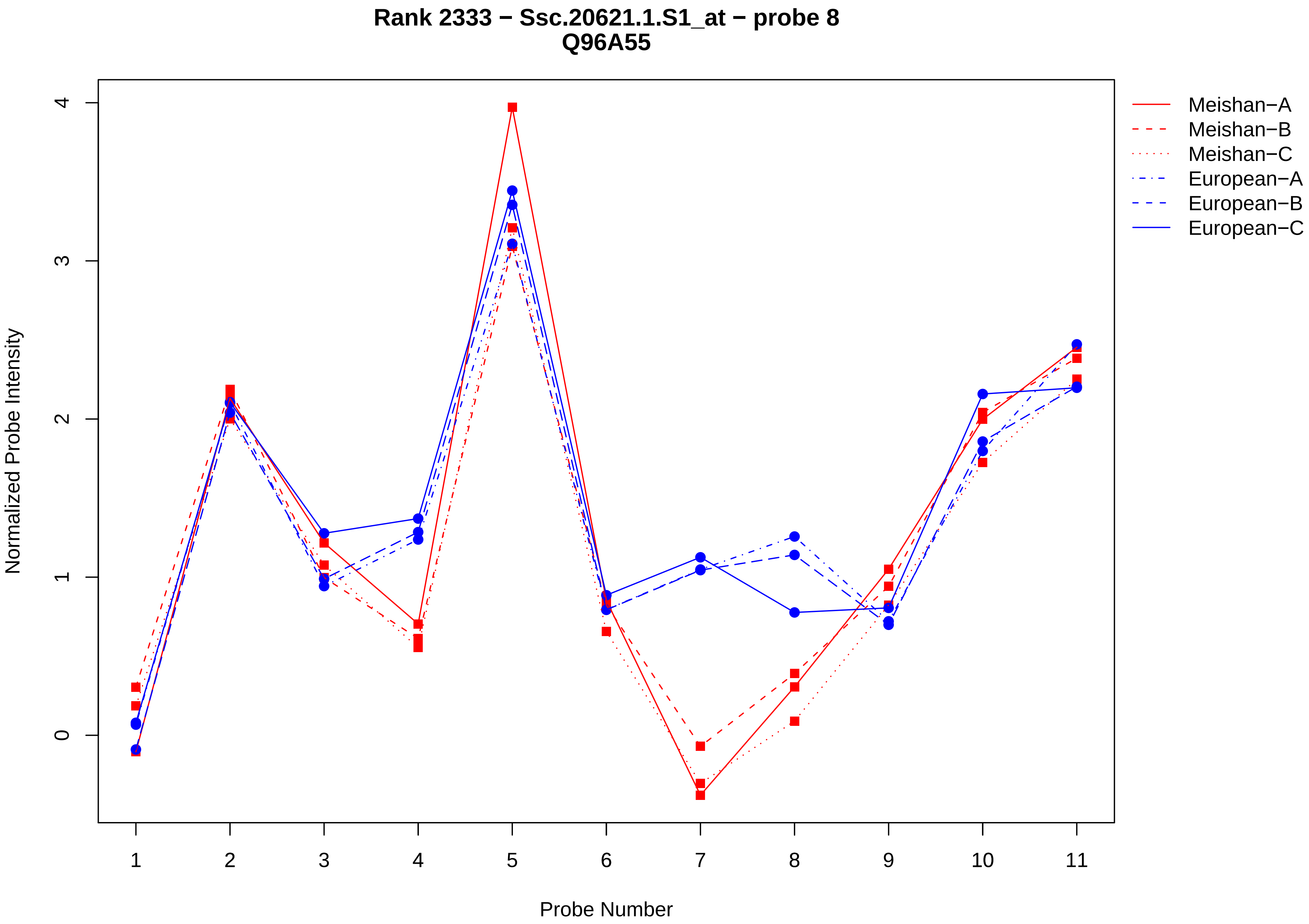
Rank 2333 − Ssc.20621.1.S1_at − probe 8
Q96A55
Normalized Probe Intensity
Probe Number
0
1
2
3
4
1
2
3
4
5
6
7
8
9
10
11
Meishan−A
Meishan−B
Meishan−C
European−A
European−B
European−C

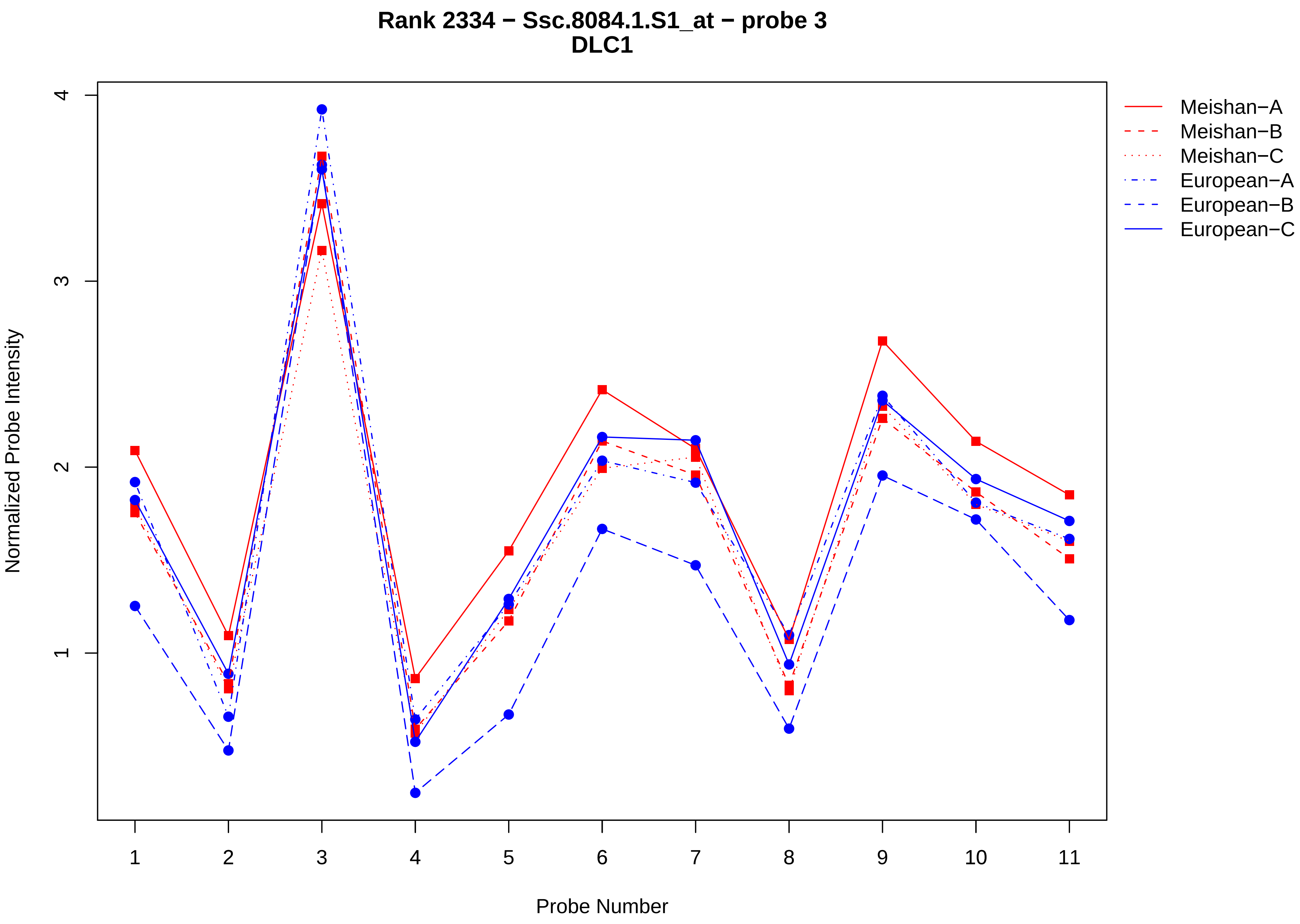
Rank 2334 − Ssc.8084.1.S1_at − probe 3
DLC1
Normalized Probe Intensity
1
2
3
4
Probe Number
1
2
3
4
5
6
7
8
9
10
11
Meishan−A
Meishan−B
Meishan−C
European−A
European−B
European−C

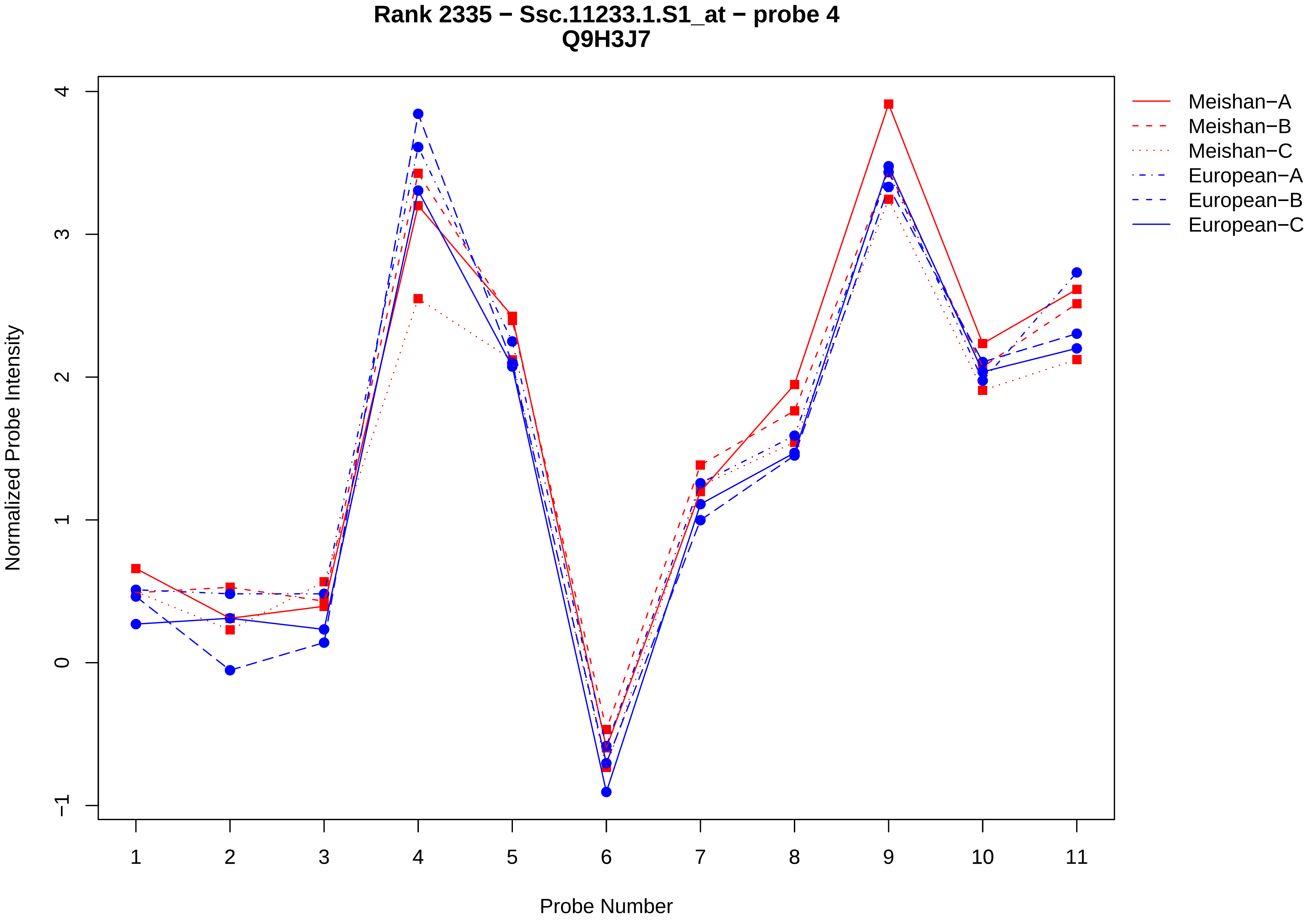

Rank 2335 − Ssc.11233.1.S1_at − probe 4
Q9H3J7
Normalized Probe Intensity
Probe Number
−1
0
1
2
3
4
1
2
3
4
5
6
7
8
9
10
11
Meishan−A
Meishan−B
Meishan−C
European−A
European−B
European−C

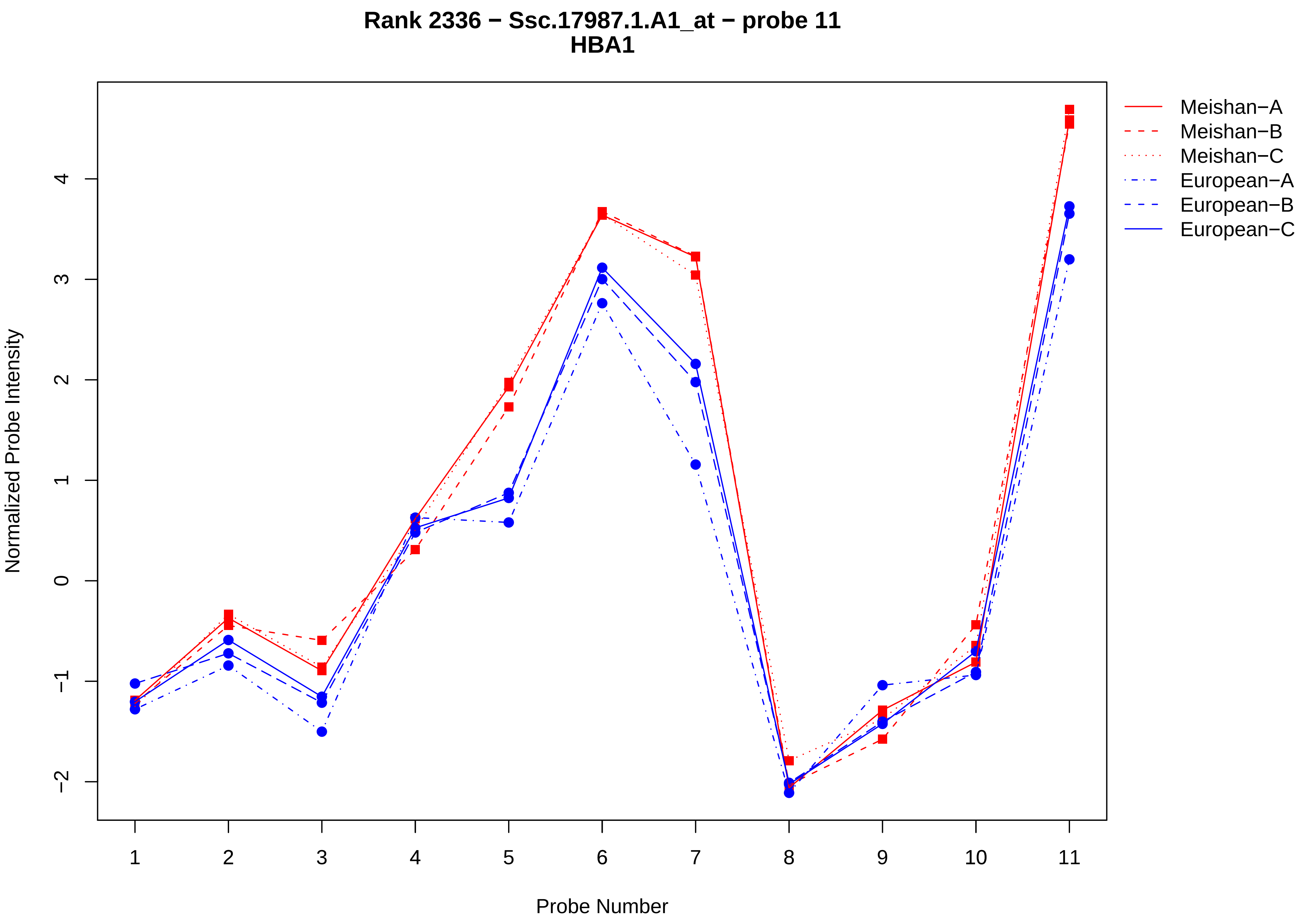

Rank 2336 − Ssc.17987.1.A1_at − probe 11
HBA1
Normalized Probe Intensity
Probe Number
Meishan−A
Meishan−B
Meishan−C
European−A
European−B
European−C
−2
−1
0
1
2
3
4
1
2
3
4
5
6
7
8
9
10
11

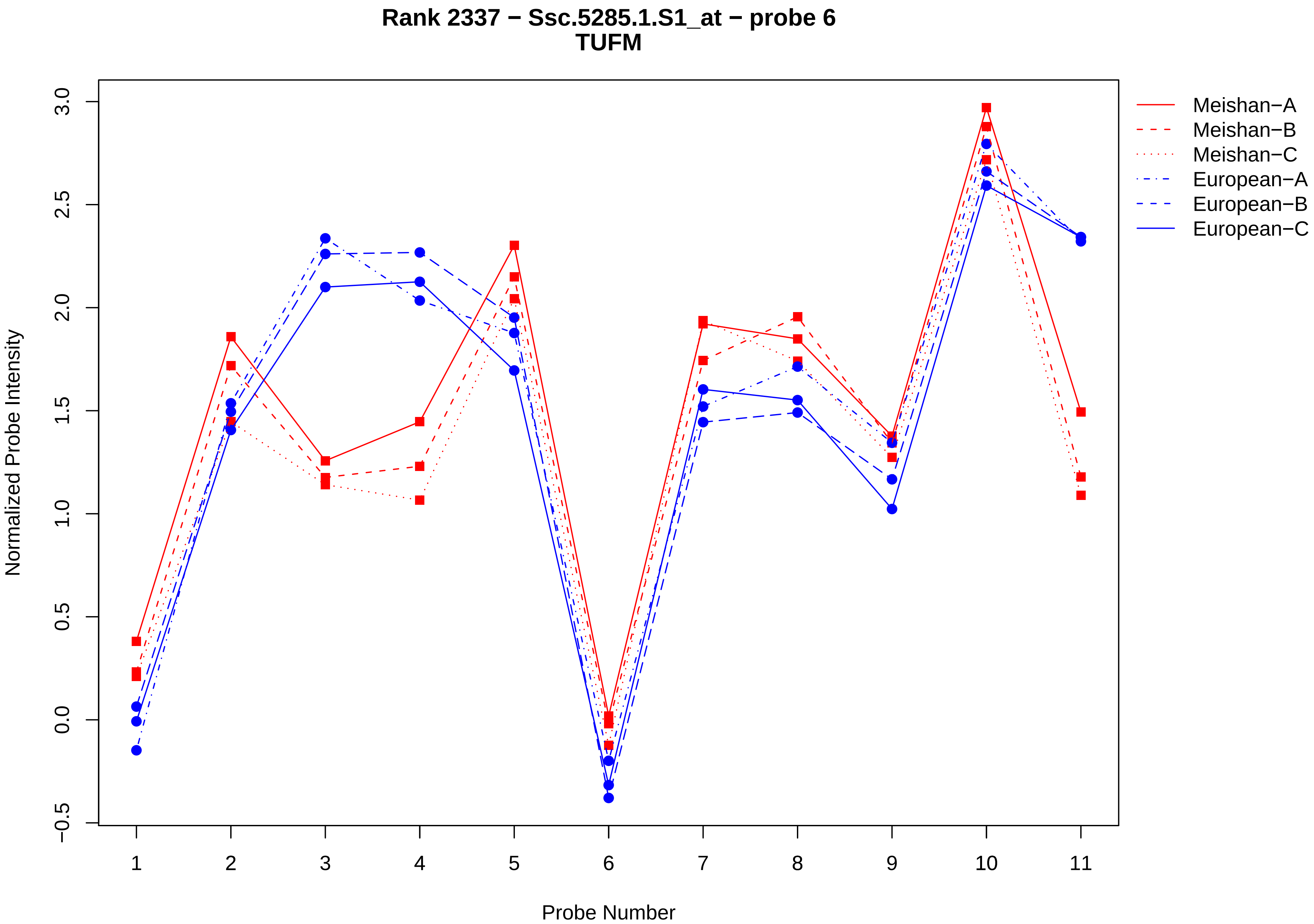

Rank 2337 − Ssc.5285.1.S1_at − probe 6
TUFM
Normalized Probe Intensity
Probe Number
−0.5
0.0
0.5
1.0
1.5
2.0
2.5
3.0
1
2
3
4
5
6
7
8
9
10
11
Meishan−A
Meishan−B
Meishan−C
European−A
European−B
European−C

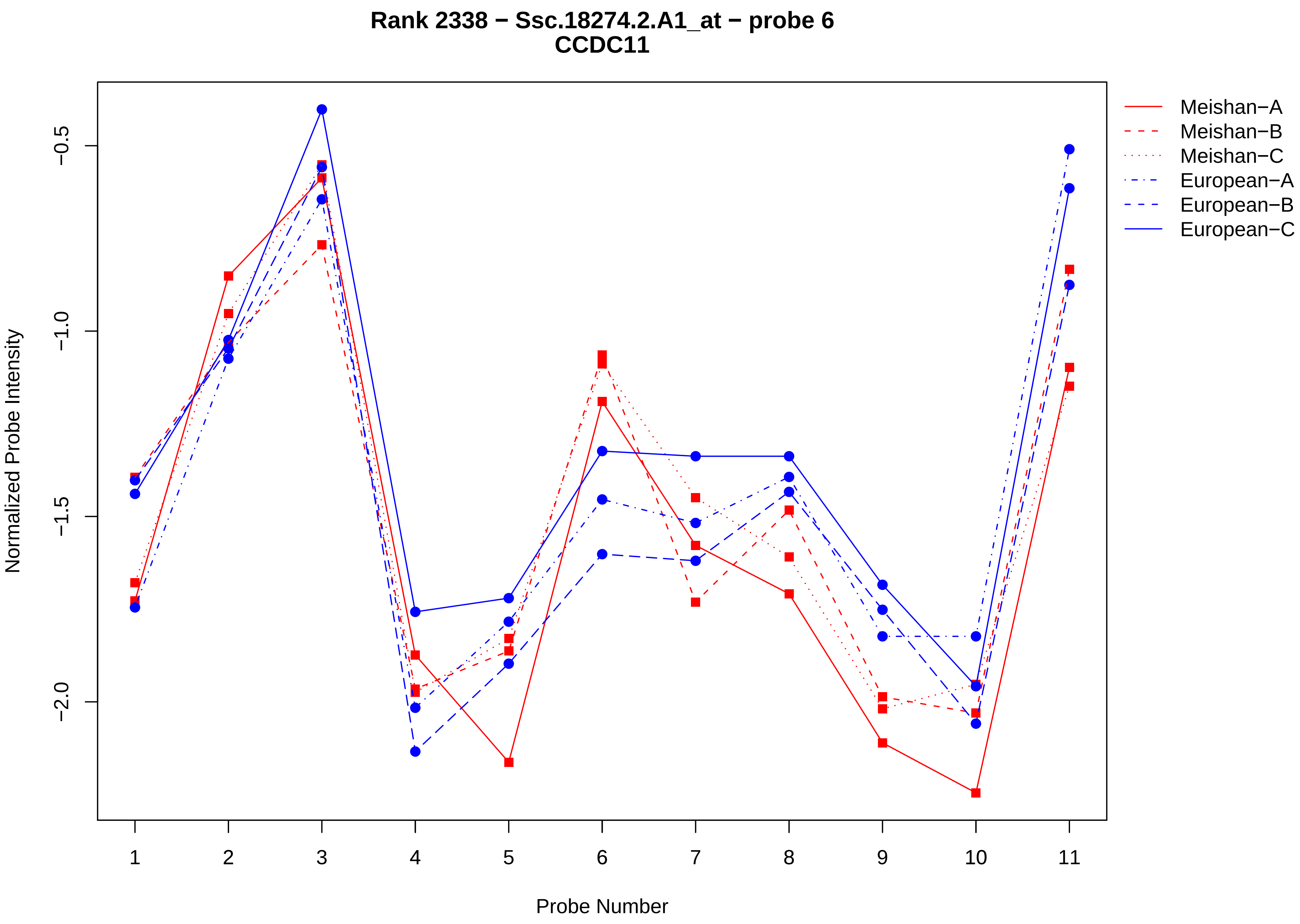
Rank 2338 − Ssc.18274.2.A1_at − probe 6
CCDC11
Normalized Probe Intensity
−0.5
−1.0
−1.5
−2.0
Probe Number
1
2
3
4
5
6
7
8
9
10
11
Meishan−A
Meishan−B
Meishan−C
European−A
European−B
European−C

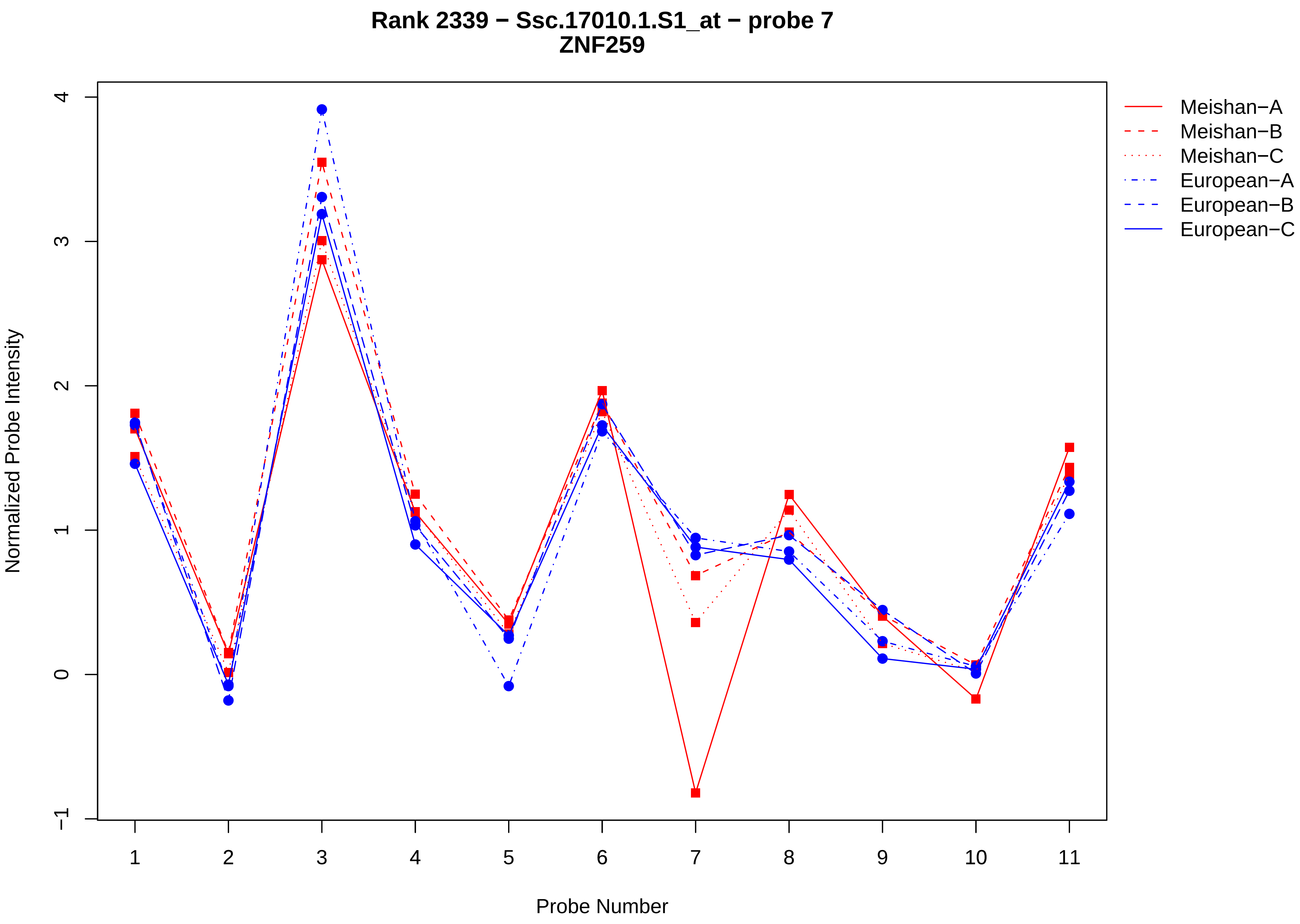

Rank 2339 − Ssc.17010.1.S1_at − probe 7
ZNF259
Normalized Probe Intensity
Probe Number
Meishan−A
Meishan−B
Meishan−C
European−A
European−B
European−C

Rank 2340 − Ssc.1527.2.A1_at − probe 6
SLC20A1

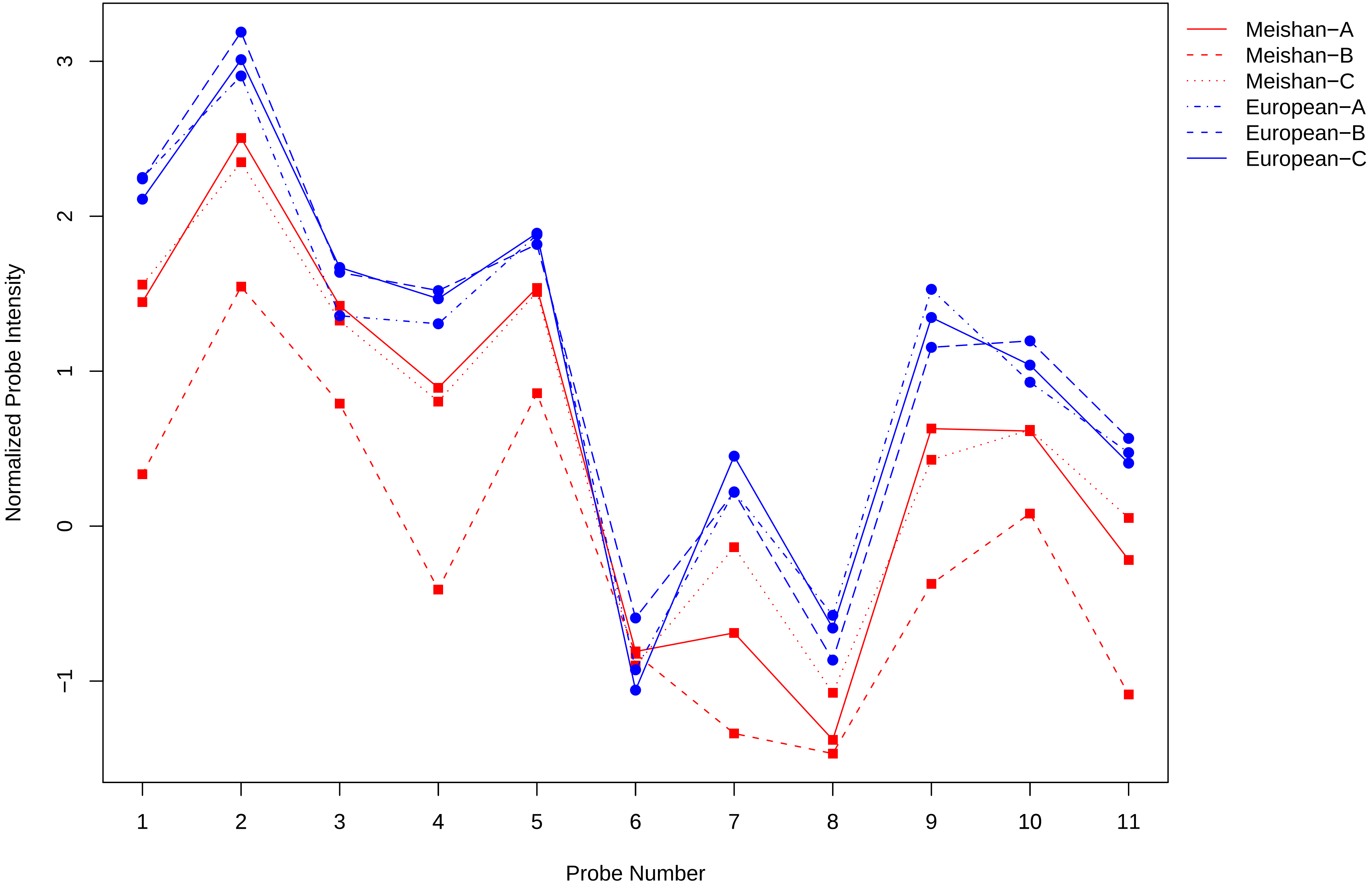

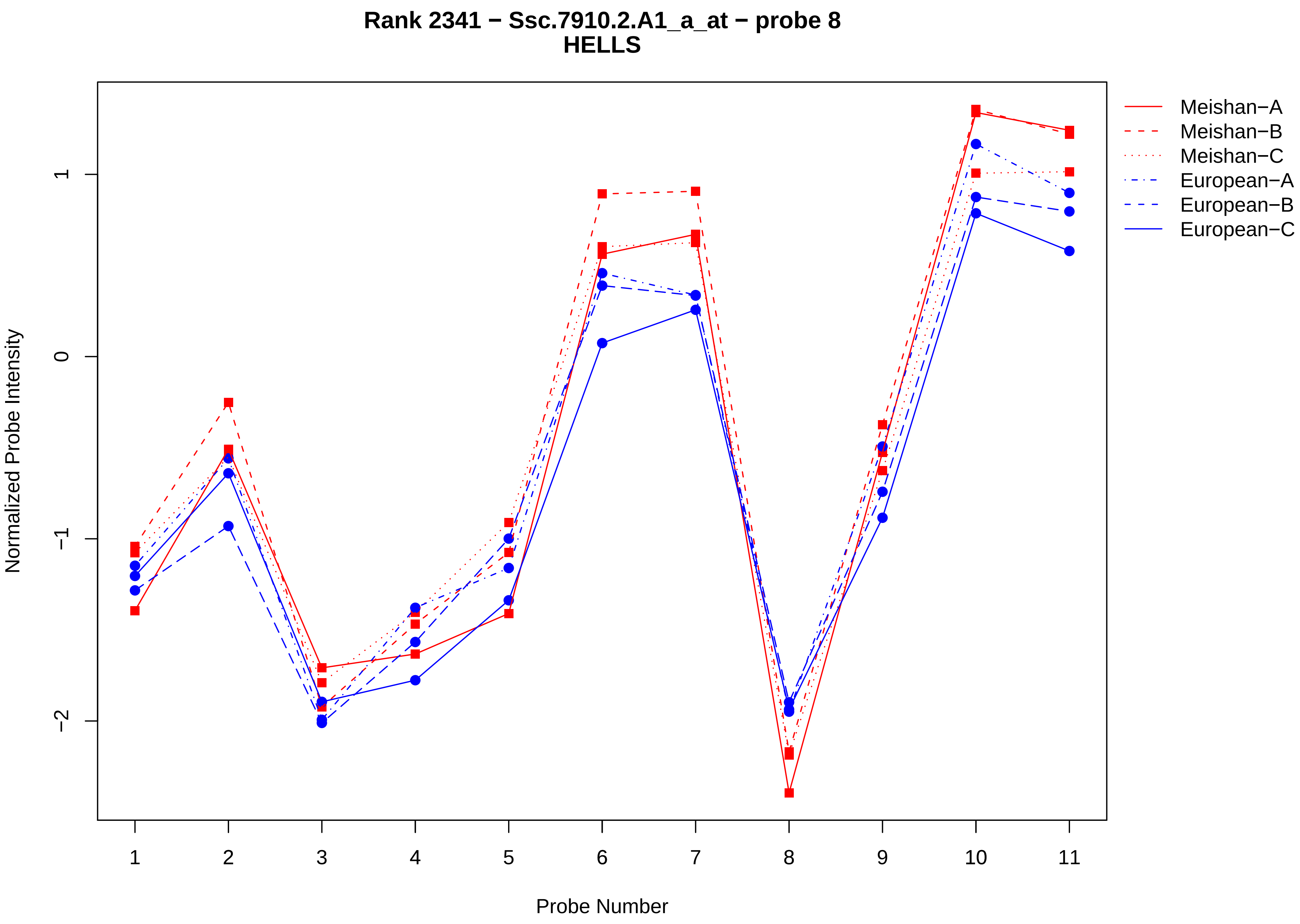

Rank 2341 − Ssc.7910.2.A1_a_at − probe 8
HELLS
Normalized Probe Intensity
Probe Number
1
0
−1
−2
1
2
3
4
5
6
7
8
9
10
11
Meishan−A
Meishan−B
Meishan−C
European−A
European−B
European−C

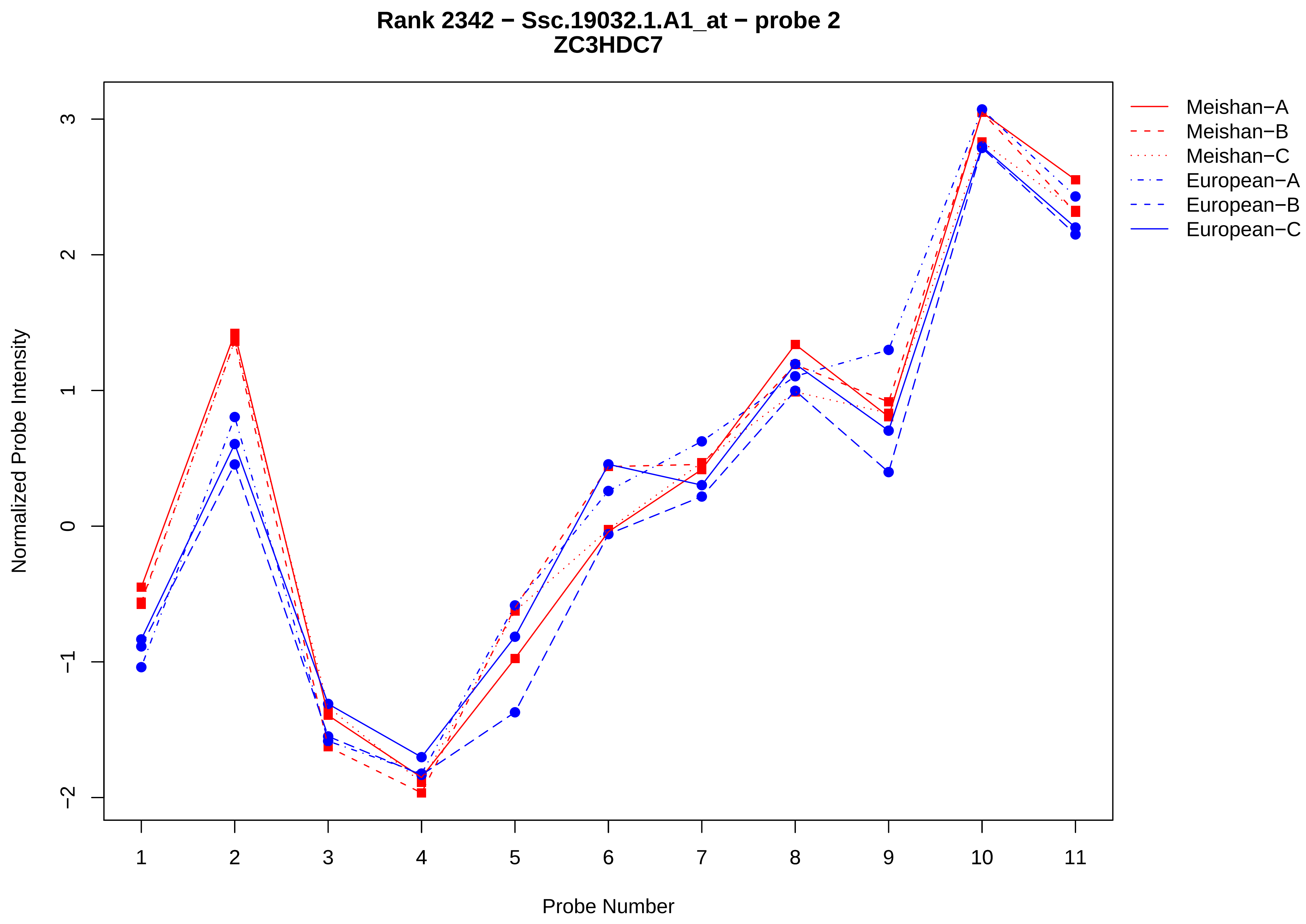

Rank 2342 − Ssc.19032.1.A1_at − probe 2
ZC3HDC7
Normalized Probe Intensity
Probe Number
Meishan−A
Meishan−B
Meishan−C
European−A
European−B
European−C

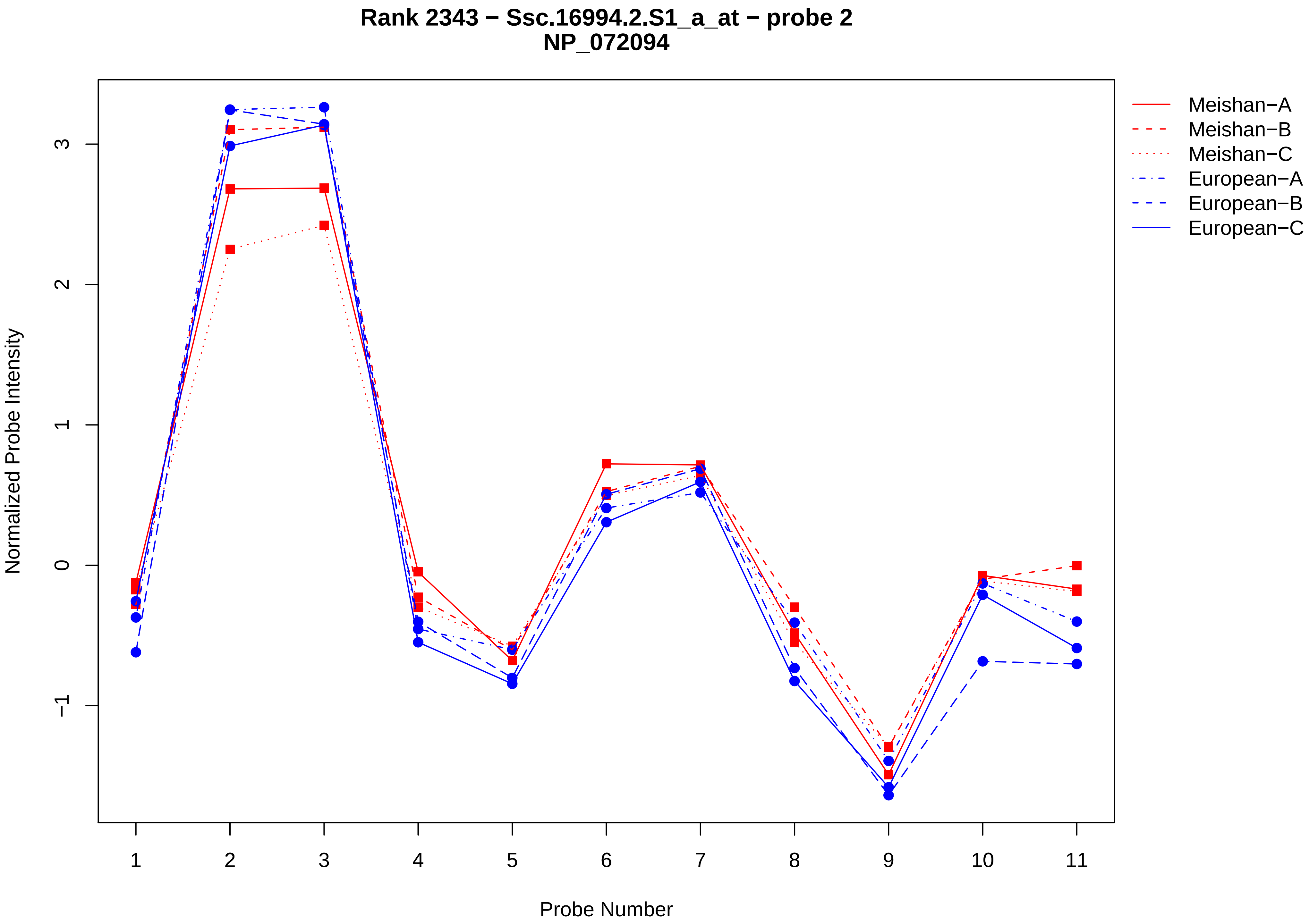

Rank 2343 − Ssc.16994.2.S1_a_at − probe 2
NP_072094
Normalized Probe Intensity
Probe Number
−1
0
1
2
3
1
2
3
4
5
6
7
8
9
10
11
Meishan−A
Meishan−B
Meishan−C
European−A
European−B
European−C

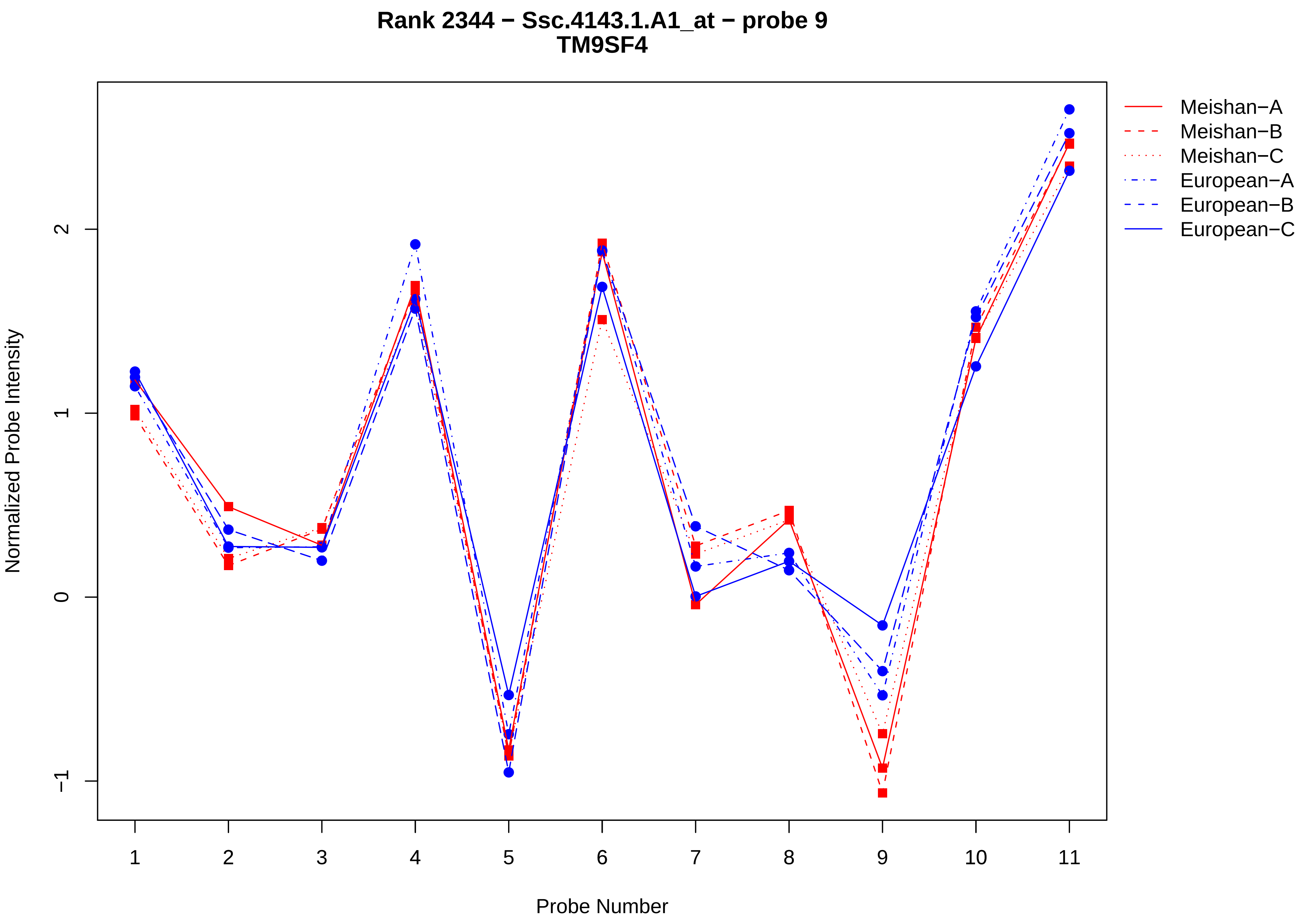
Rank 2344 − Ssc.4143.1.A1_at − probe 9
TM9SF4
Normalized Probe Intensity
Probe Number
−1
0
1
2
1
2
3
4
5
6
7
8
9
10
11
Meishan−A
Meishan−B
Meishan−C
European−A
European−B
European−C

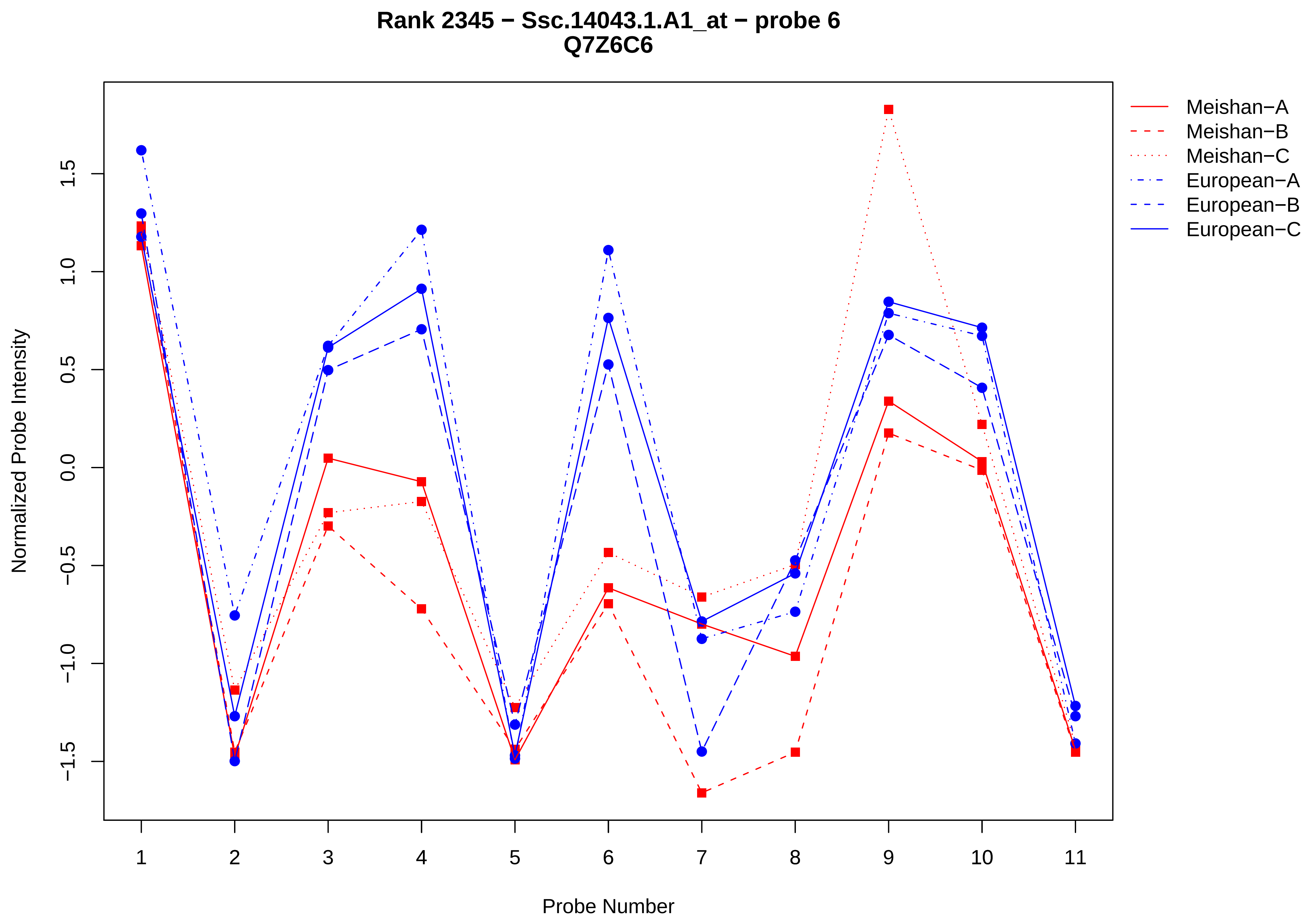

Rank 2345 − Ssc.14043.1.A1_at − probe 6
Q7Z6C6
Normalized Probe Intensity
Probe Number
Meishan−A
Meishan−B
Meishan−C
European−A
European−B
European−C
1.5
1.0
0.5
0.0
−0.5
−1.0
−1.5
1
2
3
4
5
6
7
8
9
10
11

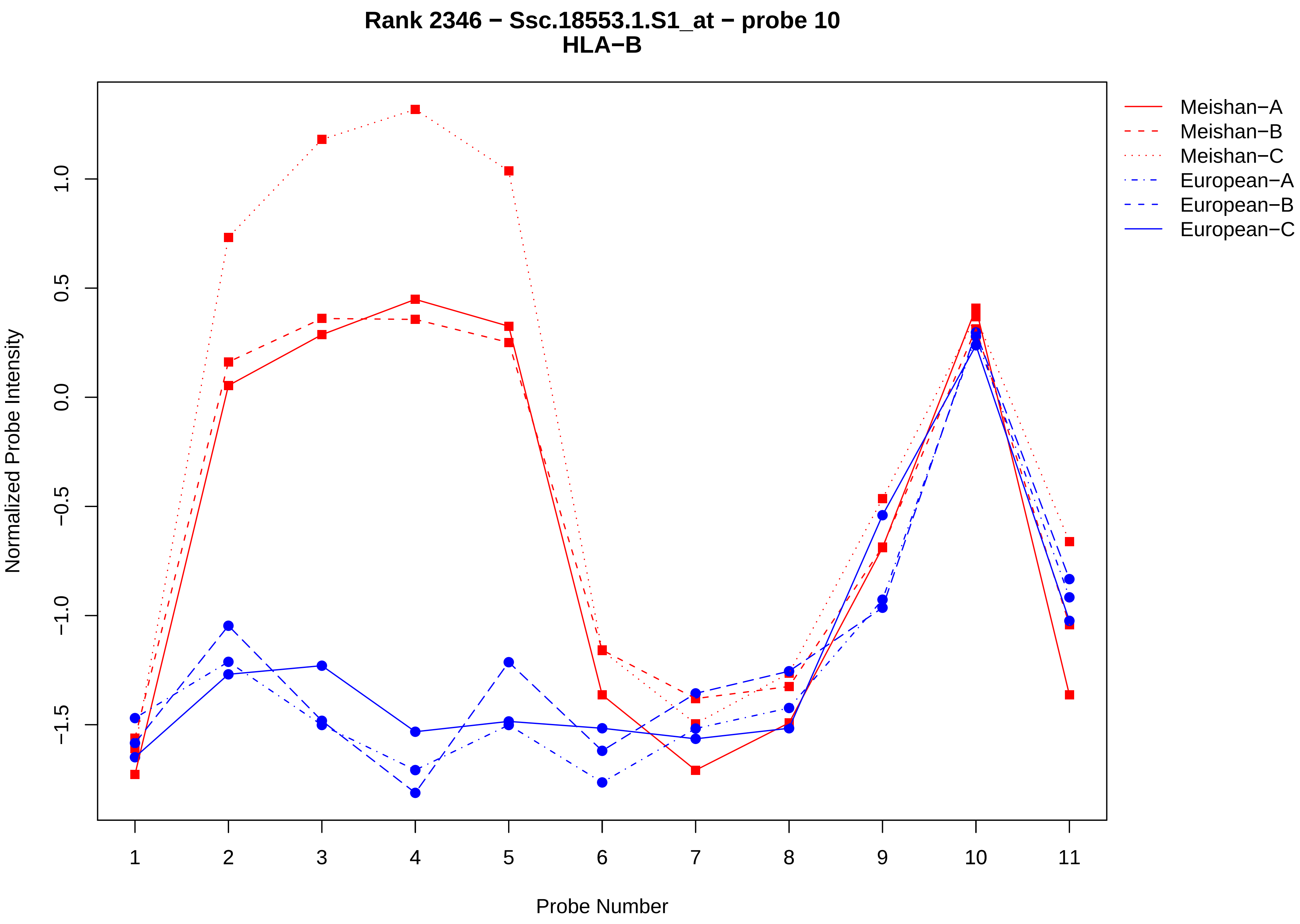

Rank 2346 − Ssc.18553.1.S1_at − probe 10
HLA−B
Normalized Probe Intensity
Probe Number
Meishan−A
Meishan−B
Meishan−C
European−A
European−B
European−C

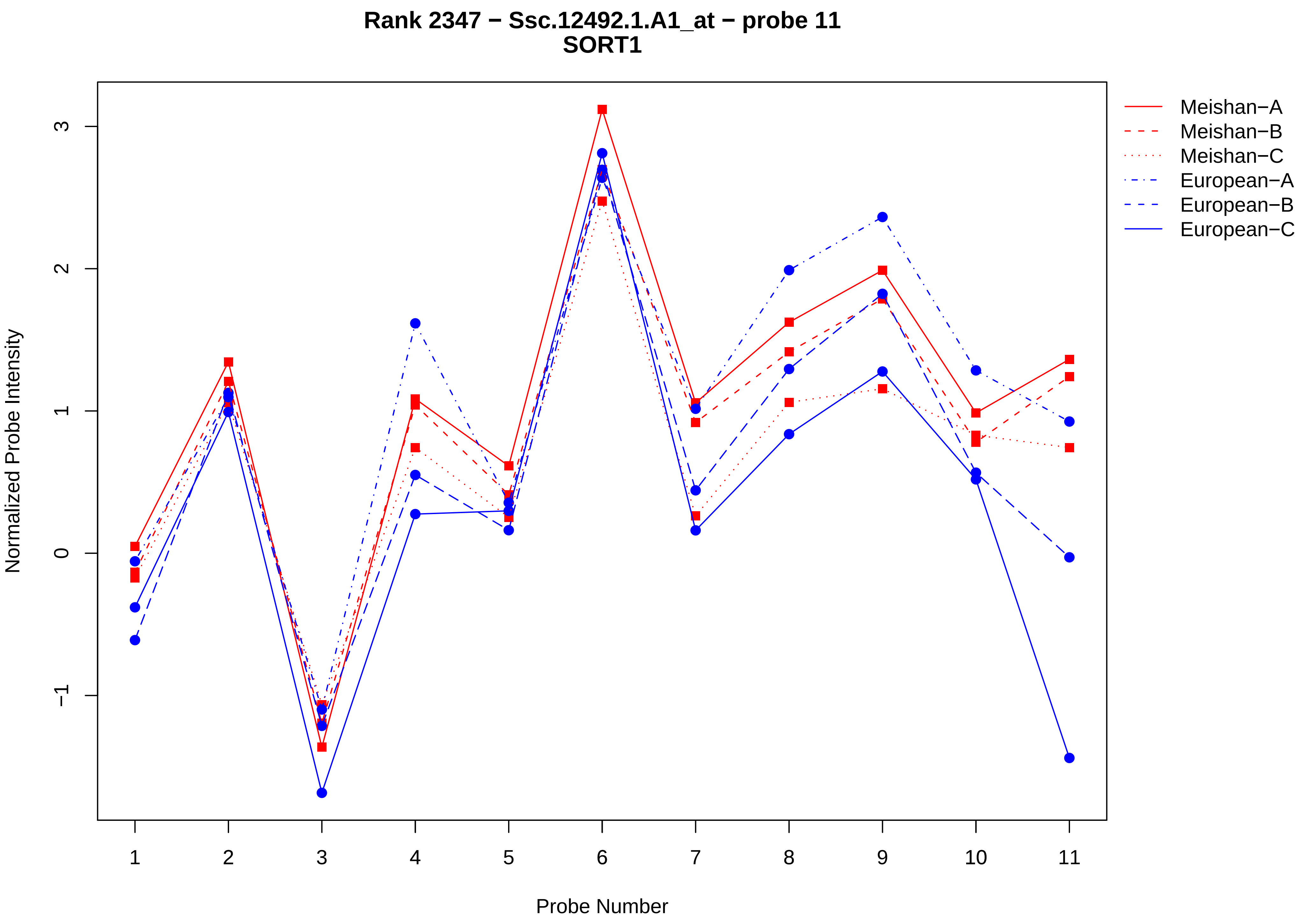

Rank 2347 − Ssc.12492.1.A1_at − probe 11
SORT1
Normalized Probe Intensity
Probe Number
−1
0
1
2
3
1
2
3
4
5
6
7
8
9
10
11
Meishan−A
Meishan−B
Meishan−C
European−A
European−B
European−C

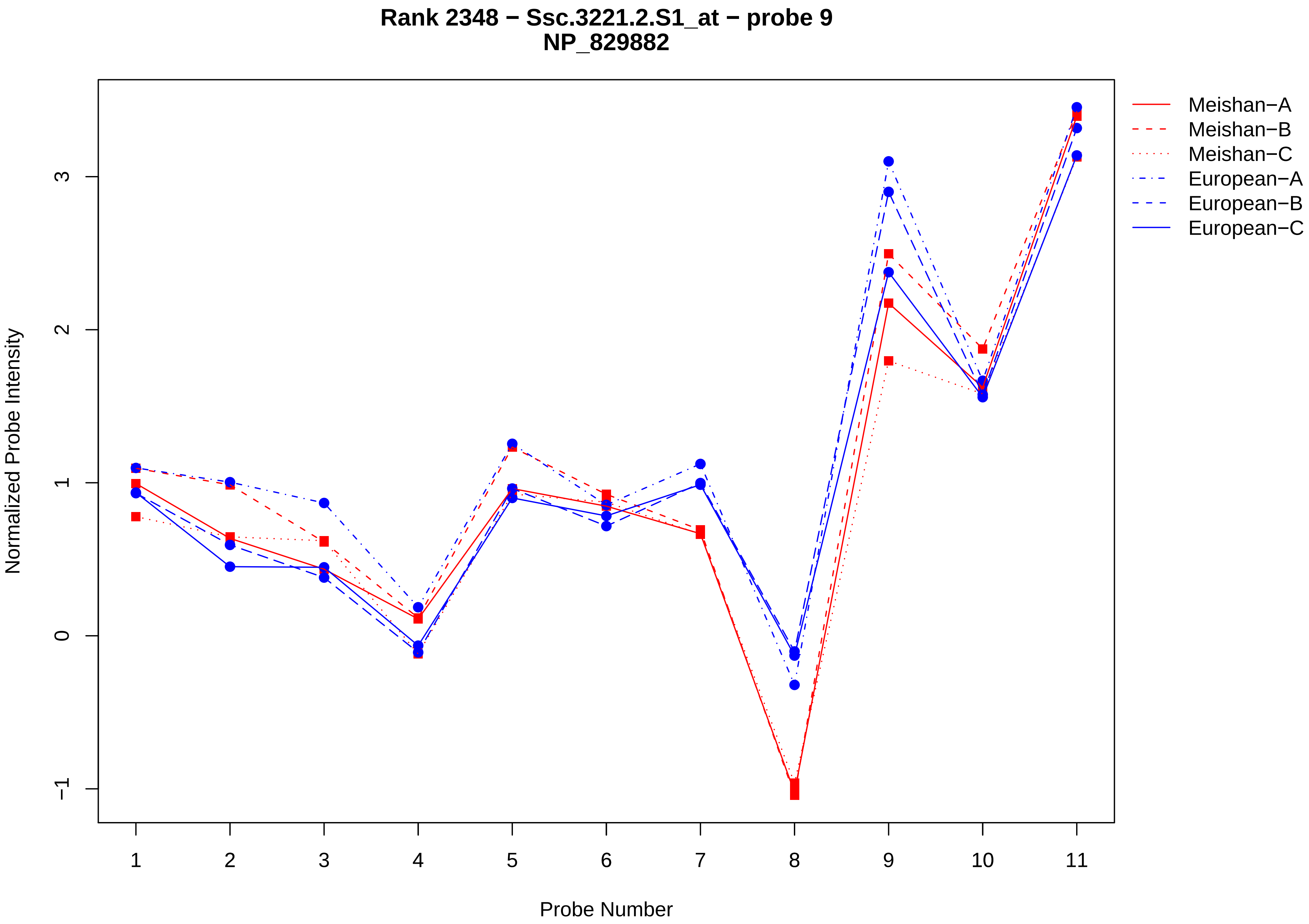

Rank 2348 − Ssc.3221.2.S1_at − probe 9
NP_829882
Normalized Probe Intensity
Probe Number
−1
0
1
2
3
1
2
3
4
5
6
7
8
9
10
11
Meishan−A
Meishan−B
Meishan−C
European−A
European−B
European−C

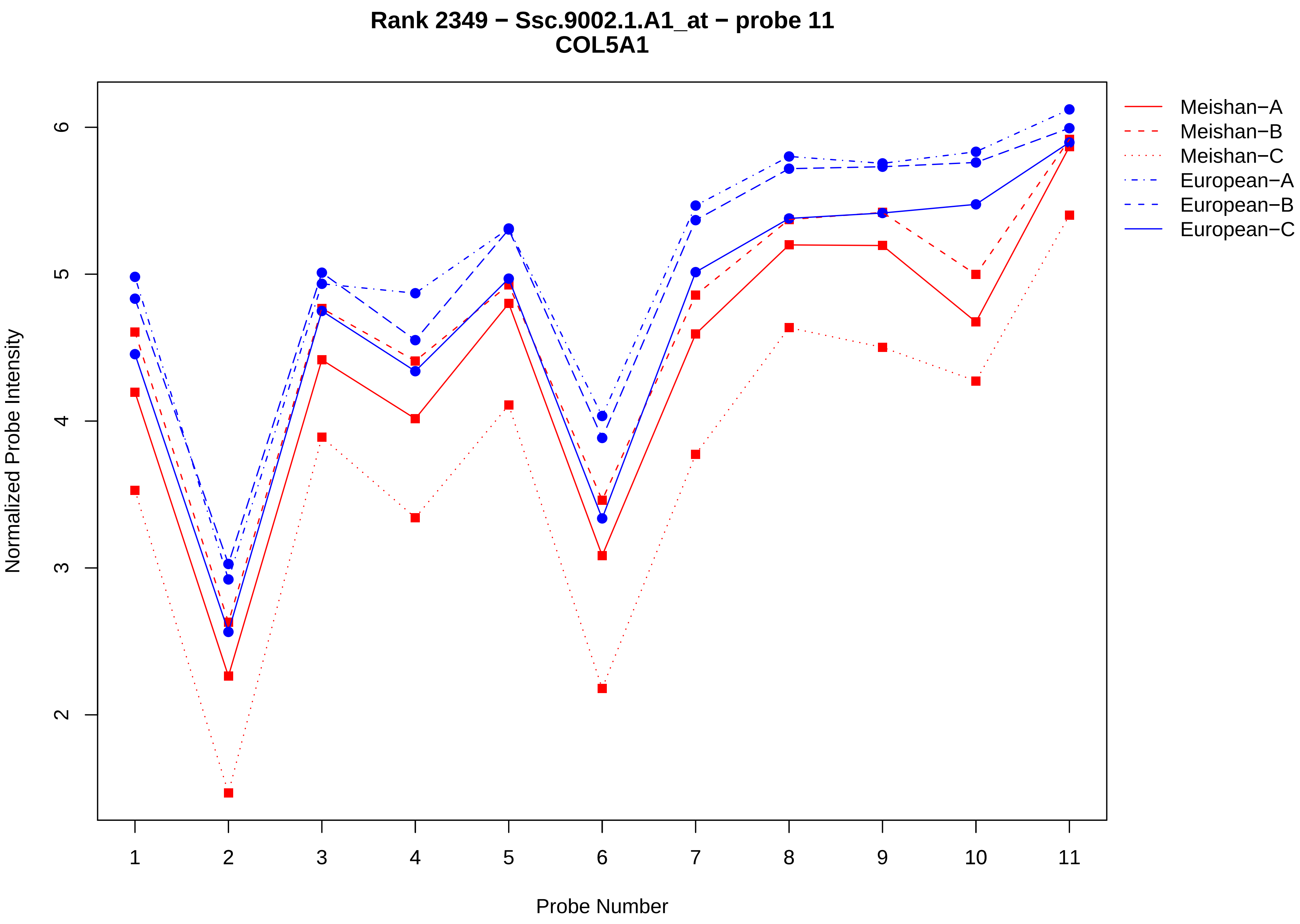
Rank 2349 − Ssc.9002.1.A1_at − probe 11
COL5A1
Normalized Probe Intensity
Probe Number
Meishan−A
Meishan−B
Meishan−C
European−A
European−B
European−C

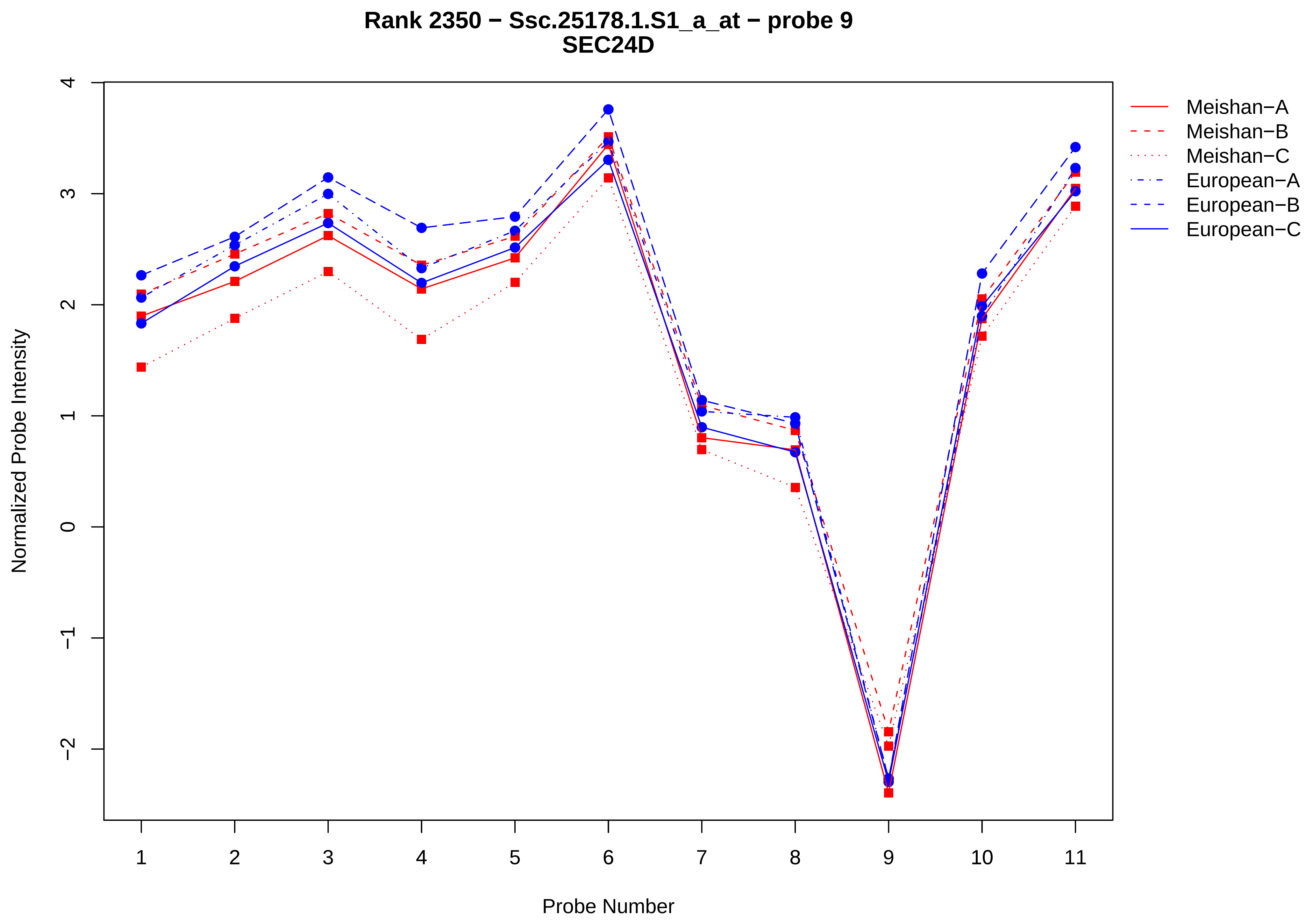

Rank 2350 − Ssc.25178.1.S1_a_at − probe 9
SEC24D
Normalized Probe Intensity
Probe Number
Meishan−A
Meishan−B
Meishan−C
European−A
European−B
European−C

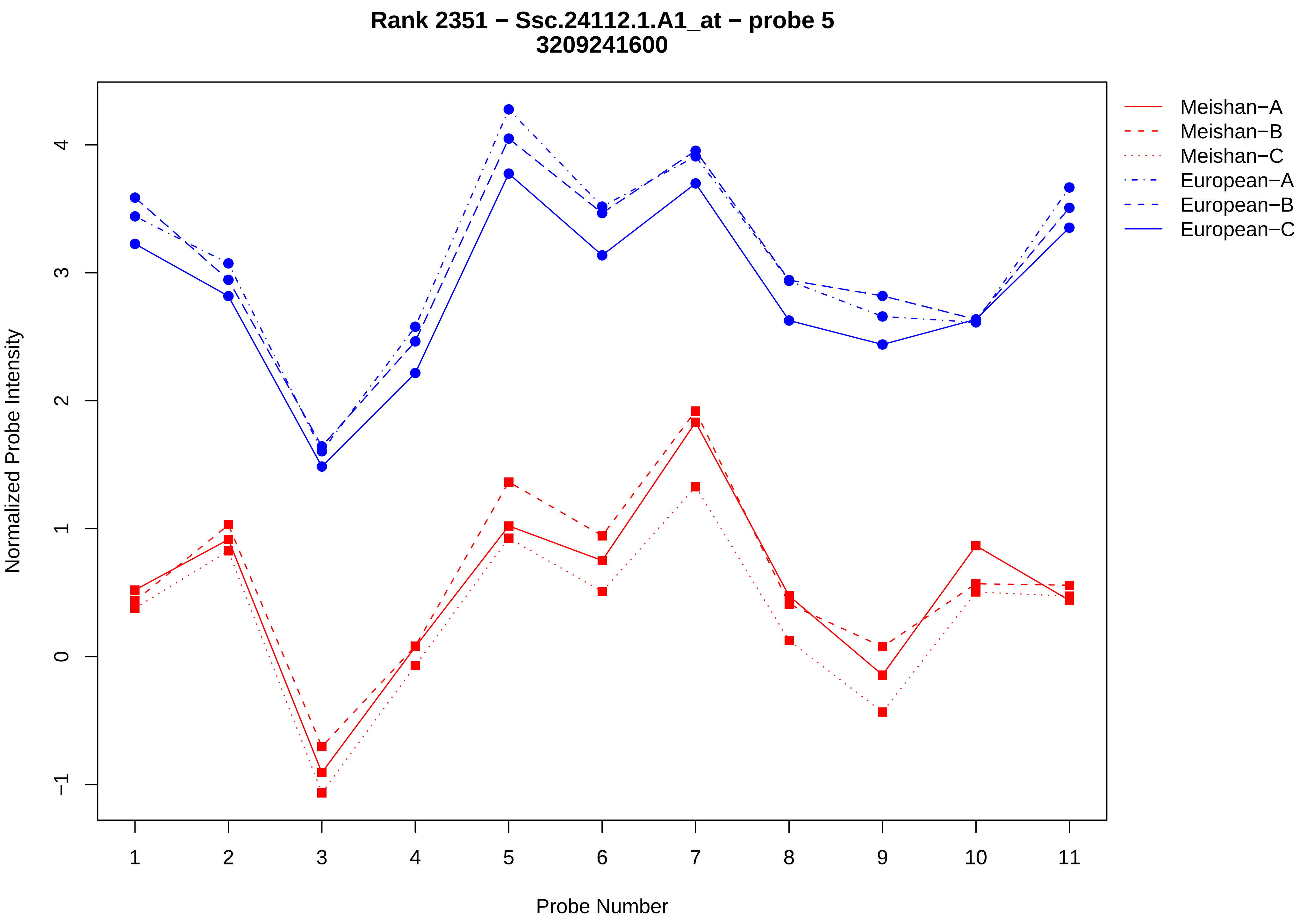
Rank 2351 − Ssc.24112.1.A1_at − probe 5
3209241600
Normalized Probe Intensity
Probe Number
Meishan−A
Meishan−B
Meishan−C
European−A
European−B
European−C
−1
0
1
2
3
4
1
2
3
4
5
6
7
8
9
10
11

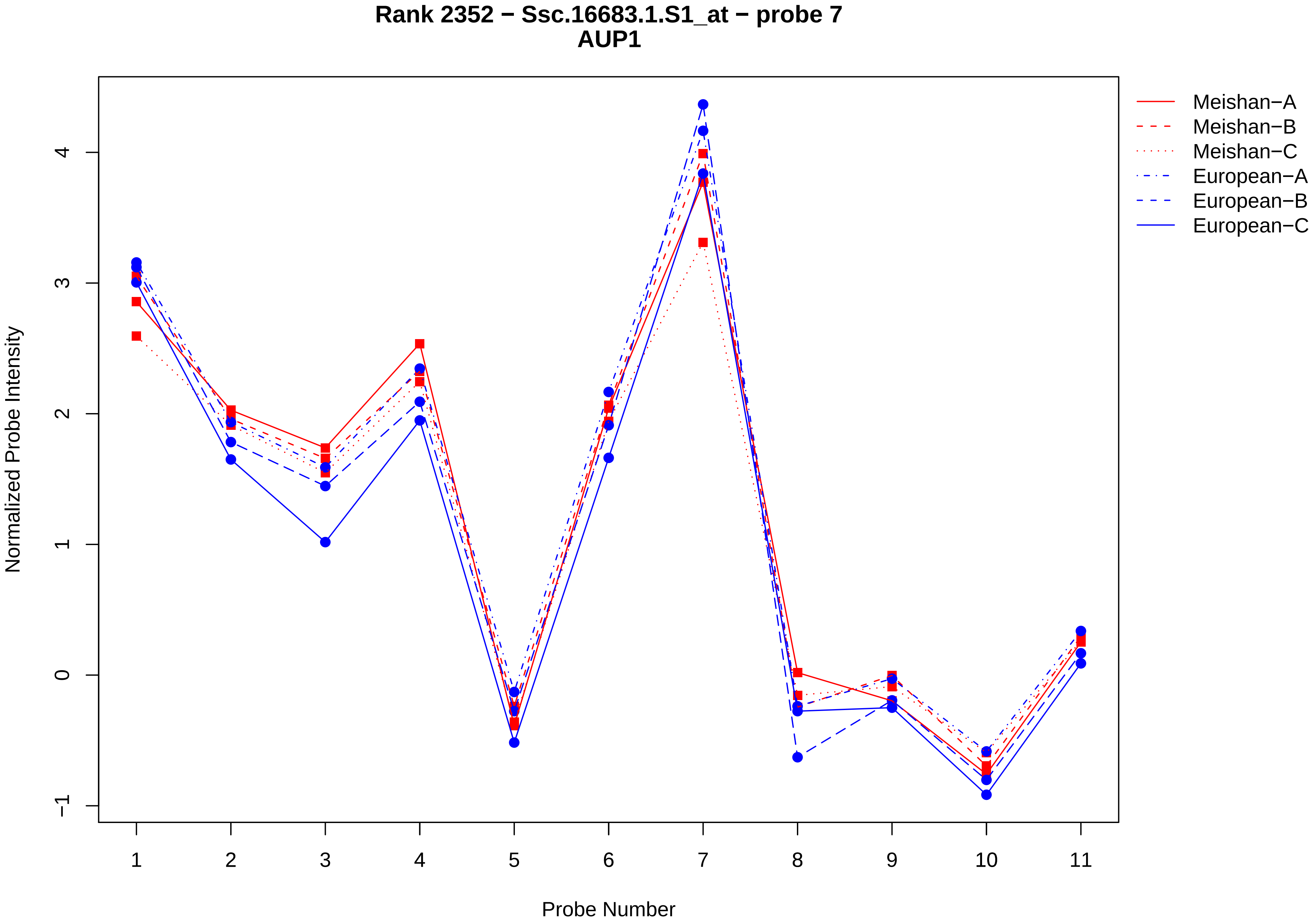

Rank 2352 − Ssc.16683.1.S1_at − probe 7
AUP1
Normalized Probe Intensity
Probe Number
Meishan−A
Meishan−B
Meishan−C
European−A
European−B
European−C
−1
0
1
2
3
4
1
2
3
4
5
6
7
8
9
10
11

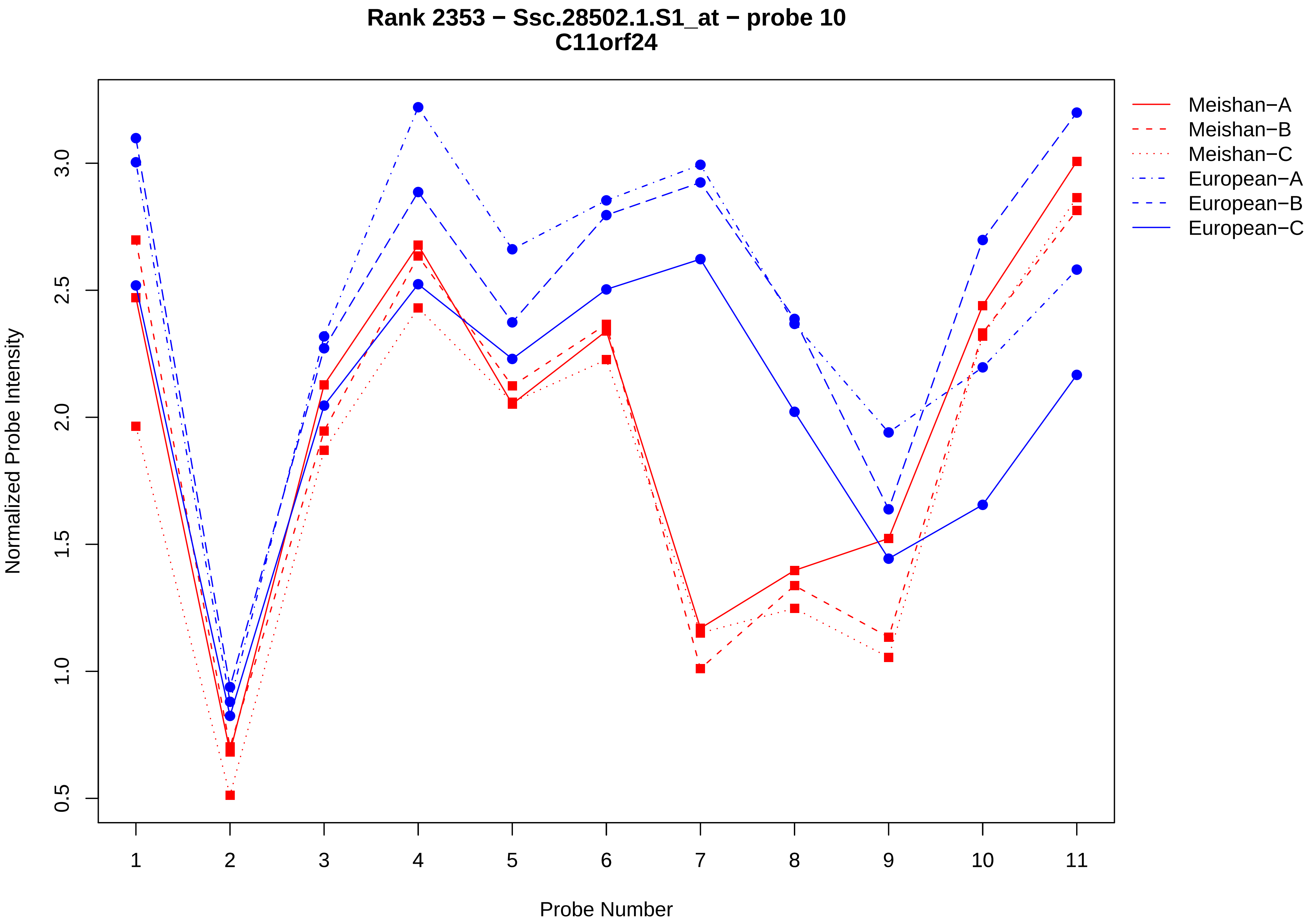

Rank 2353 − Ssc.28502.1.S1_at − probe 10
C11orf24
Normalized Probe Intensity
Probe Number
0.5
1.0
1.5
2.0
2.5
3.0
1
2
3
4
5
6
7
8
9
10
11
Meishan−A
Meishan−B
Meishan−C
European−A
European−B
European−C

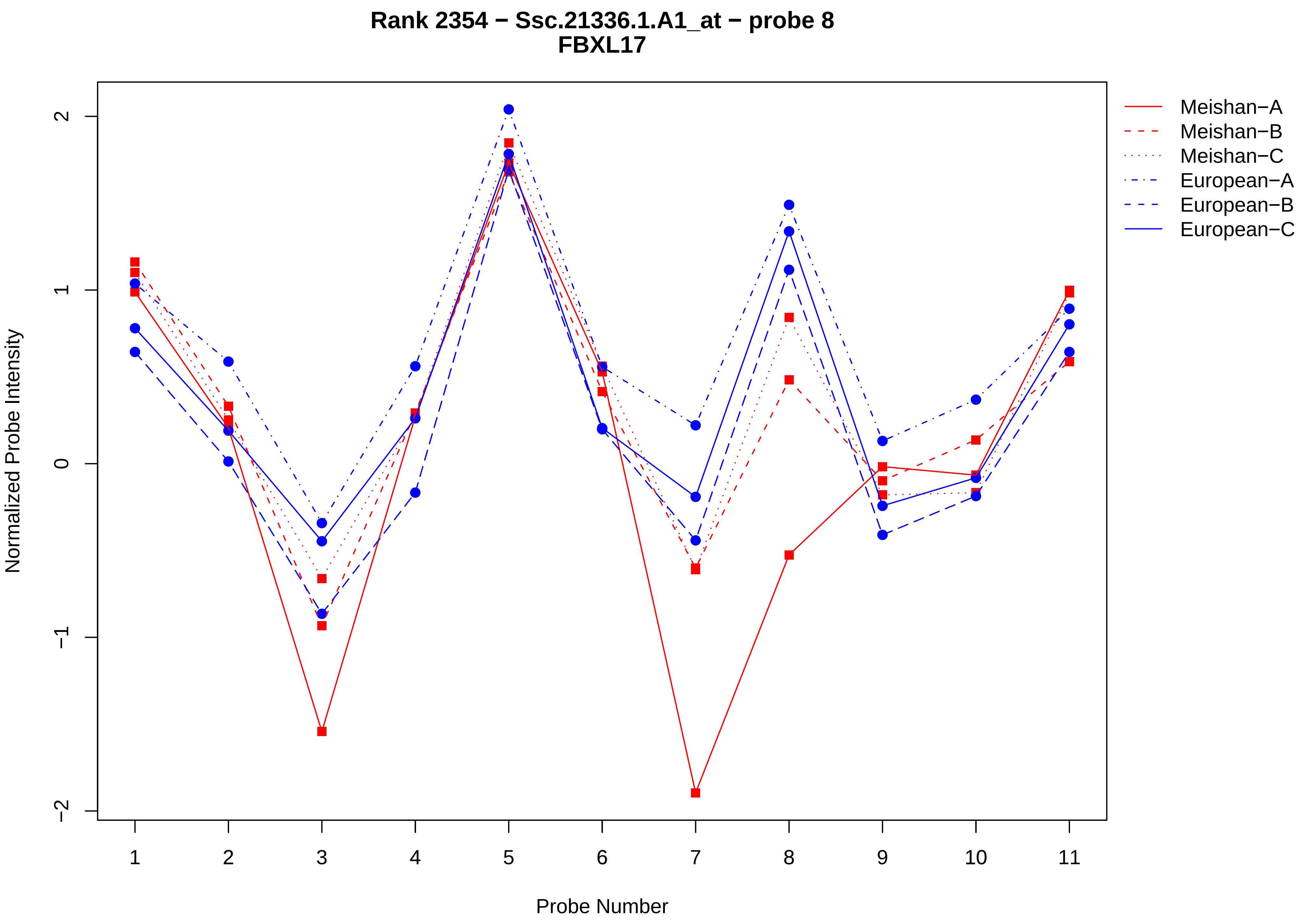

Rank 2354 − Ssc.21336.1.A1_at − probe 8
FBXL17
Normalized Probe Intensity
Probe Number
−2
−1
0
1
2
1
2
3
4
5
6
7
8
9
10
11
Meishan−A
Meishan−B
Meishan−C
European−A
European−B
European−C

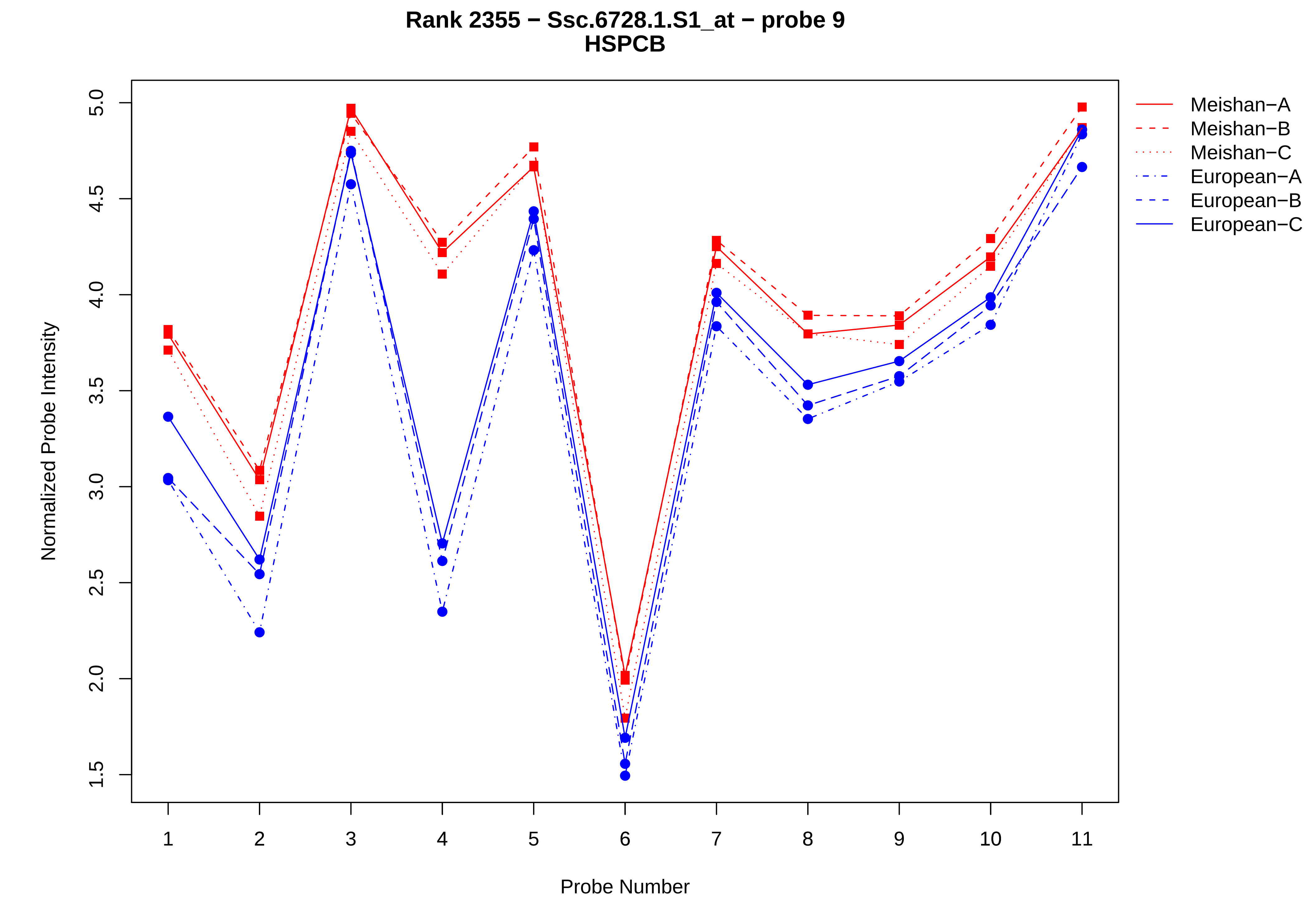
Rank 2355 − Ssc.6728.1.S1_at − probe 9
HSPCB
Normalized Probe Intensity
Probe Number
Meishan−A
Meishan−B
Meishan−C
European−A
European−B
European−C
1.5
2.0
2.5
3.0
3.5
4.0
4.5
5.0
1
2
3
4
5
6
7
8
9
10
11

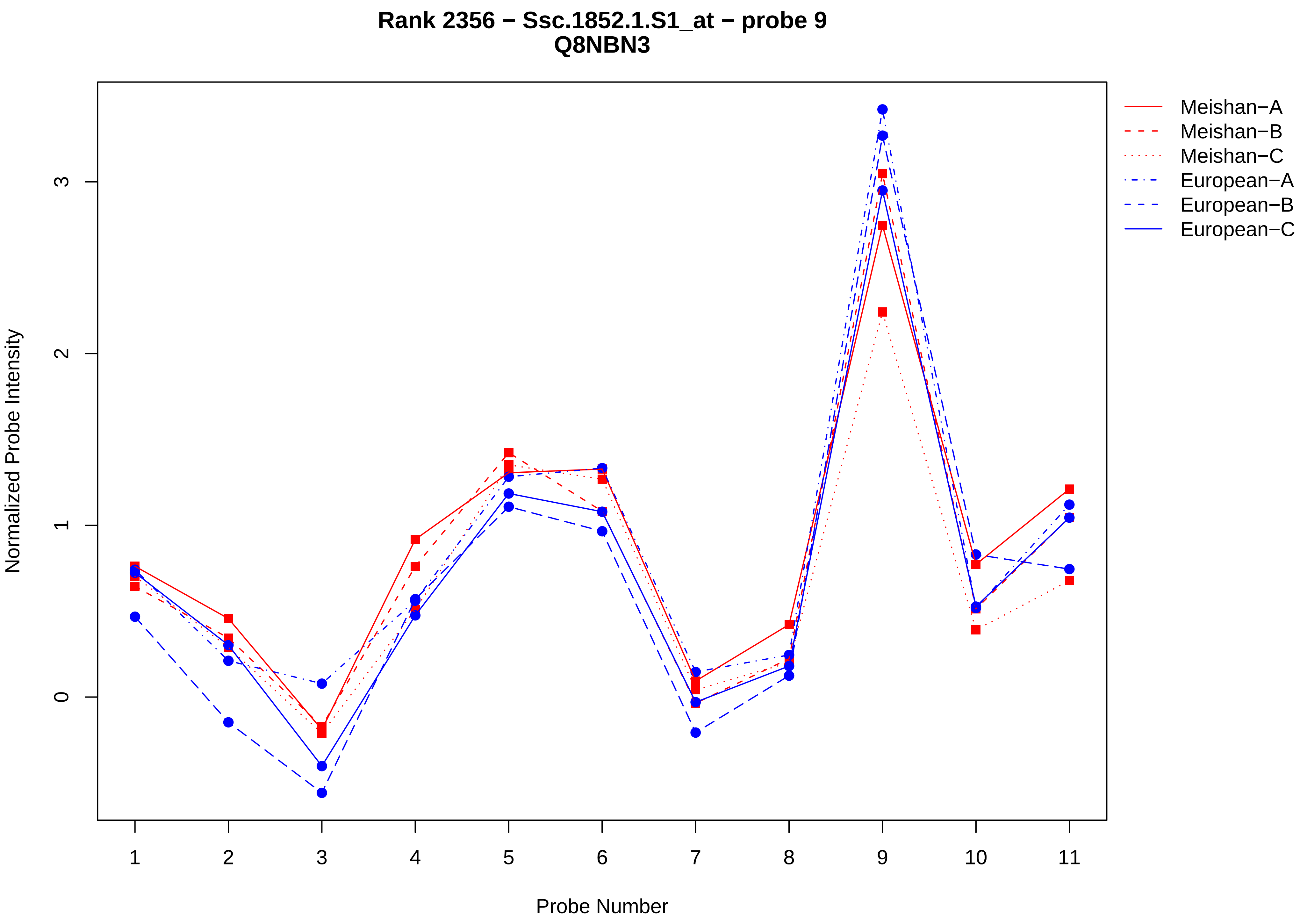

Rank 2356 − Ssc.1852.1.S1_at − probe 9
Q8NBN3
Normalized Probe Intensity
Probe Number
0
1
2
3
1
2
3
4
5
6
7
8
9
10
11
Meishan−A
Meishan−B
Meishan−C
European−A
European−B
European−C

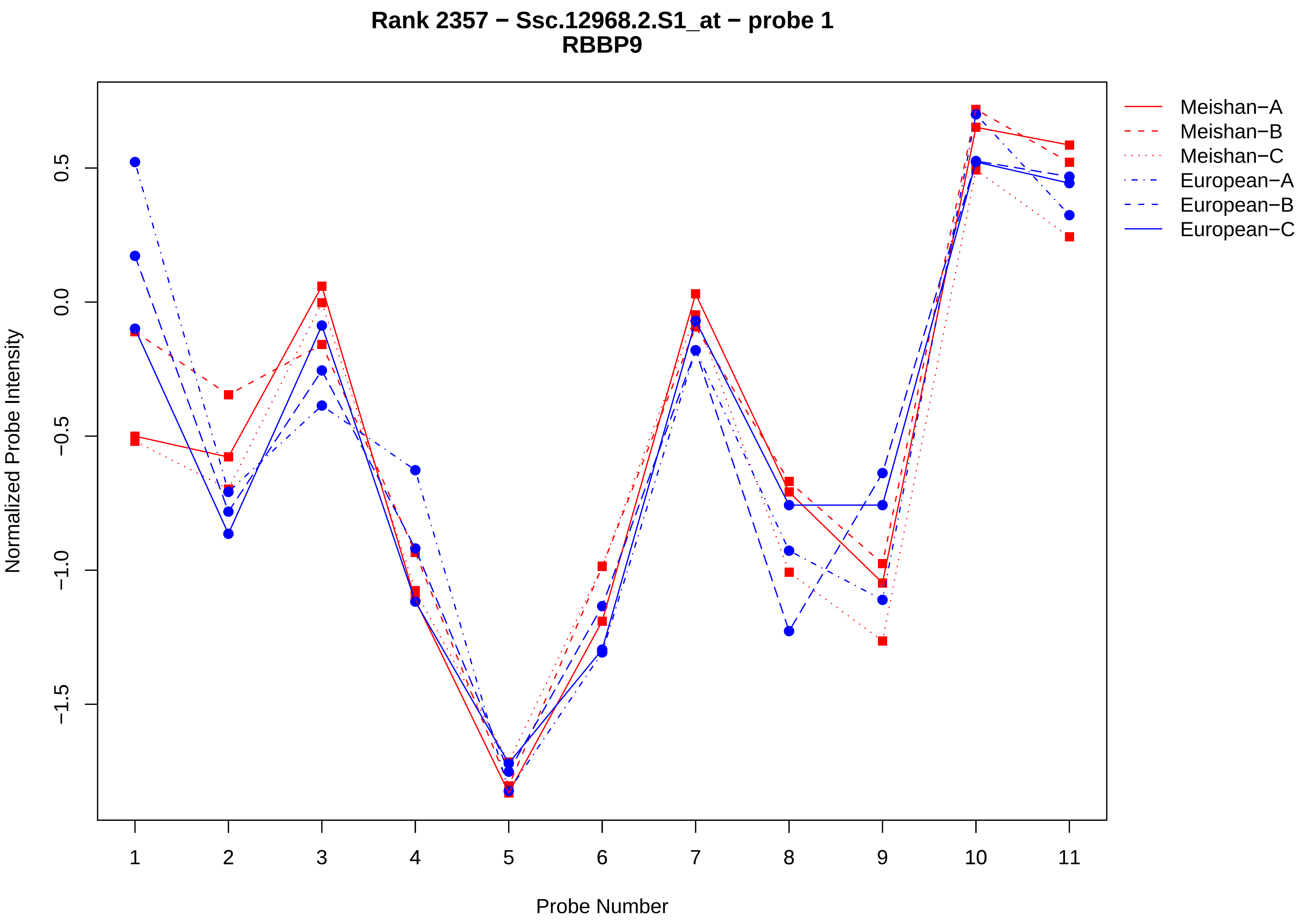

Rank 2357 − Ssc.12968.2.S1_at − probe 1
RBBP9
Normalized Probe Intensity
Probe Number
0.5
0.0
−0.5
−1.0
−1.5
1
2
3
4
5
6
7
8
9
10
11
Meishan−A
Meishan−B
Meishan−C
European−A
European−B
European−C

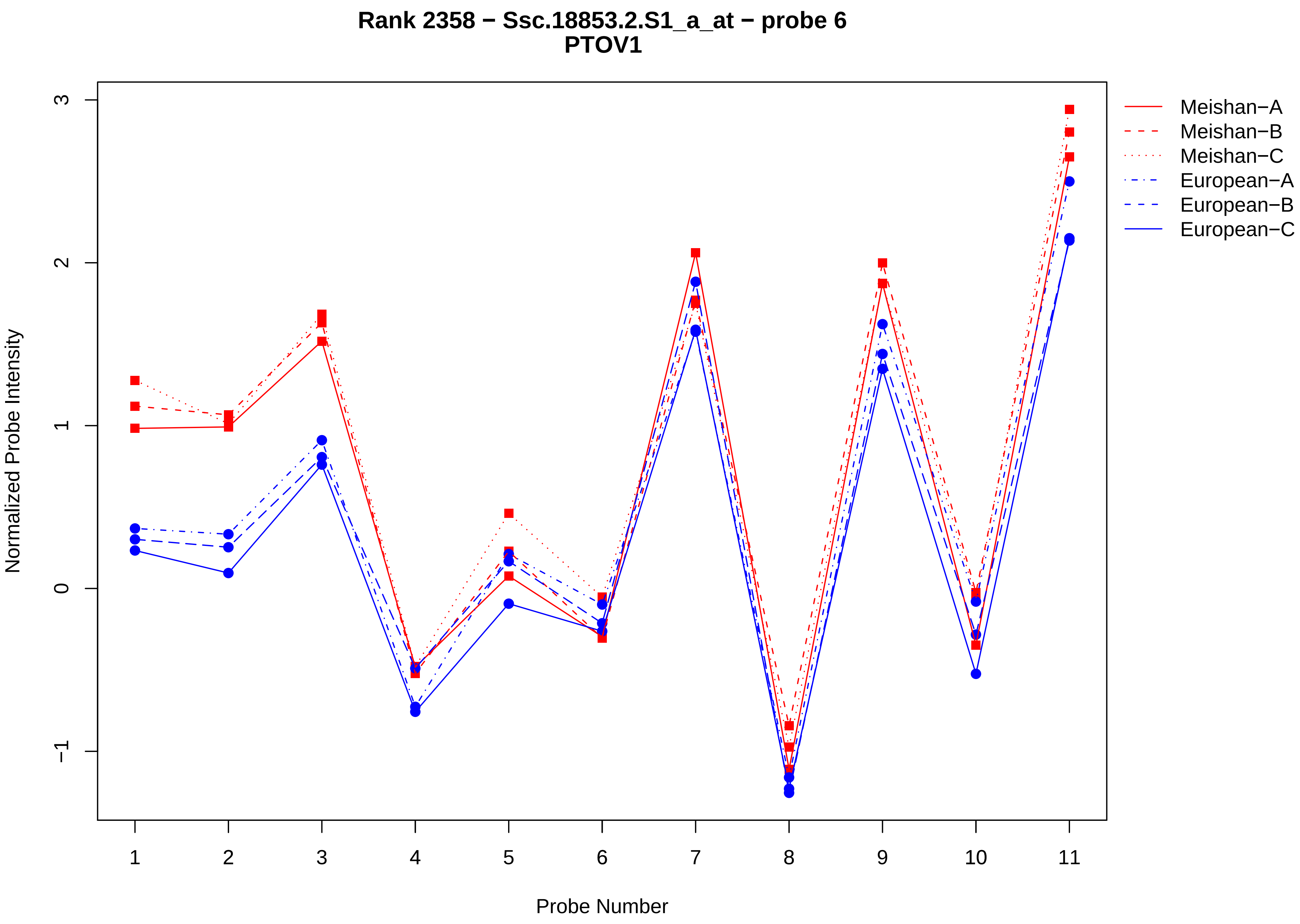

Rank 2358 − Ssc.18853.2.S1_a_at − probe 6
PTOV1
Normalized Probe Intensity
Probe Number
Meishan−A
Meishan−B
Meishan−C
European−A
European−B
European−C
−1
0
1
2
3
1
2
3
4
5
6
7
8
9
10
11

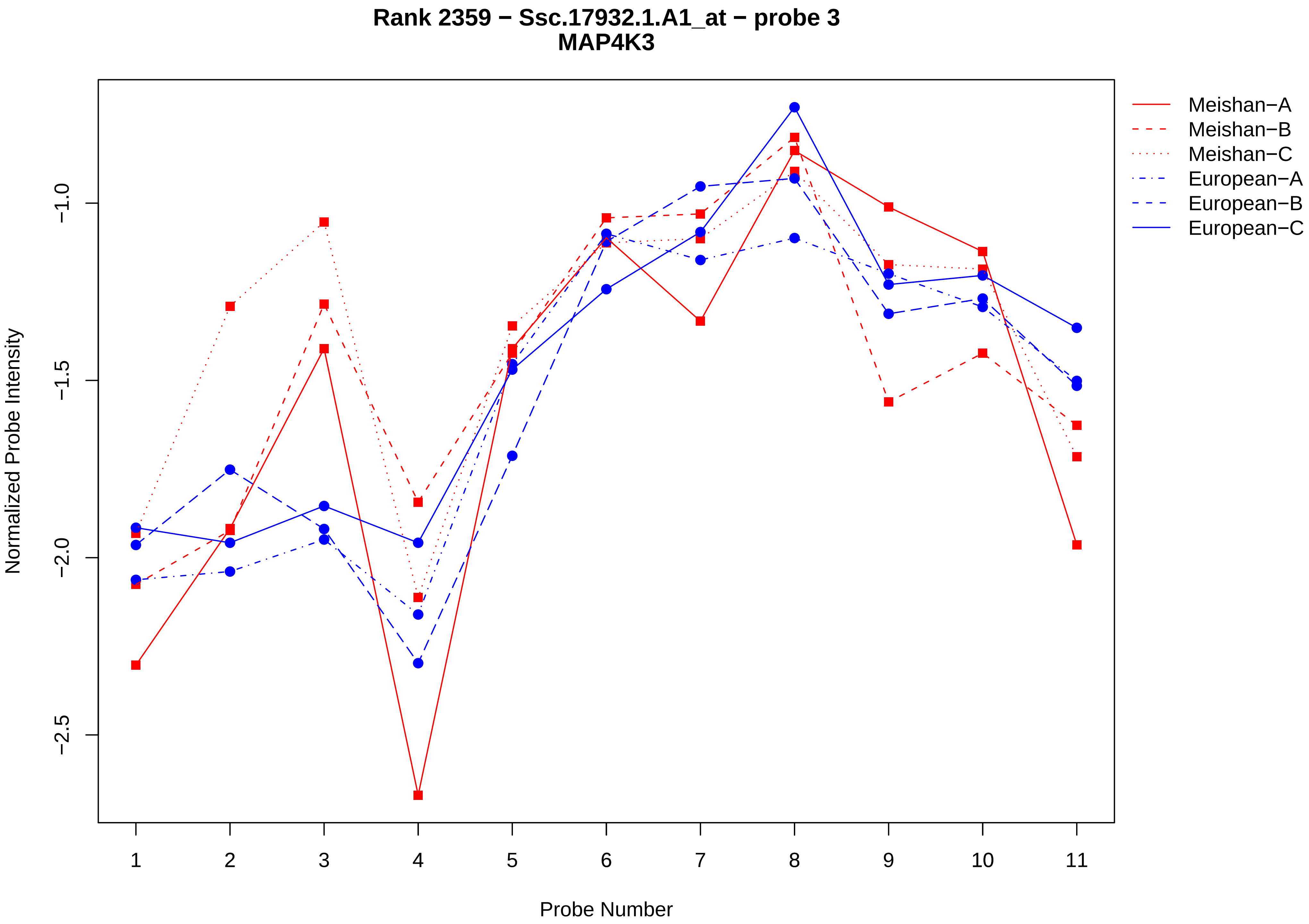

Rank 2359 − Ssc.17932.1.A1_at − probe 3
MAP4K3
Normalized Probe Intensity
Probe Number
−1.0
−1.5
−2.0
−2.5
1
2
3
4
5
6
7
8
9
10
11
Meishan−A
Meishan−B
Meishan−C
European−A
European−B
European−C

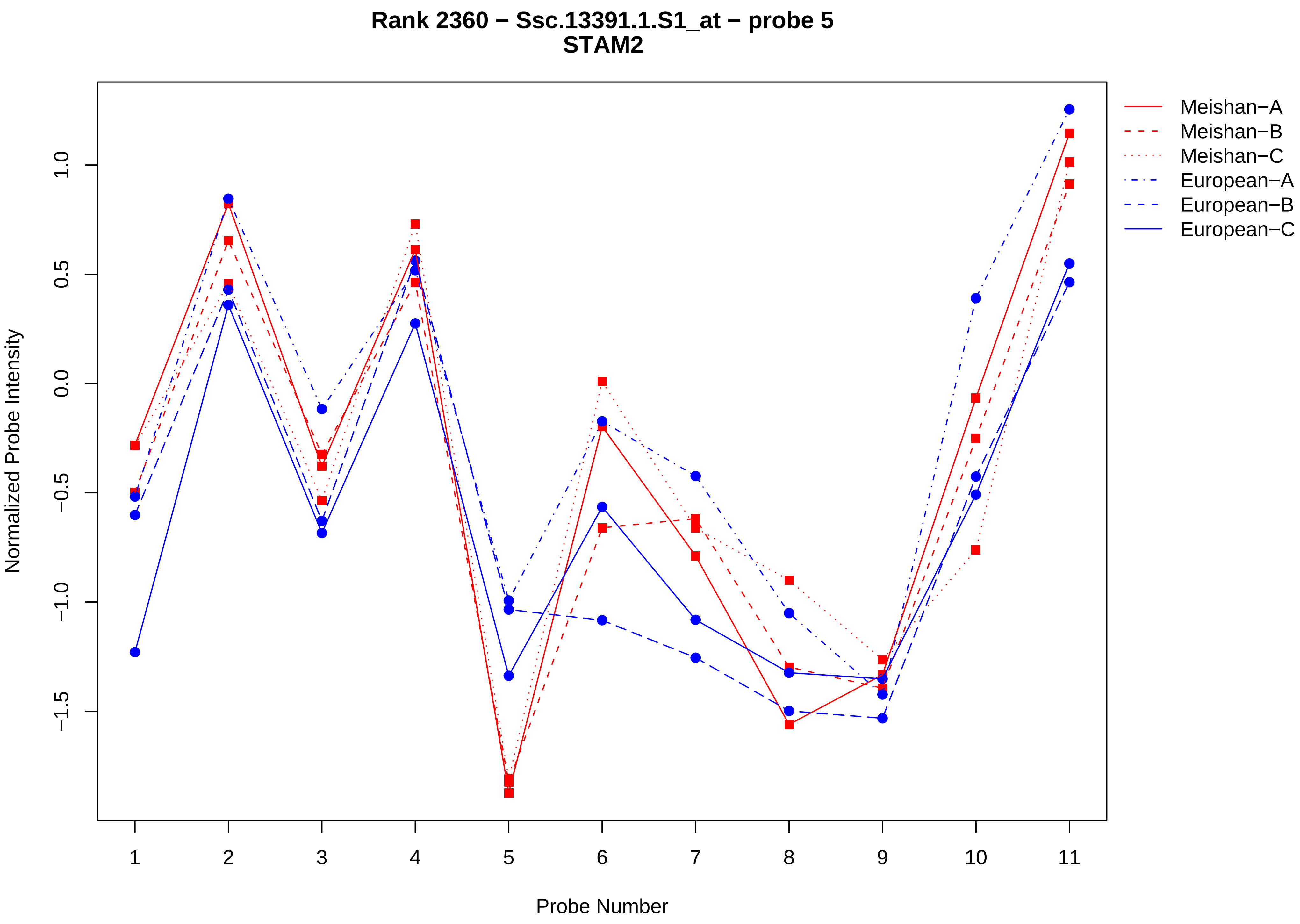

Rank 2360 − Ssc.13391.1.S1_at − probe 5
STAM2
Normalized Probe Intensity
Probe Number
1.0
0.5
0.0
−0.5
−1.0
−1.5
1
2
3
4
5
6
7
8
9
10
11
Meishan−A
Meishan−B
Meishan−C
European−A
European−B
European−C

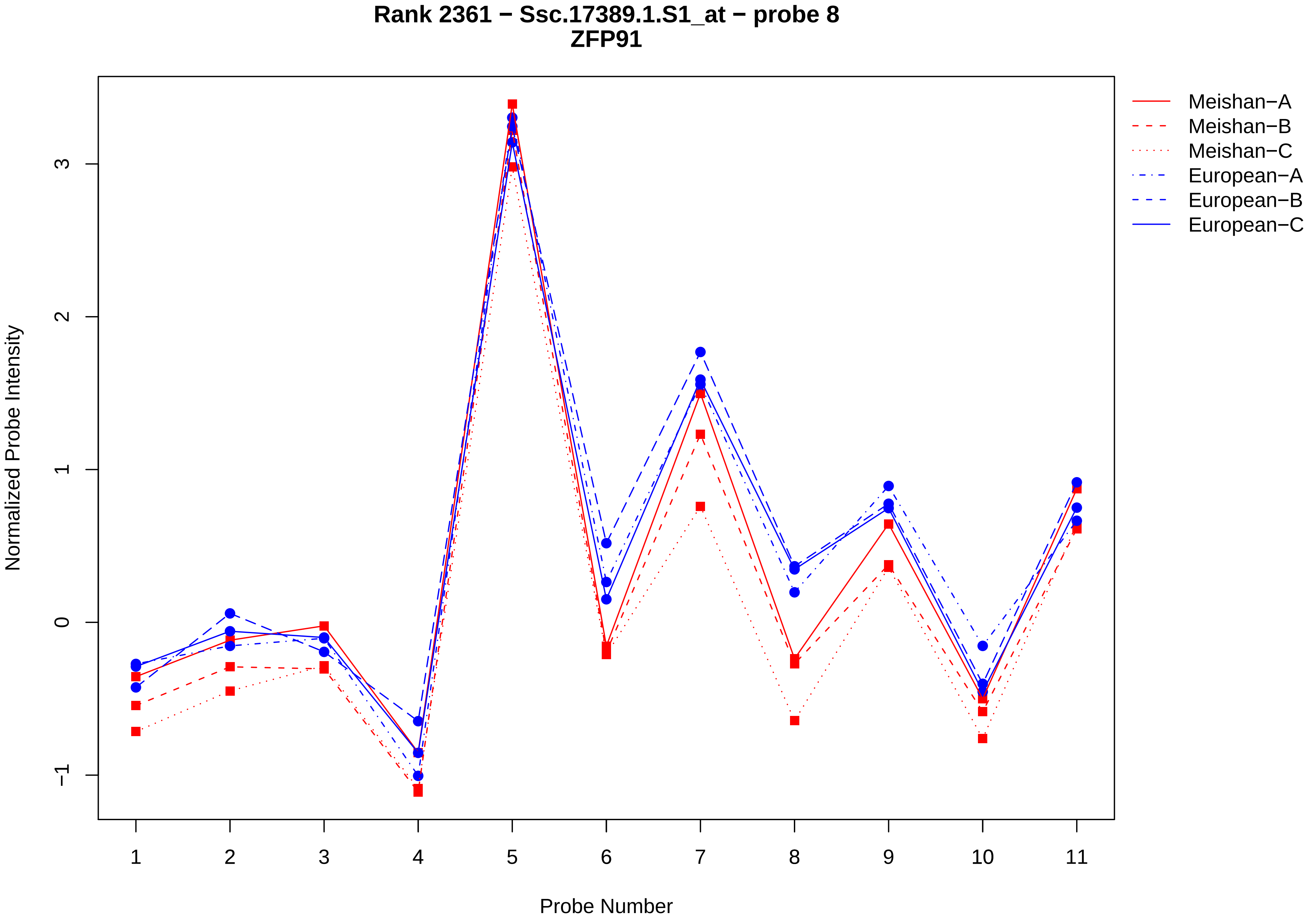

Rank 2361 − Ssc.17389.1.S1_at − probe 8
ZFP91
Normalized Probe Intensity
Probe Number
−1
0
1
2
3
1
2
3
4
5
6
7
8
9
10
11
Meishan−A
Meishan−B
Meishan−C
European−A
European−B
European−C

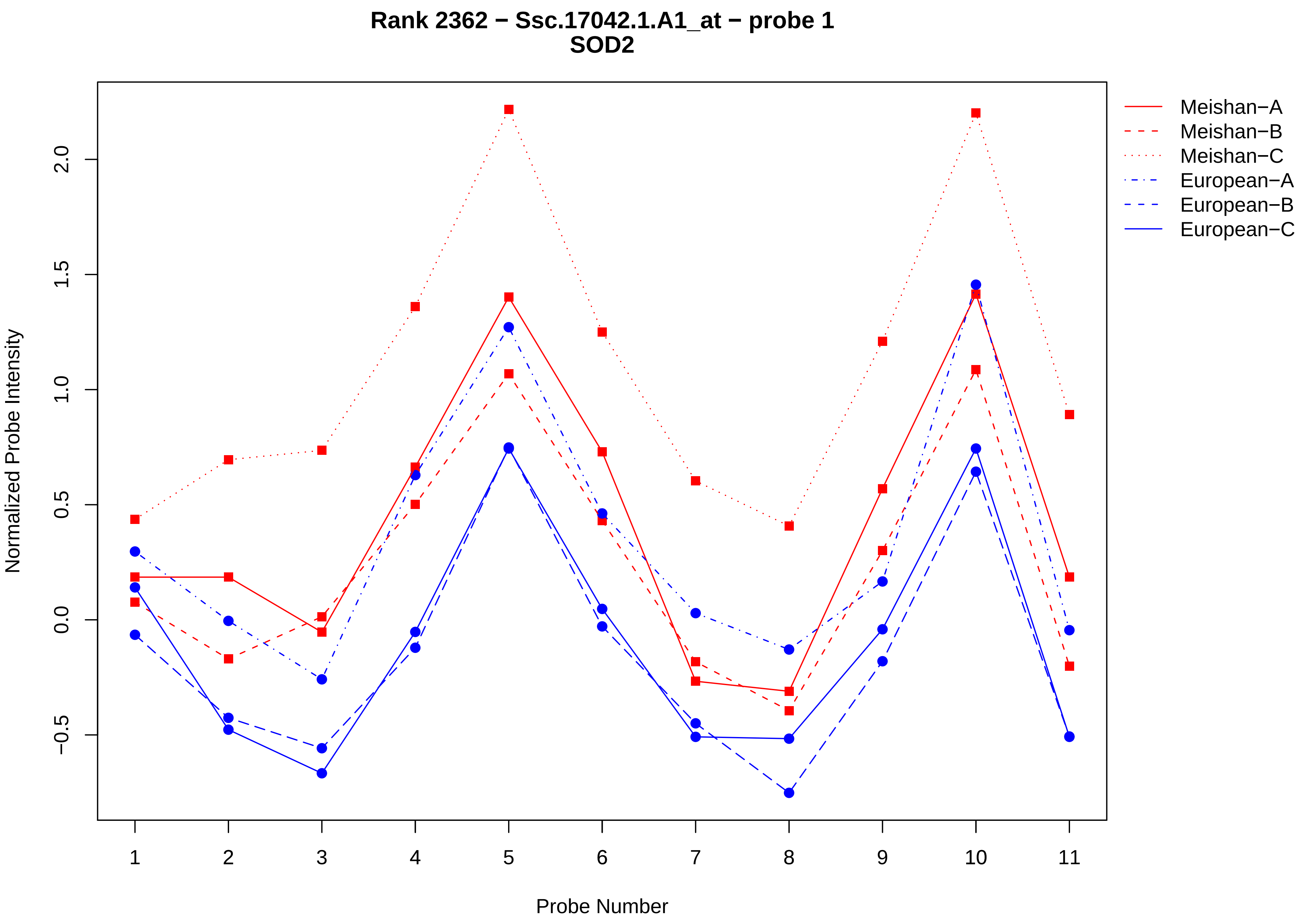
Rank 2362 − Ssc.17042.1.A1_at − probe 1
SOD2
Normalized Probe Intensity
Probe Number
Meishan−A
Meishan−B
Meishan−C
European−A
European−B
European−C
−0.5
0.0
0.5
1.0
1.5
2.0
1
2
3
4
5
6
7
8
9
10
11

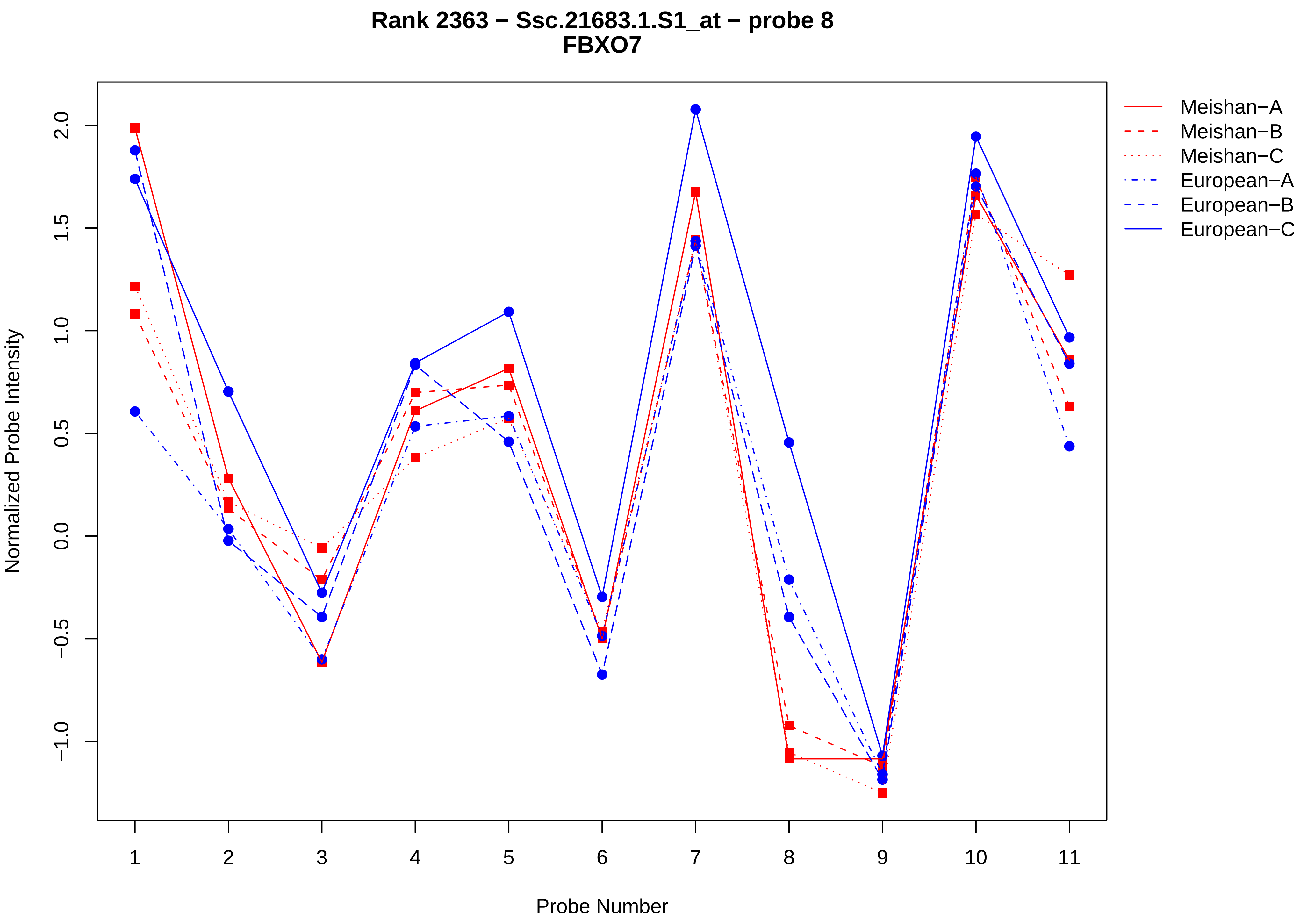

Rank 2363 − Ssc.21683.1.S1_at − probe 8
FBXO7
Normalized Probe Intensity
−1.0
−0.5
0.0
0.5
1.0
1.5
2.0
1
2
3
4
5
6
7
8
9
10
11
Probe Number
Meishan−A
Meishan−B
Meishan−C
European−A
European−B
European−C

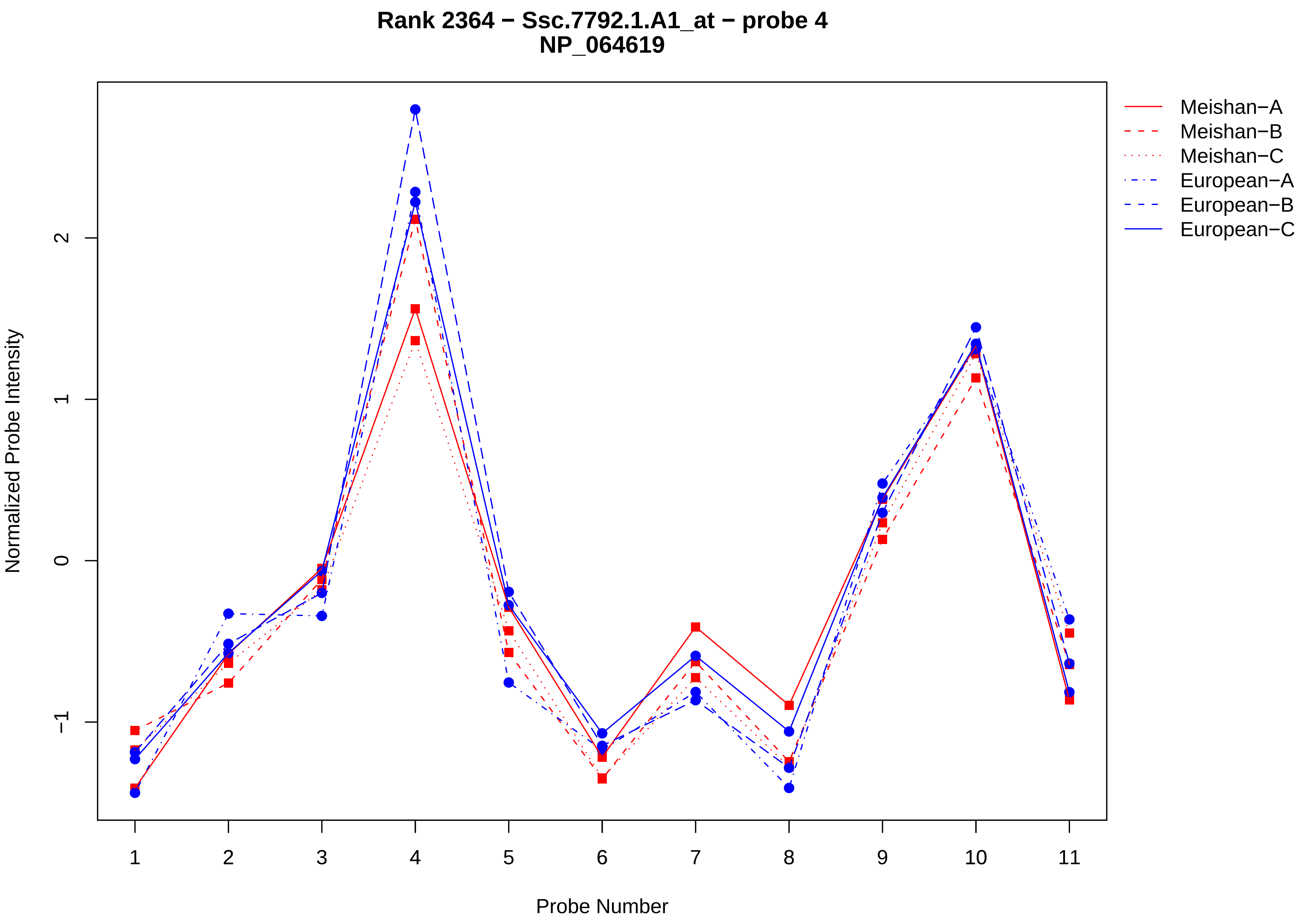
Rank 2364 − Ssc.7792.1.A1_at − probe 4
NP_064619
Normalized Probe Intensity
Probe Number
Meishan−A
Meishan−B
Meishan−C
European−A
European−B
European−C

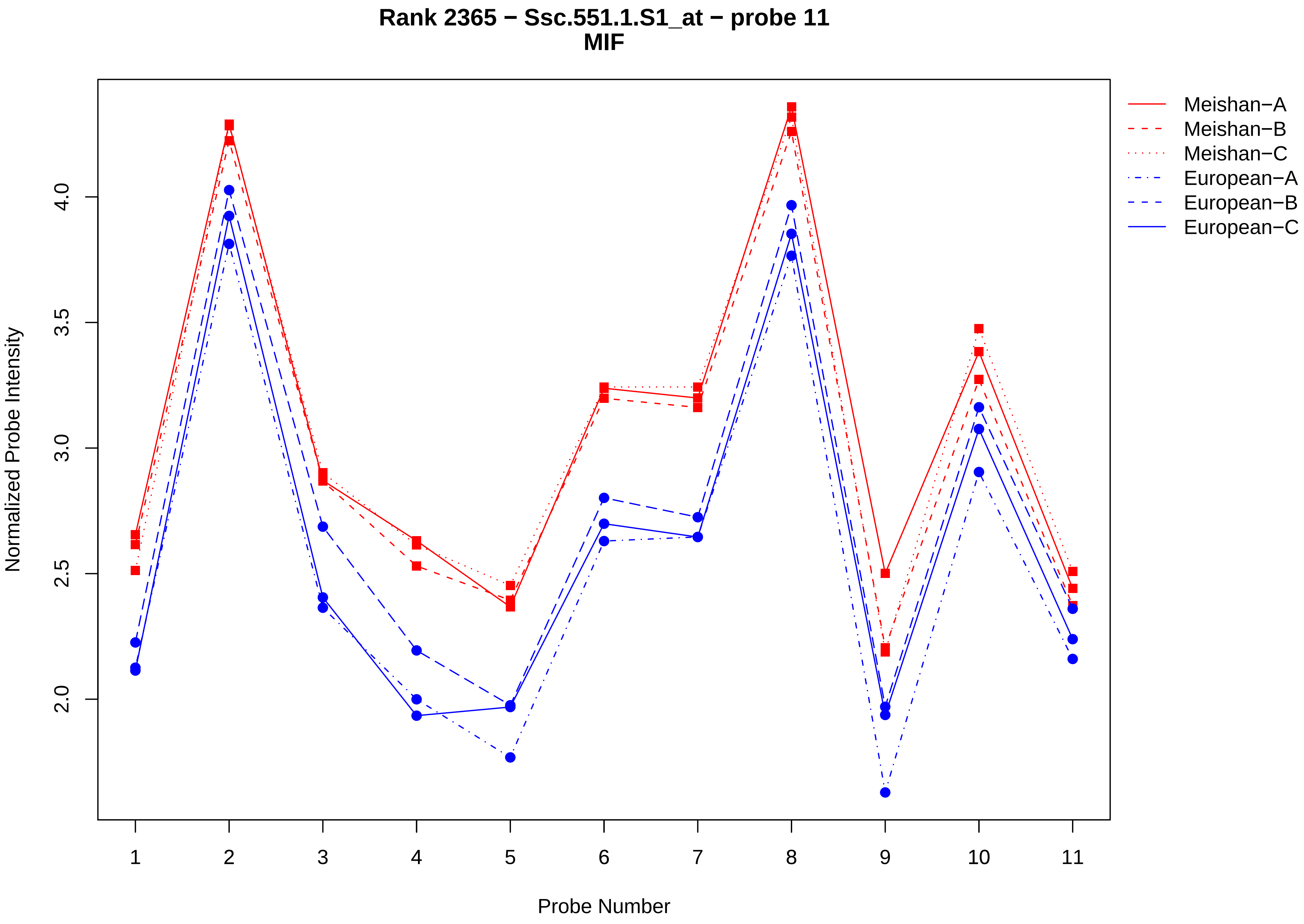

Rank 2365 − Ssc.551.1.S1_at − probe 11
MIF
Normalized Probe Intensity
Probe Number
2.0
2.5
3.0
3.5
4.0
1
2
3
4
5
6
7
8
9
10
11
Meishan−A
Meishan−B
Meishan−C
European−A
European−B
European−C

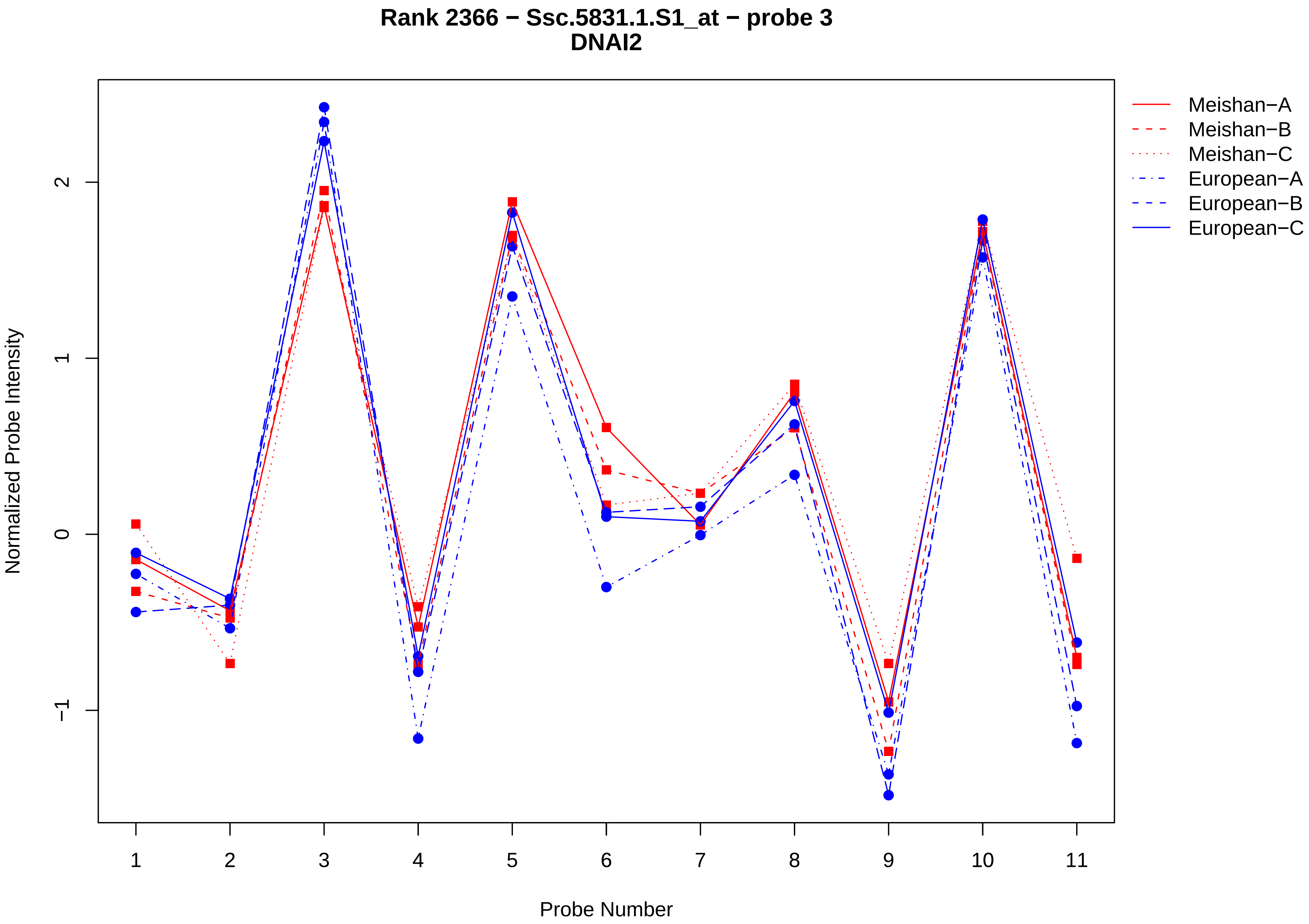

Rank 2366 − Ssc.5831.1.S1_at − probe 3
DNAI2
Normalized Probe Intensity
Probe Number
Meishan−A
Meishan−B
Meishan−C
European−A
European−B
European−C
−1
0
1
2
1
2
3
4
5
6
7
8
9
10
11

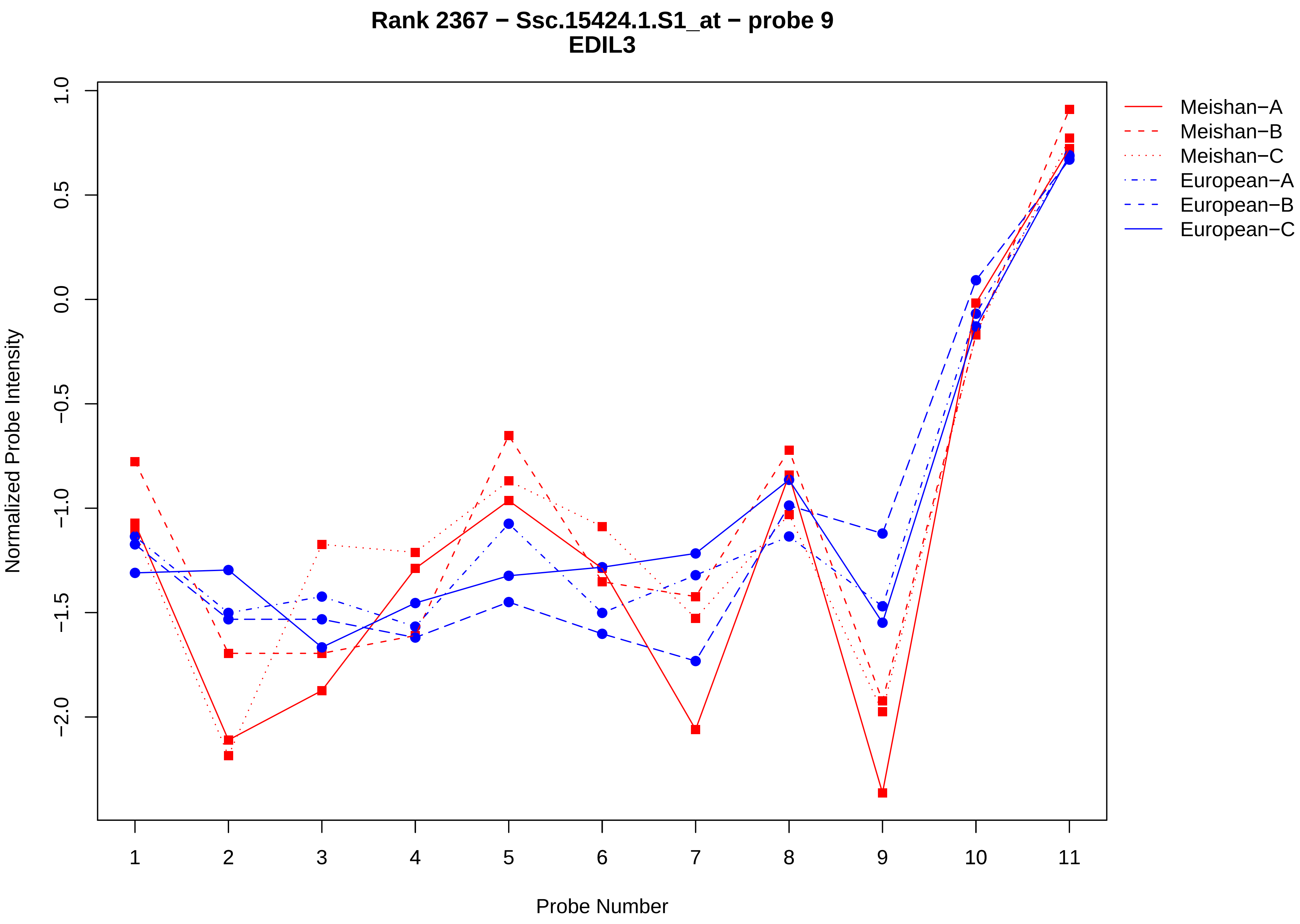

Rank 2367 − Ssc.15424.1.S1_at − probe 9
EDIL3
Normalized Probe Intensity
Probe Number
1.0
0.5
0.0
−0.5
−1.0
−1.5
−2.0
1
2
3
4
5
6
7
8
9
10
11
Meishan−A
Meishan−B
Meishan−C
European−A
European−B
European−C

Rank 2368 − Ssc.9538.1.S1_at − probe 7
Q8IWE4

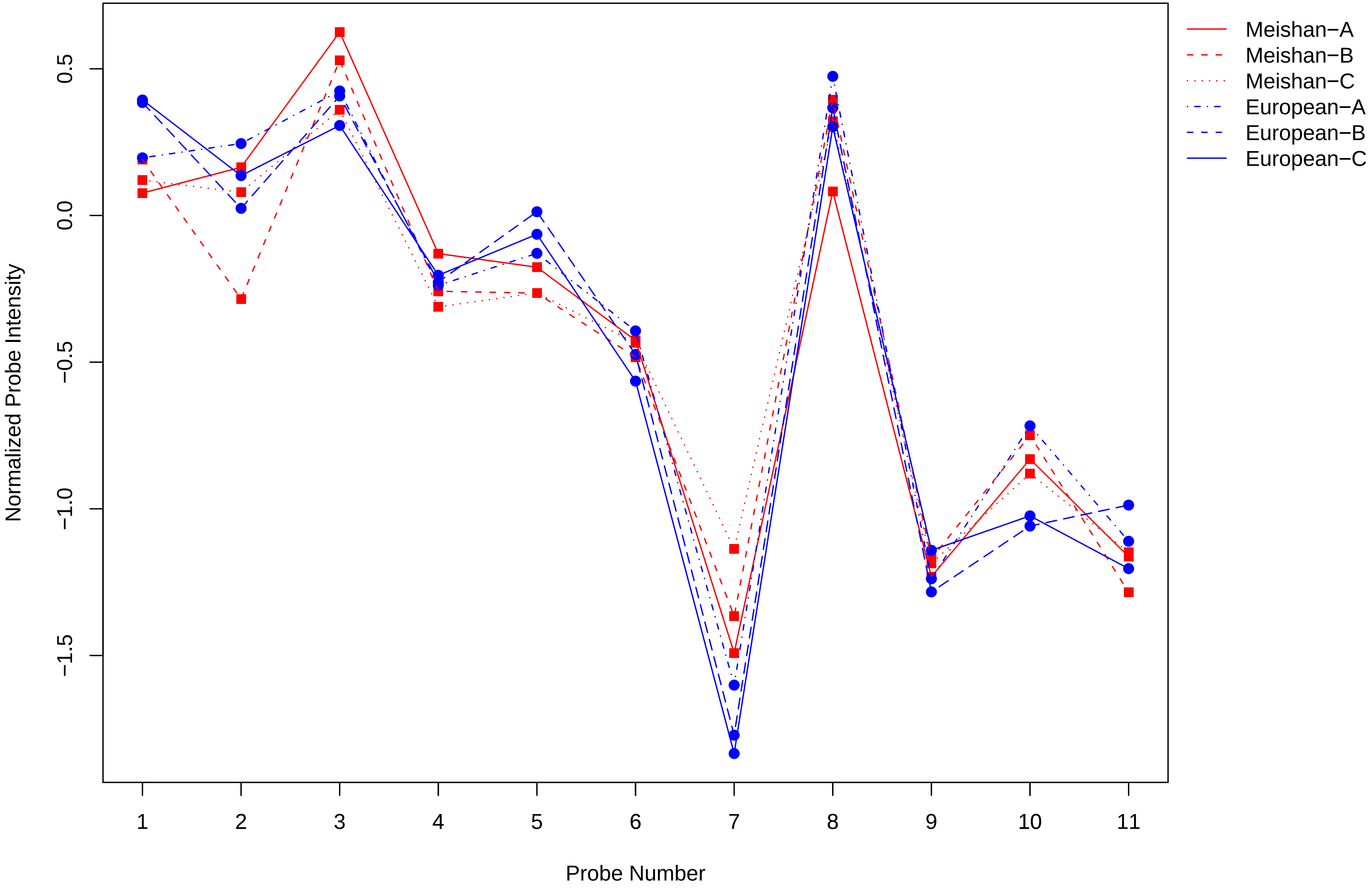

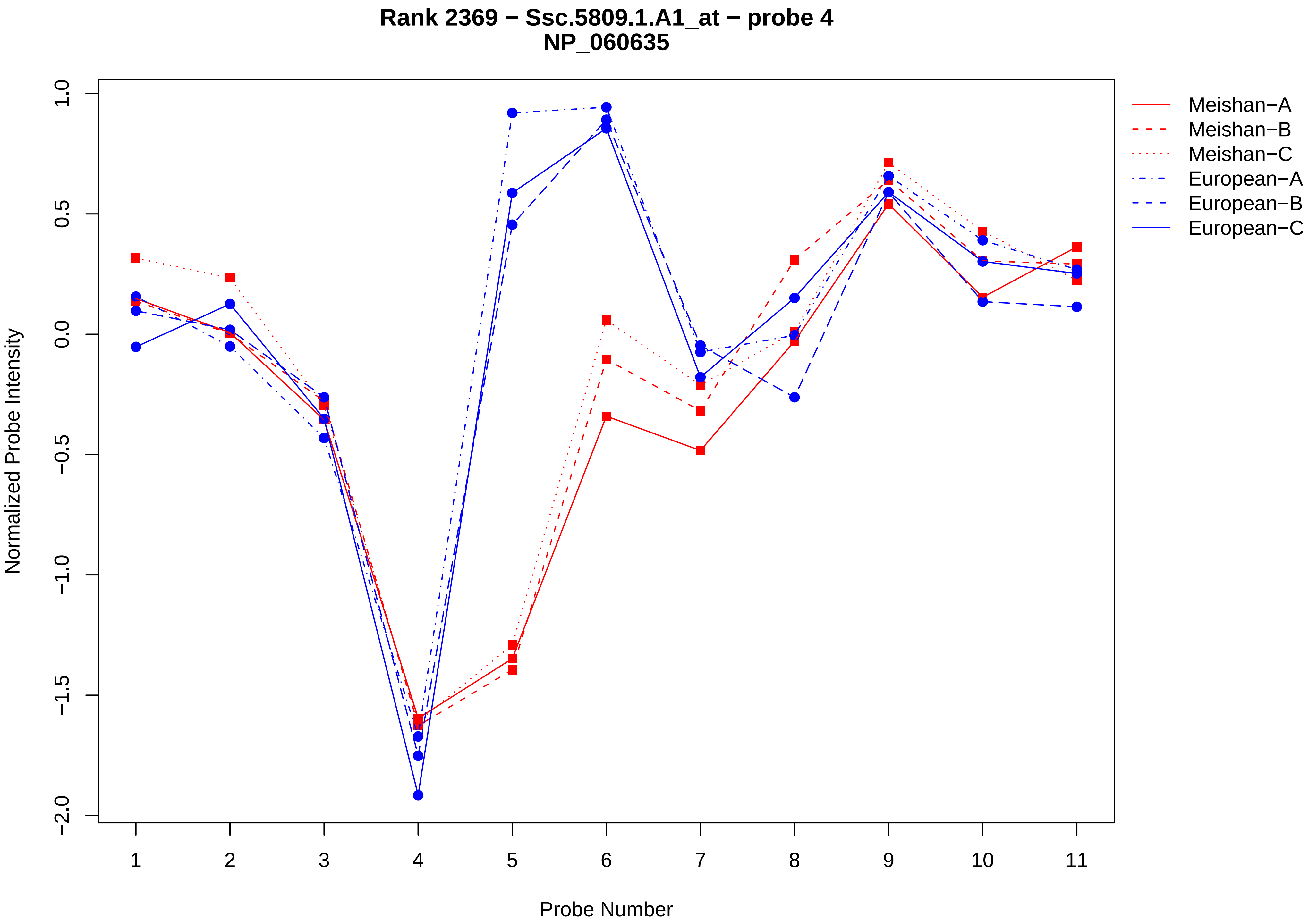

Rank 2369 − Ssc.5809.1.A1_at − probe 4
NP_060635
Normalized Probe Intensity
Probe Number
1.0
0.5
0.0
−0.5
−1.0
−1.5
−2.0
1
2
3
4
5
6
7
8
9
10
11
Meishan−A
Meishan−B
Meishan−C
European−A
European−B
European−C

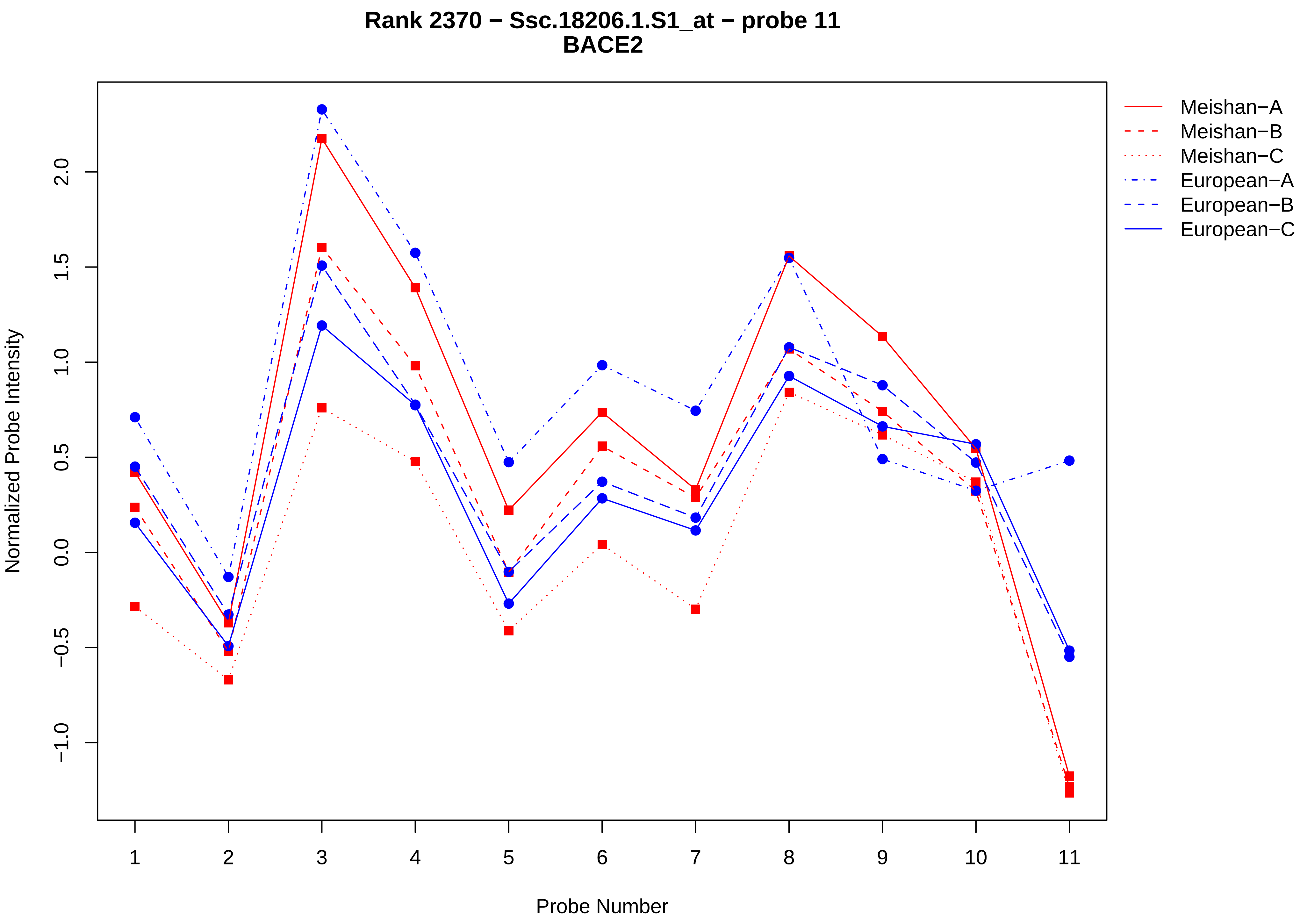
Rank 2370 − Ssc.18206.1.S1_at − probe 11
BACE2
Normalized Probe Intensity
Probe Number
−1.0
−0.5
0.0
0.5
1.0
1.5
2.0
1
2
3
4
5
6
7
8
9
10
11
Meishan−A
Meishan−B
Meishan−C
European−A
European−B
European−C

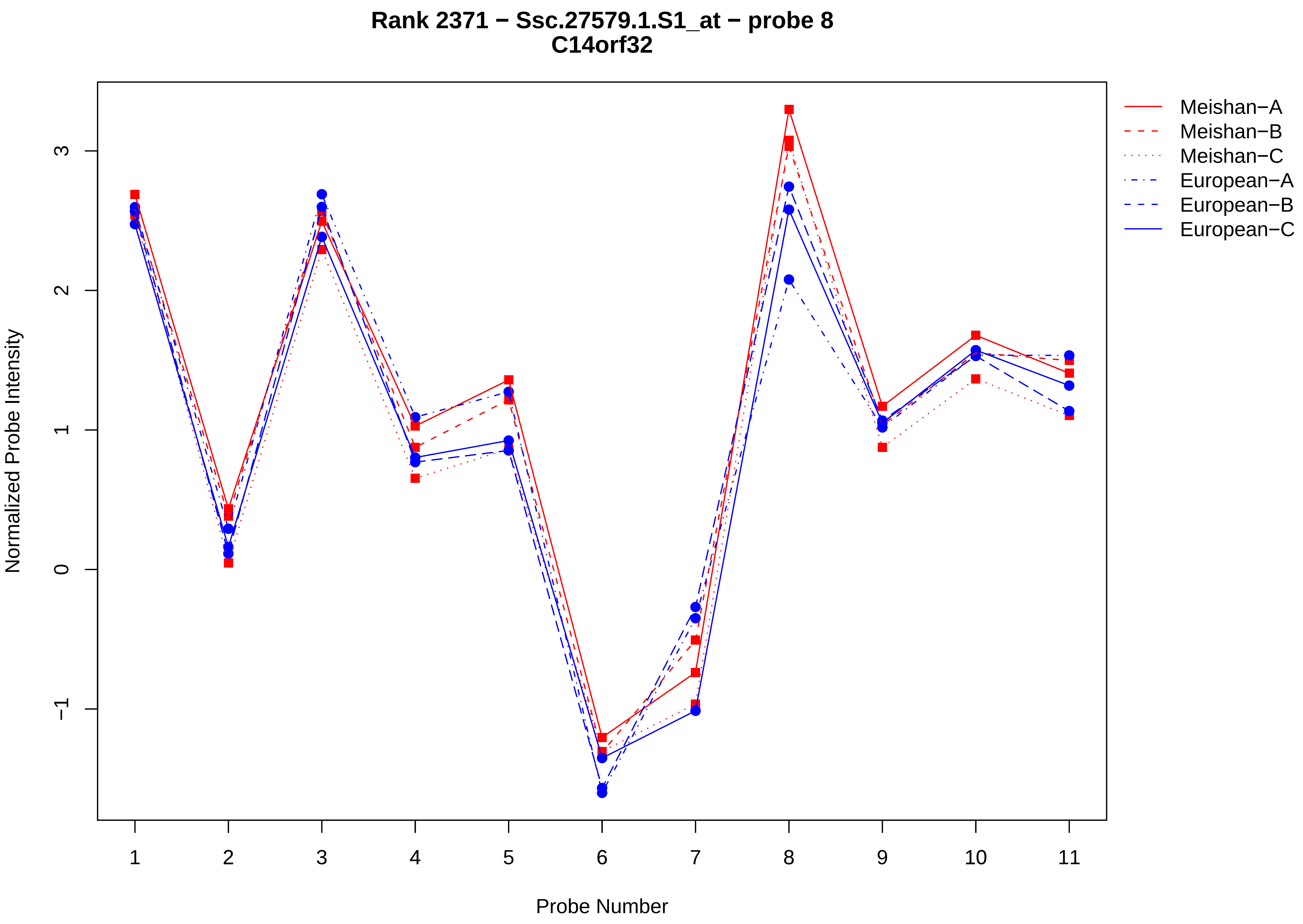
Rank 2371 − Ssc.27579.1.S1_at − probe 8
C14orf32
Normalized Probe Intensity
Probe Number
Meishan−A
Meishan−B
Meishan−C
European−A
European−B
European−C
−1
0
1
2
3
1
2
3
4
5
6
7
8
9
10
11

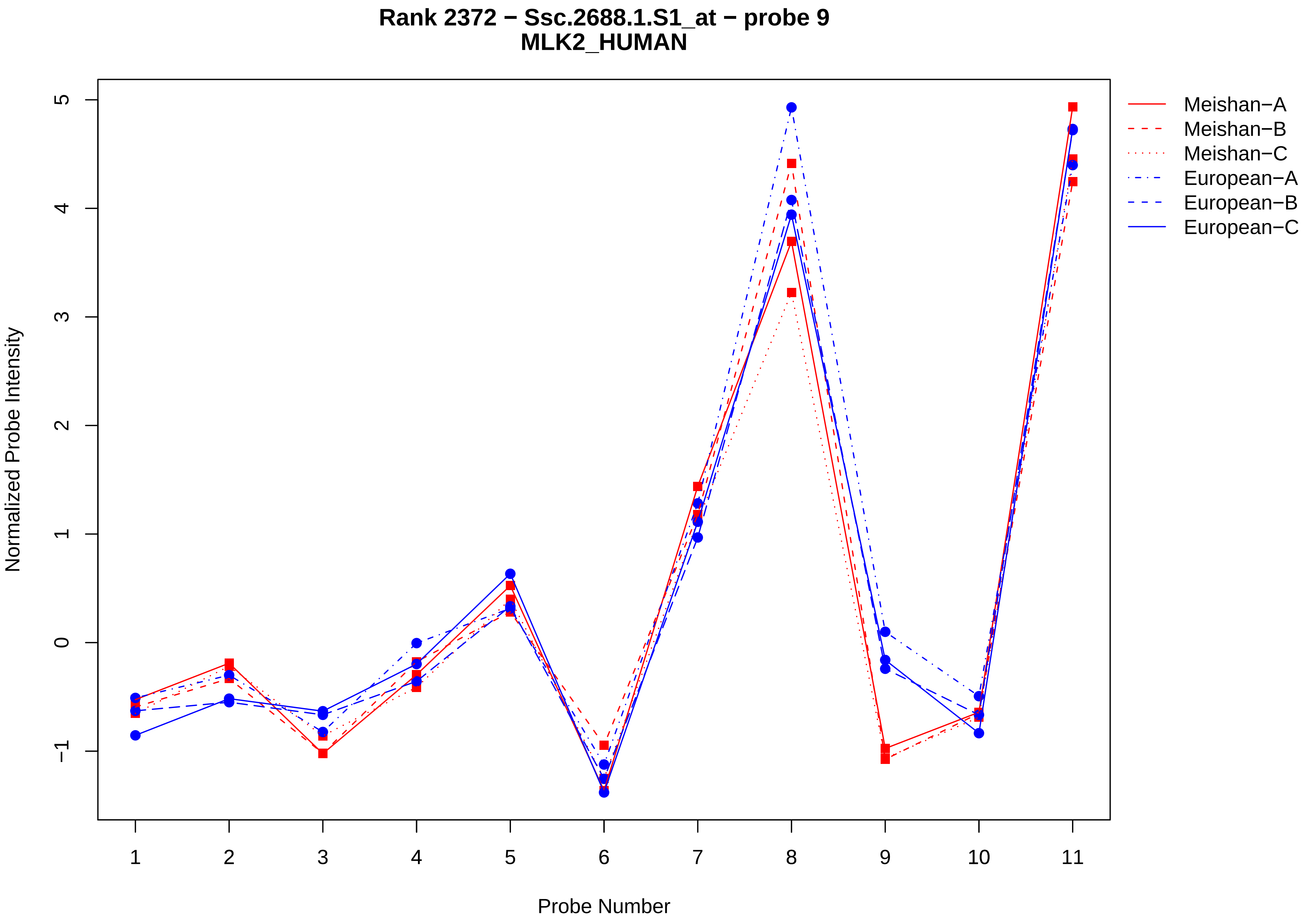

Rank 2372 − Ssc.2688.1.S1_at − probe 9
MLK2_HUMAN
Normalized Probe Intensity
Probe Number
Meishan−A
Meishan−B
Meishan−C
European−A
European−B
European−C
−1
0
1
2
3
4
5
1
2
3
4
5
6
7
8
9
10
11

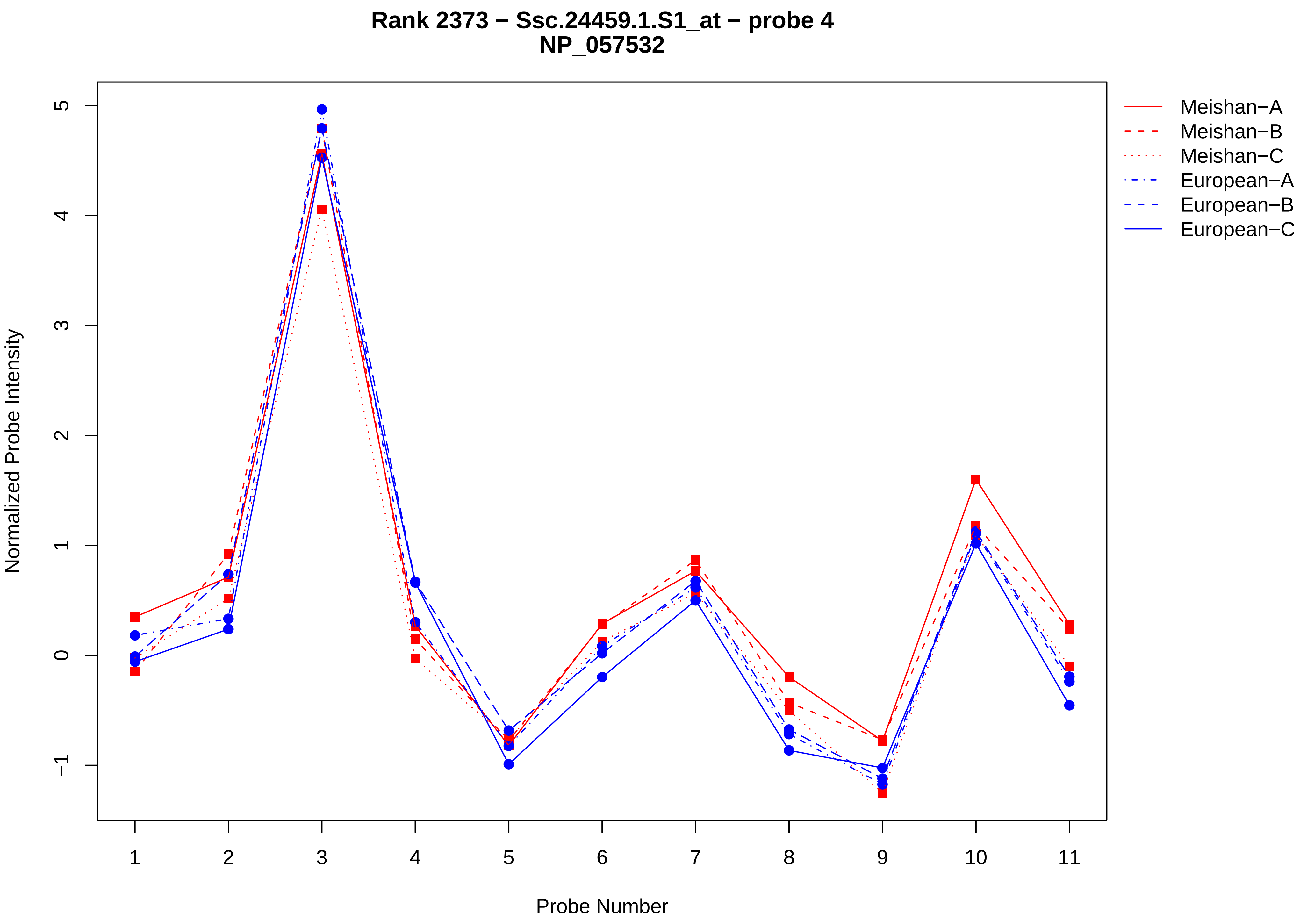

Rank 2373 − Ssc.24459.1.S1_at − probe 4
NP_057532
Normalized Probe Intensity
Probe Number
Meishan−A
Meishan−B
Meishan−C
European−A
European−B
European−C

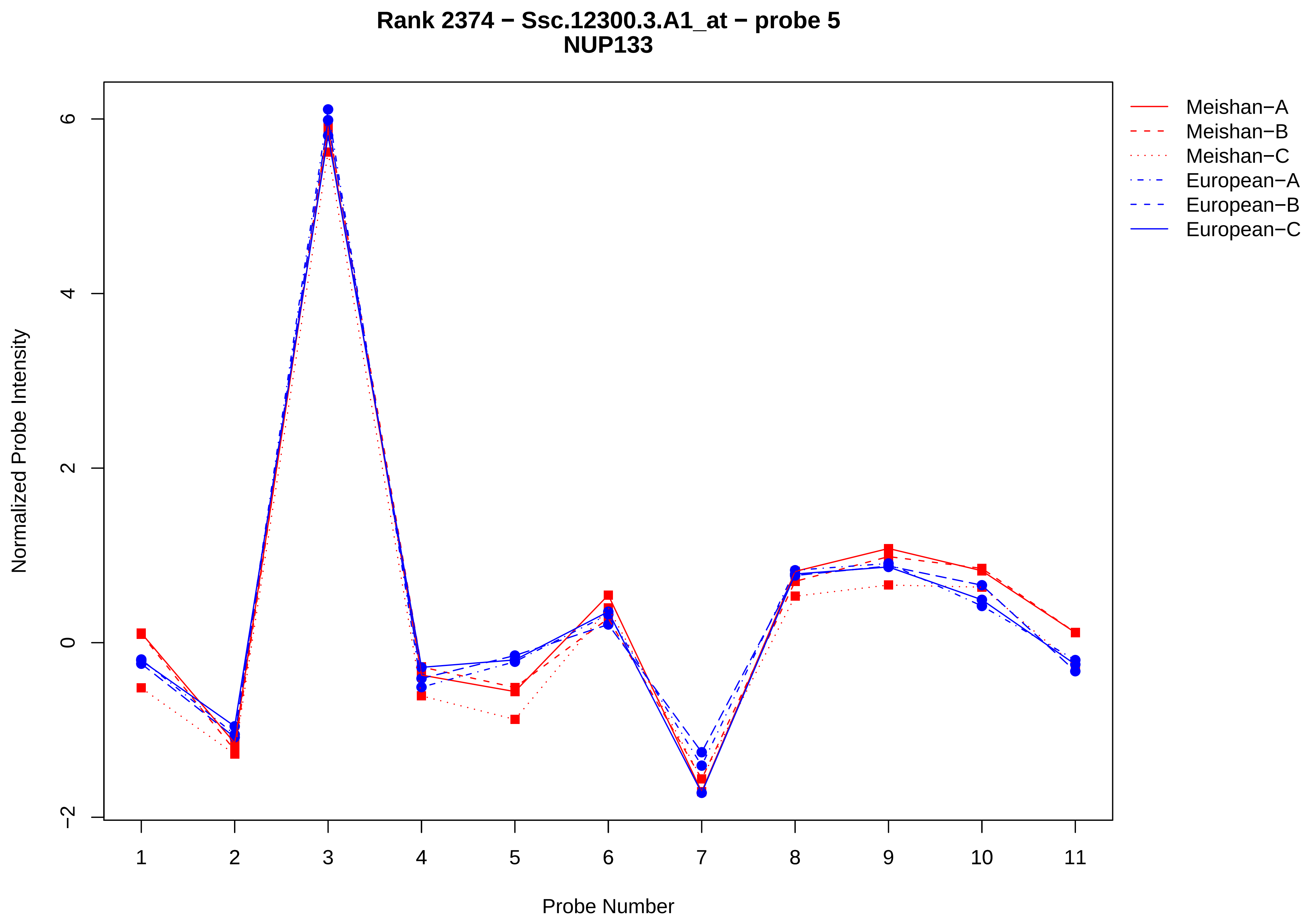

Rank 2374 − Ssc.12300.3.A1_at − probe 5
NUP133
Normalized Probe Intensity
Probe Number
Meishan−A
Meishan−B
Meishan−C
European−A
European−B
European−C

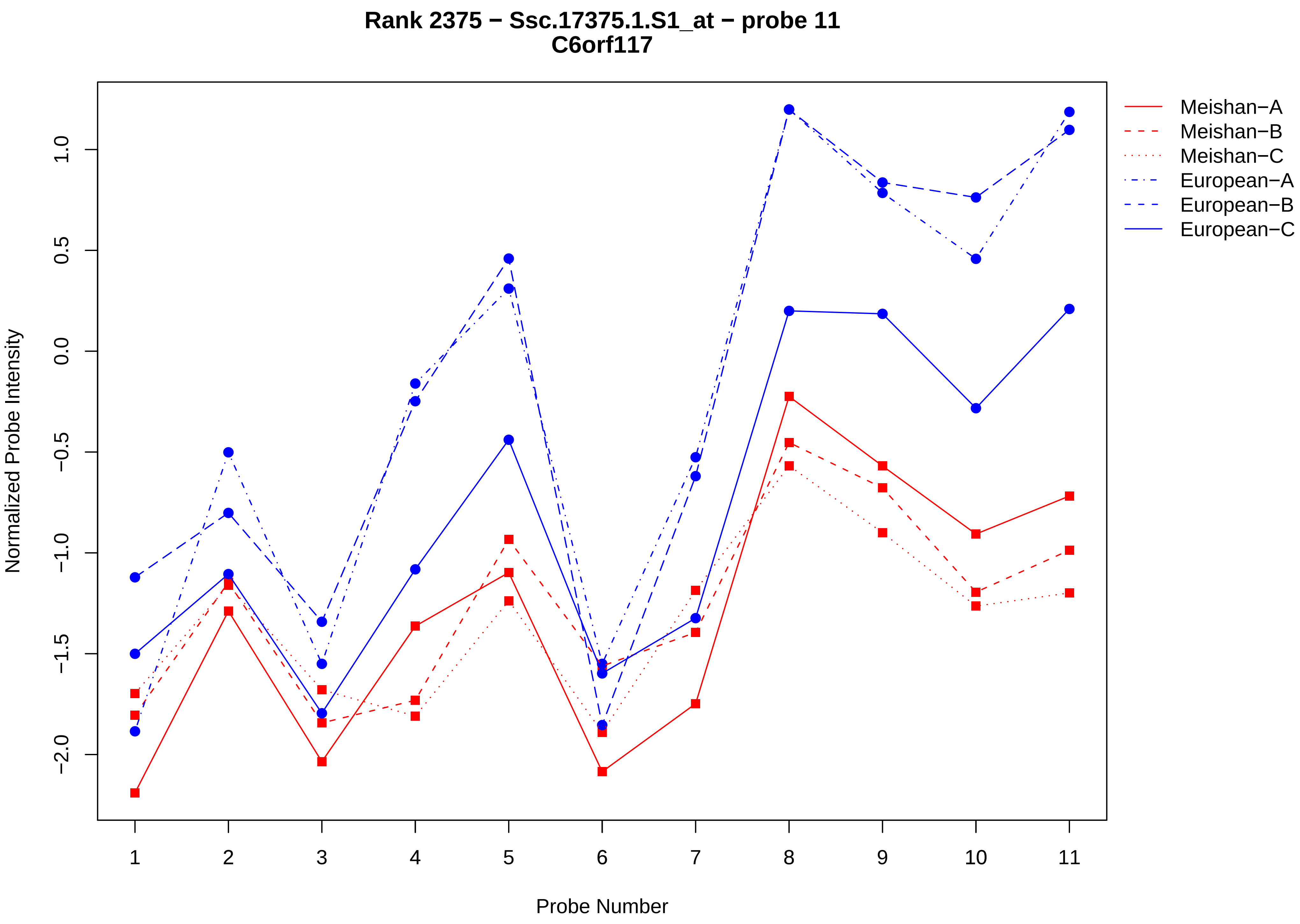
Rank 2375 − Ssc.17375.1.S1_at − probe 11
C6orf117
Normalized Probe Intensity
Probe Number
−2.0
−1.5
−1.0
−0.5
0.0
0.5
1.0
1
2
3
4
5
6
7
8
9
10
11
Meishan−A
Meishan−B
Meishan−C
European−A
European−B
European−C

Rank 2376 − Ssc.25314.1.S1_a_at − probe 1
AHCY

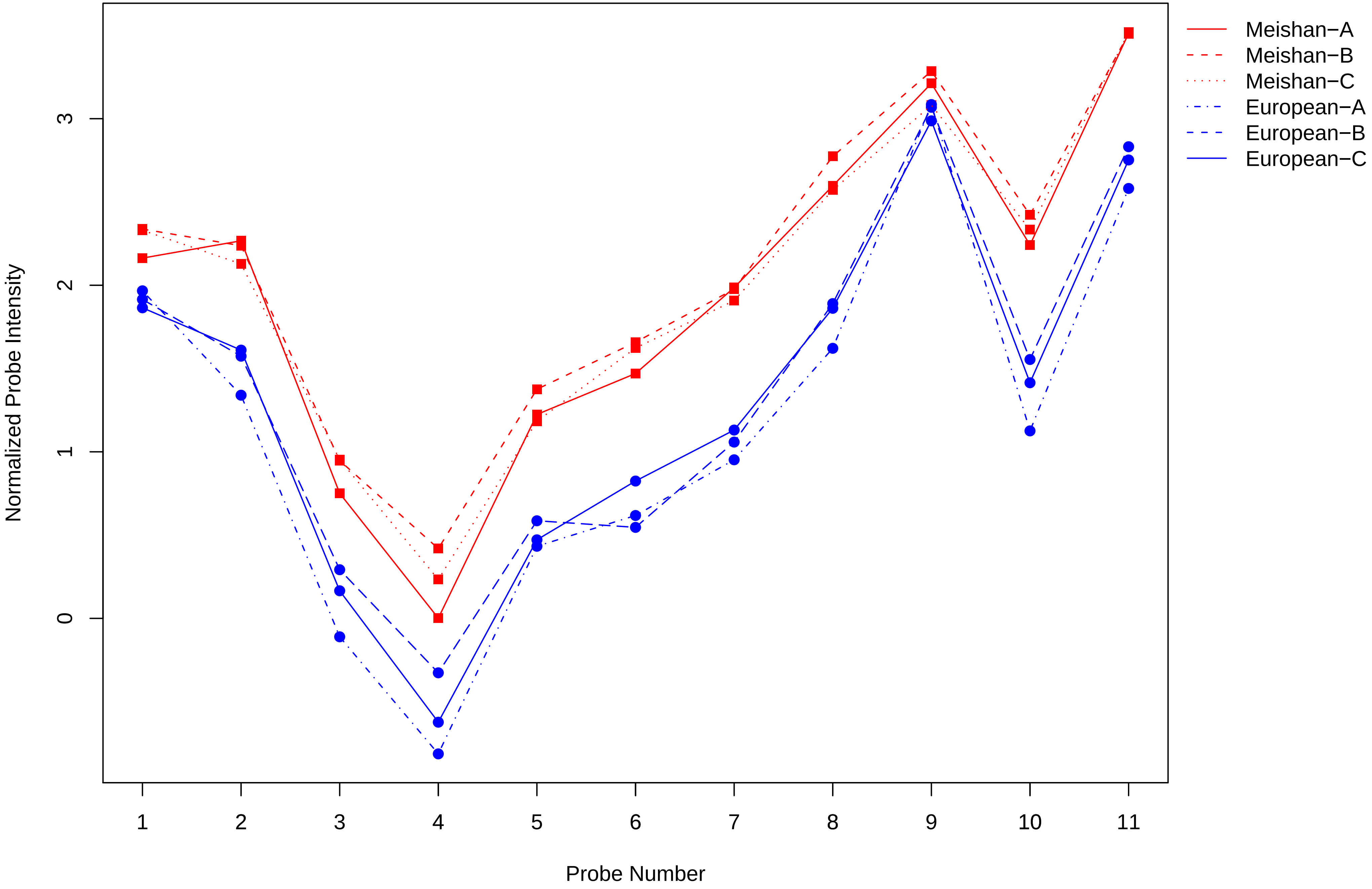

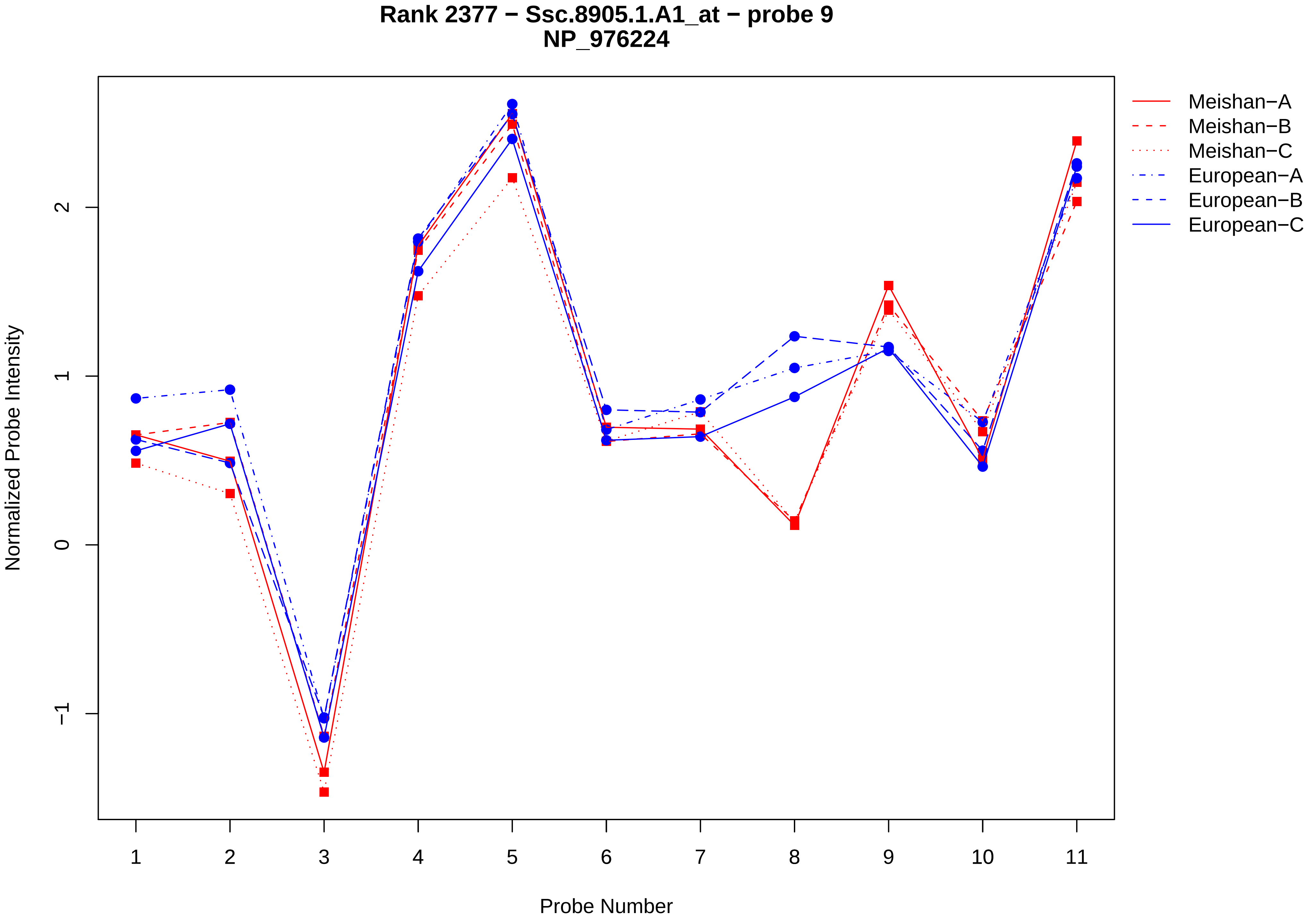

Rank 2377 − Ssc.8905.1.A1_at − probe 9
NP_976224
Normalized Probe Intensity
Probe Number
−1
0
1
2
1
2
3
4
5
6
7
8
9
10
11
Meishan−A
Meishan−B
Meishan−C
European−A
European−B
European−C

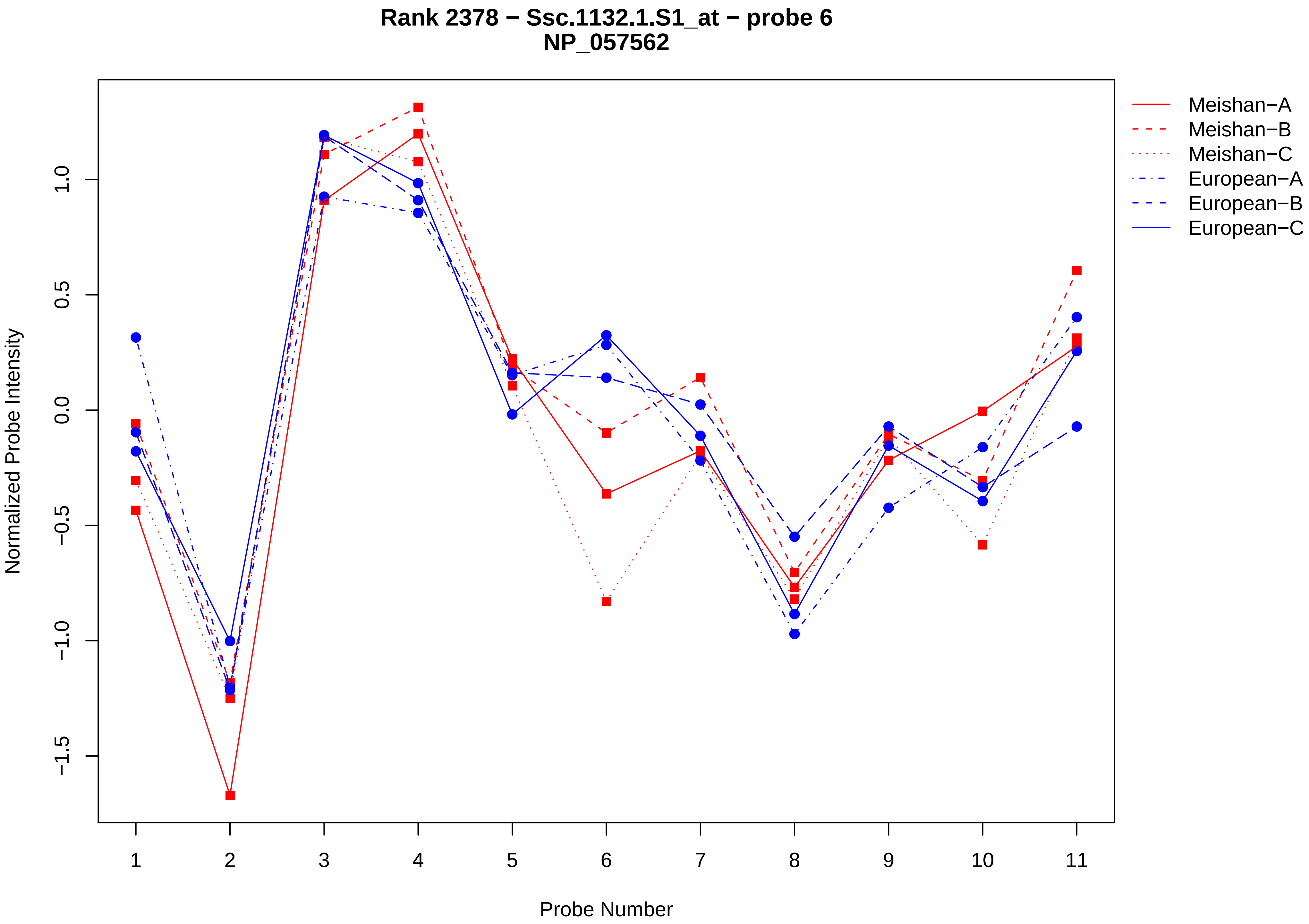

Rank 2378 − Ssc.1132.1.S1_at − probe 6
NP_057562
Normalized Probe Intensity
Probe Number
1.0
0.5
0.0
−0.5
−1.0
−1.5
1
2
3
4
5
6
7
8
9
10
11
Meishan−A
Meishan−B
Meishan−C
European−A
European−B
European−C

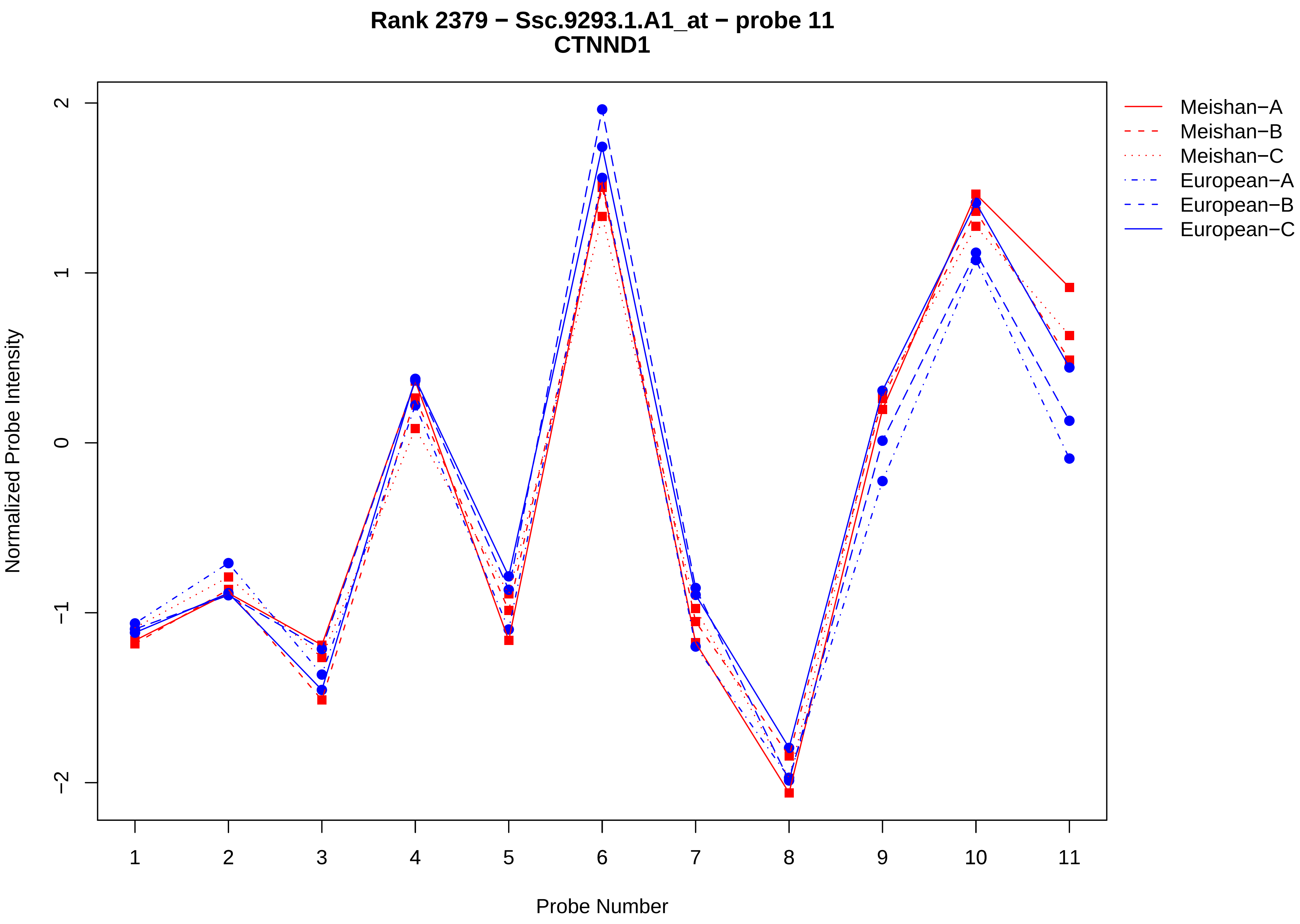

Rank 2379 − Ssc.9293.1.A1_at − probe 11
CTNND1
Normalized Probe Intensity
Probe Number
Meishan−A
Meishan−B
Meishan−C
European−A
European−B
European−C
2
1
0
−1
−2
1
2
3
4
5
6
7
8
9
10
11

Rank 2380 − Ssc.3188.1.S1_at − probe 10
Q9BRG1

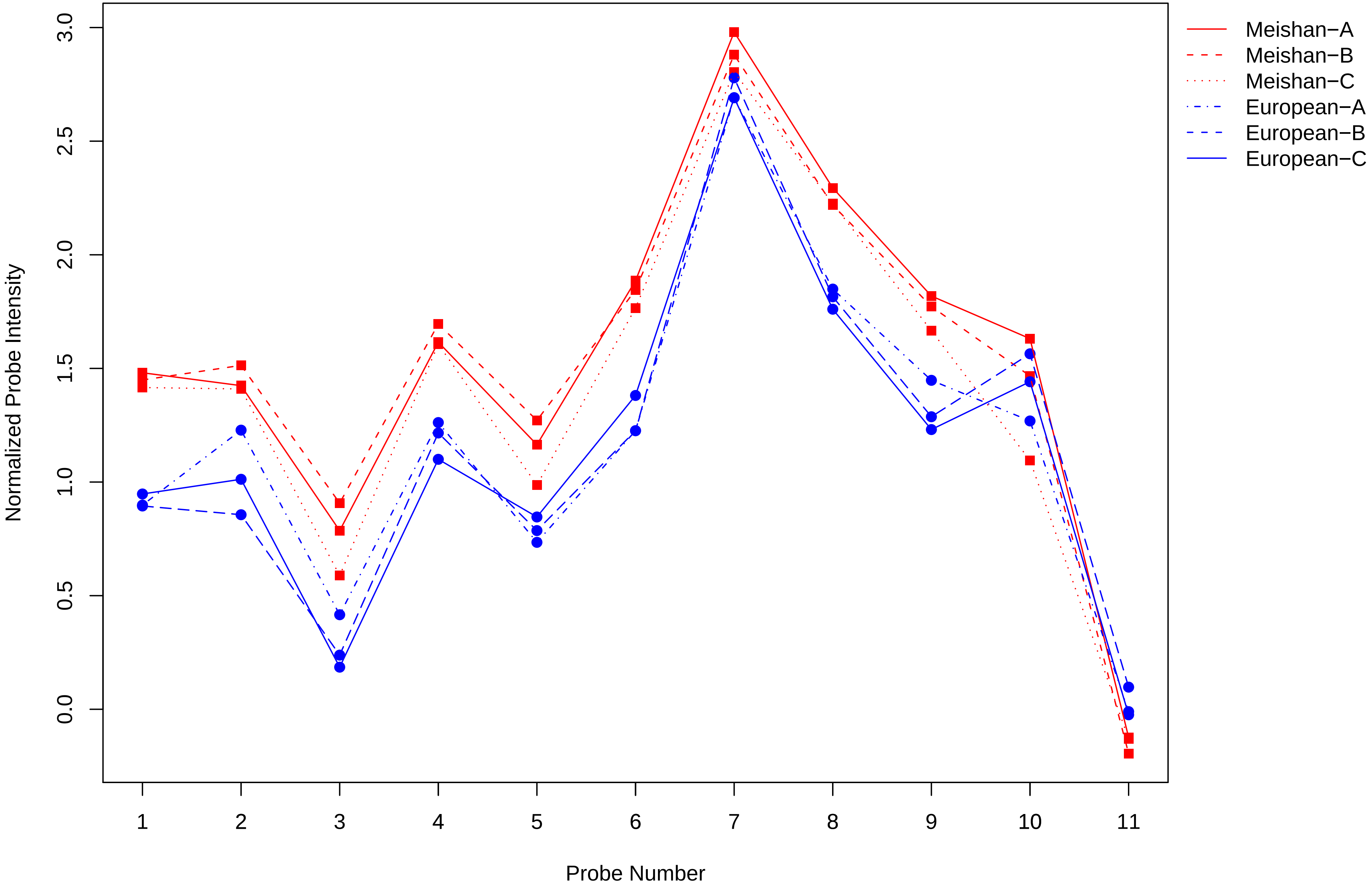

Rank 2381 − Ssc.9461.1.A1_at − probe 2
MIG6_HUMAN

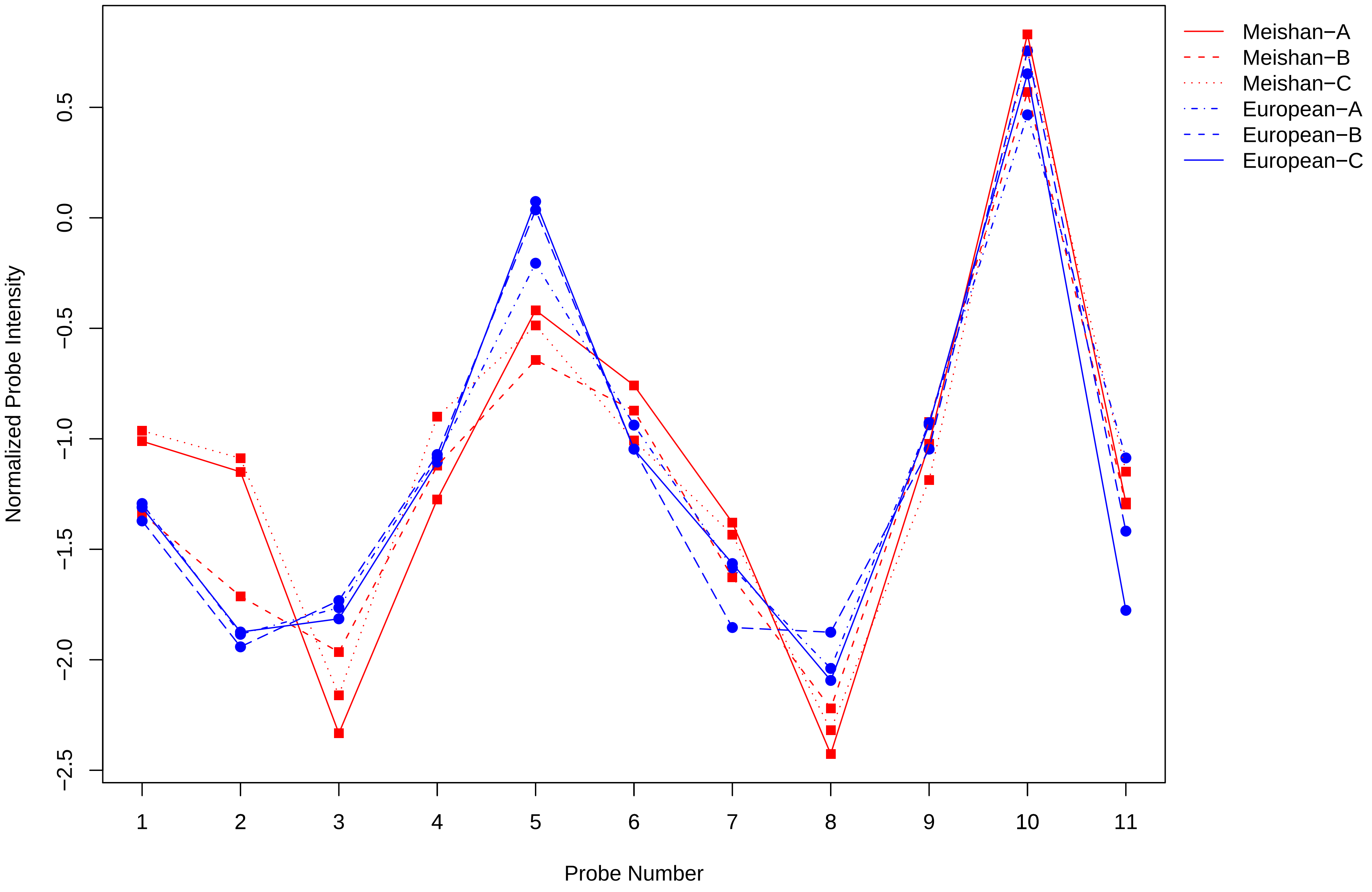

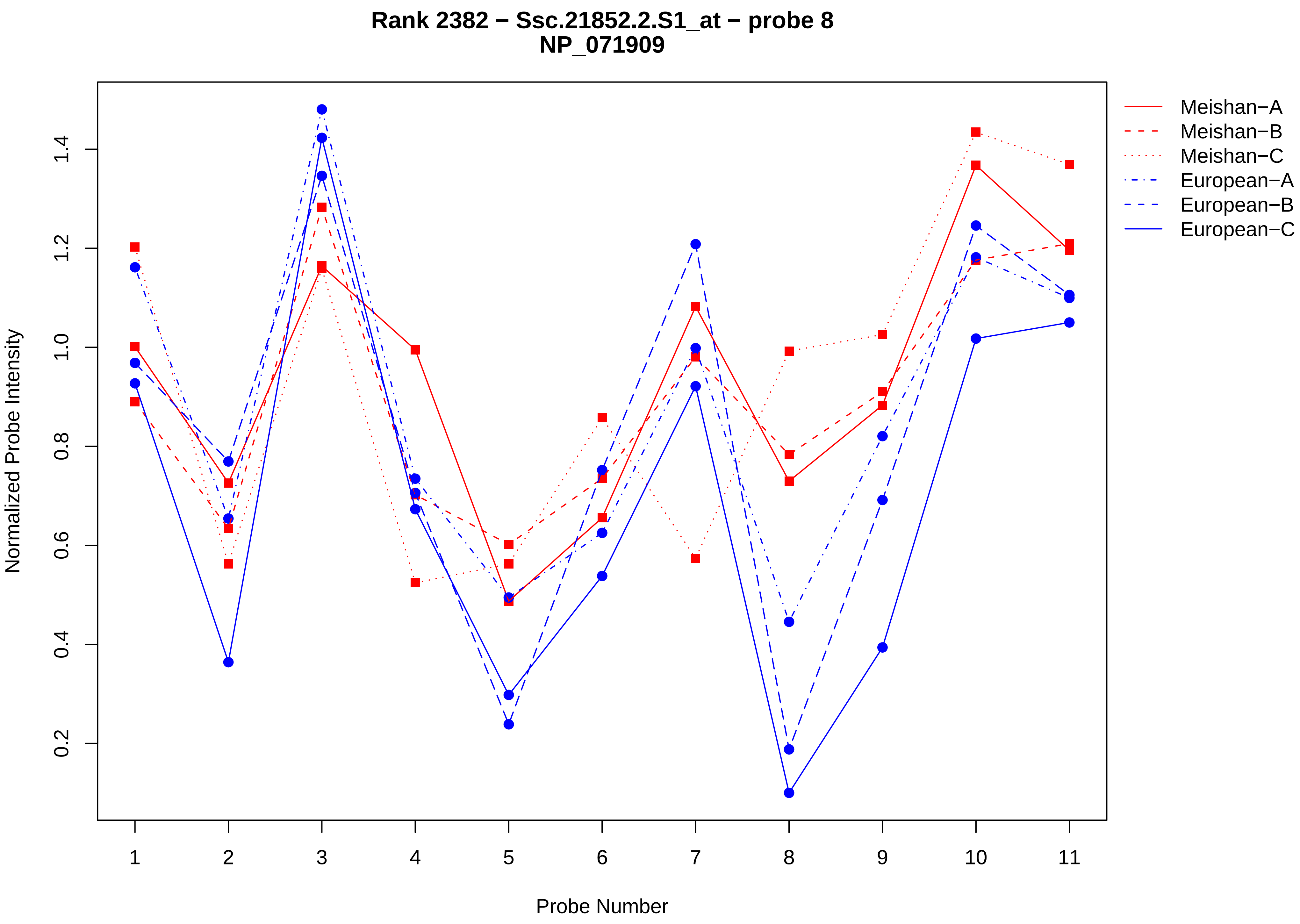

Rank 2382 − Ssc.21852.2.S1_at − probe 8
NP_071909
Normalized Probe Intensity
0.2
0.4
0.6
0.8
1.0
1.2
1.4
Probe Number
1
2
3
4
5
6
7
8
9
10
11
Meishan−A
Meishan−B
Meishan−C
European−A
European−B
European−C

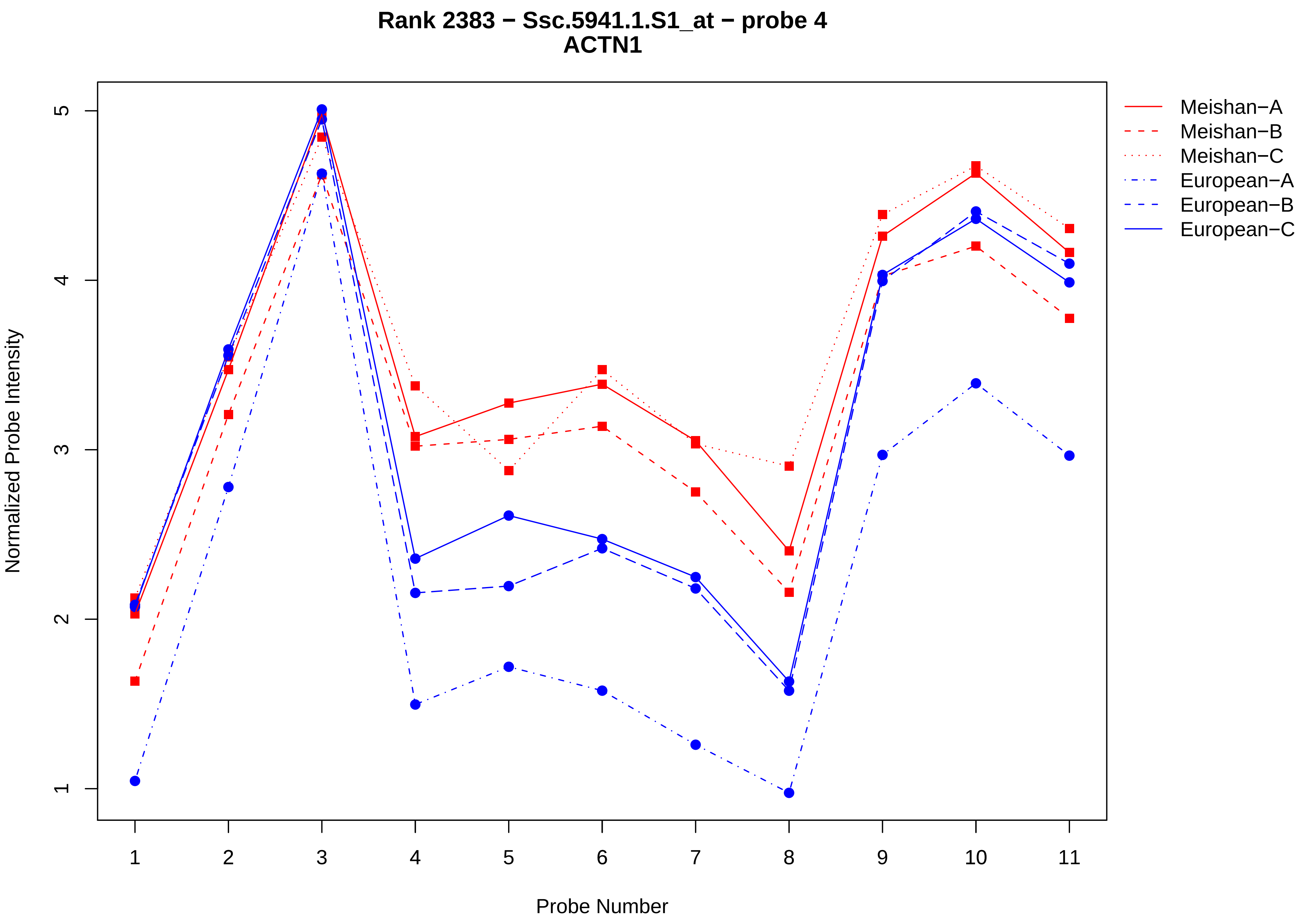

Rank 2383 − Ssc.5941.1.S1_at − probe 4
ACTN1
Normalized Probe Intensity
Probe Number
1
2
3
4
5
1
2
3
4
5
6
7
8
9
10
11
Meishan−A
Meishan−B
Meishan−C
European−A
European−B
European−C

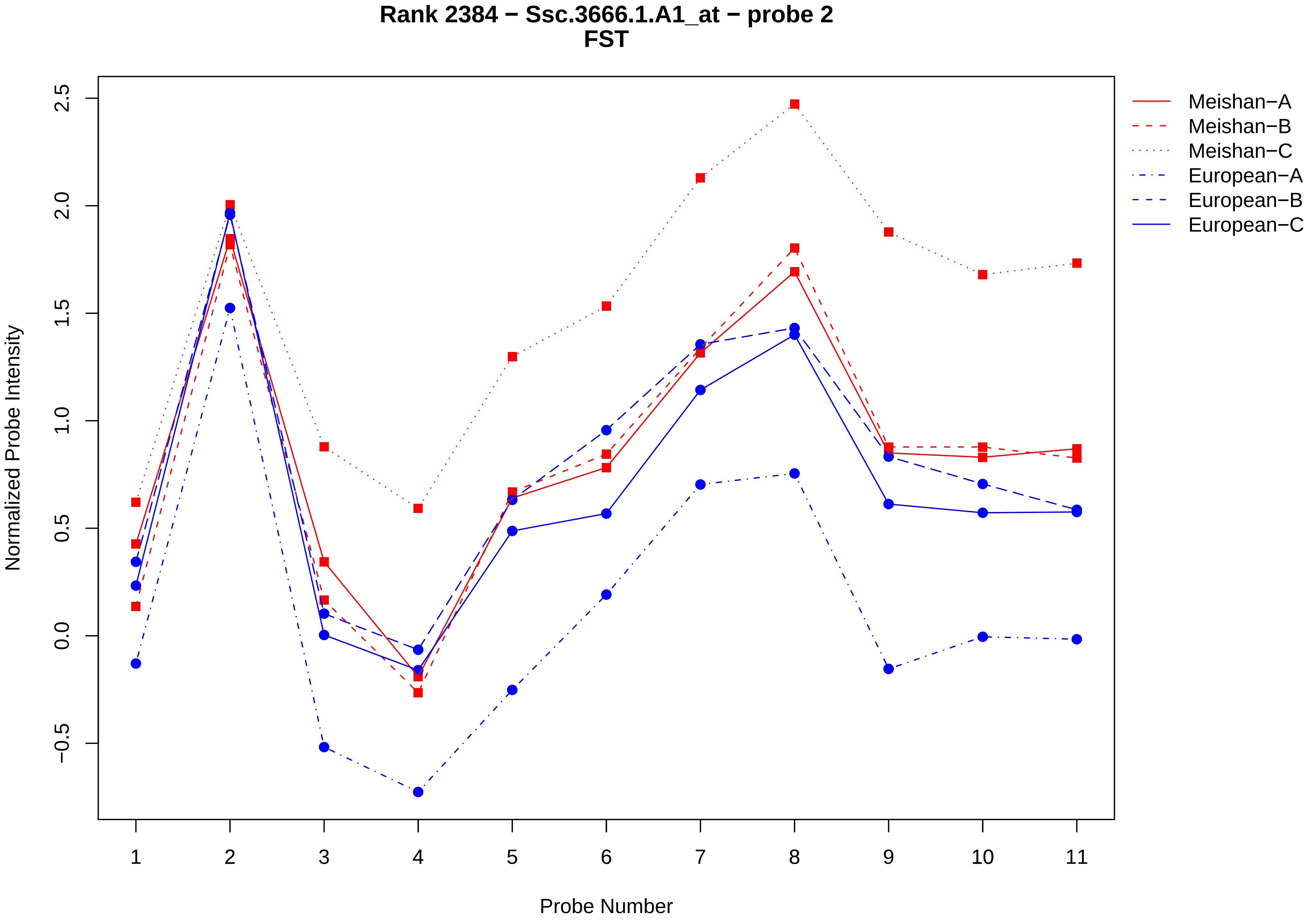

Rank 2384 − Ssc.3666.1.A1_at − probe 2
FST
Normalized Probe Intensity
Probe Number
Meishan−A
Meishan−B
Meishan−C
European−A
European−B
European−C

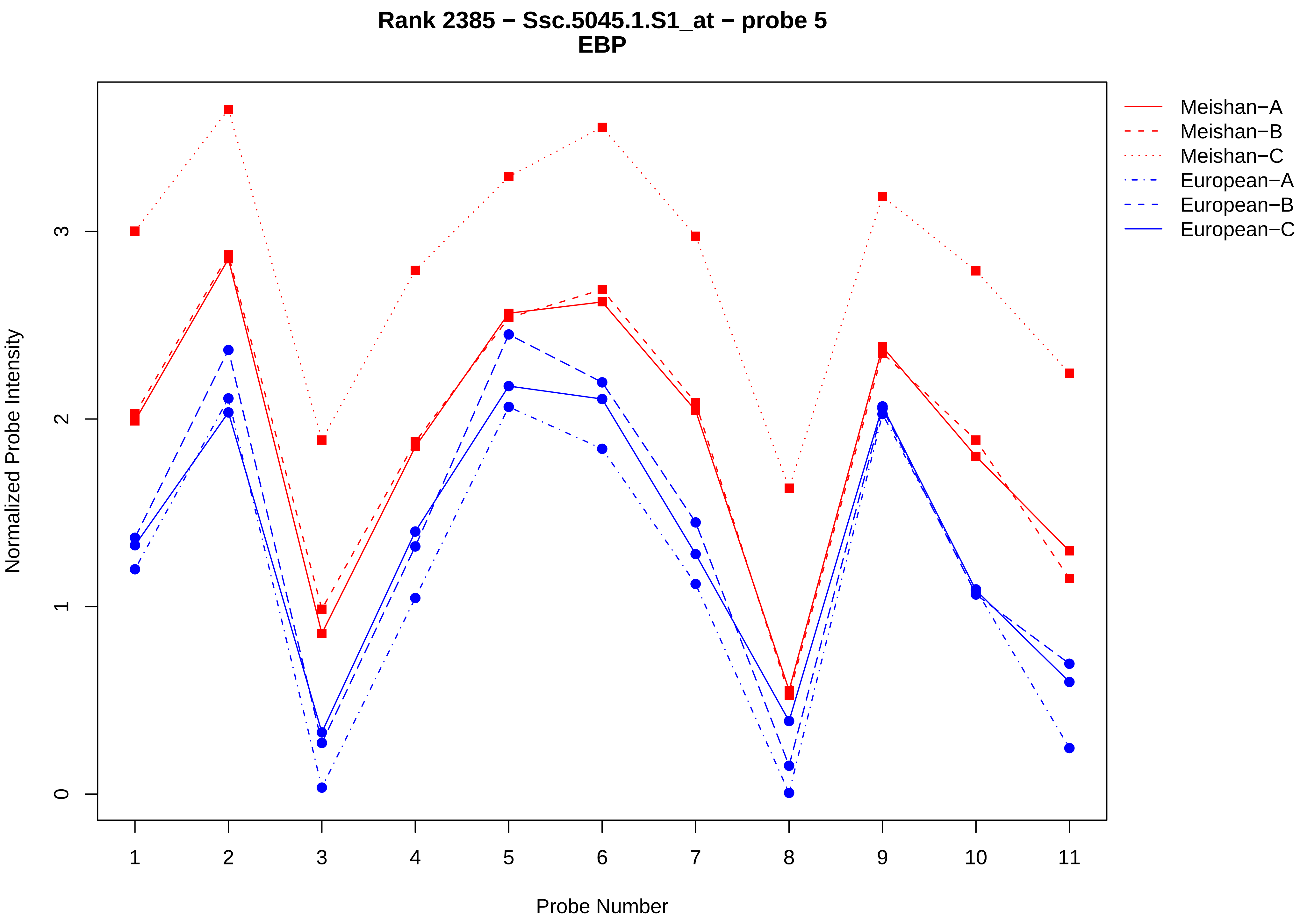

Rank 2385 − Ssc.5045.1.S1_at − probe 5
EBP
Normalized Probe Intensity
Probe Number
0
1
2
3
1
2
3
4
5
6
7
8
9
10
11
Meishan−A
Meishan−B
Meishan−C
European−A
European−B
European−C

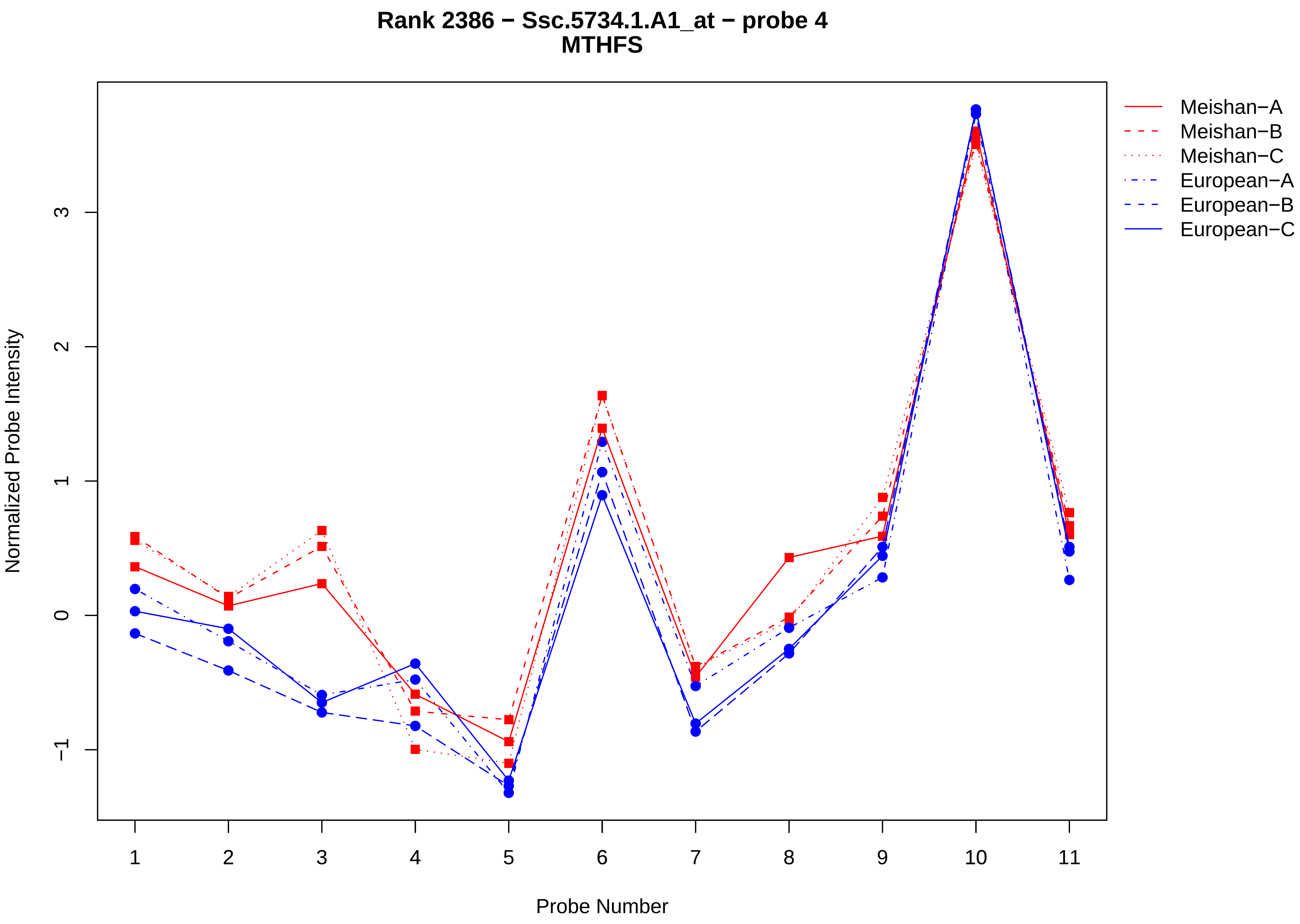

Rank 2386 − Ssc.5734.1.A1_at − probe 4
MTHFS
Normalized Probe Intensity
Probe Number
Meishan−A
Meishan−B
Meishan−C
European−A
European−B
European−C
−1
0
1
2
3
1
2
3
4
5
6
7
8
9
10
11

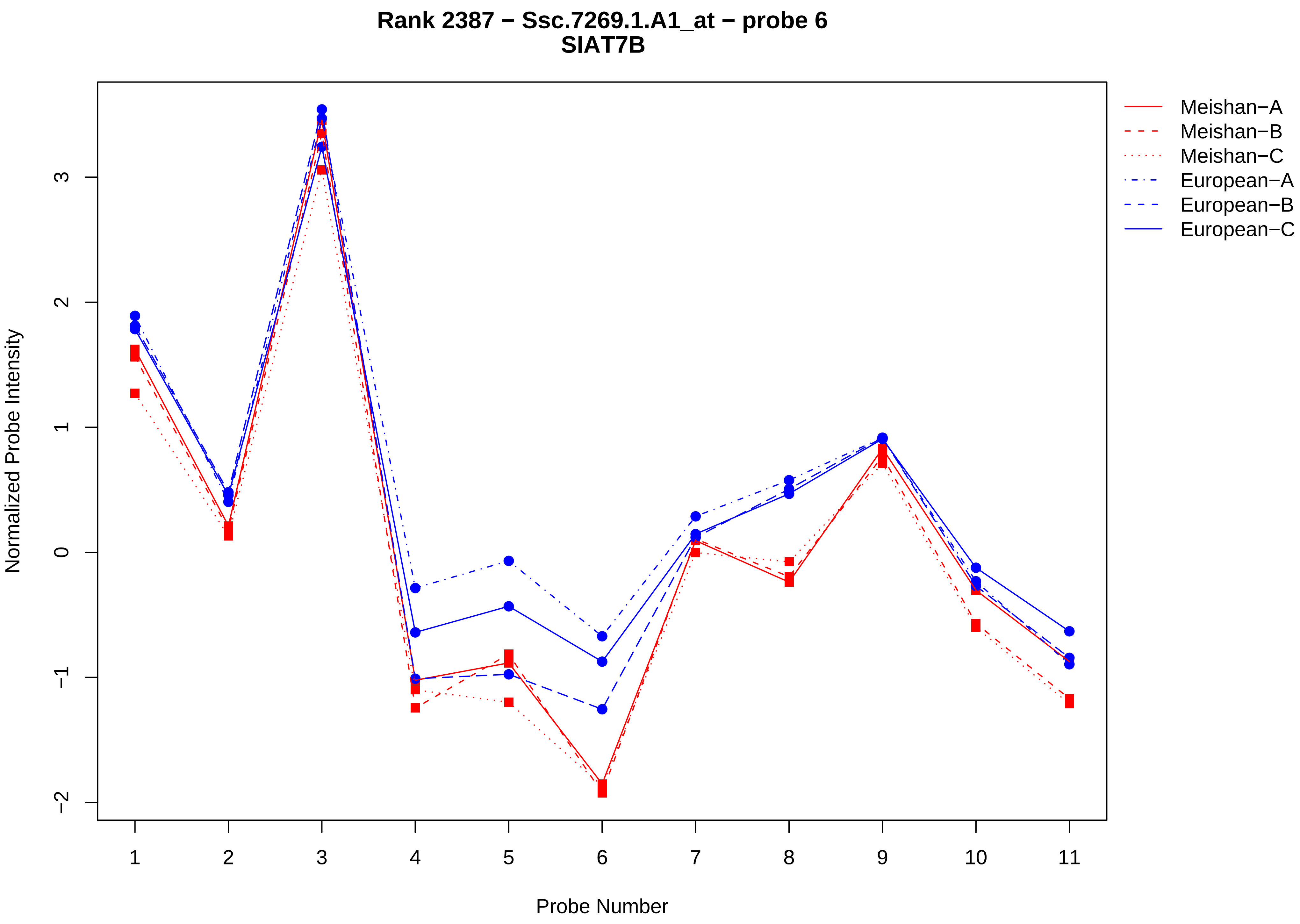

Rank 2387 − Ssc.7269.1.A1_at − probe 6
SIAT7B
Normalized Probe Intensity
Probe Number
−2
−1
0
1
2
3
1
2
3
4
5
6
7
8
9
10
11
Meishan−A
Meishan−B
Meishan−C
European−A
European−B
European−C

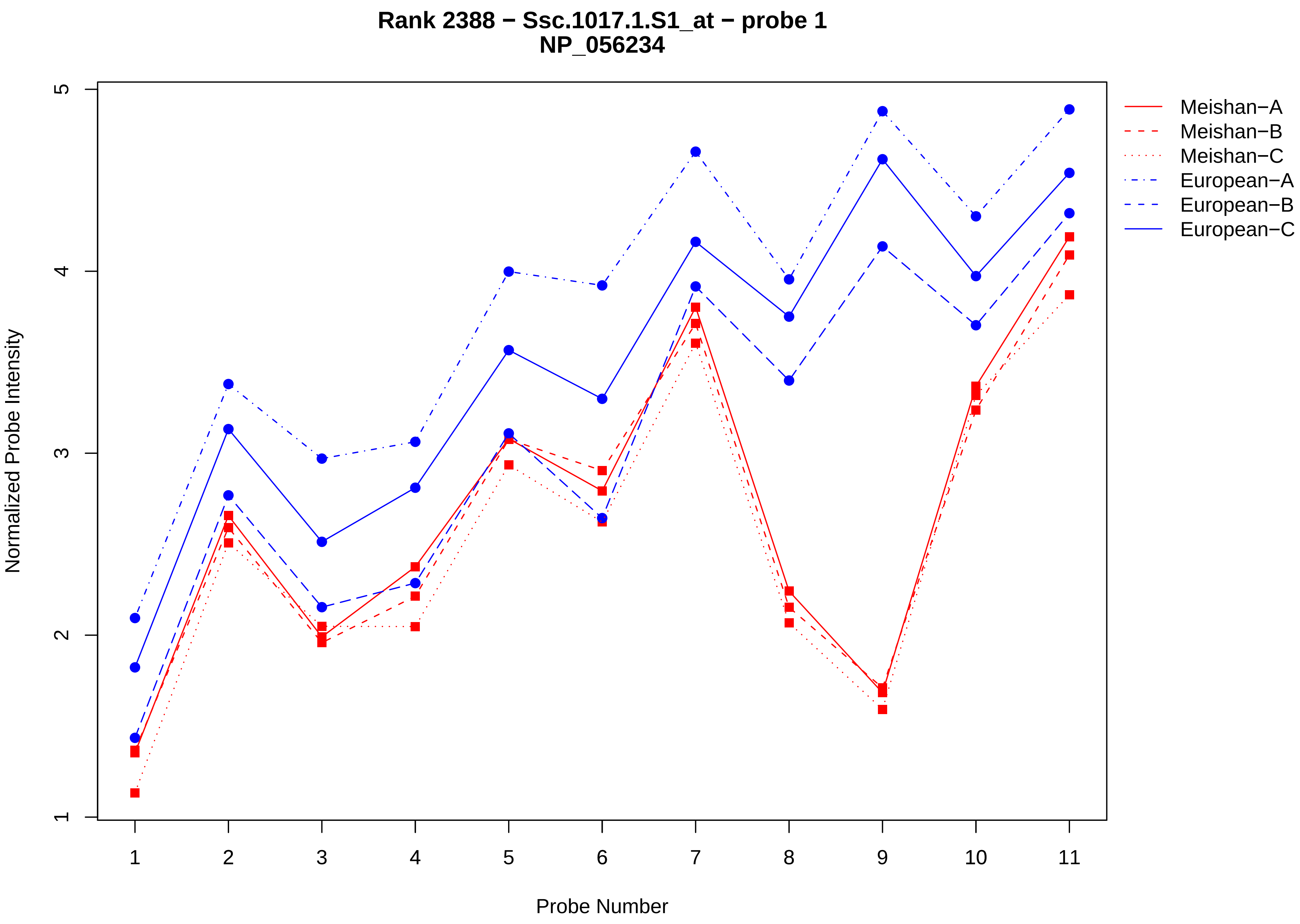
Rank 2388 − Ssc.1017.1.S1_at − probe 1
NP_056234
Normalized Probe Intensity
Probe Number
Meishan−A
Meishan−B
Meishan−C
European−A
European−B
European−C
1
2
3
4
5
6
7
8
9
10
11

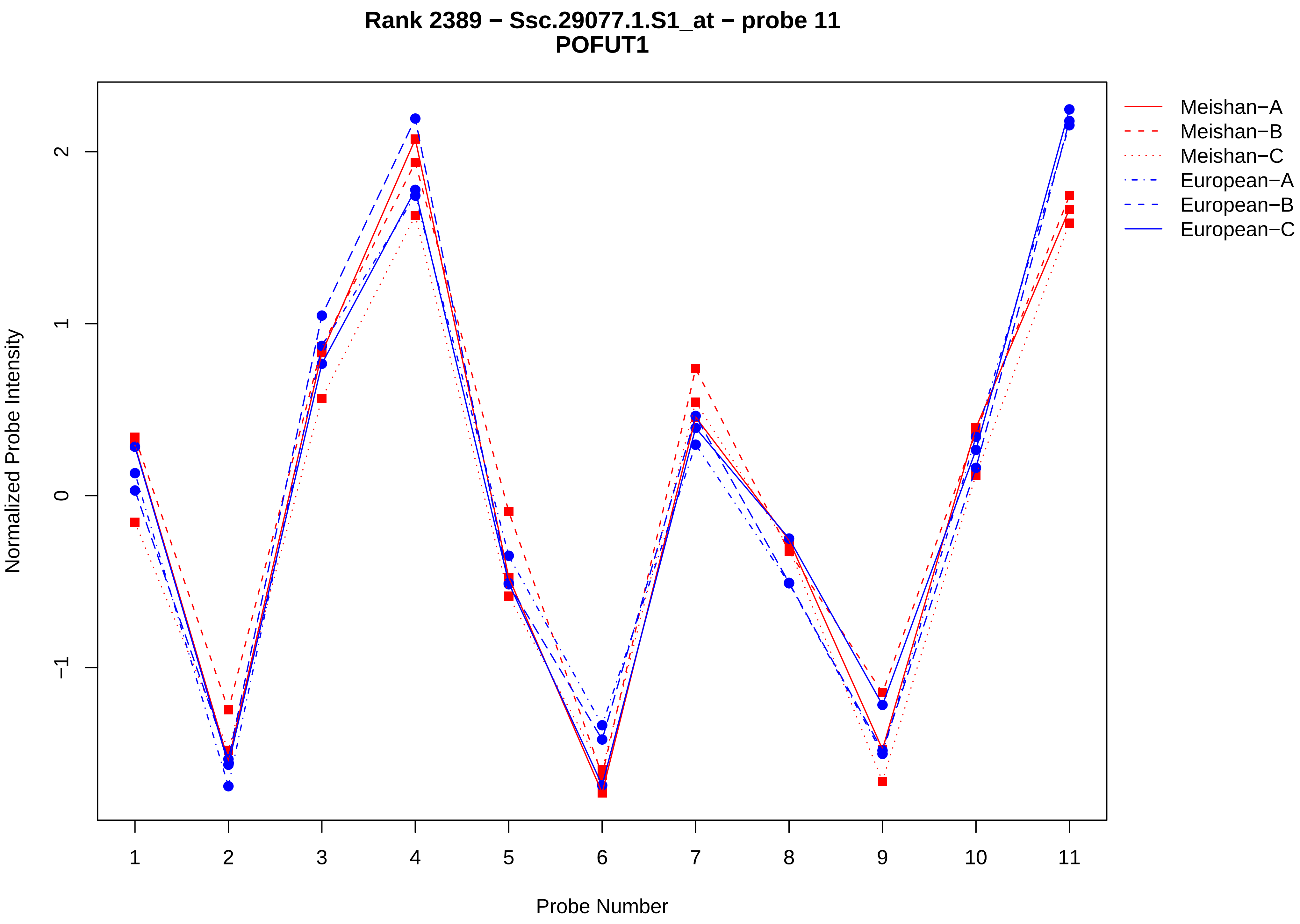

Rank 2389 − Ssc.29077.1.S1_at − probe 11
POFUT1
Normalized Probe Intensity
Probe Number
−1
0
1
2
1
2
3
4
5
6
7
8
9
10
11
Meishan−A
Meishan−B
Meishan−C
European−A
European−B
European−C

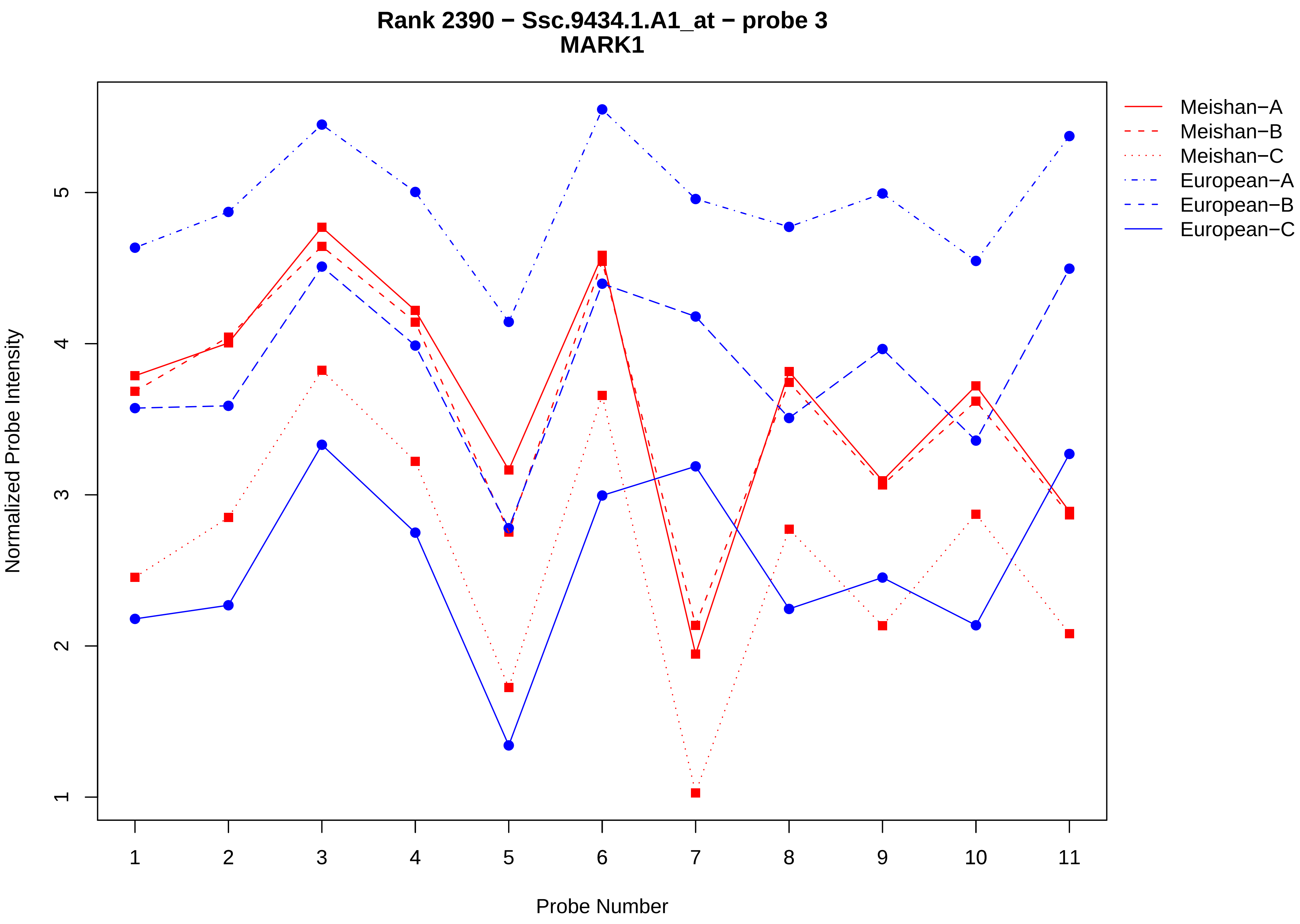

Rank 2390 − Ssc.9434.1.A1_at − probe 3
MARK1
Meishan−A
Meishan−B
Meishan−C
European−A
European−B
European−C
Normalized Probe Intensity
Probe Number
1
2
3
4
5
1
2
3
4
5
6
7
8
9
10
11

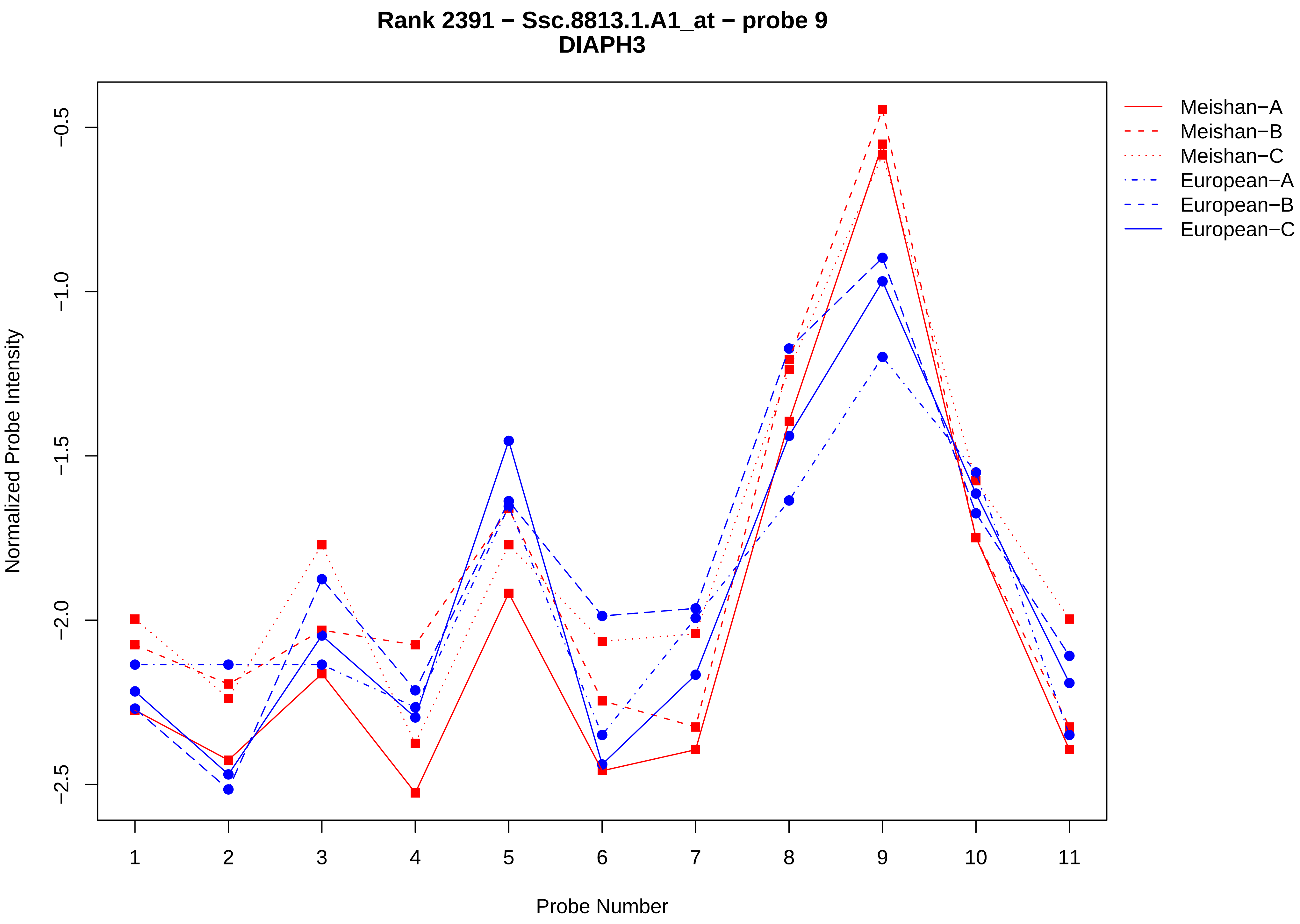

Rank 2391 − Ssc.8813.1.A1_at − probe 9
DIAPH3
Normalized Probe Intensity
−0.5
−1.0
−1.5
−2.0
−2.5
Probe Number
1
2
3
4
5
6
7
8
9
10
11
Meishan−A
Meishan−B
Meishan−C
European−A
European−B
European−C

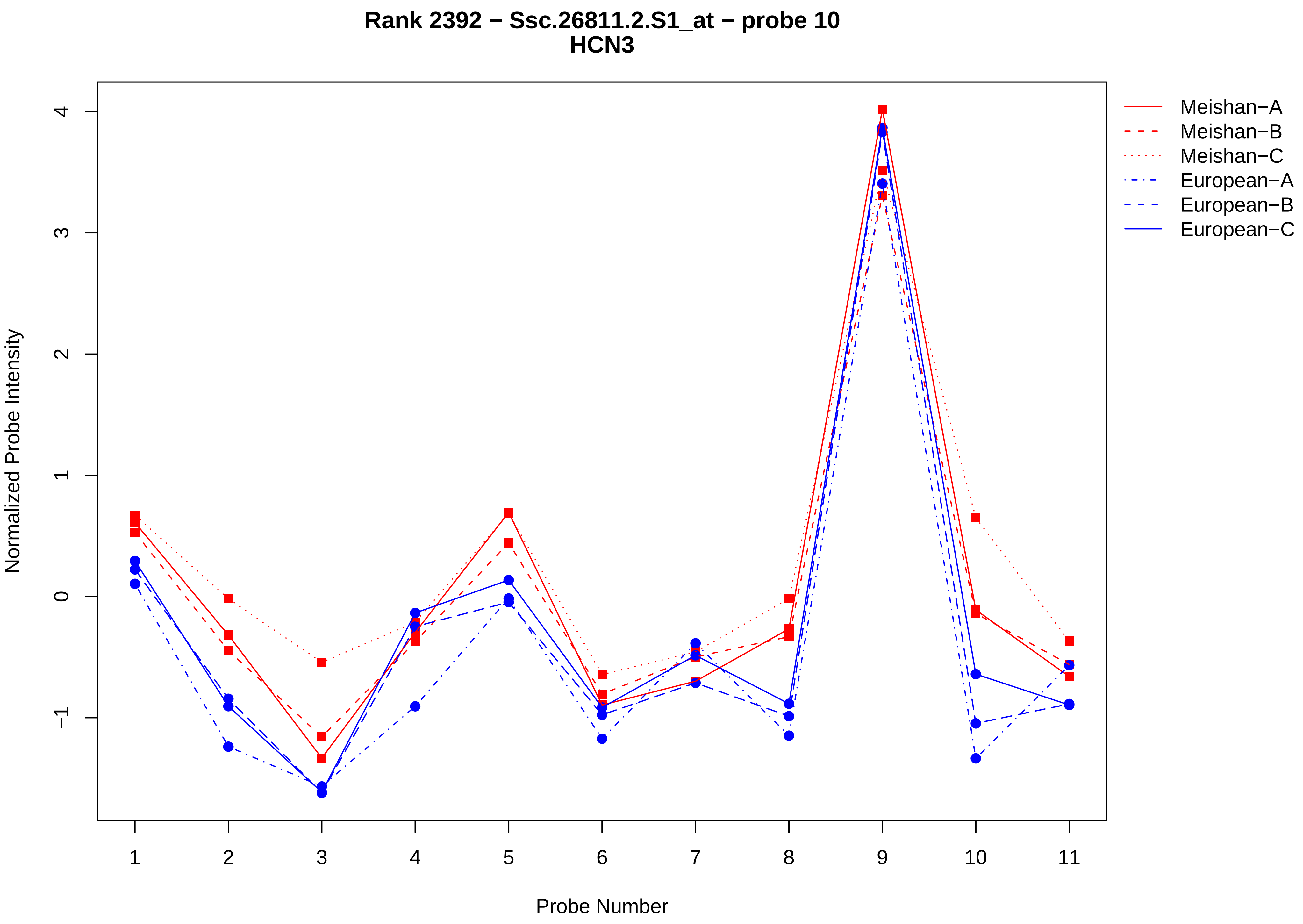
Rank 2392 − Ssc.26811.2.S1_at − probe 10
HCN3
Normalized Probe Intensity
Probe Number
Meishan−A
Meishan−B
Meishan−C
European−A
European−B
European−C
−1
0
1
2
3
4
1
2
3
4
5
6
7
8
9
10
11

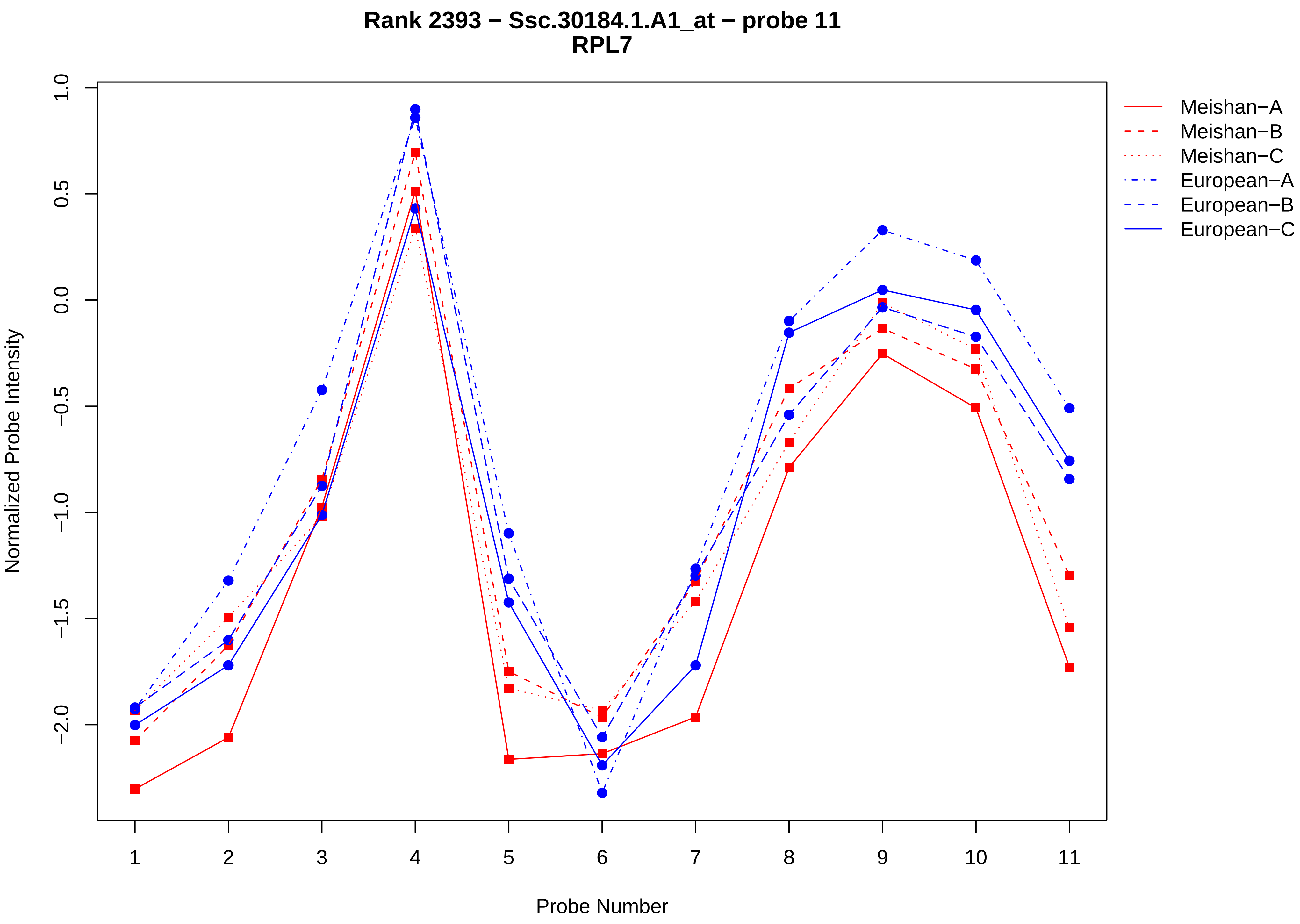
Rank 2393 − Ssc.30184.1.A1_at − probe 11
RPL7
Normalized Probe Intensity
Probe Number
Meishan−A
Meishan−B
Meishan−C
European−A
European−B
European−C
1.0
0.5
0.0
−0.5
−1.0
−1.5
−2.0
1
2
3
4
5
6
7
8
9
10
11

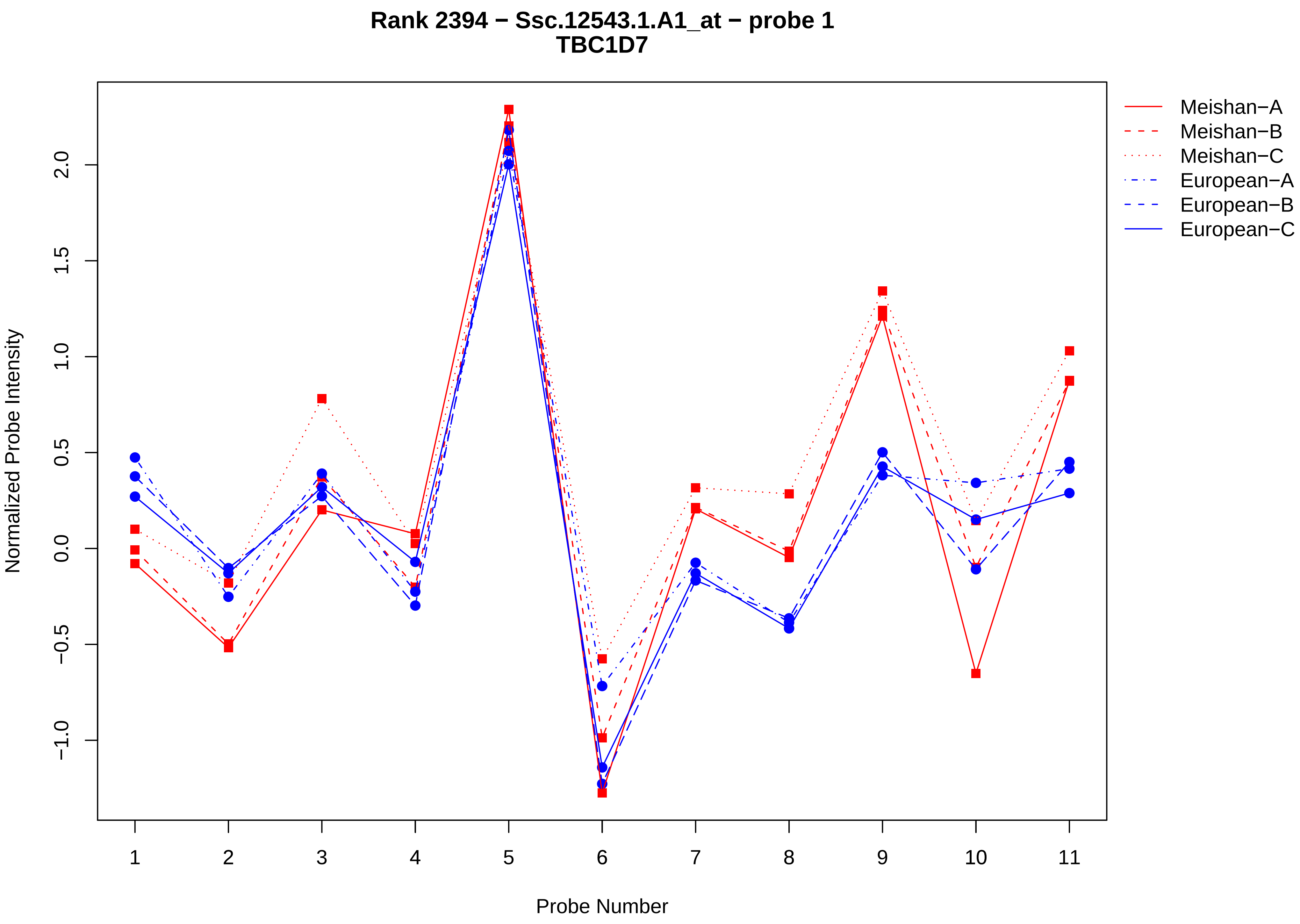
Rank 2394 − Ssc.12543.1.A1_at − probe 1
TBC1D7
Normalized Probe Intensity
Probe Number
−1.0
−0.5
0.0
0.5
1.0
1.5
2.0
1
2
3
4
5
6
7
8
9
10
11
Meishan−A
Meishan−B
Meishan−C
European−A
European−B
European−C

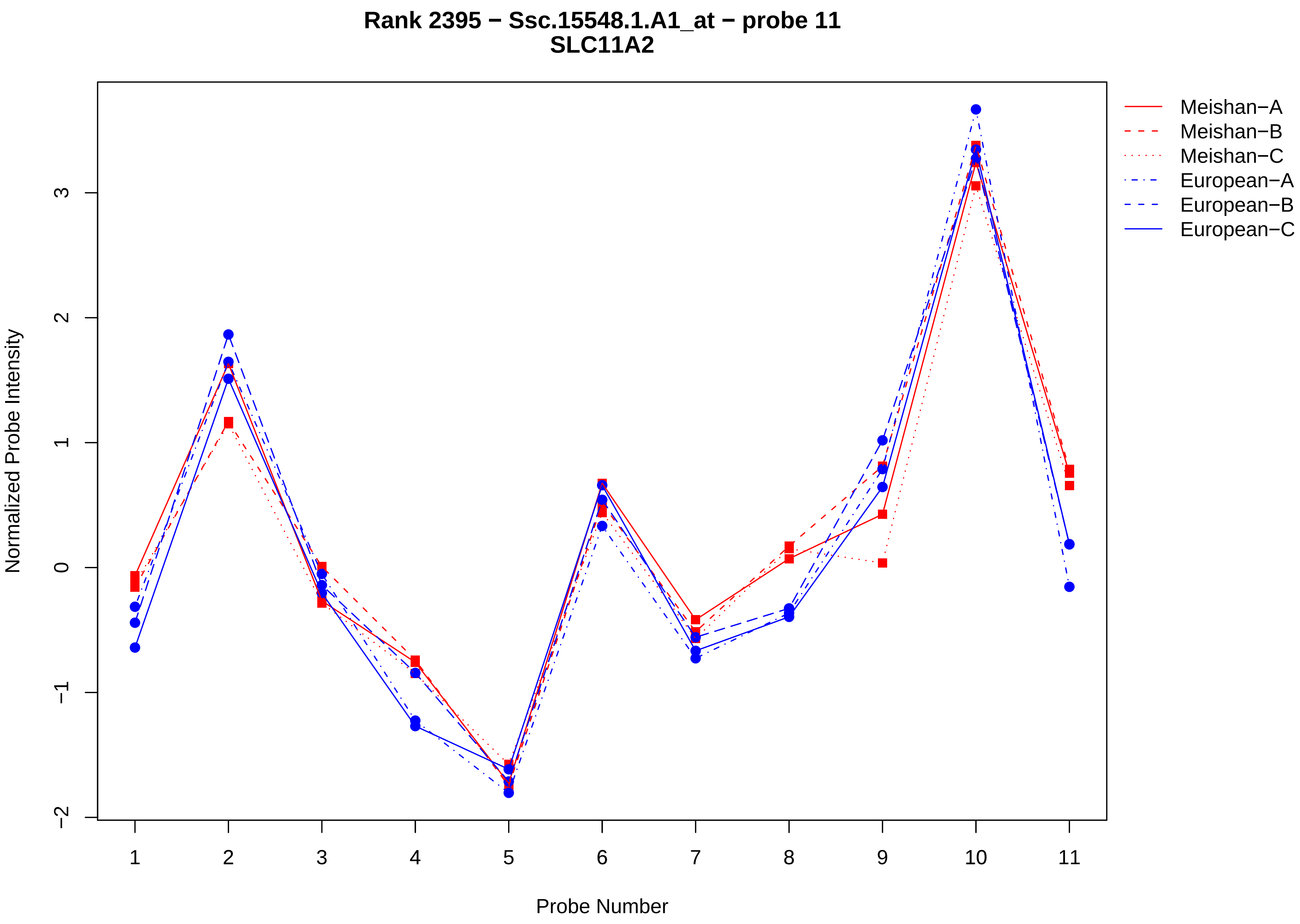
Rank 2395 − Ssc.15548.1.A1_at − probe 11
SLC11A2
Normalized Probe Intensity
Probe Number
−2
−1
0
1
2
3
1
2
3
4
5
6
7
8
9
10
11
Meishan−A
Meishan−B
Meishan−C
European−A
European−B
European−C

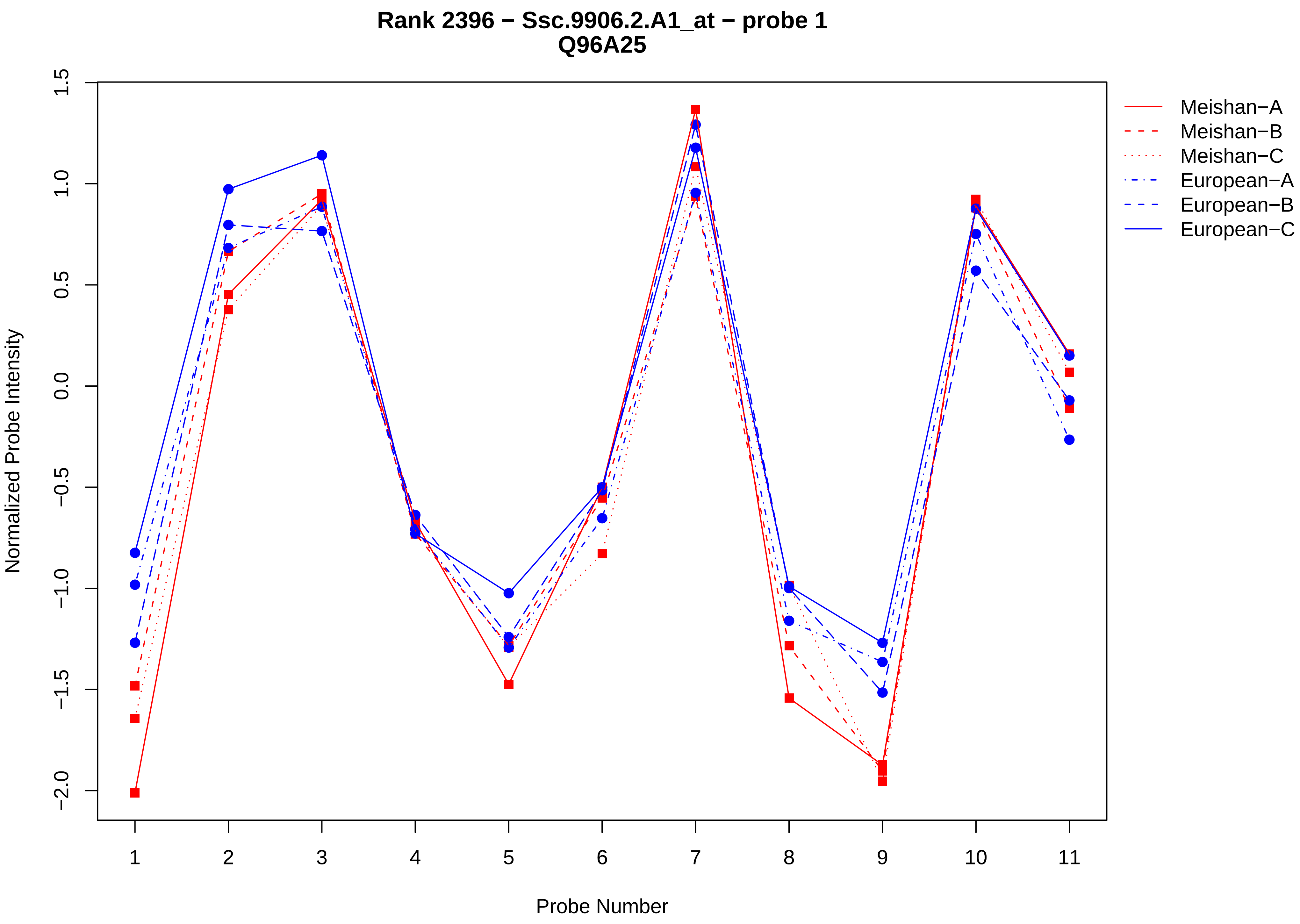

Rank 2396 − Ssc.9906.2.A1_at − probe 1
Q96A25
Normalized Probe Intensity
Probe Number
1.5
1.0
0.5
0.0
−0.5
−1.0
−1.5
−2.0
1
2
3
4
5
6
7
8
9
10
11
Meishan−A
Meishan−B
Meishan−C
European−A
European−B
European−C

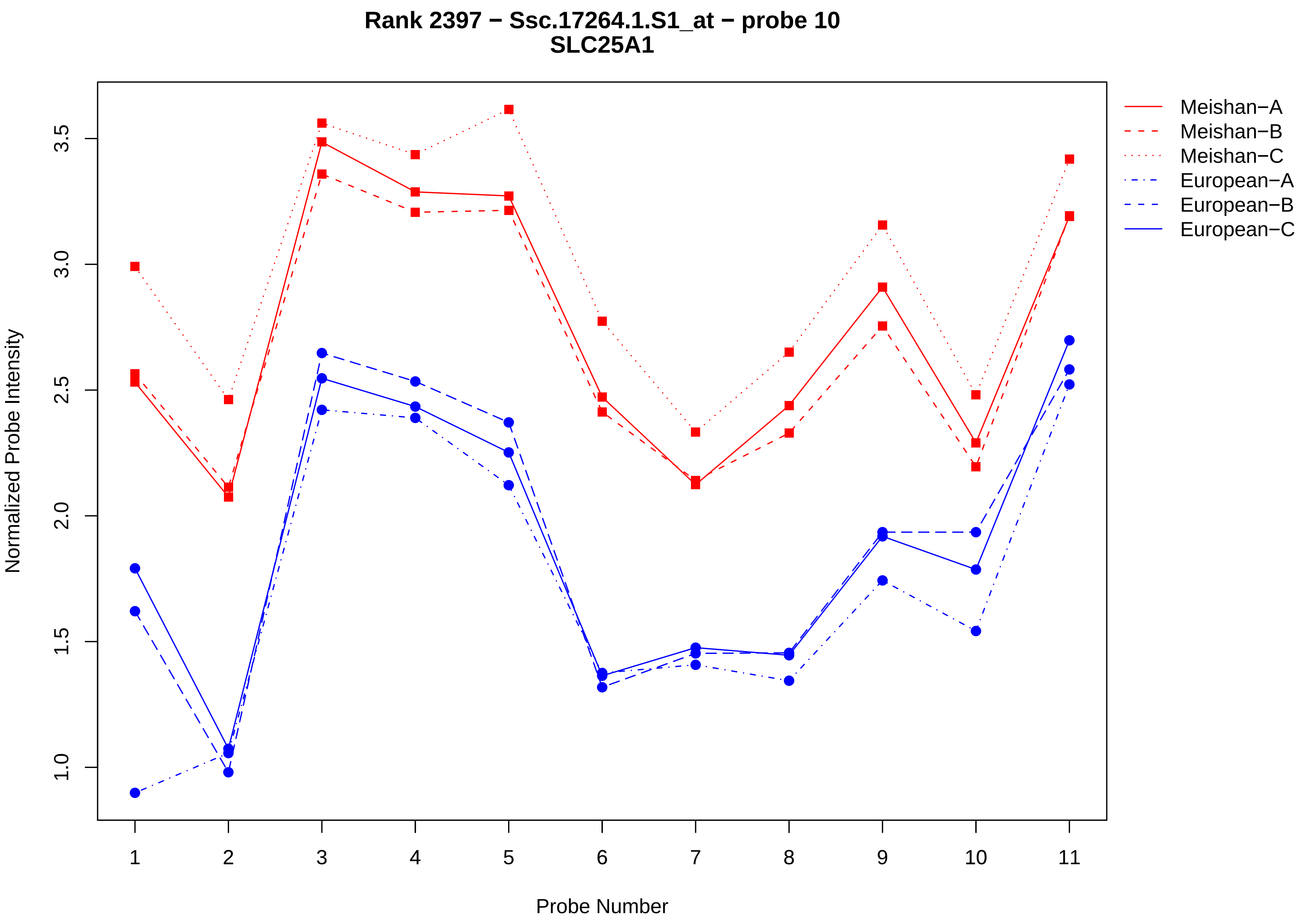
Rank 2397 − Ssc.17264.1.S1_at − probe 10
SLC25A1
Normalized Probe Intensity
Probe Number
1.0
1.5
2.0
2.5
3.0
3.5
1
2
3
4
5
6
7
8
9
10
11
Meishan−A
Meishan−B
Meishan−C
European−A
European−B
European−C

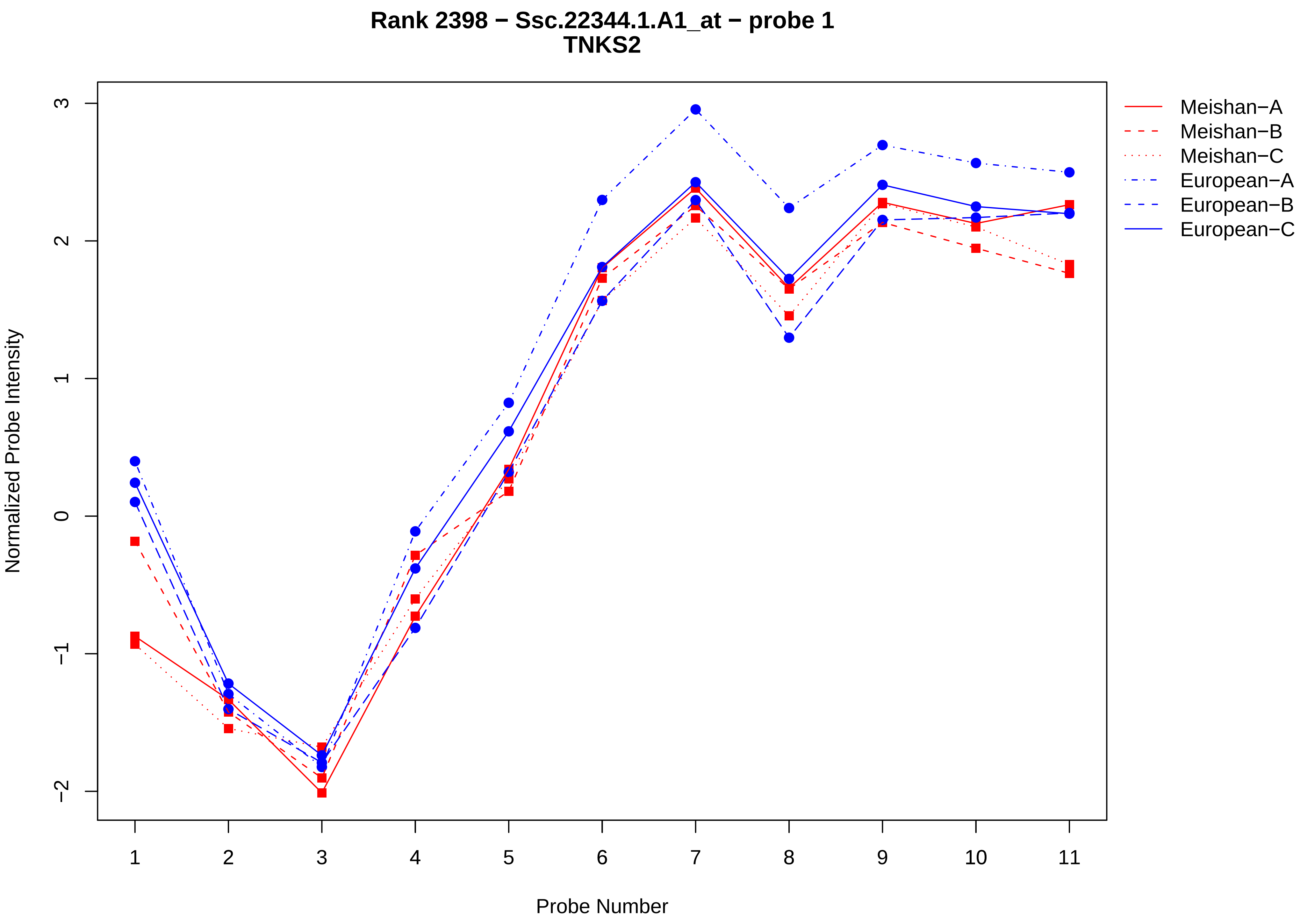

Rank 2398 − Ssc.22344.1.A1_at − probe 1
TNKS2
Normalized Probe Intensity
Probe Number
Meishan−A
Meishan−B
Meishan−C
European−A
European−B
European−C

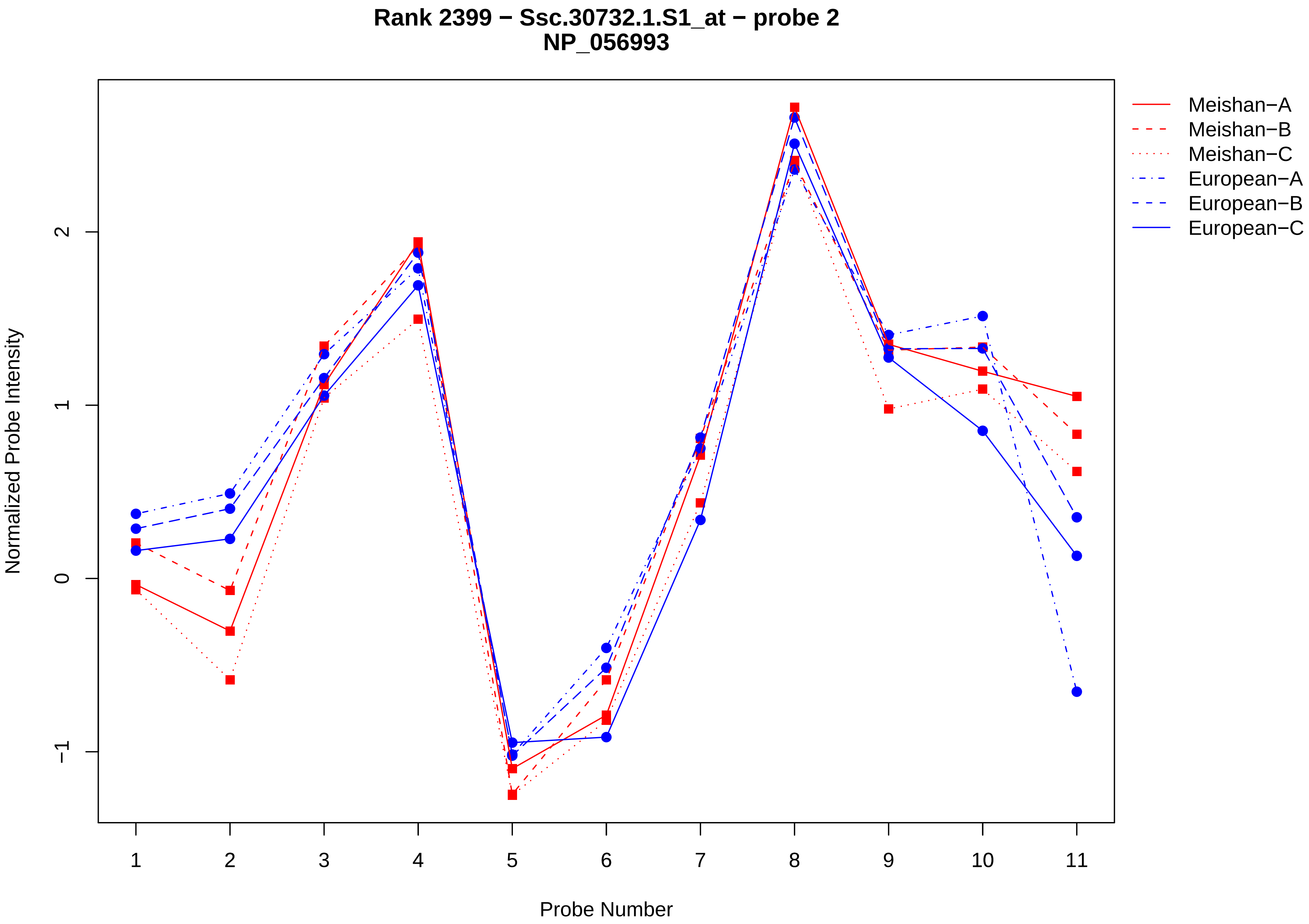
Rank 2399 − Ssc.30732.1.S1_at − probe 2
NP_056993
Normalized Probe Intensity
Probe Number
−1
0
1
2
1
2
3
4
5
6
7
8
9
10
11
Meishan−A
Meishan−B
Meishan−C
European−A
European−B
European−C

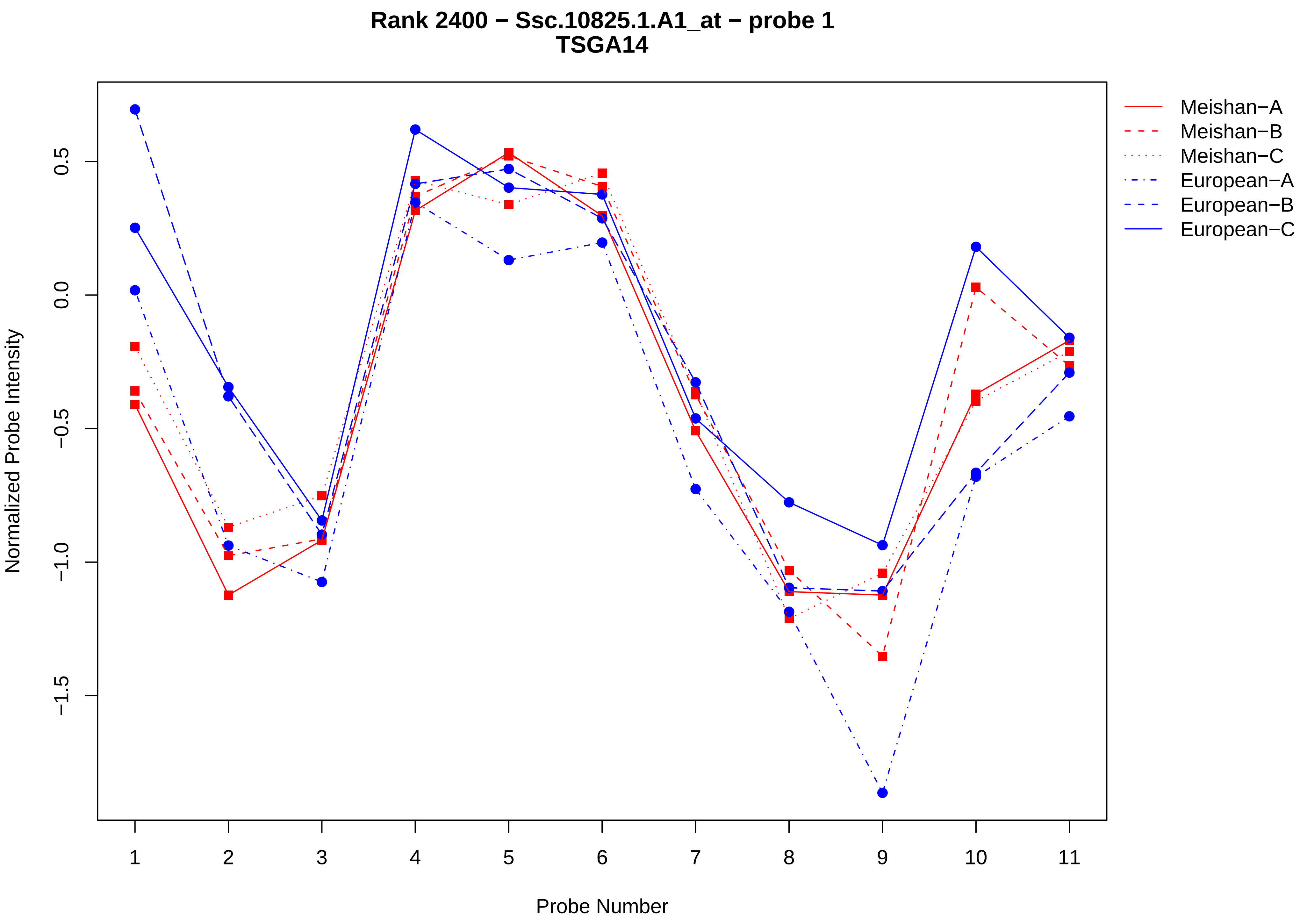

Rank 2400 − Ssc.10825.1.A1_at − probe 1
TSGA14
Normalized Probe Intensity
Probe Number
Meishan−A
Meishan−B
Meishan−C
European−A
European−B
European−C

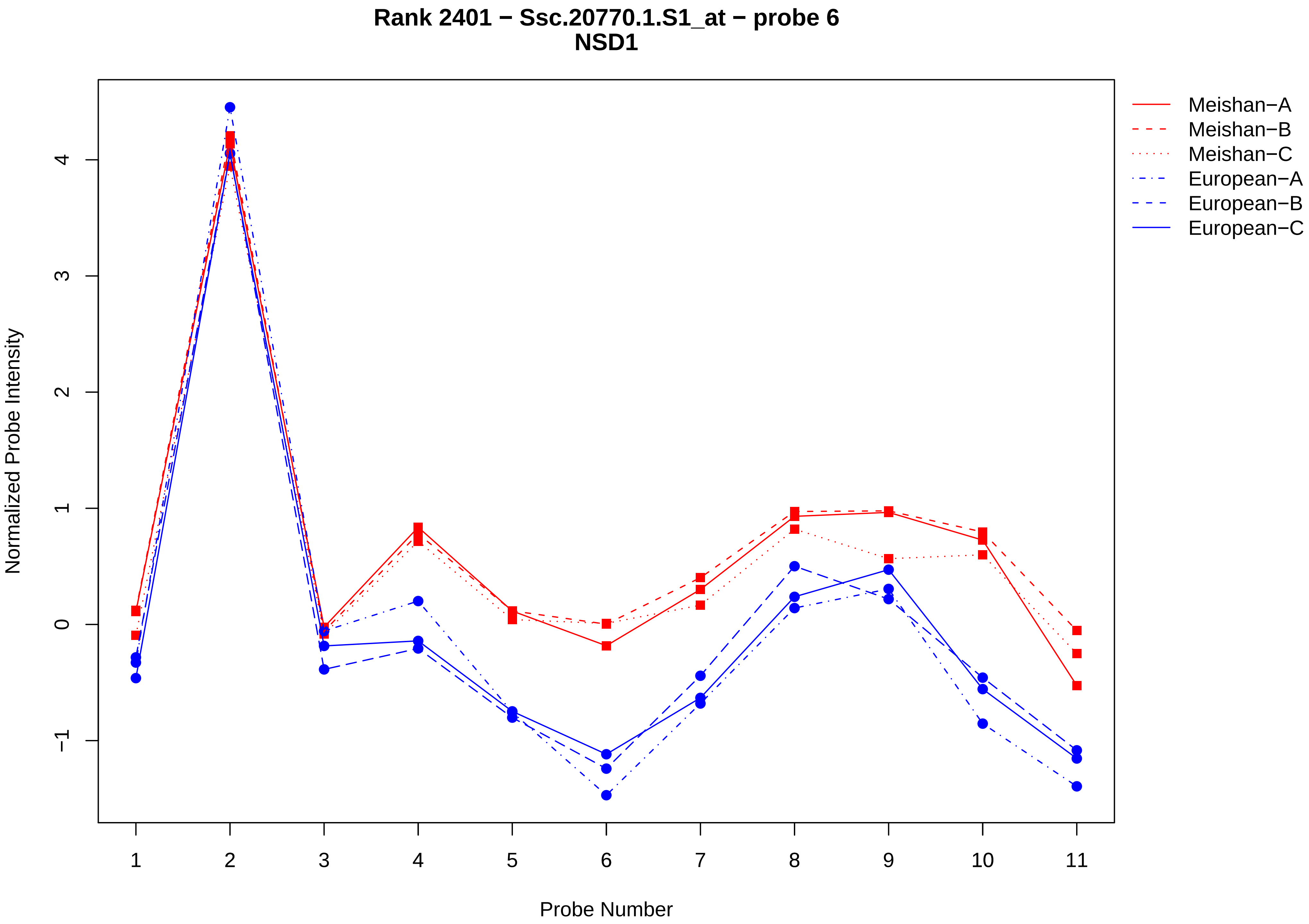
Rank 2401 − Ssc.20770.1.S1_at − probe 6
NSD1
Normalized Probe Intensity
Probe Number
Meishan−A
Meishan−B
Meishan−C
European−A
European−B
European−C
−1
0
1
2
3
4
1
2
3
4
5
6
7
8
9
10
11

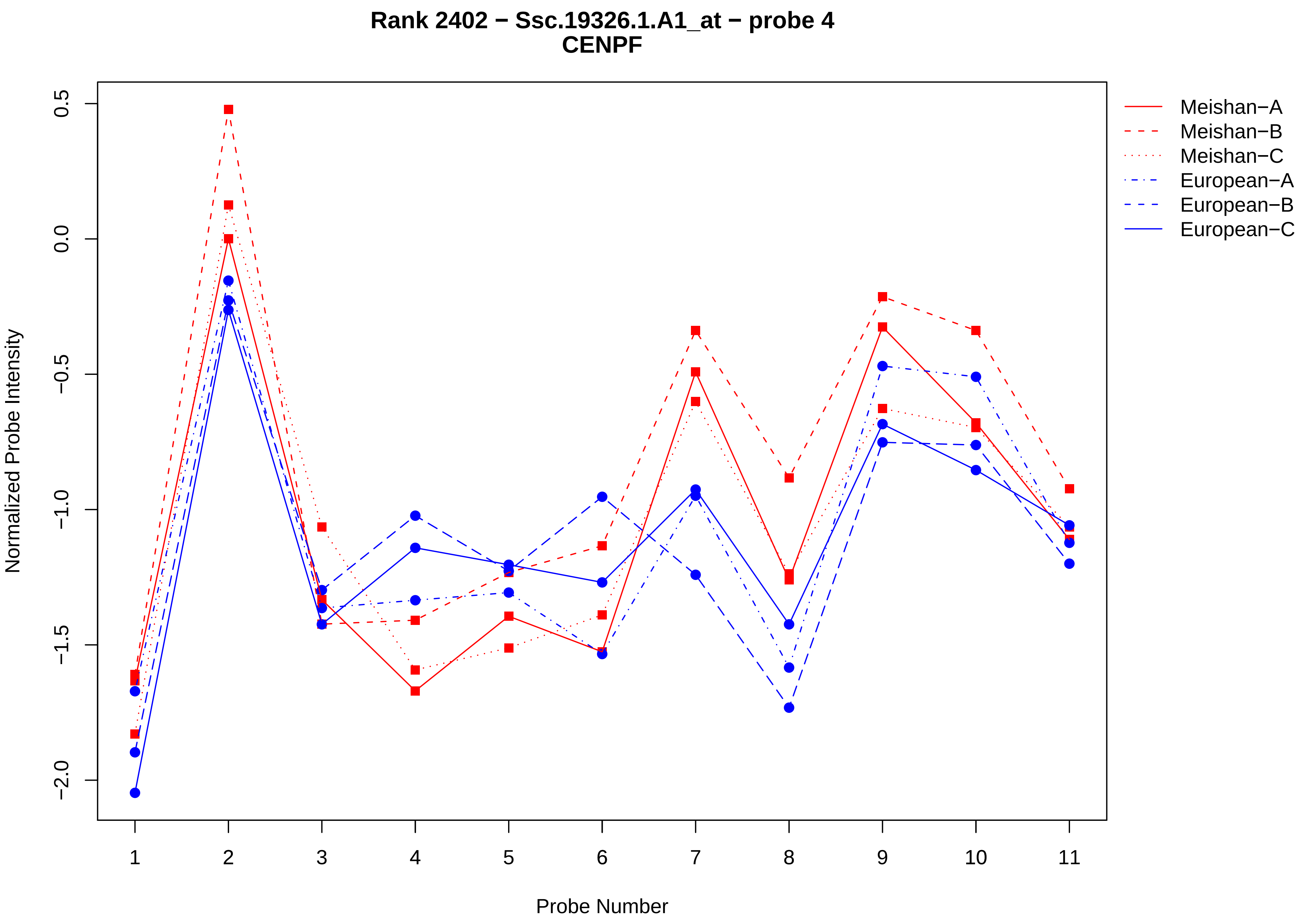

Rank 2402 − Ssc.19326.1.A1_at − probe 4
CENPF
Normalized Probe Intensity
Probe Number
0.5
0.0
−0.5
−1.0
−1.5
−2.0
1
2
3
4
5
6
7
8
9
10
11
Meishan−A
Meishan−B
Meishan−C
European−A
European−B
European−C

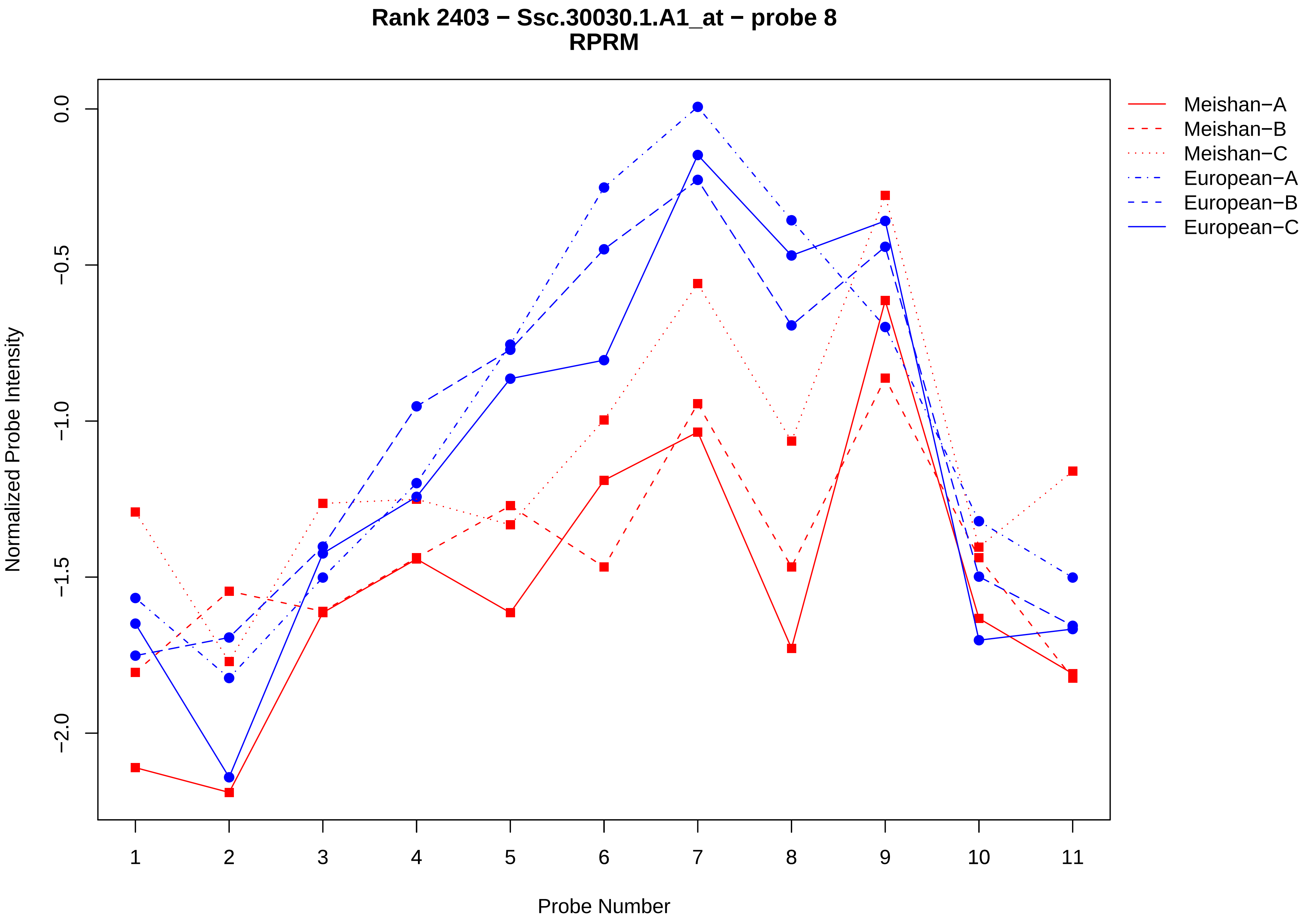

Rank 2403 − Ssc.30030.1.A1_at − probe 8
RPRM
Normalized Probe Intensity
0.0
−0.5
−1.0
−1.5
−2.0
1
2
3
4
5
6
7
8
9
10
11
Probe Number
Meishan−A
Meishan−B
Meishan−C
European−A
European−B
European−C

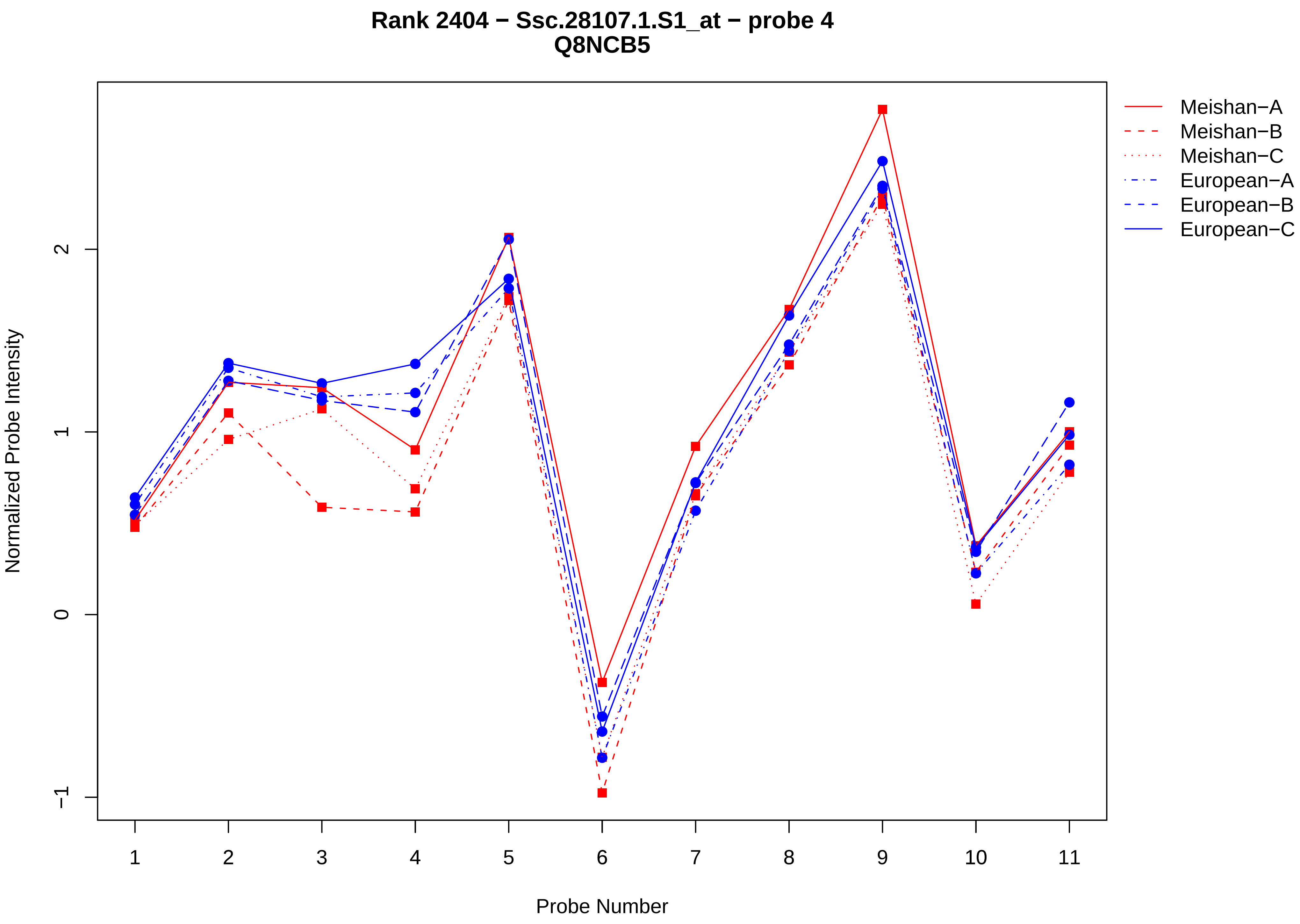

Rank 2404 − Ssc.28107.1.S1_at − probe 4
Q8NCB5
Normalized Probe Intensity
Probe Number
−1
0
1
2
1
2
3
4
5
6
7
8
9
10
11
Meishan−A
Meishan−B
Meishan−C
European−A
European−B
European−C

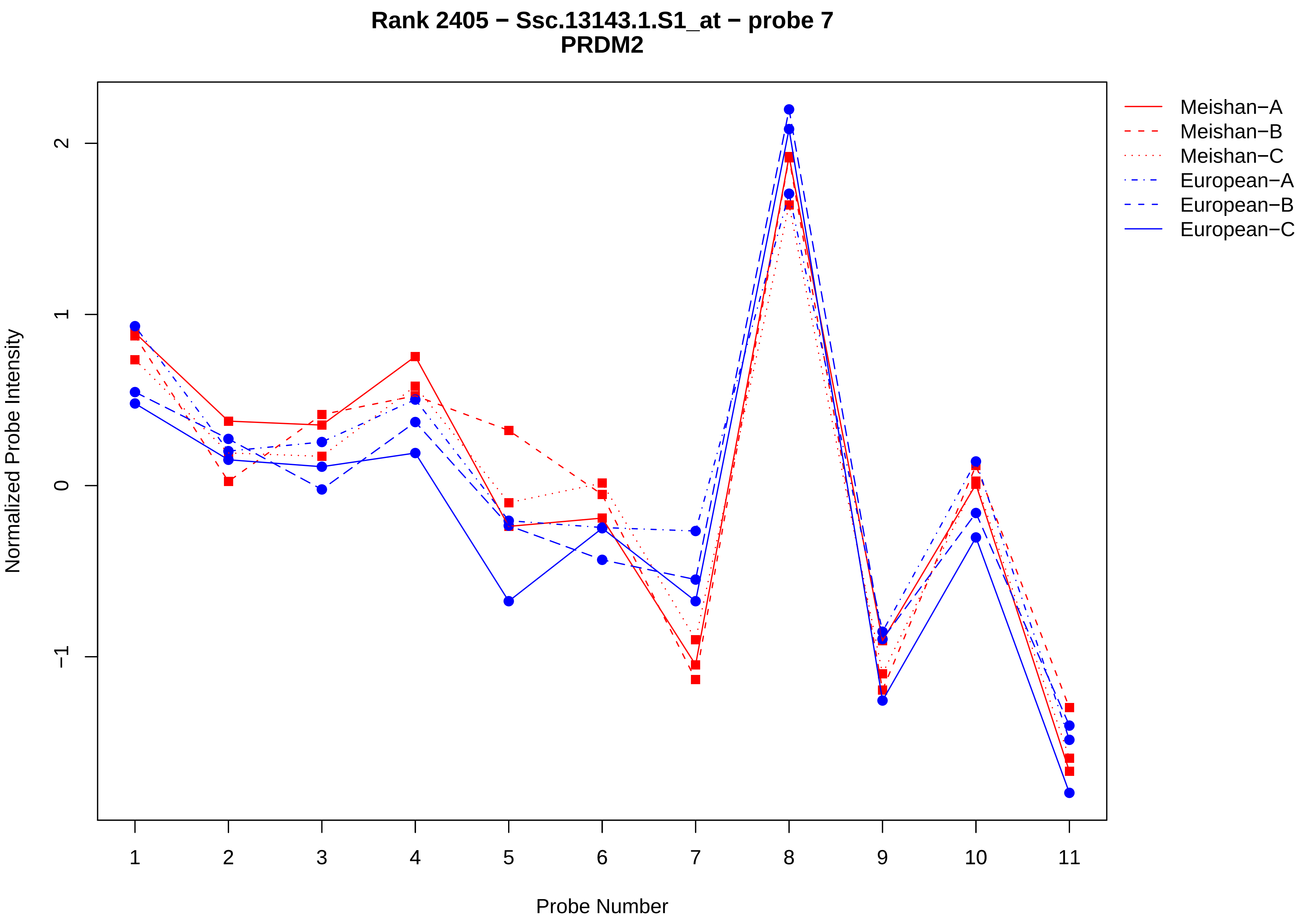

Rank 2405 − Ssc.13143.1.S1_at − probe 7
PRDM2
Normalized Probe Intensity
Probe Number
−1
0
1
2
1
2
3
4
5
6
7
8
9
10
11
Meishan−A
Meishan−B
Meishan−C
European−A
European−B
European−C

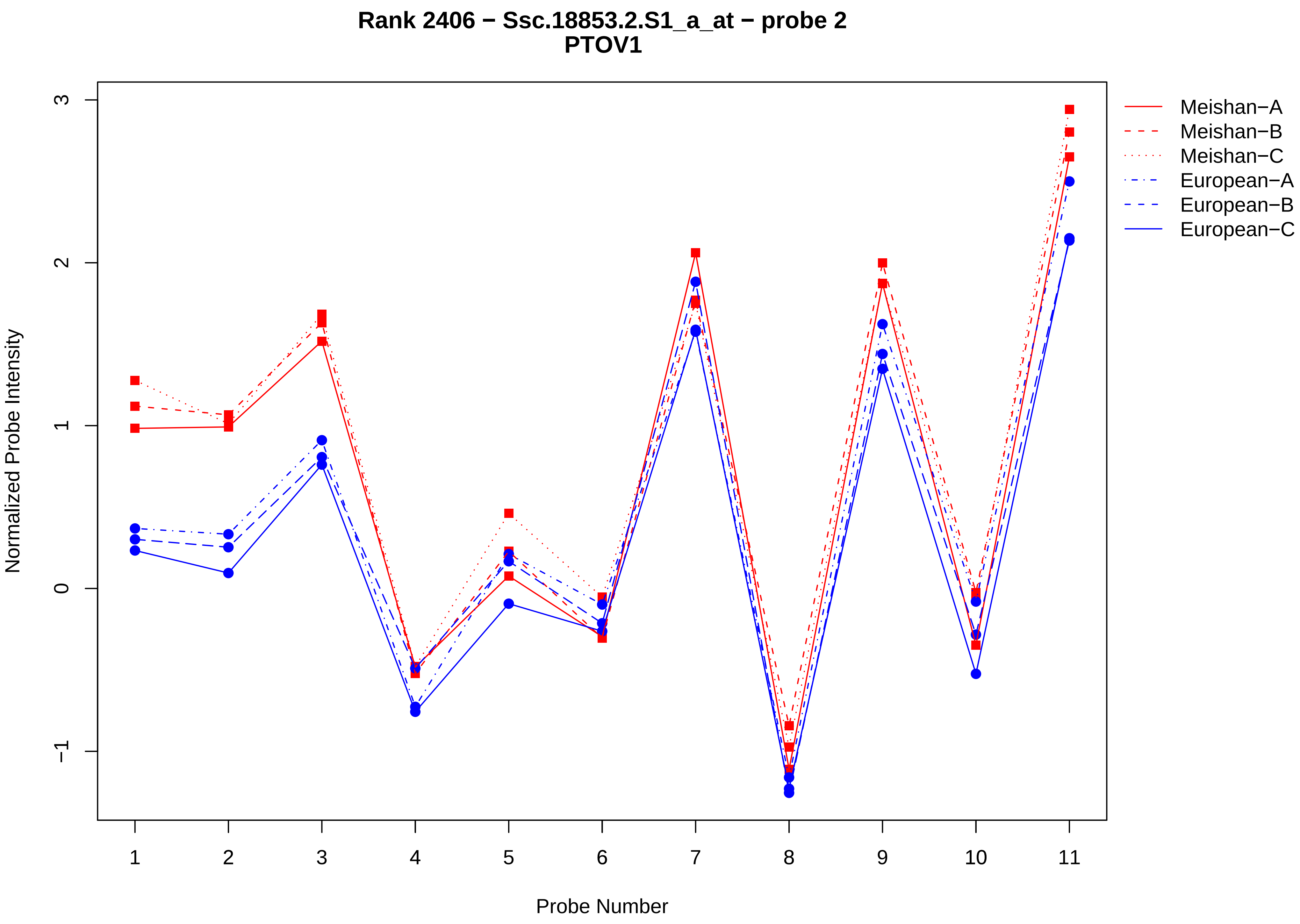

Rank 2406 − Ssc.18853.2.S1_a_at − probe 2
PTOV1
Normalized Probe Intensity
Probe Number
−1
0
1
2
3
1
2
3
4
5
6
7
8
9
10
11
Meishan−A
Meishan−B
Meishan−C
European−A
European−B
European−C

Rank 2407 − Ssc.29076.1.A1_at − probe 3
PHKA1

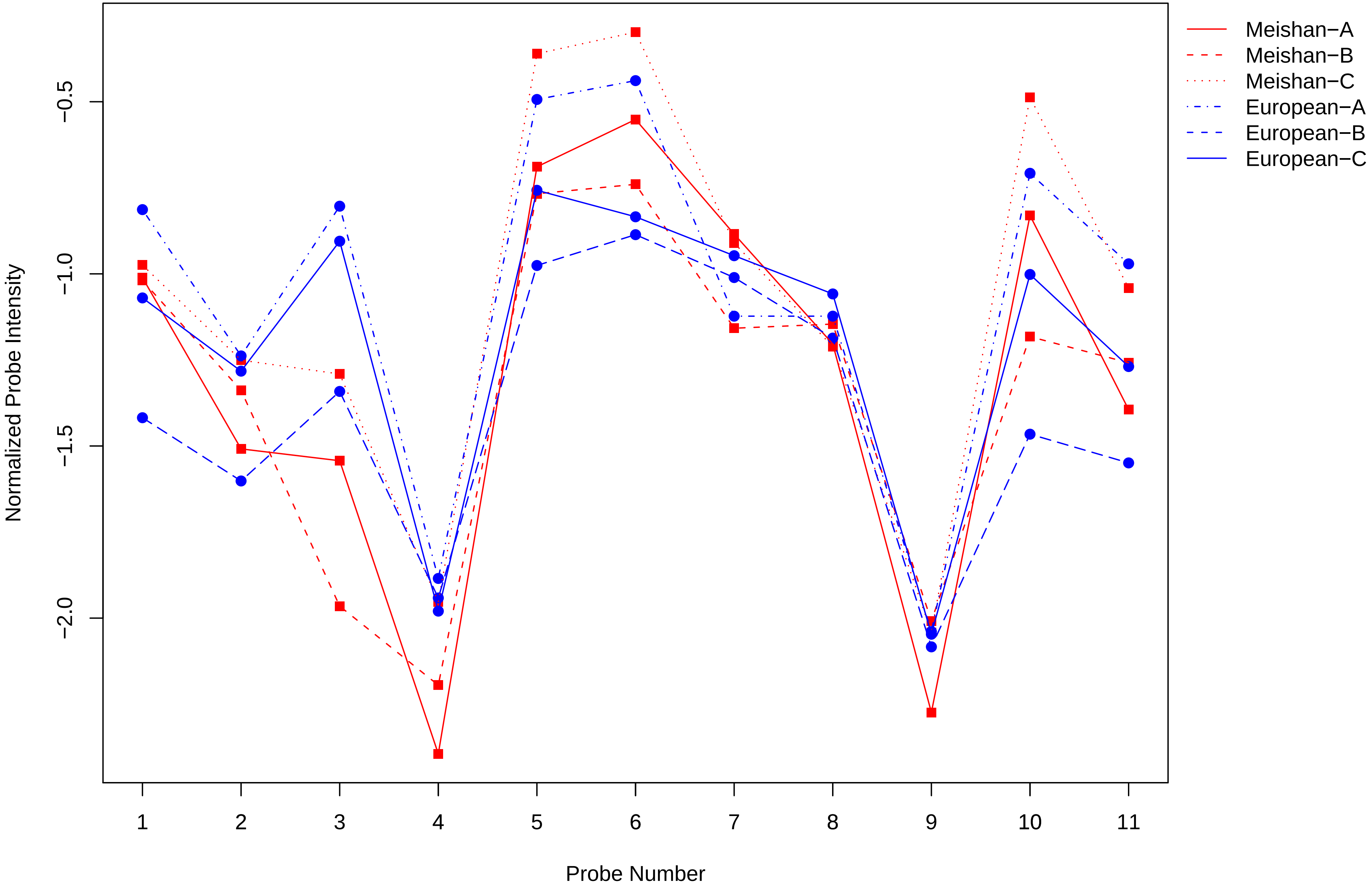

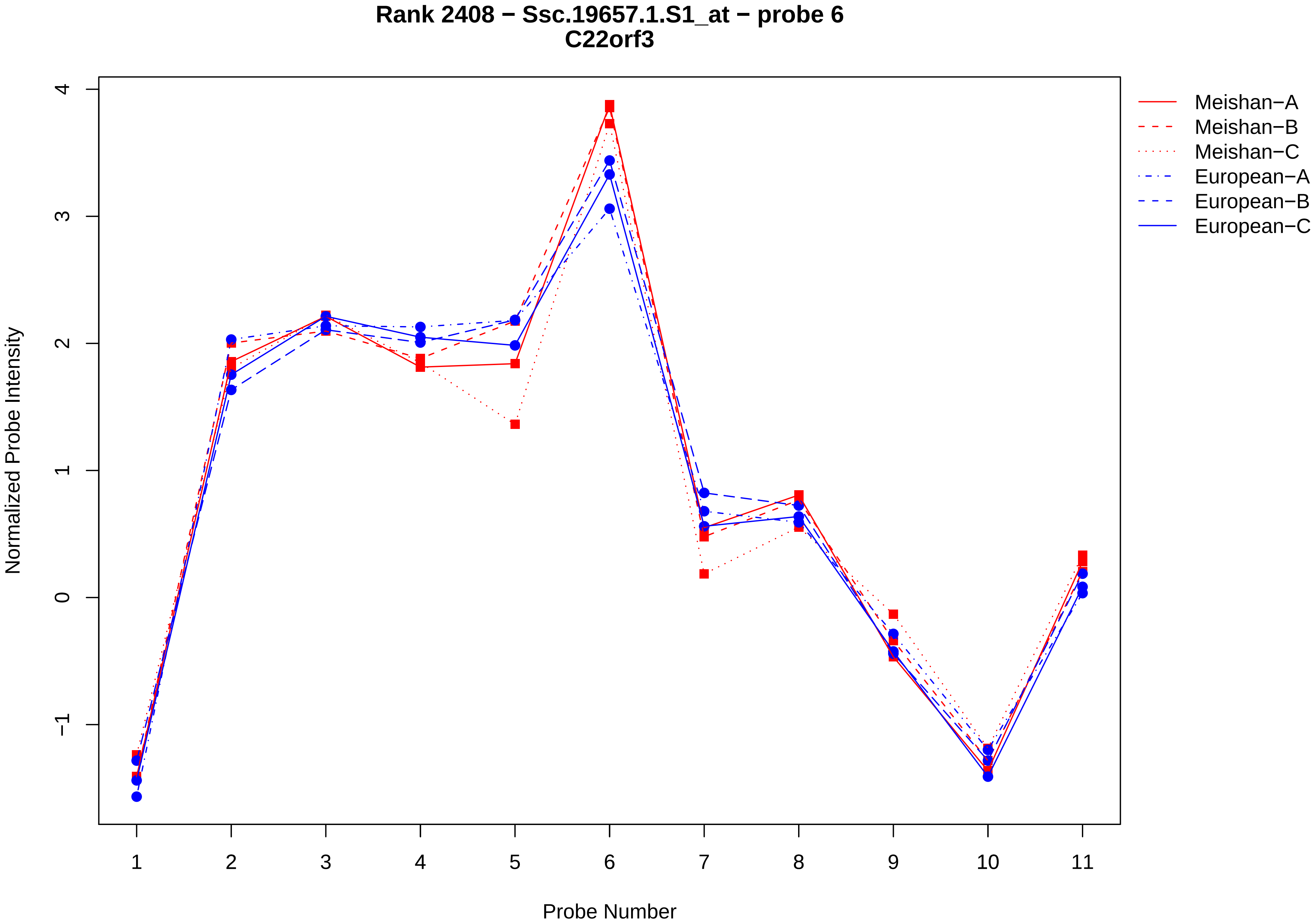
Rank 2408 − Ssc.19657.1.S1_at − probe 6
C22orf3
Normalized Probe Intensity
Probe Number
−1
0
1
2
3
4
1
2
3
4
5
6
7
8
9
10
11
Meishan−A
Meishan−B
Meishan−C
European−A
European−B
European−C

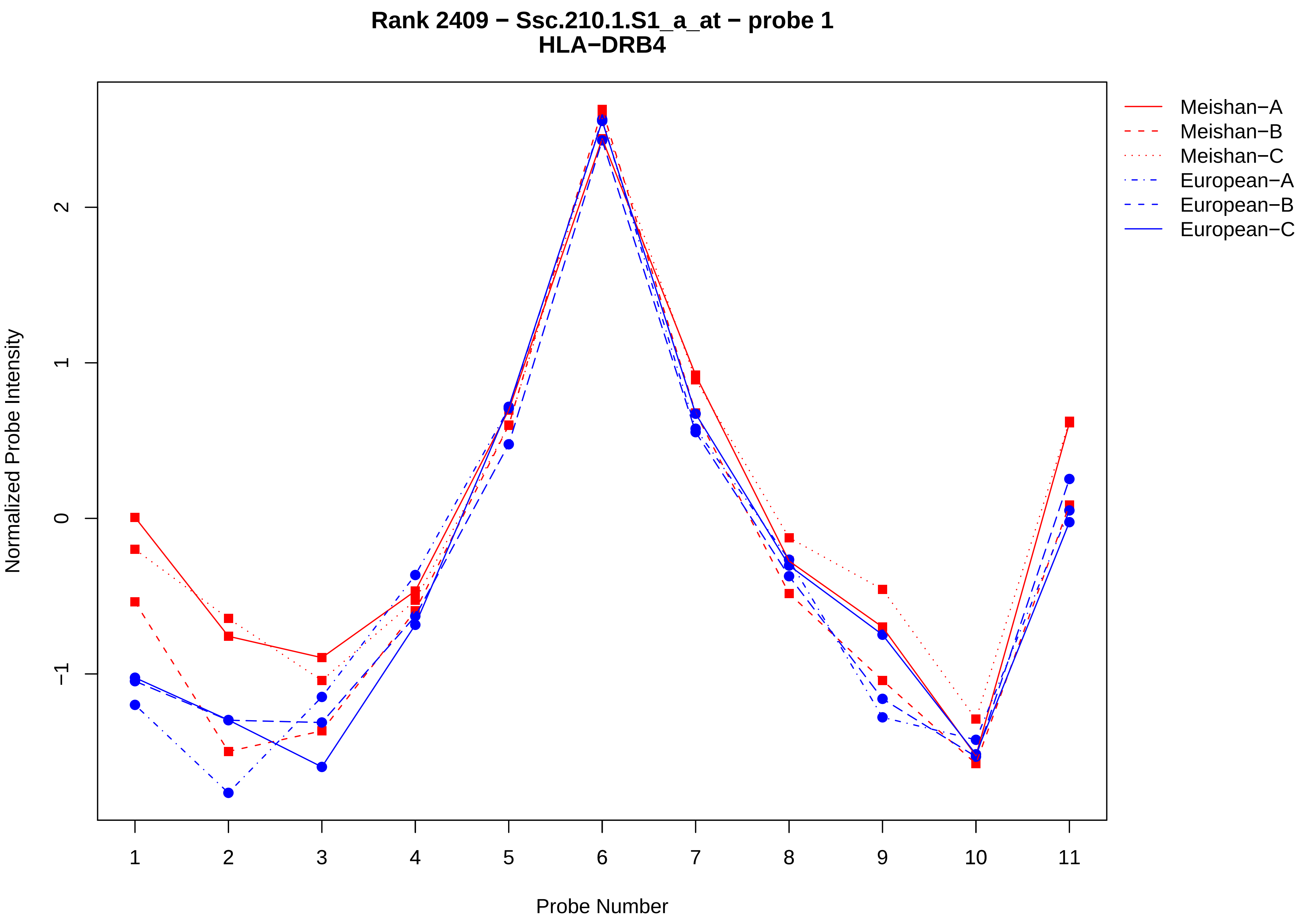

Rank 2409 − Ssc.210.1.S1_a_at − probe 1
HLA−DRB4
Normalized Probe Intensity
Probe Number
−1
0
1
2
1
2
3
4
5
6
7
8
9
10
11
Meishan−A
Meishan−B
Meishan−C
European−A
European−B
European−C

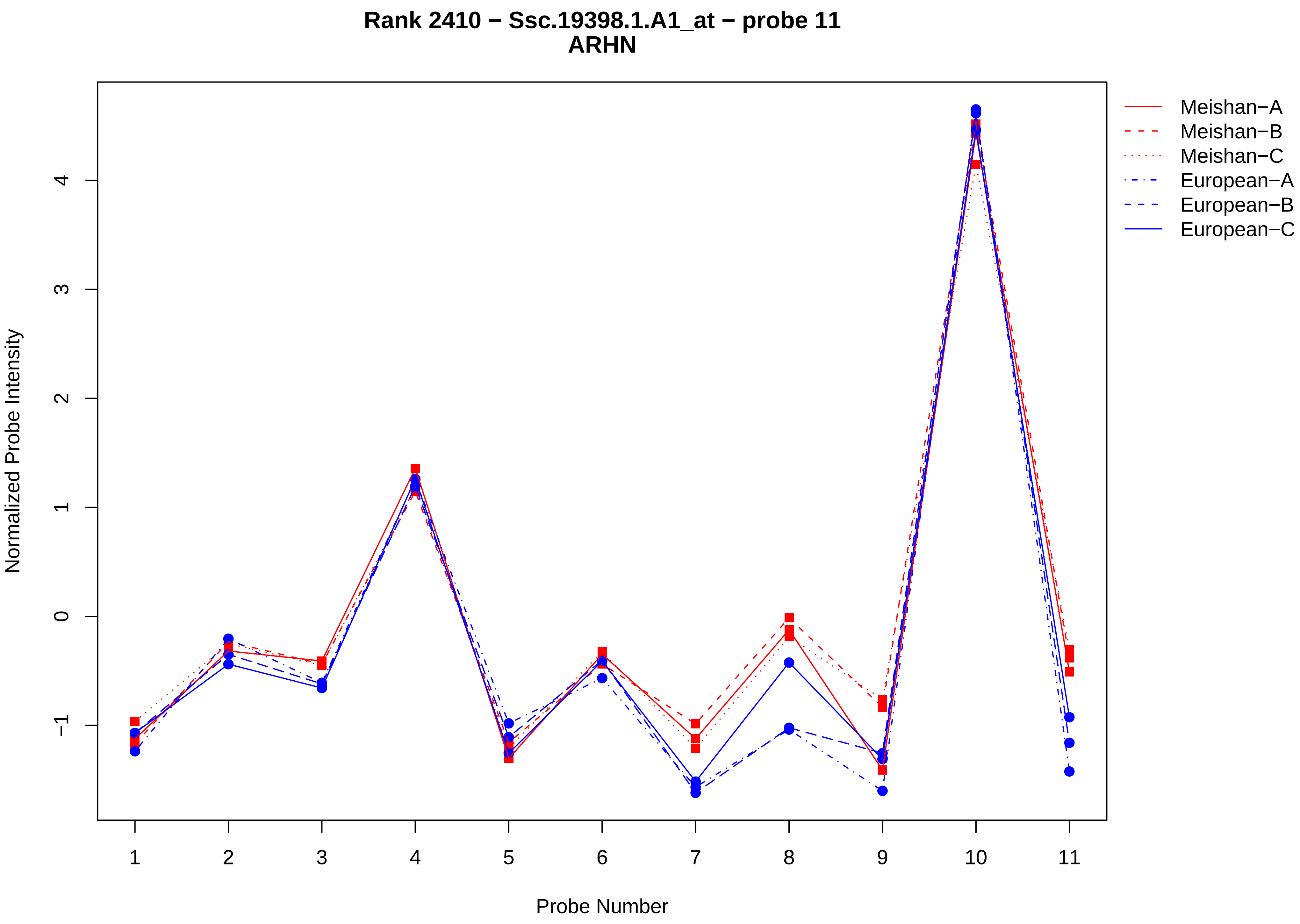

Rank 2410 − Ssc.19398.1.A1_at − probe 11
ARHN
Normalized Probe Intensity
Probe Number
Meishan−A
Meishan−B
Meishan−C
European−A
European−B
European−C

Rank 2411 − Ssc.13311.1.A1_at − probe 5
Q6ZV73

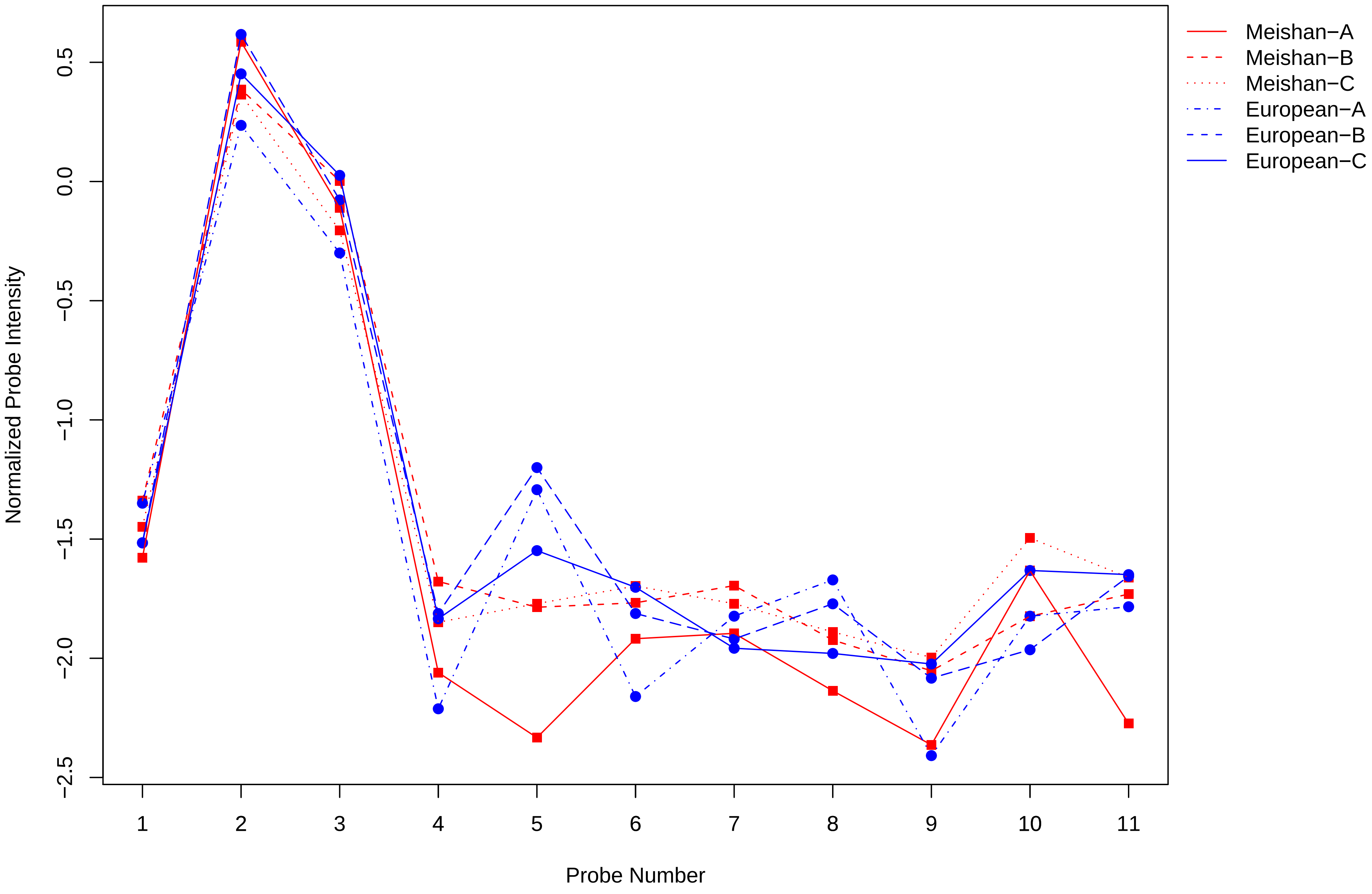

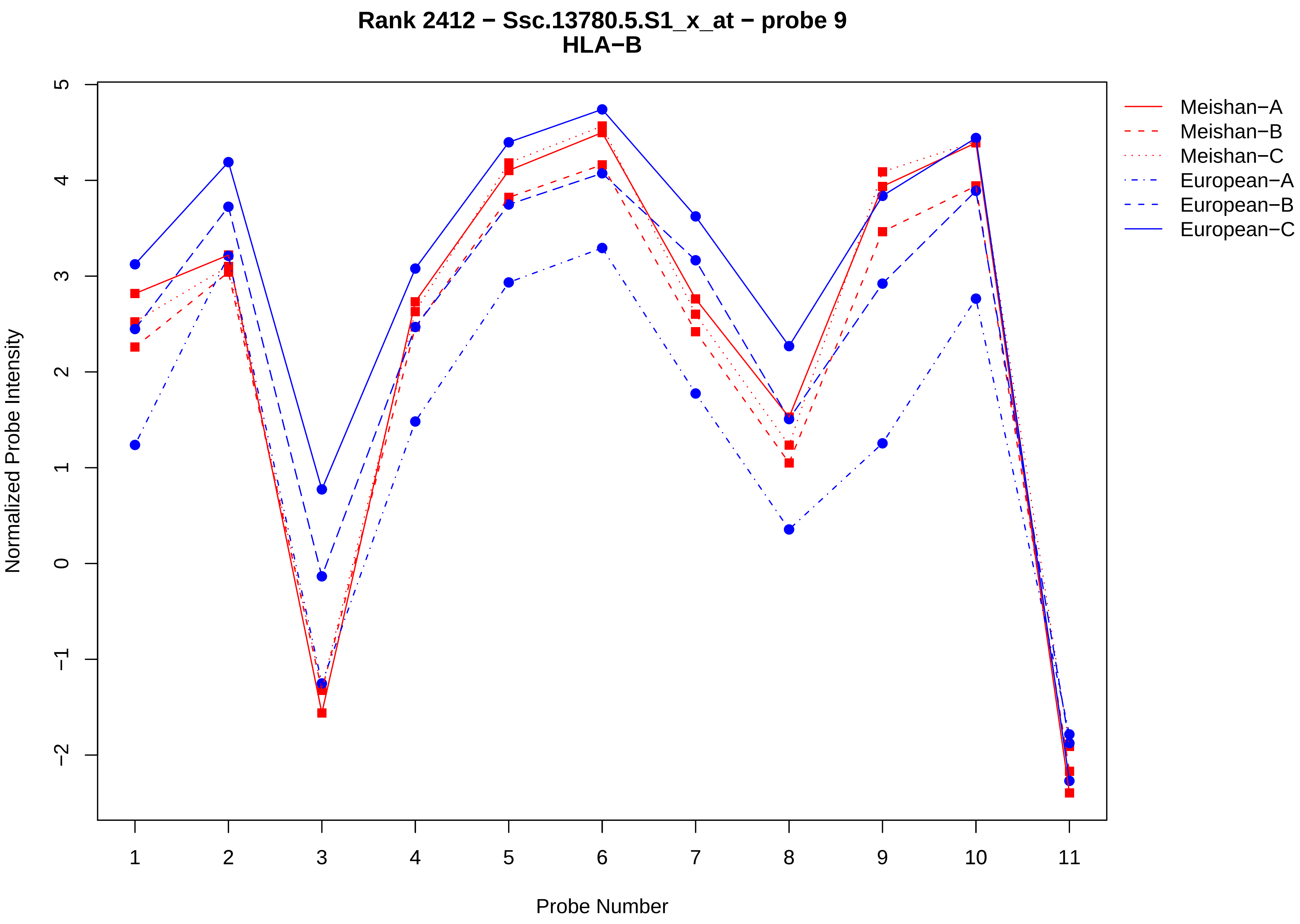

Rank 2412 − Ssc.13780.5.S1_x_at − probe 9
HLA−B
Normalized Probe Intensity
Probe Number
Meishan−A
Meishan−B
Meishan−C
European−A
European−B
European−C

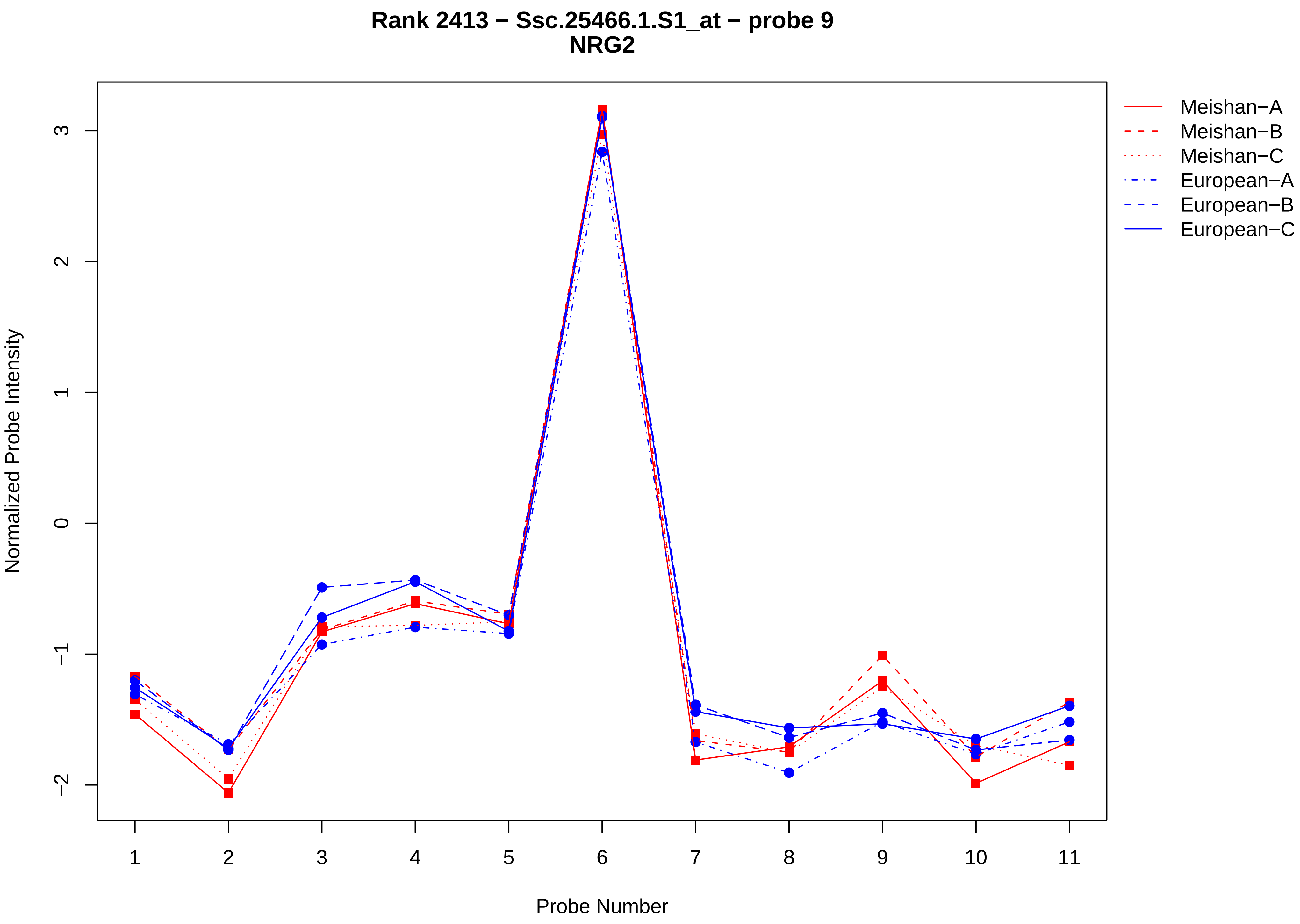

Rank 2413 − Ssc.25466.1.S1_at − probe 9
NRG2
Normalized Probe Intensity
Probe Number
−2
−1
0
1
2
3
1
2
3
4
5
6
7
8
9
10
11
Meishan−A
Meishan−B
Meishan−C
European−A
European−B
European−C

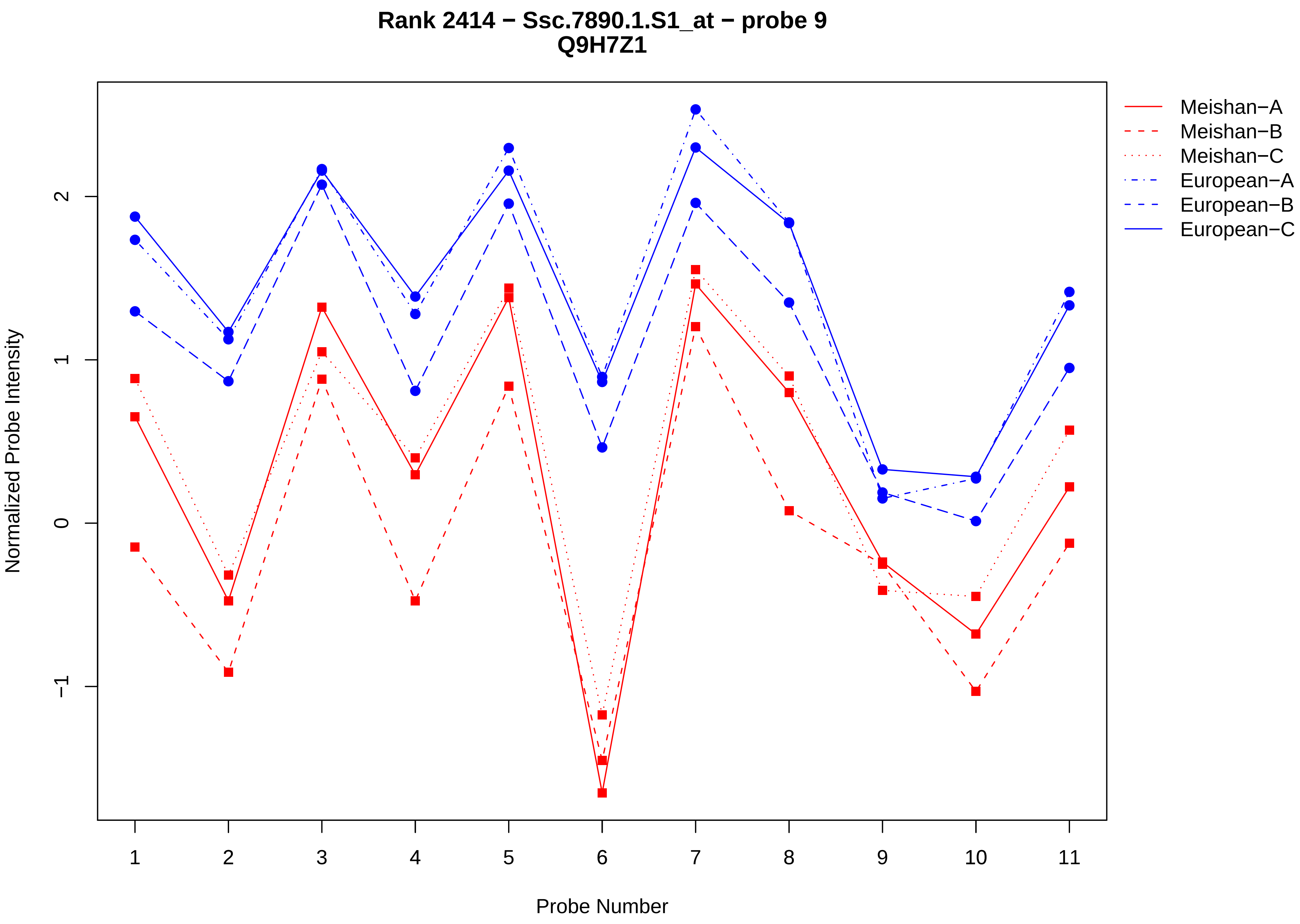
Rank 2414 − Ssc.7890.1.S1_at − probe 9
Q9H7Z1
Normalized Probe Intensity
Probe Number
Meishan−A
Meishan−B
Meishan−C
European−A
European−B
European−C
−1
0
1
2
1
2
3
4
5
6
7
8
9
10
11

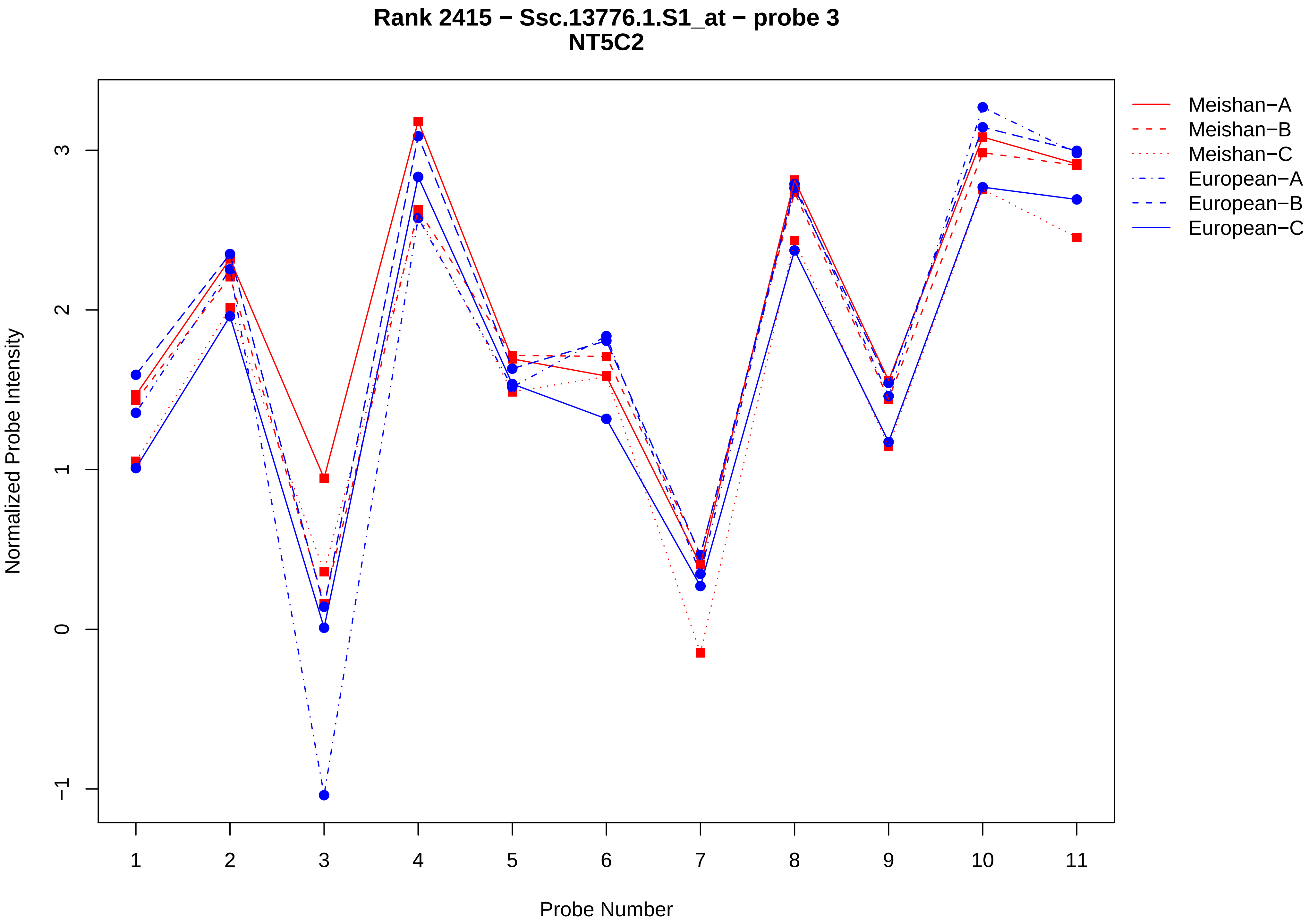

Rank 2415 − Ssc.13776.1.S1_at − probe 3
NT5C2
Normalized Probe Intensity
Probe Number
Meishan−A
Meishan−B
Meishan−C
European−A
European−B
European−C
−1
0
1
2
3
1
2
3
4
5
6
7
8
9
10
11

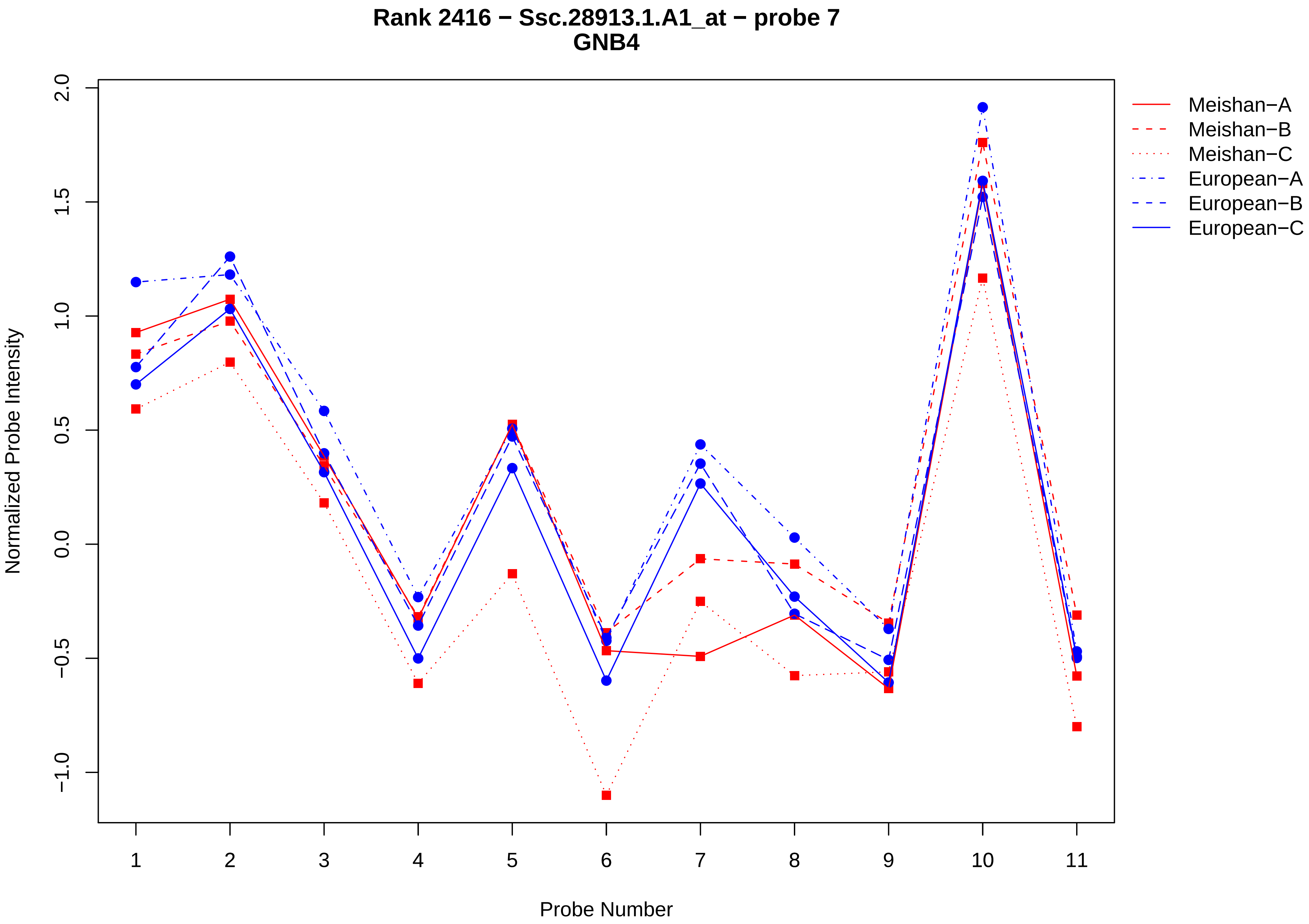

Rank 2416 − Ssc.28913.1.A1_at − probe 7
GNB4
Normalized Probe Intensity
Probe Number
2.0
1.5
1.0
0.5
0.0
−0.5
−1.0
1
2
3
4
5
6
7
8
9
10
11
Meishan−A
Meishan−B
Meishan−C
European−A
European−B
European−C

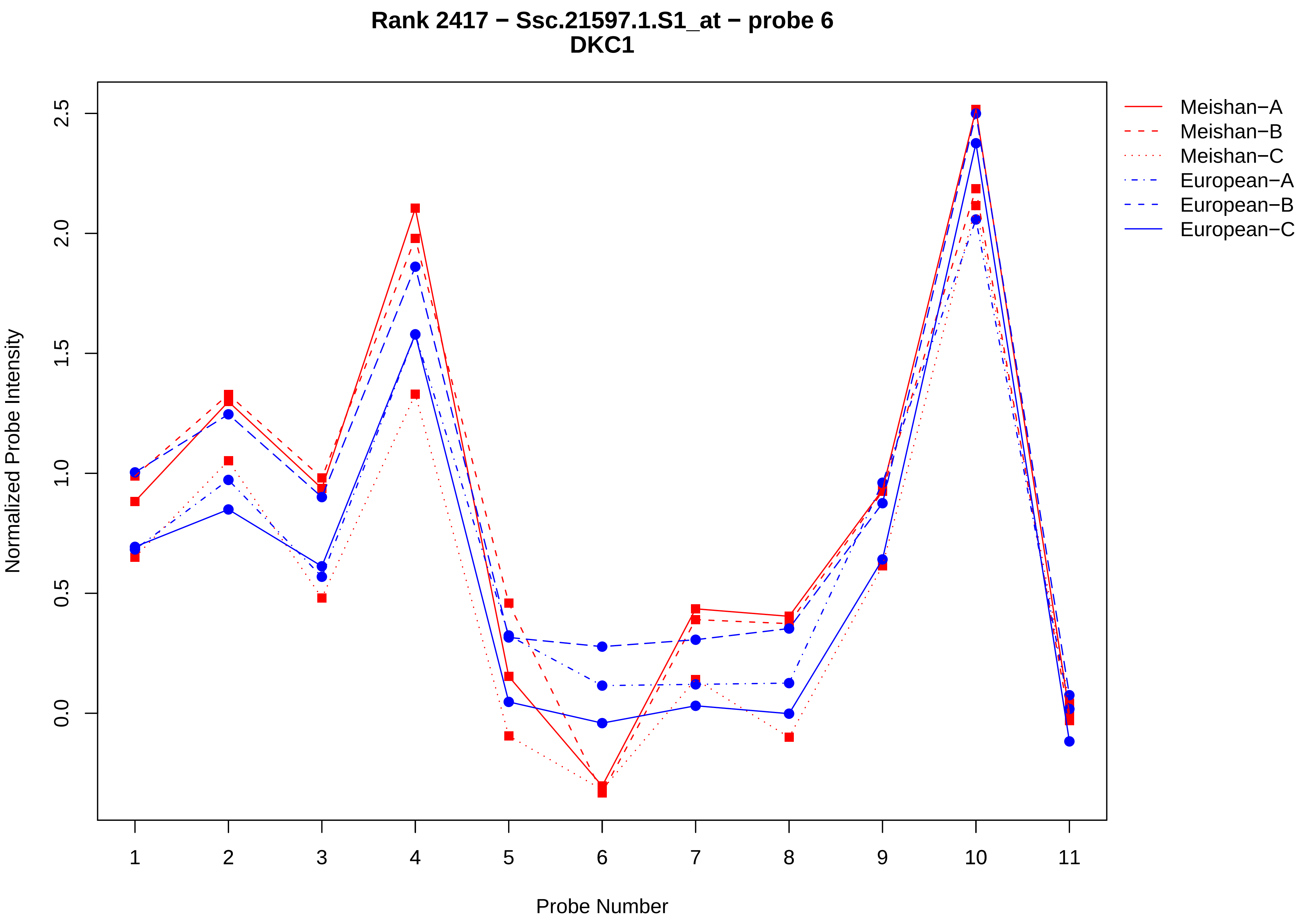

Rank 2417 − Ssc.21597.1.S1_at − probe 6
DKC1
Normalized Probe Intensity
Probe Number
0.0
0.5
1.0
1.5
2.0
2.5
1
2
3
4
5
6
7
8
9
10
11
Meishan−A
Meishan−B
Meishan−C
European−A
European−B
European−C

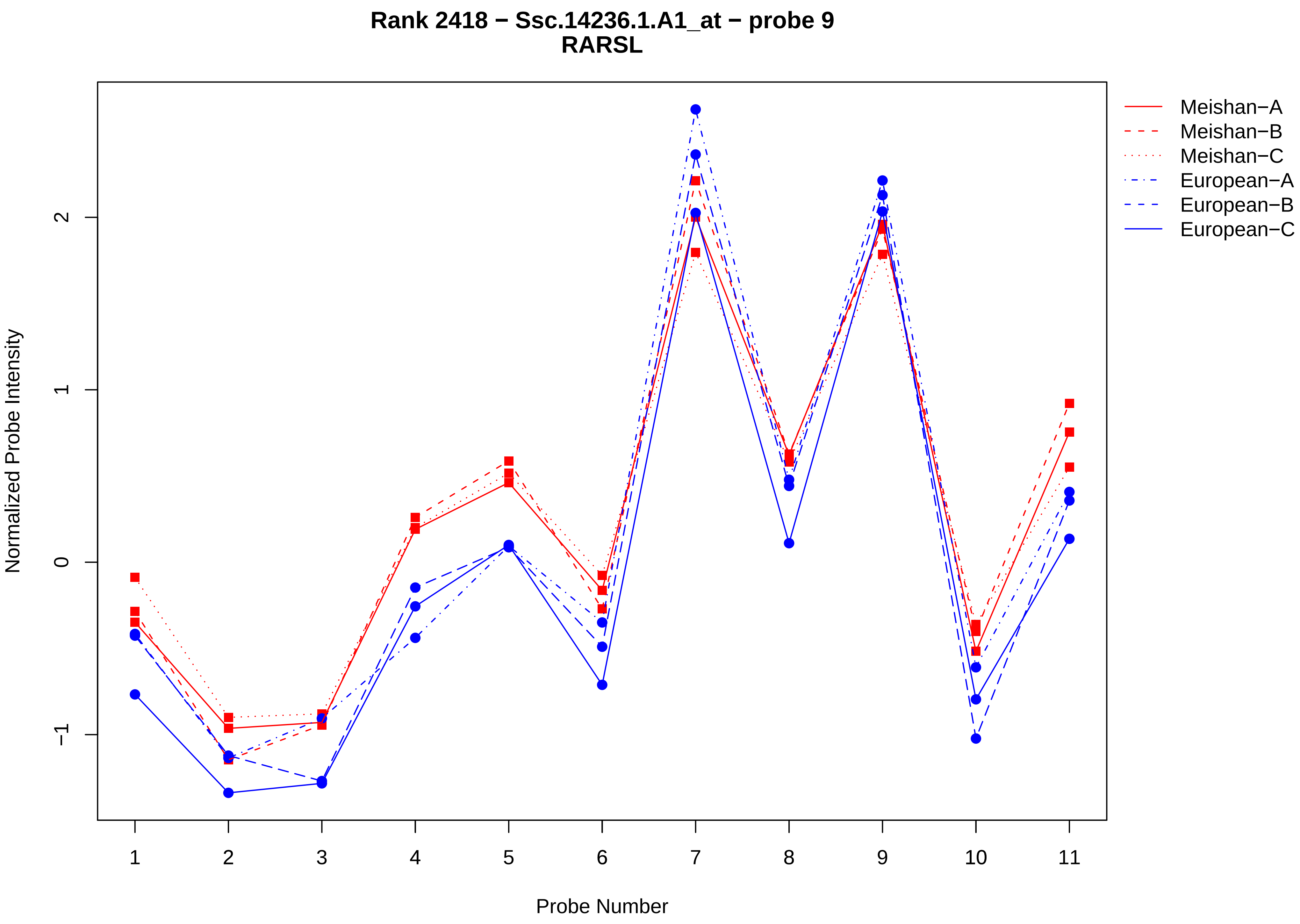

Rank 2418 − Ssc.14236.1.A1_at − probe 9
RARSL
Normalized Probe Intensity
Probe Number
−1
0
1
2
1
2
3
4
5
6
7
8
9
10
11
Meishan−A
Meishan−B
Meishan−C
European−A
European−B
European−C

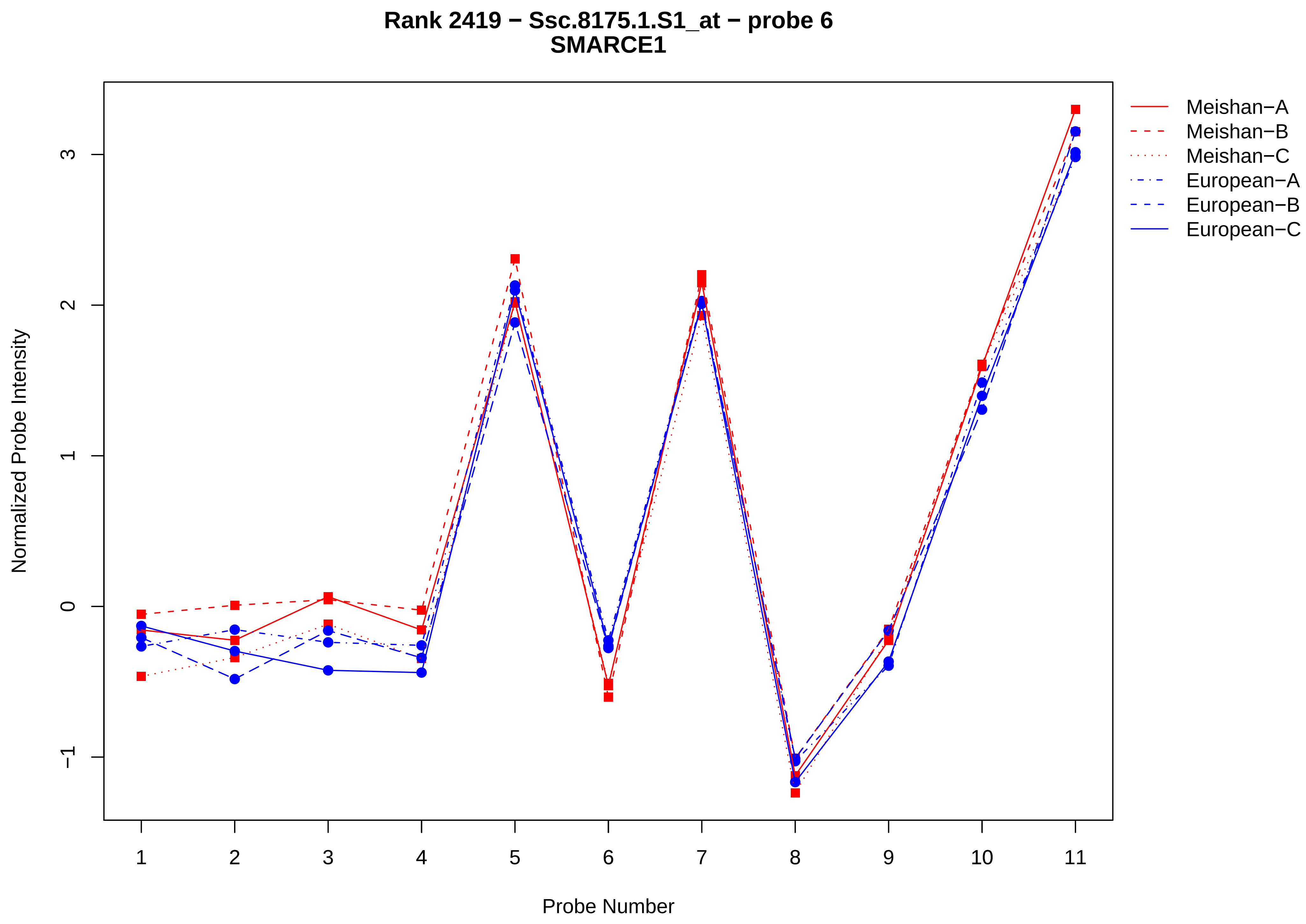

Rank 2419 − Ssc.8175.1.S1_at − probe 6
SMARCE1
Normalized Probe Intensity
Probe Number
−1
0
1
2
3
1
2
3
4
5
6
7
8
9
10
11
Meishan−A
Meishan−B
Meishan−C
European−A
European−B
European−C

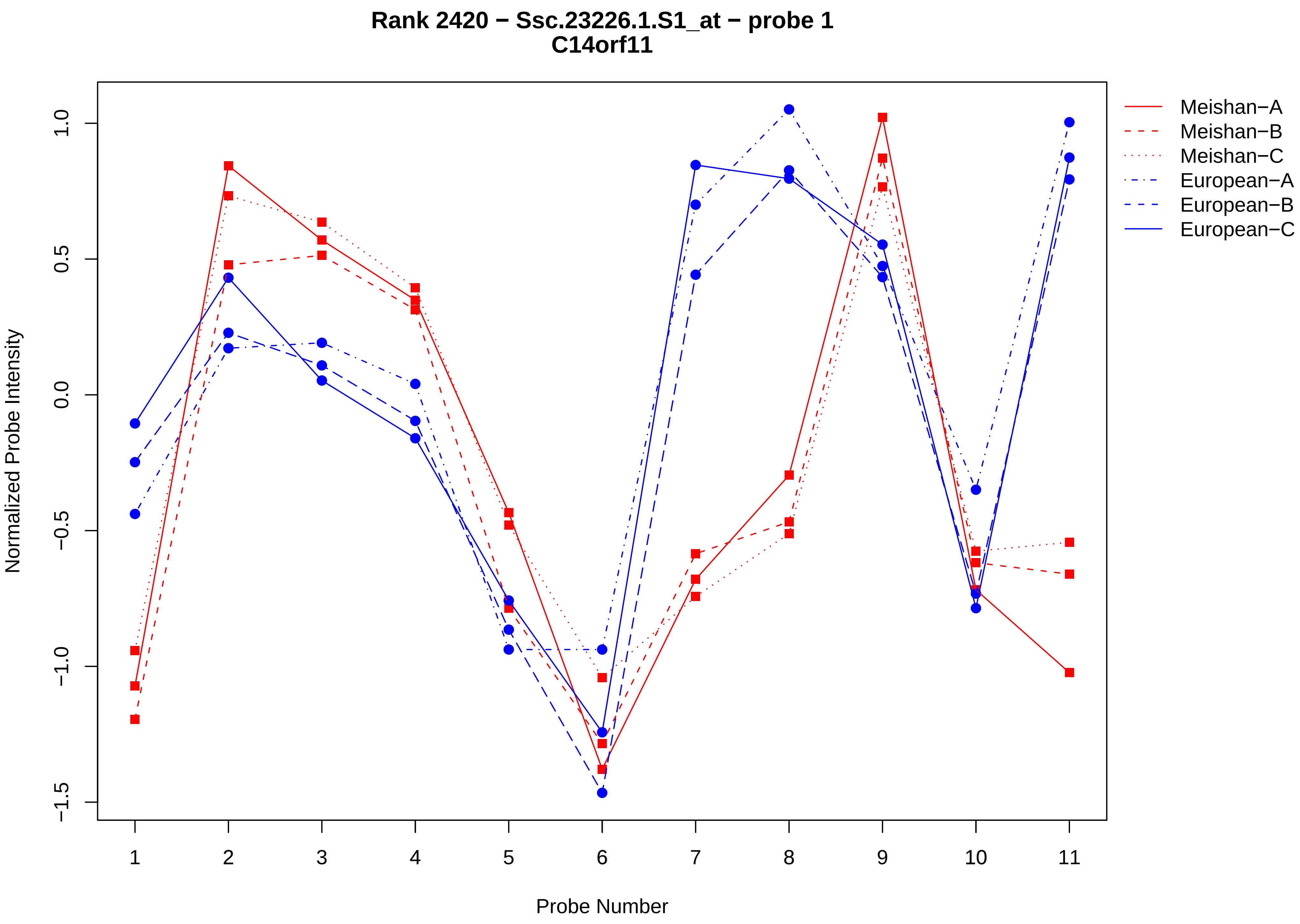

Rank 2420 − Ssc.23226.1.S1_at − probe 1
C14orf11
Normalized Probe Intensity
Probe Number
1.0
0.5
0.0
−0.5
−1.0
−1.5
1
2
3
4
5
6
7
8
9
10
11
Meishan−A
Meishan−B
Meishan−C
European−A
European−B
European−C

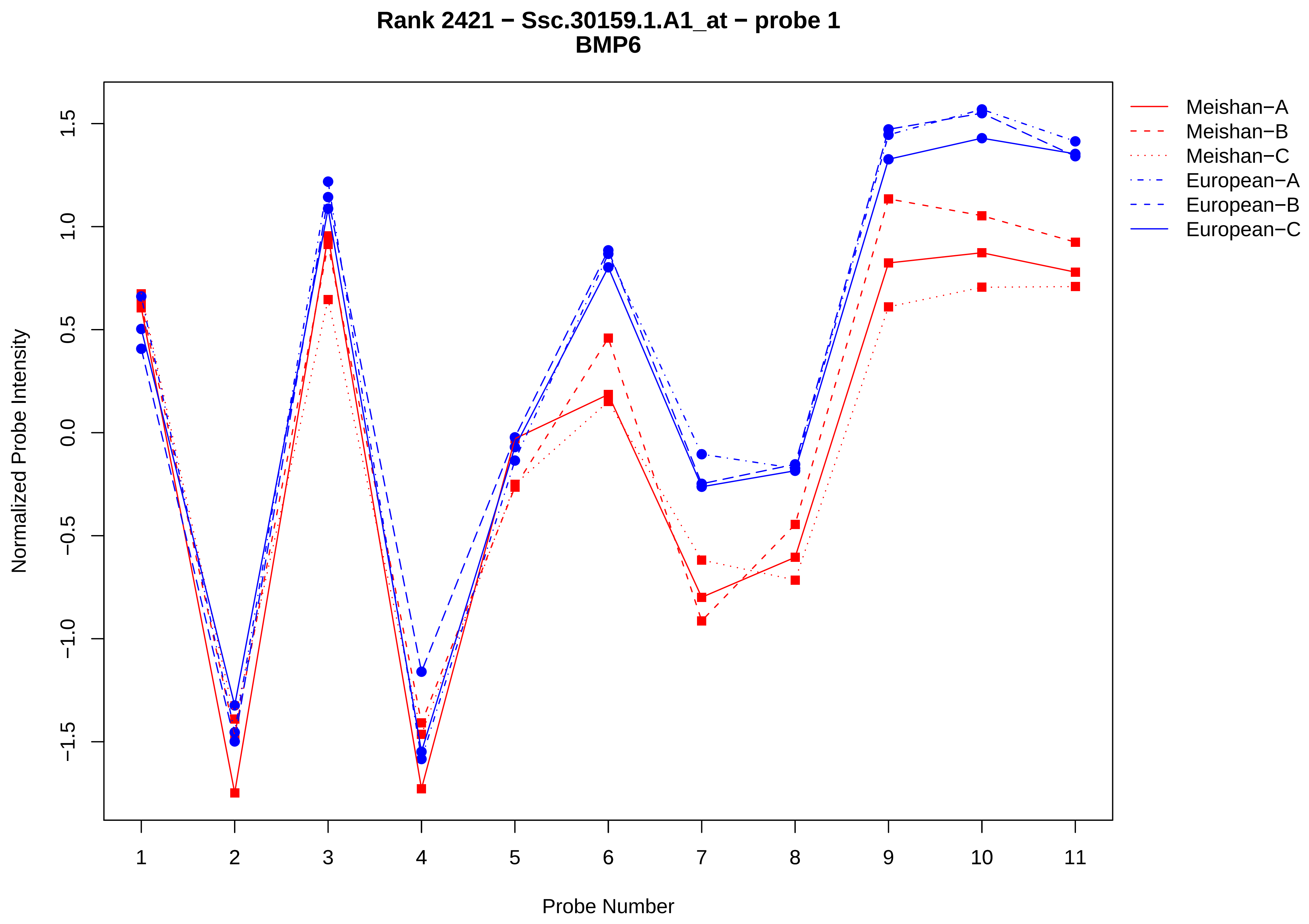

Rank 2421 − Ssc.30159.1.A1_at − probe 1
BMP6
Normalized Probe Intensity
Probe Number
−1.5
−1.0
−0.5
0.0
0.5
1.0
1.5
1
2
3
4
5
6
7
8
9
10
11
Meishan−A
Meishan−B
Meishan−C
European−A
European−B
European−C

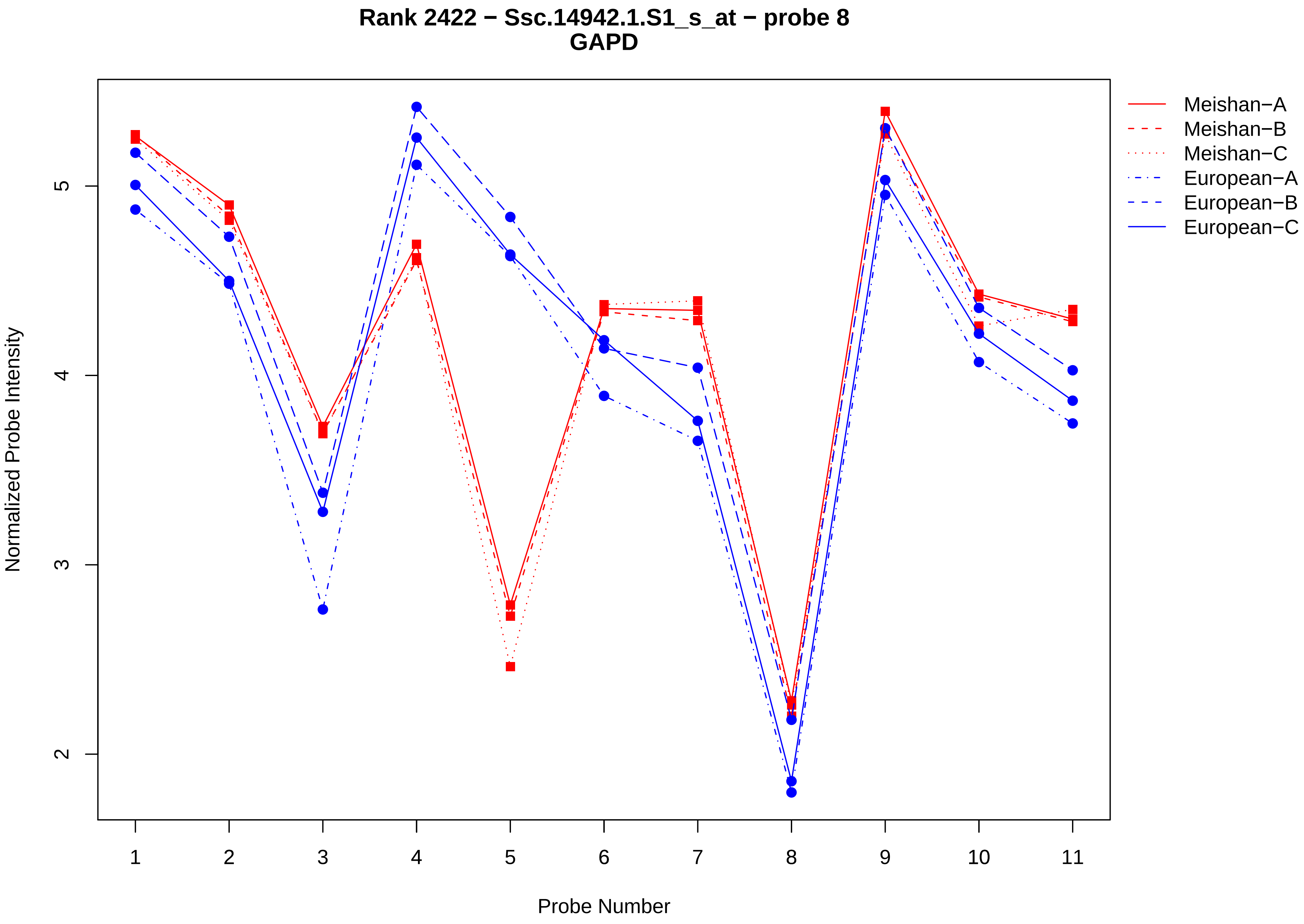
Rank 2422 − Ssc.14942.1.S1_s_at − probe 8
GAPD
Normalized Probe Intensity
Probe Number
2
3
4
5
1
2
3
4
5
6
7
8
9
10
11
Meishan−A
Meishan−B
Meishan−C
European−A
European−B
European−C

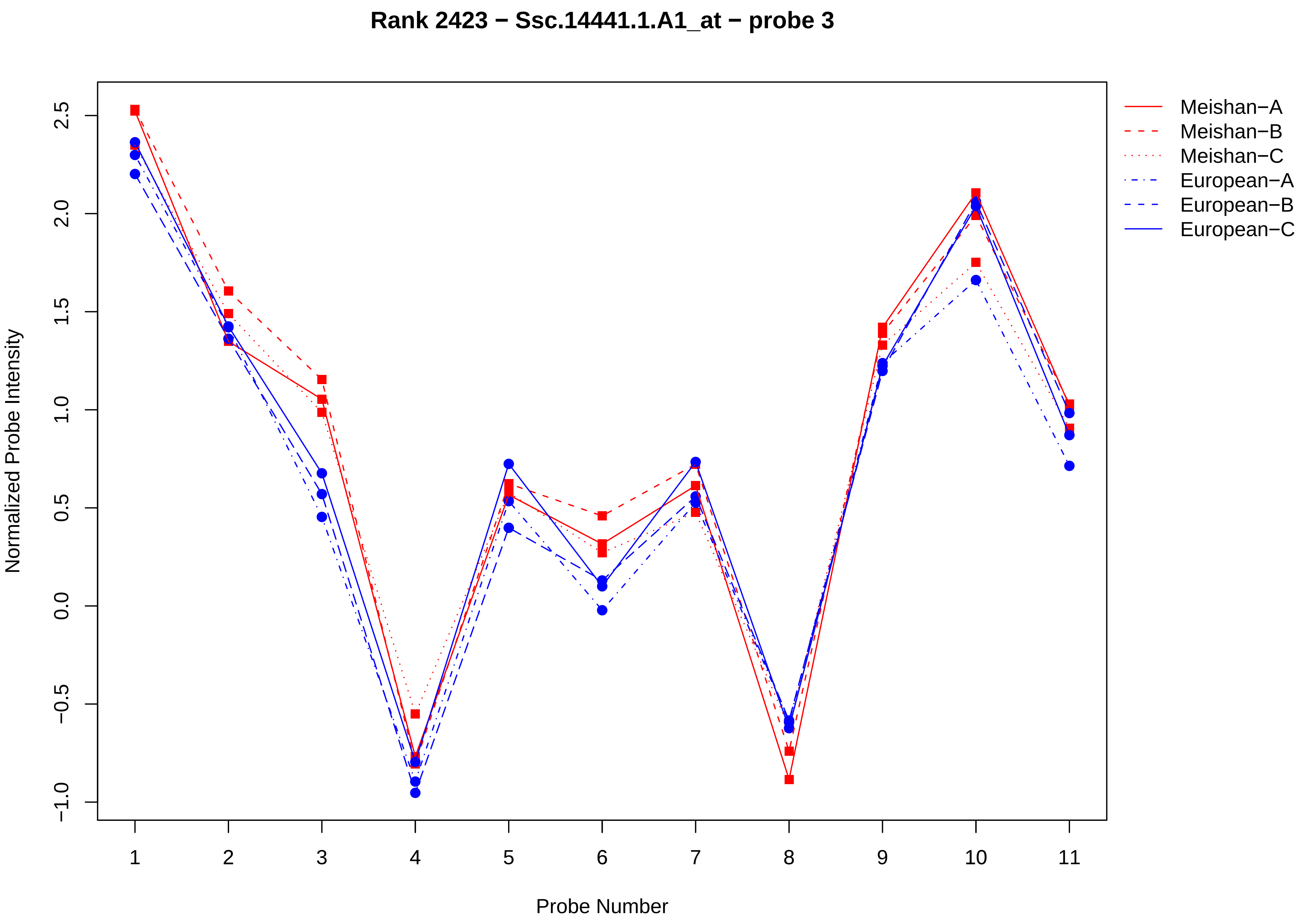

Rank 2423 − Ssc.14441.1.A1_at − probe 3
Normalized Probe Intensity
Probe Number
Meishan−A
Meishan−B
Meishan−C
European−A
European−B
European−C

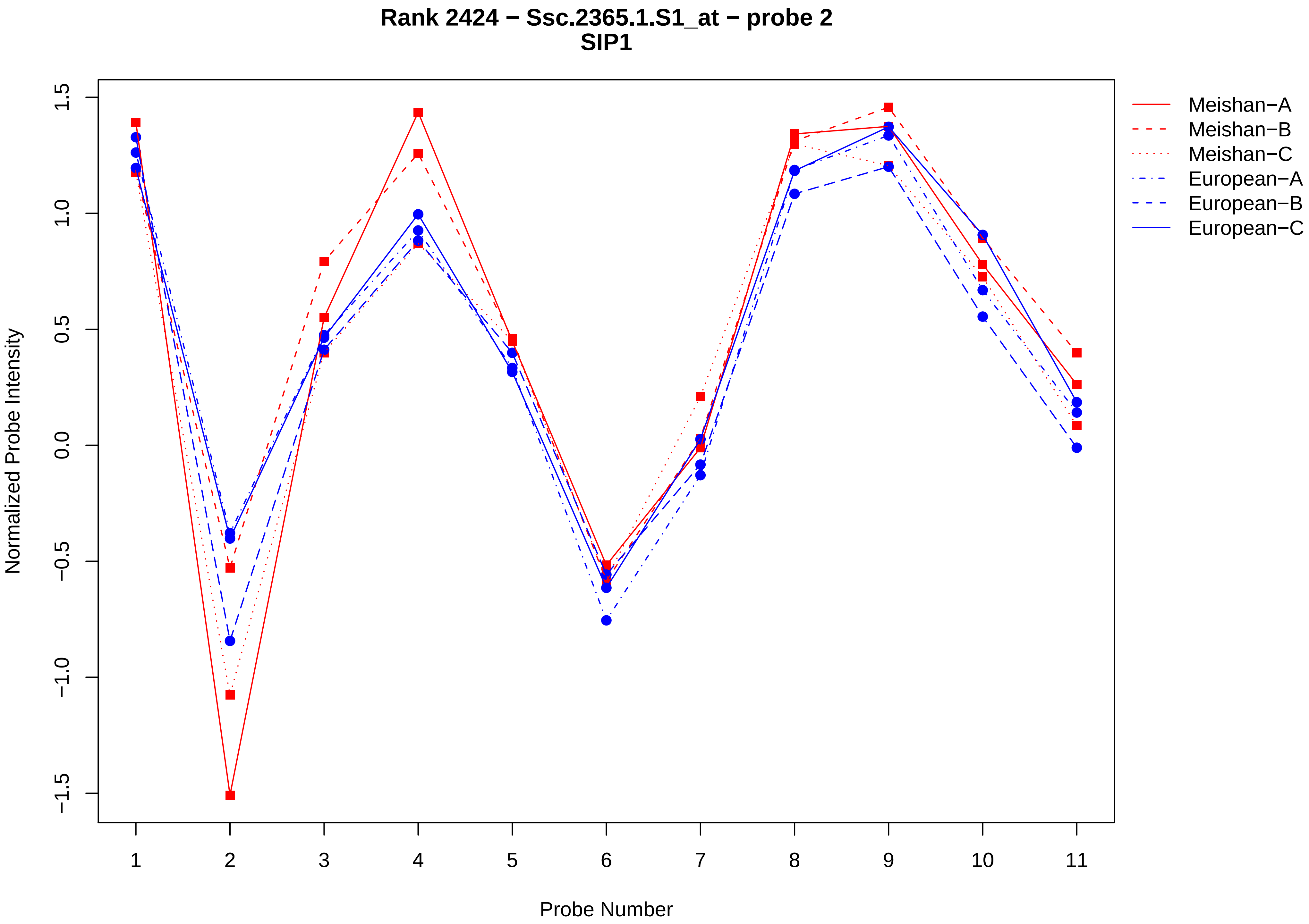

Rank 2424 − Ssc.2365.1.S1_at − probe 2
SIP1
Normalized Probe Intensity
Probe Number
−1.5
−1.0
−0.5
0.0
0.5
1.0
1.5
1
2
3
4
5
6
7
8
9
10
11
Meishan−A
Meishan−B
Meishan−C
European−A
European−B
European−C

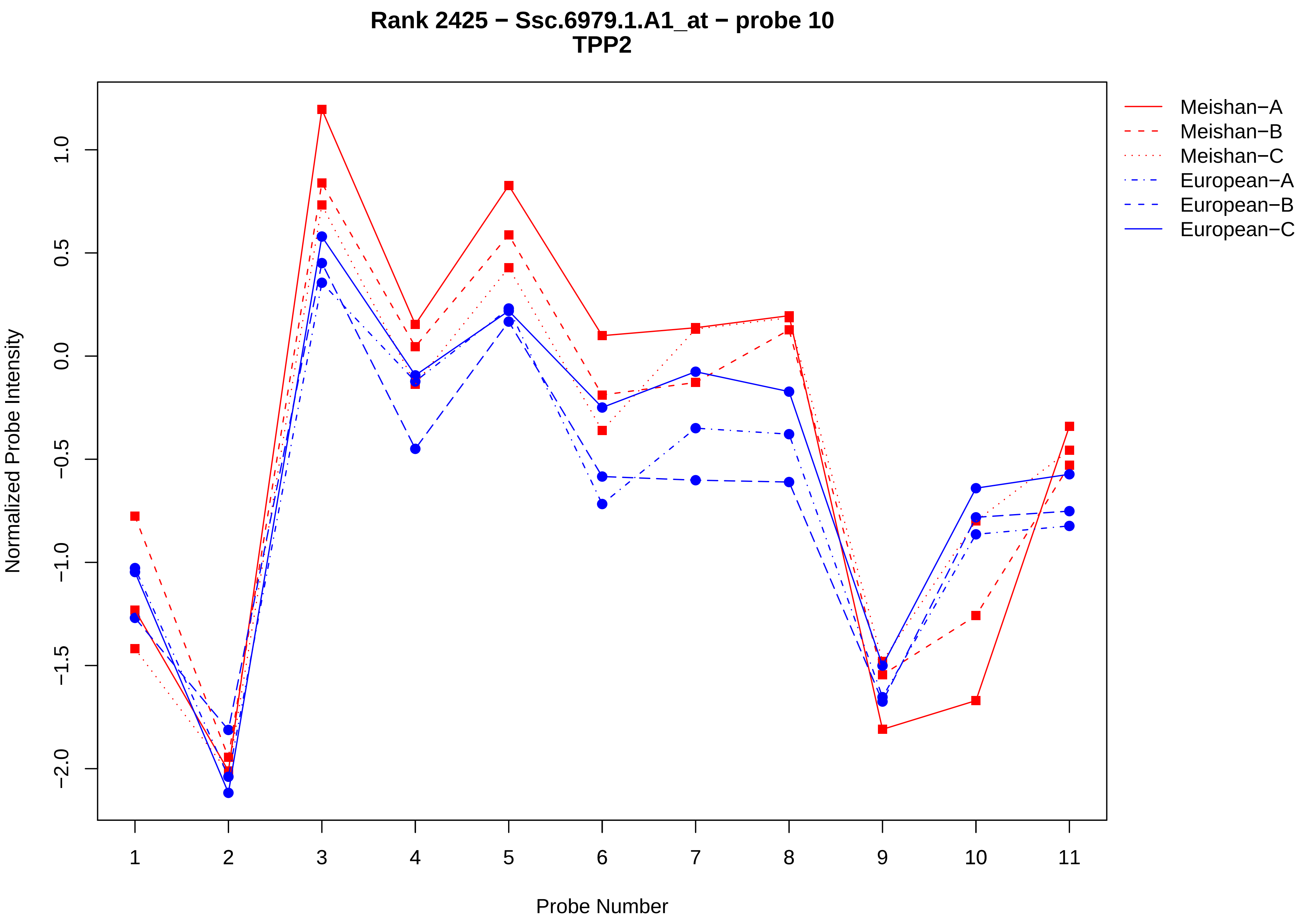
Rank 2425 − Ssc.6979.1.A1_at − probe 10
TPP2
Normalized Probe Intensity
Probe Number
1.0
0.5
0.0
−0.5
−1.0
−1.5
−2.0
1
2
3
4
5
6
7
8
9
10
11
Meishan−A
Meishan−B
Meishan−C
European−A
European−B
European−C

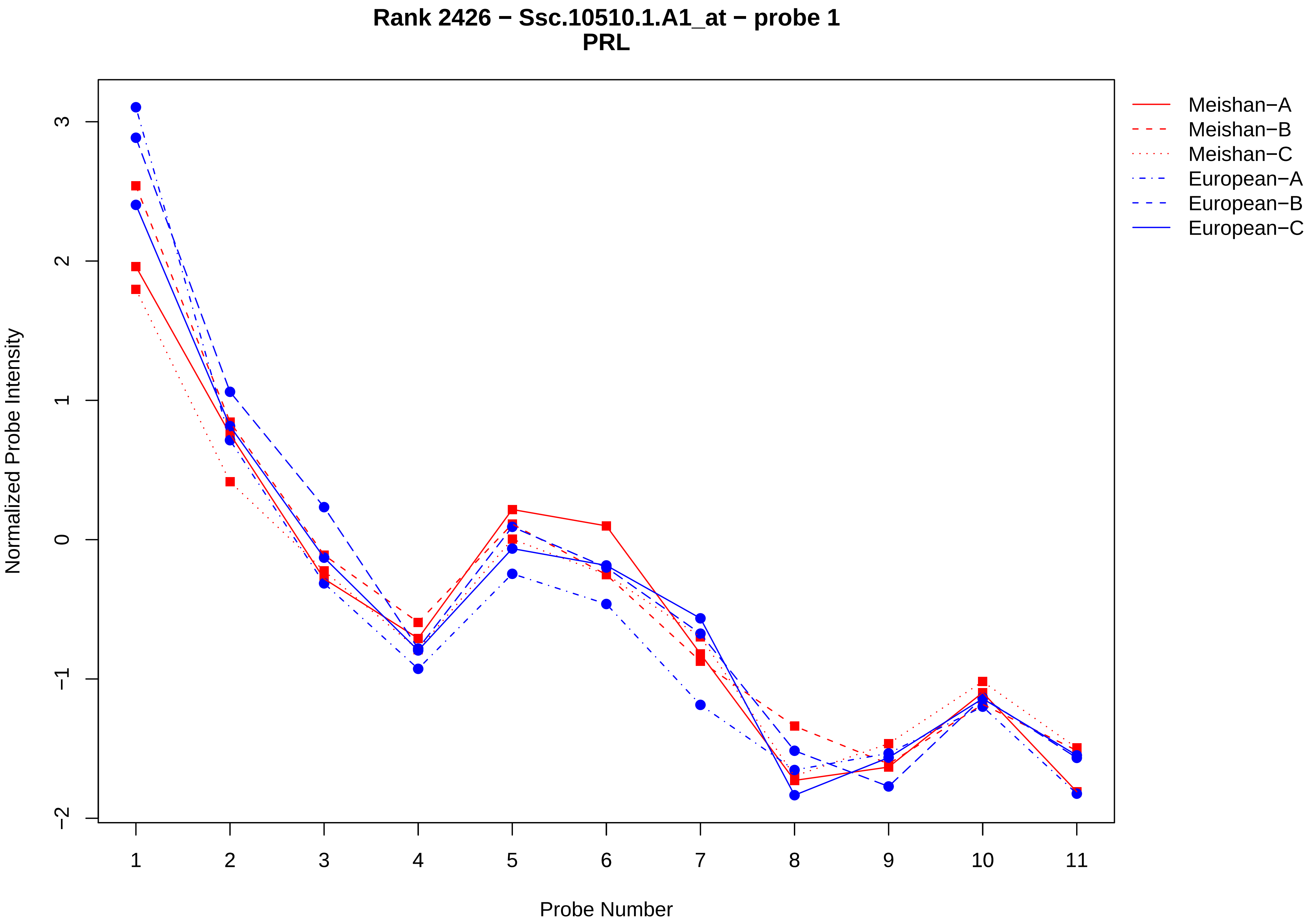

Rank 2426 − Ssc.10510.1.A1_at − probe 1
PRL
Normalized Probe Intensity
Probe Number
−2
−1
0
1
2
3
1
2
3
4
5
6
7
8
9
10
11
Meishan−A
Meishan−B
Meishan−C
European−A
European−B
European−C

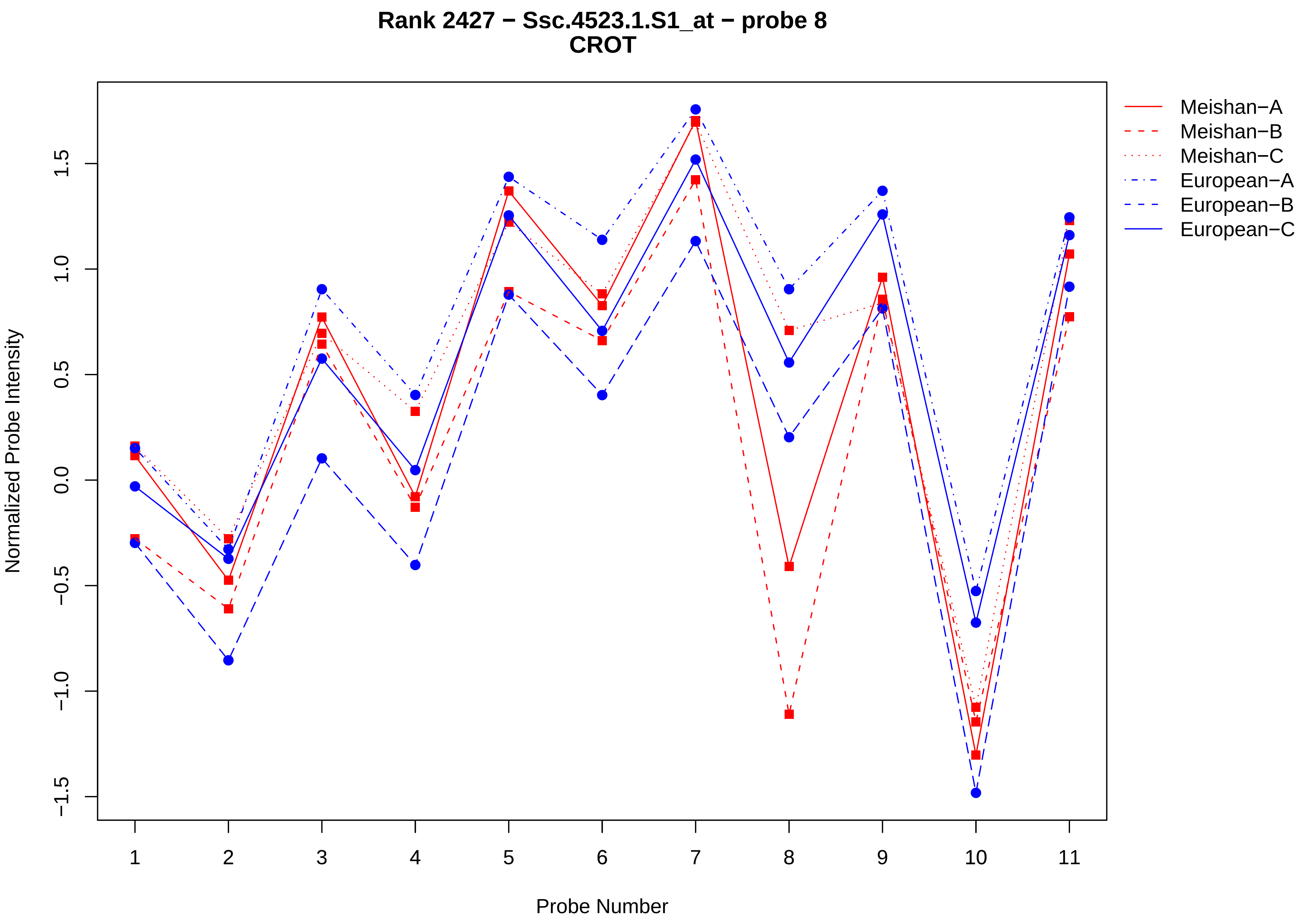
Rank 2427 − Ssc.4523.1.S1_at − probe 8
CROT
Normalized Probe Intensity
Probe Number
Meishan−A
Meishan−B
Meishan−C
European−A
European−B
European−C
1.5
1.0
0.5
0.0
−0.5
−1.0
−1.5
1
2
3
4
5
6
7
8
9
10
11

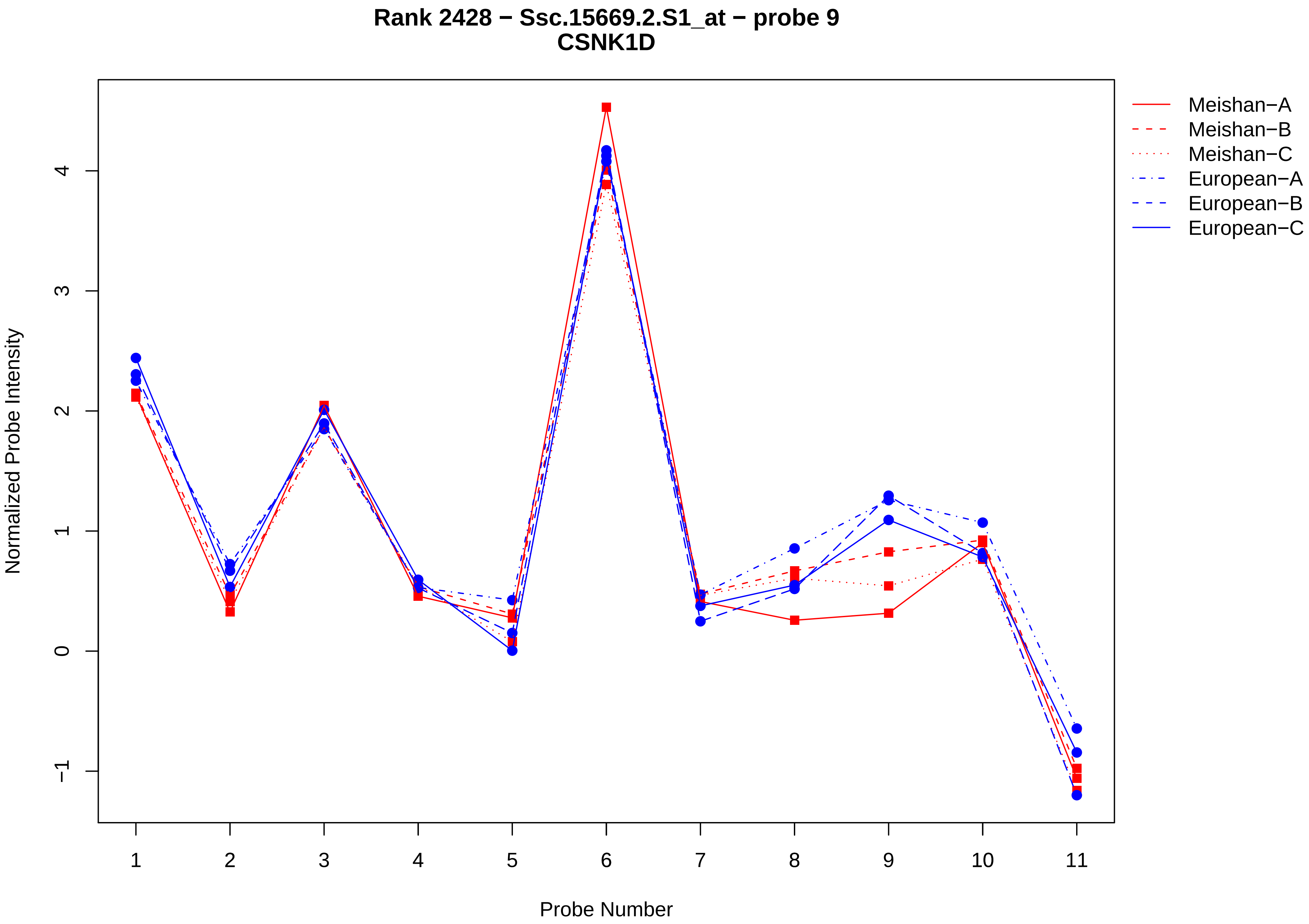

Rank 2428 − Ssc.15669.2.S1_at − probe 9
CSNK1D
Normalized Probe Intensity
Probe Number
Meishan−A
Meishan−B
Meishan−C
European−A
European−B
European−C
−1
0
1
2
3
4
1
2
3
4
5
6
7
8
9
10
11

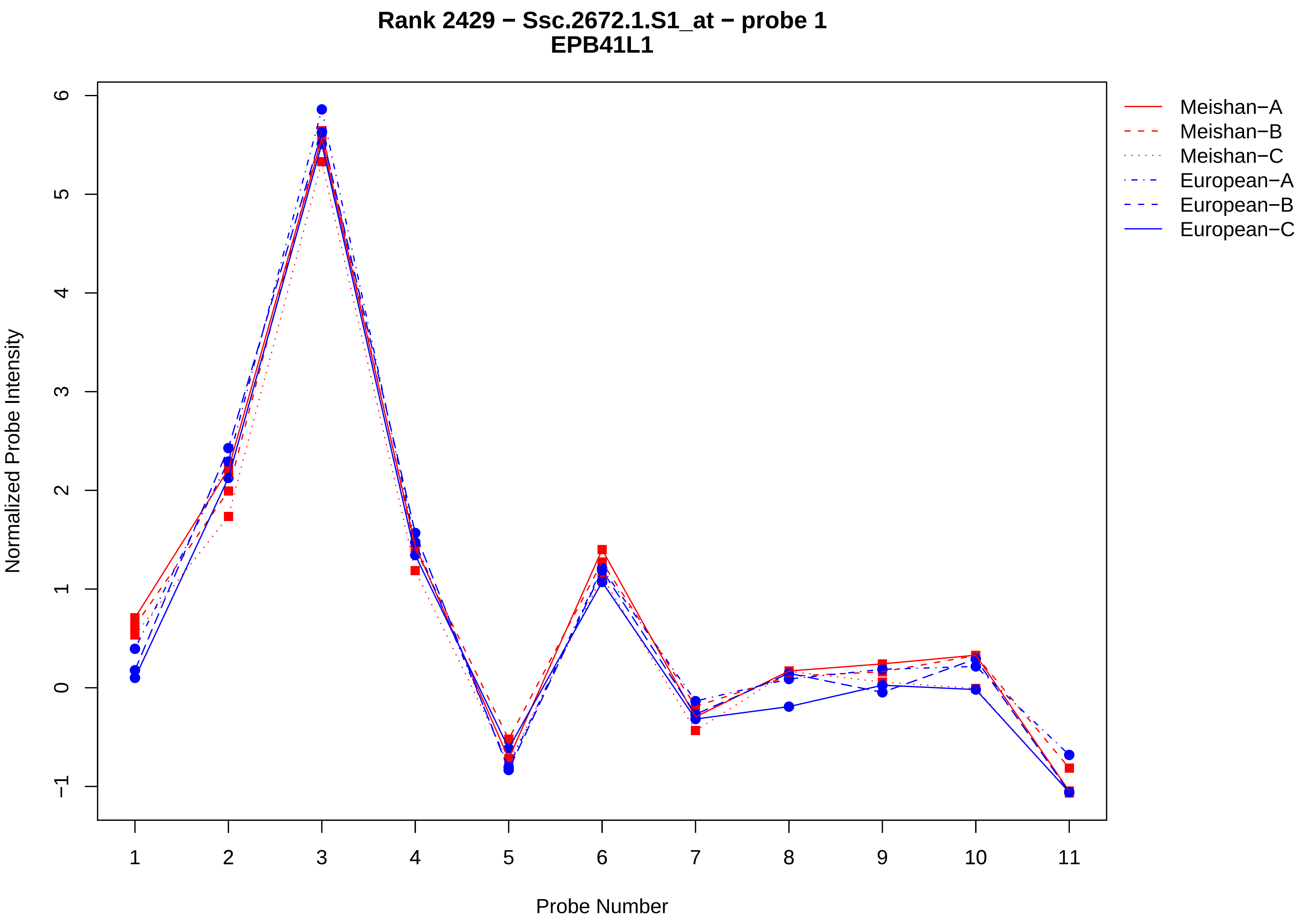

Rank 2429 − Ssc.2672.1.S1_at − probe 1
EPB41L1
Normalized Probe Intensity
Probe Number
−1
0
1
2
3
4
5
6
1
2
3
4
5
6
7
8
9
10
11
Meishan−A
Meishan−B
Meishan−C
European−A
European−B
European−C

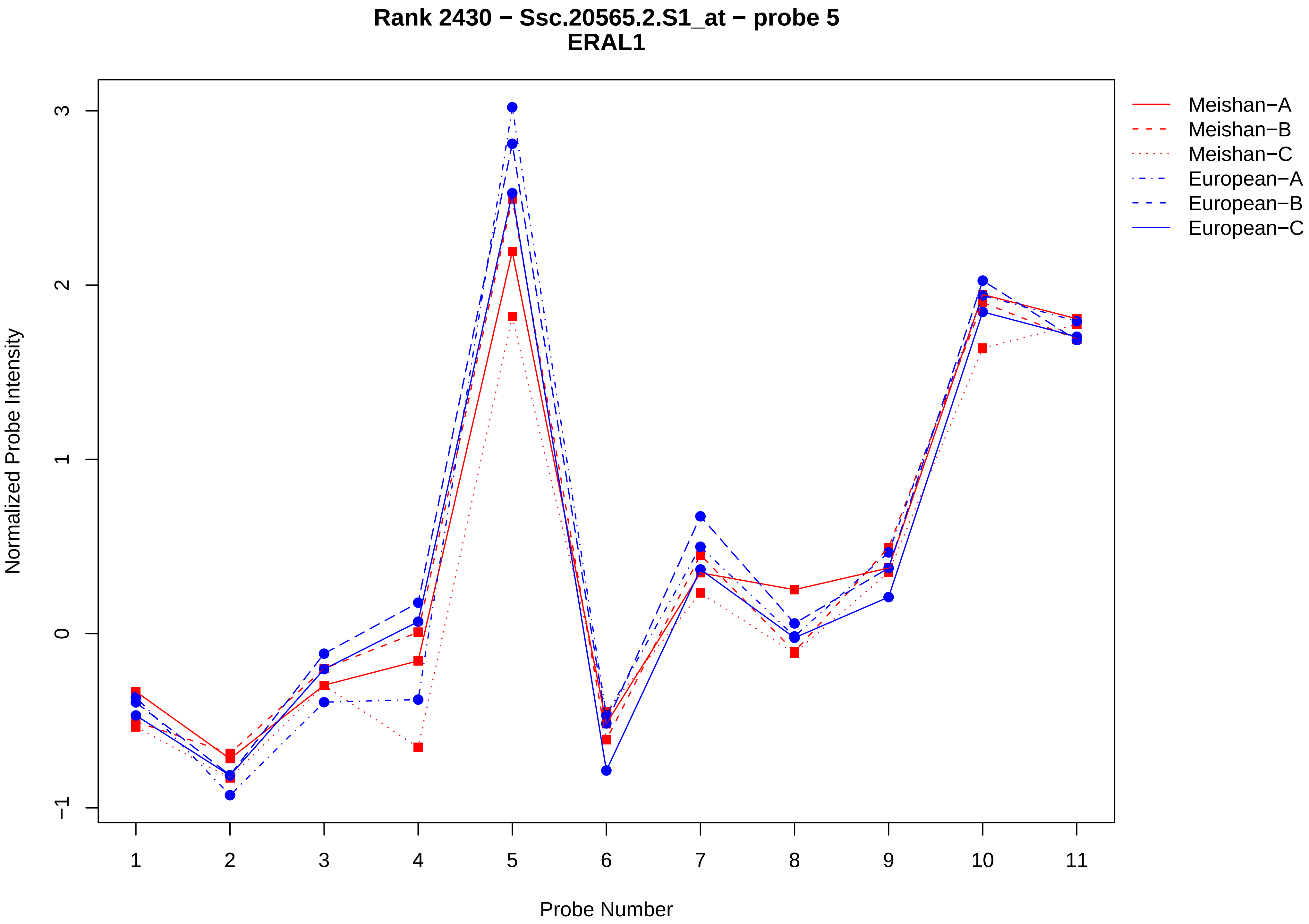

Rank 2430 − Ssc.20565.2.S1_at − probe 5
ERAL1
Meishan−A
Meishan−B
Meishan−C
European−A
European−B
European−C
Normalized Probe Intensity
Probe Number
−1
0
1
2
3
1
2
3
4
5
6
7
8
9
10
11

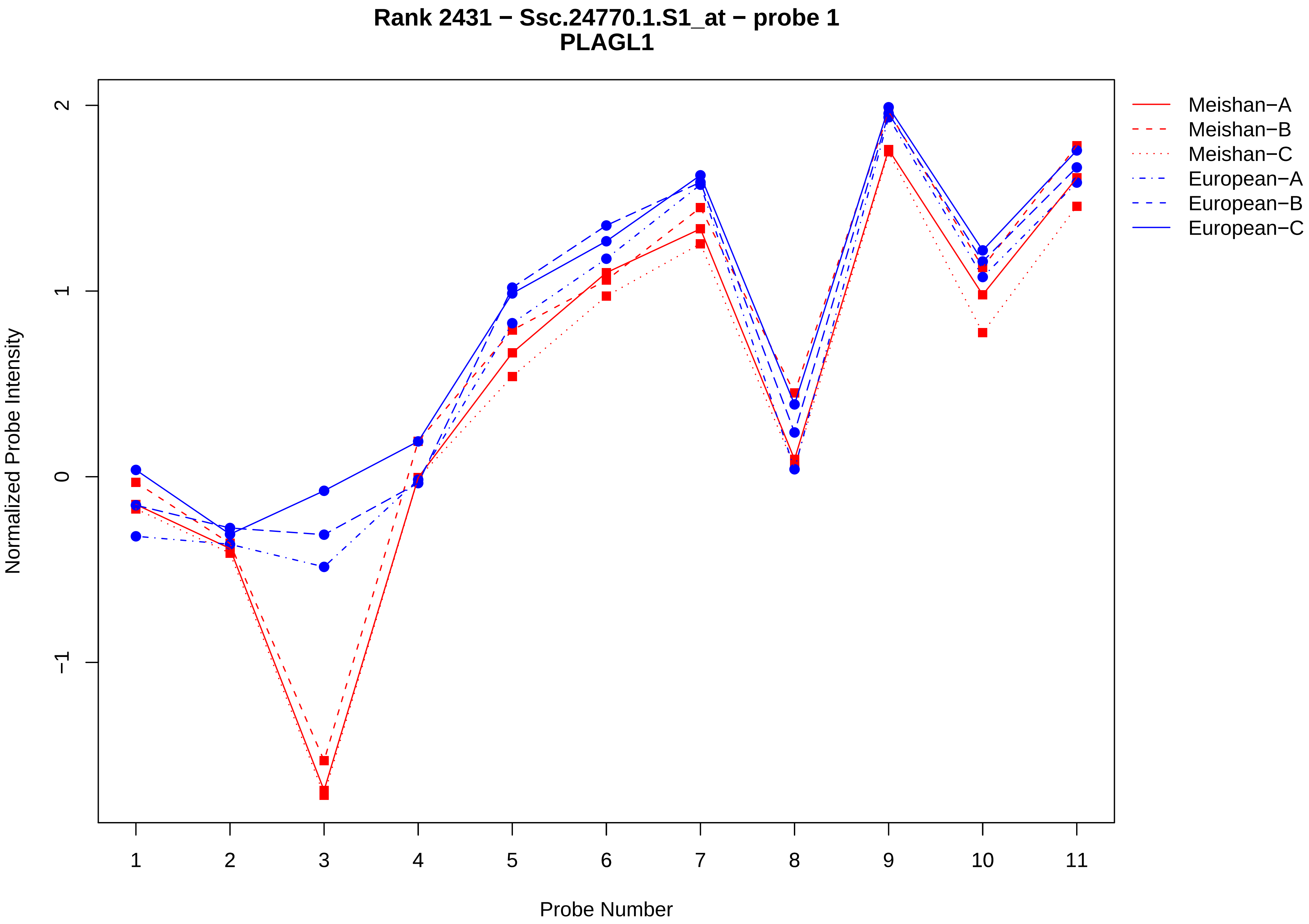

Rank 2431 − Ssc.24770.1.S1_at − probe 1
PLAGL1
Normalized Probe Intensity
Probe Number
2
1
0
−1
1
2
3
4
5
6
7
8
9
10
11
Meishan−A
Meishan−B
Meishan−C
European−A
European−B
European−C

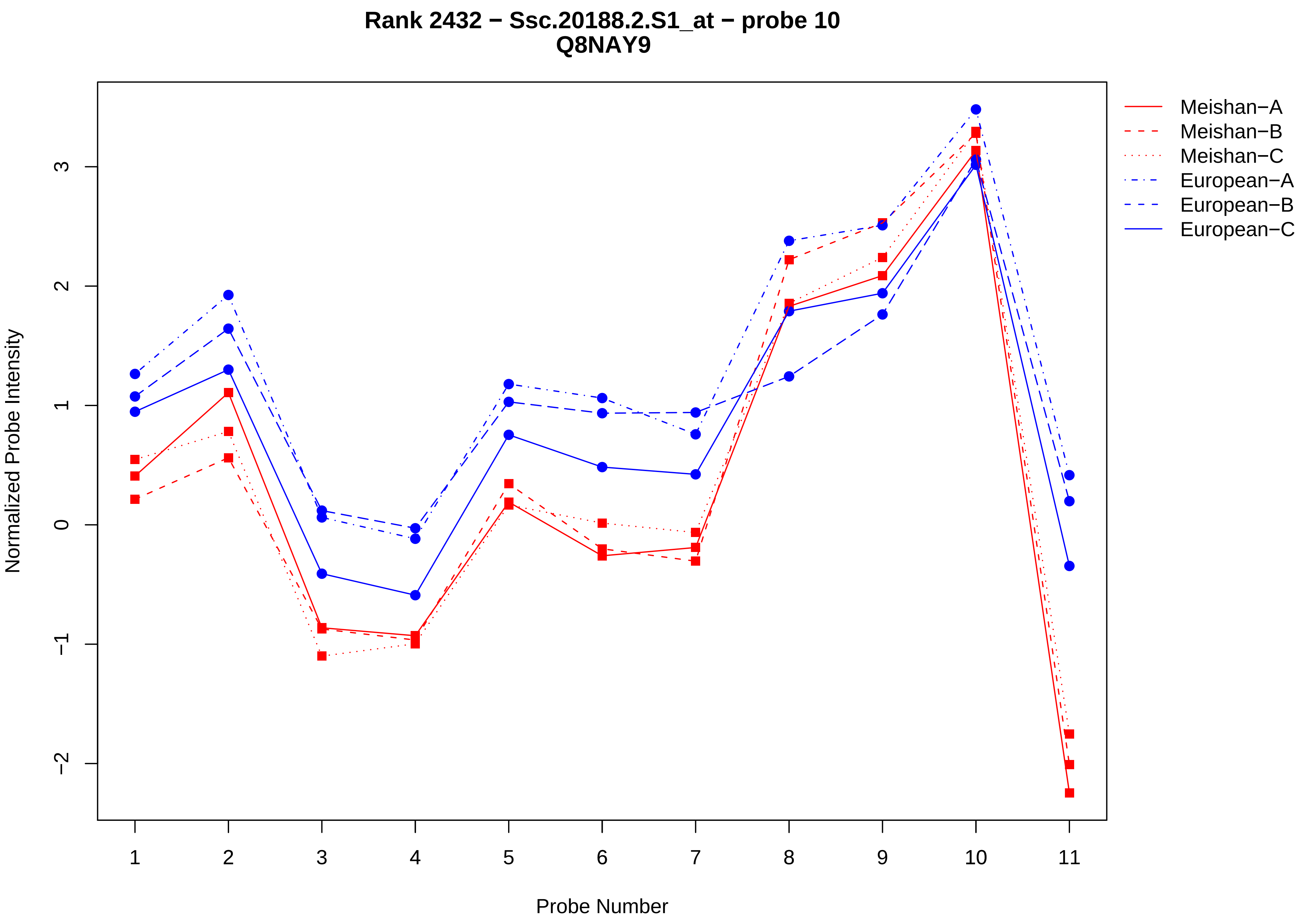
Rank 2432 − Ssc.20188.2.S1_at − probe 10
Q8NAY9
Meishan−A
Meishan−B
Meishan−C
European−A
European−B
European−C
Normalized Probe Intensity
Probe Number
−2
−1
0
1
2
3
1
2
3
4
5
6
7
8
9
10
11

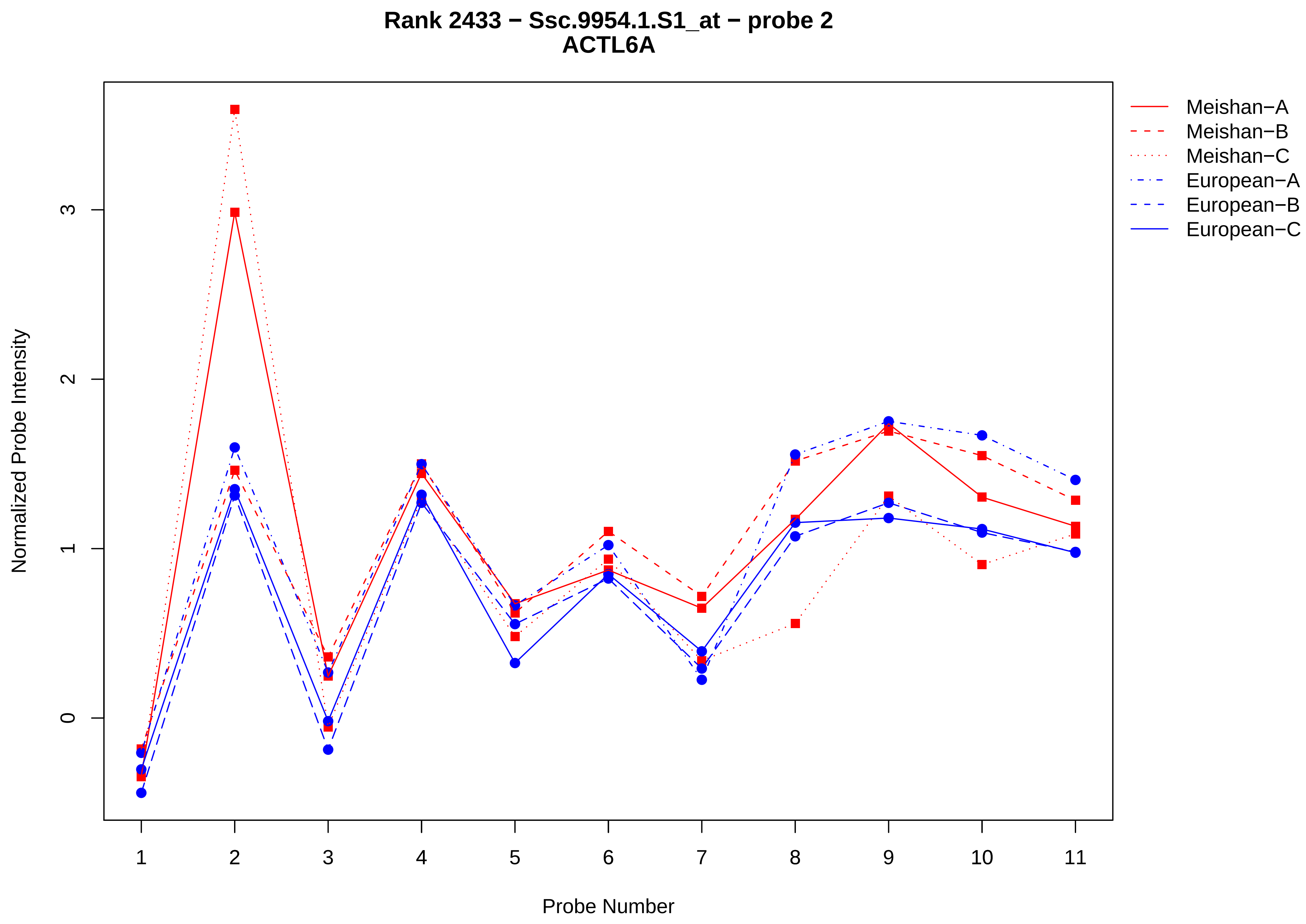

Rank 2433 − Ssc.9954.1.S1_at − probe 2
ACTL6A
Normalized Probe Intensity
0
1
2
3
1
2
3
4
5
6
7
8
9
10
11
Probe Number
Meishan−A
Meishan−B
Meishan−C
European−A
European−B
European−C

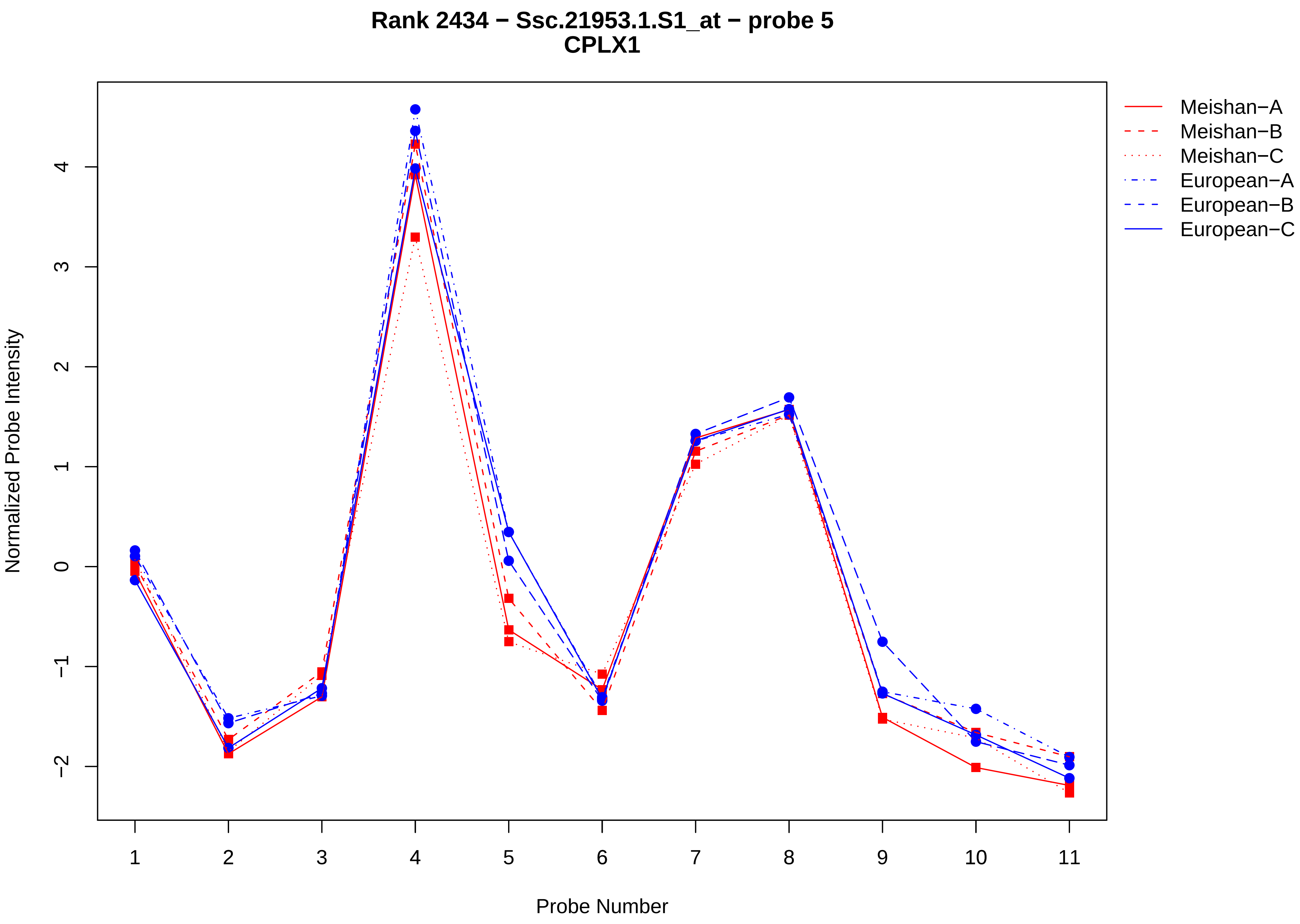

Rank 2434 − Ssc.21953.1.S1_at − probe 5
CPLX1
Normalized Probe Intensity
Probe Number
−2
−1
0
1
2
3
4
1
2
3
4
5
6
7
8
9
10
11
Meishan−A
Meishan−B
Meishan−C
European−A
European−B
European−C

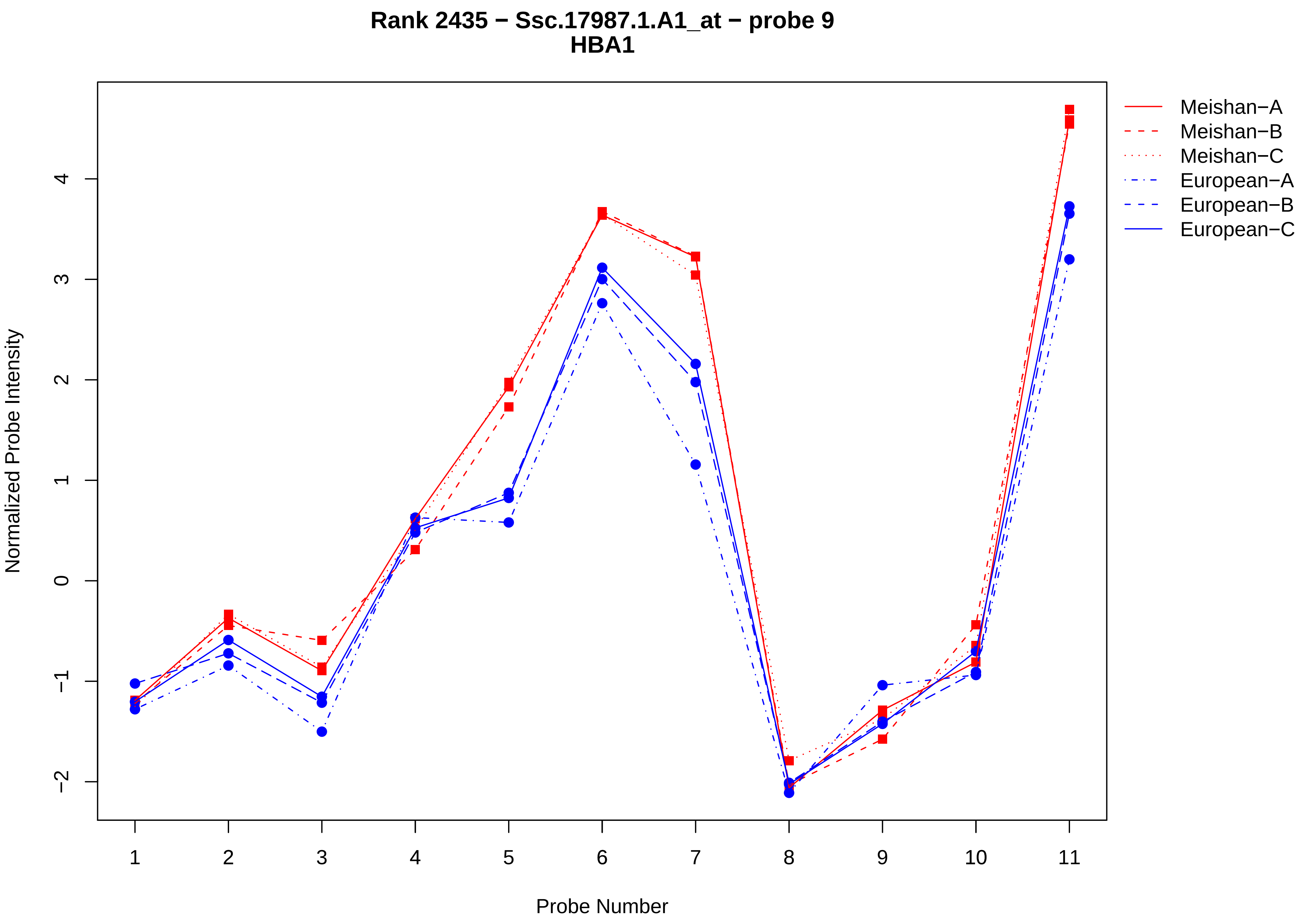

Rank 2435 − Ssc.17987.1.A1_at − probe 9
HBA1
Normalized Probe Intensity
Probe Number
−2
−1
0
1
2
3
4
1
2
3
4
5
6
7
8
9
10
11
Meishan−A
Meishan−B
Meishan−C
European−A
European−B
European−C

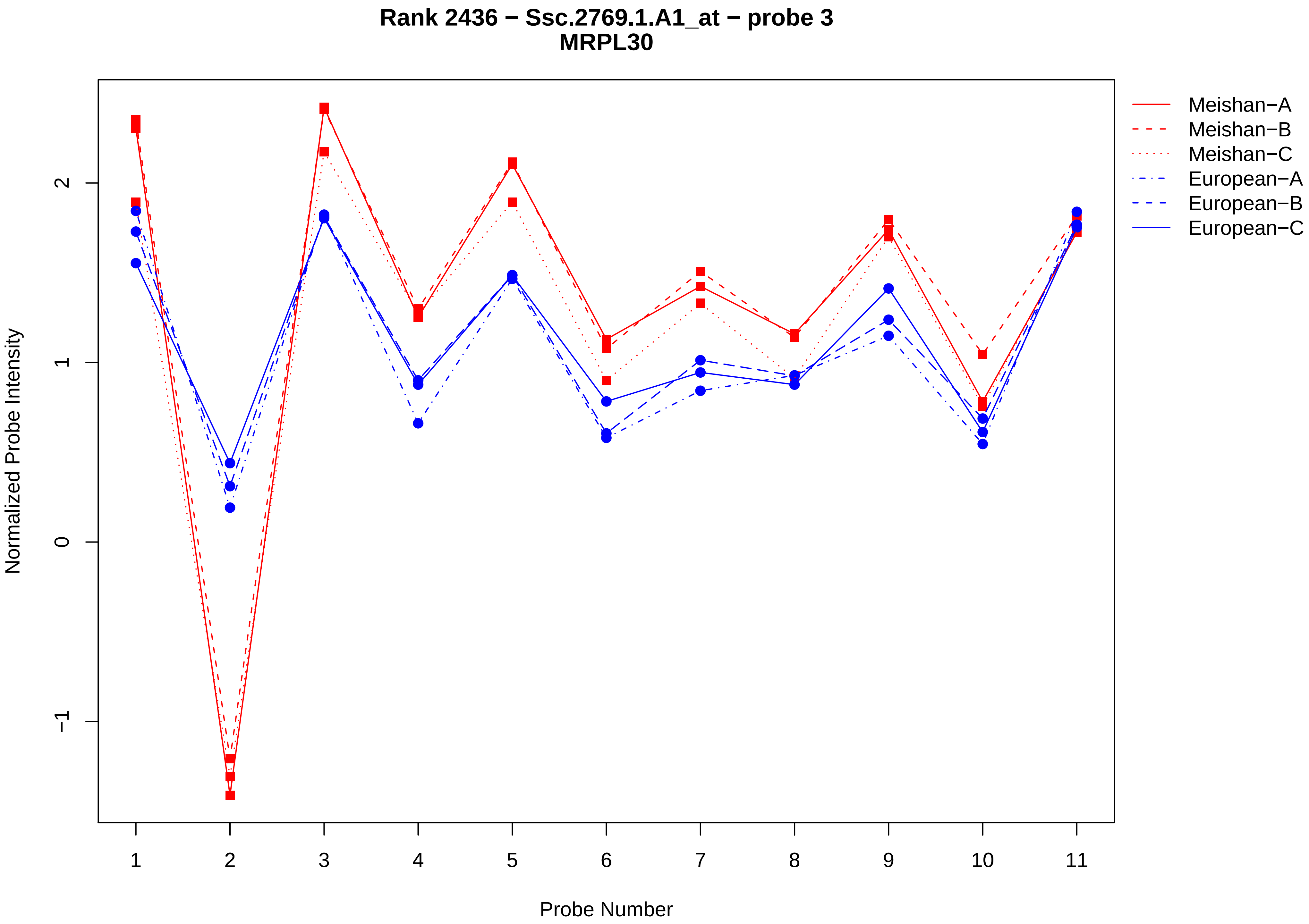

Rank 2436 − Ssc.2769.1.A1_at − probe 3
MRPL30
Normalized Probe Intensity
Probe Number
−1
0
1
2
1
2
3
4
5
6
7
8
9
10
11
Meishan−A
Meishan−B
Meishan−C
European−A
European−B
European−C

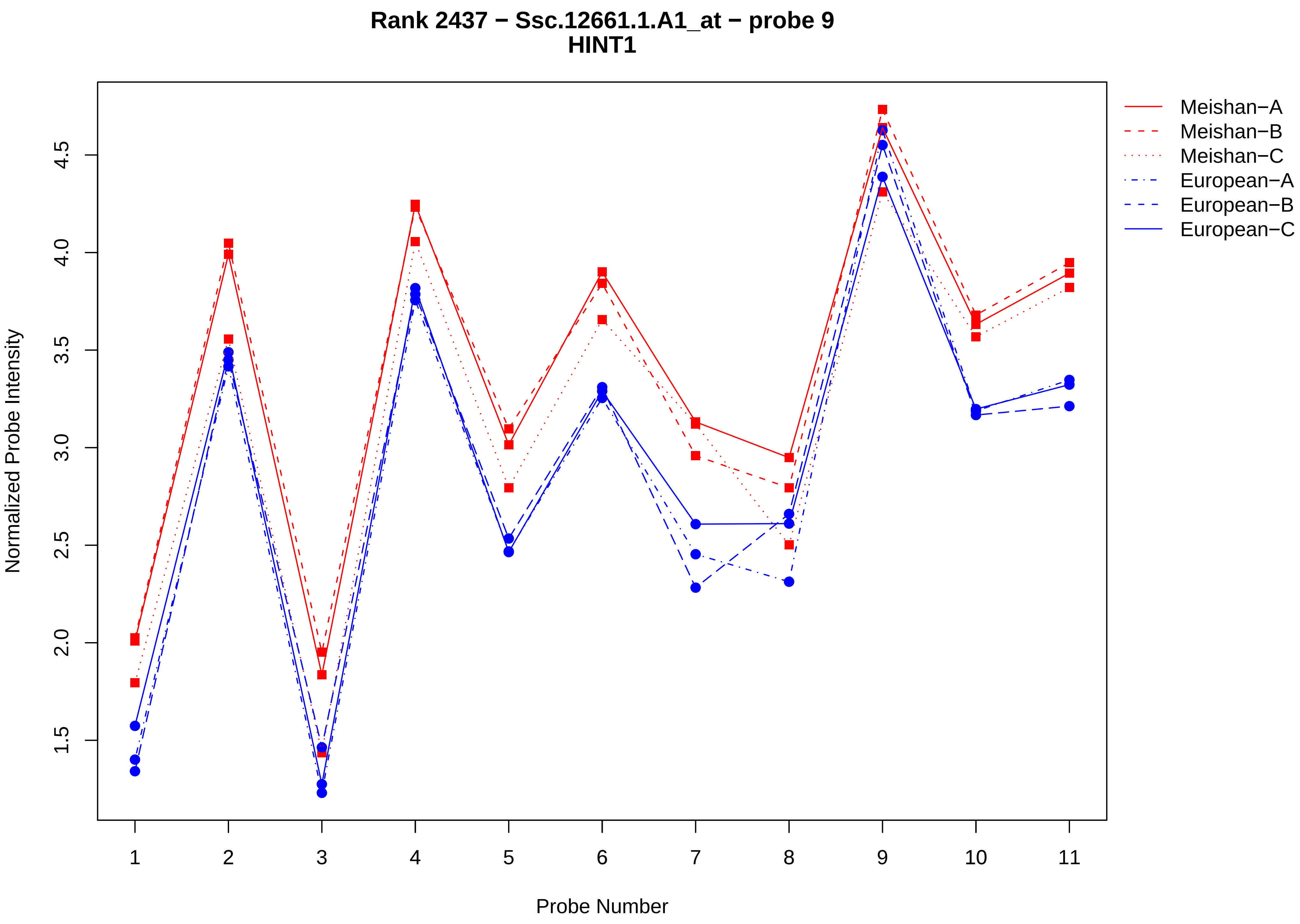

Rank 2437 − Ssc.12661.1.A1_at − probe 9
HINT1
Normalized Probe Intensity
Probe Number
1.5
2.0
2.5
3.0
3.5
4.0
4.5
1
2
3
4
5
6
7
8
9
10
11
Meishan−A
Meishan−B
Meishan−C
European−A
European−B
European−C

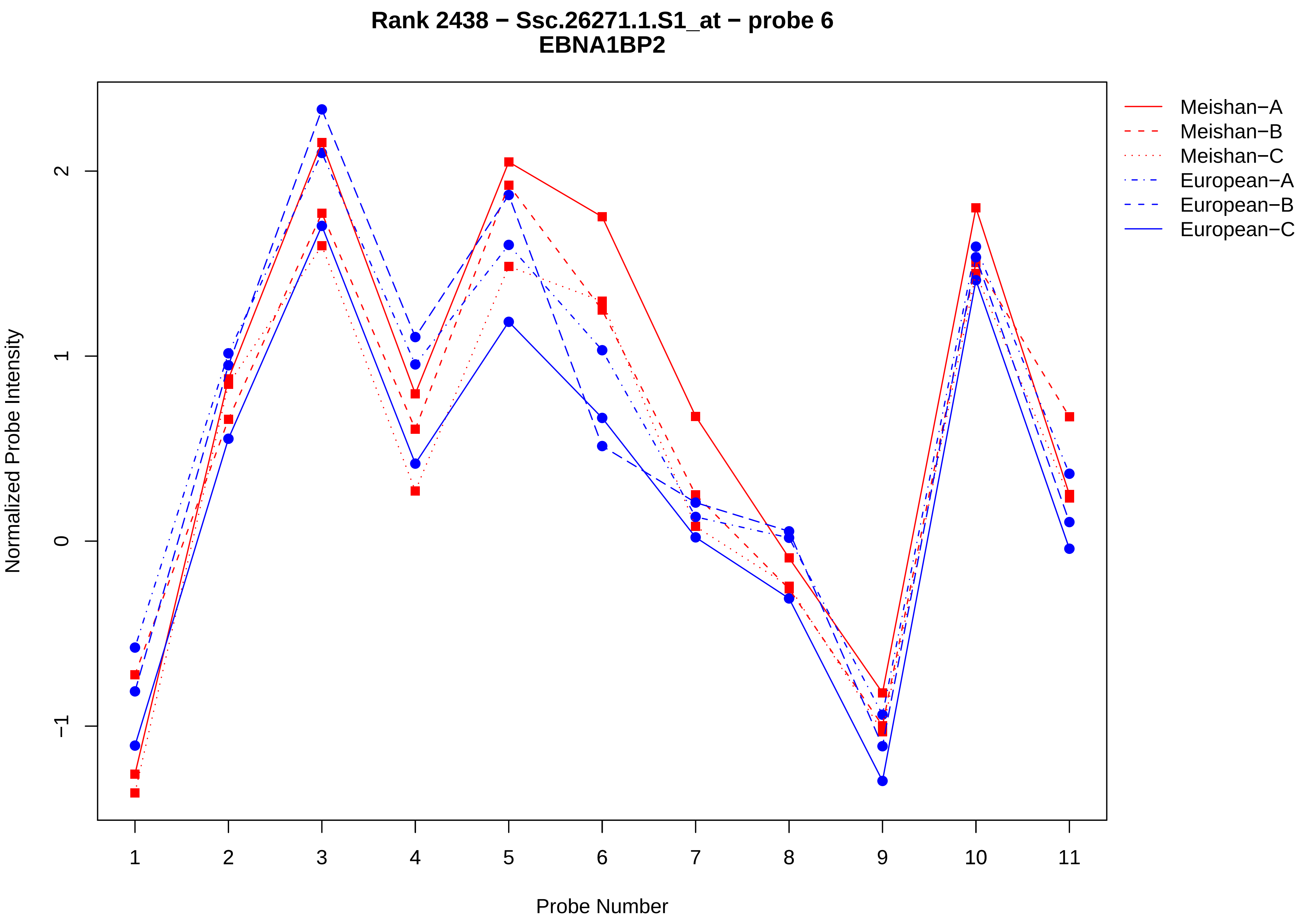

Rank 2438 − Ssc.26271.1.S1_at − probe 6
EBNA1BP2
Normalized Probe Intensity
Probe Number
−1
0
1
2
1
2
3
4
5
6
7
8
9
10
11
Meishan−A
Meishan−B
Meishan−C
European−A
European−B
European−C

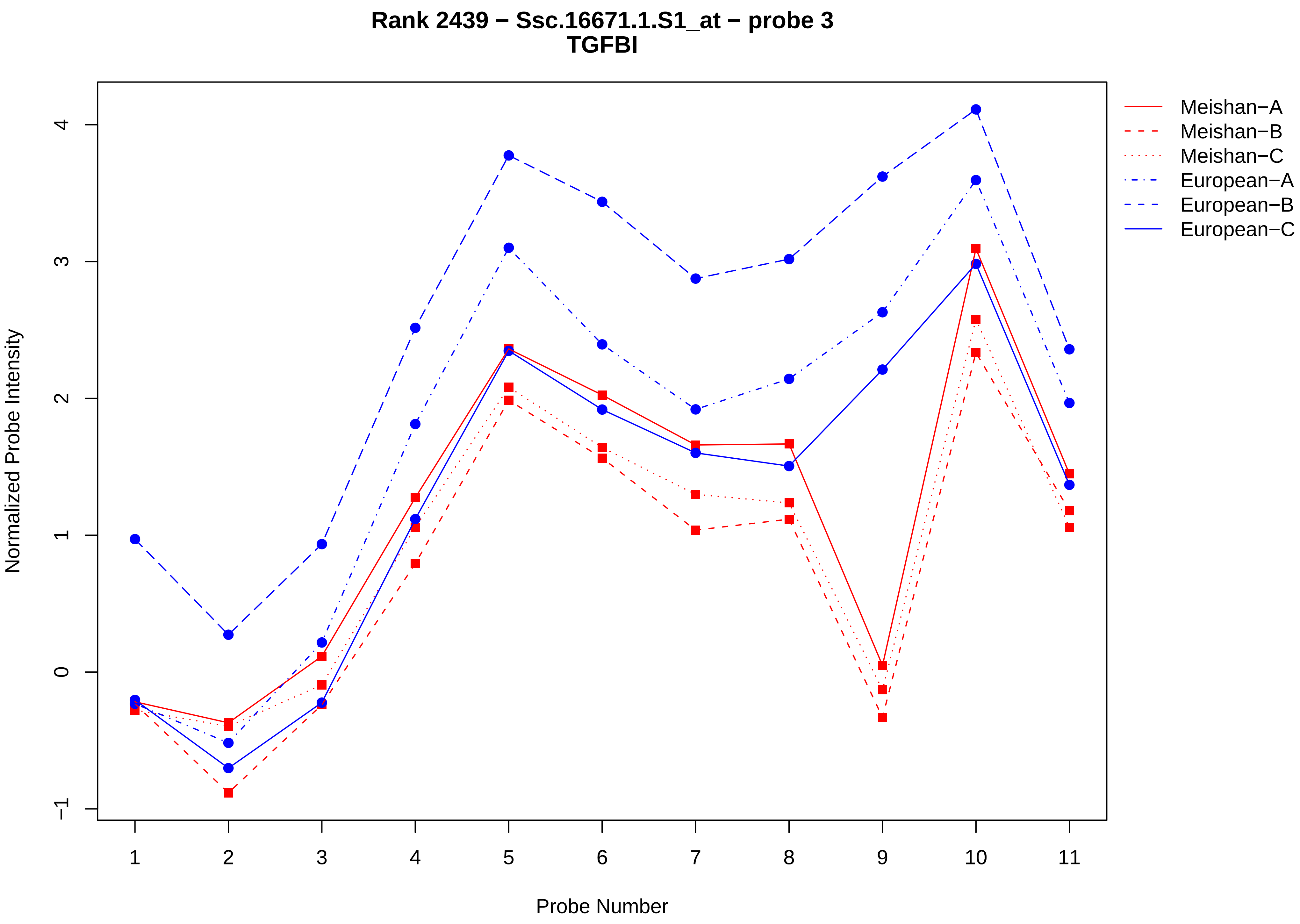

Rank 2439 − Ssc.16671.1.S1_at − probe 3
TGFBI
Normalized Probe Intensity
Probe Number
−1
0
1
2
3
4
1
2
3
4
5
6
7
8
9
10
11
Meishan−A
Meishan−B
Meishan−C
European−A
European−B
European−C

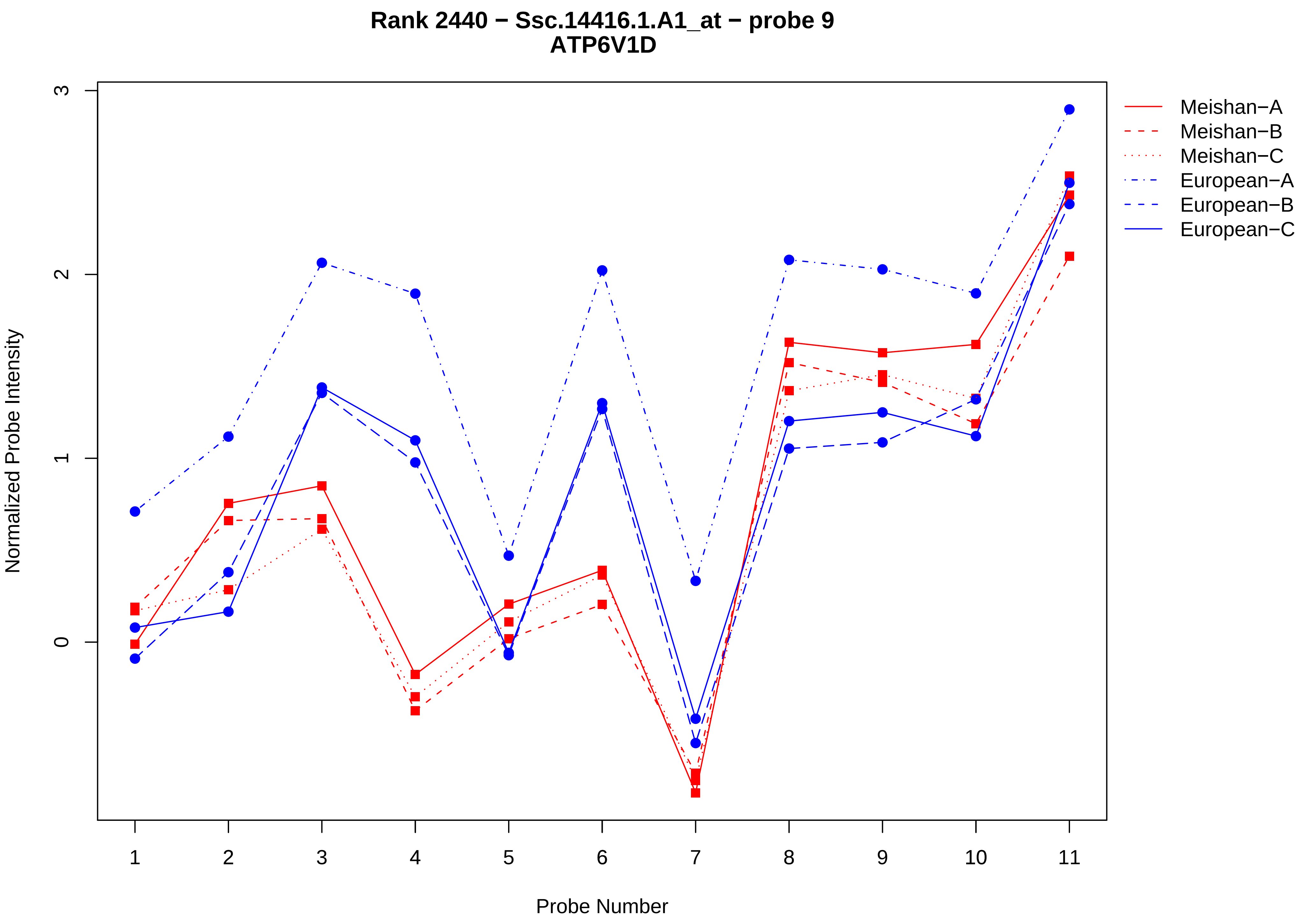
Rank 2440 − Ssc.14416.1.A1_at − probe 9
ATP6V1D
Normalized Probe Intensity
Probe Number
0
1
2
3
1
2
3
4
5
6
7
8
9
10
11
Meishan−A
Meishan−B
Meishan−C
European−A
European−B
European−C

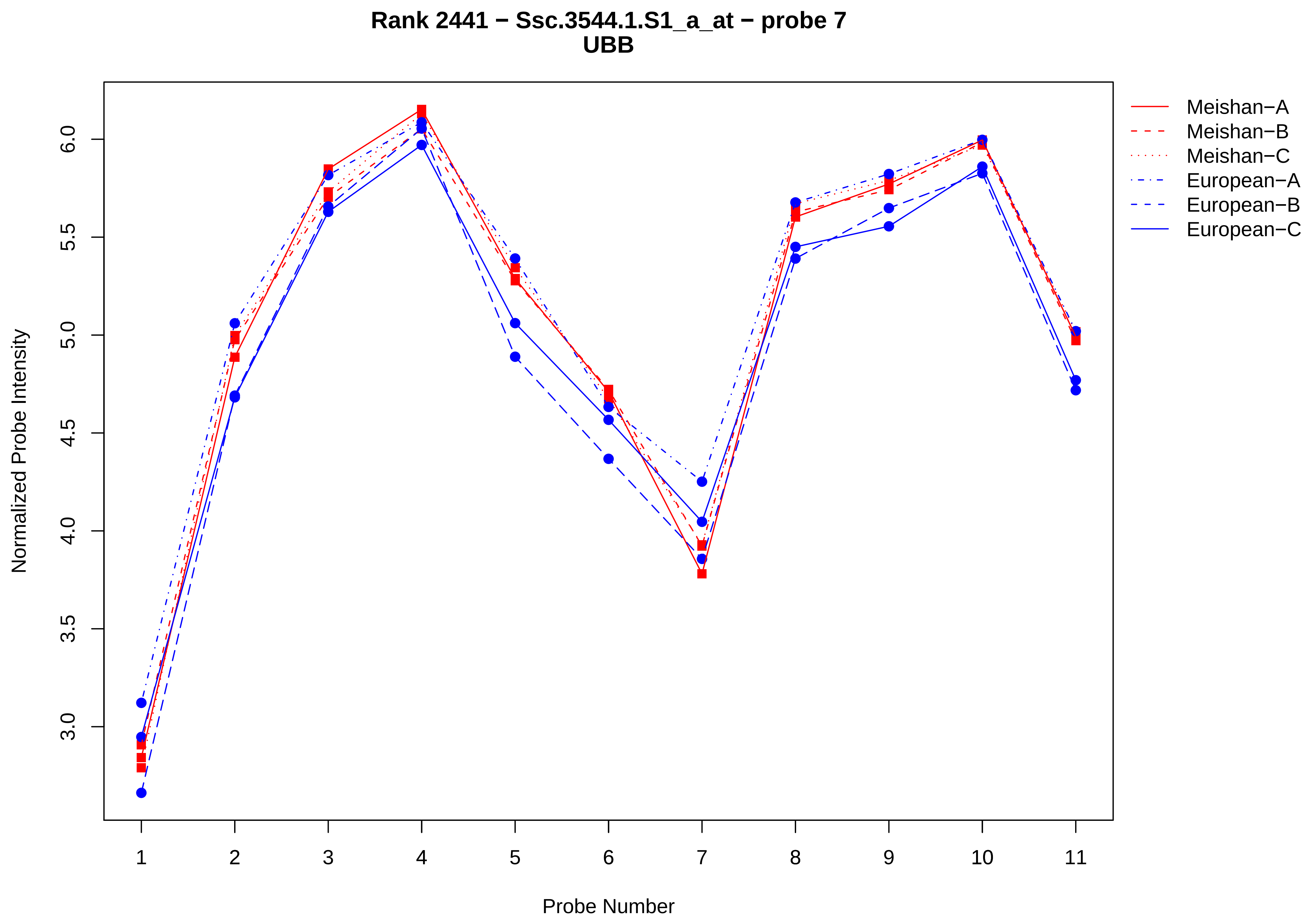

Rank 2441 − Ssc.3544.1.S1_a_at − probe 7
UBB
Normalized Probe Intensity
3.0
3.5
4.0
4.5
5.0
5.5
6.0
Probe Number
1
2
3
4
5
6
7
8
9
10
11
Meishan−A
Meishan−B
Meishan−C
European−A
European−B
European−C

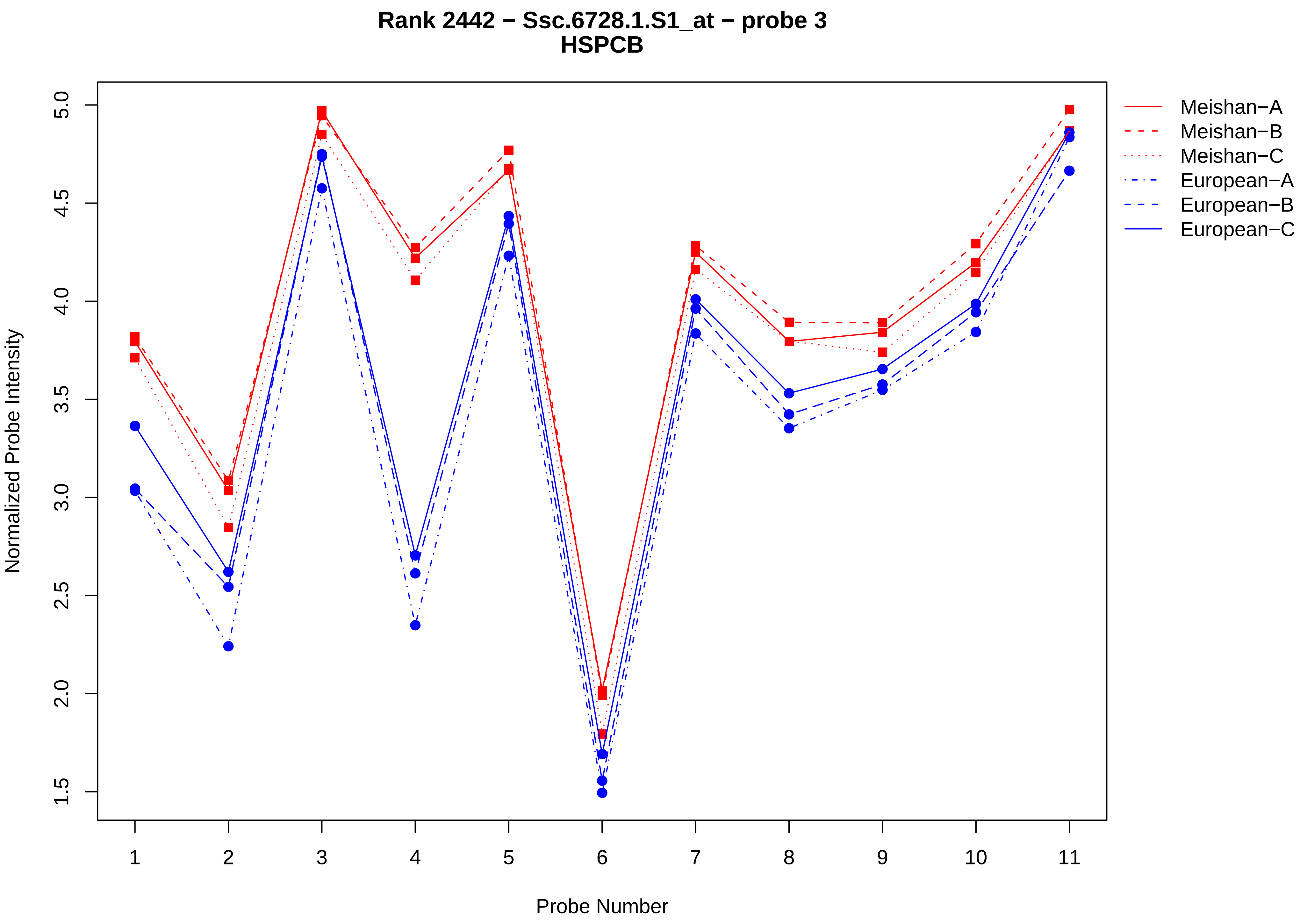

Rank 2442 − Ssc.6728.1.S1_at − probe 3
HSPCB
Normalized Probe Intensity
1.5
2.0
2.5
3.0
3.5
4.0
4.5
5.0
1
2
3
4
5
6
7
8
9
10
11
Probe Number
Meishan−A
Meishan−B
Meishan−C
European−A
European−B
European−C

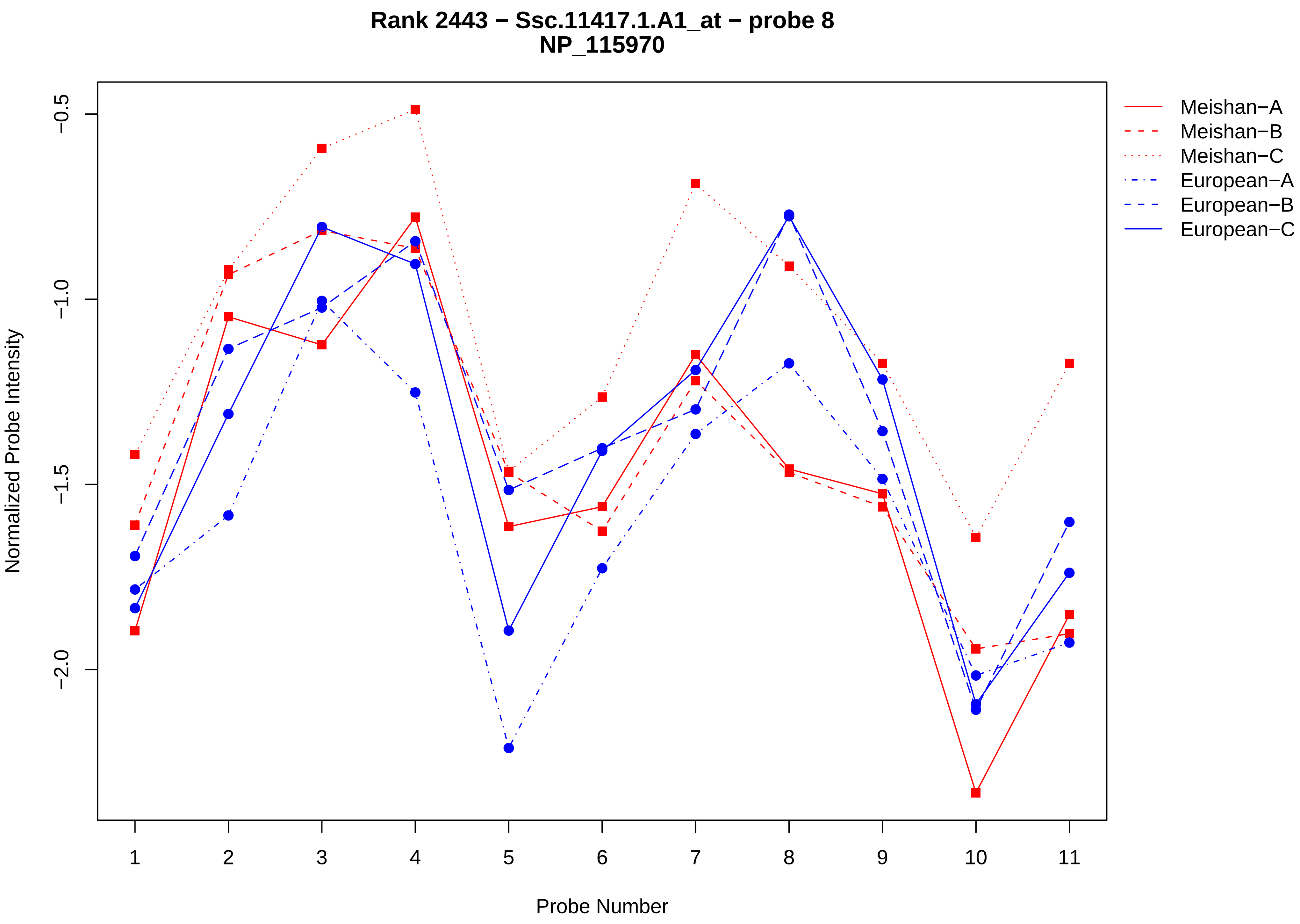

Rank 2443 − Ssc.11417.1.A1_at − probe 8
NP_115970
Normalized Probe Intensity
Probe Number
−0.5
−1.0
−1.5
−2.0
1
2
3
4
5
6
7
8
9
10
11
Meishan−A
Meishan−B
Meishan−C
European−A
European−B
European−C

Rank 2444 − Ssc.3216.2.A1_at − probe 6
HNRPH2

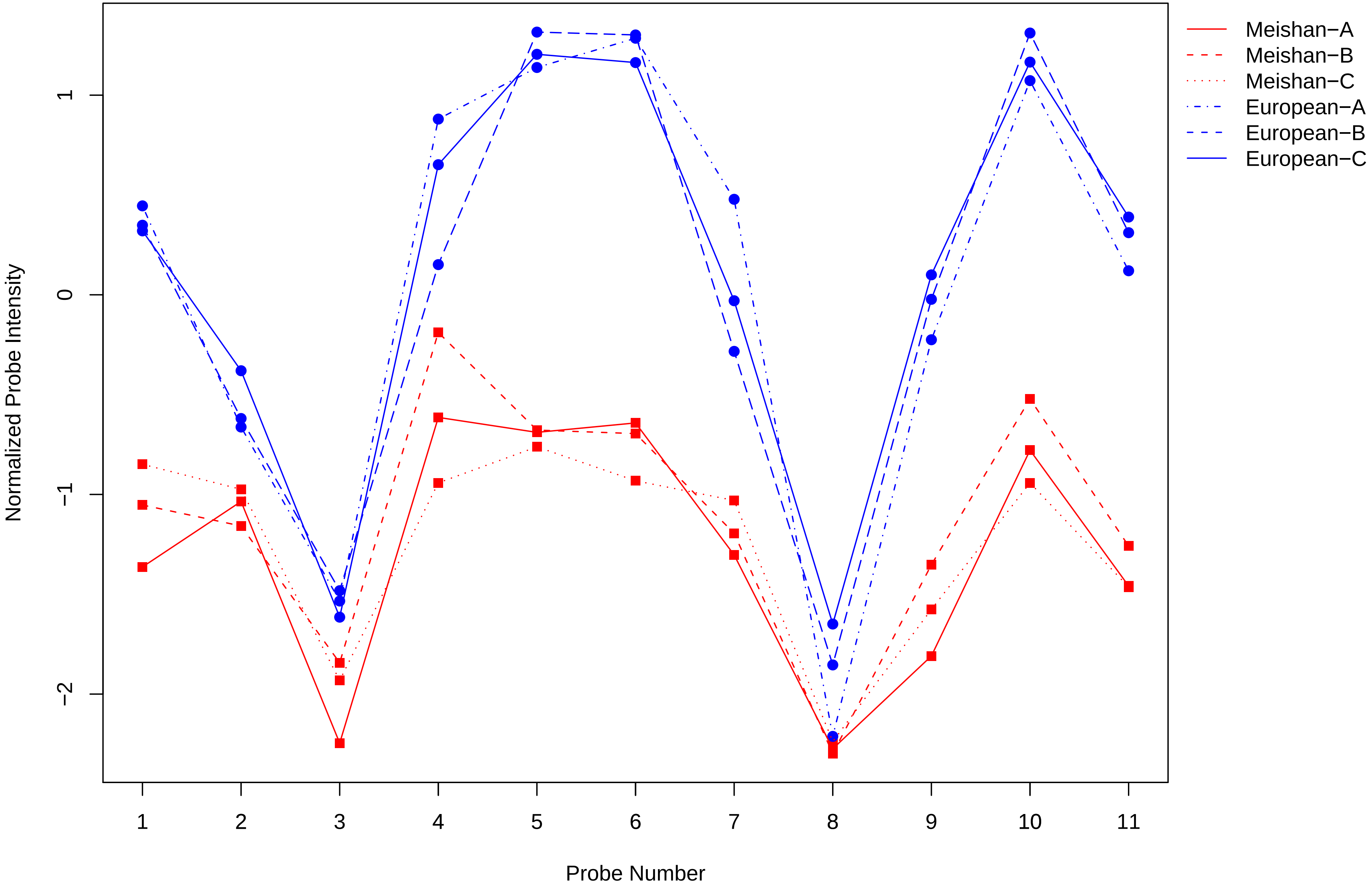

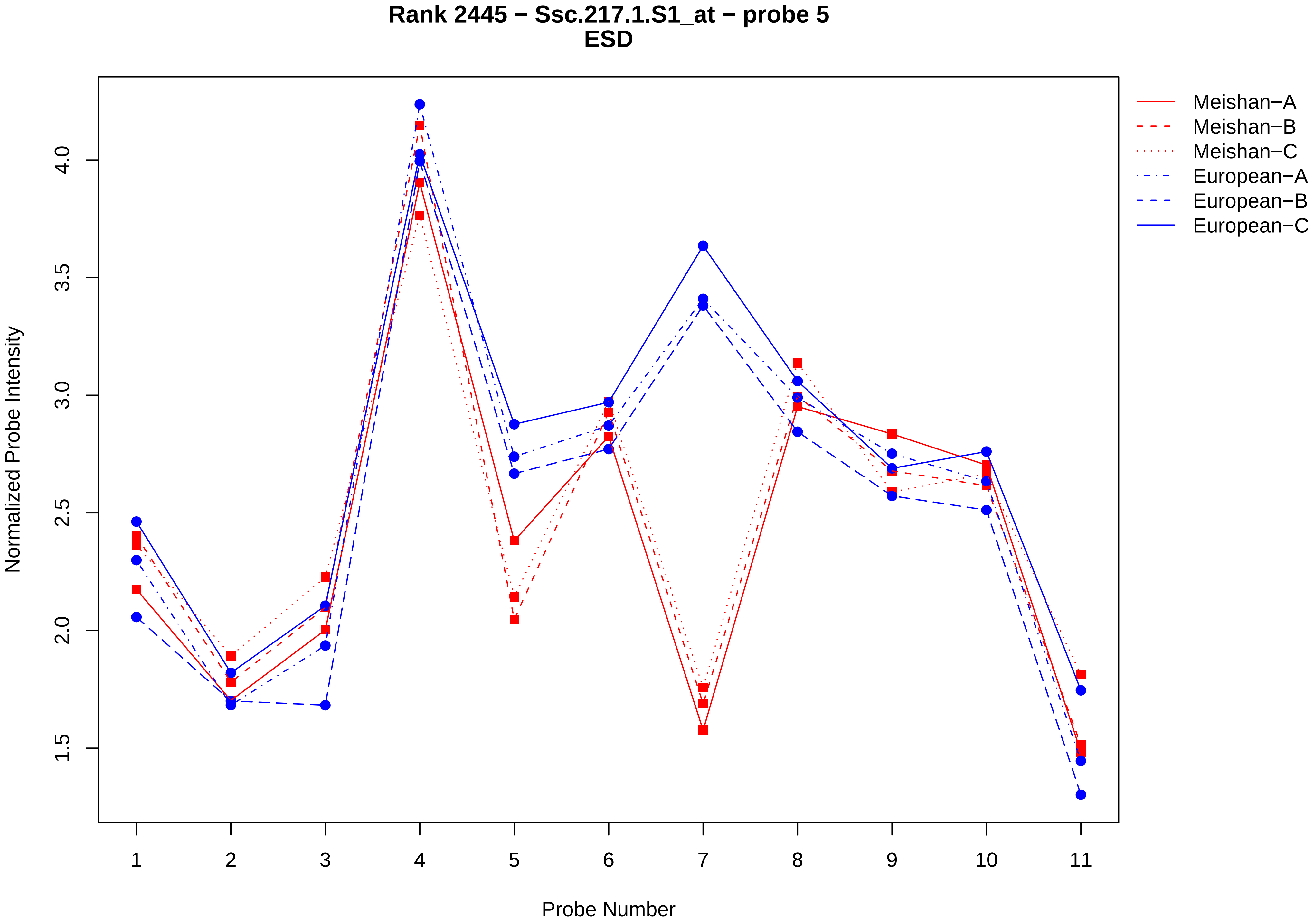

Rank 2445 − Ssc.217.1.S1_at − probe 5
ESD
Normalized Probe Intensity
Probe Number
1.5
2.0
2.5
3.0
3.5
4.0
1
2
3
4
5
6
7
8
9
10
11
Meishan−A
Meishan−B
Meishan−C
European−A
European−B
European−C

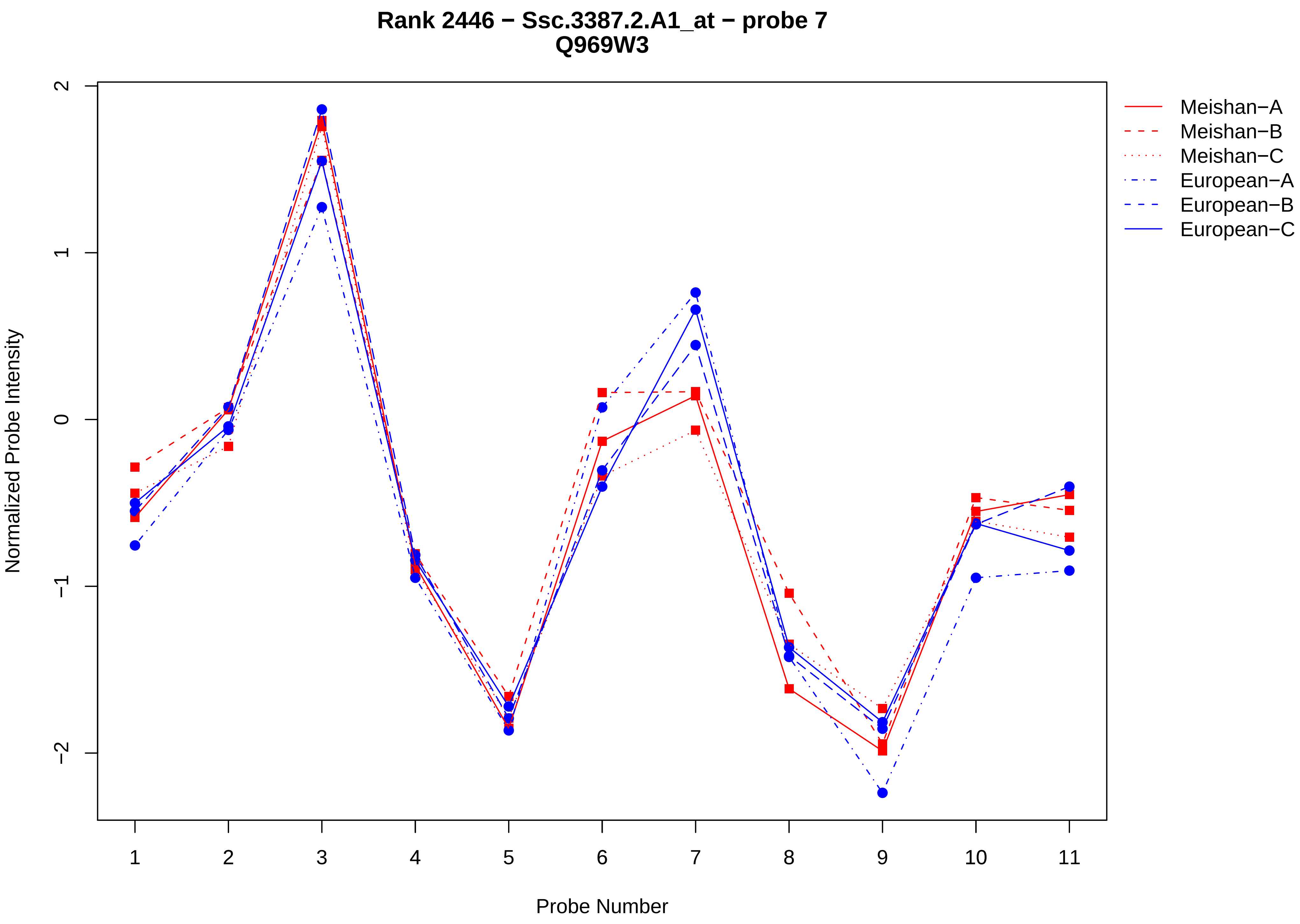

Rank 2446 − Ssc.3387.2.A1_at − probe 7
Q969W3
Normalized Probe Intensity
Probe Number
−2
−1
0
1
2
1
2
3
4
5
6
7
8
9
10
11
Meishan−A
Meishan−B
Meishan−C
European−A
European−B
European−C

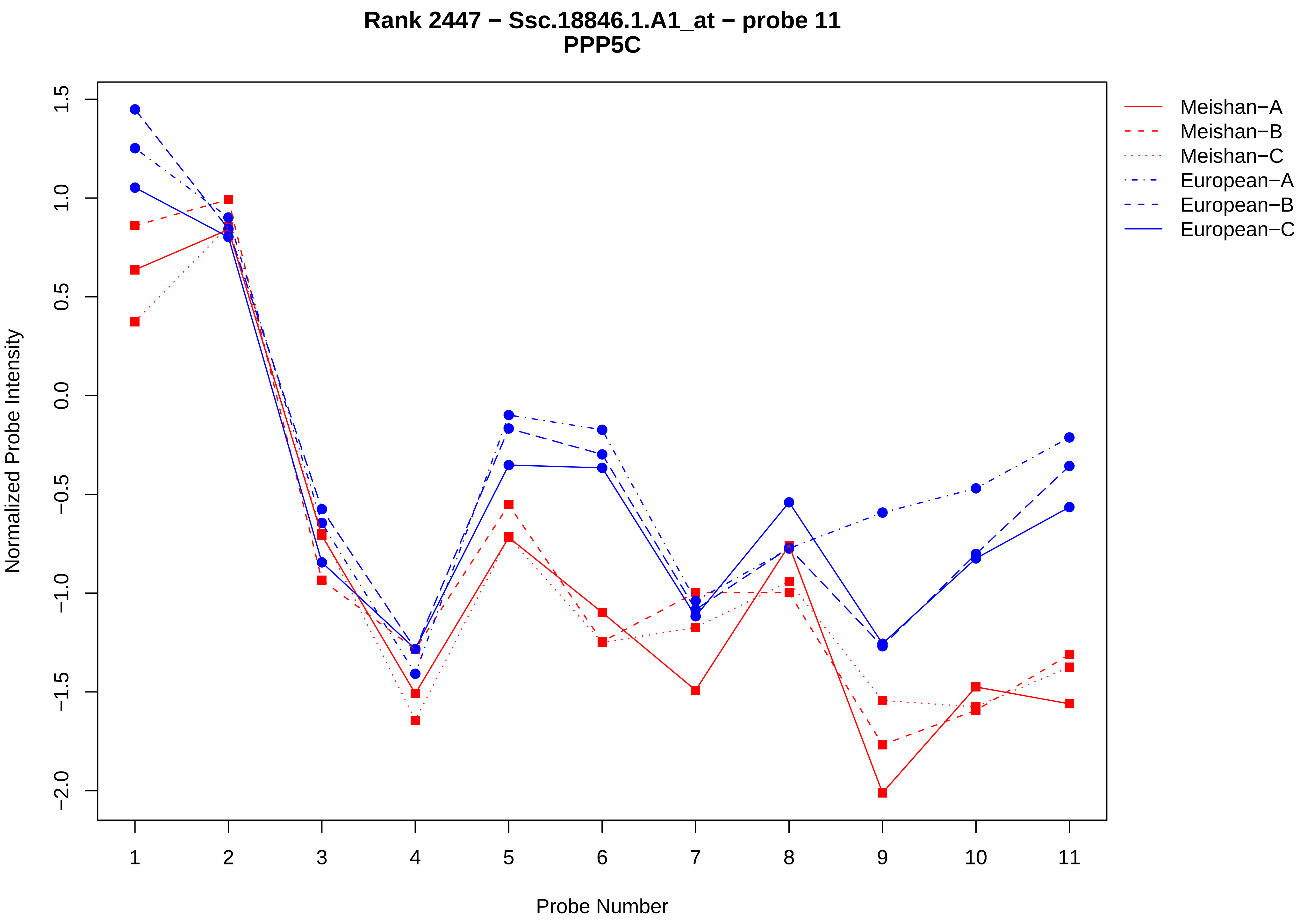

Rank 2447 − Ssc.18846.1.A1_at − probe 11
PPP5C
Normalized Probe Intensity
Probe Number
Meishan−A
Meishan−B
Meishan−C
European−A
European−B
European−C

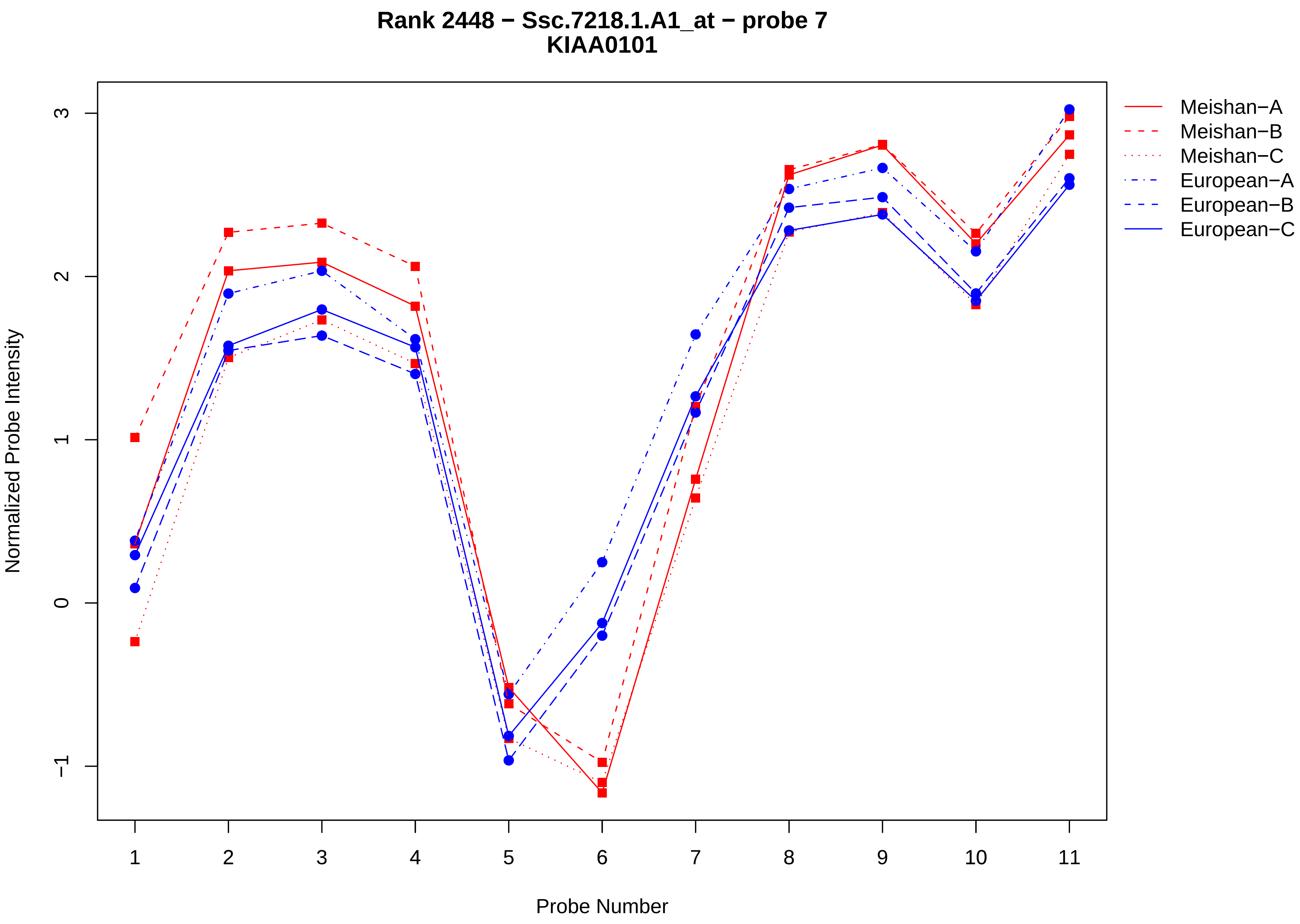
Rank 2448 − Ssc.7218.1.A1_at − probe 7
KIAA0101
Normalized Probe Intensity
Probe Number
Meishan−A
Meishan−B
Meishan−C
European−A
European−B
European−C
−1
0
1
2
3
1
2
3
4
5
6
7
8
9
10
11

Rank 2449 − Ssc.9715.1.A1_at − probe 4
C21orf107

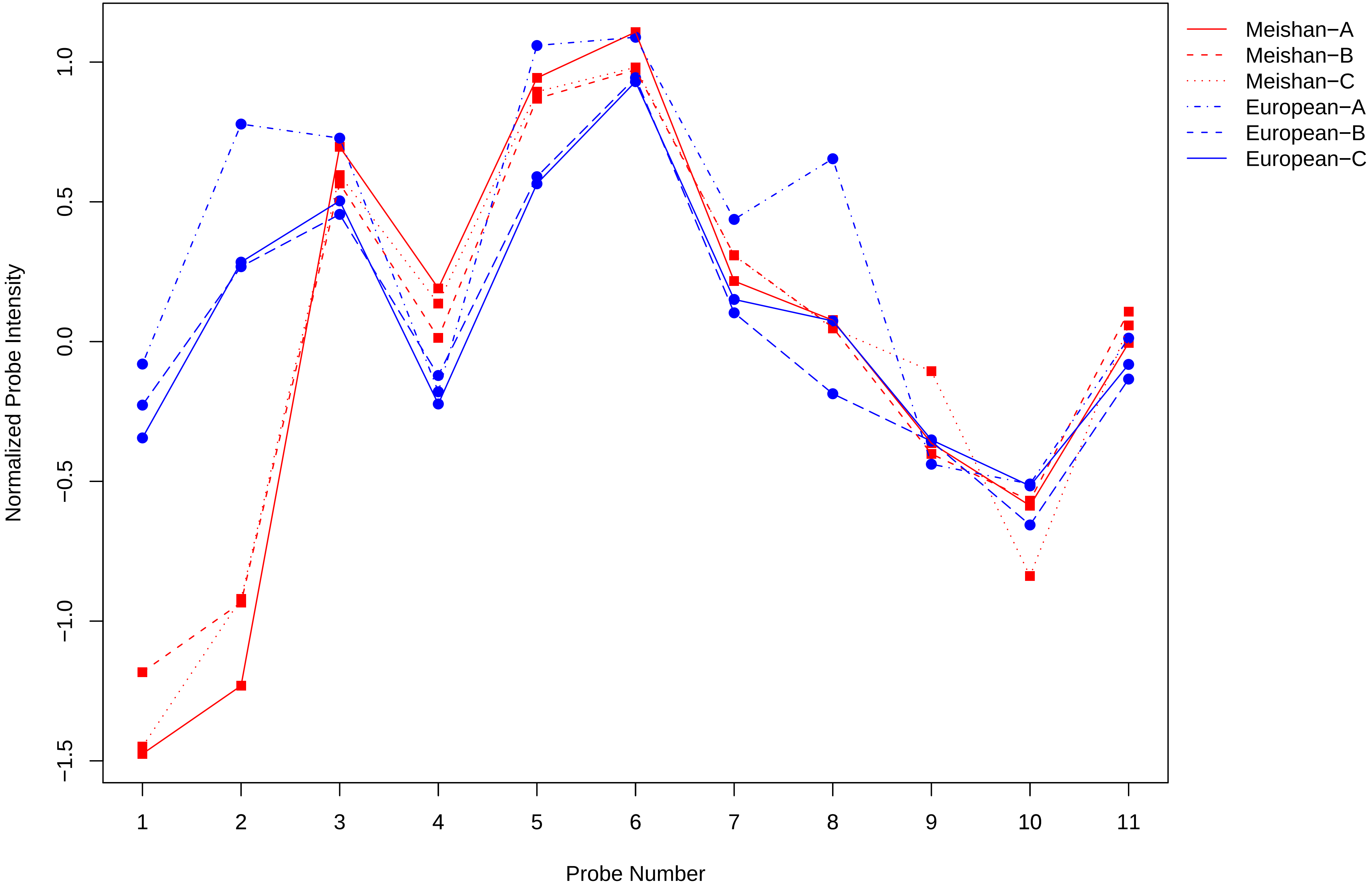

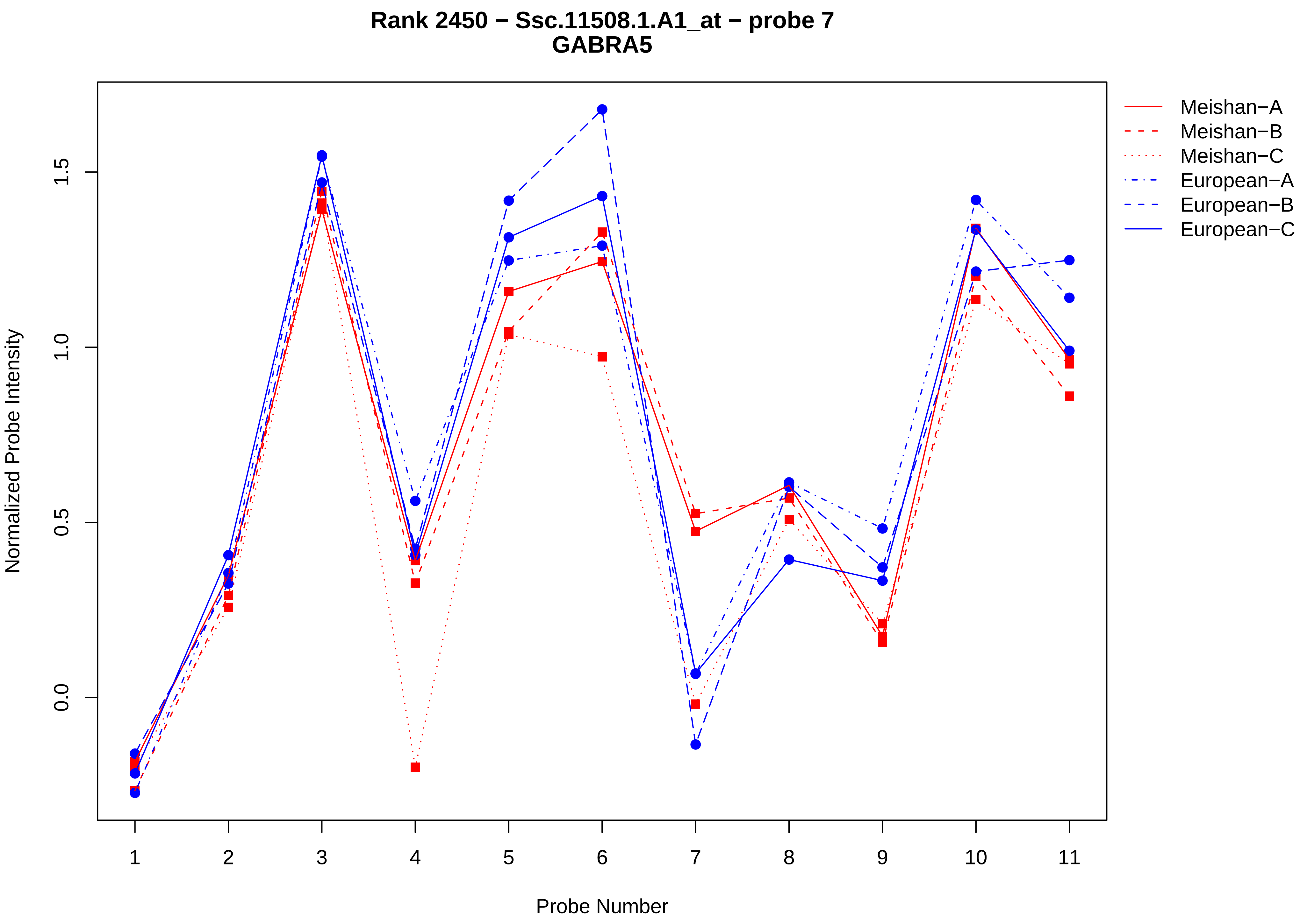

Rank 2450 − Ssc.11508.1.A1_at − probe 7
GABRA5
Normalized Probe Intensity
0.0
0.5
1.0
1.5
1
2
3
4
5
6
7
8
9
10
11
Probe Number
Meishan−A
Meishan−B
Meishan−C
European−A
European−B
European−C

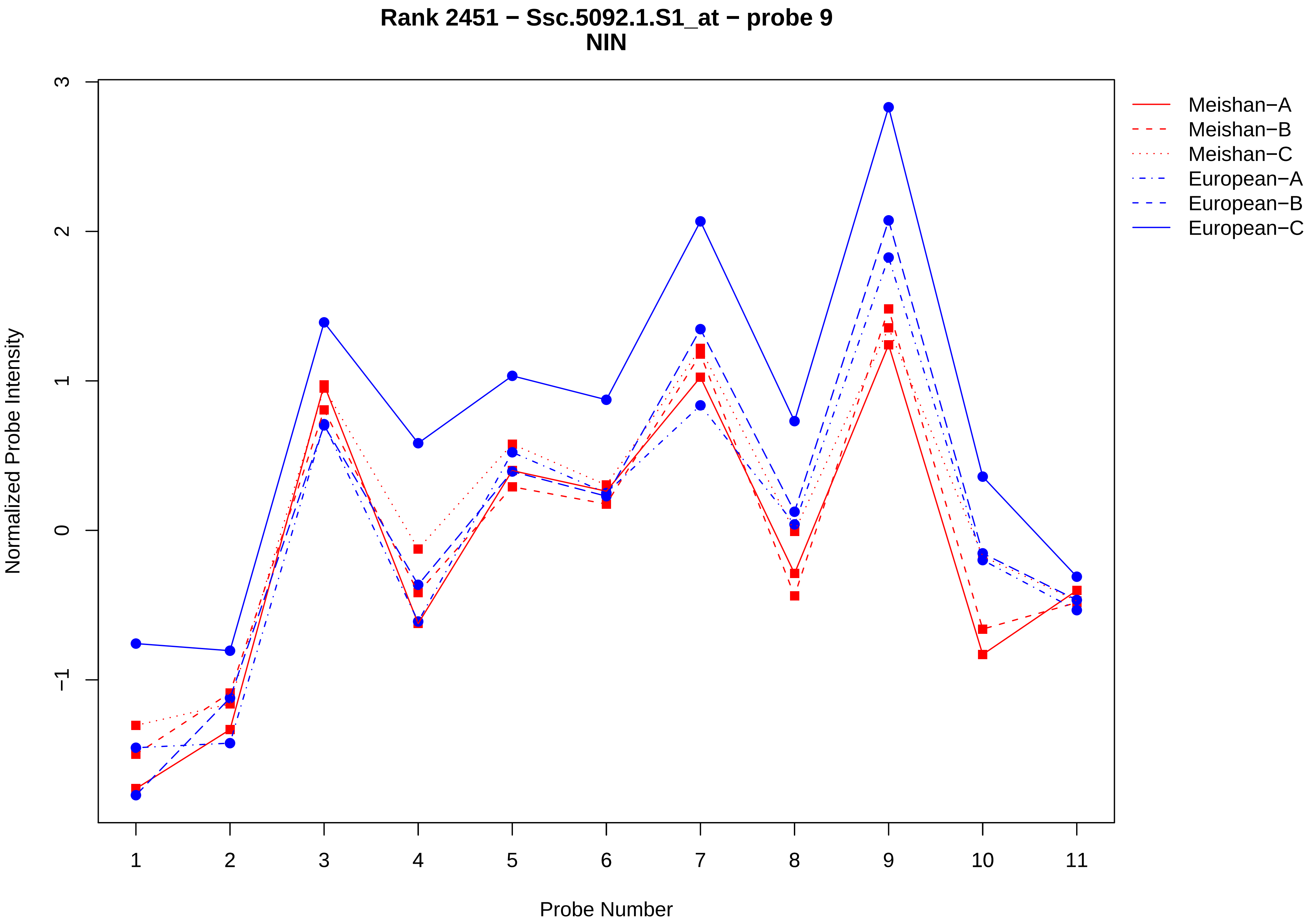

Rank 2451 − Ssc.5092.1.S1_at − probe 9
NIN
Normalized Probe Intensity
Probe Number
−1
0
1
2
3
1
2
3
4
5
6
7
8
9
10
11
Meishan−A
Meishan−B
Meishan−C
European−A
European−B
European−C

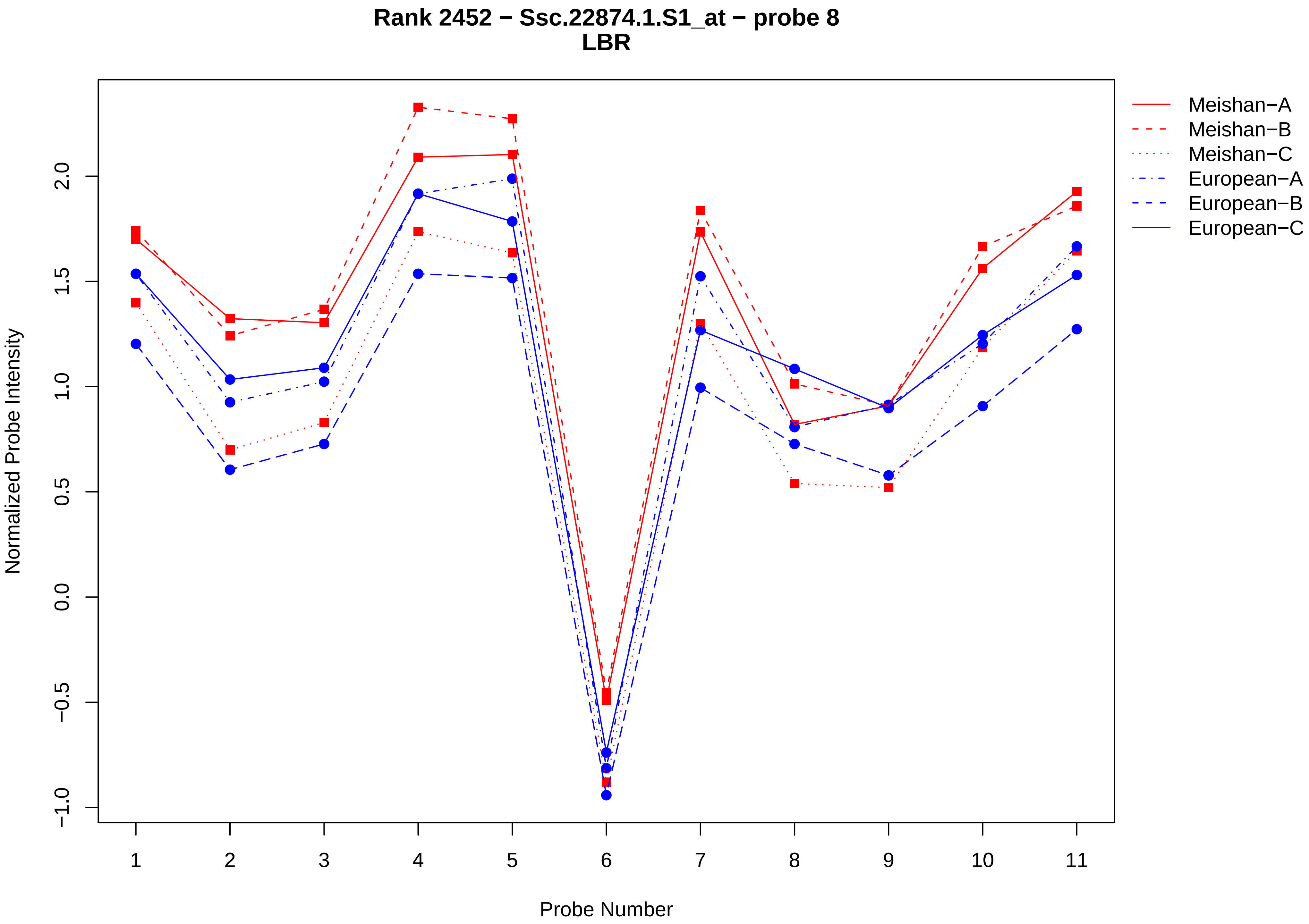
Rank 2452 − Ssc.22874.1.S1_at − probe 8
LBR
Normalized Probe Intensity
Probe Number
−1.0
−0.5
0.0
0.5
1.0
1.5
2.0
1
2
3
4
5
6
7
8
9
10
11
Meishan−A
Meishan−B
Meishan−C
European−A
European−B
European−C

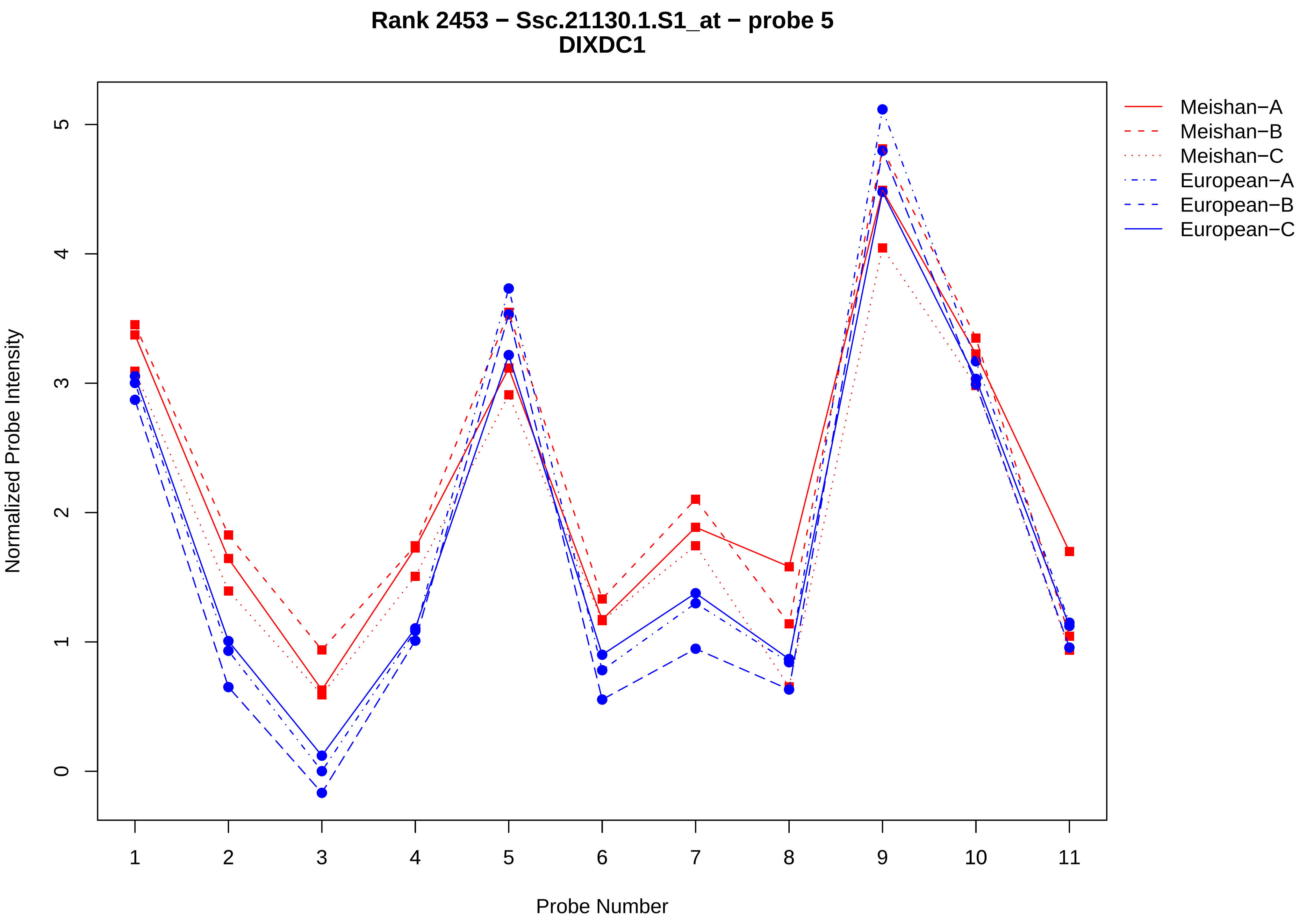

Rank 2453 − Ssc.21130.1.S1_at − probe 5
DIXDC1
Normalized Probe Intensity
Probe Number
0
1
2
3
4
5
1
2
3
4
5
6
7
8
9
10
11
Meishan−A
Meishan−B
Meishan−C
European−A
European−B
European−C

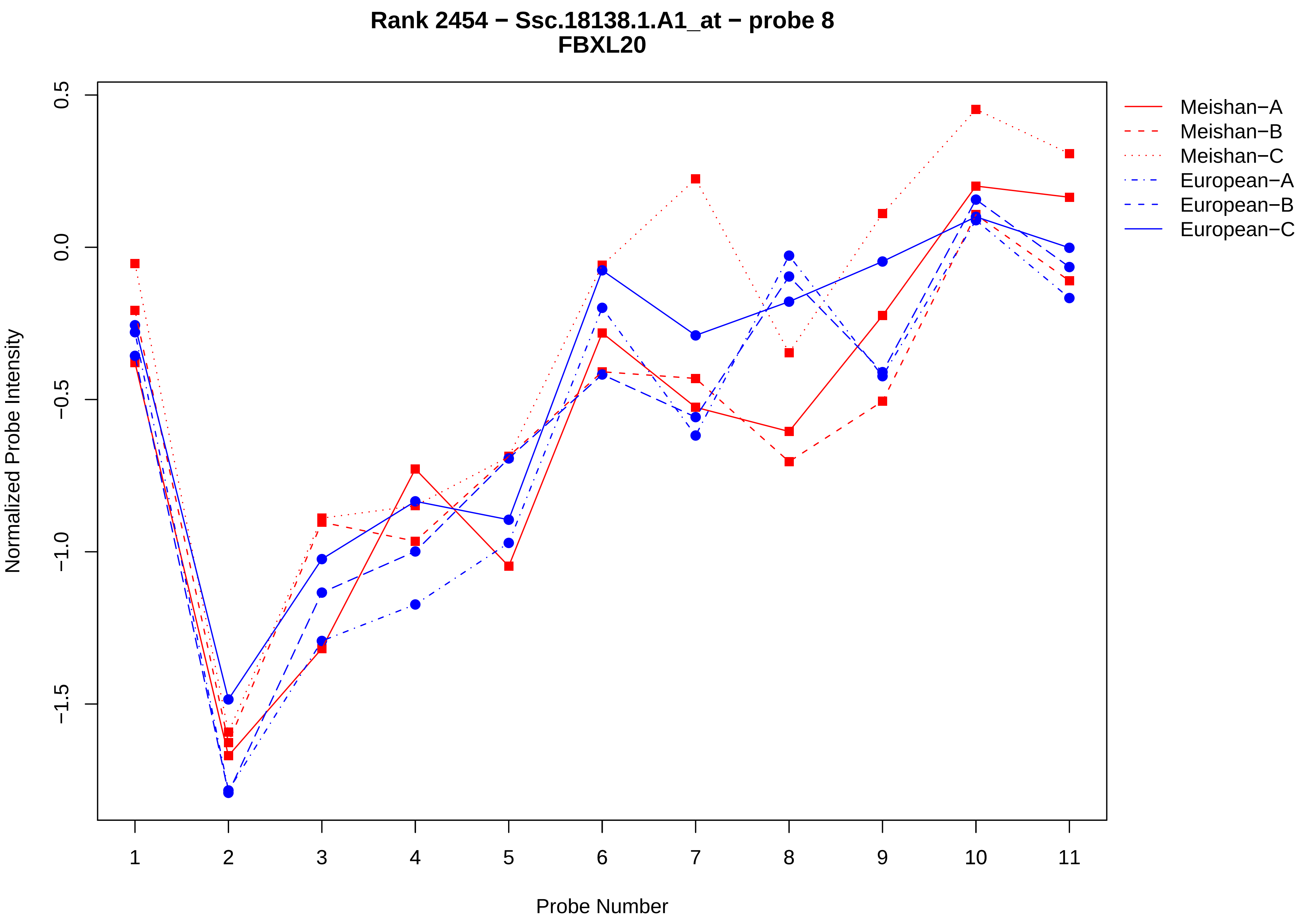

Rank 2454 − Ssc.18138.1.A1_at − probe 8
FBXL20
Normalized Probe Intensity
Probe Number
0.5
0.0
−0.5
−1.0
−1.5
1
2
3
4
5
6
7
8
9
10
11
Meishan−A
Meishan−B
Meishan−C
European−A
European−B
European−C

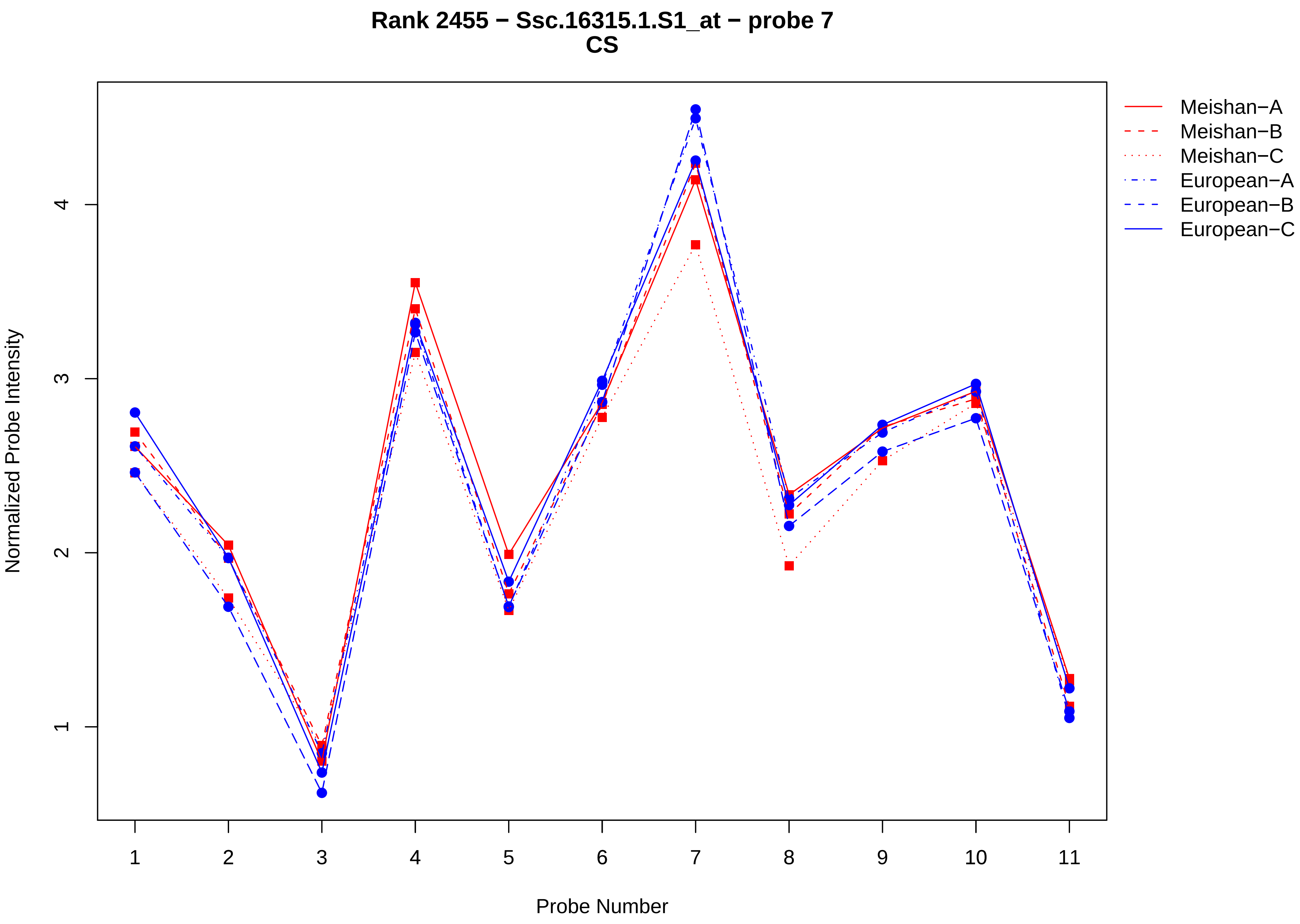

Rank 2455 − Ssc.16315.1.S1_at − probe 7
CS
Normalized Probe Intensity
Probe Number
1
2
3
4
5
6
7
8
9
10
11
Meishan−A
Meishan−B
Meishan−C
European−A
European−B
European−C

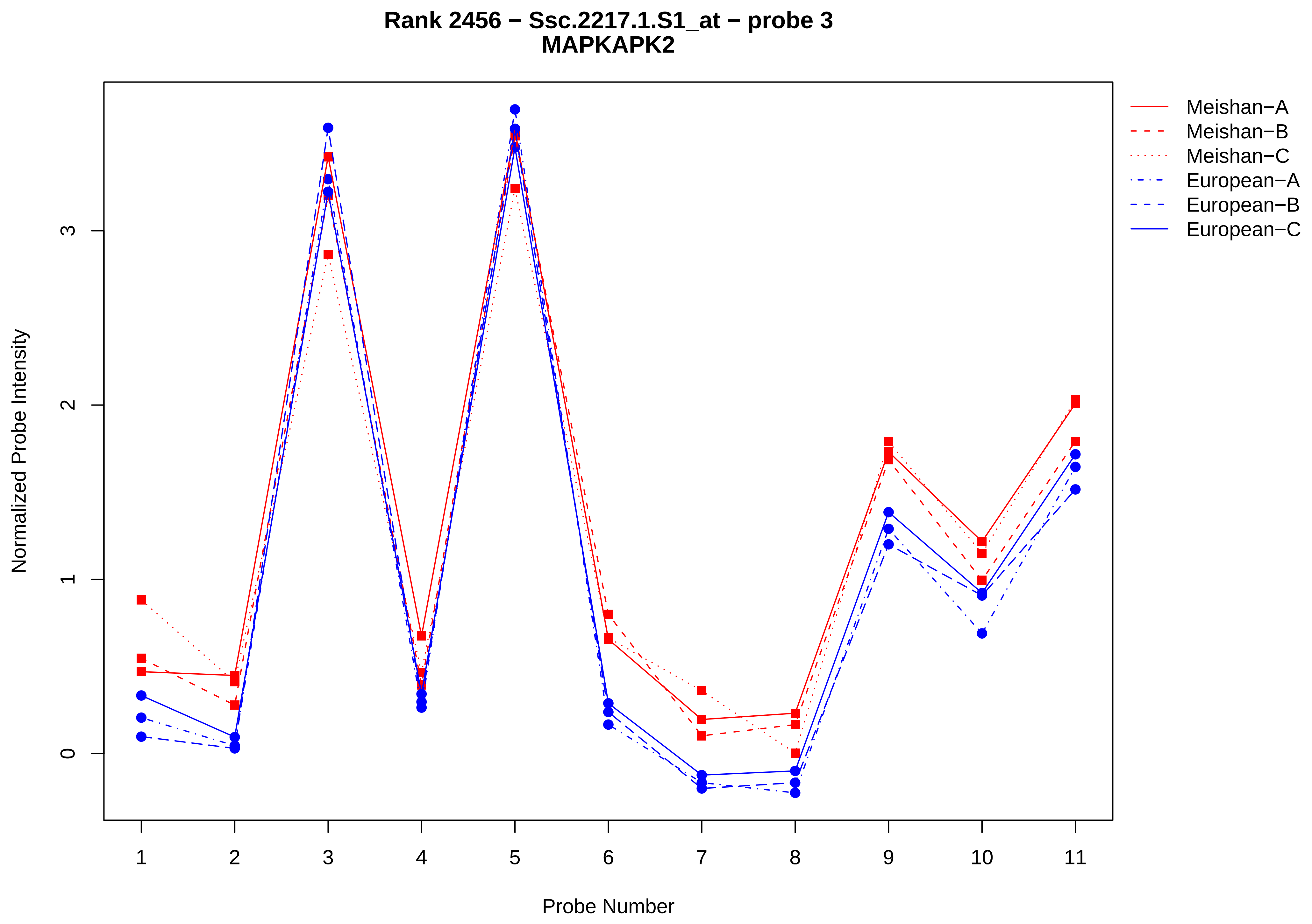

Rank 2456 − Ssc.2217.1.S1_at − probe 3
MAPKAPK2
Normalized Probe Intensity
Probe Number
0
1
2
3
1
2
3
4
5
6
7
8
9
10
11
Meishan−A
Meishan−B
Meishan−C
European−A
European−B
European−C

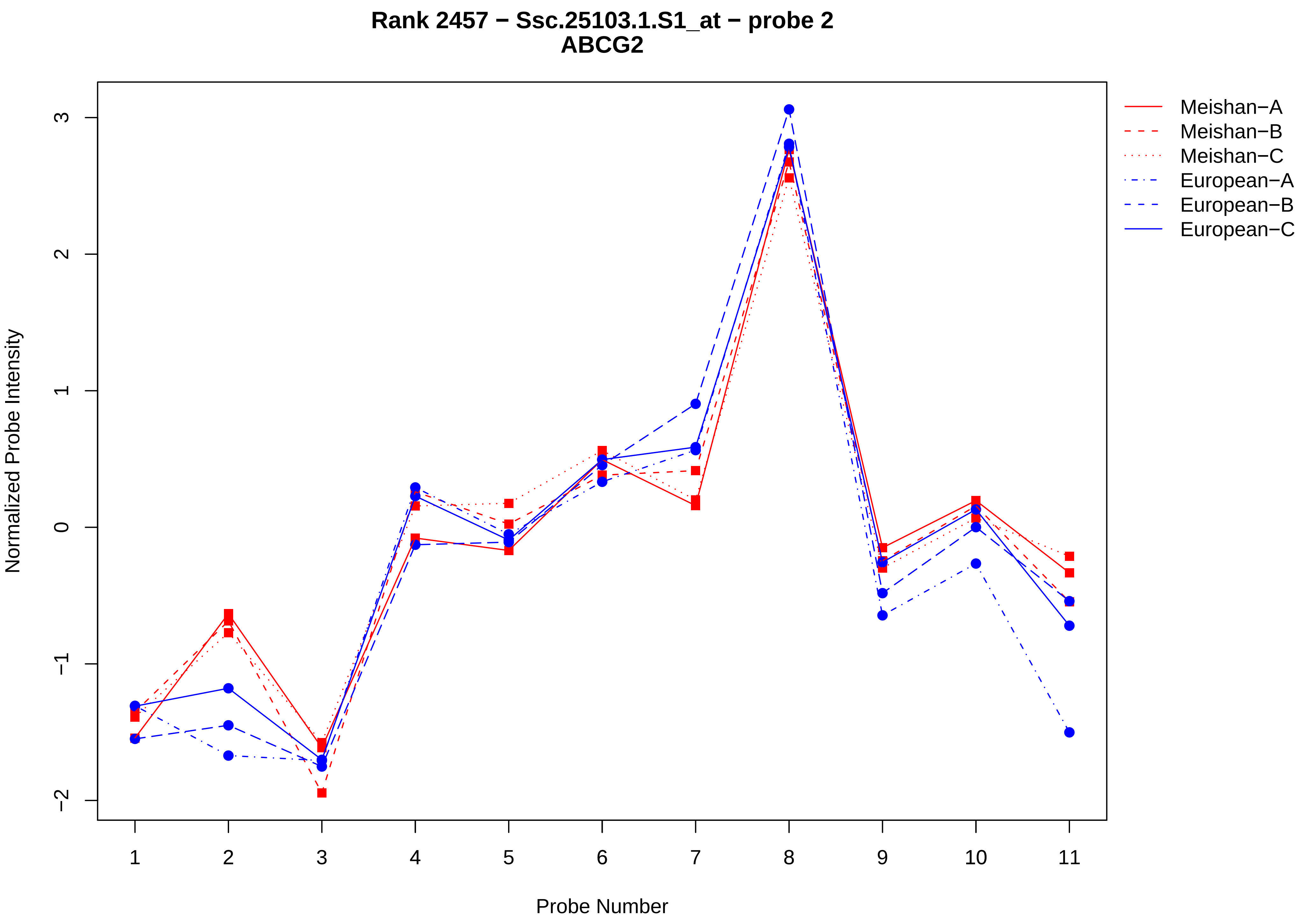

Rank 2457 − Ssc.25103.1.S1_at − probe 2
ABCG2
Normalized Probe Intensity
Probe Number
−2
−1
0
1
2
3
1
2
3
4
5
6
7
8
9
10
11
Meishan−A
Meishan−B
Meishan−C
European−A
European−B
European−C

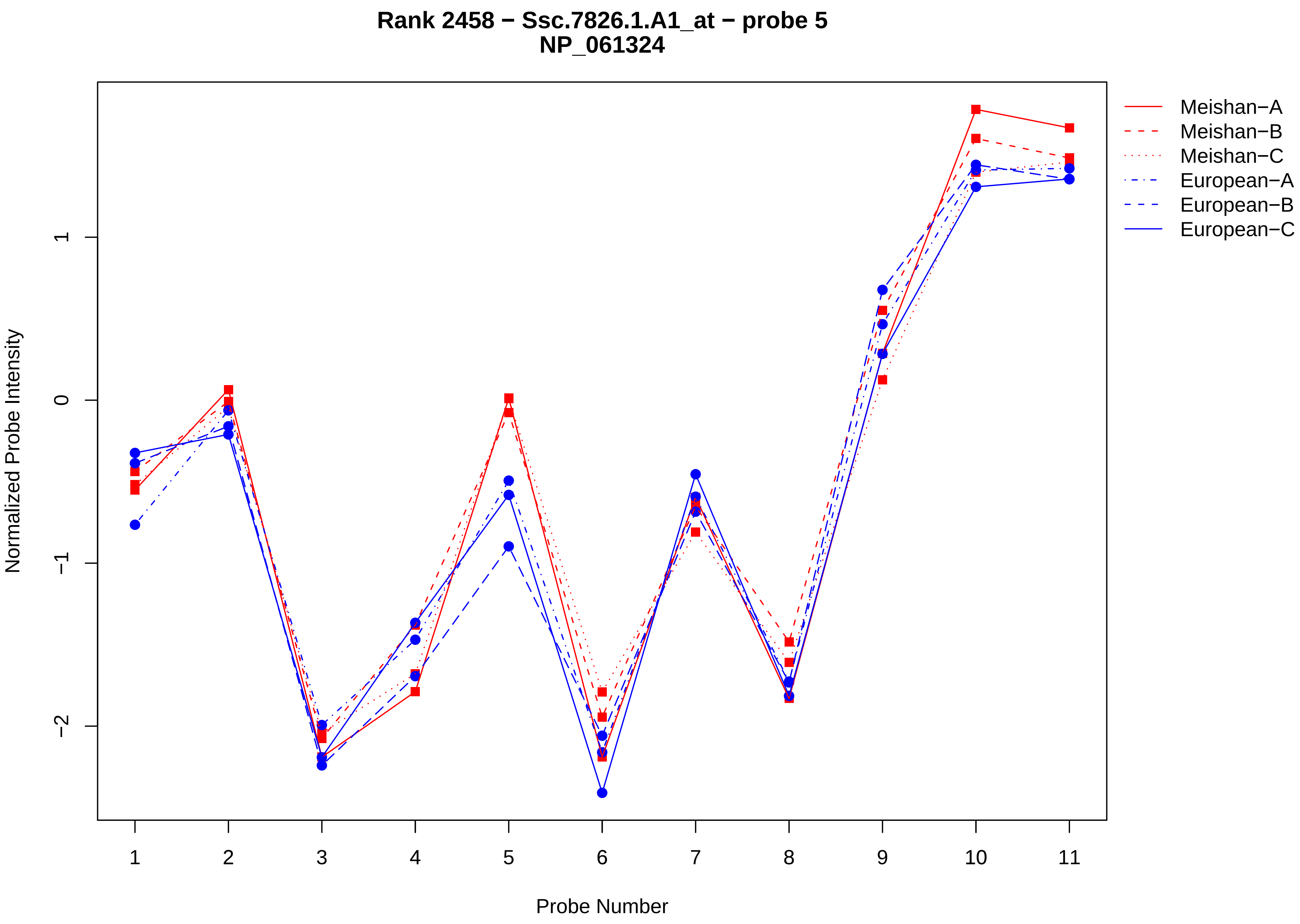
Rank 2458 − Ssc.7826.1.A1_at − probe 5
NP_061324
Normalized Probe Intensity
Probe Number
Meishan−A
Meishan−B
Meishan−C
European−A
European−B
European−C

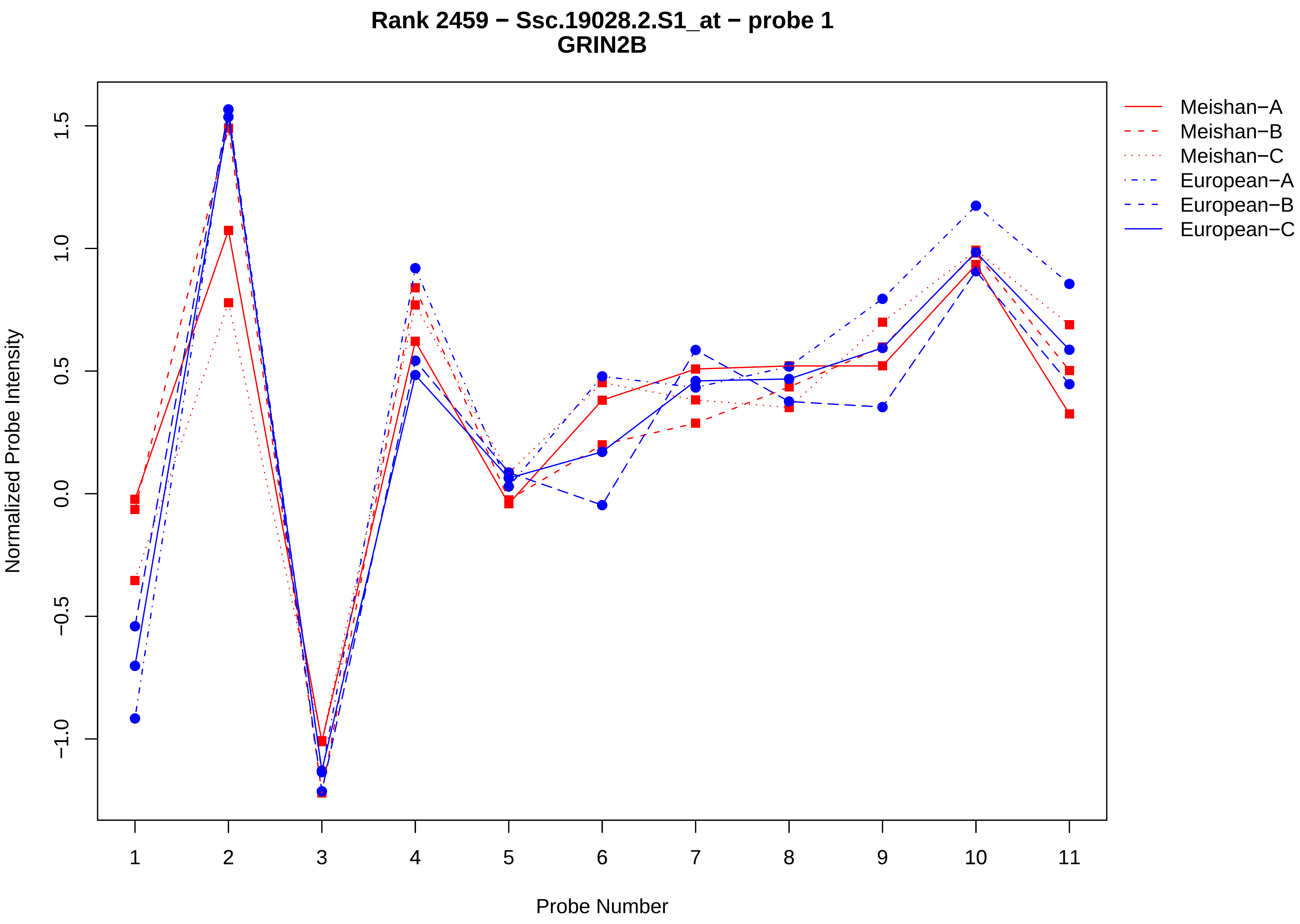

Rank 2459 − Ssc.19028.2.S1_at − probe 1
GRIN2B
Normalized Probe Intensity
Probe Number
Meishan−A
Meishan−B
Meishan−C
European−A
European−B
European−C

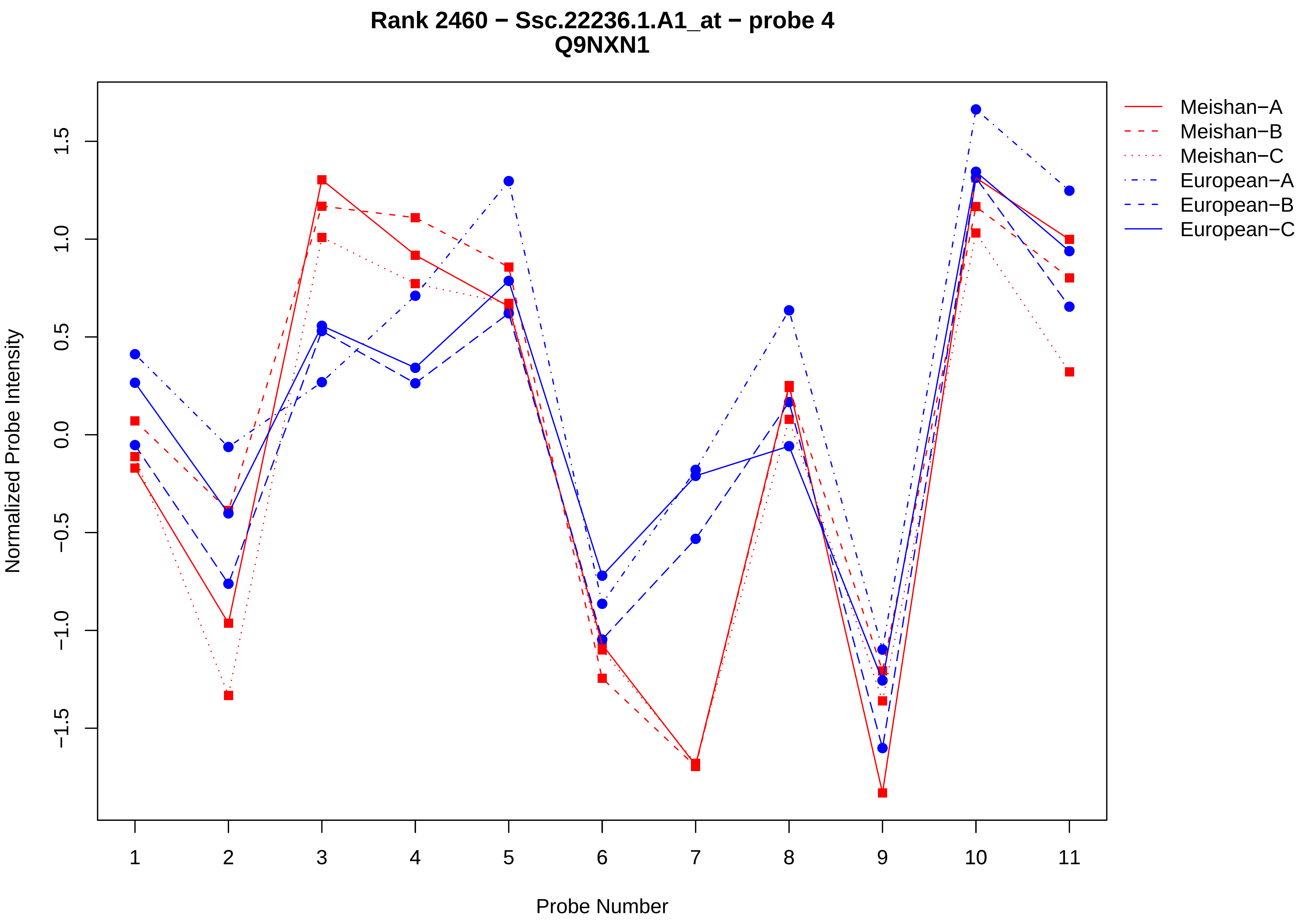

Rank 2460 − Ssc.22236.1.A1_at − probe 4
Q9NXN1
Normalized Probe Intensity
Probe Number
1.5
1.0
0.5
0.0
−0.5
−1.0
−1.5
1 2 3 4 5 6 7 8 9 10 11
Meishan−A
Meishan−B
Meishan−C
European−A
European−B
European−C

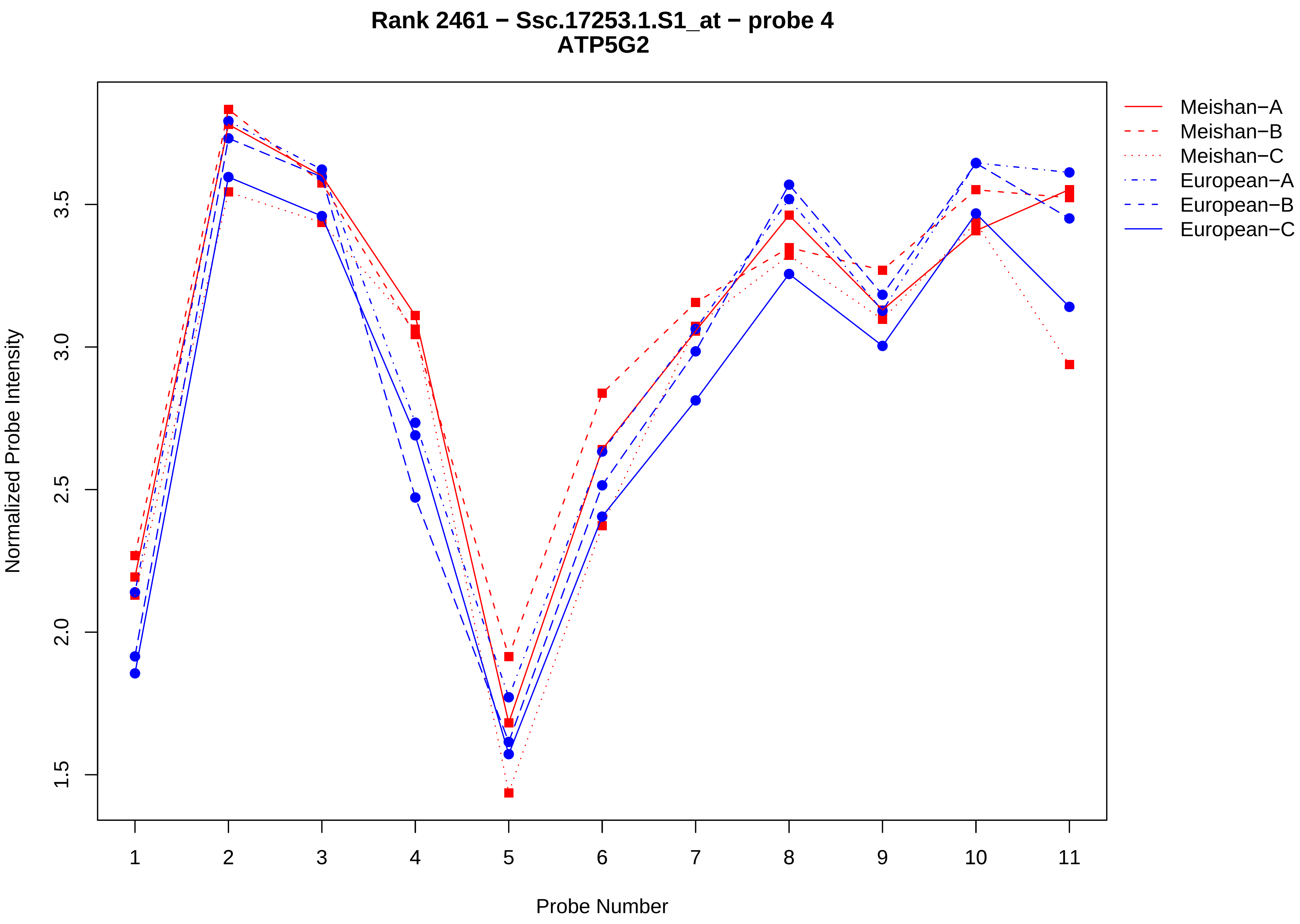

Rank 2461 − Ssc.17253.1.S1_at − probe 4
ATP5G2
Normalized Probe Intensity
Probe Number
1.5
2.0
2.5
3.0
3.5
1
2
3
4
5
6
7
8
9
10
11
Meishan−A
Meishan−B
Meishan−C
European−A
European−B
European−C

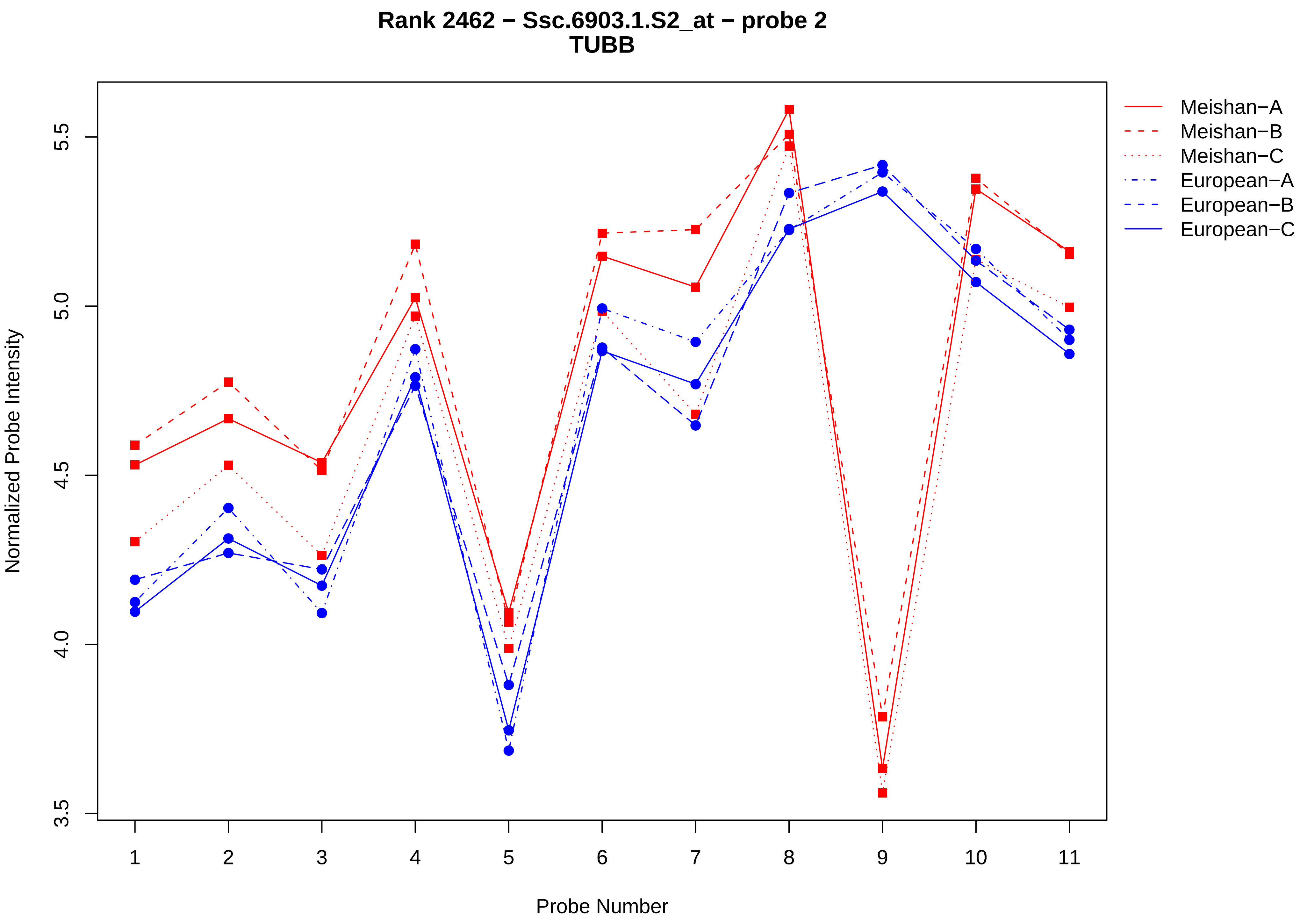

Rank 2462 − Ssc.6903.1.S2_at − probe 2
TUBB
Normalized Probe Intensity
Probe Number
3.5
4.0
4.5
5.0
5.5
1
2
3
4
5
6
7
8
9
10
11
Meishan−A
Meishan−B
Meishan−C
European−A
European−B
European−C

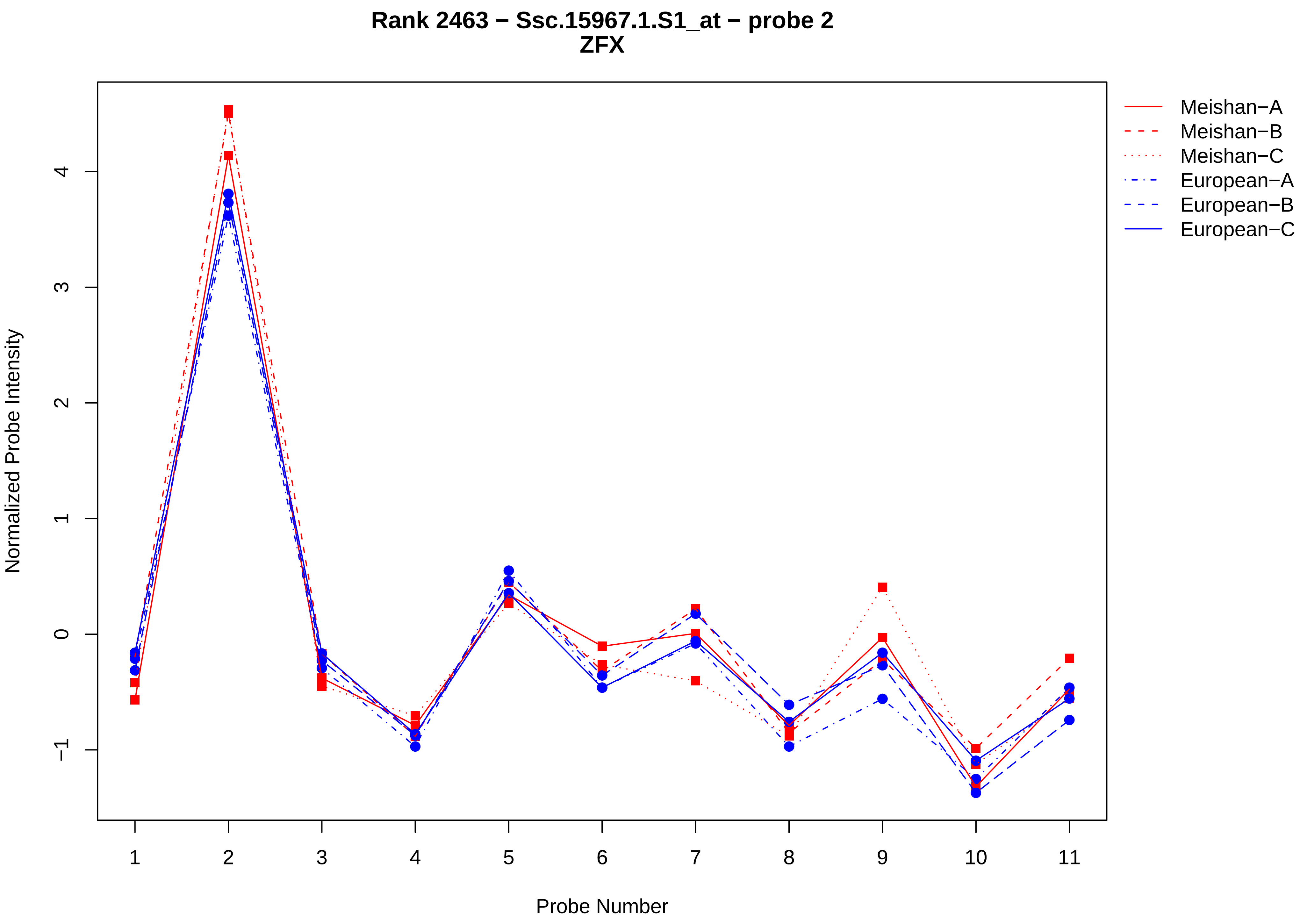

Rank 2463 − Ssc.15967.1.S1_at − probe 2
ZFX
Normalized Probe Intensity
Probe Number
−1
0
1
2
3
4
1
2
3
4
5
6
7
8
9
10
11
Meishan−A
Meishan−B
Meishan−C
European−A
European−B
European−C

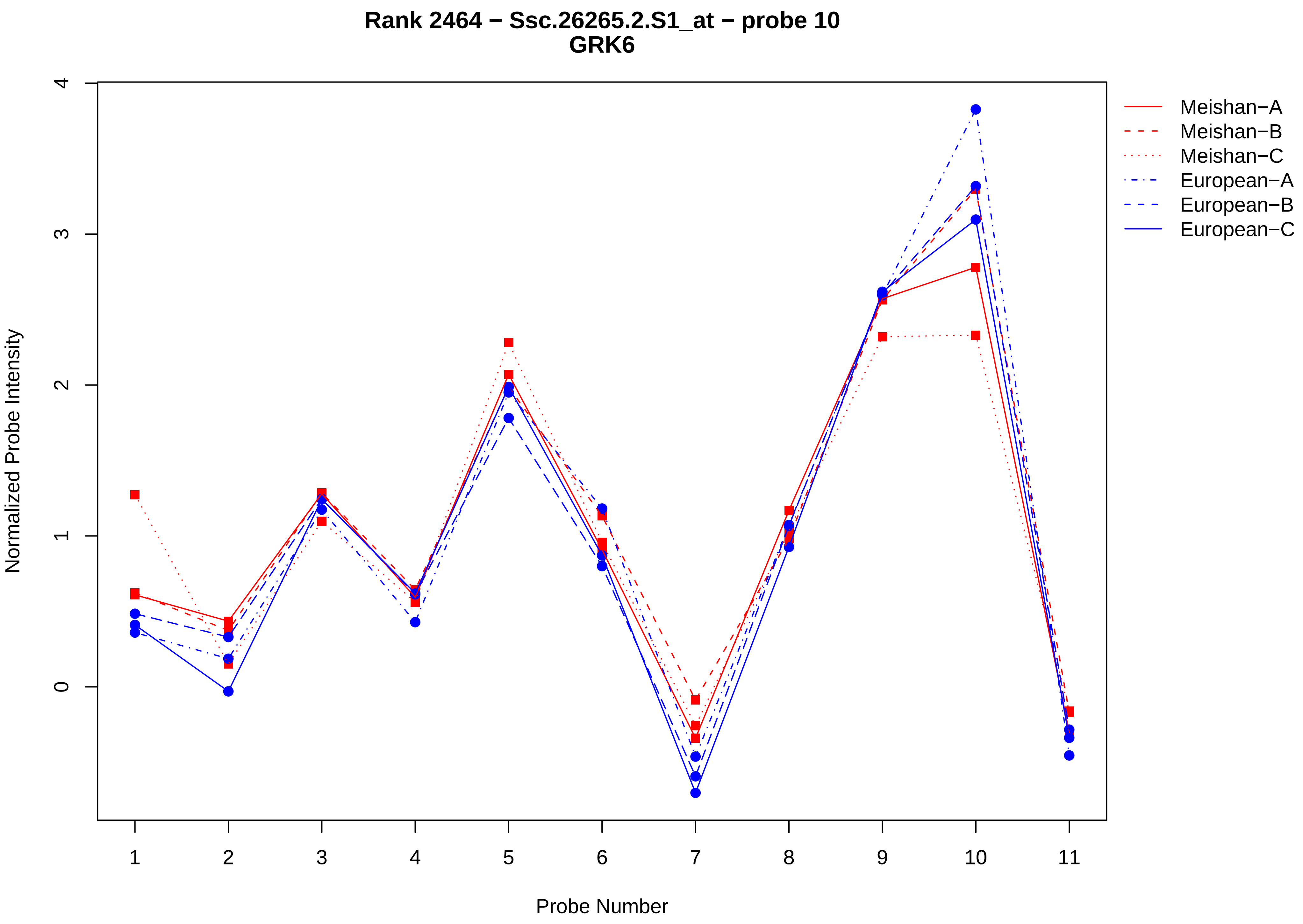

Rank 2464 − Ssc.26265.2.S1_at − probe 10
GRK6
Normalized Probe Intensity
Probe Number
0
1
2
3
4
1
2
3
4
5
6
7
8
9
10
11
Meishan−A
Meishan−B
Meishan−C
European−A
European−B
European−C

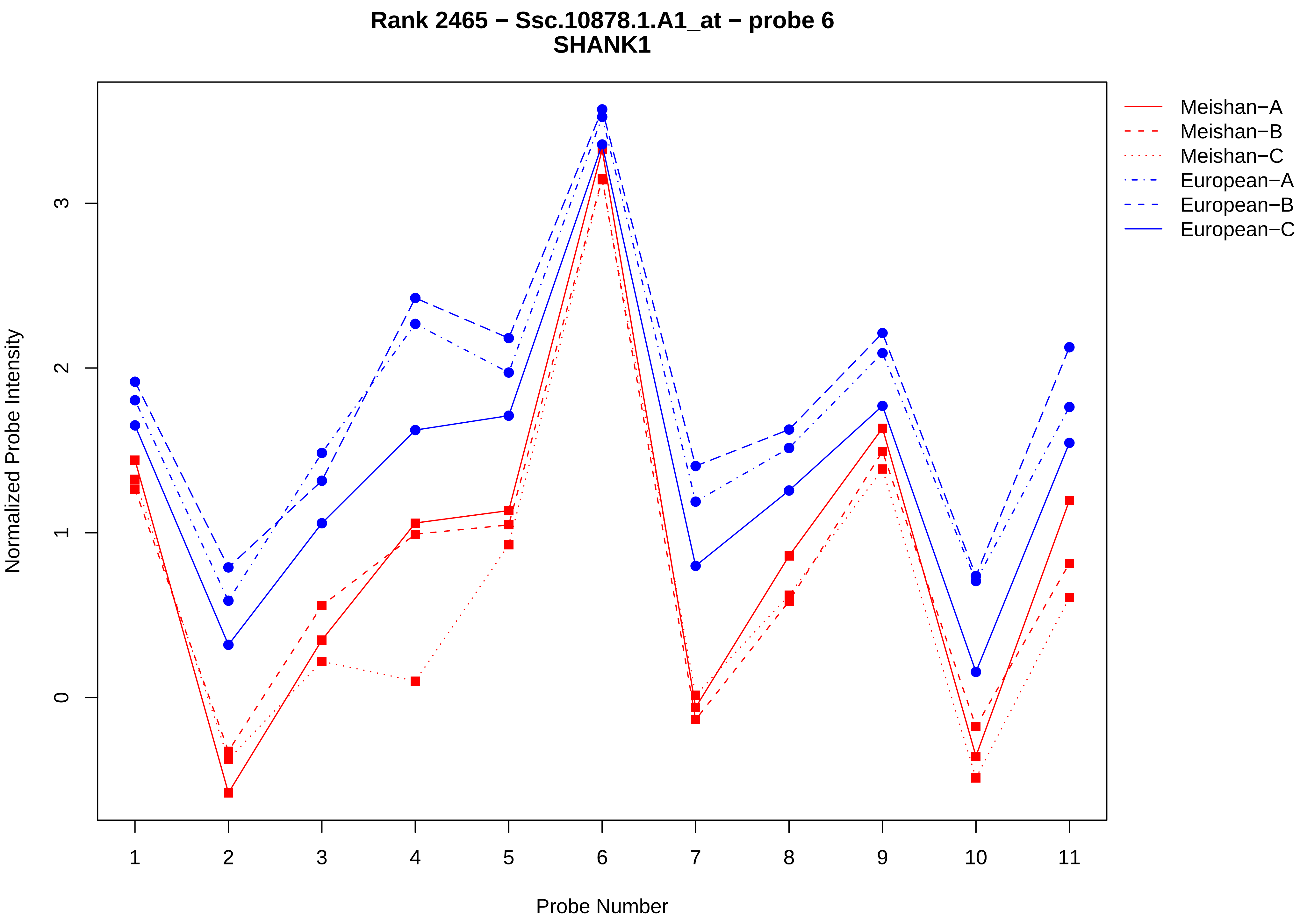

Rank 2465 − Ssc.10878.1.A1_at − probe 6
SHANK1
Normalized Probe Intensity
Probe Number
0
1
2
3
1
2
3
4
5
6
7
8
9
10
11
Meishan−A
Meishan−B
Meishan−C
European−A
European−B
European−C

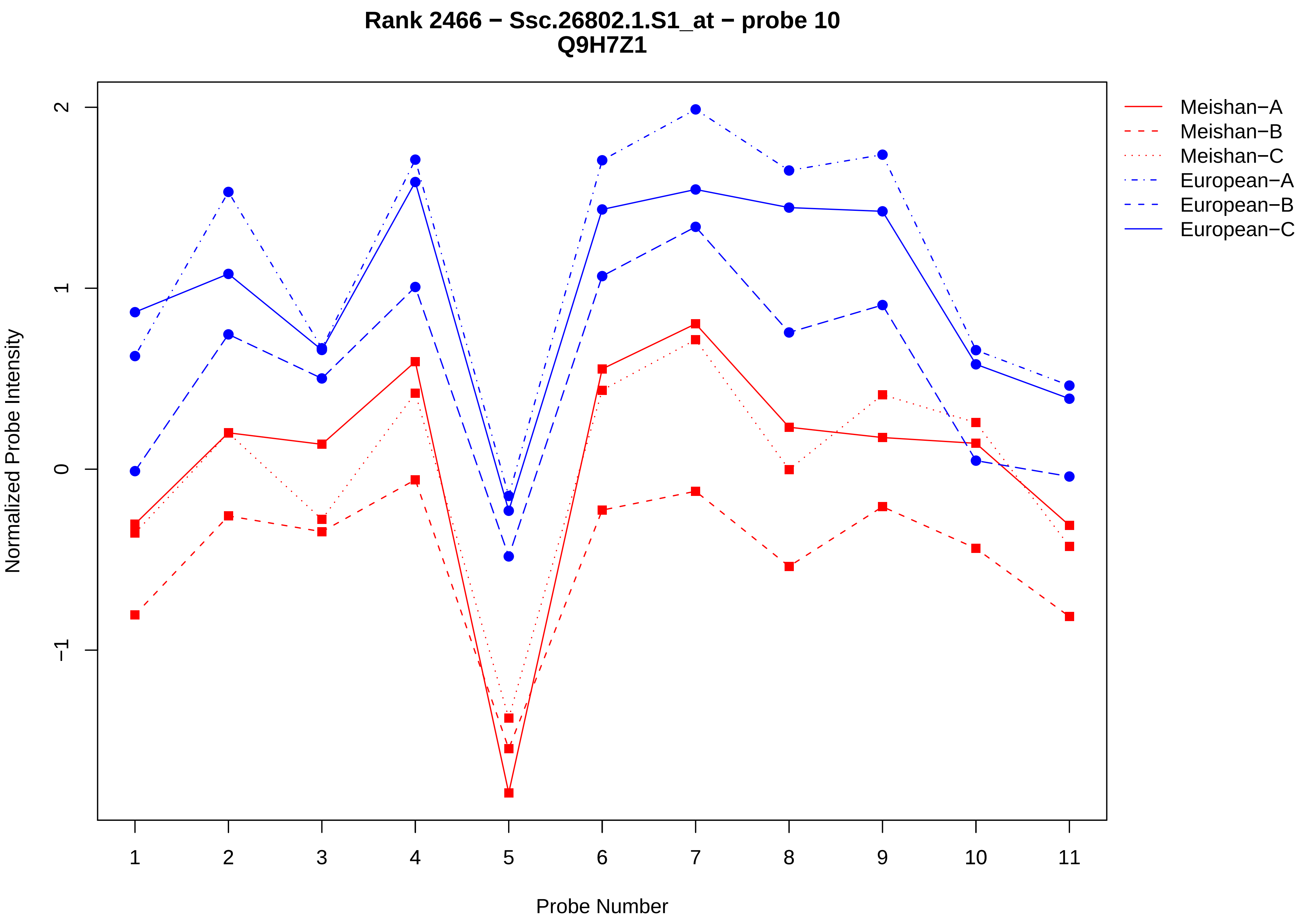

Rank 2466 − Ssc.26802.1.S1_at − probe 10
Q9H7Z1
Normalized Probe Intensity
Probe Number
−1
0
1
2
1
2
3
4
5
6
7
8
9
10
11
Meishan−A
Meishan−B
Meishan−C
European−A
European−B
European−C

Rank 2467 − Ssc.20915.1.S1_at − probe 4
PSMA2

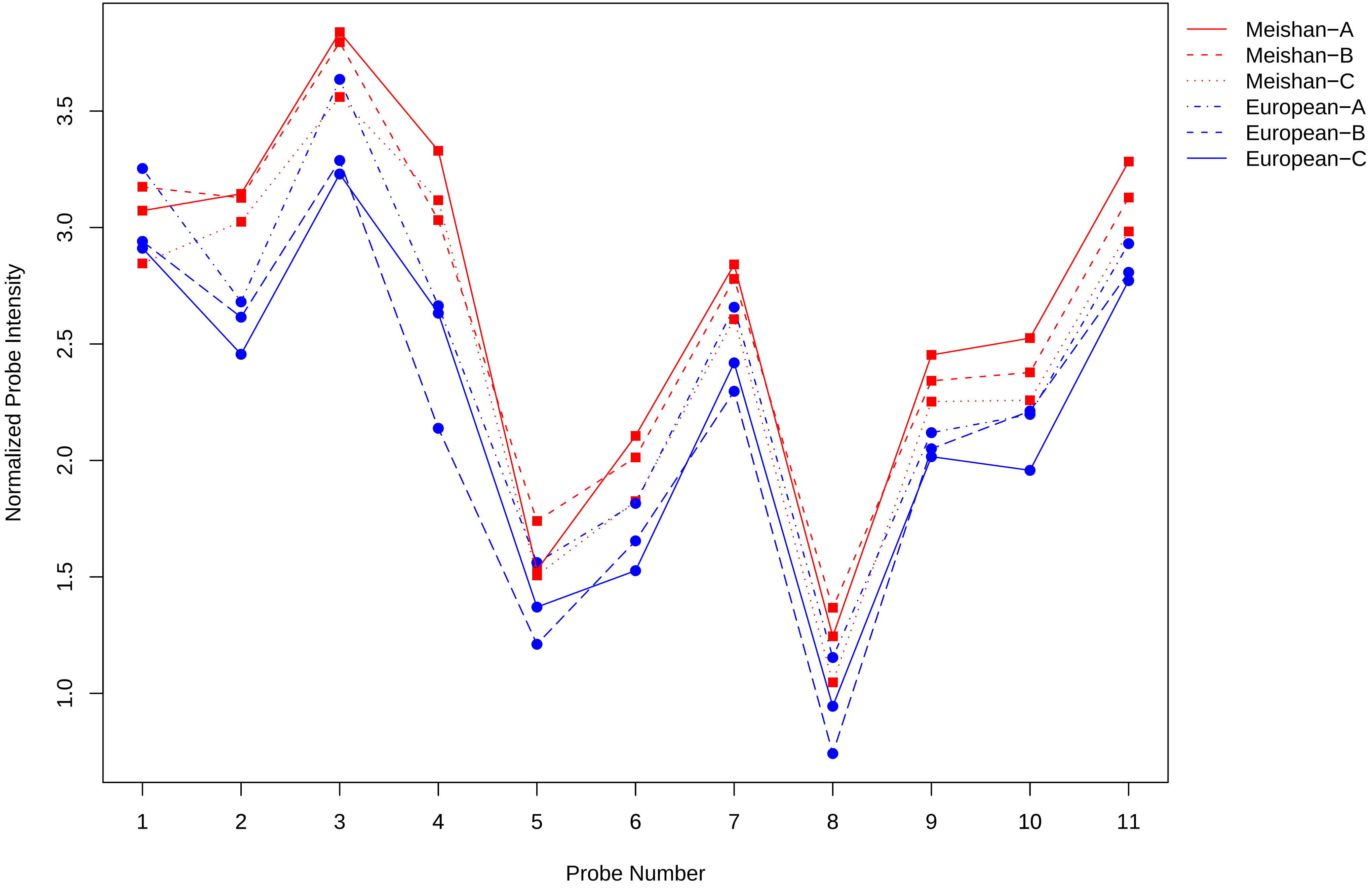

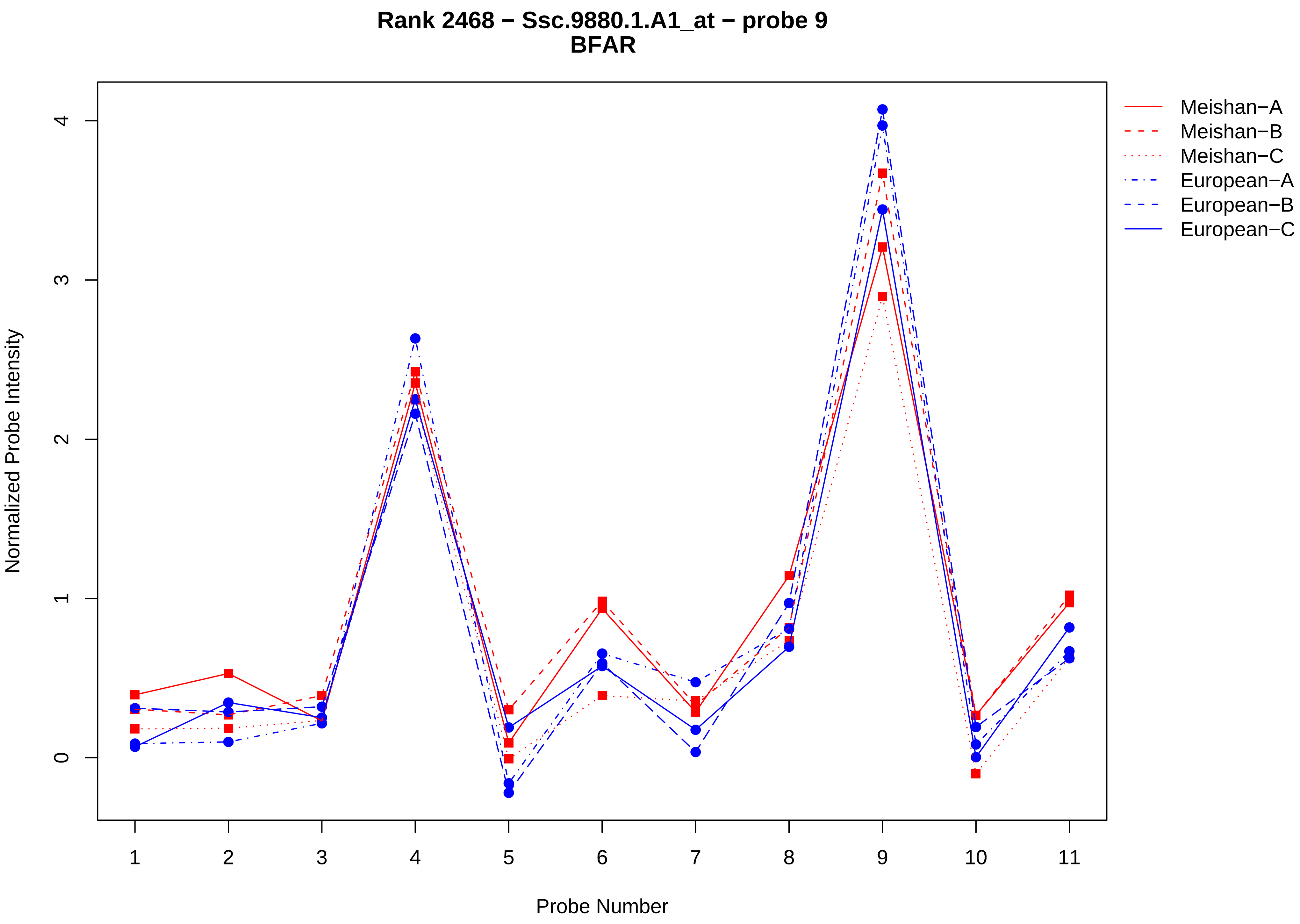

Rank 2468 − Ssc.9880.1.A1_at − probe 9
BFAR
Normalized Probe Intensity
Probe Number
0
1
2
3
4
1
2
3
4
5
6
7
8
9
10
11
Meishan−A
Meishan−B
Meishan−C
European−A
European−B
European−C

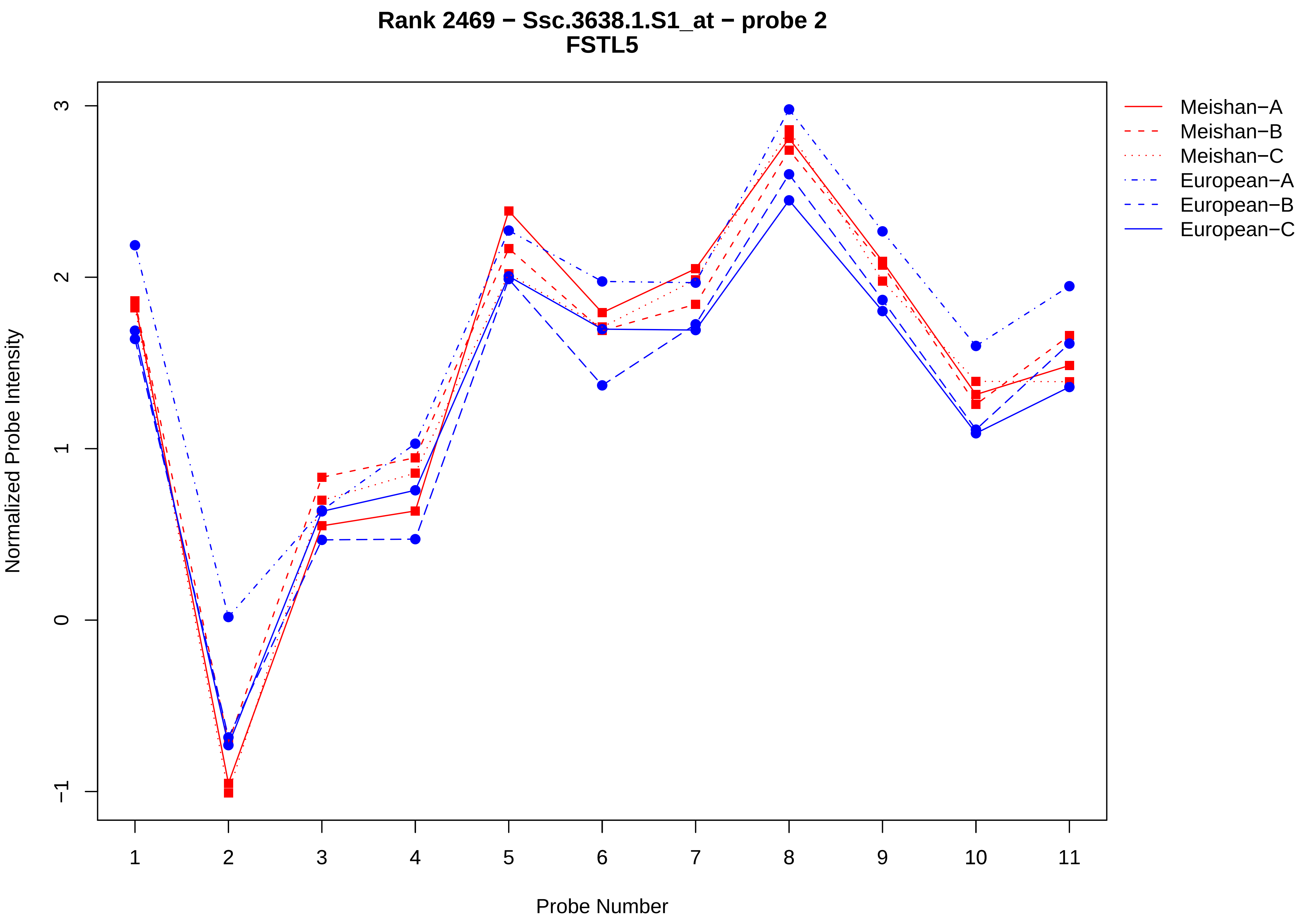

Rank 2469 − Ssc.3638.1.S1_at − probe 2
FSTL5
Normalized Probe Intensity
Probe Number
−1
0
1
2
3
1
2
3
4
5
6
7
8
9
10
11
Meishan−A
Meishan−B
Meishan−C
European−A
European−B
European−C

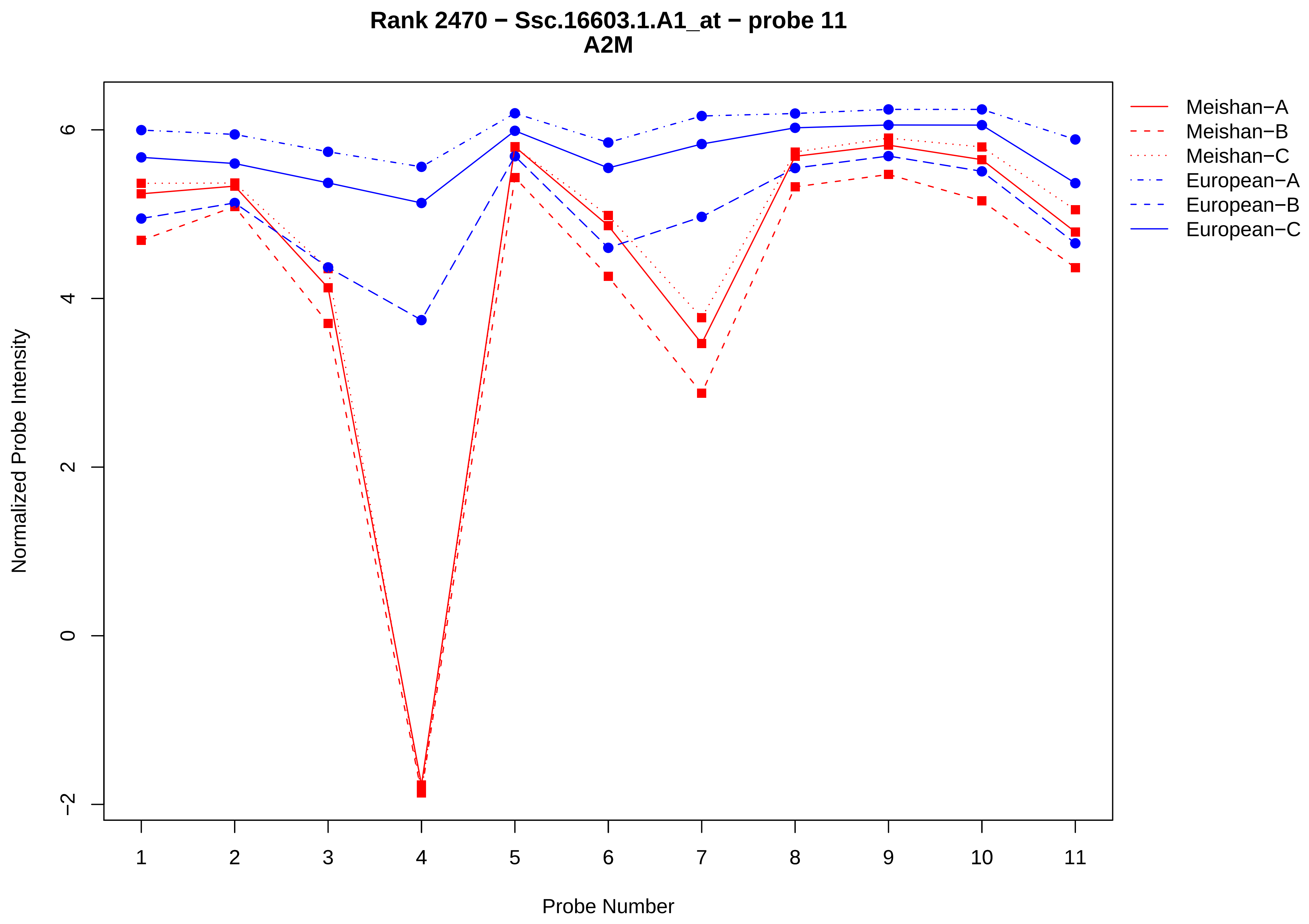

Rank 2470 − Ssc.16603.1.A1_at − probe 11
A2M
Normalized Probe Intensity
Probe Number
Meishan−A
Meishan−B
Meishan−C
European−A
European−B
European−C

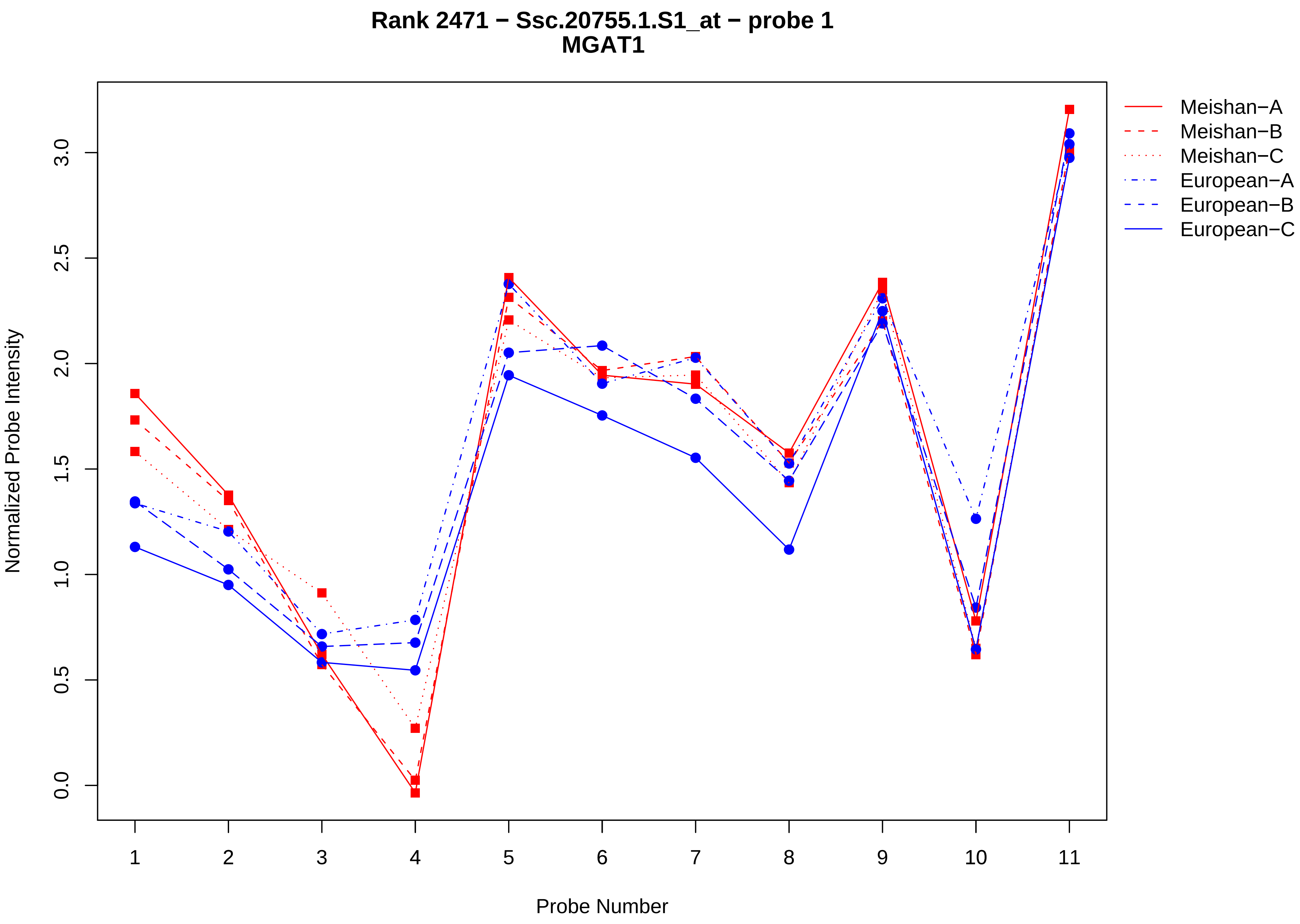

Rank 2471 − Ssc.20755.1.S1_at − probe 1
MGAT1
Normalized Probe Intensity
0.0
0.5
1.0
1.5
2.0
2.5
3.0
1
2
3
4
5
6
7
8
9
10
11
Probe Number
Meishan−A
Meishan−B
Meishan−C
European−A
European−B
European−C

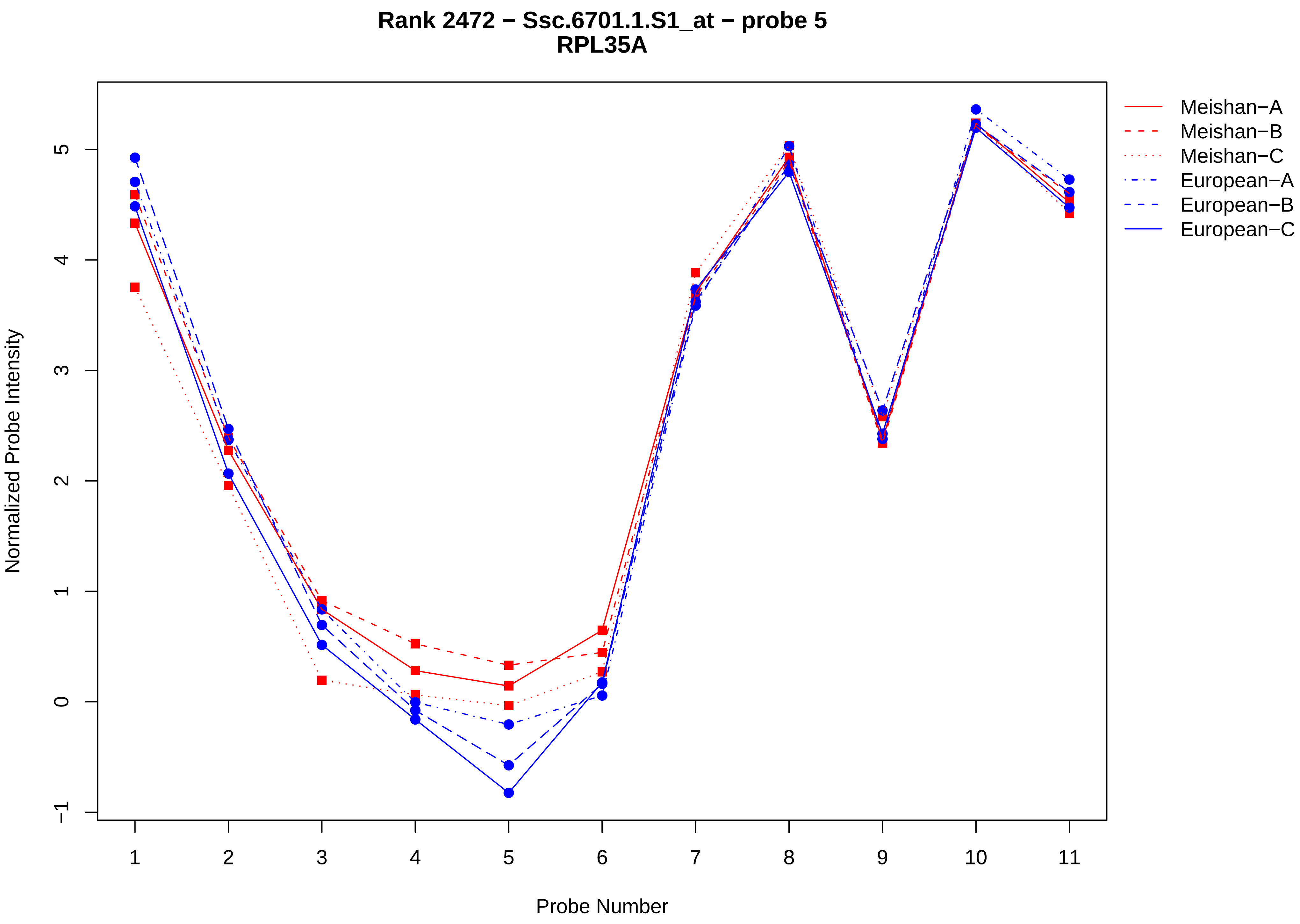
Rank 2472 − Ssc.6701.1.S1_at − probe 5
RPL35A
Normalized Probe Intensity
Probe Number
Meishan−A
Meishan−B
Meishan−C
European−A
European−B
European−C
−1
0
1
2
3
4
5
1
2
3
4
5
6
7
8
9
10
11

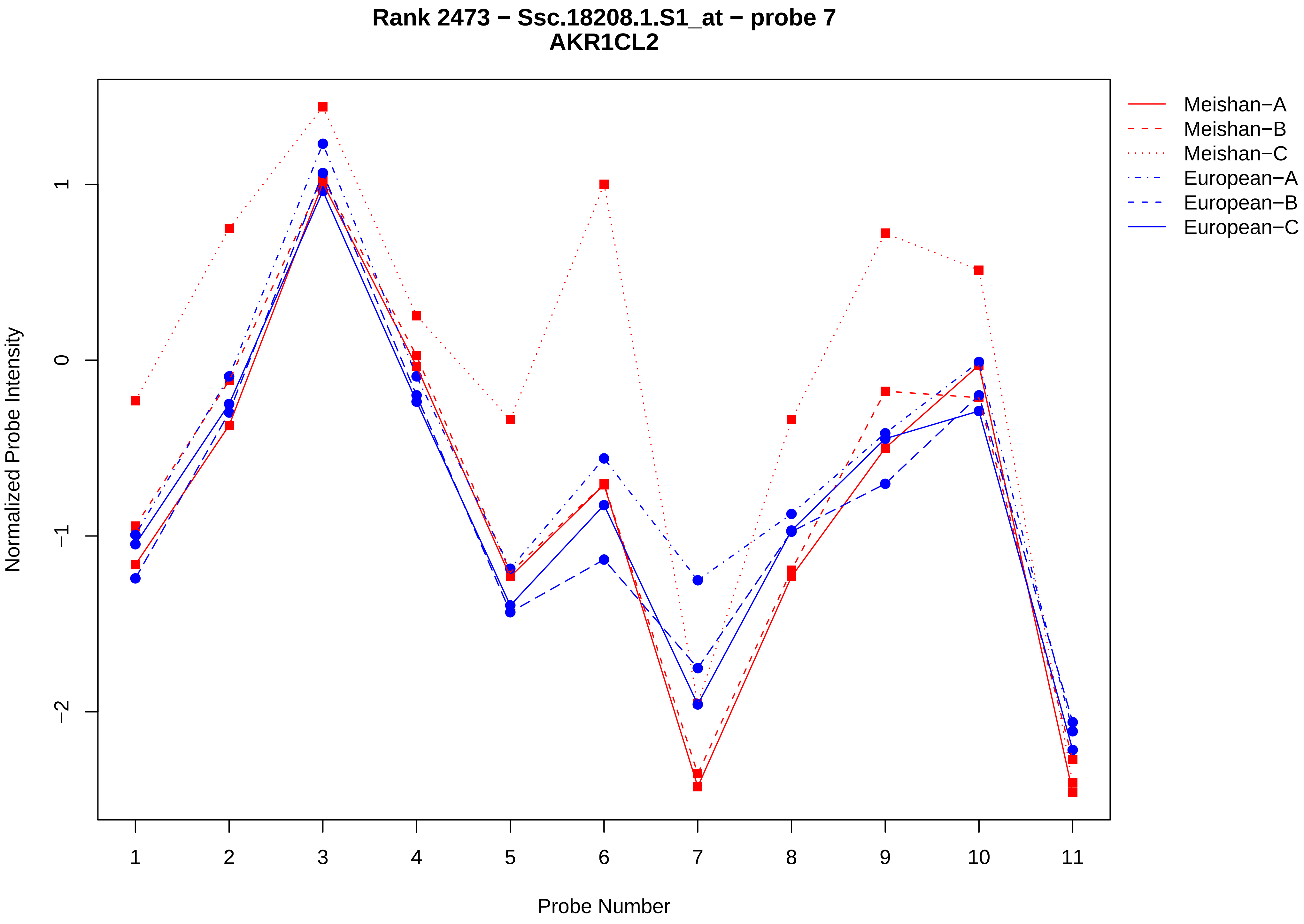

Rank 2473 − Ssc.18208.1.S1_at − probe 7
AKR1CL2
Normalized Probe Intensity
Probe Number
Meishan−A
Meishan−B
Meishan−C
European−A
European−B
European−C
−2
−1
0
1
1
2
3
4
5
6
7
8
9
10
11

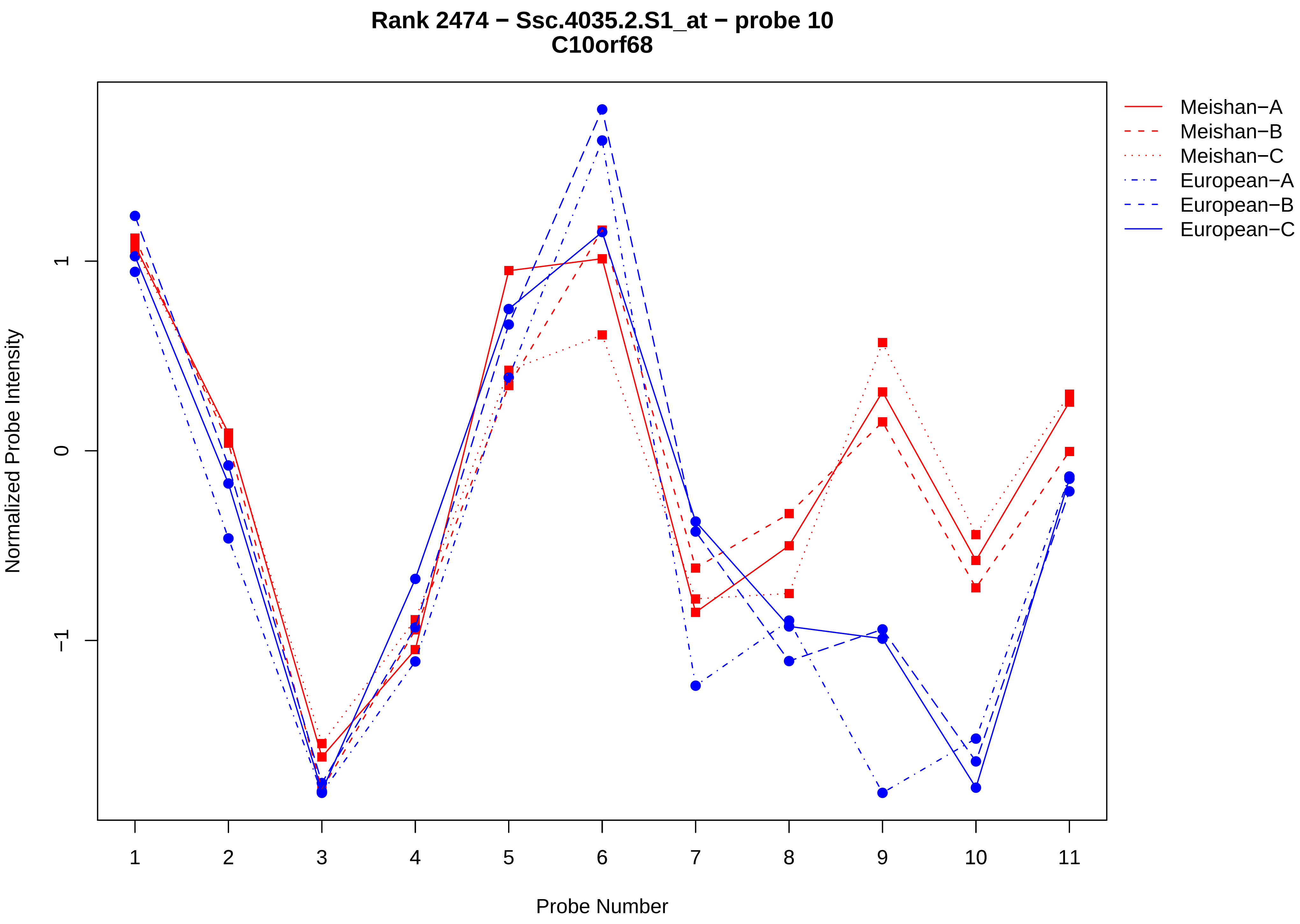

Rank 2474 − Ssc.4035.2.S1_at − probe 10
C10orf68
Normalized Probe Intensity
Probe Number
1
0
−1
1
2
3
4
5
6
7
8
9
10
11
Meishan−A
Meishan−B
Meishan−C
European−A
European−B
European−C

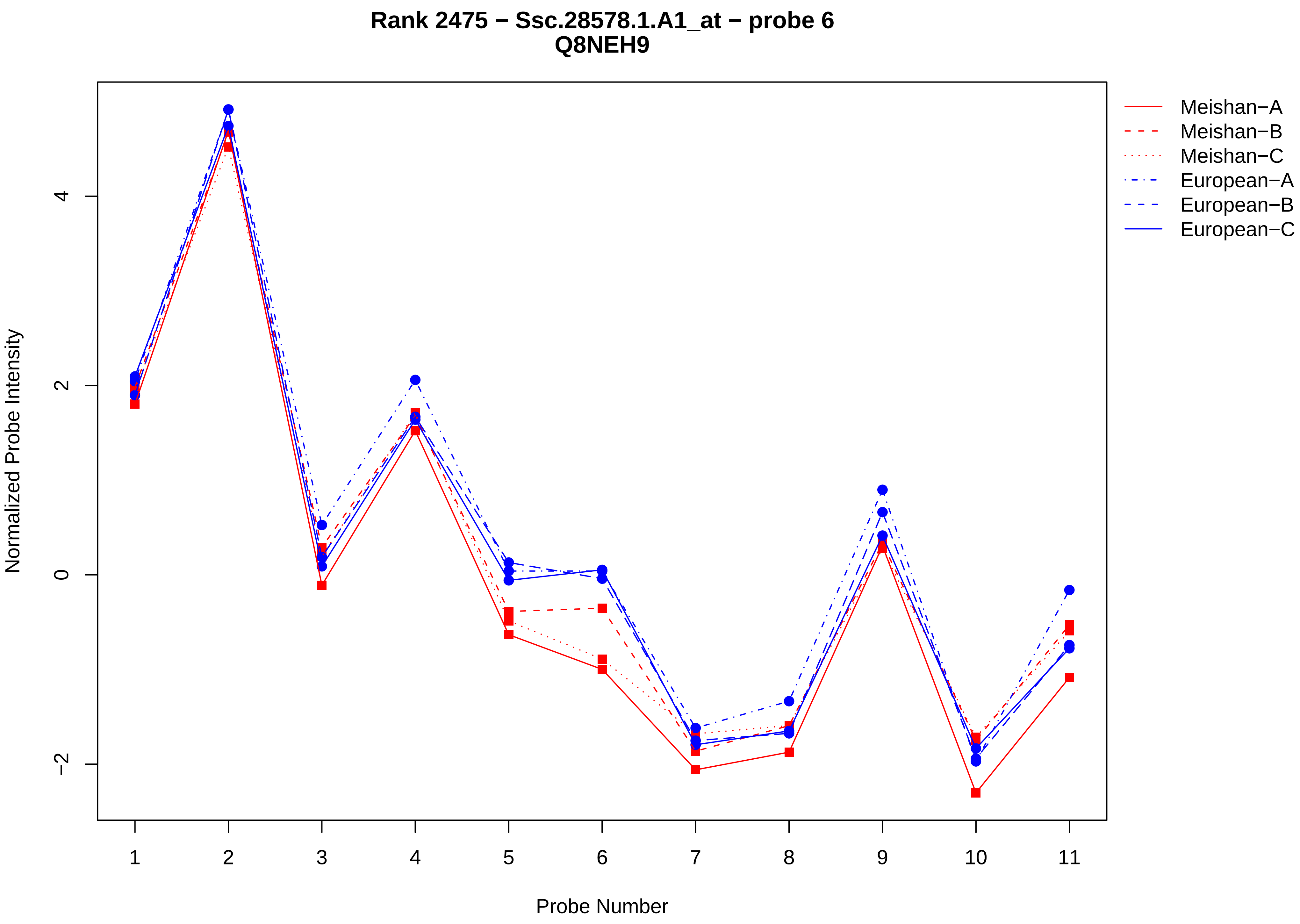
Rank 2475 − Ssc.28578.1.A1_at − probe 6
Q8NEH9
Normalized Probe Intensity
Probe Number
−2
0
2
4
1
2
3
4
5
6
7
8
9
10
11
Meishan−A
Meishan−B
Meishan−C
European−A
European−B
European−C

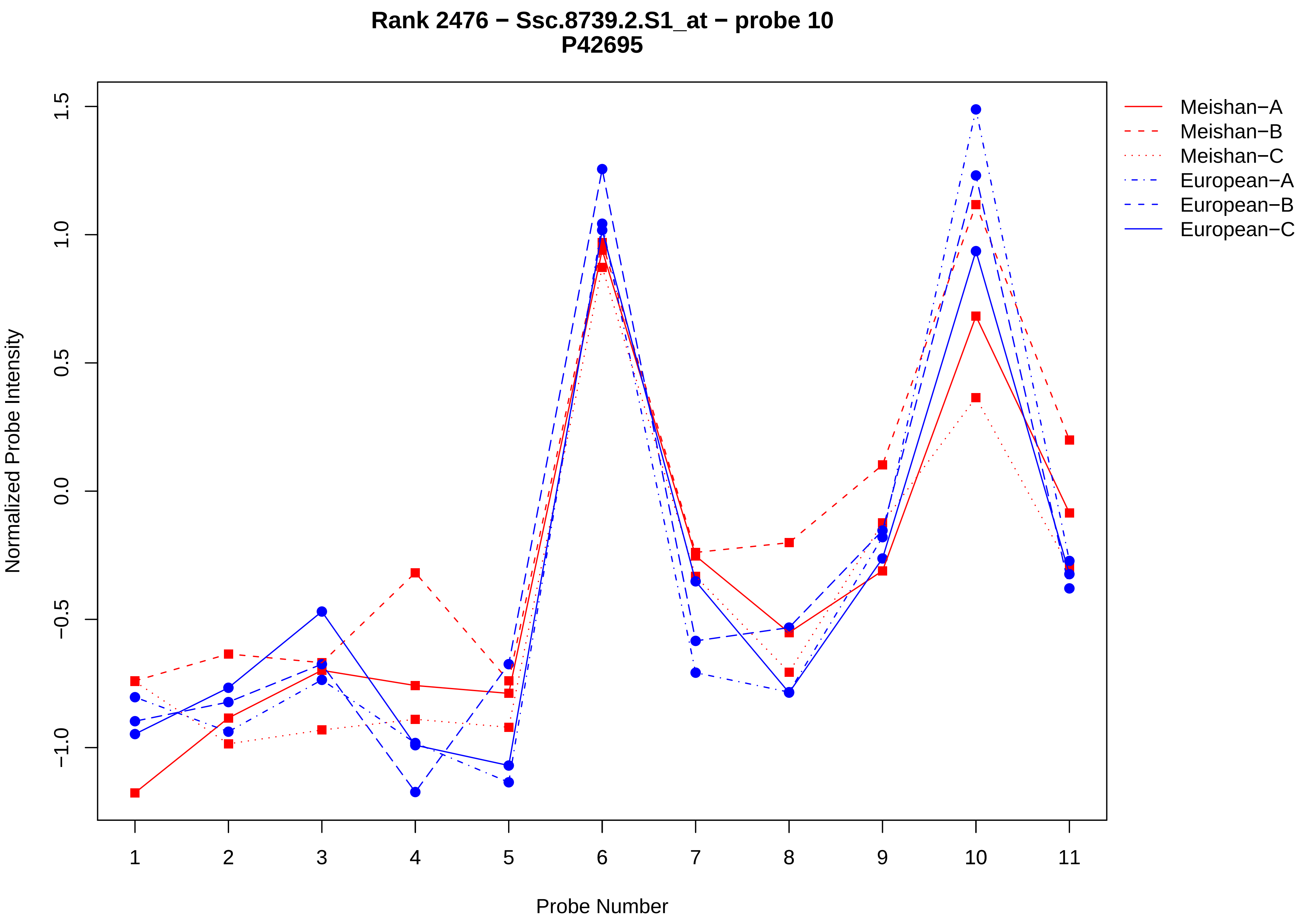
Rank 2476 − Ssc.8739.2.S1_at − probe 10
P42695
Normalized Probe Intensity
Probe Number
Meishan−A
Meishan−B
Meishan−C
European−A
European−B
European−C
1.5
1.0
0.5
0.0
−0.5
−1.0
1
2
3
4
5
6
7
8
9
10
11

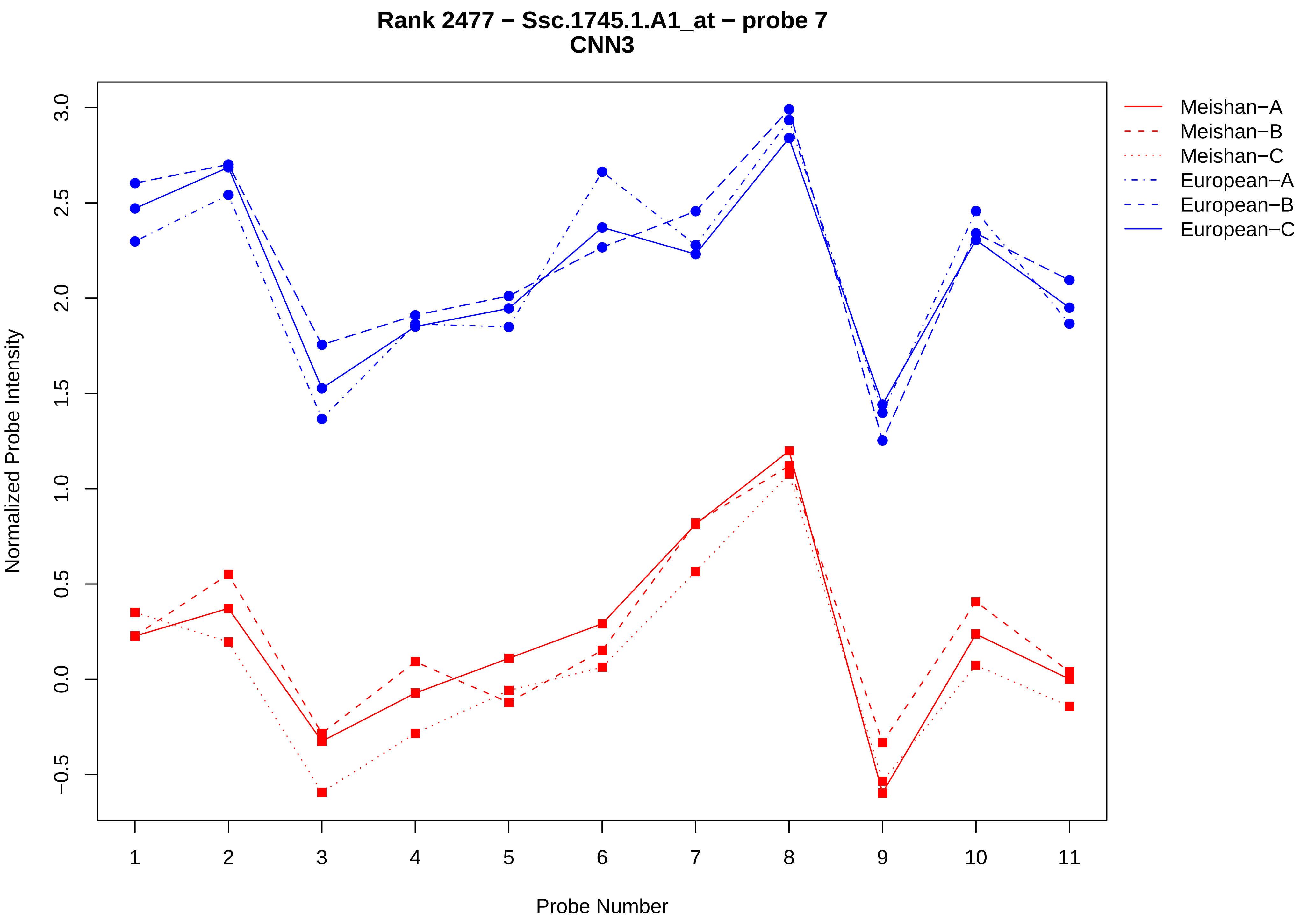

Rank 2477 − Ssc.1745.1.A1_at − probe 7
CNN3
Normalized Probe Intensity
−0.5
0.0
0.5
1.0
1.5
2.0
2.5
3.0
Probe Number
1
2
3
4
5
6
7
8
9
10
11
Meishan−A
Meishan−B
Meishan−C
European−A
European−B
European−C

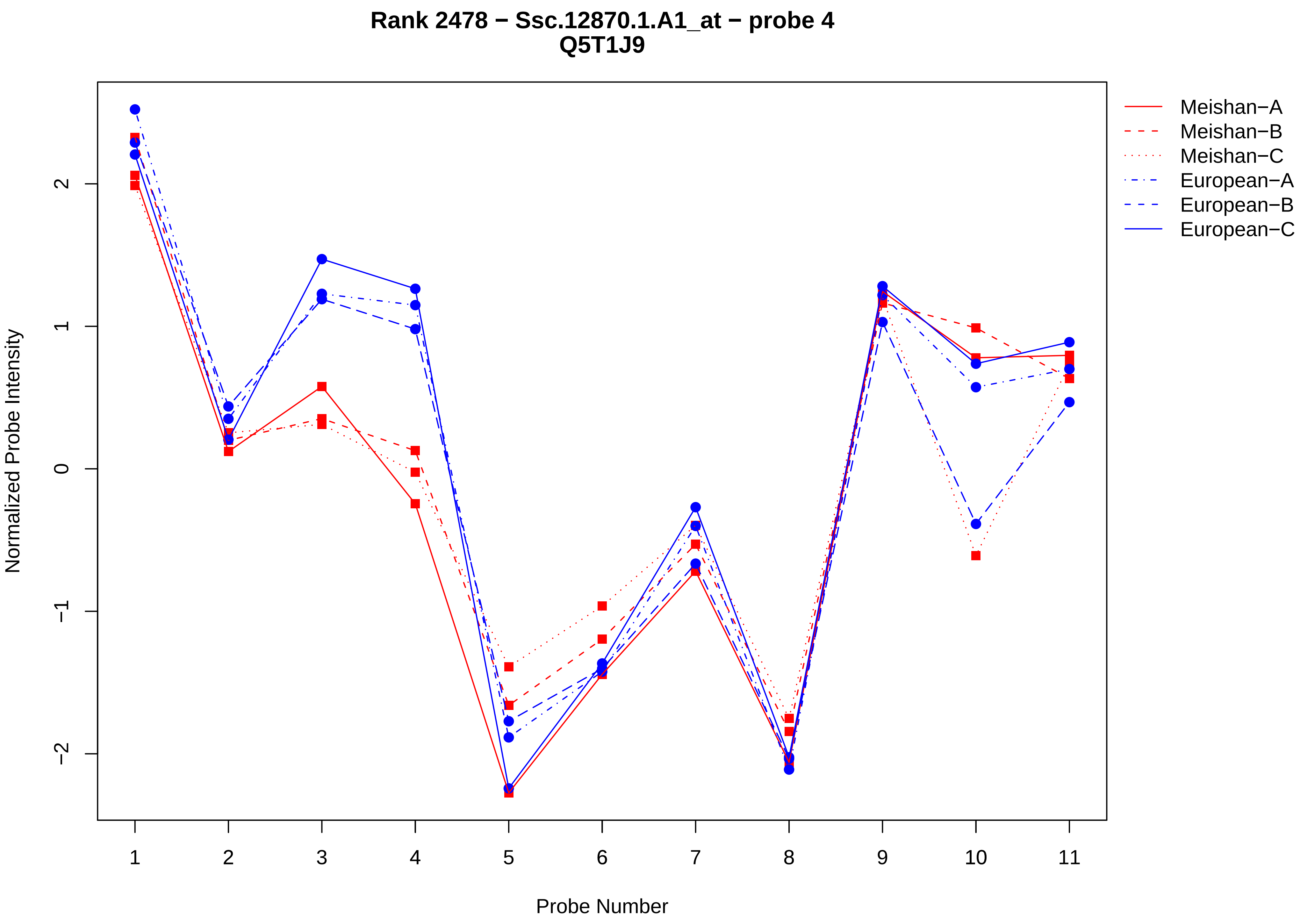
Rank 2478 − Ssc.12870.1.A1_at − probe 4
Q5T1J9
Normalized Probe Intensity
Probe Number
−2
−1
0
1
2
1
2
3
4
5
6
7
8
9
10
11
Meishan−A
Meishan−B
Meishan−C
European−A
European−B
European−C

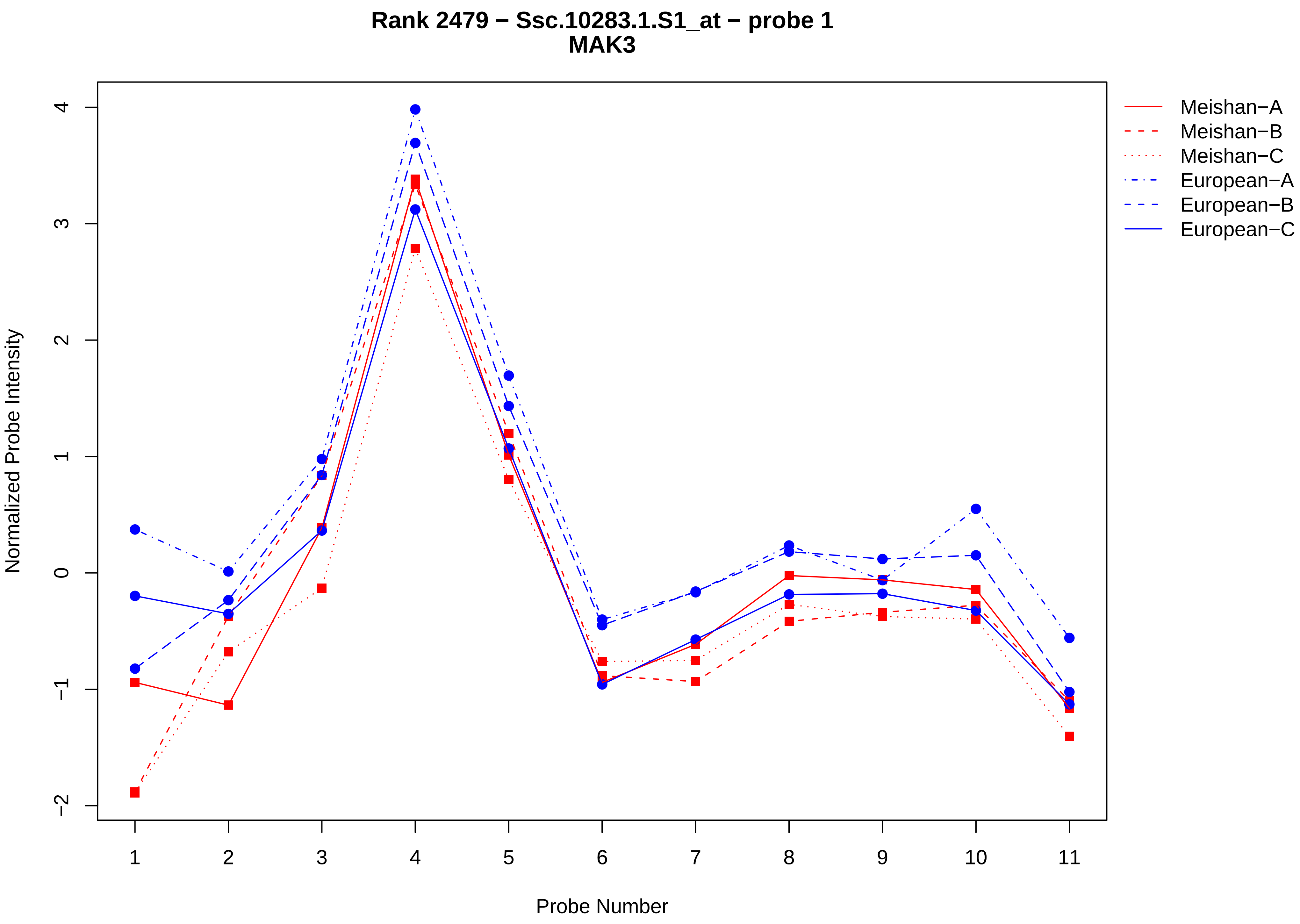

Rank 2479 − Ssc.10283.1.S1_at − probe 1
MAK3
Normalized Probe Intensity
Probe Number
Meishan−A
Meishan−B
Meishan−C
European−A
European−B
European−C
−2
−1
0
1
2
3
4
1
2
3
4
5
6
7
8
9
10
11

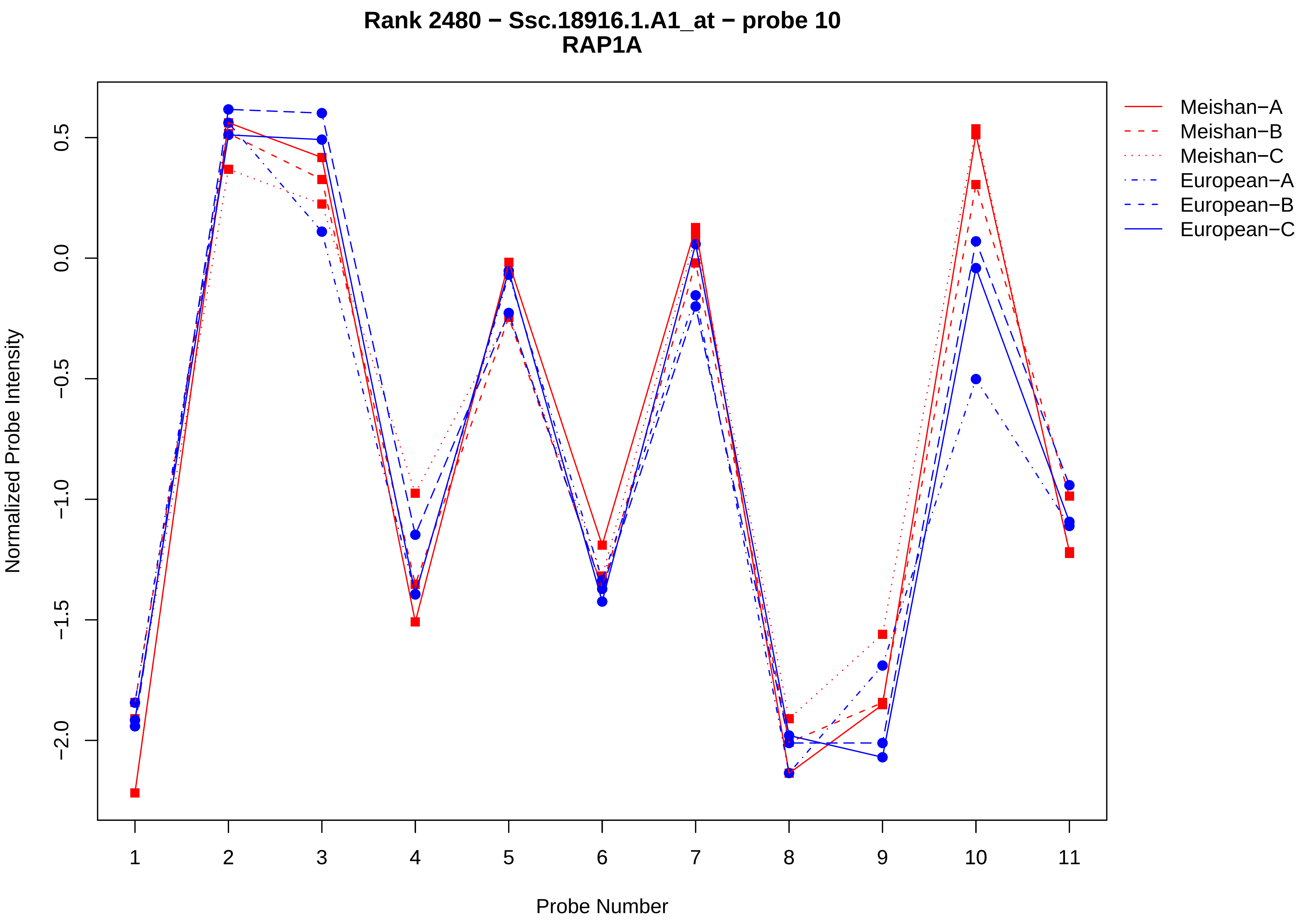
Rank 2480 − Ssc.18916.1.A1_at − probe 10
RAP1A
Normalized Probe Intensity
Probe Number
0.5
0.0
−0.5
−1.0
−1.5
−2.0
1
2
3
4
5
6
7
8
9
10
11
Meishan−A
Meishan−B
Meishan−C
European−A
European−B
European−C

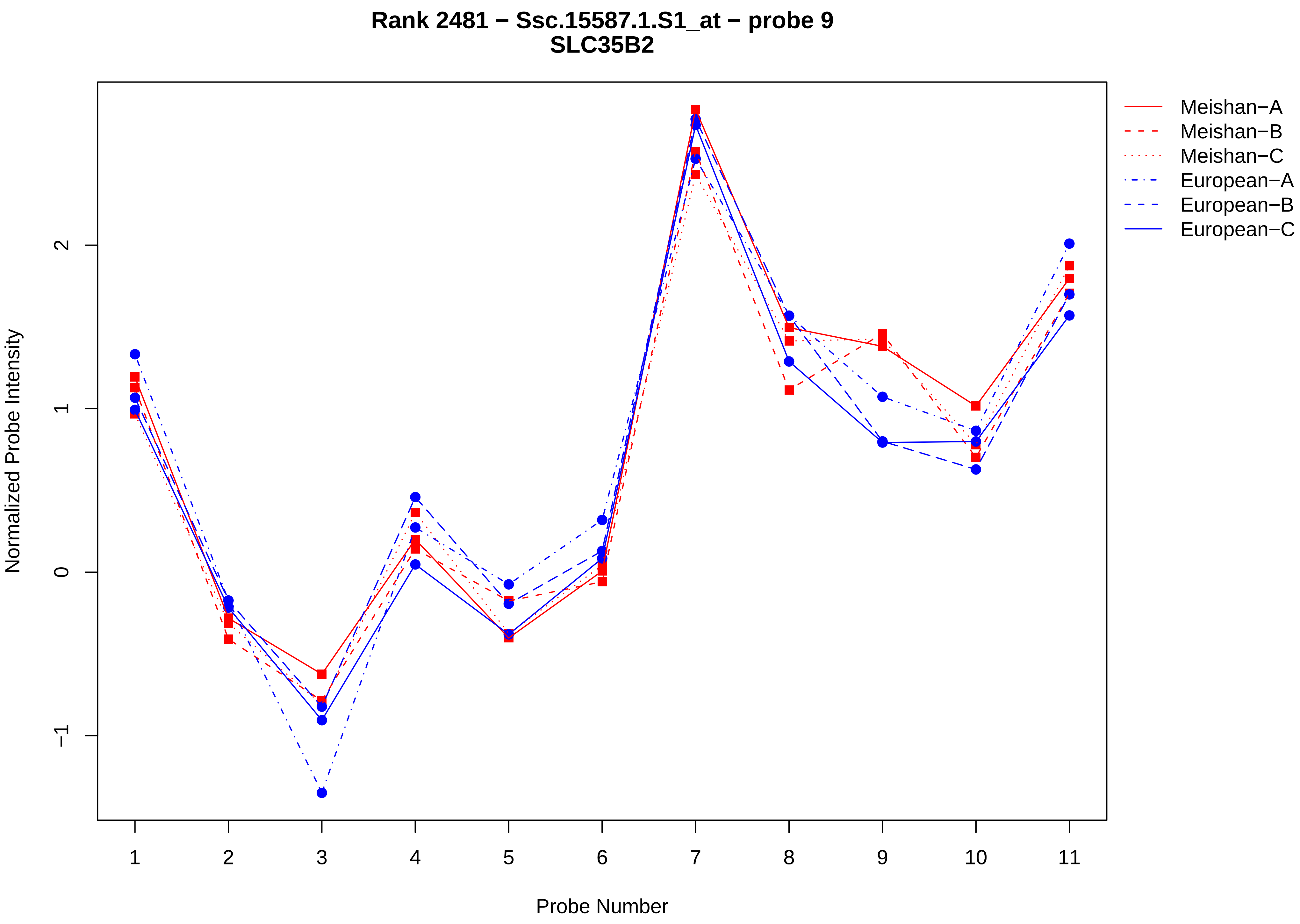
Rank 2481 − Ssc.15587.1.S1_at − probe 9
SLC35B2
Normalized Probe Intensity
Probe Number
Meishan−A
Meishan−B
Meishan−C
European−A
European−B
European−C
−1
0
1
2
1
2
3
4
5
6
7
8
9
10
11

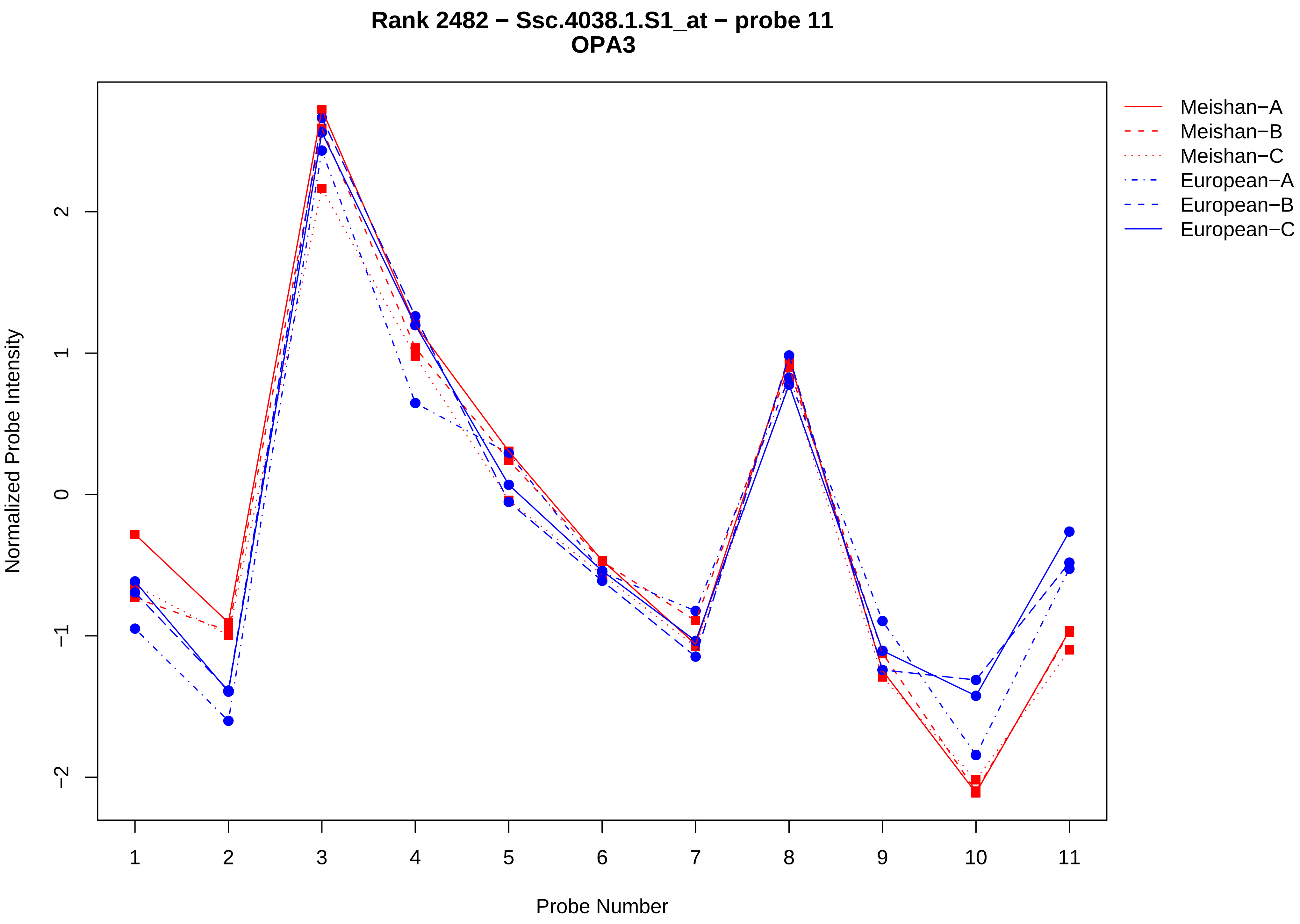

Rank 2482 − Ssc.4038.1.S1_at − probe 11
OPA3
Normalized Probe Intensity
Probe Number
Meishan−A
Meishan−B
Meishan−C
European−A
European−B
European−C

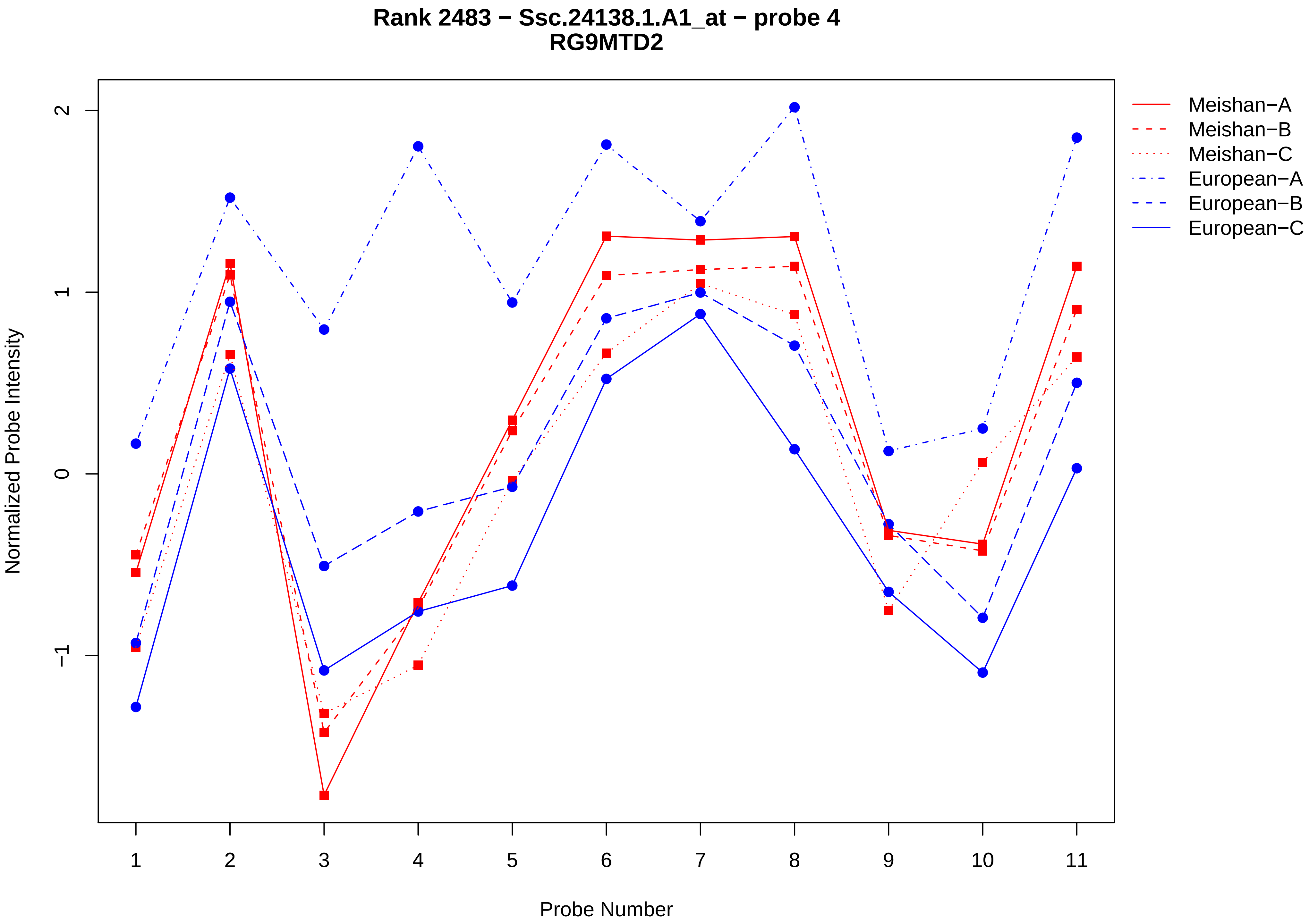

Rank 2483 − Ssc.24138.1.A1_at − probe 4
RG9MTD2
Normalized Probe Intensity
Probe Number
Meishan−A
Meishan−B
Meishan−C
European−A
European−B
European−C

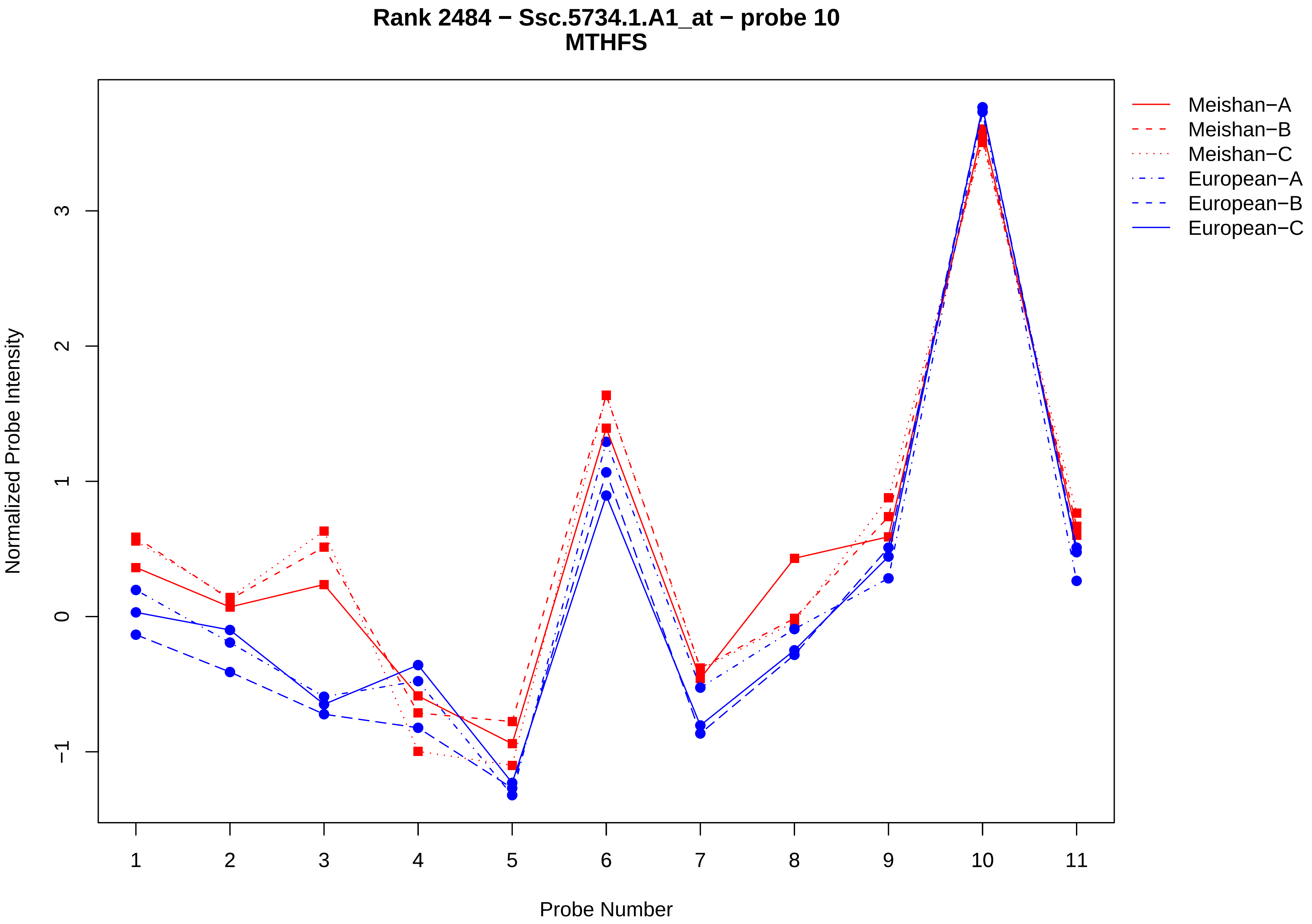

Rank 2484 − Ssc.5734.1.A1_at − probe 10
MTHFS
Normalized Probe Intensity
Probe Number
−1
0
1
2
3
1
2
3
4
5
6
7
8
9
10
11
Meishan−A
Meishan−B
Meishan−C
European−A
European−B
European−C

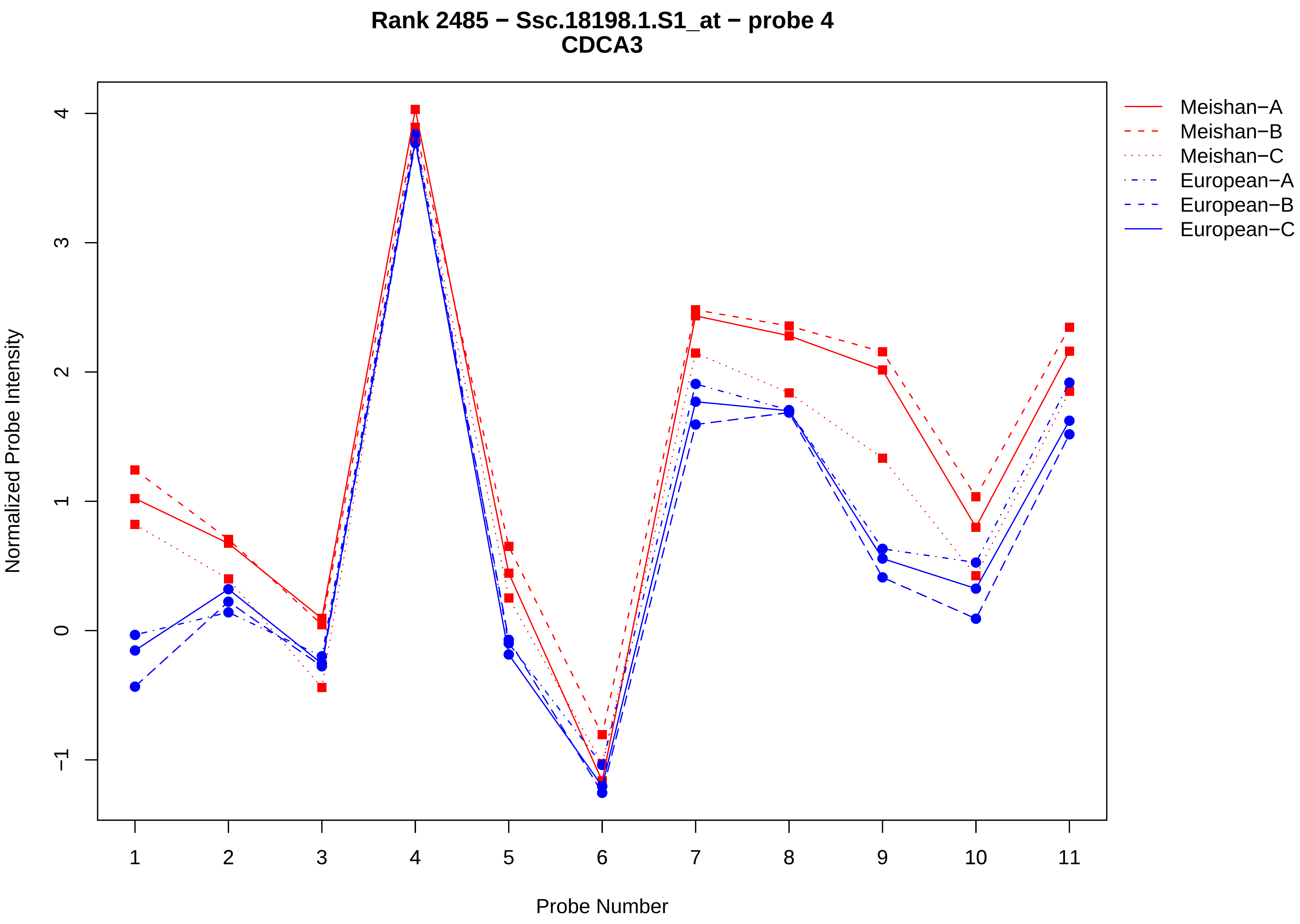

Rank 2485 − Ssc.18198.1.S1_at − probe 4
CDCA3
Normalized Probe Intensity
Probe Number
Meishan−A
Meishan−B
Meishan−C
European−A
European−B
European−C
−1
0
1
2
3
4
1
2
3
4
5
6
7
8
9
10
11

Rank 2486 − Ssc.6512.1.S1_at − probe 7
Q6ZMJ2

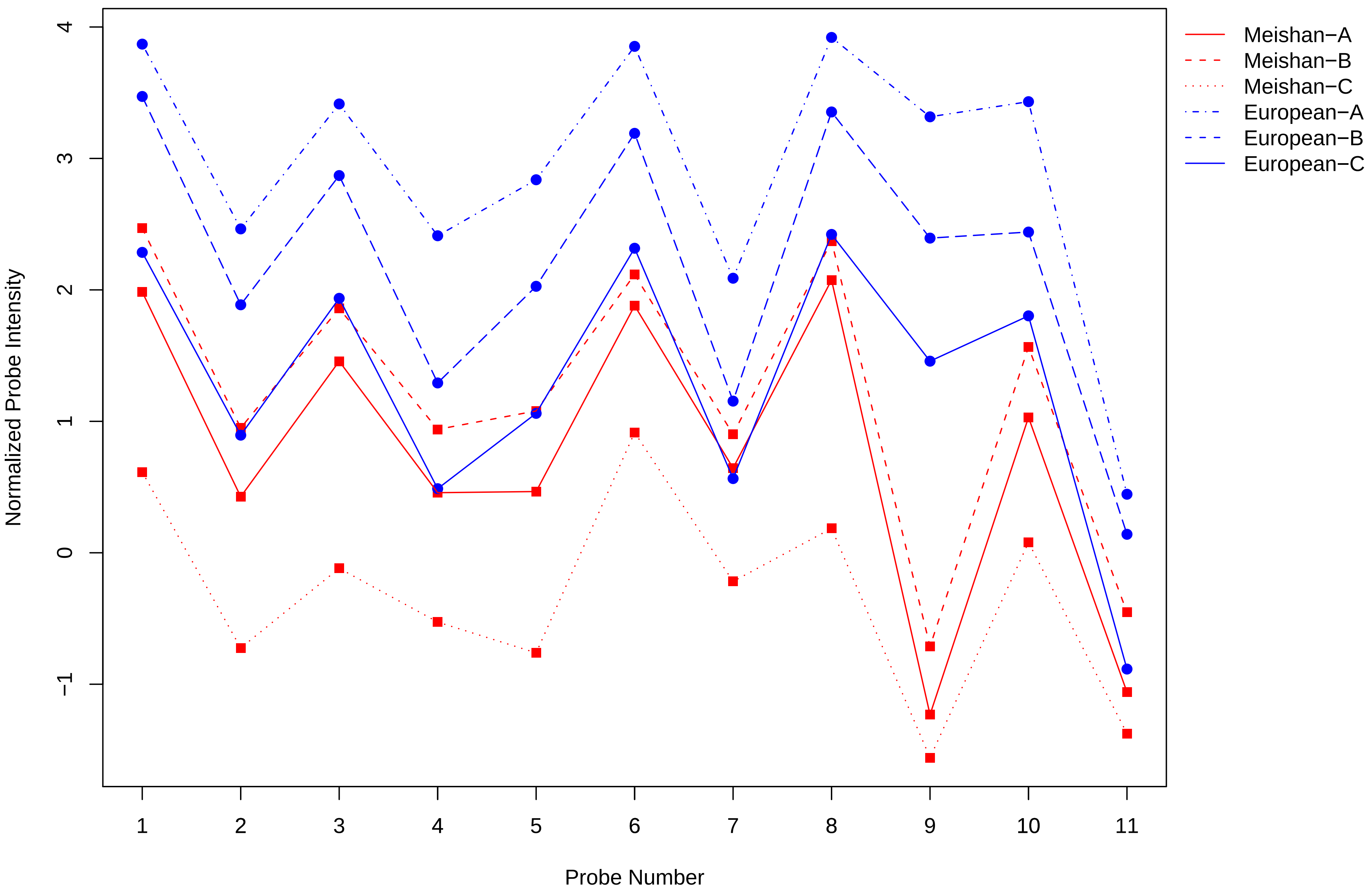

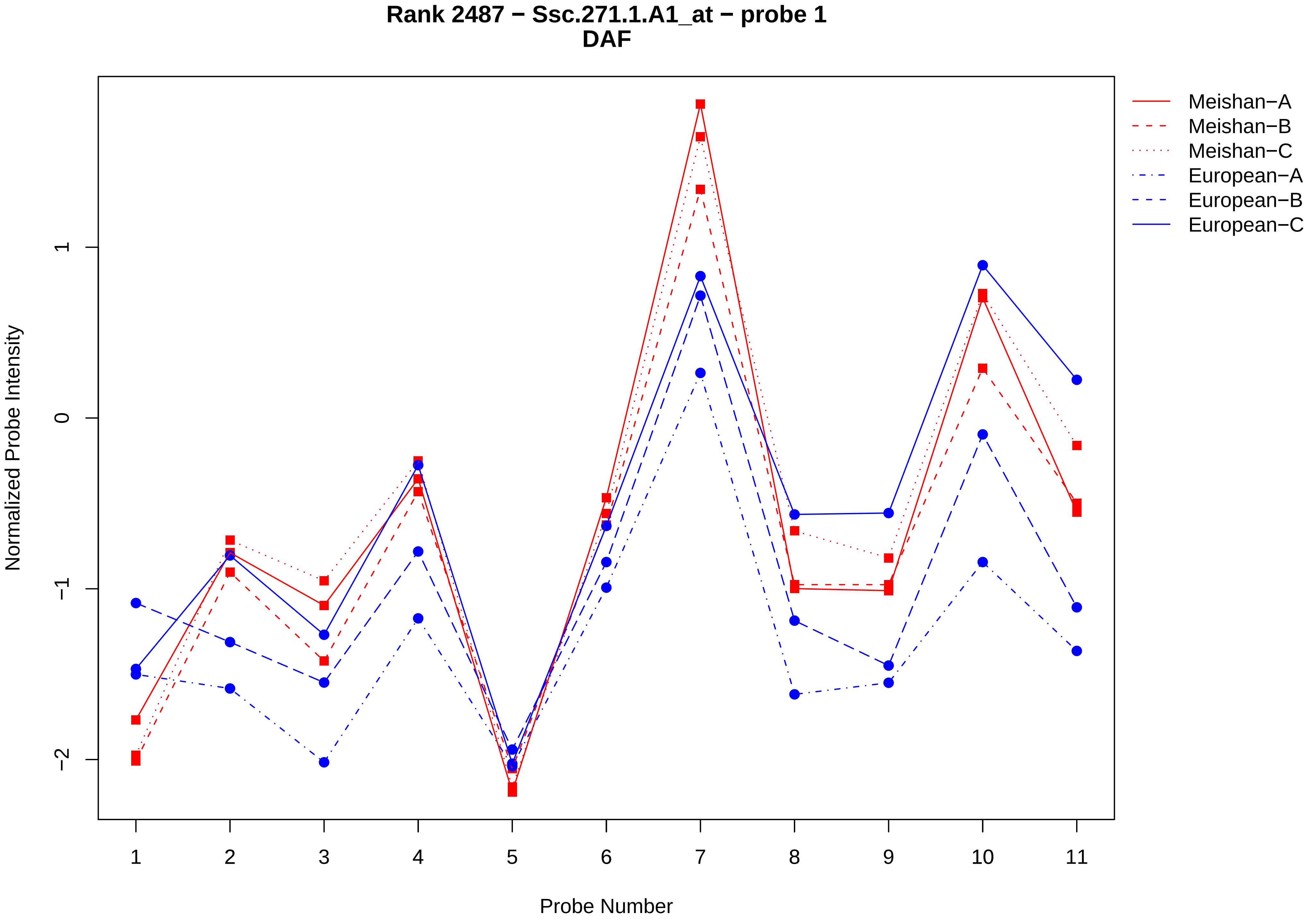

Rank 2487 − Ssc.271.1.A1_at − probe 1
DAF
Normalized Probe Intensity
Probe Number
−2
−1
0
1
1
2
3
4
5
6
7
8
9
10
11
Meishan−A
Meishan−B
Meishan−C
European−A
European−B
European−C

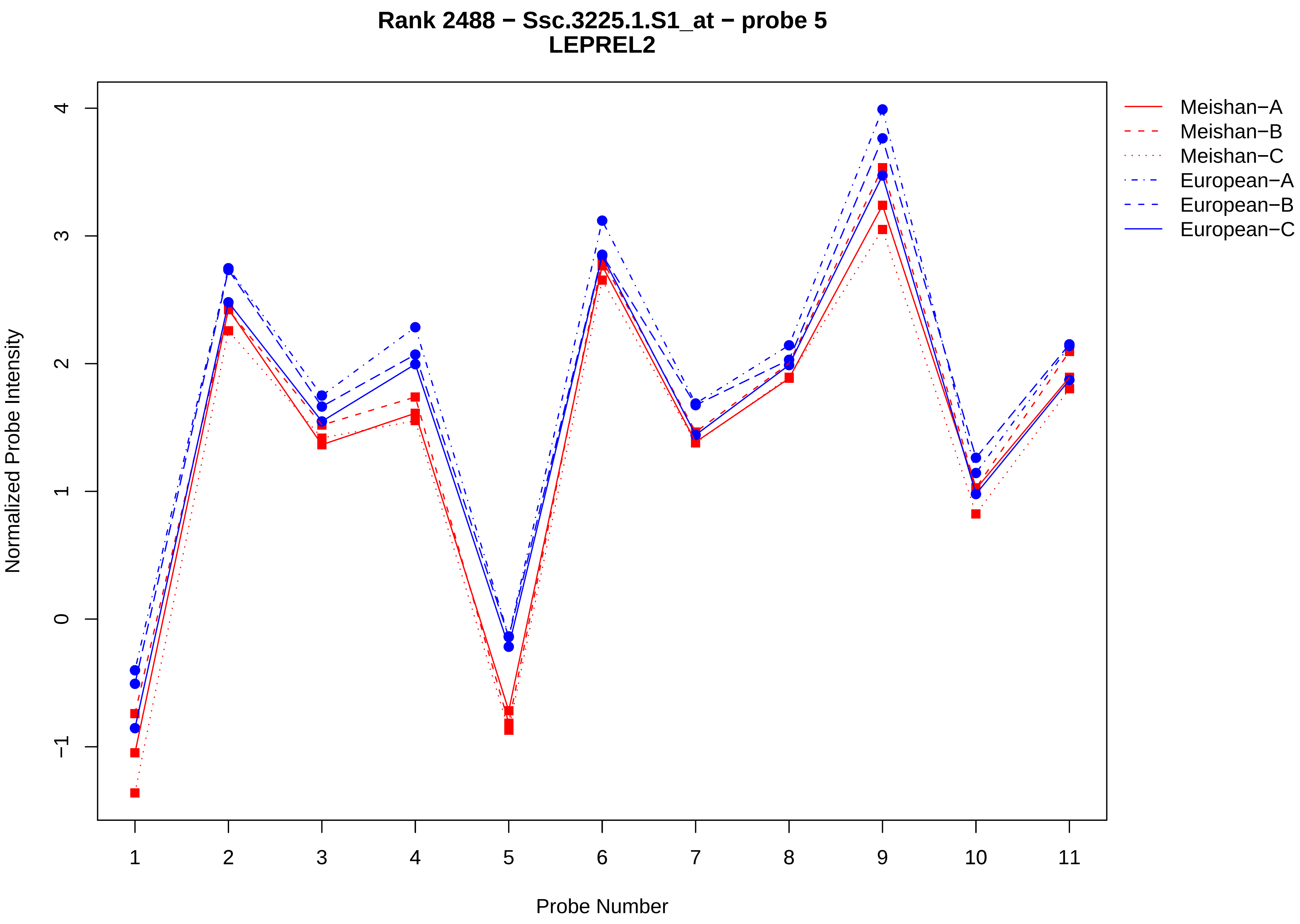
Rank 2488 − Ssc.3225.1.S1_at − probe 5
LEPREL2
Normalized Probe Intensity
Probe Number
Meishan−A
Meishan−B
Meishan−C
European−A
European−B
European−C
−1
0
1
2
3
4
1
2
3
4
5
6
7
8
9
10
11

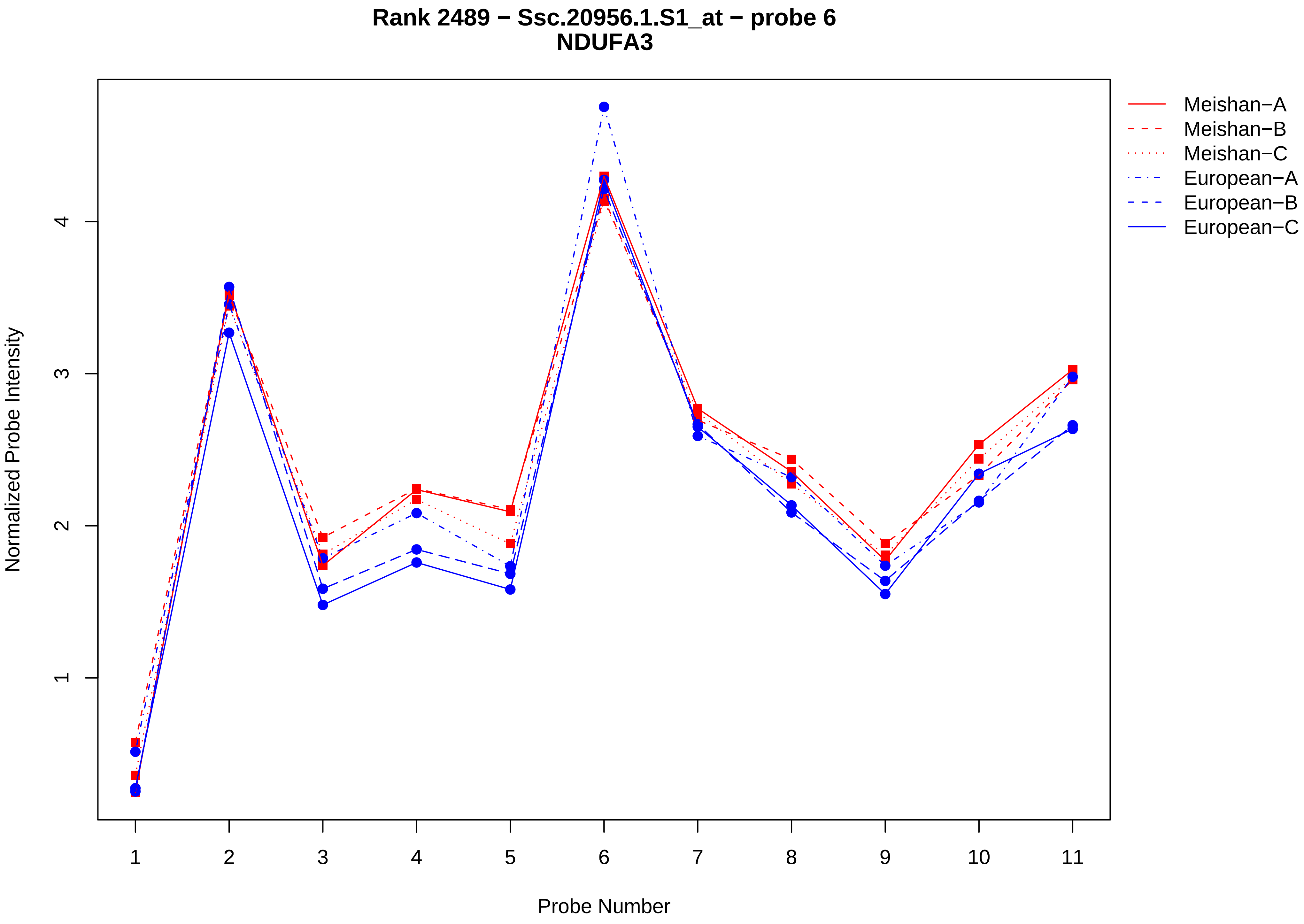
Rank 2489 − Ssc.20956.1.S1_at − probe 6
NDUFA3
Normalized Probe Intensity
Probe Number
Meishan−A
Meishan−B
Meishan−C
European−A
European−B
European−C
1
2
3
4
1
2
3
4
5
6
7
8
9
10
11

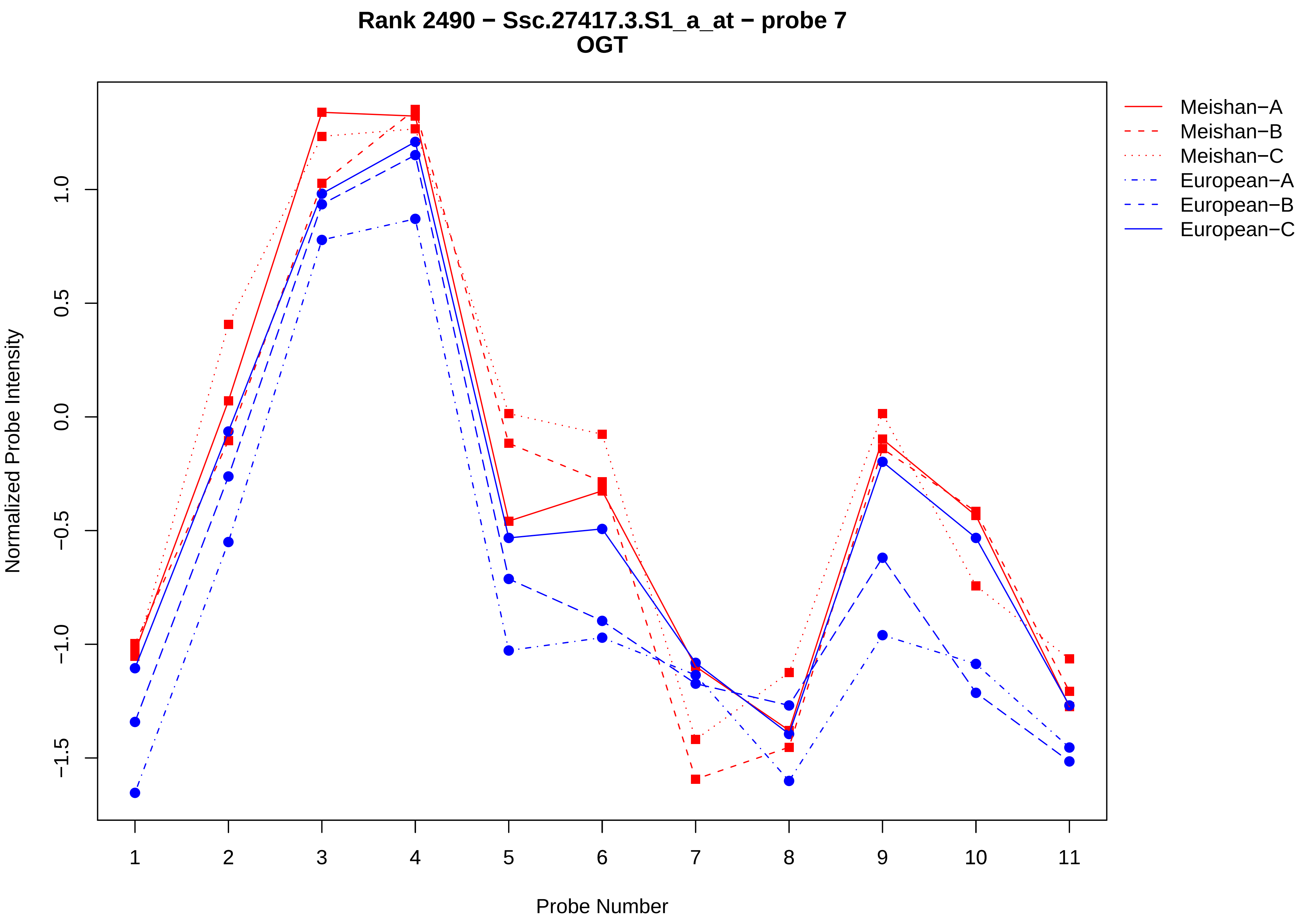

Rank 2490 − Ssc.27417.3.S1_a_at − probe 7
OGT
Normalized Probe Intensity
Probe Number
−1.5
−1.0
−0.5
0.0
0.5
1.0
1
2
3
4
5
6
7
8
9
10
11
Meishan−A
Meishan−B
Meishan−C
European−A
European−B
European−C

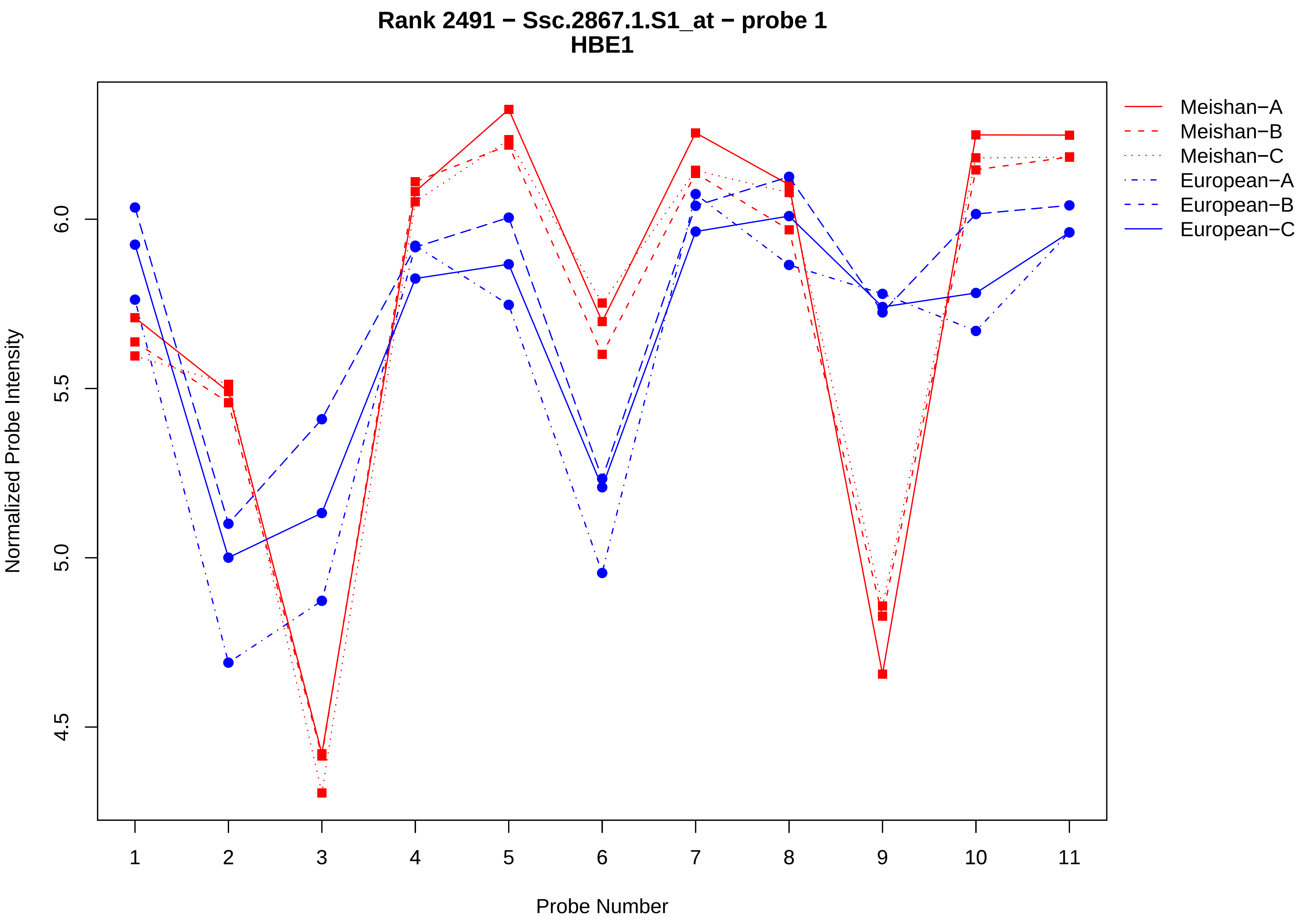

Rank 2491 − Ssc.2867.1.S1_at − probe 1
HBE1
Normalized Probe Intensity
4.5
5.0
5.5
6.0
1
2
3
4
5
6
7
8
9
10
11
Probe Number
Meishan−A
Meishan−B
Meishan−C
European−A
European−B
European−C

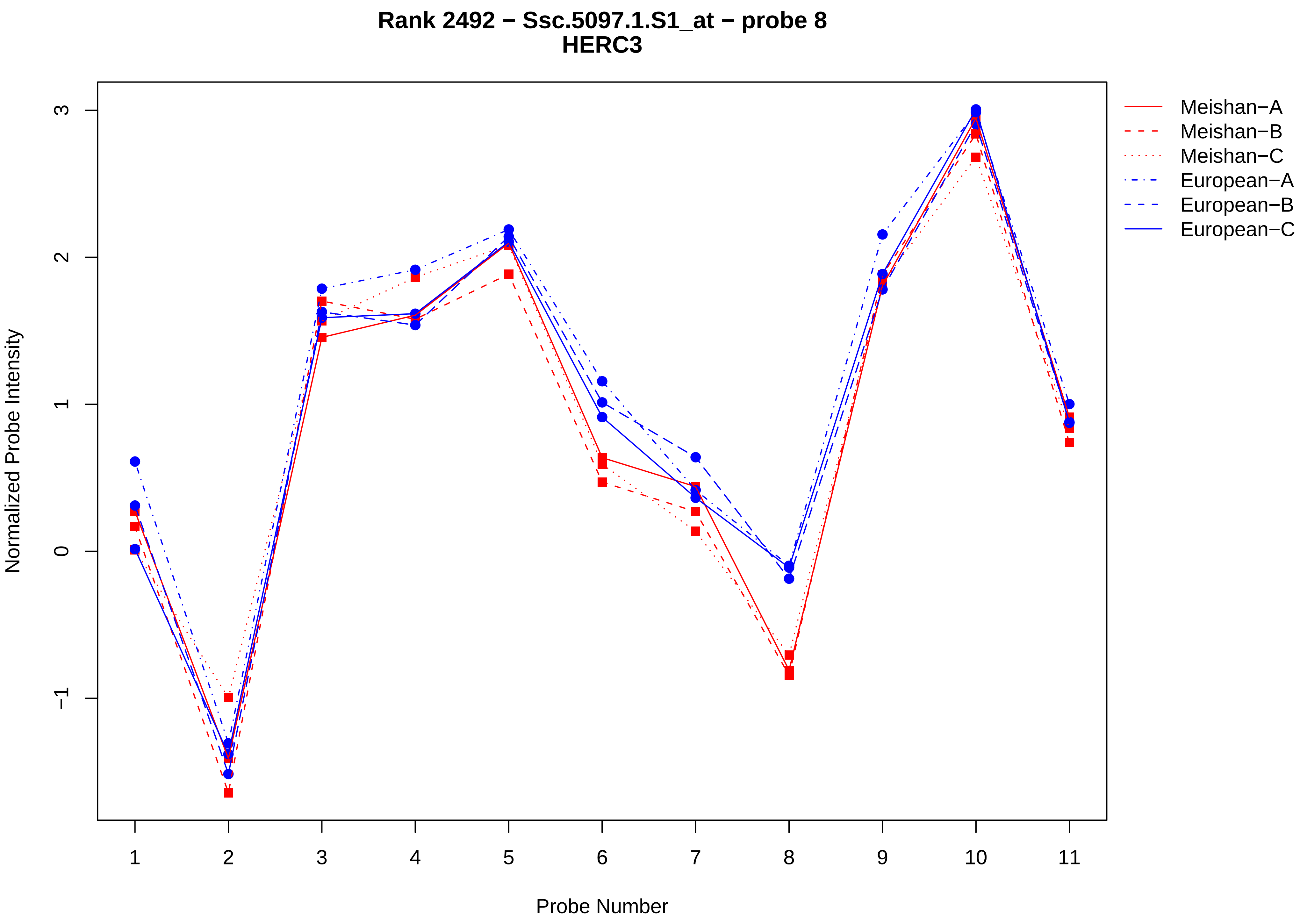

Rank 2492 − Ssc.5097.1.S1_at − probe 8
HERC3
Normalized Probe Intensity
Probe Number
−1
0
1
2
3
1
2
3
4
5
6
7
8
9
10
11
Meishan−A
Meishan−B
Meishan−C
European−A
European−B
European−C

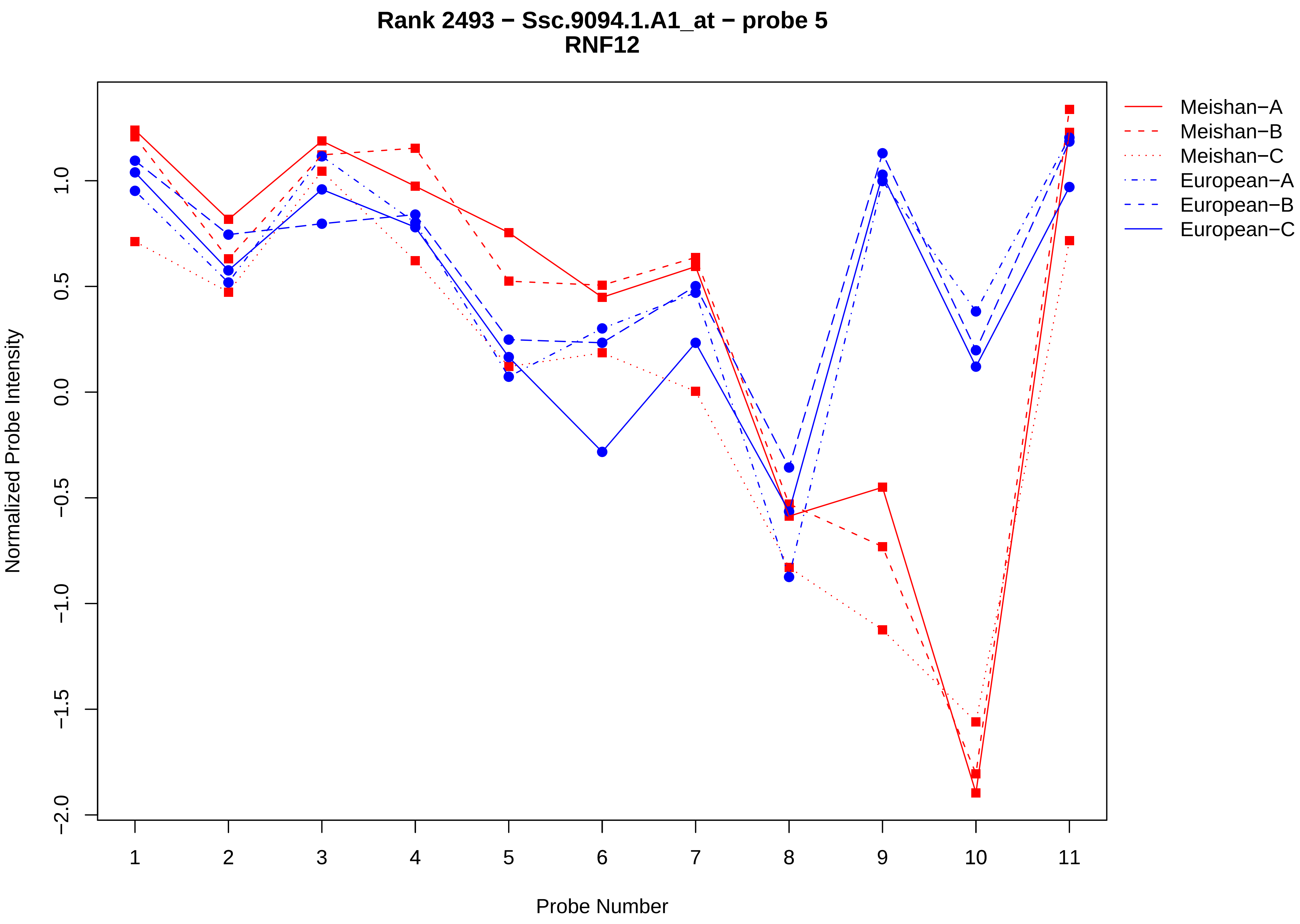

Rank 2493 − Ssc.9094.1.A1_at − probe 5
RNF12
Normalized Probe Intensity
Probe Number
1.0
0.5
0.0
−0.5
−1.0
−1.5
−2.0
1
2
3
4
5
6
7
8
9
10
11
Meishan−A
Meishan−B
Meishan−C
European−A
European−B
European−C

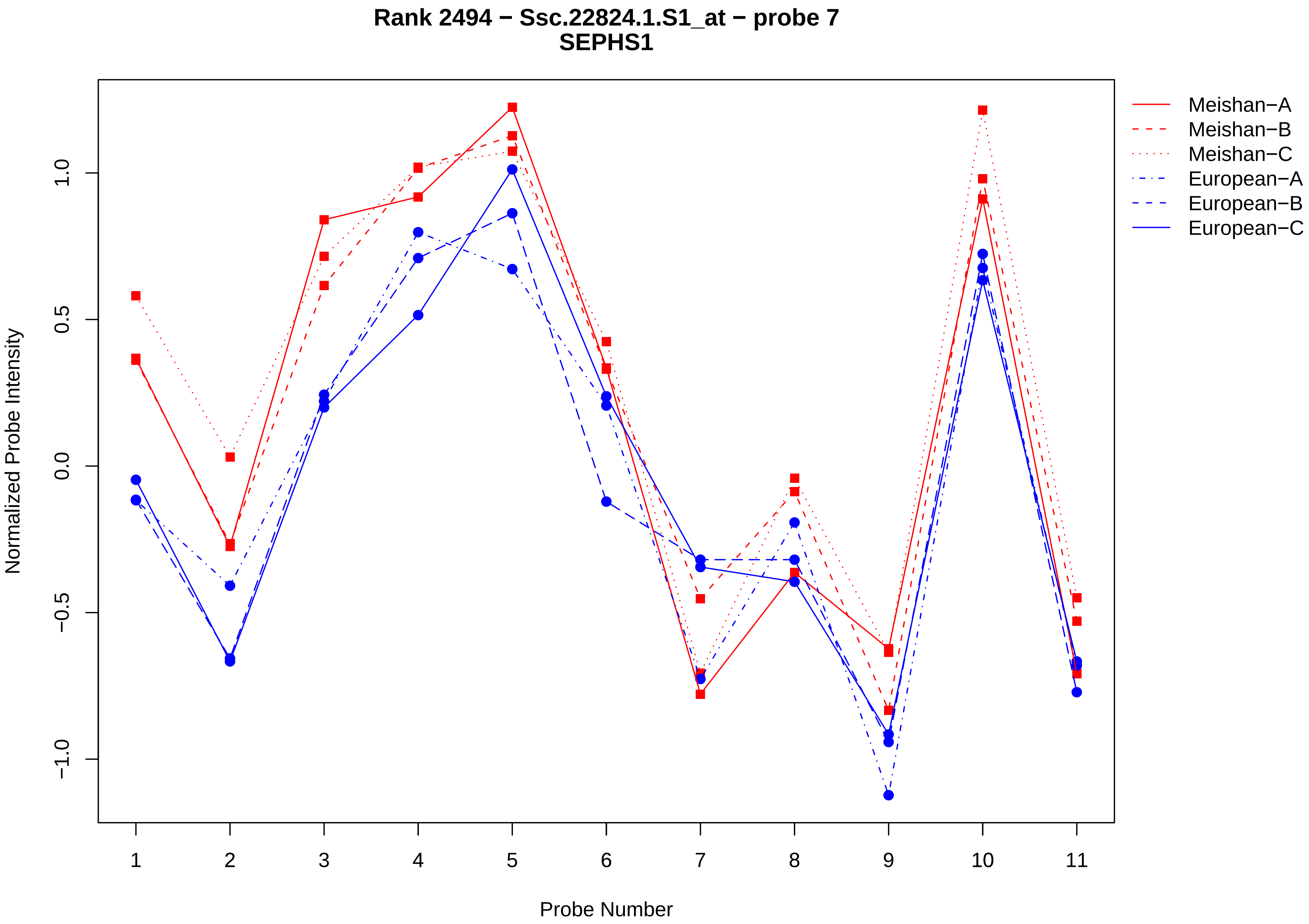

Rank 2494 − Ssc.22824.1.S1_at − probe 7
SEPHS1
Normalized Probe Intensity
Probe Number
1.0
0.5
0.0
−0.5
−1.0
1
2
3
4
5
6
7
8
9
10
11
Meishan−A
Meishan−B
Meishan−C
European−A
European−B
European−C

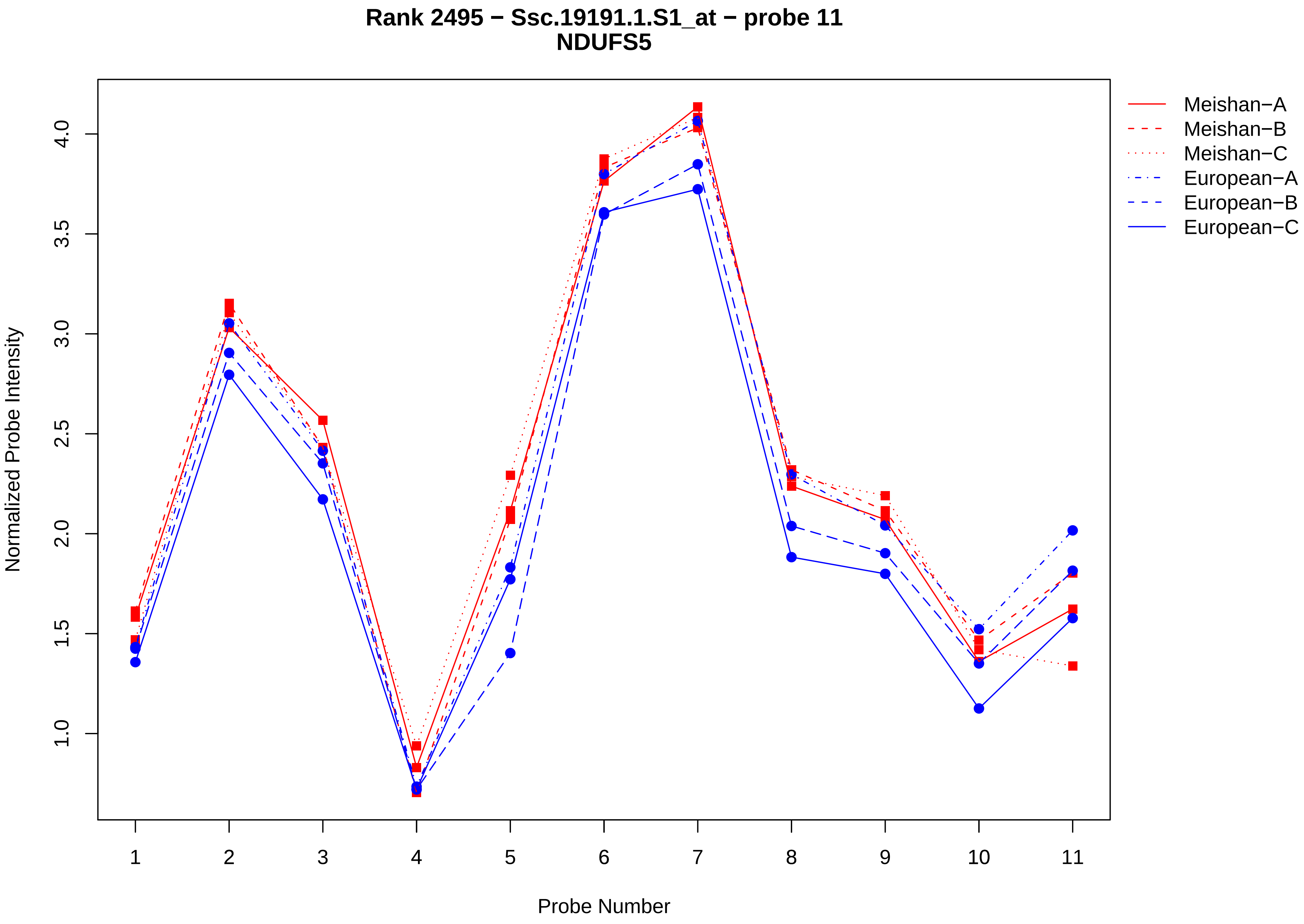

Rank 2495 − Ssc.19191.1.S1_at − probe 11
NDUFS5
Normalized Probe Intensity
Probe Number
1.0
1.5
2.0
2.5
3.0
3.5
4.0
1
2
3
4
5
6
7
8
9
10
11
Meishan−A
Meishan−B
Meishan−C
European−A
European−B
European−C

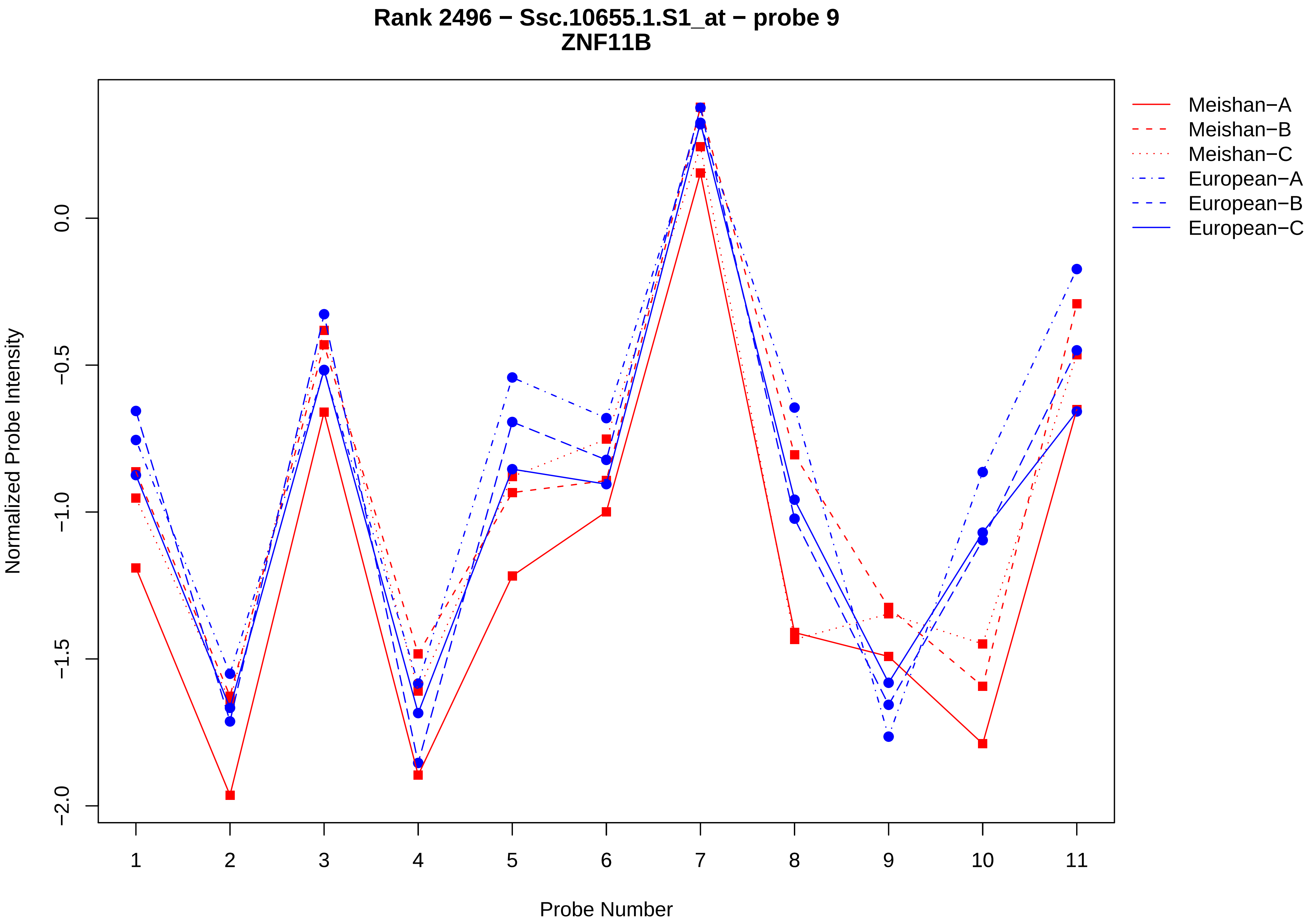
Rank 2496 − Ssc.10655.1.S1_at − probe 9
ZNF11B
Normalized Probe Intensity
Probe Number
0.0
−0.5
−1.0
−1.5
−2.0
1
2
3
4
5
6
7
8
9
10
11
Meishan−A
Meishan−B
Meishan−C
European−A
European−B
European−C

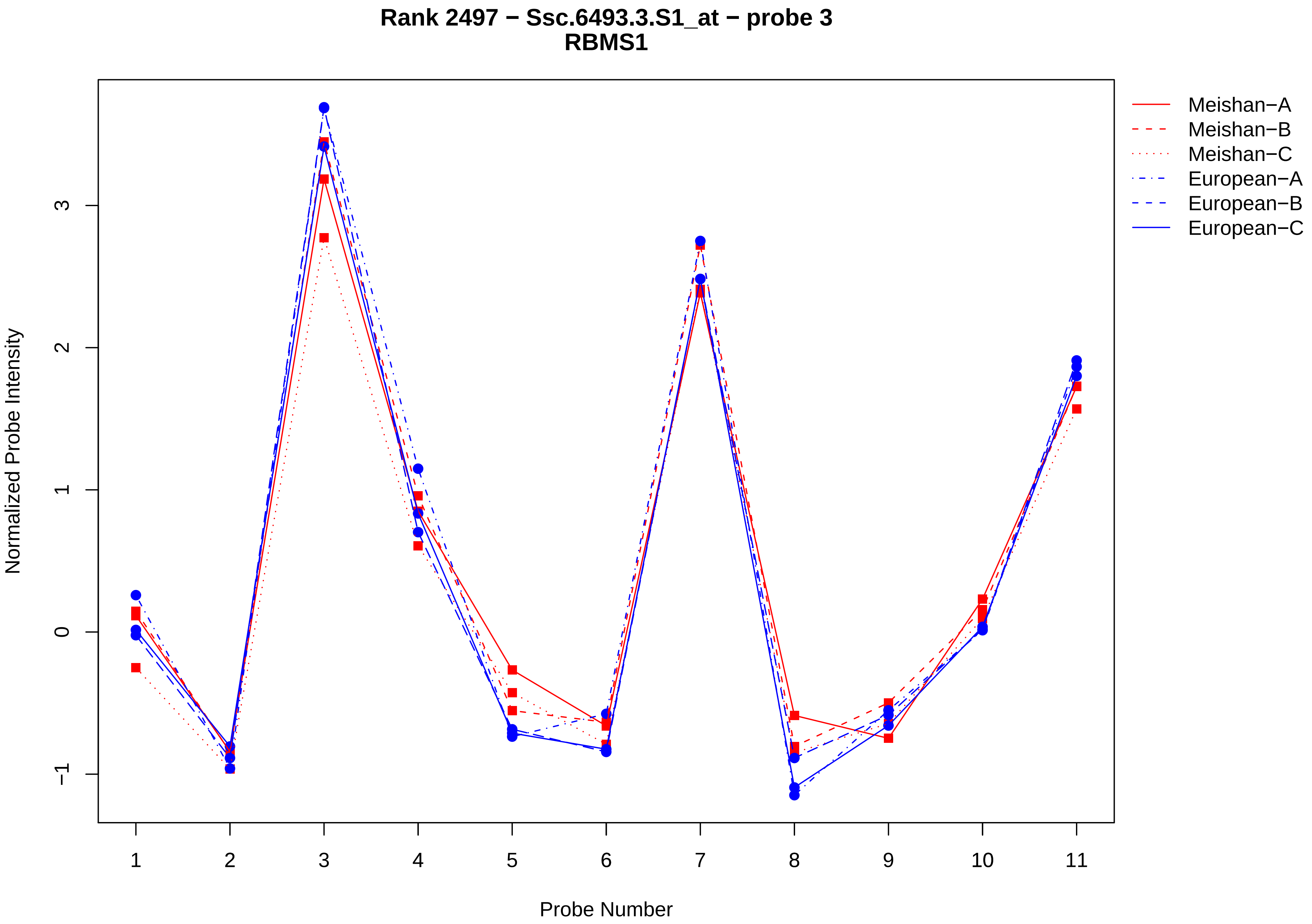

Rank 2497 − Ssc.6493.3.S1_at − probe 3
RBMS1
Normalized Probe Intensity
Probe Number
−1
0
1
2
3
1
2
3
4
5
6
7
8
9
10
11
Meishan−A
Meishan−B
Meishan−C
European−A
European−B
European−C

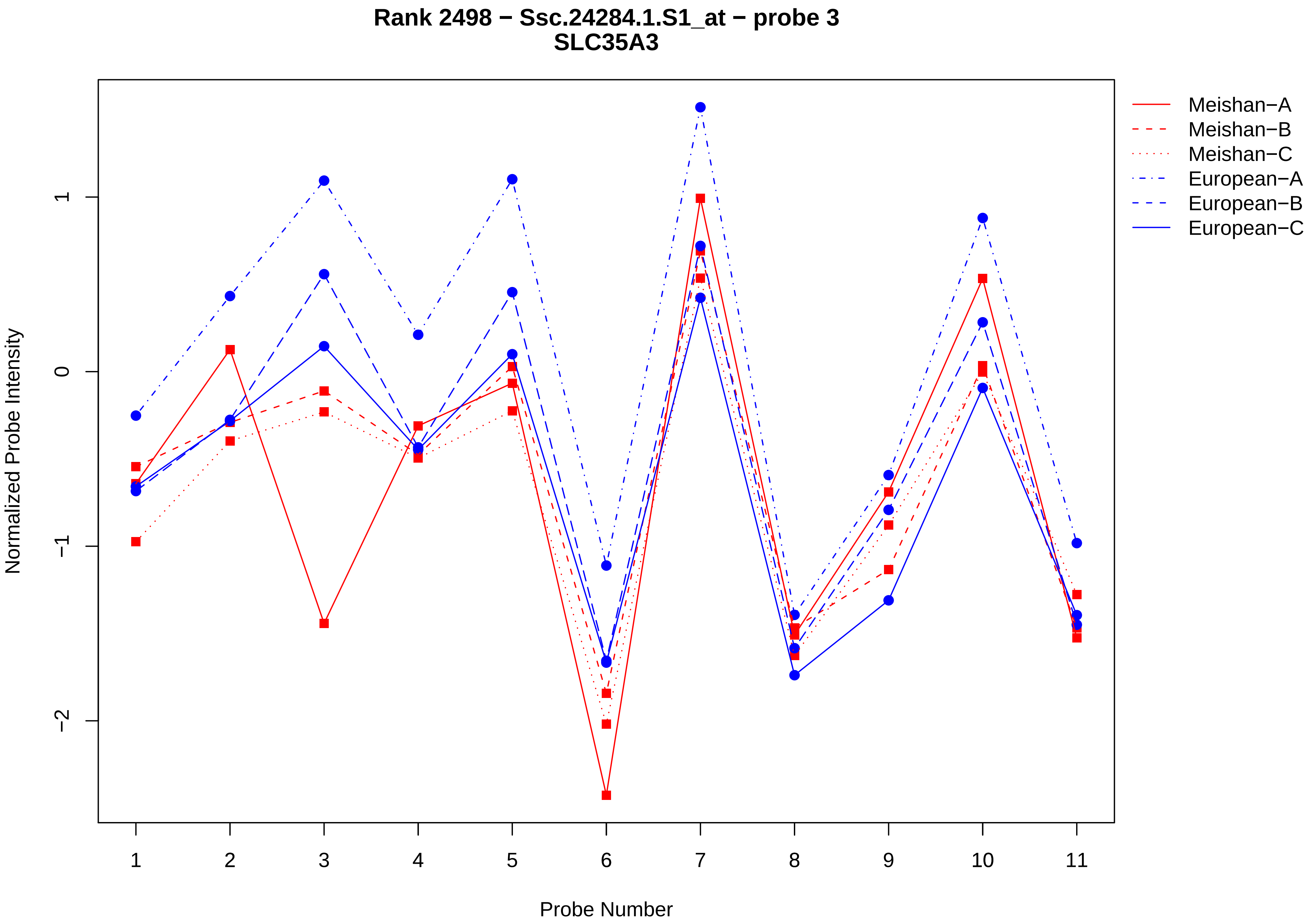

Rank 2498 − Ssc.24284.1.S1_at − probe 3
SLC35A3
Normalized Probe Intensity
Probe Number
Meishan−A
Meishan−B
Meishan−C
European−A
European−B
European−C
1
0
−1
−2
1
2
3
4
5
6
7
8
9
10
11

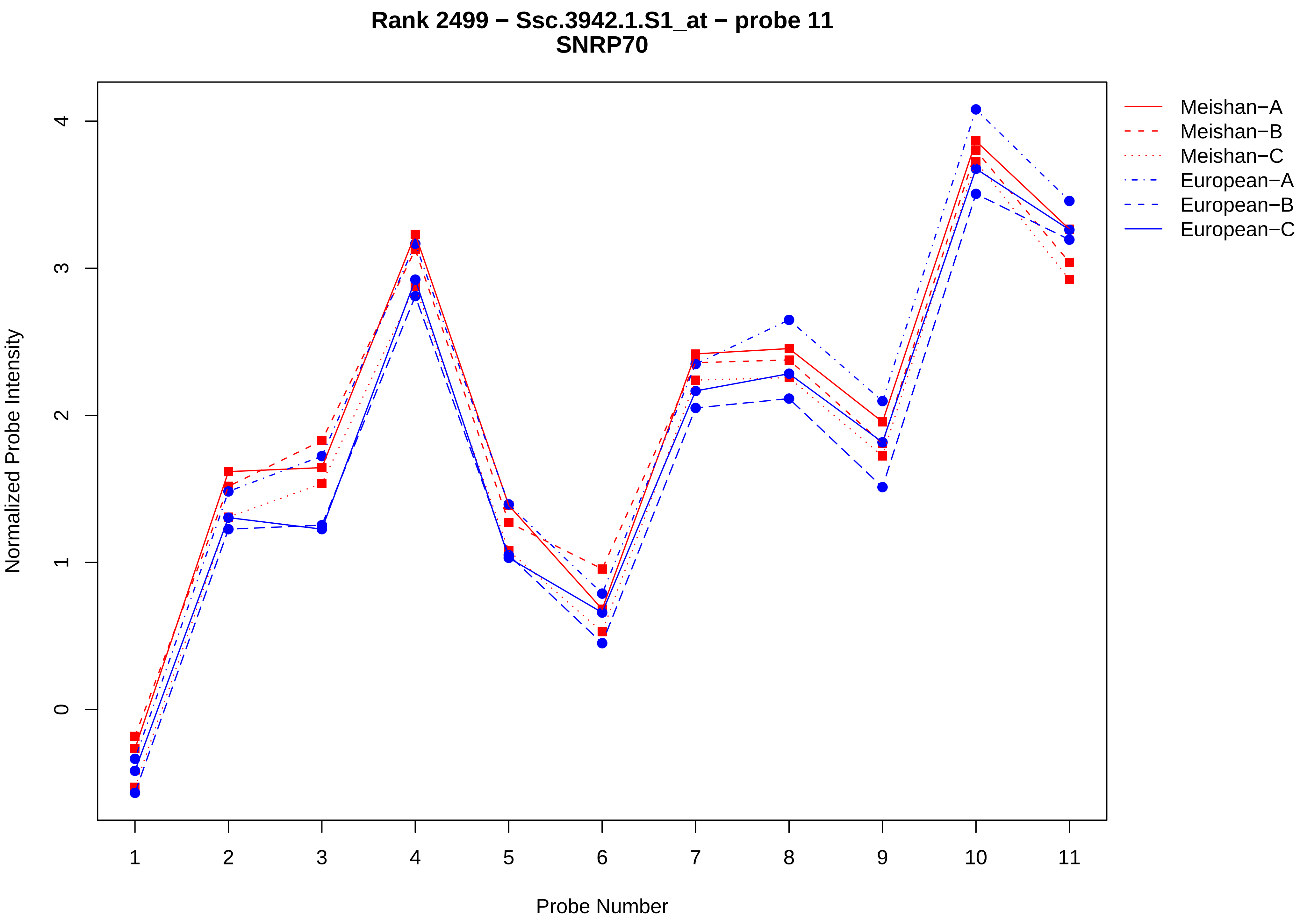

Rank 2499 − Ssc.3942.1.S1_at − probe 11
SNRP70
Normalized Probe Intensity
Probe Number
0
1
2
3
4
1
2
3
4
5
6
7
8
9
10
11
Meishan−A
Meishan−B
Meishan−C
European−A
European−B
European−C

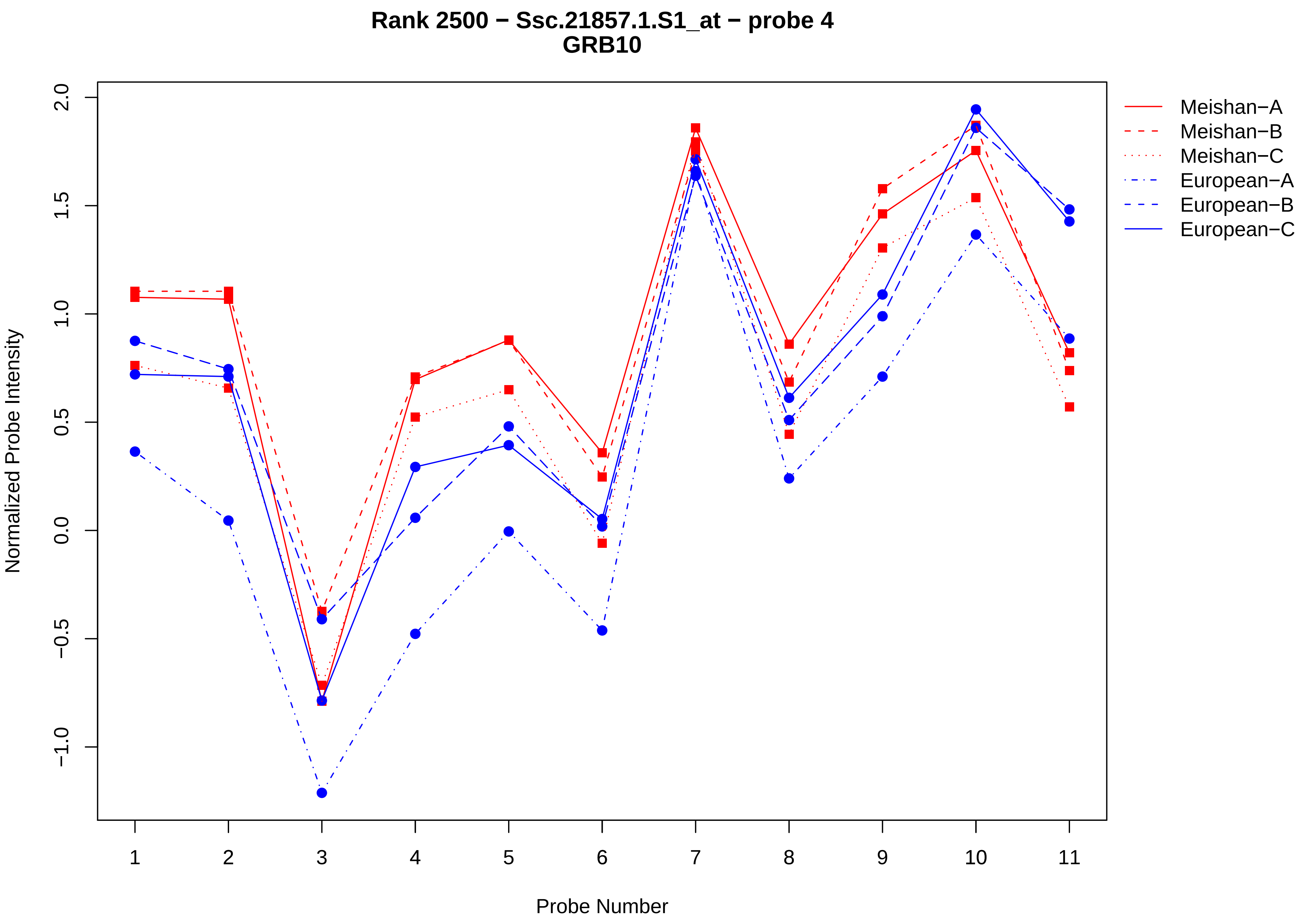
Rank 2500 − Ssc.21857.1.S1_at − probe 4
GRB10
Normalized Probe Intensity
−1.0
−0.5
0.0
0.5
1.0
1.5
2.0
Probe Number
1
2
3
4
5
6
7
8
9
10
11
Meishan−A
Meishan−B
Meishan−C
European−A
European−B
European−C

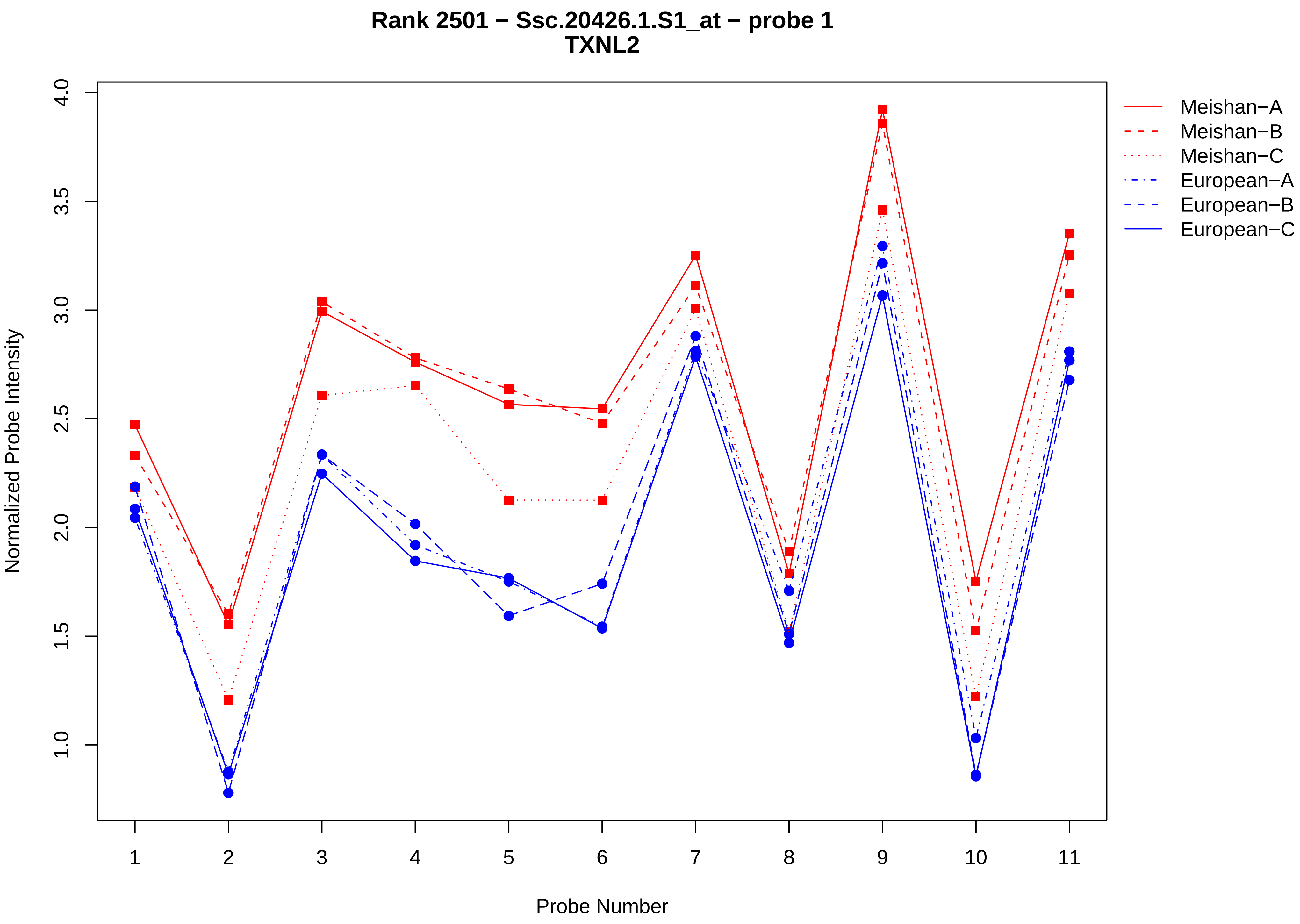
Rank 2501 − Ssc.20426.1.S1_at − probe 1
TXNL2
Normalized Probe Intensity
Probe Number
Meishan−A
Meishan−B
Meishan−C
European−A
European−B
European−C

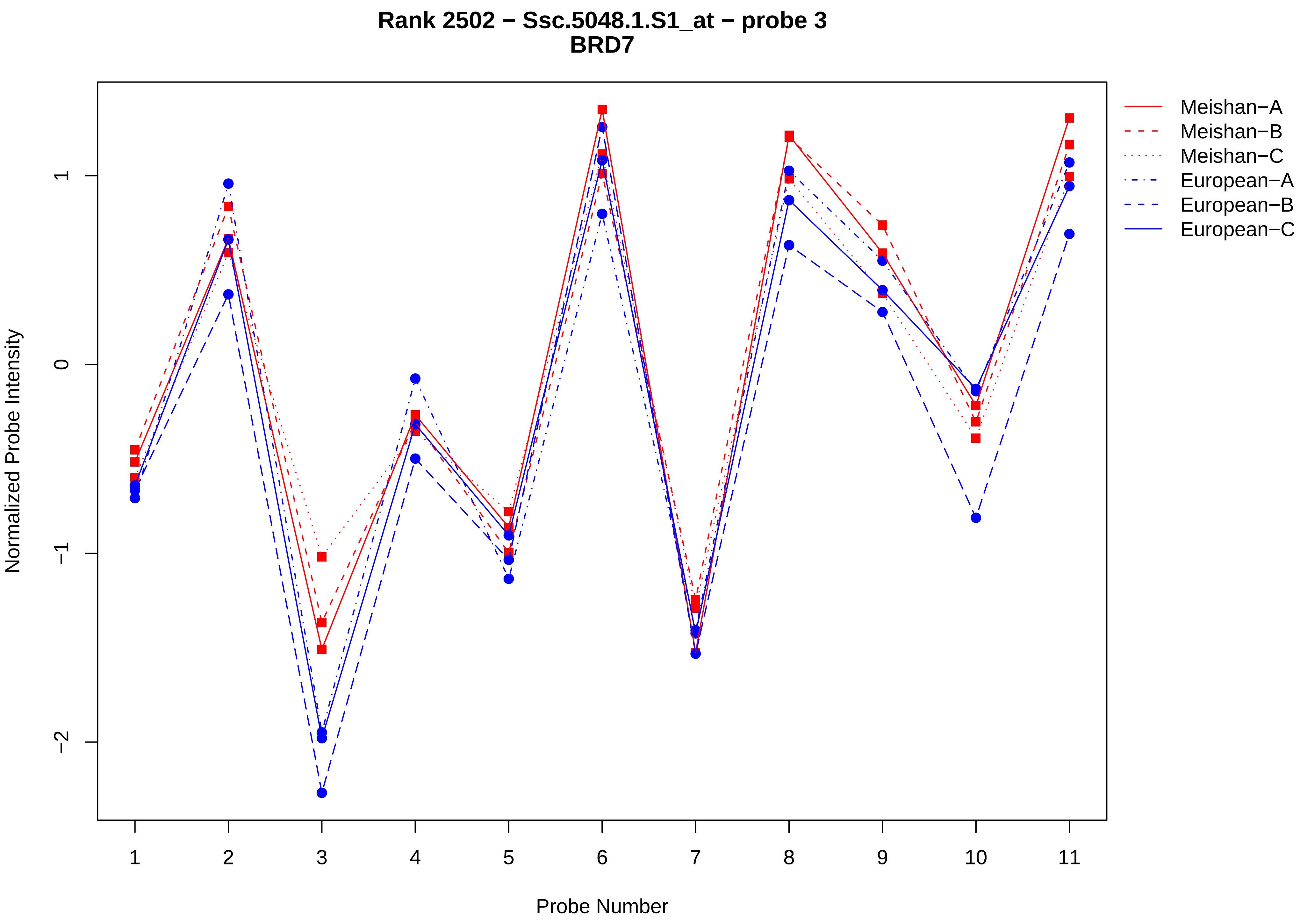
Rank 2502 − Ssc.5048.1.S1_at − probe 3
BRD7
Normalized Probe Intensity
Probe Number
Meishan−A
Meishan−B
Meishan−C
European−A
European−B
European−C
−2
−1
0
1
1
2
3
4
5
6
7
8
9
10
11

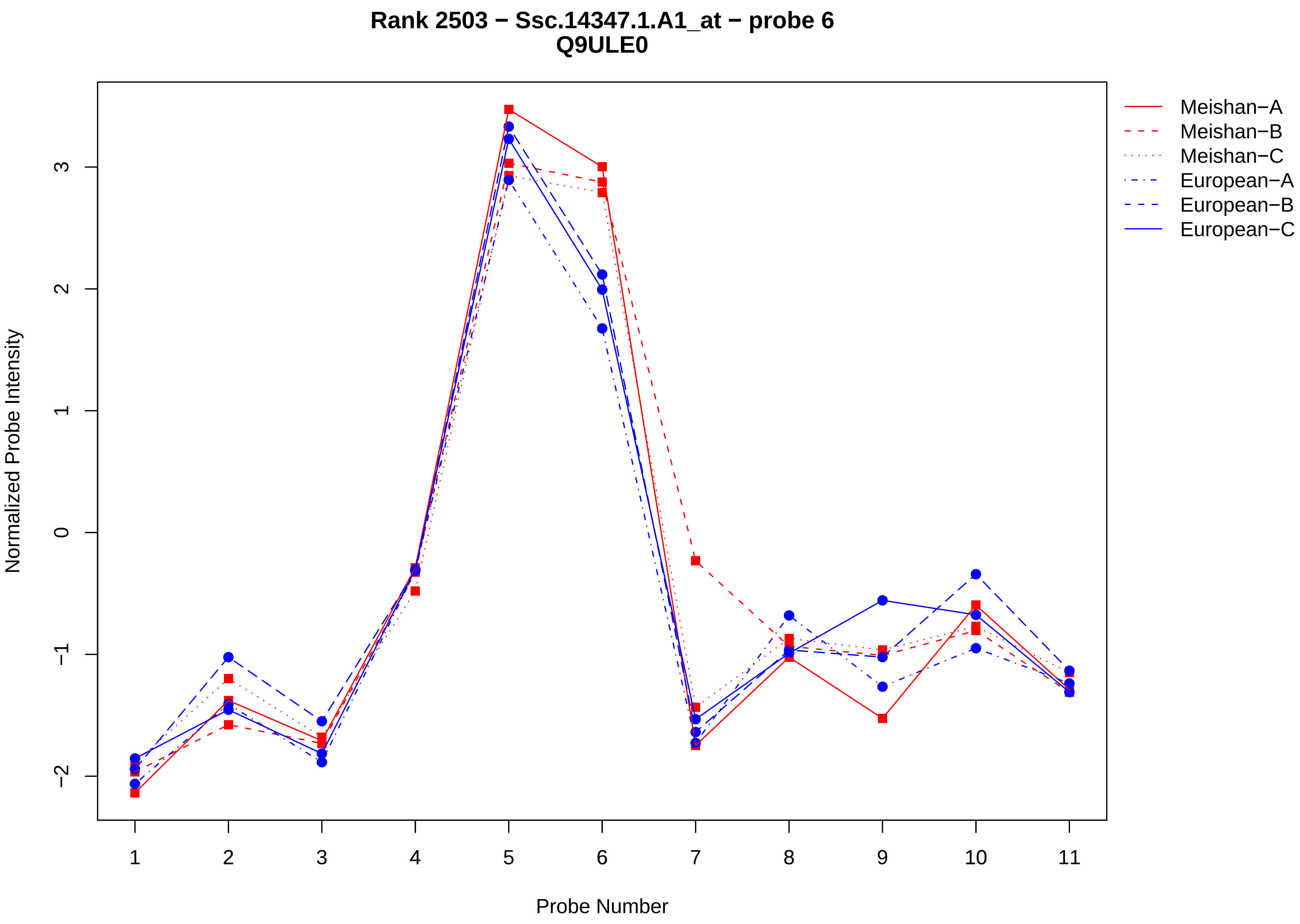

Rank 2503 − Ssc.14347.1.A1_at − probe 6
Q9ULE0
Normalized Probe Intensity
Probe Number
Meishan−A
Meishan−B
Meishan−C
European−A
European−B
European−C

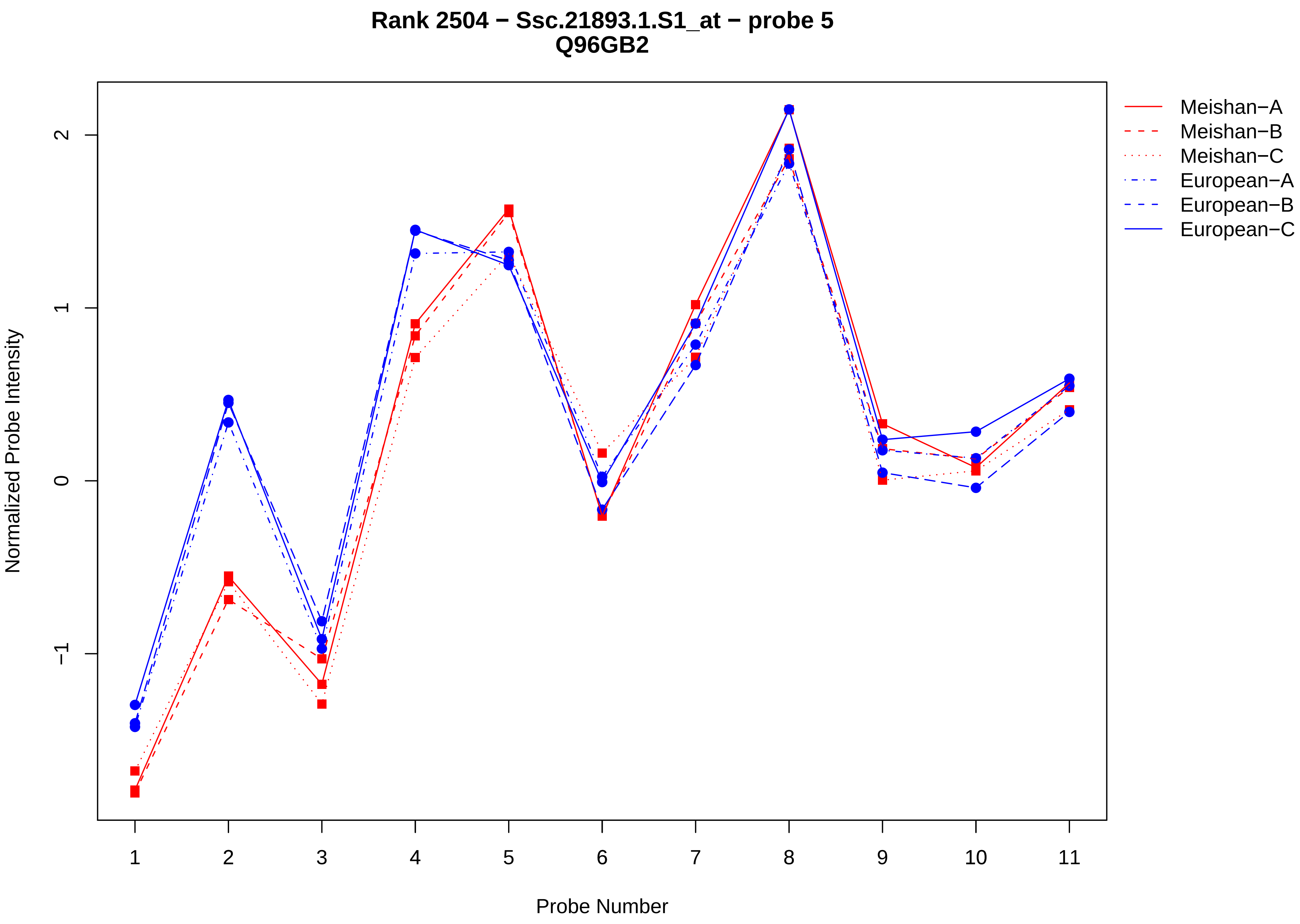
Rank 2504 − Ssc.21893.1.S1_at − probe 5
Q96GB2
Normalized Probe Intensity
Probe Number
Meishan−A
Meishan−B
Meishan−C
European−A
European−B
European−C
−1
0
1
2
1
2
3
4
5
6
7
8
9
10
11

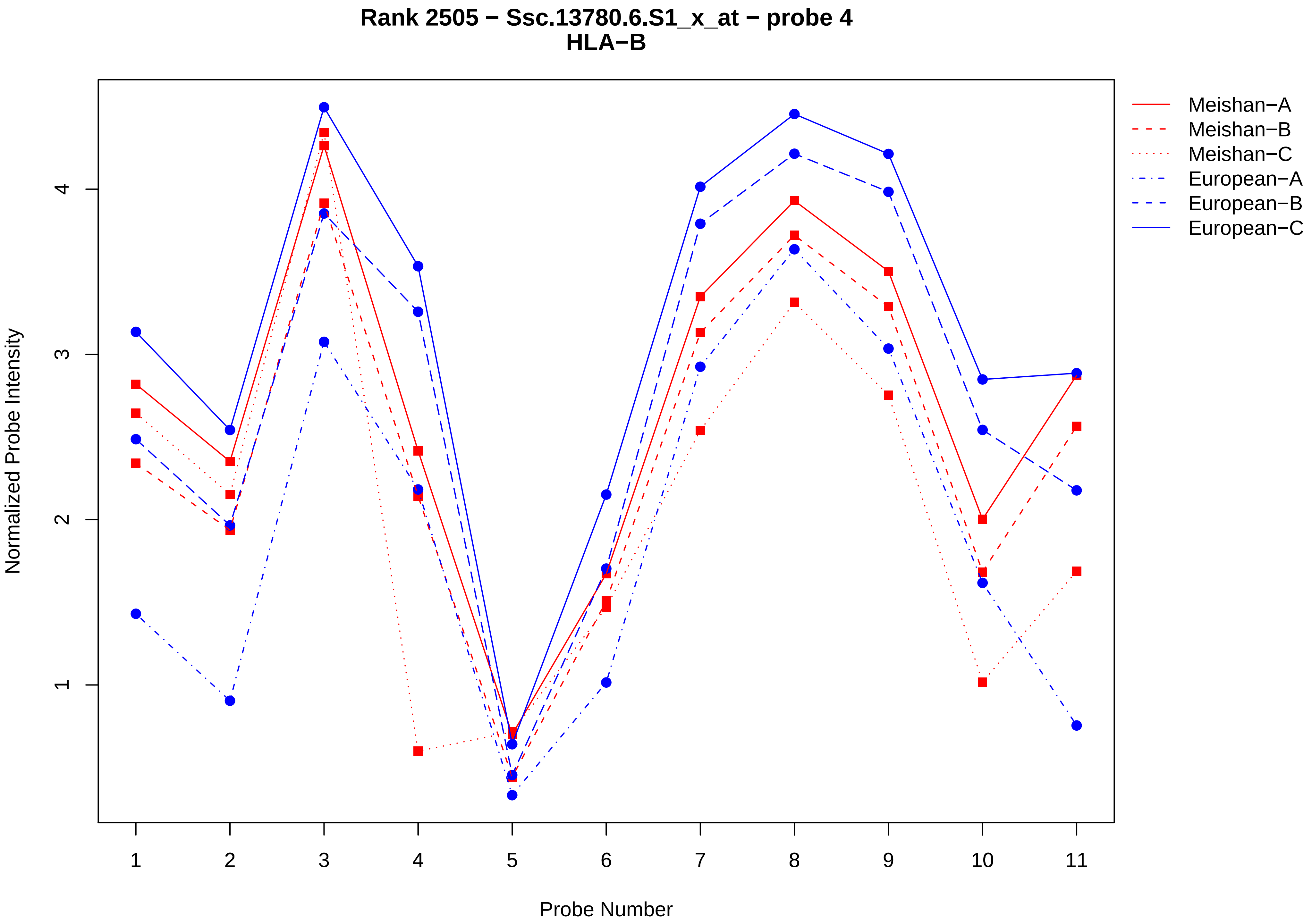

Rank 2505 − Ssc.13780.6.S1_x_at − probe 4
HLA−B
Normalized Probe Intensity
Probe Number
1
2
3
4
5
6
7
8
9
10
11
Meishan−A
Meishan−B
Meishan−C
European−A
European−B
European−C

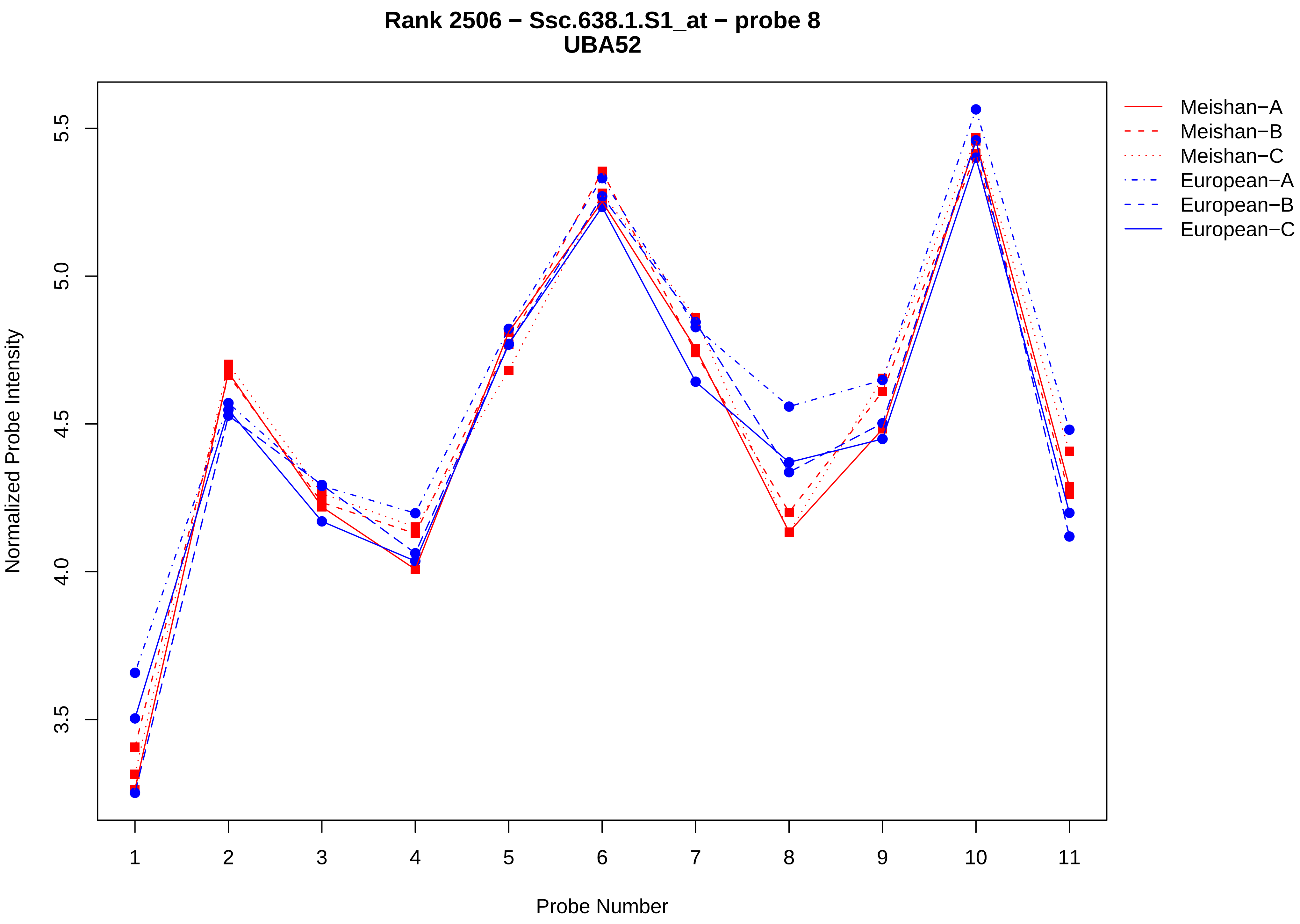

Rank 2506 − Ssc.638.1.S1_at − probe 8
UBA52
Normalized Probe Intensity
3.5
4.0
4.5
5.0
5.5
1
2
3
4
5
6
7
8
9
10
11
Probe Number
Meishan−A
Meishan−B
Meishan−C
European−A
European−B
European−C

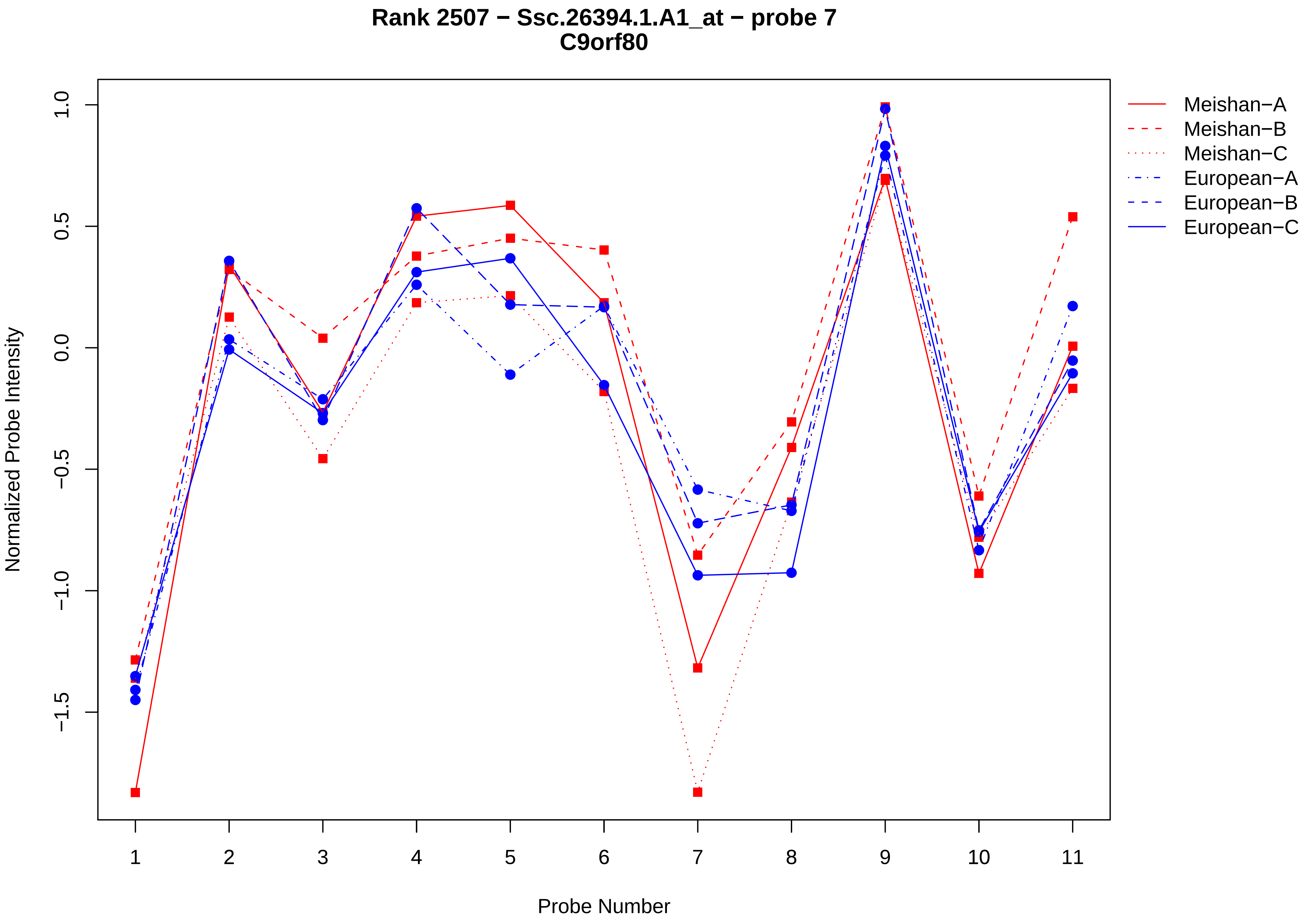

Rank 2507 − Ssc.26394.1.A1_at − probe 7
C9orf80
Normalized Probe Intensity
Probe Number
Meishan−A
Meishan−B
Meishan−C
European−A
European−B
European−C

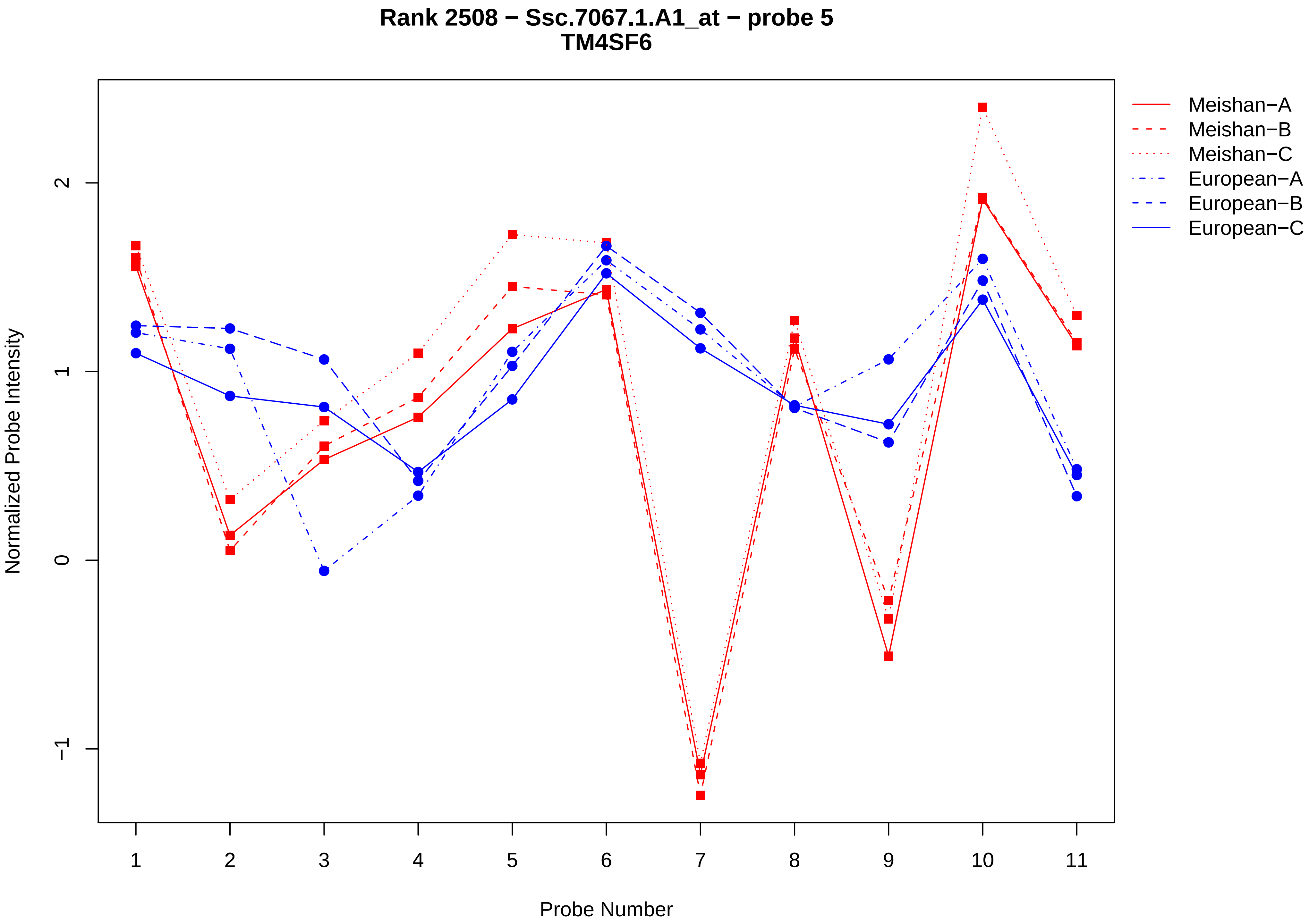

Rank 2508 − Ssc.7067.1.A1_at − probe 5
TM4SF6
Normalized Probe Intensity
Probe Number
−1
0
1
2
1
2
3
4
5
6
7
8
9
10
11
Meishan−A
Meishan−B
Meishan−C
European−A
European−B
European−C

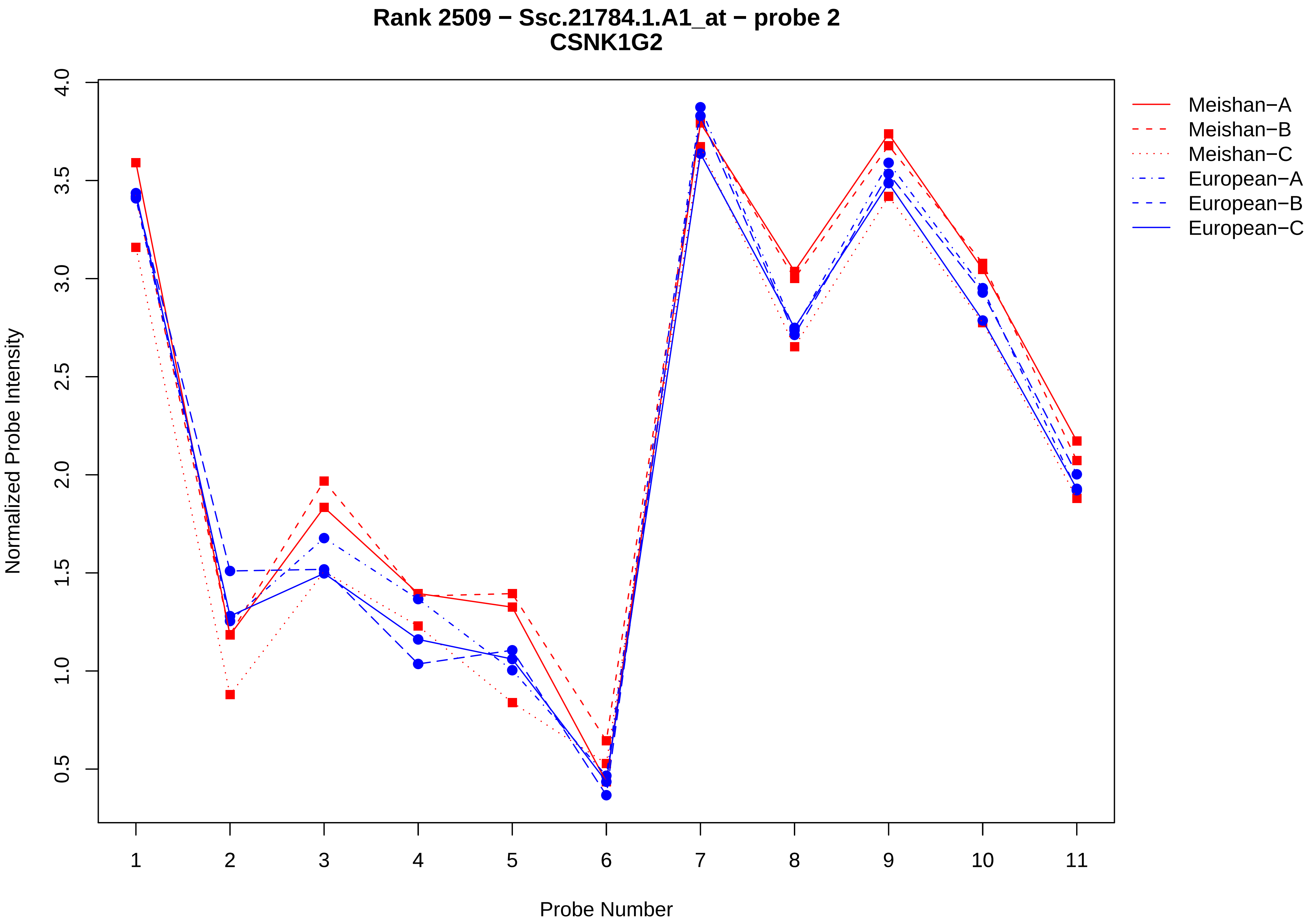
Rank 2509 − Ssc.21784.1.A1_at − probe 2
CSNK1G2
Normalized Probe Intensity
Probe Number
0.5
1.0
1.5
2.0
2.5
3.0
3.5
4.0
1
2
3
4
5
6
7
8
9
10
11
Meishan−A
Meishan−B
Meishan−C
European−A
European−B
European−C

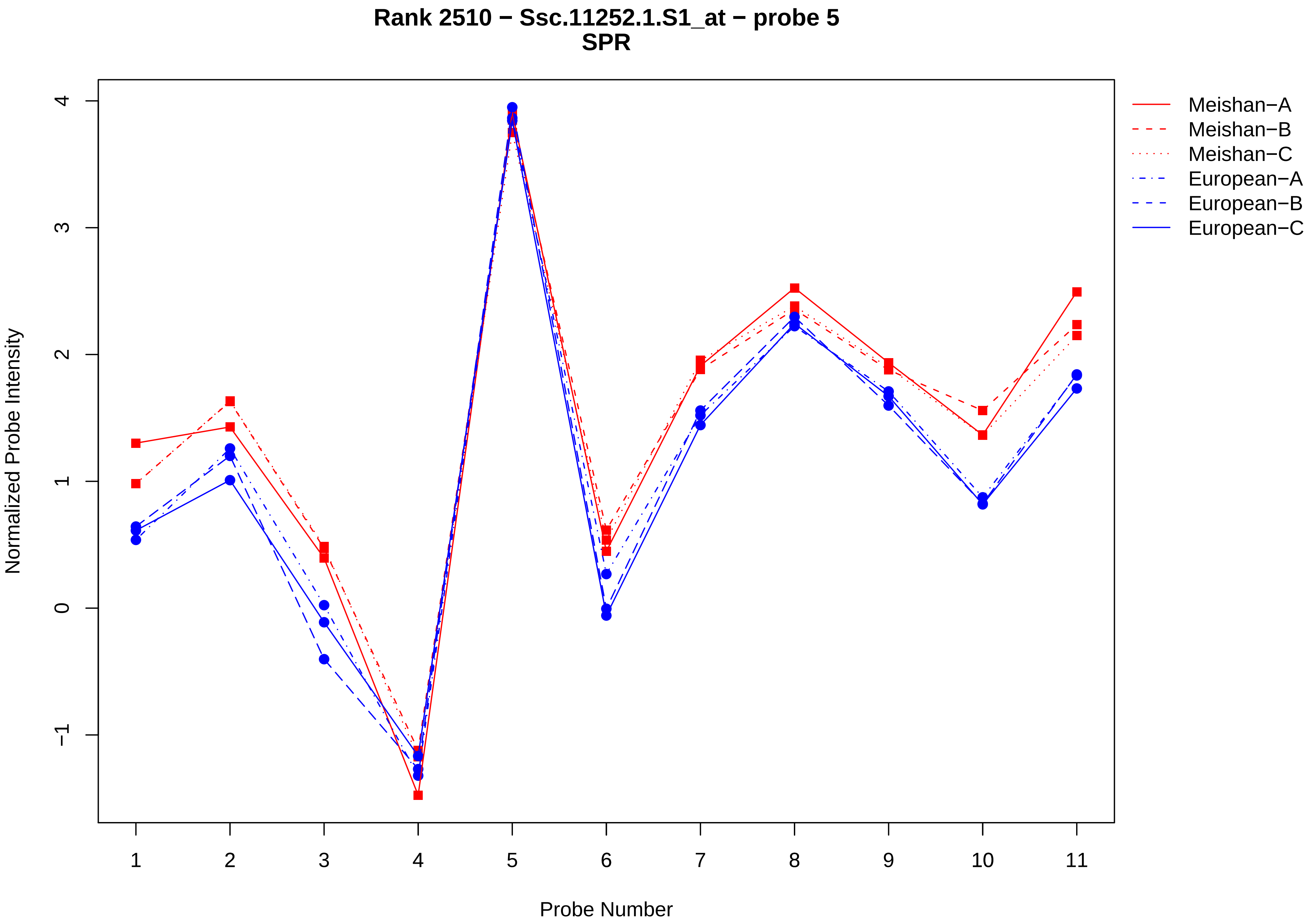

Rank 2510 − Ssc.11252.1.S1_at − probe 5
SPR
Normalized Probe Intensity
Probe Number
−1
0
1
2
3
4
1
2
3
4
5
6
7
8
9
10
11
Meishan−A
Meishan−B
Meishan−C
European−A
European−B
European−C

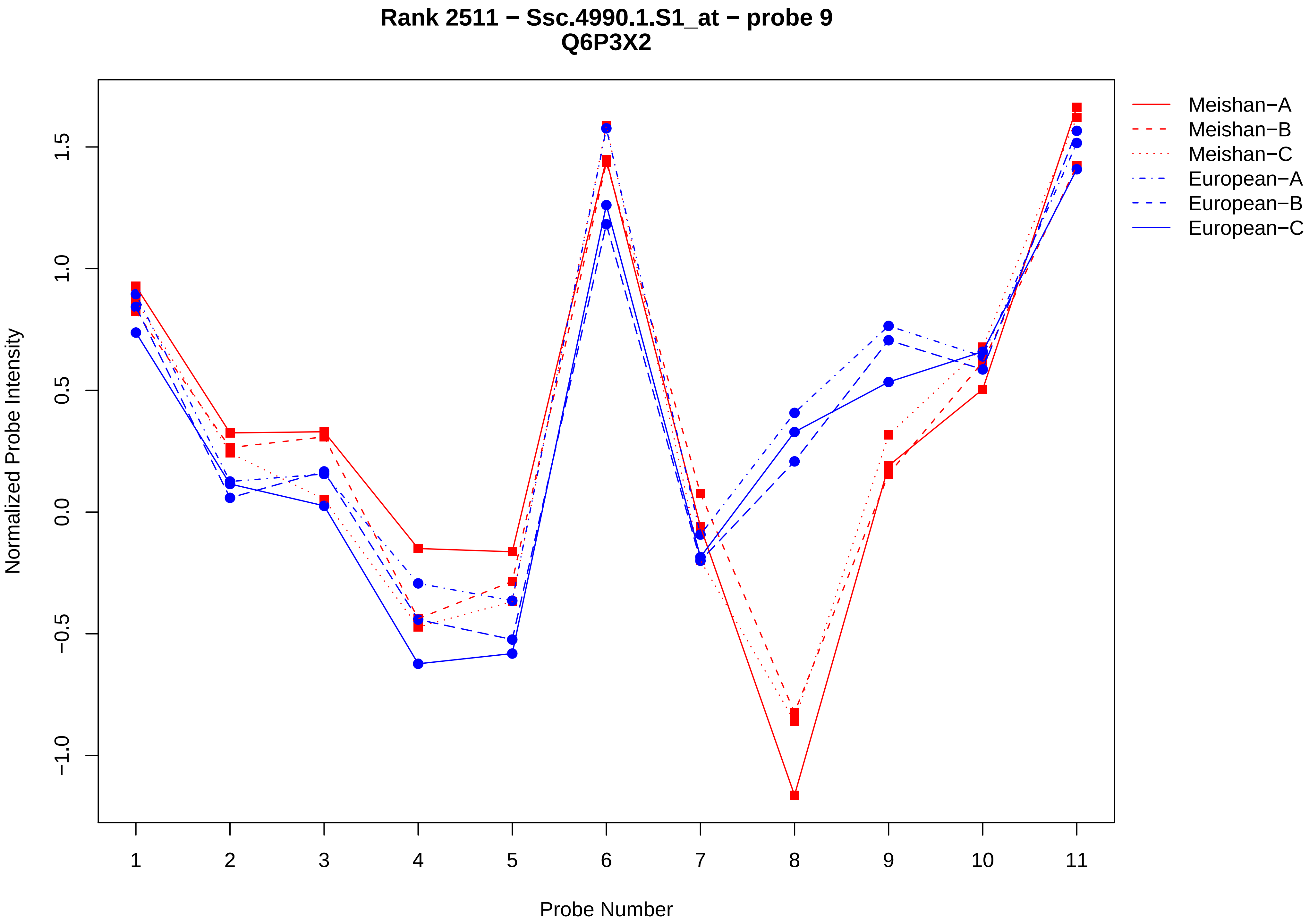
Rank 2511 − Ssc.4990.1.S1_at − probe 9
Q6P3X2
Normalized Probe Intensity
Probe Number
−1.0
−0.5
0.0
0.5
1.0
1.5
1
2
3
4
5
6
7
8
9
10
11
Meishan−A
Meishan−B
Meishan−C
European−A
European−B
European−C

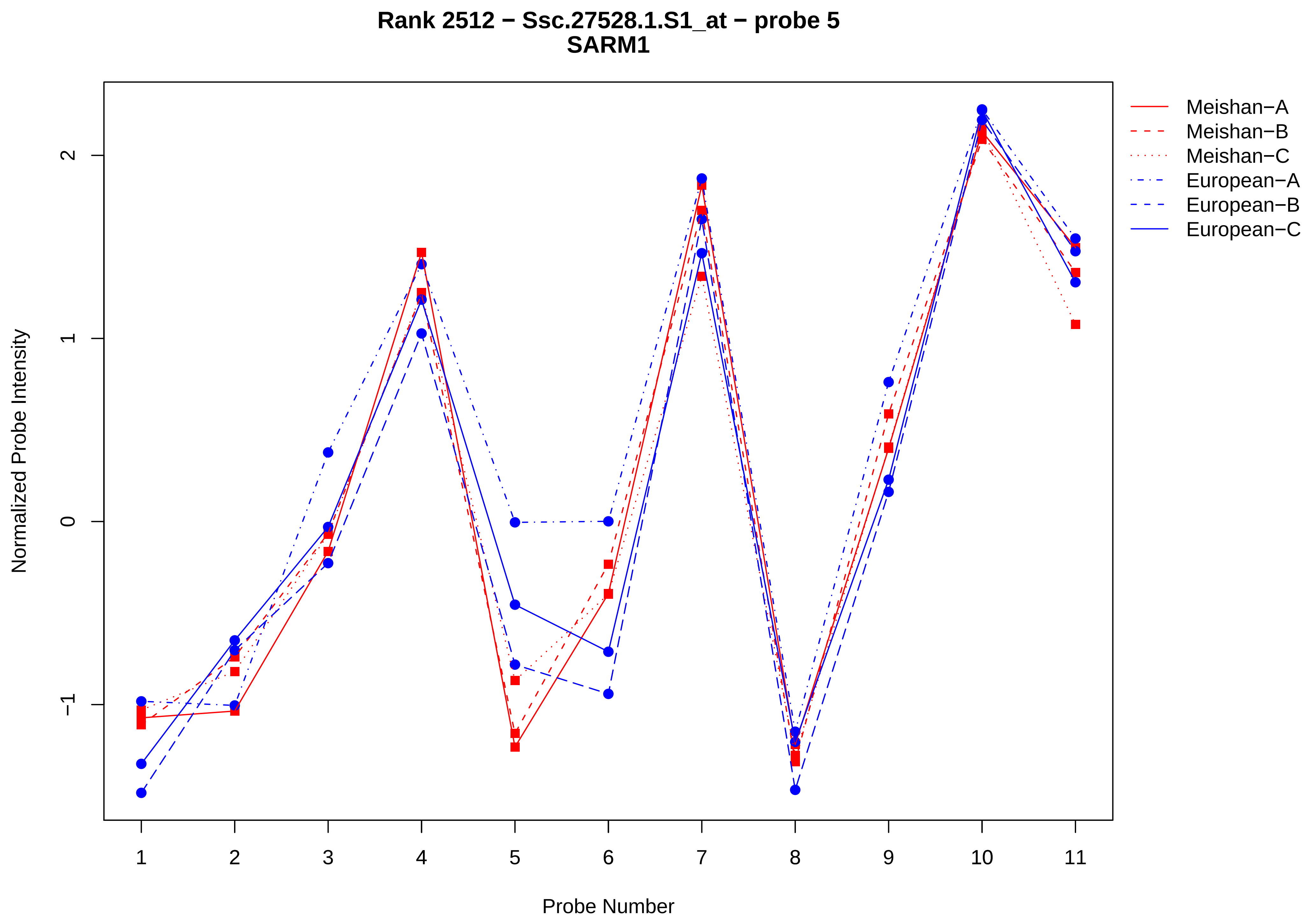

Rank 2512 − Ssc.27528.1.S1_at − probe 5
SARM1
Normalized Probe Intensity
Probe Number
−1
0
1
2
1
2
3
4
5
6
7
8
9
10
11
Meishan−A
Meishan−B
Meishan−C
European−A
European−B
European−C

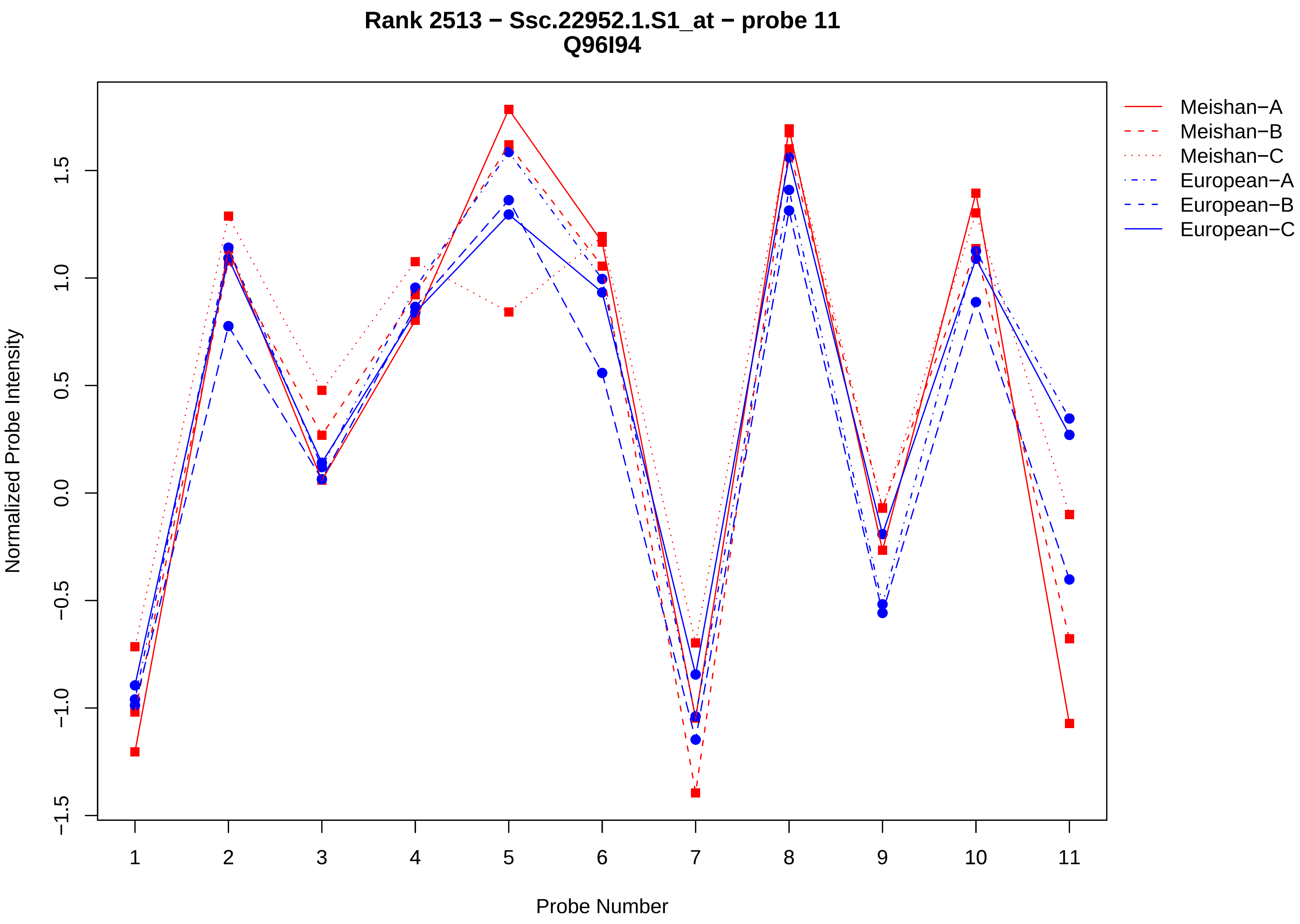

Rank 2513 − Ssc.22952.1.S1_at − probe 11
Q96I94
Normalized Probe Intensity
Probe Number
Meishan−A
Meishan−B
Meishan−C
European−A
European−B
European−C
−1.5
−1.0
−0.5
0.0
0.5
1.0
1.5
1
2
3
4
5
6
7
8
9
10
11

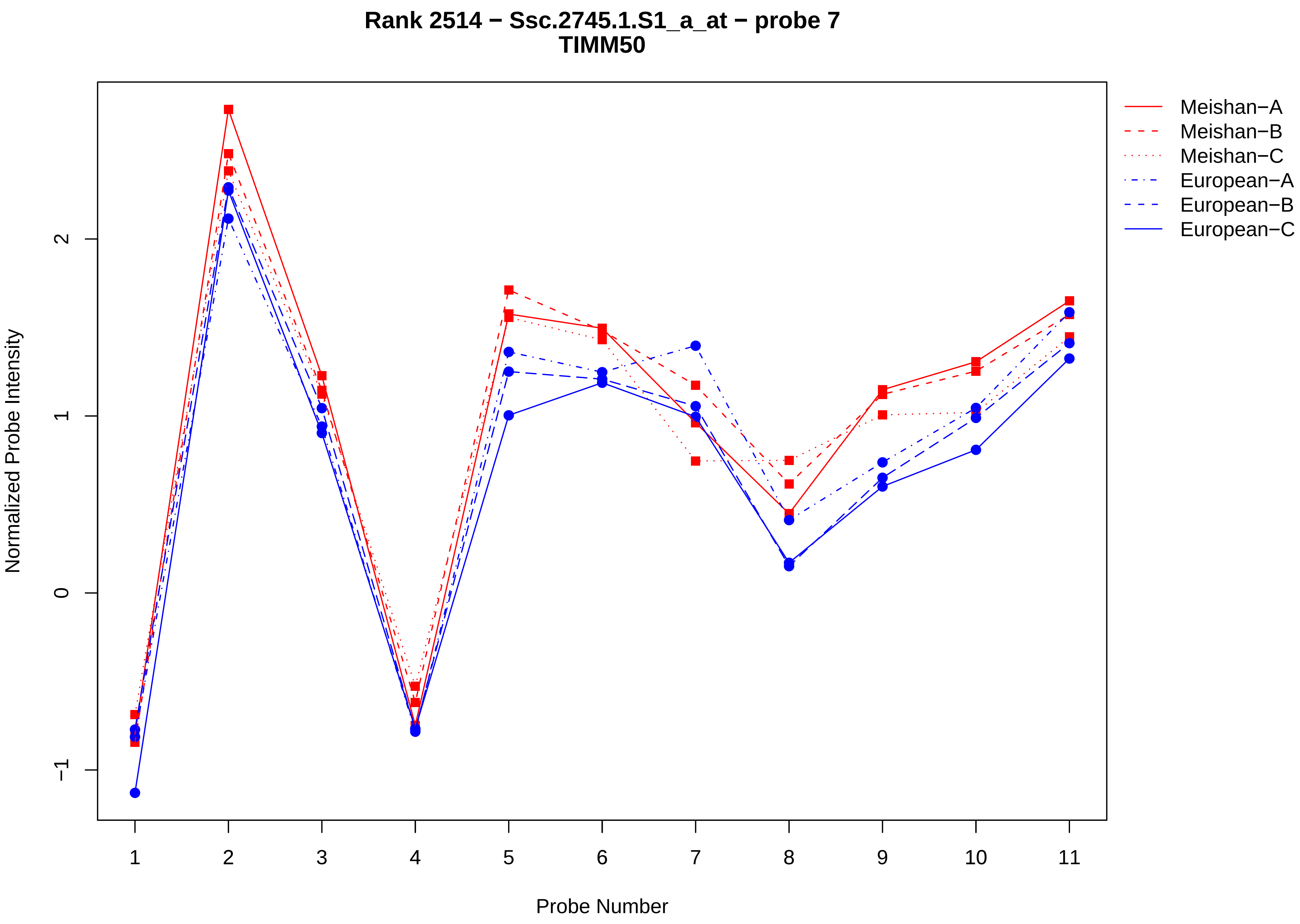
Rank 2514 − Ssc.2745.1.S1_a_at − probe 7
TIMM50
Normalized Probe Intensity
Probe Number
−1
0
1
2
1
2
3
4
5
6
7
8
9
10
11
Meishan−A
Meishan−B
Meishan−C
European−A
European−B
European−C

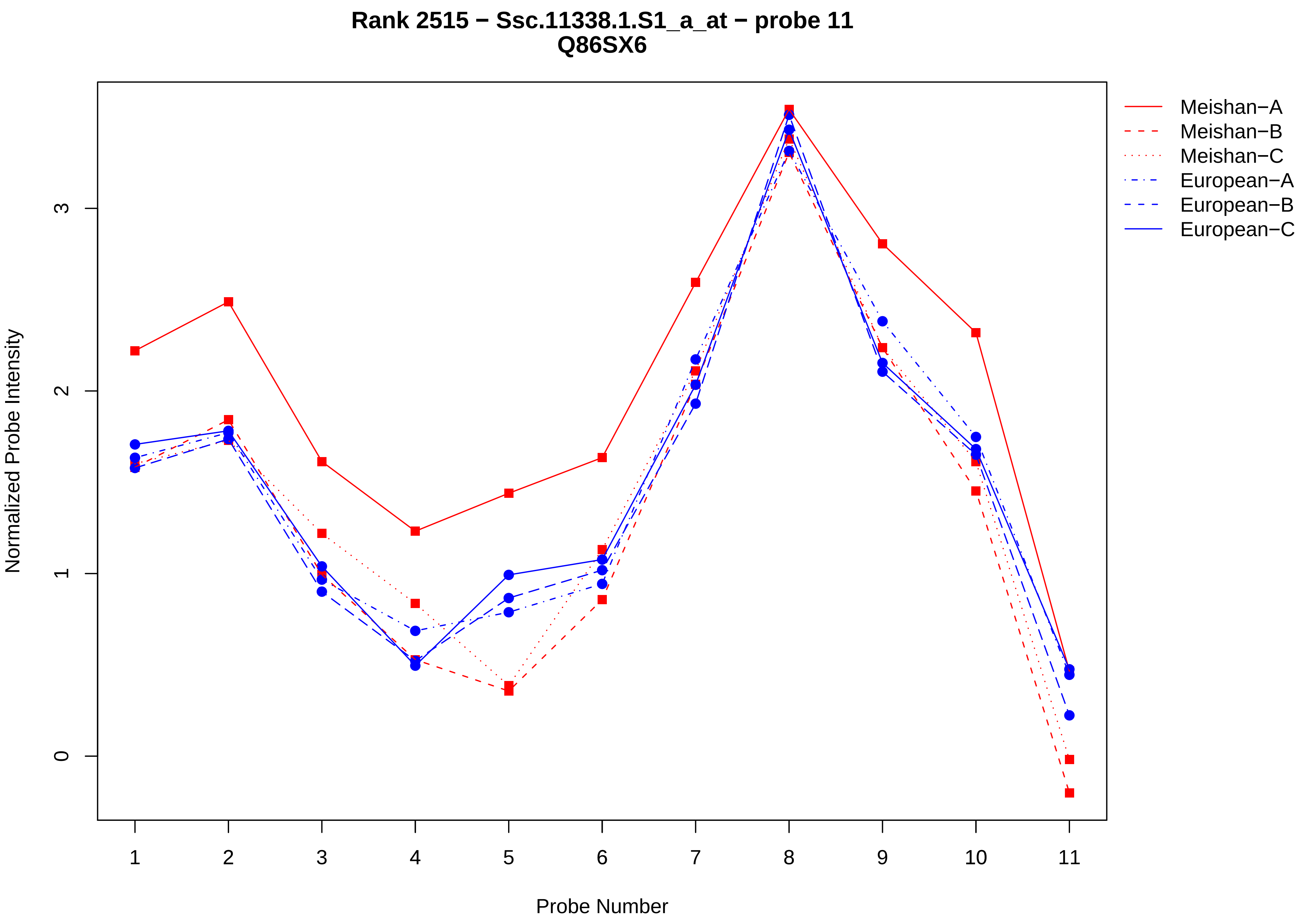

Rank 2515 − Ssc.11338.1.S1_a_at − probe 11
Q86SX6
Normalized Probe Intensity
Probe Number
0
1
2
3
1
2
3
4
5
6
7
8
9
10
11
Meishan−A
Meishan−B
Meishan−C
European−A
European−B
European−C

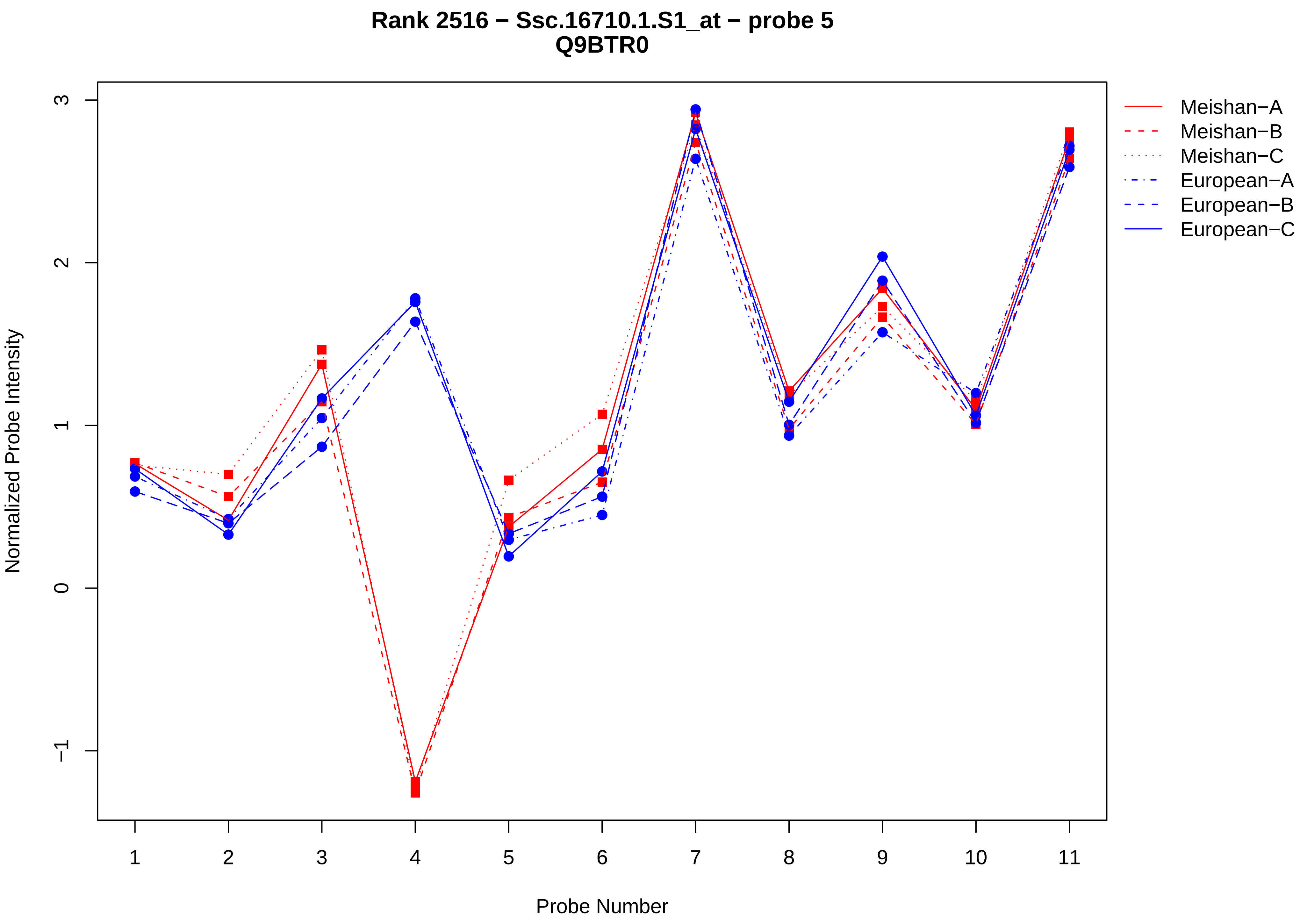

Rank 2516 − Ssc.16710.1.S1_at − probe 5
Q9BTR0
Normalized Probe Intensity
Probe Number
−1
0
1
2
3
1
2
3
4
5
6
7
8
9
10
11
Meishan−A
Meishan−B
Meishan−C
European−A
European−B
European−C

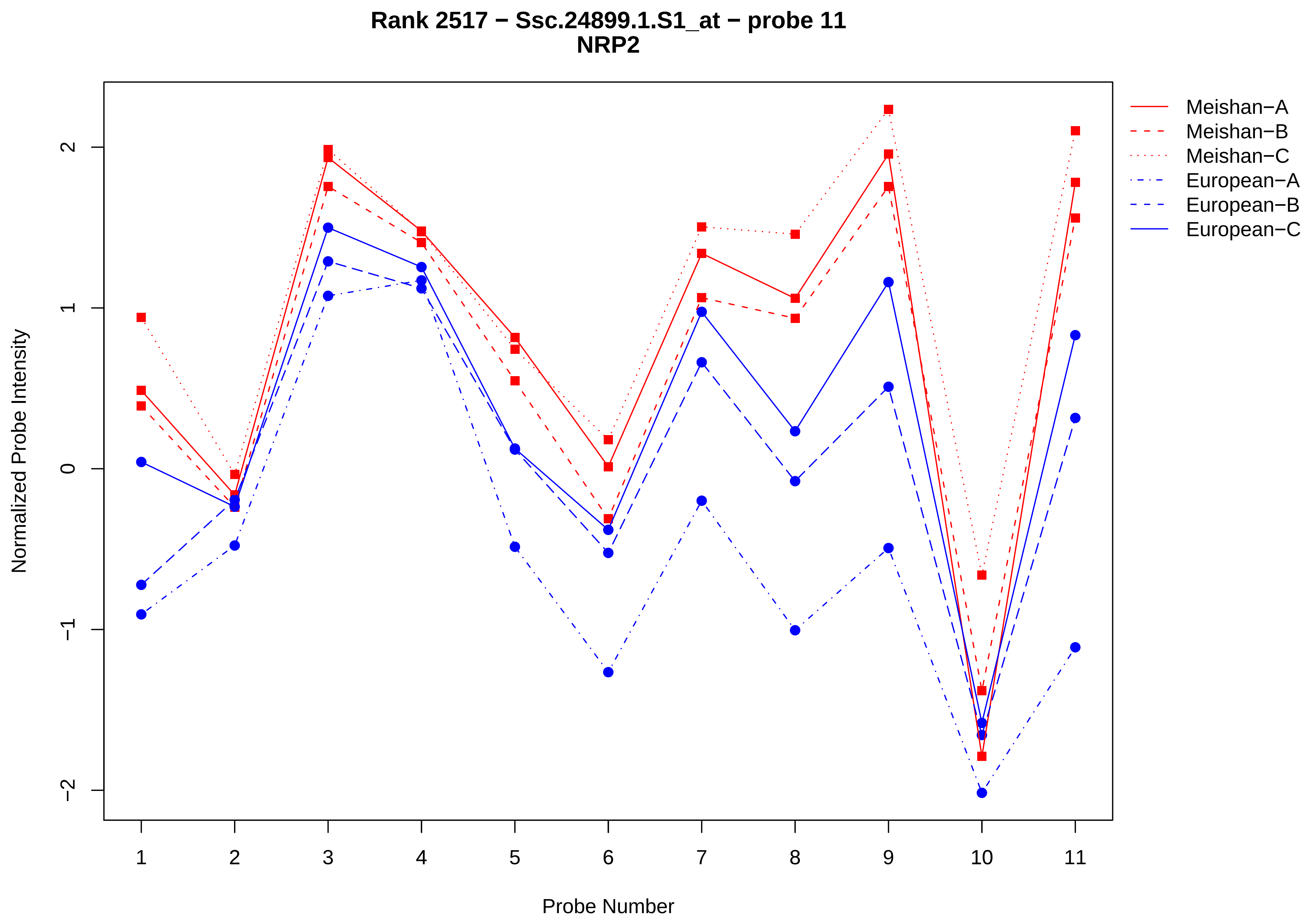
Rank 2517 − Ssc.24899.1.S1_at − probe 11
NRP2
Normalized Probe Intensity
Probe Number
Meishan−A
Meishan−B
Meishan−C
European−A
European−B
European−C
−2
−1
0
1
2
1
2
3
4
5
6
7
8
9
10
11

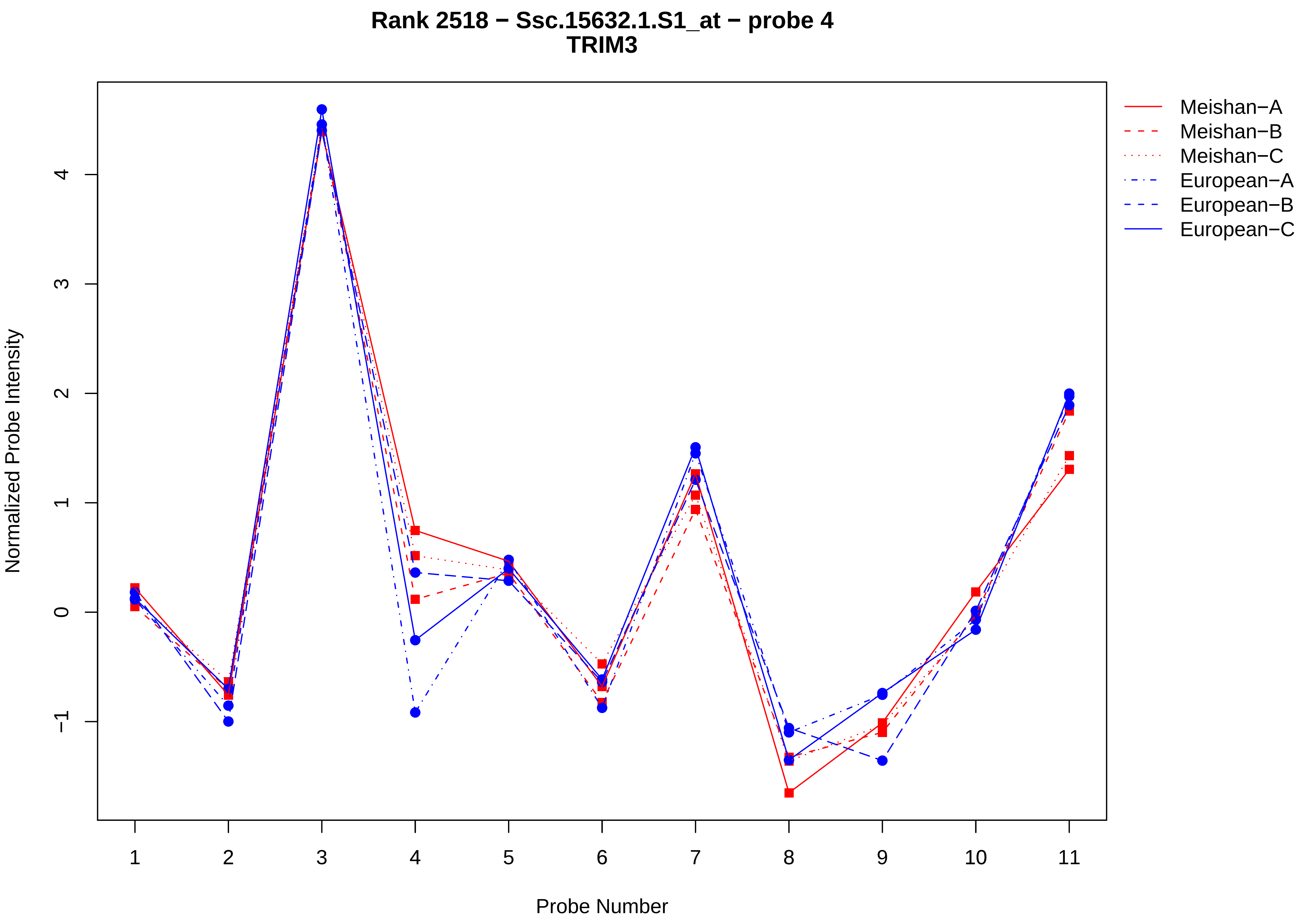

Rank 2518 − Ssc.15632.1.S1_at − probe 4
TRIM3
Normalized Probe Intensity
Probe Number
Meishan−A
Meishan−B
Meishan−C
European−A
European−B
European−C

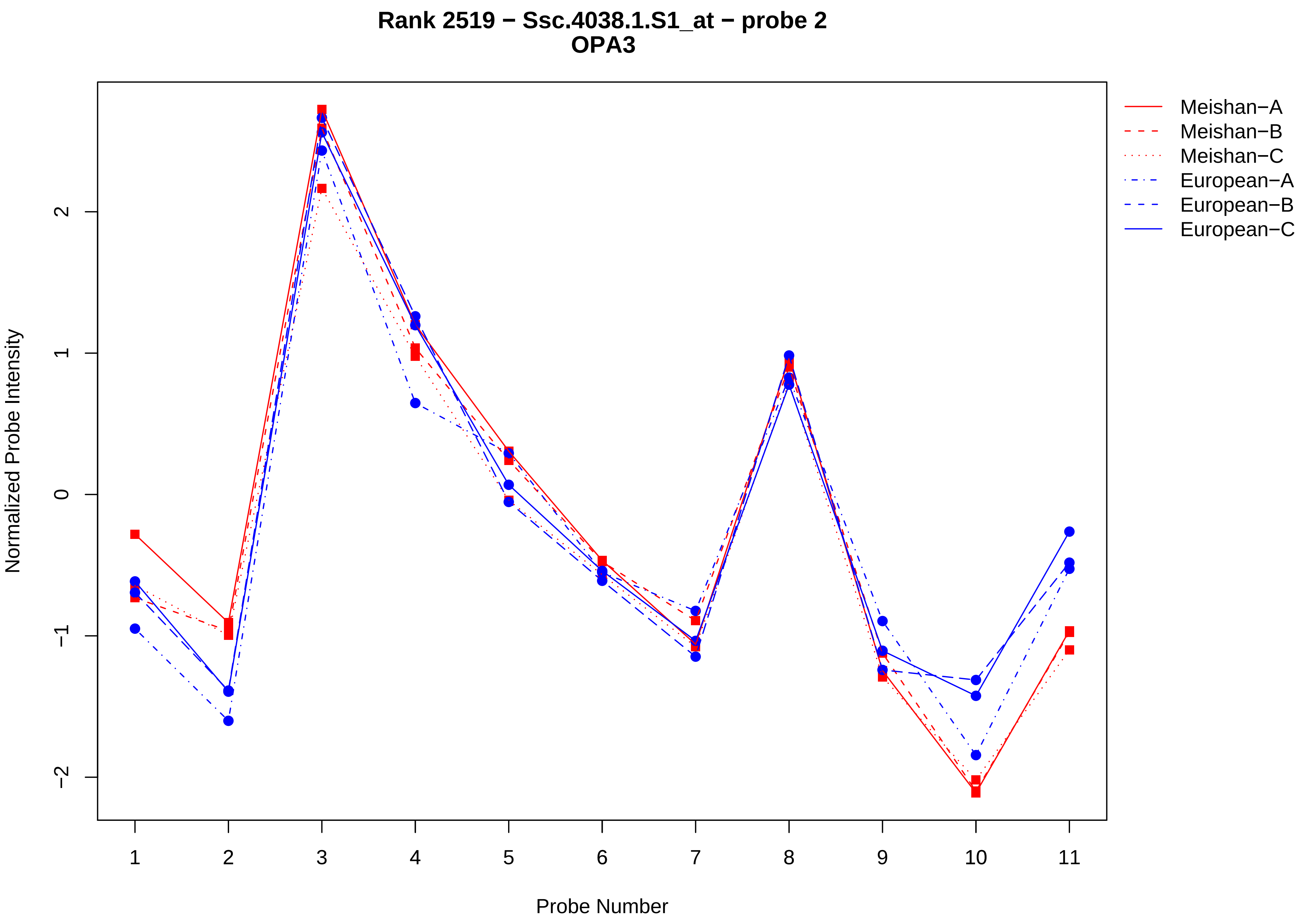
Rank 2519 − Ssc.4038.1.S1_at − probe 2
OPA3
Normalized Probe Intensity
Probe Number
Meishan−A
Meishan−B
Meishan−C
European−A
European−B
European−C
−2
−1
0
1
2
1
2
3
4
5
6
7
8
9
10
11

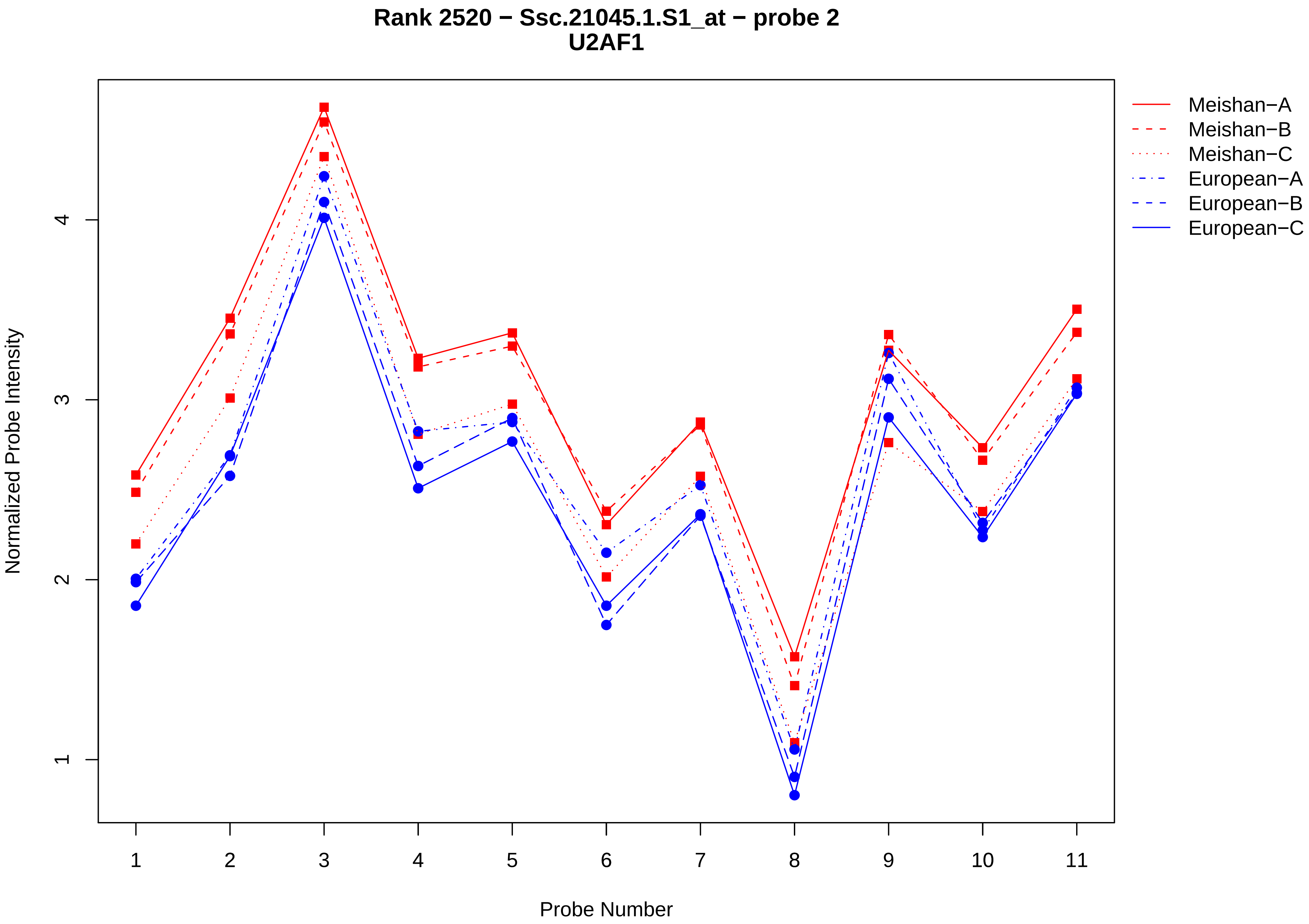

Rank 2520 − Ssc.21045.1.S1_at − probe 2
U2AF1
Normalized Probe Intensity
Probe Number
1
2
3
4
5
6
7
8
9
10
11
Meishan−A
Meishan−B
Meishan−C
European−A
European−B
European−C

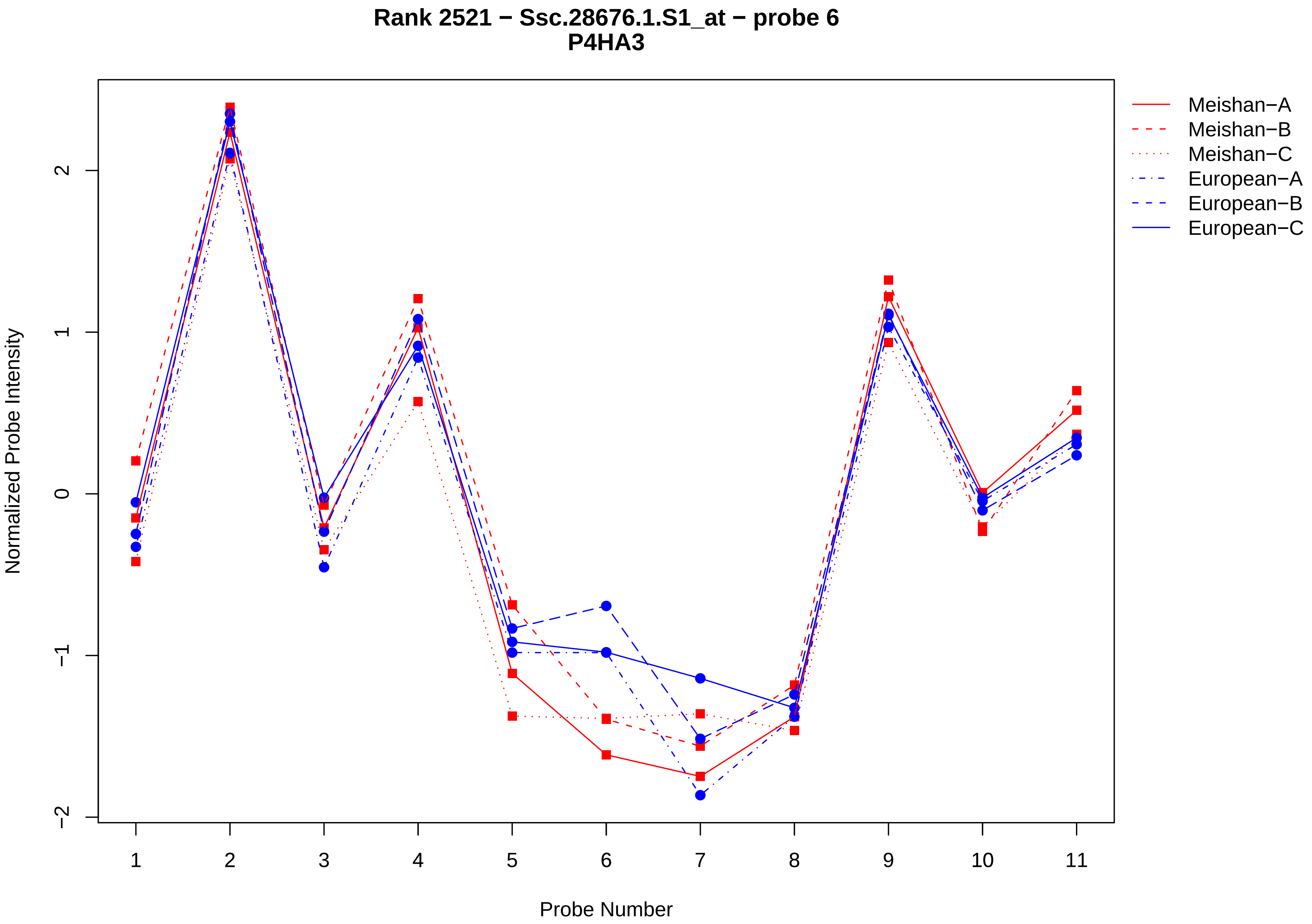

Rank 2521 − Ssc.28676.1.S1_at − probe 6
P4HA3
Normalized Probe Intensity
Probe Number
−2
−1
0
1
2
1
2
3
4
5
6
7
8
9
10
11
Meishan−A
Meishan−B
Meishan−C
European−A
European−B
European−C

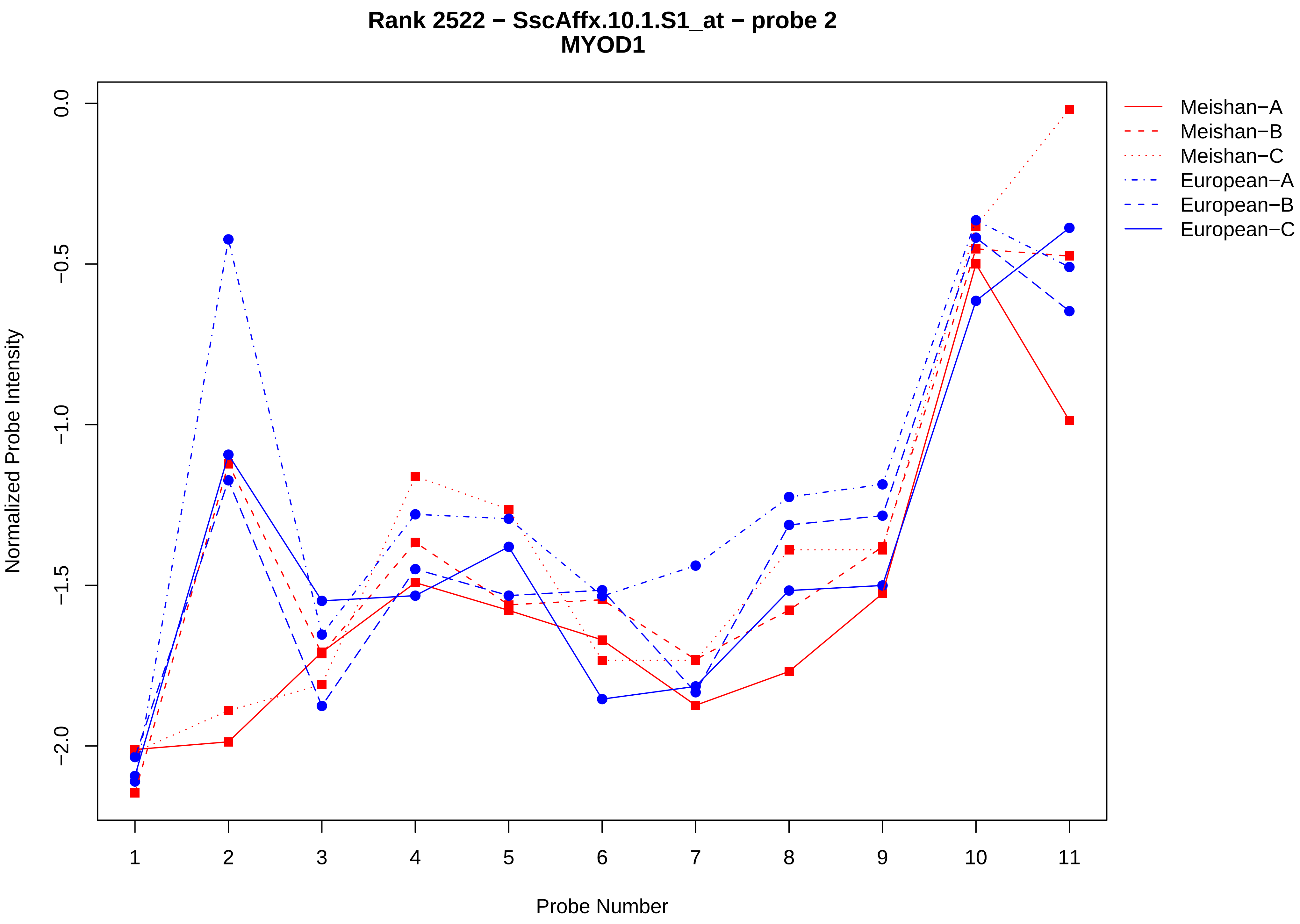
Rank 2522 − SscAffx.10.1.S1_at − probe 2
MYOD1
Normalized Probe Intensity
0.0
−0.5
−1.0
−1.5
−2.0
Probe Number
1
2
3
4
5
6
7
8
9
10
11
Meishan−A
Meishan−B
Meishan−C
European−A
European−B
European−C

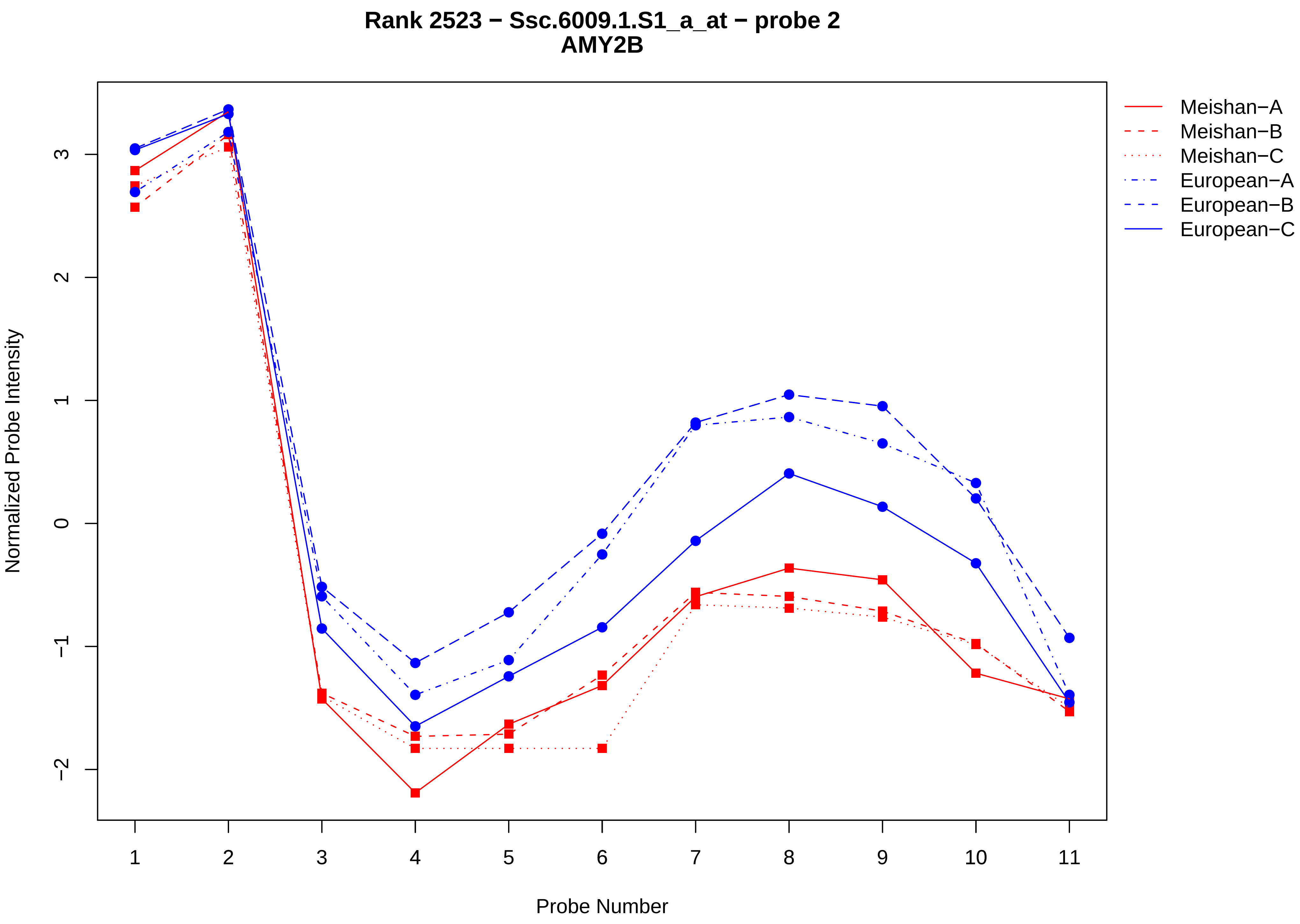

Rank 2523 − Ssc.6009.1.S1_a_at − probe 2
AMY2B
Normalized Probe Intensity
Probe Number
Meishan−A
Meishan−B
Meishan−C
European−A
European−B
European−C

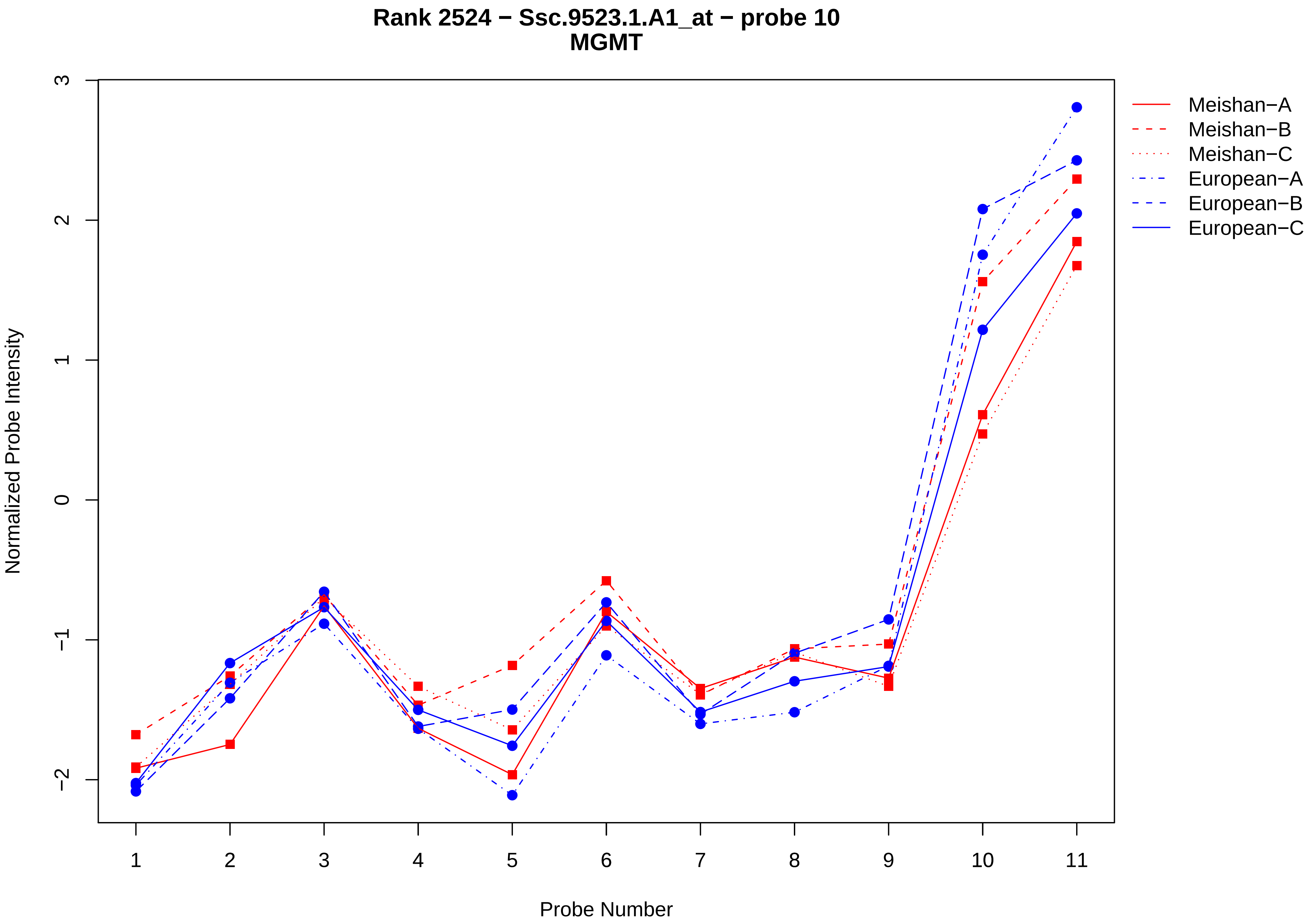

Rank 2524 − Ssc.9523.1.A1_at − probe 10
MGMT
Normalized Probe Intensity
Probe Number
−2
−1
0
1
2
3
1
2
3
4
5
6
7
8
9
10
11
Meishan−A
Meishan−B
Meishan−C
European−A
European−B
European−C

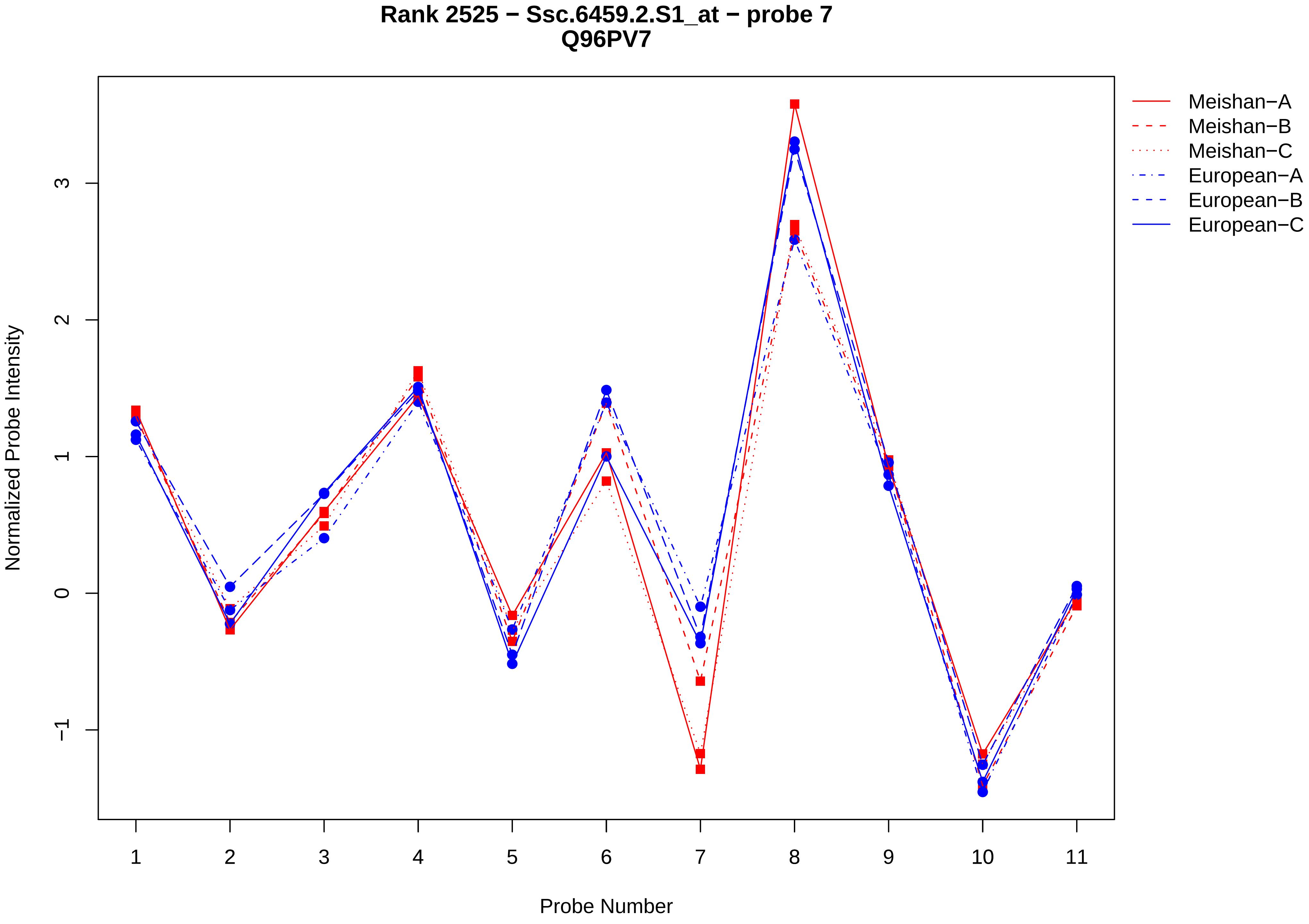
Rank 2525 − Ssc.6459.2.S1_at − probe 7
Q96PV7
Normalized Probe Intensity
Probe Number
Meishan−A
Meishan−B
Meishan−C
European−A
European−B
European−C

Rank 2526 − Ssc.7546.1.A1_at − probe 3
NP_955389

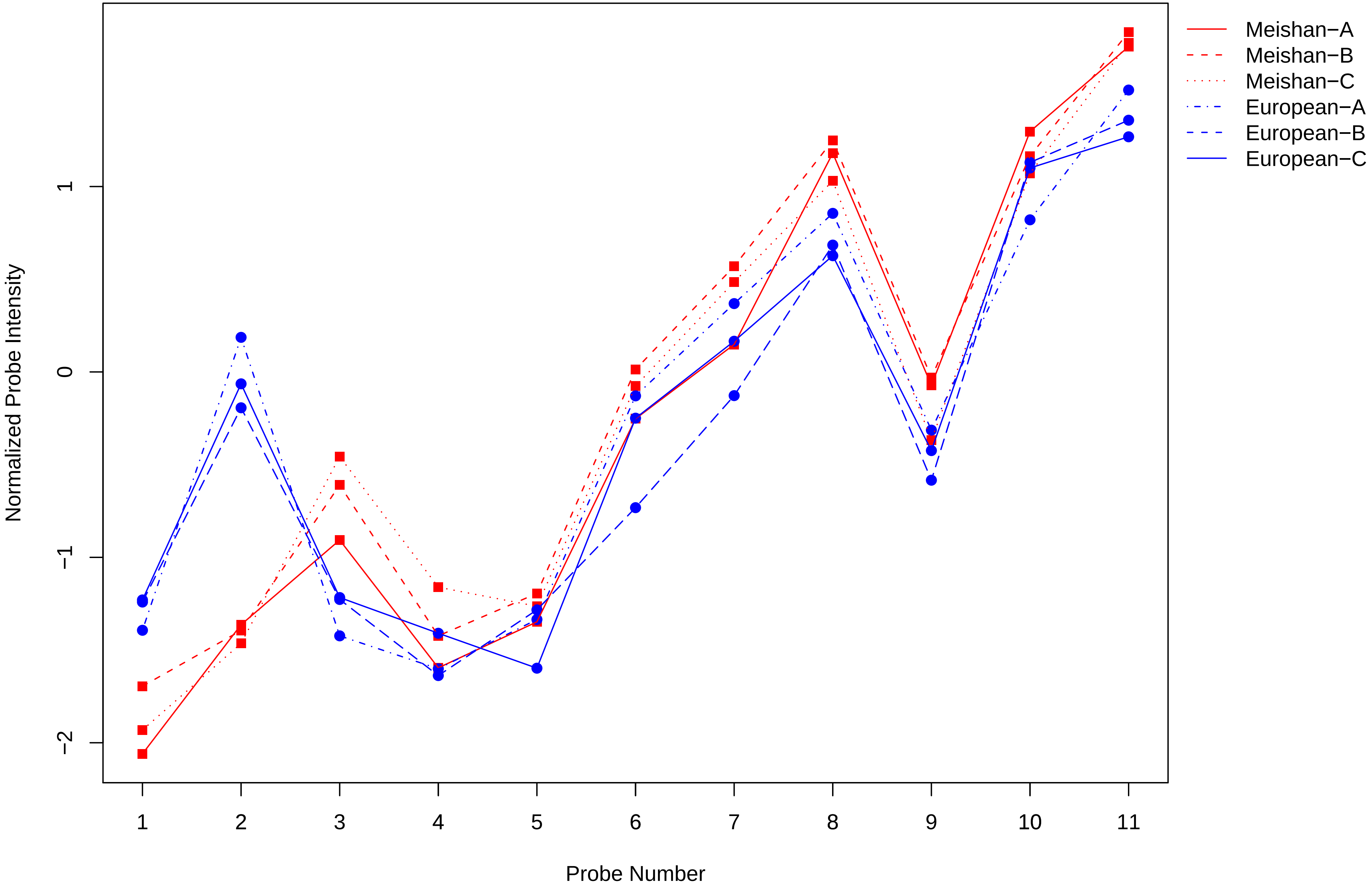

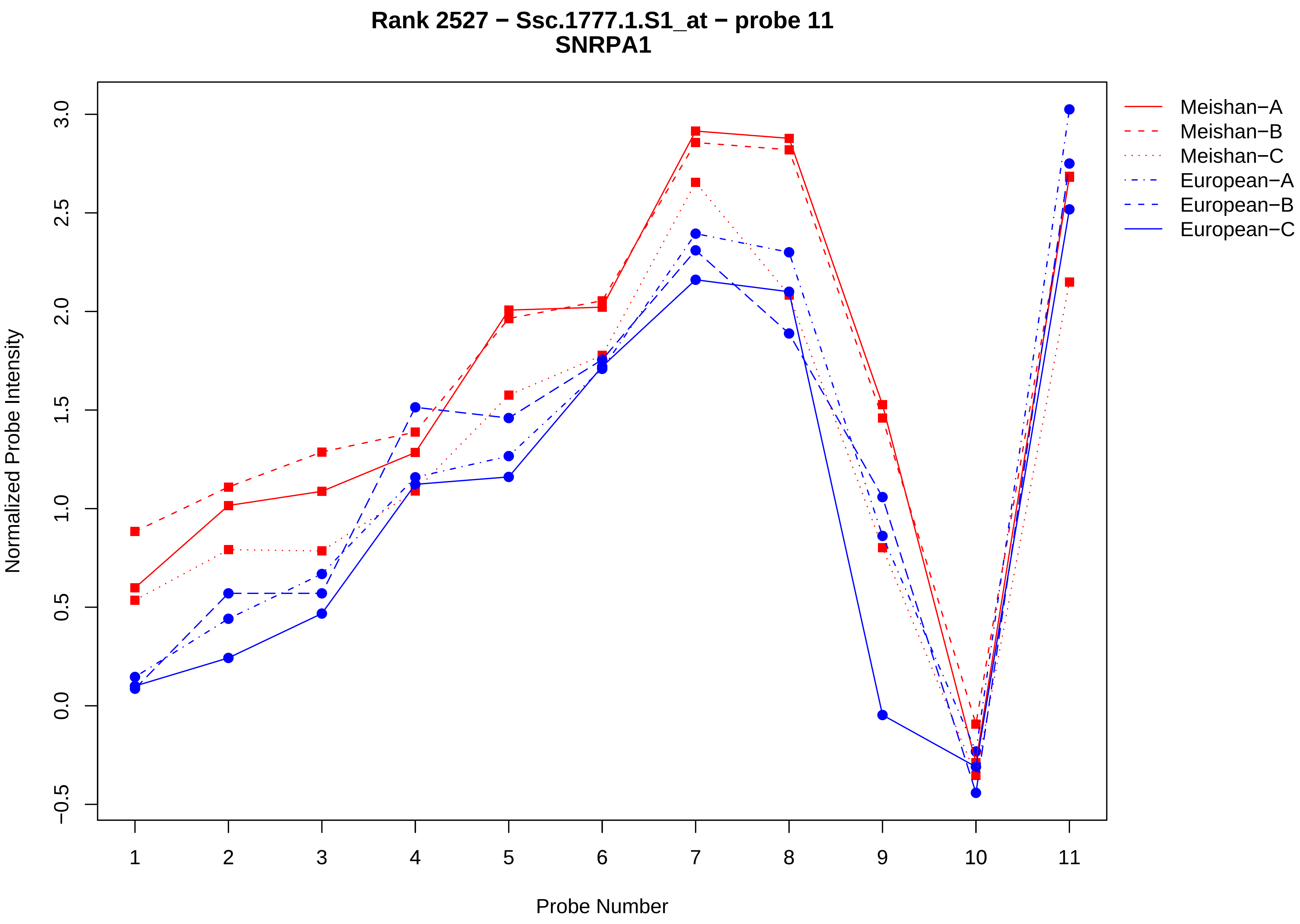

Rank 2527 − Ssc.1777.1.S1_at − probe 11
SNRPA1
Normalized Probe Intensity
Probe Number
Meishan−A
Meishan−B
Meishan−C
European−A
European−B
European−C

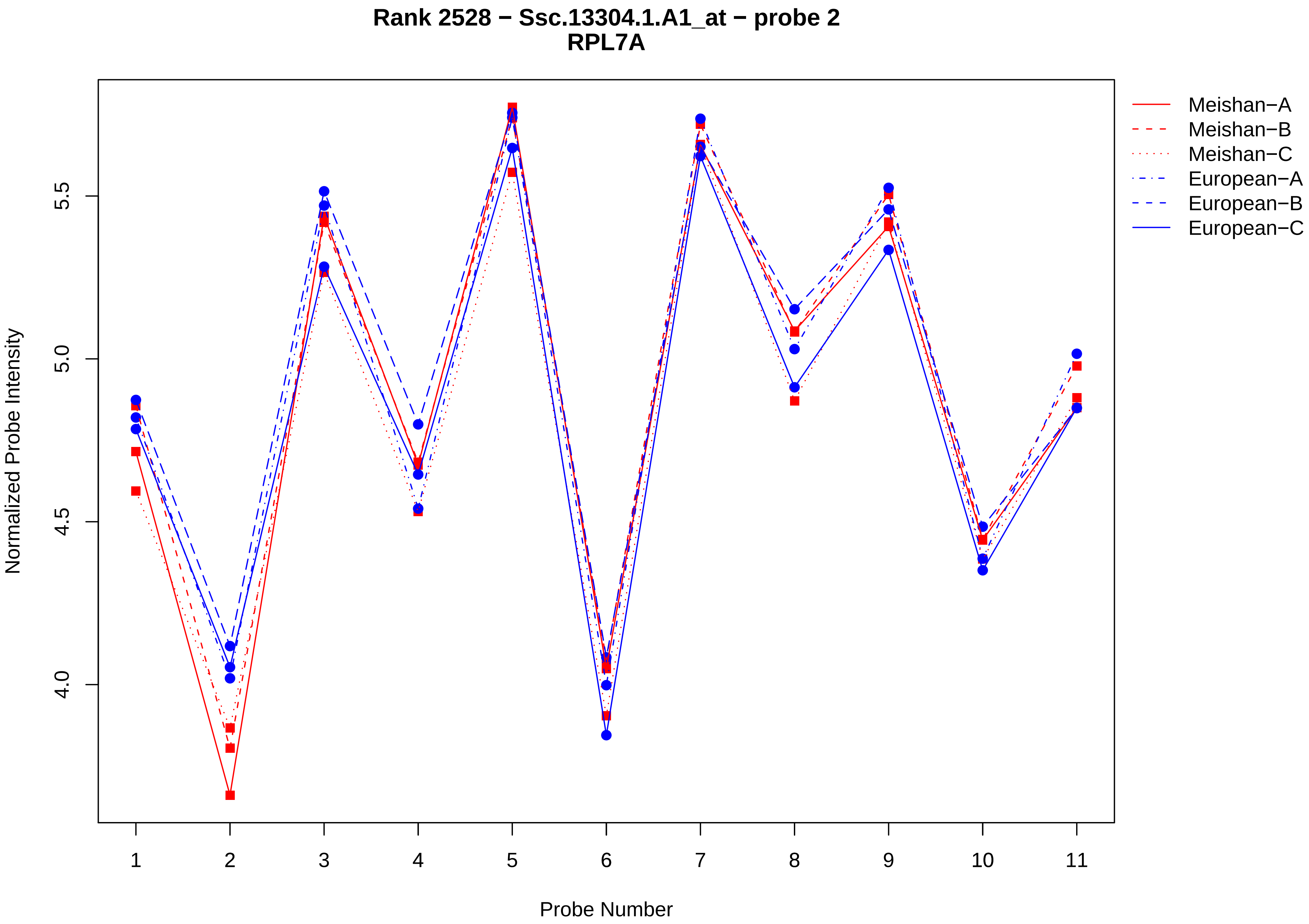

Rank 2528 − Ssc.13304.1.A1_at − probe 2
RPL7A
Normalized Probe Intensity
4.0
4.5
5.0
5.5
1
2
3
4
5
6
7
8
9
10
11
Probe Number
Meishan−A
Meishan−B
Meishan−C
European−A
European−B
European−C

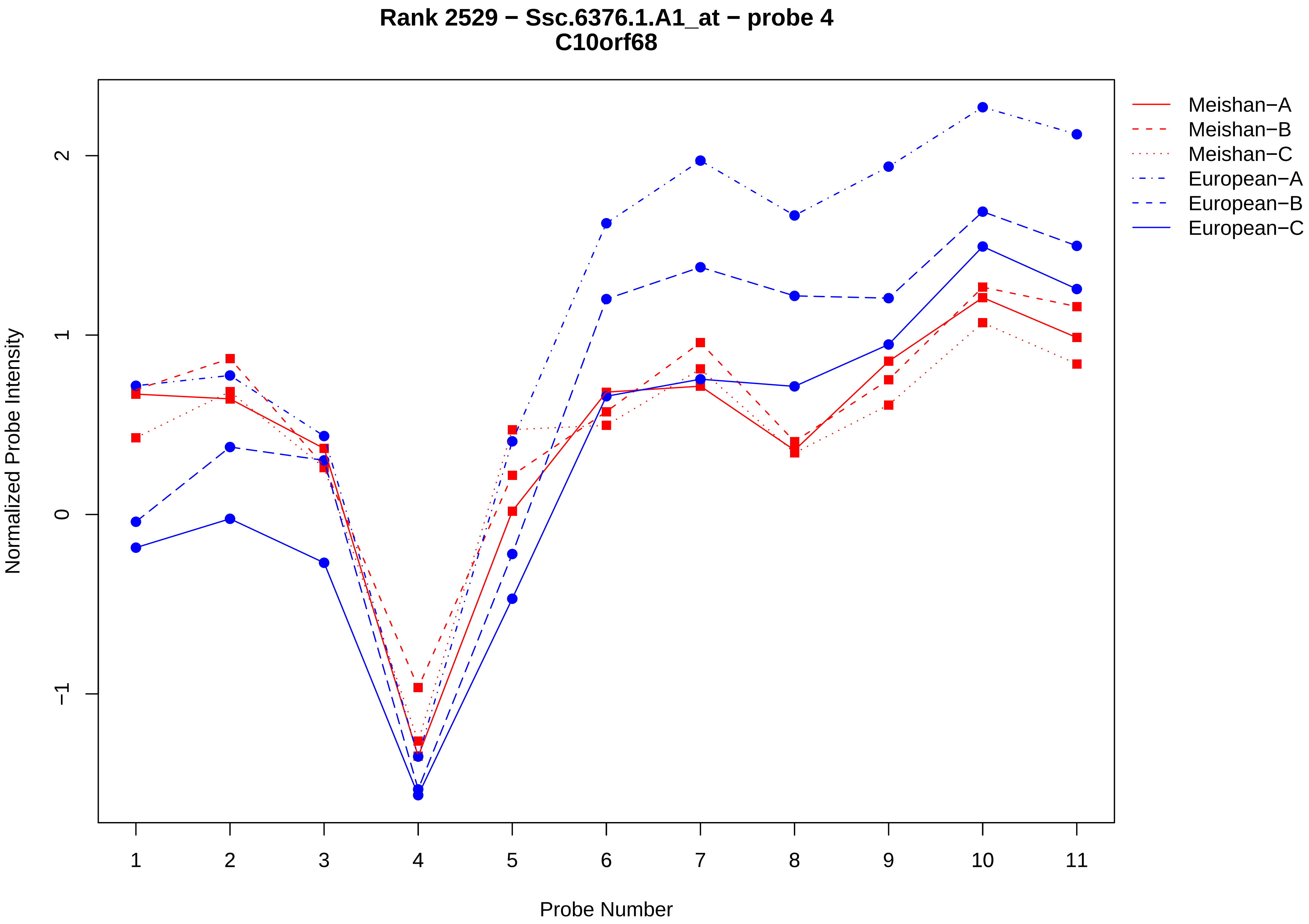
Rank 2529 − Ssc.6376.1.A1_at − probe 4
C10orf68
Normalized Probe Intensity
−1
0
1
2
1
2
3
4
5
6
7
8
9
10
11
Probe Number
Meishan−A
Meishan−B
Meishan−C
European−A
European−B
European−C

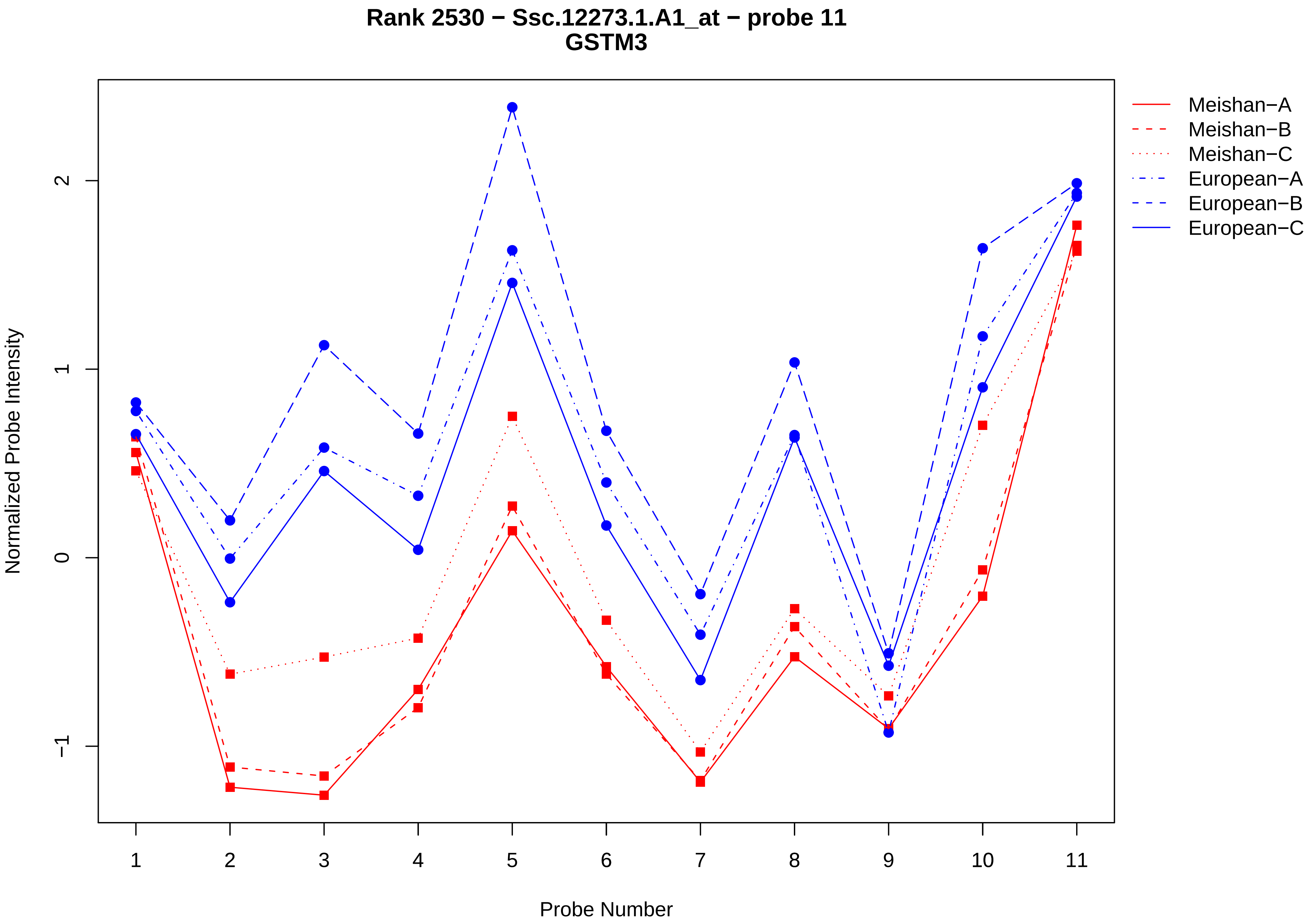
Rank 2530 − Ssc.12273.1.A1_at − probe 11
GSTM3
Normalized Probe Intensity
Probe Number
−1
0
1
2
1
2
3
4
5
6
7
8
9
10
11
Meishan−A
Meishan−B
Meishan−C
European−A
European−B
European−C

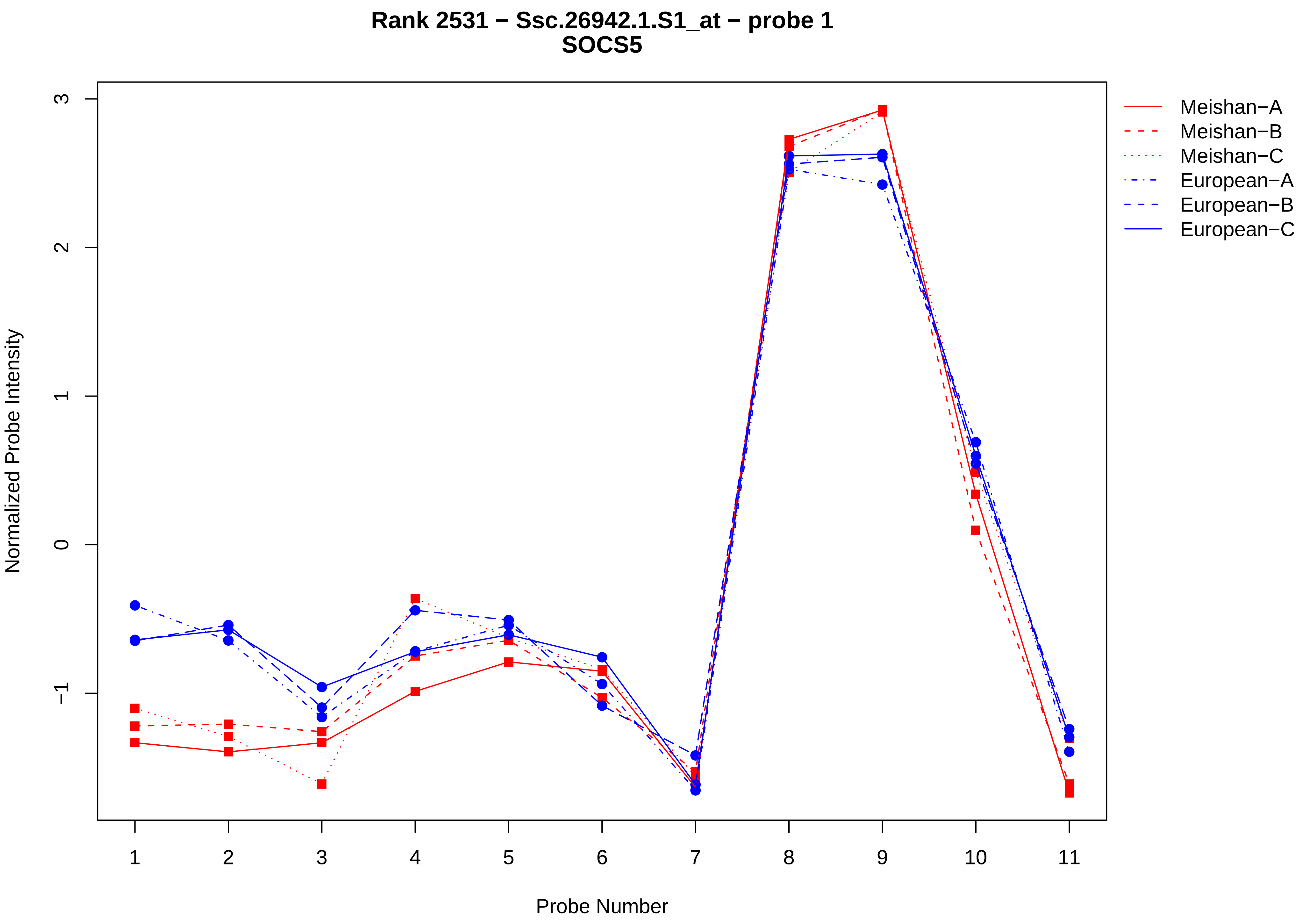

Rank 2531 − Ssc.26942.1.S1_at − probe 1
SOCS5
Normalized Probe Intensity
Probe Number
3
2
1
0
−1
1
2
3
4
5
6
7
8
9
10
11
Meishan−A
Meishan−B
Meishan−C
European−A
European−B
European−C

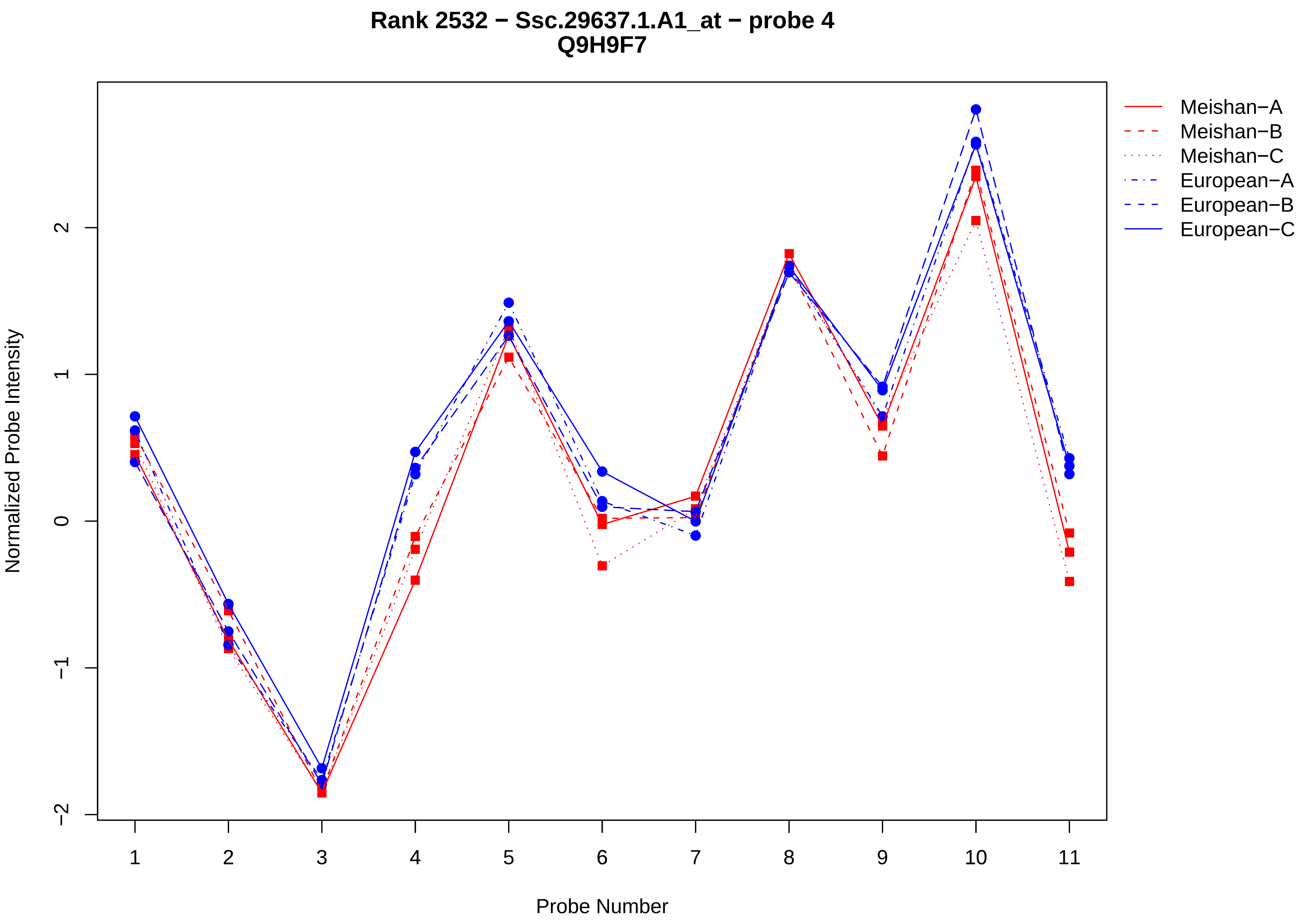

Rank 2532 − Ssc.29637.1.A1_at − probe 4
Q9H9F7
Normalized Probe Intensity
Probe Number
−2
−1
0
1
2
1
2
3
4
5
6
7
8
9
10
11
Meishan−A
Meishan−B
Meishan−C
European−A
European−B
European−C

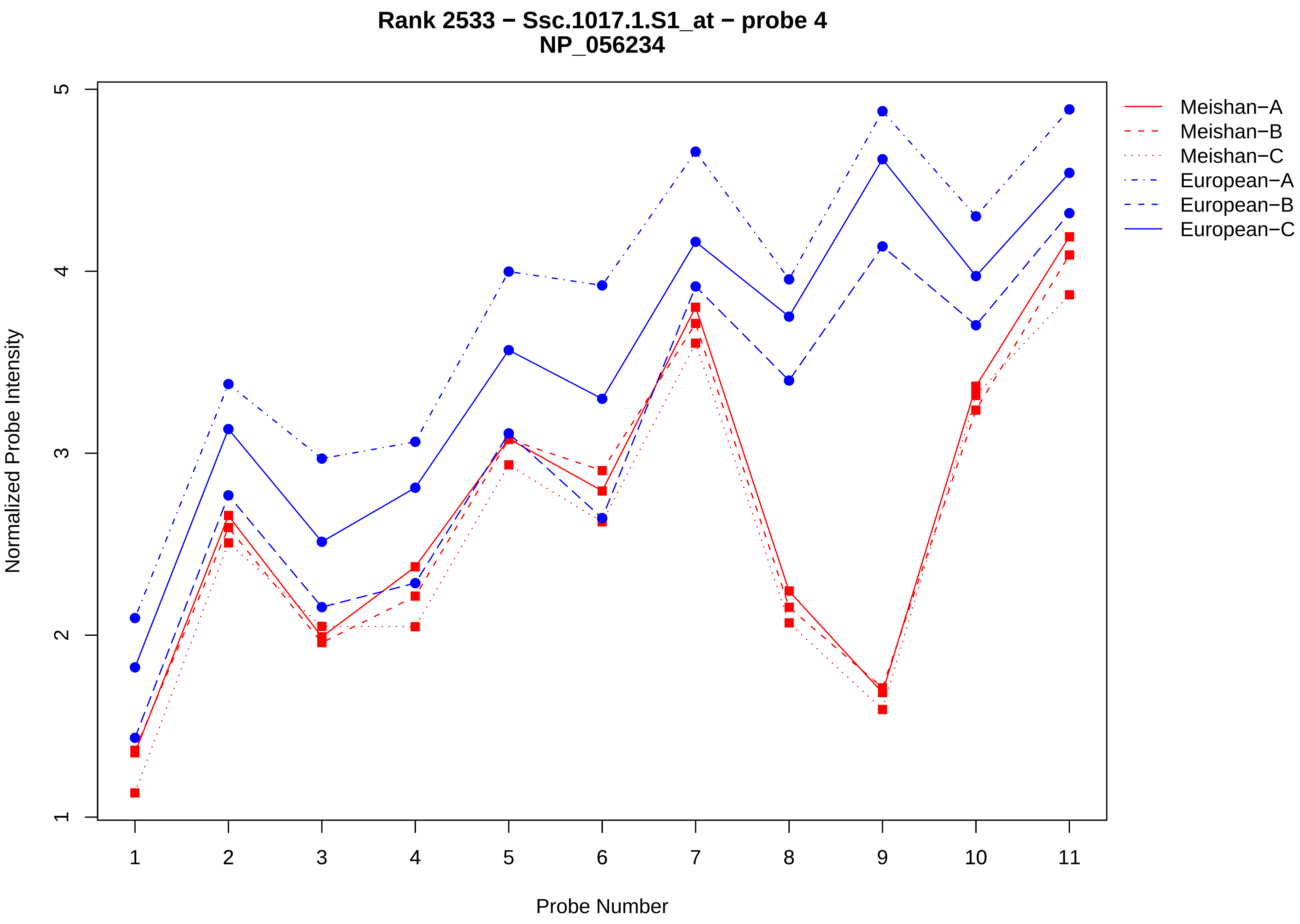

Rank 2533 − Ssc.1017.1.S1_at − probe 4
NP_056234
Normalized Probe Intensity
Probe Number
1
2
3
4
5
1
2
3
4
5
6
7
8
9
10
11
Meishan−A
Meishan−B
Meishan−C
European−A
European−B
European−C

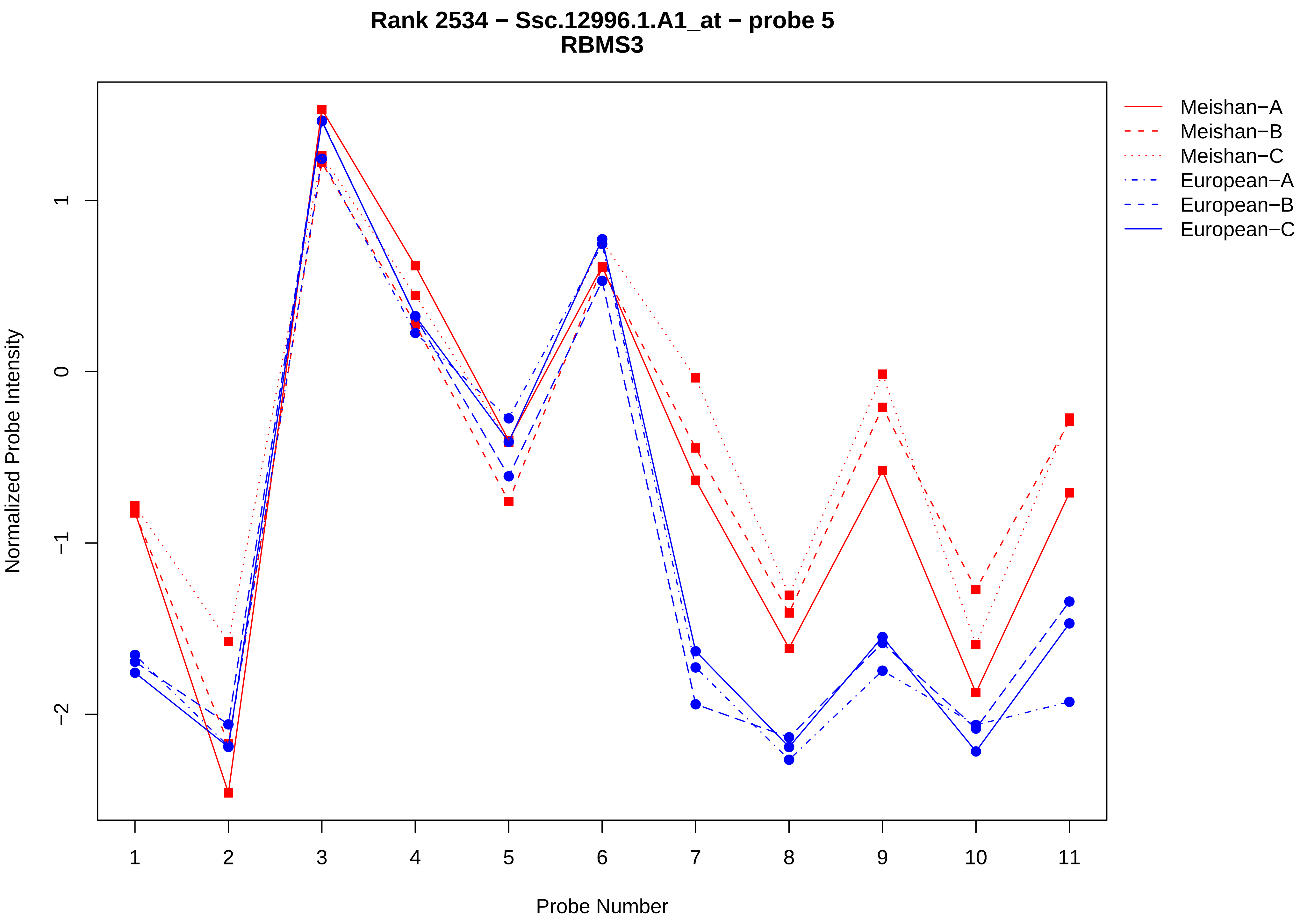

Rank 2534 − Ssc.12996.1.A1_at − probe 5
RBMS3
Normalized Probe Intensity
Probe Number
1
0
−1
−2
1
2
3
4
5
6
7
8
9
10
11
Meishan−A
Meishan−B
Meishan−C
European−A
European−B
European−C

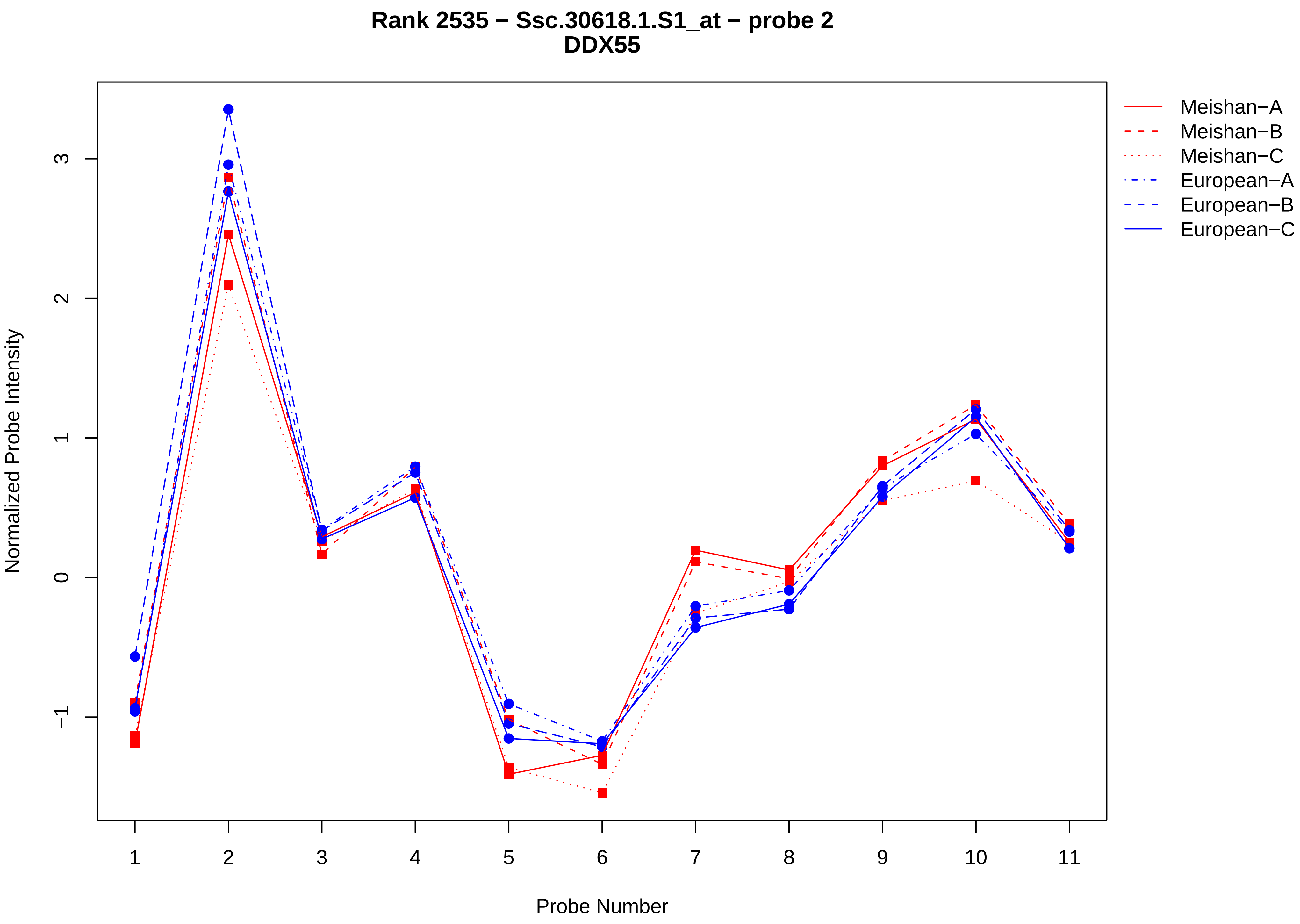

Rank 2535 − Ssc.30618.1.S1_at − probe 2
DDX55
Normalized Probe Intensity
Probe Number
−1
0
1
2
3
1
2
3
4
5
6
7
8
9
10
11
Meishan−A
Meishan−B
Meishan−C
European−A
European−B
European−C

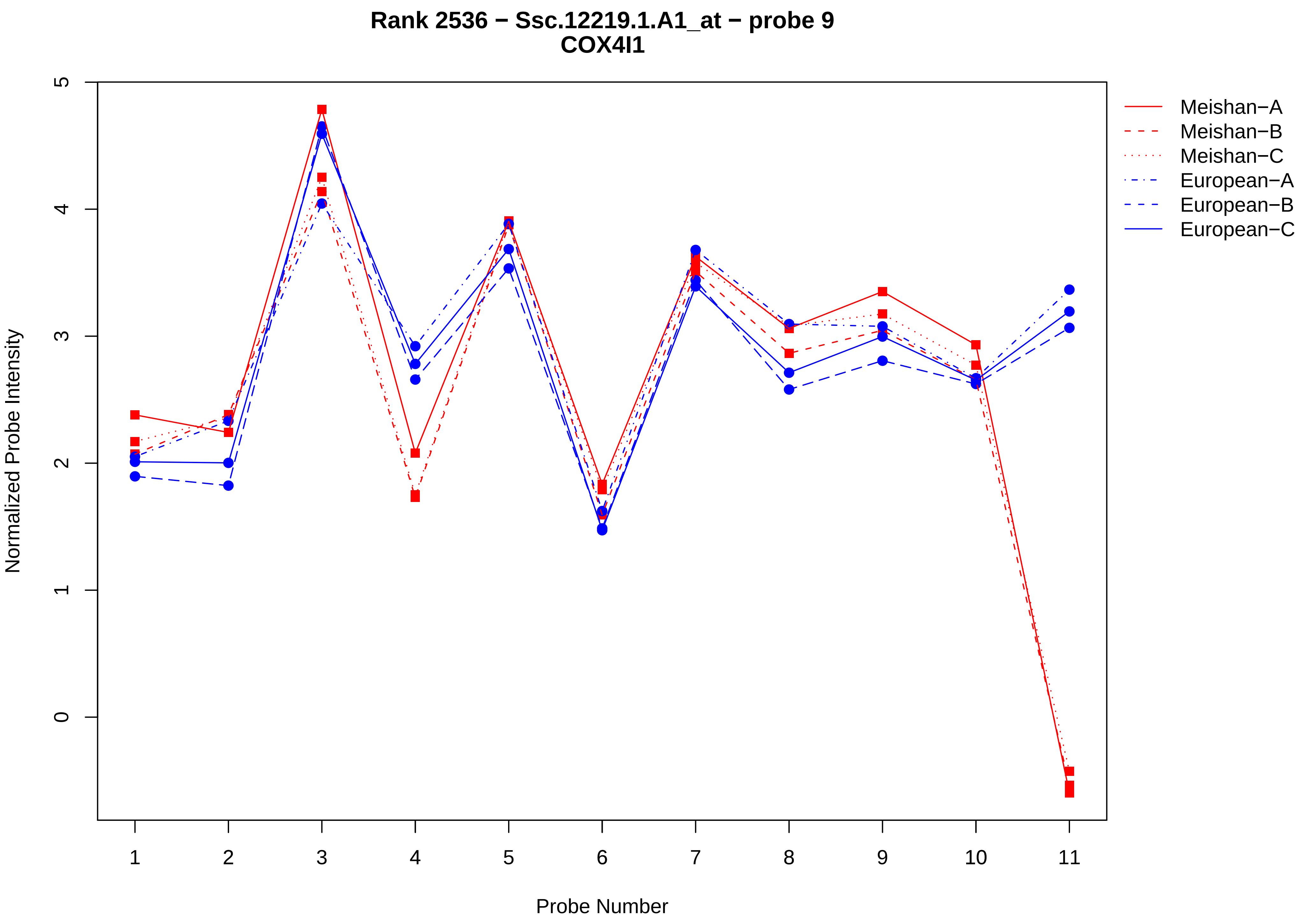

Rank 2536 − Ssc.12219.1.A1_at − probe 9
COX4I1
Normalized Probe Intensity
Probe Number
0
1
2
3
4
5
1
2
3
4
5
6
7
8
9
10
11
Meishan−A
Meishan−B
Meishan−C
European−A
European−B
European−C

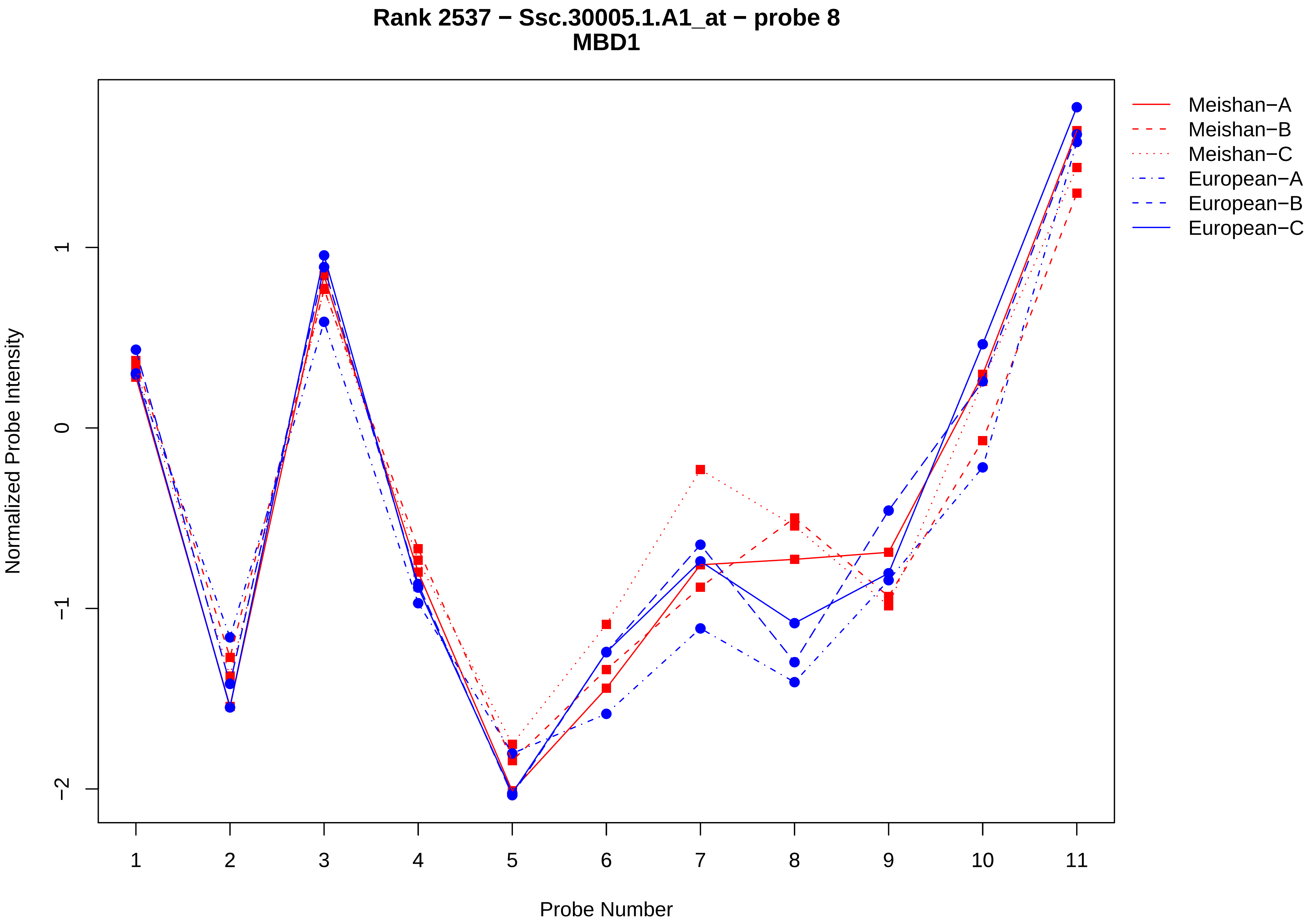

Rank 2537 − Ssc.30005.1.A1_at − probe 8
MBD1
Normalized Probe Intensity
Probe Number
Meishan−A
Meishan−B
Meishan−C
European−A
European−B
European−C
−2
−1
0
1
1
2
3
4
5
6
7
8
9
10
11

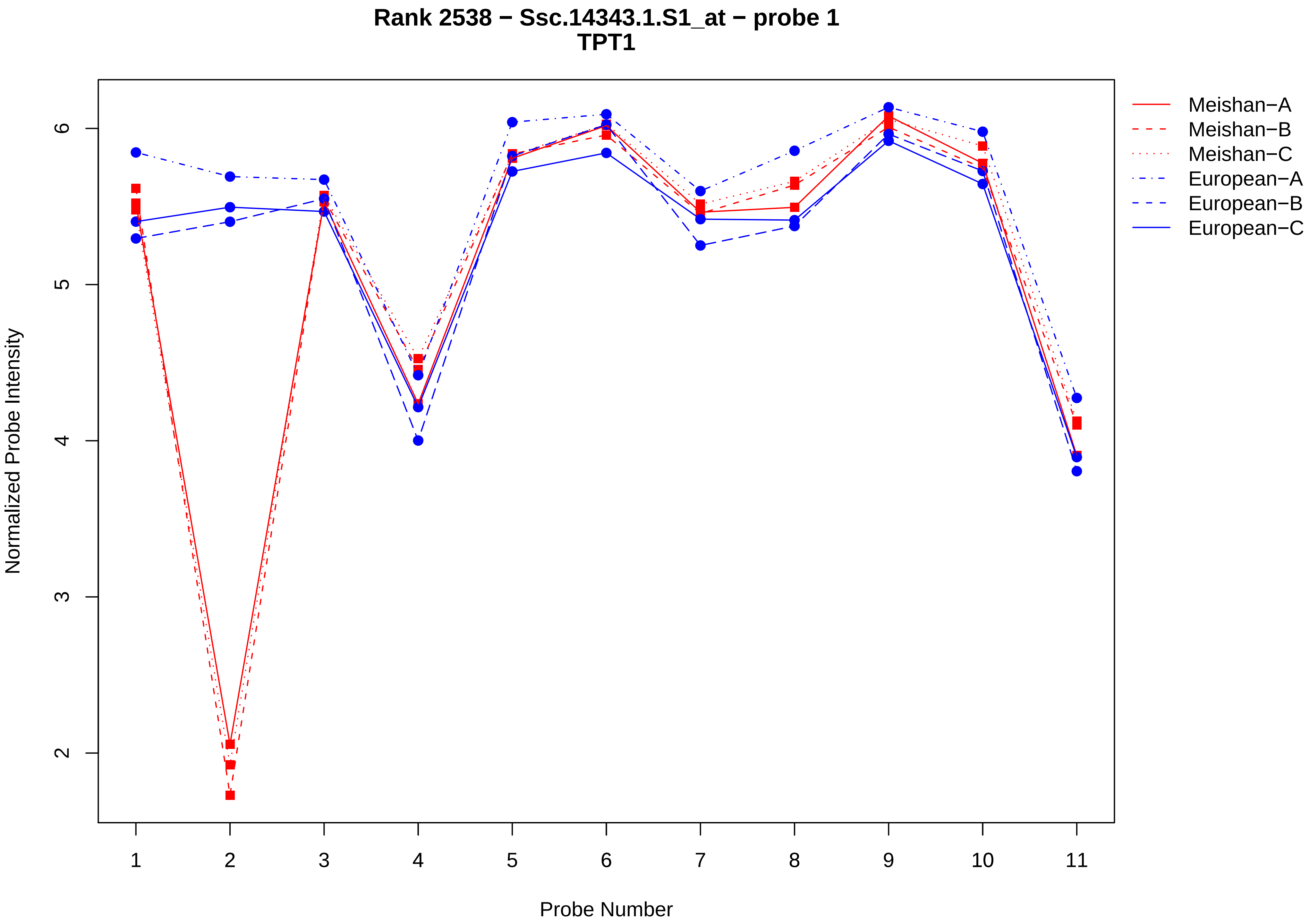

Rank 2538 − Ssc.14343.1.S1_at − probe 1
TPT1
Normalized Probe Intensity
Probe Number
2
3
4
5
6
1
2
3
4
5
6
7
8
9
10
11
Meishan−A
Meishan−B
Meishan−C
European−A
European−B
European−C

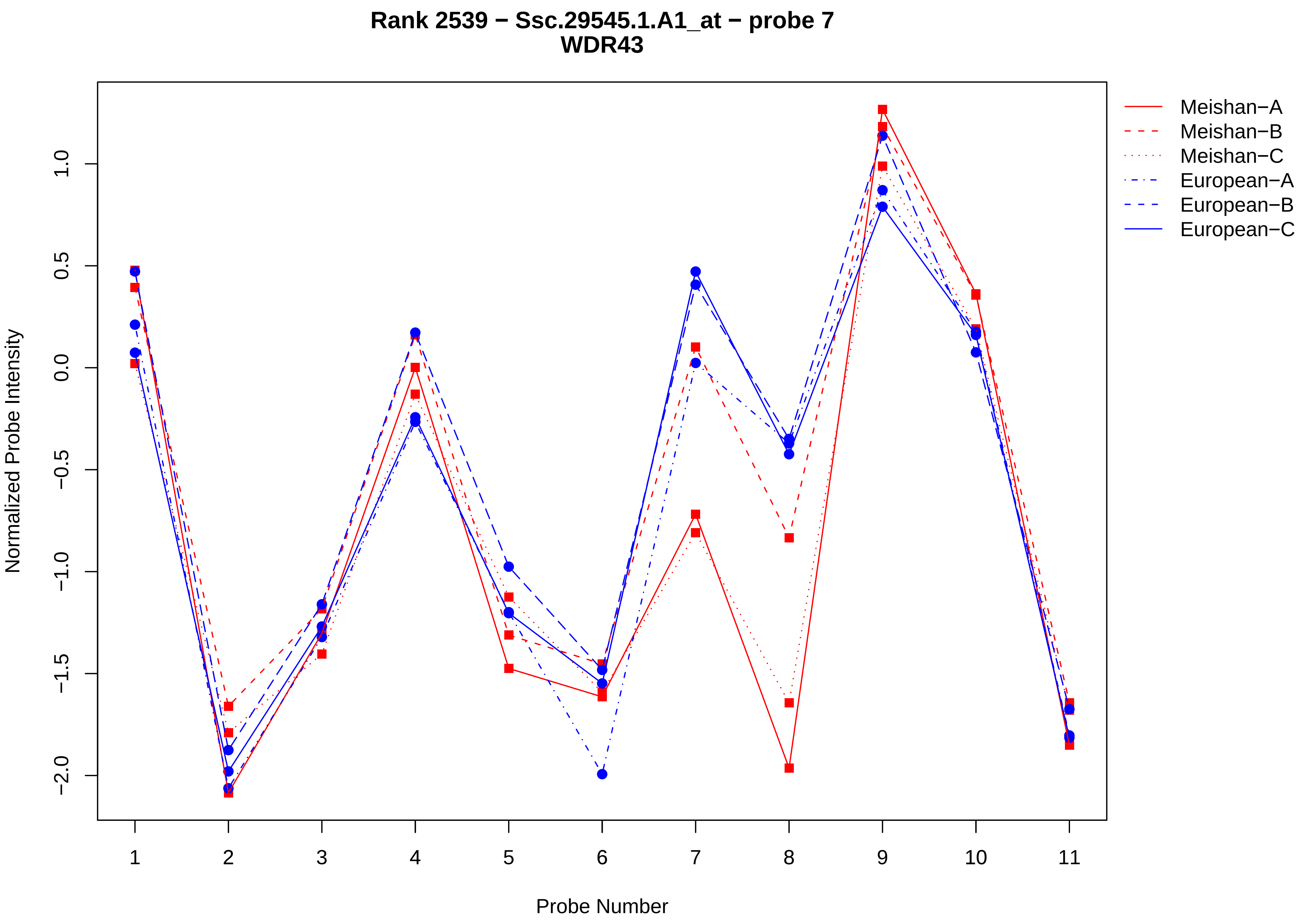

Rank 2539 − Ssc.29545.1.A1_at − probe 7
WDR43
Normalized Probe Intensity
Probe Number
Meishan−A
Meishan−B
Meishan−C
European−A
European−B
European−C
1.0
0.5
0.0
−0.5
−1.0
−1.5
−2.0
1
2
3
4
5
6
7
8
9
10
11

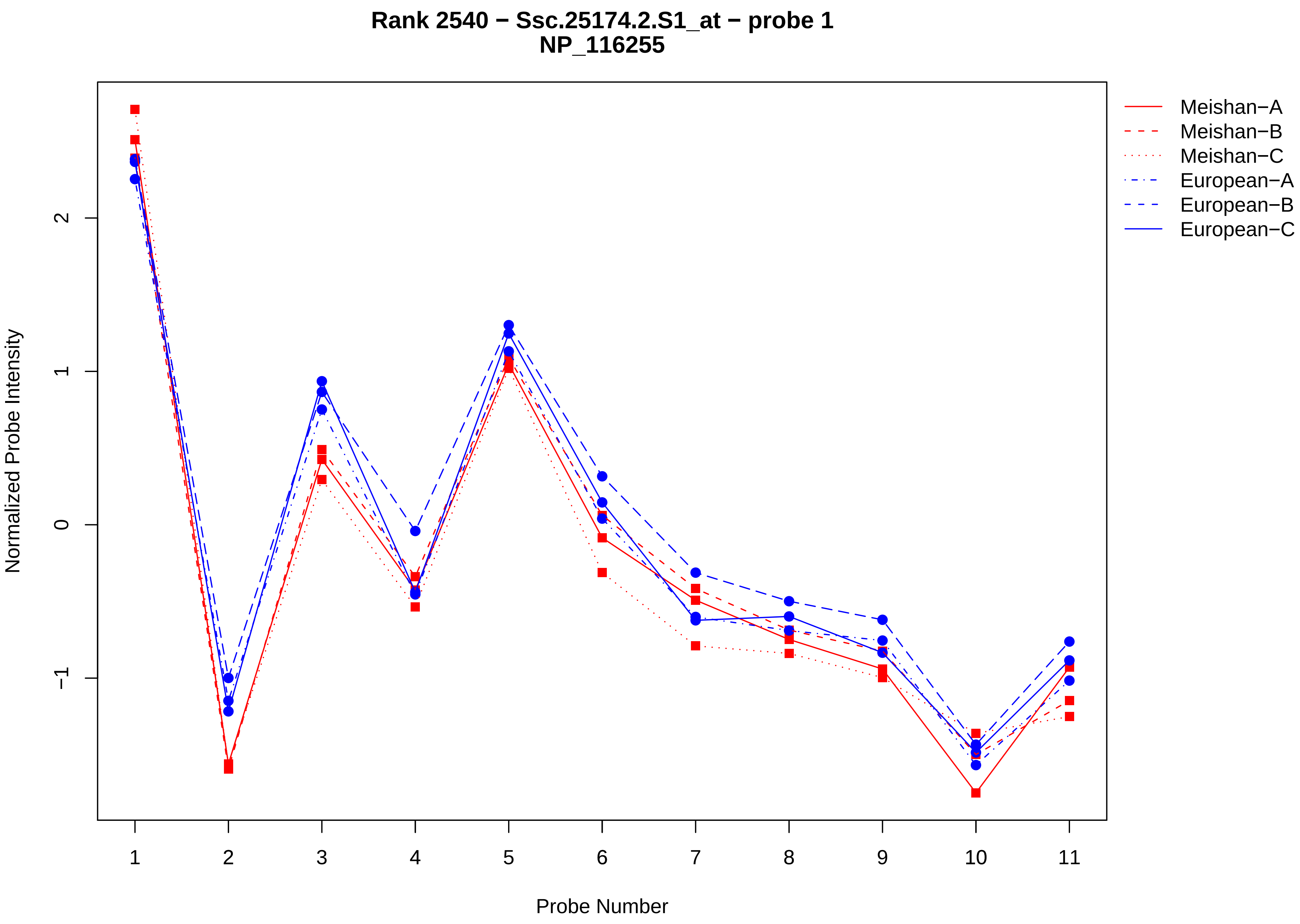

Rank 2540 − Ssc.25174.2.S1_at − probe 1
NP_116255
Normalized Probe Intensity
Probe Number
Meishan−A
Meishan−B
Meishan−C
European−A
European−B
European−C
−1
0
1
2
1
2
3
4
5
6
7
8
9
10
11

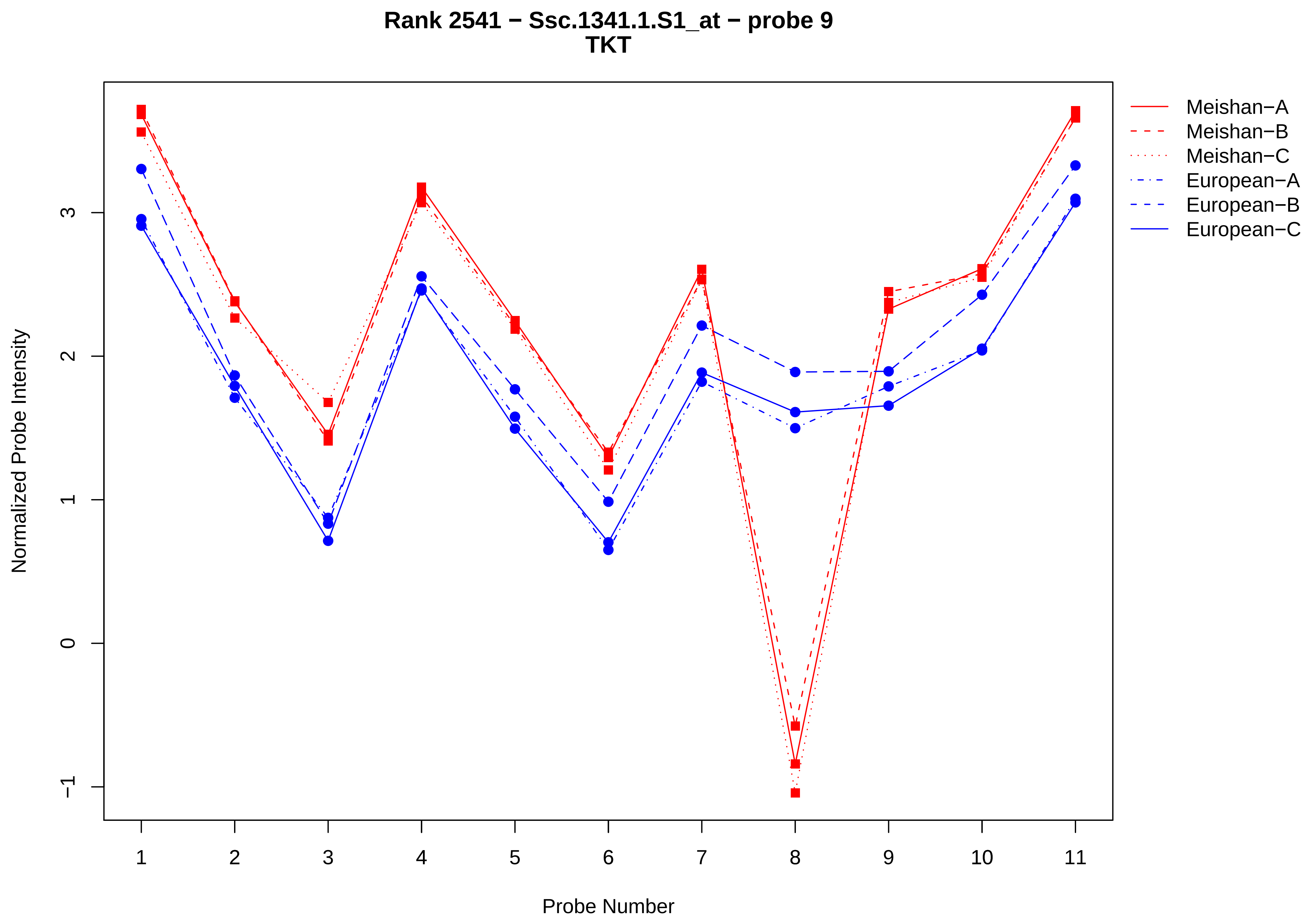
Rank 2541 − Ssc.1341.1.S1_at − probe 9
TKT
Normalized Probe Intensity
Probe Number
−1
0
1
2
3
1
2
3
4
5
6
7
8
9
10
11
Meishan−A
Meishan−B
Meishan−C
European−A
European−B
European−C

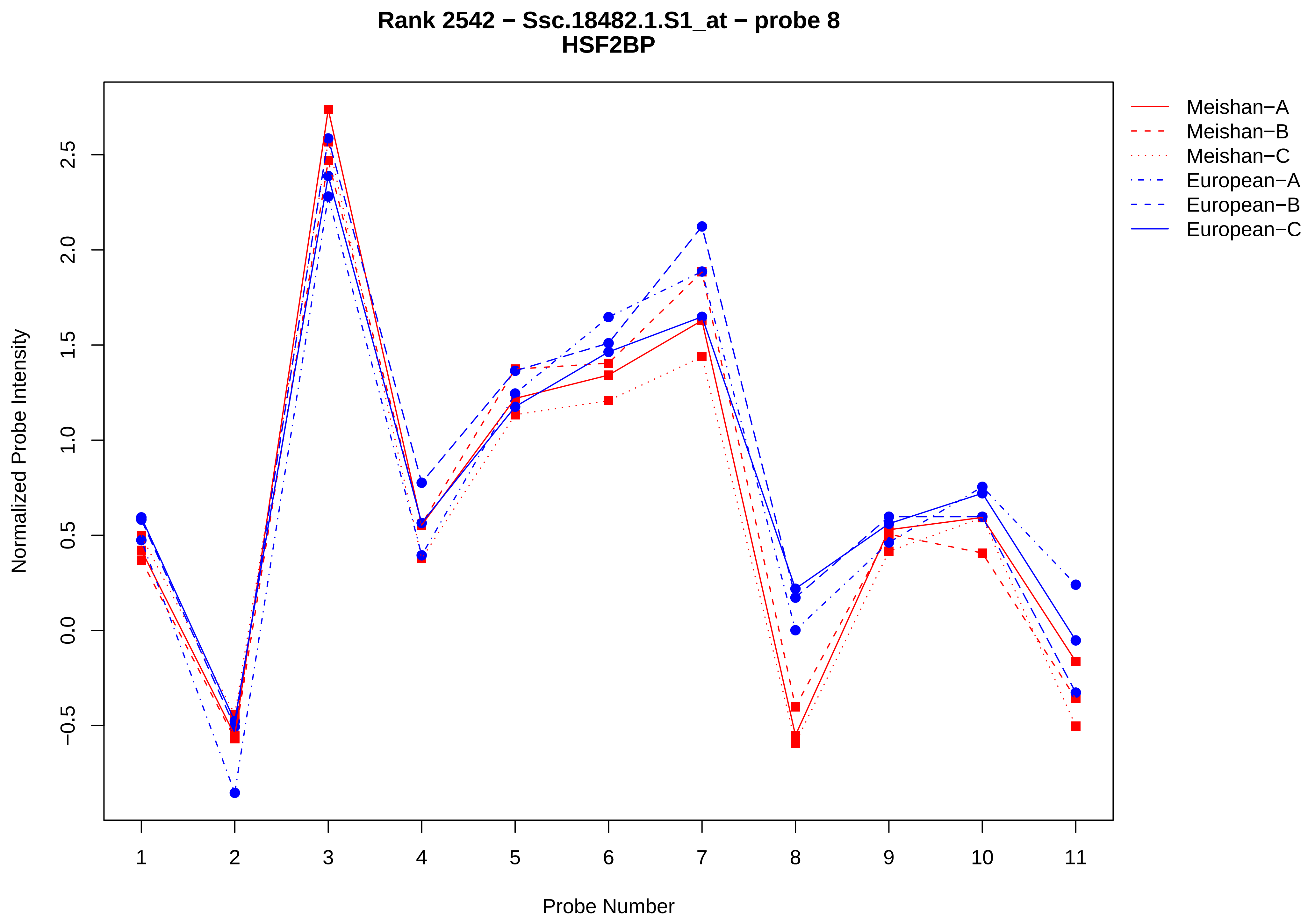

Rank 2542 − Ssc.18482.1.S1_at − probe 8
HSF2BP
Normalized Probe Intensity
Probe Number
−0.5
0.0
0.5
1.0
1.5
2.0
2.5
1
2
3
4
5
6
7
8
9
10
11
Meishan−A
Meishan−B
Meishan−C
European−A
European−B
European−C

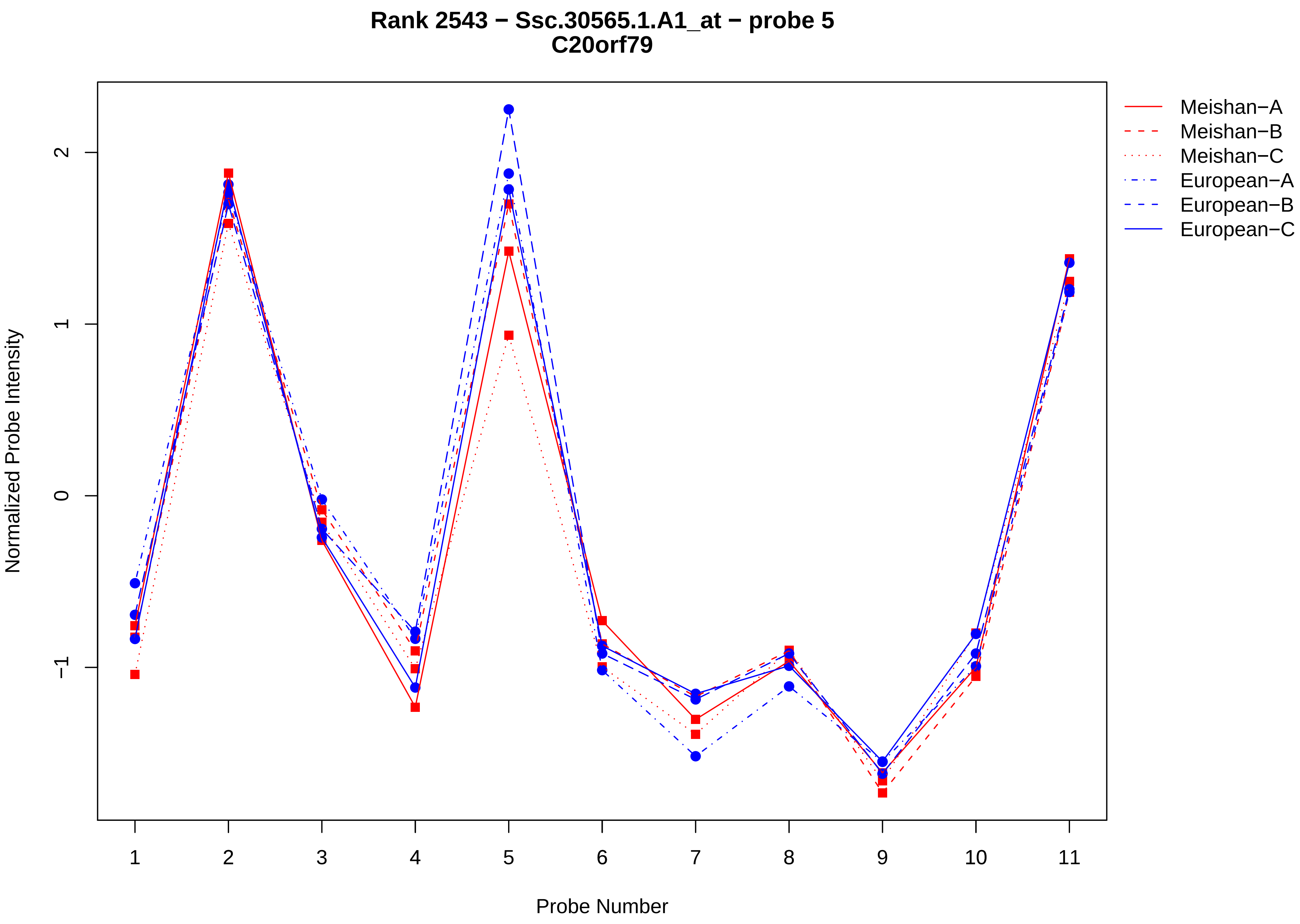
Rank 2543 − Ssc.30565.1.A1_at − probe 5
C20orf79
Normalized Probe Intensity
Probe Number
−1
0
1
2
1
2
3
4
5
6
7
8
9
10
11
Meishan−A
Meishan−B
Meishan−C
European−A
European−B
European−C

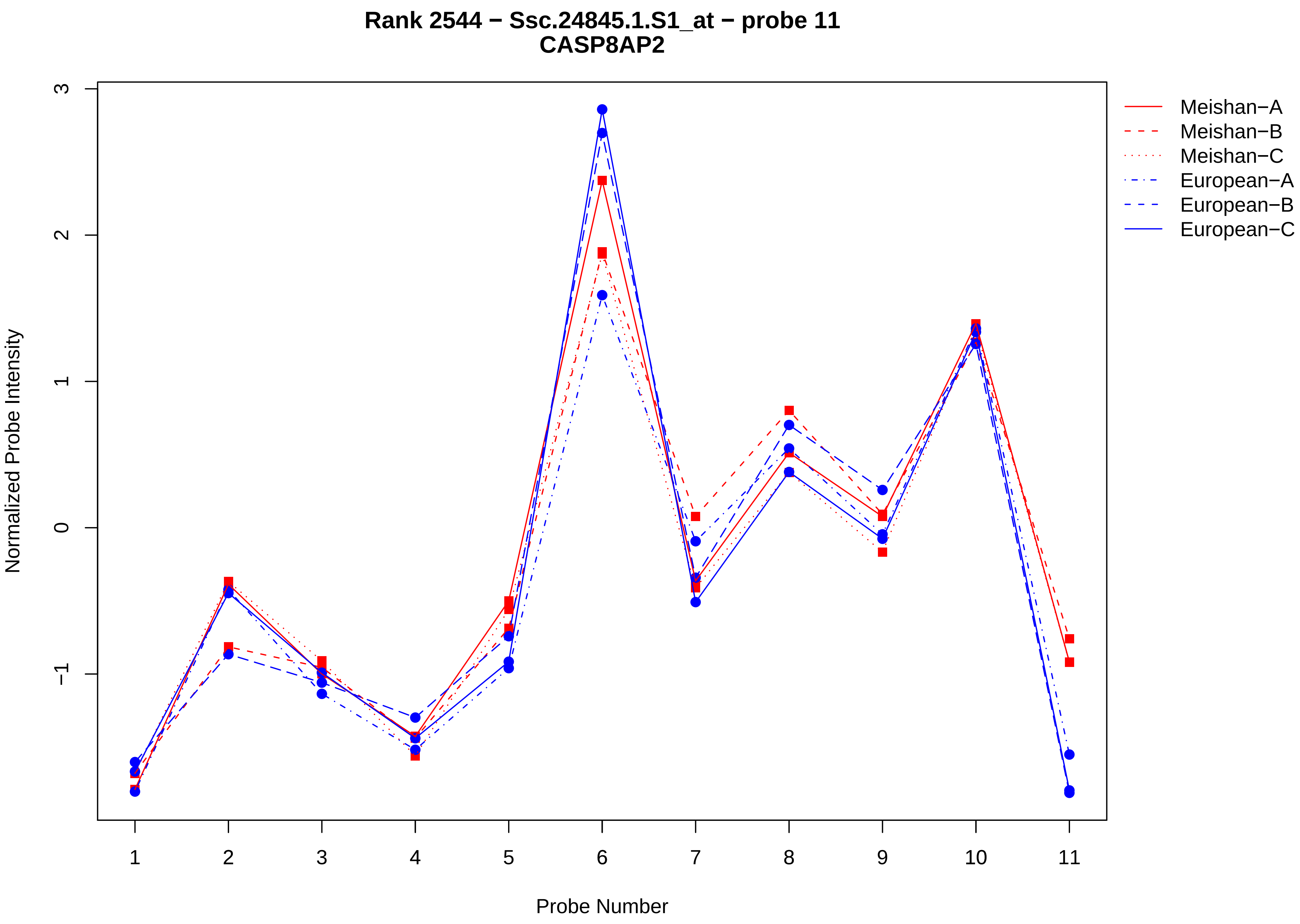

Rank 2544 − Ssc.24845.1.S1_at − probe 11
CASP8AP2
Normalized Probe Intensity
Probe Number
Meishan−A
Meishan−B
Meishan−C
European−A
European−B
European−C

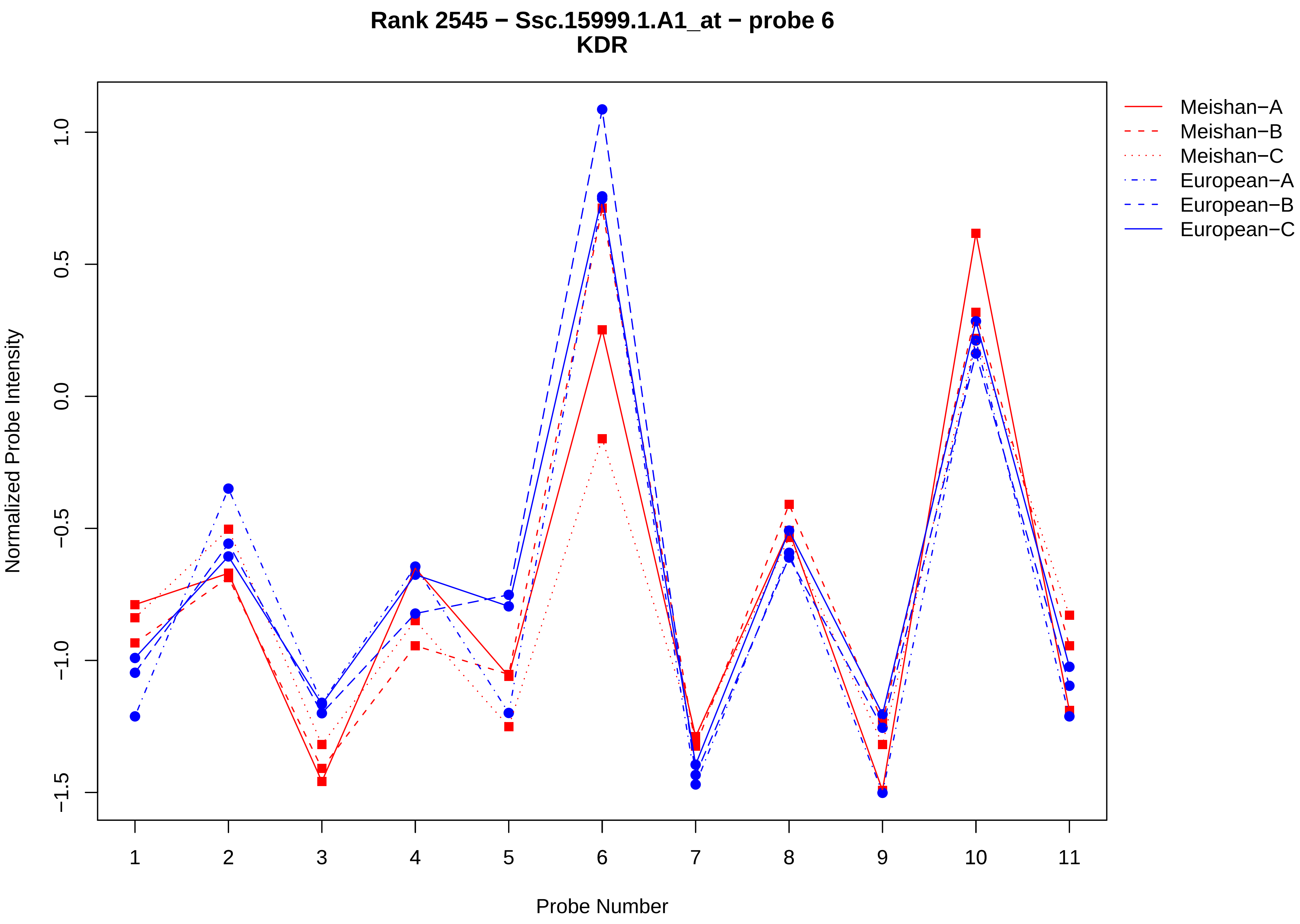
Rank 2545 − Ssc.15999.1.A1_at − probe 6
KDR
Normalized Probe Intensity
Probe Number
Meishan−A
Meishan−B
Meishan−C
European−A
European−B
European−C
1.0
0.5
0.0
−0.5
−1.0
−1.5
1
2
3
4
5
6
7
8
9
10
11

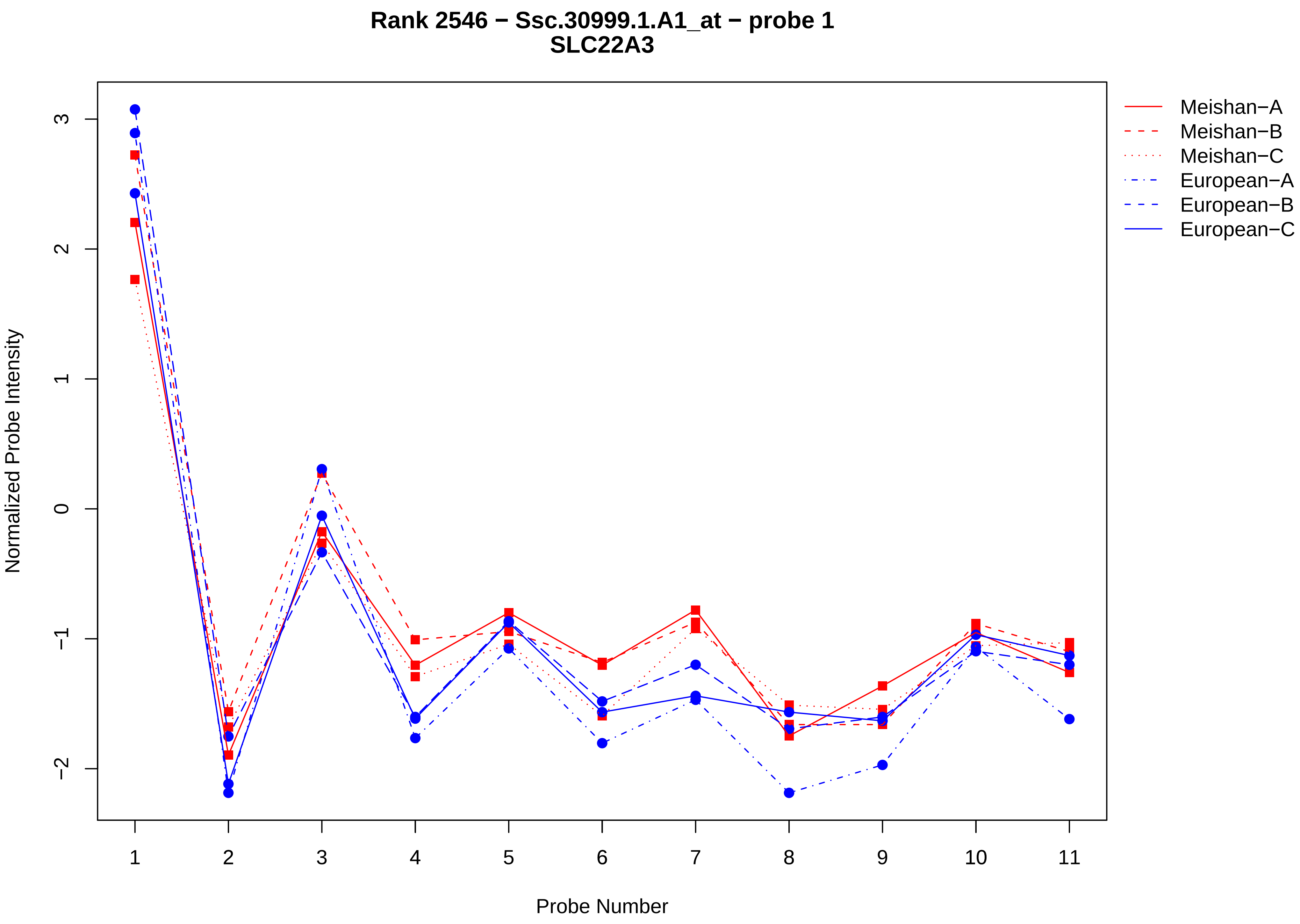

Rank 2546 − Ssc.30999.1.A1_at − probe 1
SLC22A3
Normalized Probe Intensity
Probe Number
−2
−1
0
1
2
3
1
2
3
4
5
6
7
8
9
10
11
Meishan−A
Meishan−B
Meishan−C
European−A
European−B
European−C

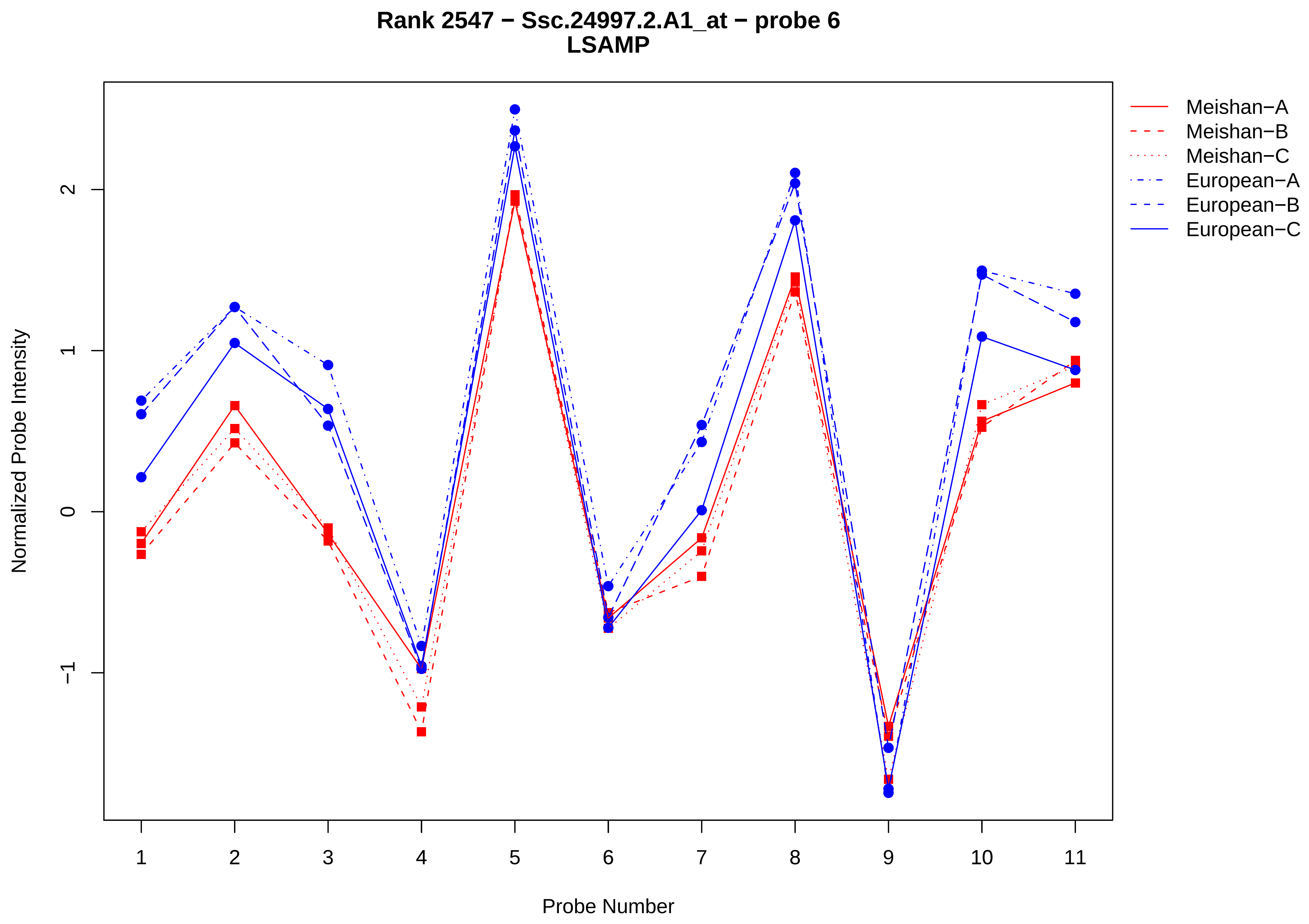

Rank 2547 − Ssc.24997.2.A1_at − probe 6
LSAMP
Normalized Probe Intensity
Probe Number
Meishan−A
Meishan−B
Meishan−C
European−A
European−B
European−C

Rank 2548 − Ssc.6830.1.A1_at − probe 9
PSMA1

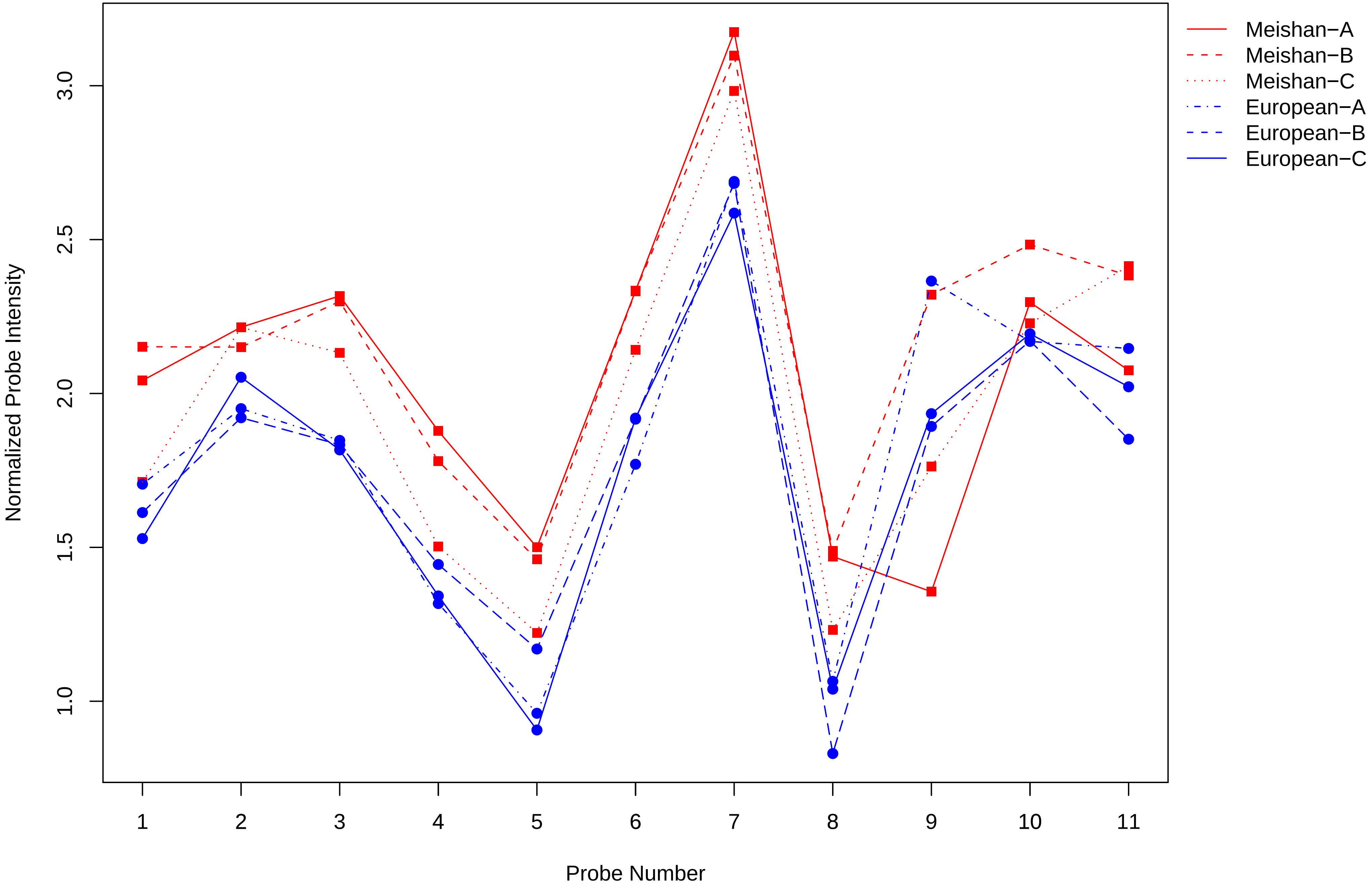

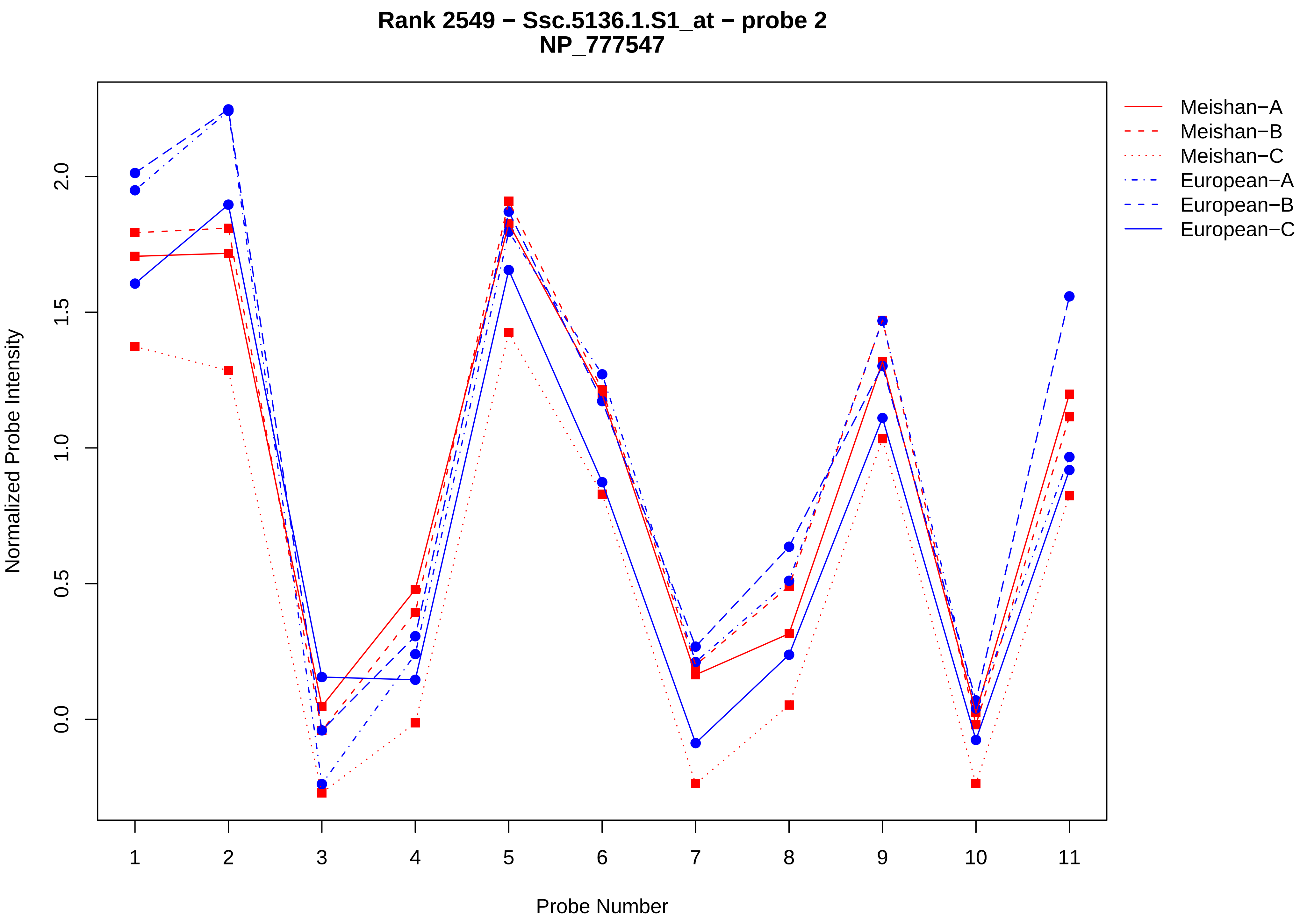

Rank 2549 − Ssc.5136.1.S1_at − probe 2
NP_777547
Normalized Probe Intensity
Probe Number
0.0
0.5
1.0
1.5
2.0
1
2
3
4
5
6
7
8
9
10
11
Meishan−A
Meishan−B
Meishan−C
European−A
European−B
European−C

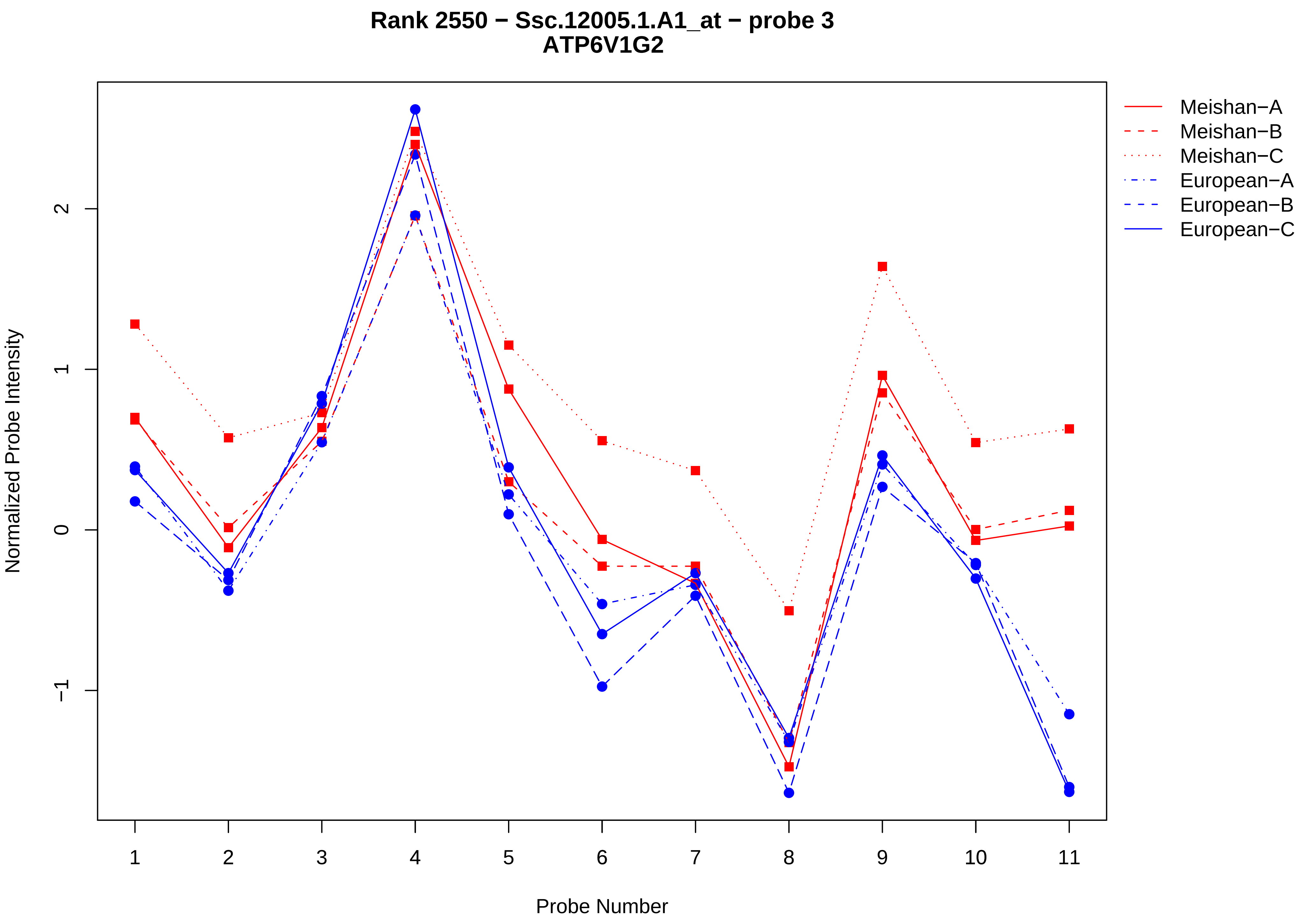
Rank 2550 − Ssc.12005.1.A1_at − probe 3
ATP6V1G2
Normalized Probe Intensity
Probe Number
−1
0
1
2
1
2
3
4
5
6
7
8
9
10
11
Meishan−A
Meishan−B
Meishan−C
European−A
European−B
European−C

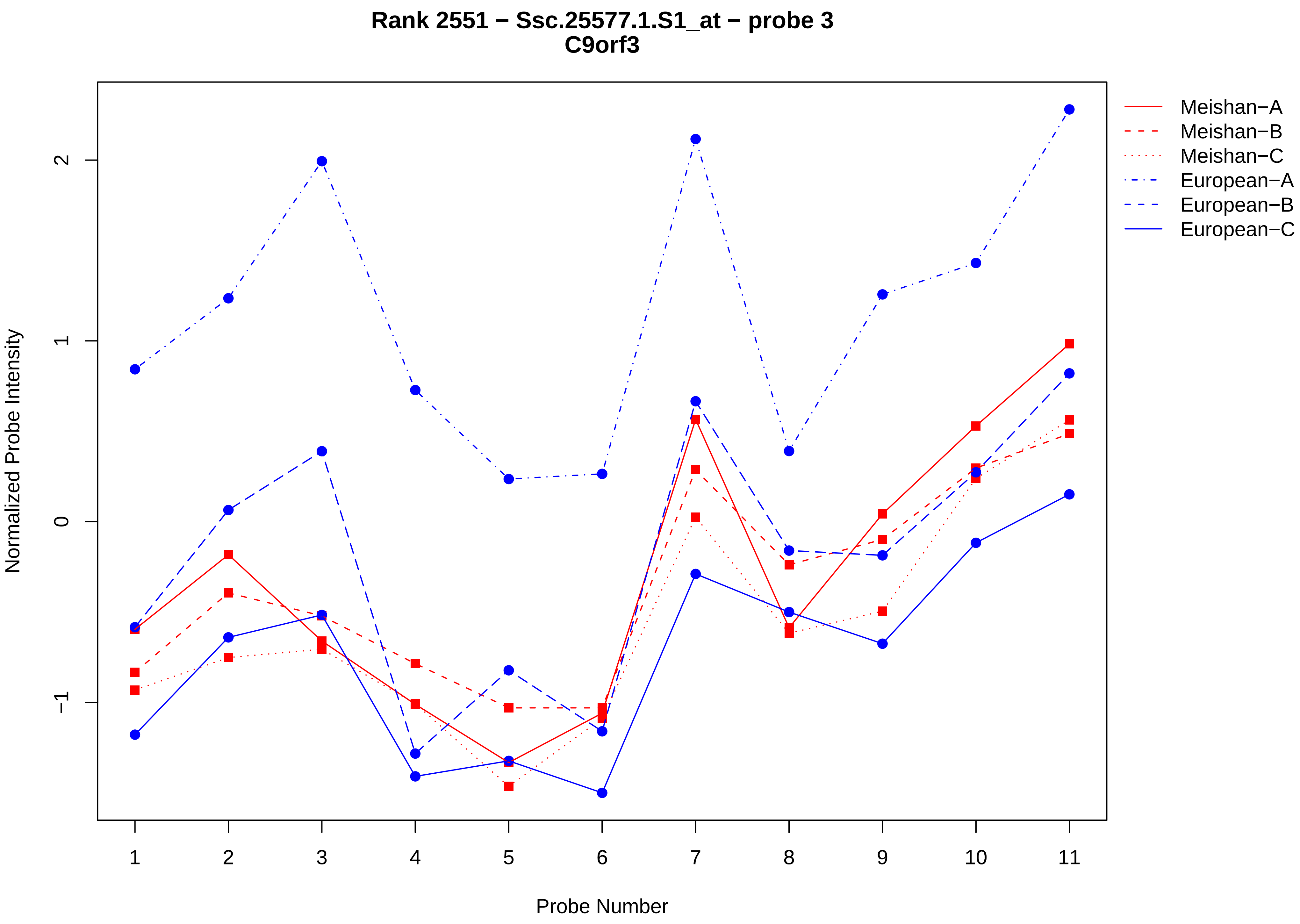

Rank 2551 − Ssc.25577.1.S1_at − probe 3
C9orf3
Normalized Probe Intensity
Probe Number
−1
0
1
2
1
2
3
4
5
6
7
8
9
10
11
Meishan−A
Meishan−B
Meishan−C
European−A
European−B
European−C

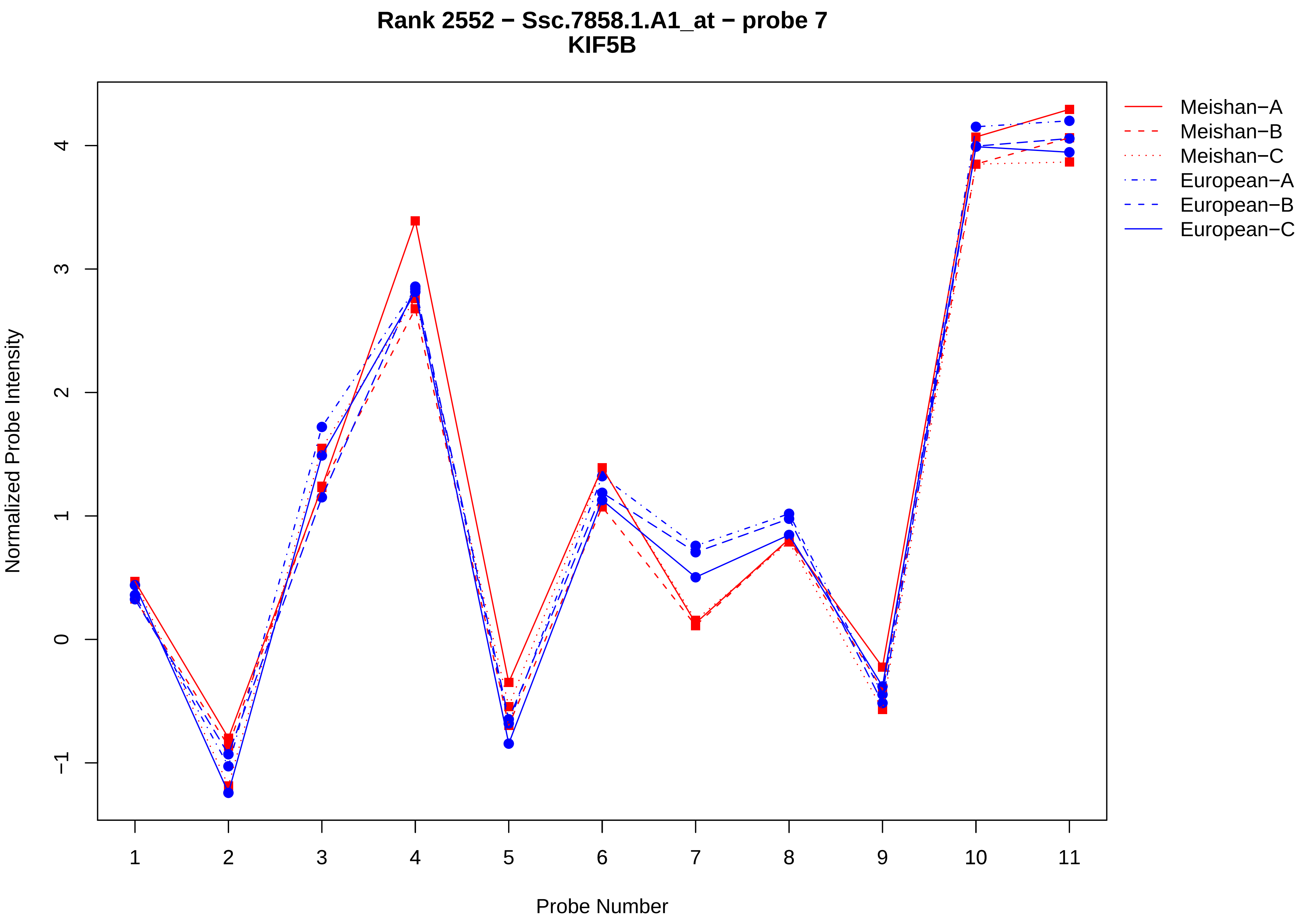

Rank 2552 − Ssc.7858.1.A1_at − probe 7
KIF5B
Normalized Probe Intensity
Probe Number
−1
0
1
2
3
4
1
2
3
4
5
6
7
8
9
10
11
Meishan−A
Meishan−B
Meishan−C
European−A
European−B
European−C

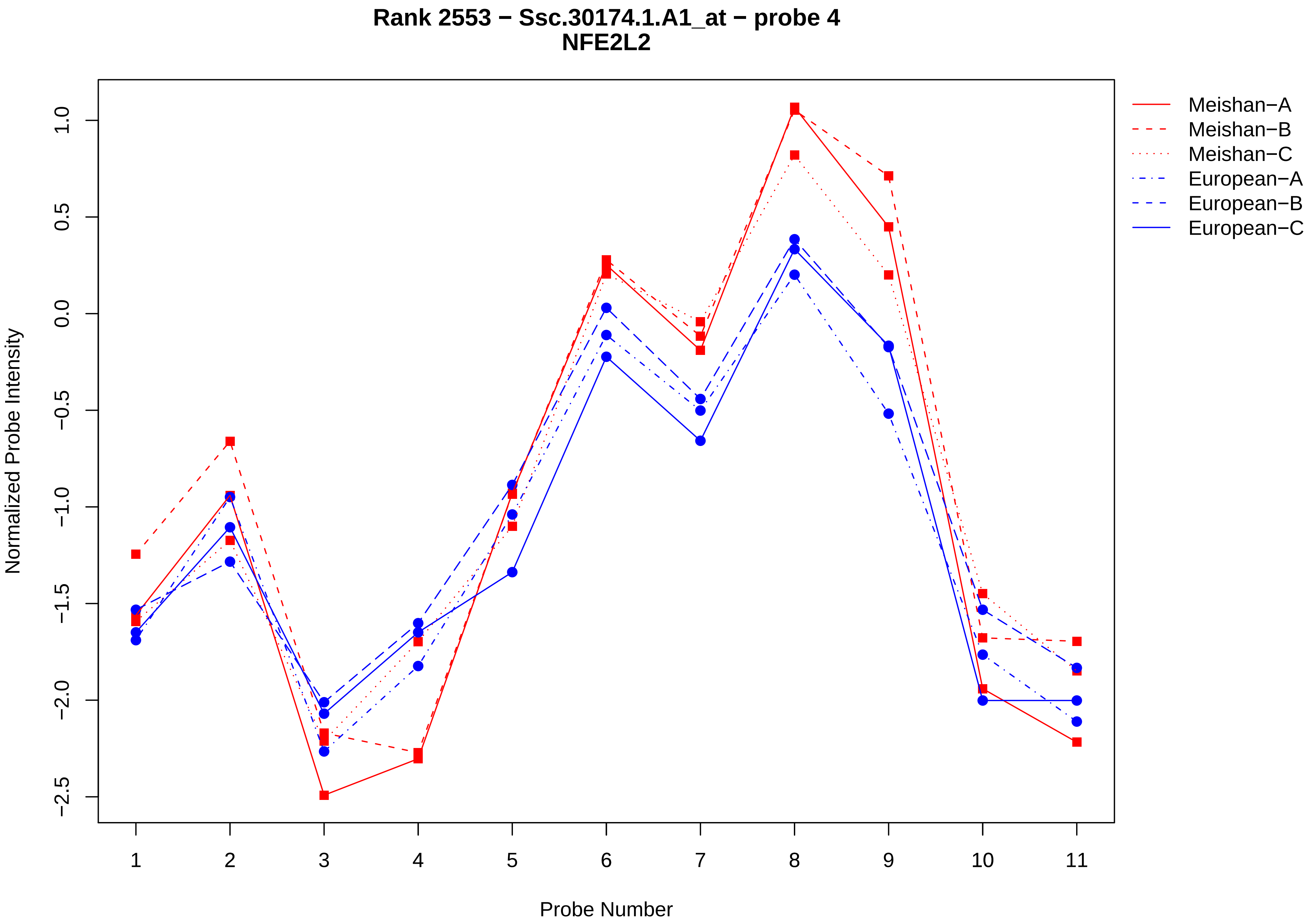

Rank 2553 − Ssc.30174.1.A1_at − probe 4
NFE2L2
Normalized Probe Intensity
Probe Number
1.0
0.5
0.0
−0.5
−1.0
−1.5
−2.0
−2.5
1
2
3
4
5
6
7
8
9
10
11
Meishan−A
Meishan−B
Meishan−C
European−A
European−B
European−C

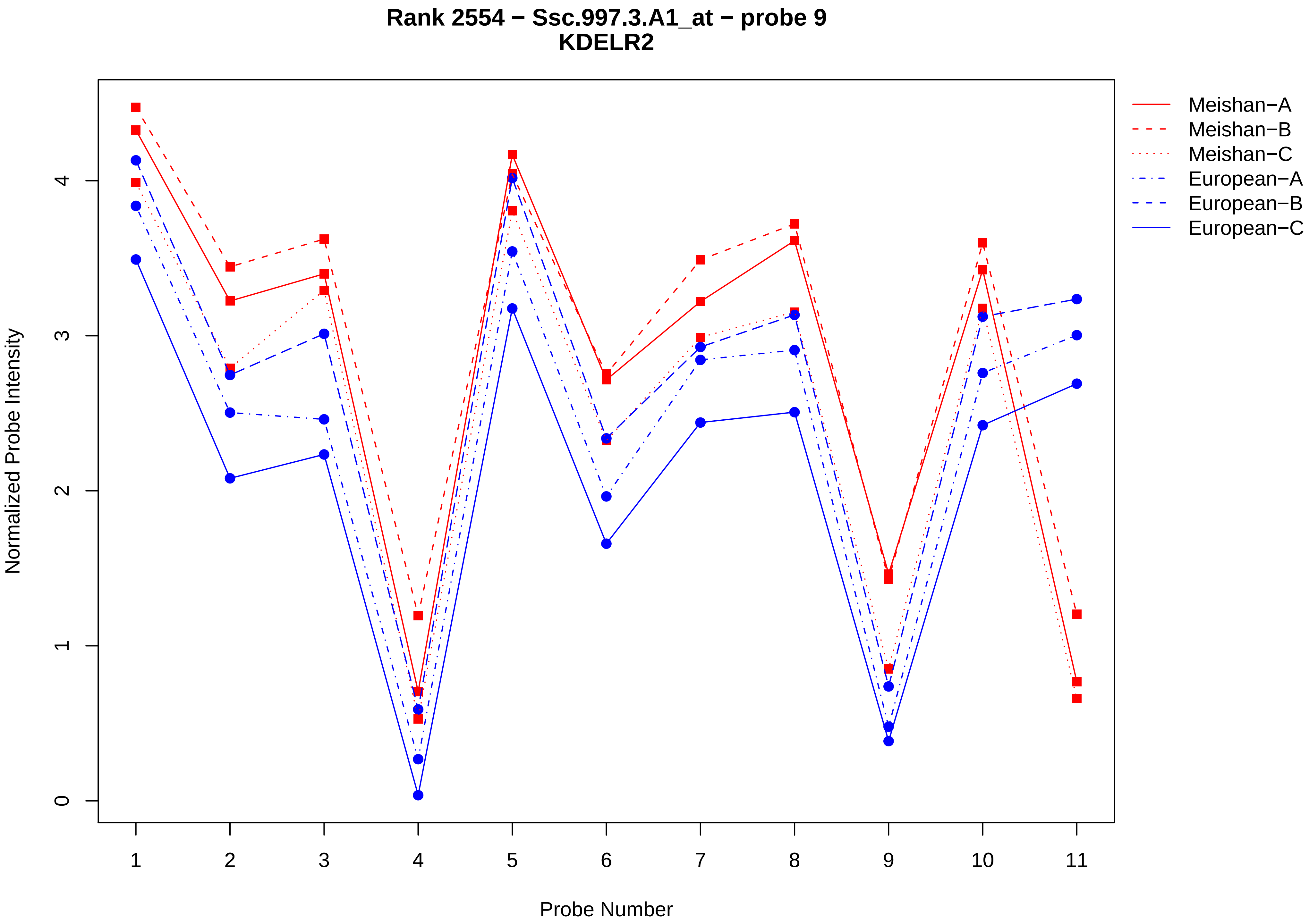

Rank 2554 − Ssc.997.3.A1_at − probe 9
KDELR2
Normalized Probe Intensity
Probe Number
0
1
2
3
4
1
2
3
4
5
6
7
8
9
10
11
Meishan−A
Meishan−B
Meishan−C
European−A
European−B
European−C

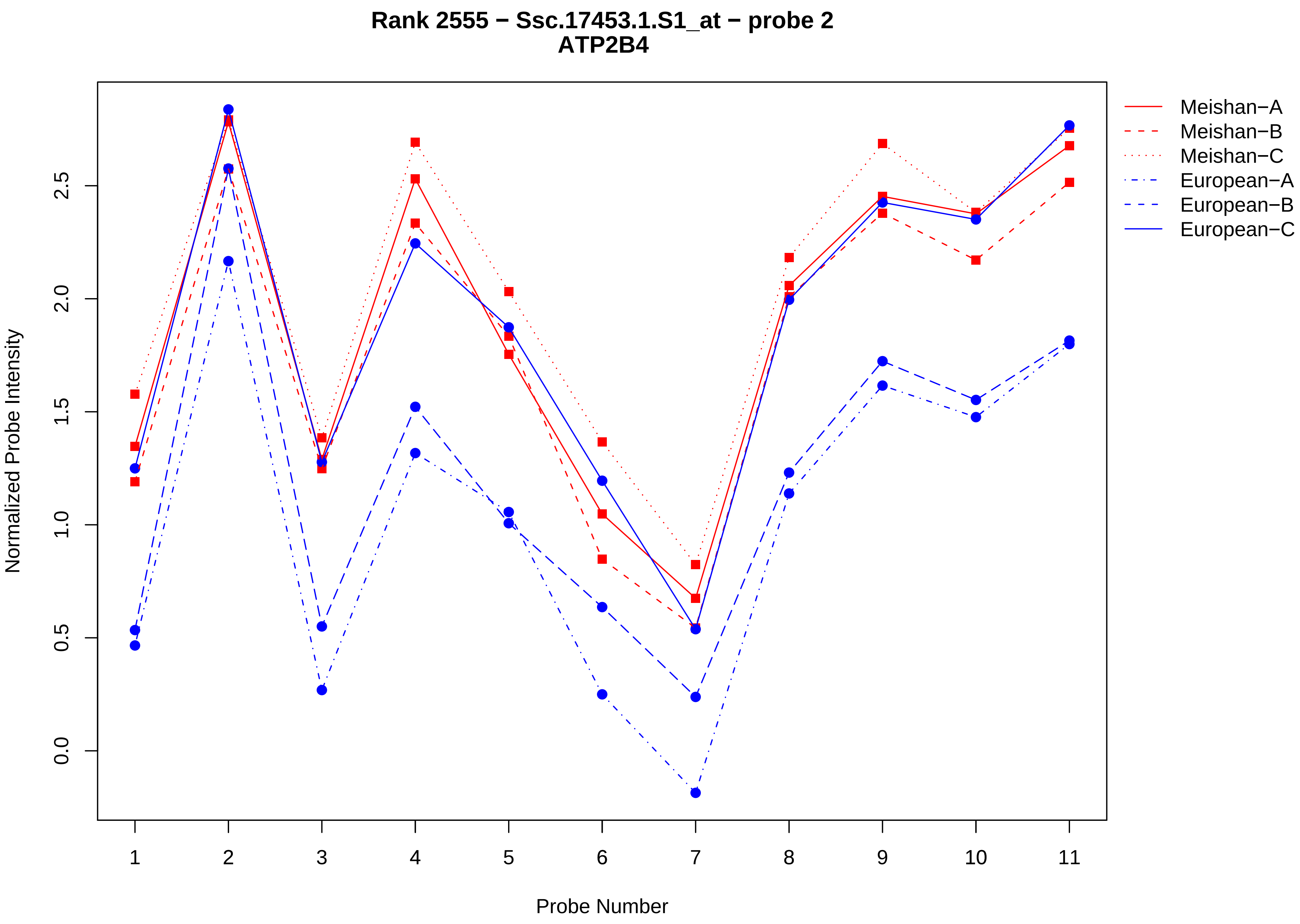
Rank 2555 − Ssc.17453.1.S1_at − probe 2
ATP2B4
Normalized Probe Intensity
Probe Number
0.0
0.5
1.0
1.5
2.0
2.5
1
2
3
4
5
6
7
8
9
10
11
Meishan−A
Meishan−B
Meishan−C
European−A
European−B
European−C

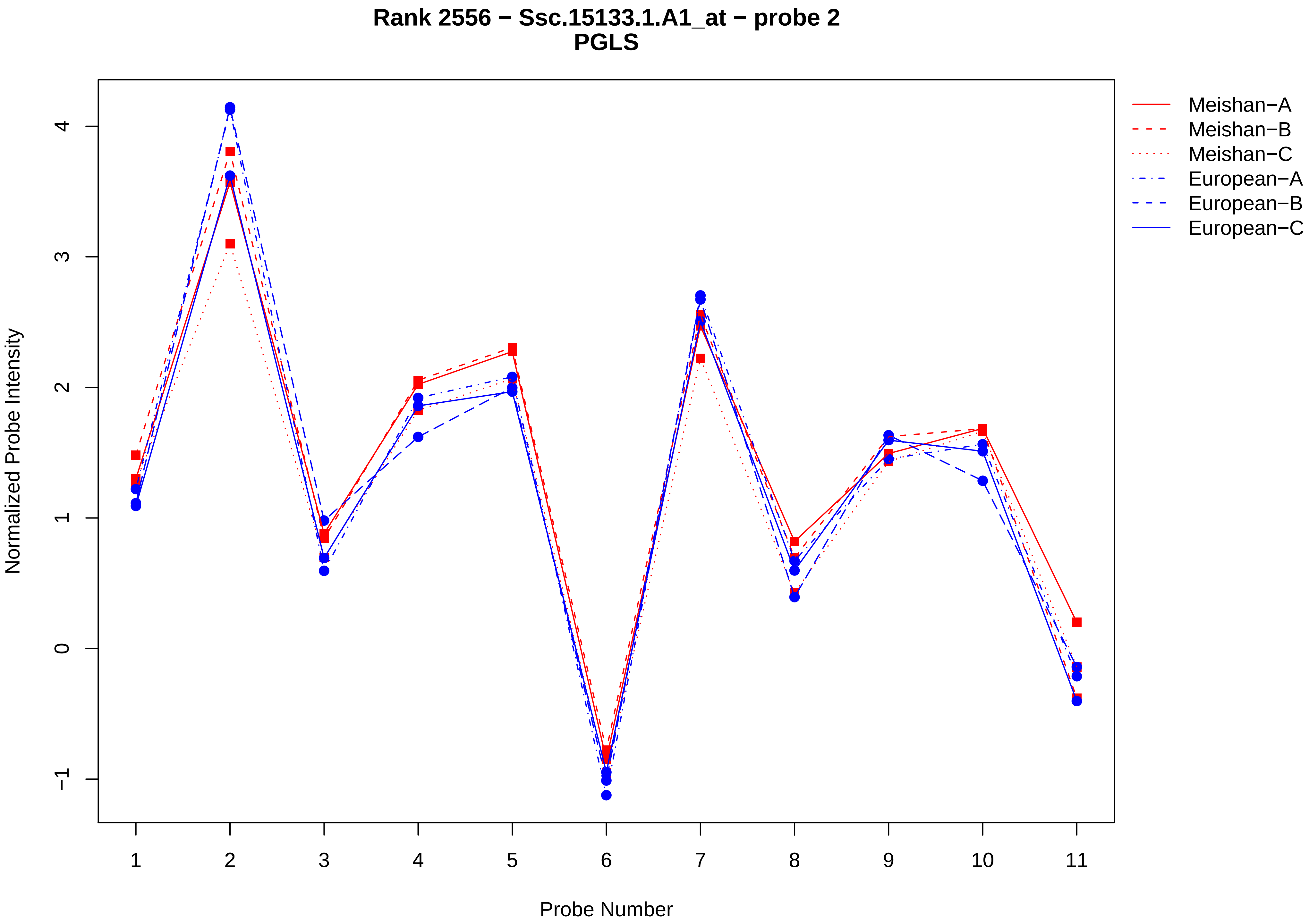
Rank 2556 − Ssc.15133.1.A1_at − probe 2
PGLS
Normalized Probe Intensity
Probe Number
Meishan−A
Meishan−B
Meishan−C
European−A
European−B
European−C
−1
0
1
2
3
4
1
2
3
4
5
6
7
8
9
10
11

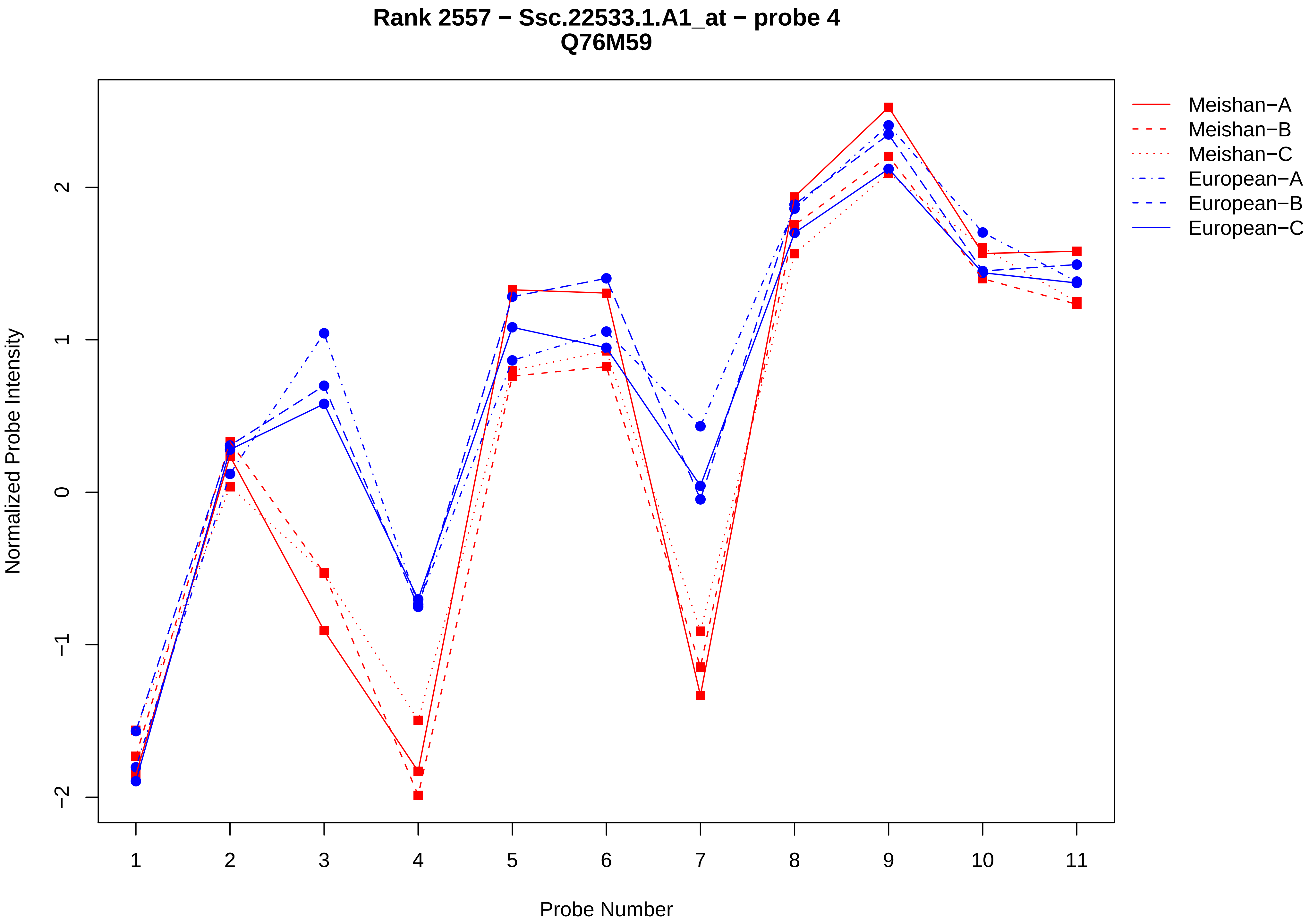

Rank 2557 − Ssc.22533.1.A1_at − probe 4
Q76M59
Normalized Probe Intensity
Probe Number
−2
−1
0
1
2
1
2
3
4
5
6
7
8
9
10
11
Meishan−A
Meishan−B
Meishan−C
European−A
European−B
European−C

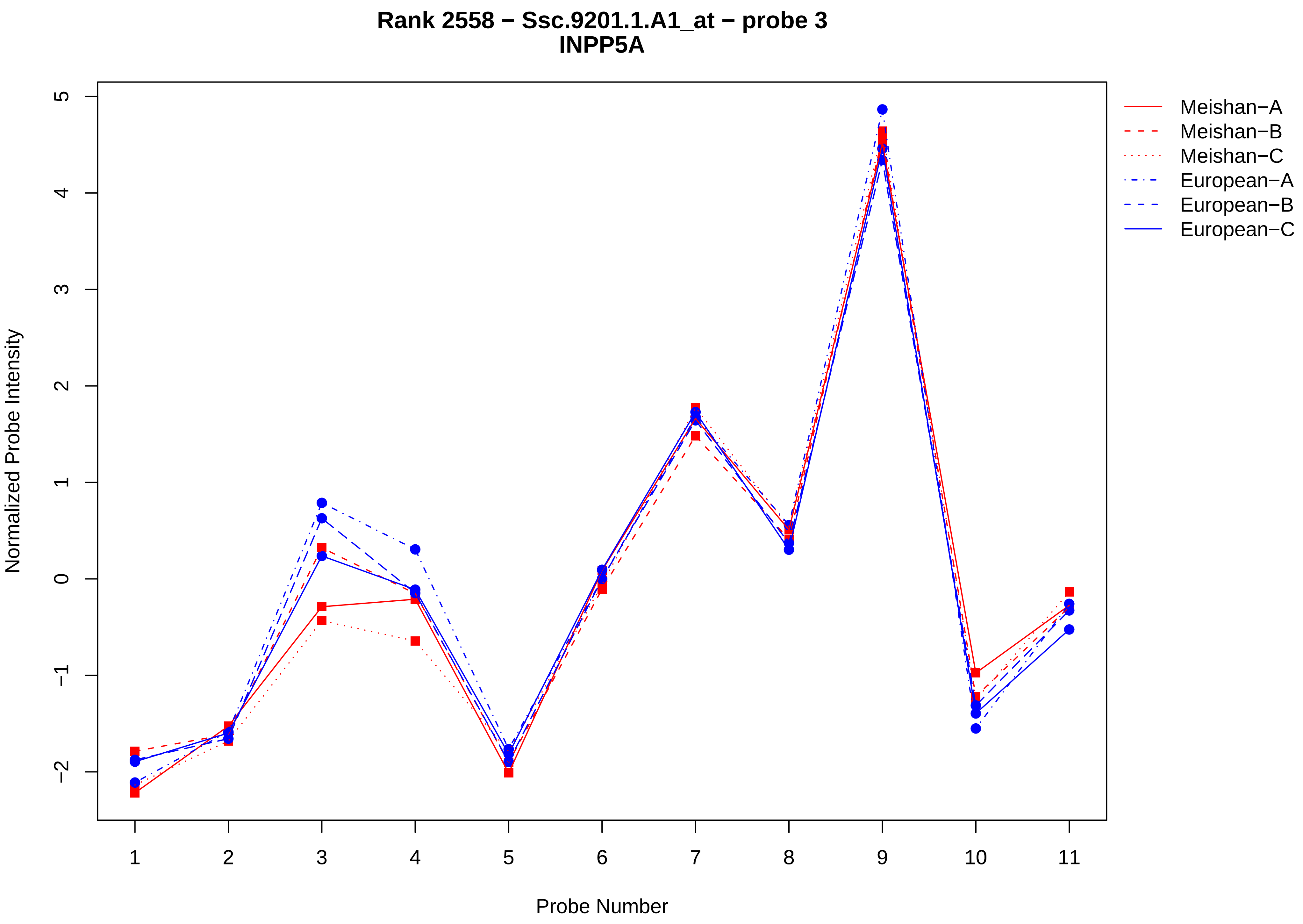

Rank 2558 − Ssc.9201.1.A1_at − probe 3
INPP5A
Normalized Probe Intensity
Probe Number
Meishan−A
Meishan−B
Meishan−C
European−A
European−B
European−C

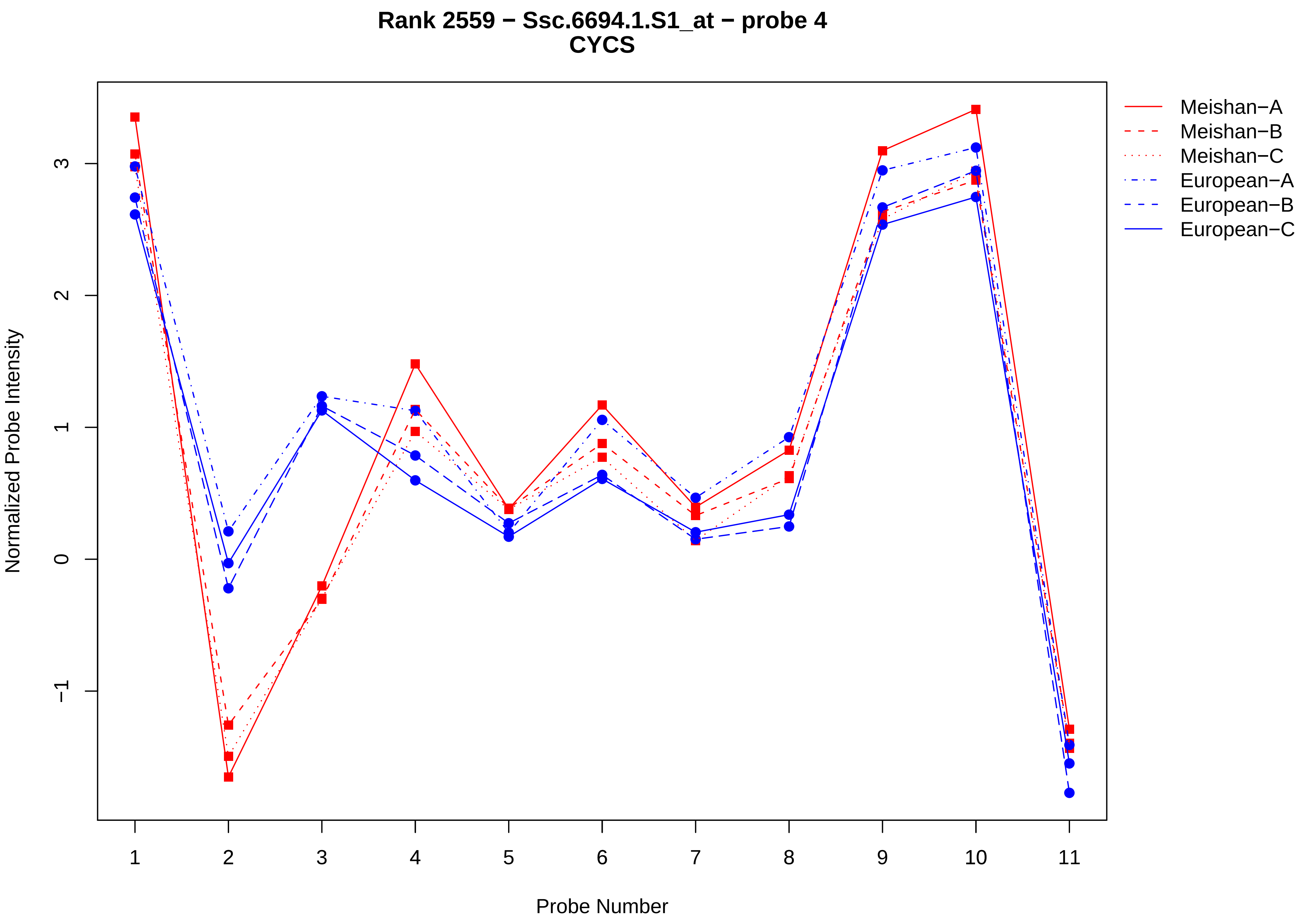

Rank 2559 − Ssc.6694.1.S1_at − probe 4
CYCS
Normalized Probe Intensity
Probe Number
−1
0
1
2
3
1
2
3
4
5
6
7
8
9
10
11
Meishan−A
Meishan−B
Meishan−C
European−A
European−B
European−C

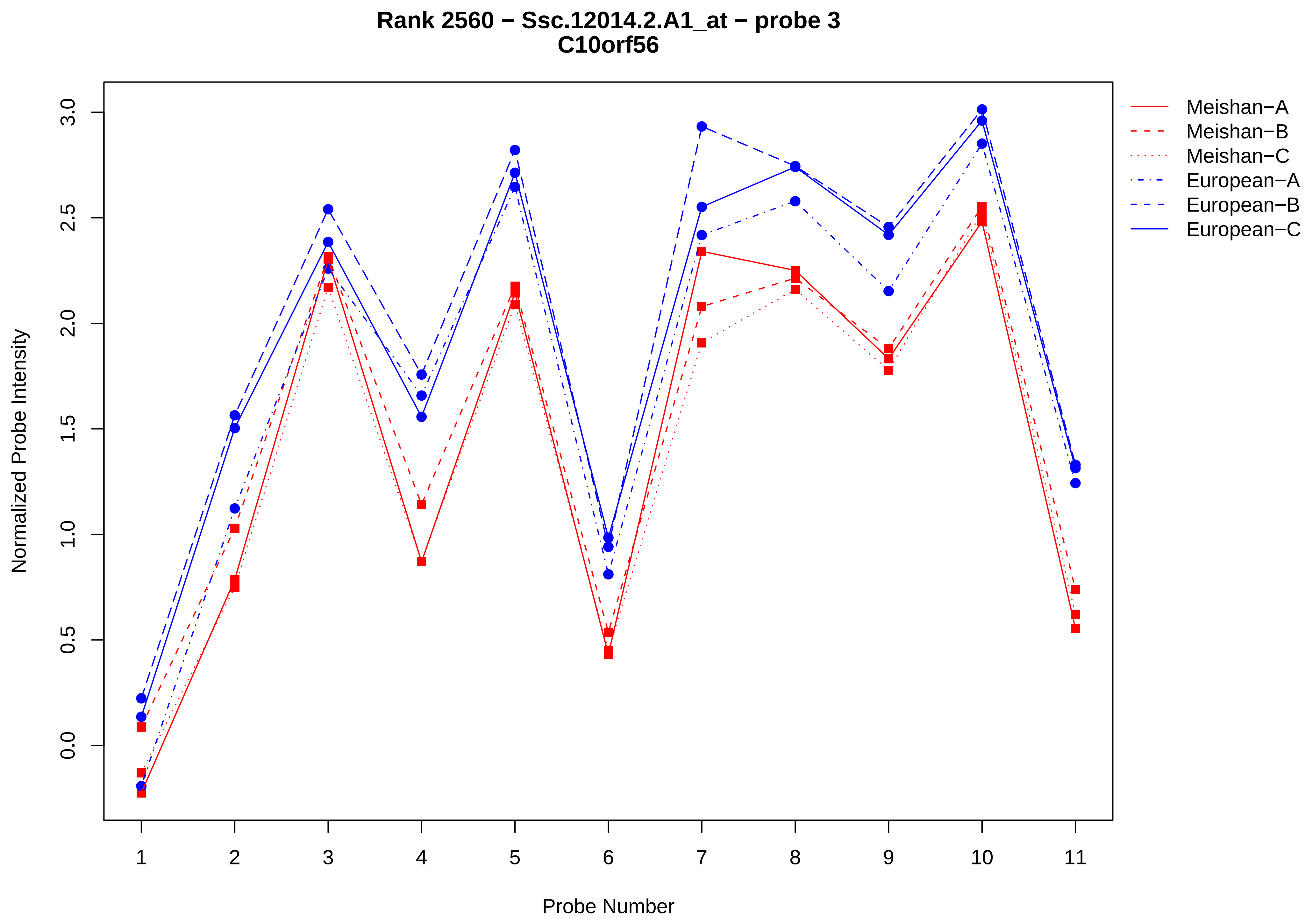

Rank 2560 − Ssc.12014.2.A1_at − probe 3
C10orf56
Normalized Probe Intensity
Probe Number
0.0
0.5
1.0
1.5
2.0
2.5
3.0
1
2
3
4
5
6
7
8
9
10
11
Meishan−A
Meishan−B
Meishan−C
European−A
European−B
European−C

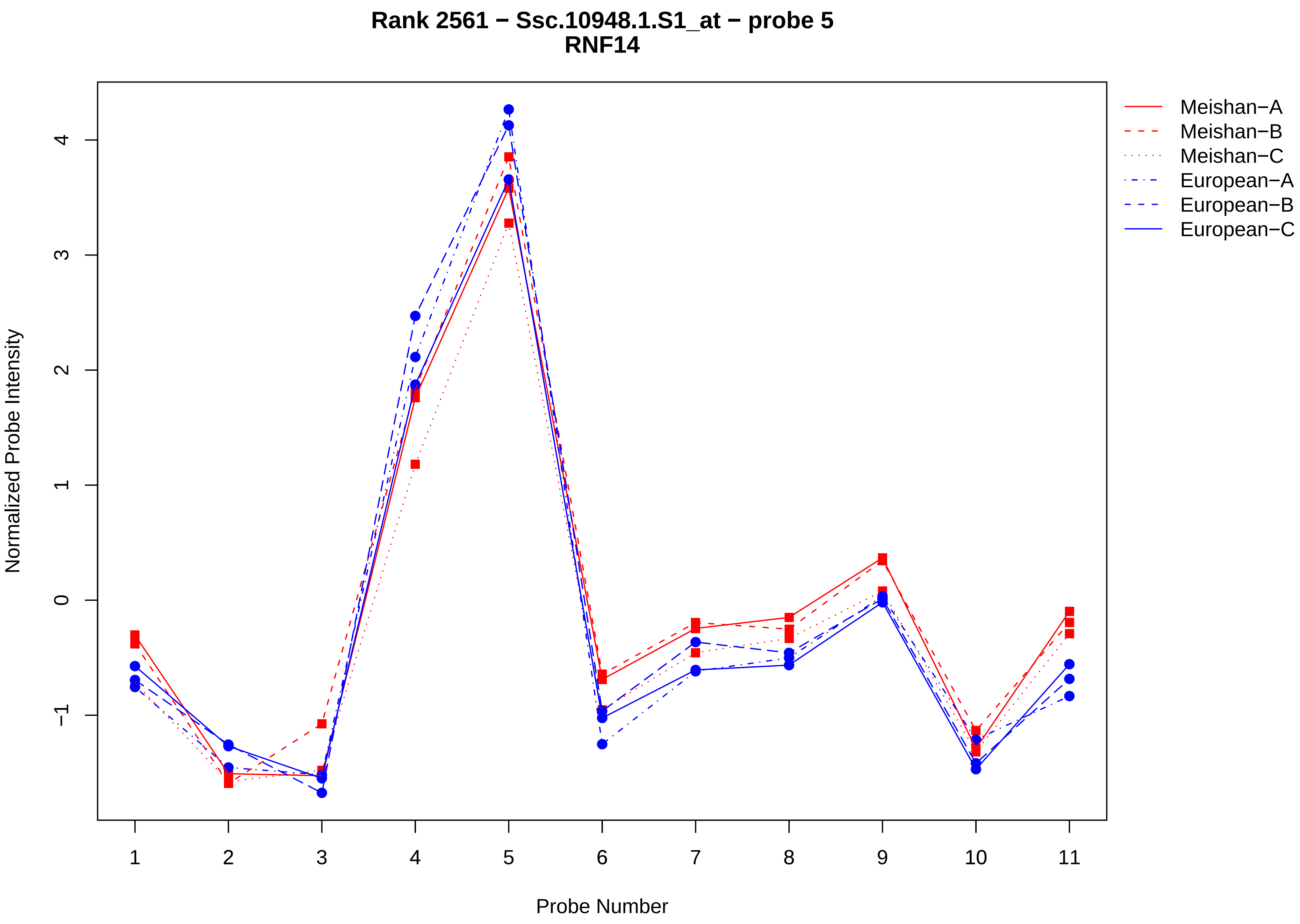

Rank 2561 − Ssc.10948.1.S1_at − probe 5
RNF14
Normalized Probe Intensity
Probe Number
−1
0
1
2
3
4
1
2
3
4
5
6
7
8
9
10
11
Meishan−A
Meishan−B
Meishan−C
European−A
European−B
European−C

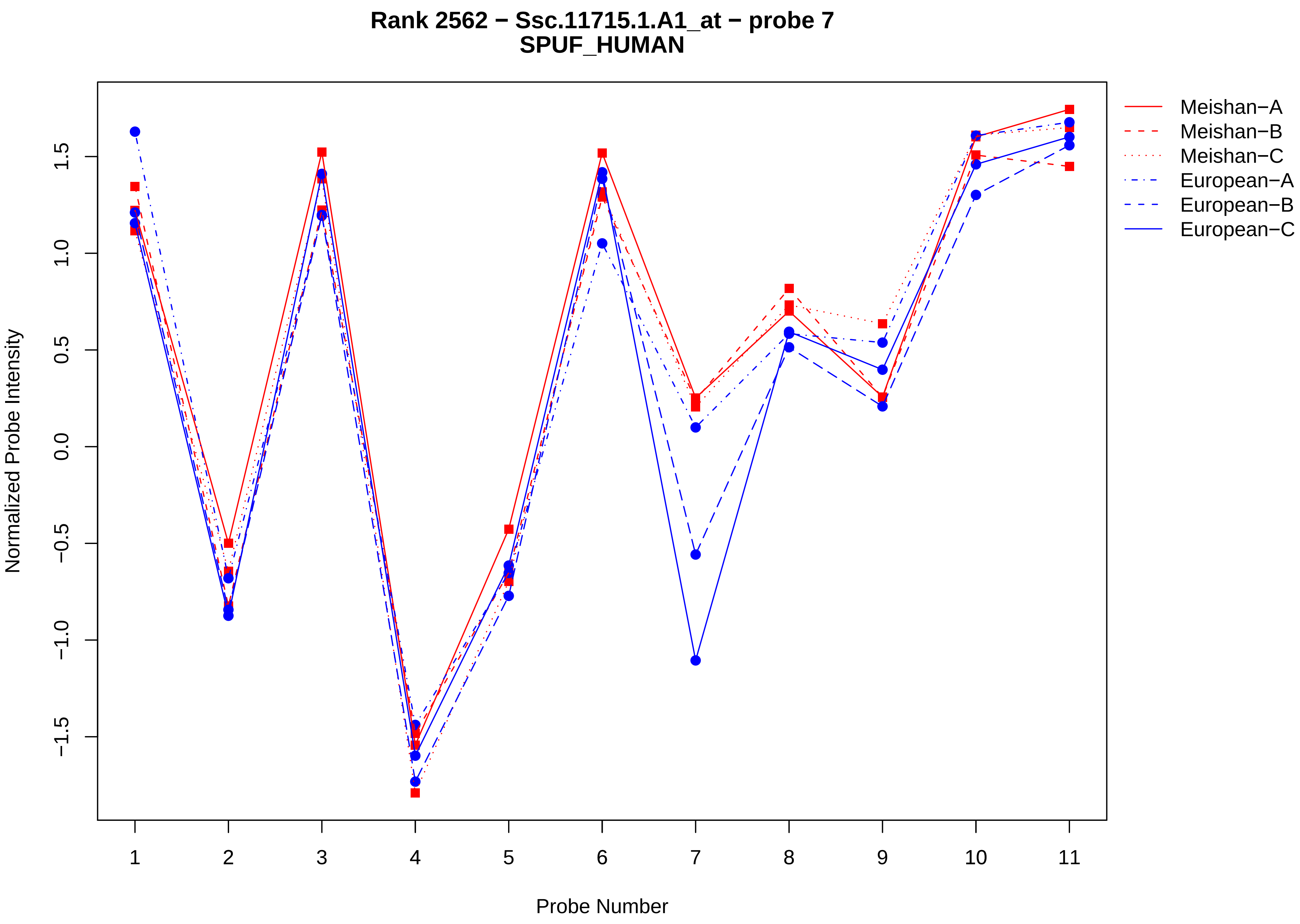

Rank 2562 − Ssc.11715.1.A1_at − probe 7
SPUF_HUMAN
Normalized Probe Intensity
Probe Number
Meishan−A
Meishan−B
Meishan−C
European−A
European−B
European−C
1.5
1.0
0.5
0.0
−0.5
−1.0
−1.5
1
2
3
4
5
6
7
8
9
10
11

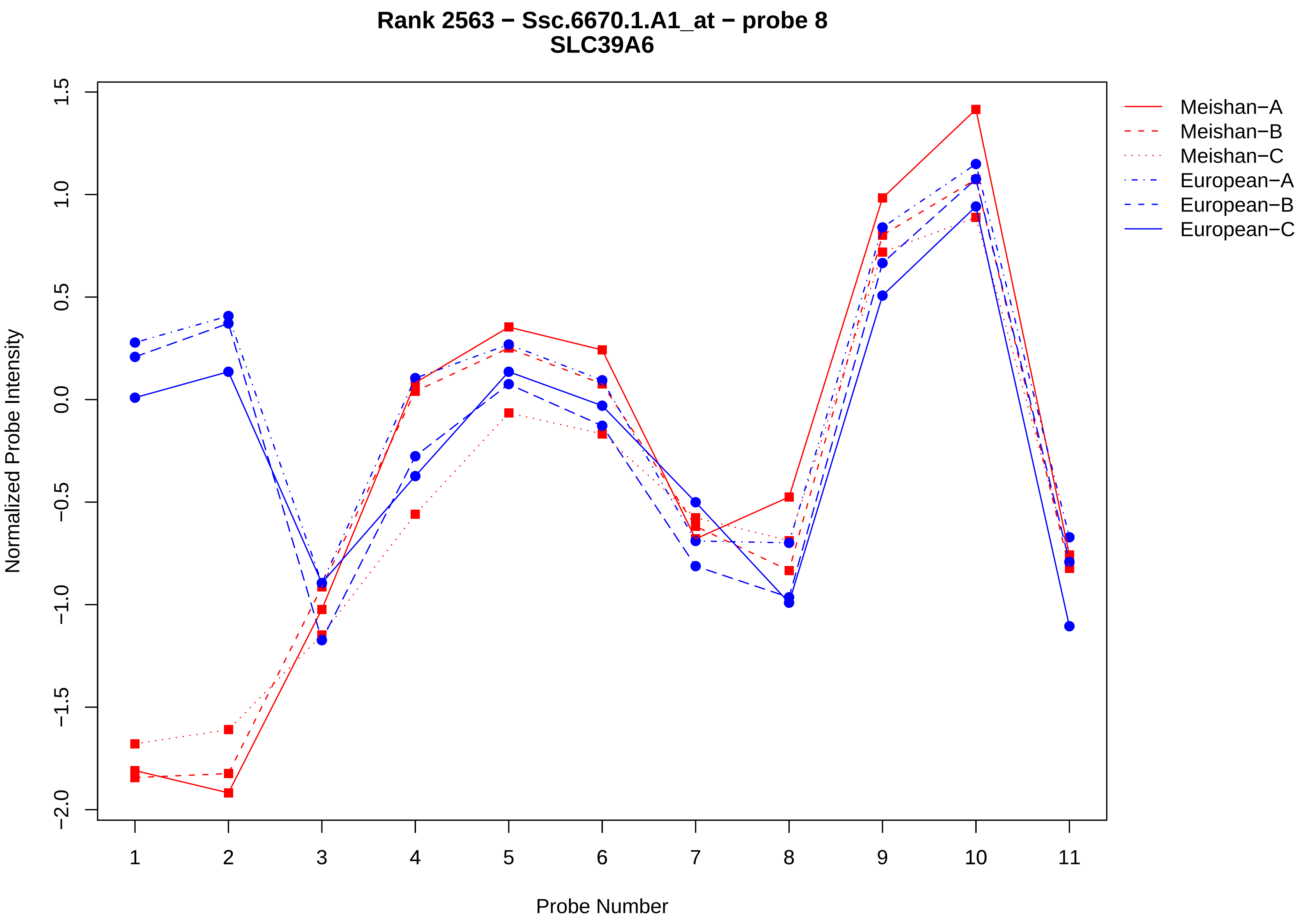
Rank 2563 − Ssc.6670.1.A1_at − probe 8
SLC39A6
Normalized Probe Intensity
Probe Number
1.5
1.0
0.5
0.0
−0.5
−1.0
−1.5
−2.0
1
2
3
4
5
6
7
8
9
10
11
Meishan−A
Meishan−B
Meishan−C
European−A
European−B
European−C

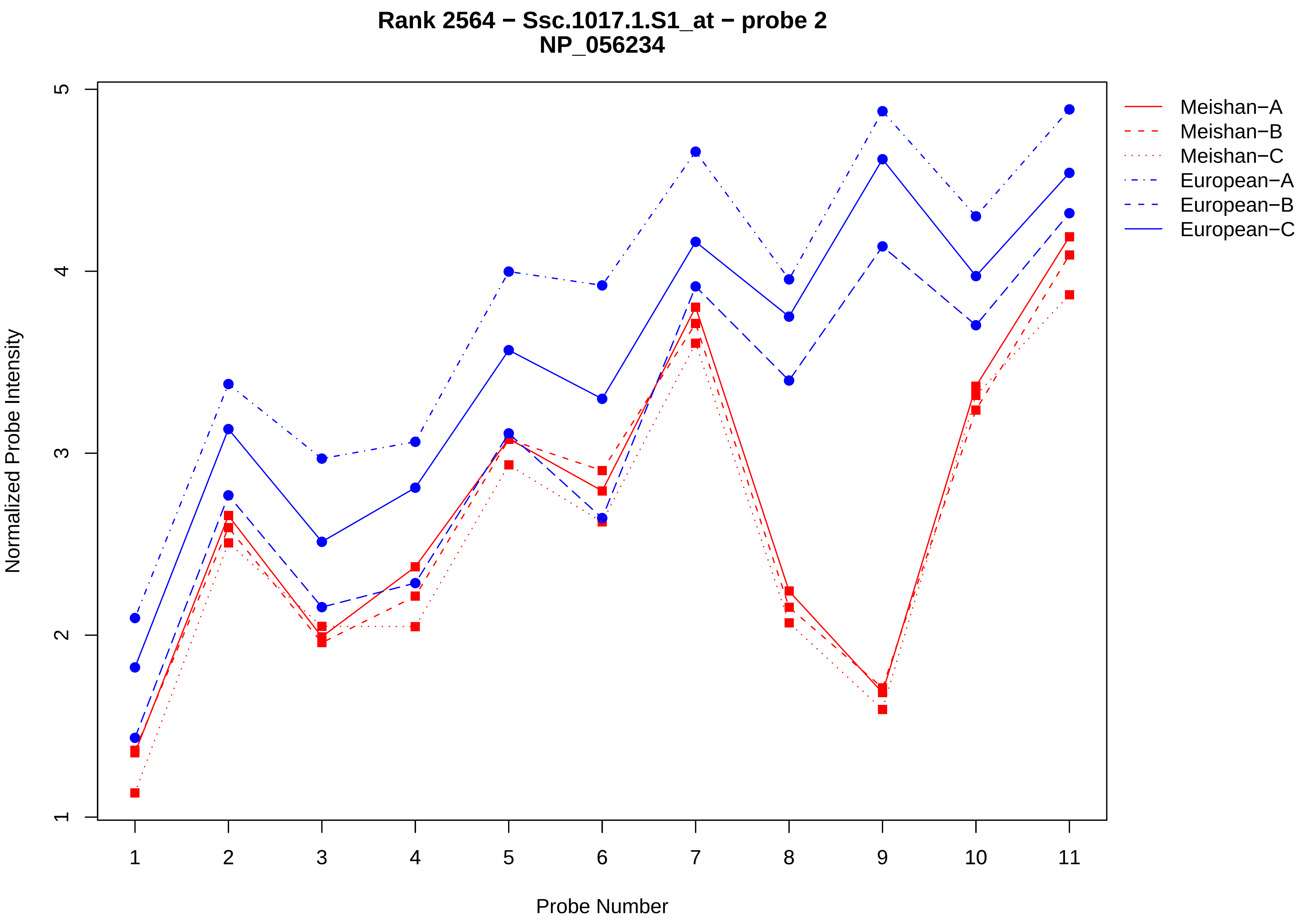

Rank 2564 − Ssc.1017.1.S1_at − probe 2
NP_056234
Normalized Probe Intensity
Probe Number
1
2
3
4
5
1
2
3
4
5
6
7
8
9
10
11
Meishan−A
Meishan−B
Meishan−C
European−A
European−B
European−C

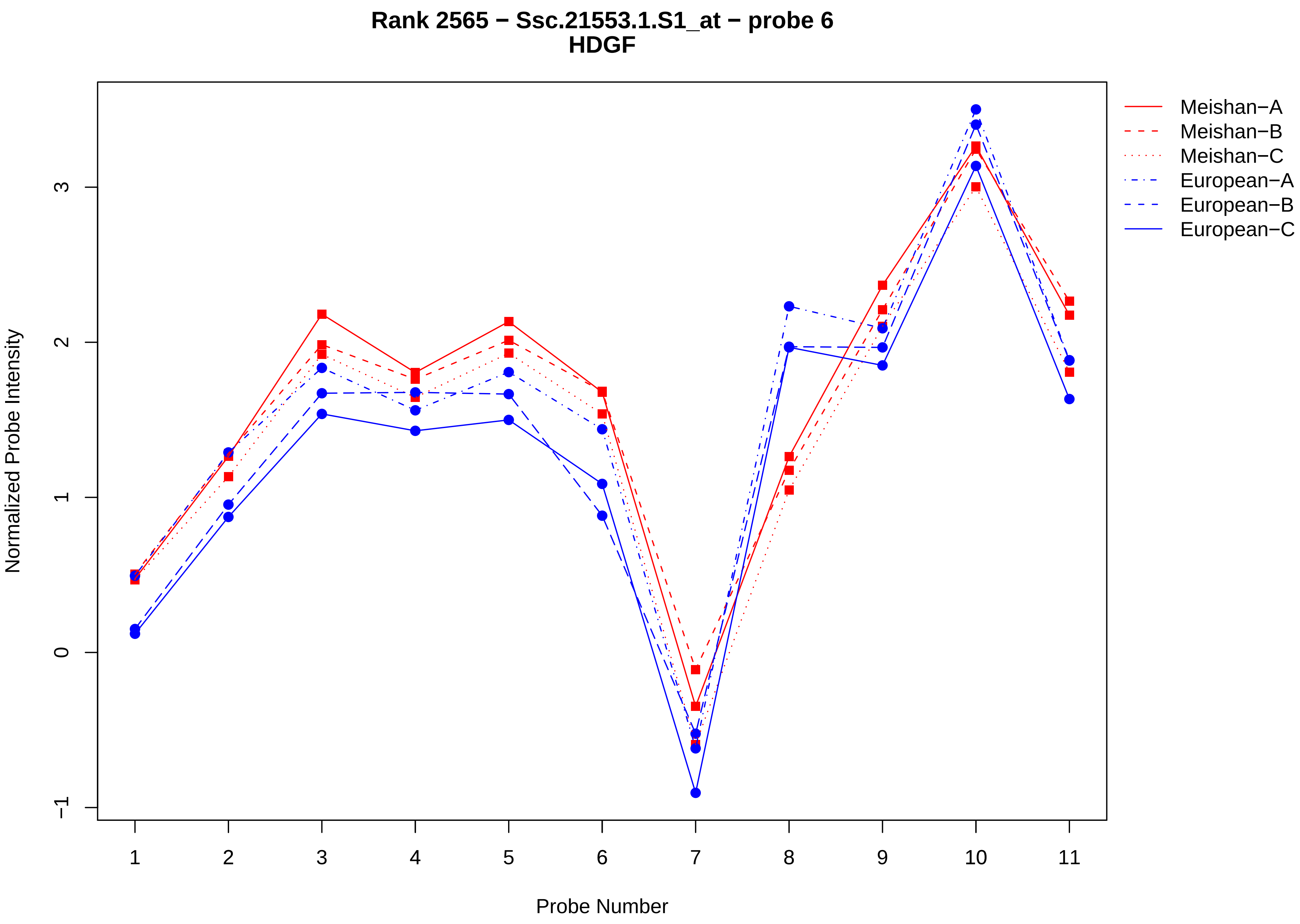

Rank 2565 − Ssc.21553.1.S1_at − probe 6
HDGF
Normalized Probe Intensity
Probe Number
−1
0
1
2
3
1
2
3
4
5
6
7
8
9
10
11
Meishan−A
Meishan−B
Meishan−C
European−A
European−B
European−C

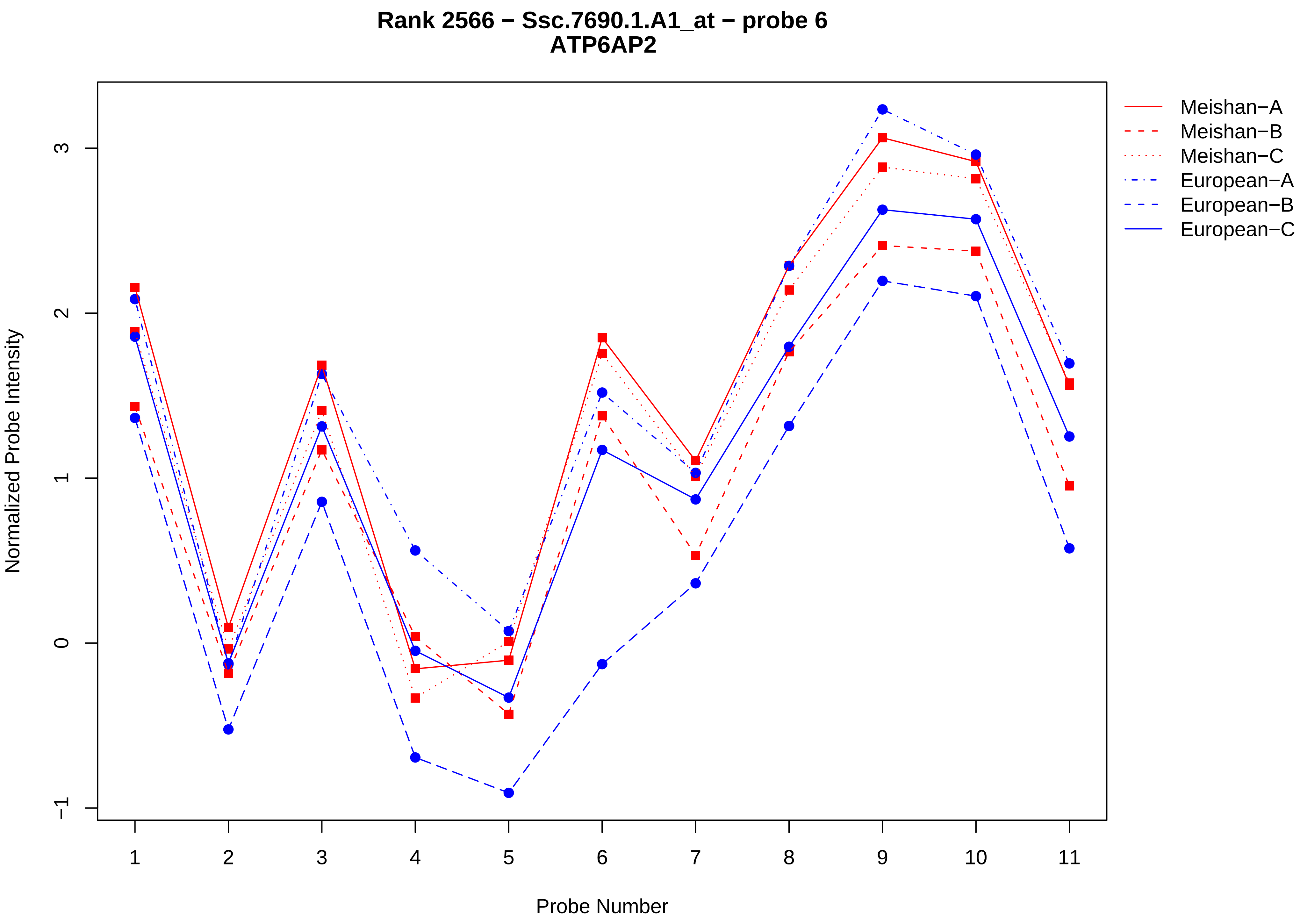
Rank 2566 − Ssc.7690.1.A1_at − probe 6
ATP6AP2
Normalized Probe Intensity
−1
0
1
2
3
1
2
3
4
5
6
7
8
9
10
11
Probe Number
Meishan−A
Meishan−B
Meishan−C
European−A
European−B
European−C

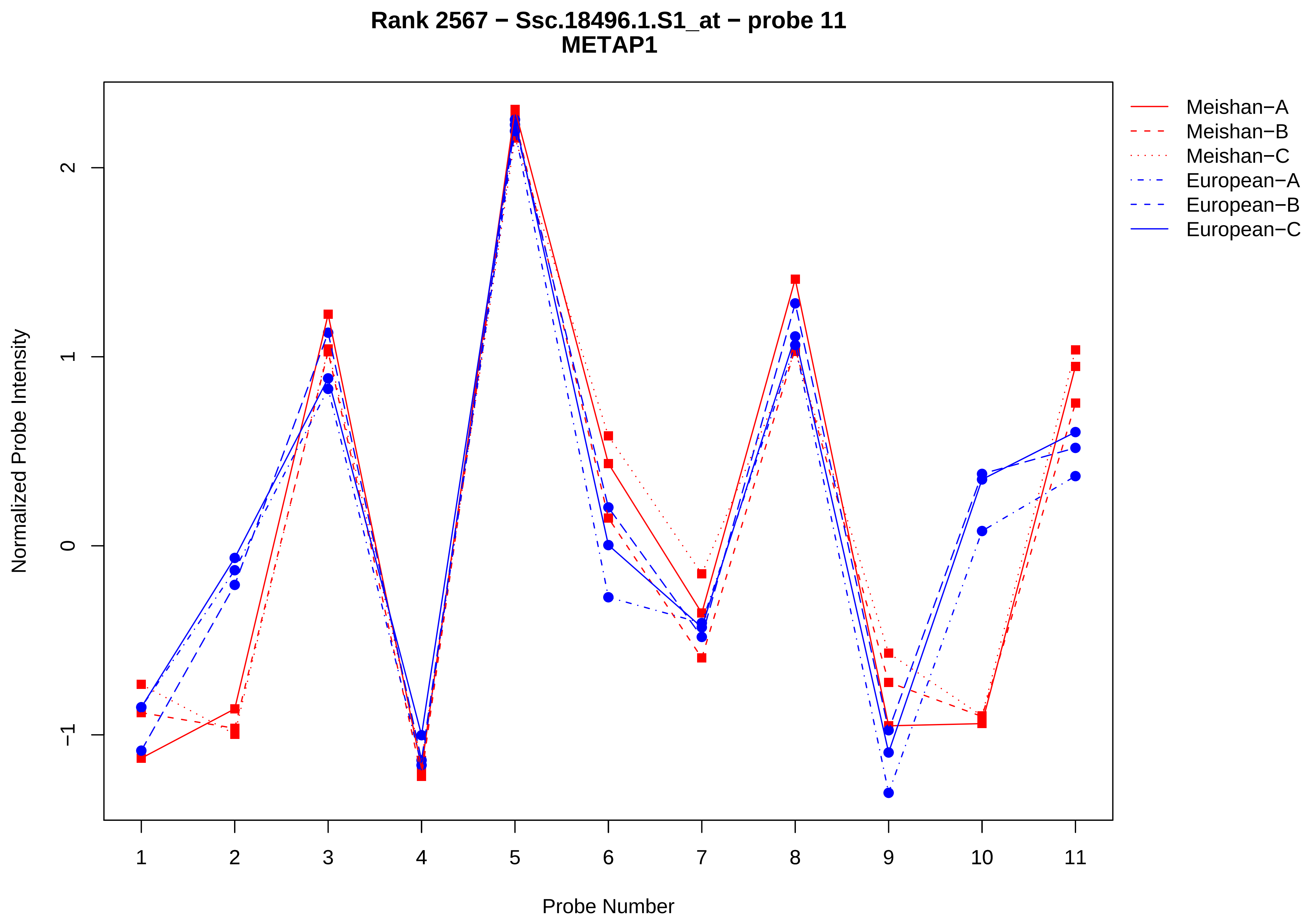

Rank 2567 − Ssc.18496.1.S1_at − probe 11
METAP1
Normalized Probe Intensity
Probe Number
−1
0
1
2
1
2
3
4
5
6
7
8
9
10
11
Meishan−A
Meishan−B
Meishan−C
European−A
European−B
European−C

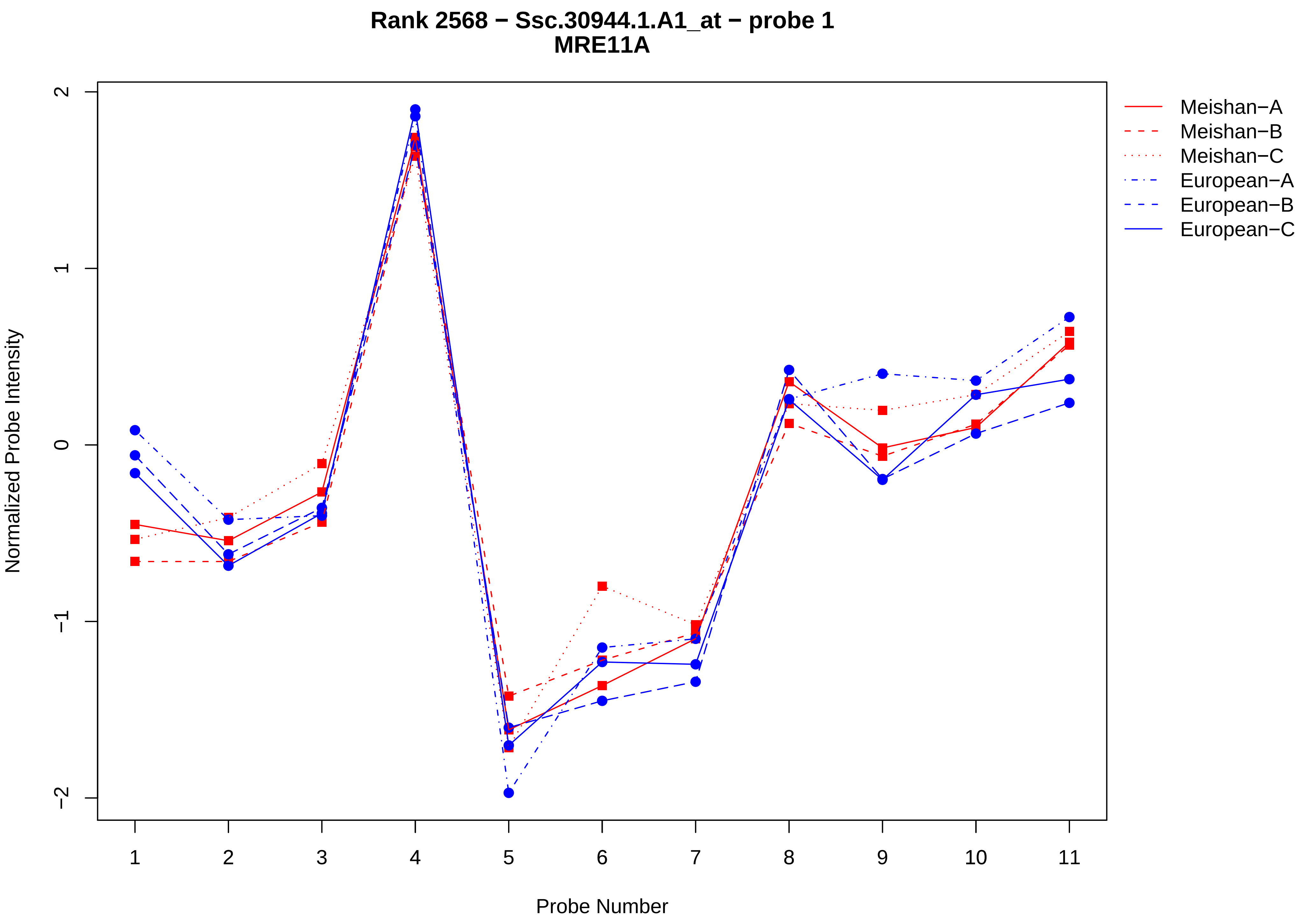

Rank 2568 − Ssc.30944.1.A1_at − probe 1
MRE11A
Normalized Probe Intensity
Probe Number
Meishan−A
Meishan−B
Meishan−C
European−A
European−B
European−C

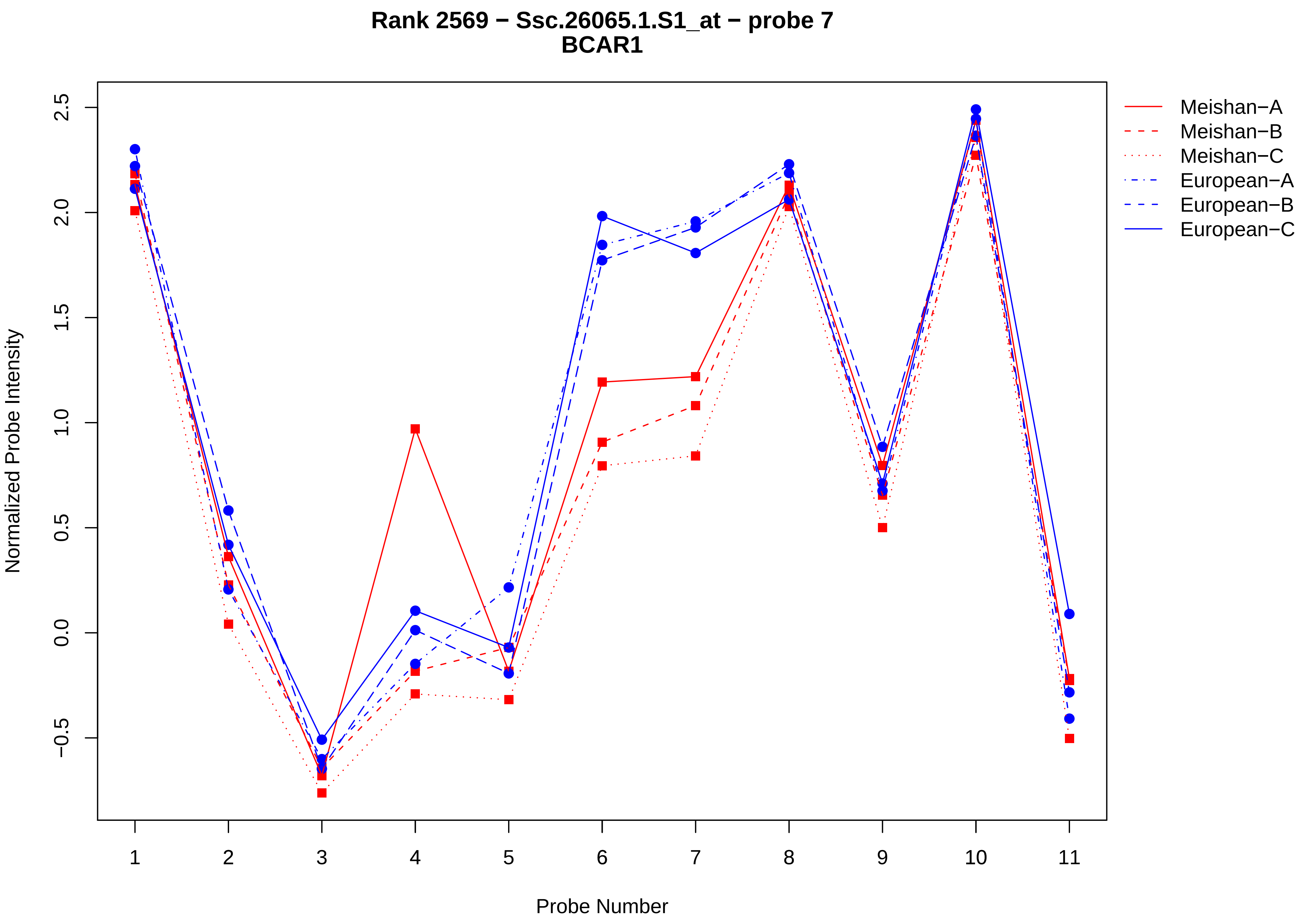

Rank 2569 − Ssc.26065.1.S1_at − probe 7
BCAR1
Normalized Probe Intensity
Probe Number
Meishan−A
Meishan−B
Meishan−C
European−A
European−B
European−C

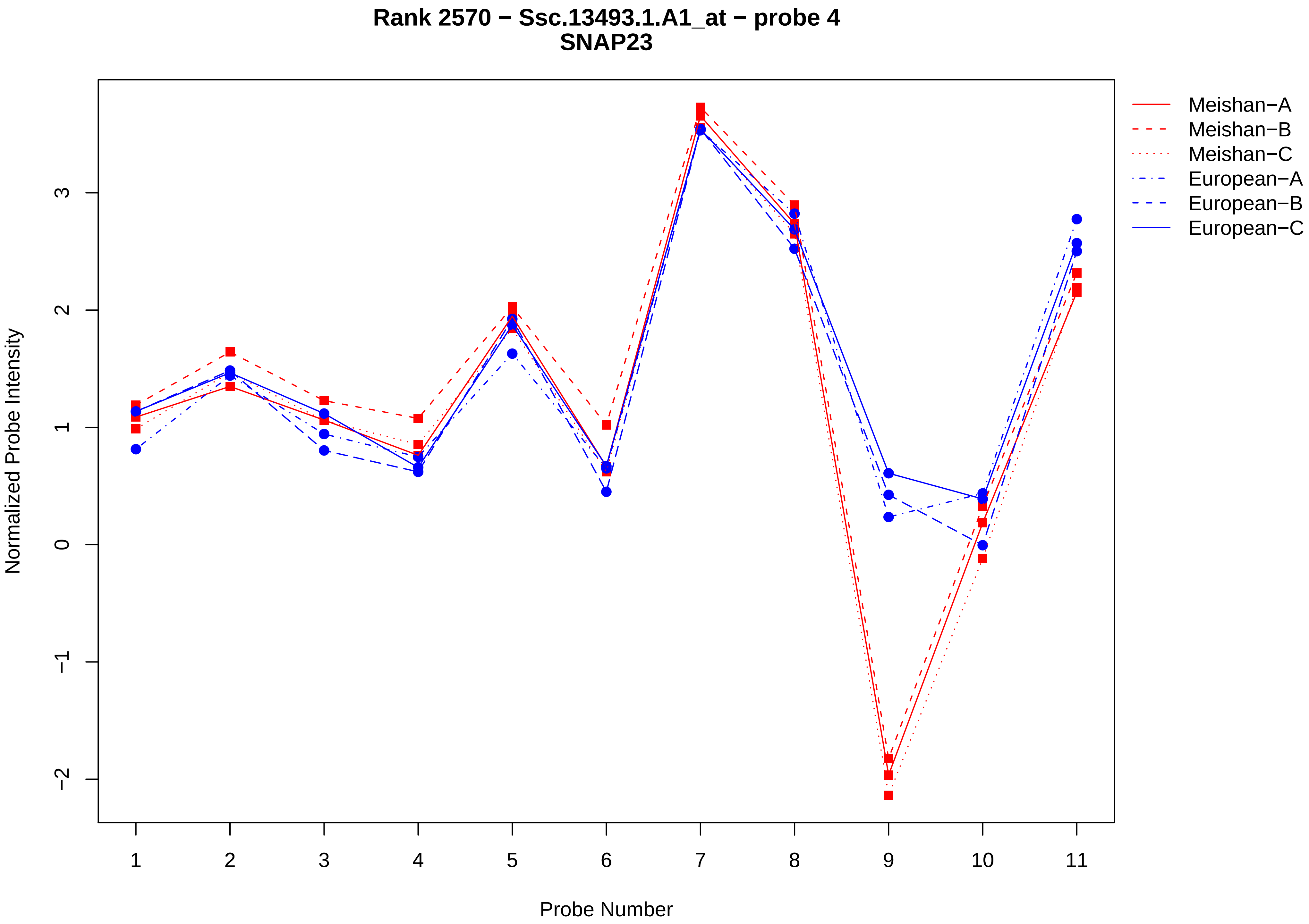

Rank 2570 − Ssc.13493.1.A1_at − probe 4
SNAP23
Normalized Probe Intensity
Probe Number
−2
−1
0
1
2
3
1
2
3
4
5
6
7
8
9
10
11
Meishan−A
Meishan−B
Meishan−C
European−A
European−B
European−C

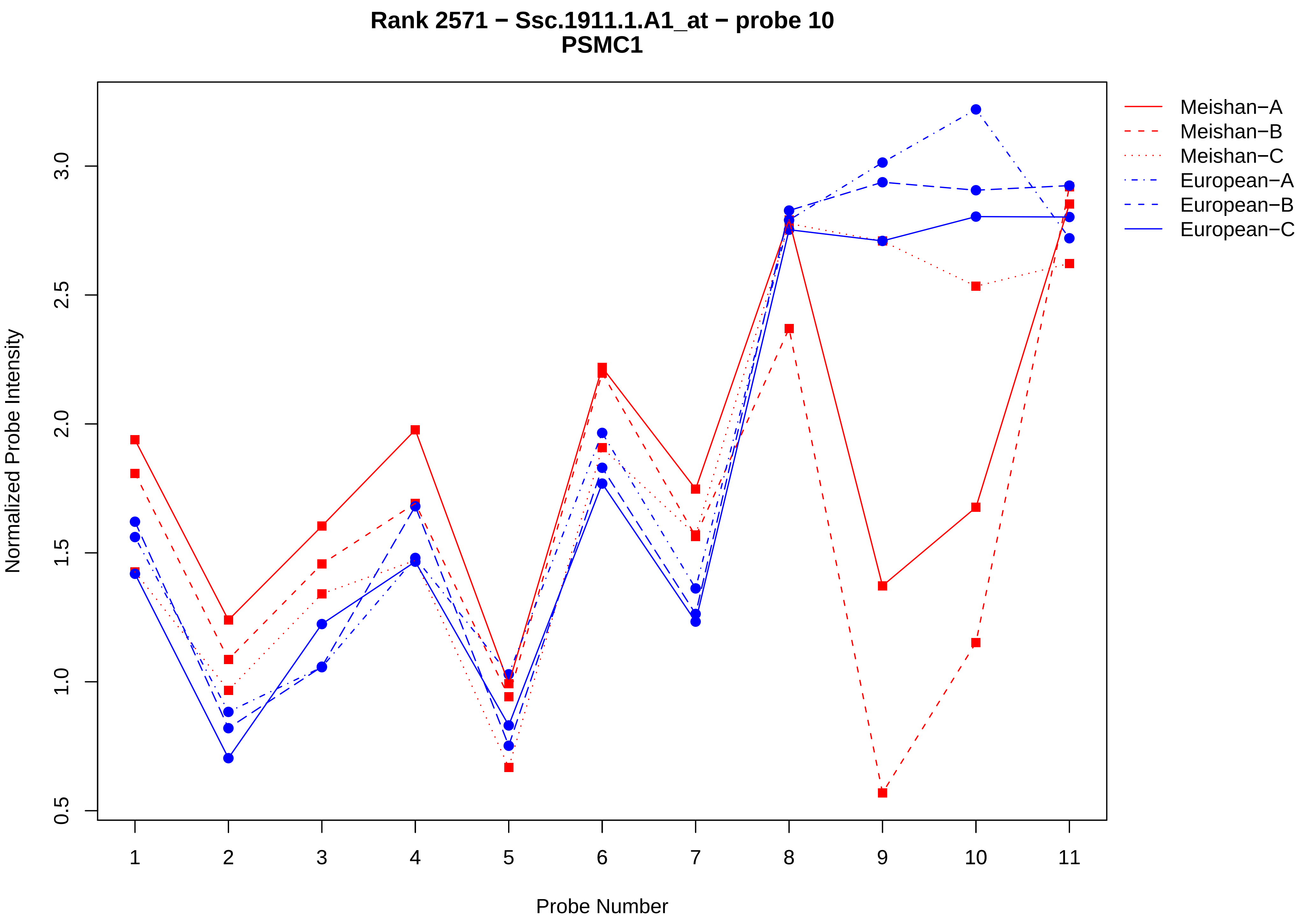

Rank 2571 − Ssc.1911.1.A1_at − probe 10
PSMC1
Normalized Probe Intensity
Probe Number
0.5
1.0
1.5
2.0
2.5
3.0
1
2
3
4
5
6
7
8
9
10
11
Meishan−A
Meishan−B
Meishan−C
European−A
European−B
European−C

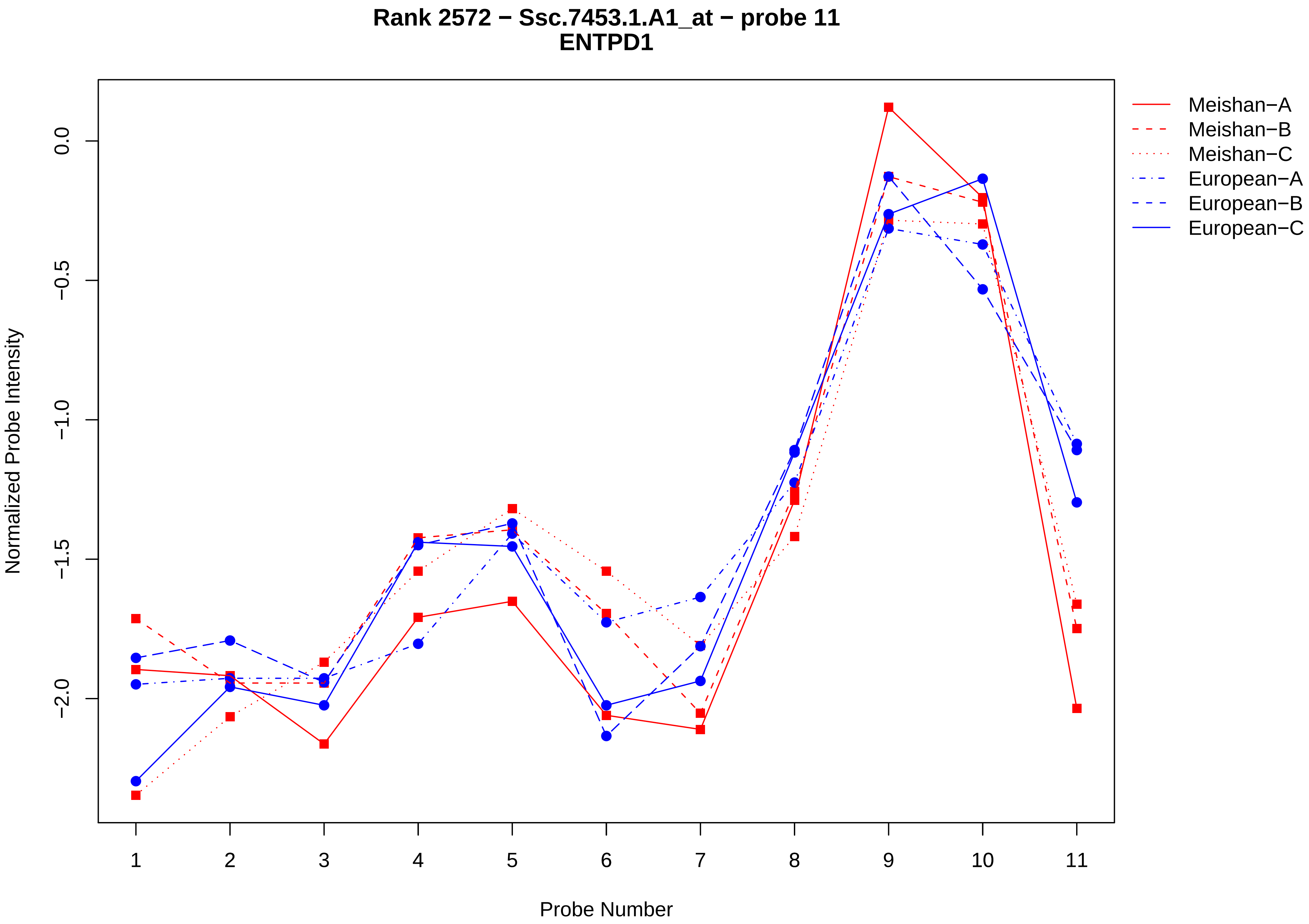

Rank 2572 − Ssc.7453.1.A1_at − probe 11
ENTPD1
Normalized Probe Intensity
Probe Number
0.0
−0.5
−1.0
−1.5
−2.0
1
2
3
4
5
6
7
8
9
10
11
Meishan−A
Meishan−B
Meishan−C
European−A
European−B
European−C

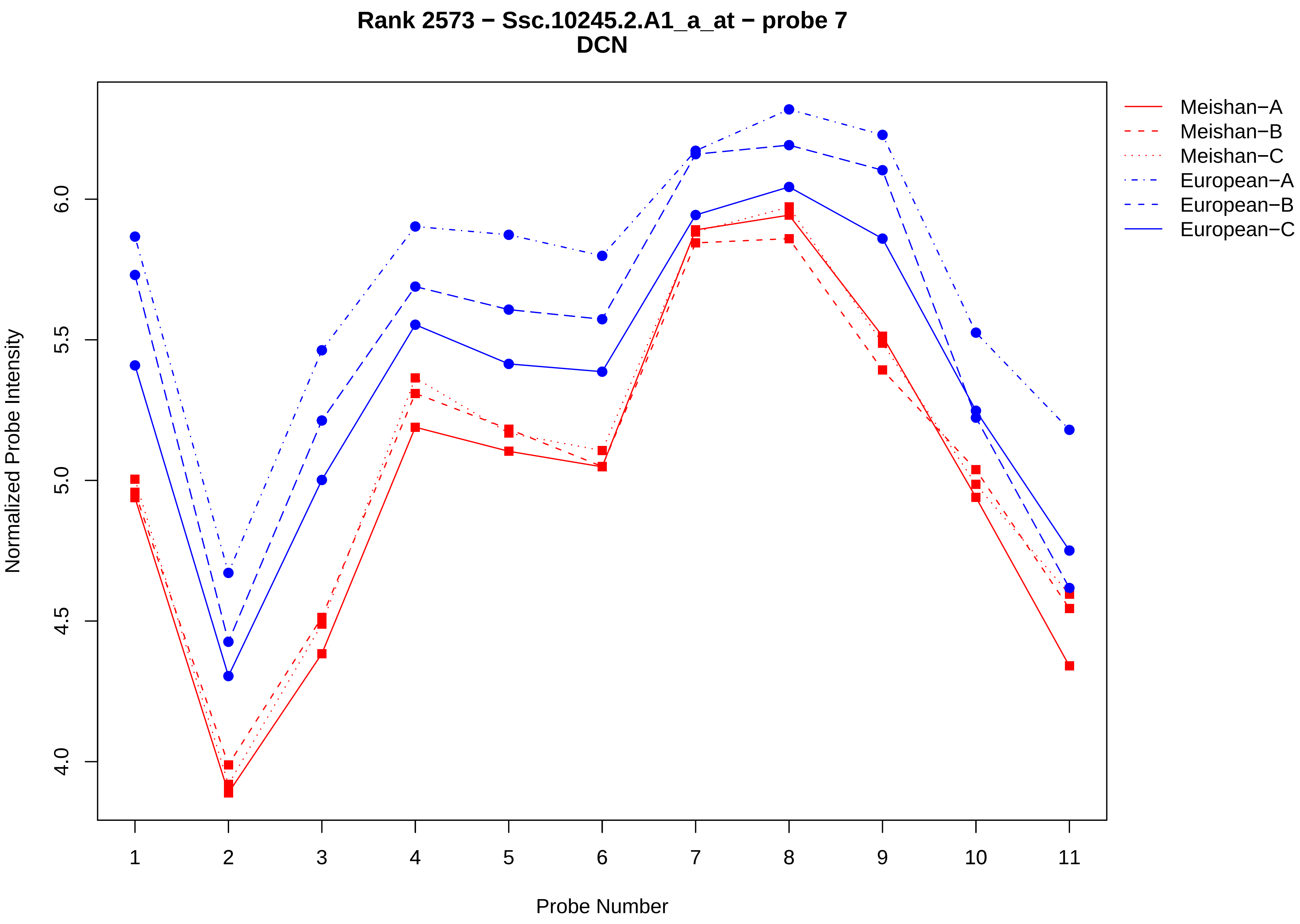

Rank 2573 − Ssc.10245.2.A1_a_at − probe 7
DCN
Normalized Probe Intensity
Probe Number
4.0
4.5
5.0
5.5
6.0
1
2
3
4
5
6
7
8
9
10
11
Meishan−A
Meishan−B
Meishan−C
European−A
European−B
European−C

Rank 2574 − Ssc.3278.1.A1_at − probe 4
PRDM4

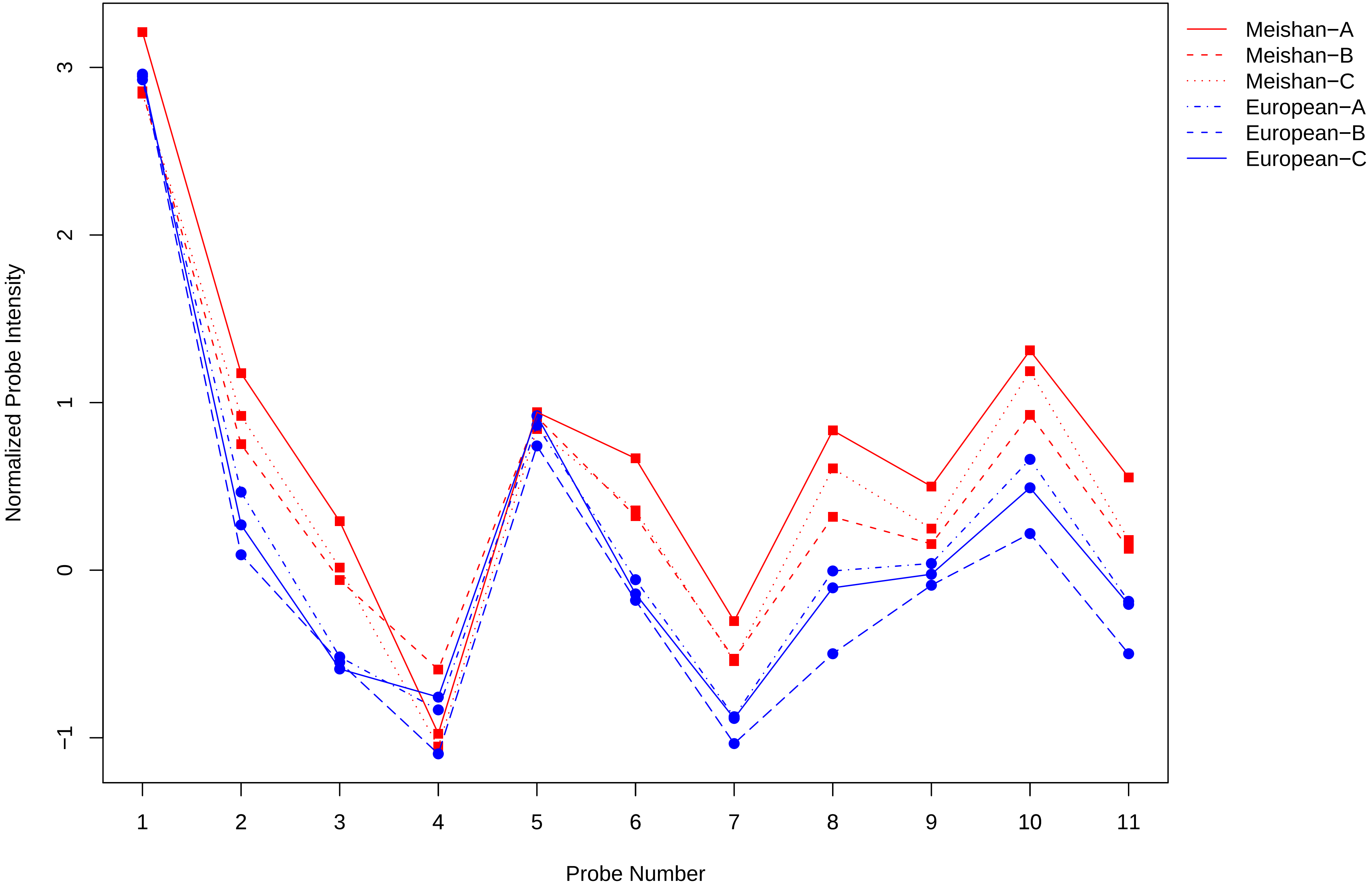

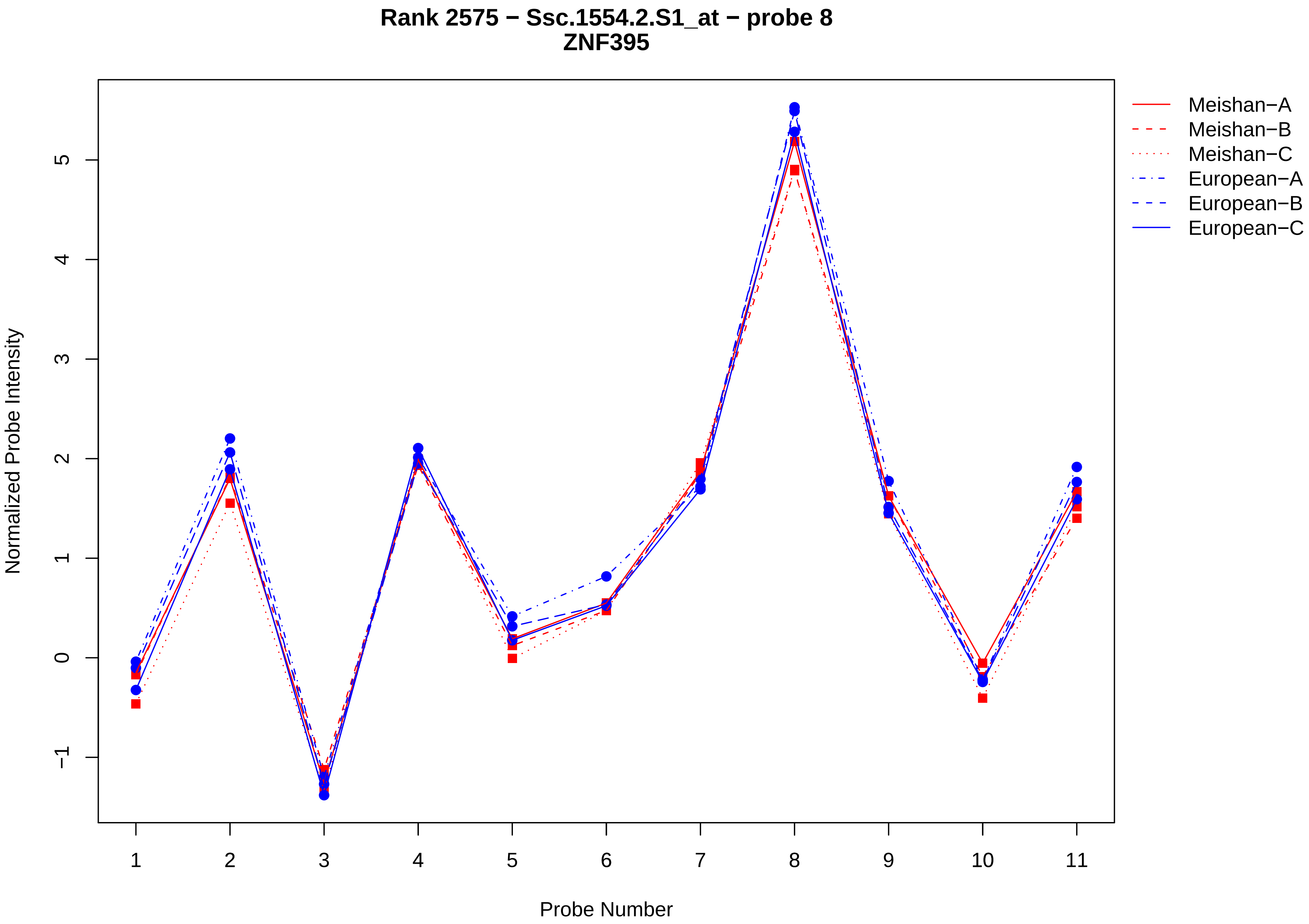

Rank 2575 − Ssc.1554.2.S1_at − probe 8
ZNF395
Normalized Probe Intensity
Probe Number
−1
0
1
2
3
4
5
1
2
3
4
5
6
7
8
9
10
11
Meishan−A
Meishan−B
Meishan−C
European−A
European−B
European−C

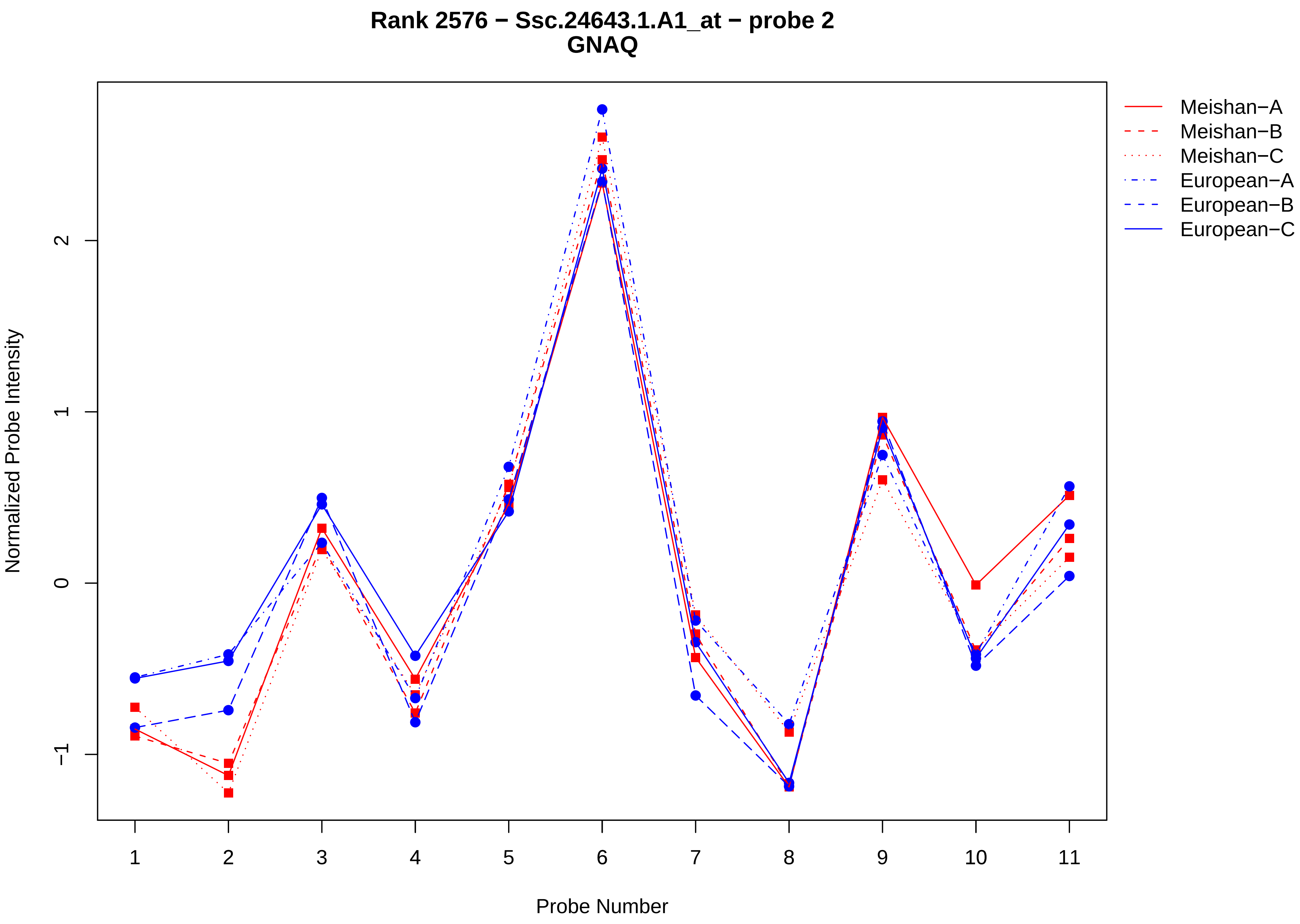

Rank 2576 − Ssc.24643.1.A1_at − probe 2
GNAQ
Normalized Probe Intensity
Probe Number
Meishan−A
Meishan−B
Meishan−C
European−A
European−B
European−C
−1
0
1
2
1
2
3
4
5
6
7
8
9
10
11

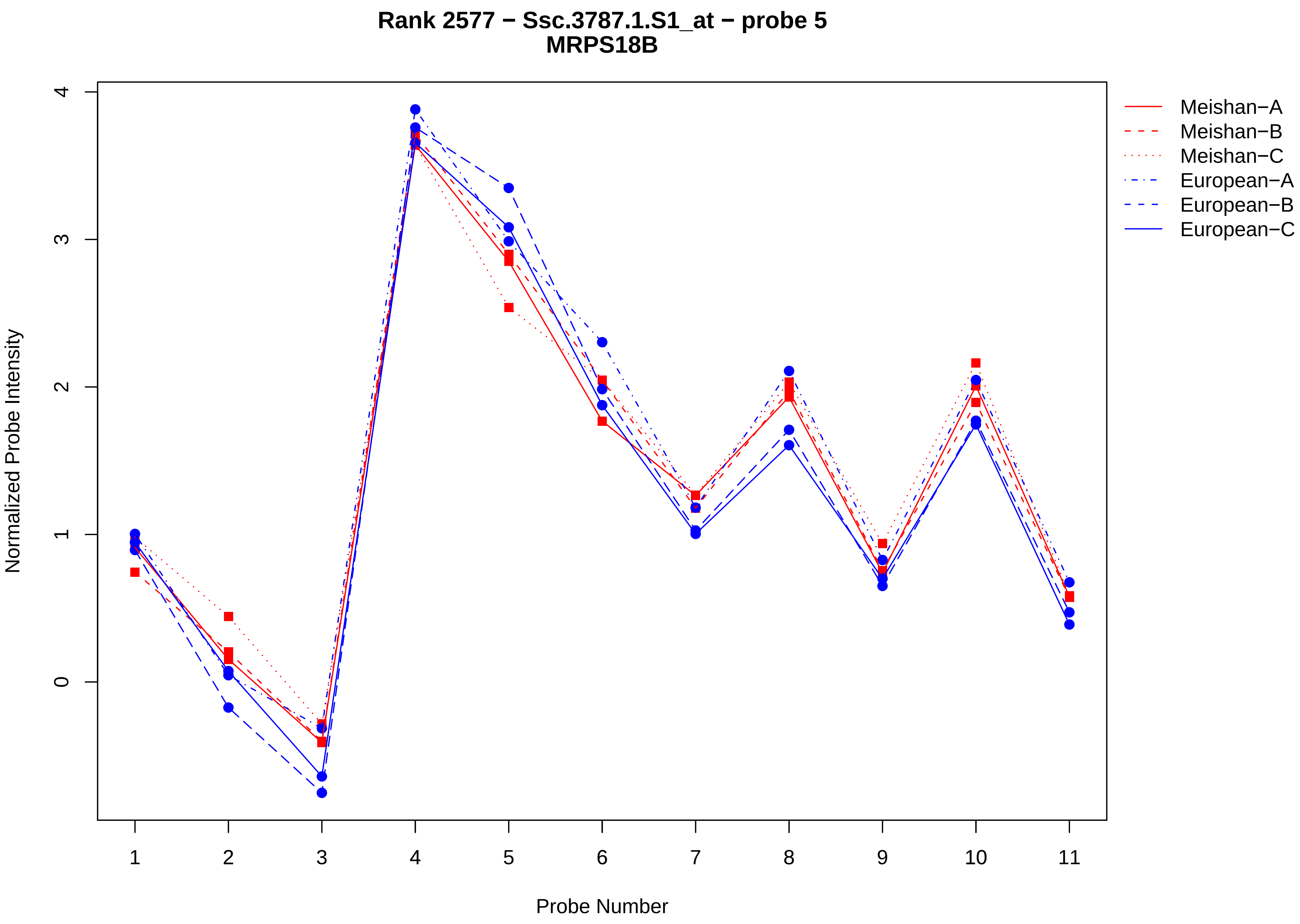

Rank 2577 − Ssc.3787.1.S1_at − probe 5
MRPS18B
Normalized Probe Intensity
Probe Number
0
1
2
3
4
1
2
3
4
5
6
7
8
9
10
11
Meishan−A
Meishan−B
Meishan−C
European−A
European−B
European−C

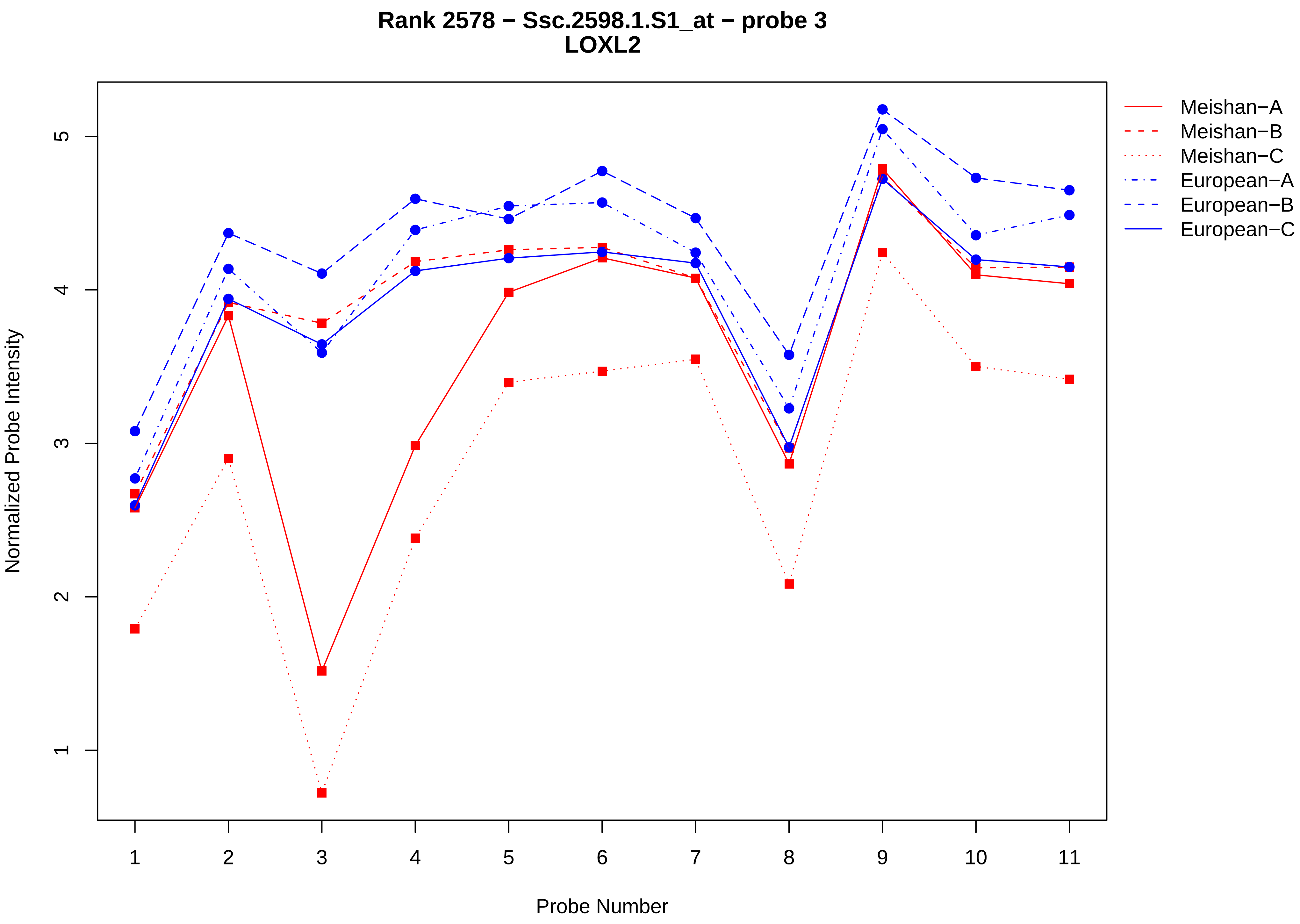
Rank 2578 − Ssc.2598.1.S1_at − probe 3
LOXL2
Normalized Probe Intensity
Probe Number
1
2
3
4
5
1
2
3
4
5
6
7
8
9
10
11
Meishan−A
Meishan−B
Meishan−C
European−A
European−B
European−C

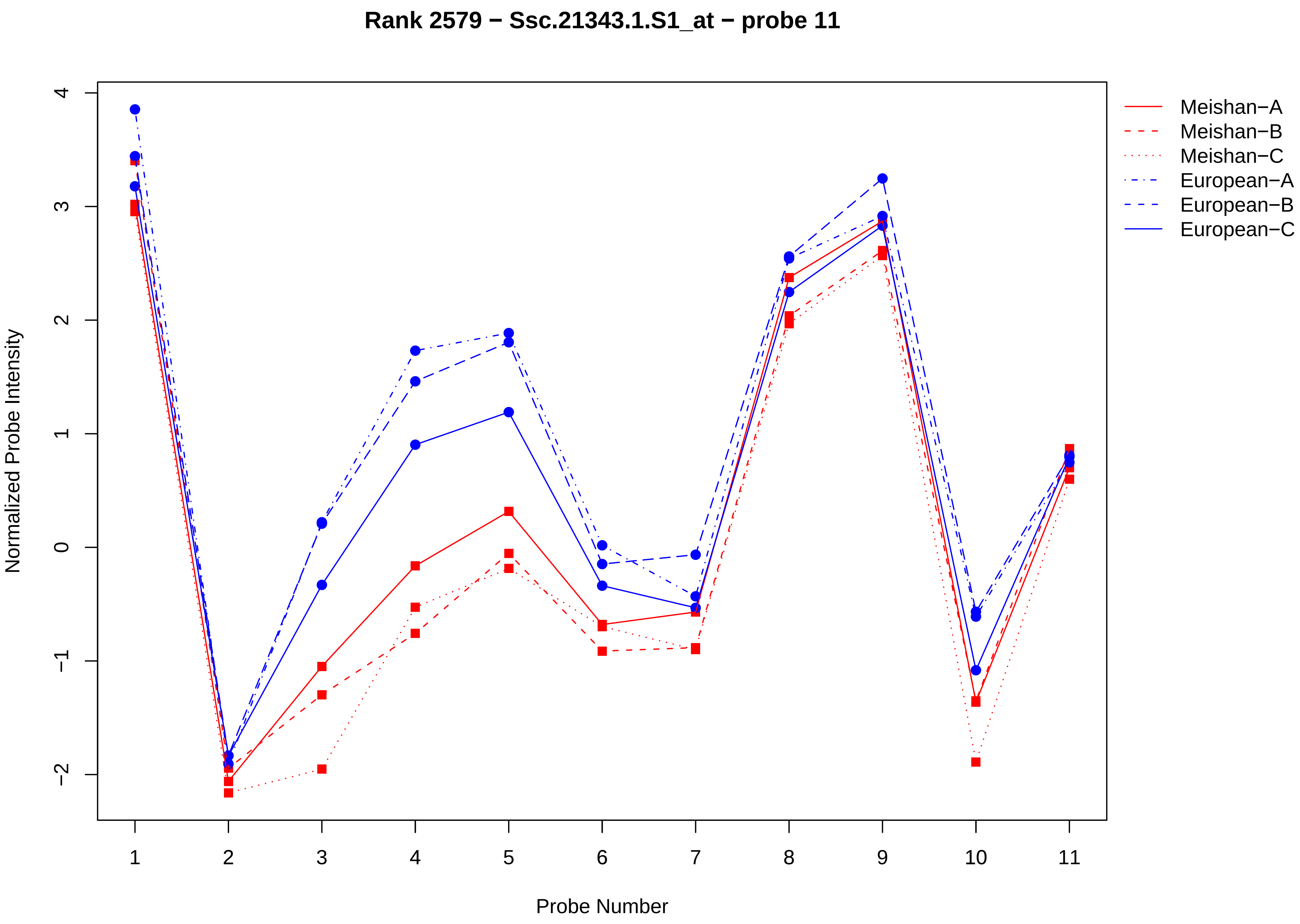

Rank 2579 − Ssc.21343.1.S1_at − probe 11
Normalized Probe Intensity
Probe Number
−2
−1
0
1
2
3
4
1
2
3
4
5
6
7
8
9
10
11
Meishan−A
Meishan−B
Meishan−C
European−A
European−B
European−C

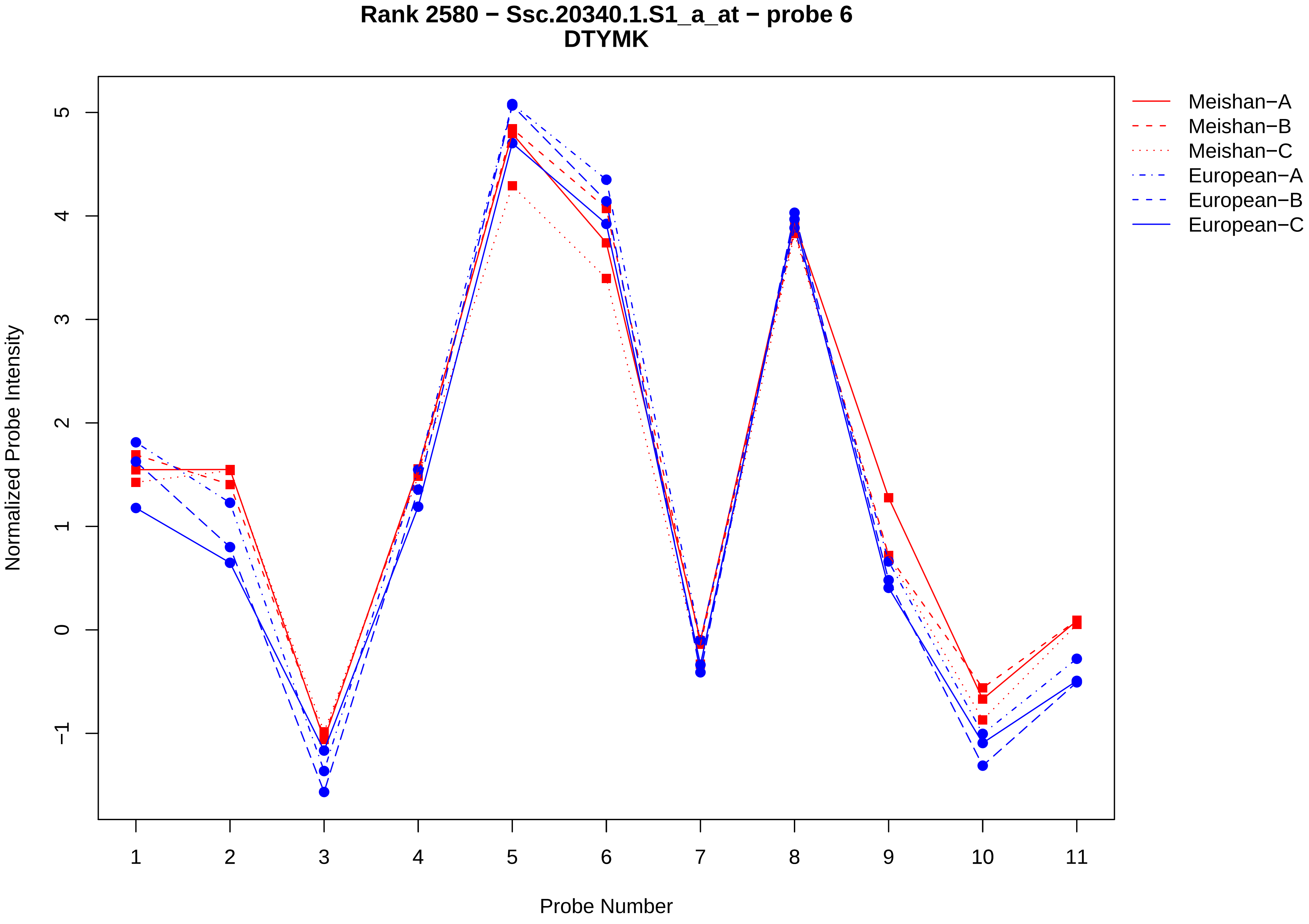

Rank 2580 − Ssc.20340.1.S1_a_at − probe 6
DTYMK
Normalized Probe Intensity
Probe Number
−1
0
1
2
3
4
5
1
2
3
4
5
6
7
8
9
10
11
Meishan−A
Meishan−B
Meishan−C
European−A
European−B
European−C

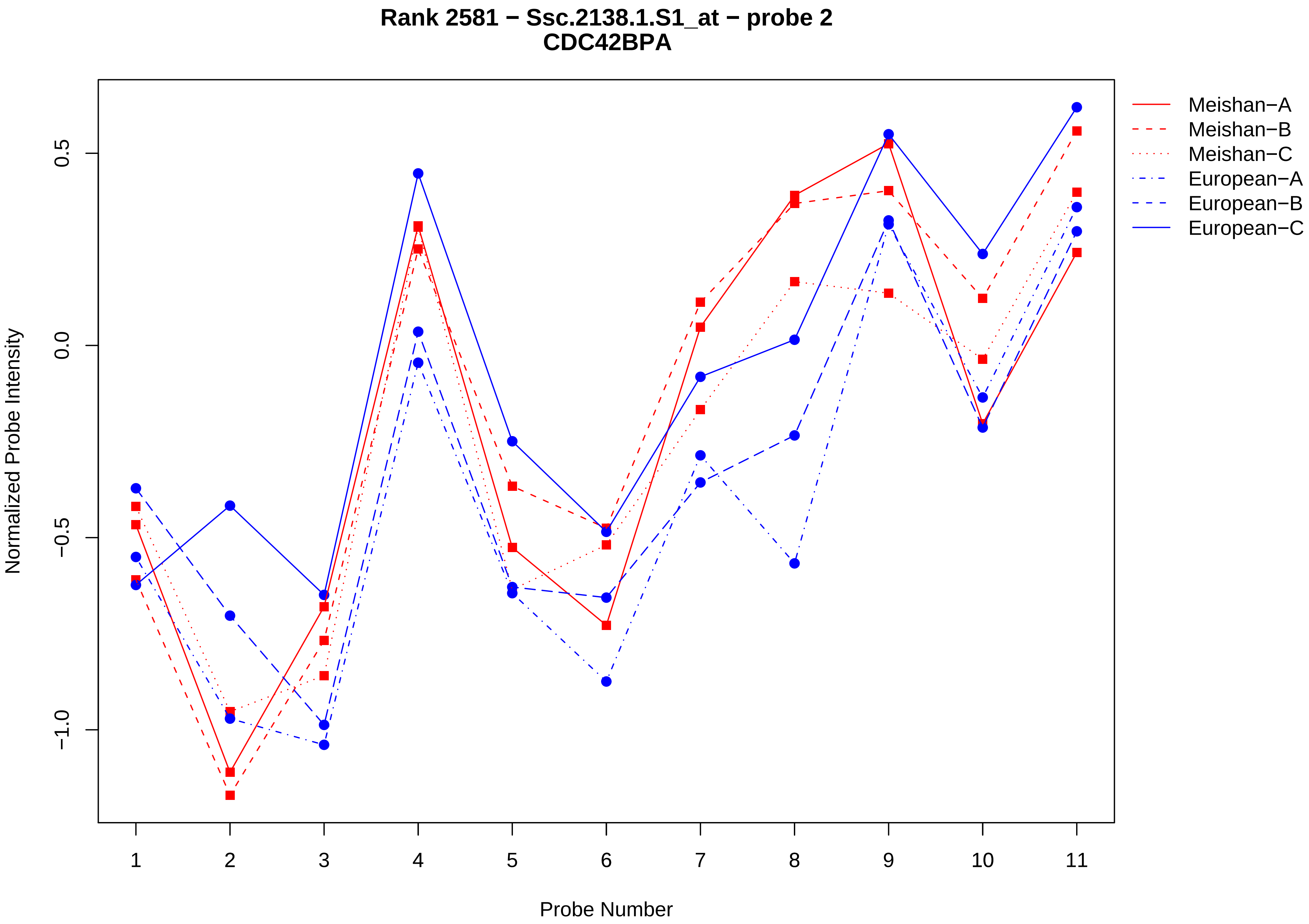

Rank 2581 − Ssc.2138.1.S1_at − probe 2
CDC42BPA
Normalized Probe Intensity
Probe Number
0.5
0.0
−0.5
−1.0
1
2
3
4
5
6
7
8
9
10
11
Meishan−A
Meishan−B
Meishan−C
European−A
European−B
European−C

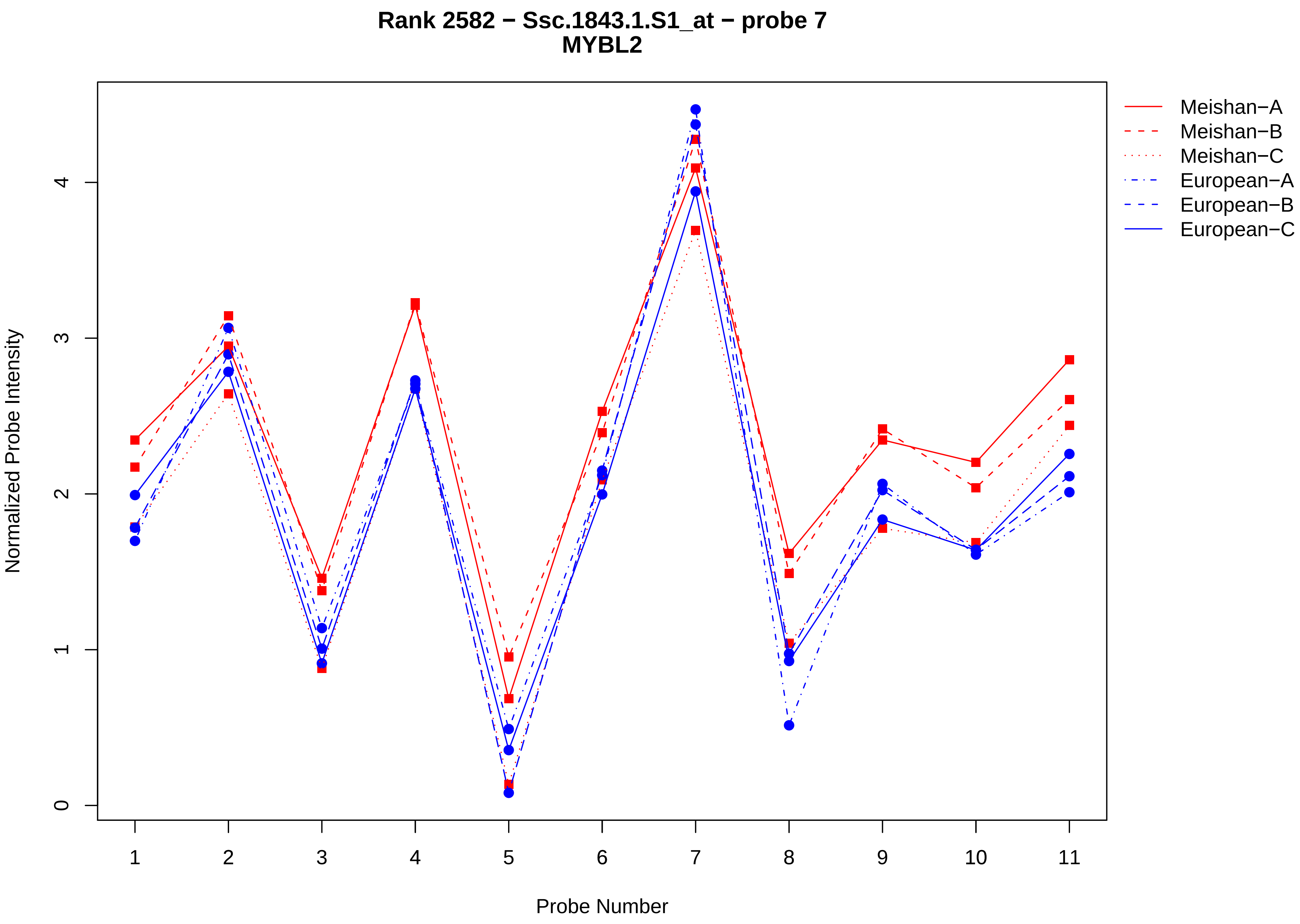

Rank 2582 − Ssc.1843.1.S1_at − probe 7
MYBL2
Normalized Probe Intensity
Probe Number
0
1
2
3
4
1
2
3
4
5
6
7
8
9
10
11
Meishan−A
Meishan−B
Meishan−C
European−A
European−B
European−C

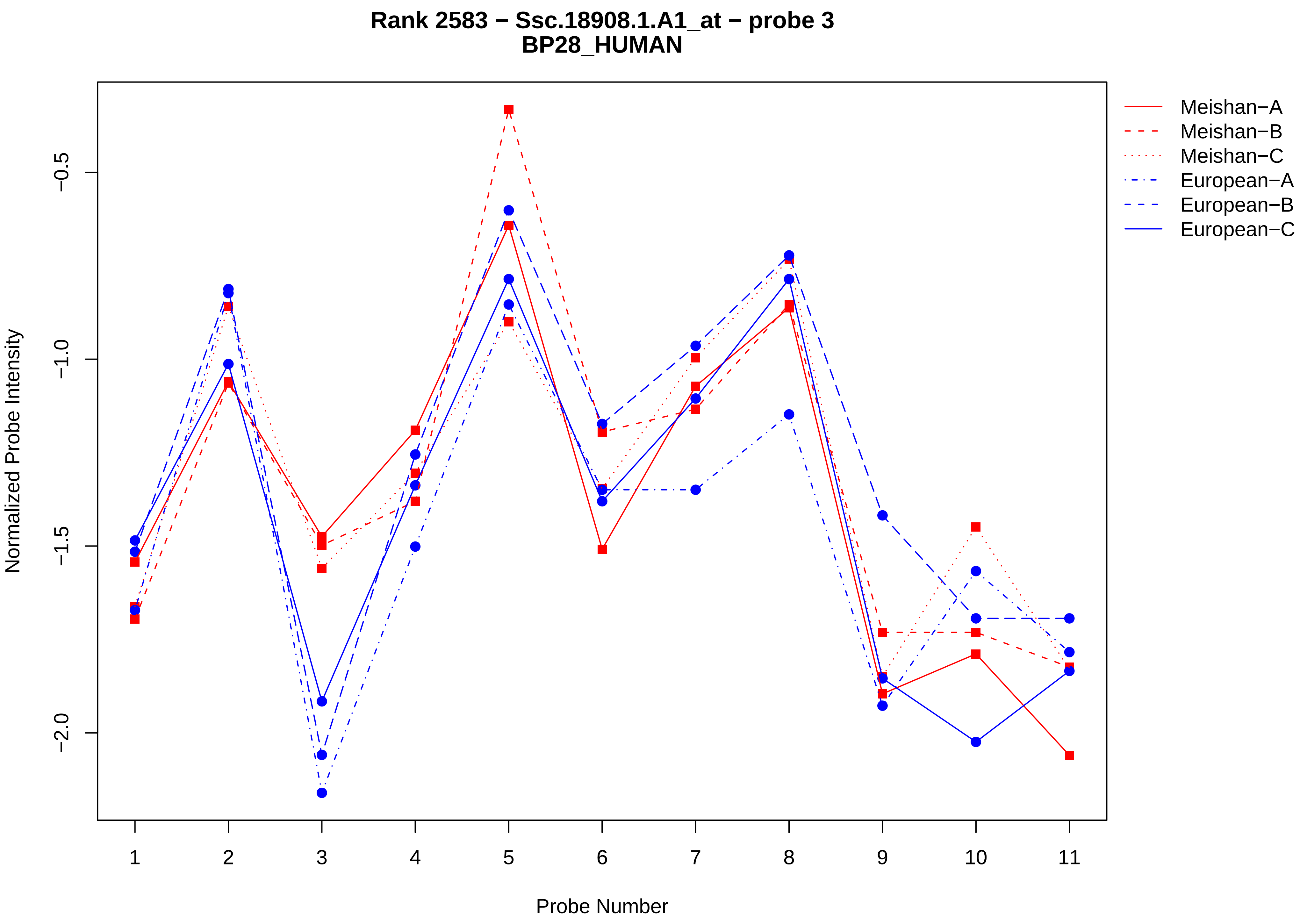

Rank 2583 − Ssc.18908.1.A1_at − probe 3
BP28_HUMAN
Normalized Probe Intensity
−0.5
−1.0
−1.5
−2.0
1
2
3
4
5
6
7
8
9
10
11
Probe Number
Meishan−A
Meishan−B
Meishan−C
European−A
European−B
European−C

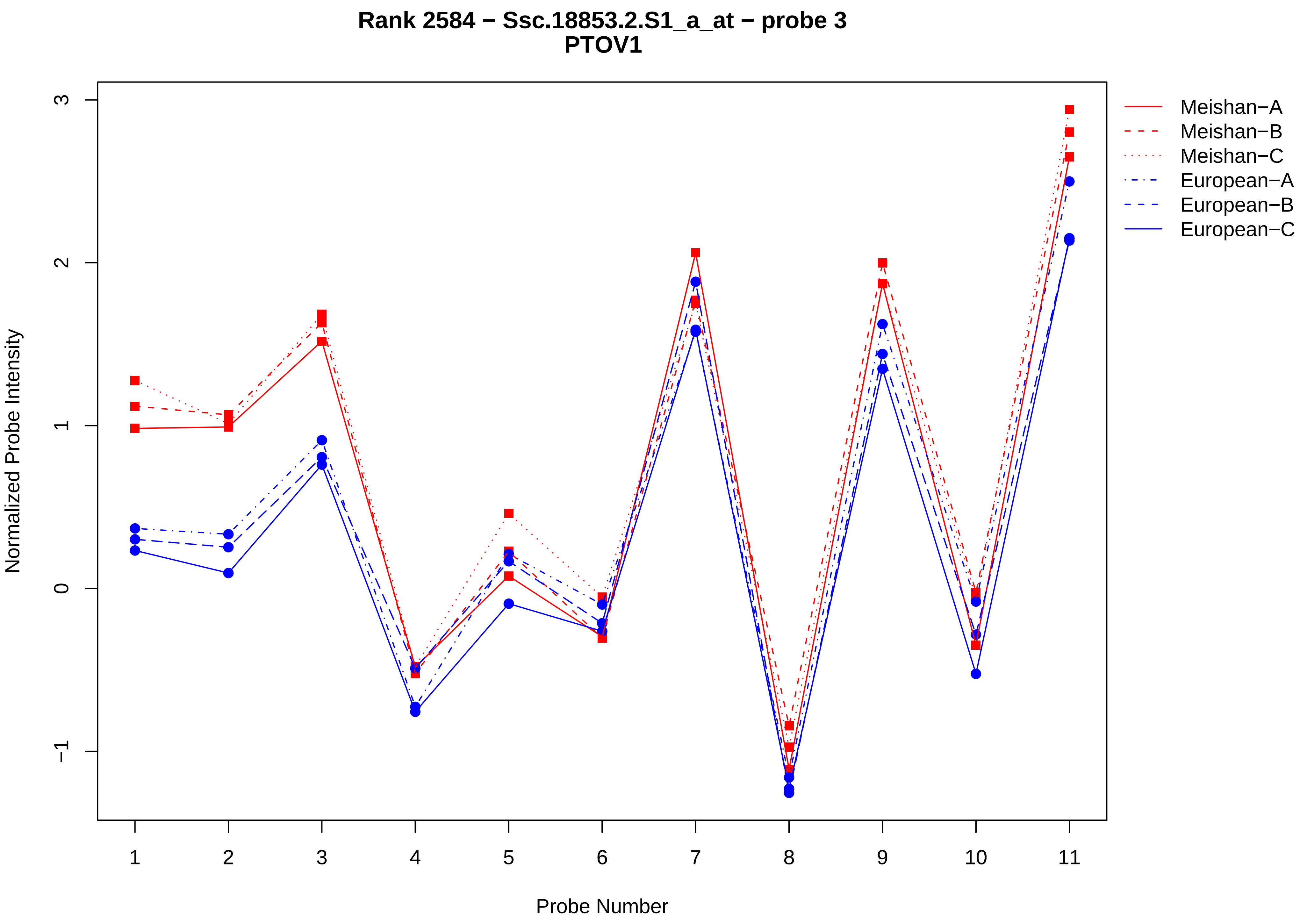

Rank 2584 − Ssc.18853.2.S1_a_at − probe 3
PTOV1
Normalized Probe Intensity
Probe Number
Meishan−A
Meishan−B
Meishan−C
European−A
European−B
European−C
−1
0
1
2
3
1
2
3
4
5
6
7
8
9
10
11

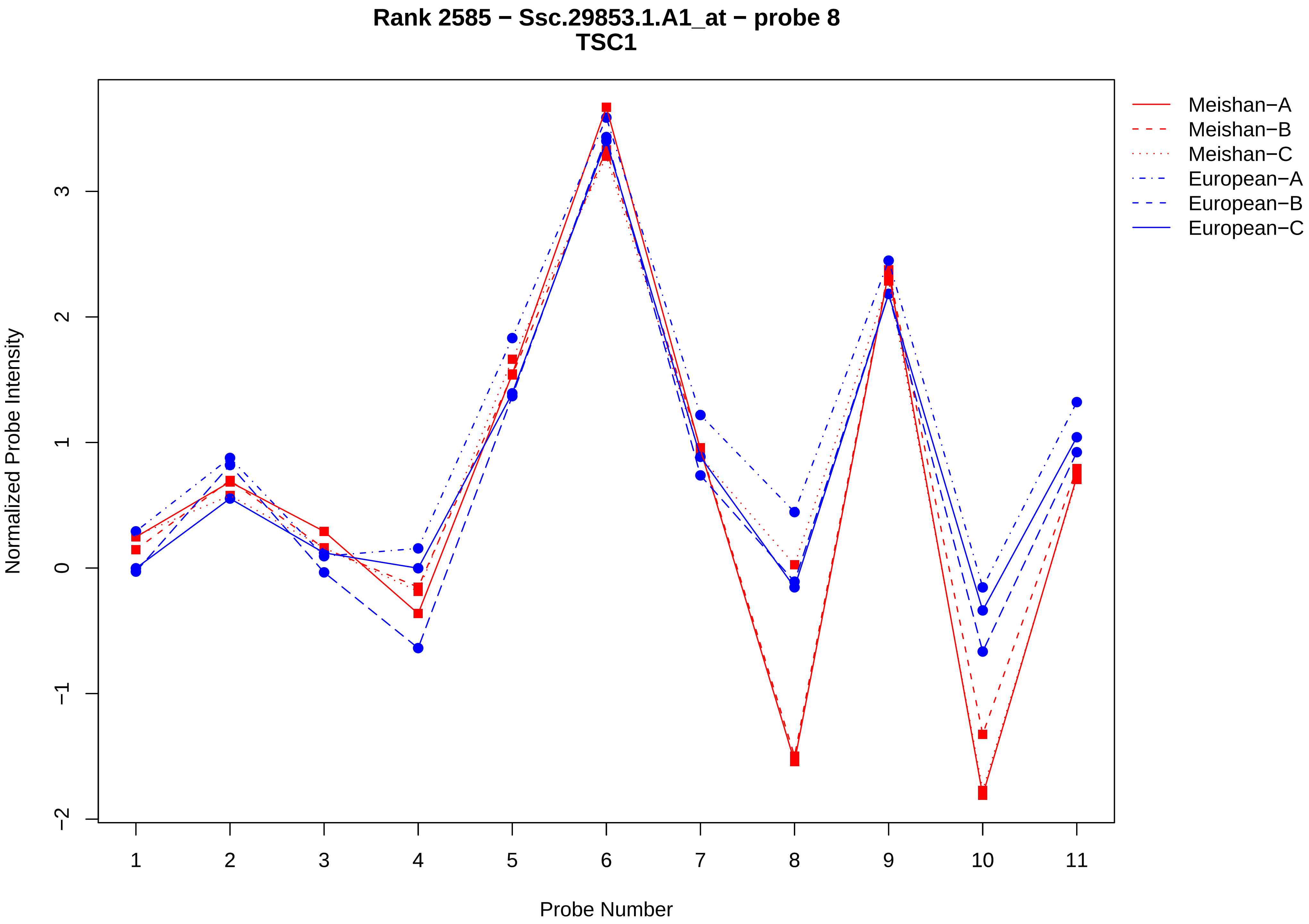

Rank 2585 − Ssc.29853.1.A1_at − probe 8
TSC1
Normalized Probe Intensity
Probe Number
−2
−1
0
1
2
3
1
2
3
4
5
6
7
8
9
10
11
Meishan−A
Meishan−B
Meishan−C
European−A
European−B
European−C

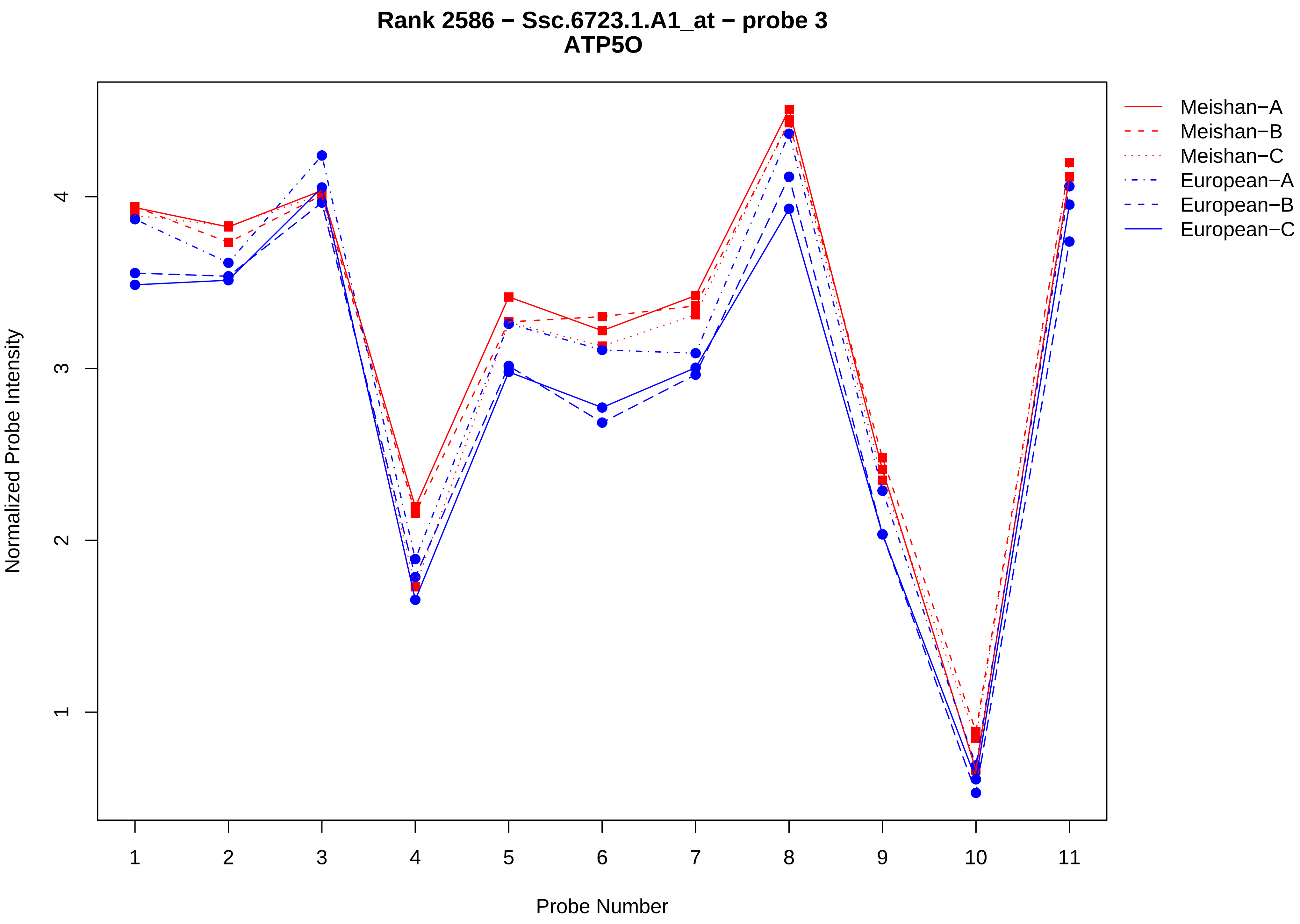
Rank 2586 − Ssc.6723.1.A1_at − probe 3
ATP5O
Normalized Probe Intensity
Probe Number
1
2
3
4
1
2
3
4
5
6
7
8
9
10
11
Meishan−A
Meishan−B
Meishan−C
European−A
European−B
European−C

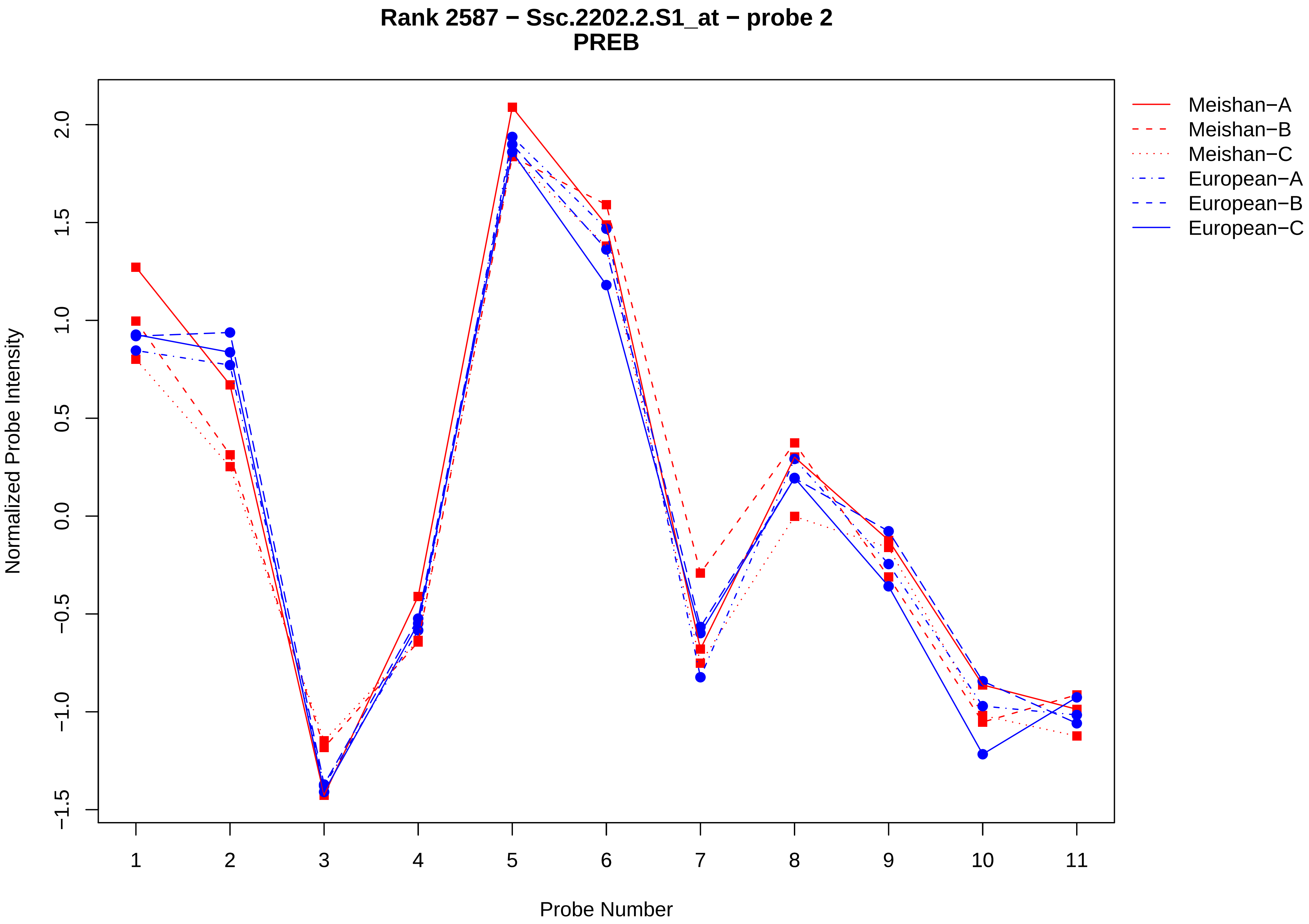

Rank 2587 − Ssc.2202.2.S1_at − probe 2
PREB
Normalized Probe Intensity
Probe Number
Meishan−A
Meishan−B
Meishan−C
European−A
European−B
European−C
−1.5
−1.0
−0.5
0.0
0.5
1.0
1.5
2.0
1
2
3
4
5
6
7
8
9
10
11

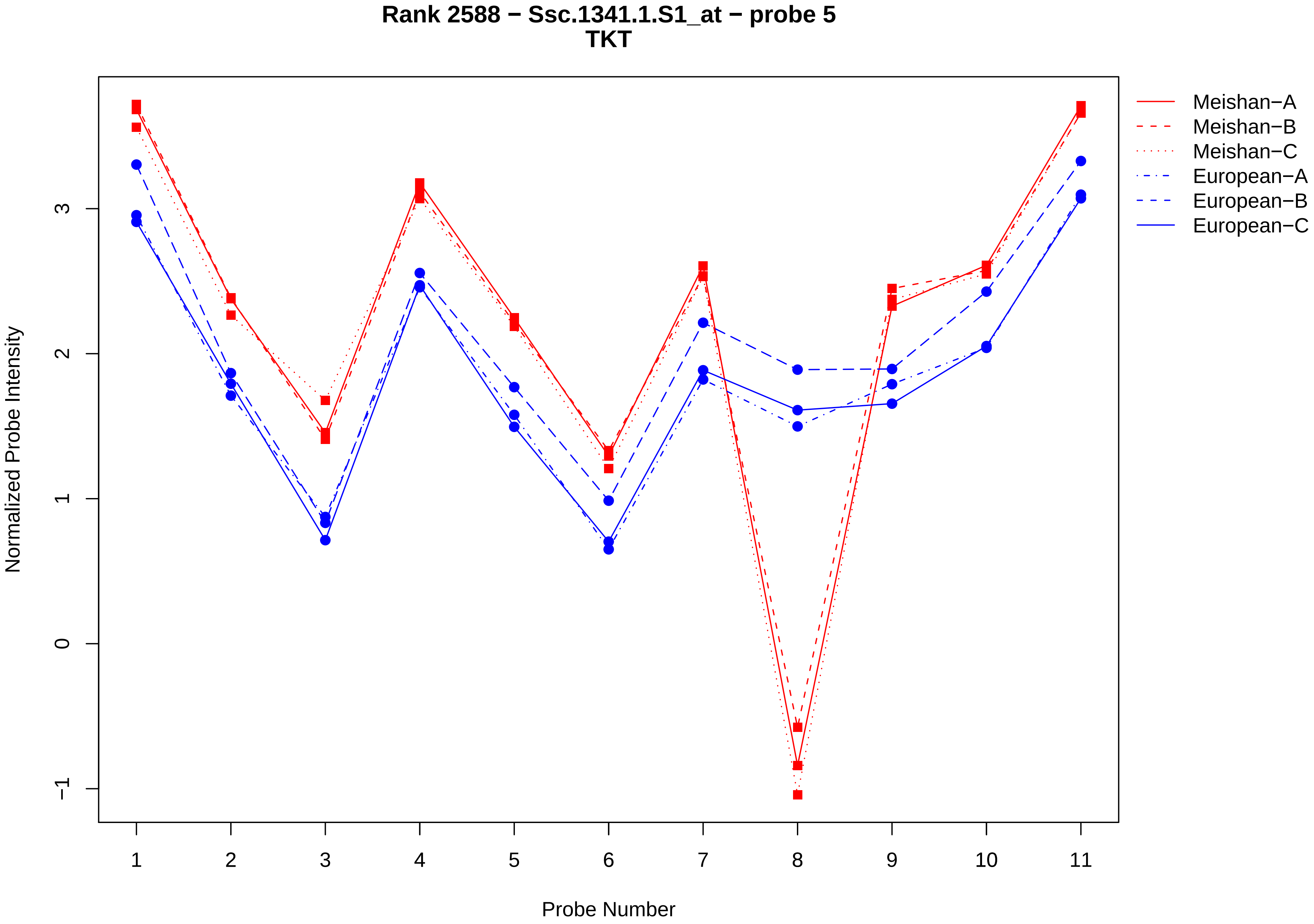

Rank 2588 − Ssc.1341.1.S1_at − probe 5
TKT
Normalized Probe Intensity
Probe Number
Meishan−A
Meishan−B
Meishan−C
European−A
European−B
European−C
−1
0
1
2
3
1
2
3
4
5
6
7
8
9
10
11

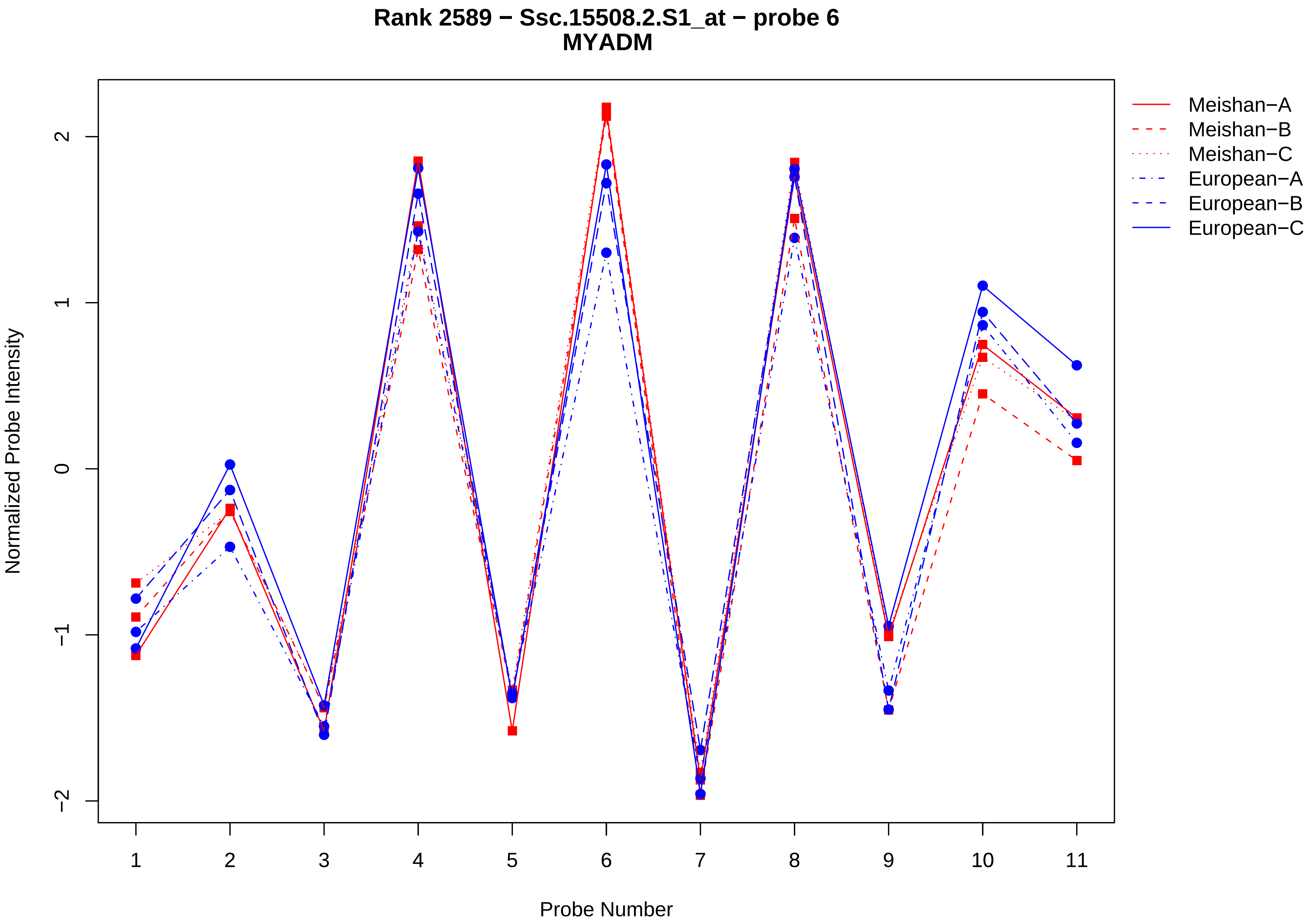

Rank 2589 − Ssc.15508.2.S1_at − probe 6
MYADM
Normalized Probe Intensity
Probe Number
−2
−1
0
1
2
1
2
3
4
5
6
7
8
9
10
11
Meishan−A
Meishan−B
Meishan−C
European−A
European−B
European−C

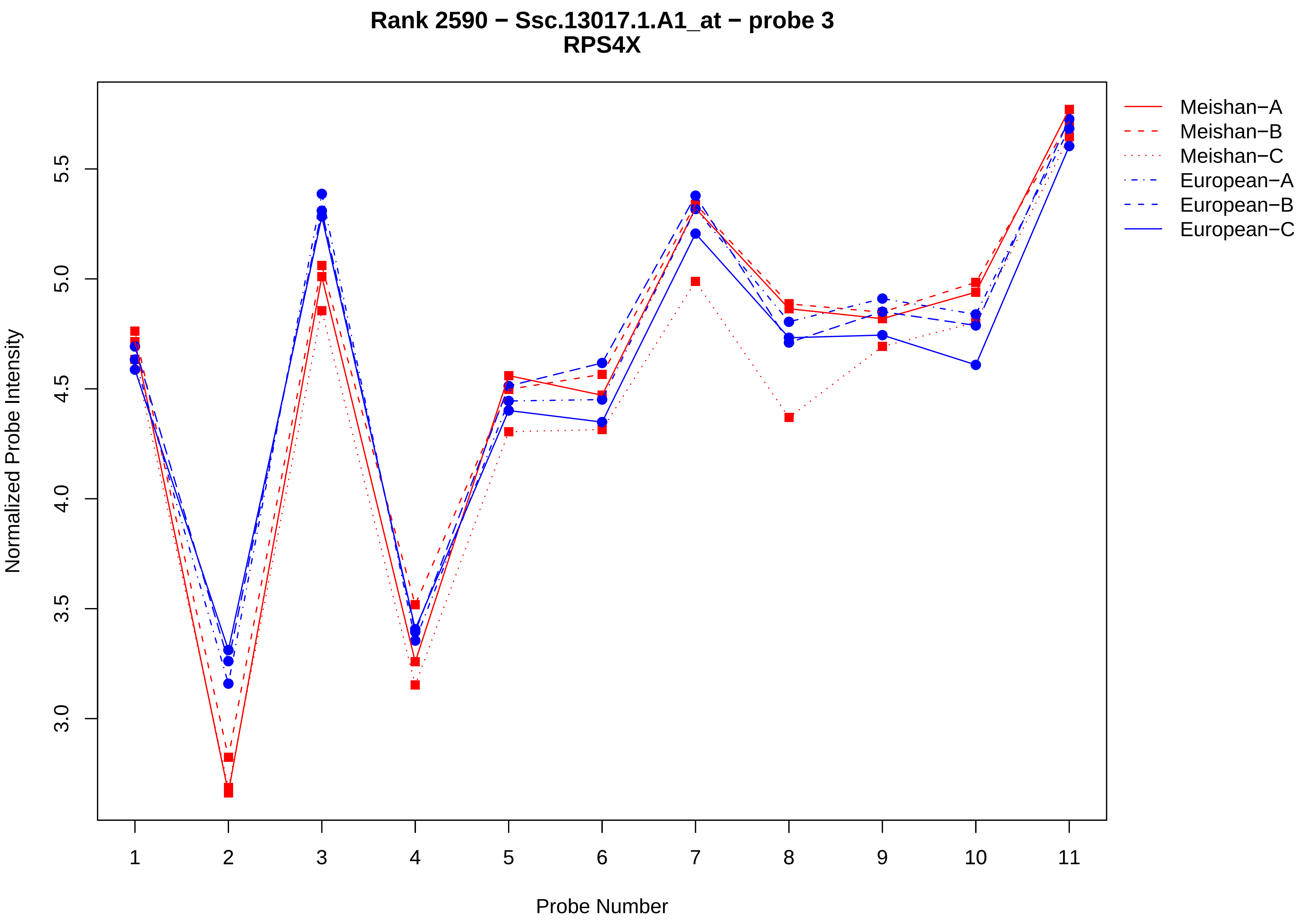

Rank 2590 − Ssc.13017.1.A1_at − probe 3
RPS4X
Normalized Probe Intensity
Probe Number
3.0
3.5
4.0
4.5
5.0
5.5
1
2
3
4
5
6
7
8
9
10
11
Meishan−A
Meishan−B
Meishan−C
European−A
European−B
European−C

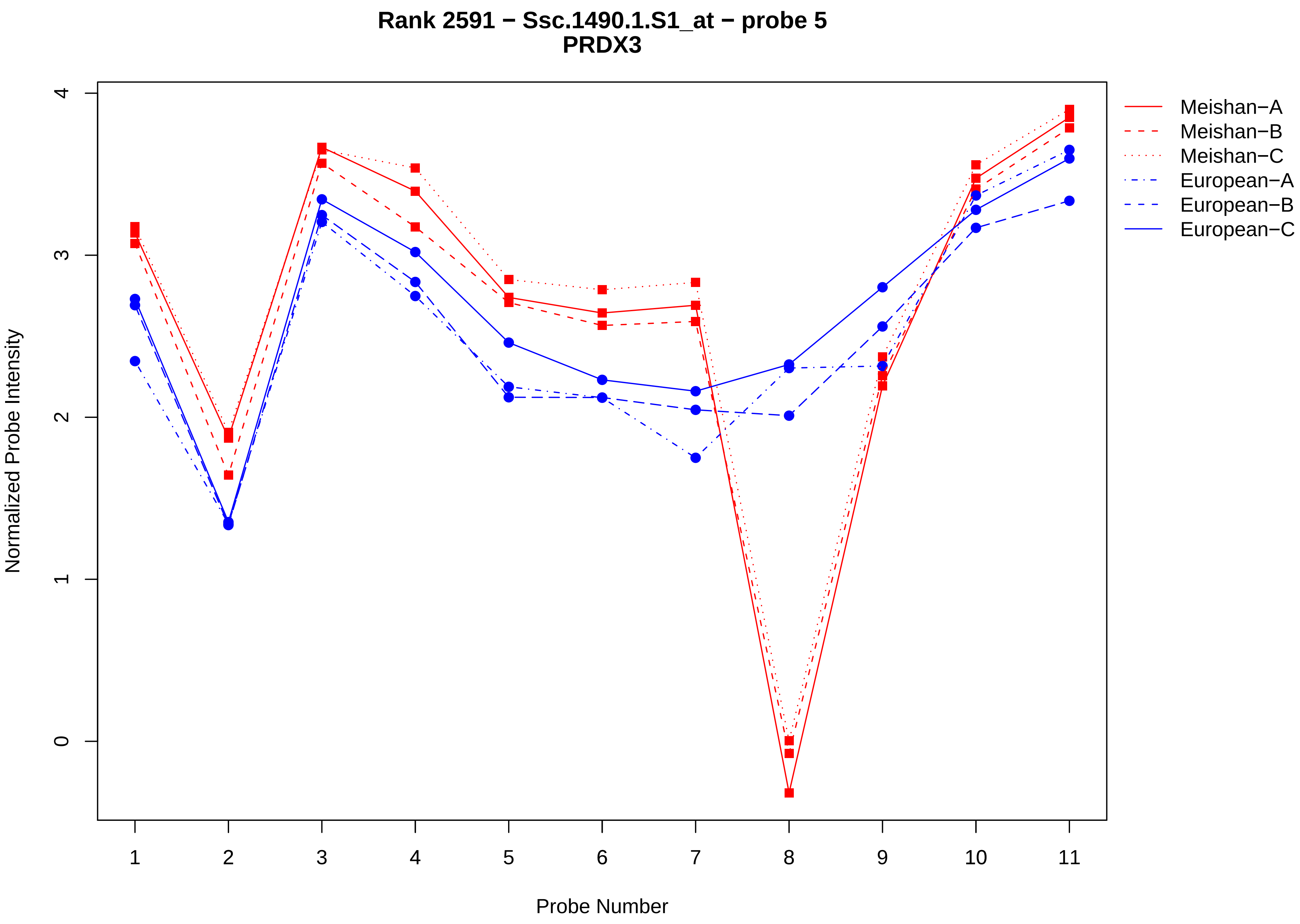

Rank 2591 − Ssc.1490.1.S1_at − probe 5
PRDX3
Normalized Probe Intensity
Probe Number
0
1
2
3
4
1
2
3
4
5
6
7
8
9
10
11
Meishan−A
Meishan−B
Meishan−C
European−A
European−B
European−C

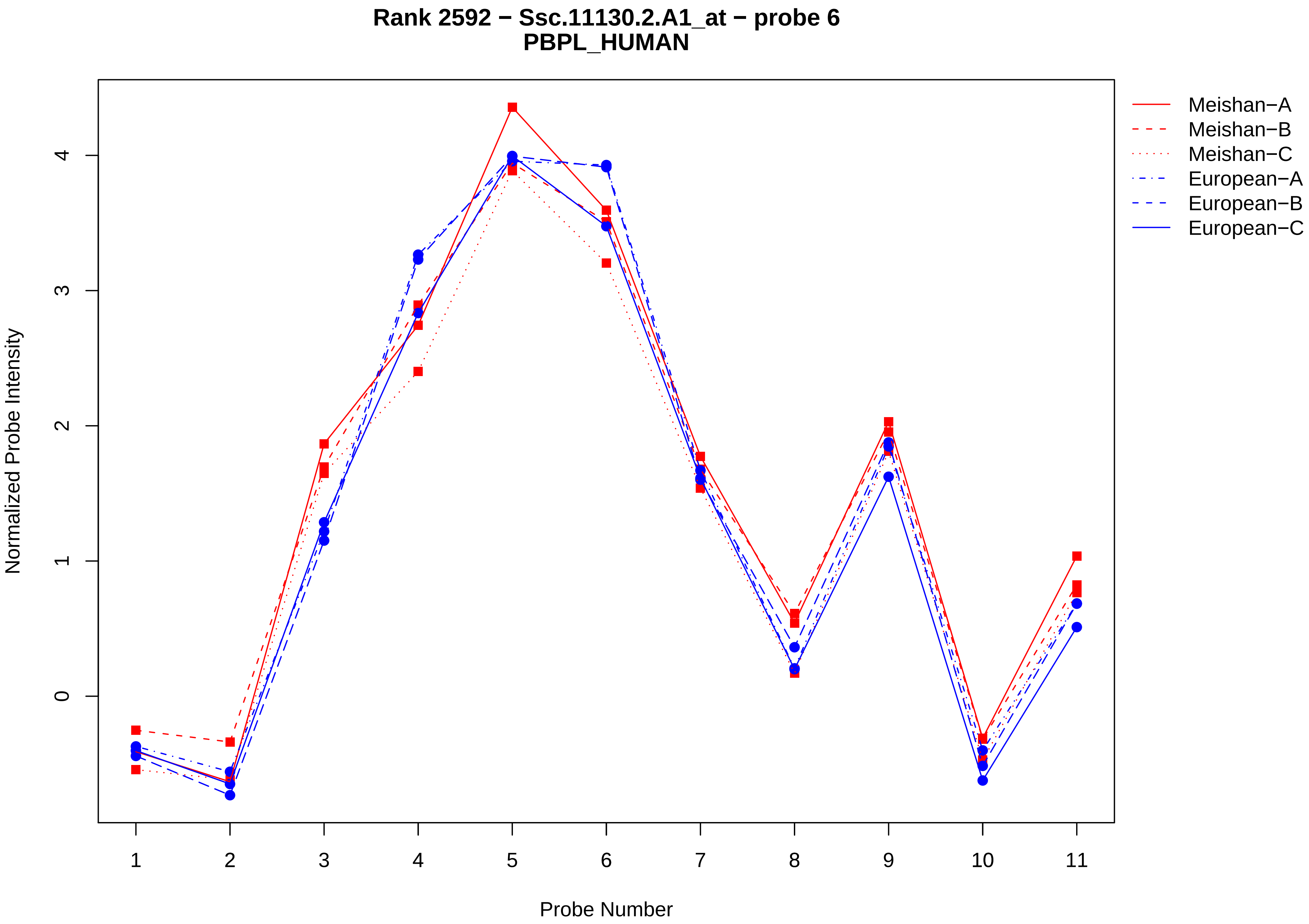

Rank 2592 − Ssc.11130.2.A1_at − probe 6
PBPL_HUMAN
Normalized Probe Intensity
Probe Number
0
1
2
3
4
1
2
3
4
5
6
7
8
9
10
11
Meishan−A
Meishan−B
Meishan−C
European−A
European−B
European−C

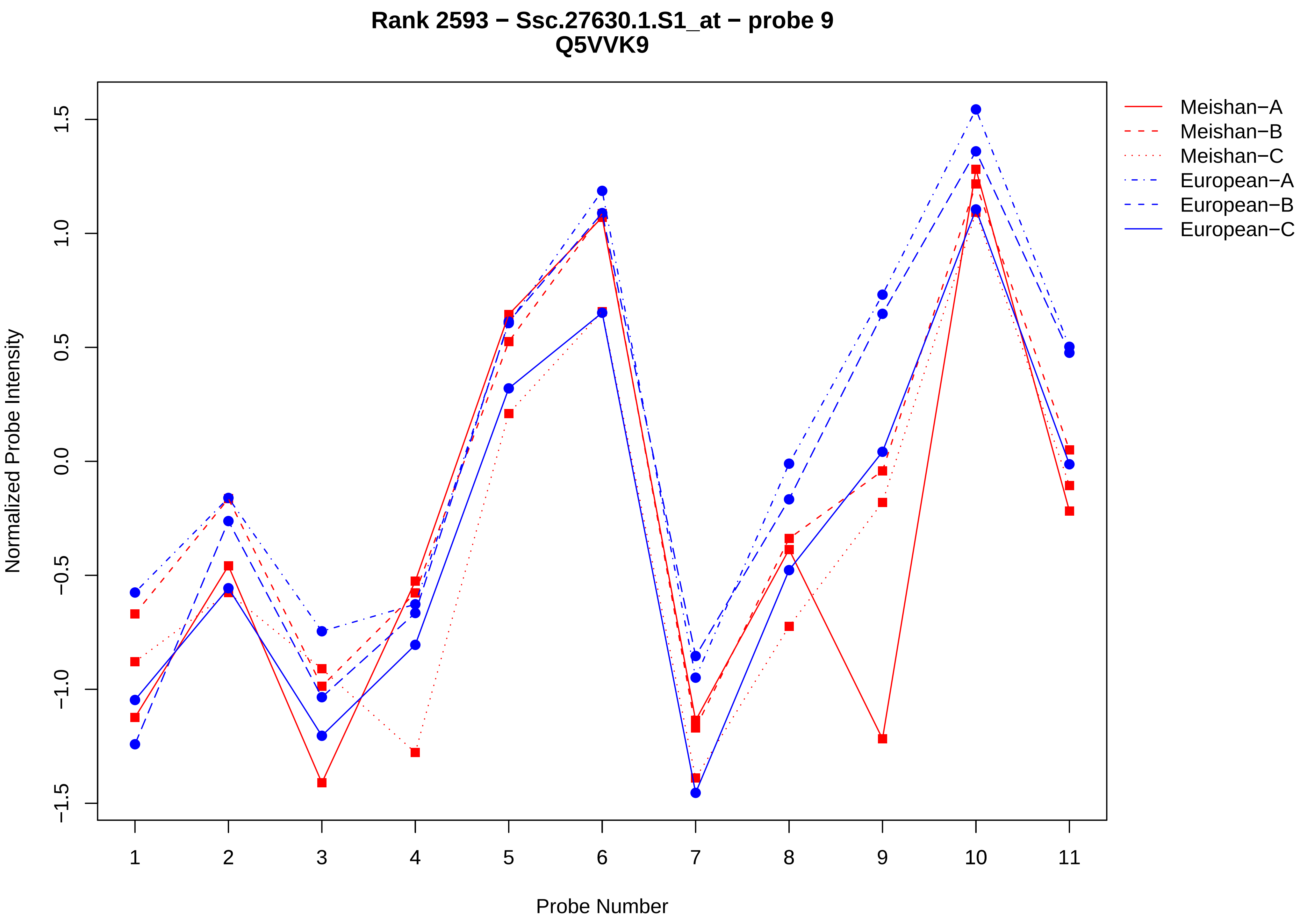

Rank 2593 − Ssc.27630.1.S1_at − probe 9
Q5VVK9
Normalized Probe Intensity
Probe Number
Meishan−A
Meishan−B
Meishan−C
European−A
European−B
European−C
1.5
1.0
0.5
0.0
−0.5
−1.0
−1.5
1
2
3
4
5
6
7
8
9
10
11

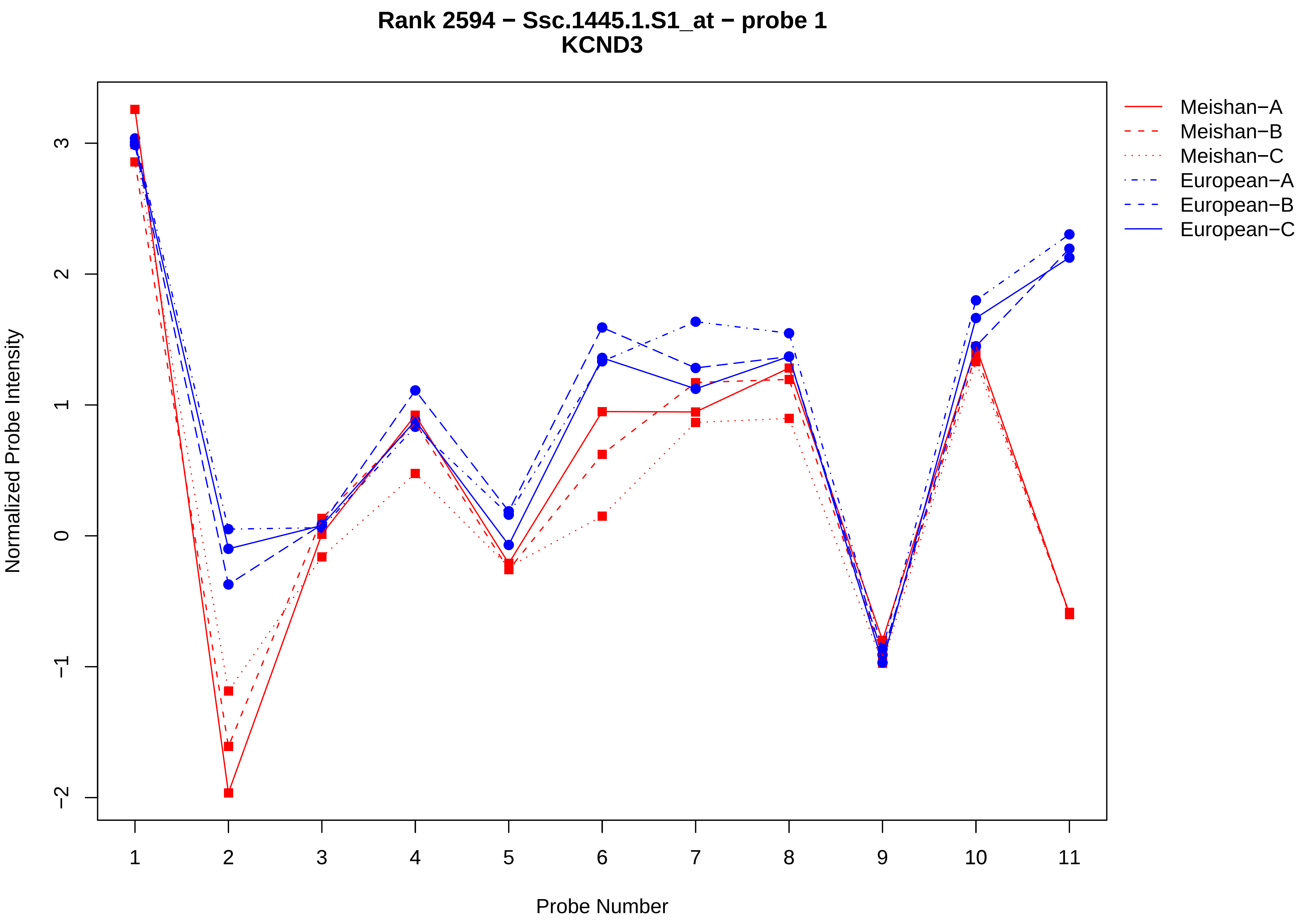

Rank 2594 − Ssc.1445.1.S1_at − probe 1
KCND3
Normalized Probe Intensity
Probe Number
−2
−1
0
1
2
3
1
2
3
4
5
6
7
8
9
10
11
Meishan−A
Meishan−B
Meishan−C
European−A
European−B
European−C

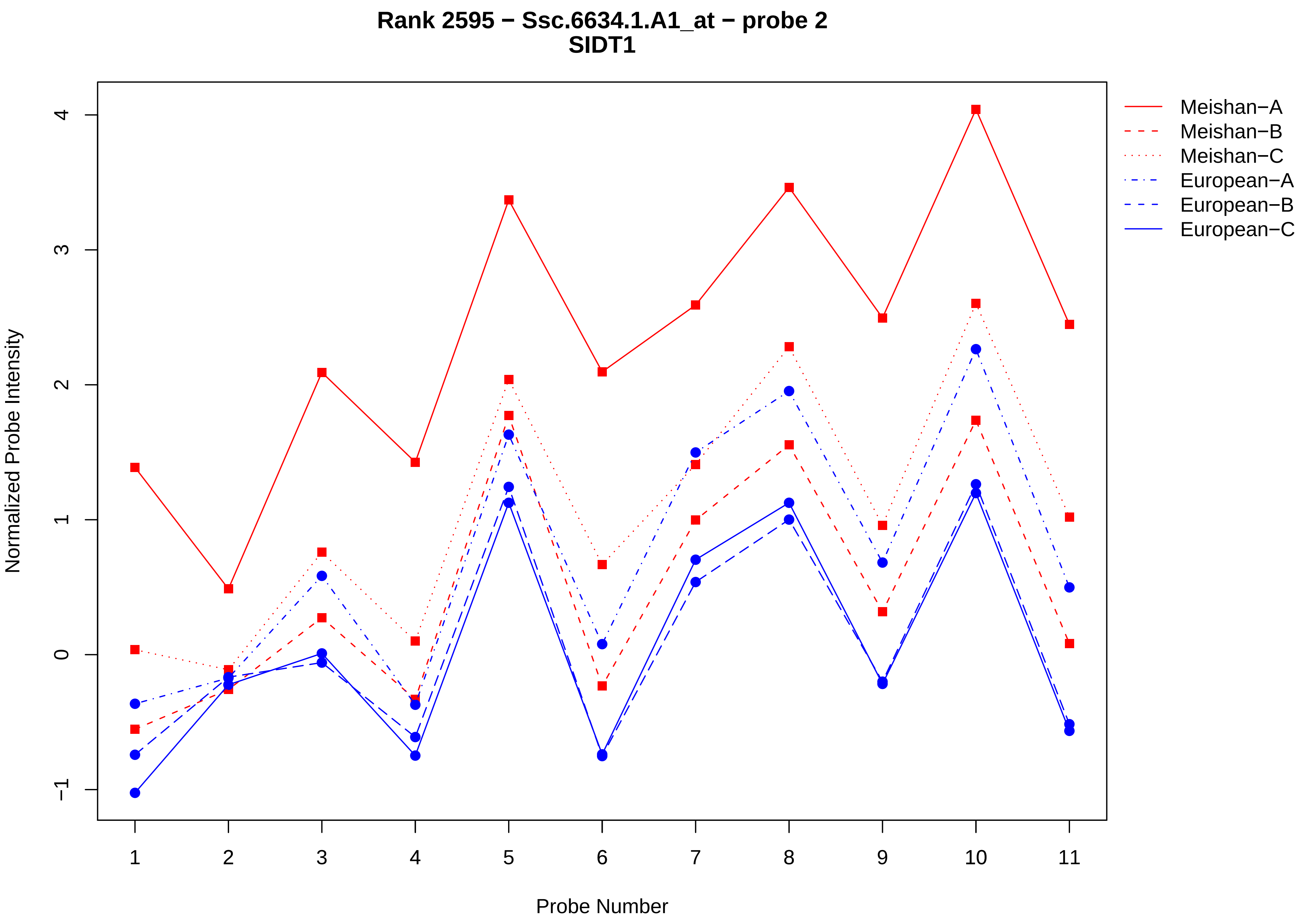

Rank 2595 − Ssc.6634.1.A1_at − probe 2
SIDT1
Normalized Probe Intensity
Probe Number
−1
0
1
2
3
4
1
2
3
4
5
6
7
8
9
10
11
Meishan−A
Meishan−B
Meishan−C
European−A
European−B
European−C

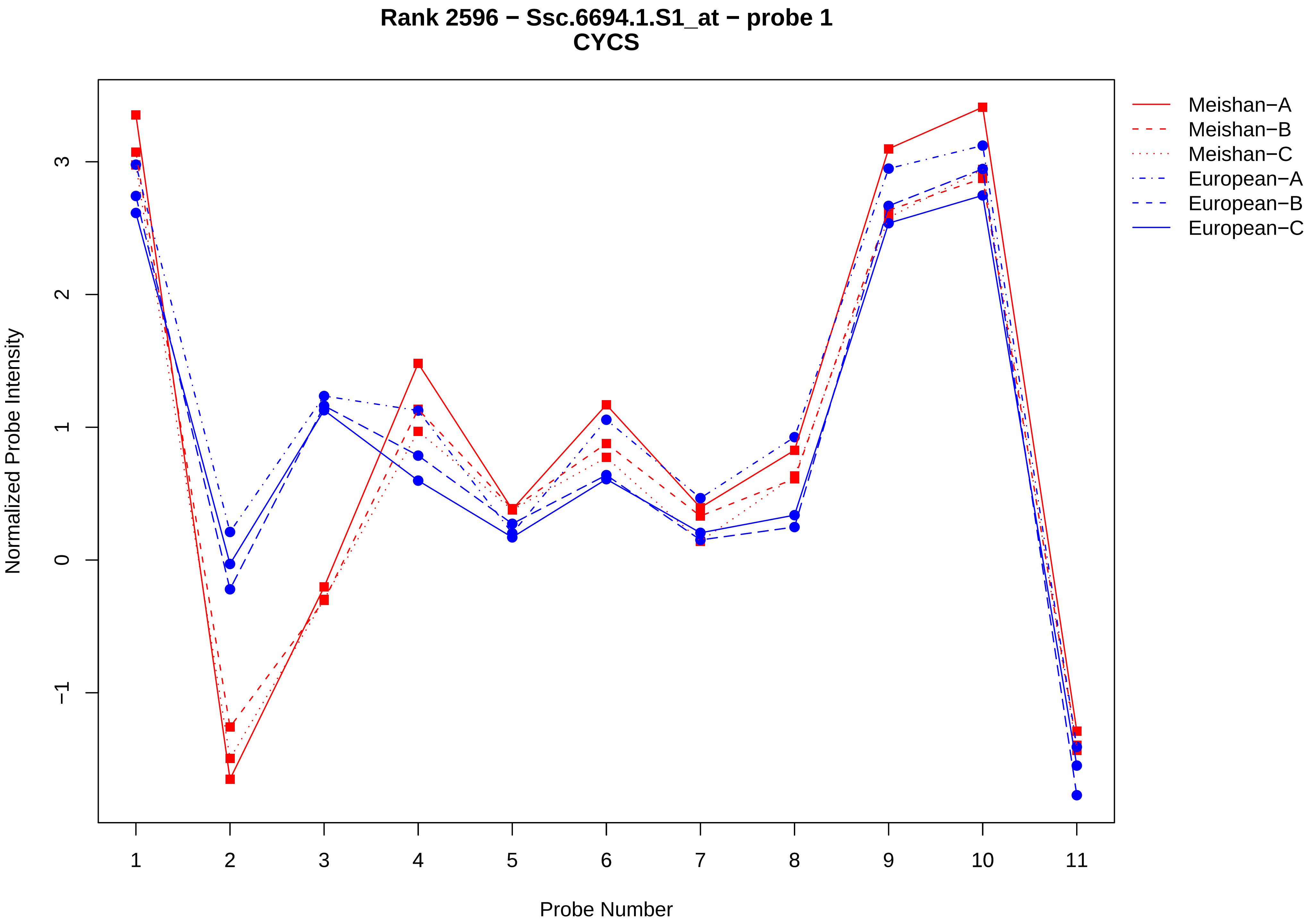

Rank 2596 − Ssc.6694.1.S1_at − probe 1
CYCS
Meishan−A
Meishan−B
Meishan−C
European−A
European−B
European−C
Normalized Probe Intensity
Probe Number
−1
0
1
2
3
1
2
3
4
5
6
7
8
9
10
11

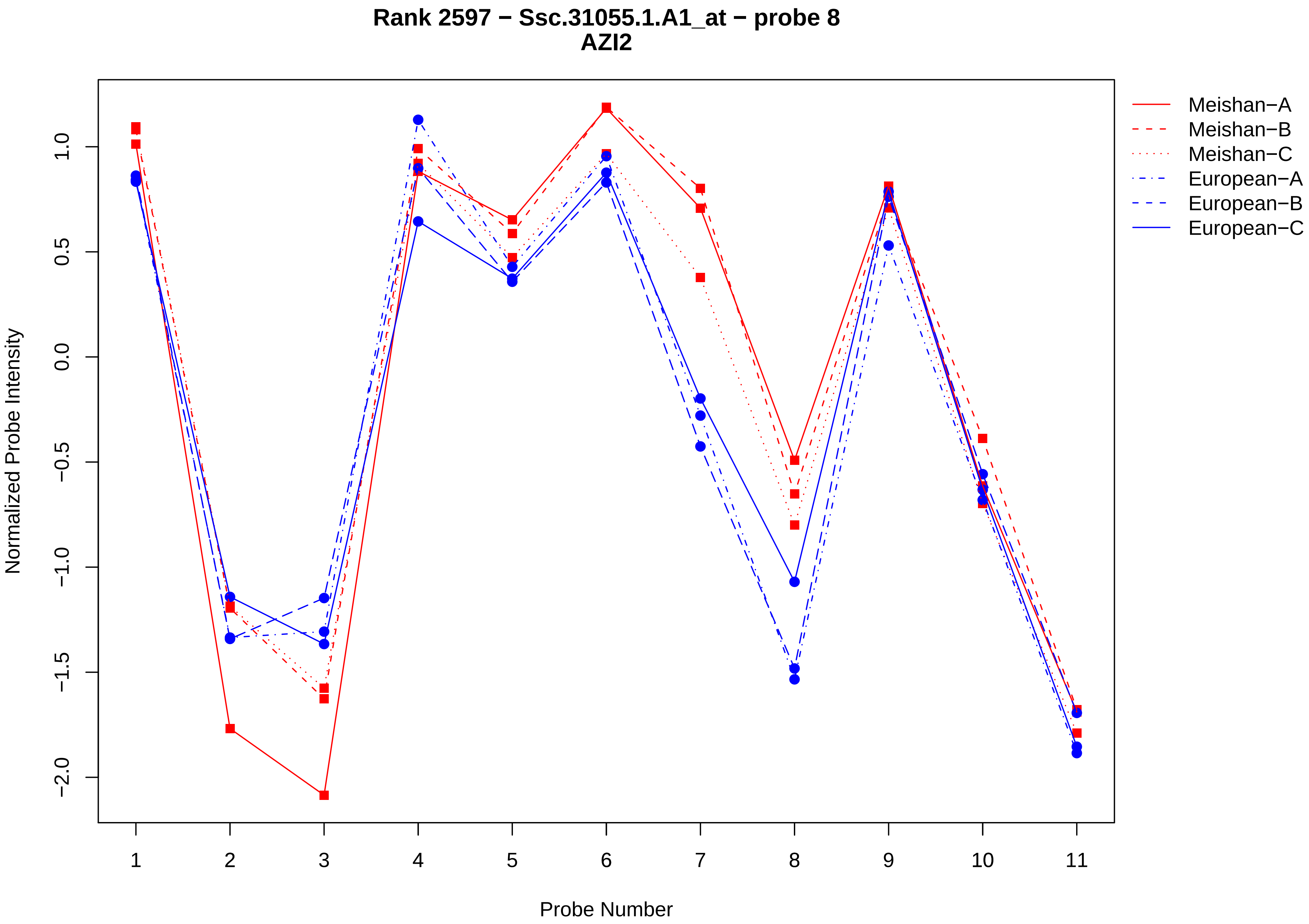

Rank 2597 − Ssc.31055.1.A1_at − probe 8
AZI2
Normalized Probe Intensity
Probe Number
1.0
0.5
0.0
−0.5
−1.0
−1.5
−2.0
1
2
3
4
5
6
7
8
9
10
11
Meishan−A
Meishan−B
Meishan−C
European−A
European−B
European−C

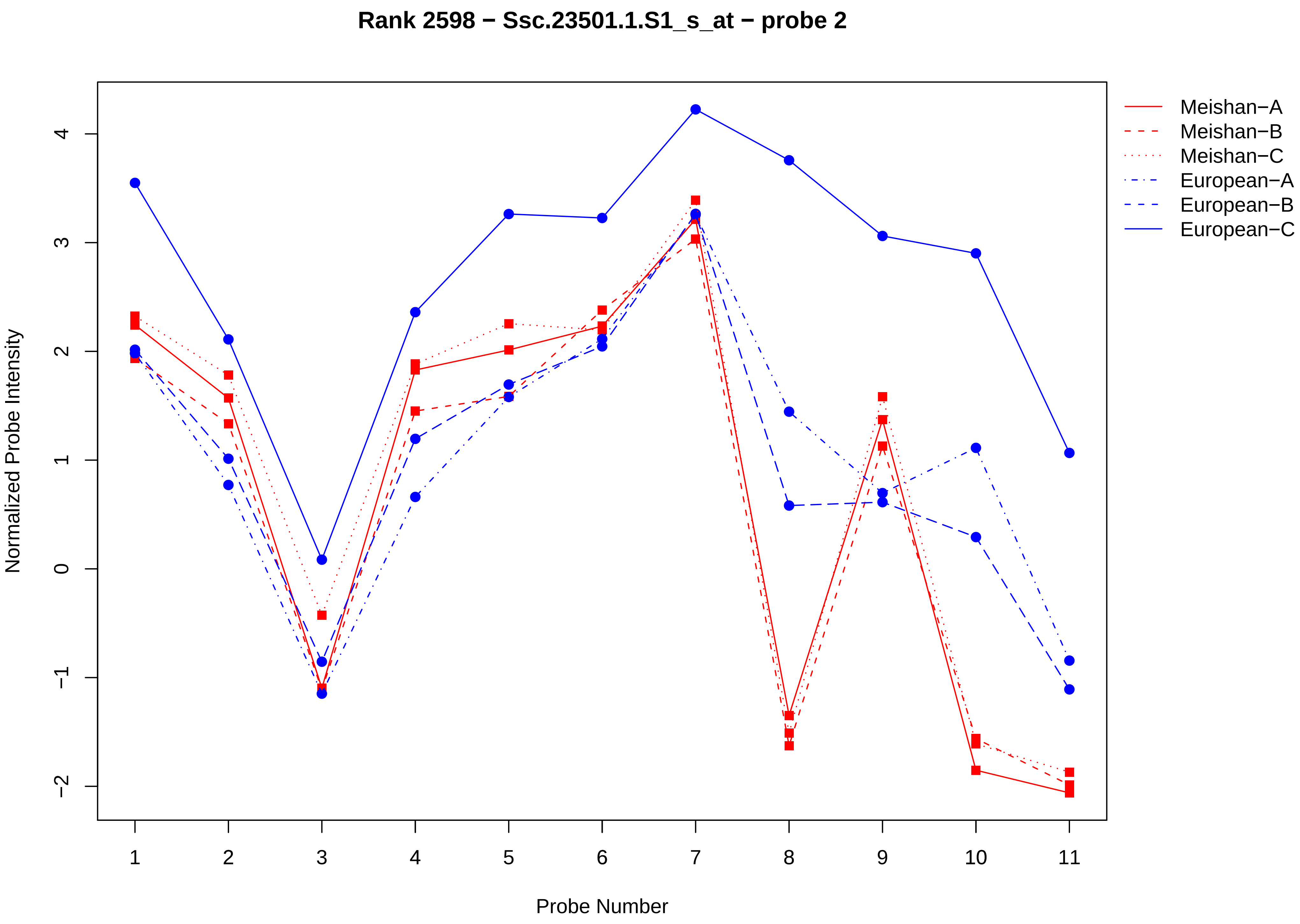

Rank 2598 − Ssc.23501.1.S1_s_at − probe 2
Normalized Probe Intensity
Probe Number
−2
−1
0
1
2
3
4
1
2
3
4
5
6
7
8
9
10
11
Meishan−A
Meishan−B
Meishan−C
European−A
European−B
European−C

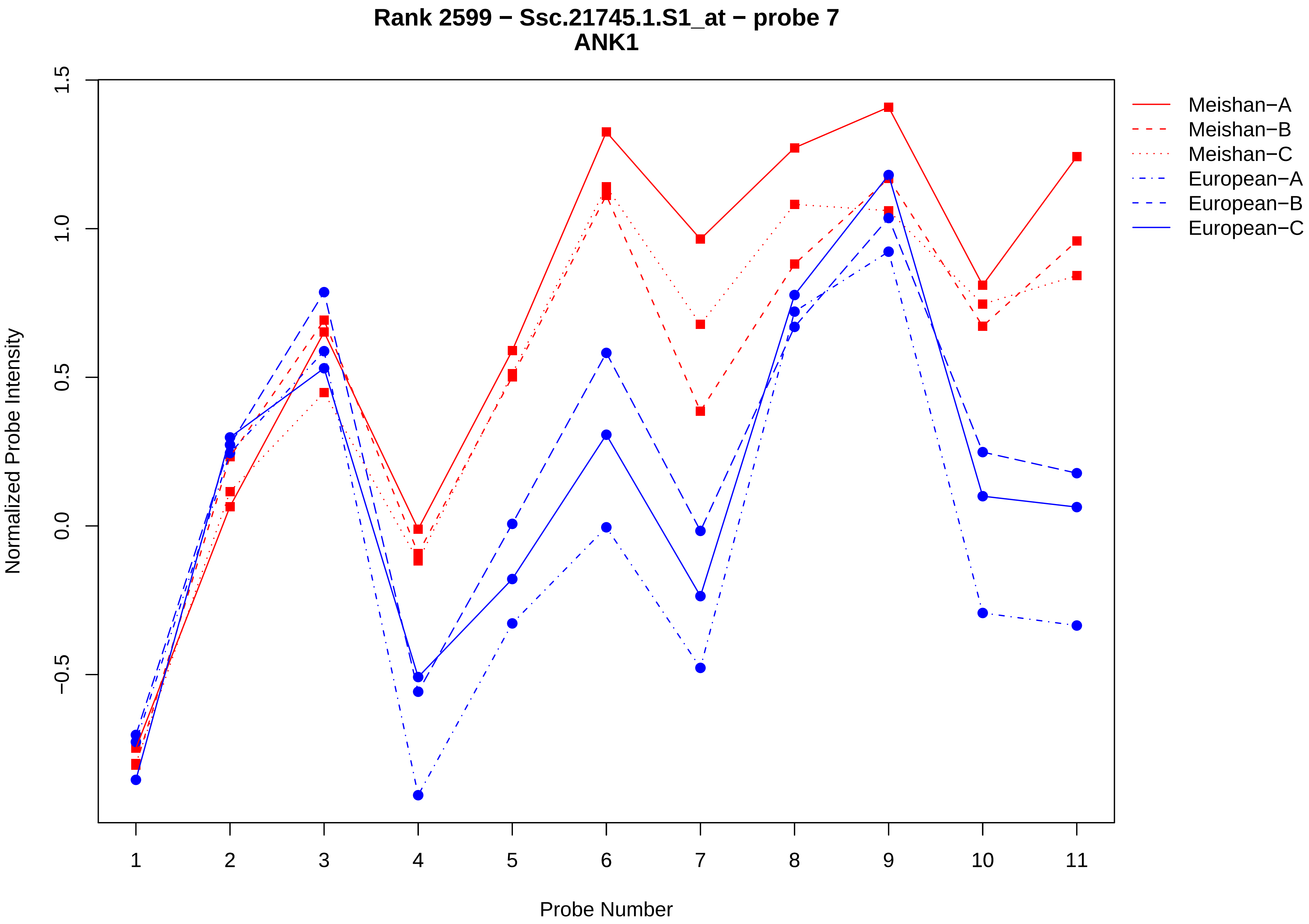
Rank 2599 − Ssc.21745.1.S1_at − probe 7
ANK1
Normalized Probe Intensity
Probe Number
1.5
1.0
0.5
0.0
−0.5
1
2
3
4
5
6
7
8
9
10
11
Meishan−A
Meishan−B
Meishan−C
European−A
European−B
European−C

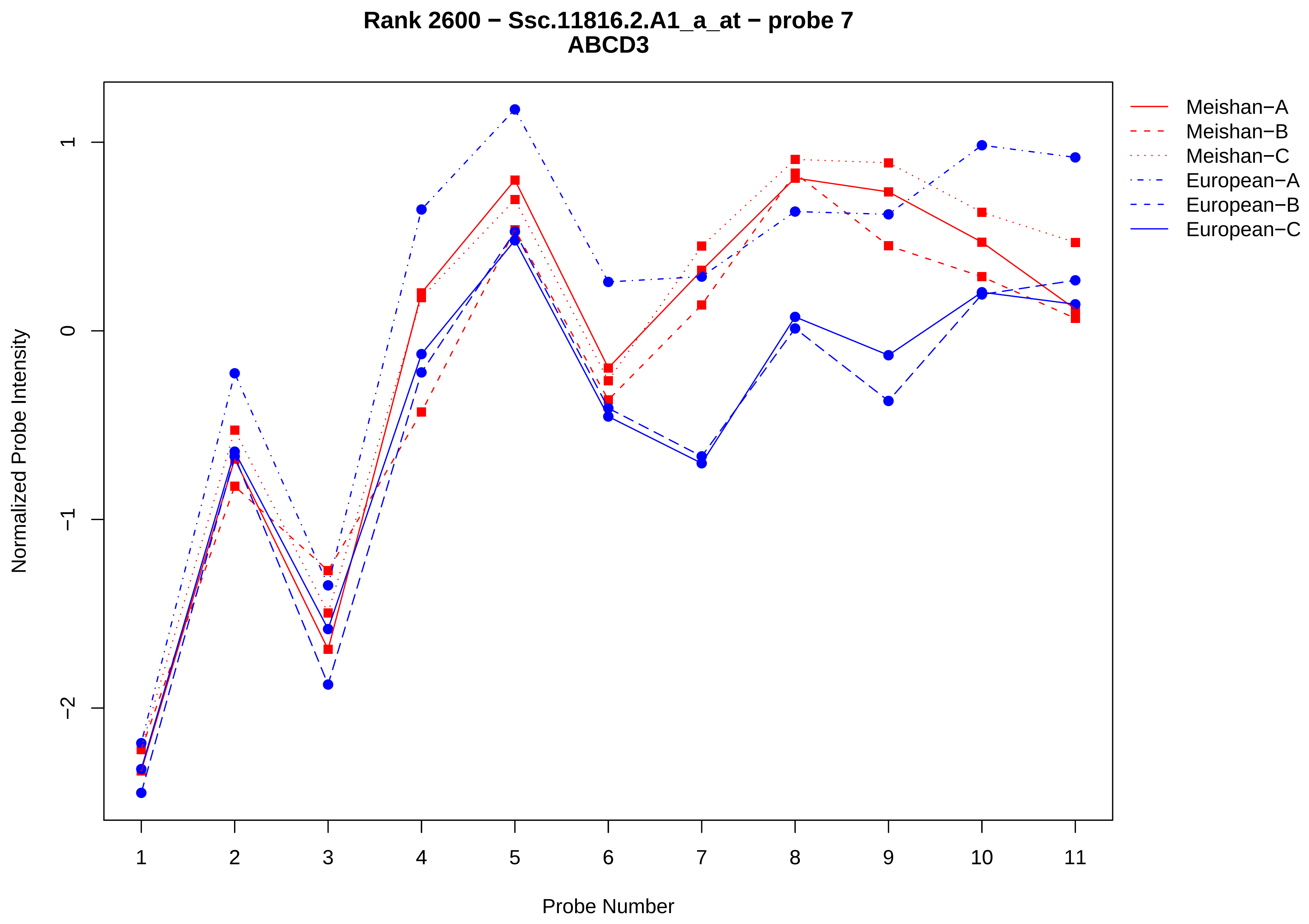

Rank 2600 − Ssc.11816.2.A1_a_at − probe 7
ABCD3
Normalized Probe Intensity
Probe Number
1
0
−1
−2
1
2
3
4
5
6
7
8
9
10
11
Meishan−A
Meishan−B
Meishan−C
European−A
European−B
European−C

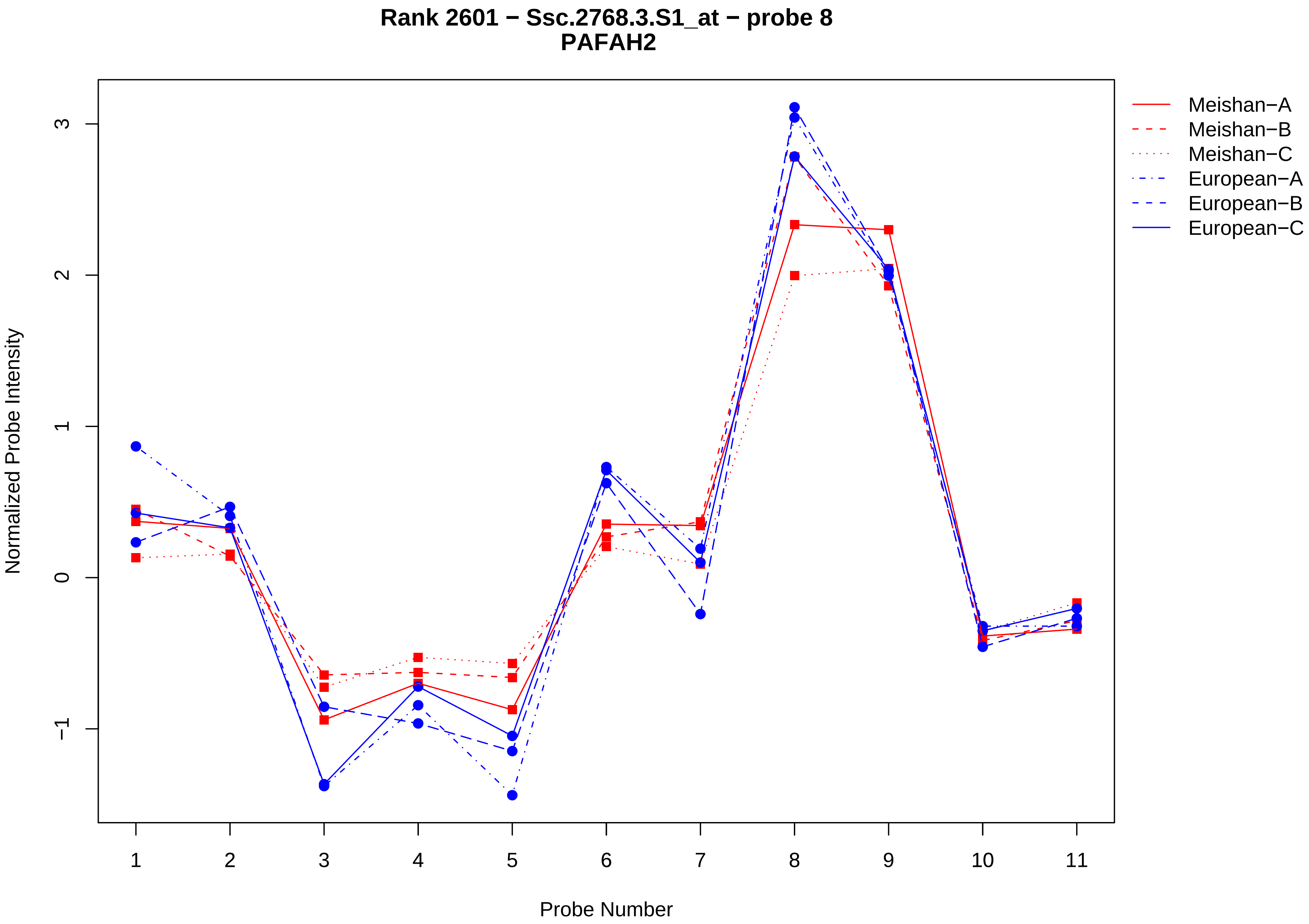

Rank 2601 − Ssc.2768.3.S1_at − probe 8
PAFAH2
Normalized Probe Intensity
Probe Number
−1
0
1
2
3
1
2
3
4
5
6
7
8
9
10
11
Meishan−A
Meishan−B
Meishan−C
European−A
European−B
European−C

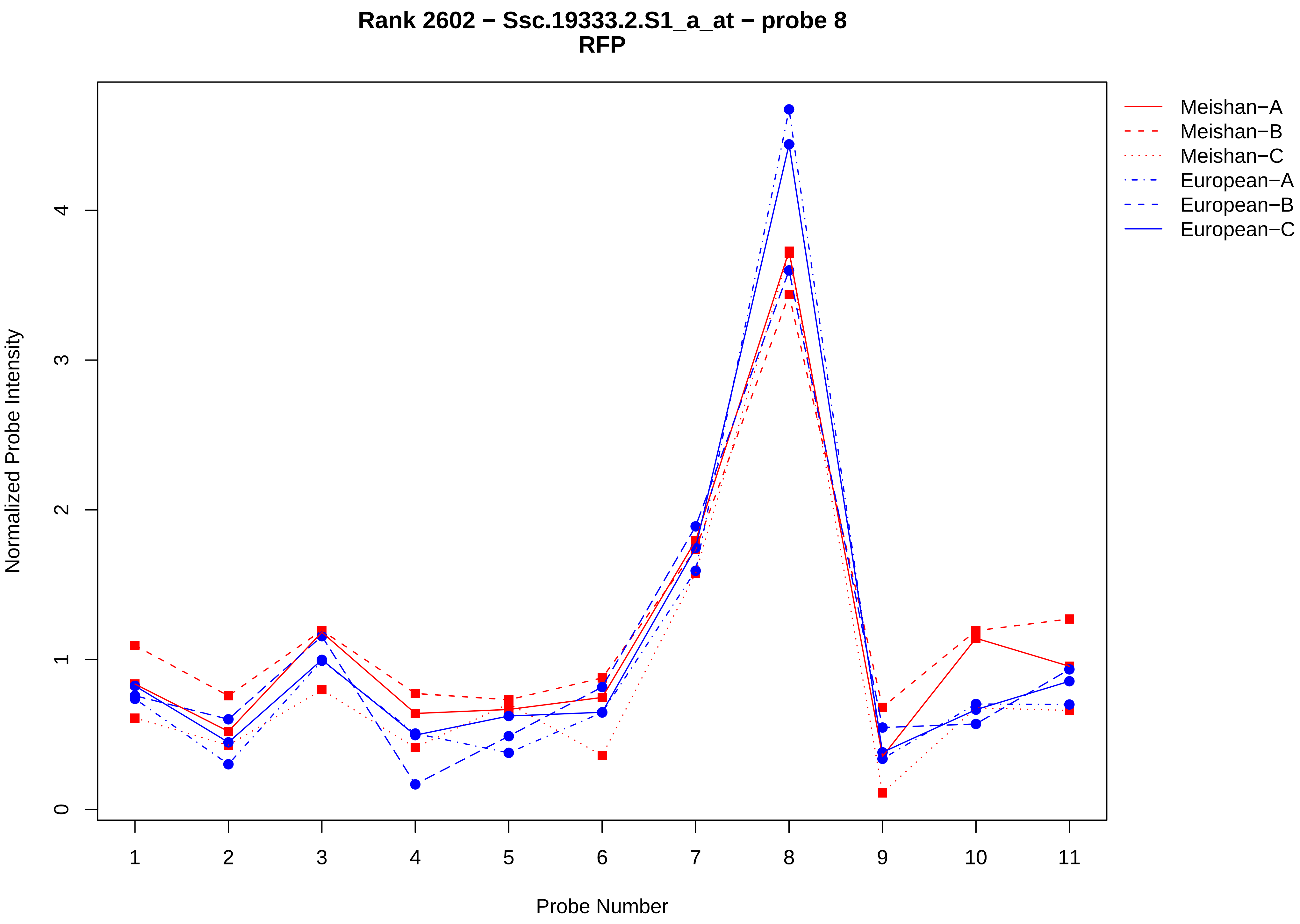
Rank 2602 − Ssc.19333.2.S1_a_at − probe 8
RFP
Normalized Probe Intensity
Probe Number
0
1
2
3
4
1
2
3
4
5
6
7
8
9
10
11
Meishan−A
Meishan−B
Meishan−C
European−A
European−B
European−C

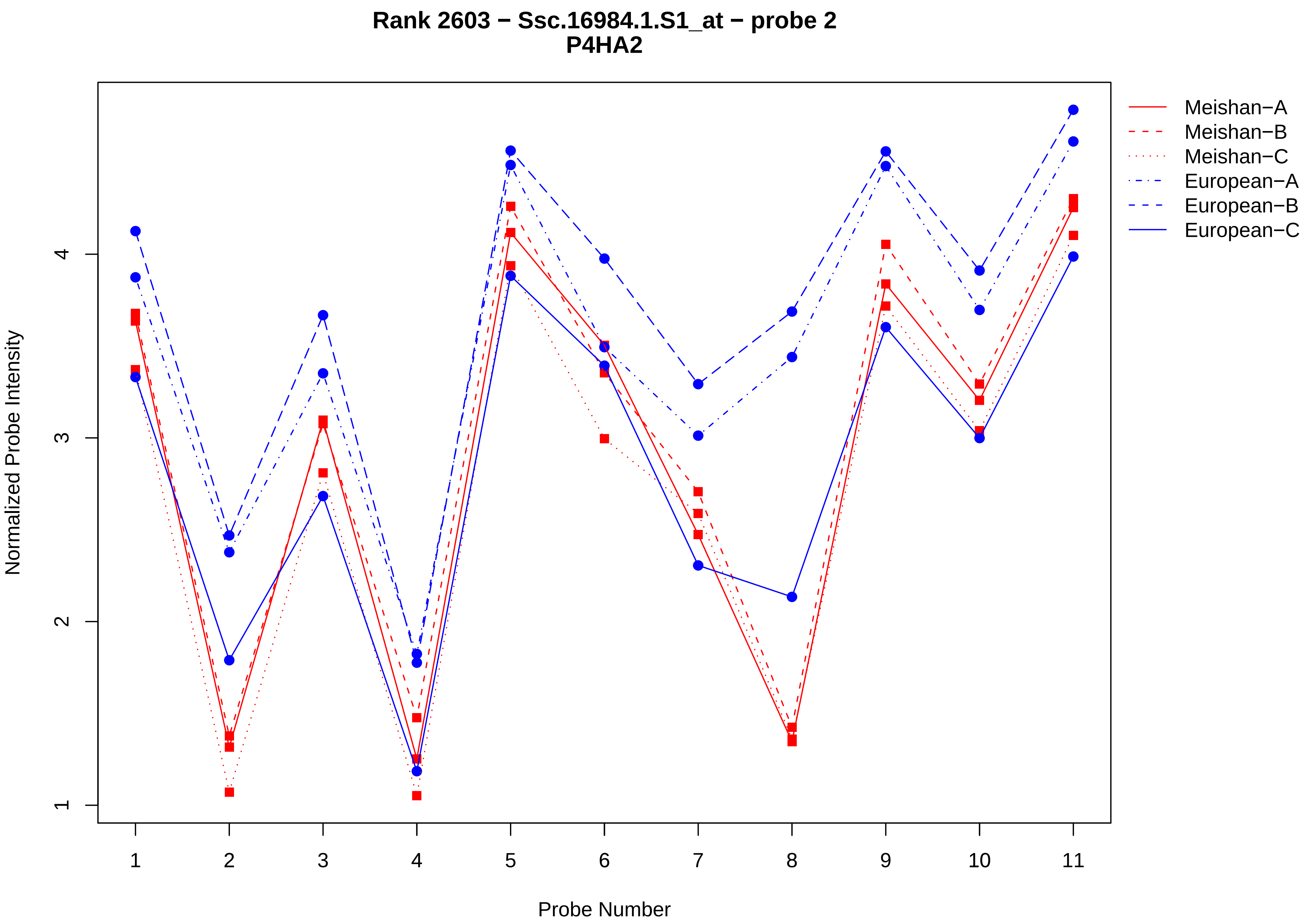
Rank 2603 − Ssc.16984.1.S1_at − probe 2
P4HA2
Normalized Probe Intensity
Probe Number
1
2
3
4
5
6
7
8
9
10
11
Meishan−A
Meishan−B
Meishan−C
European−A
European−B
European−C

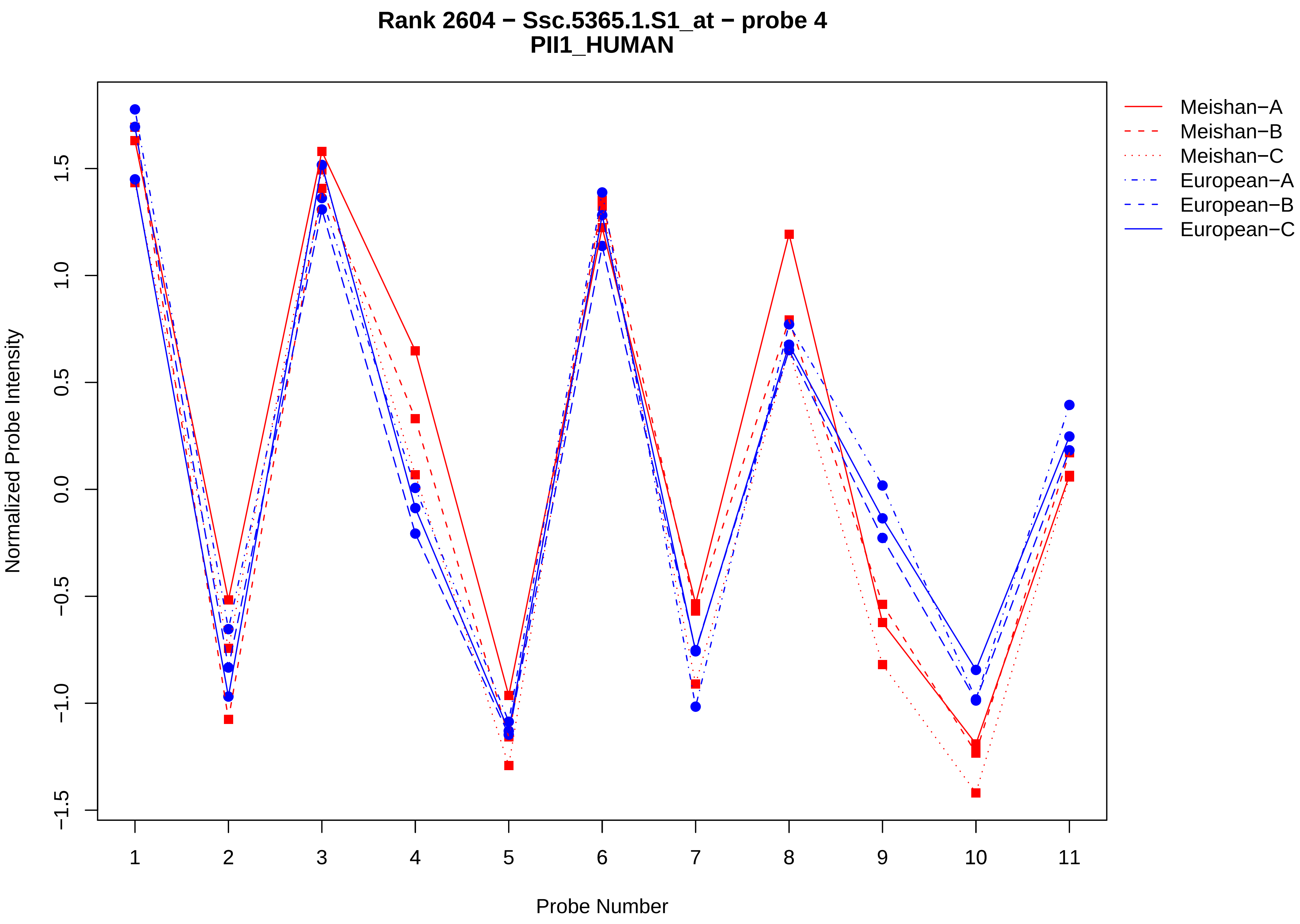

Rank 2604 − Ssc.5365.1.S1_at − probe 4
PII1_HUMAN
Normalized Probe Intensity
Probe Number
−1.5
−1.0
−0.5
0.0
0.5
1.0
1.5
1
2
3
4
5
6
7
8
9
10
11
Meishan−A
Meishan−B
Meishan−C
European−A
European−B
European−C

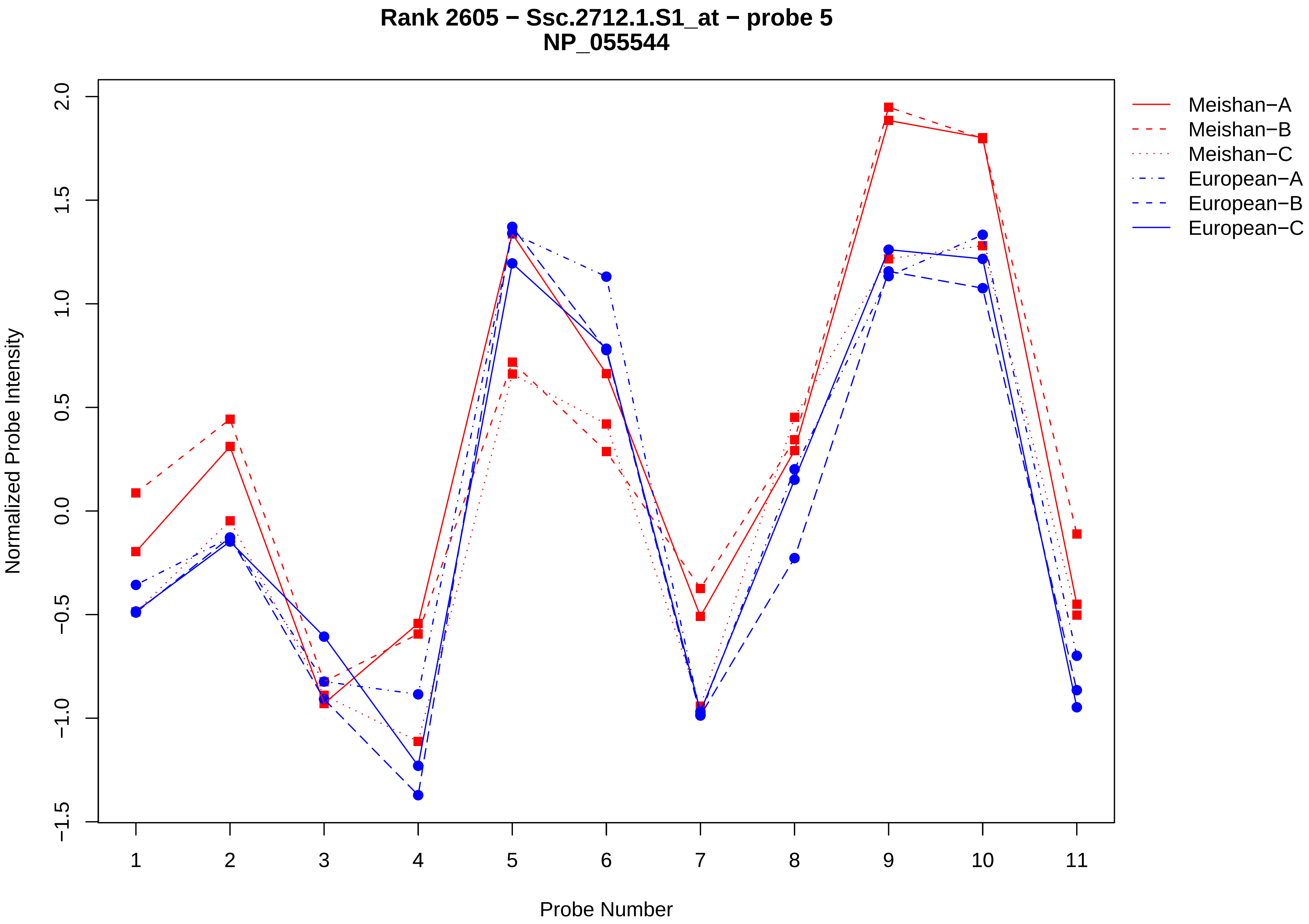

Rank 2605 − Ssc.2712.1.S1_at − probe 5
NP_055544
Normalized Probe Intensity
Probe Number
−1.5
−1.0
−0.5
0.0
0.5
1.0
1.5
2.0
1
2
3
4
5
6
7
8
9
10
11
Meishan−A
Meishan−B
Meishan−C
European−A
European−B
European−C

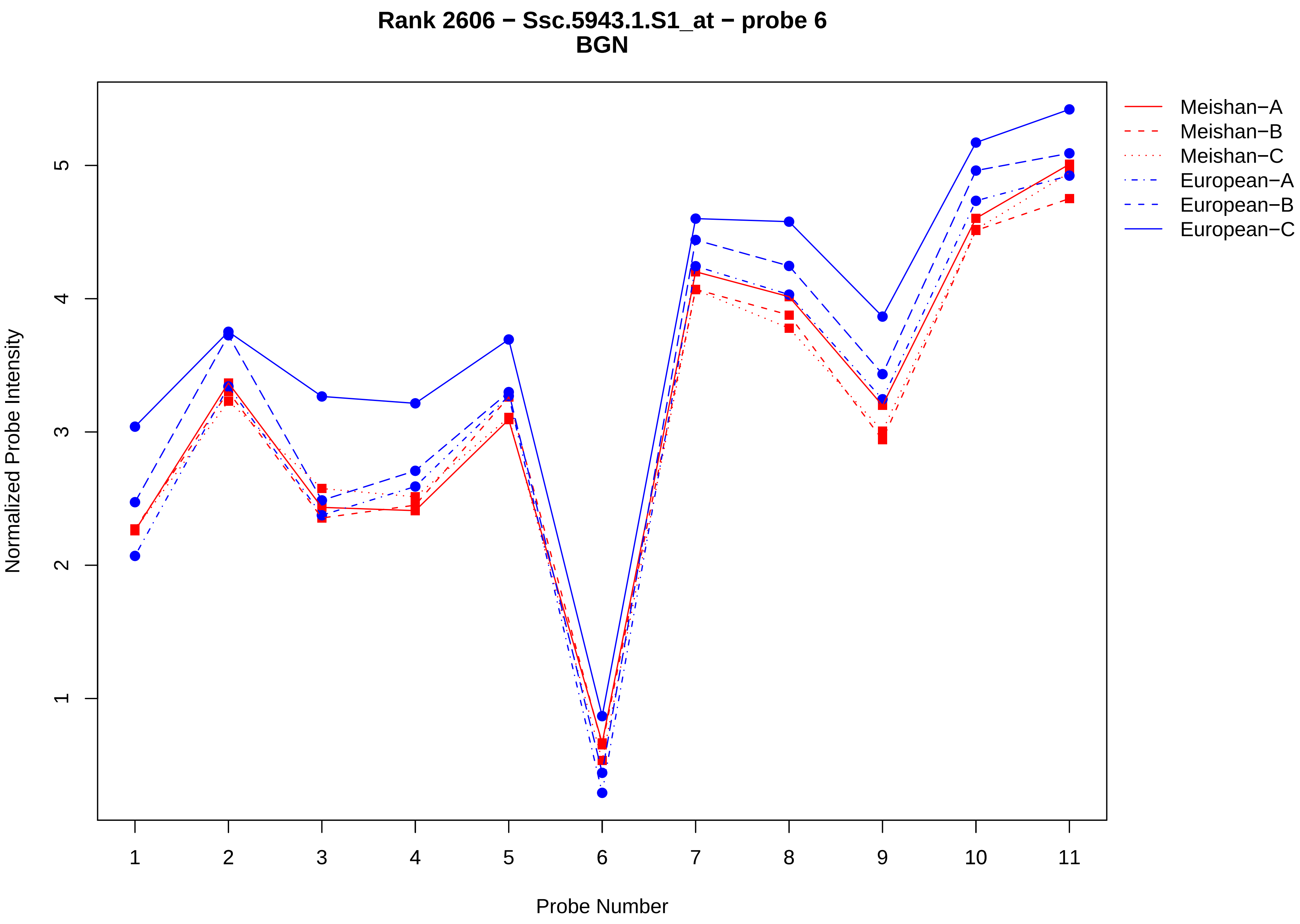

Rank 2606 − Ssc.5943.1.S1_at − probe 6
BGN
Normalized Probe Intensity
Probe Number
1
2
3
4
5
1
2
3
4
5
6
7
8
9
10
11
Meishan−A
Meishan−B
Meishan−C
European−A
European−B
European−C

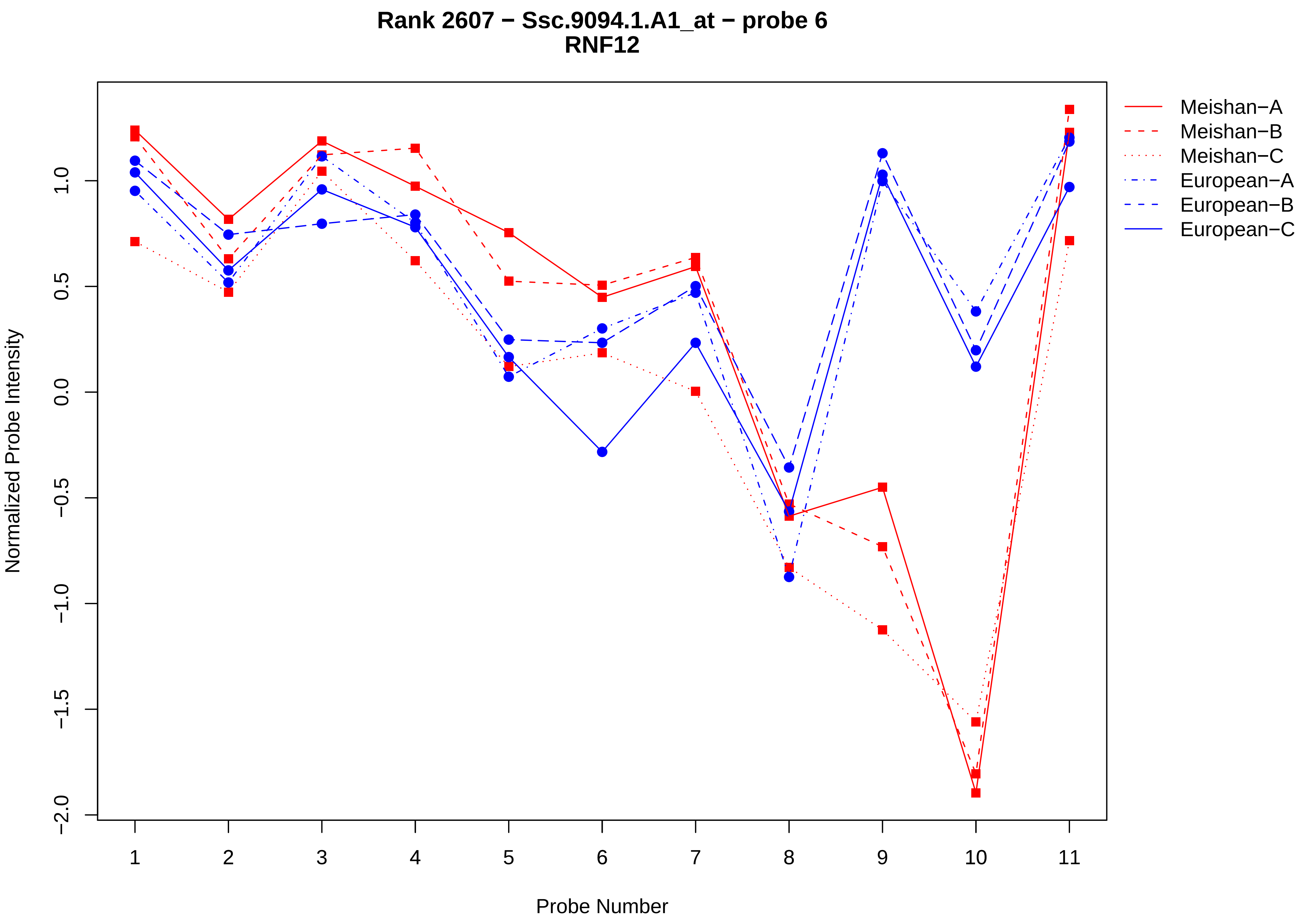

Rank 2607 − Ssc.9094.1.A1_at − probe 6
RNF12
Normalized Probe Intensity
−2.0
−1.5
−1.0
−0.5
0.0
0.5
1.0
1
2
3
4
5
6
7
8
9
10
11
Probe Number
Meishan−A
Meishan−B
Meishan−C
European−A
European−B
European−C

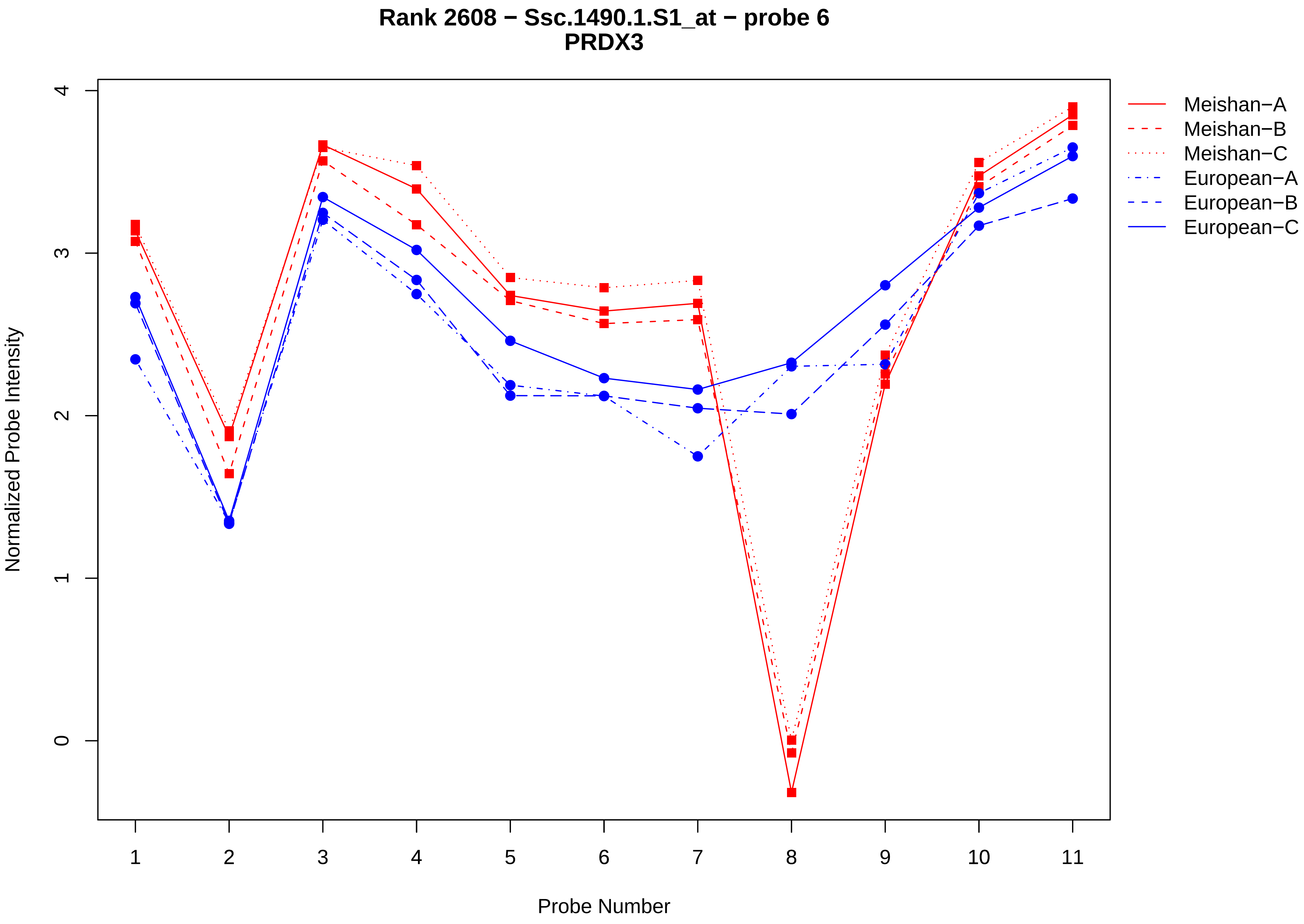

Rank 2608 − Ssc.1490.1.S1_at − probe 6
PRDX3
Normalized Probe Intensity
Probe Number
0
1
2
3
4
1
2
3
4
5
6
7
8
9
10
11
Meishan−A
Meishan−B
Meishan−C
European−A
European−B
European−C

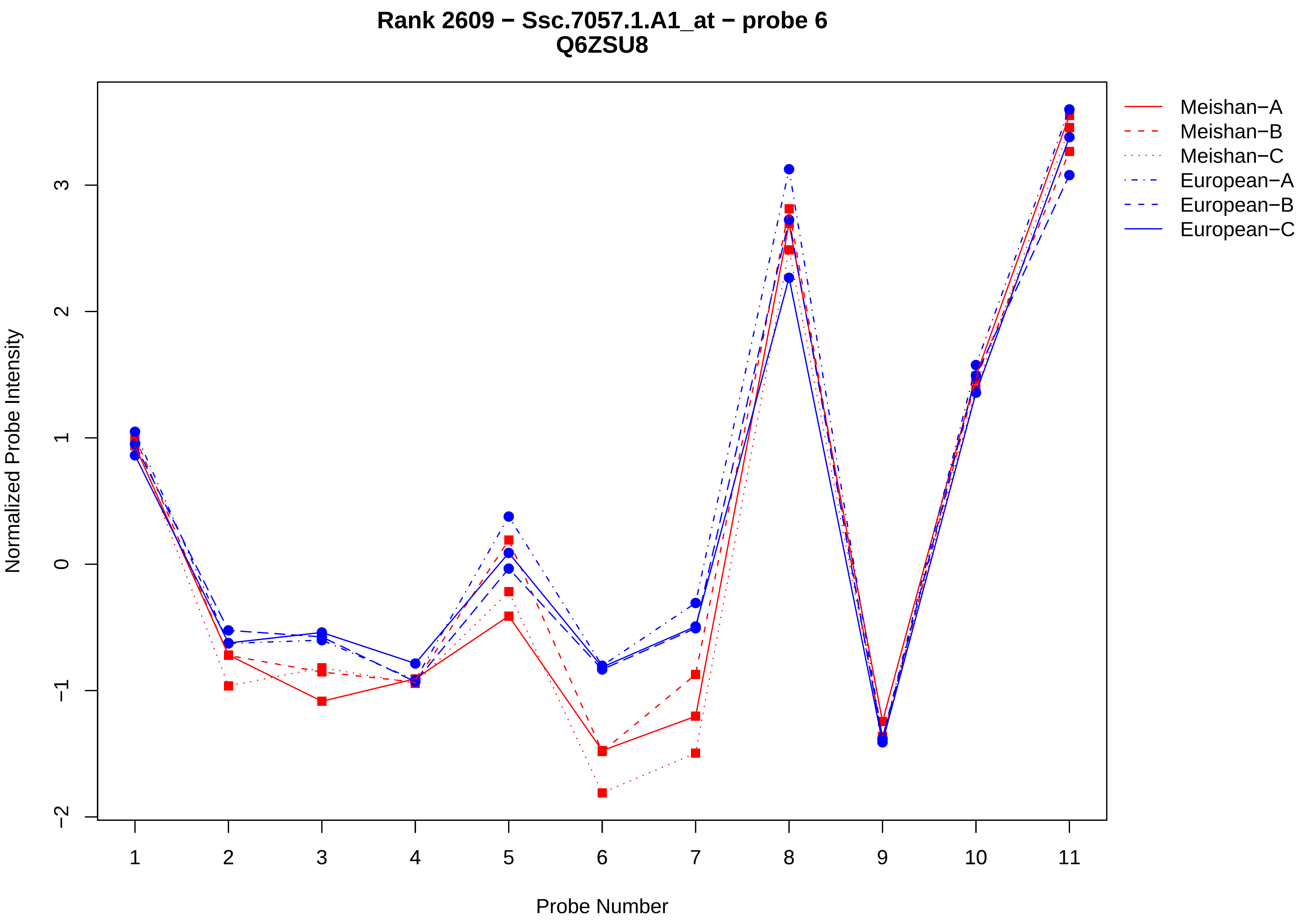

Rank 2609 − Ssc.7057.1.A1_at − probe 6
Q6ZSU8
Normalized Probe Intensity
Probe Number
−2
−1
0
1
2
3
1
2
3
4
5
6
7
8
9
10
11
Meishan−A
Meishan−B
Meishan−C
European−A
European−B
European−C

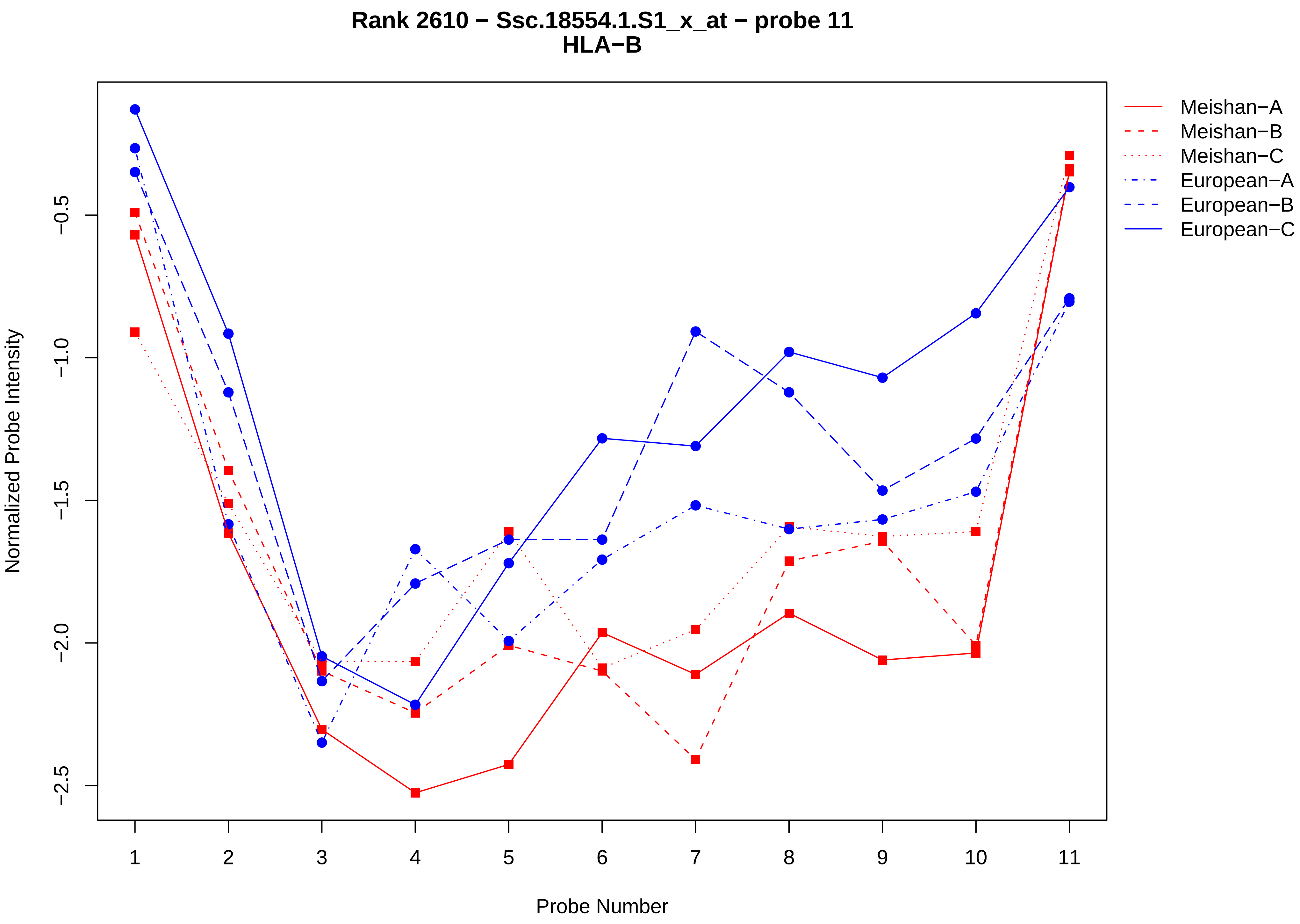
Rank 2610 − Ssc.18554.1.S1_x_at − probe 11
HLA−B
Normalized Probe Intensity
−0.5
−1.0
−1.5
−2.0
−2.5
Probe Number
1
2
3
4
5
6
7
8
9
10
11
Meishan−A
Meishan−B
Meishan−C
European−A
European−B
European−C

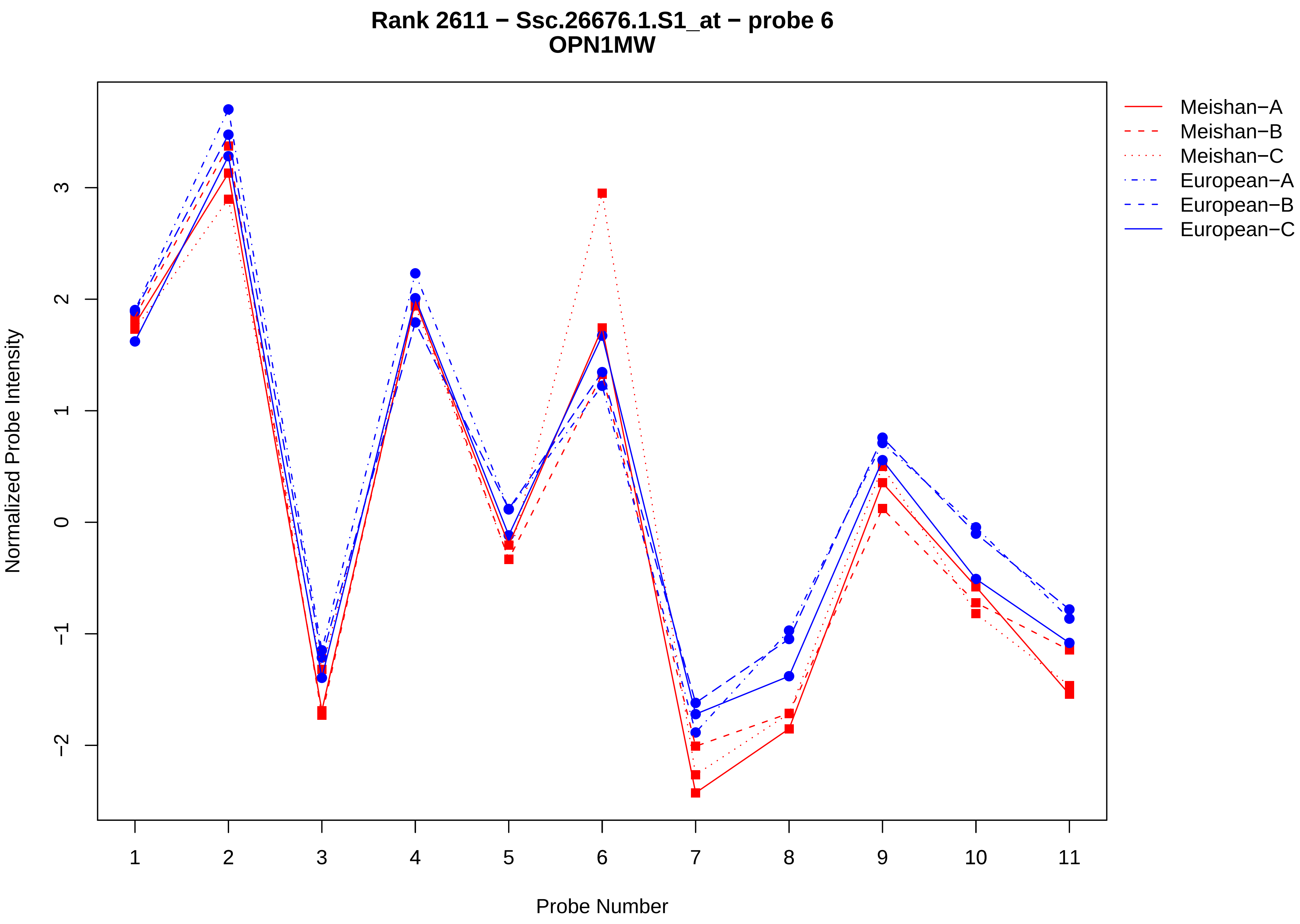
Rank 2611 − Ssc.26676.1.S1_at − probe 6
OPN1MW
Normalized Probe Intensity
Probe Number
Meishan−A
Meishan−B
Meishan−C
European−A
European−B
European−C
−2
−1
0
1
2
3
1
2
3
4
5
6
7
8
9
10
11

Rank 2612 − Ssc.24103.1.A1_s_at − probe 9
IGHM

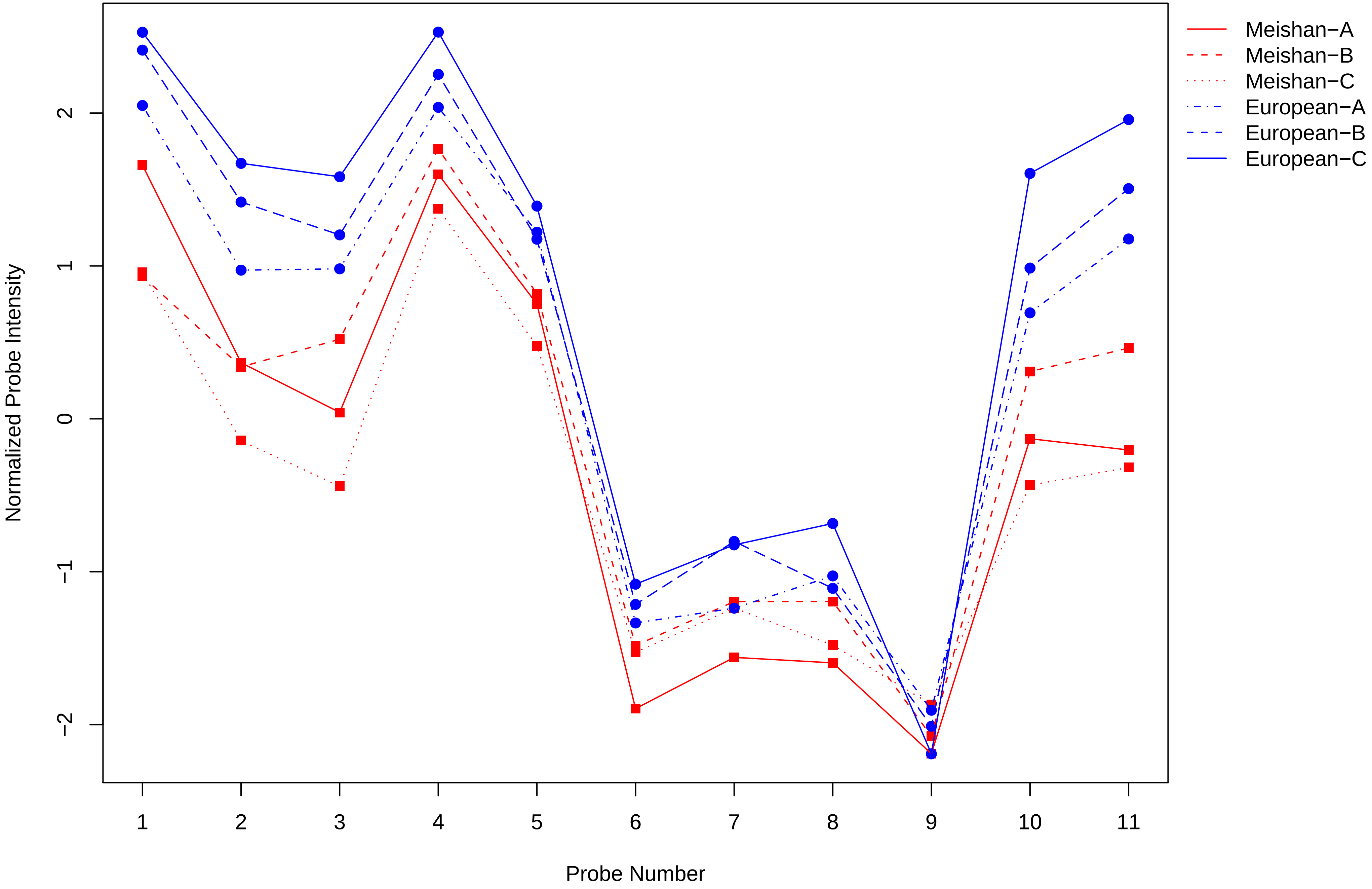

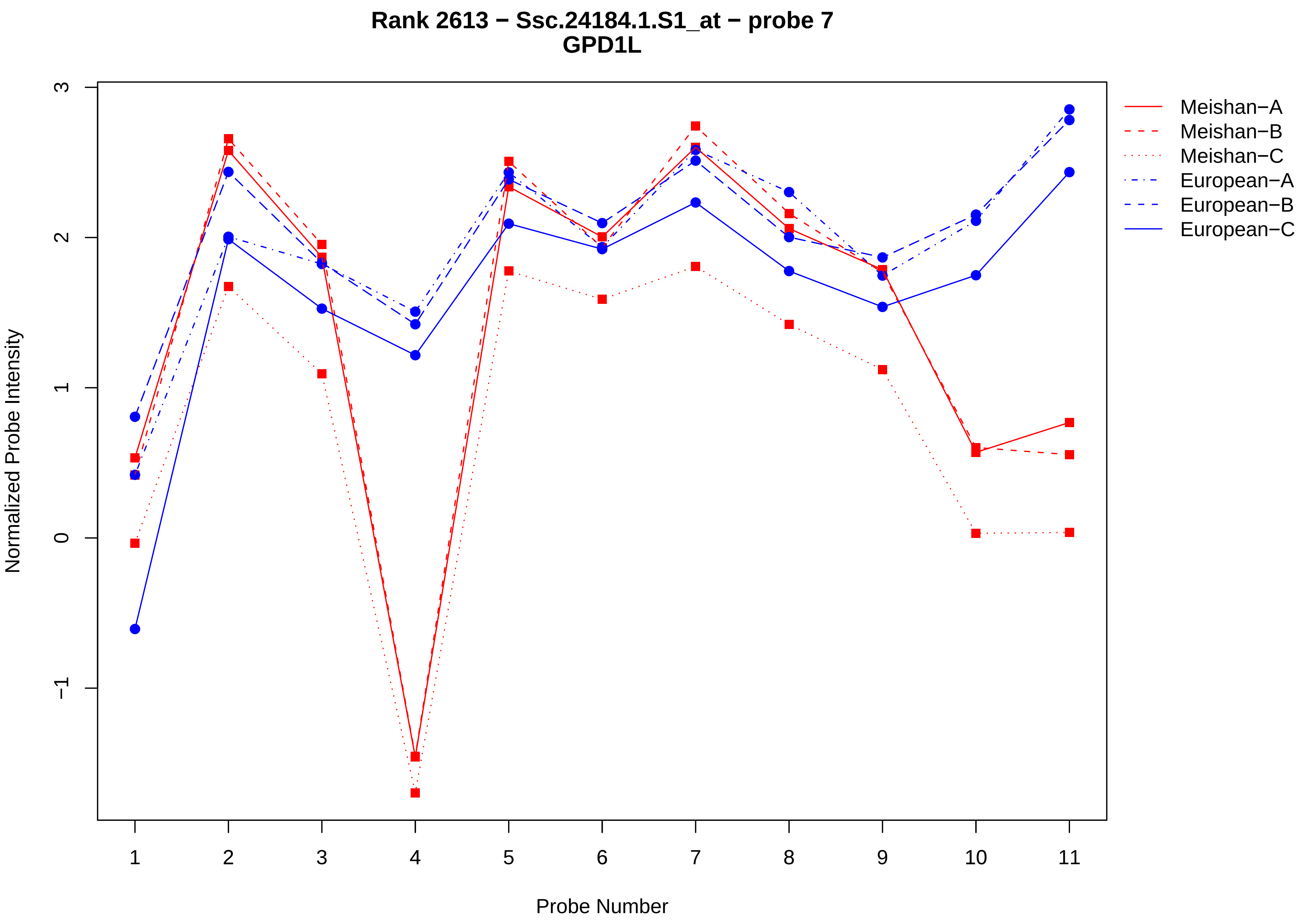

Rank 2613 − Ssc.24184.1.S1_at − probe 7
GPD1L
Normalized Probe Intensity
Probe Number
−1
0
1
2
3
1
2
3
4
5
6
7
8
9
10
11
Meishan−A
Meishan−B
Meishan−C
European−A
European−B
European−C

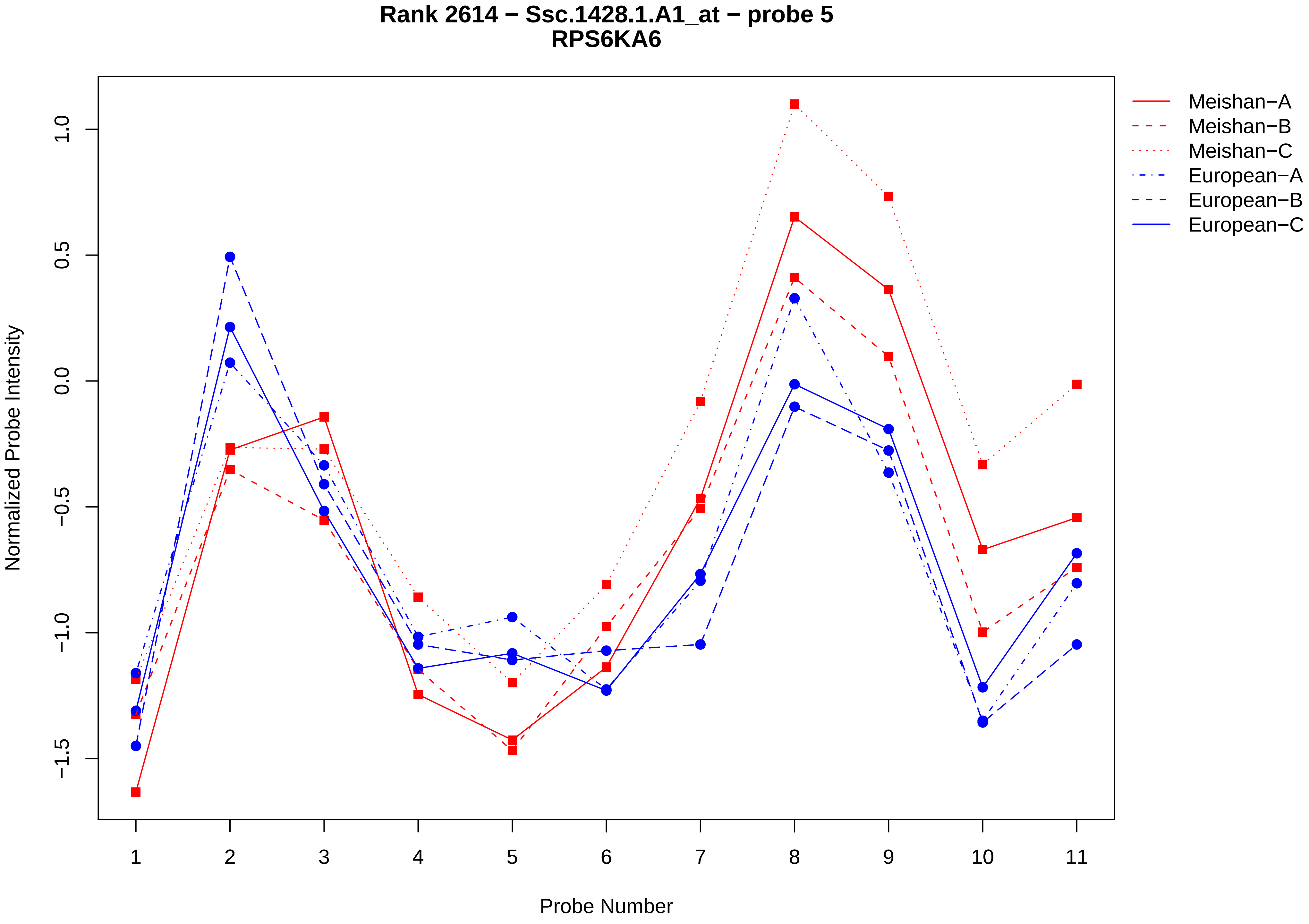

Rank 2614 − Ssc.1428.1.A1_at − probe 5
RPS6KA6
Normalized Probe Intensity
Probe Number
Meishan−A
Meishan−B
Meishan−C
European−A
European−B
European−C

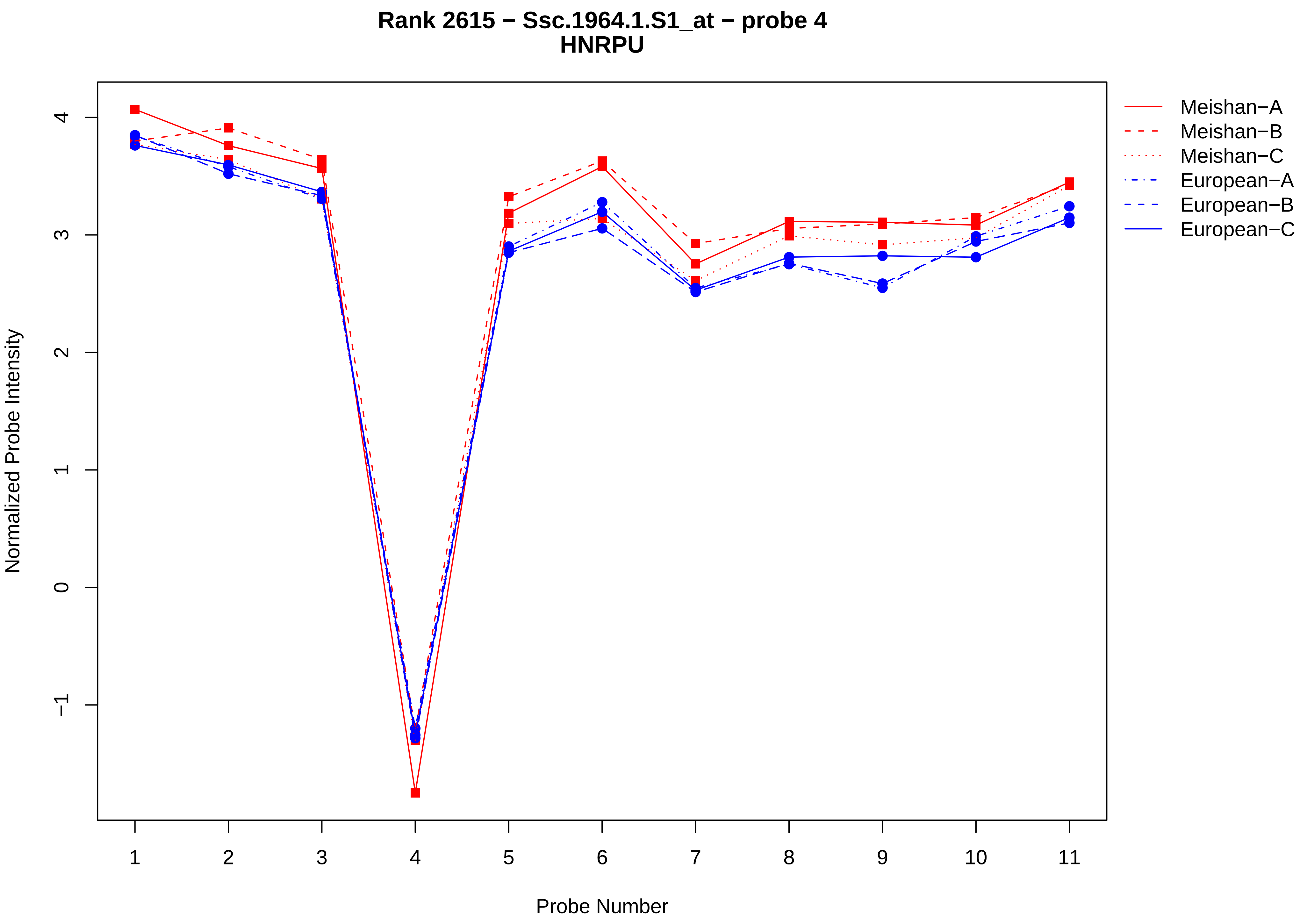
Rank 2615 − Ssc.1964.1.S1_at − probe 4
HNRPU
Normalized Probe Intensity
Probe Number
−1
0
1
2
3
4
1
2
3
4
5
6
7
8
9
10
11
Meishan−A
Meishan−B
Meishan−C
European−A
European−B
European−C

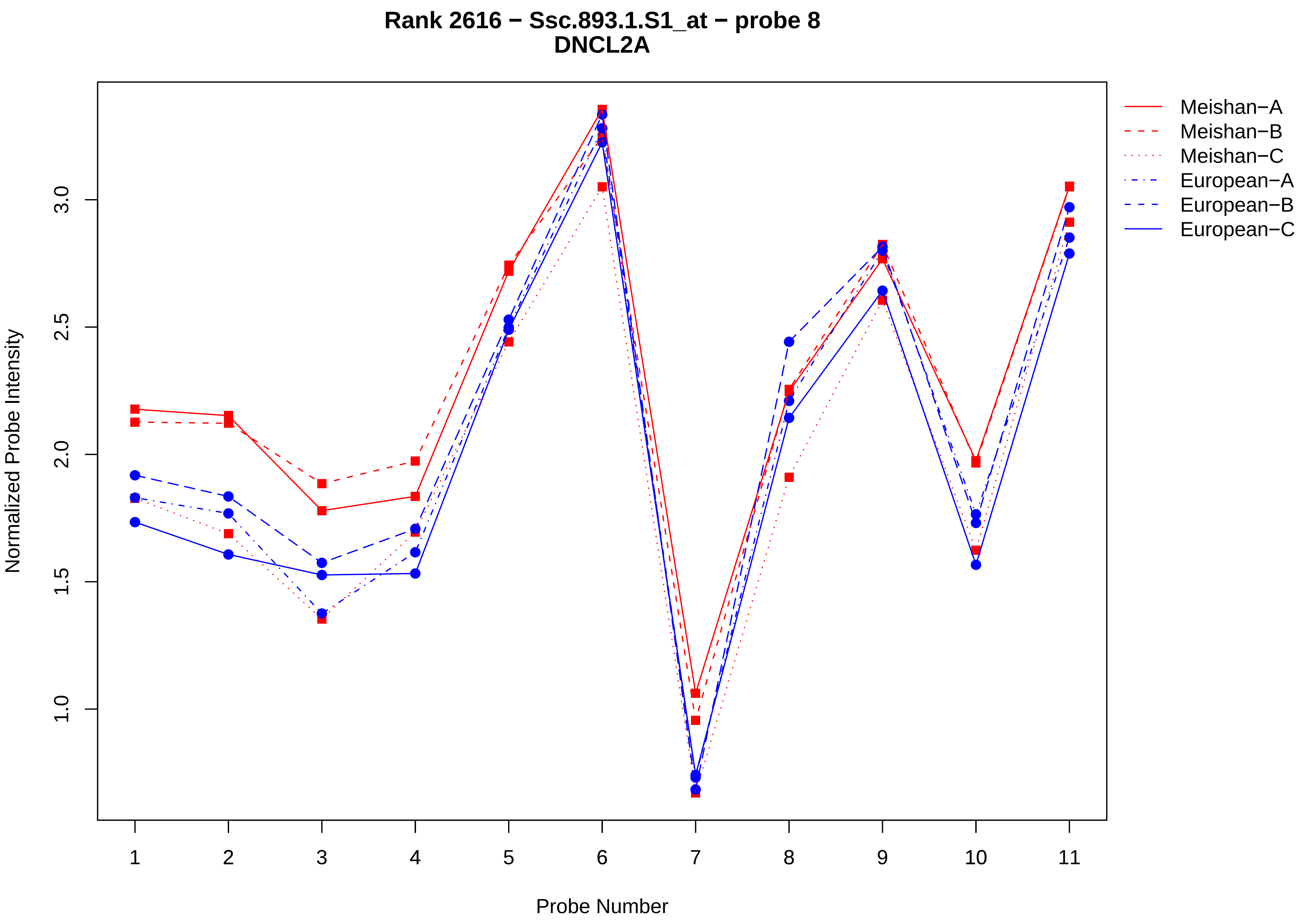

Rank 2616 − Ssc.893.1.S1_at − probe 8
DNCL2A
Normalized Probe Intensity
Probe Number
1.0
1.5
2.0
2.5
3.0
1
2
3
4
5
6
7
8
9
10
11
Meishan−A
Meishan−B
Meishan−C
European−A
European−B
European−C

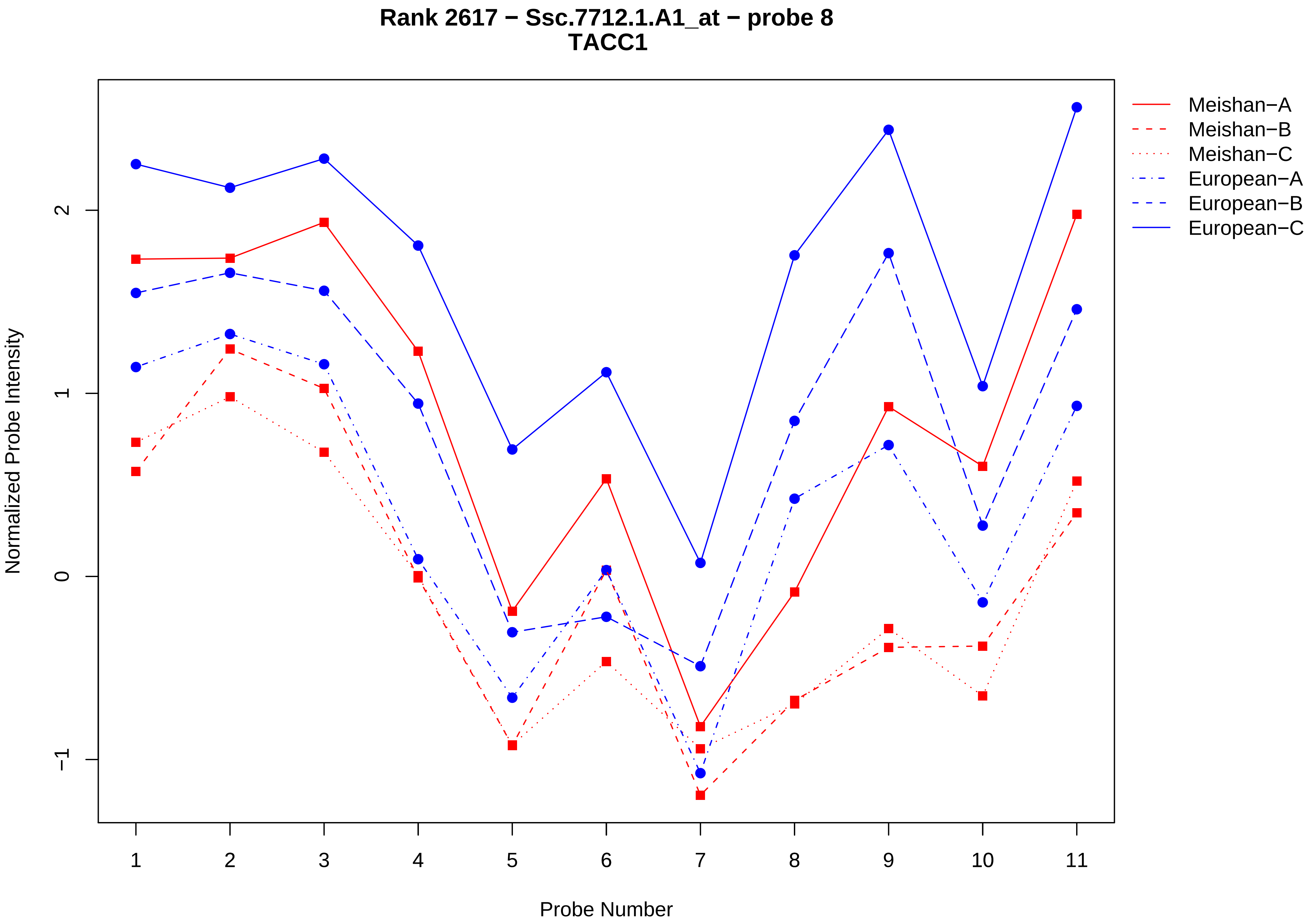

Rank 2617 − Ssc.7712.1.A1_at − probe 8
TACC1
Normalized Probe Intensity
Probe Number
−1
0
1
2
1
2
3
4
5
6
7
8
9
10
11
Meishan−A
Meishan−B
Meishan−C
European−A
European−B
European−C

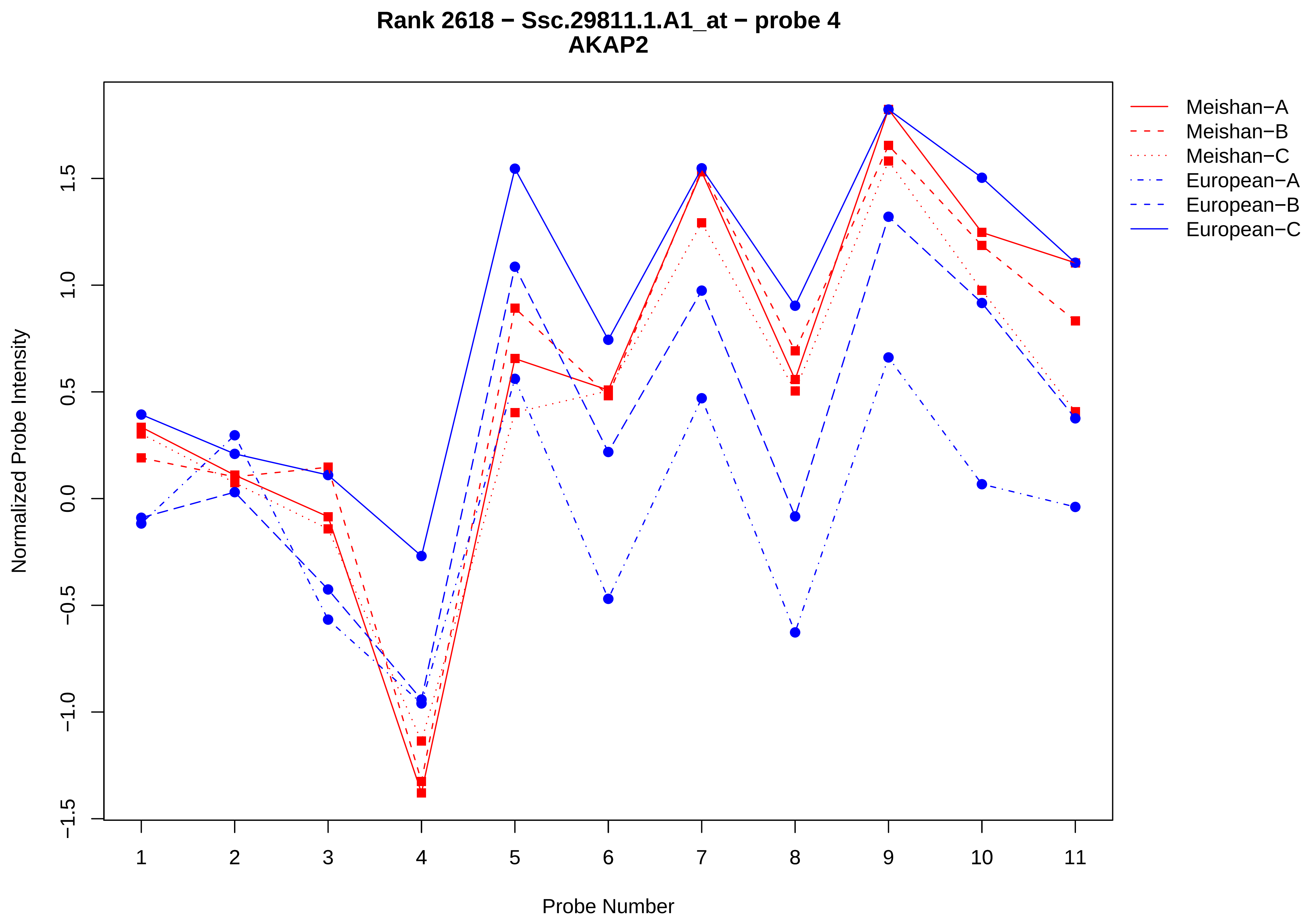
Rank 2618 − Ssc.29811.1.A1_at − probe 4
AKAP2
Normalized Probe Intensity
Probe Number
−1.5
−1.0
−0.5
0.0
0.5
1.0
1.5
1
2
3
4
5
6
7
8
9
10
11
Meishan−A
Meishan−B
Meishan−C
European−A
European−B
European−C

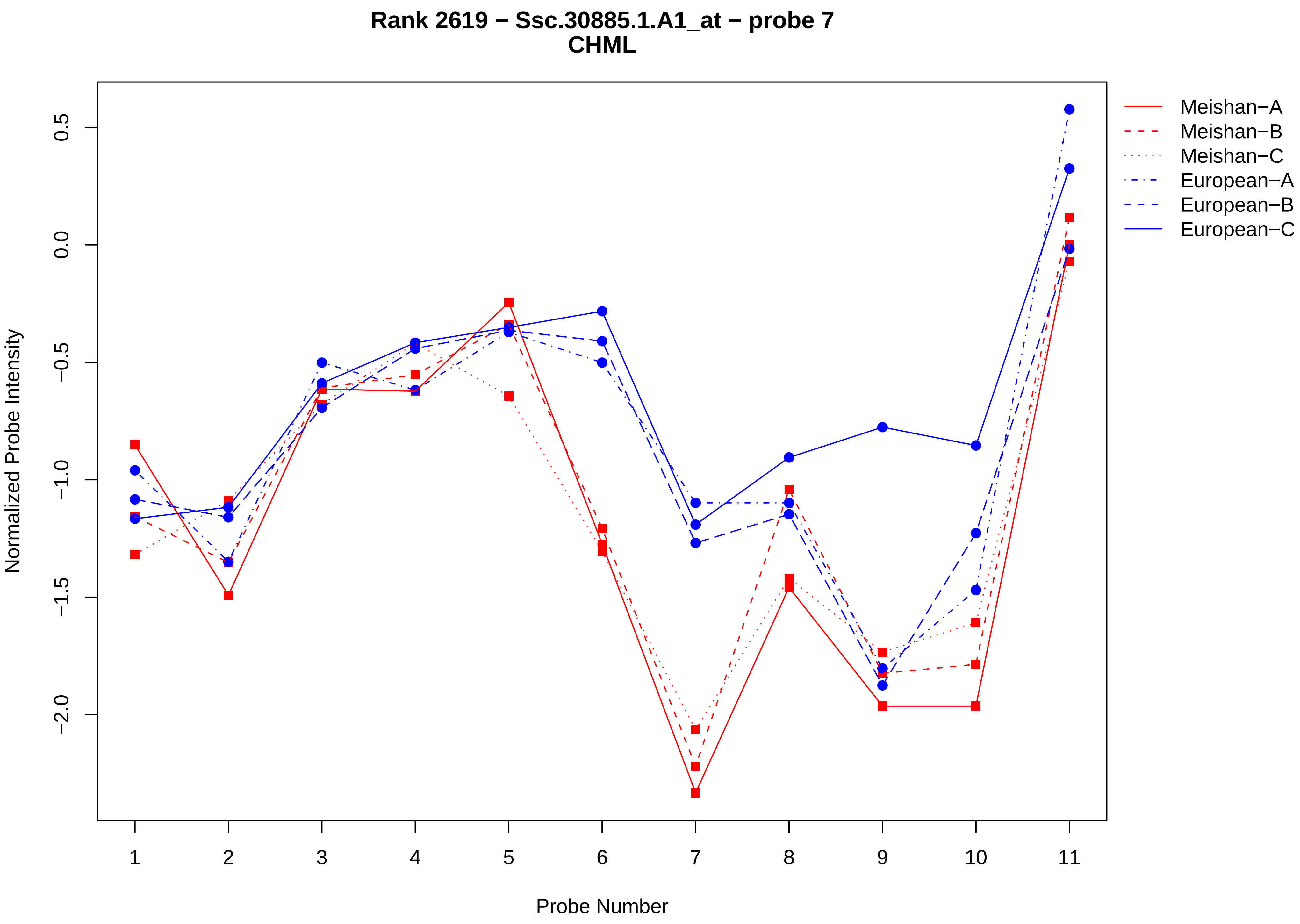

Rank 2619 − Ssc.30885.1.A1_at − probe 7
CHML
Normalized Probe Intensity
Probe Number
0.5
0.0
−0.5
−1.0
−1.5
−2.0
1
2
3
4
5
6
7
8
9
10
11
Meishan−A
Meishan−B
Meishan−C
European−A
European−B
European−C

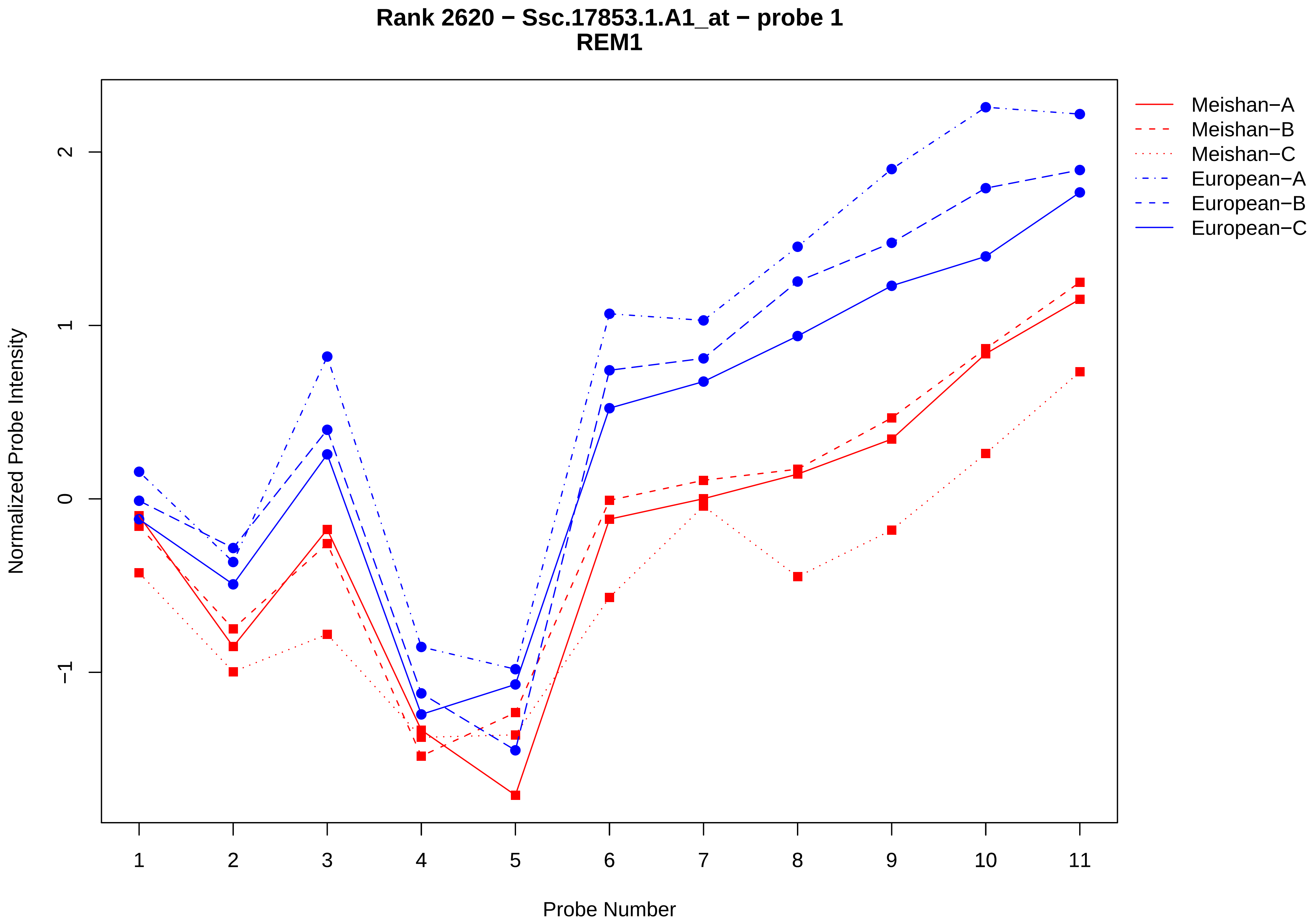
Rank 2620 − Ssc.17853.1.A1_at − probe 1
REM1
Normalized Probe Intensity
Probe Number
−1
0
1
2
1
2
3
4
5
6
7
8
9
10
11
Meishan−A
Meishan−B
Meishan−C
European−A
European−B
European−C

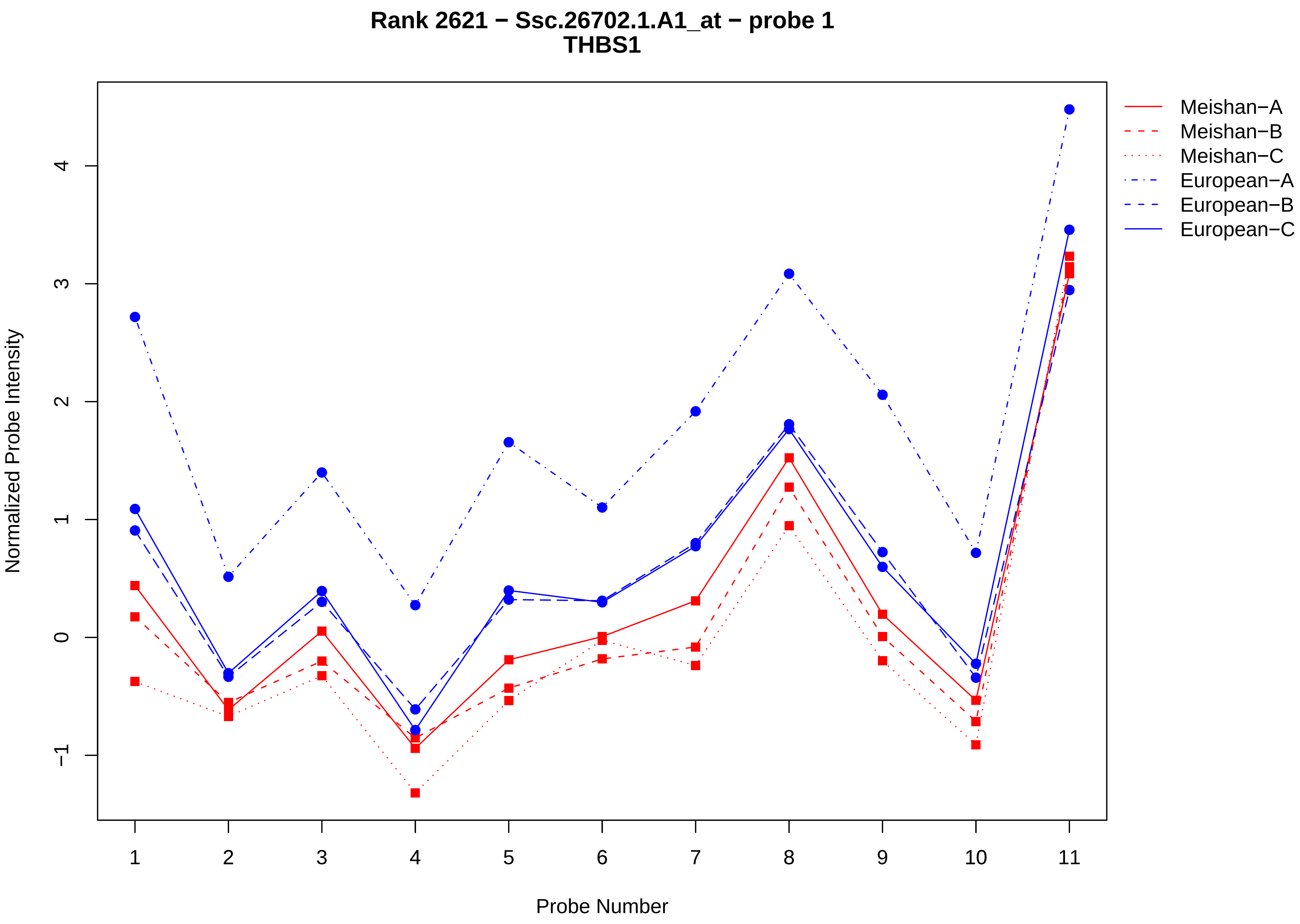

Rank 2621 − Ssc.26702.1.A1_at − probe 1
THBS1
Normalized Probe Intensity
Probe Number
Meishan−A
Meishan−B
Meishan−C
European−A
European−B
European−C
−1
0
1
2
3
4
1
2
3
4
5
6
7
8
9
10
11

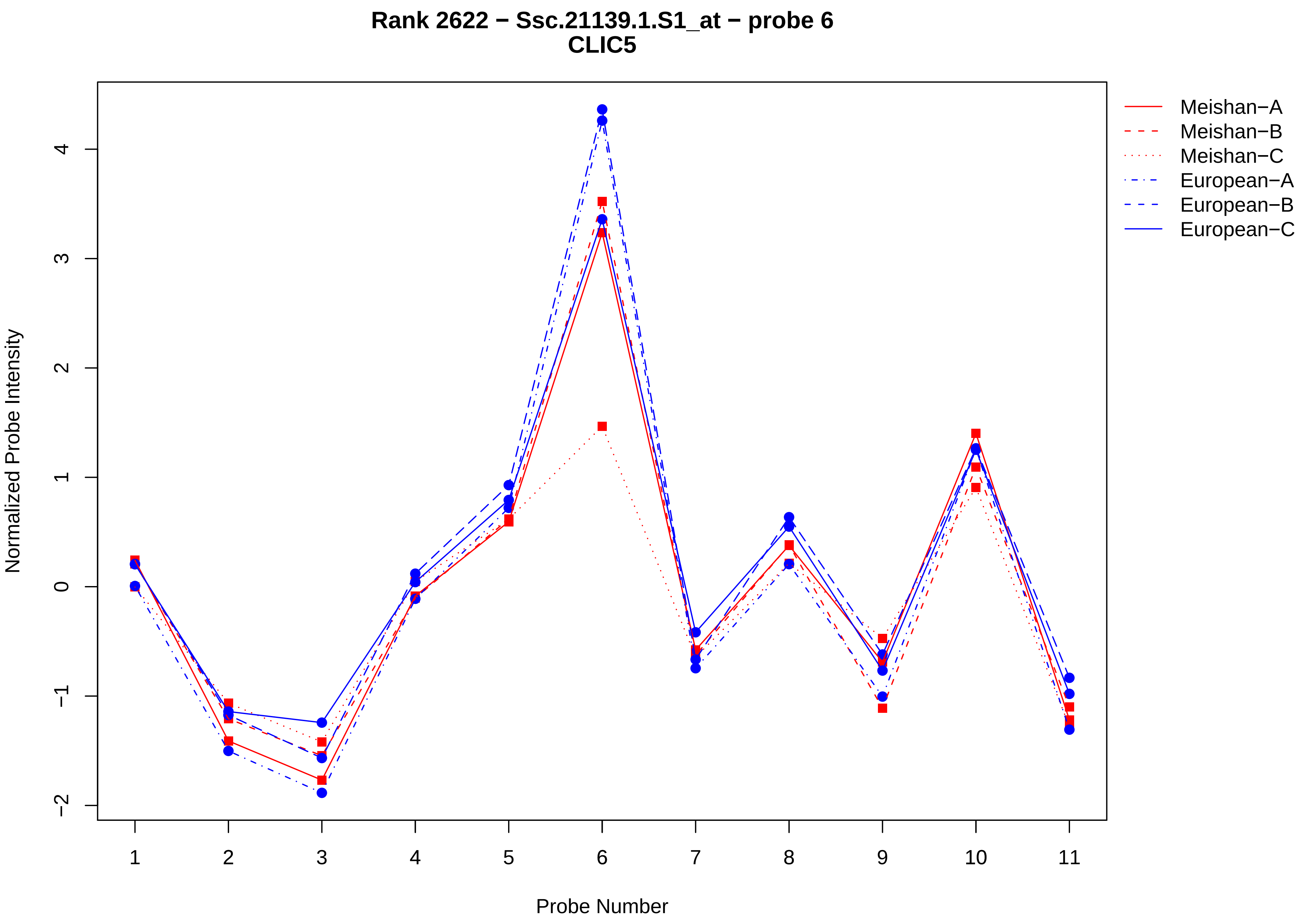

Rank 2622 − Ssc.21139.1.S1_at − probe 6
CLIC5
Normalized Probe Intensity
Probe Number
−2
−1
0
1
2
3
4
1
2
3
4
5
6
7
8
9
10
11
Meishan−A
Meishan−B
Meishan−C
European−A
European−B
European−C

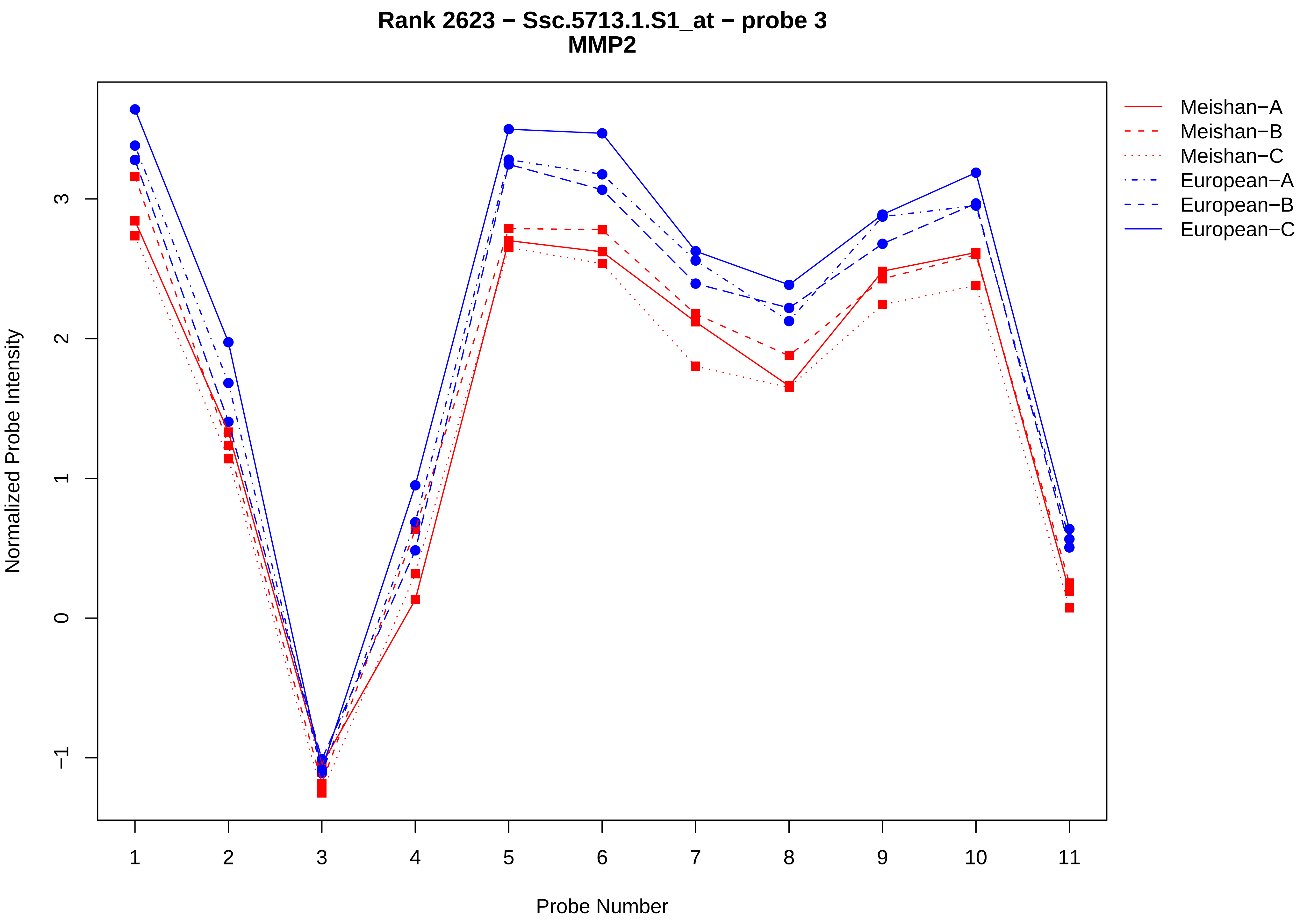

Rank 2623 − Ssc.5713.1.S1_at − probe 3
MMP2
Normalized Probe Intensity
Probe Number
−1
0
1
2
3
1
2
3
4
5
6
7
8
9
10
11
Meishan−A
Meishan−B
Meishan−C
European−A
European−B
European−C

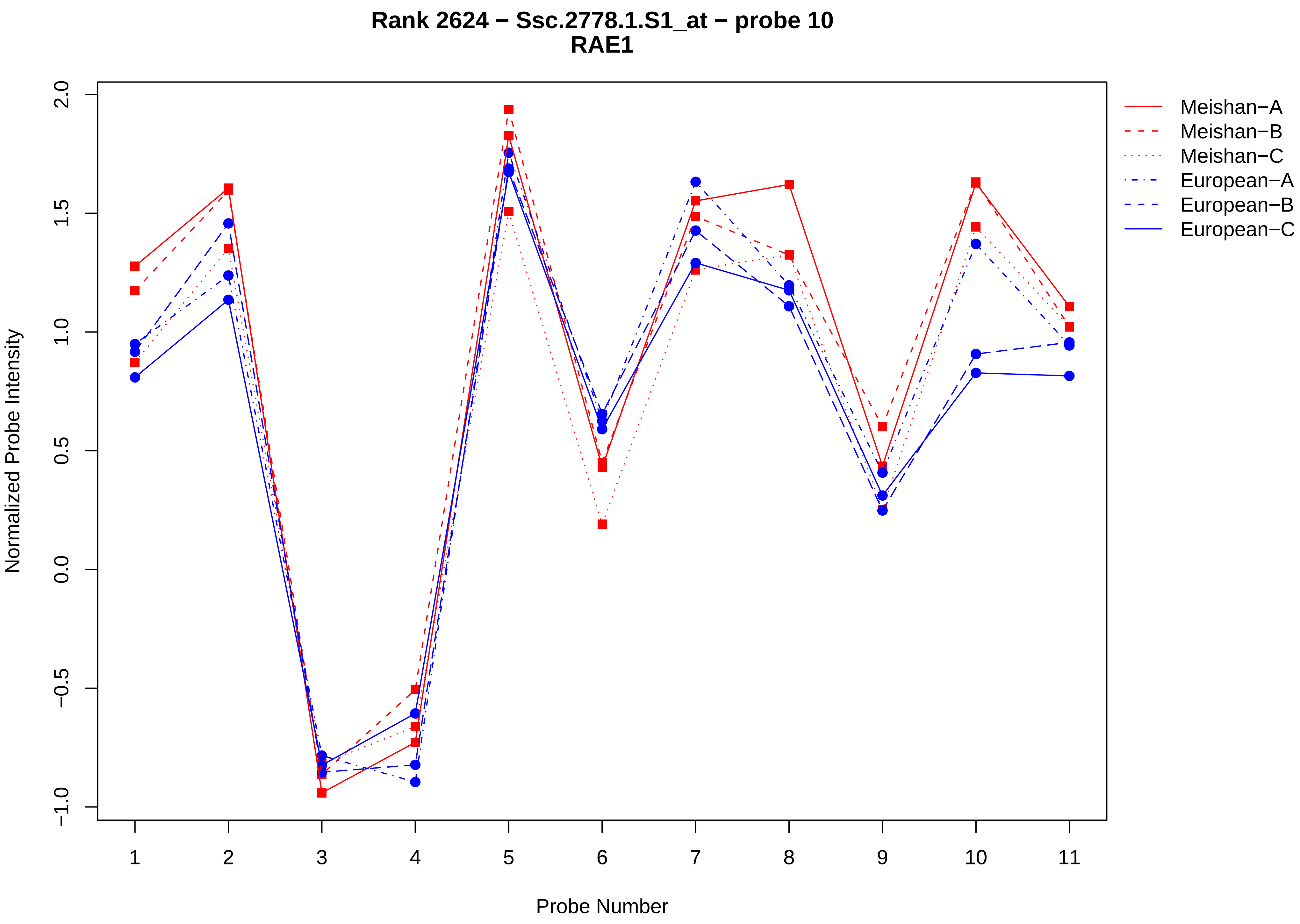

Rank 2624 − Ssc.2778.1.S1_at − probe 10
RAE1
Normalized Probe Intensity
Probe Number
−1.0
−0.5
0.0
0.5
1.0
1.5
2.0
1
2
3
4
5
6
7
8
9
10
11
Meishan−A
Meishan−B
Meishan−C
European−A
European−B
European−C

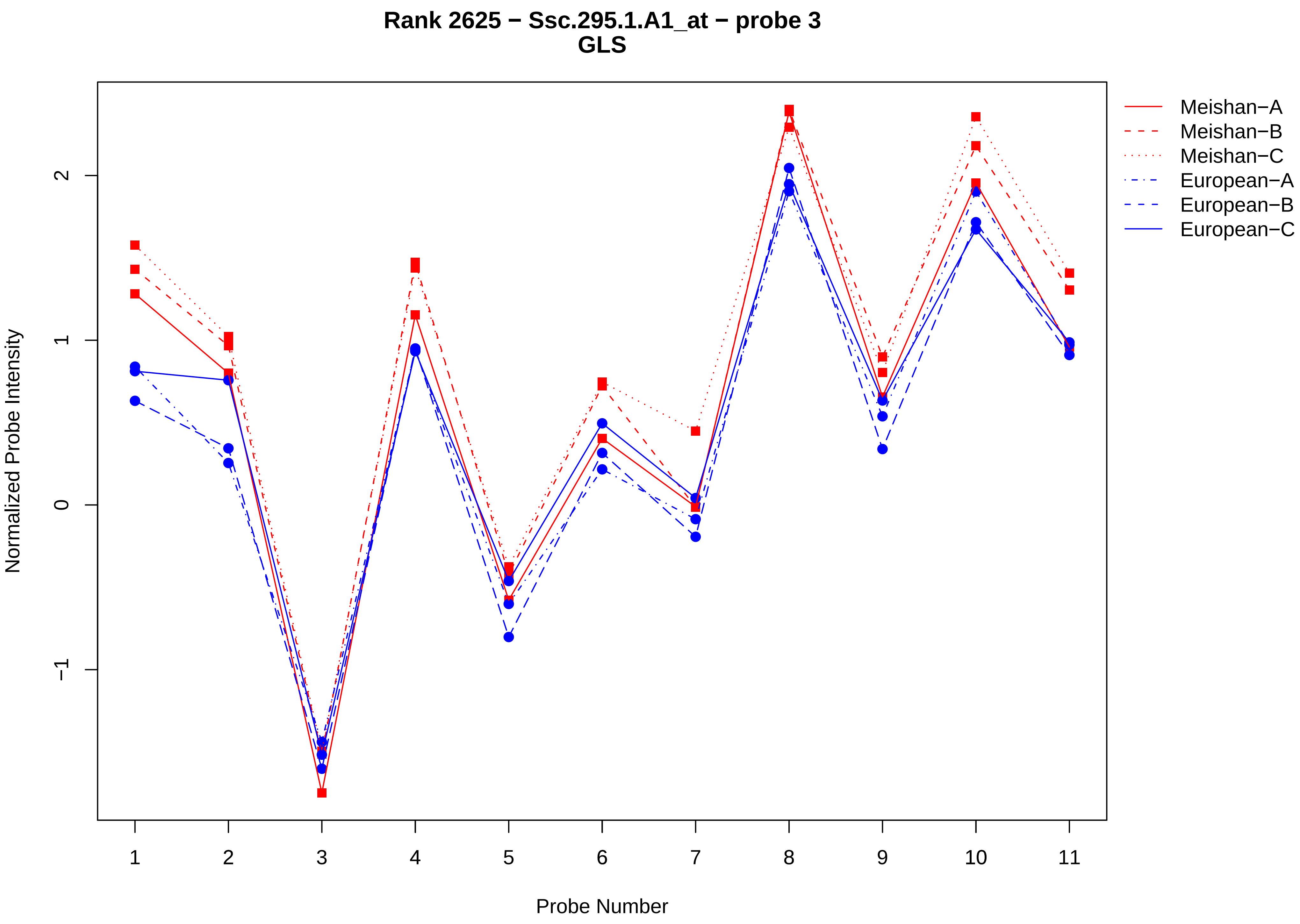

Rank 2625 − Ssc.295.1.A1_at − probe 3
GLS
Normalized Probe Intensity
Probe Number
−1
0
1
2
1
2
3
4
5
6
7
8
9
10
11
Meishan−A
Meishan−B
Meishan−C
European−A
European−B
European−C

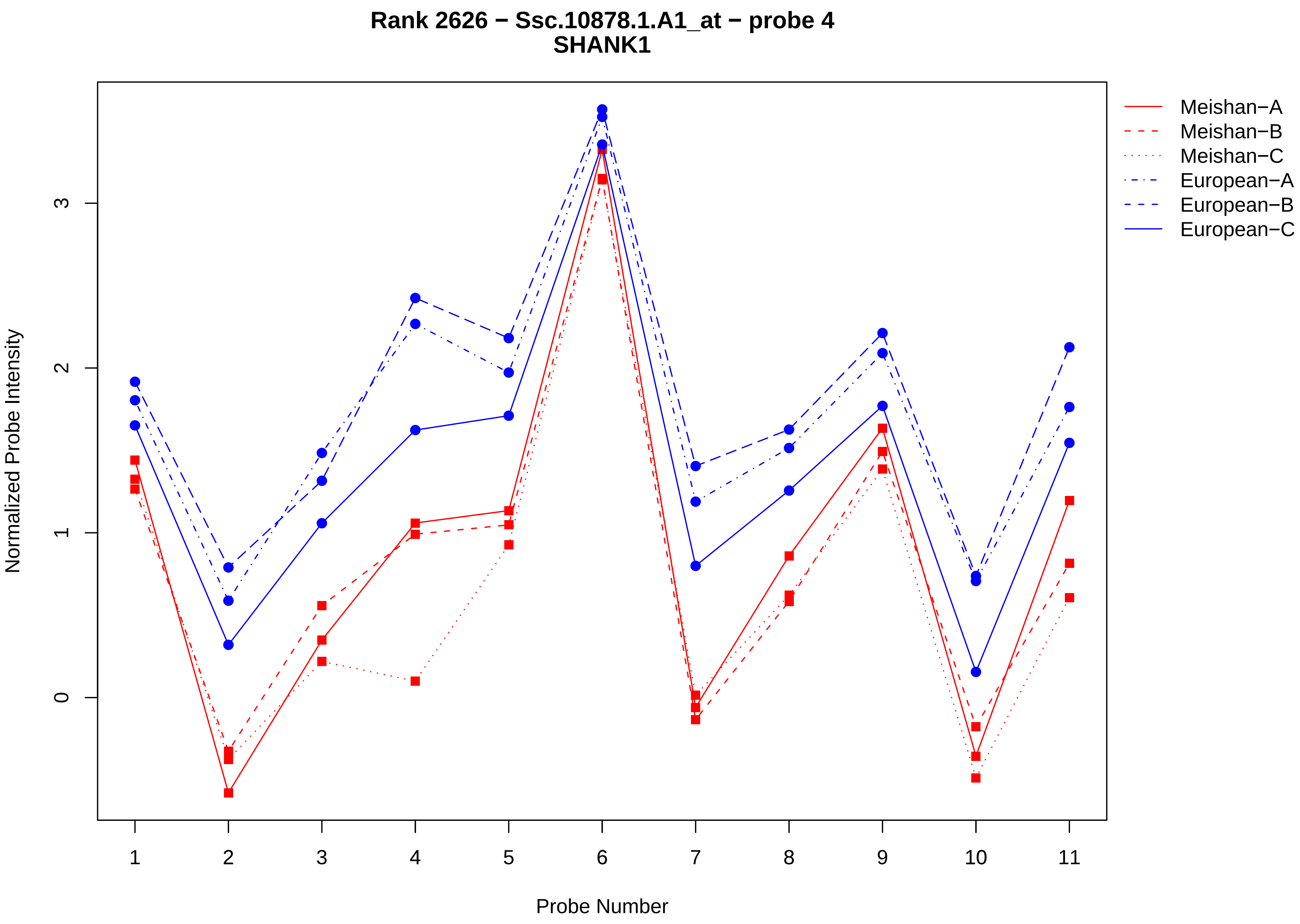
Rank 2626 − Ssc.10878.1.A1_at − probe 4
SHANK1
Normalized Probe Intensity
Probe Number
0
1
2
3
1
2
3
4
5
6
7
8
9
10
11
Meishan−A
Meishan−B
Meishan−C
European−A
European−B
European−C

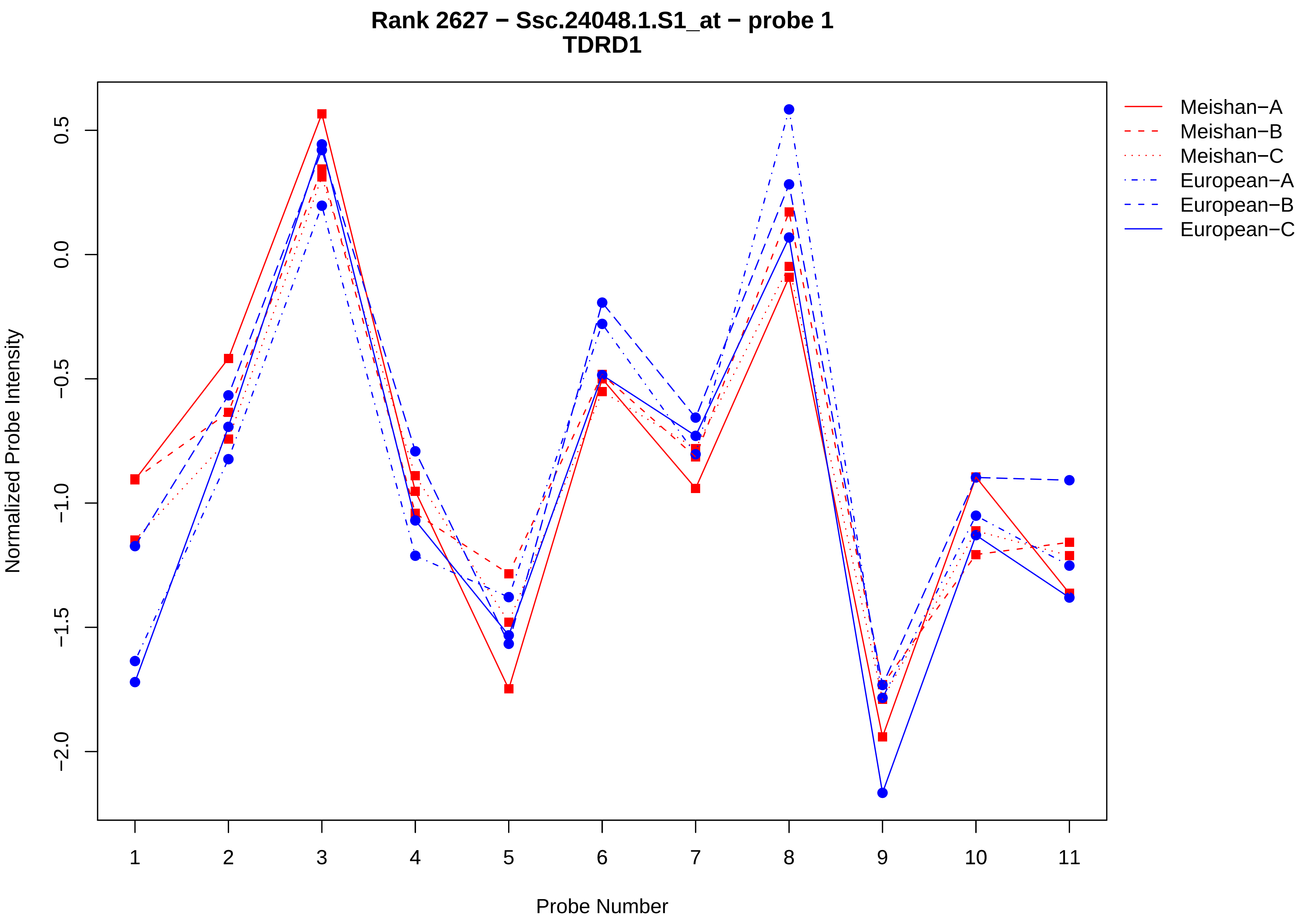

Rank 2627 − Ssc.24048.1.S1_at − probe 1
TDRD1
Normalized Probe Intensity
Probe Number
0.5
0.0
−0.5
−1.0
−1.5
−2.0
1
2
3
4
5
6
7
8
9
10
11
Meishan−A
Meishan−B
Meishan−C
European−A
European−B
European−C

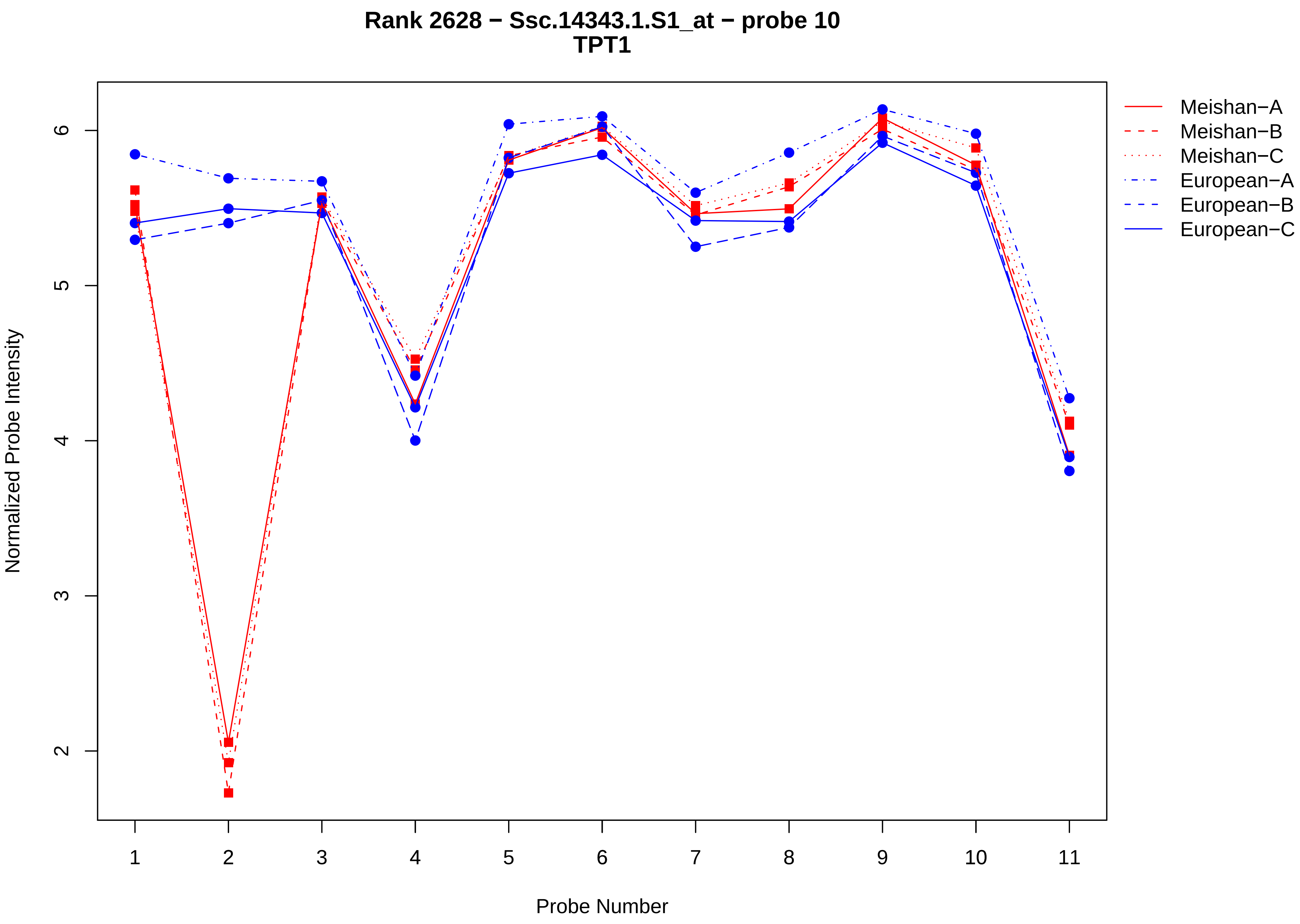

Rank 2628 − Ssc.14343.1.S1_at − probe 10
TPT1
Normalized Probe Intensity
Probe Number
Meishan−A
Meishan−B
Meishan−C
European−A
European−B
European−C

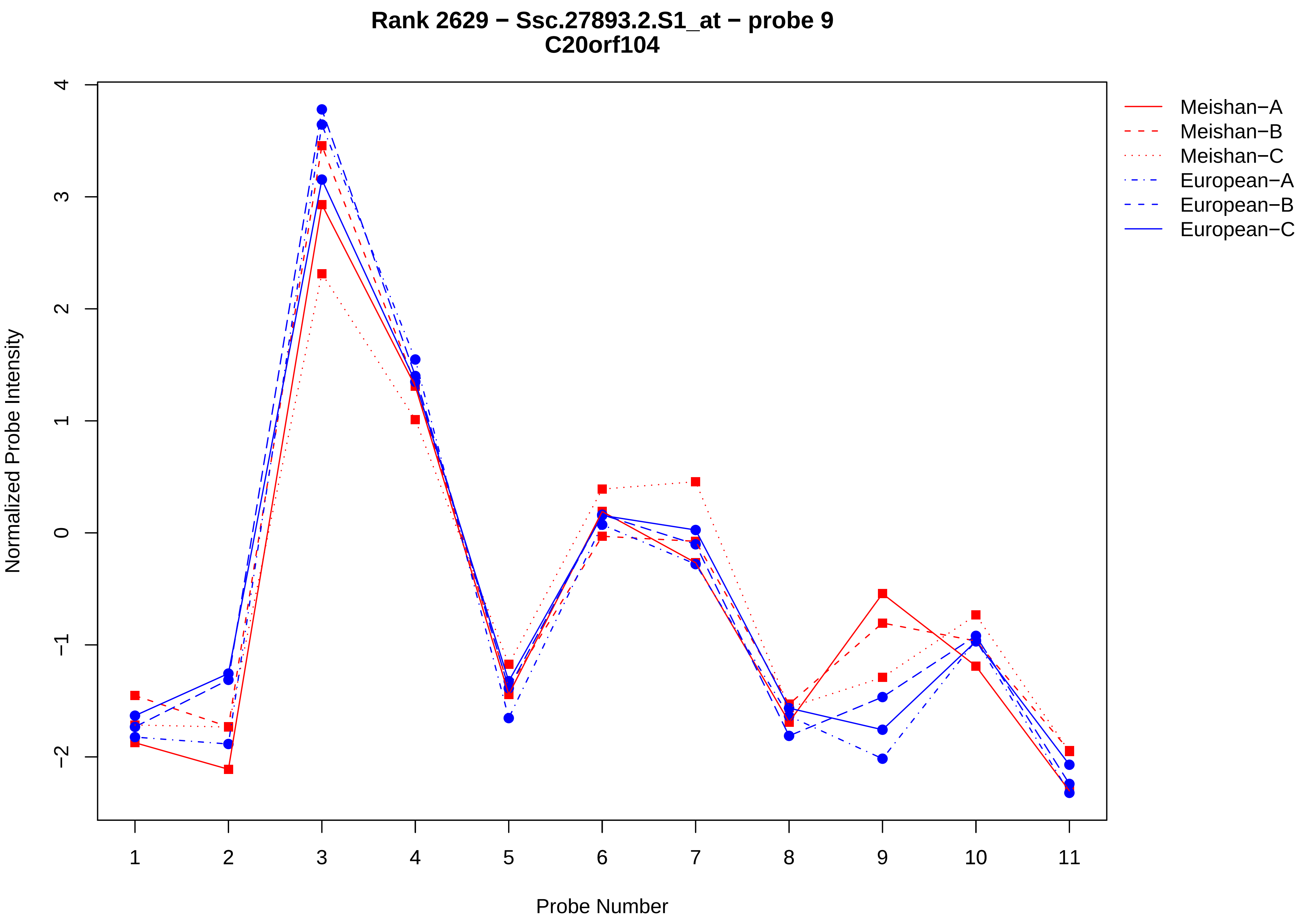

Rank 2629 − Ssc.27893.2.S1_at − probe 9
C20orf104
Normalized Probe Intensity
Probe Number
Meishan−A
Meishan−B
Meishan−C
European−A
European−B
European−C

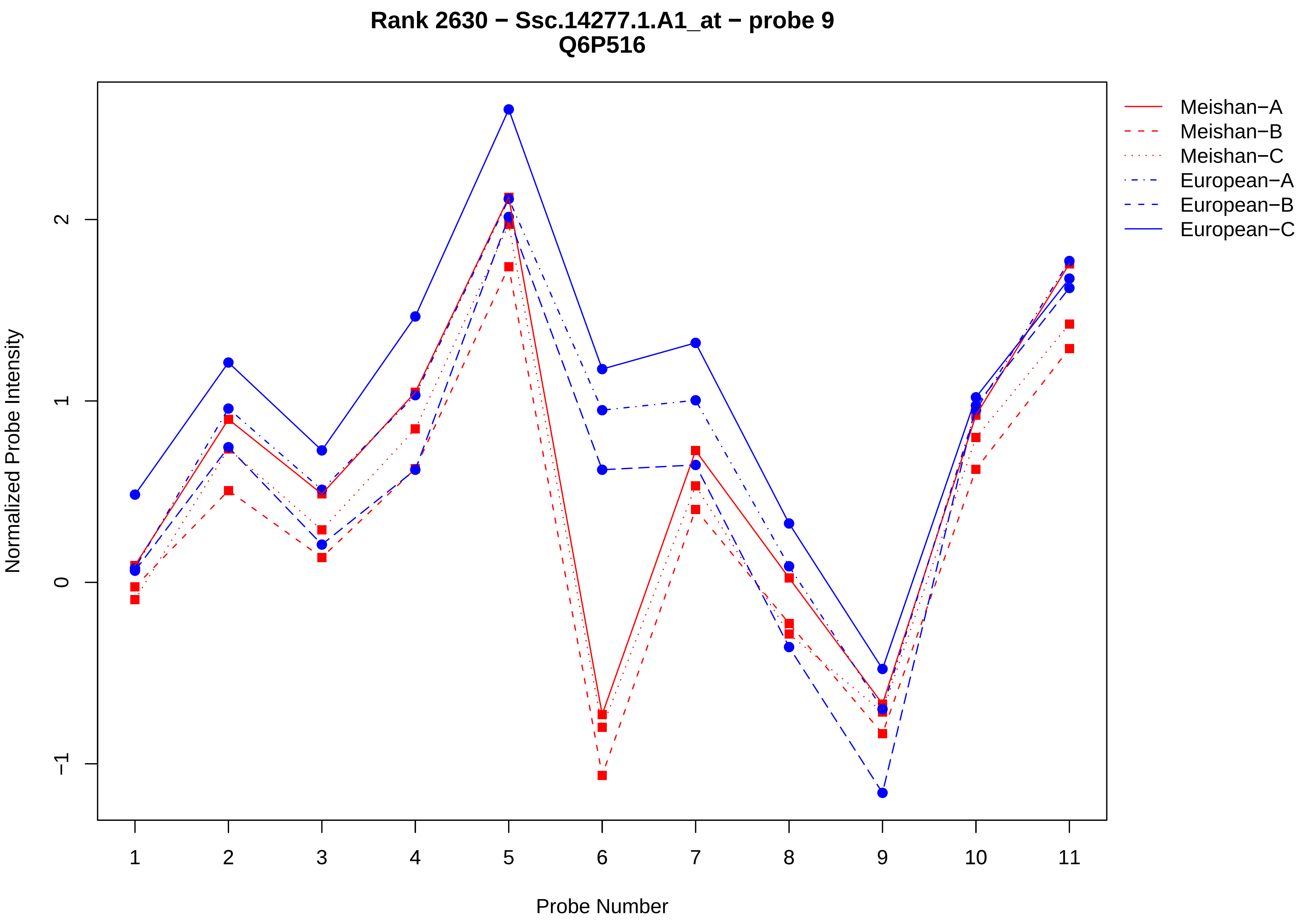

Rank 2630 − Ssc.14277.1.A1_at − probe 9
Q6P516
Normalized Probe Intensity
Probe Number
−1
0
1
2
1
2
3
4
5
6
7
8
9
10
11
Meishan−A
Meishan−B
Meishan−C
European−A
European−B
European−C

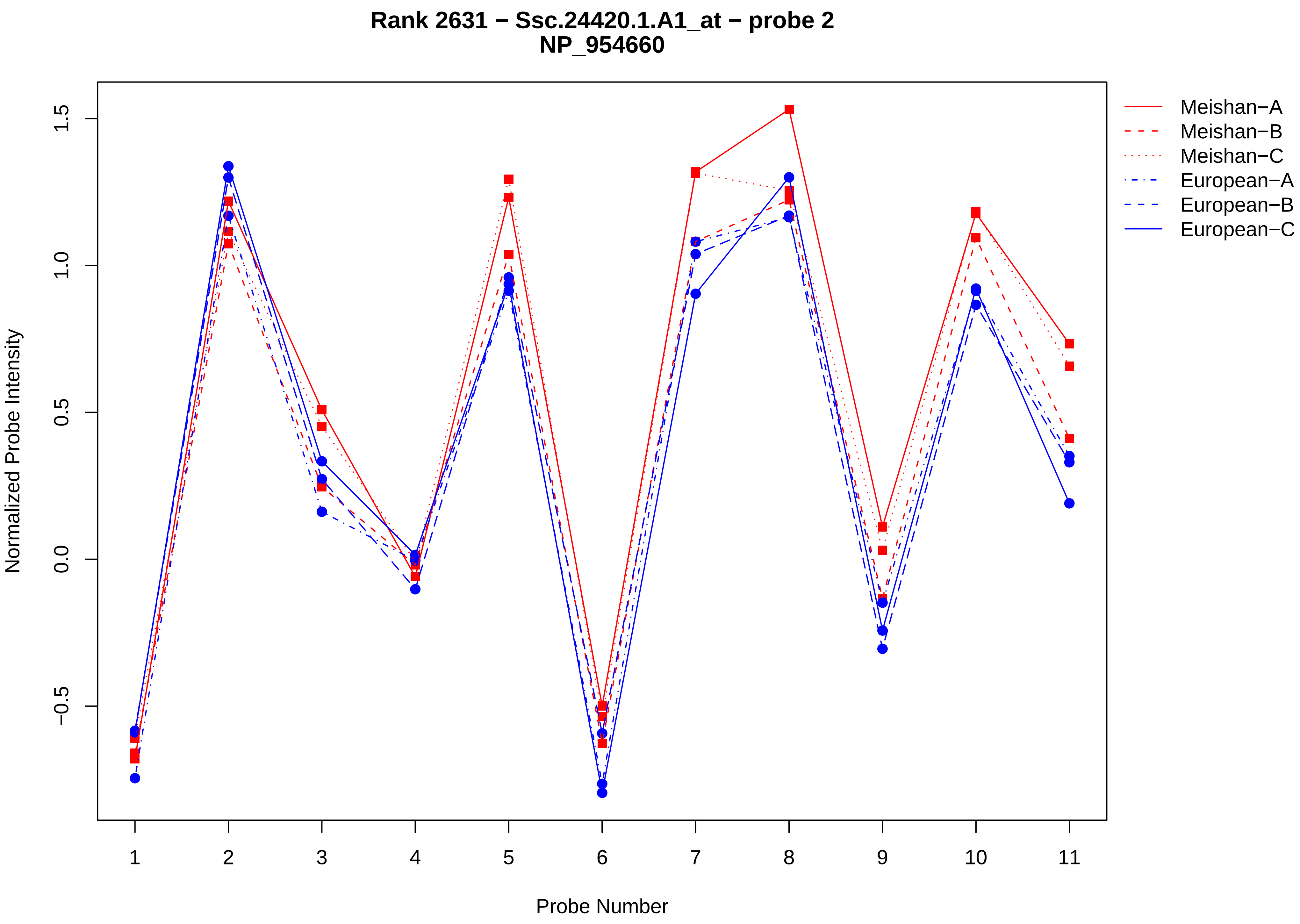

Rank 2631 − Ssc.24420.1.A1_at − probe 2
NP_954660
Normalized Probe Intensity
Probe Number
Meishan−A
Meishan−B
Meishan−C
European−A
European−B
European−C
−0.5
0.0
0.5
1.0
1.5
1
2
3
4
5
6
7
8
9
10
11

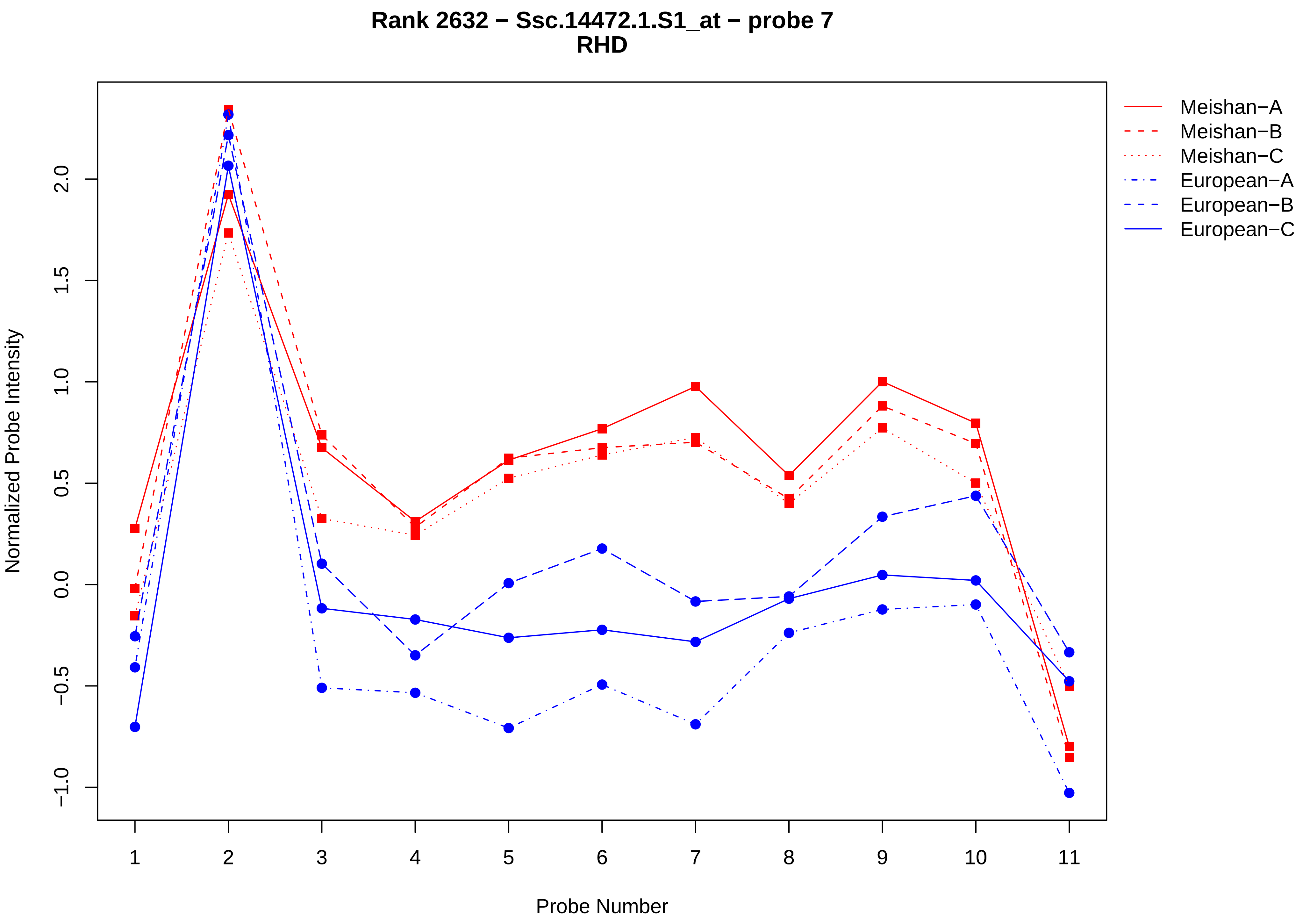

Rank 2632 − Ssc.14472.1.S1_at − probe 7
RHD
Normalized Probe Intensity
Probe Number
−1.0
−0.5
0.0
0.5
1.0
1.5
2.0
1
2
3
4
5
6
7
8
9
10
11
Meishan−A
Meishan−B
Meishan−C
European−A
European−B
European−C

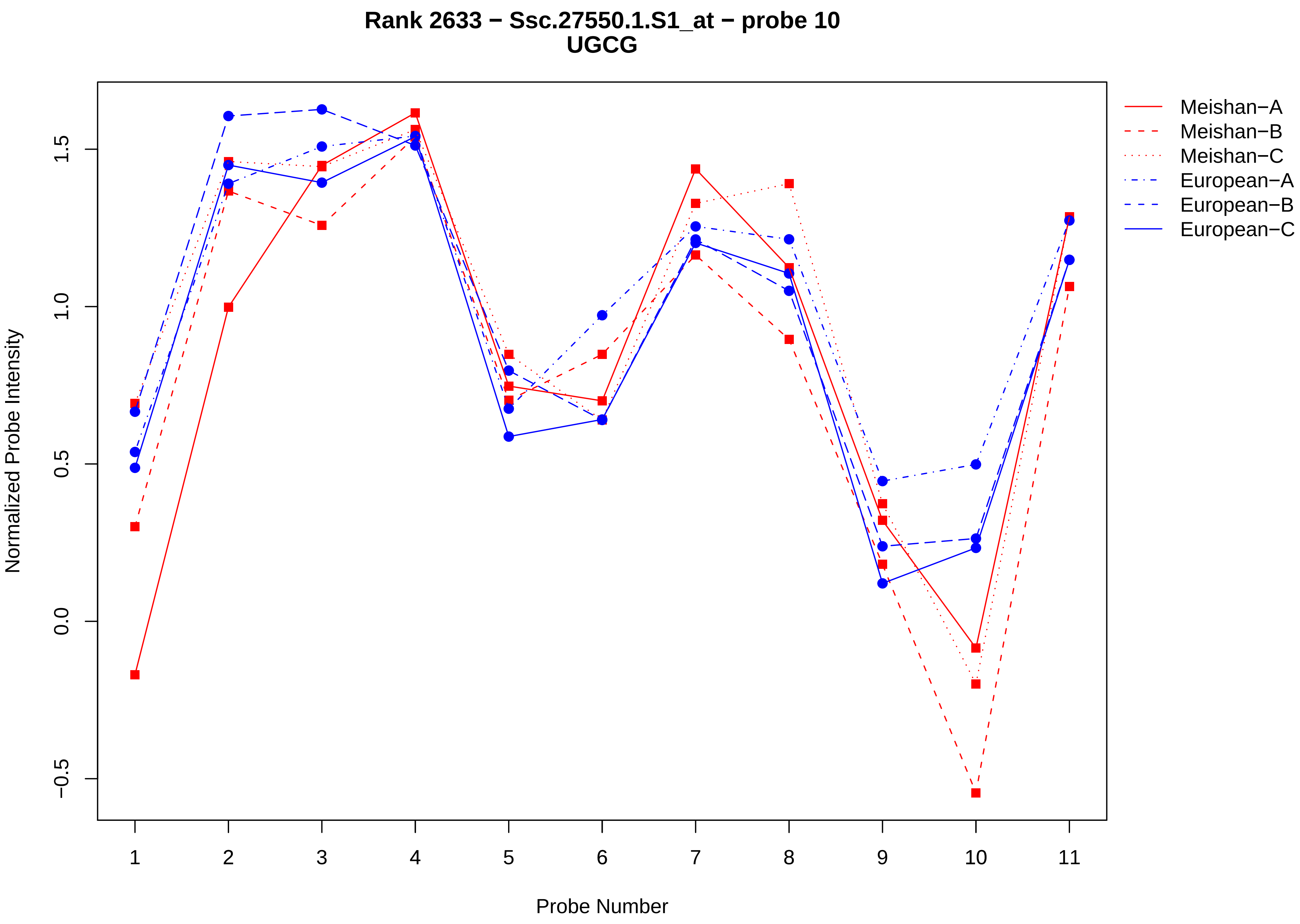
Rank 2633 − Ssc.27550.1.S1_at − probe 10
UGCG
Normalized Probe Intensity
Probe Number
Meishan−A
Meishan−B
Meishan−C
European−A
European−B
European−C
−0.5
0.0
0.5
1.0
1.5
1
2
3
4
5
6
7
8
9
10
11

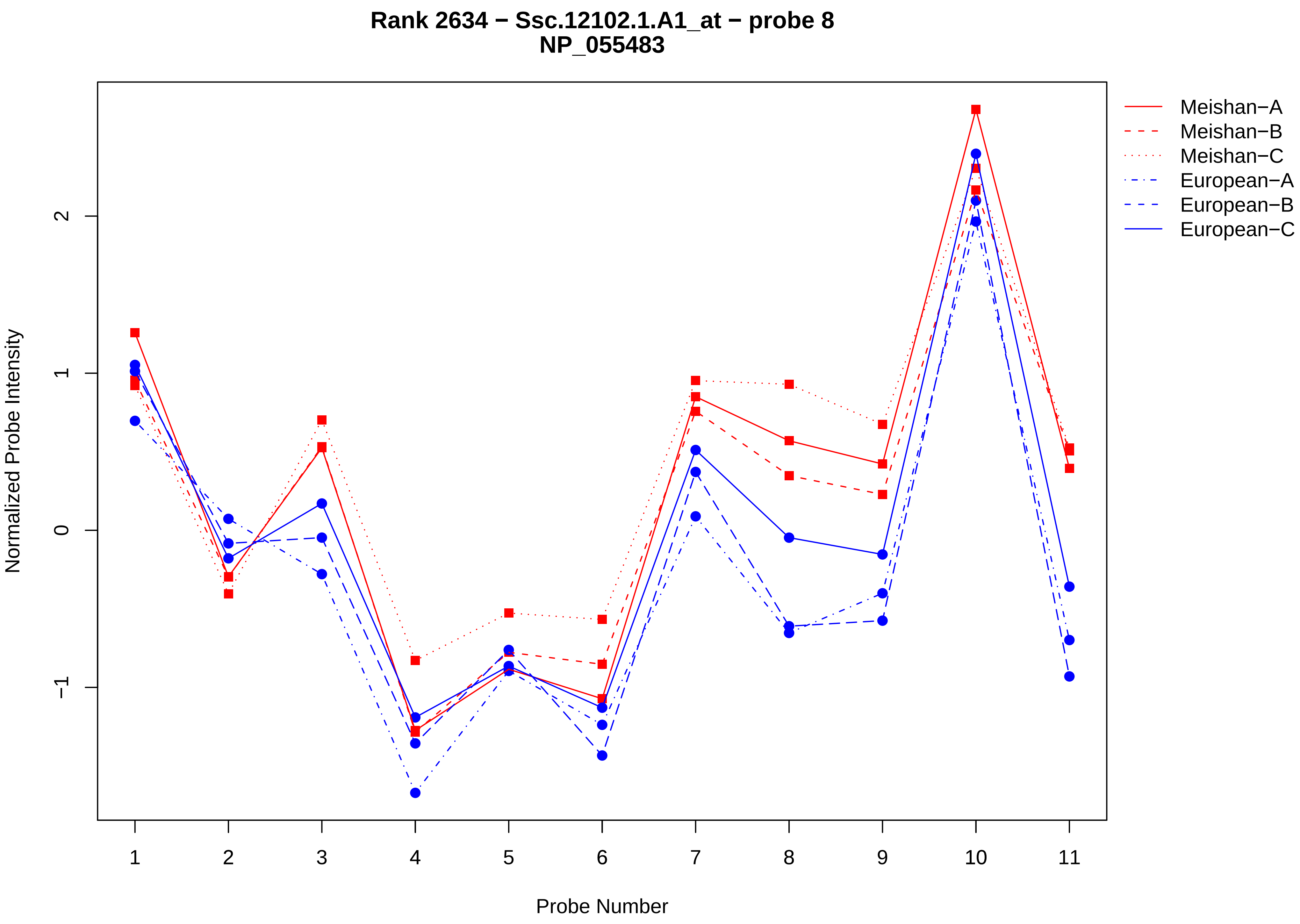

Rank 2634 − Ssc.12102.1.A1_at − probe 8
NP_055483
Normalized Probe Intensity
Probe Number
−1
0
1
2
1
2
3
4
5
6
7
8
9
10
11
Meishan−A
Meishan−B
Meishan−C
European−A
European−B
European−C

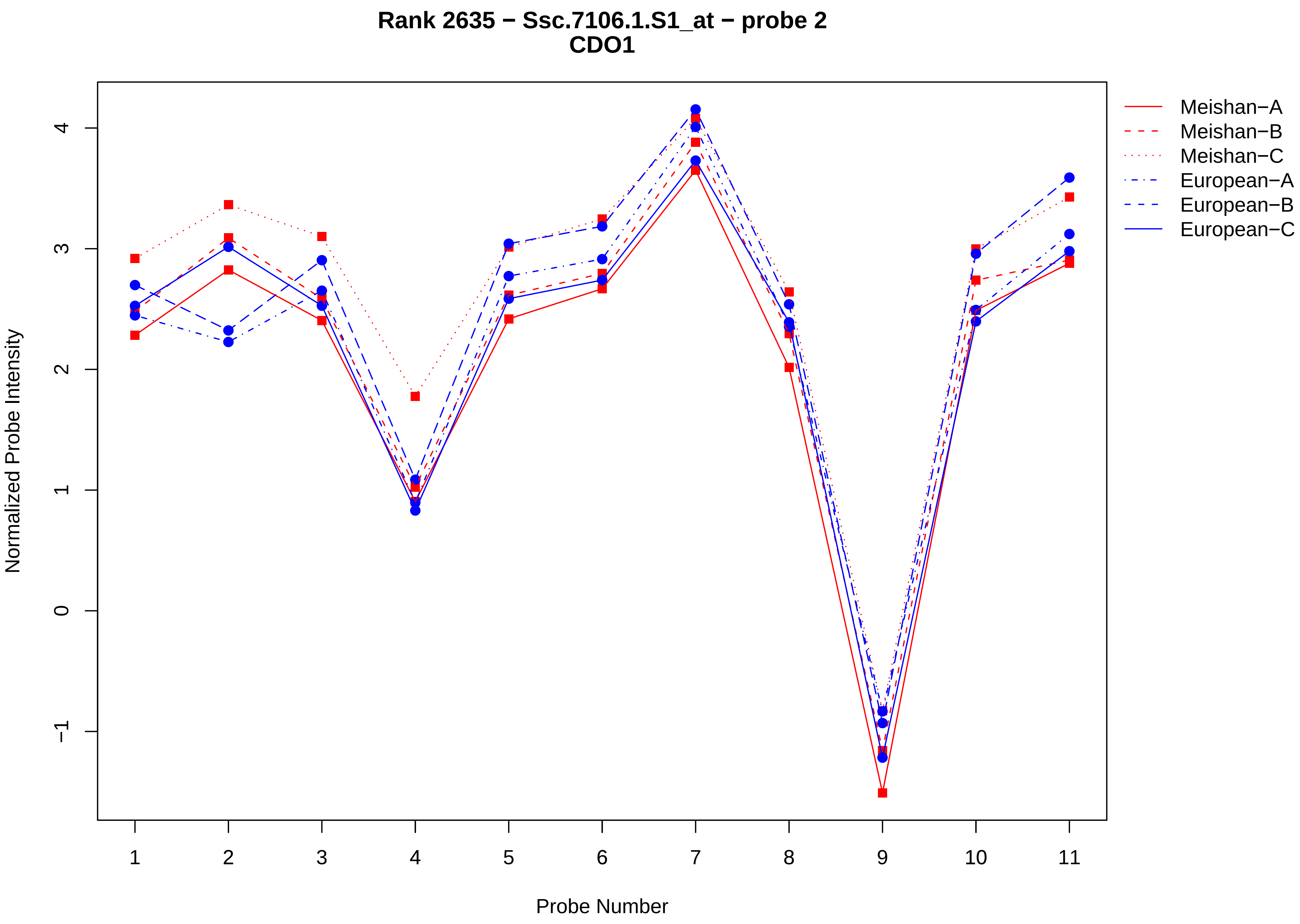

Rank 2635 − Ssc.7106.1.S1_at − probe 2
CDO1
Normalized Probe Intensity
Probe Number
Meishan−A
Meishan−B
Meishan−C
European−A
European−B
European−C
−1
0
1
2
3
4
1
2
3
4
5
6
7
8
9
10
11

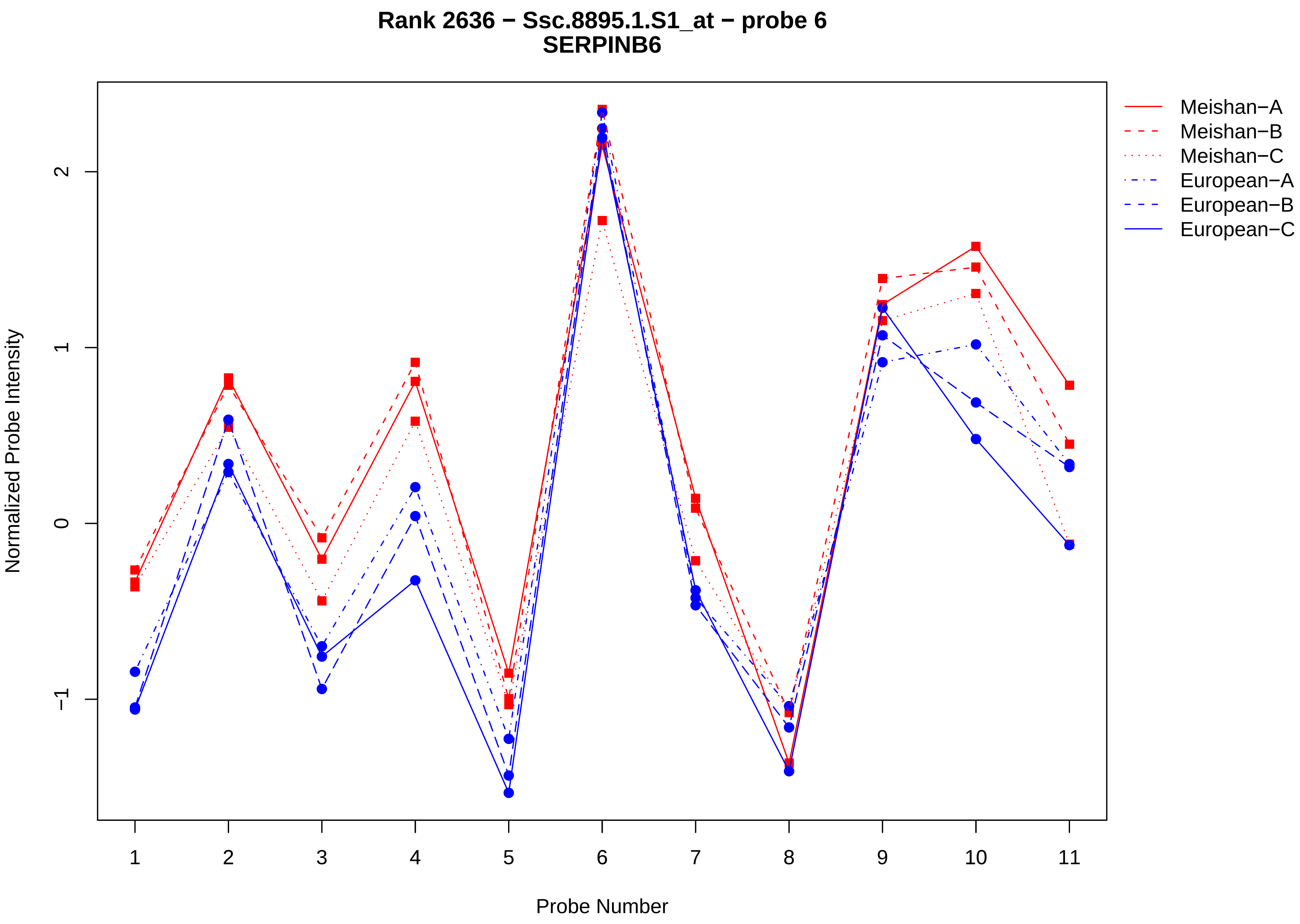

Rank 2636 − Ssc.8895.1.S1_at − probe 6
SERPINB6
Normalized Probe Intensity
Probe Number
Meishan−A
Meishan−B
Meishan−C
European−A
European−B
European−C

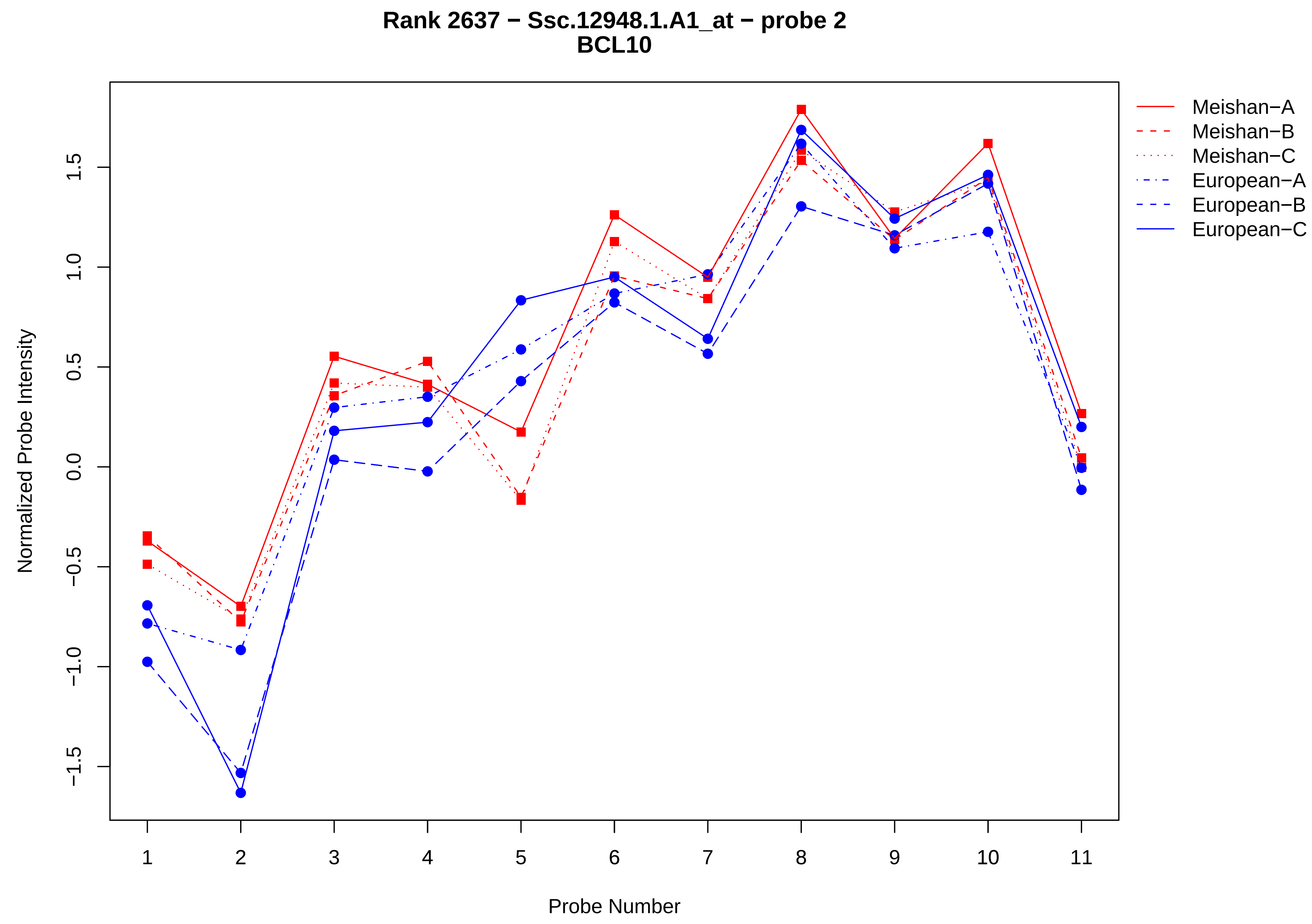
Rank 2637 − Ssc.12948.1.A1_at − probe 2
BCL10
Normalized Probe Intensity
Probe Number
−1.5
−1.0
−0.5
0.0
0.5
1.0
1.5
1
2
3
4
5
6
7
8
9
10
11
Meishan−A
Meishan−B
Meishan−C
European−A
European−B
European−C

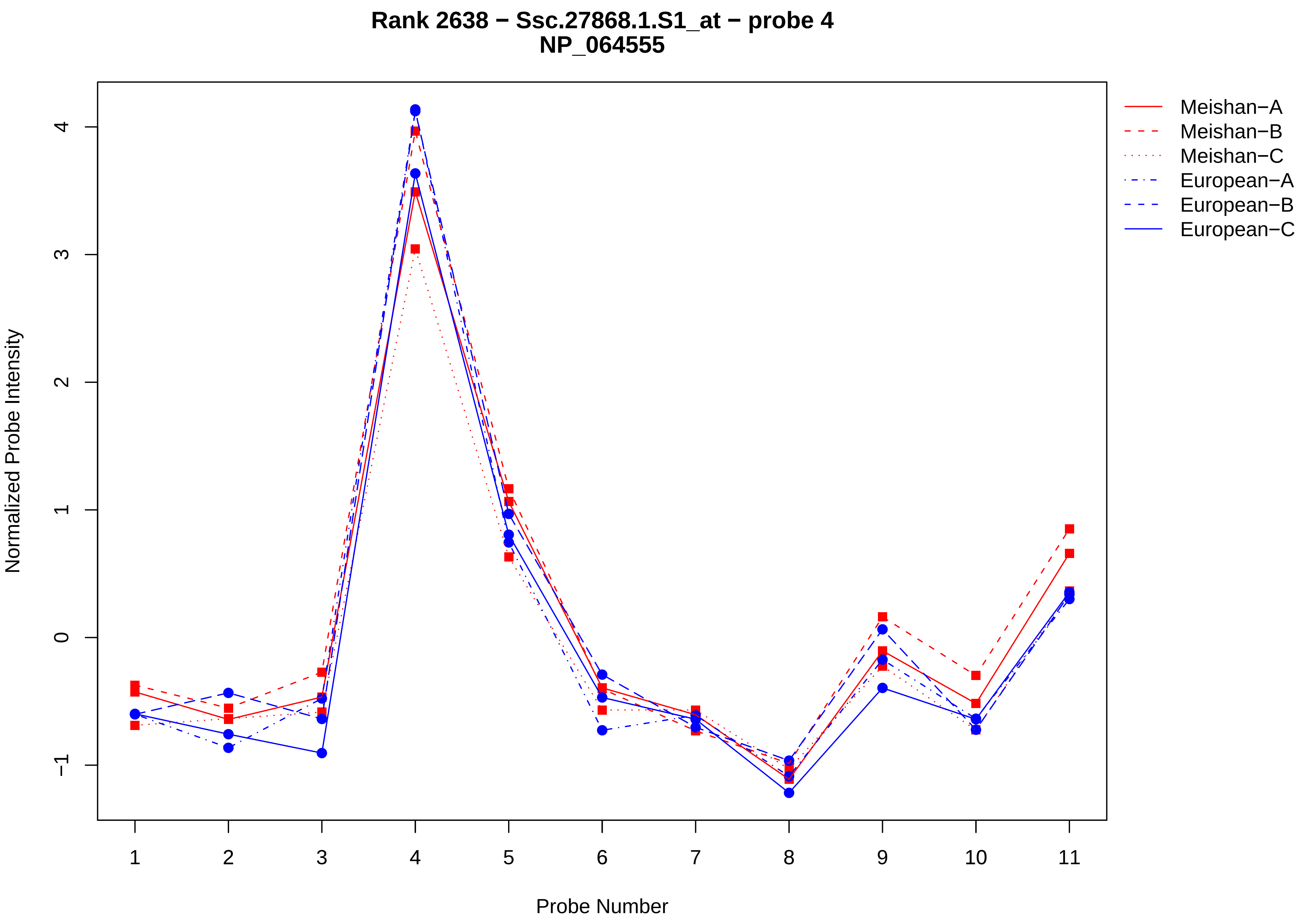
Rank 2638 − Ssc.27868.1.S1_at − probe 4
NP_064555
Normalized Probe Intensity
Probe Number
−1
0
1
2
3
4
1
2
3
4
5
6
7
8
9
10
11
Meishan−A
Meishan−B
Meishan−C
European−A
European−B
European−C

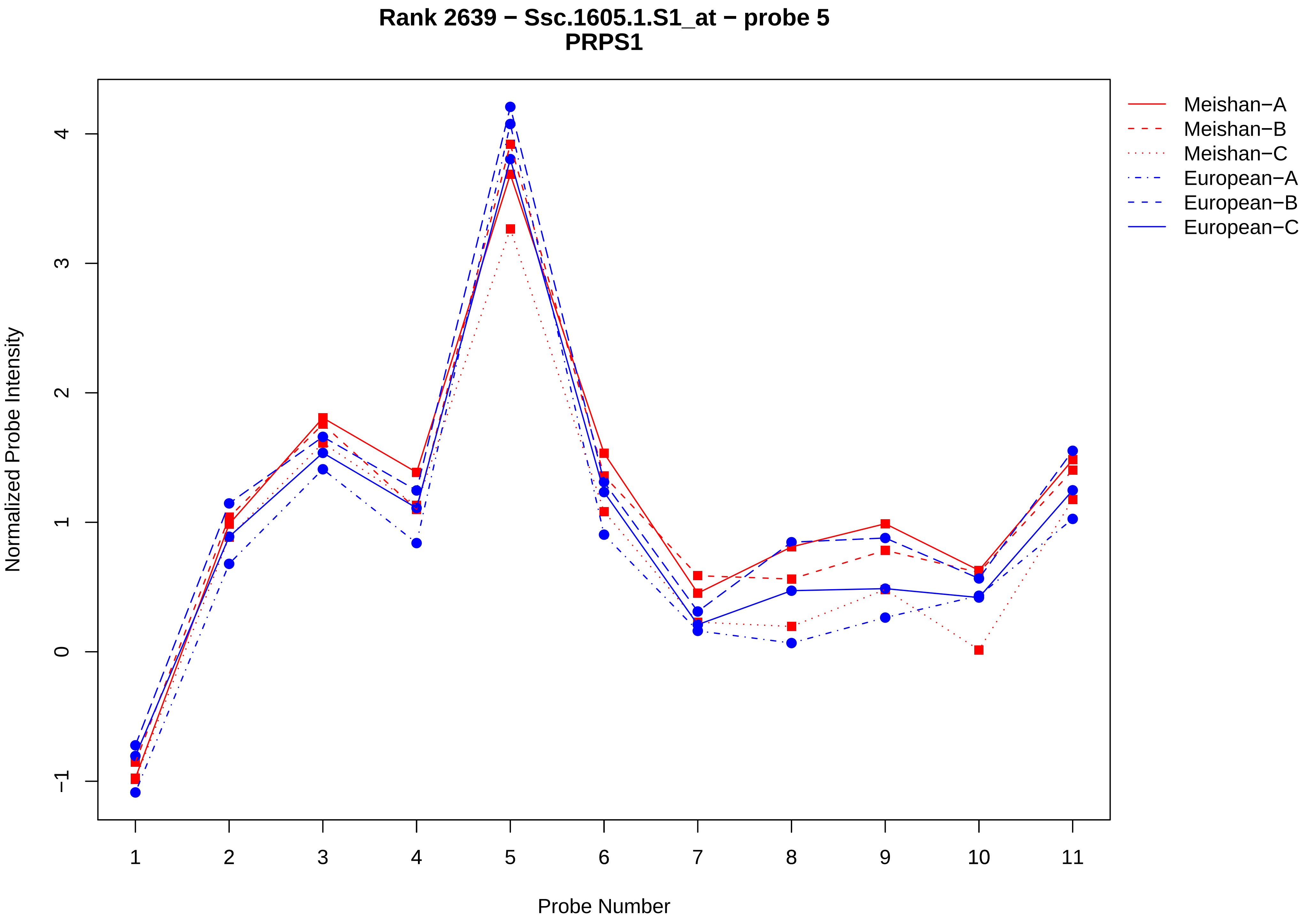

Rank 2639 − Ssc.1605.1.S1_at − probe 5
PRPS1
Normalized Probe Intensity
Probe Number
−1
0
1
2
3
4
1
2
3
4
5
6
7
8
9
10
11
Meishan−A
Meishan−B
Meishan−C
European−A
European−B
European−C

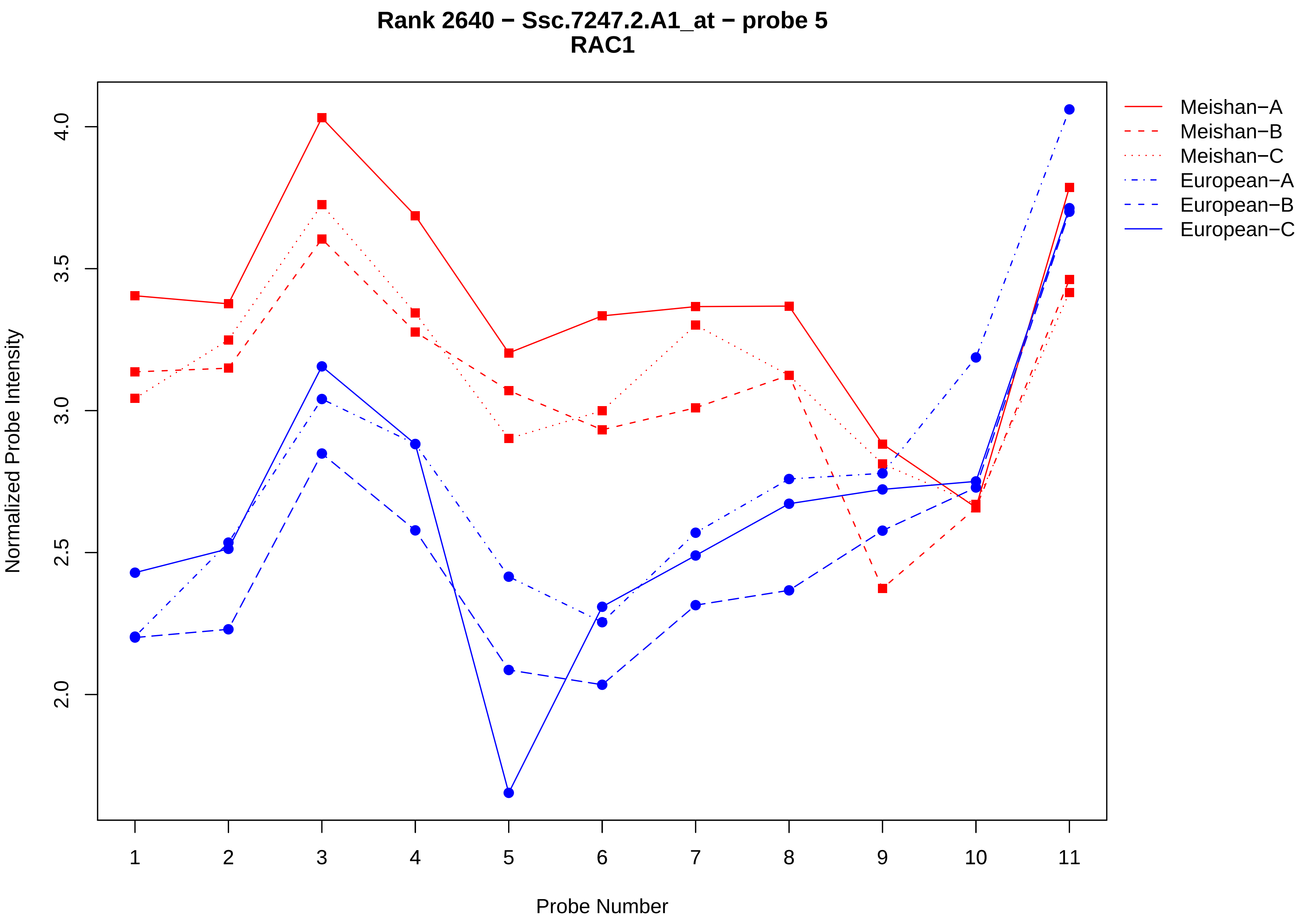

Rank 2640 − Ssc.7247.2.A1_at − probe 5
RAC1
Normalized Probe Intensity
Probe Number
2.0
2.5
3.0
3.5
4.0
1
2
3
4
5
6
7
8
9
10
11
Meishan−A
Meishan−B
Meishan−C
European−A
European−B
European−C

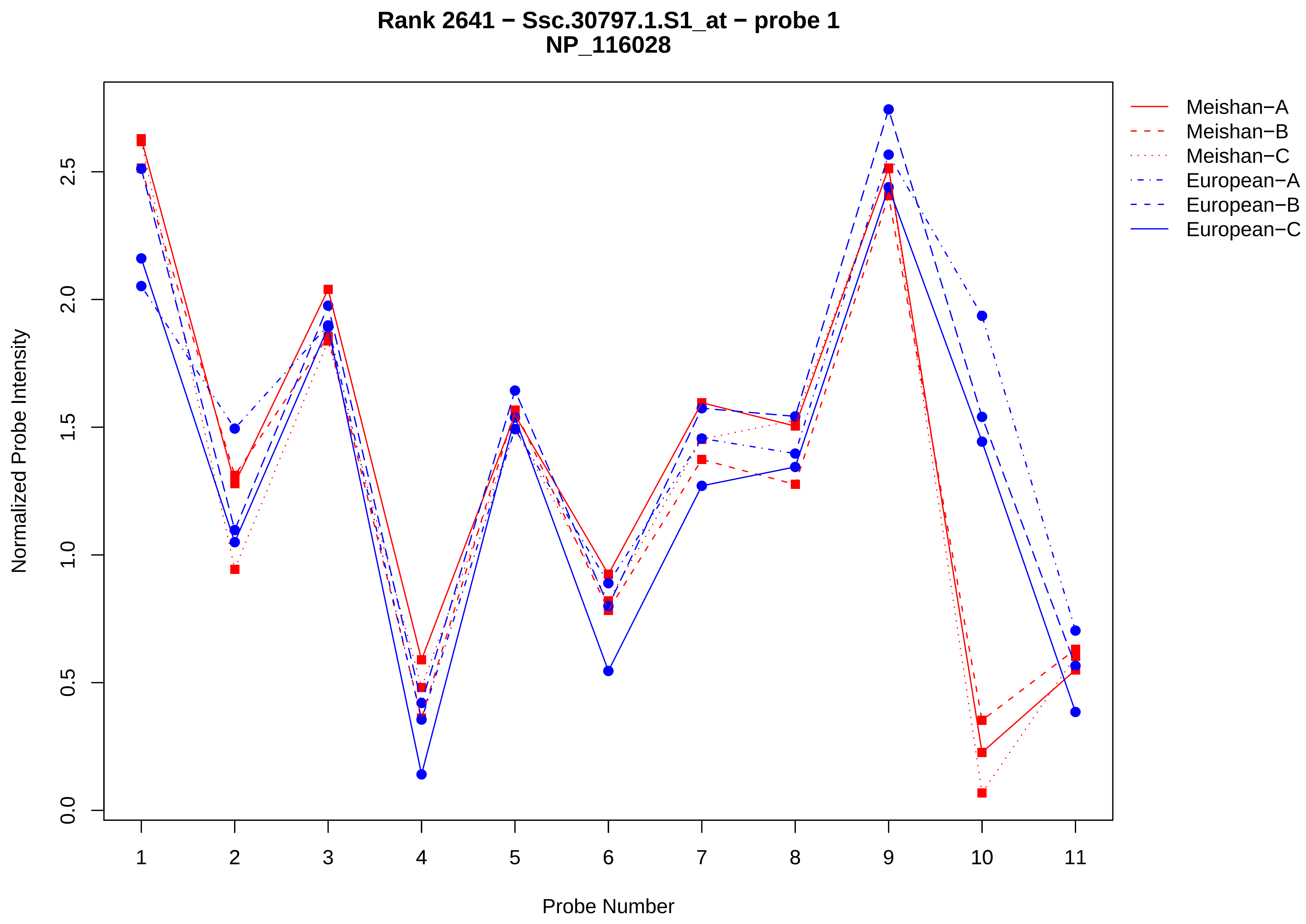

Rank 2641 − Ssc.30797.1.S1_at − probe 1
NP_116028
Normalized Probe Intensity
0.0
0.5
1.0
1.5
2.0
2.5
Probe Number
1
2
3
4
5
6
7
8
9
10
11
Meishan−A
Meishan−B
Meishan−C
European−A
European−B
European−C

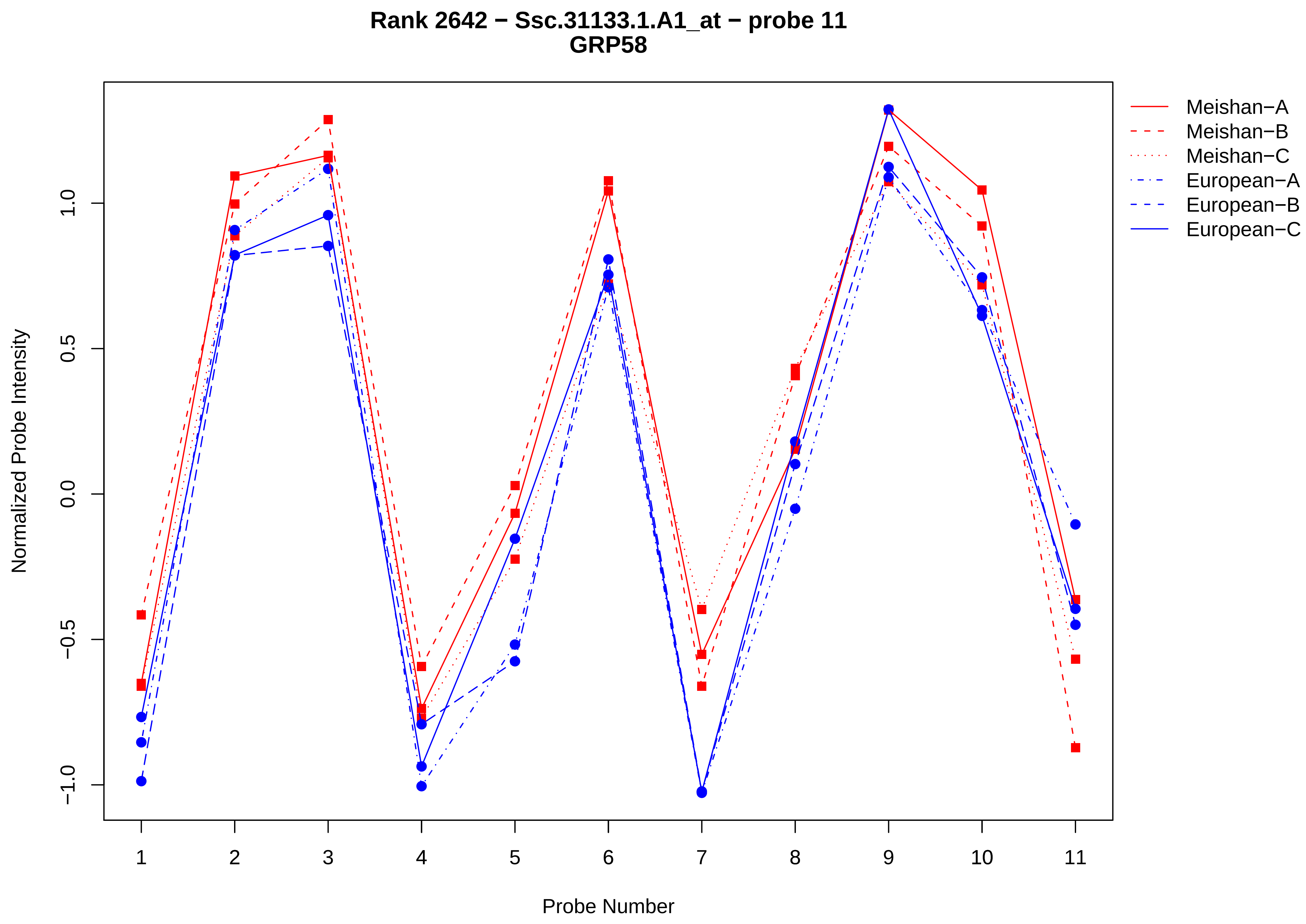

Rank 2642 − Ssc.31133.1.A1_at − probe 11
GRP58
Normalized Probe Intensity
Probe Number
−1.0
−0.5
0.0
0.5
1.0
1
2
3
4
5
6
7
8
9
10
11
Meishan−A
Meishan−B
Meishan−C
European−A
European−B
European−C

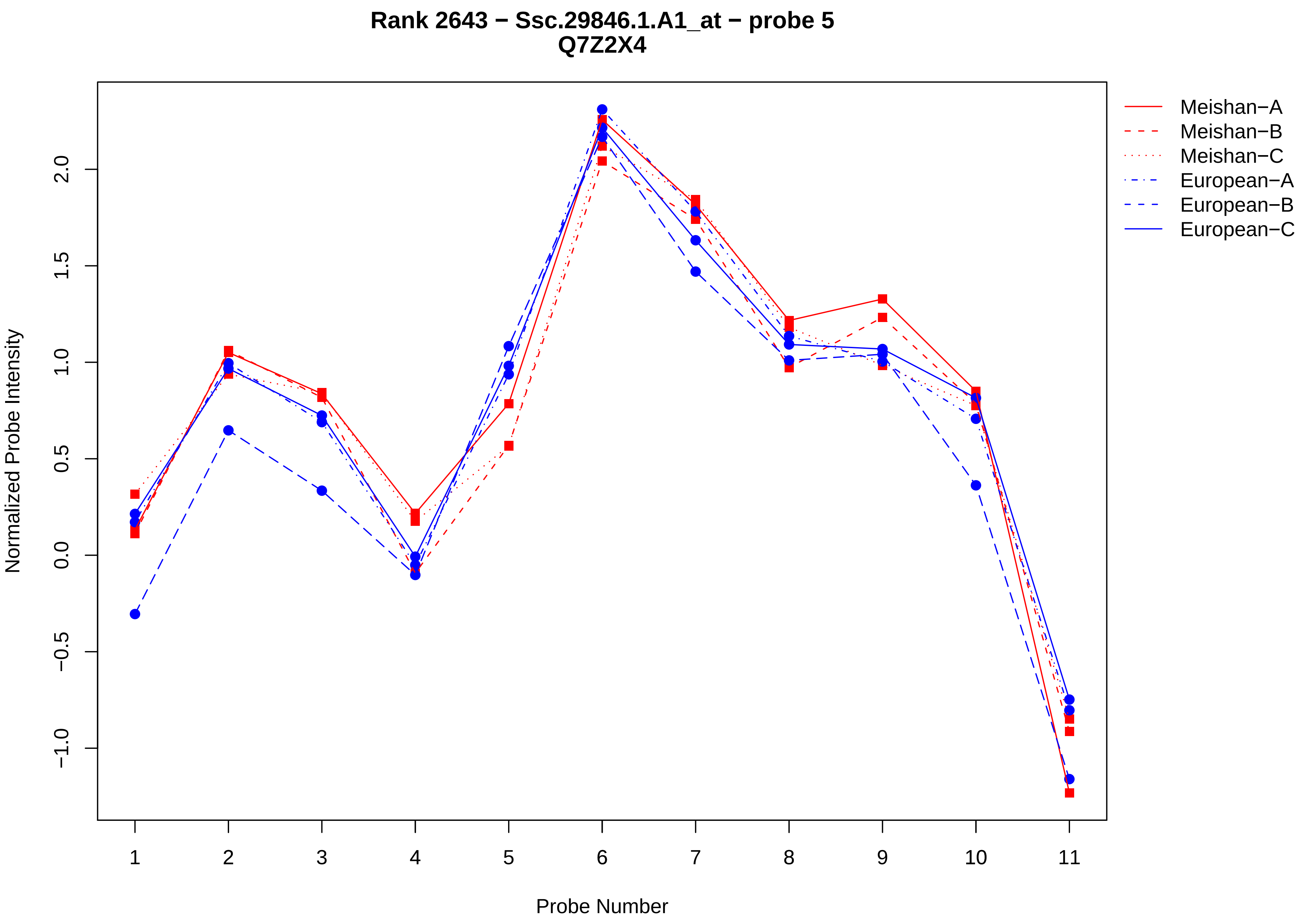
Rank 2643 − Ssc.29846.1.A1_at − probe 5
Q7Z2X4
Normalized Probe Intensity
Probe Number
−1.0
−0.5
0.0
0.5
1.0
1.5
2.0
1
2
3
4
5
6
7
8
9
10
11
Meishan−A
Meishan−B
Meishan−C
European−A
European−B
European−C

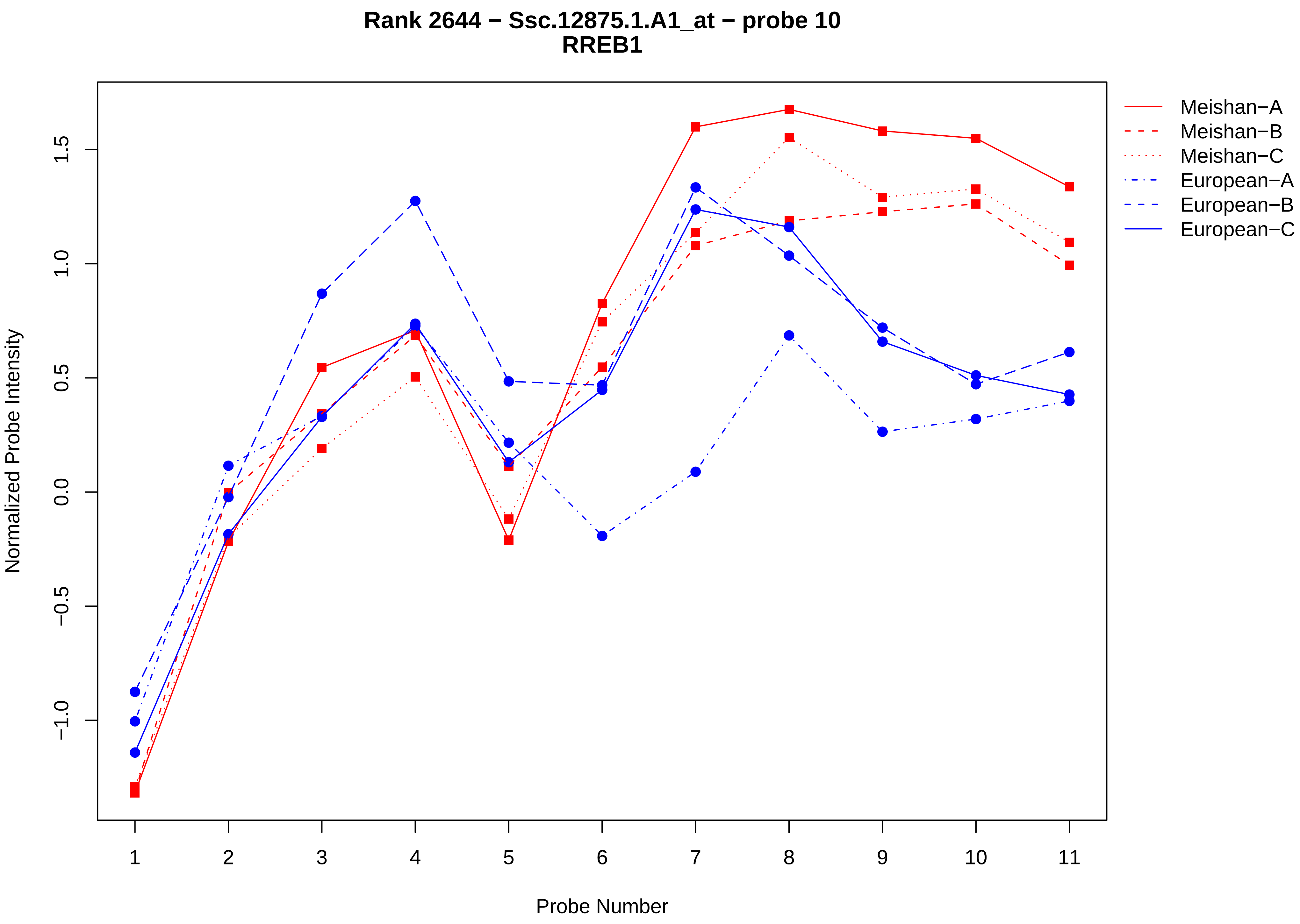

Rank 2644 − Ssc.12875.1.A1_at − probe 10
RREB1
Normalized Probe Intensity
Probe Number
−1.0
−0.5
0.0
0.5
1.0
1.5
1
2
3
4
5
6
7
8
9
10
11
Meishan−A
Meishan−B
Meishan−C
European−A
European−B
European−C

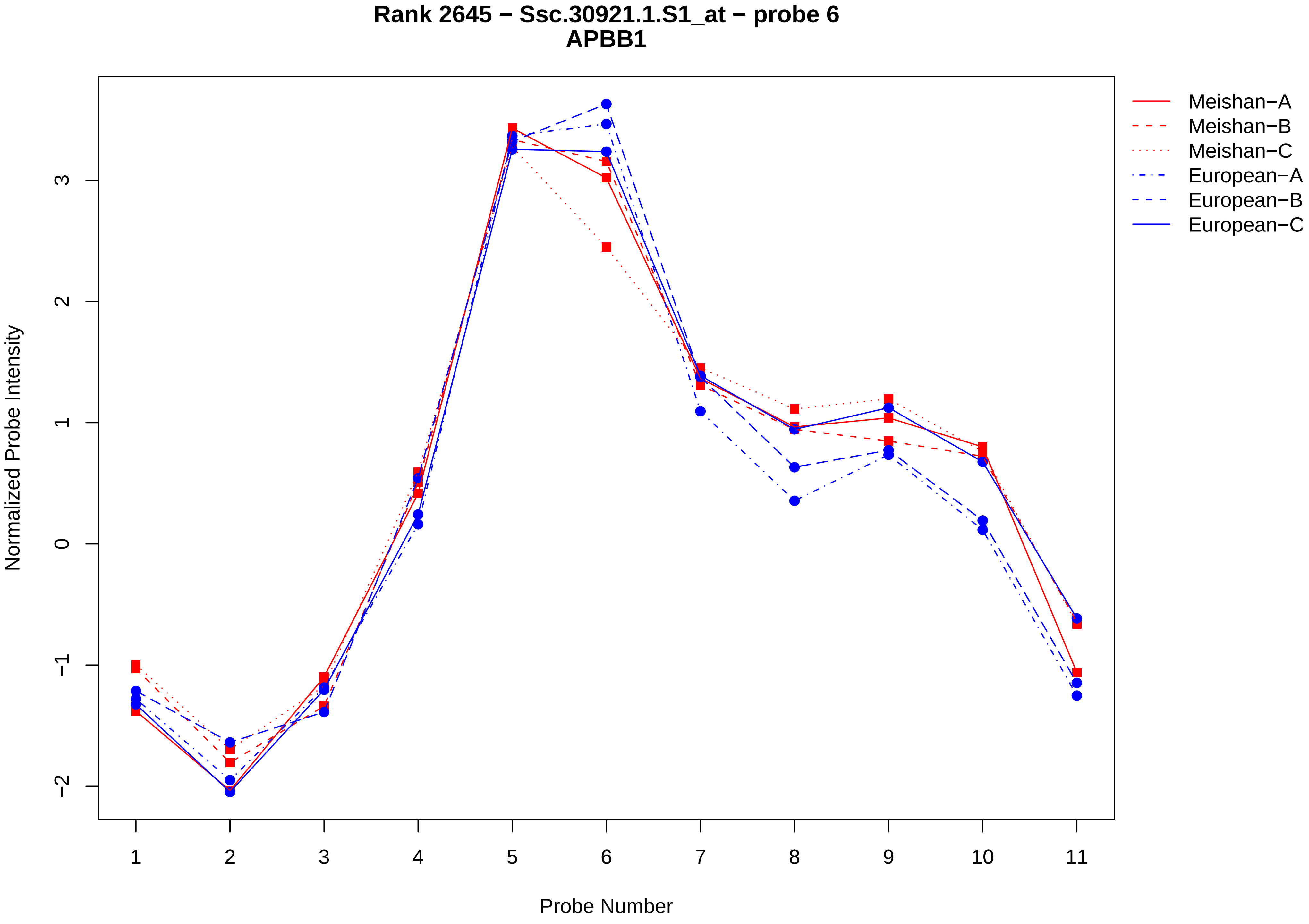

Rank 2645 − Ssc.30921.1.S1_at − probe 6
APBB1
Normalized Probe Intensity
Probe Number
Meishan−A
Meishan−B
Meishan−C
European−A
European−B
European−C

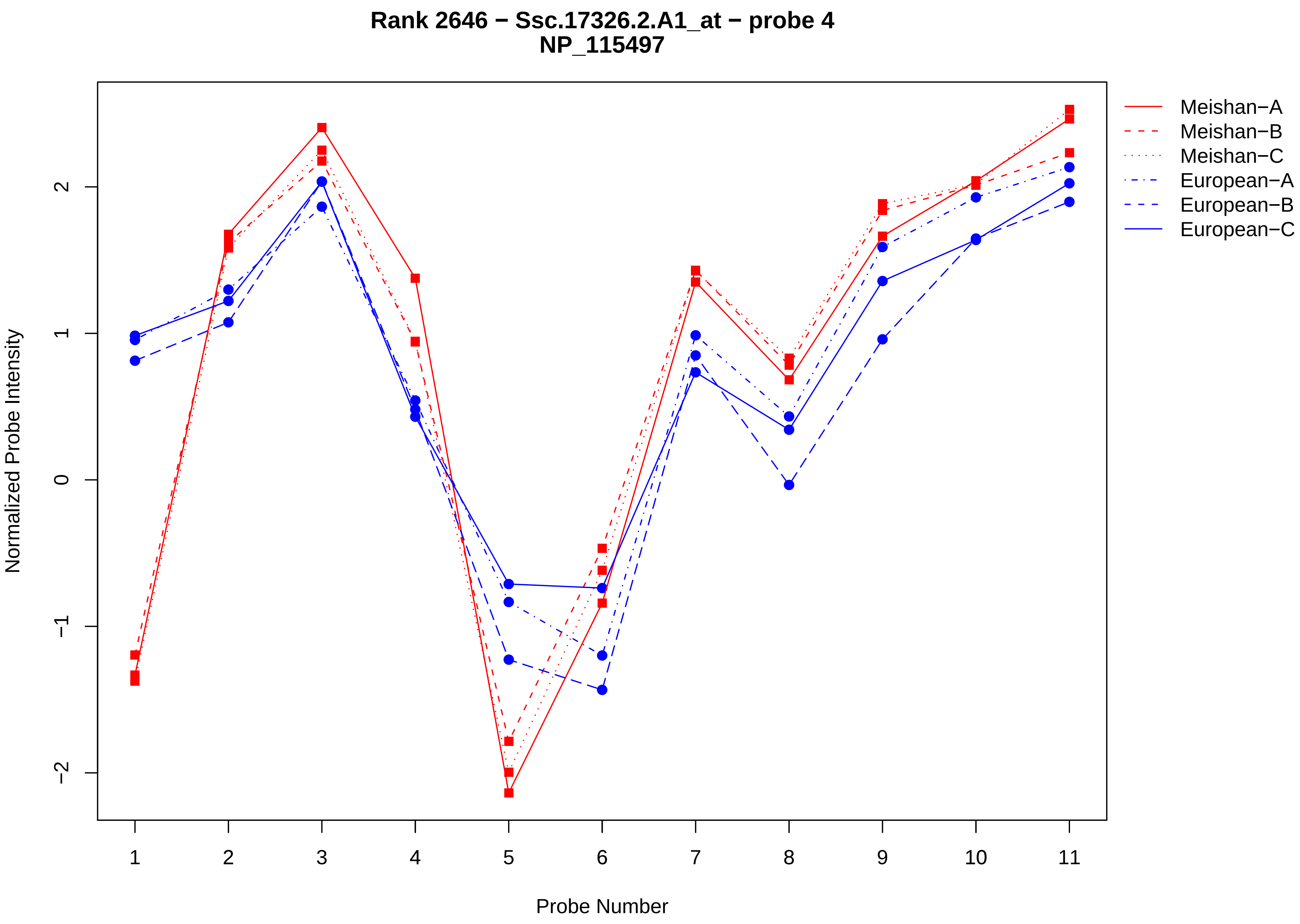

Rank 2646 − Ssc.17326.2.A1_at − probe 4
NP_115497
Normalized Probe Intensity
Probe Number
−2
−1
0
1
2
1
2
3
4
5
6
7
8
9
10
11
Meishan−A
Meishan−B
Meishan−C
European−A
European−B
European−C

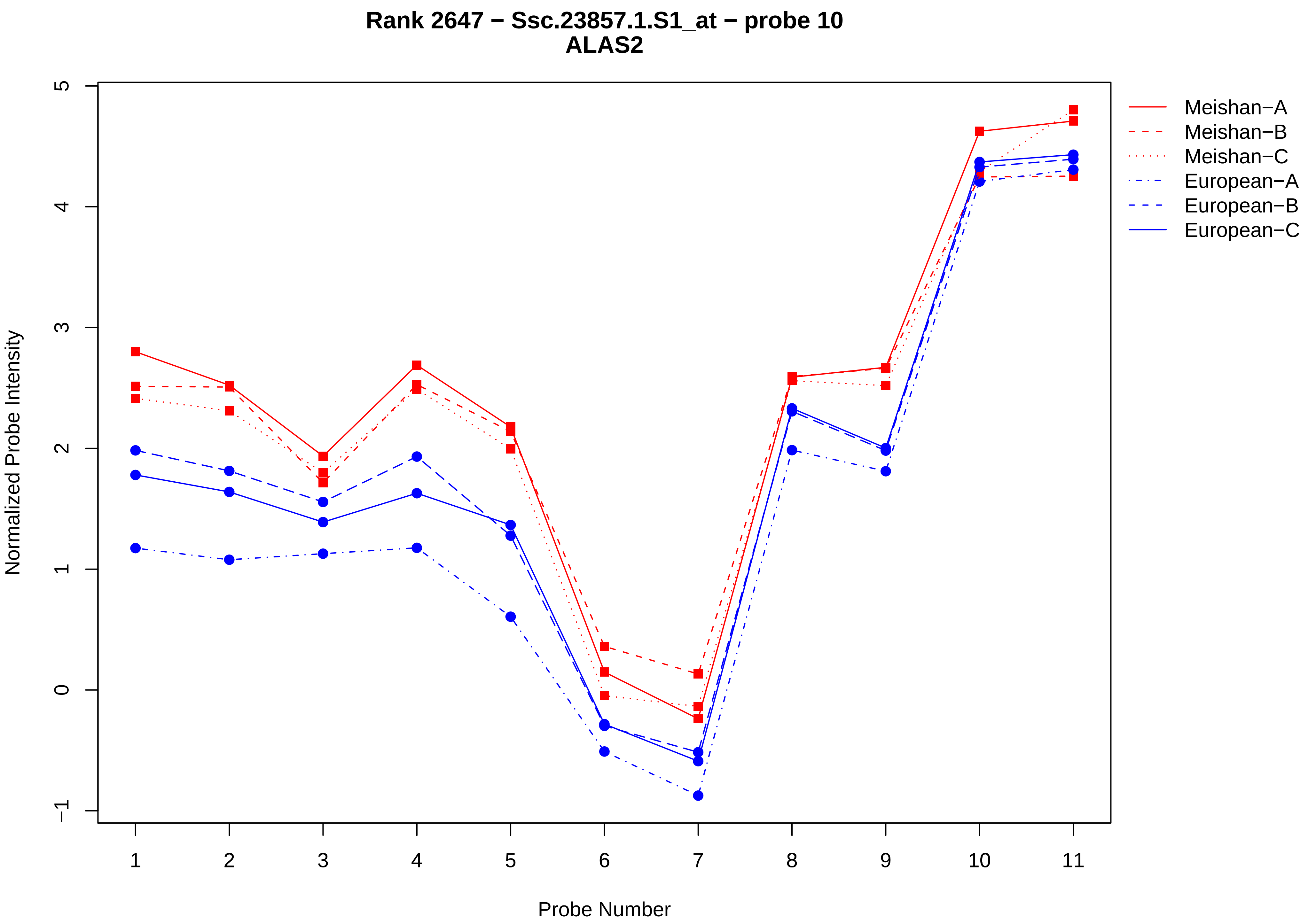

Rank 2647 − Ssc.23857.1.S1_at − probe 10
ALAS2
Normalized Probe Intensity
Probe Number
Meishan−A
Meishan−B
Meishan−C
European−A
European−B
European−C

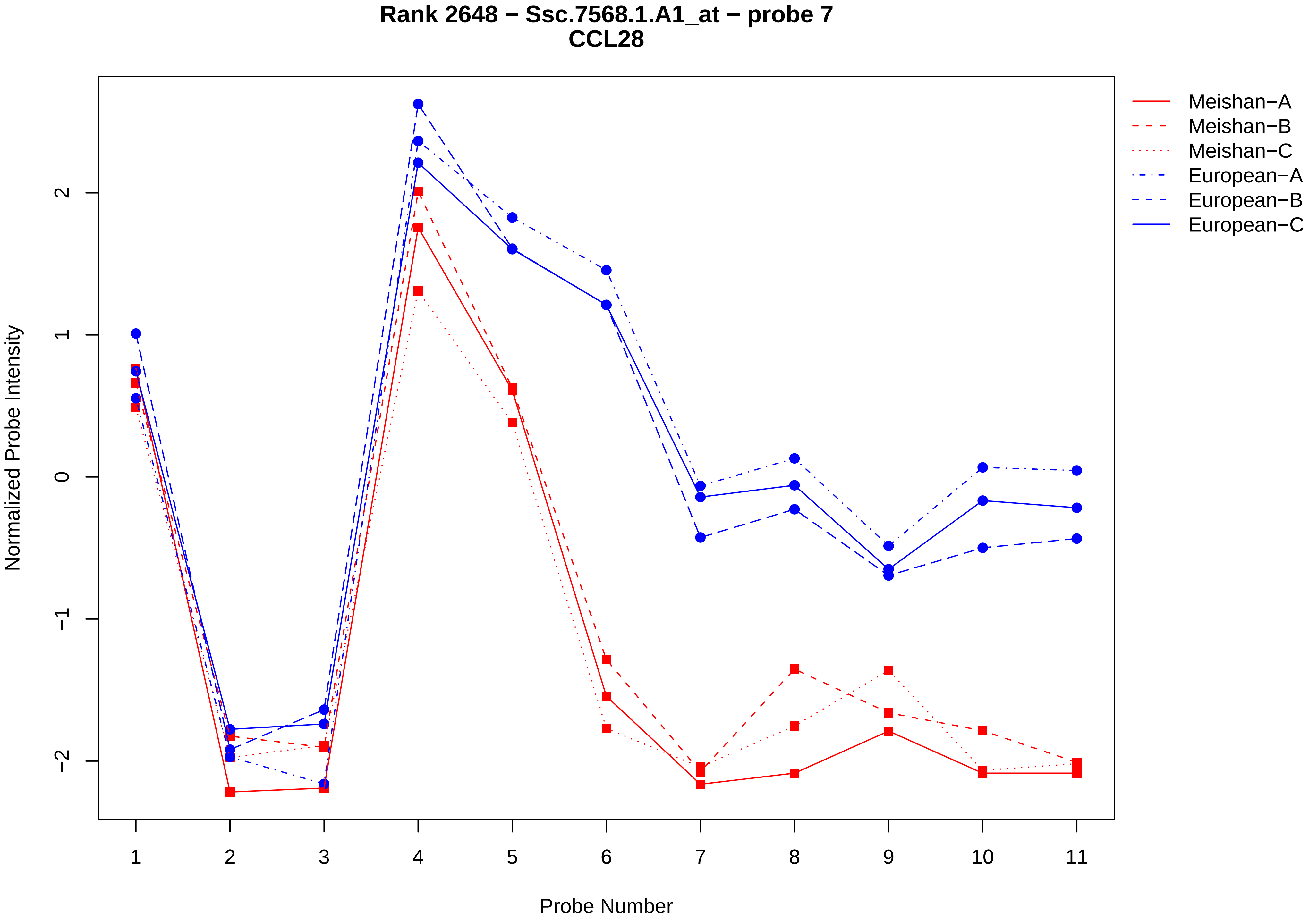

Rank 2648 − Ssc.7568.1.A1_at − probe 7
CCL28
Normalized Probe Intensity
Probe Number
Meishan−A
Meishan−B
Meishan−C
European−A
European−B
European−C
−2
−1
0
1
2
1
2
3
4
5
6
7
8
9
10
11

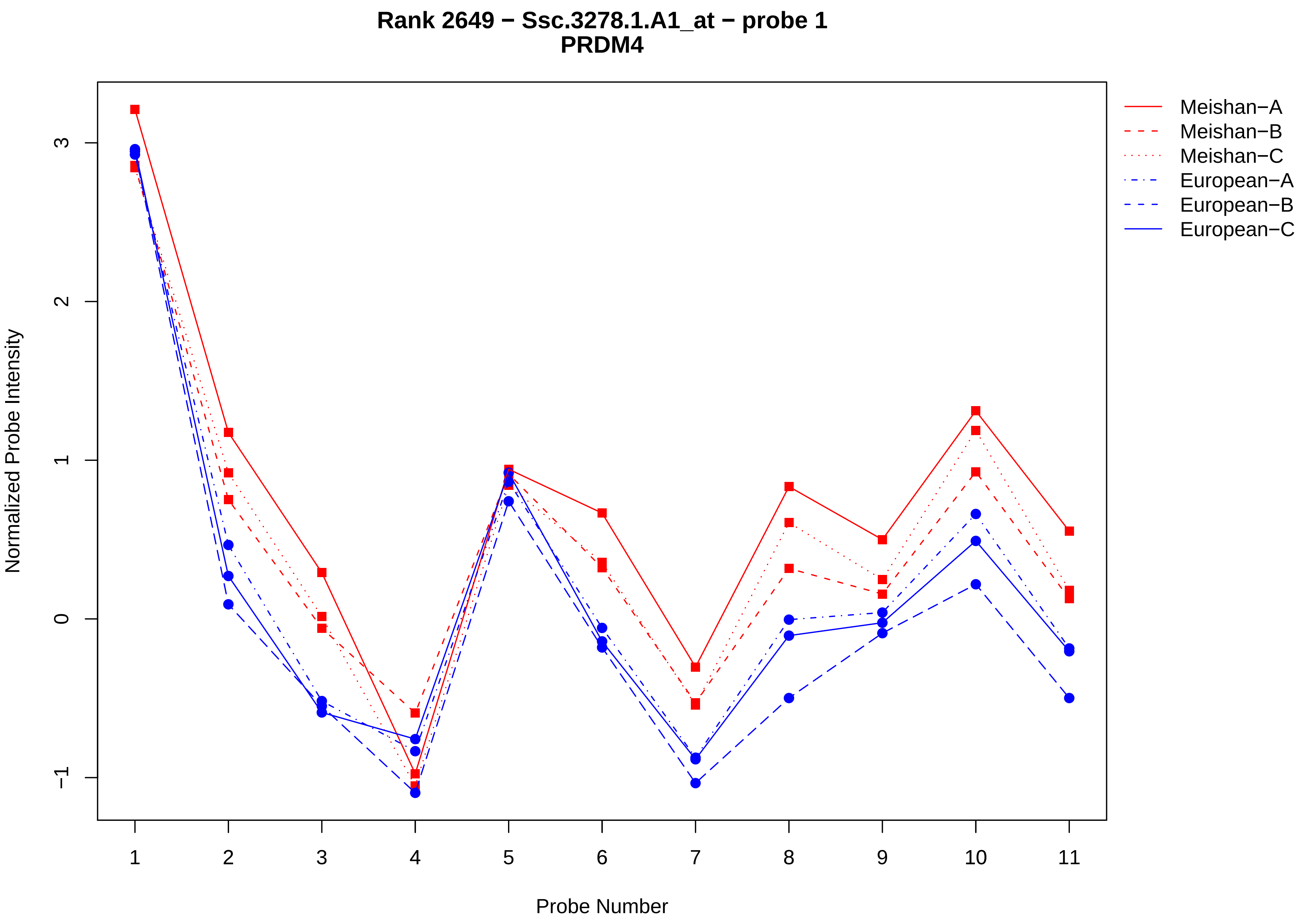

Rank 2649 − Ssc.3278.1.A1_at − probe 1
PRDM4
Normalized Probe Intensity
Probe Number
−1
0
1
2
3
1
2
3
4
5
6
7
8
9
10
11
Meishan−A
Meishan−B
Meishan−C
European−A
European−B
European−C

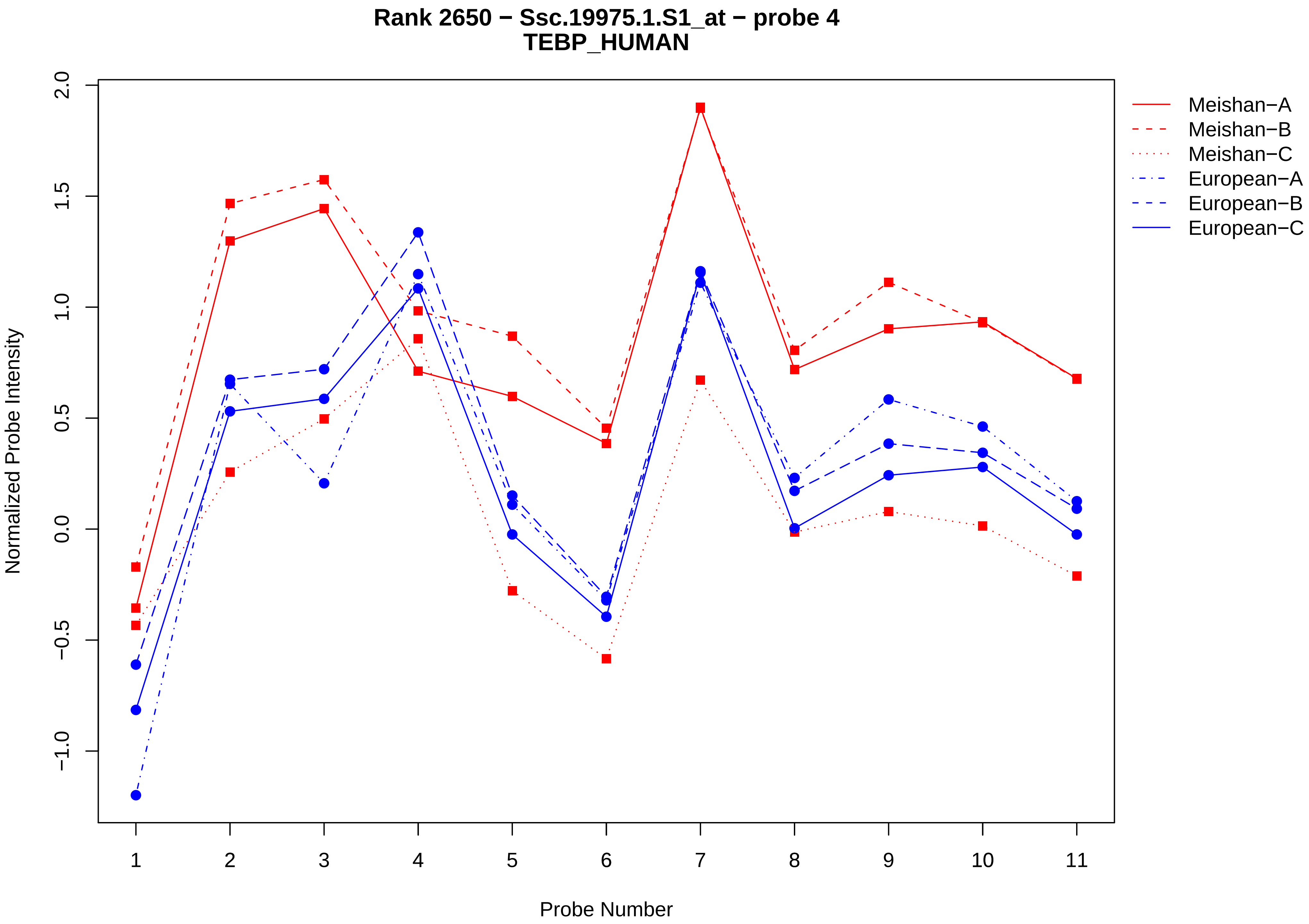

Rank 2650 − Ssc.19975.1.S1_at − probe 4
TEBP_HUMAN
Normalized Probe Intensity
Probe Number
−1.0
−0.5
0.0
0.5
1.0
1.5
2.0
1
2
3
4
5
6
7
8
9
10
11
Meishan−A
Meishan−B
Meishan−C
European−A
European−B
European−C

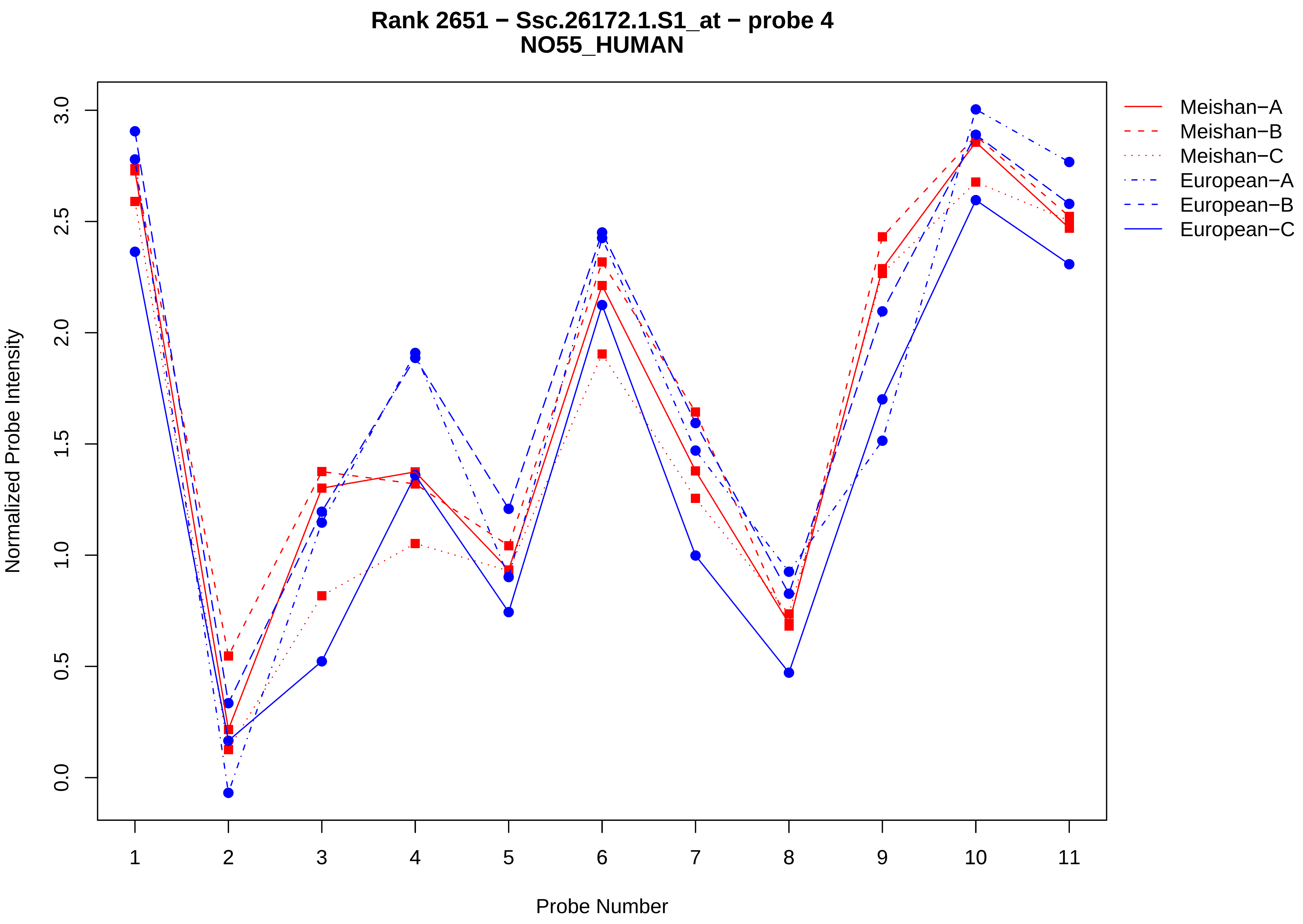

Rank 2651 − Ssc.26172.1.S1_at − probe 4
NO55_HUMAN
Normalized Probe Intensity
Probe Number
0.0
0.5
1.0
1.5
2.0
2.5
3.0
1
2
3
4
5
6
7
8
9
10
11
Meishan−A
Meishan−B
Meishan−C
European−A
European−B
European−C

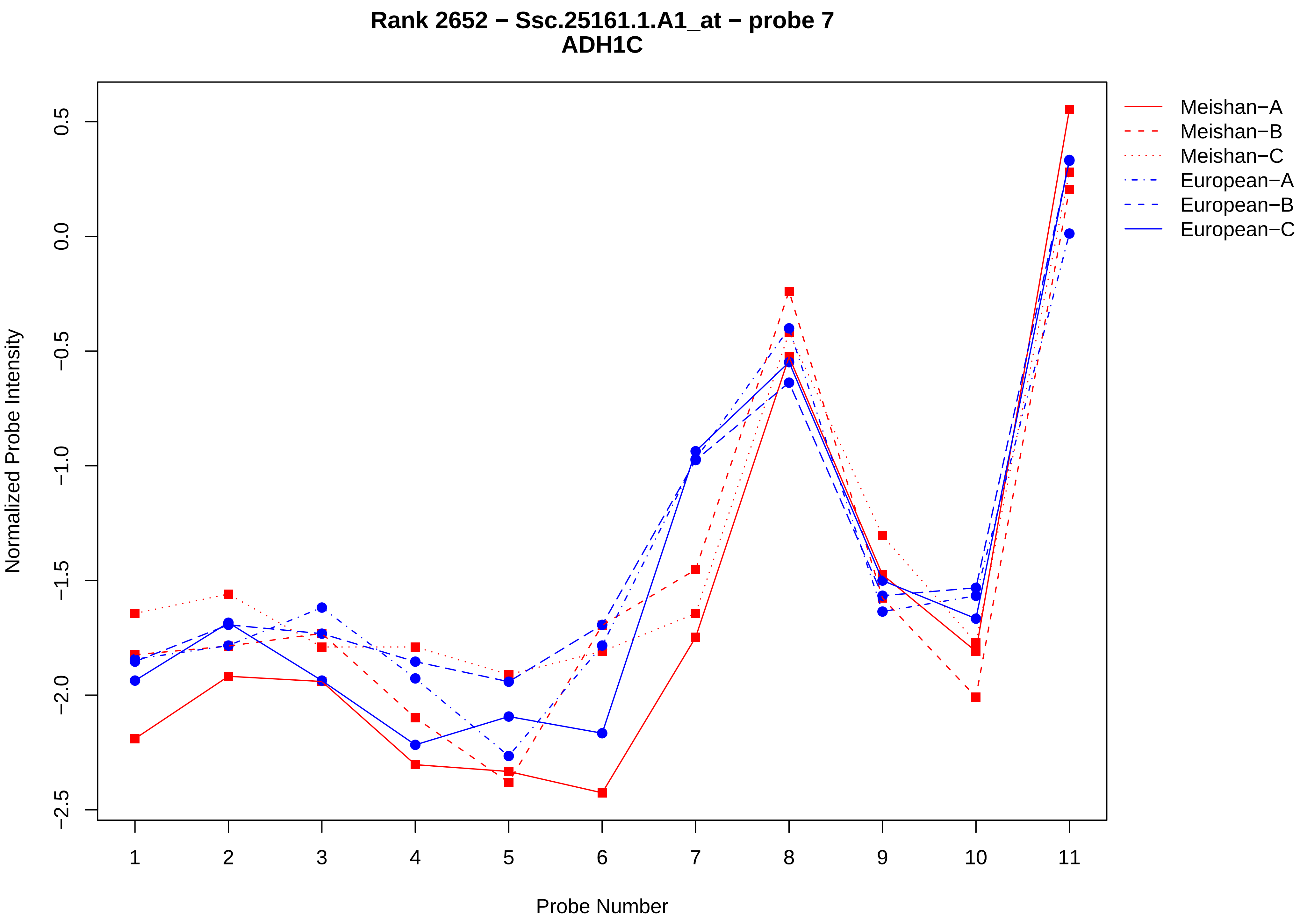

Rank 2652 − Ssc.25161.1.A1_at − probe 7
ADH1C
Normalized Probe Intensity
Probe Number
0.5
0.0
−0.5
−1.0
−1.5
−2.0
−2.5
1
2
3
4
5
6
7
8
9
10
11
Meishan−A
Meishan−B
Meishan−C
European−A
European−B
European−C

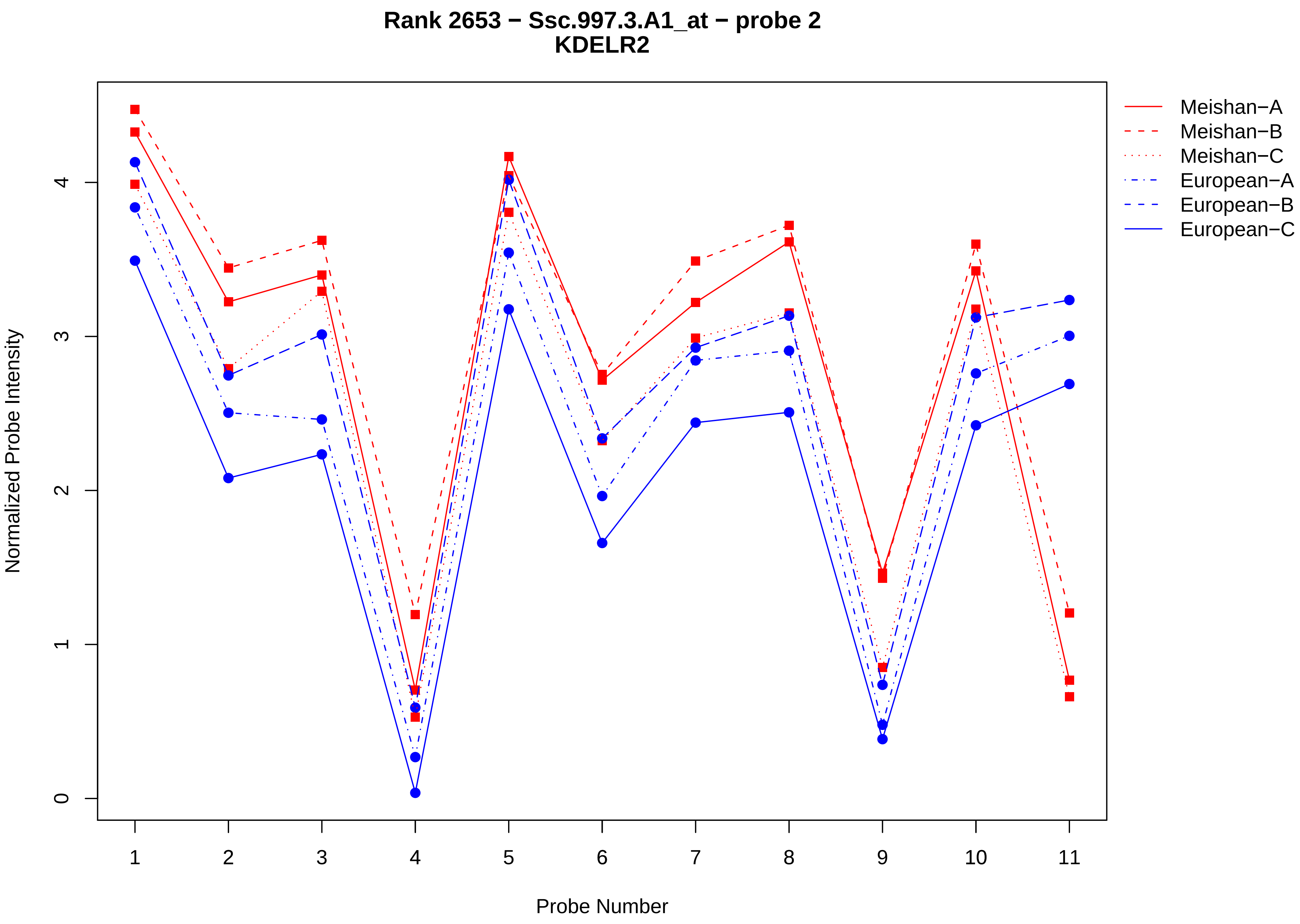
Rank 2653 − Ssc.997.3.A1_at − probe 2
KDELR2
Normalized Probe Intensity
0
1
2
3
4
1
2
3
4
5
6
7
8
9
10
11
Probe Number
Meishan−A
Meishan−B
Meishan−C
European−A
European−B
European−C

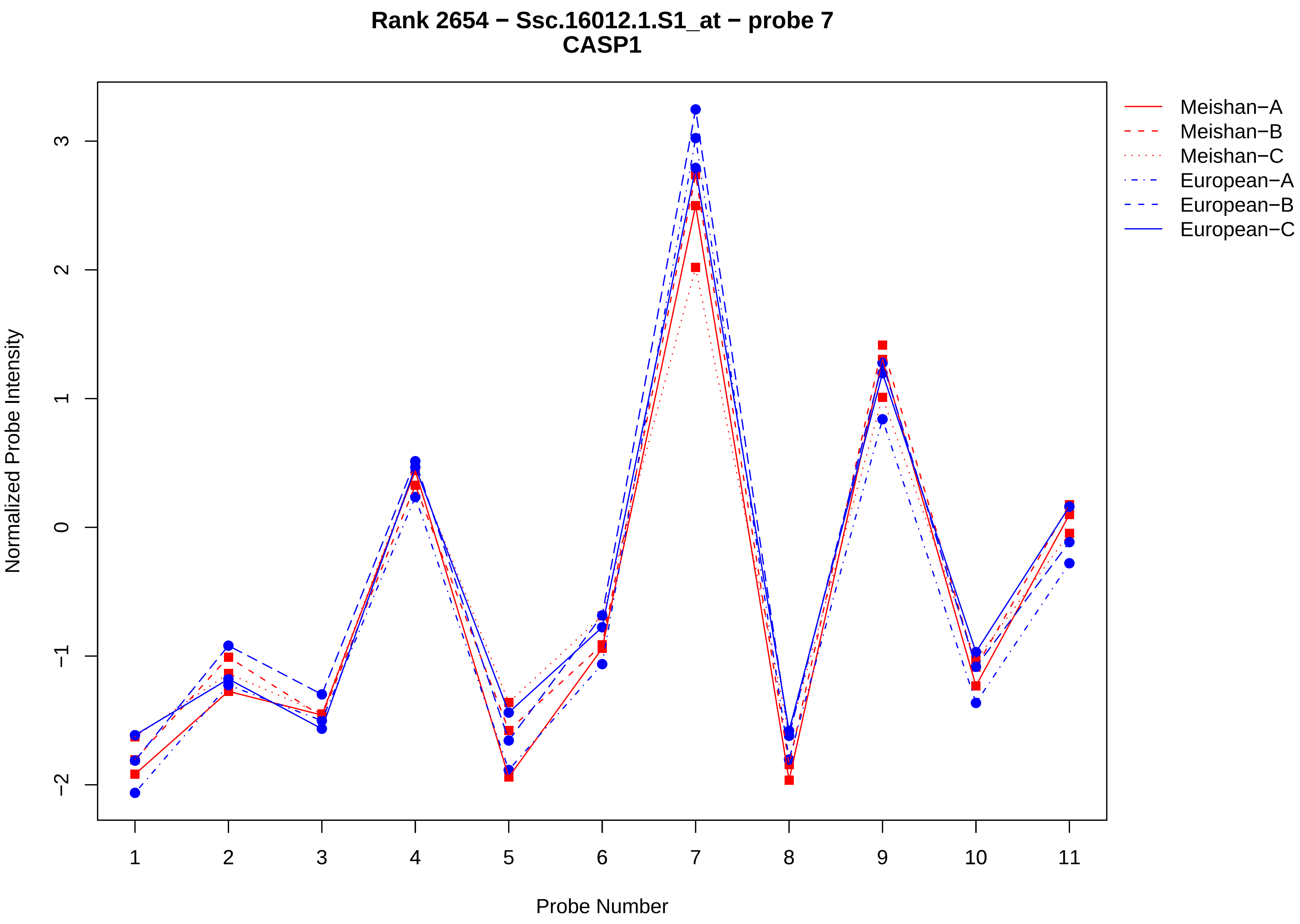

Rank 2654 − Ssc.16012.1.S1_at − probe 7
CASP1
Normalized Probe Intensity
Probe Number
−2
−1
0
1
2
3
1
2
3
4
5
6
7
8
9
10
11
Meishan−A
Meishan−B
Meishan−C
European−A
European−B
European−C

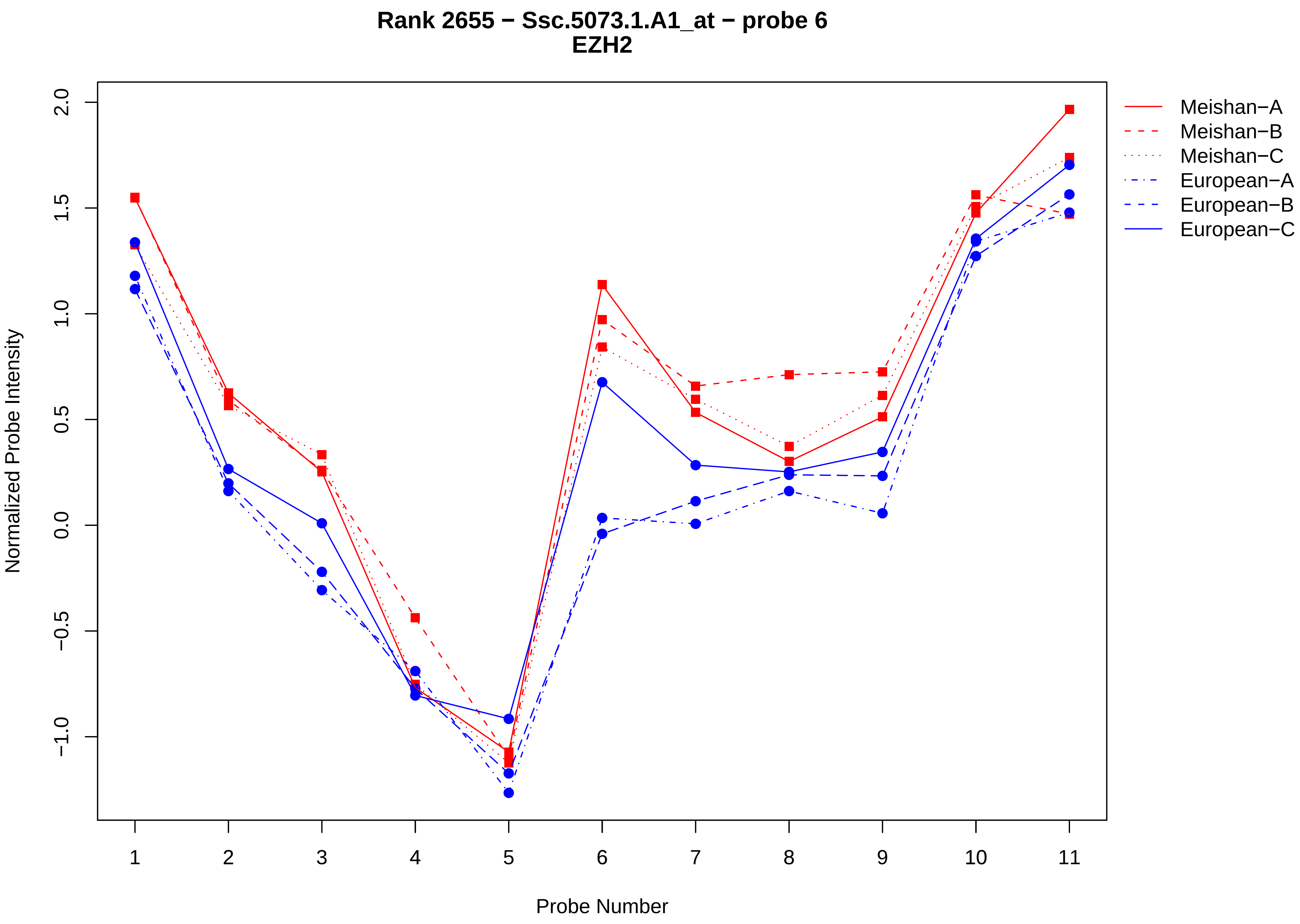

Rank 2655 − Ssc.5073.1.A1_at − probe 6
EZH2
Normalized Probe Intensity
Probe Number
−1.0
−0.5
0.0
0.5
1.0
1.5
2.0
1
2
3
4
5
6
7
8
9
10
11
Meishan−A
Meishan−B
Meishan−C
European−A
European−B
European−C

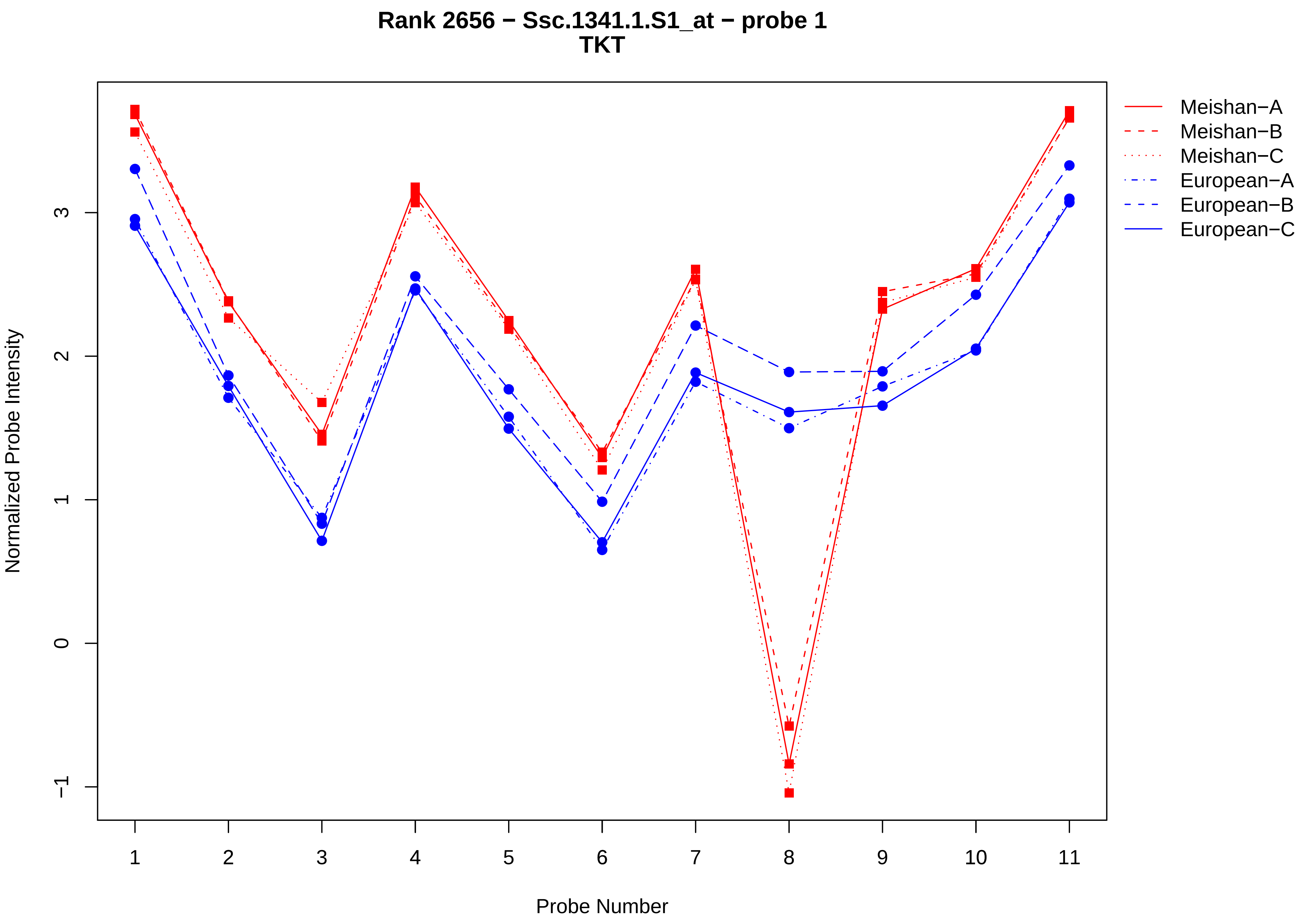
Rank 2656 − Ssc.1341.1.S1_at − probe 1
TKT
Normalized Probe Intensity
Probe Number
−1
0
1
2
3
1
2
3
4
5
6
7
8
9
10
11
Meishan−A
Meishan−B
Meishan−C
European−A
European−B
European−C

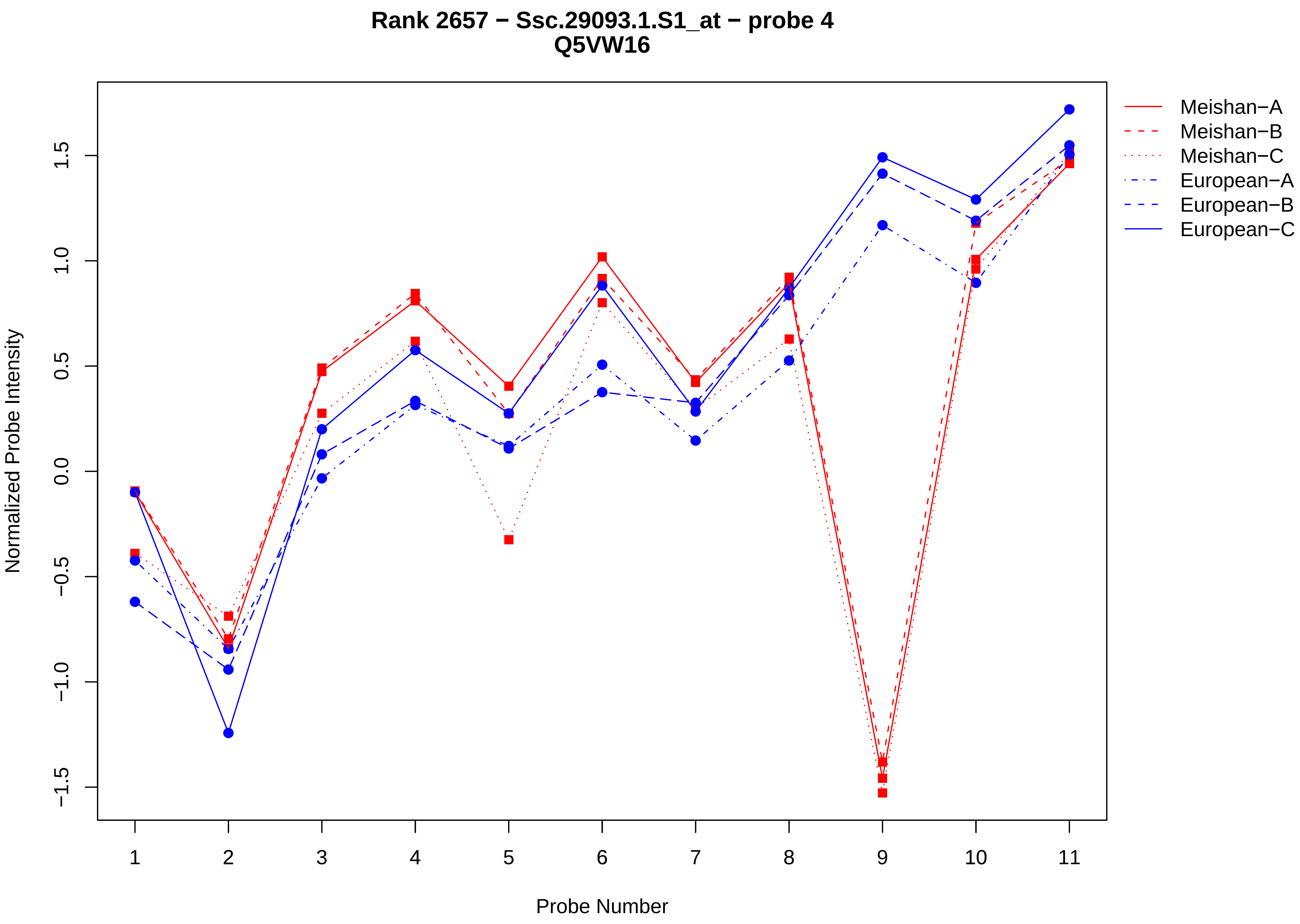

Rank 2657 − Ssc.29093.1.S1_at − probe 4
Q5VW16
Normalized Probe Intensity
Probe Number
Meishan−A
Meishan−B
Meishan−C
European−A
European−B
European−C
−1.5
−1.0
−0.5
0.0
0.5
1.0
1.5
1
2
3
4
5
6
7
8
9
10
11

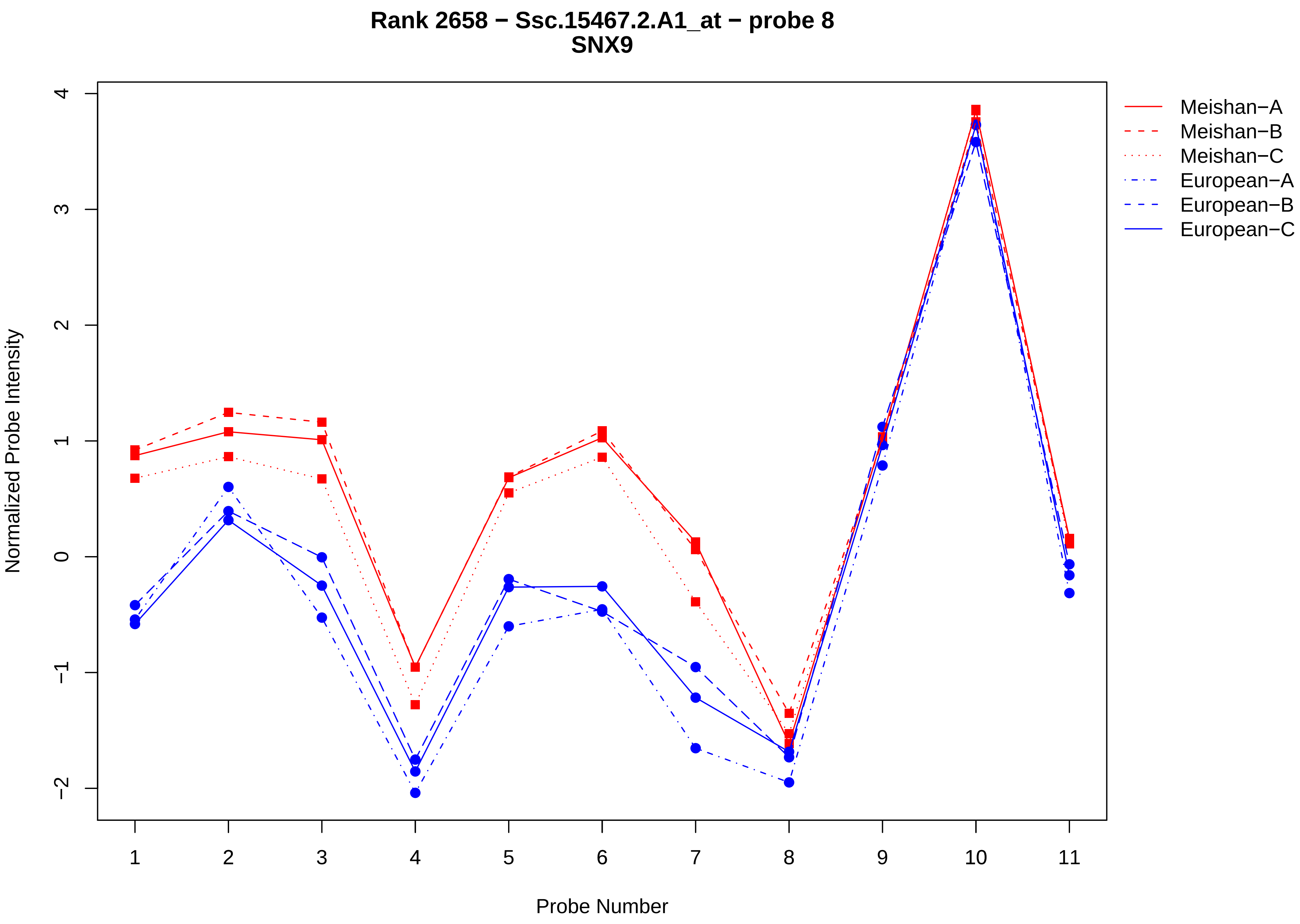

Rank 2658 − Ssc.15467.2.A1_at − probe 8
SNX9
Normalized Probe Intensity
Probe Number
−2
−1
0
1
2
3
4
1
2
3
4
5
6
7
8
9
10
11
Meishan−A
Meishan−B
Meishan−C
European−A
European−B
European−C

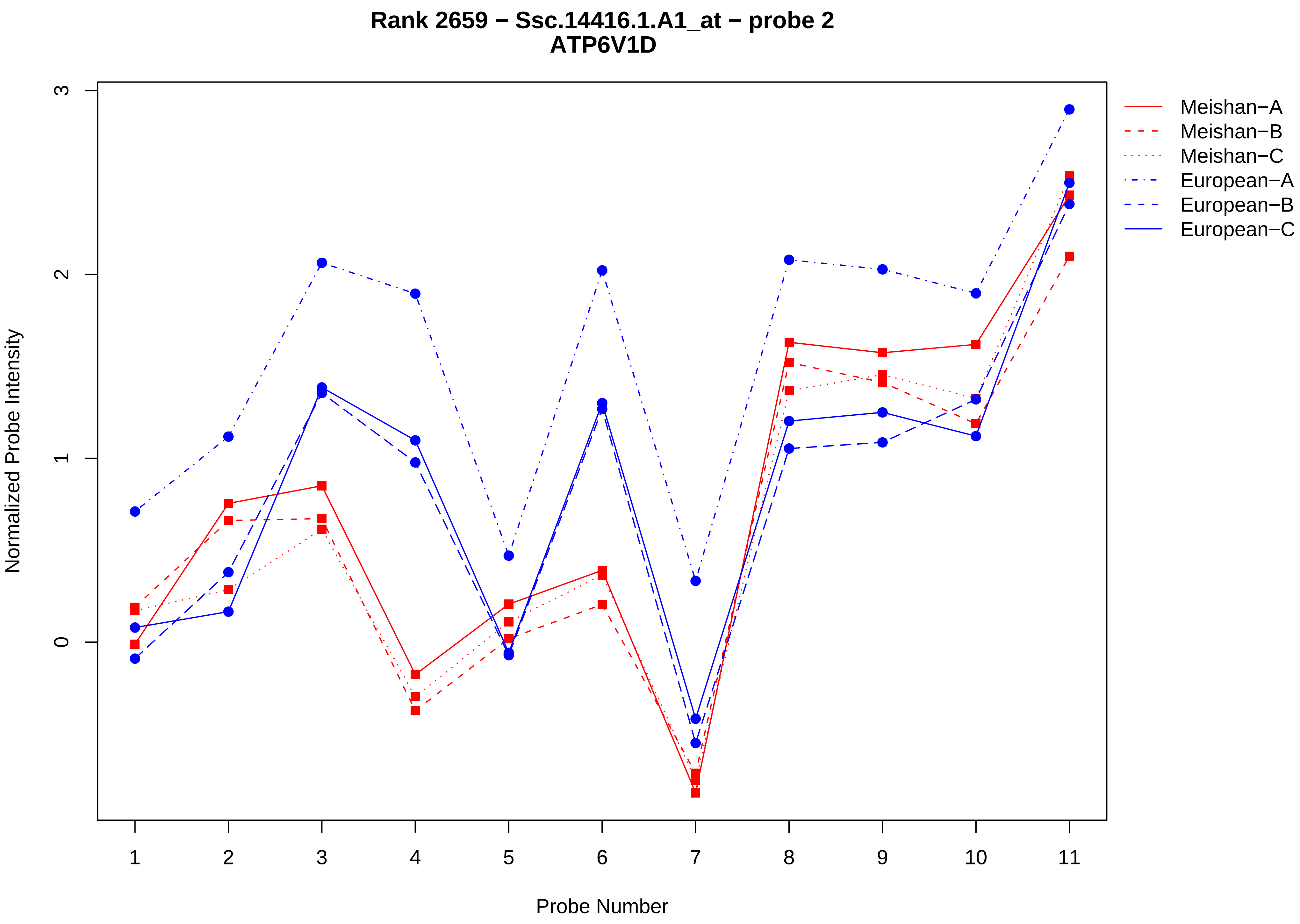

Rank 2659 − Ssc.14416.1.A1_at − probe 2
ATP6V1D
Normalized Probe Intensity
Probe Number
0
1
2
3
1
2
3
4
5
6
7
8
9
10
11
Meishan−A
Meishan−B
Meishan−C
European−A
European−B
European−C

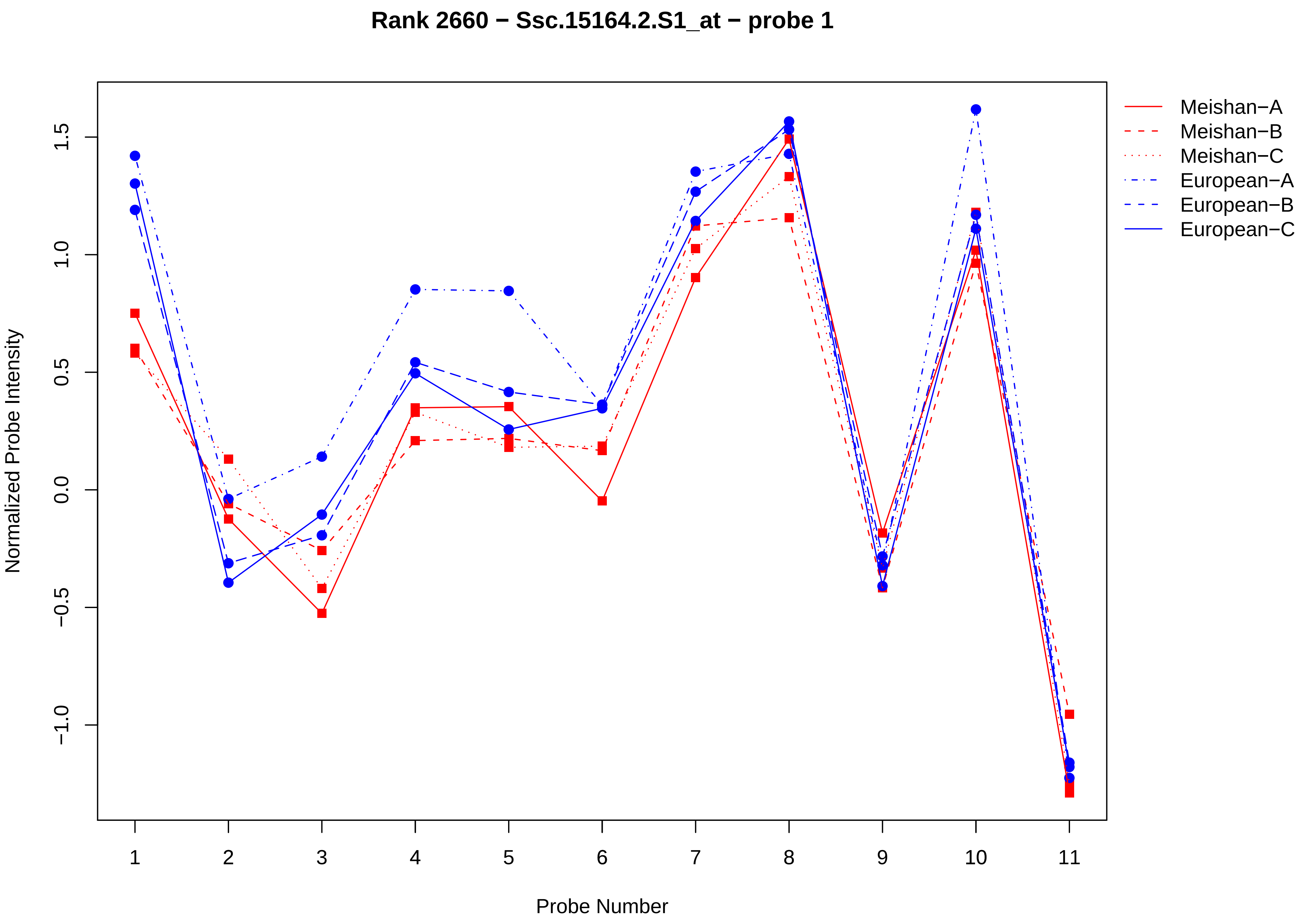

Rank 2660 − Ssc.15164.2.S1_at − probe 1
Normalized Probe Intensity
Probe Number
−1.0
−0.5
0.0
0.5
1.0
1.5
1
2
3
4
5
6
7
8
9
10
11
Meishan−A
Meishan−B
Meishan−C
European−A
European−B
European−C

Rank 2661 − Ssc.6093.2.A1_at − probe 5
MAP1B

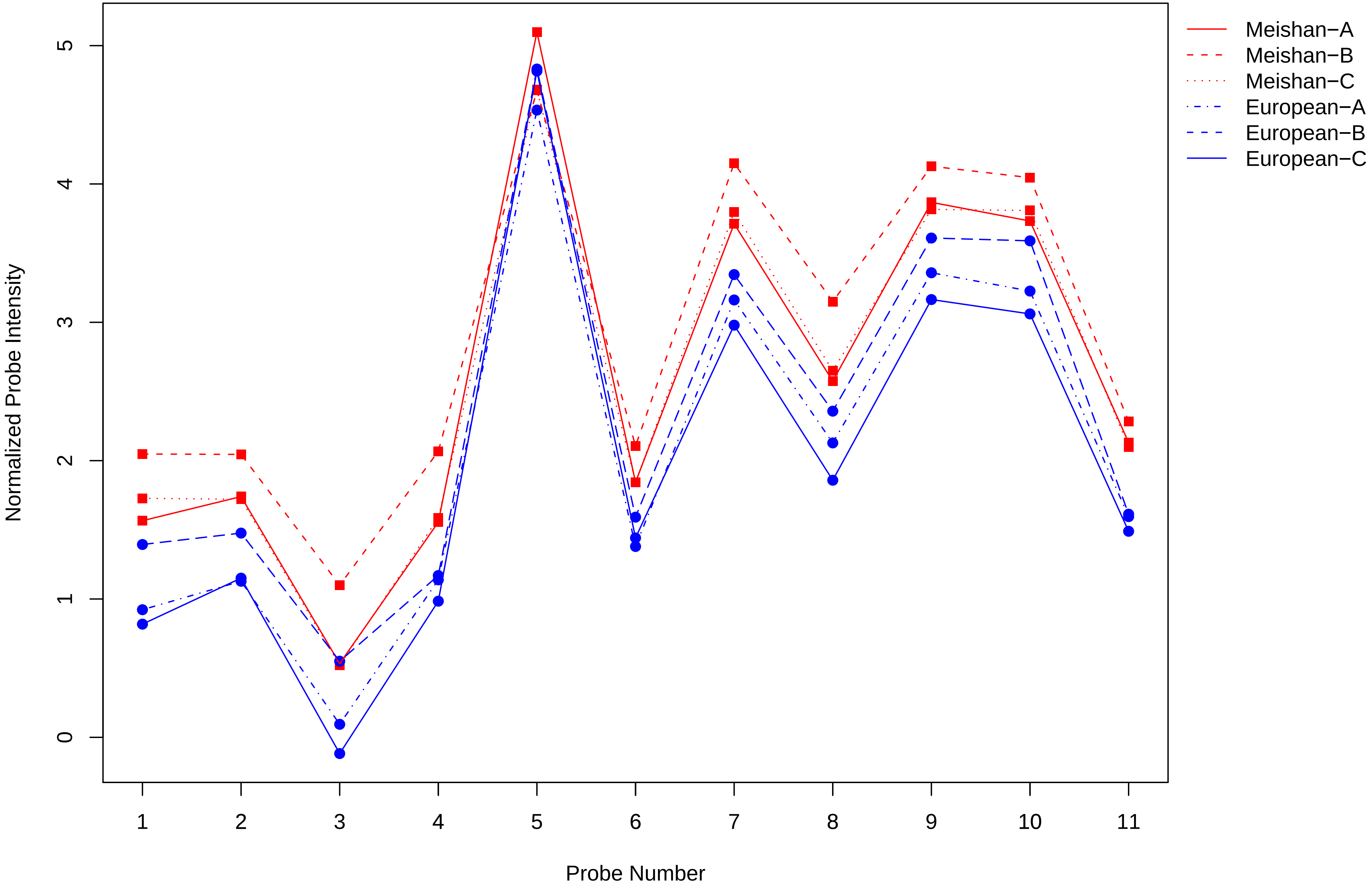

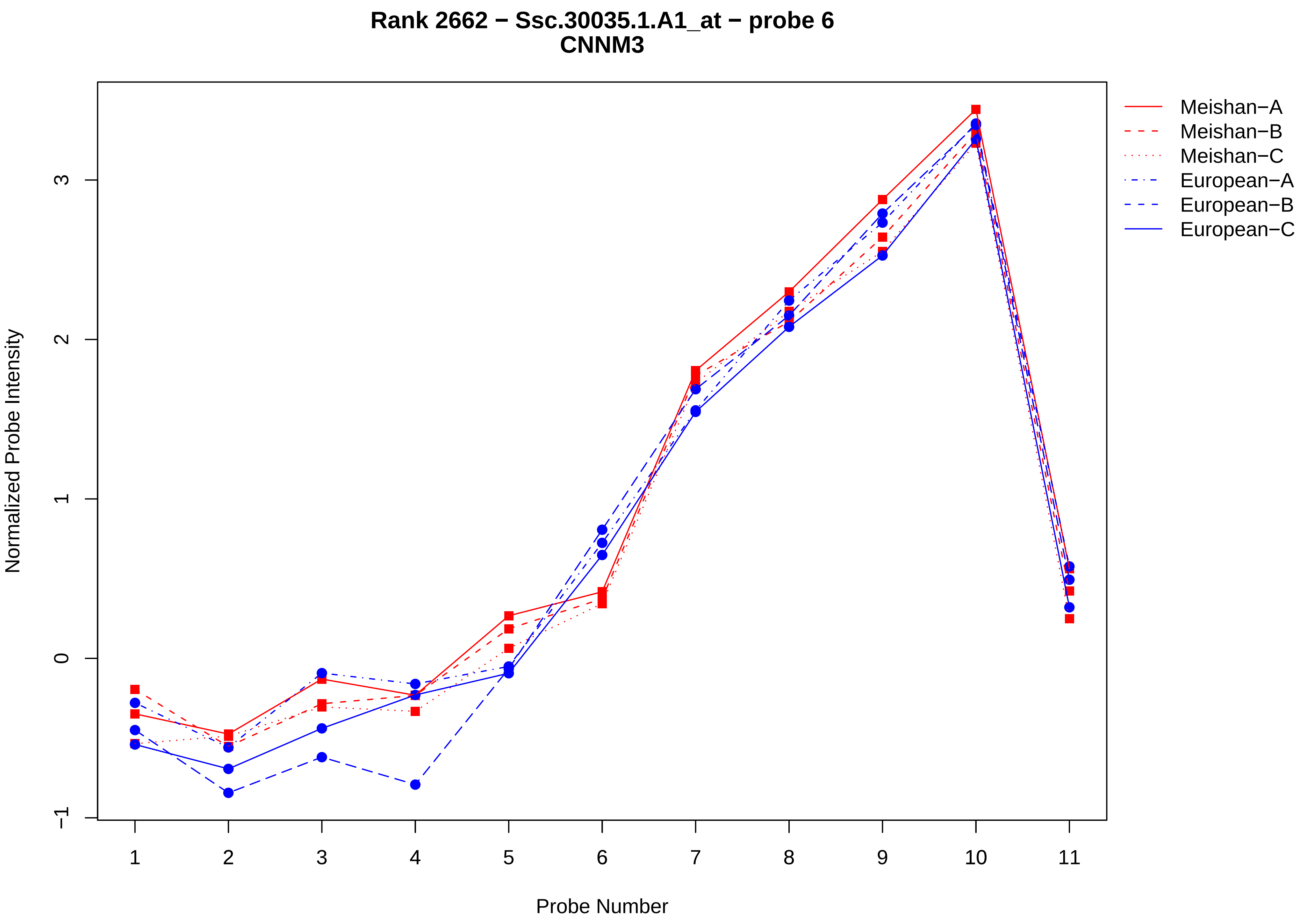

Rank 2662 − Ssc.30035.1.A1_at − probe 6
CNNM3
Normalized Probe Intensity
Probe Number
−1
0
1
2
3
1
2
3
4
5
6
7
8
9
10
11
Meishan−A
Meishan−B
Meishan−C
European−A
European−B
European−C

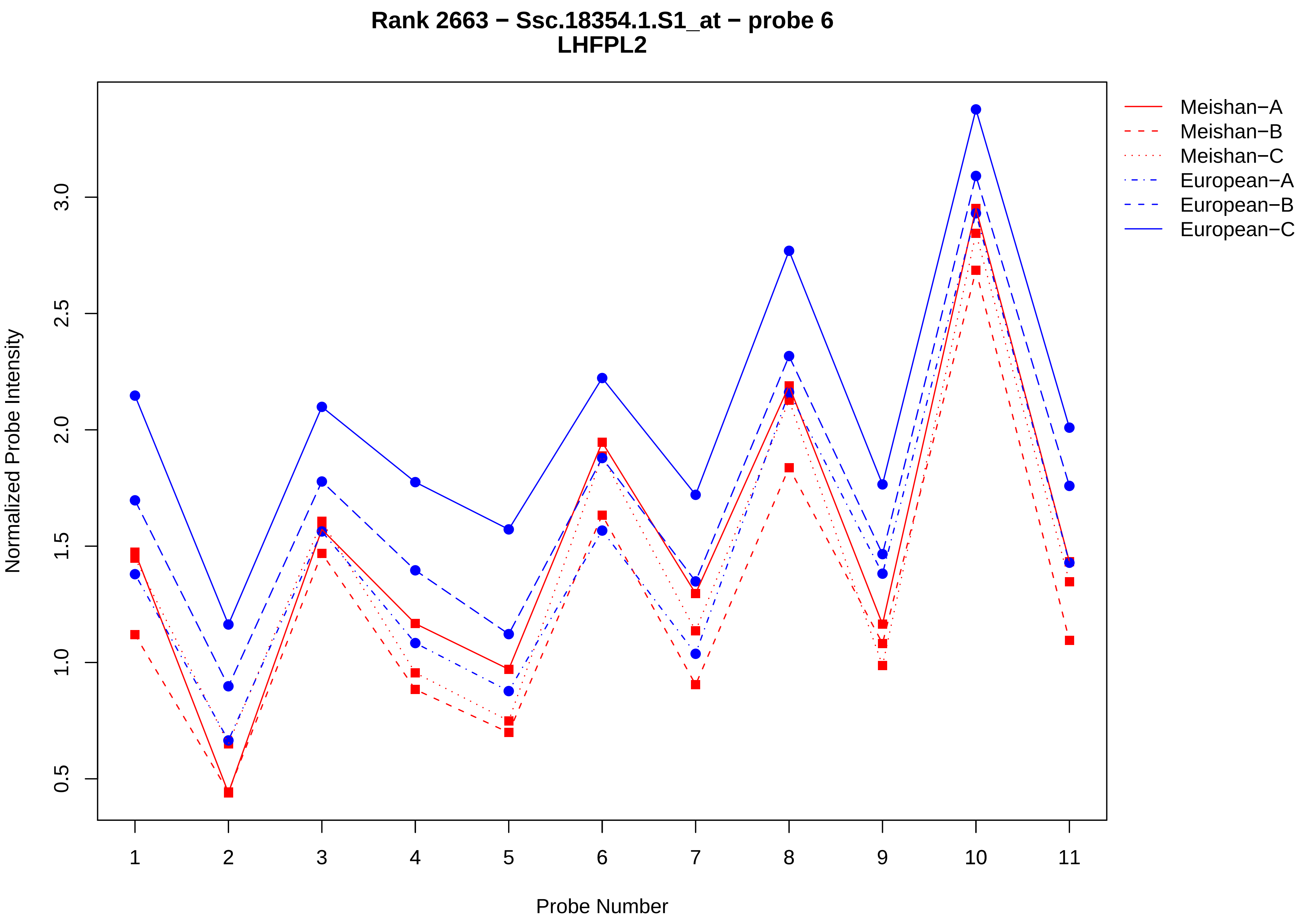

Rank 2663 − Ssc.18354.1.S1_at − probe 6
LHFPL2
Normalized Probe Intensity
Probe Number
0.5
1.0
1.5
2.0
2.5
3.0
1
2
3
4
5
6
7
8
9
10
11
Meishan−A
Meishan−B
Meishan−C
European−A
European−B
European−C

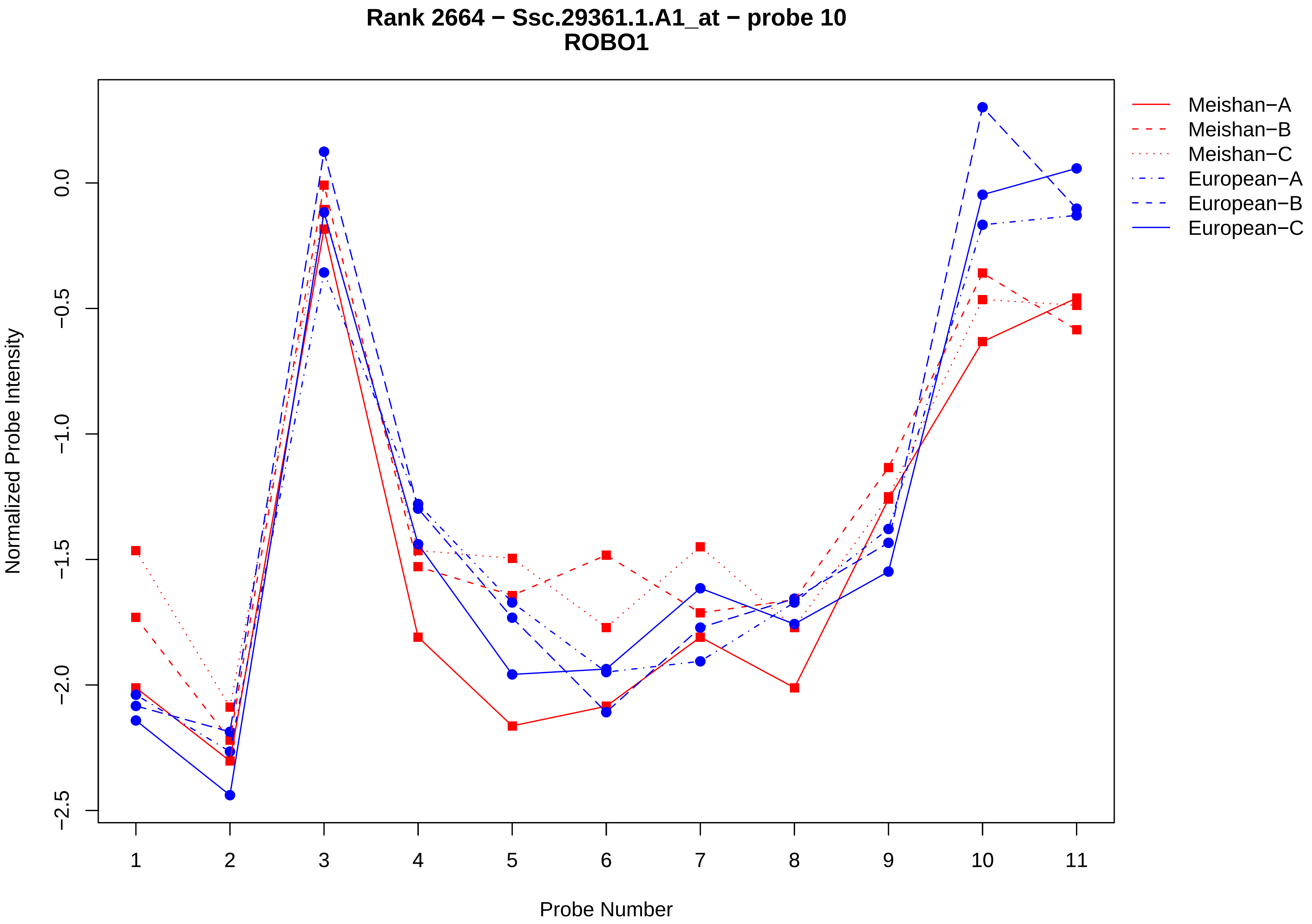

Rank 2664 − Ssc.29361.1.A1_at − probe 10
ROBO1
Normalized Probe Intensity
Probe Number
0.0
−0.5
−1.0
−1.5
−2.0
−2.5
1
2
3
4
5
6
7
8
9
10
11
Meishan−A
Meishan−B
Meishan−C
European−A
European−B
European−C

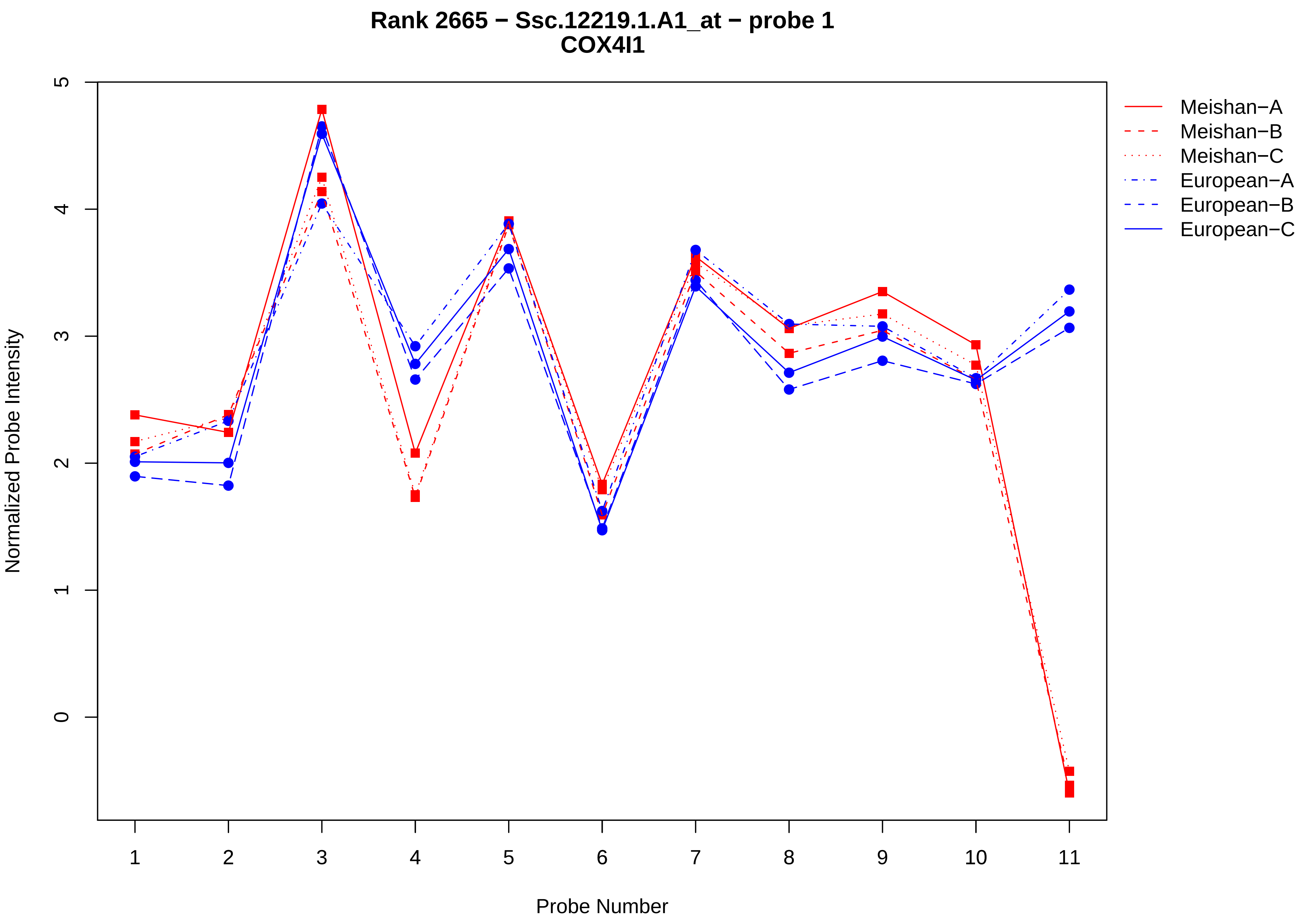

Rank 2665 − Ssc.12219.1.A1_at − probe 1
COX4I1
Normalized Probe Intensity
Probe Number
0
1
2
3
4
5
1
2
3
4
5
6
7
8
9
10
11
Meishan−A
Meishan−B
Meishan−C
European−A
European−B
European−C

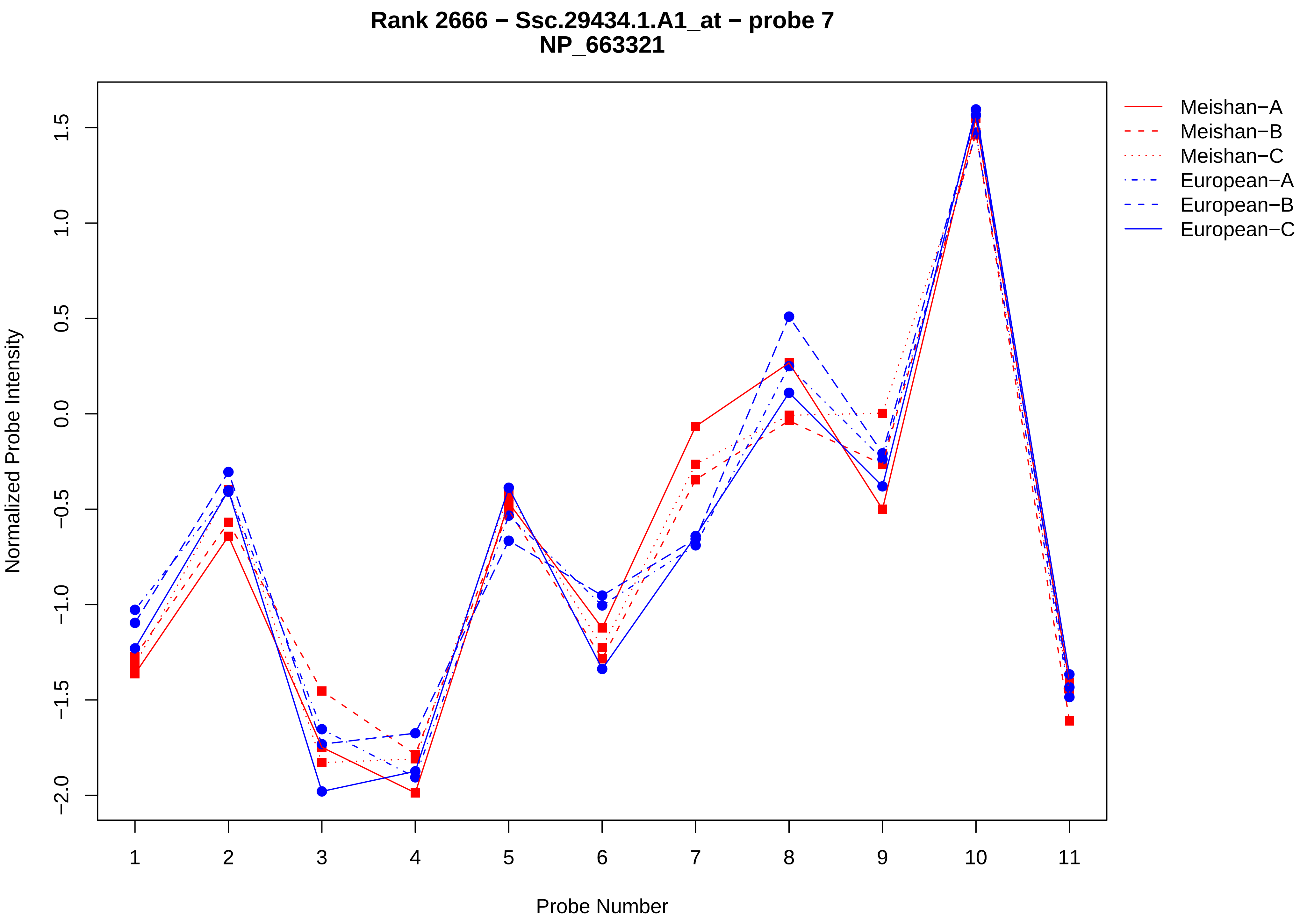
Rank 2666 − Ssc.29434.1.A1_at − probe 7
NP_663321
Normalized Probe Intensity
Probe Number
Meishan−A
Meishan−B
Meishan−C
European−A
European−B
European−C
1.5
1.0
0.5
0.0
−0.5
−1.0
−1.5
−2.0
1
2
3
4
5
6
7
8
9
10
11

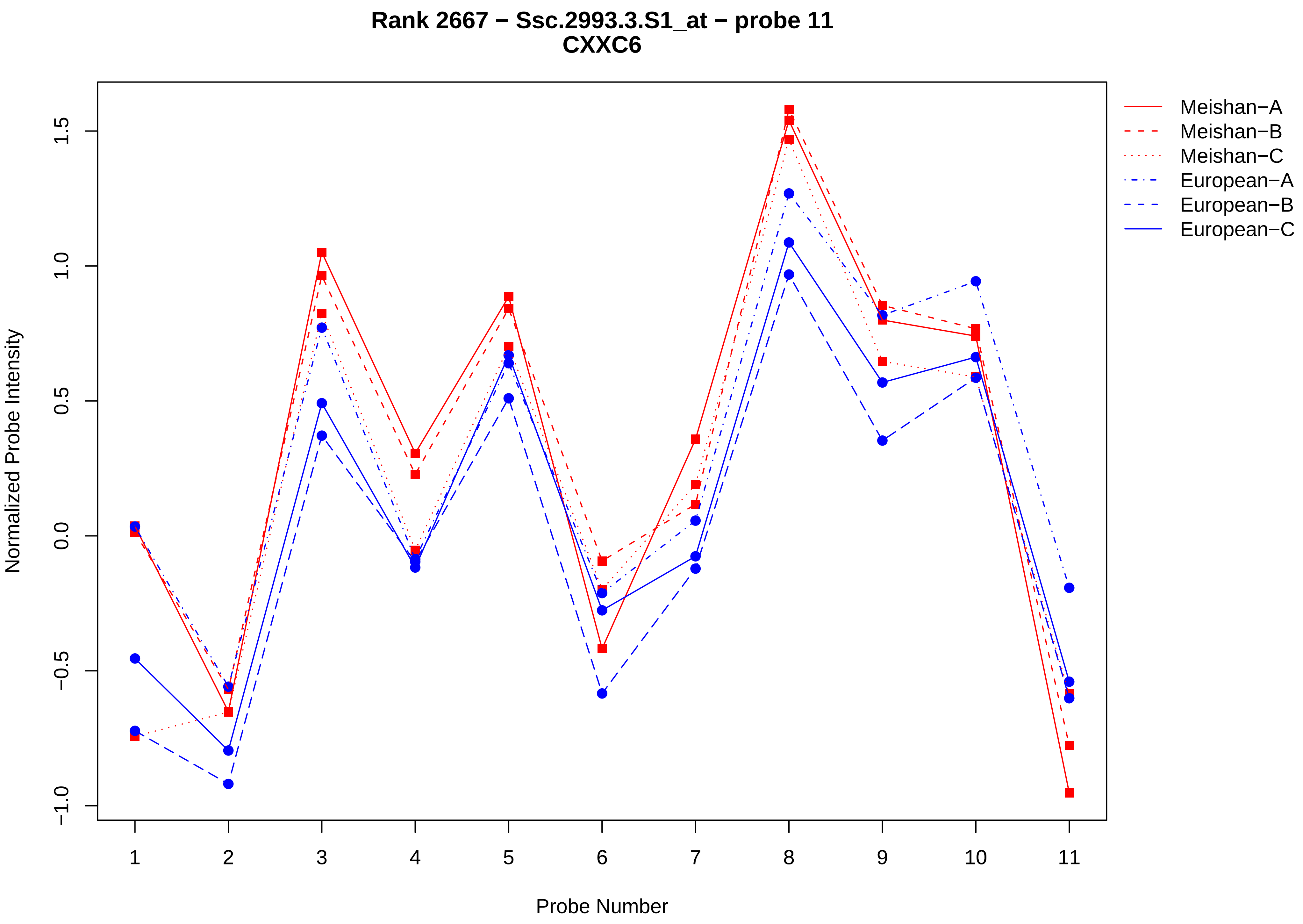

Rank 2667 − Ssc.2993.3.S1_at − probe 11
CXXC6
Normalized Probe Intensity
Probe Number
−1.0
−0.5
0.0
0.5
1.0
1.5
1
2
3
4
5
6
7
8
9
10
11
Meishan−A
Meishan−B
Meishan−C
European−A
European−B
European−C

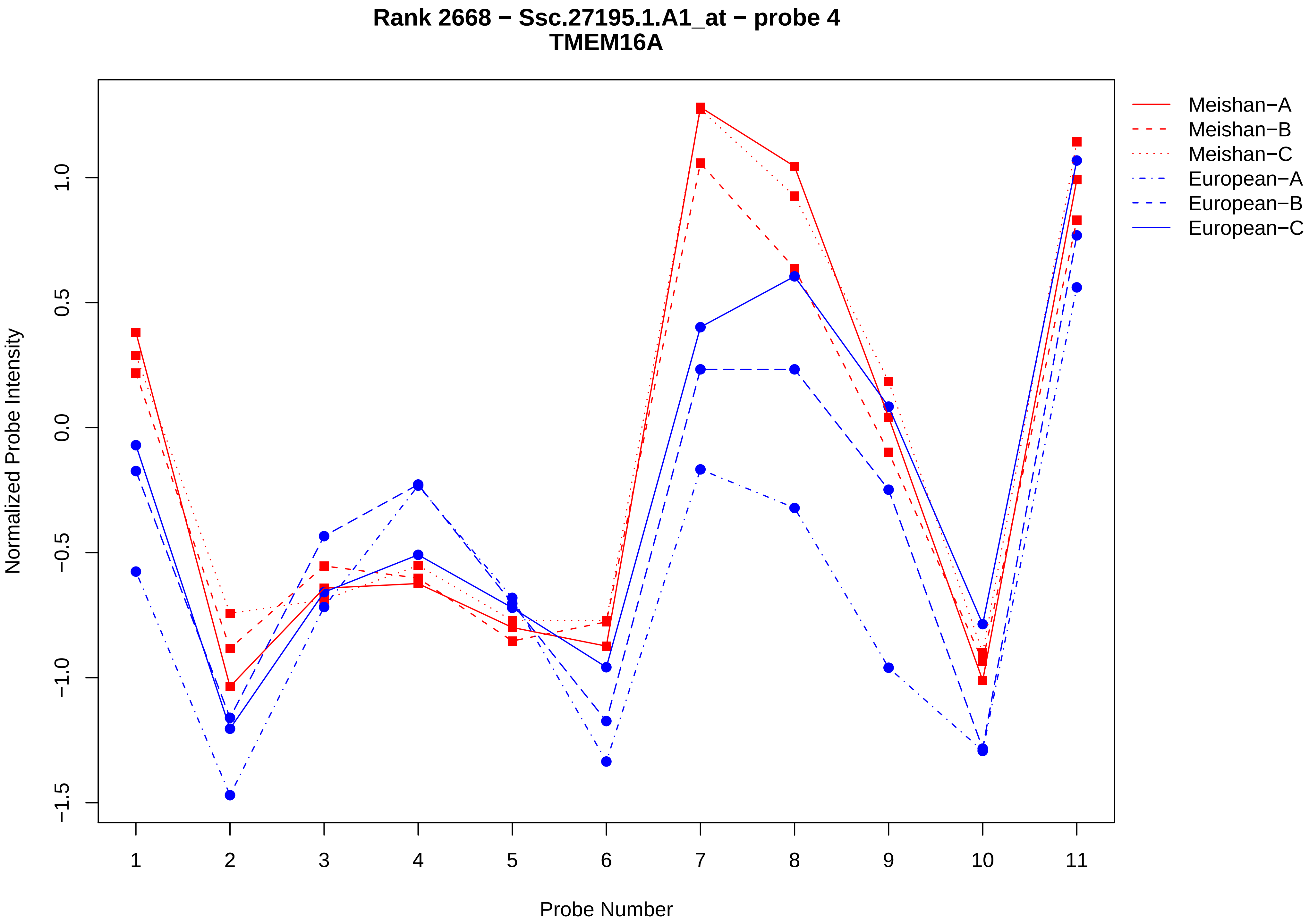

Rank 2668 − Ssc.27195.1.A1_at − probe 4
TMEM16A
Normalized Probe Intensity
Probe Number
Meishan−A
Meishan−B
Meishan−C
European−A
European−B
European−C
−1.5
−1.0
−0.5
0.0
0.5
1.0
1
2
3
4
5
6
7
8
9
10
11

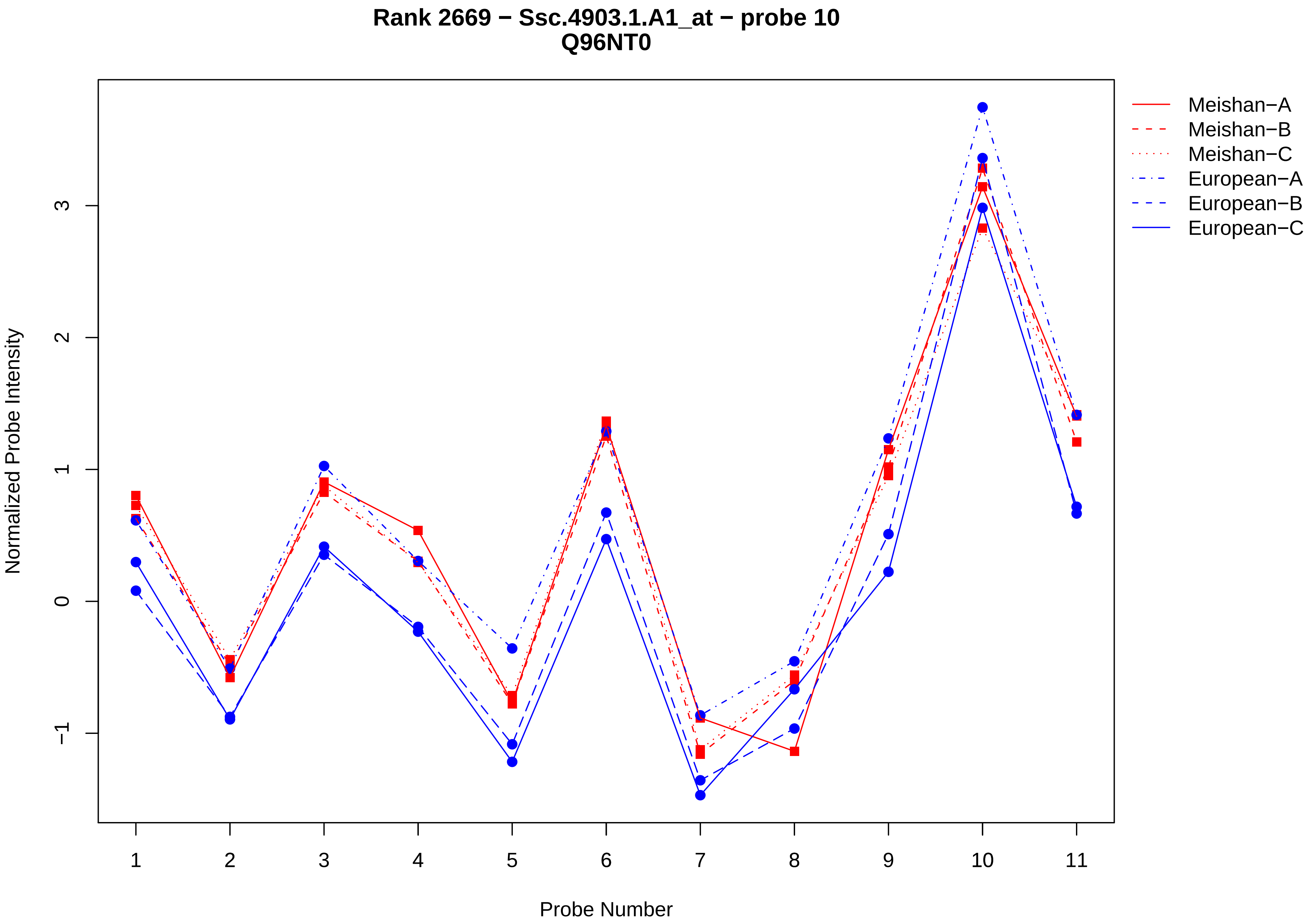

Rank 2669 − Ssc.4903.1.A1_at − probe 10
Q96NT0
Normalized Probe Intensity
Probe Number
Meishan−A
Meishan−B
Meishan−C
European−A
European−B
European−C
−1
0
1
2
3
1
2
3
4
5
6
7
8
9
10
11

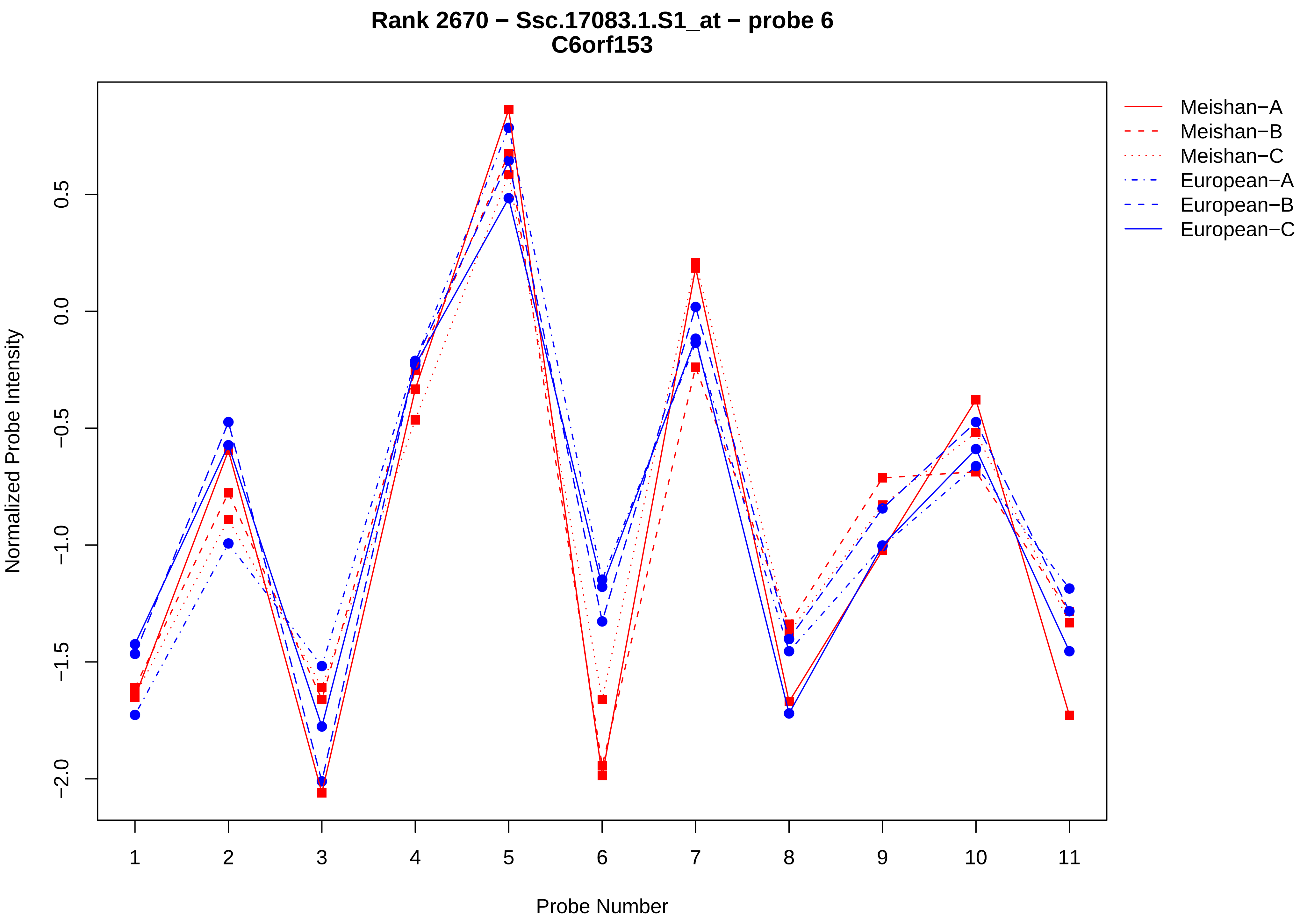

Rank 2670 − Ssc.17083.1.S1_at − probe 6
C6orf153
Normalized Probe Intensity
Probe Number
Meishan−A
Meishan−B
Meishan−C
European−A
European−B
European−C
0.5
0.0
−0.5
−1.0
−1.5
−2.0
1
2
3
4
5
6
7
8
9
10
11

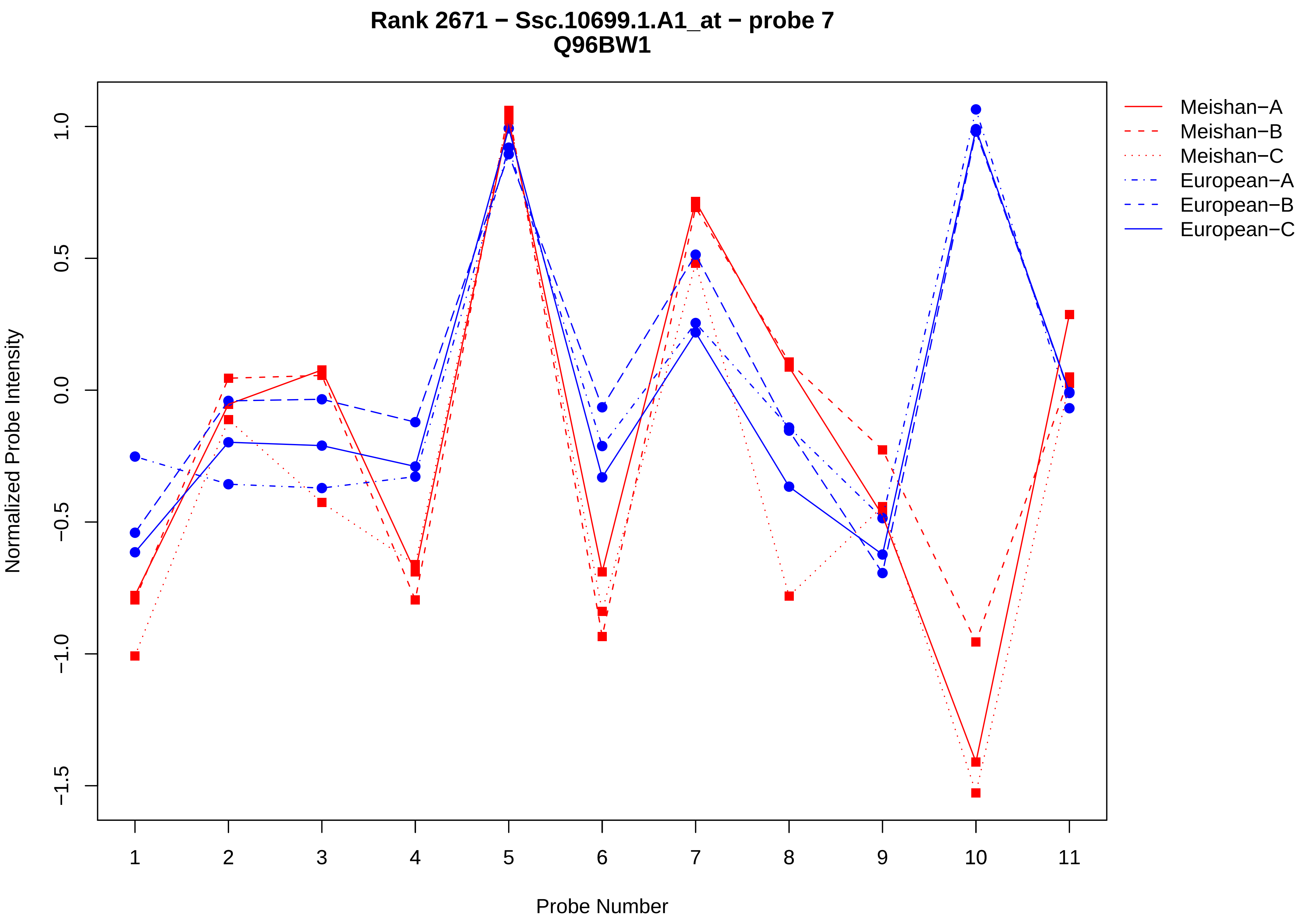
Rank 2671 − Ssc.10699.1.A1_at − probe 7
Q96BW1
Normalized Probe Intensity
Probe Number
Meishan−A
Meishan−B
Meishan−C
European−A
European−B
European−C
1.0
0.5
0.0
−0.5
−1.0
−1.5
1
2
3
4
5
6
7
8
9
10
11

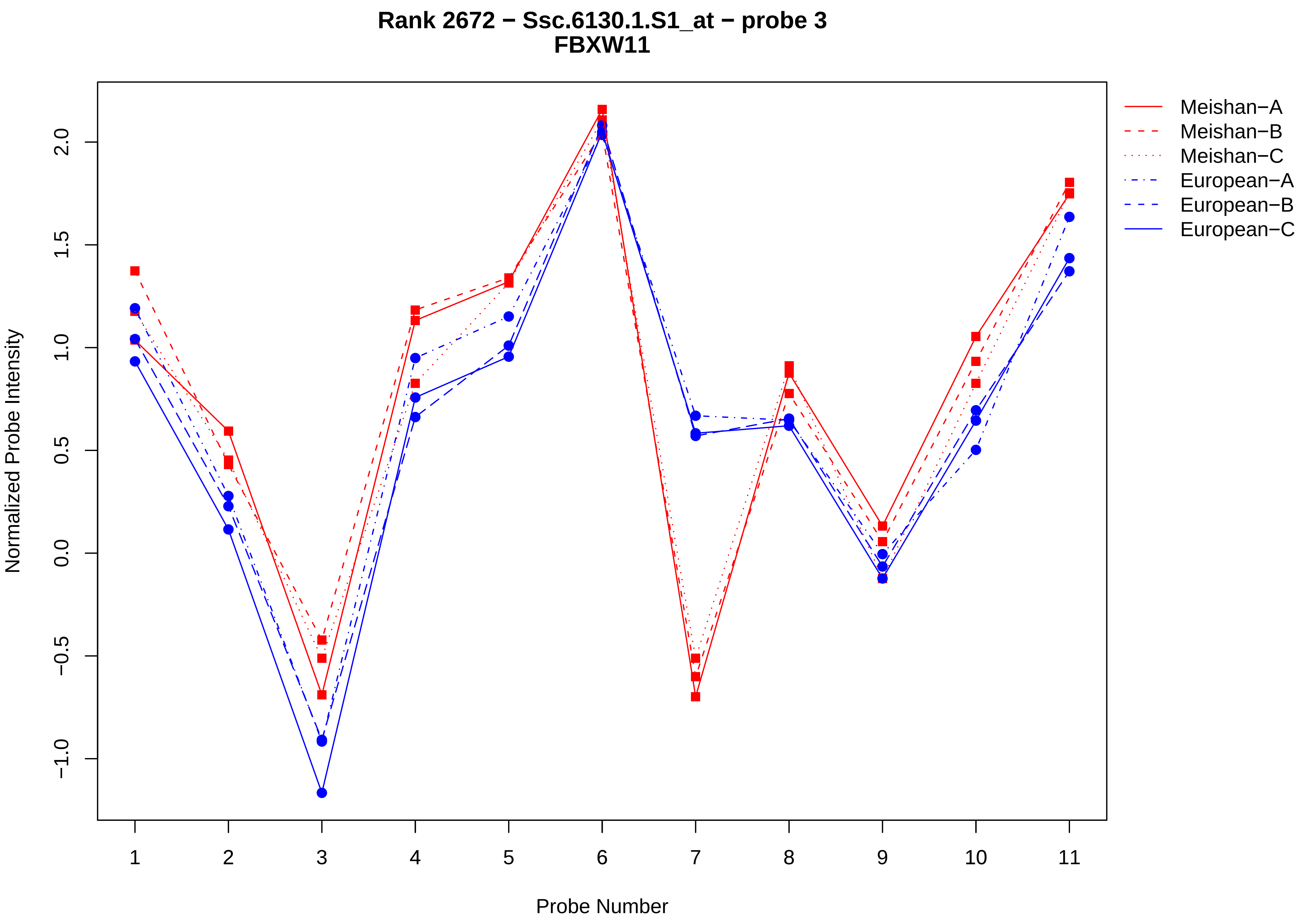

Rank 2672 − Ssc.6130.1.S1_at − probe 3
FBXW11
Normalized Probe Intensity
Probe Number
Meishan−A
Meishan−B
Meishan−C
European−A
European−B
European−C

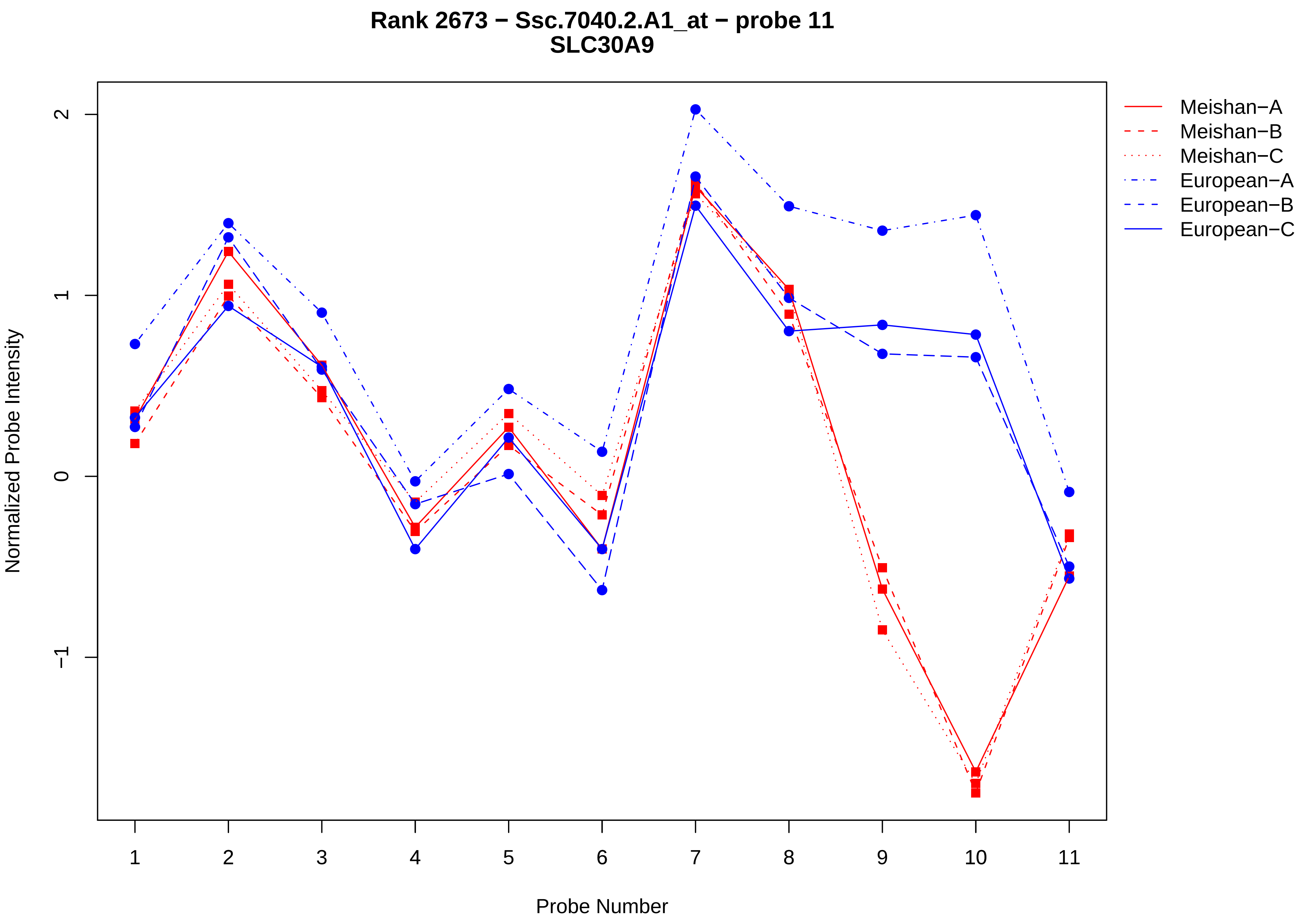

Rank 2673 − Ssc.7040.2.A1_at − probe 11
SLC30A9
Normalized Probe Intensity
Probe Number
−1
0
1
2
1
2
3
4
5
6
7
8
9
10
11
Meishan−A
Meishan−B
Meishan−C
European−A
European−B
European−C

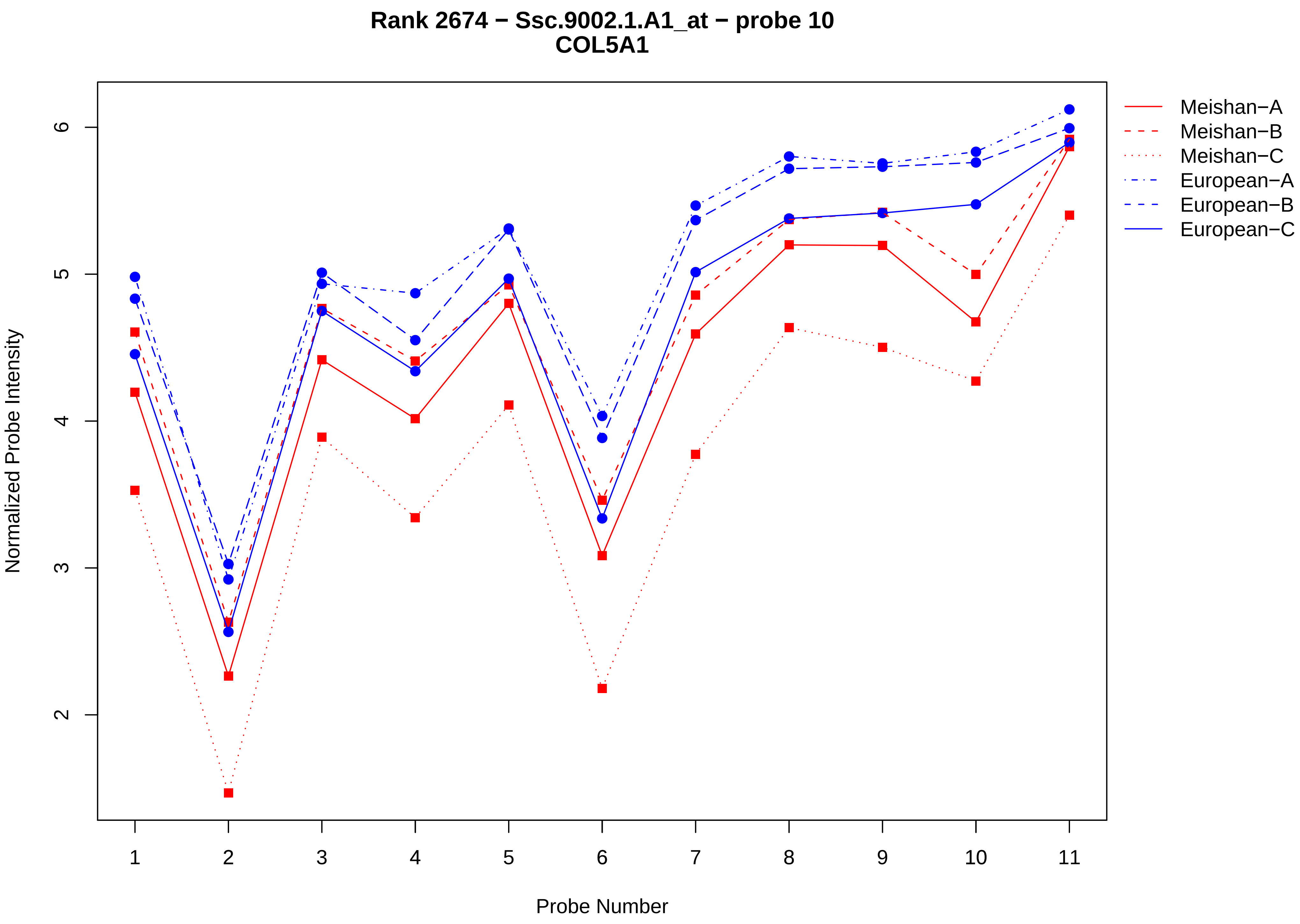

Rank 2674 − Ssc.9002.1.A1_at − probe 10
COL5A1
Normalized Probe Intensity
Probe Number
2
3
4
5
6
1
2
3
4
5
6
7
8
9
10
11
Meishan−A
Meishan−B
Meishan−C
European−A
European−B
European−C

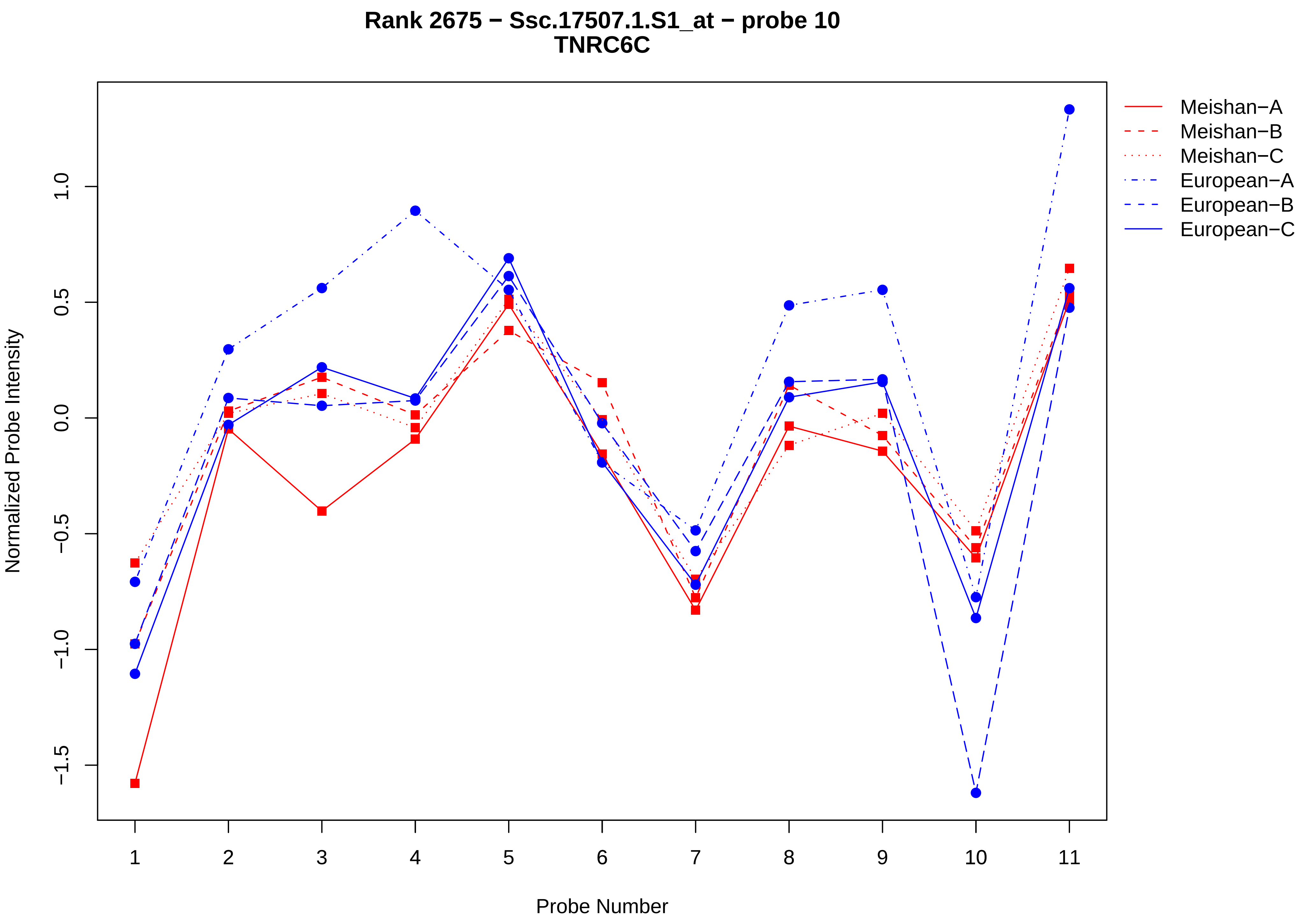

Rank 2675 − Ssc.17507.1.S1_at − probe 10
TNRC6C
Normalized Probe Intensity
Probe Number
Meishan−A
Meishan−B
Meishan−C
European−A
European−B
European−C
1.0
0.5
0.0
−0.5
−1.0
−1.5
1
2
3
4
5
6
7
8
9
10
11

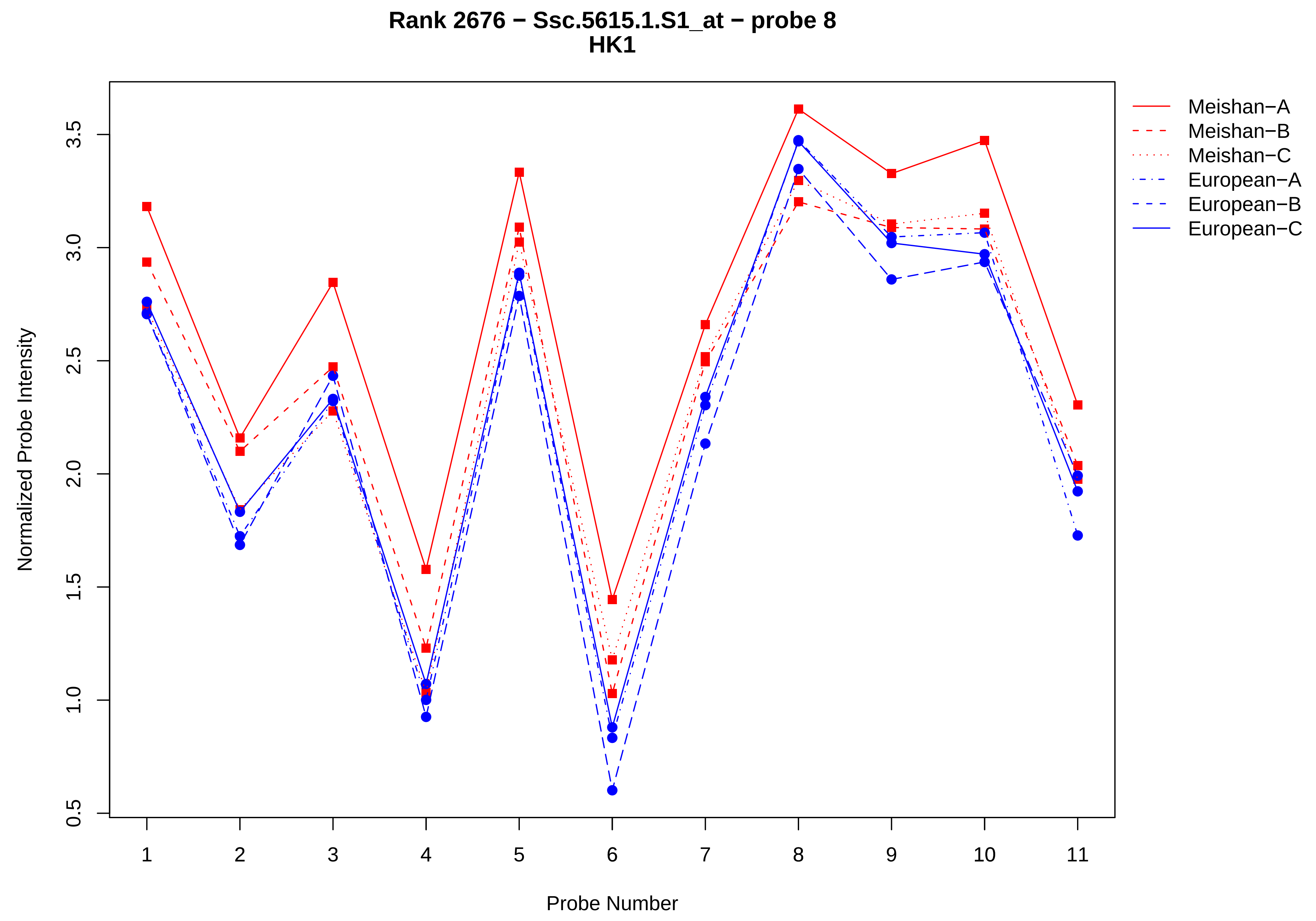

Rank 2676 − Ssc.5615.1.S1_at − probe 8
HK1
Normalized Probe Intensity
Probe Number
0.5
1.0
1.5
2.0
2.5
3.0
3.5
1
2
3
4
5
6
7
8
9
10
11
Meishan−A
Meishan−B
Meishan−C
European−A
European−B
European−C

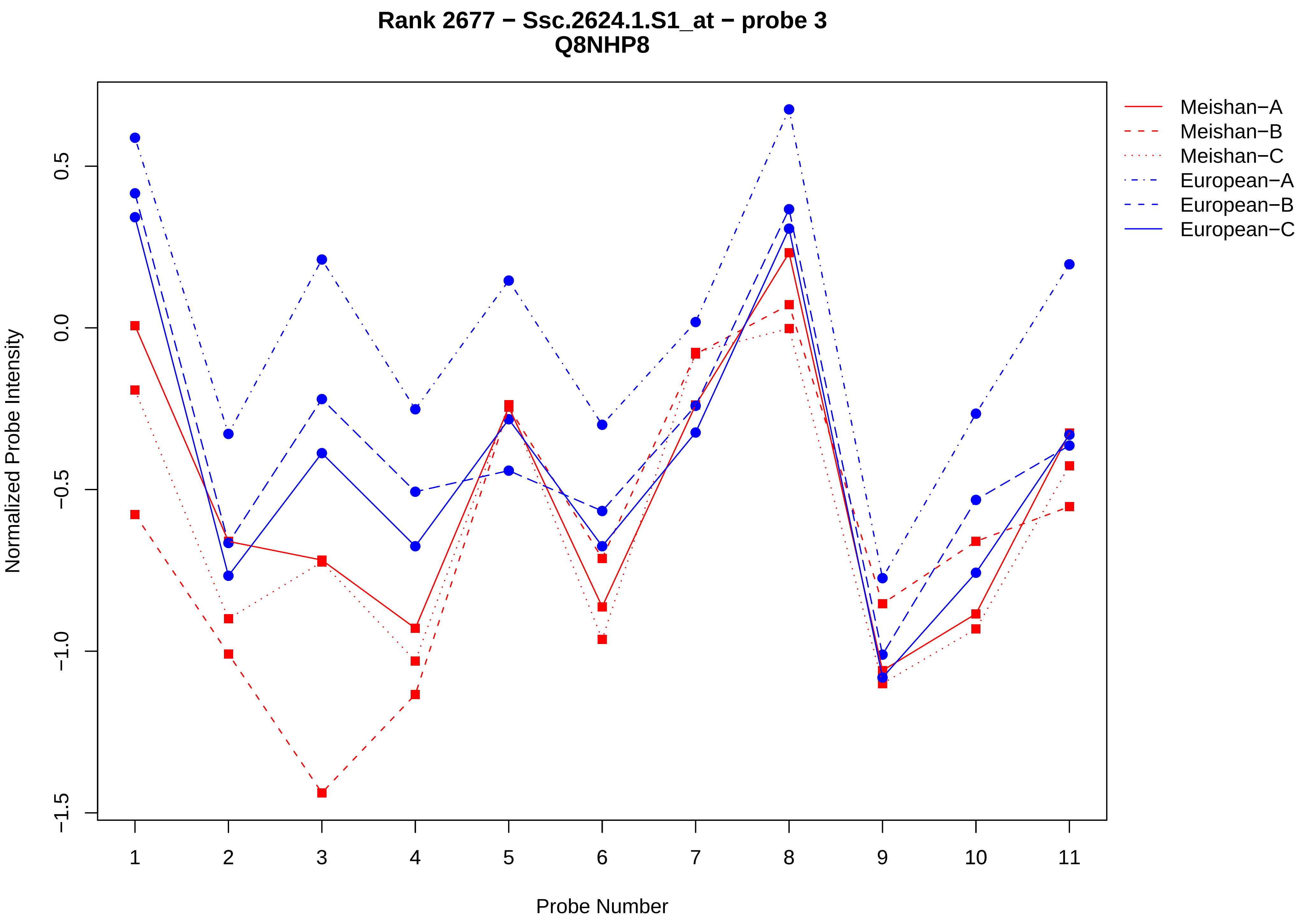

Rank 2677 − Ssc.2624.1.S1_at − probe 3
Q8NHP8
Normalized Probe Intensity
Probe Number
0.5
0.0
−0.5
−1.0
−1.5
1
2
3
4
5
6
7
8
9
10
11
Meishan−A
Meishan−B
Meishan−C
European−A
European−B
European−C

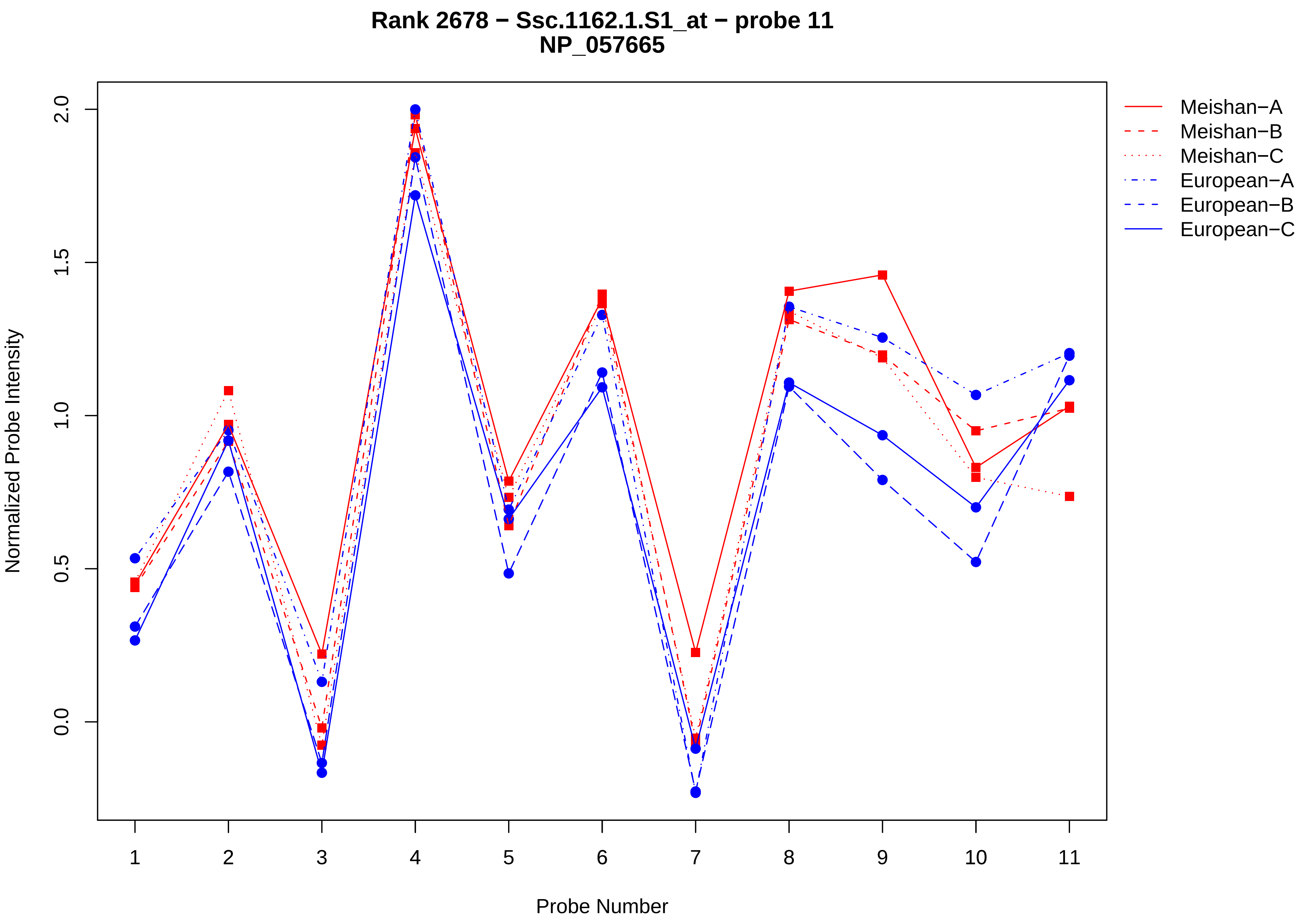

Rank 2678 − Ssc.1162.1.S1_at − probe 11
NP_057665
Normalized Probe Intensity
Probe Number
0.0
0.5
1.0
1.5
2.0
1
2
3
4
5
6
7
8
9
10
11
Meishan−A
Meishan−B
Meishan−C
European−A
European−B
European−C

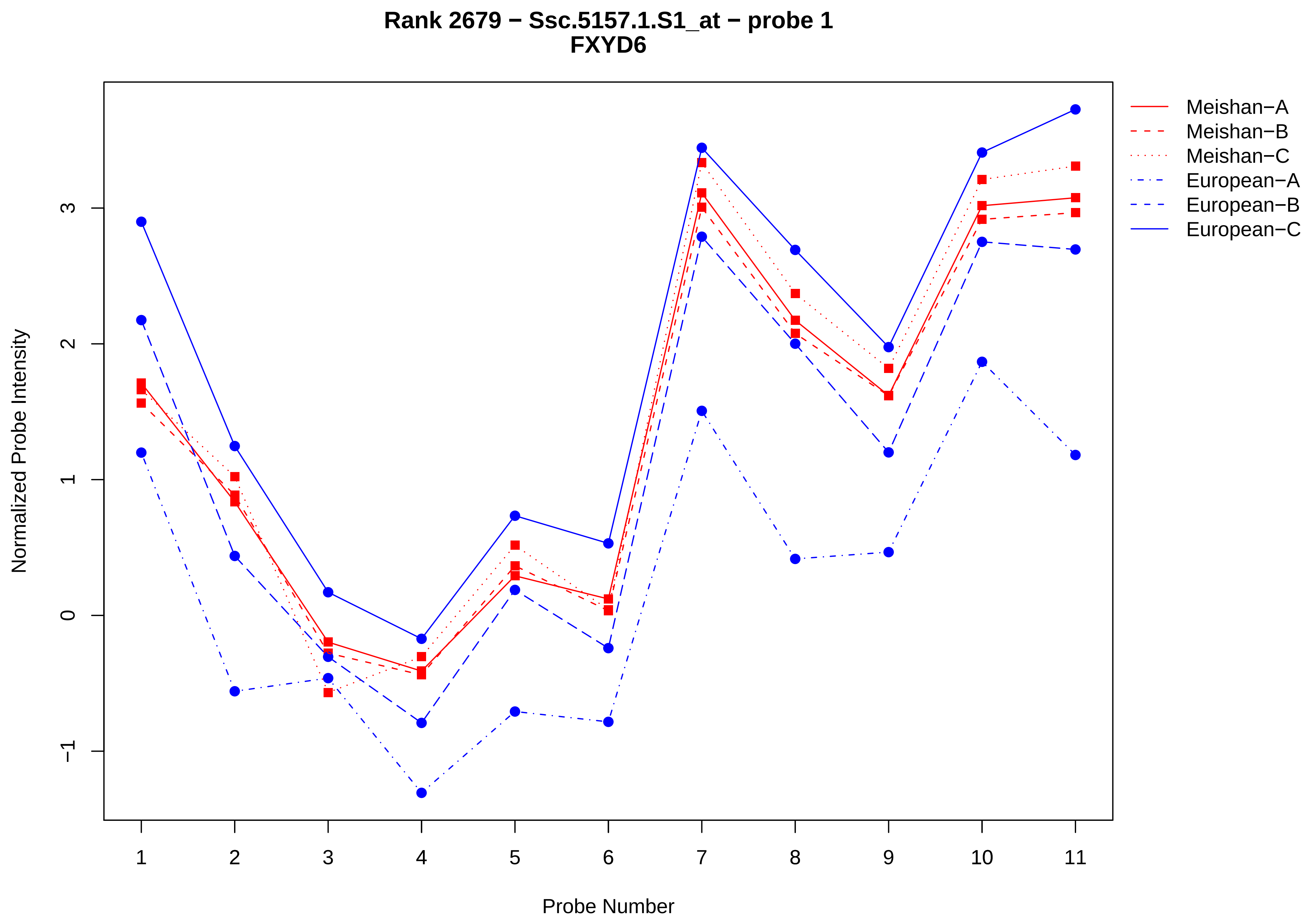

Rank 2679 − Ssc.5157.1.S1_at − probe 1
FXYD6
Normalized Probe Intensity
Probe Number
−1
0
1
2
3
1
2
3
4
5
6
7
8
9
10
11
Meishan−A
Meishan−B
Meishan−C
European−A
European−B
European−C

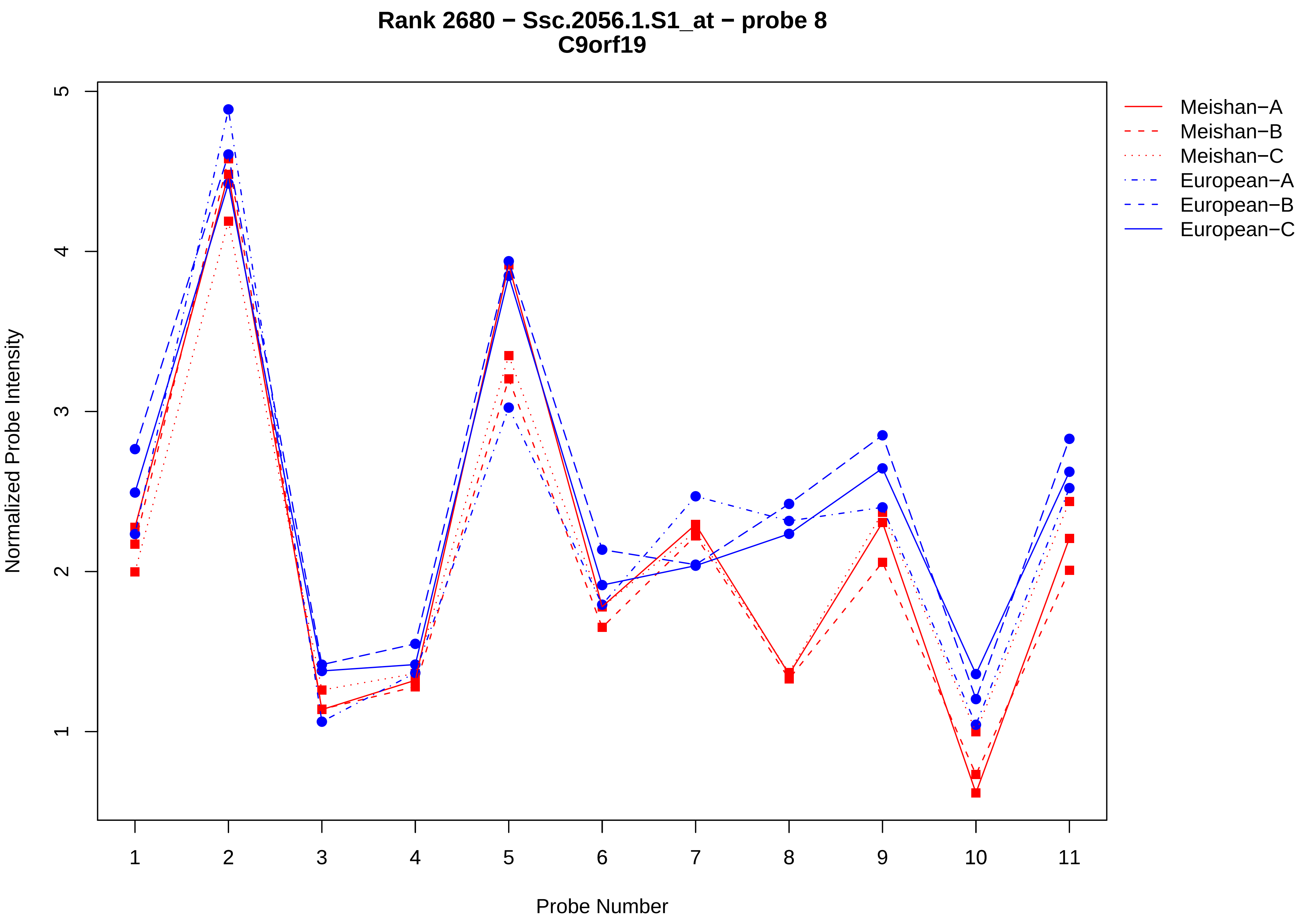
Rank 2680 − Ssc.2056.1.S1_at − probe 8
C9orf19
Normalized Probe Intensity
Probe Number
1
2
3
4
5
1
2
3
4
5
6
7
8
9
10
11
Meishan−A
Meishan−B
Meishan−C
European−A
European−B
European−C

Rank 2681 − Ssc.7229.1.S1_at − probe 3
SH3BGRL

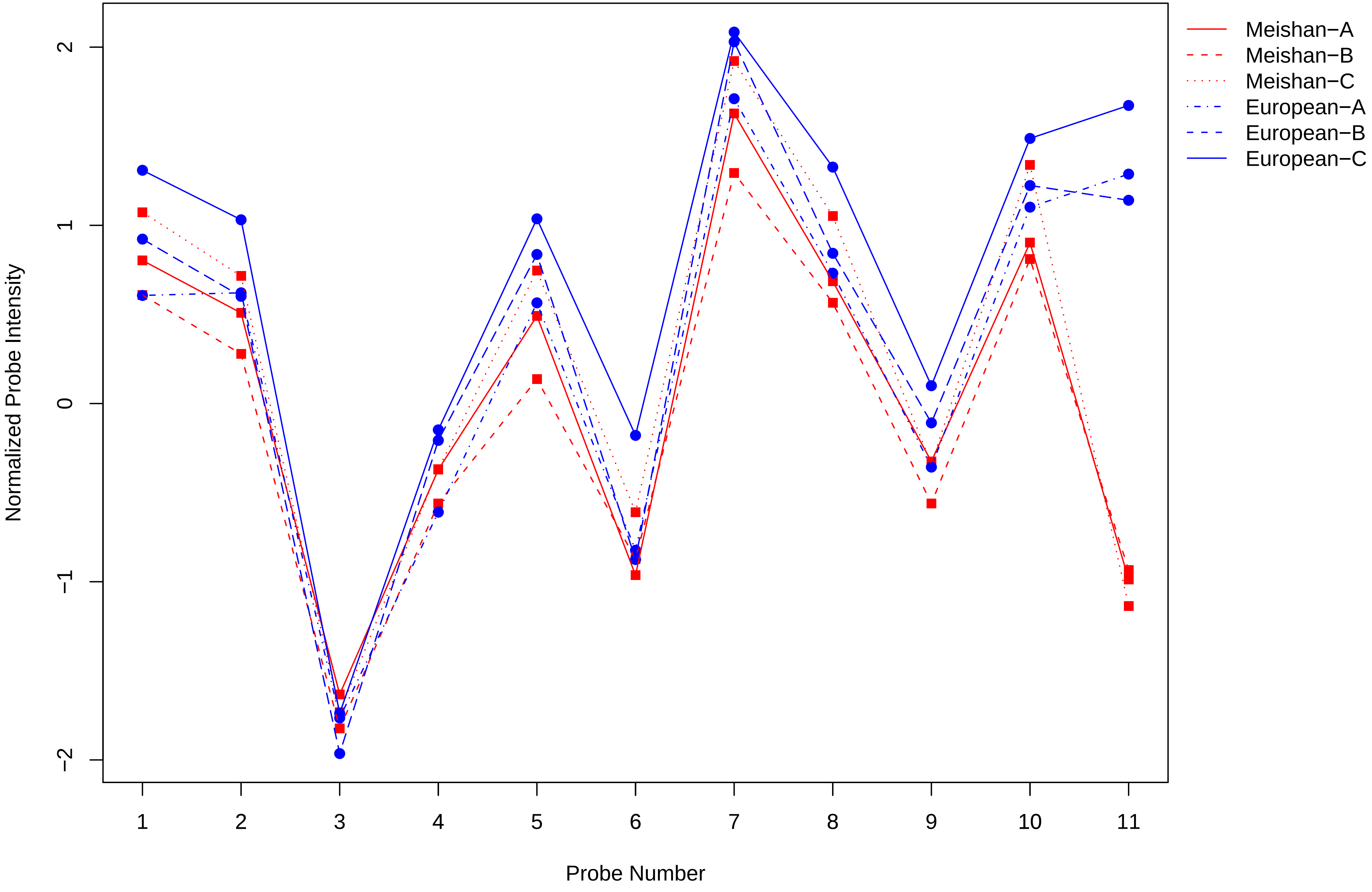

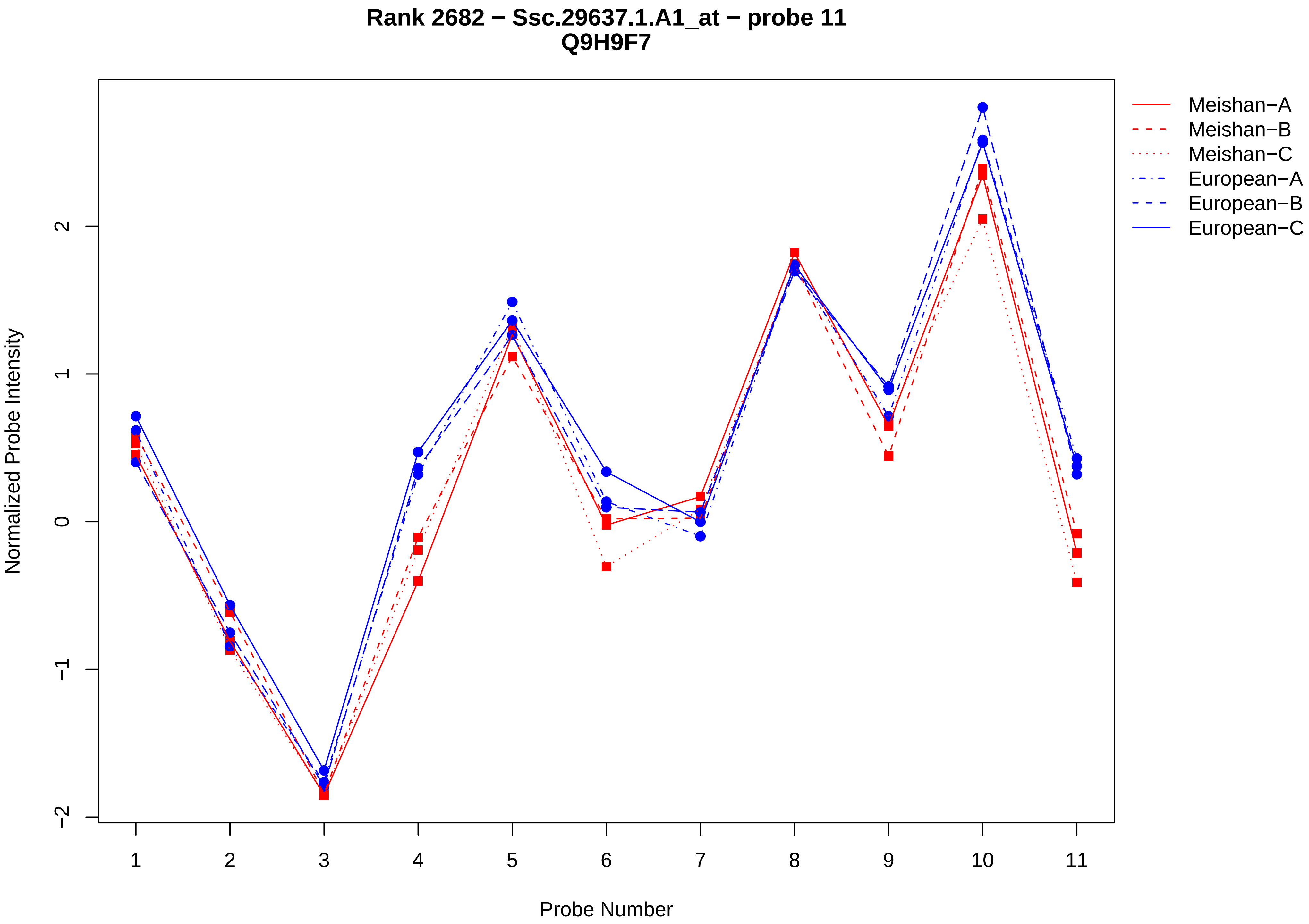
Rank 2682 − Ssc.29637.1.A1_at − probe 11
Q9H9F7
Normalized Probe Intensity
Probe Number
−2
−1
0
1
2
1
2
3
4
5
6
7
8
9
10
11
Meishan−A
Meishan−B
Meishan−C
European−A
European−B
European−C

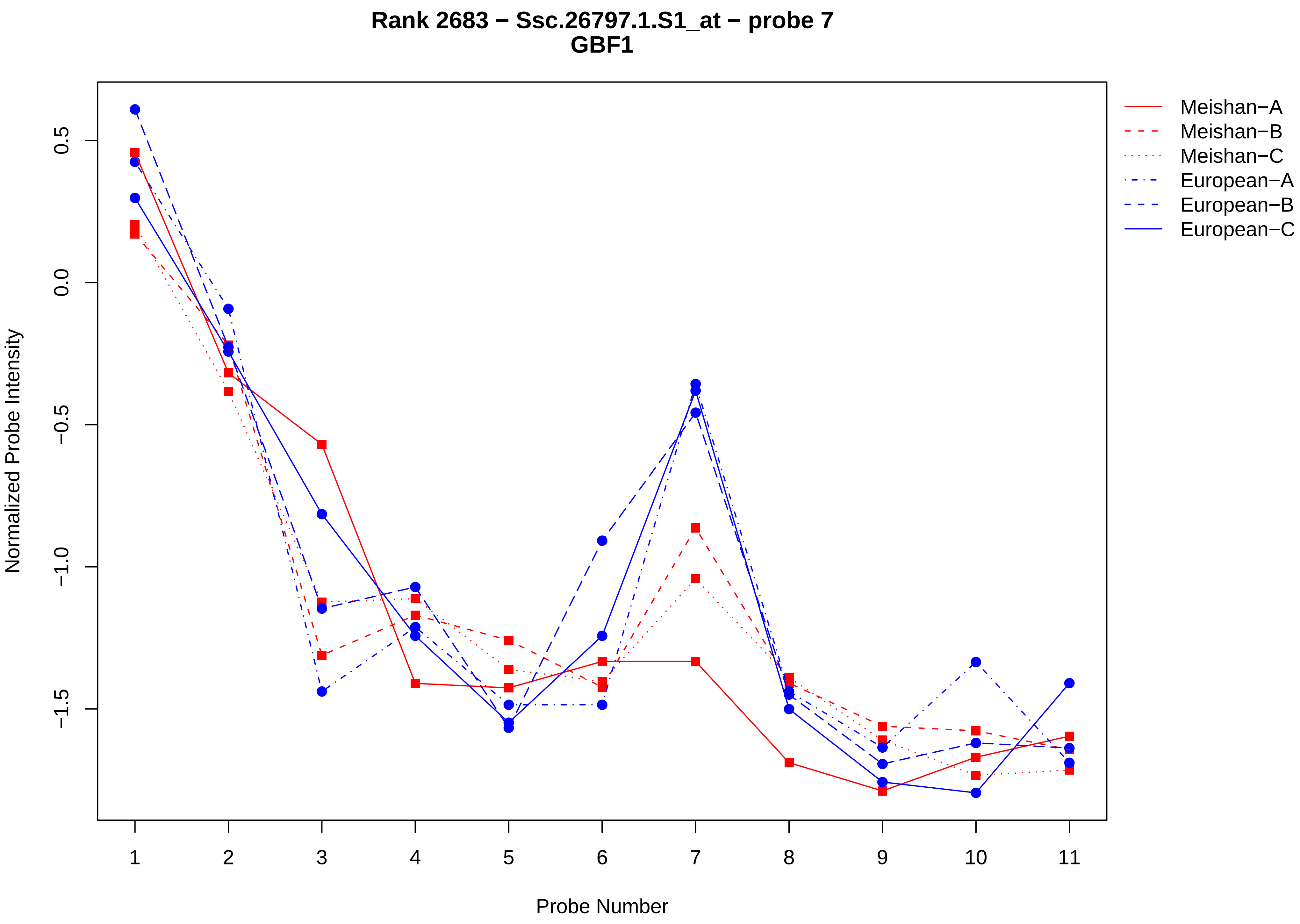

Rank 2683 − Ssc.26797.1.S1_at − probe 7
GBF1
Normalized Probe Intensity
Probe Number
0.5
0.0
−0.5
−1.0
−1.5
1
2
3
4
5
6
7
8
9
10
11
Meishan−A
Meishan−B
Meishan−C
European−A
European−B
European−C

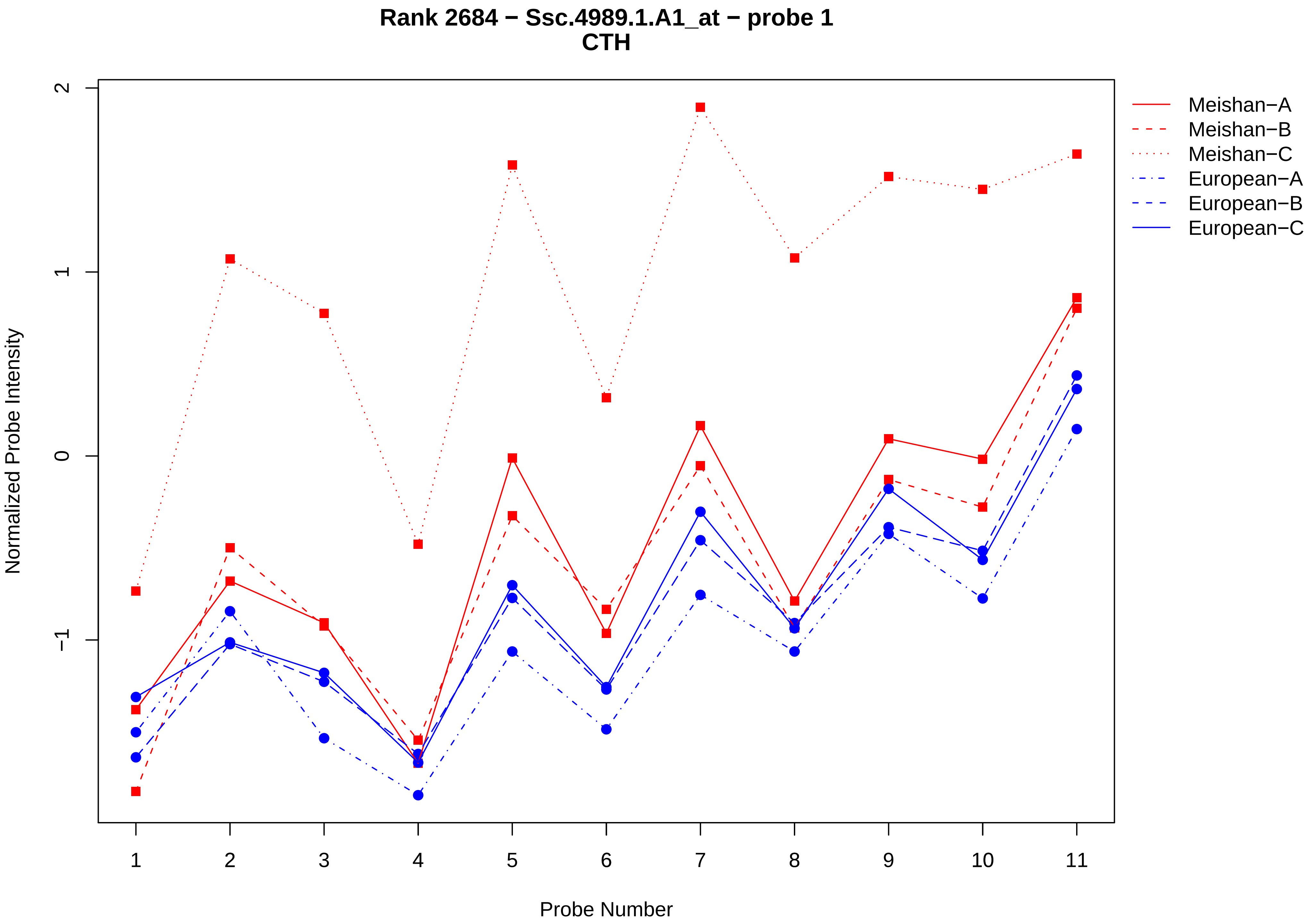

Rank 2684 − Ssc.4989.1.A1_at − probe 1
CTH
Normalized Probe Intensity
Probe Number
2
1
0
−1
1
2
3
4
5
6
7
8
9
10
11
Meishan−A
Meishan−B
Meishan−C
European−A
European−B
European−C

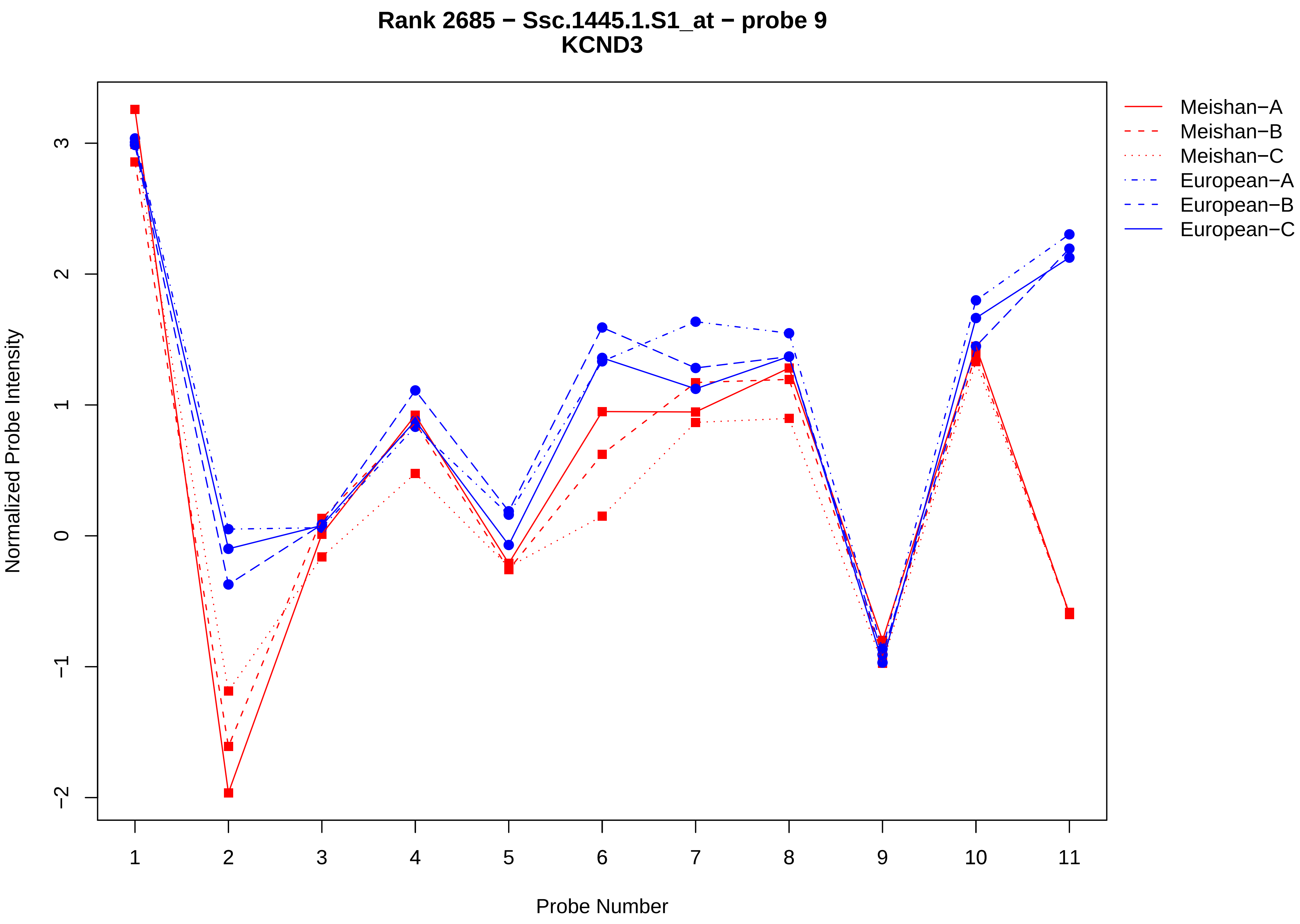

Rank 2685 − Ssc.1445.1.S1_at − probe 9
KCND3
Normalized Probe Intensity
Probe Number
Meishan−A
Meishan−B
Meishan−C
European−A
European−B
European−C

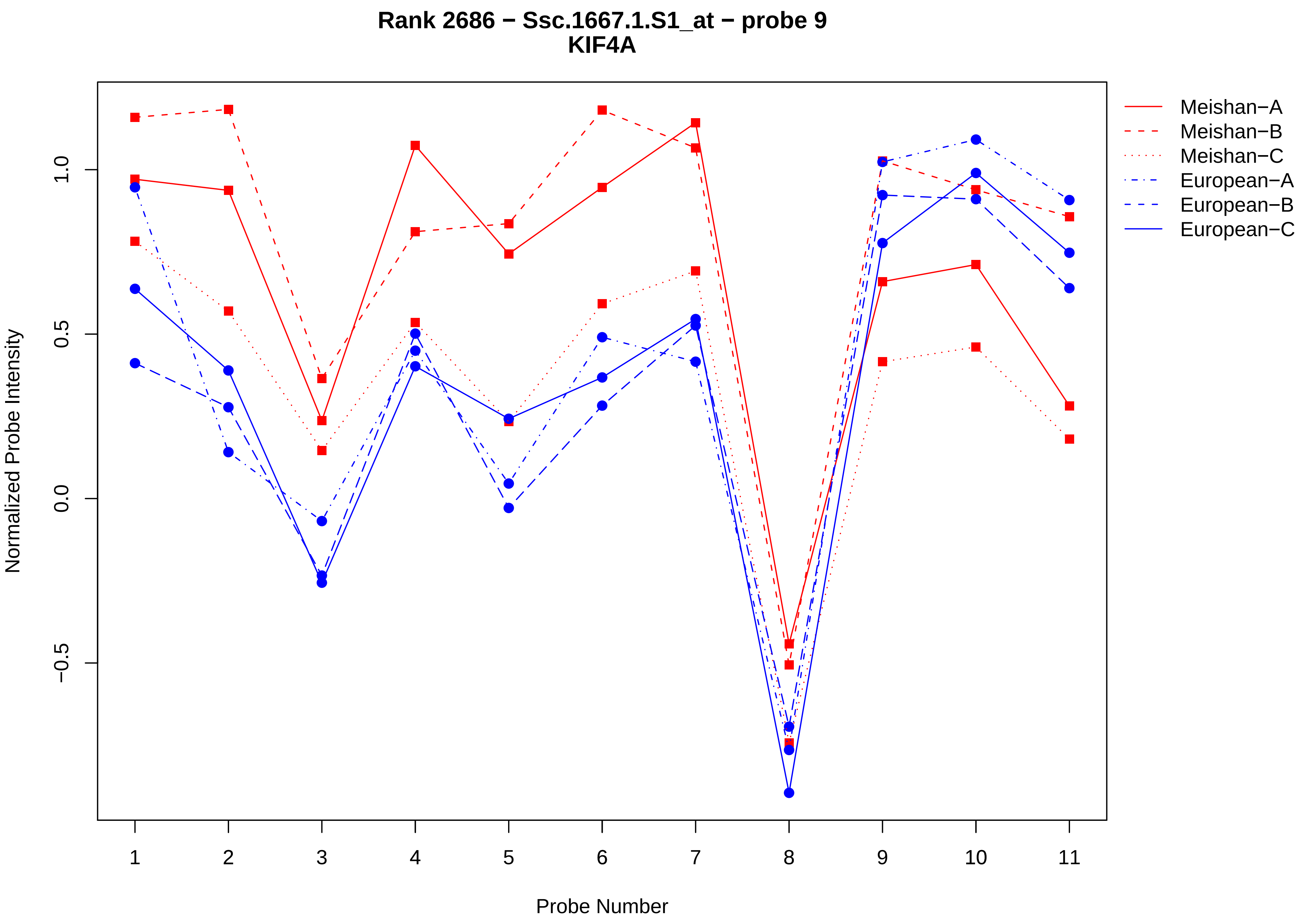

Rank 2686 − Ssc.1667.1.S1_at − probe 9
KIF4A
Normalized Probe Intensity
Probe Number
−0.5
0.0
0.5
1.0
1
2
3
4
5
6
7
8
9
10
11
Meishan−A
Meishan−B
Meishan−C
European−A
European−B
European−C

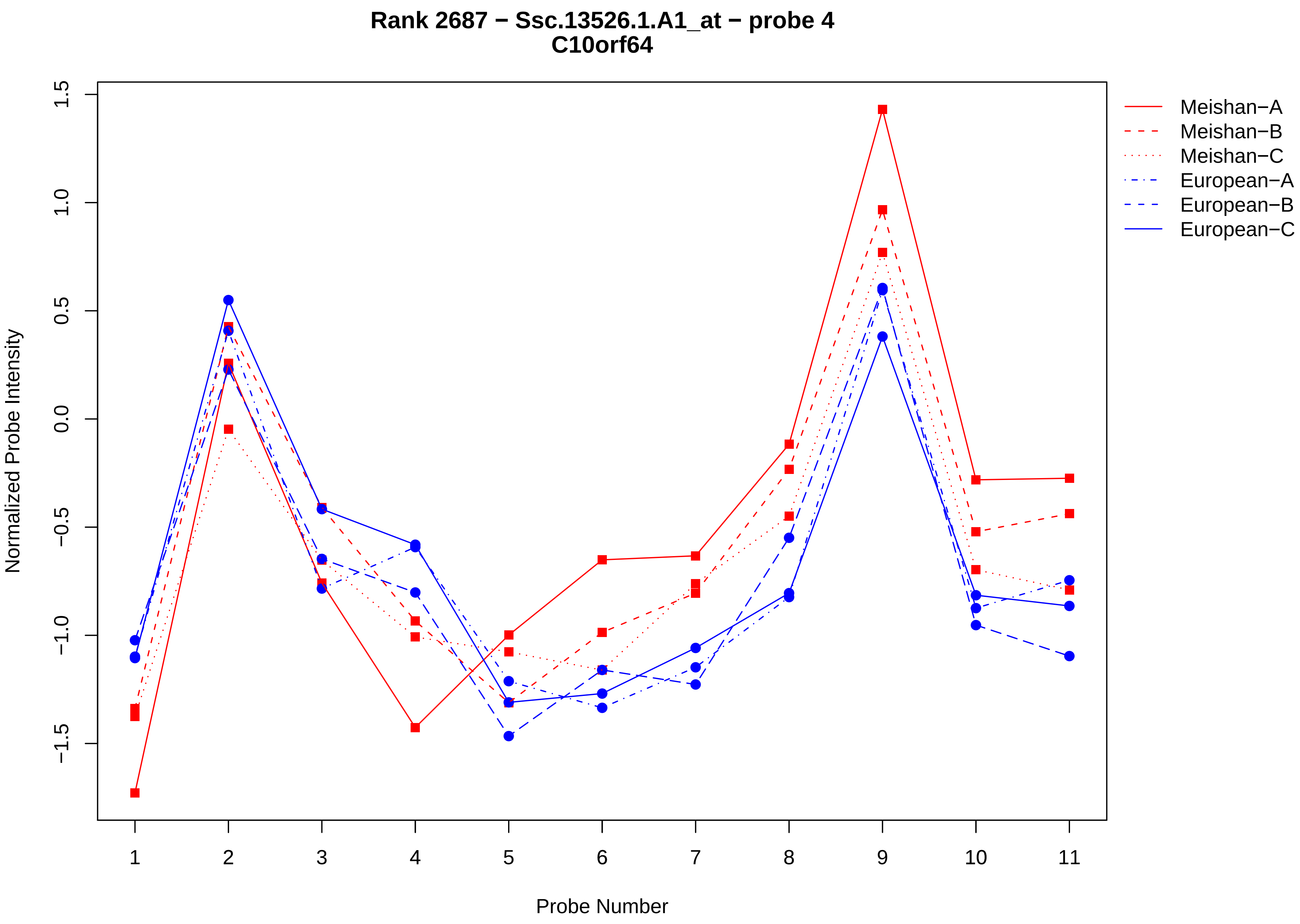

Rank 2687 − Ssc.13526.1.A1_at − probe 4
C10orf64
Normalized Probe Intensity
Probe Number
Meishan−A
Meishan−B
Meishan−C
European−A
European−B
European−C

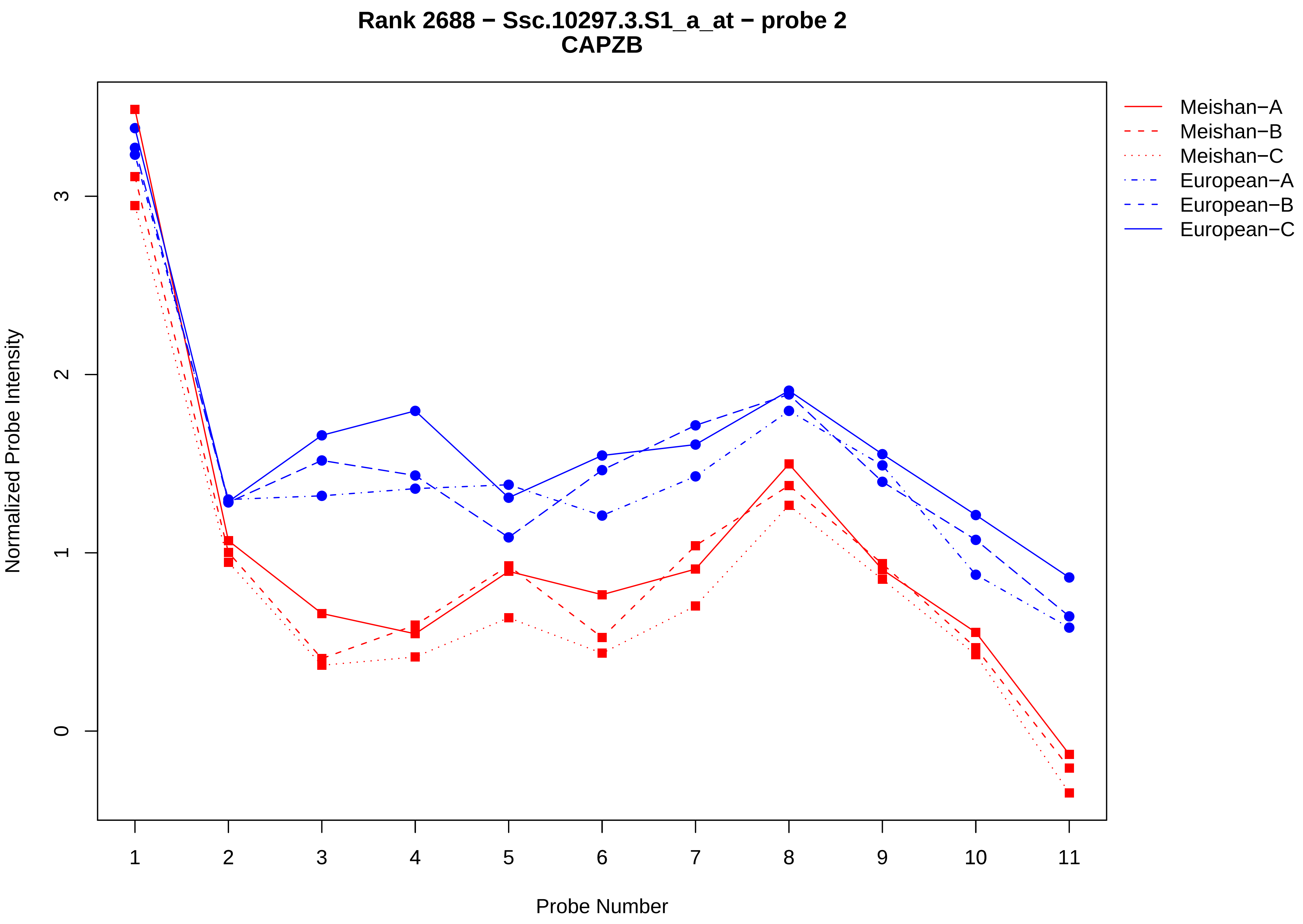

Rank 2688 − Ssc.10297.3.S1_a_at − probe 2
CAPZB
Normalized Probe Intensity
Probe Number
0
1
2
3
1
2
3
4
5
6
7
8
9
10
11
Meishan−A
Meishan−B
Meishan−C
European−A
European−B
European−C

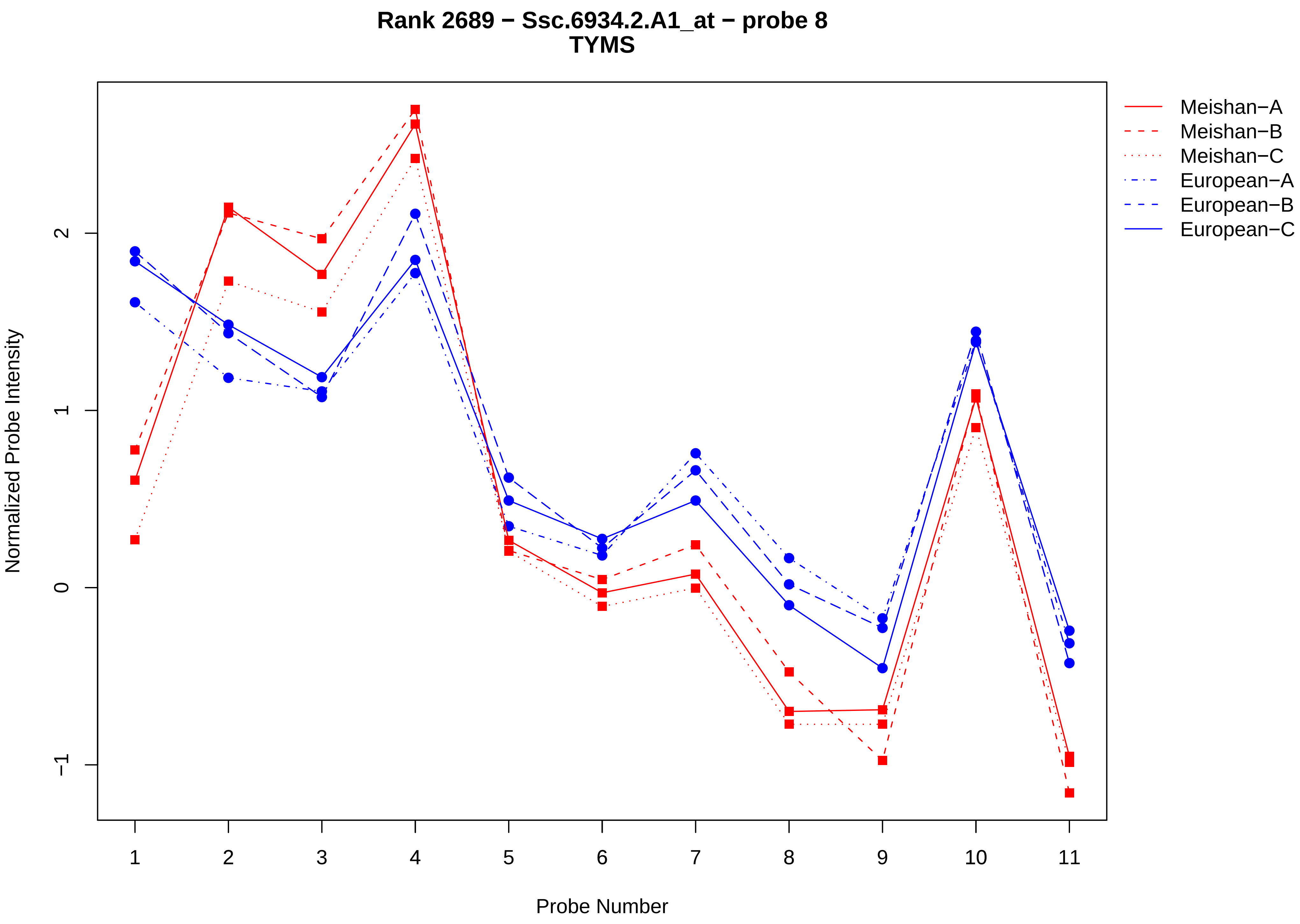

Rank 2689 − Ssc.6934.2.A1_at − probe 8
TYMS
Normalized Probe Intensity
Probe Number
−1
0
1
2
1
2
3
4
5
6
7
8
9
10
11
Meishan−A
Meishan−B
Meishan−C
European−A
European−B
European−C

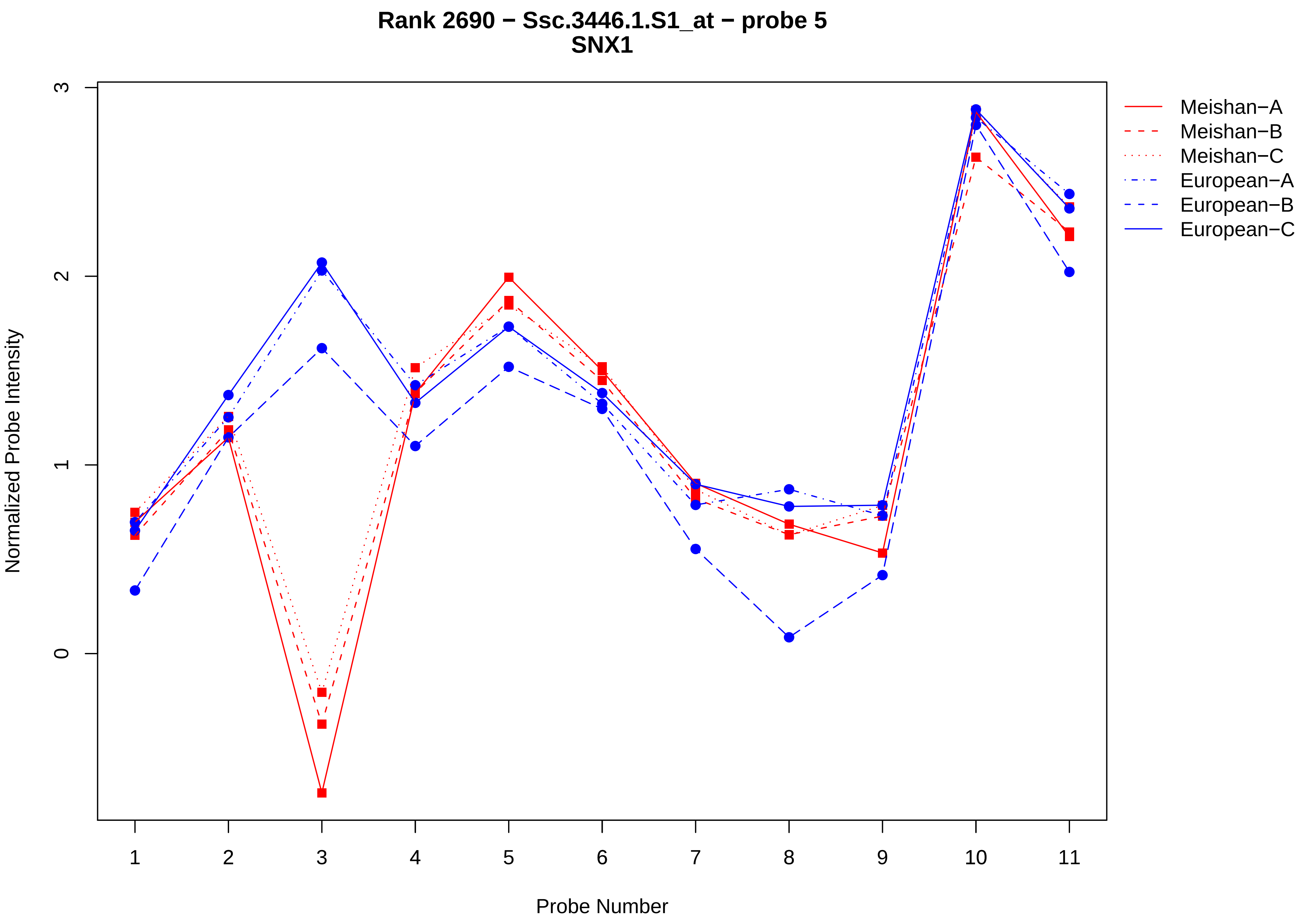

Rank 2690 − Ssc.3446.1.S1_at − probe 5
SNX1
Normalized Probe Intensity
Probe Number
0
1
2
3
1
2
3
4
5
6
7
8
9
10
11
Meishan−A
Meishan−B
Meishan−C
European−A
European−B
European−C

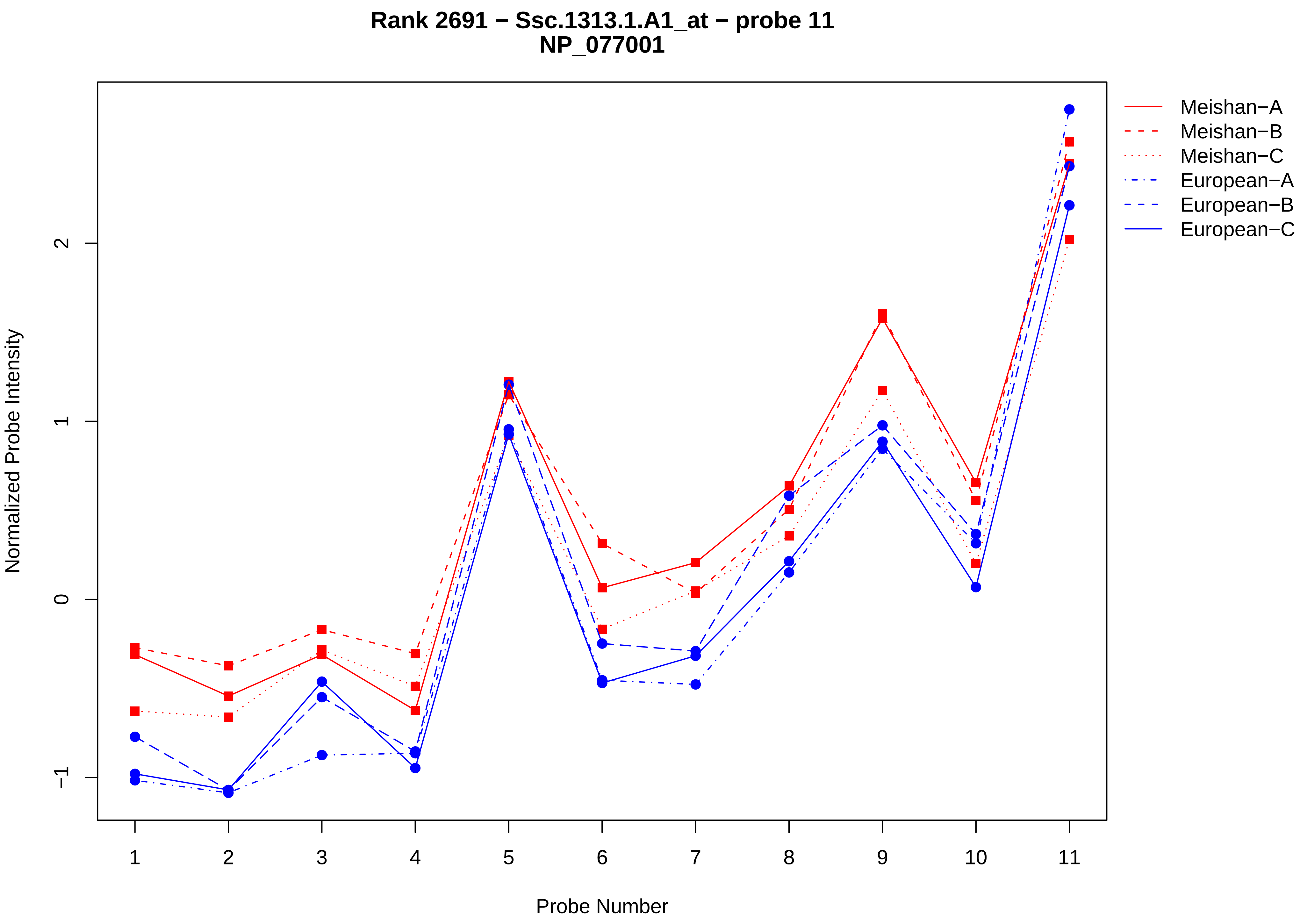

Rank 2691 − Ssc.1313.1.A1_at − probe 11
NP_077001
Normalized Probe Intensity
Probe Number
−1
0
1
2
1
2
3
4
5
6
7
8
9
10
11
Meishan−A
Meishan−B
Meishan−C
European−A
European−B
European−C

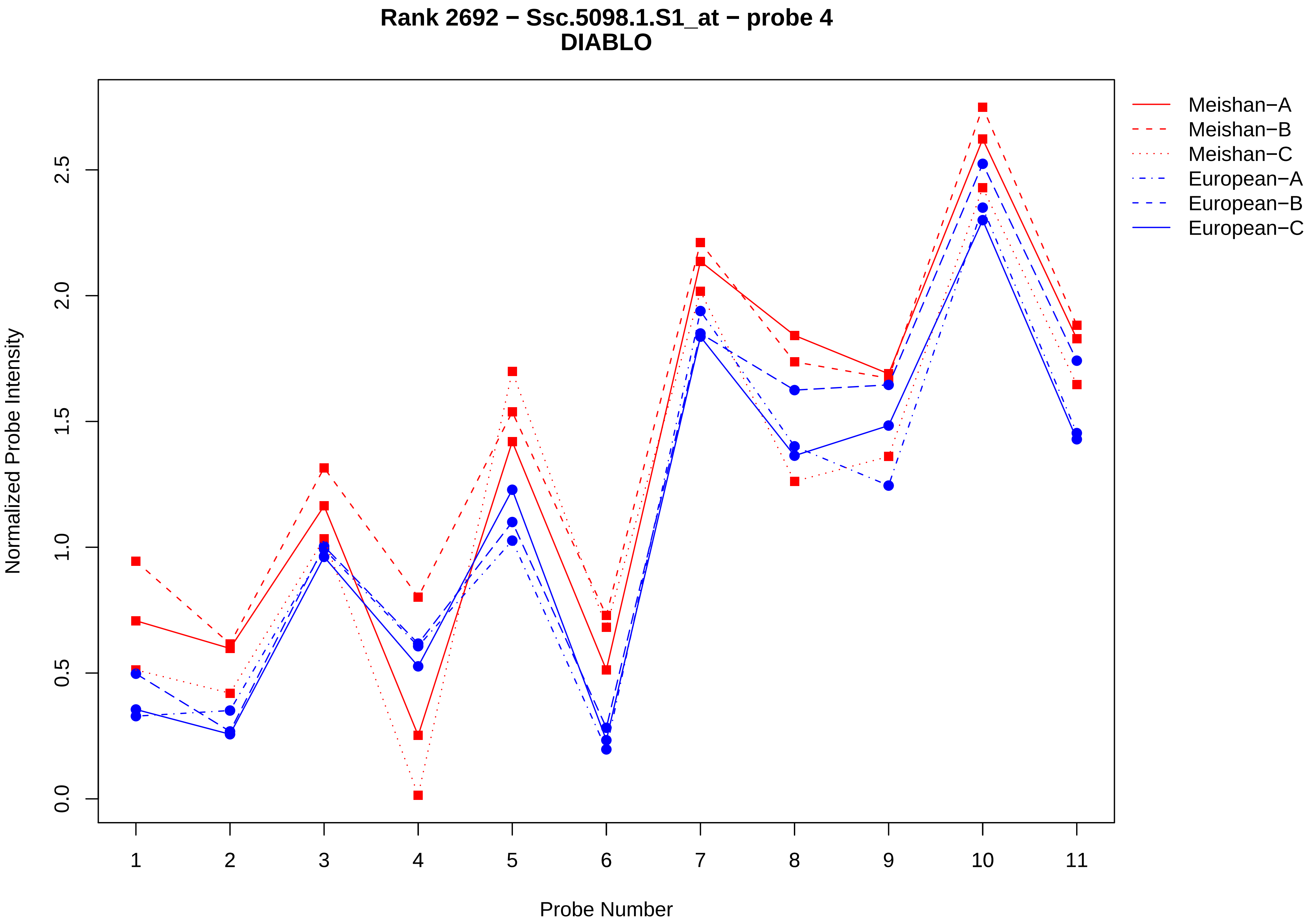

Rank 2692 − Ssc.5098.1.S1_at − probe 4
DIABLO
Normalized Probe Intensity
Probe Number
0.0
0.5
1.0
1.5
2.0
2.5
1
2
3
4
5
6
7
8
9
10
11
Meishan−A
Meishan−B
Meishan−C
European−A
European−B
European−C

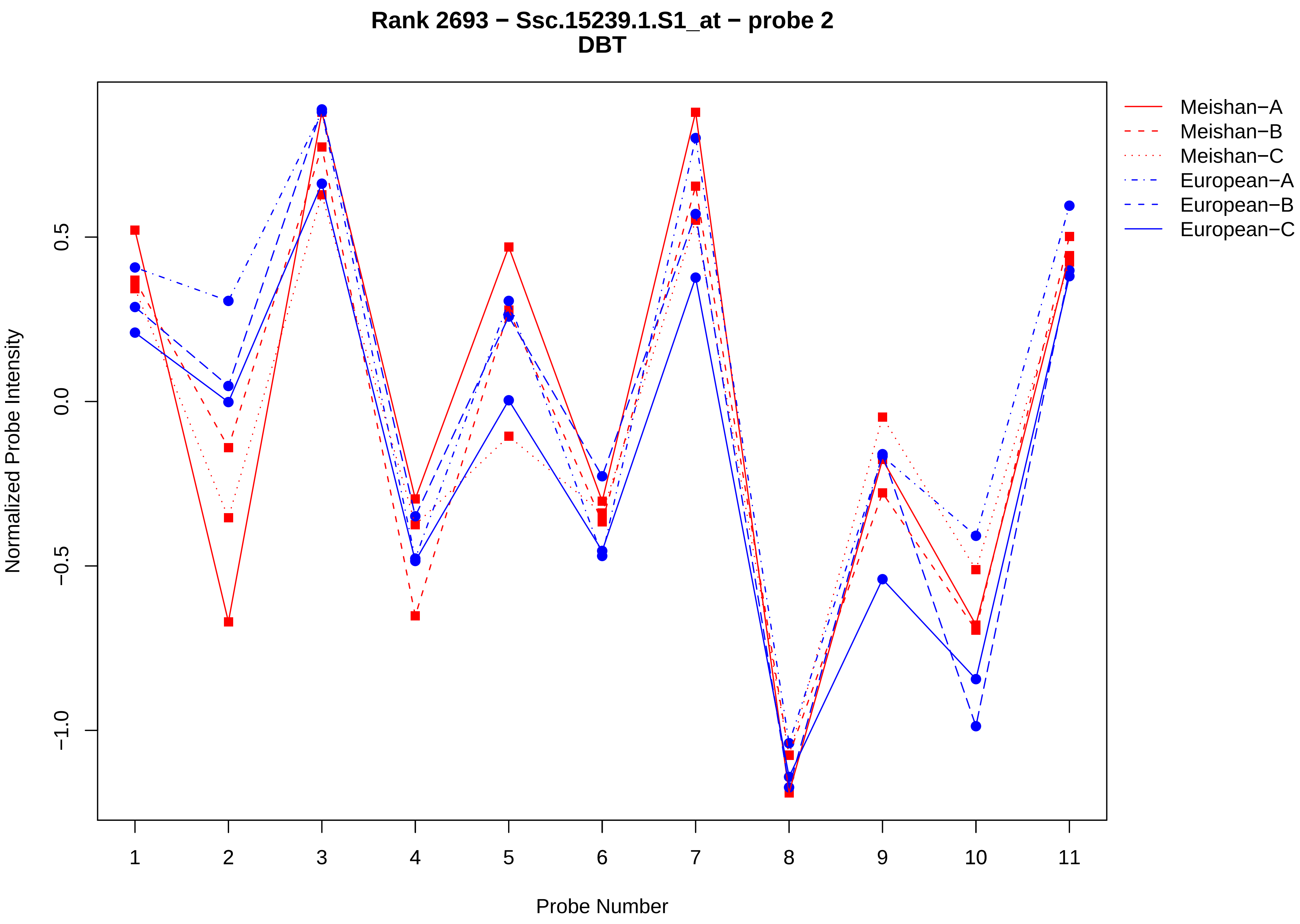

Rank 2693 − Ssc.15239.1.S1_at − probe 2
DBT
Normalized Probe Intensity
Probe Number
0.5
0.0
−0.5
−1.0
1
2
3
4
5
6
7
8
9
10
11
Meishan−A
Meishan−B
Meishan−C
European−A
European−B
European−C

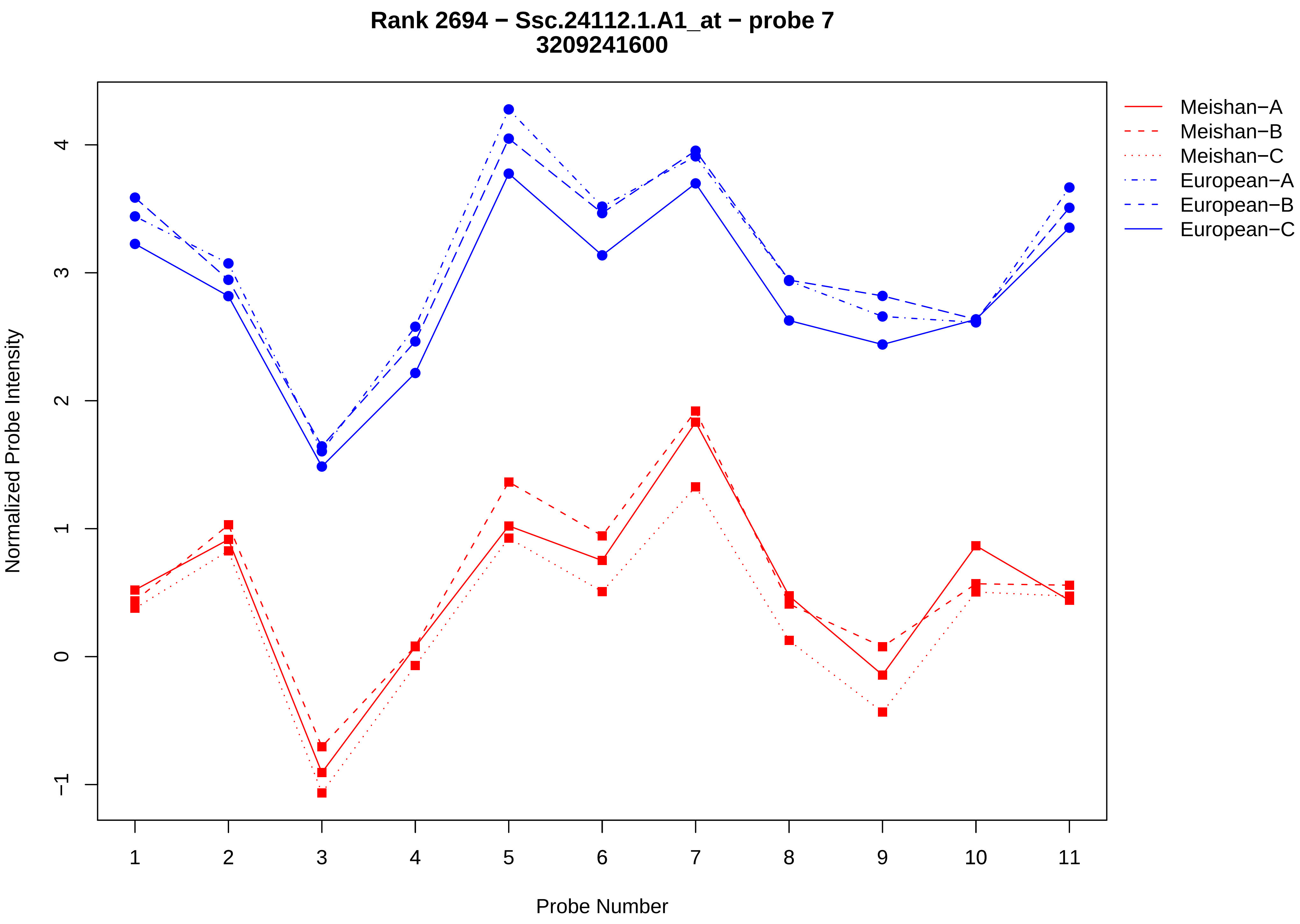

Rank 2694 − Ssc.24112.1.A1_at − probe 7
3209241600
Normalized Probe Intensity
Probe Number
Meishan−A
Meishan−B
Meishan−C
European−A
European−B
European−C
−1
0
1
2
3
4
1
2
3
4
5
6
7
8
9
10
11

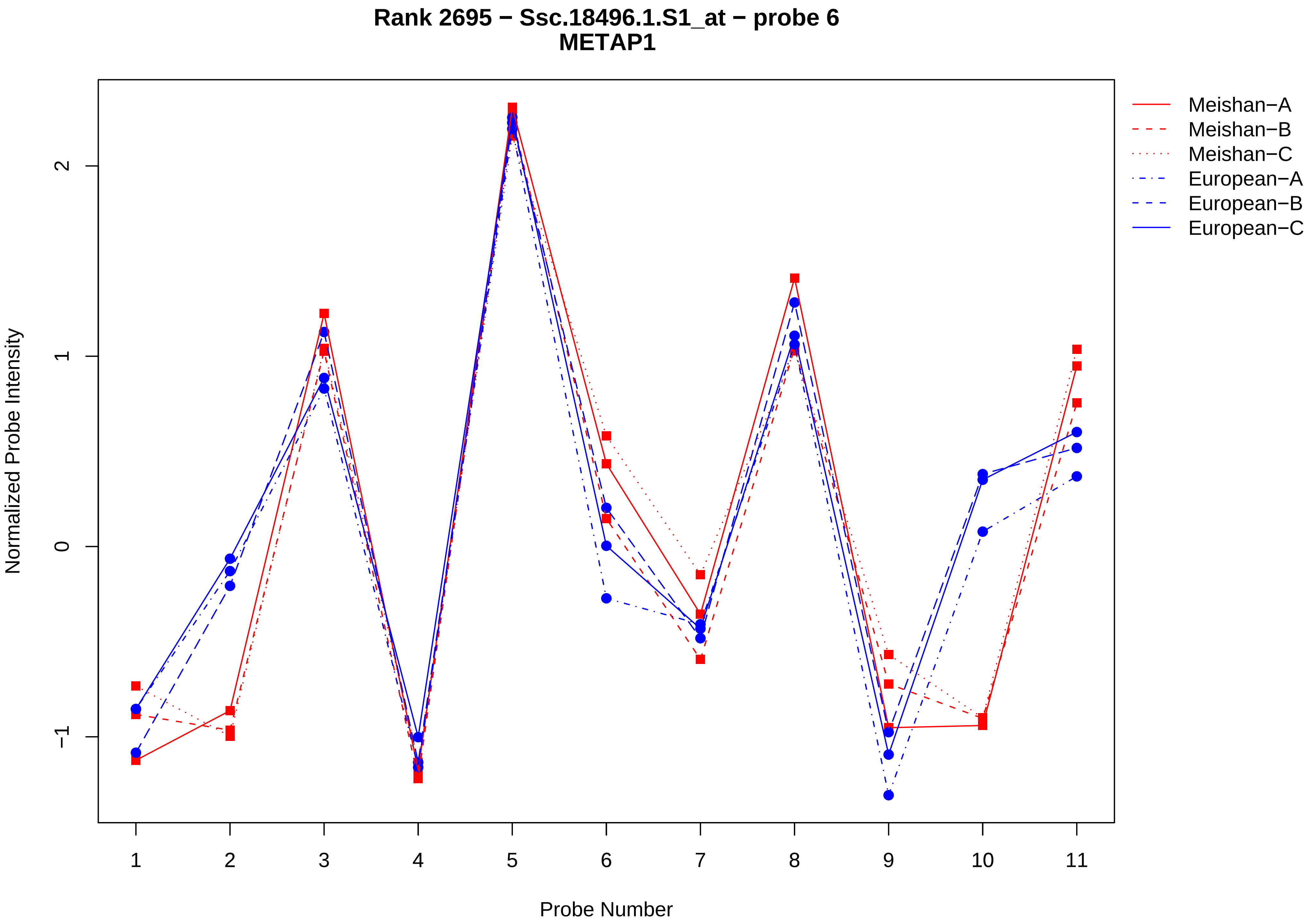
Rank 2695 − Ssc.18496.1.S1_at − probe 6
METAP1
Normalized Probe Intensity
Probe Number
Meishan−A
Meishan−B
Meishan−C
European−A
European−B
European−C
−1
0
1
2
1
2
3
4
5
6
7
8
9
10
11

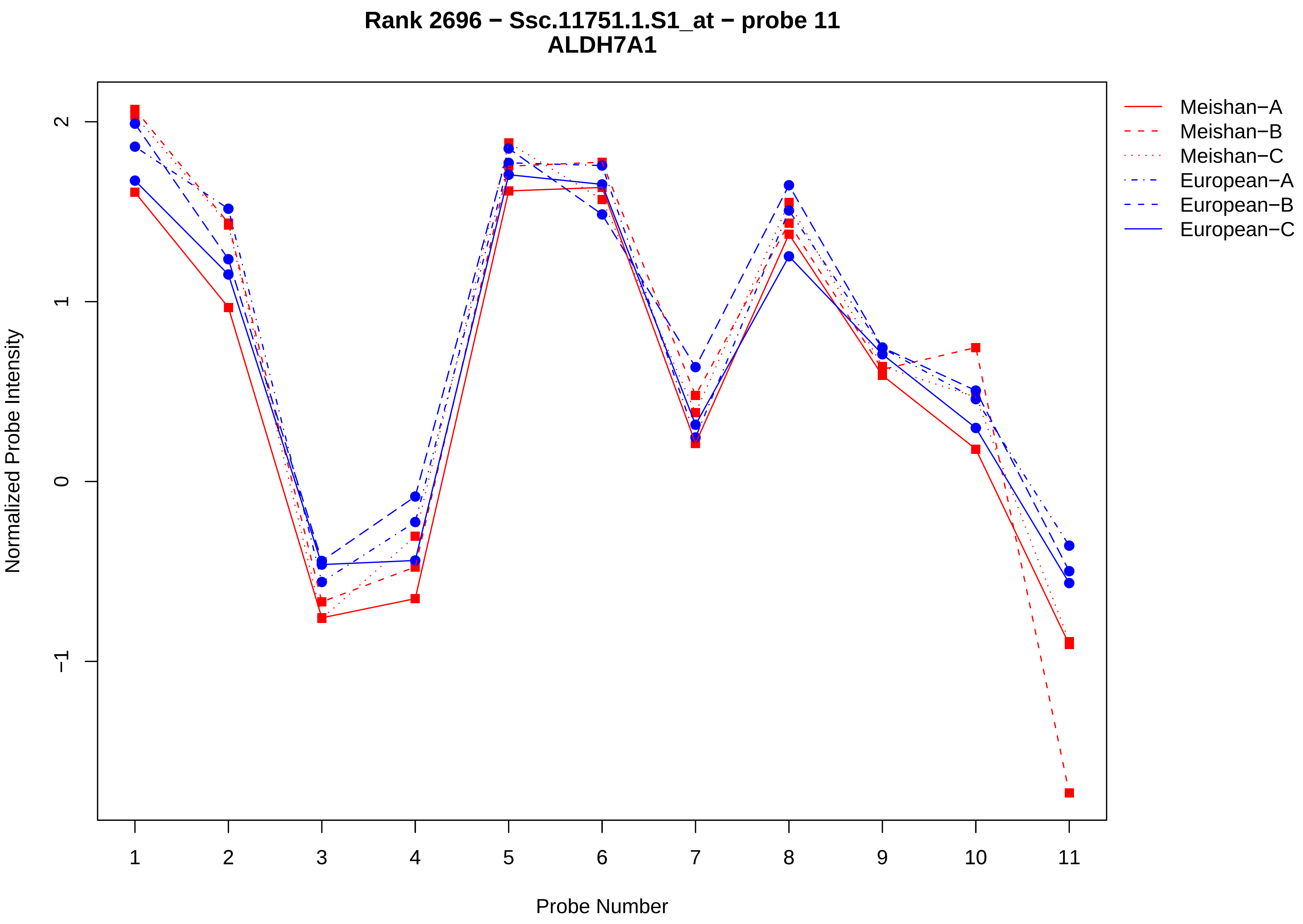

Rank 2696 − Ssc.11751.1.S1_at − probe 11
ALDH7A1
Normalized Probe Intensity
Probe Number
−1
0
1
2
1
2
3
4
5
6
7
8
9
10
11
Meishan−A
Meishan−B
Meishan−C
European−A
European−B
European−C

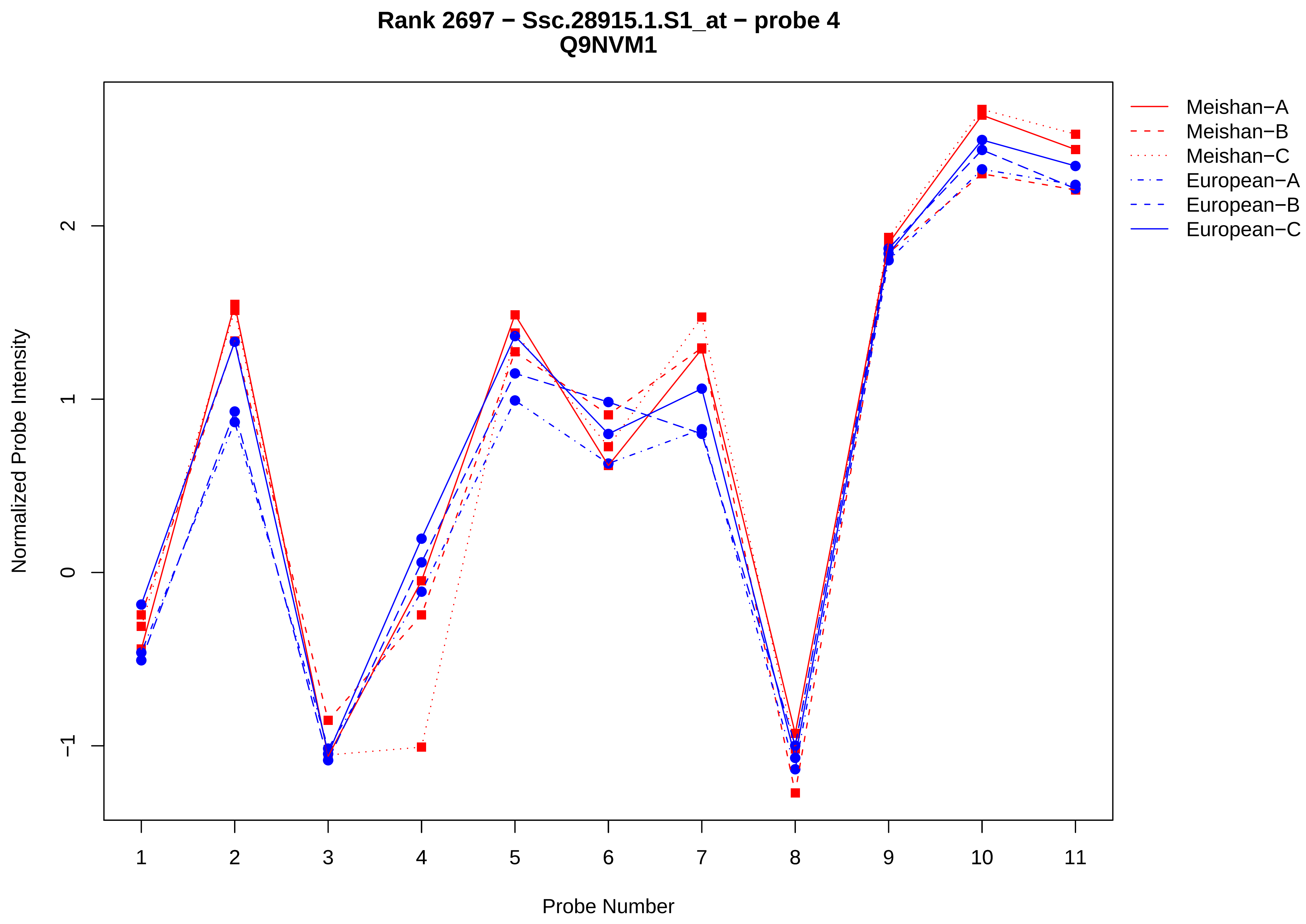

Rank 2697 − Ssc.28915.1.S1_at − probe 4
Q9NVM1
Normalized Probe Intensity
Probe Number
−1
0
1
2
1
2
3
4
5
6
7
8
9
10
11
Meishan−A
Meishan−B
Meishan−C
European−A
European−B
European−C

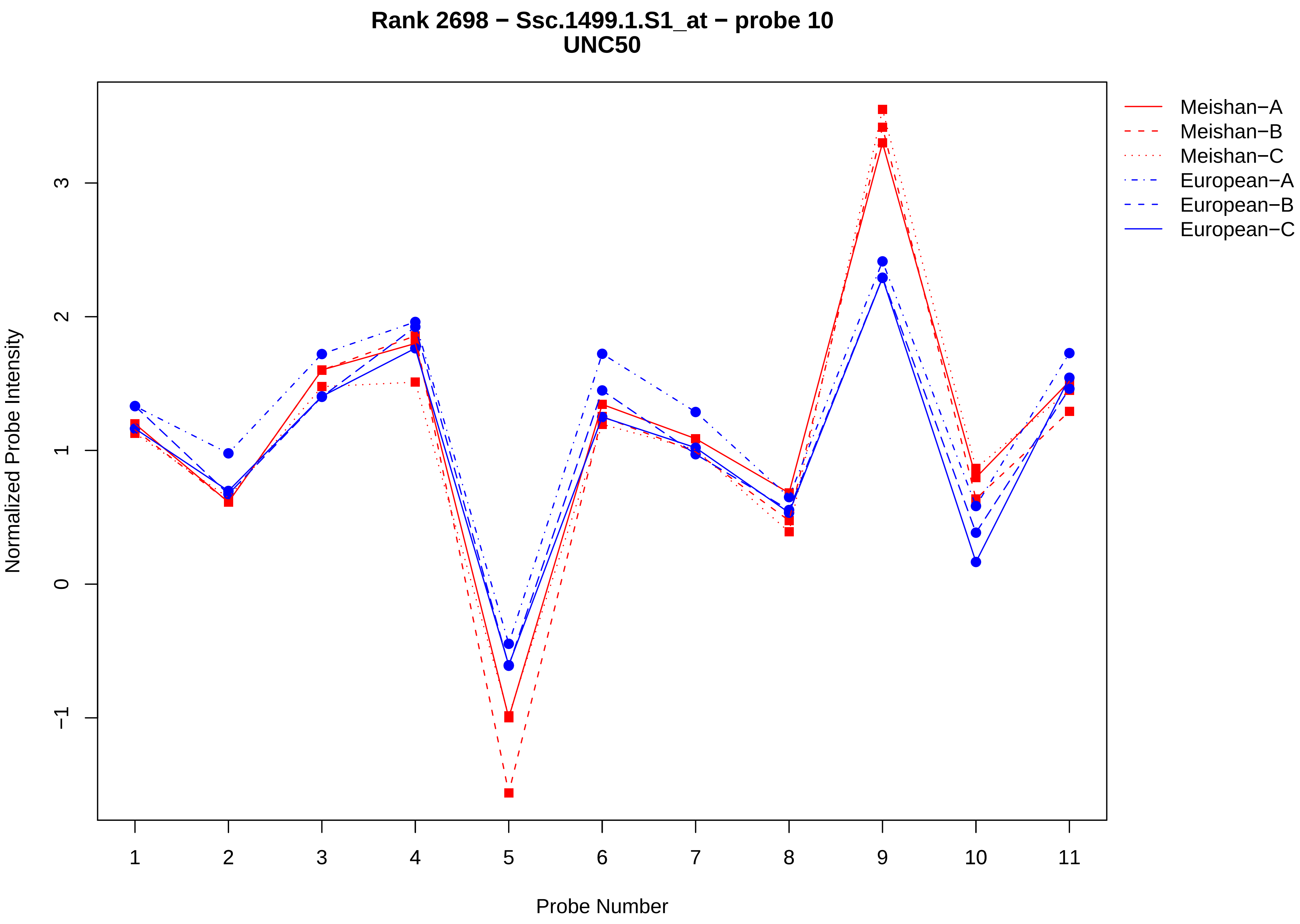

Rank 2698 − Ssc.1499.1.S1_at − probe 10
UNC50
Normalized Probe Intensity
Probe Number
−1
0
1
2
3
1
2
3
4
5
6
7
8
9
10
11
Meishan−A
Meishan−B
Meishan−C
European−A
European−B
European−C

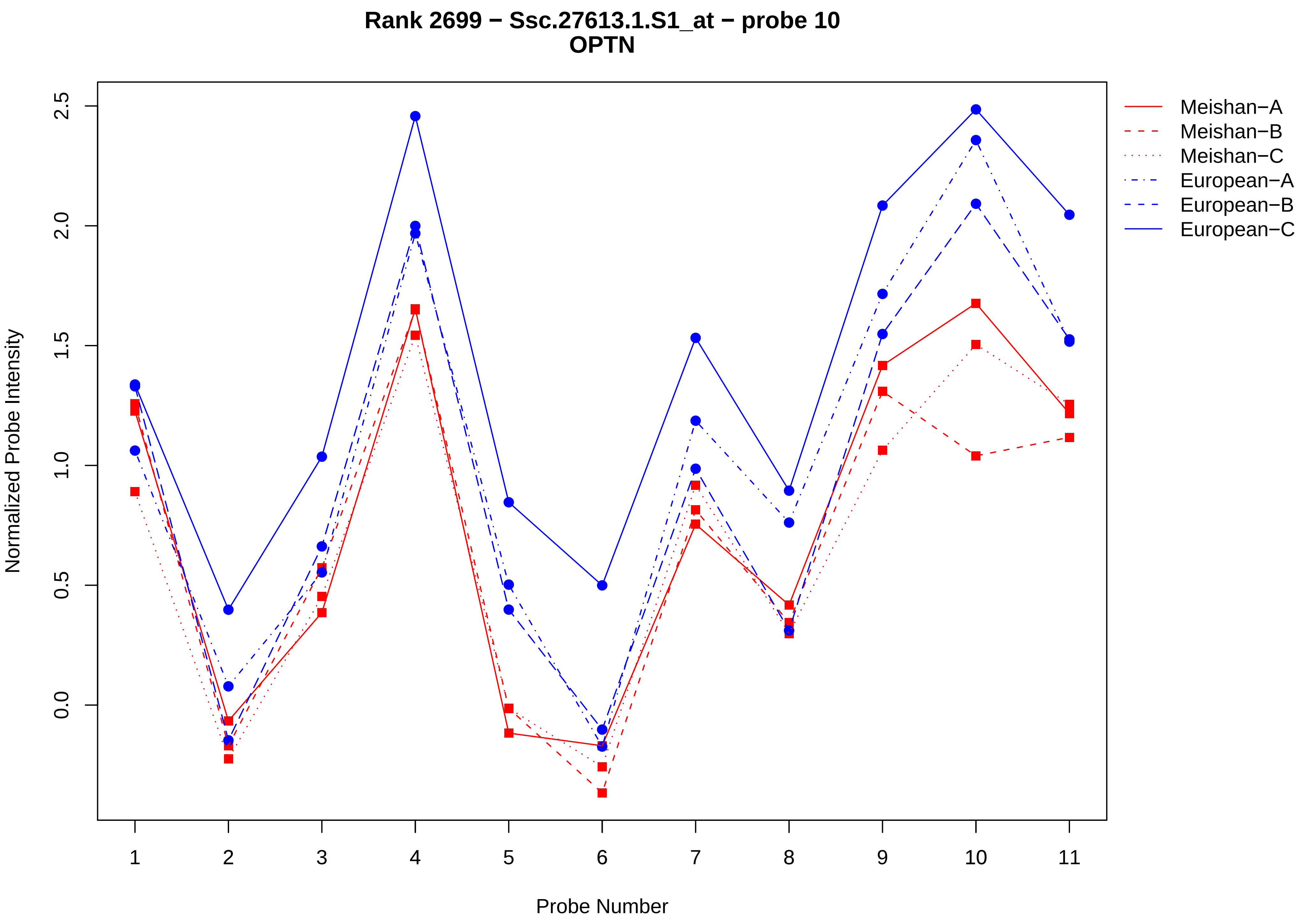
Rank 2699 − Ssc.27613.1.S1_at − probe 10
OPTN
Normalized Probe Intensity
Probe Number
0.0
0.5
1.0
1.5
2.0
2.5
1
2
3
4
5
6
7
8
9
10
11
Meishan−A
Meishan−B
Meishan−C
European−A
European−B
European−C

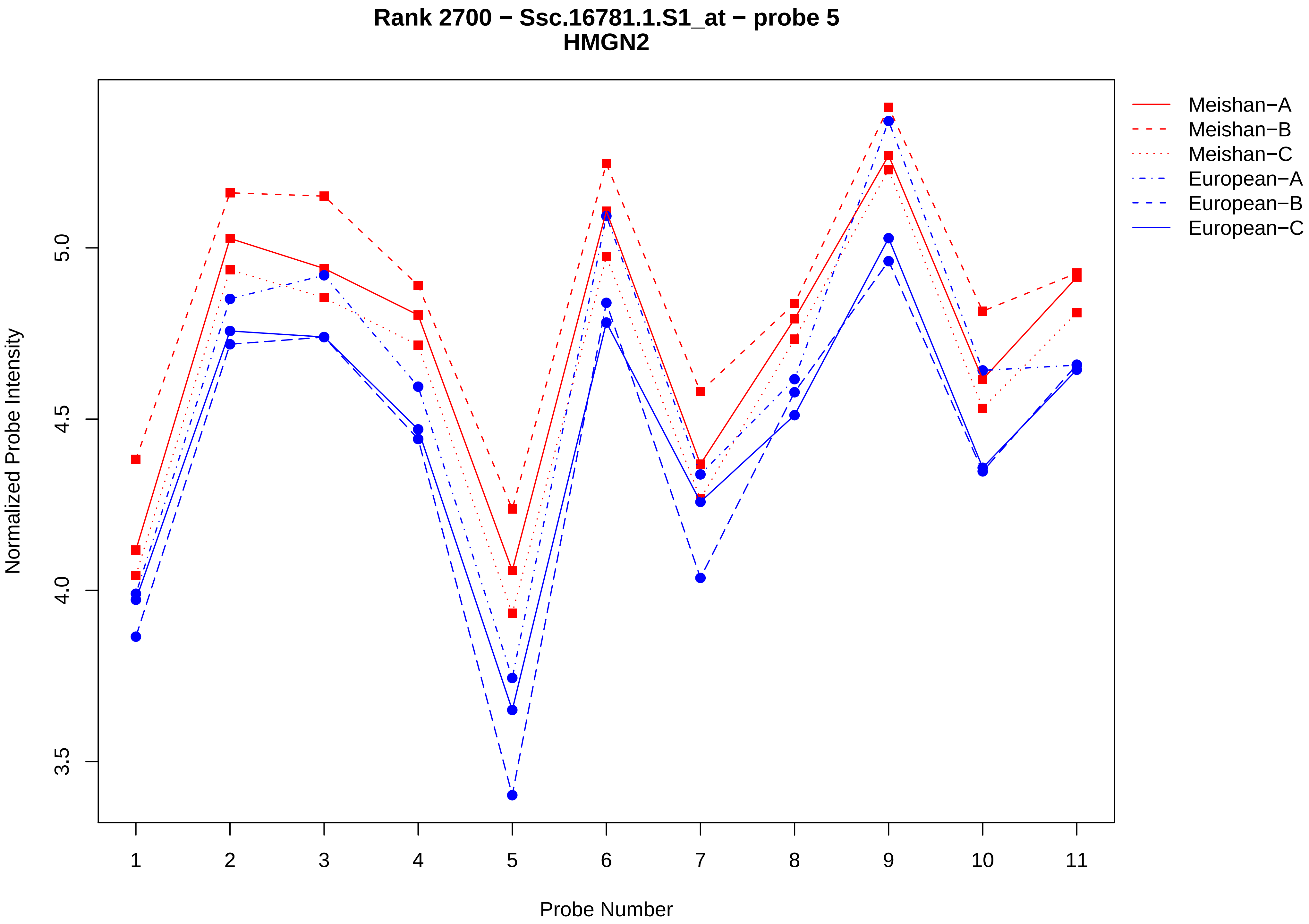

Rank 2700 − Ssc.16781.1.S1_at − probe 5
HMGN2
Normalized Probe Intensity
Probe Number
3.5
4.0
4.5
5.0
1
2
3
4
5
6
7
8
9
10
11
Meishan−A
Meishan−B
Meishan−C
European−A
European−B
European−C

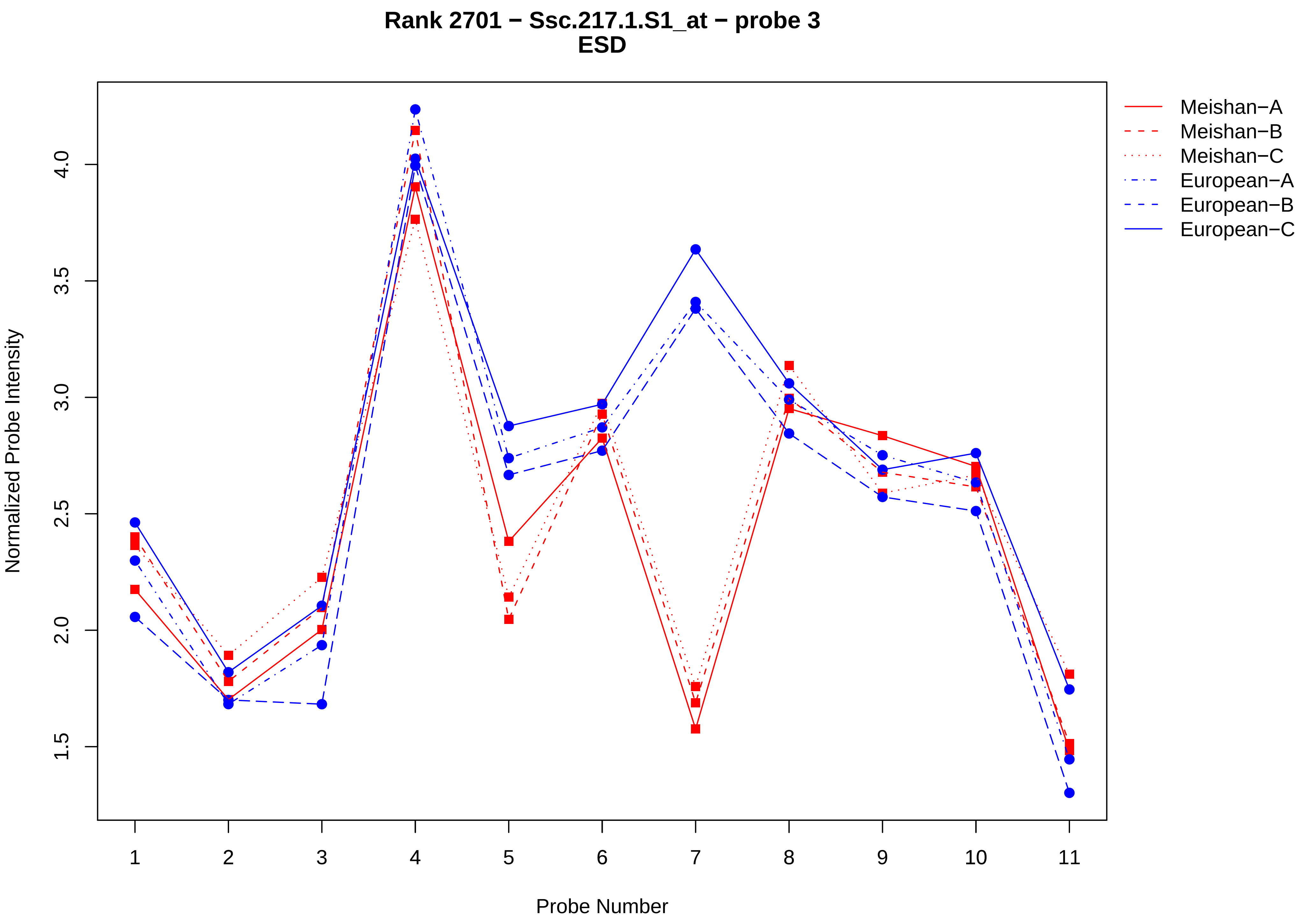

Rank 2701 − Ssc.217.1.S1_at − probe 3
ESD
Normalized Probe Intensity
Probe Number
1.5
2.0
2.5
3.0
3.5
4.0
1
2
3
4
5
6
7
8
9
10
11
Meishan−A
Meishan−B
Meishan−C
European−A
European−B
European−C

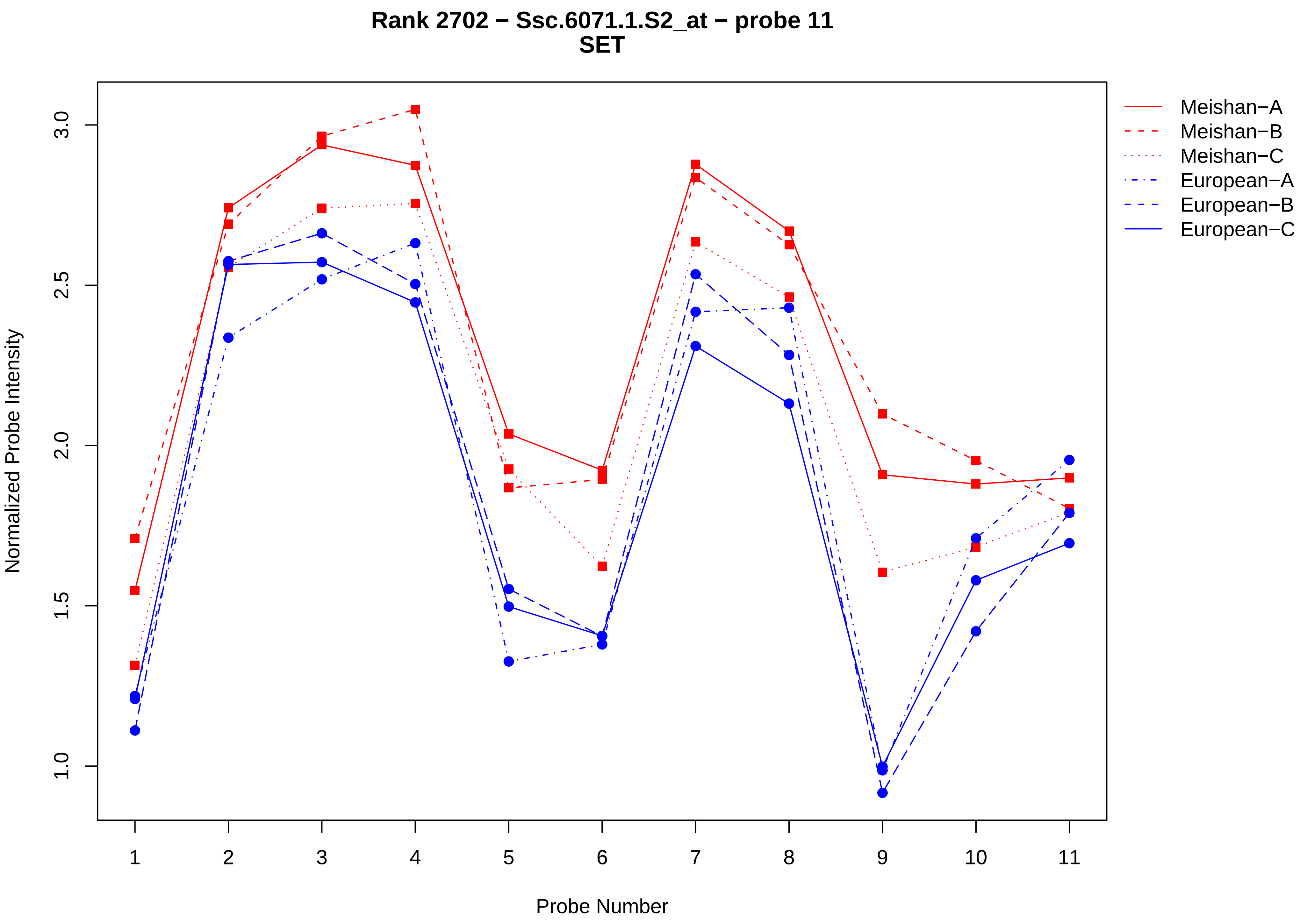

Rank 2702 − Ssc.6071.1.S2_at − probe 11
SET
Normalized Probe Intensity
Probe Number
1.0
1.5
2.0
2.5
3.0
1
2
3
4
5
6
7
8
9
10
11
Meishan−A
Meishan−B
Meishan−C
European−A
European−B
European−C

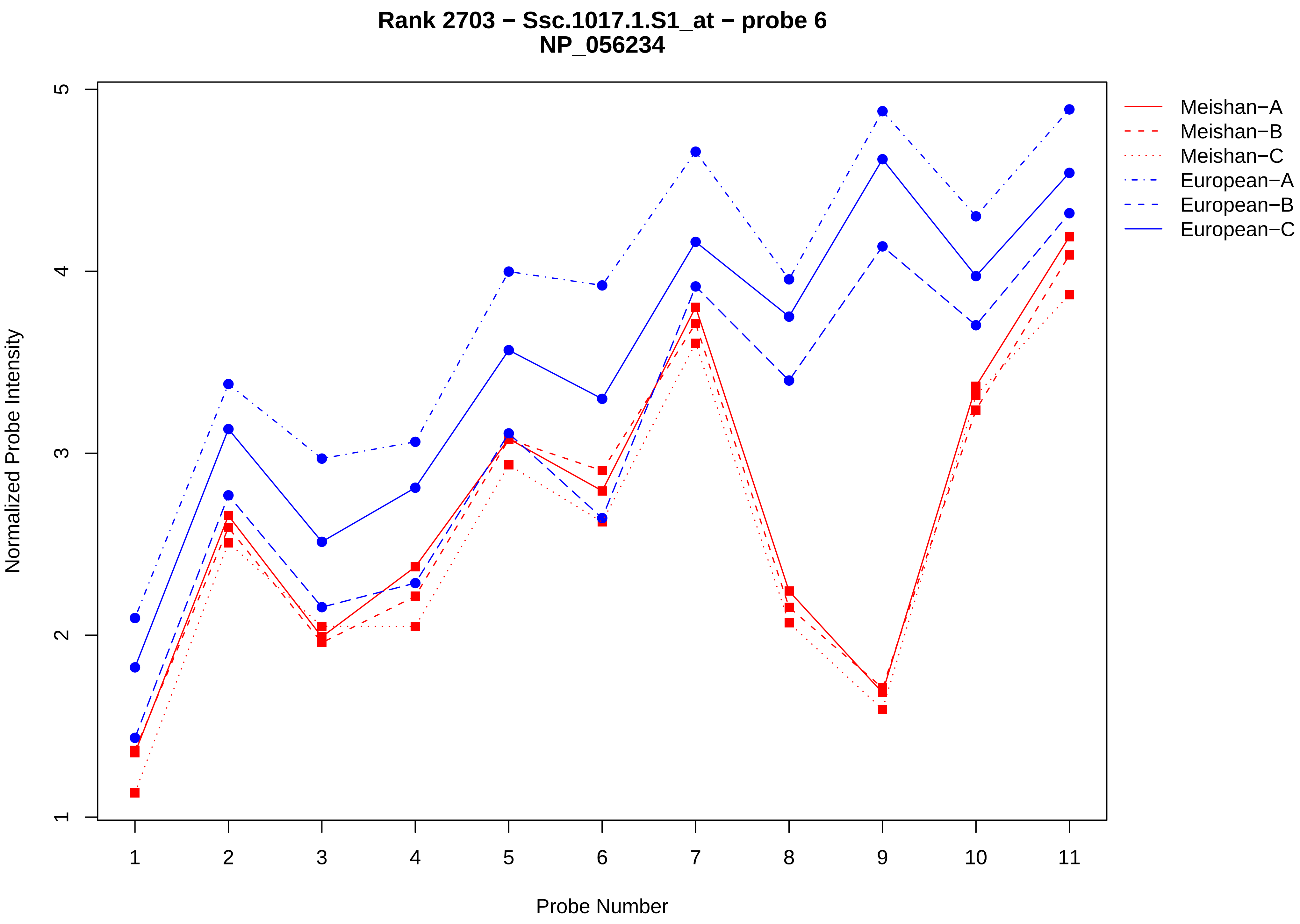

Rank 2703 − Ssc.1017.1.S1_at − probe 6
NP_056234
Normalized Probe Intensity
1
2
3
4
5
Probe Number
1
2
3
4
5
6
7
8
9
10
11
Meishan−A
Meishan−B
Meishan−C
European−A
European−B
European−C

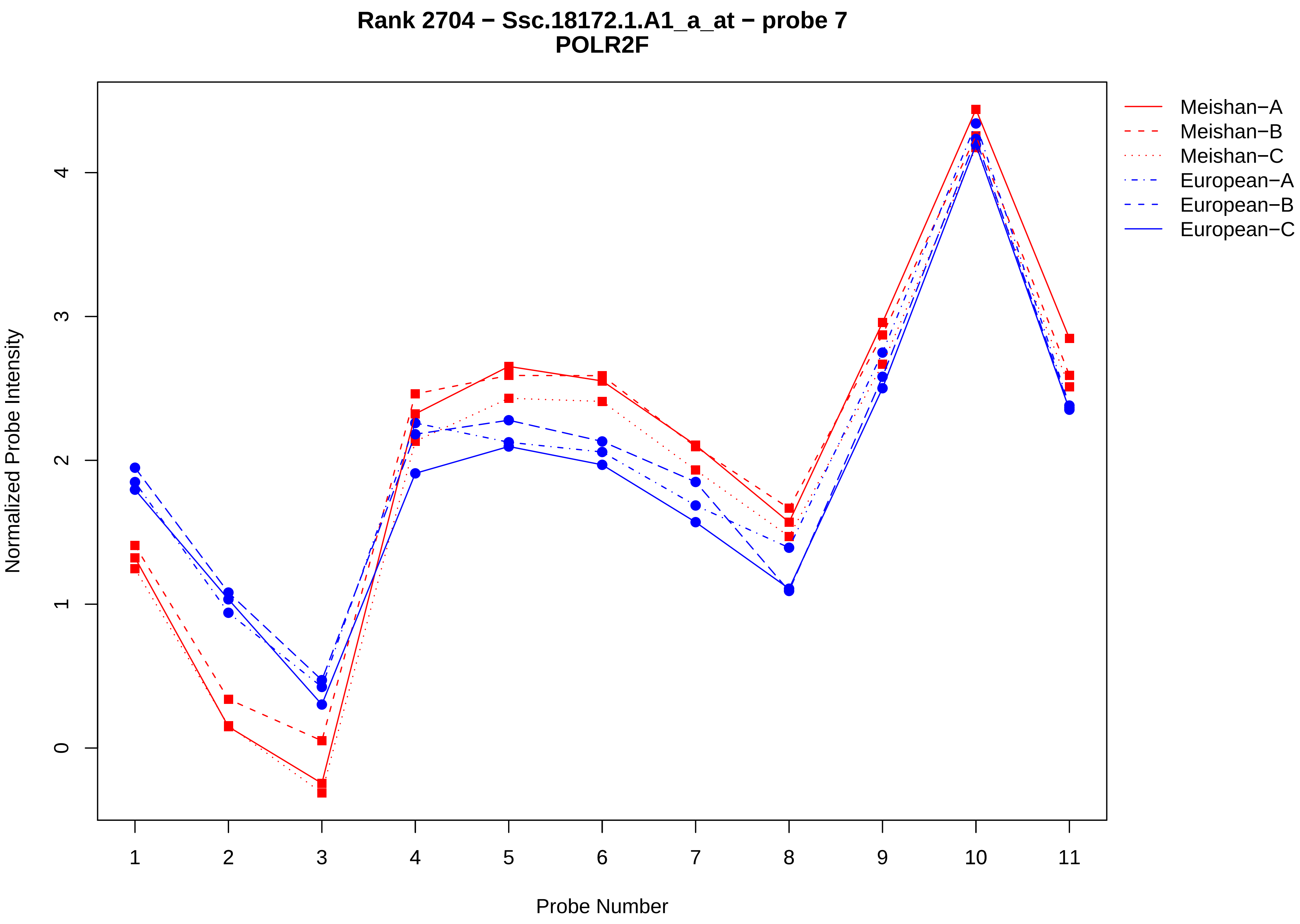

Rank 2704 − Ssc.18172.1.A1_a_at − probe 7
POLR2F
Normalized Probe Intensity
Probe Number
0
1
2
3
4
1
2
3
4
5
6
7
8
9
10
11
Meishan−A
Meishan−B
Meishan−C
European−A
European−B
European−C

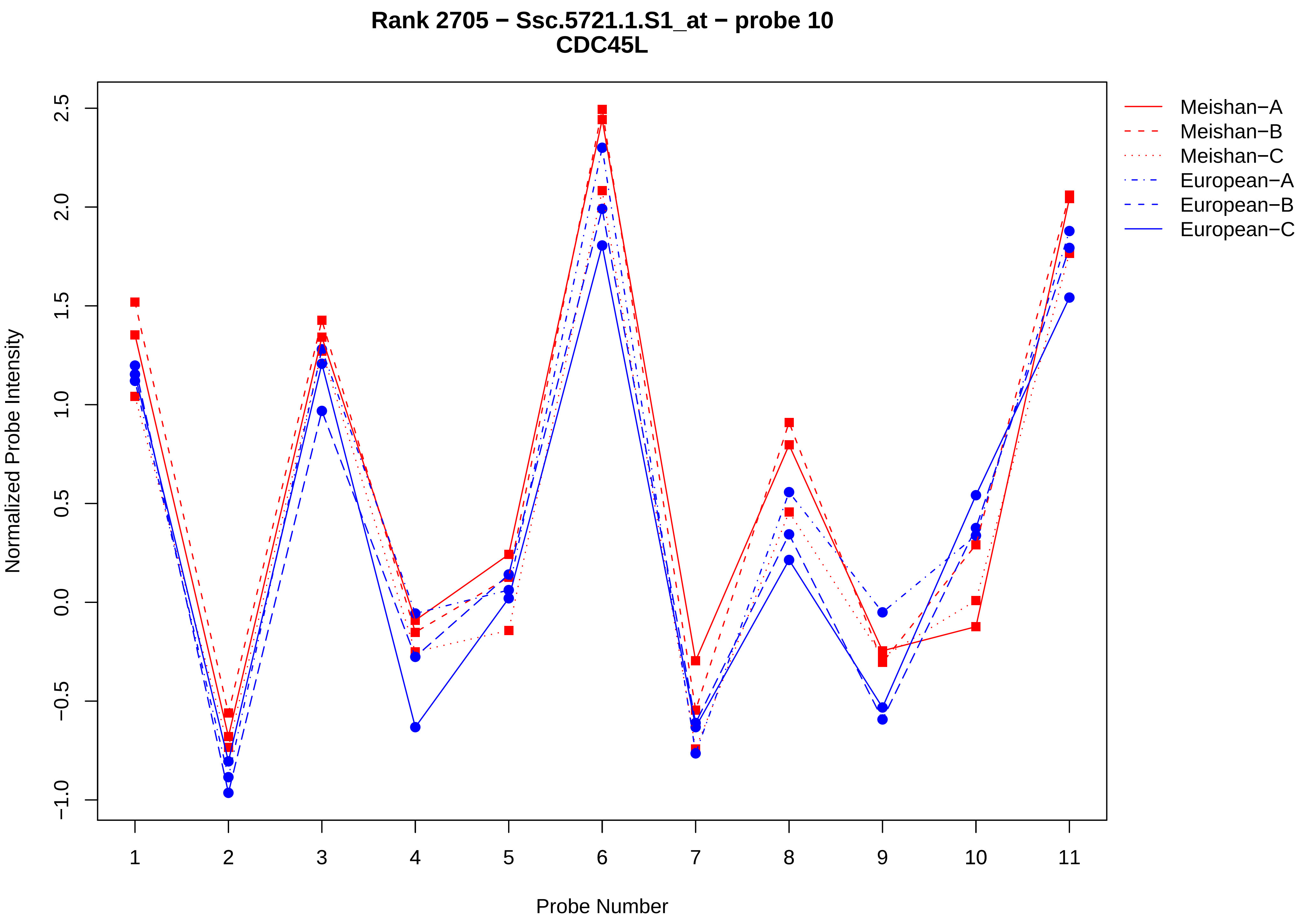

Rank 2705 − Ssc.5721.1.S1_at − probe 10
CDC45L
Normalized Probe Intensity
Probe Number
Meishan−A
Meishan−B
Meishan−C
European−A
European−B
European−C

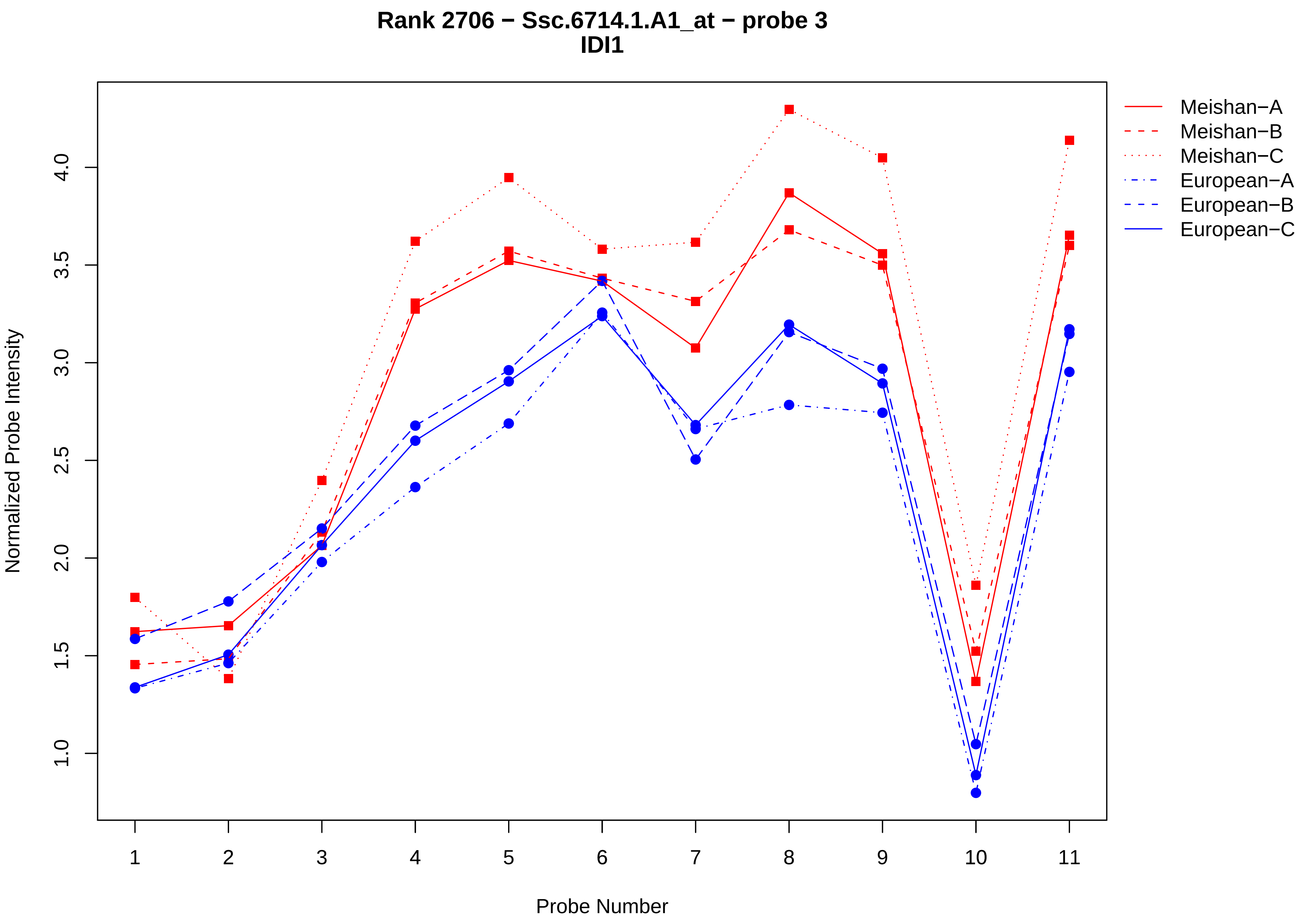

Rank 2706 − Ssc.6714.1.A1_at − probe 3
IDI1
Normalized Probe Intensity
Probe Number
Meishan−A
Meishan−B
Meishan−C
European−A
European−B
European−C
1.0
1.5
2.0
2.5
3.0
3.5
4.0
1
2
3
4
5
6
7
8
9
10
11

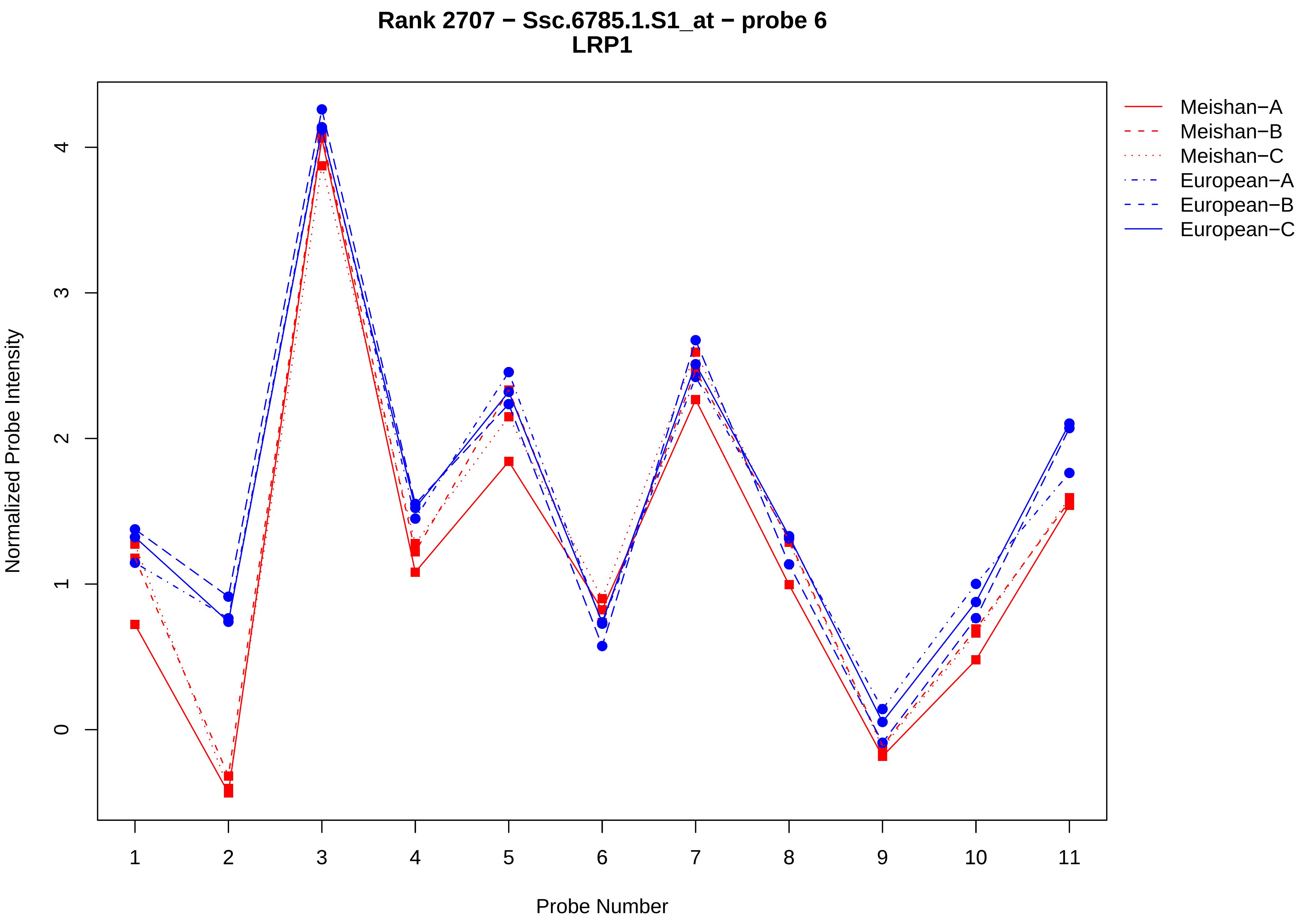

Rank 2707 − Ssc.6785.1.S1_at − probe 6
LRP1
Normalized Probe Intensity
Probe Number
0
1
2
3
4
1
2
3
4
5
6
7
8
9
10
11
Meishan−A
Meishan−B
Meishan−C
European−A
European−B
European−C

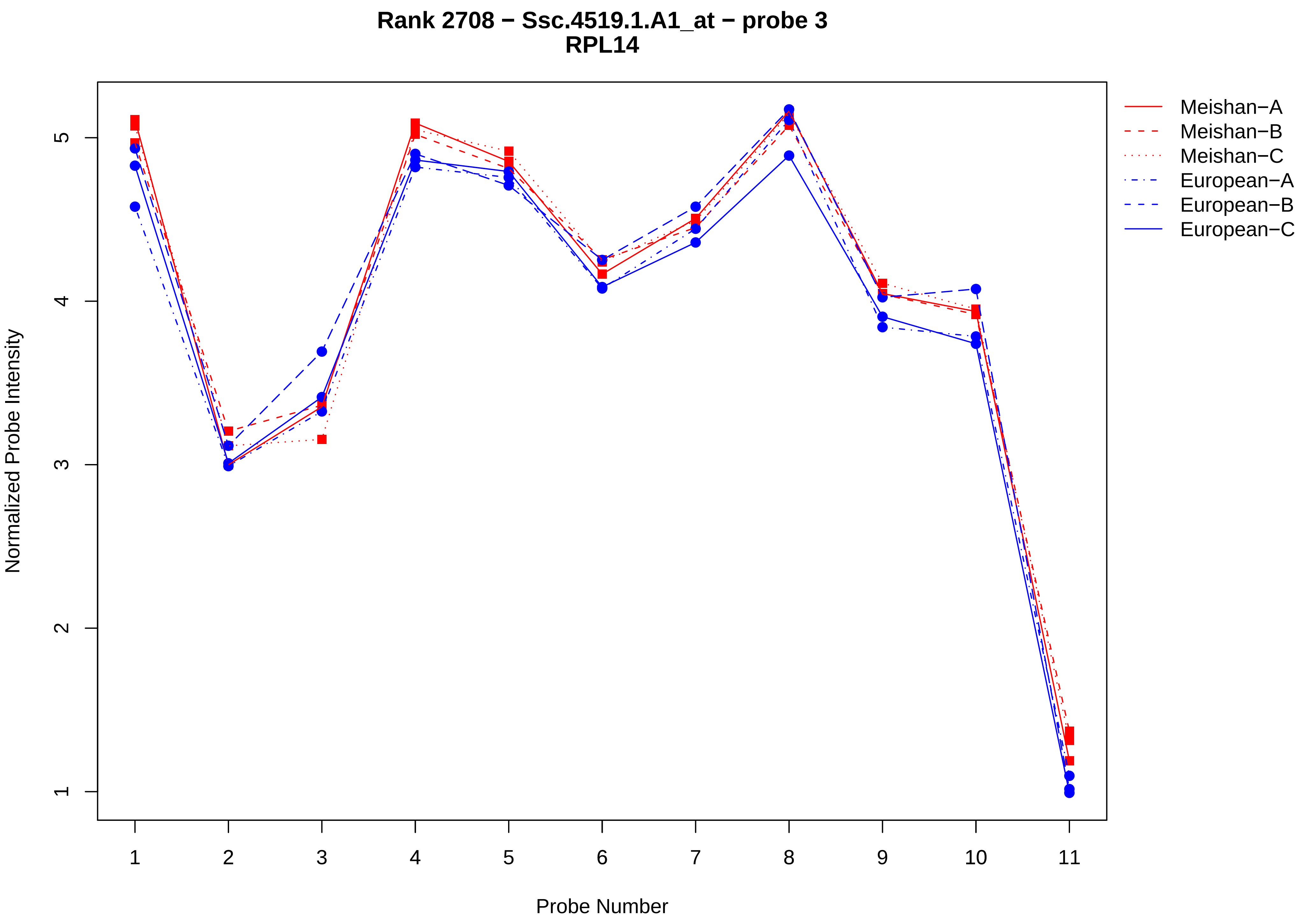
Rank 2708 − Ssc.4519.1.A1_at − probe 3
RPL14
Normalized Probe Intensity
Probe Number
1
2
3
4
5
1
2
3
4
5
6
7
8
9
10
11
Meishan−A
Meishan−B
Meishan−C
European−A
European−B
European−C

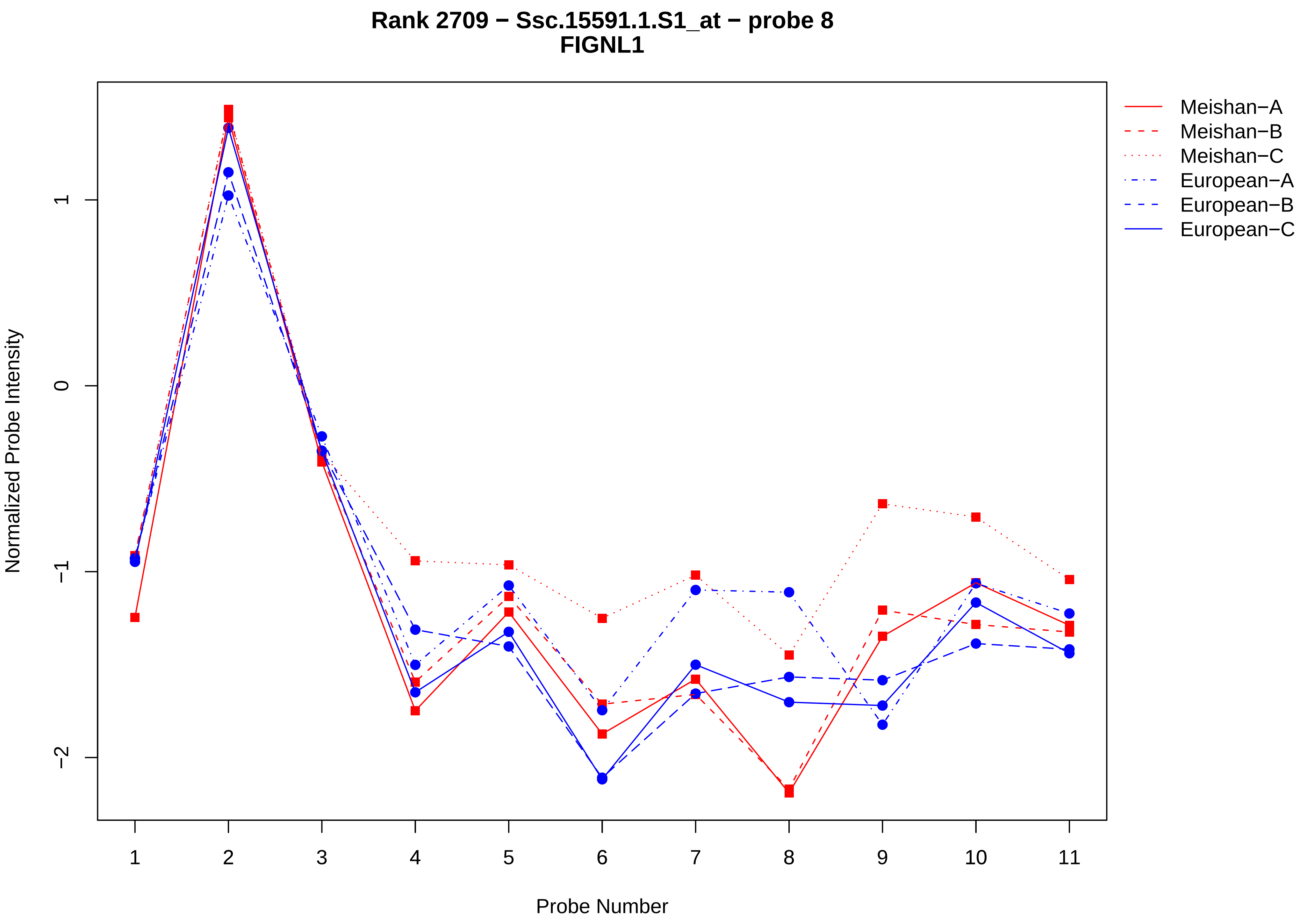

Rank 2709 − Ssc.15591.1.S1_at − probe 8
FIGNL1
Normalized Probe Intensity
Probe Number
1
0
−1
−2
1
2
3
4
5
6
7
8
9
10
11
Meishan−A
Meishan−B
Meishan−C
European−A
European−B
European−C

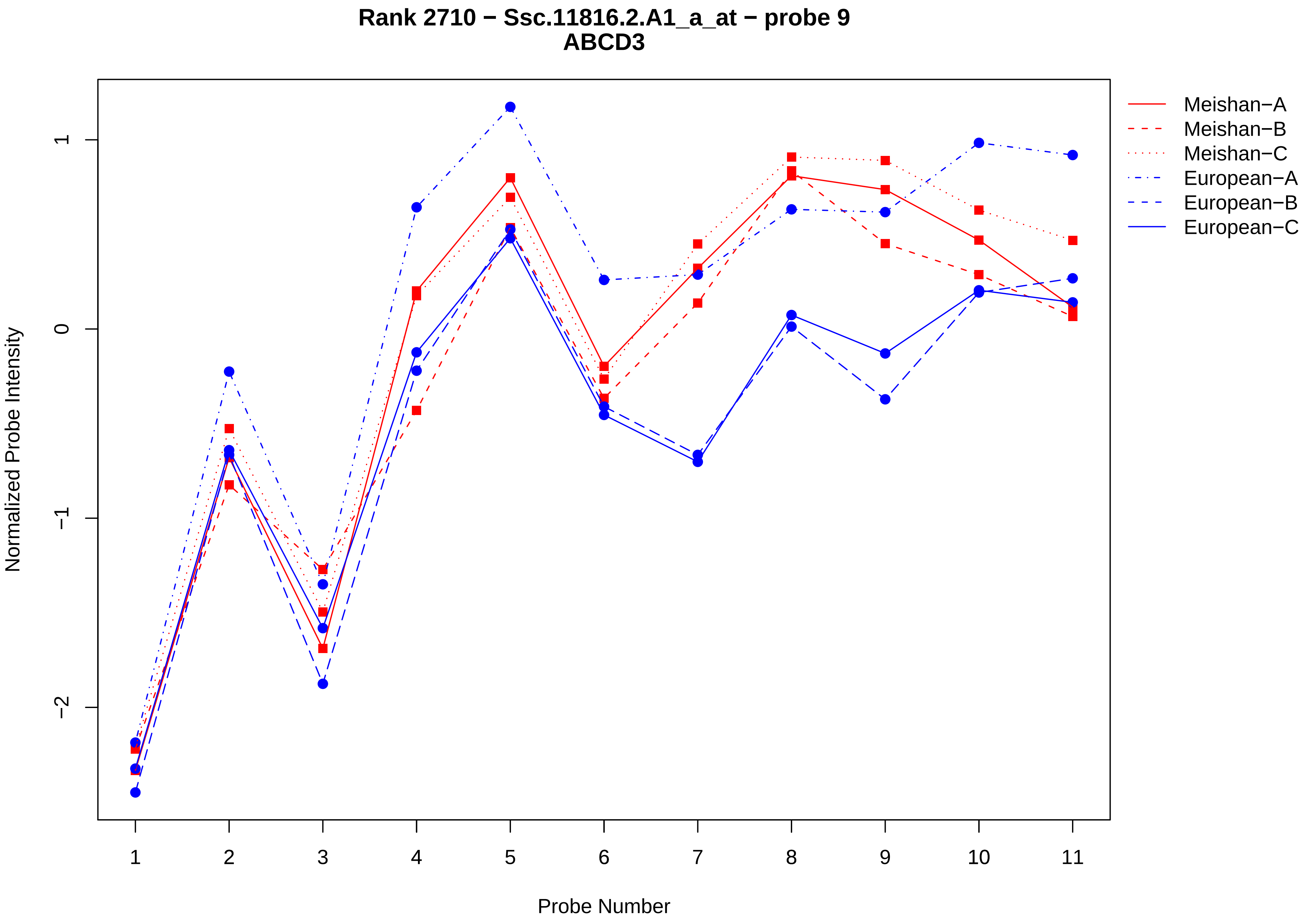

Rank 2710 − Ssc.11816.2.A1_a_at − probe 9
ABCD3
Normalized Probe Intensity
Probe Number
Meishan−A
Meishan−B
Meishan−C
European−A
European−B
European−C

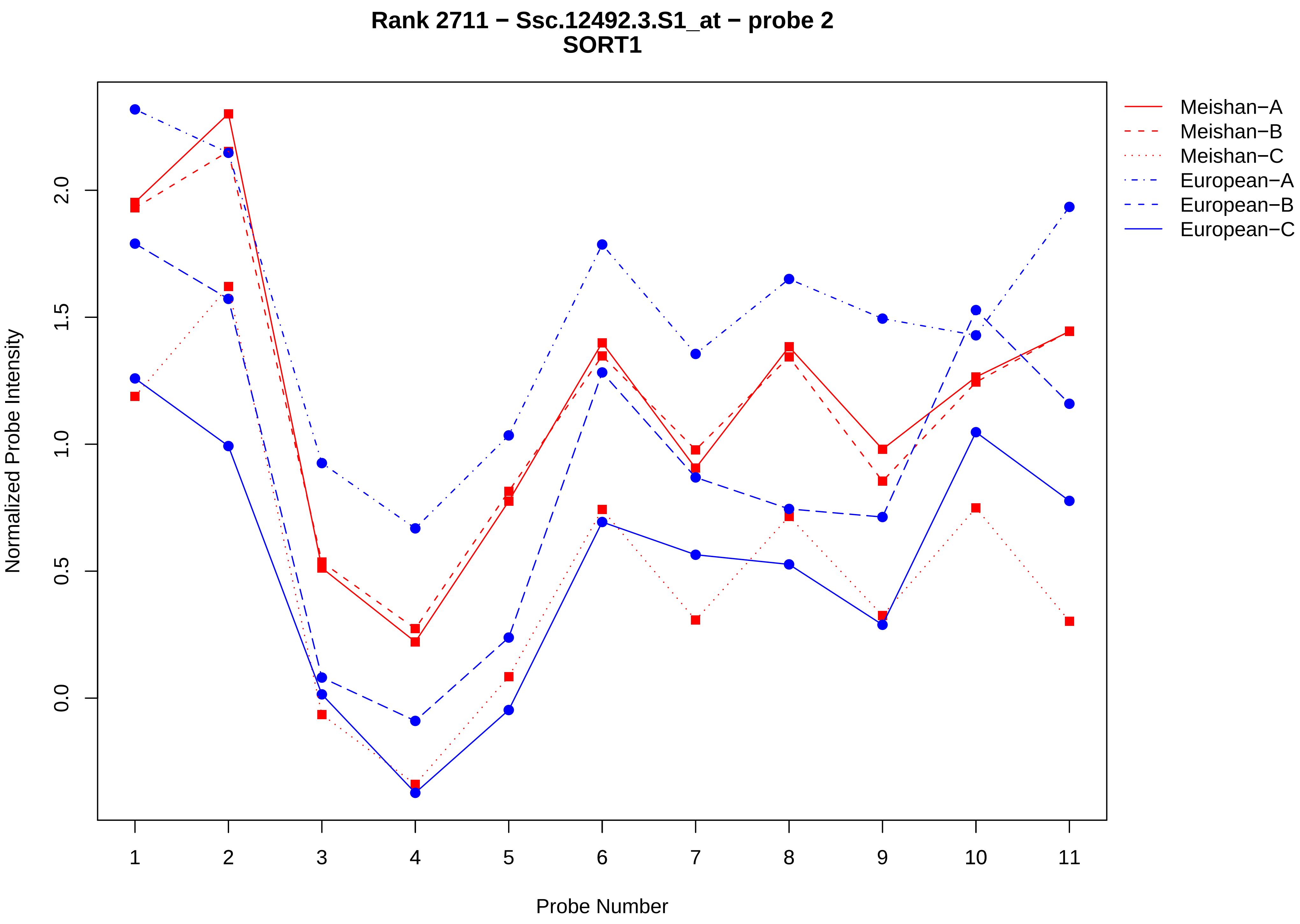

Rank 2711 − Ssc.12492.3.S1_at − probe 2
SORT1
Normalized Probe Intensity
0.0
0.5
1.0
1.5
2.0
1
2
3
4
5
6
7
8
9
10
11
Probe Number
Meishan−A
Meishan−B
Meishan−C
European−A
European−B
European−C

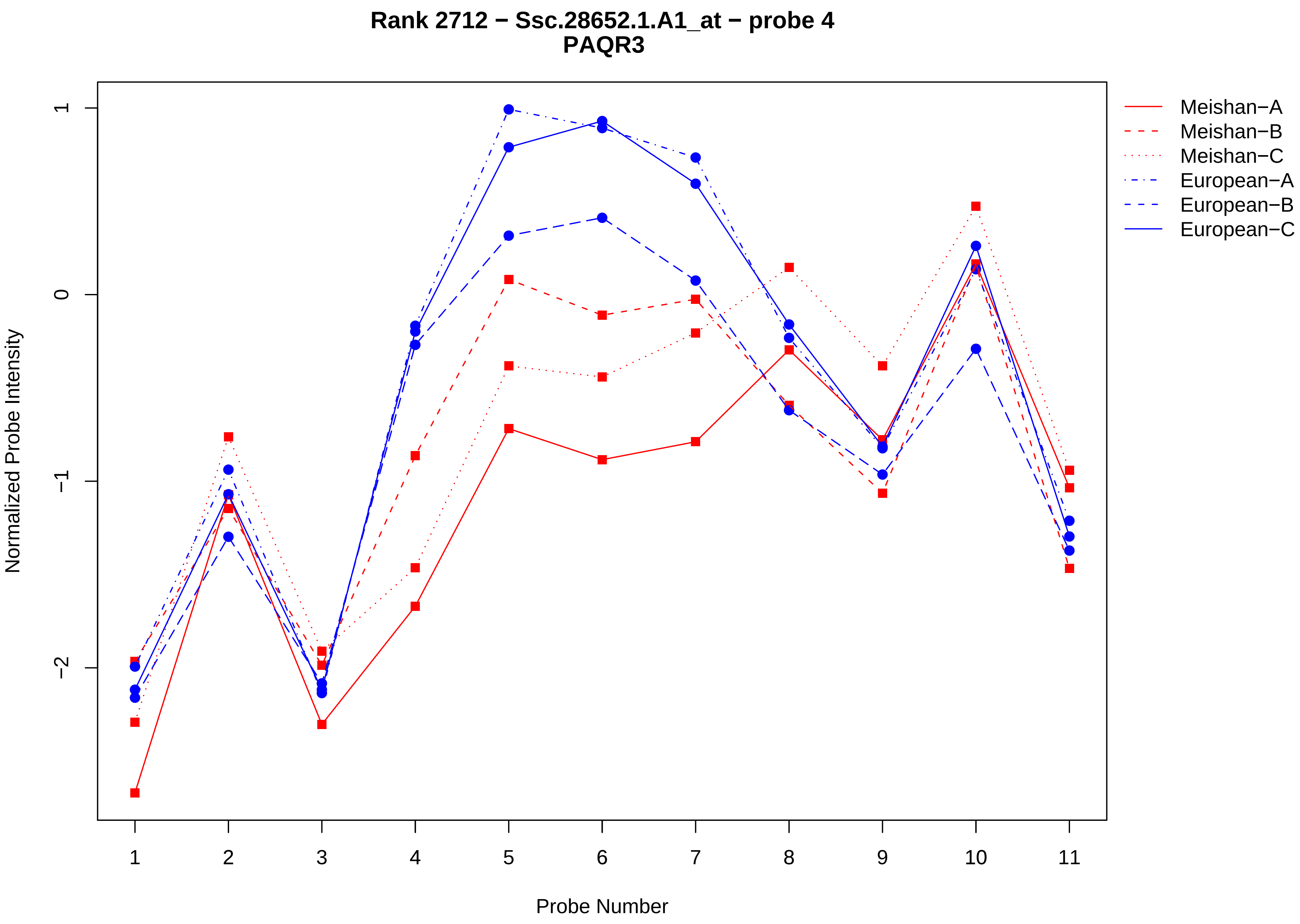

Rank 2712 − Ssc.28652.1.A1_at − probe 4
PAQR3
Normalized Probe Intensity
Probe Number
1
0
−1
−2
1
2
3
4
5
6
7
8
9
10
11
Meishan−A
Meishan−B
Meishan−C
European−A
European−B
European−C

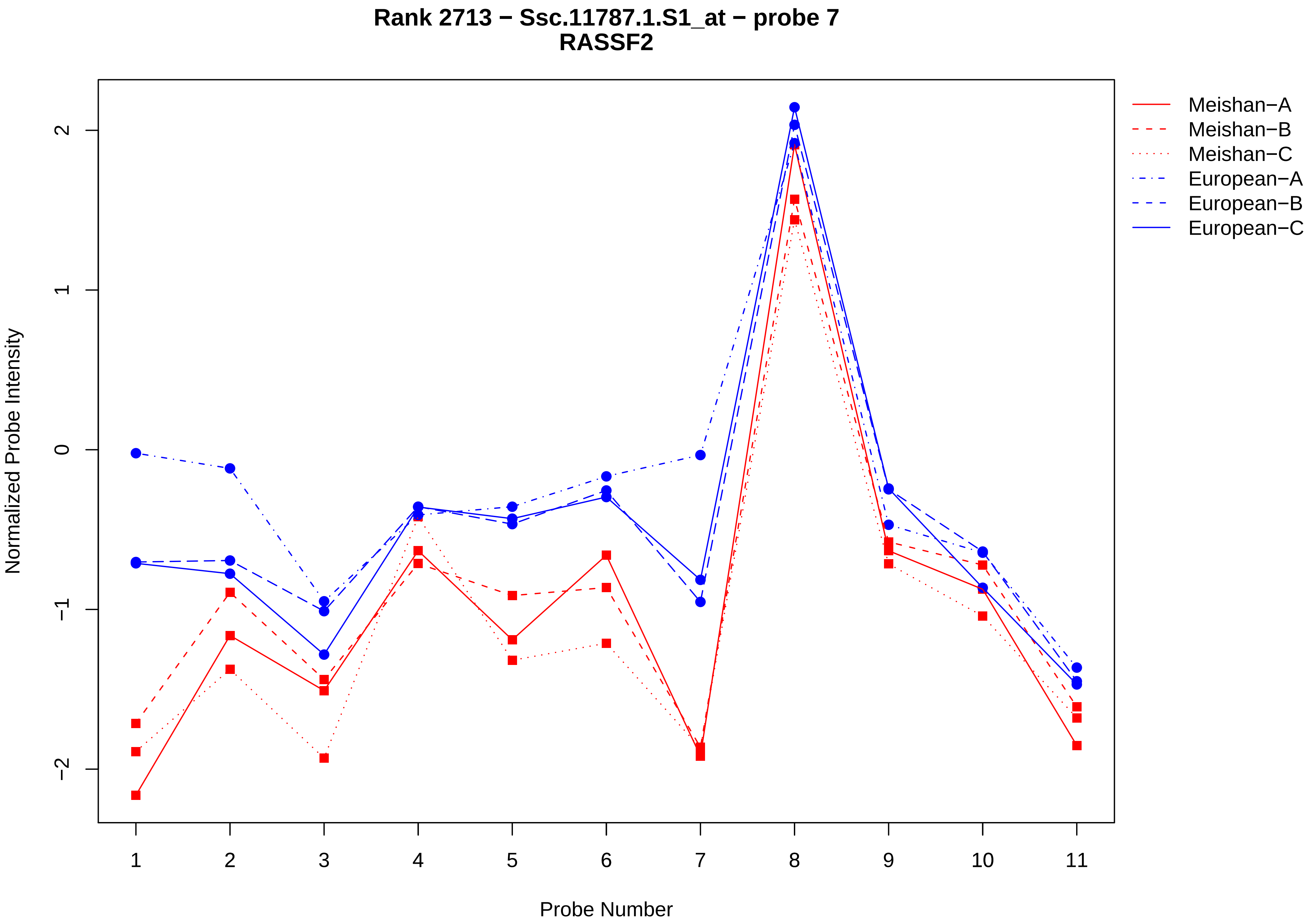

Rank 2713 − Ssc.11787.1.S1_at − probe 7
RASSF2
Normalized Probe Intensity
Probe Number
−2
−1
0
1
2
1
2
3
4
5
6
7
8
9
10
11
Meishan−A
Meishan−B
Meishan−C
European−A
European−B
European−C

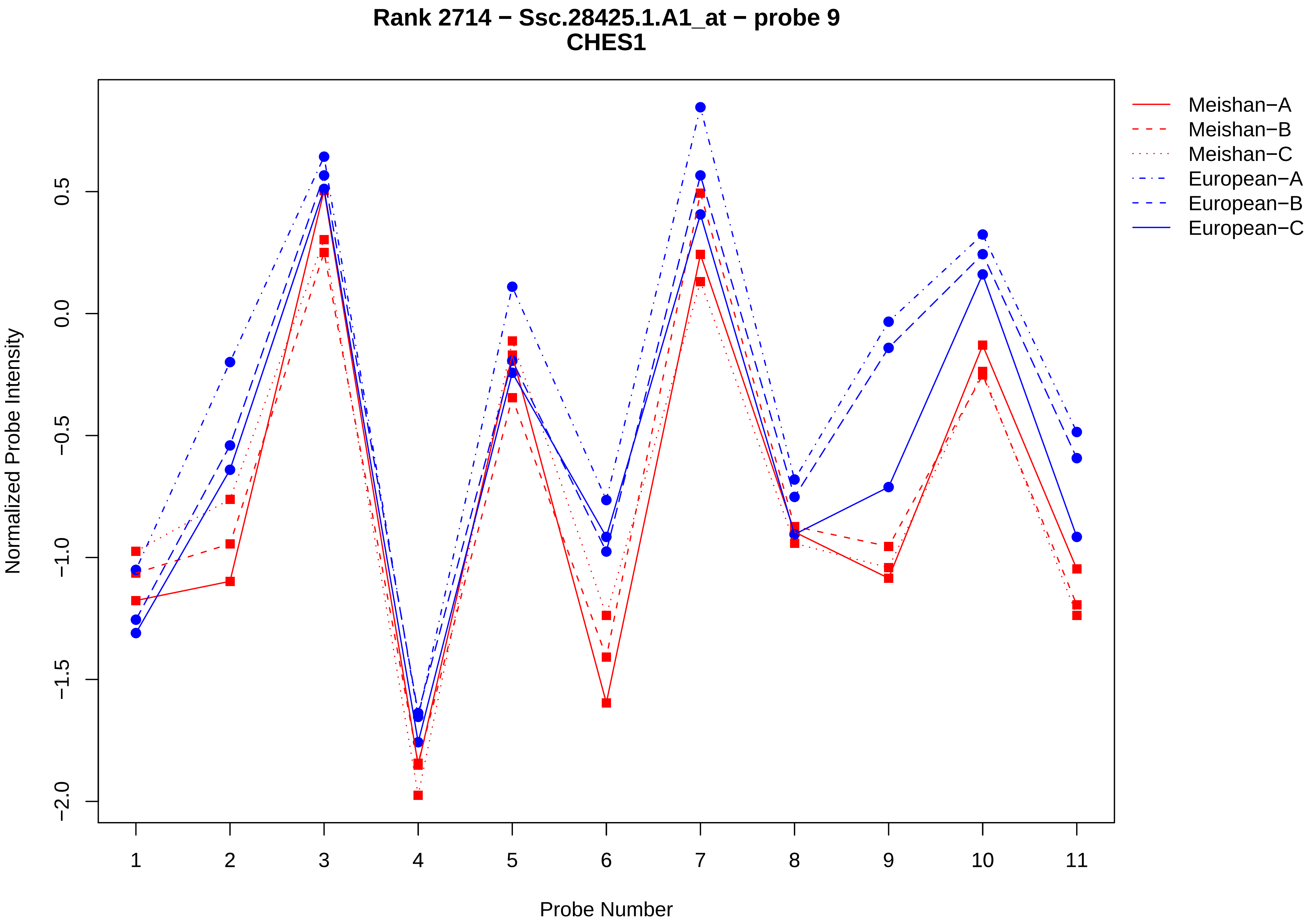
Rank 2714 − Ssc.28425.1.A1_at − probe 9
CHES1
Normalized Probe Intensity
Probe Number
−2.0
−1.5
−1.0
−0.5
0.0
0.5
1
2
3
4
5
6
7
8
9
10
11
Meishan−A
Meishan−B
Meishan−C
European−A
European−B
European−C

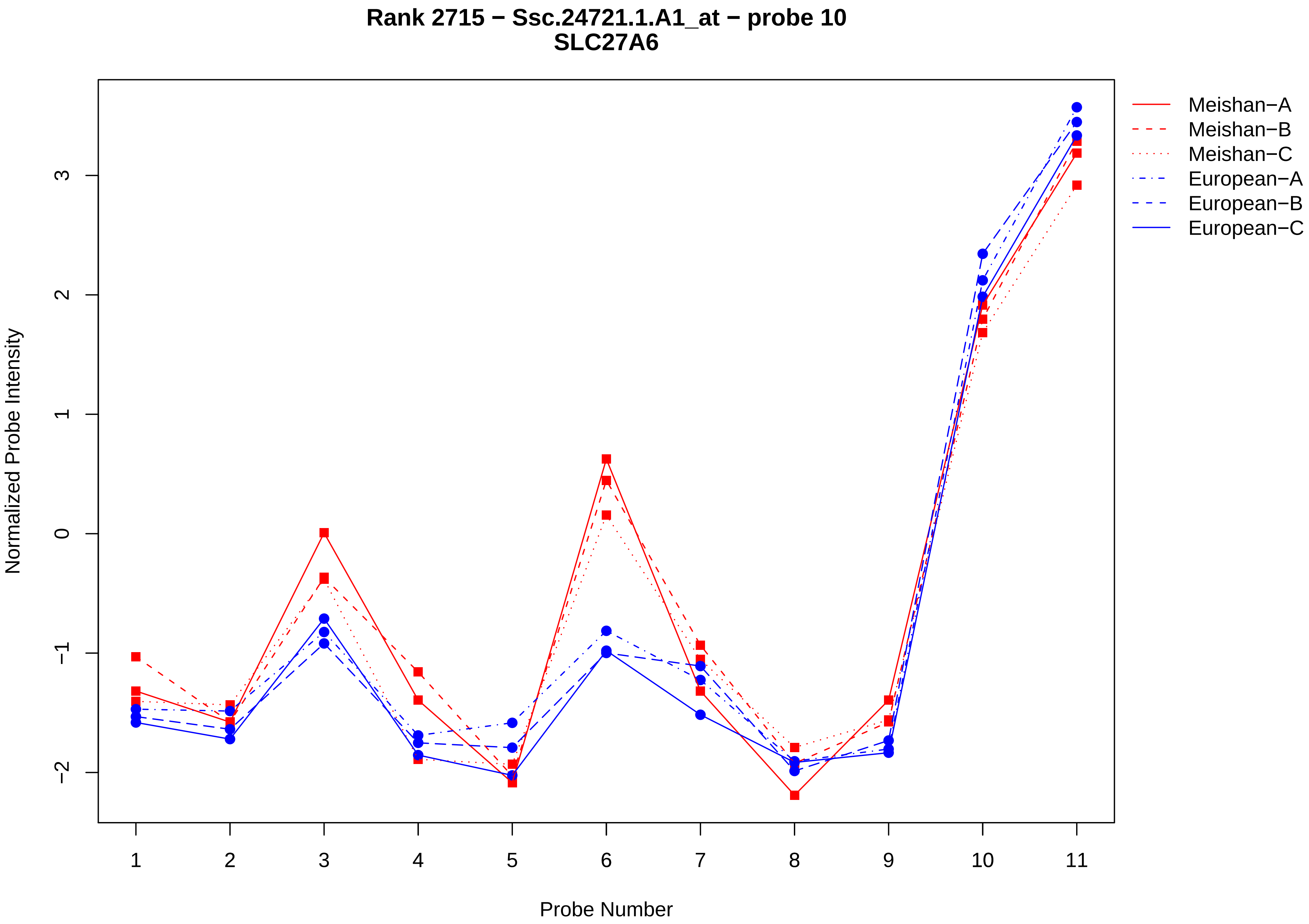

Rank 2715 − Ssc.24721.1.A1_at − probe 10
SLC27A6
Normalized Probe Intensity
Probe Number
Meishan−A
Meishan−B
Meishan−C
European−A
European−B
European−C
−2
−1
0
1
2
3
1
2
3
4
5
6
7
8
9
10
11

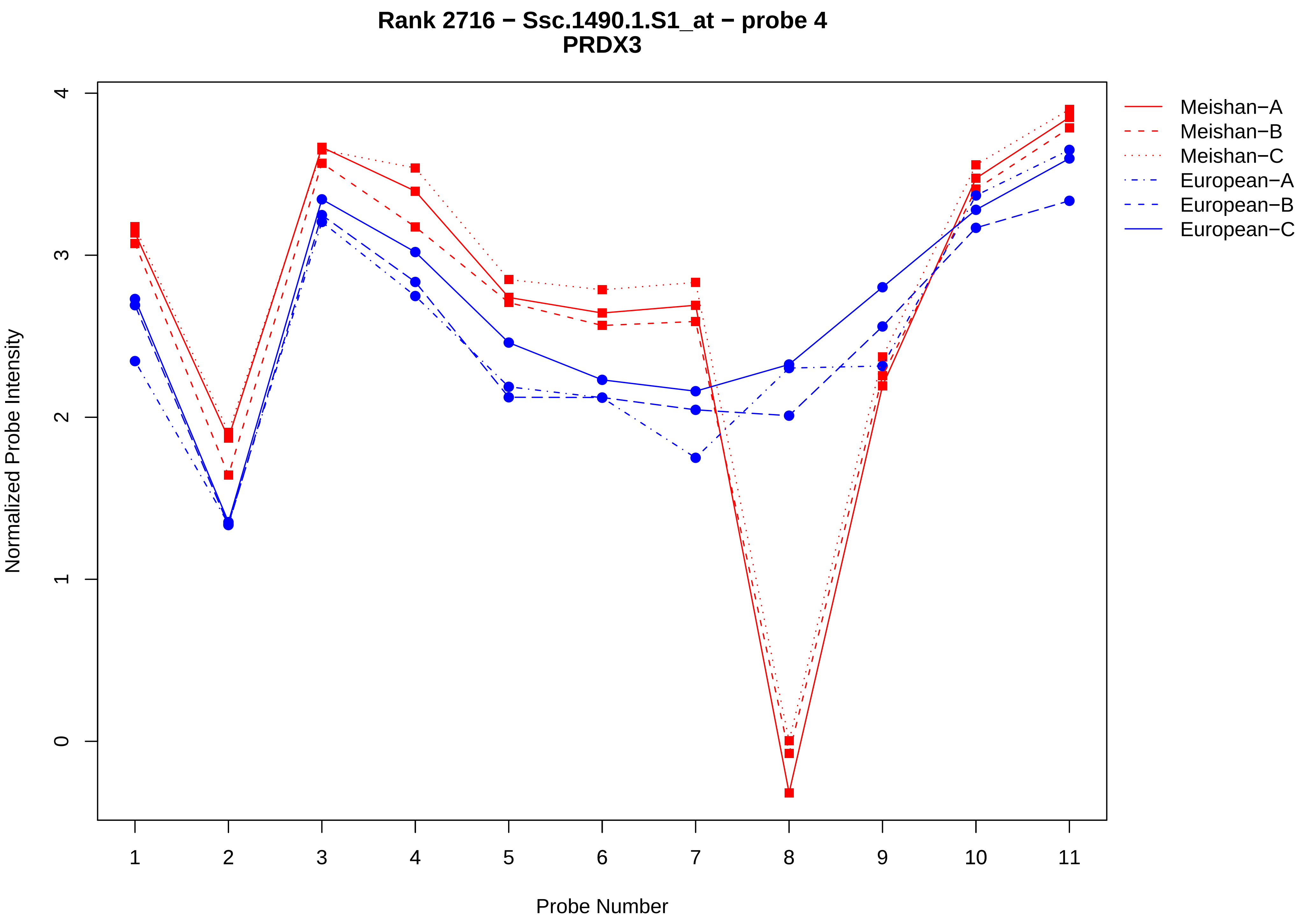

Rank 2716 − Ssc.1490.1.S1_at − probe 4
PRDX3
Normalized Probe Intensity
Probe Number
0
1
2
3
4
1
2
3
4
5
6
7
8
9
10
11
Meishan−A
Meishan−B
Meishan−C
European−A
European−B
European−C

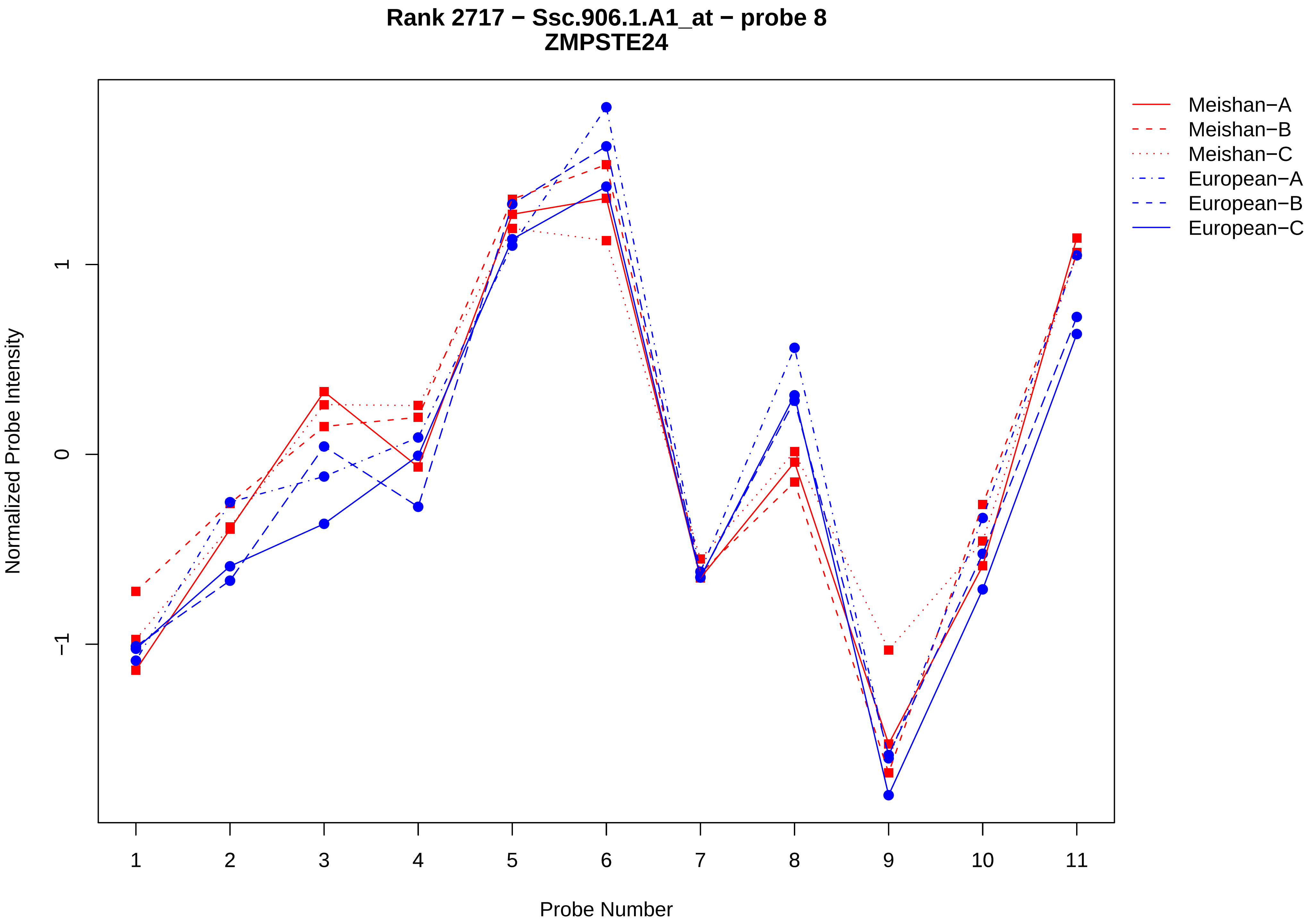

Rank 2717 − Ssc.906.1.A1_at − probe 8
ZMPSTE24
Normalized Probe Intensity
Probe Number
−1
0
1
1
2
3
4
5
6
7
8
9
10
11
Meishan−A
Meishan−B
Meishan−C
European−A
European−B
European−C

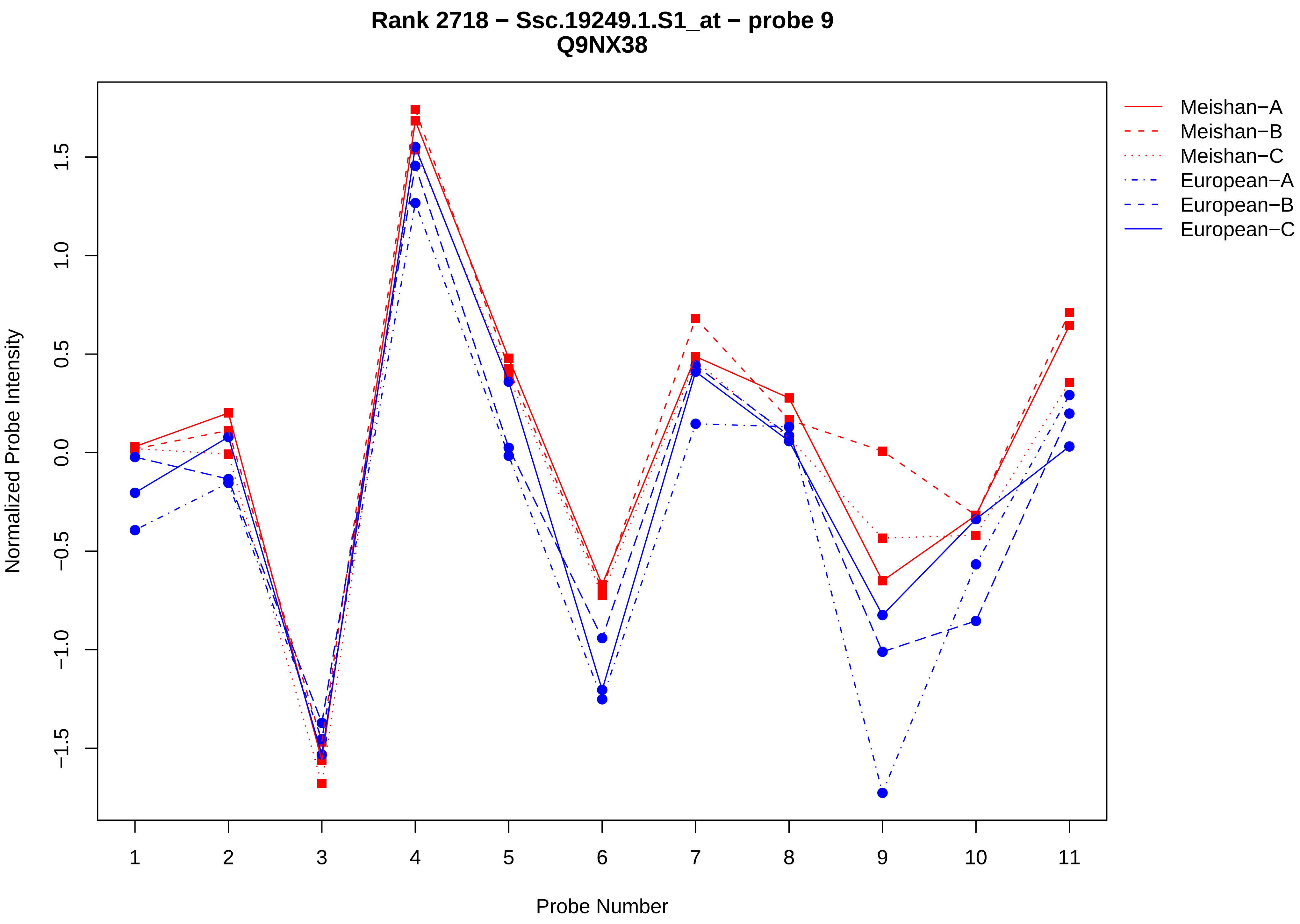

Rank 2718 − Ssc.19249.1.S1_at − probe 9
Q9NX38
Normalized Probe Intensity
Probe Number
Meishan−A
Meishan−B
Meishan−C
European−A
European−B
European−C
−1.5
−1.0
−0.5
0.0
0.5
1.0
1.5
1
2
3
4
5
6
7
8
9
10
11

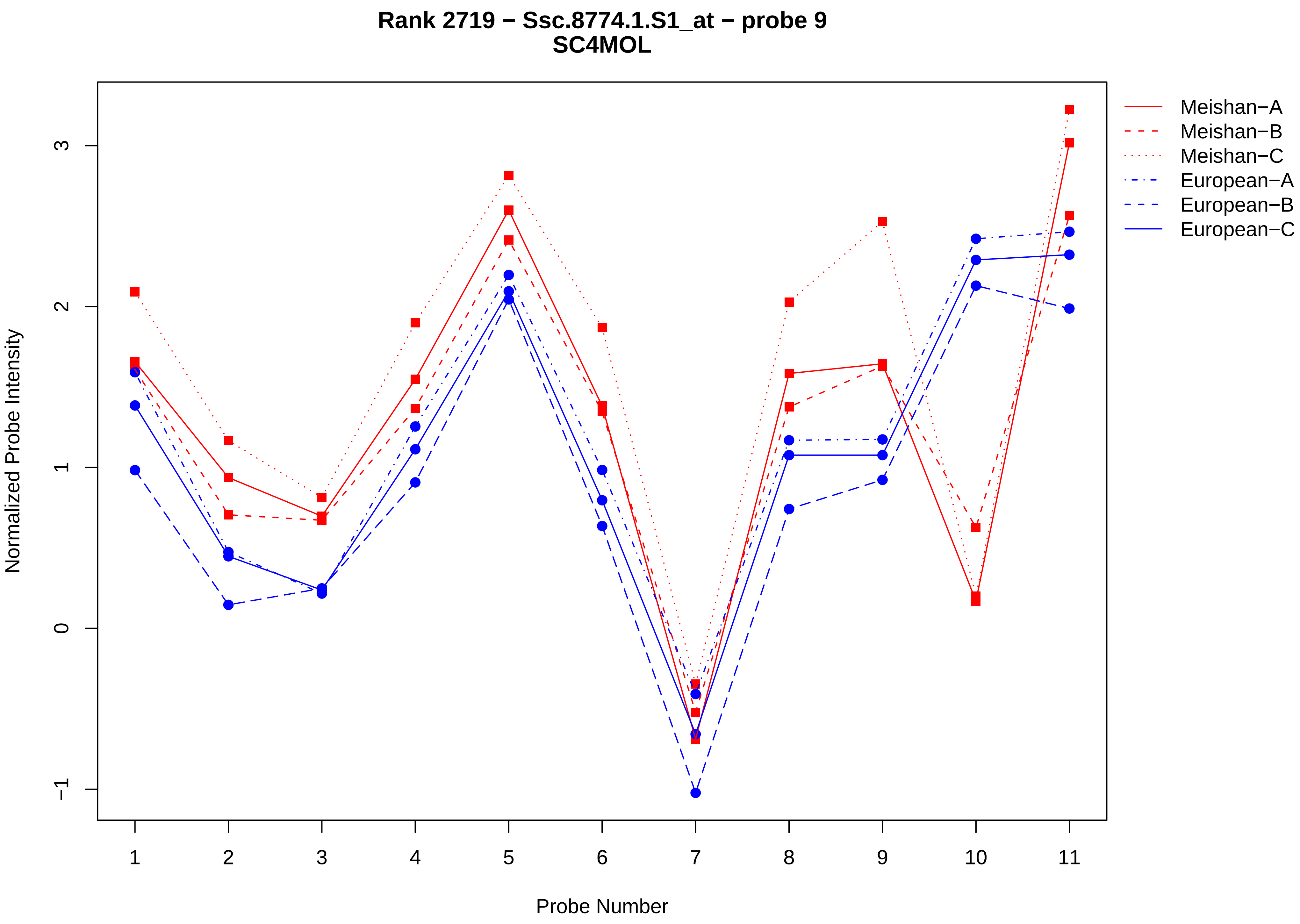
Rank 2719 − Ssc.8774.1.S1_at − probe 9
SC4MOL
Normalized Probe Intensity
Probe Number
−1
0
1
2
3
1
2
3
4
5
6
7
8
9
10
11
Meishan−A
Meishan−B
Meishan−C
European−A
European−B
European−C

Rank 2720 − Ssc.18180.1.A1_at − probe 1
NP_872628

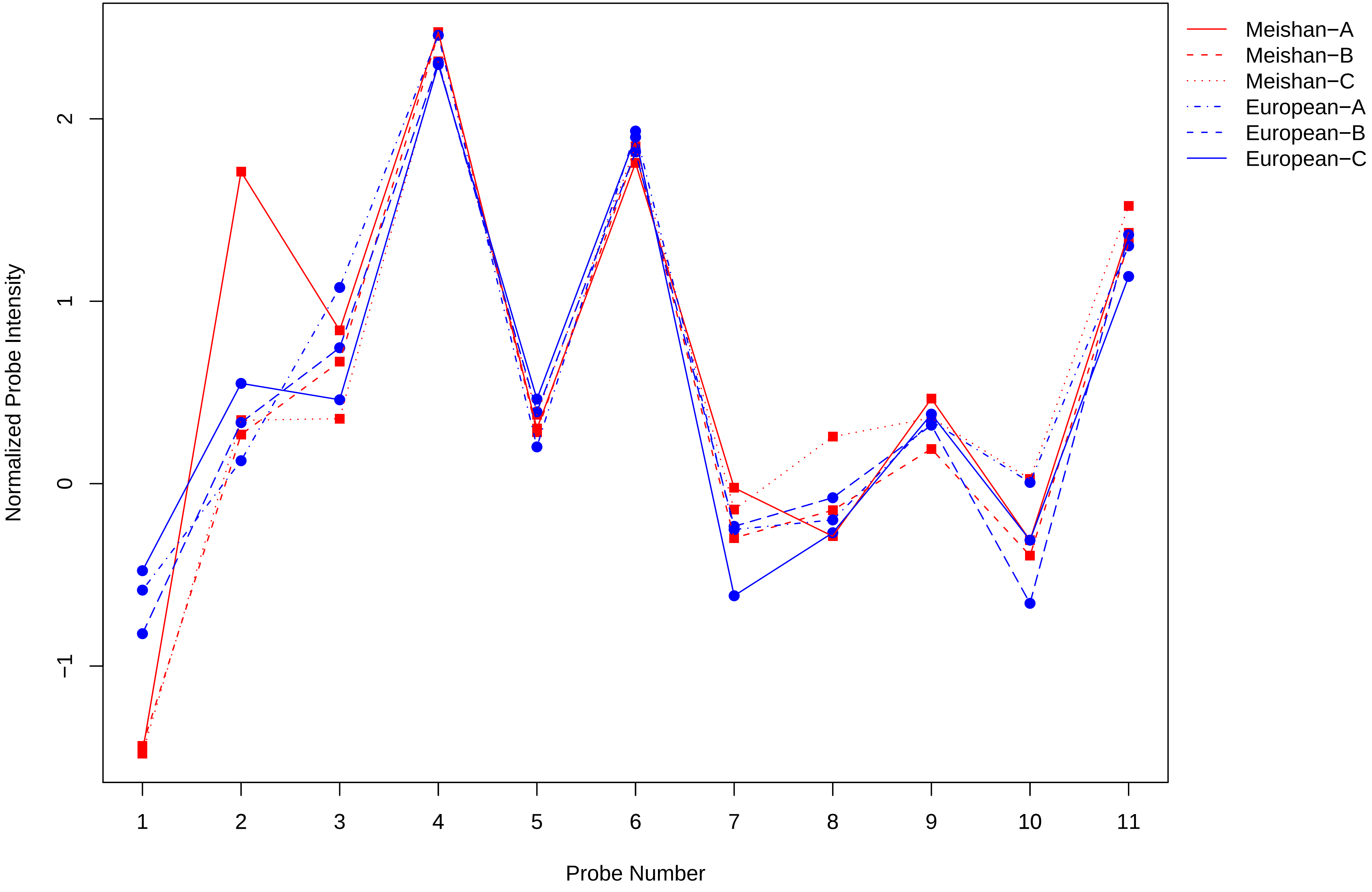

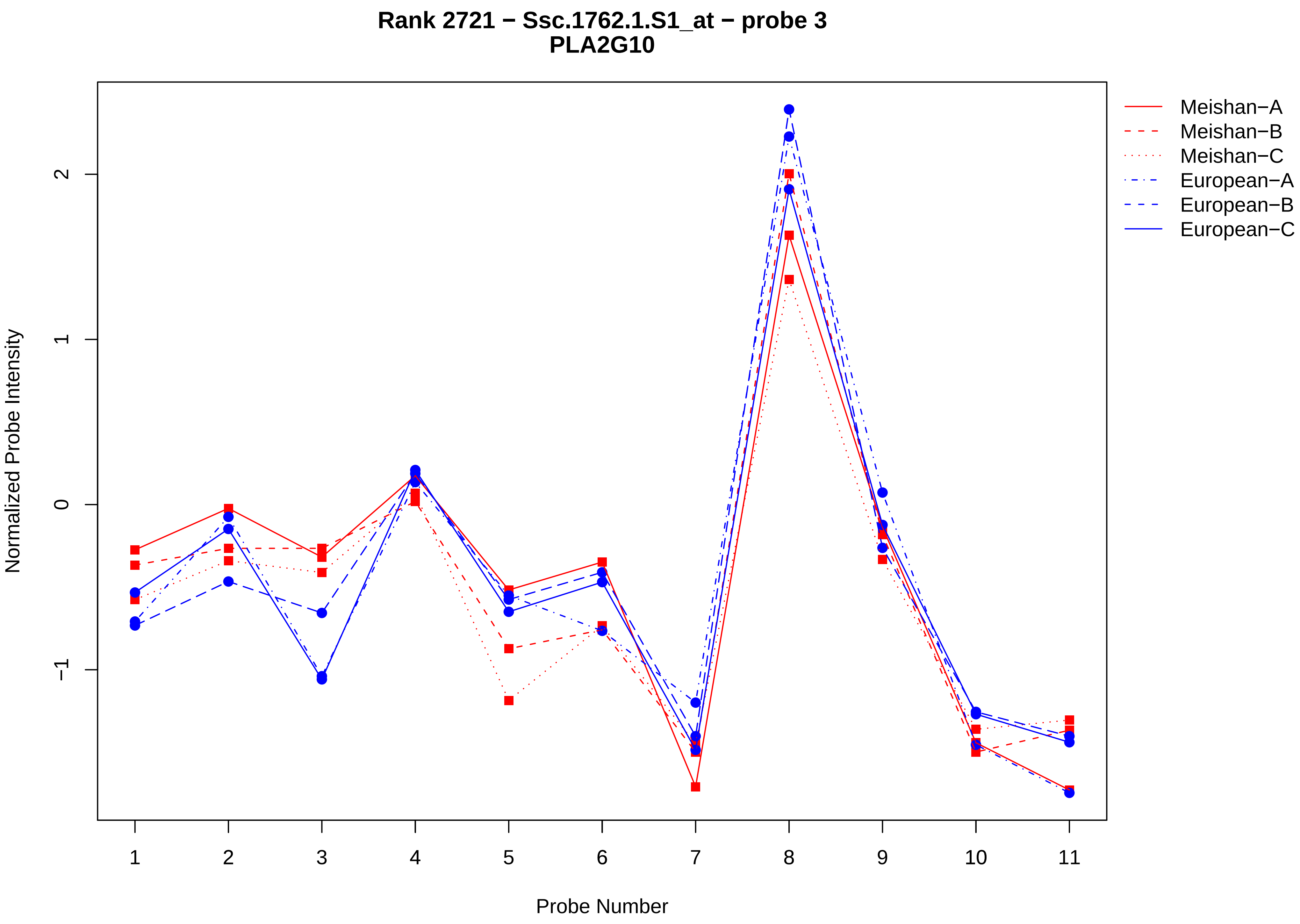

Rank 2721 − Ssc.1762.1.S1_at − probe 3
PLA2G10
Normalized Probe Intensity
−1
0
1
2
1
2
3
4
5
6
7
8
9
10
11
Probe Number
Meishan−A
Meishan−B
Meishan−C
European−A
European−B
European−C

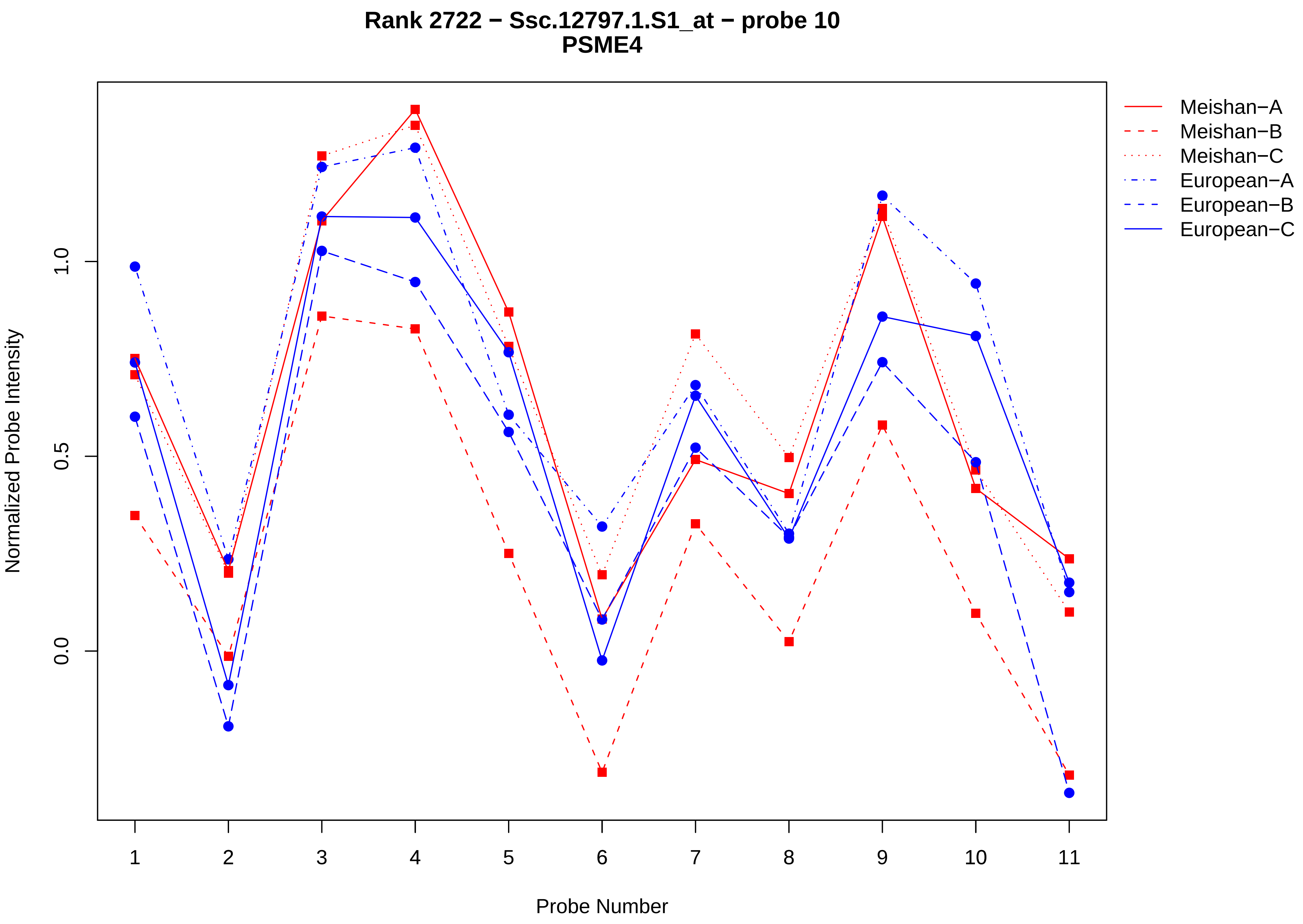

Rank 2722 − Ssc.12797.1.S1_at − probe 10
PSME4
Normalized Probe Intensity
0.0
0.5
1.0
Probe Number
1
2
3
4
5
6
7
8
9
10
11
Meishan−A
Meishan−B
Meishan−C
European−A
European−B
European−C

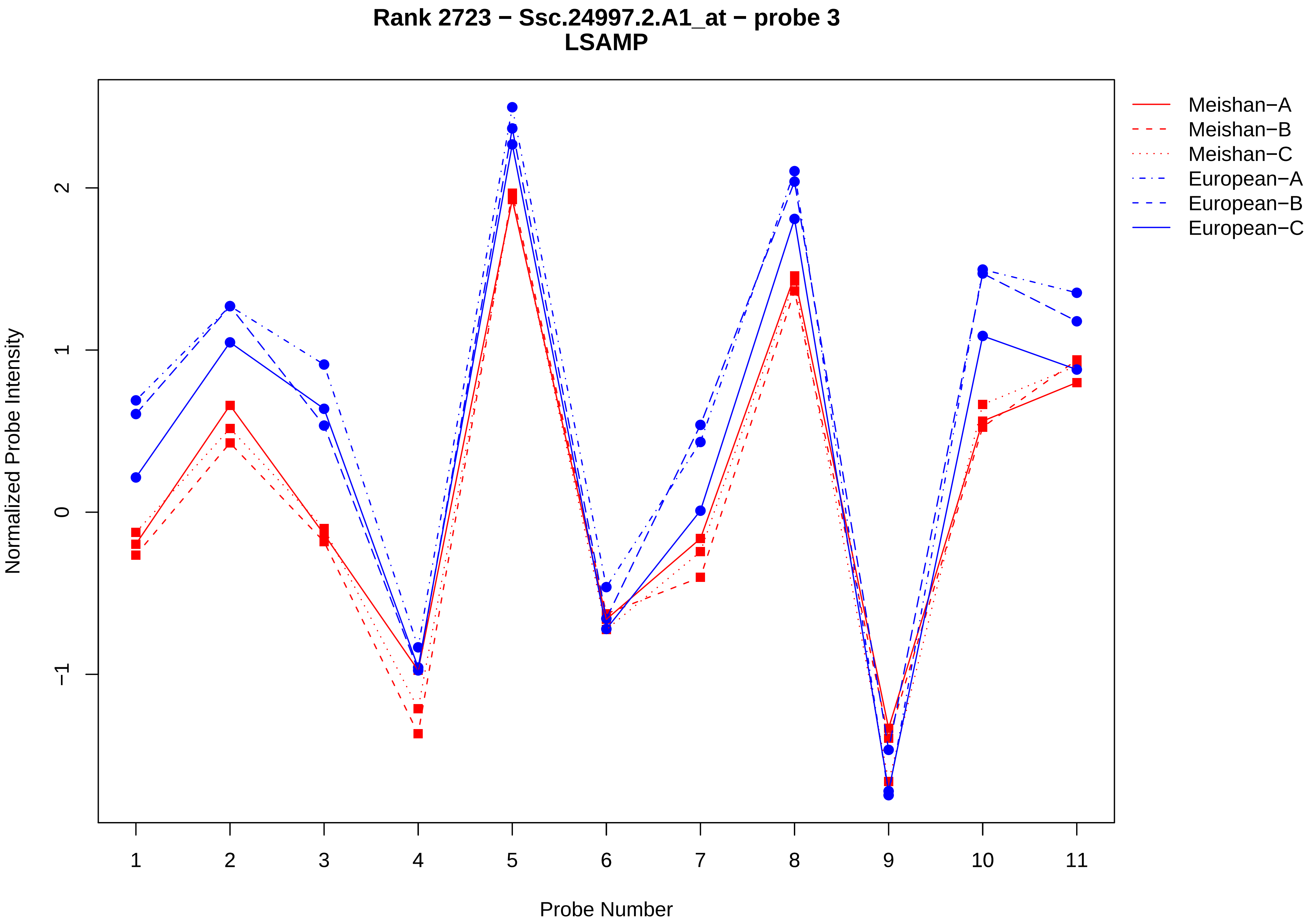
Rank 2723 − Ssc.24997.2.A1_at − probe 3
LSAMP
Normalized Probe Intensity
Probe Number
Meishan−A
Meishan−B
Meishan−C
European−A
European−B
European−C

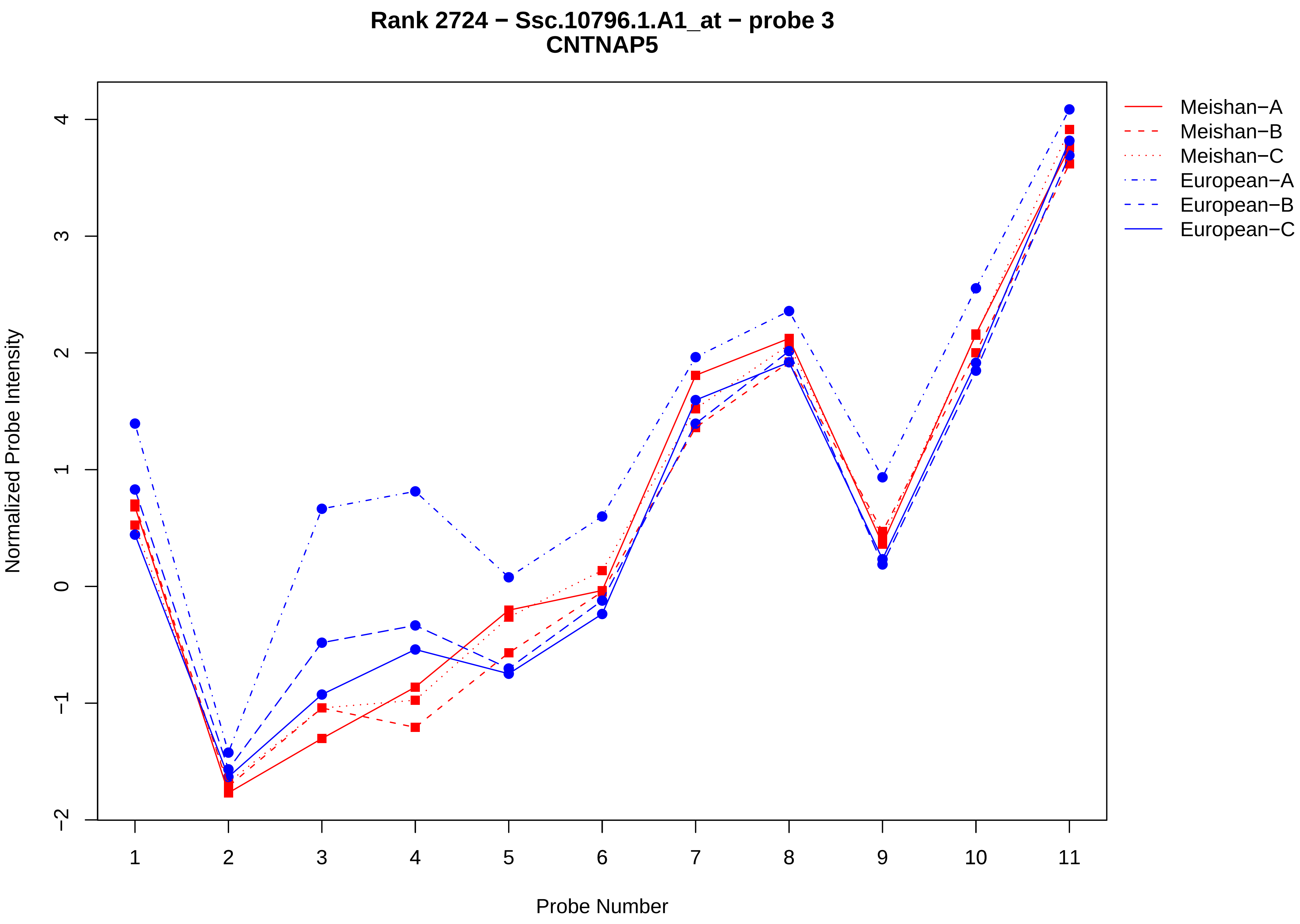
Rank 2724 − Ssc.10796.1.A1_at − probe 3
CNTNAP5
Normalized Probe Intensity
Probe Number
−2
−1
0
1
2
3
4
1
2
3
4
5
6
7
8
9
10
11
Meishan−A
Meishan−B
Meishan−C
European−A
European−B
European−C

Rank 2725 − Ssc.8929.1.S1_at − probe 5
NP_699164

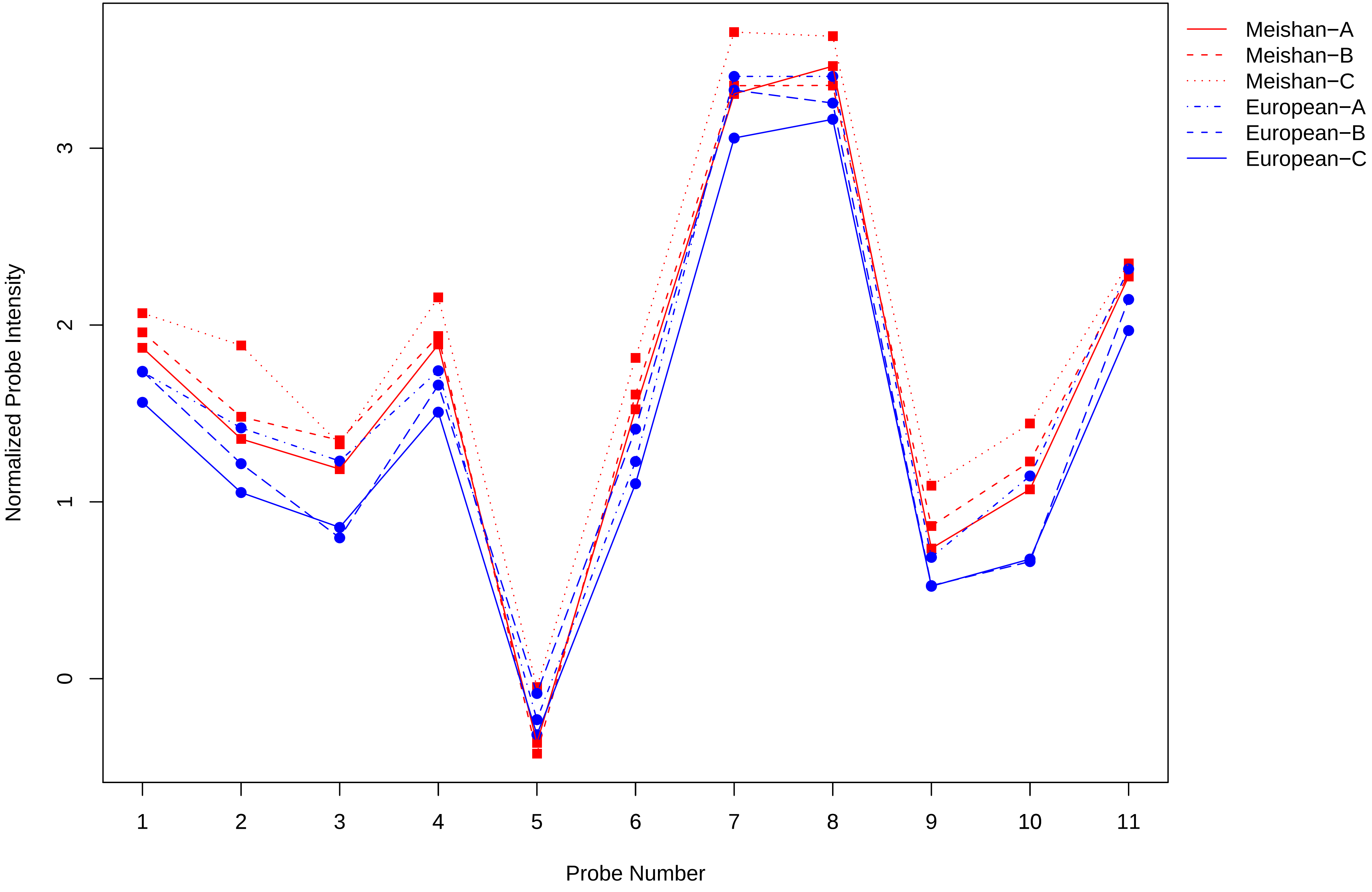

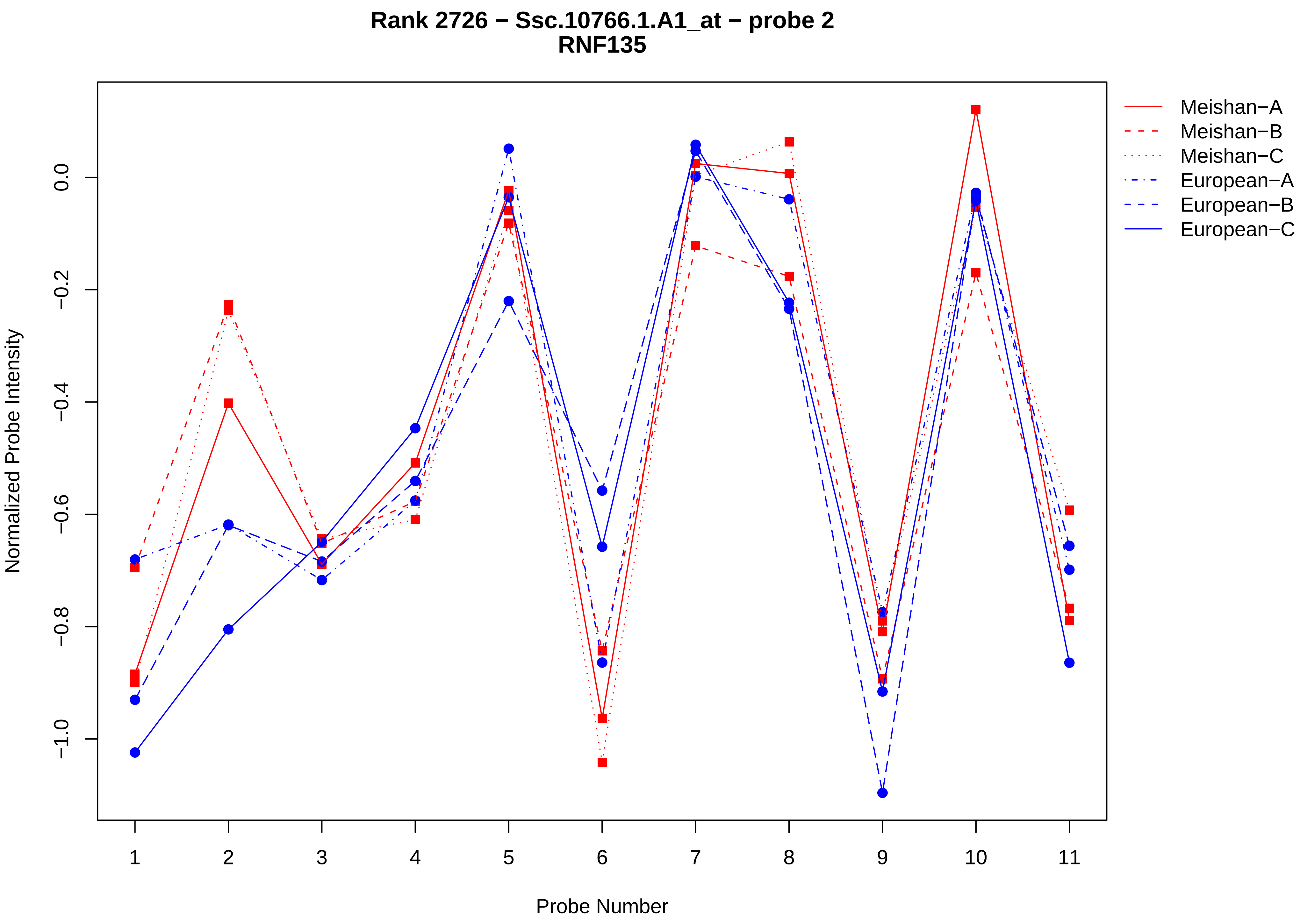

Rank 2726 − Ssc.10766.1.A1_at − probe 2
RNF135
Normalized Probe Intensity
Probe Number
0.0
−0.2
−0.4
−0.6
−0.8
−1.0
1
2
3
4
5
6
7
8
9
10
11
Meishan−A
Meishan−B
Meishan−C
European−A
European−B
European−C

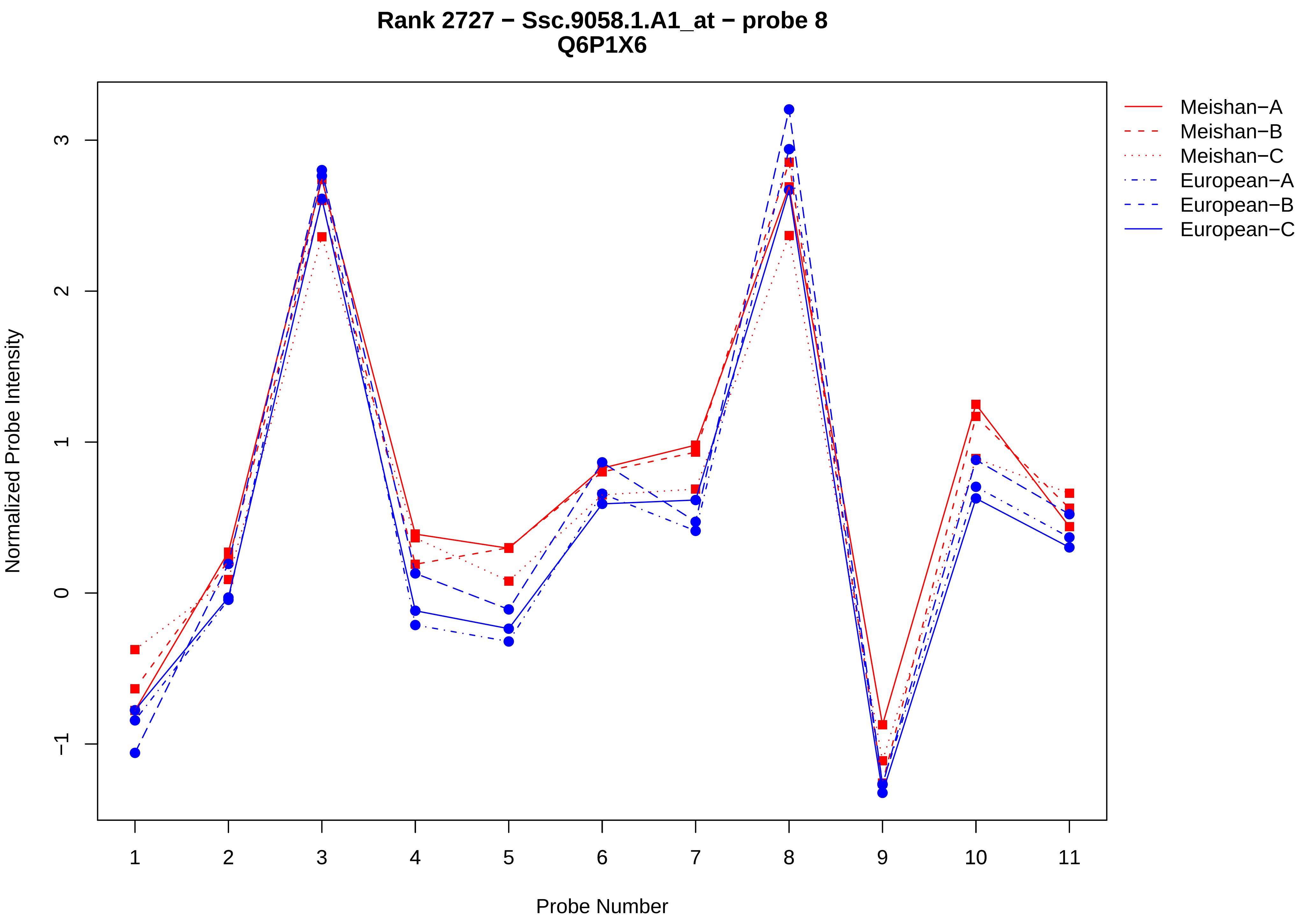

Rank 2727 − Ssc.9058.1.A1_at − probe 8
Q6P1X6
Normalized Probe Intensity
Probe Number
−1
0
1
2
3
1
2
3
4
5
6
7
8
9
10
11
Meishan−A
Meishan−B
Meishan−C
European−A
European−B
European−C

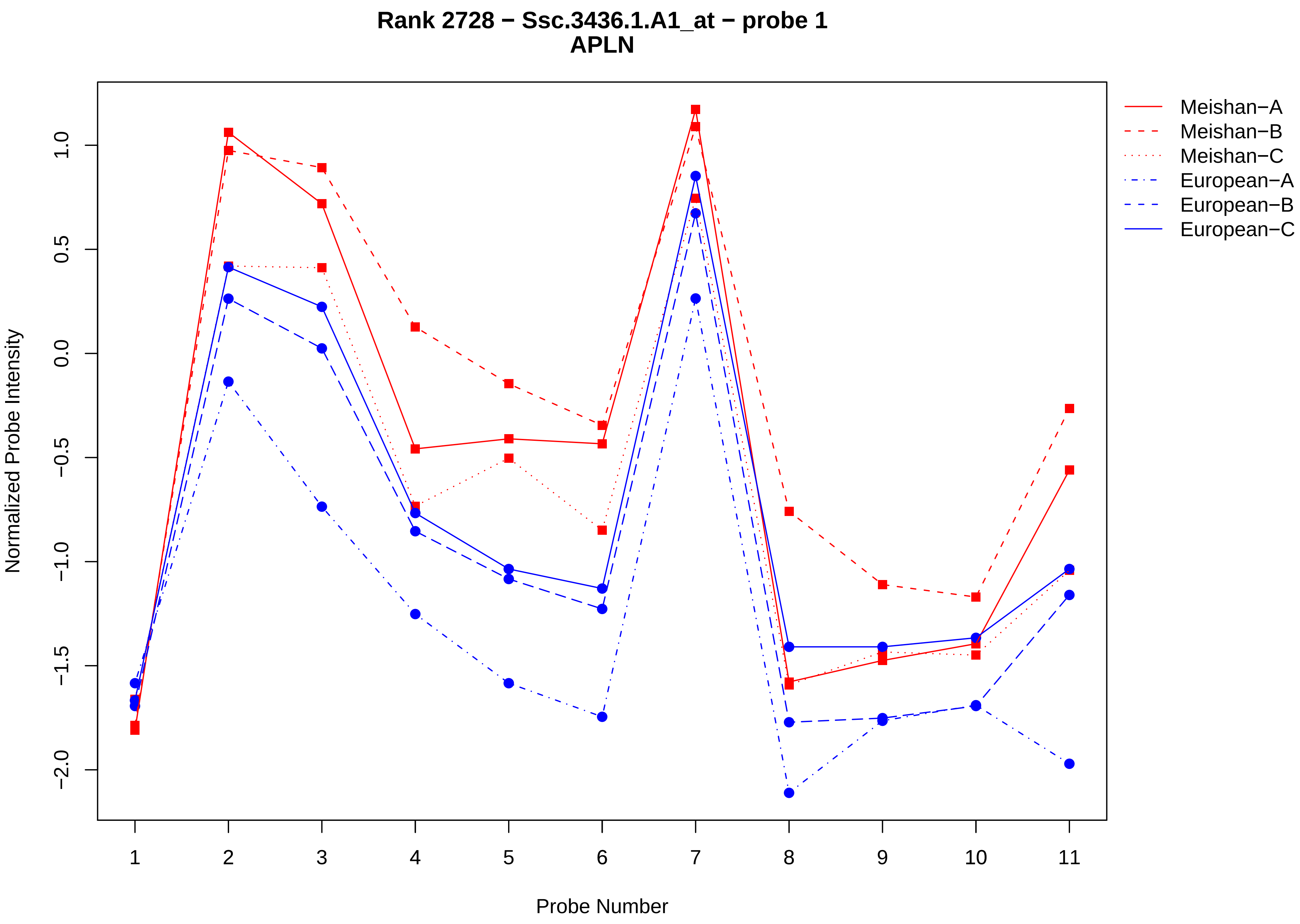

Rank 2728 − Ssc.3436.1.A1_at − probe 1
APLN
Normalized Probe Intensity
Probe Number
1.0
0.5
0.0
−0.5
−1.0
−1.5
−2.0
1
2
3
4
5
6
7
8
9
10
11
Meishan−A
Meishan−B
Meishan−C
European−A
European−B
European−C

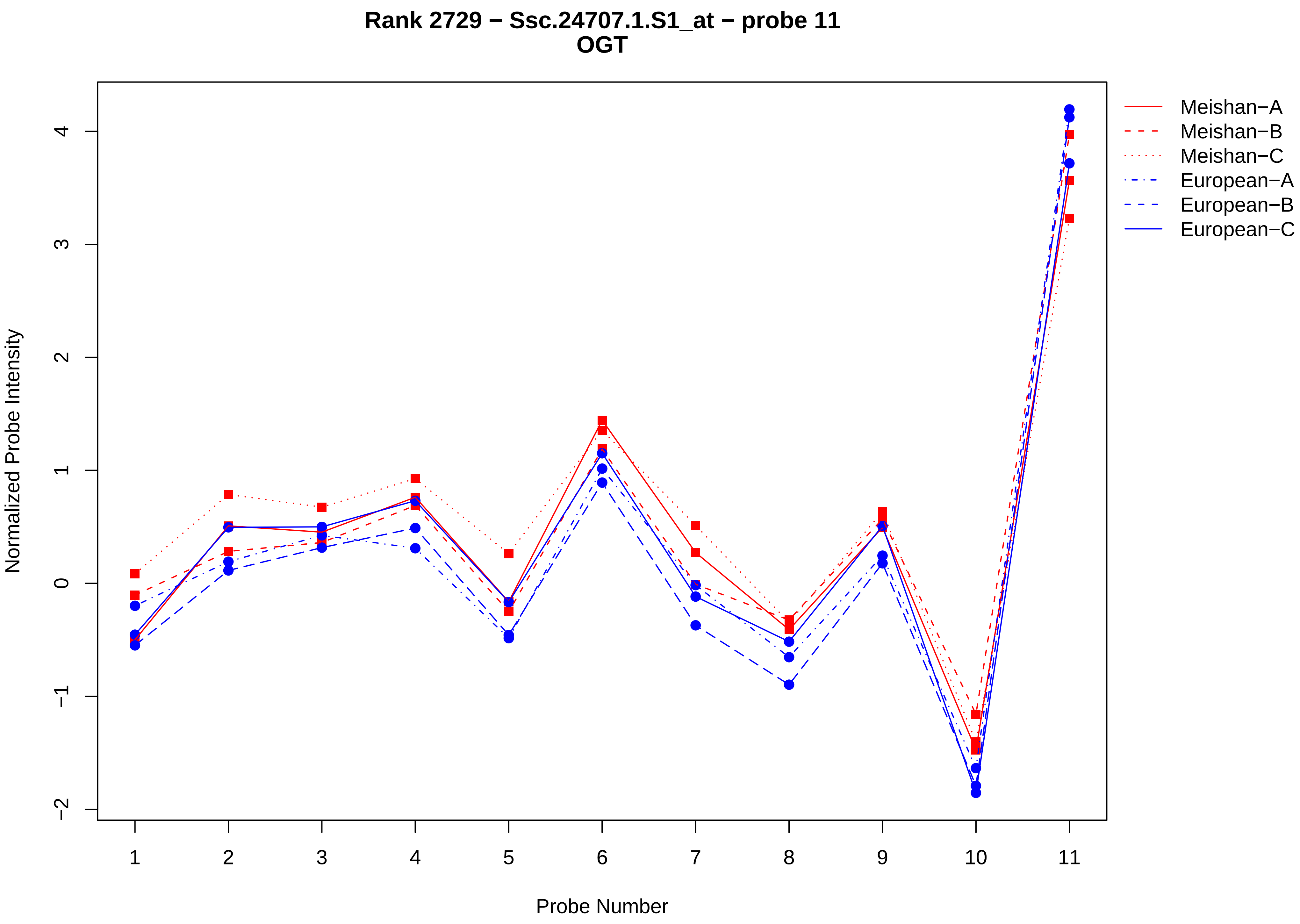

Rank 2729 − Ssc.24707.1.S1_at − probe 11
OGT
Normalized Probe Intensity
Probe Number
Meishan−A
Meishan−B
Meishan−C
European−A
European−B
European−C

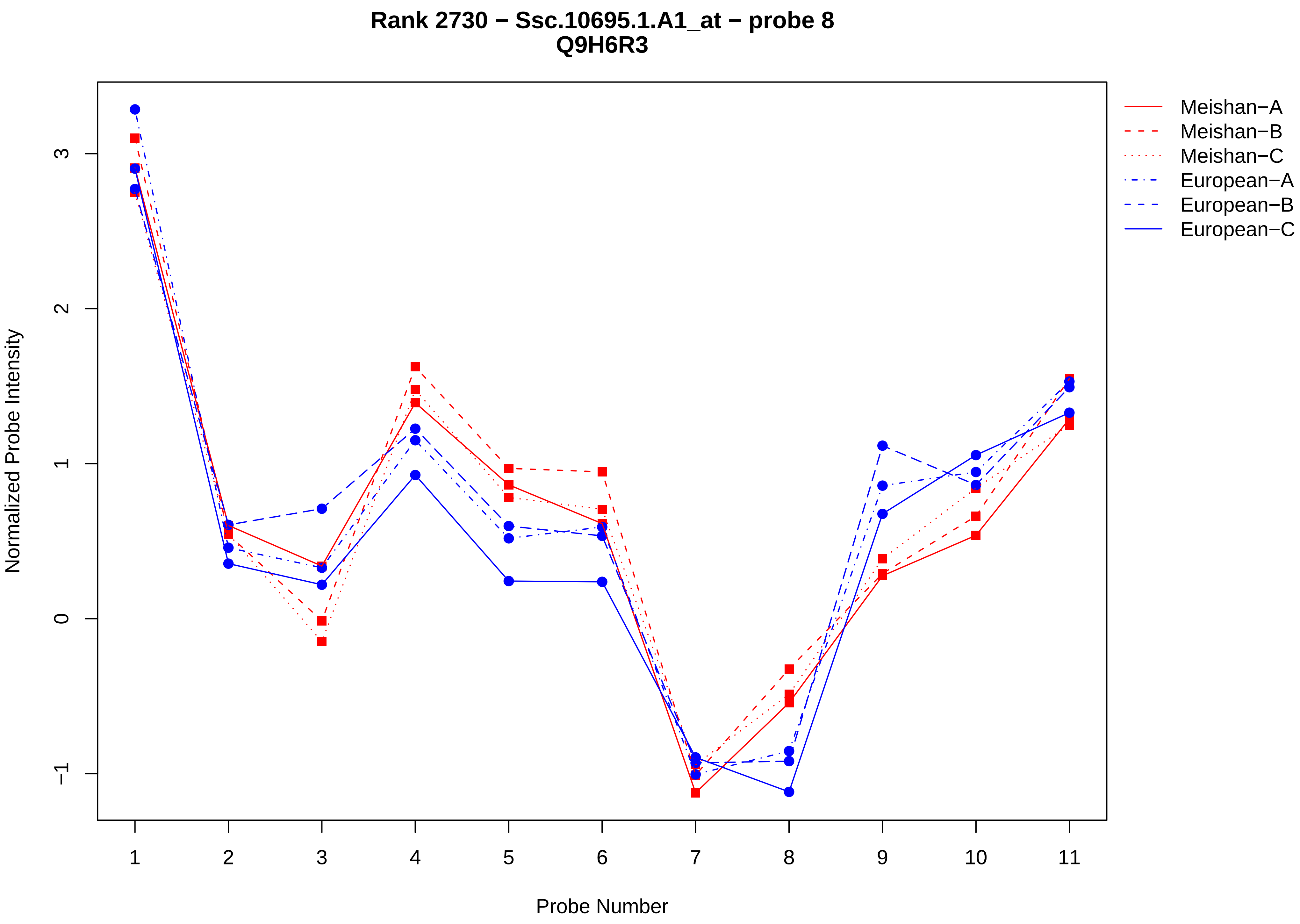
Rank 2730 − Ssc.10695.1.A1_at − probe 8
Q9H6R3
Normalized Probe Intensity
Probe Number
Meishan−A
Meishan−B
Meishan−C
European−A
European−B
European−C
−1
0
1
2
3
1
2
3
4
5
6
7
8
9
10
11

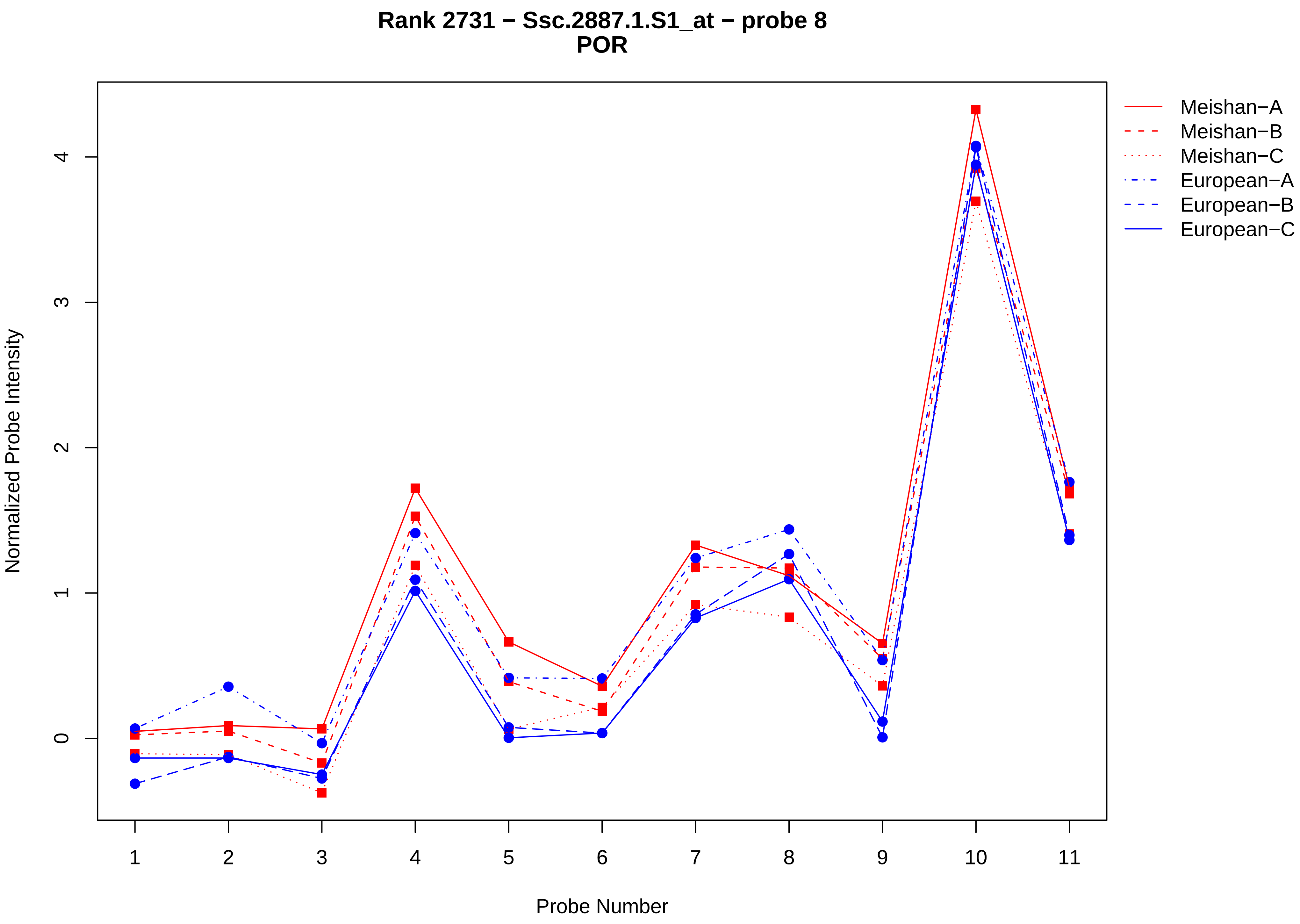

Rank 2731 − Ssc.2887.1.S1_at − probe 8
POR
Normalized Probe Intensity
Probe Number
0
1
2
3
4
1
2
3
4
5
6
7
8
9
10
11
Meishan−A
Meishan−B
Meishan−C
European−A
European−B
European−C

Rank 2732 − Ssc.12138.1.A1_at − probe 11
ROBO1

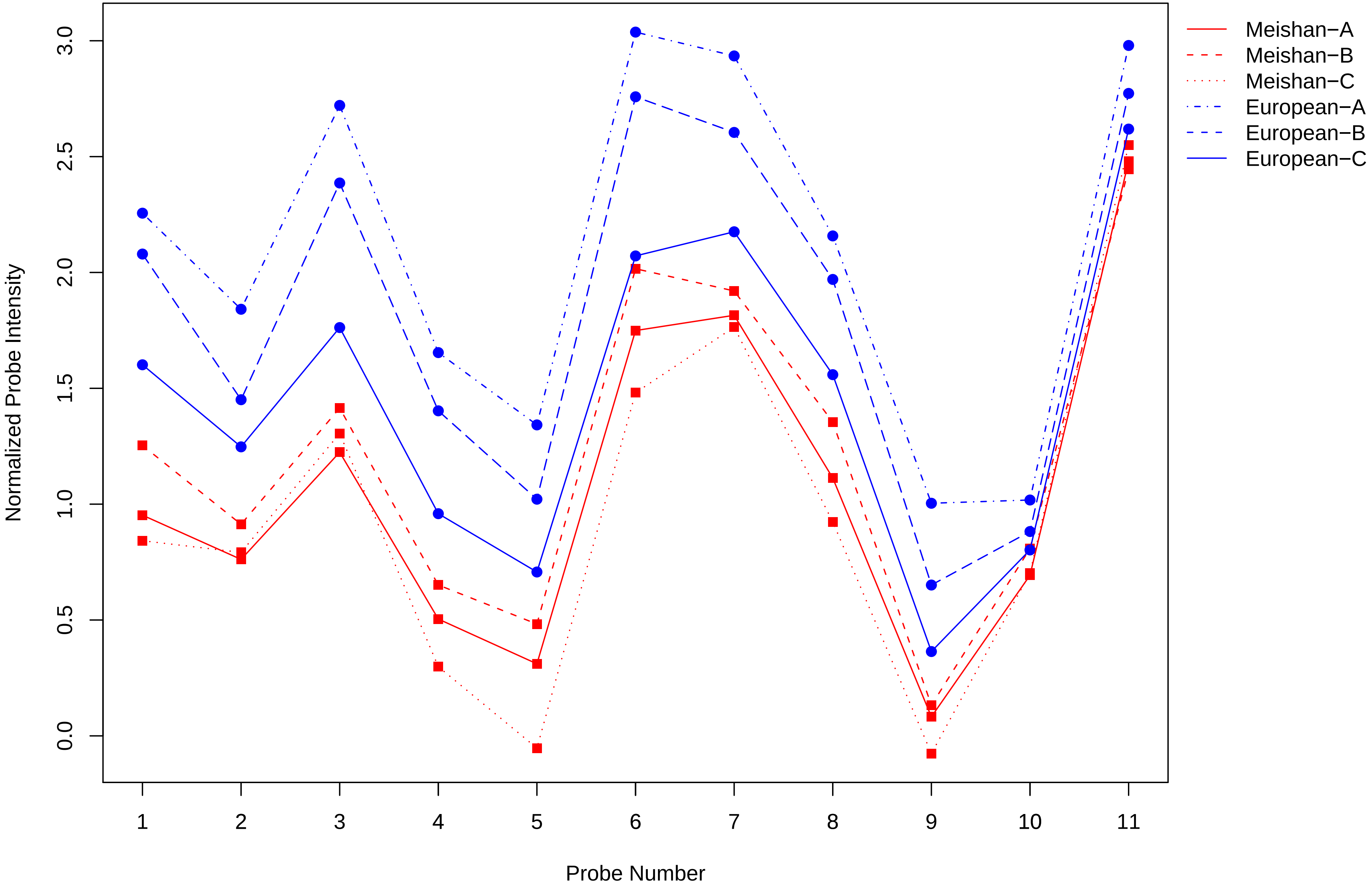

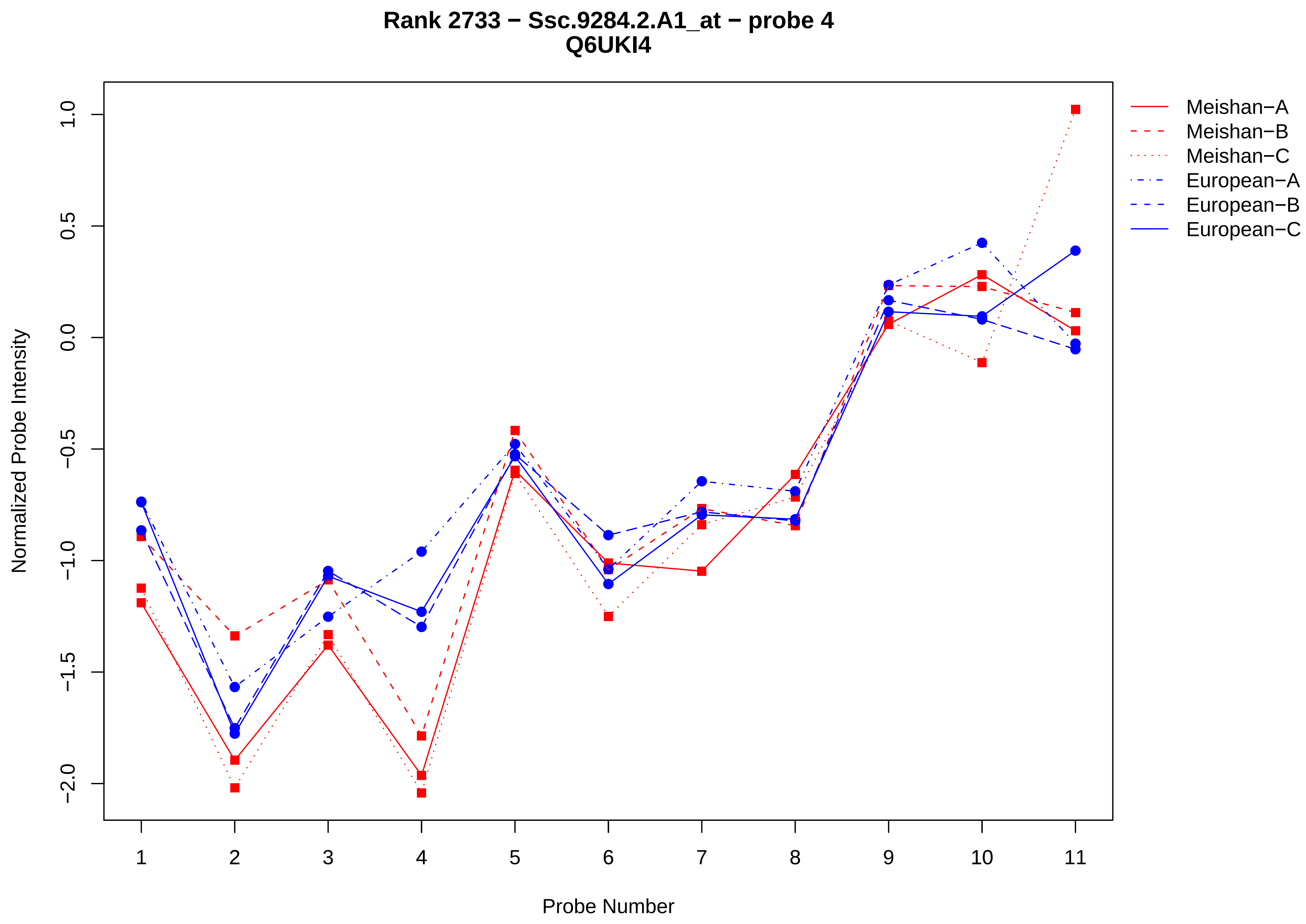

Rank 2733 − Ssc.9284.2.A1_at − probe 4
Q6UKI4
Normalized Probe Intensity
Probe Number
1.0
0.5
0.0
−0.5
−1.0
−1.5
−2.0
1
2
3
4
5
6
7
8
9
10
11
Meishan−A
Meishan−B
Meishan−C
European−A
European−B
European−C

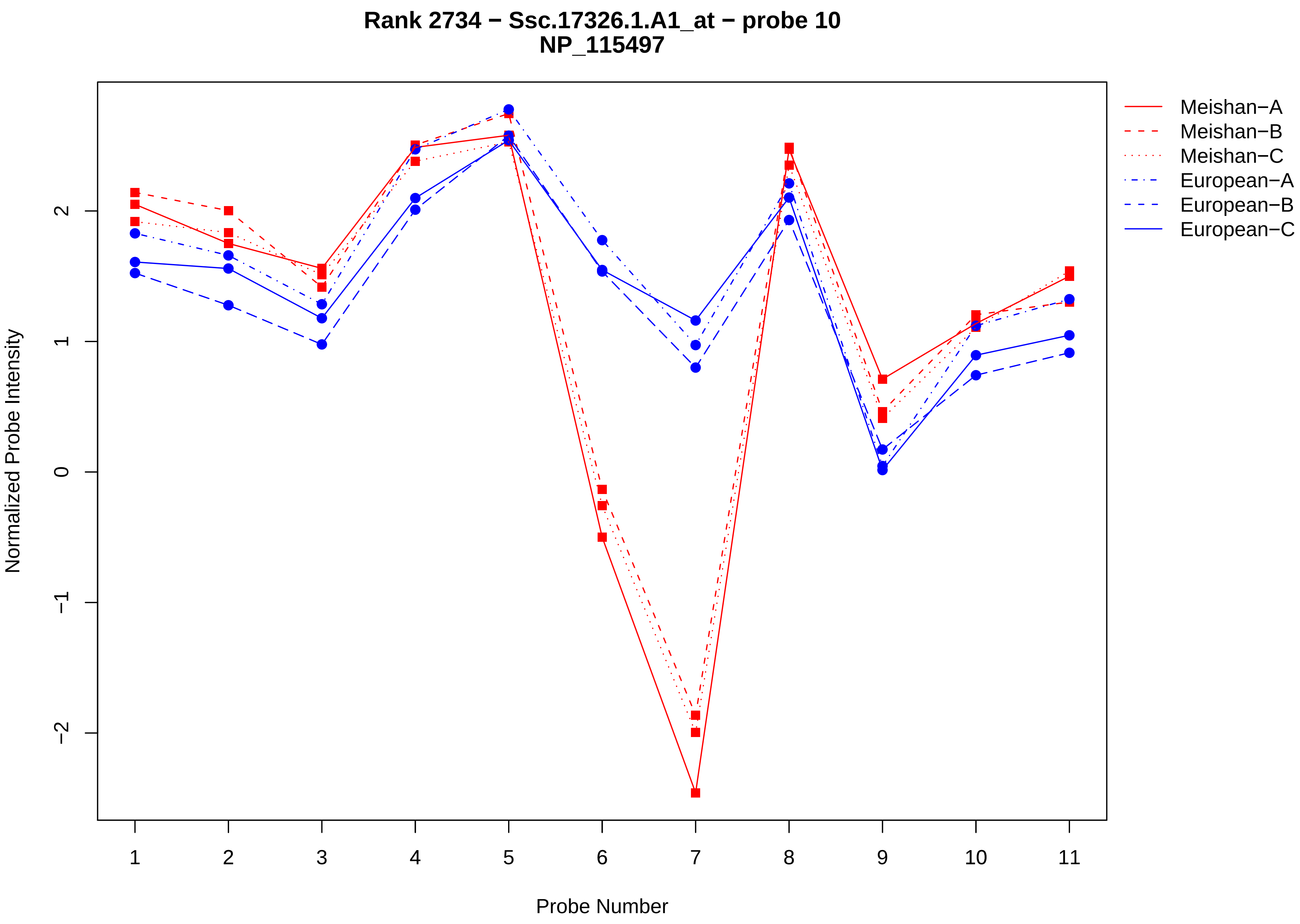
Rank 2734 − Ssc.17326.1.A1_at − probe 10
NP_115497
Normalized Probe Intensity
Probe Number
−2
−1
0
1
2
1
2
3
4
5
6
7
8
9
10
11
Meishan−A
Meishan−B
Meishan−C
European−A
European−B
European−C

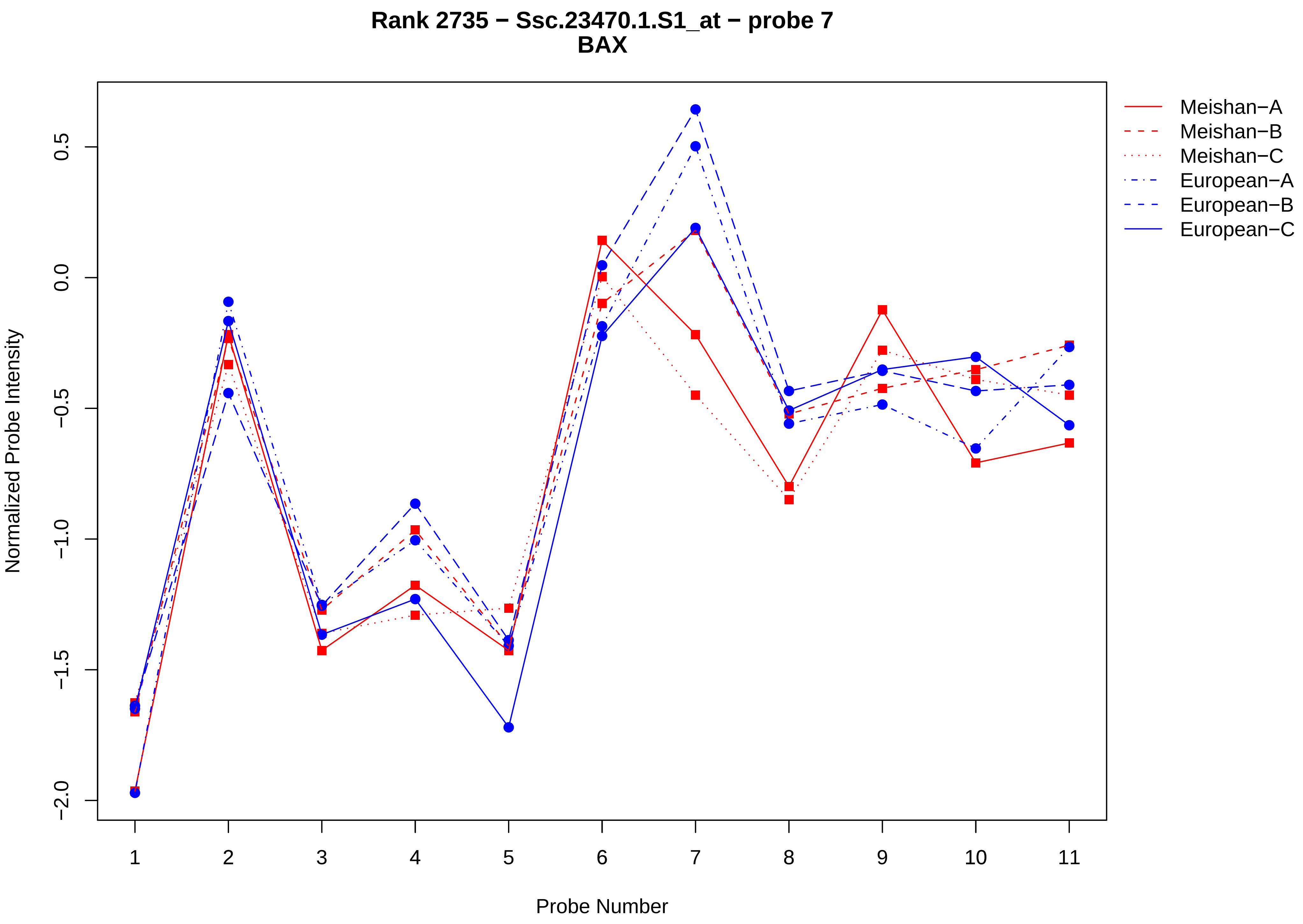

Rank 2735 − Ssc.23470.1.S1_at − probe 7
BAX
Normalized Probe Intensity
Probe Number
0.5
0.0
−0.5
−1.0
−1.5
−2.0
1
2
3
4
5
6
7
8
9
10
11
Meishan−A
Meishan−B
Meishan−C
European−A
European−B
European−C

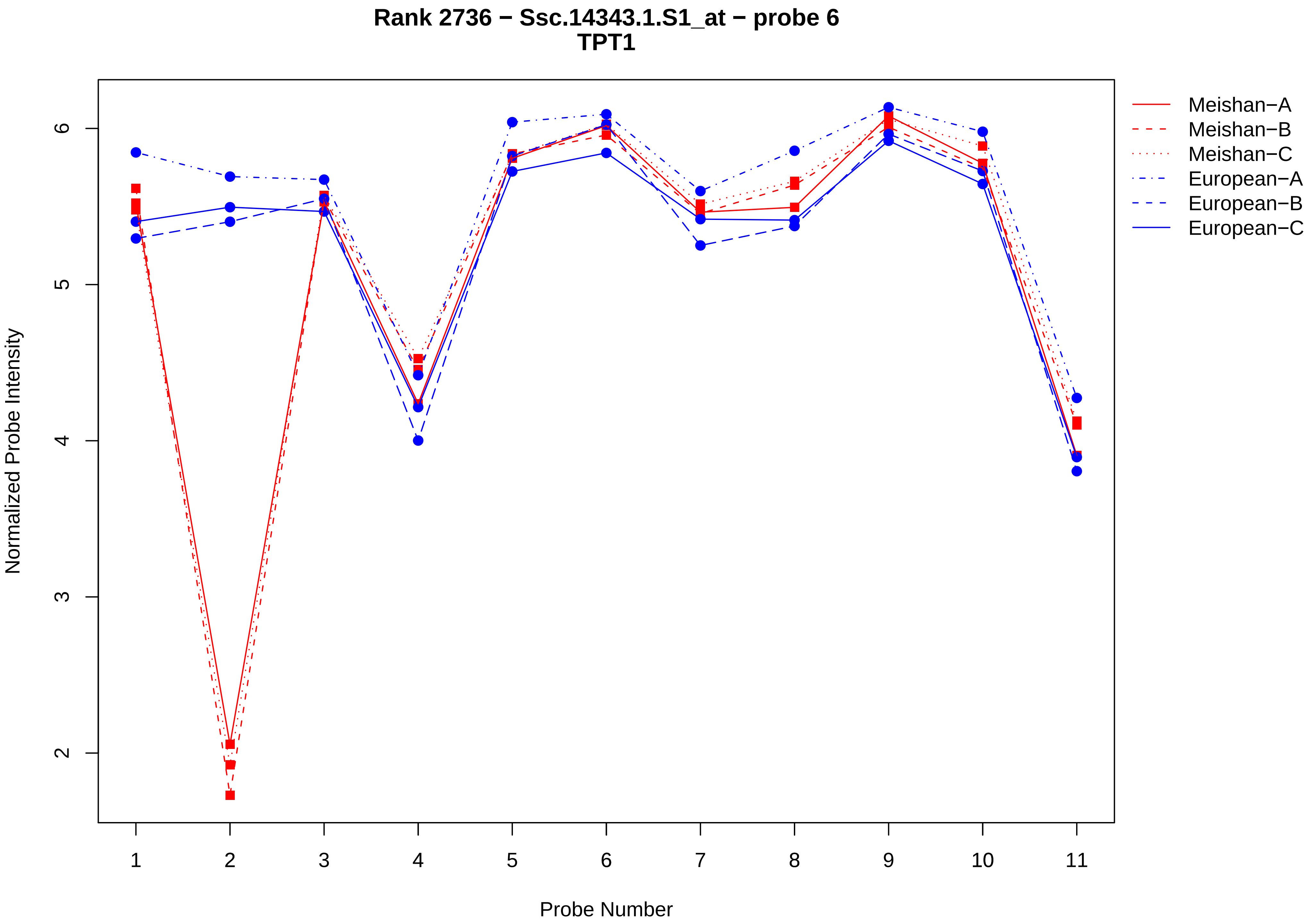

Rank 2736 − Ssc.14343.1.S1_at − probe 6
TPT1
Normalized Probe Intensity
Probe Number
Meishan−A
Meishan−B
Meishan−C
European−A
European−B
European−C

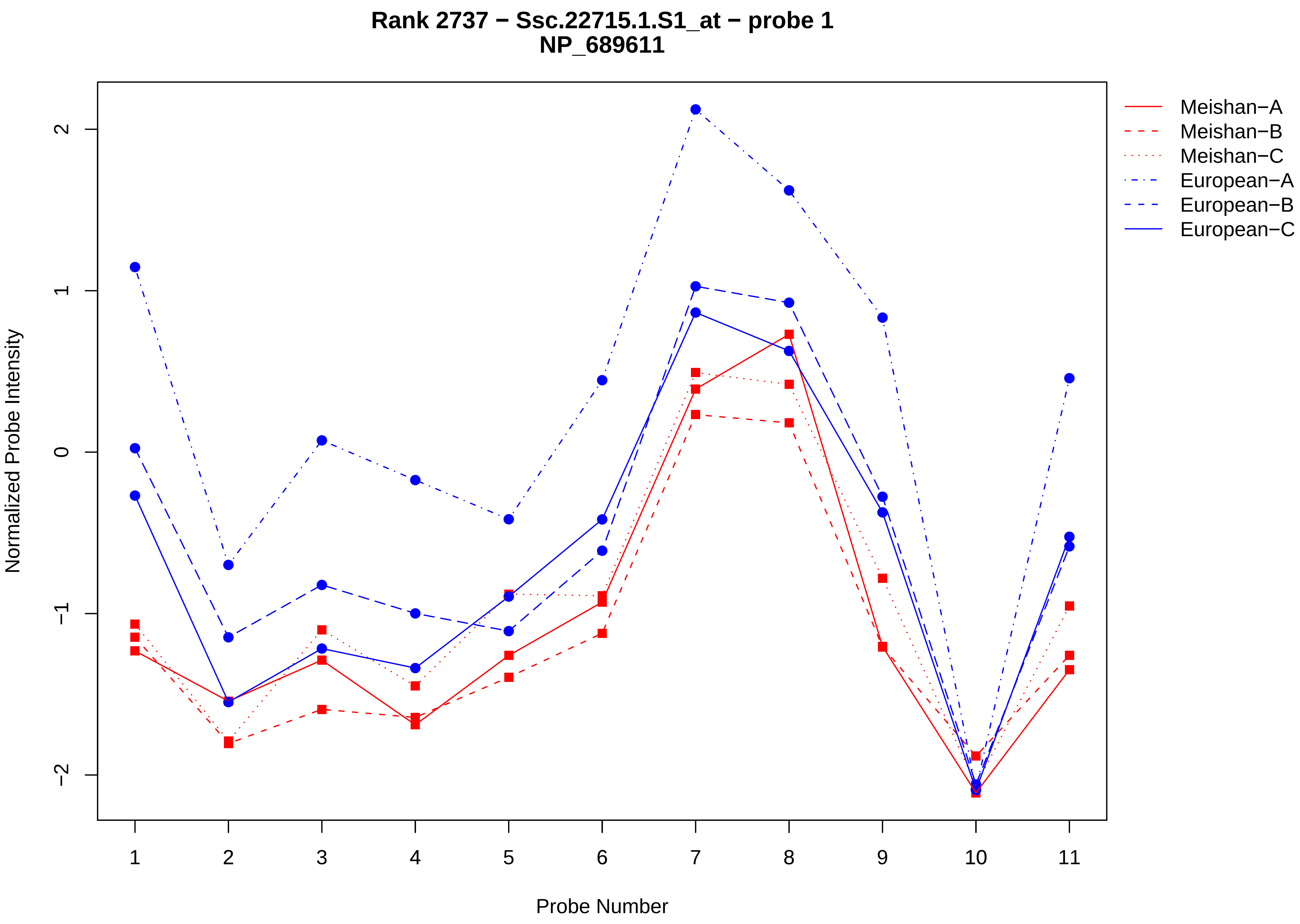

Rank 2737 − Ssc.22715.1.S1_at − probe 1
NP_689611
Normalized Probe Intensity
Probe Number
2
1
0
−1
−2
1
2
3
4
5
6
7
8
9
10
11
Meishan−A
Meishan−B
Meishan−C
European−A
European−B
European−C

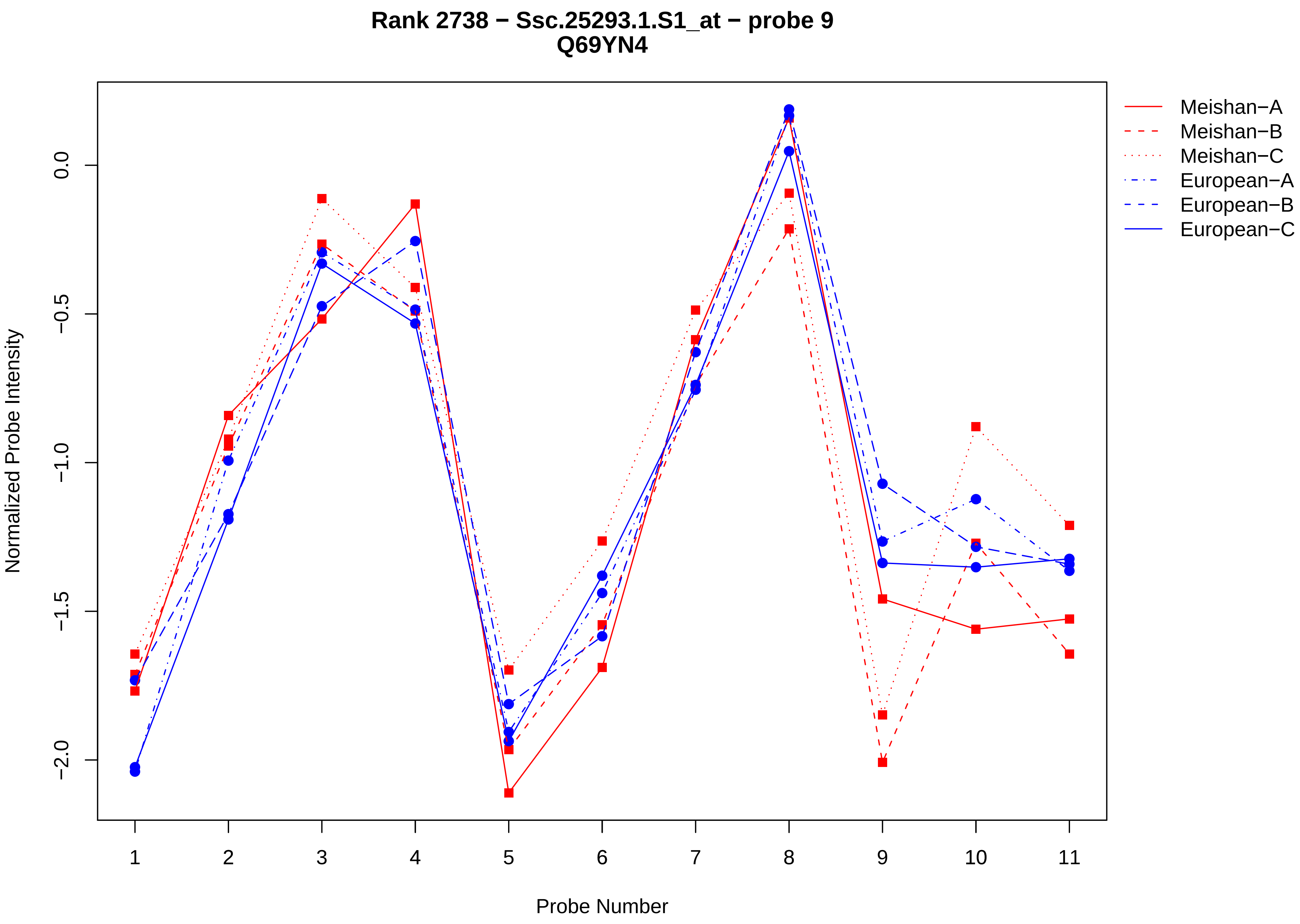

Rank 2738 − Ssc.25293.1.S1_at − probe 9
Q69YN4
Normalized Probe Intensity
Probe Number
0.0
−0.5
−1.0
−1.5
−2.0
1
2
3
4
5
6
7
8
9
10
11
Meishan−A
Meishan−B
Meishan−C
European−A
European−B
European−C

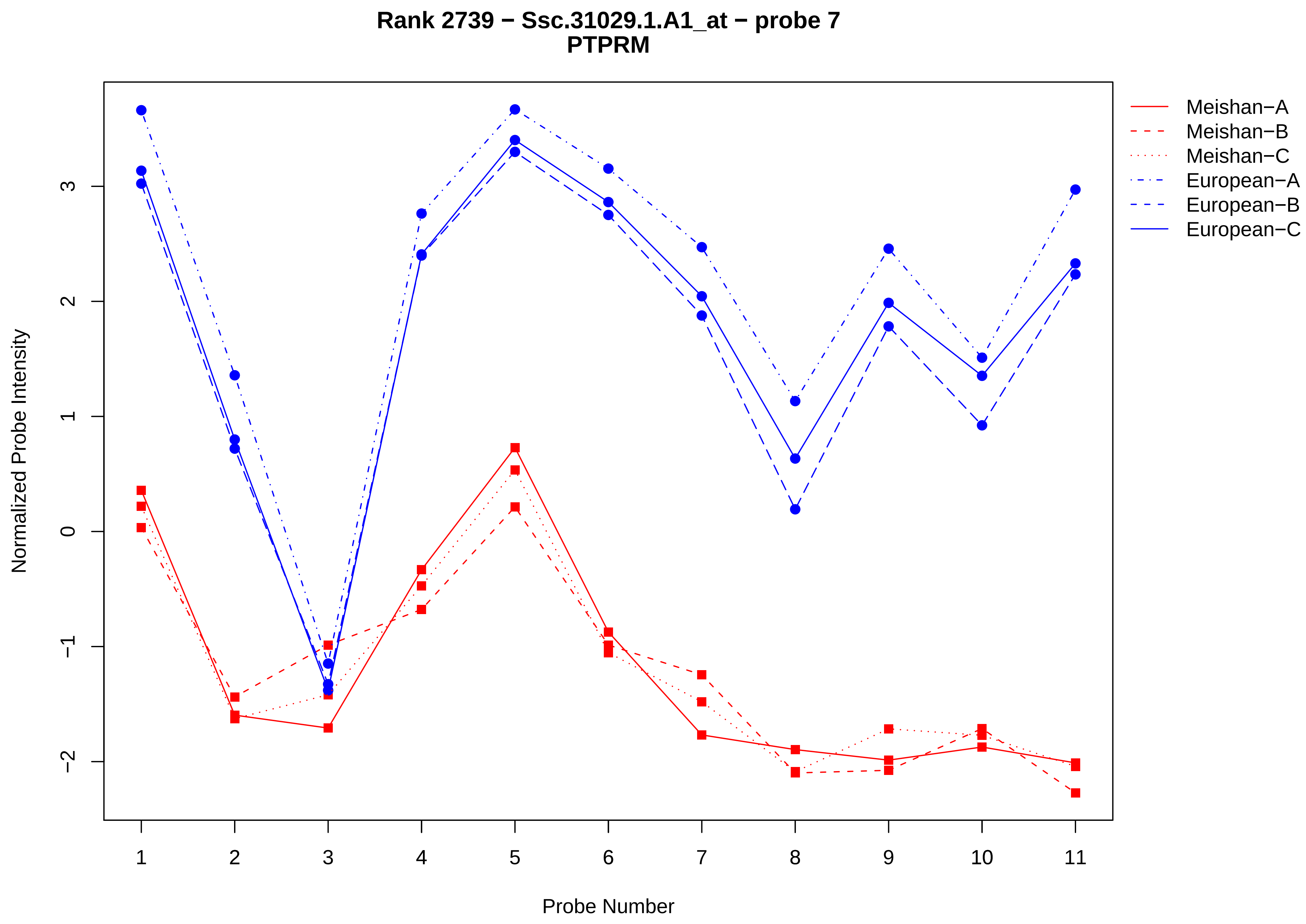

Rank 2739 − Ssc.31029.1.A1_at − probe 7
PTPRM
Normalized Probe Intensity
Probe Number
−2
−1
0
1
2
3
1
2
3
4
5
6
7
8
9
10
11
Meishan−A
Meishan−B
Meishan−C
European−A
European−B
European−C

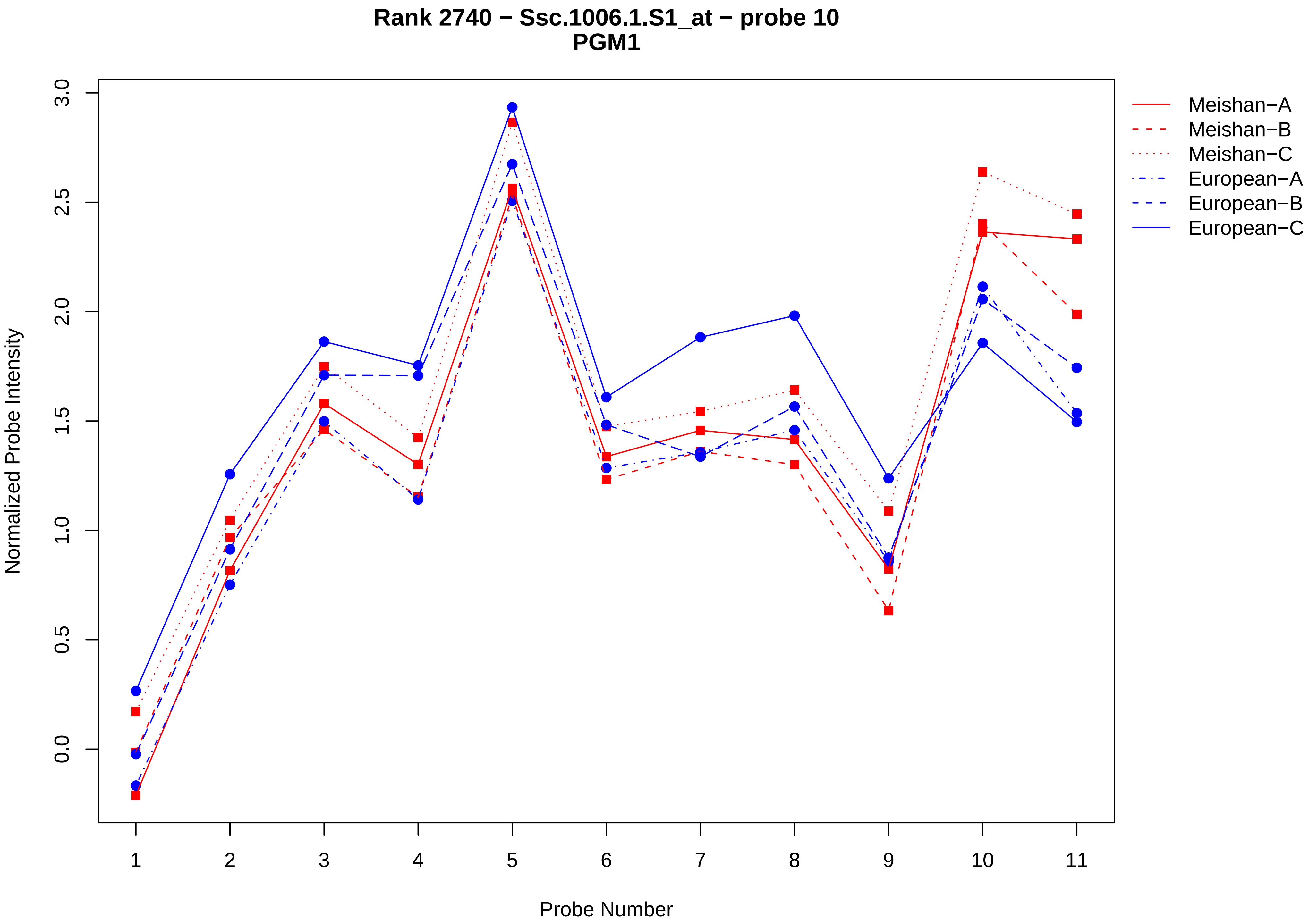

Rank 2740 − Ssc.1006.1.S1_at − probe 10
PGM1
Normalized Probe Intensity
Probe Number
0.0
0.5
1.0
1.5
2.0
2.5
3.0
1
2
3
4
5
6
7
8
9
10
11
Meishan−A
Meishan−B
Meishan−C
European−A
European−B
European−C

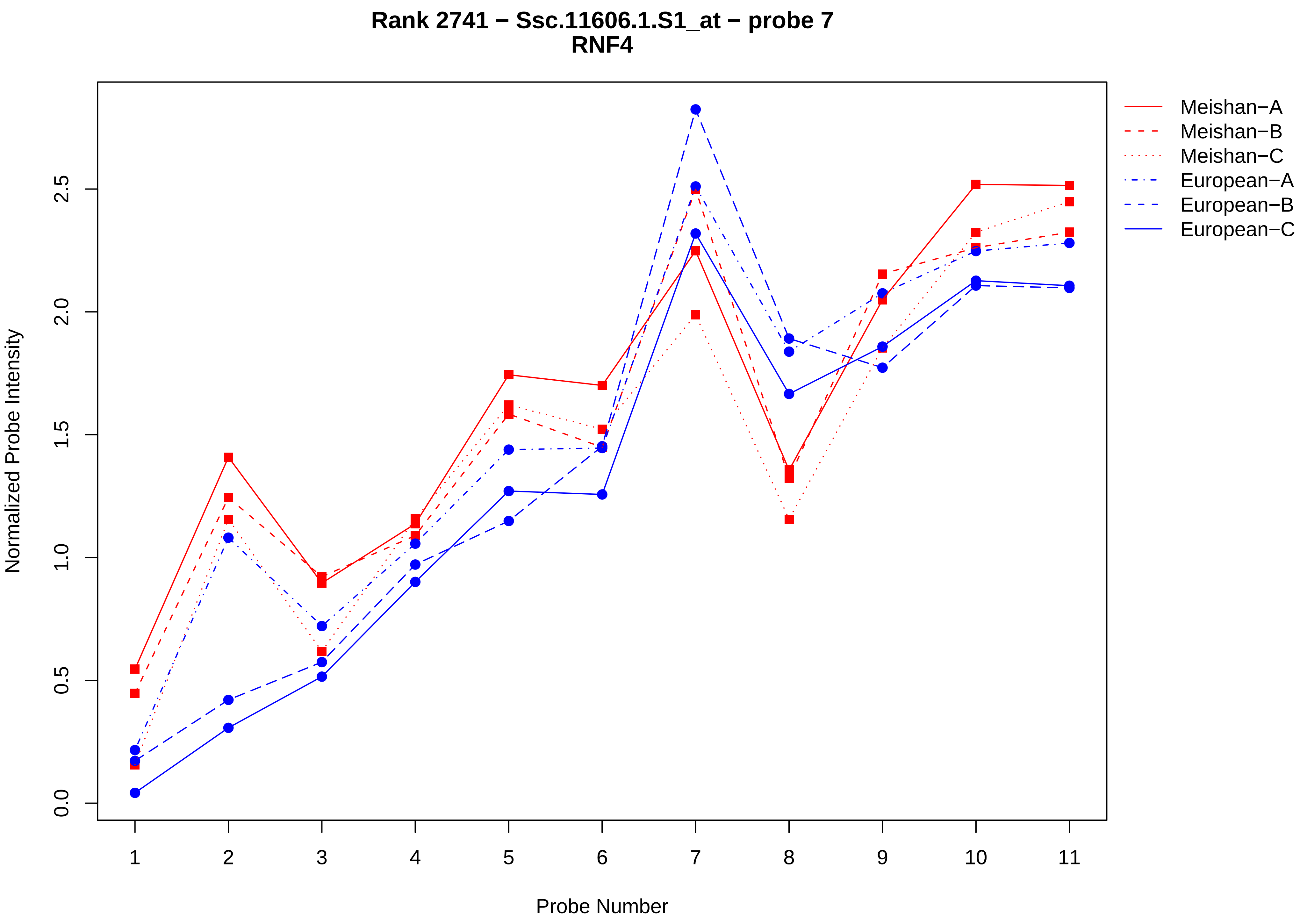

Rank 2741 − Ssc.11606.1.S1_at − probe 7
RNF4
Normalized Probe Intensity
0.0
0.5
1.0
1.5
2.0
2.5
1
2
3
4
5
6
7
8
9
10
11
Probe Number
Meishan−A
Meishan−B
Meishan−C
European−A
European−B
European−C

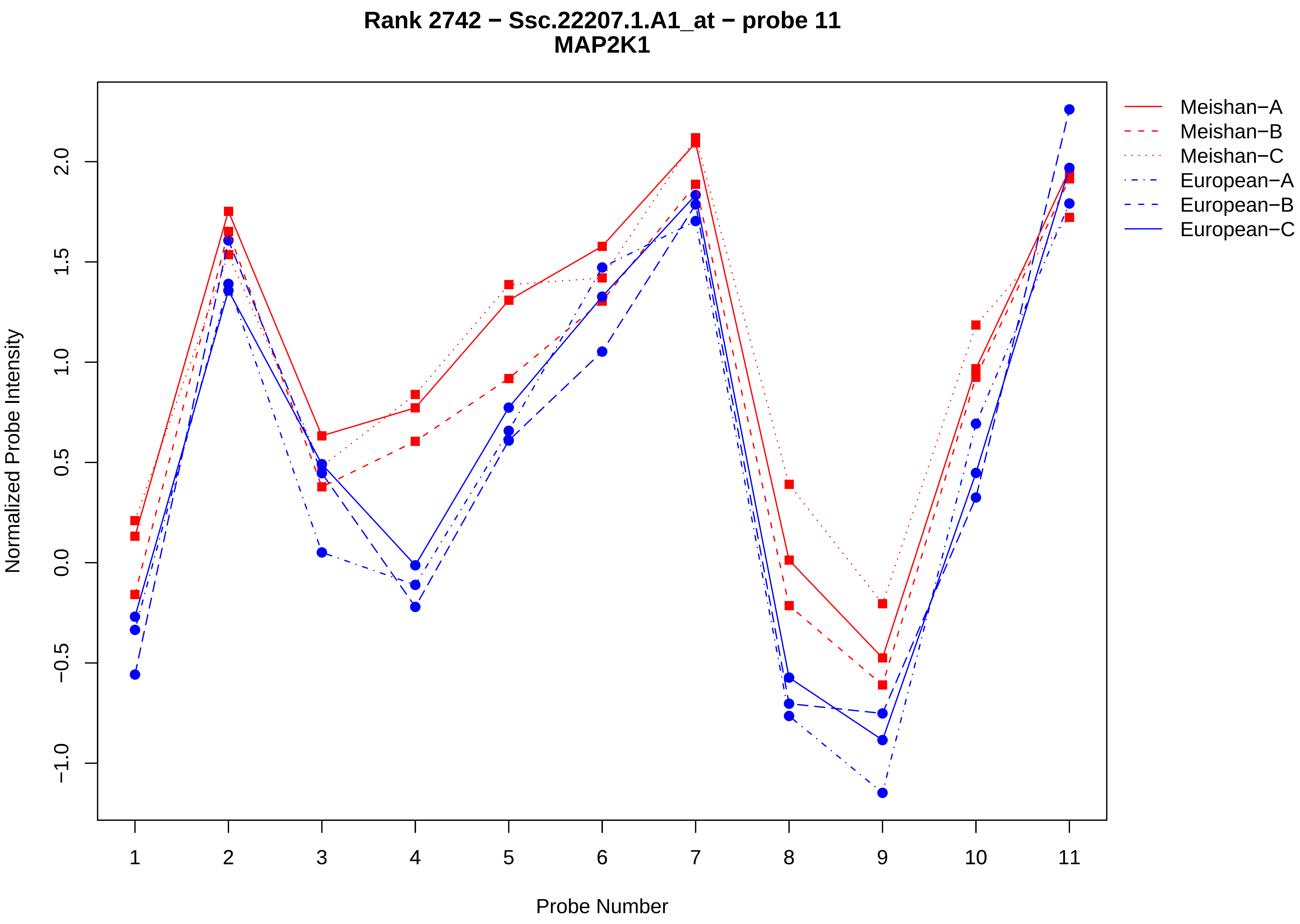
Rank 2742 − Ssc.22207.1.A1_at − probe 11
MAP2K1
Normalized Probe Intensity
Probe Number
−1.0
−0.5
0.0
0.5
1.0
1.5
2.0
1
2
3
4
5
6
7
8
9
10
11
Meishan−A
Meishan−B
Meishan−C
European−A
European−B
European−C

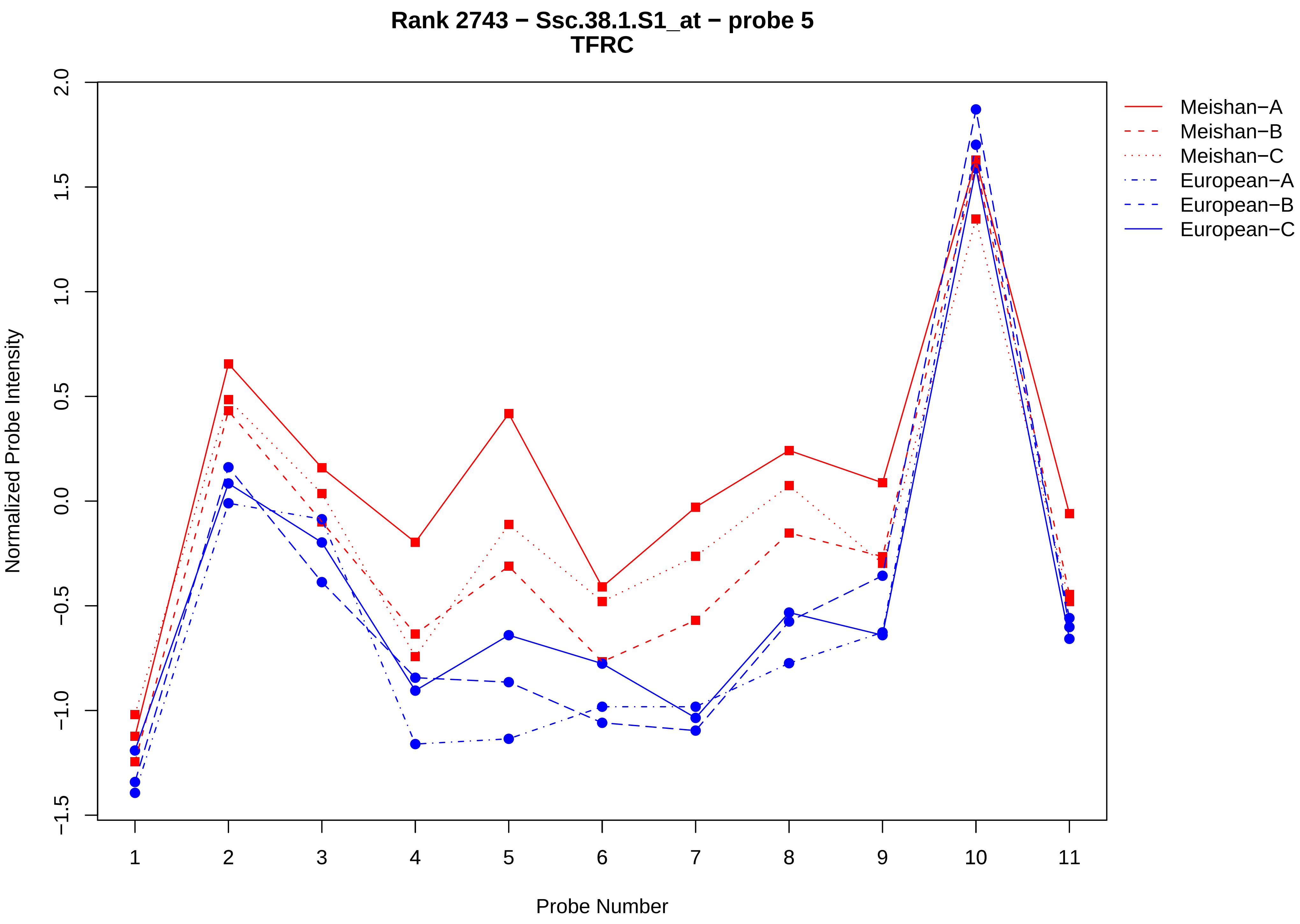
Rank 2743 − Ssc.38.1.S1_at − probe 5
TFRC
Normalized Probe Intensity
Probe Number
2.0
1.5
1.0
0.5
0.0
−0.5
−1.0
−1.5
1
2
3
4
5
6
7
8
9
10
11
Meishan−A
Meishan−B
Meishan−C
European−A
European−B
European−C

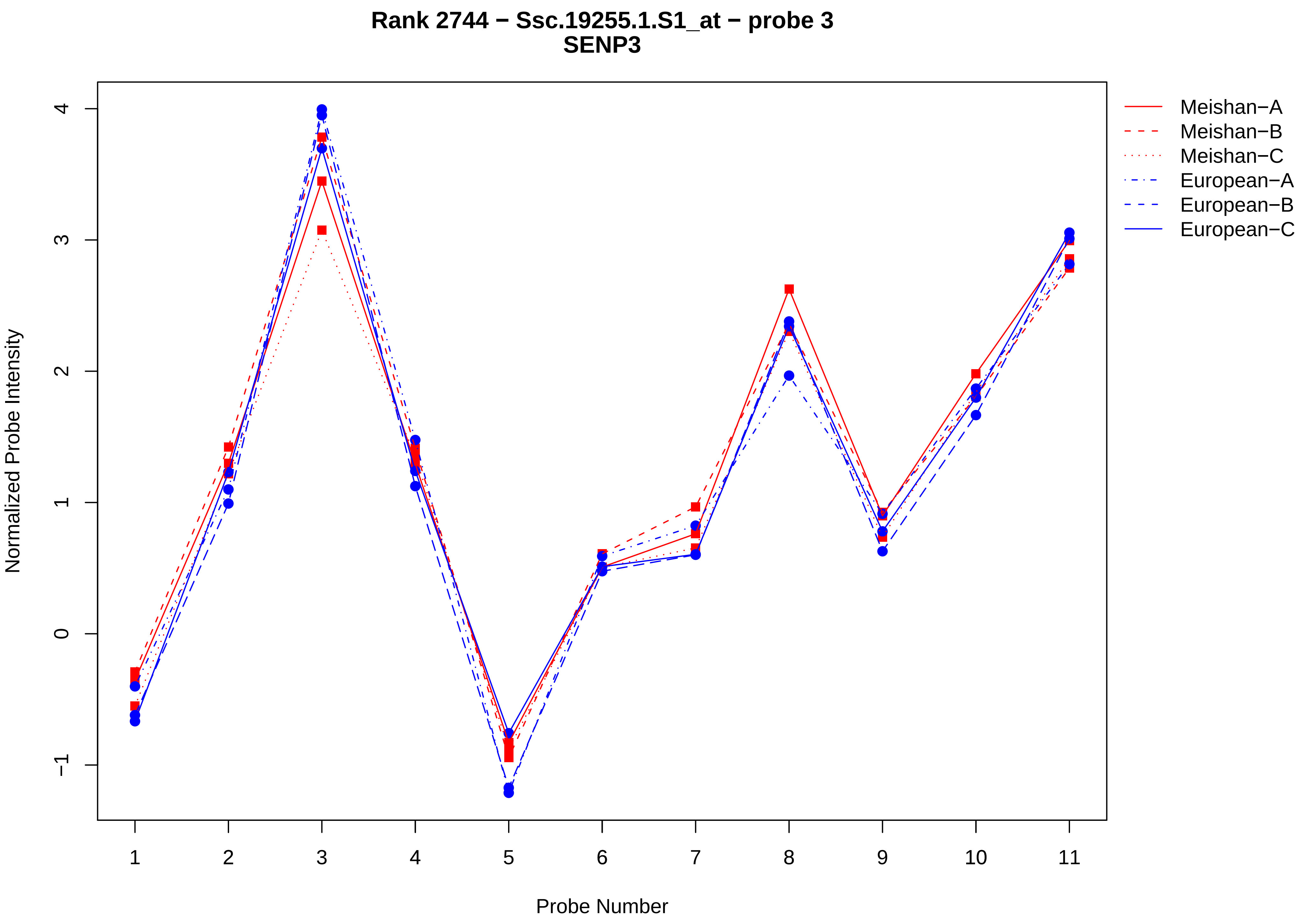

Rank 2744 − Ssc.19255.1.S1_at − probe 3
SENP3
Normalized Probe Intensity
Probe Number
−1
0
1
2
3
4
1
2
3
4
5
6
7
8
9
10
11
Meishan−A
Meishan−B
Meishan−C
European−A
European−B
European−C

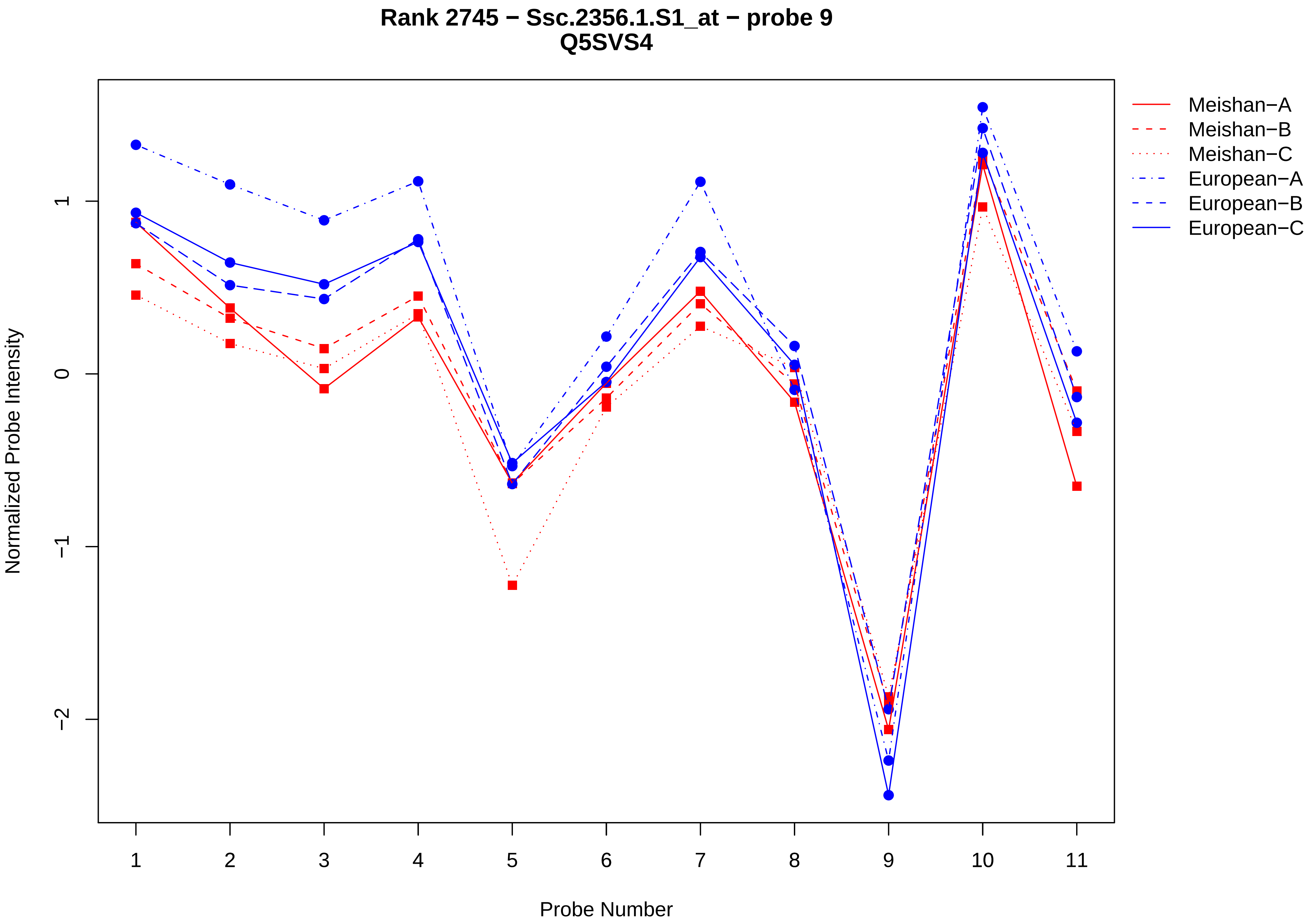

Rank 2745 − Ssc.2356.1.S1_at − probe 9
Q5SVS4
Normalized Probe Intensity
Probe Number
Meishan−A
Meishan−B
Meishan−C
European−A
European−B
European−C
−2
−1
0
1
1
2
3
4
5
6
7
8
9
10
11

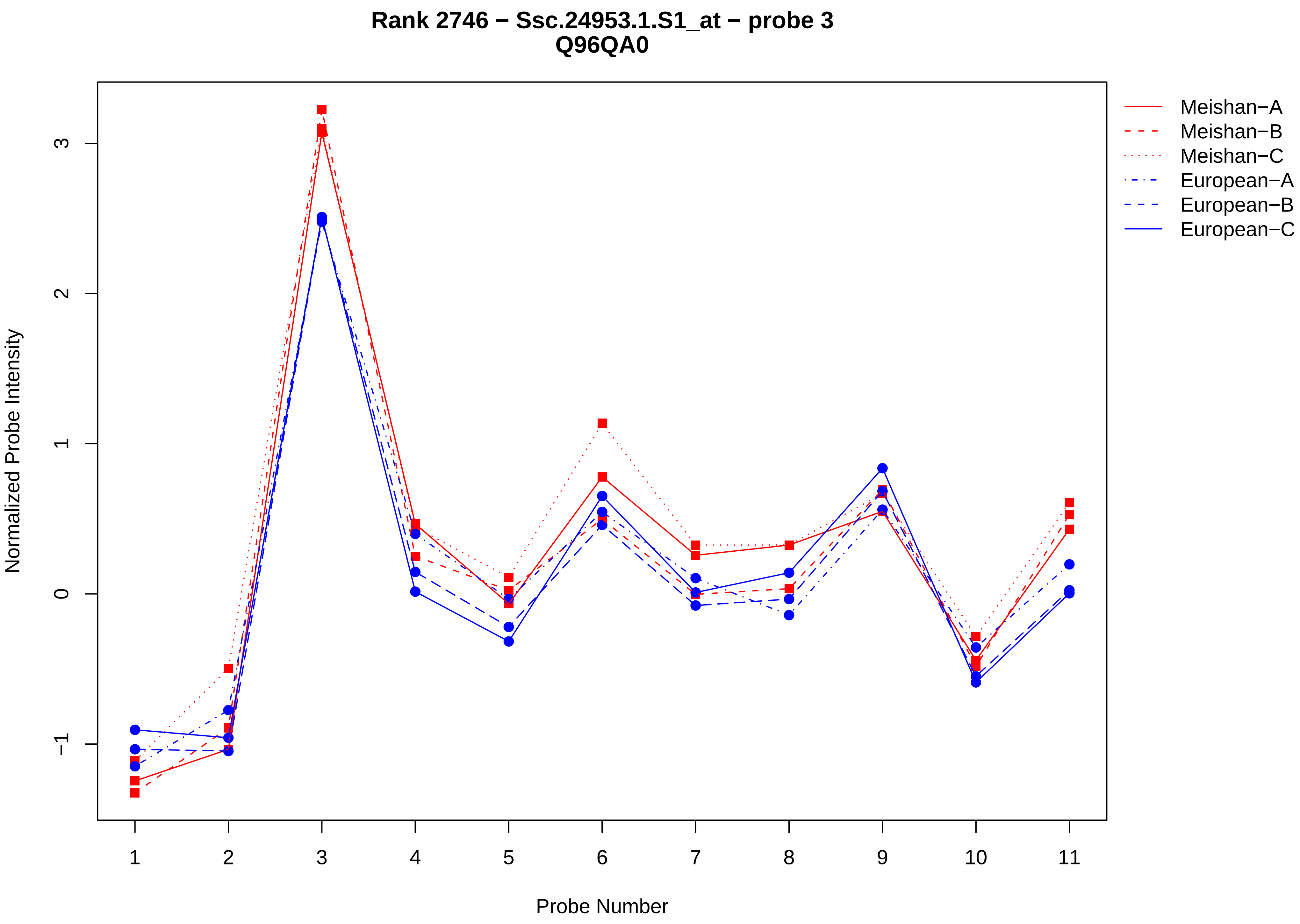

Rank 2746 − Ssc.24953.1.S1_at − probe 3
Q96QA0
Normalized Probe Intensity
Probe Number
−1
0
1
2
3
1
2
3
4
5
6
7
8
9
10
11
Meishan−A
Meishan−B
Meishan−C
European−A
European−B
European−C

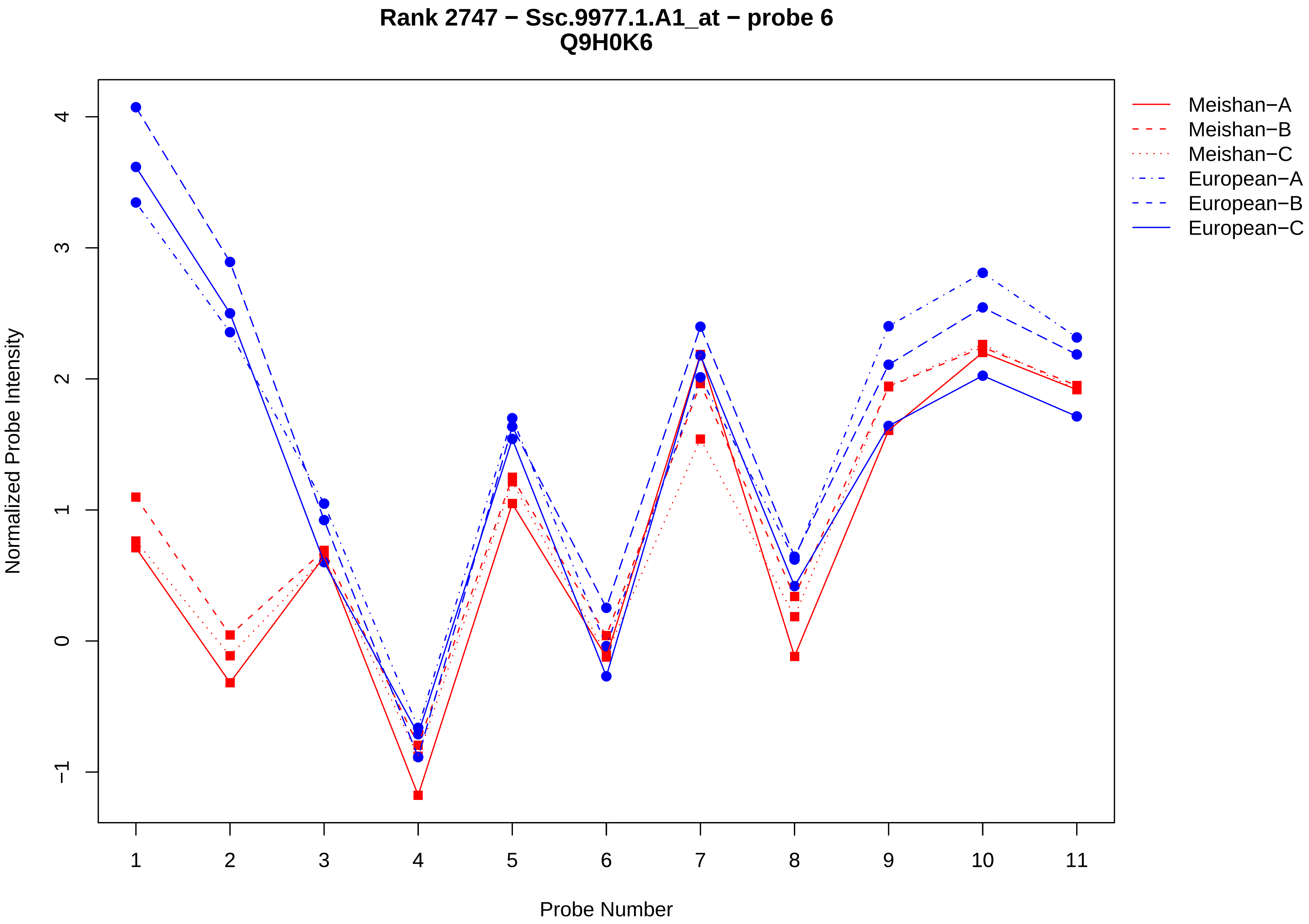

Rank 2747 − Ssc.9977.1.A1_at − probe 6
Q9H0K6
Normalized Probe Intensity
Probe Number
−1
0
1
2
3
4
1
2
3
4
5
6
7
8
9
10
11
Meishan−A
Meishan−B
Meishan−C
European−A
European−B
European−C

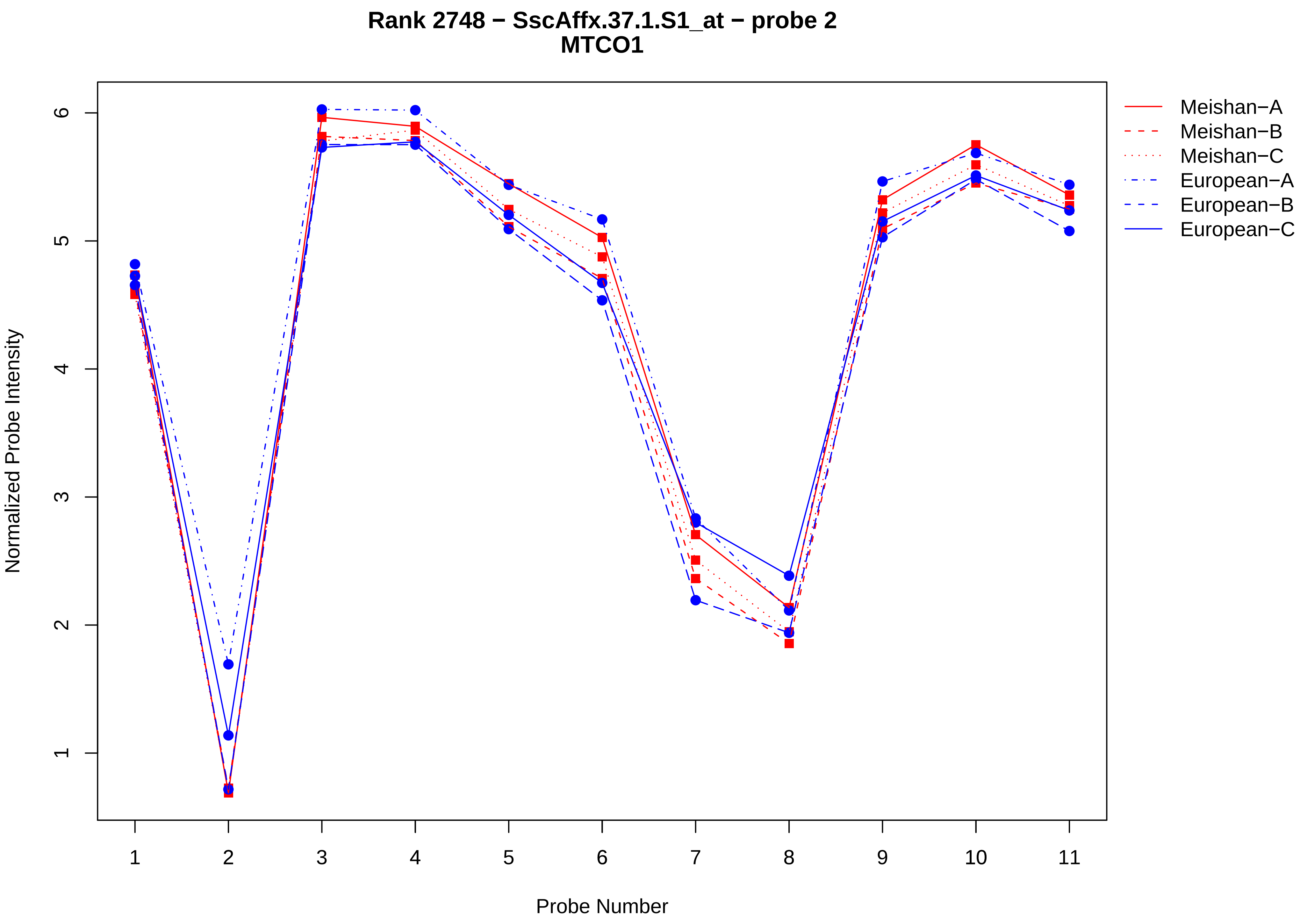
Rank 2748 − SscAffx.37.1.S1_at − probe 2
MTCO1
Normalized Probe Intensity
Probe Number
1
2
3
4
5
6
7
8
9
10
11
Meishan−A
Meishan−B
Meishan−C
European−A
European−B
European−C

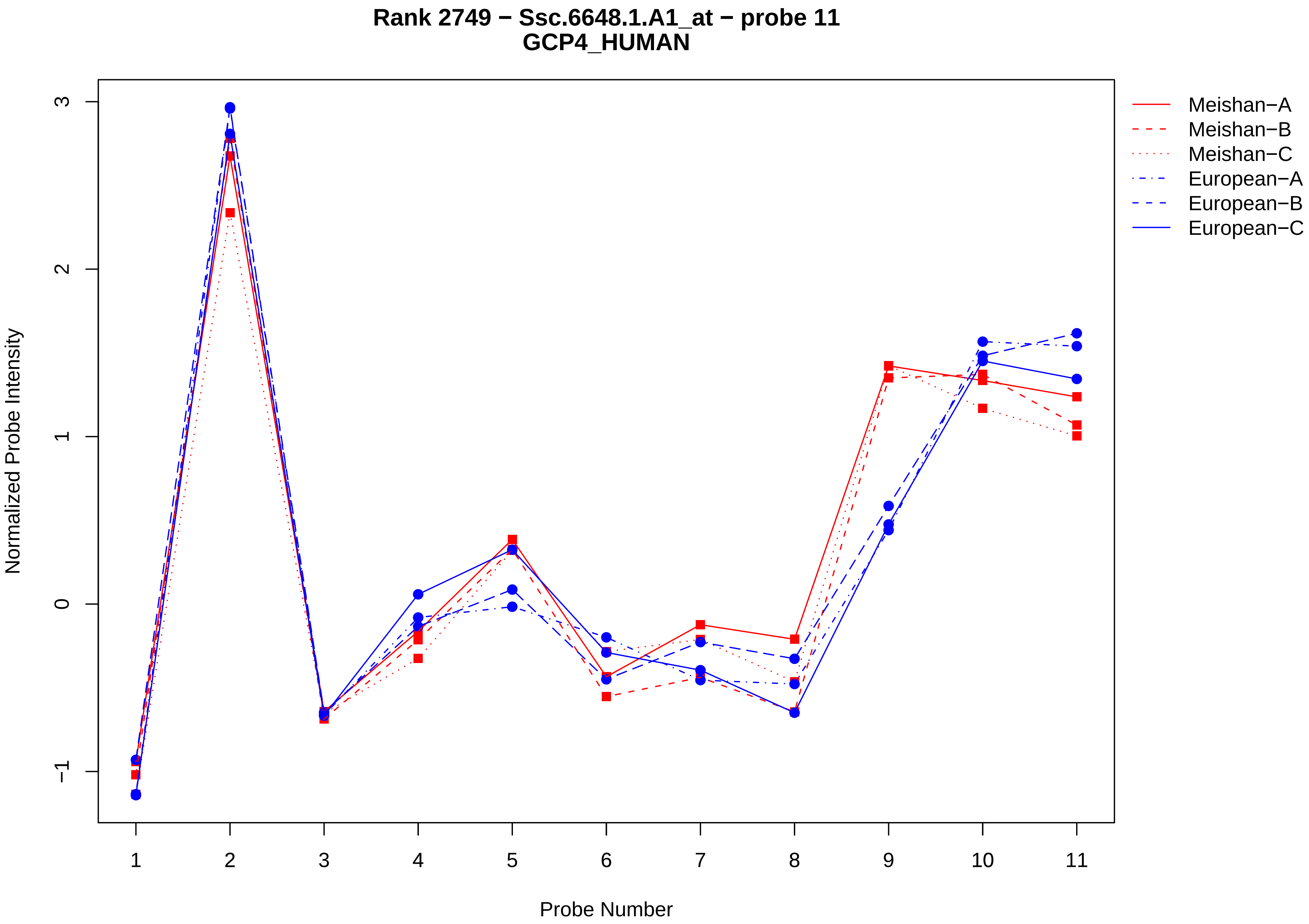

Rank 2749 − Ssc.6648.1.A1_at − probe 11
GCP4_HUMAN
Normalized Probe Intensity
Probe Number
Meishan−A
Meishan−B
Meishan−C
European−A
European−B
European−C
−1
0
1
2
3
1
2
3
4
5
6
7
8
9
10
11

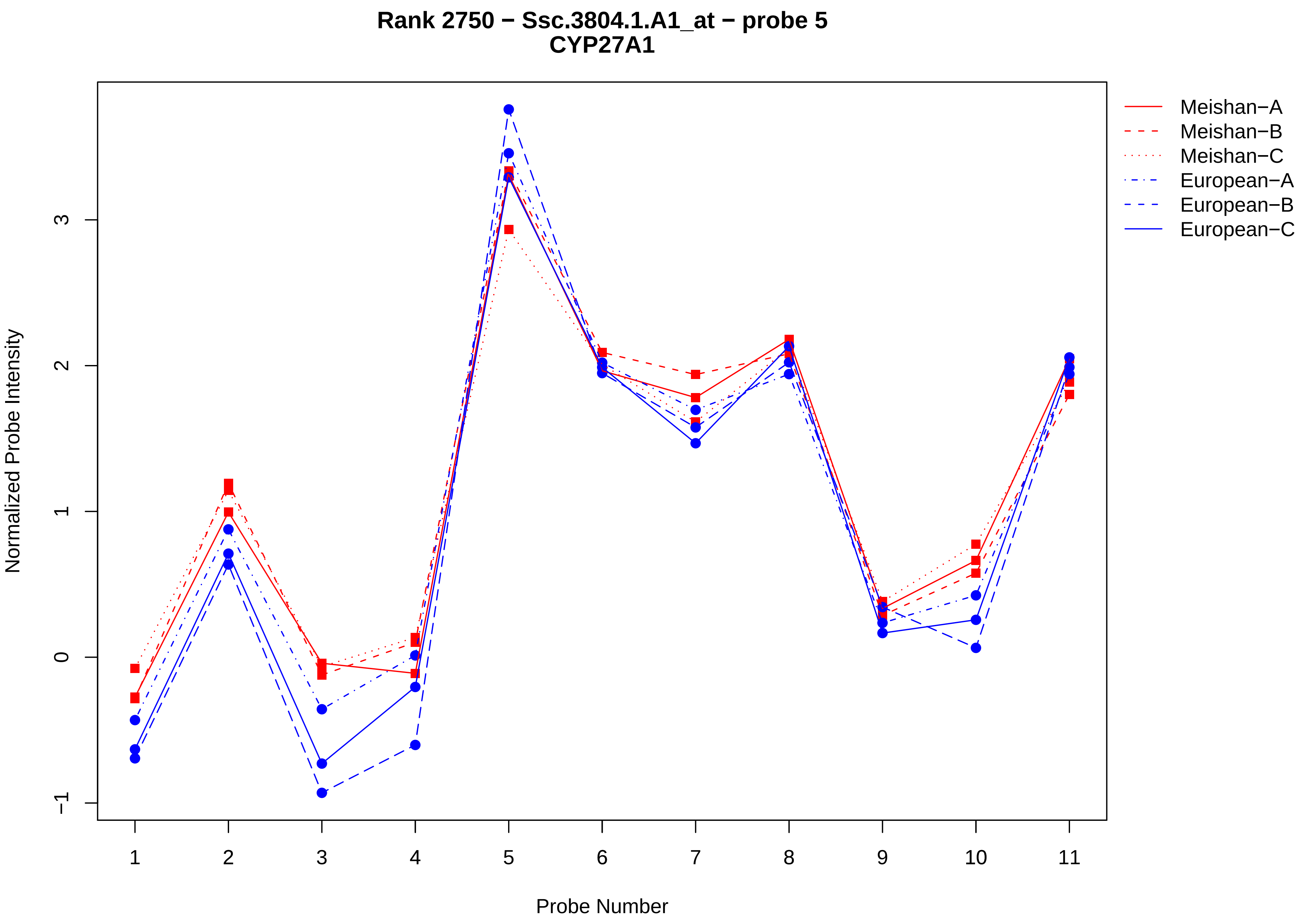

Rank 2750 − Ssc.3804.1.A1_at − probe 5
CYP27A1
Normalized Probe Intensity
Probe Number
Meishan−A
Meishan−B
Meishan−C
European−A
European−B
European−C

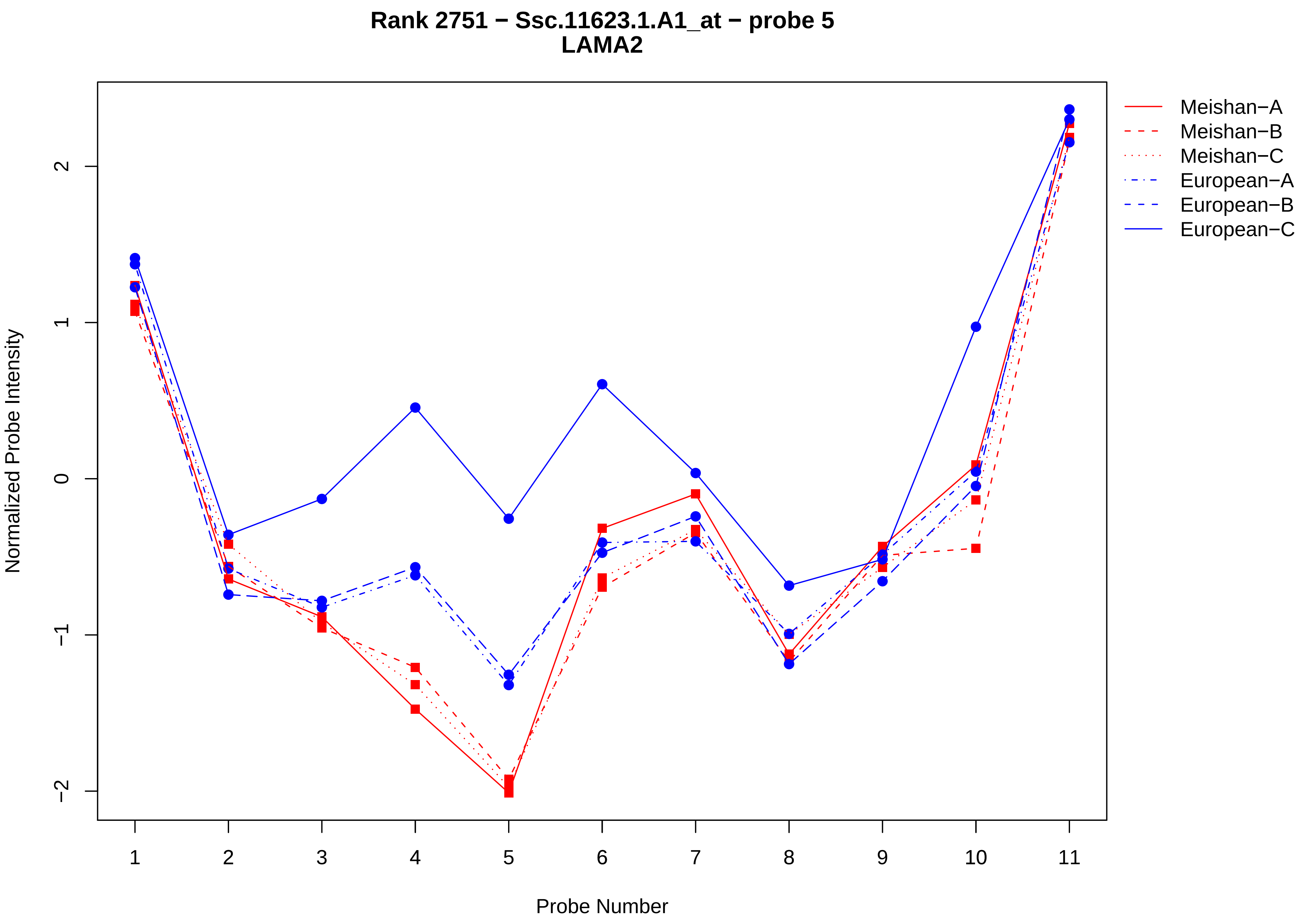

Rank 2751 − Ssc.11623.1.A1_at − probe 5
LAMA2
Normalized Probe Intensity
Probe Number
−2
−1
0
1
2
1
2
3
4
5
6
7
8
9
10
11
Meishan−A
Meishan−B
Meishan−C
European−A
European−B
European−C

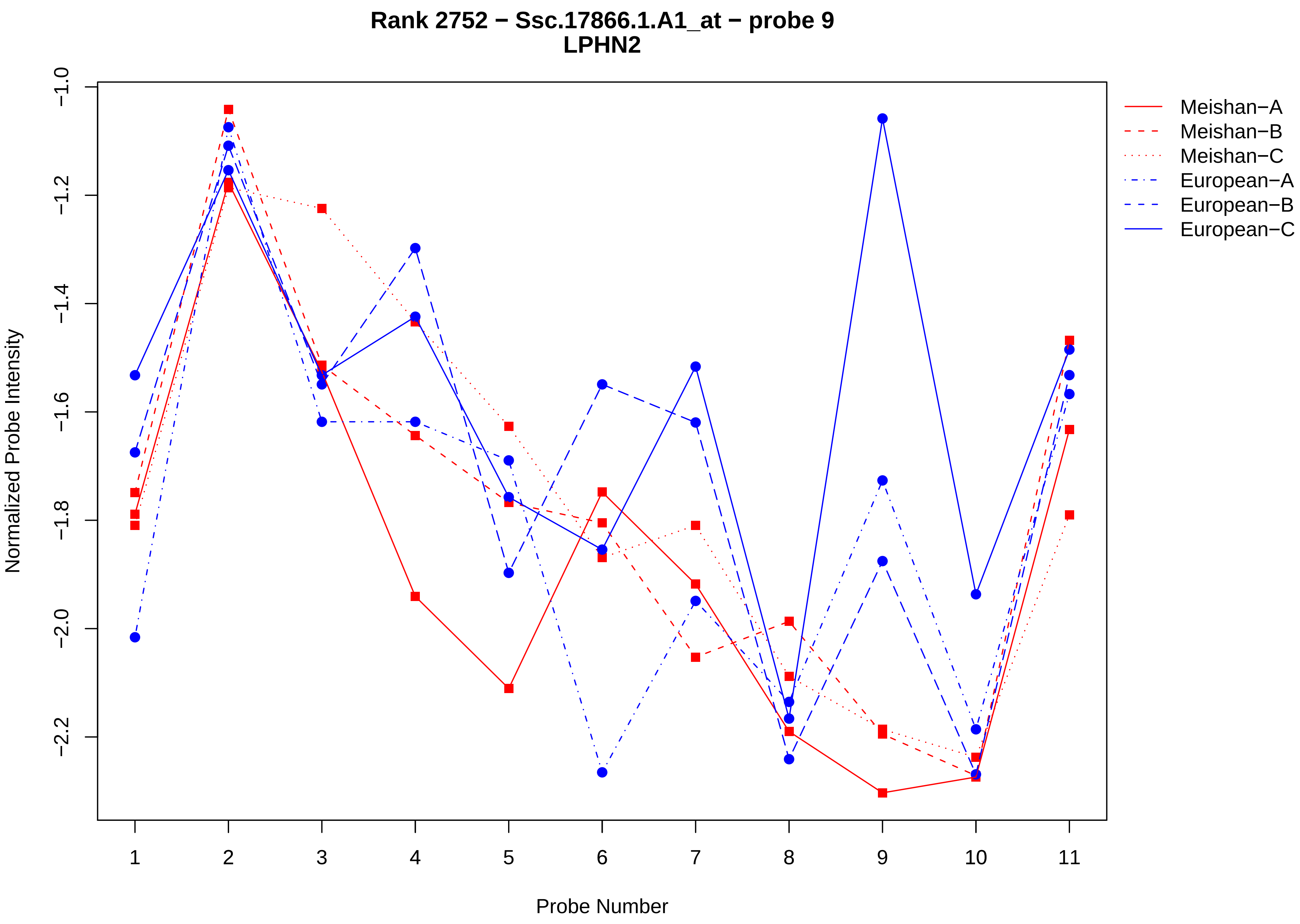

Rank 2752 − Ssc.17866.1.A1_at − probe 9
LPHN2
Normalized Probe Intensity
Probe Number
−1.0
−1.2
−1.4
−1.6
−1.8
−2.0
−2.2
1
2
3
4
5
6
7
8
9
10
11
Meishan−A
Meishan−B
Meishan−C
European−A
European−B
European−C

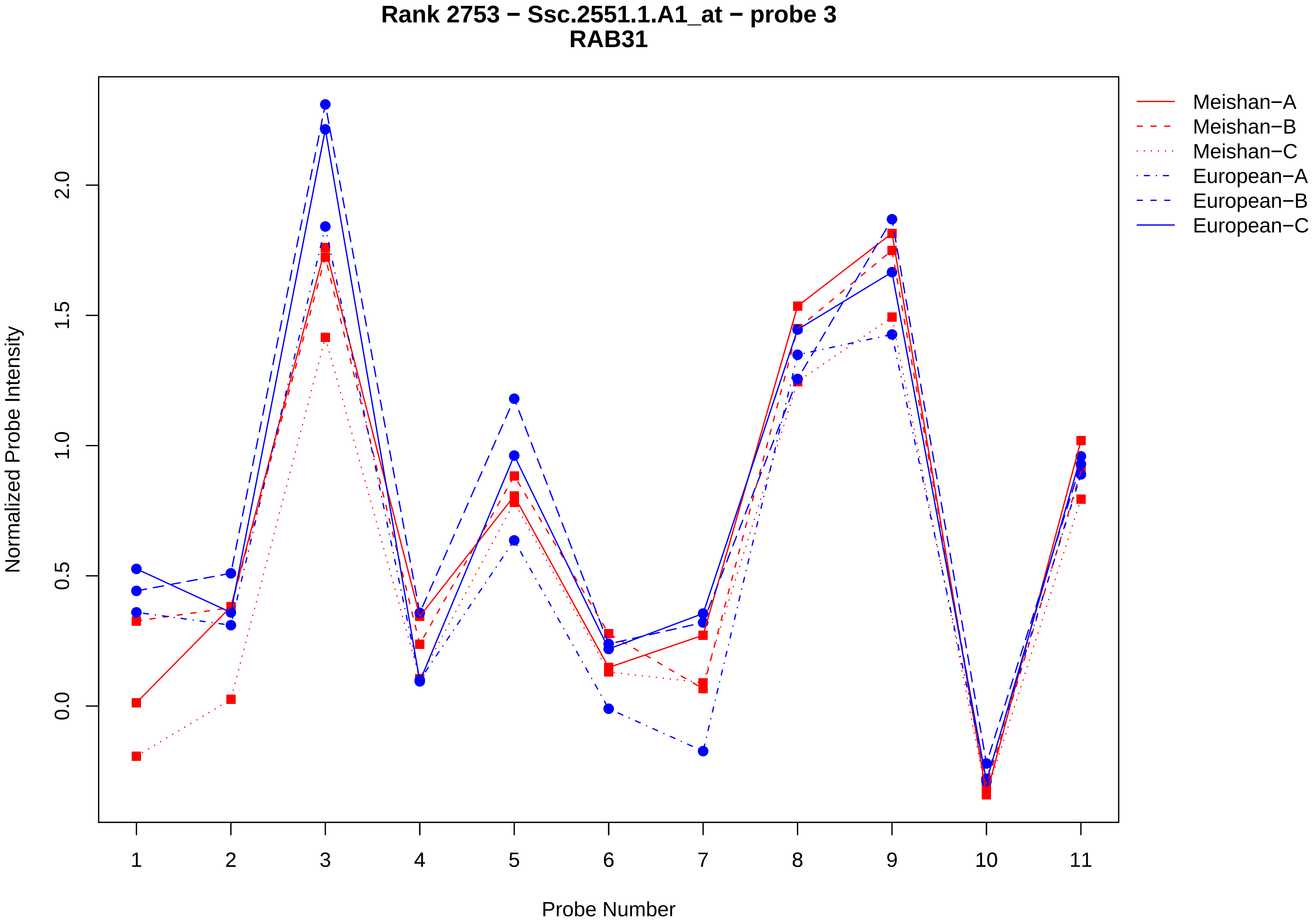

Rank 2753 − Ssc.2551.1.A1_at − probe 3
RAB31
Normalized Probe Intensity
0.0
0.5
1.0
1.5
2.0
1
2
3
4
5
6
7
8
9
10
11
Probe Number
Meishan−A
Meishan−B
Meishan−C
European−A
European−B
European−C

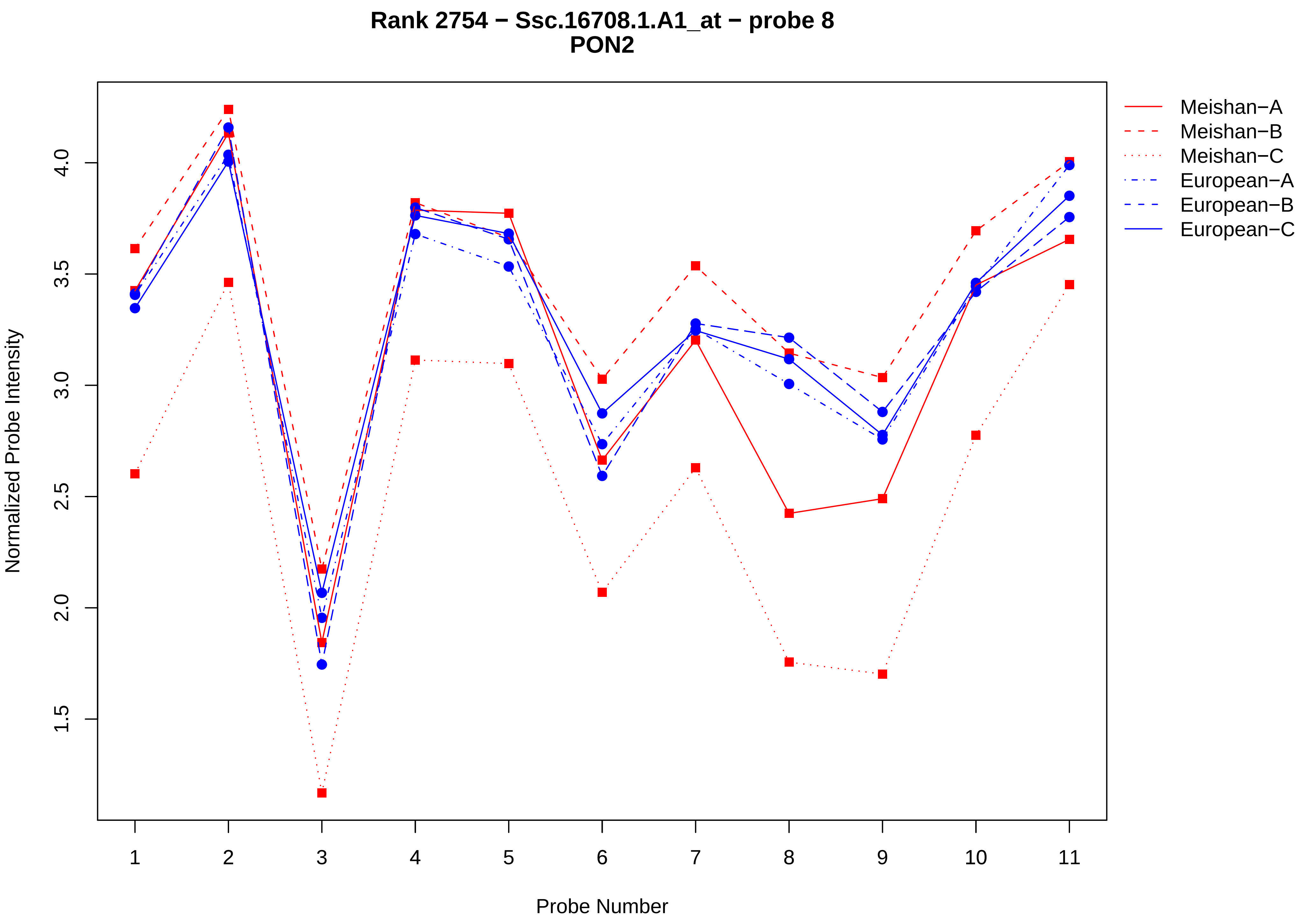

Rank 2754 − Ssc.16708.1.A1_at − probe 8
PON2
Normalized Probe Intensity
Probe Number
1.5
2.0
2.5
3.0
3.5
4.0
1
2
3
4
5
6
7
8
9
10
11
Meishan−A
Meishan−B
Meishan−C
European−A
European−B
European−C

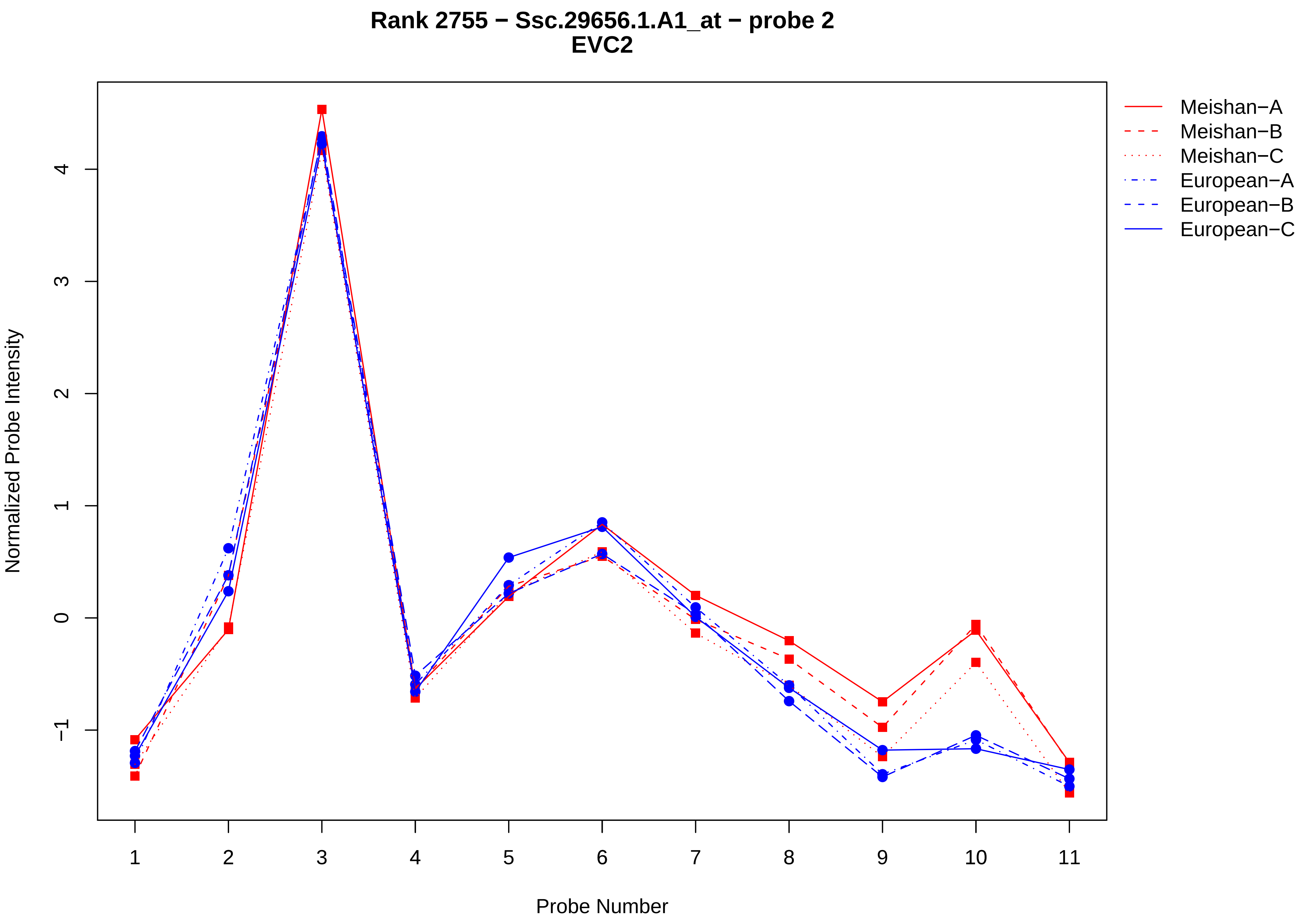

Rank 2755 − Ssc.29656.1.A1_at − probe 2
EVC2
Normalized Probe Intensity
Probe Number
Meishan−A
Meishan−B
Meishan−C
European−A
European−B
European−C
−1
0
1
2
3
4
1
2
3
4
5
6
7
8
9
10
11

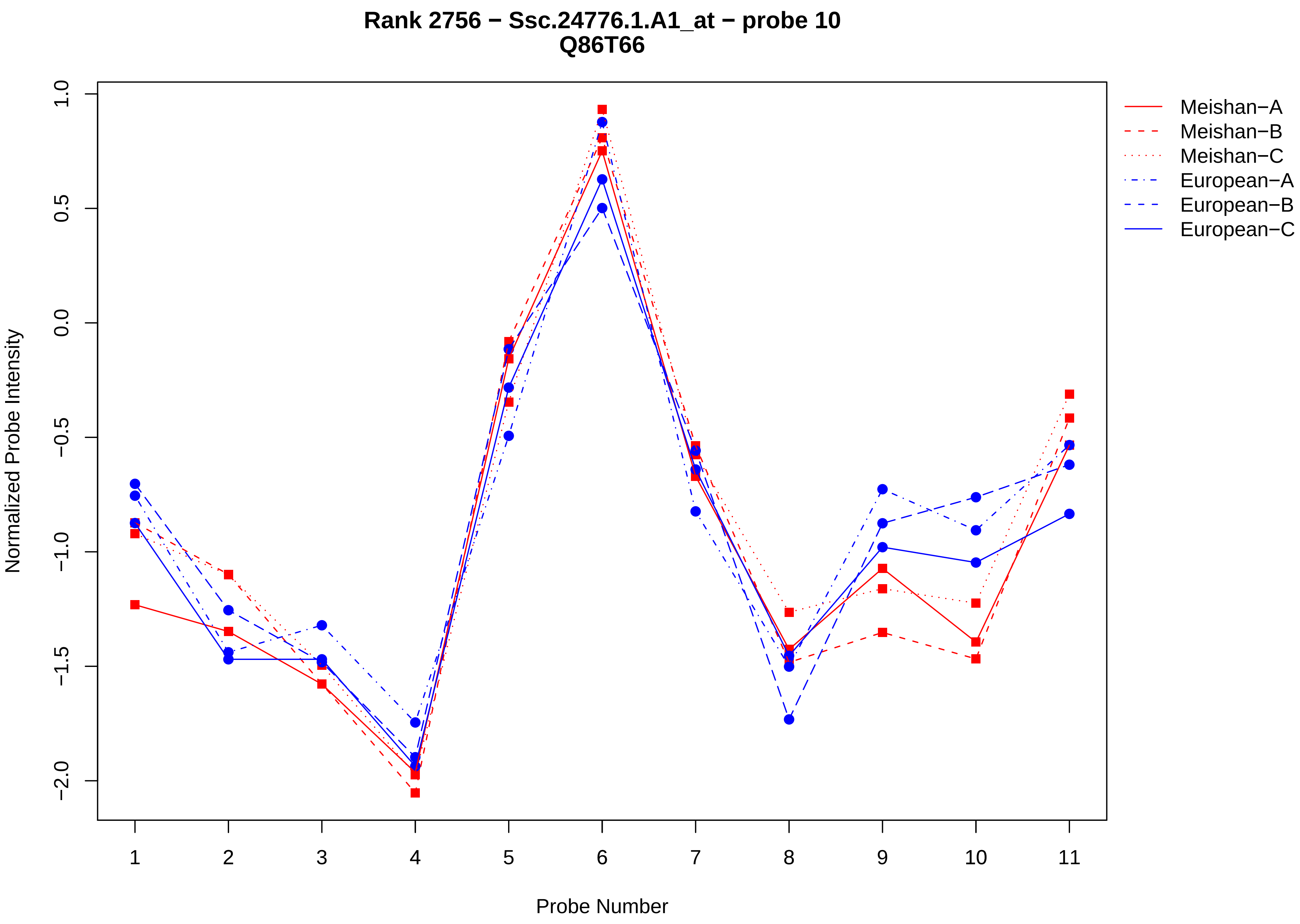

Rank 2756 − Ssc.24776.1.A1_at − probe 10
Q86T66
Normalized Probe Intensity
1.0
0.5
0.0
−0.5
−1.0
−1.5
−2.0
1
2
3
4
5
6
7
8
9
10
11
Probe Number
Meishan−A
Meishan−B
Meishan−C
European−A
European−B
European−C

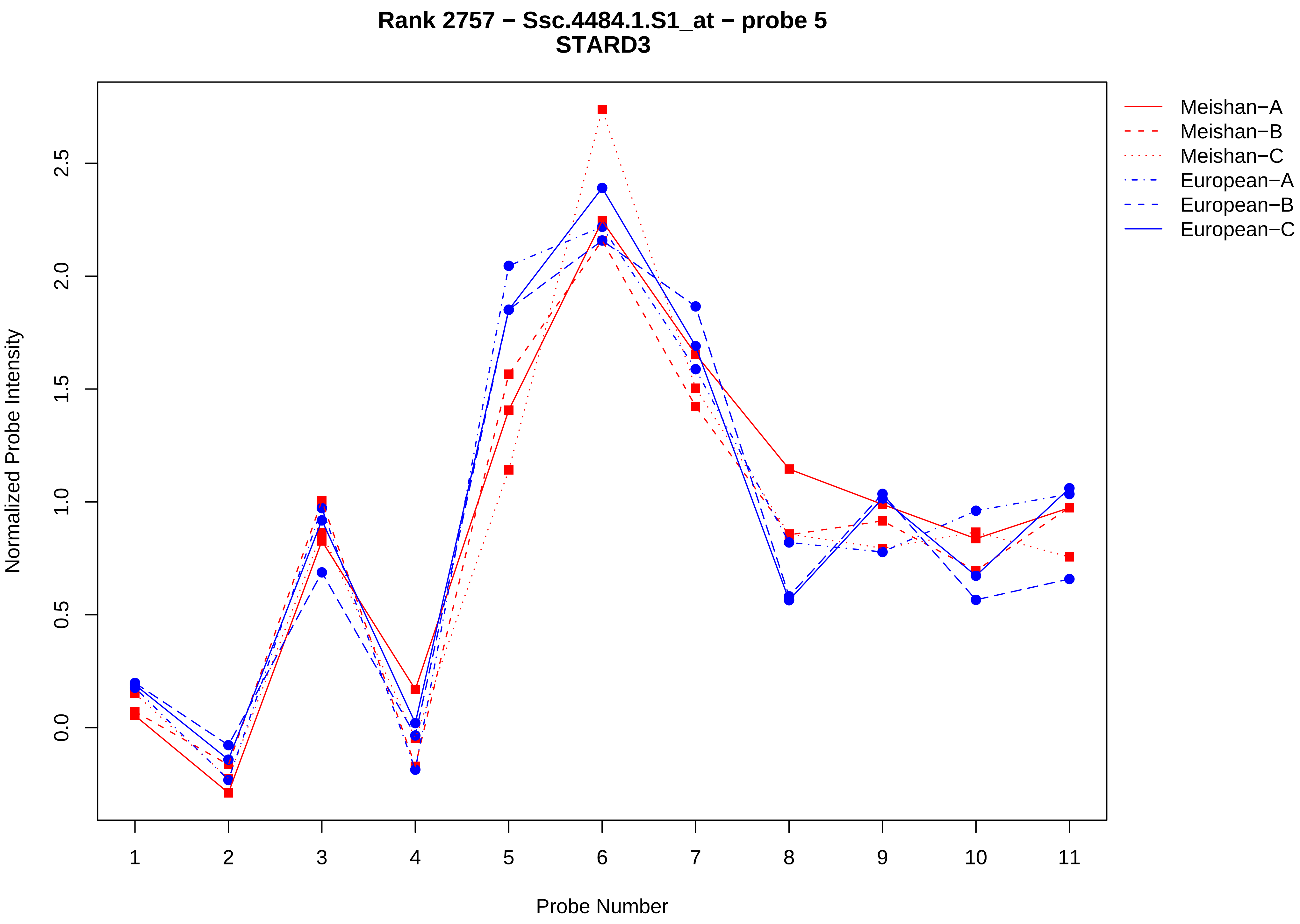

Rank 2757 − Ssc.4484.1.S1_at − probe 5
STARD3
Normalized Probe Intensity
0.0
0.5
1.0
1.5
2.0
2.5
1
2
3
4
5
6
7
8
9
10
11
Probe Number
Meishan−A
Meishan−B
Meishan−C
European−A
European−B
European−C

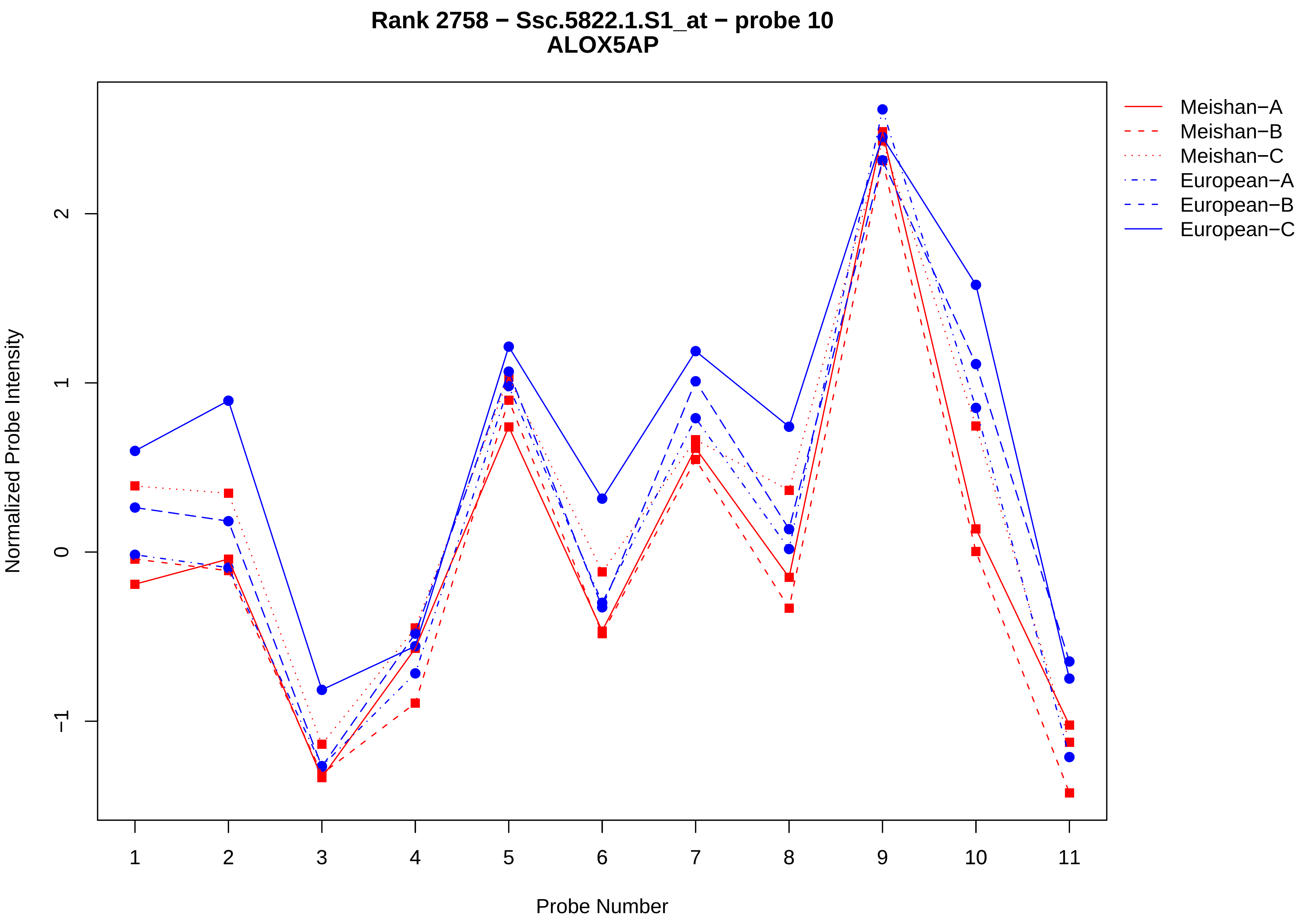
Rank 2758 − Ssc.5822.1.S1_at − probe 10
ALOX5AP
Normalized Probe Intensity
Probe Number
−1
0
1
2
1
2
3
4
5
6
7
8
9
10
11
Meishan−A
Meishan−B
Meishan−C
European−A
European−B
European−C

Rank 2759 − Ssc.26734.1.A1_at − probe 2
SIDT1

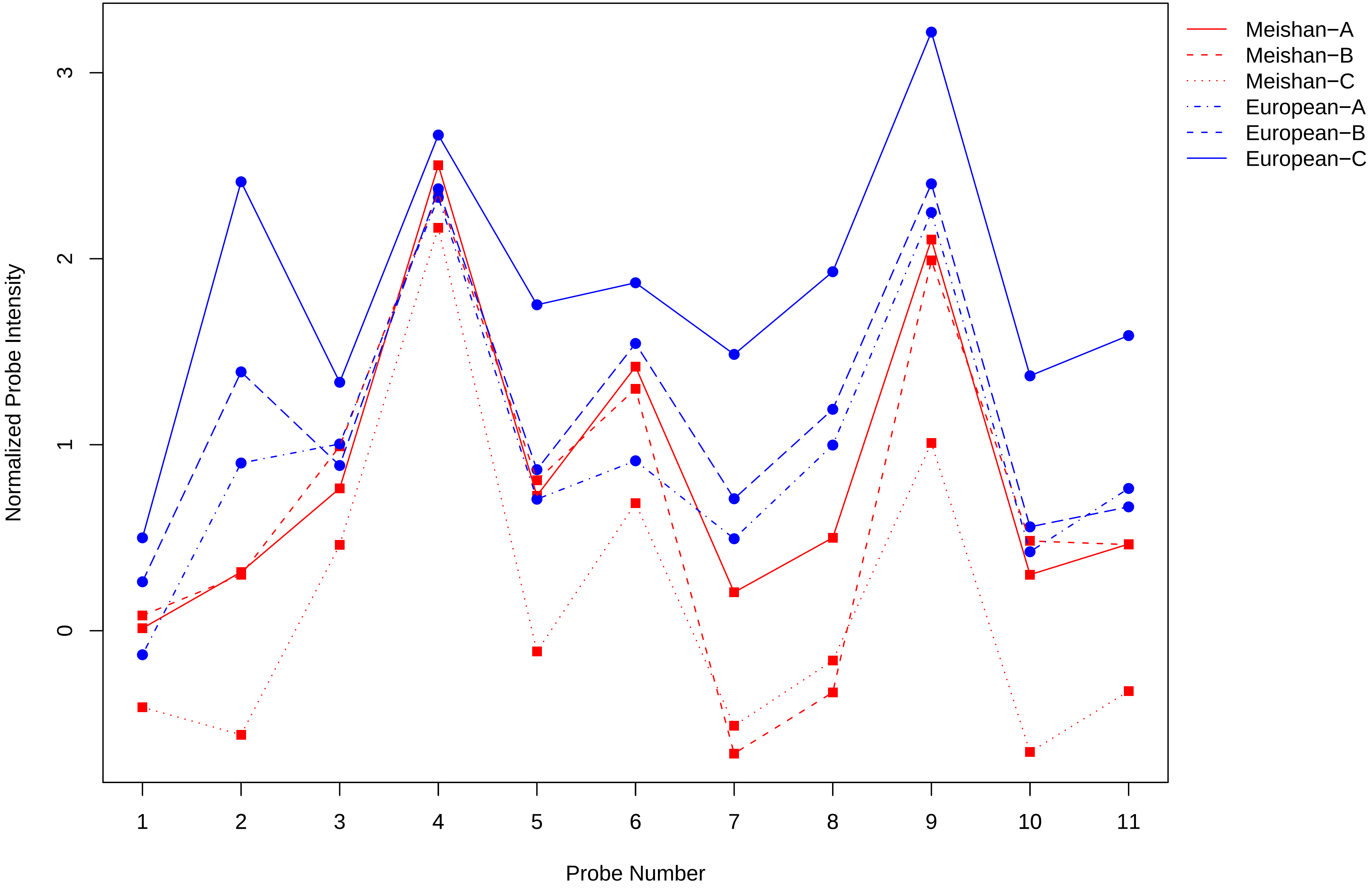

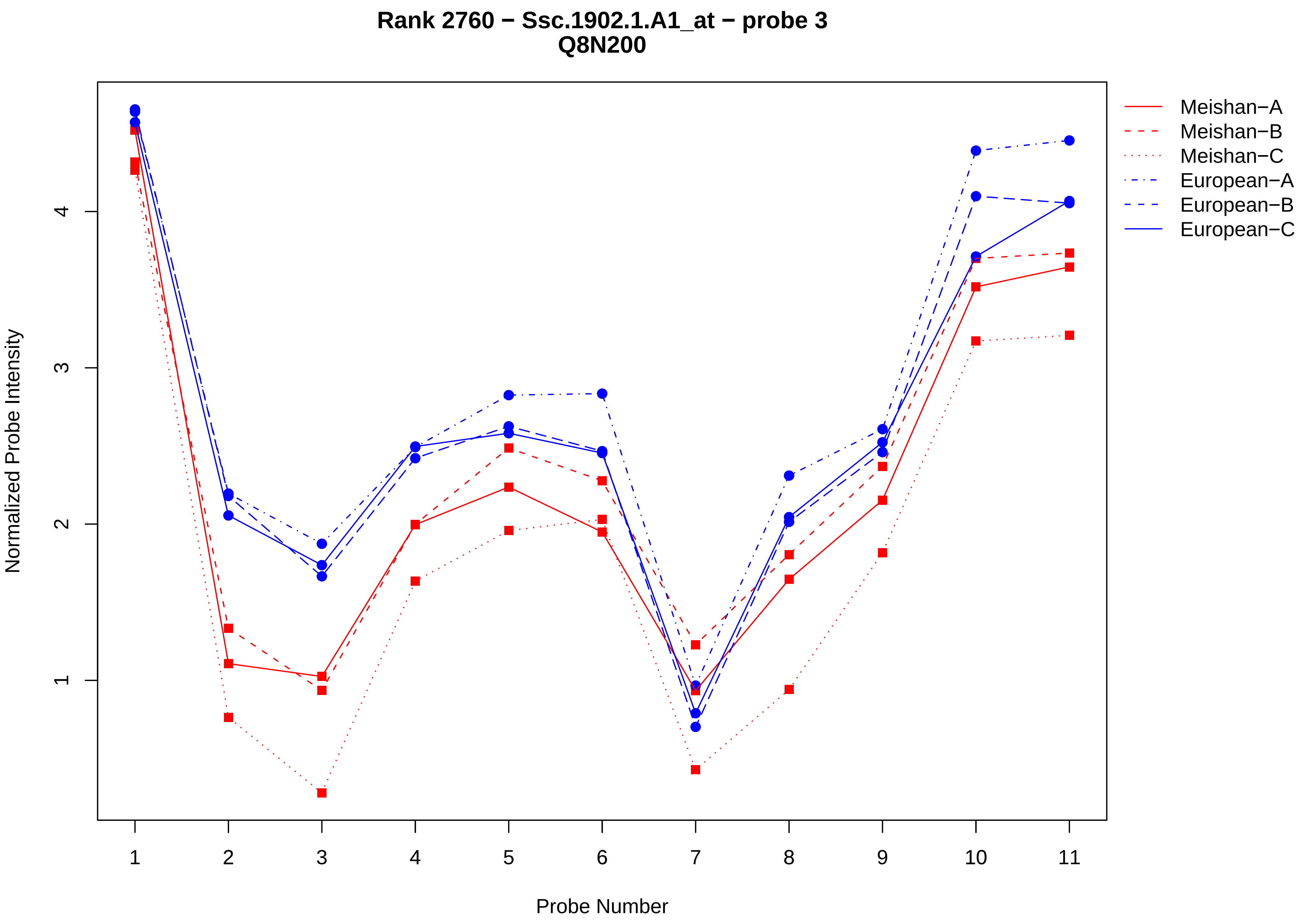

Rank 2760 − Ssc.1902.1.A1_at − probe 3
Q8N200
Normalized Probe Intensity
Probe Number
1
2
3
4
1
2
3
4
5
6
7
8
9
10
11
Meishan−A
Meishan−B
Meishan−C
European−A
European−B
European−C

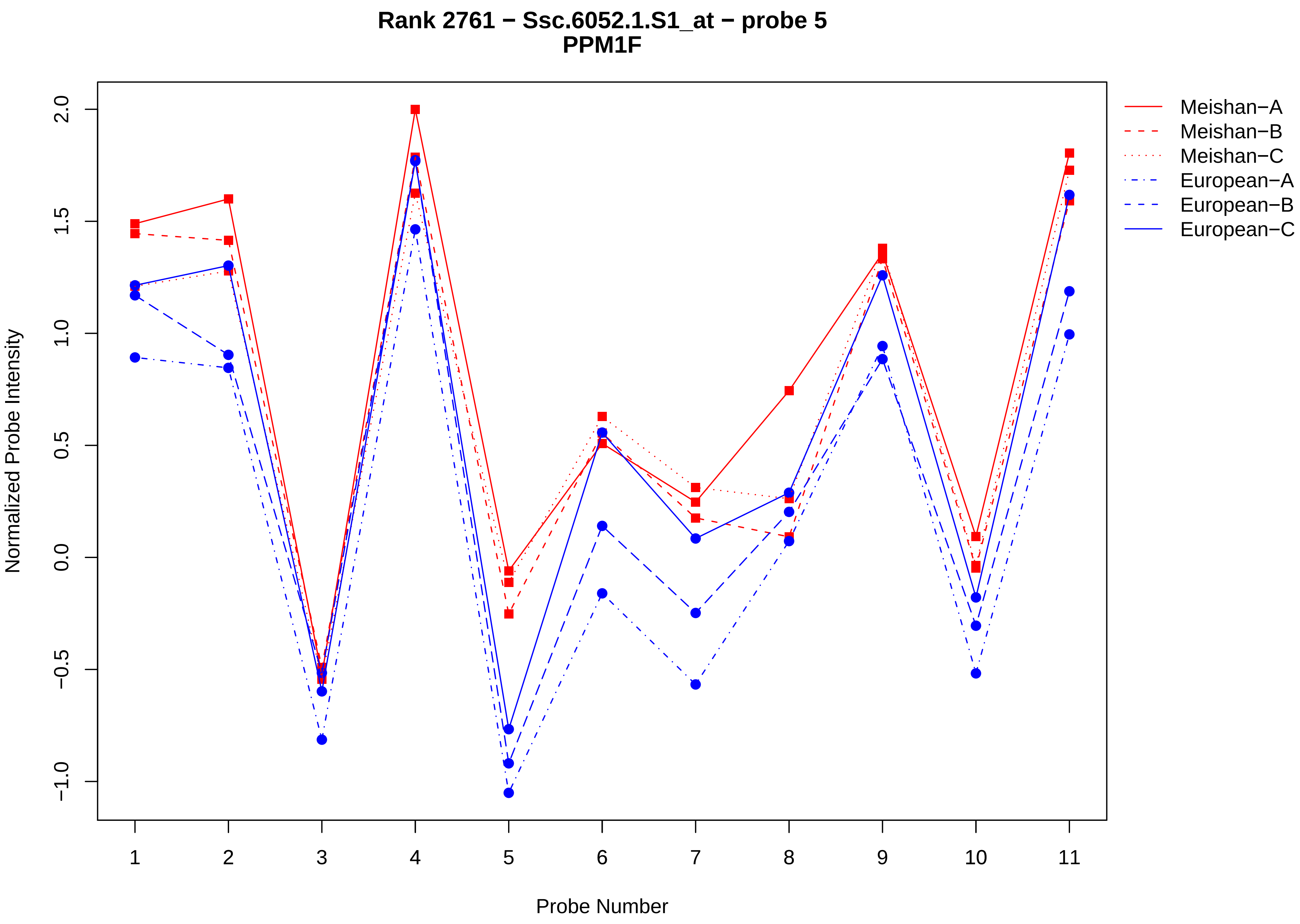

Rank 2761 − Ssc.6052.1.S1_at − probe 5
PPM1F
Normalized Probe Intensity
Probe Number
−1.0
−0.5
0.0
0.5
1.0
1.5
2.0
1
2
3
4
5
6
7
8
9
10
11
Meishan−A
Meishan−B
Meishan−C
European−A
European−B
European−C

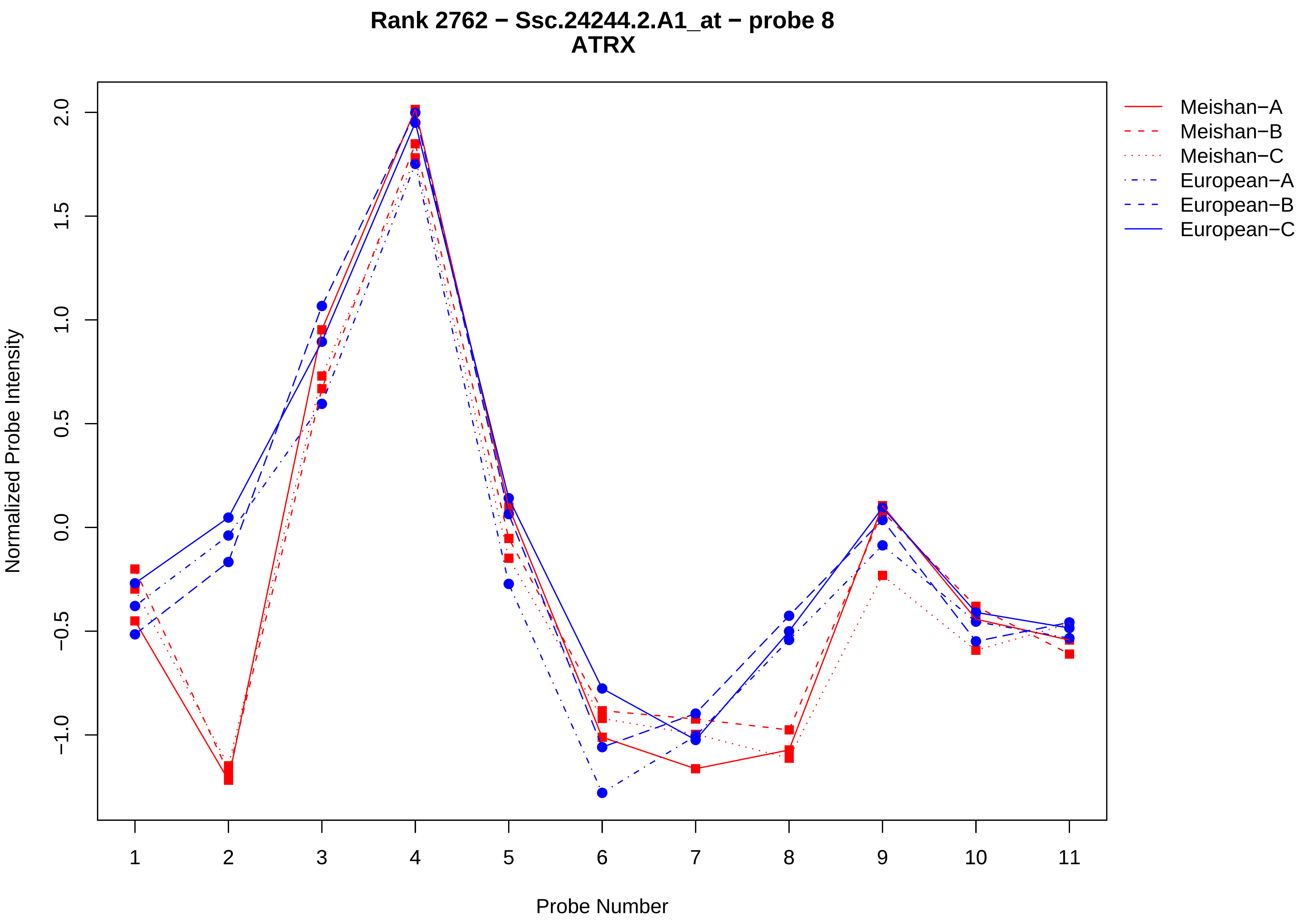
Rank 2762 − Ssc.24244.2.A1_at − probe 8
ATRX
Normalized Probe Intensity
Probe Number
2.0
1.5
1.0
0.5
0.0
−0.5
−1.0
1
2
3
4
5
6
7
8
9
10
11
Meishan−A
Meishan−B
Meishan−C
European−A
European−B
European−C

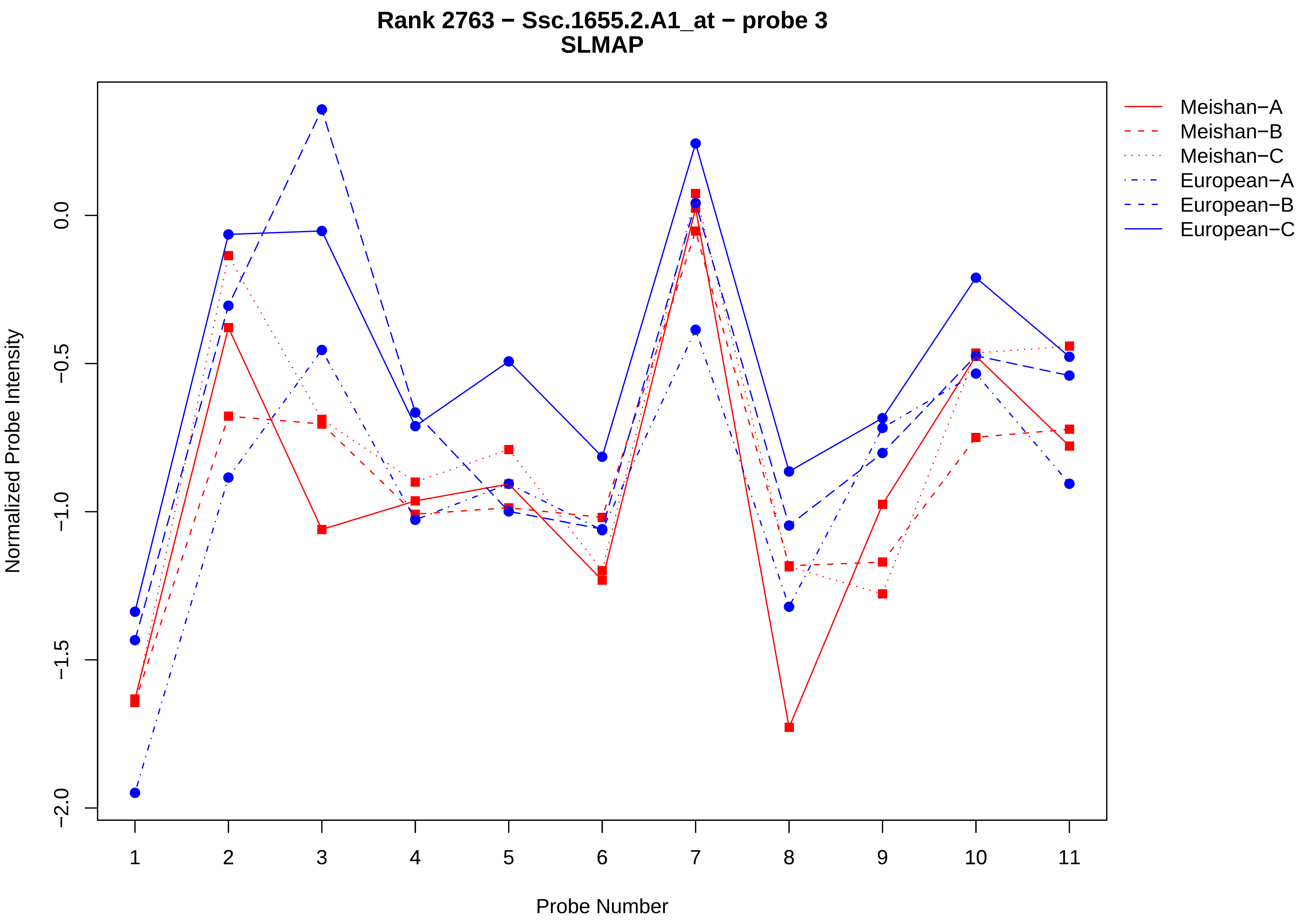

Rank 2763 − Ssc.1655.2.A1_at − probe 3
SLMAP
Normalized Probe Intensity
Probe Number
0.0
−0.5
−1.0
−1.5
−2.0
1
2
3
4
5
6
7
8
9
10
11
Meishan−A
Meishan−B
Meishan−C
European−A
European−B
European−C

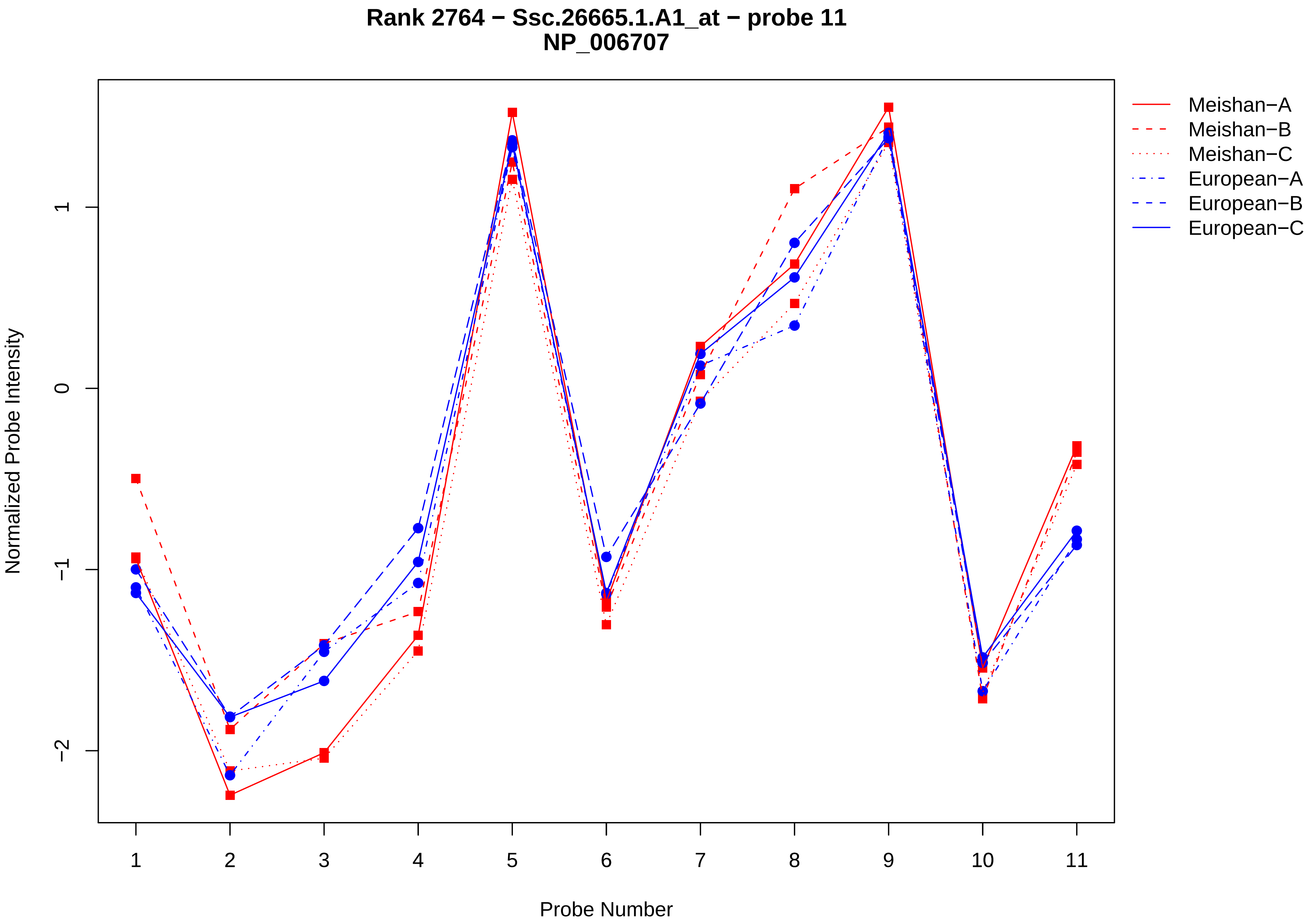

Rank 2764 − Ssc.26665.1.A1_at − probe 11
NP_006707
Normalized Probe Intensity
Probe Number
Meishan−A
Meishan−B
Meishan−C
European−A
European−B
European−C
−2
−1
0
1
1
2
3
4
5
6
7
8
9
10
11

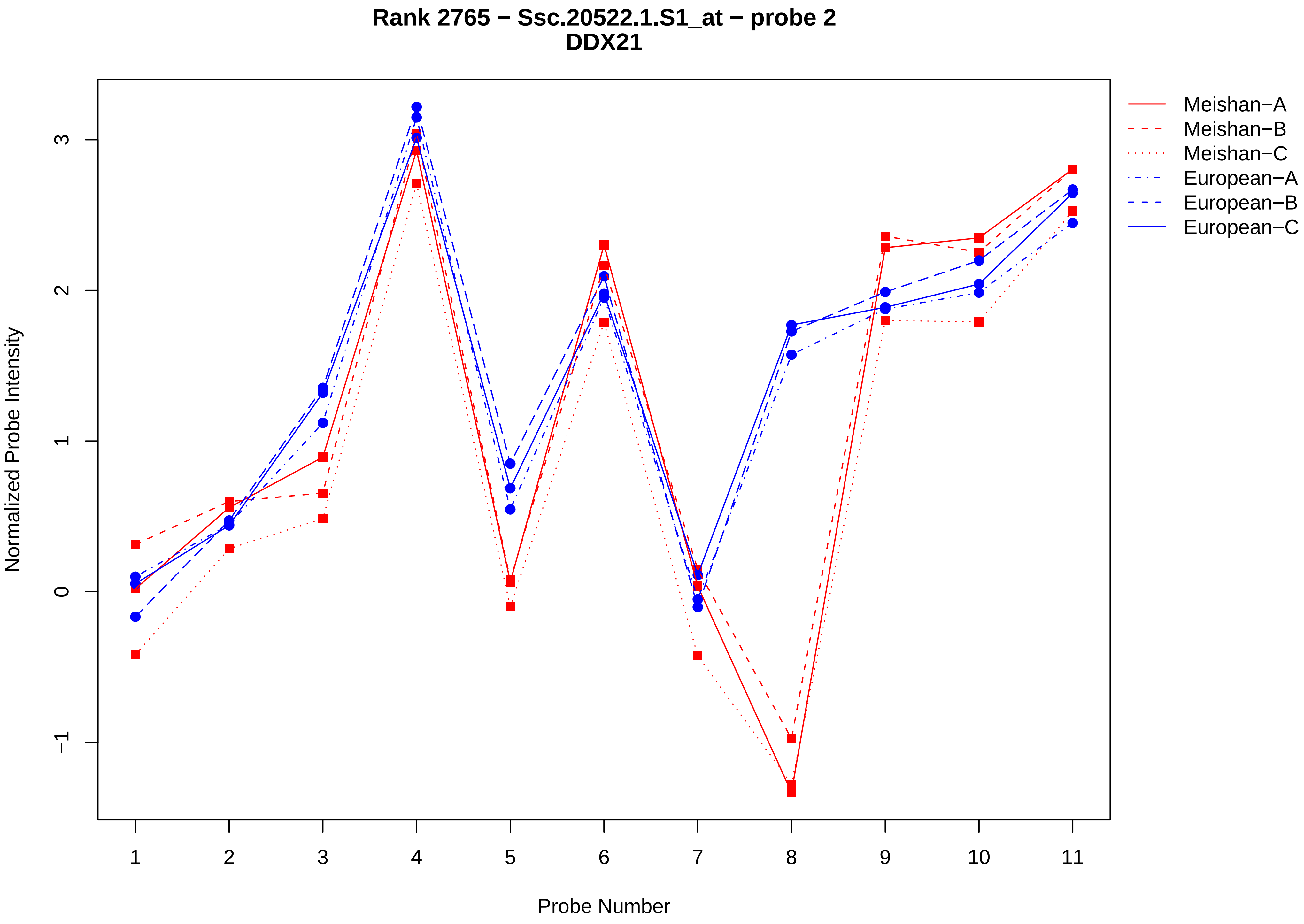

Rank 2765 − Ssc.20522.1.S1_at − probe 2
DDX21
Normalized Probe Intensity
Probe Number
Meishan−A
Meishan−B
Meishan−C
European−A
European−B
European−C
−1
0
1
2
3
1
2
3
4
5
6
7
8
9
10
11

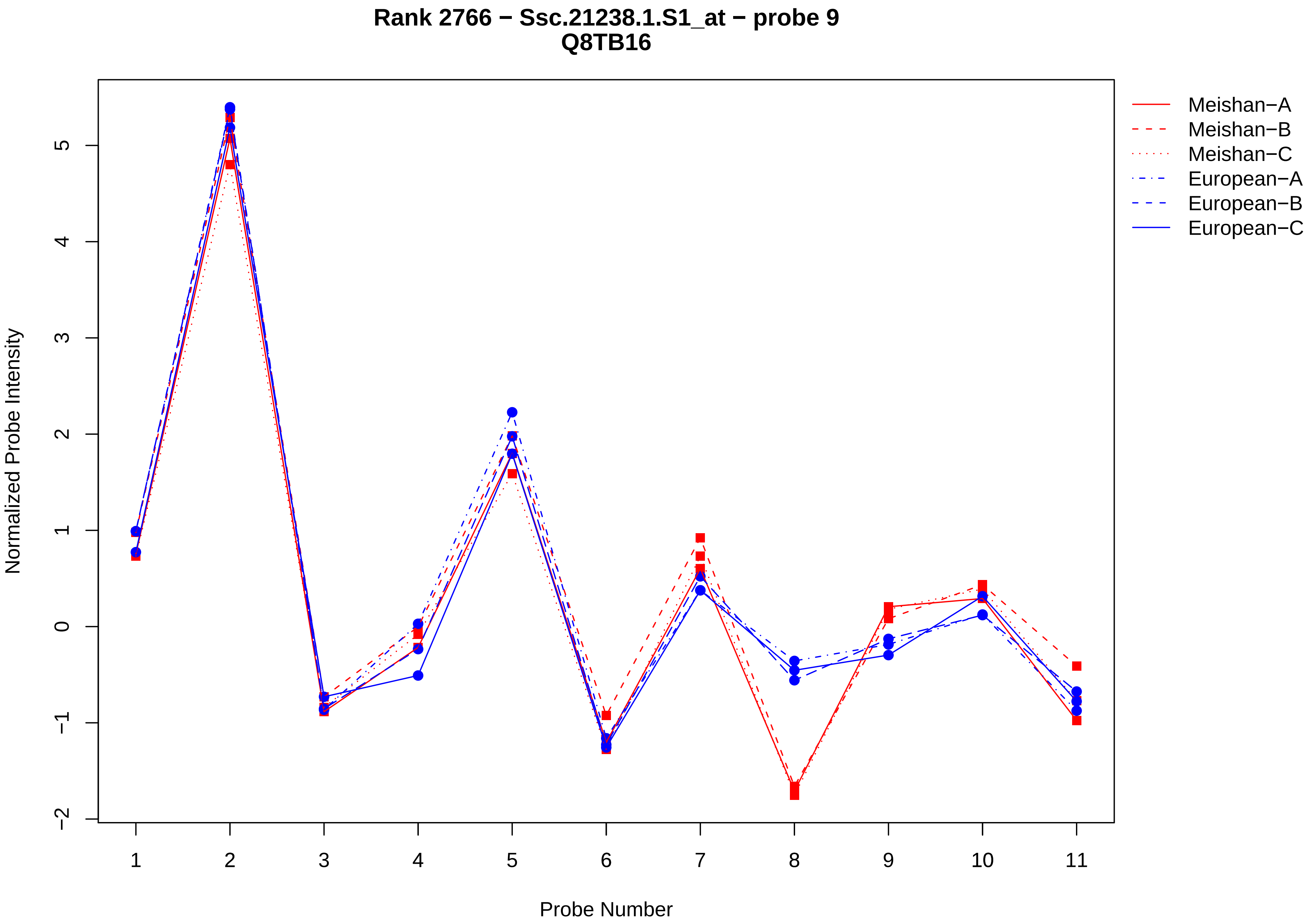
Rank 2766 − Ssc.21238.1.S1_at − probe 9
Q8TB16
Normalized Probe Intensity
Probe Number
Meishan−A
Meishan−B
Meishan−C
European−A
European−B
European−C

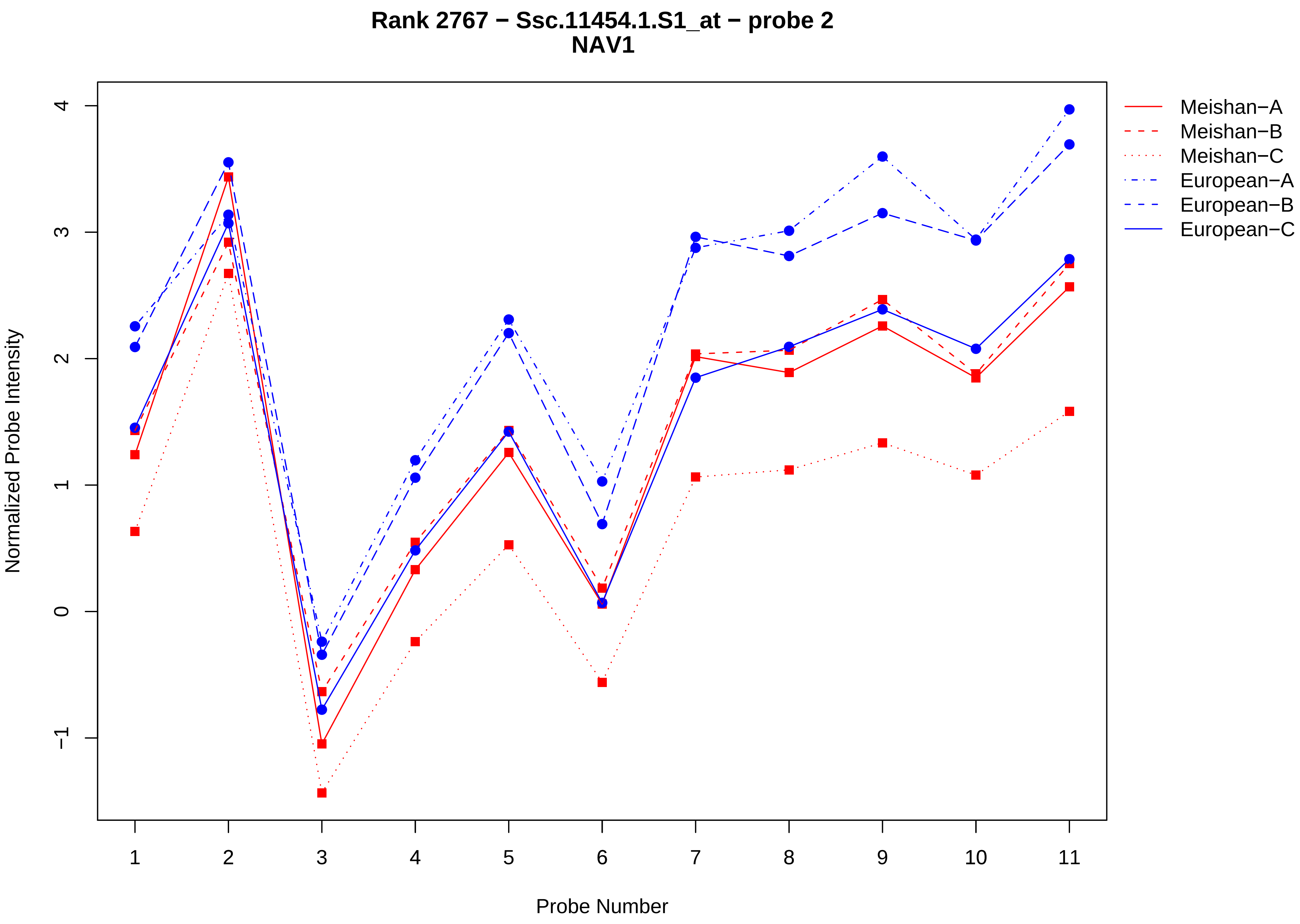

Rank 2767 − Ssc.11454.1.S1_at − probe 2
NAV1
Normalized Probe Intensity
Probe Number
−1
0
1
2
3
4
1
2
3
4
5
6
7
8
9
10
11
Meishan−A
Meishan−B
Meishan−C
European−A
European−B
European−C

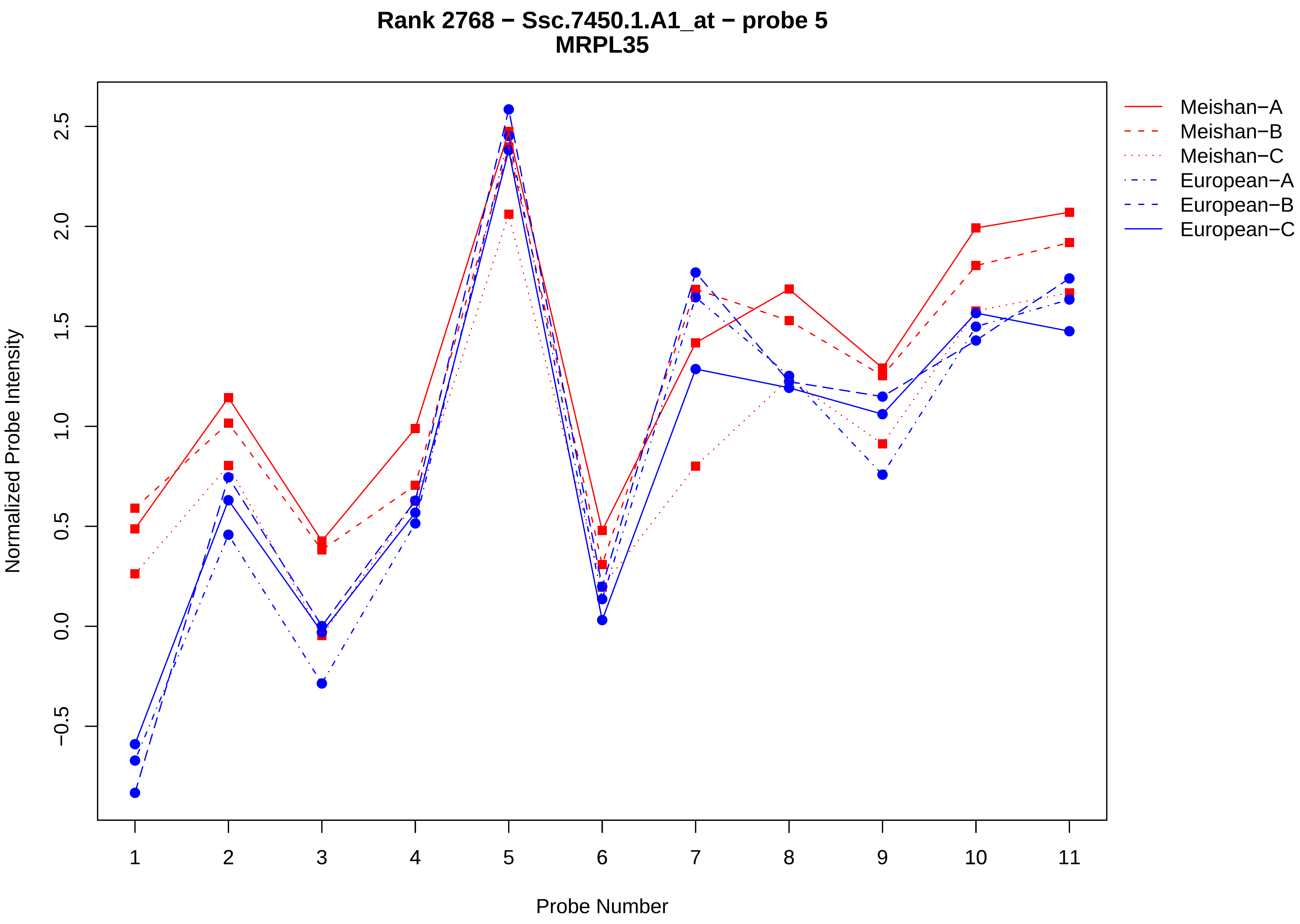

Rank 2768 − Ssc.7450.1.A1_at − probe 5
MRPL35
Normalized Probe Intensity
Probe Number
−0.5
0.0
0.5
1.0
1.5
2.0
2.5
1
2
3
4
5
6
7
8
9
10
11
Meishan−A
Meishan−B
Meishan−C
European−A
European−B
European−C

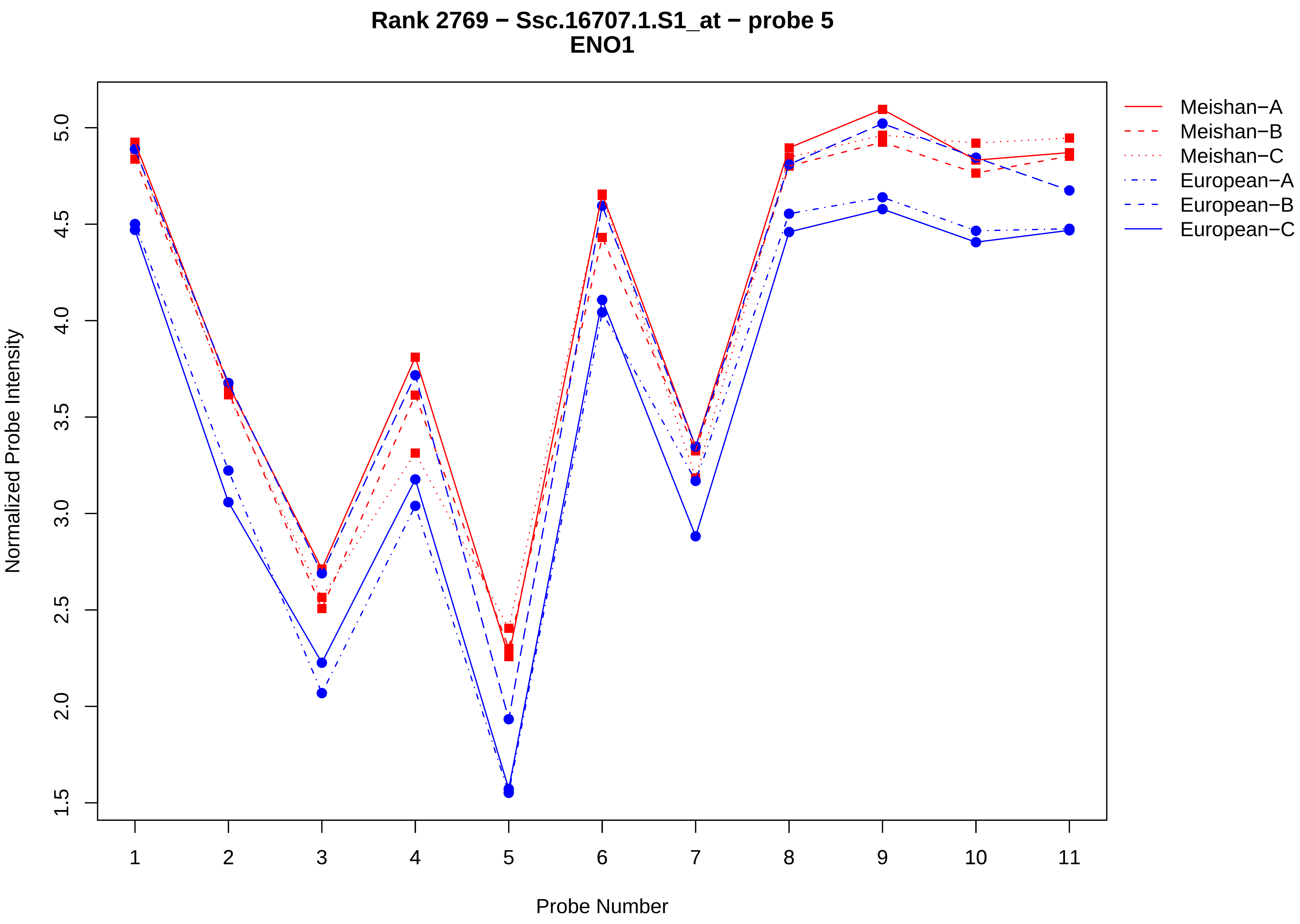

Rank 2769 − Ssc.16707.1.S1_at − probe 5
ENO1
Normalized Probe Intensity
Probe Number
1.5
2.0
2.5
3.0
3.5
4.0
4.5
5.0
1
2
3
4
5
6
7
8
9
10
11
Meishan−A
Meishan−B
Meishan−C
European−A
European−B
European−C

Rank 2770 − Ssc.6009.1.S1_at − probe 8
AMY2B

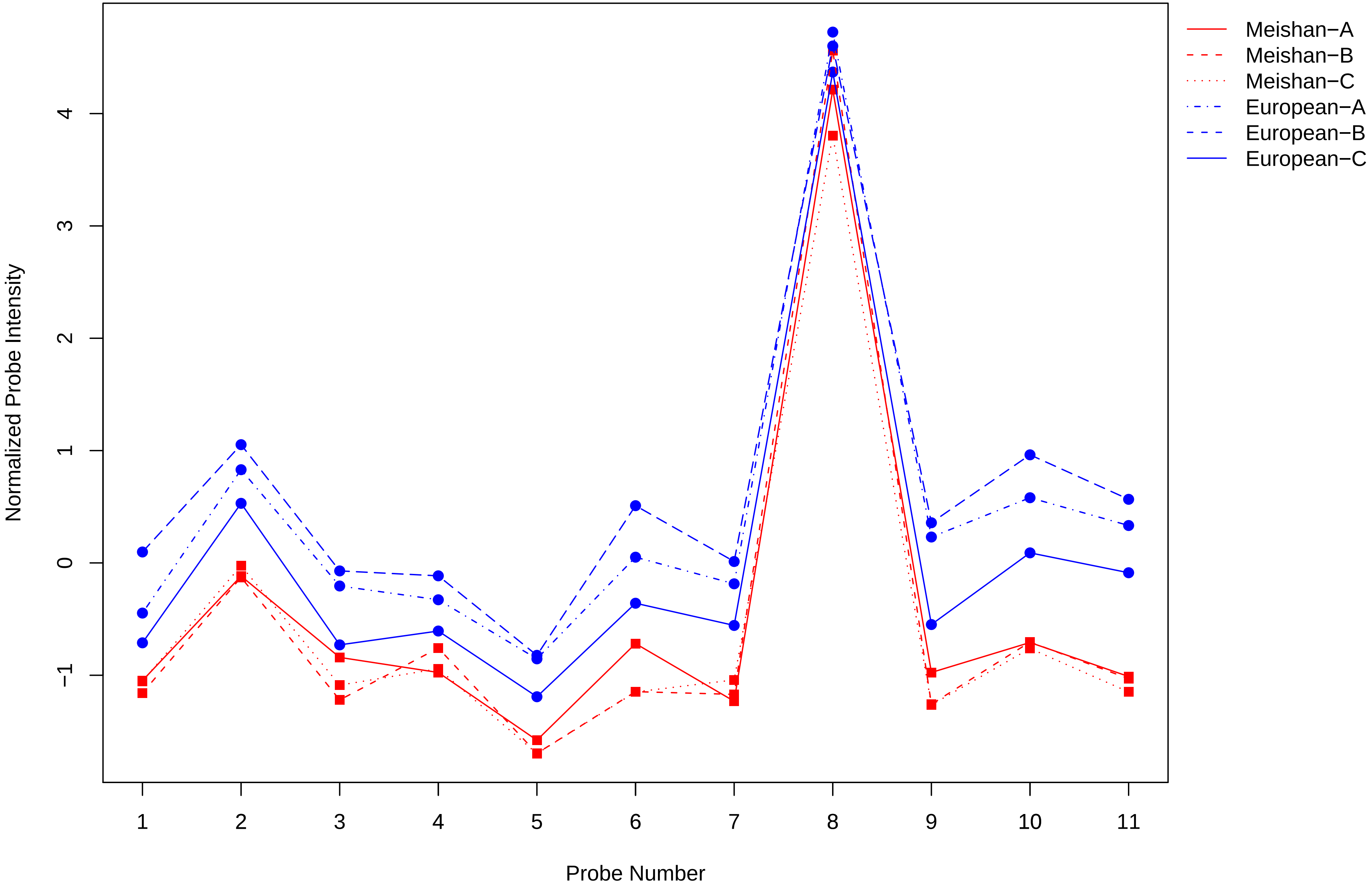

Rank 2771 − Ssc.27398.1.S1_at − probe 10
C21orf108

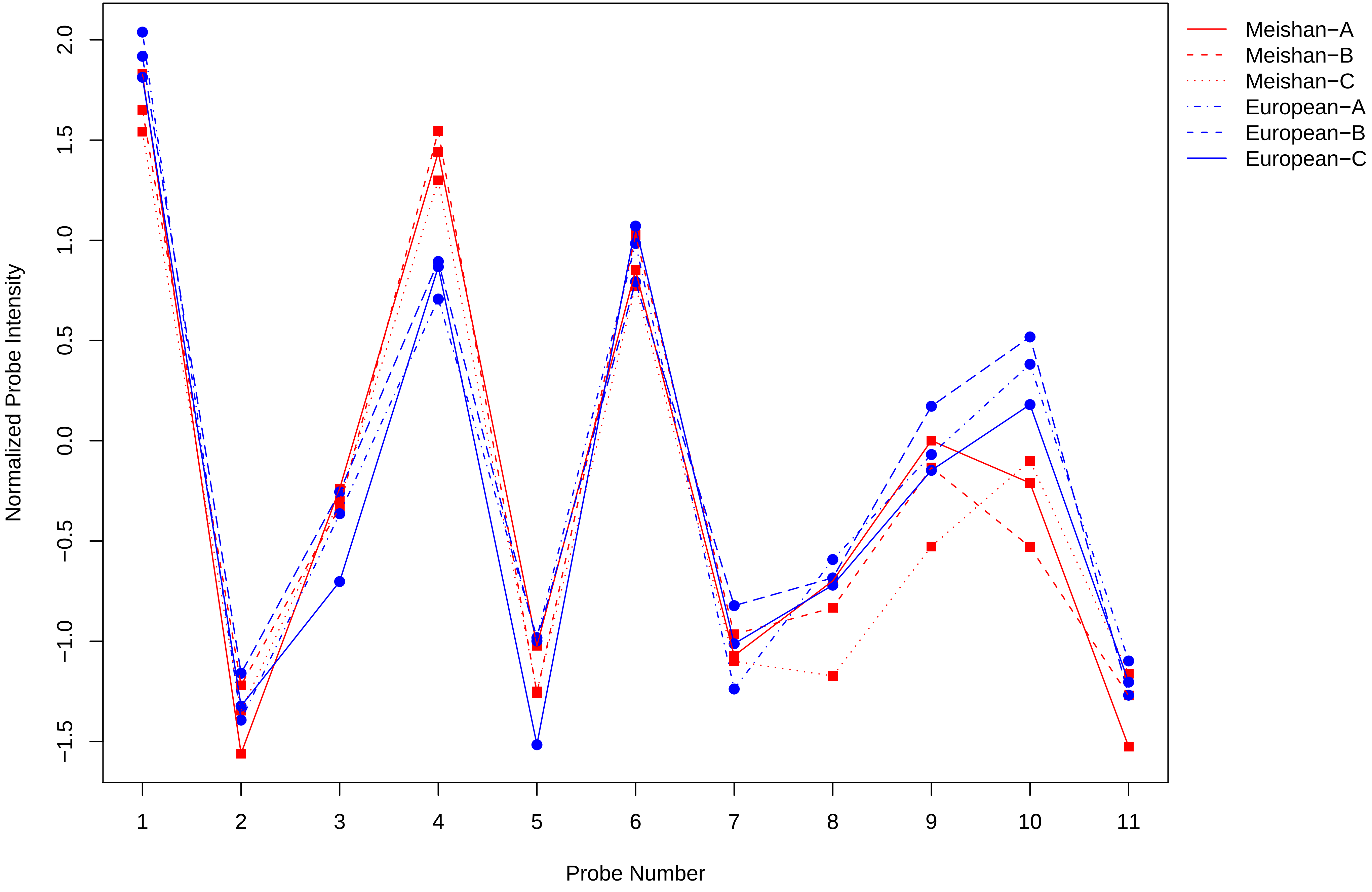

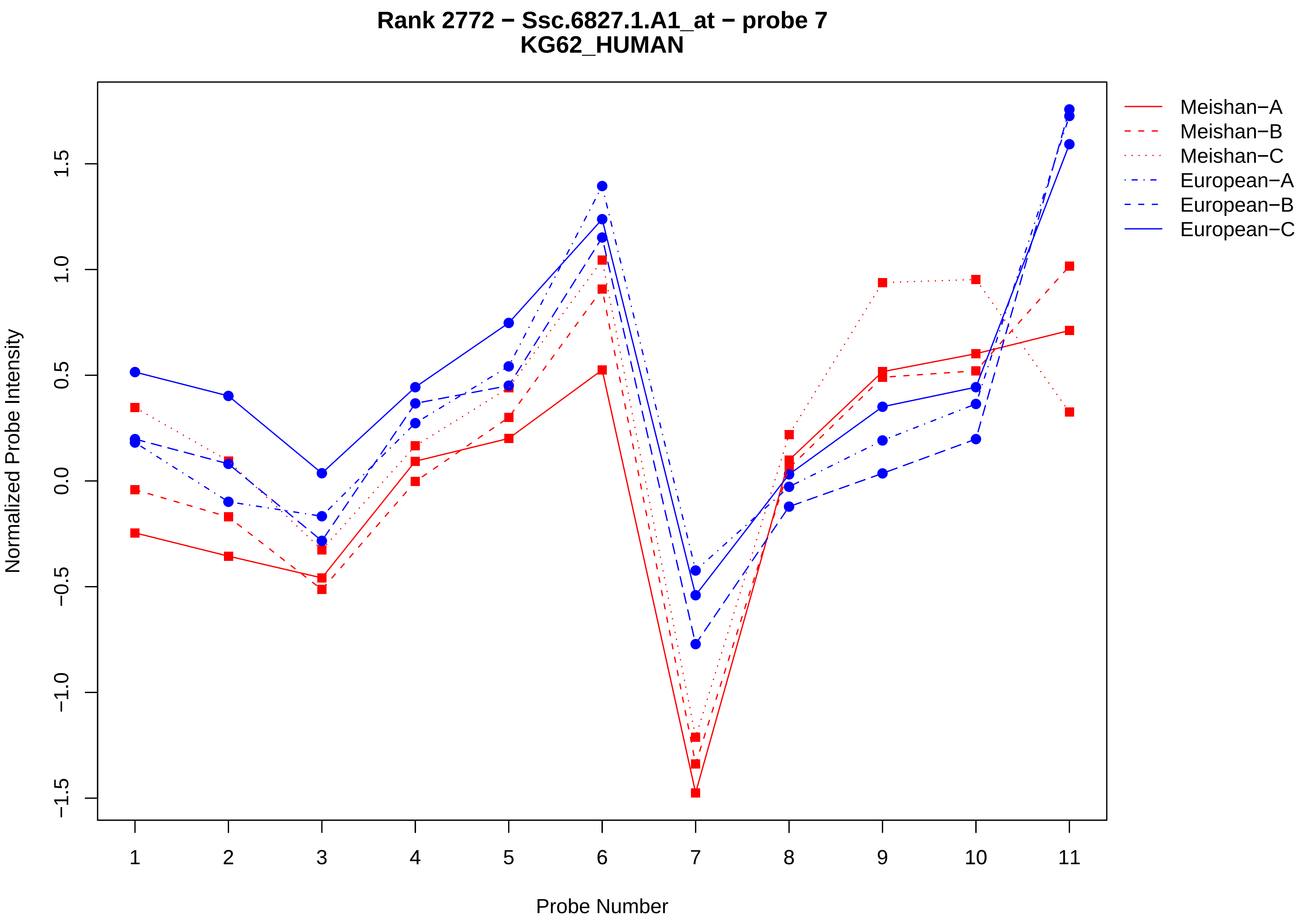

Rank 2772 − Ssc.6827.1.A1_at − probe 7
KG62_HUMAN
Normalized Probe Intensity
Probe Number
−1.5
−1.0
−0.5
0.0
0.5
1.0
1.5
1
2
3
4
5
6
7
8
9
10
11
Meishan−A
Meishan−B
Meishan−C
European−A
European−B
European−C

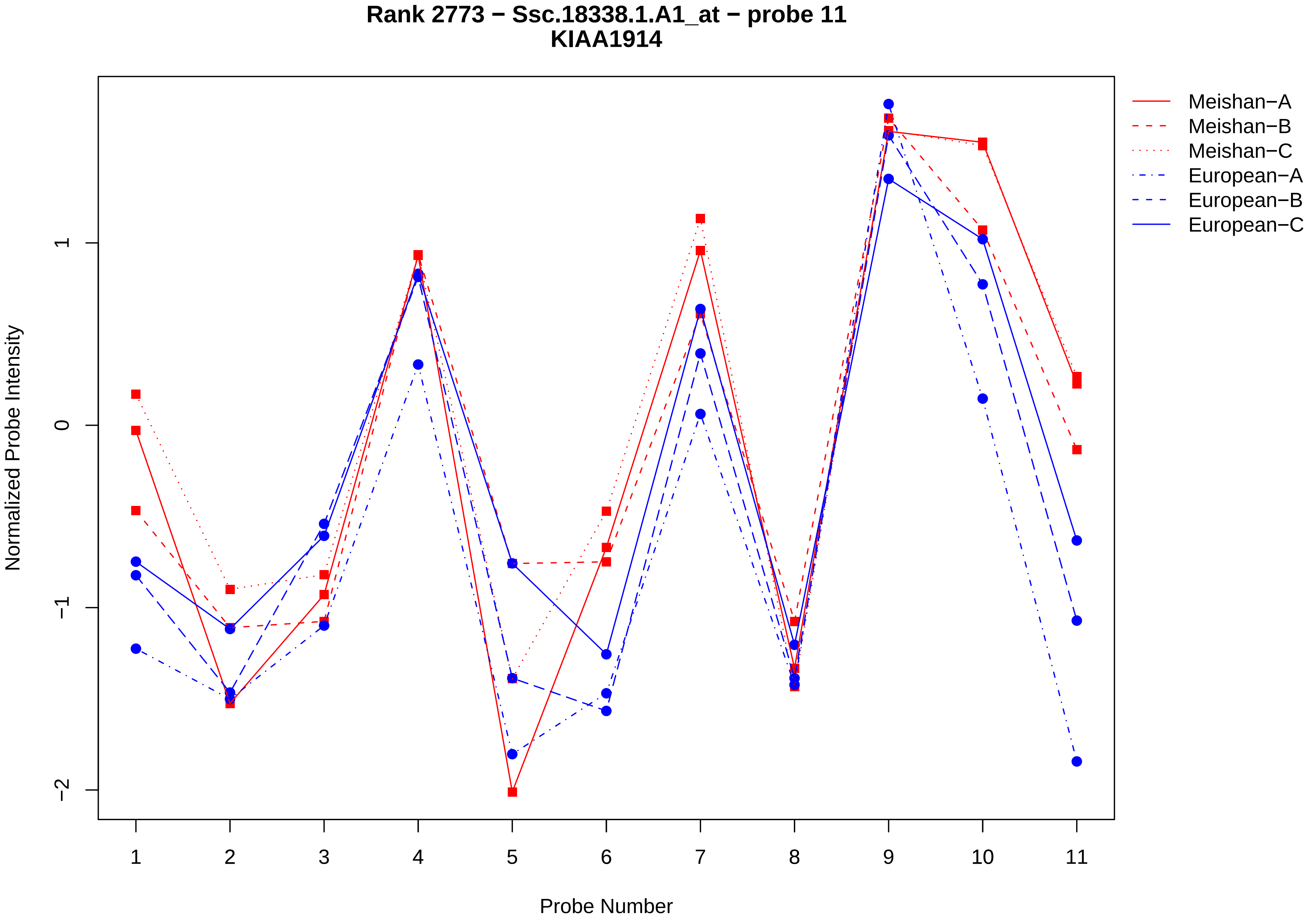

Rank 2773 − Ssc.18338.1.A1_at − probe 11
KIAA1914
Normalized Probe Intensity
Probe Number
−2
−1
0
1
1
2
3
4
5
6
7
8
9
10
11
Meishan−A
Meishan−B
Meishan−C
European−A
European−B
European−C

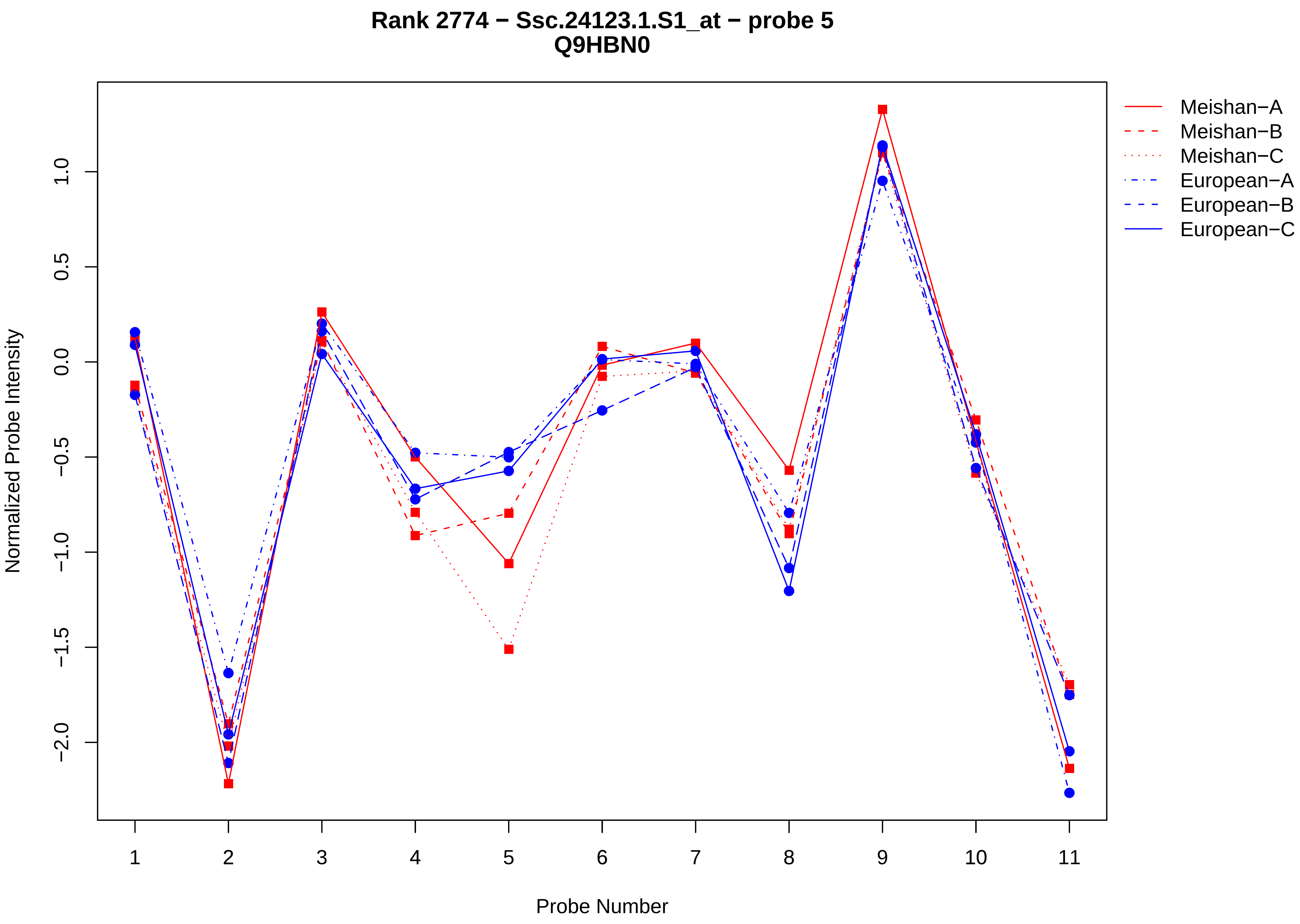

Rank 2774 − Ssc.24123.1.S1_at − probe 5
Q9HBN0
Normalized Probe Intensity
Probe Number
Meishan−A
Meishan−B
Meishan−C
European−A
European−B
European−C

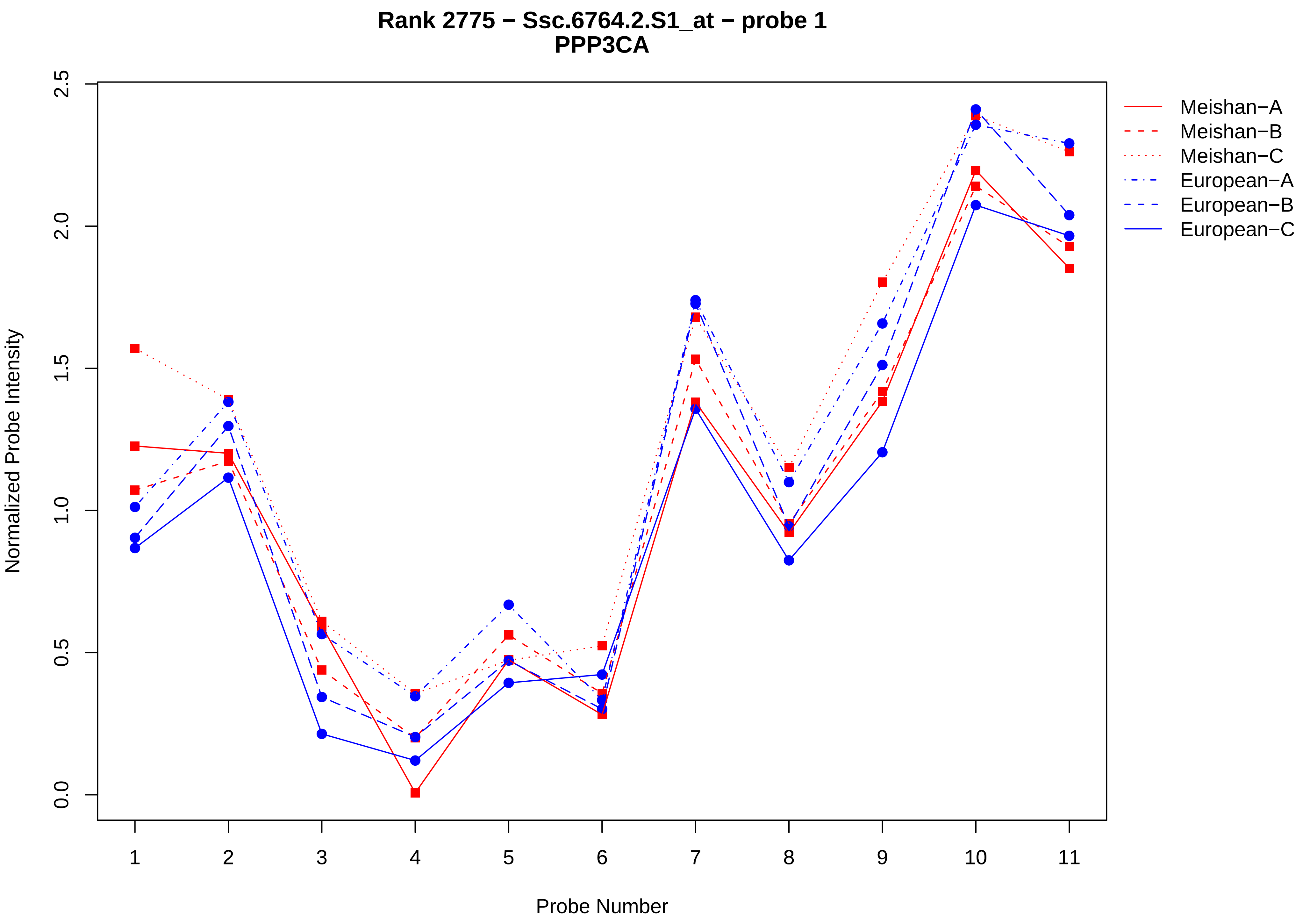

Rank 2775 − Ssc.6764.2.S1_at − probe 1
PPP3CA
Normalized Probe Intensity
0.0
0.5
1.0
1.5
2.0
2.5
1
2
3
4
5
6
7
8
9
10
11
Probe Number
Meishan−A
Meishan−B
Meishan−C
European−A
European−B
European−C

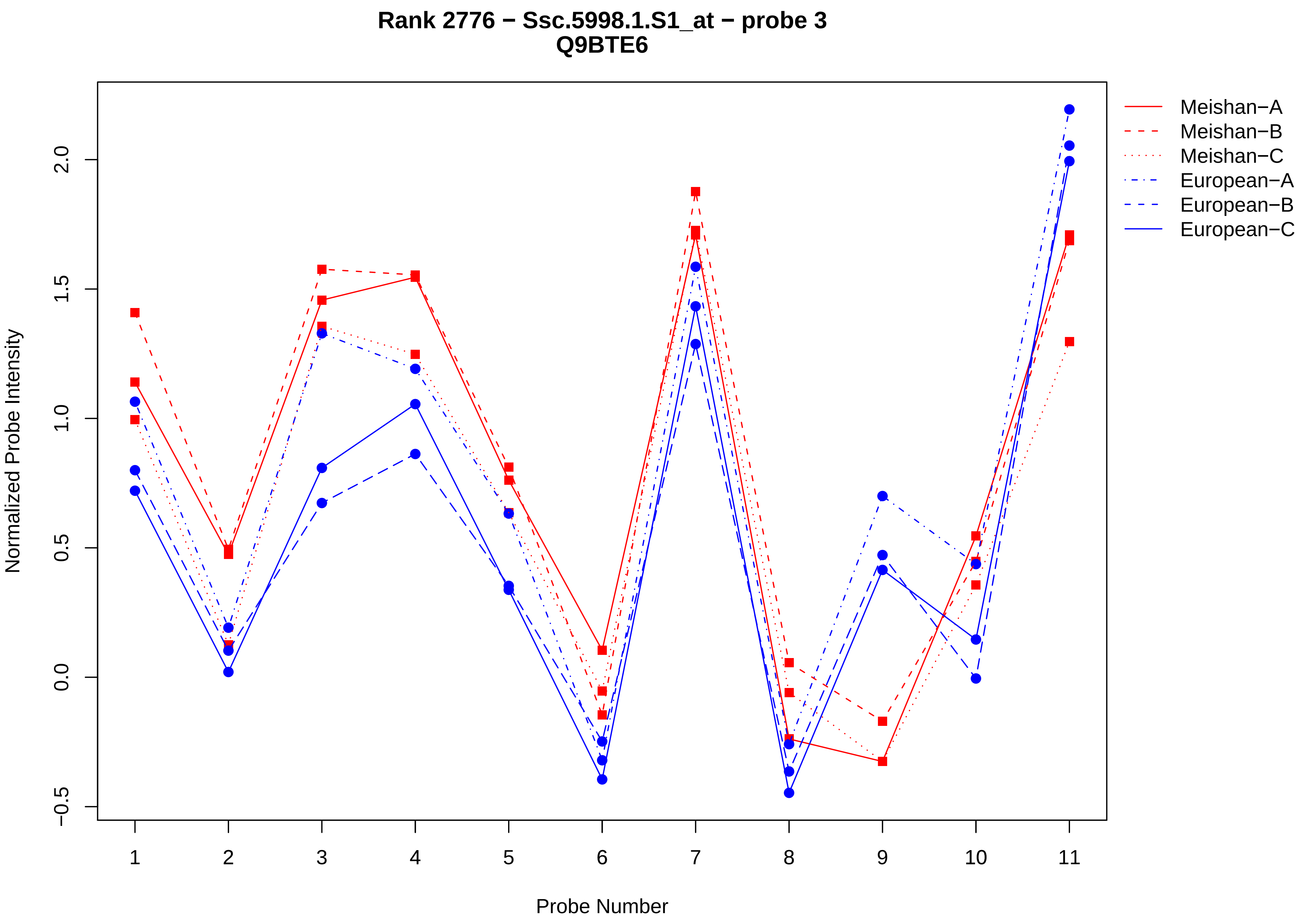

Rank 2776 − Ssc.5998.1.S1_at − probe 3
Q9BTE6
Normalized Probe Intensity
−0.5
0.0
0.5
1.0
1.5
2.0
1
2
3
4
5
6
7
8
9
10
11
Probe Number
Meishan−A
Meishan−B
Meishan−C
European−A
European−B
European−C

Rank 2777 − Ssc.28164.2.S1_at − probe 9
INCENP

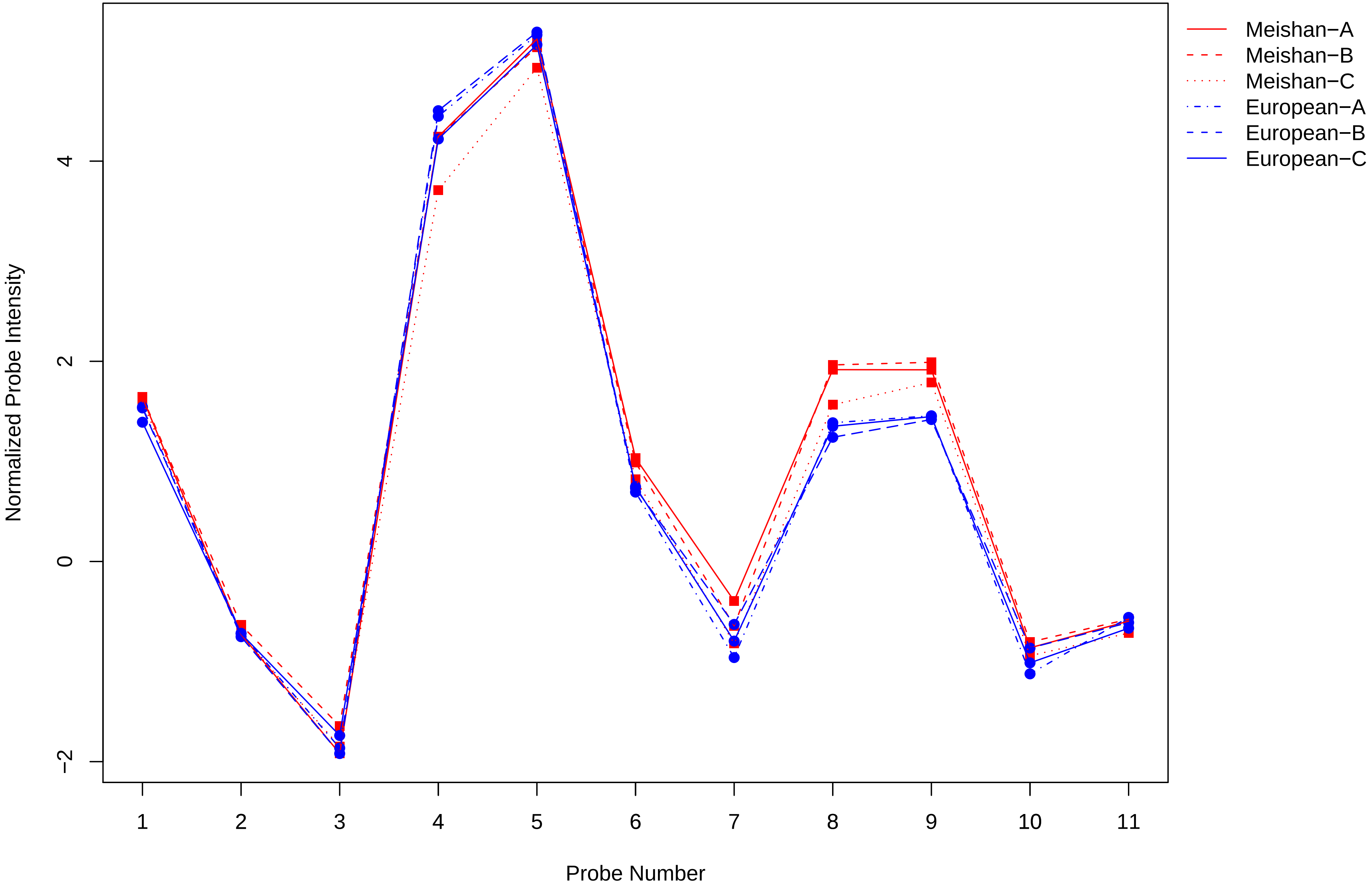

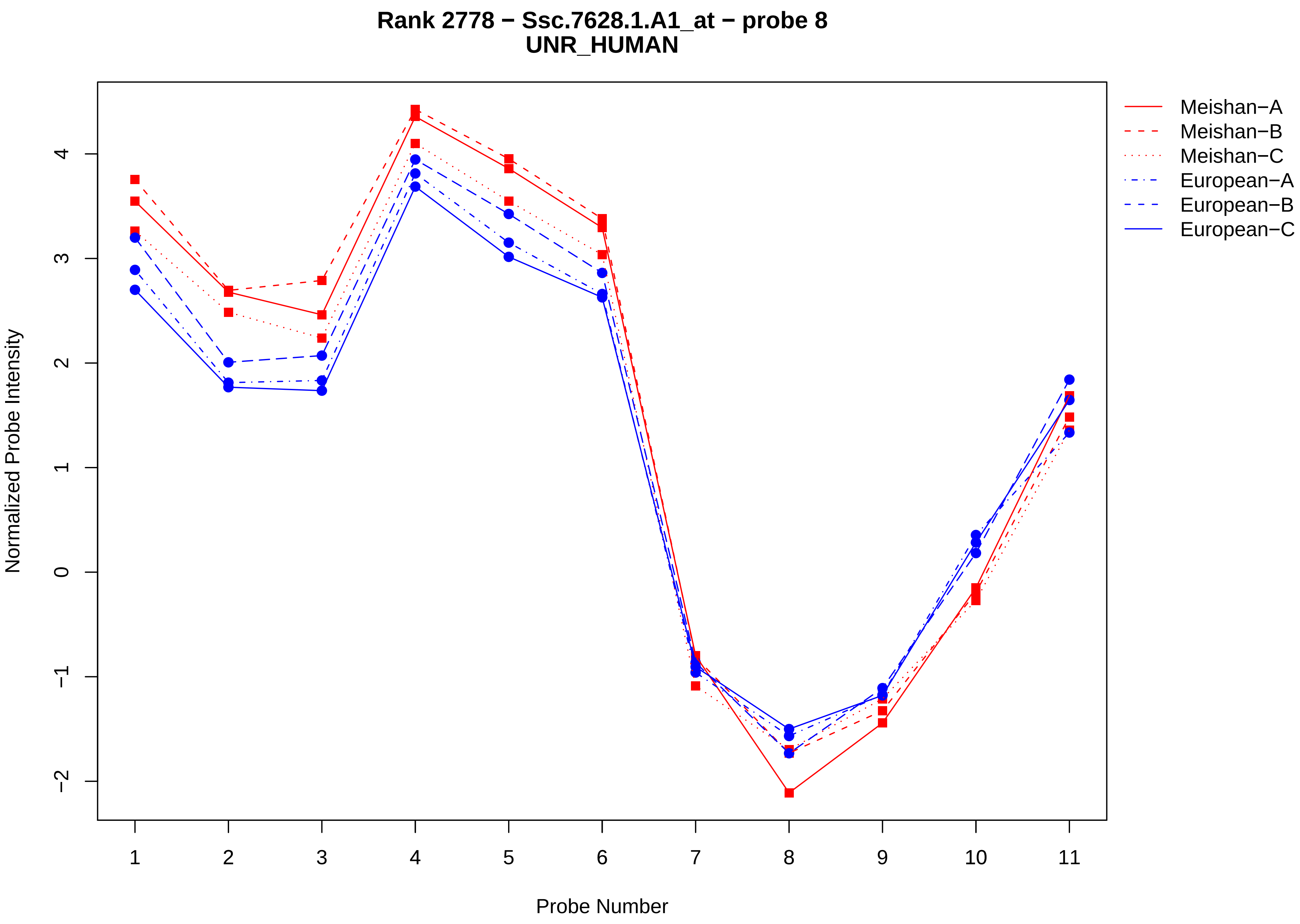

Rank 2778 − Ssc.7628.1.A1_at − probe 8
UNR_HUMAN
Normalized Probe Intensity
Probe Number
−2
−1
0
1
2
3
4
1
2
3
4
5
6
7
8
9
10
11
Meishan−A
Meishan−B
Meishan−C
European−A
European−B
European−C

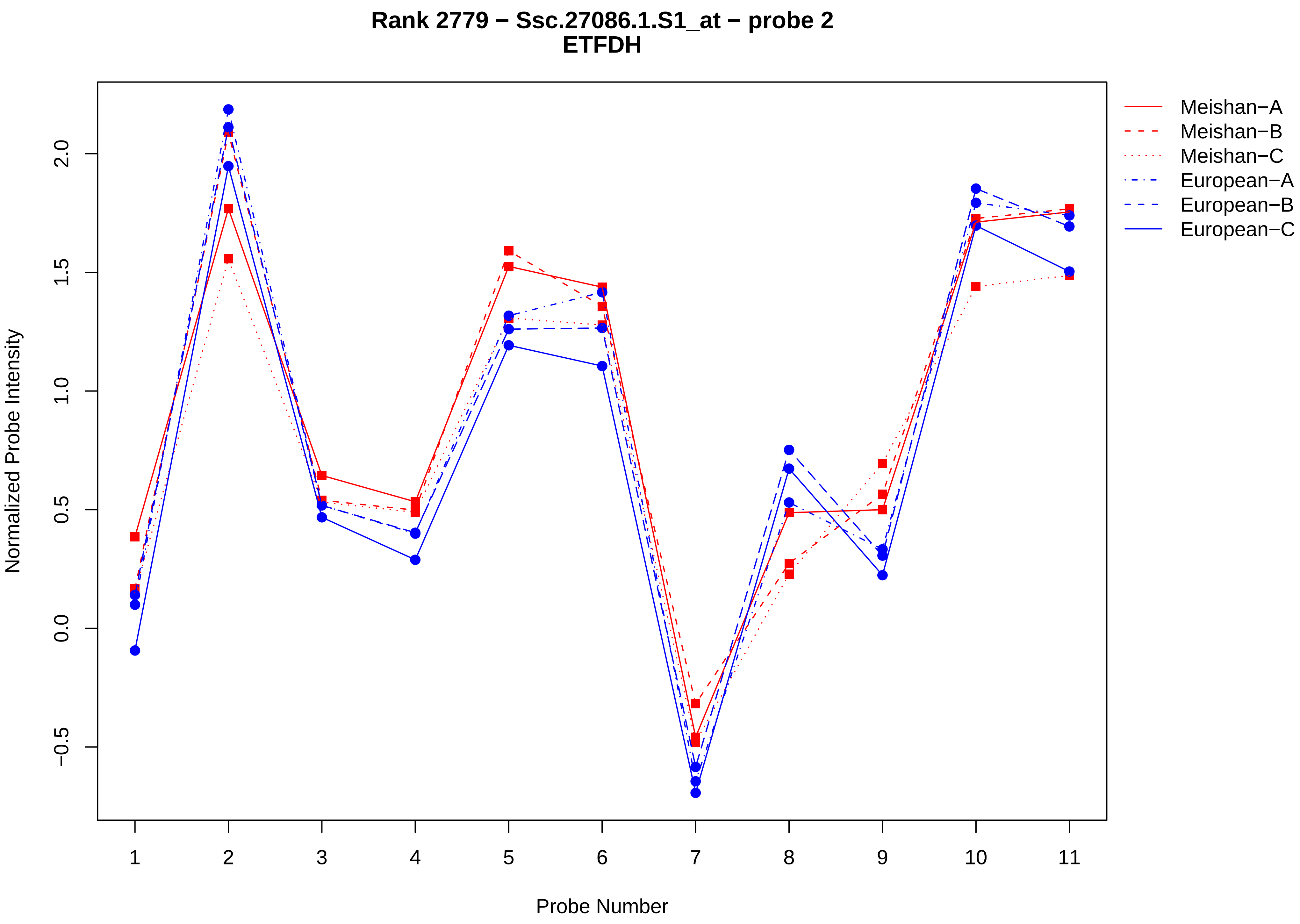
Rank 2779 − Ssc.27086.1.S1_at − probe 2
ETFDH
Normalized Probe Intensity
Probe Number
−0.5
0.0
0.5
1.0
1.5
2.0
1
2
3
4
5
6
7
8
9
10
11
Meishan−A
Meishan−B
Meishan−C
European−A
European−B
European−C

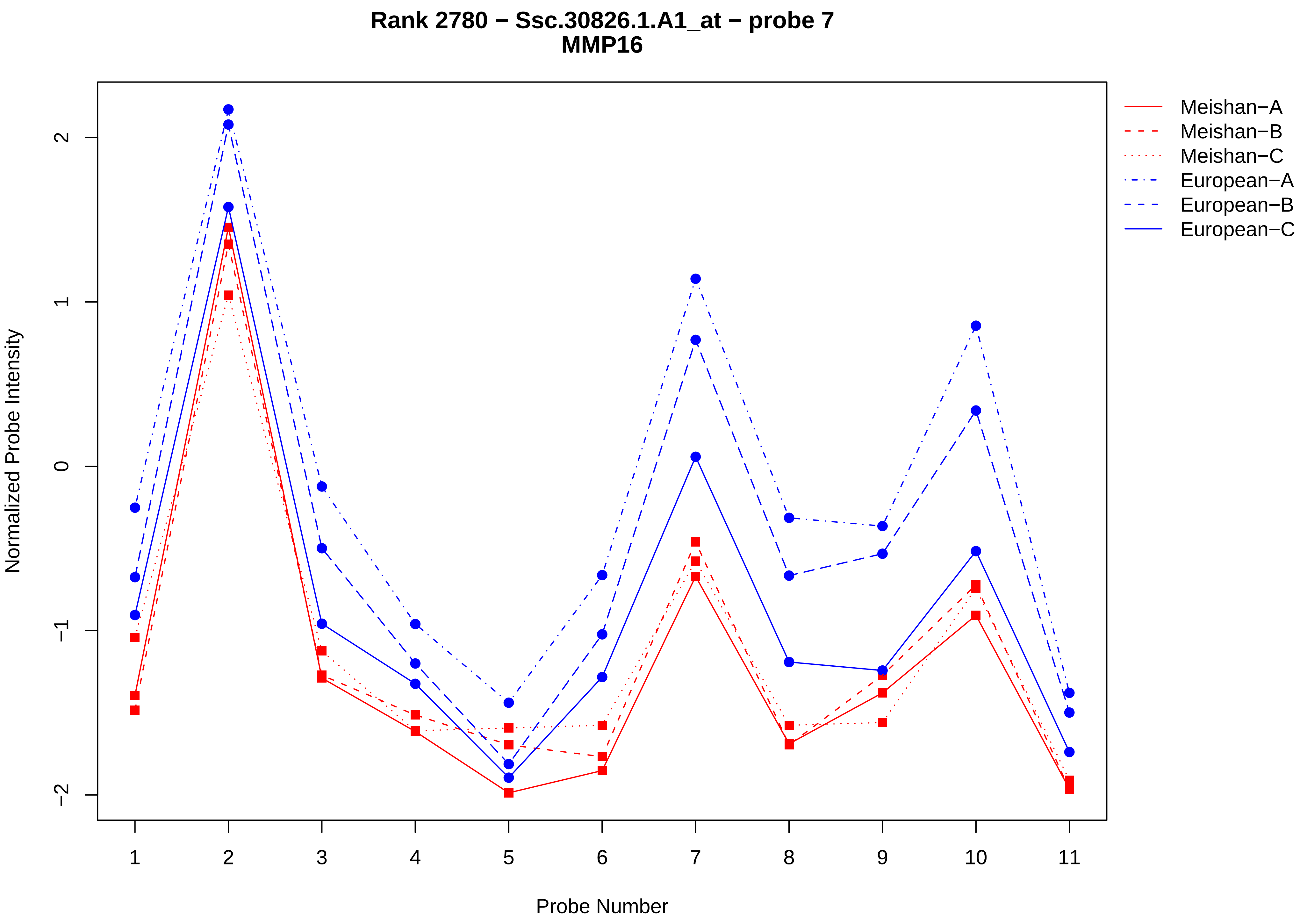

Rank 2780 − Ssc.30826.1.A1_at − probe 7
MMP16
Normalized Probe Intensity
Probe Number
Meishan−A
Meishan−B
Meishan−C
European−A
European−B
European−C

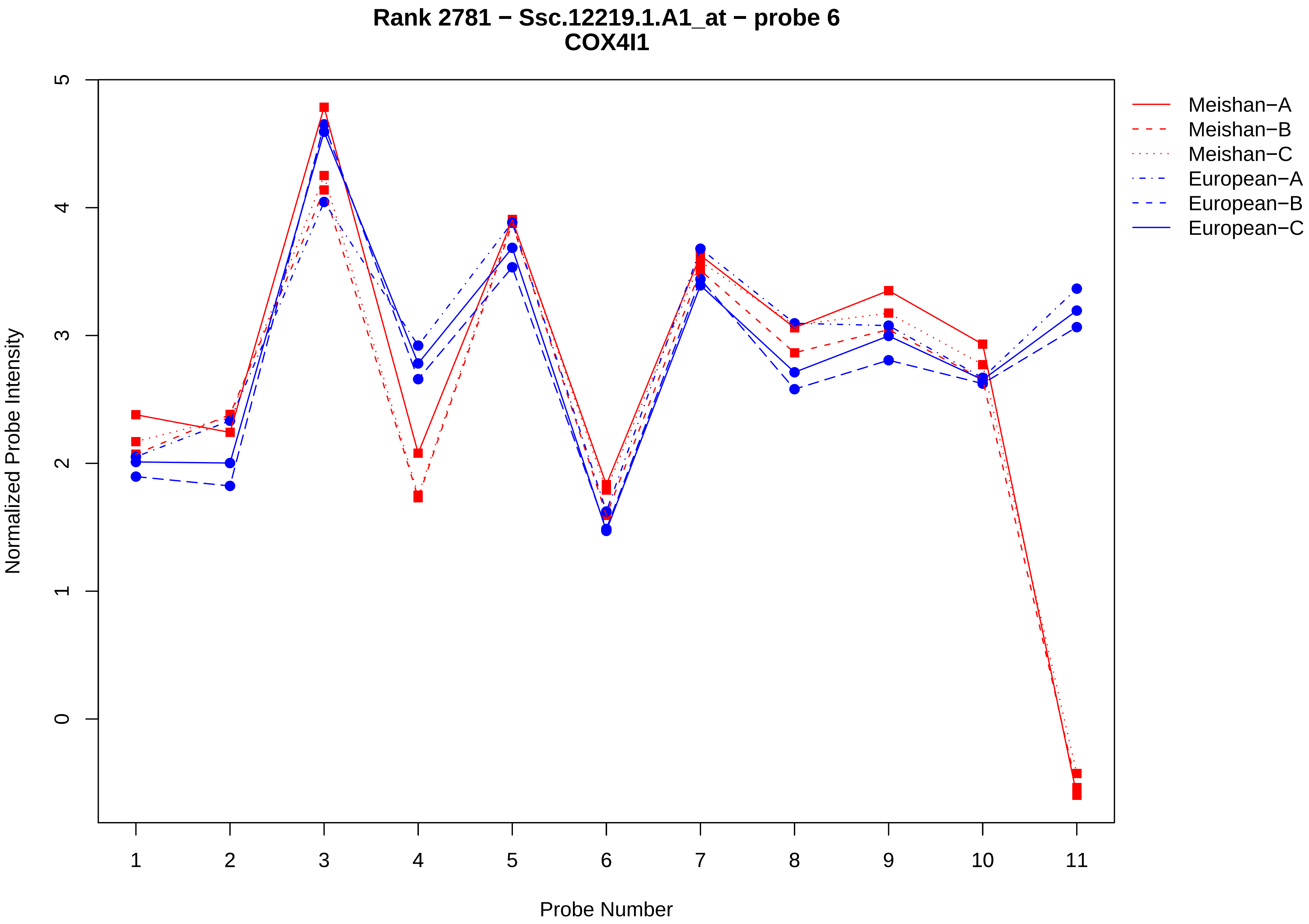

Rank 2781 − Ssc.12219.1.A1_at − probe 6
COX4I1
Normalized Probe Intensity
Probe Number
0
1
2
3
4
5
1
2
3
4
5
6
7
8
9
10
11
Meishan−A
Meishan−B
Meishan−C
European−A
European−B
European−C

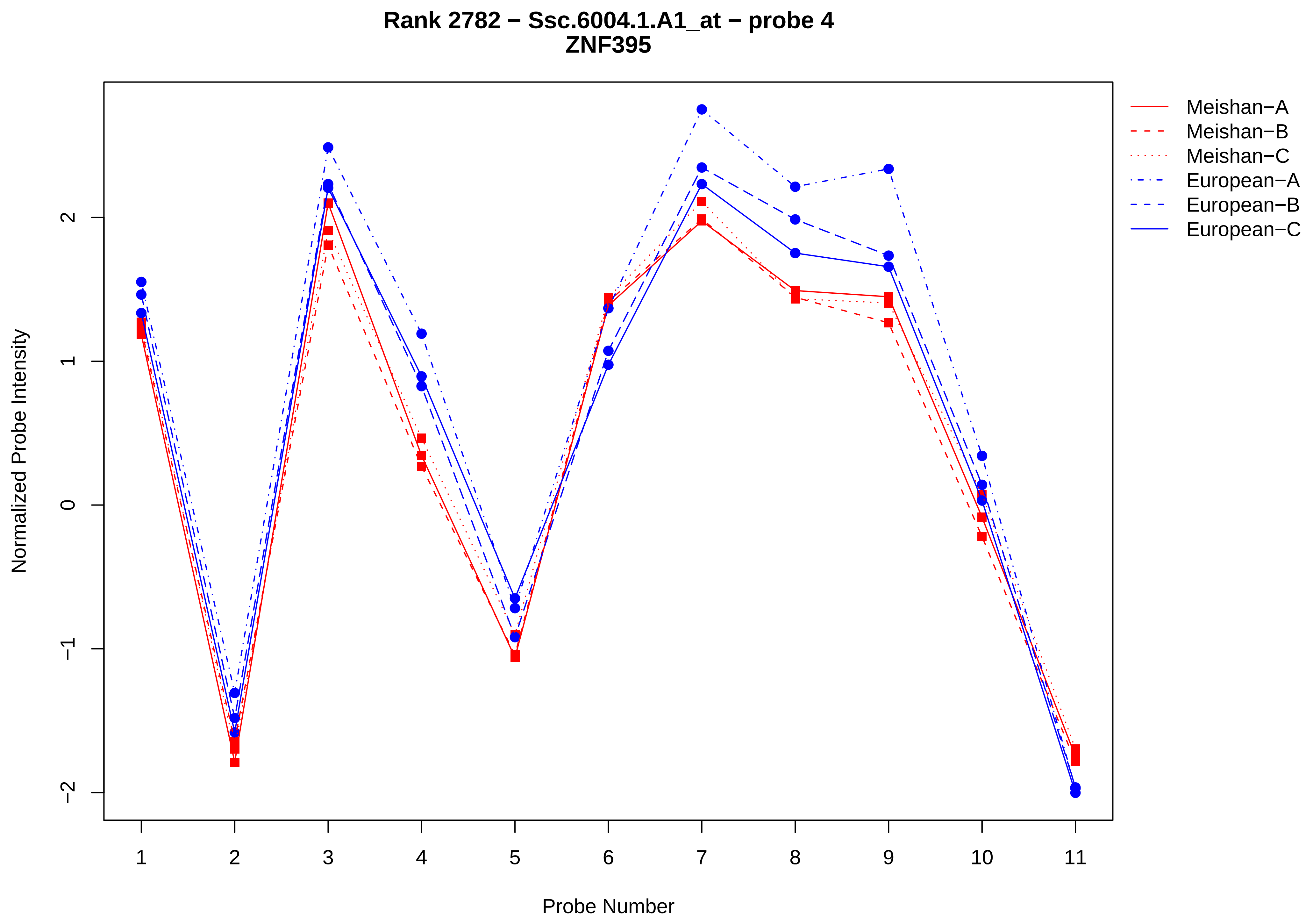

Rank 2782 − Ssc.6004.1.A1_at − probe 4
ZNF395
Normalized Probe Intensity
Probe Number
−2
−1
0
1
2
1
2
3
4
5
6
7
8
9
10
11
Meishan−A
Meishan−B
Meishan−C
European−A
European−B
European−C

Rank 2783 − Ssc.18894.1.S1_a_at − probe 9
Q96GX8

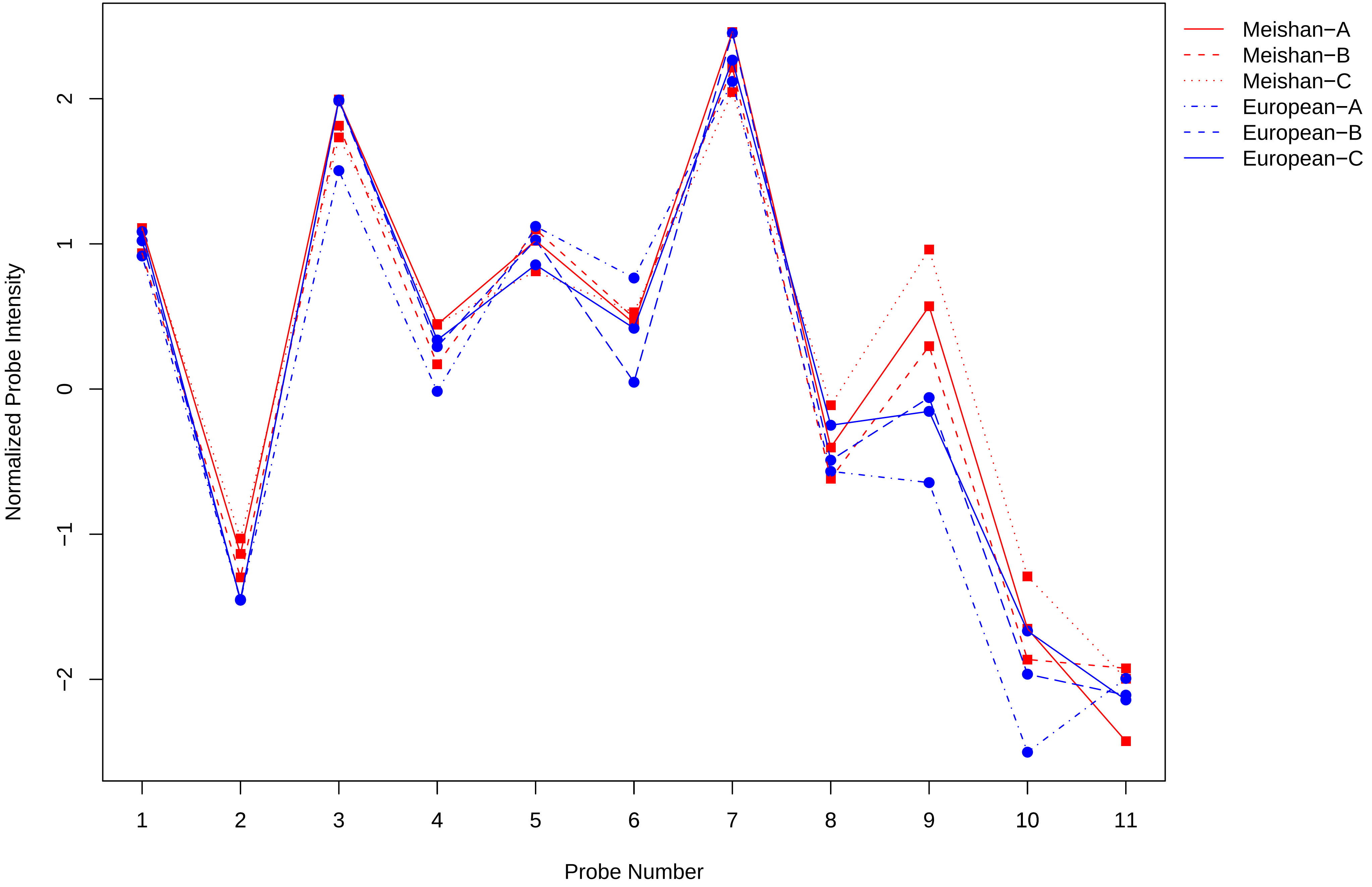

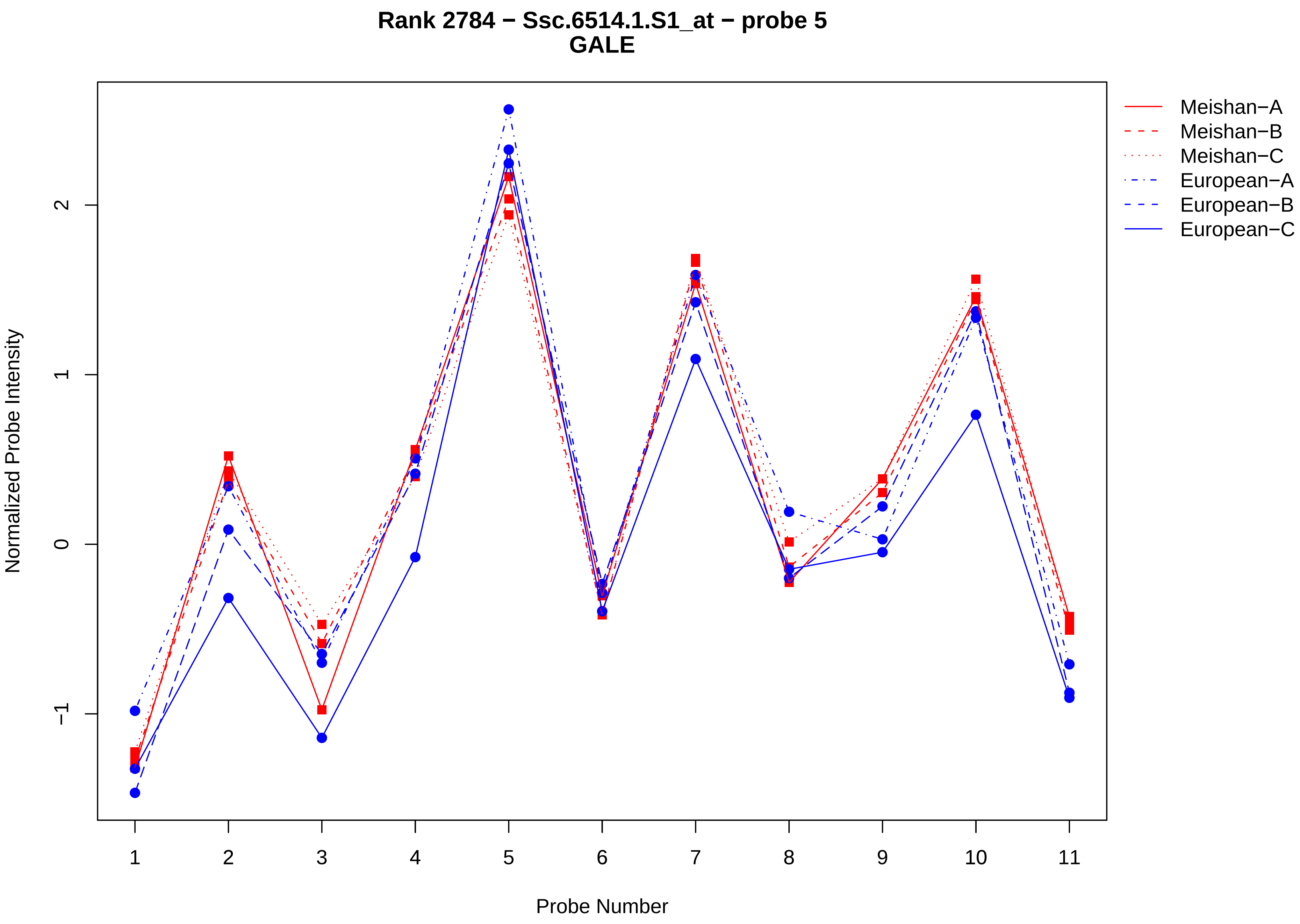

Rank 2784 − Ssc.6514.1.S1_at − probe 5
GALE
Normalized Probe Intensity
Probe Number
Meishan−A
Meishan−B
Meishan−C
European−A
European−B
European−C
−1
0
1
2
1
2
3
4
5
6
7
8
9
10
11

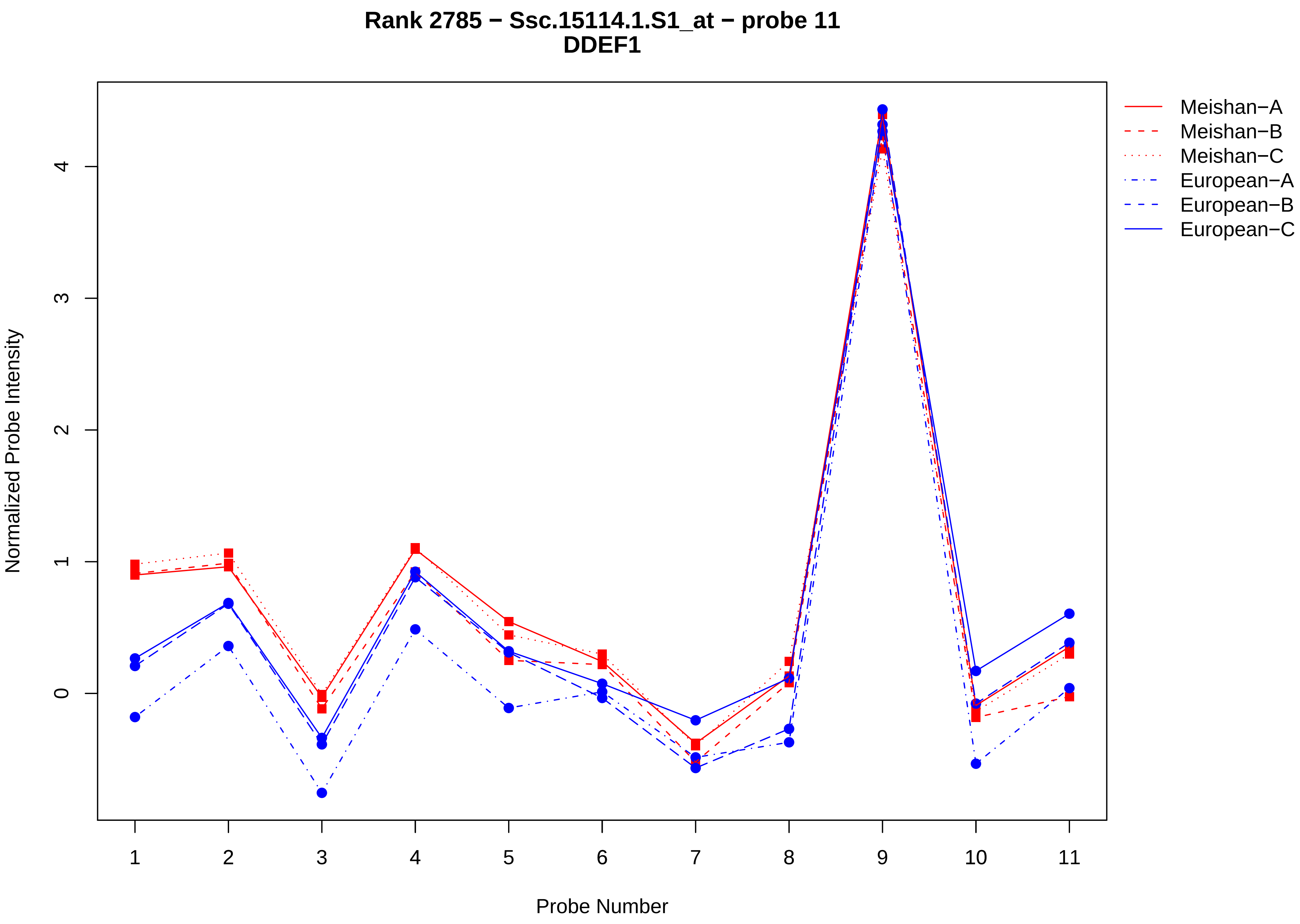

Rank 2785 − Ssc.15114.1.S1_at − probe 11
DDEF1
Normalized Probe Intensity
0
1
2
3
4
Probe Number
1
2
3
4
5
6
7
8
9
10
11
Meishan−A
Meishan−B
Meishan−C
European−A
European−B
European−C

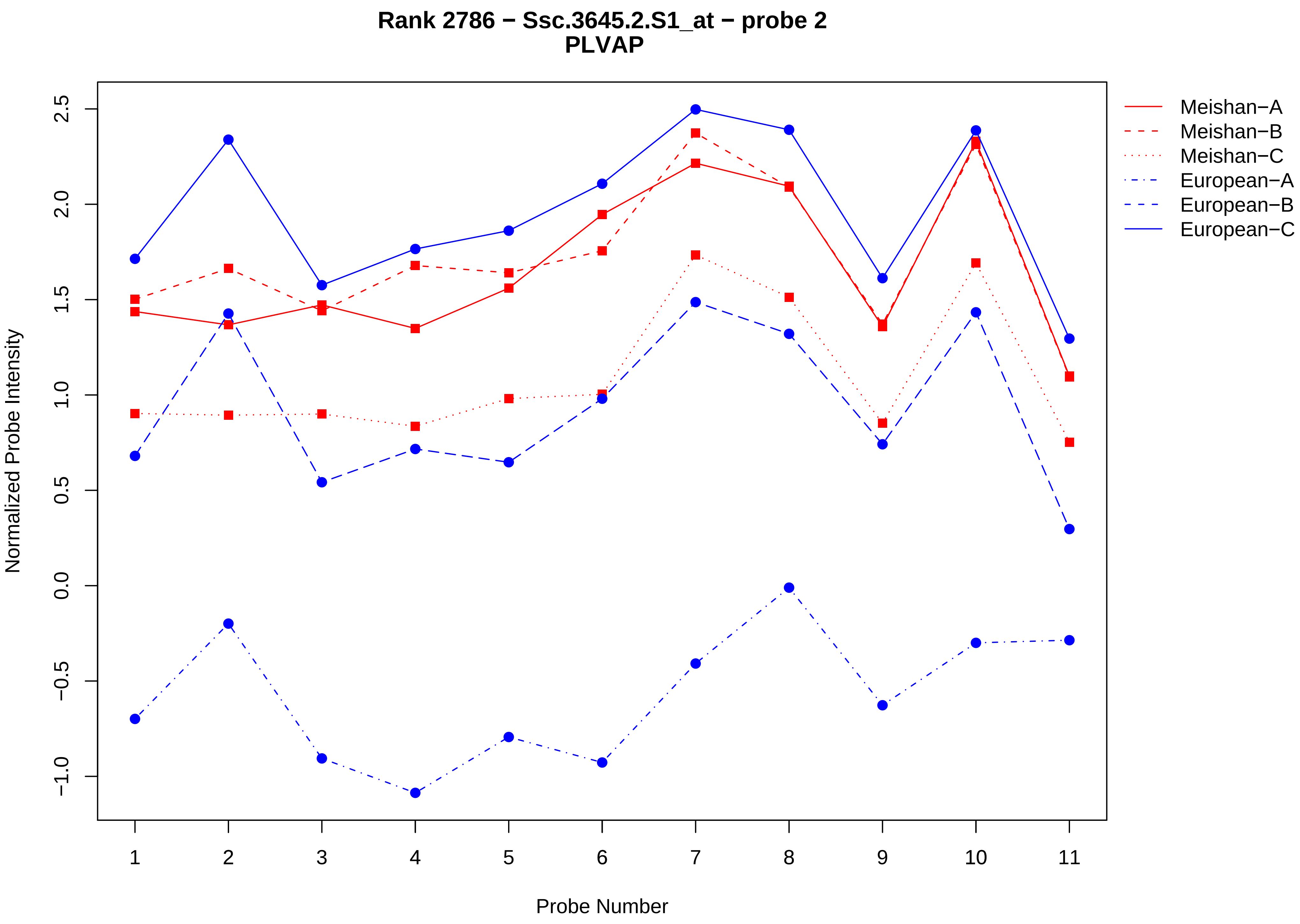
Rank 2786 − Ssc.3645.2.S1_at − probe 2
PLVAP
Normalized Probe Intensity
Probe Number
Meishan−A
Meishan−B
Meishan−C
European−A
European−B
European−C

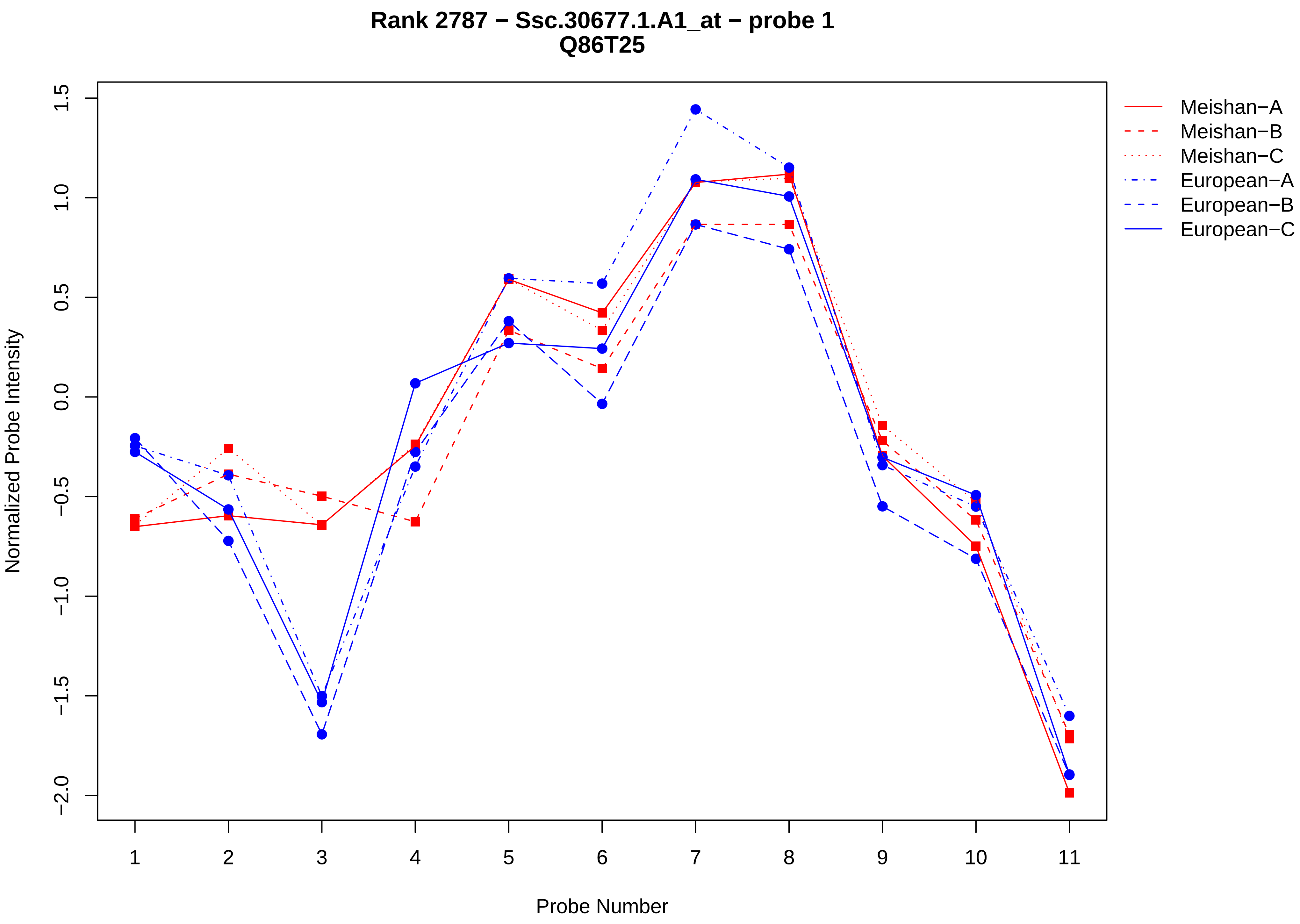

Rank 2787 − Ssc.30677.1.A1_at − probe 1
Q86T25
Normalized Probe Intensity
Probe Number
1.5
1.0
0.5
0.0
−0.5
−1.0
−1.5
−2.0
1
2
3
4
5
6
7
8
9
10
11
Meishan−A
Meishan−B
Meishan−C
European−A
European−B
European−C

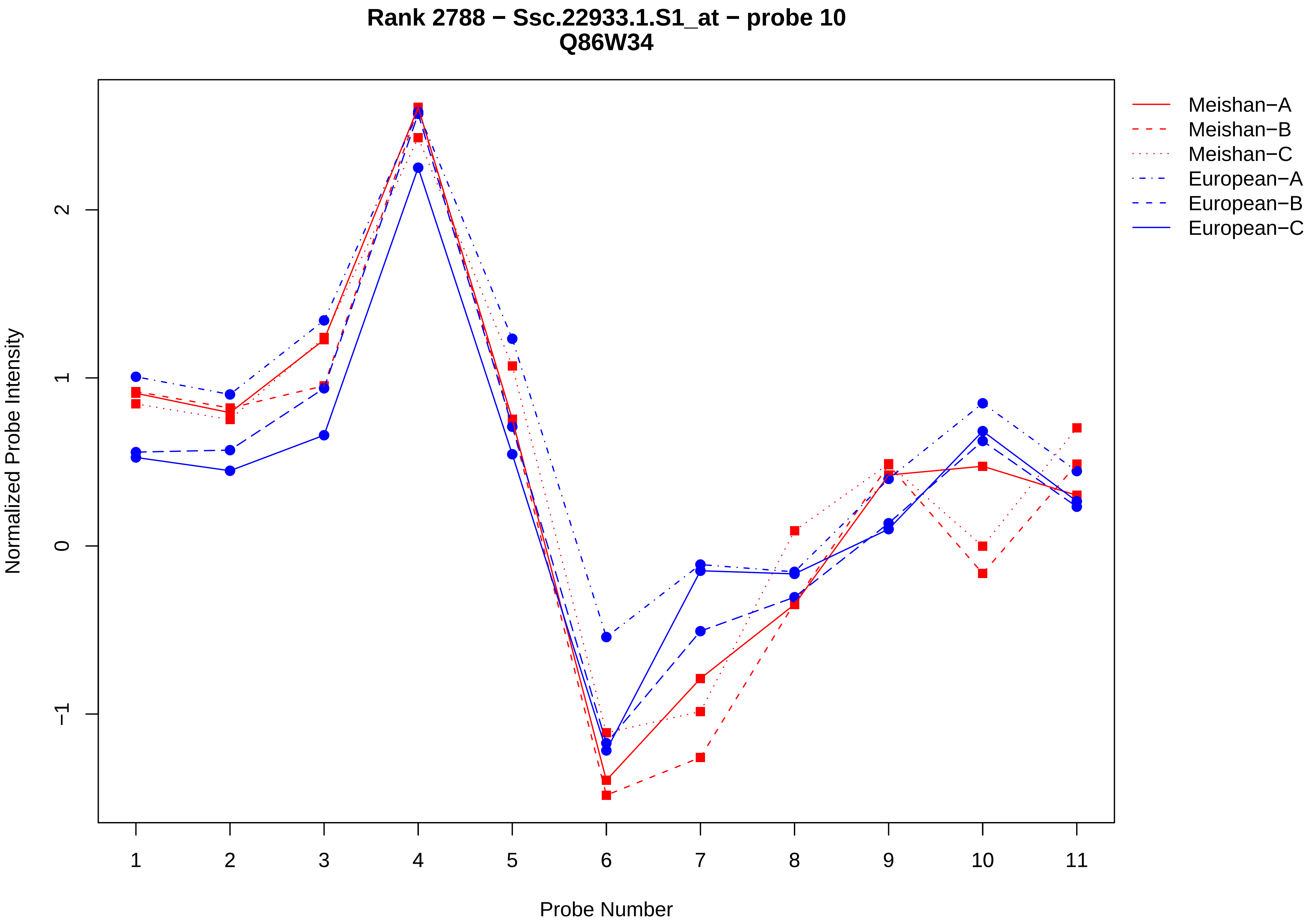

Rank 2788 − Ssc.22933.1.S1_at − probe 10
Q86W34
Normalized Probe Intensity
Probe Number
−1
0
1
2
1
2
3
4
5
6
7
8
9
10
11
Meishan−A
Meishan−B
Meishan−C
European−A
European−B
European−C

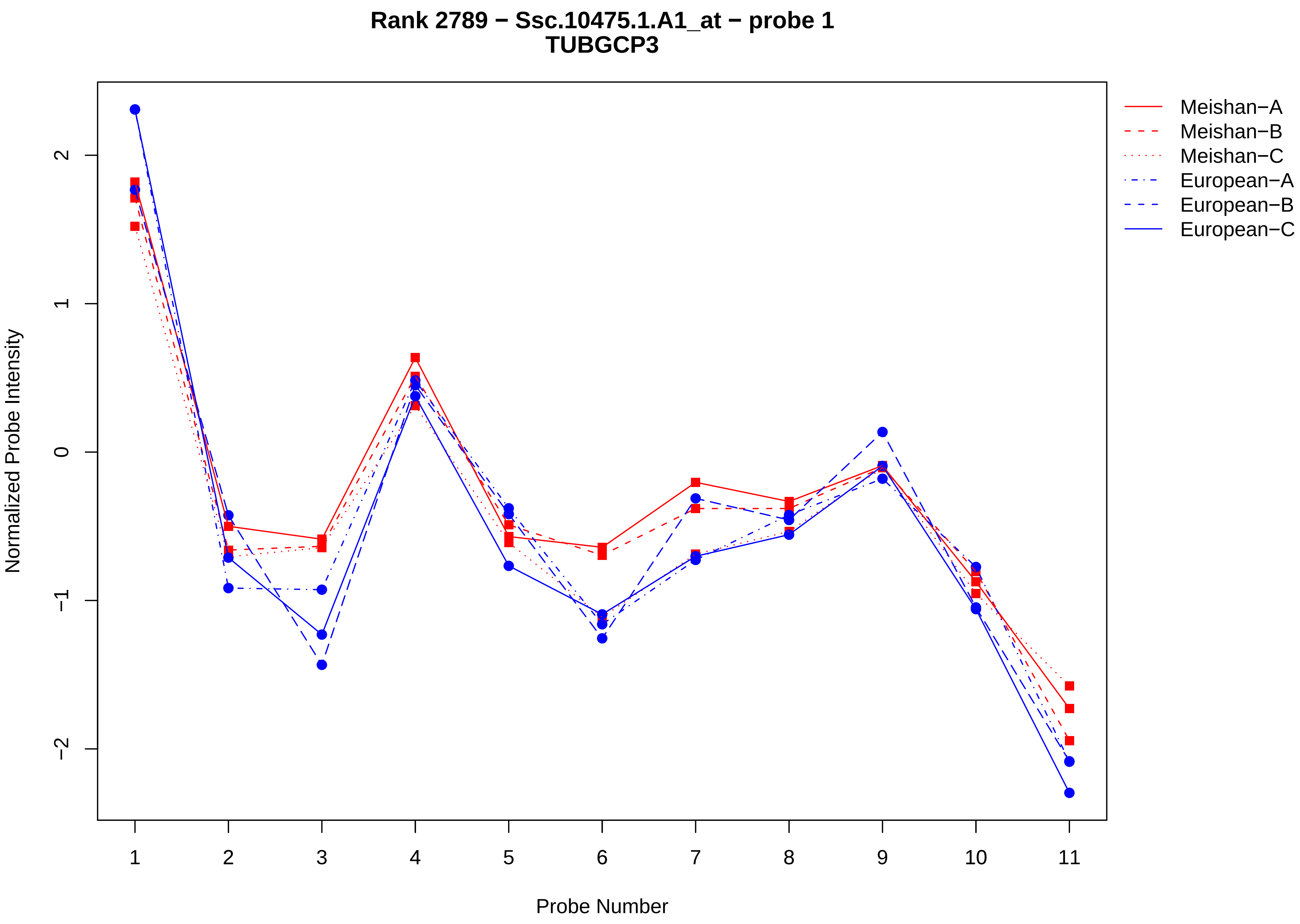

Rank 2789 − Ssc.10475.1.A1_at − probe 1
TUBGCP3
Normalized Probe Intensity
Probe Number
Meishan−A
Meishan−B
Meishan−C
European−A
European−B
European−C
2
1
0
−1
−2
1
2
3
4
5
6
7
8
9
10
11

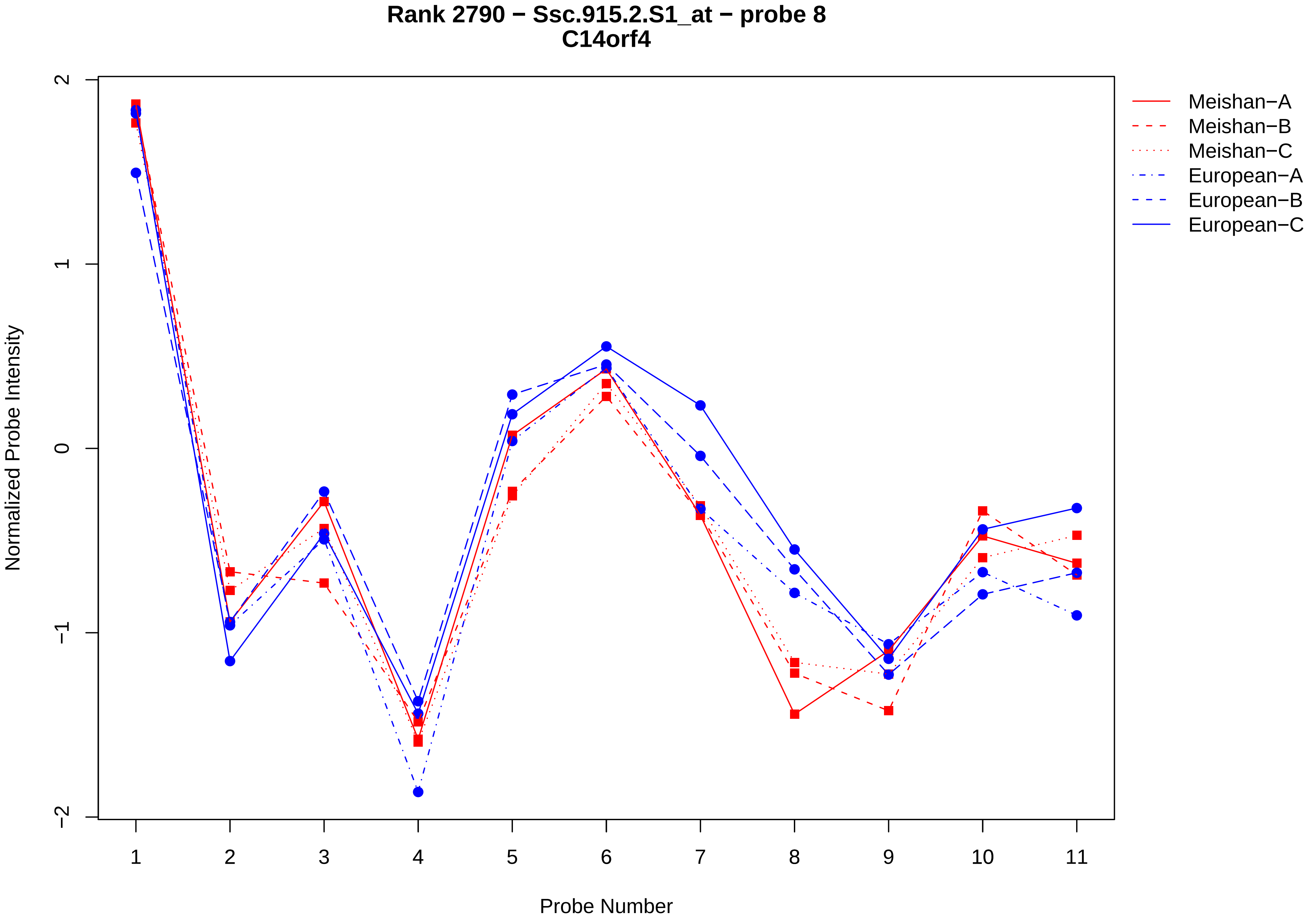

Rank 2790 − Ssc.915.2.S1_at − probe 8
C14orf4
Normalized Probe Intensity
Probe Number
−2
−1
0
1
2
1
2
3
4
5
6
7
8
9
10
11
Meishan−A
Meishan−B
Meishan−C
European−A
European−B
European−C

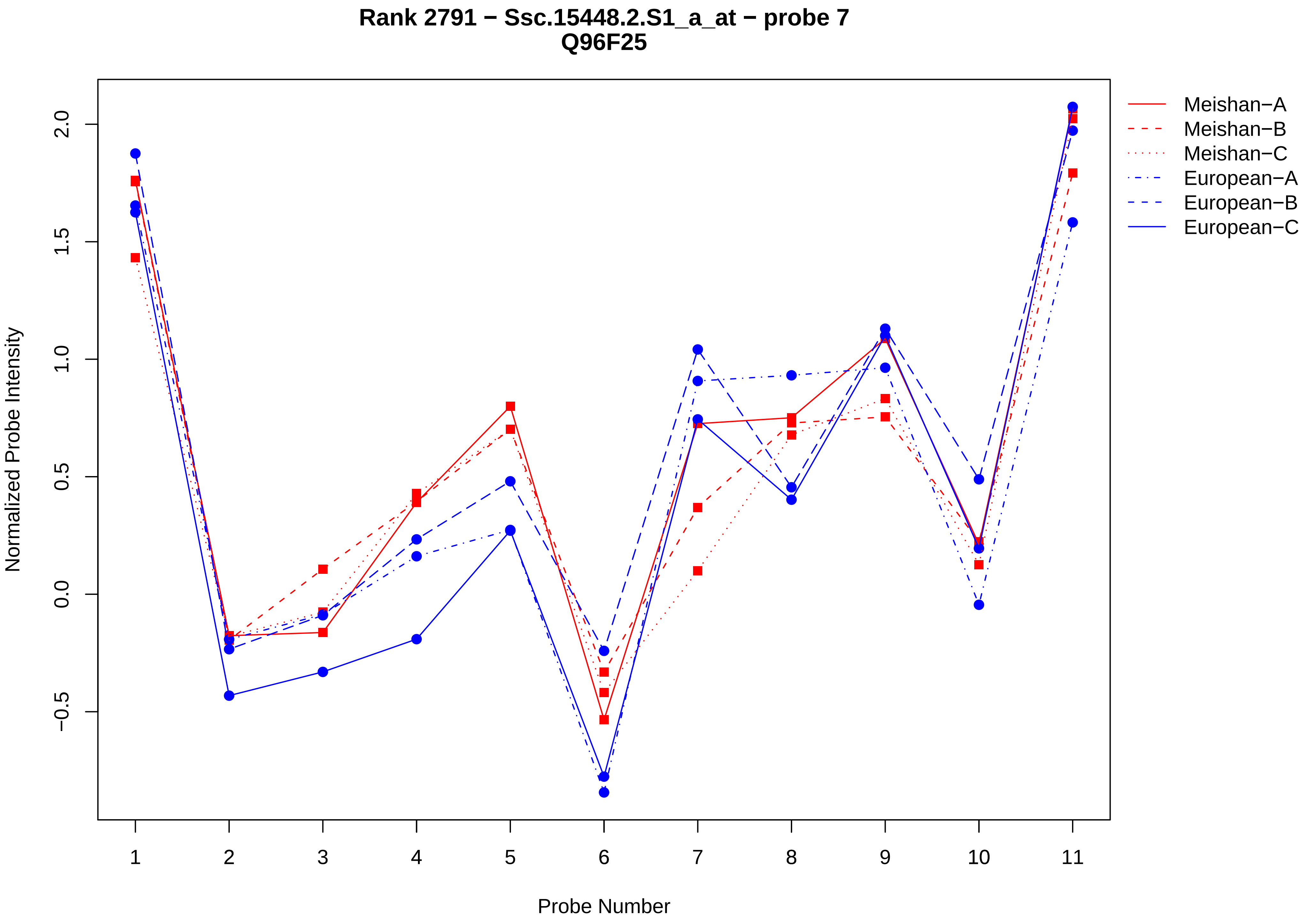

Rank 2791 − Ssc.15448.2.S1_a_at − probe 7
Q96F25
Normalized Probe Intensity
Probe Number
−0.5
0.0
0.5
1.0
1.5
2.0
1
2
3
4
5
6
7
8
9
10
11
Meishan−A
Meishan−B
Meishan−C
European−A
European−B
European−C

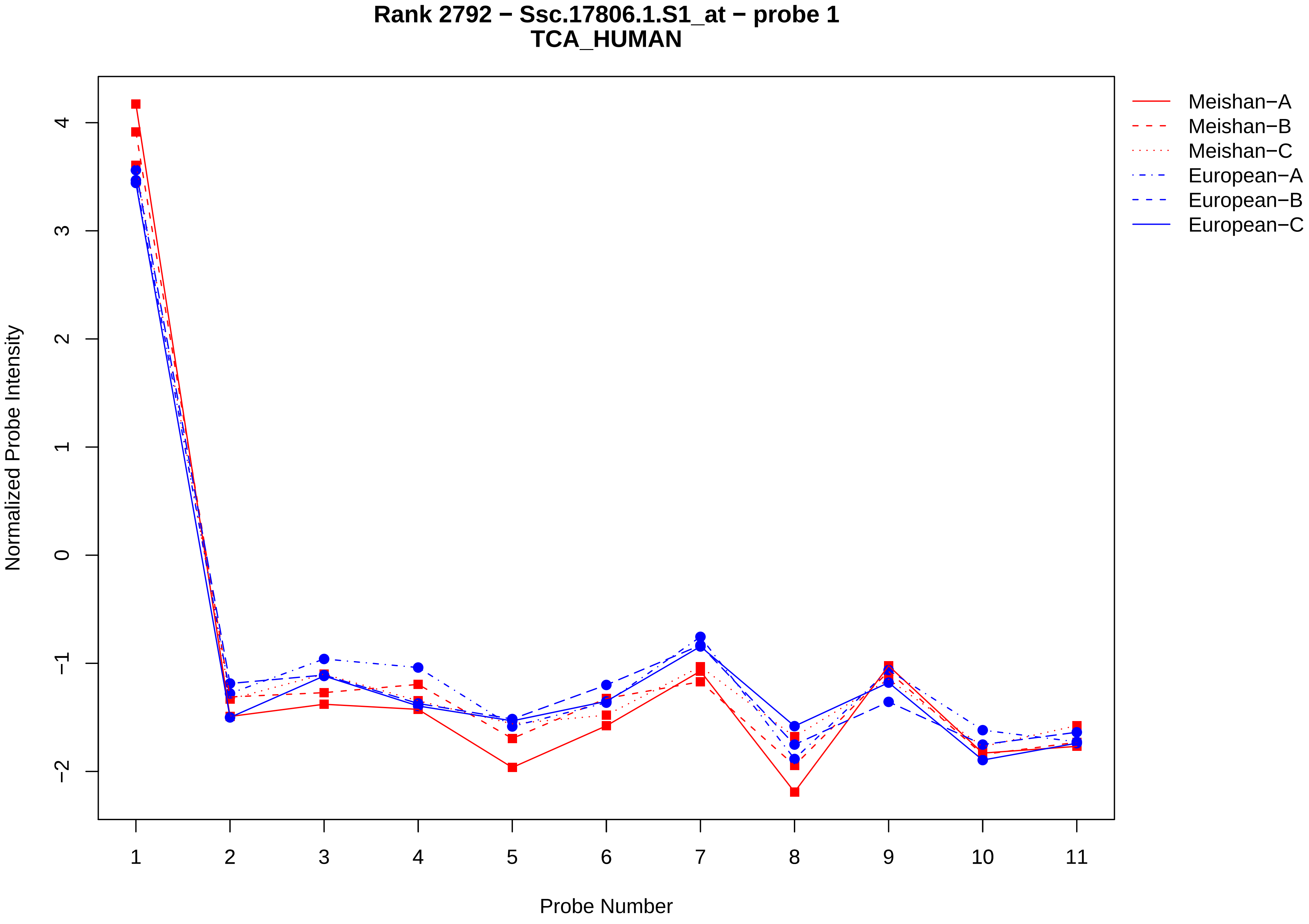

Rank 2792 − Ssc.17806.1.S1_at − probe 1
TCA_HUMAN
Normalized Probe Intensity
Probe Number
Meishan−A
Meishan−B
Meishan−C
European−A
European−B
European−C
4
3
2
1
0
−1
−2
1
2
3
4
5
6
7
8
9
10
11

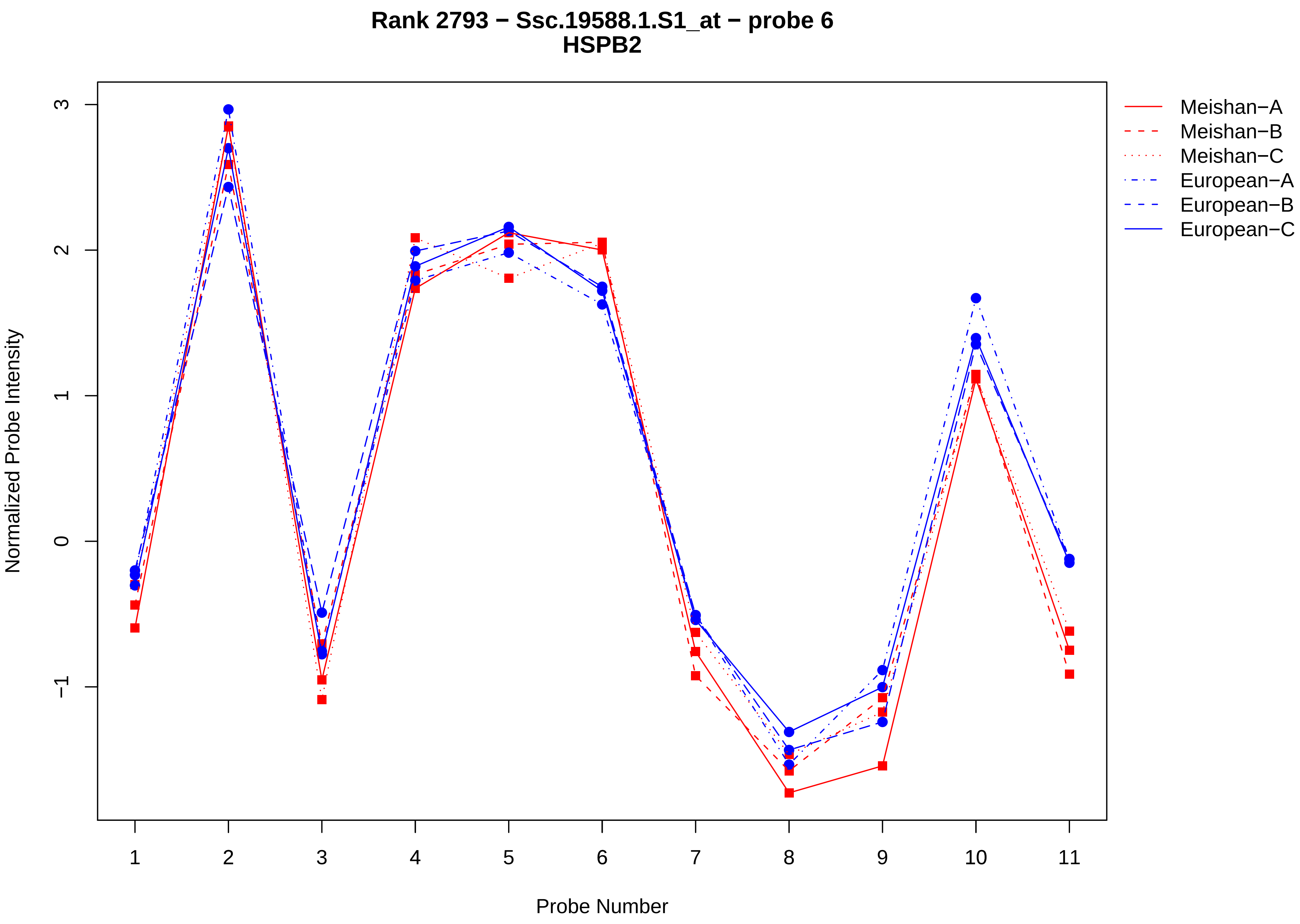
Rank 2793 − Ssc.19588.1.S1_at − probe 6
HSPB2
Normalized Probe Intensity
Probe Number
−1
0
1
2
3
1
2
3
4
5
6
7
8
9
10
11
Meishan−A
Meishan−B
Meishan−C
European−A
European−B
European−C

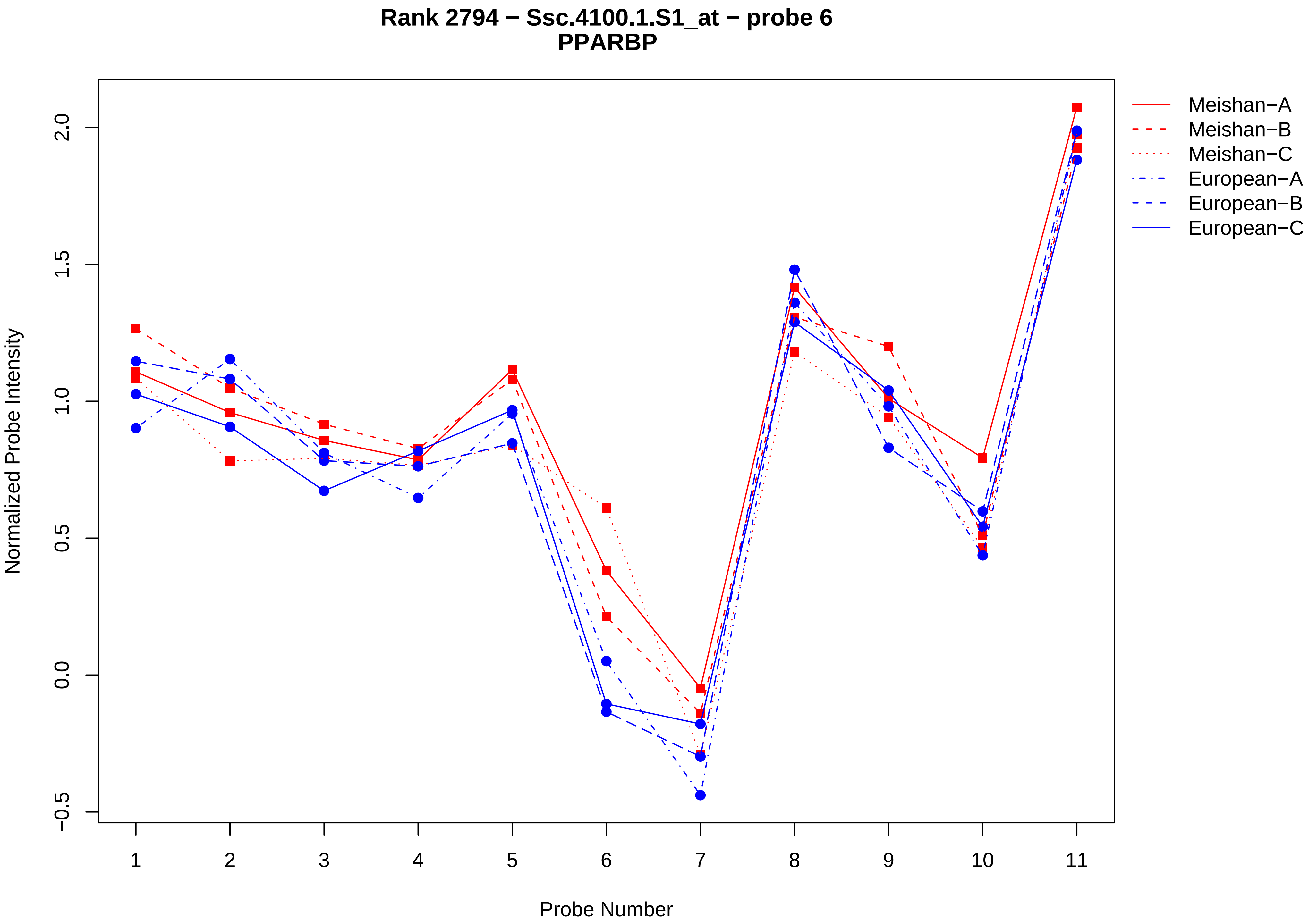

Rank 2794 − Ssc.4100.1.S1_at − probe 6
PPARBP
Normalized Probe Intensity
Probe Number
−0.5
0.0
0.5
1.0
1.5
2.0
1
2
3
4
5
6
7
8
9
10
11
Meishan−A
Meishan−B
Meishan−C
European−A
European−B
European−C

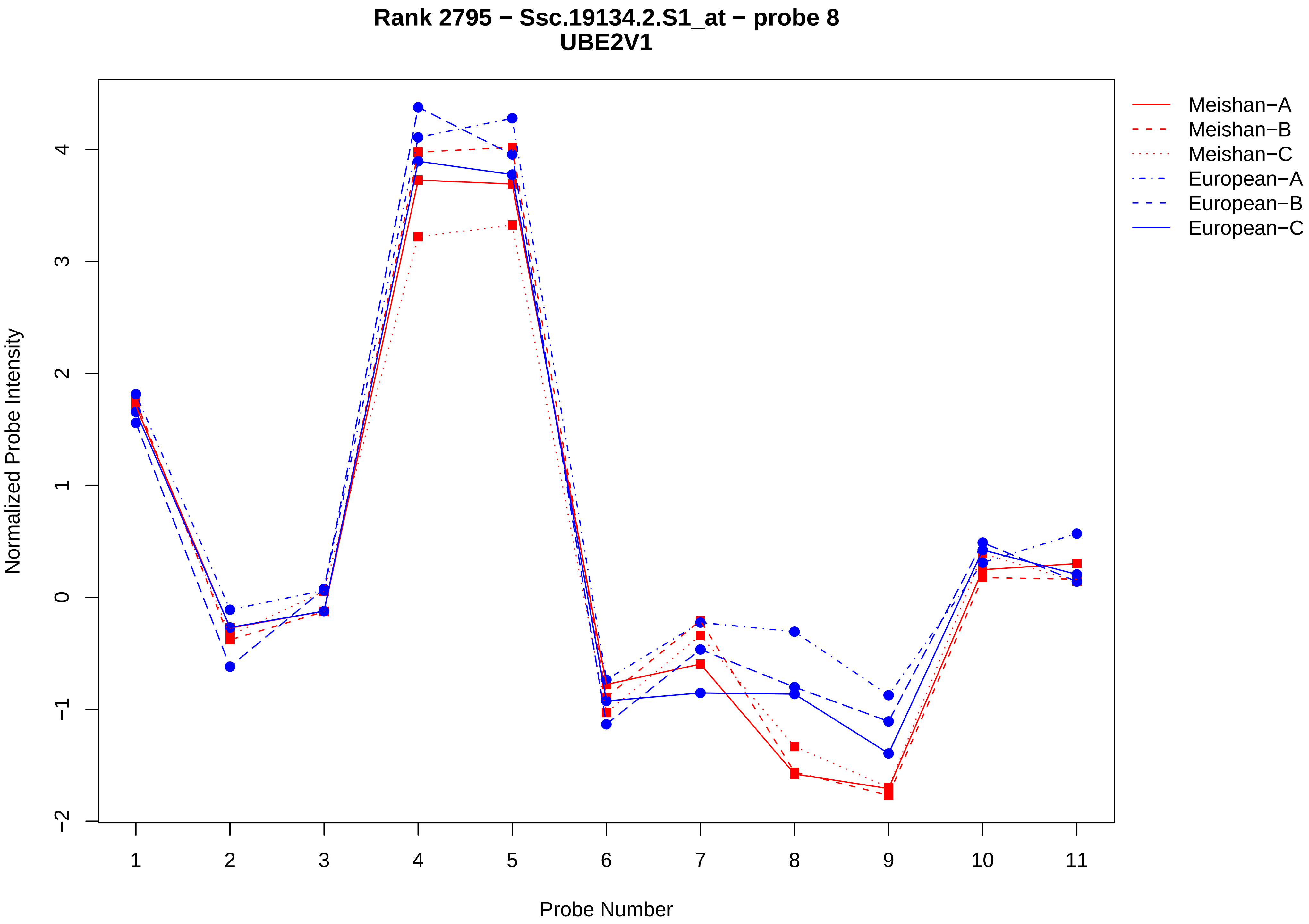
Rank 2795 − Ssc.19134.2.S1_at − probe 8
UBE2V1
Normalized Probe Intensity
Probe Number
Meishan−A
Meishan−B
Meishan−C
European−A
European−B
European−C
−2
−1
0
1
2
3
4
1
2
3
4
5
6
7
8
9
10
11

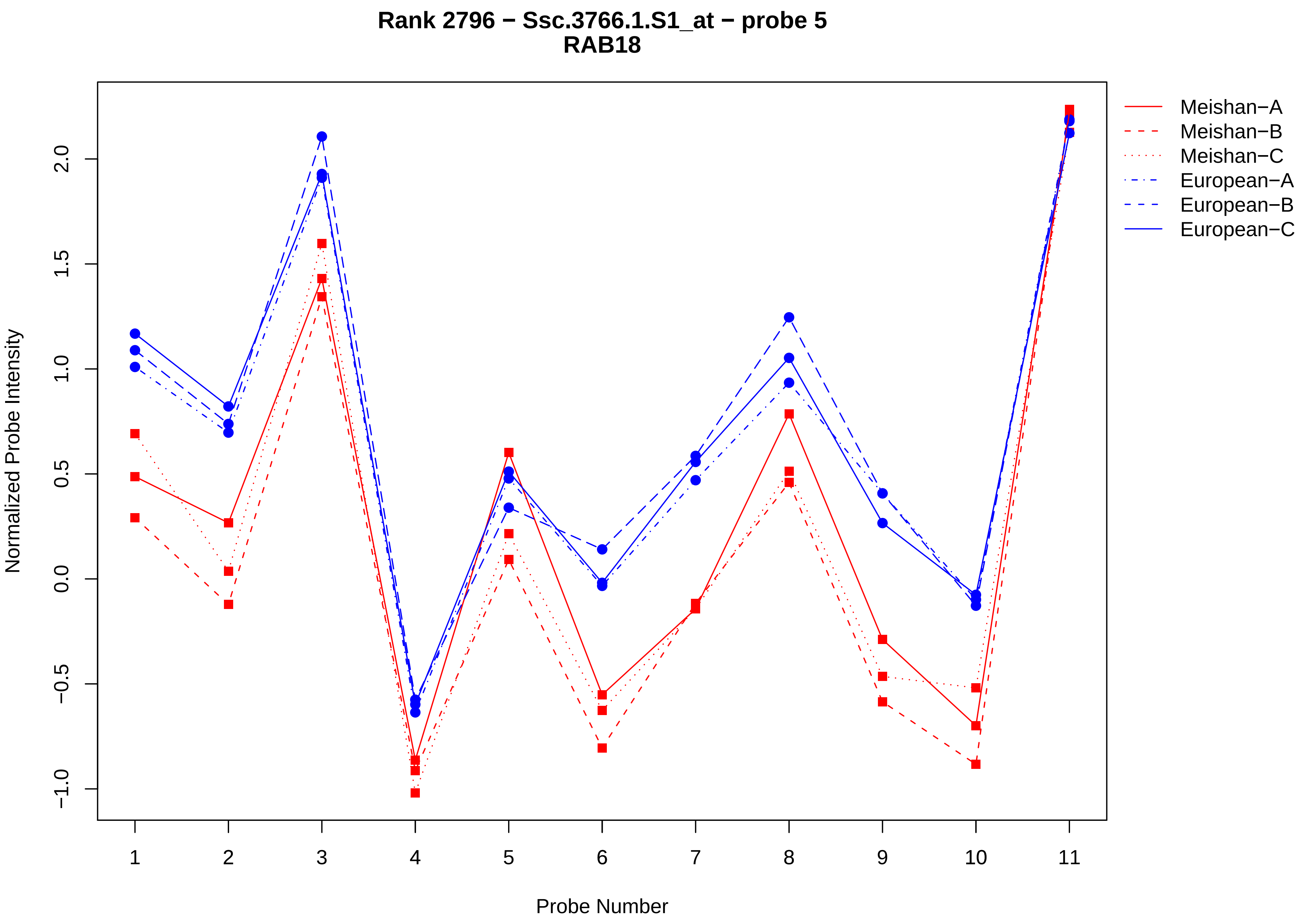

Rank 2796 − Ssc.3766.1.S1_at − probe 5
RAB18
Normalized Probe Intensity
Probe Number
Meishan−A
Meishan−B
Meishan−C
European−A
European−B
European−C

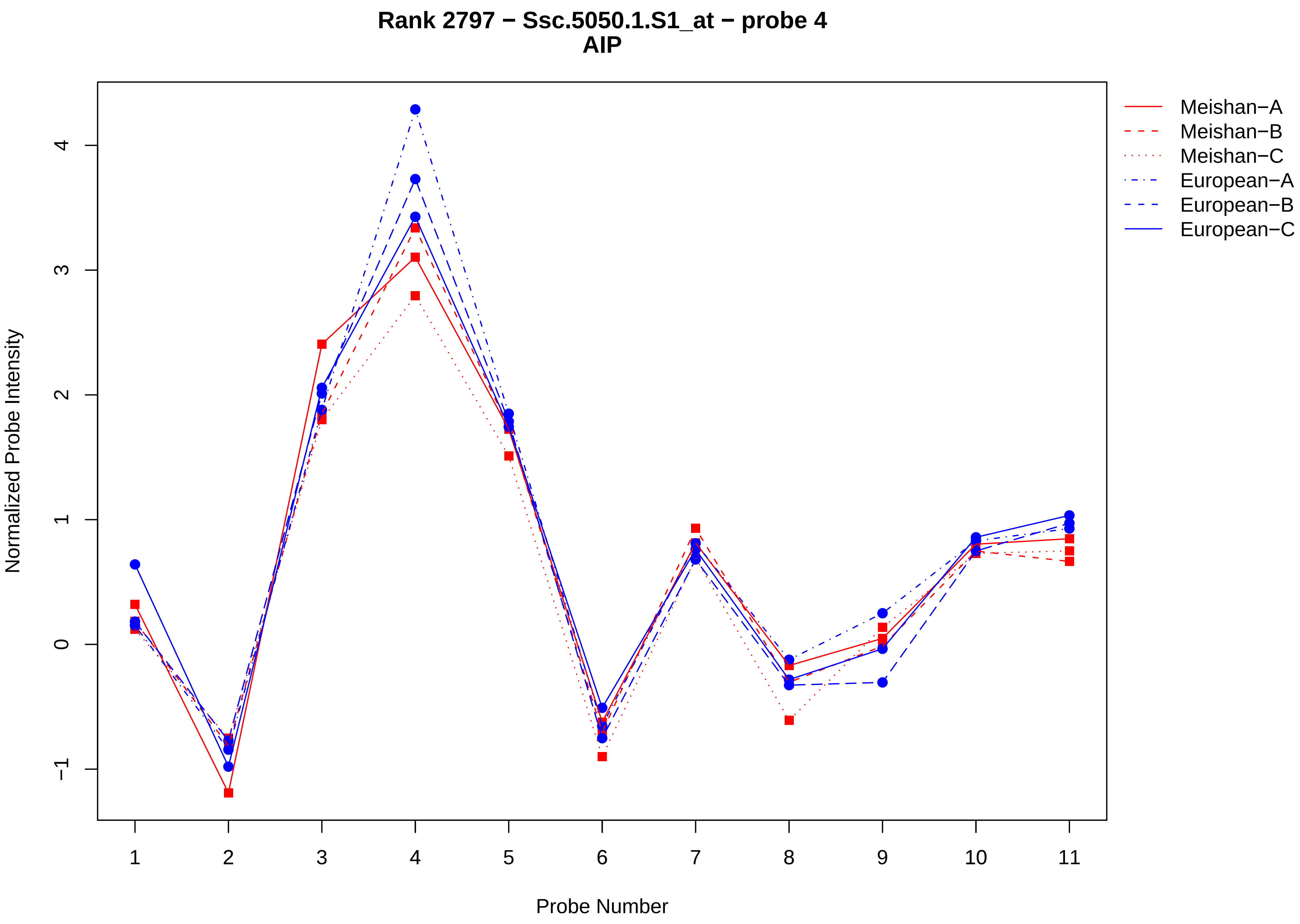

Rank 2797 − Ssc.5050.1.S1_at − probe 4
AIP
Normalized Probe Intensity
Probe Number
−1
0
1
2
3
4
1
2
3
4
5
6
7
8
9
10
11
Meishan−A
Meishan−B
Meishan−C
European−A
European−B
European−C

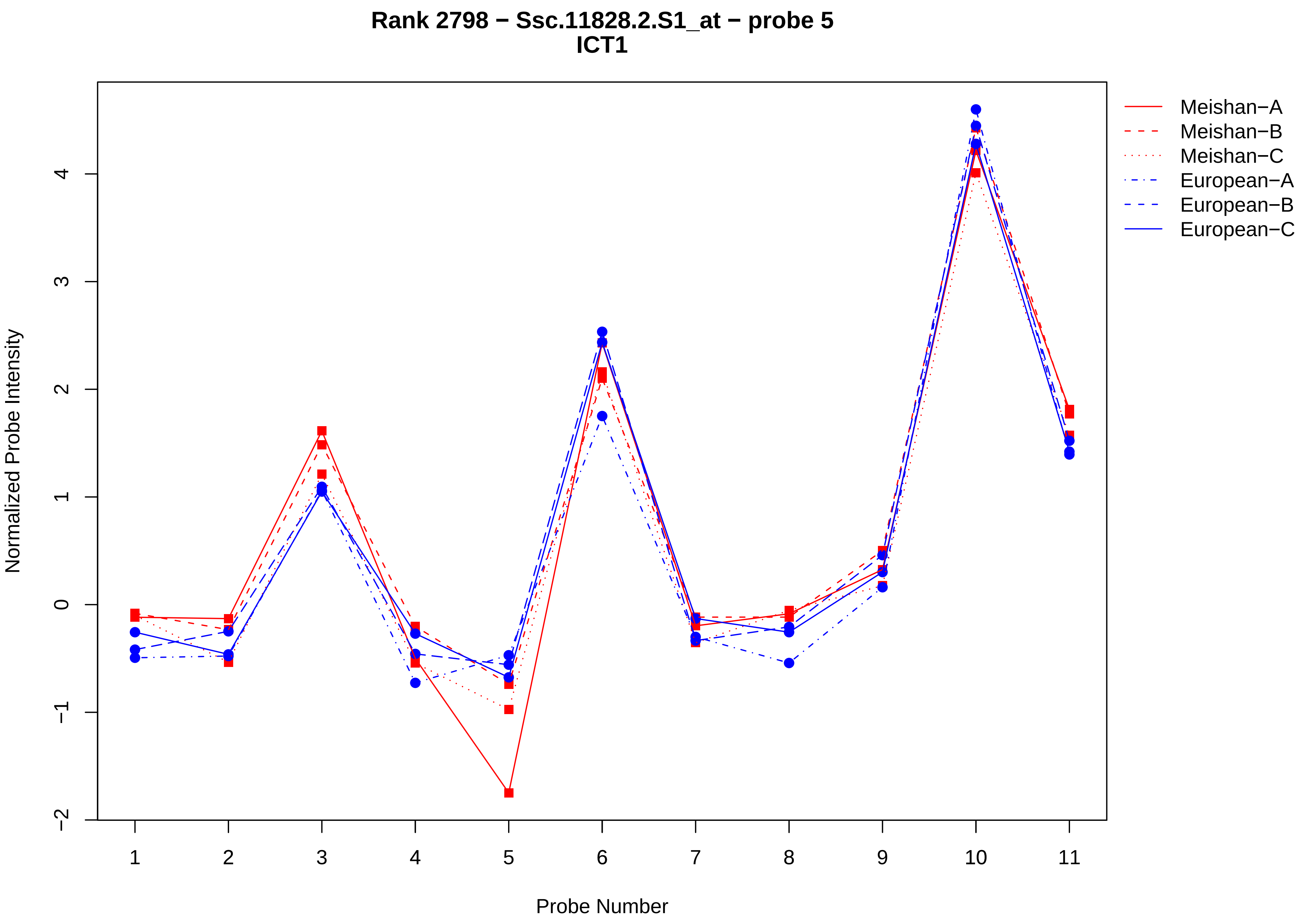

Rank 2798 − Ssc.11828.2.S1_at − probe 5
ICT1
Normalized Probe Intensity
Probe Number
−2
−1
0
1
2
3
4
1
2
3
4
5
6
7
8
9
10
11
Meishan−A
Meishan−B
Meishan−C
European−A
European−B
European−C

Rank 2799 − Ssc.29090.1.A1_at − probe 6
SASH1

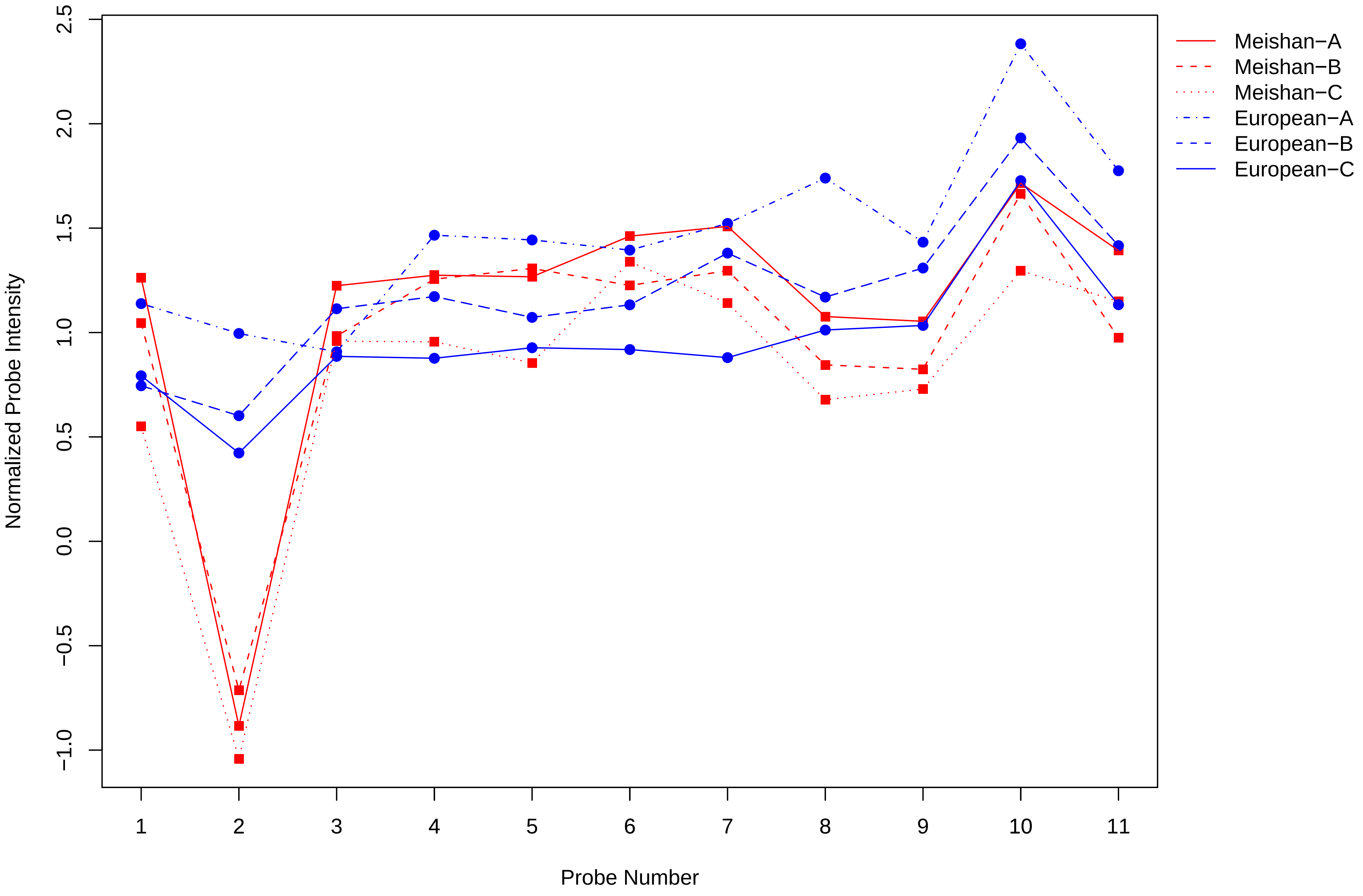

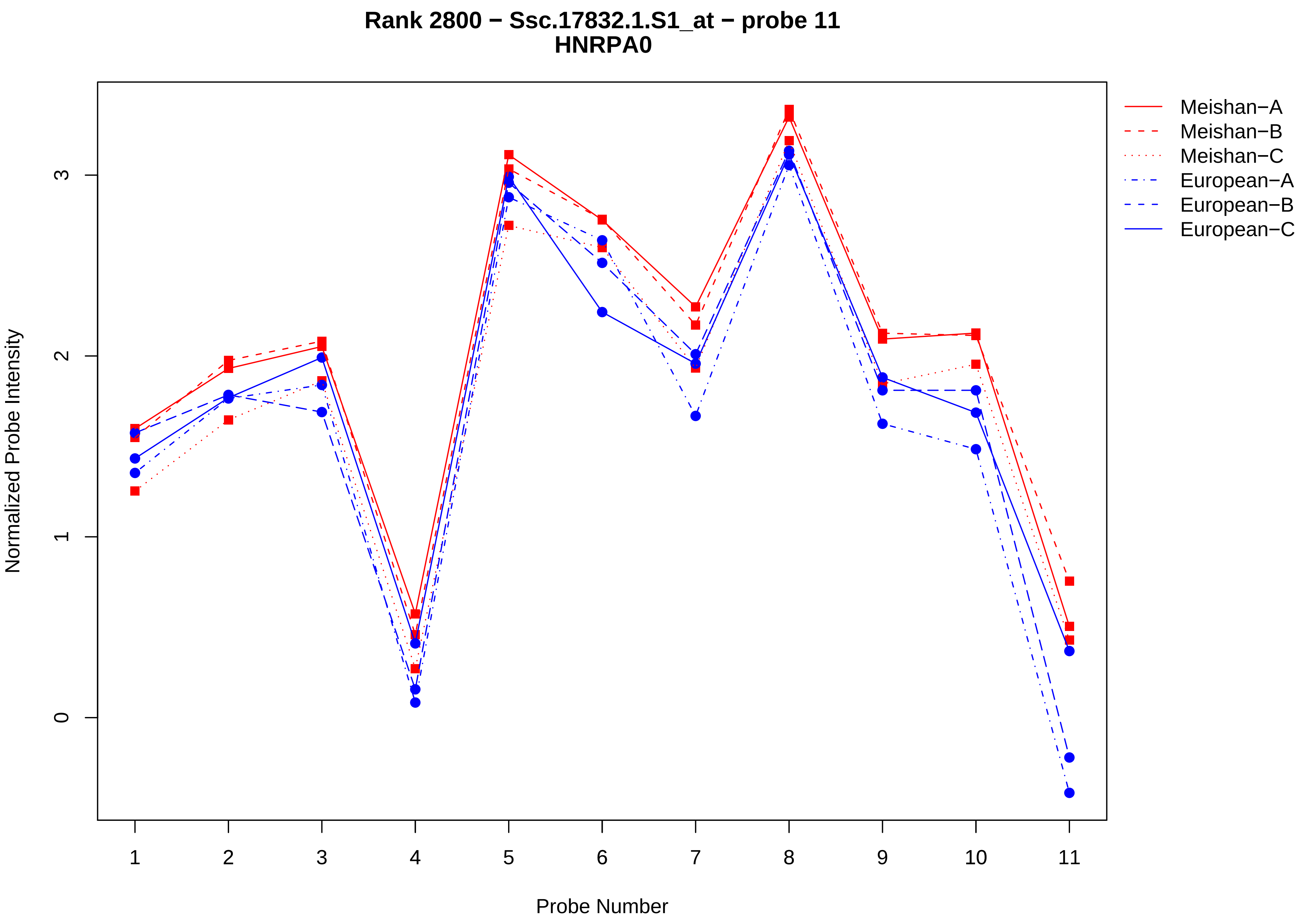
Rank 2800 − Ssc.17832.1.S1_at − probe 11
HNRPA0
Normalized Probe Intensity
Probe Number
0
1
2
3
1
2
3
4
5
6
7
8
9
10
11
Meishan−A
Meishan−B
Meishan−C
European−A
European−B
European−C

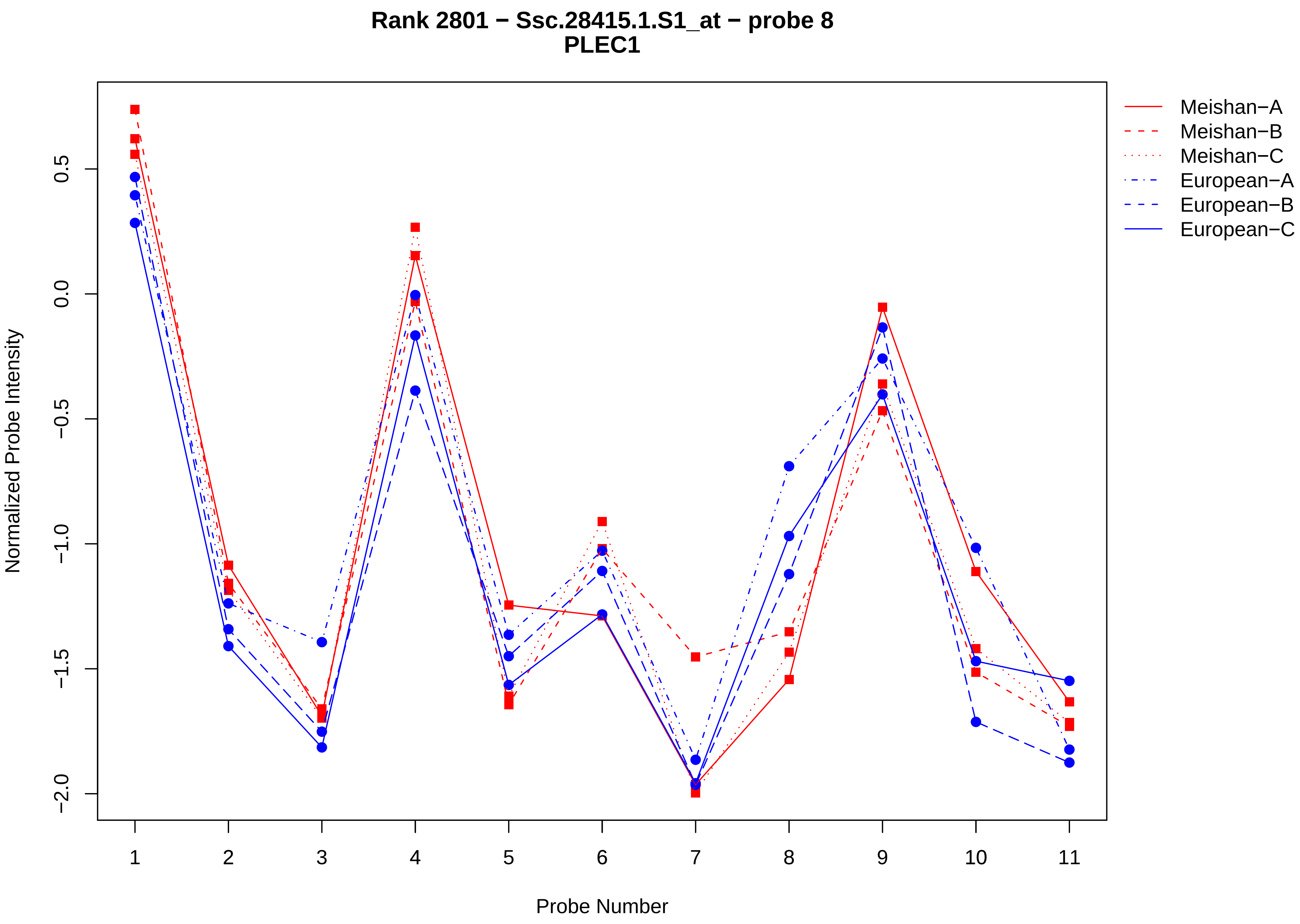

Rank 2801 − Ssc.28415.1.S1_at − probe 8
PLEC1
Normalized Probe Intensity
Probe Number
0.5
0.0
−0.5
−1.0
−1.5
−2.0
1
2
3
4
5
6
7
8
9
10
11
Meishan−A
Meishan−B
Meishan−C
European−A
European−B
European−C

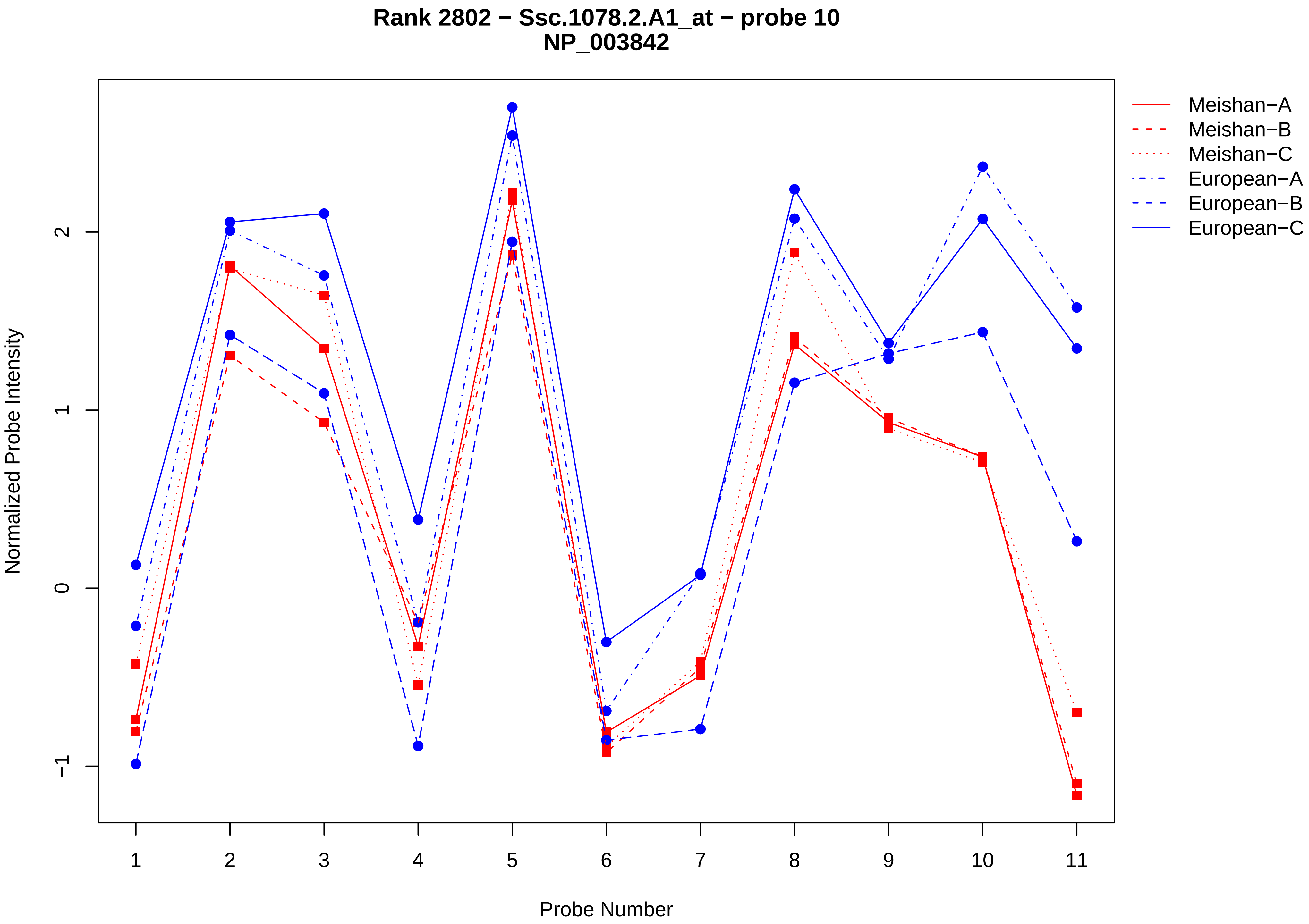

Rank 2802 − Ssc.1078.2.A1_at − probe 10
NP_003842
Normalized Probe Intensity
Probe Number
−1
0
1
2
1
2
3
4
5
6
7
8
9
10
11
Meishan−A
Meishan−B
Meishan−C
European−A
European−B
European−C

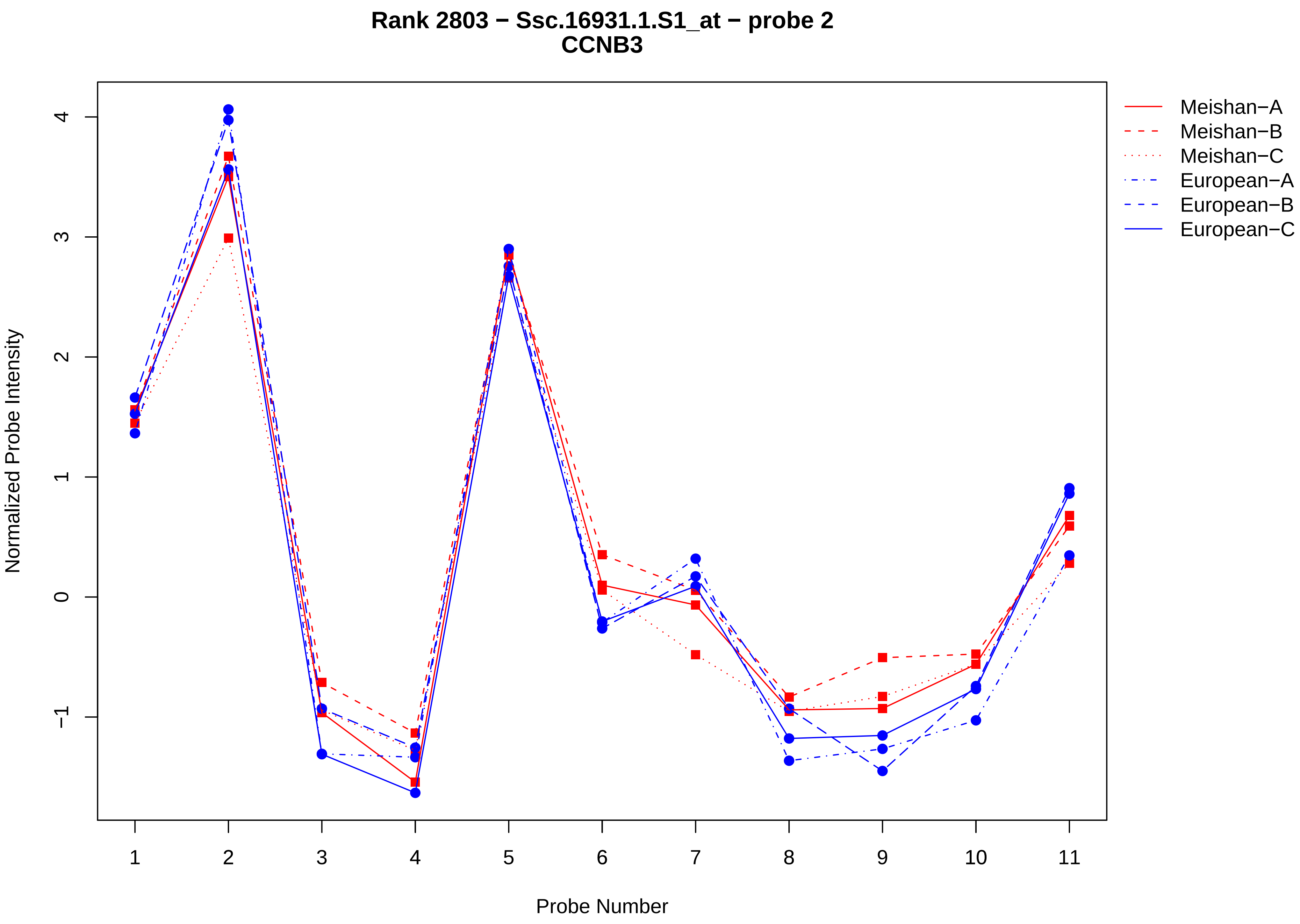

Rank 2803 − Ssc.16931.1.S1_at − probe 2
CCNB3
Normalized Probe Intensity
Probe Number
−1
0
1
2
3
4
1
2
3
4
5
6
7
8
9
10
11
Meishan−A
Meishan−B
Meishan−C
European−A
European−B
European−C

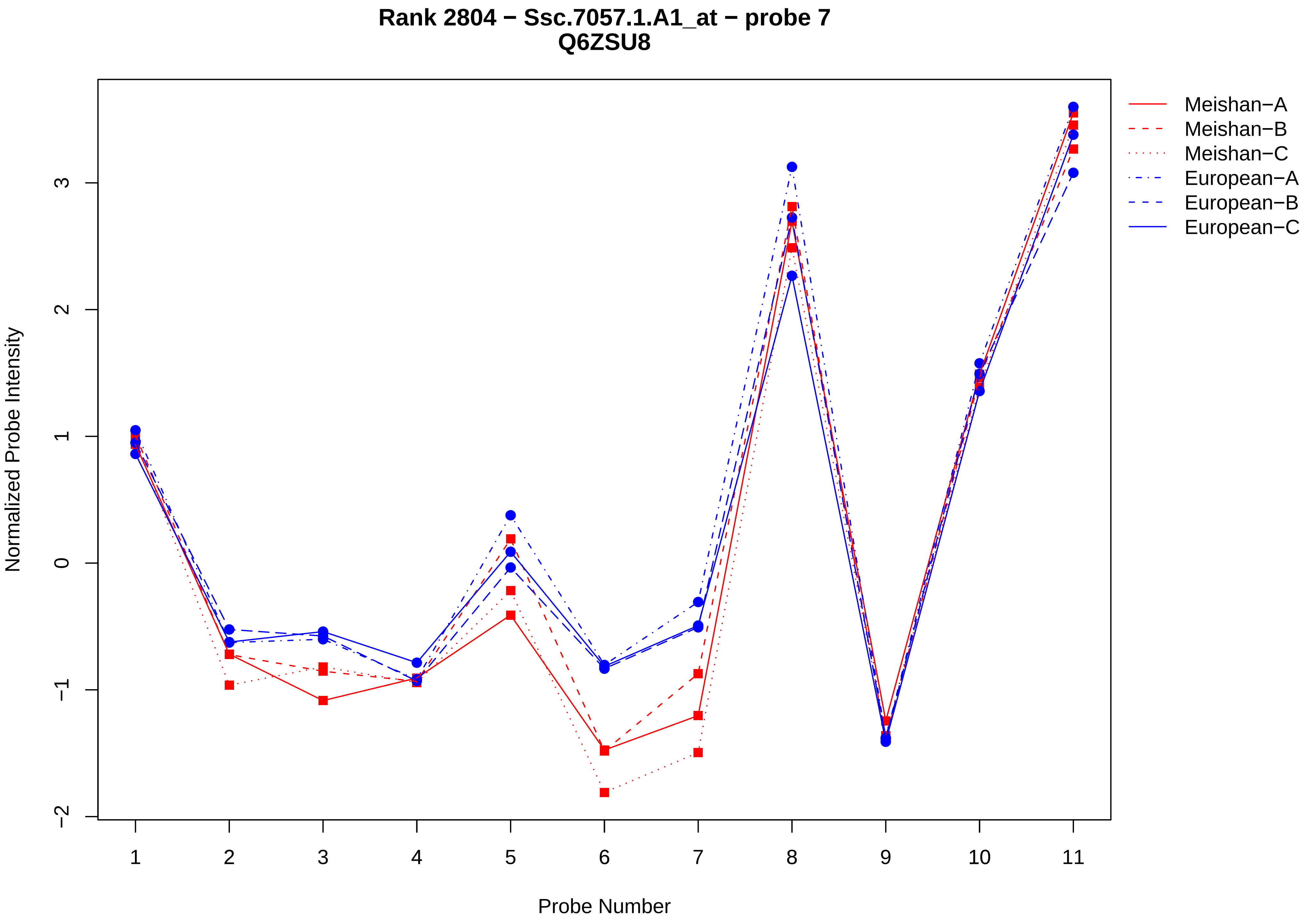

Rank 2804 − Ssc.7057.1.A1_at − probe 7
Q6ZSU8
Normalized Probe Intensity
Probe Number
−2
−1
0
1
2
3
1
2
3
4
5
6
7
8
9
10
11
Meishan−A
Meishan−B
Meishan−C
European−A
European−B
European−C

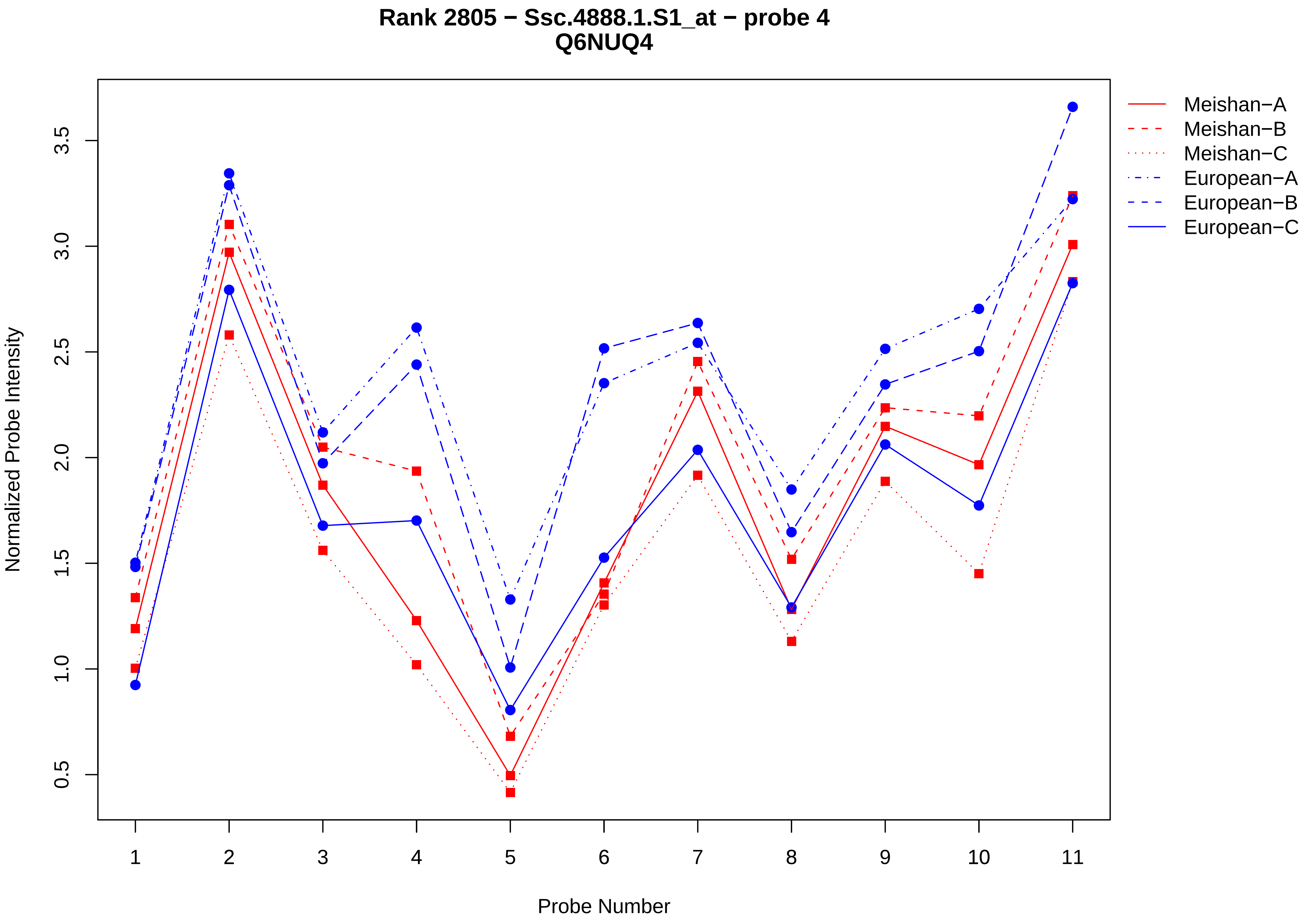

Rank 2805 − Ssc.4888.1.S1_at − probe 4
Q6NUQ4
Normalized Probe Intensity
Probe Number
0.5
1.0
1.5
2.0
2.5
3.0
3.5
1
2
3
4
5
6
7
8
9
10
11
Meishan−A
Meishan−B
Meishan−C
European−A
European−B
European−C

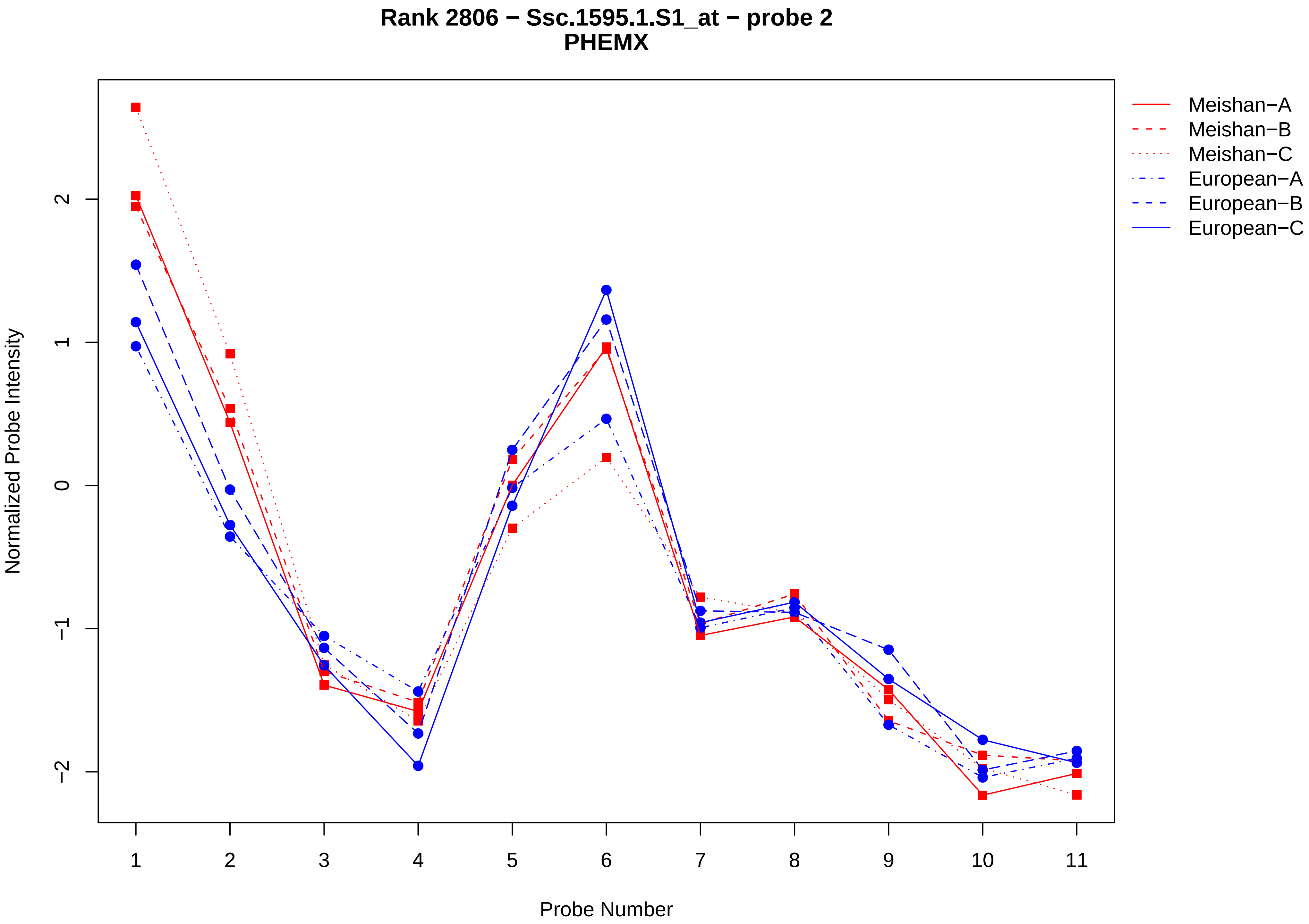

Rank 2806 − Ssc.1595.1.S1_at − probe 2
PHEMX
Normalized Probe Intensity
Probe Number
−2
−1
0
1
2
1
2
3
4
5
6
7
8
9
10
11
Meishan−A
Meishan−B
Meishan−C
European−A
European−B
European−C

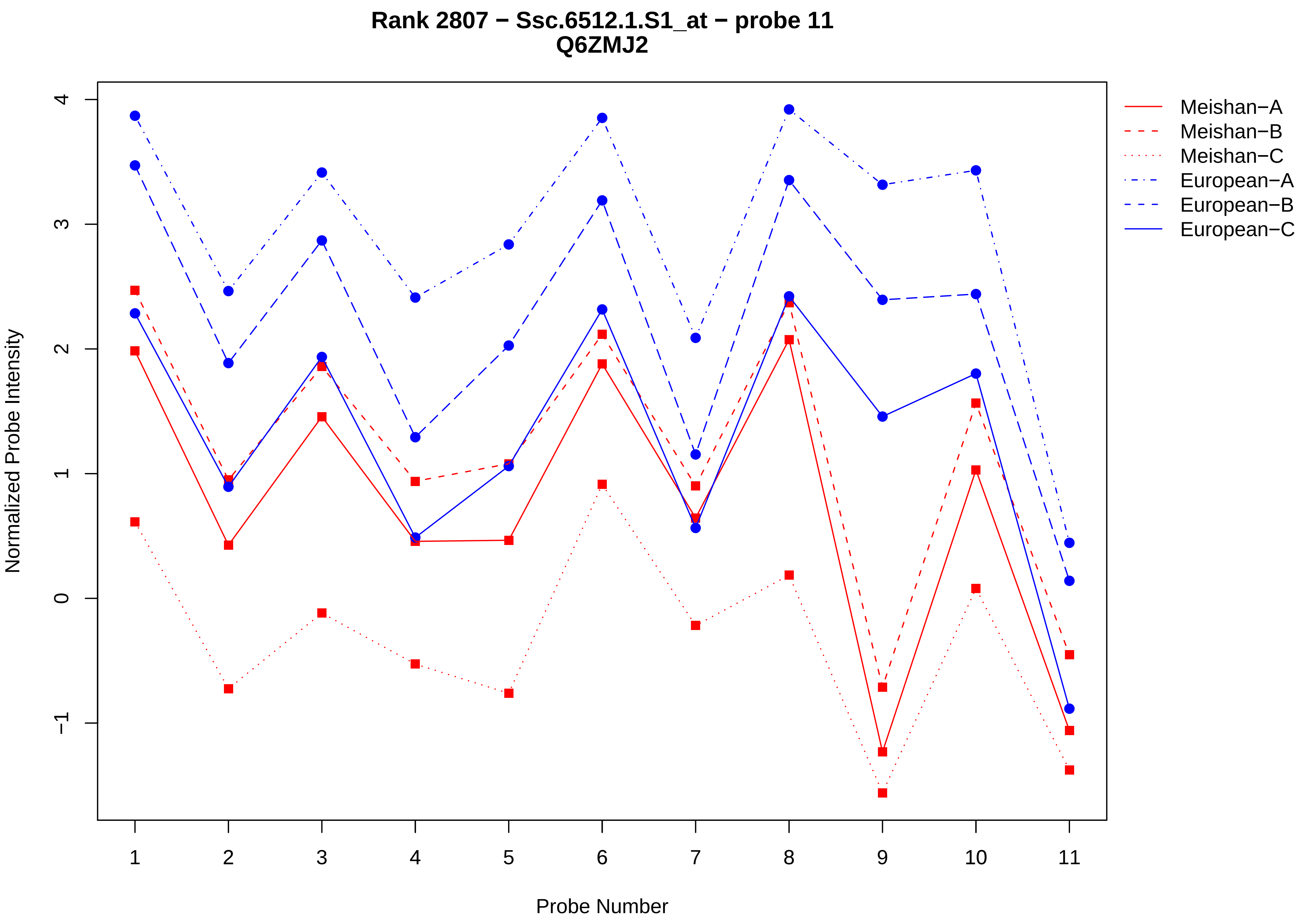

Rank 2807 − Ssc.6512.1.S1_at − probe 11
Q6ZMJ2
Normalized Probe Intensity
Probe Number
Meishan−A
Meishan−B
Meishan−C
European−A
European−B
European−C

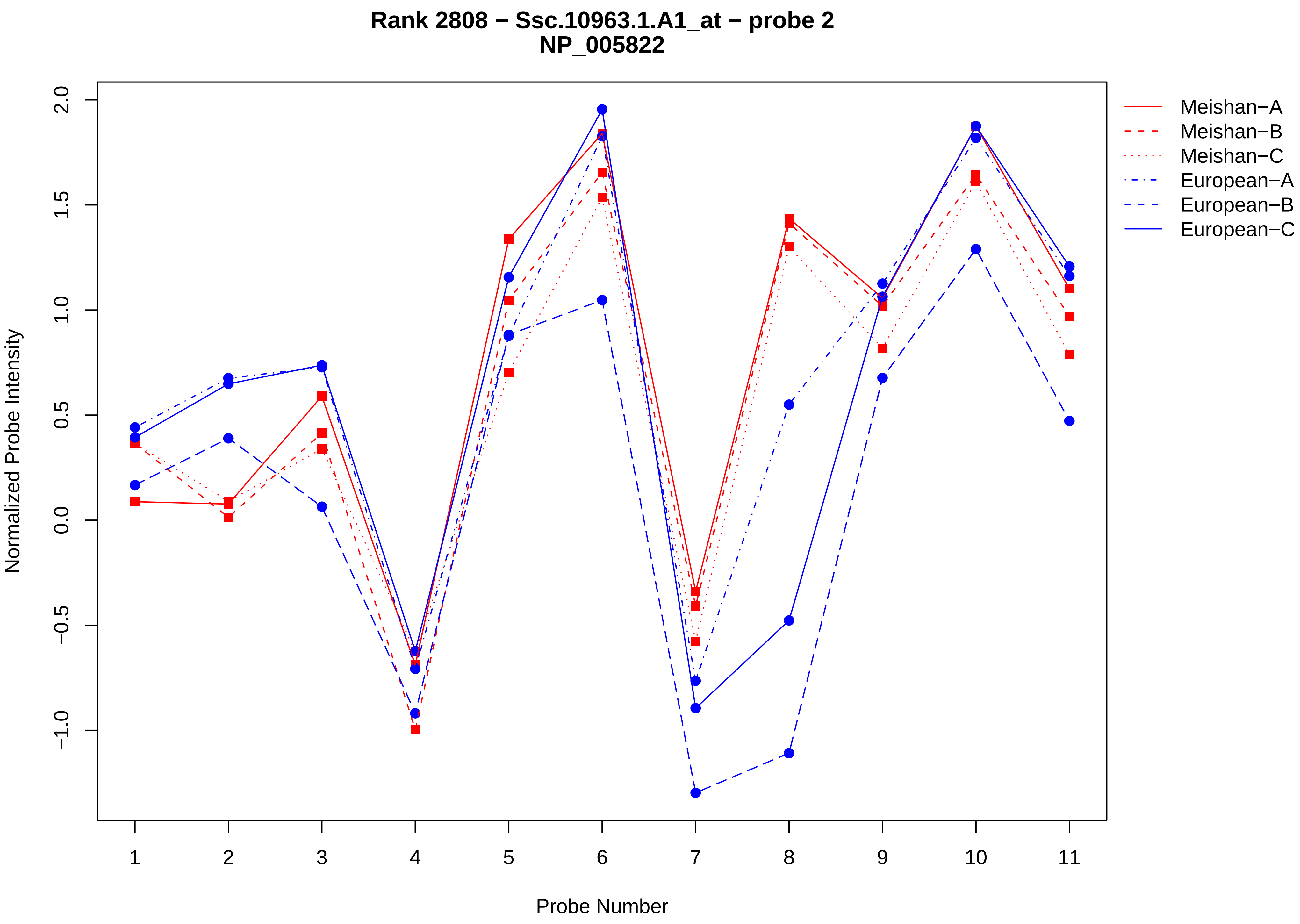

Rank 2808 − Ssc.10963.1.A1_at − probe 2
NP_005822
Normalized Probe Intensity
Probe Number
Meishan−A
Meishan−B
Meishan−C
European−A
European−B
European−C

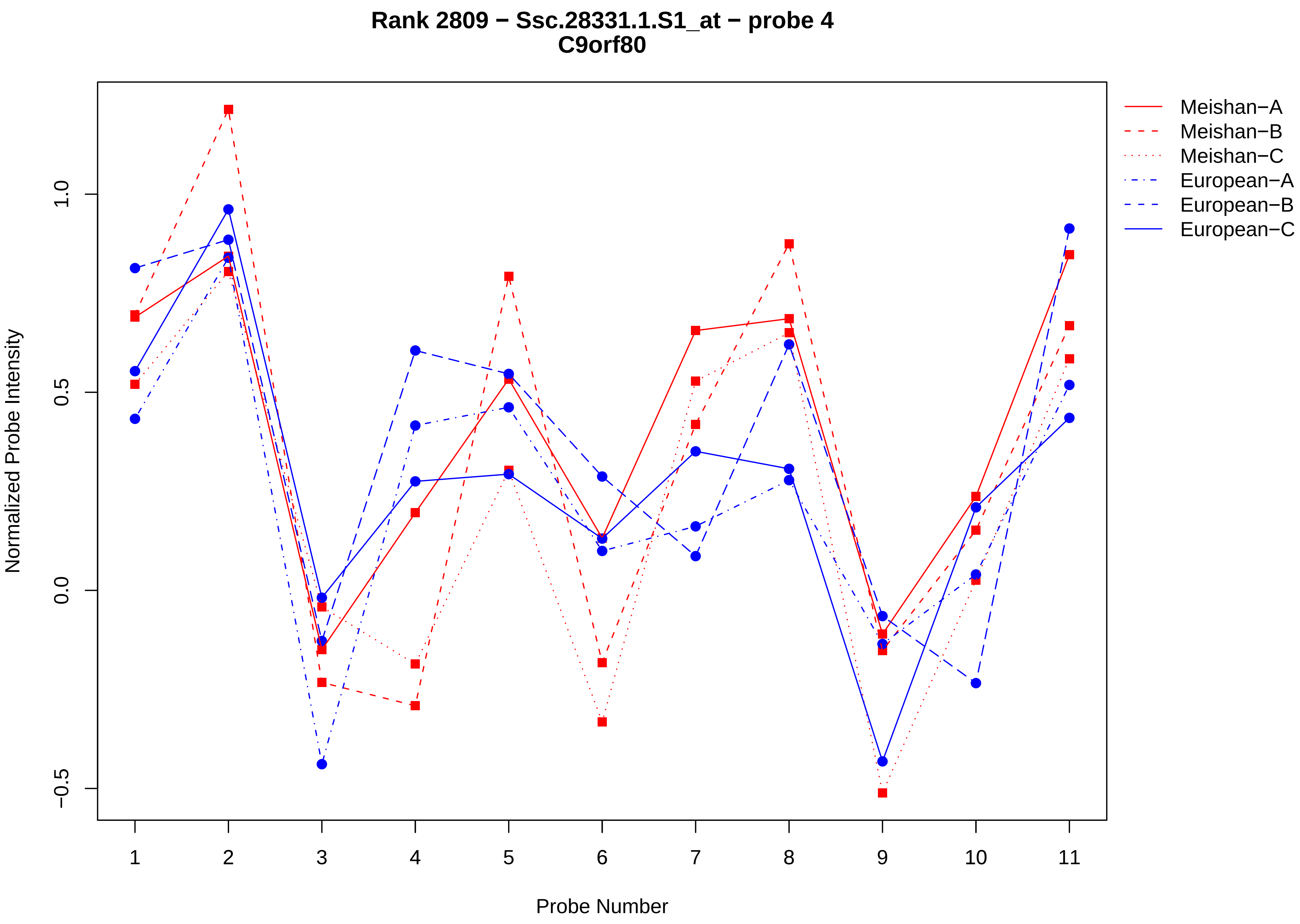

Rank 2809 − Ssc.28331.1.S1_at − probe 4
C9orf80
Normalized Probe Intensity
Probe Number
−0.5
0.0
0.5
1.0
1
2
3
4
5
6
7
8
9
10
11
Meishan−A
Meishan−B
Meishan−C
European−A
European−B
European−C

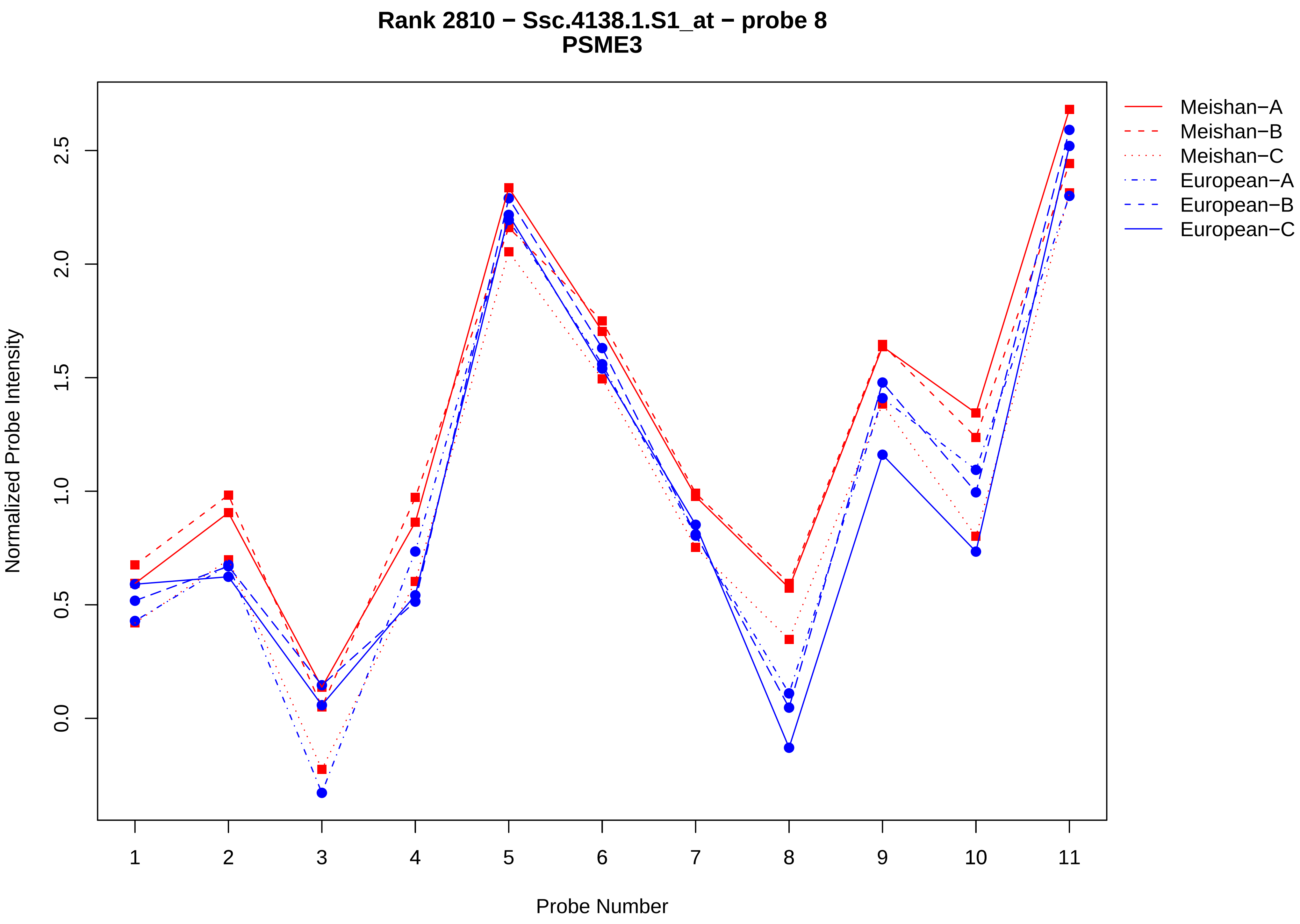

Rank 2810 − Ssc.4138.1.S1_at − probe 8
PSME3
Normalized Probe Intensity
Probe Number
0.0
0.5
1.0
1.5
2.0
2.5
1
2
3
4
5
6
7
8
9
10
11
Meishan−A
Meishan−B
Meishan−C
European−A
European−B
European−C

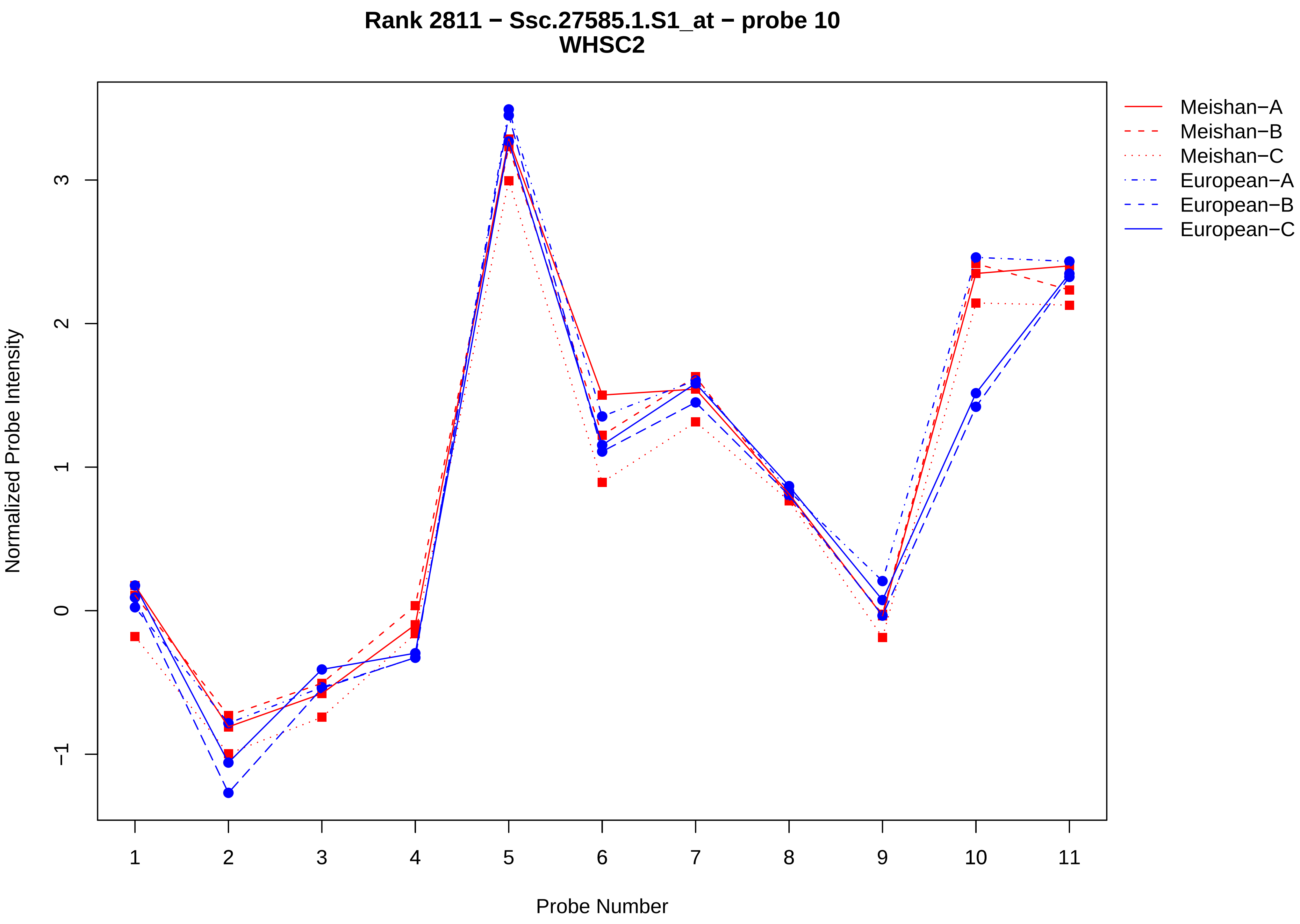
Rank 2811 − Ssc.27585.1.S1_at − probe 10
WHSC2
Normalized Probe Intensity
Probe Number
−1
0
1
2
3
1
2
3
4
5
6
7
8
9
10
11
Meishan−A
Meishan−B
Meishan−C
European−A
European−B
European−C

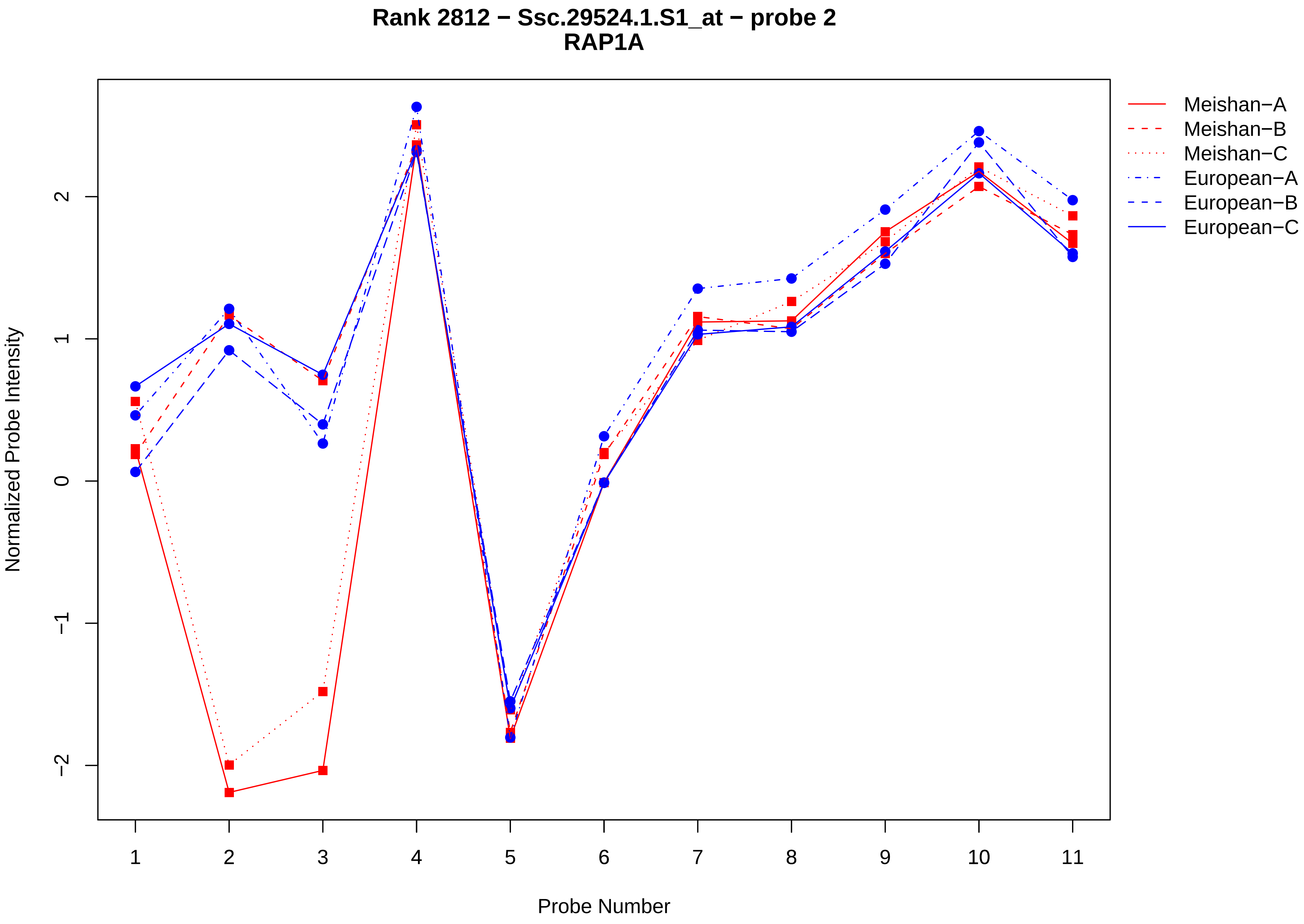

Rank 2812 − Ssc.29524.1.S1_at − probe 2
RAP1A
Normalized Probe Intensity
Probe Number
−2
−1
0
1
2
1
2
3
4
5
6
7
8
9
10
11
Meishan−A
Meishan−B
Meishan−C
European−A
European−B
European−C

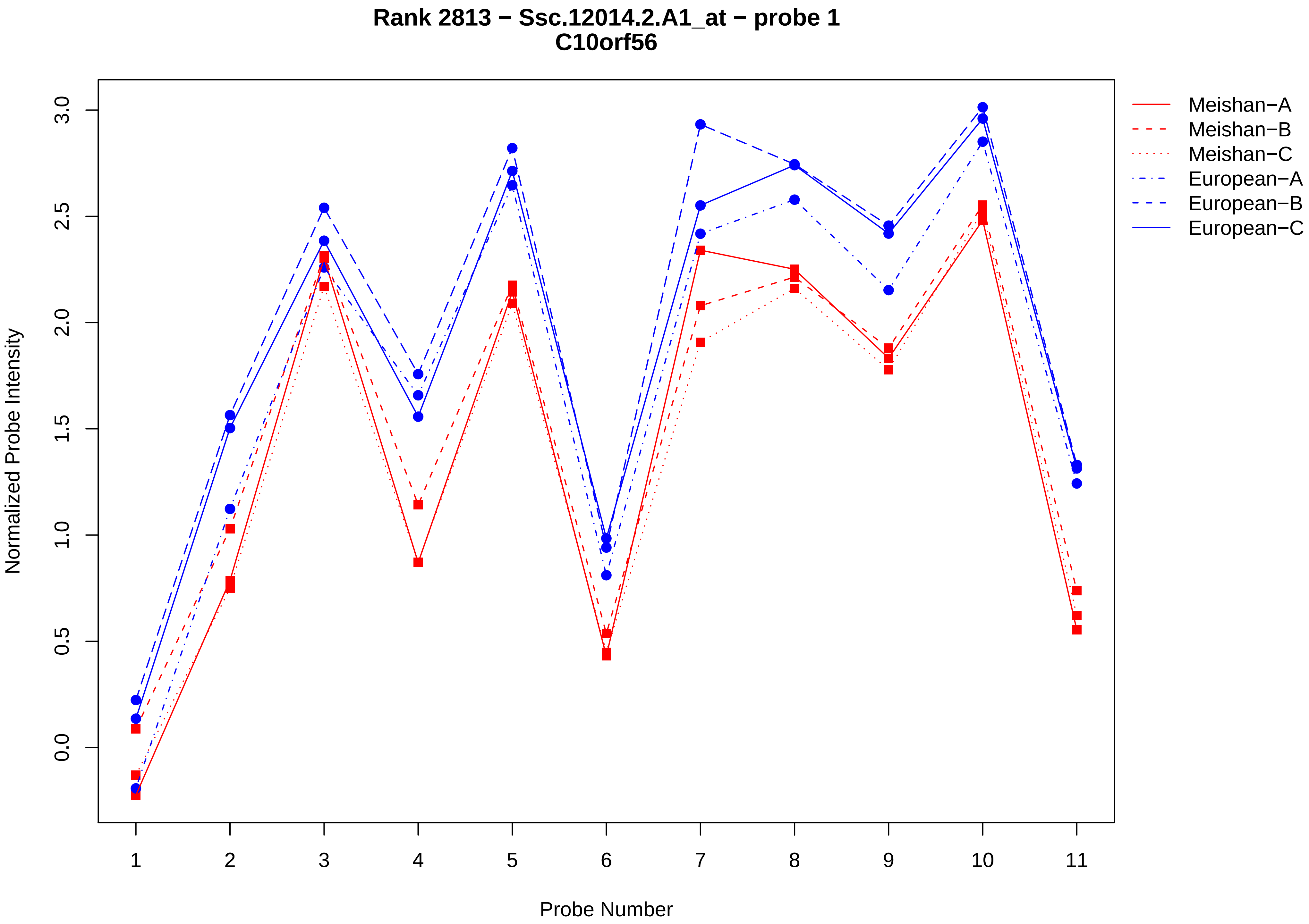

Rank 2813 − Ssc.12014.2.A1_at − probe 1
C10orf56
Normalized Probe Intensity
Probe Number
0.0
0.5
1.0
1.5
2.0
2.5
3.0
1
2
3
4
5
6
7
8
9
10
11
Meishan−A
Meishan−B
Meishan−C
European−A
European−B
European−C

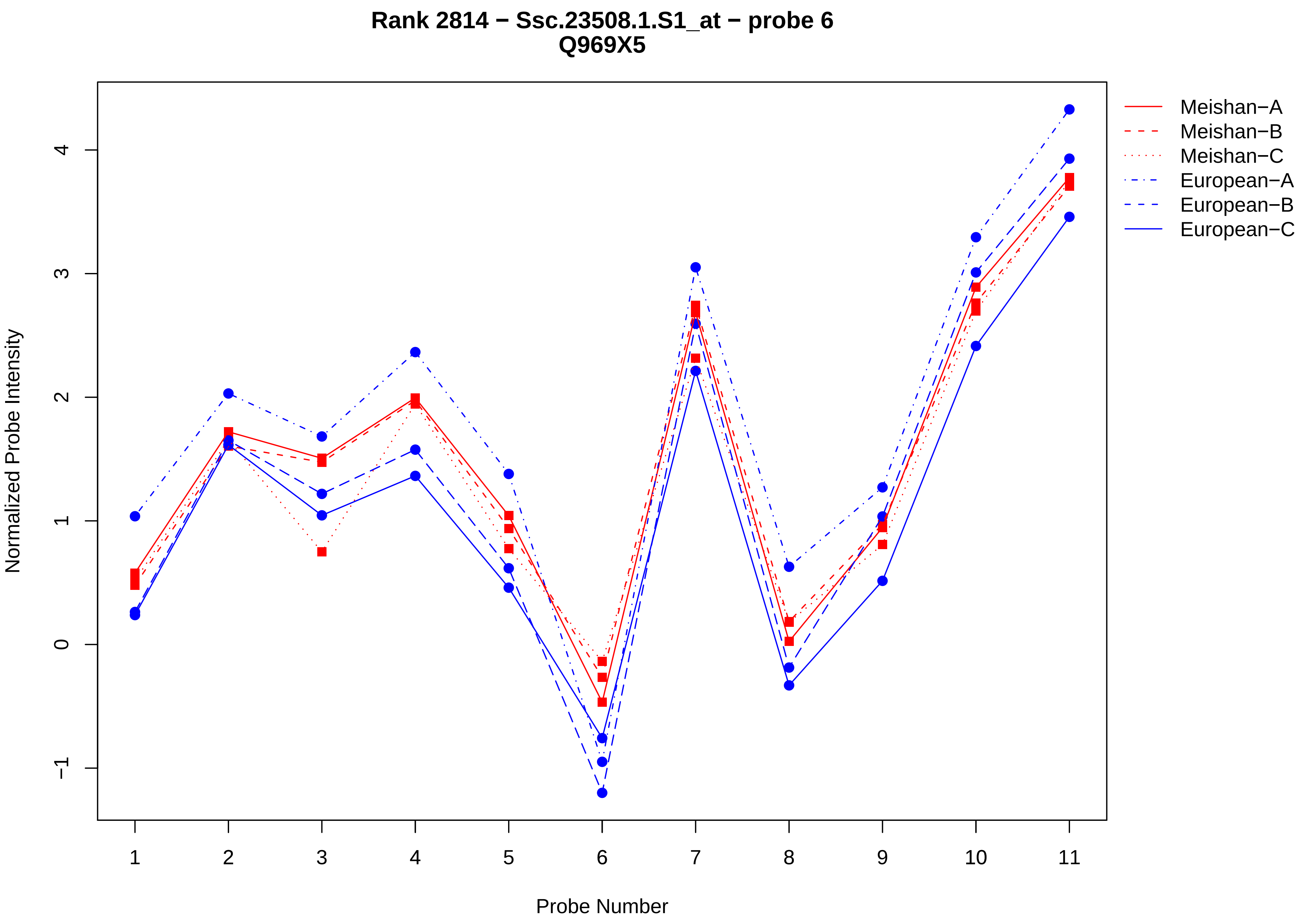

Rank 2814 − Ssc.23508.1.S1_at − probe 6
Q969X5
Normalized Probe Intensity
Probe Number
−1
0
1
2
3
4
1
2
3
4
5
6
7
8
9
10
11
Meishan−A
Meishan−B
Meishan−C
European−A
European−B
European−C

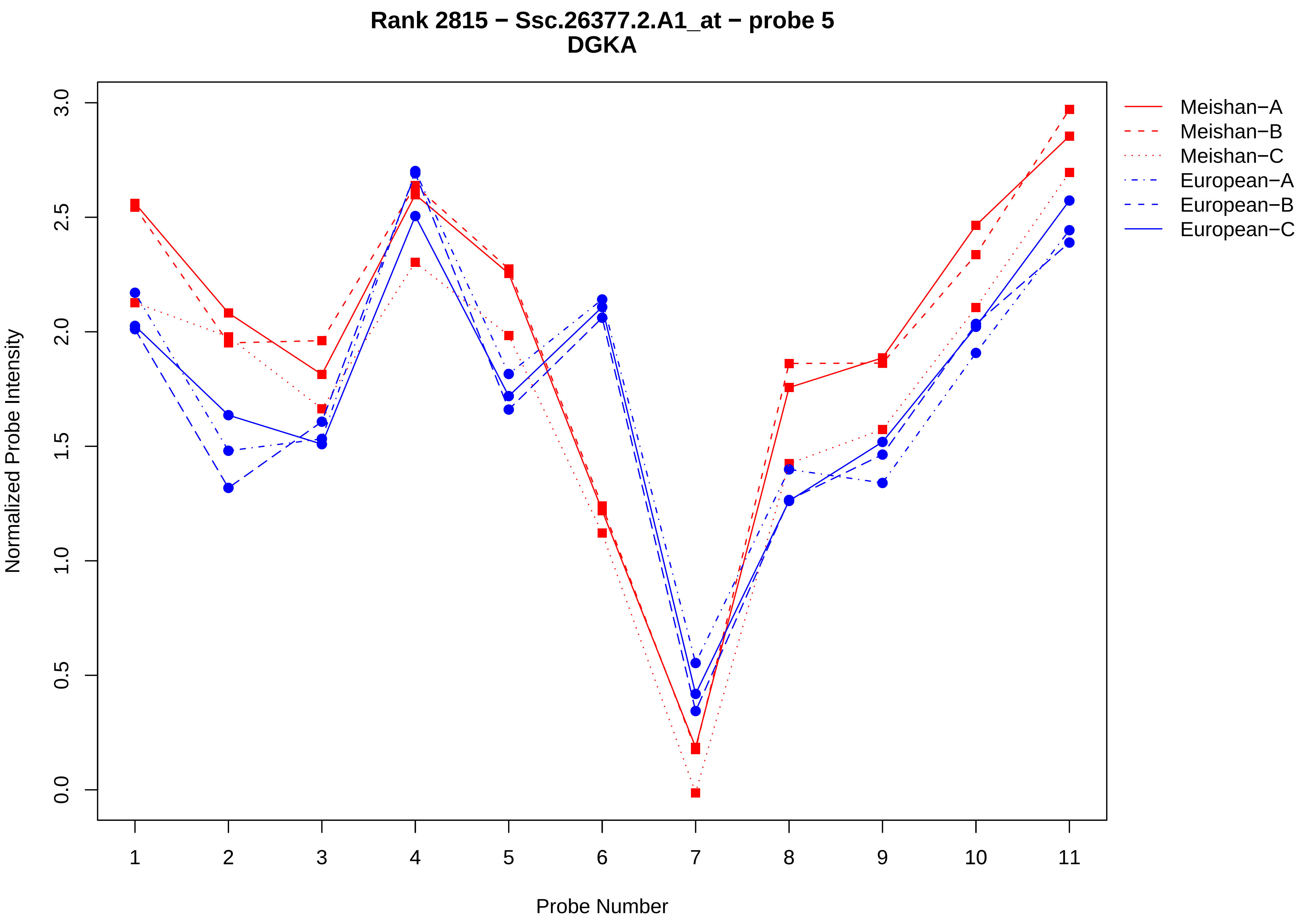

Rank 2815 − Ssc.26377.2.A1_at − probe 5
DGKA
Normalized Probe Intensity
0.0
0.5
1.0
1.5
2.0
2.5
3.0
1
2
3
4
5
6
7
8
9
10
11
Probe Number
Meishan−A
Meishan−B
Meishan−C
European−A
European−B
European−C

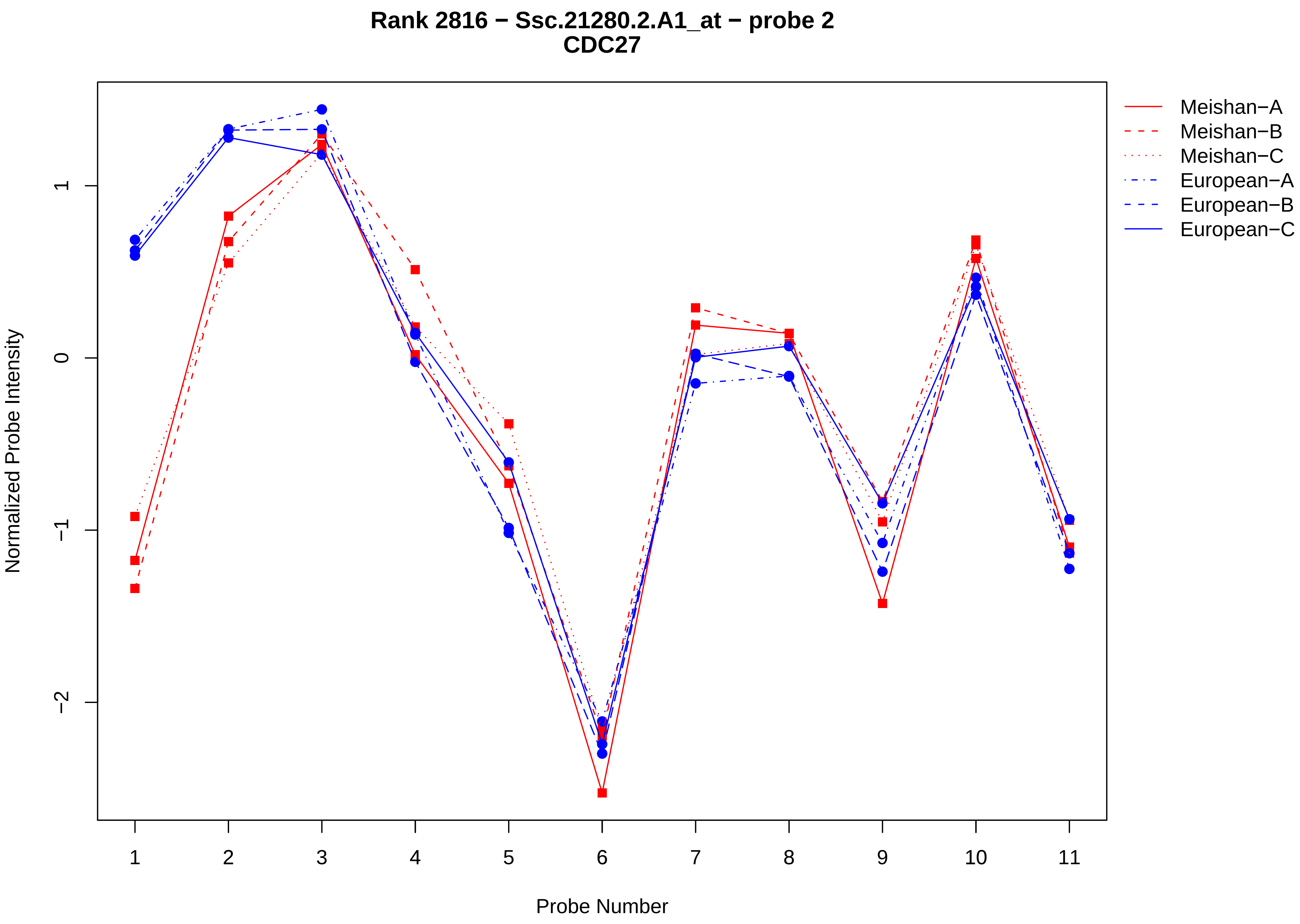

Rank 2816 − Ssc.21280.2.A1_at − probe 2
CDC27
Normalized Probe Intensity
Probe Number
1
0
−1
−2
1
2
3
4
5
6
7
8
9
10
11
Meishan−A
Meishan−B
Meishan−C
European−A
European−B
European−C

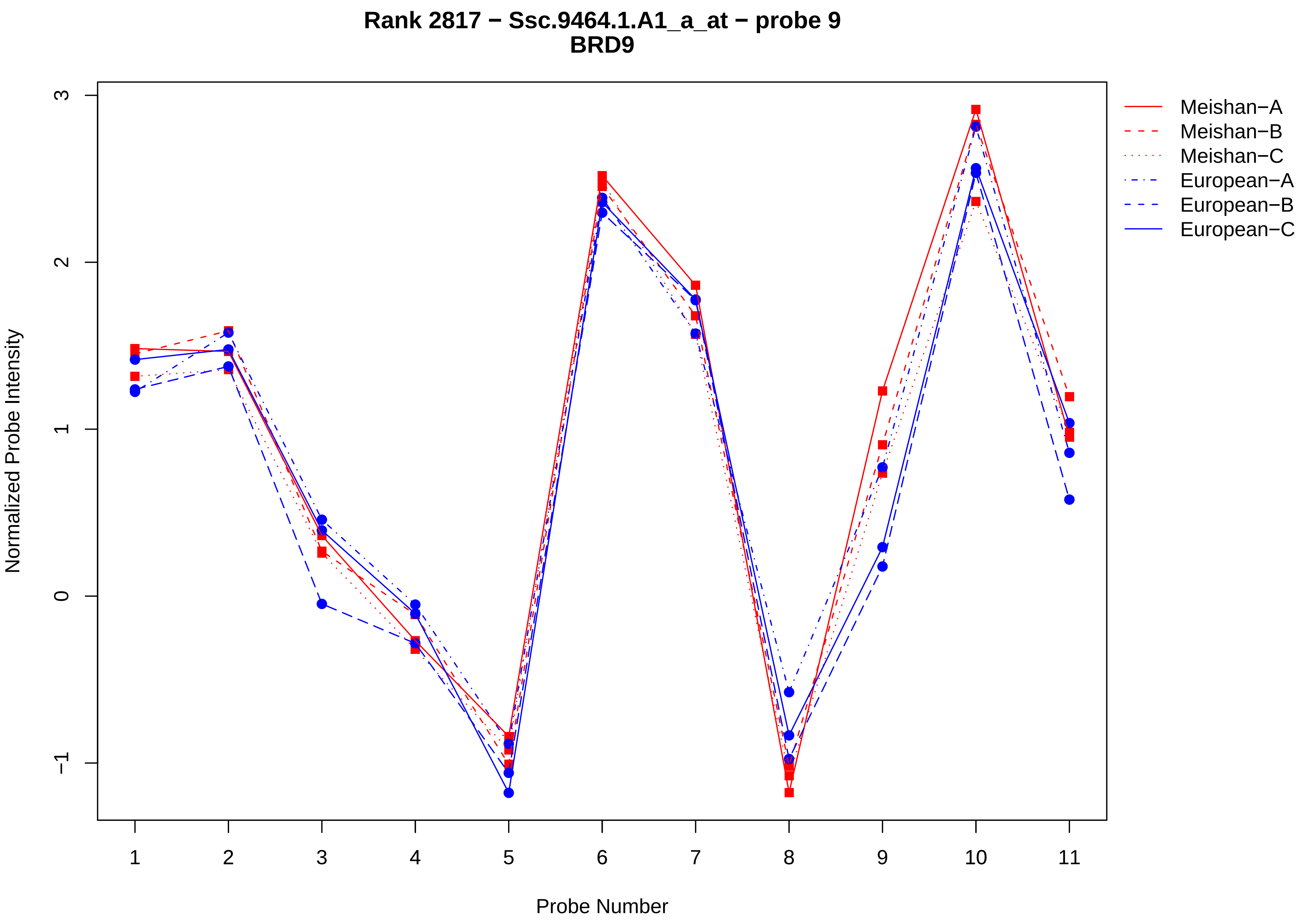

Rank 2817 − Ssc.9464.1.A1_a_at − probe 9
BRD9
Normalized Probe Intensity
Probe Number
Meishan−A
Meishan−B
Meishan−C
European−A
European−B
European−C

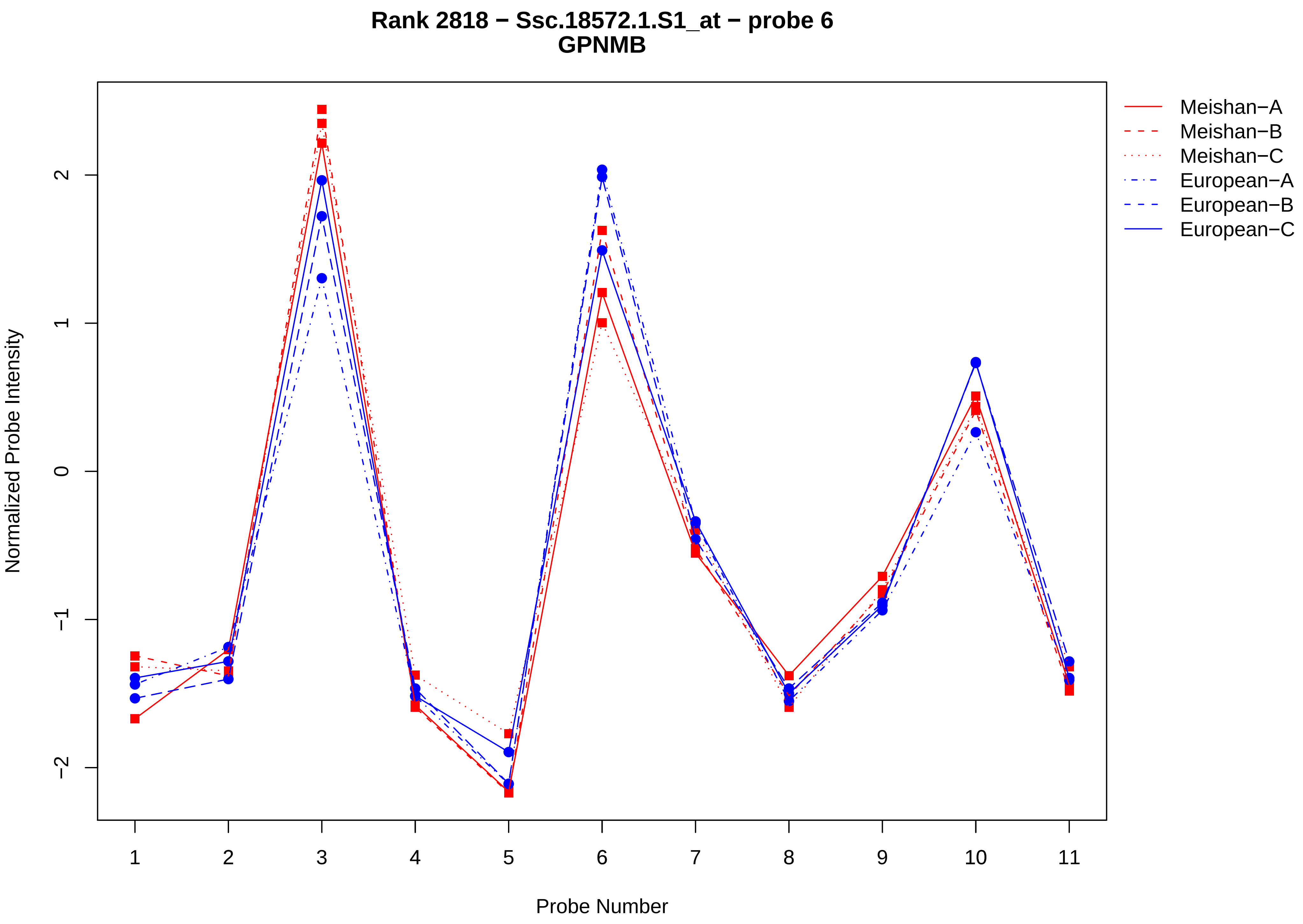

Rank 2818 − Ssc.18572.1.S1_at − probe 6
GPNMB
Normalized Probe Intensity
Probe Number
Meishan−A
Meishan−B
Meishan−C
European−A
European−B
European−C

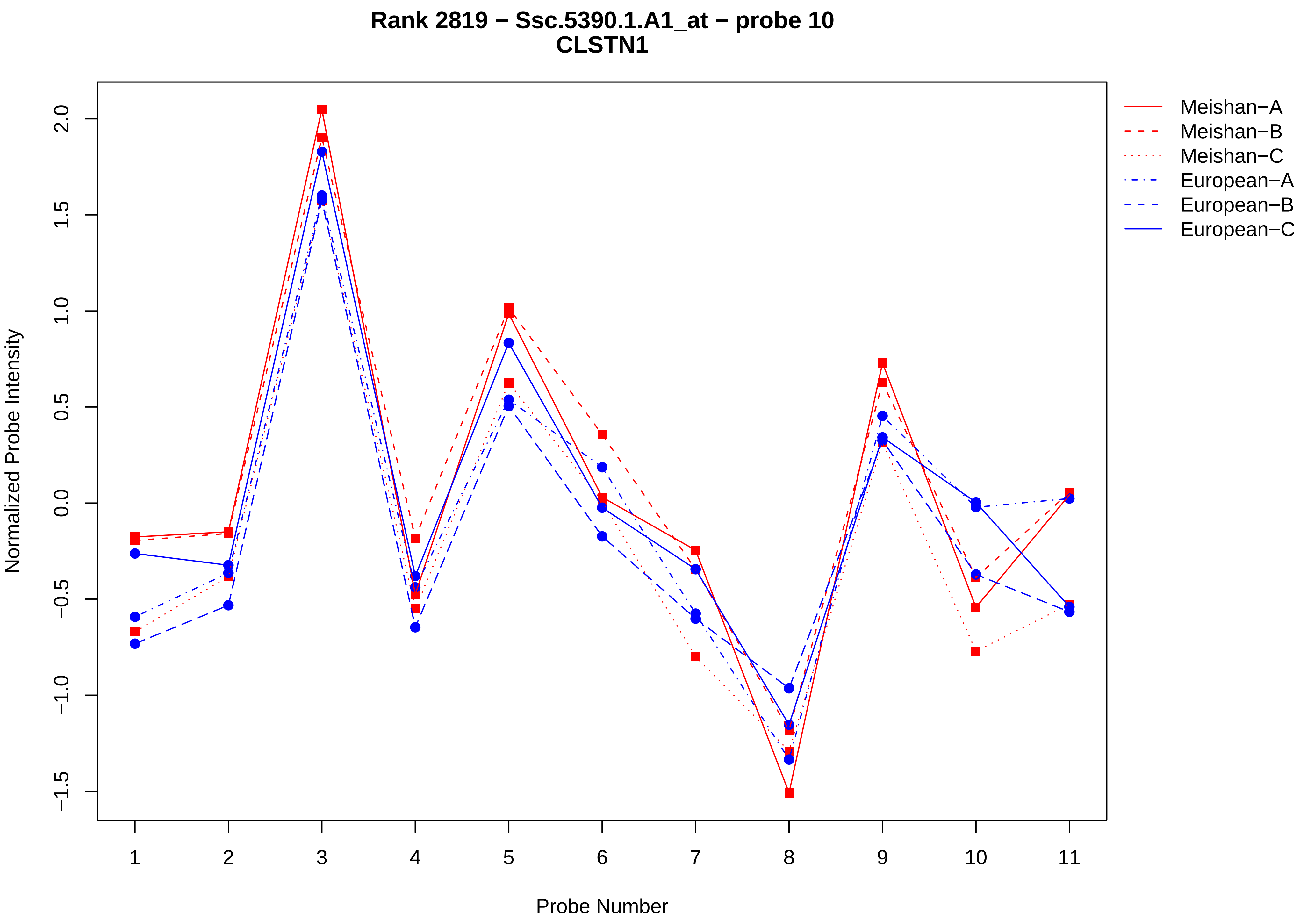

Rank 2819 − Ssc.5390.1.A1_at − probe 10
CLSTN1
Normalized Probe Intensity
Probe Number
Meishan−A
Meishan−B
Meishan−C
European−A
European−B
European−C
−1.5
−1.0
−0.5
0.0
0.5
1.0
1.5
2.0
1
2
3
4
5
6
7
8
9
10
11

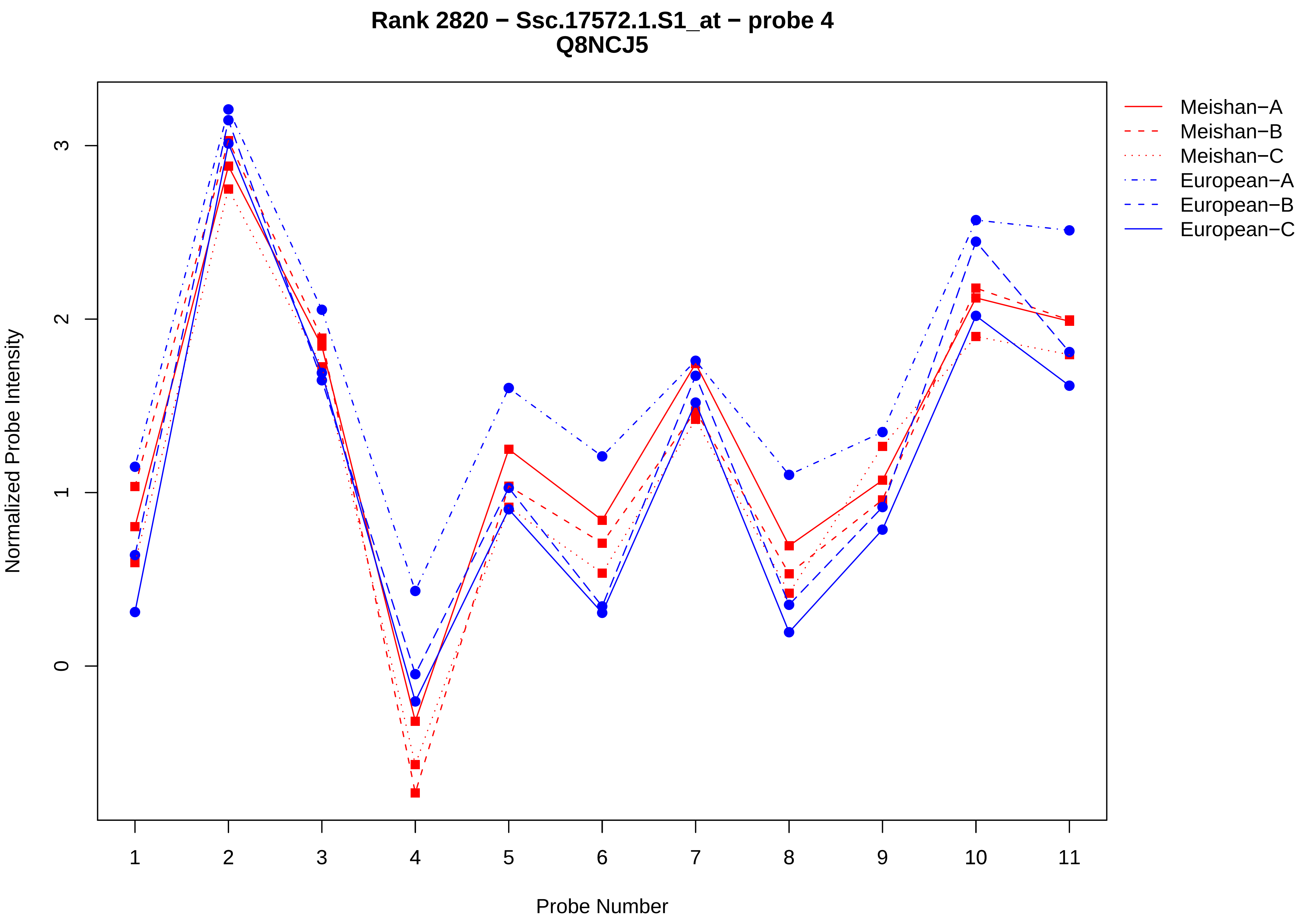

Rank 2820 − Ssc.17572.1.S1_at − probe 4
Q8NCJ5
Normalized Probe Intensity
Probe Number
0
1
2
3
1
2
3
4
5
6
7
8
9
10
11
Meishan−A
Meishan−B
Meishan−C
European−A
European−B
European−C

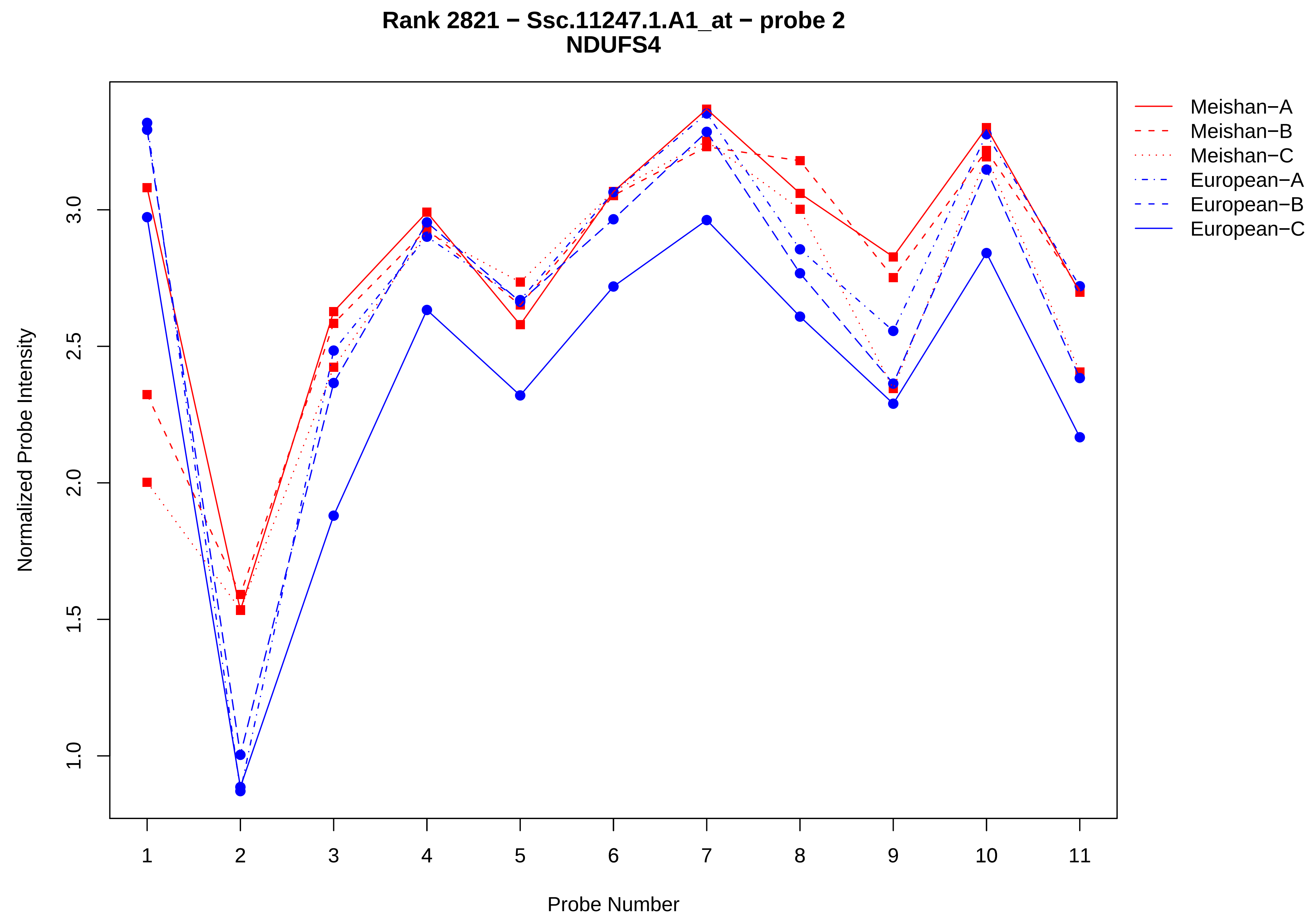

Rank 2821 − Ssc.11247.1.A1_at − probe 2
NDUFS4
Normalized Probe Intensity
1.0
1.5
2.0
2.5
3.0
Probe Number
1
2
3
4
5
6
7
8
9
10
11
Meishan−A
Meishan−B
Meishan−C
European−A
European−B
European−C

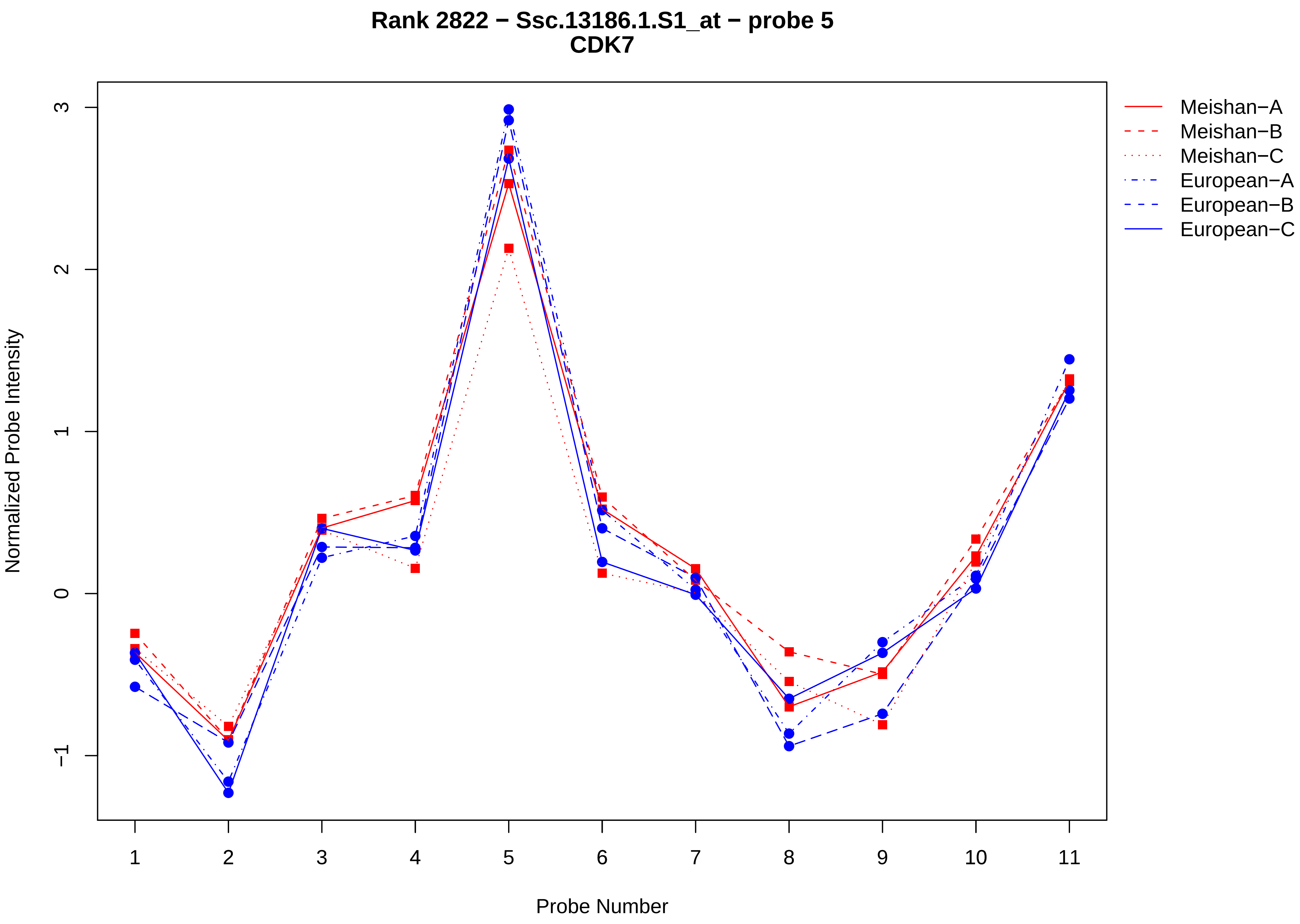

Rank 2822 − Ssc.13186.1.S1_at − probe 5
CDK7
Normalized Probe Intensity
Probe Number
Meishan−A
Meishan−B
Meishan−C
European−A
European−B
European−C

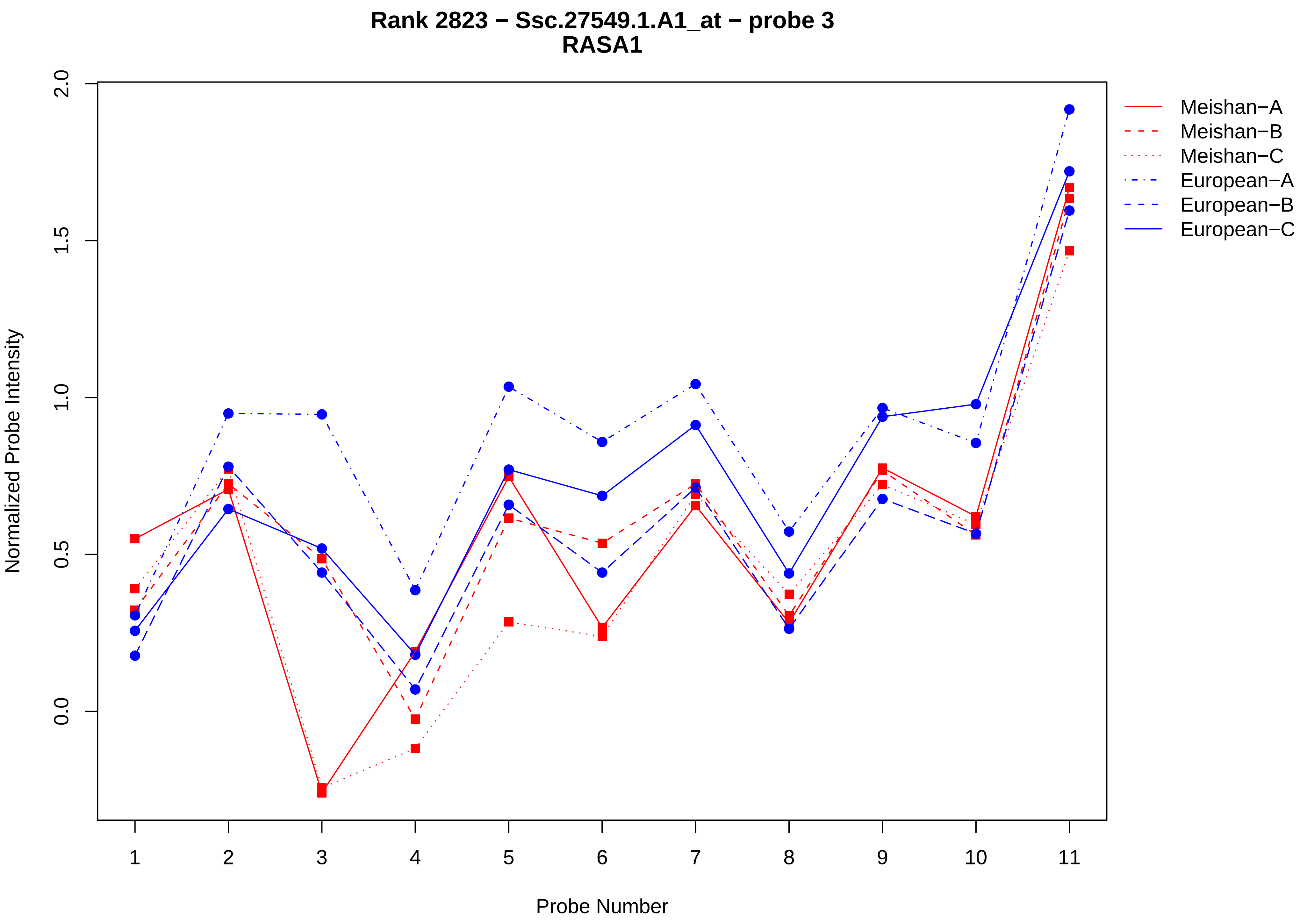

Rank 2823 − Ssc.27549.1.A1_at − probe 3
RASA1
Normalized Probe Intensity
Probe Number
0.0
0.5
1.0
1.5
2.0
1
2
3
4
5
6
7
8
9
10
11
Meishan−A
Meishan−B
Meishan−C
European−A
European−B
European−C

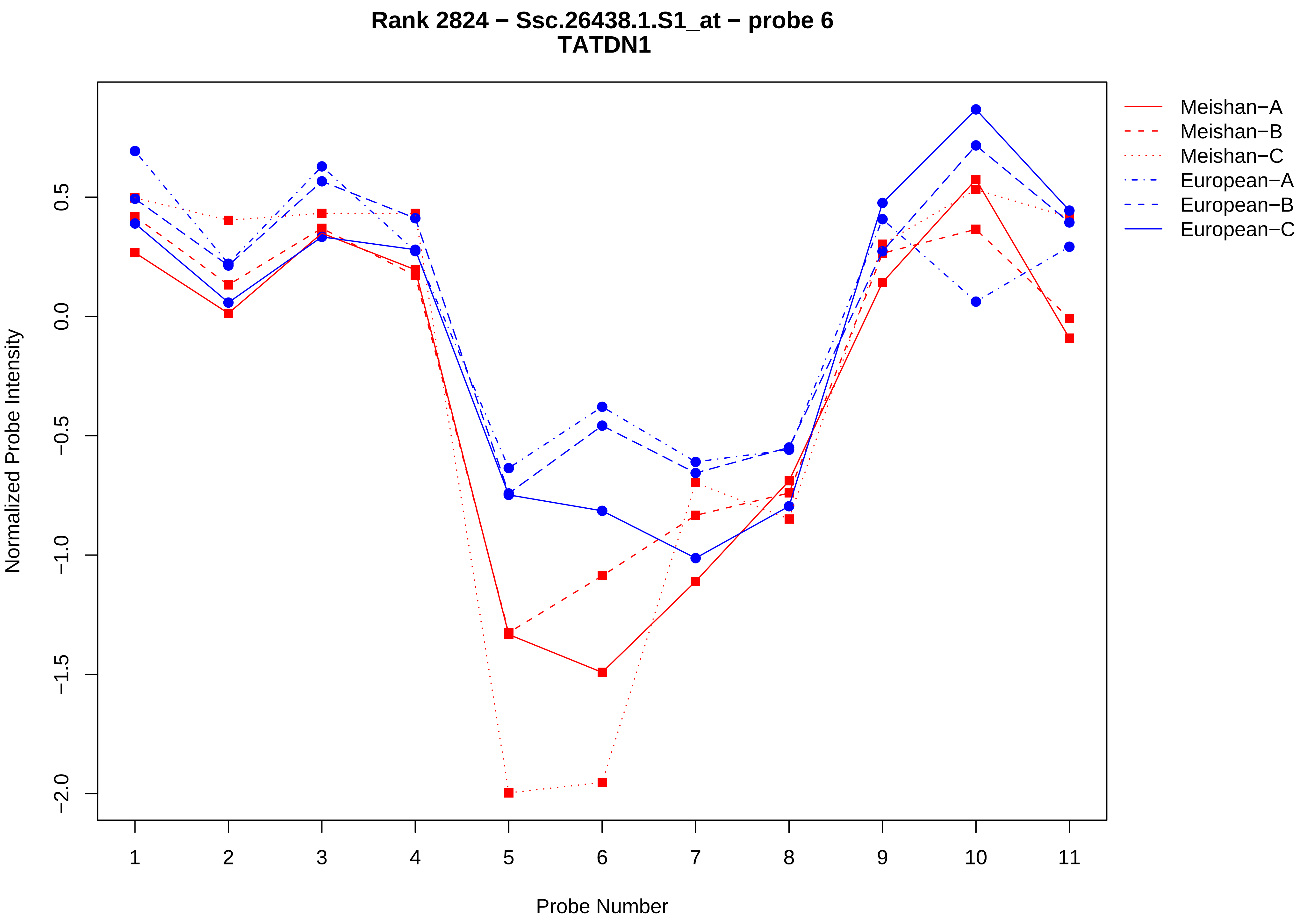

Rank 2824 − Ssc.26438.1.S1_at − probe 6
TATDN1
Normalized Probe Intensity
Probe Number
−2.0
−1.5
−1.0
−0.5
0.0
0.5
1
2
3
4
5
6
7
8
9
10
11
Meishan−A
Meishan−B
Meishan−C
European−A
European−B
European−C

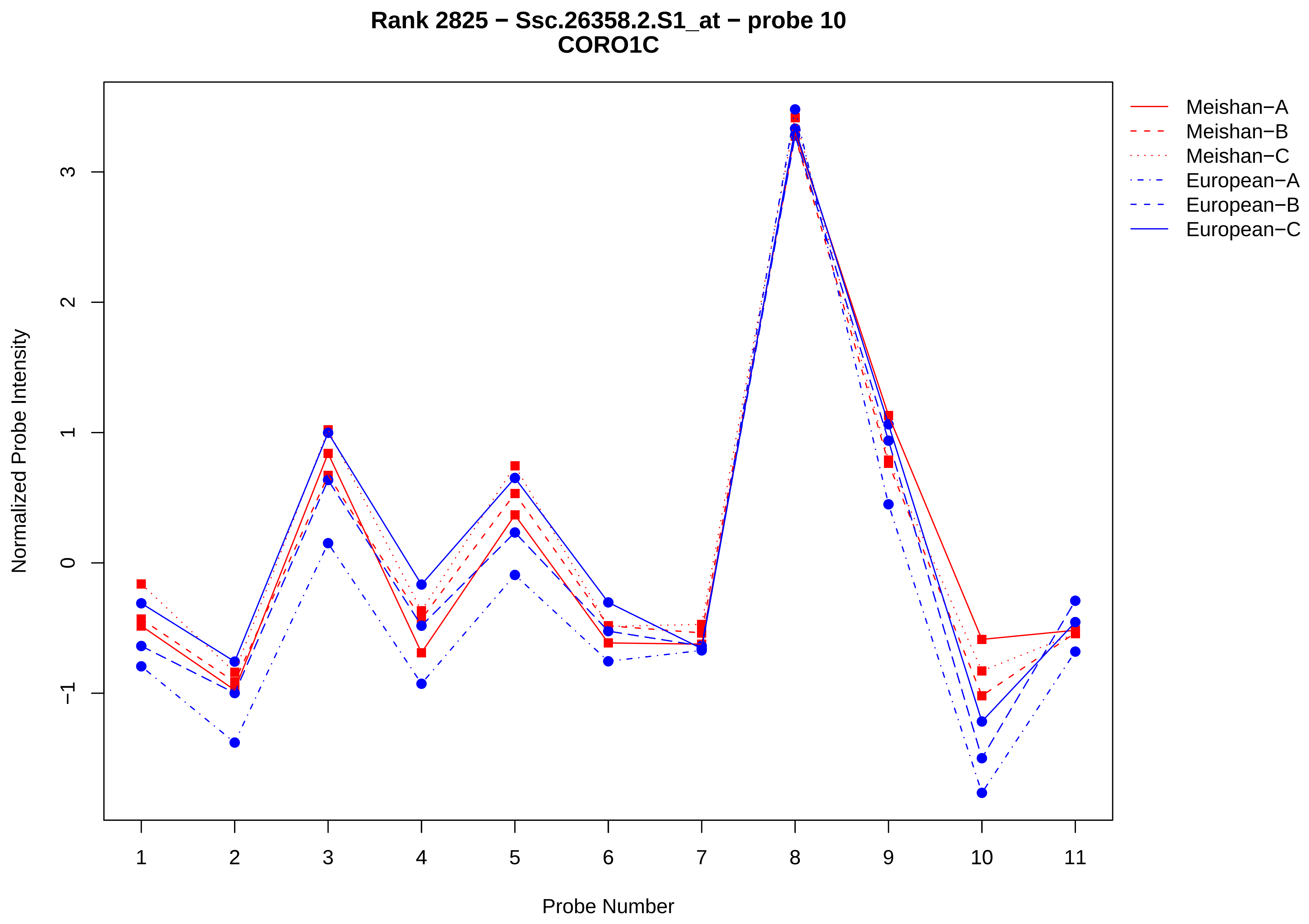

Rank 2825 − Ssc.26358.2.S1_at − probe 10
CORO1C
Normalized Probe Intensity
Probe Number
−1
0
1
2
3
1
2
3
4
5
6
7
8
9
10
11
Meishan−A
Meishan−B
Meishan−C
European−A
European−B
European−C

Rank 2826 − Ssc.27248.1.S1_at − probe 11
Q9BSE4

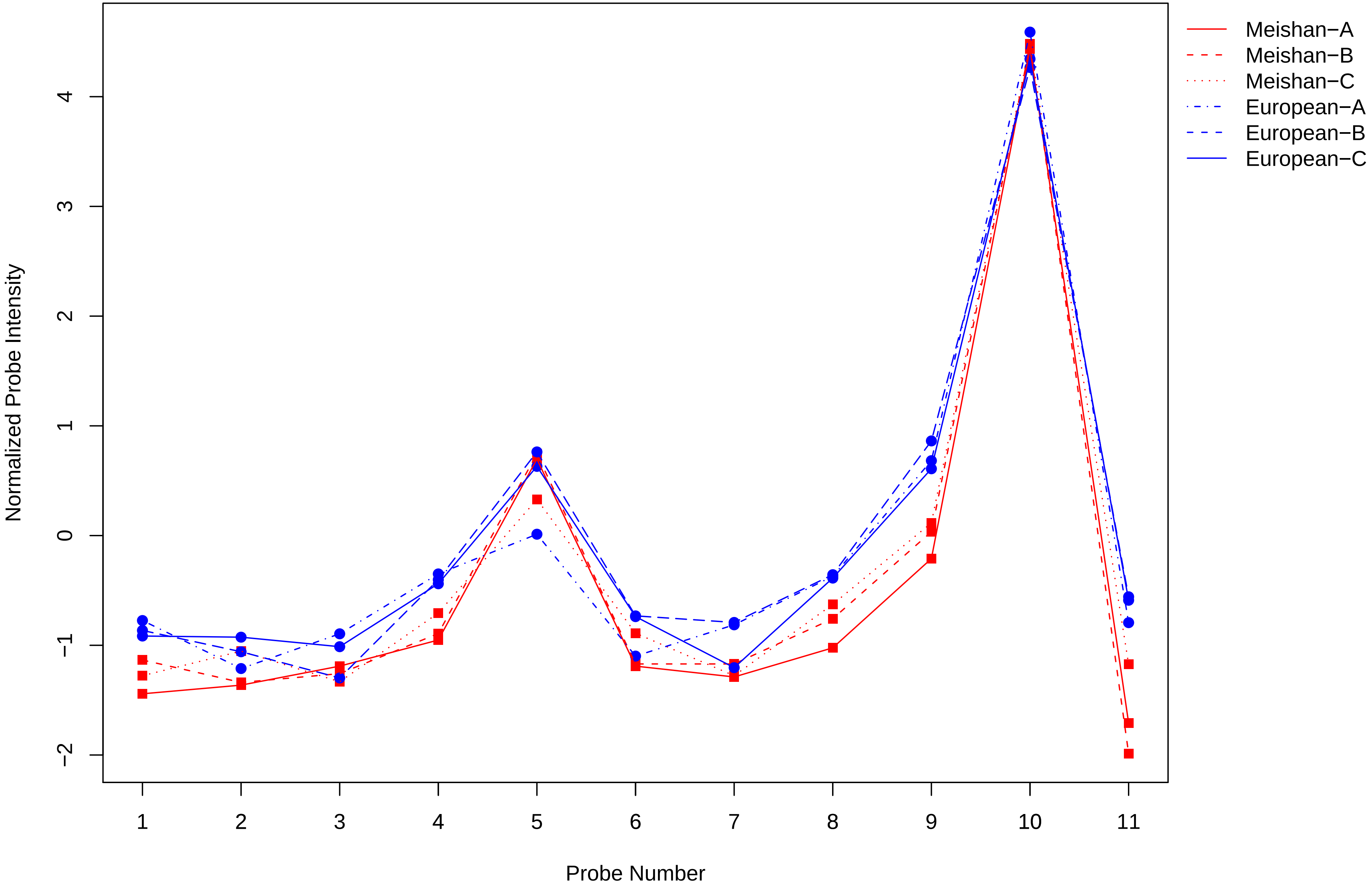

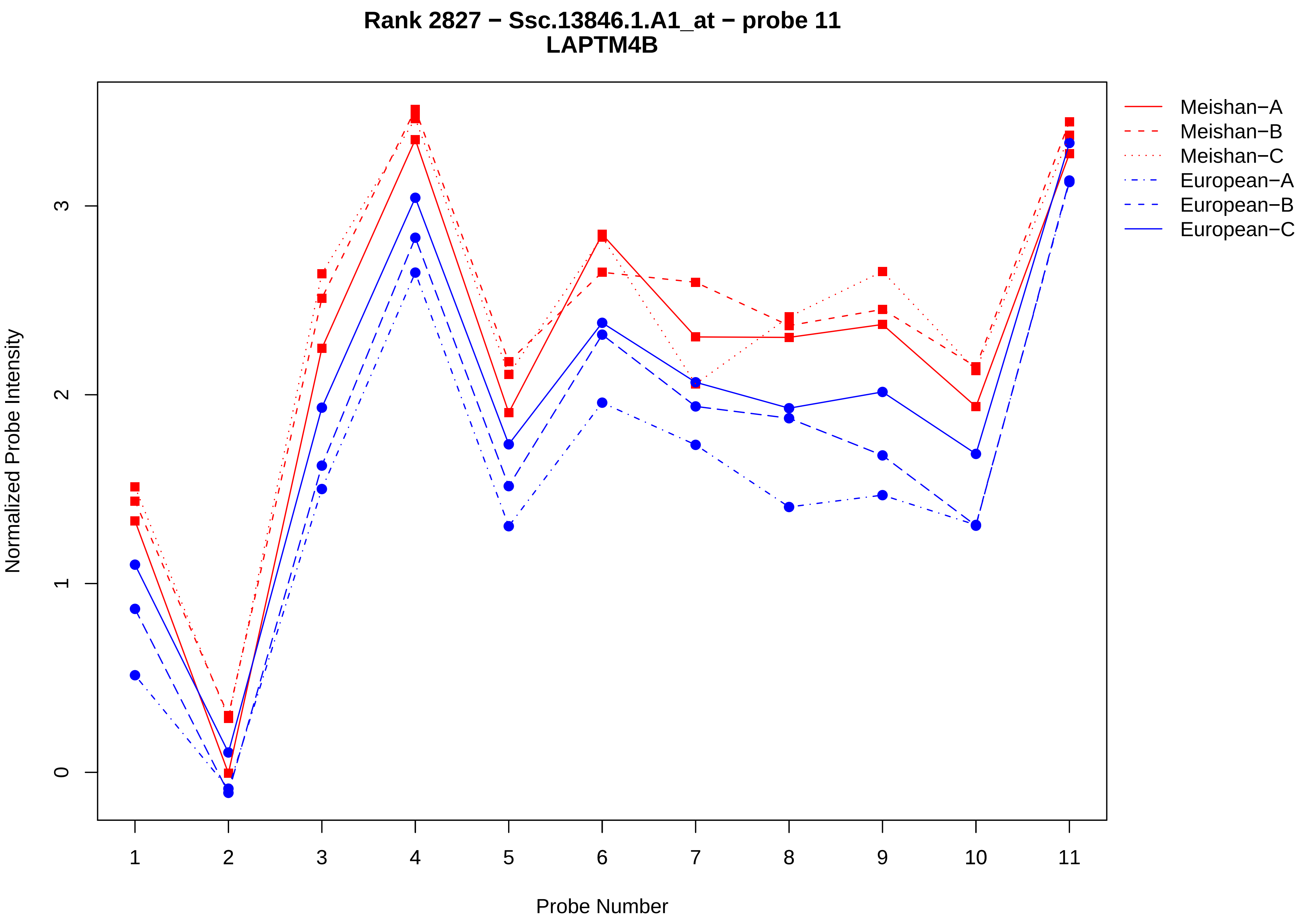

Rank 2827 − Ssc.13846.1.A1_at − probe 11
LAPTM4B
Normalized Probe Intensity
0
1
2
3
1
2
3
4
5
6
7
8
9
10
11
Probe Number
Meishan−A
Meishan−B
Meishan−C
European−A
European−B
European−C

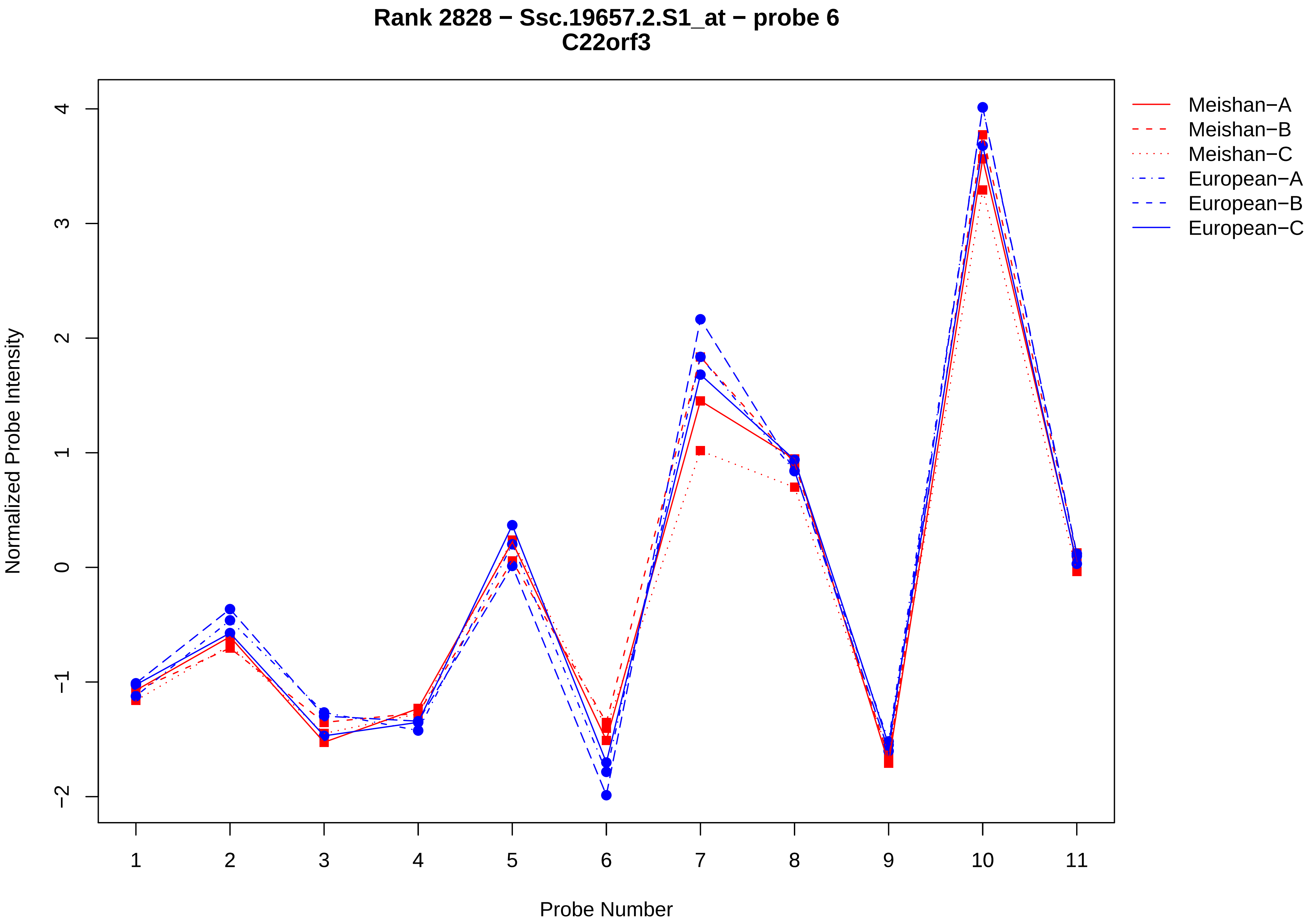

Rank 2828 − Ssc.19657.2.S1_at − probe 6
C22orf3
Normalized Probe Intensity
Probe Number
−2
−1
0
1
2
3
4
1
2
3
4
5
6
7
8
9
10
11
Meishan−A
Meishan−B
Meishan−C
European−A
European−B
European−C

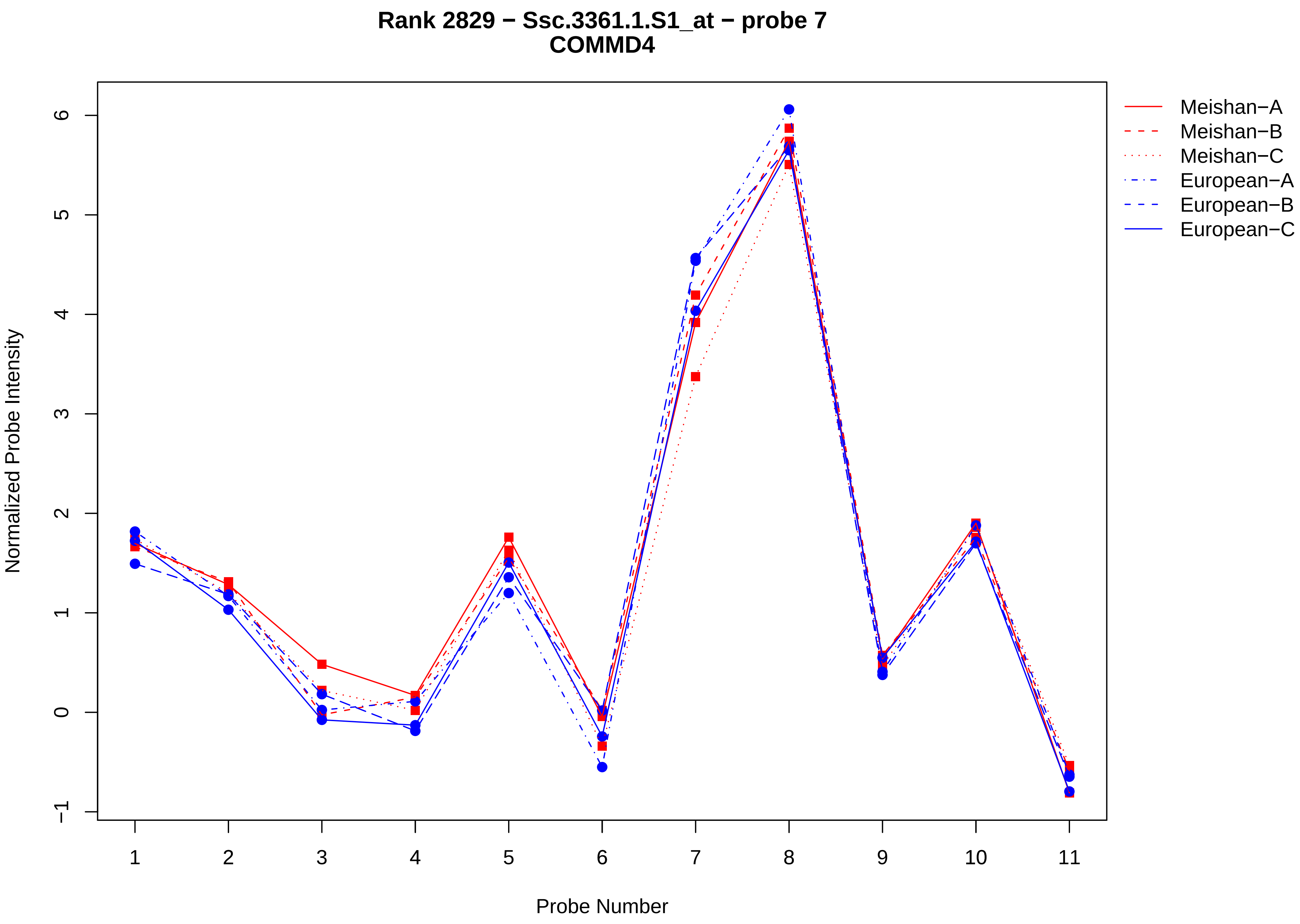
Rank 2829 − Ssc.3361.1.S1_at − probe 7
COMMD4
Normalized Probe Intensity
Probe Number
Meishan−A
Meishan−B
Meishan−C
European−A
European−B
European−C

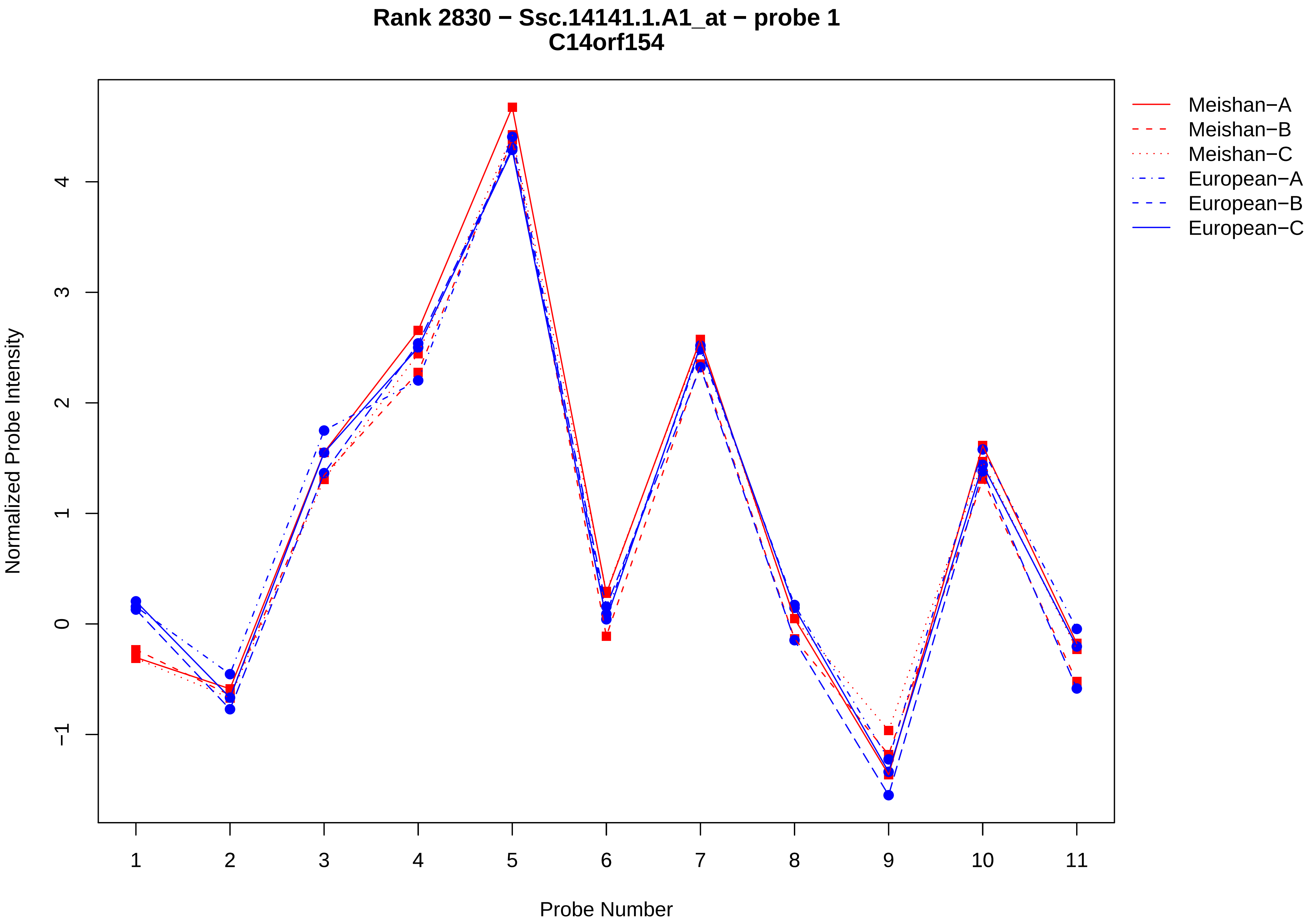

Rank 2830 − Ssc.14141.1.A1_at − probe 1
C14orf154
Normalized Probe Intensity
Probe Number
Meishan−A
Meishan−B
Meishan−C
European−A
European−B
European−C
−1
0
1
2
3
4
1
2
3
4
5
6
7
8
9
10
11

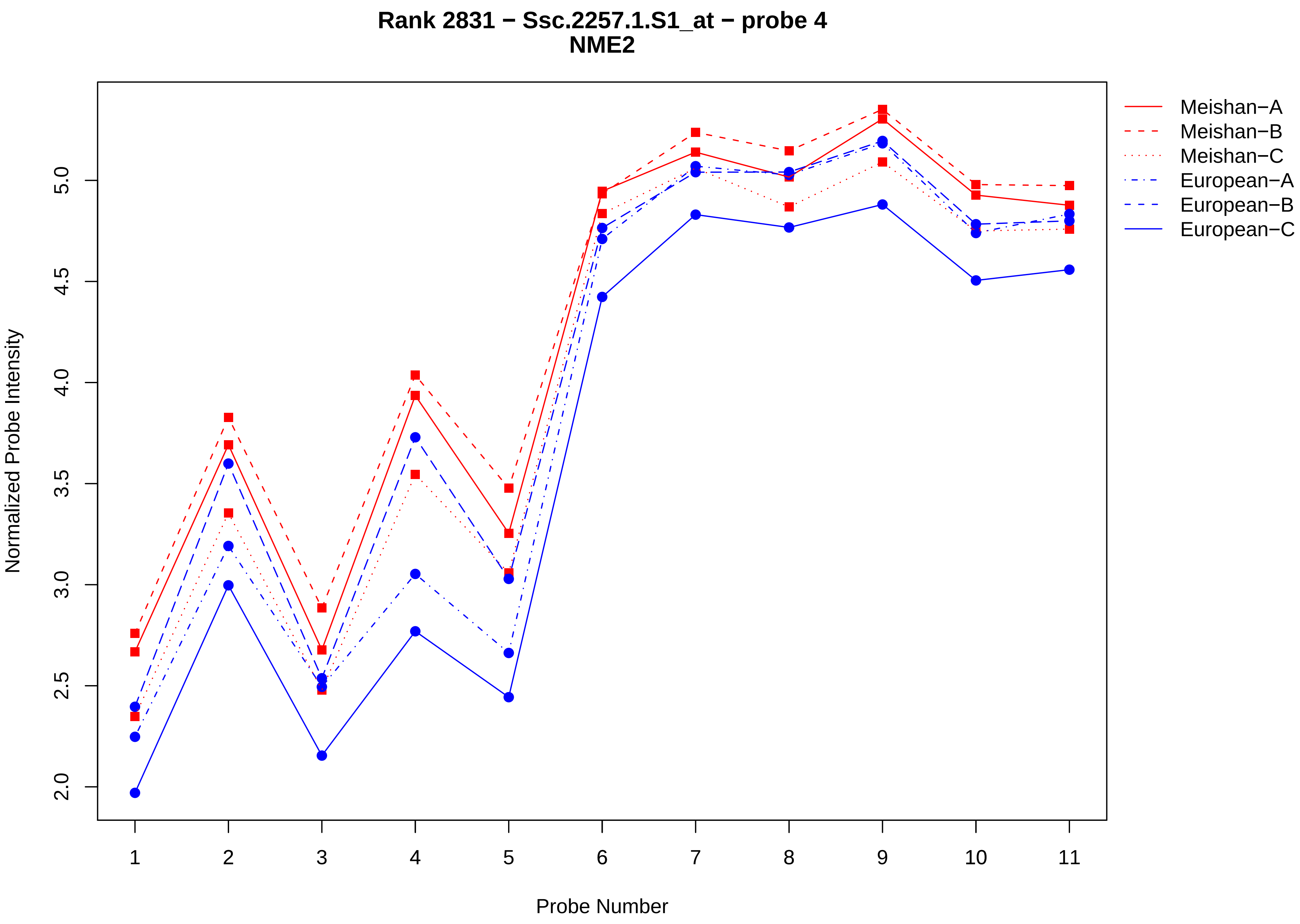
Rank 2831 − Ssc.2257.1.S1_at − probe 4
NME2
Normalized Probe Intensity
Probe Number
2.0
2.5
3.0
3.5
4.0
4.5
5.0
1
2
3
4
5
6
7
8
9
10
11
Meishan−A
Meishan−B
Meishan−C
European−A
European−B
European−C

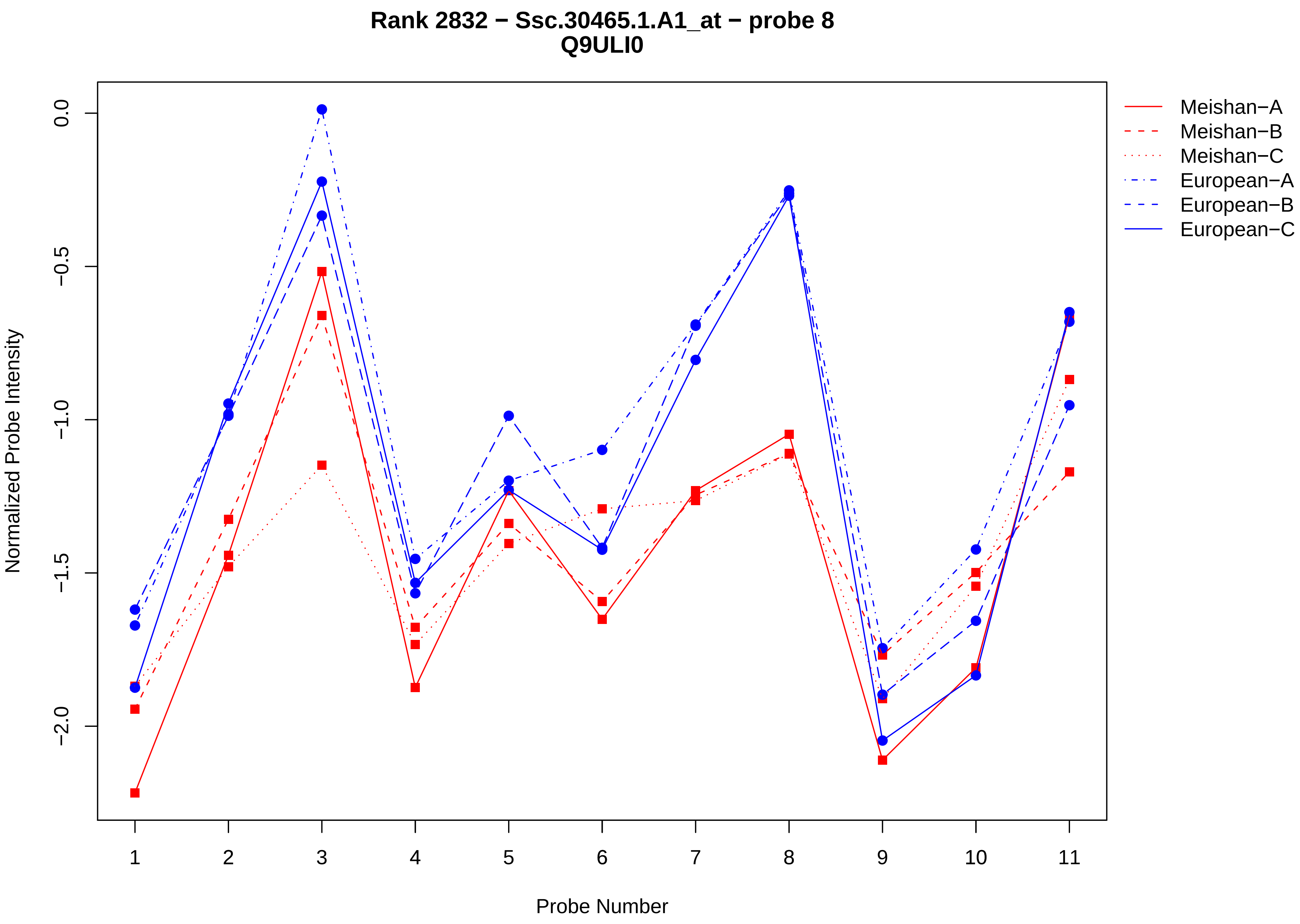
Rank 2832 − Ssc.30465.1.A1_at − probe 8
Q9ULI0
Normalized Probe Intensity
Probe Number
0.0
−0.5
−1.0
−1.5
−2.0
1
2
3
4
5
6
7
8
9
10
11
Meishan−A
Meishan−B
Meishan−C
European−A
European−B
European−C

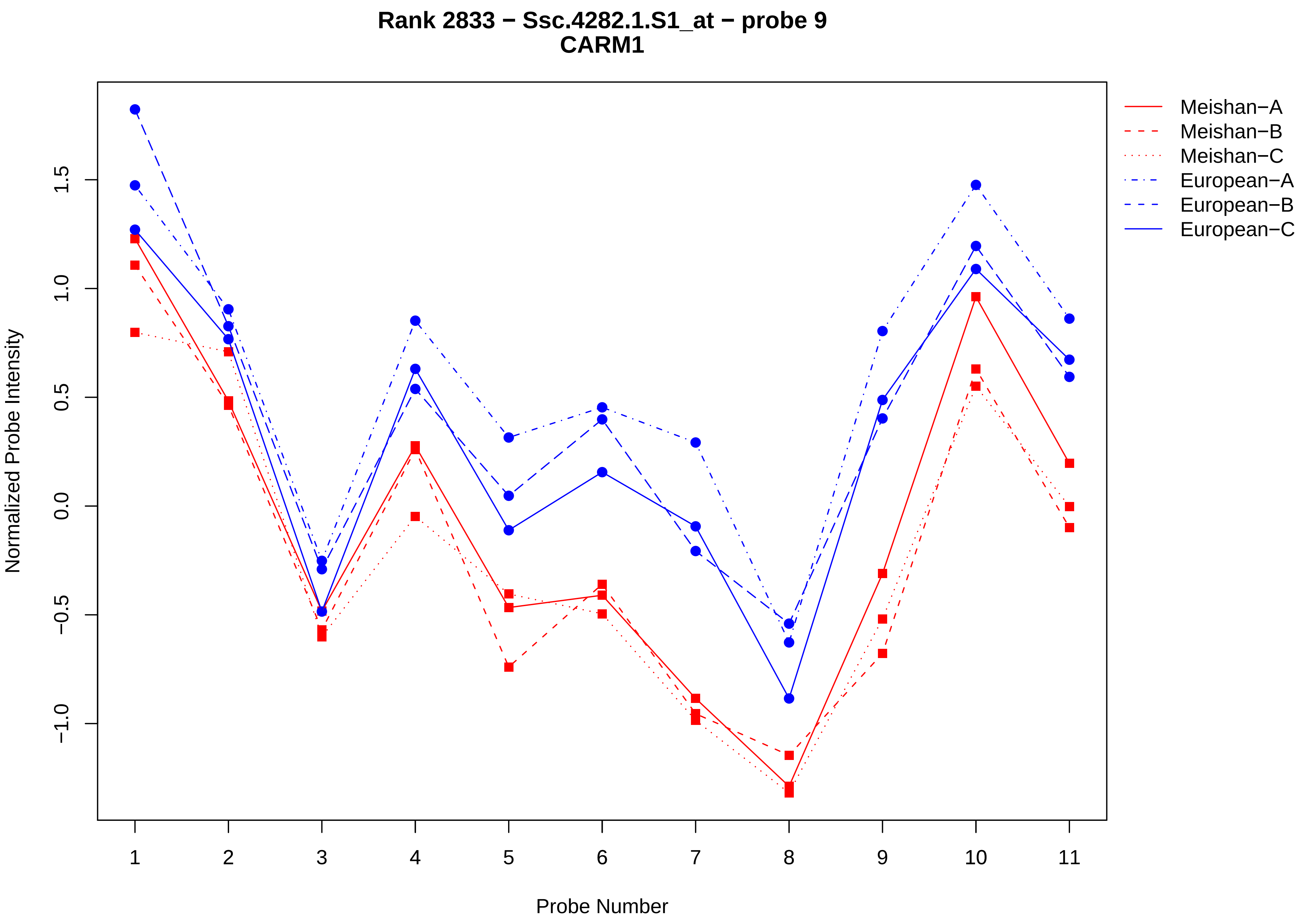

Rank 2833 − Ssc.4282.1.S1_at − probe 9
CARM1
Normalized Probe Intensity
Probe Number
−1.0
−0.5
0.0
0.5
1.0
1.5
1
2
3
4
5
6
7
8
9
10
11
Meishan−A
Meishan−B
Meishan−C
European−A
European−B
European−C

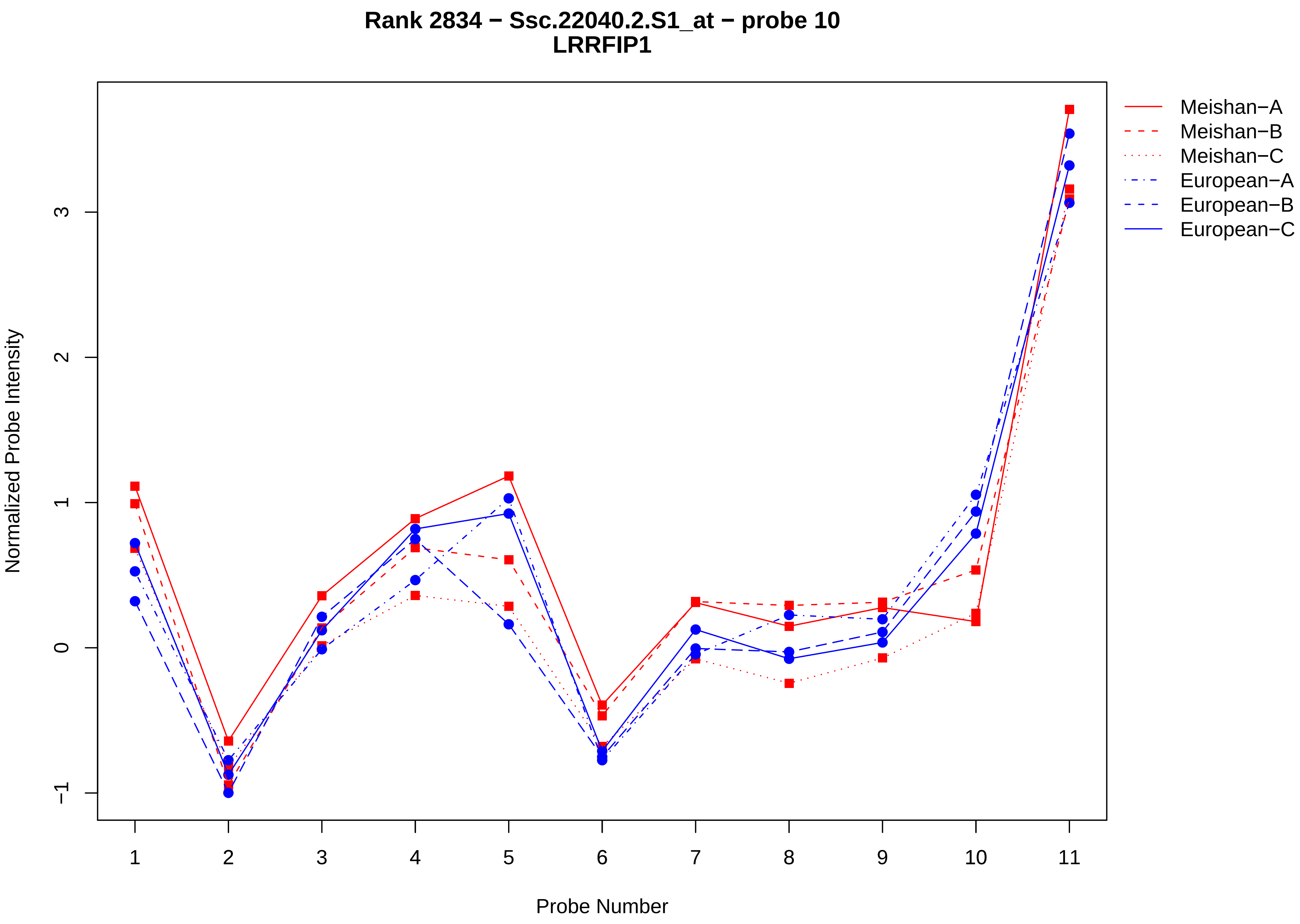

Rank 2834 − Ssc.22040.2.S1_at − probe 10
LRRFIP1
Normalized Probe Intensity
Probe Number
−1
0
1
2
3
1
2
3
4
5
6
7
8
9
10
11
Meishan−A
Meishan−B
Meishan−C
European−A
European−B
European−C

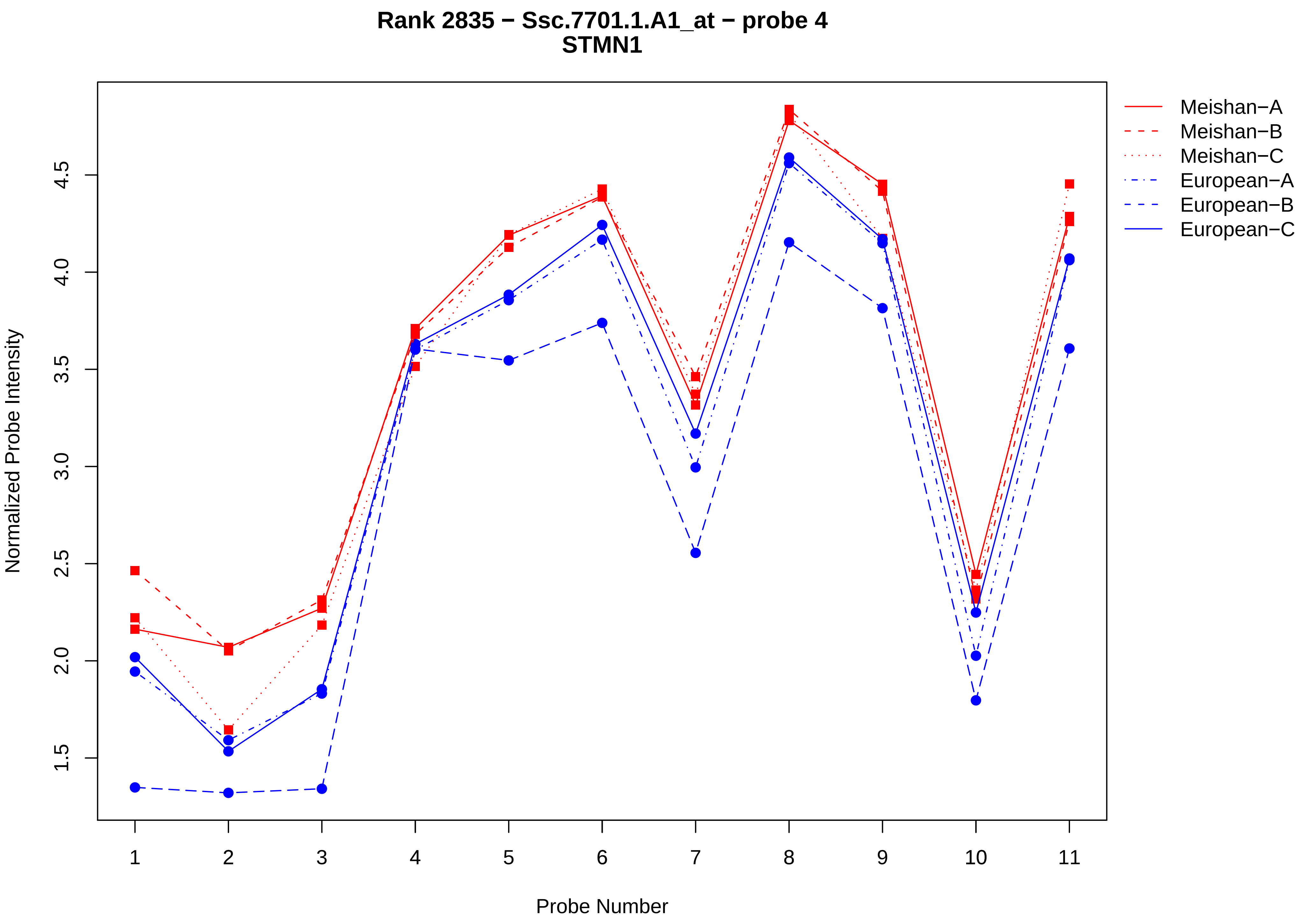

Rank 2835 − Ssc.7701.1.A1_at − probe 4
STMN1
Normalized Probe Intensity
Probe Number
1.5
2.0
2.5
3.0
3.5
4.0
4.5
1
2
3
4
5
6
7
8
9
10
11
Meishan−A
Meishan−B
Meishan−C
European−A
European−B
European−C

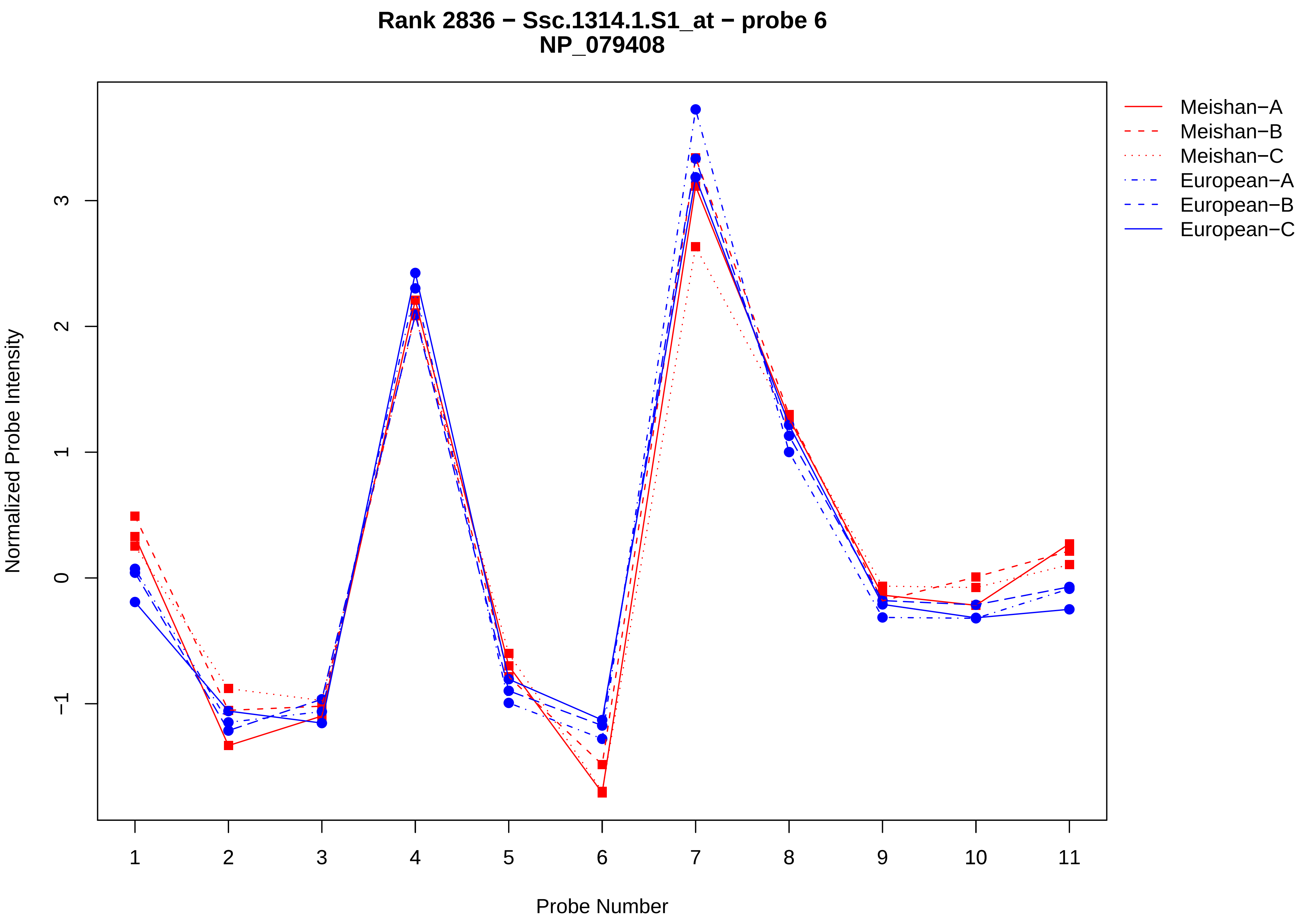

Rank 2836 − Ssc.1314.1.S1_at − probe 6
NP_079408
Normalized Probe Intensity
Probe Number
Meishan−A
Meishan−B
Meishan−C
European−A
European−B
European−C

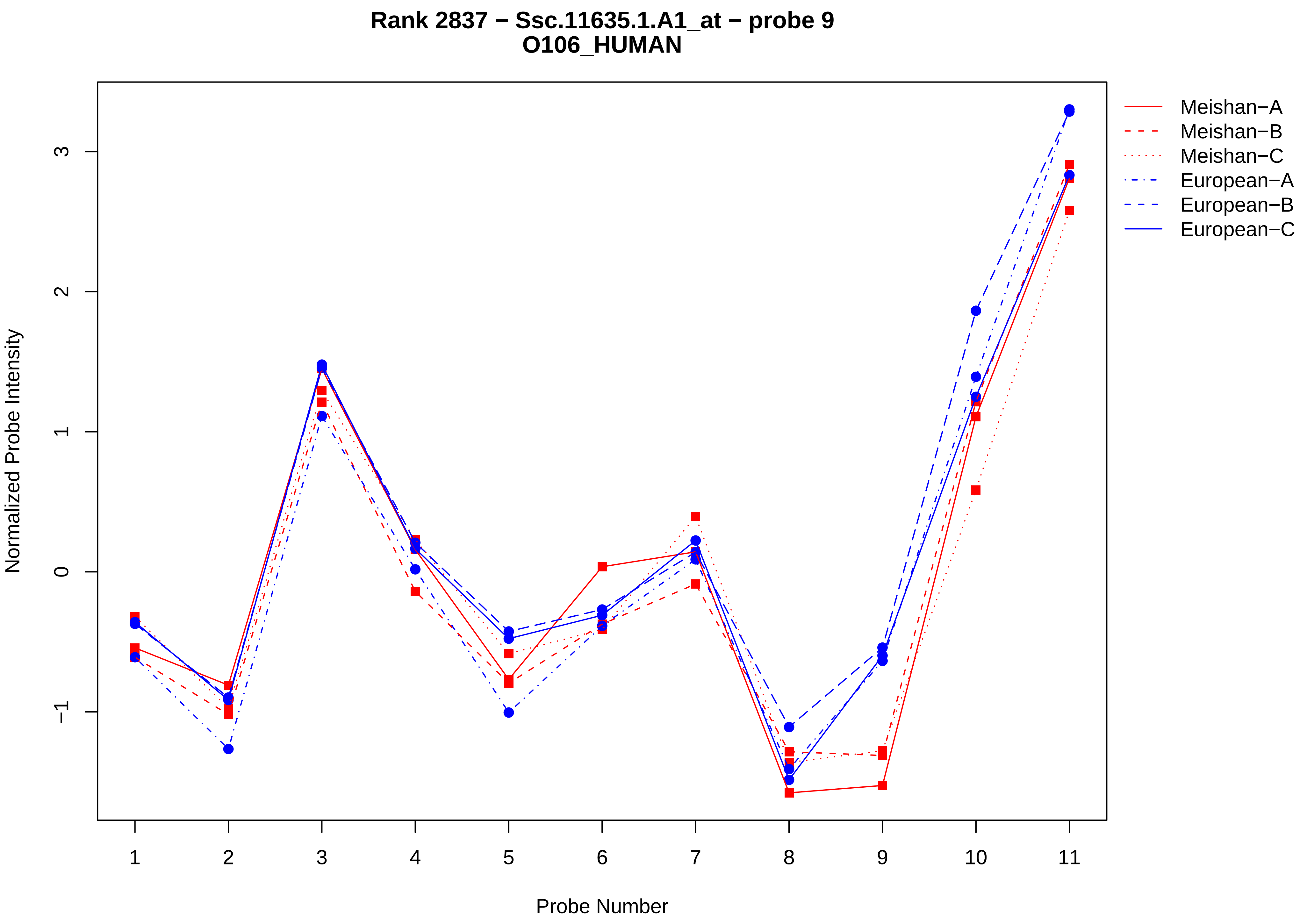

Rank 2837 − Ssc.11635.1.A1_at − probe 9
O106_HUMAN
Normalized Probe Intensity
Probe Number
Meishan−A
Meishan−B
Meishan−C
European−A
European−B
European−C

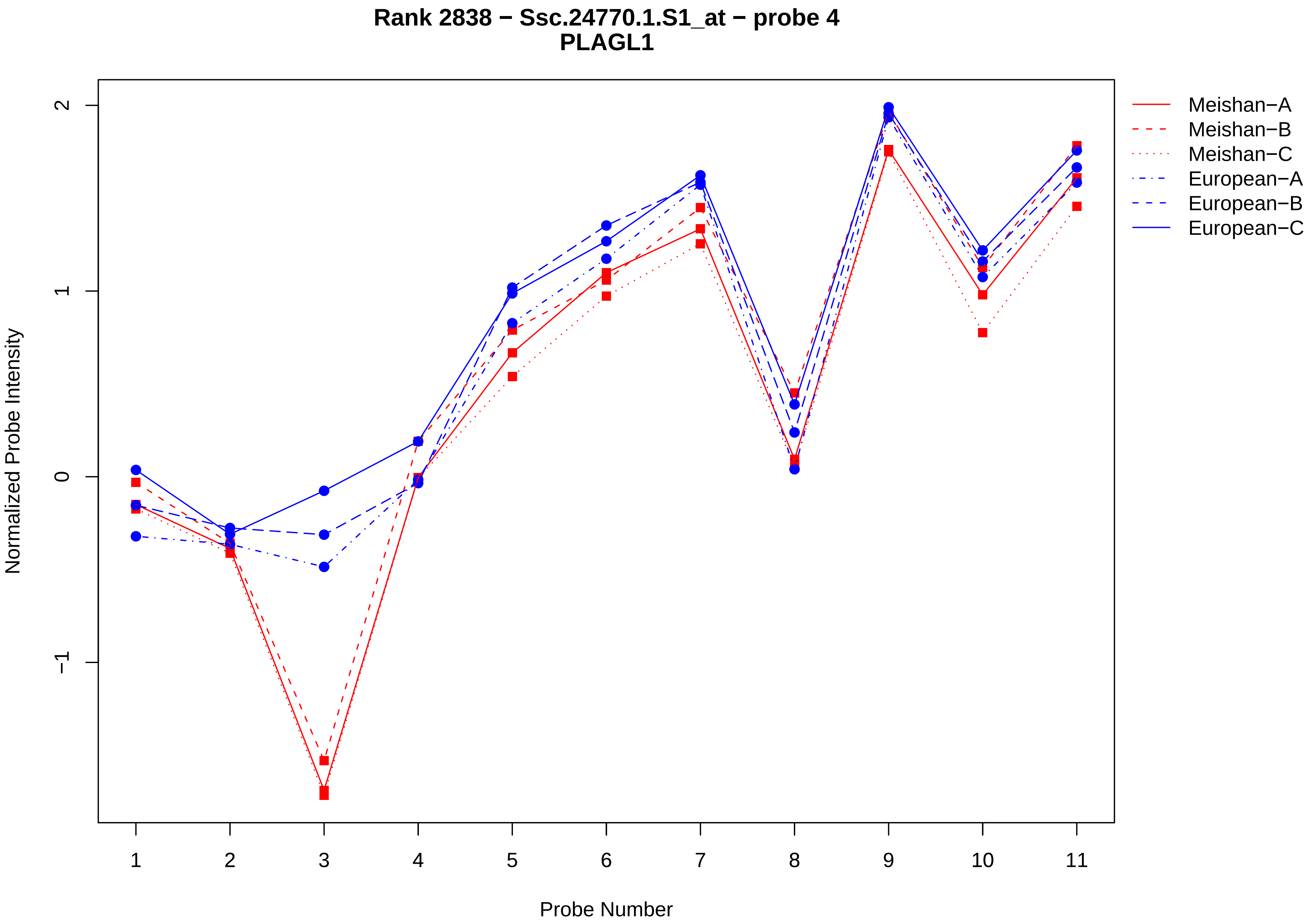

Rank 2838 − Ssc.24770.1.S1_at − probe 4
PLAGL1
Normalized Probe Intensity
Probe Number
−1
0
1
2
1
2
3
4
5
6
7
8
9
10
11
Meishan−A
Meishan−B
Meishan−C
European−A
European−B
European−C

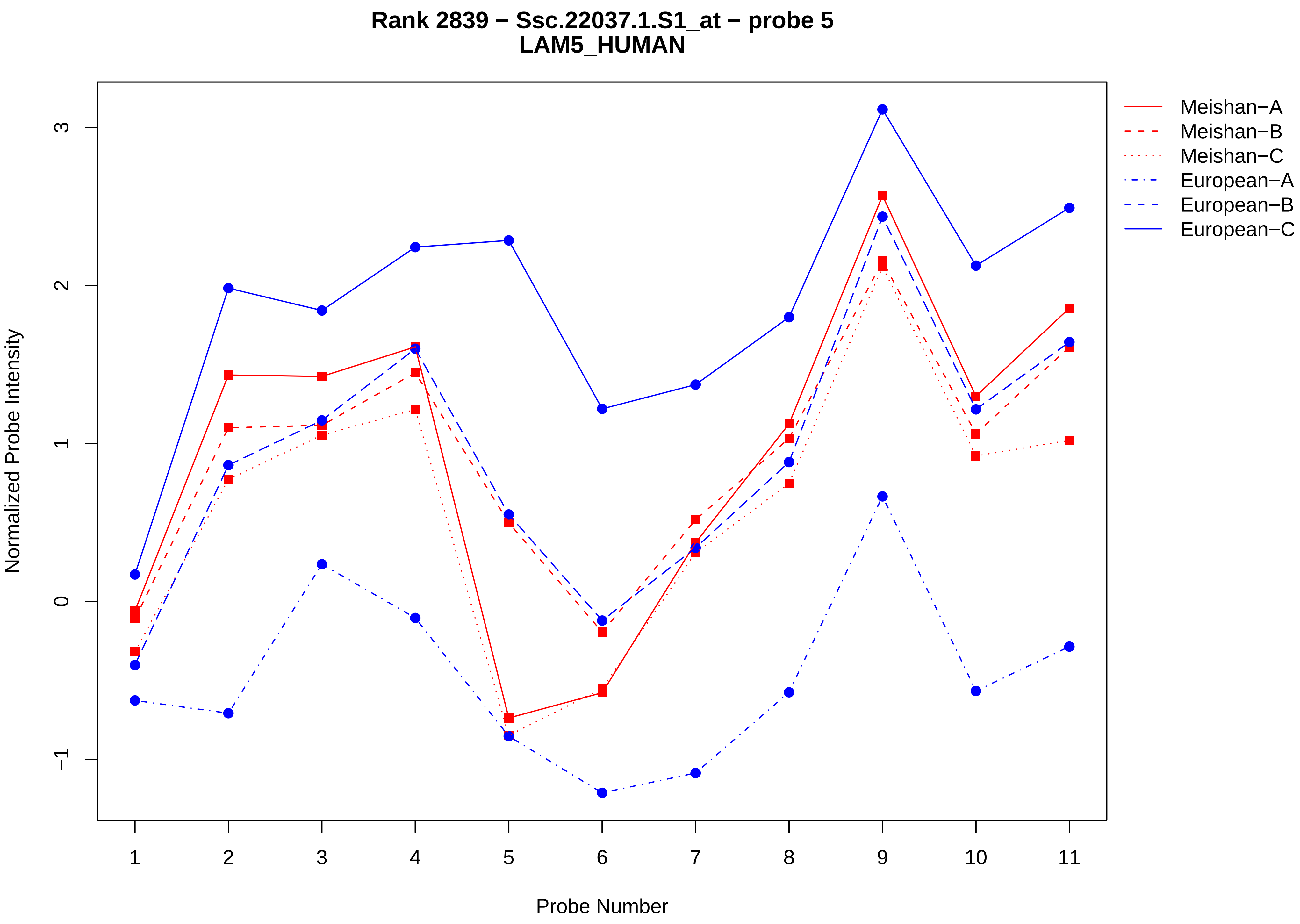

Rank 2839 − Ssc.22037.1.S1_at − probe 5
LAM5_HUMAN
Normalized Probe Intensity
Probe Number
−1
0
1
2
3
1
2
3
4
5
6
7
8
9
10
11
Meishan−A
Meishan−B
Meishan−C
European−A
European−B
European−C

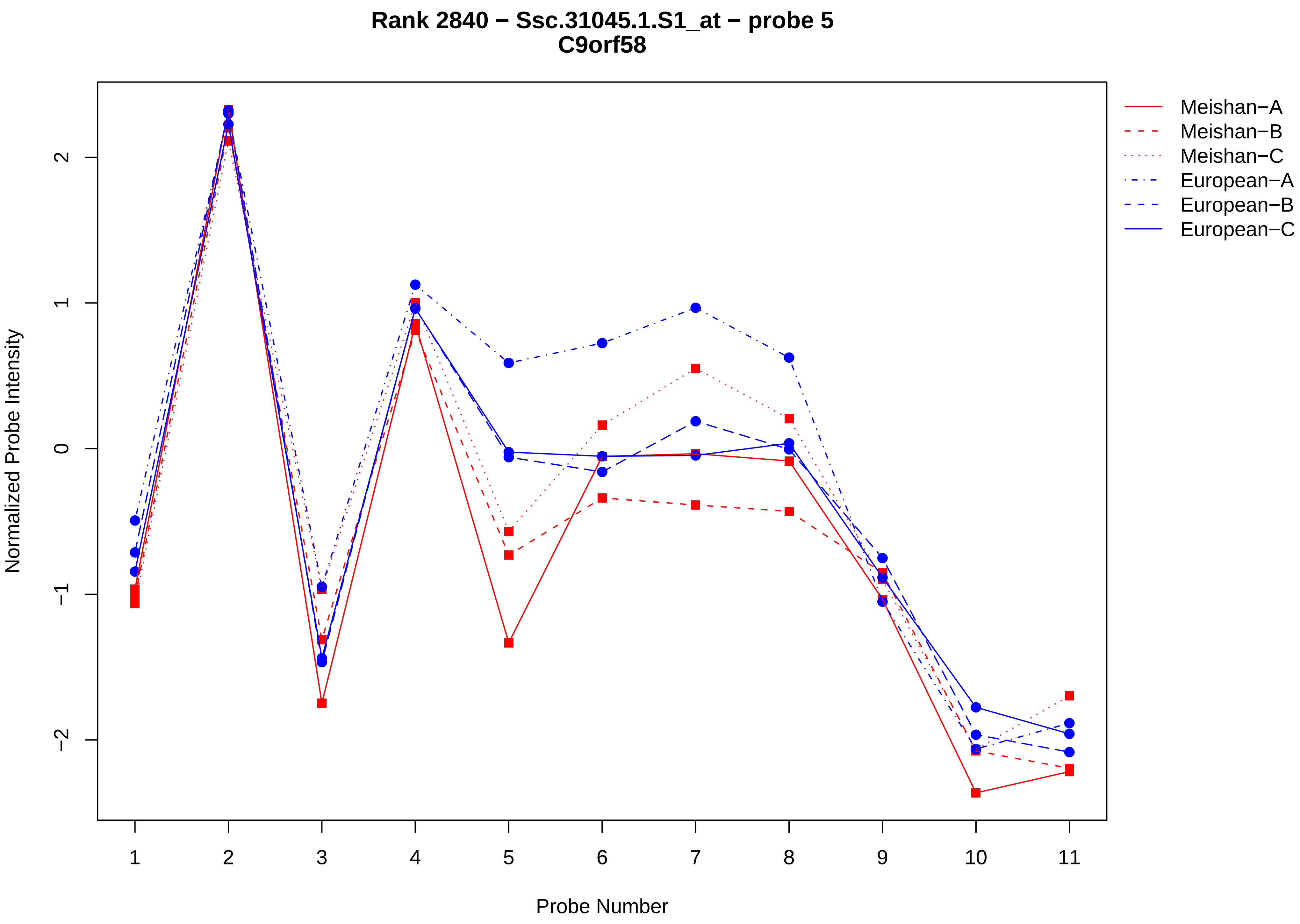
Rank 2840 − Ssc.31045.1.S1_at − probe 5
C9orf58
Normalized Probe Intensity
Probe Number
−2
−1
0
1
2
1
2
3
4
5
6
7
8
9
10
11
Meishan−A
Meishan−B
Meishan−C
European−A
European−B
European−C

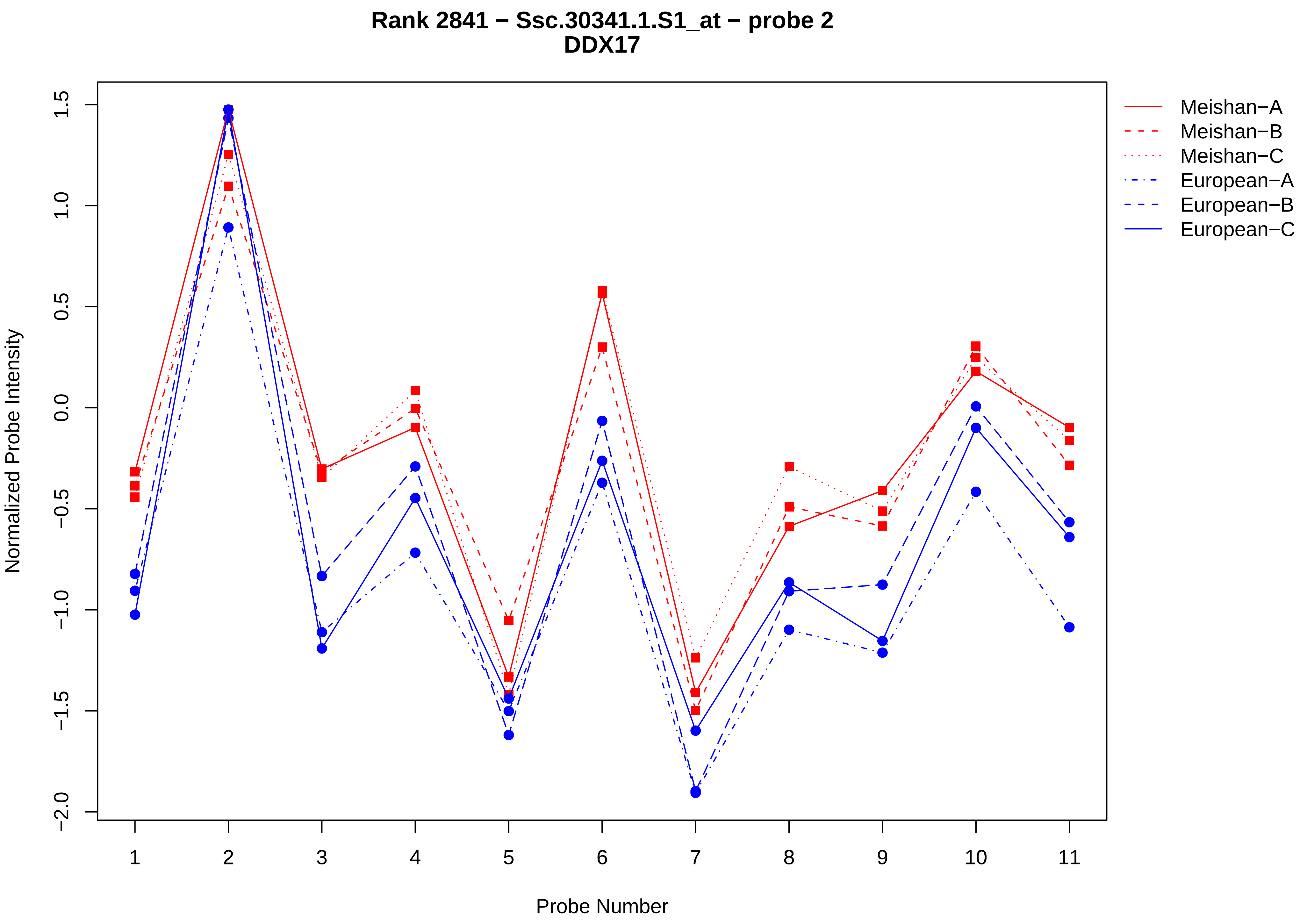
Rank 2841 − Ssc.30341.1.S1_at − probe 2
DDX17
Normalized Probe Intensity
Probe Number
1.5
1.0
0.5
0.0
−0.5
−1.0
−1.5
−2.0
1
2
3
4
5
6
7
8
9
10
11
Meishan−A
Meishan−B
Meishan−C
European−A
European−B
European−C

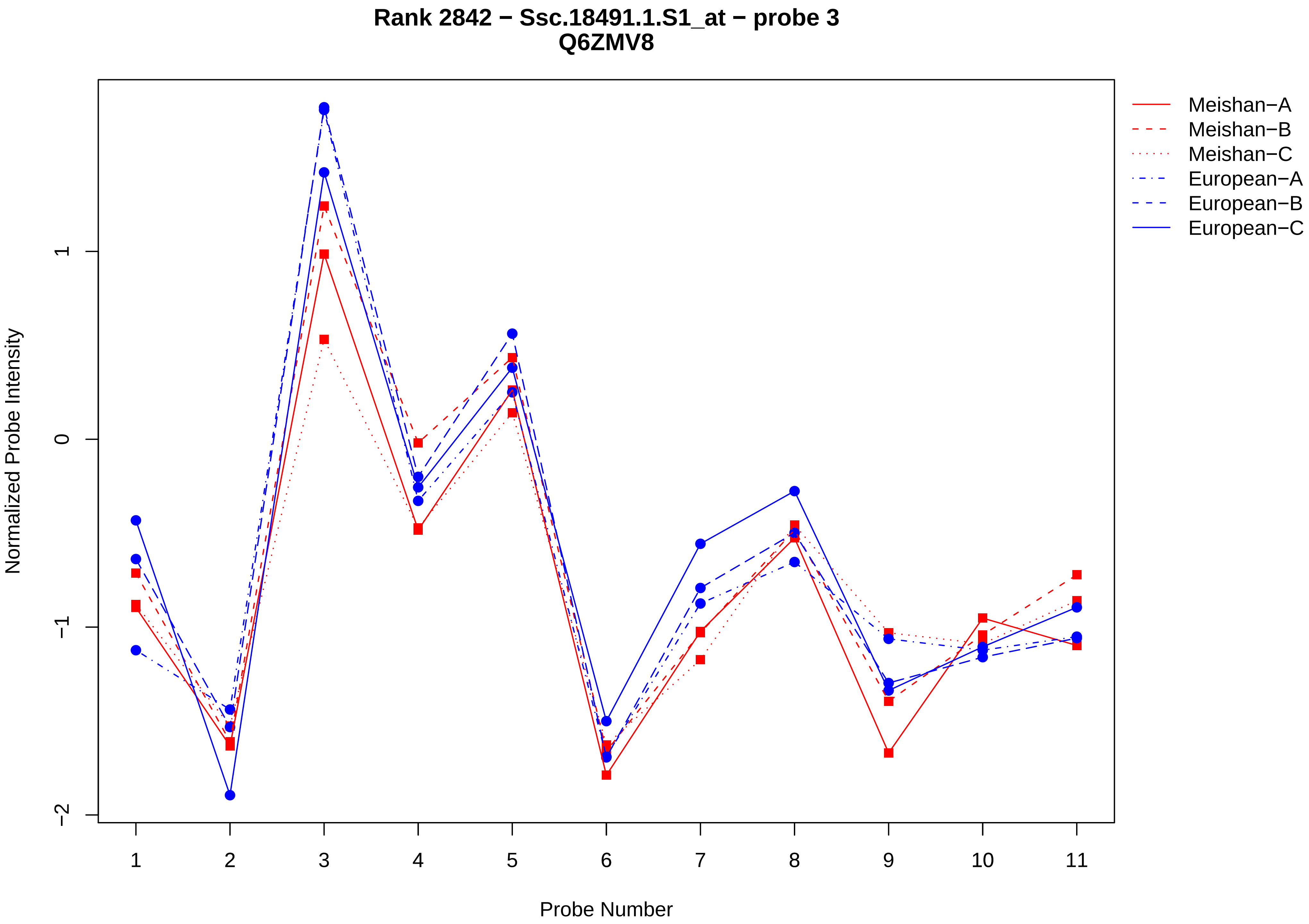
Rank 2842 − Ssc.18491.1.S1_at − probe 3
Q6ZMV8
Normalized Probe Intensity
Probe Number
−2
−1
0
1
1
2
3
4
5
6
7
8
9
10
11
Meishan−A
Meishan−B
Meishan−C
European−A
European−B
European−C

Rank 2843 − Ssc.16994.2.S1_a_at − probe 3
NP_072094

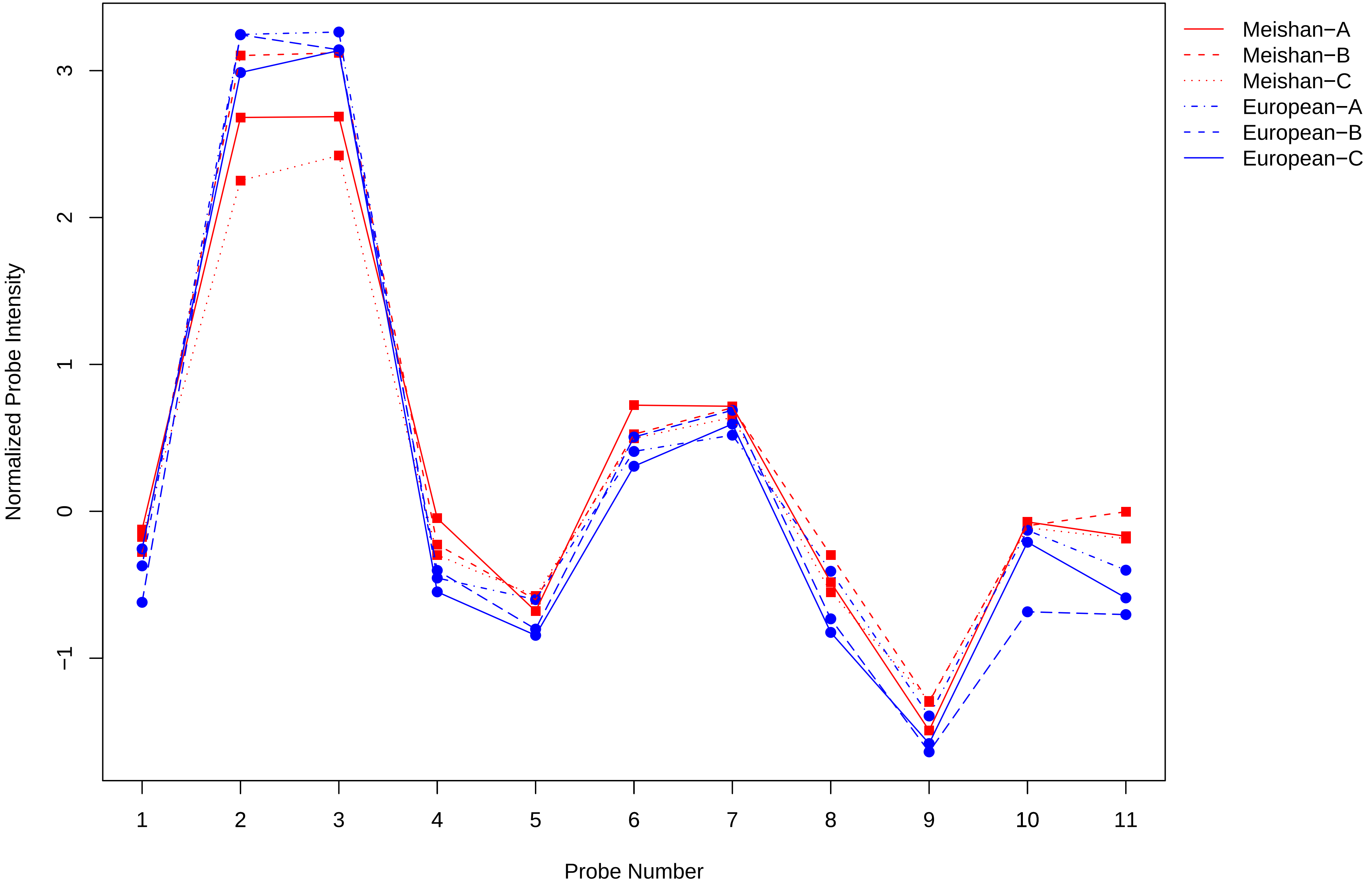

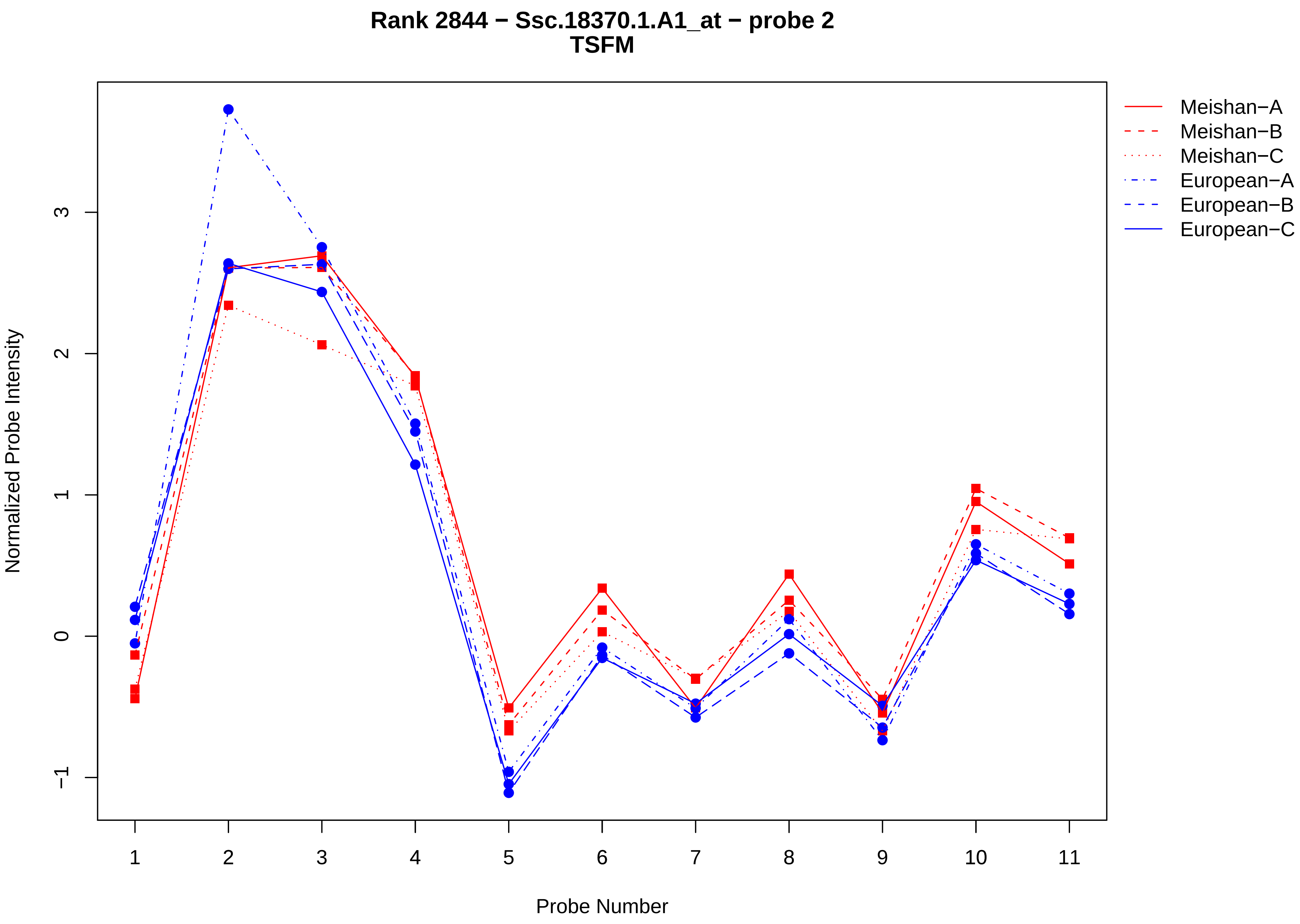

Rank 2844 − Ssc.18370.1.A1_at − probe 2
TSFM
Normalized Probe Intensity
Probe Number
−1
0
1
2
3
1
2
3
4
5
6
7
8
9
10
11
Meishan−A
Meishan−B
Meishan−C
European−A
European−B
European−C

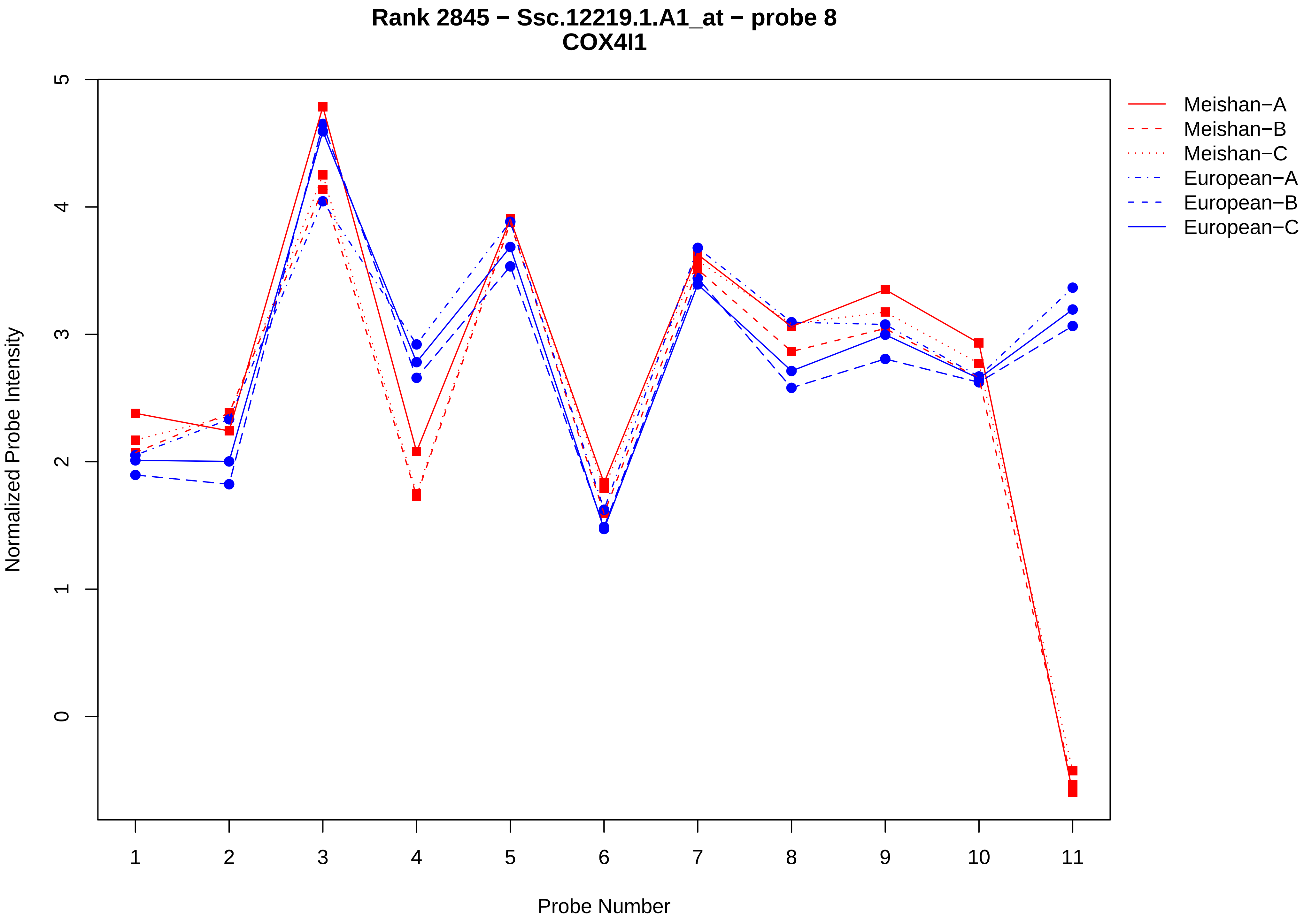

Rank 2845 − Ssc.12219.1.A1_at − probe 8
COX4I1
Normalized Probe Intensity
Probe Number
0
1
2
3
4
5
1
2
3
4
5
6
7
8
9
10
11
Meishan−A
Meishan−B
Meishan−C
European−A
European−B
European−C

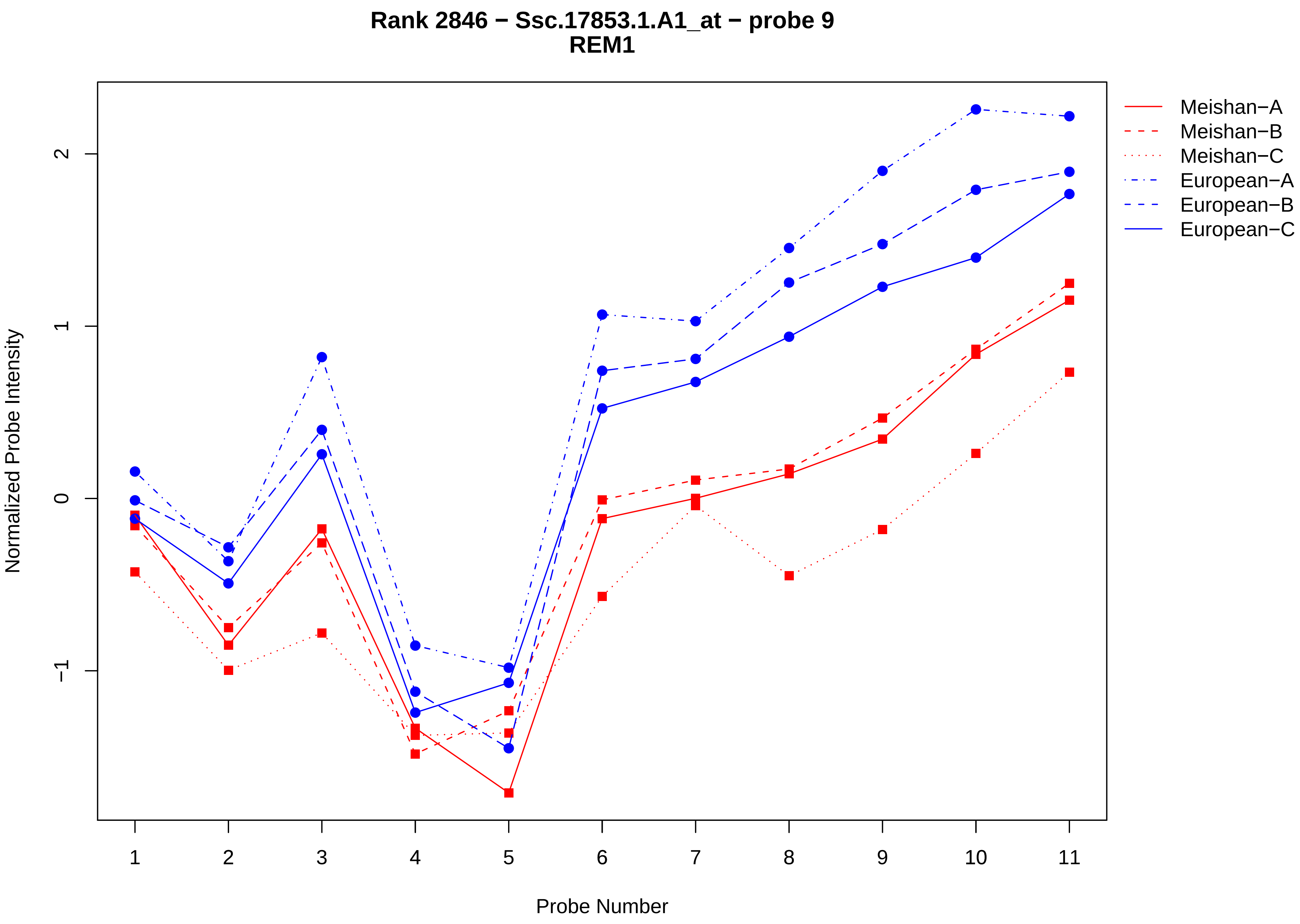

Rank 2846 − Ssc.17853.1.A1_at − probe 9
REM1
Normalized Probe Intensity
Probe Number
−1
0
1
2
1
2
3
4
5
6
7
8
9
10
11
Meishan−A
Meishan−B
Meishan−C
European−A
European−B
European−C

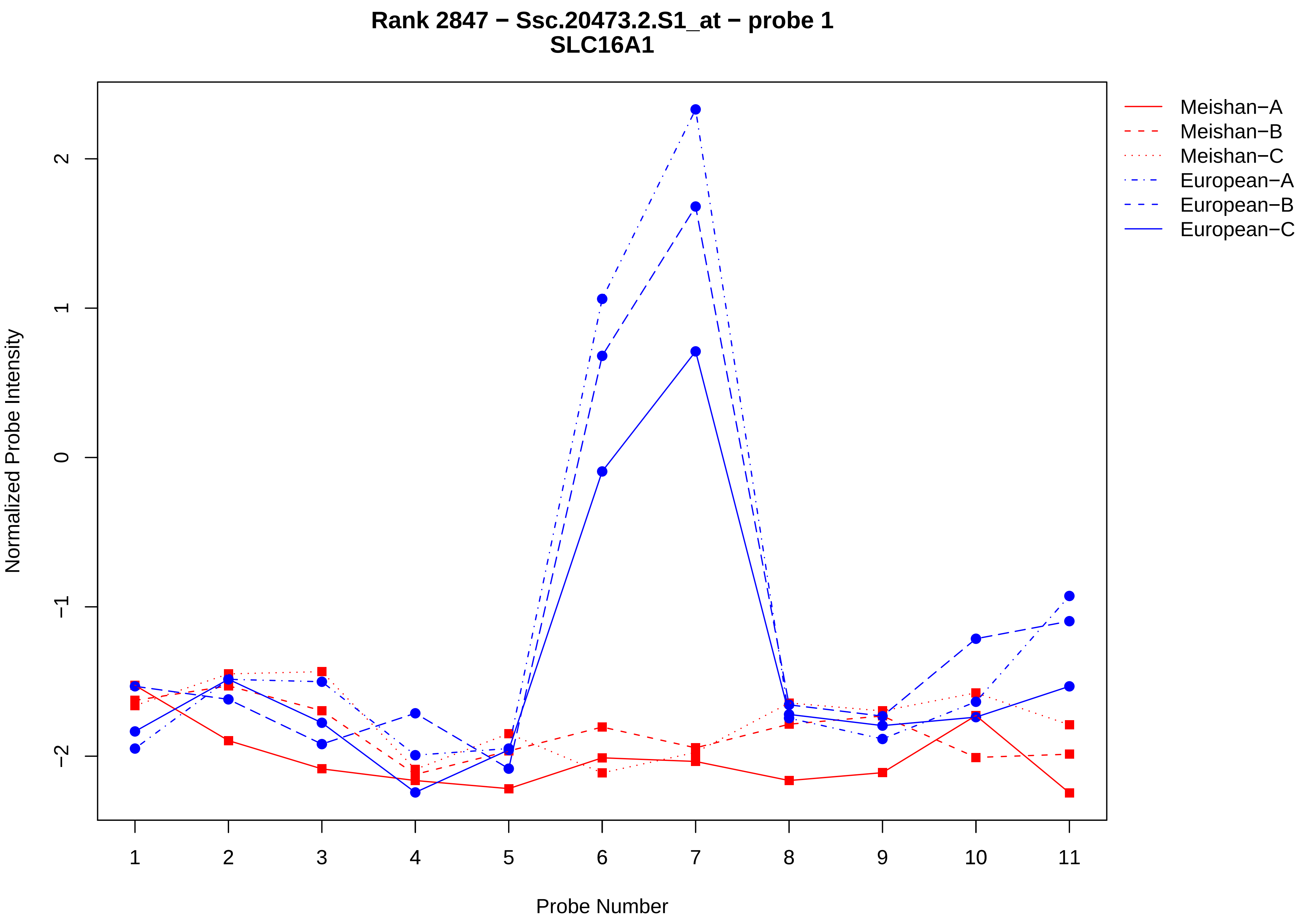
Rank 2847 − Ssc.20473.2.S1_at − probe 1
SLC16A1
Normalized Probe Intensity
Probe Number
−2
−1
0
1
2
1
2
3
4
5
6
7
8
9
10
11
Meishan−A
Meishan−B
Meishan−C
European−A
European−B
European−C

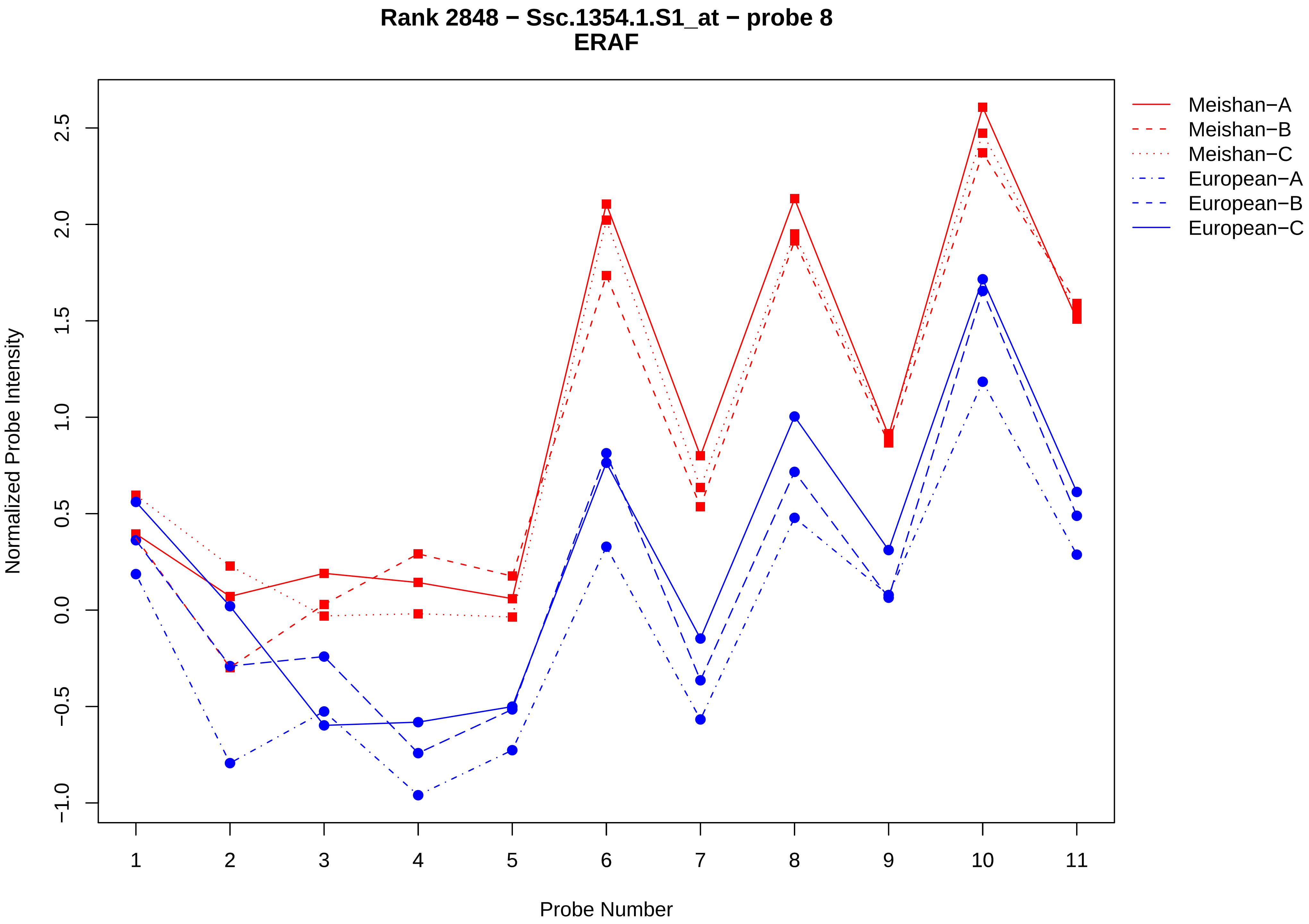
Rank 2848 − Ssc.1354.1.S1_at − probe 8
ERAF
Normalized Probe Intensity
Probe Number
Meishan−A
Meishan−B
Meishan−C
European−A
European−B
European−C
−1.0
−0.5
0.0
0.5
1.0
1.5
2.0
2.5
1
2
3
4
5
6
7
8
9
10
11

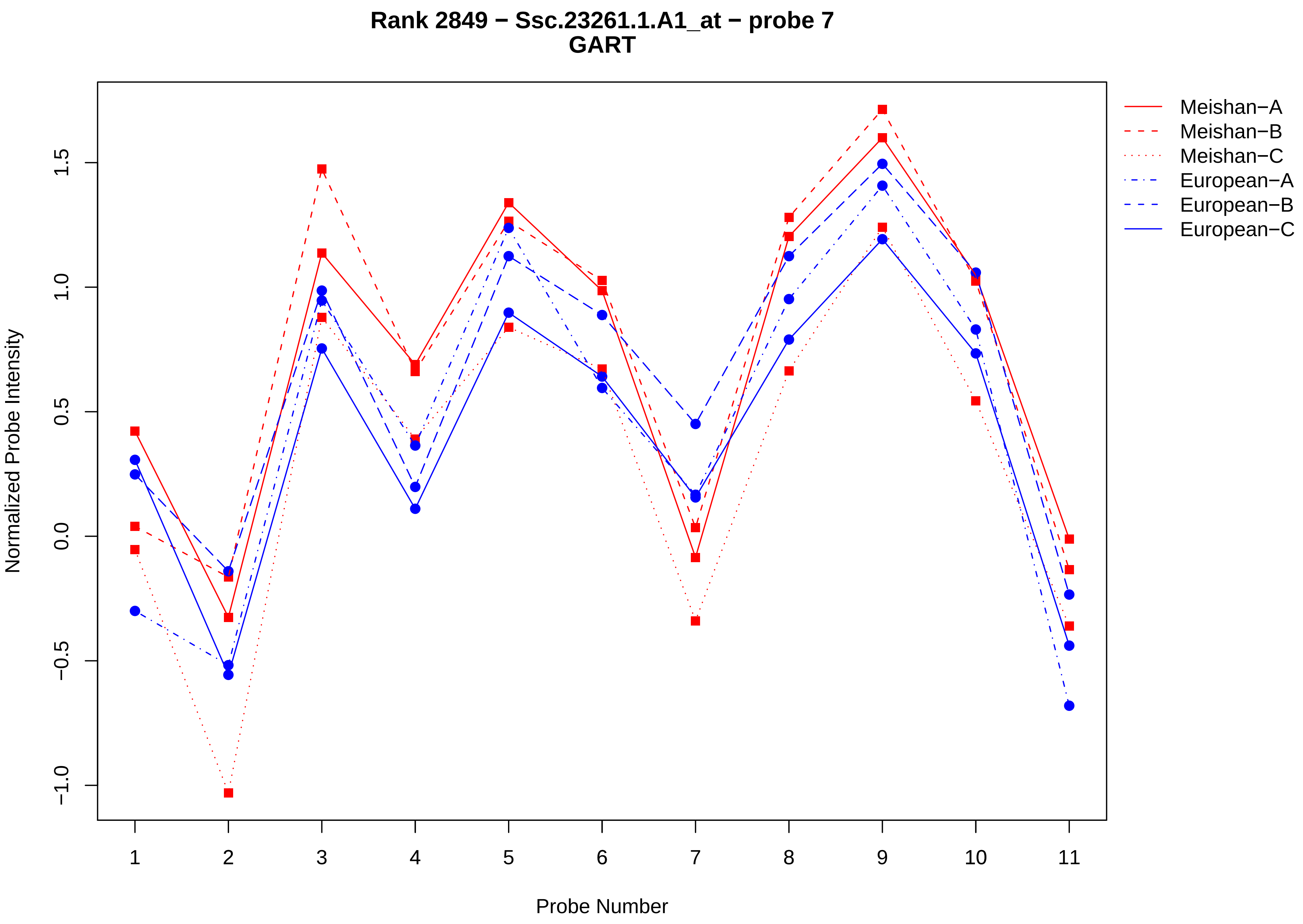

Rank 2849 − Ssc.23261.1.A1_at − probe 7
GART
Meishan−A
Meishan−B
Meishan−C
European−A
European−B
European−C
Normalized Probe Intensity
Probe Number
−1.0
−0.5
0.0
0.5
1.0
1.5
1
2
3
4
5
6
7
8
9
10
11

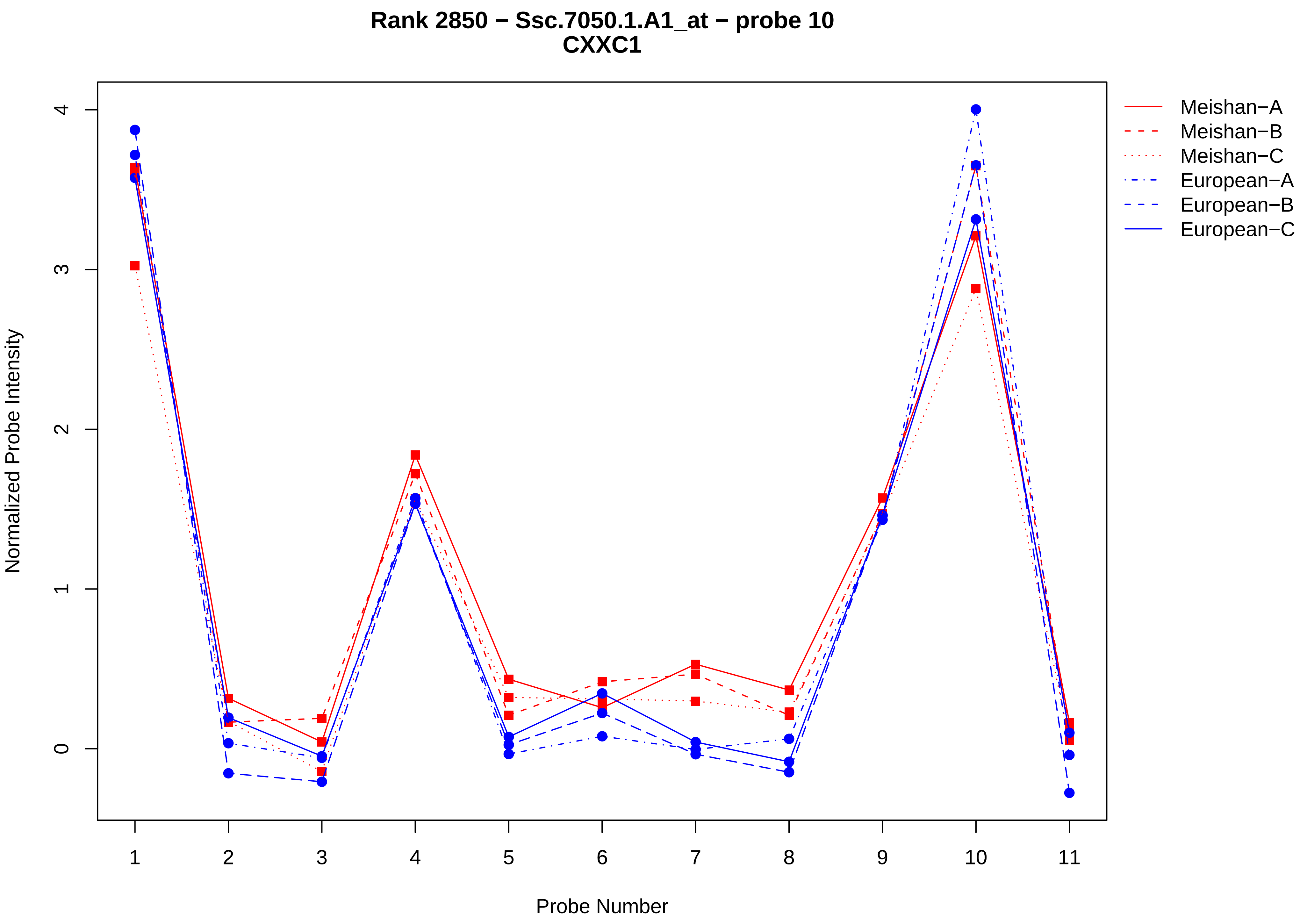

Rank 2850 − Ssc.7050.1.A1_at − probe 10
CXXC1
Normalized Probe Intensity
Probe Number
0
1
2
3
4
1
2
3
4
5
6
7
8
9
10
11
Meishan−A
Meishan−B
Meishan−C
European−A
European−B
European−C

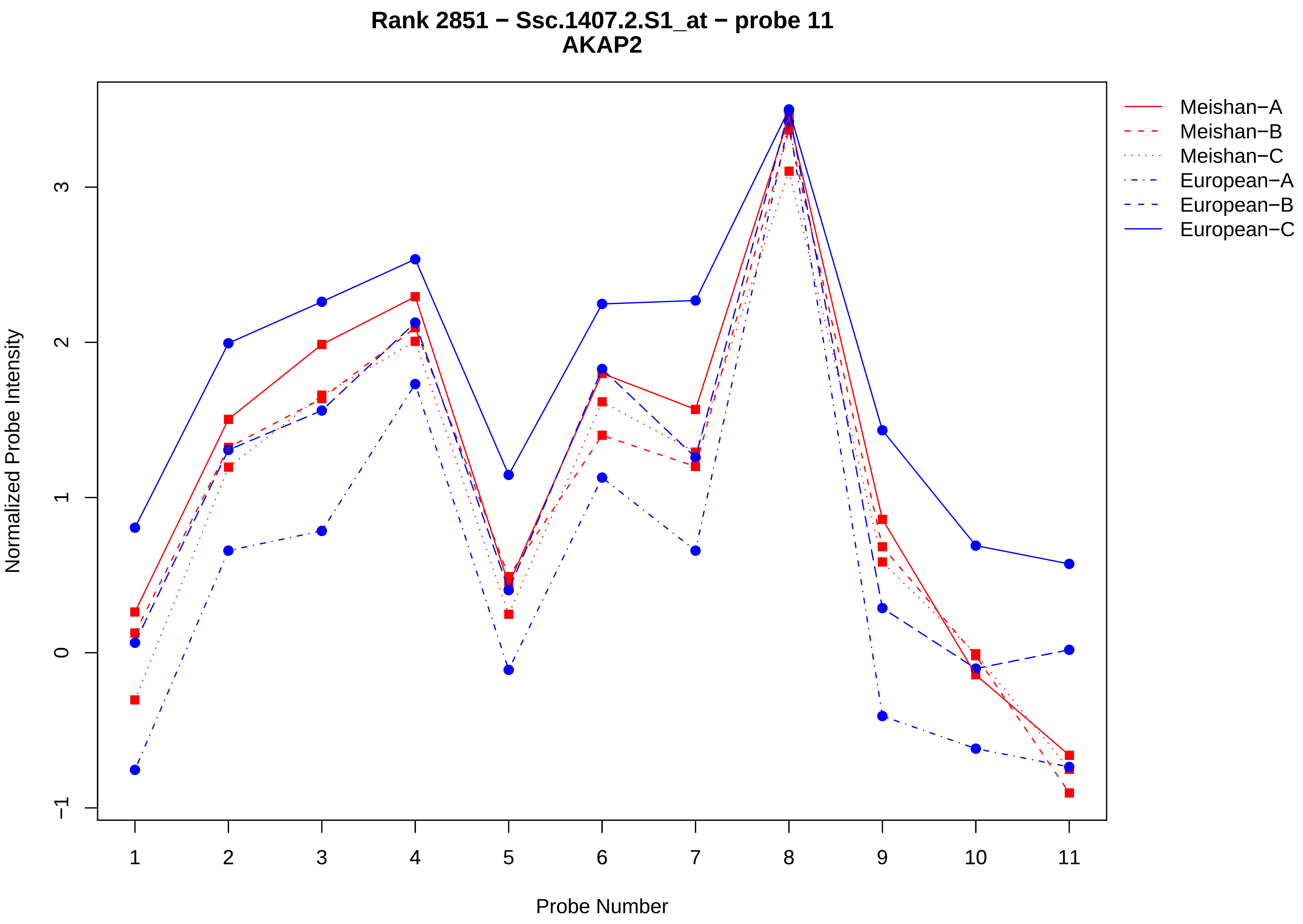

Rank 2851 − Ssc.1407.2.S1_at − probe 11
AKAP2
Normalized Probe Intensity
Probe Number
−1
0
1
2
3
1
2
3
4
5
6
7
8
9
10
11
Meishan−A
Meishan−B
Meishan−C
European−A
European−B
European−C

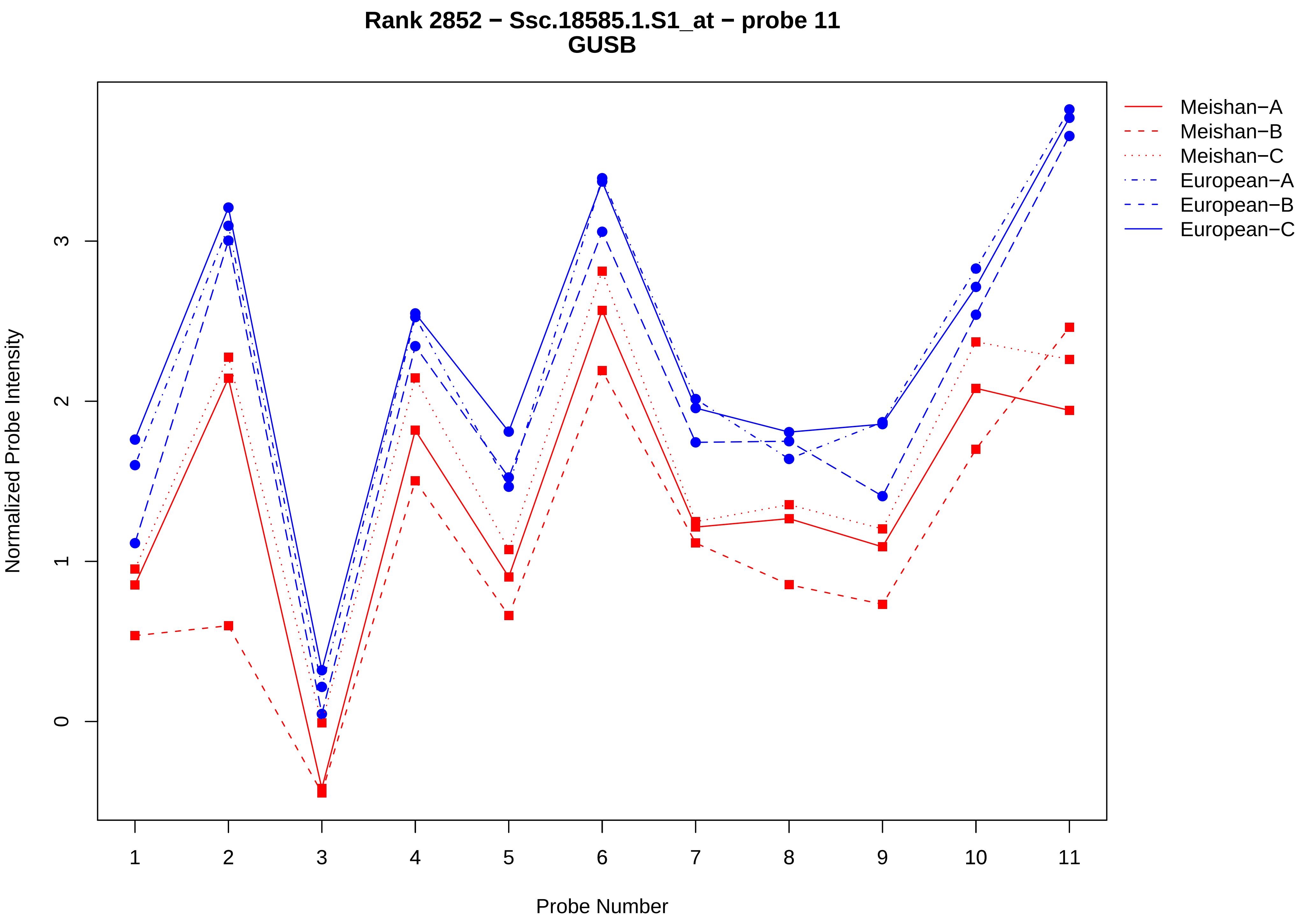

Rank 2852 − Ssc.18585.1.S1_at − probe 11
GUSB
Normalized Probe Intensity
0
1
2
3
1
2
3
4
5
6
7
8
9
10
11
Probe Number
Meishan−A
Meishan−B
Meishan−C
European−A
European−B
European−C

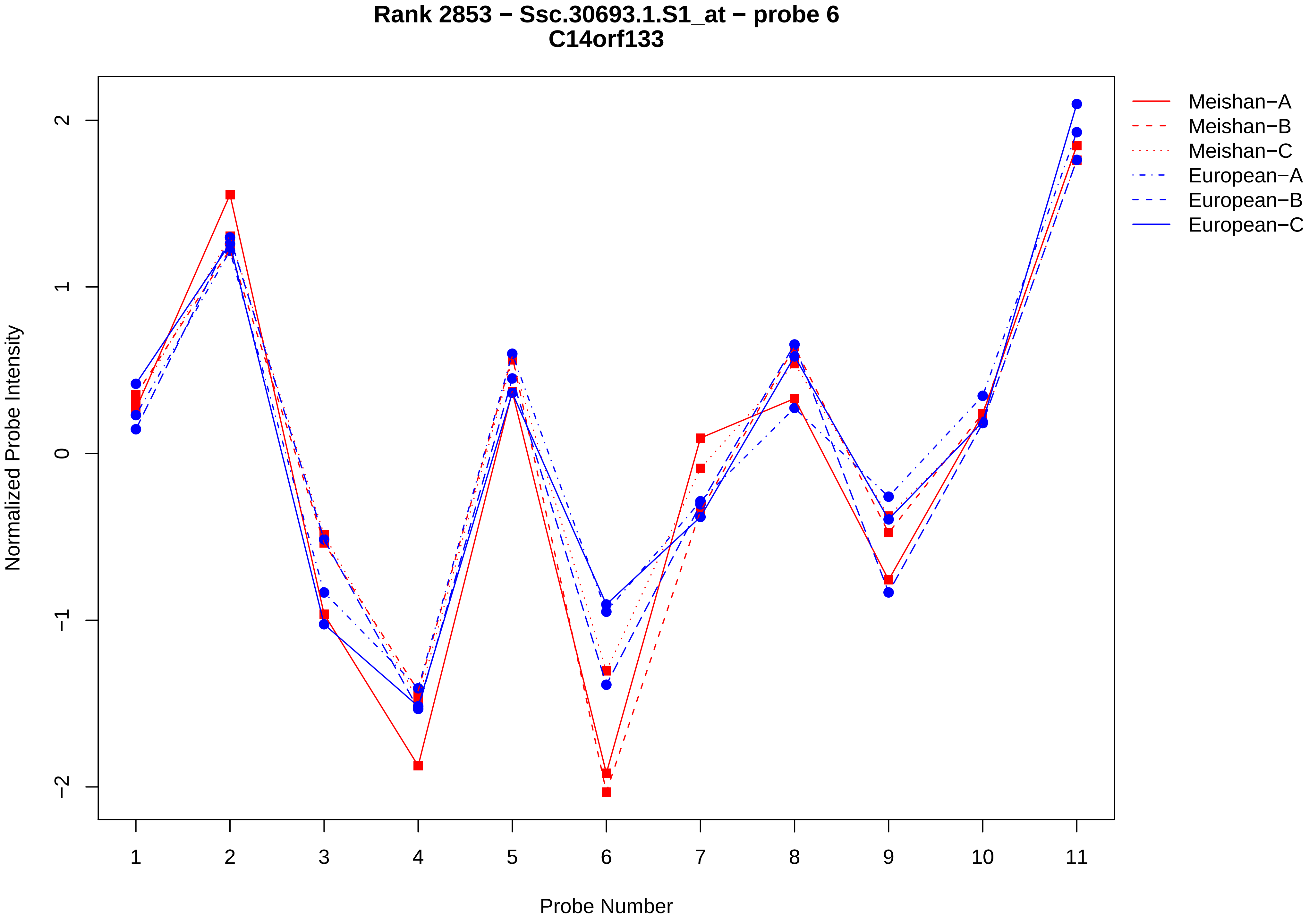
Rank 2853 − Ssc.30693.1.S1_at − probe 6
C14orf133
Normalized Probe Intensity
Probe Number
−2
−1
0
1
2
1
2
3
4
5
6
7
8
9
10
11
Meishan−A
Meishan−B
Meishan−C
European−A
European−B
European−C

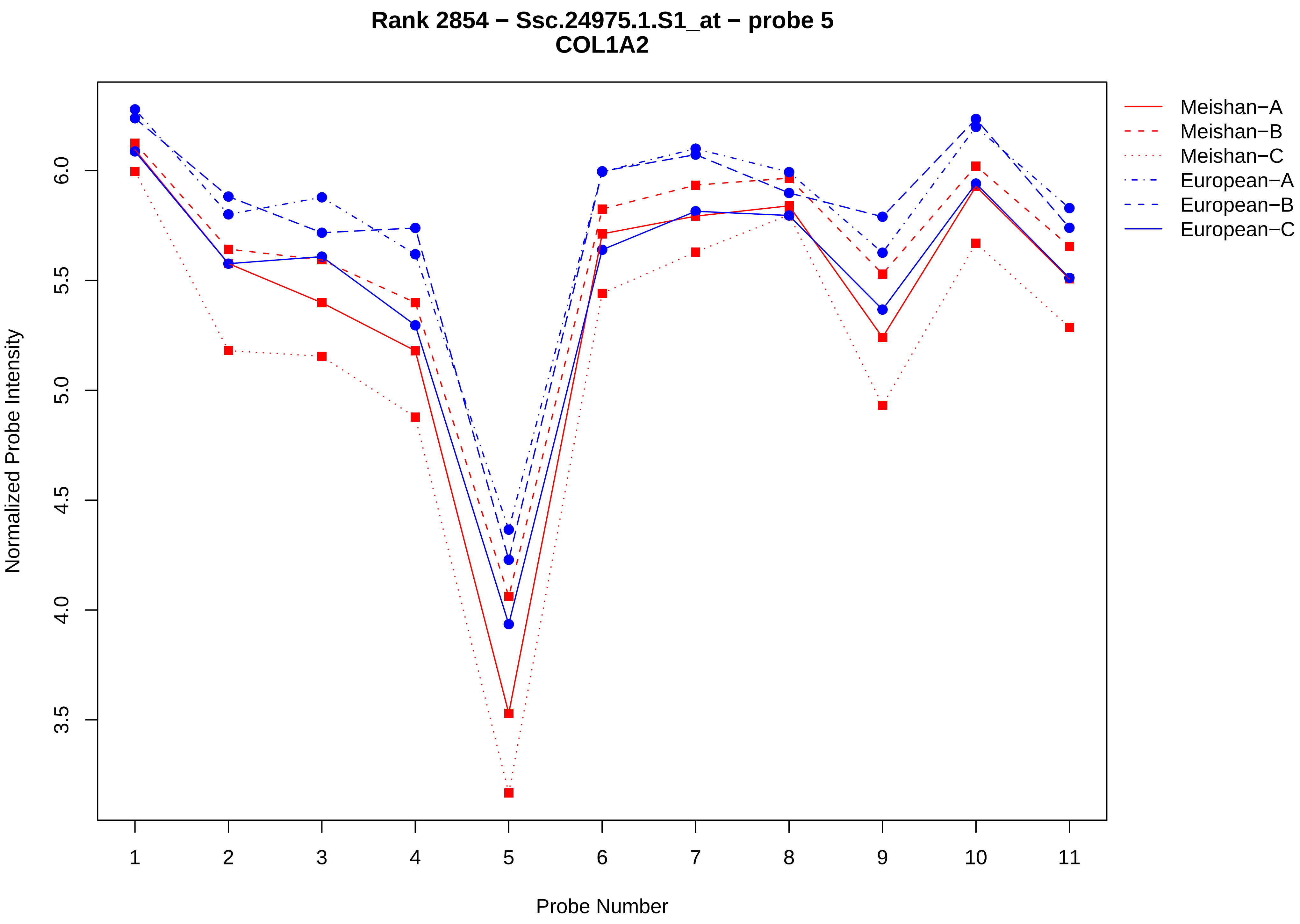
Rank 2854 − Ssc.24975.1.S1_at − probe 5
COL1A2
Normalized Probe Intensity
3.5
4.0
4.5
5.0
5.5
6.0
Probe Number
1
2
3
4
5
6
7
8
9
10
11
Meishan−A
Meishan−B
Meishan−C
European−A
European−B
European−C

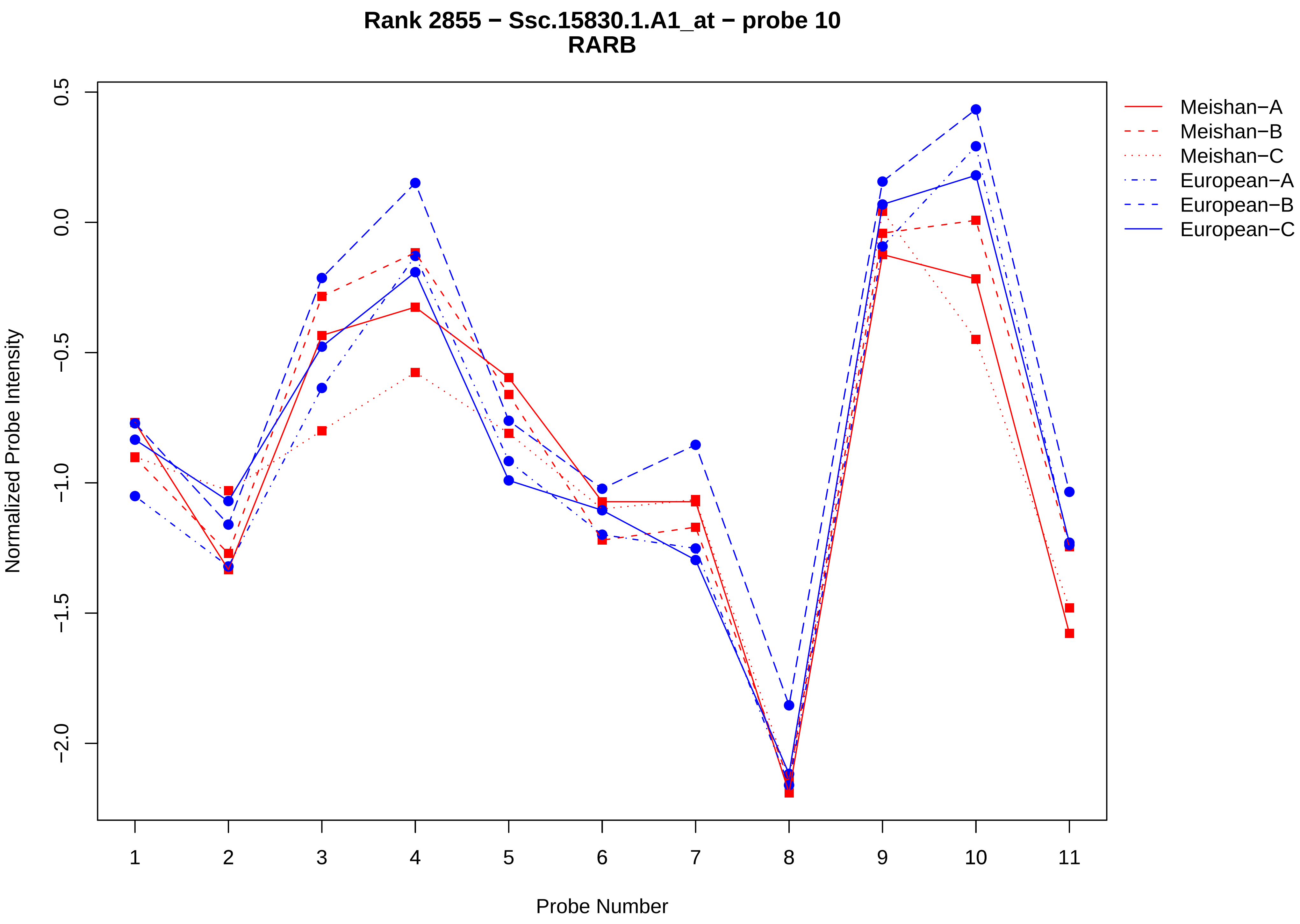

Rank 2855 − Ssc.15830.1.A1_at − probe 10
RARB
Normalized Probe Intensity
Probe Number
0.5
0.0
−0.5
−1.0
−1.5
−2.0
1
2
3
4
5
6
7
8
9
10
11
Meishan−A
Meishan−B
Meishan−C
European−A
European−B
European−C

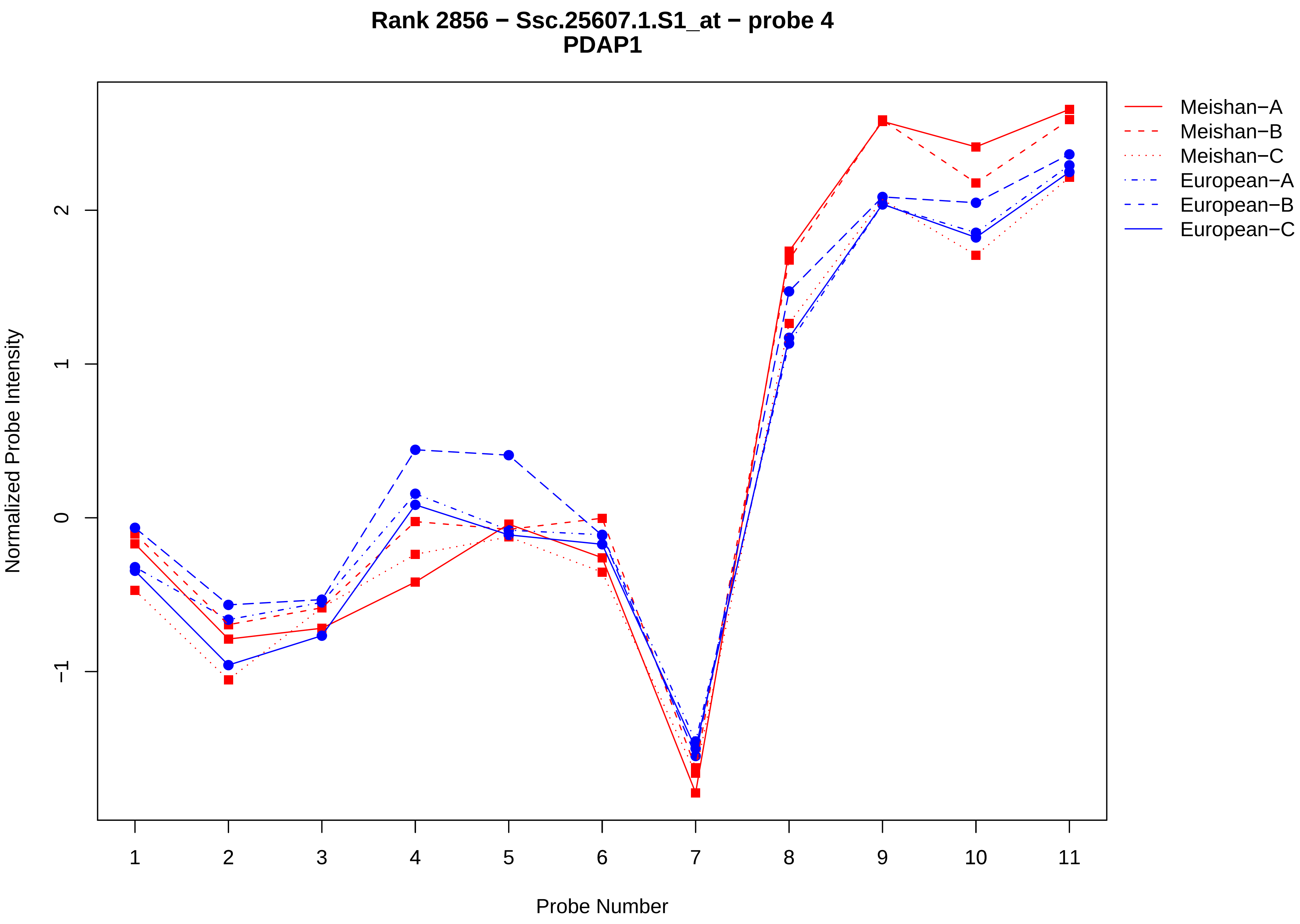

Rank 2856 − Ssc.25607.1.S1_at − probe 4
PDAP1
Normalized Probe Intensity
Probe Number
−1
0
1
2
1
2
3
4
5
6
7
8
9
10
11
Meishan−A
Meishan−B
Meishan−C
European−A
European−B
European−C

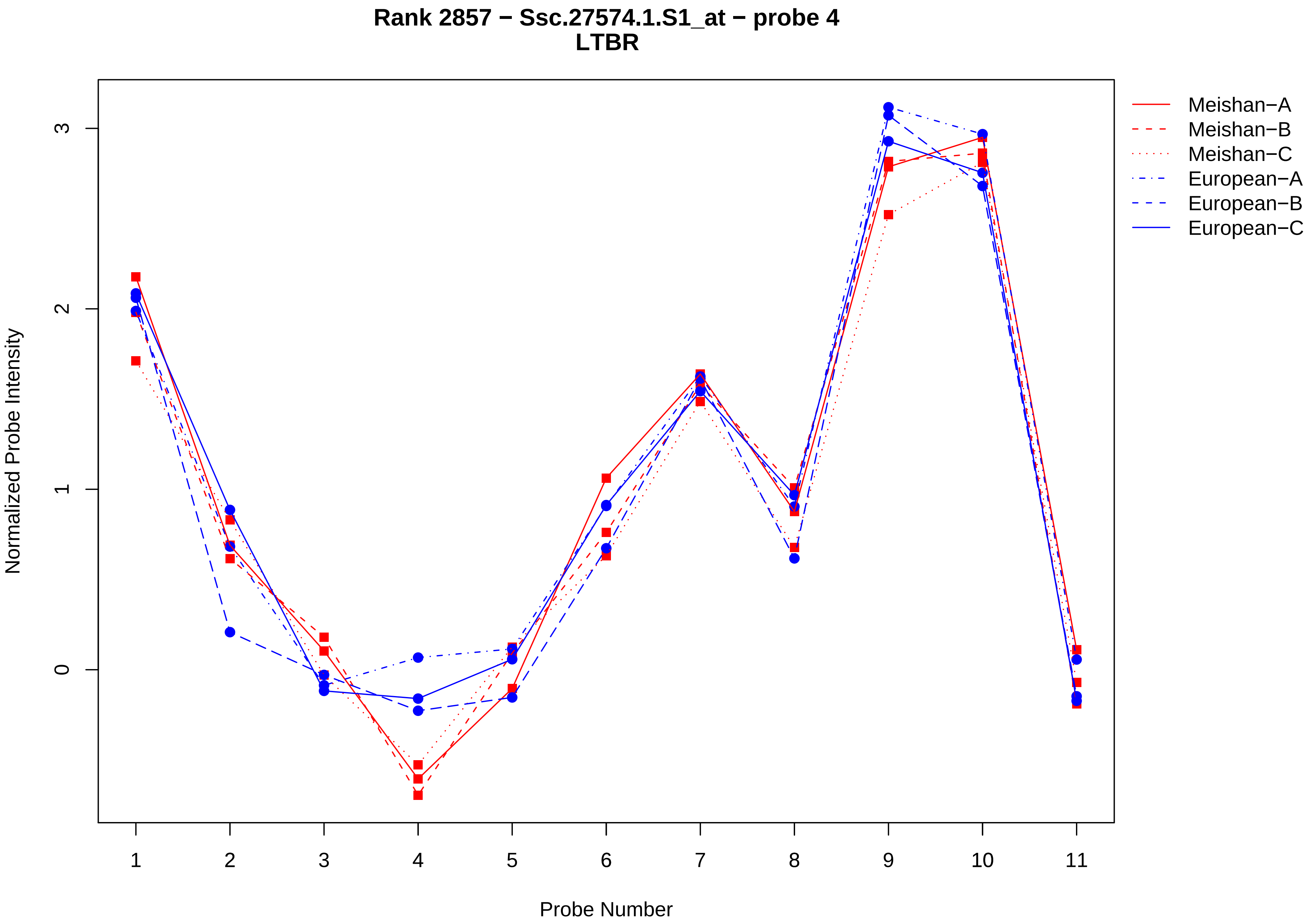

Rank 2857 − Ssc.27574.1.S1_at − probe 4
LTBR
Normalized Probe Intensity
Probe Number
Meishan−A
Meishan−B
Meishan−C
European−A
European−B
European−C
0
1
2
3
1
2
3
4
5
6
7
8
9
10
11

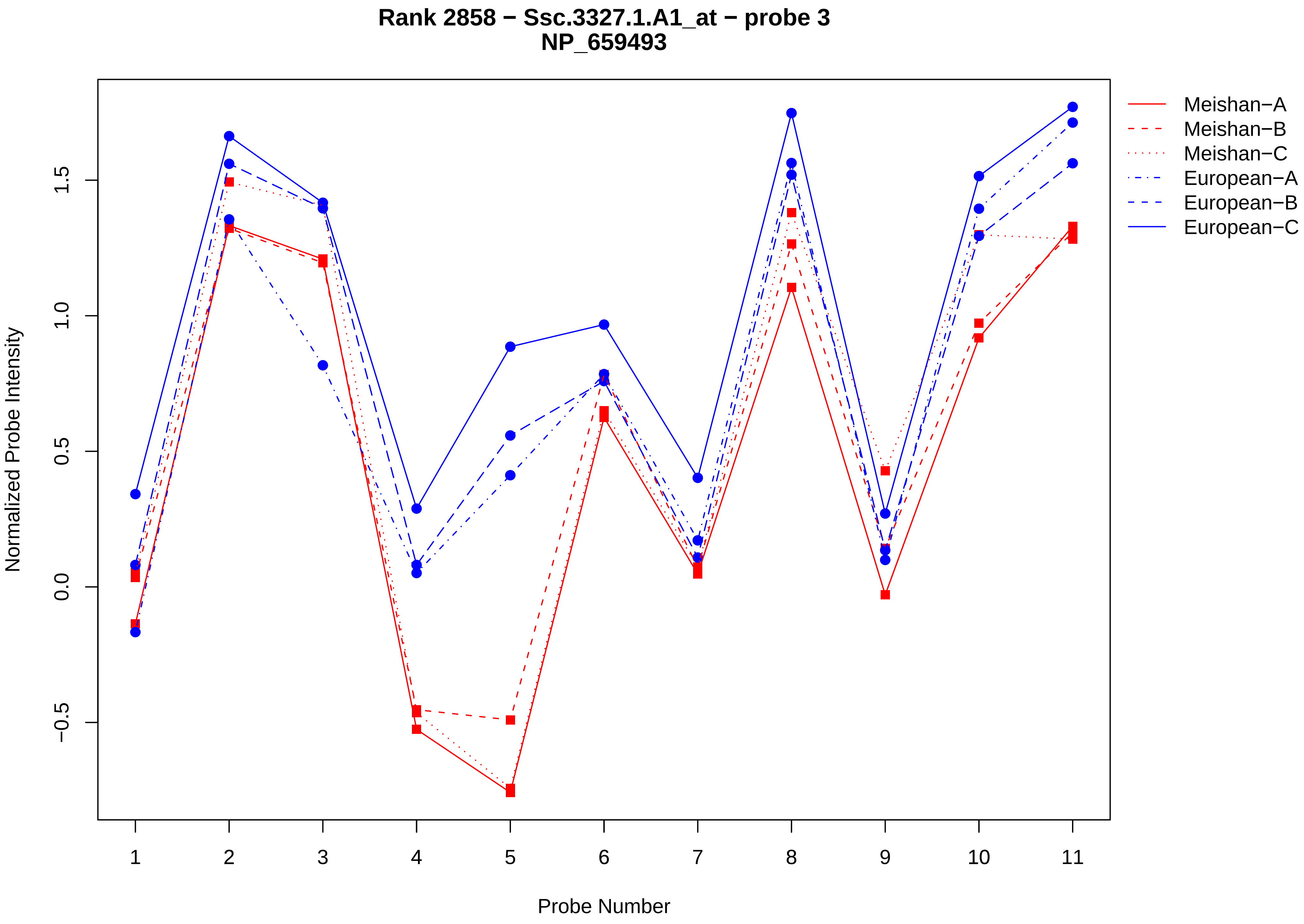

Rank 2858 − Ssc.3327.1.A1_at − probe 3
NP_659493
Normalized Probe Intensity
Probe Number
−0.5
0.0
0.5
1.0
1.5
1
2
3
4
5
6
7
8
9
10
11
Meishan−A
Meishan−B
Meishan−C
European−A
European−B
European−C

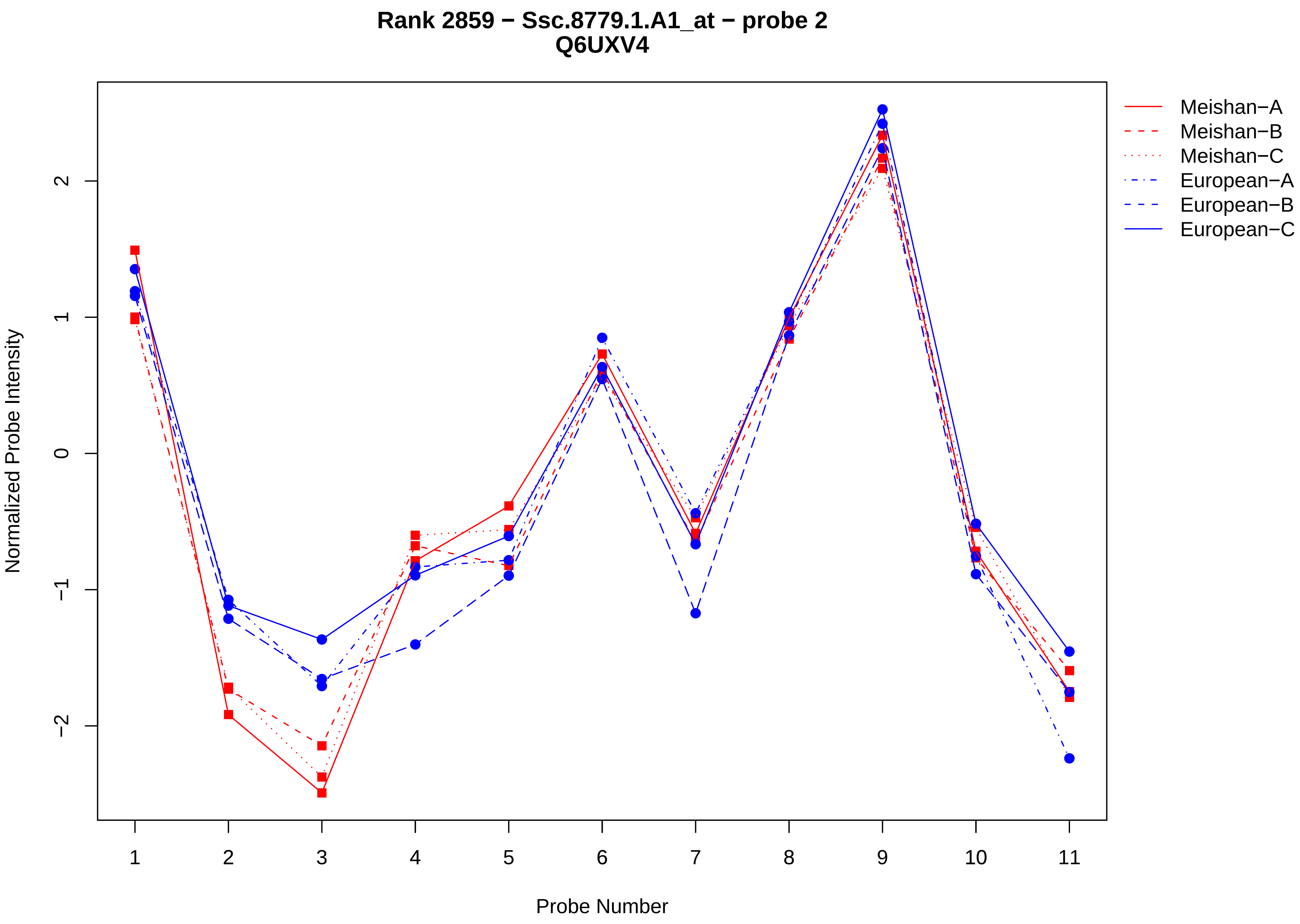

Rank 2859 − Ssc.8779.1.A1_at − probe 2
Q6UXV4
Normalized Probe Intensity
Probe Number
−2
−1
0
1
2
1
2
3
4
5
6
7
8
9
10
11
Meishan−A
Meishan−B
Meishan−C
European−A
European−B
European−C

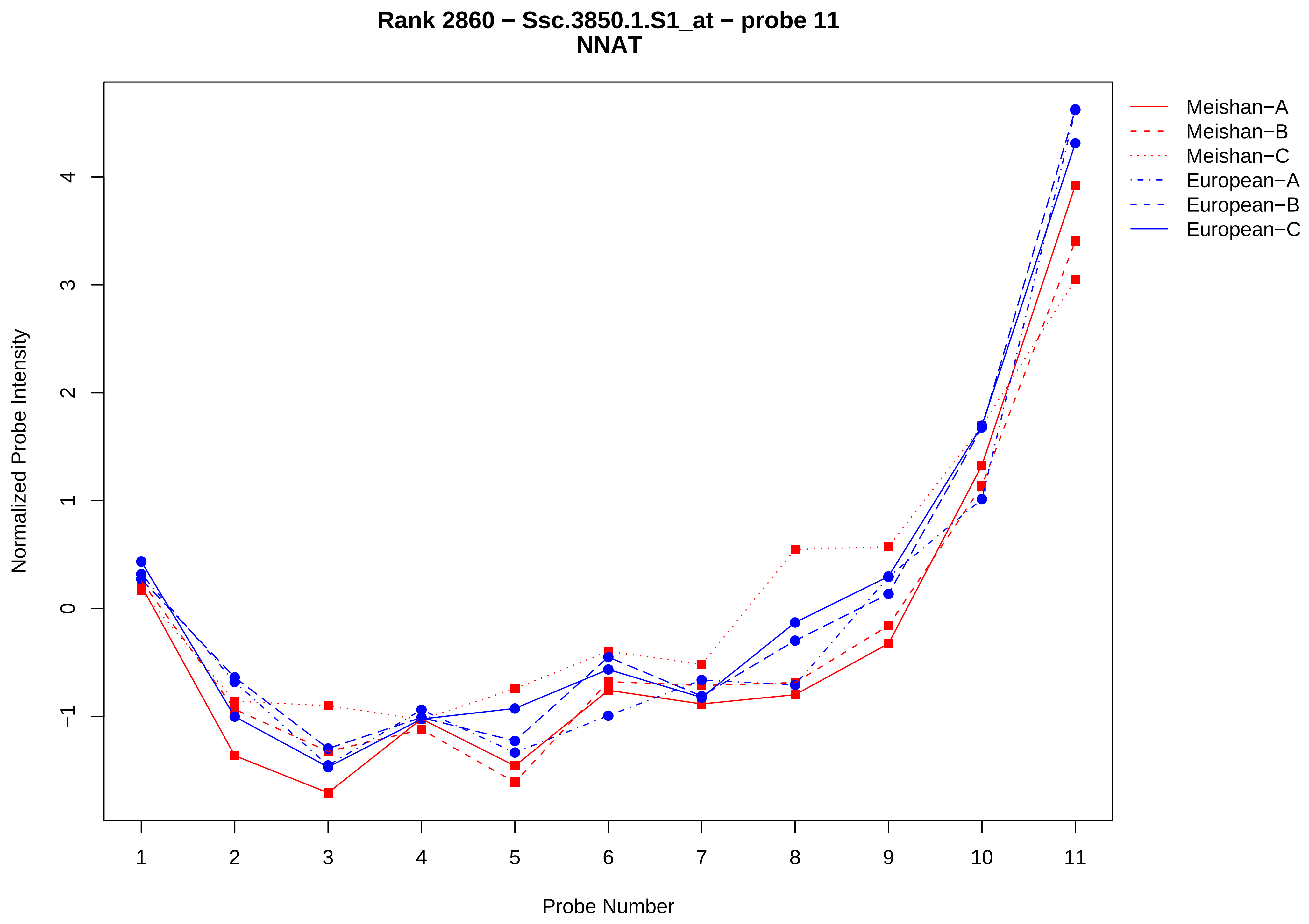

Rank 2860 − Ssc.3850.1.S1_at − probe 11
NNAT
Normalized Probe Intensity
Probe Number
Meishan−A
Meishan−B
Meishan−C
European−A
European−B
European−C

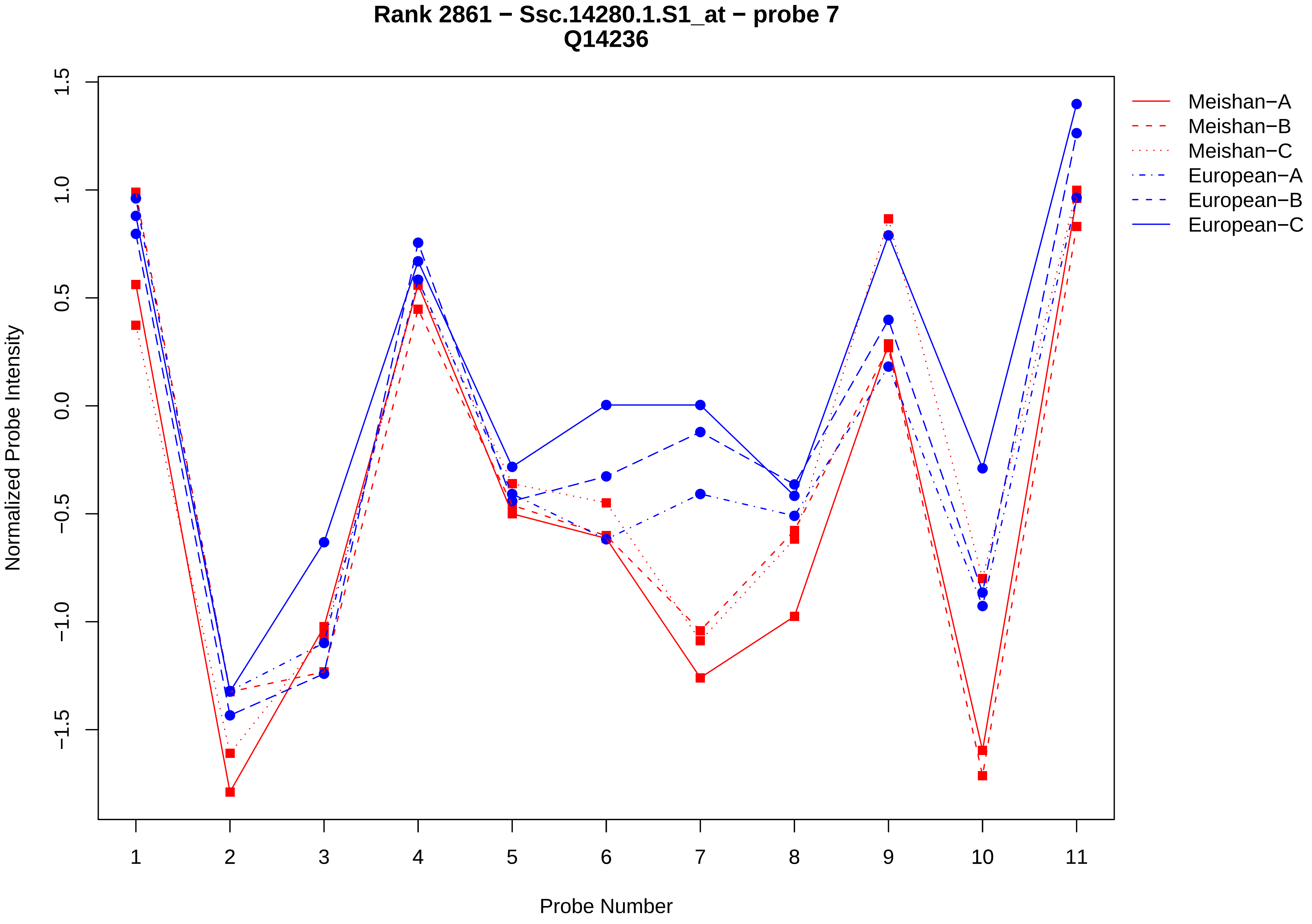
Rank 2861 − Ssc.14280.1.S1_at − probe 7
Q14236
Normalized Probe Intensity
Probe Number
Meishan−A
Meishan−B
Meishan−C
European−A
European−B
European−C
1.5
1.0
0.5
0.0
−0.5
−1.0
−1.5
1
2
3
4
5
6
7
8
9
10
11

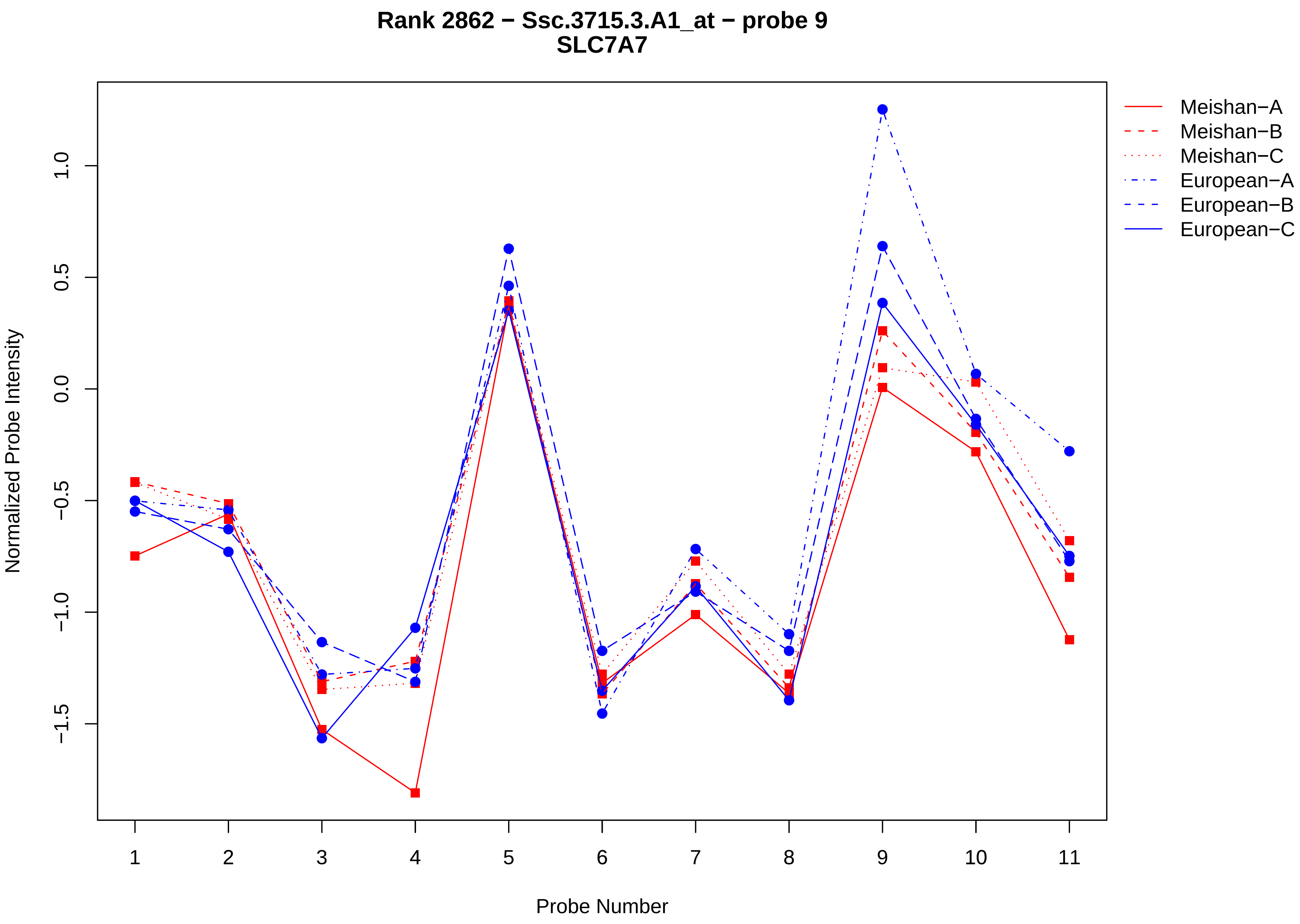

Rank 2862 − Ssc.3715.3.A1_at − probe 9
SLC7A7
Normalized Probe Intensity
Probe Number
1.0
0.5
0.0
−0.5
−1.0
−1.5
1
2
3
4
5
6
7
8
9
10
11
Meishan−A
Meishan−B
Meishan−C
European−A
European−B
European−C

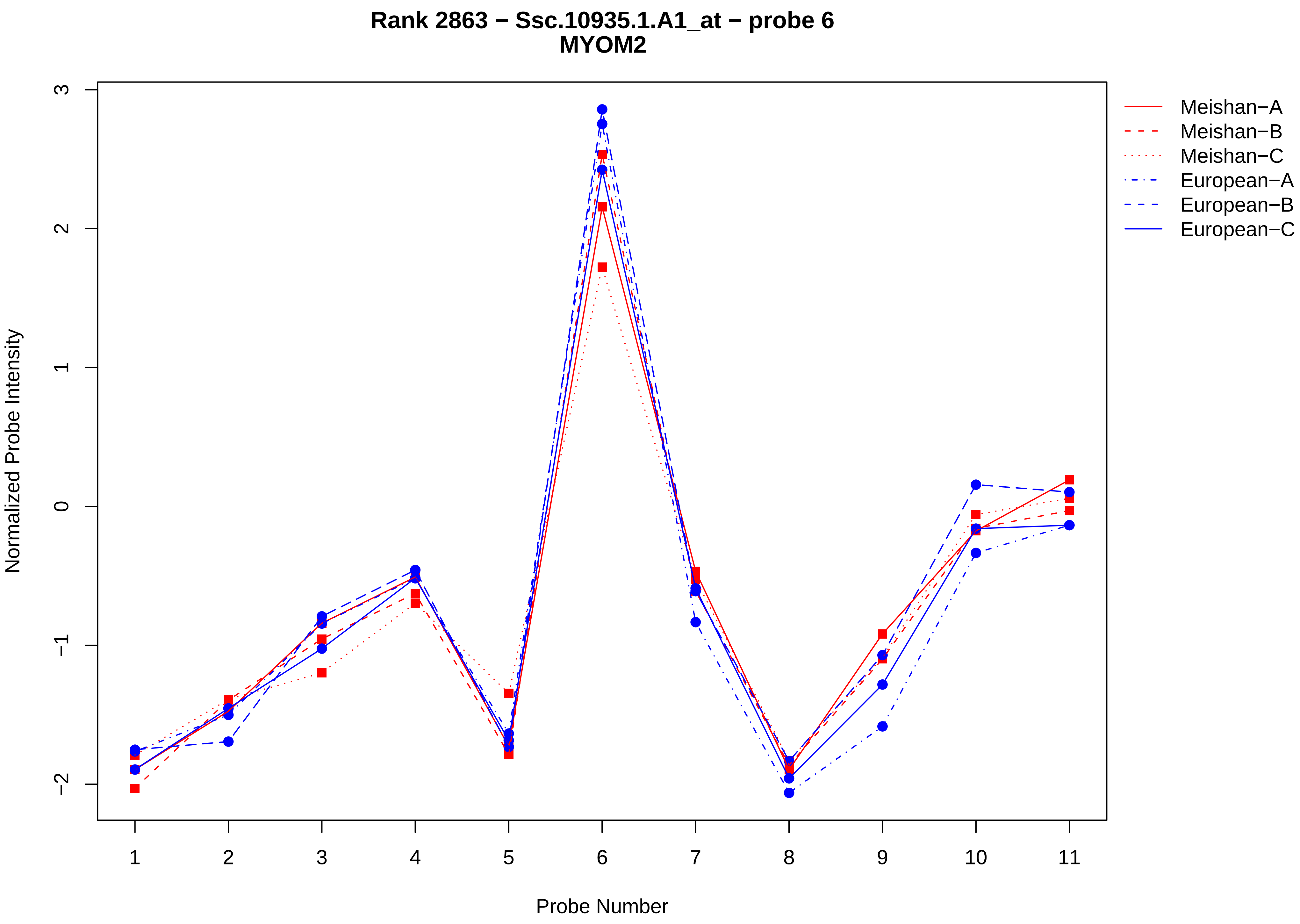

Rank 2863 − Ssc.10935.1.A1_at − probe 6
MYOM2
Normalized Probe Intensity
Probe Number
Meishan−A
Meishan−B
Meishan−C
European−A
European−B
European−C

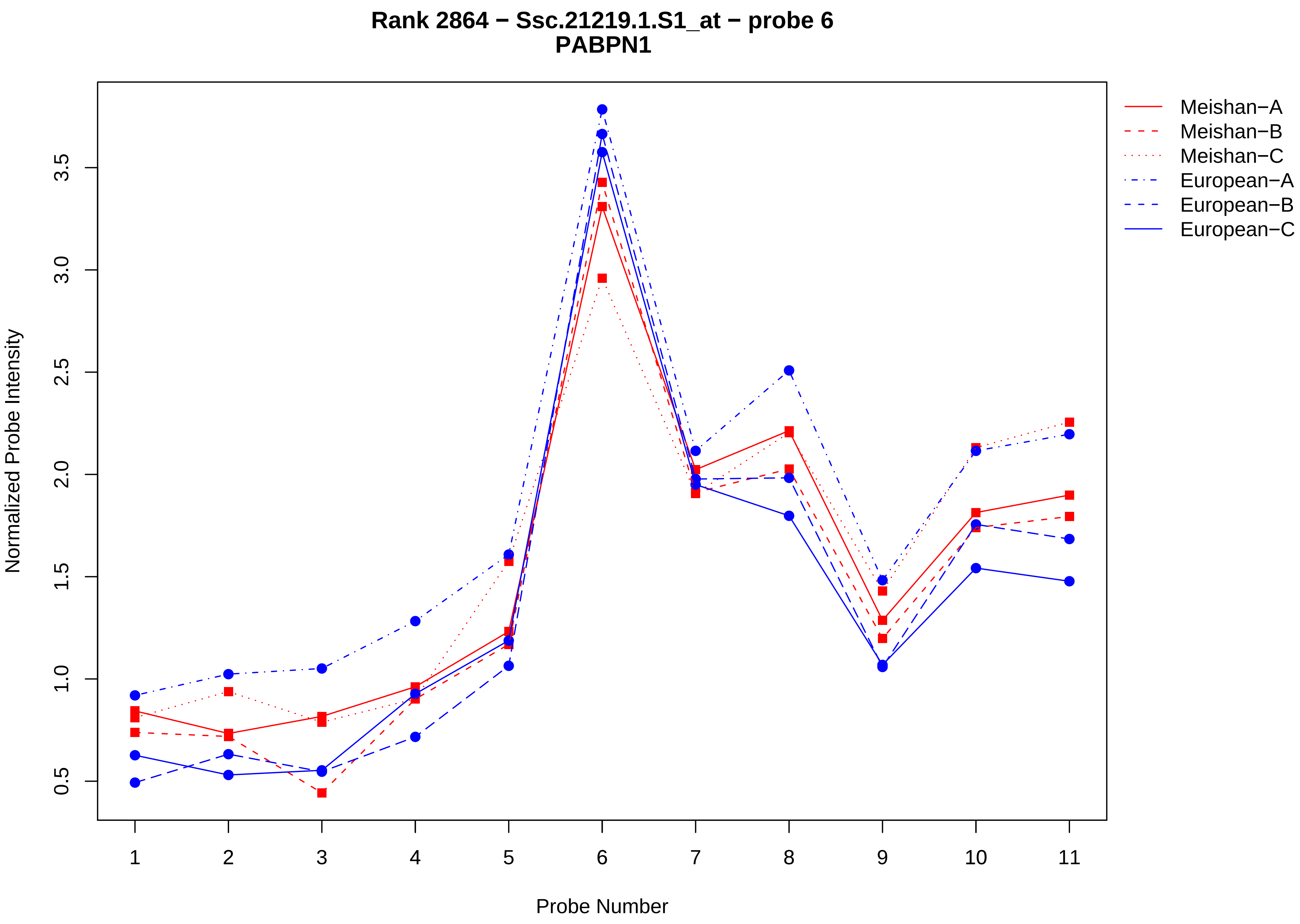

Rank 2864 − Ssc.21219.1.S1_at − probe 6
PABPN1
Normalized Probe Intensity
0.5
1.0
1.5
2.0
2.5
3.0
3.5
Probe Number
1
2
3
4
5
6
7
8
9
10
11
Meishan−A
Meishan−B
Meishan−C
European−A
European−B
European−C

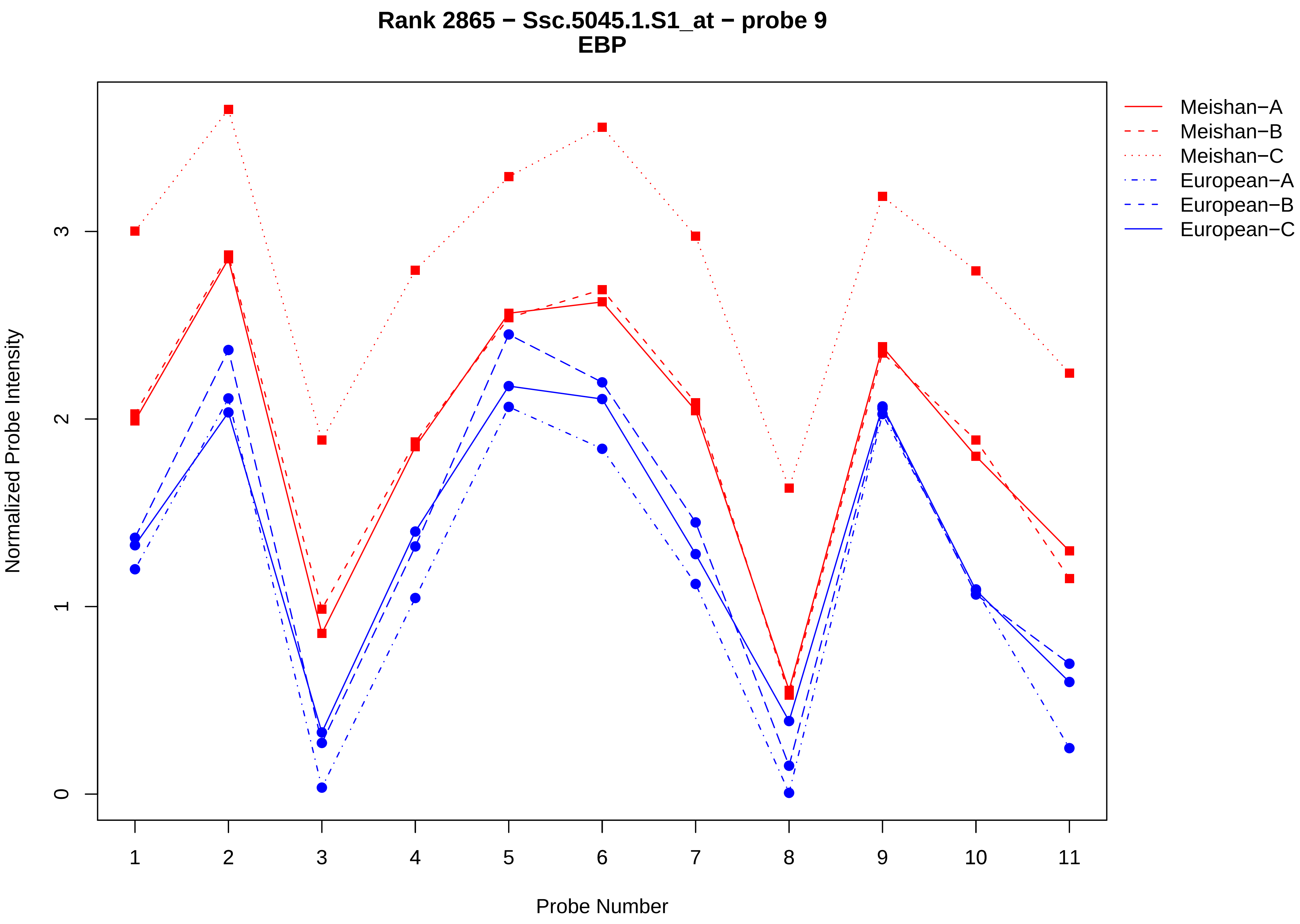

Rank 2865 − Ssc.5045.1.S1_at − probe 9
EBP
Normalized Probe Intensity
Probe Number
0
1
2
3
1
2
3
4
5
6
7
8
9
10
11
Meishan−A
Meishan−B
Meishan−C
European−A
European−B
European−C

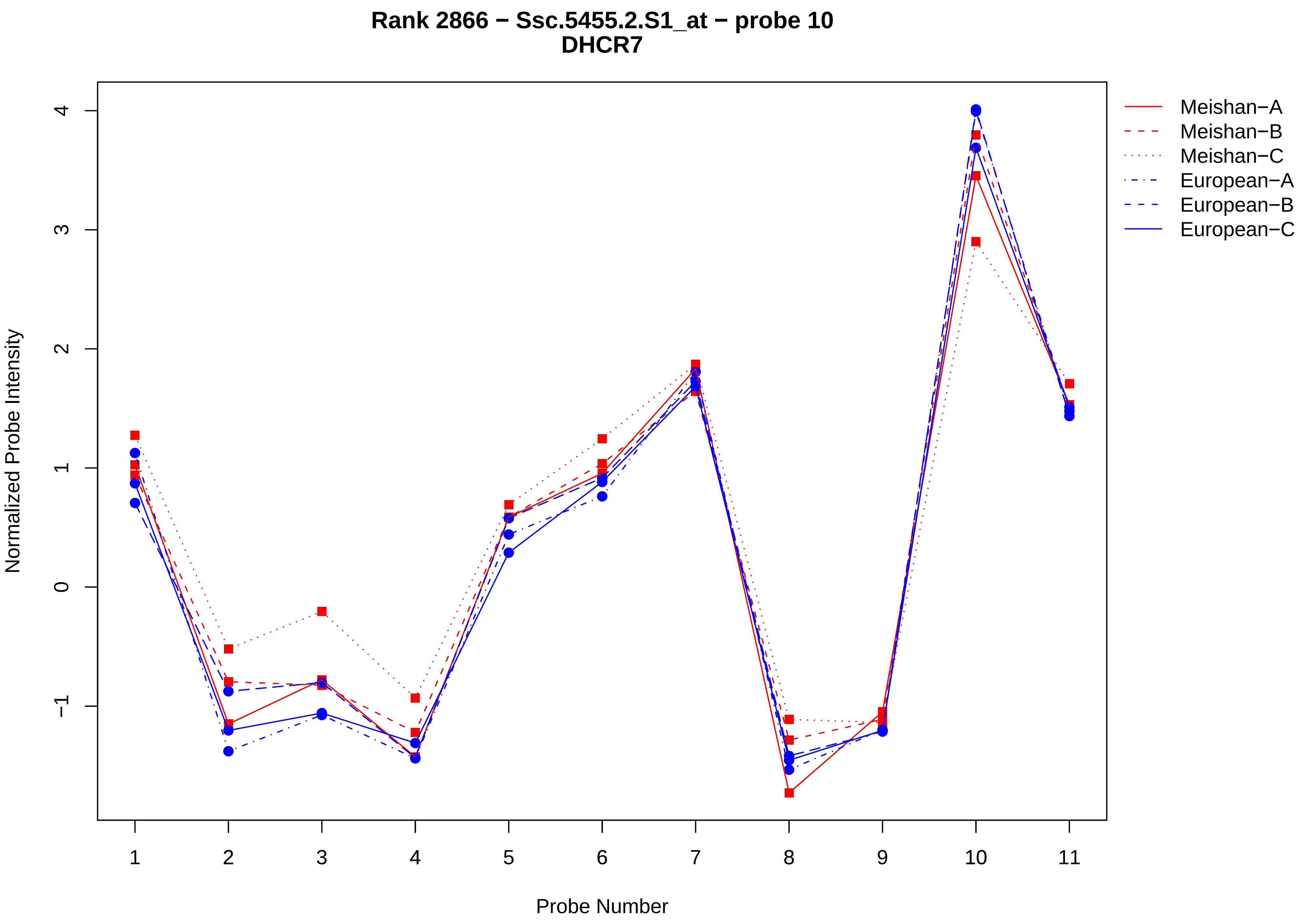

Rank 2866 − Ssc.5455.2.S1_at − probe 10
DHCR7
Normalized Probe Intensity
Probe Number
4
3
2
1
0
−1
1
2
3
4
5
6
7
8
9
10
11
Meishan−A
Meishan−B
Meishan−C
European−A
European−B
European−C

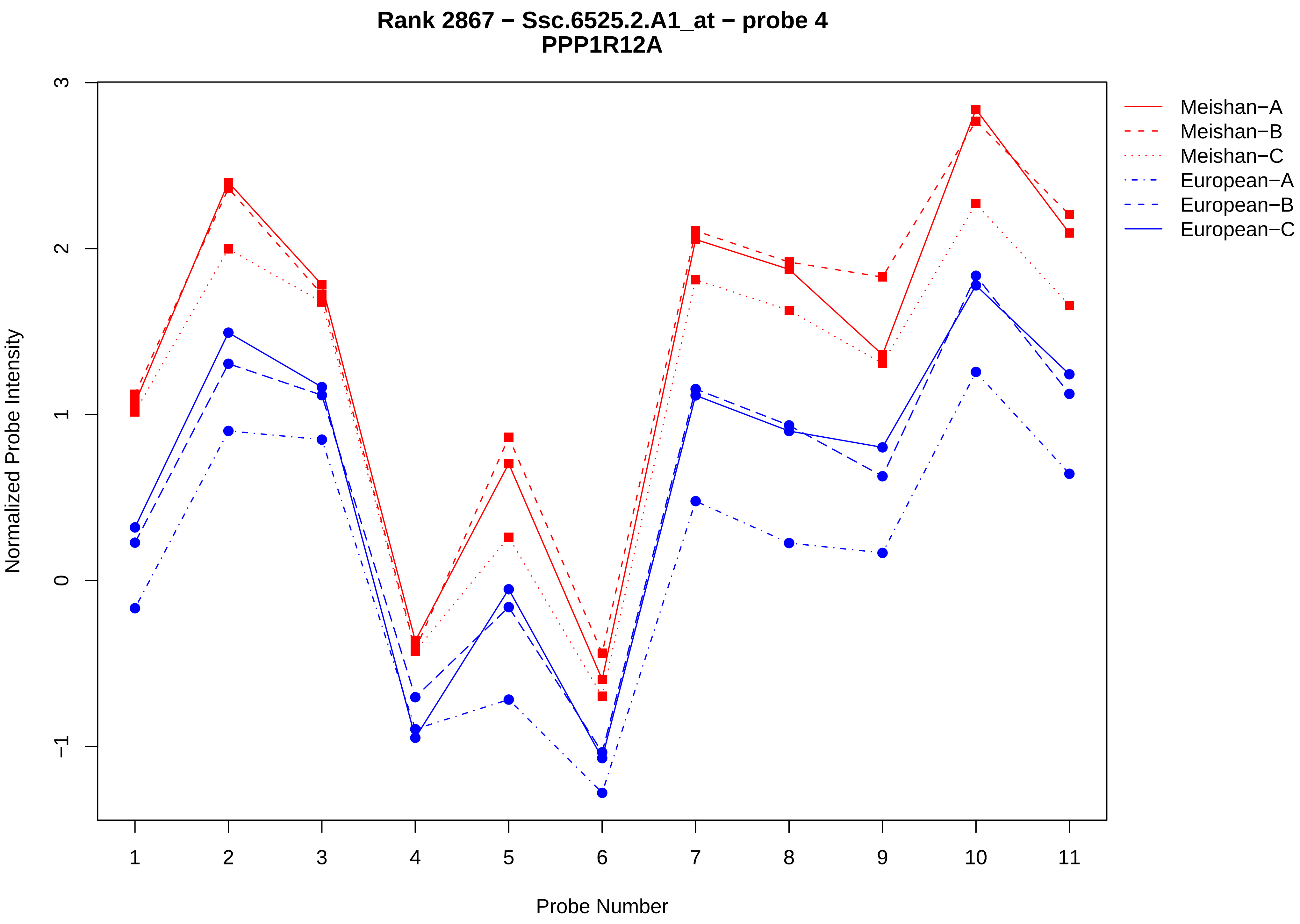

Rank 2867 − Ssc.6525.2.A1_at − probe 4
PPP1R12A
Normalized Probe Intensity
Probe Number
−1
0
1
2
3
1
2
3
4
5
6
7
8
9
10
11
Meishan−A
Meishan−B
Meishan−C
European−A
European−B
European−C

Rank 2868 − Ssc.23301.1.S1_at − probe 8
SLC39A1

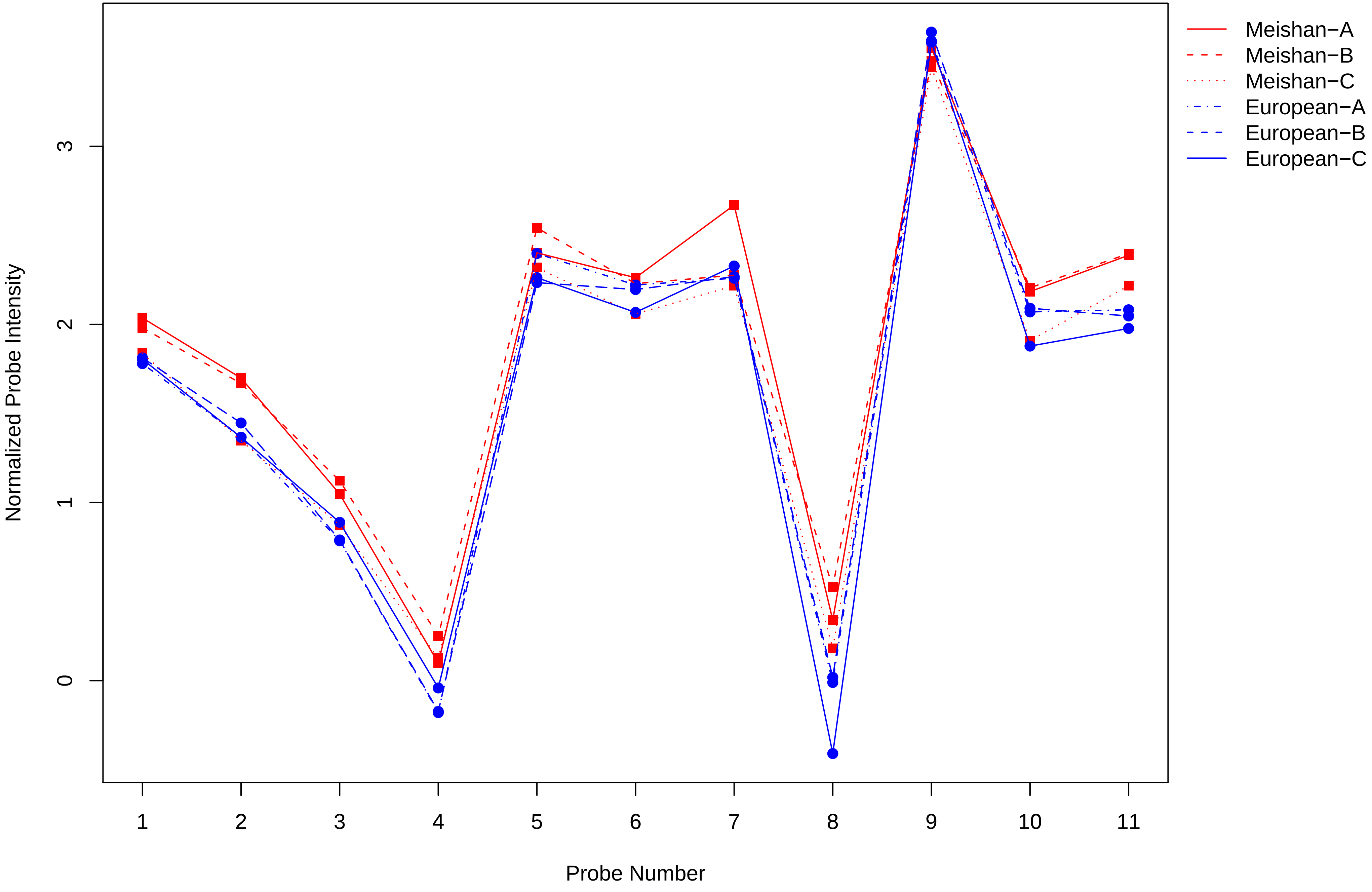

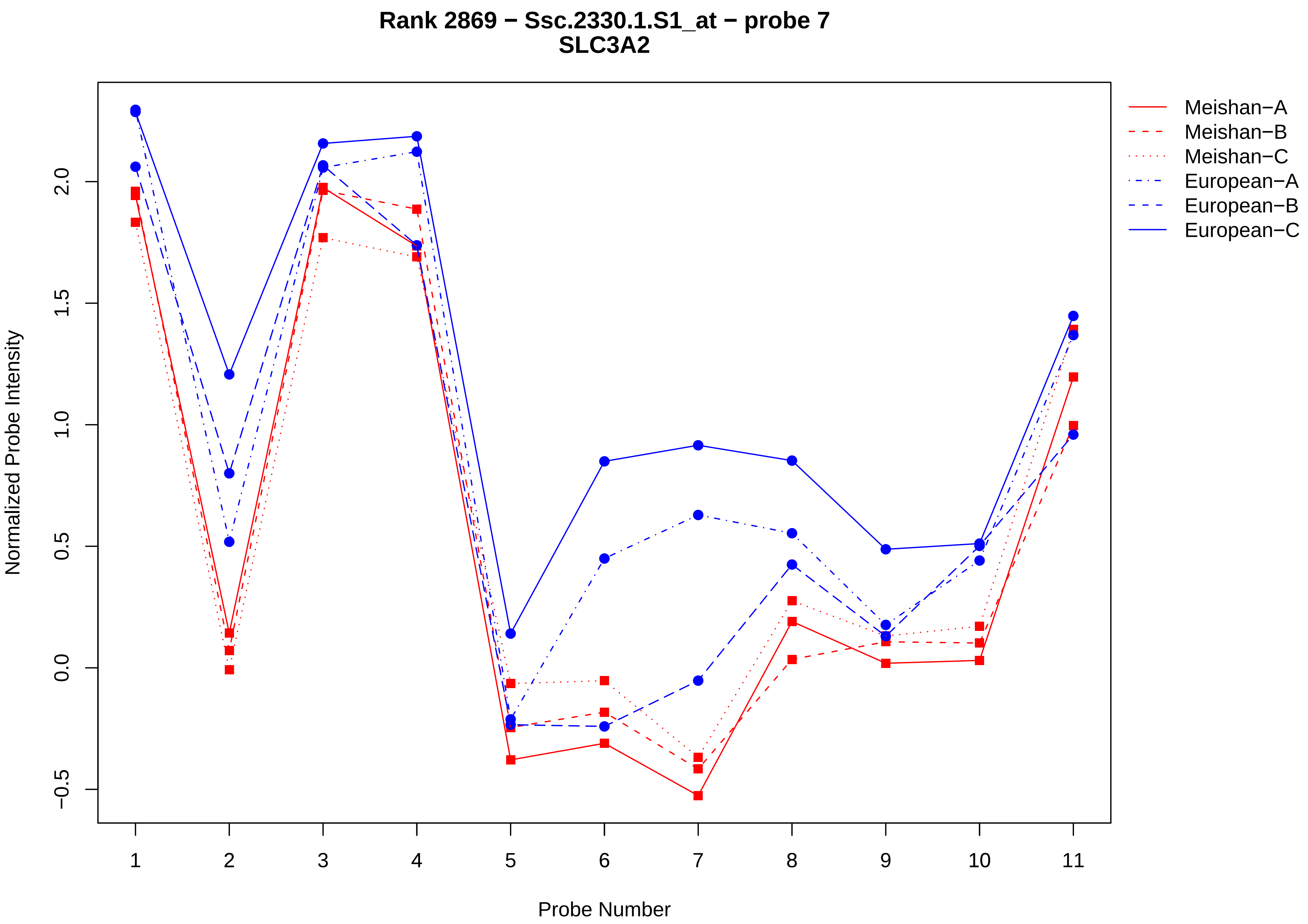

Rank 2869 − Ssc.2330.1.S1_at − probe 7
SLC3A2
Normalized Probe Intensity
Probe Number
−0.5
0.0
0.5
1.0
1.5
2.0
1
2
3
4
5
6
7
8
9
10
11
Meishan−A
Meishan−B
Meishan−C
European−A
European−B
European−C

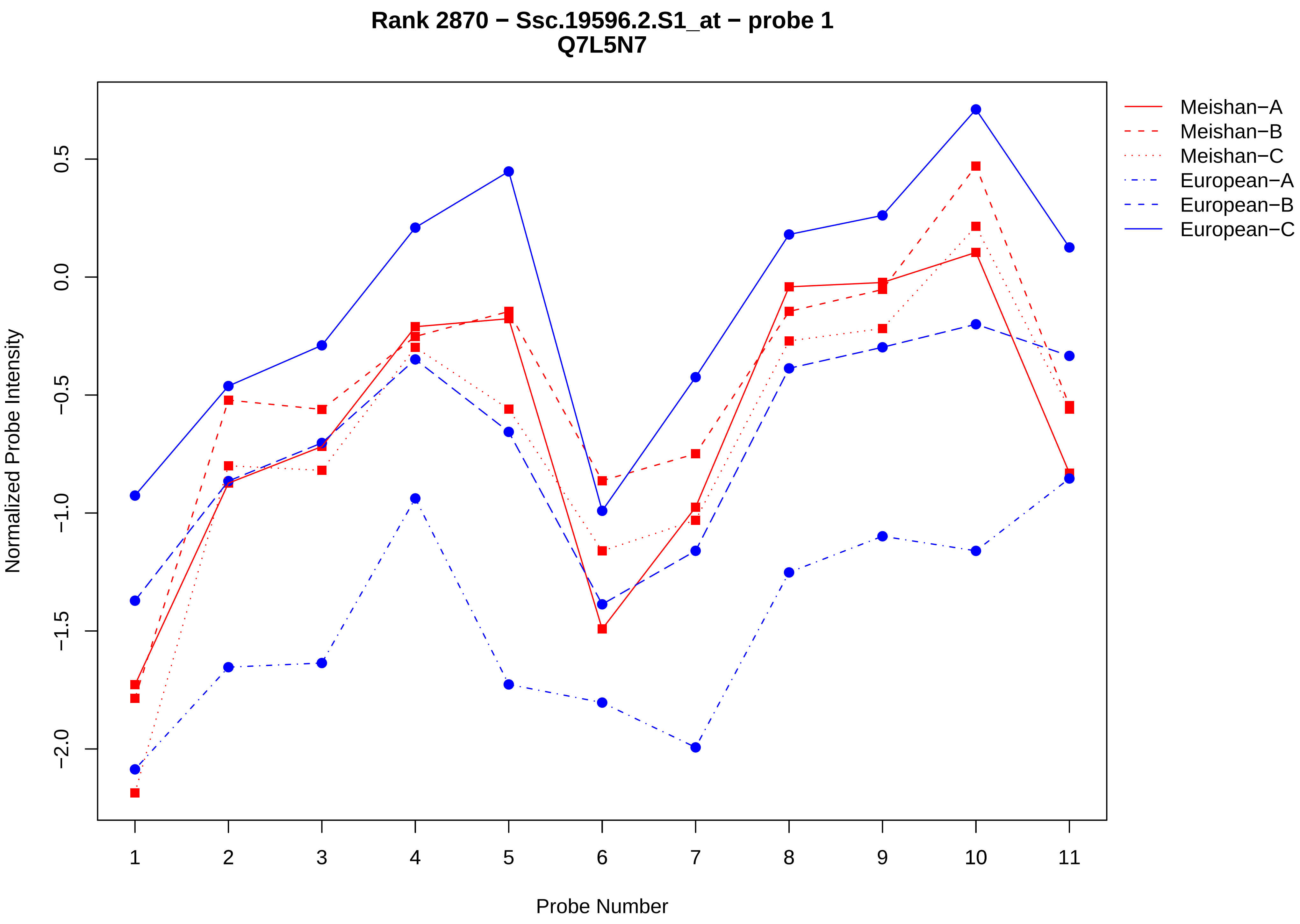
Rank 2870 − Ssc.19596.2.S1_at − probe 1
Q7L5N7
Normalized Probe Intensity
Probe Number
0.5
0.0
−0.5
−1.0
−1.5
−2.0
1
2
3
4
5
6
7
8
9
10
11
Meishan−A
Meishan−B
Meishan−C
European−A
European−B
European−C

Rank 2871 − Ssc.3627.1.A1_at − probe 5
Q7RTP2

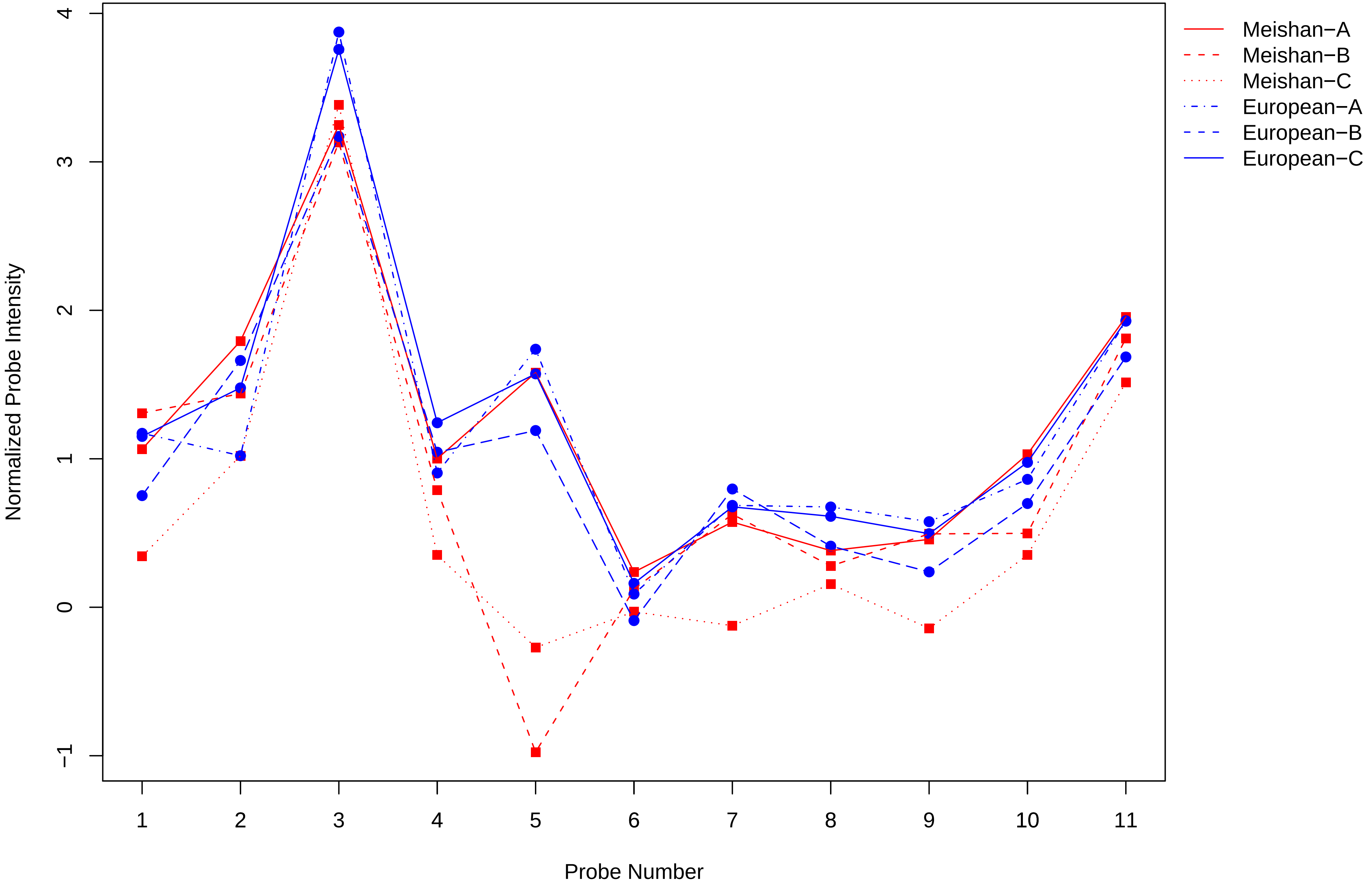

Rank 2872 − Ssc.28431.1.A1_at − probe 11
PIN4

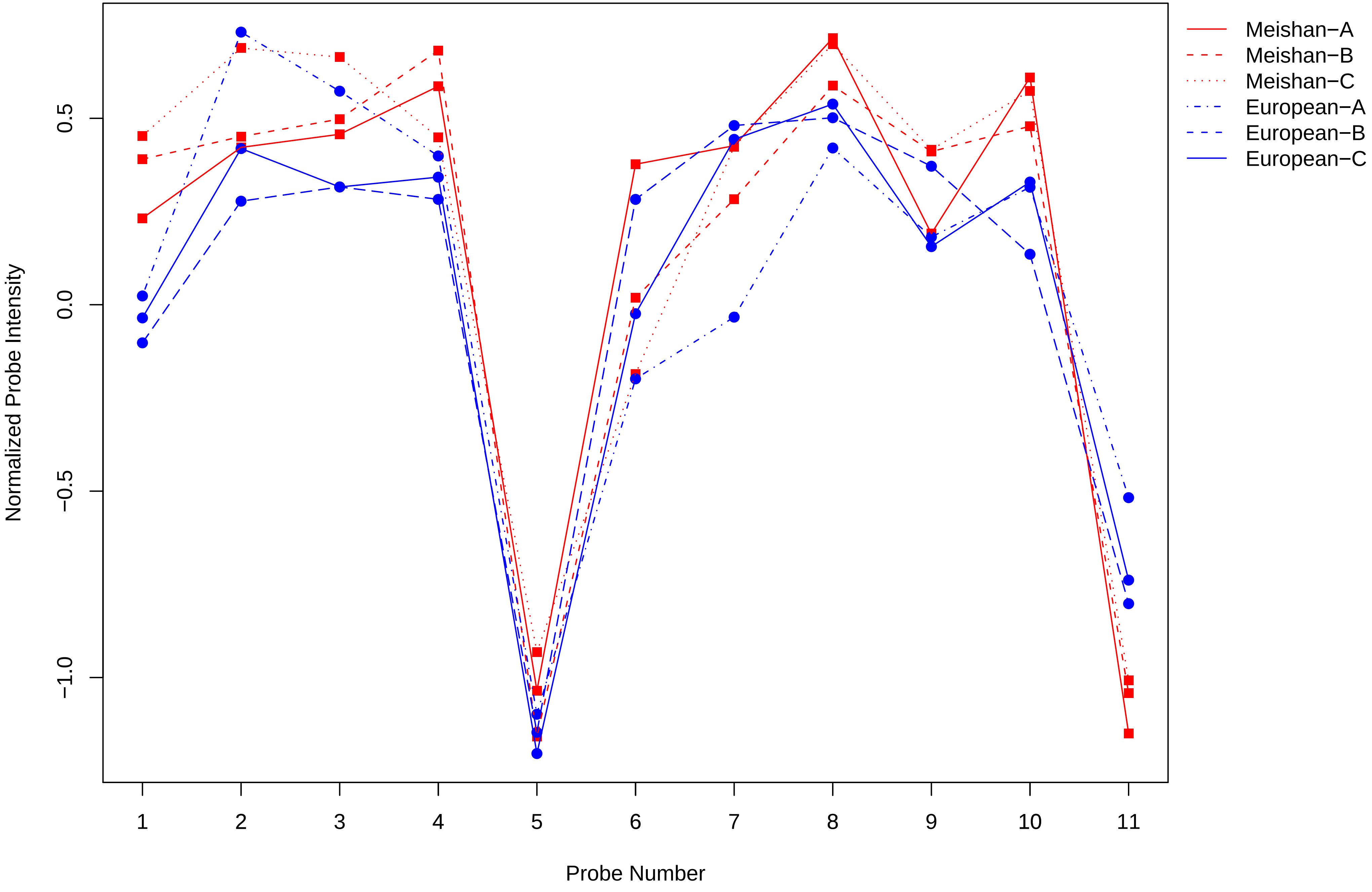

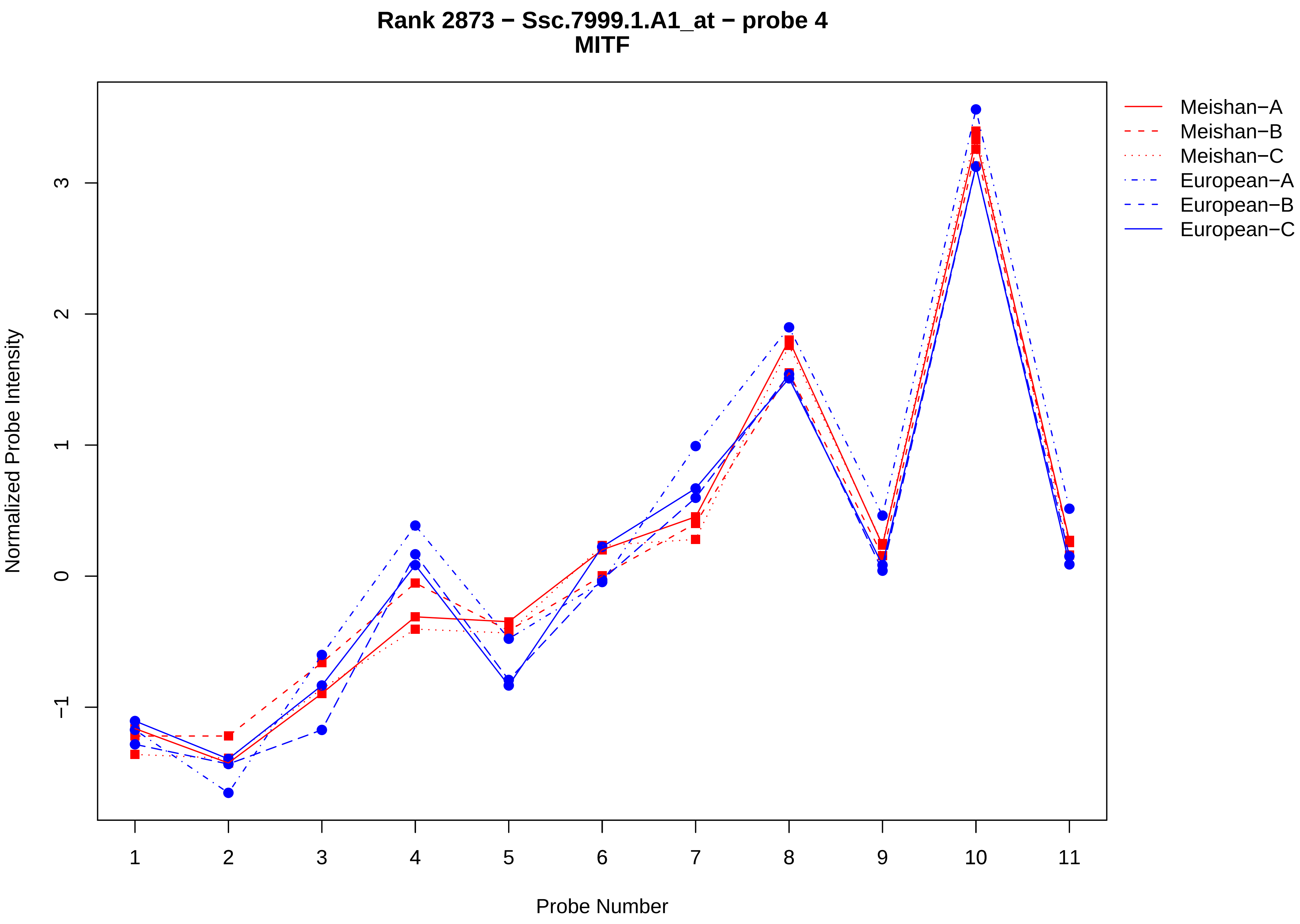

Rank 2873 − Ssc.7999.1.A1_at − probe 4
MITF
Normalized Probe Intensity
Probe Number
−1
0
1
2
3
1
2
3
4
5
6
7
8
9
10
11
Meishan−A
Meishan−B
Meishan−C
European−A
European−B
European−C

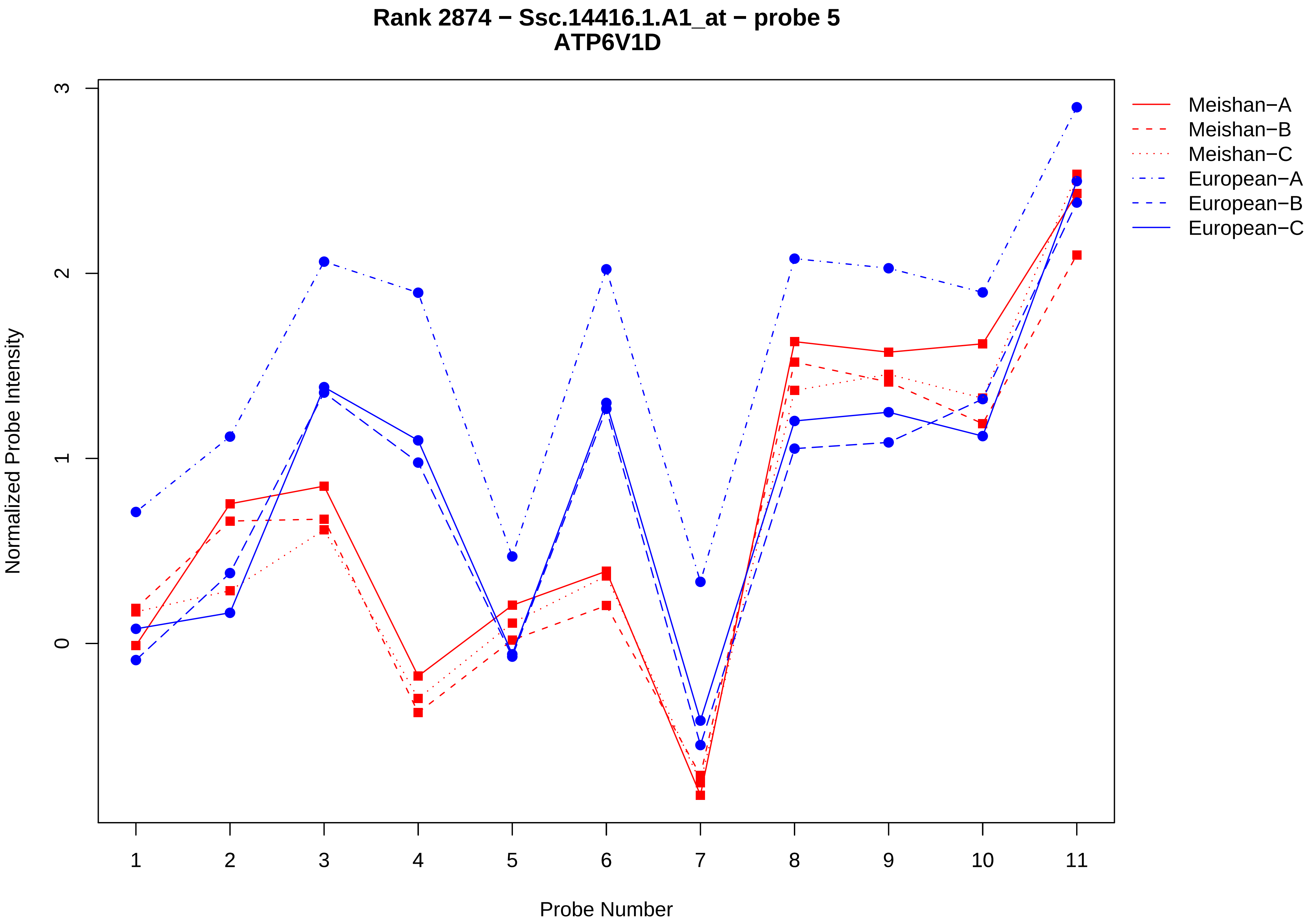

Rank 2874 − Ssc.14416.1.A1_at − probe 5
ATP6V1D
Normalized Probe Intensity
Probe Number
0
1
2
3
1
2
3
4
5
6
7
8
9
10
11
Meishan−A
Meishan−B
Meishan−C
European−A
European−B
European−C

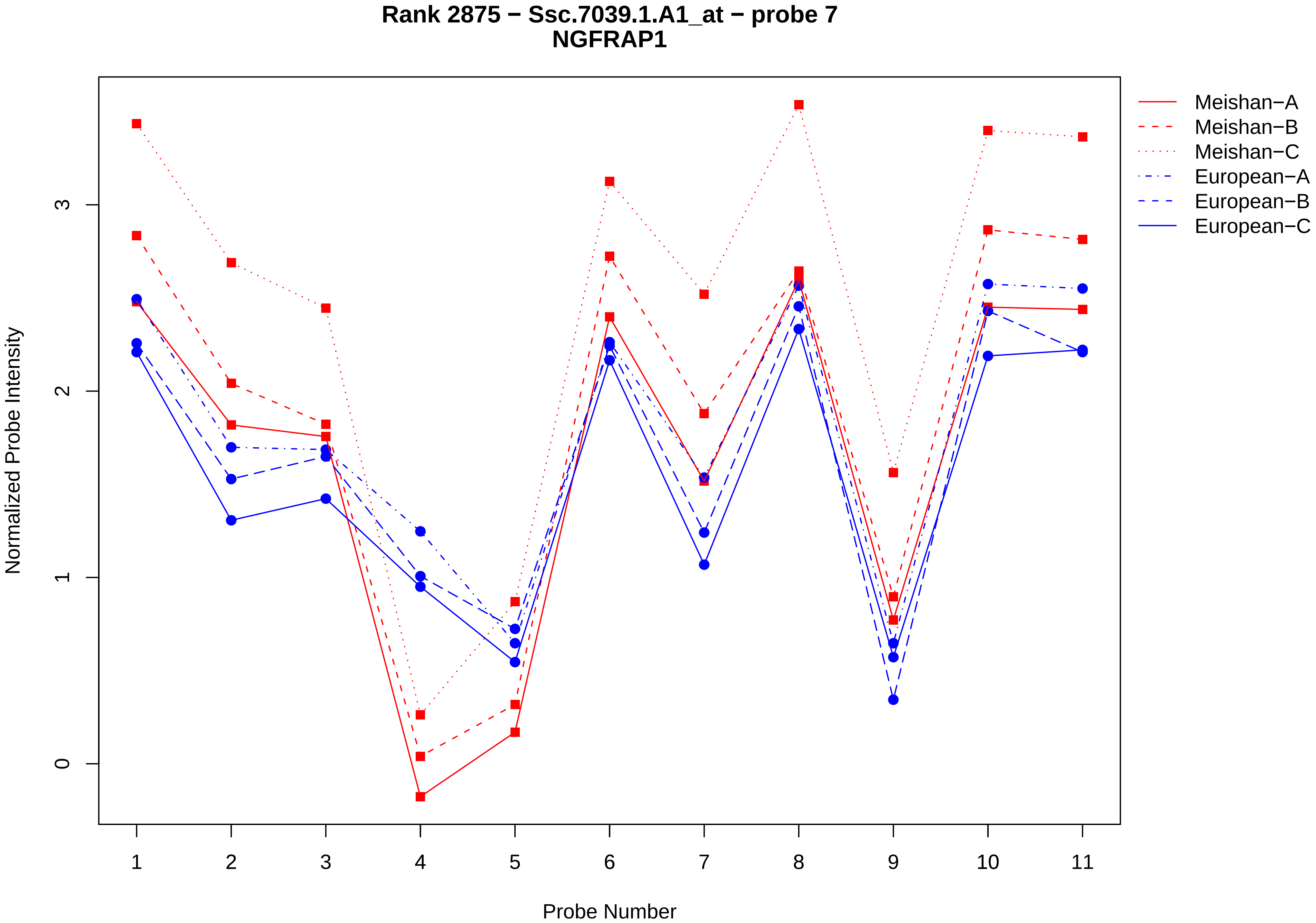
Rank 2875 − Ssc.7039.1.A1_at − probe 7
NGFRAP1
Normalized Probe Intensity
Probe Number
0
1
2
3
1
2
3
4
5
6
7
8
9
10
11
Meishan−A
Meishan−B
Meishan−C
European−A
European−B
European−C

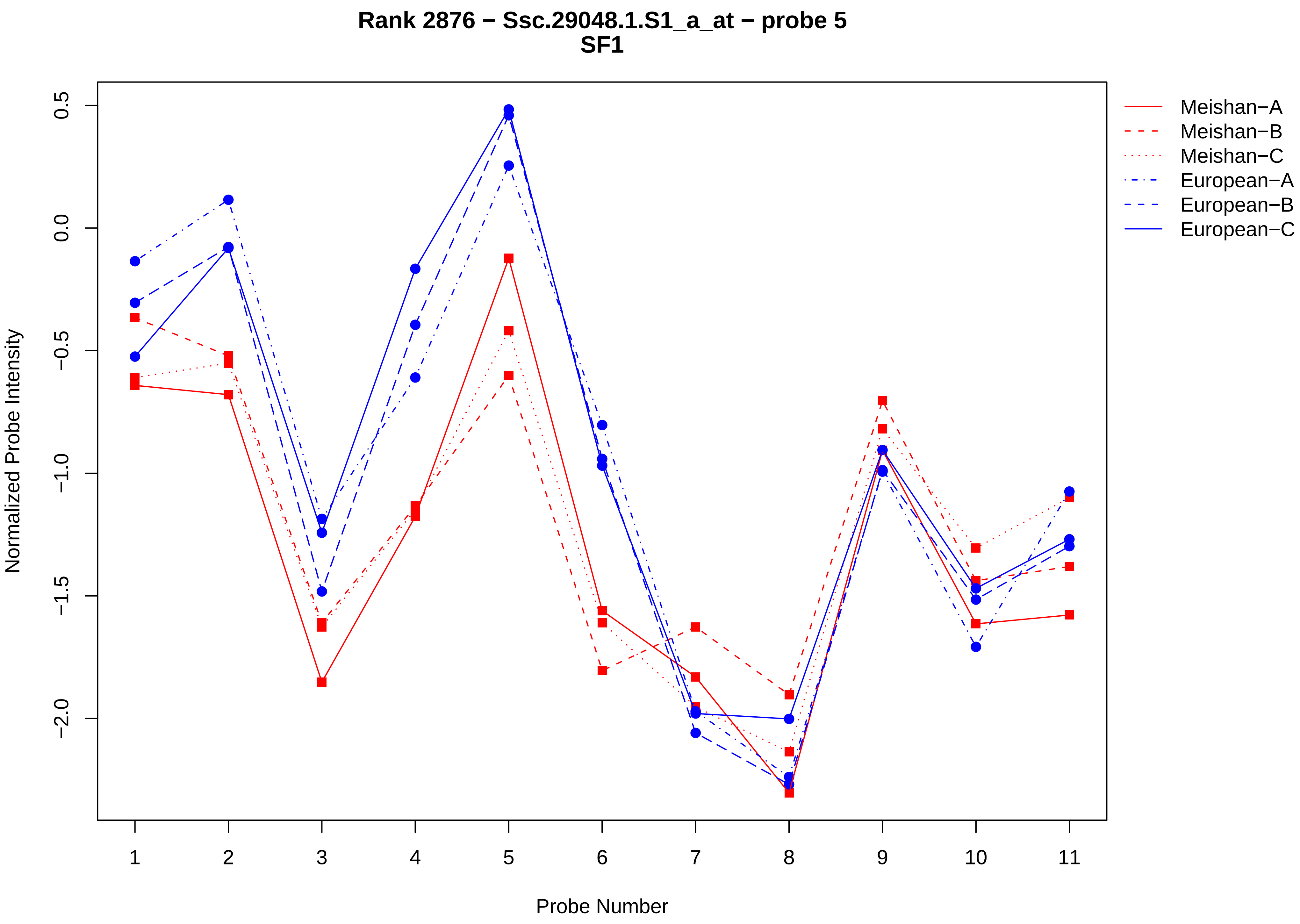

Rank 2876 − Ssc.29048.1.S1_a_at − probe 5
SF1
Normalized Probe Intensity
Probe Number
0.5
0.0
−0.5
−1.0
−1.5
−2.0
1
2
3
4
5
6
7
8
9
10
11
Meishan−A
Meishan−B
Meishan−C
European−A
European−B
European−C

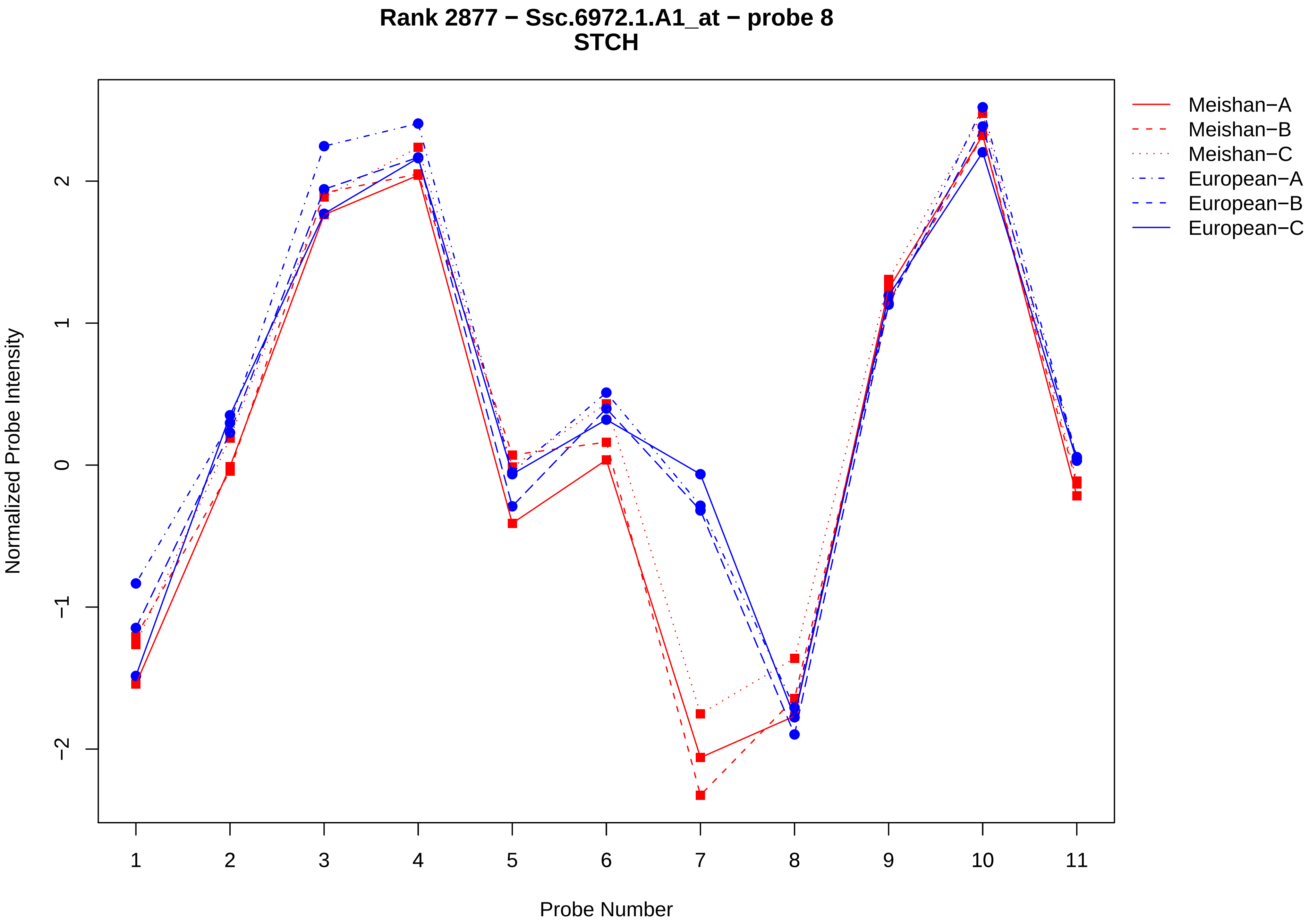

Rank 2877 − Ssc.6972.1.A1_at − probe 8
STCH
Normalized Probe Intensity
Probe Number
Meishan−A
Meishan−B
Meishan−C
European−A
European−B
European−C
−2
−1
0
1
2
1
2
3
4
5
6
7
8
9
10
11

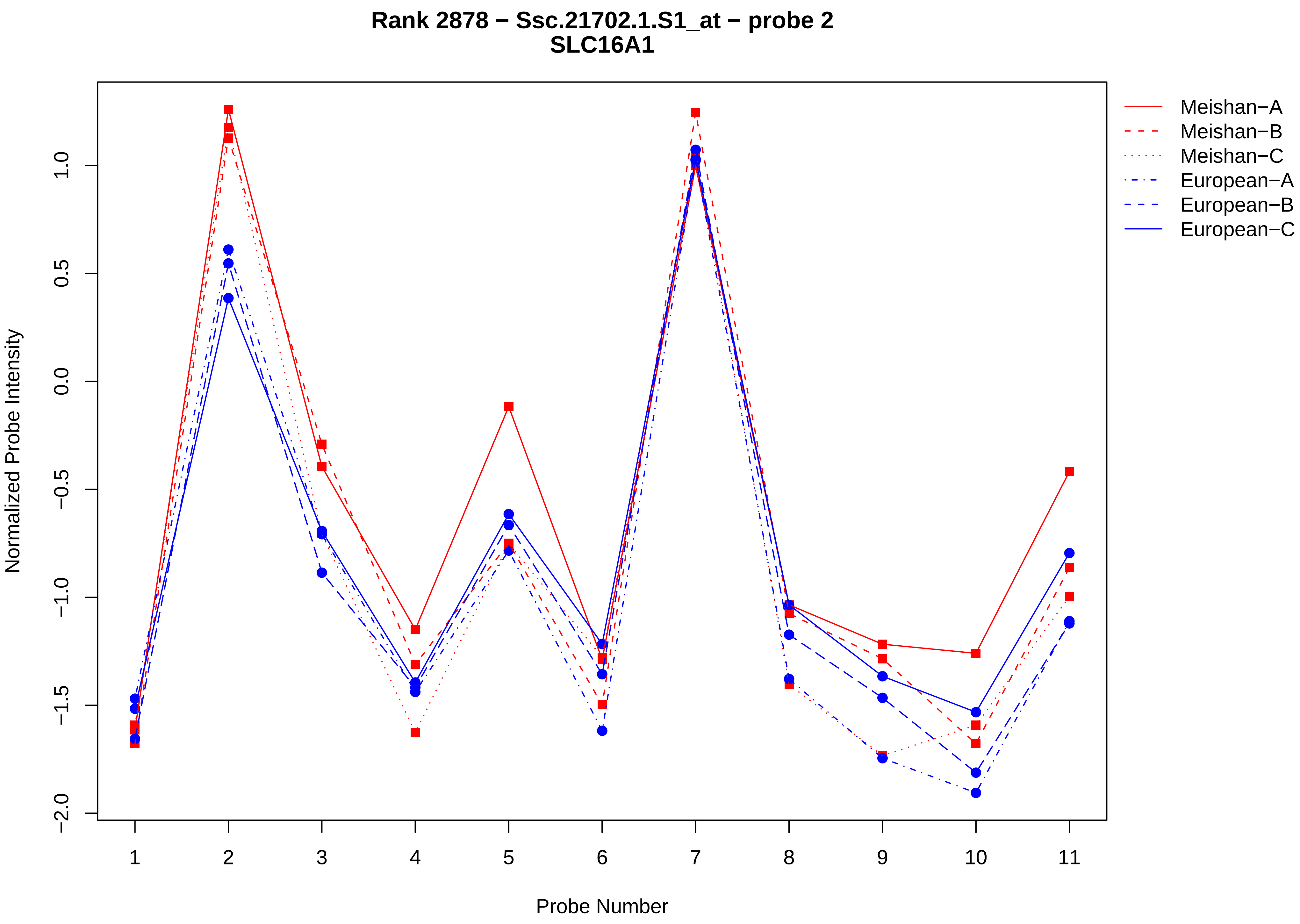

Rank 2878 − Ssc.21702.1.S1_at − probe 2
SLC16A1
Normalized Probe Intensity
Probe Number
1.0
0.5
0.0
−0.5
−1.0
−1.5
−2.0
1
2
3
4
5
6
7
8
9
10
11
Meishan−A
Meishan−B
Meishan−C
European−A
European−B
European−C

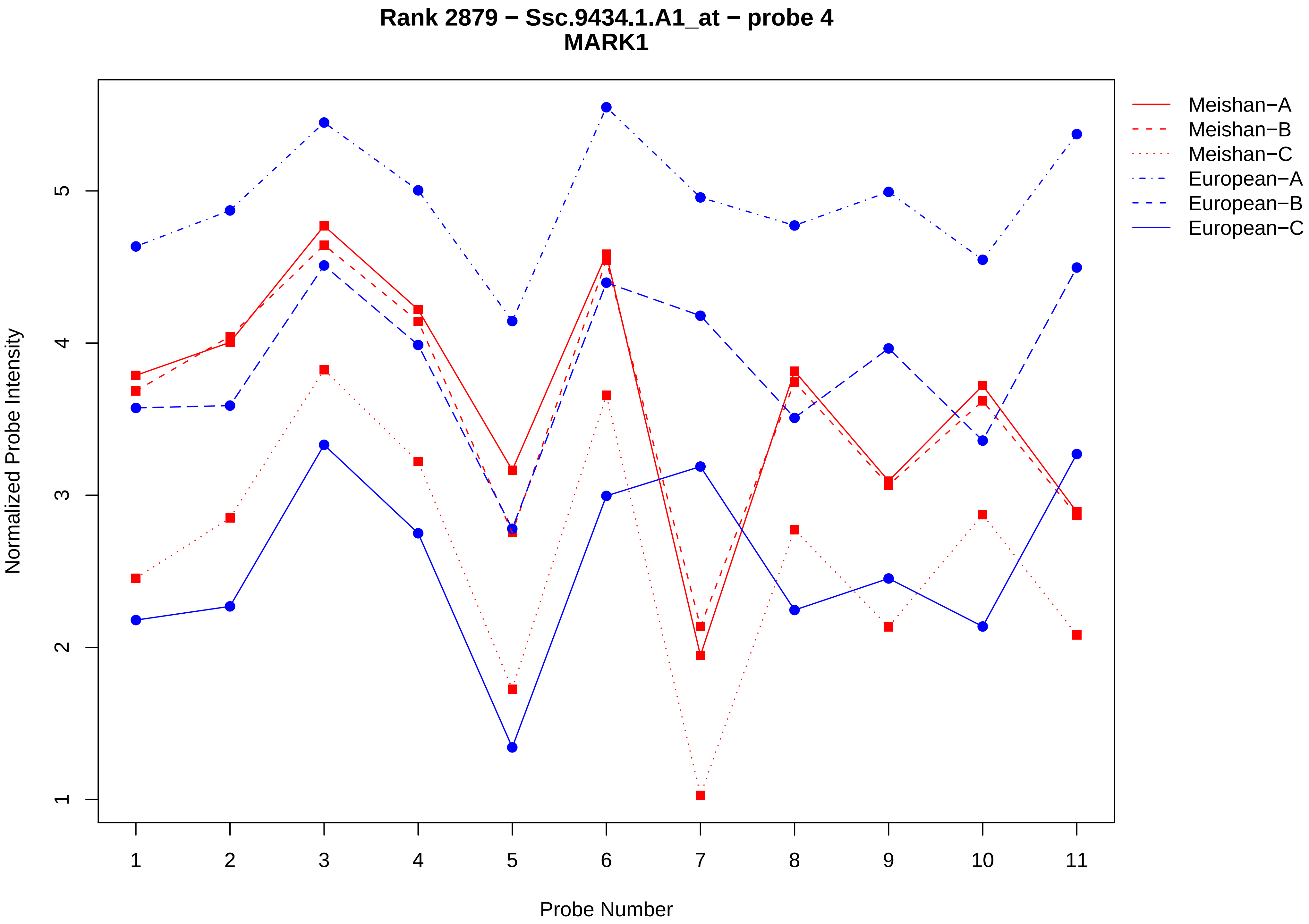

Rank 2879 − Ssc.9434.1.A1_at − probe 4
MARK1
Normalized Probe Intensity
Probe Number
1
2
3
4
5
1
2
3
4
5
6
7
8
9
10
11
Meishan−A
Meishan−B
Meishan−C
European−A
European−B
European−C

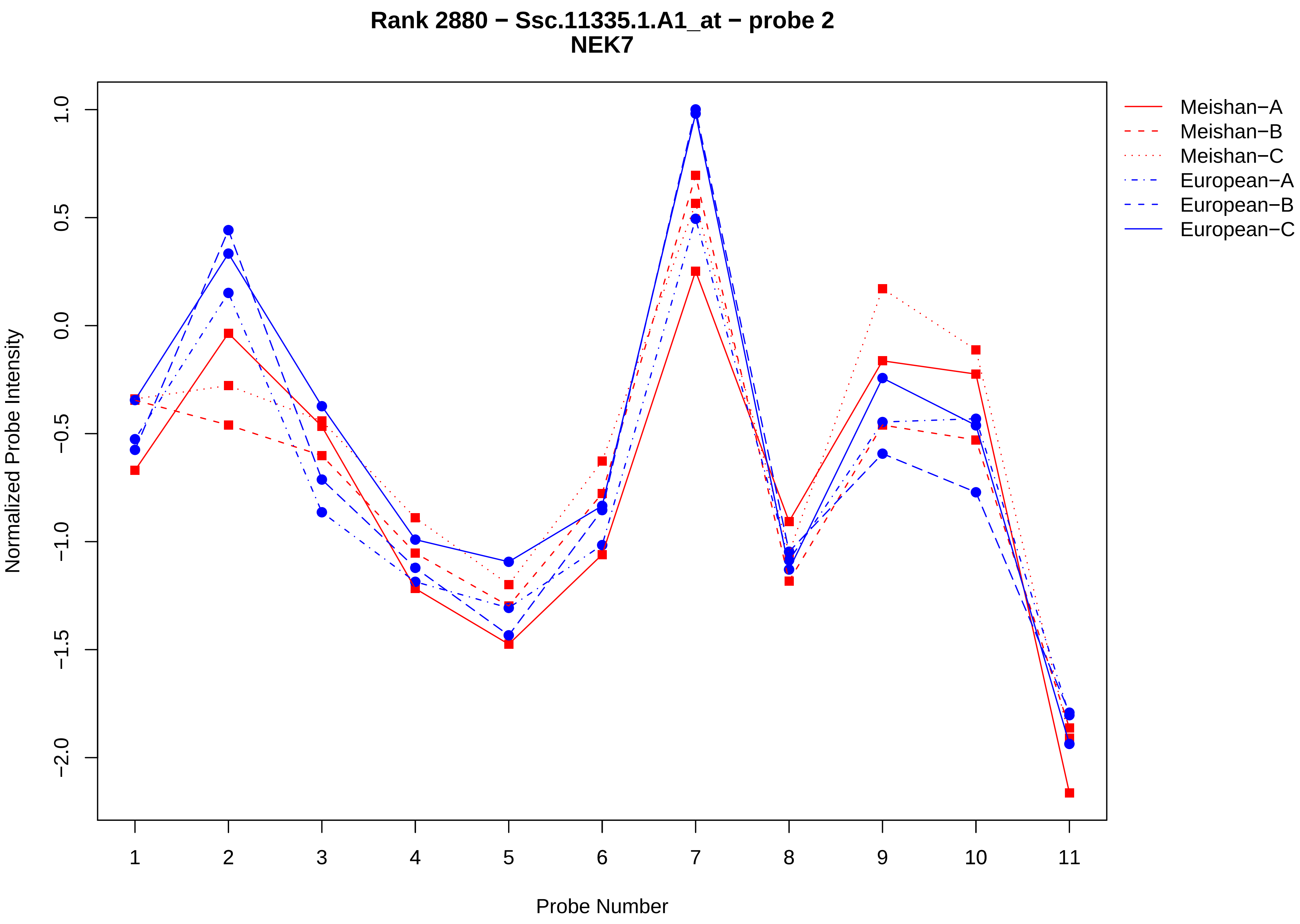

Rank 2880 − Ssc.11335.1.A1_at − probe 2
NEK7
Normalized Probe Intensity
Probe Number
Meishan−A
Meishan−B
Meishan−C
European−A
European−B
European−C
1.0
0.5
0.0
−0.5
−1.0
−1.5
−2.0
1
2
3
4
5
6
7
8
9
10
11

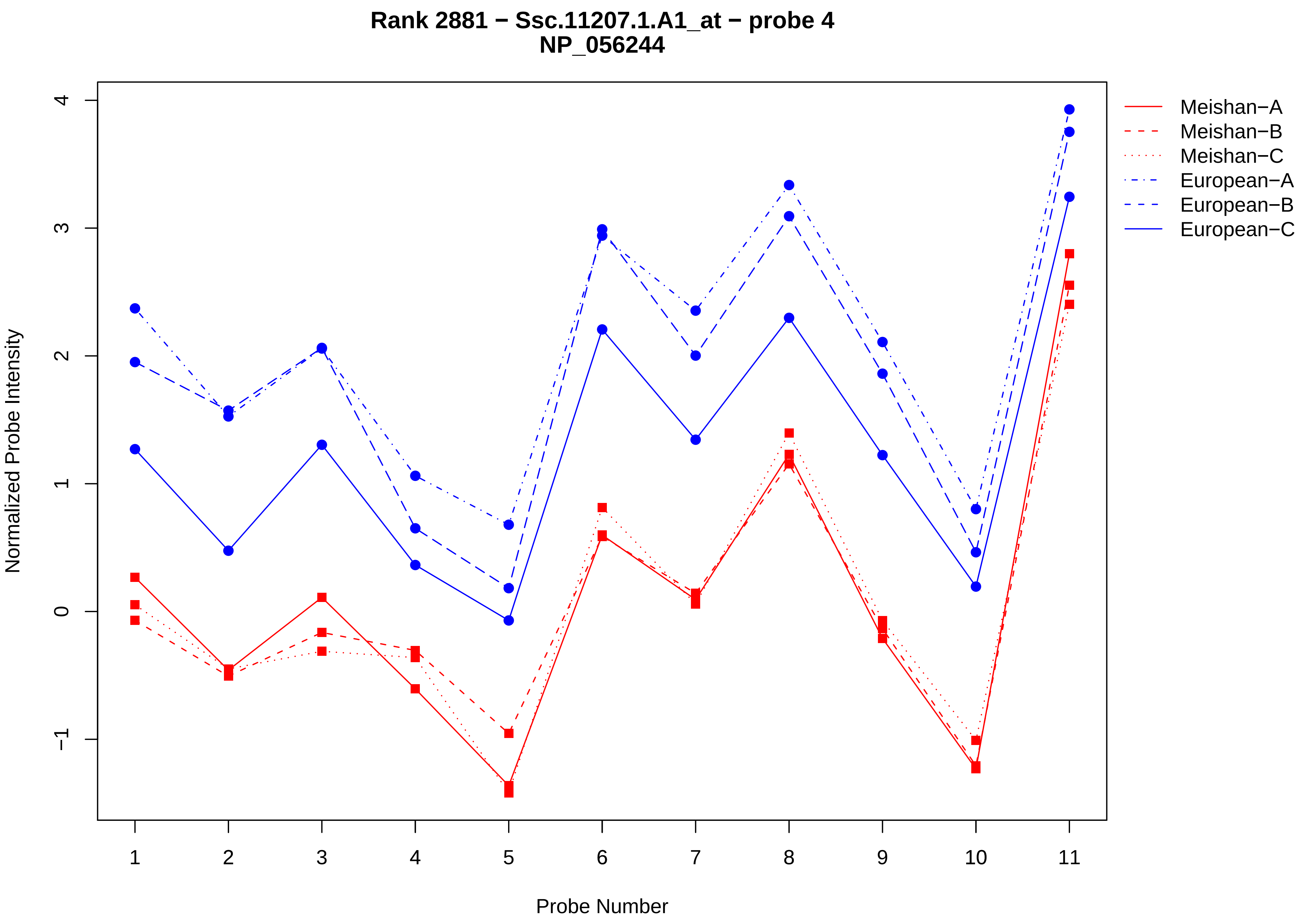

Rank 2881 − Ssc.11207.1.A1_at − probe 4
NP_056244
Normalized Probe Intensity
Probe Number
−1
0
1
2
3
4
1
2
3
4
5
6
7
8
9
10
11
Meishan−A
Meishan−B
Meishan−C
European−A
European−B
European−C

Rank 2882 − Ssc.24103.1.A1_s_at − probe 11
IGHM

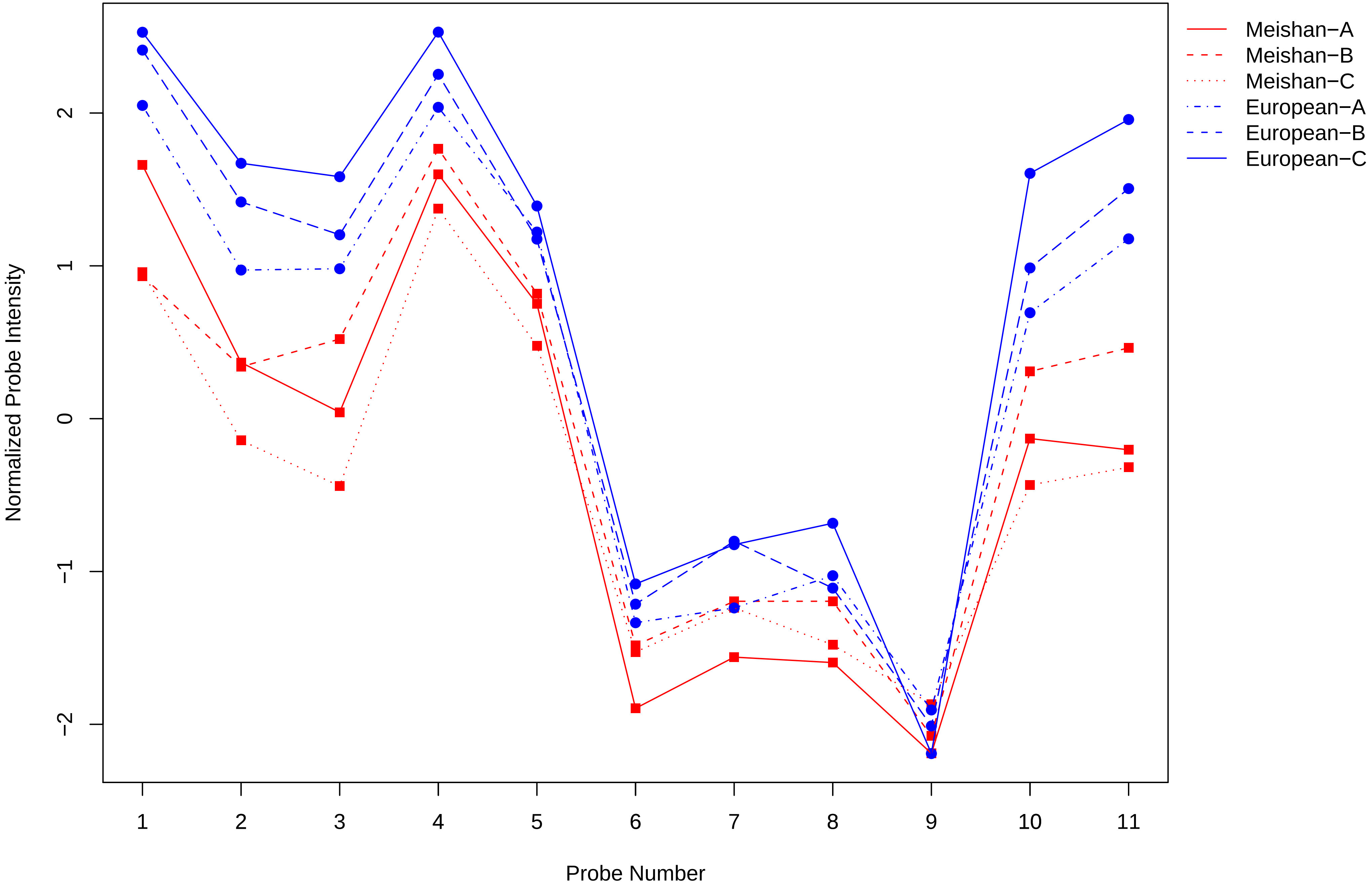

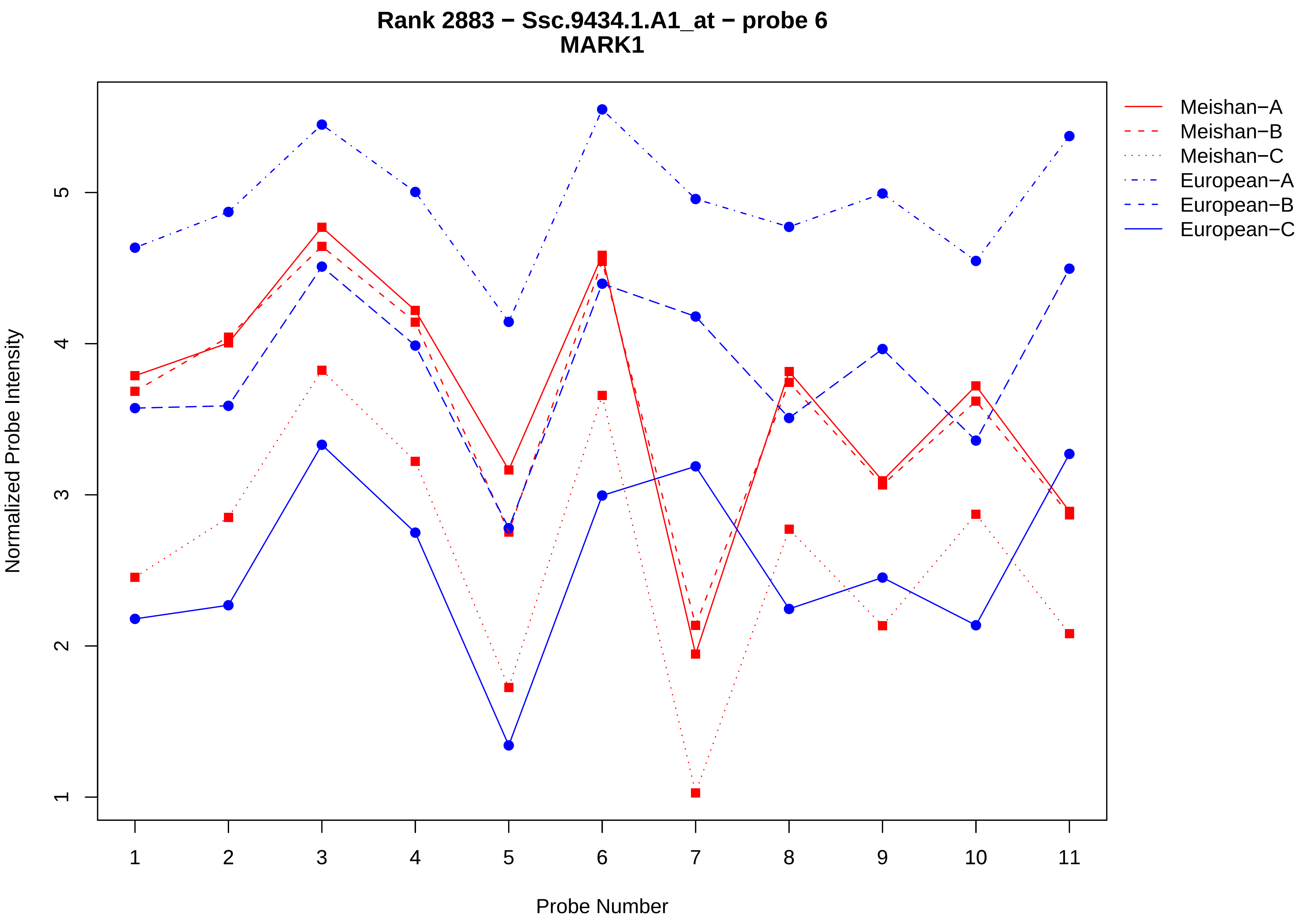

Rank 2883 − Ssc.9434.1.A1_at − probe 6
MARK1
Normalized Probe Intensity
1
2
3
4
5
Probe Number
1
2
3
4
5
6
7
8
9
10
11
Meishan−A
Meishan−B
Meishan−C
European−A
European−B
European−C

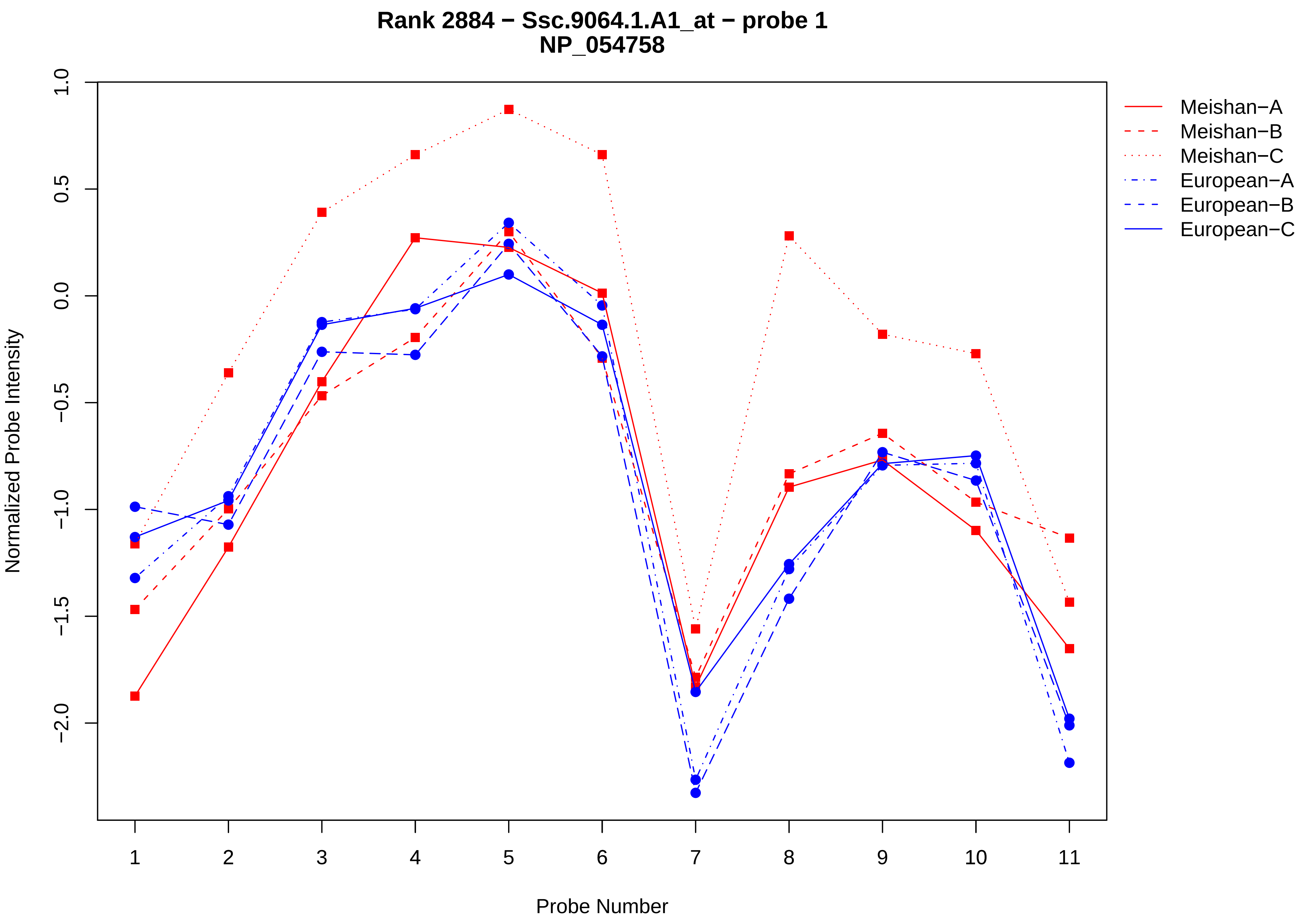

Rank 2884 − Ssc.9064.1.A1_at − probe 1
NP_054758
Normalized Probe Intensity
Probe Number
1.0
0.5
0.0
−0.5
−1.0
−1.5
−2.0
1
2
3
4
5
6
7
8
9
10
11
Meishan−A
Meishan−B
Meishan−C
European−A
European−B
European−C

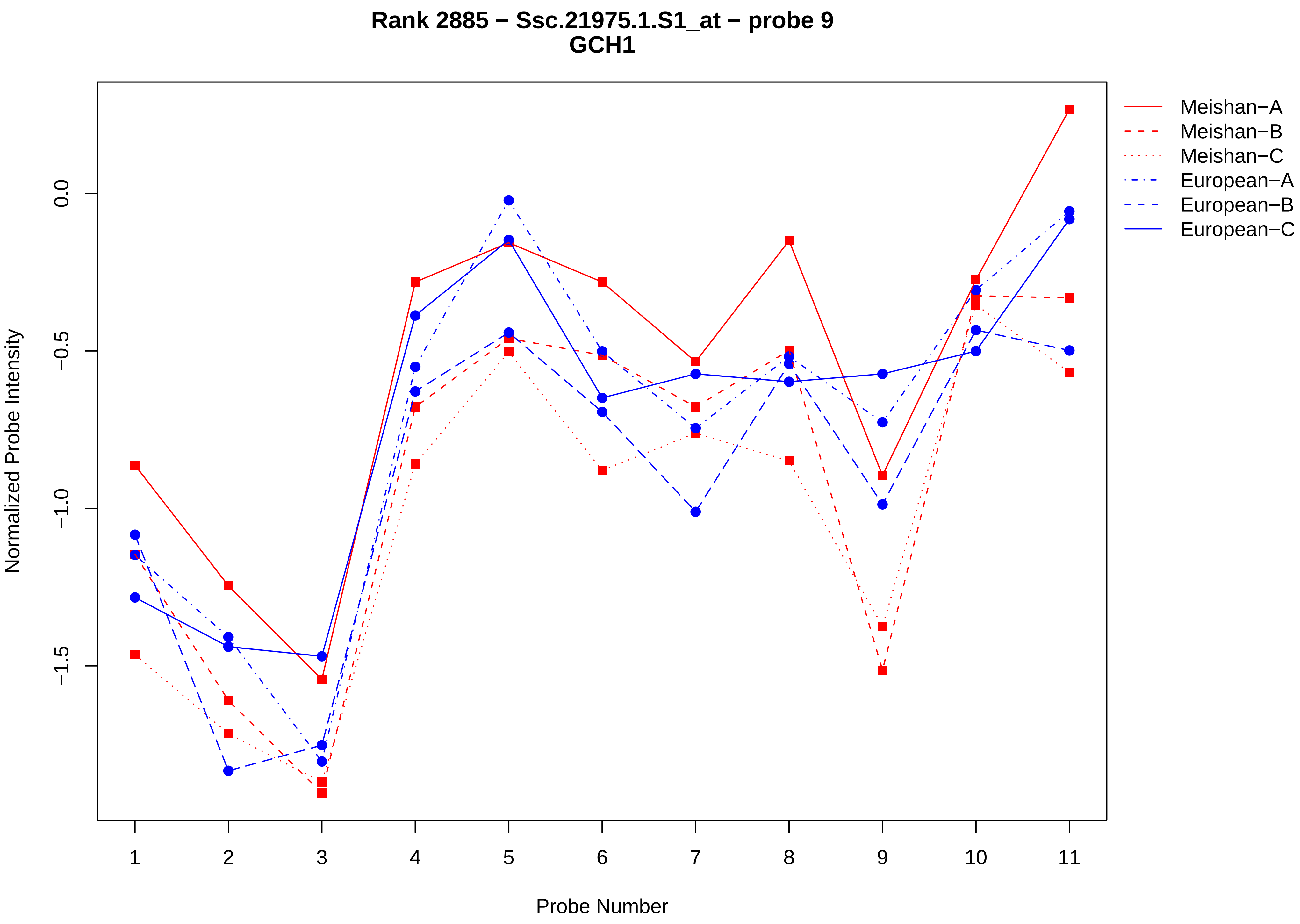

Rank 2885 − Ssc.21975.1.S1_at − probe 9
GCH1
Normalized Probe Intensity
Probe Number
0.0
−0.5
−1.0
−1.5
1
2
3
4
5
6
7
8
9
10
11
Meishan−A
Meishan−B
Meishan−C
European−A
European−B
European−C

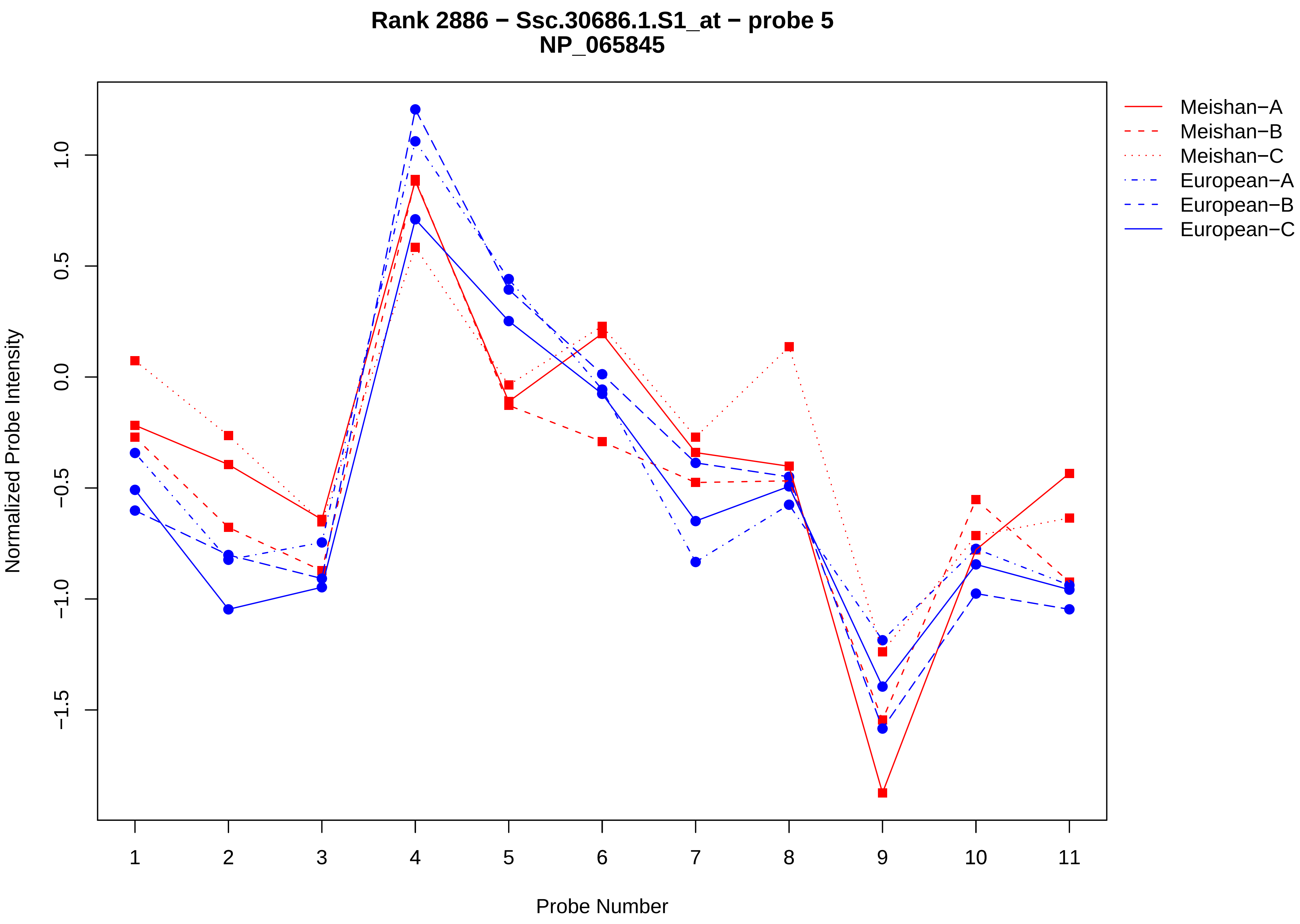

Rank 2886 − Ssc.30686.1.S1_at − probe 5
NP_065845
Normalized Probe Intensity
Probe Number
Meishan−A
Meishan−B
Meishan−C
European−A
European−B
European−C
1.0
0.5
0.0
−0.5
−1.0
−1.5
1
2
3
4
5
6
7
8
9
10
11

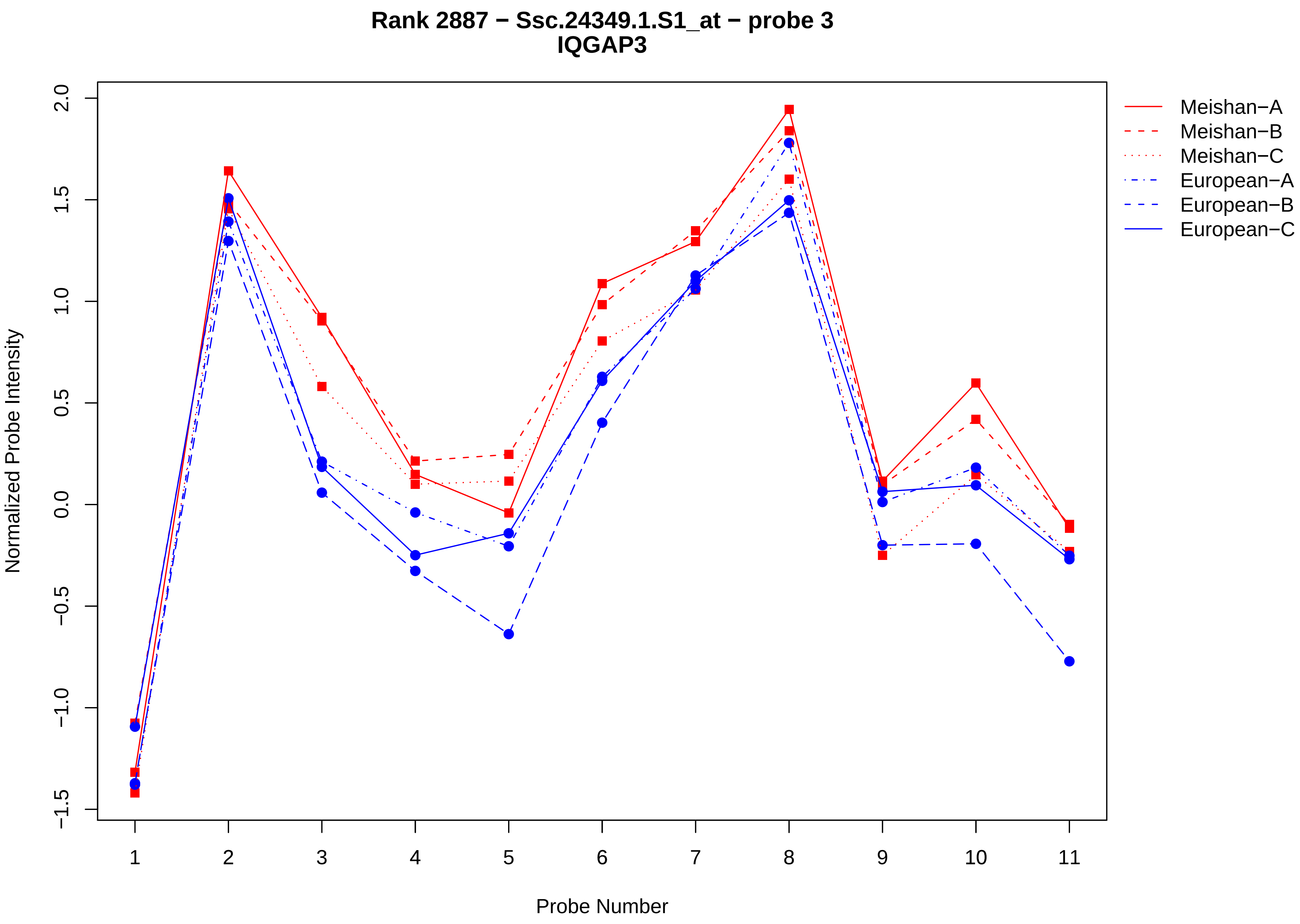

Rank 2887 − Ssc.24349.1.S1_at − probe 3
IQGAP3
Normalized Probe Intensity
Probe Number
−1.5
−1.0
−0.5
0.0
0.5
1.0
1.5
2.0
1
2
3
4
5
6
7
8
9
10
11
Meishan−A
Meishan−B
Meishan−C
European−A
European−B
European−C

Rank 2888 − Ssc.26067.1.S1_at − probe 9
PODXL

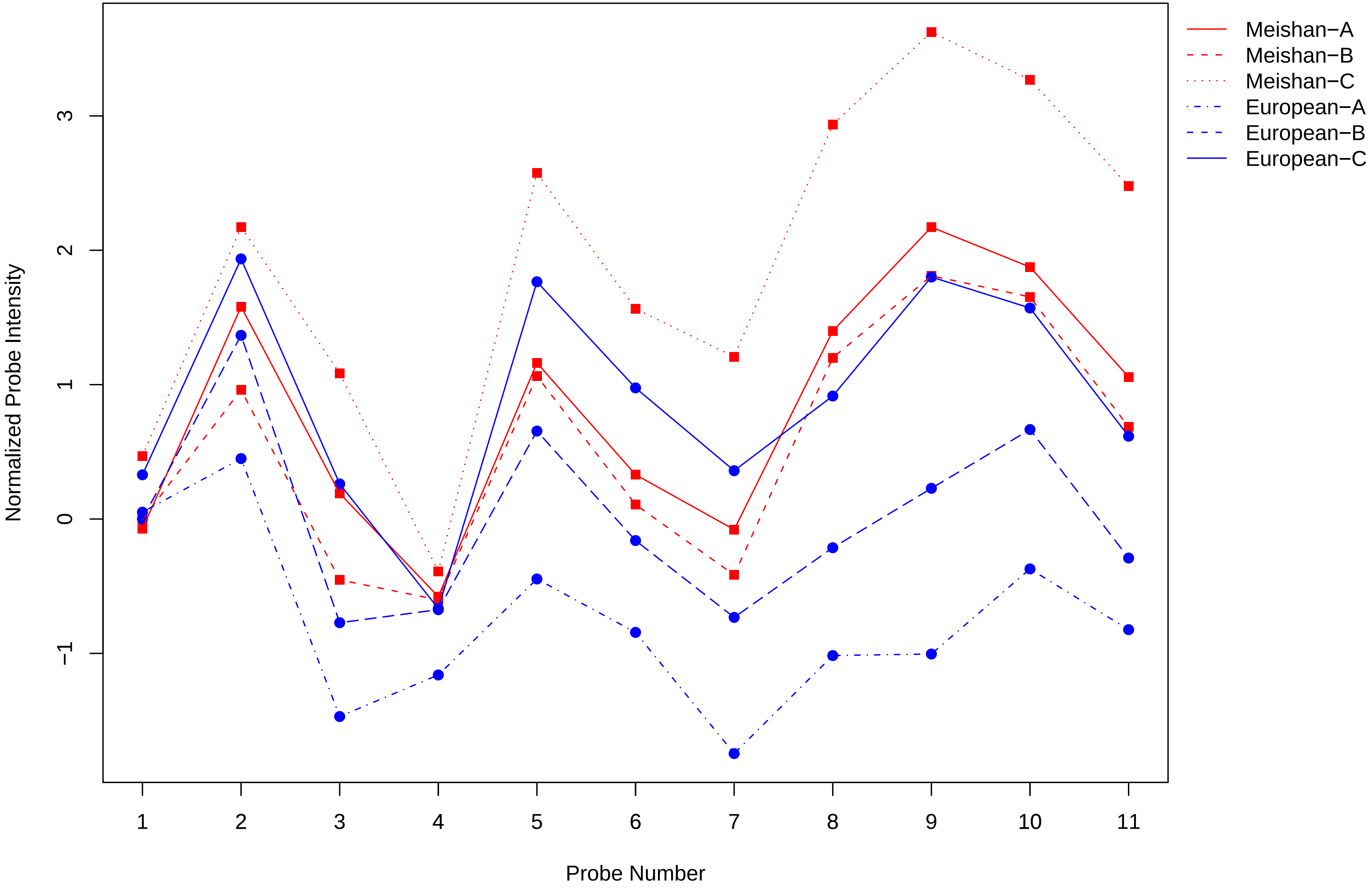

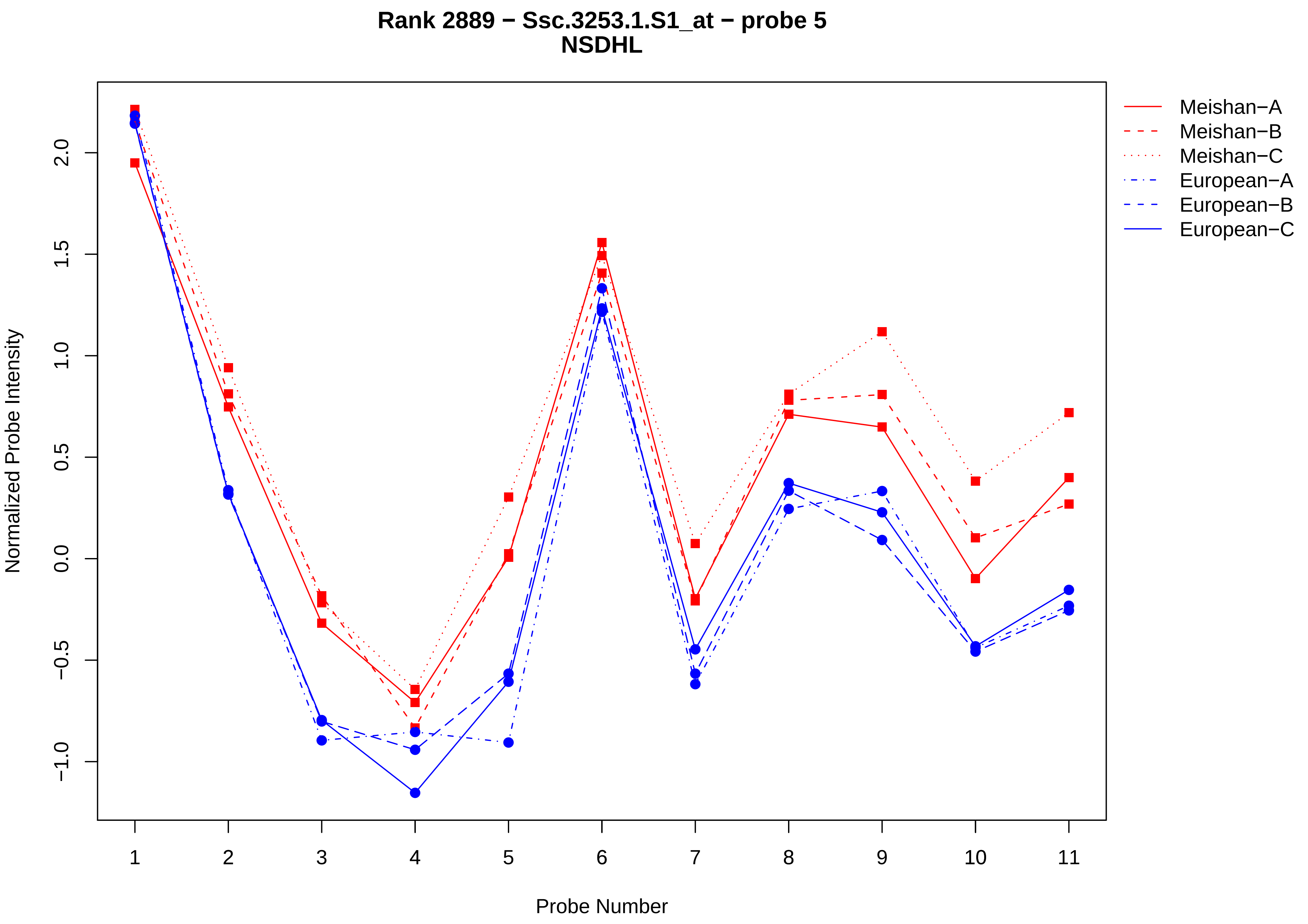

Rank 2889 − Ssc.3253.1.S1_at − probe 5
NSDHL
Normalized Probe Intensity
Probe Number
Meishan−A
Meishan−B
Meishan−C
European−A
European−B
European−C

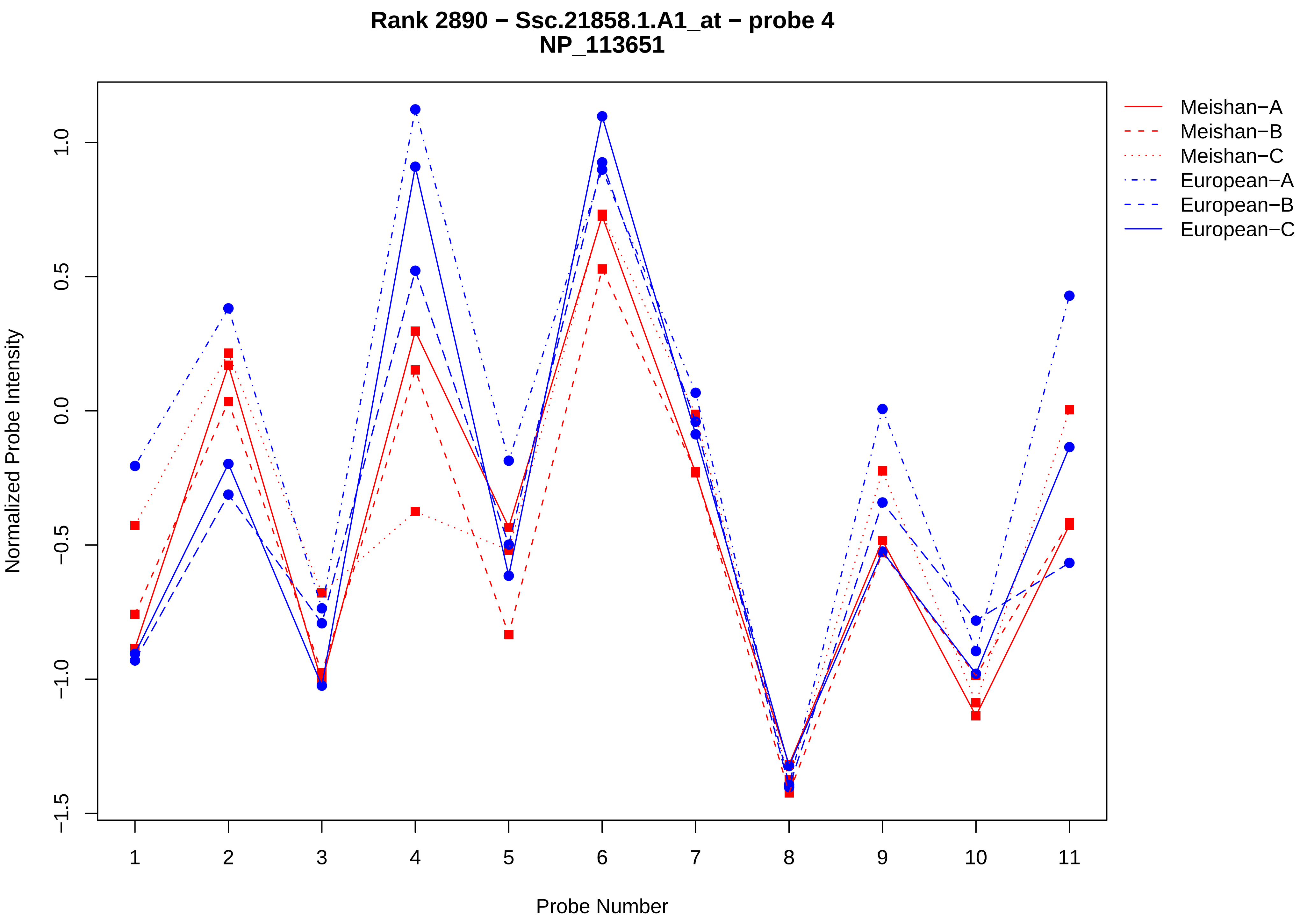

Rank 2890 − Ssc.21858.1.A1_at − probe 4
NP_113651
Normalized Probe Intensity
Probe Number
1.0
0.5
0.0
−0.5
−1.0
−1.5
1
2
3
4
5
6
7
8
9
10
11
Meishan−A
Meishan−B
Meishan−C
European−A
European−B
European−C

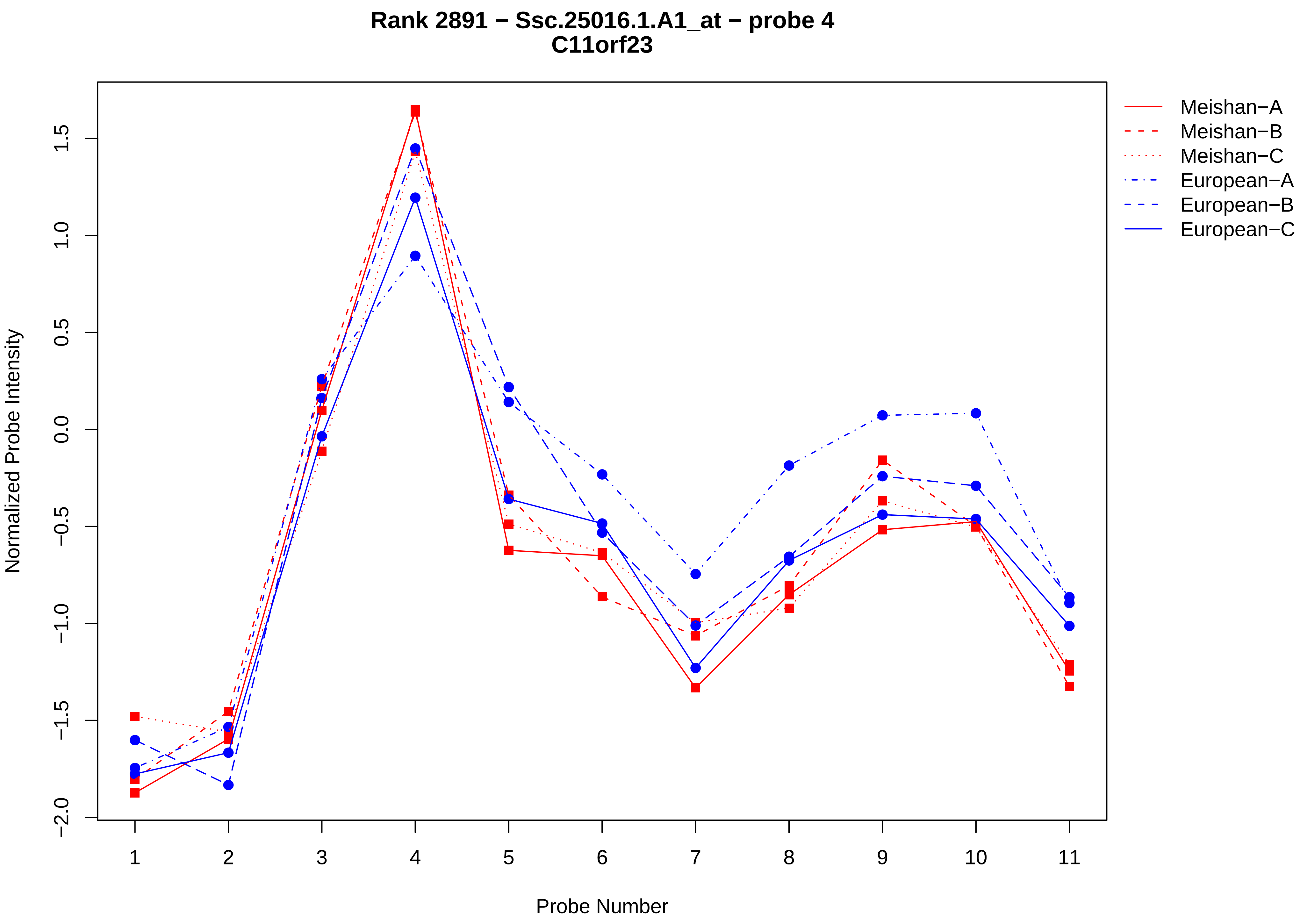

Rank 2891 − Ssc.25016.1.A1_at − probe 4
C11orf23
Normalized Probe Intensity
Probe Number
−2.0
−1.5
−1.0
−0.5
0.0
0.5
1.0
1.5
1
2
3
4
5
6
7
8
9
10
11
Meishan−A
Meishan−B
Meishan−C
European−A
European−B
European−C

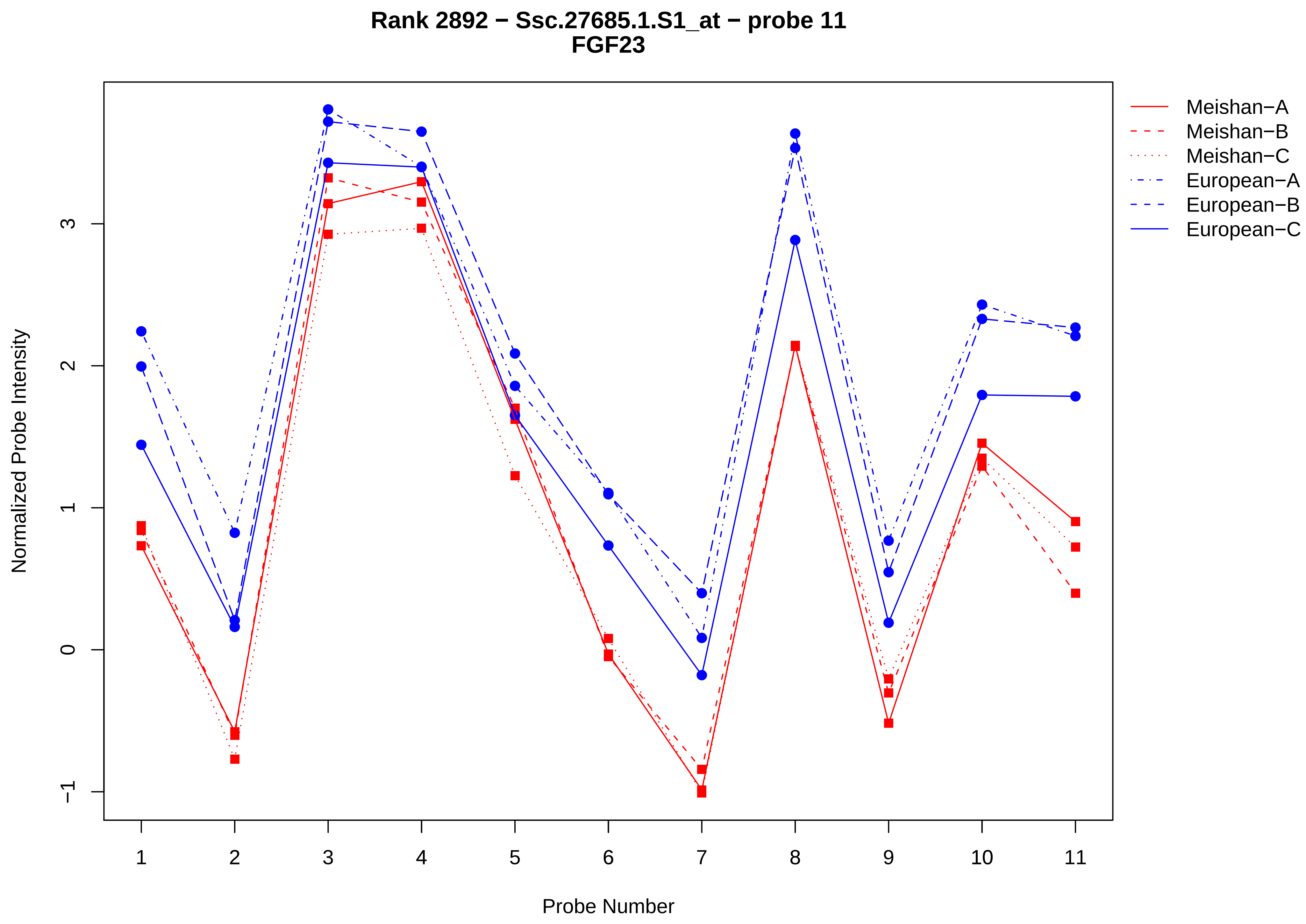

Rank 2892 − Ssc.27685.1.S1_at − probe 11
FGF23
Normalized Probe Intensity
Probe Number
−1
0
1
2
3
1
2
3
4
5
6
7
8
9
10
11
Meishan−A
Meishan−B
Meishan−C
European−A
European−B
European−C

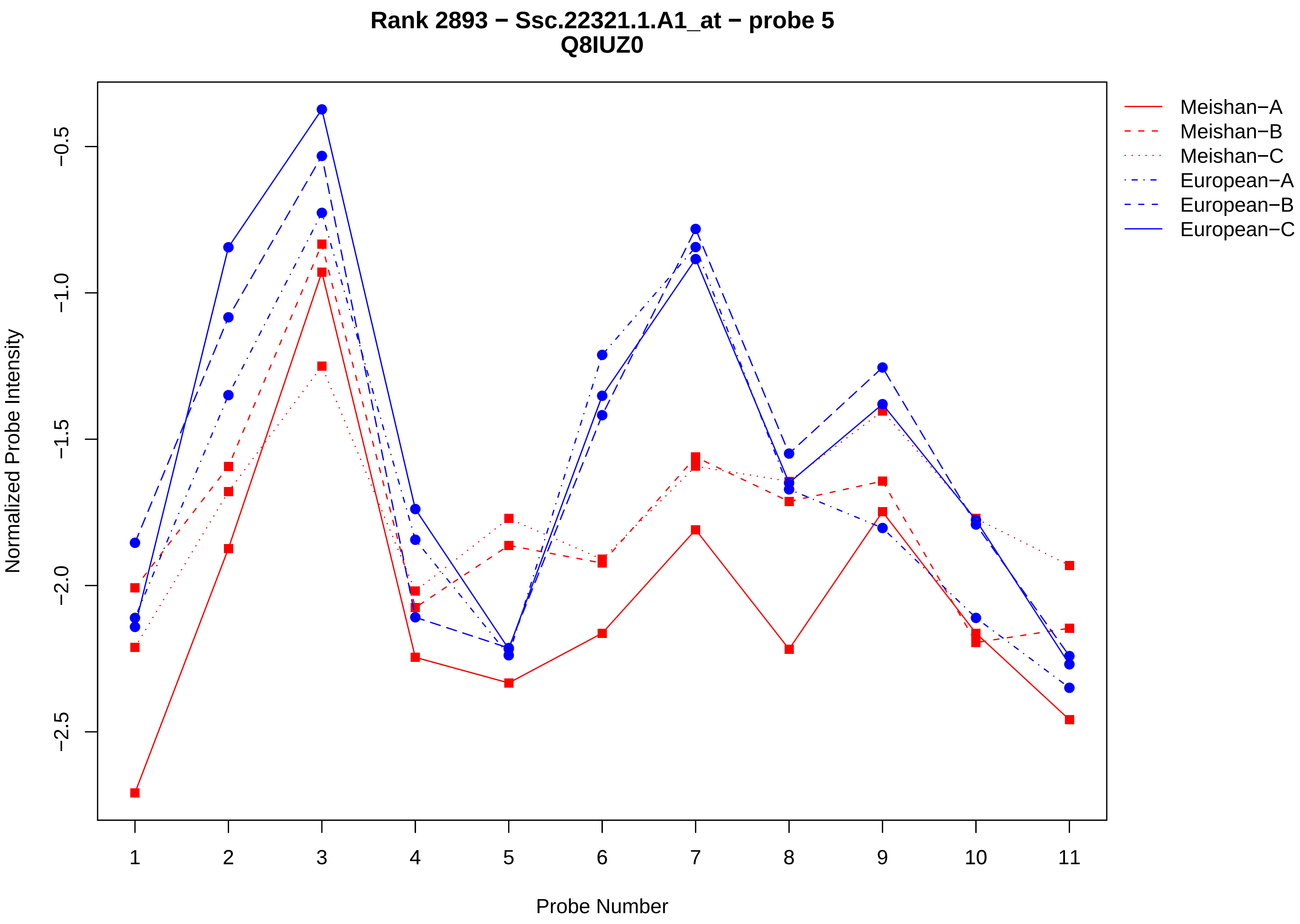

Rank 2893 − Ssc.22321.1.A1_at − probe 5
Q8IUZ0
Normalized Probe Intensity
−0.5
−1.0
−1.5
−2.0
−2.5
1
2
3
4
5
6
7
8
9
10
11
Probe Number
Meishan−A
Meishan−B
Meishan−C
European−A
European−B
European−C

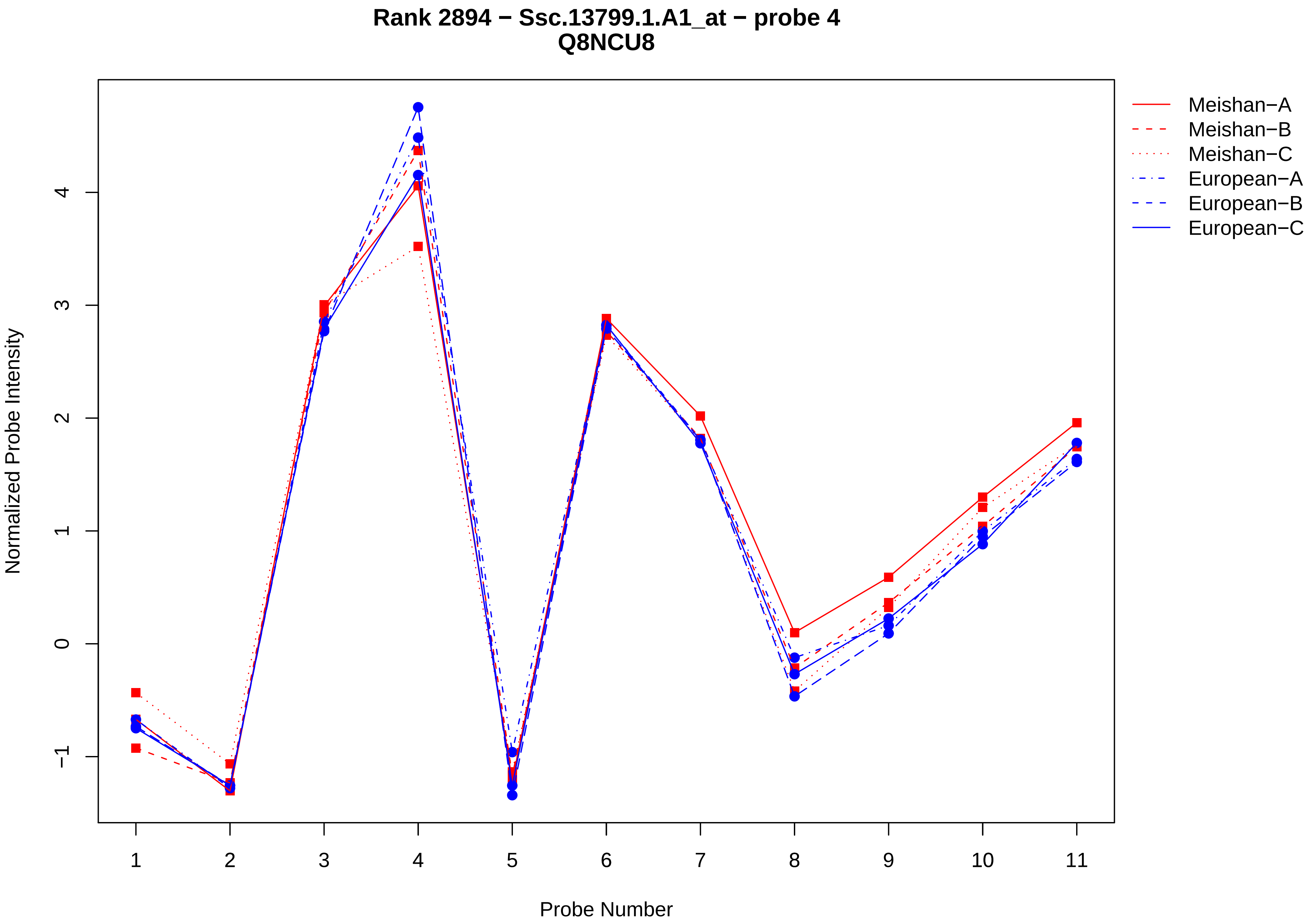

Rank 2894 − Ssc.13799.1.A1_at − probe 4
Q8NCU8
Normalized Probe Intensity
Probe Number
Meishan−A
Meishan−B
Meishan−C
European−A
European−B
European−C
−1
0
1
2
3
4
1
2
3
4
5
6
7
8
9
10
11

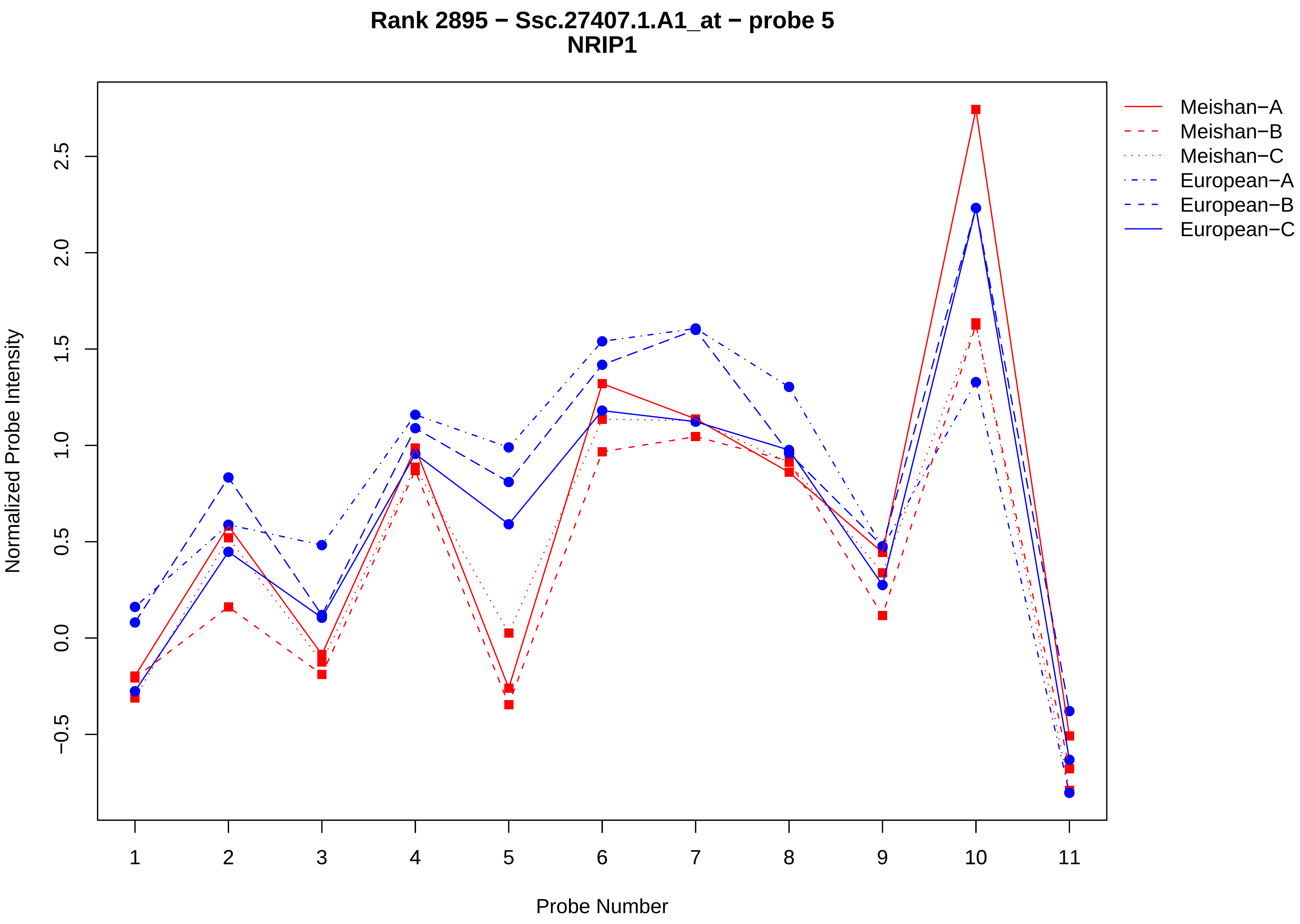

Rank 2895 − Ssc.27407.1.A1_at − probe 5
NRIP1
Normalized Probe Intensity
Probe Number
−0.5
0.0
0.5
1.0
1.5
2.0
2.5
1
2
3
4
5
6
7
8
9
10
11
Meishan−A
Meishan−B
Meishan−C
European−A
European−B
European−C

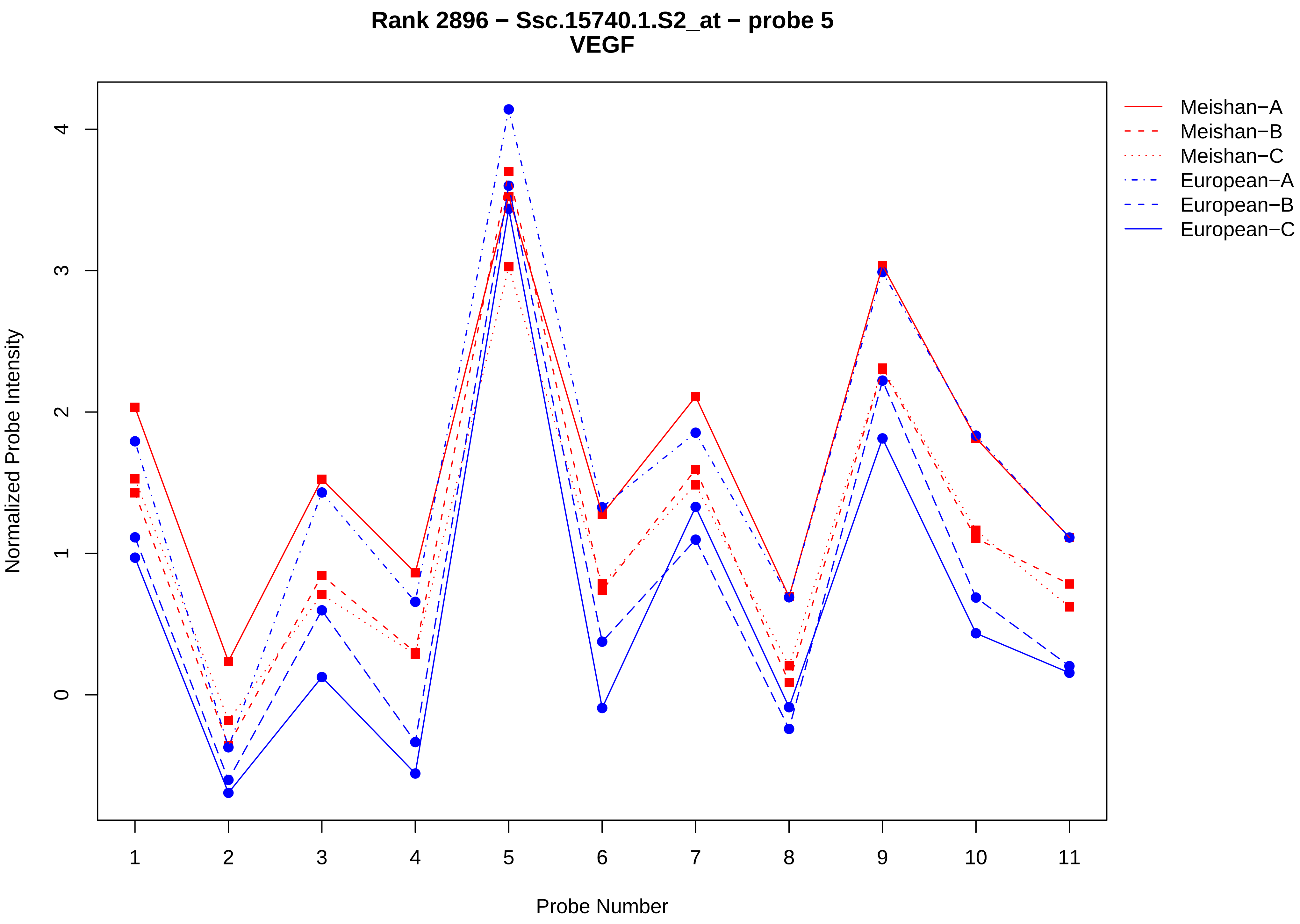

Rank 2896 − Ssc.15740.1.S2_at − probe 5
VEGF
Normalized Probe Intensity
Probe Number
0
1
2
3
4
1
2
3
4
5
6
7
8
9
10
11
Meishan−A
Meishan−B
Meishan−C
European−A
European−B
European−C

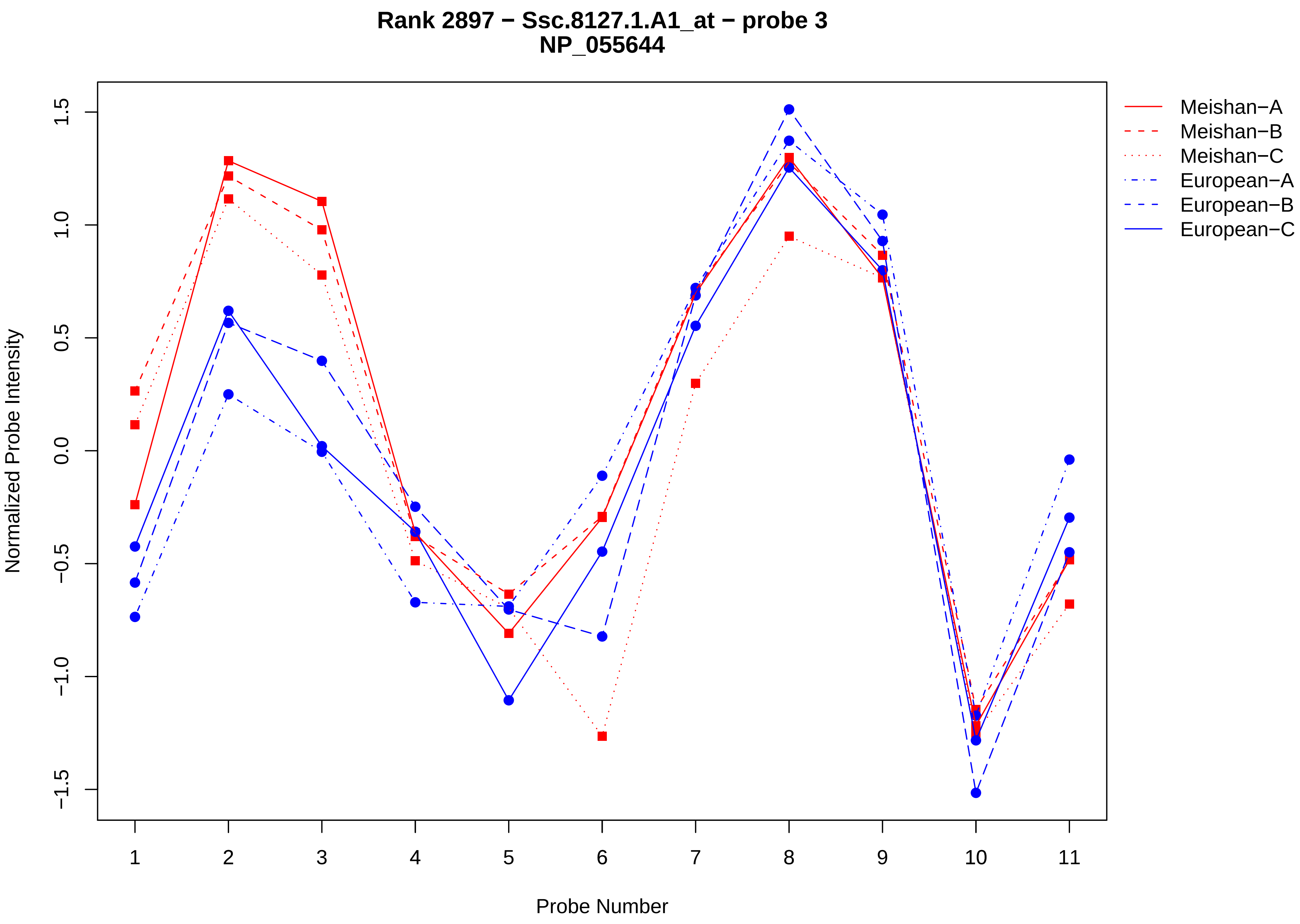

Rank 2897 − Ssc.8127.1.A1_at − probe 3
NP_055644
Normalized Probe Intensity
Probe Number
Meishan−A
Meishan−B
Meishan−C
European−A
European−B
European−C
1.5
1.0
0.5
0.0
−0.5
−1.0
−1.5
1
2
3
4
5
6
7
8
9
10
11

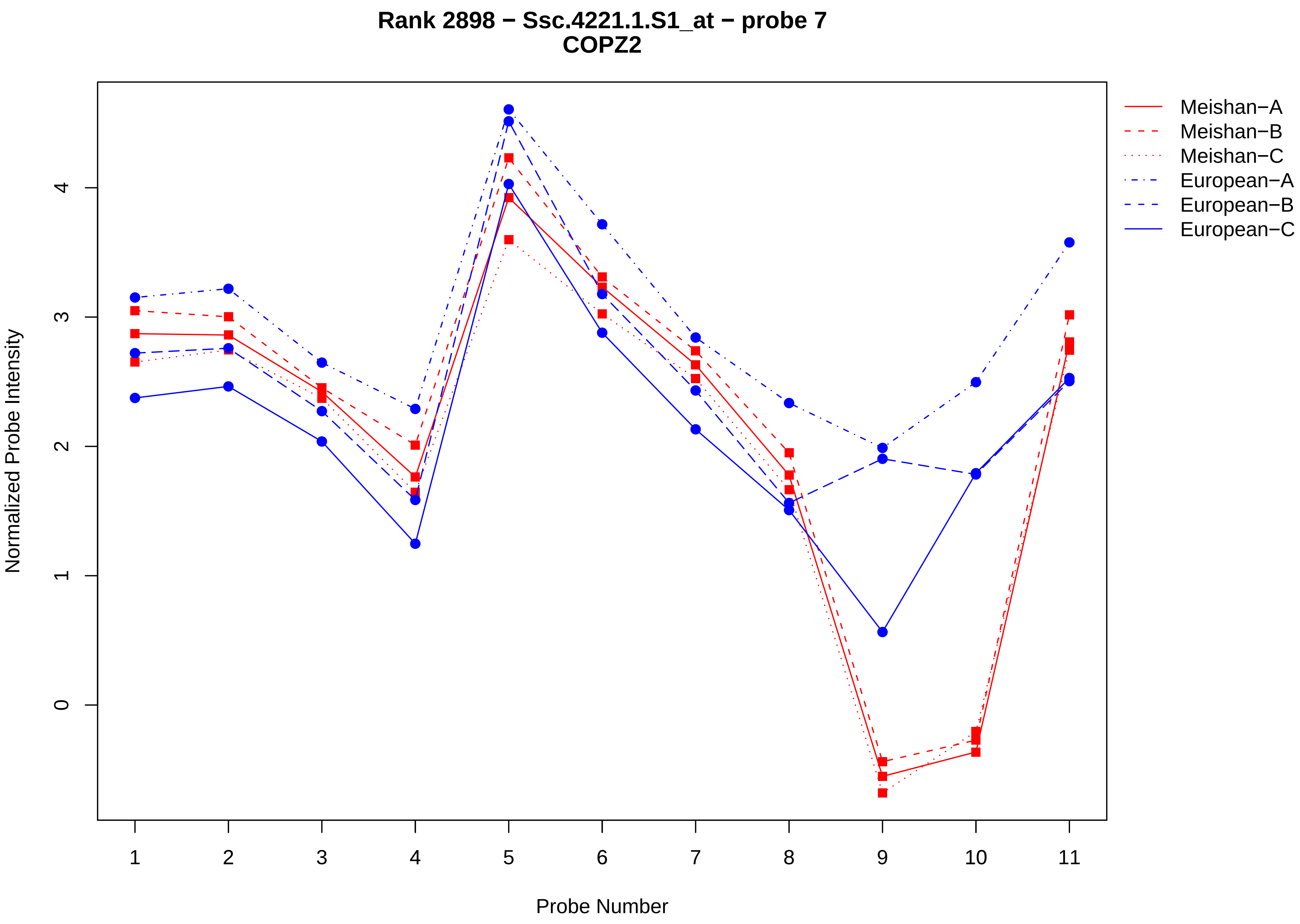
Rank 2898 − Ssc.4221.1.S1_at − probe 7
COPZ2
Normalized Probe Intensity
Probe Number
0
1
2
3
4
1
2
3
4
5
6
7
8
9
10
11
Meishan−A
Meishan−B
Meishan−C
European−A
European−B
European−C

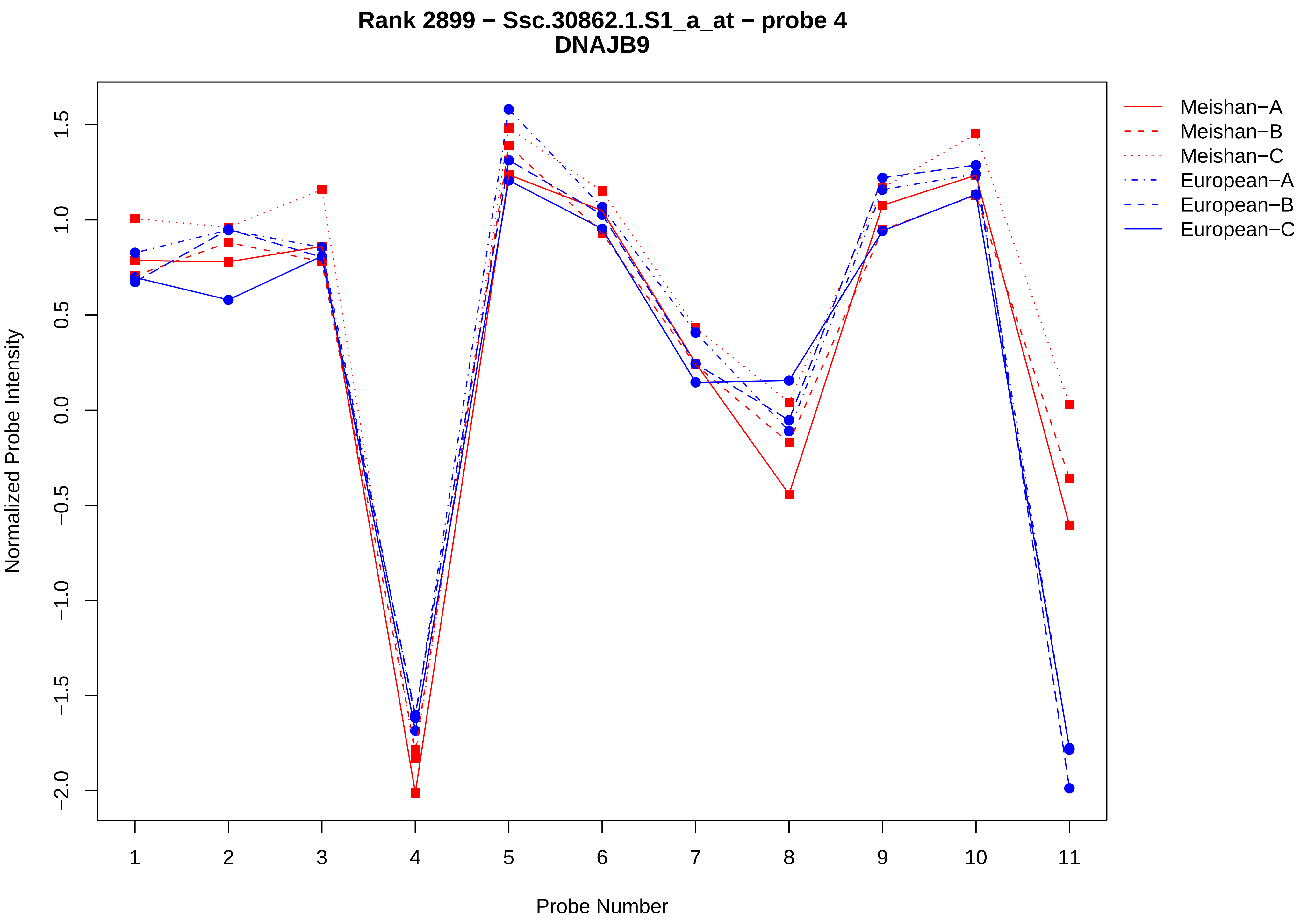

Rank 2899 − Ssc.30862.1.S1_a_at − probe 4
DNAJB9
Normalized Probe Intensity
Probe Number
1.5
1.0
0.5
0.0
−0.5
−1.0
−1.5
−2.0
1
2
3
4
5
6
7
8
9
10
11
Meishan−A
Meishan−B
Meishan−C
European−A
European−B
European−C

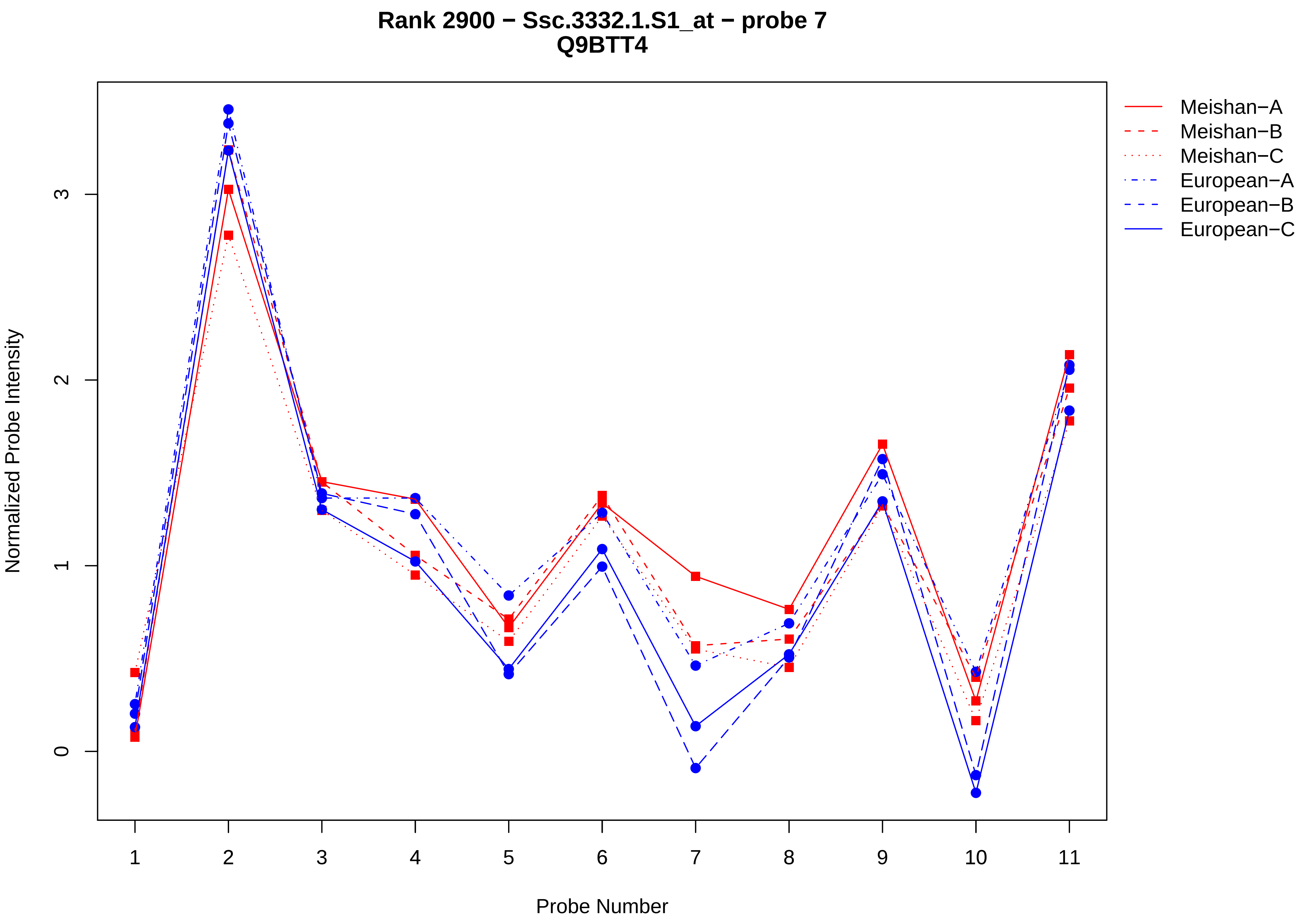
Rank 2900 − Ssc.3332.1.S1_at − probe 7
Q9BTT4
Normalized Probe Intensity
0
1
2
3
1
2
3
4
5
6
7
8
9
10
11
Probe Number
Meishan−A
Meishan−B
Meishan−C
European−A
European−B
European−C

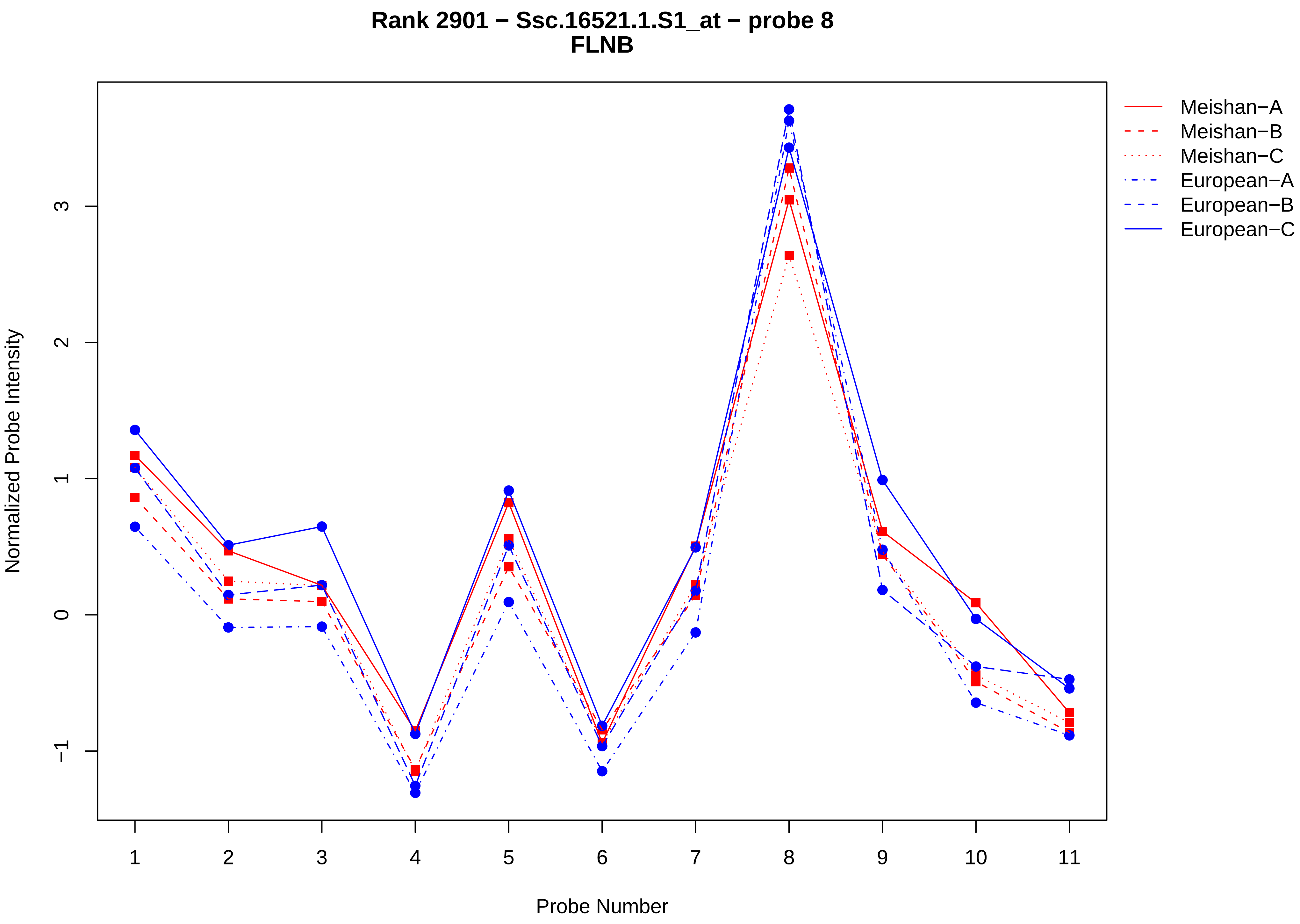

Rank 2901 − Ssc.16521.1.S1_at − probe 8
FLNB
Normalized Probe Intensity
Probe Number
−1
0
1
2
3
1
2
3
4
5
6
7
8
9
10
11
Meishan−A
Meishan−B
Meishan−C
European−A
European−B
European−C

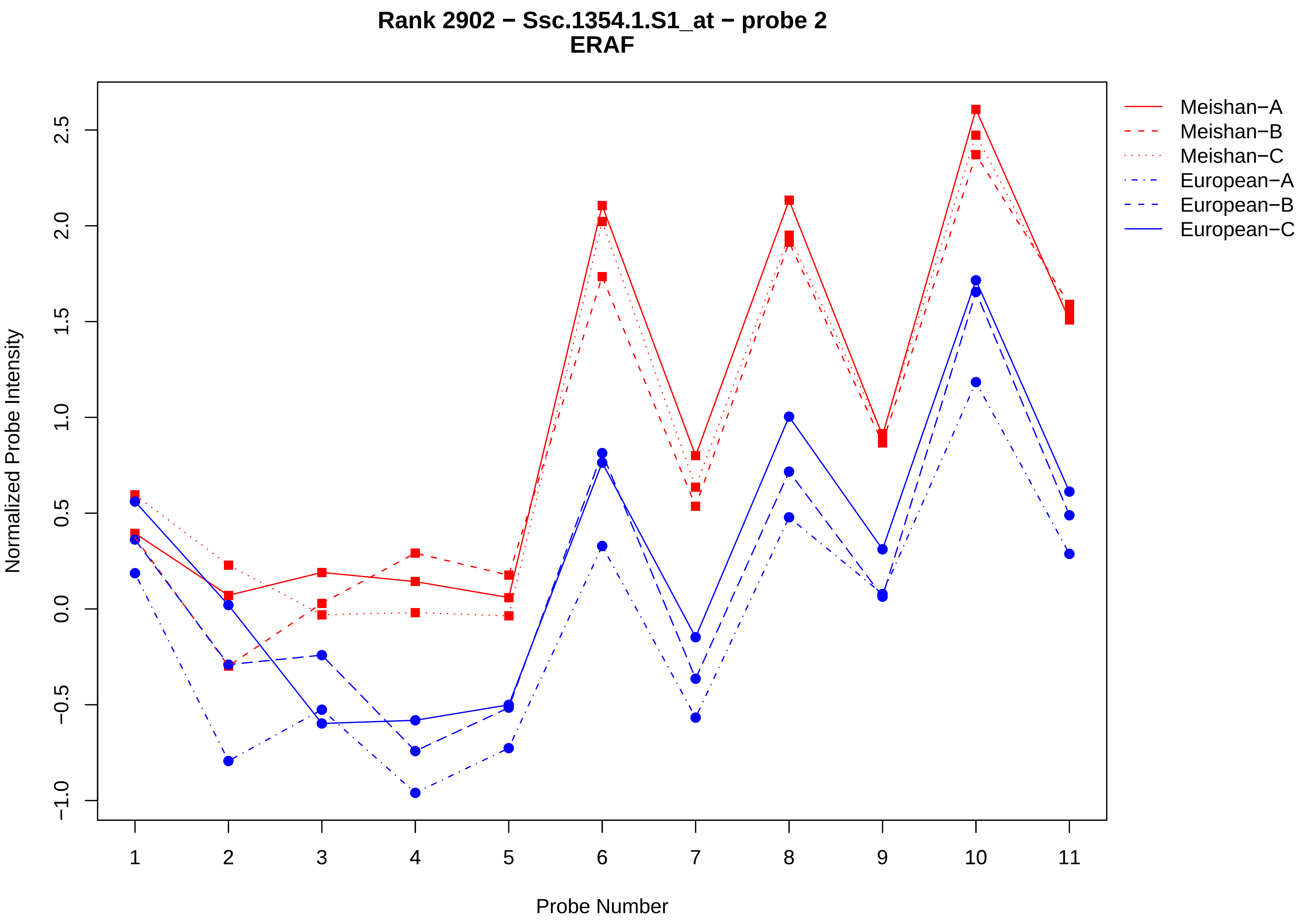

Rank 2902 − Ssc.1354.1.S1_at − probe 2
ERAF
Normalized Probe Intensity
Probe Number
Meishan−A
Meishan−B
Meishan−C
European−A
European−B
European−C
−1.0
−0.5
0.0
0.5
1.0
1.5
2.0
2.5
1
2
3
4
5
6
7
8
9
10
11

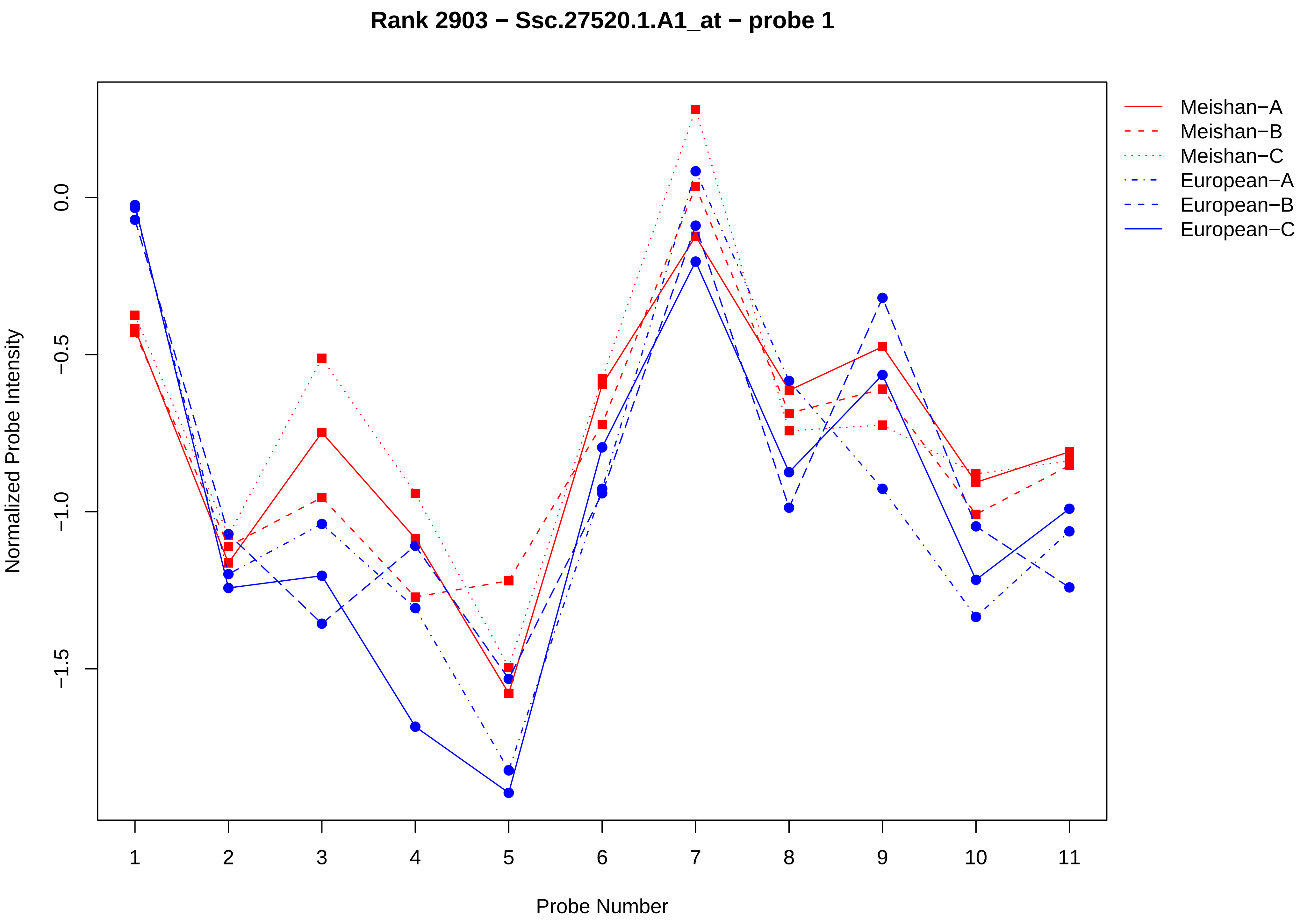
Rank 2903 − Ssc.27520.1.A1_at − probe 1
Normalized Probe Intensity
0.0
−0.5
−1.0
−1.5
1
2
3
4
5
6
7
8
9
10
11
Probe Number
Meishan−A
Meishan−B
Meishan−C
European−A
European−B
European−C

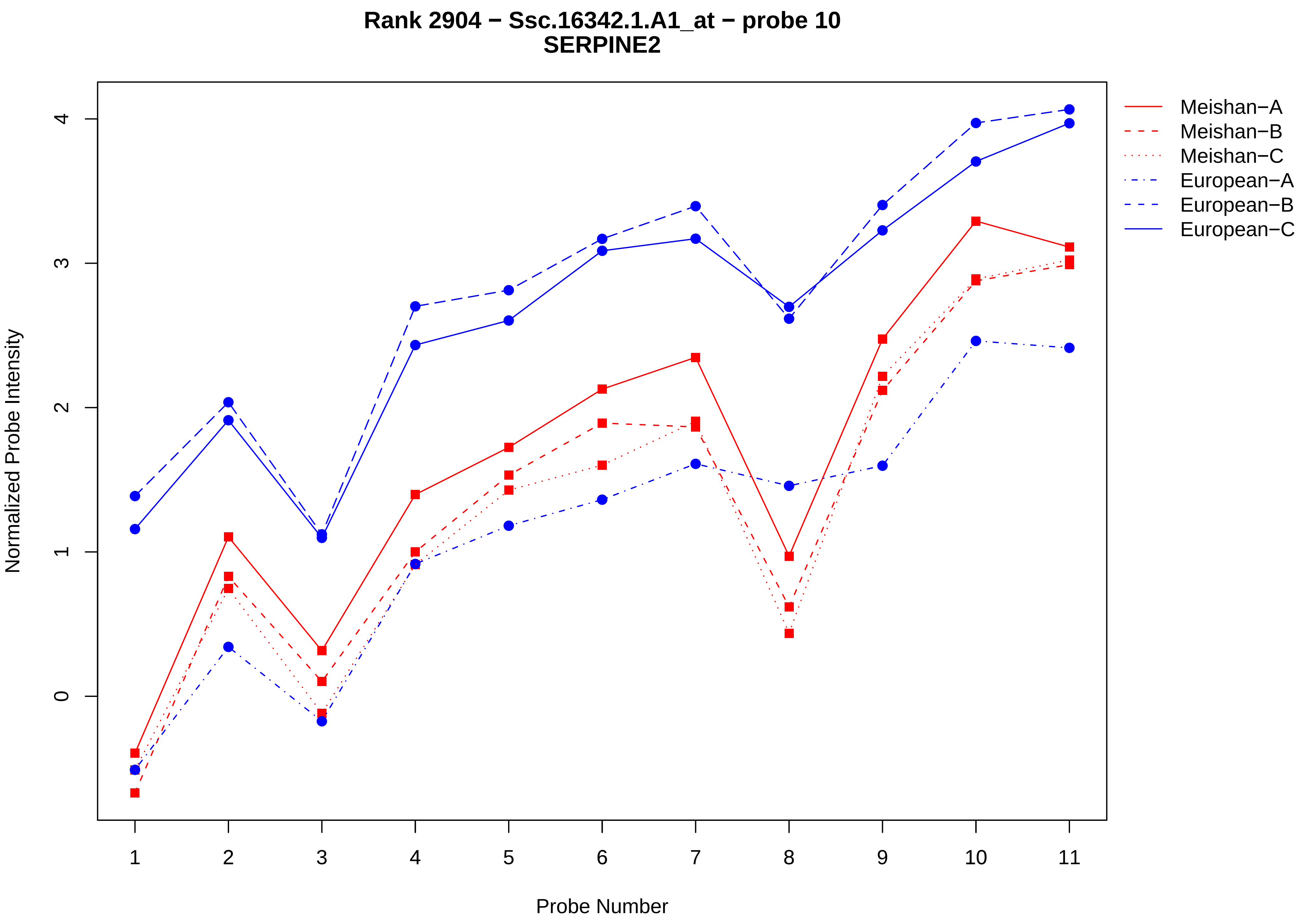
Rank 2904 − Ssc.16342.1.A1_at − probe 10
SERPINE2
Normalized Probe Intensity
Probe Number
0
1
2
3
4
1
2
3
4
5
6
7
8
9
10
11
Meishan−A
Meishan−B
Meishan−C
European−A
European−B
European−C

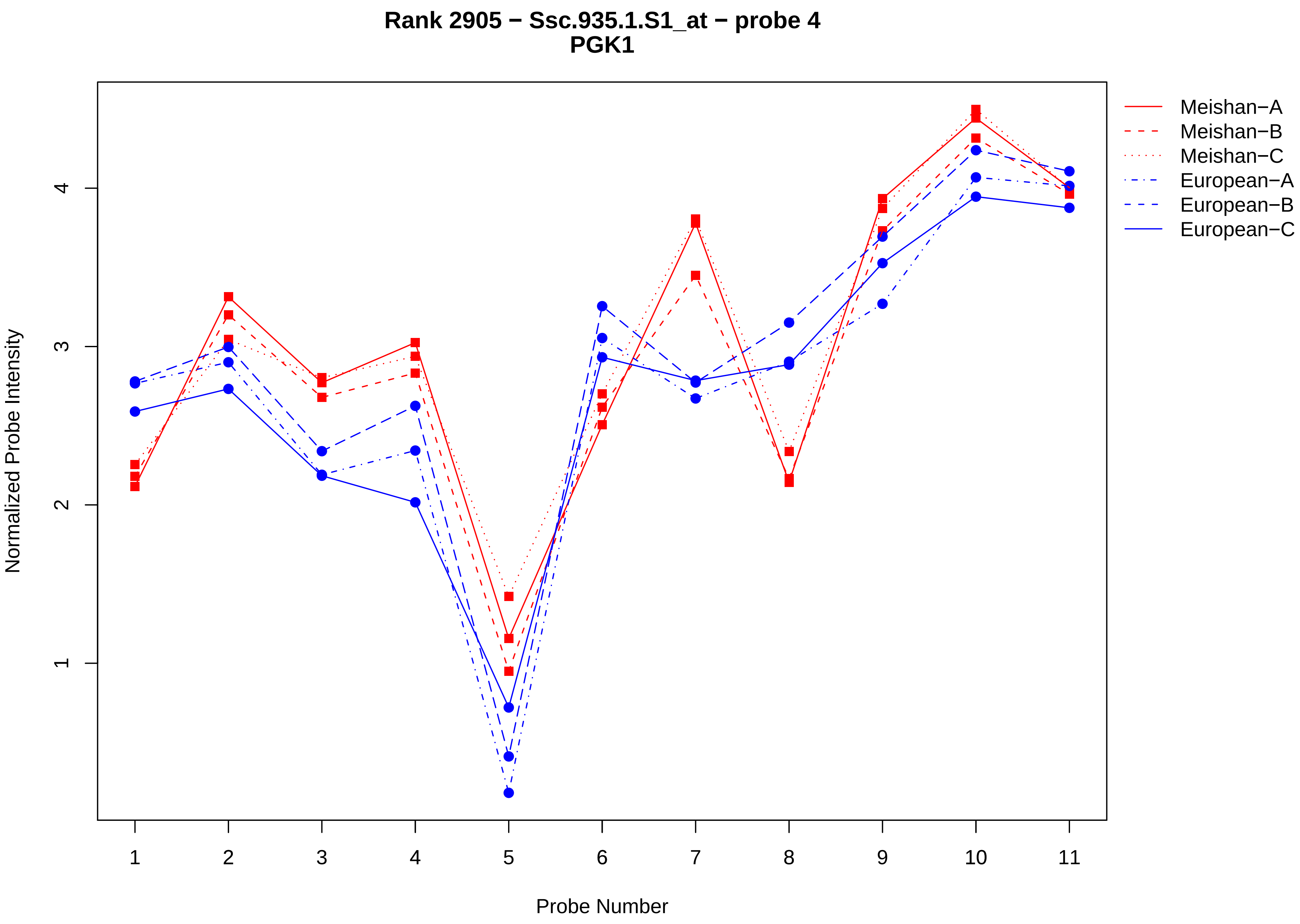

Rank 2905 − Ssc.935.1.S1_at − probe 4
PGK1
Normalized Probe Intensity
Probe Number
1
2
3
4
1 2 3 4 5 6 7 8 9 10 11
Meishan−A
Meishan−B
Meishan−C
European−A
European−B
European−C

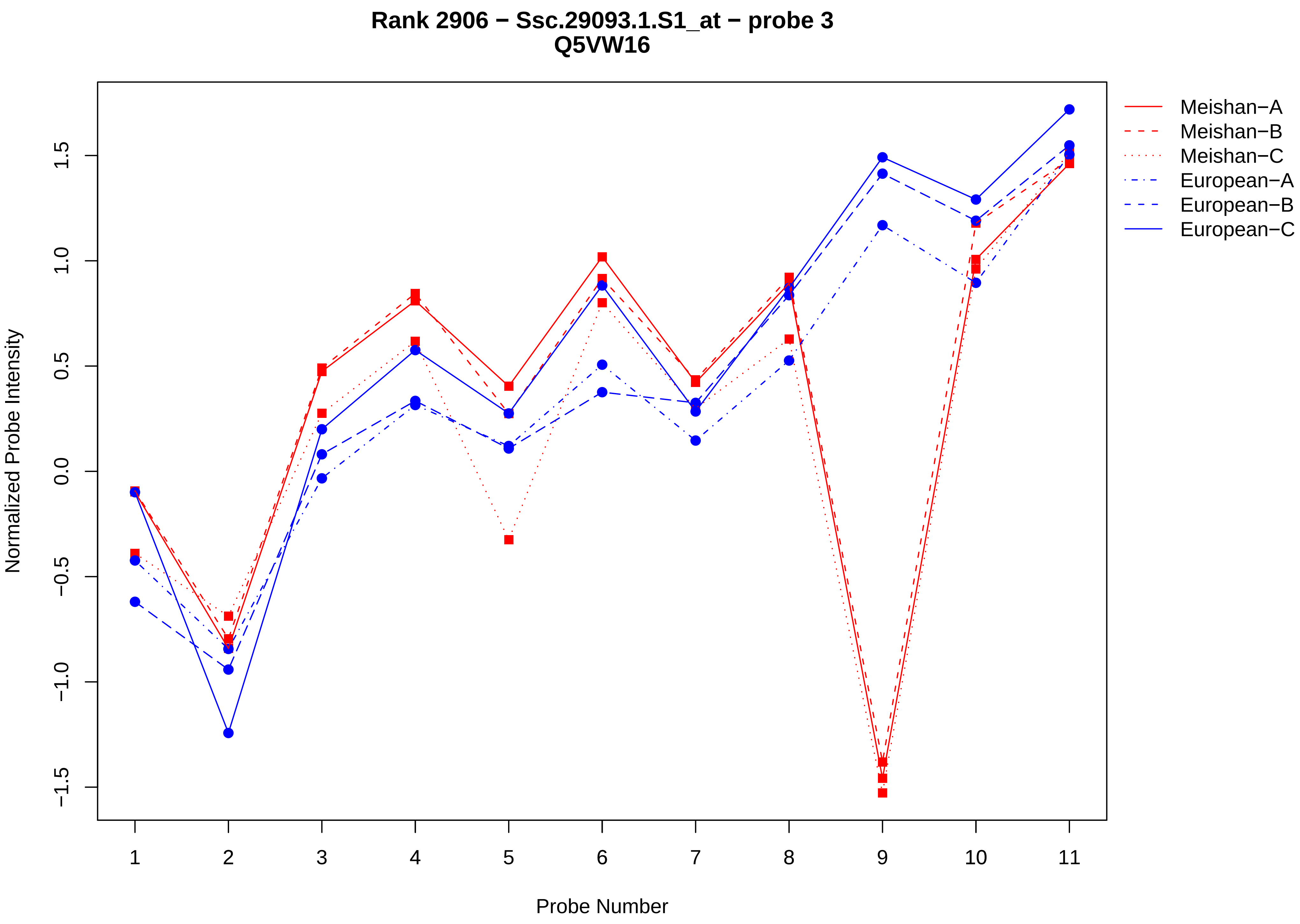

Rank 2906 − Ssc.29093.1.S1_at − probe 3
Q5VW16
Normalized Probe Intensity
Probe Number
Meishan−A
Meishan−B
Meishan−C
European−A
European−B
European−C
−1.5
−1.0
−0.5
0.0
0.5
1.0
1.5
1
2
3
4
5
6
7
8
9
10
11

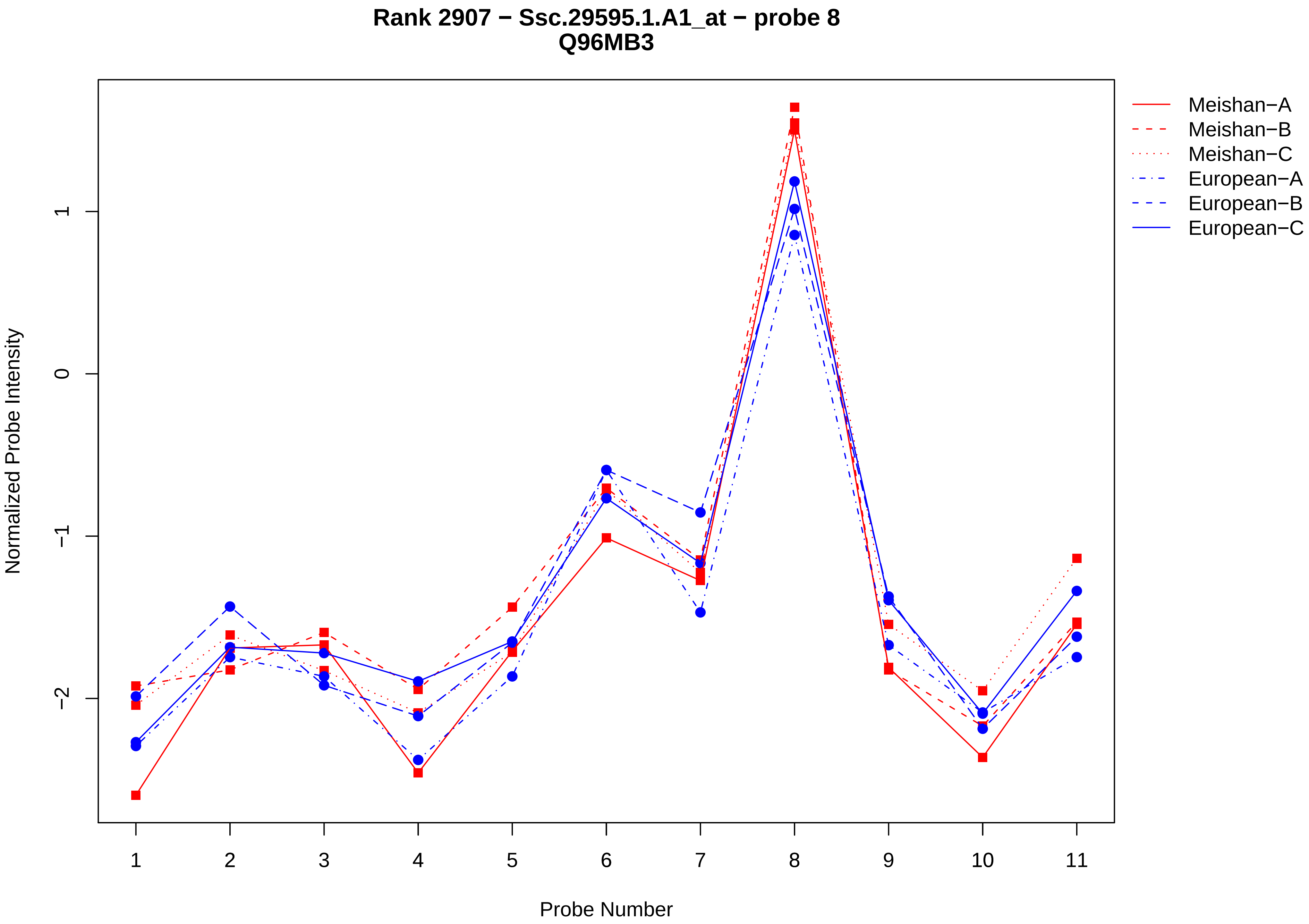

Rank 2907 − Ssc.29595.1.A1_at − probe 8
Q96MB3
Normalized Probe Intensity
Probe Number
1
0
−1
−2
1
2
3
4
5
6
7
8
9
10
11
Meishan−A
Meishan−B
Meishan−C
European−A
European−B
European−C

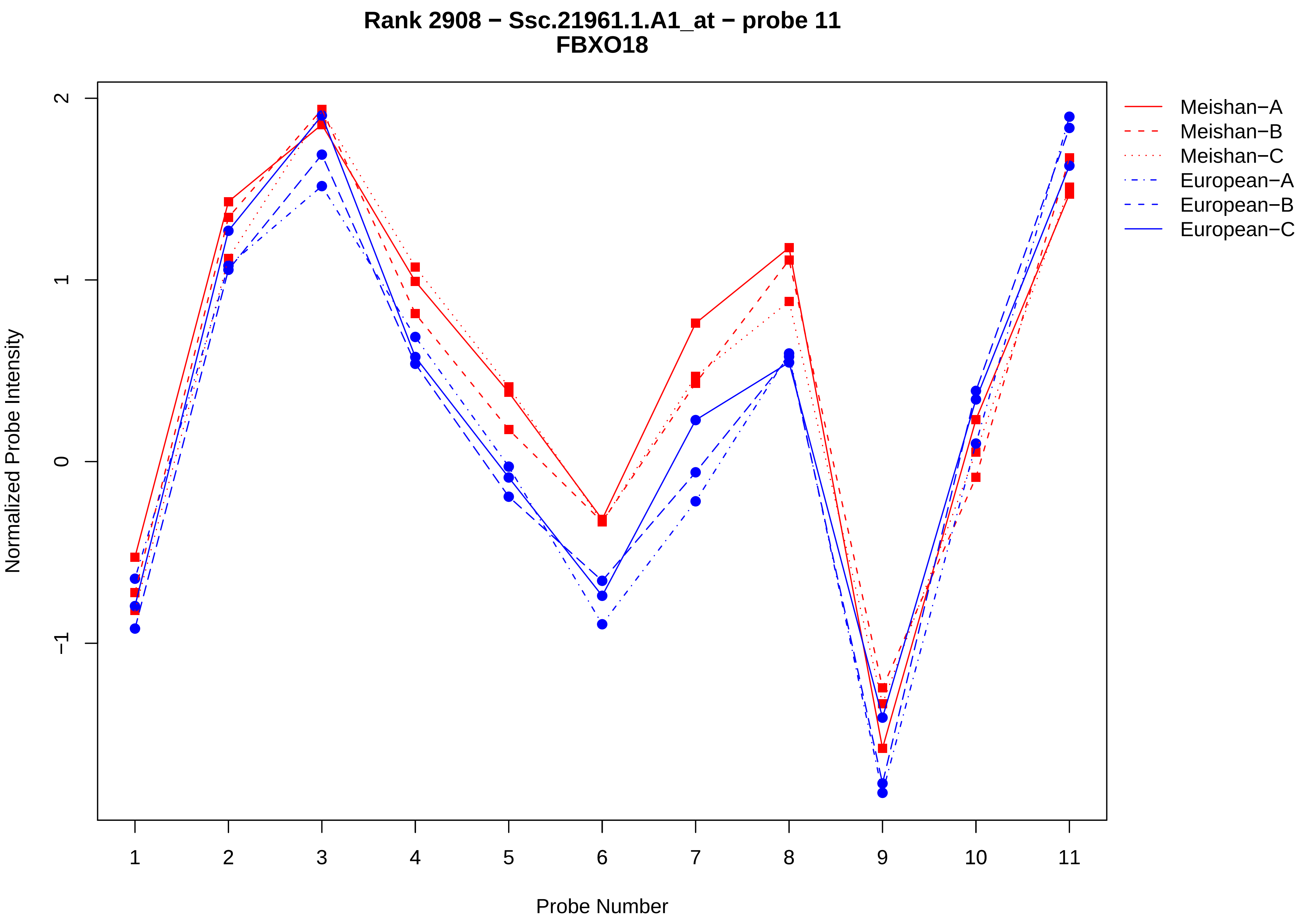

Rank 2908 − Ssc.21961.1.A1_at − probe 11
FBXO18
Normalized Probe Intensity
Probe Number
Meishan−A
Meishan−B
Meishan−C
European−A
European−B
European−C
2
1
0
−1
1
2
3
4
5
6
7
8
9
10
11

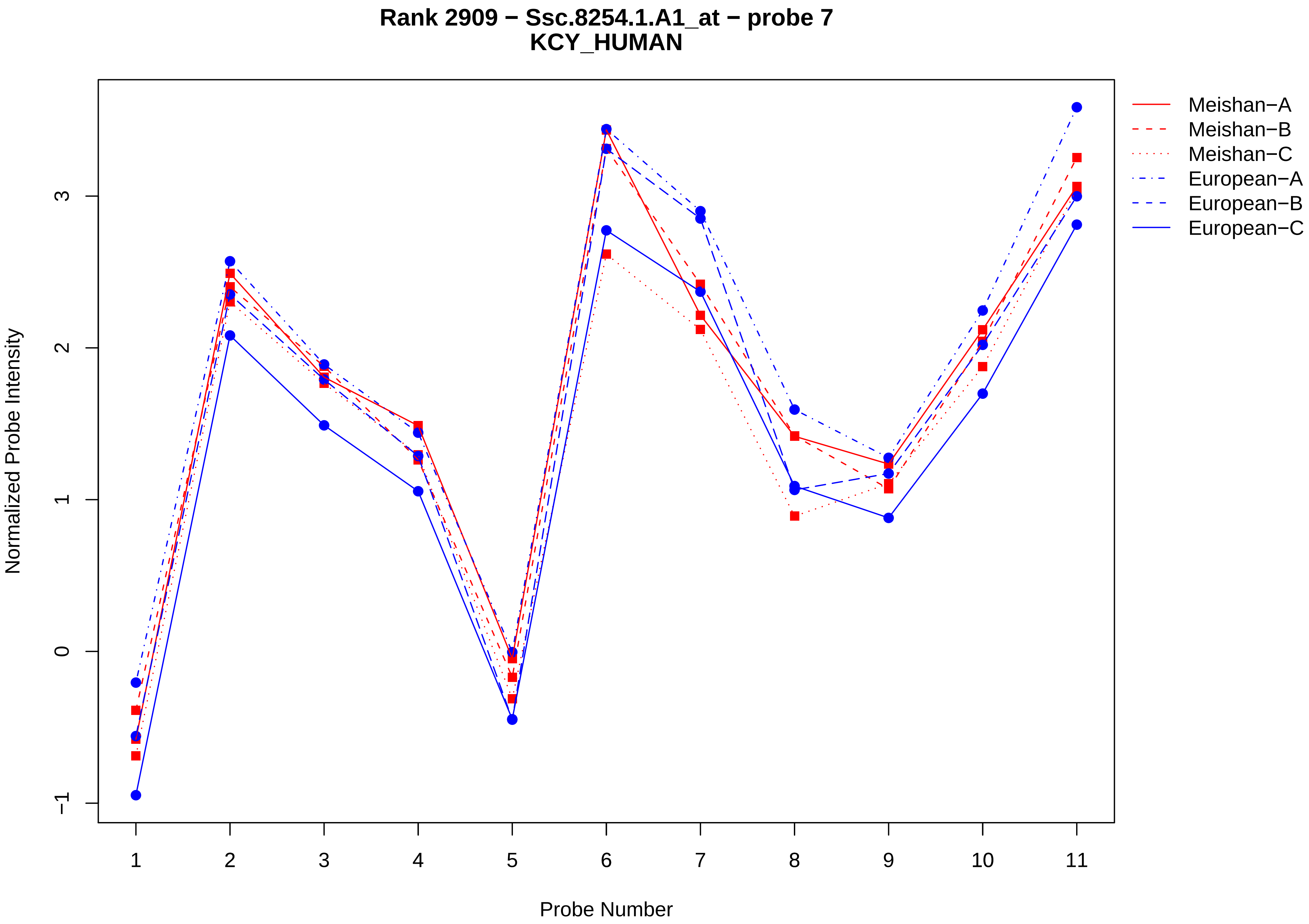

Rank 2909 − Ssc.8254.1.A1_at − probe 7
KCY_HUMAN
Normalized Probe Intensity
Probe Number
−1
0
1
2
3
1
2
3
4
5
6
7
8
9
10
11
Meishan−A
Meishan−B
Meishan−C
European−A
European−B
European−C

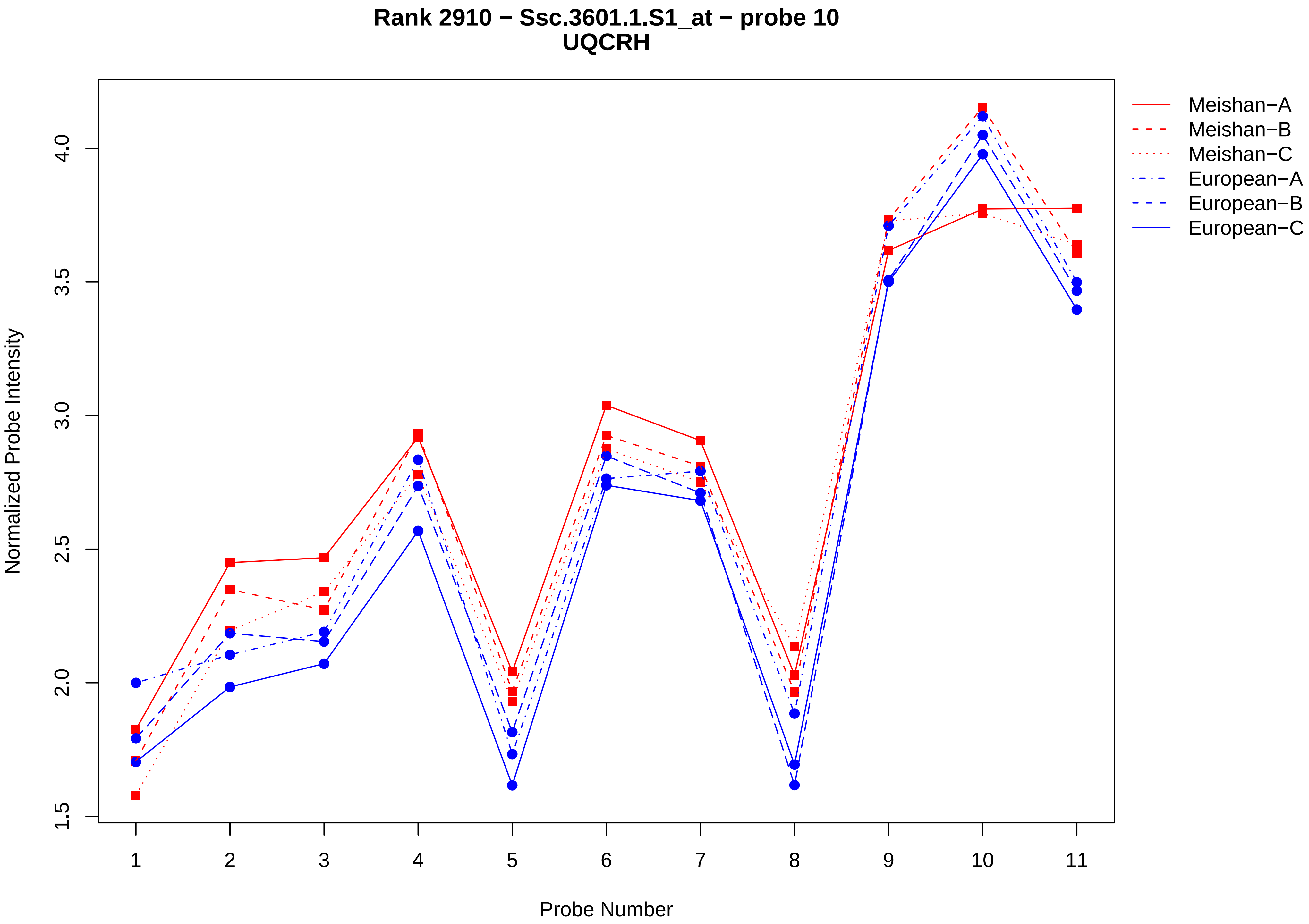

Rank 2910 − Ssc.3601.1.S1_at − probe 10
UQCRH
Normalized Probe Intensity
Probe Number
1.5
2.0
2.5
3.0
3.5
4.0
1
2
3
4
5
6
7
8
9
10
11
Meishan−A
Meishan−B
Meishan−C
European−A
European−B
European−C

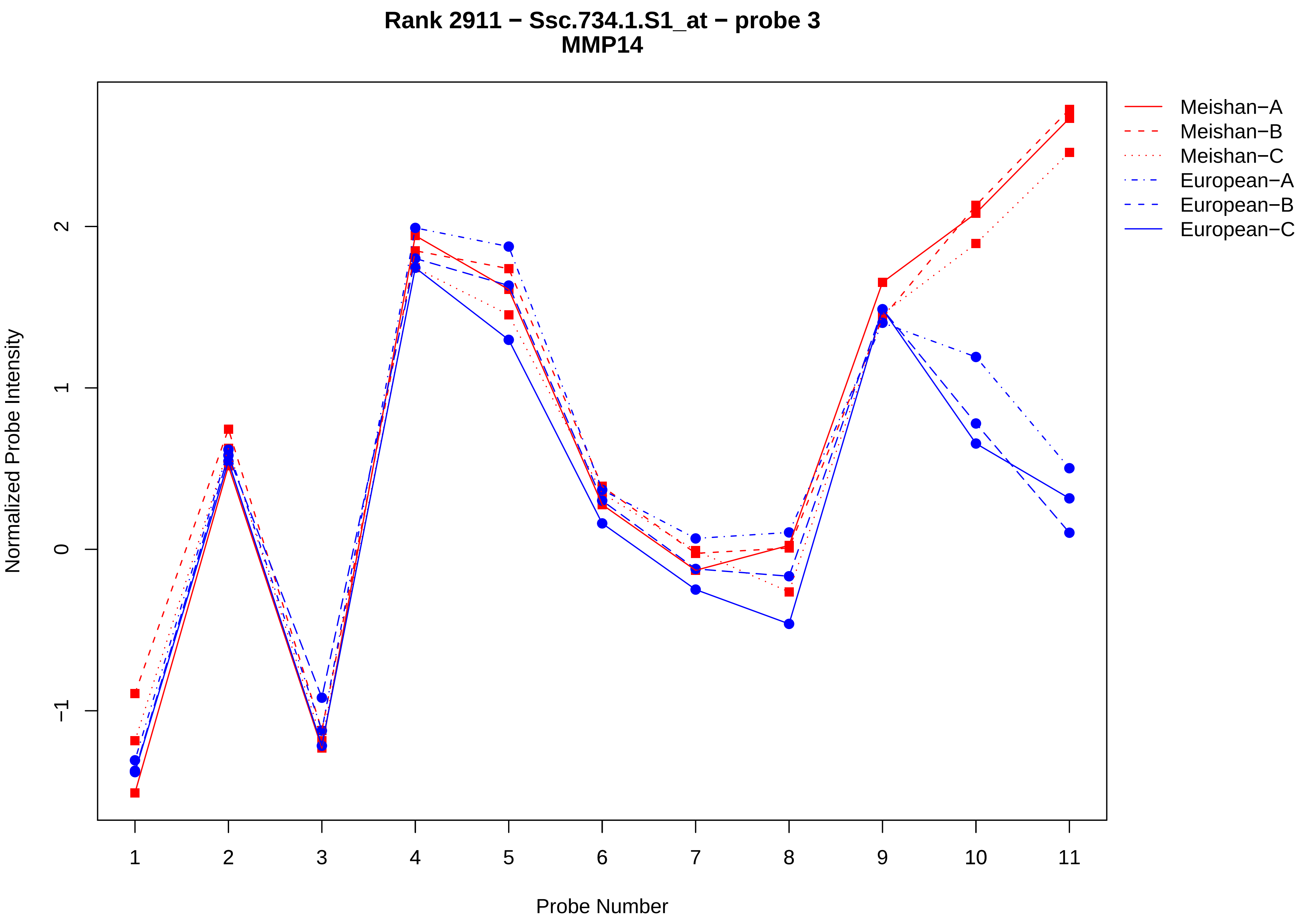

Rank 2911 − Ssc.734.1.S1_at − probe 3
MMP14
Normalized Probe Intensity
Probe Number
−1
0
1
2
1
2
3
4
5
6
7
8
9
10
11
Meishan−A
Meishan−B
Meishan−C
European−A
European−B
European−C

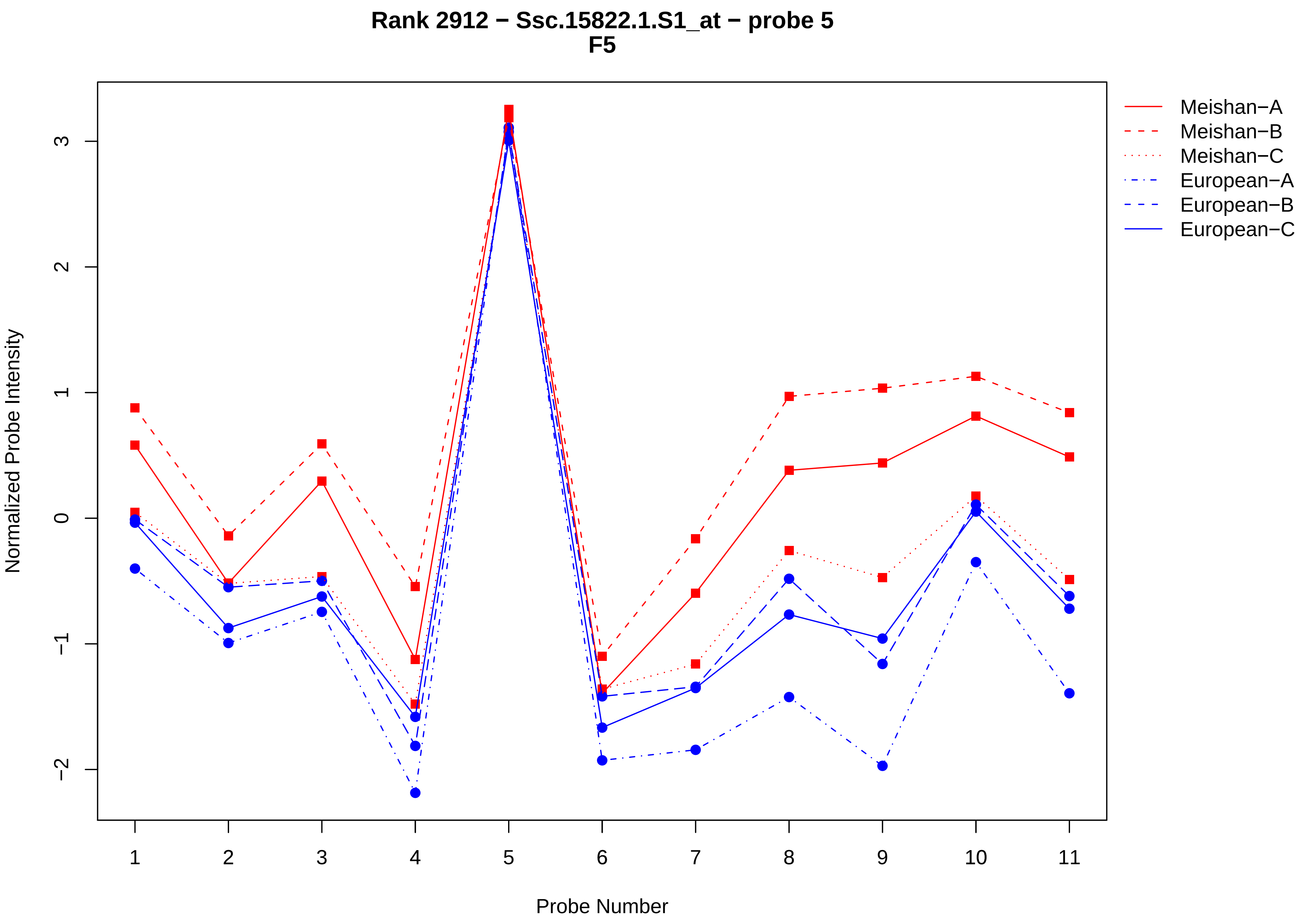

Rank 2912 − Ssc.15822.1.S1_at − probe 5
F5
Normalized Probe Intensity
Probe Number
Meishan−A
Meishan−B
Meishan−C
European−A
European−B
European−C
−2
−1
0
1
2
3
1
2
3
4
5
6
7
8
9
10
11

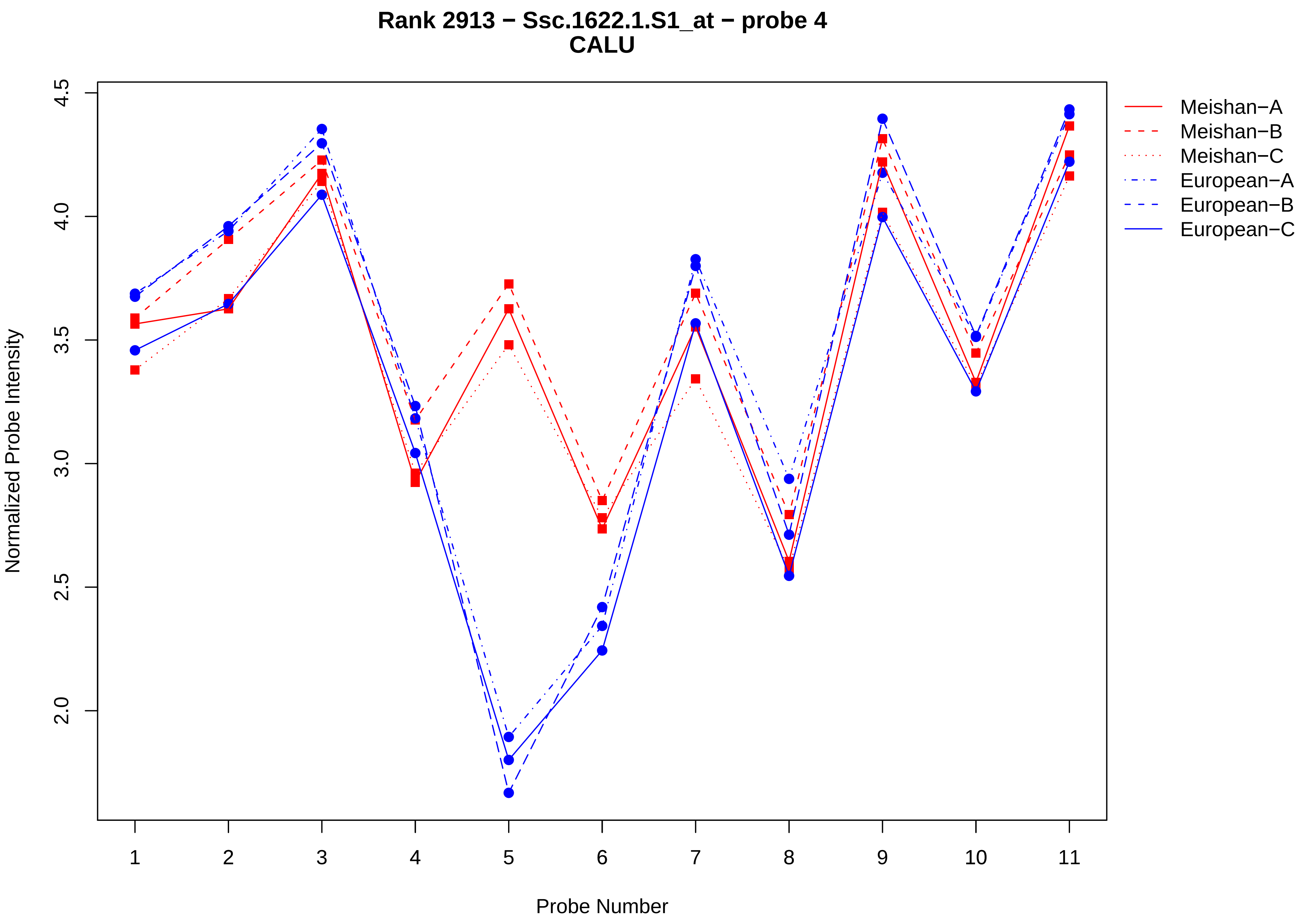

Rank 2913 − Ssc.1622.1.S1_at − probe 4
CALU
Normalized Probe Intensity
Probe Number
4.5
4.0
3.5
3.0
2.5
2.0
1
2
3
4
5
6
7
8
9
10
11
Meishan−A
Meishan−B
Meishan−C
European−A
European−B
European−C

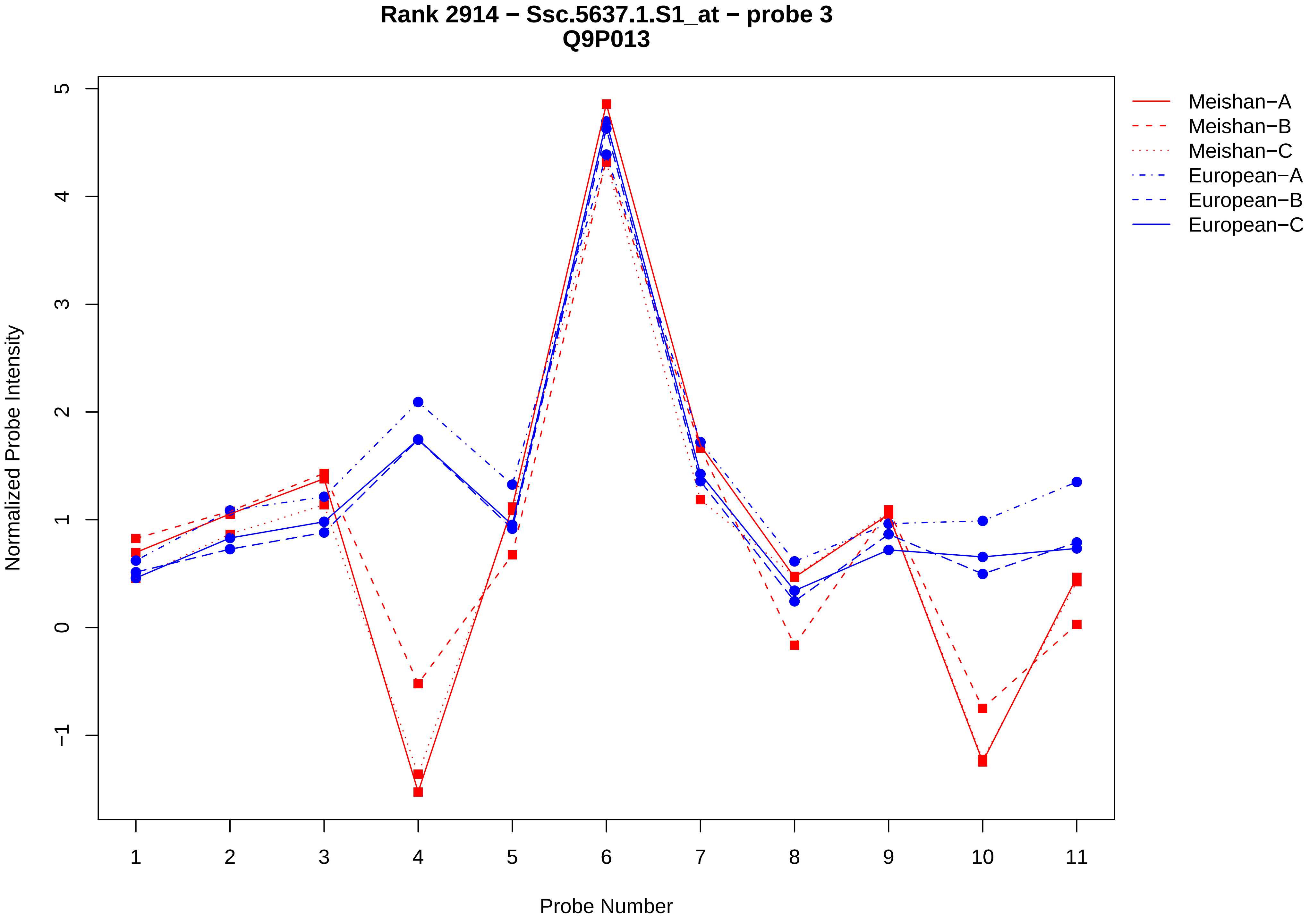

Rank 2914 − Ssc.5637.1.S1_at − probe 3
Q9P013
Normalized Probe Intensity
Probe Number
Meishan−A
Meishan−B
Meishan−C
European−A
European−B
European−C
−1
0
1
2
3
4
5
1
2
3
4
5
6
7
8
9
10
11

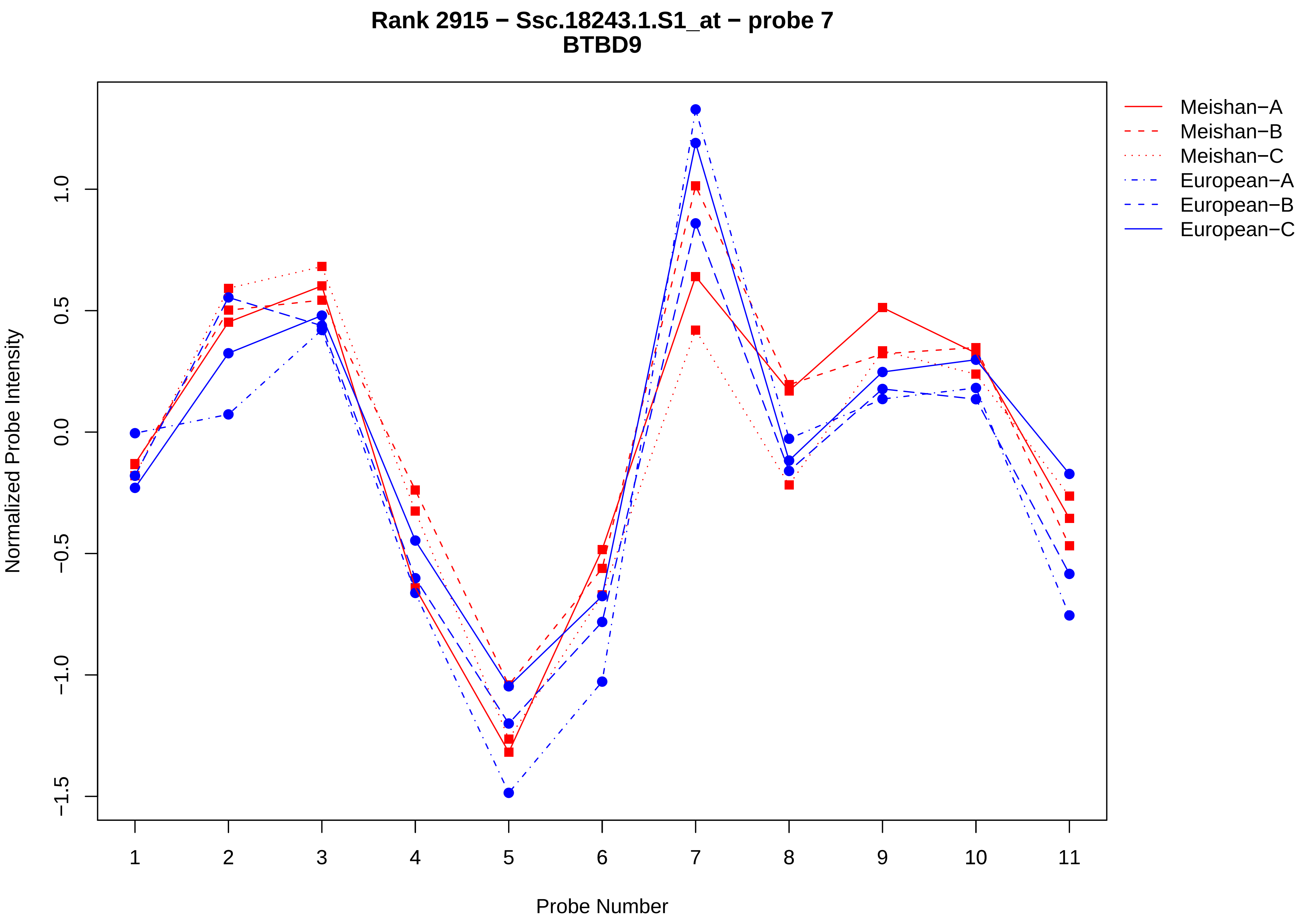

Rank 2915 − Ssc.18243.1.S1_at − probe 7
BTBD9
Normalized Probe Intensity
Probe Number
−1.5
−1.0
−0.5
0.0
0.5
1.0
1
2
3
4
5
6
7
8
9
10
11
Meishan−A
Meishan−B
Meishan−C
European−A
European−B
European−C

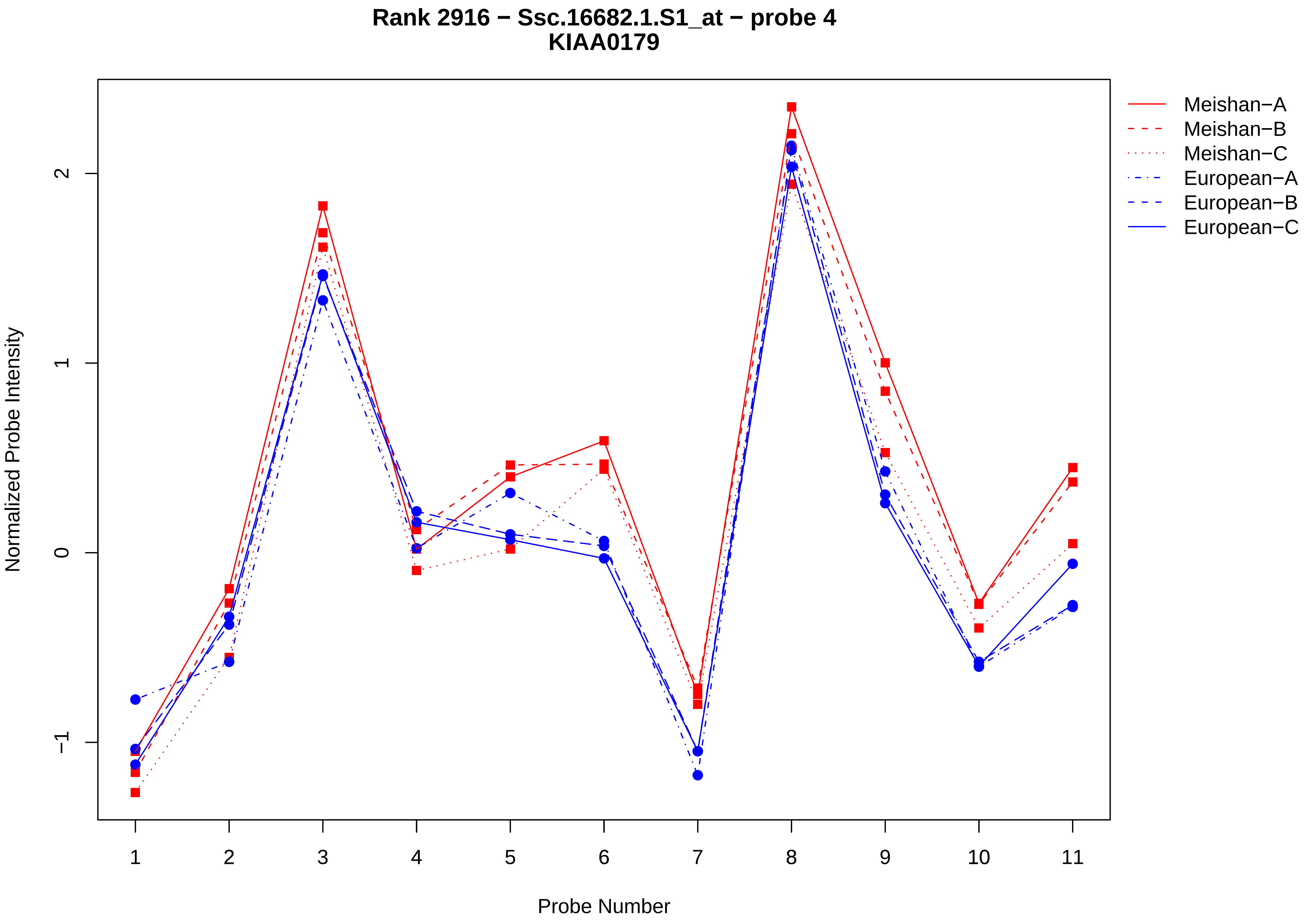

Rank 2916 − Ssc.16682.1.S1_at − probe 4
KIAA0179
Normalized Probe Intensity
Probe Number
Meishan−A
Meishan−B
Meishan−C
European−A
European−B
European−C
−1
0
1
2
1
2
3
4
5
6
7
8
9
10
11

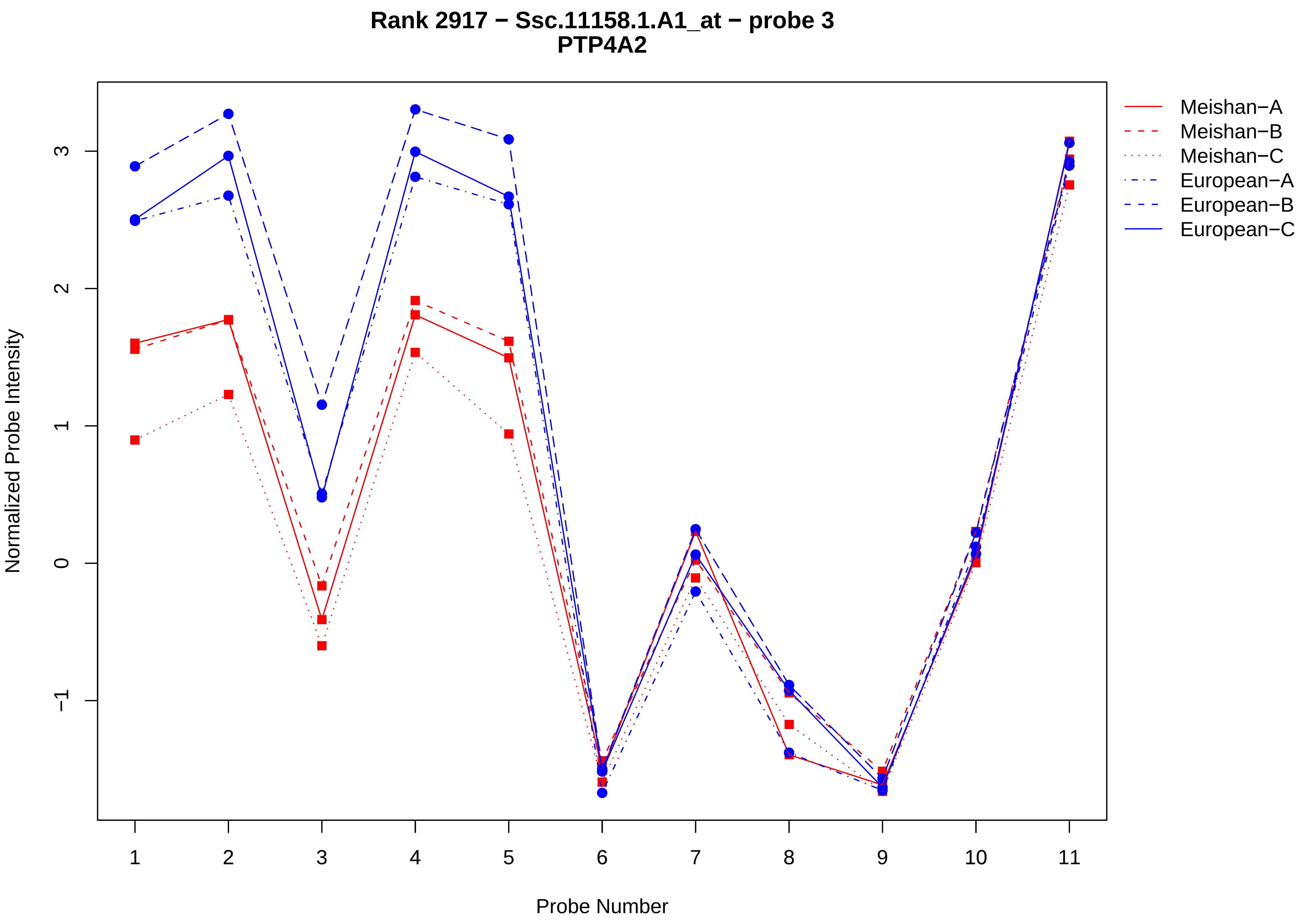
Rank 2917 − Ssc.11158.1.A1_at − probe 3
PTP4A2
Normalized Probe Intensity
−1
0
1
2
3
1
2
3
4
5
6
7
8
9
10
11
Probe Number
Meishan−A
Meishan−B
Meishan−C
European−A
European−B
European−C

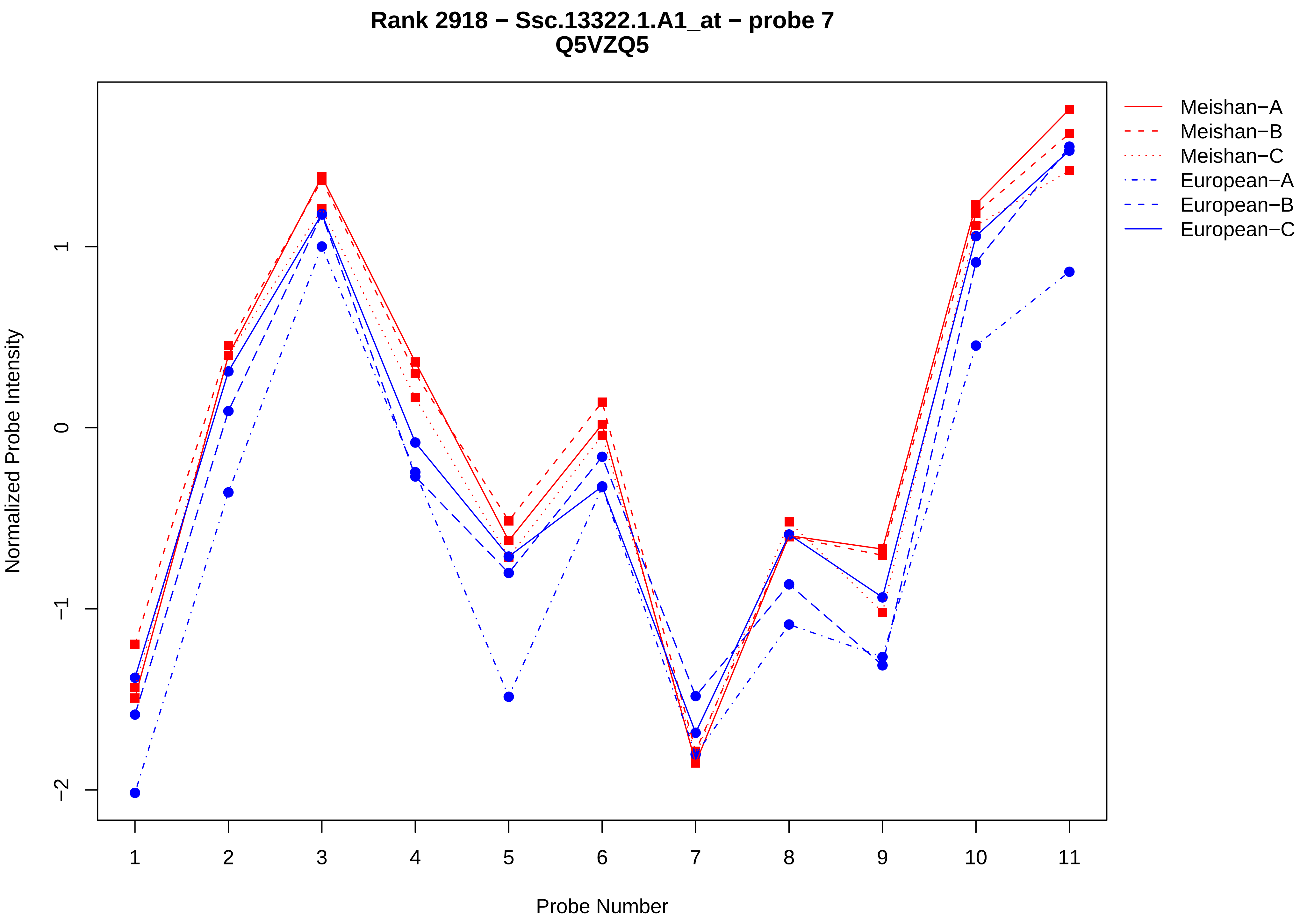

Rank 2918 − Ssc.13322.1.A1_at − probe 7
Q5VZQ5
Normalized Probe Intensity
Probe Number
−2
−1
0
1
1
2
3
4
5
6
7
8
9
10
11
Meishan−A
Meishan−B
Meishan−C
European−A
European−B
European−C

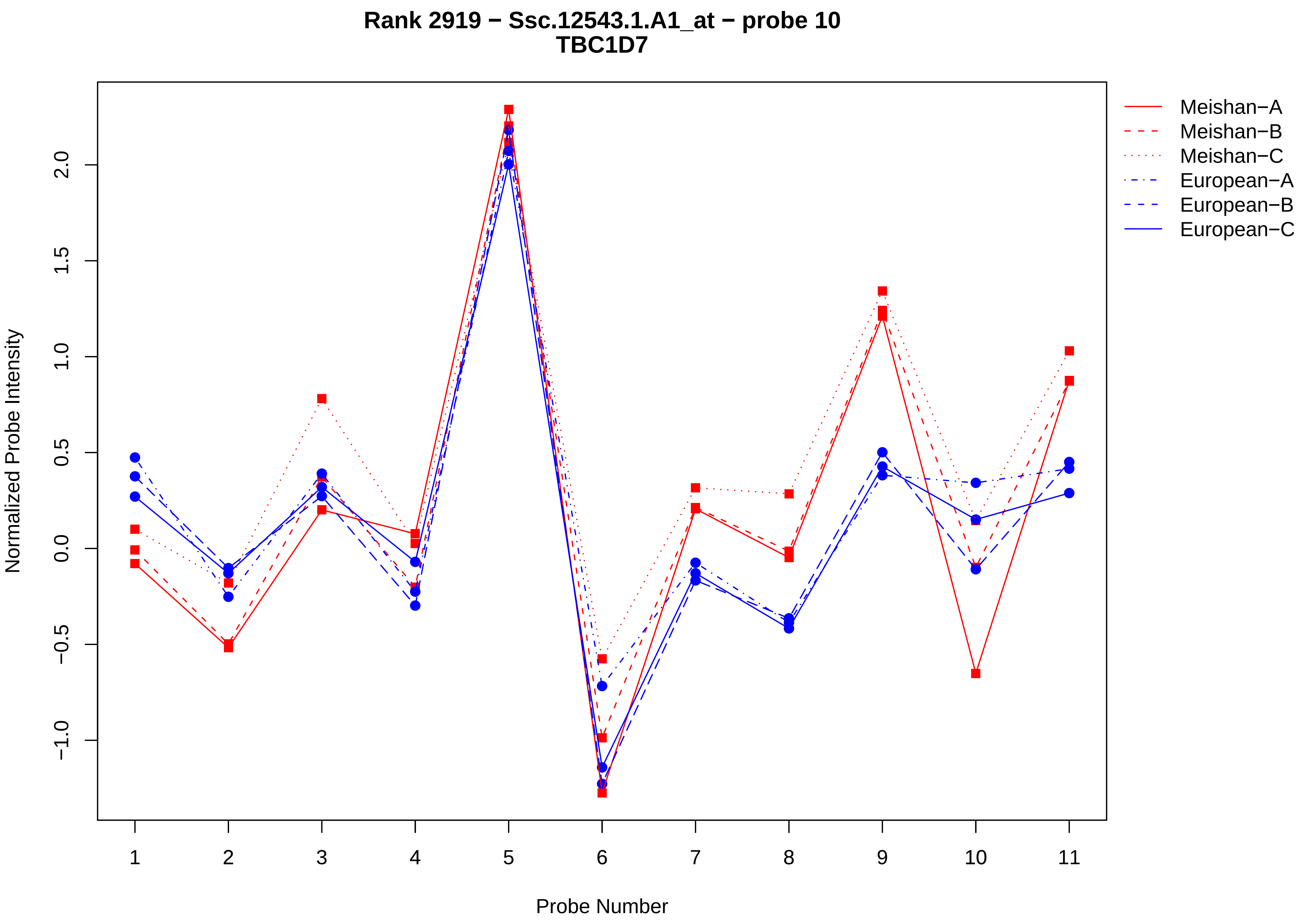

Rank 2919 − Ssc.12543.1.A1_at − probe 10
TBC1D7
Normalized Probe Intensity
Probe Number
Meishan−A
Meishan−B
Meishan−C
European−A
European−B
European−C

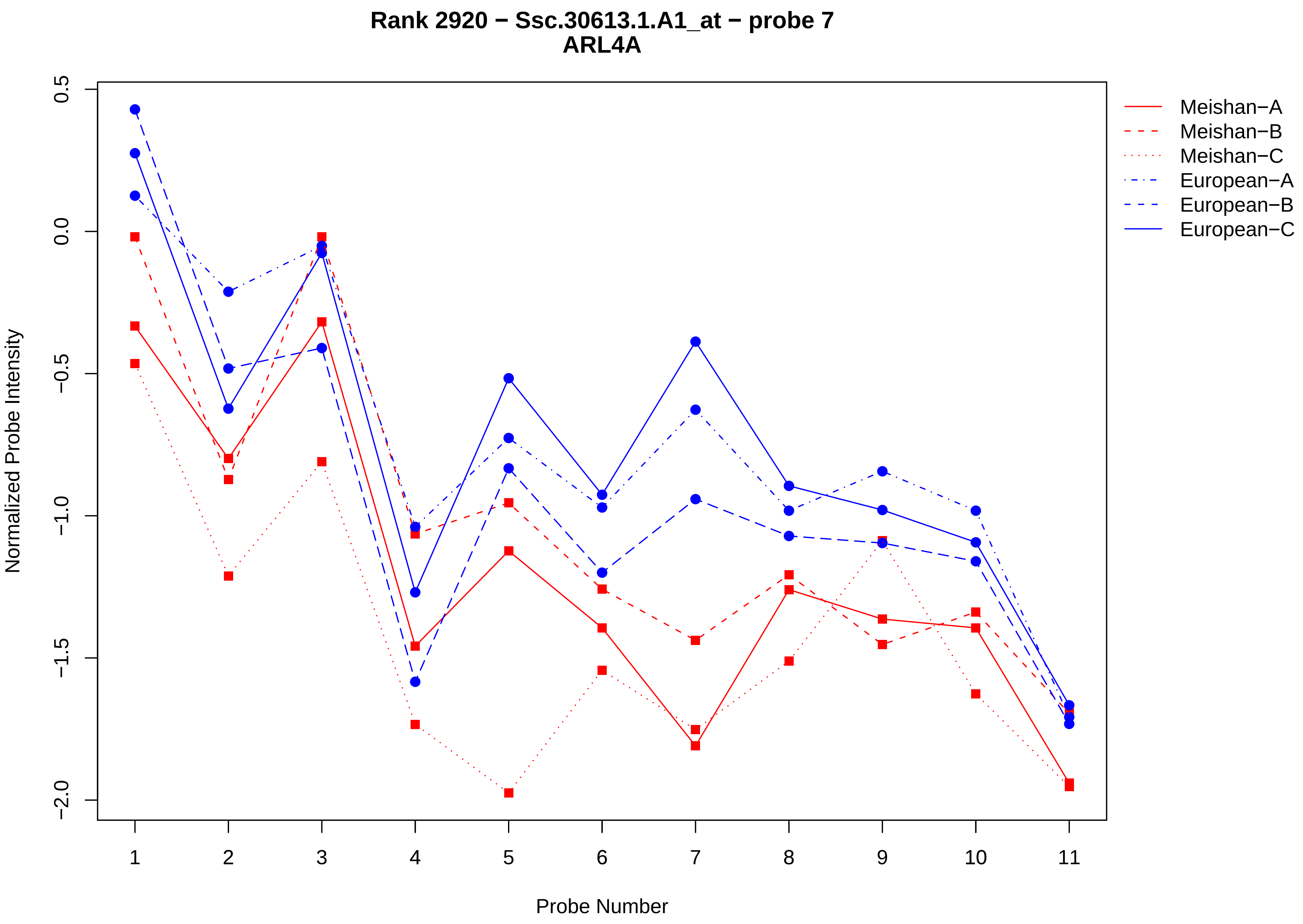

Rank 2920 − Ssc.30613.1.A1_at − probe 7
ARL4A
Normalized Probe Intensity
Probe Number
0.5
0.0
−0.5
−1.0
−1.5
−2.0
1
2
3
4
5
6
7
8
9
10
11
Meishan−A
Meishan−B
Meishan−C
European−A
European−B
European−C

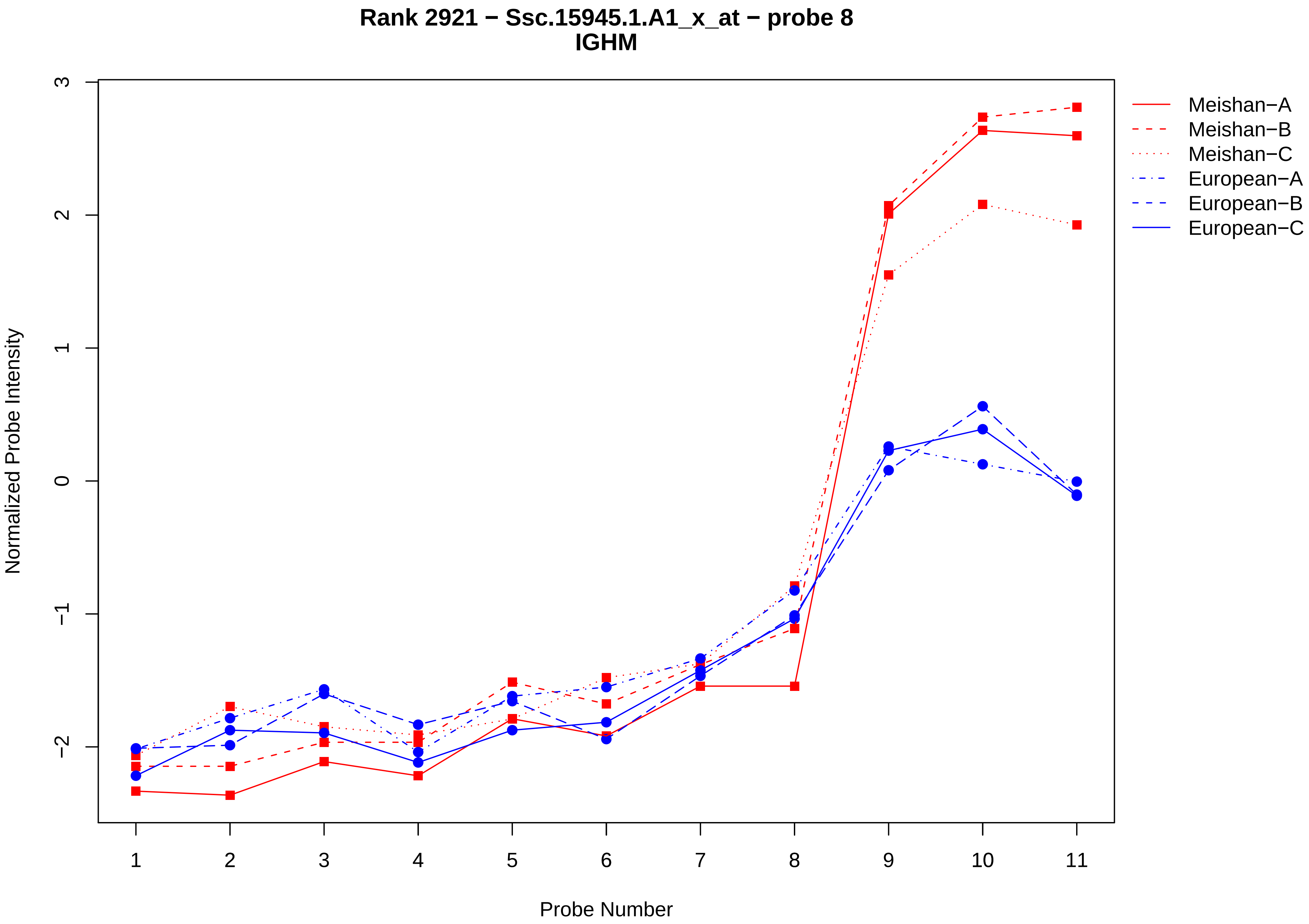
Rank 2921 − Ssc.15945.1.A1_x_at − probe 8
IGHM
Normalized Probe Intensity
Probe Number
Meishan−A
Meishan−B
Meishan−C
European−A
European−B
European−C
3
2
1
0
−1
−2
1
2
3
4
5
6
7
8
9
10
11

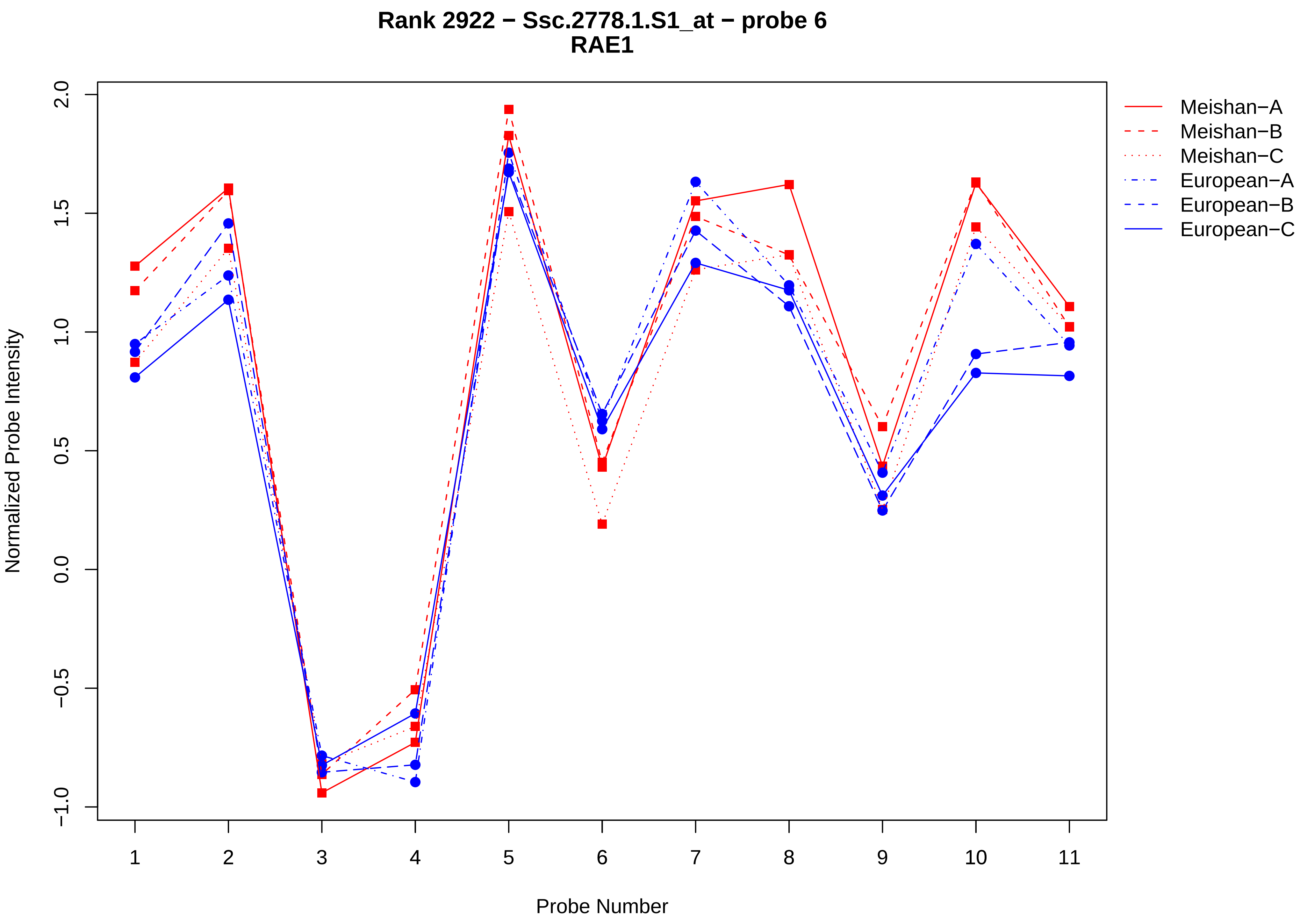

Rank 2922 − Ssc.2778.1.S1_at − probe 6
RAE1
Normalized Probe Intensity
Probe Number
−1.0
−0.5
0.0
0.5
1.0
1.5
2.0
1
2
3
4
5
6
7
8
9
10
11
Meishan−A
Meishan−B
Meishan−C
European−A
European−B
European−C

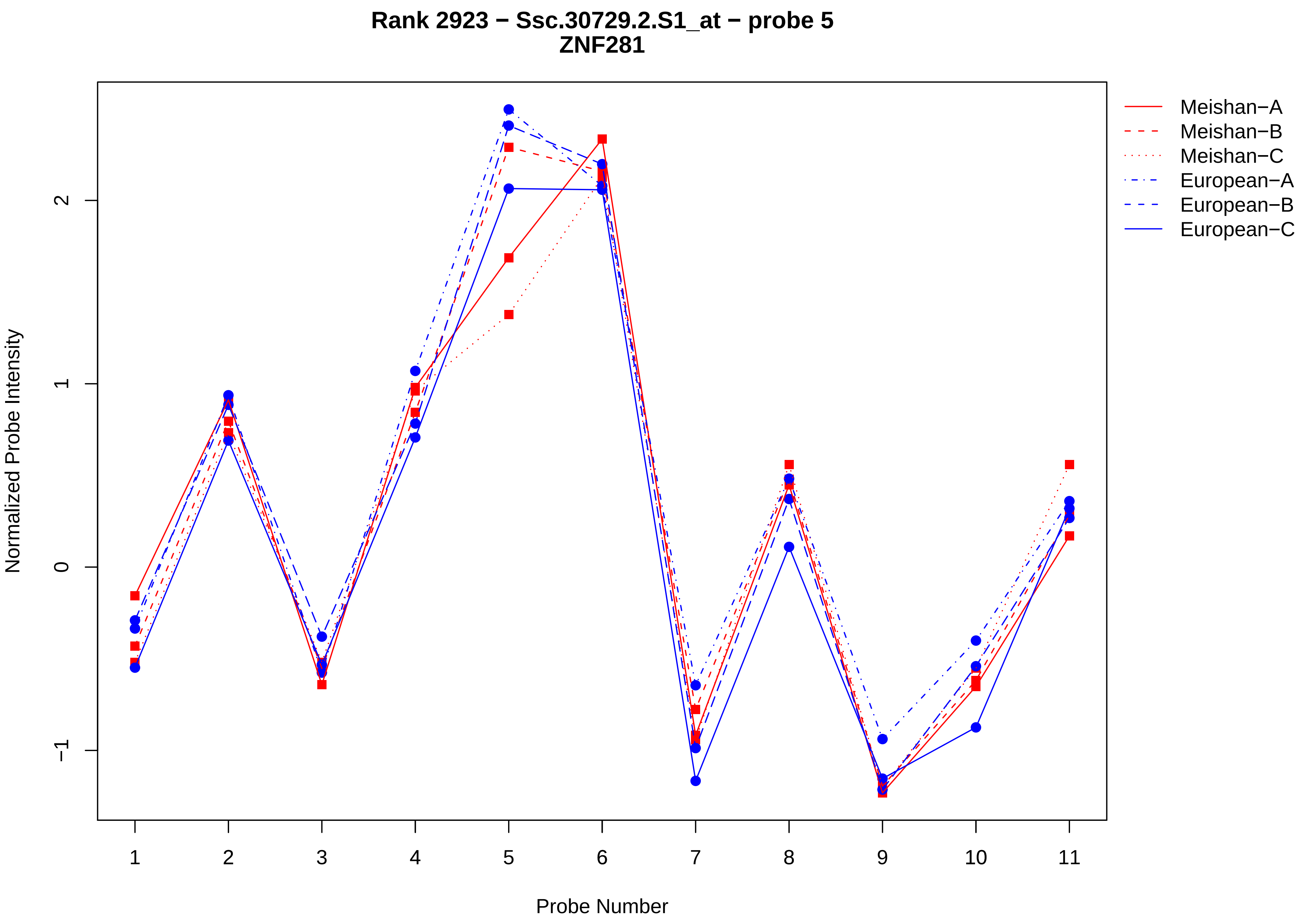
Rank 2923 − Ssc.30729.2.S1_at − probe 5
ZNF281
Normalized Probe Intensity
Probe Number
−1
0
1
2
1
2
3
4
5
6
7
8
9
10
11
Meishan−A
Meishan−B
Meishan−C
European−A
European−B
European−C

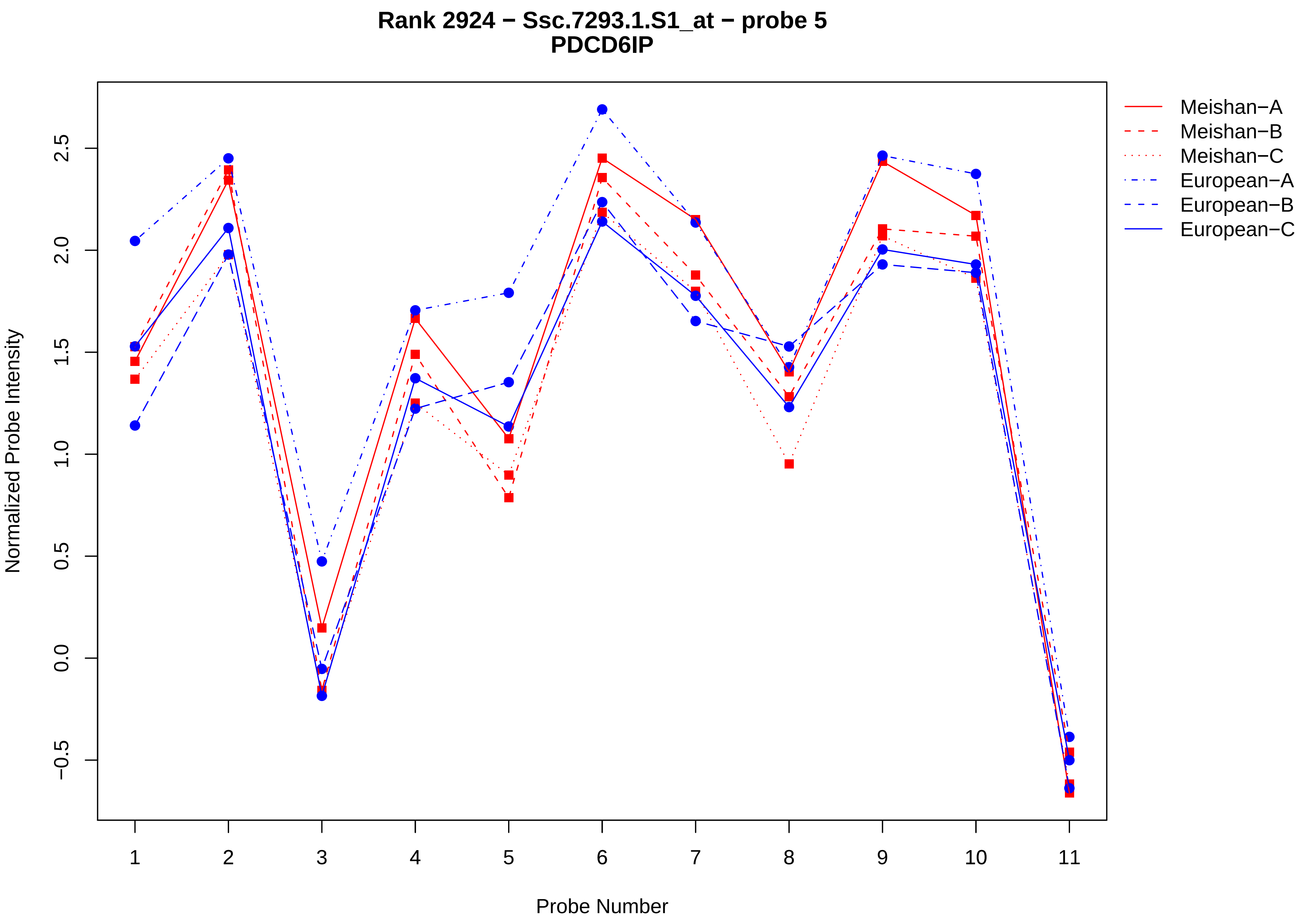
Rank 2924 − Ssc.7293.1.S1_at − probe 5
PDCD6IP
Normalized Probe Intensity
Probe Number
−0.5
0.0
0.5
1.0
1.5
2.0
2.5
1
2
3
4
5
6
7
8
9
10
11
Meishan−A
Meishan−B
Meishan−C
European−A
European−B
European−C

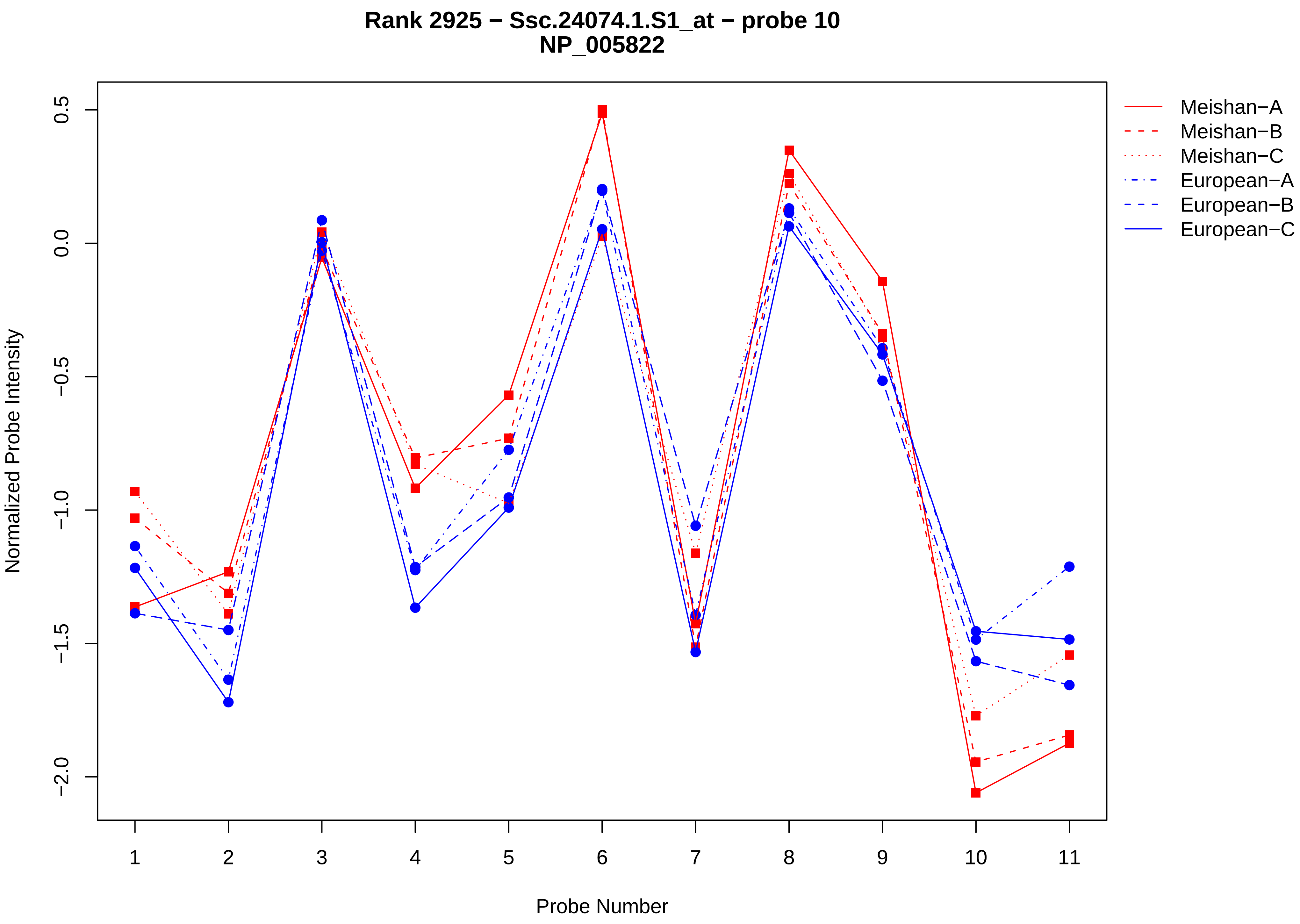
Rank 2925 − Ssc.24074.1.S1_at − probe 10
NP_005822
Normalized Probe Intensity
Probe Number
Meishan−A
Meishan−B
Meishan−C
European−A
European−B
European−C
0.5
0.0
−0.5
−1.0
−1.5
−2.0
1
2
3
4
5
6
7
8
9
10
11

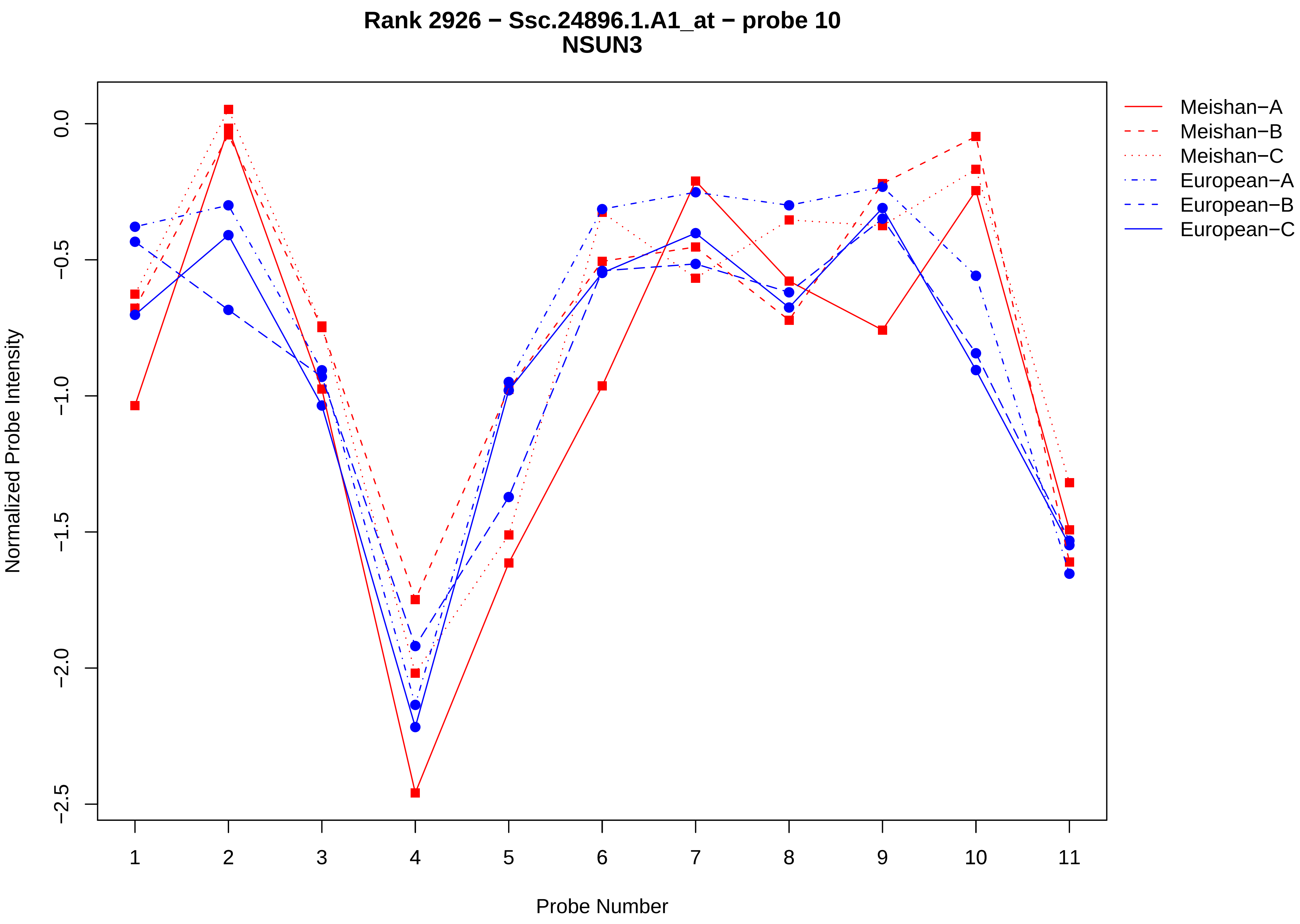

Rank 2926 − Ssc.24896.1.A1_at − probe 10
NSUN3
Normalized Probe Intensity
Probe Number
0.0
−0.5
−1.0
−1.5
−2.0
−2.5
1
2
3
4
5
6
7
8
9
10
11
Meishan−A
Meishan−B
Meishan−C
European−A
European−B
European−C

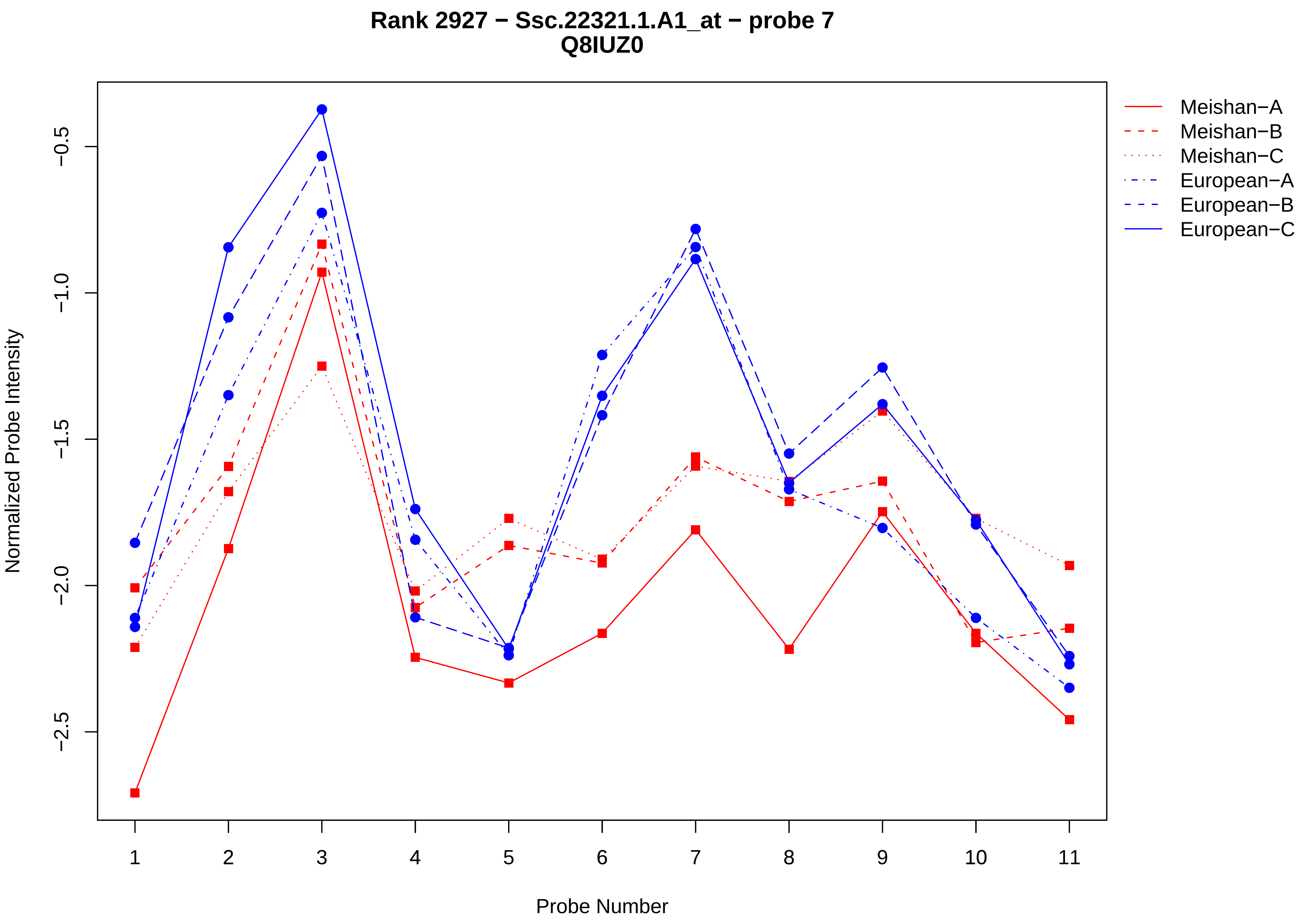

Rank 2927 − Ssc.22321.1.A1_at − probe 7
Q8IUZ0
Normalized Probe Intensity
Probe Number
−0.5
−1.0
−1.5
−2.0
−2.5
1
2
3
4
5
6
7
8
9
10
11
Meishan−A
Meishan−B
Meishan−C
European−A
European−B
European−C

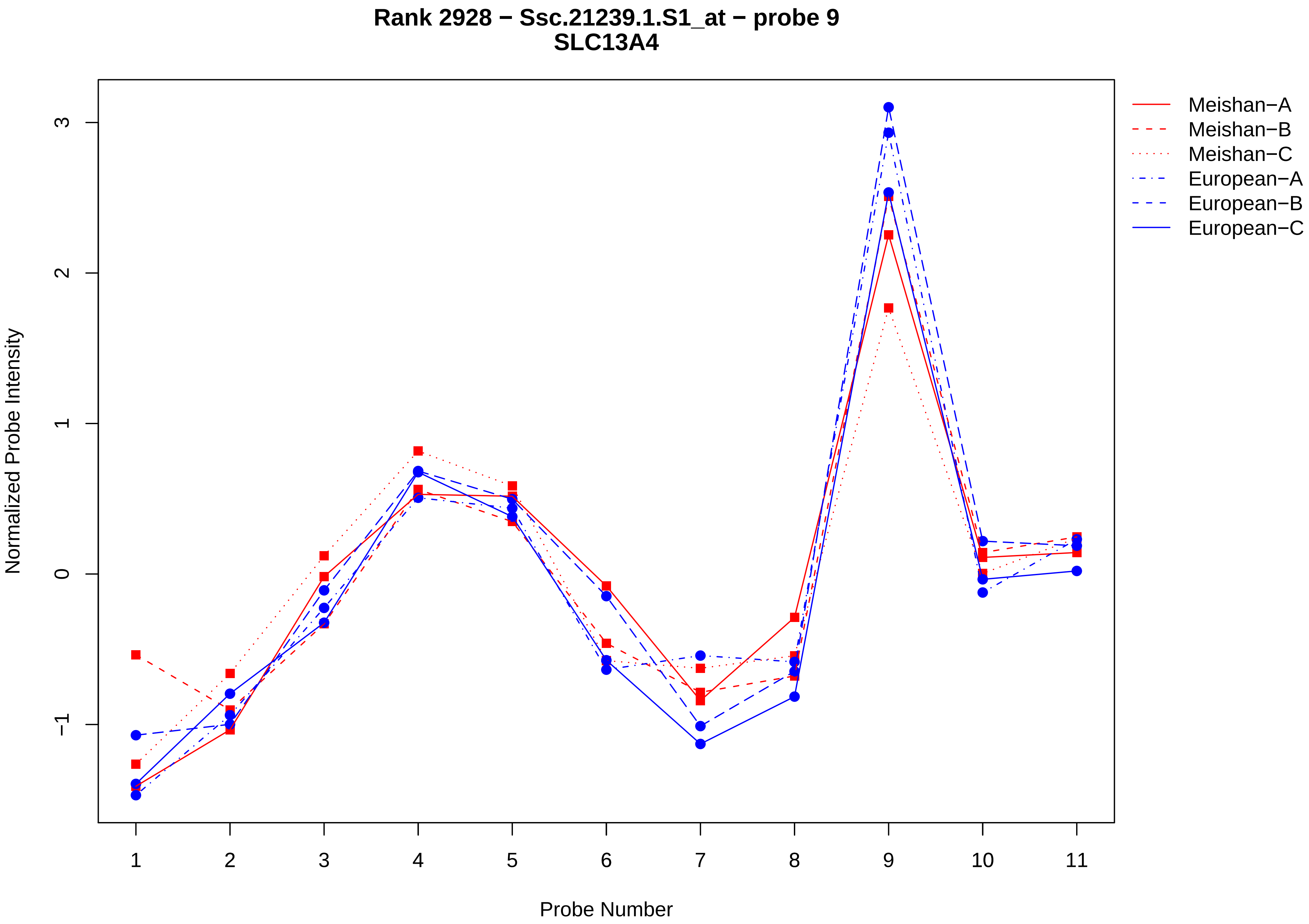

Rank 2928 − Ssc.21239.1.S1_at − probe 9
SLC13A4
Normalized Probe Intensity
Probe Number
Meishan−A
Meishan−B
Meishan−C
European−A
European−B
European−C

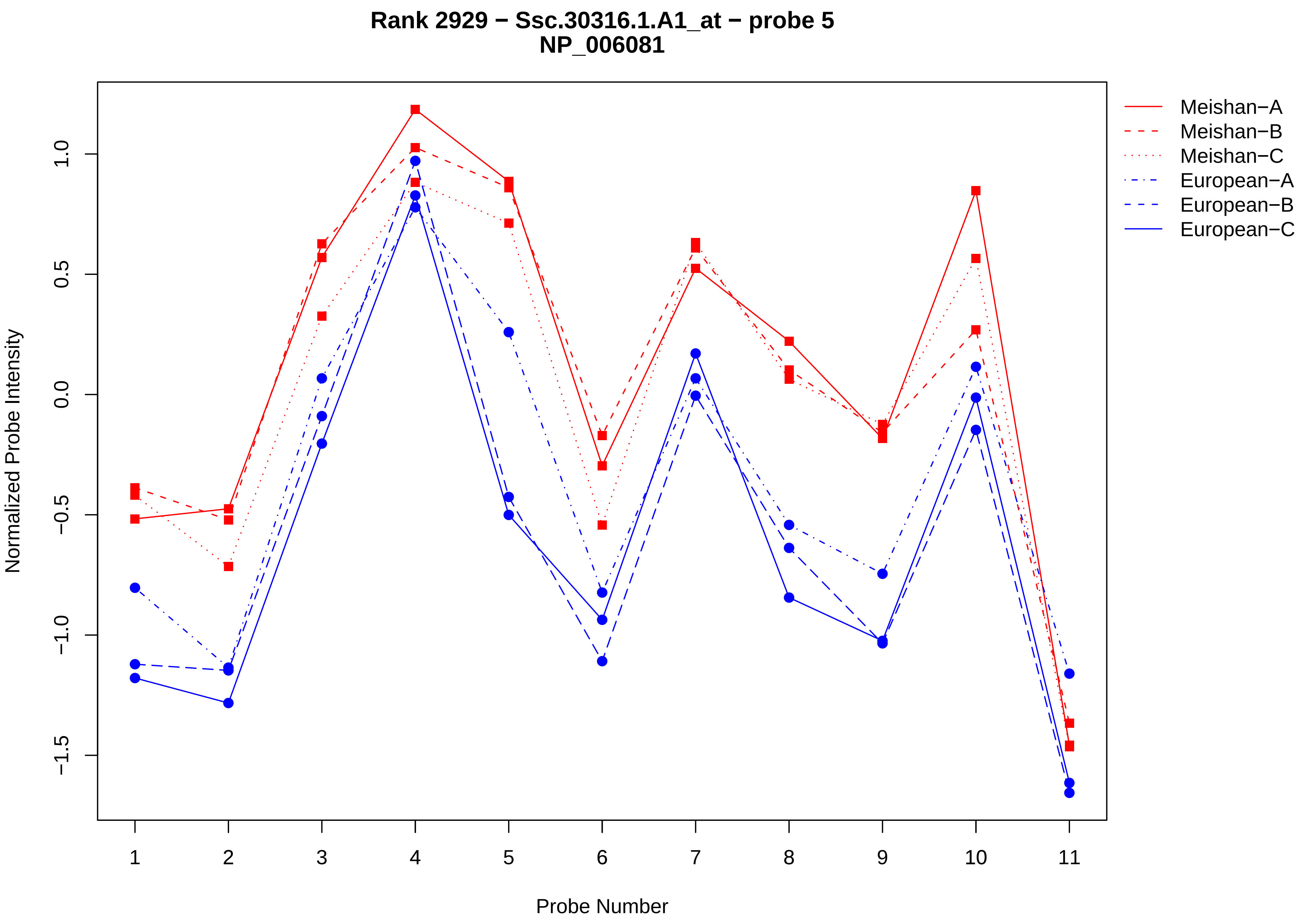

Rank 2929 − Ssc.30316.1.A1_at − probe 5
NP_006081
Normalized Probe Intensity
Probe Number
1.0
0.5
0.0
−0.5
−1.0
−1.5
1
2
3
4
5
6
7
8
9
10
11
Meishan−A
Meishan−B
Meishan−C
European−A
European−B
European−C

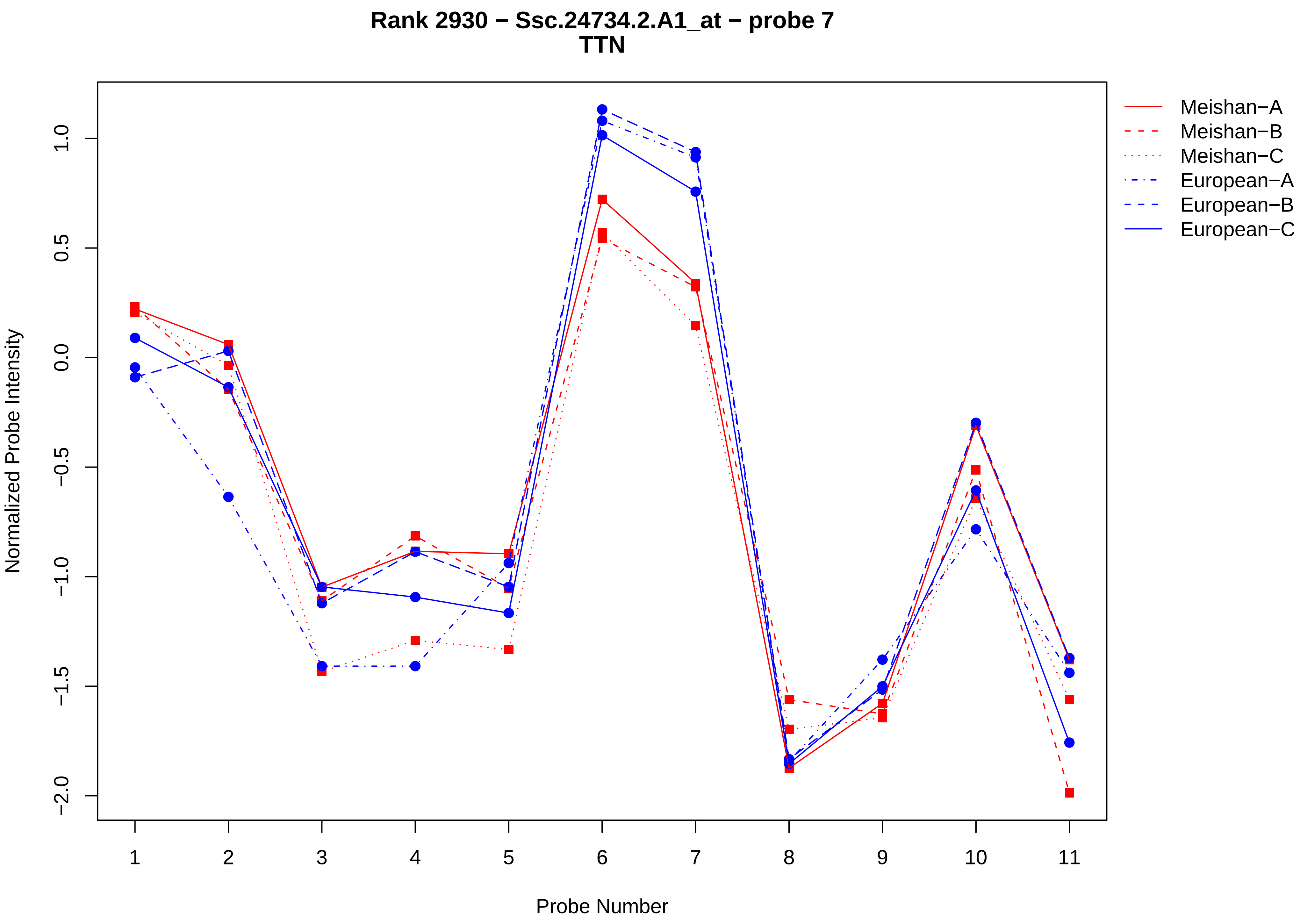

Rank 2930 − Ssc.24734.2.A1_at − probe 7
TTN
Normalized Probe Intensity
Probe Number
1.0
0.5
0.0
−0.5
−1.0
−1.5
−2.0
1
2
3
4
5
6
7
8
9
10
11
Meishan−A
Meishan−B
Meishan−C
European−A
European−B
European−C

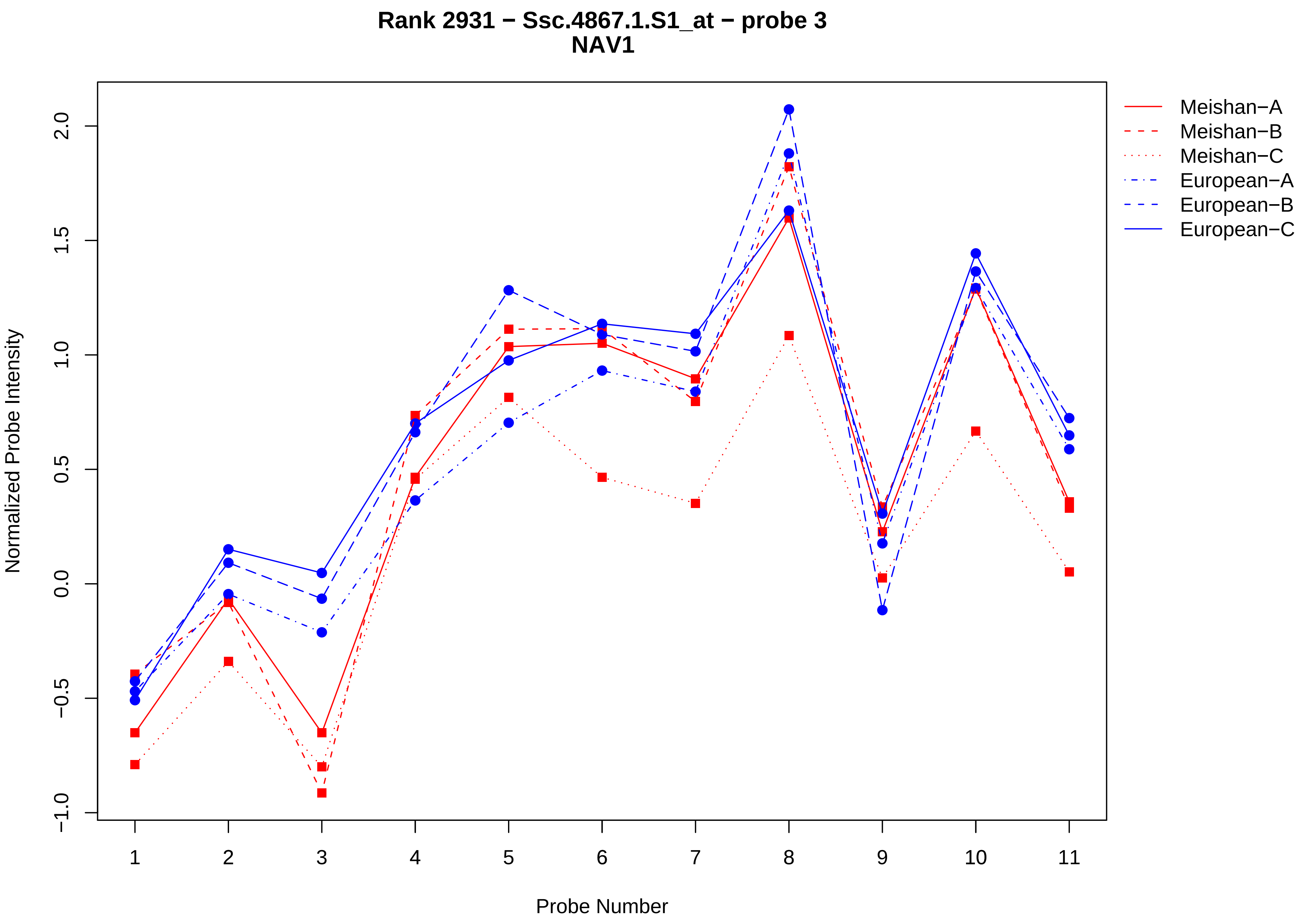
Rank 2931 − Ssc.4867.1.S1_at − probe 3
NAV1
Normalized Probe Intensity
Probe Number
Meishan−A
Meishan−B
Meishan−C
European−A
European−B
European−C
−1.0
−0.5
0.0
0.5
1.0
1.5
2.0
1
2
3
4
5
6
7
8
9
10
11

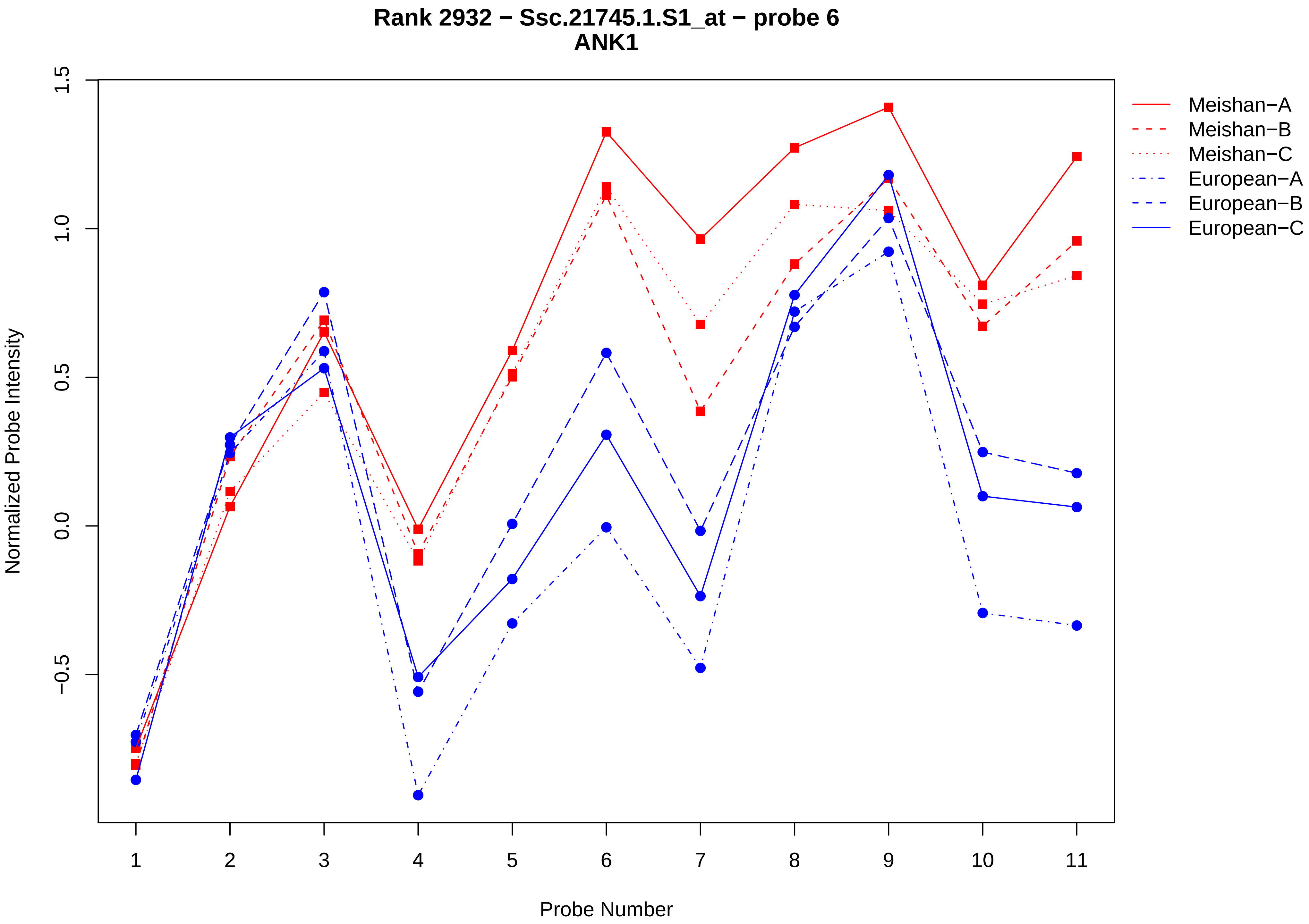

Rank 2932 − Ssc.21745.1.S1_at − probe 6
ANK1
Normalized Probe Intensity
Probe Number
1.5
1.0
0.5
0.0
−0.5
1
2
3
4
5
6
7
8
9
10
11
Meishan−A
Meishan−B
Meishan−C
European−A
European−B
European−C

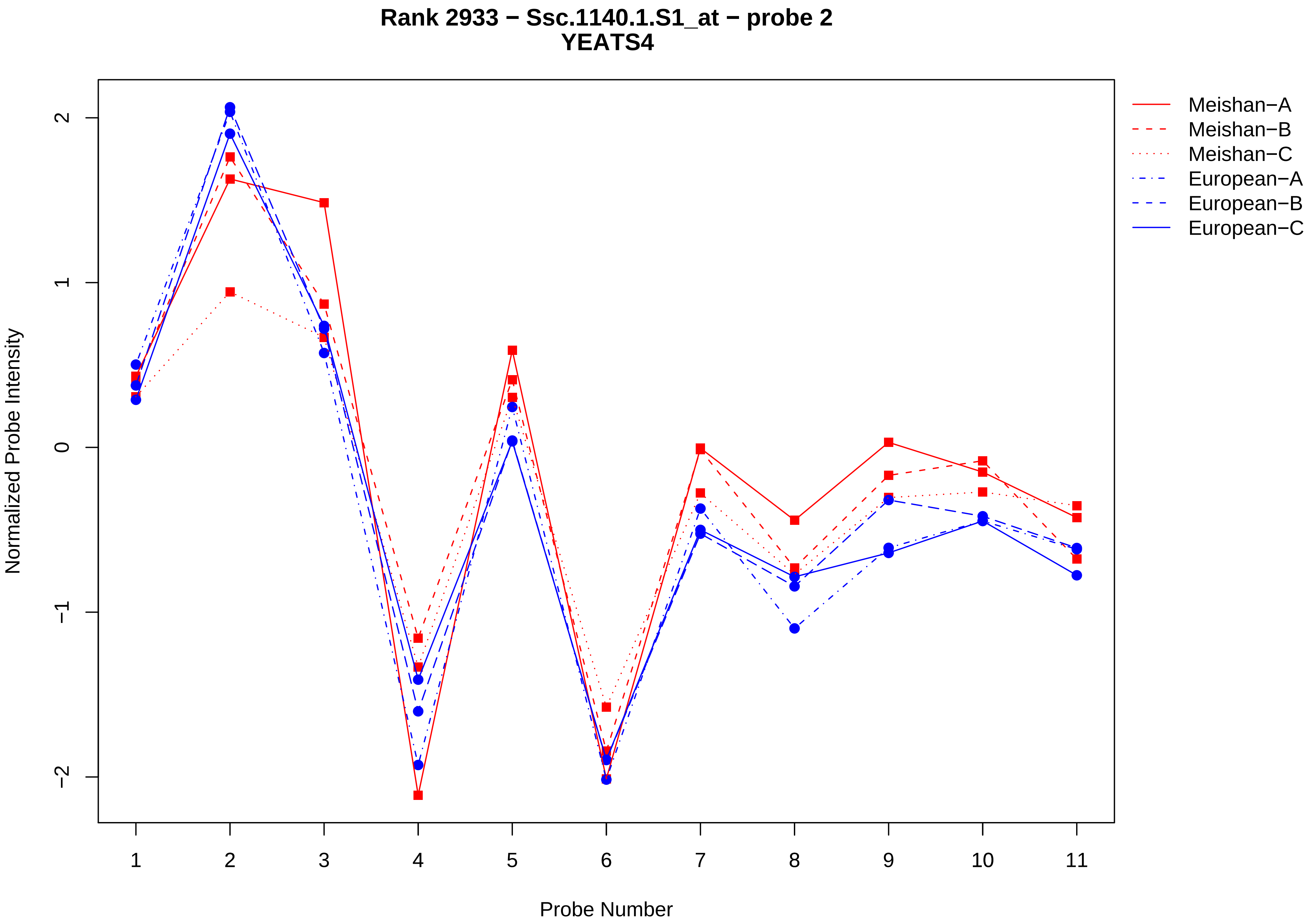

Rank 2933 − Ssc.1140.1.S1_at − probe 2
YEATS4
Normalized Probe Intensity
Probe Number
Meishan−A
Meishan−B
Meishan−C
European−A
European−B
European−C

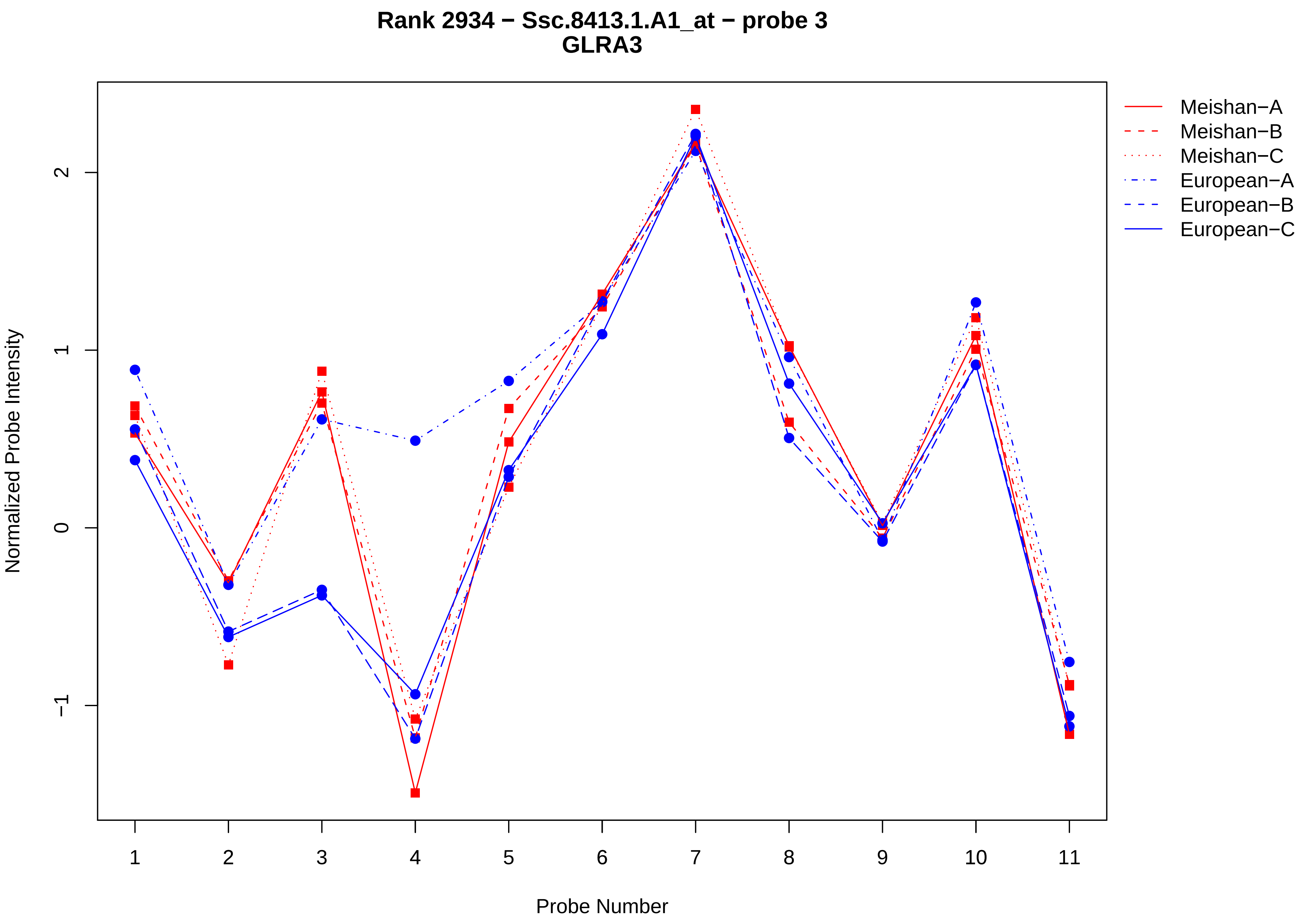

Rank 2934 − Ssc.8413.1.A1_at − probe 3
GLRA3
Normalized Probe Intensity
Probe Number
Meishan−A
Meishan−B
Meishan−C
European−A
European−B
European−C

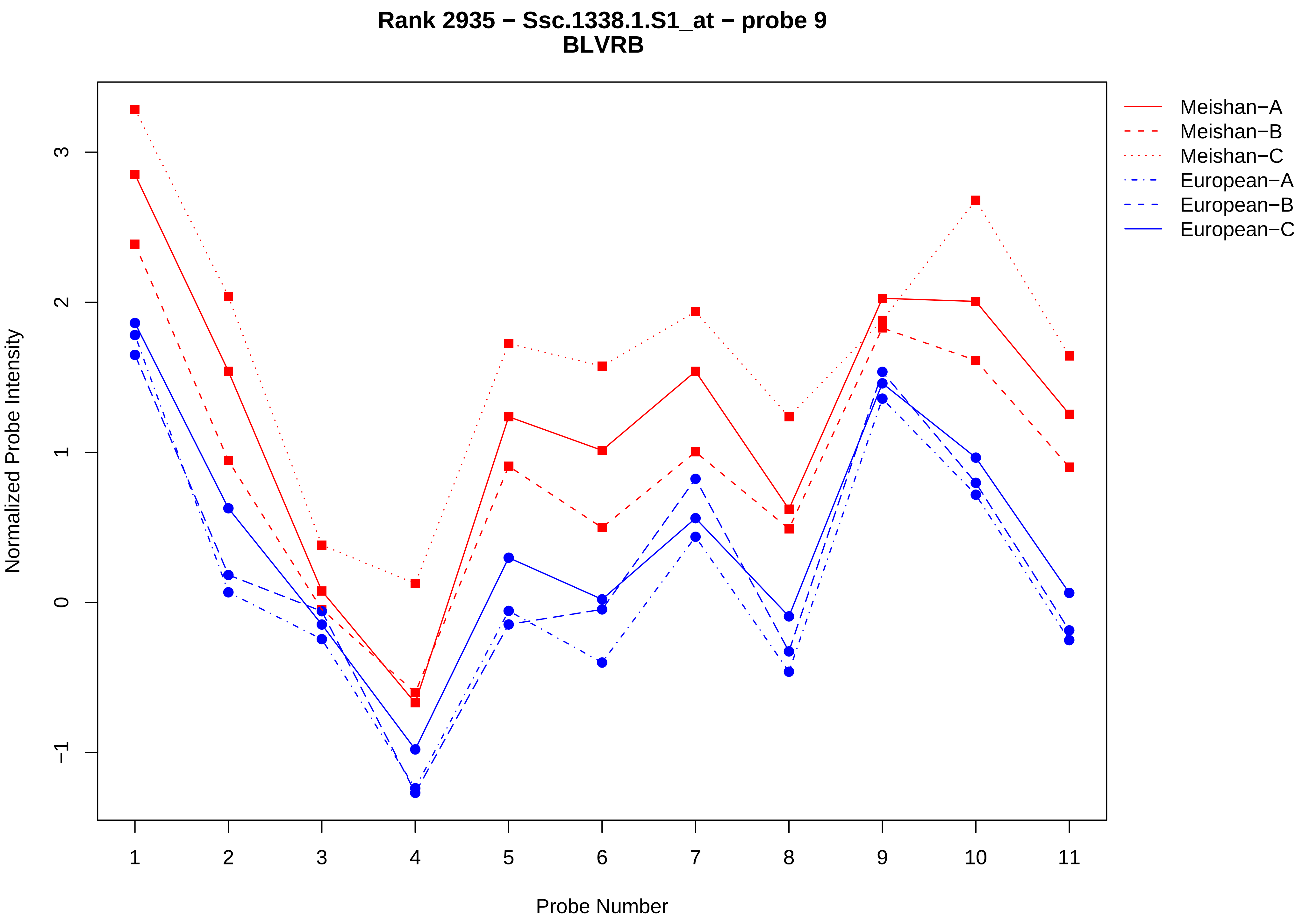
Rank 2935 − Ssc.1338.1.S1_at − probe 9
BLVRB
Normalized Probe Intensity
Probe Number
−1
0
1
2
3
1
2
3
4
5
6
7
8
9
10
11
Meishan−A
Meishan−B
Meishan−C
European−A
European−B
European−C

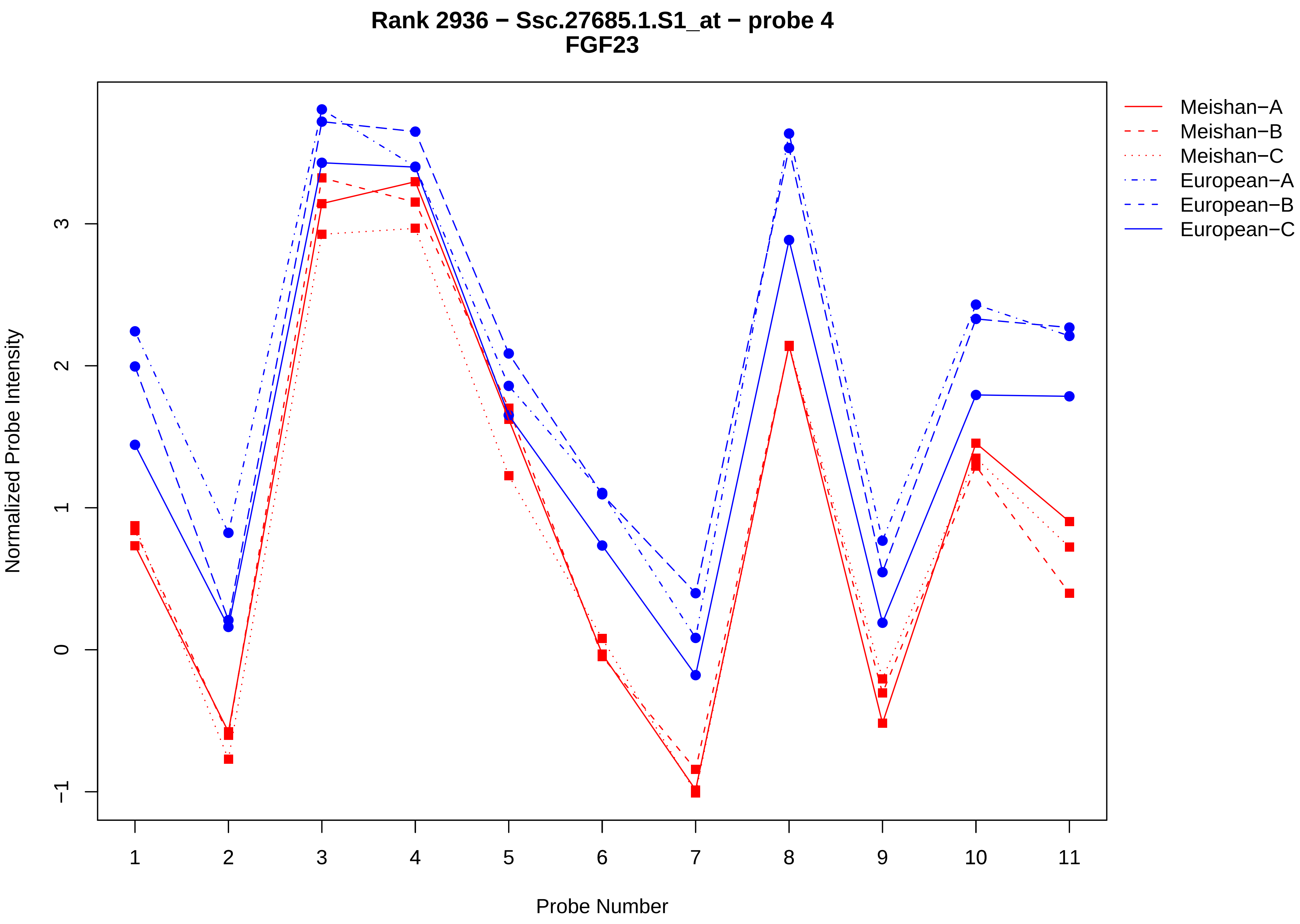

Rank 2936 − Ssc.27685.1.S1_at − probe 4
FGF23
Normalized Probe Intensity
Probe Number
−1
0
1
2
3
1
2
3
4
5
6
7
8
9
10
11
Meishan−A
Meishan−B
Meishan−C
European−A
European−B
European−C

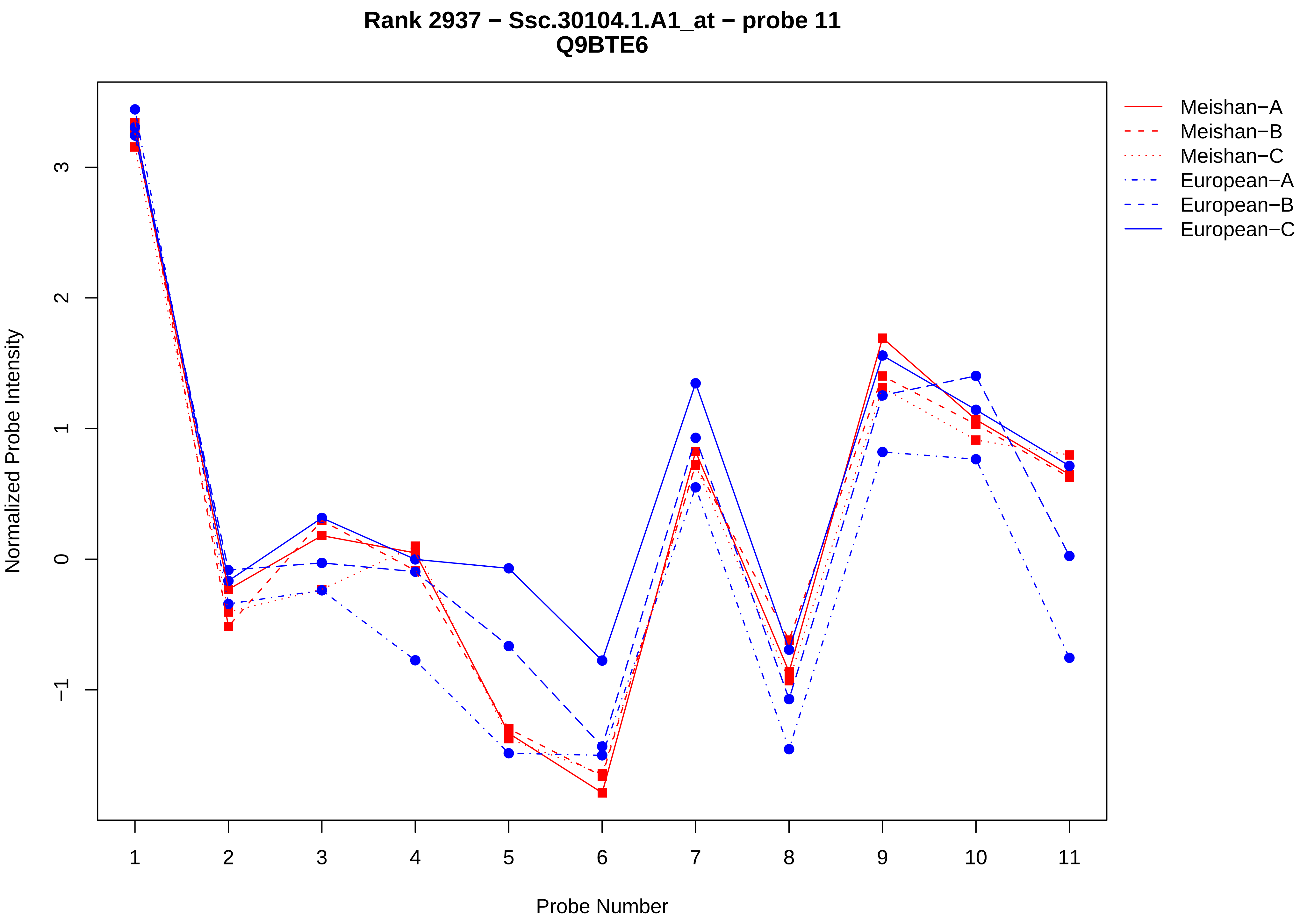
Rank 2937 − Ssc.30104.1.A1_at − probe 11
Q9BTE6
Normalized Probe Intensity
Probe Number
−1
0
1
2
3
1
2
3
4
5
6
7
8
9
10
11
Meishan−A
Meishan−B
Meishan−C
European−A
European−B
European−C

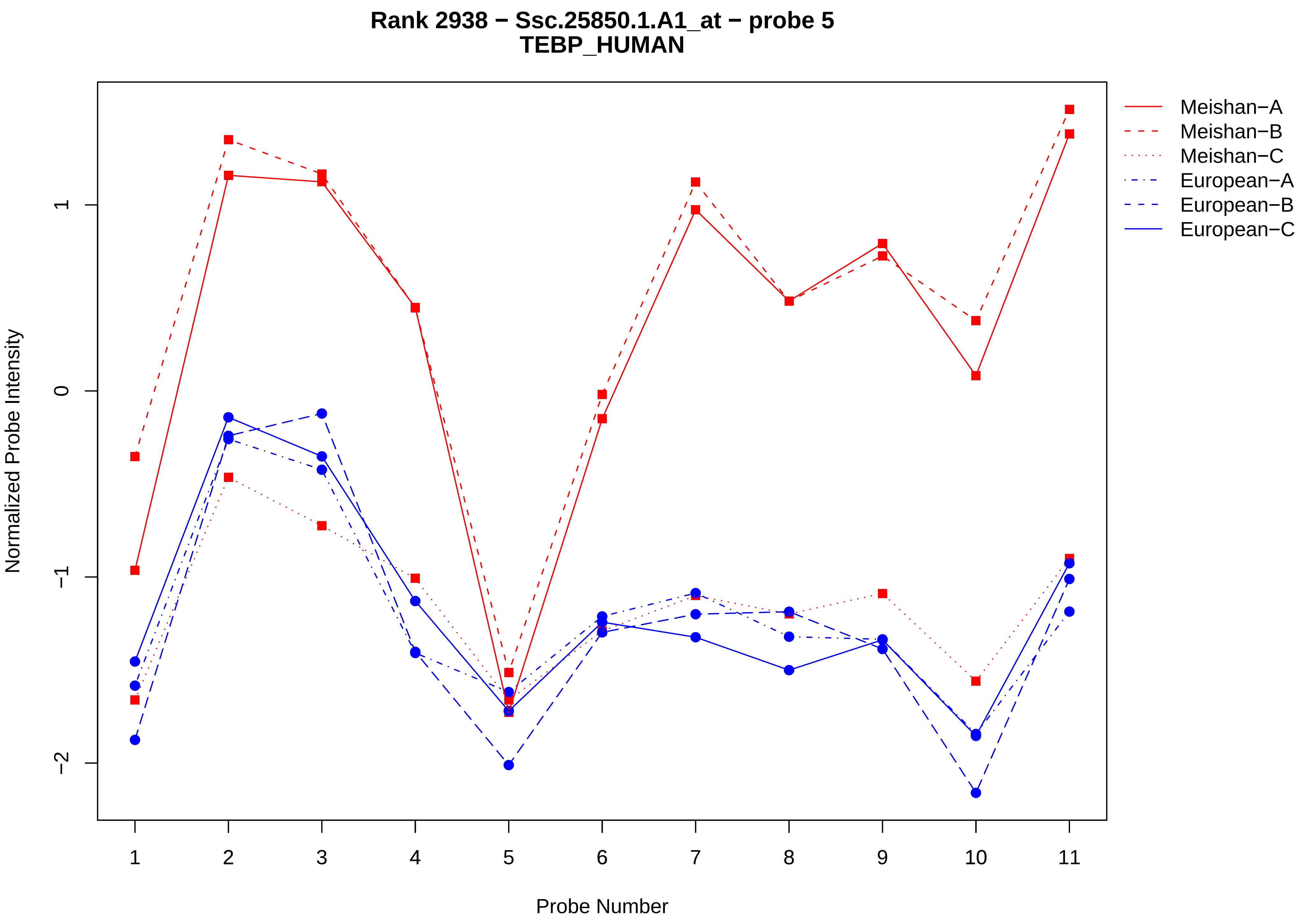

Rank 2938 − Ssc.25850.1.A1_at − probe 5
TEBP_HUMAN
Normalized Probe Intensity
Probe Number
1
0
−1
−2
1
2
3
4
5
6
7
8
9
10
11
Meishan−A
Meishan−B
Meishan−C
European−A
European−B
European−C

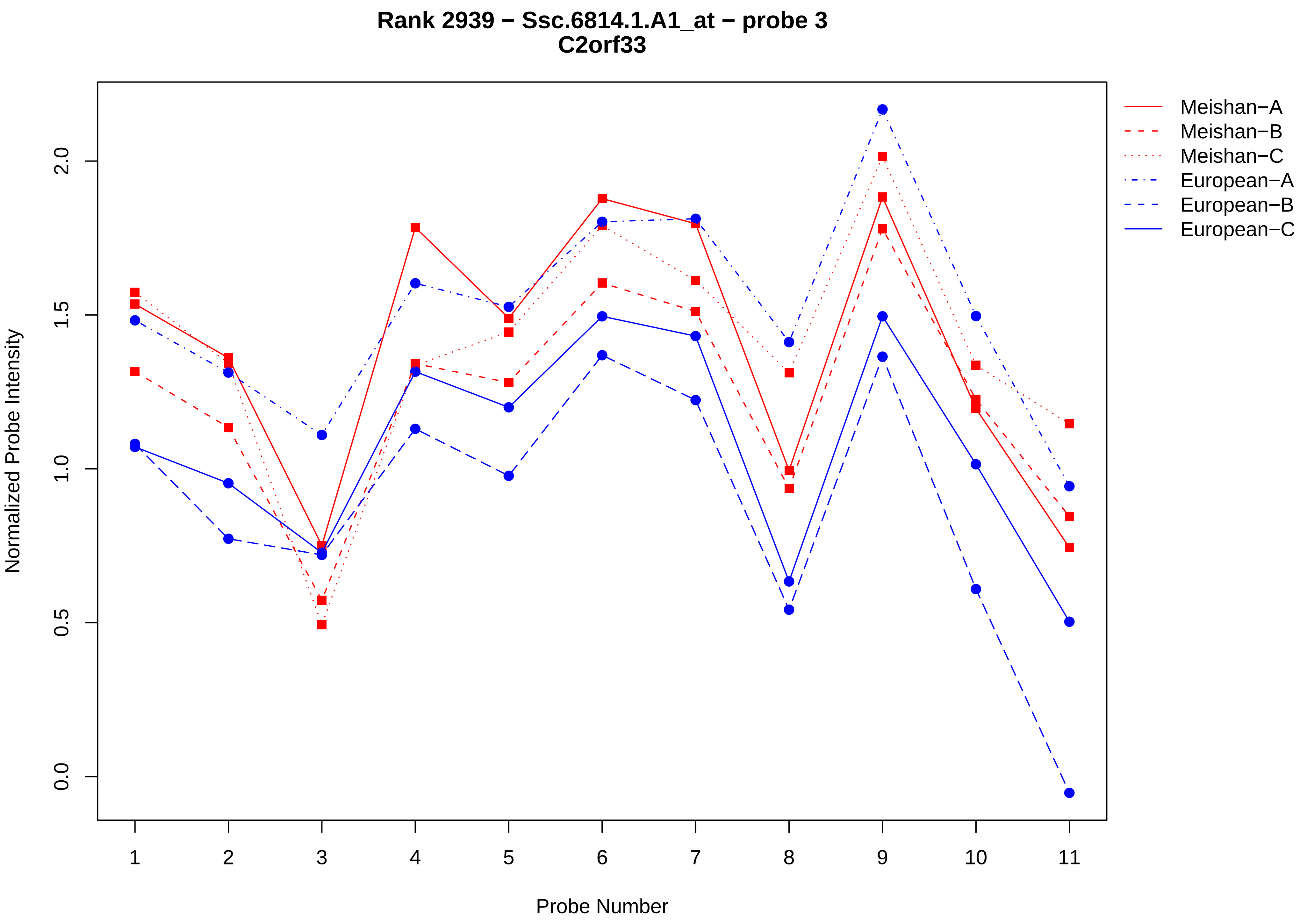
Rank 2939 − Ssc.6814.1.A1_at − probe 3
C2orf33
Normalized Probe Intensity
Probe Number
0.0
0.5
1.0
1.5
2.0
1
2
3
4
5
6
7
8
9
10
11
Meishan−A
Meishan−B
Meishan−C
European−A
European−B
European−C

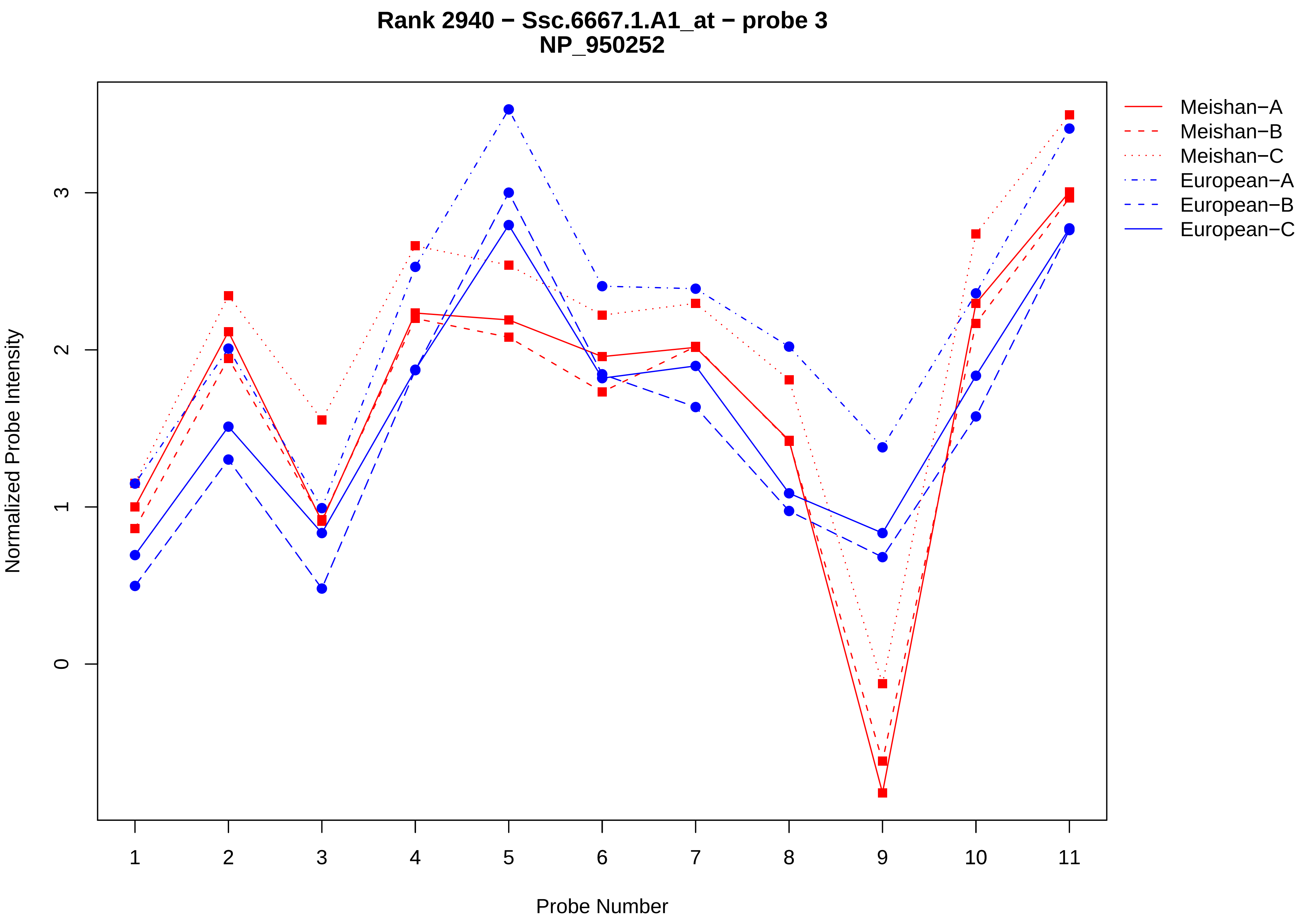
Rank 2940 − Ssc.6667.1.A1_at − probe 3
NP_950252
Normalized Probe Intensity
Probe Number
0
1
2
3
1
2
3
4
5
6
7
8
9
10
11
Meishan−A
Meishan−B
Meishan−C
European−A
European−B
European−C

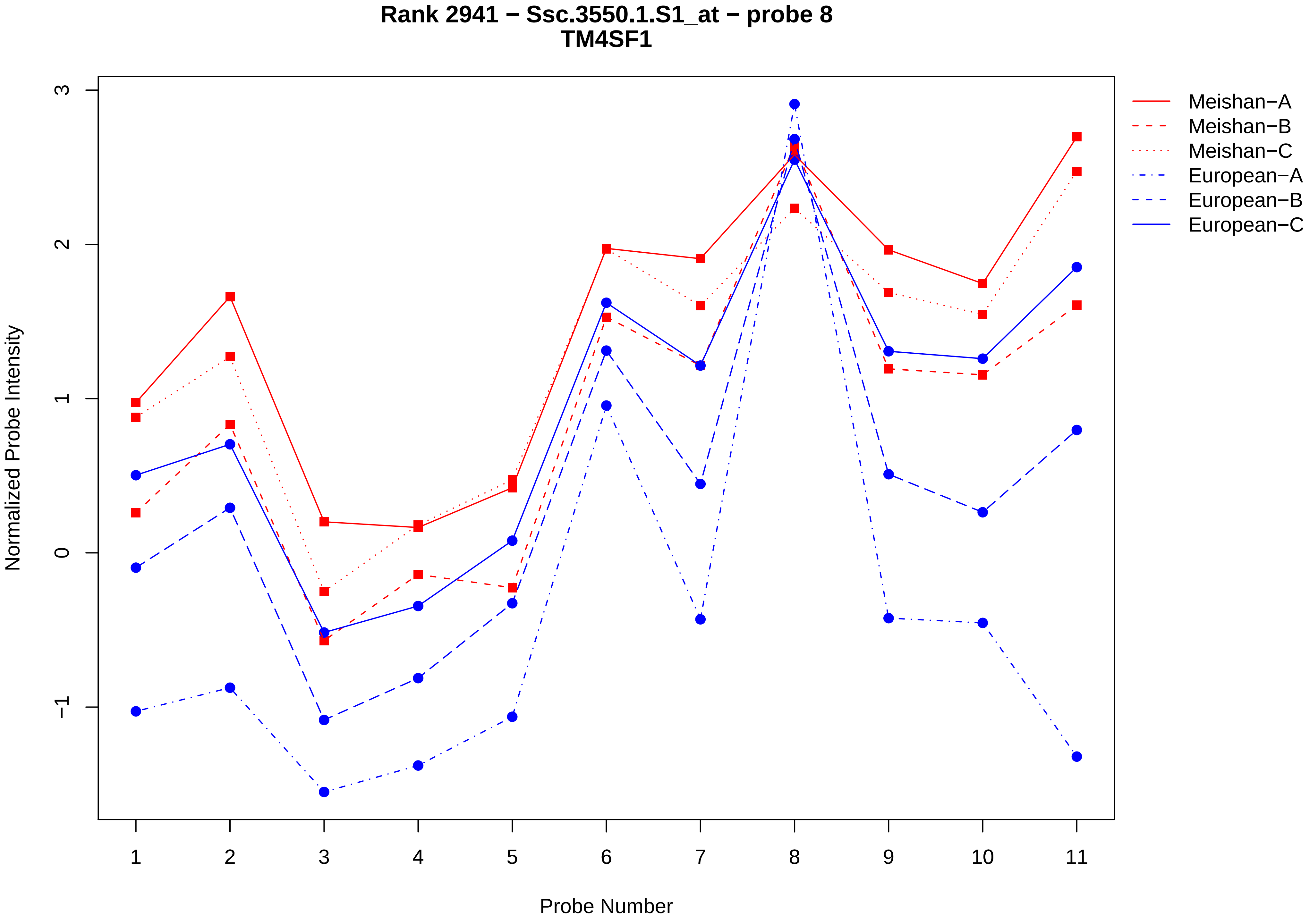

Rank 2941 − Ssc.3550.1.S1_at − probe 8
TM4SF1
Normalized Probe Intensity
Probe Number
Meishan−A
Meishan−B
Meishan−C
European−A
European−B
European−C
−1
0
1
2
3
1
2
3
4
5
6
7
8
9
10
11

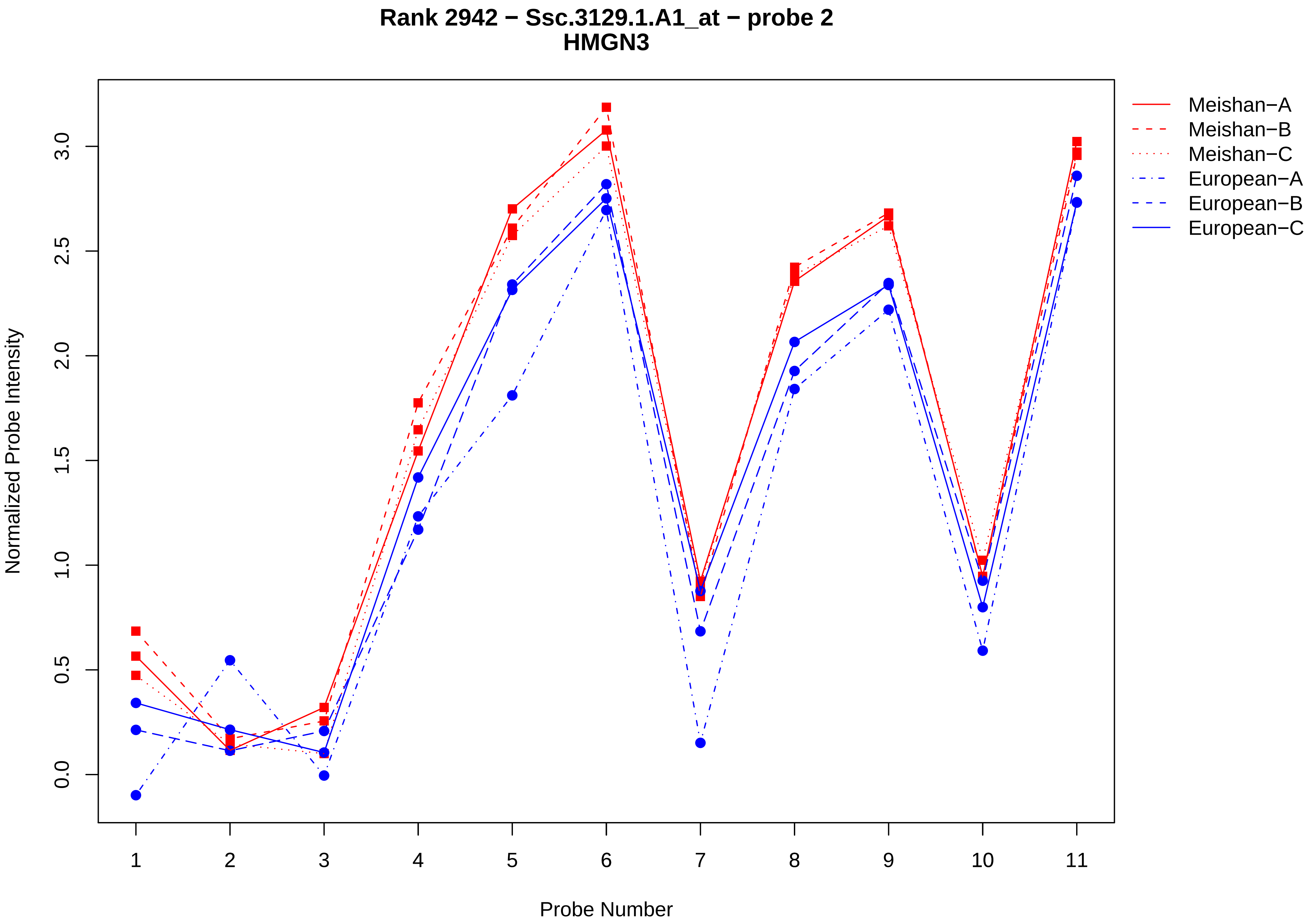
Rank 2942 − Ssc.3129.1.A1_at − probe 2
HMGN3
Normalized Probe Intensity
Probe Number
0.0
0.5
1.0
1.5
2.0
2.5
3.0
1
2
3
4
5
6
7
8
9
10
11
Meishan−A
Meishan−B
Meishan−C
European−A
European−B
European−C

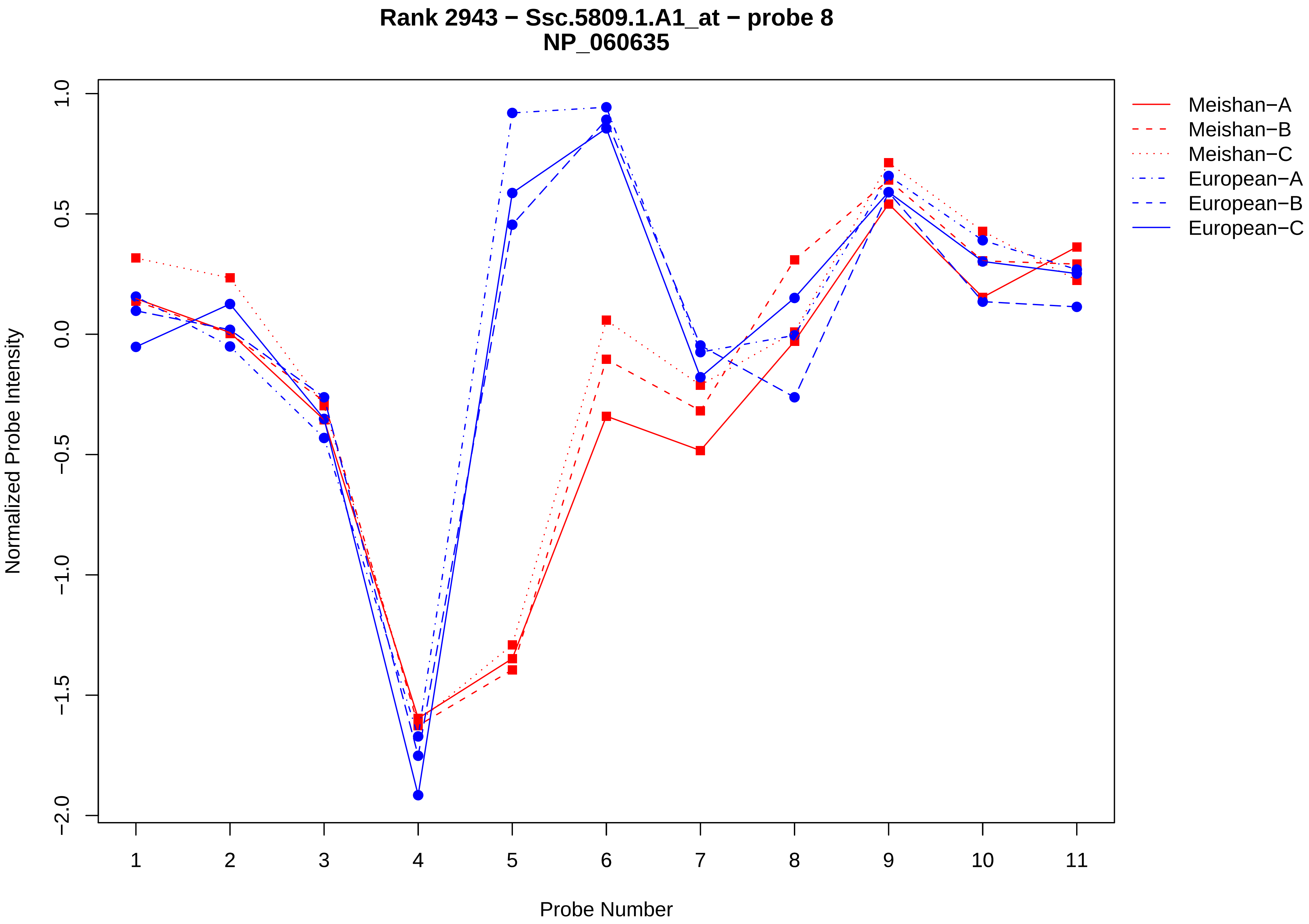

Rank 2943 − Ssc.5809.1.A1_at − probe 8
NP_060635
Normalized Probe Intensity
Probe Number
1.0
0.5
0.0
−0.5
−1.0
−1.5
−2.0
1
2
3
4
5
6
7
8
9
10
11
Meishan−A
Meishan−B
Meishan−C
European−A
European−B
European−C

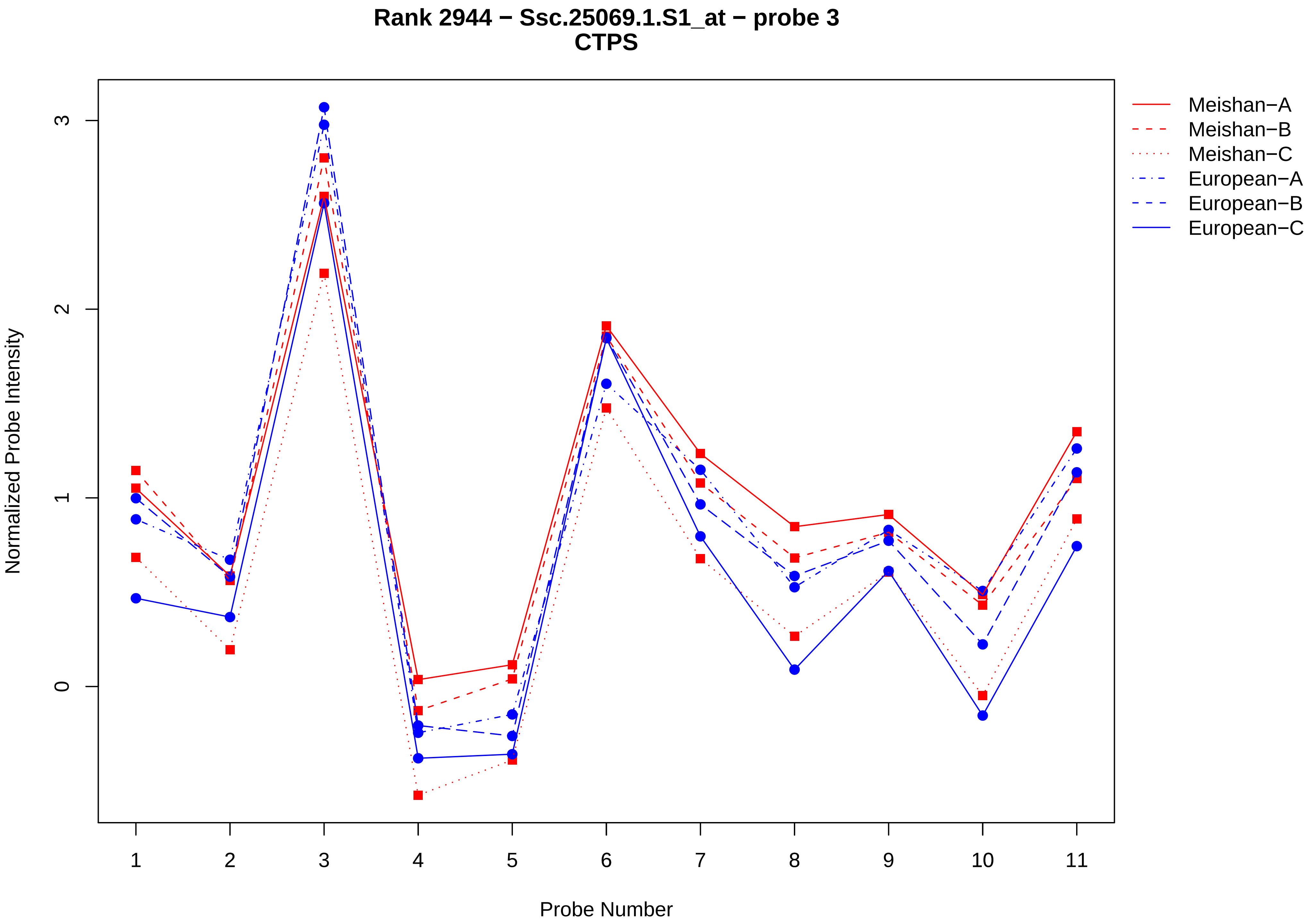

Rank 2944 − Ssc.25069.1.S1_at − probe 3
CTPS
Normalized Probe Intensity
Probe Number
0
1
2
3
1
2
3
4
5
6
7
8
9
10
11
Meishan−A
Meishan−B
Meishan−C
European−A
European−B
European−C

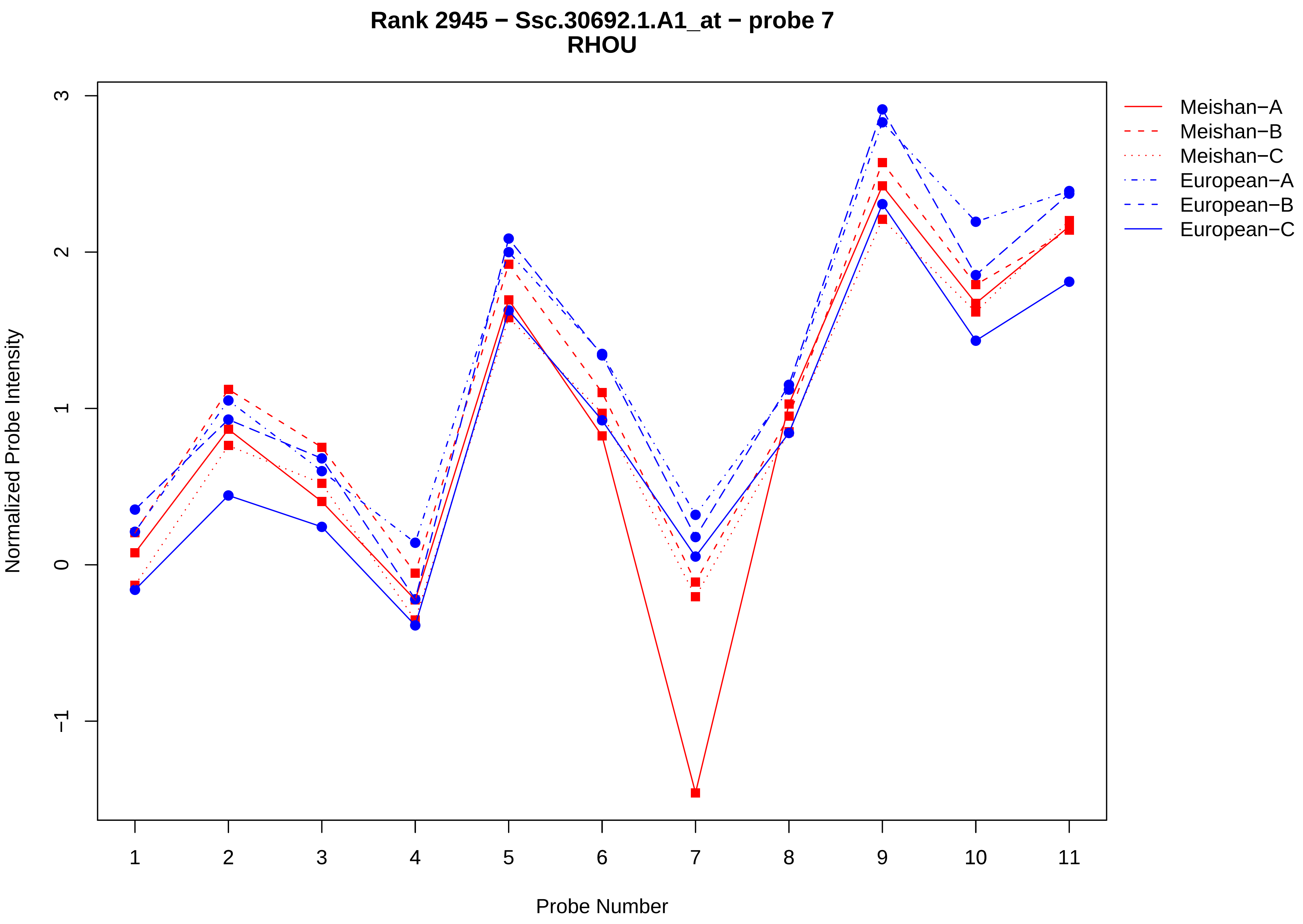

Rank 2945 − Ssc.30692.1.A1_at − probe 7
RHOU
Normalized Probe Intensity
Probe Number
Meishan−A
Meishan−B
Meishan−C
European−A
European−B
European−C

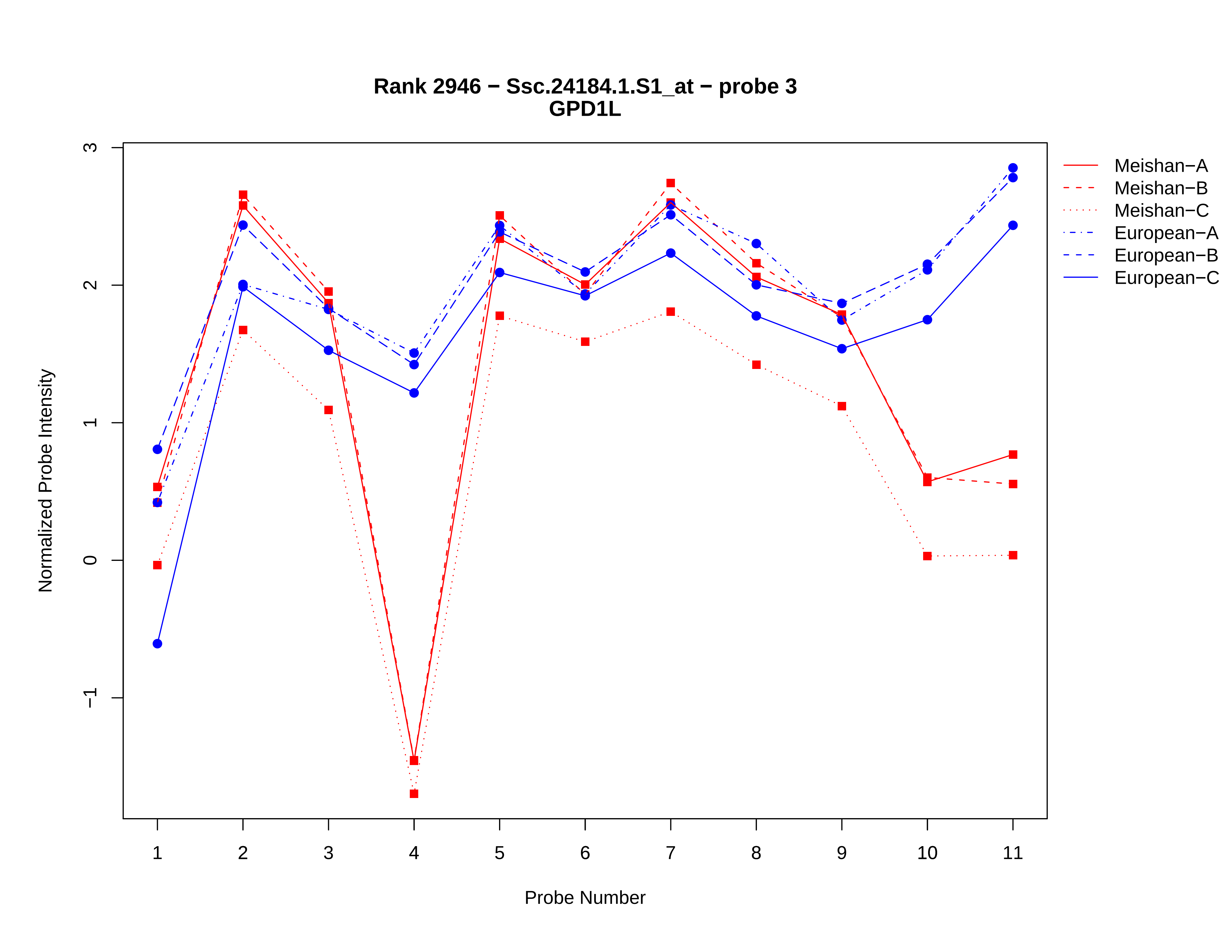

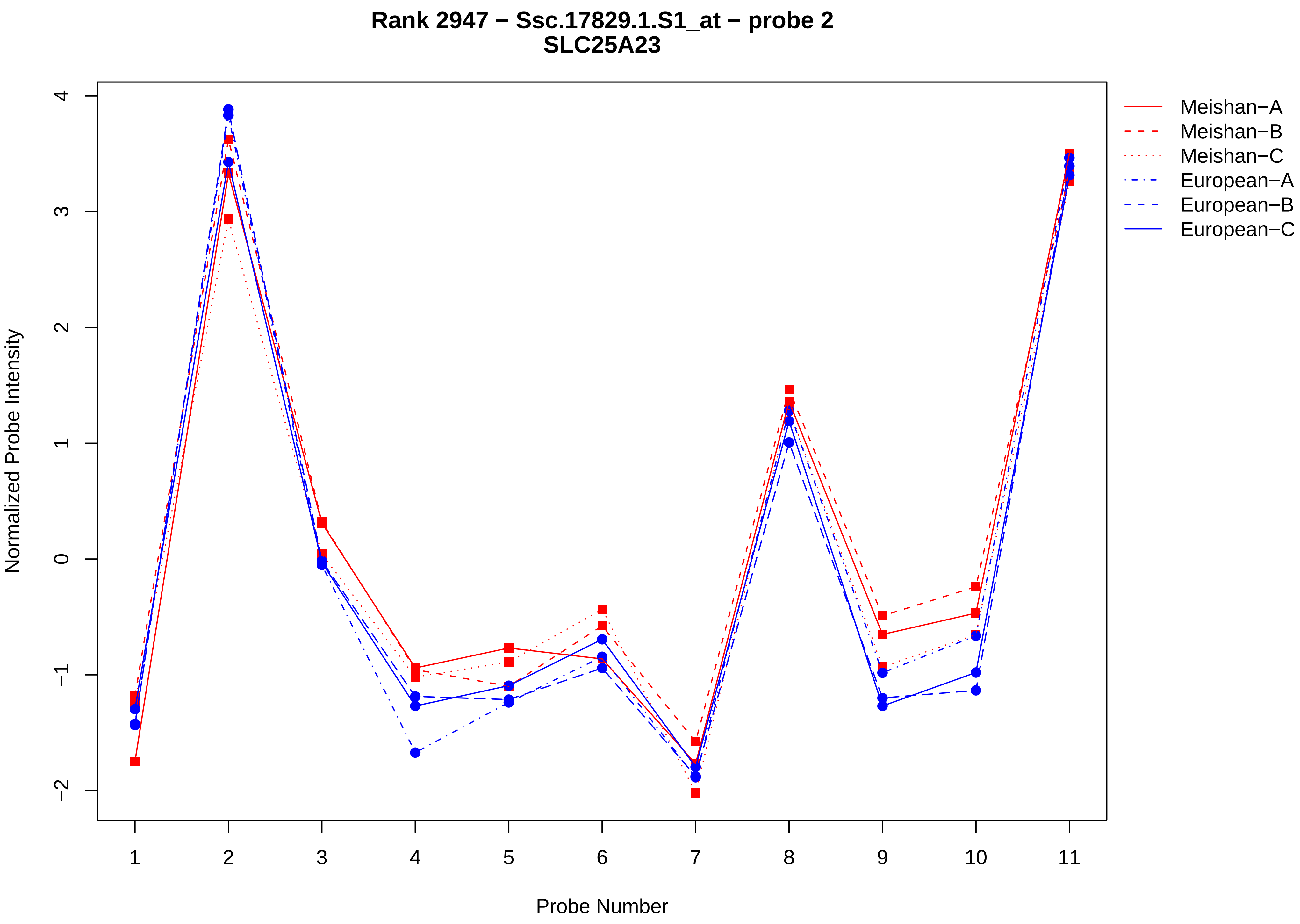

Rank 2947 − Ssc.17829.1.S1_at − probe 2
SLC25A23
Normalized Probe Intensity
Probe Number
−2
−1
0
1
2
3
4
1
2
3
4
5
6
7
8
9
10
11
Meishan−A
Meishan−B
Meishan−C
European−A
European−B
European−C

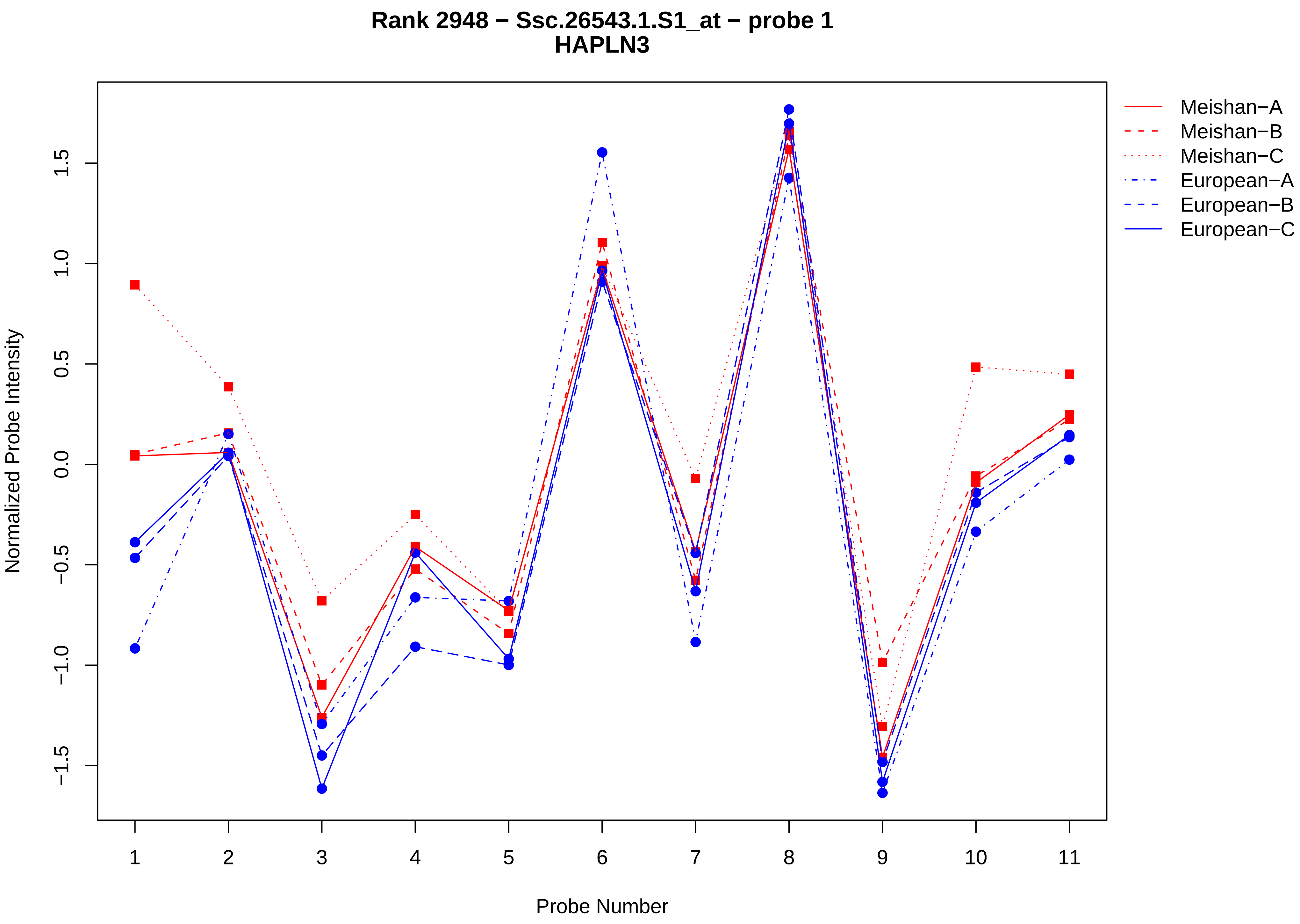

Rank 2948 − Ssc.26543.1.S1_at − probe 1
HAPLN3
Normalized Probe Intensity
Probe Number
Meishan−A
Meishan−B
Meishan−C
European−A
European−B
European−C
−1.5
−1.0
−0.5
0.0
0.5
1.0
1.5
1
2
3
4
5
6
7
8
9
10
11

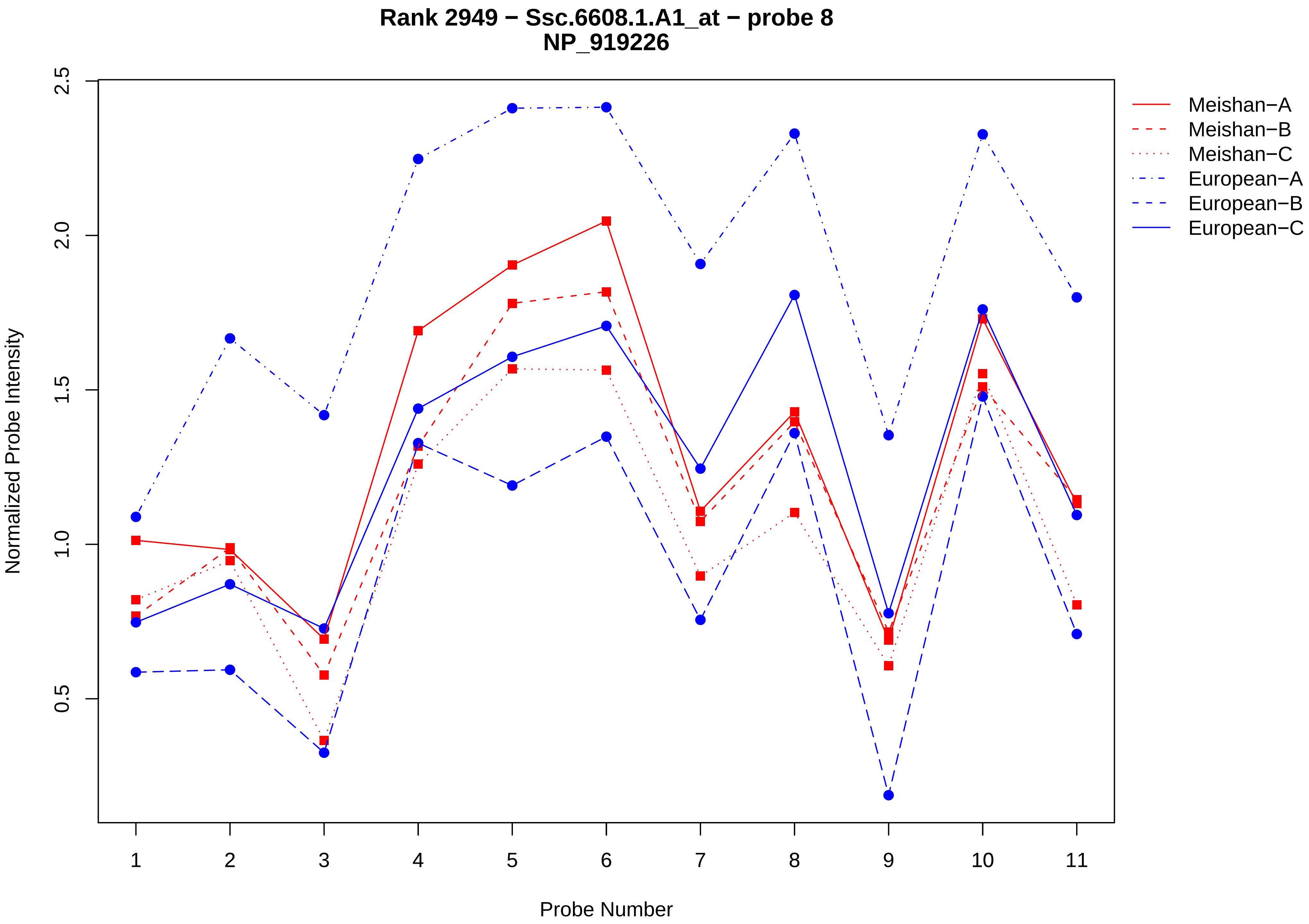
Rank 2949 − Ssc.6608.1.A1_at − probe 8
NP_919226
Normalized Probe Intensity
Probe Number
0.5
1.0
1.5
2.0
2.5
1
2
3
4
5
6
7
8
9
10
11
Meishan−A
Meishan−B
Meishan−C
European−A
European−B
European−C

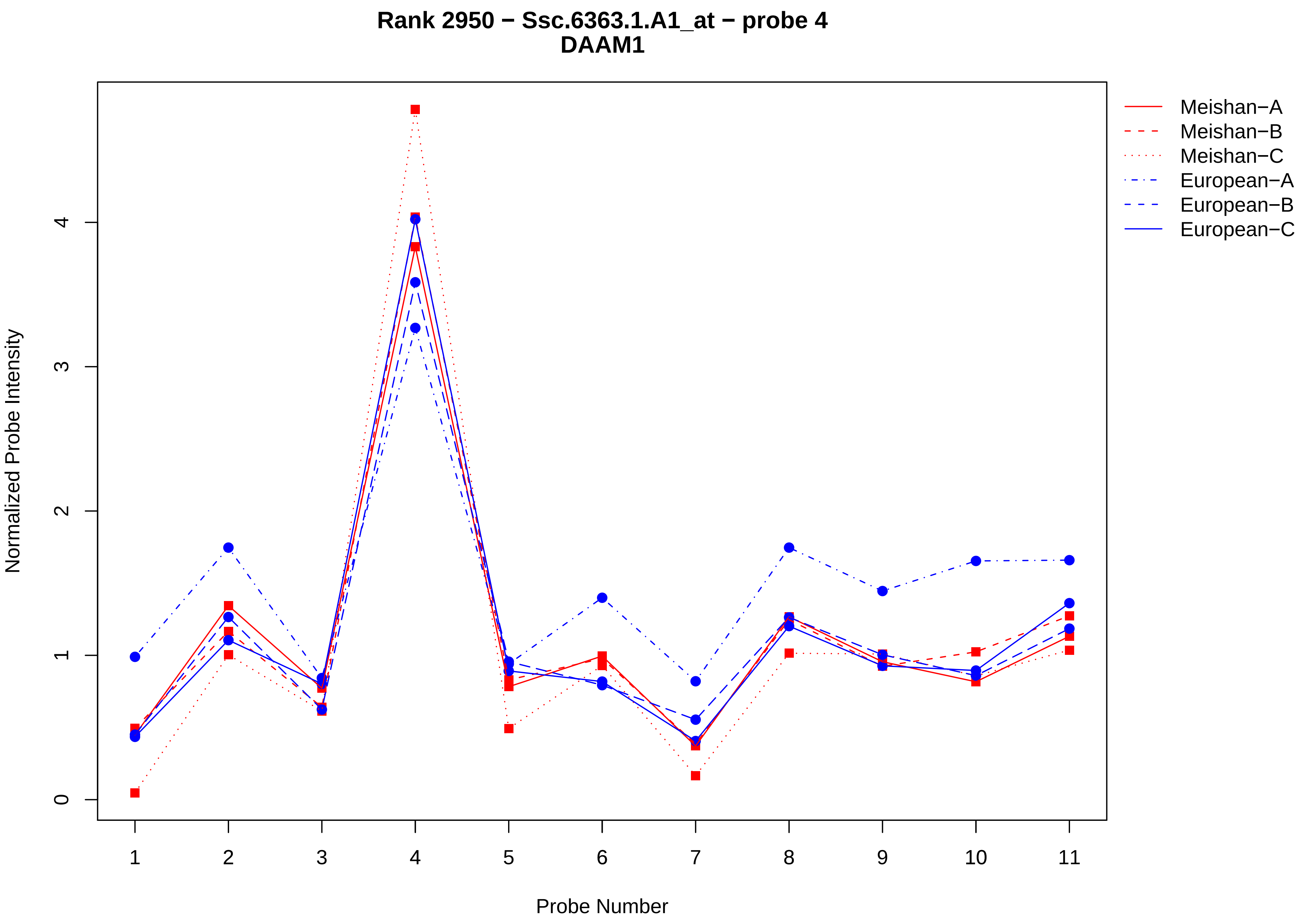

Rank 2950 − Ssc.6363.1.A1_at − probe 4
DAAM1
Normalized Probe Intensity
0
1
2
3
4
1
2
3
4
5
6
7
8
9
10
11
Probe Number
Meishan−A
Meishan−B
Meishan−C
European−A
European−B
European−C

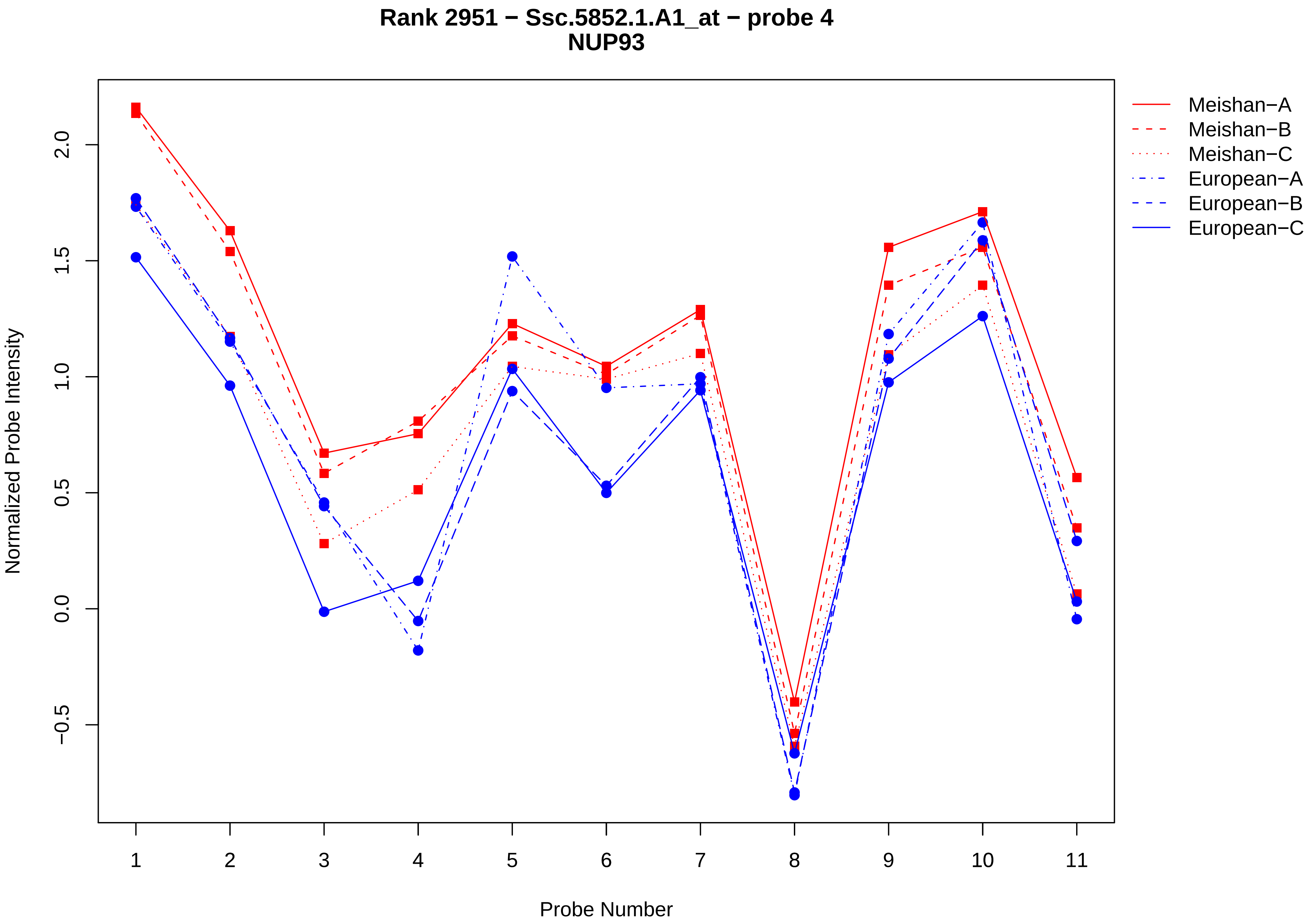

Rank 2951 − Ssc.5852.1.A1_at − probe 4
NUP93
Normalized Probe Intensity
Probe Number
Meishan−A
Meishan−B
Meishan−C
European−A
European−B
European−C

Rank 2952 − Ssc.1504.2.S1_at − probe 4
ABCF1

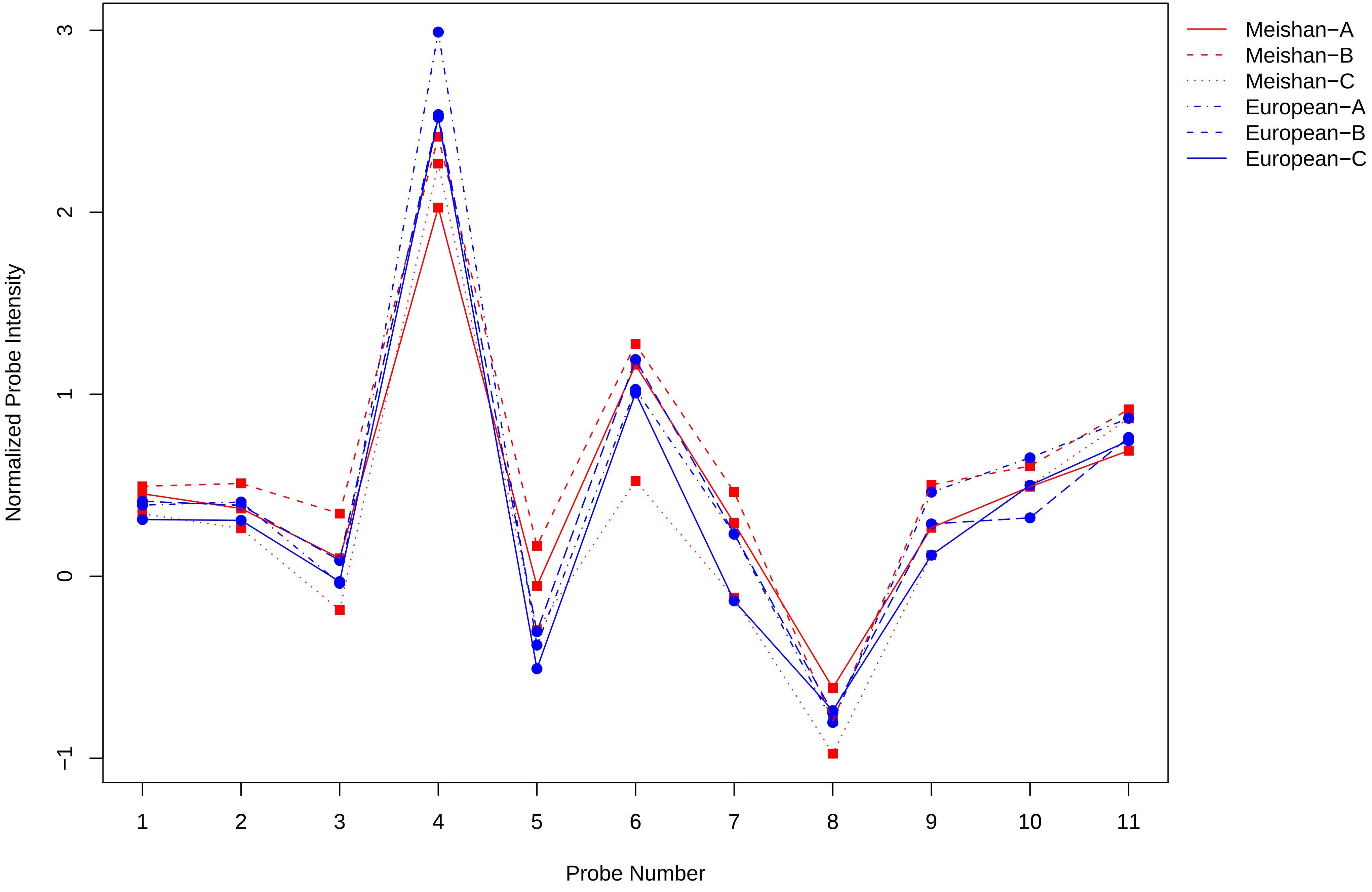

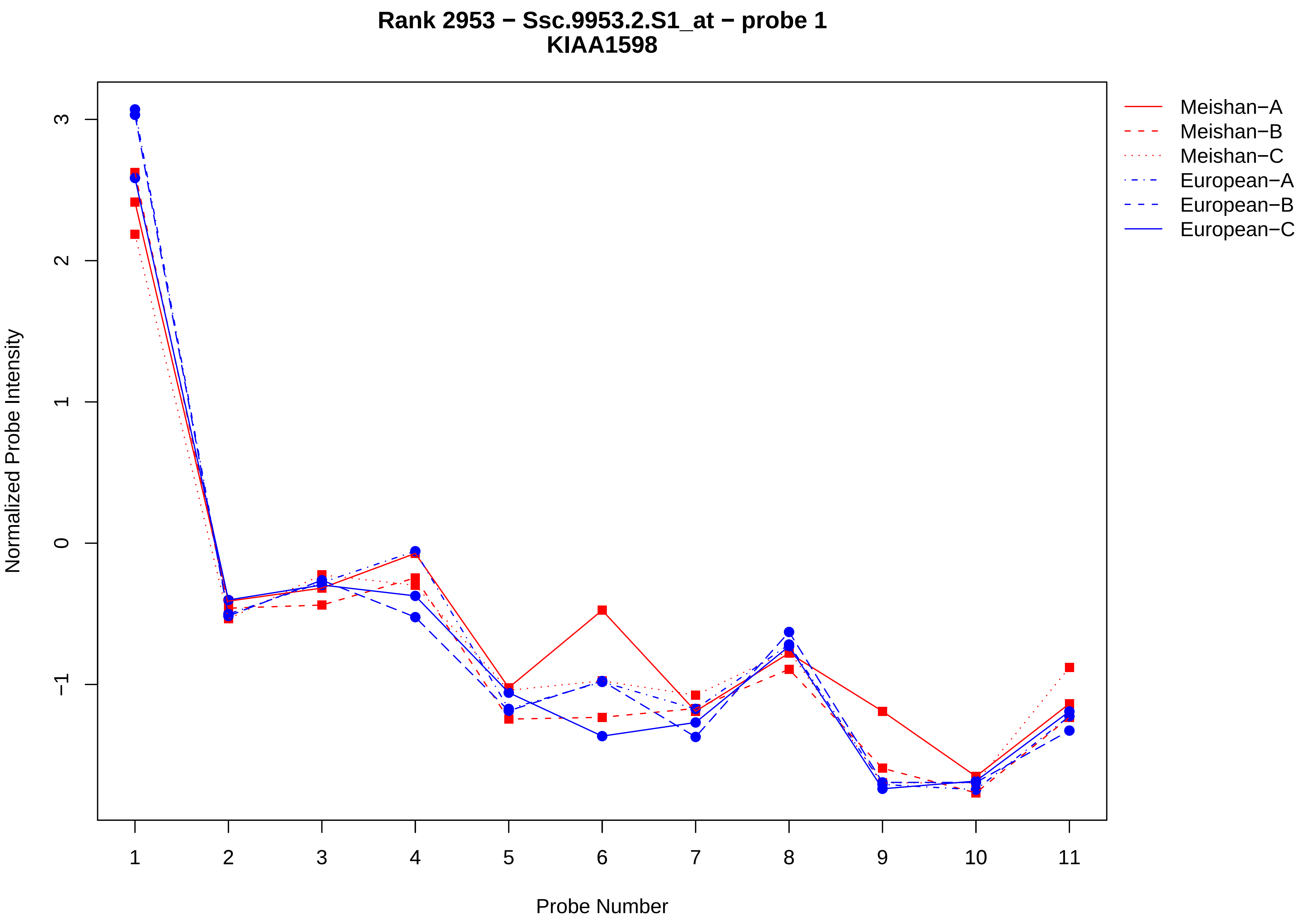

Rank 2953 − Ssc.9953.2.S1_at − probe 1
KIAA1598
Normalized Probe Intensity
Probe Number
Meishan−A
Meishan−B
Meishan−C
European−A
European−B
European−C

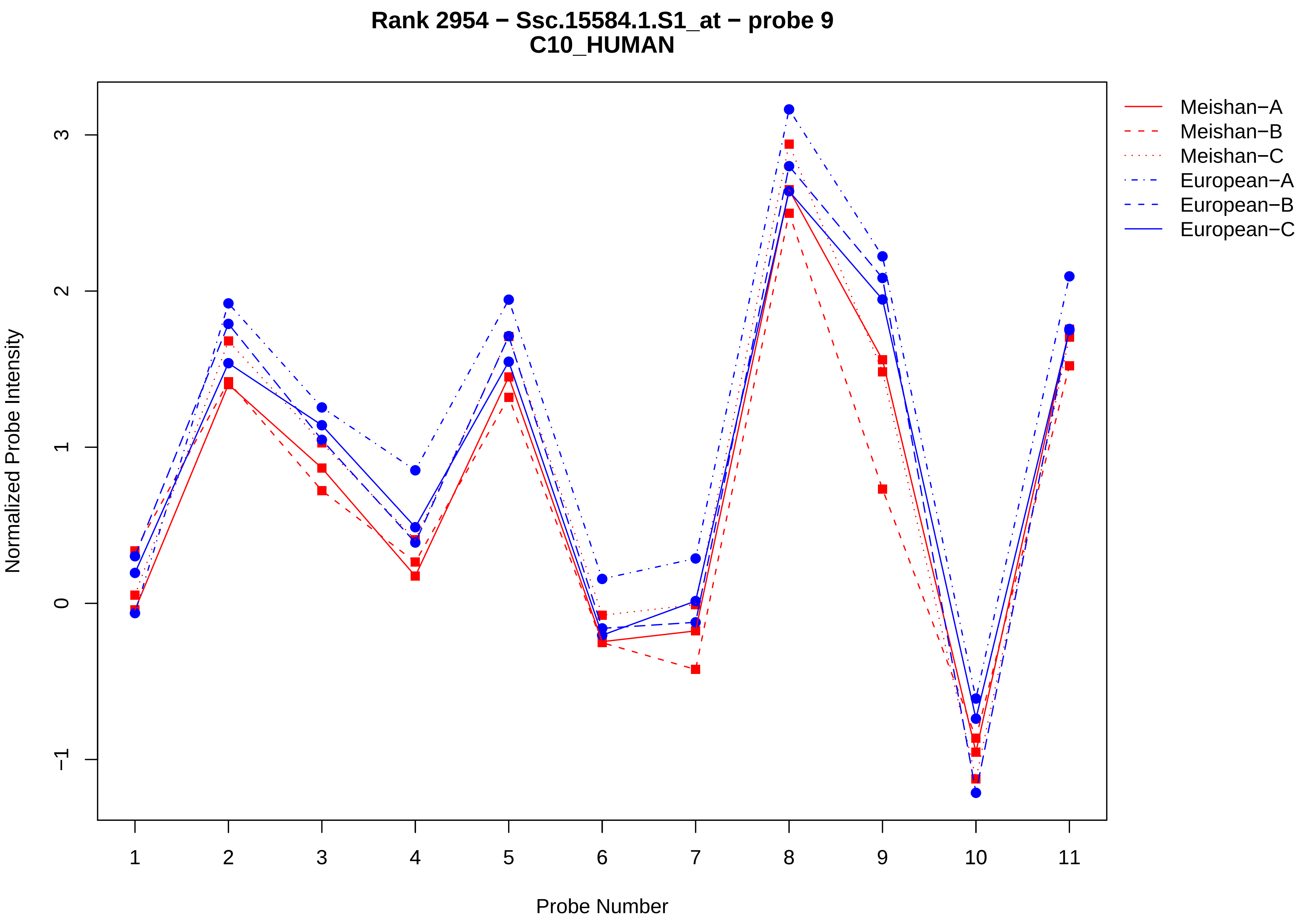

Rank 2954 − Ssc.15584.1.S1_at − probe 9
C10_HUMAN
Normalized Probe Intensity
Probe Number
−1
0
1
2
3
1
2
3
4
5
6
7
8
9
10
11
Meishan−A
Meishan−B
Meishan−C
European−A
European−B
European−C

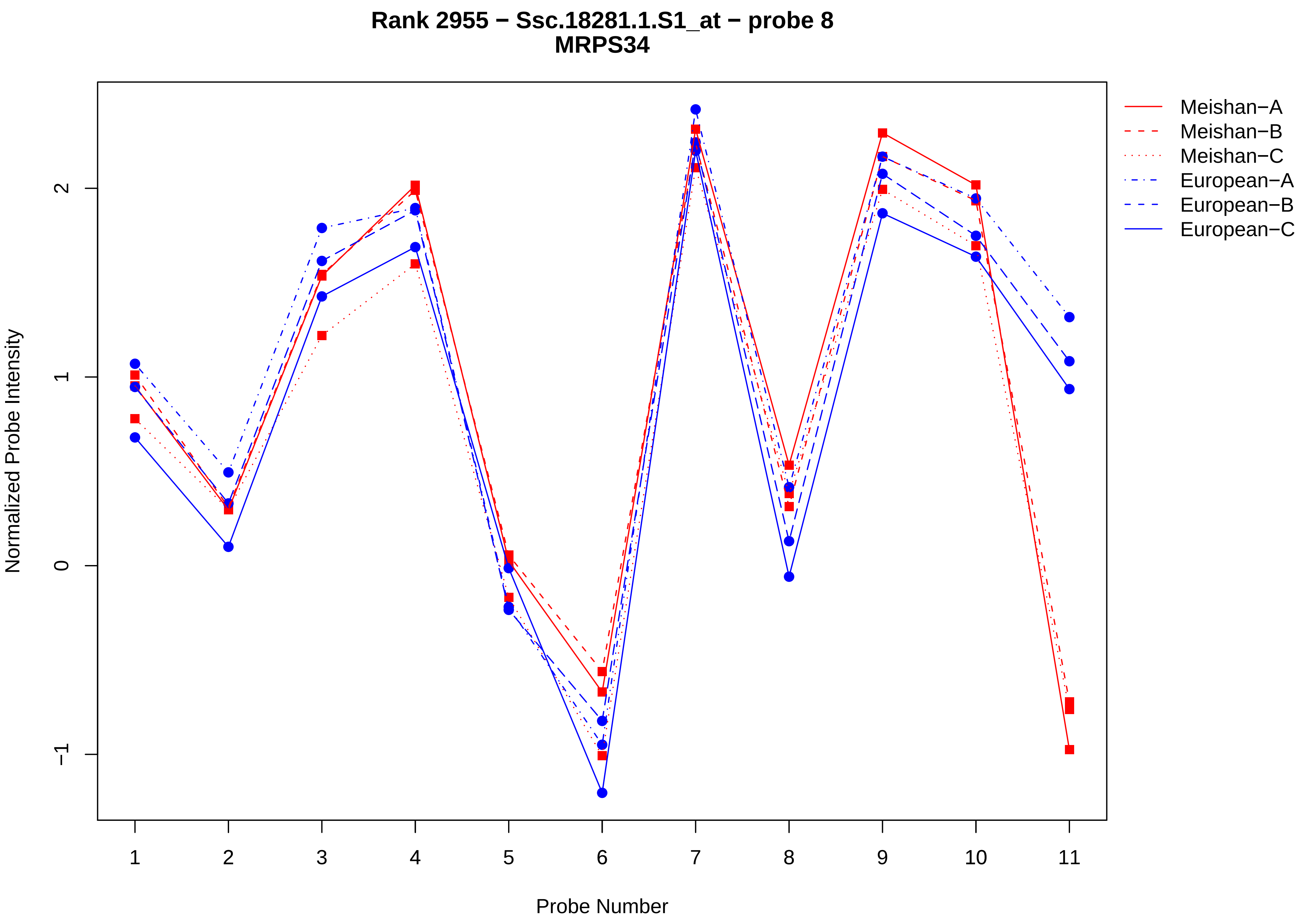

Rank 2955 − Ssc.18281.1.S1_at − probe 8
MRPS34
Normalized Probe Intensity
Probe Number
−1
0
1
2
1
2
3
4
5
6
7
8
9
10
11
Meishan−A
Meishan−B
Meishan−C
European−A
European−B
European−C

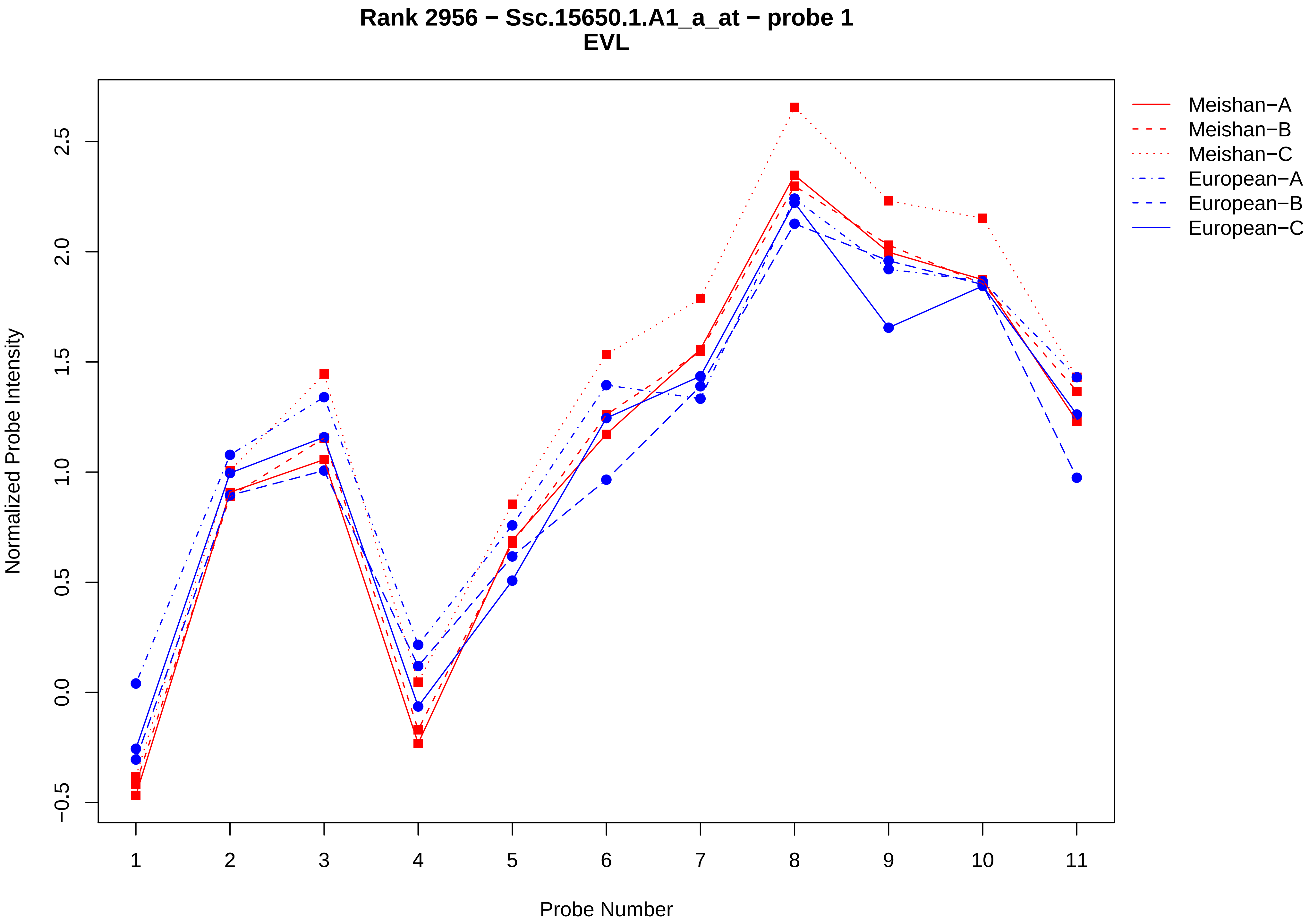
Rank 2956 − Ssc.15650.1.A1_a_at − probe 1
EVL
Normalized Probe Intensity
Probe Number
−0.5
0.0
0.5
1.0
1.5
2.0
2.5
1
2
3
4
5
6
7
8
9
10
11
Meishan−A
Meishan−B
Meishan−C
European−A
European−B
European−C

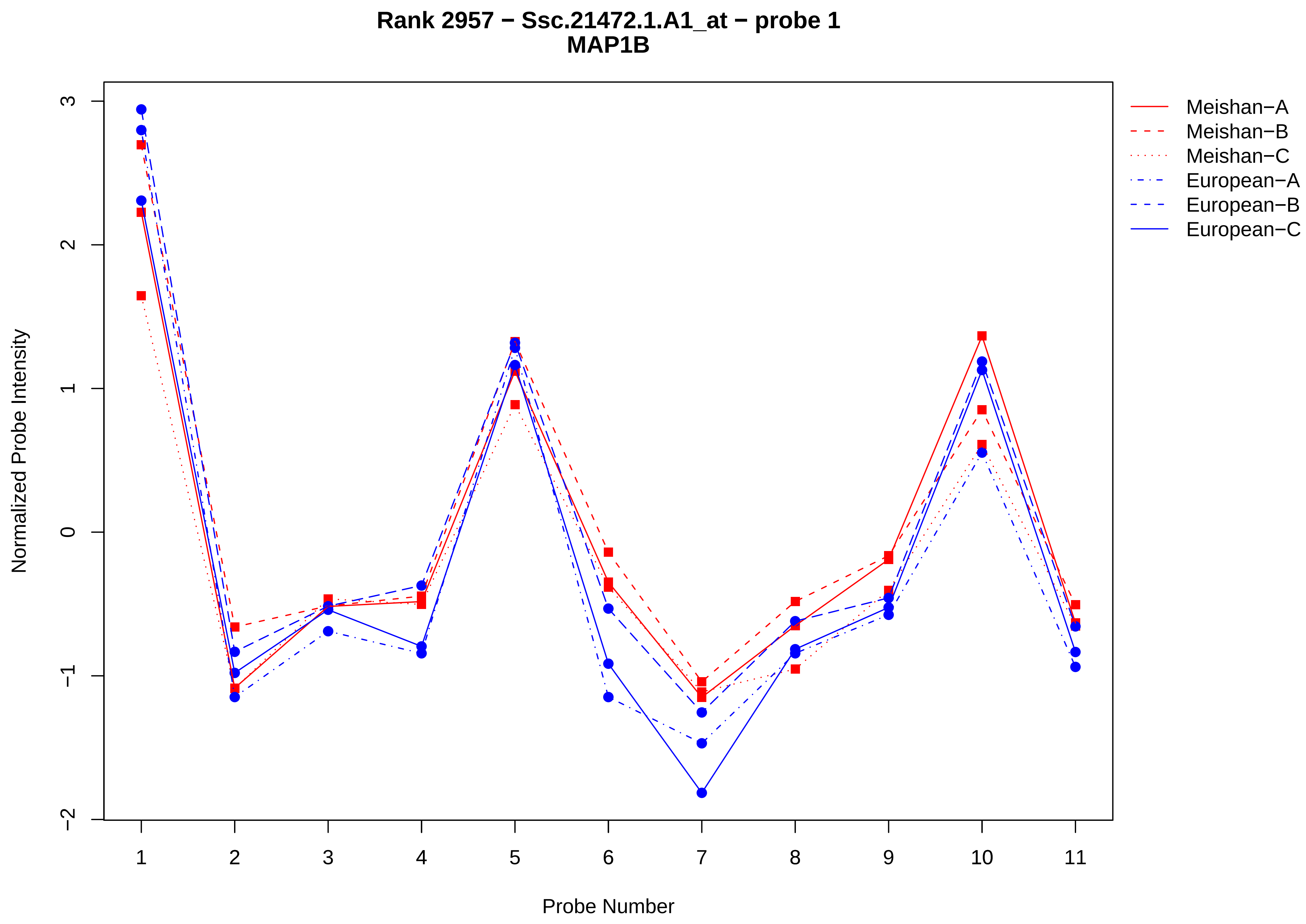

Rank 2957 − Ssc.21472.1.A1_at − probe 1
MAP1B
Normalized Probe Intensity
Probe Number
Meishan−A
Meishan−B
Meishan−C
European−A
European−B
European−C
−2
−1
0
1
2
3
1
2
3
4
5
6
7
8
9
10
11

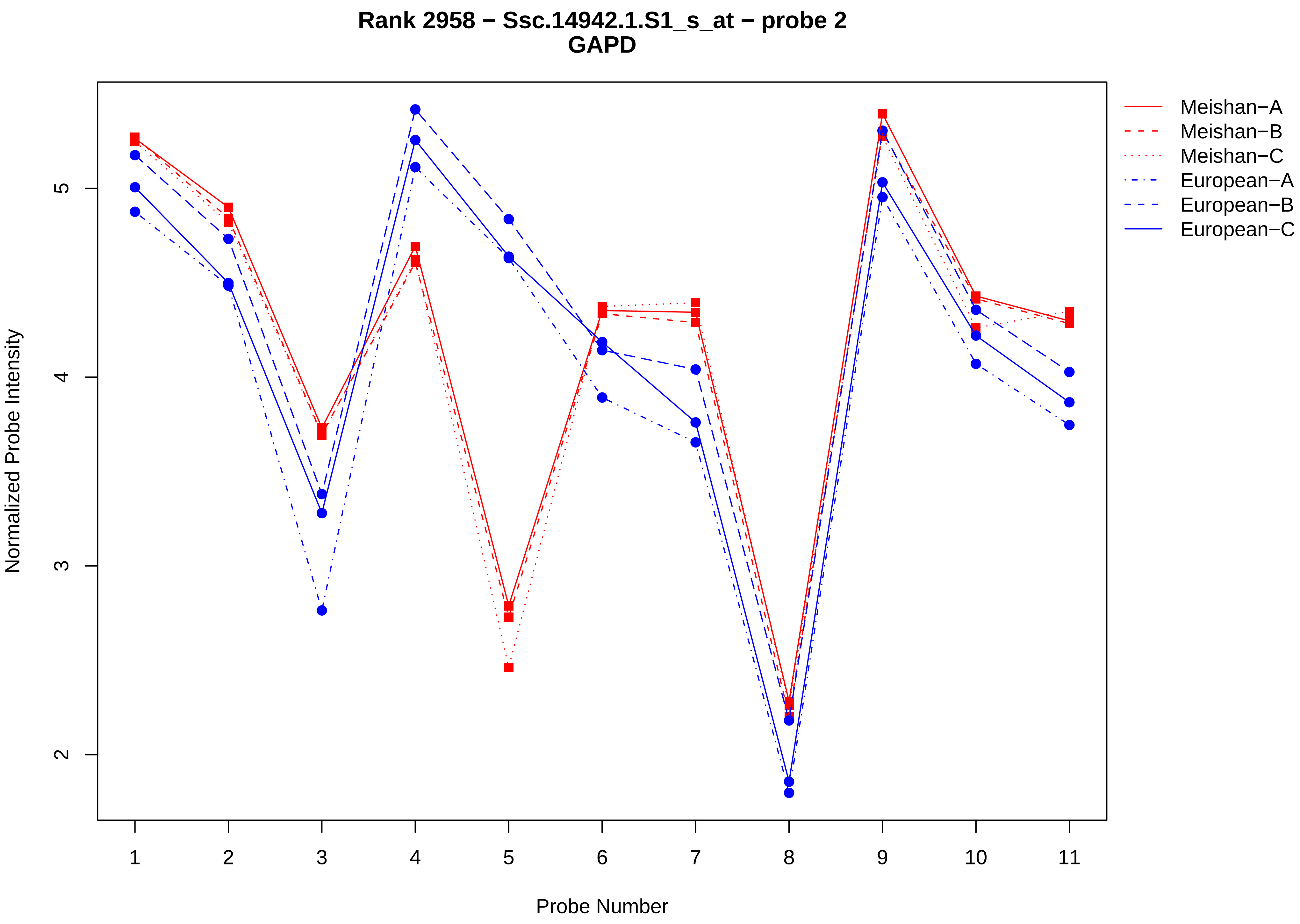

Rank 2958 − Ssc.14942.1.S1_s_at − probe 2
GAPD
Normalized Probe Intensity
Probe Number
Meishan−A
Meishan−B
Meishan−C
European−A
European−B
European−C

Rank 2959 − Ssc.30786.1.A1_at − probe 7
NP_660158

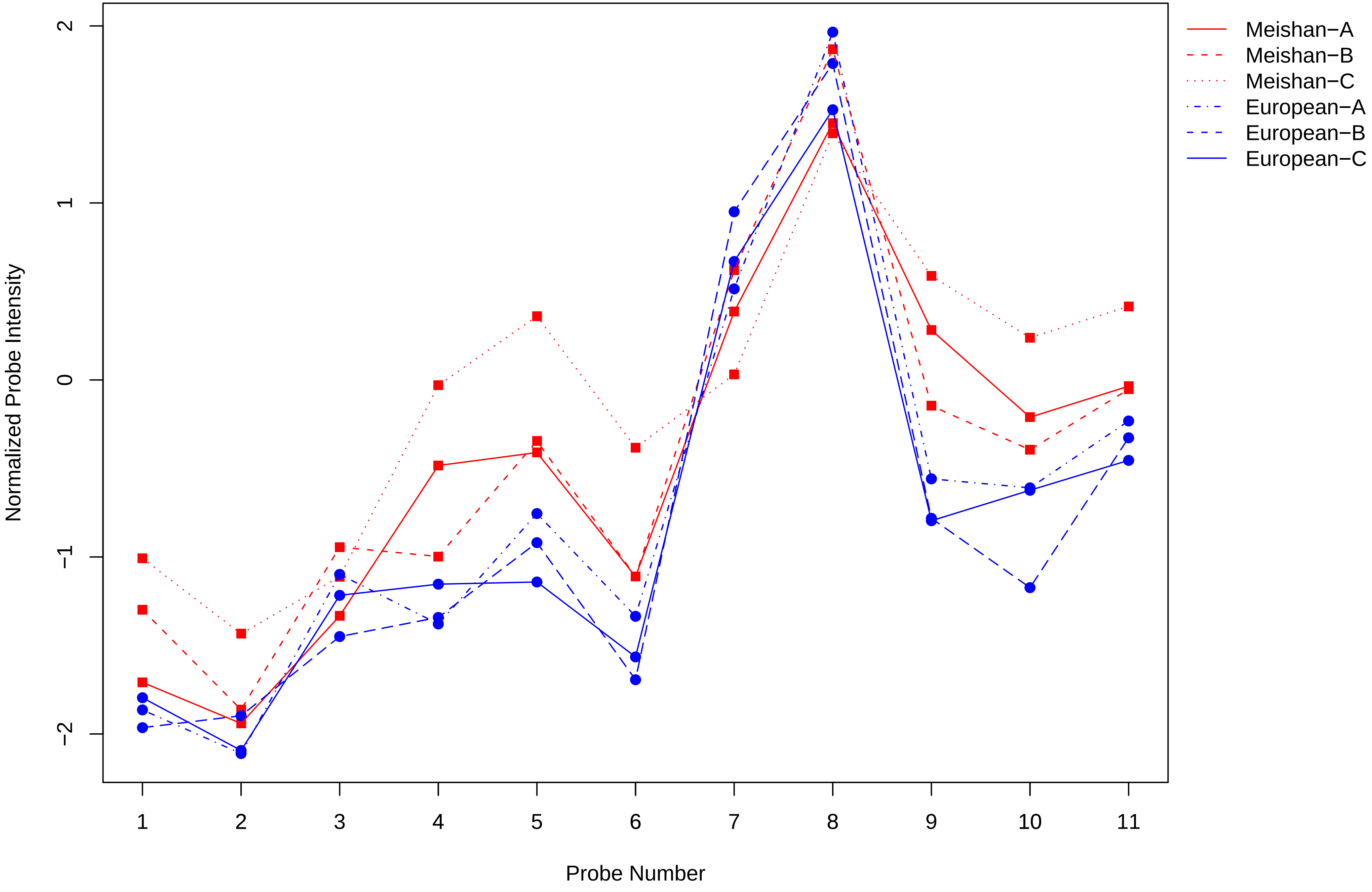

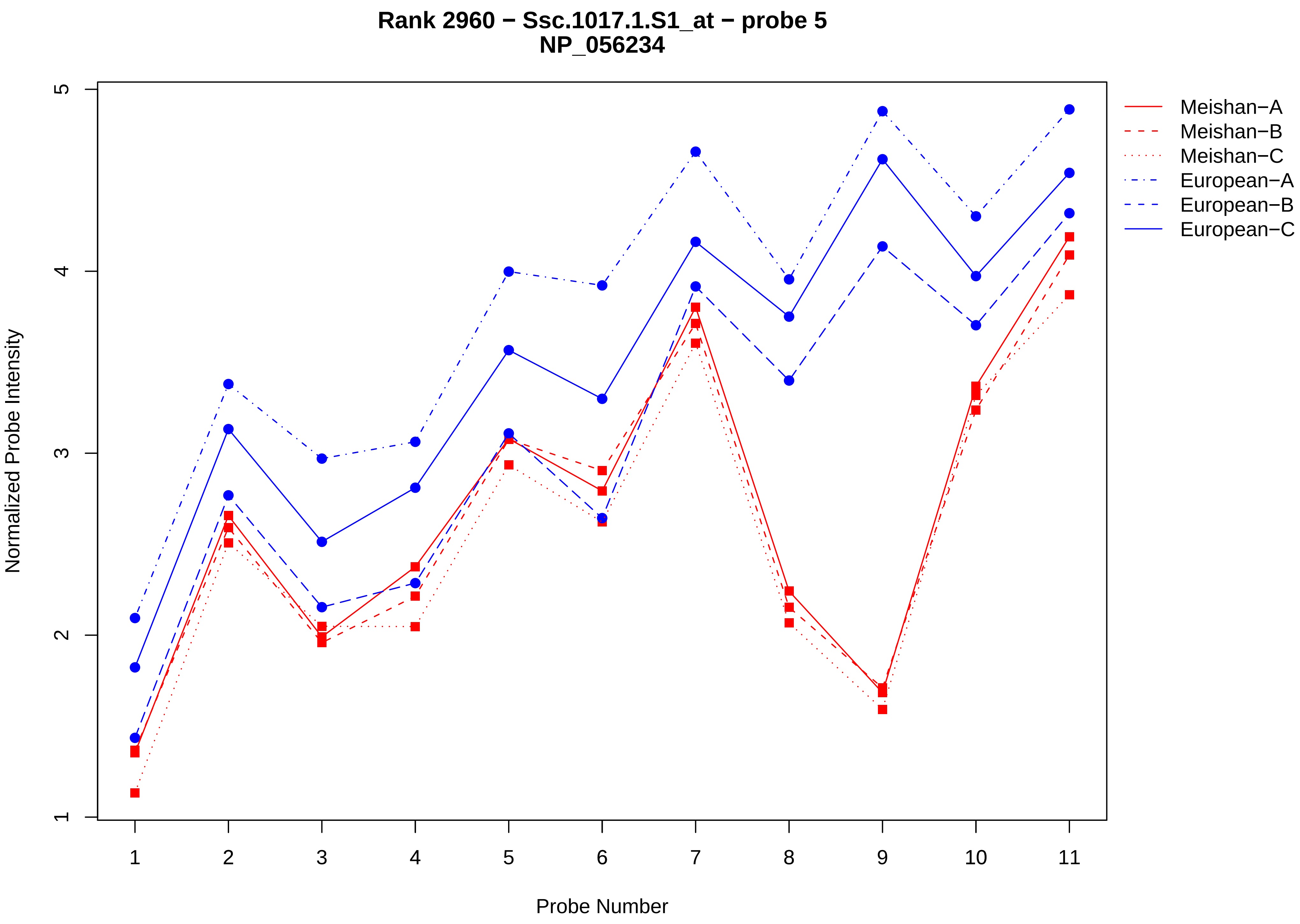
Rank 2960 − Ssc.1017.1.S1_at − probe 5
NP_056234
Normalized Probe Intensity
1
2
3
4
5
Probe Number
1
2
3
4
5
6
7
8
9
10
11
Meishan−A
Meishan−B
Meishan−C
European−A
European−B
European−C

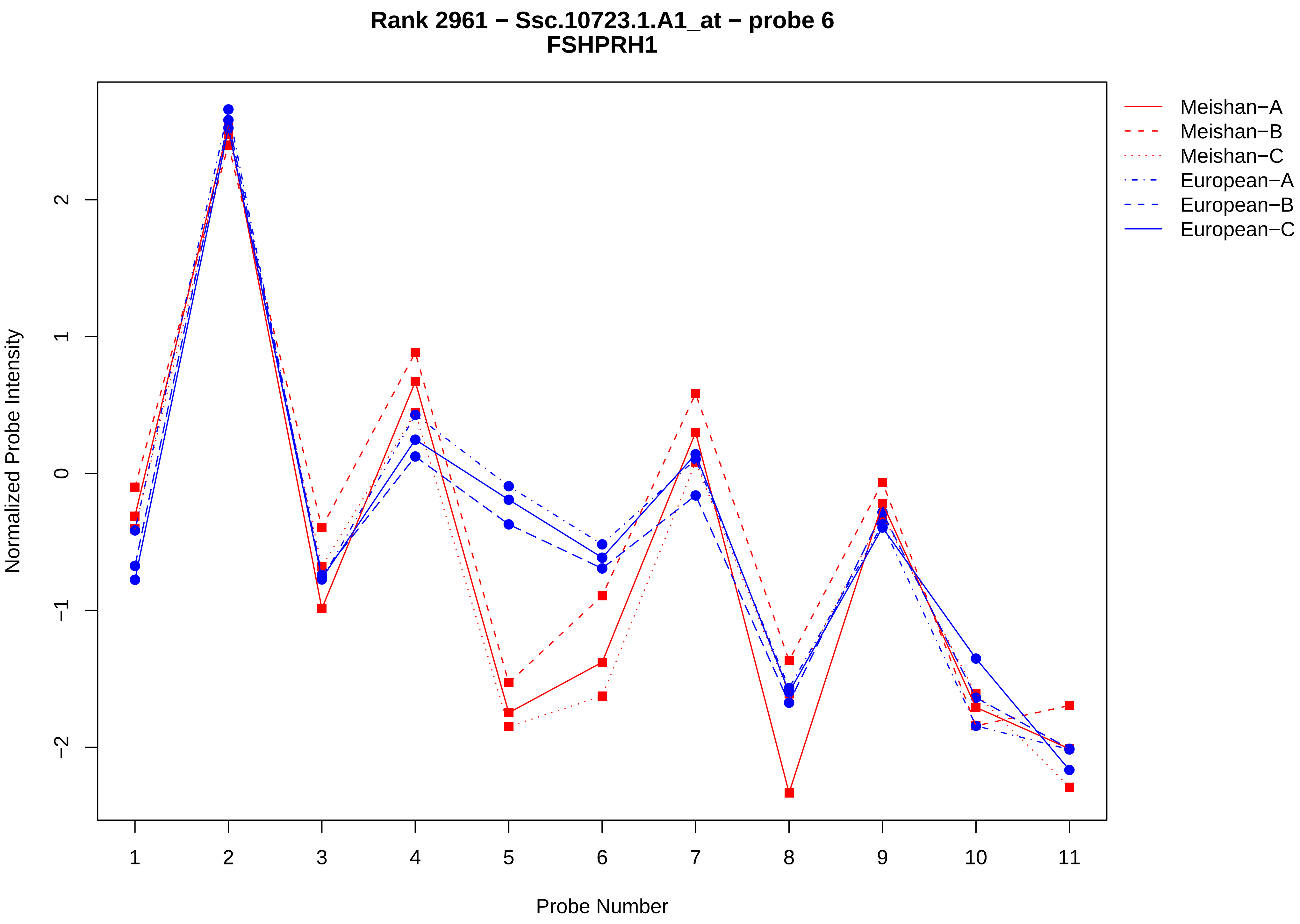

Rank 2961 − Ssc.10723.1.A1_at − probe 6
FSHPRH1
Normalized Probe Intensity
Probe Number
−2
−1
0
1
2
1
2
3
4
5
6
7
8
9
10
11
Meishan−A
Meishan−B
Meishan−C
European−A
European−B
European−C

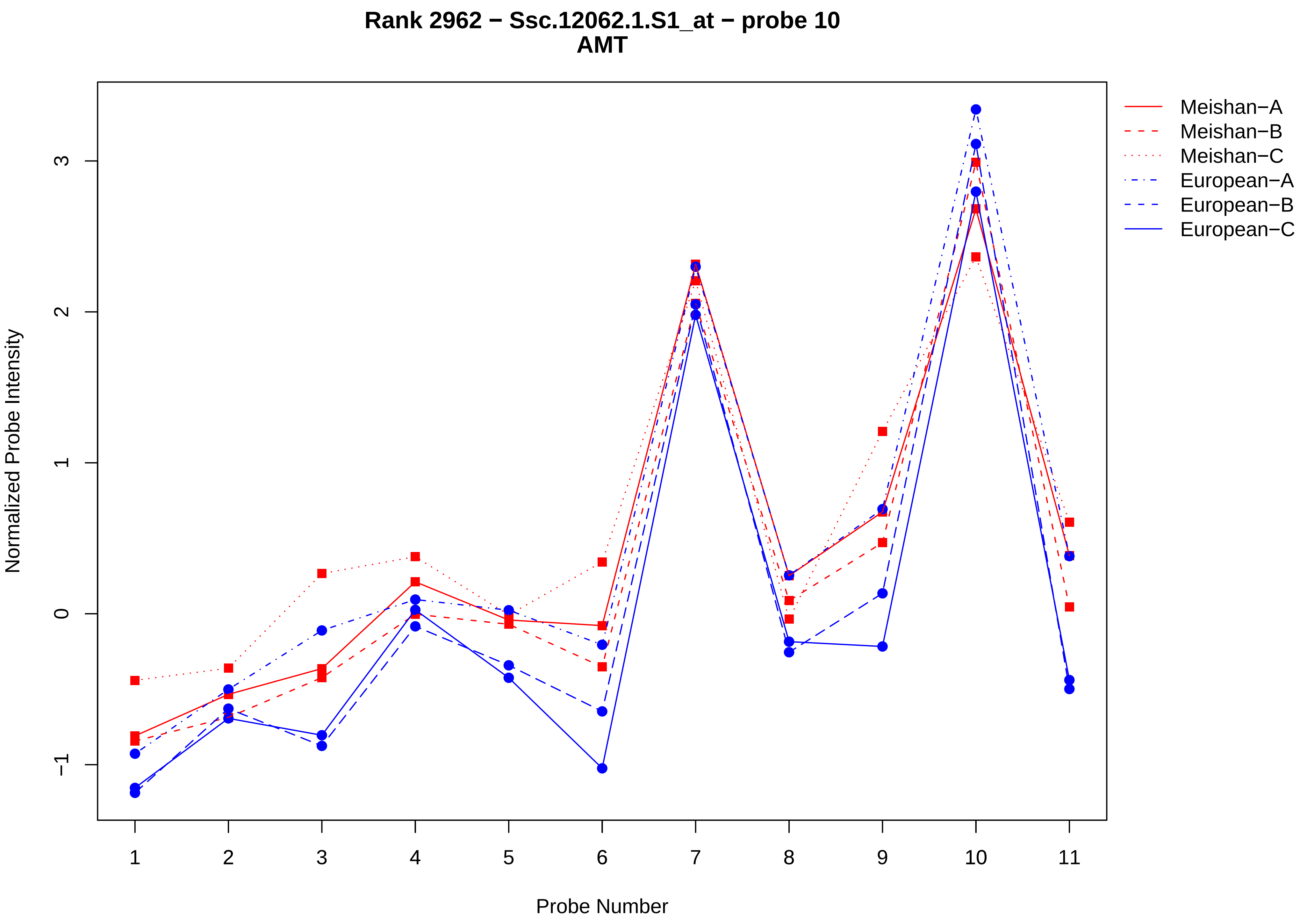

Rank 2962 − Ssc.12062.1.S1_at − probe 10
AMT
Normalized Probe Intensity
Probe Number
−1
0
1
2
3
1
2
3
4
5
6
7
8
9
10
11
Meishan−A
Meishan−B
Meishan−C
European−A
European−B
European−C

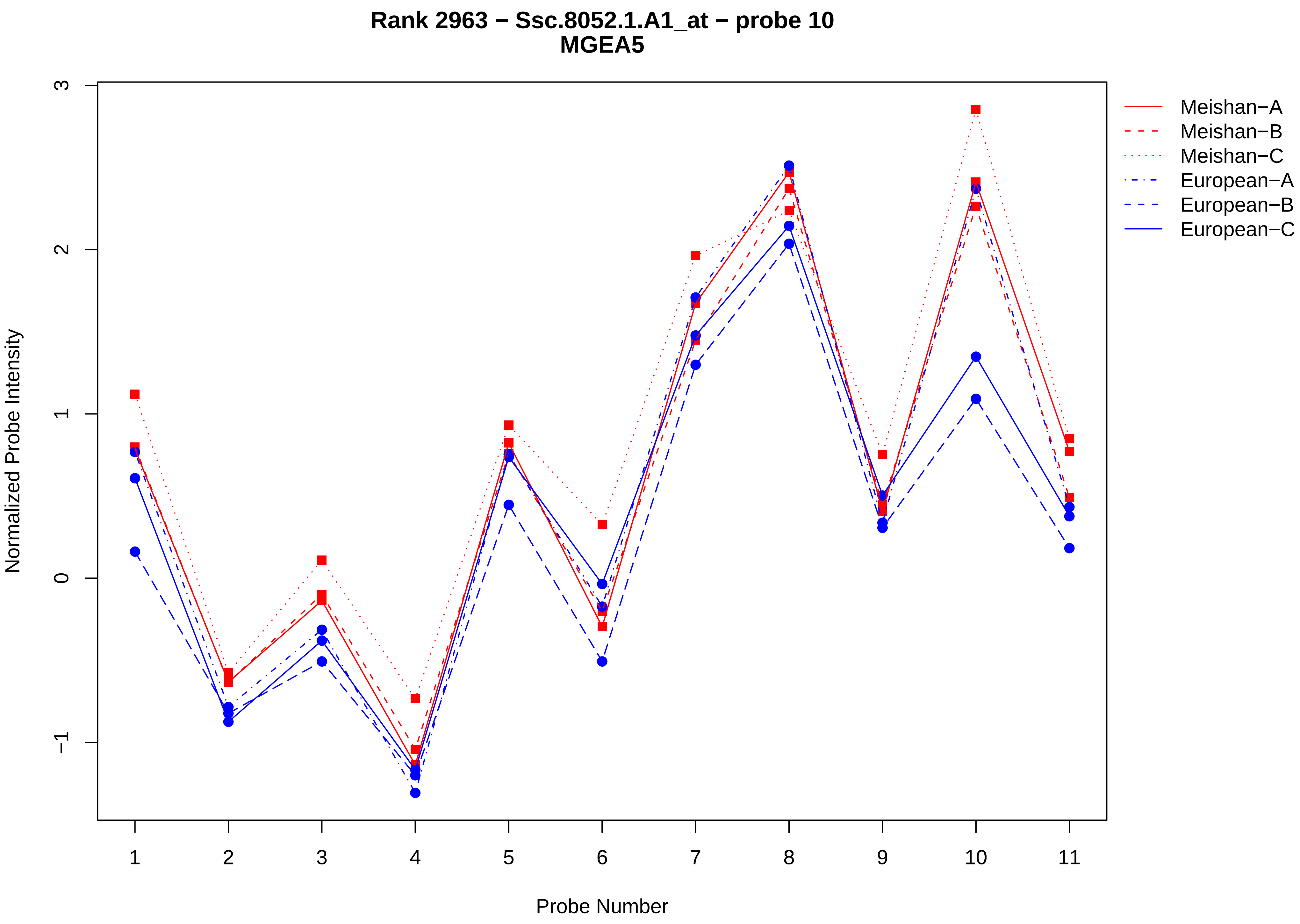

Rank 2963 − Ssc.8052.1.A1_at − probe 10
MGEA5
Normalized Probe Intensity
Probe Number
Meishan−A
Meishan−B
Meishan−C
European−A
European−B
European−C
−1
0
1
2
3
1
2
3
4
5
6
7
8
9
10
11

Rank 2964 − Ssc.10219.1.A1_at − probe 3
SLC1A6

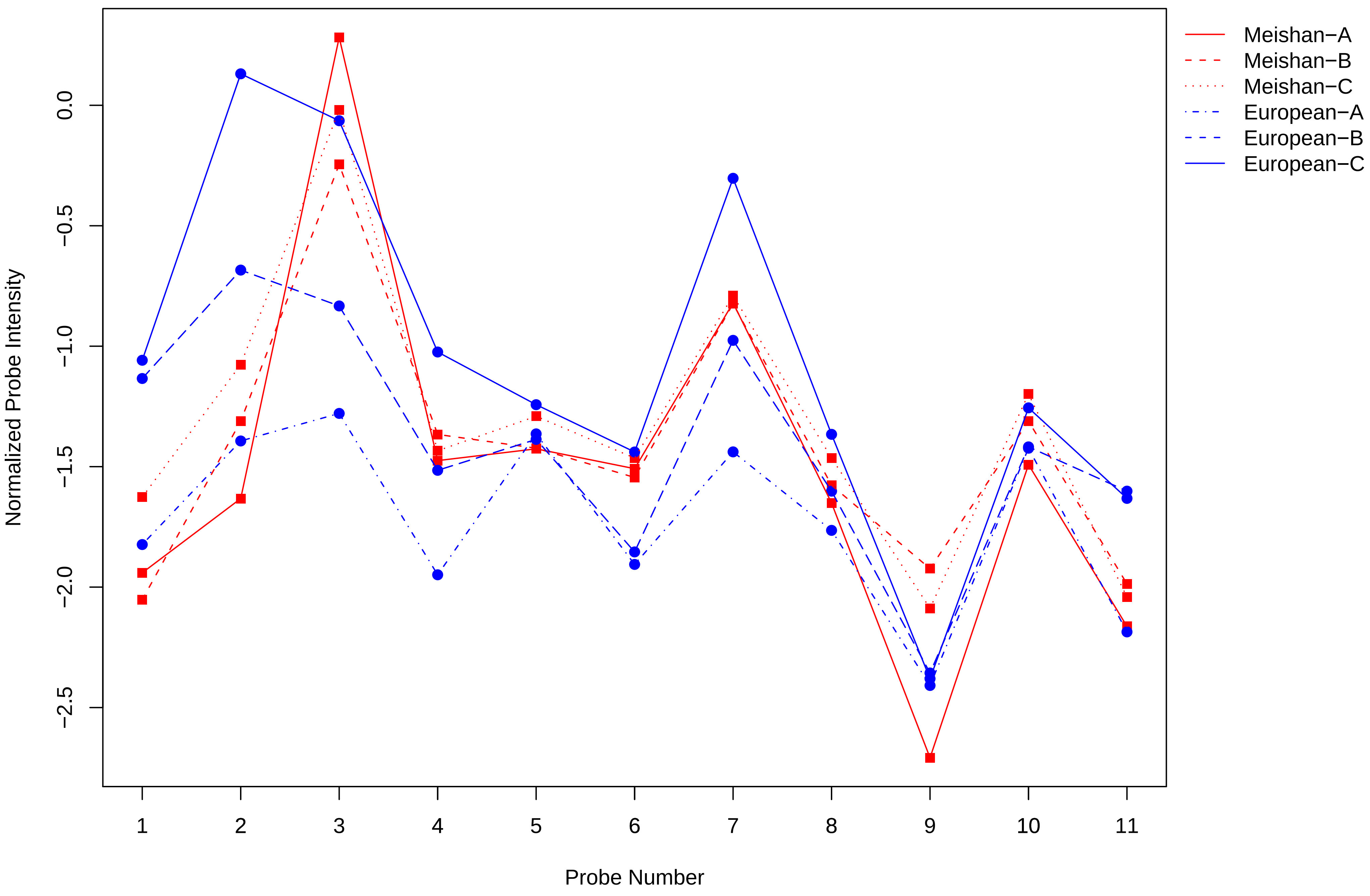

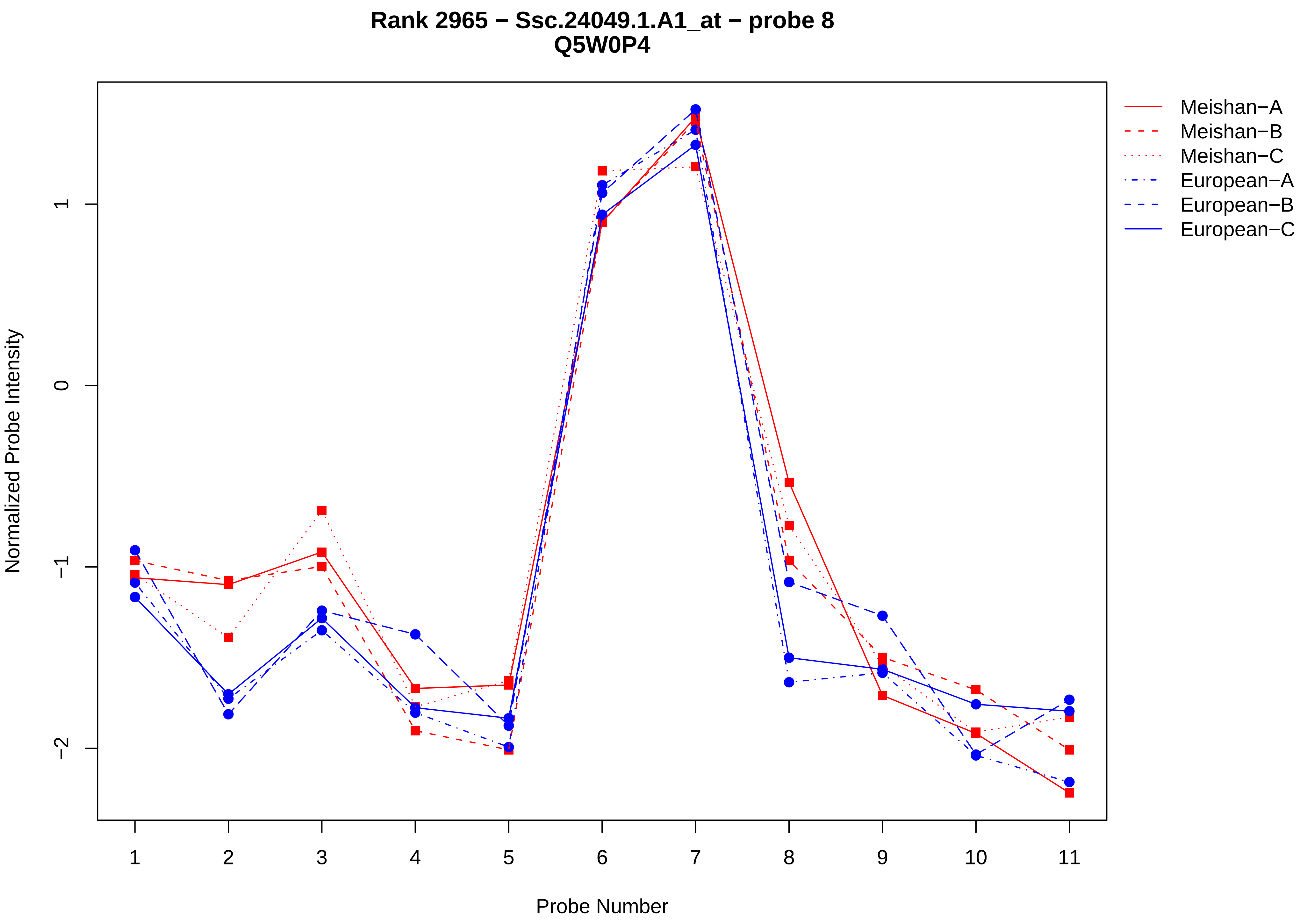

Rank 2965 − Ssc.24049.1.A1_at − probe 8
Q5W0P4
Normalized Probe Intensity
Probe Number
1
0
−1
−2
1 2 3 4 5 6 7 8 9 10 11
Meishan−A
Meishan−B
Meishan−C
European−A
European−B
European−C

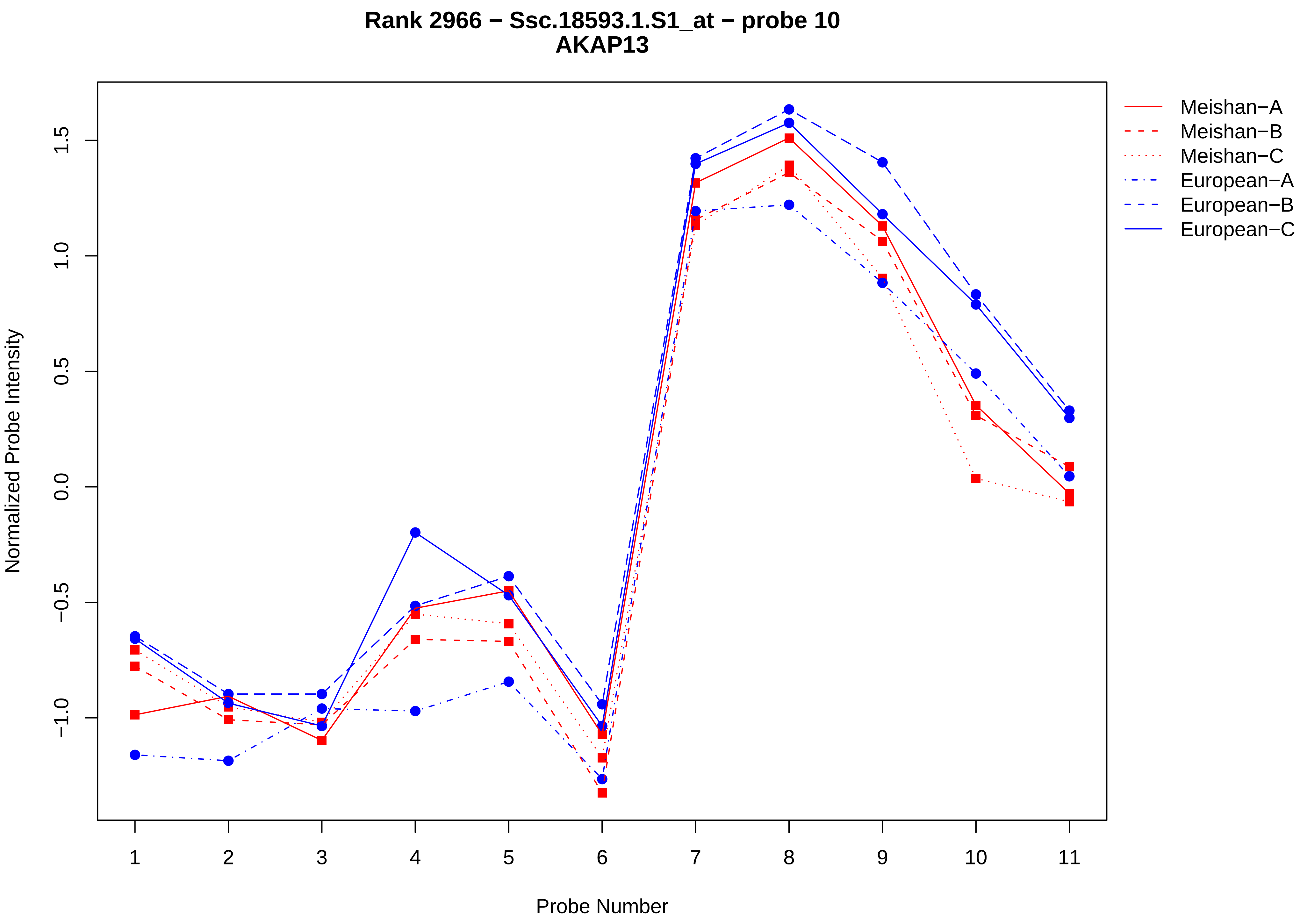

Rank 2966 − Ssc.18593.1.S1_at − probe 10
AKAP13
Normalized Probe Intensity
Probe Number
Meishan−A
Meishan−B
Meishan−C
European−A
European−B
European−C
1.5
1.0
0.5
0.0
−0.5
−1.0
1
2
3
4
5
6
7
8
9
10
11

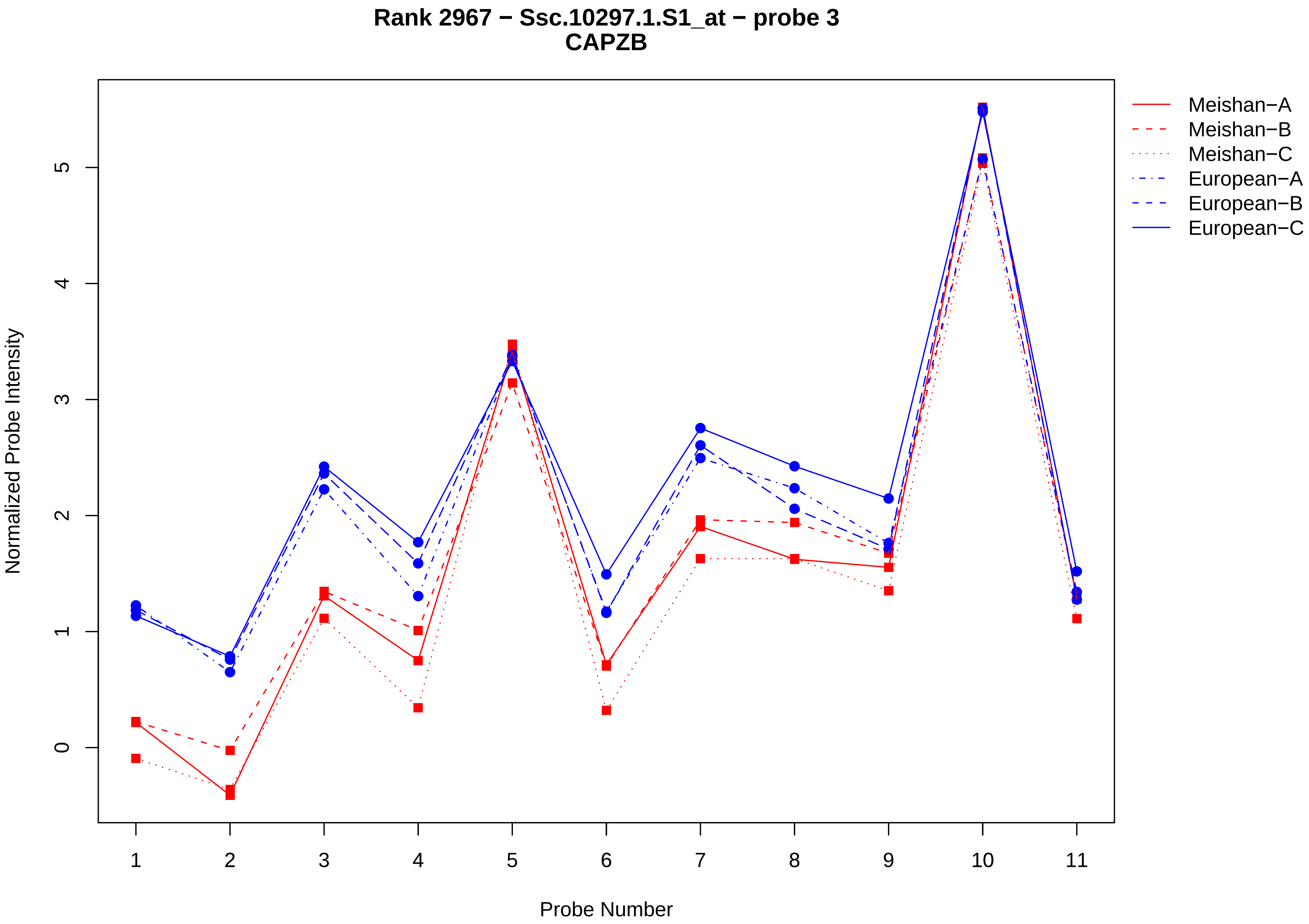

Rank 2967 − Ssc.10297.1.S1_at − probe 3
CAPZB
Normalized Probe Intensity
Probe Number
0
1
2
3
4
5
1
2
3
4
5
6
7
8
9
10
11
Meishan−A
Meishan−B
Meishan−C
European−A
European−B
European−C

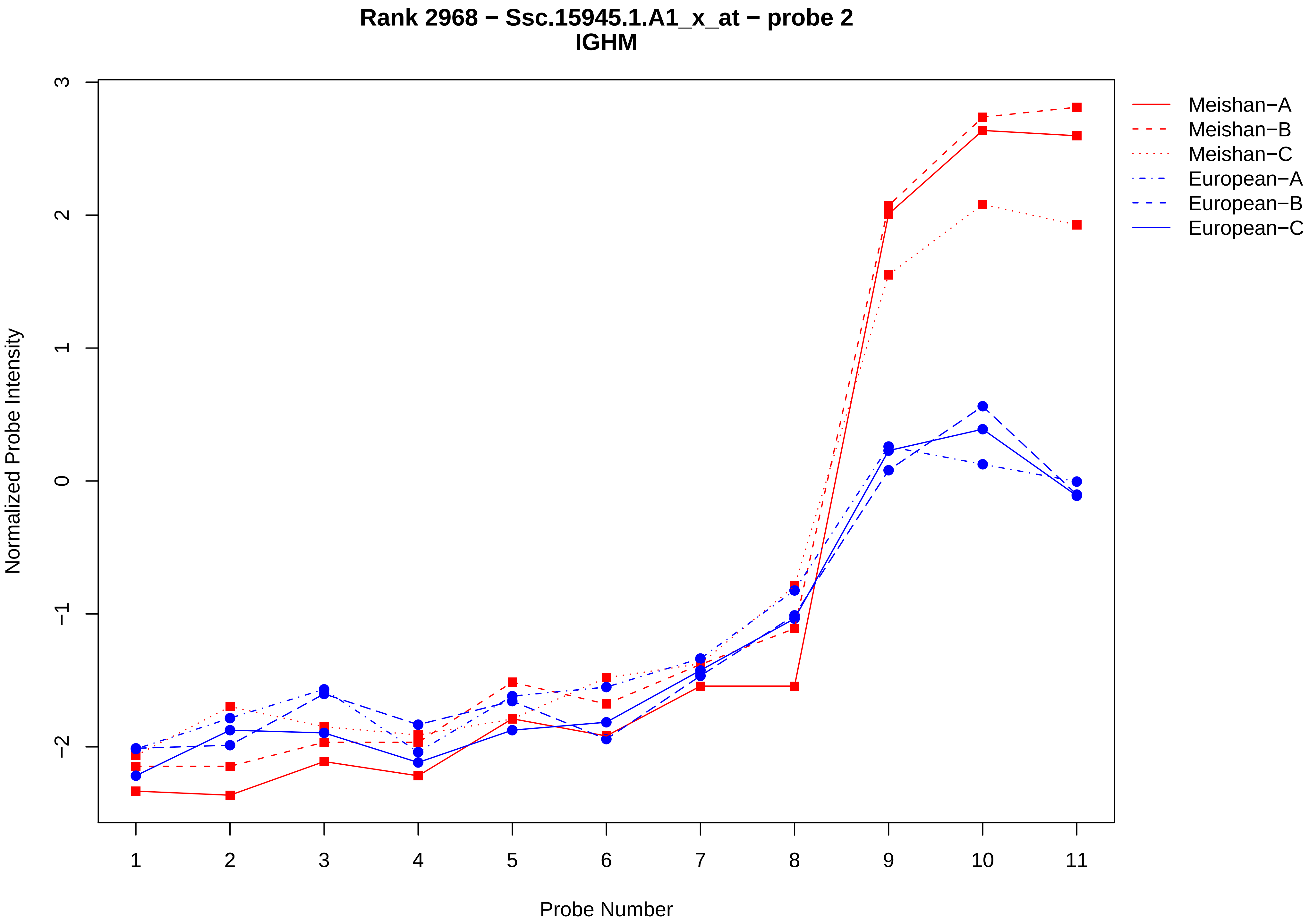

Rank 2968 − Ssc.15945.1.A1_x_at − probe 2
IGHM
Normalized Probe Intensity
Probe Number
−2
−1
0
1
2
3
1
2
3
4
5
6
7
8
9
10
11
Meishan−A
Meishan−B
Meishan−C
European−A
European−B
European−C

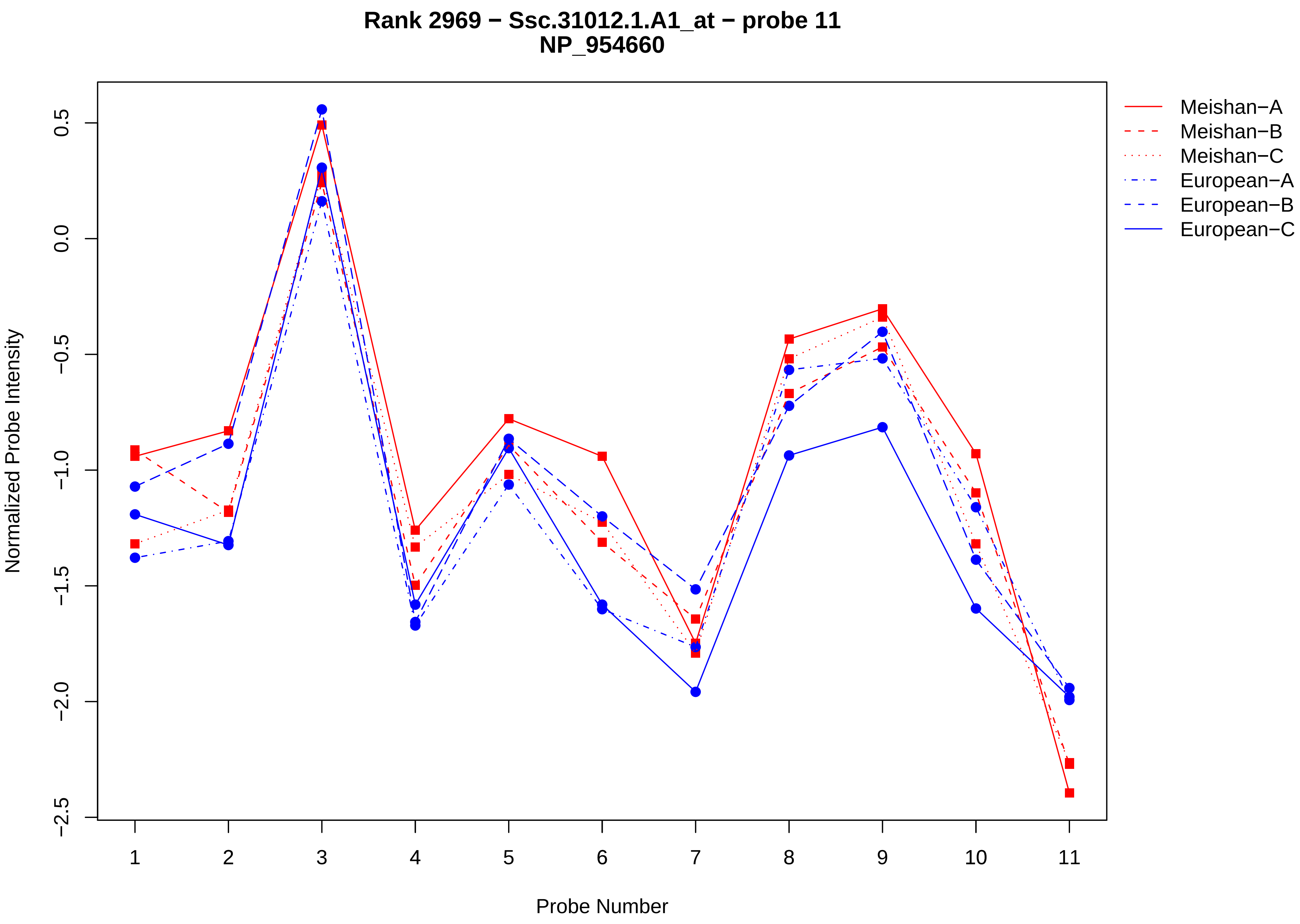

Rank 2969 − Ssc.31012.1.A1_at − probe 11
NP_954660
Normalized Probe Intensity
Probe Number
0.5
0.0
−0.5
−1.0
−1.5
−2.0
−2.5
1
2
3
4
5
6
7
8
9
10
11
Meishan−A
Meishan−B
Meishan−C
European−A
European−B
European−C

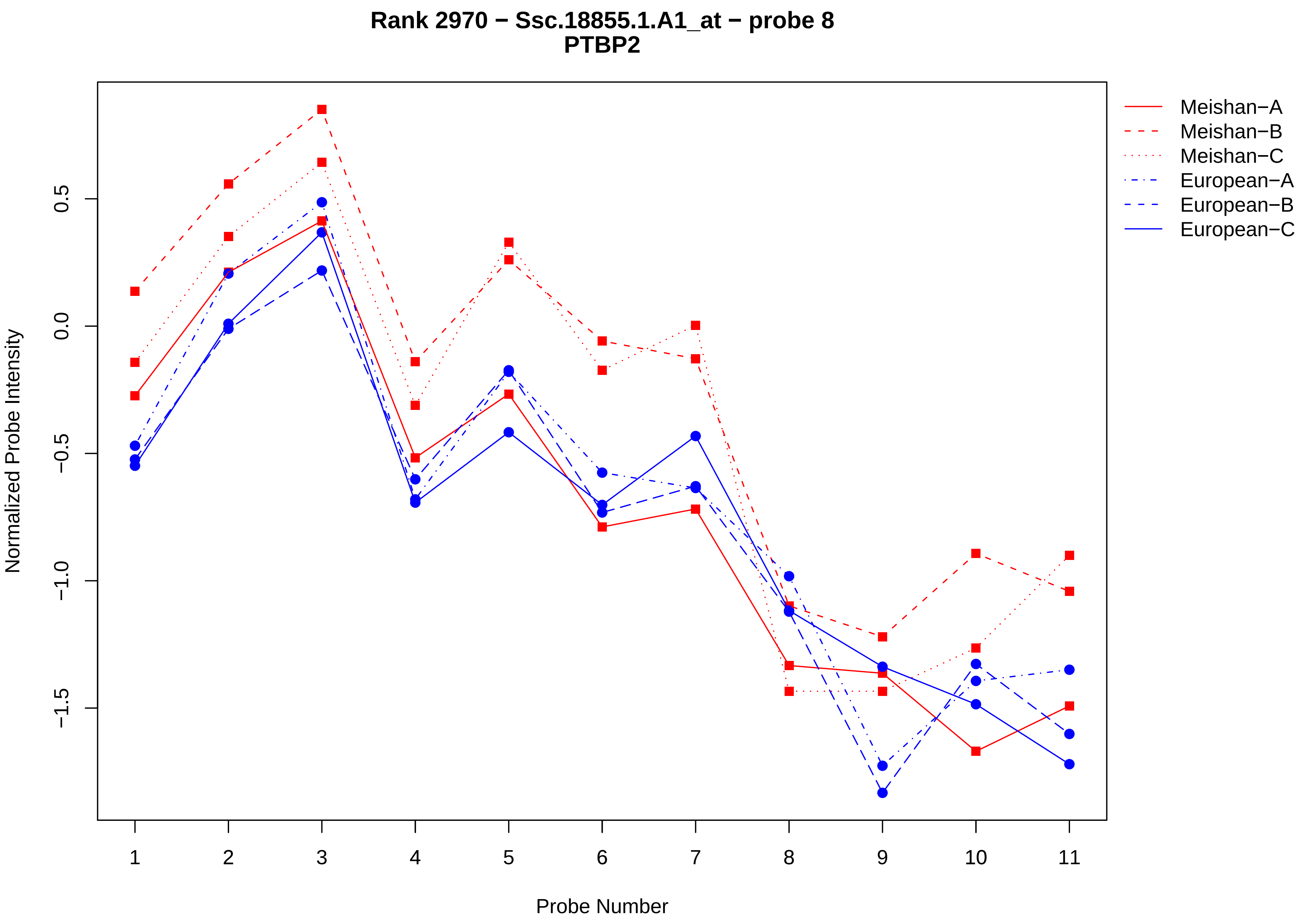

Rank 2970 − Ssc.18855.1.A1_at − probe 8
PTBP2
Normalized Probe Intensity
Probe Number
0.5
0.0
−0.5
−1.0
−1.5
1
2
3
4
5
6
7
8
9
10
11
Meishan−A
Meishan−B
Meishan−C
European−A
European−B
European−C

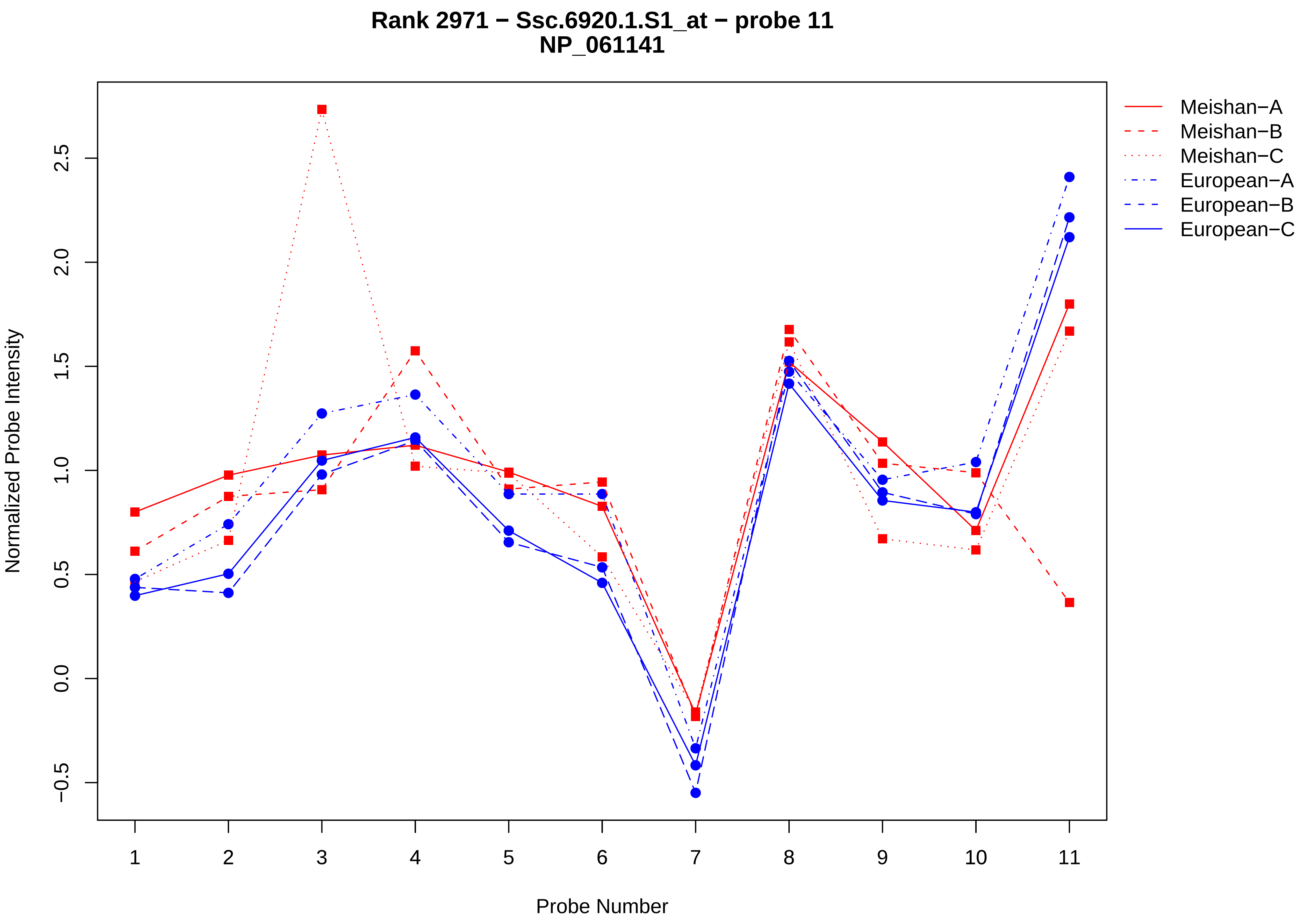
Rank 2971 − Ssc.6920.1.S1_at − probe 11
NP_061141
Normalized Probe Intensity
Probe Number
−0.5
0.0
0.5
1.0
1.5
2.0
2.5
1
2
3
4
5
6
7
8
9
10
11
Meishan−A
Meishan−B
Meishan−C
European−A
European−B
European−C

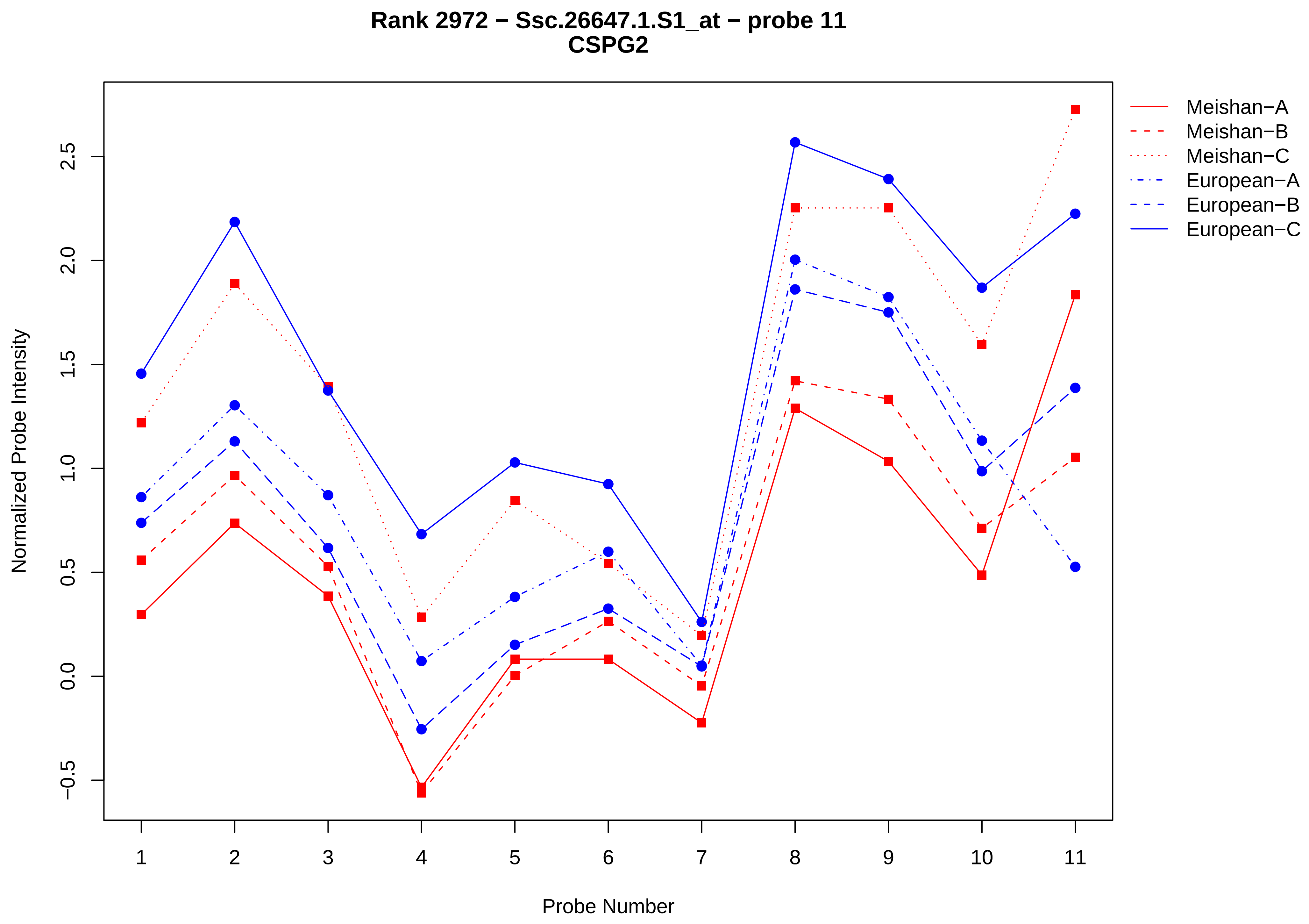

Rank 2972 − Ssc.26647.1.S1_at − probe 11
CSPG2
Normalized Probe Intensity
Probe Number
−0.5
0.0
0.5
1.0
1.5
2.0
2.5
1
2
3
4
5
6
7
8
9
10
11
Meishan−A
Meishan−B
Meishan−C
European−A
European−B
European−C

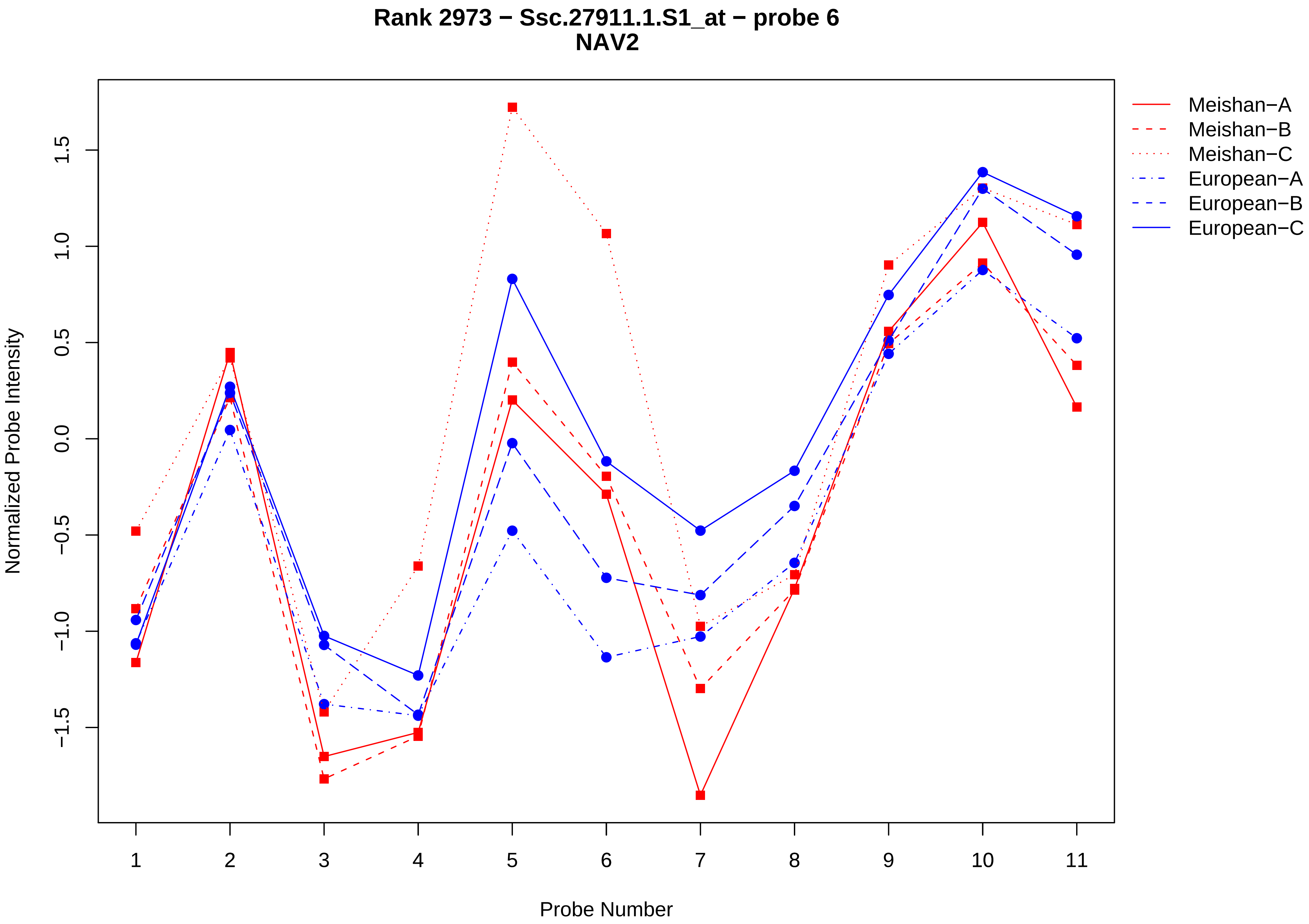

Rank 2973 − Ssc.27911.1.S1_at − probe 6
NAV2
Normalized Probe Intensity
Probe Number
1.5
1.0
0.5
0.0
−0.5
−1.0
−1.5
1
2
3
4
5
6
7
8
9
10
11
Meishan−A
Meishan−B
Meishan−C
European−A
European−B
European−C

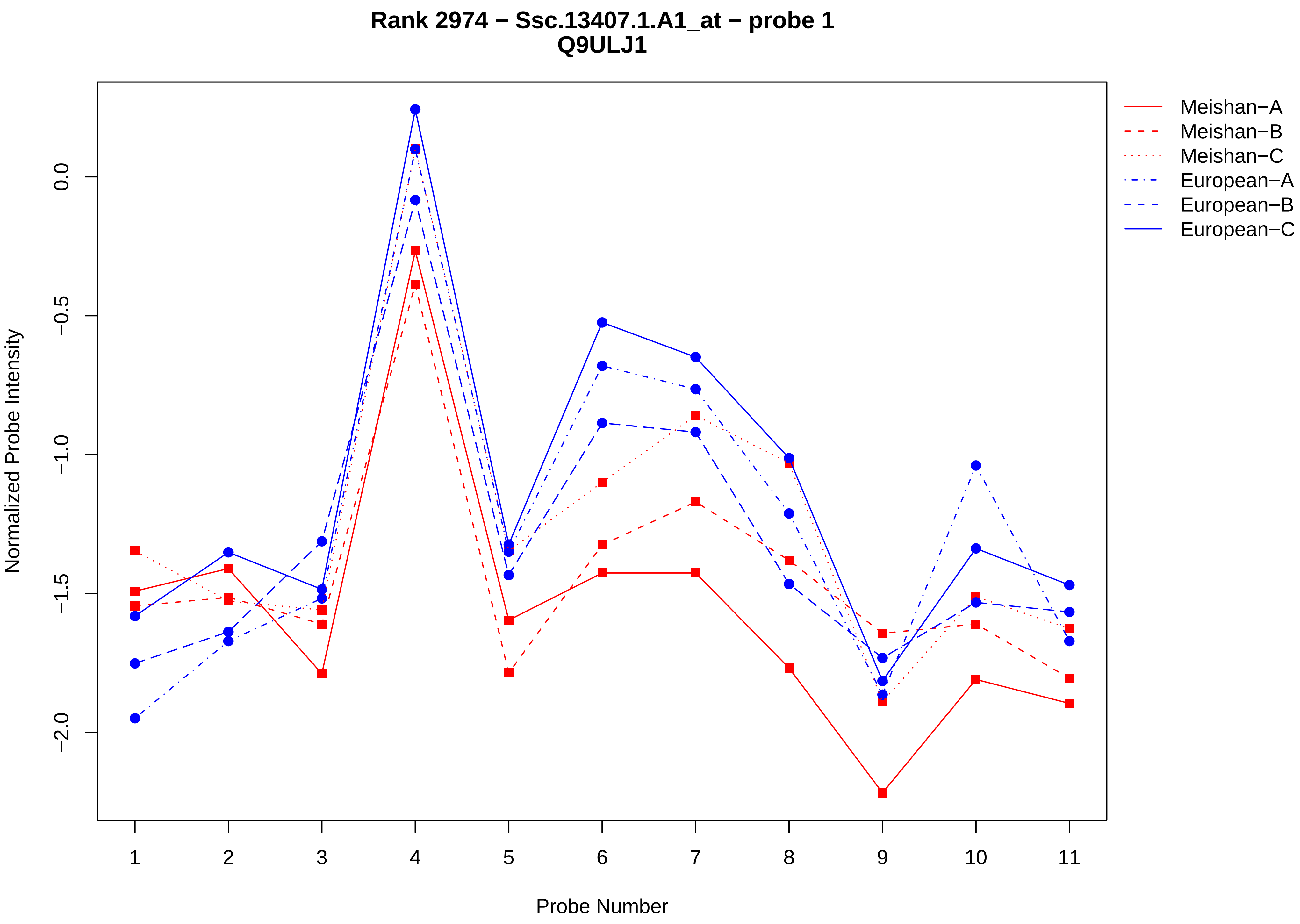

Rank 2974 − Ssc.13407.1.A1_at − probe 1
Q9ULJ1
Normalized Probe Intensity
Probe Number
0.0
−0.5
−1.0
−1.5
−2.0
1
2
3
4
5
6
7
8
9
10
11
Meishan−A
Meishan−B
Meishan−C
European−A
European−B
European−C

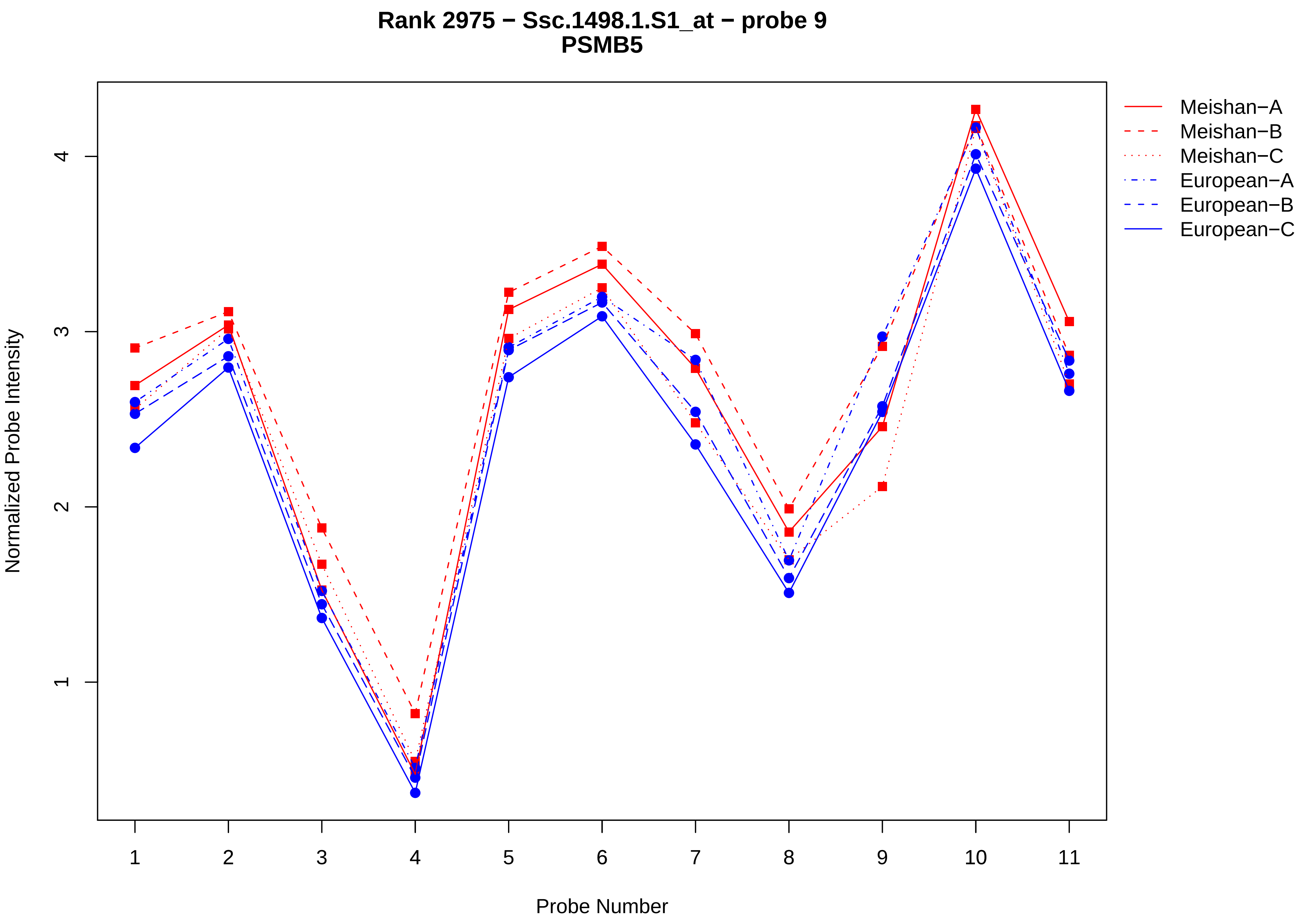

Rank 2975 − Ssc.1498.1.S1_at − probe 9
PSMB5
Normalized Probe Intensity
Probe Number
1
2
3
4
1
2
3
4
5
6
7
8
9
10
11
Meishan−A
Meishan−B
Meishan−C
European−A
European−B
European−C

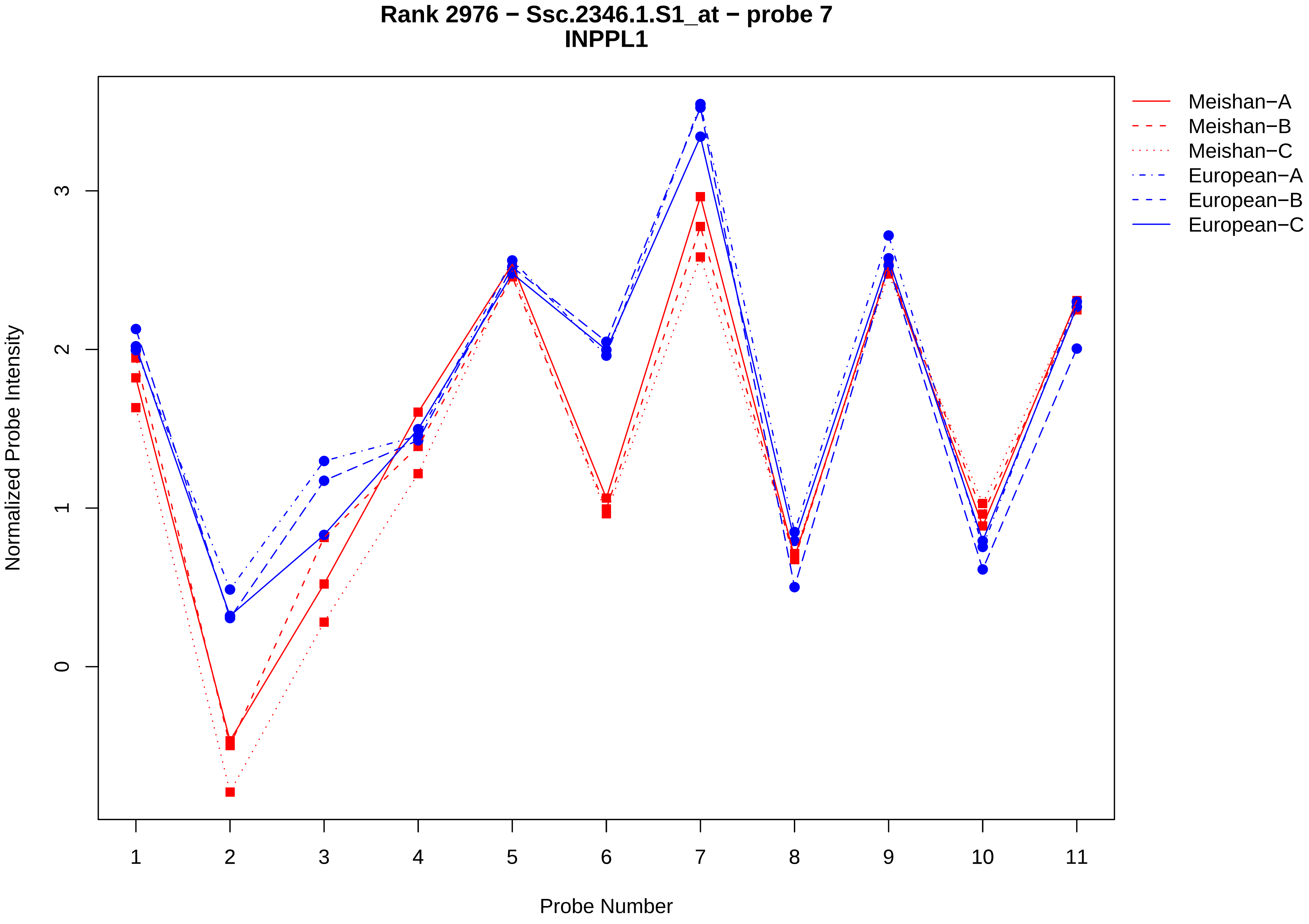
Rank 2976 − Ssc.2346.1.S1_at − probe 7
INPPL1
Normalized Probe Intensity
Probe Number
0
1
2
3
1
2
3
4
5
6
7
8
9
10
11
Meishan−A
Meishan−B
Meishan−C
European−A
European−B
European−C

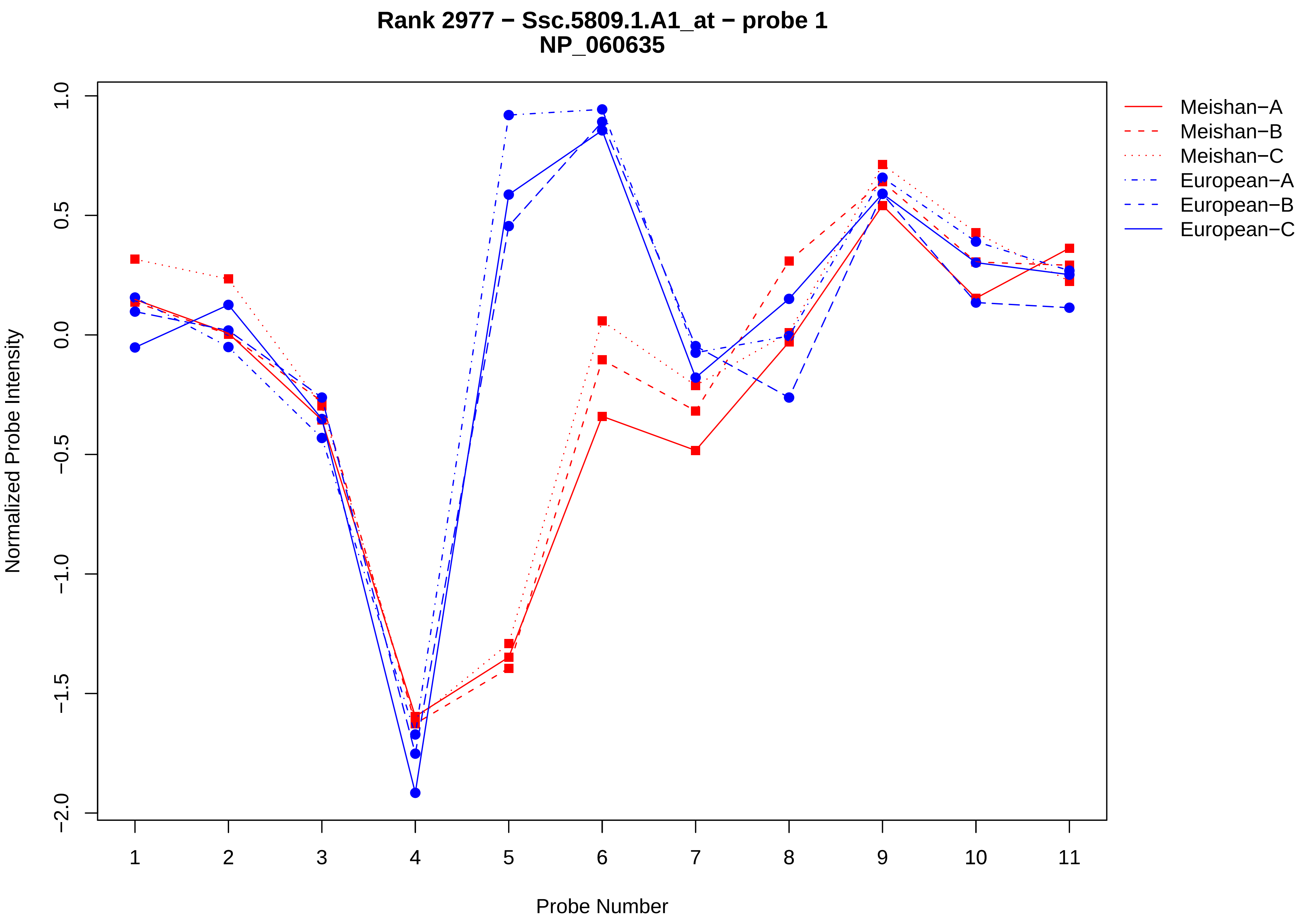

Rank 2977 − Ssc.5809.1.A1_at − probe 1
NP_060635
Normalized Probe Intensity
Probe Number
1.0
0.5
0.0
−0.5
−1.0
−1.5
−2.0
1
2
3
4
5
6
7
8
9
10
11
Meishan−A
Meishan−B
Meishan−C
European−A
European−B
European−C

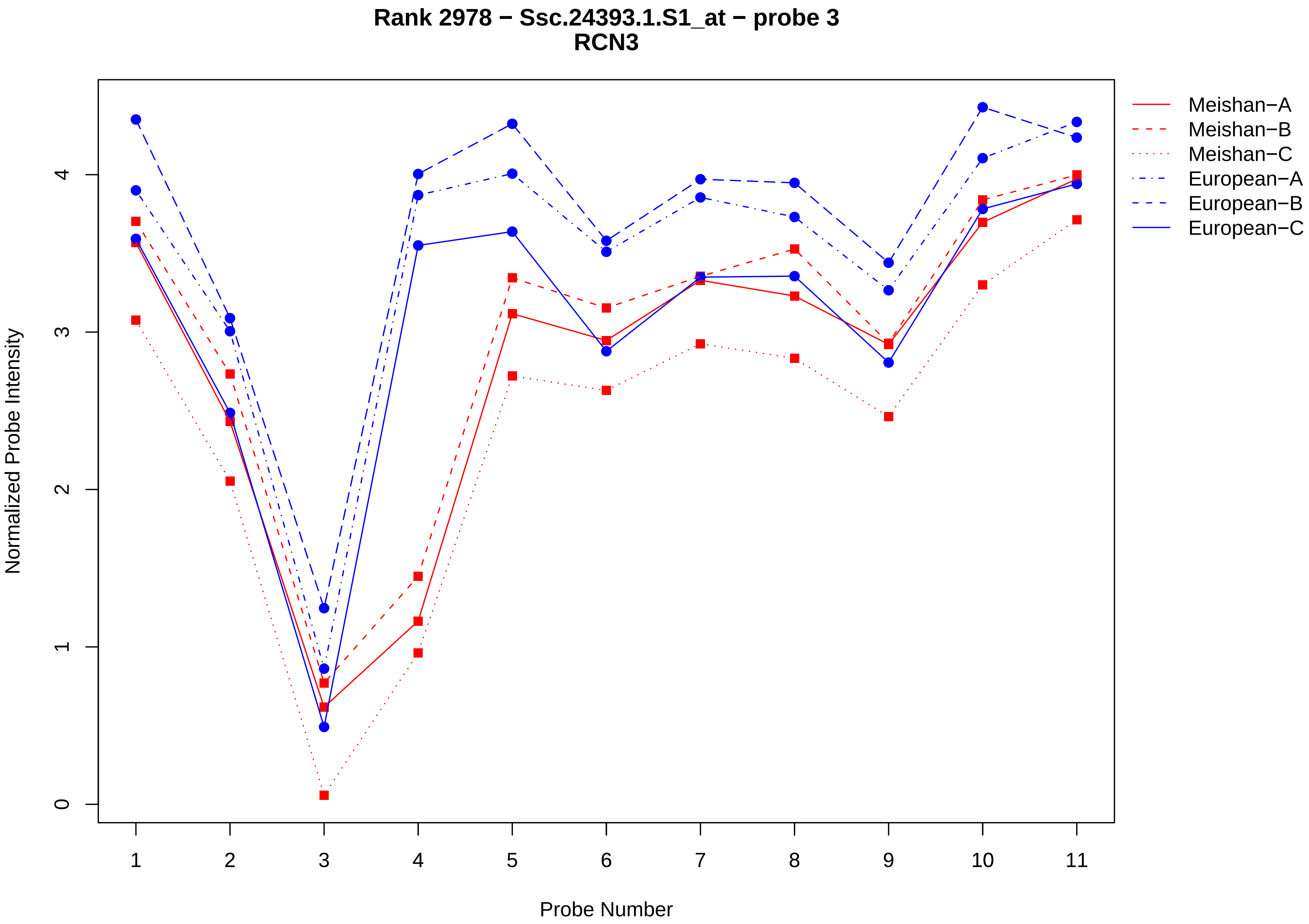

Rank 2978 − Ssc.24393.1.S1_at − probe 3
RCN3
Normalized Probe Intensity
Probe Number
0
1
2
3
4
1
2
3
4
5
6
7
8
9
10
11
Meishan−A
Meishan−B
Meishan−C
European−A
European−B
European−C

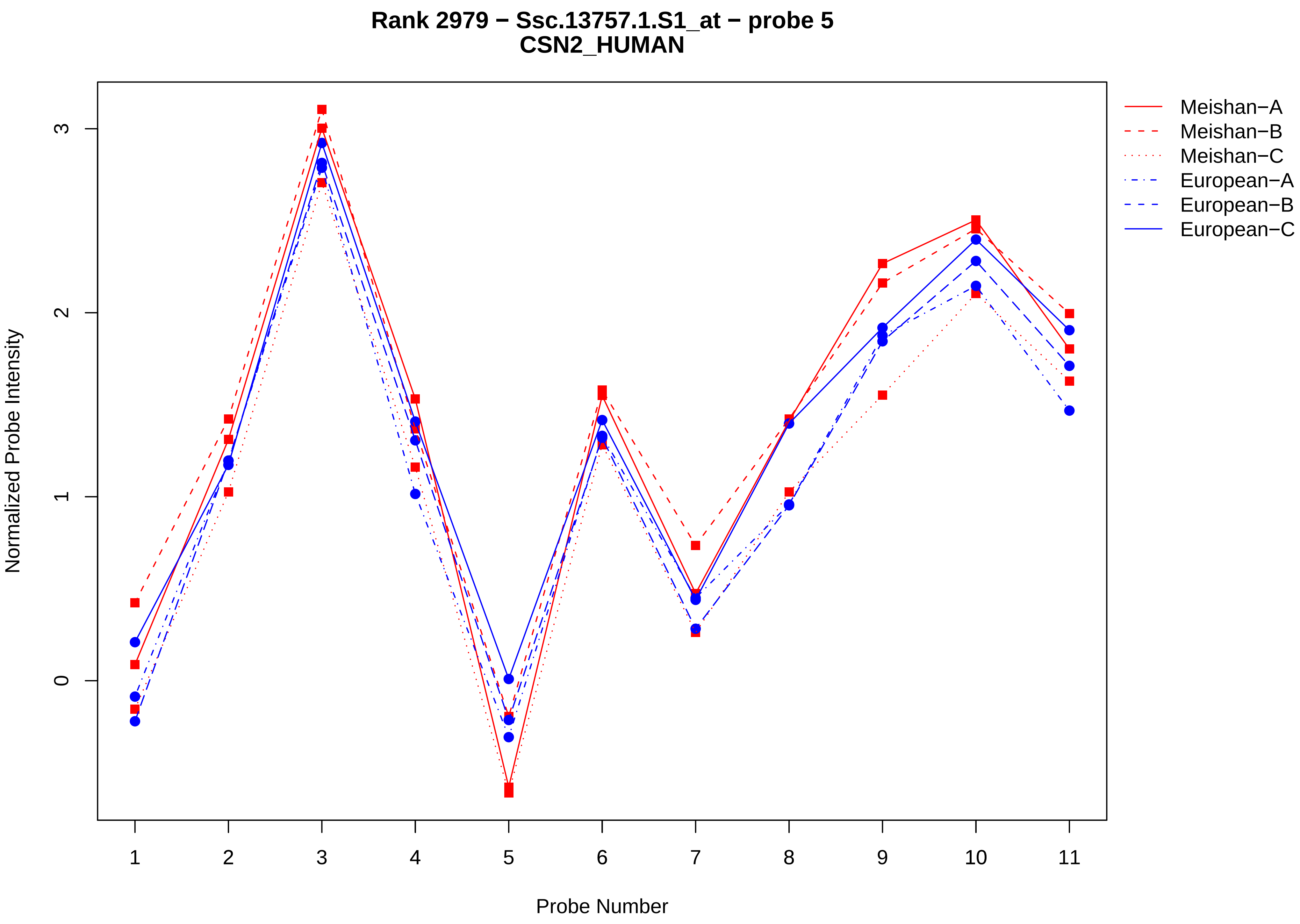

Rank 2979 − Ssc.13757.1.S1_at − probe 5
CSN2_HUMAN
Normalized Probe Intensity
Probe Number
0
1
2
3
1
2
3
4
5
6
7
8
9
10
11
Meishan−A
Meishan−B
Meishan−C
European−A
European−B
European−C

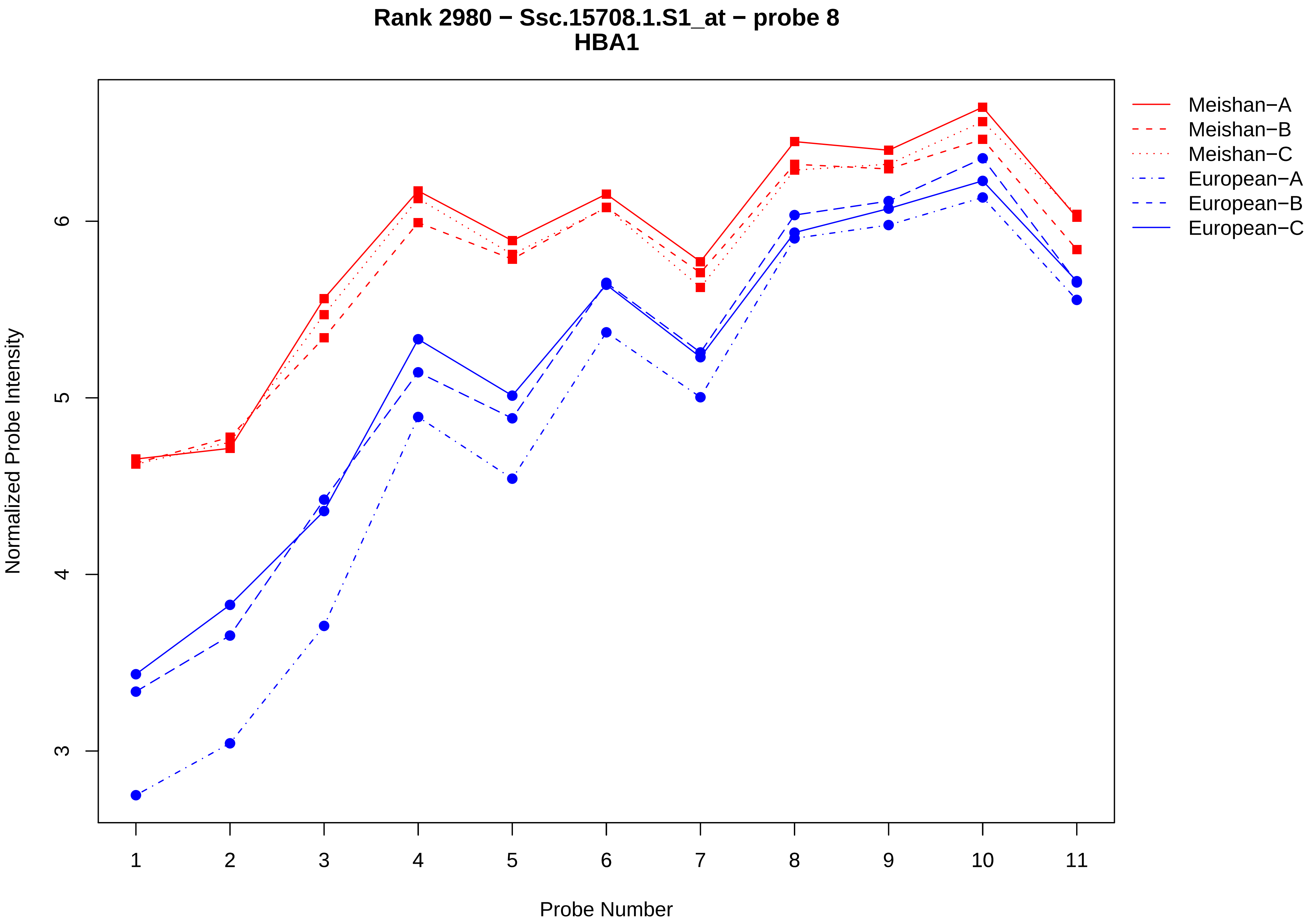

Rank 2980 − Ssc.15708.1.S1_at − probe 8
HBA1
Normalized Probe Intensity
Probe Number
3
4
5
6
1
2
3
4
5
6
7
8
9
10
11
Meishan−A
Meishan−B
Meishan−C
European−A
European−B
European−C

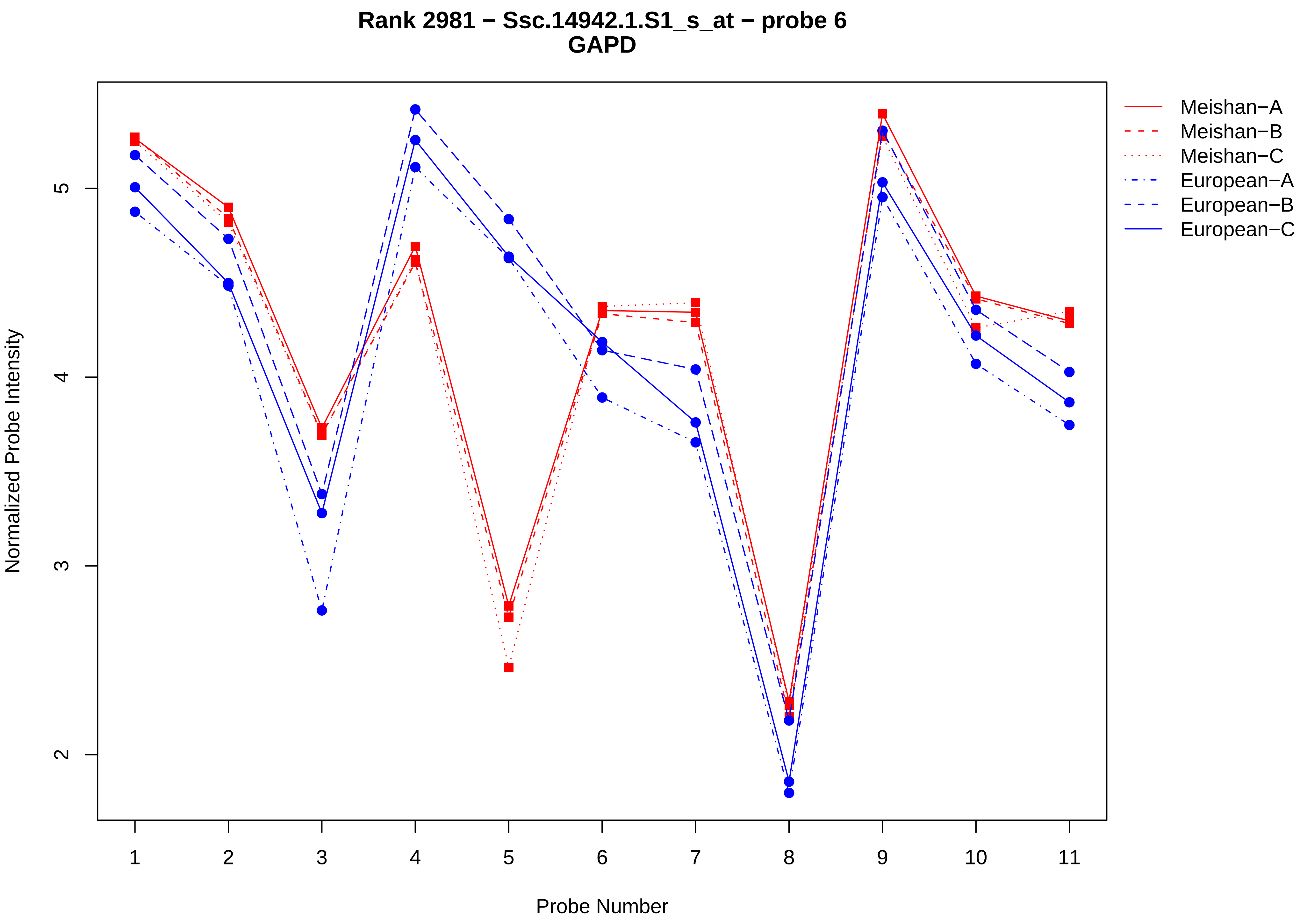

Rank 2981 − Ssc.14942.1.S1_s_at − probe 6
GAPD
Normalized Probe Intensity
Probe Number
2
3
4
5
1
2
3
4
5
6
7
8
9
10
11
Meishan−A
Meishan−B
Meishan−C
European−A
European−B
European−C

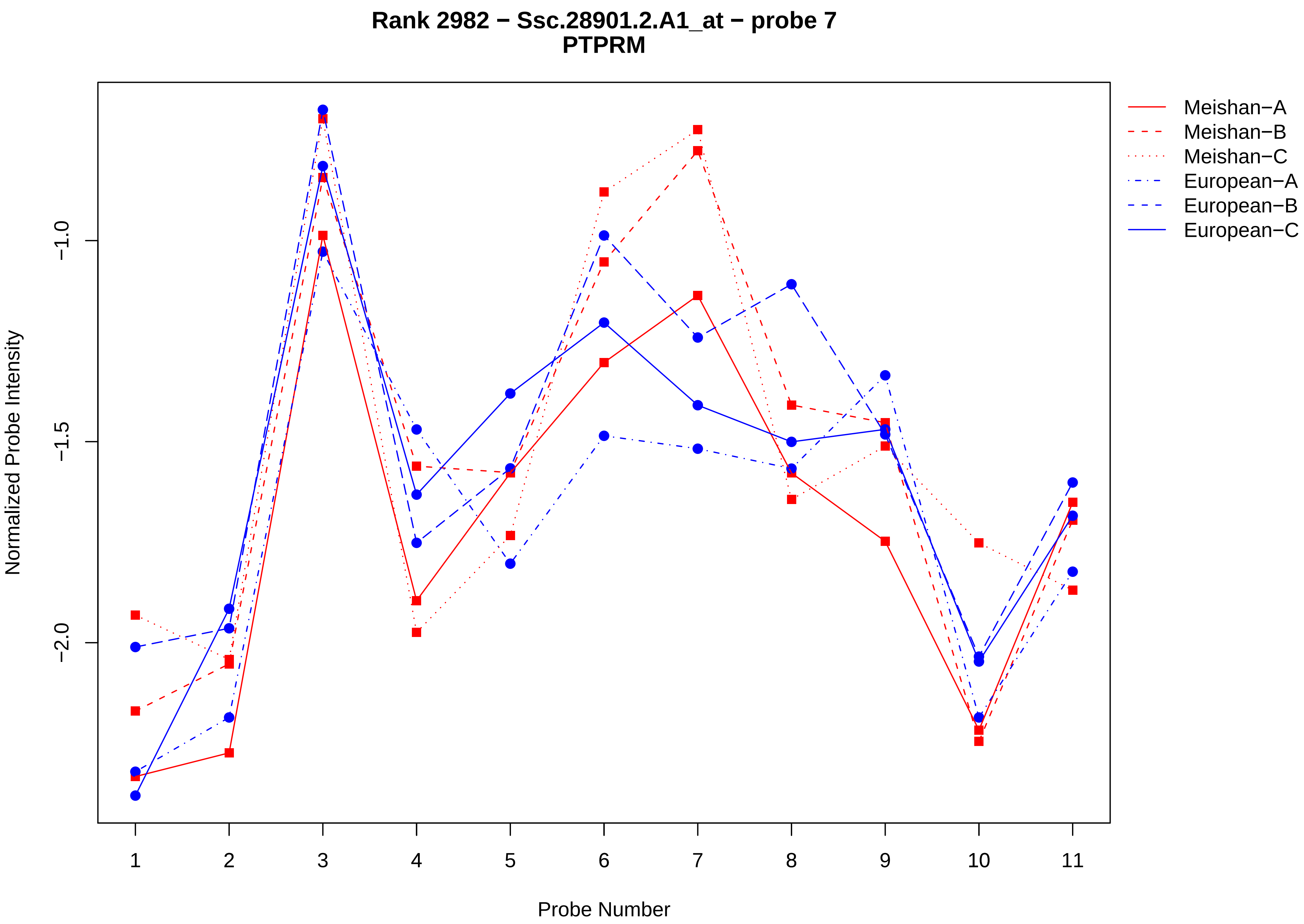

Rank 2982 − Ssc.28901.2.A1_at − probe 7
PTPRM
Normalized Probe Intensity
−1.0
−1.5
−2.0
1
2
3
4
5
6
7
8
9
10
11
Probe Number
Meishan−A
Meishan−B
Meishan−C
European−A
European−B
European−C

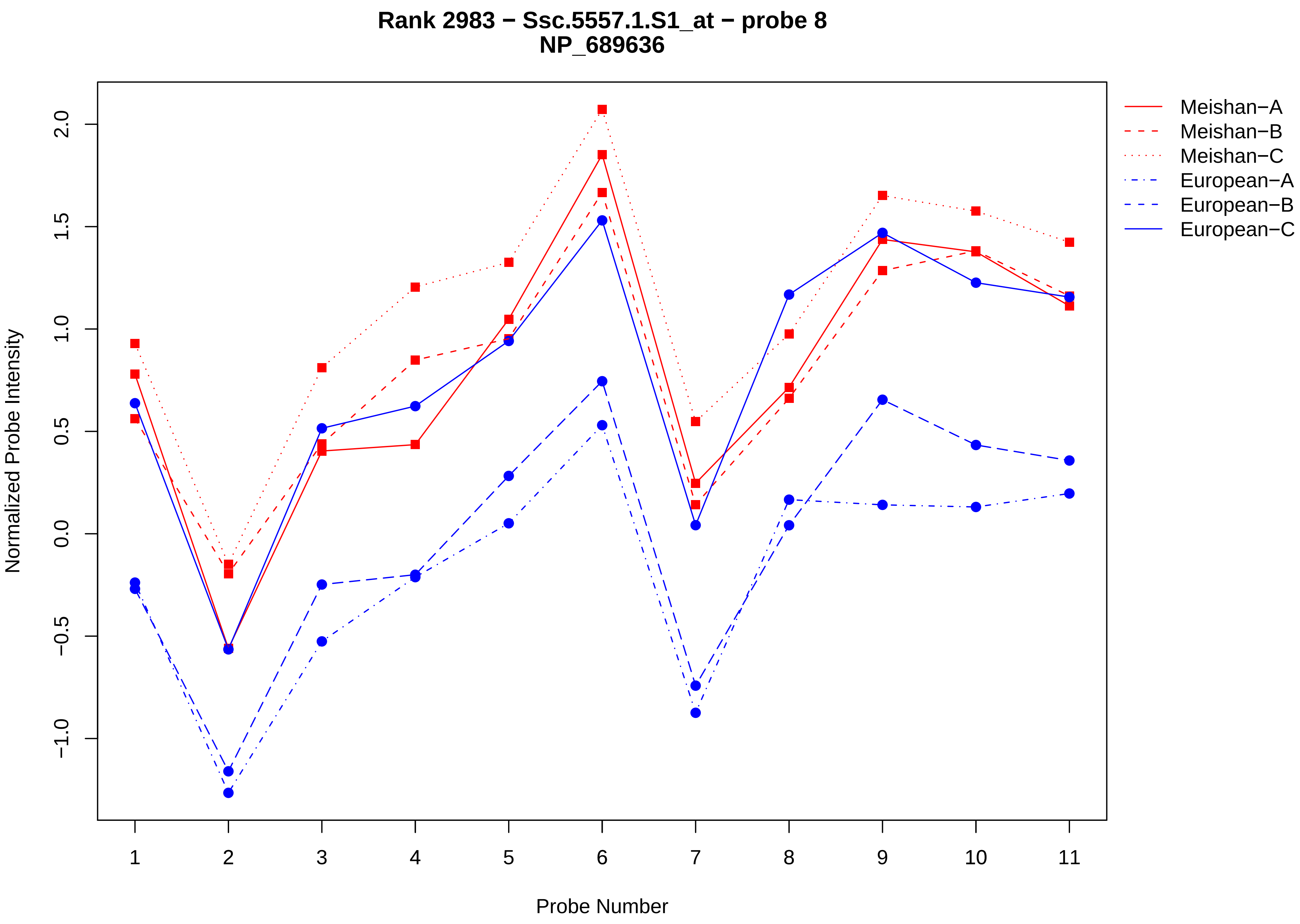

Rank 2983 − Ssc.5557.1.S1_at − probe 8
NP_689636
Normalized Probe Intensity
Probe Number
−1.0
−0.5
0.0
0.5
1.0
1.5
2.0
1
2
3
4
5
6
7
8
9
10
11
Meishan−A
Meishan−B
Meishan−C
European−A
European−B
European−C

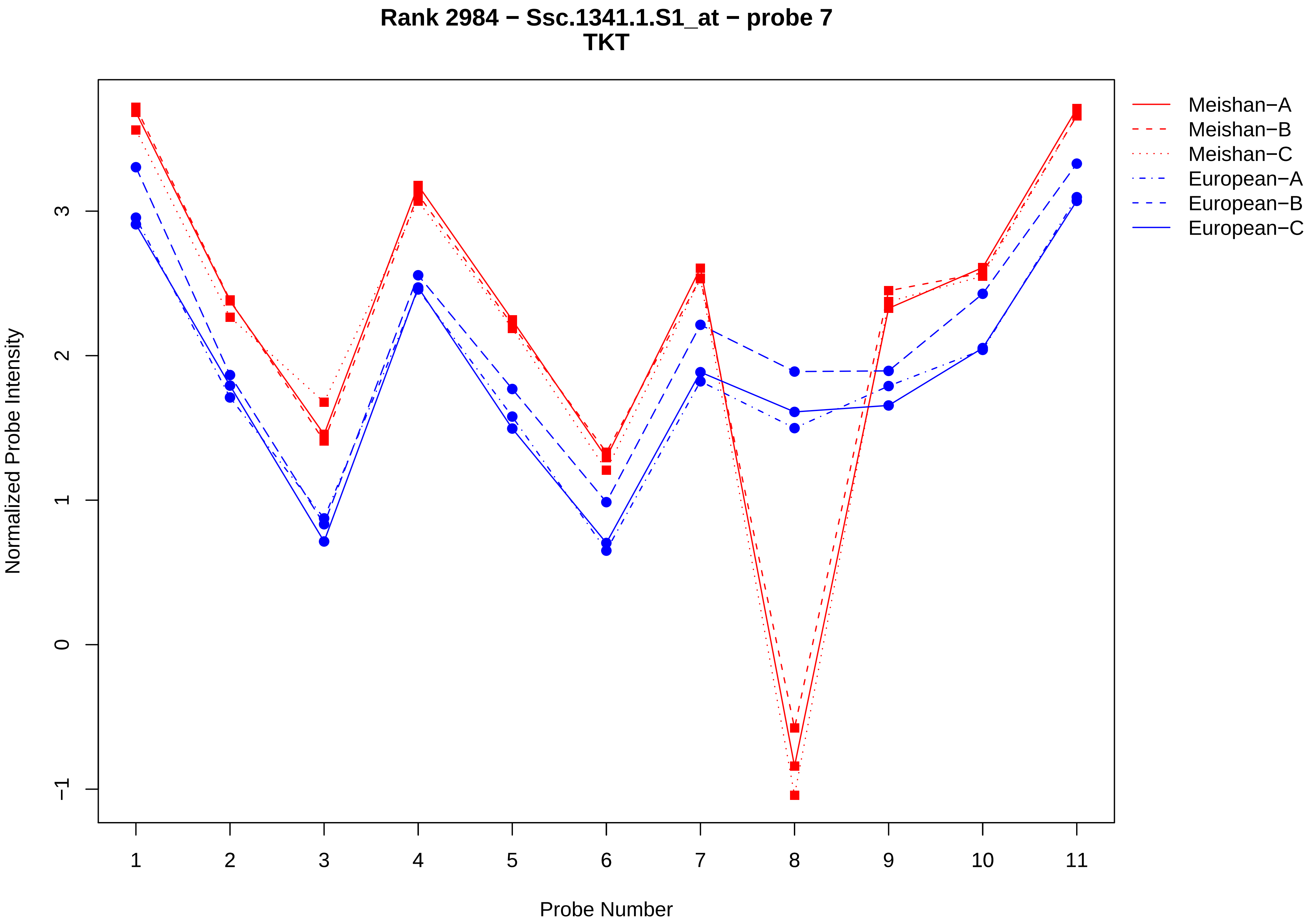

Rank 2984 − Ssc.1341.1.S1_at − probe 7
TKT
Normalized Probe Intensity
Probe Number
−1
0
1
2
3
1
2
3
4
5
6
7
8
9
10
11
Meishan−A
Meishan−B
Meishan−C
European−A
European−B
European−C

Rank 2985 − Ssc.30946.1.A1_at − probe 10
C10orf9

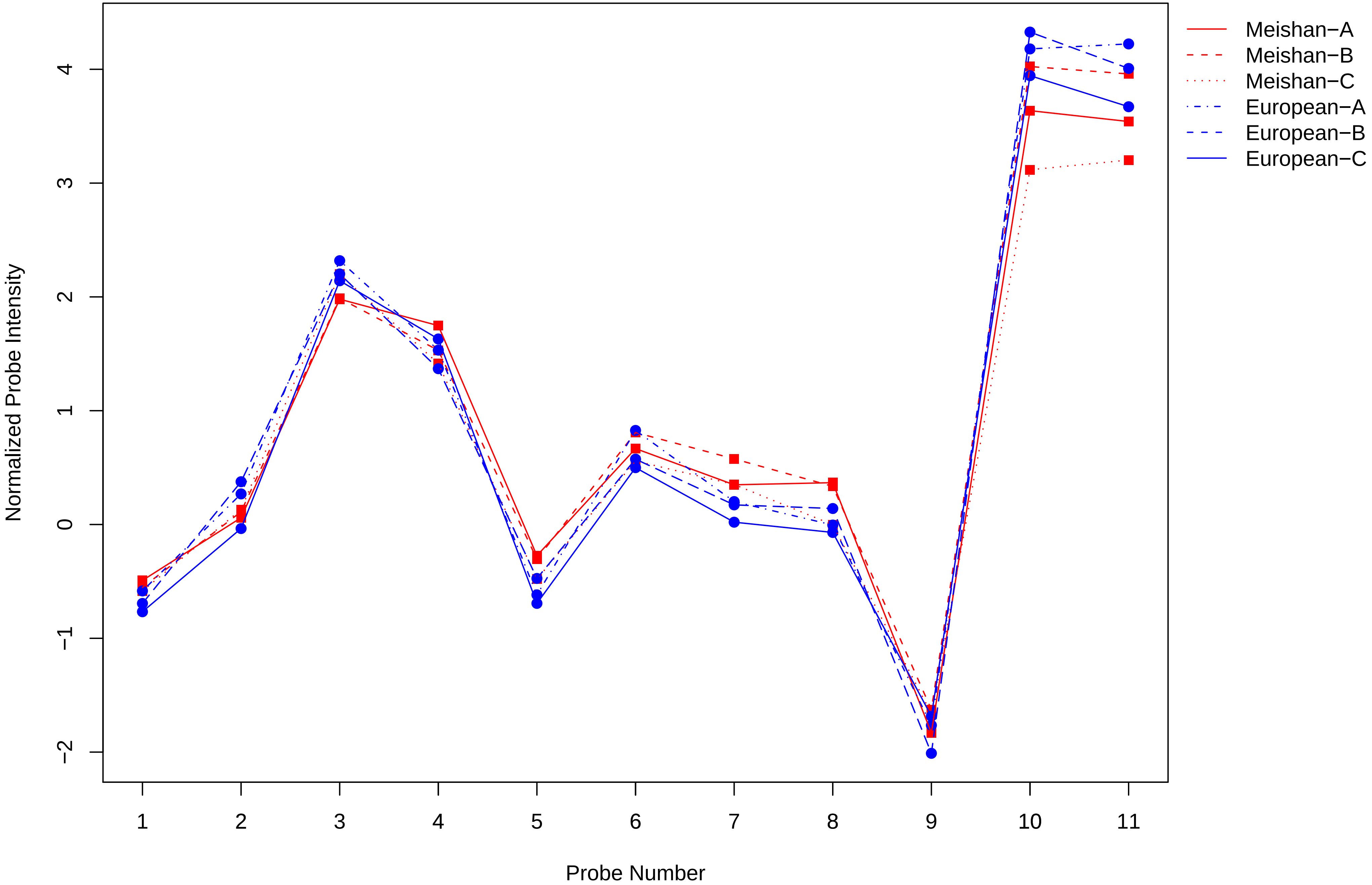

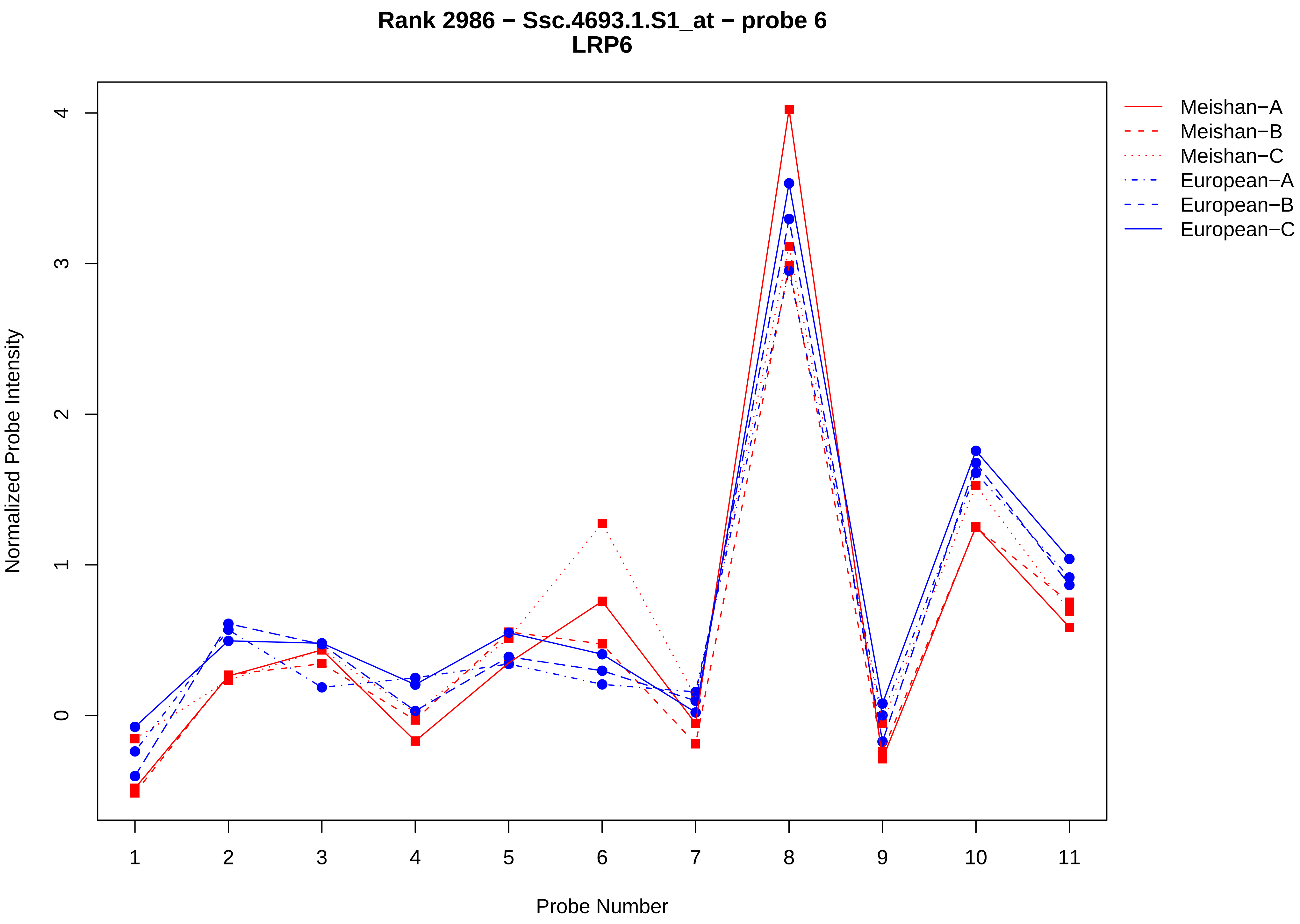

Rank 2986 − Ssc.4693.1.S1_at − probe 6
LRP6
Normalized Probe Intensity
Probe Number
0
1
2
3
4
1
2
3
4
5
6
7
8
9
10
11
Meishan−A
Meishan−B
Meishan−C
European−A
European−B
European−C

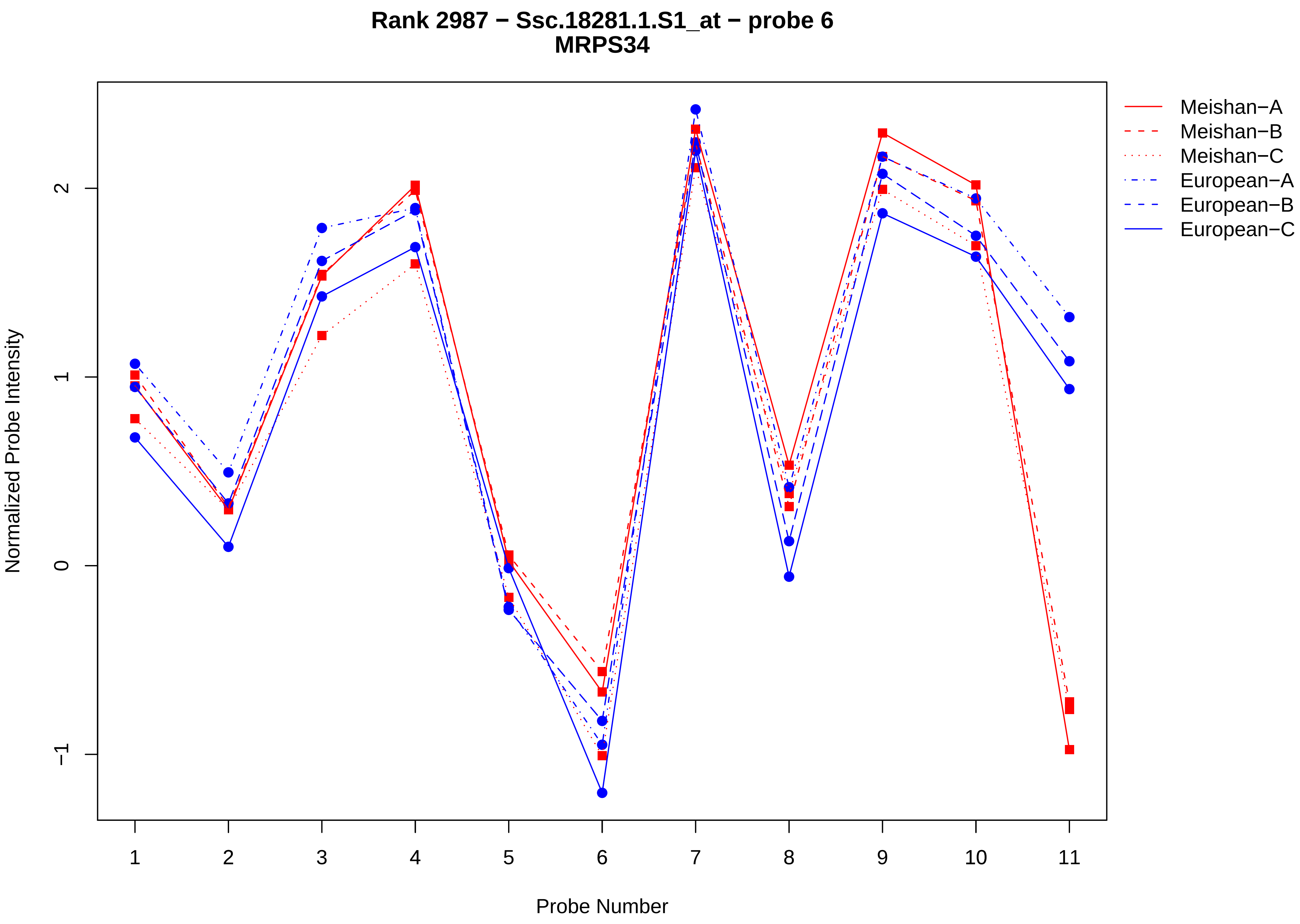
Rank 2987 − Ssc.18281.1.S1_at − probe 6
MRPS34
Normalized Probe Intensity
Probe Number
Meishan−A
Meishan−B
Meishan−C
European−A
European−B
European−C
−1
0
1
2
1
2
3
4
5
6
7
8
9
10
11

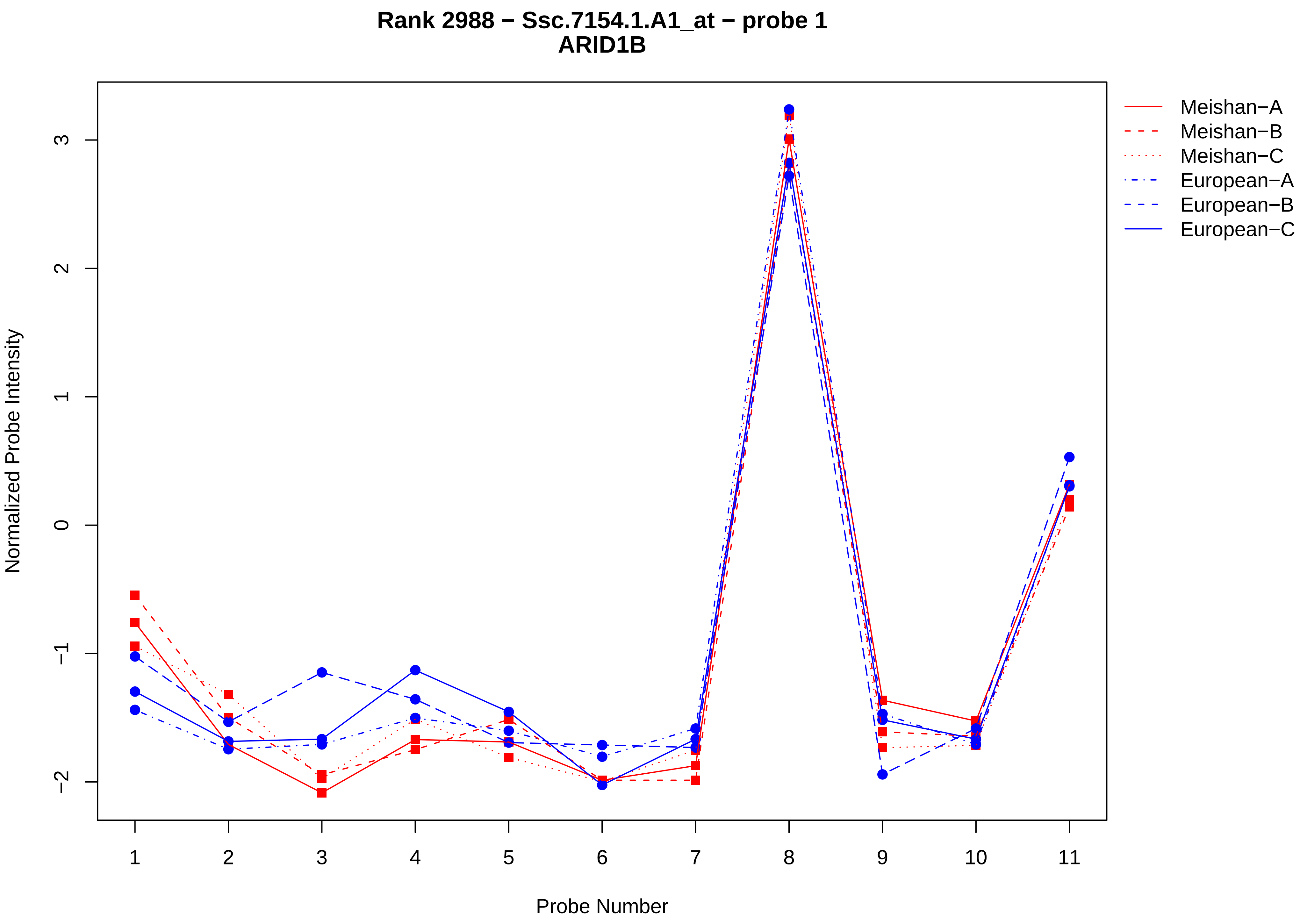

Rank 2988 − Ssc.7154.1.A1_at − probe 1
ARID1B
Normalized Probe Intensity
Probe Number
−2
−1
0
1
2
3
1
2
3
4
5
6
7
8
9
10
11
Meishan−A
Meishan−B
Meishan−C
European−A
European−B
European−C

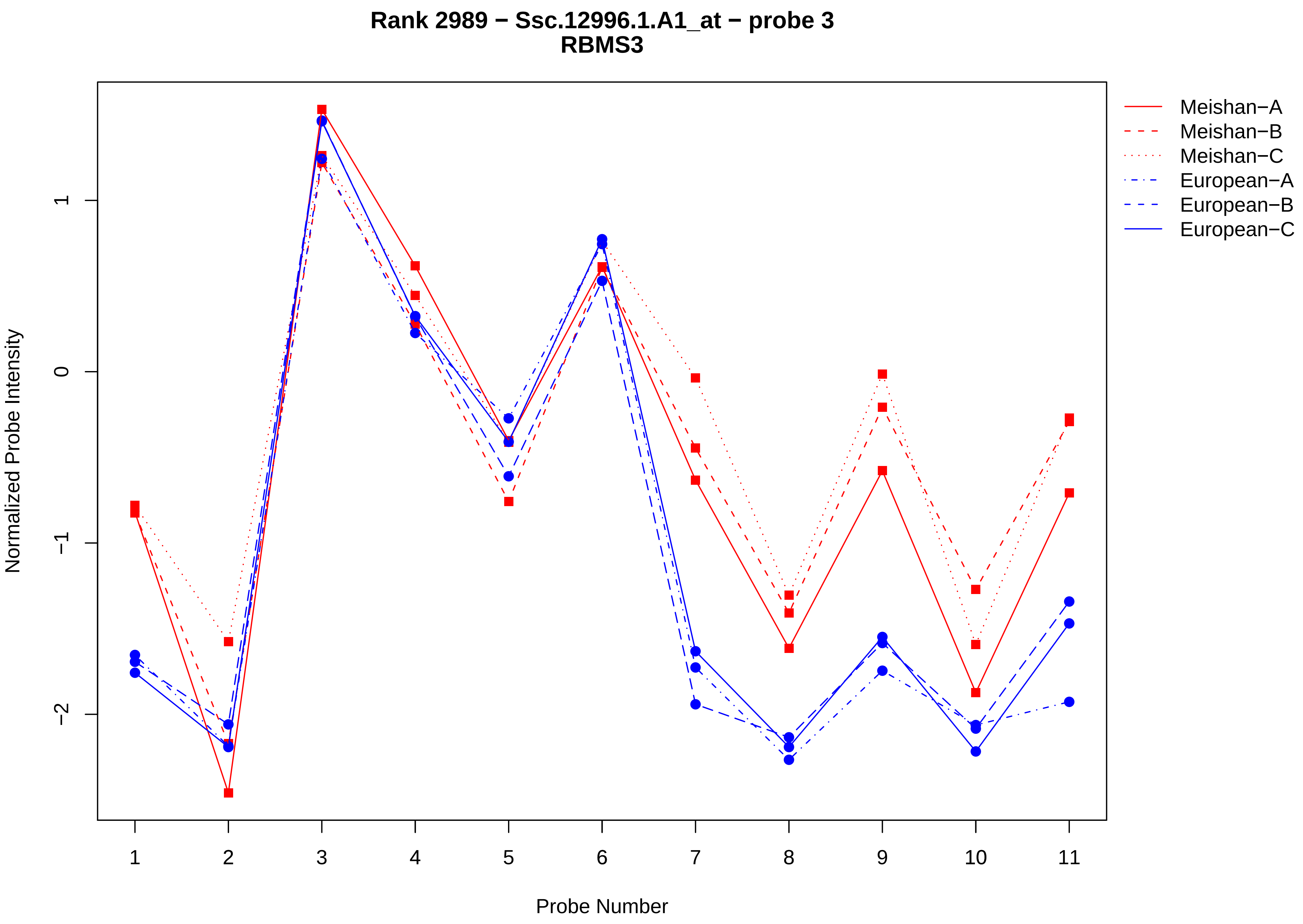

Rank 2989 − Ssc.12996.1.A1_at − probe 3
RBMS3
Normalized Probe Intensity
Probe Number
1
0
−1
−2
1
2
3
4
5
6
7
8
9
10
11
Meishan−A
Meishan−B
Meishan−C
European−A
European−B
European−C

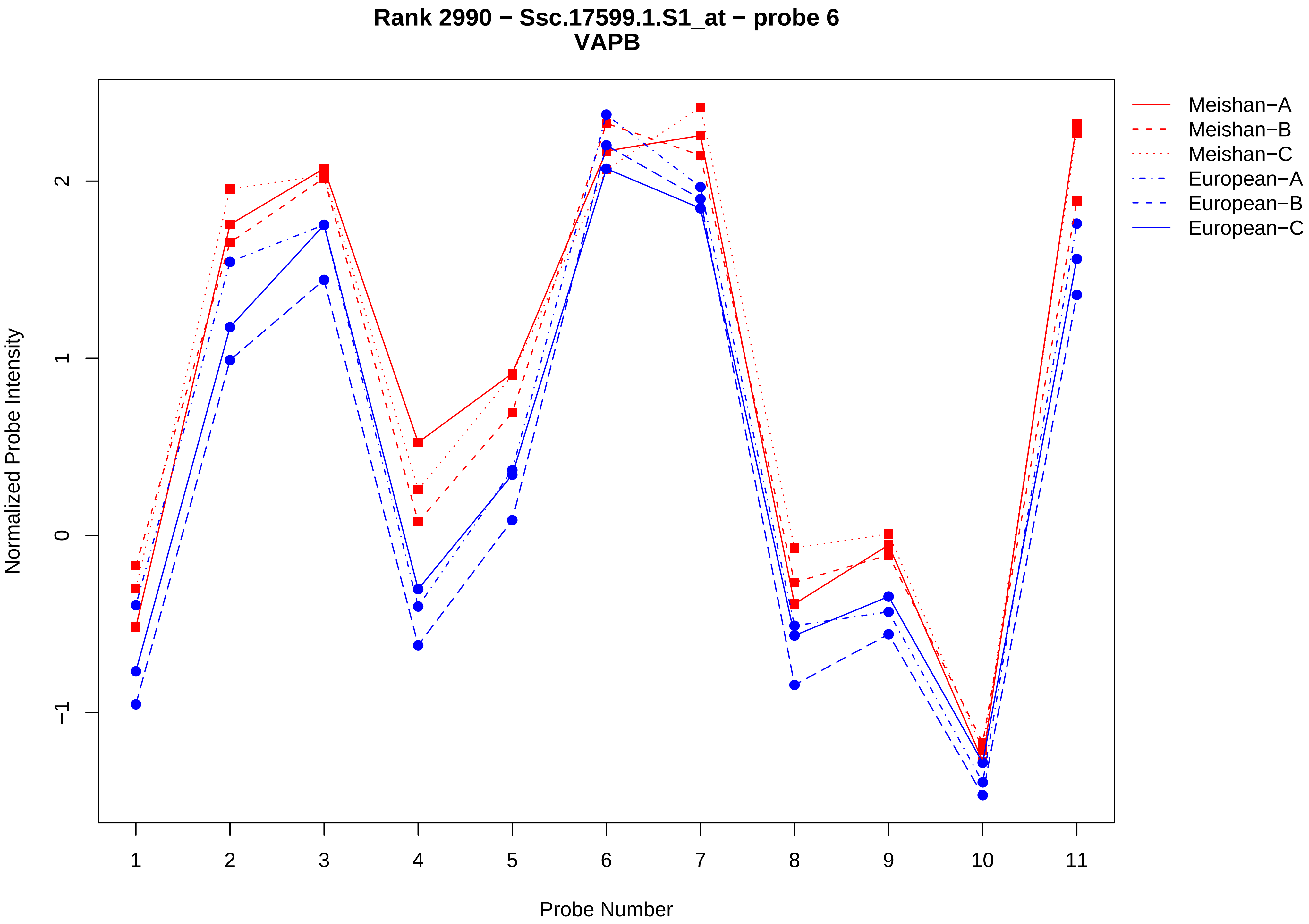

Rank 2990 − Ssc.17599.1.S1_at − probe 6
VAPB
Normalized Probe Intensity
Probe Number
−1
0
1
2
1
2
3
4
5
6
7
8
9
10
11
Meishan−A
Meishan−B
Meishan−C
European−A
European−B
European−C

Rank 2991 − Ssc.22958.1.S1_a_at − probe 2
BTBD1

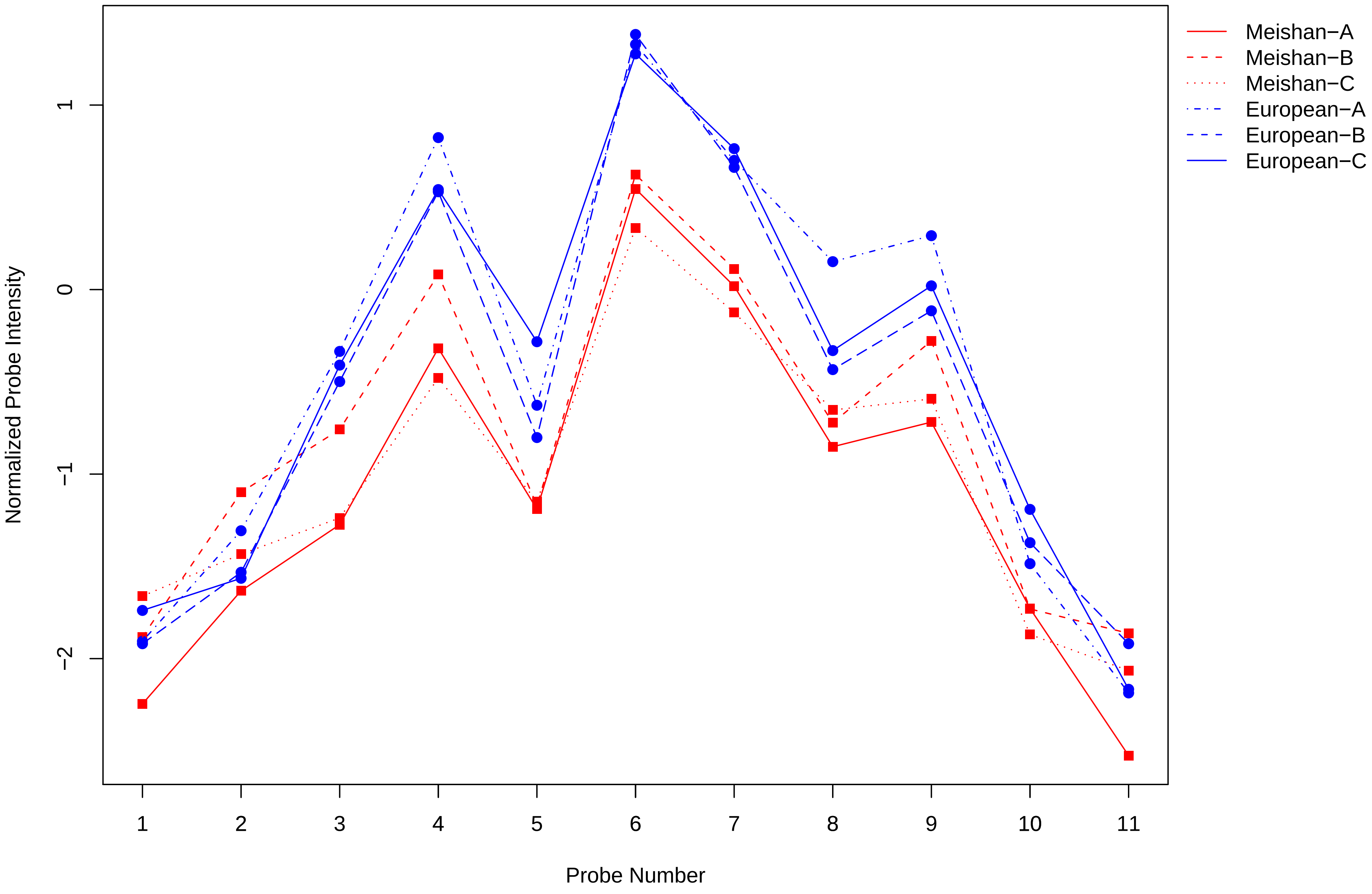

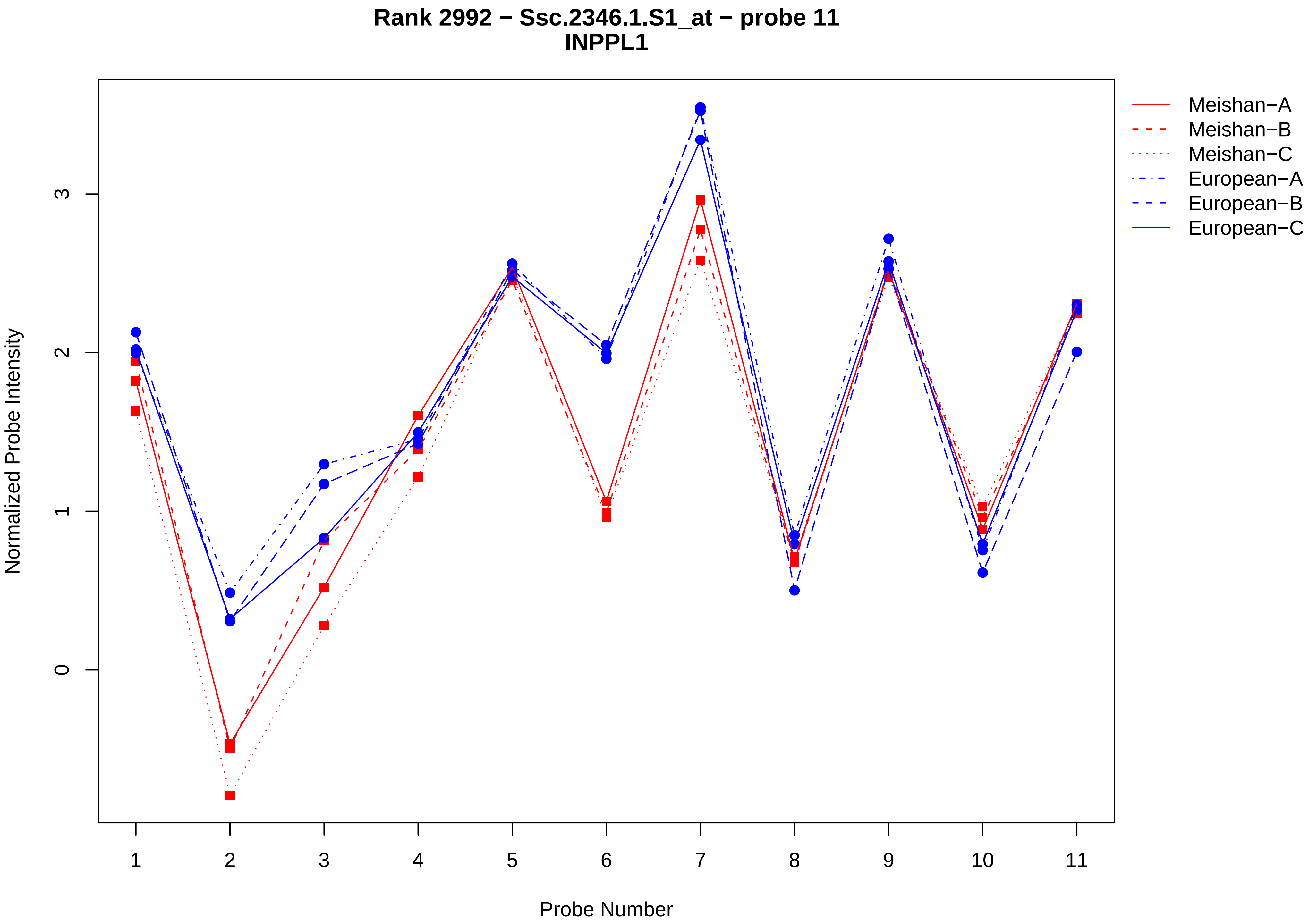
Rank 2992 − Ssc.2346.1.S1_at − probe 11
INPPL1
Normalized Probe Intensity
Probe Number
0
1
2
3
1
2
3
4
5
6
7
8
9
10
11
Meishan−A
Meishan−B
Meishan−C
European−A
European−B
European−C

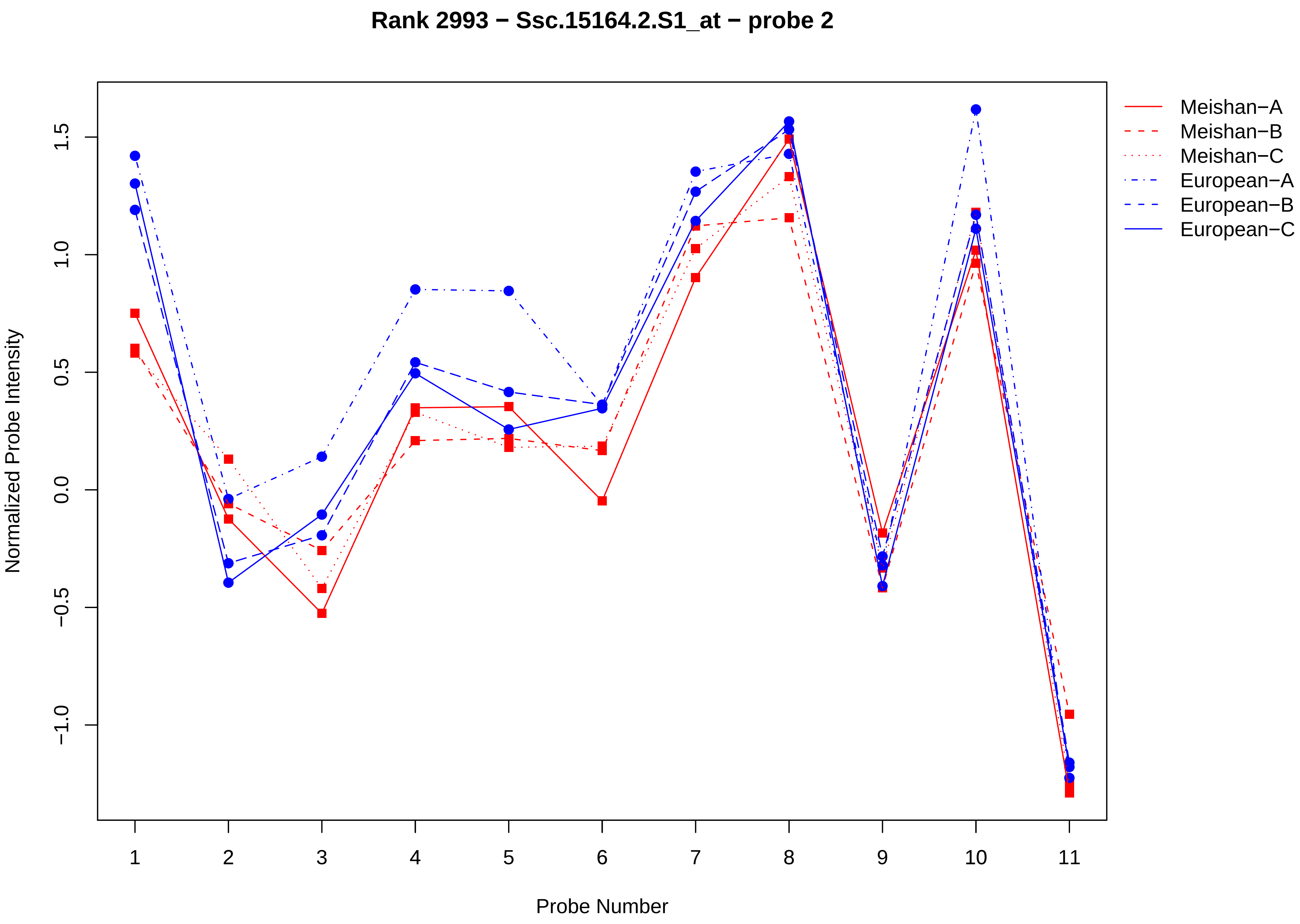

Rank 2993 − Ssc.15164.2.S1_at − probe 2
Normalized Probe Intensity
Probe Number
−1.0
−0.5
0.0
0.5
1.0
1.5
1
2
3
4
5
6
7
8
9
10
11
Meishan−A
Meishan−B
Meishan−C
European−A
European−B
European−C

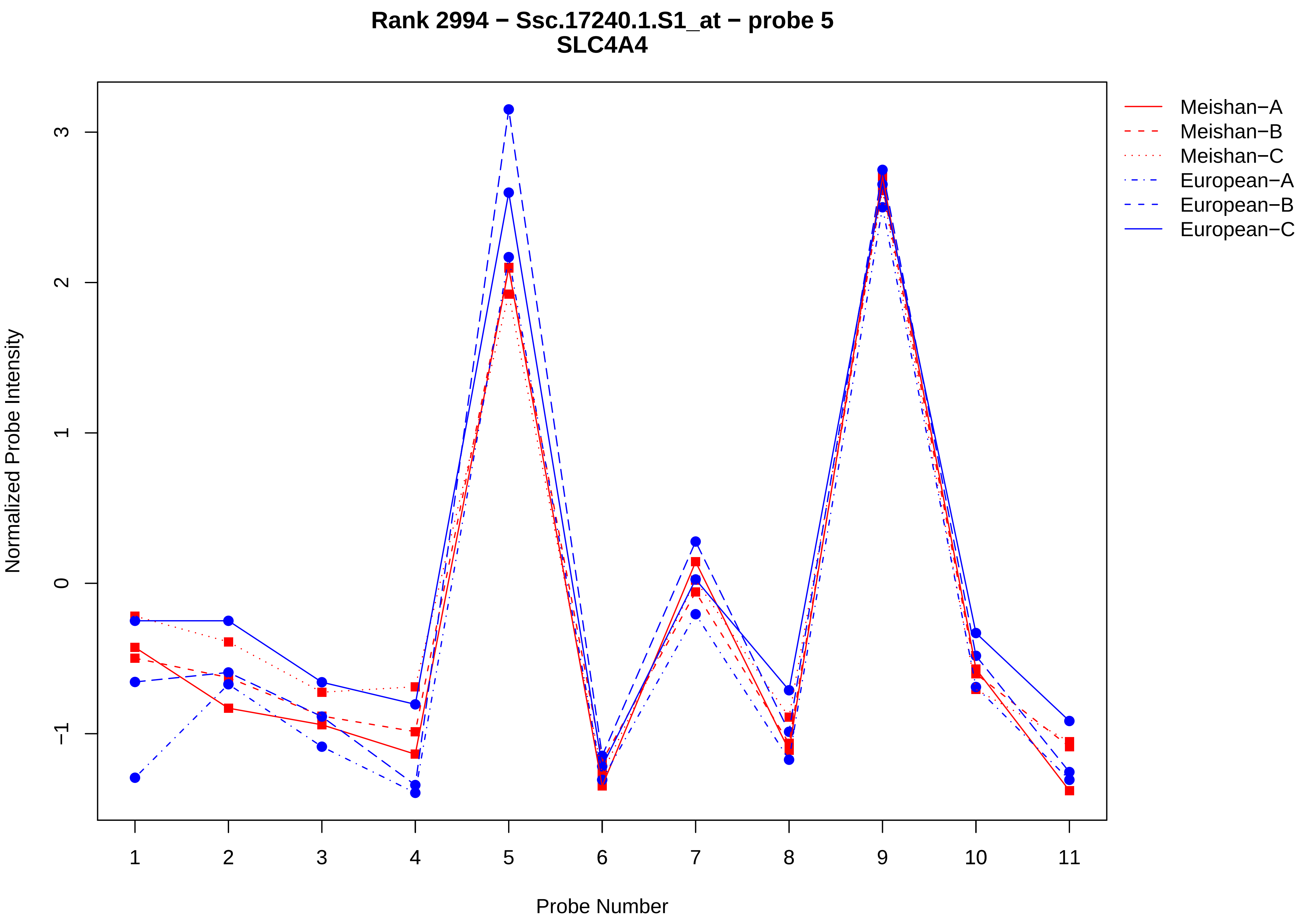

Rank 2994 − Ssc.17240.1.S1_at − probe 5
SLC4A4
Normalized Probe Intensity
Probe Number
−1
0
1
2
3
1
2
3
4
5
6
7
8
9
10
11
Meishan−A
Meishan−B
Meishan−C
European−A
European−B
European−C

Rank 2995 − SscAffx.34.1.S1_at − probe 7
MTCO1

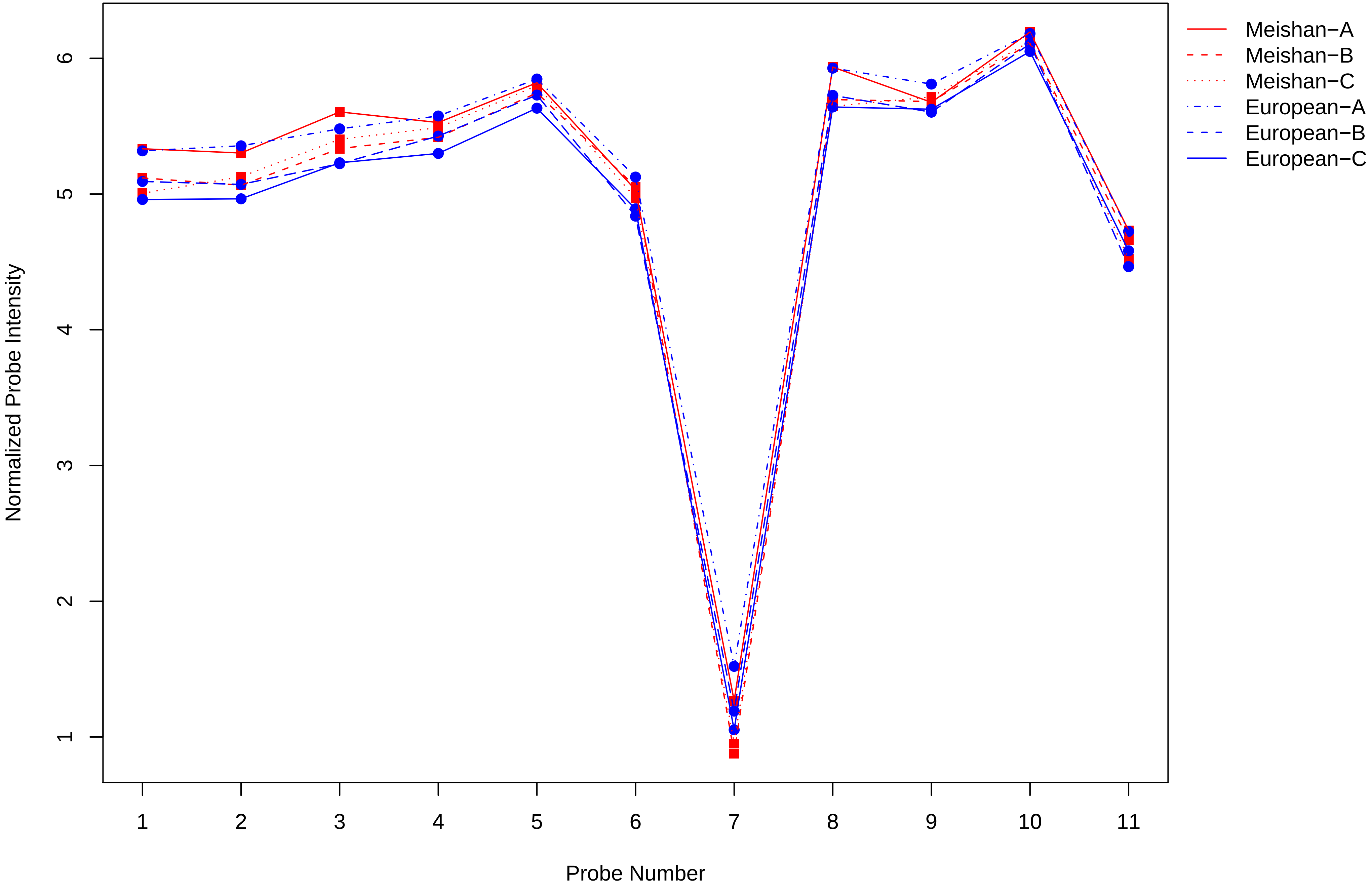

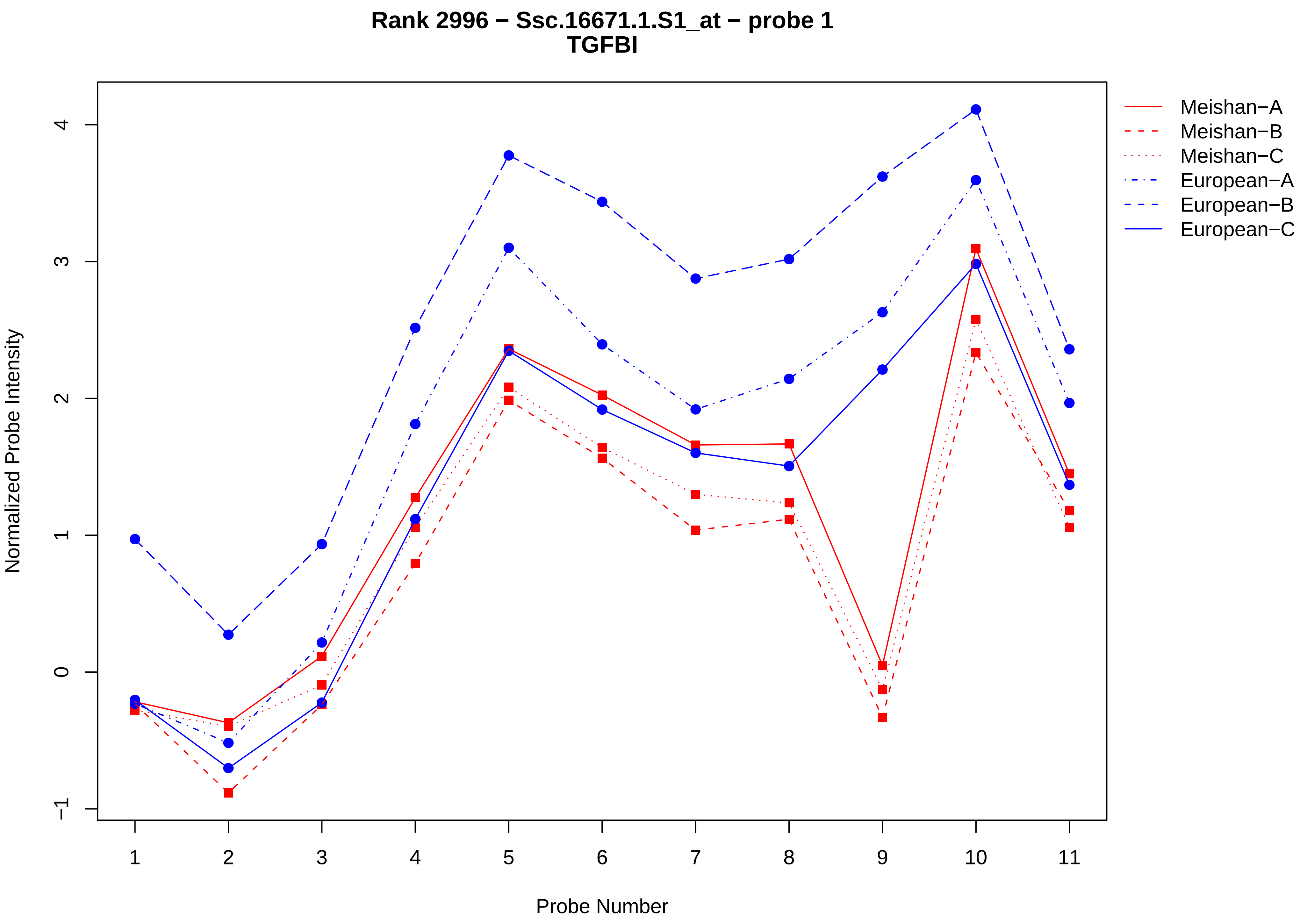

Rank 2996 − Ssc.16671.1.S1_at − probe 1
TGFBI
Normalized Probe Intensity
Probe Number
−1
0
1
2
3
4
1
2
3
4
5
6
7
8
9
10
11
Meishan−A
Meishan−B
Meishan−C
European−A
European−B
European−C

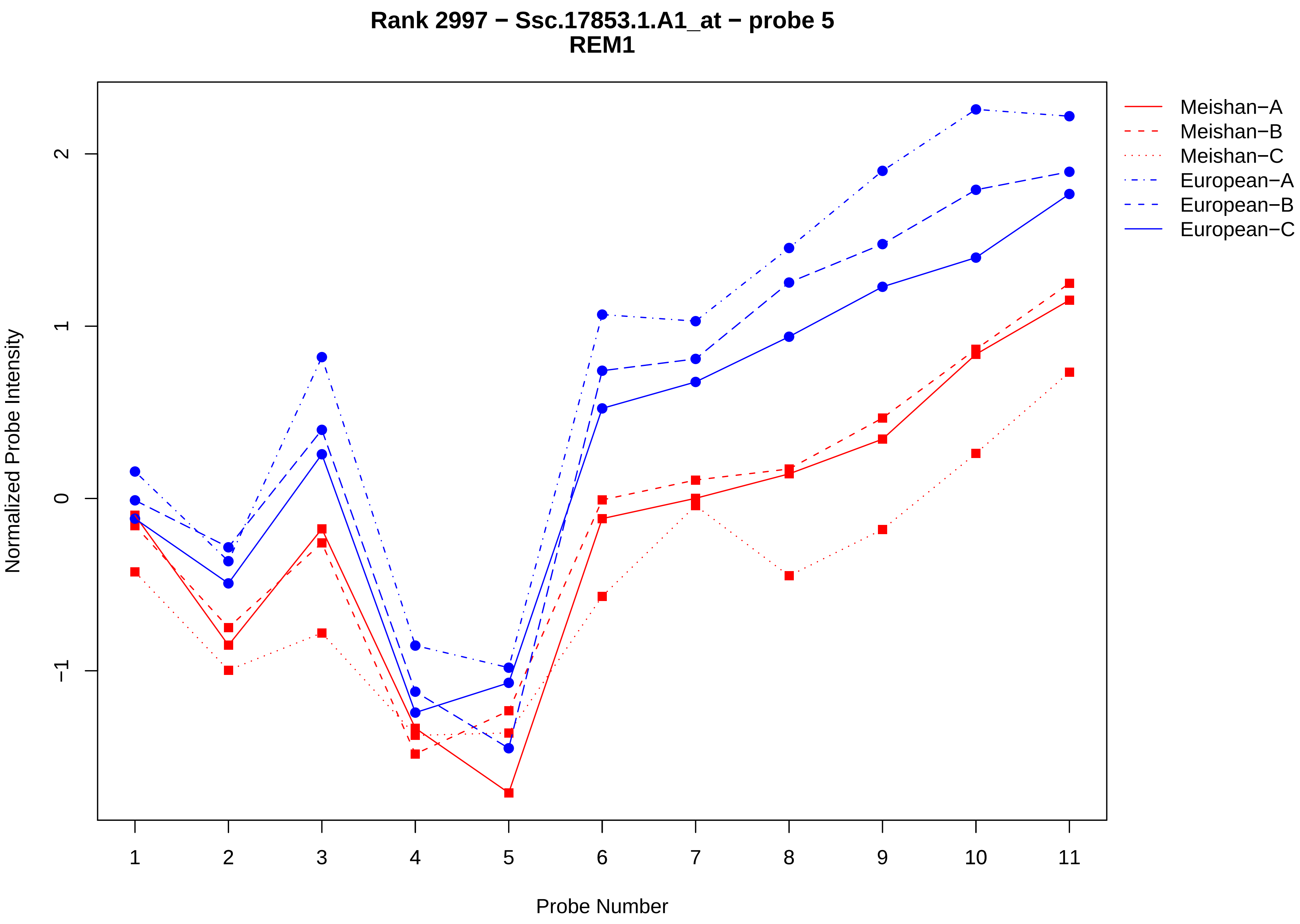

Rank 2997 − Ssc.17853.1.A1_at − probe 5
REM1
Normalized Probe Intensity
Probe Number
−1
0
1
2
1
2
3
4
5
6
7
8
9
10
11
Meishan−A
Meishan−B
Meishan−C
European−A
European−B
European−C

Rank 2998 − Ssc.25561.1.A1_at − probe 11
PWP1_HUMAN

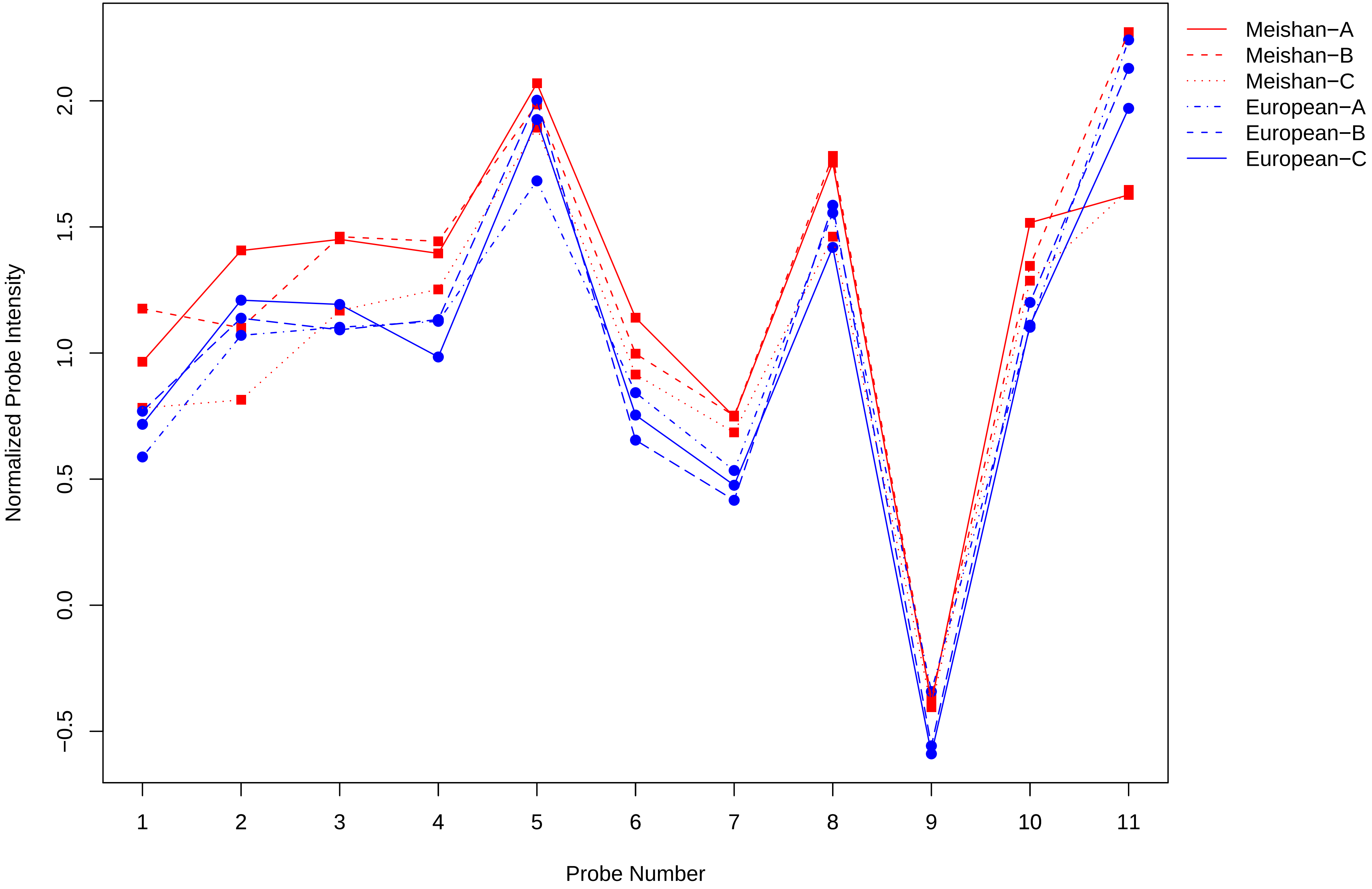

Rank 2999 − Ssc.27685.1.S1_at − probe 5
FGF23

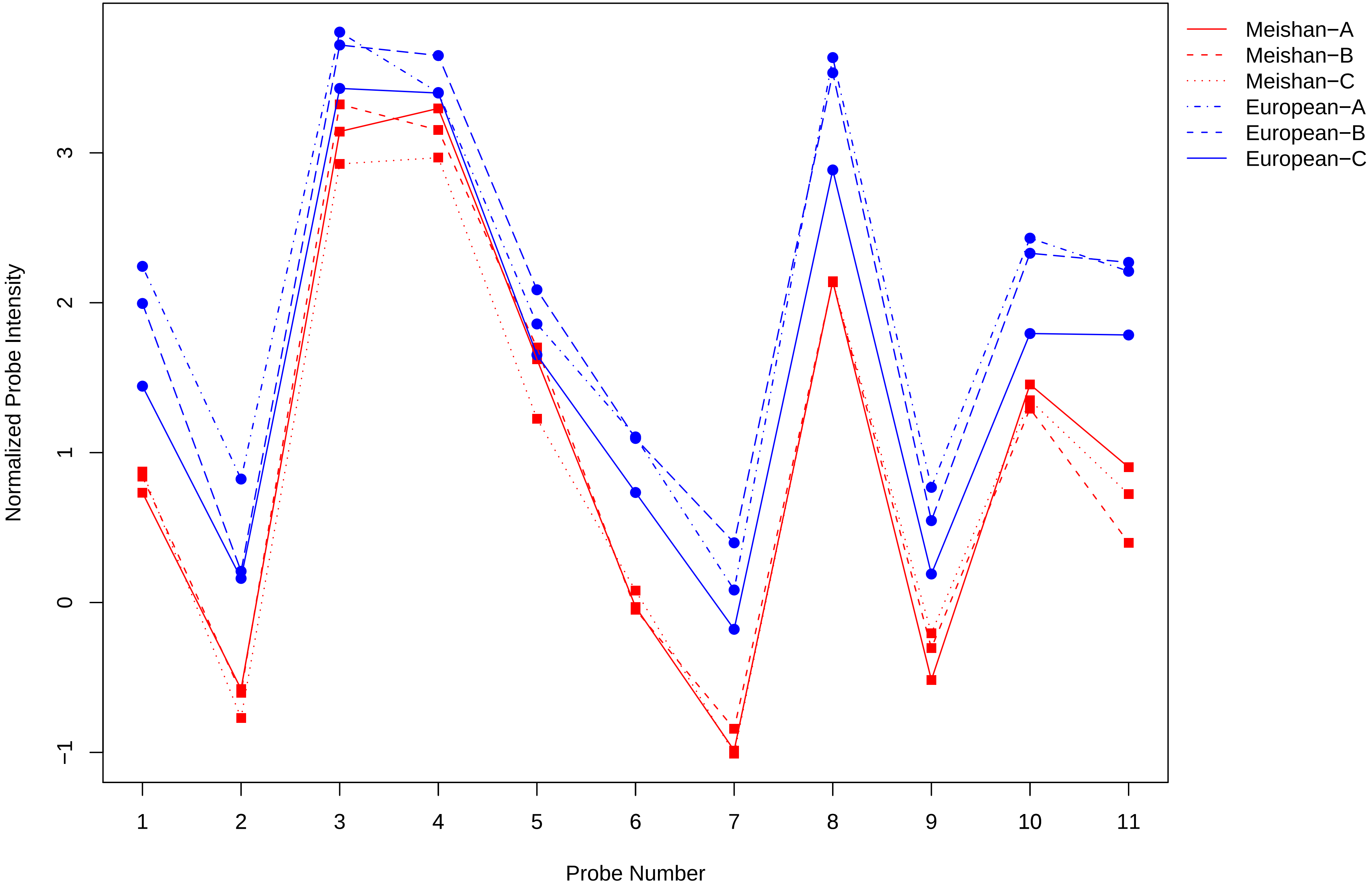

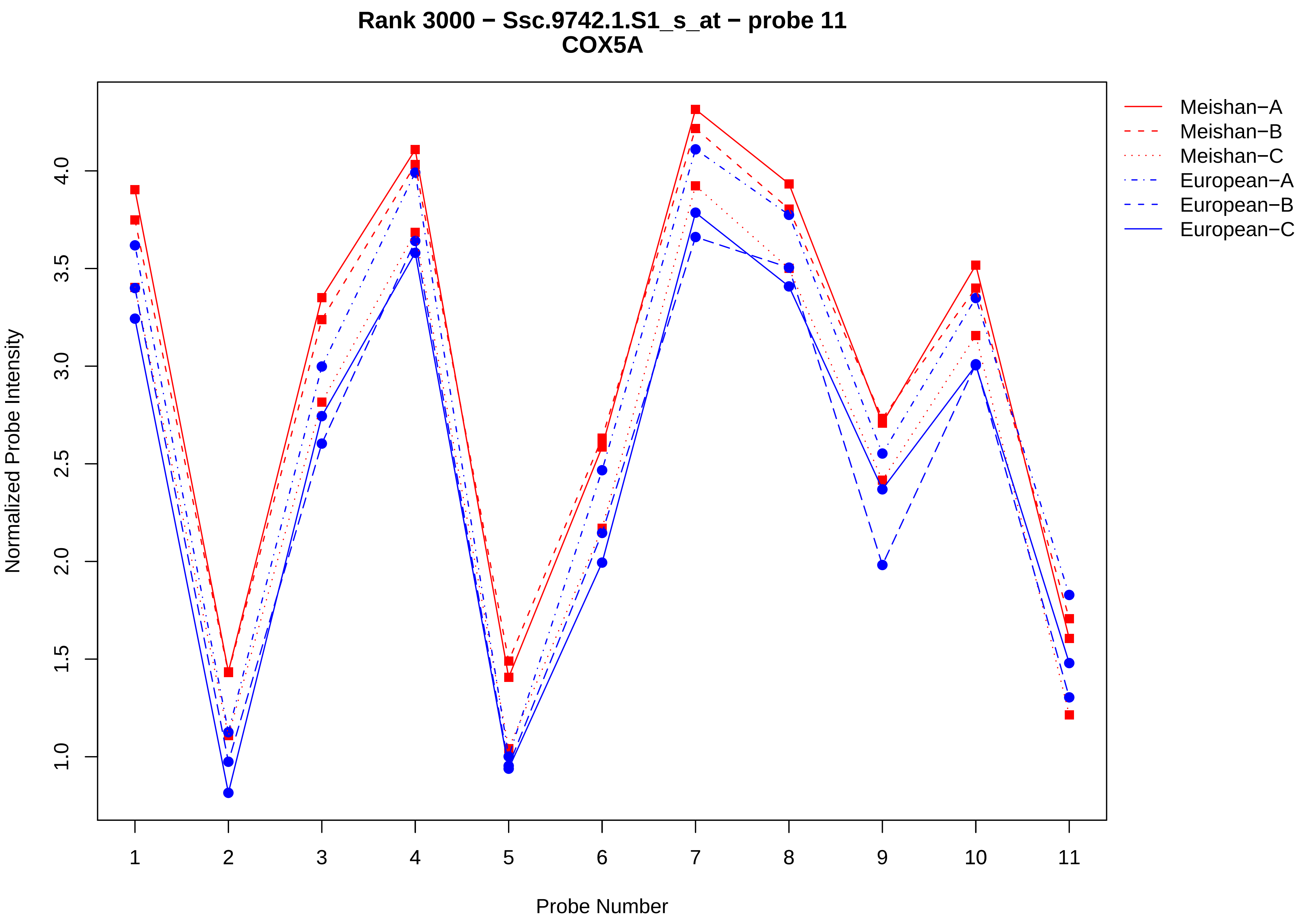

Rank 3000 − Ssc.9742.1.S1_s_at − probe 11
COX5A
Normalized Probe Intensity
1.0
1.5
2.0
2.5
3.0
3.5
4.0
Probe Number
1
2
3
4
5
6
7
8
9
10
11
Meishan−A
Meishan−B
Meishan−C
European−A
European−B
European−C

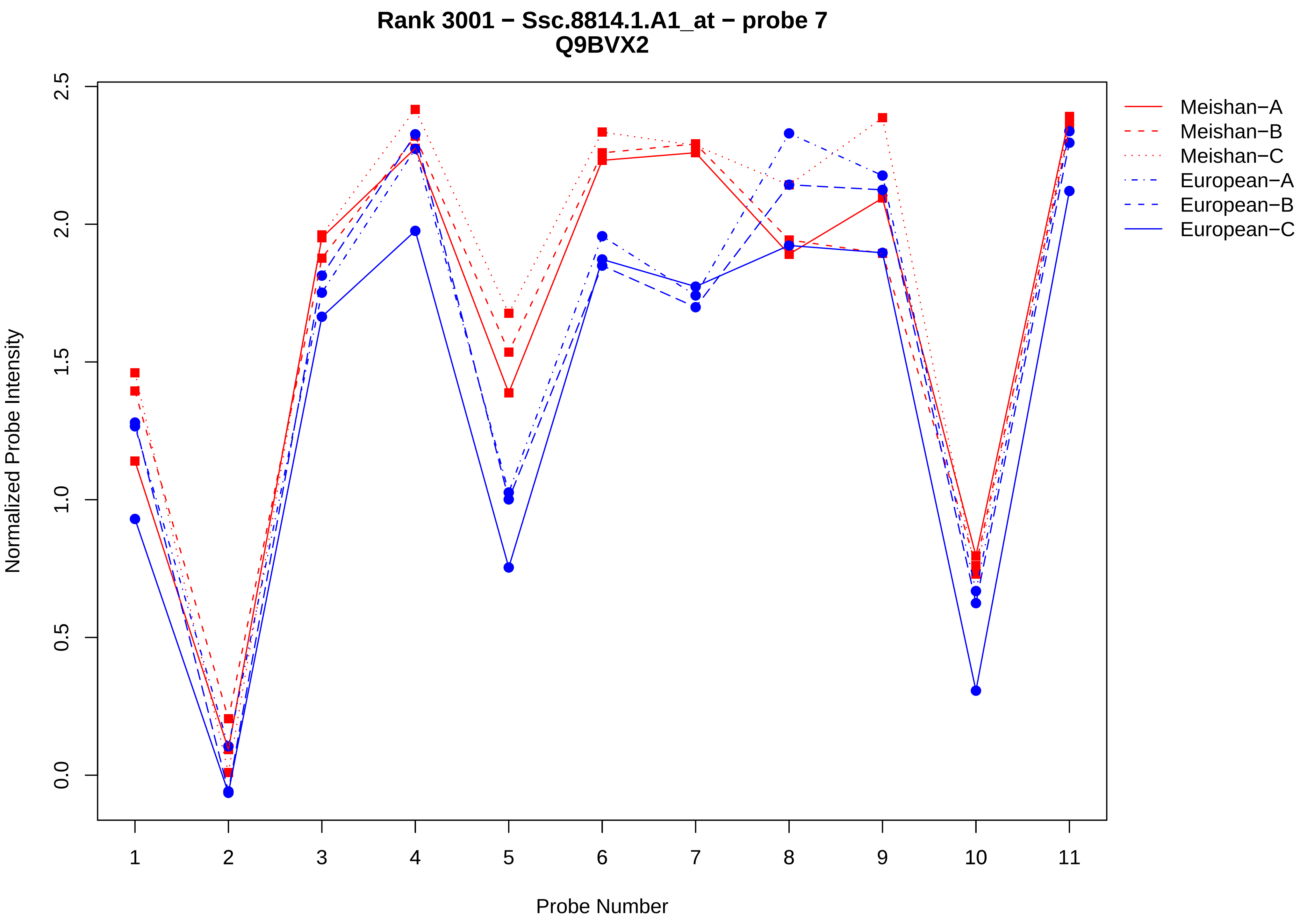

Rank 3001 − Ssc.8814.1.A1_at − probe 7
Q9BVX2
Normalized Probe Intensity
0.0
0.5
1.0
1.5
2.0
2.5
Probe Number
1
2
3
4
5
6
7
8
9
10
11
Meishan−A
Meishan−B
Meishan−C
European−A
European−B
European−C

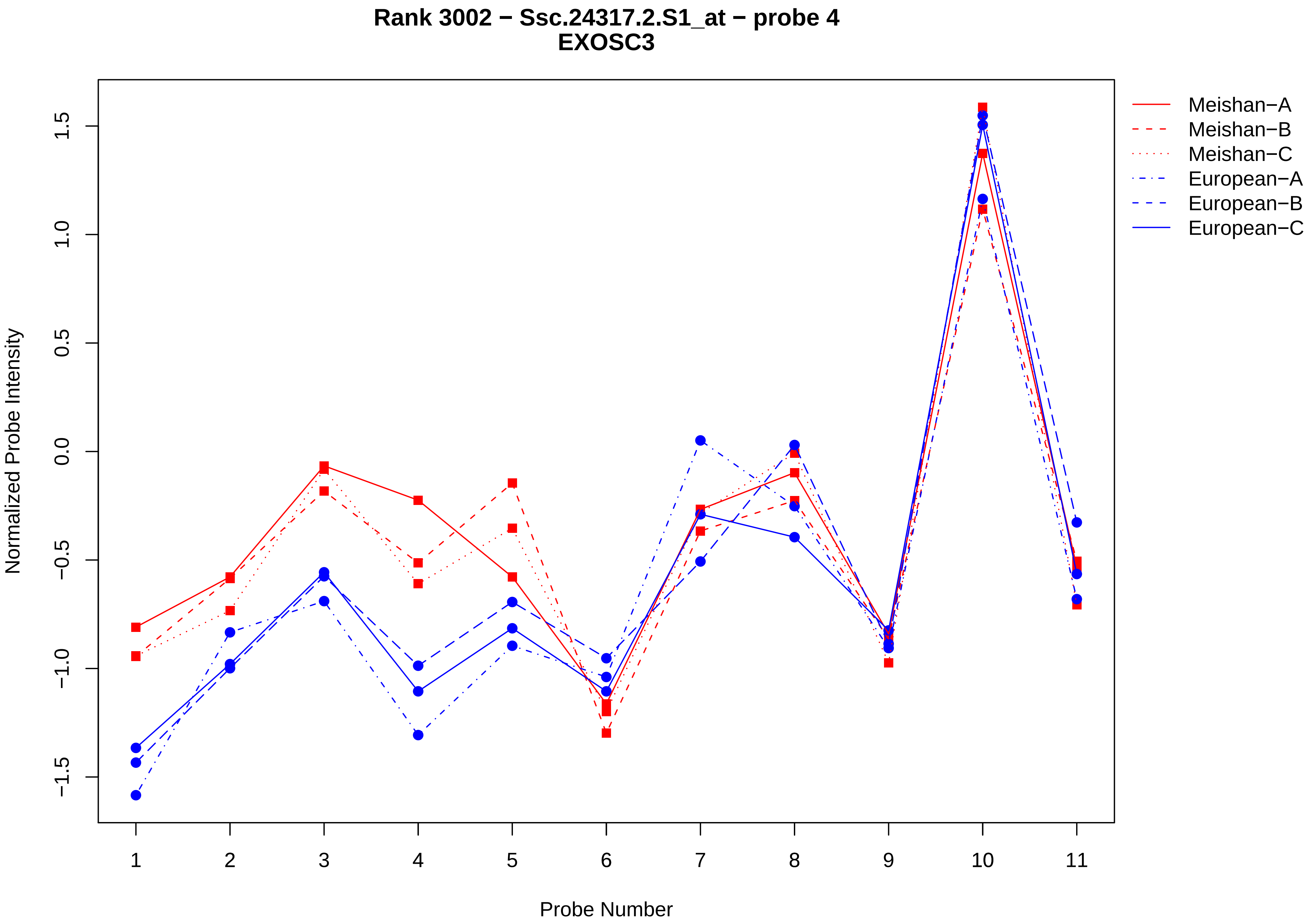

Rank 3002 − Ssc.24317.2.S1_at − probe 4
EXOSC3
Normalized Probe Intensity
Probe Number
Meishan−A
Meishan−B
Meishan−C
European−A
European−B
European−C
−1.5
−1.0
−0.5
0.0
0.5
1.0
1.5
1
2
3
4
5
6
7
8
9
10
11

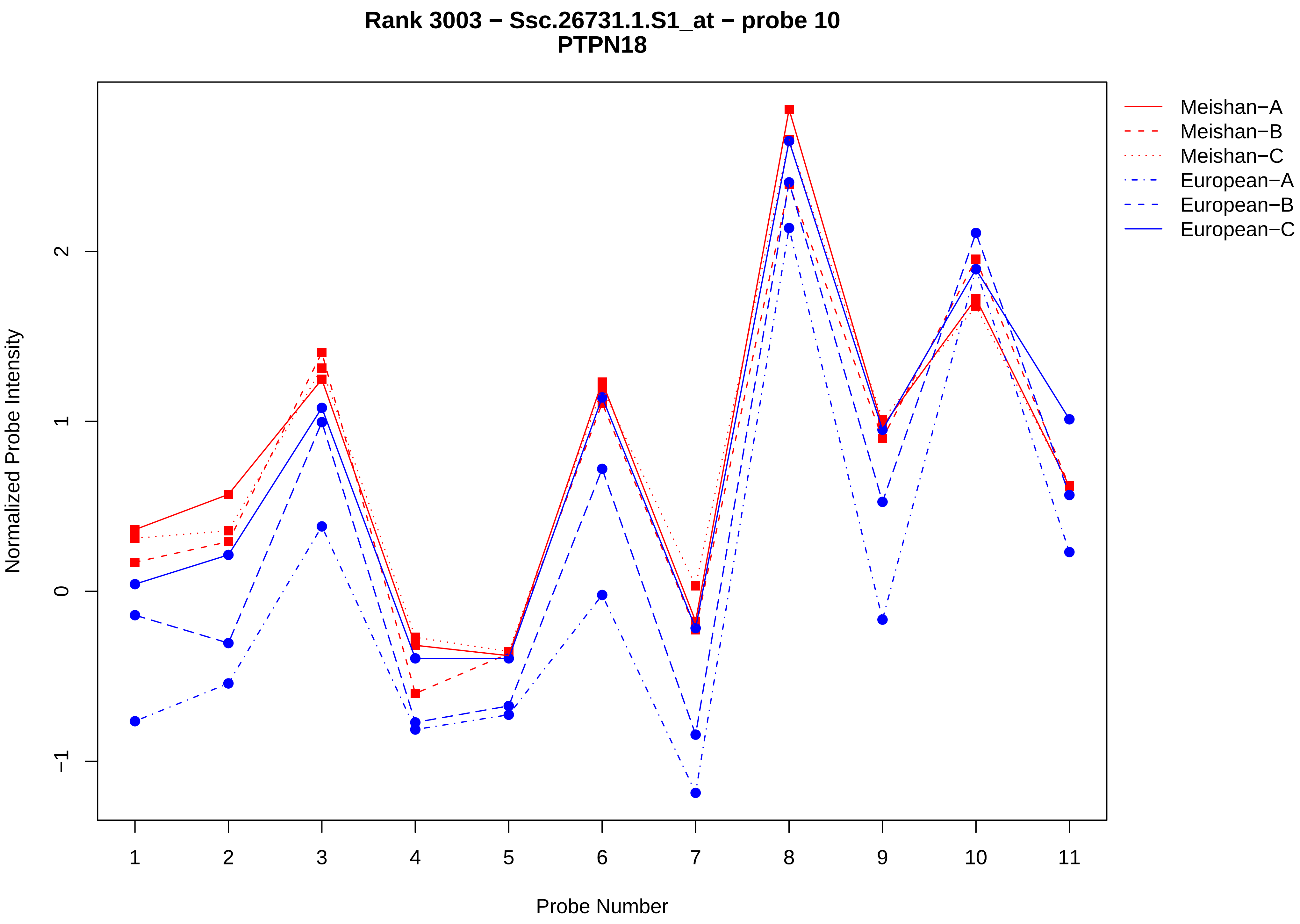

Rank 3003 − Ssc.26731.1.S1_at − probe 10
PTPN18
Normalized Probe Intensity
Probe Number
−1
0
1
2
1
2
3
4
5
6
7
8
9
10
11
Meishan−A
Meishan−B
Meishan−C
European−A
European−B
European−C

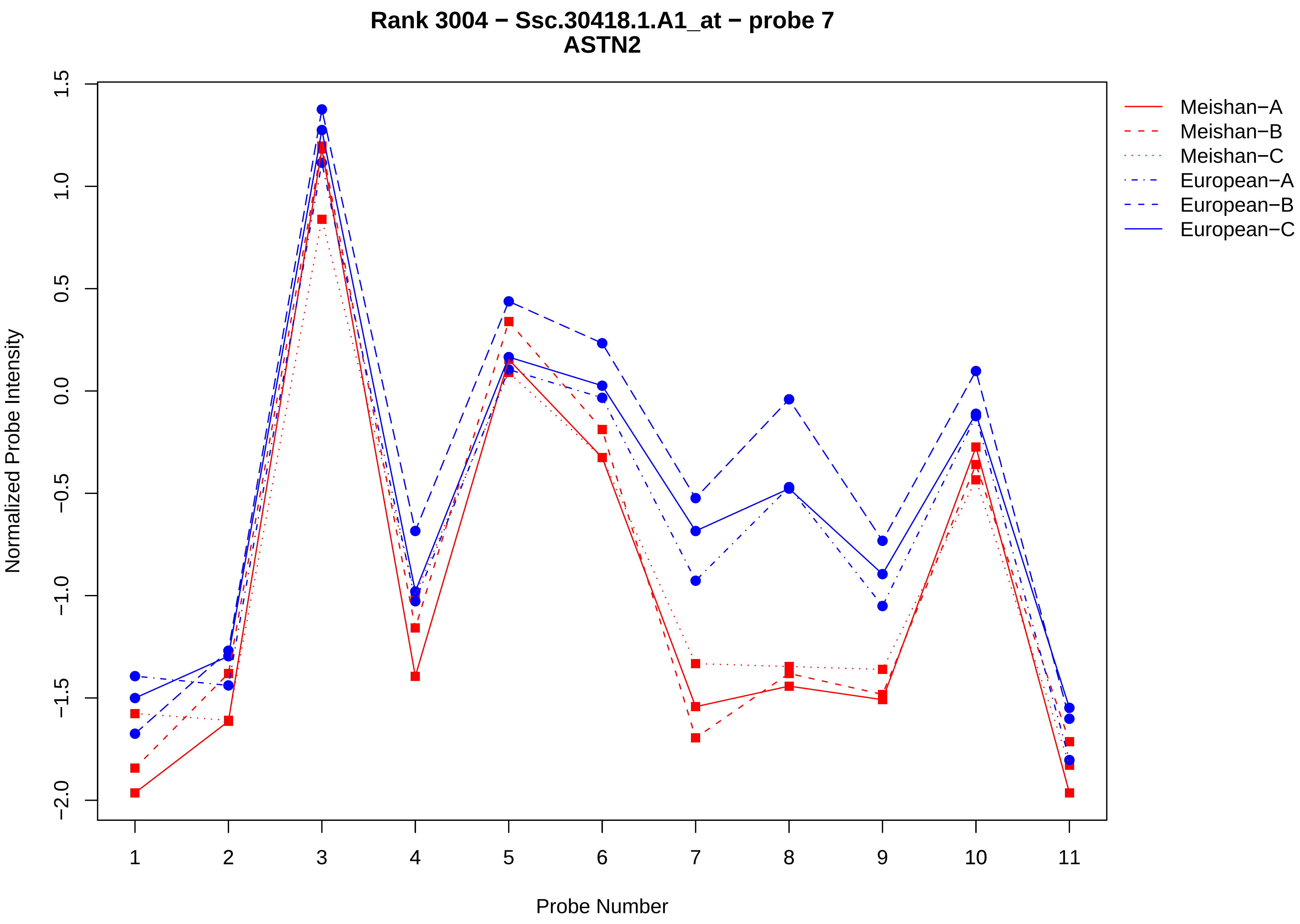

Rank 3004 − Ssc.30418.1.A1_at − probe 7
ASTN2
Normalized Probe Intensity
Probe Number
1.5
1.0
0.5
0.0
−0.5
−1.0
−1.5
−2.0
1
2
3
4
5
6
7
8
9
10
11
Meishan−A
Meishan−B
Meishan−C
European−A
European−B
European−C

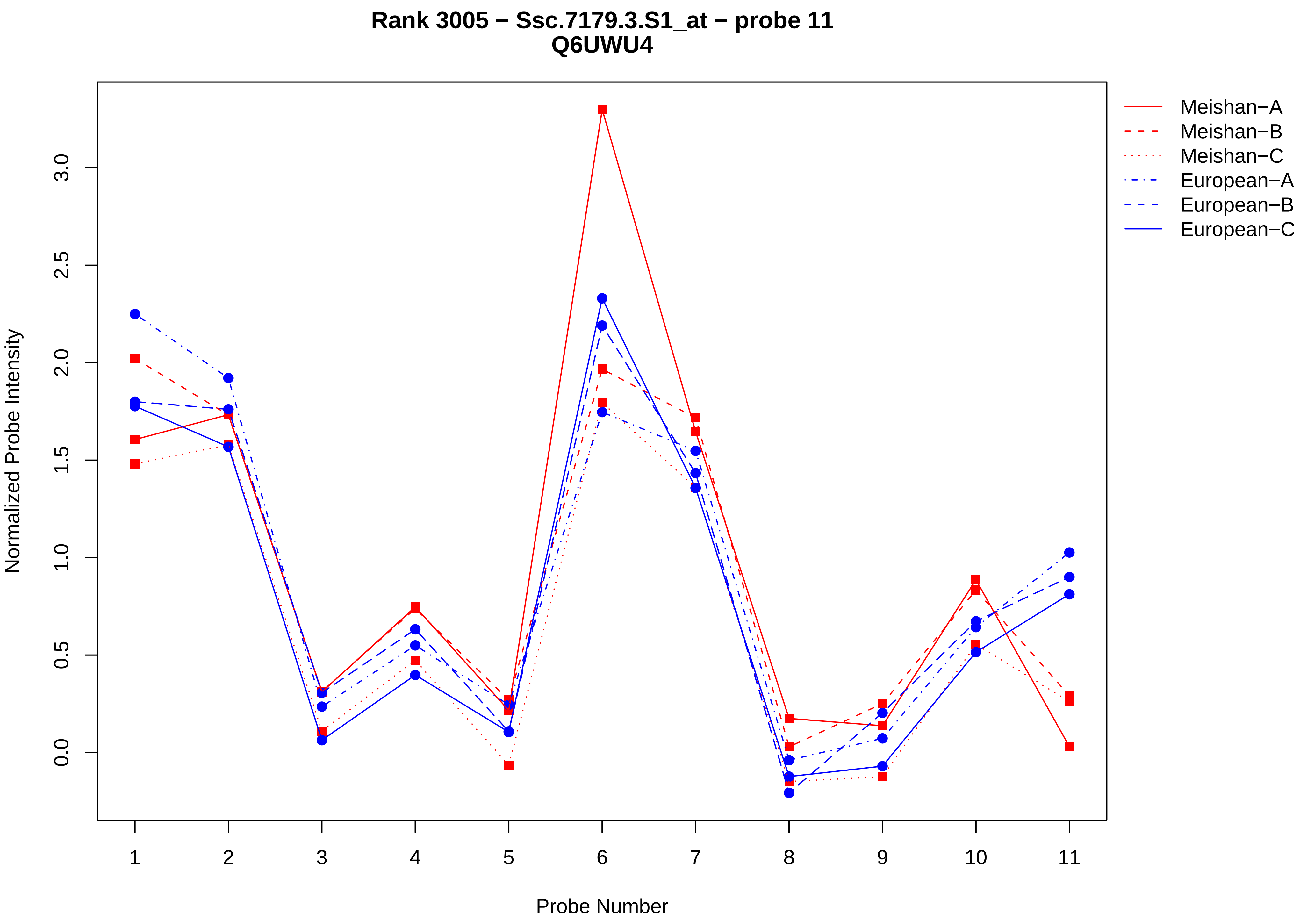

Rank 3005 − Ssc.7179.3.S1_at − probe 11
Q6UWU4
Normalized Probe Intensity
Probe Number
0.0
0.5
1.0
1.5
2.0
2.5
3.0
1
2
3
4
5
6
7
8
9
10
11
Meishan−A
Meishan−B
Meishan−C
European−A
European−B
European−C

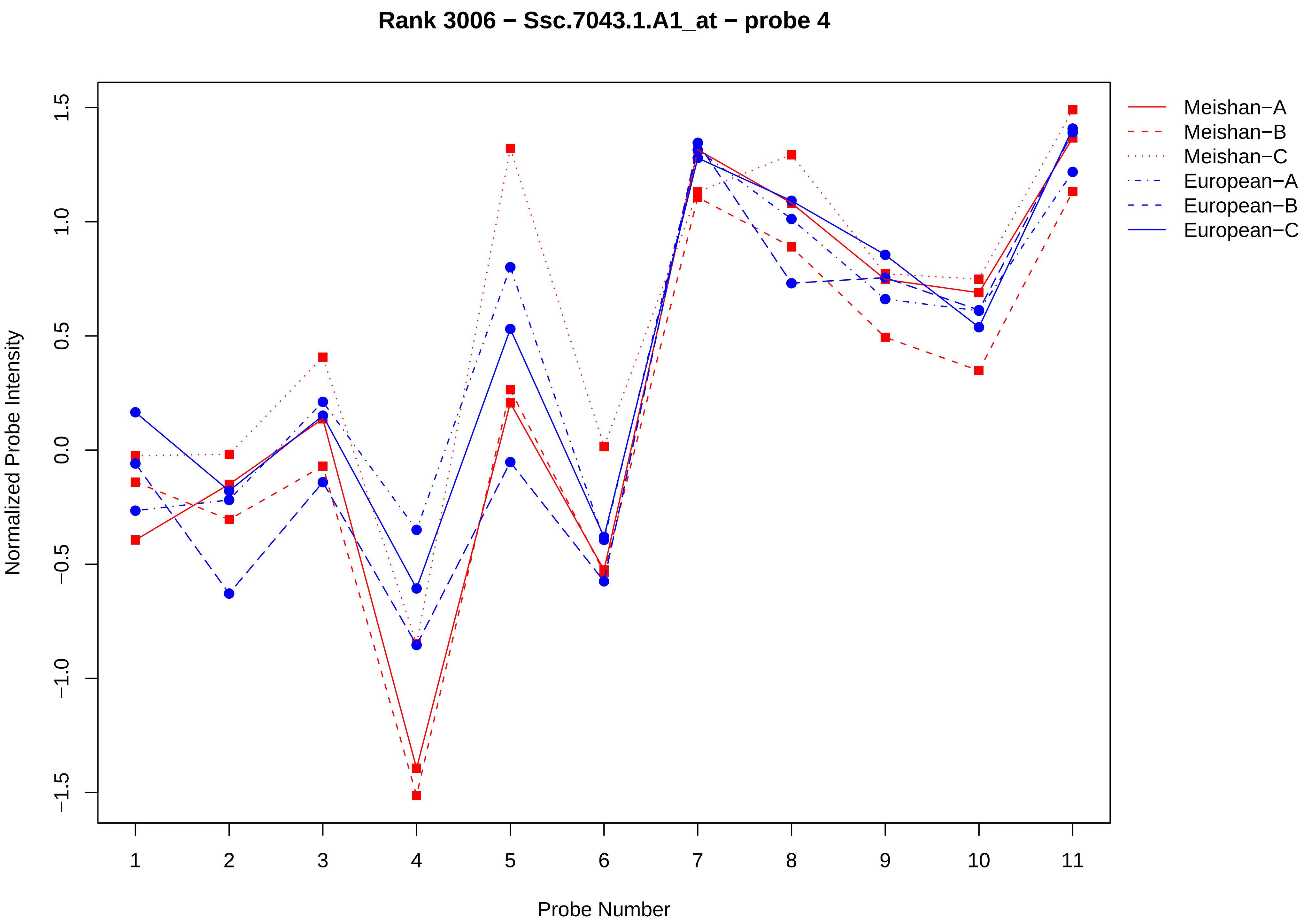

Rank 3006 − Ssc.7043.1.A1_at − probe 4
Normalized Probe Intensity
−1.5
−1.0
−0.5
0.0
0.5
1.0
1.5
1
2
3
4
5
6
7
8
9
10
11
Probe Number
Meishan−A
Meishan−B
Meishan−C
European−A
European−B
European−C

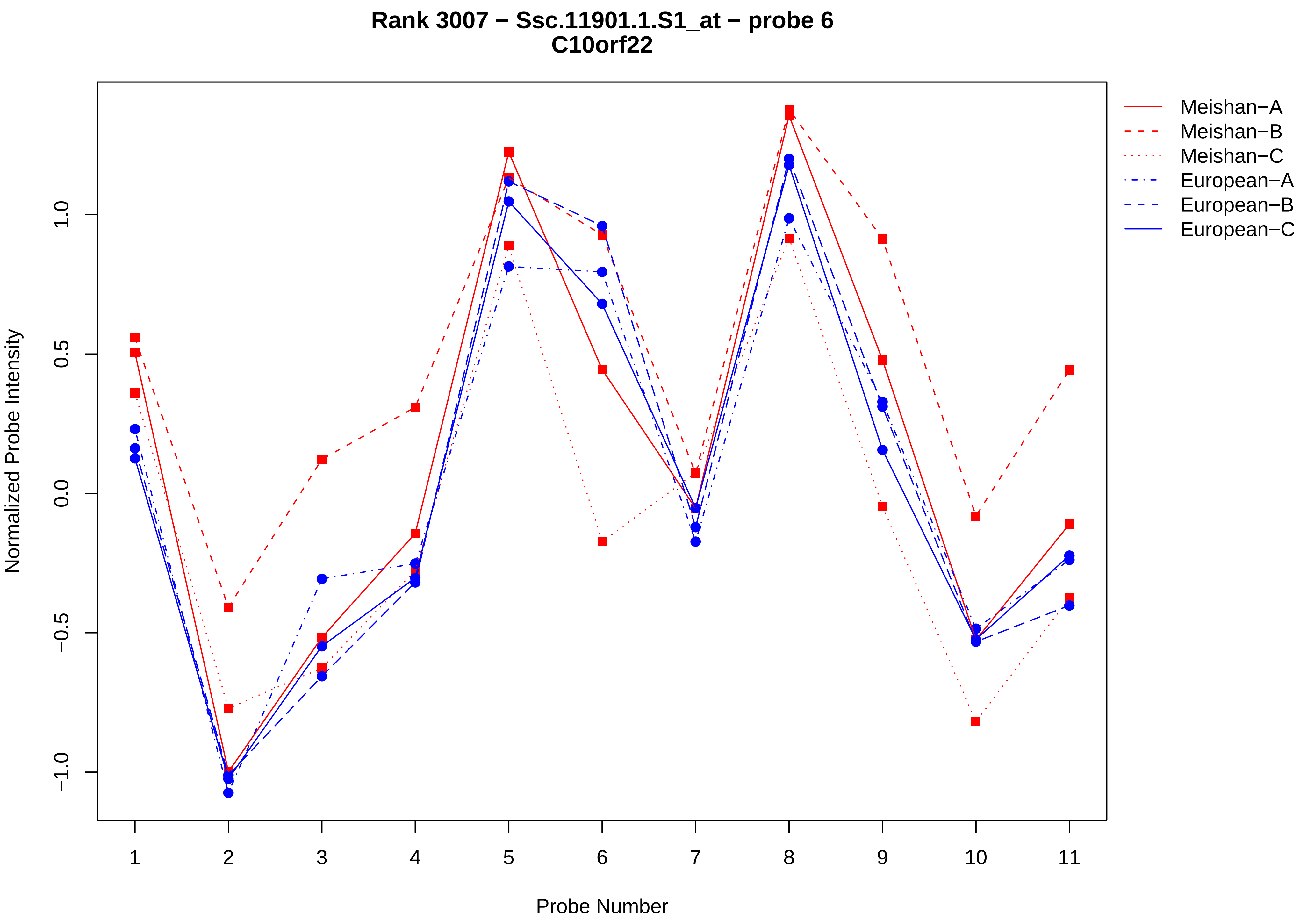
Rank 3007 − Ssc.11901.1.S1_at − probe 6
C10orf22
Normalized Probe Intensity
Probe Number
−1.0
−0.5
0.0
0.5
1.0
1
2
3
4
5
6
7
8
9
10
11
Meishan−A
Meishan−B
Meishan−C
European−A
European−B
European−C

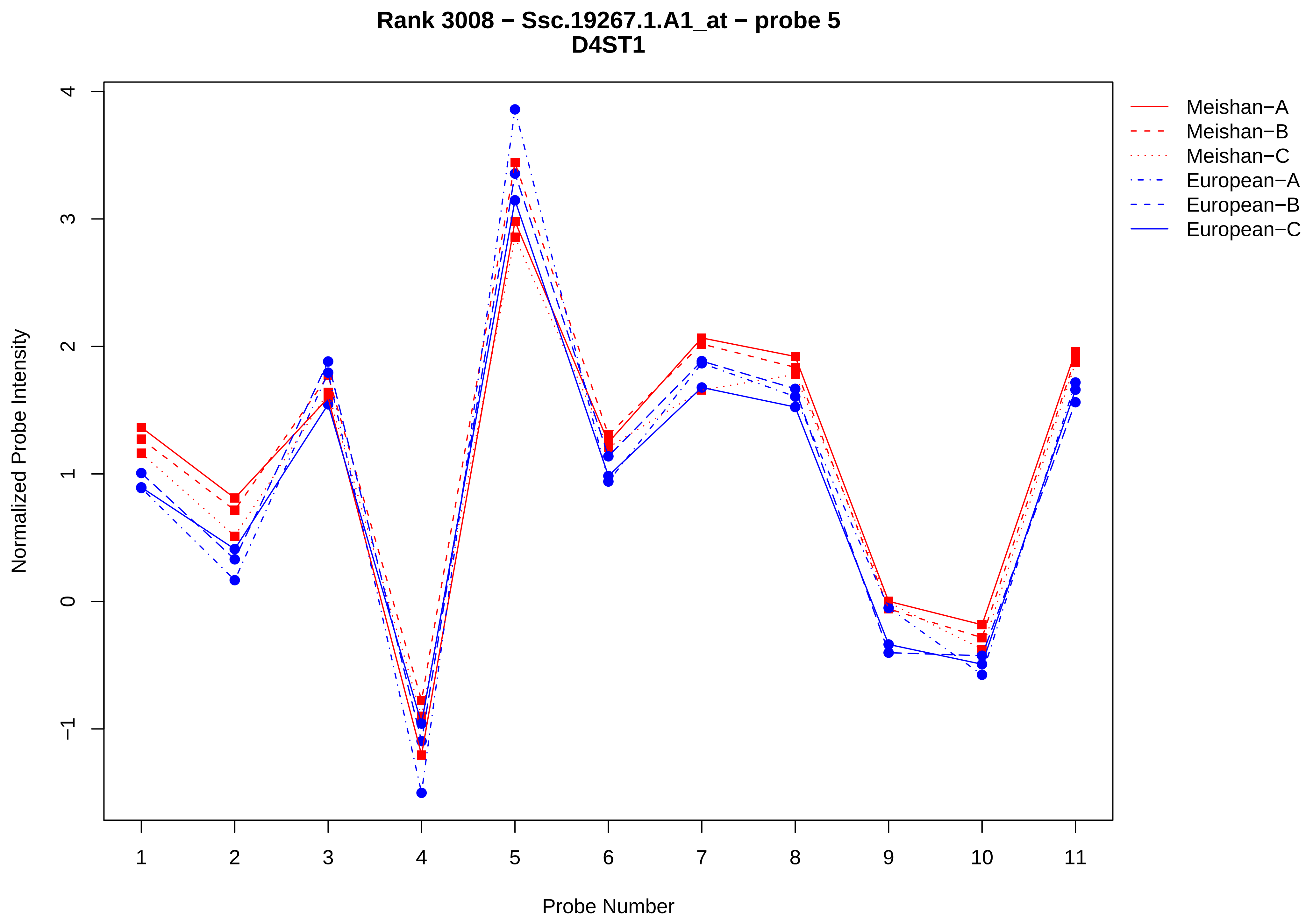

Rank 3008 − Ssc.19267.1.A1_at − probe 5
D4ST1
Normalized Probe Intensity
Probe Number
Meishan−A
Meishan−B
Meishan−C
European−A
European−B
European−C

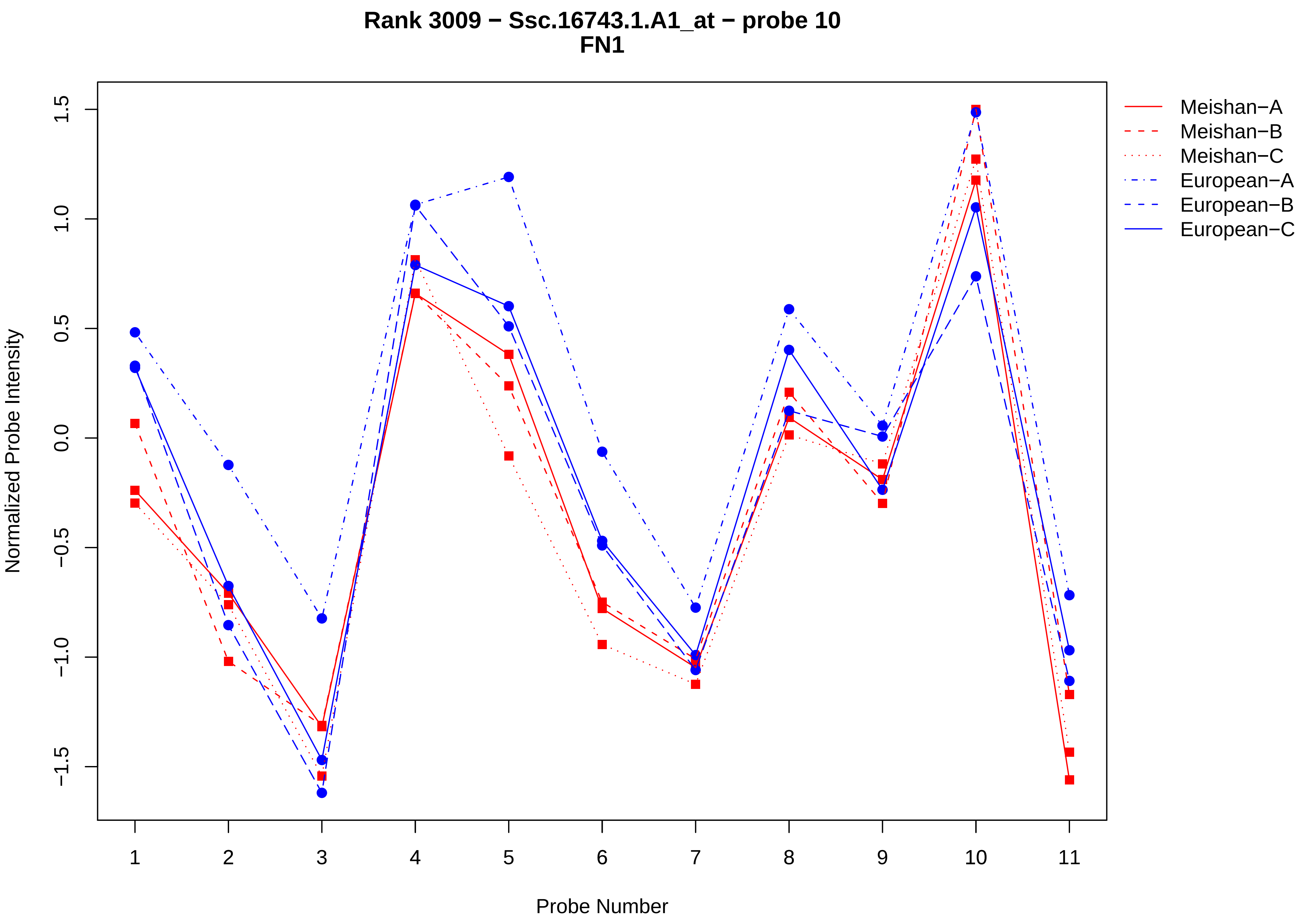

Rank 3009 − Ssc.16743.1.A1_at − probe 10
FN1
Normalized Probe Intensity
Probe Number
−1.5
−1.0
−0.5
0.0
0.5
1.0
1.5
1
2
3
4
5
6
7
8
9
10
11
Meishan−A
Meishan−B
Meishan−C
European−A
European−B
European−C

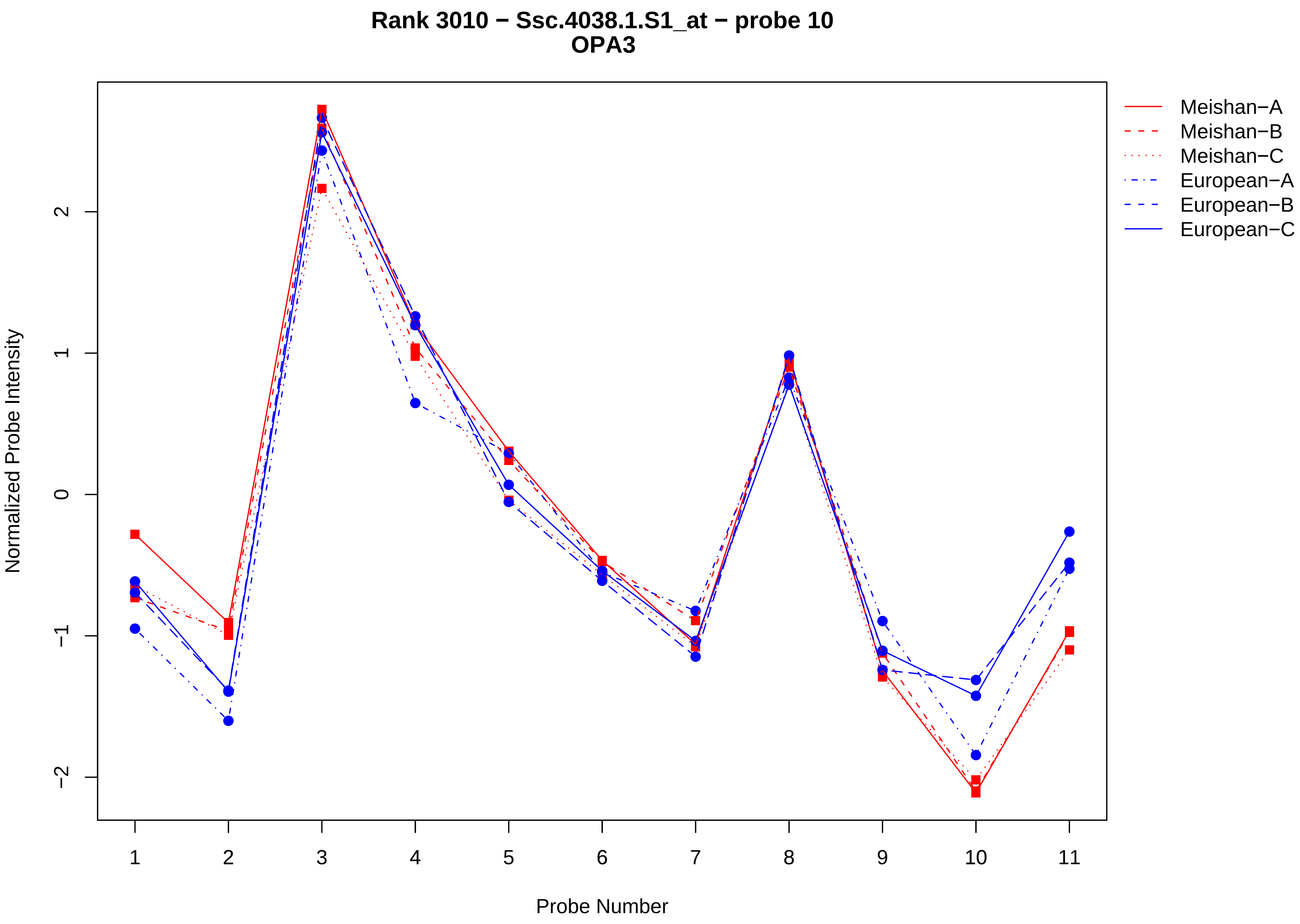

Rank 3010 − Ssc.4038.1.S1_at − probe 10
OPA3
Normalized Probe Intensity
Probe Number
−2
−1
0
1
2
1
2
3
4
5
6
7
8
9
10
11
Meishan−A
Meishan−B
Meishan−C
European−A
European−B
European−C

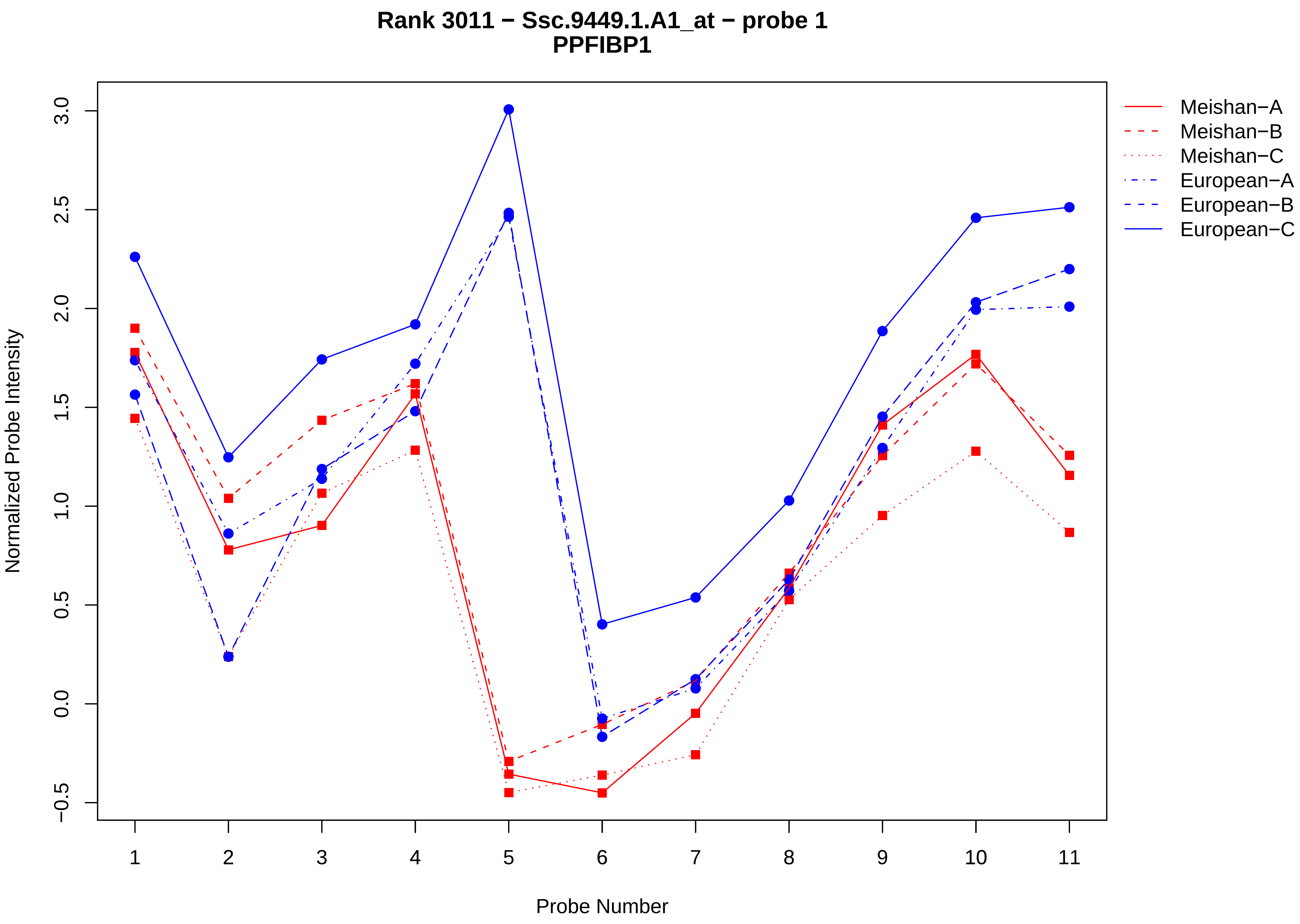

Rank 3011 − Ssc.9449.1.A1_at − probe 1
PPFIBP1
Normalized Probe Intensity
Probe Number
−0.5
0.0
0.5
1.0
1.5
2.0
2.5
3.0
1
2
3
4
5
6
7
8
9
10
11
Meishan−A
Meishan−B
Meishan−C
European−A
European−B
European−C

Rank 3012 − Ssc.6482.2.S1_at − probe 6
GCL1_HUMAN

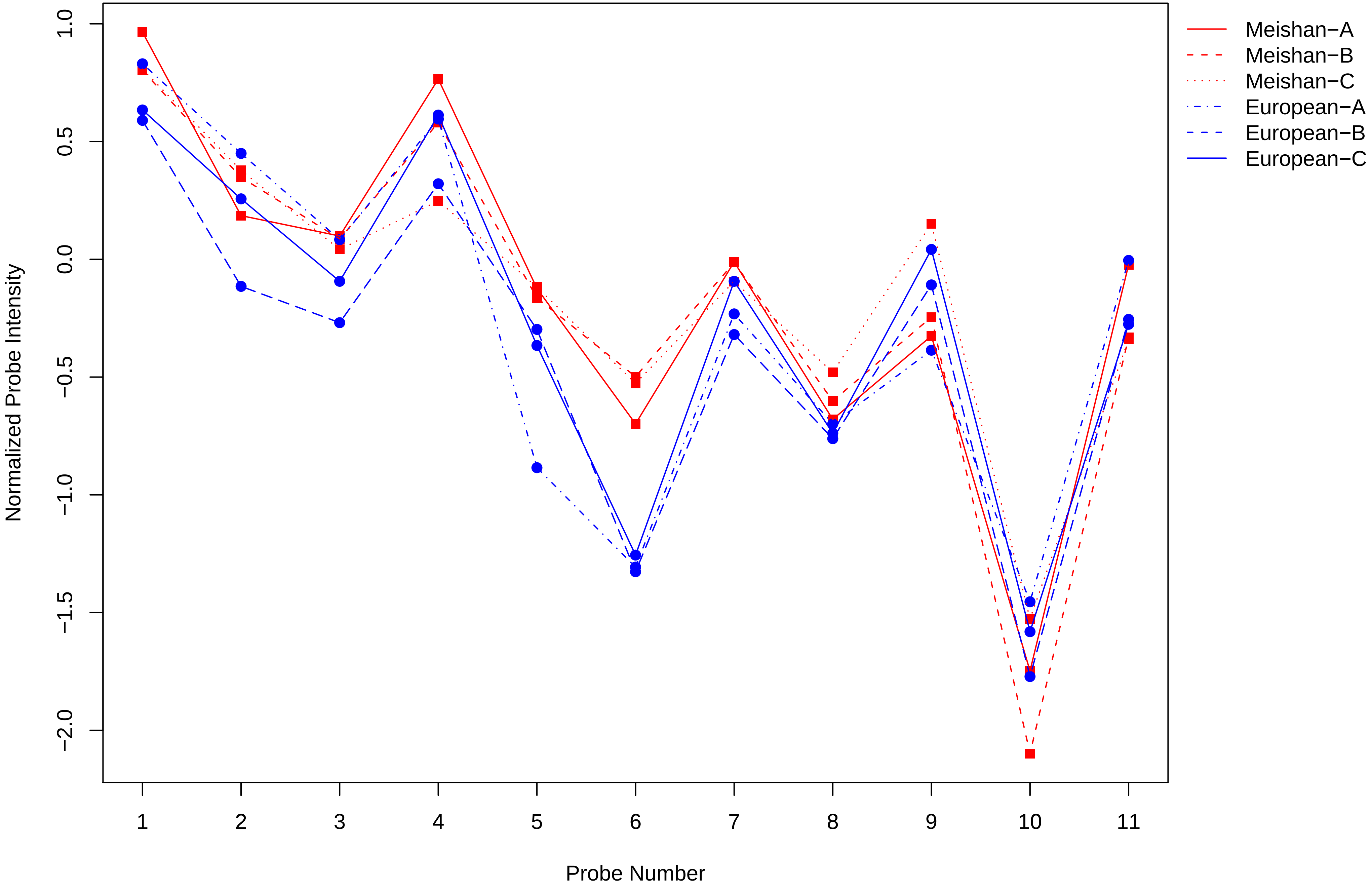

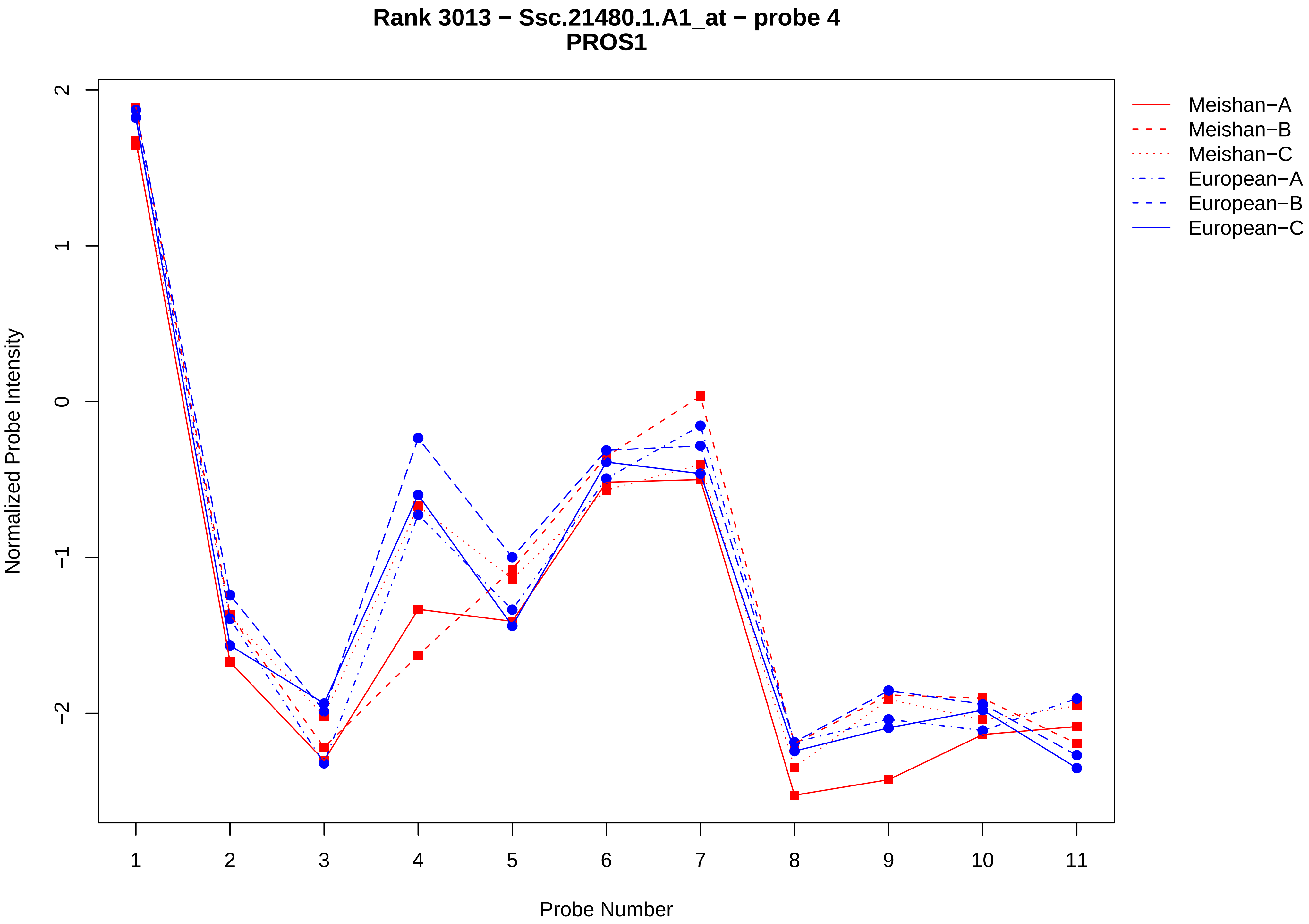

Rank 3013 − Ssc.21480.1.A1_at − probe 4
PROS1
Normalized Probe Intensity
Probe Number
Meishan−A
Meishan−B
Meishan−C
European−A
European−B
European−C
2
1
0
−1
−2
1
2
3
4
5
6
7
8
9
10
11

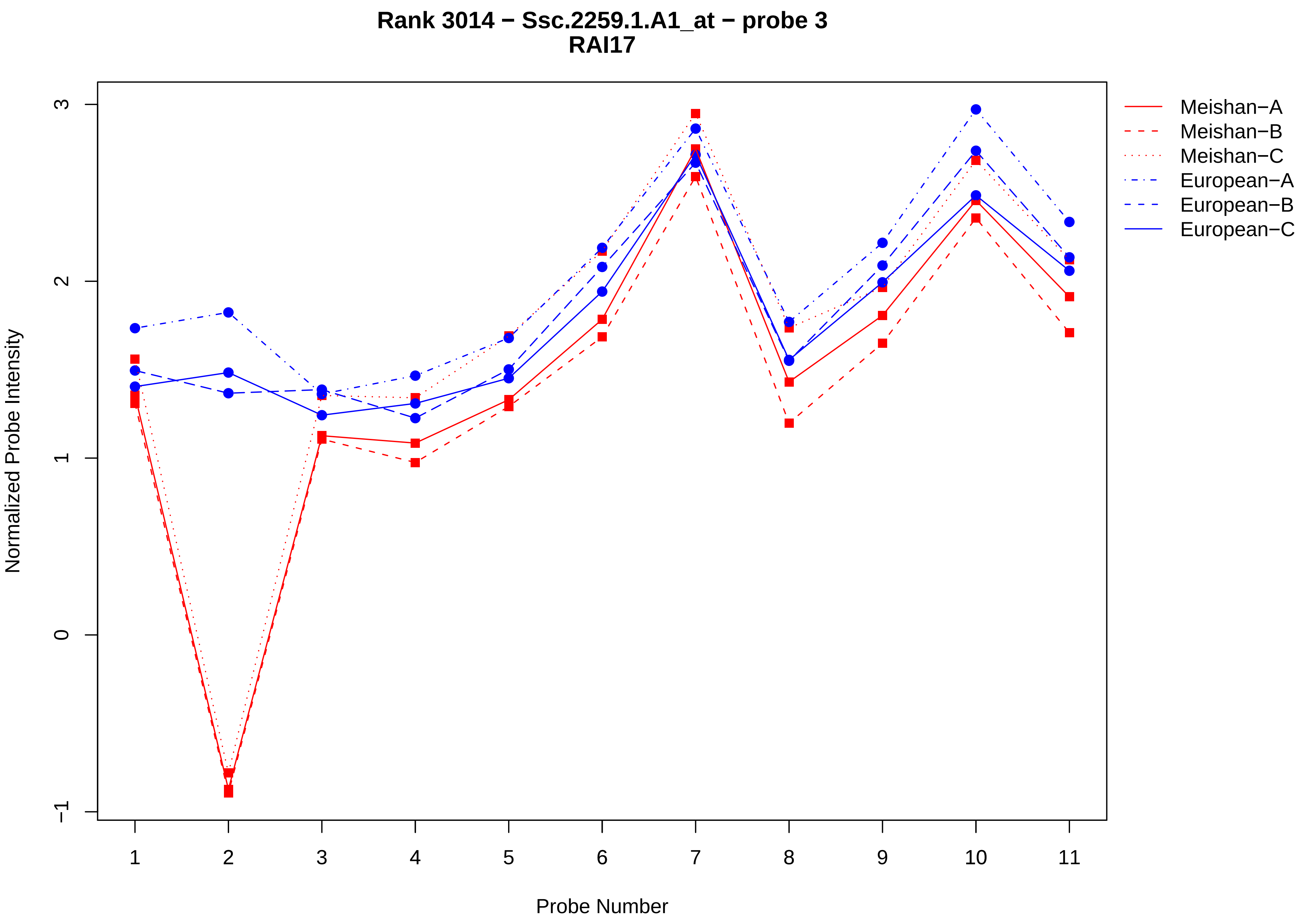

Rank 3014 − Ssc.2259.1.A1_at − probe 3
RAI17
Normalized Probe Intensity
Probe Number
−1
0
1
2
3
1
2
3
4
5
6
7
8
9
10
11
Meishan−A
Meishan−B
Meishan−C
European−A
European−B
European−C

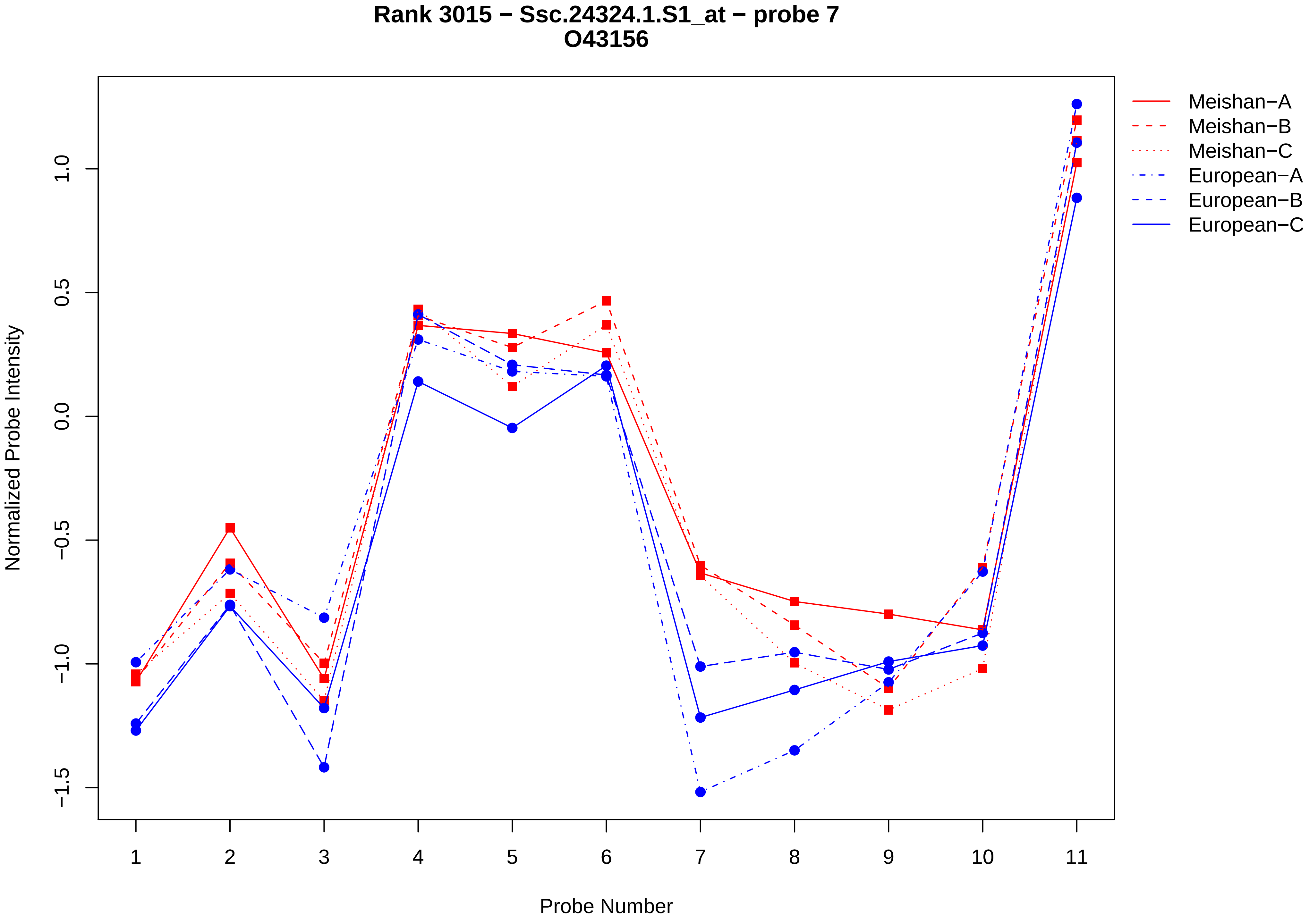

Rank 3015 − Ssc.24324.1.S1_at − probe 7
O43156
Normalized Probe Intensity
−1.5
−1.0
−0.5
0.0
0.5
1.0
Probe Number
1
2
3
4
5
6
7
8
9
10
11
Meishan−A
Meishan−B
Meishan−C
European−A
European−B
European−C

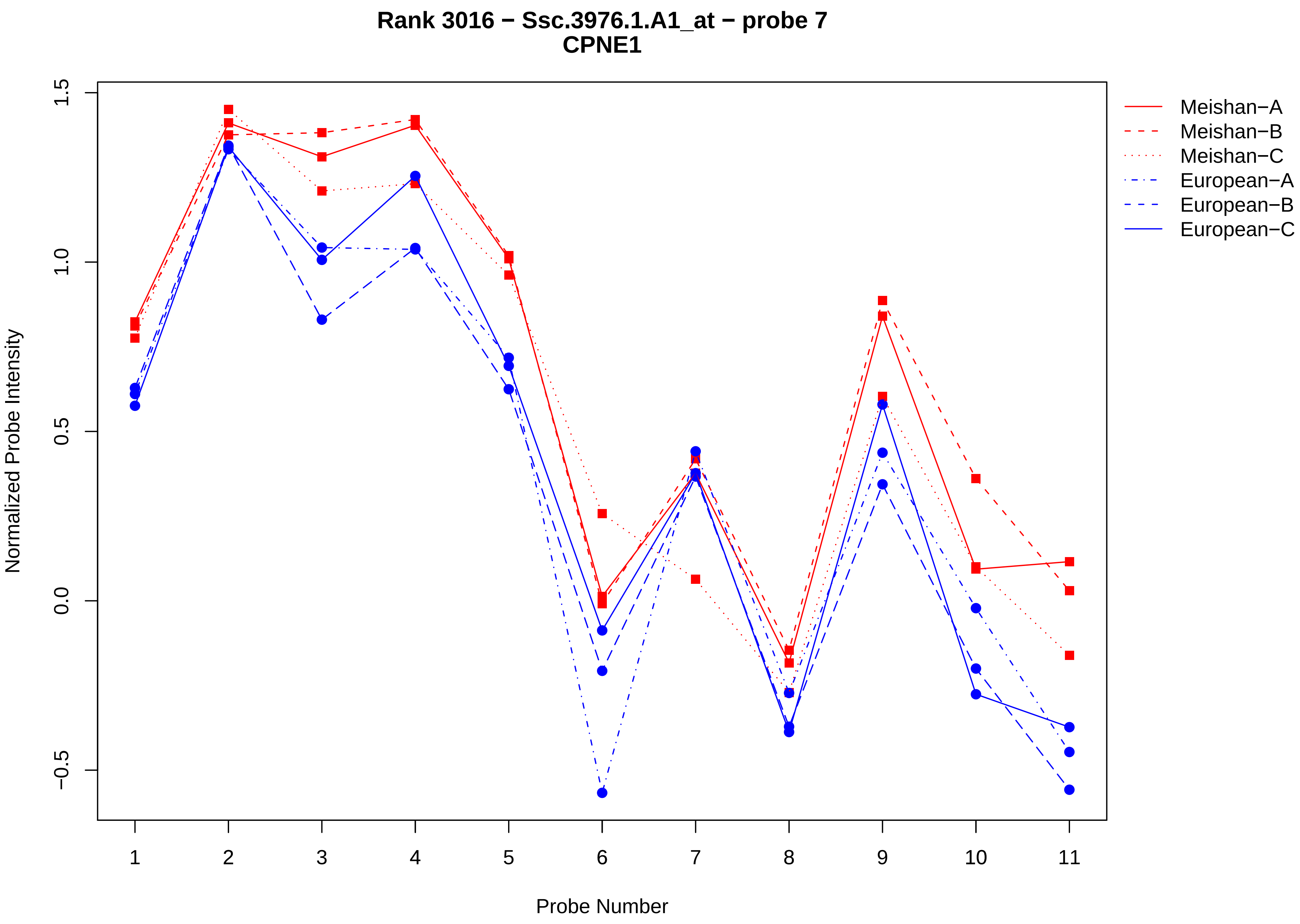

Rank 3016 − Ssc.3976.1.A1_at − probe 7
CPNE1
Normalized Probe Intensity
Probe Number
1.5
1.0
0.5
0.0
−0.5
1
2
3
4
5
6
7
8
9
10
11
Meishan−A
Meishan−B
Meishan−C
European−A
European−B
European−C

Rank 3017 − Ssc.27885.1.S2_at − probe 1
HNRPA2B1

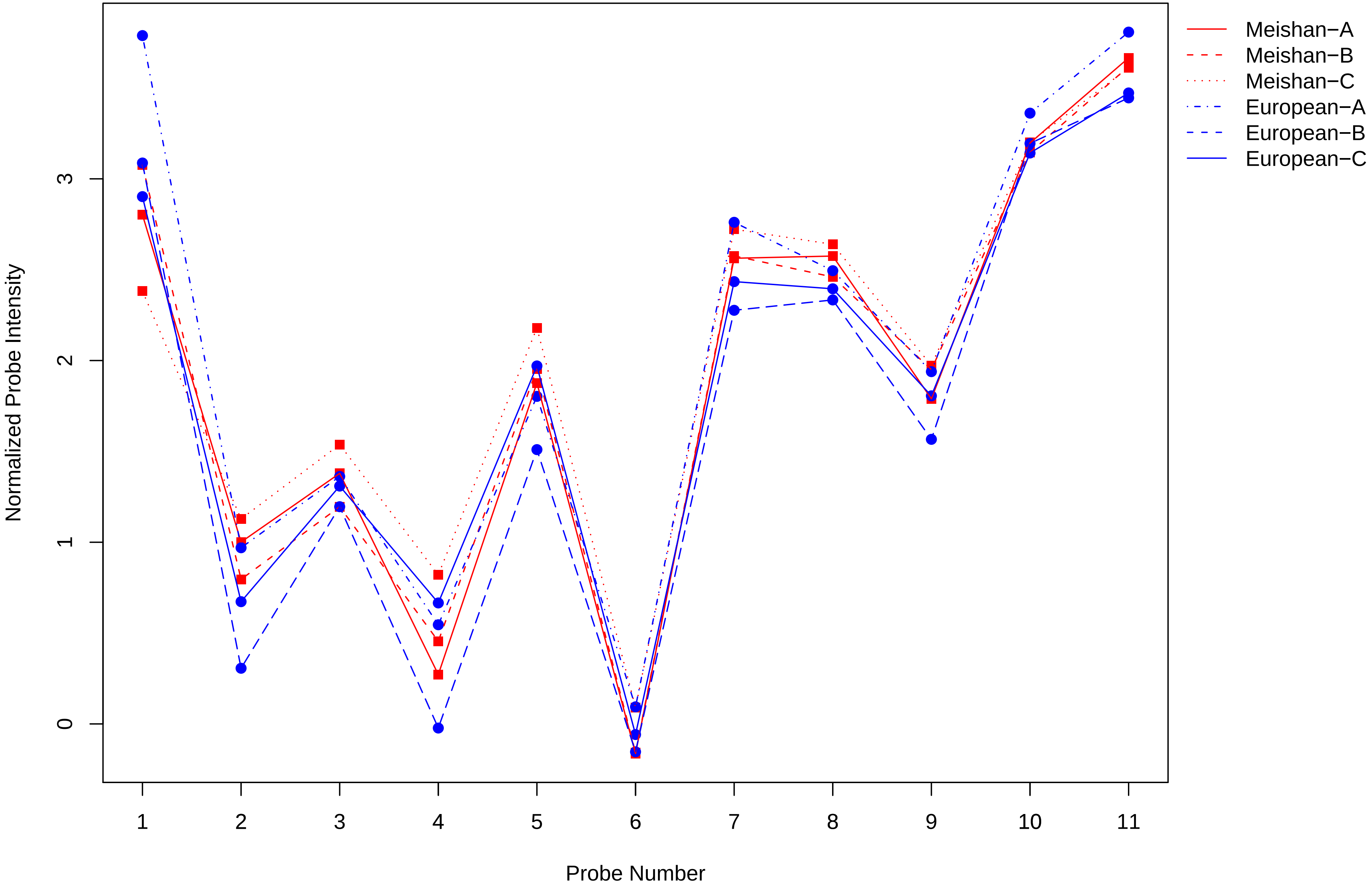

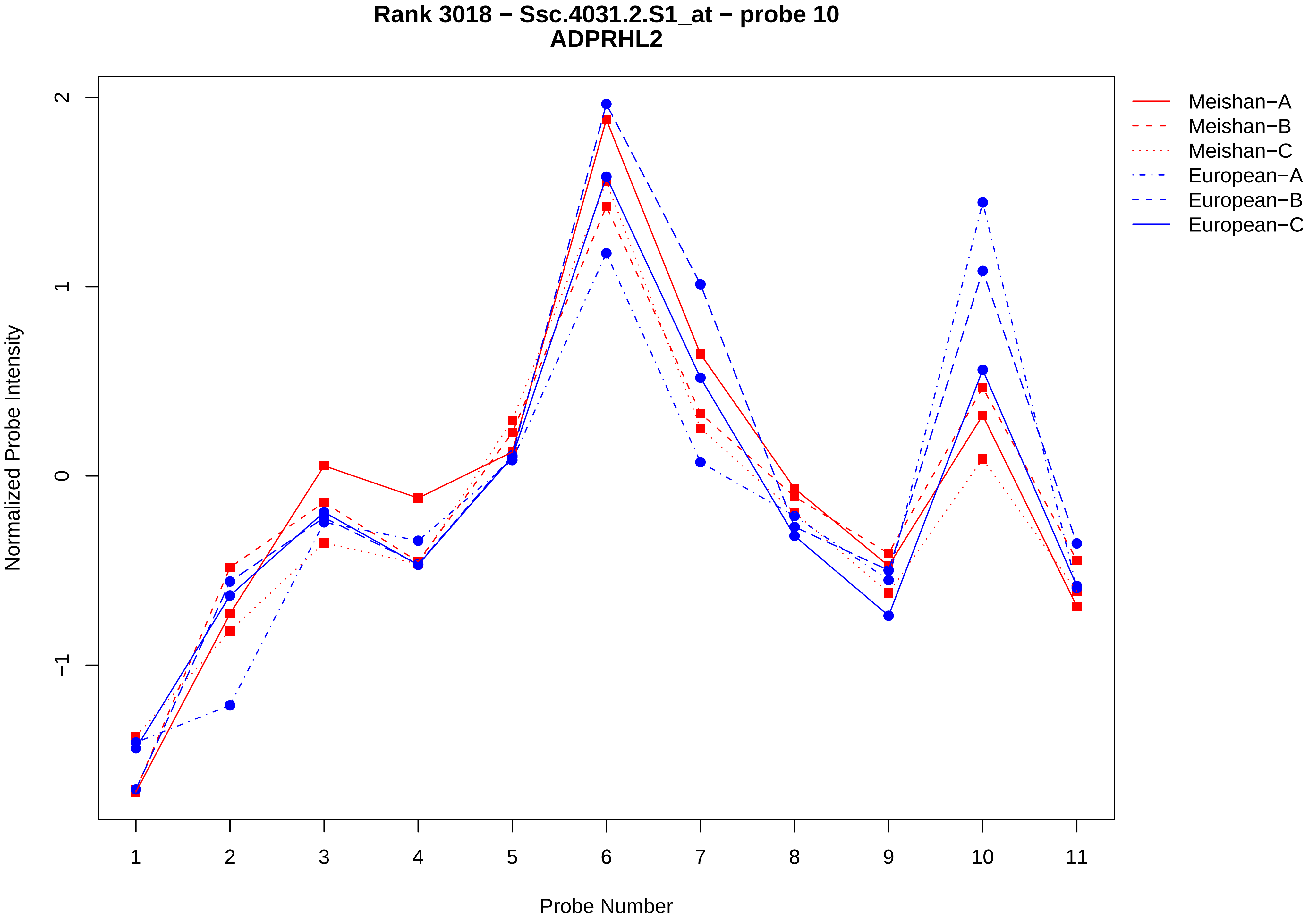

Rank 3018 − Ssc.4031.2.S1_at − probe 10
ADPRHL2
Normalized Probe Intensity
Probe Number
Meishan−A
Meishan−B
Meishan−C
European−A
European−B
European−C
2
1
0
−1
1
2
3
4
5
6
7
8
9
10
11

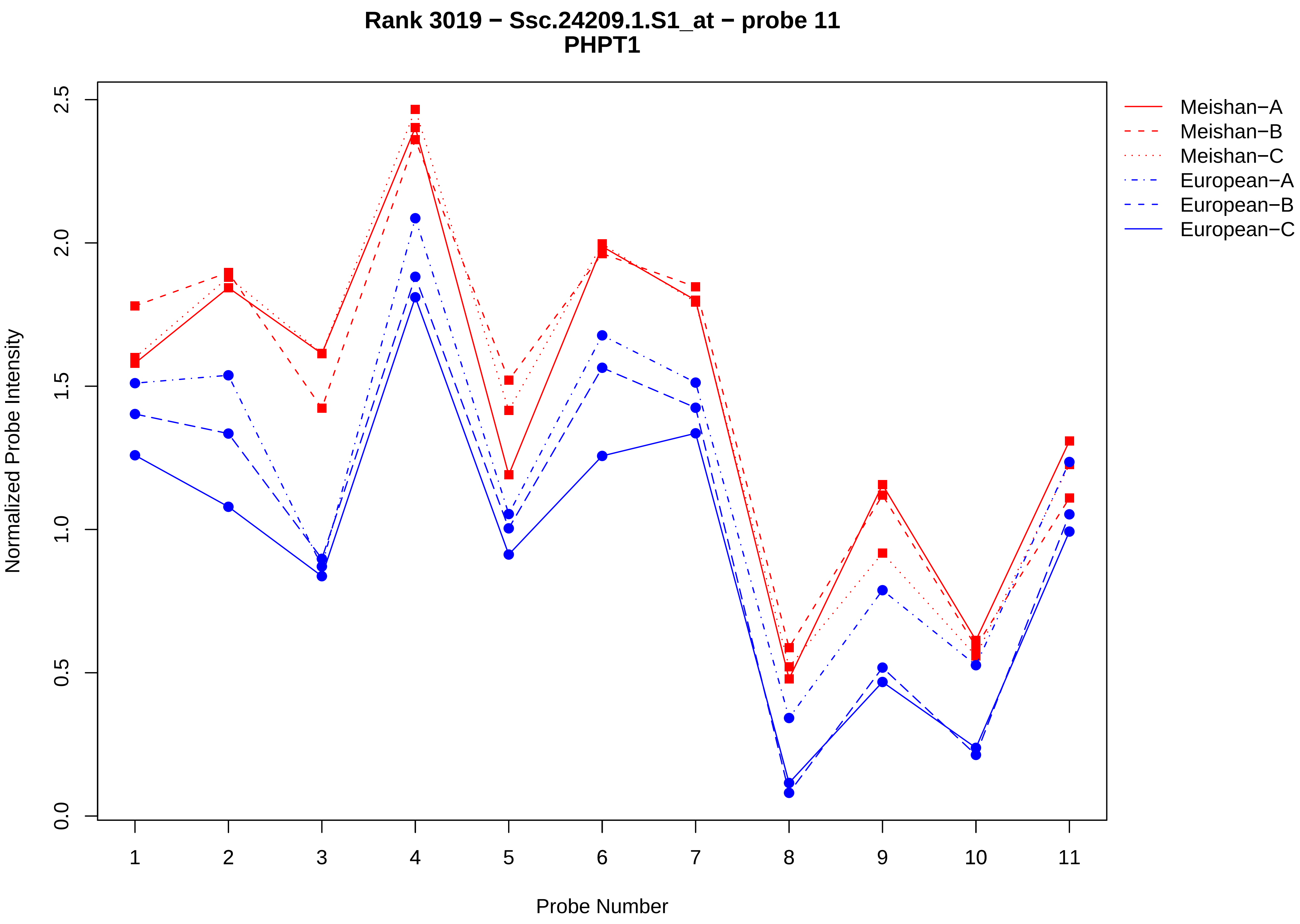

Rank 3019 − Ssc.24209.1.S1_at − probe 11
PHPT1
Normalized Probe Intensity
0.0
0.5
1.0
1.5
2.0
2.5
Probe Number
1
2
3
4
5
6
7
8
9
10
11
Meishan−A
Meishan−B
Meishan−C
European−A
European−B
European−C

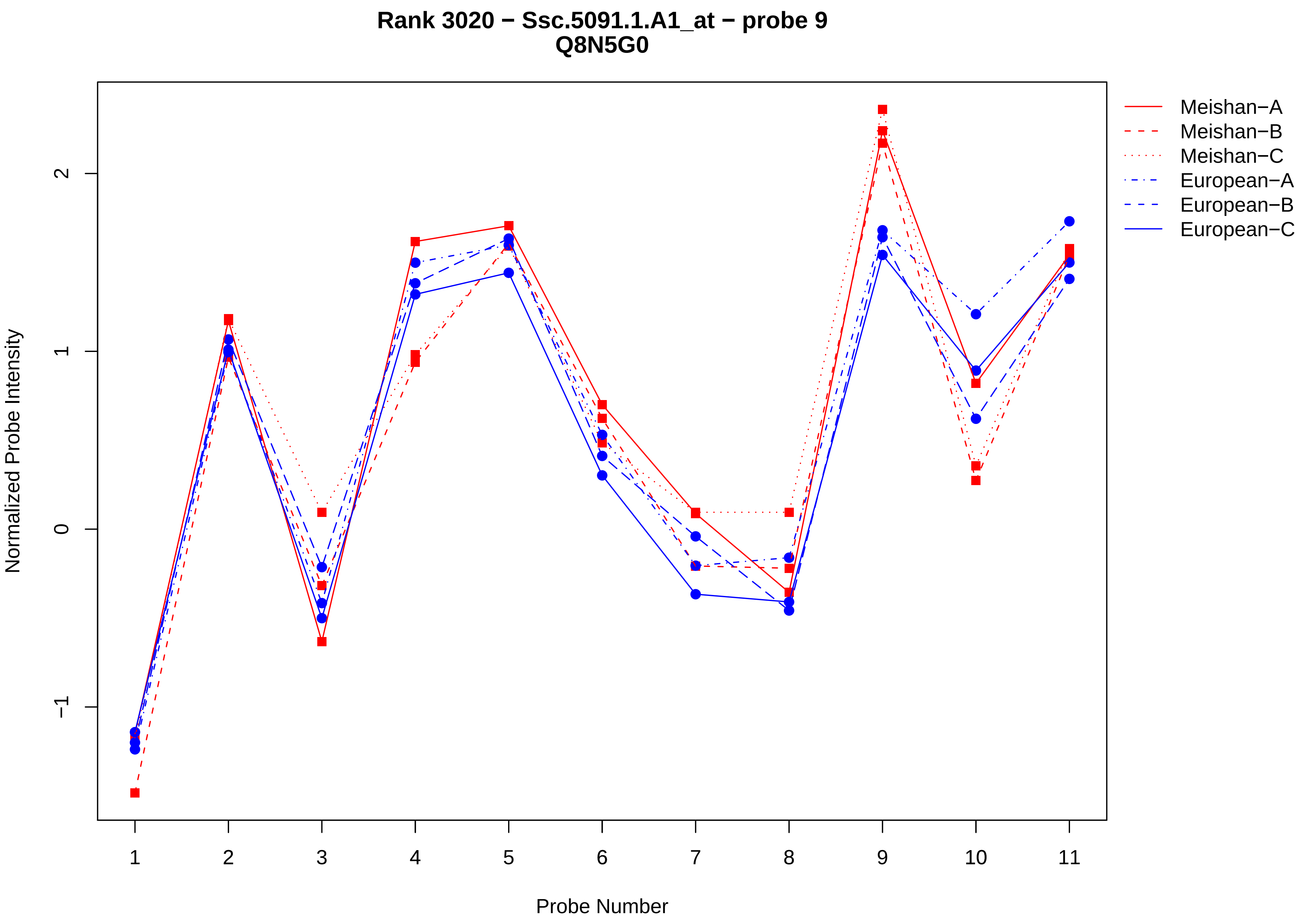

Rank 3020 − Ssc.5091.1.A1_at − probe 9
Q8N5G0
Normalized Probe Intensity
Probe Number
−1
0
1
2
1
2
3
4
5
6
7
8
9
10
11
Meishan−A
Meishan−B
Meishan−C
European−A
European−B
European−C

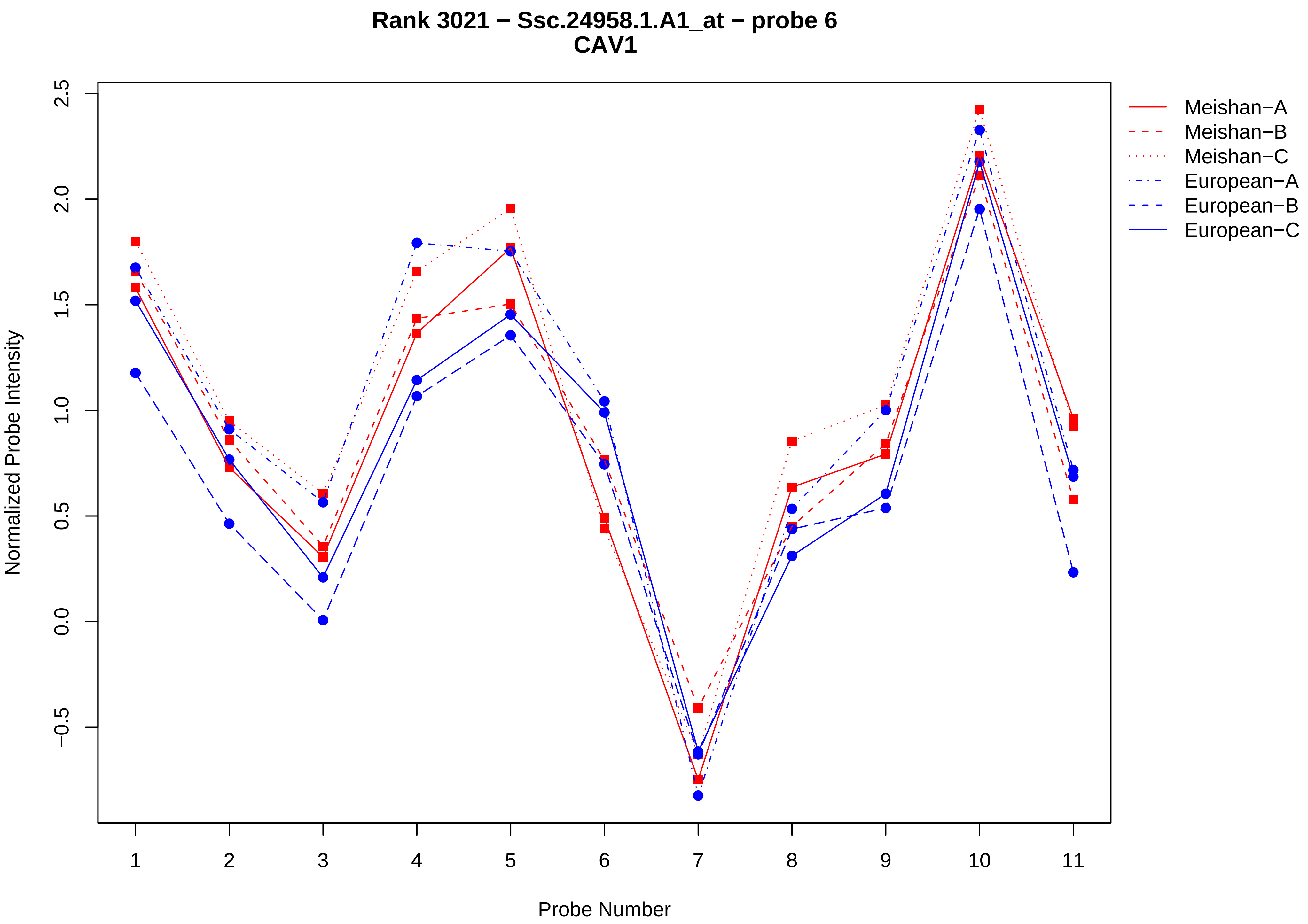

Rank 3021 − Ssc.24958.1.A1_at − probe 6
CAV1
Normalized Probe Intensity
Probe Number
−0.5
0.0
0.5
1.0
1.5
2.0
2.5
1
2
3
4
5
6
7
8
9
10
11
Meishan−A
Meishan−B
Meishan−C
European−A
European−B
European−C

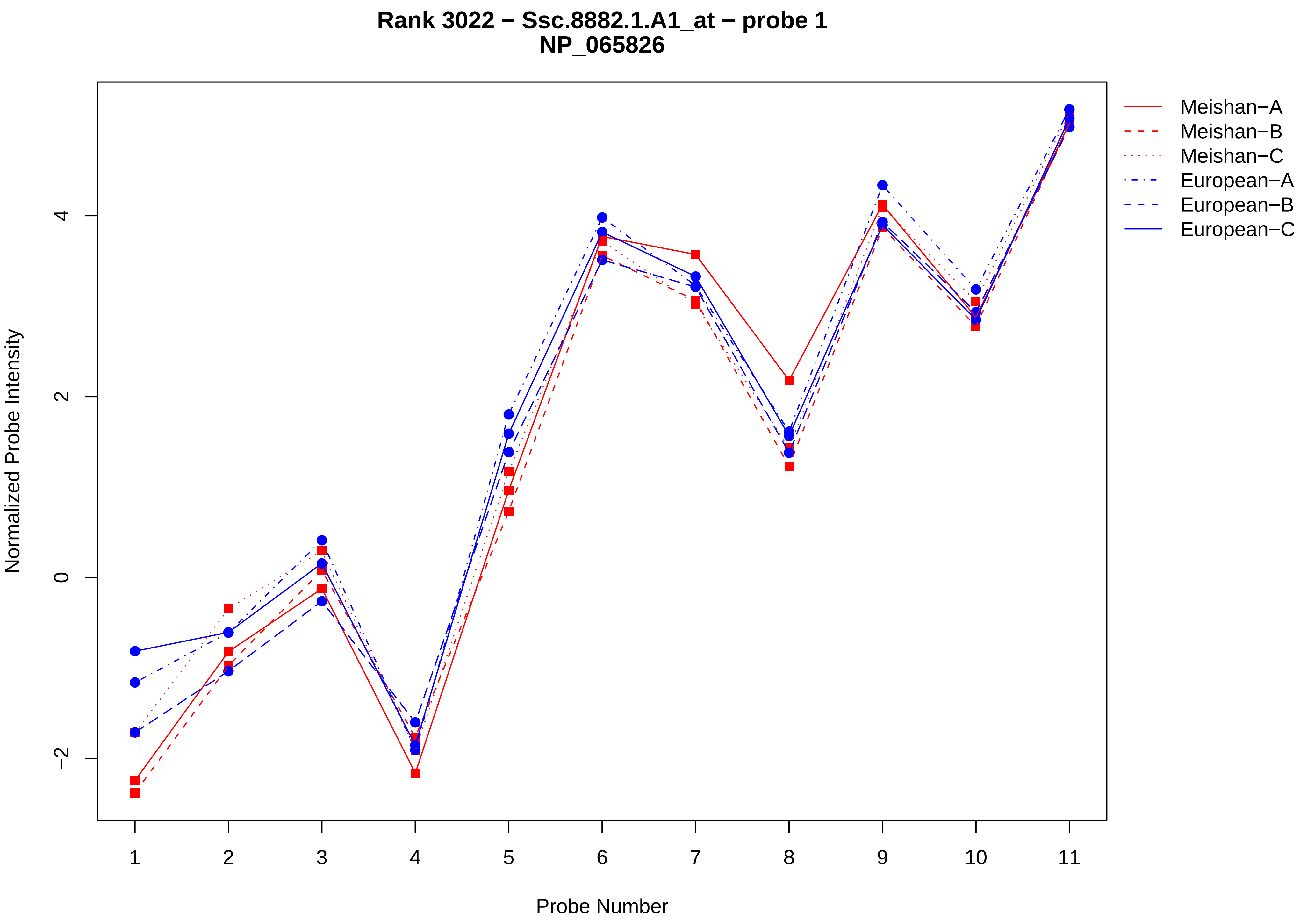

Rank 3022 − Ssc.8882.1.A1_at − probe 1
NP_065826
Normalized Probe Intensity
Probe Number
−2
0
2
4
1
2
3
4
5
6
7
8
9
10
11
Meishan−A
Meishan−B
Meishan−C
European−A
European−B
European−C

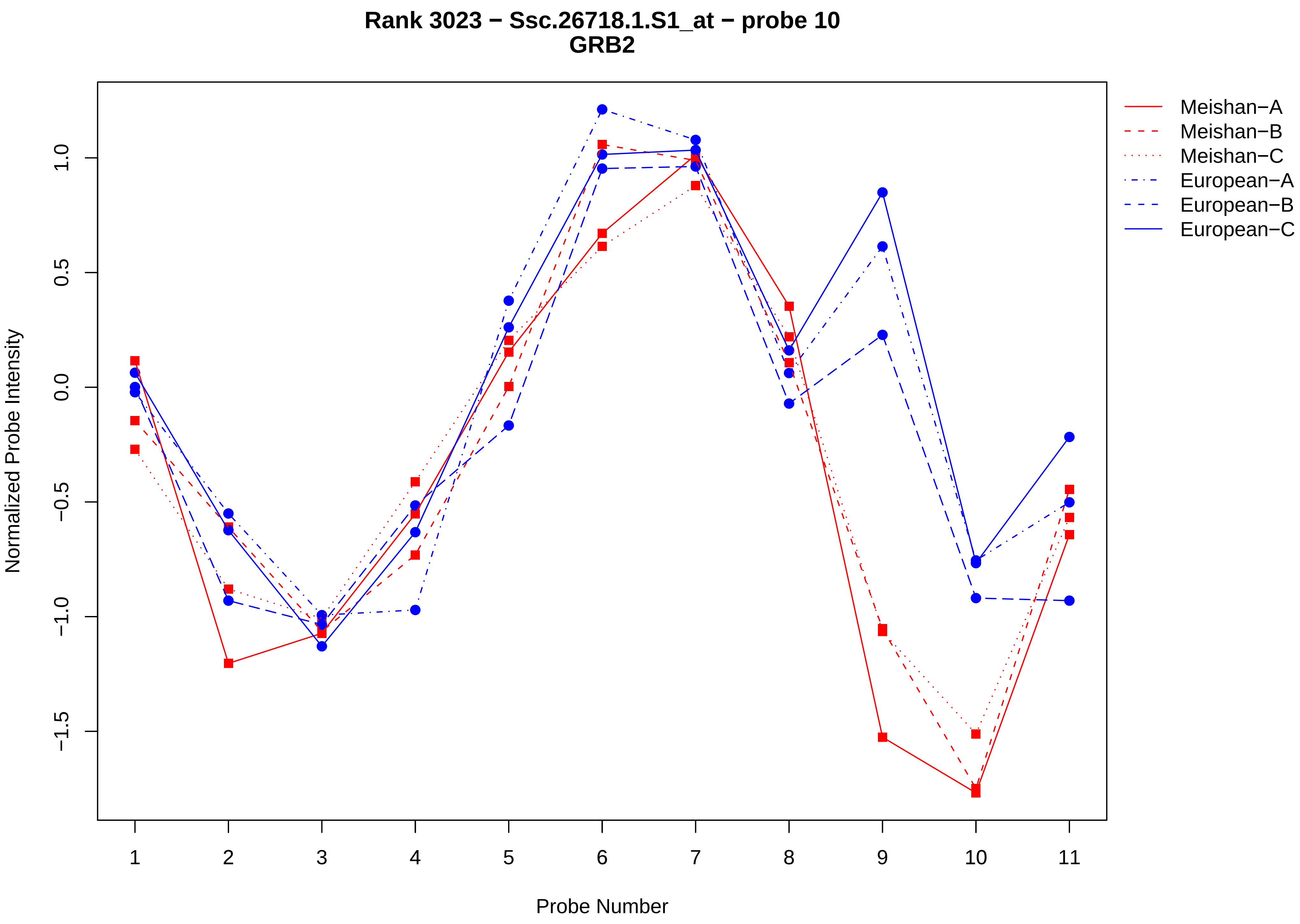
Rank 3023 − Ssc.26718.1.S1_at − probe 10
GRB2
Normalized Probe Intensity
Probe Number
1.0
0.5
0.0
−0.5
−1.0
−1.5
1
2
3
4
5
6
7
8
9
10
11
Meishan−A
Meishan−B
Meishan−C
European−A
European−B
European−C

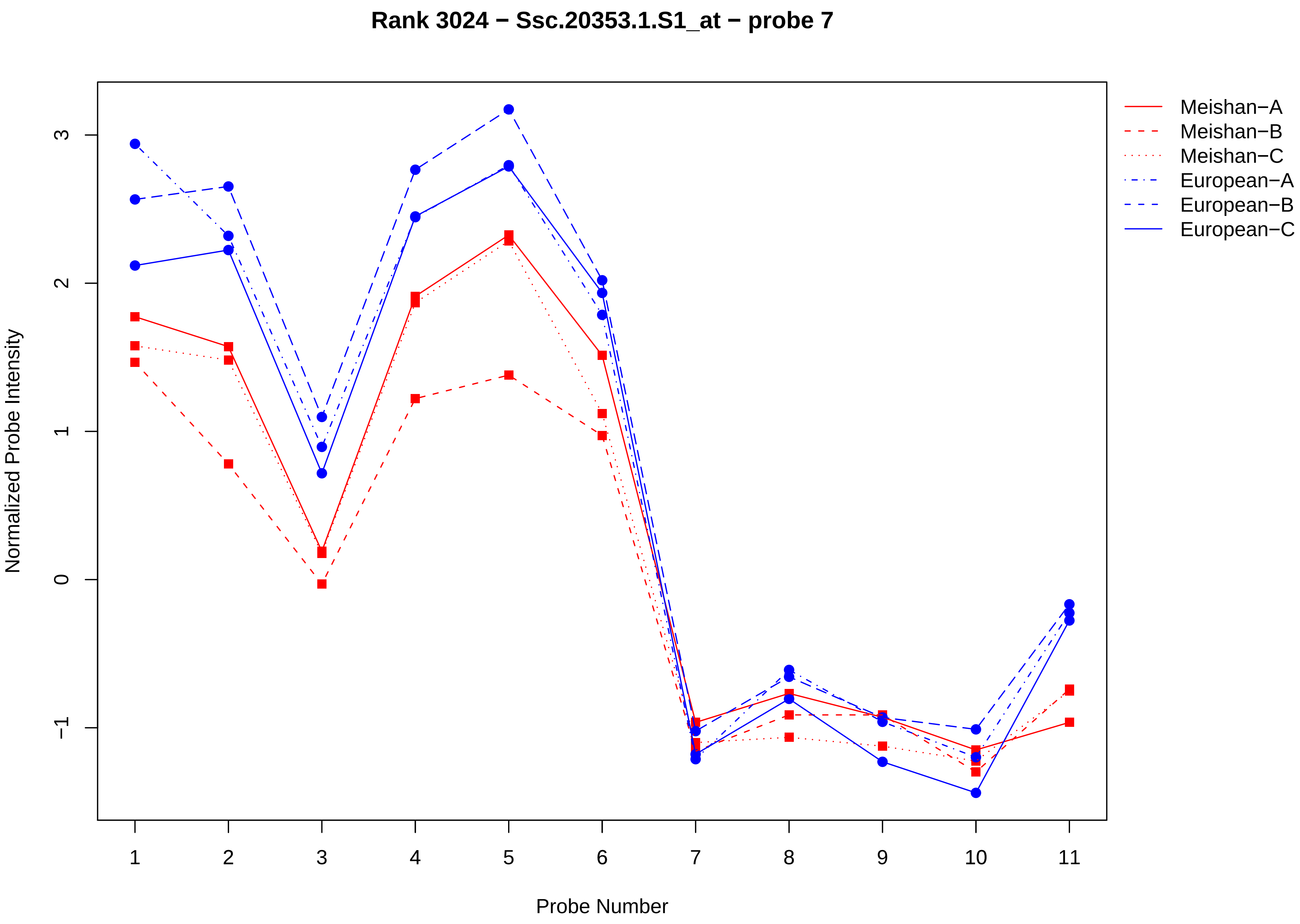

Rank 3024 − Ssc.20353.1.S1_at − probe 7
Normalized Probe Intensity
Probe Number
−1
0
1
2
3
1
2
3
4
5
6
7
8
9
10
11
Meishan−A
Meishan−B
Meishan−C
European−A
European−B
European−C

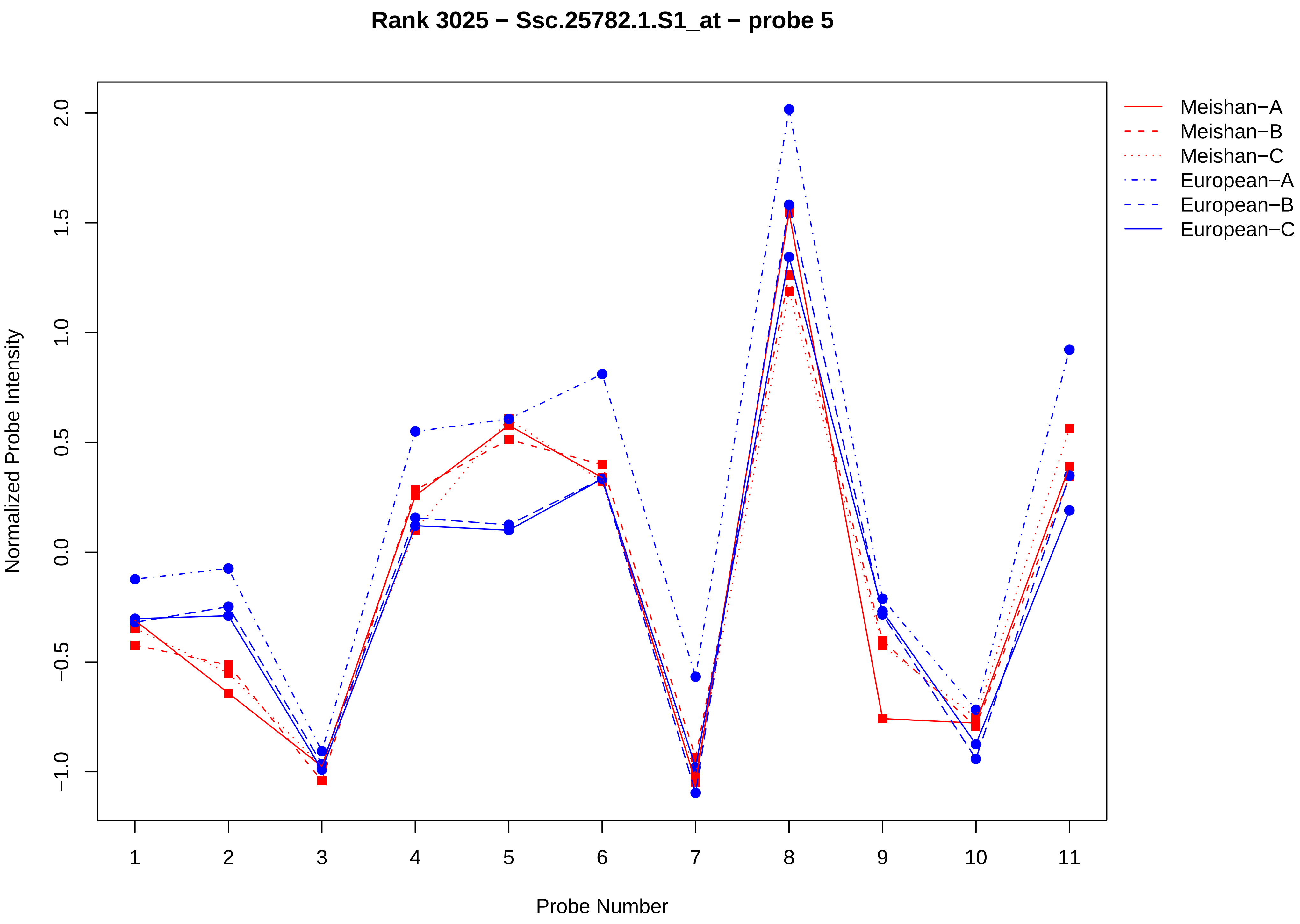

Rank 3025 − Ssc.25782.1.S1_at − probe 5
Normalized Probe Intensity
2.0
1.5
1.0
0.5
0.0
−0.5
−1.0
Probe Number
1
2
3
4
5
6
7
8
9
10
11
Meishan−A
Meishan−B
Meishan−C
European−A
European−B
European−C

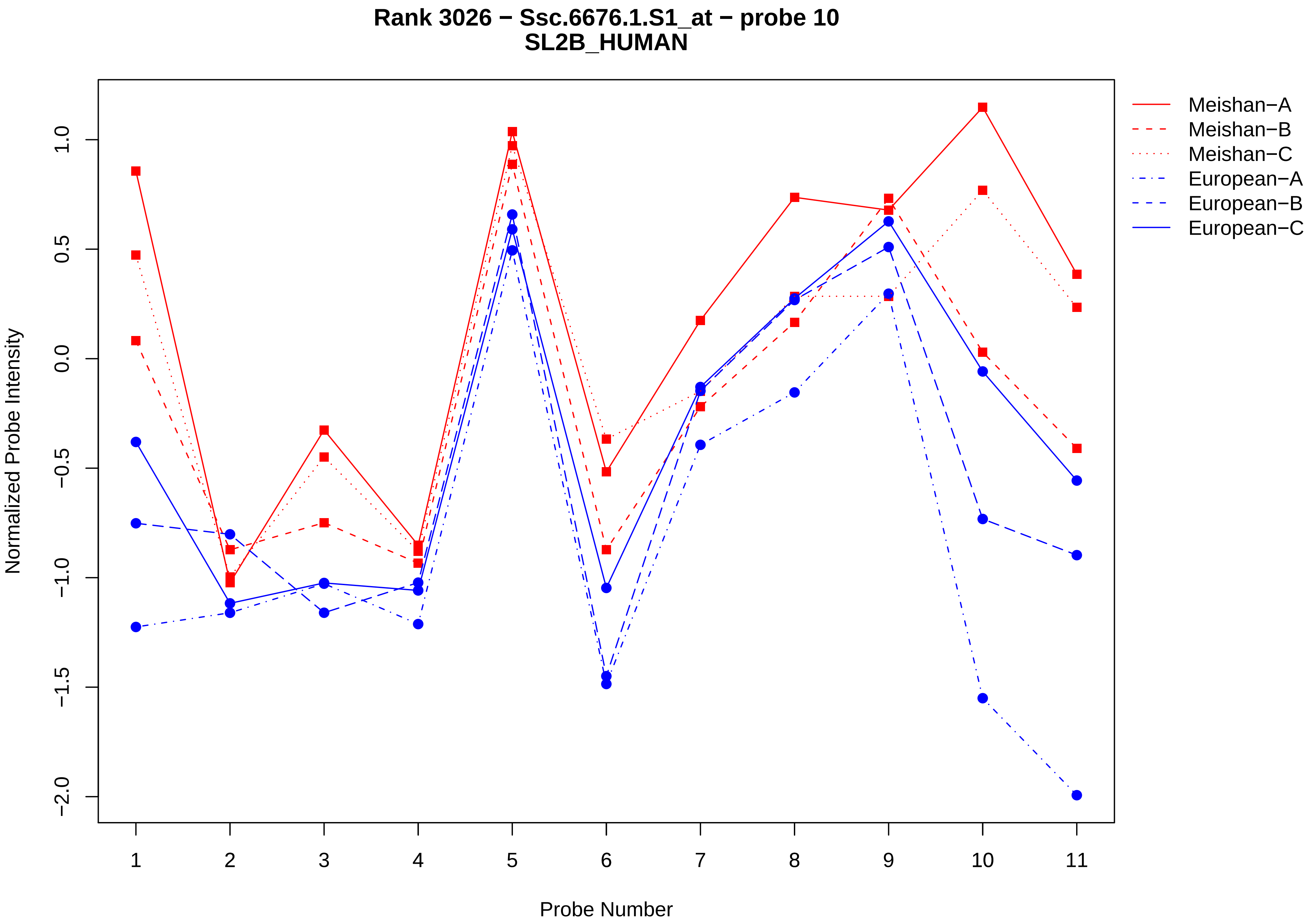

Rank 3026 − Ssc.6676.1.S1_at − probe 10
SL2B_HUMAN
Normalized Probe Intensity
Probe Number
1.0
0.5
0.0
−0.5
−1.0
−1.5
−2.0
1
2
3
4
5
6
7
8
9
10
11
Meishan−A
Meishan−B
Meishan−C
European−A
European−B
European−C

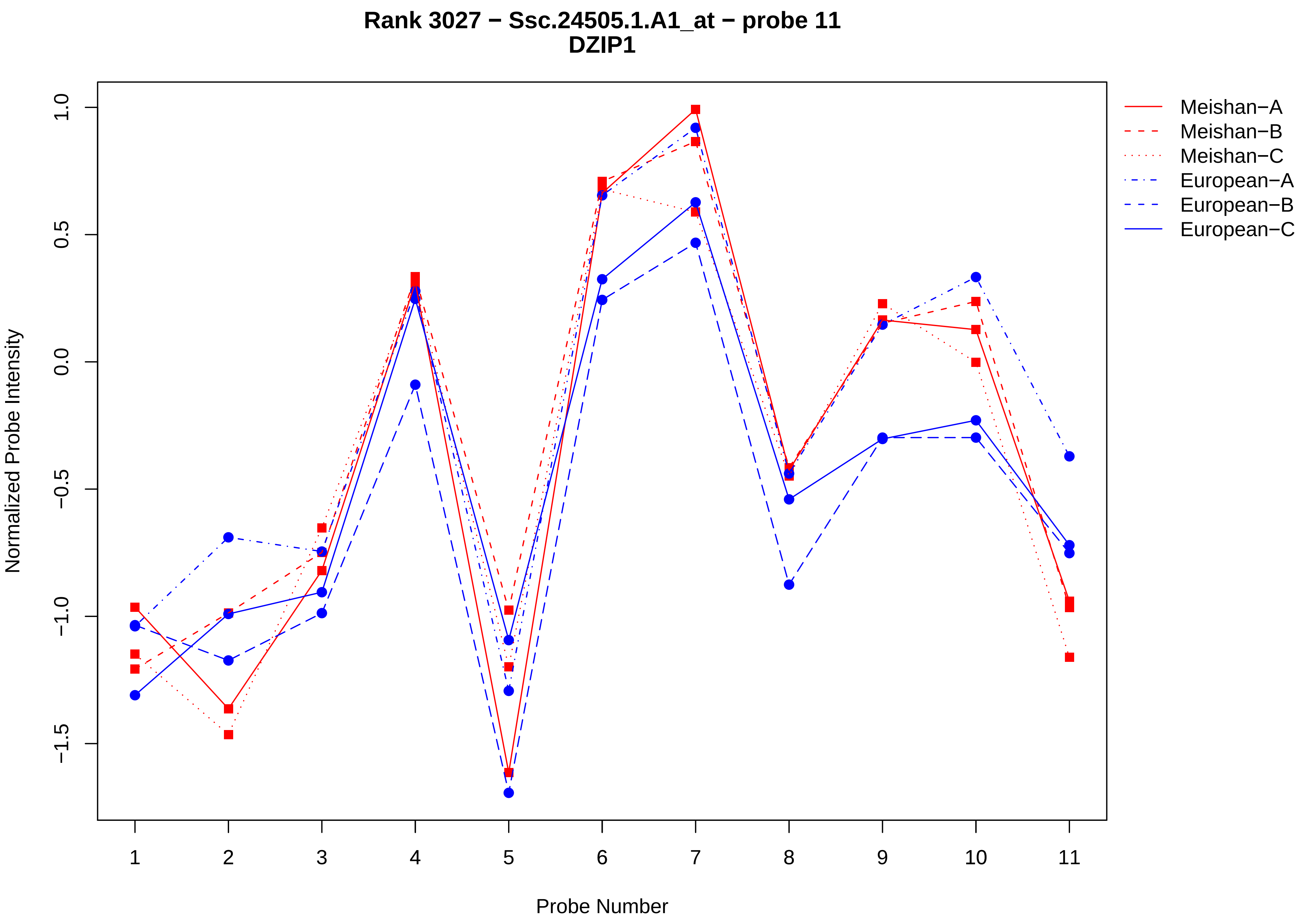
Rank 3027 − Ssc.24505.1.A1_at − probe 11
DZIP1
Normalized Probe Intensity
Probe Number
1.0
0.5
0.0
−0.5
−1.0
−1.5
1
2
3
4
5
6
7
8
9
10
11
Meishan−A
Meishan−B
Meishan−C
European−A
European−B
European−C

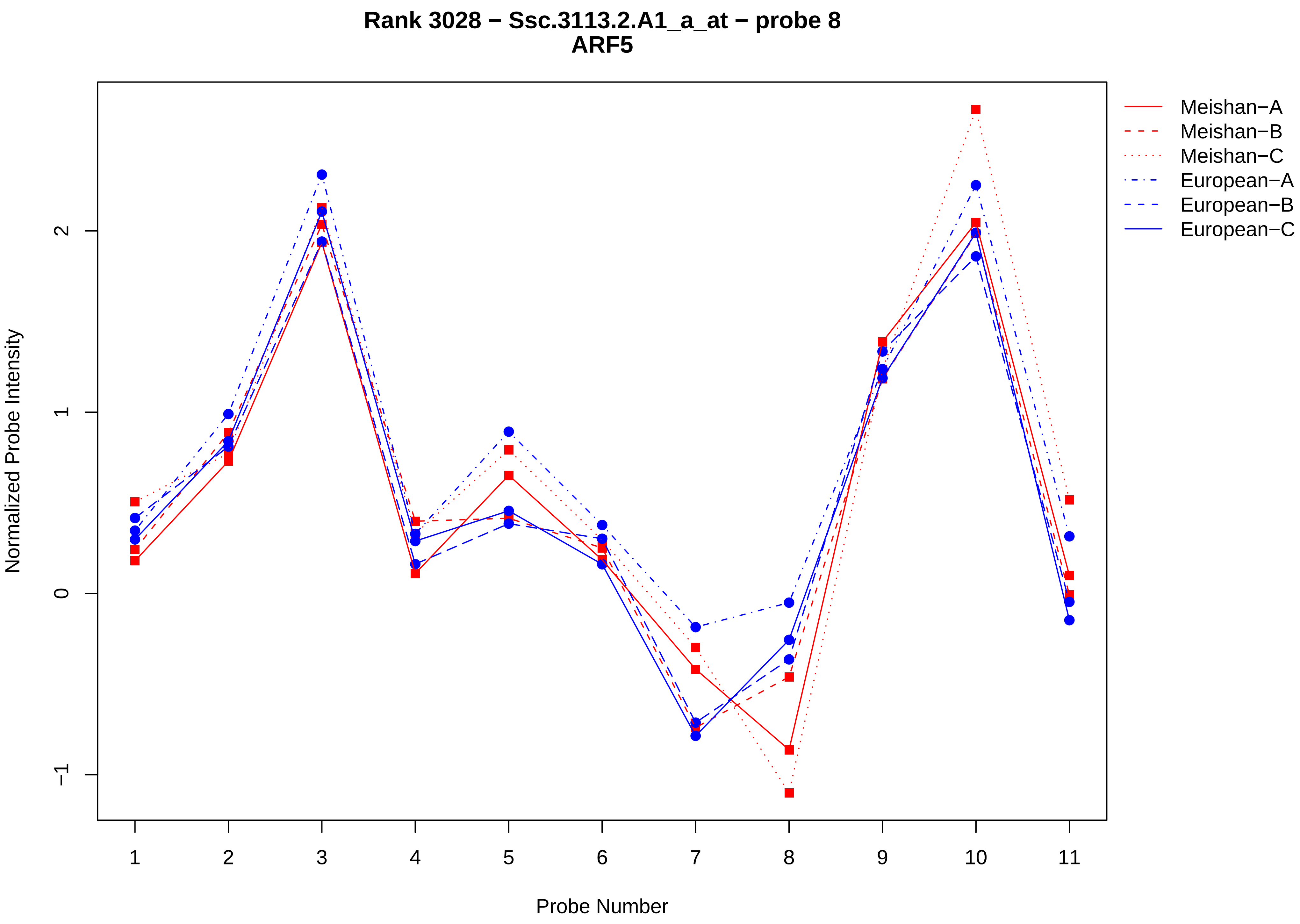

Rank 3028 − Ssc.3113.2.A1_a_at − probe 8
ARF5
Normalized Probe Intensity
Probe Number
−1
0
1
2
1
2
3
4
5
6
7
8
9
10
11
Meishan−A
Meishan−B
Meishan−C
European−A
European−B
European−C

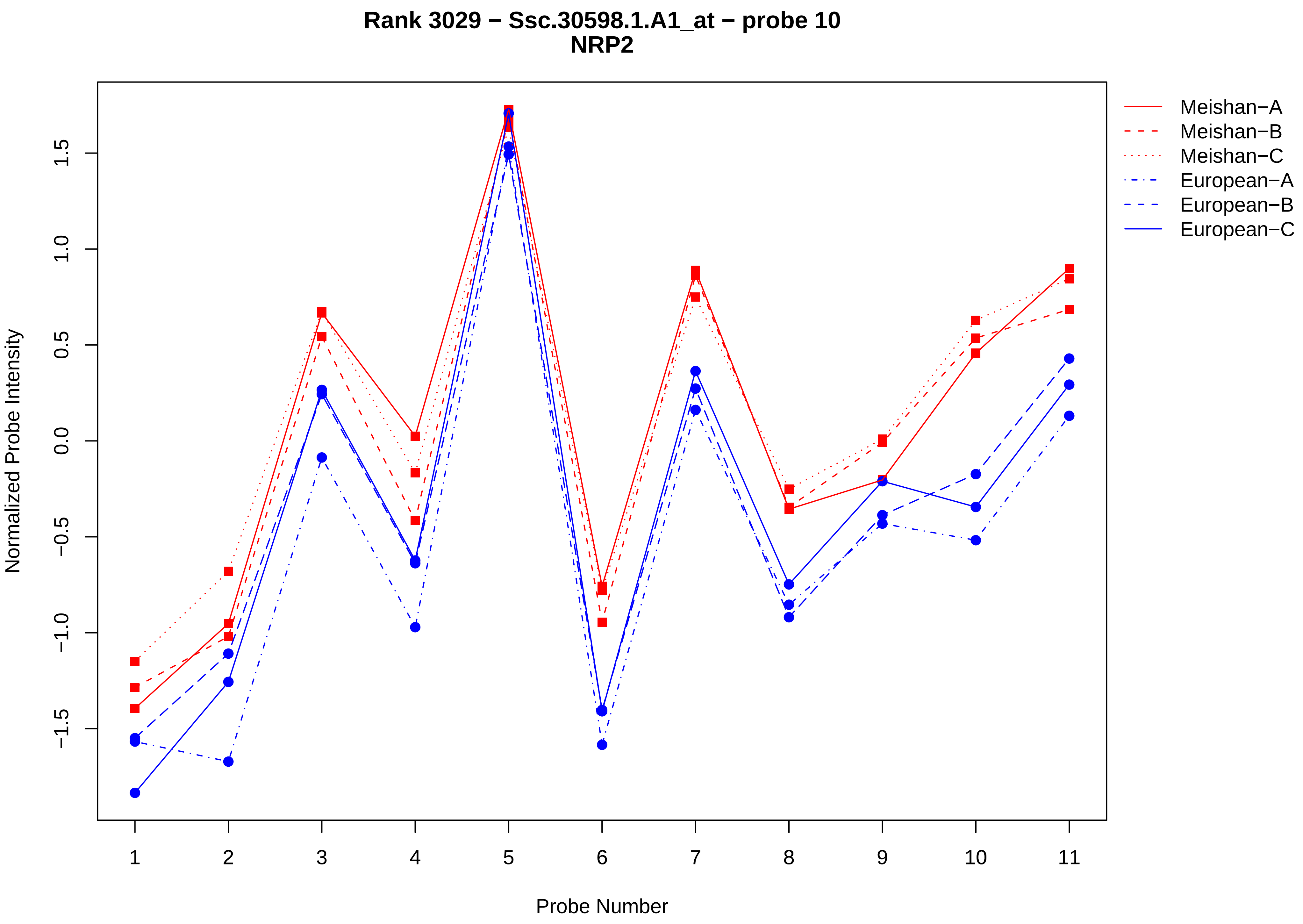

Rank 3029 − Ssc.30598.1.A1_at − probe 10
NRP2
Normalized Probe Intensity
Probe Number
Meishan−A
Meishan−B
Meishan−C
European−A
European−B
European−C
1.5
1.0
0.5
0.0
−0.5
−1.0
−1.5
1
2
3
4
5
6
7
8
9
10
11

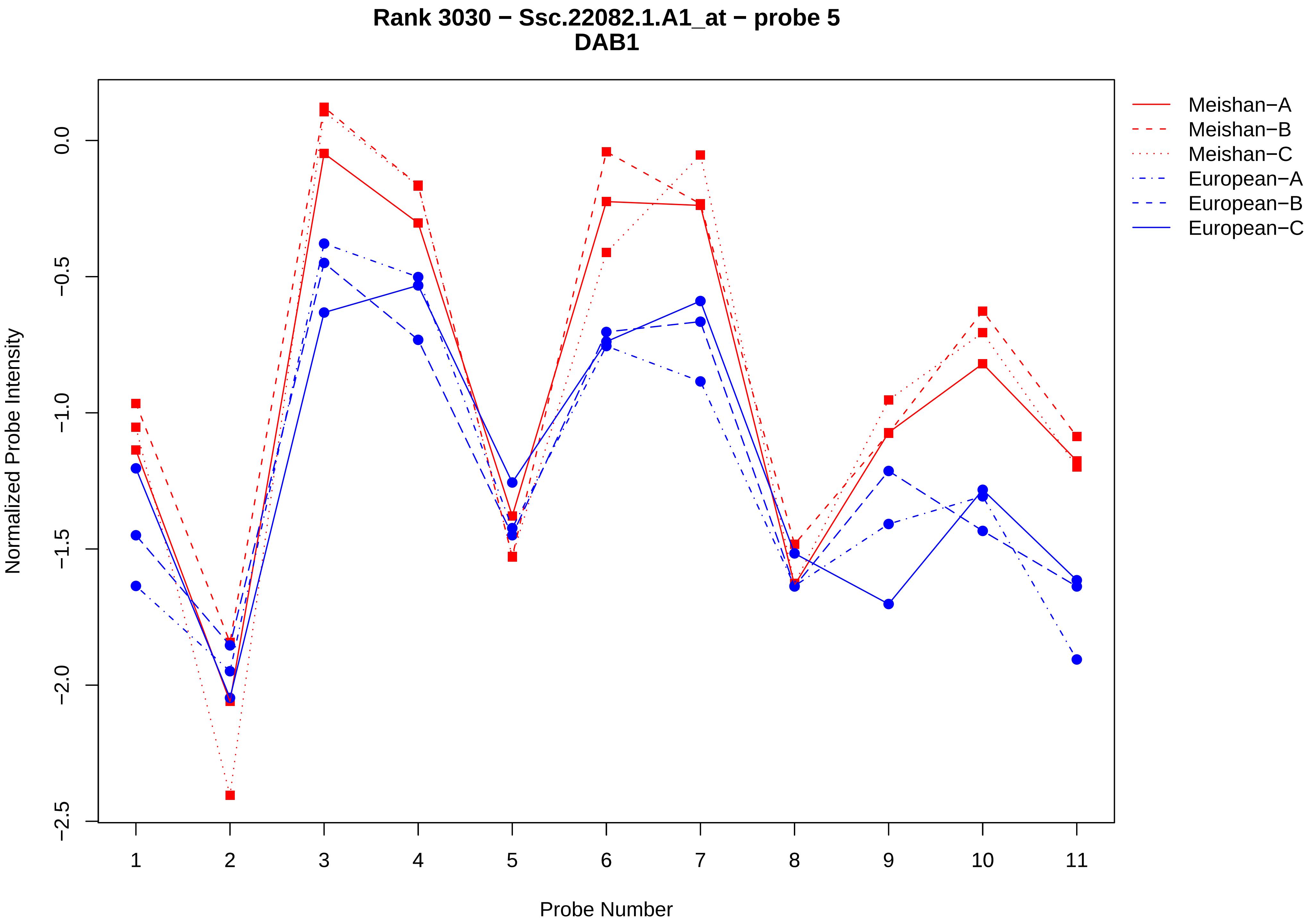

Rank 3030 − Ssc.22082.1.A1_at − probe 5
DAB1
Normalized Probe Intensity
Probe Number
0.0
−0.5
−1.0
−1.5
−2.0
−2.5
1
2
3
4
5
6
7
8
9
10
11
Meishan−A
Meishan−B
Meishan−C
European−A
European−B
European−C

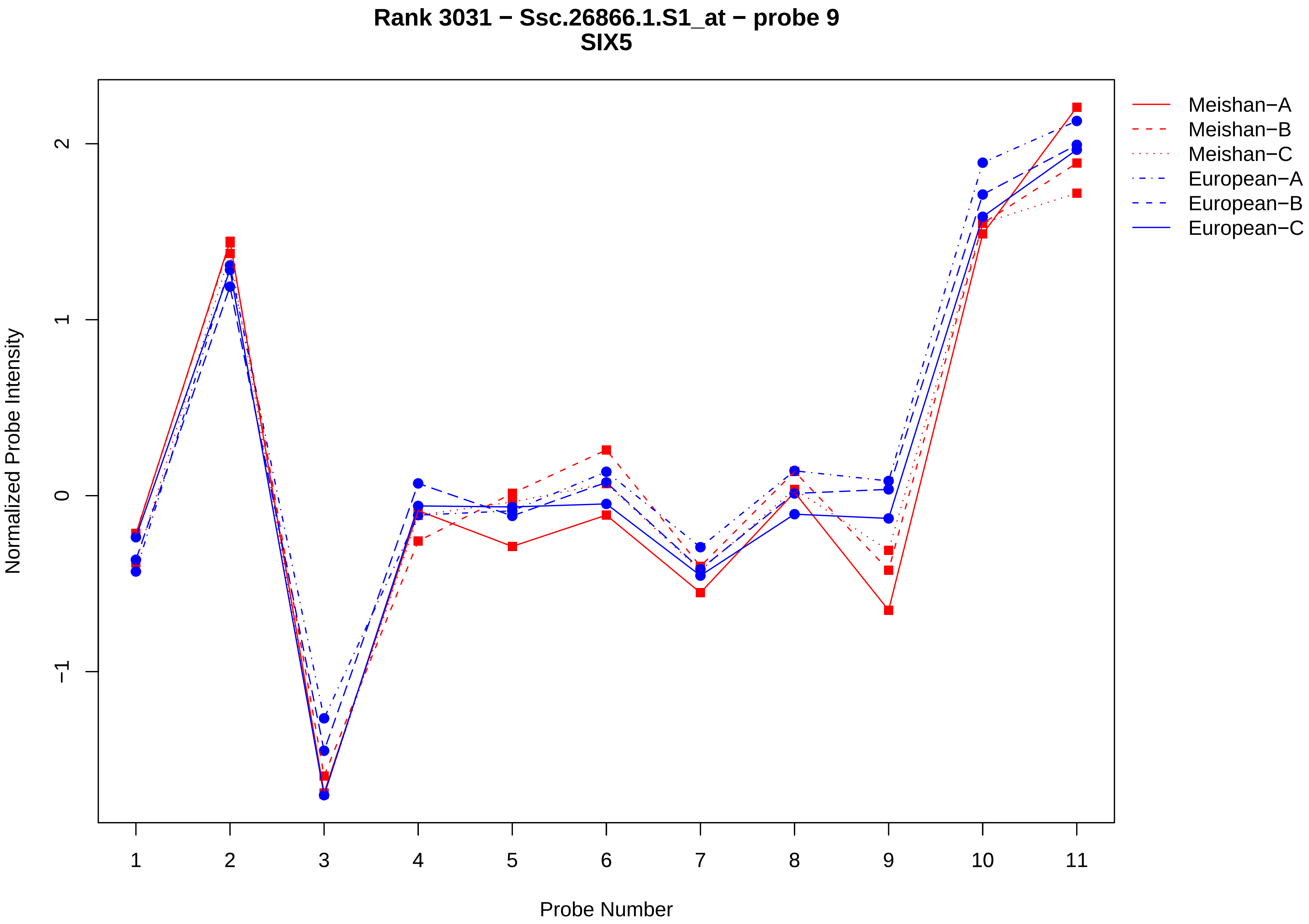
Rank 3031 − Ssc.26866.1.S1_at − probe 9
SIX5
Normalized Probe Intensity
Probe Number
Meishan−A
Meishan−B
Meishan−C
European−A
European−B
European−C
−1
0
1
2
1
2
3
4
5
6
7
8
9
10
11

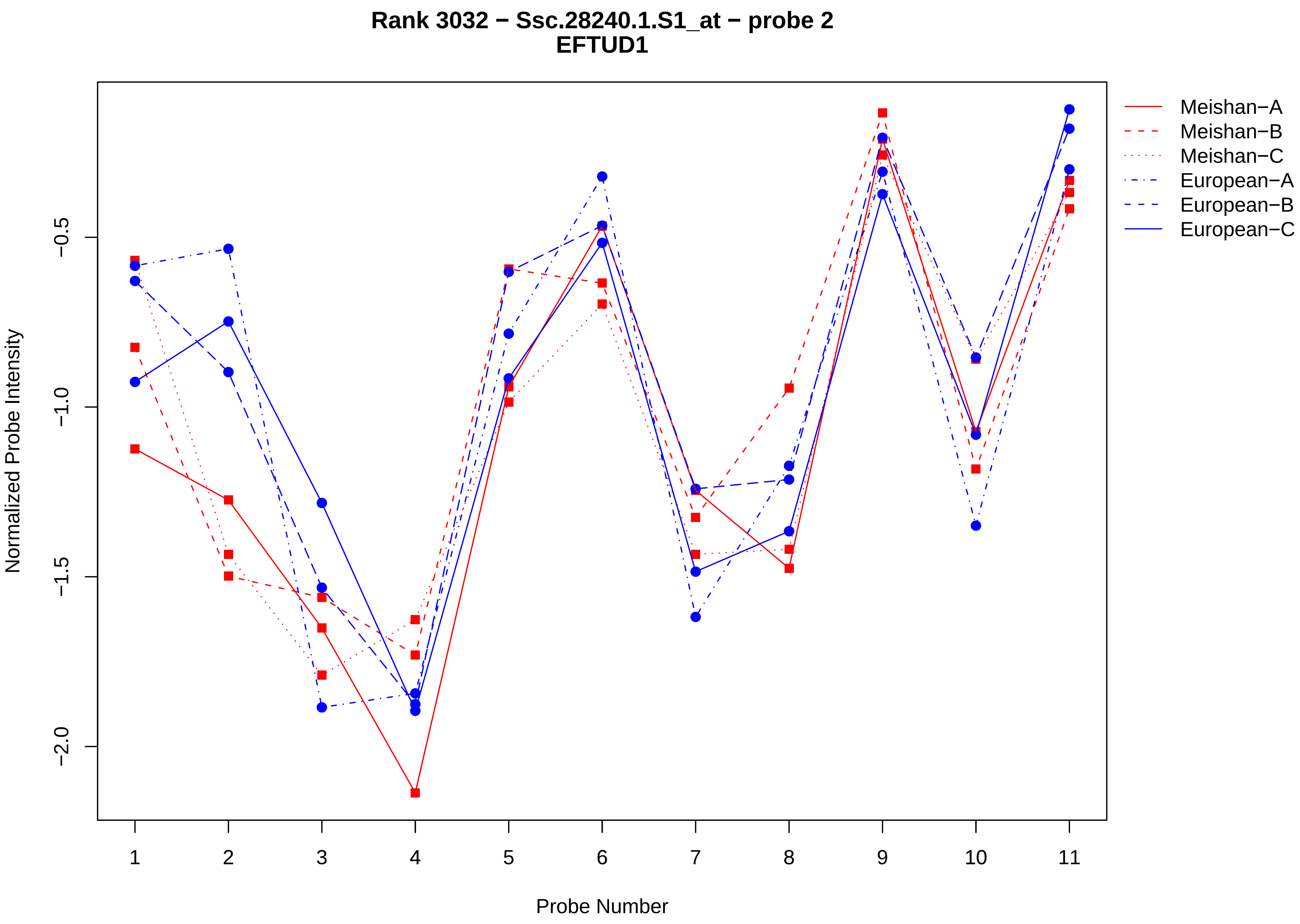

Rank 3032 − Ssc.28240.1.S1_at − probe 2
EFTUD1
Normalized Probe Intensity
−0.5
−1.0
−1.5
−2.0
1
2
3
4
5
6
7
8
9
10
11
Probe Number
Meishan−A
Meishan−B
Meishan−C
European−A
European−B
European−C

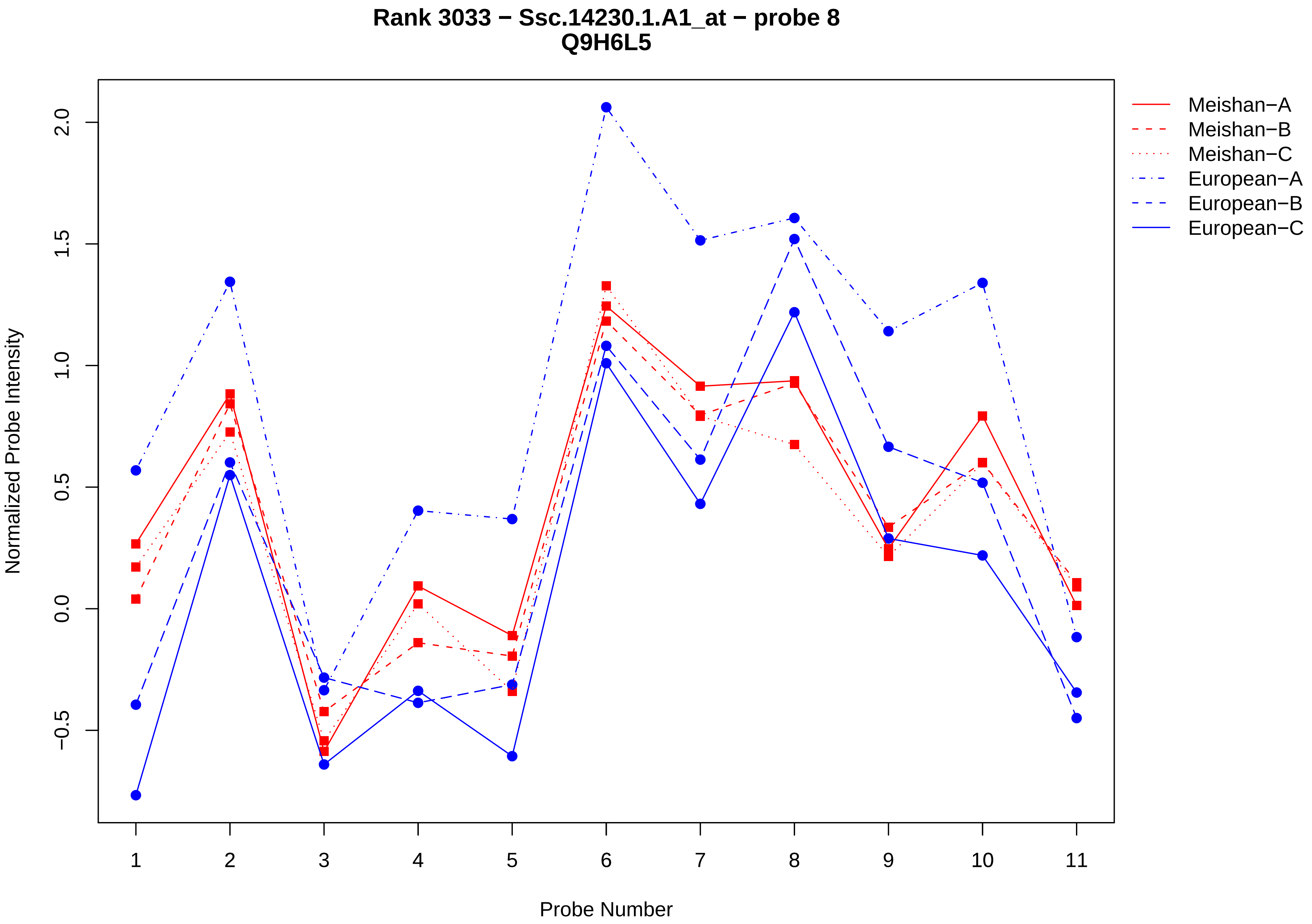

Rank 3033 − Ssc.14230.1.A1_at − probe 8
Q9H6L5
Normalized Probe Intensity
Probe Number
Meishan−A
Meishan−B
Meishan−C
European−A
European−B
European−C
−0.5
0.0
0.5
1.0
1.5
2.0
1
2
3
4
5
6
7
8
9
10
11

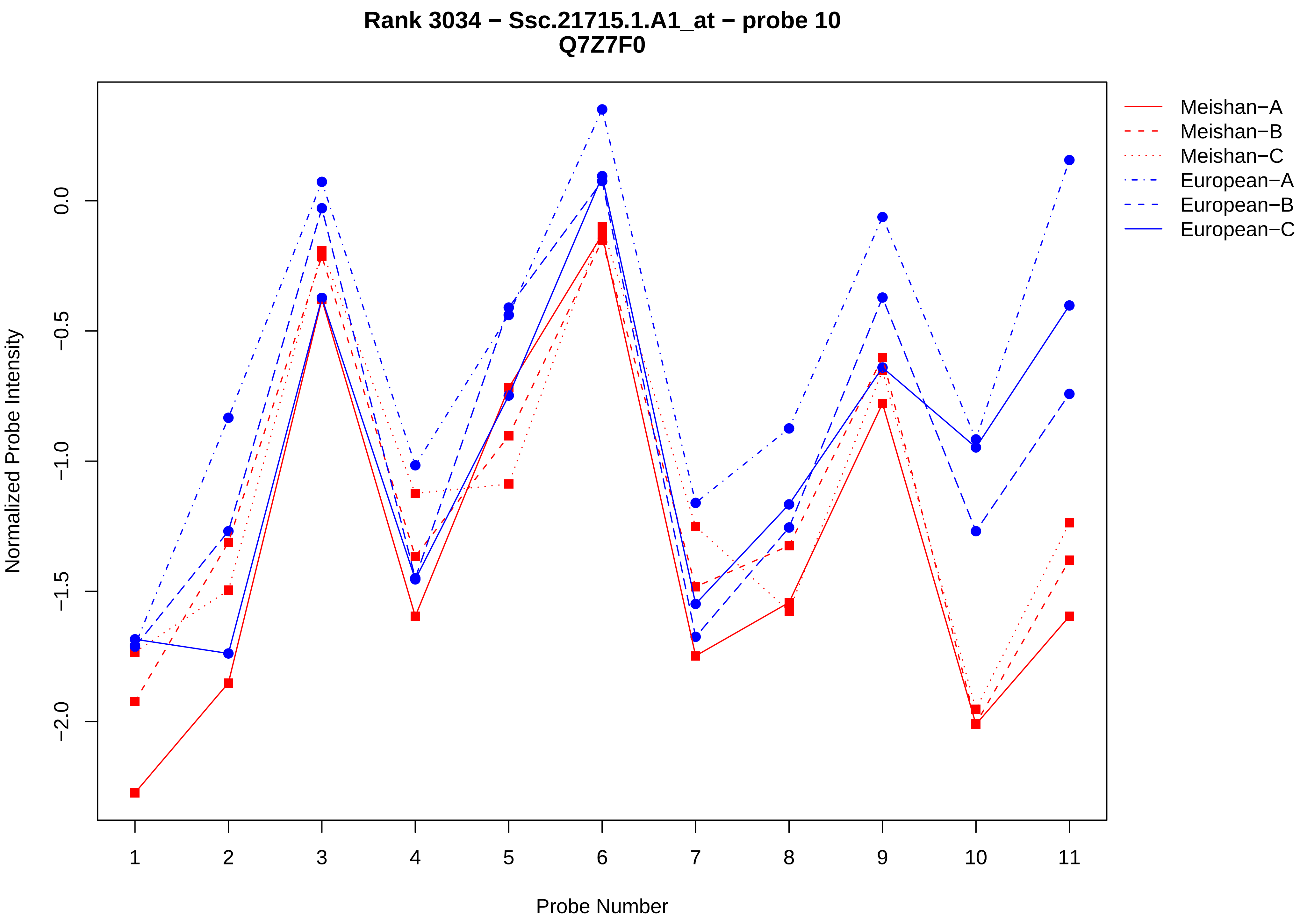

Rank 3034 − Ssc.21715.1.A1_at − probe 10
Q7Z7F0
Normalized Probe Intensity
0.0
−0.5
−1.0
−1.5
−2.0
1
2
3
4
5
6
7
8
9
10
11
Probe Number
Meishan−A
Meishan−B
Meishan−C
European−A
European−B
European−C

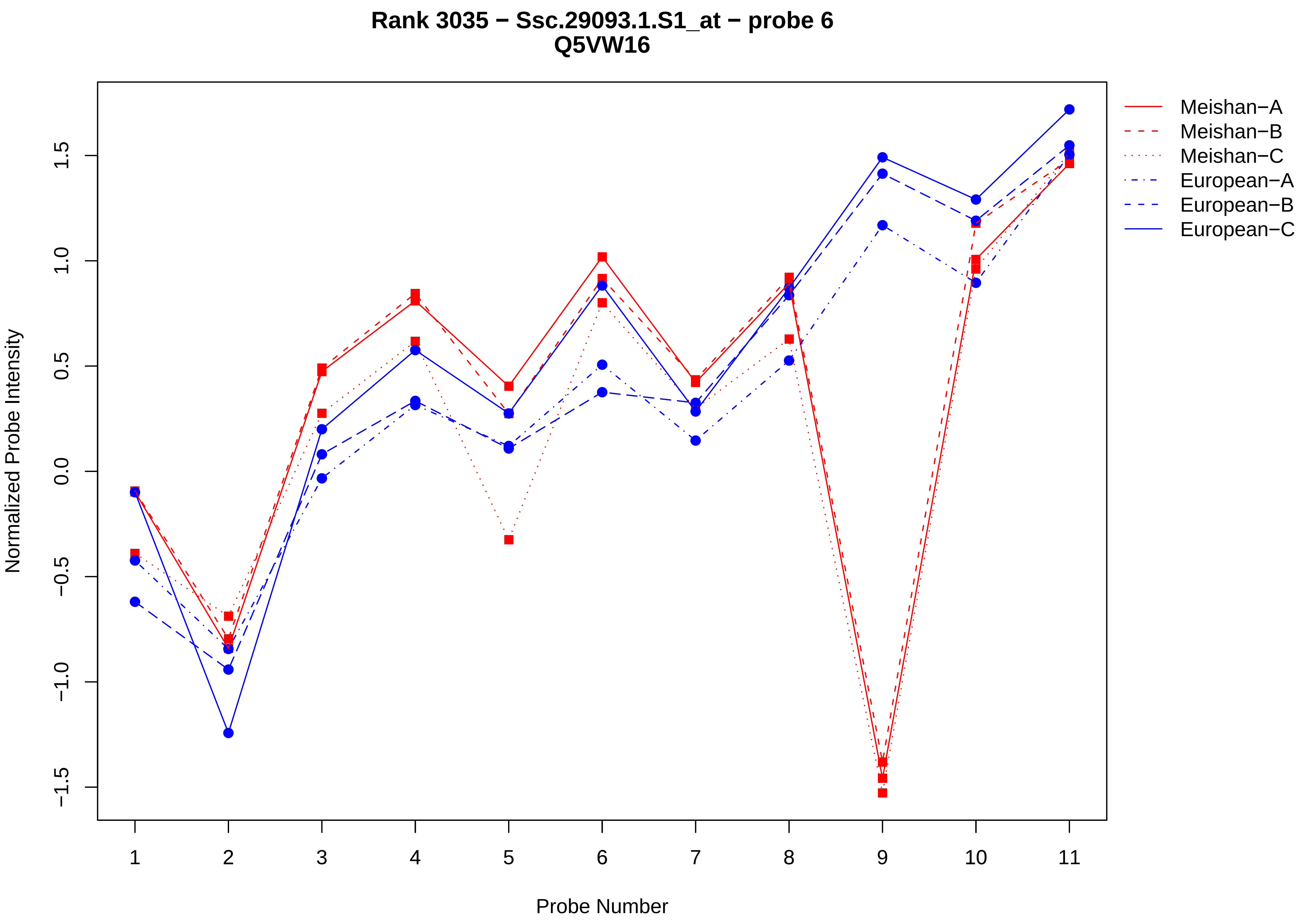

Rank 3035 − Ssc.29093.1.S1_at − probe 6
Q5VW16
Normalized Probe Intensity
Probe Number
Meishan−A
Meishan−B
Meishan−C
European−A
European−B
European−C

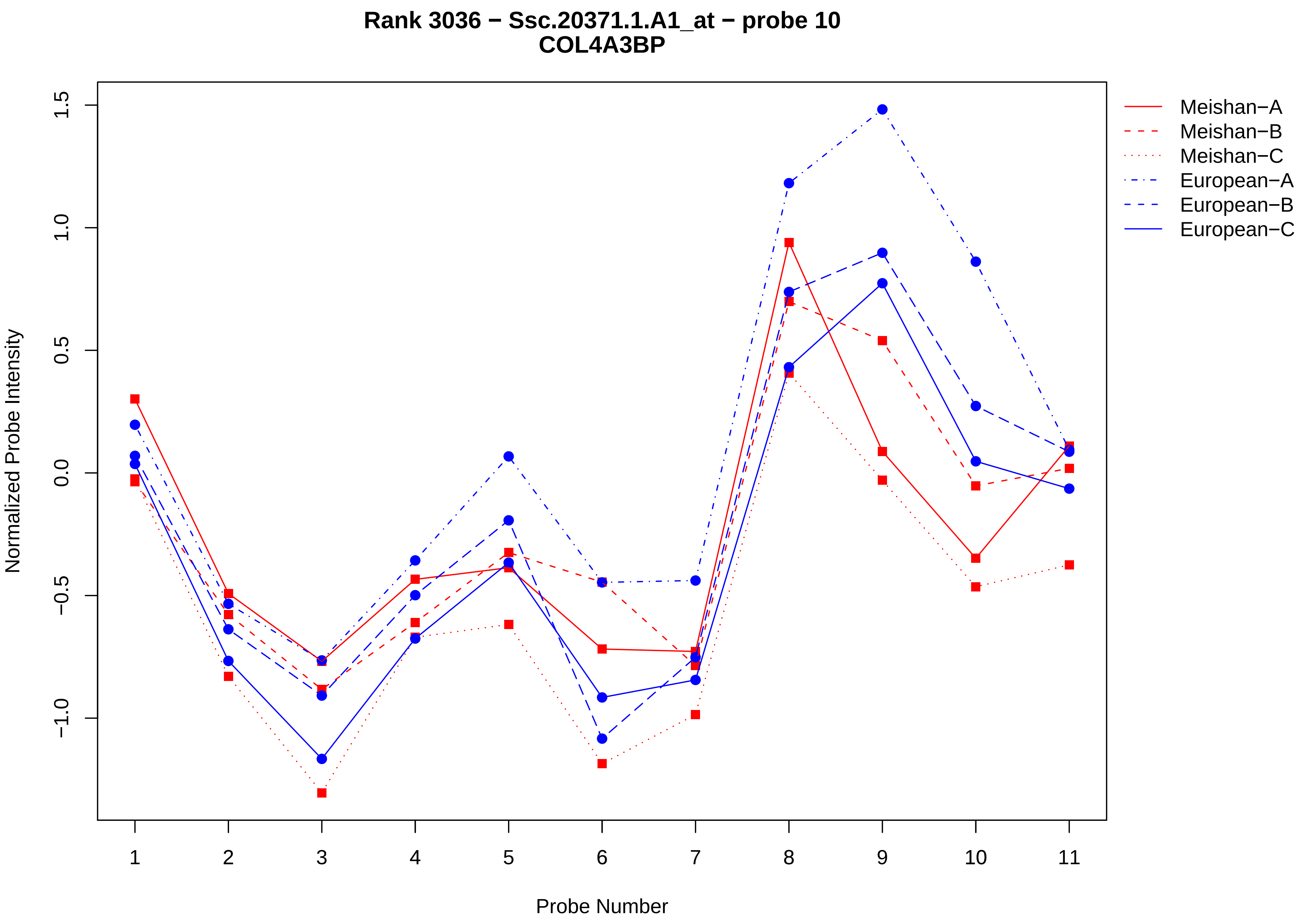
Rank 3036 − Ssc.20371.1.A1_at − probe 10
COL4A3BP
Normalized Probe Intensity
Probe Number
1.5
1.0
0.5
0.0
−0.5
−1.0
1
2
3
4
5
6
7
8
9
10
11
Meishan−A
Meishan−B
Meishan−C
European−A
European−B
European−C

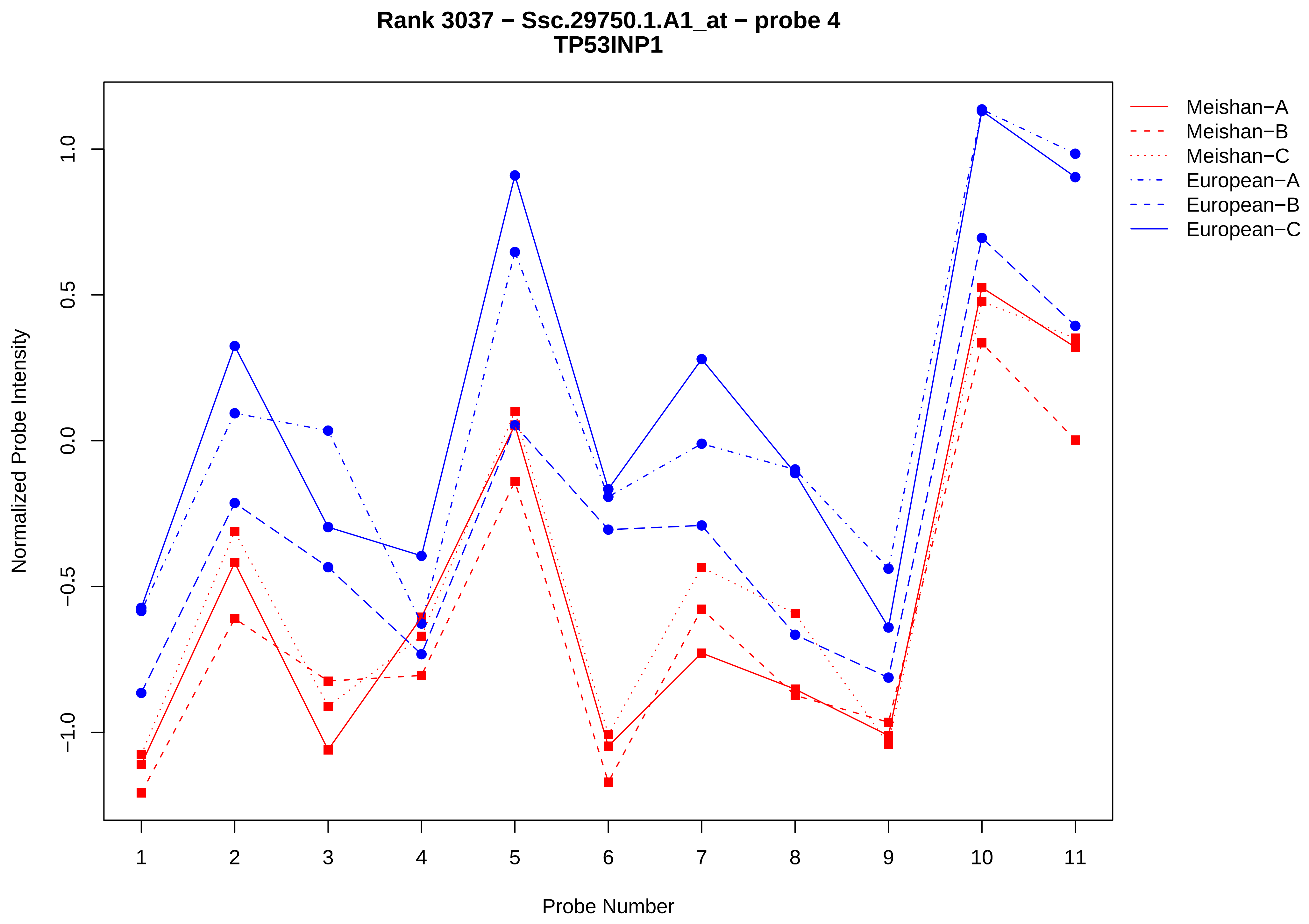

Rank 3037 − Ssc.29750.1.A1_at − probe 4
TP53INP1
Normalized Probe Intensity
Probe Number
1.0
0.5
0.0
−0.5
−1.0
1
2
3
4
5
6
7
8
9
10
11
Meishan−A
Meishan−B
Meishan−C
European−A
European−B
European−C

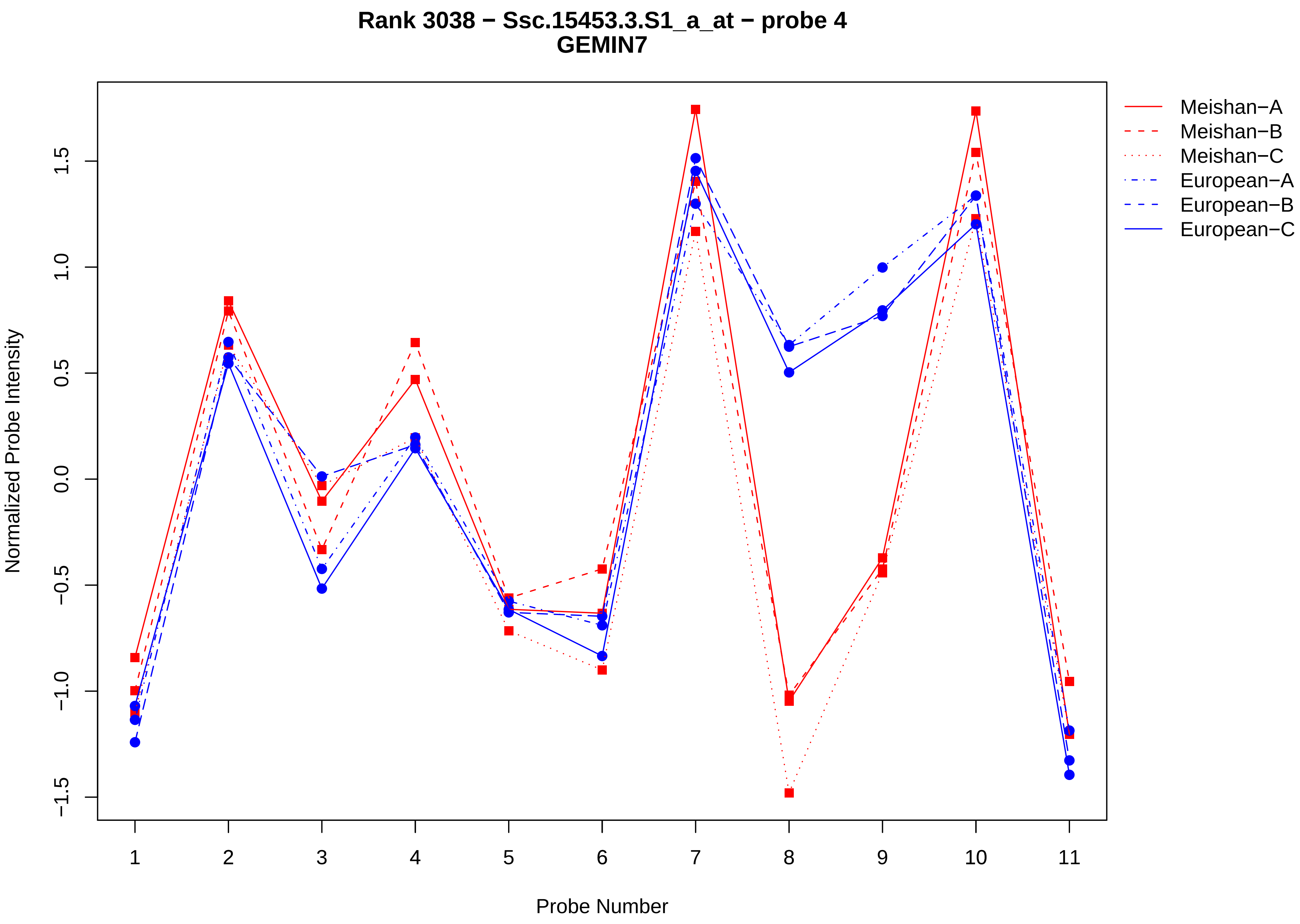

Rank 3038 − Ssc.15453.3.S1_a_at − probe 4
GEMIN7
Normalized Probe Intensity
Probe Number
−1.5
−1.0
−0.5
0.0
0.5
1.0
1.5
1
2
3
4
5
6
7
8
9
10
11
Meishan−A
Meishan−B
Meishan−C
European−A
European−B
European−C

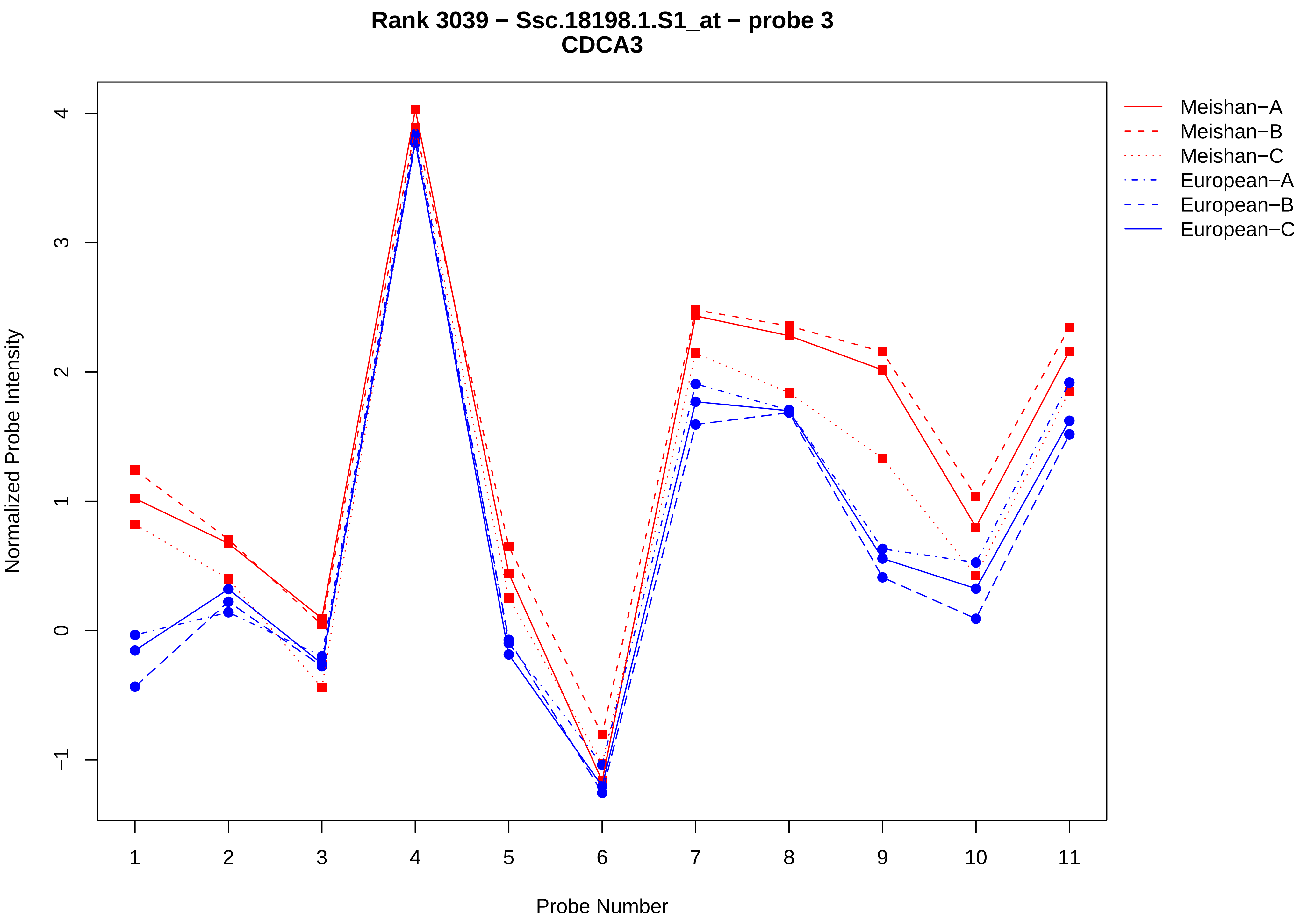

Rank 3039 − Ssc.18198.1.S1_at − probe 3
CDCA3
Normalized Probe Intensity
Probe Number
−1
0
1
2
3
4
1
2
3
4
5
6
7
8
9
10
11
Meishan−A
Meishan−B
Meishan−C
European−A
European−B
European−C

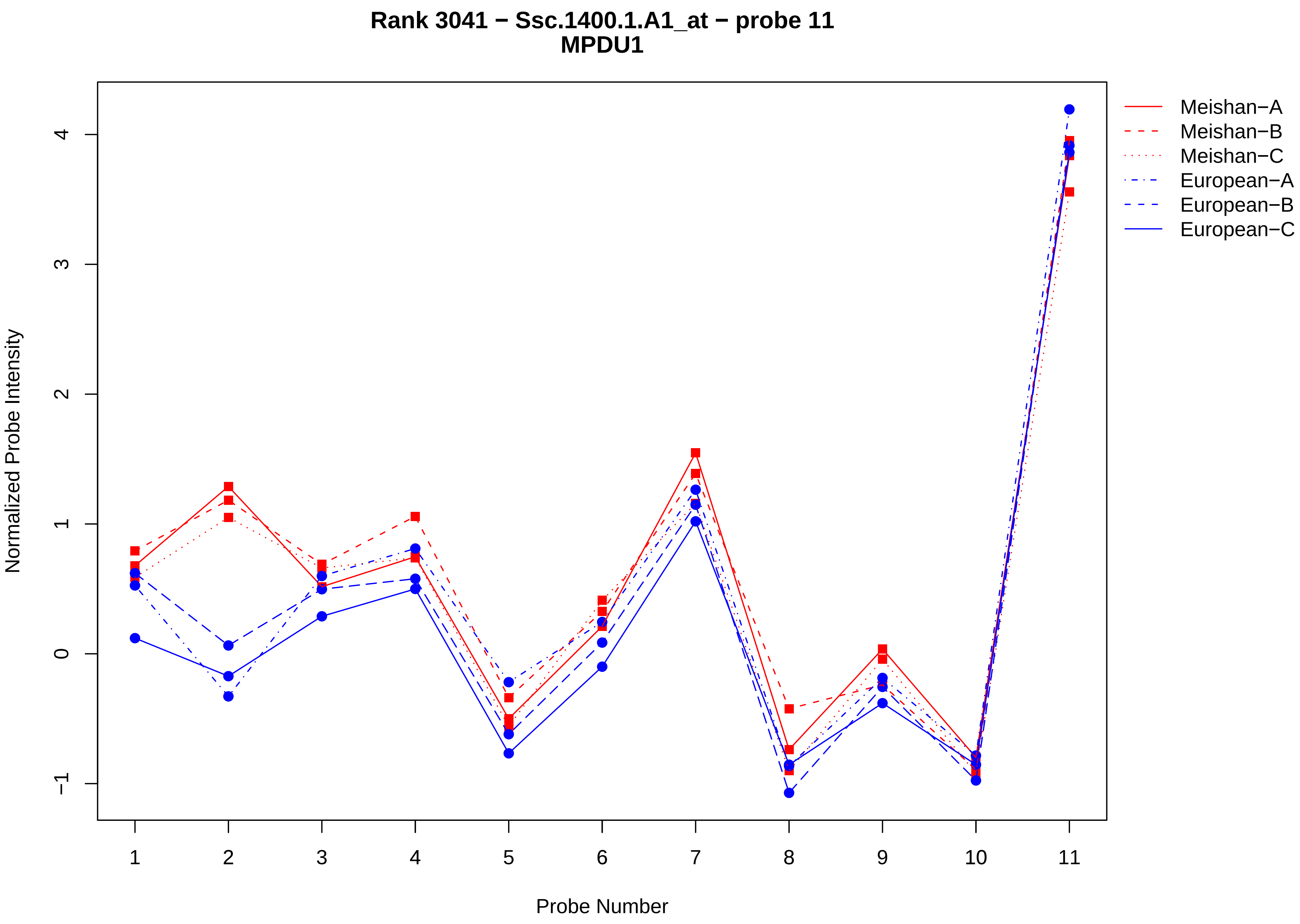

Rank 3041 − Ssc.1400.1.A1_at − probe 11
MPDU1
Normalized Probe Intensity
Probe Number
−1
0
1
2
3
4
1
2
3
4
5
6
7
8
9
10
11
Meishan−A
Meishan−B
Meishan−C
European−A
European−B
European−C

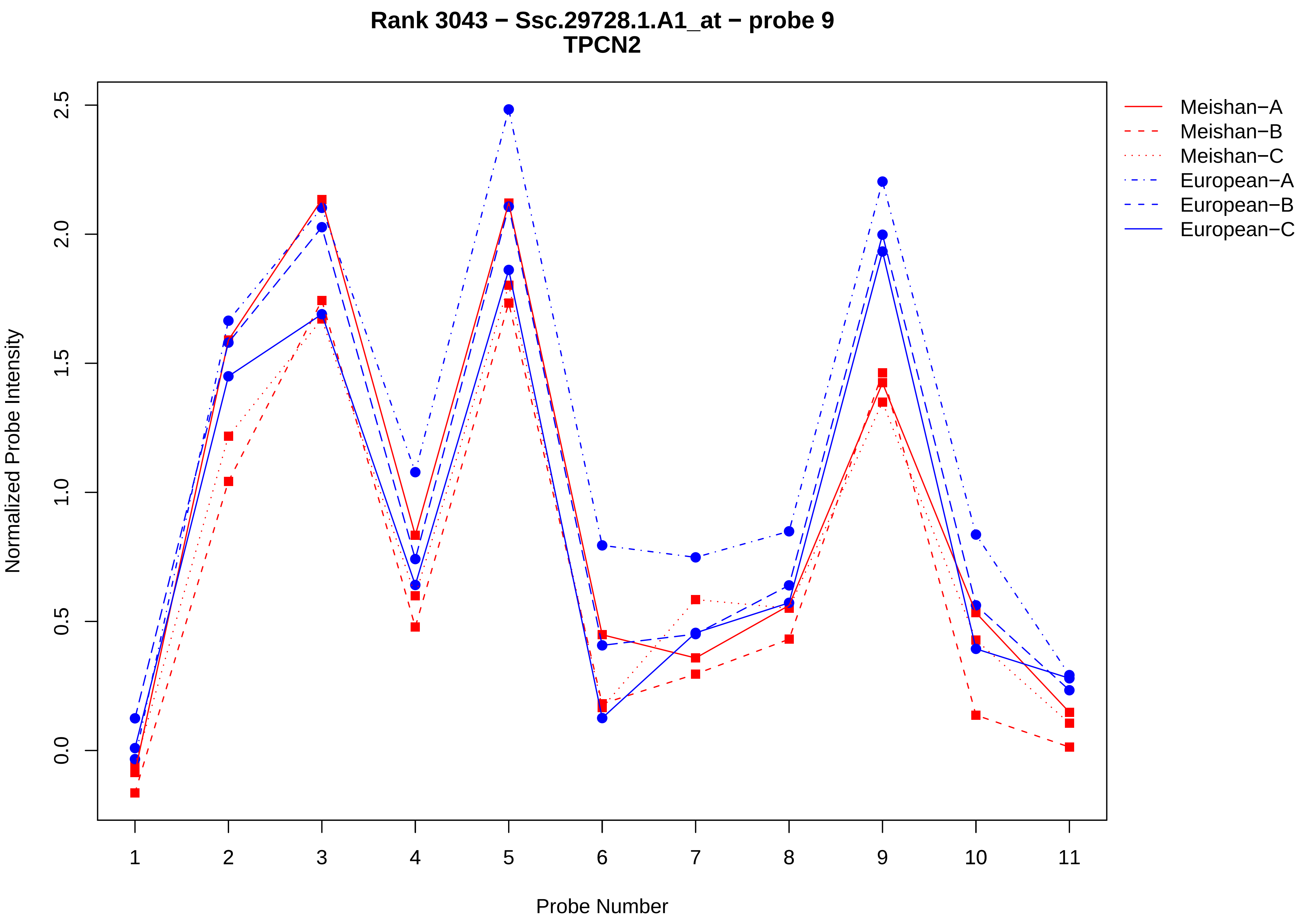

Rank 3043 − Ssc.29728.1.A1_at − probe 9
TPCN2
Normalized Probe Intensity
Probe Number
0.0
0.5
1.0
1.5
2.0
2.5
1
2
3
4
5
6
7
8
9
10
11
Meishan−A
Meishan−B
Meishan−C
European−A
European−B
European−C

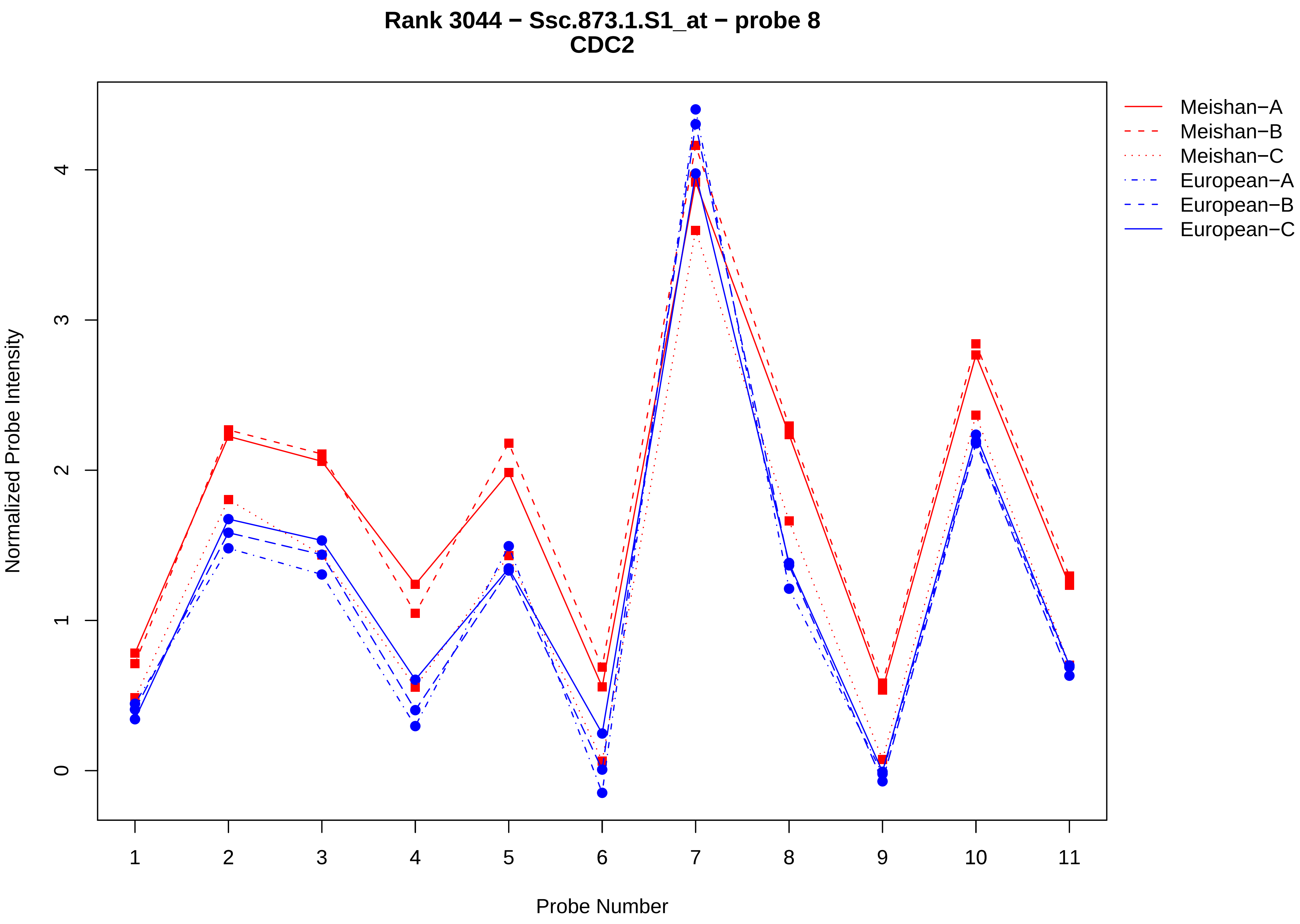

Rank 3044 − Ssc.873.1.S1_at − probe 8
CDC2
Normalized Probe Intensity
Probe Number
0
1
2
3
4
1
2
3
4
5
6
7
8
9
10
11
Meishan−A
Meishan−B
Meishan−C
European−A
European−B
European−C

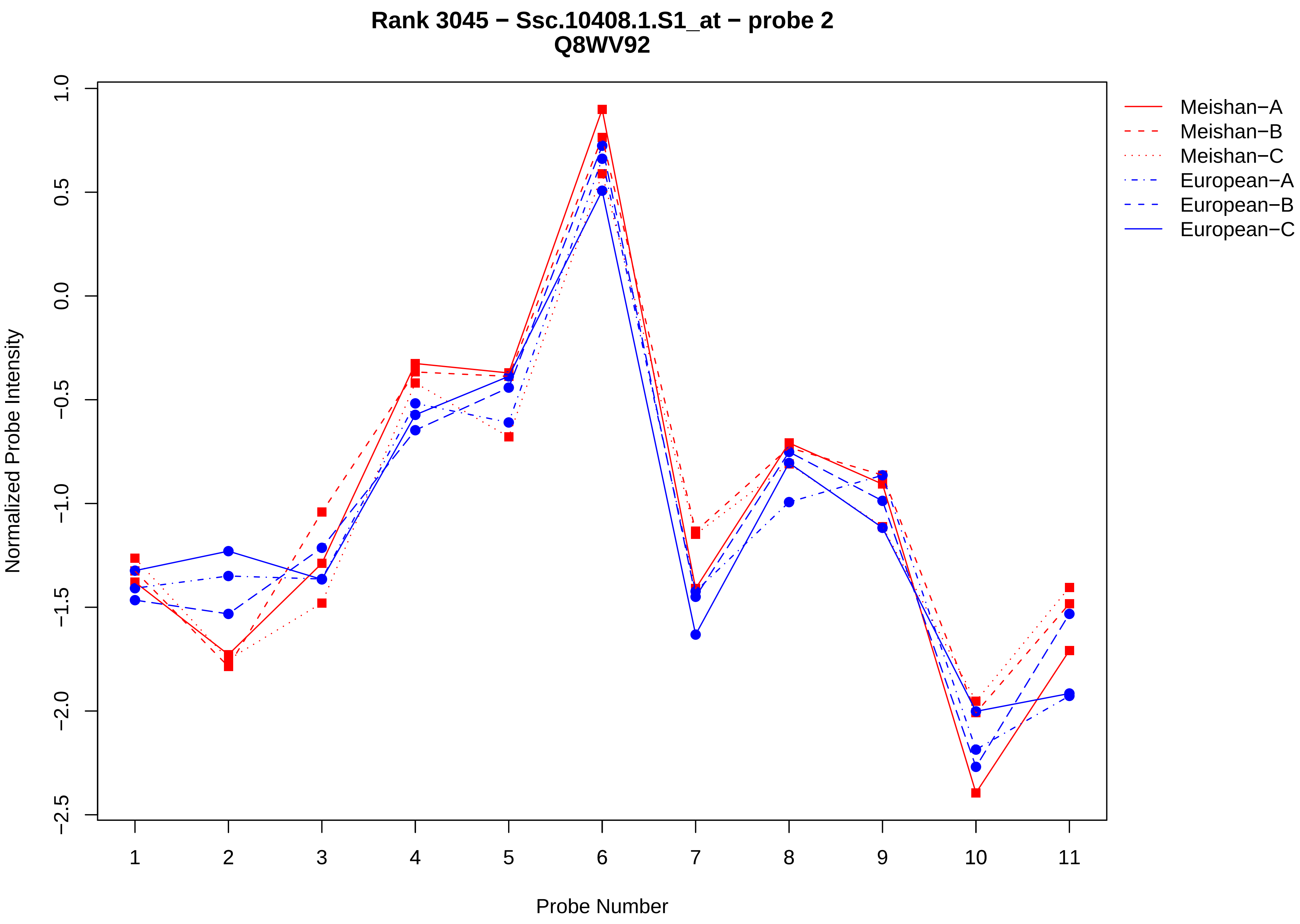

Rank 3045 − Ssc.10408.1.S1_at − probe 2
Q8WV92
Normalized Probe Intensity
Probe Number
1.0
0.5
0.0
−0.5
−1.0
−1.5
−2.0
−2.5
1
2
3
4
5
6
7
8
9
10
11
Meishan−A
Meishan−B
Meishan−C
European−A
European−B
European−C

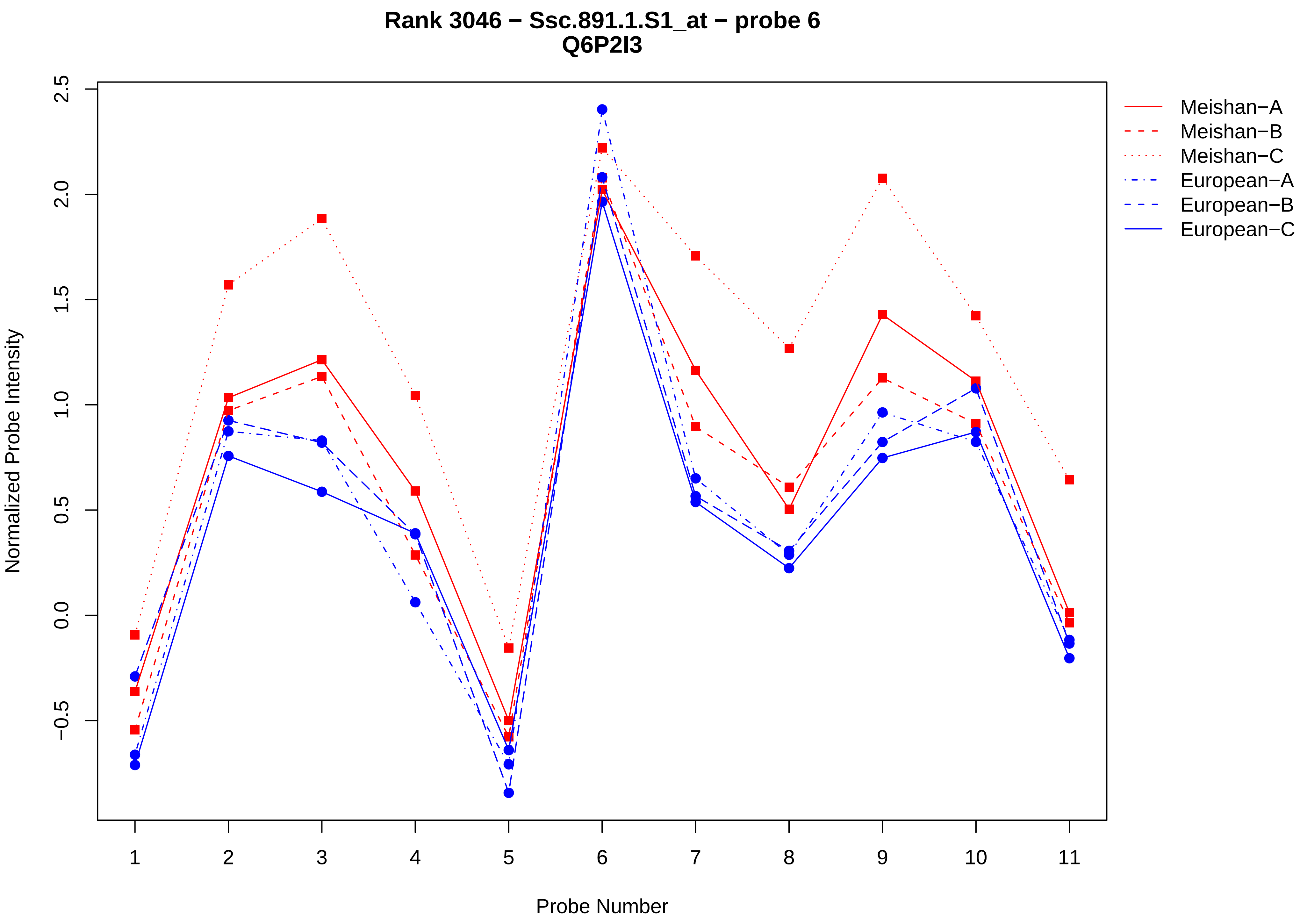

Rank 3046 − Ssc.891.1.S1_at − probe 6
Q6P2I3
Normalized Probe Intensity
Probe Number
−0.5
0.0
0.5
1.0
1.5
2.0
2.5
1
2
3
4
5
6
7
8
9
10
11
Meishan−A
Meishan−B
Meishan−C
European−A
European−B
European−C

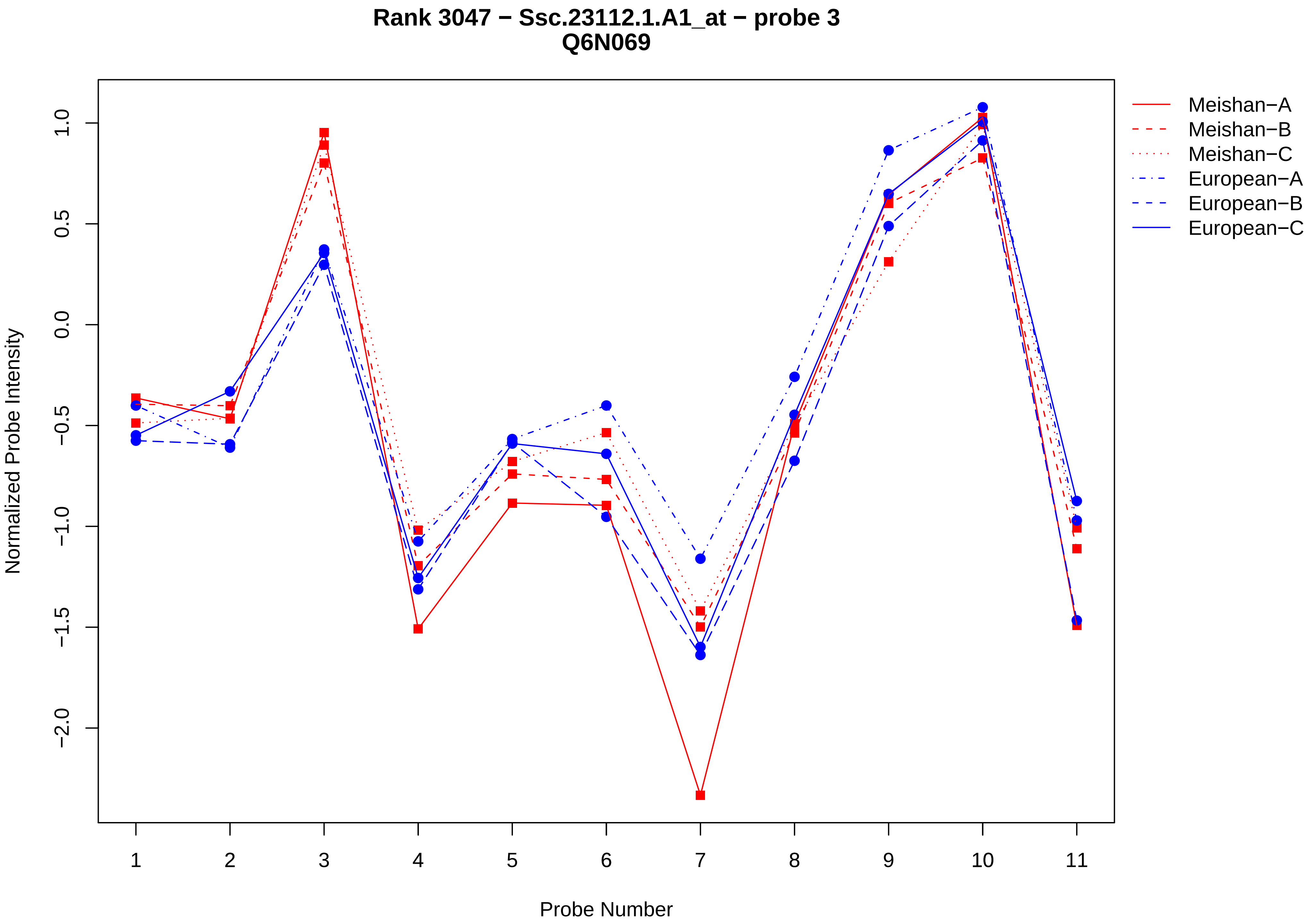

Rank 3047 − Ssc.23112.1.A1_at − probe 3
Q6N069
Normalized Probe Intensity
Probe Number
1.0
0.5
0.0
−0.5
−1.0
−1.5
−2.0
1
2
3
4
5
6
7
8
9
10
11
Meishan−A
Meishan−B
Meishan−C
European−A
European−B
European−C

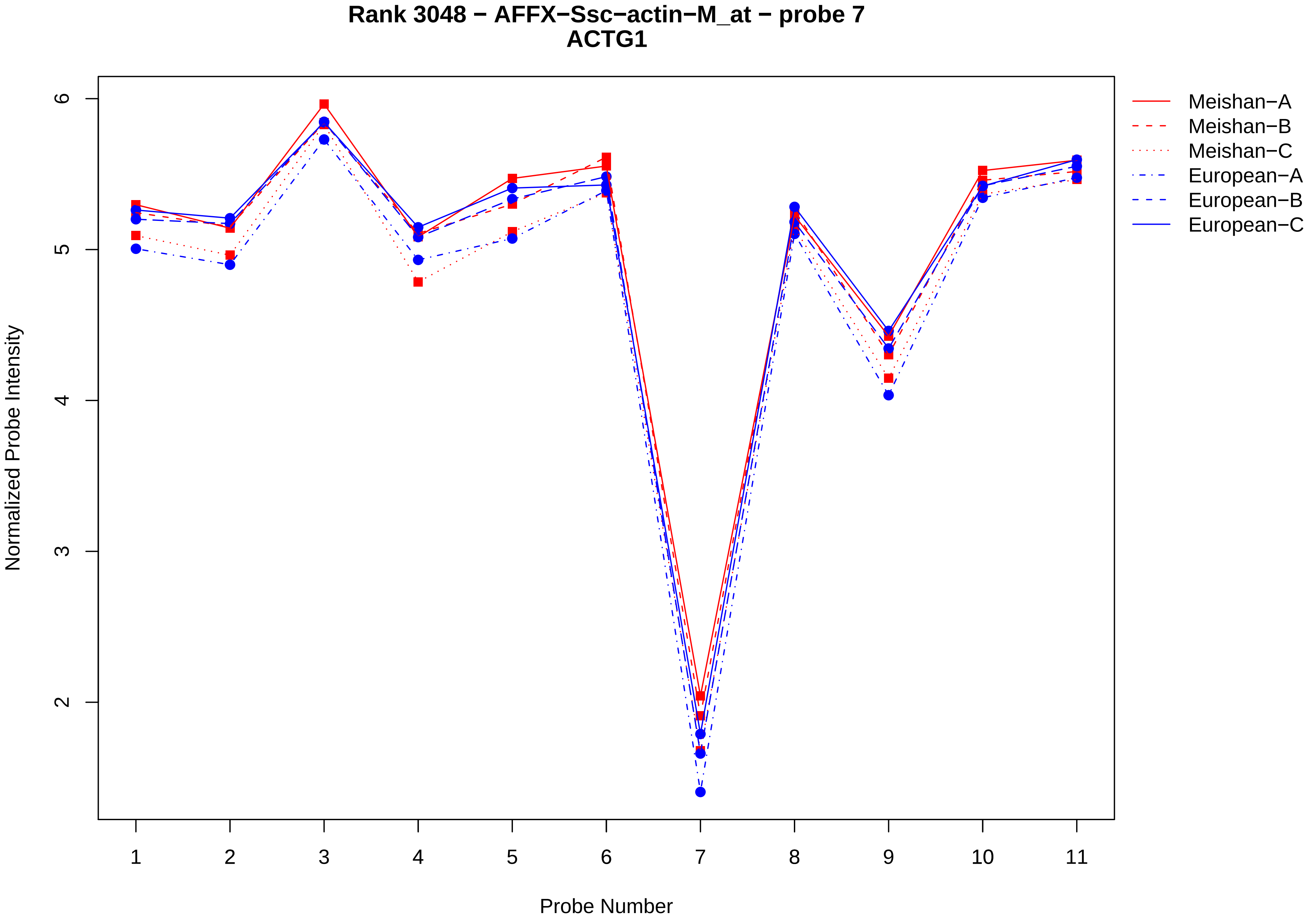

Rank 3048 − AFFX−Ssc−actin−M_at − probe 7
ACTG1
Normalized Probe Intensity
Probe Number
Meishan−A
Meishan−B
Meishan−C
European−A
European−B
European−C

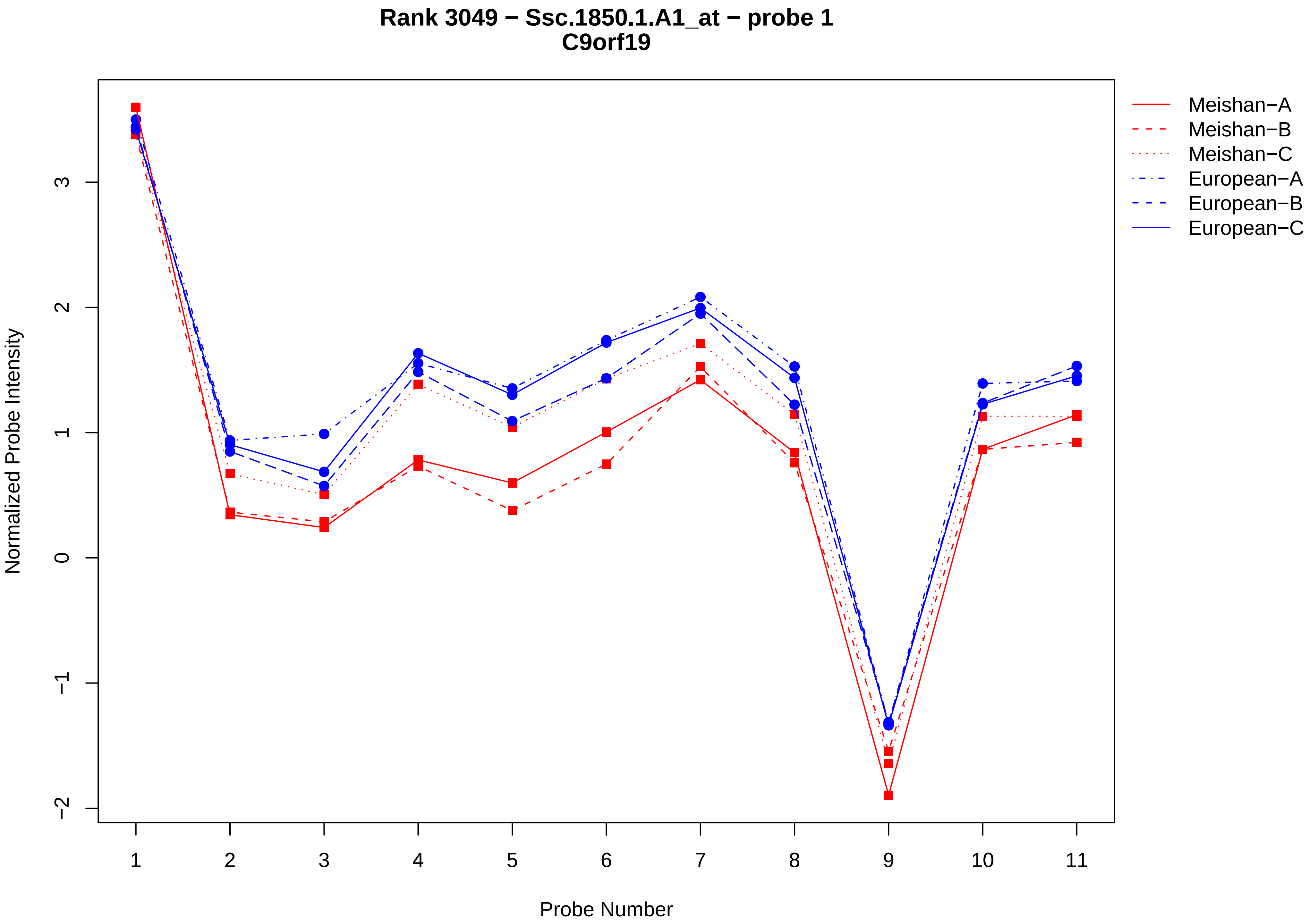

Rank 3049 − Ssc.1850.1.A1_at − probe 1
C9orf19
Normalized Probe Intensity
Probe Number
−2
−1
0
1
2
3
1
2
3
4
5
6
7
8
9
10
11
Meishan−A
Meishan−B
Meishan−C
European−A
European−B
European−C

Rank 3050 − Ssc.30513.1.A1_at − probe 11
CADPS2

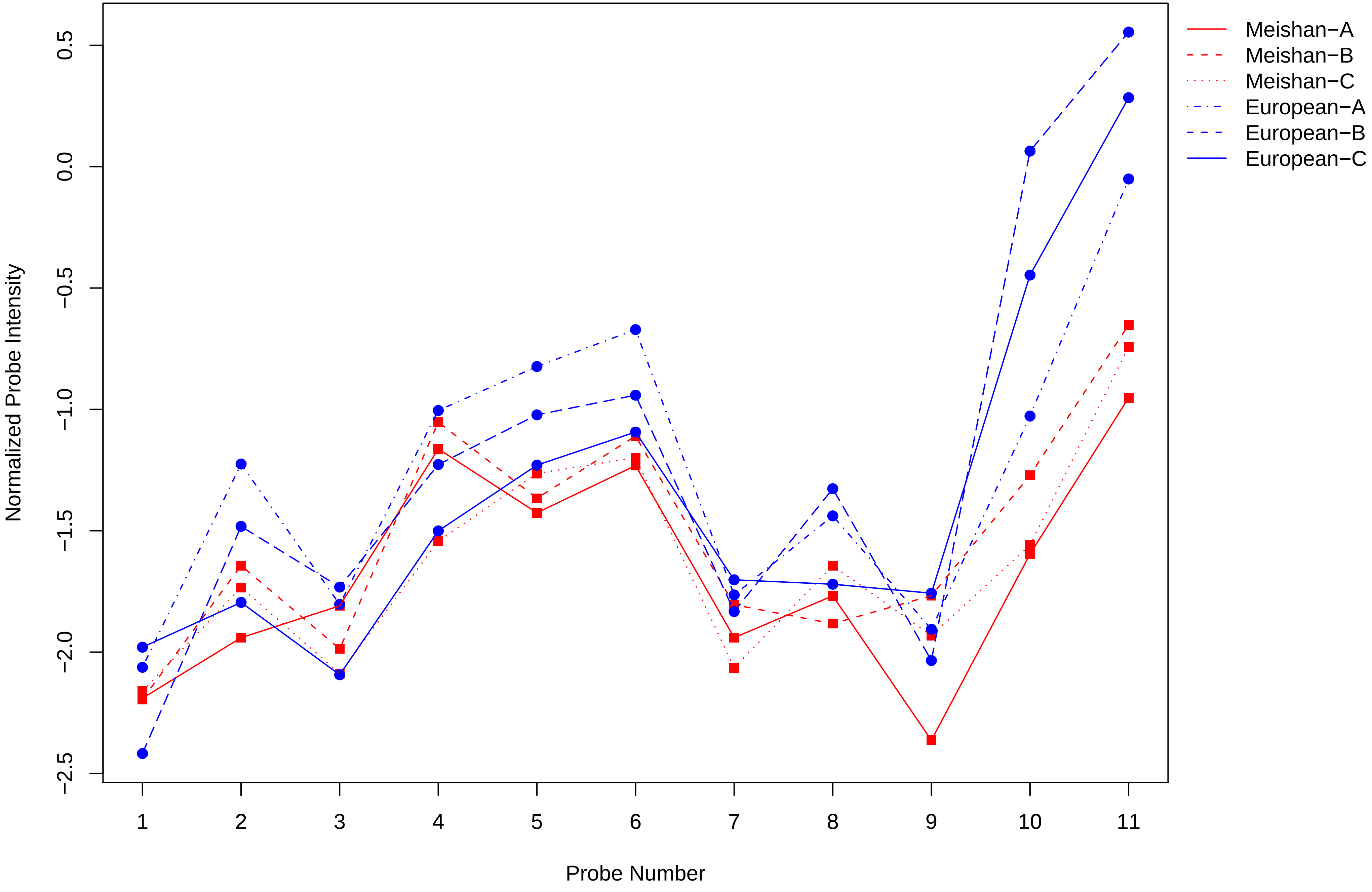

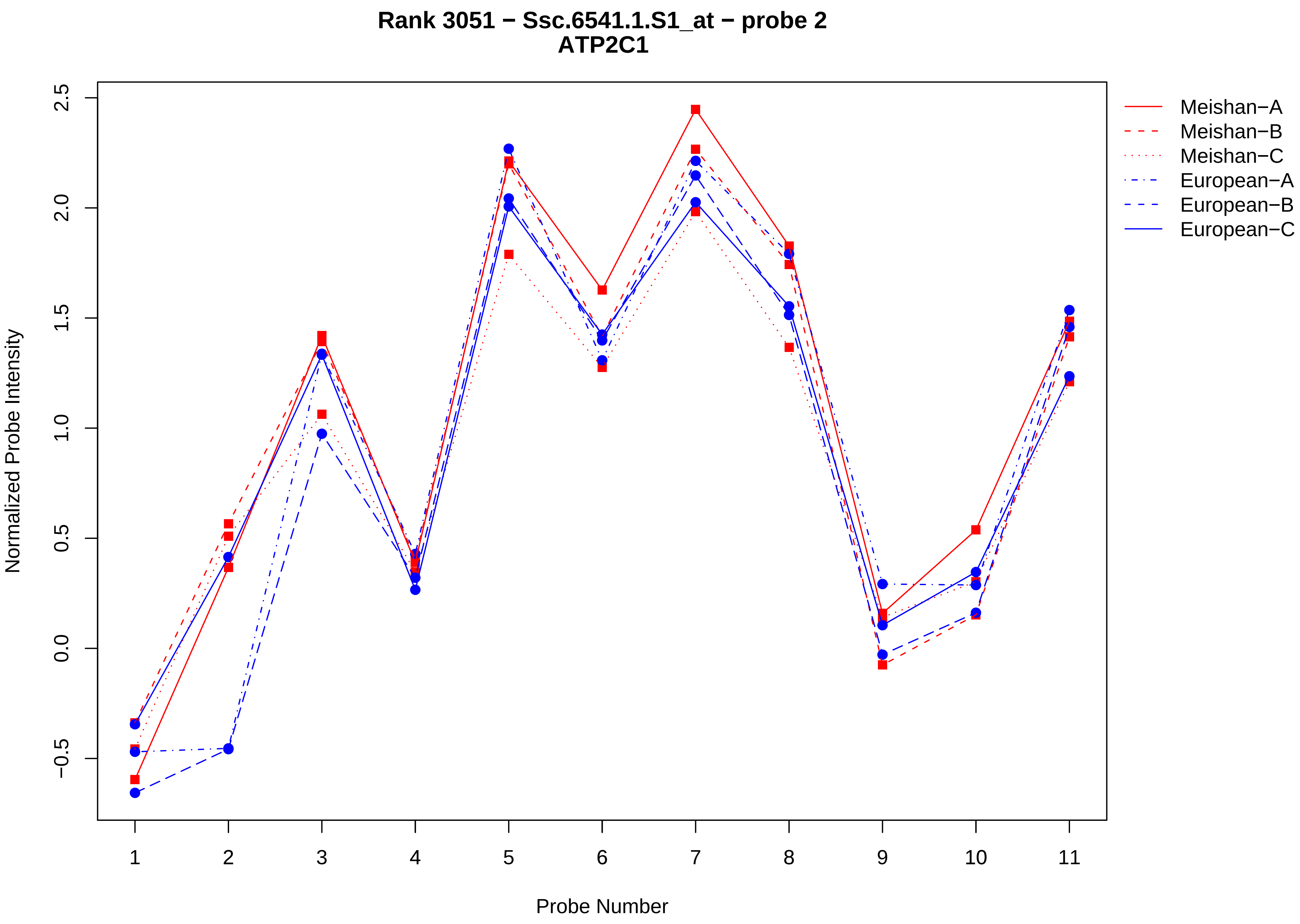
Rank 3051 − Ssc.6541.1.S1_at − probe 2
ATP2C1
Normalized Probe Intensity
Probe Number
−0.5
0.0
0.5
1.0
1.5
2.0
2.5
1
2
3
4
5
6
7
8
9
10
11
Meishan−A
Meishan−B
Meishan−C
European−A
European−B
European−C

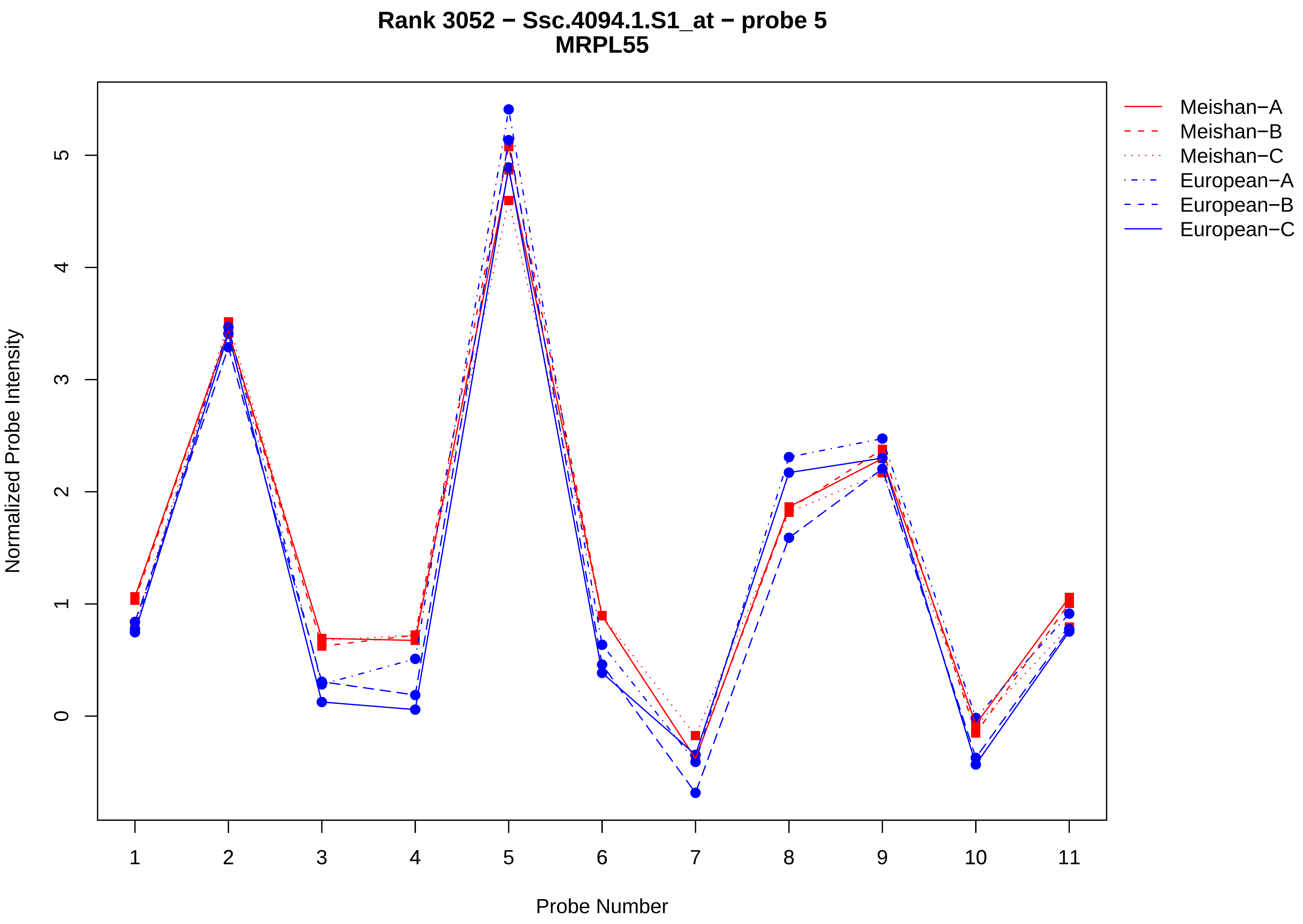

Rank 3052 − Ssc.4094.1.S1_at − probe 5
MRPL55
Normalized Probe Intensity
Probe Number
0
1
2
3
4
5
1
2
3
4
5
6
7
8
9
10
11
Meishan−A
Meishan−B
Meishan−C
European−A
European−B
European−C

Rank 3053 − Ssc.24170.1.A1_at − probe 9
Q9BVC6

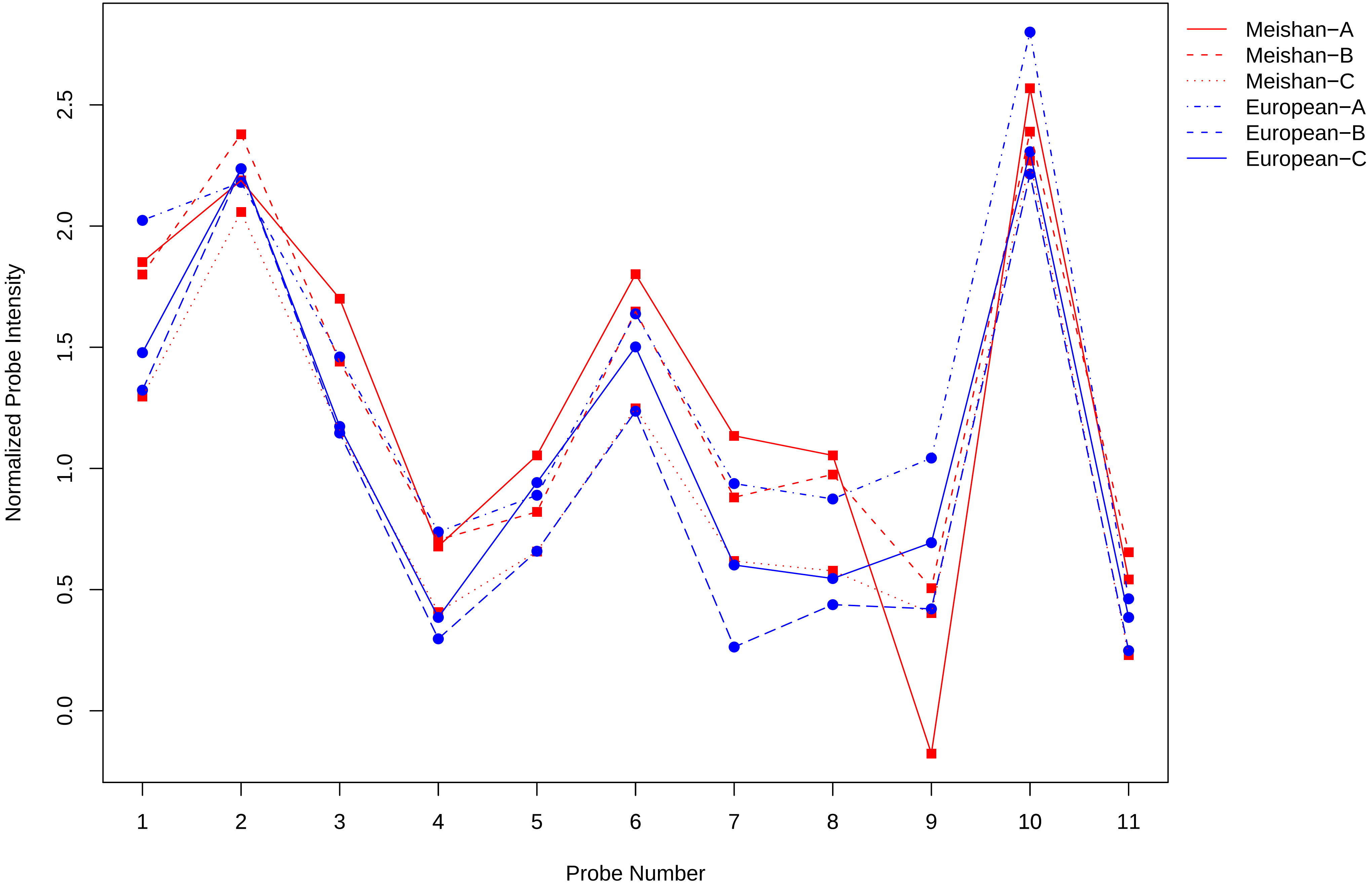

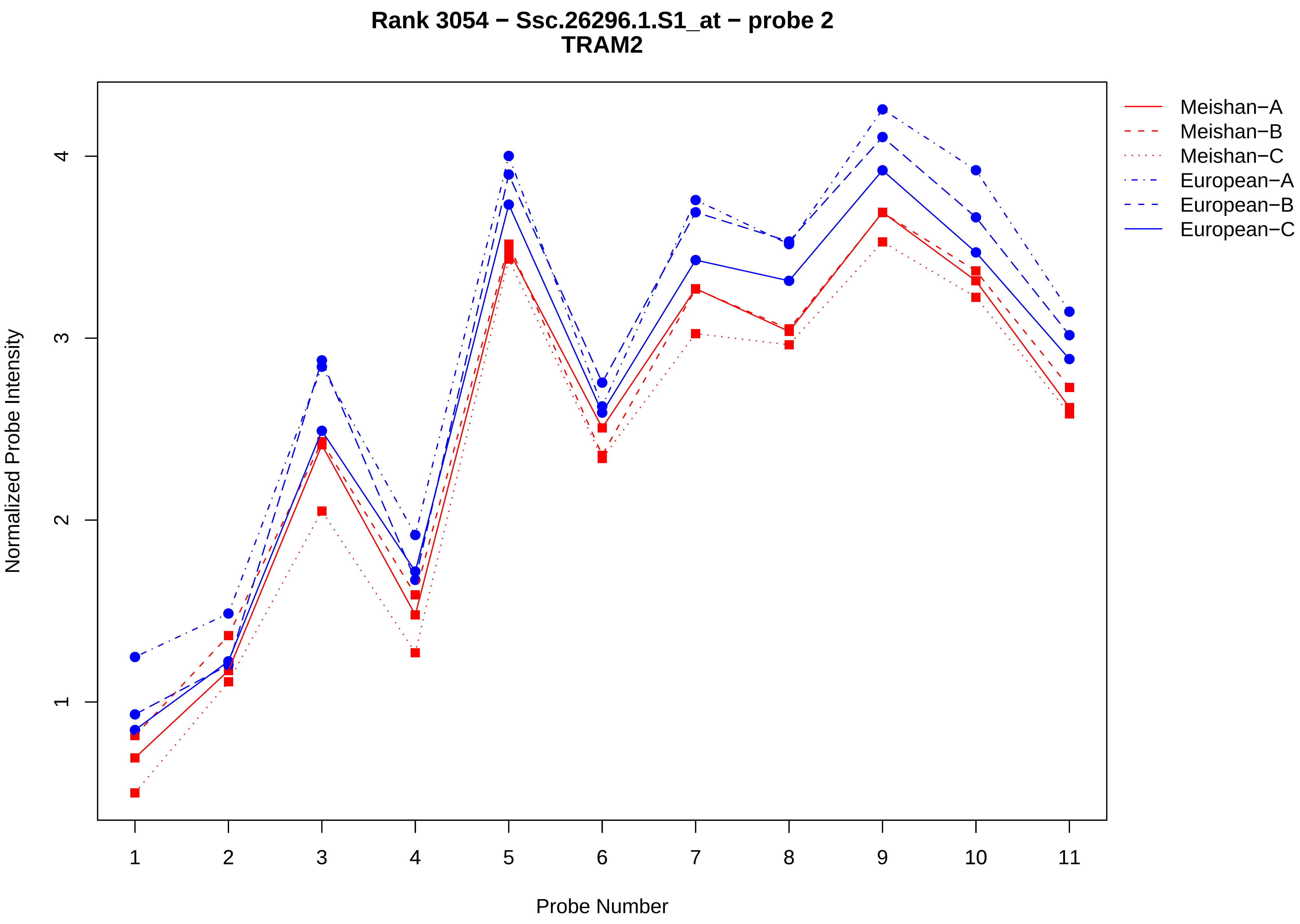

Rank 3054 − Ssc.26296.1.S1_at − probe 2
TRAM2
Normalized Probe Intensity
Probe Number
1
2
3
4
5
6
7
8
9
10
11
Meishan−A
Meishan−B
Meishan−C
European−A
European−B
European−C

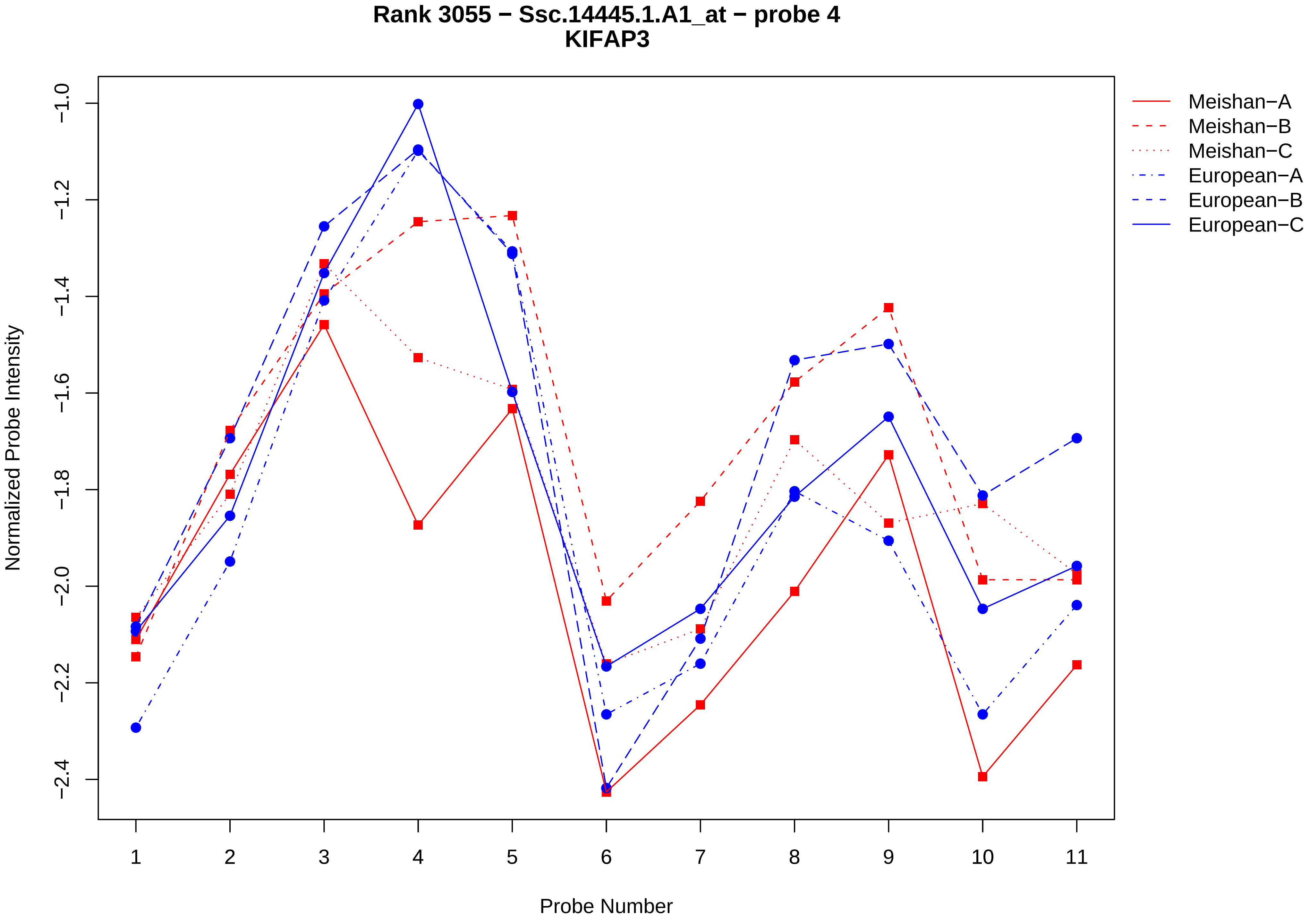

Rank 3055 − Ssc.14445.1.A1_at − probe 4
KIFAP3
Normalized Probe Intensity
Probe Number
−1.0
−1.2
−1.4
−1.6
−1.8
−2.0
−2.2
−2.4
1
2
3
4
5
6
7
8
9
10
11
Meishan−A
Meishan−B
Meishan−C
European−A
European−B
European−C

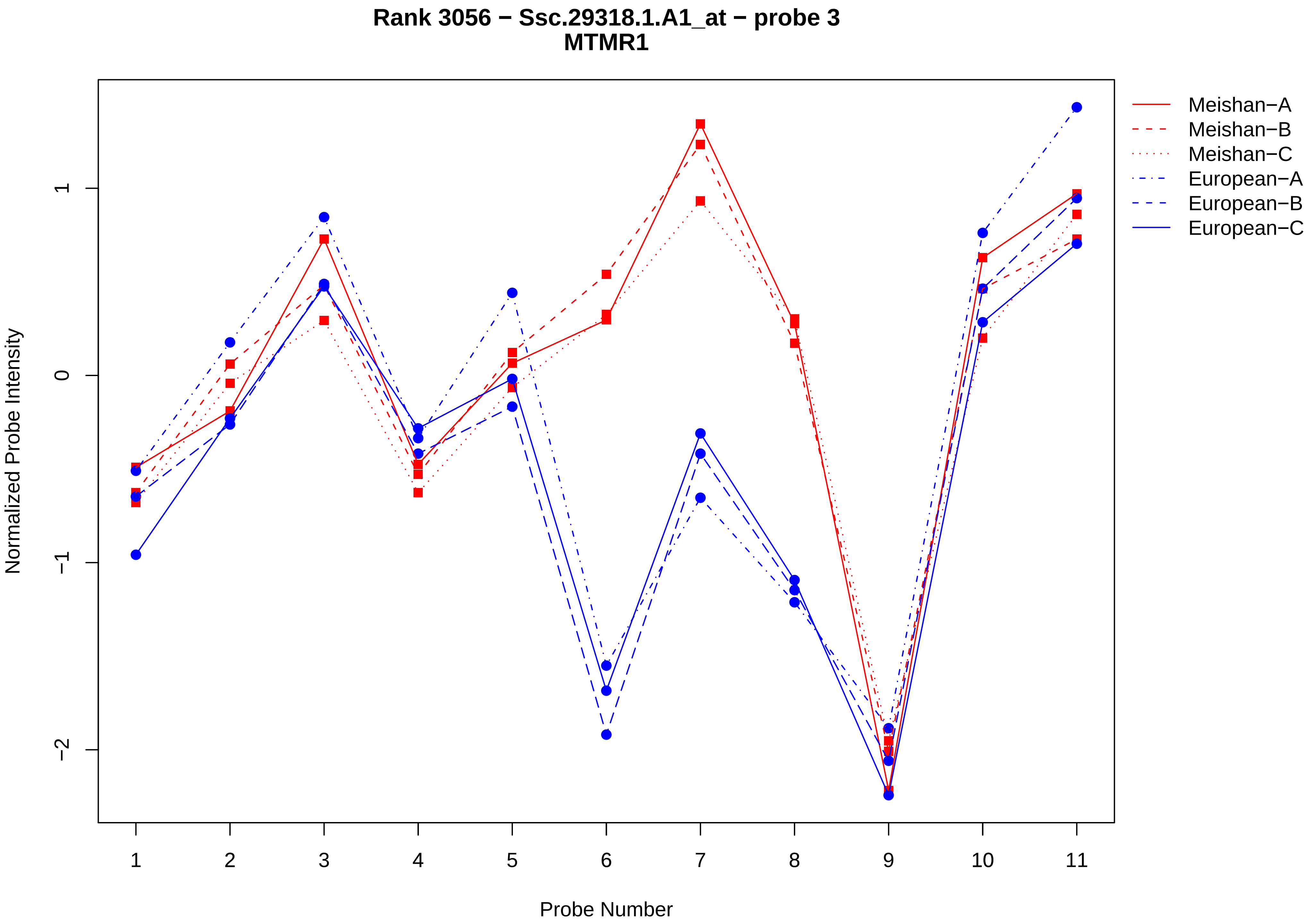

Rank 3056 − Ssc.29318.1.A1_at − probe 3
MTMR1
Normalized Probe Intensity
Probe Number
Meishan−A
Meishan−B
Meishan−C
European−A
European−B
European−C
−2
−1
0
1
1
2
3
4
5
6
7
8
9
10
11

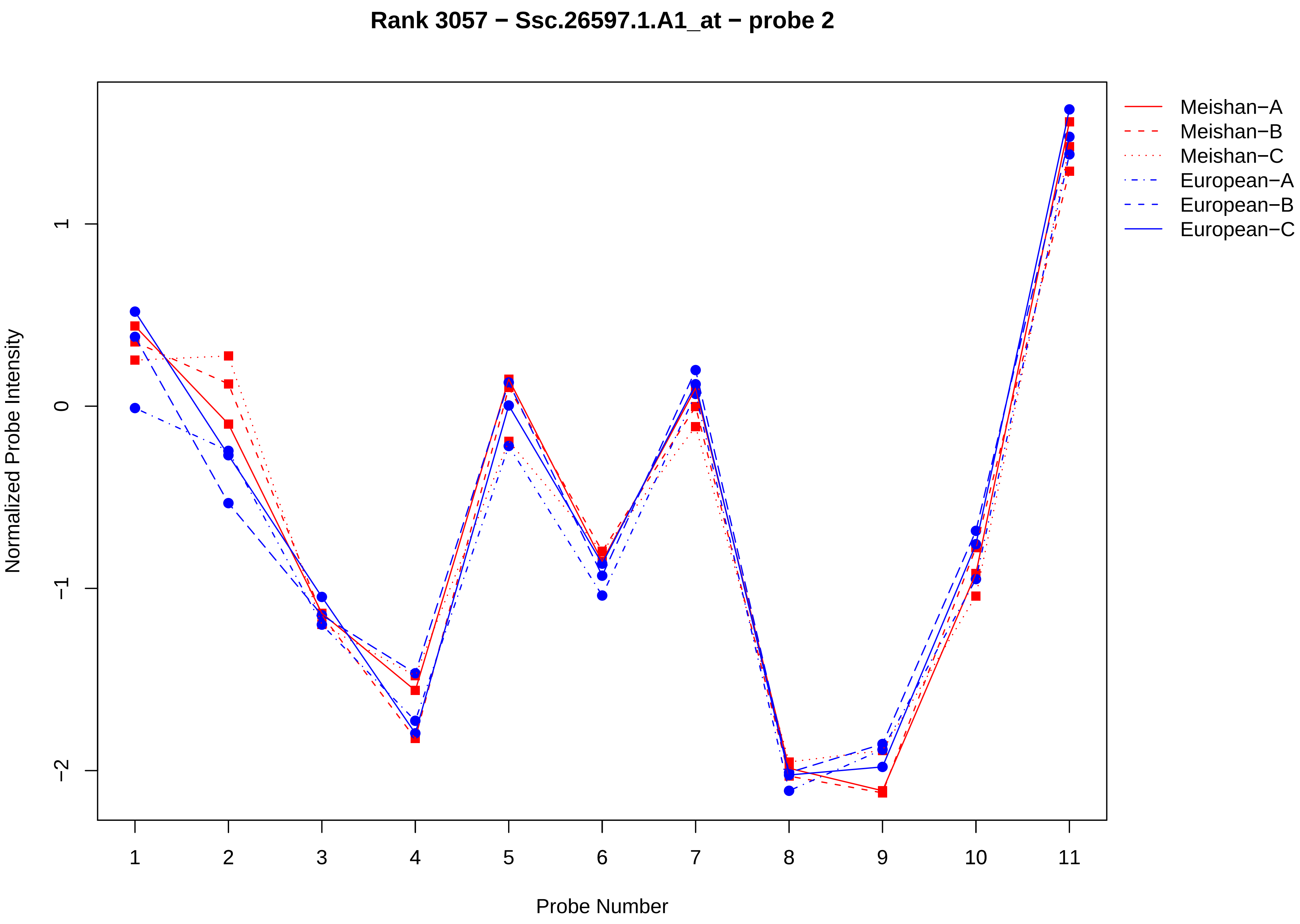

Rank 3057 − Ssc.26597.1.A1_at − probe 2
Normalized Probe Intensity
Probe Number
1
0
−1
−2
1
2
3
4
5
6
7
8
9
10
11
Meishan−A
Meishan−B
Meishan−C
European−A
European−B
European−C

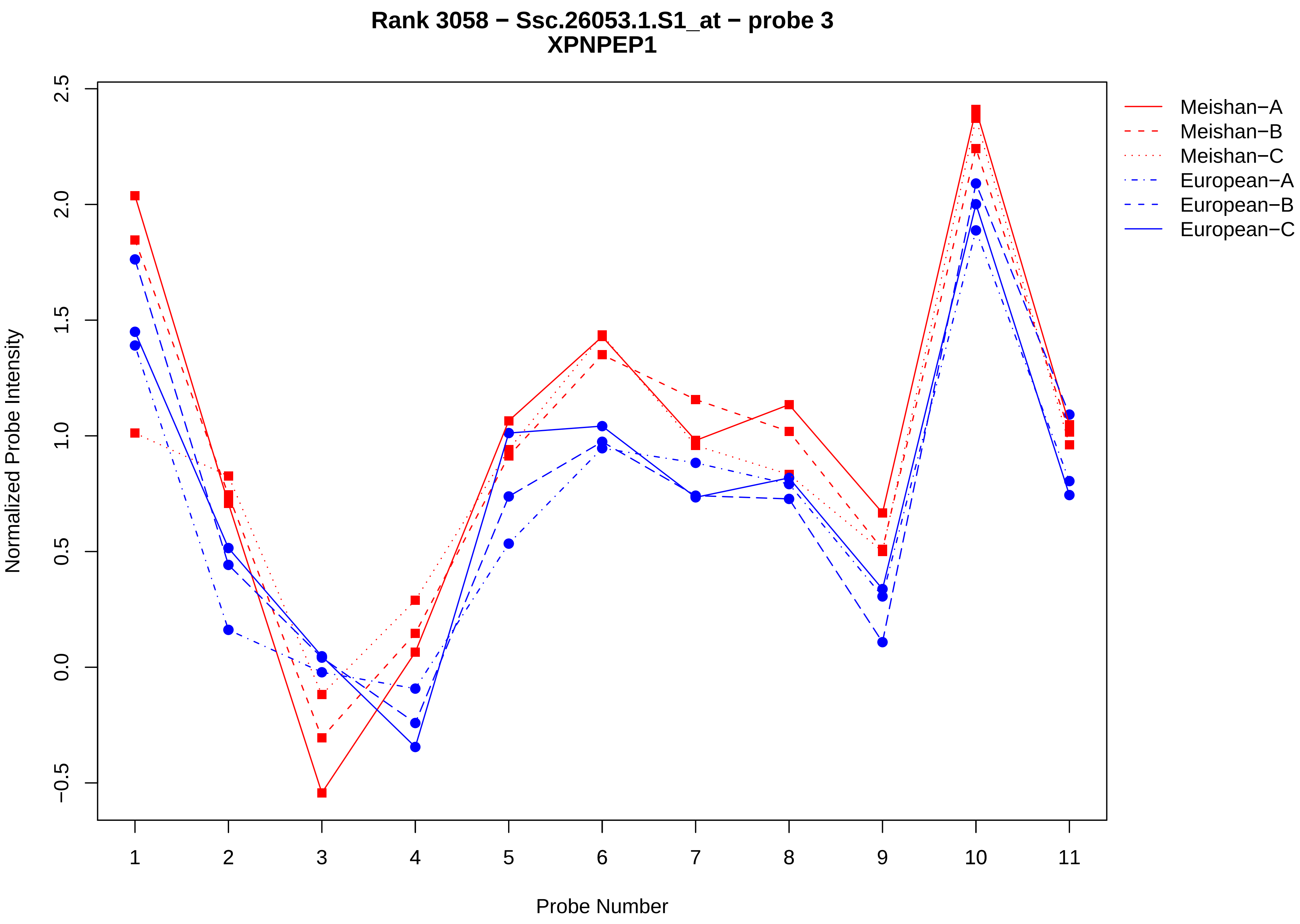

Rank 3058 − Ssc.26053.1.S1_at − probe 3
XPNPEP1
Normalized Probe Intensity
Probe Number
Meishan−A
Meishan−B
Meishan−C
European−A
European−B
European−C
−0.5
0.0
0.5
1.0
1.5
2.0
2.5
1
2
3
4
5
6
7
8
9
10
11

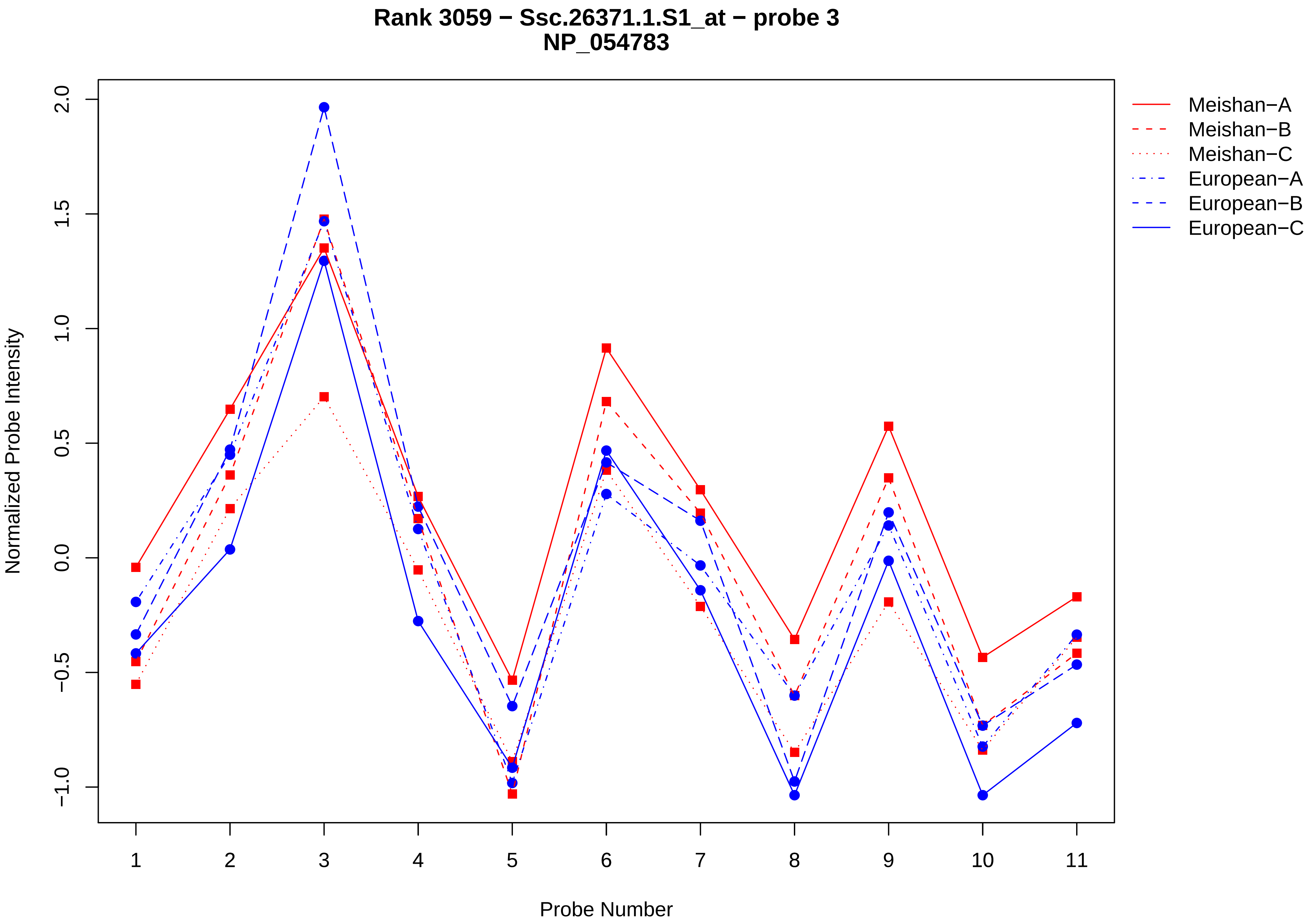

Rank 3059 − Ssc.26371.1.S1_at − probe 3
NP_054783
Normalized Probe Intensity
Probe Number
−1.0
−0.5
0.0
0.5
1.0
1.5
2.0
1
2
3
4
5
6
7
8
9
10
11
Meishan−A
Meishan−B
Meishan−C
European−A
European−B
European−C

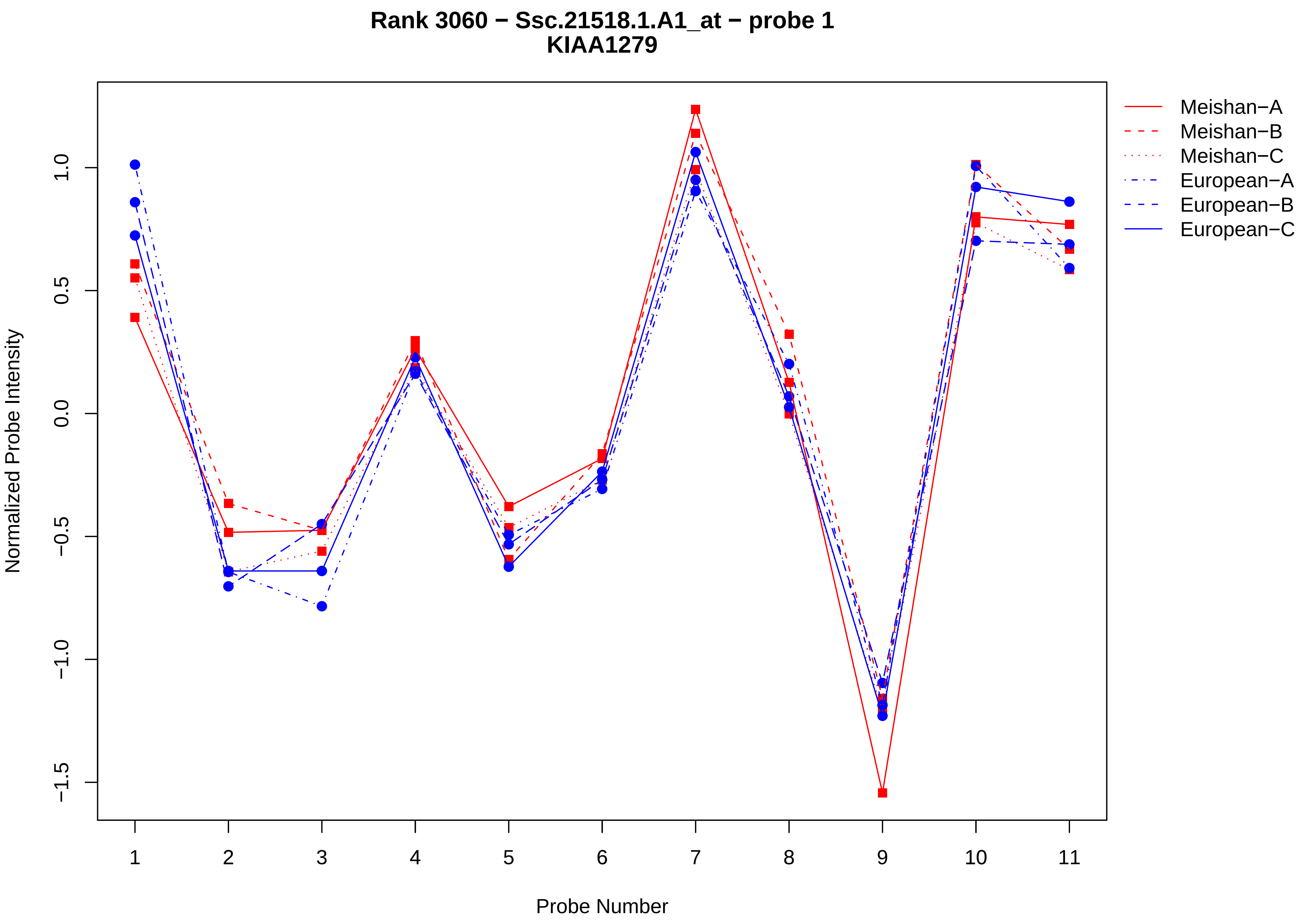

Rank 3060 − Ssc.21518.1.A1_at − probe 1
KIAA1279
Normalized Probe Intensity
Probe Number
Meishan−A
Meishan−B
Meishan−C
European−A
European−B
European−C

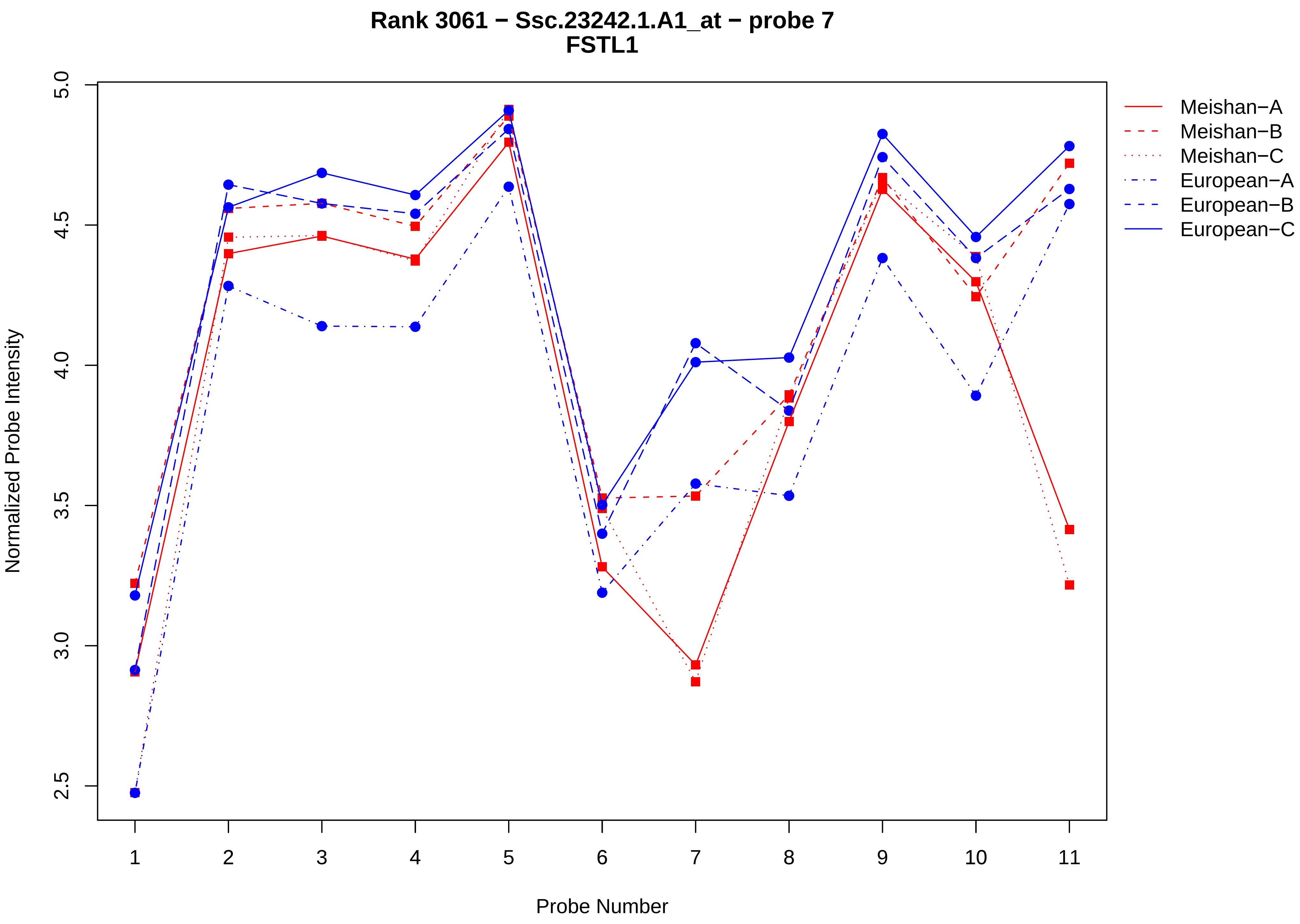
Rank 3061 − Ssc.23242.1.A1_at − probe 7
FSTL1
Normalized Probe Intensity
Probe Number
5.0
4.5
4.0
3.5
3.0
2.5
1
2
3
4
5
6
7
8
9
10
11
Meishan−A
Meishan−B
Meishan−C
European−A
European−B
European−C

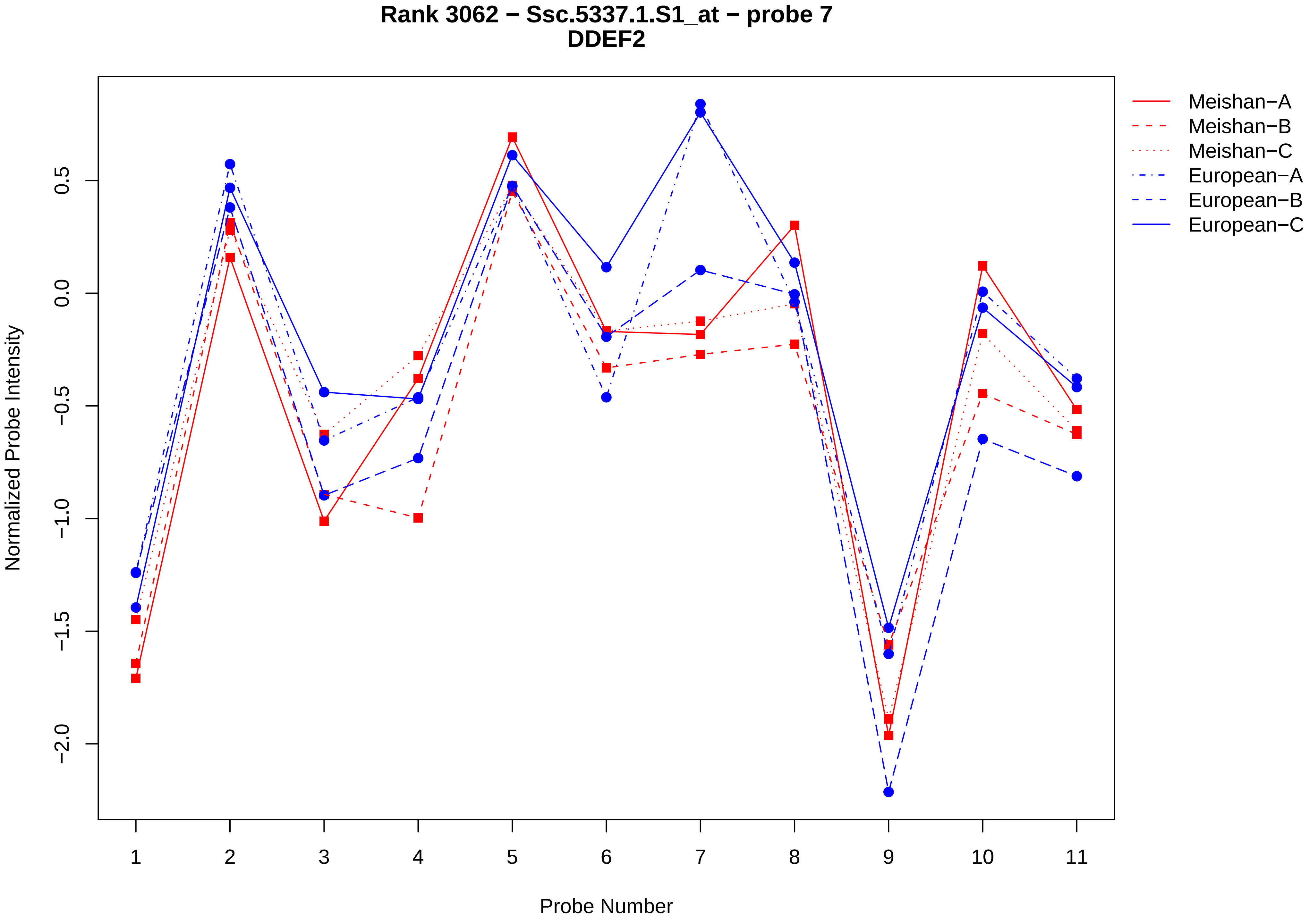

Rank 3062 − Ssc.5337.1.S1_at − probe 7
DDEF2
Normalized Probe Intensity
Probe Number
−2.0
−1.5
−1.0
−0.5
0.0
0.5
1 2 3 4 5 6 7 8 9 10 11
Meishan−A
Meishan−B
Meishan−C
European−A
European−B
European−C

Rank 3063 − Ssc.17449.1.S1_at − probe 2
OLFM1

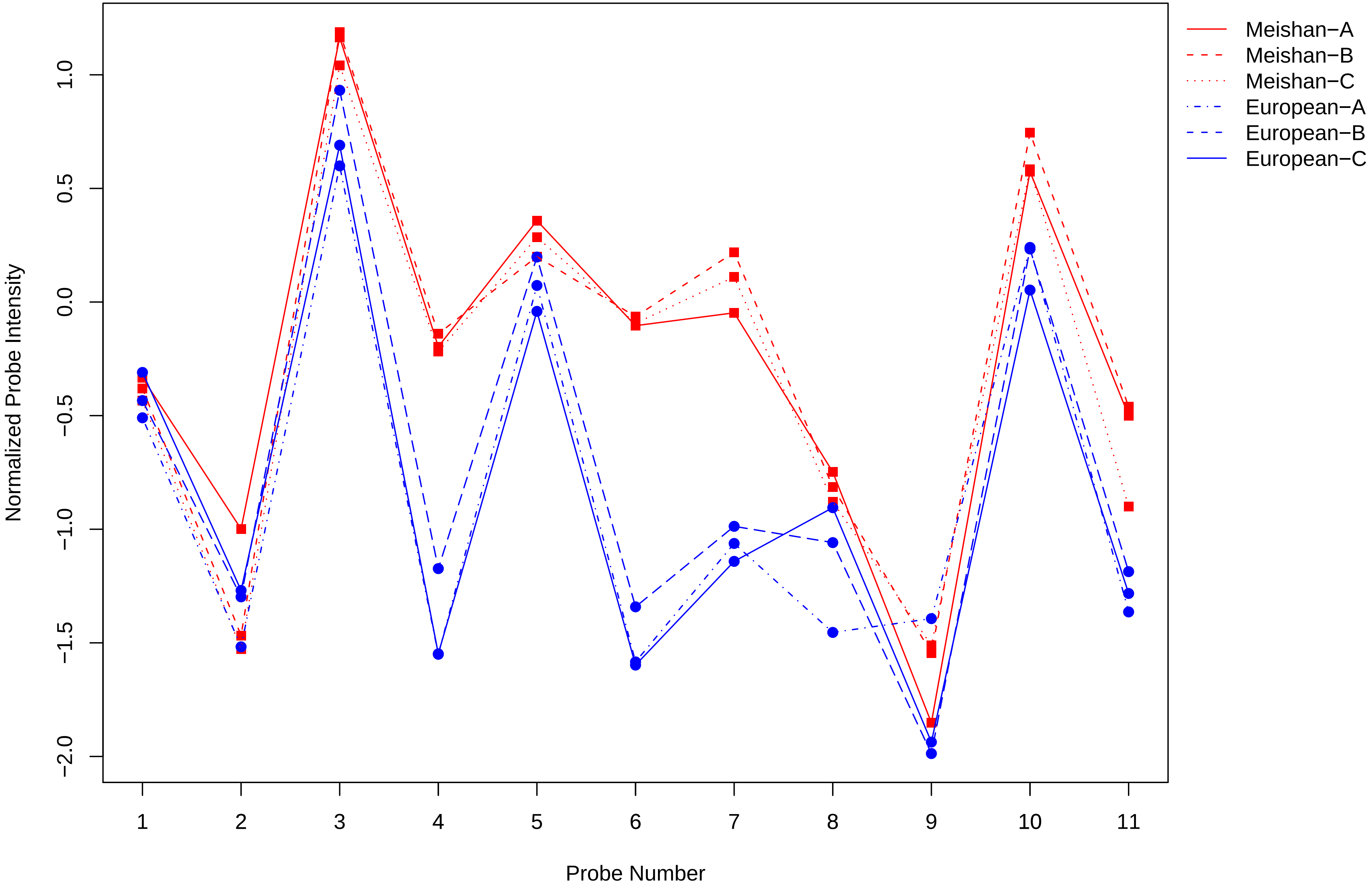

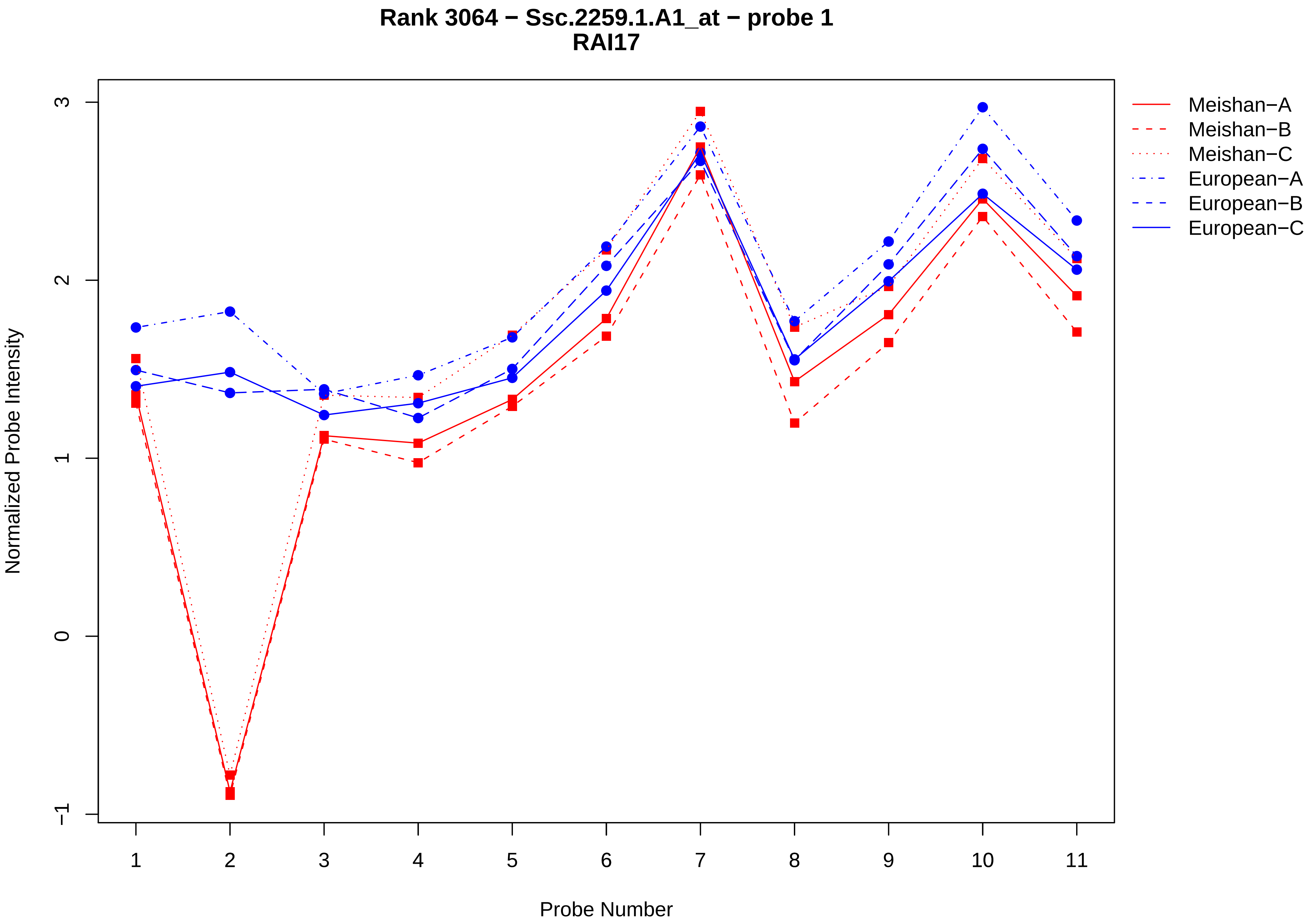
Rank 3064 − Ssc.2259.1.A1_at − probe 1
RAI17
Normalized Probe Intensity
Probe Number
−1
0
1
2
3
1
2
3
4
5
6
7
8
9
10
11
Meishan−A
Meishan−B
Meishan−C
European−A
European−B
European−C

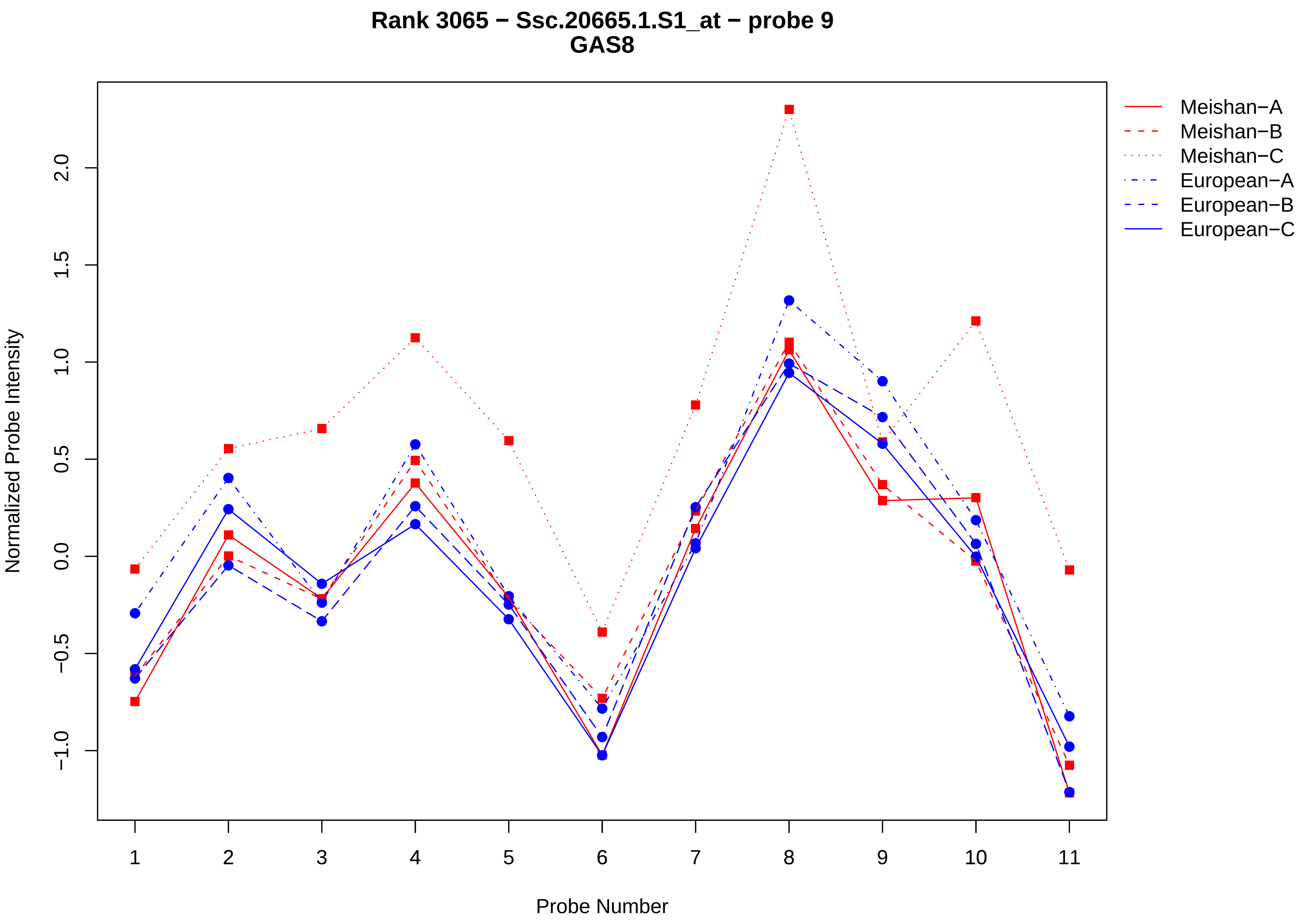

Rank 3065 − Ssc.20665.1.S1_at − probe 9
GAS8
Normalized Probe Intensity
Probe Number
Meishan−A
Meishan−B
Meishan−C
European−A
European−B
European−C
−1.0
−0.5
0.0
0.5
1.0
1.5
2.0
1
2
3
4
5
6
7
8
9
10
11

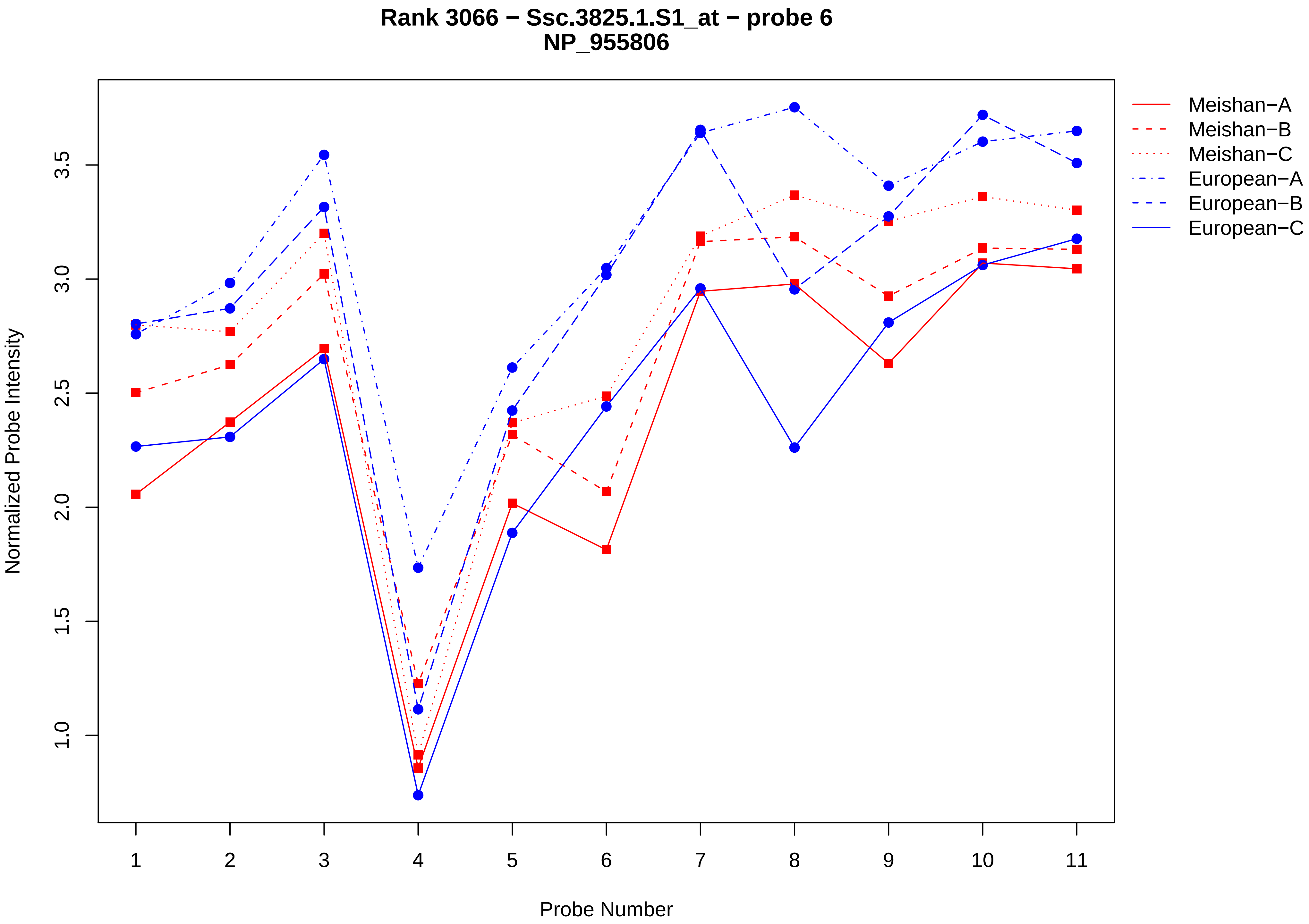

Rank 3066 − Ssc.3825.1.S1_at − probe 6
NP_955806
Normalized Probe Intensity
1.0
1.5
2.0
2.5
3.0
3.5
1
2
3
4
5
6
7
8
9
10
11
Probe Number
Meishan−A
Meishan−B
Meishan−C
European−A
European−B
European−C

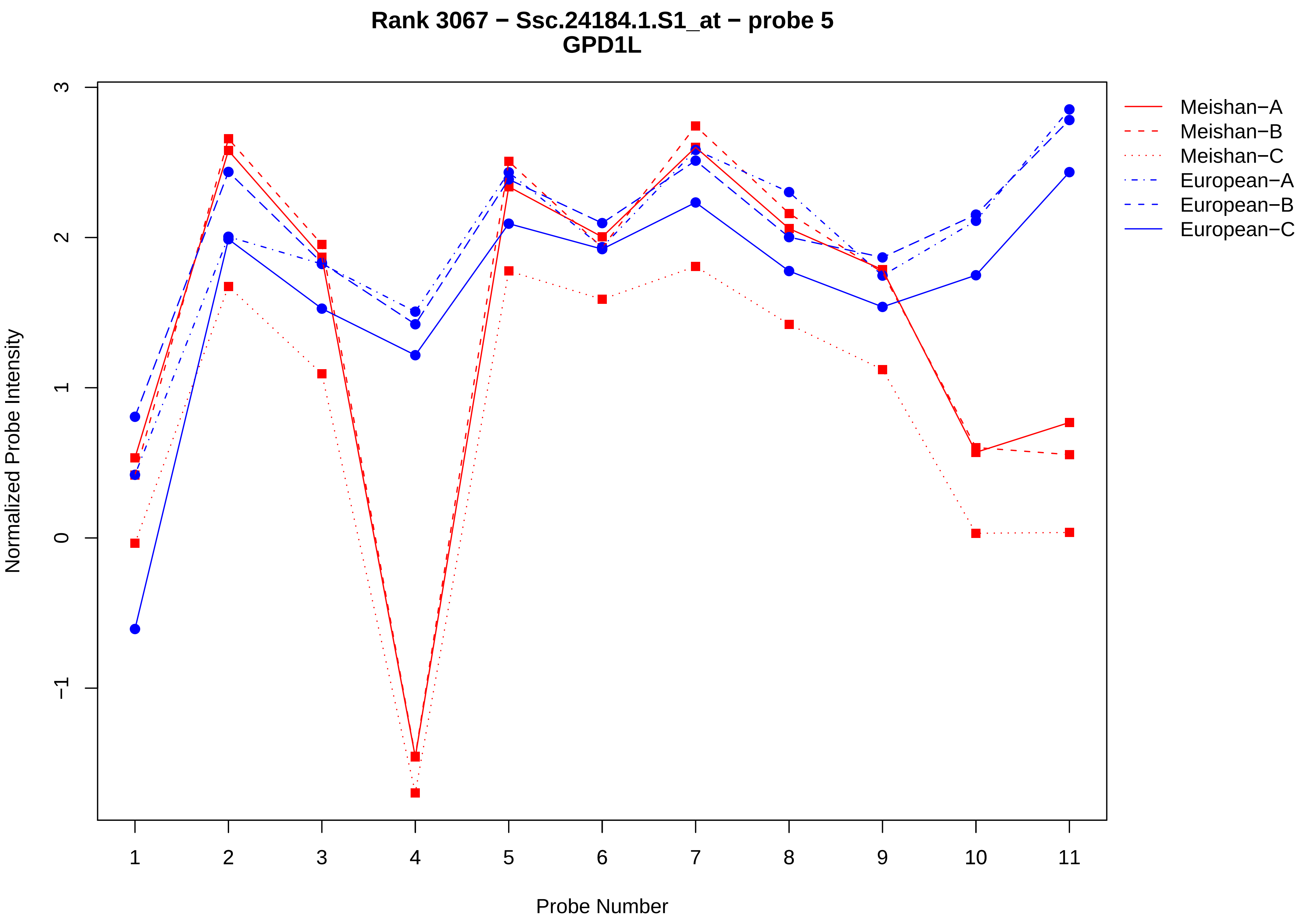

Rank 3067 − Ssc.24184.1.S1_at − probe 5
GPD1L
Normalized Probe Intensity
Probe Number
Meishan−A
Meishan−B
Meishan−C
European−A
European−B
European−C

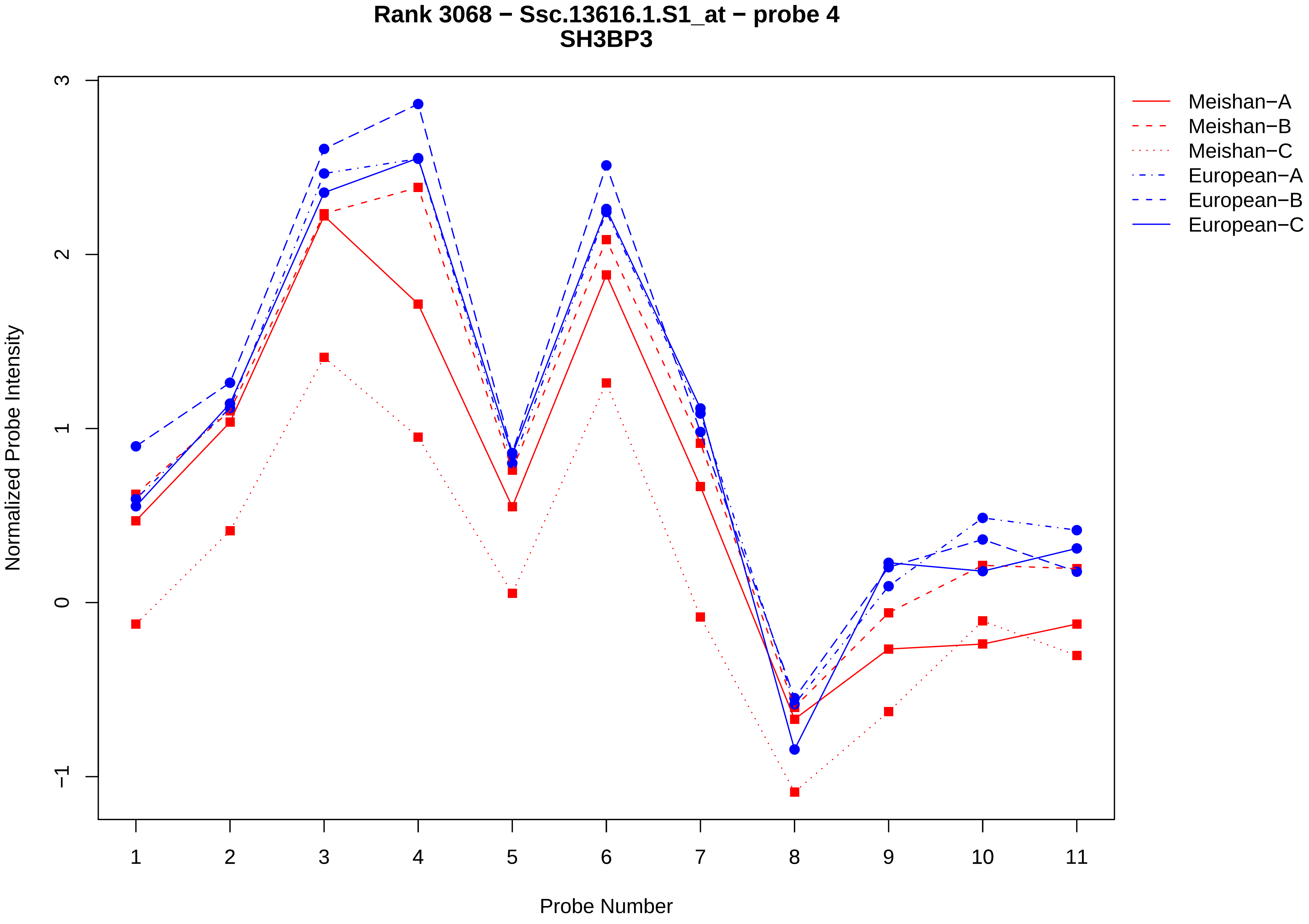

Rank 3068 − Ssc.13616.1.S1_at − probe 4
SH3BP3
Normalized Probe Intensity
Probe Number
−1
0
1
2
3
1
2
3
4
5
6
7
8
9
10
11
Meishan−A
Meishan−B
Meishan−C
European−A
European−B
European−C

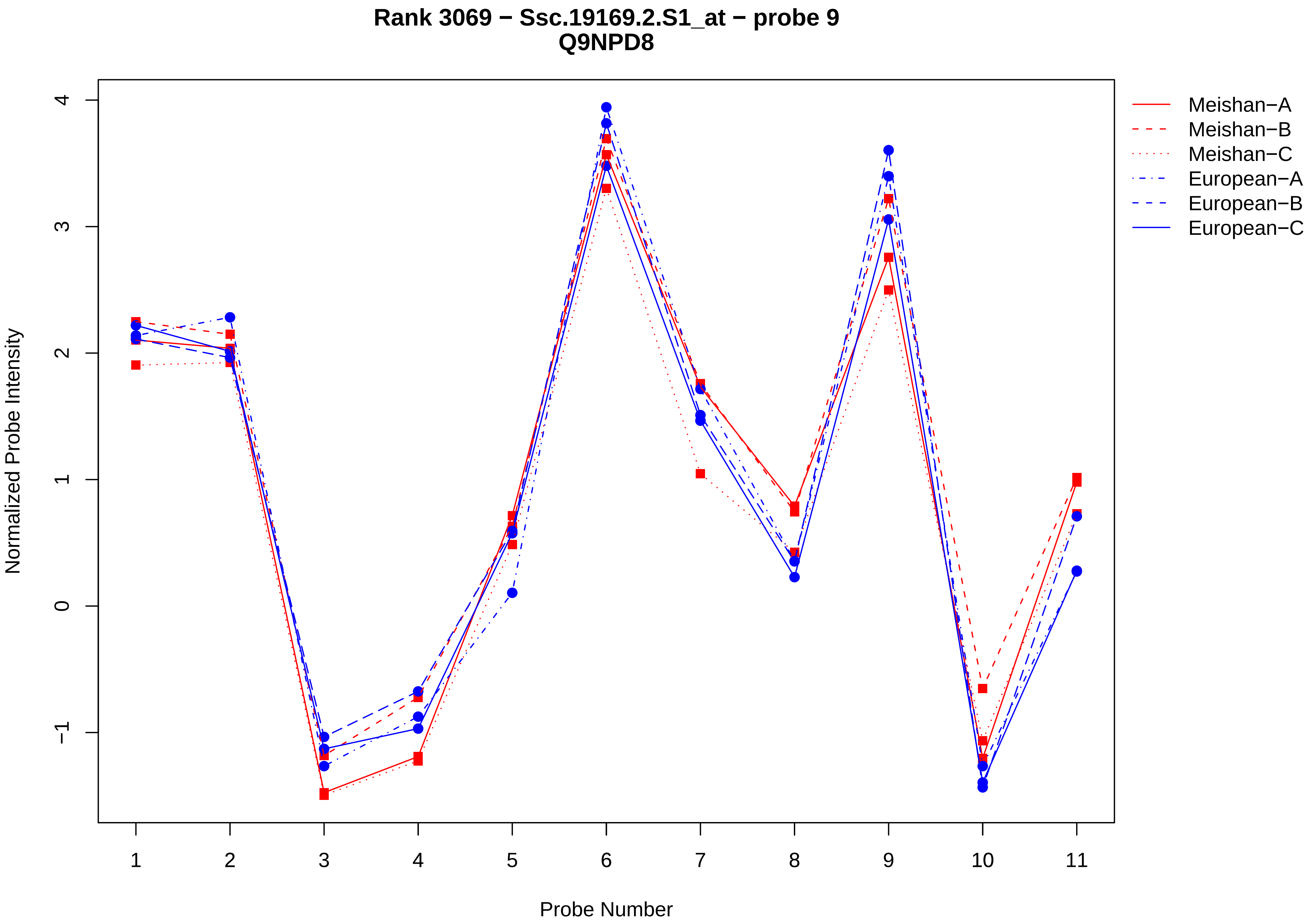

Rank 3069 − Ssc.19169.2.S1_at − probe 9
Q9NPD8
Normalized Probe Intensity
Probe Number
Meishan−A
Meishan−B
Meishan−C
European−A
European−B
European−C
−1
0
1
2
3
4
1
2
3
4
5
6
7
8
9
10
11

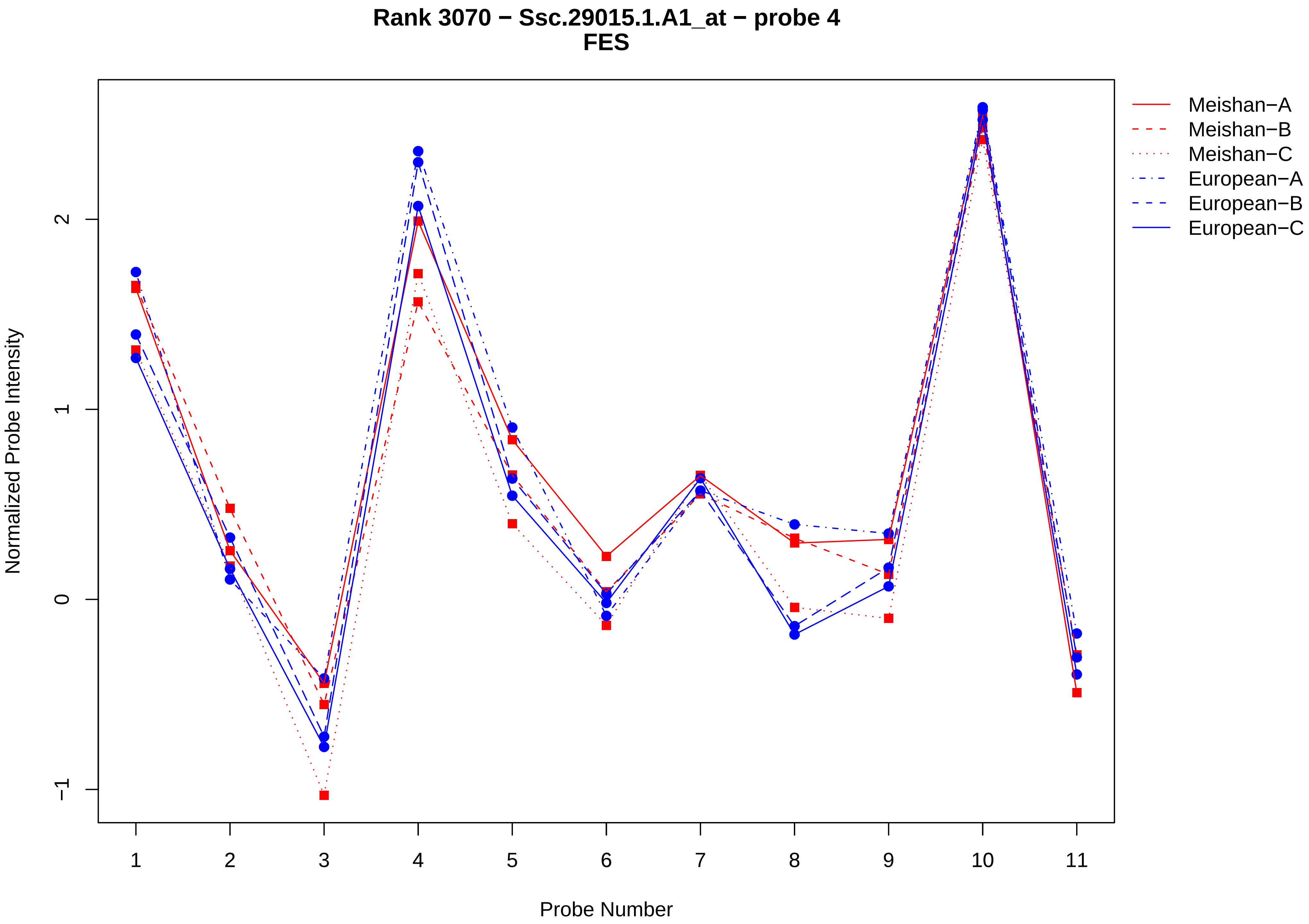

Rank 3070 − Ssc.29015.1.A1_at − probe 4
FES
Normalized Probe Intensity
Probe Number
−1
0
1
2
1
2
3
4
5
6
7
8
9
10
11
Meishan−A
Meishan−B
Meishan−C
European−A
European−B
European−C

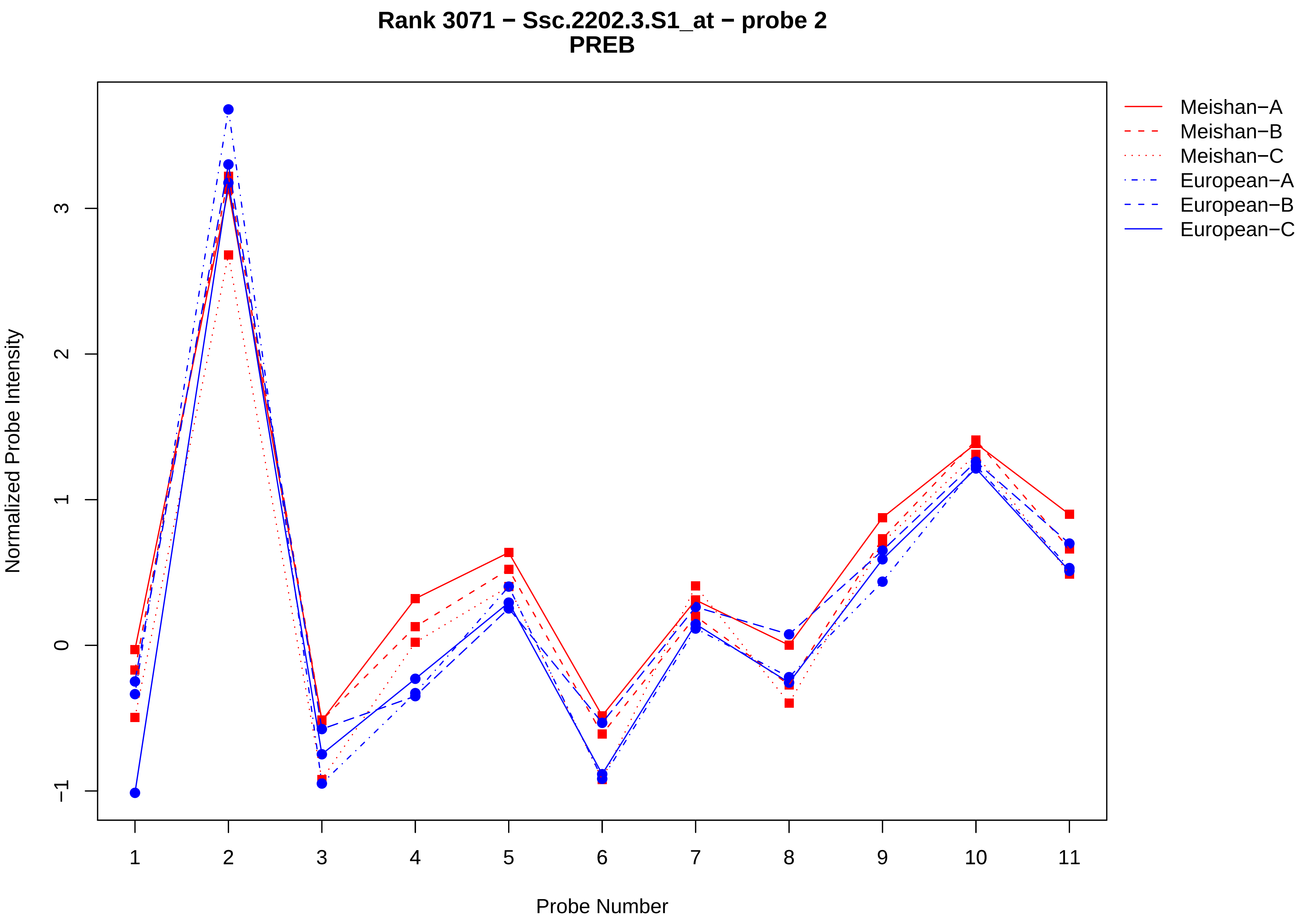

Rank 3071 − Ssc.2202.3.S1_at − probe 2
PREB
Normalized Probe Intensity
Probe Number
−1
0
1
2
3
1
2
3
4
5
6
7
8
9
10
11
Meishan−A
Meishan−B
Meishan−C
European−A
European−B
European−C

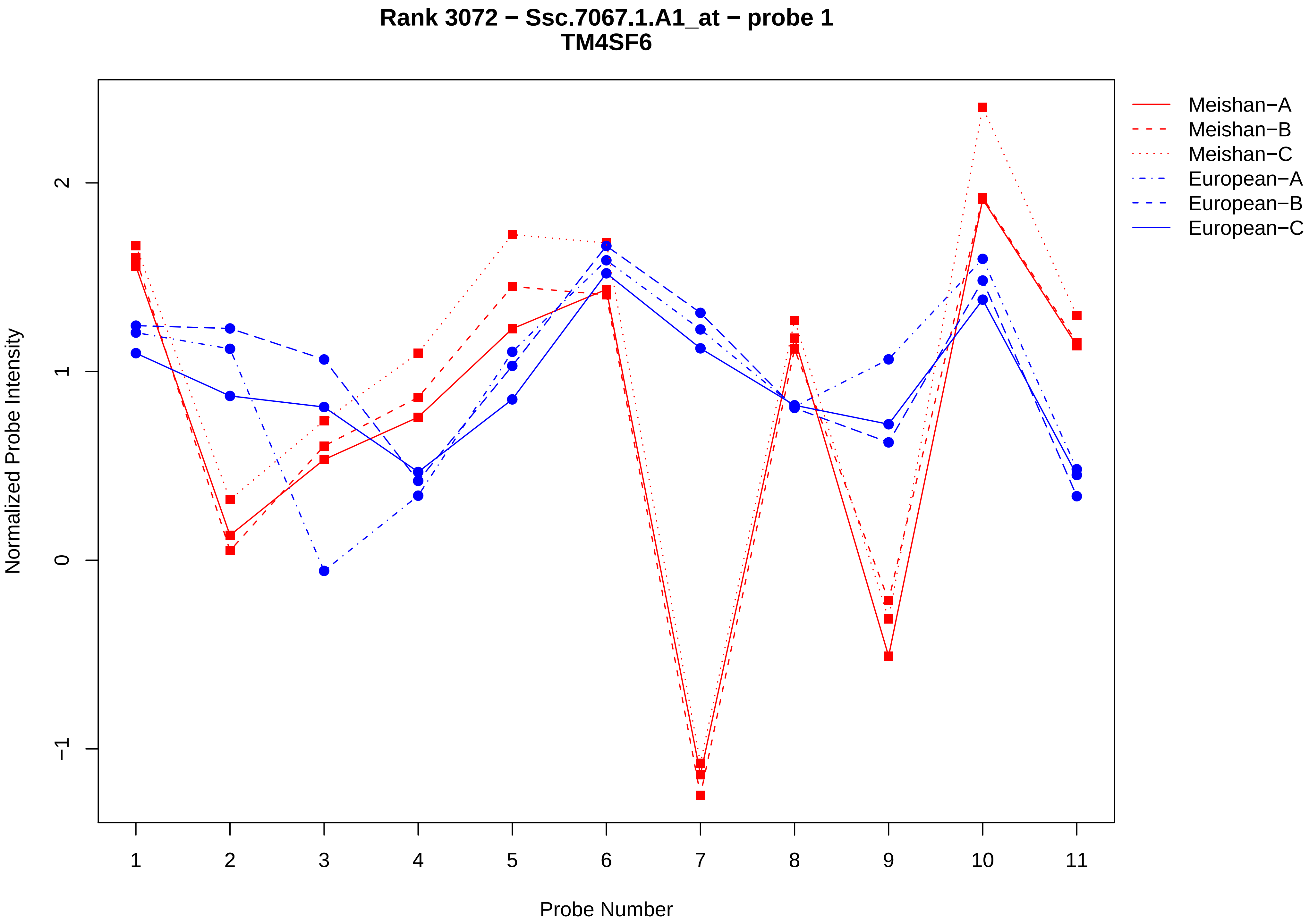

Rank 3072 − Ssc.7067.1.A1_at − probe 1
TM4SF6
Normalized Probe Intensity
Probe Number
−1
0
1
2
1
2
3
4
5
6
7
8
9
10
11
Meishan−A
Meishan−B
Meishan−C
European−A
European−B
European−C

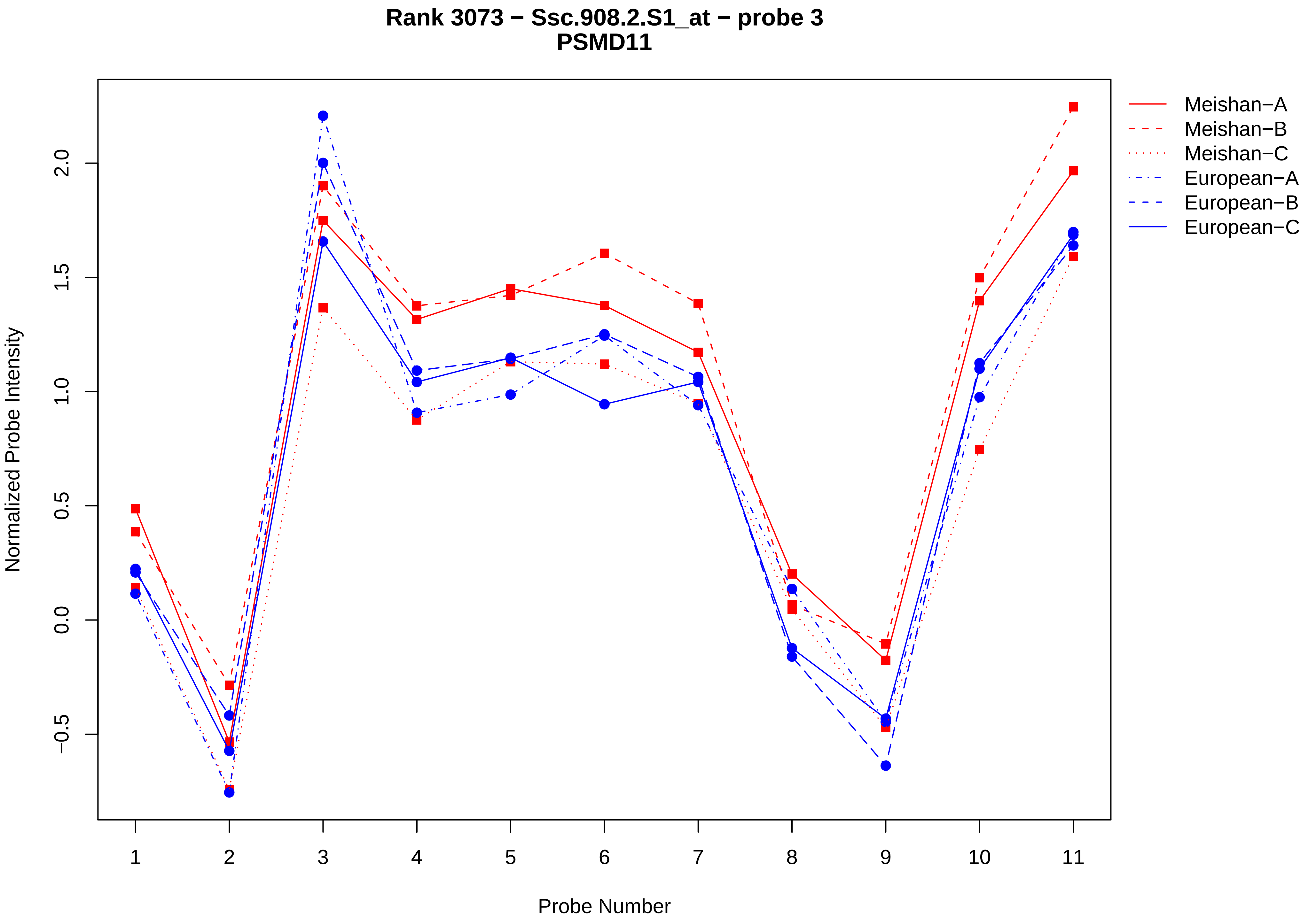
Rank 3073 − Ssc.908.2.S1_at − probe 3
PSMD11
Normalized Probe Intensity
Probe Number
−0.5
0.0
0.5
1.0
1.5
2.0
1
2
3
4
5
6
7
8
9
10
11
Meishan−A
Meishan−B
Meishan−C
European−A
European−B
European−C

Rank 3074 − Ssc.7336.1.A1_at − probe 1
RPL41

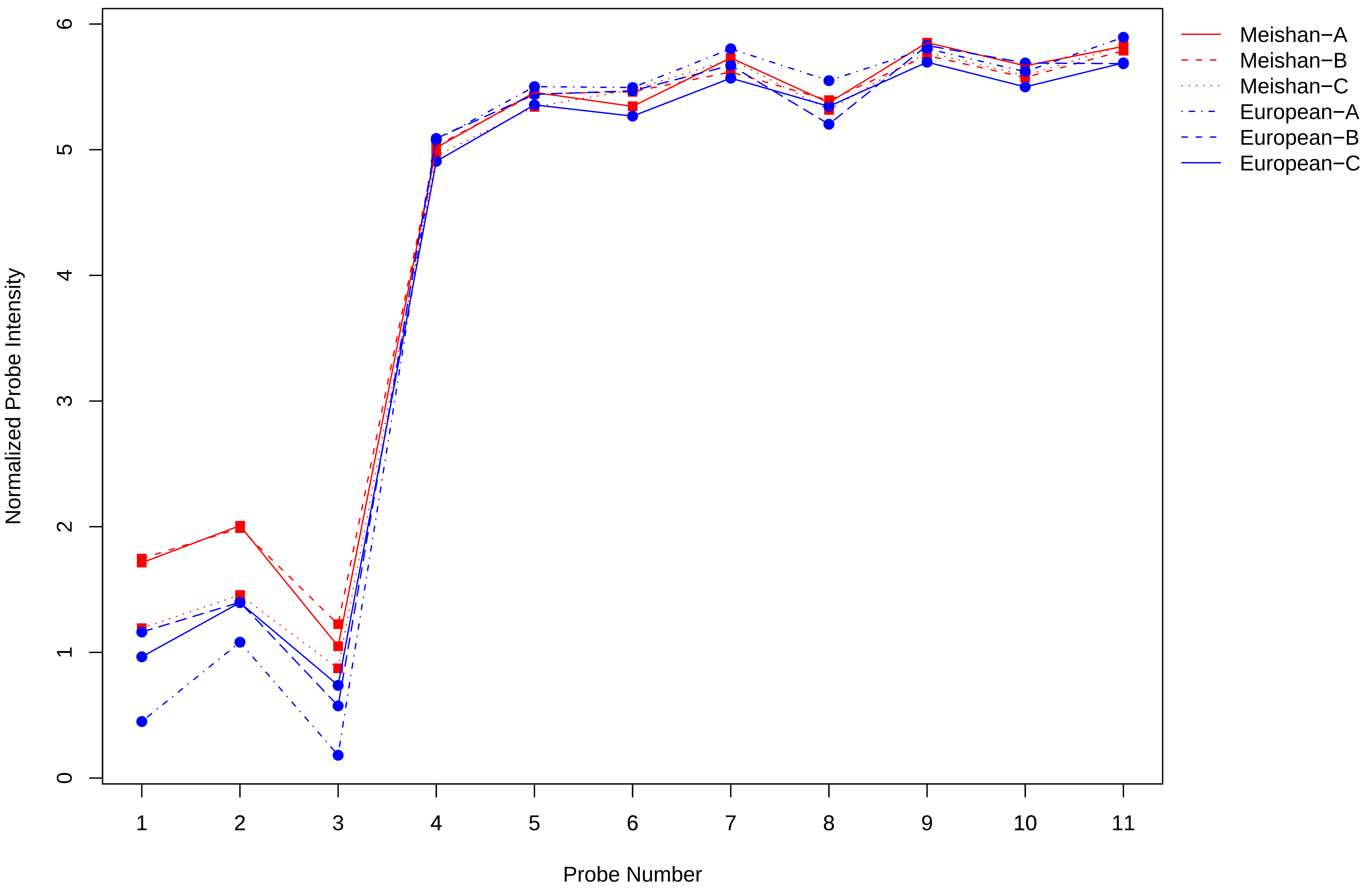

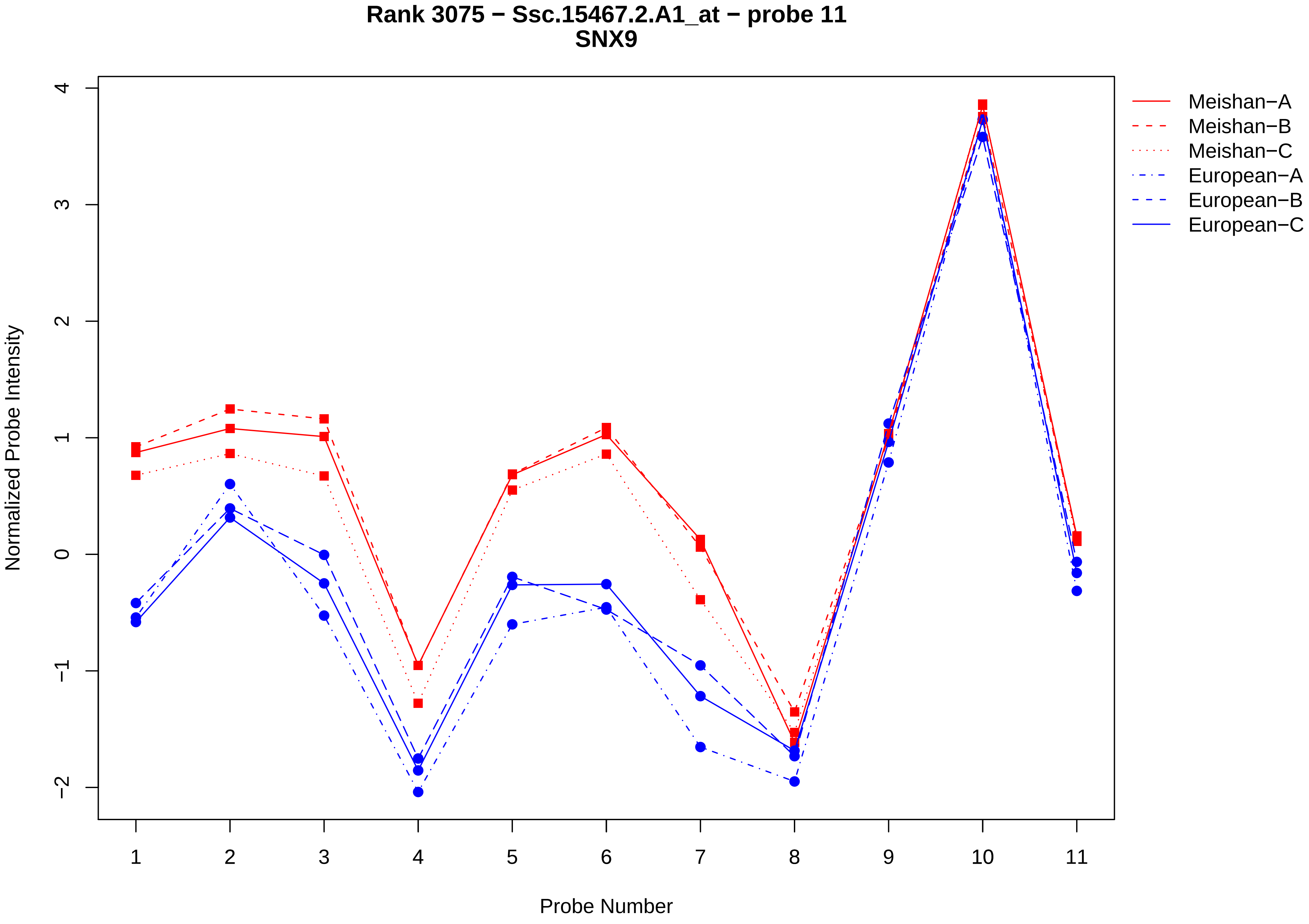

Rank 3075 − Ssc.15467.2.A1_at − probe 11
SNX9
Normalized Probe Intensity
Probe Number
Meishan−A
Meishan−B
Meishan−C
European−A
European−B
European−C

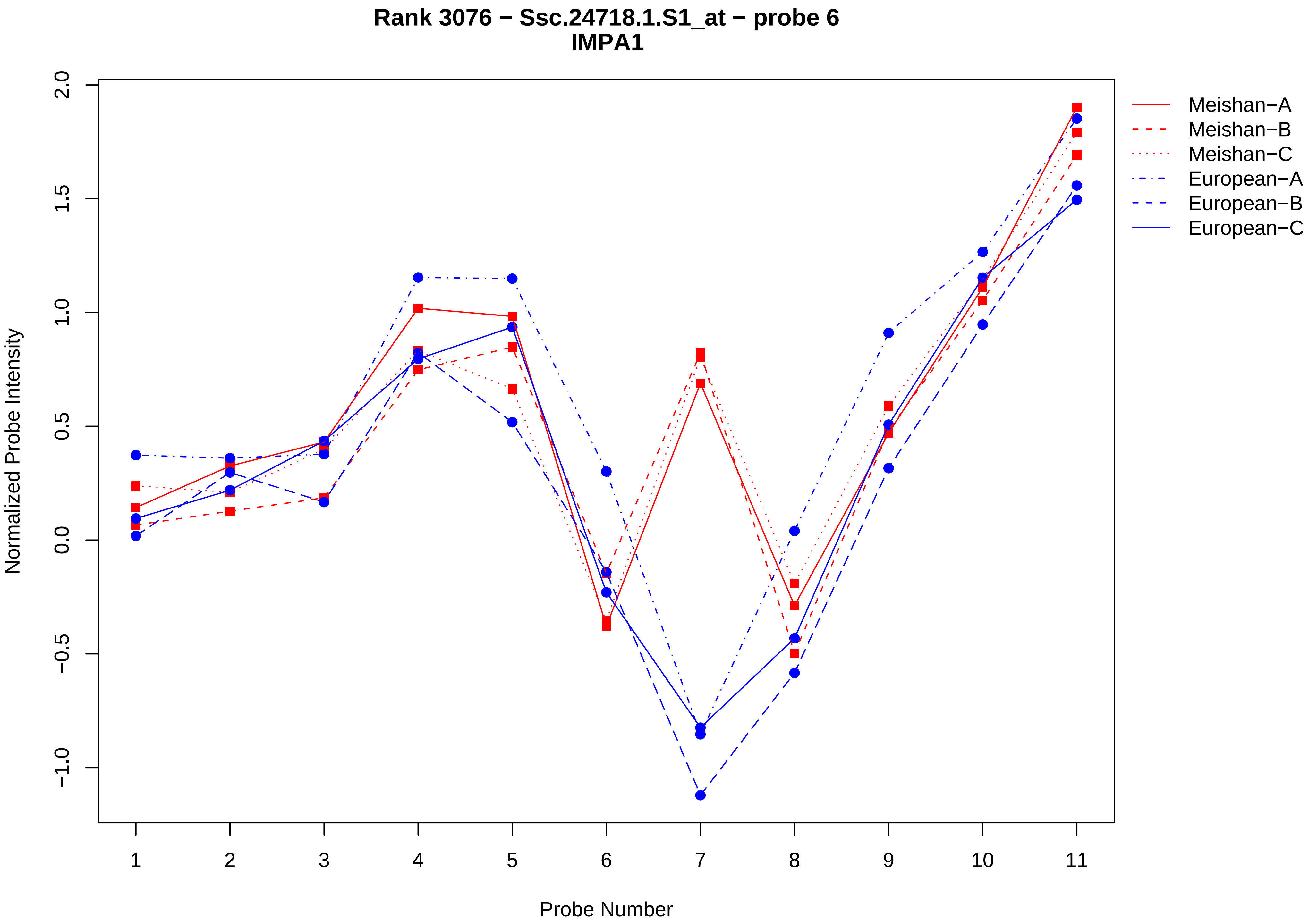

Rank 3076 − Ssc.24718.1.S1_at − probe 6
IMPA1
Normalized Probe Intensity
Probe Number
Meishan−A
Meishan−B
Meishan−C
European−A
European−B
European−C
−1.0
−0.5
0.0
0.5
1.0
1.5
2.0
1
2
3
4
5
6
7
8
9
10
11

Rank 3077 − Ssc.21714.1.S1_at − probe 1
Q96A72

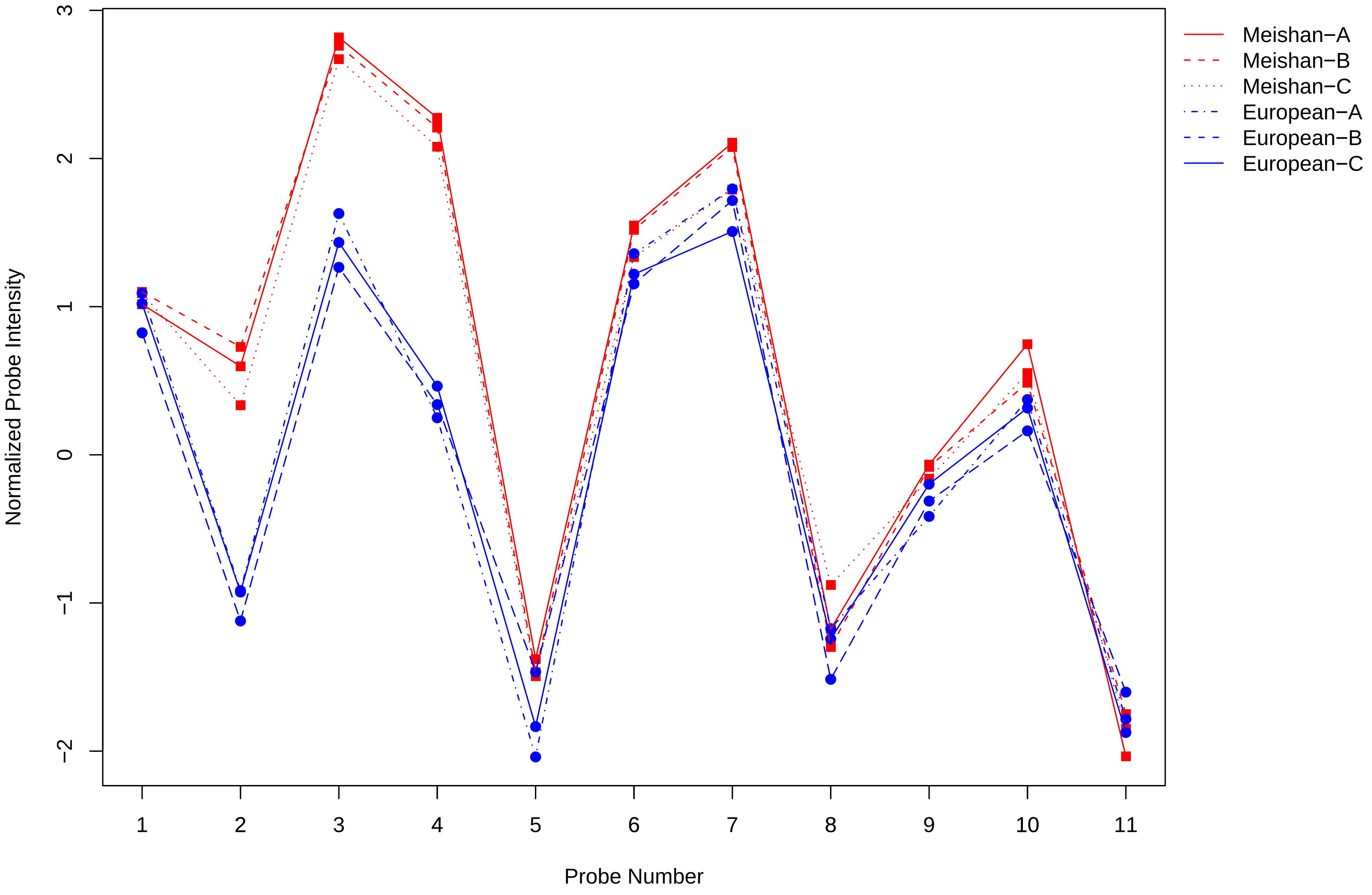

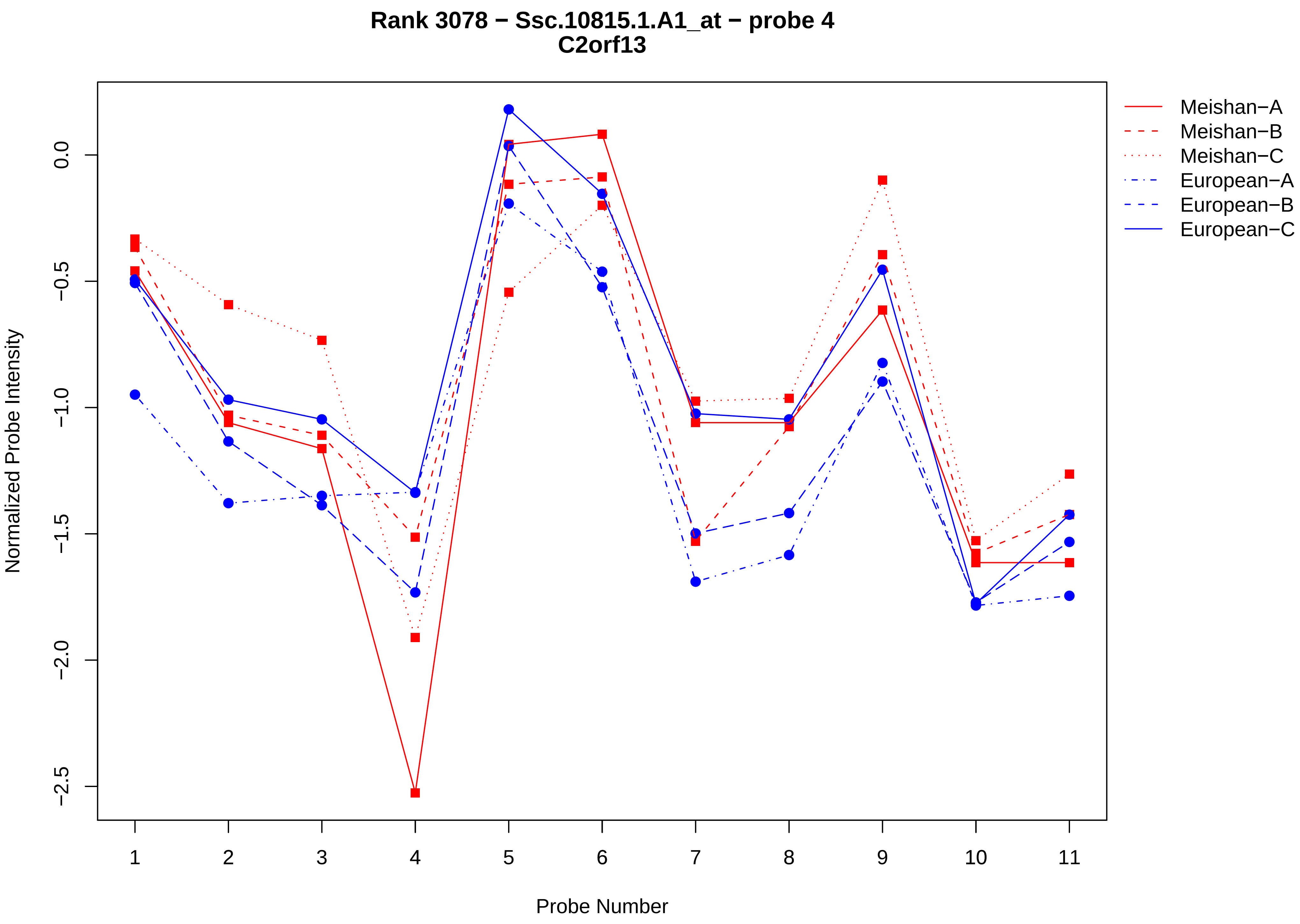

Rank 3078 − Ssc.10815.1.A1_at − probe 4
C2orf13
Normalized Probe Intensity
Probe Number
0.0
−0.5
−1.0
−1.5
−2.0
−2.5
1
2
3
4
5
6
7
8
9
10
11
Meishan−A
Meishan−B
Meishan−C
European−A
European−B
European−C

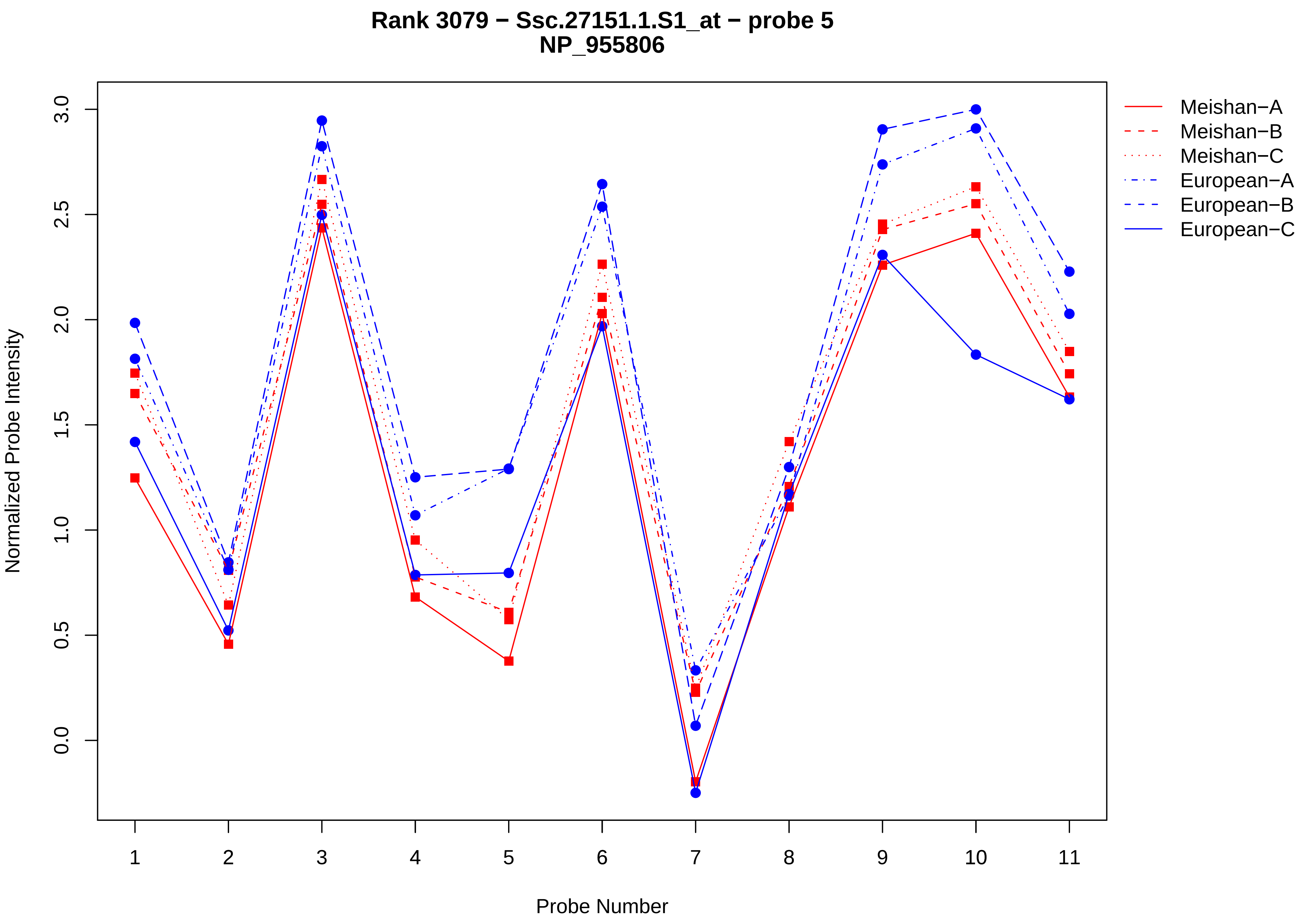

Rank 3079 − Ssc.27151.1.S1_at − probe 5
NP_955806
Normalized Probe Intensity
Probe Number
0.0
0.5
1.0
1.5
2.0
2.5
3.0
1
2
3
4
5
6
7
8
9
10
11
Meishan−A
Meishan−B
Meishan−C
European−A
European−B
European−C

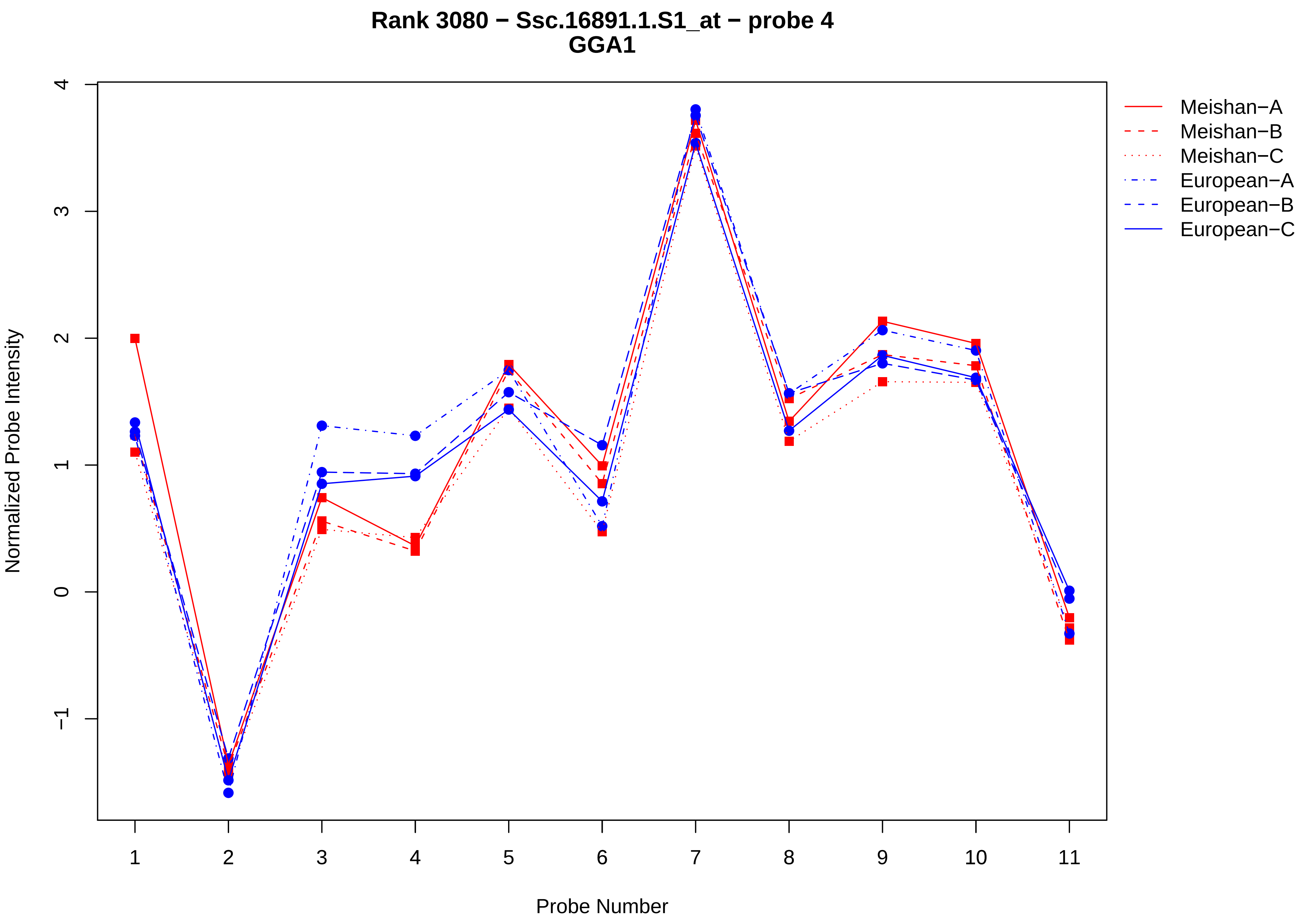

Rank 3080 − Ssc.16891.1.S1_at − probe 4
GGA1
Normalized Probe Intensity
Probe Number
−1
0
1
2
3
4
1
2
3
4
5
6
7
8
9
10
11
Meishan−A
Meishan−B
Meishan−C
European−A
European−B
European−C

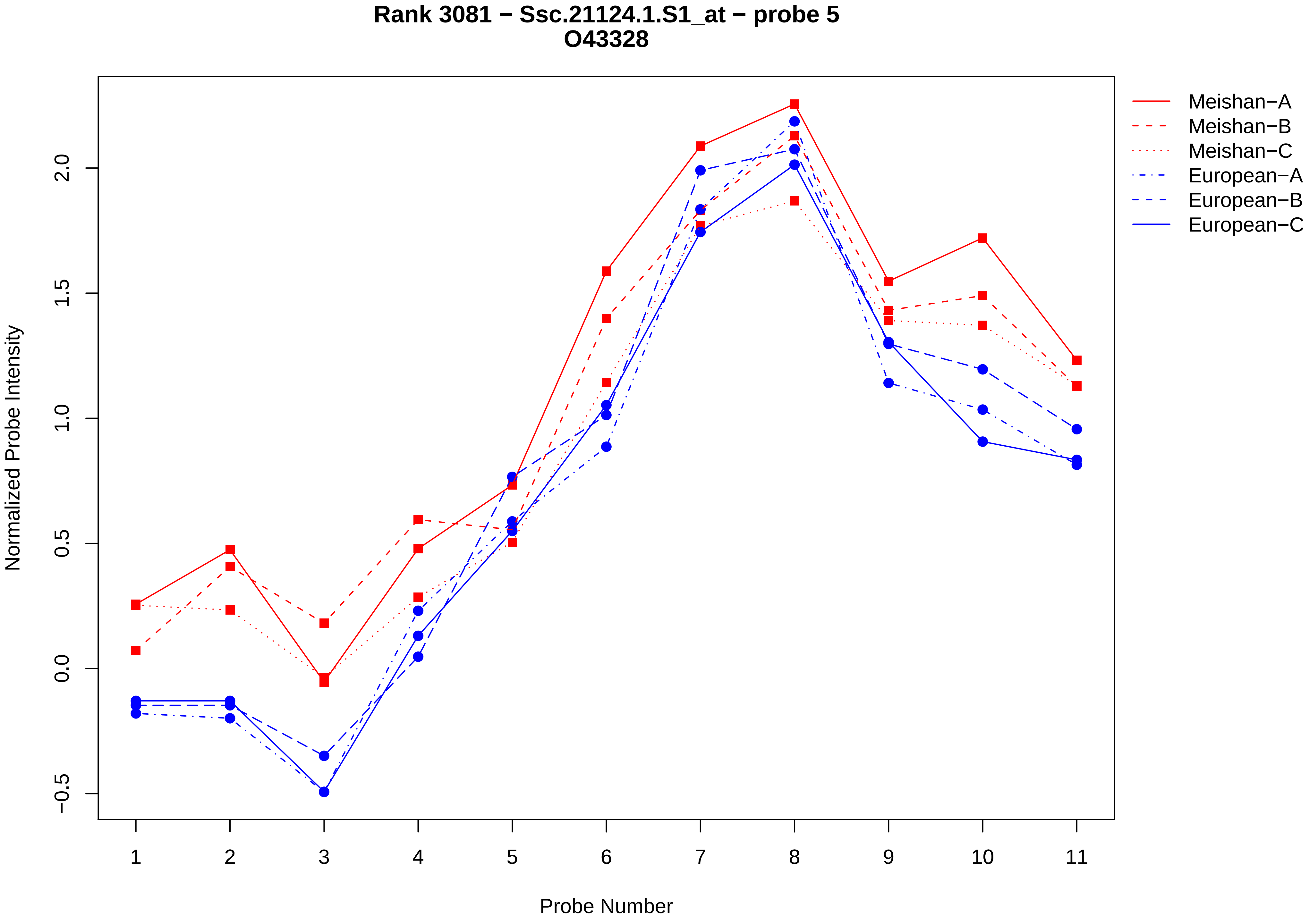

Rank 3081 − Ssc.21124.1.S1_at − probe 5
O43328
Normalized Probe Intensity
−0.5
0.0
0.5
1.0
1.5
2.0
1
2
3
4
5
6
7
8
9
10
11
Probe Number
Meishan−A
Meishan−B
Meishan−C
European−A
European−B
European−C

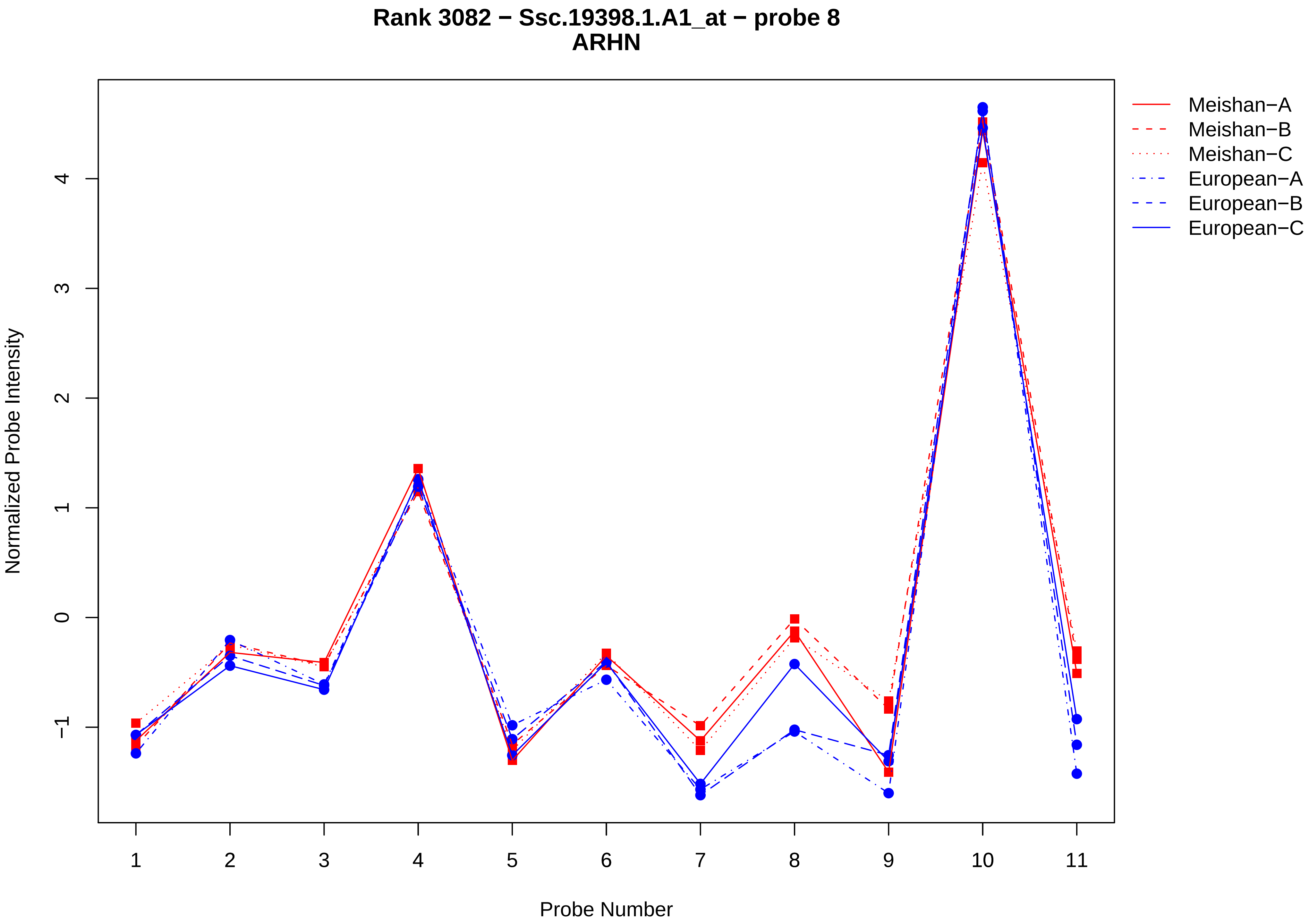

Rank 3082 − Ssc.19398.1.A1_at − probe 8
ARHN
Normalized Probe Intensity
Probe Number
−1
0
1
2
3
4
1
2
3
4
5
6
7
8
9
10
11
Meishan−A
Meishan−B
Meishan−C
European−A
European−B
European−C

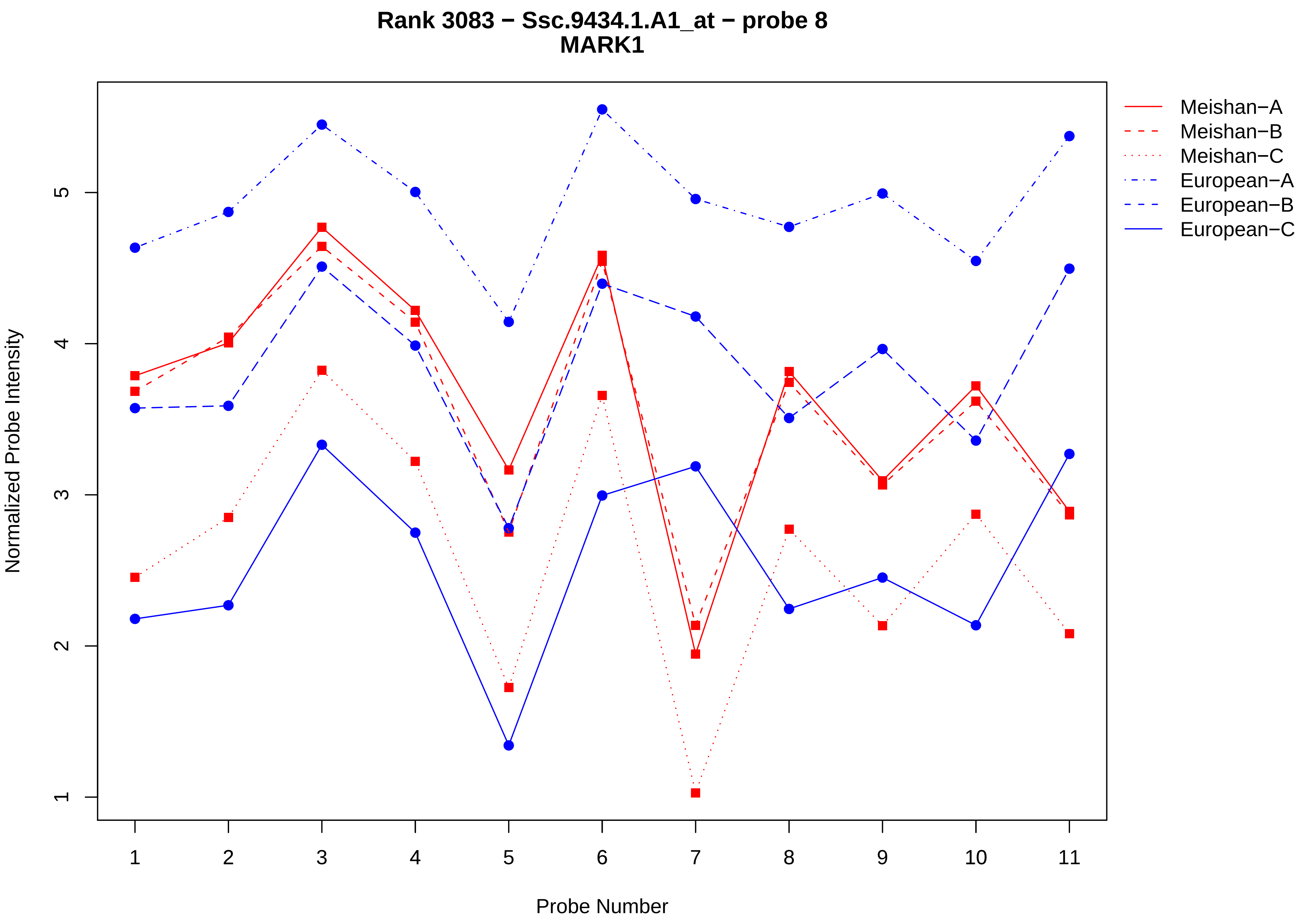

Rank 3083 − Ssc.9434.1.A1_at − probe 8
MARK1
Normalized Probe Intensity
Probe Number
1
2
3
4
5
6
7
8
9
10
11
Meishan−A
Meishan−B
Meishan−C
European−A
European−B
European−C

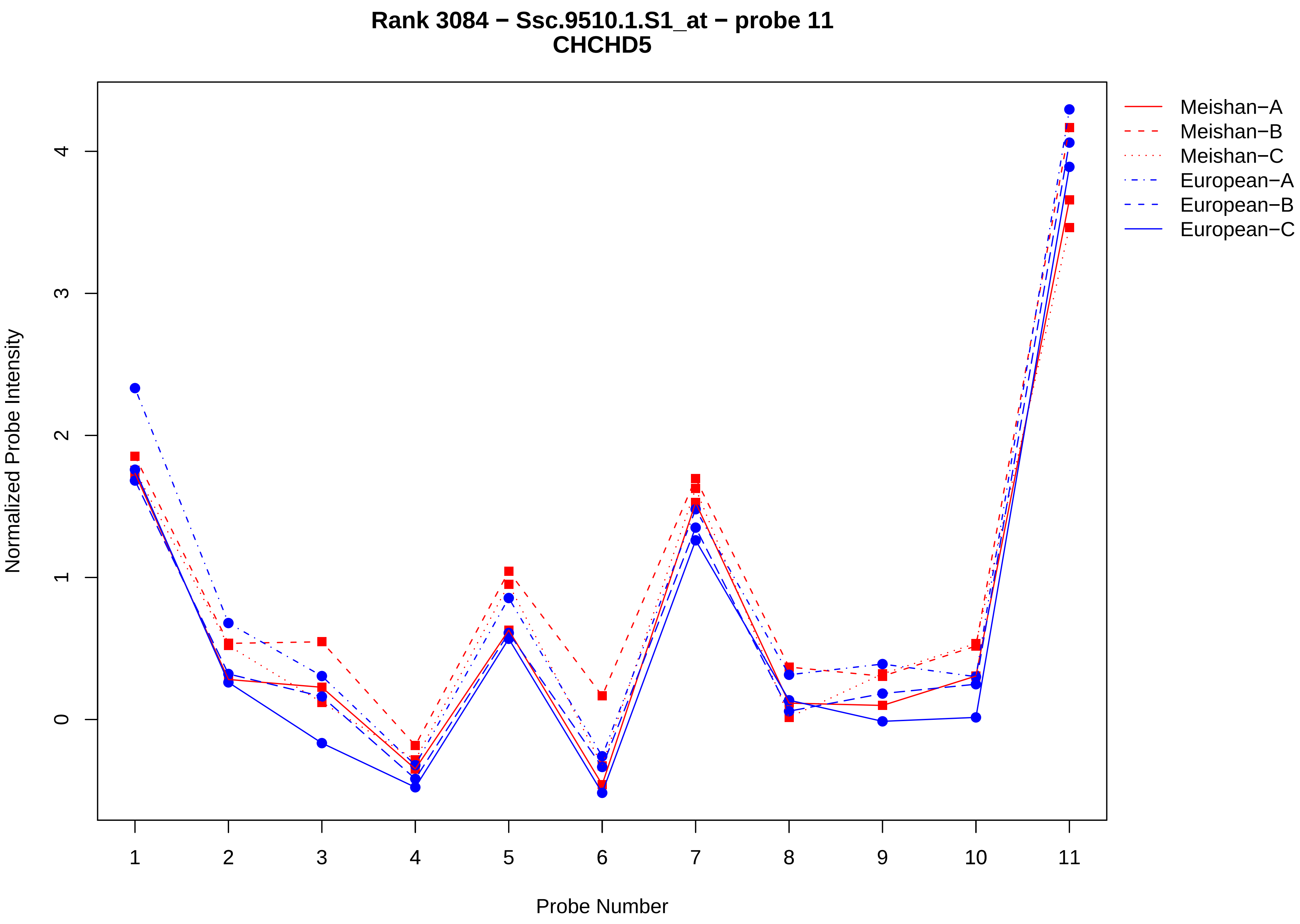
Rank 3084 − Ssc.9510.1.S1_at − probe 11
CHCHD5
Normalized Probe Intensity
Probe Number
0
1
2
3
4
1
2
3
4
5
6
7
8
9
10
11
Meishan−A
Meishan−B
Meishan−C
European−A
European−B
European−C

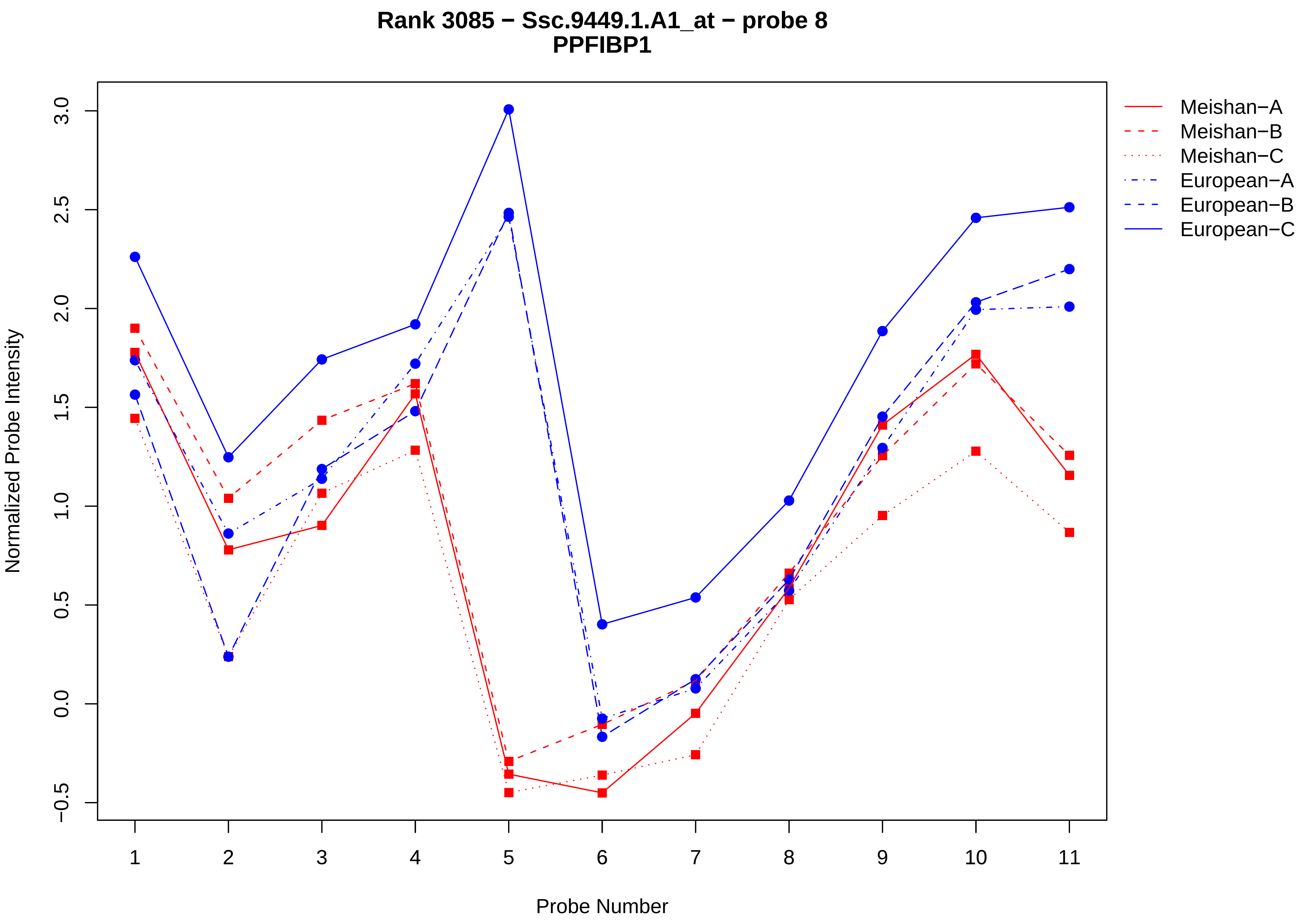

Rank 3085 − Ssc.9449.1.A1_at − probe 8
PPFIBP1
Normalized Probe Intensity
Probe Number
−0.5
0.0
0.5
1.0
1.5
2.0
2.5
3.0
1
2
3
4
5
6
7
8
9
10
11
Meishan−A
Meishan−B
Meishan−C
European−A
European−B
European−C

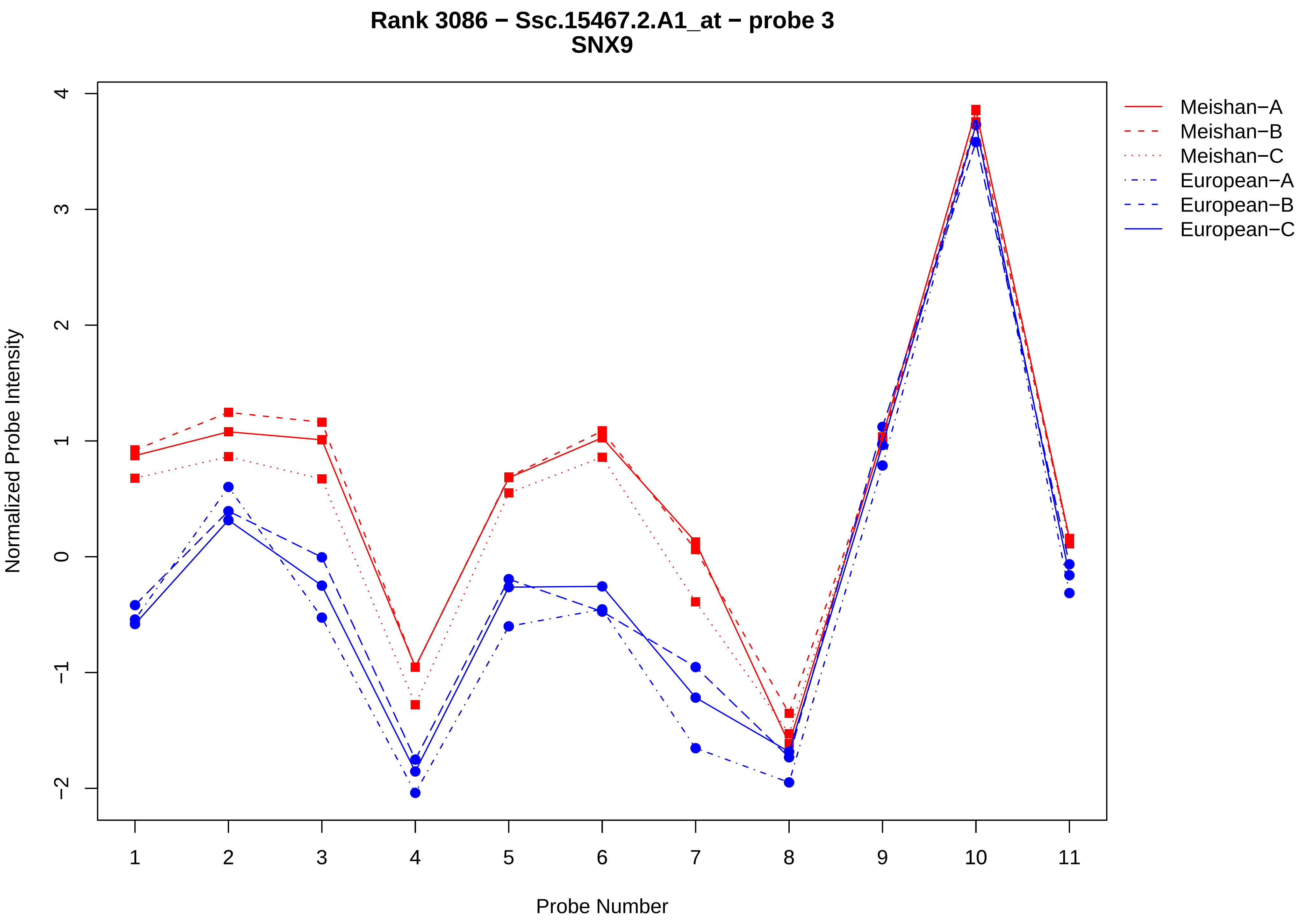

Rank 3086 − Ssc.15467.2.A1_at − probe 3
SNX9
Normalized Probe Intensity
Probe Number
Meishan−A
Meishan−B
Meishan−C
European−A
European−B
European−C

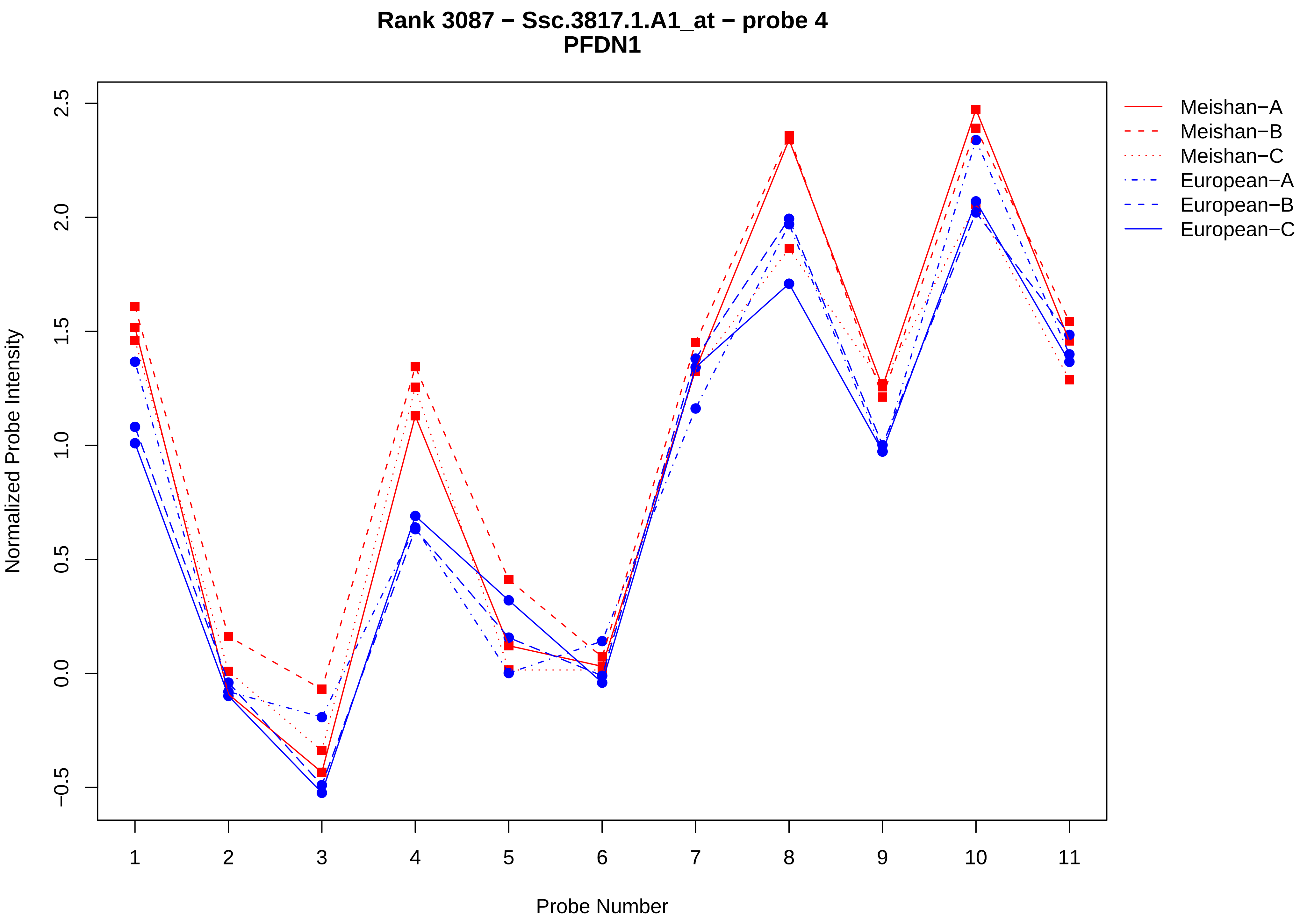

Rank 3087 − Ssc.3817.1.A1_at − probe 4
PFDN1
Normalized Probe Intensity
−0.5
0.0
0.5
1.0
1.5
2.0
2.5
1
2
3
4
5
6
7
8
9
10
11
Probe Number
Meishan−A
Meishan−B
Meishan−C
European−A
European−B
European−C

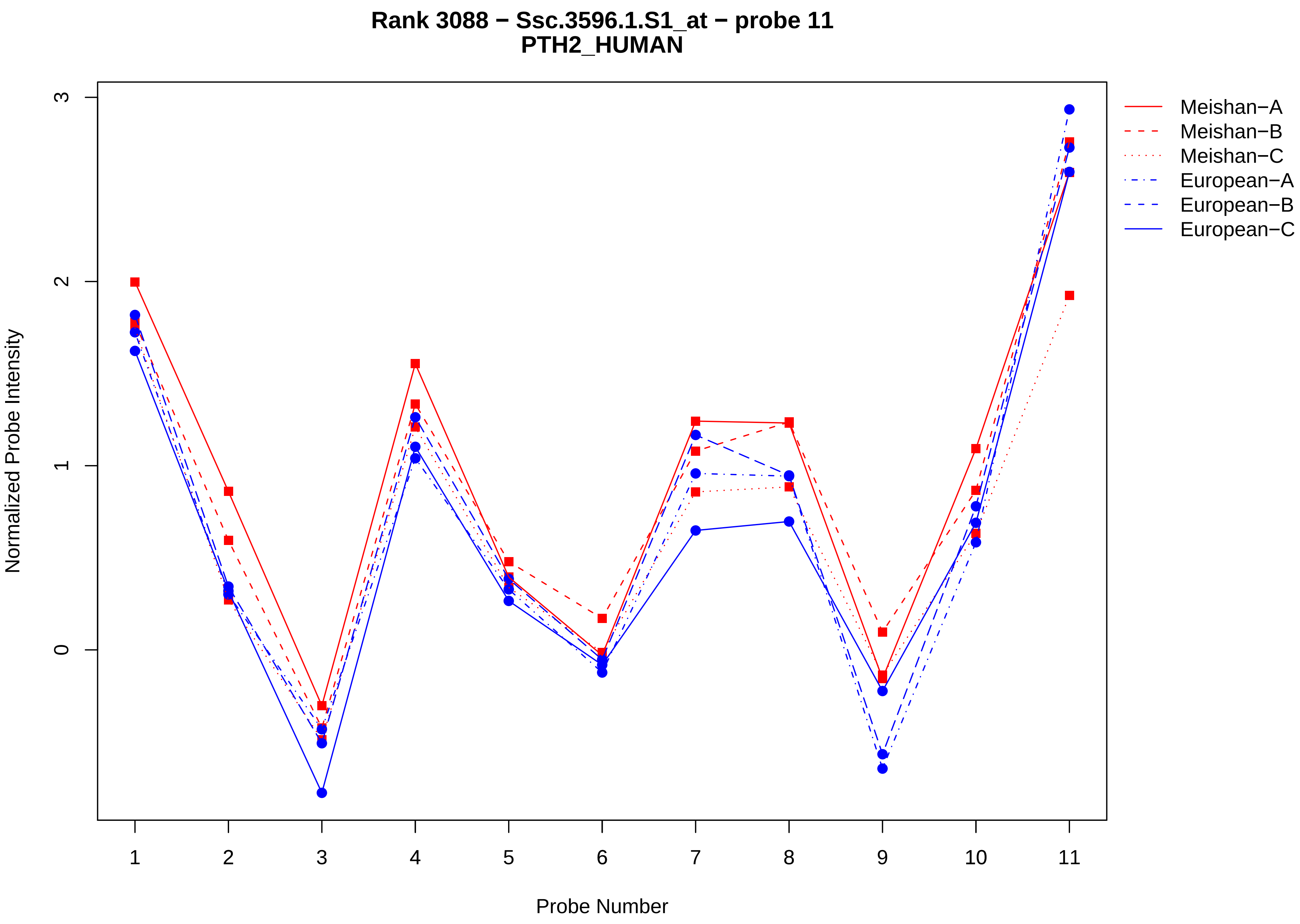

Rank 3088 − Ssc.3596.1.S1_at − probe 11
PTH2_HUMAN
Normalized Probe Intensity
Probe Number
0
1
2
3
1
2
3
4
5
6
7
8
9
10
11
Meishan−A
Meishan−B
Meishan−C
European−A
European−B
European−C

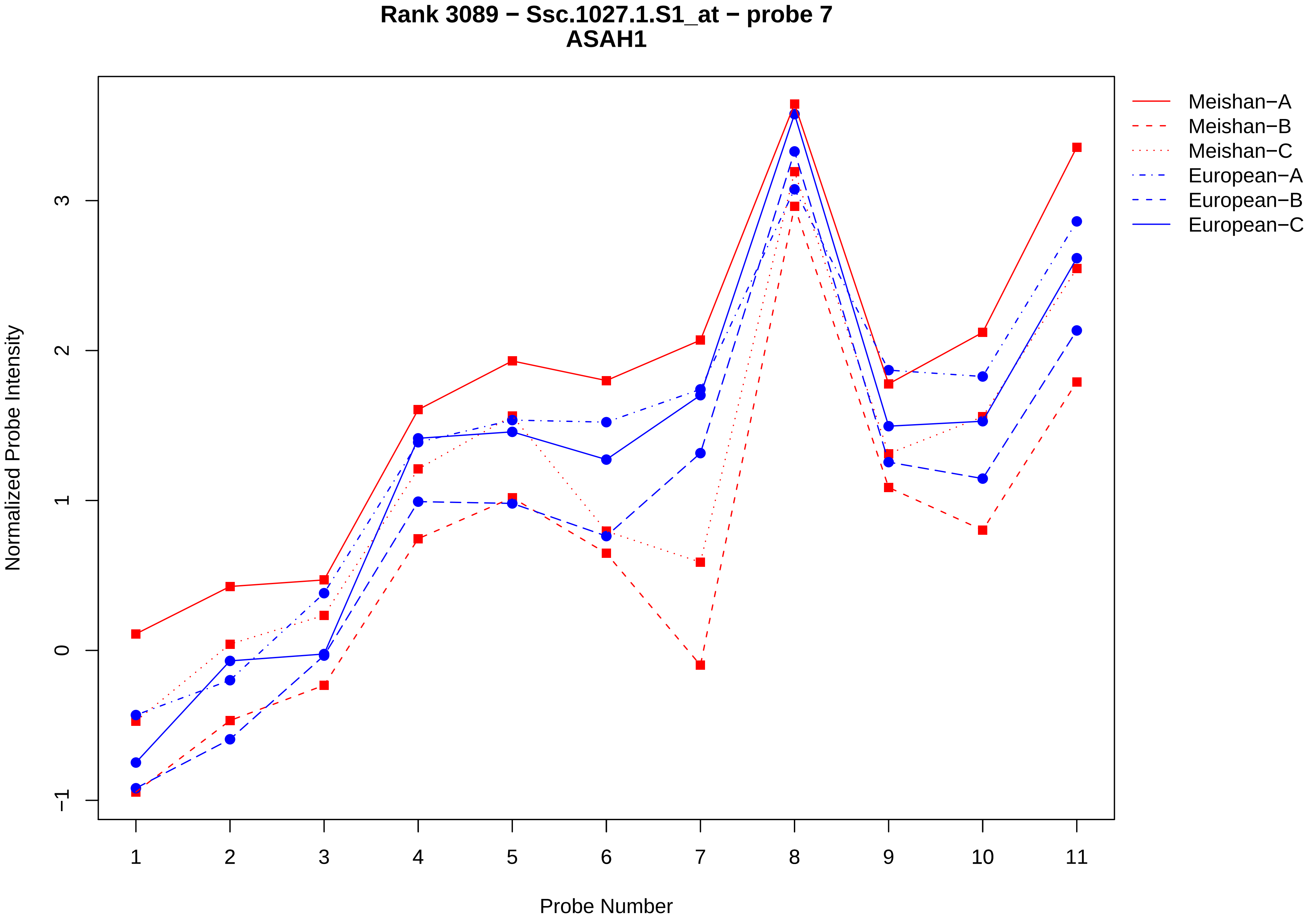
Rank 3089 − Ssc.1027.1.S1_at − probe 7
ASAH1
Normalized Probe Intensity
Probe Number
−1
0
1
2
3
1
2
3
4
5
6
7
8
9
10
11
Meishan−A
Meishan−B
Meishan−C
European−A
European−B
European−C

Rank 3090 − Ssc.19486.1.S1_at − probe 5
CCNB1IP1

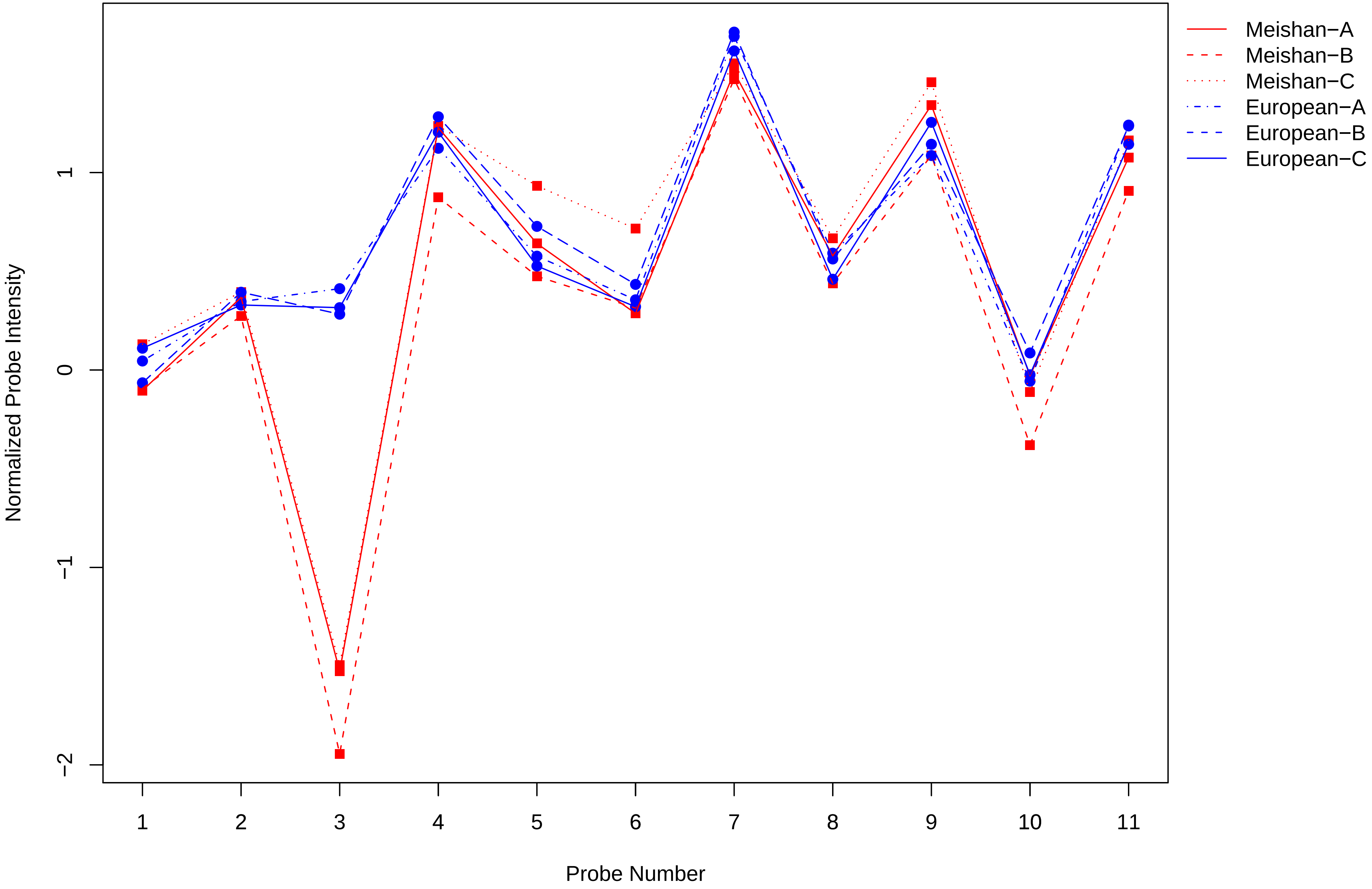

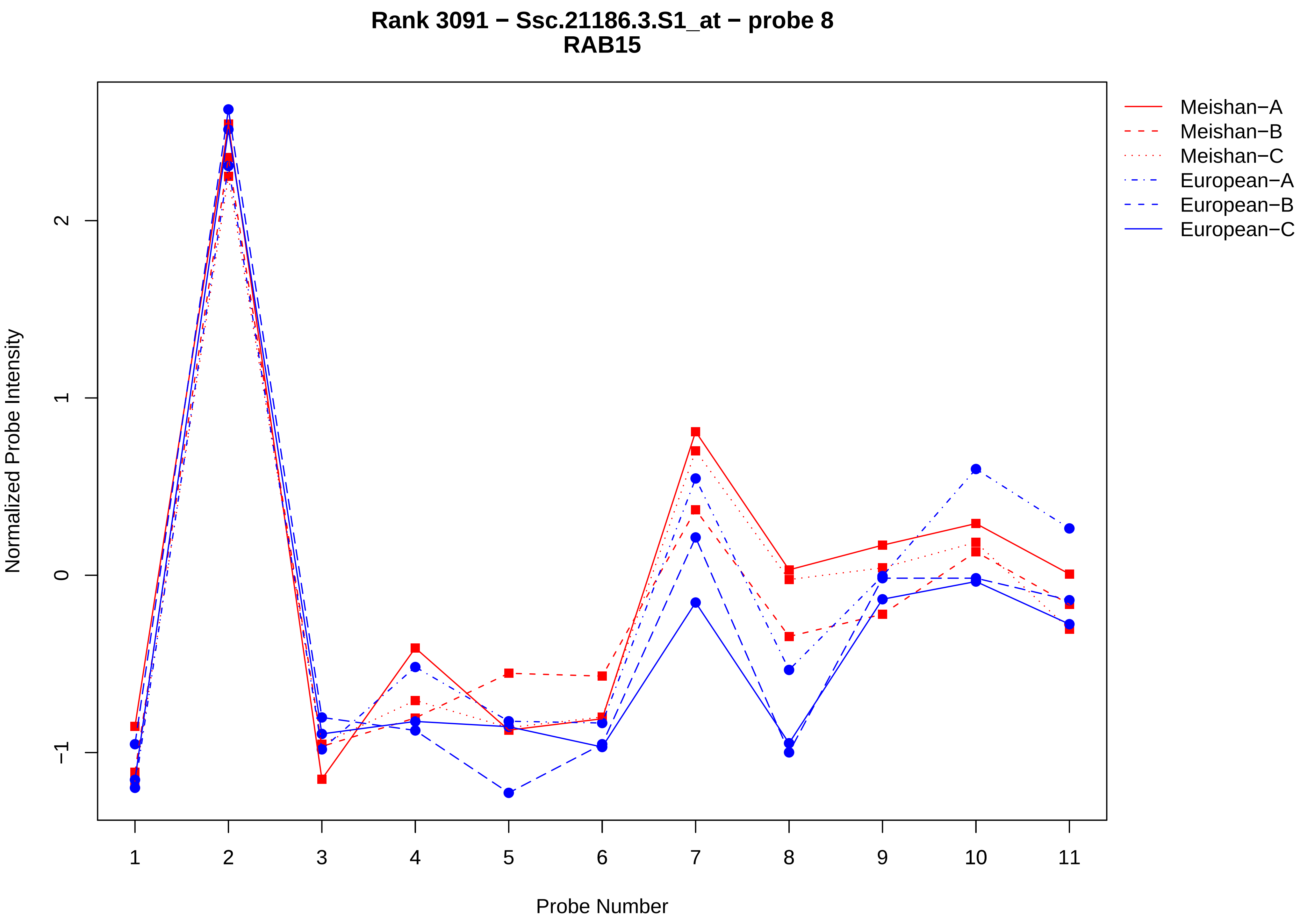

Rank 3091 − Ssc.21186.3.S1_at − probe 8
RAB15
Normalized Probe Intensity
Probe Number
Meishan−A
Meishan−B
Meishan−C
European−A
European−B
European−C

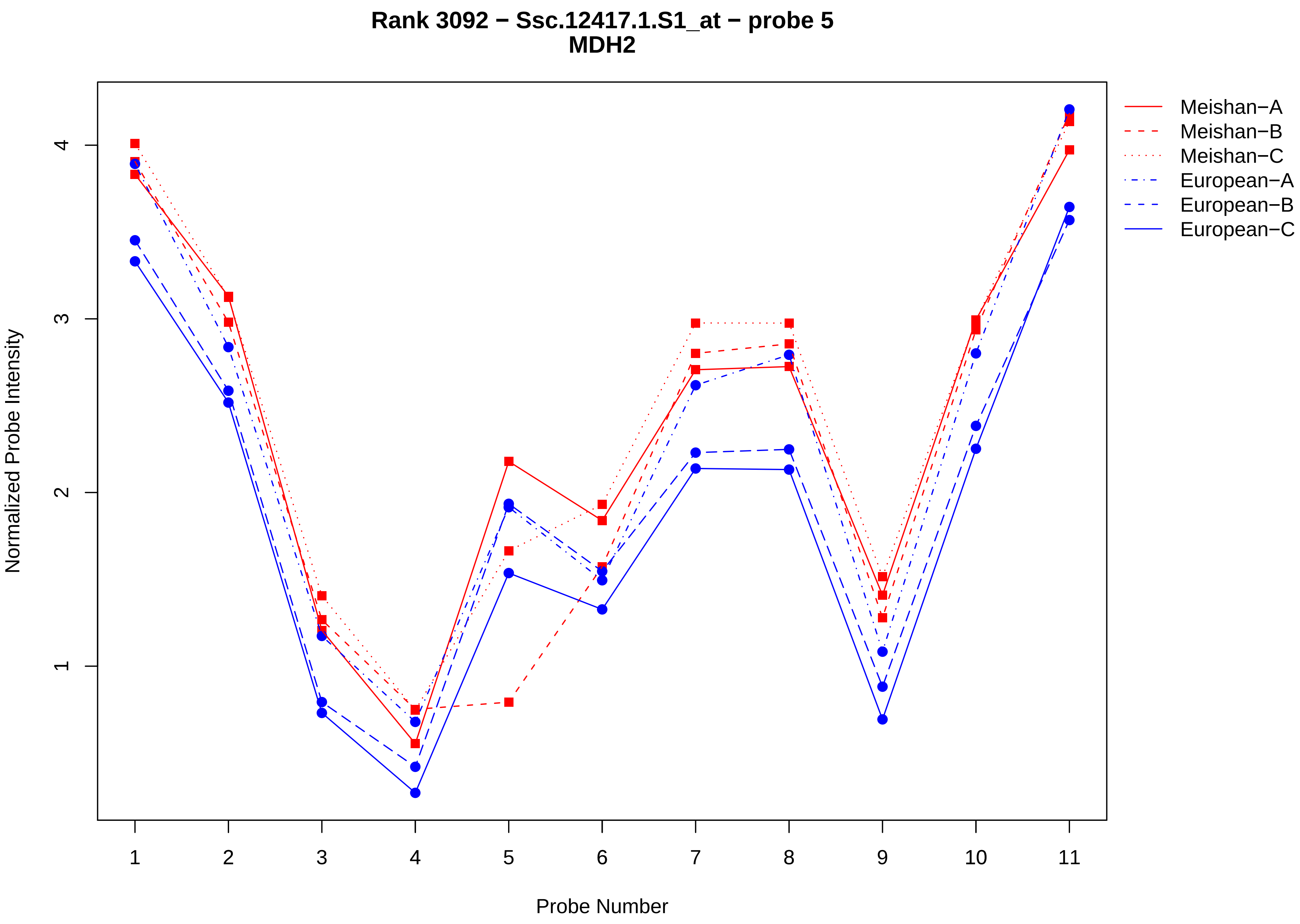

Rank 3092 − Ssc.12417.1.S1_at − probe 5
MDH2
Normalized Probe Intensity
Probe Number
1
2
3
4
1
2
3
4
5
6
7
8
9
10
11
Meishan−A
Meishan−B
Meishan−C
European−A
European−B
European−C

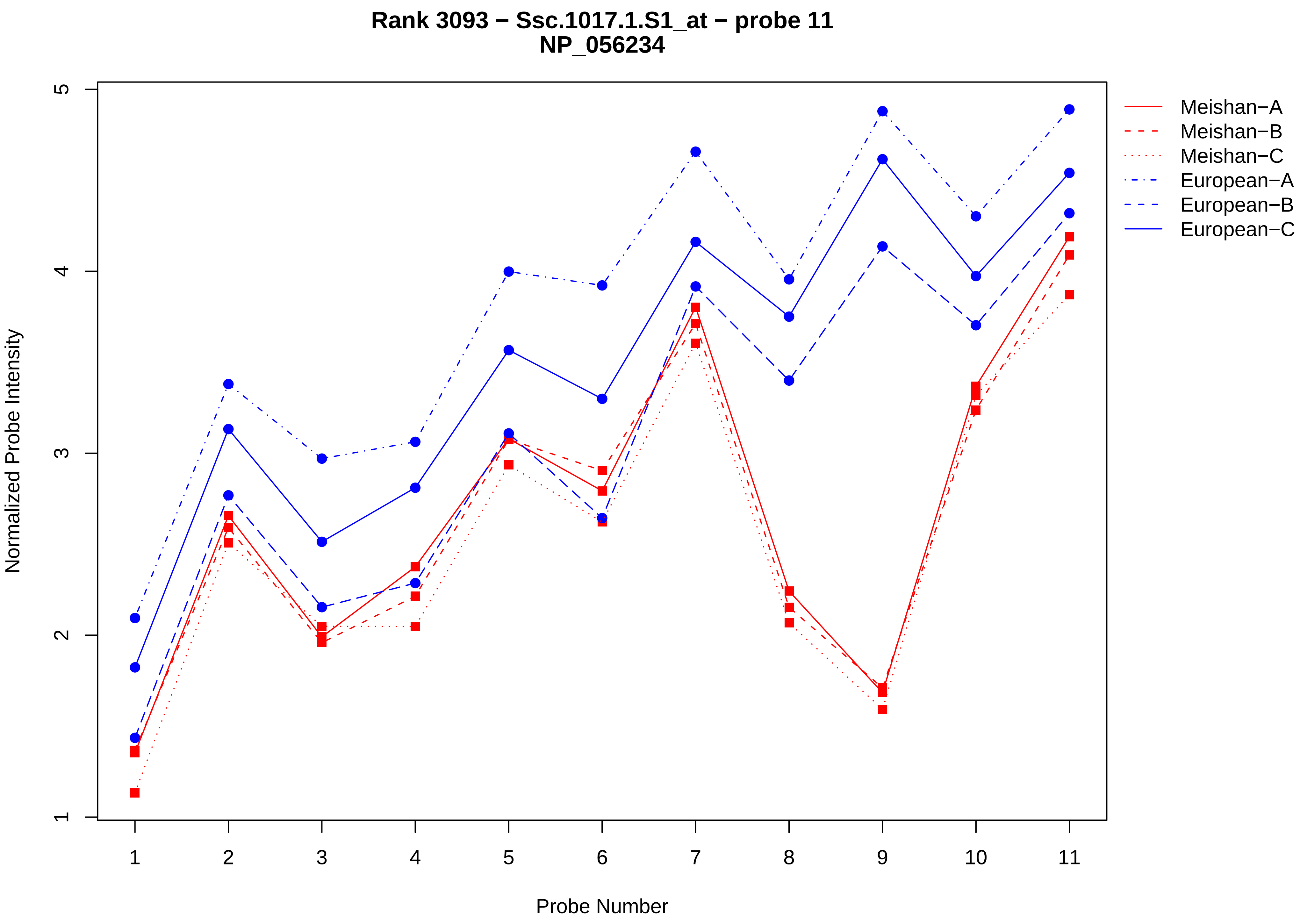

Rank 3093 − Ssc.1017.1.S1_at − probe 11
NP_056234
Normalized Probe Intensity
Probe Number
1
2
3
4
5
1
2
3
4
5
6
7
8
9
10
11
Meishan−A
Meishan−B
Meishan−C
European−A
European−B
European−C

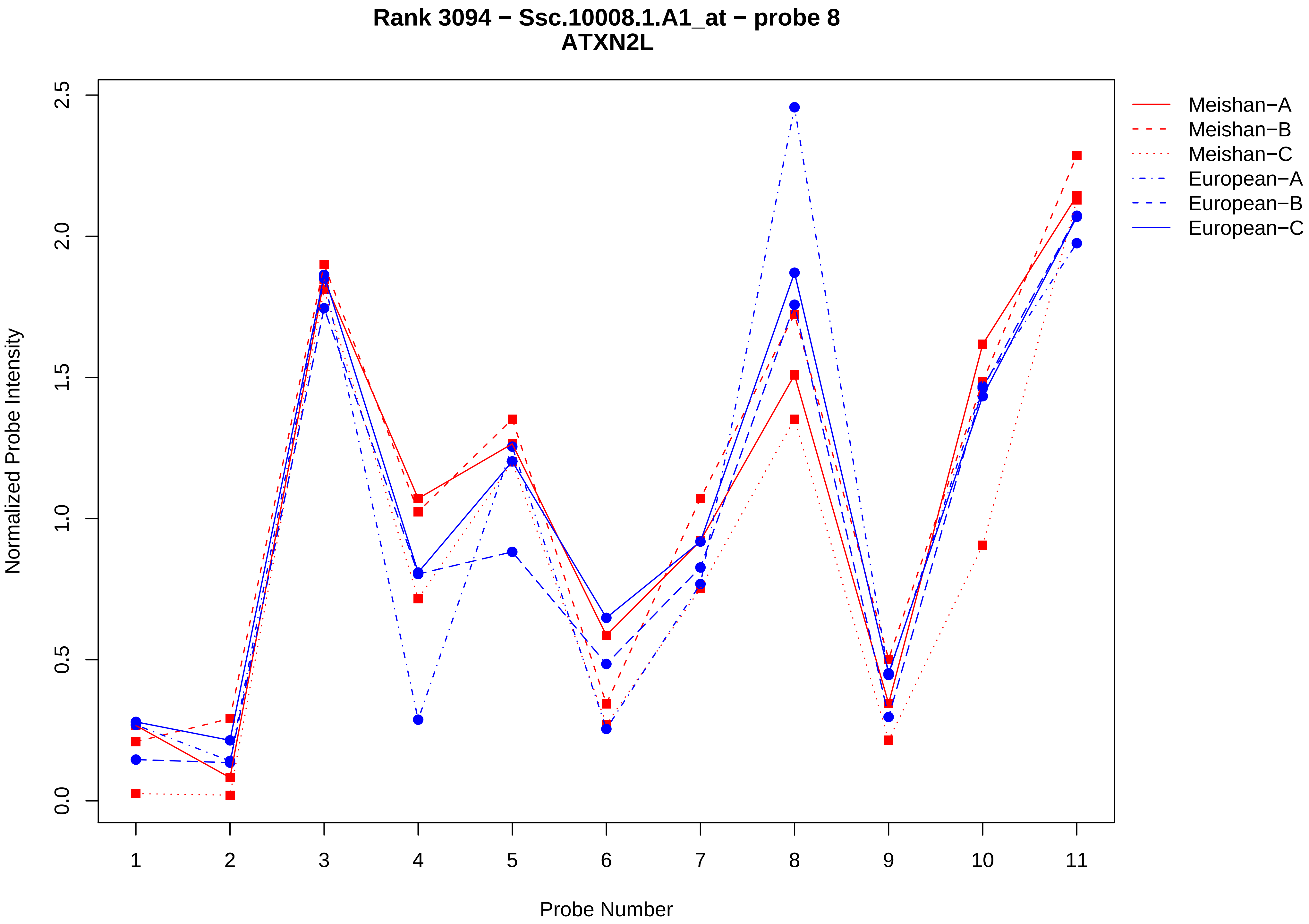
Rank 3094 − Ssc.10008.1.A1_at − probe 8
ATXN2L
Normalized Probe Intensity
0.0
0.5
1.0
1.5
2.0
2.5
Probe Number
1
2
3
4
5
6
7
8
9
10
11
Meishan−A
Meishan−B
Meishan−C
European−A
European−B
European−C

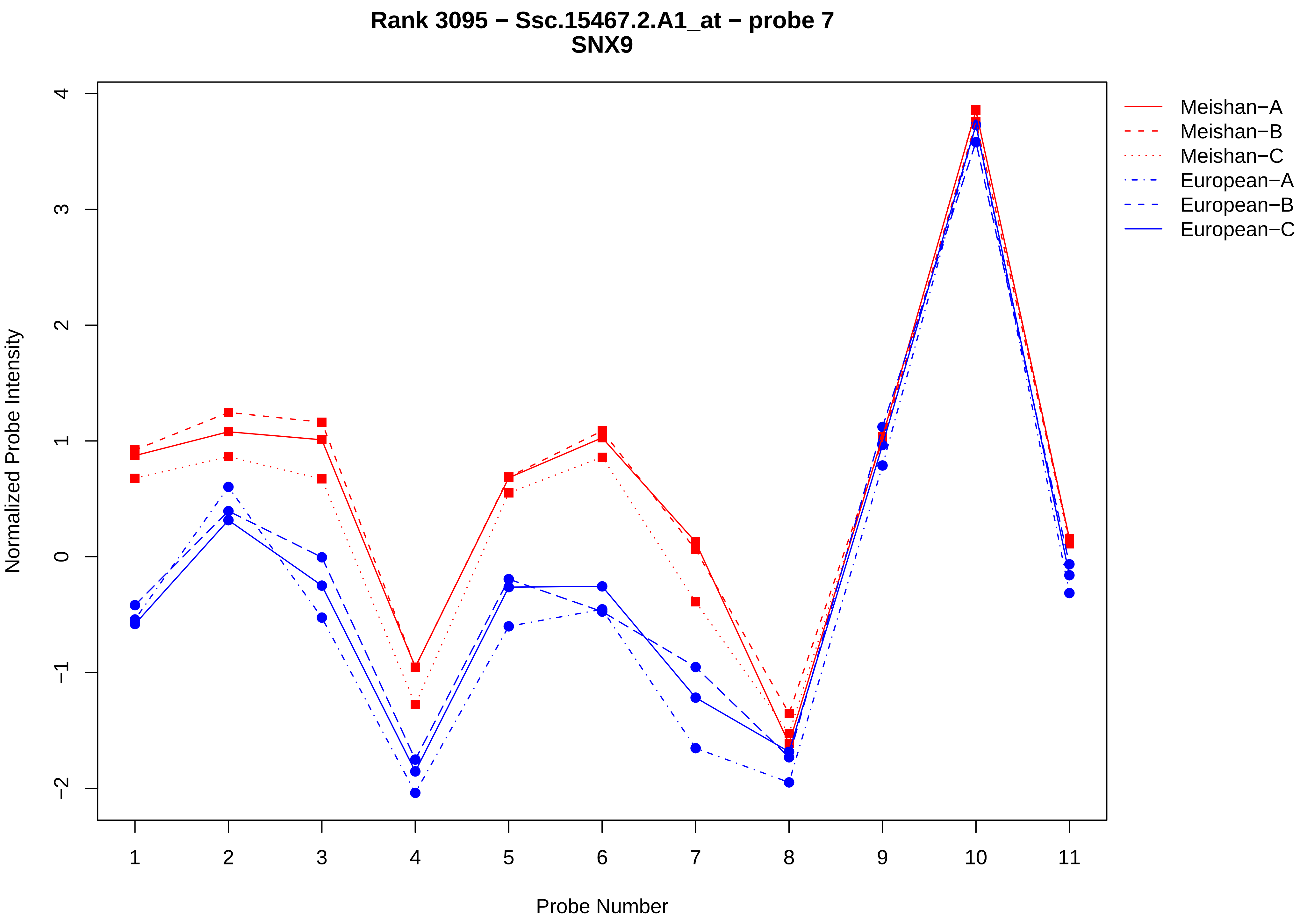

Rank 3095 − Ssc.15467.2.A1_at − probe 7
SNX9
Normalized Probe Intensity
Probe Number
Meishan−A
Meishan−B
Meishan−C
European−A
European−B
European−C
−2
−1
0
1
2
3
4
1
2
3
4
5
6
7
8
9
10
11

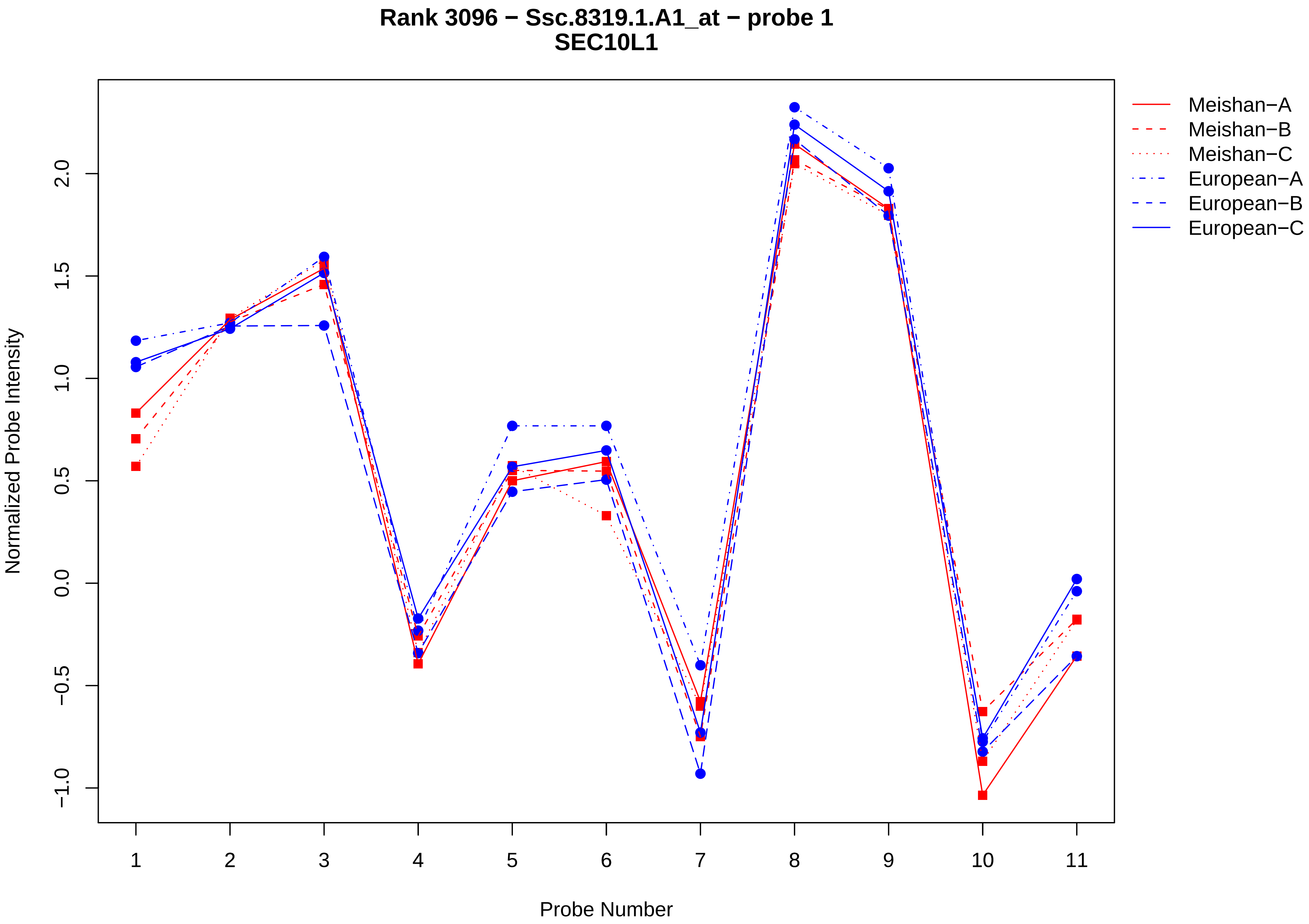

Rank 3096 − Ssc.8319.1.A1_at − probe 1
SEC10L1
Normalized Probe Intensity
Probe Number
−1.0
−0.5
0.0
0.5
1.0
1.5
2.0
1
2
3
4
5
6
7
8
9
10
11
Meishan−A
Meishan−B
Meishan−C
European−A
European−B
European−C

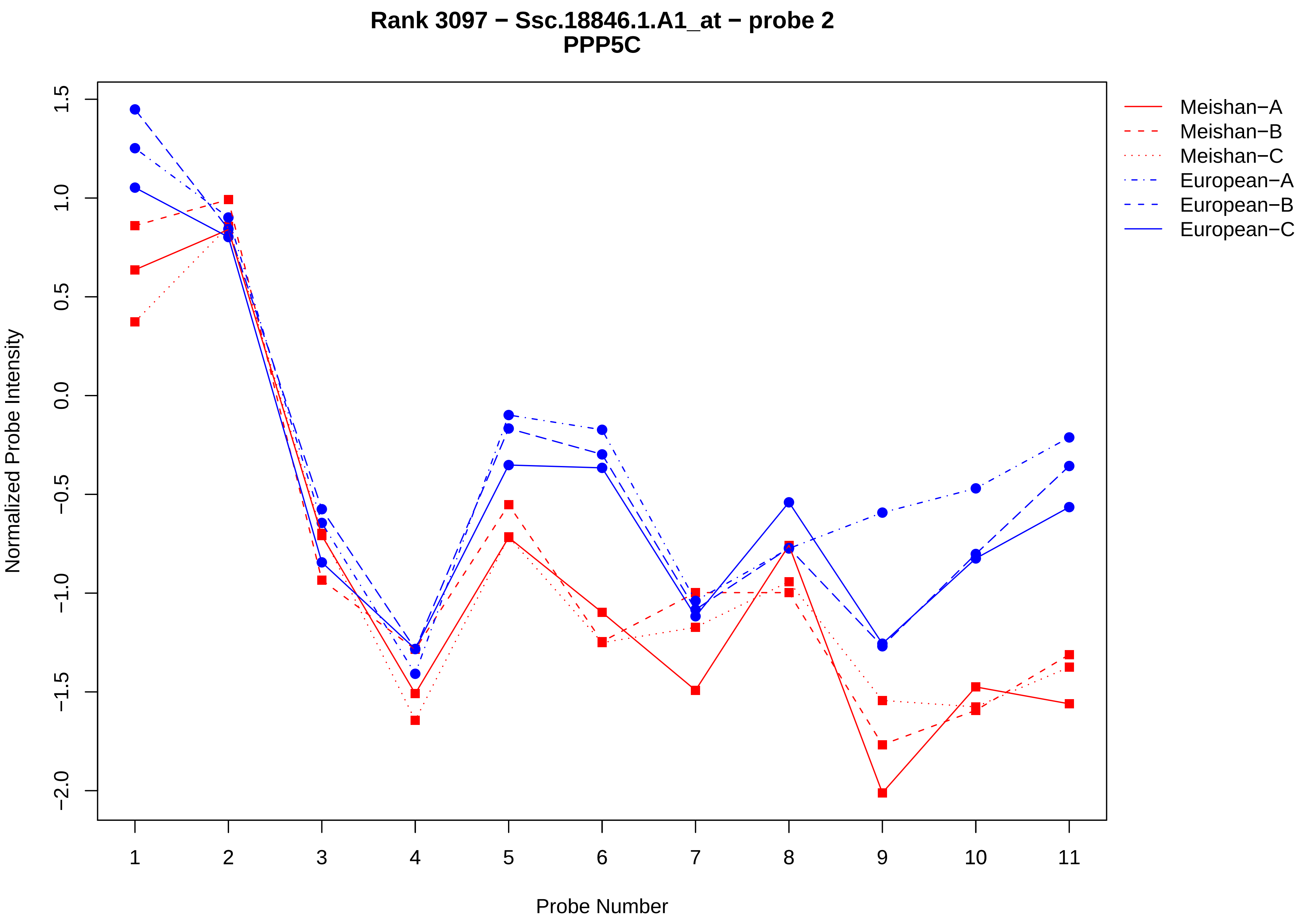

Rank 3097 − Ssc.18846.1.A1_at − probe 2
PPP5C
Normalized Probe Intensity
Probe Number
1.5
1.0
0.5
0.0
−0.5
−1.0
−1.5
−2.0
1
2
3
4
5
6
7
8
9
10
11
Meishan−A
Meishan−B
Meishan−C
European−A
European−B
European−C

Rank 3098 − Ssc.16422.2.A1_at − probe 9
PLAA

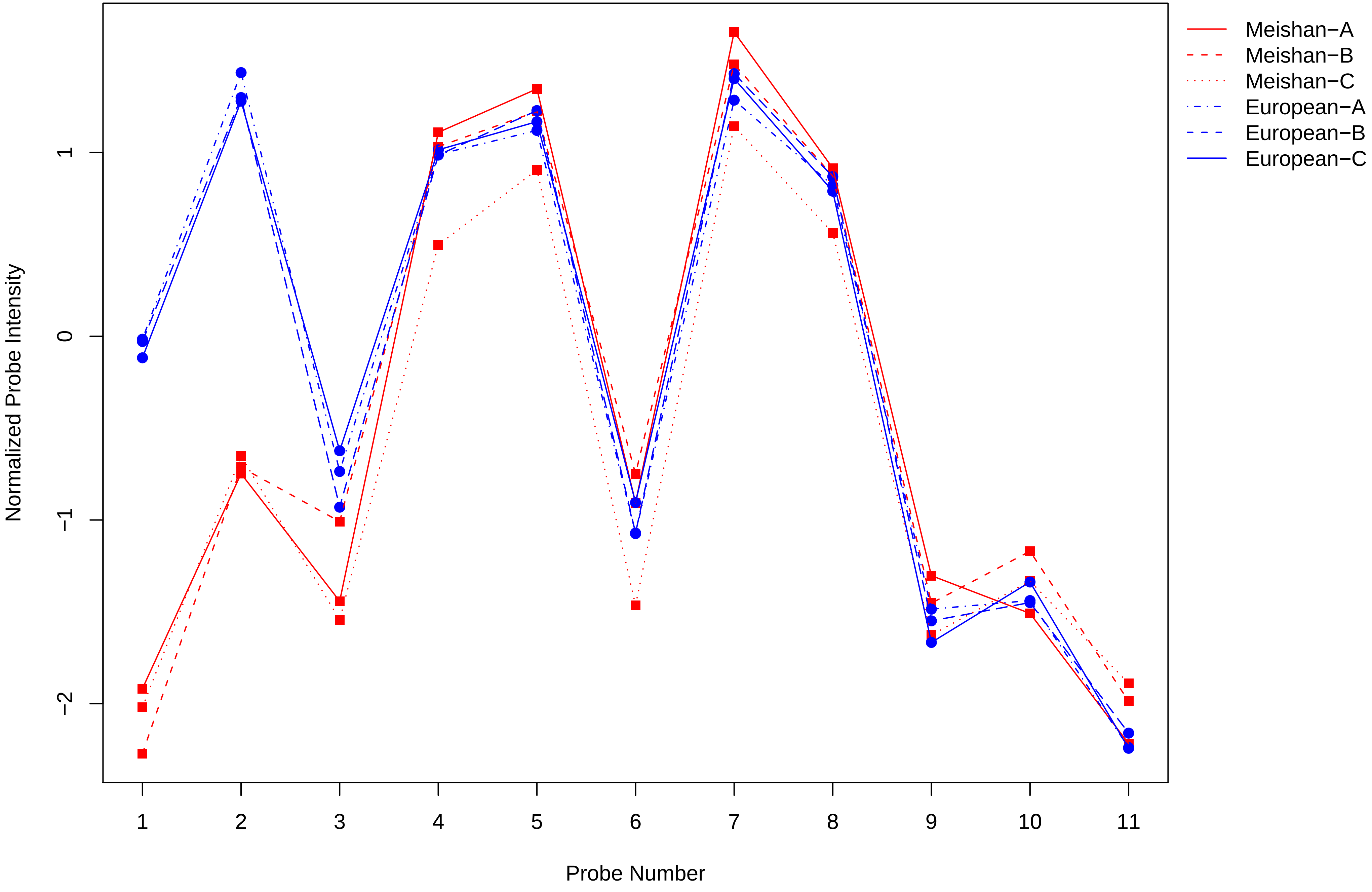

Rank 3099 − Ssc.9673.1.A1_at − probe 4
CASP2

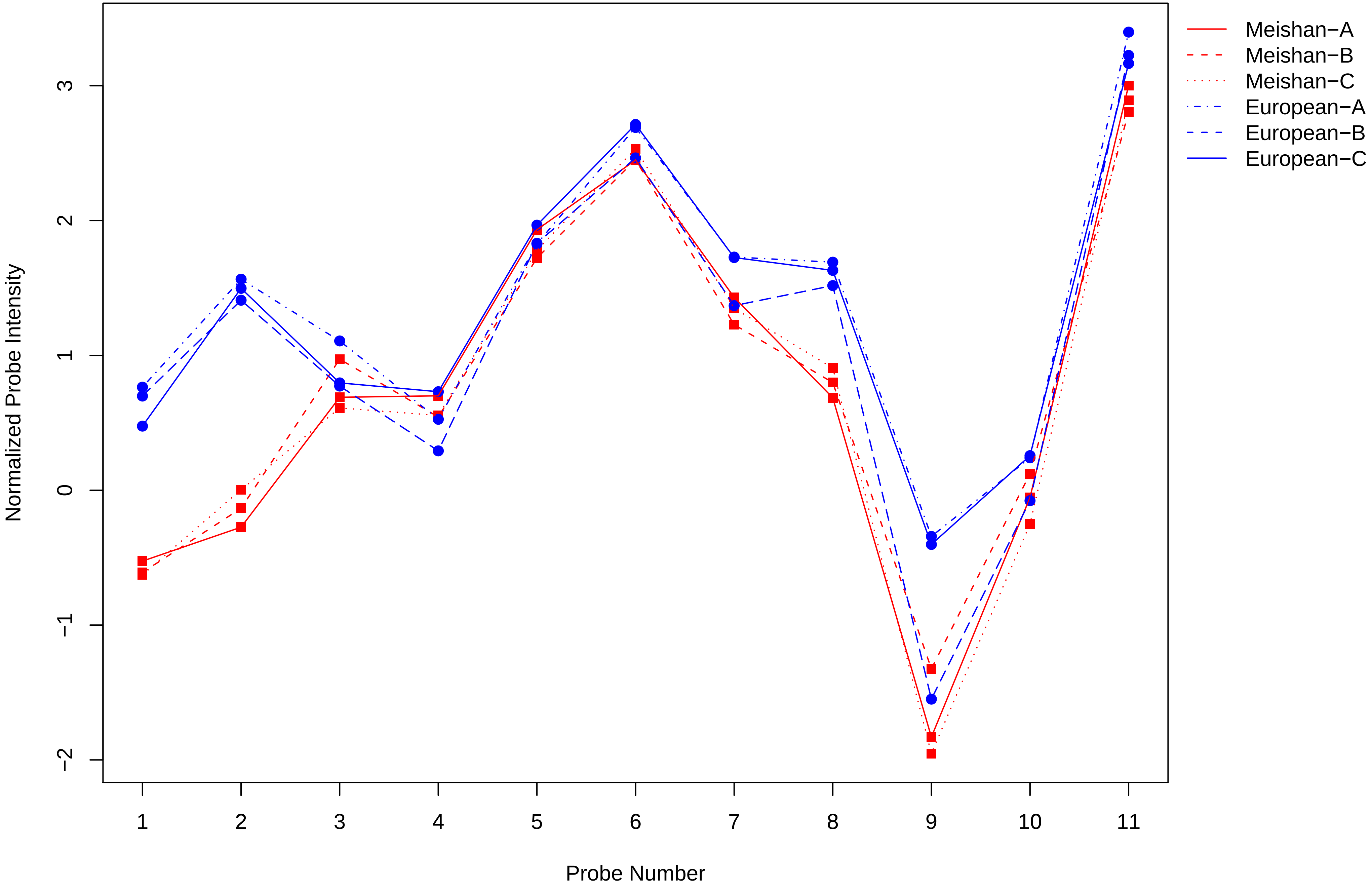

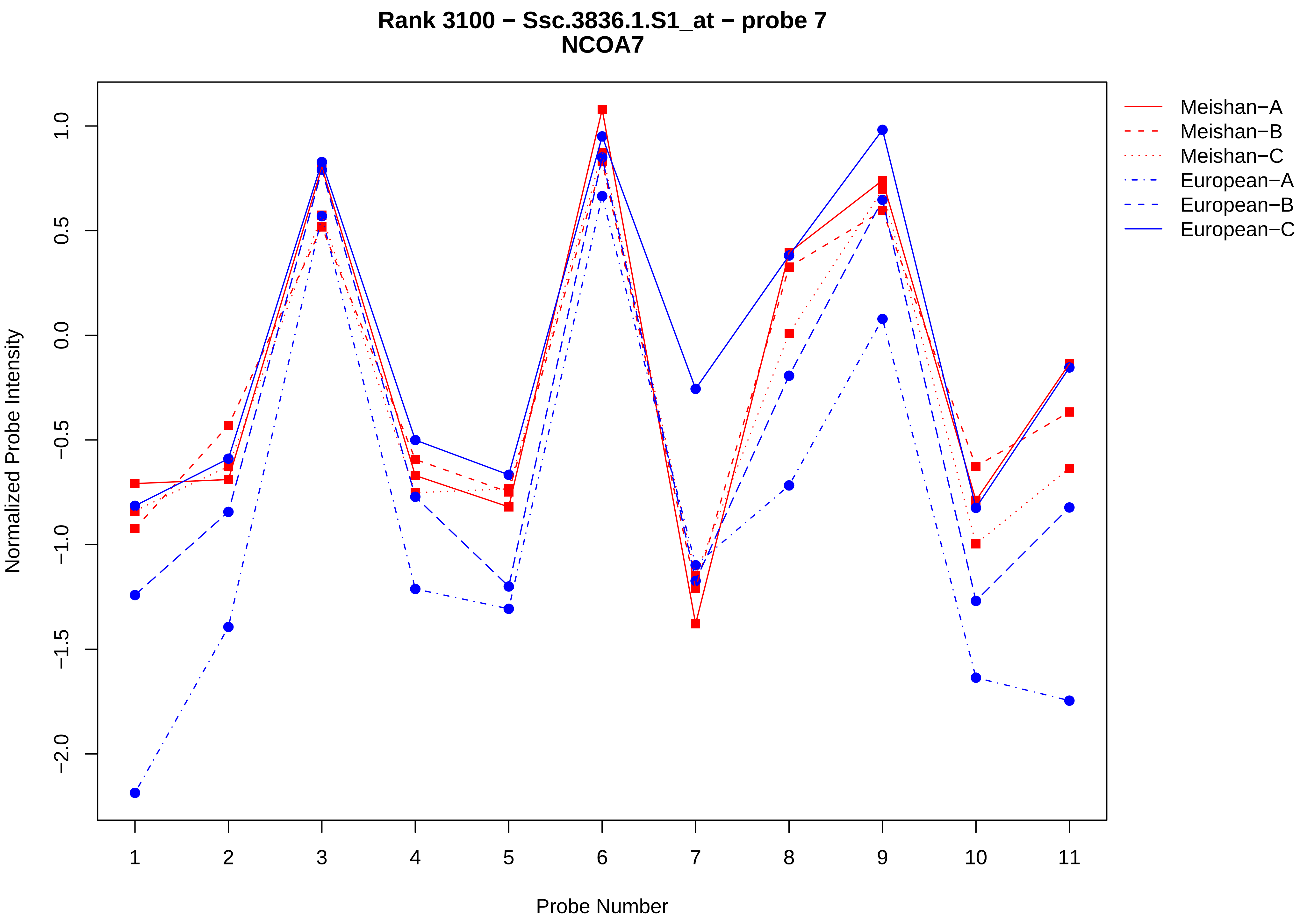

Rank 3100 − Ssc.3836.1.S1_at − probe 7
NCOA7
Normalized Probe Intensity
Probe Number
Meishan−A
Meishan−B
Meishan−C
European−A
European−B
European−C

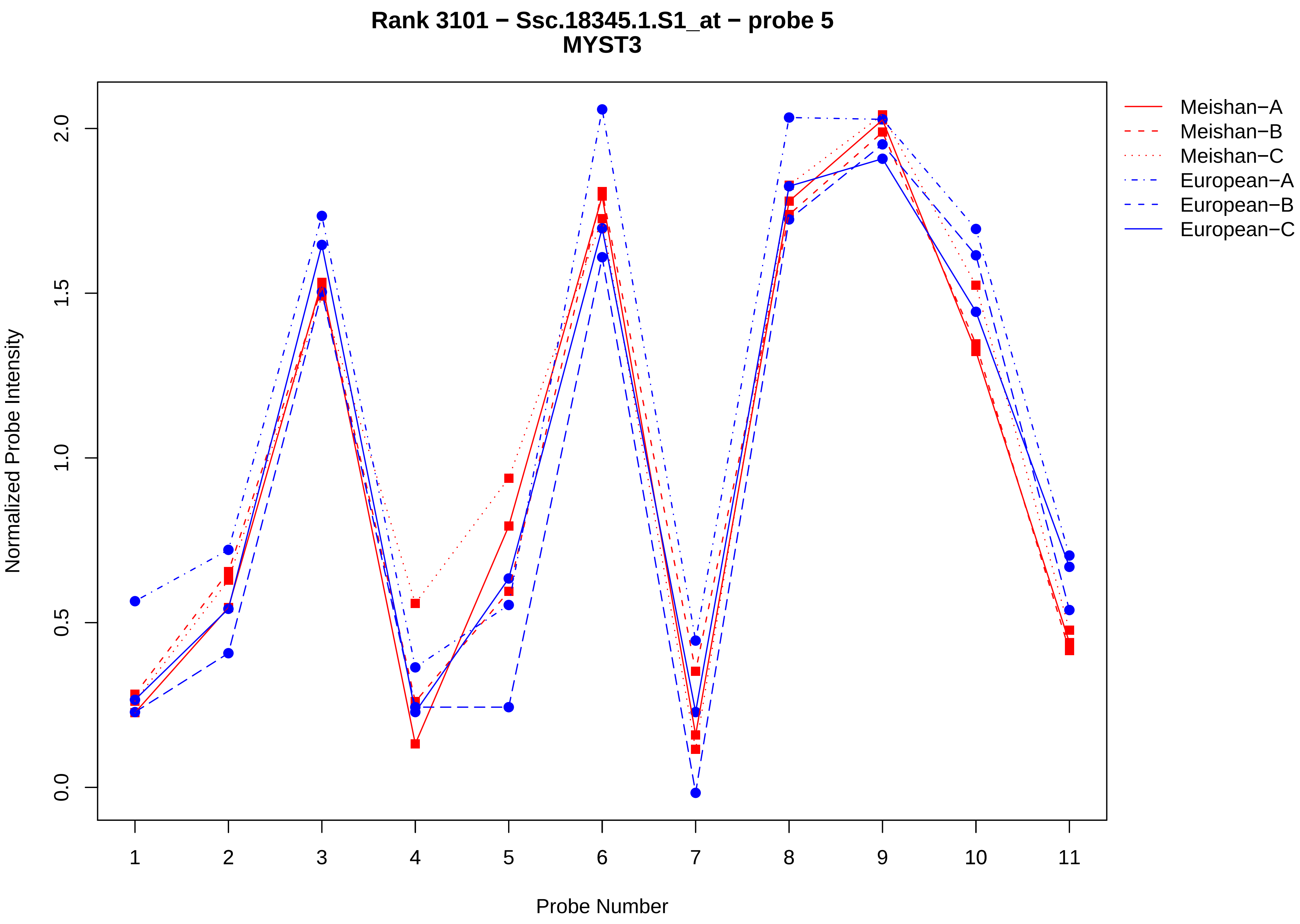
Rank 3101 − Ssc.18345.1.S1_at − probe 5
MYST3
Normalized Probe Intensity
Probe Number
0.0
0.5
1.0
1.5
2.0
1
2
3
4
5
6
7
8
9
10
11
Meishan−A
Meishan−B
Meishan−C
European−A
European−B
European−C

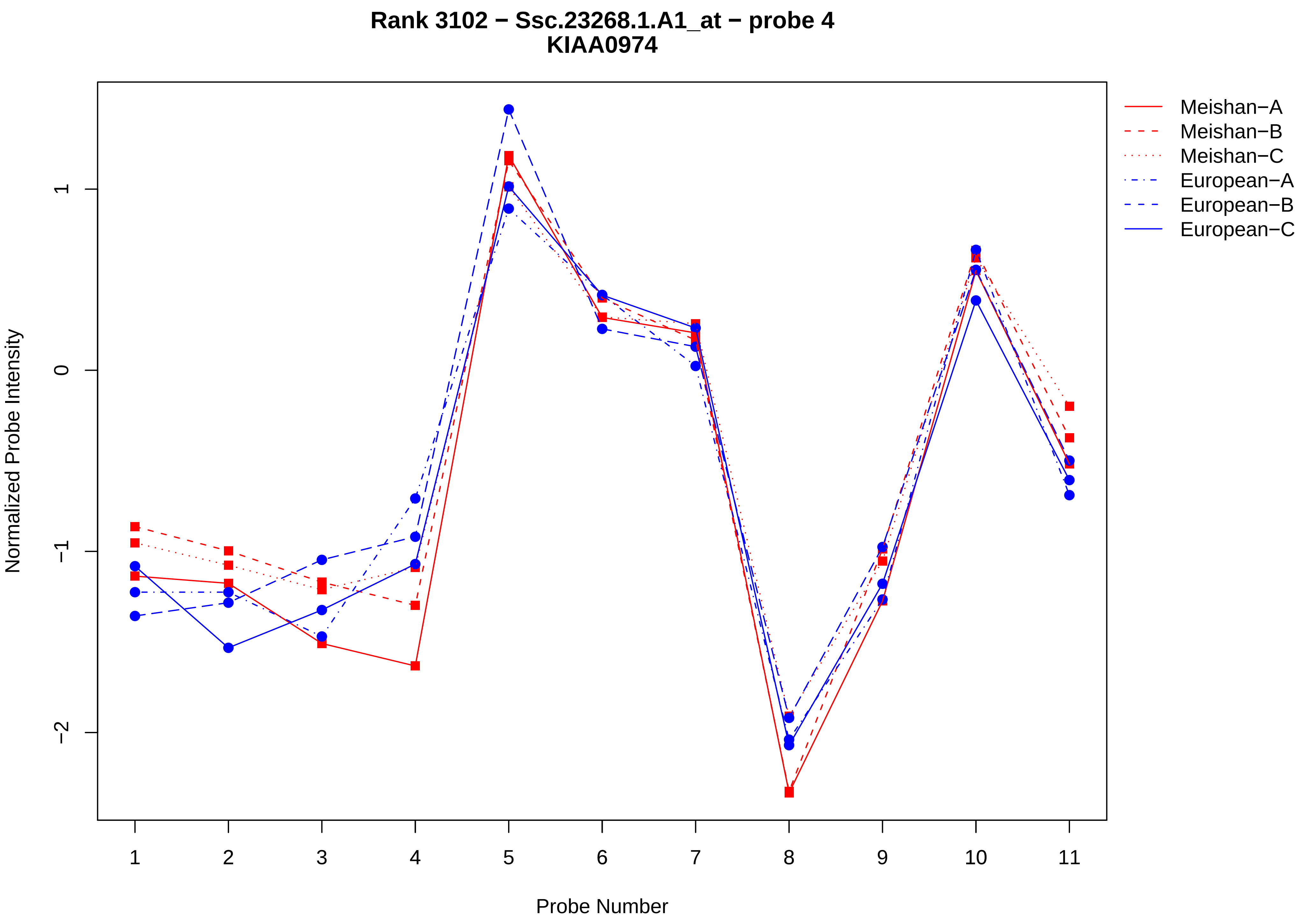

Rank 3102 − Ssc.23268.1.A1_at − probe 4
KIAA0974
Normalized Probe Intensity
Probe Number
−2
−1
0
1
1
2
3
4
5
6
7
8
9
10
11
Meishan−A
Meishan−B
Meishan−C
European−A
European−B
European−C

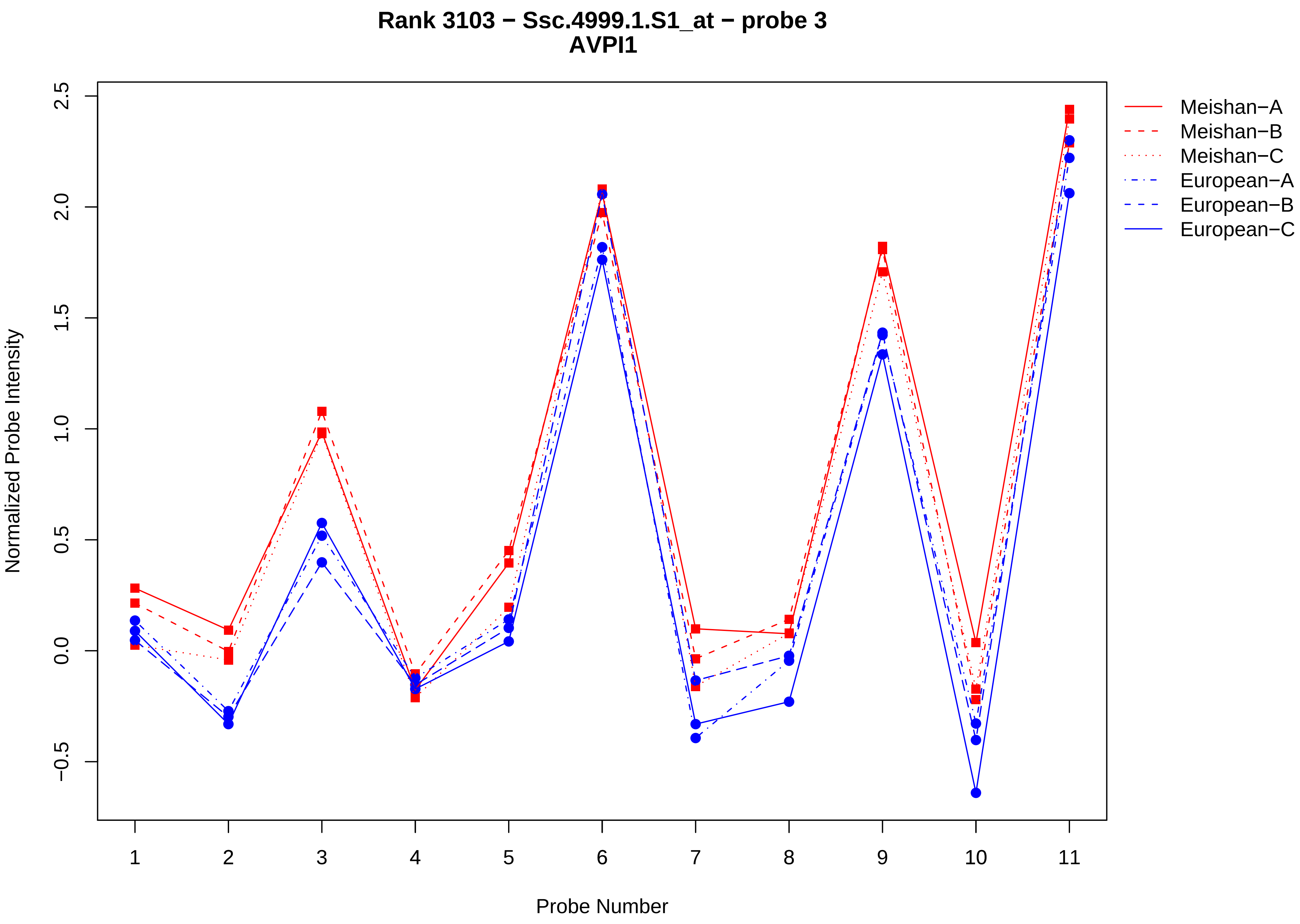
Rank 3103 − Ssc.4999.1.S1_at − probe 3
AVPI1
Normalized Probe Intensity
Probe Number
−0.5
0.0
0.5
1.0
1.5
2.0
2.5
1
2
3
4
5
6
7
8
9
10
11
Meishan−A
Meishan−B
Meishan−C
European−A
European−B
European−C

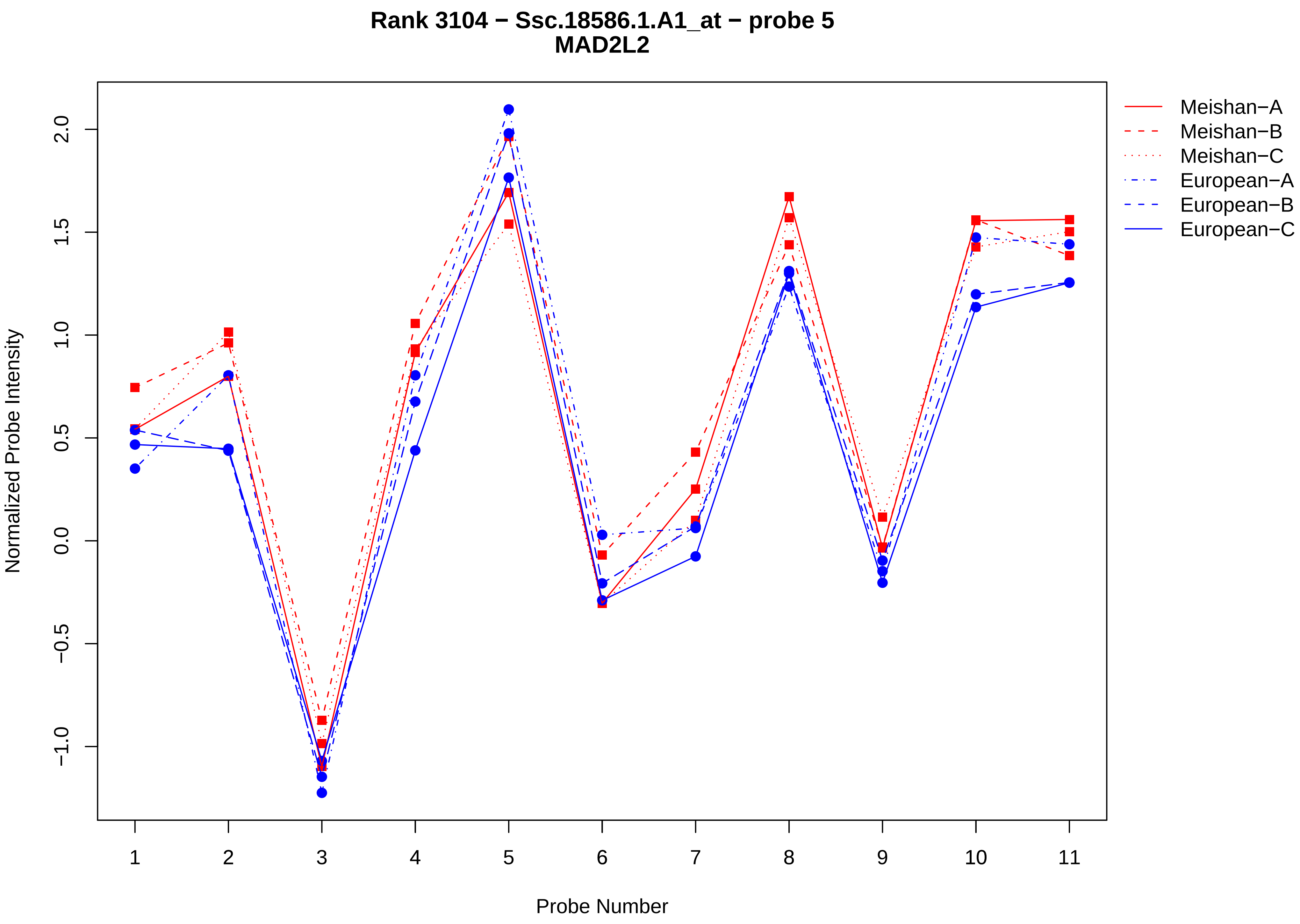
Rank 3104 − Ssc.18586.1.A1_at − probe 5
MAD2L2
Normalized Probe Intensity
Probe Number
−1.0
−0.5
0.0
0.5
1.0
1.5
2.0
1
2
3
4
5
6
7
8
9
10
11
Meishan−A
Meishan−B
Meishan−C
European−A
European−B
European−C

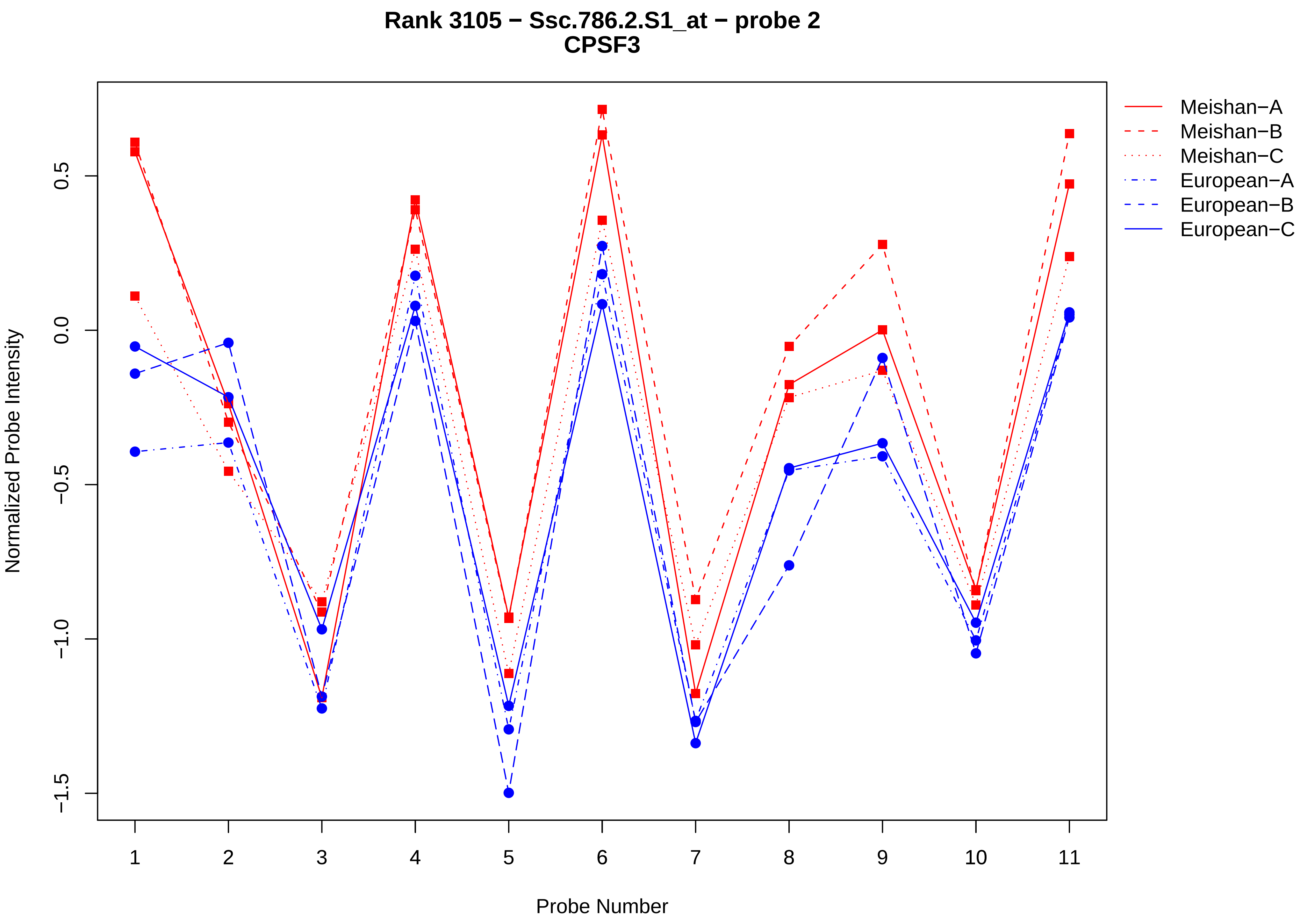

Rank 3105 − Ssc.786.2.S1_at − probe 2
CPSF3
Normalized Probe Intensity
Probe Number
0.5
0.0
−0.5
−1.0
−1.5
1
2
3
4
5
6
7
8
9
10
11
Meishan−A
Meishan−B
Meishan−C
European−A
European−B
European−C

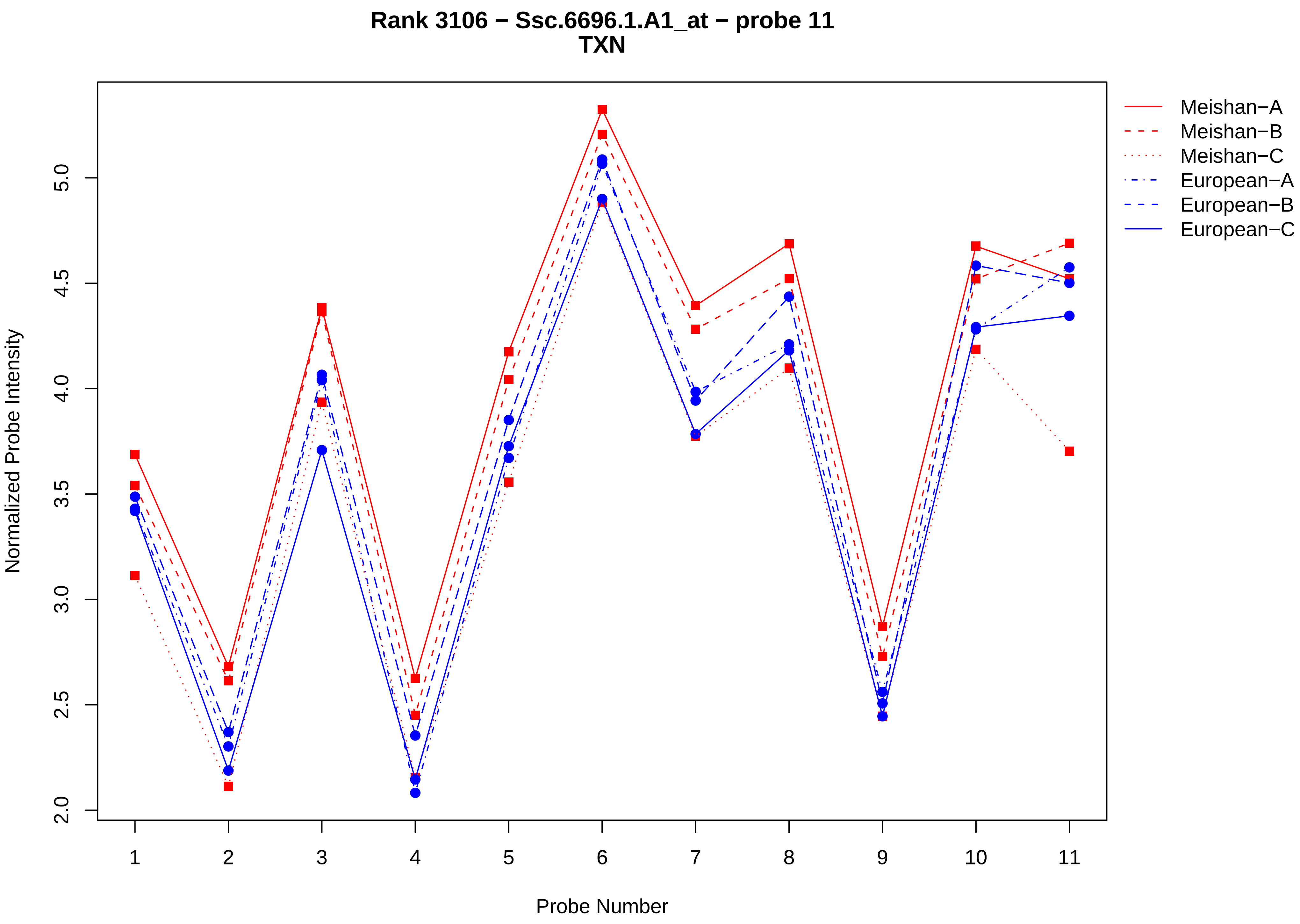

Rank 3106 − Ssc.6696.1.A1_at − probe 11
TXN
Normalized Probe Intensity
2.0
2.5
3.0
3.5
4.0
4.5
5.0
Probe Number
1
2
3
4
5
6
7
8
9
10
11
Meishan−A
Meishan−B
Meishan−C
European−A
European−B
European−C

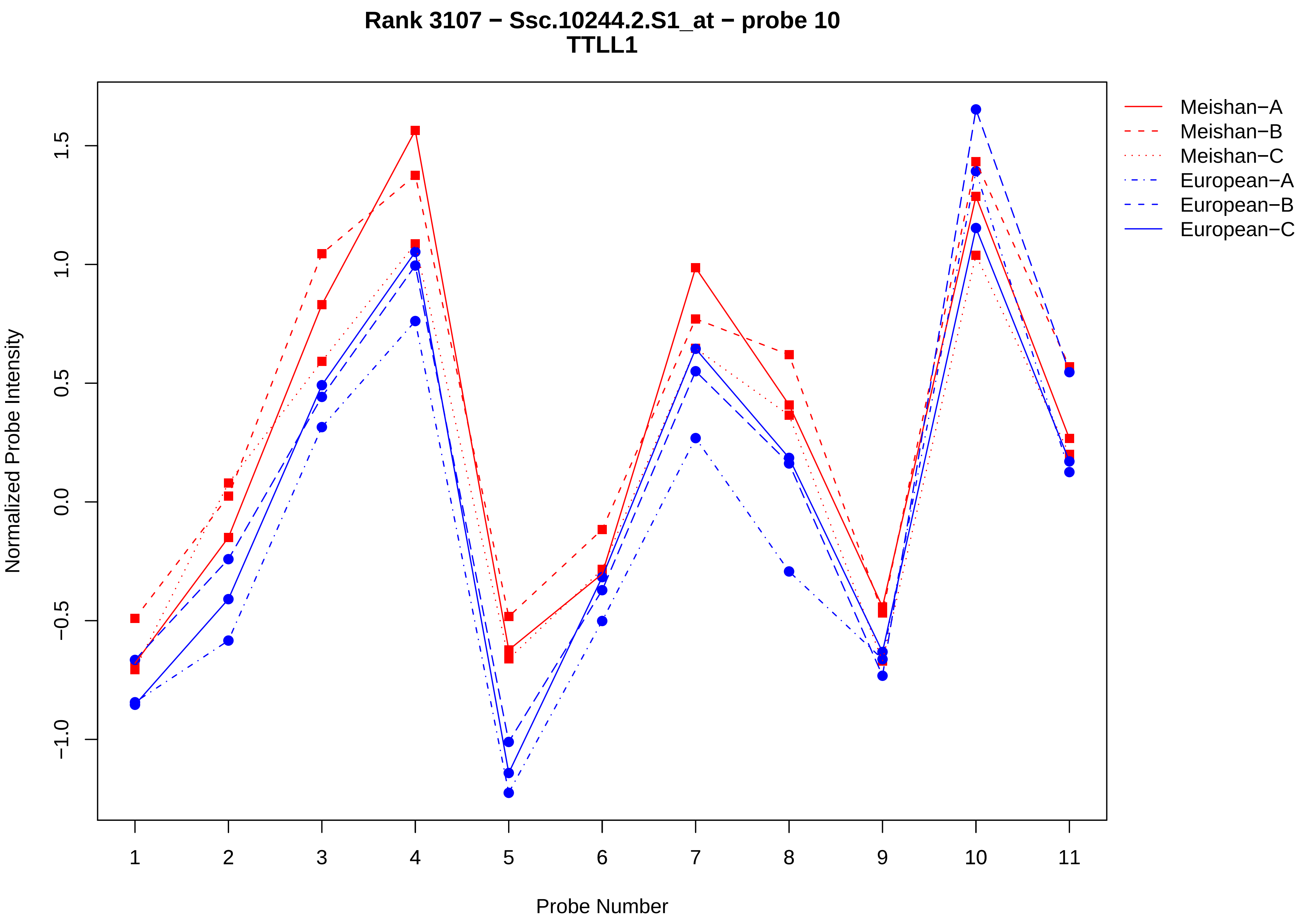

Rank 3107 − Ssc.10244.2.S1_at − probe 10
TTLL1
Normalized Probe Intensity
−1.0
−0.5
0.0
0.5
1.0
1.5
1
2
3
4
5
6
7
8
9
10
11
Probe Number
Meishan−A
Meishan−B
Meishan−C
European−A
European−B
European−C

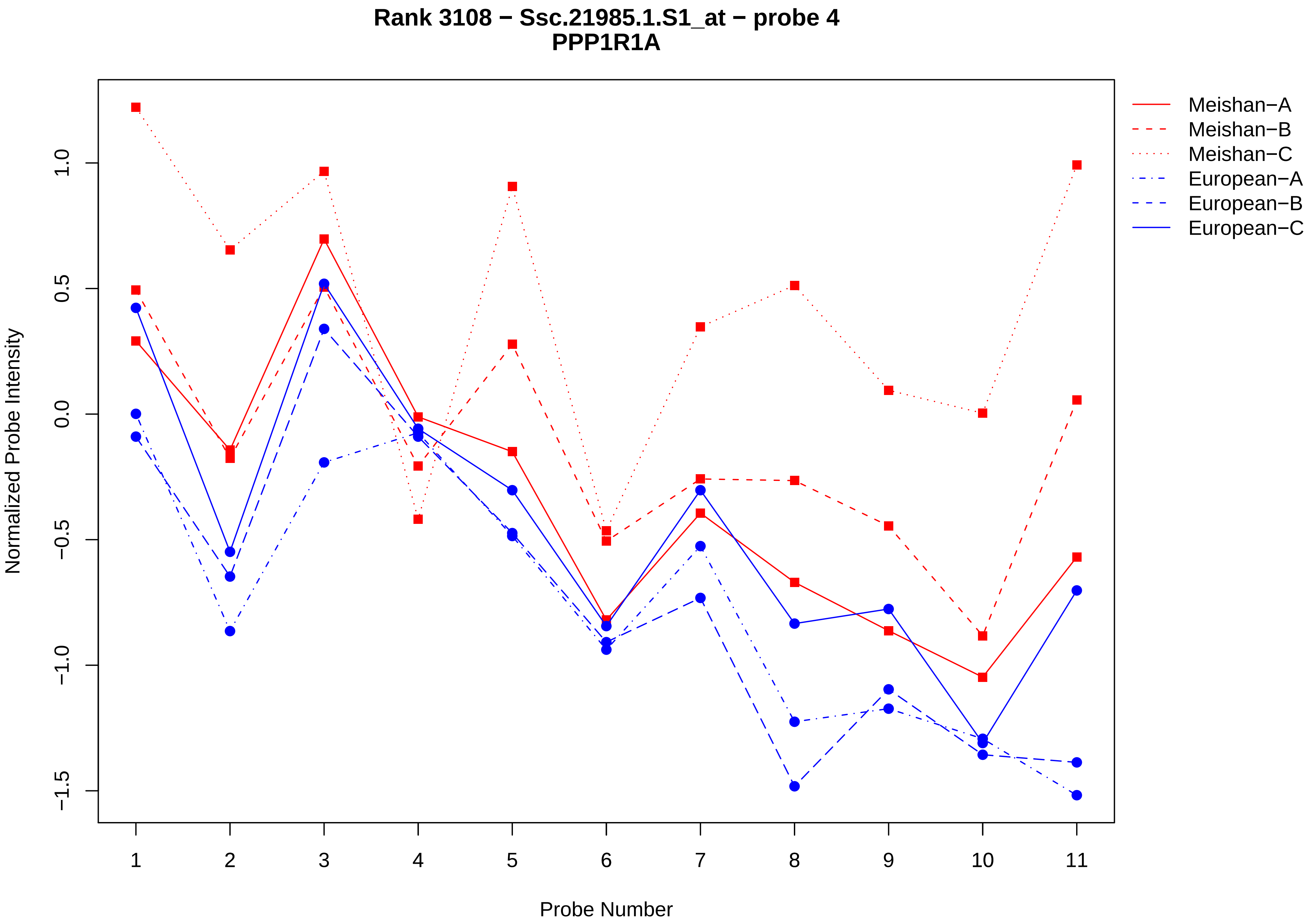

Rank 3108 − Ssc.21985.1.S1_at − probe 4
PPP1R1A
Normalized Probe Intensity
Probe Number
1.0
0.5
0.0
−0.5
−1.0
−1.5
1
2
3
4
5
6
7
8
9
10
11
Meishan−A
Meishan−B
Meishan−C
European−A
European−B
European−C

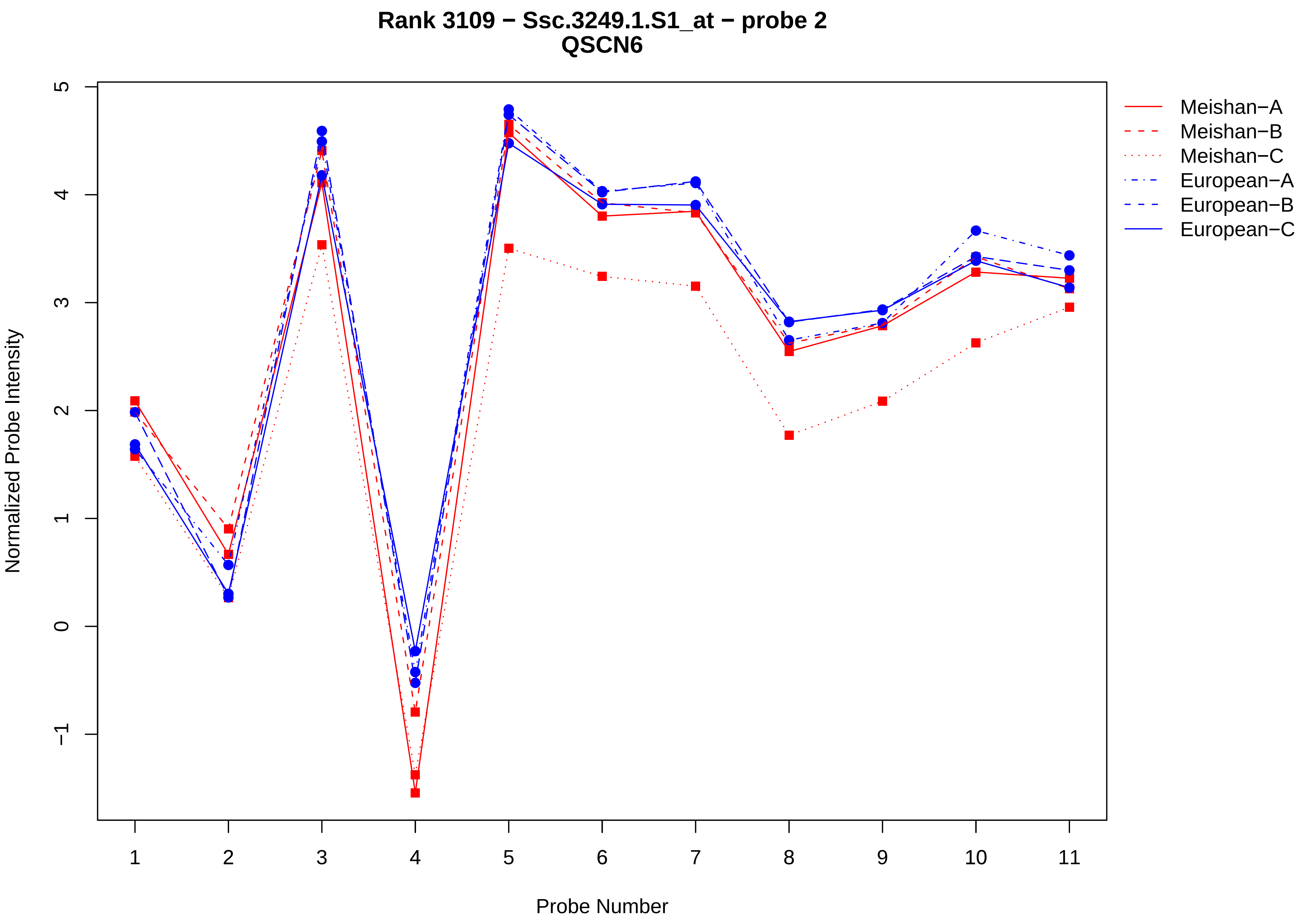
Rank 3109 − Ssc.3249.1.S1_at − probe 2
QSCN6
Normalized Probe Intensity
Probe Number
−1
0
1
2
3
4
5
1
2
3
4
5
6
7
8
9
10
11
Meishan−A
Meishan−B
Meishan−C
European−A
European−B
European−C

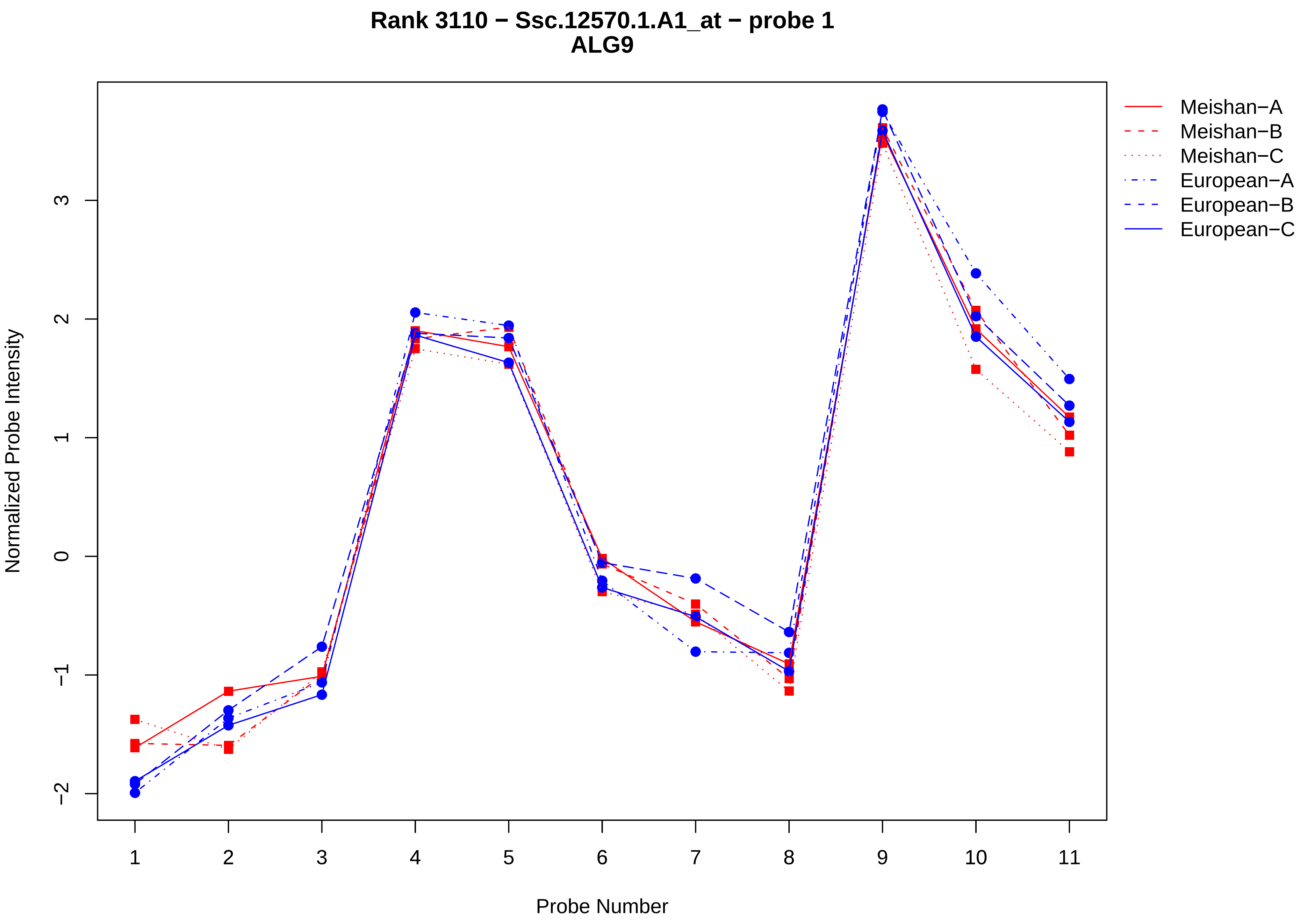
Rank 3110 − Ssc.12570.1.A1_at − probe 1
ALG9
Normalized Probe Intensity
Probe Number
−2
−1
0
1
2
3
1
2
3
4
5
6
7
8
9
10
11
Meishan−A
Meishan−B
Meishan−C
European−A
European−B
European−C

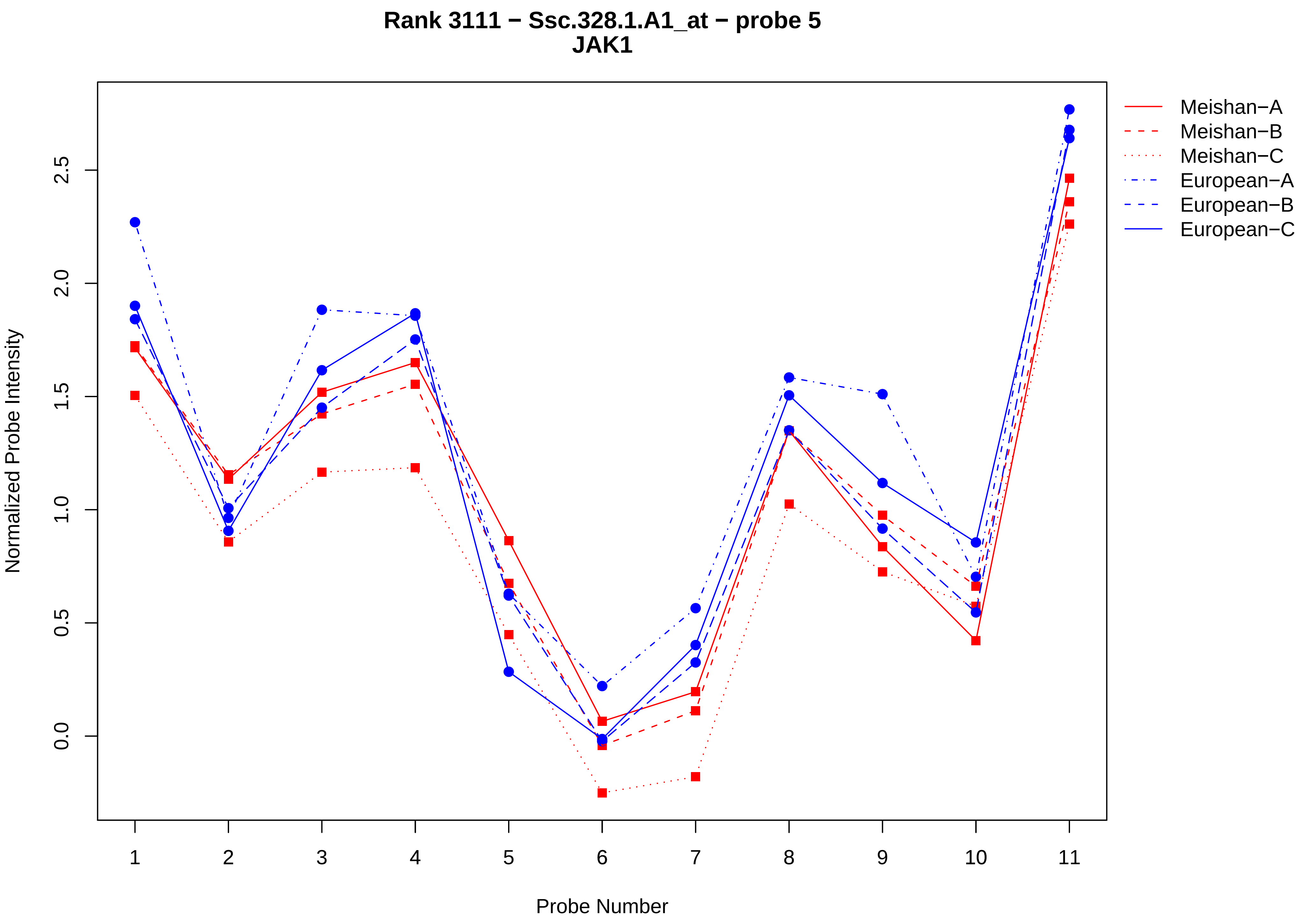
Rank 3111 − Ssc.328.1.A1_at − probe 5
JAK1
Normalized Probe Intensity
Probe Number
0.0
0.5
1.0
1.5
2.0
2.5
1
2
3
4
5
6
7
8
9
10
11
Meishan−A
Meishan−B
Meishan−C
European−A
European−B
European−C

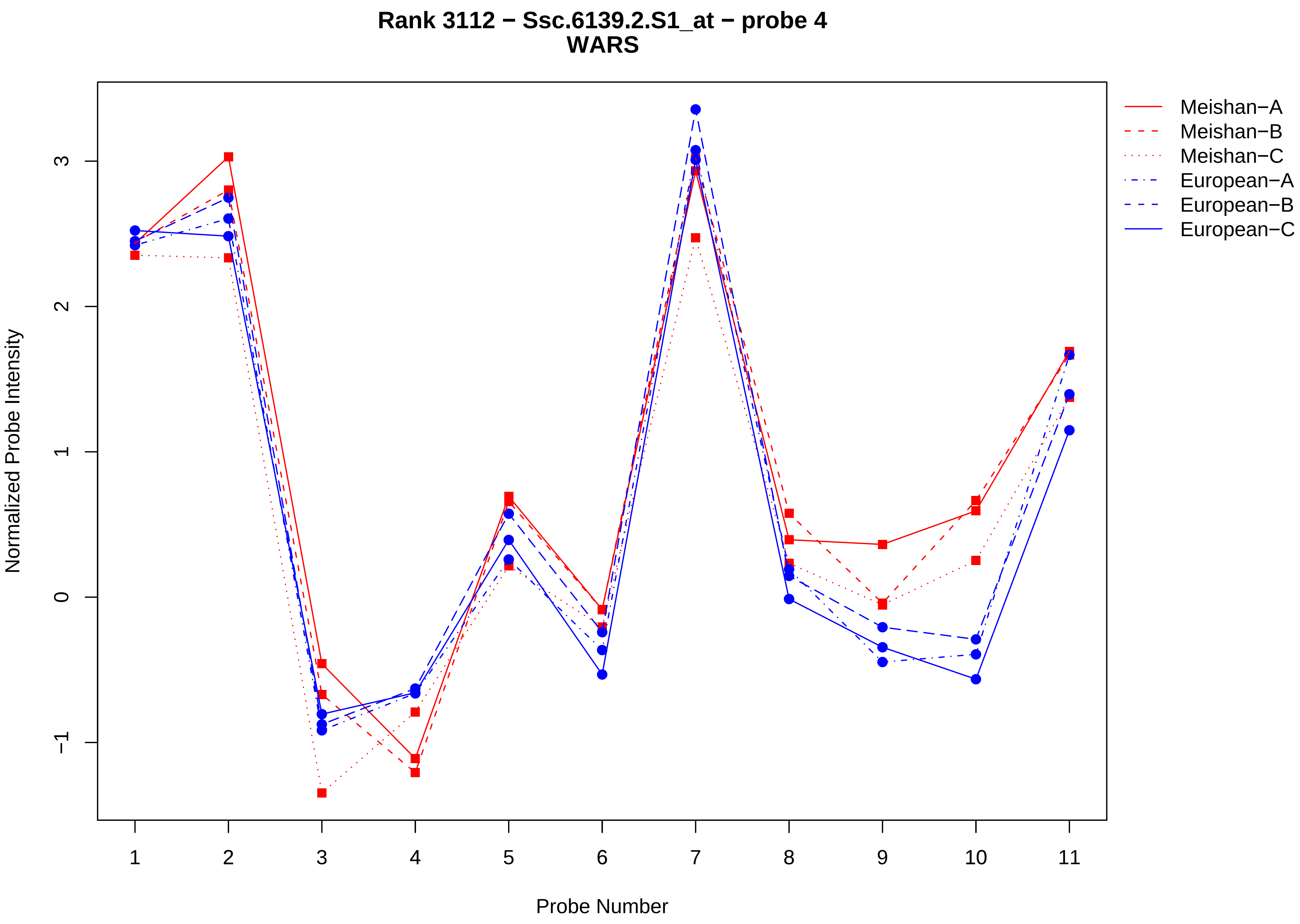

Rank 3112 − Ssc.6139.2.S1_at − probe 4
WARS
Normalized Probe Intensity
Probe Number
Meishan−A
Meishan−B
Meishan−C
European−A
European−B
European−C
−1
0
1
2
3
1
2
3
4
5
6
7
8
9
10
11

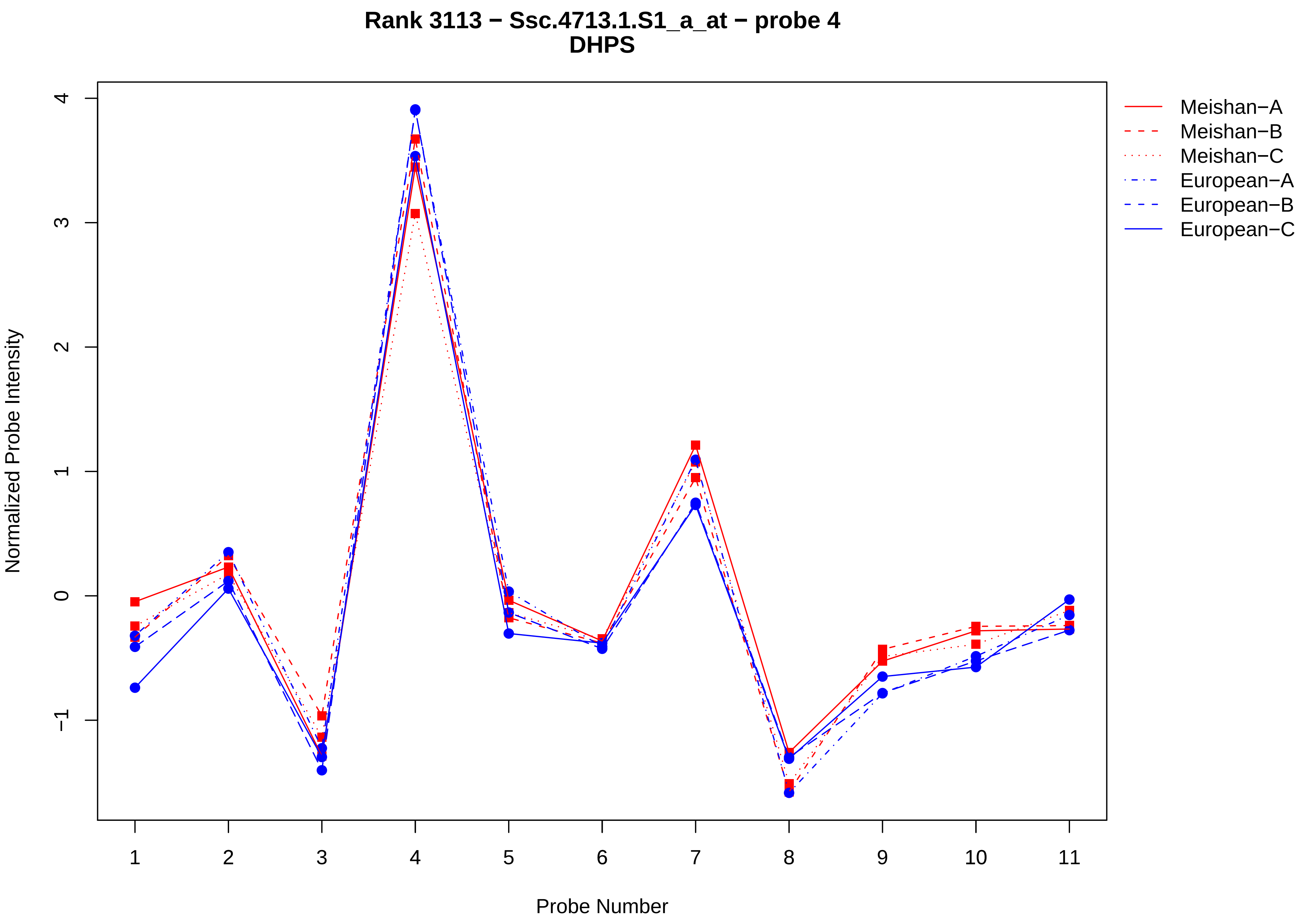

Rank 3113 − Ssc.4713.1.S1_a_at − probe 4
DHPS
Normalized Probe Intensity
Probe Number
Meishan−A
Meishan−B
Meishan−C
European−A
European−B
European−C

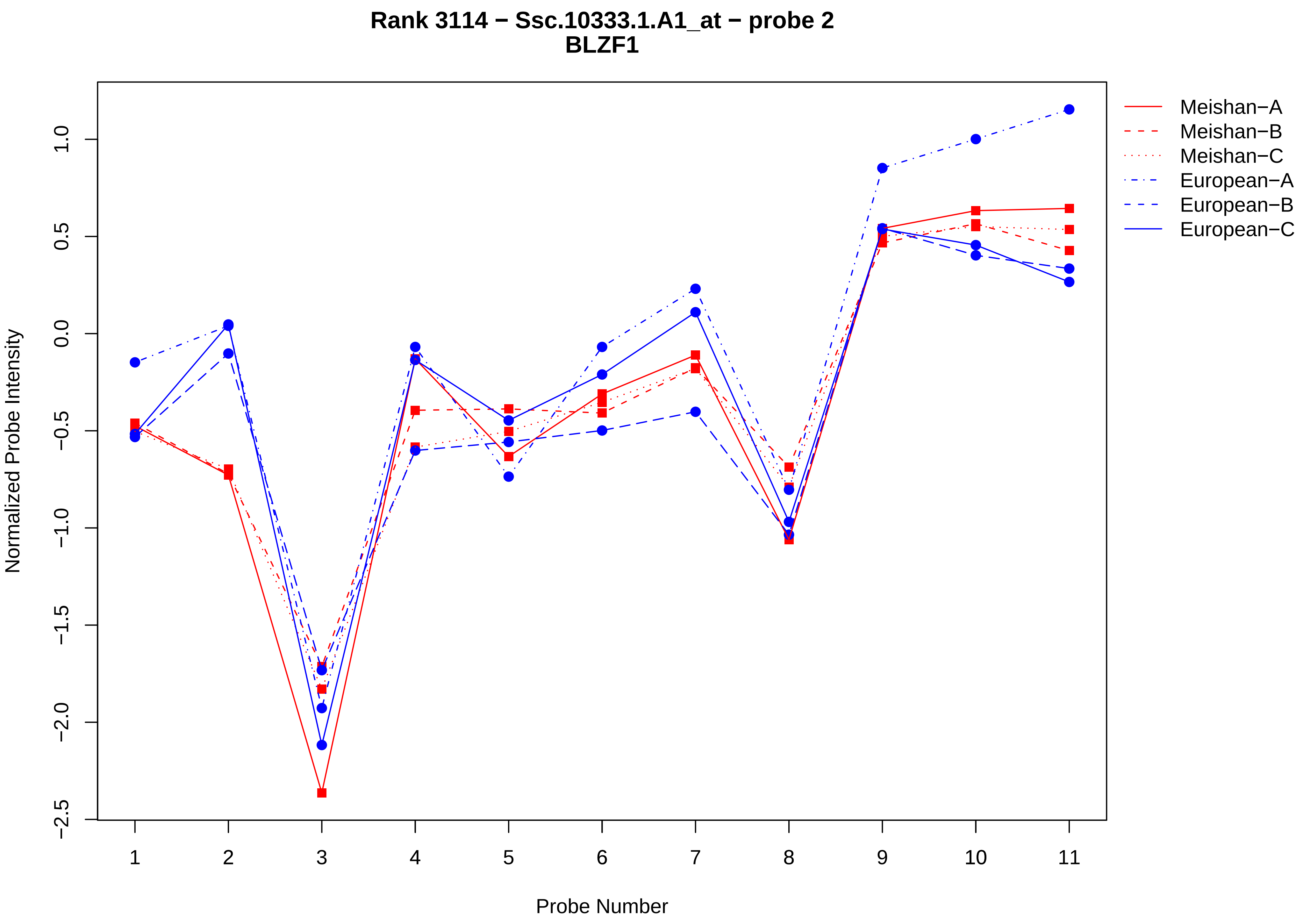

Rank 3114 − Ssc.10333.1.A1_at − probe 2
BLZF1
Normalized Probe Intensity
Probe Number
1.0
0.5
0.0
−0.5
−1.0
−1.5
−2.0
−2.5
1
2
3
4
5
6
7
8
9
10
11
Meishan−A
Meishan−B
Meishan−C
European−A
European−B
European−C

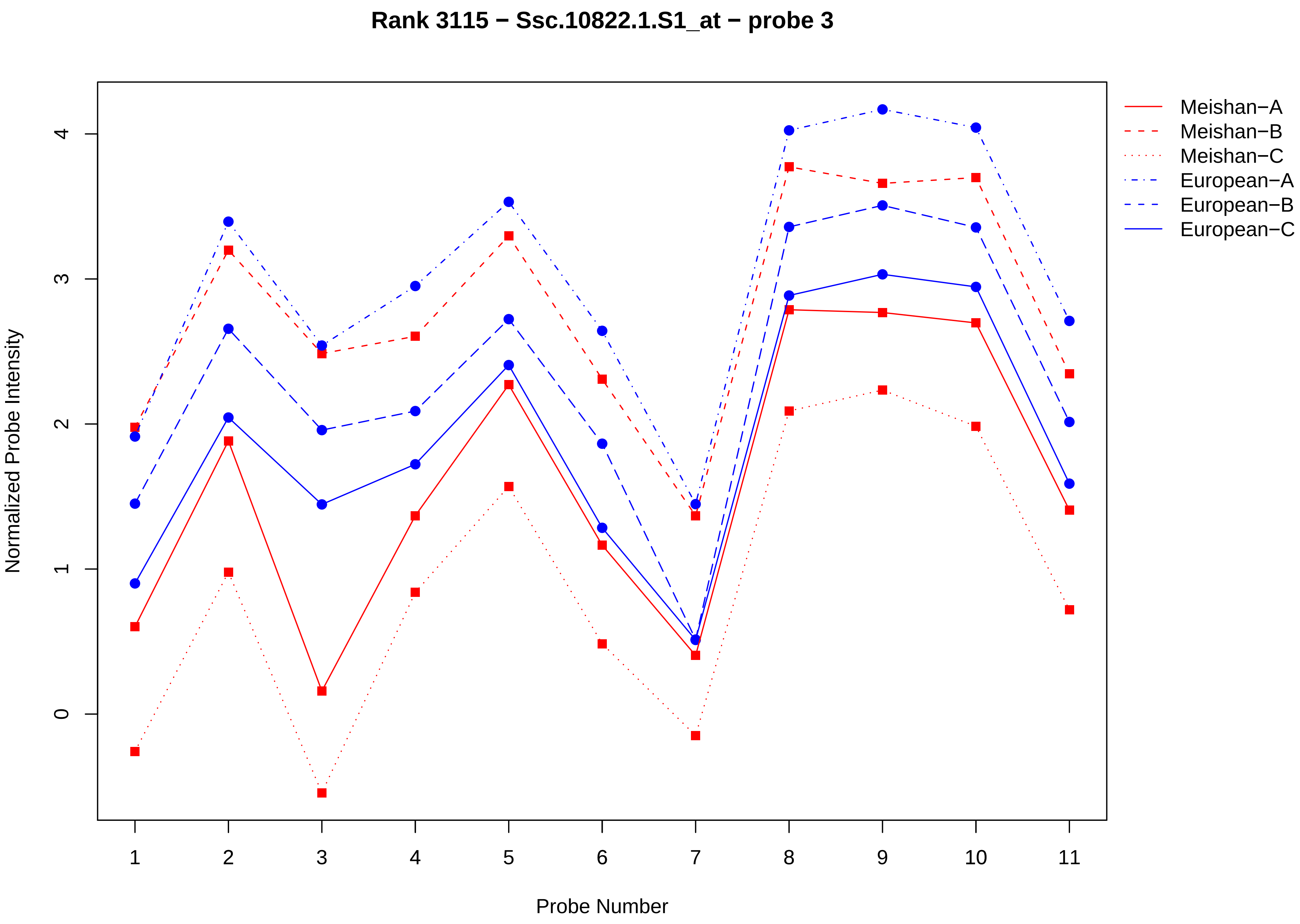

Rank 3115 − Ssc.10822.1.S1_at − probe 3
Normalized Probe Intensity
Probe Number
0
1
2
3
4
1
2
3
4
5
6
7
8
9
10
11
Meishan−A
Meishan−B
Meishan−C
European−A
European−B
European−C

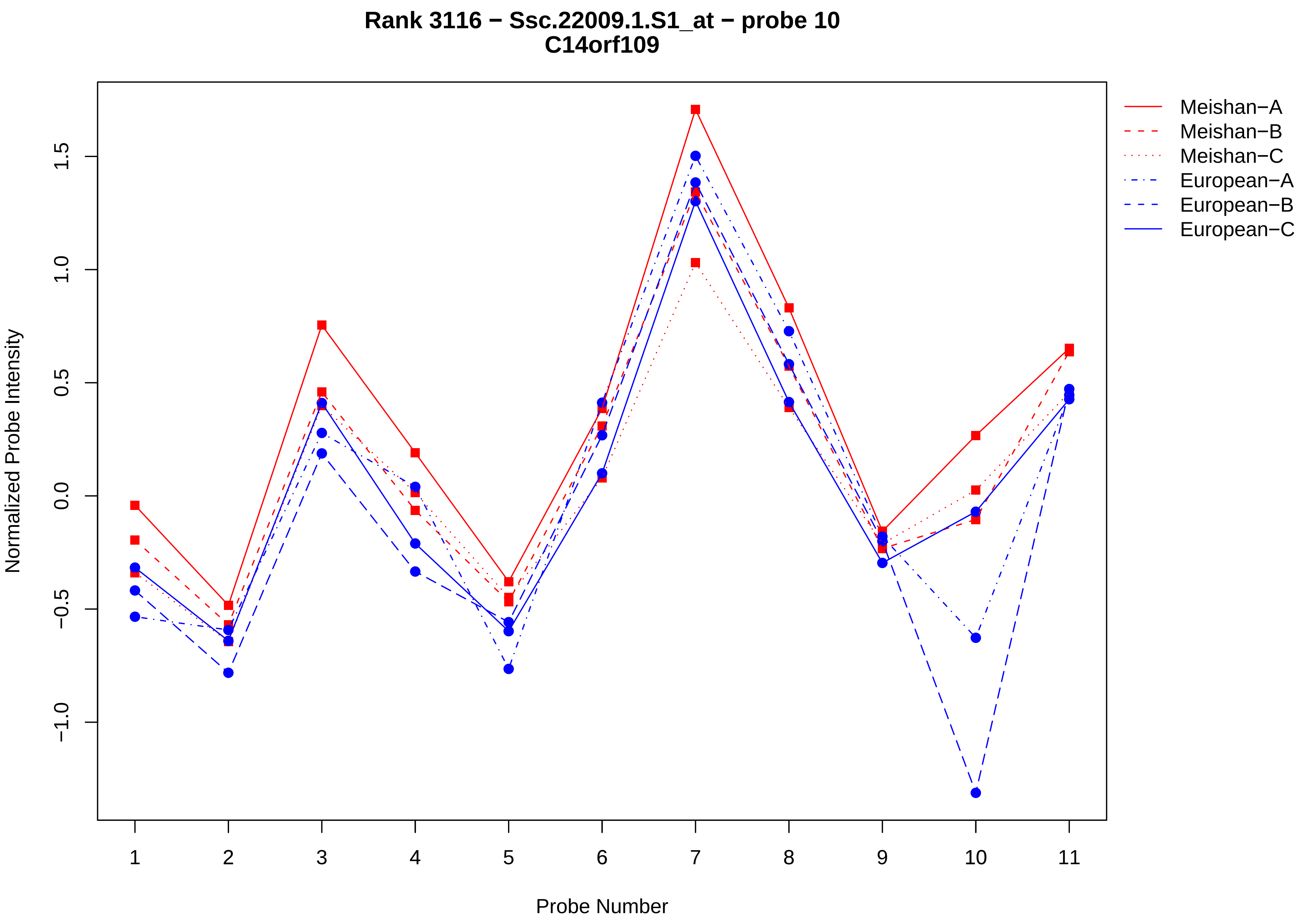

Rank 3116 − Ssc.22009.1.S1_at − probe 10
C14orf109
Normalized Probe Intensity
Probe Number
Meishan−A
Meishan−B
Meishan−C
European−A
European−B
European−C
−1.0
−0.5
0.0
0.5
1.0
1.5
1
2
3
4
5
6
7
8
9
10
11

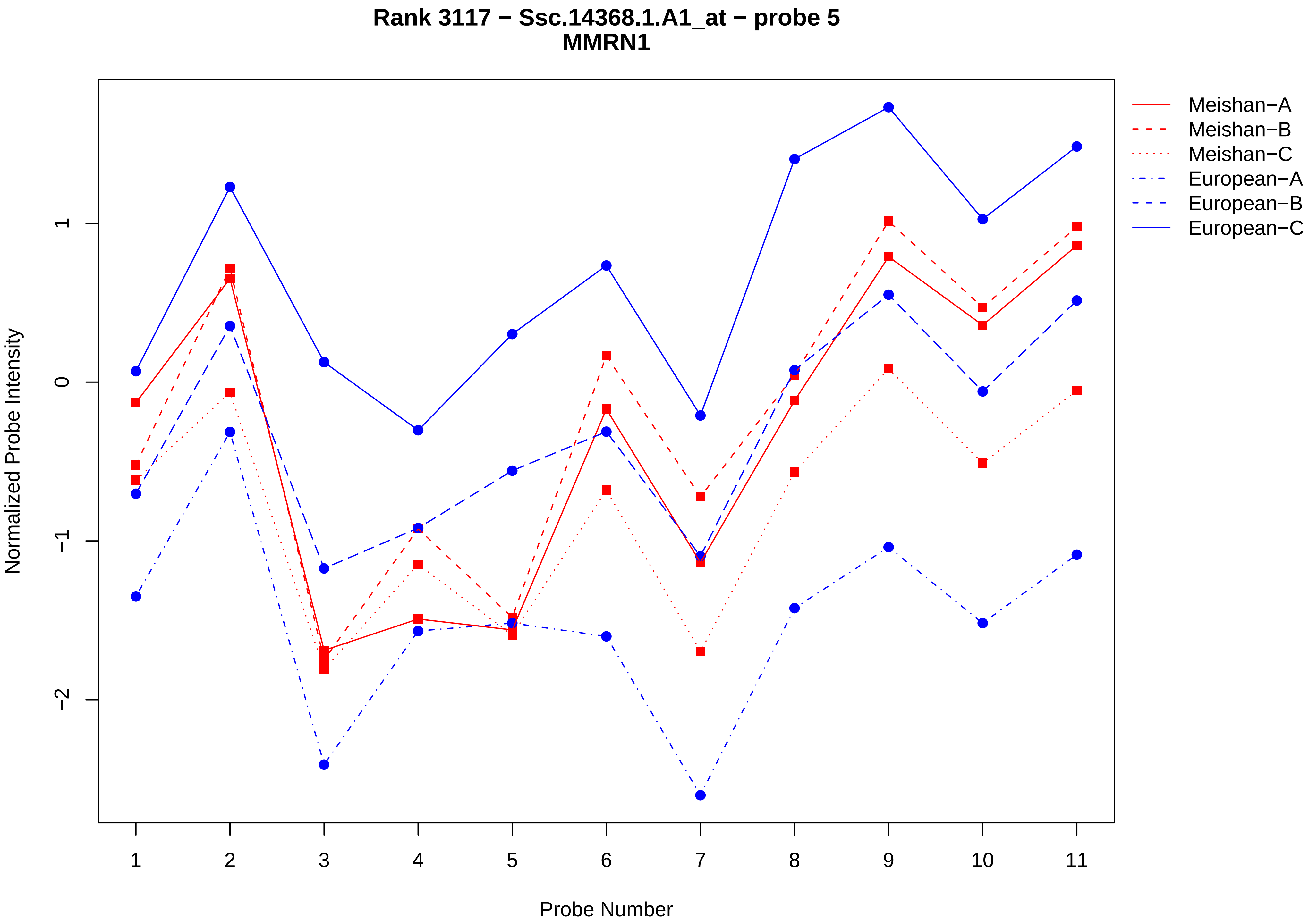

Rank 3117 − Ssc.14368.1.A1_at − probe 5
MMRN1
Normalized Probe Intensity
Probe Number
1
0
−1
−2
1
2
3
4
5
6
7
8
9
10
11
Meishan−A
Meishan−B
Meishan−C
European−A
European−B
European−C

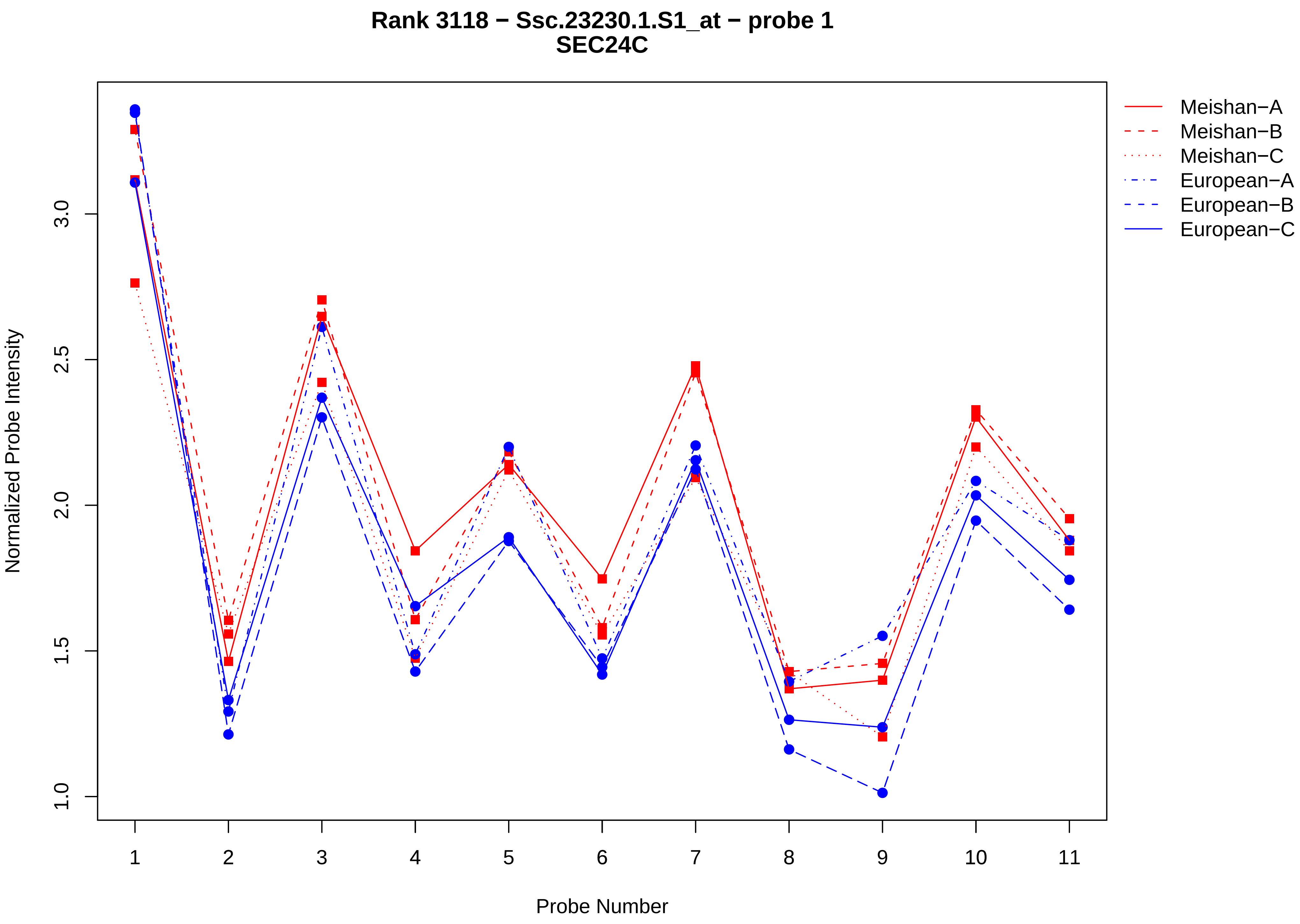

Rank 3118 − Ssc.23230.1.S1_at − probe 1
SEC24C
Normalized Probe Intensity
1.0
1.5
2.0
2.5
3.0
Probe Number
1
2
3
4
5
6
7
8
9
10
11
Meishan−A
Meishan−B
Meishan−C
European−A
European−B
European−C

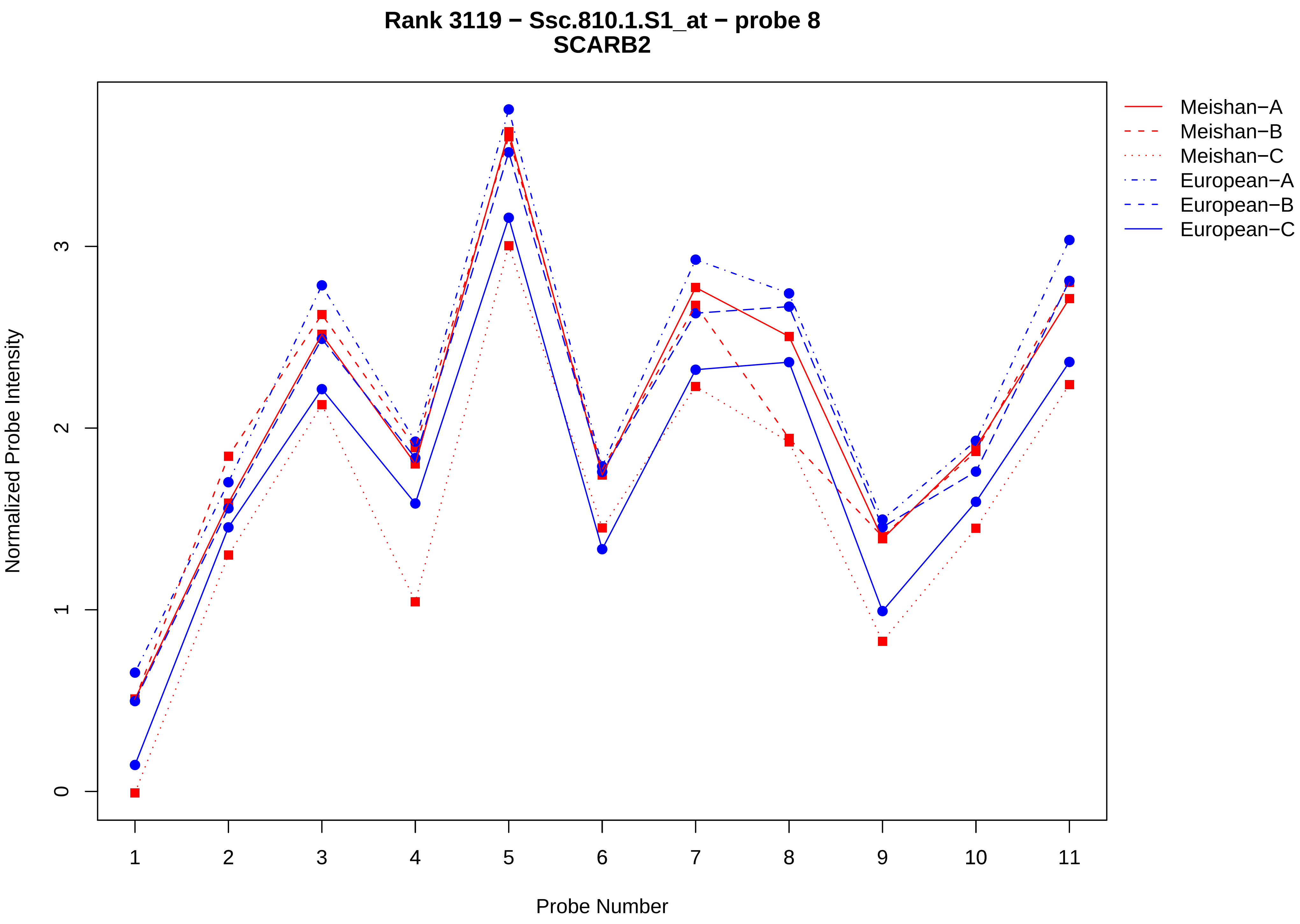

Rank 3119 − Ssc.810.1.S1_at − probe 8
SCARB2
Normalized Probe Intensity
0
1
2
3
1
2
3
4
5
6
7
8
9
10
11
Probe Number
Meishan−A
Meishan−B
Meishan−C
European−A
European−B
European−C

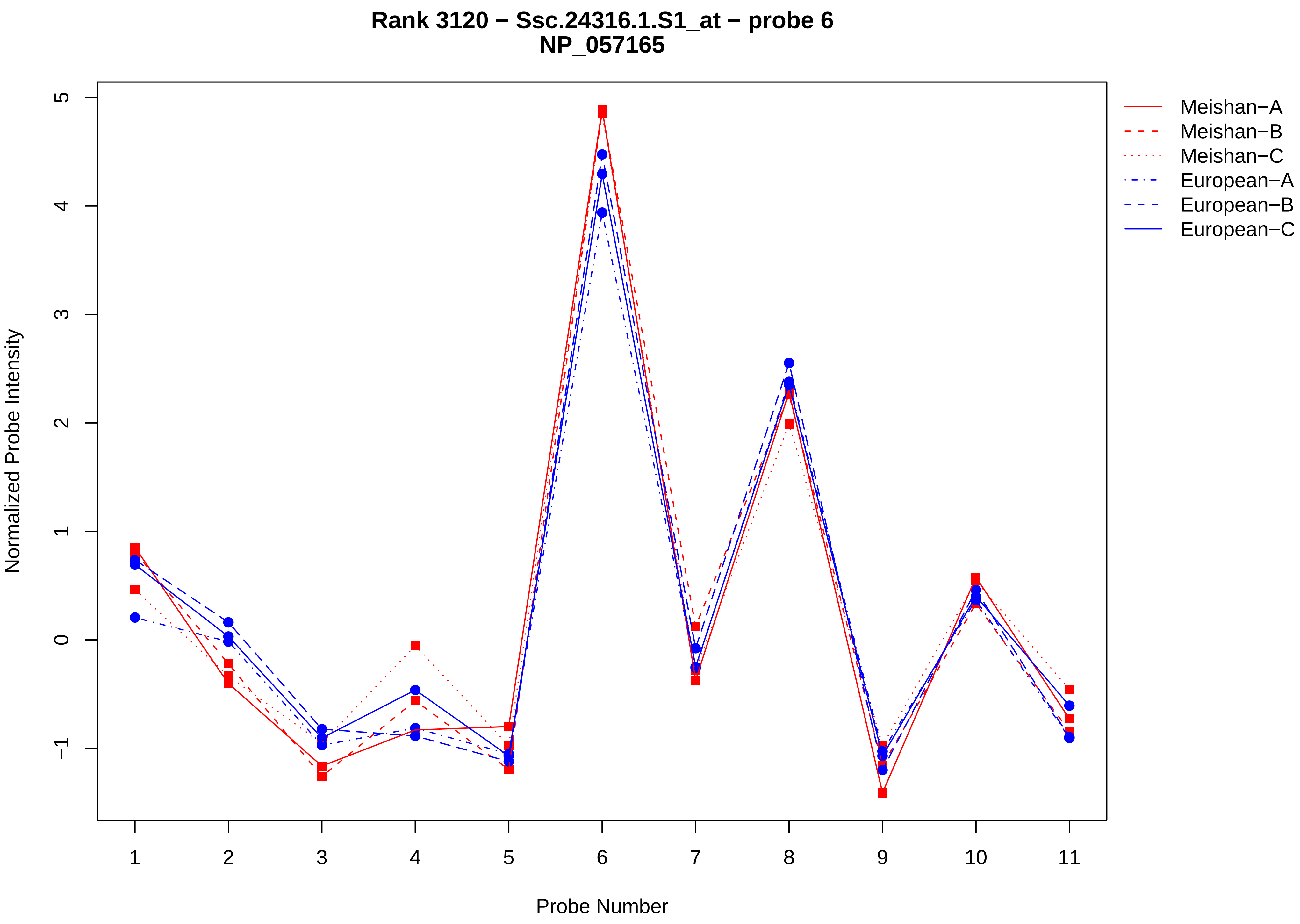

Rank 3120 − Ssc.24316.1.S1_at − probe 6
NP_057165
Normalized Probe Intensity
Probe Number
Meishan−A
Meishan−B
Meishan−C
European−A
European−B
European−C

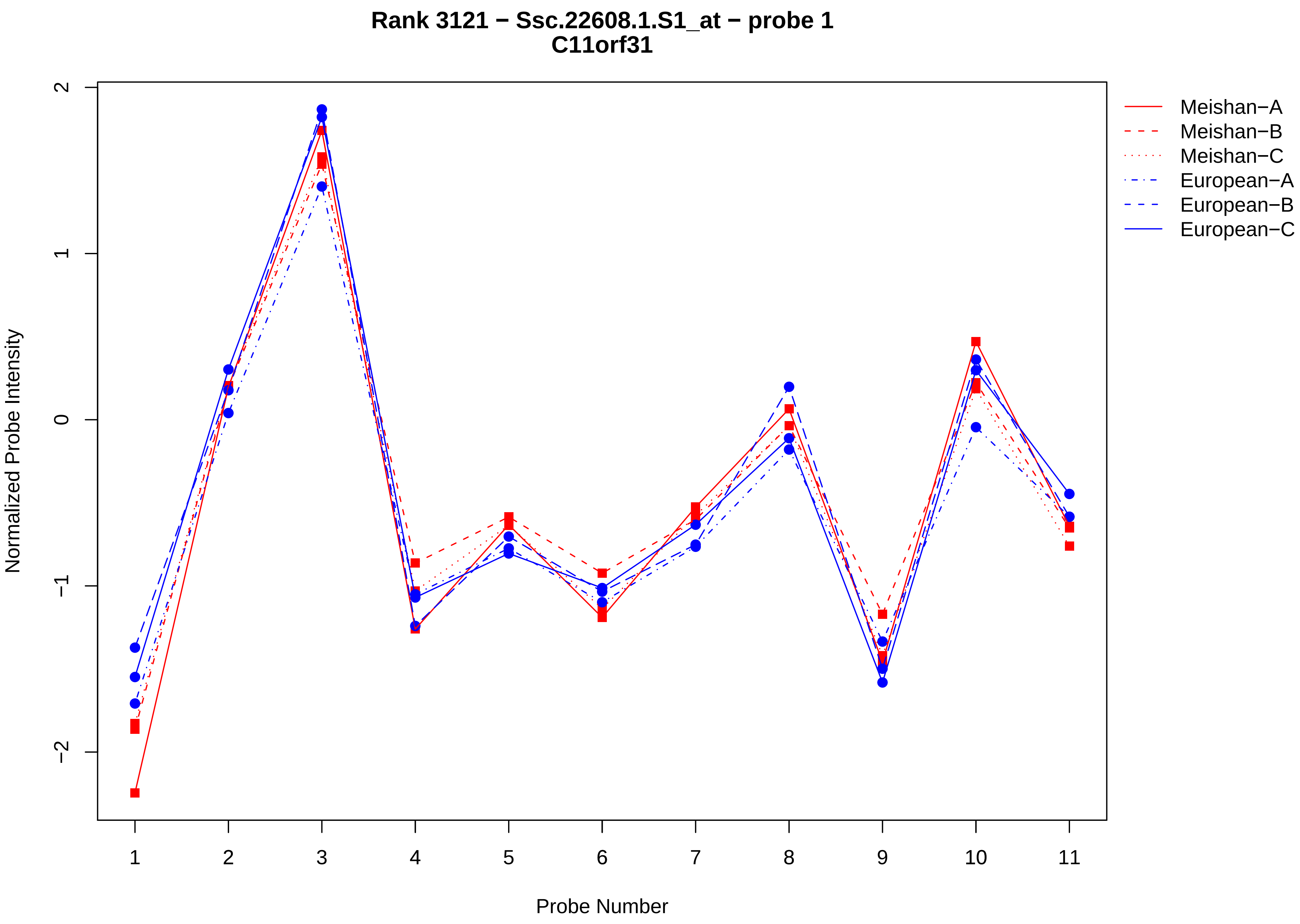

Rank 3121 − Ssc.22608.1.S1_at − probe 1
C11orf31
Normalized Probe Intensity
Probe Number
−2
−1
0
1
2
1
2
3
4
5
6
7
8
9
10
11
Meishan−A
Meishan−B
Meishan−C
European−A
European−B
European−C

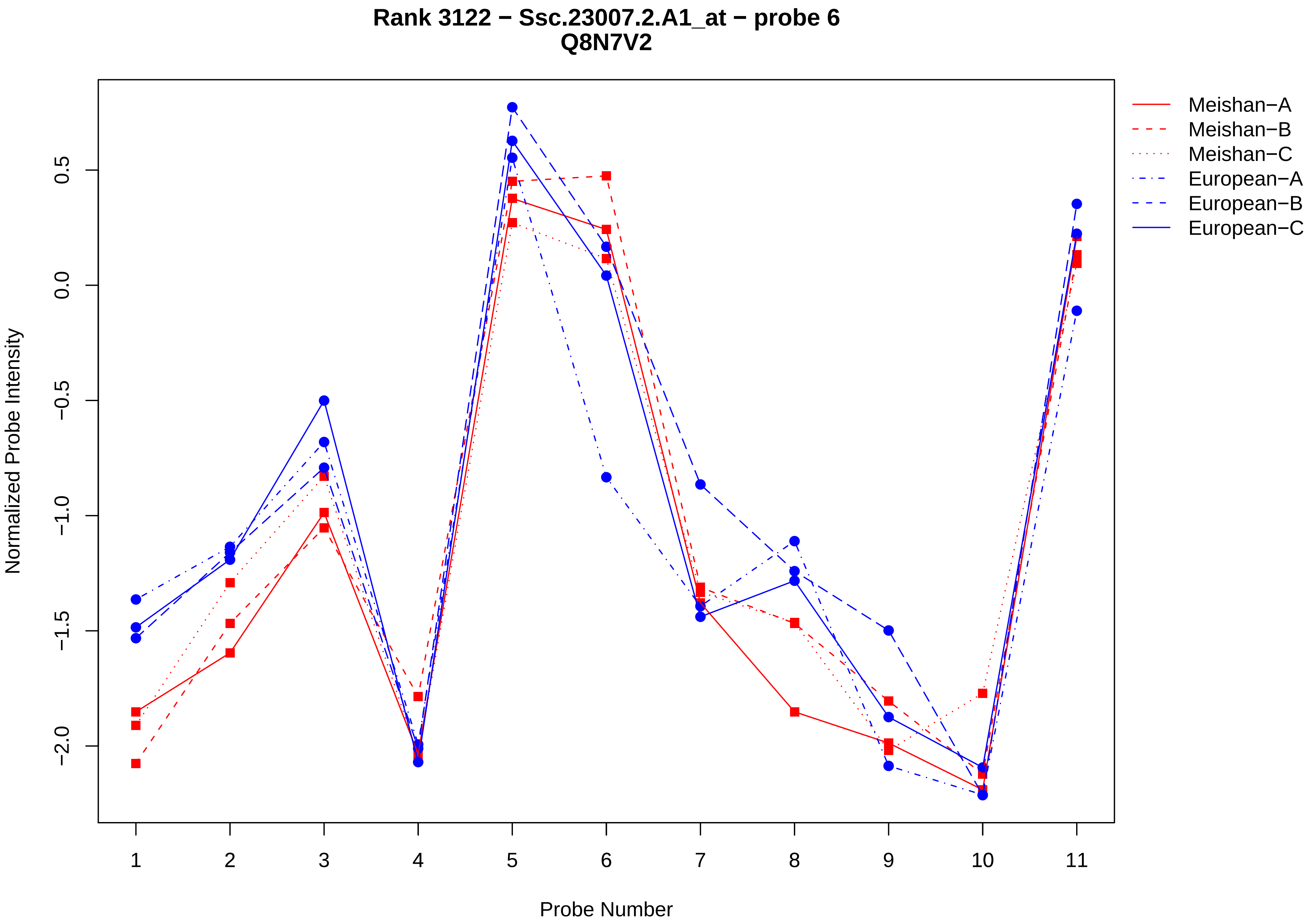

Rank 3122 − Ssc.23007.2.A1_at − probe 6
Q8N7V2
Normalized Probe Intensity
Probe Number
0.5
0.0
−0.5
−1.0
−1.5
−2.0
1
2
3
4
5
6
7
8
9
10
11
Meishan−A
Meishan−B
Meishan−C
European−A
European−B
European−C

Rank 3123 − Ssc.4609.1.S1_at − probe 8
O94958

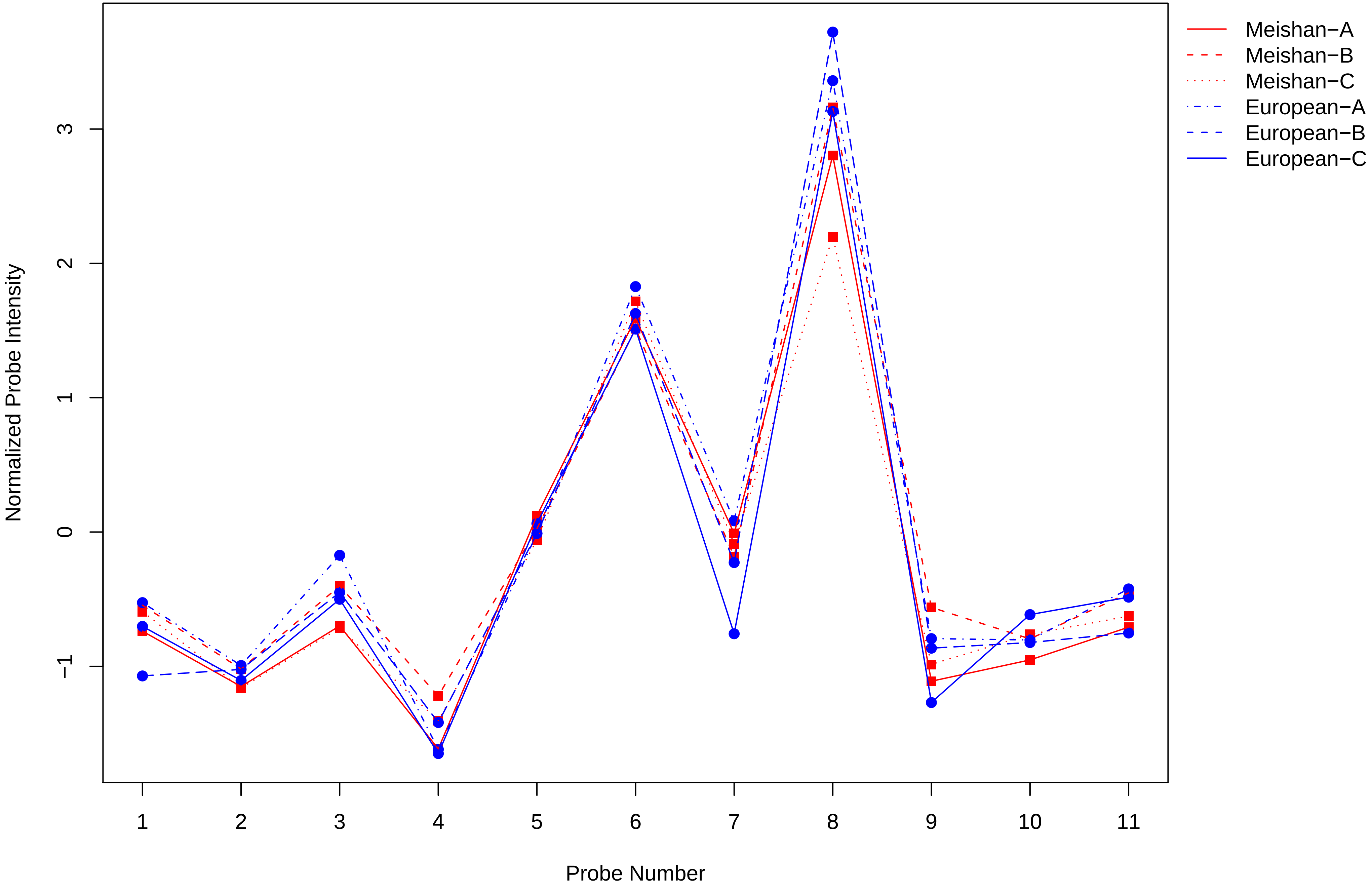

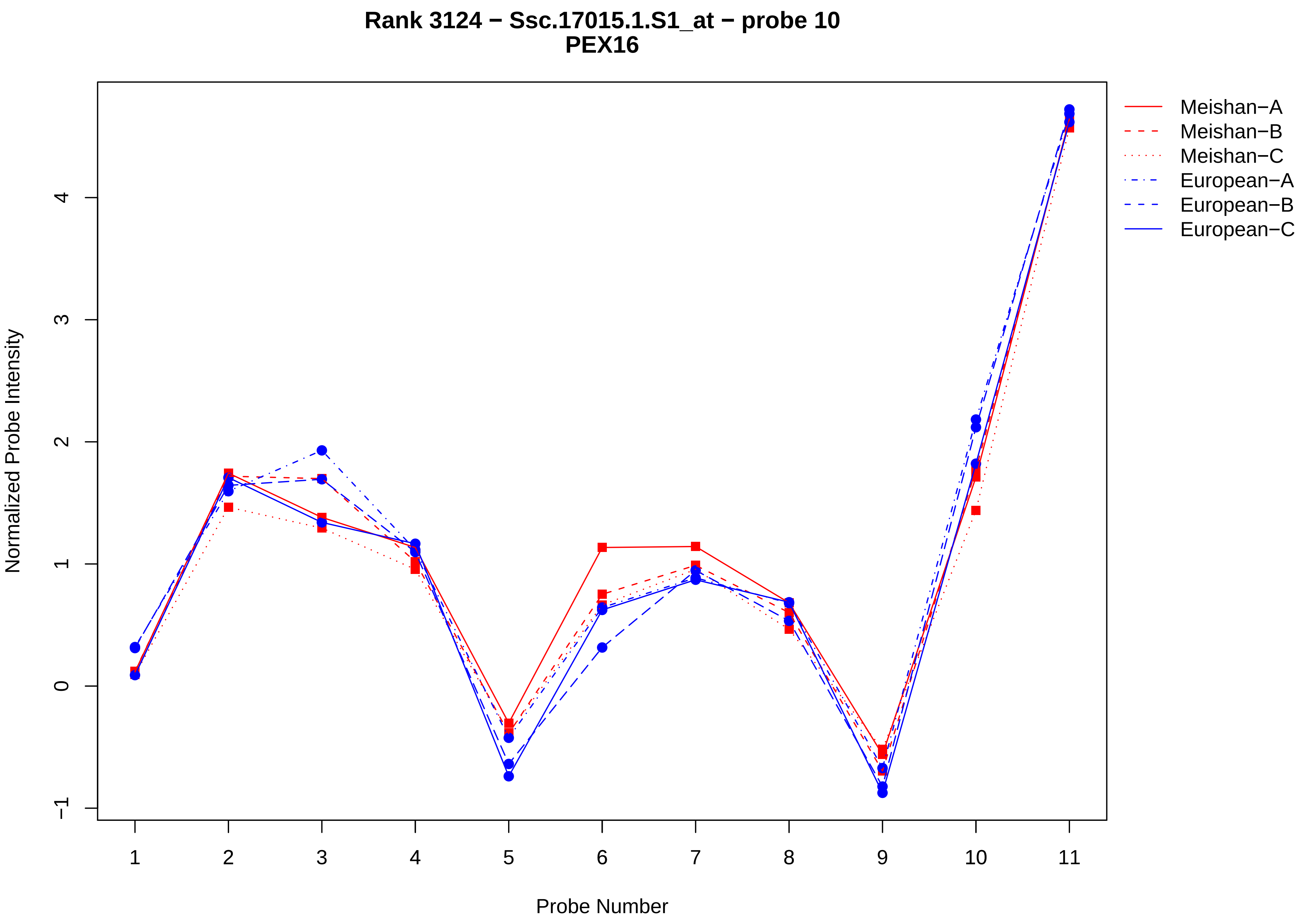

Rank 3124 − Ssc.17015.1.S1_at − probe 10
PEX16
Normalized Probe Intensity
Probe Number
−1
0
1
2
3
4
1
2
3
4
5
6
7
8
9
10
11
Meishan−A
Meishan−B
Meishan−C
European−A
European−B
European−C

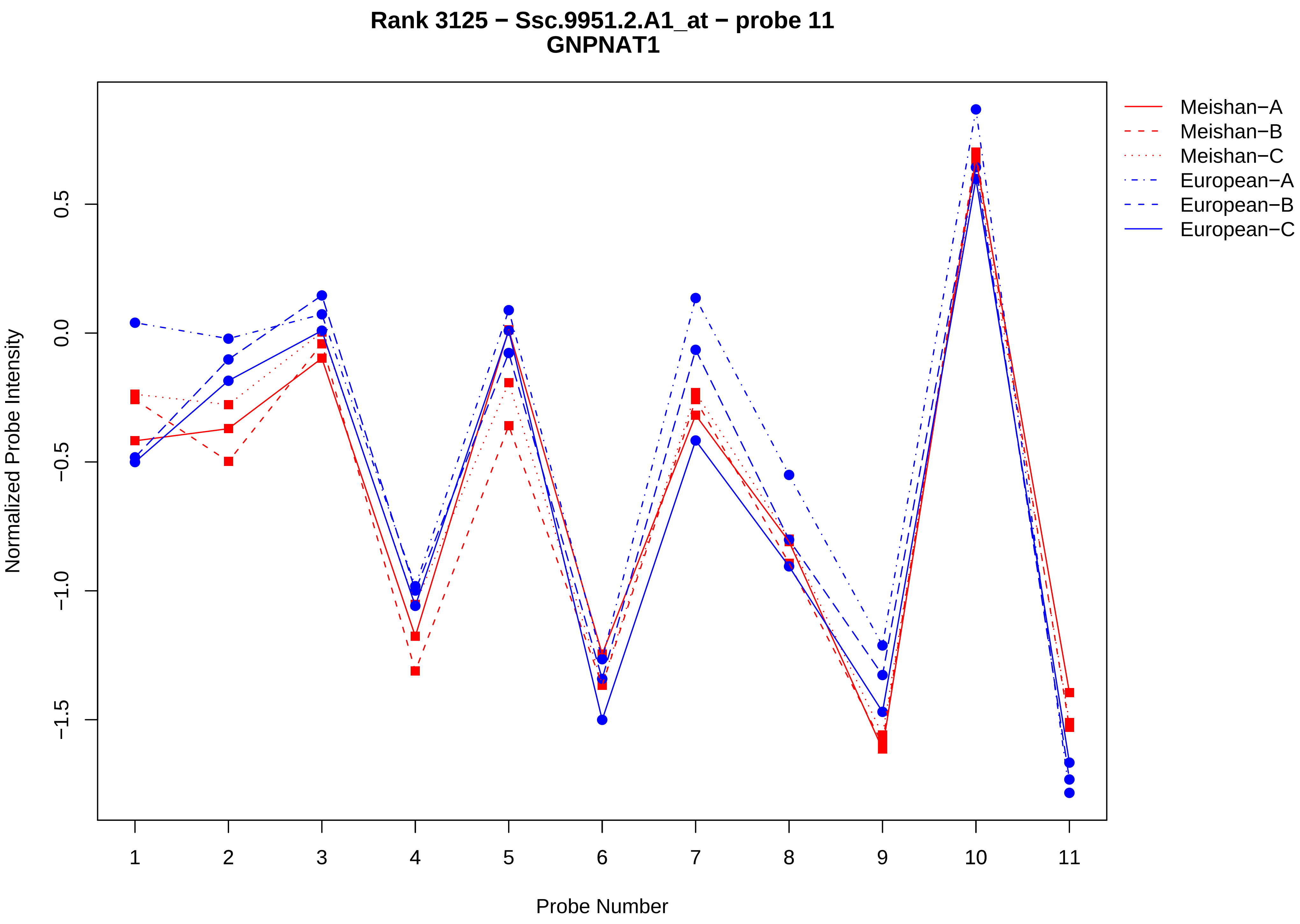

Rank 3125 − Ssc.9951.2.A1_at − probe 11
GNPNAT1
Normalized Probe Intensity
Probe Number
Meishan−A
Meishan−B
Meishan−C
European−A
European−B
European−C
0.5
0.0
−0.5
−1.0
−1.5
1
2
3
4
5
6
7
8
9
10
11

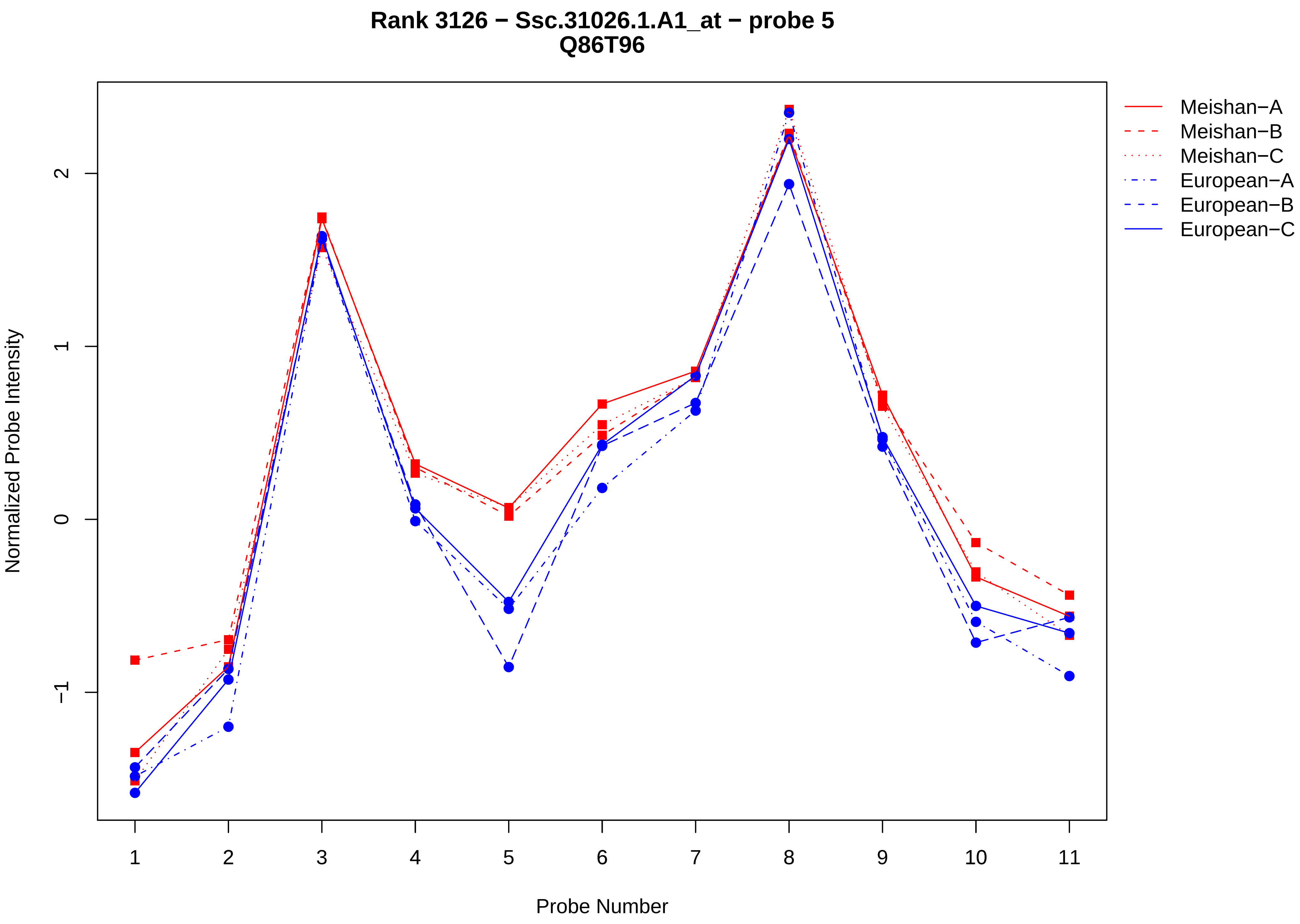
Rank 3126 − Ssc.31026.1.A1_at − probe 5
Q86T96
Normalized Probe Intensity
Probe Number
Meishan−A
Meishan−B
Meishan−C
European−A
European−B
European−C
−1
0
1
2
1
2
3
4
5
6
7
8
9
10
11

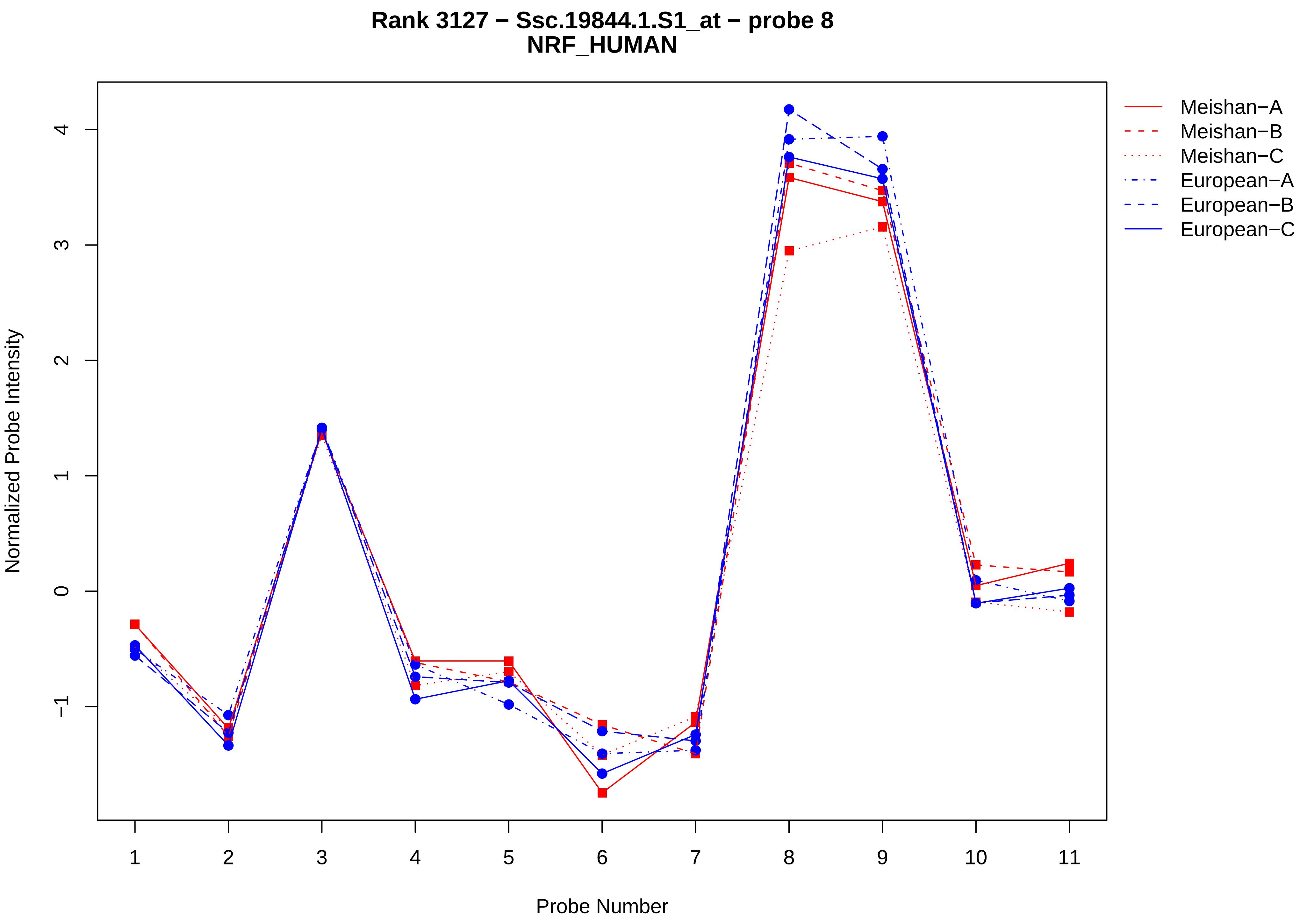

Rank 3127 − Ssc.19844.1.S1_at − probe 8
NRF_HUMAN
Normalized Probe Intensity
Probe Number
−1
0
1
2
3
4
1
2
3
4
5
6
7
8
9
10
11
Meishan−A
Meishan−B
Meishan−C
European−A
European−B
European−C

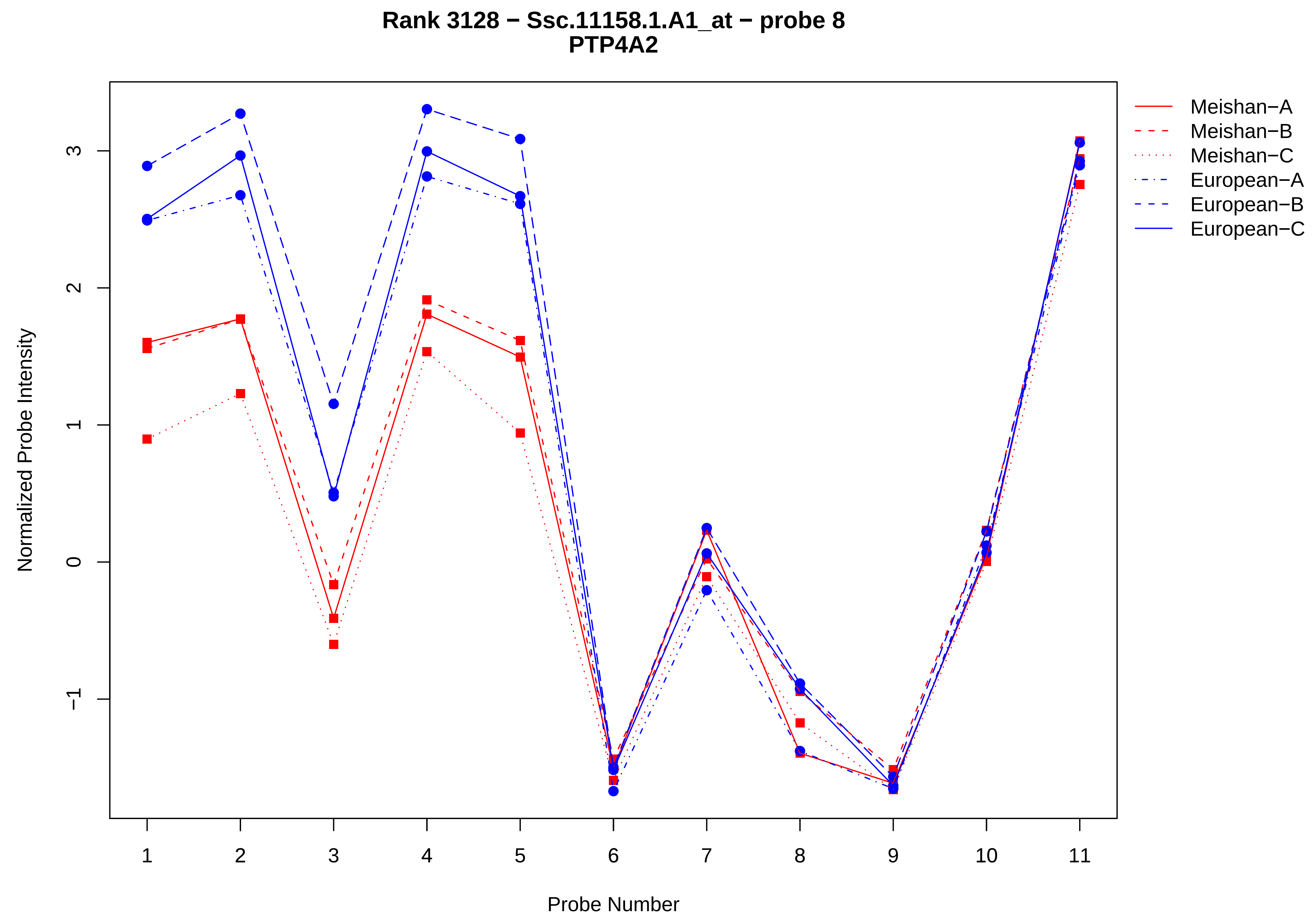

Rank 3128 − Ssc.11158.1.A1_at − probe 8
PTP4A2
Normalized Probe Intensity
Probe Number
−1
0
1
2
3
1
2
3
4
5
6
7
8
9
10
11
Meishan−A
Meishan−B
Meishan−C
European−A
European−B
European−C

Rank 3129 − Ssc.19041.2.S1_at − probe 10
ABCB6

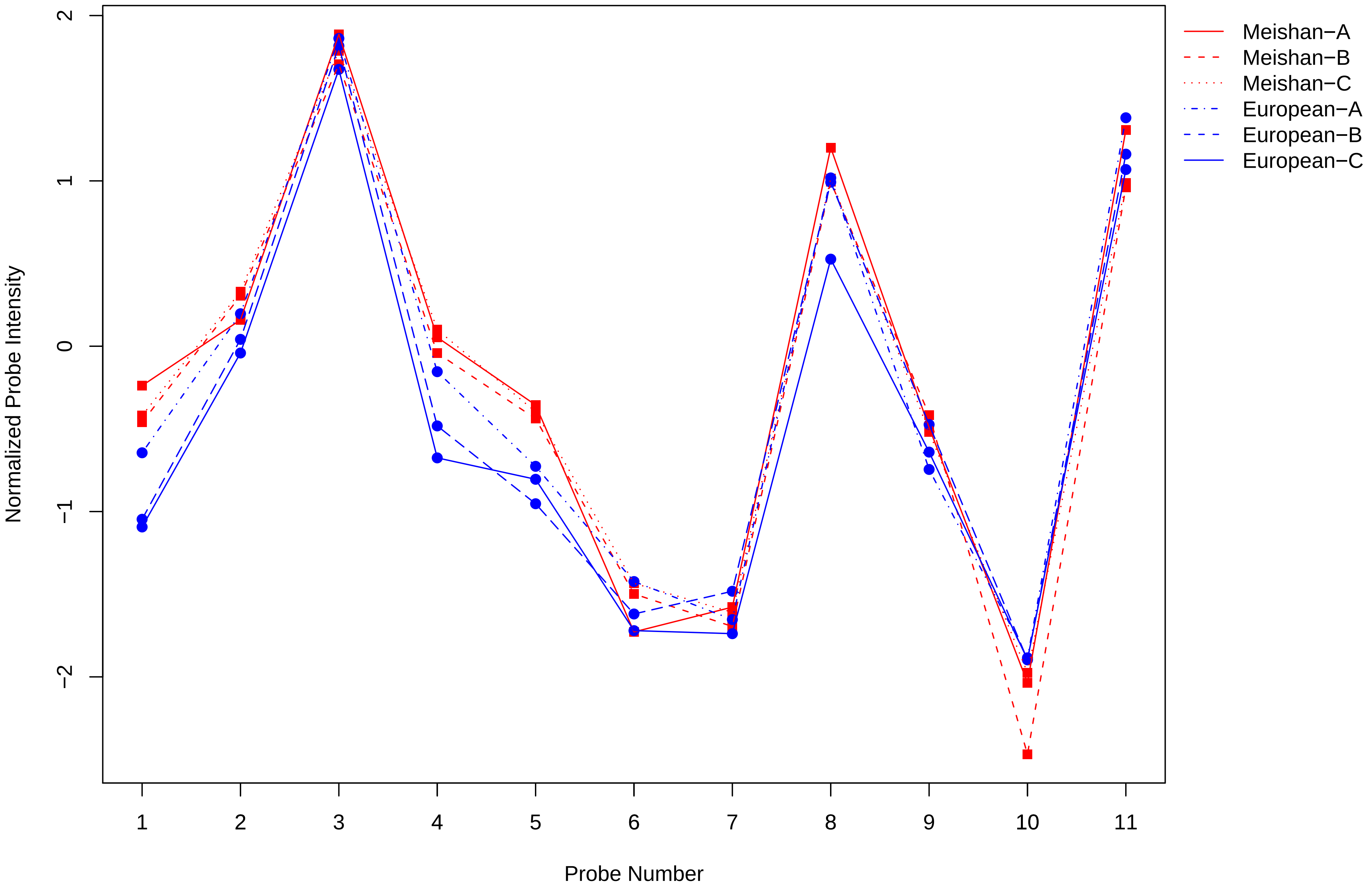

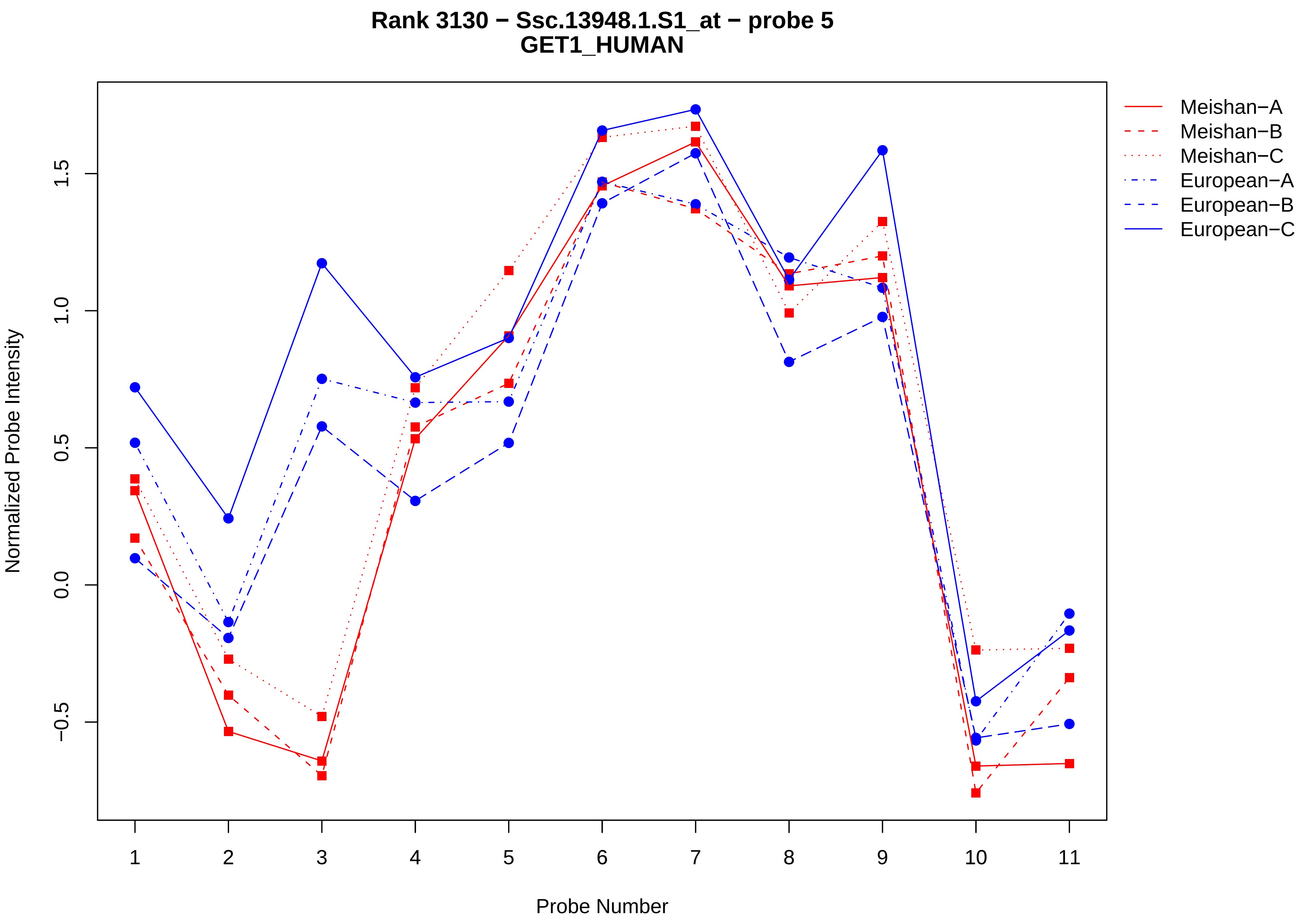
Rank 3130 − Ssc.13948.1.S1_at − probe 5
GET1_HUMAN
Normalized Probe Intensity
Probe Number
−0.5
0.0
0.5
1.0
1.5
1
2
3
4
5
6
7
8
9
10
11
Meishan−A
Meishan−B
Meishan−C
European−A
European−B
European−C

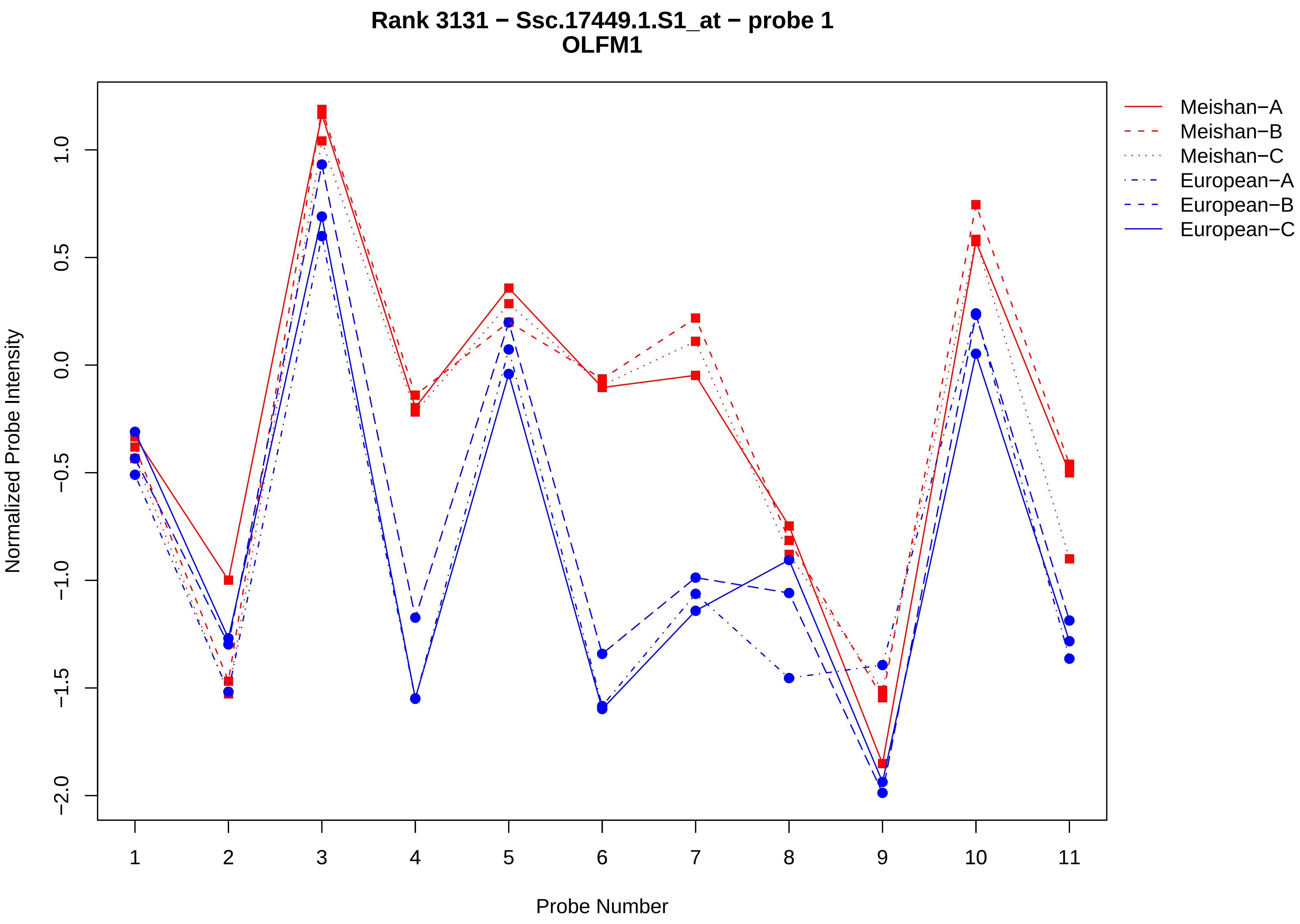

Rank 3131 − Ssc.17449.1.S1_at − probe 1
OLFM1
Normalized Probe Intensity
1.0
0.5
0.0
−0.5
−1.0
−1.5
−2.0
1
2
3
4
5
6
7
8
9
10
11
Probe Number
Meishan−A
Meishan−B
Meishan−C
European−A
European−B
European−C

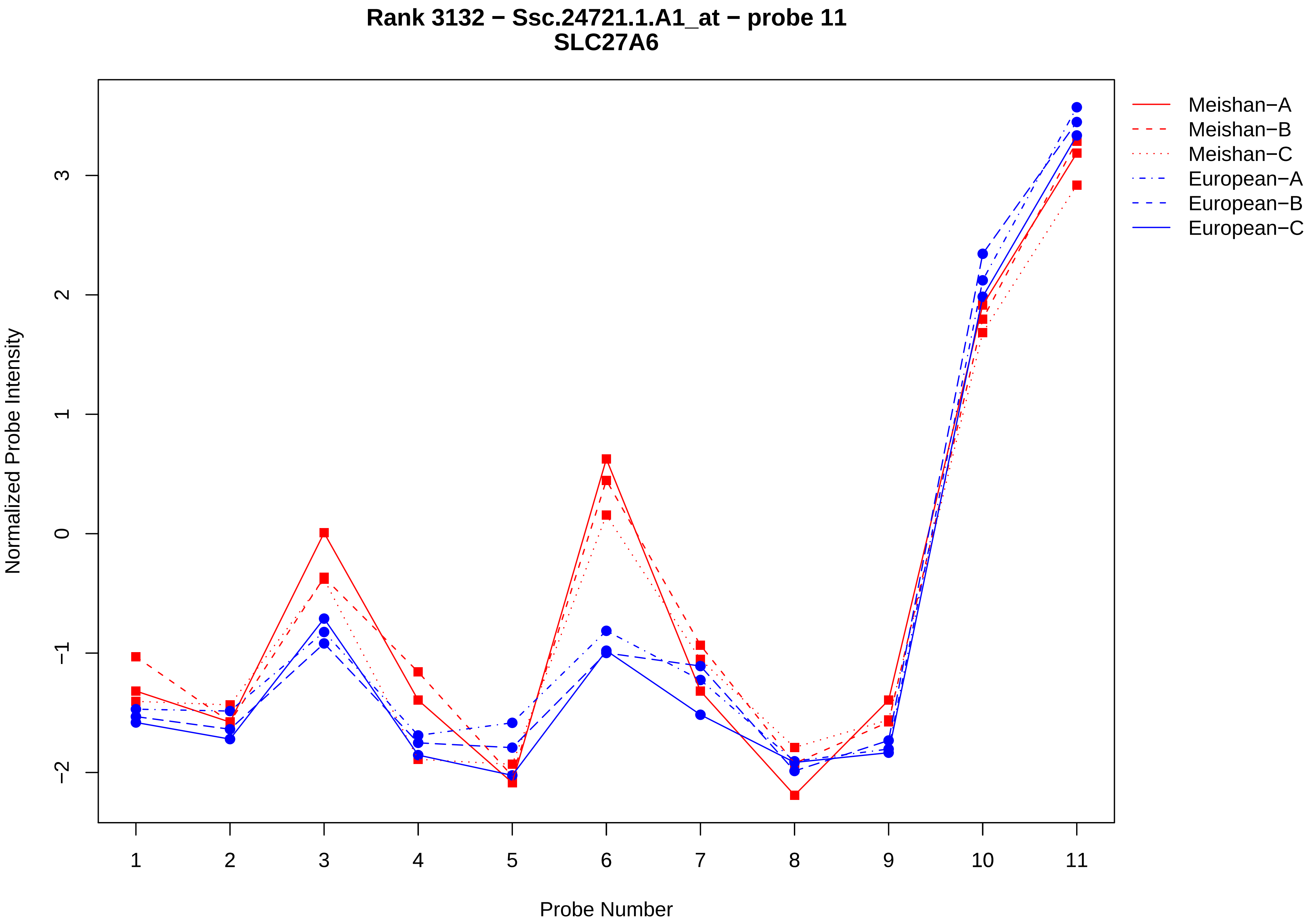

Rank 3132 − Ssc.24721.1.A1_at − probe 11
SLC27A6
Normalized Probe Intensity
Probe Number
Meishan−A
Meishan−B
Meishan−C
European−A
European−B
European−C
−2
−1
0
1
2
3
1
2
3
4
5
6
7
8
9
10
11

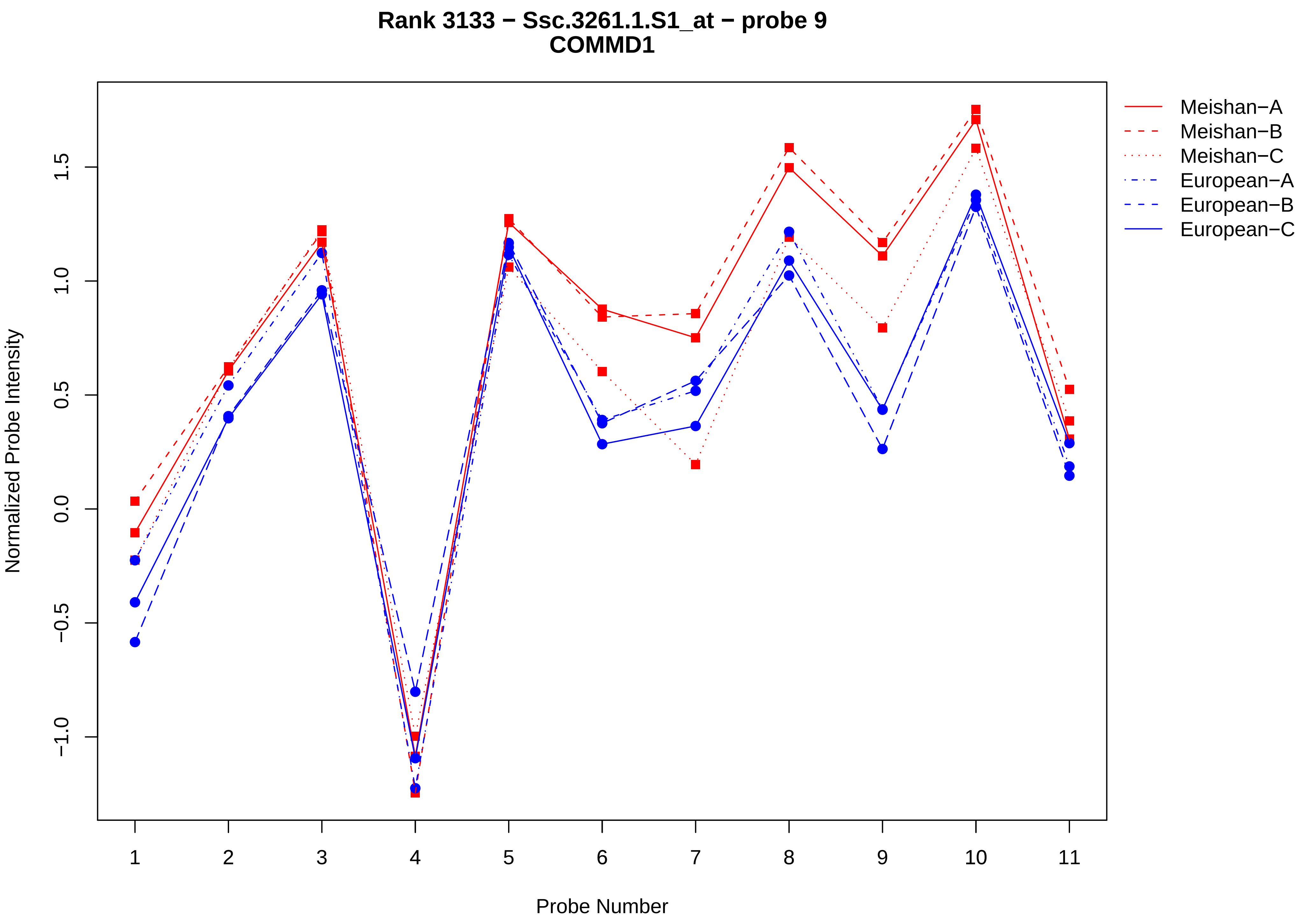

Rank 3133 − Ssc.3261.1.S1_at − probe 9
COMMD1
Normalized Probe Intensity
Probe Number
−1.0
−0.5
0.0
0.5
1.0
1.5
1
2
3
4
5
6
7
8
9
10
11
Meishan−A
Meishan−B
Meishan−C
European−A
European−B
European−C

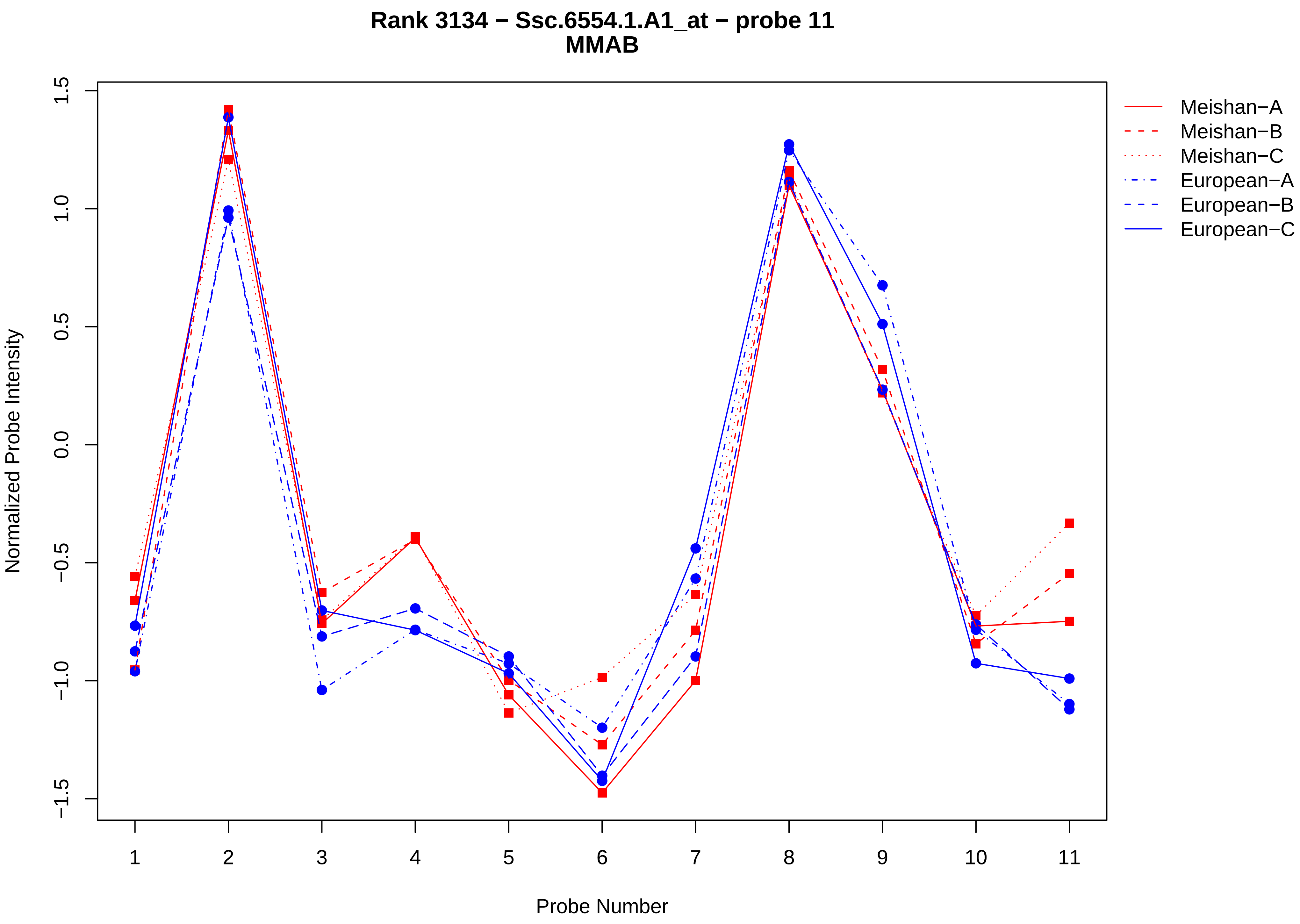
Rank 3134 − Ssc.6554.1.A1_at − probe 11
MMAB
Normalized Probe Intensity
Probe Number
Meishan−A
Meishan−B
Meishan−C
European−A
European−B
European−C
1.5
1.0
0.5
0.0
−0.5
−1.0
−1.5
1
2
3
4
5
6
7
8
9
10
11

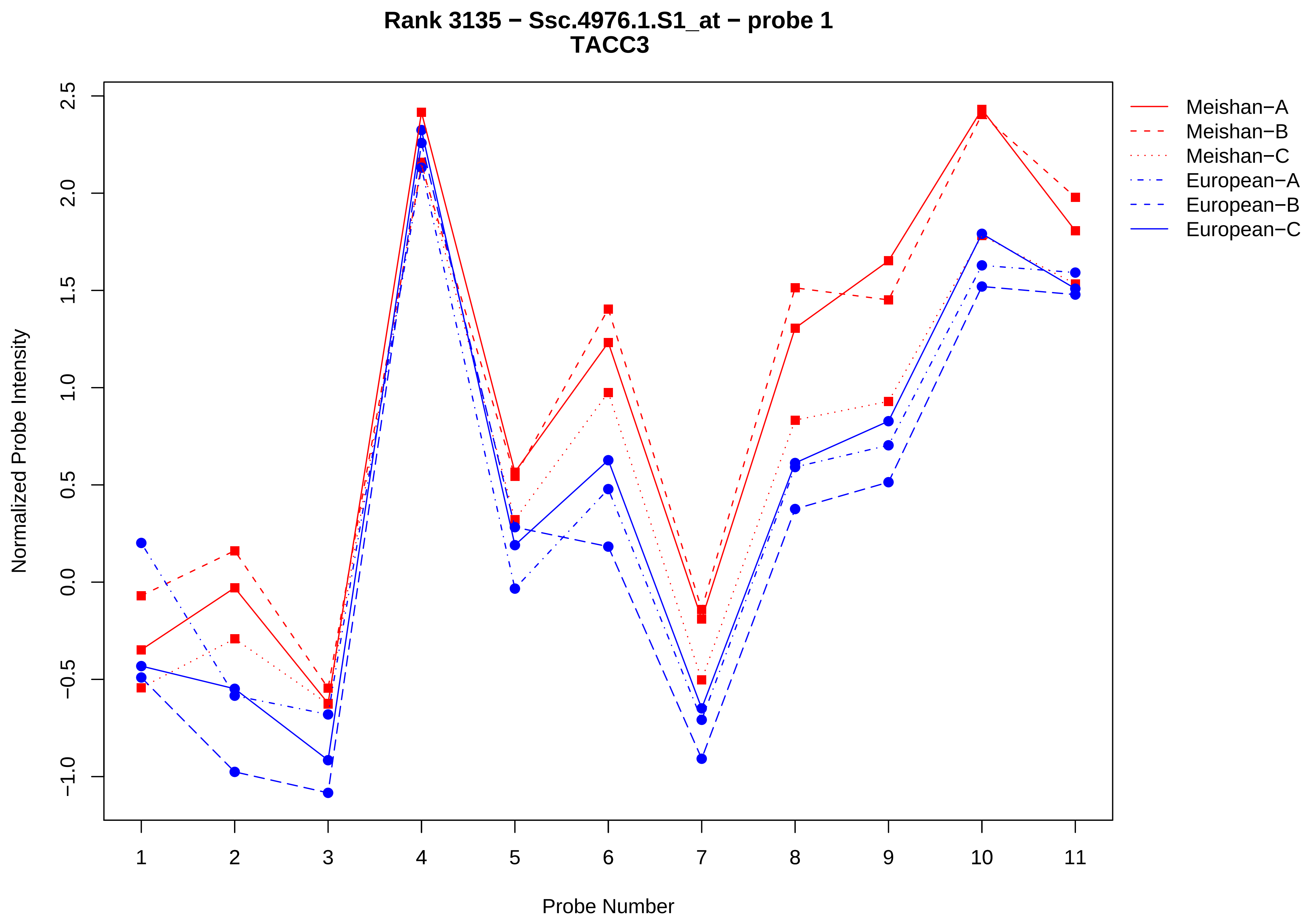

Rank 3135 − Ssc.4976.1.S1_at − probe 1
TACC3
Normalized Probe Intensity
Probe Number
−1.0
−0.5
0.0
0.5
1.0
1.5
2.0
2.5
1
2
3
4
5
6
7
8
9
10
11
Meishan−A
Meishan−B
Meishan−C
European−A
European−B
European−C

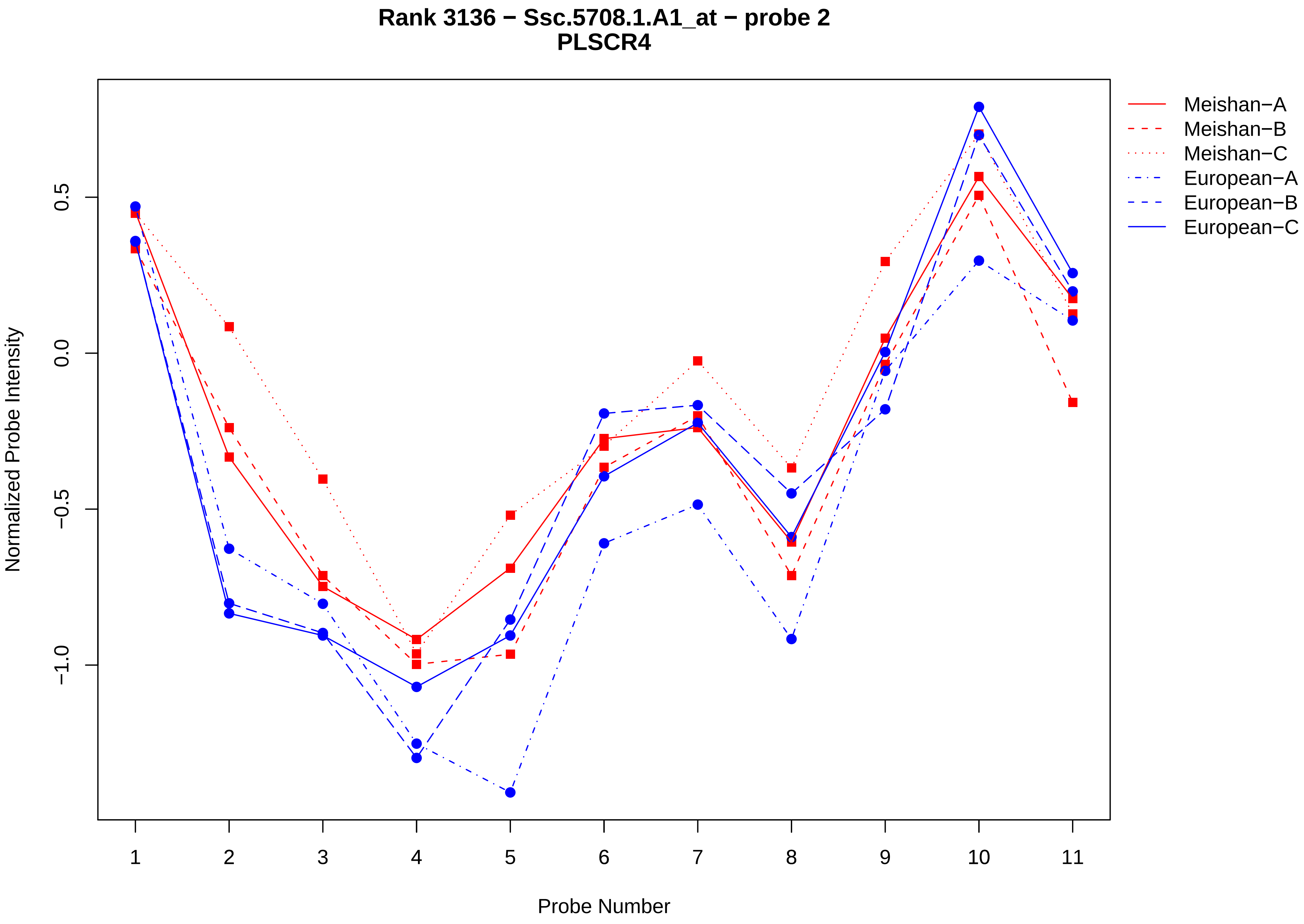

Rank 3136 − Ssc.5708.1.A1_at − probe 2
PLSCR4
Normalized Probe Intensity
Probe Number
0.5
0.0
−0.5
−1.0
1
2
3
4
5
6
7
8
9
10
11
Meishan−A
Meishan−B
Meishan−C
European−A
European−B
European−C

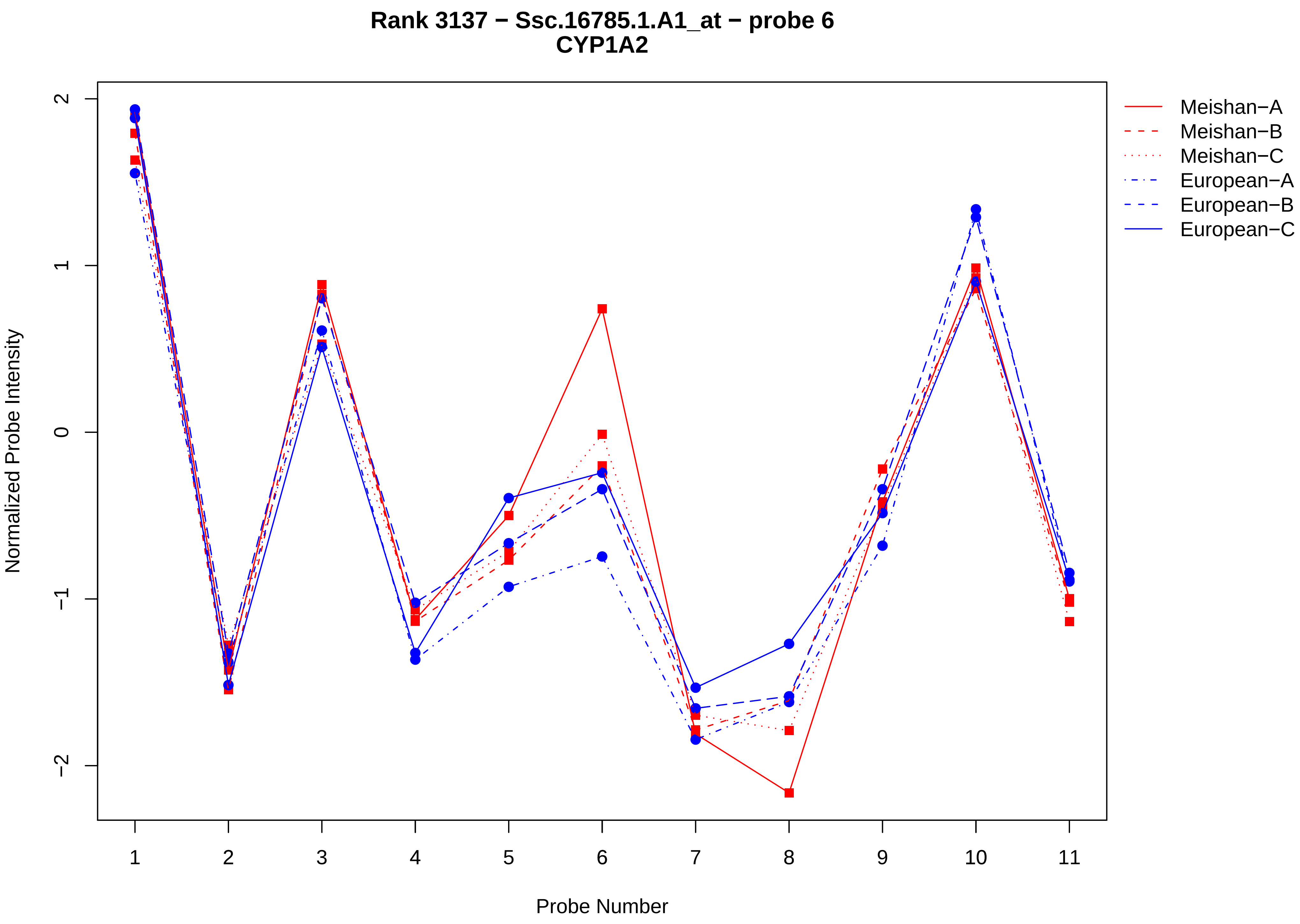

Rank 3137 − Ssc.16785.1.A1_at − probe 6
CYP1A2
Normalized Probe Intensity
Probe Number
Meishan−A
Meishan−B
Meishan−C
European−A
European−B
European−C

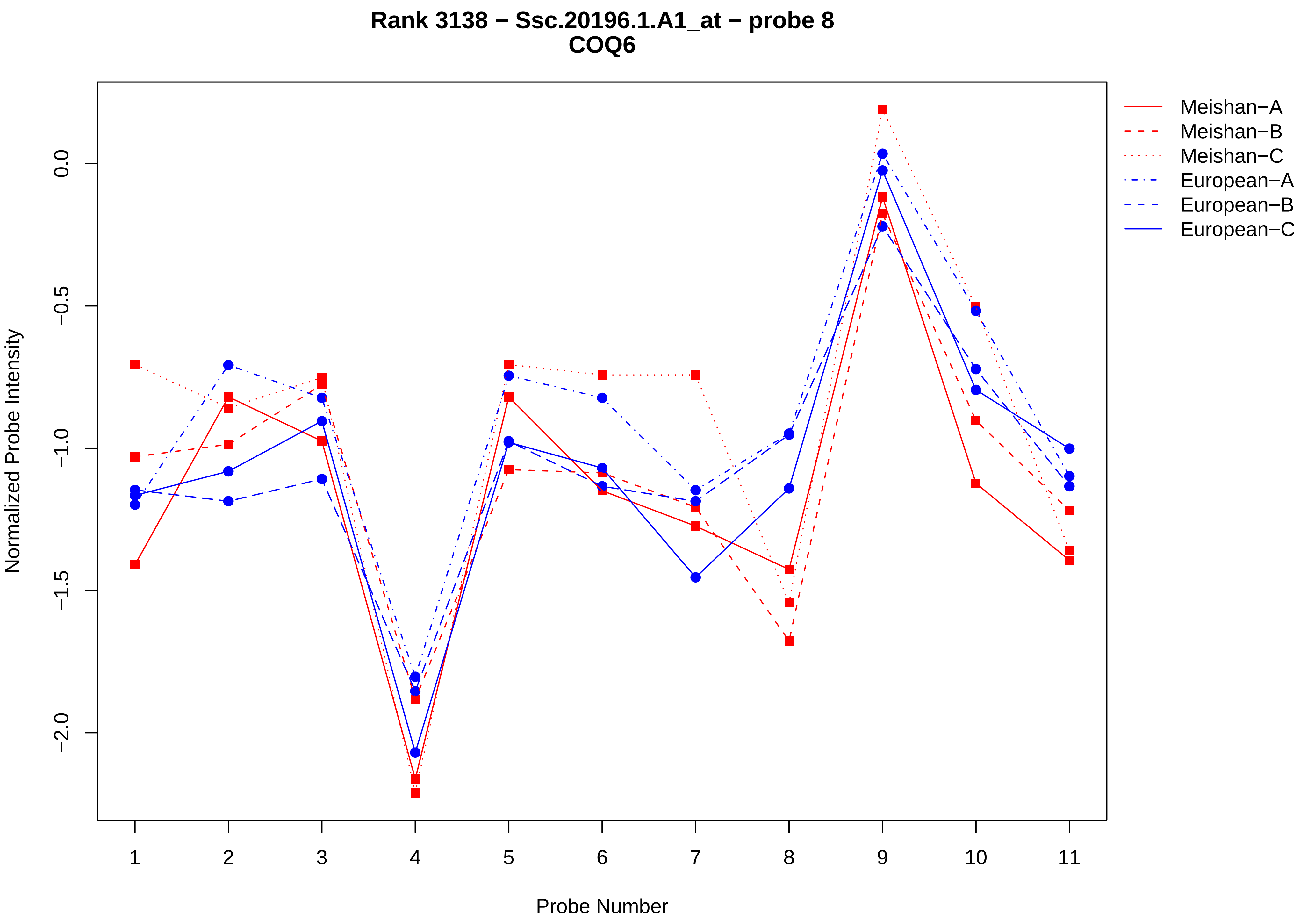

Rank 3138 − Ssc.20196.1.A1_at − probe 8
COQ6
Normalized Probe Intensity
Probe Number
0.0
−0.5
−1.0
−1.5
−2.0
1
2
3
4
5
6
7
8
9
10
11
Meishan−A
Meishan−B
Meishan−C
European−A
European−B
European−C

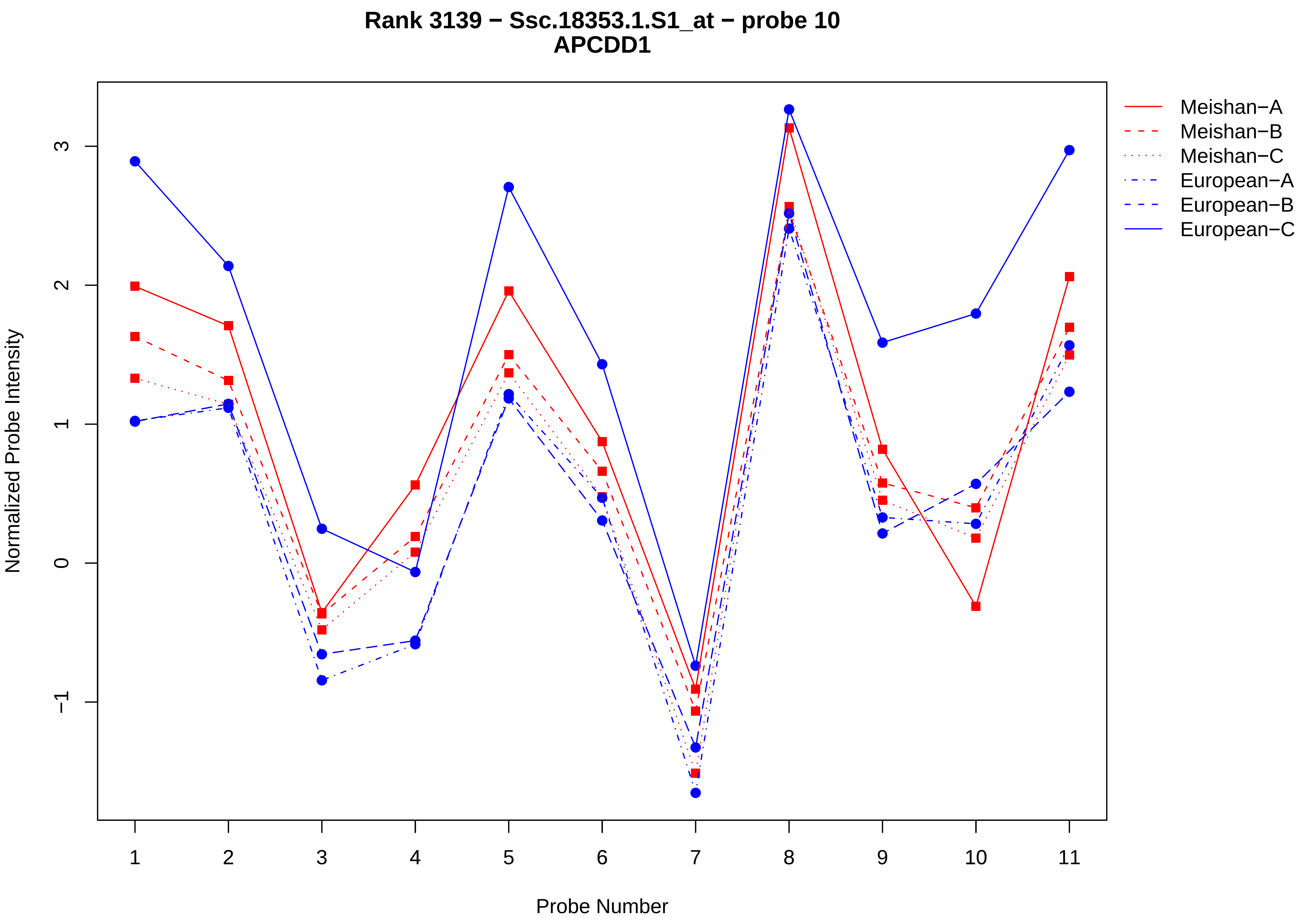

Rank 3139 − Ssc.18353.1.S1_at − probe 10
APCDD1
Normalized Probe Intensity
Probe Number
Meishan−A
Meishan−B
Meishan−C
European−A
European−B
European−C
−1
0
1
2
3
1
2
3
4
5
6
7
8
9
10
11

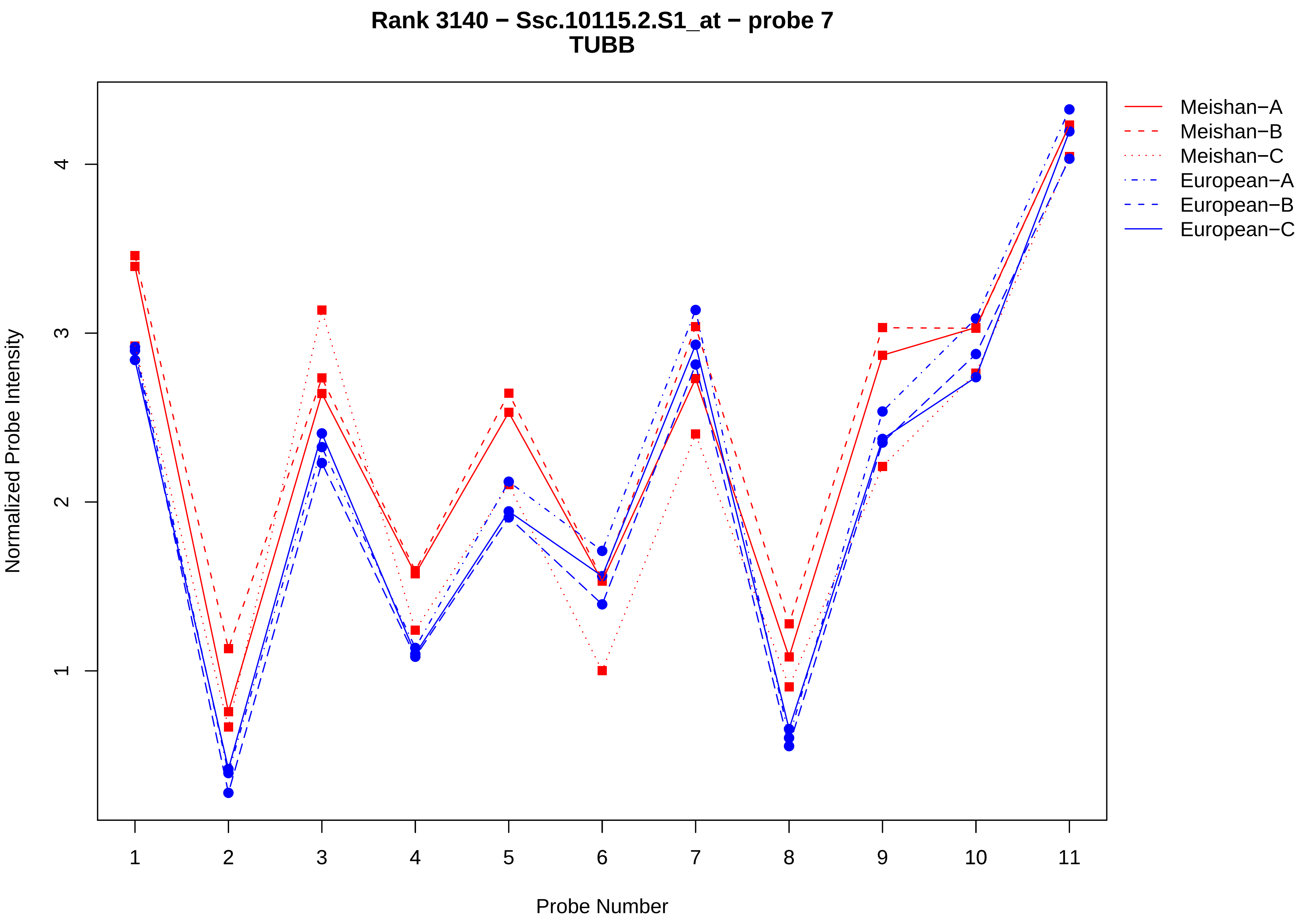

Rank 3140 − Ssc.10115.2.S1_at − probe 7
TUBB
Normalized Probe Intensity
Probe Number
1
2
3
4
1
2
3
4
5
6
7
8
9
10
11
Meishan−A
Meishan−B
Meishan−C
European−A
European−B
European−C

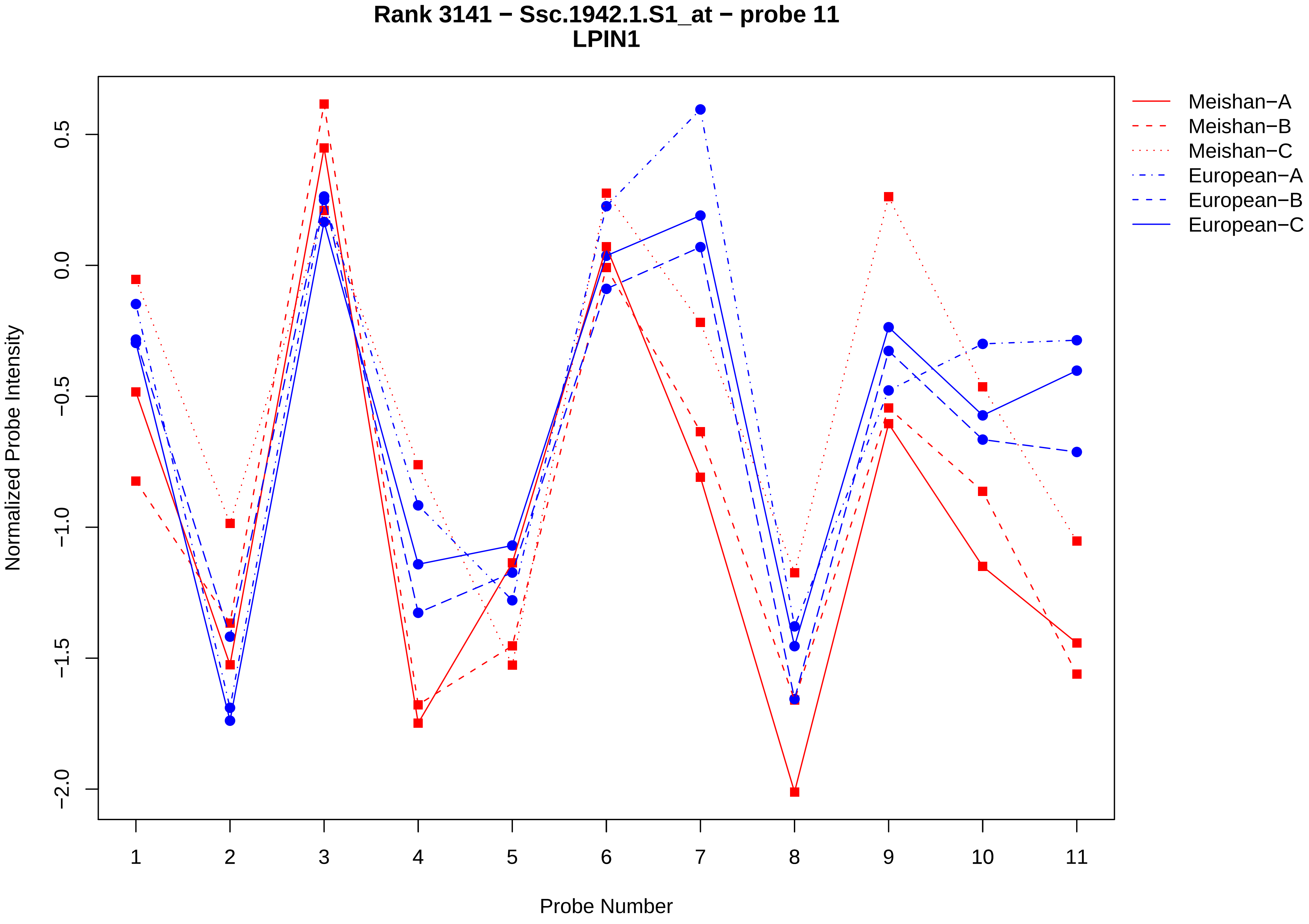

Rank 3141 − Ssc.1942.1.S1_at − probe 11
LPIN1
Normalized Probe Intensity
Probe Number
Meishan−A
Meishan−B
Meishan−C
European−A
European−B
European−C
0.5
0.0
−0.5
−1.0
−1.5
−2.0
1
2
3
4
5
6
7
8
9
10
11

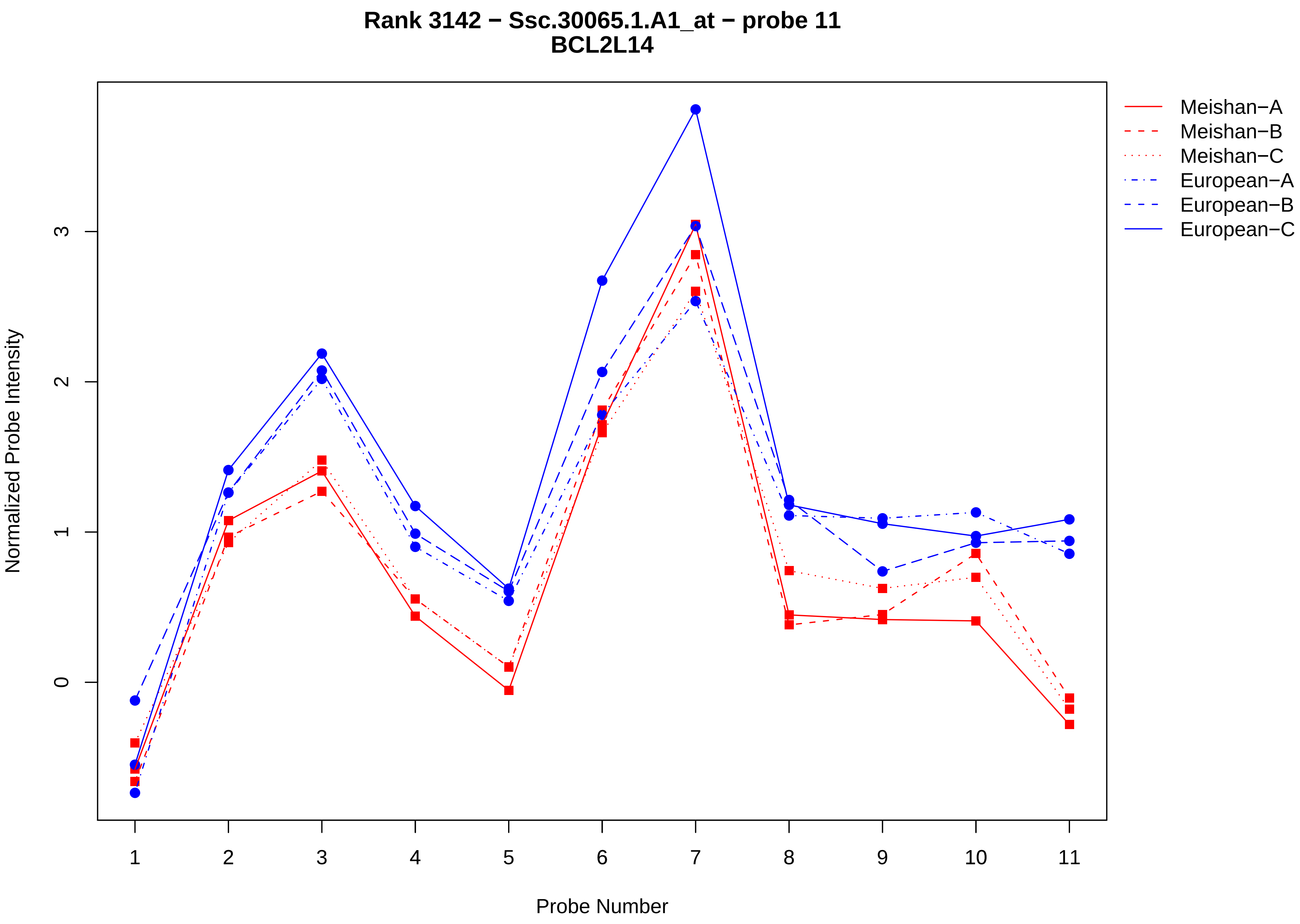
Rank 3142 − Ssc.30065.1.A1_at − probe 11
BCL2L14
Normalized Probe Intensity
0
1
2
3
1
2
3
4
5
6
7
8
9
10
11
Probe Number
Meishan−A
Meishan−B
Meishan−C
European−A
European−B
European−C

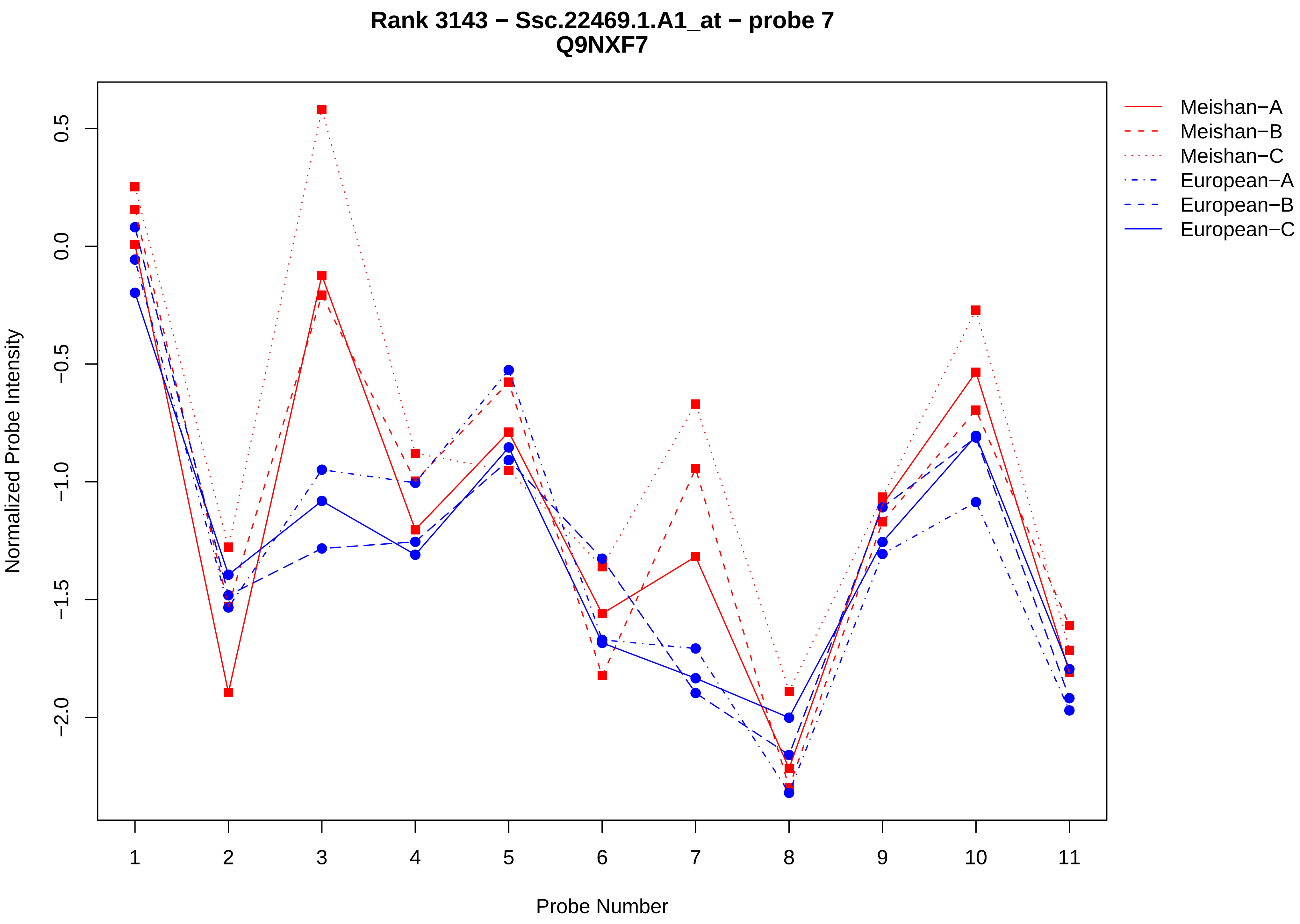
Rank 3143 − Ssc.22469.1.A1_at − probe 7
Q9NXF7
Normalized Probe Intensity
0.5
0.0
−0.5
−1.0
−1.5
−2.0
1
2
3
4
5
6
7
8
9
10
11
Probe Number
Meishan−A
Meishan−B
Meishan−C
European−A
European−B
European−C

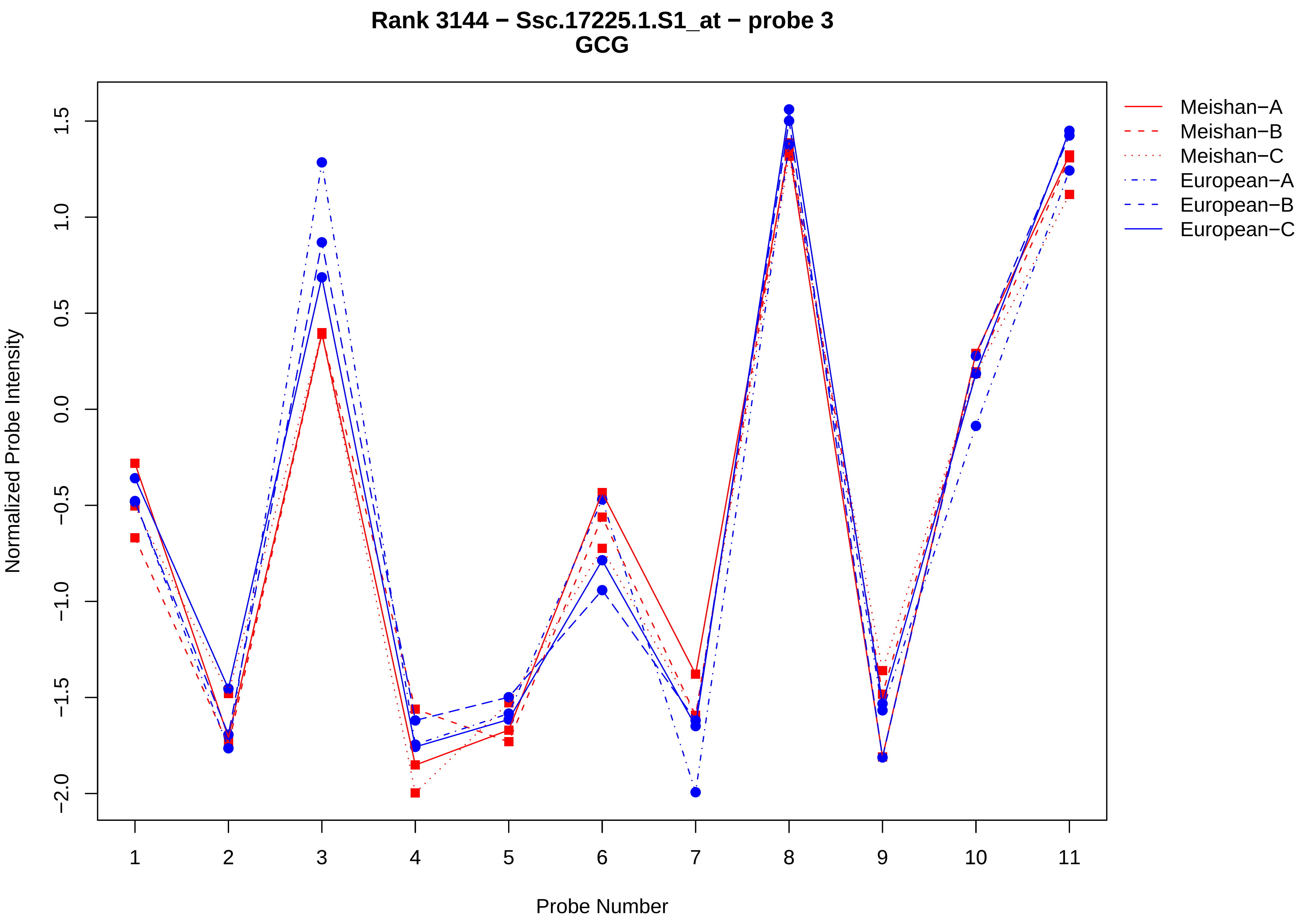

Rank 3144 − Ssc.17225.1.S1_at − probe 3
GCG
Normalized Probe Intensity
Probe Number
1.5
1.0
0.5
0.0
−0.5
−1.0
−1.5
−2.0
1
2
3
4
5
6
7
8
9
10
11
Meishan−A
Meishan−B
Meishan−C
European−A
European−B
European−C

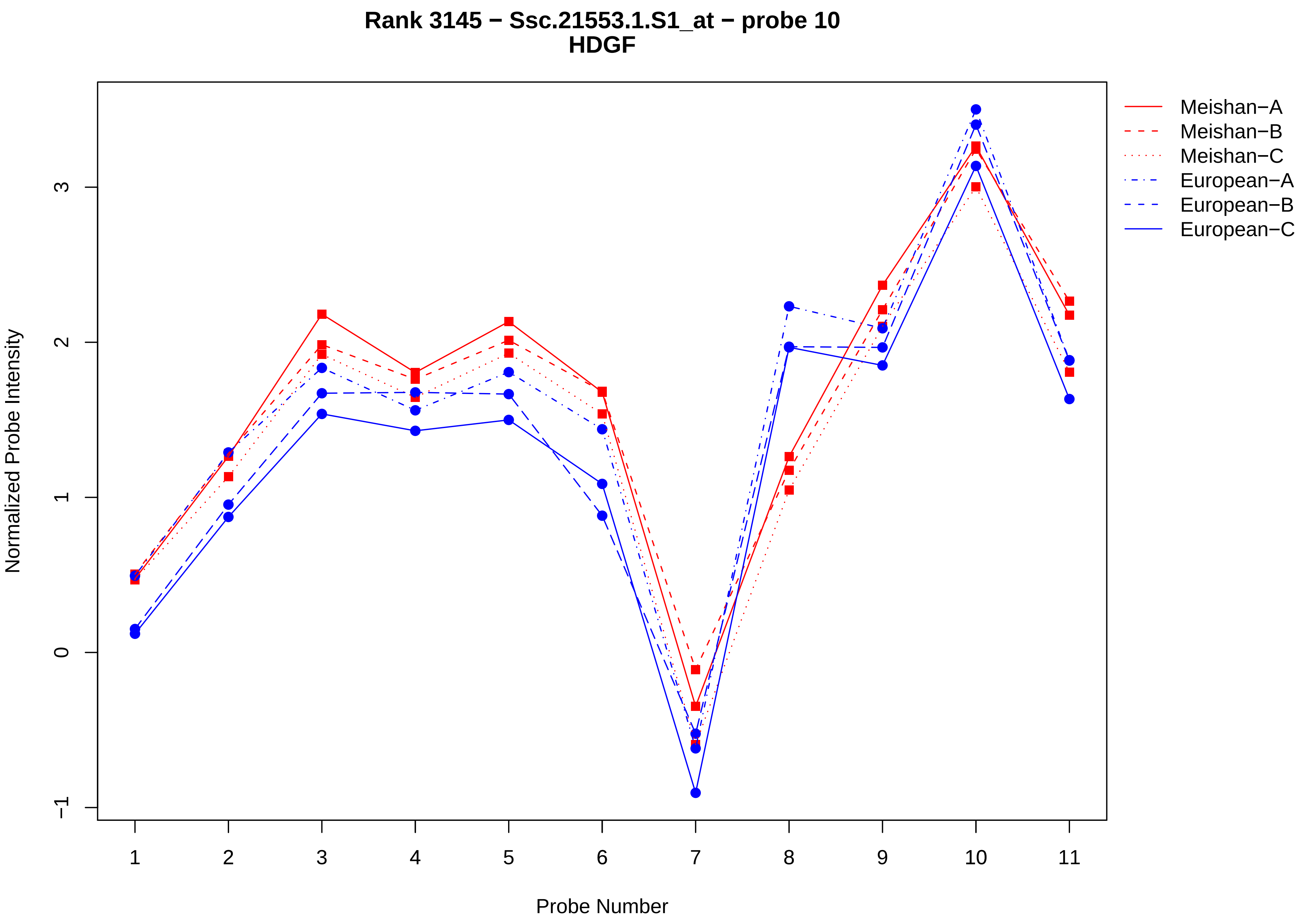
Rank 3145 − Ssc.21553.1.S1_at − probe 10
HDGF
Normalized Probe Intensity
Probe Number
−1
0
1
2
3
1
2
3
4
5
6
7
8
9
10
11
Meishan−A
Meishan−B
Meishan−C
European−A
European−B
European−C

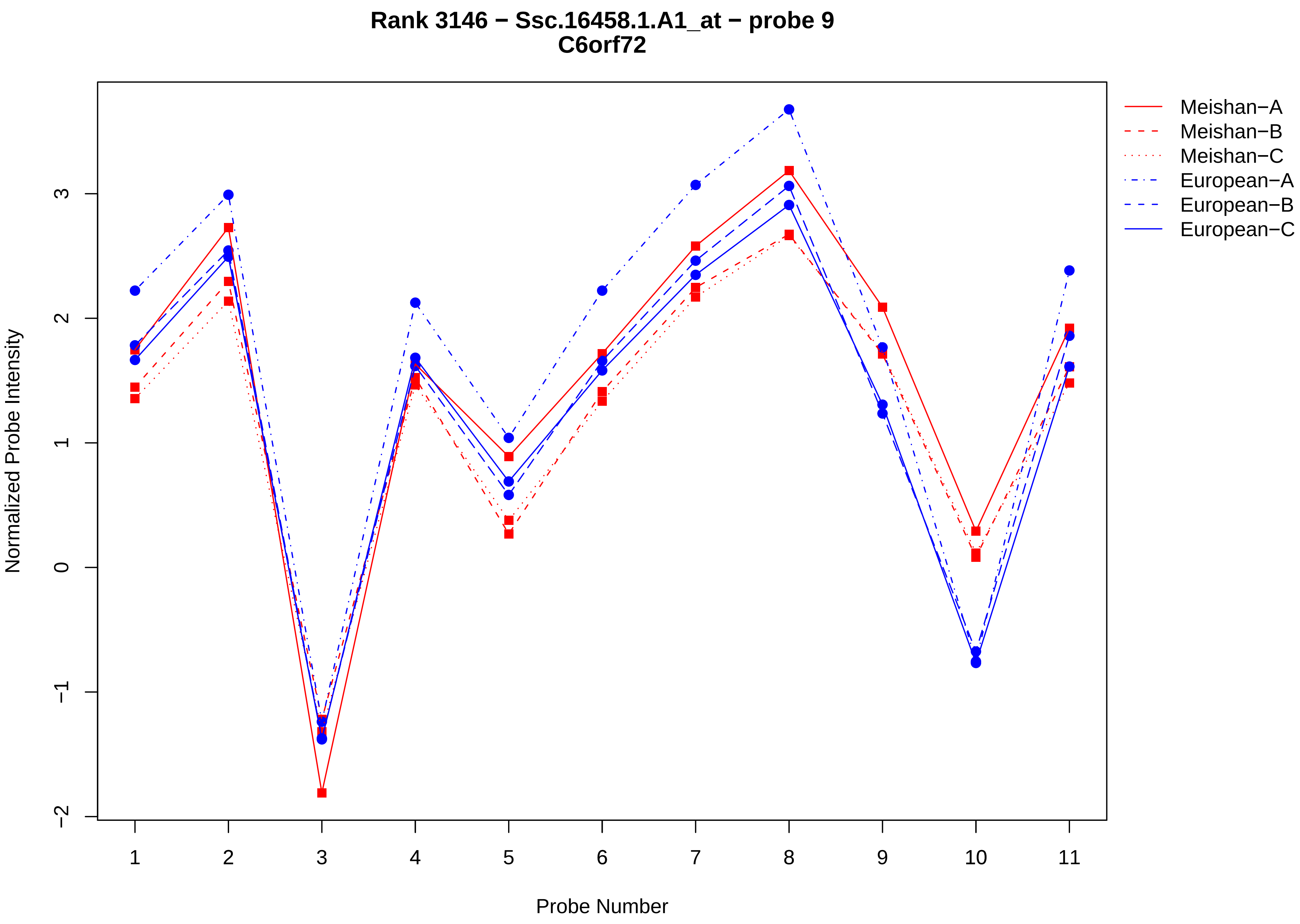

Rank 3146 − Ssc.16458.1.A1_at − probe 9
C6orf72
Normalized Probe Intensity
Probe Number
Meishan−A
Meishan−B
Meishan−C
European−A
European−B
European−C
−2
−1
0
1
2
3
1
2
3
4
5
6
7
8
9
10
11

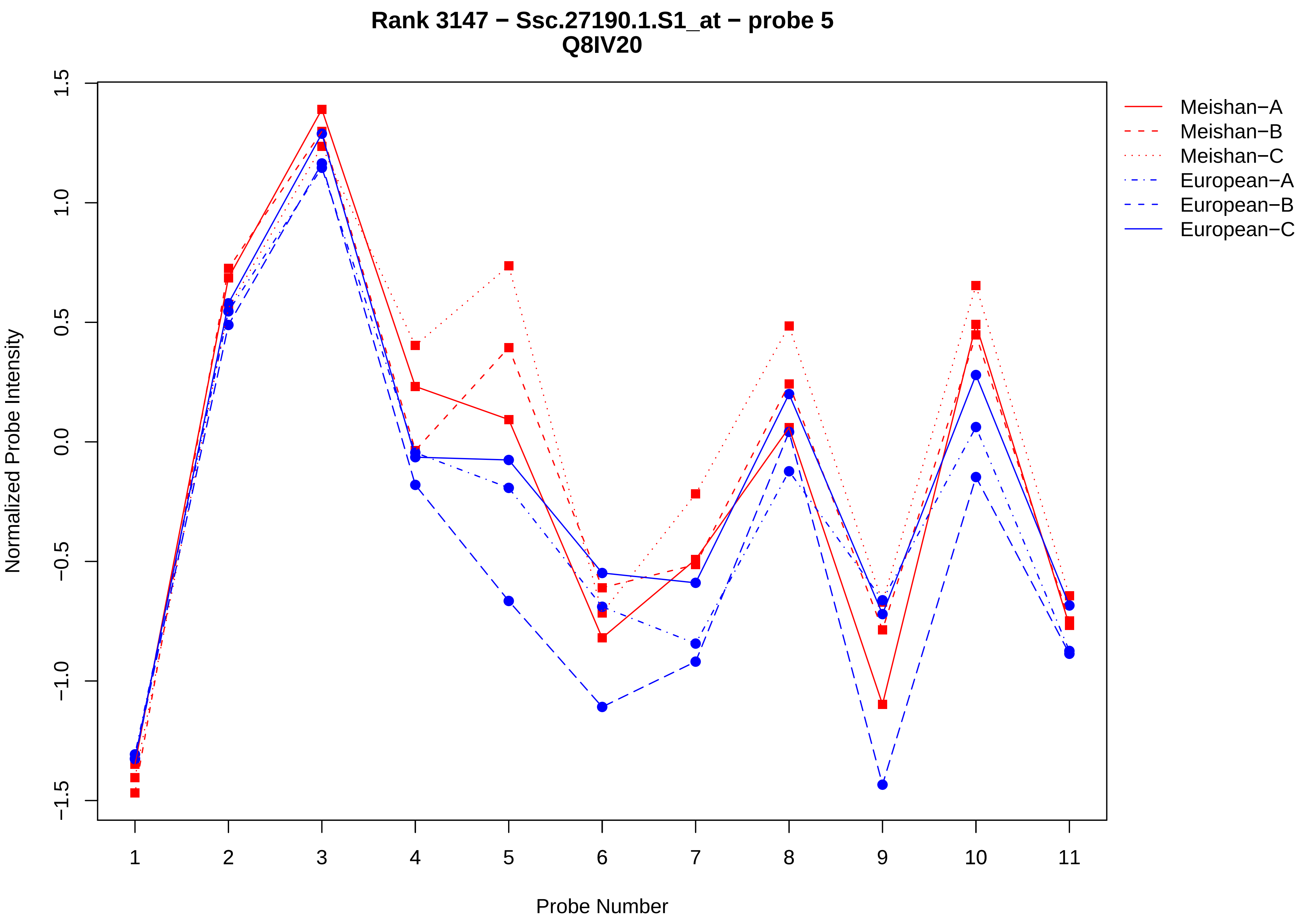

Rank 3147 − Ssc.27190.1.S1_at − probe 5
Q8IV20
Normalized Probe Intensity
Probe Number
1.5
1.0
0.5
0.0
−0.5
−1.0
−1.5
1
2
3
4
5
6
7
8
9
10
11
Meishan−A
Meishan−B
Meishan−C
European−A
European−B
European−C

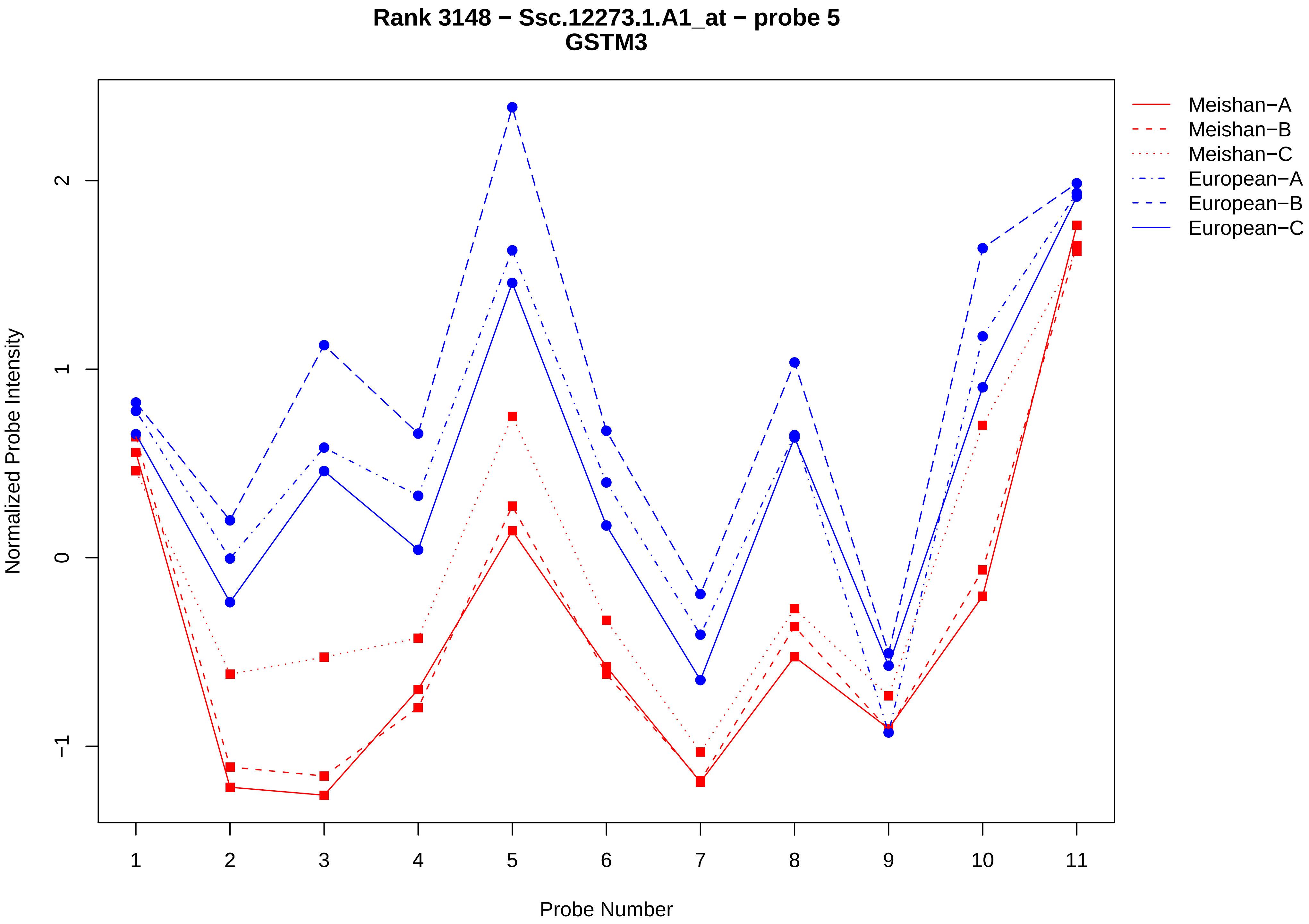

Rank 3148 − Ssc.12273.1.A1_at − probe 5
GSTM3
Normalized Probe Intensity
Probe Number
−1
0
1
2
1
2
3
4
5
6
7
8
9
10
11
Meishan−A
Meishan−B
Meishan−C
European−A
European−B
European−C

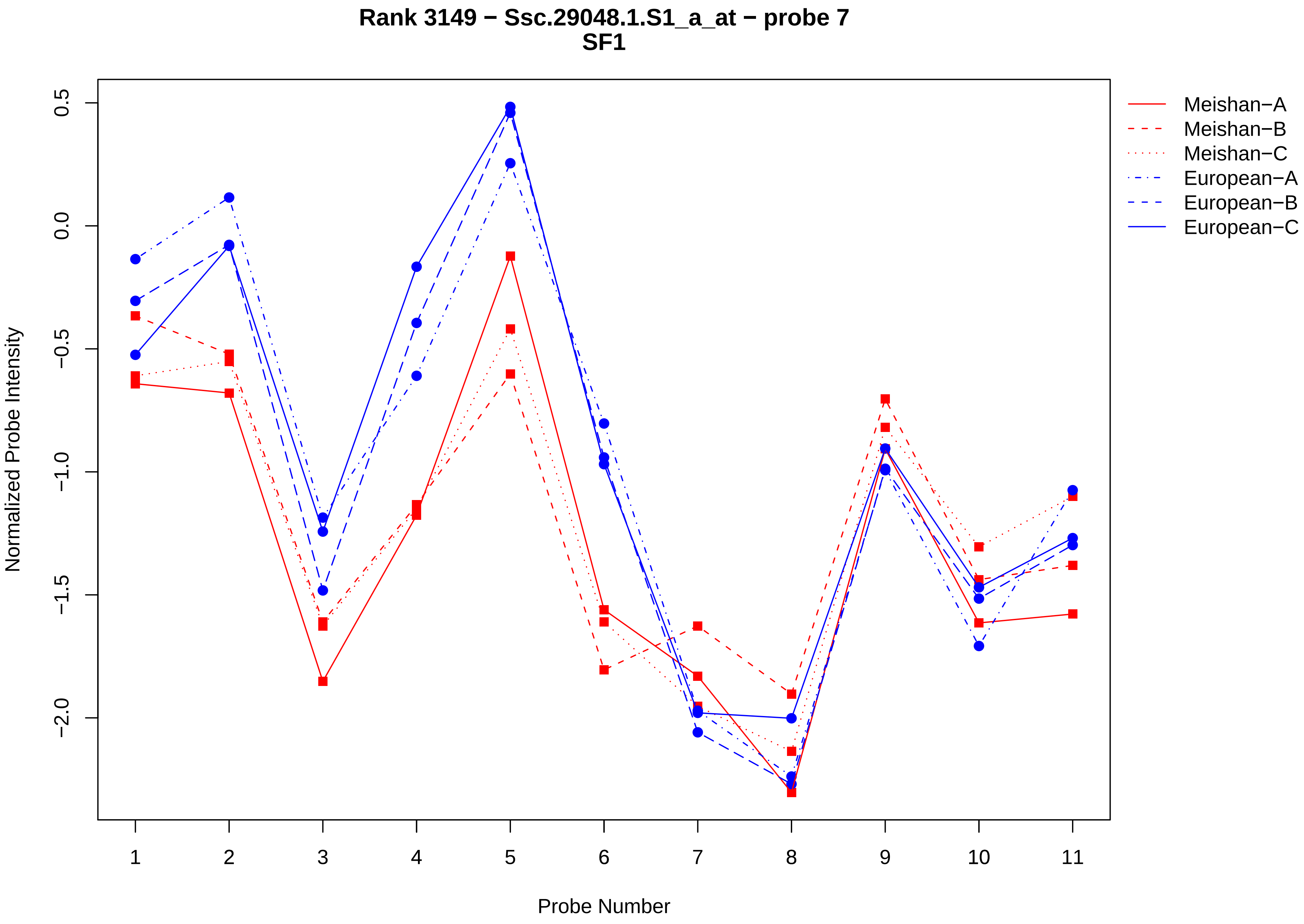

Rank 3149 − Ssc.29048.1.S1_a_at − probe 7
SF1
Normalized Probe Intensity
Probe Number
0.5
0.0
−0.5
−1.0
−1.5
−2.0
1
2
3
4
5
6
7
8
9
10
11
Meishan−A
Meishan−B
Meishan−C
European−A
European−B
European−C

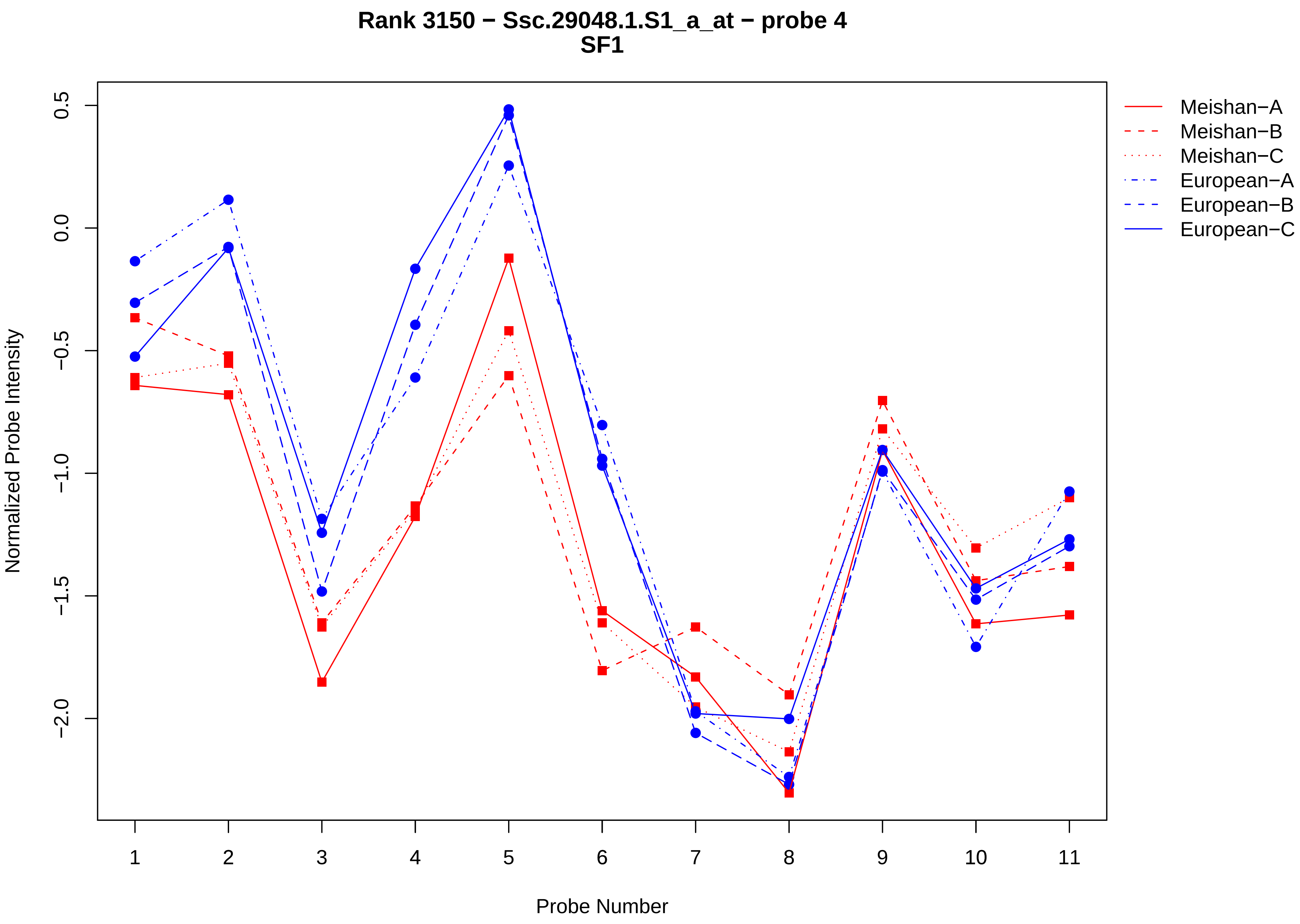
Rank 3150 − Ssc.29048.1.S1_a_at − probe 4
SF1
Normalized Probe Intensity
0.5
0.0
−0.5
−1.0
−1.5
−2.0
Probe Number
1
2
3
4
5
6
7
8
9
10
11
Meishan−A
Meishan−B
Meishan−C
European−A
European−B
European−C

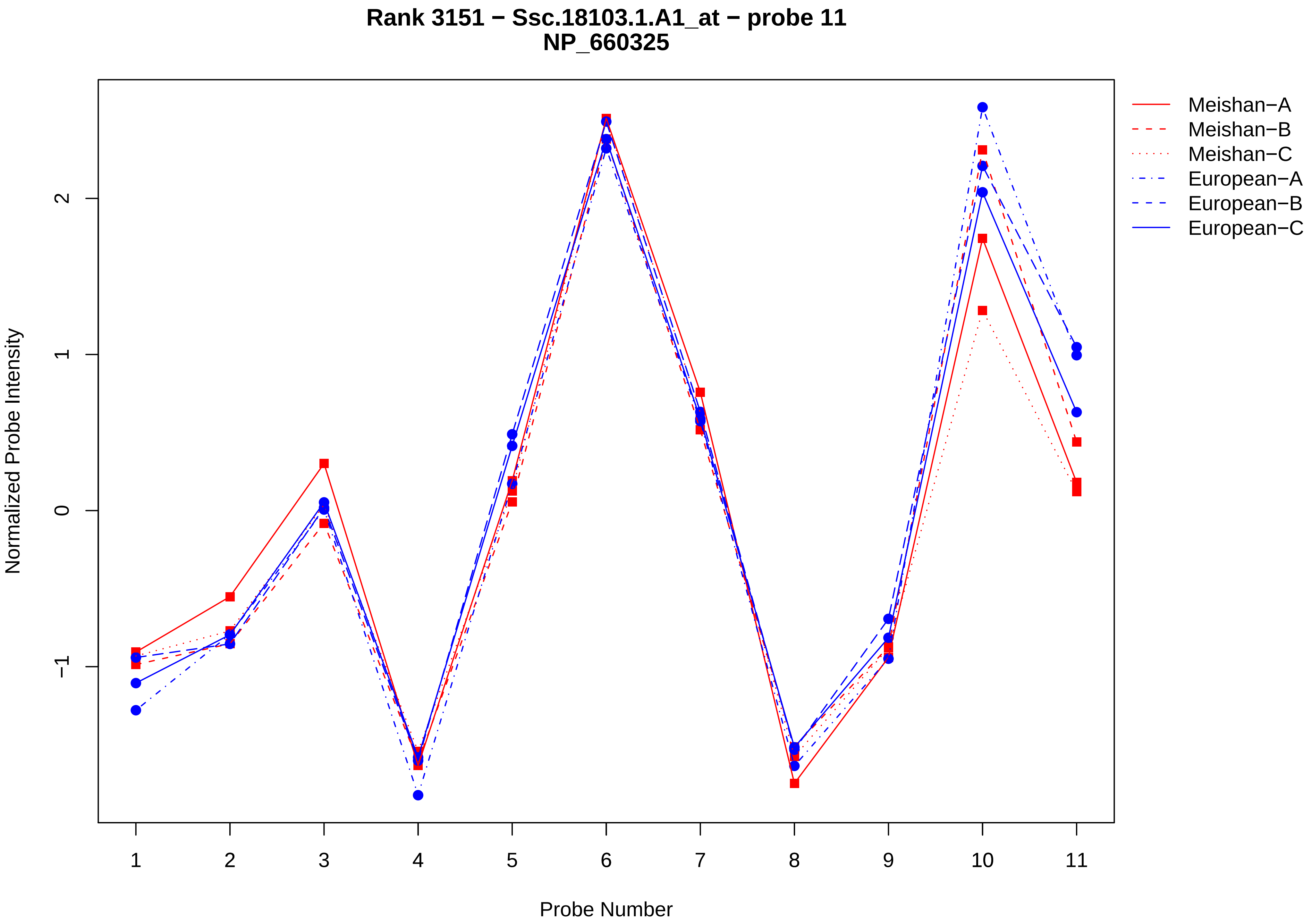

Rank 3151 − Ssc.18103.1.A1_at − probe 11
NP_660325
Normalized Probe Intensity
−1
0
1
2
1
2
3
4
5
6
7
8
9
10
11
Probe Number
Meishan−A
Meishan−B
Meishan−C
European−A
European−B
European−C

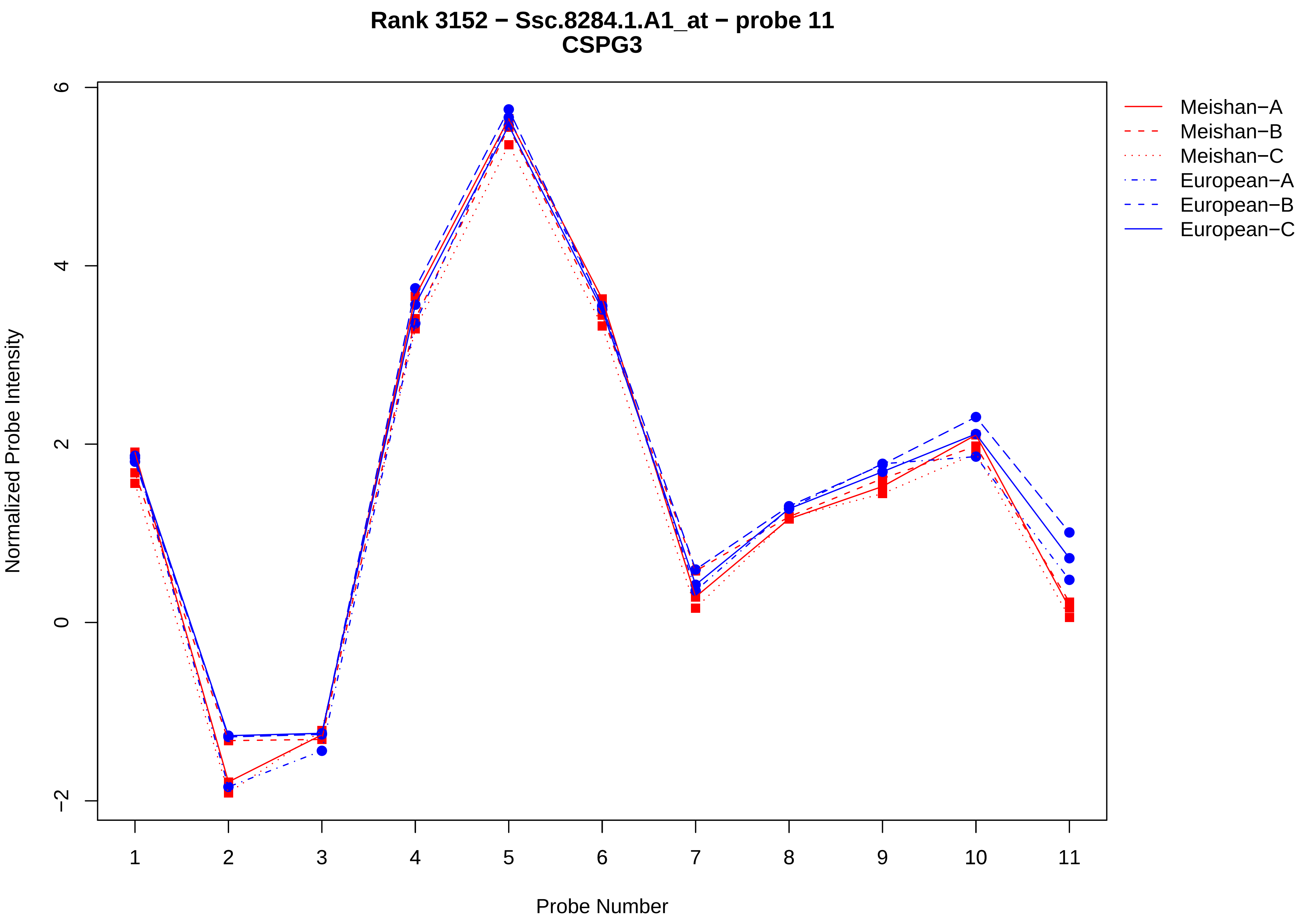

Rank 3152 − Ssc.8284.1.A1_at − probe 11
CSPG3
Normalized Probe Intensity
Probe Number
−2
0
2
4
6
1
2
3
4
5
6
7
8
9
10
11
Meishan−A
Meishan−B
Meishan−C
European−A
European−B
European−C

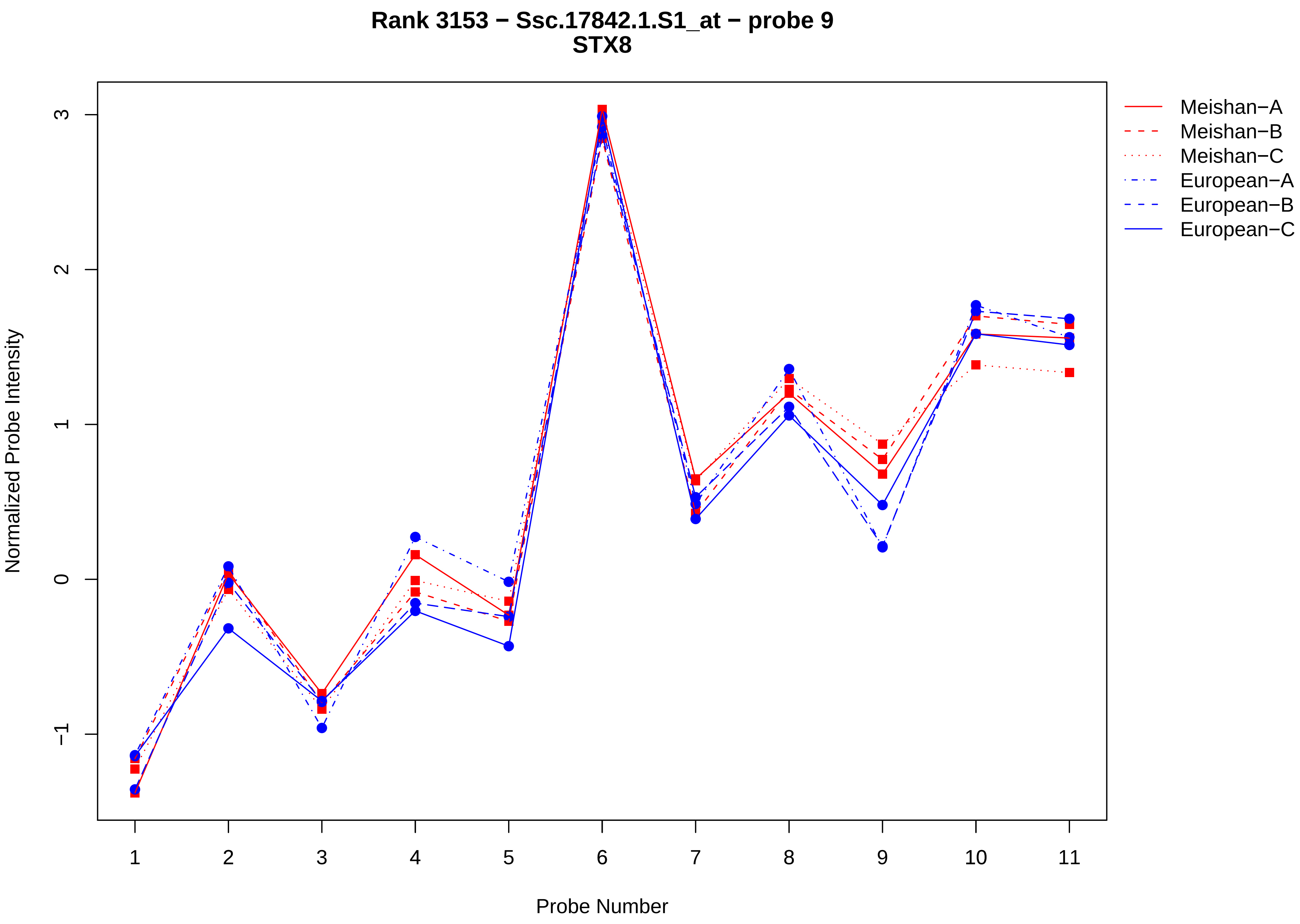

Rank 3153 − Ssc.17842.1.S1_at − probe 9
STX8
Normalized Probe Intensity
Probe Number
−1
0
1
2
3
1
2
3
4
5
6
7
8
9
10
11
Meishan−A
Meishan−B
Meishan−C
European−A
European−B
European−C

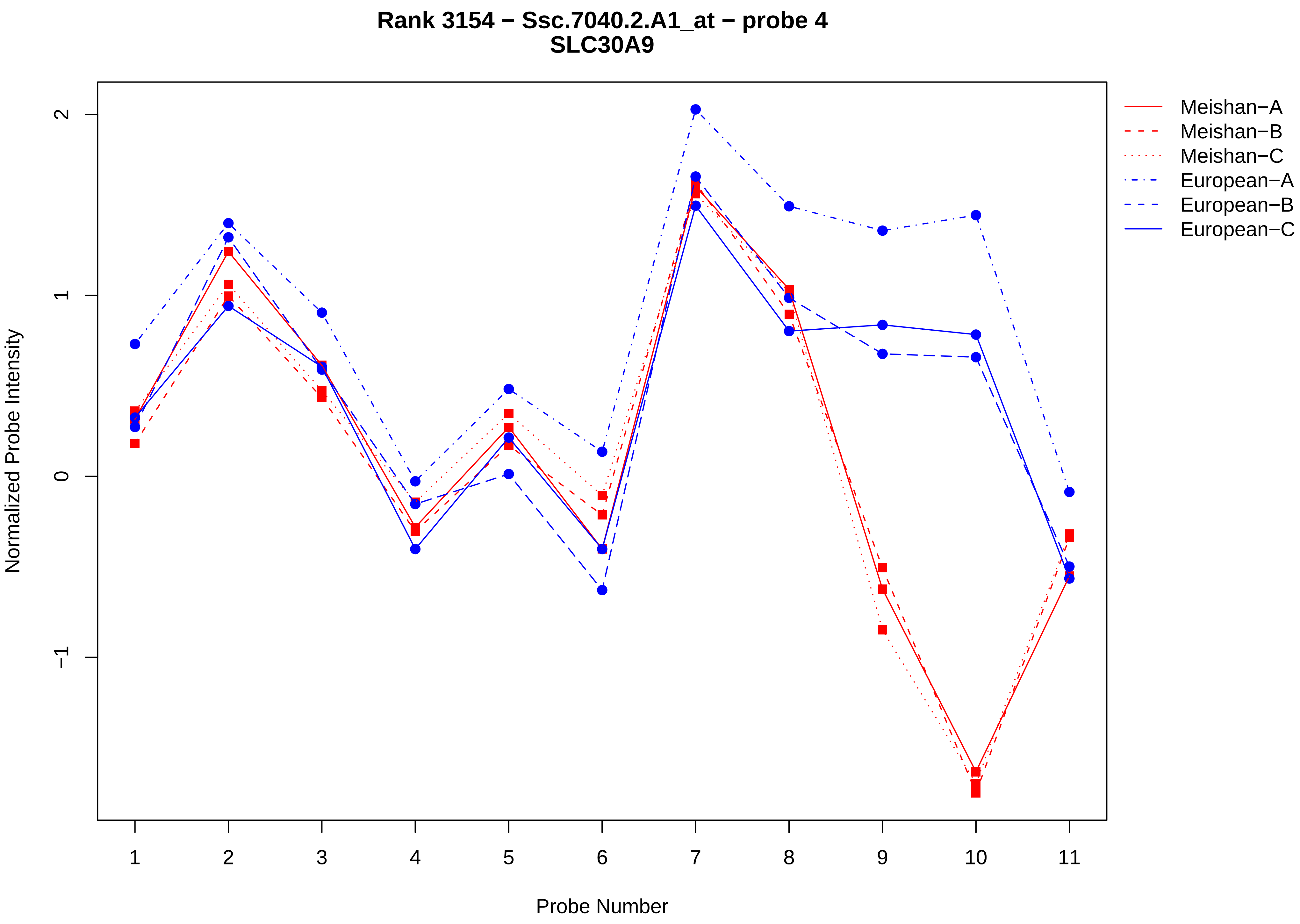

Rank 3154 − Ssc.7040.2.A1_at − probe 4
SLC30A9
Normalized Probe Intensity
Probe Number
−1
0
1
2
1
2
3
4
5
6
7
8
9
10
11
Meishan−A
Meishan−B
Meishan−C
European−A
European−B
European−C

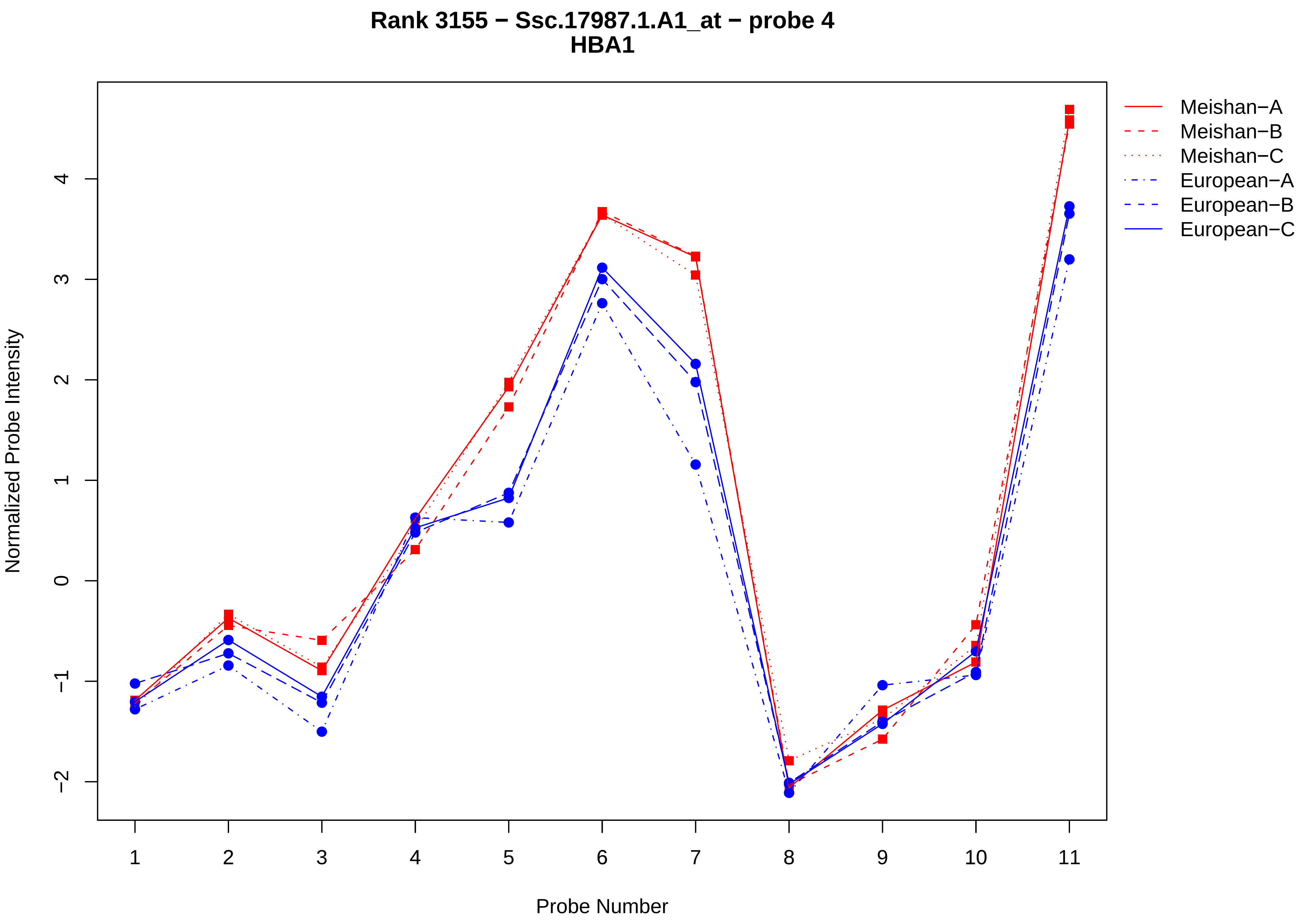
Rank 3155 − Ssc.17987.1.A1_at − probe 4
HBA1
Normalized Probe Intensity
Probe Number
−2
−1
0
1
2
3
4
1
2
3
4
5
6
7
8
9
10
11
Meishan−A
Meishan−B
Meishan−C
European−A
European−B
European−C

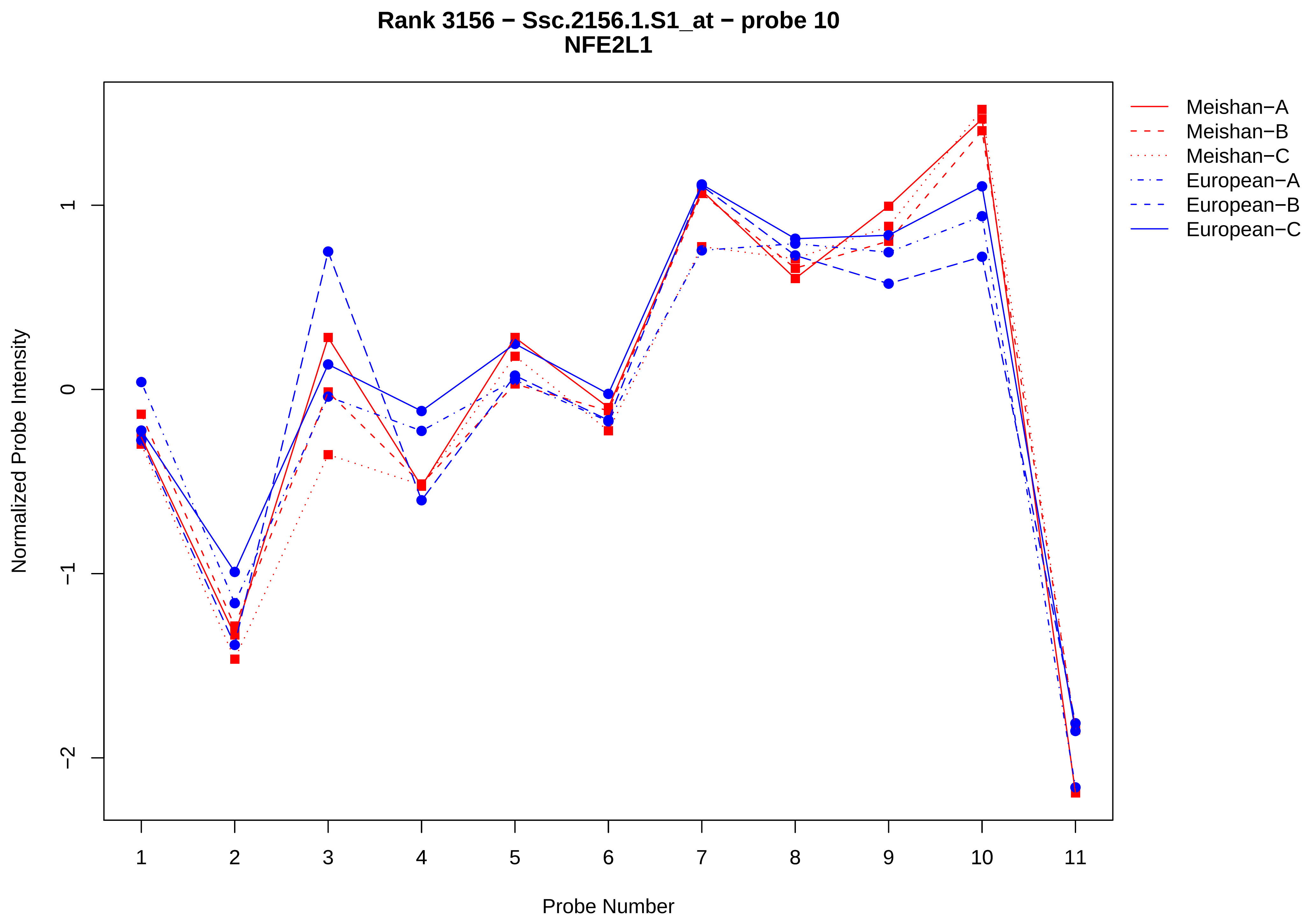

Rank 3156 − Ssc.2156.1.S1_at − probe 10
NFE2L1
Normalized Probe Intensity
Probe Number
1
0
−1
−2
1
2
3
4
5
6
7
8
9
10
11
Meishan−A
Meishan−B
Meishan−C
European−A
European−B
European−C

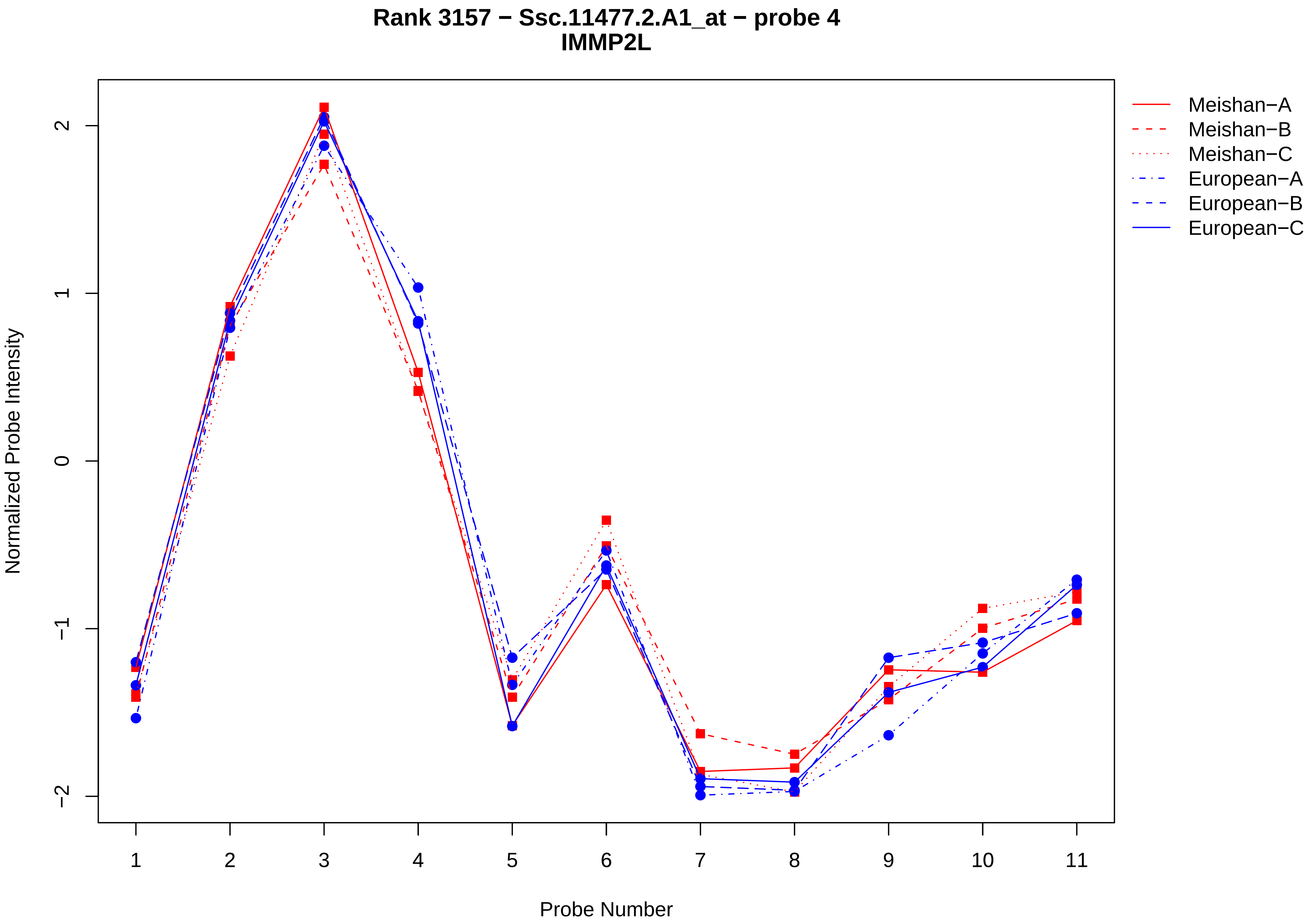

Rank 3157 − Ssc.11477.2.A1_at − probe 4
IMMP2L
Normalized Probe Intensity
Probe Number
Meishan−A
Meishan−B
Meishan−C
European−A
European−B
European−C
2
1
0
−1
−2
1
2
3
4
5
6
7
8
9
10
11

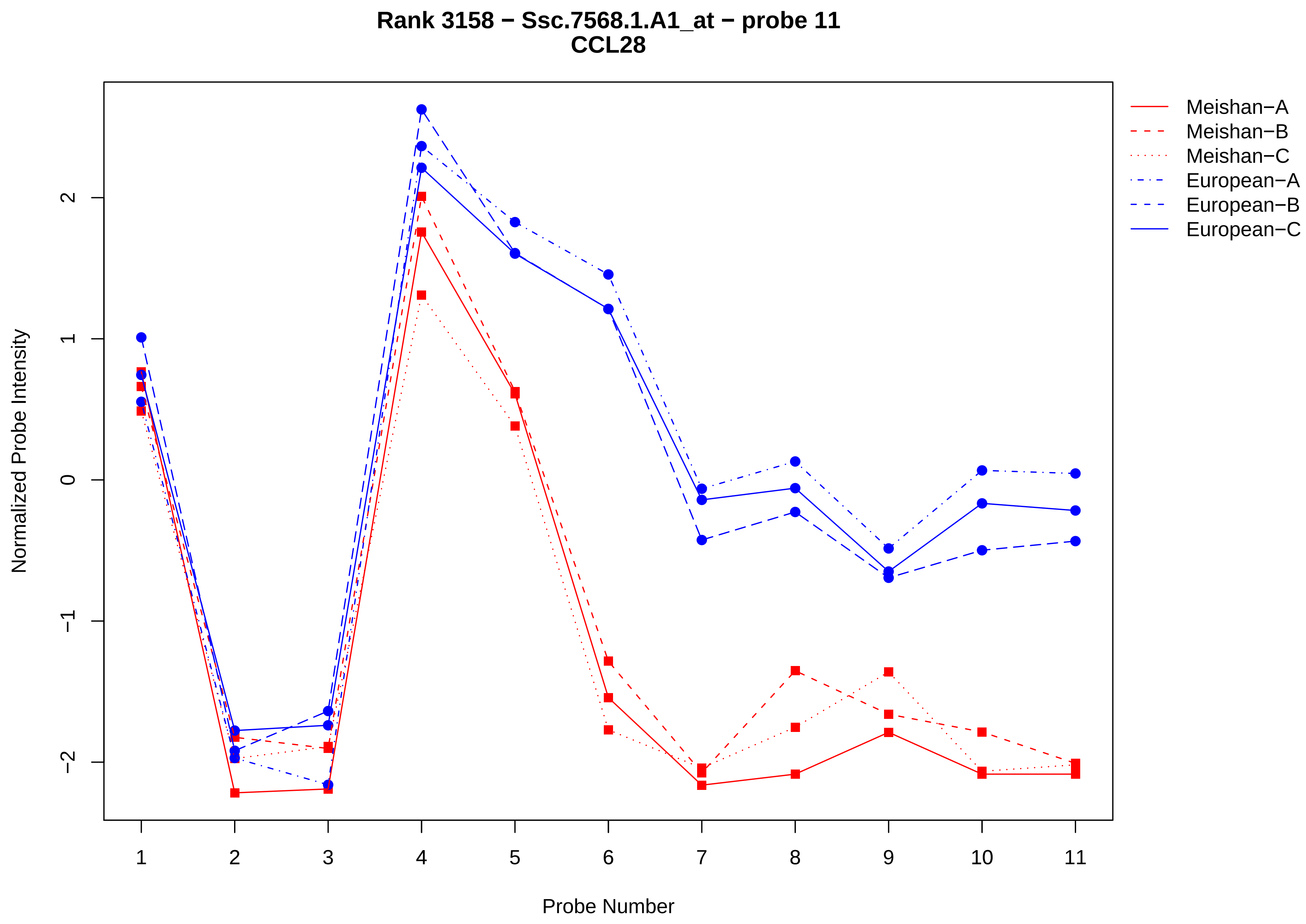

Rank 3158 − Ssc.7568.1.A1_at − probe 11
CCL28
Normalized Probe Intensity
Probe Number
−2
−1
0
1
2
1
2
3
4
5
6
7
8
9
10
11
Meishan−A
Meishan−B
Meishan−C
European−A
European−B
European−C

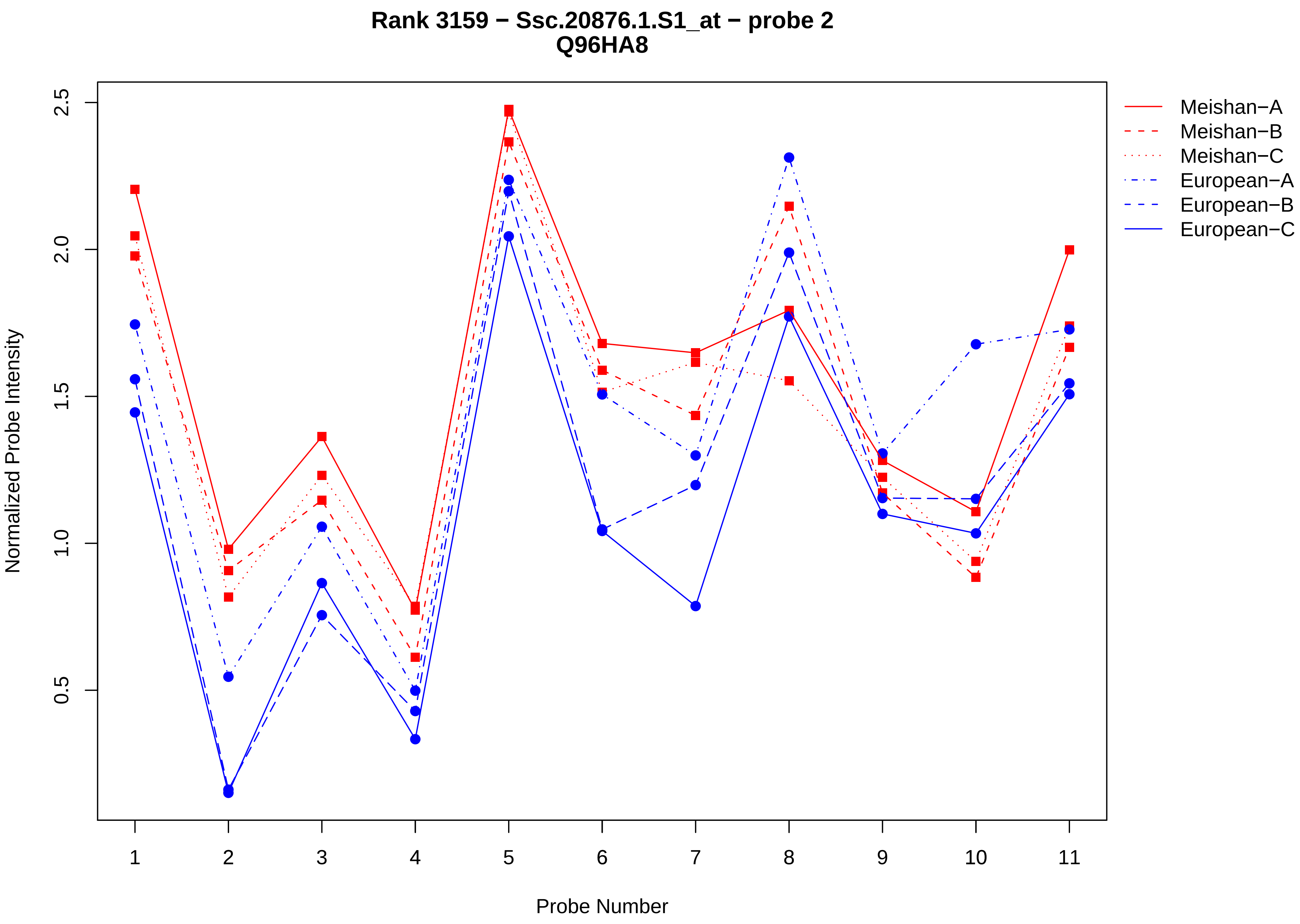

Rank 3159 − Ssc.20876.1.S1_at − probe 2
Q96HA8
Normalized Probe Intensity
0.5
1.0
1.5
2.0
2.5
1
2
3
4
5
6
7
8
9
10
11
Probe Number
Meishan−A
Meishan−B
Meishan−C
European−A
European−B
European−C

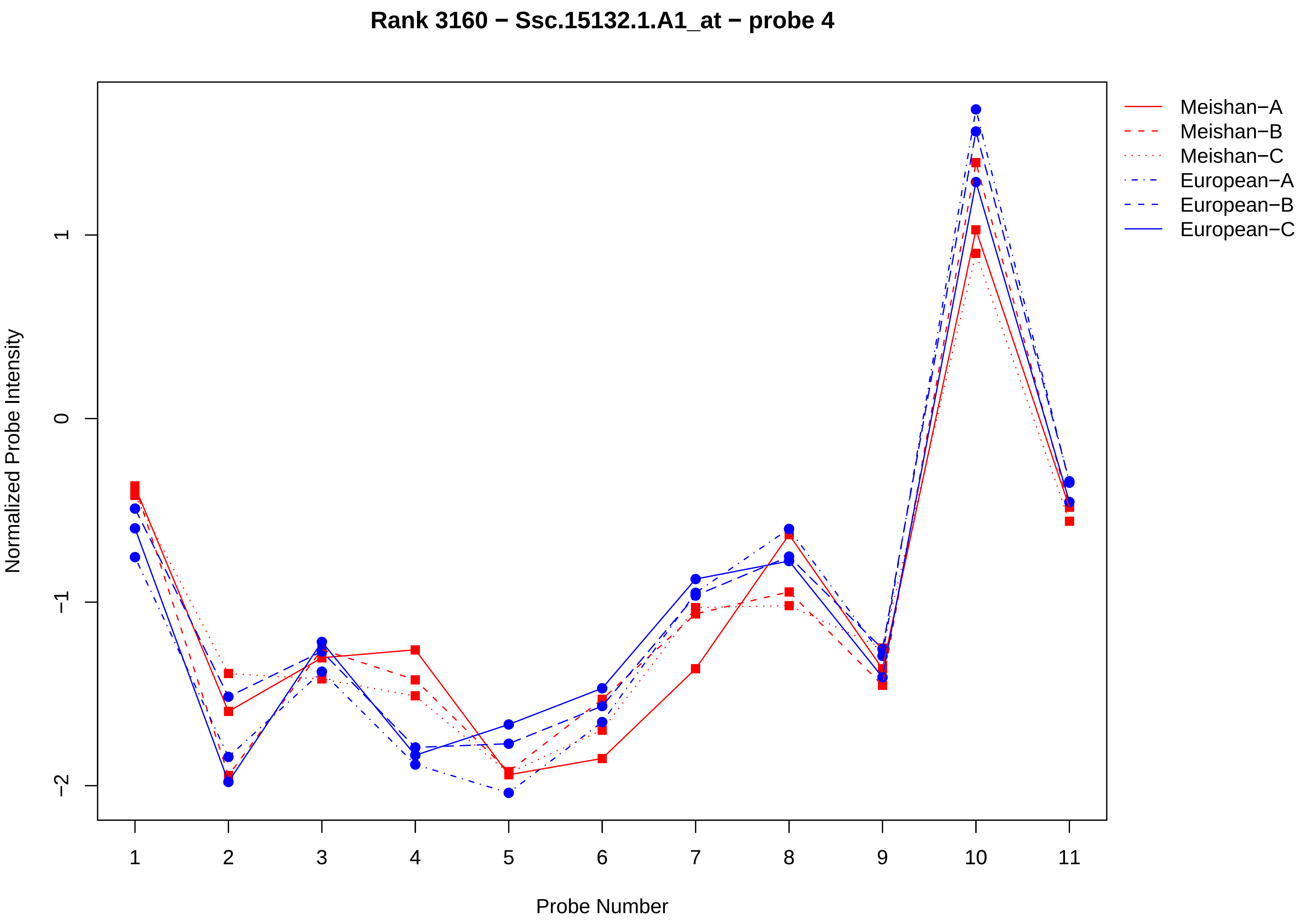
Rank 3160 − Ssc.15132.1.A1_at − probe 4
Normalized Probe Intensity
Probe Number
Meishan−A
Meishan−B
Meishan−C
European−A
European−B
European−C
1
0
−1
−2
1
2
3
4
5
6
7
8
9
10
11

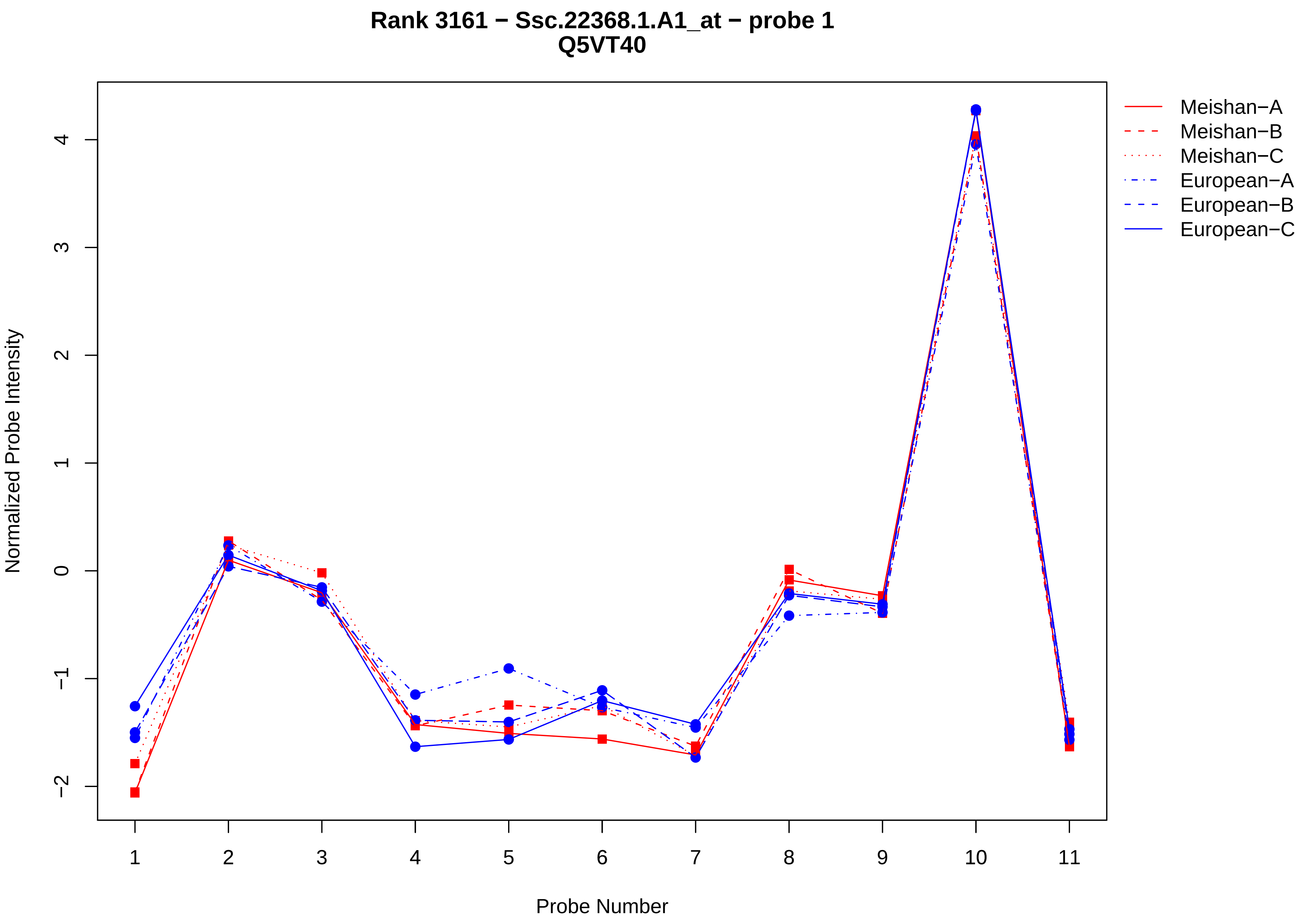

Rank 3161 − Ssc.22368.1.A1_at − probe 1
Q5VT40
Normalized Probe Intensity
Probe Number
Meishan−A
Meishan−B
Meishan−C
European−A
European−B
European−C
−2
−1
0
1
2
3
4
1
2
3
4
5
6
7
8
9
10
11

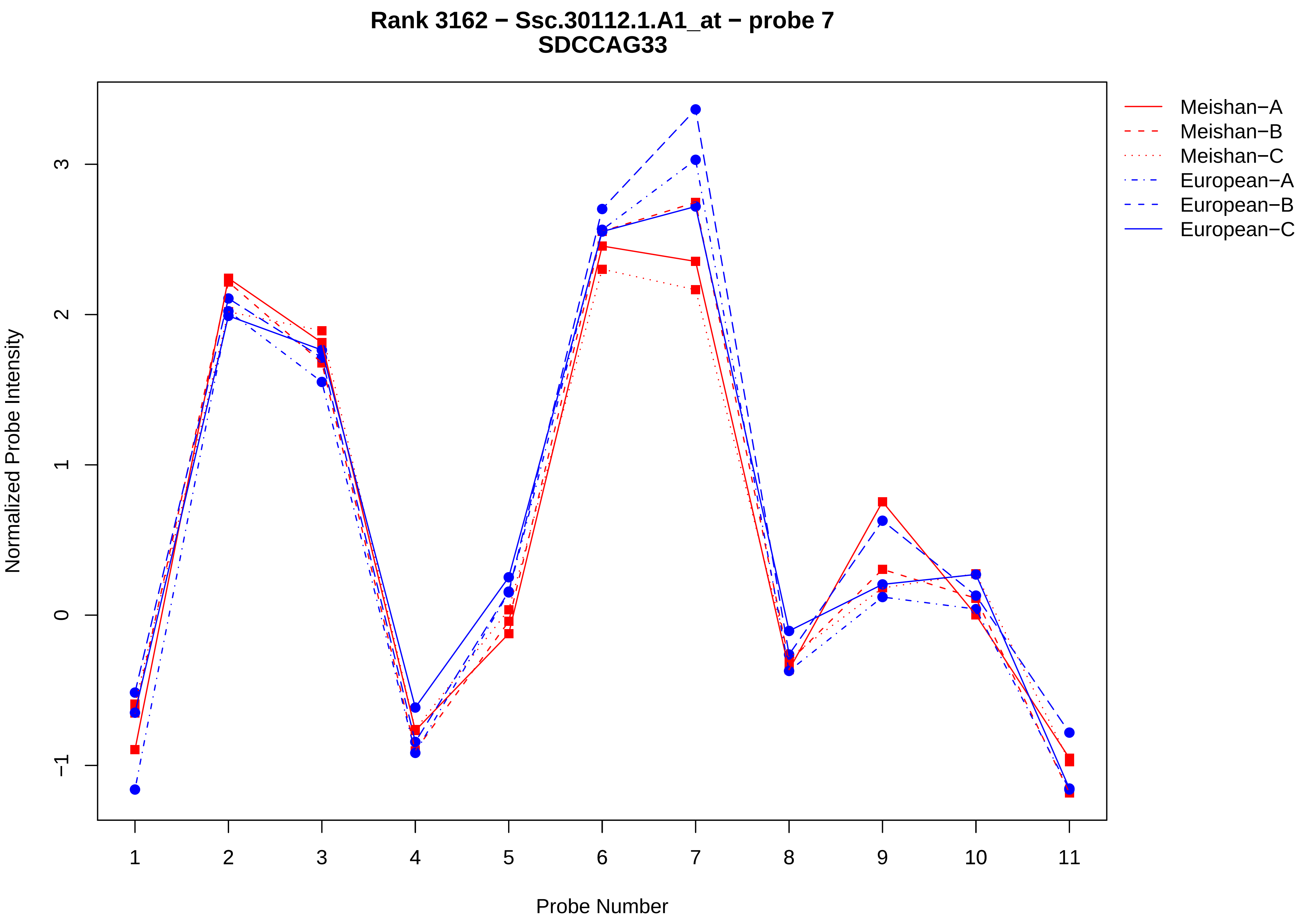

Rank 3162 − Ssc.30112.1.A1_at − probe 7
SDCCAG33
Normalized Probe Intensity
Probe Number
−1
0
1
2
3
1
2
3
4
5
6
7
8
9
10
11
Meishan−A
Meishan−B
Meishan−C
European−A
European−B
European−C

Rank 3163 − Ssc.30598.1.A1_at − probe 5
NRP2

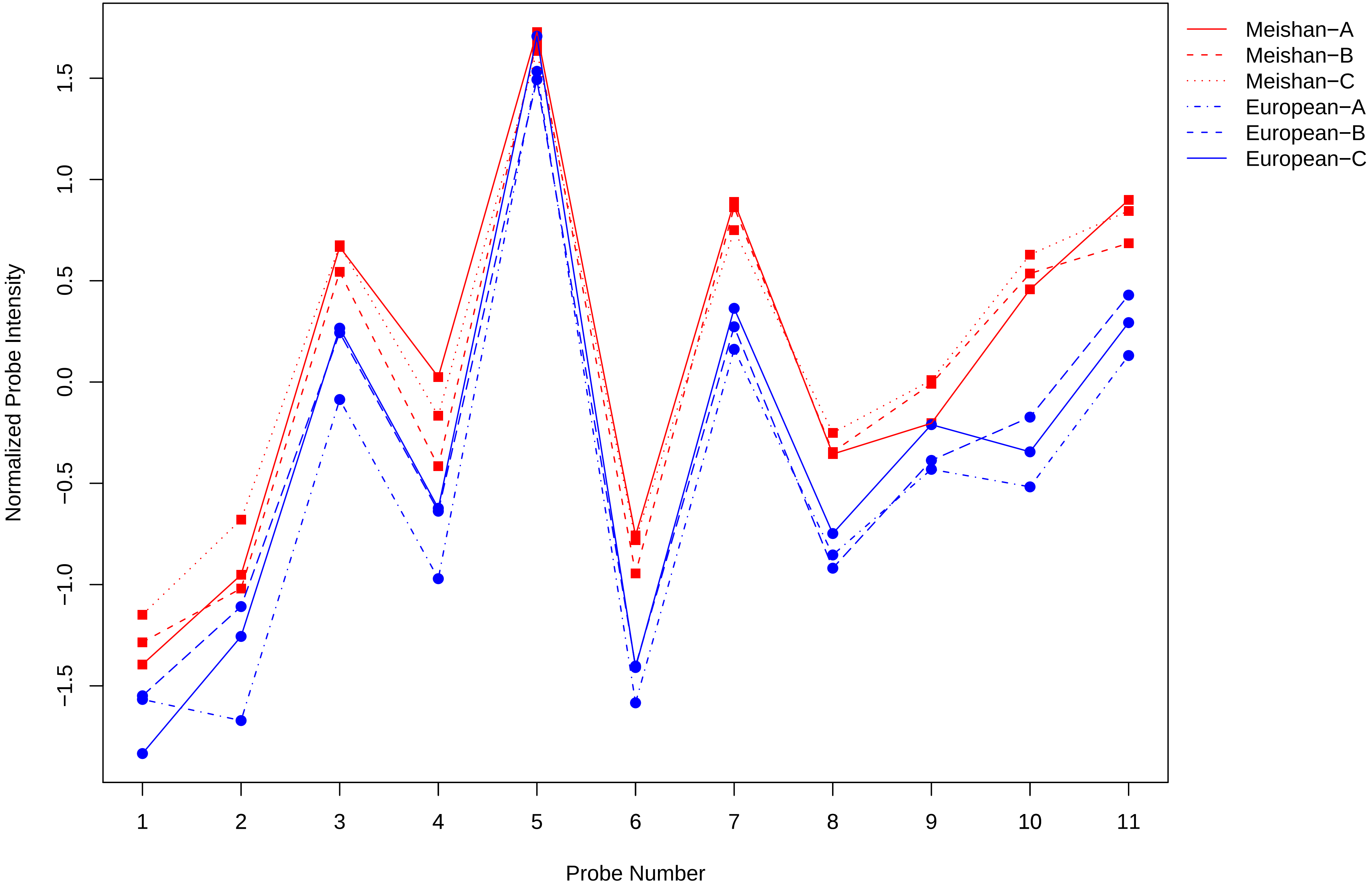

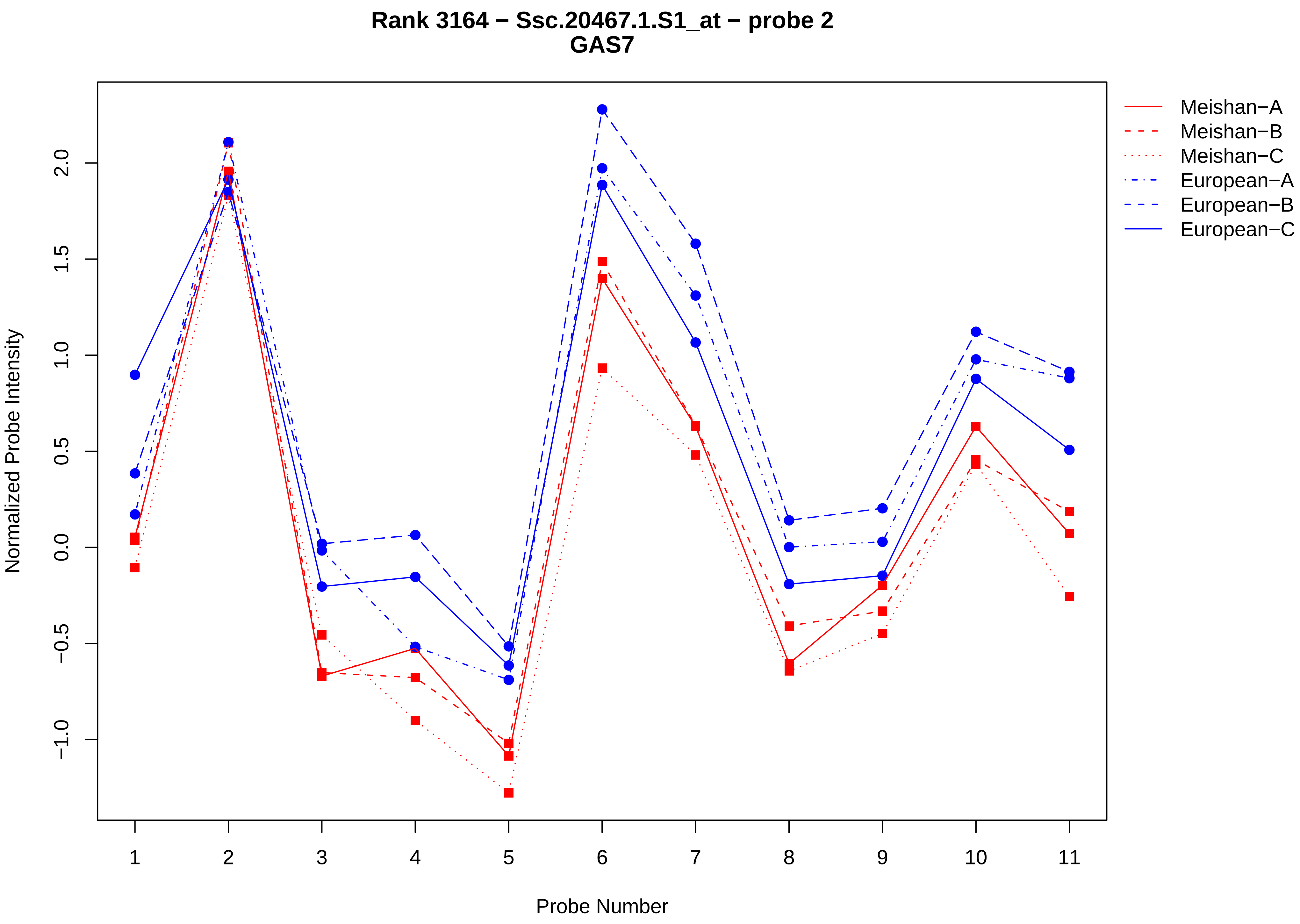

Rank 3164 − Ssc.20467.1.S1_at − probe 2
GAS7
Normalized Probe Intensity
Probe Number
−1.0
−0.5
0.0
0.5
1.0
1.5
2.0
1
2
3
4
5
6
7
8
9
10
11
Meishan−A
Meishan−B
Meishan−C
European−A
European−B
European−C

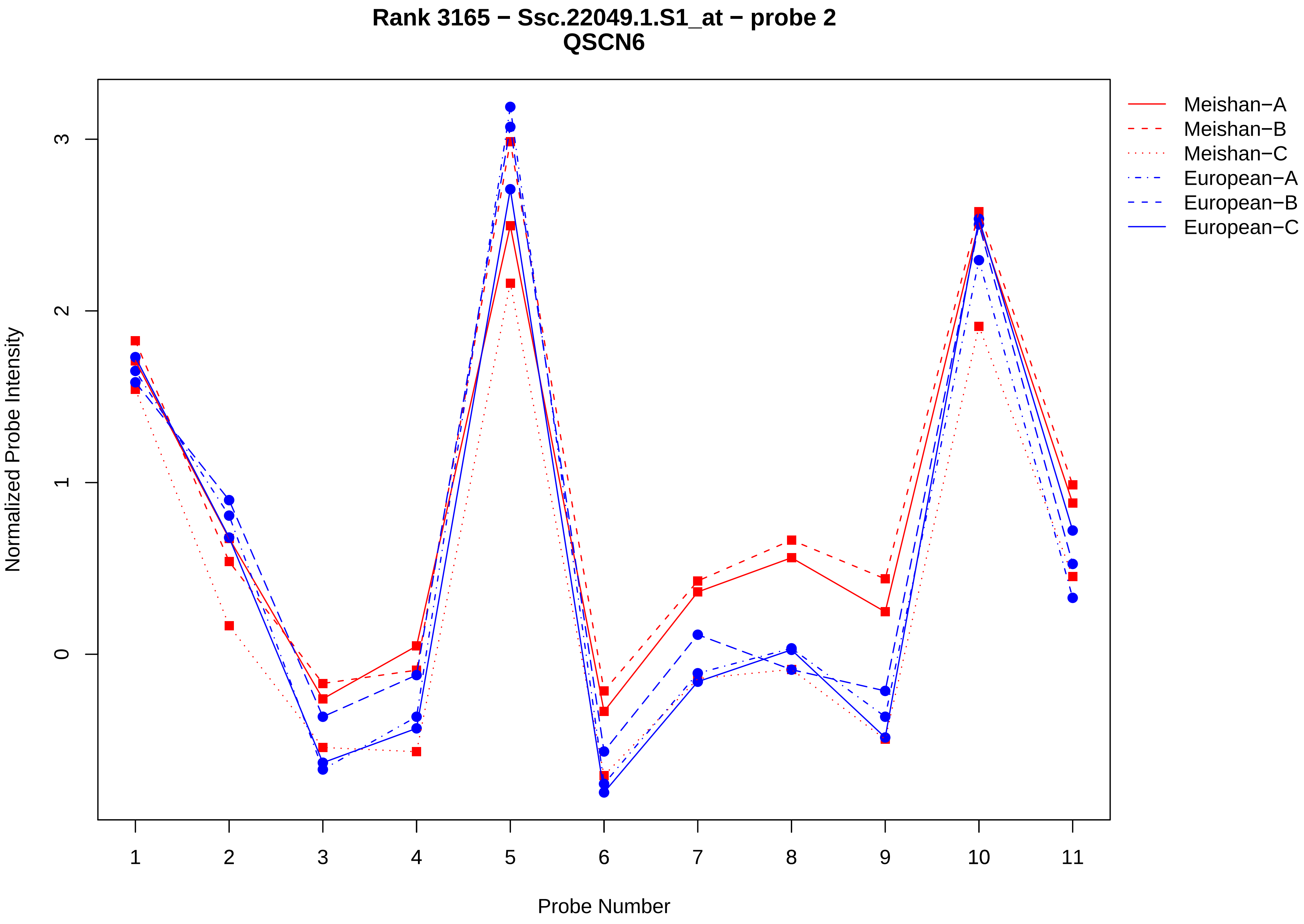

Rank 3165 − Ssc.22049.1.S1_at − probe 2
QSCN6
Normalized Probe Intensity
Probe Number
0
1
2
3
1
2
3
4
5
6
7
8
9
10
11
Meishan−A
Meishan−B
Meishan−C
European−A
European−B
European−C

Rank 3166 − Ssc.27882.1.A1_at − probe 7
TCEAL4

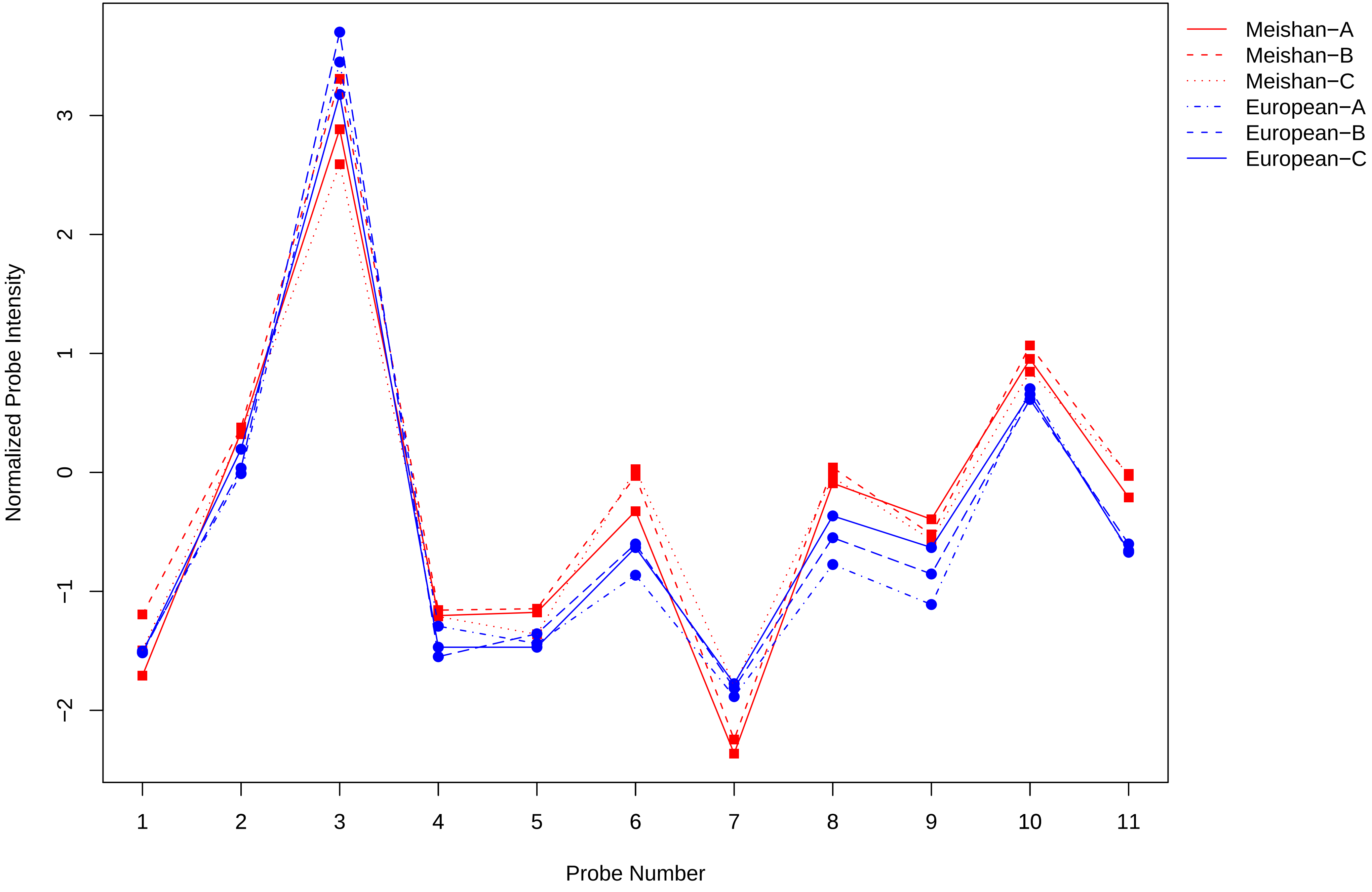

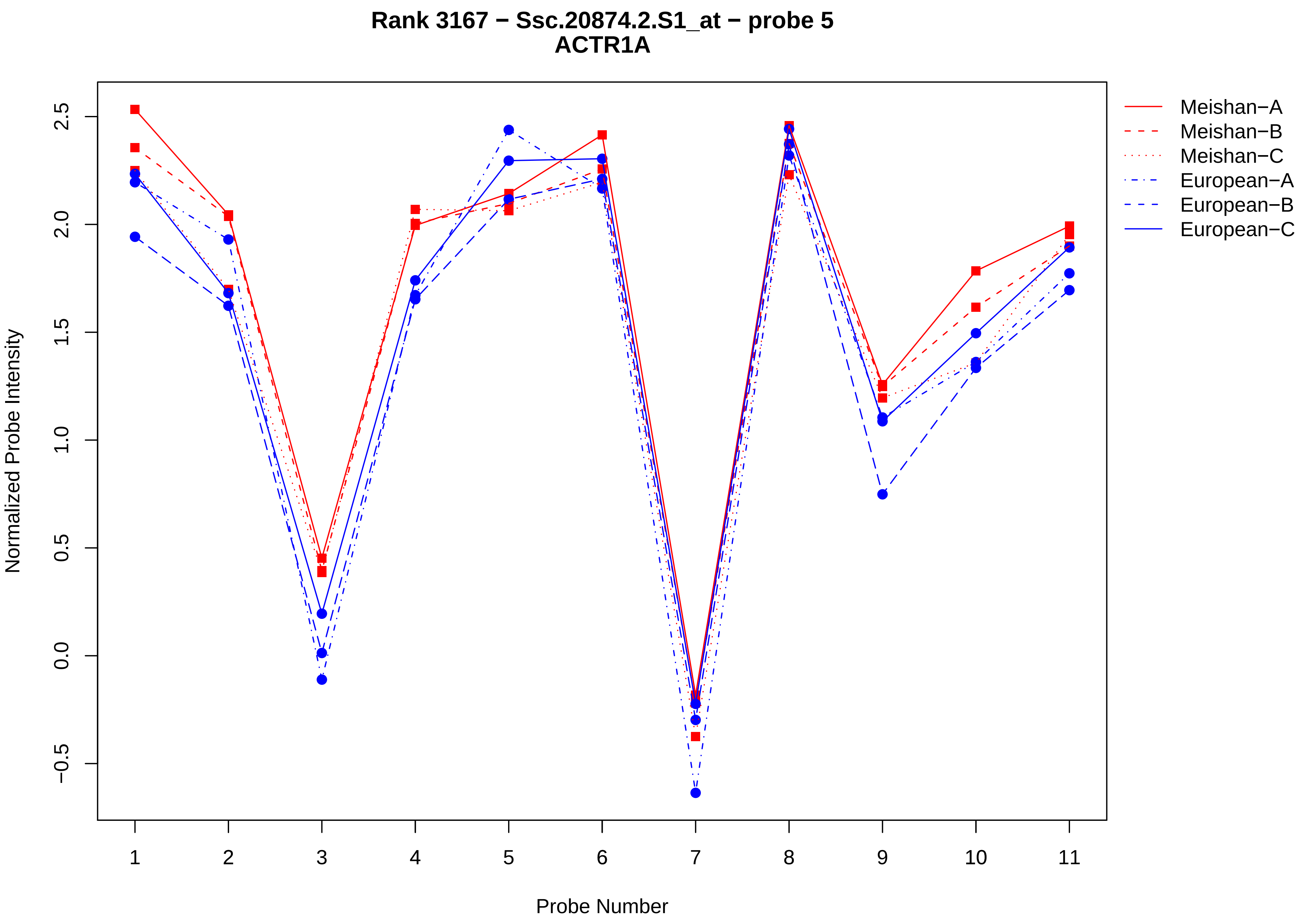

Rank 3167 − Ssc.20874.2.S1_at − probe 5
ACTR1A
Normalized Probe Intensity
Probe Number
−0.5
0.0
0.5
1.0
1.5
2.0
2.5
1
2
3
4
5
6
7
8
9
10
11
Meishan−A
Meishan−B
Meishan−C
European−A
European−B
European−C

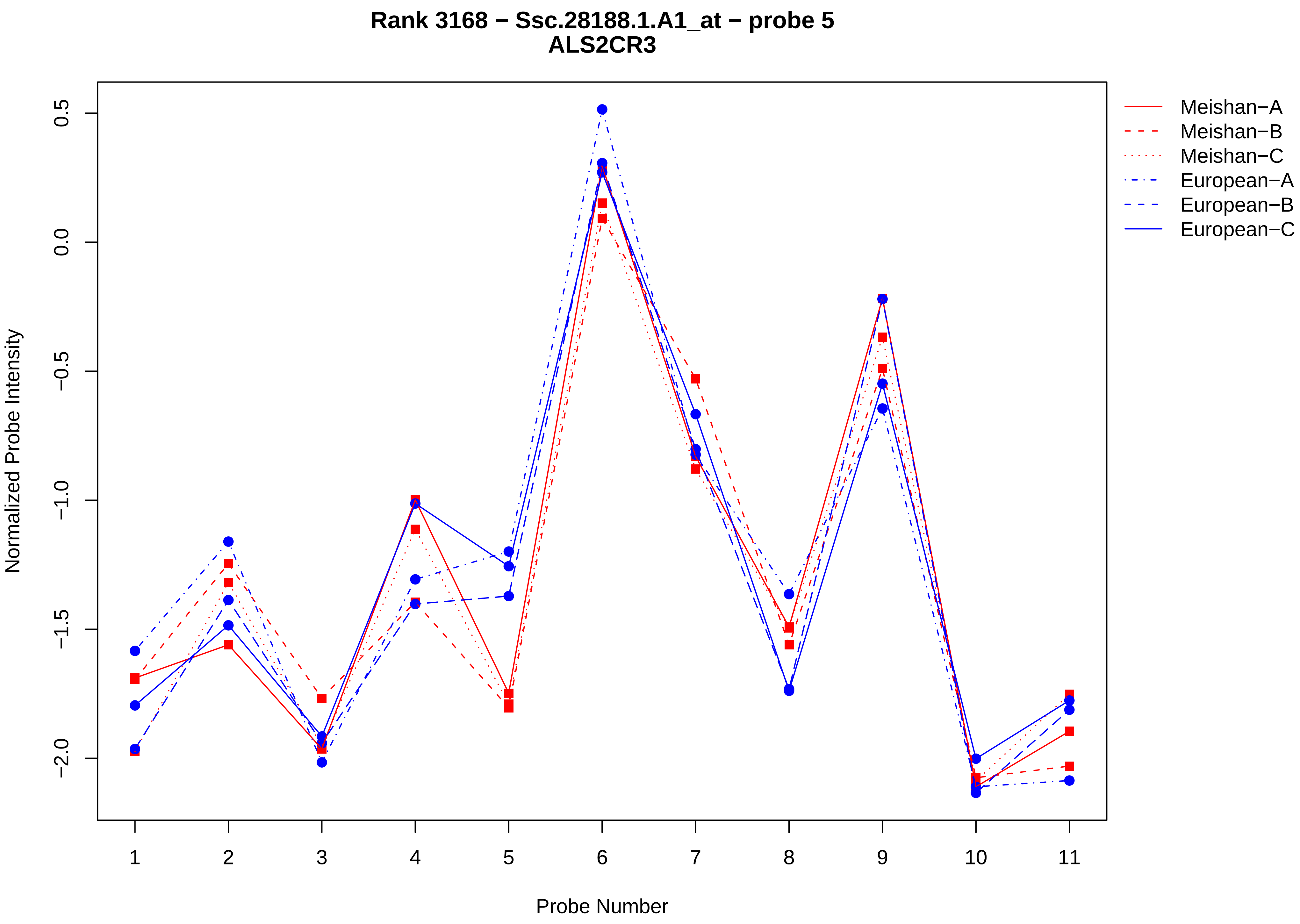

Rank 3168 − Ssc.28188.1.A1_at − probe 5
ALS2CR3
Normalized Probe Intensity
Probe Number
0.5
0.0
−0.5
−1.0
−1.5
−2.0
1
2
3
4
5
6
7
8
9
10
11
Meishan−A
Meishan−B
Meishan−C
European−A
European−B
European−C

Rank 3169 − Ssc.1524.1.S1_at − probe 5
Q8IUY4

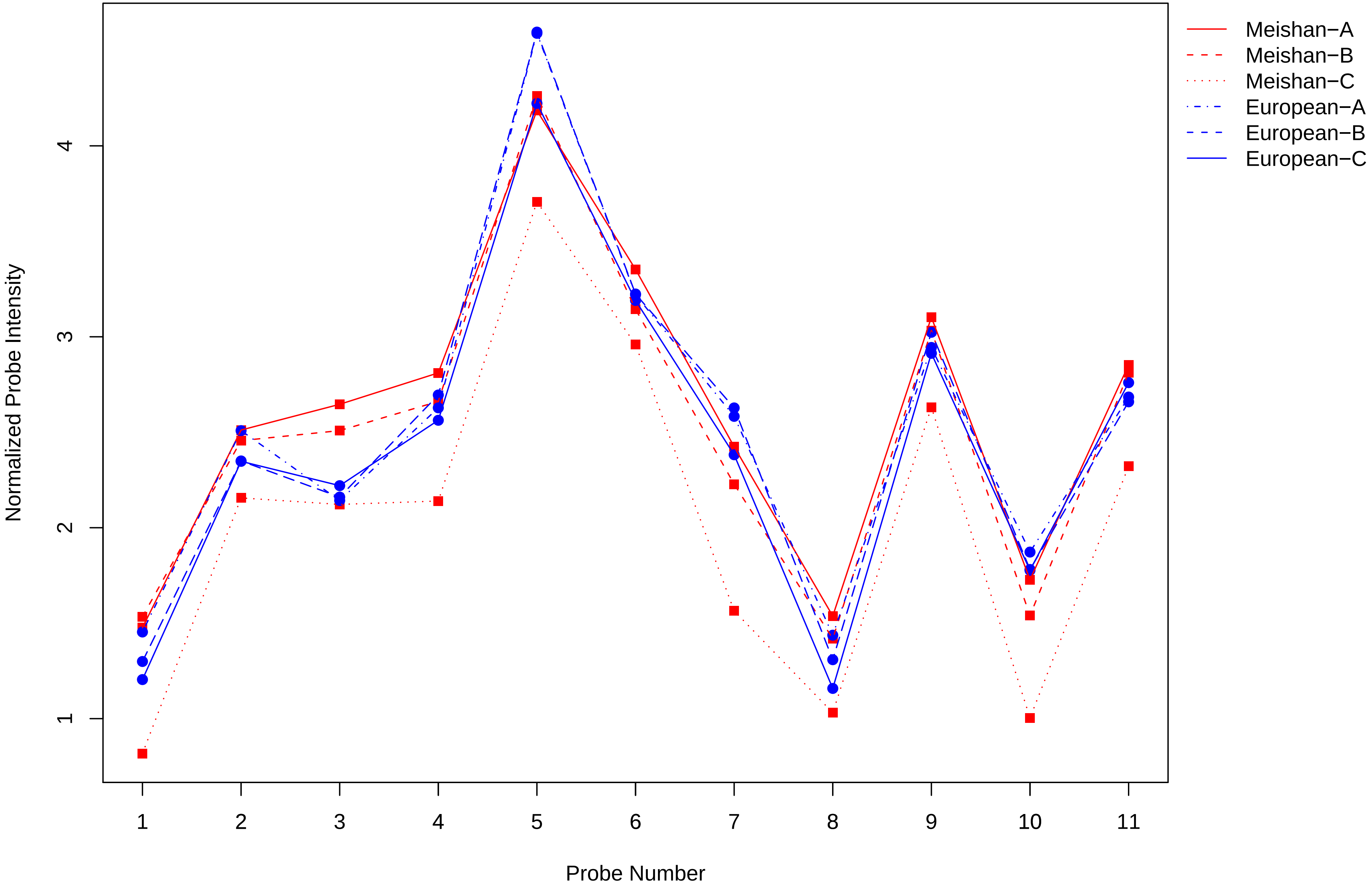

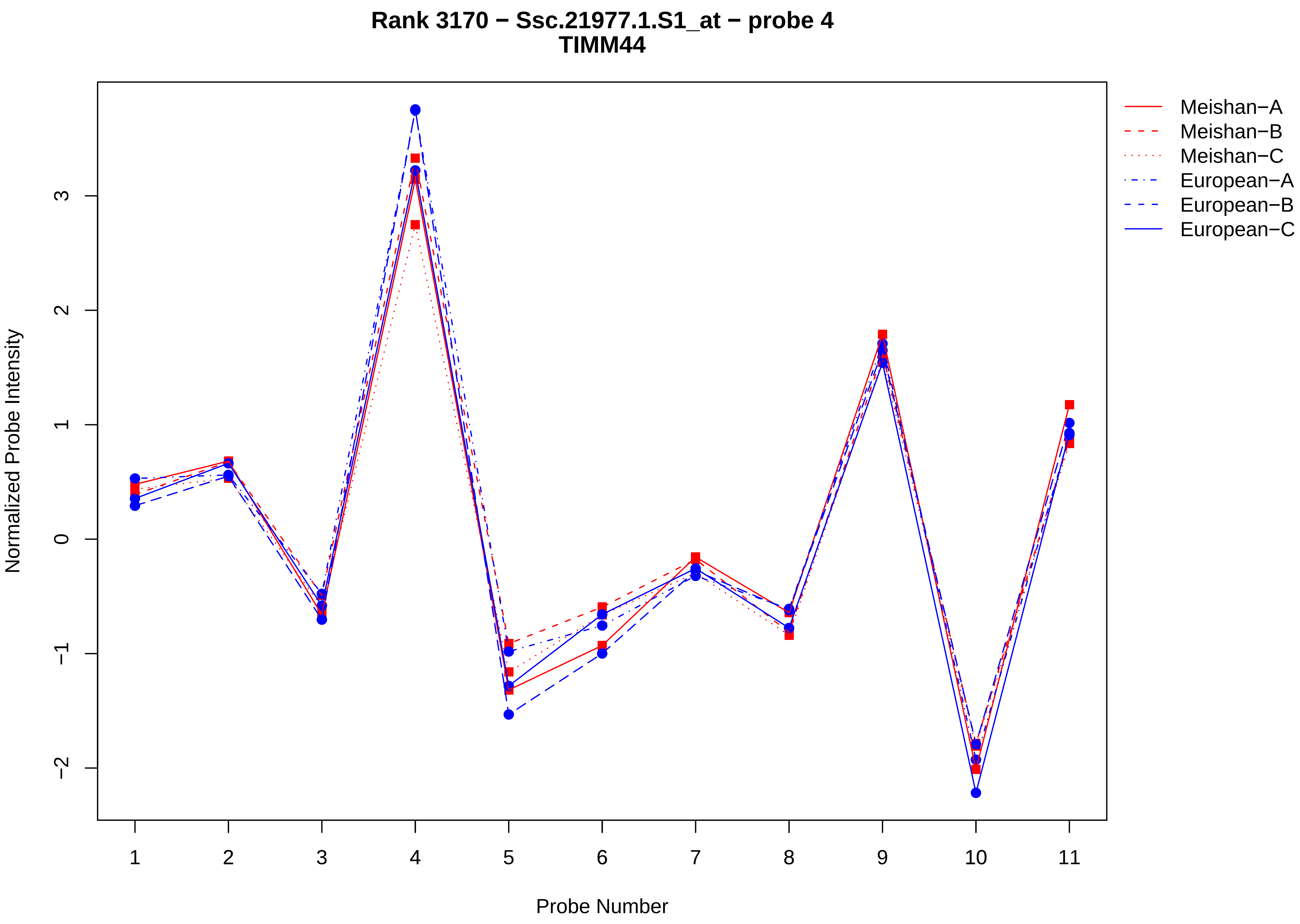

Rank 3170 − Ssc.21977.1.S1_at − probe 4
TIMM44
Normalized Probe Intensity
Probe Number
Meishan−A
Meishan−B
Meishan−C
European−A
European−B
European−C
−2
−1
0
1
2
3
1
2
3
4
5
6
7
8
9
10
11

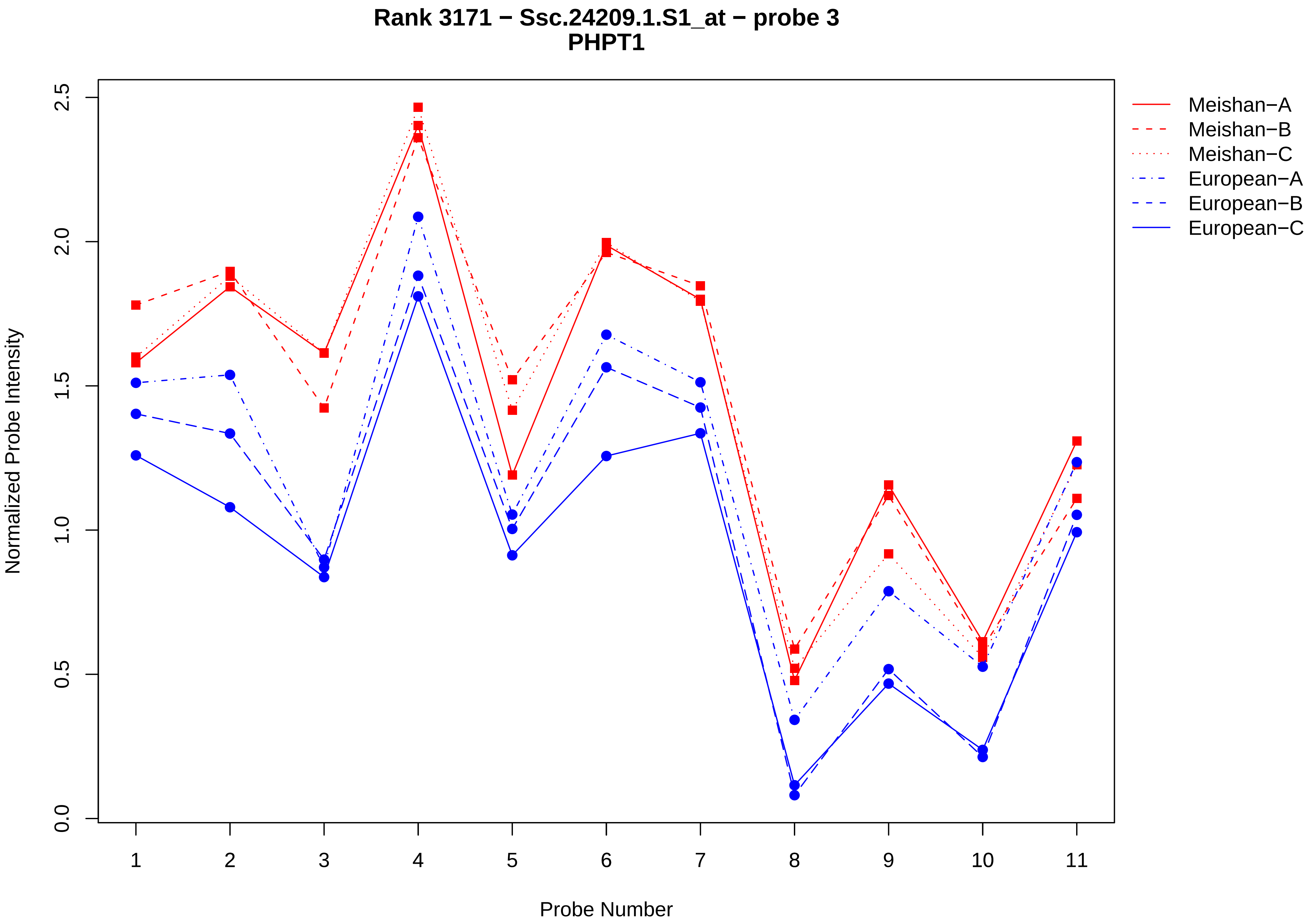
Rank 3171 − Ssc.24209.1.S1_at − probe 3
PHPT1
Normalized Probe Intensity
0.0
0.5
1.0
1.5
2.0
2.5
1
2
3
4
5
6
7
8
9
10
11
Probe Number
Meishan−A
Meishan−B
Meishan−C
European−A
European−B
European−C

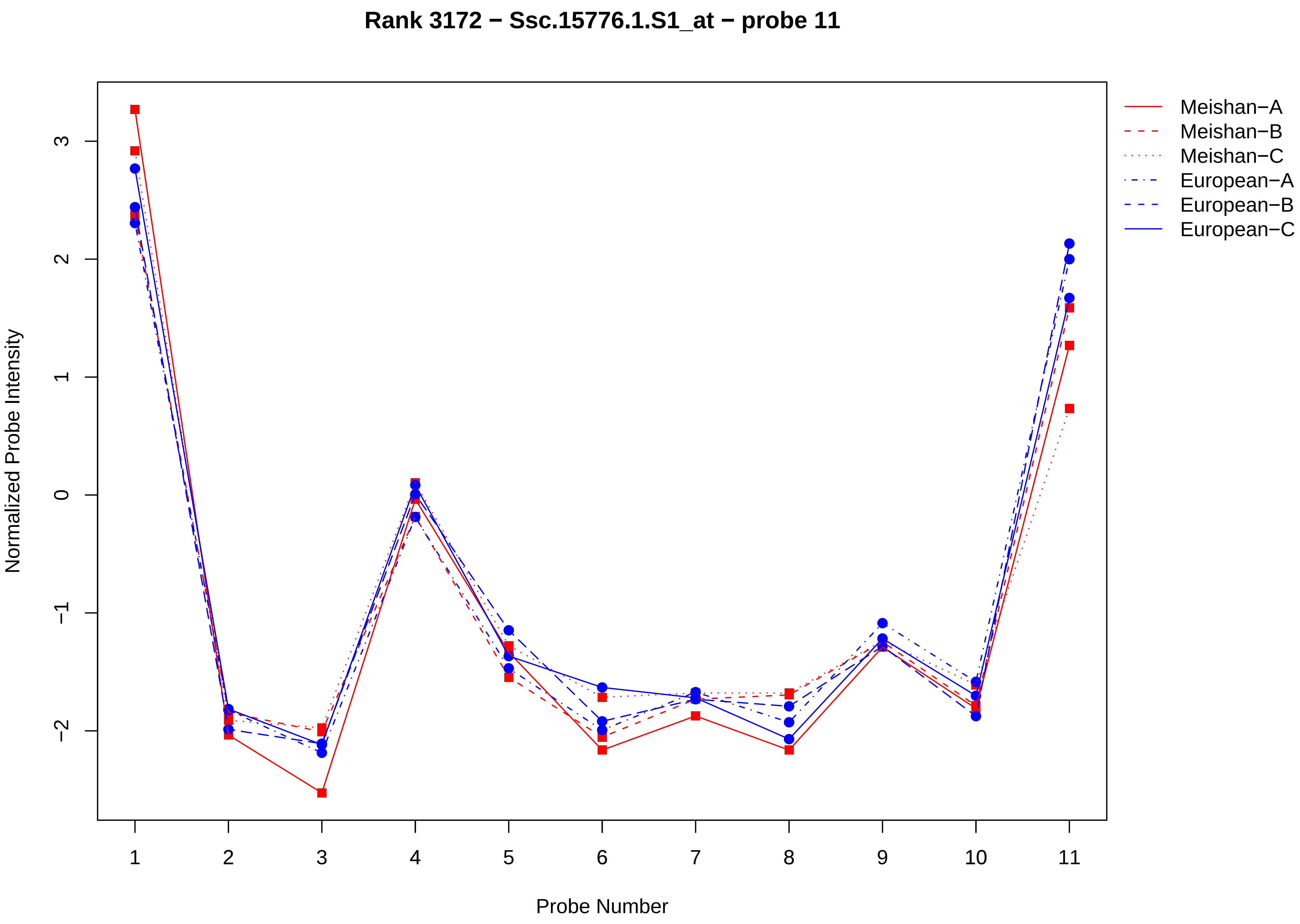

Rank 3172 − Ssc.15776.1.S1_at − probe 11
Normalized Probe Intensity
Probe Number
Meishan−A
Meishan−B
Meishan−C
European−A
European−B
European−C
−2
−1
0
1
2
3
1
2
3
4
5
6
7
8
9
10
11

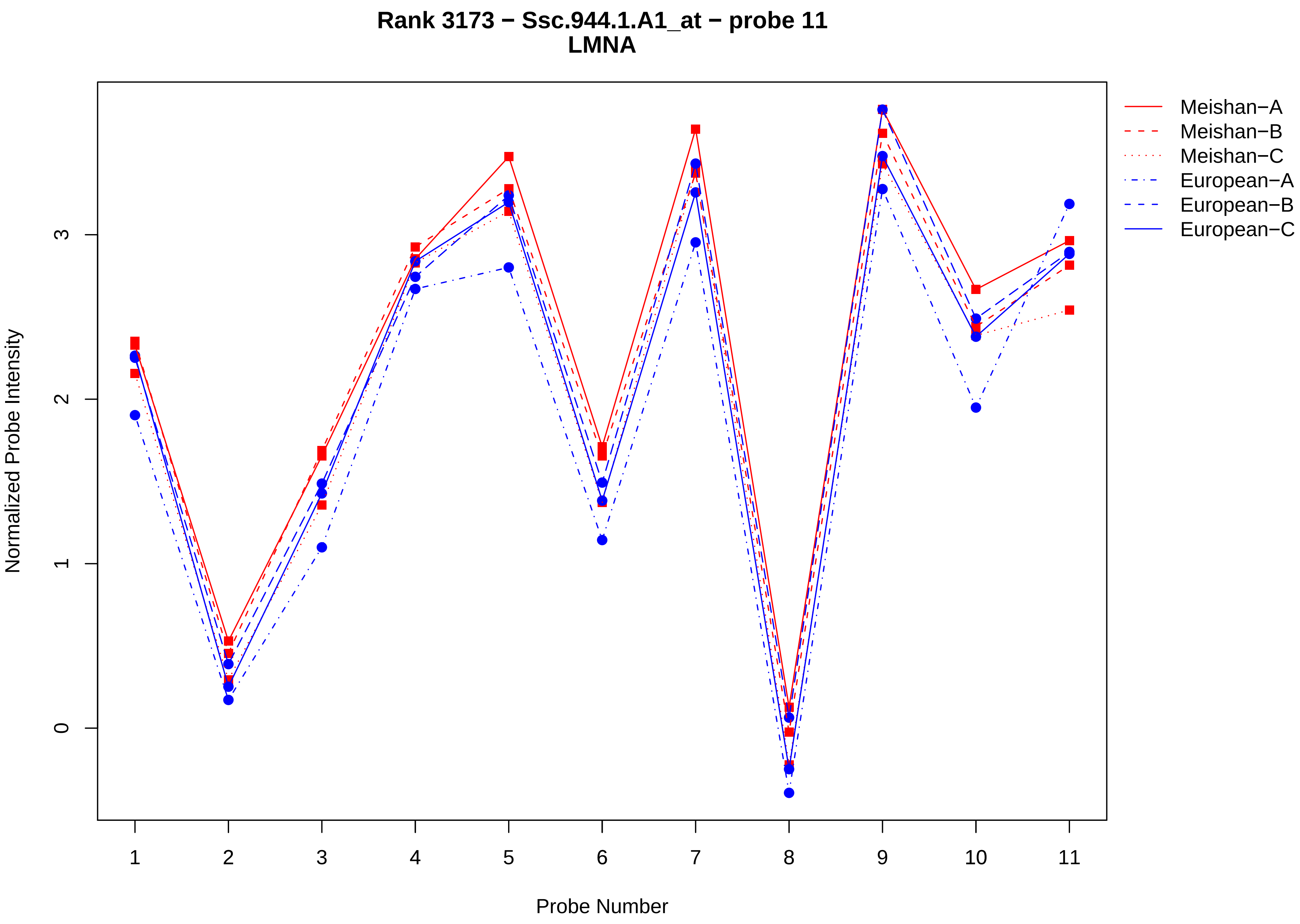

Rank 3173 − Ssc.944.1.A1_at − probe 11
LMNA
Normalized Probe Intensity
Probe Number
0
1
2
3
1
2
3
4
5
6
7
8
9
10
11
Meishan−A
Meishan−B
Meishan−C
European−A
European−B
European−C

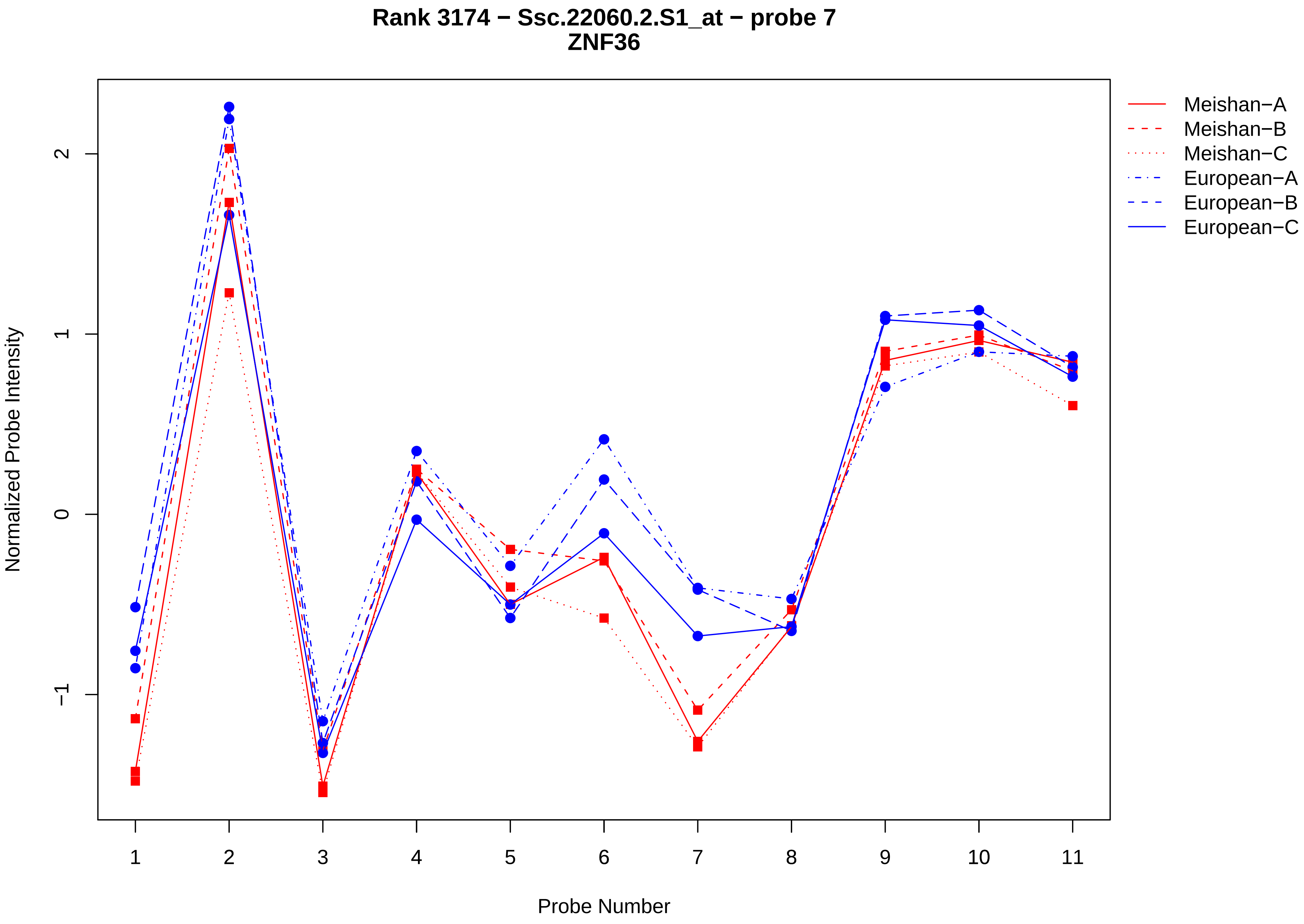

Rank 3174 − Ssc.22060.2.S1_at − probe 7
ZNF36
Normalized Probe Intensity
Probe Number
−1
0
1
2
1
2
3
4
5
6
7
8
9
10
11
Meishan−A
Meishan−B
Meishan−C
European−A
European−B
European−C

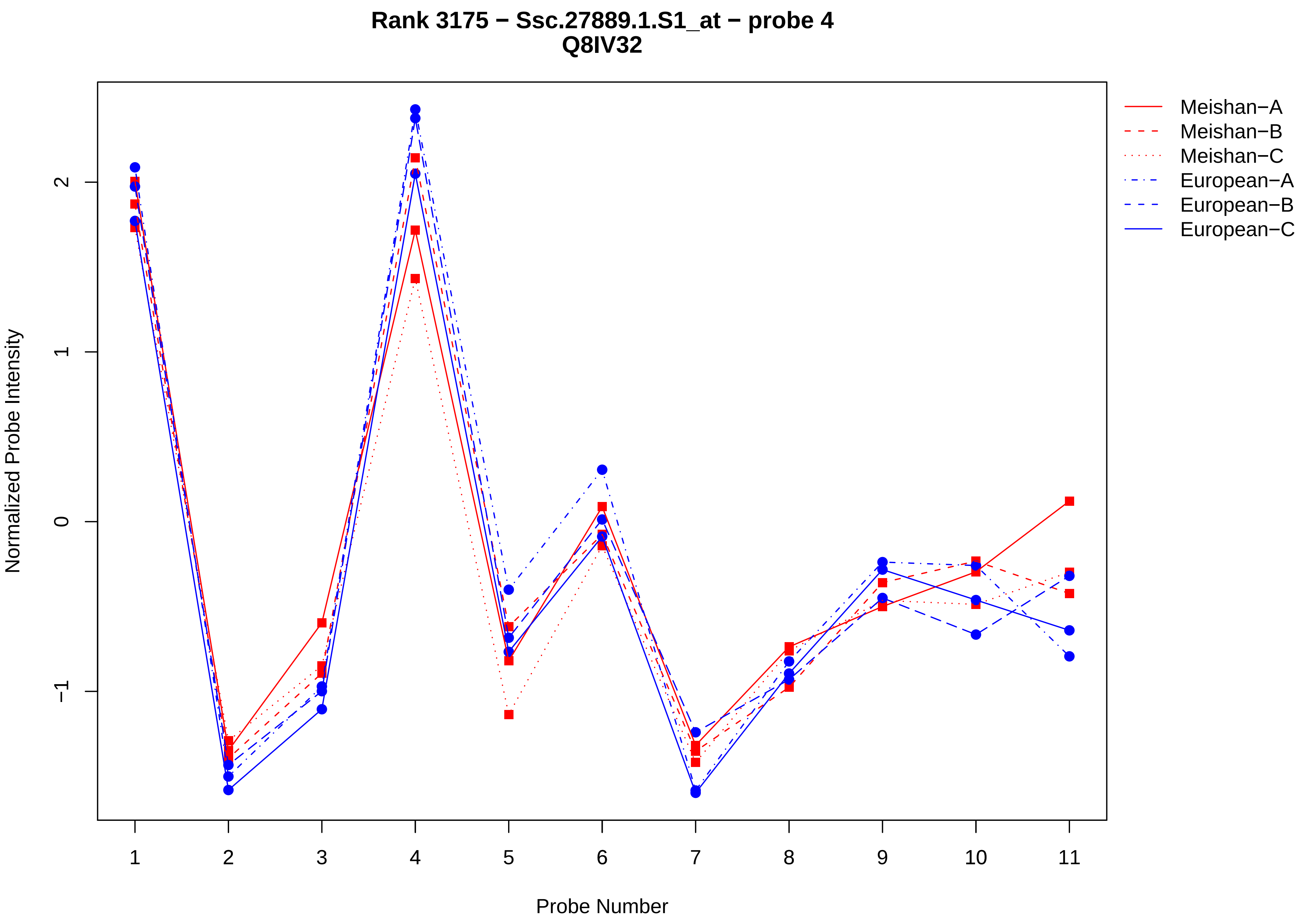

Rank 3175 − Ssc.27889.1.S1_at − probe 4
Q8IV32
Normalized Probe Intensity
Probe Number
−1
0
1
2
1
2
3
4
5
6
7
8
9
10
11
Meishan−A
Meishan−B
Meishan−C
European−A
European−B
European−C

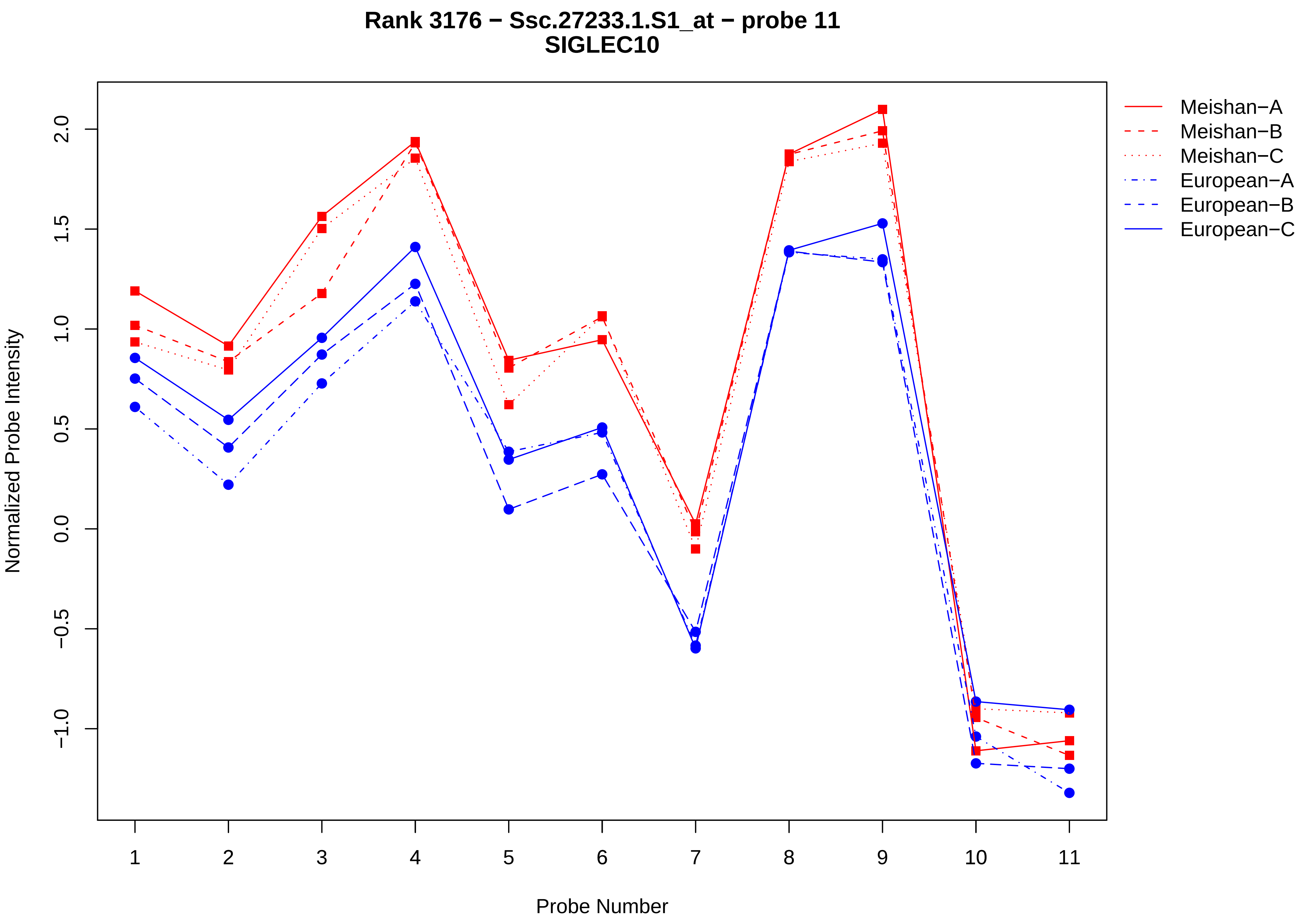

Rank 3176 − Ssc.27233.1.S1_at − probe 11
SIGLEC10
Normalized Probe Intensity
Probe Number
Meishan−A
Meishan−B
Meishan−C
European−A
European−B
European−C
−1.0
−0.5
0.0
0.5
1.0
1.5
2.0
1
2
3
4
5
6
7
8
9
10
11

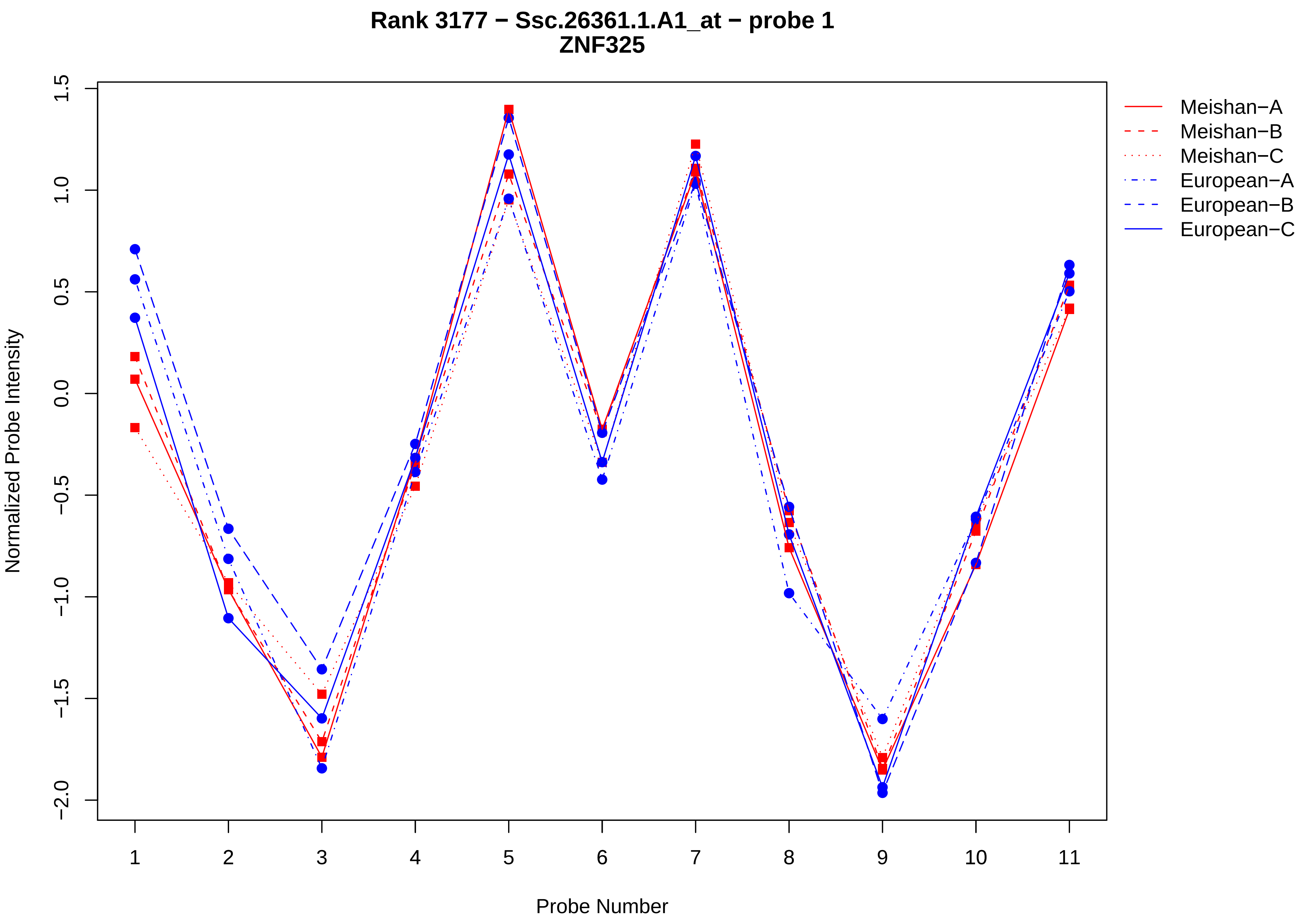

Rank 3177 − Ssc.26361.1.A1_at − probe 1
ZNF325
Normalized Probe Intensity
Probe Number
1.5
1.0
0.5
0.0
−0.5
−1.0
−1.5
−2.0
1
2
3
4
5
6
7
8
9
10
11
Meishan−A
Meishan−B
Meishan−C
European−A
European−B
European−C

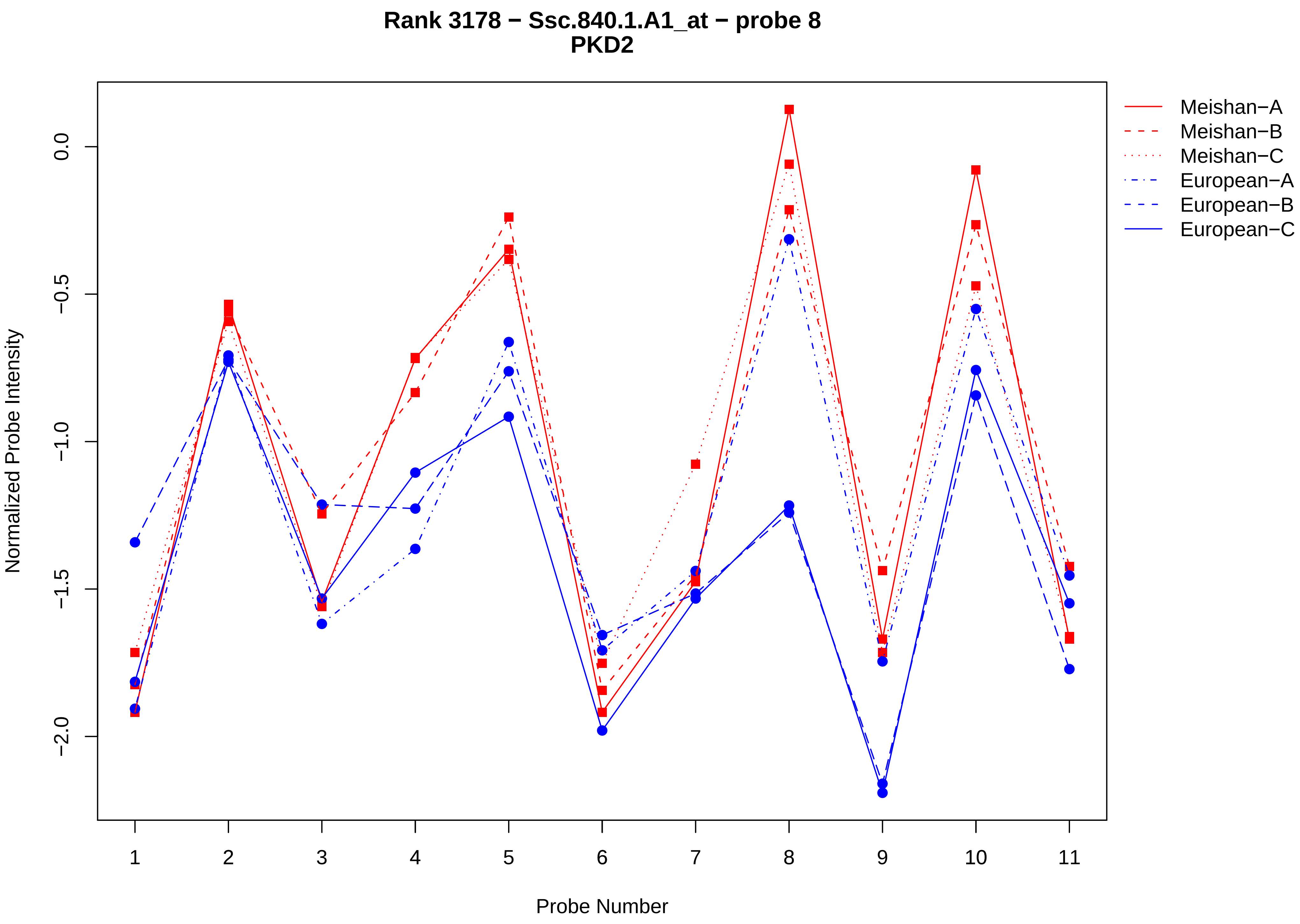

Rank 3178 − Ssc.840.1.A1_at − probe 8
PKD2
Normalized Probe Intensity
Probe Number
0.0
−0.5
−1.0
−1.5
−2.0
1
2
3
4
5
6
7
8
9
10
11
Meishan−A
Meishan−B
Meishan−C
European−A
European−B
European−C

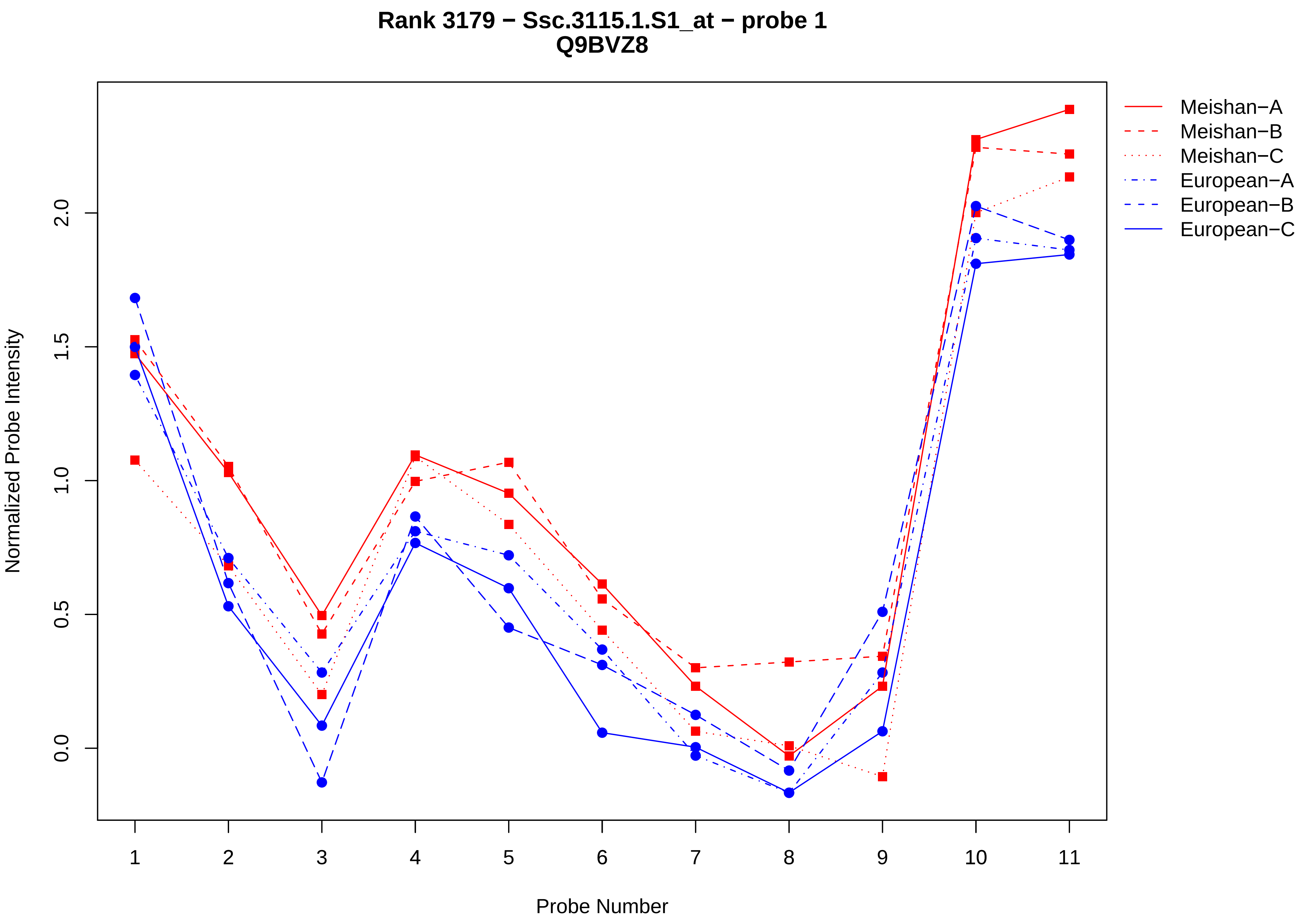

Rank 3179 − Ssc.3115.1.S1_at − probe 1
Q9BVZ8
Normalized Probe Intensity
Probe Number
0.0
0.5
1.0
1.5
2.0
1
2
3
4
5
6
7
8
9
10
11
Meishan−A
Meishan−B
Meishan−C
European−A
European−B
European−C

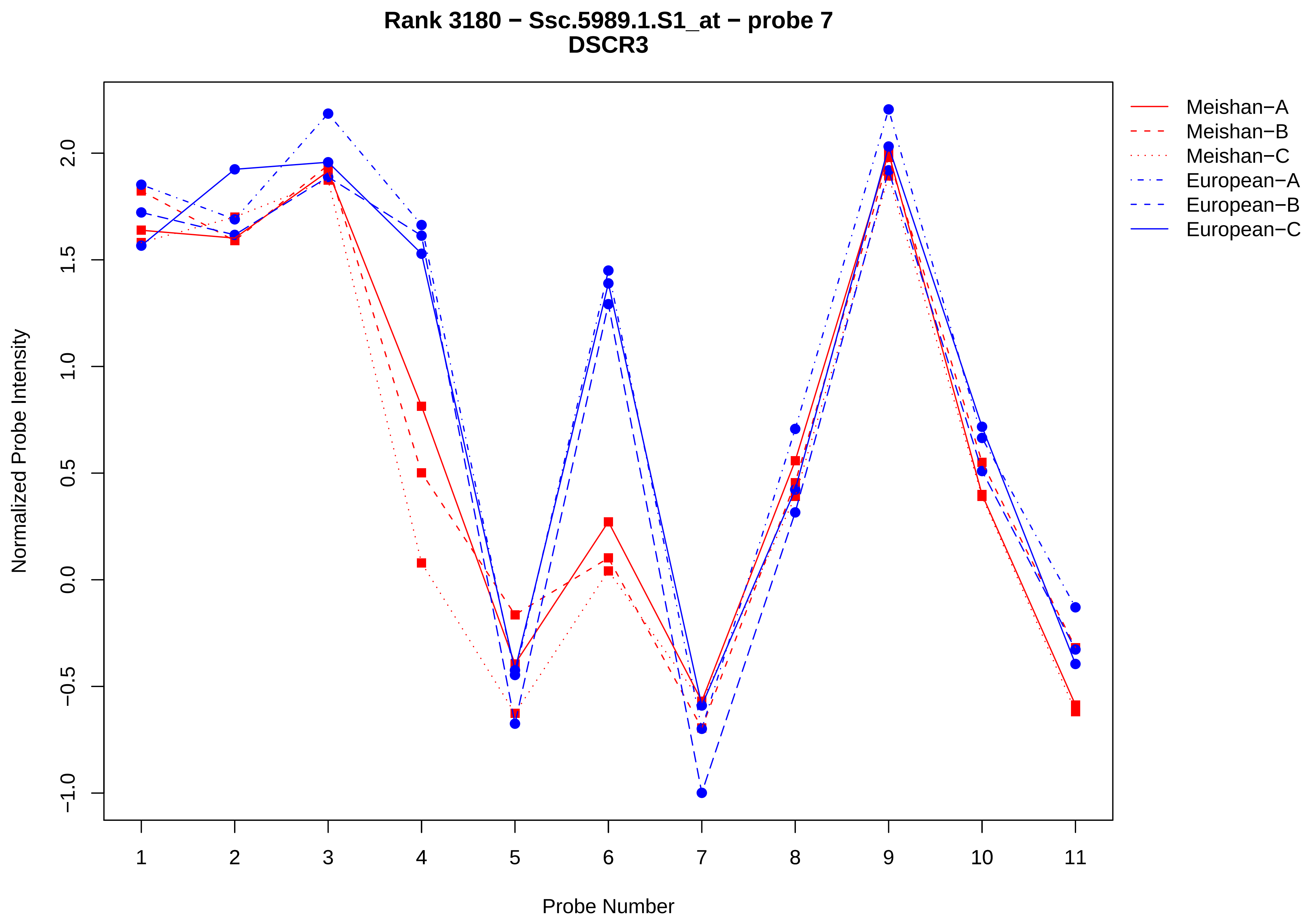
Rank 3180 − Ssc.5989.1.S1_at − probe 7
DSCR3
Normalized Probe Intensity
Probe Number
−1.0
−0.5
0.0
0.5
1.0
1.5
2.0
1
2
3
4
5
6
7
8
9
10
11
Meishan−A
Meishan−B
Meishan−C
European−A
European−B
European−C

Rank 3181 − Ssc.4942.2.S1_at − probe 6
C1IB_HUMAN

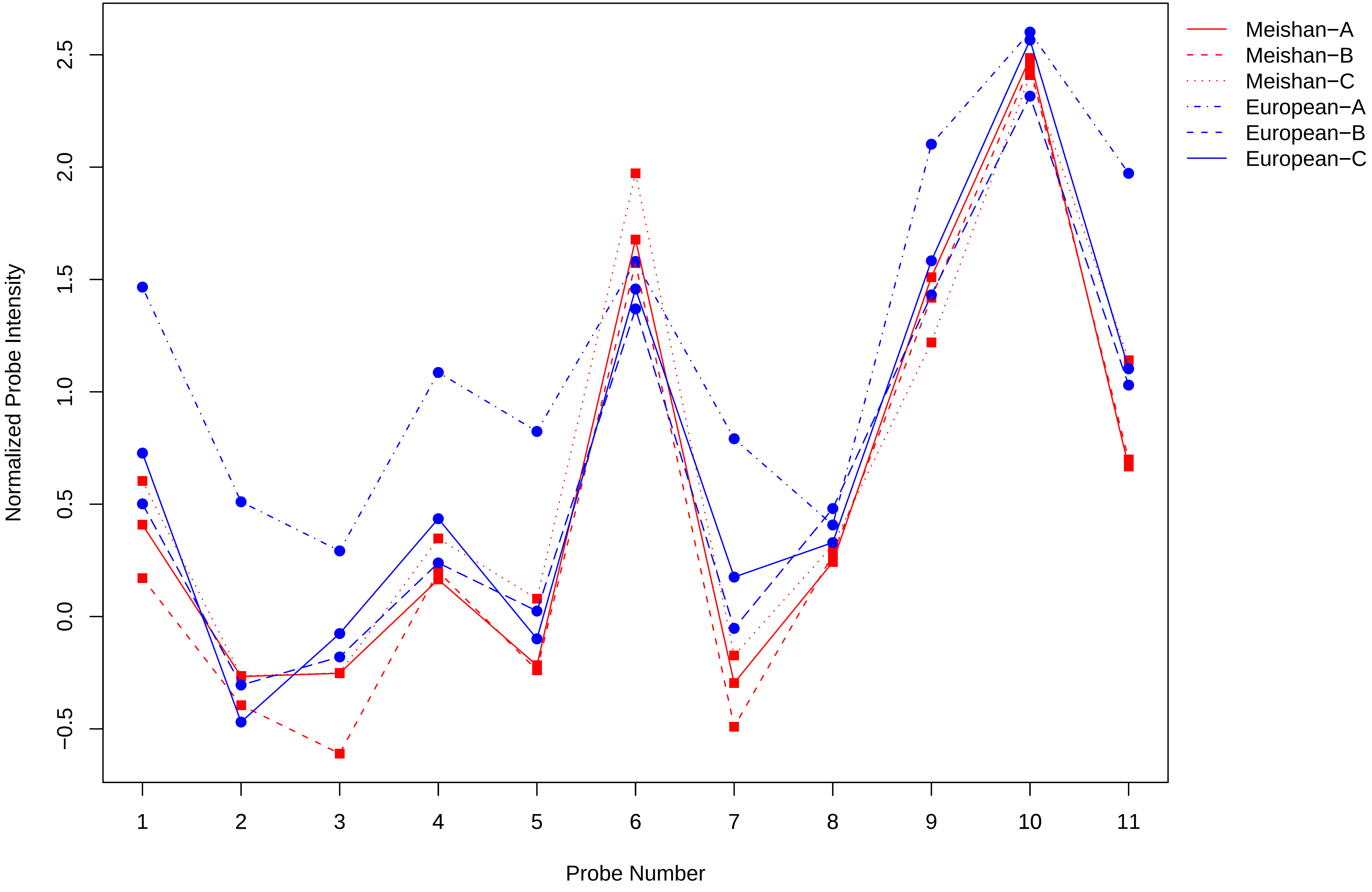

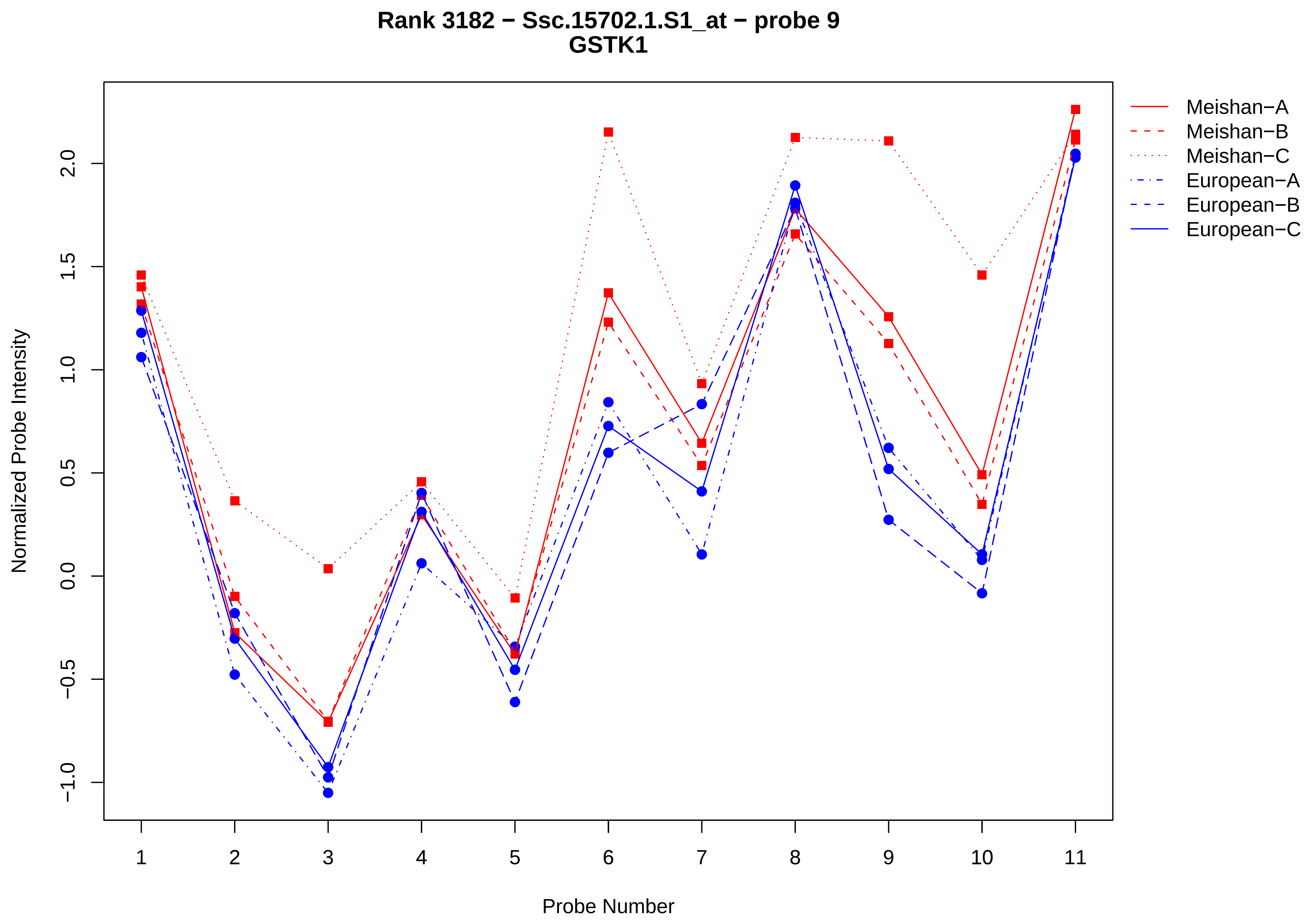

Rank 3182 − Ssc.15702.1.S1_at − probe 9
GSTK1
Normalized Probe Intensity
Probe Number
Meishan−A
Meishan−B
Meishan−C
European−A
European−B
European−C
−1.0
−0.5
0.0
0.5
1.0
1.5
2.0
1
2
3
4
5
6
7
8
9
10
11

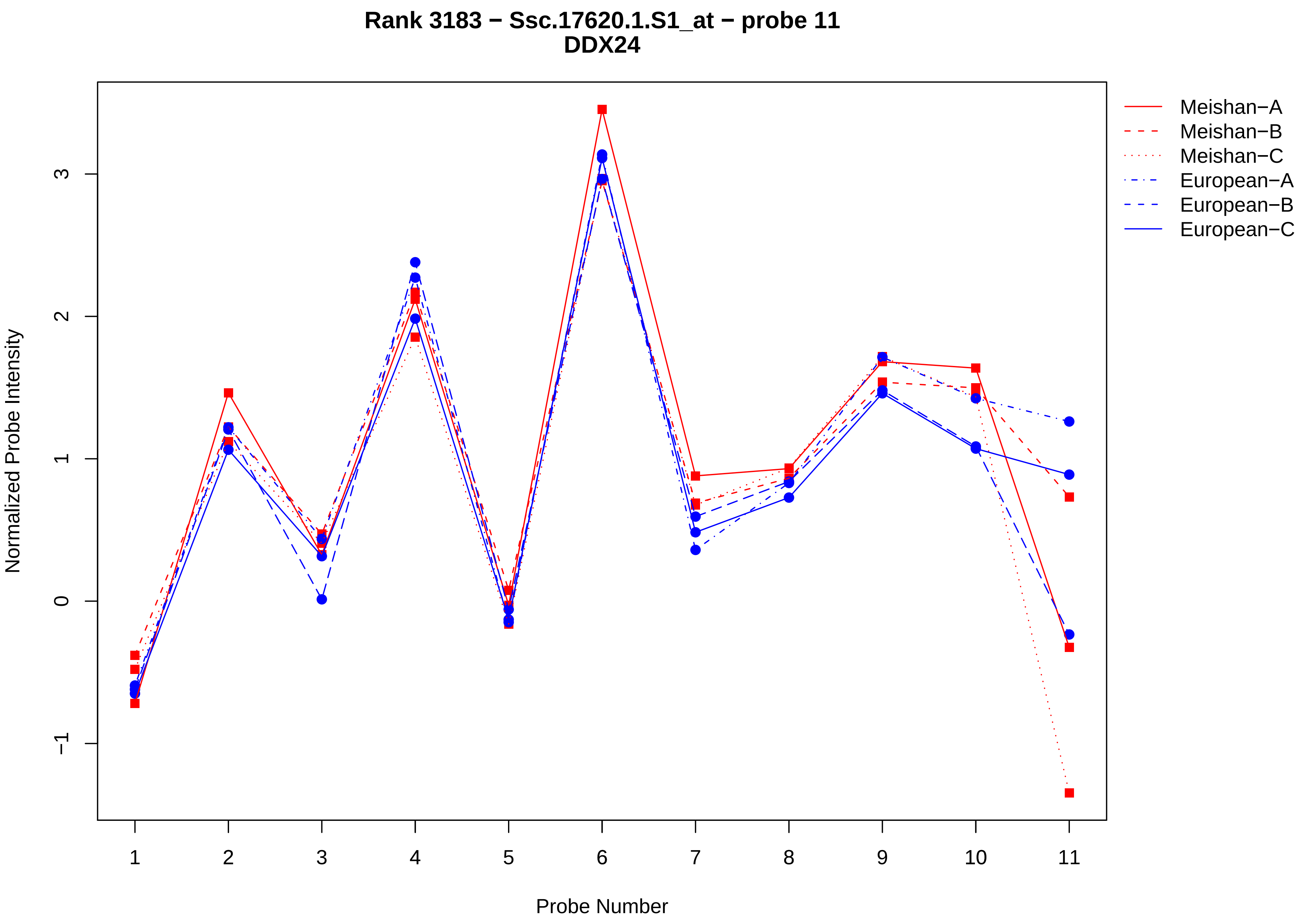

Rank 3183 − Ssc.17620.1.S1_at − probe 11
DDX24
Normalized Probe Intensity
Probe Number
−1
0
1
2
3
1
2
3
4
5
6
7
8
9
10
11
Meishan−A
Meishan−B
Meishan−C
European−A
European−B
European−C

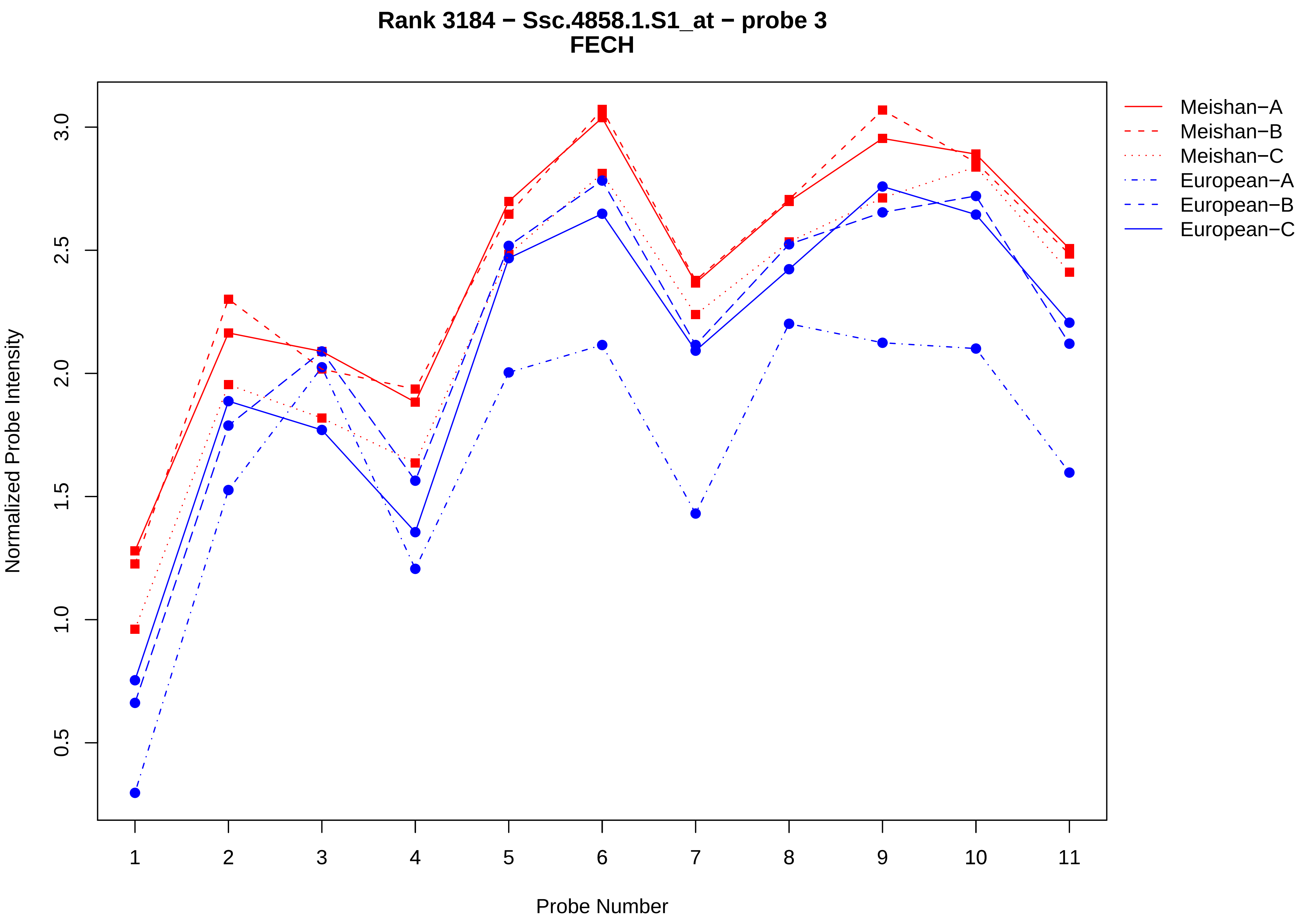

Rank 3184 − Ssc.4858.1.S1_at − probe 3
FECH
Normalized Probe Intensity
0.5
1.0
1.5
2.0
2.5
3.0
1
2
3
4
5
6
7
8
9
10
11
Probe Number
Meishan−A
Meishan−B
Meishan−C
European−A
European−B
European−C

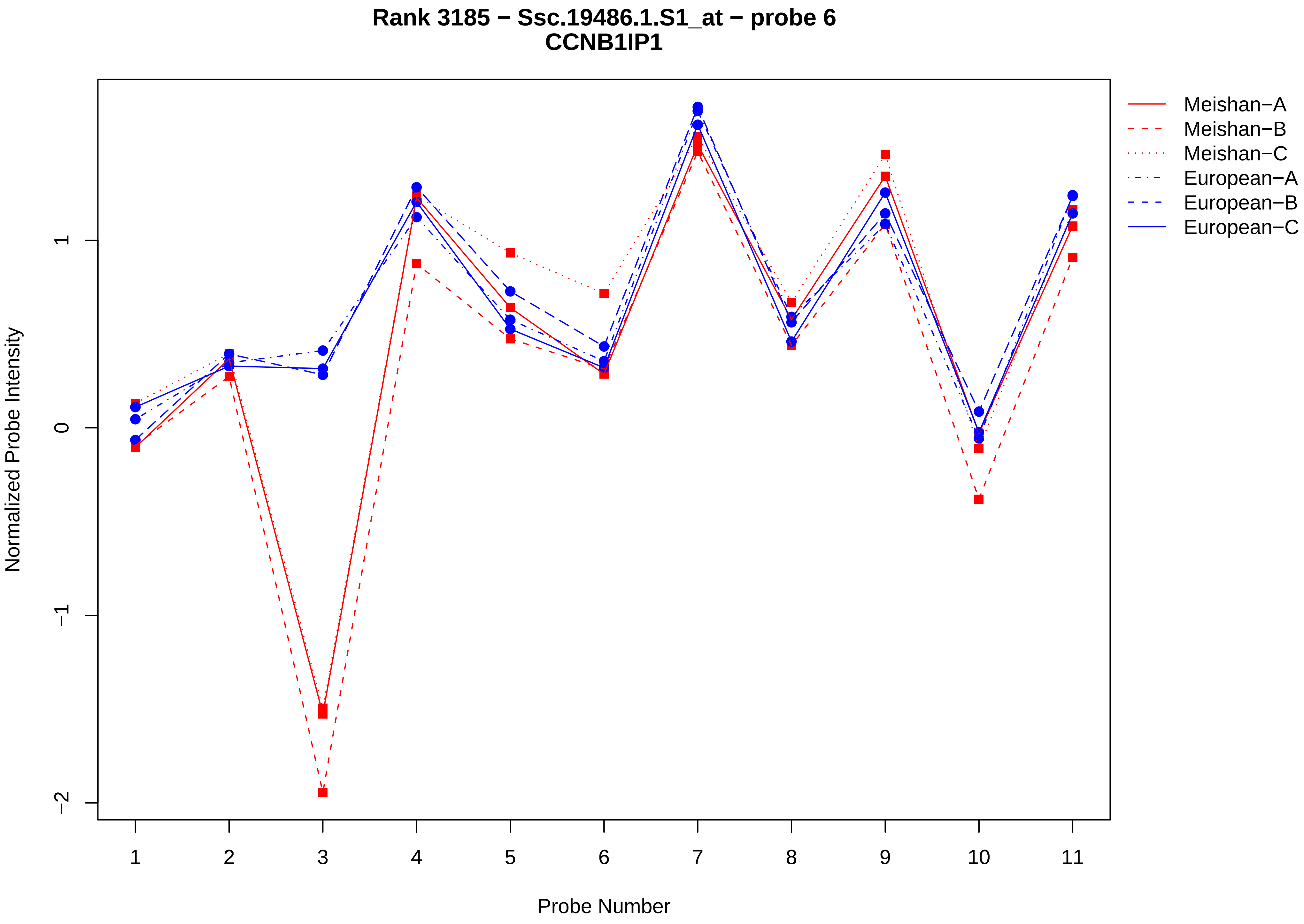

Rank 3185 − Ssc.19486.1.S1_at − probe 6
CCNB1IP1
Normalized Probe Intensity
Probe Number
Meishan−A
Meishan−B
Meishan−C
European−A
European−B
European−C
−2
−1
0
1
1
2
3
4
5
6
7
8
9
10
11

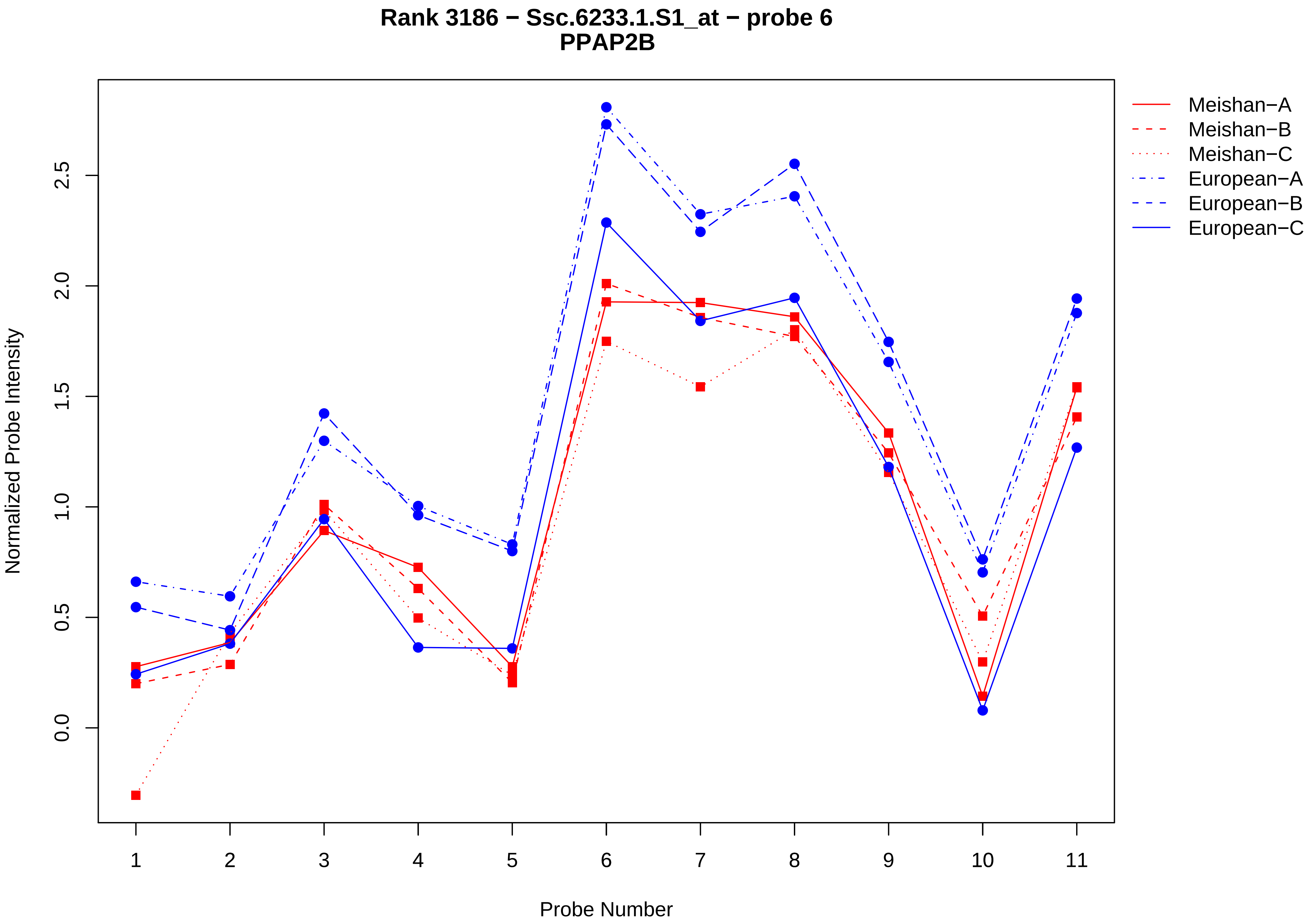

Rank 3186 − Ssc.6233.1.S1_at − probe 6
PPAP2B
Normalized Probe Intensity
0.0
0.5
1.0
1.5
2.0
2.5
1
2
3
4
5
6
7
8
9
10
11
Probe Number
Meishan−A
Meishan−B
Meishan−C
European−A
European−B
European−C

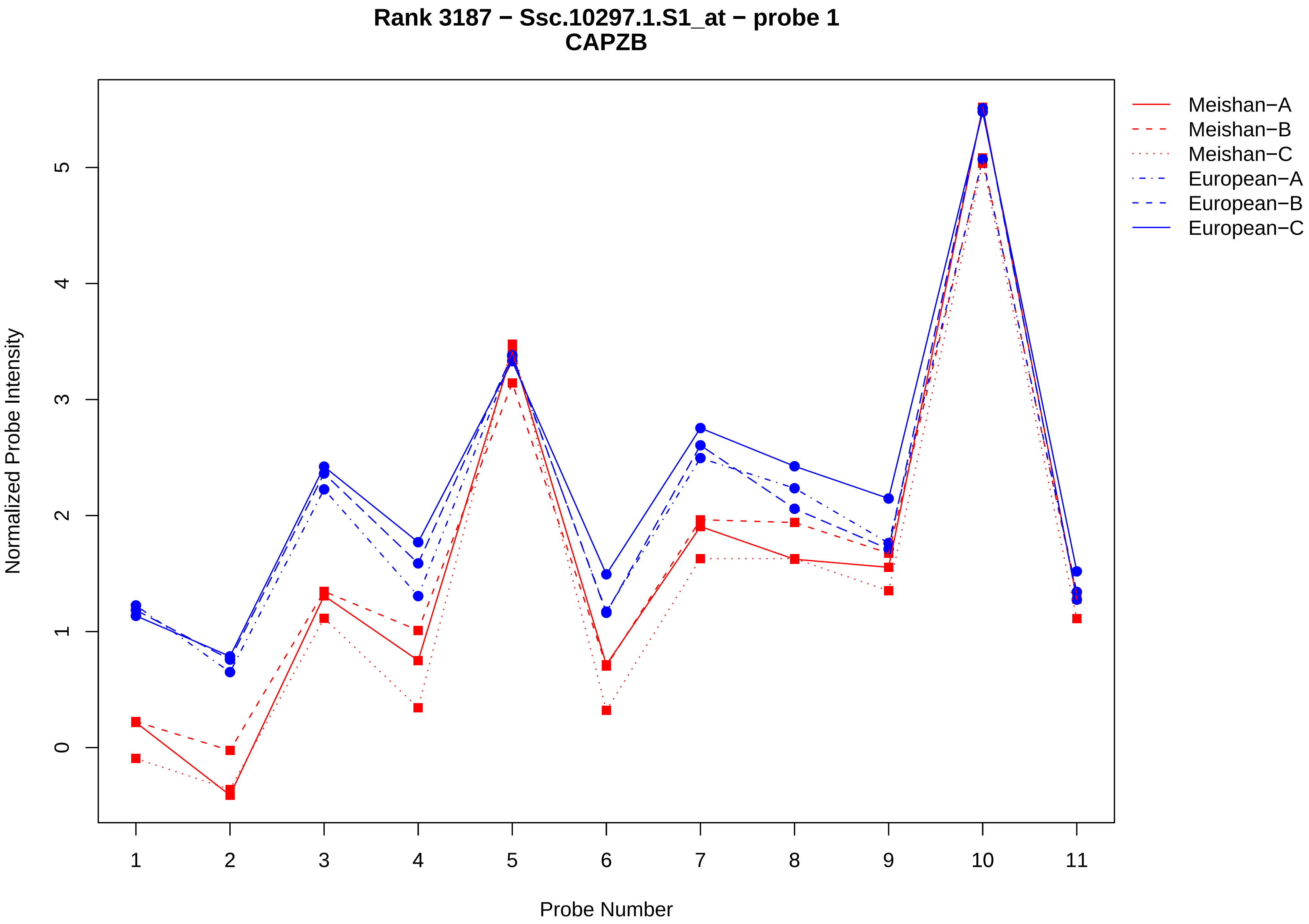

Rank 3187 − Ssc.10297.1.S1_at − probe 1
CAPZB
Normalized Probe Intensity
Probe Number
0
1
2
3
4
5
1
2
3
4
5
6
7
8
9
10
11
Meishan−A
Meishan−B
Meishan−C
European−A
European−B
European−C

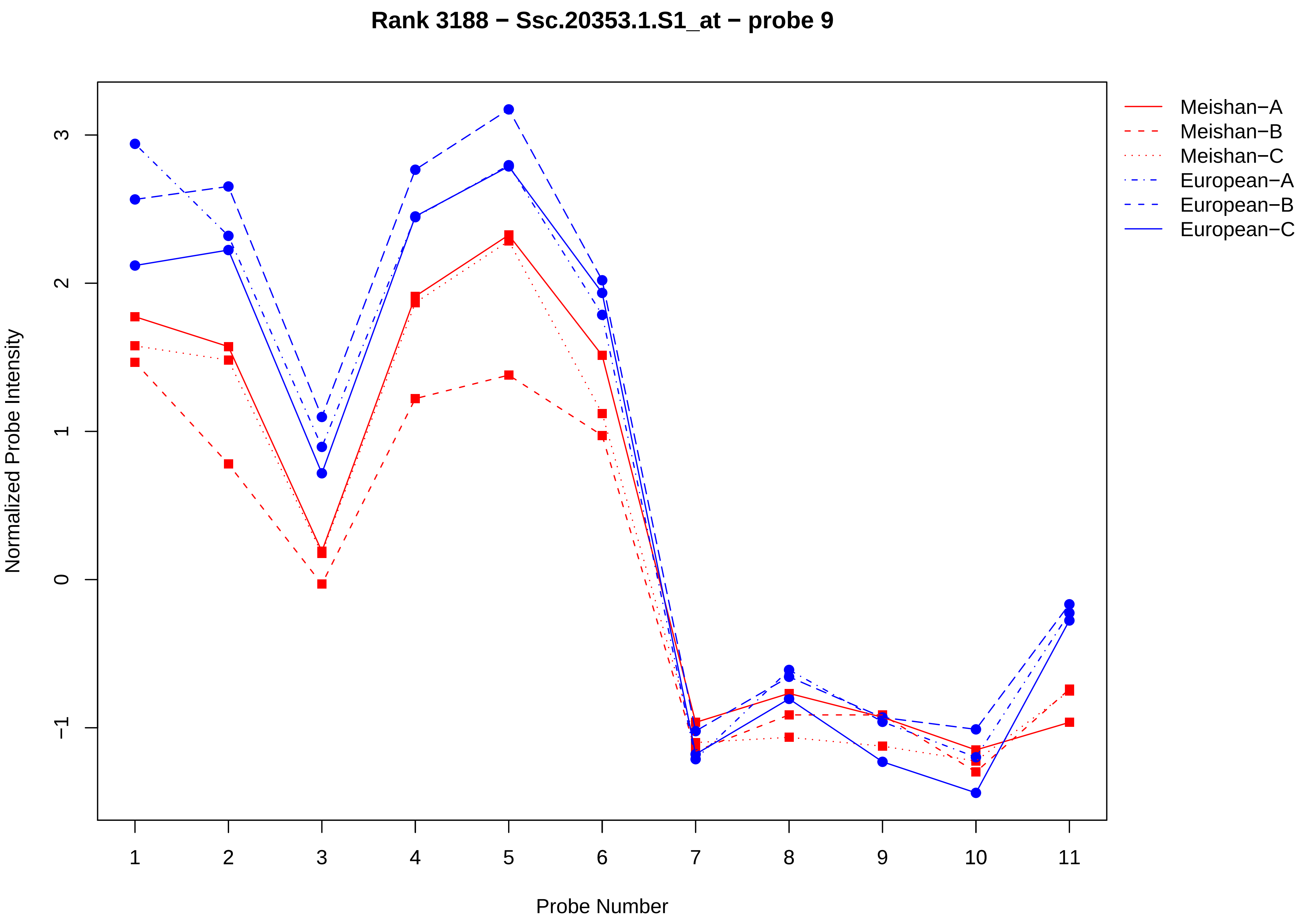

Rank 3188 − Ssc.20353.1.S1_at − probe 9
Normalized Probe Intensity
Probe Number
Meishan−A
Meishan−B
Meishan−C
European−A
European−B
European−C

Rank 3189 − Ssc.6330.1.A1_at − probe 3
NP_071926

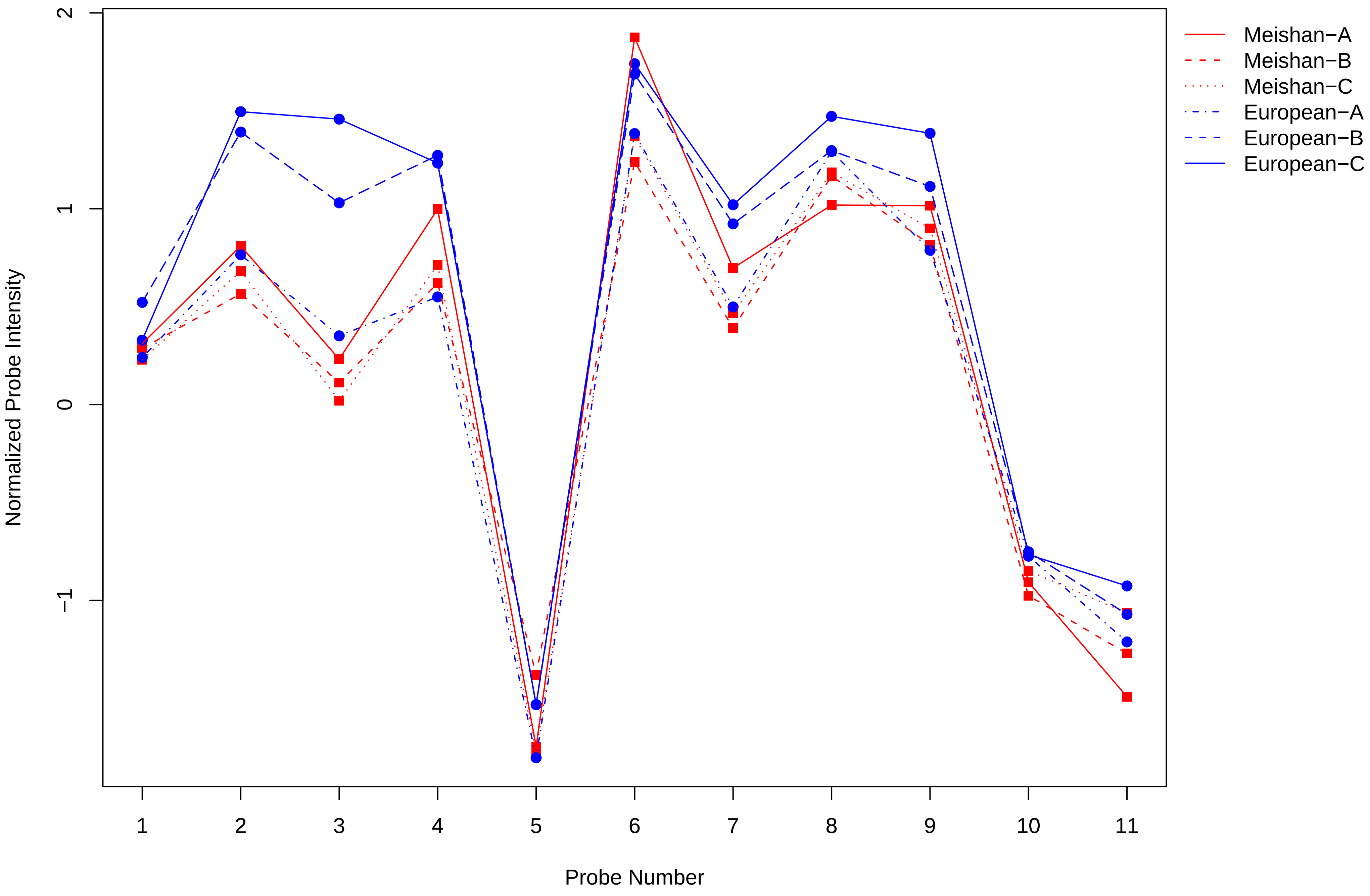

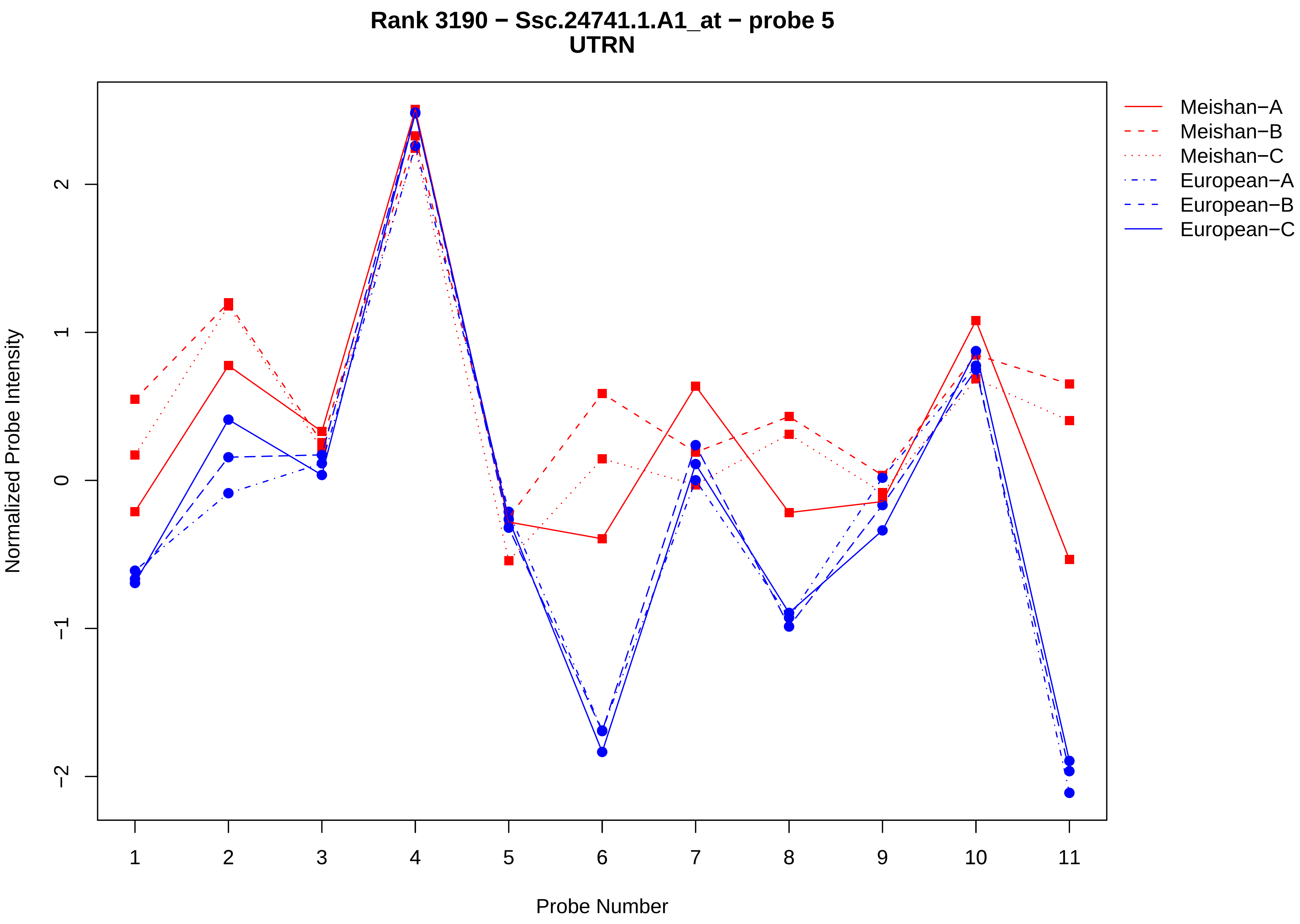

Rank 3190 − Ssc.24741.1.A1_at − probe 5
UTRN
Normalized Probe Intensity
Probe Number
−2
−1
0
1
2
1
2
3
4
5
6
7
8
9
10
11
Meishan−A
Meishan−B
Meishan−C
European−A
European−B
European−C

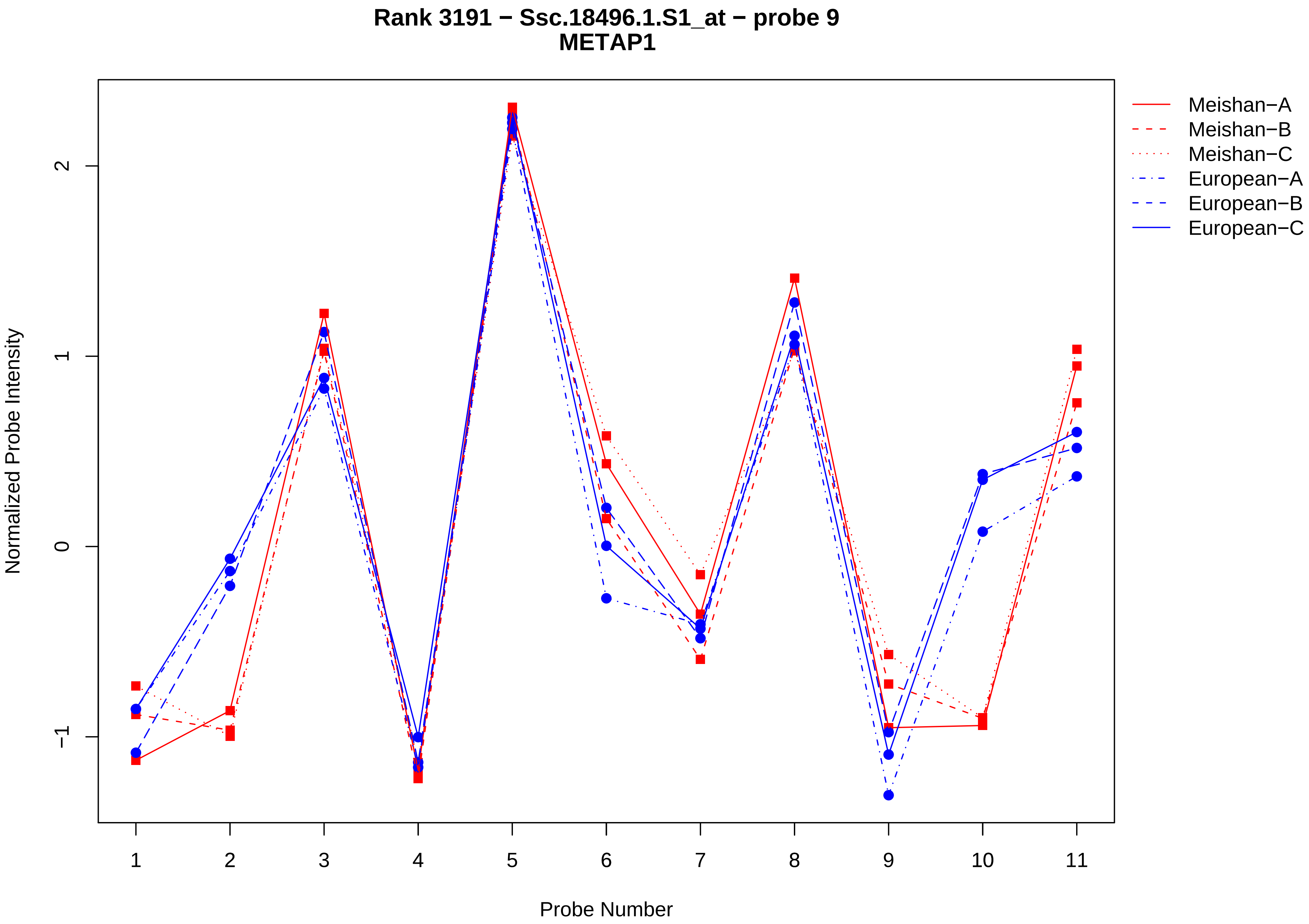

Rank 3191 − Ssc.18496.1.S1_at − probe 9
METAP1
Normalized Probe Intensity
Probe Number
−1
0
1
2
1
2
3
4
5
6
7
8
9
10
11
Meishan−A
Meishan−B
Meishan−C
European−A
European−B
European−C

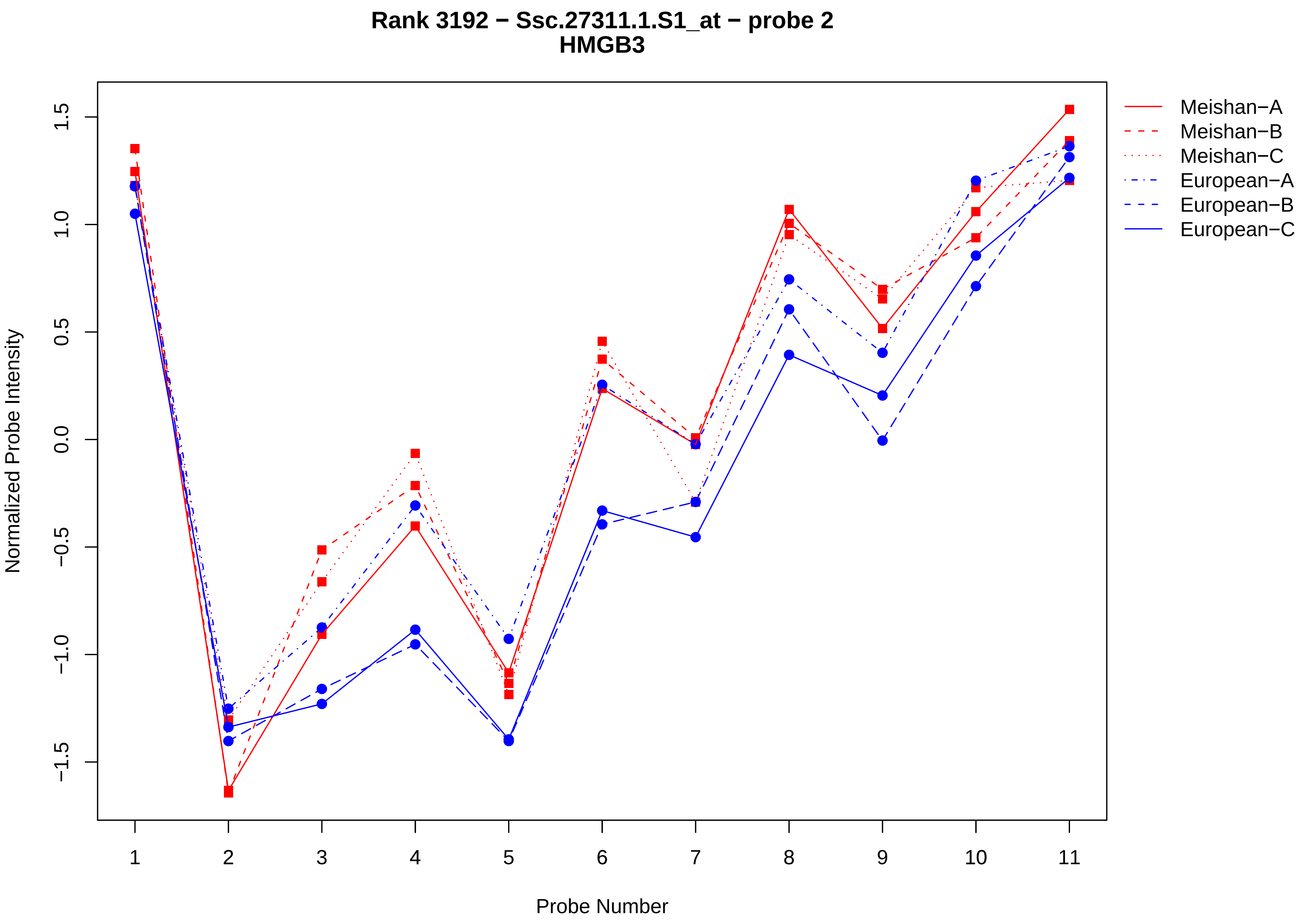
Rank 3192 − Ssc.27311.1.S1_at − probe 2
HMGB3
Normalized Probe Intensity
Probe Number
Meishan−A
Meishan−B
Meishan−C
European−A
European−B
European−C

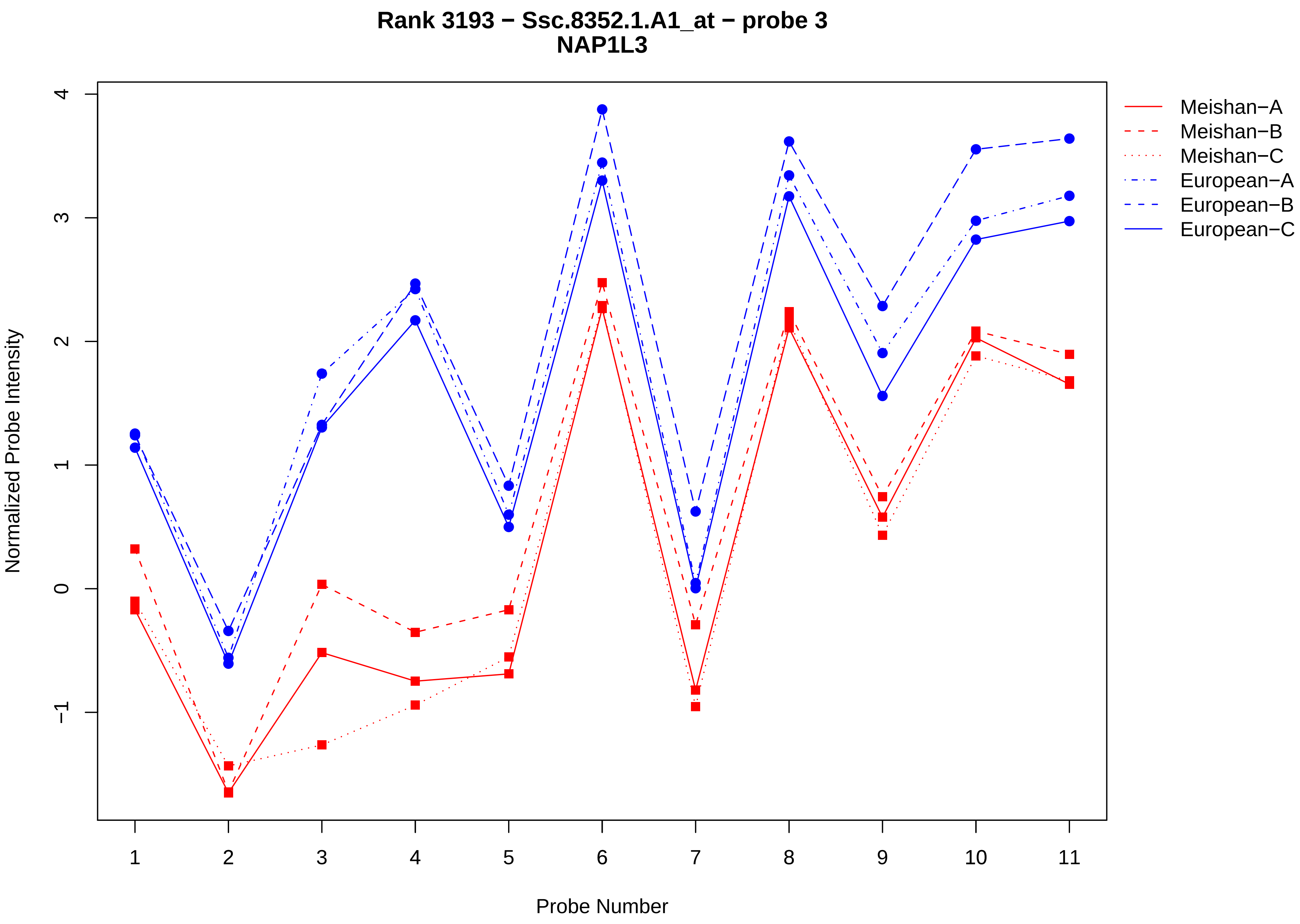

Rank 3193 − Ssc.8352.1.A1_at − probe 3
NAP1L3
Normalized Probe Intensity
Probe Number
Meishan−A
Meishan−B
Meishan−C
European−A
European−B
European−C

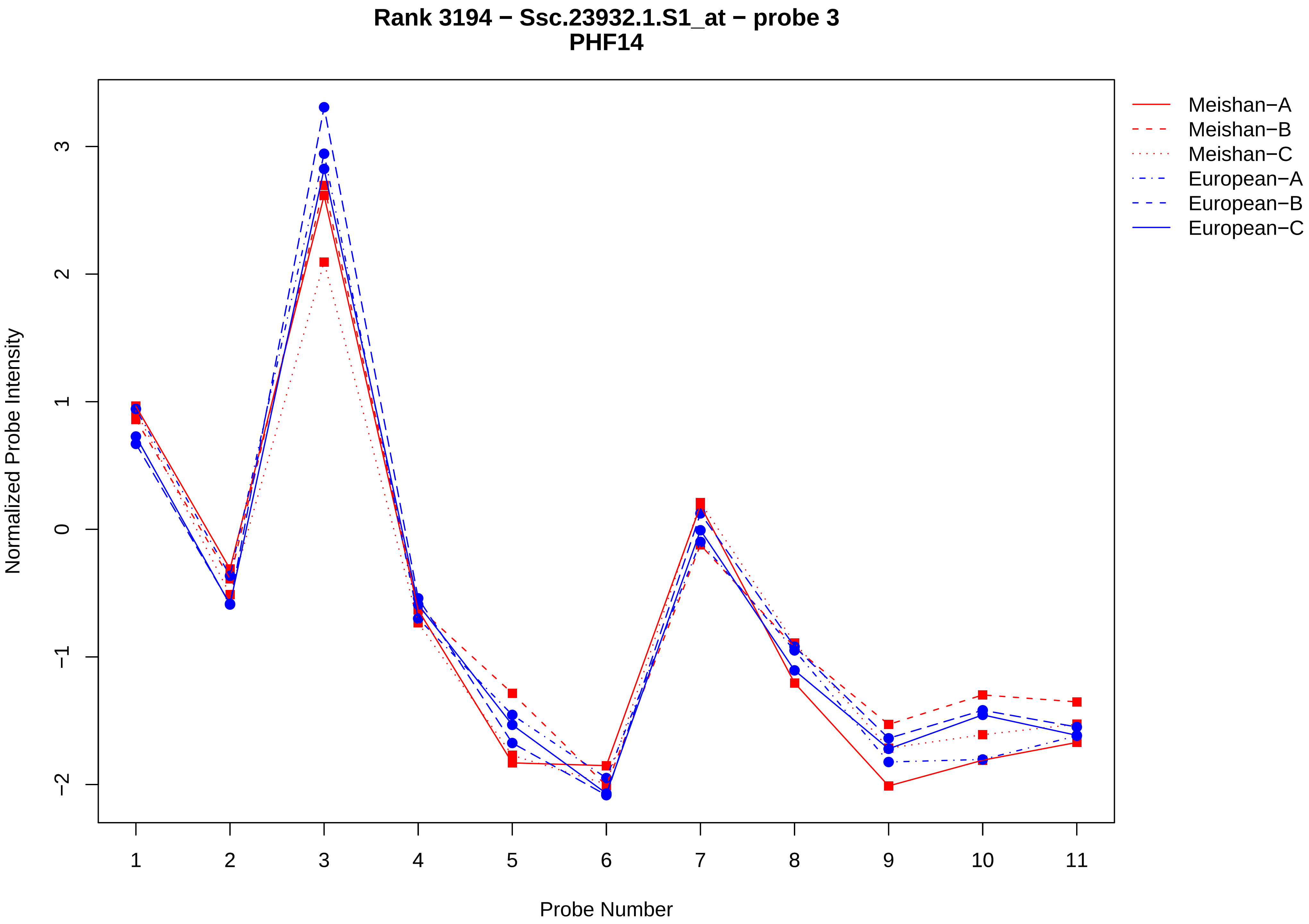

Rank 3194 − Ssc.23932.1.S1_at − probe 3
PHF14
Normalized Probe Intensity
Probe Number
Meishan−A
Meishan−B
Meishan−C
European−A
European−B
European−C
−2
−1
0
1
2
3
1
2
3
4
5
6
7
8
9
10
11

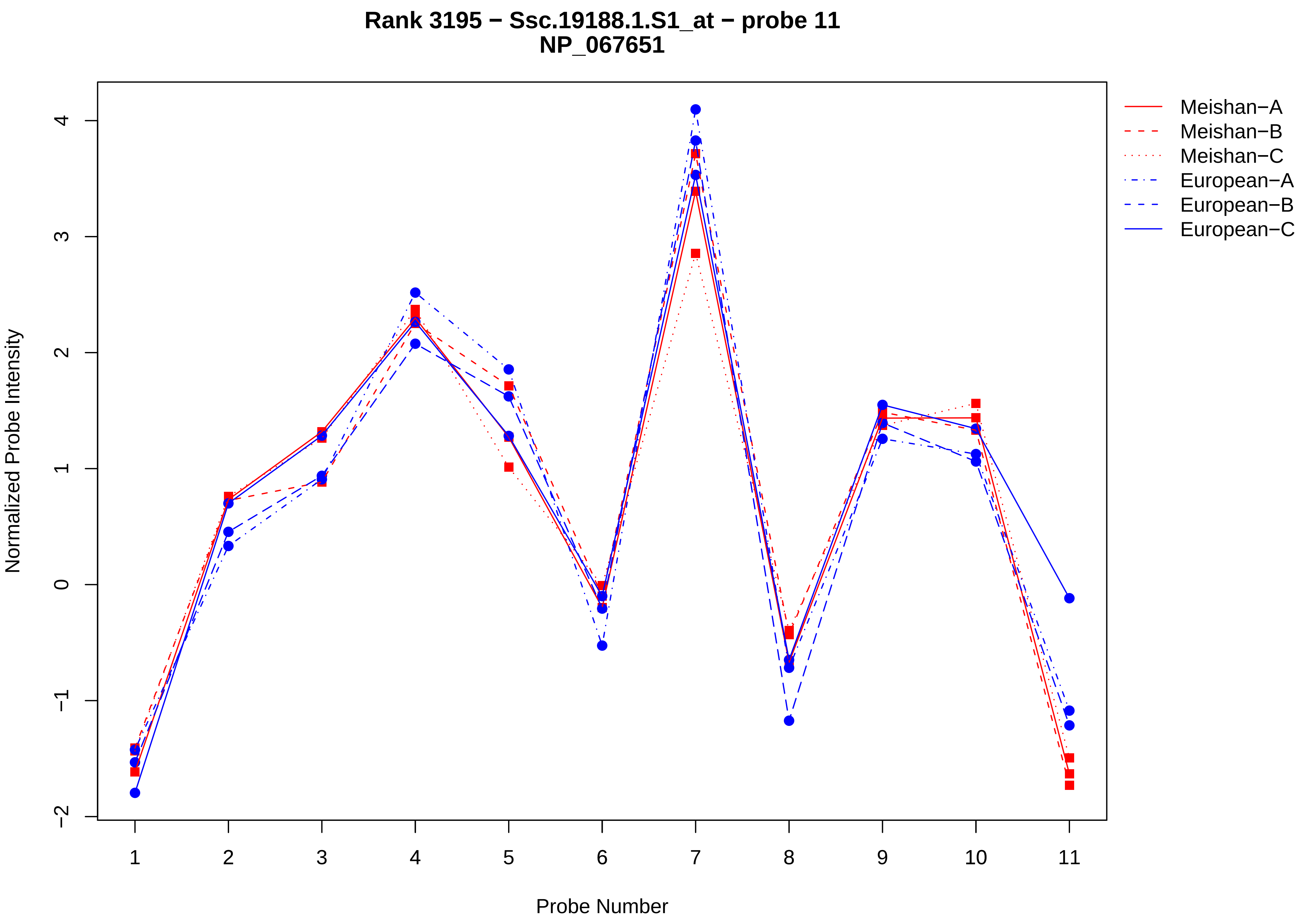

Rank 3195 − Ssc.19188.1.S1_at − probe 11
NP_067651
Normalized Probe Intensity
Probe Number
Meishan−A
Meishan−B
Meishan−C
European−A
European−B
European−C

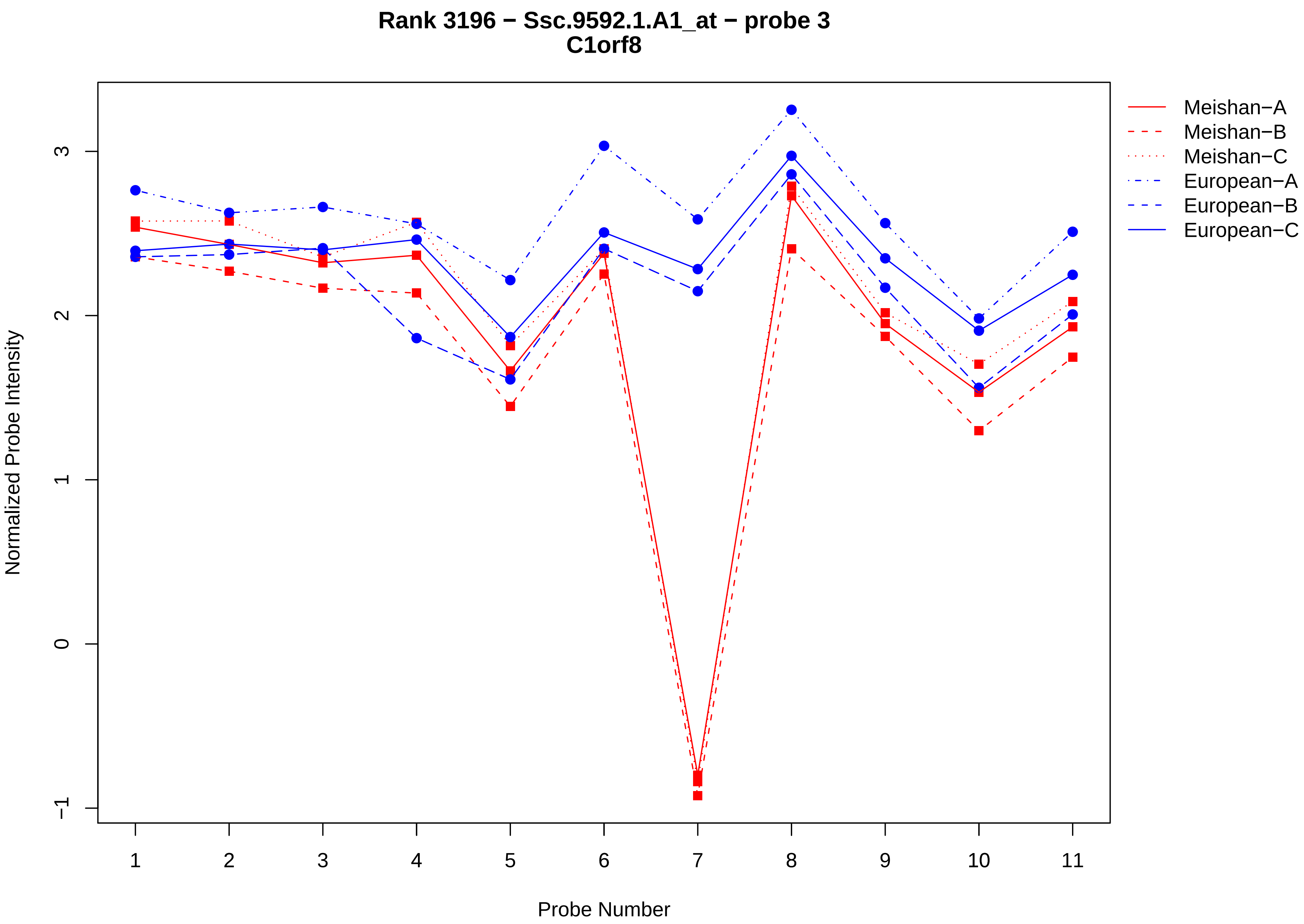
Rank 3196 − Ssc.9592.1.A1_at − probe 3
C1orf8
Normalized Probe Intensity
Probe Number
−1
0
1
2
3
1
2
3
4
5
6
7
8
9
10
11
Meishan−A
Meishan−B
Meishan−C
European−A
European−B
European−C

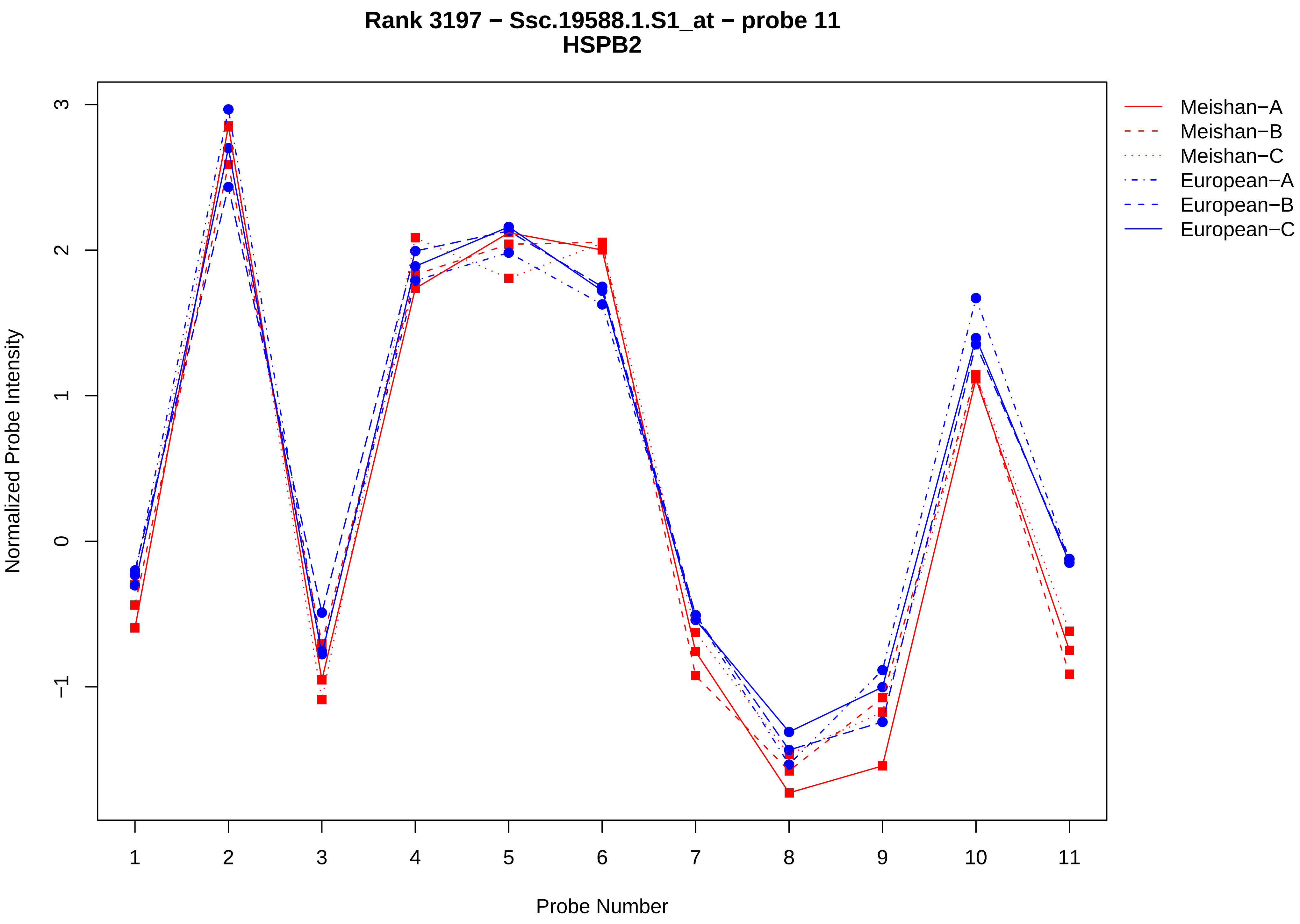

Rank 3197 − Ssc.19588.1.S1_at − probe 11
HSPB2
Normalized Probe Intensity
−1
0
1
2
3
1
2
3
4
5
6
7
8
9
10
11
Probe Number
Meishan−A
Meishan−B
Meishan−C
European−A
European−B
European−C

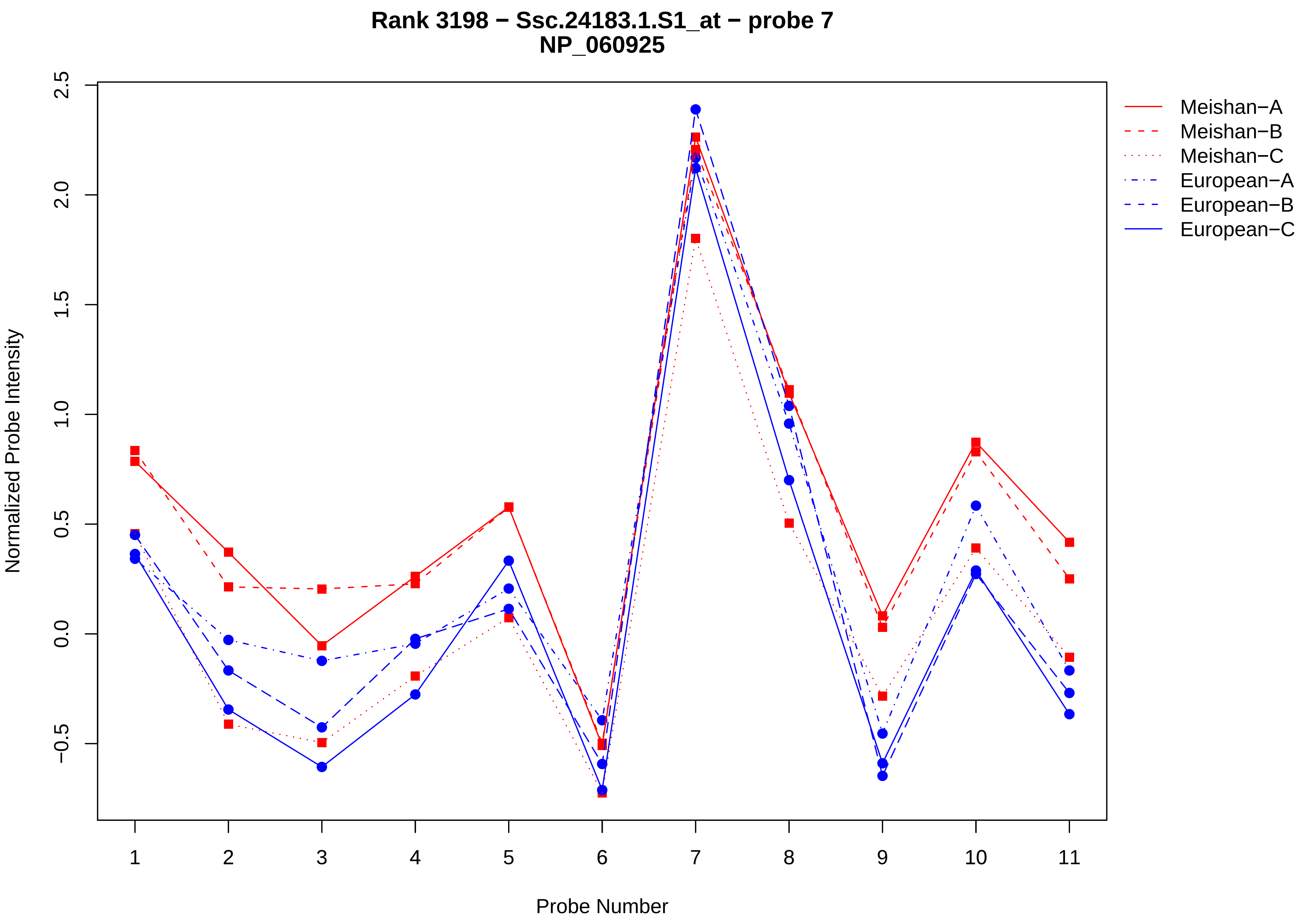
Rank 3198 − Ssc.24183.1.S1_at − probe 7
NP_060925
Normalized Probe Intensity
Probe Number
Meishan−A
Meishan−B
Meishan−C
European−A
European−B
European−C
−0.5
0.0
0.5
1.0
1.5
2.0
2.5
1
2
3
4
5
6
7
8
9
10
11

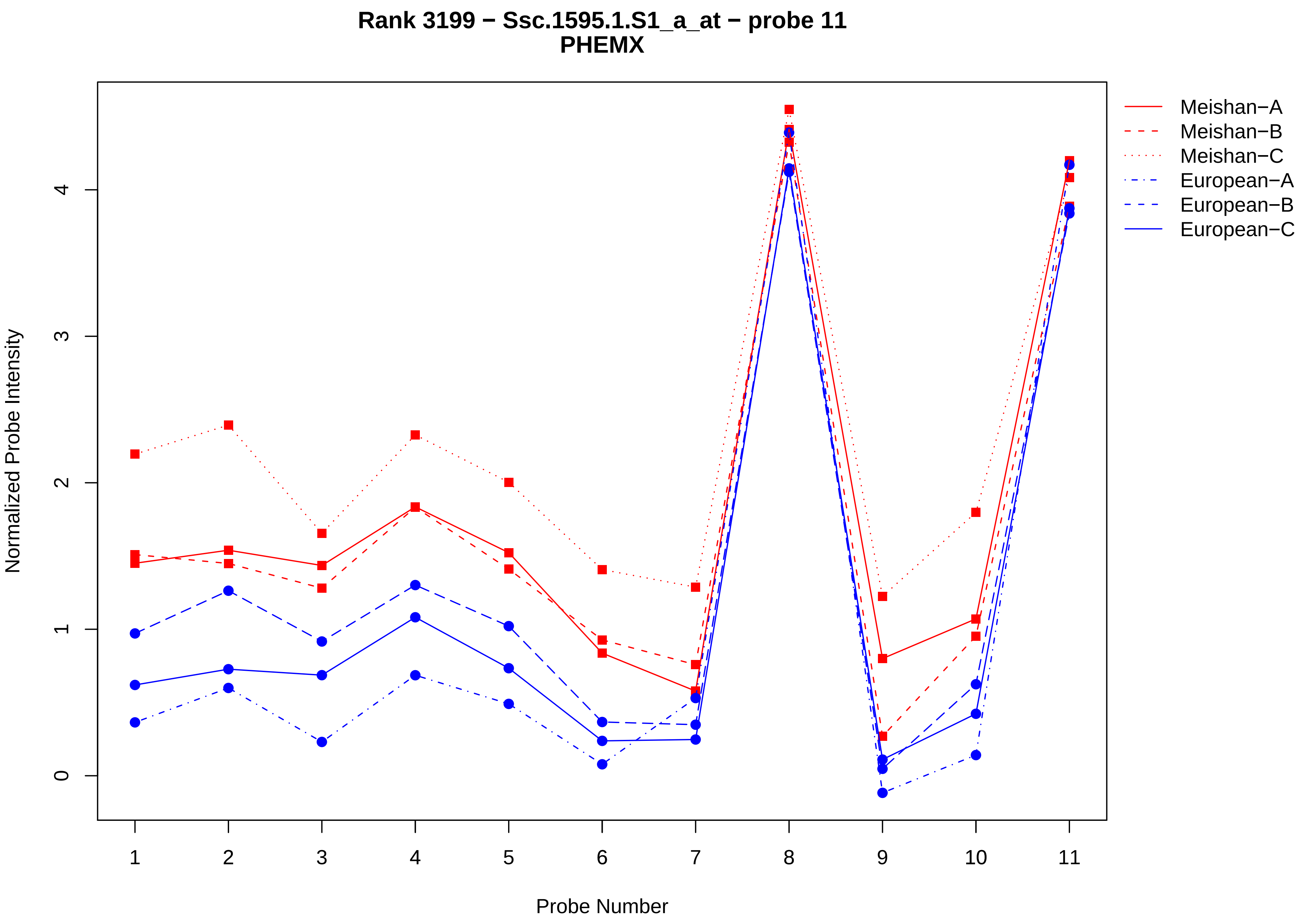

Rank 3199 − Ssc.1595.1.S1_a_at − probe 11
PHEMX
Normalized Probe Intensity
0
1
2
3
4
Probe Number
1
2
3
4
5
6
7
8
9
10
11
Meishan−A
Meishan−B
Meishan−C
European−A
European−B
European−C

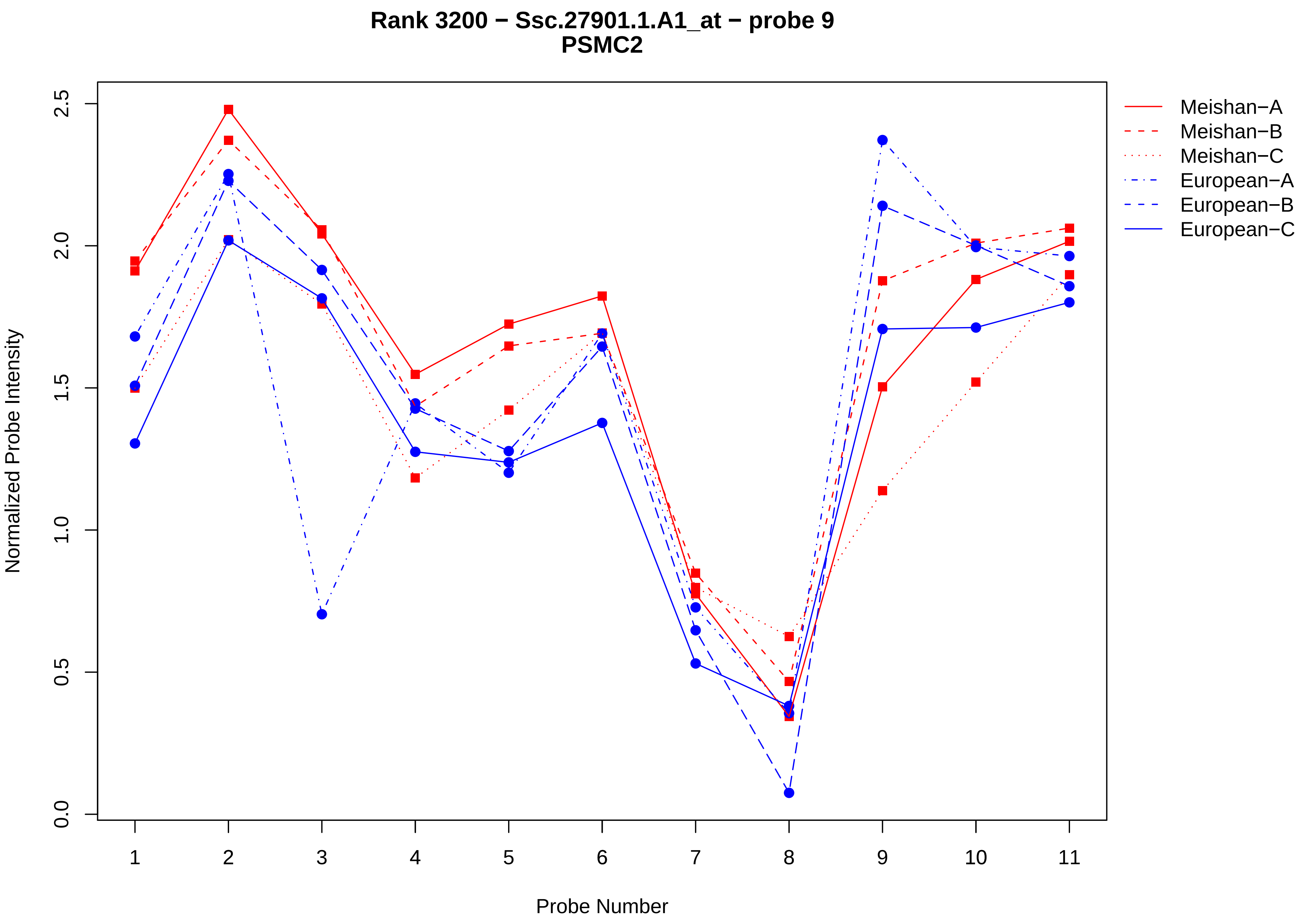

Rank 3200 − Ssc.27901.1.A1_at − probe 9
PSMC2
Normalized Probe Intensity
Probe Number
0.0
0.5
1.0
1.5
2.0
2.5
1
2
3
4
5
6
7
8
9
10
11
Meishan−A
Meishan−B
Meishan−C
European−A
European−B
European−C

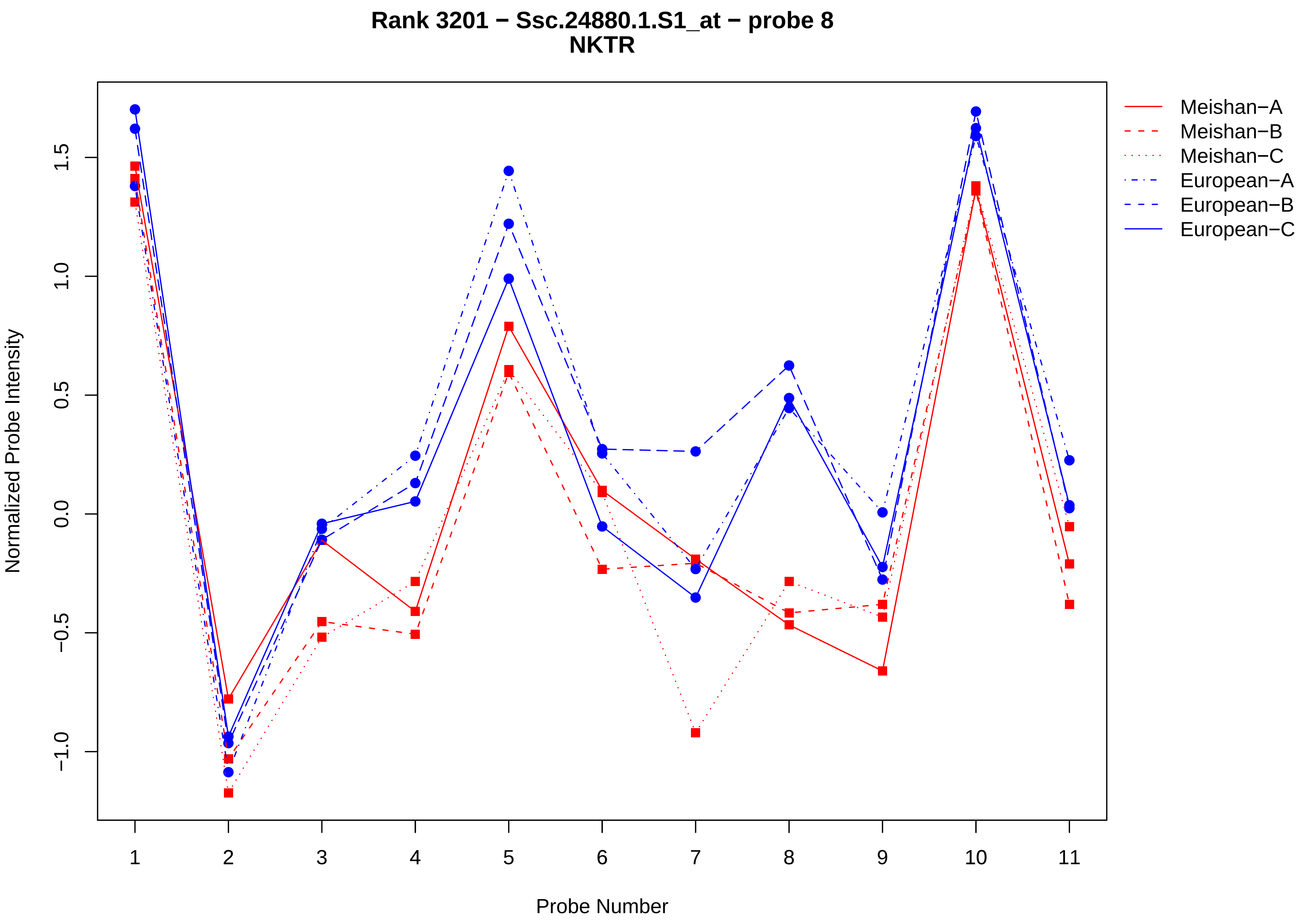

Rank 3201 − Ssc.24880.1.S1_at − probe 8
NKTR
Normalized Probe Intensity
Probe Number
−1.0
−0.5
0.0
0.5
1.0
1.5
1
2
3
4
5
6
7
8
9
10
11
Meishan−A
Meishan−B
Meishan−C
European−A
European−B
European−C

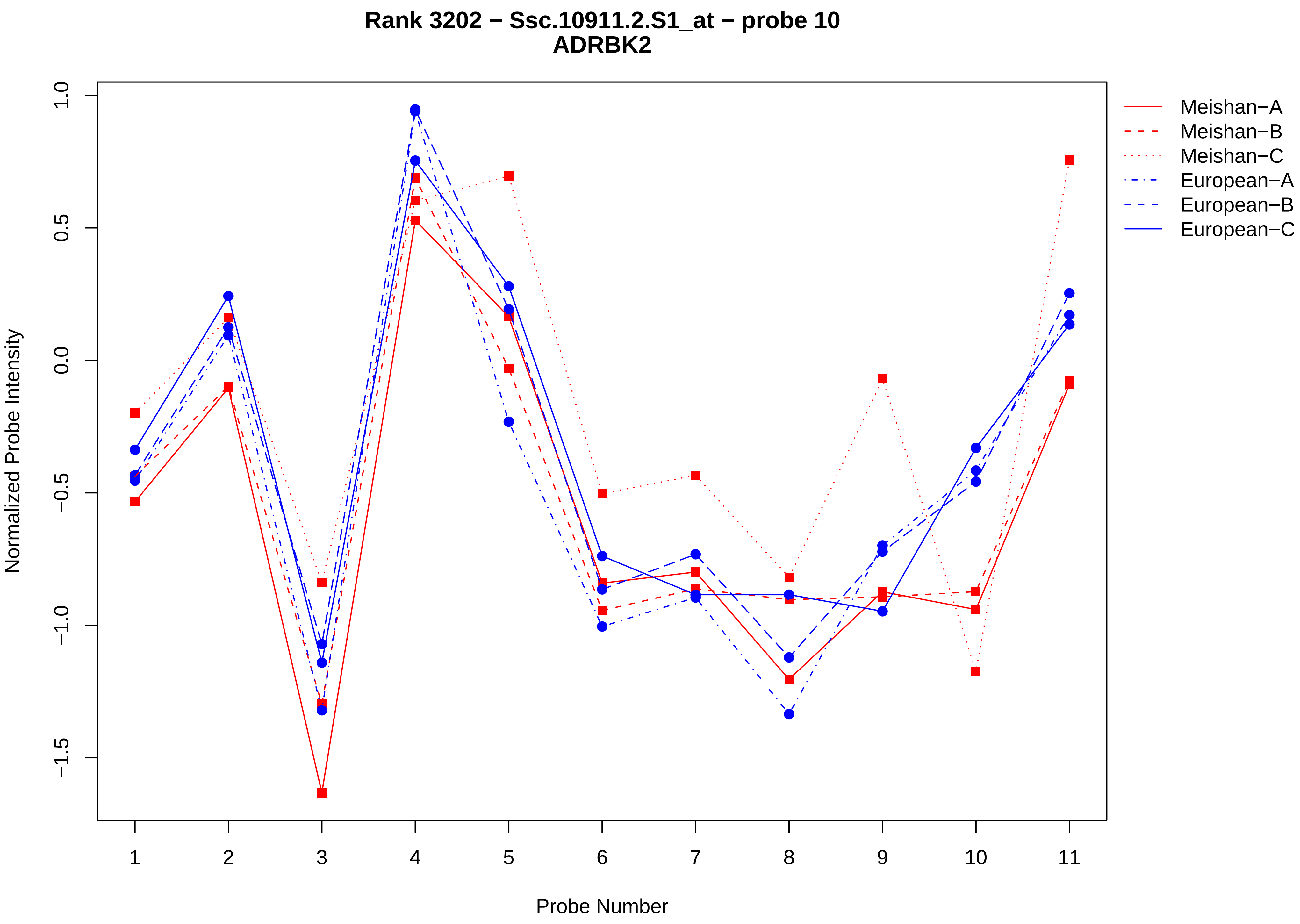

Rank 3202 − Ssc.10911.2.S1_at − probe 10
ADRBK2
Normalized Probe Intensity
Probe Number
1.0
0.5
0.0
−0.5
−1.0
−1.5
1
2
3
4
5
6
7
8
9
10
11
Meishan−A
Meishan−B
Meishan−C
European−A
European−B
European−C

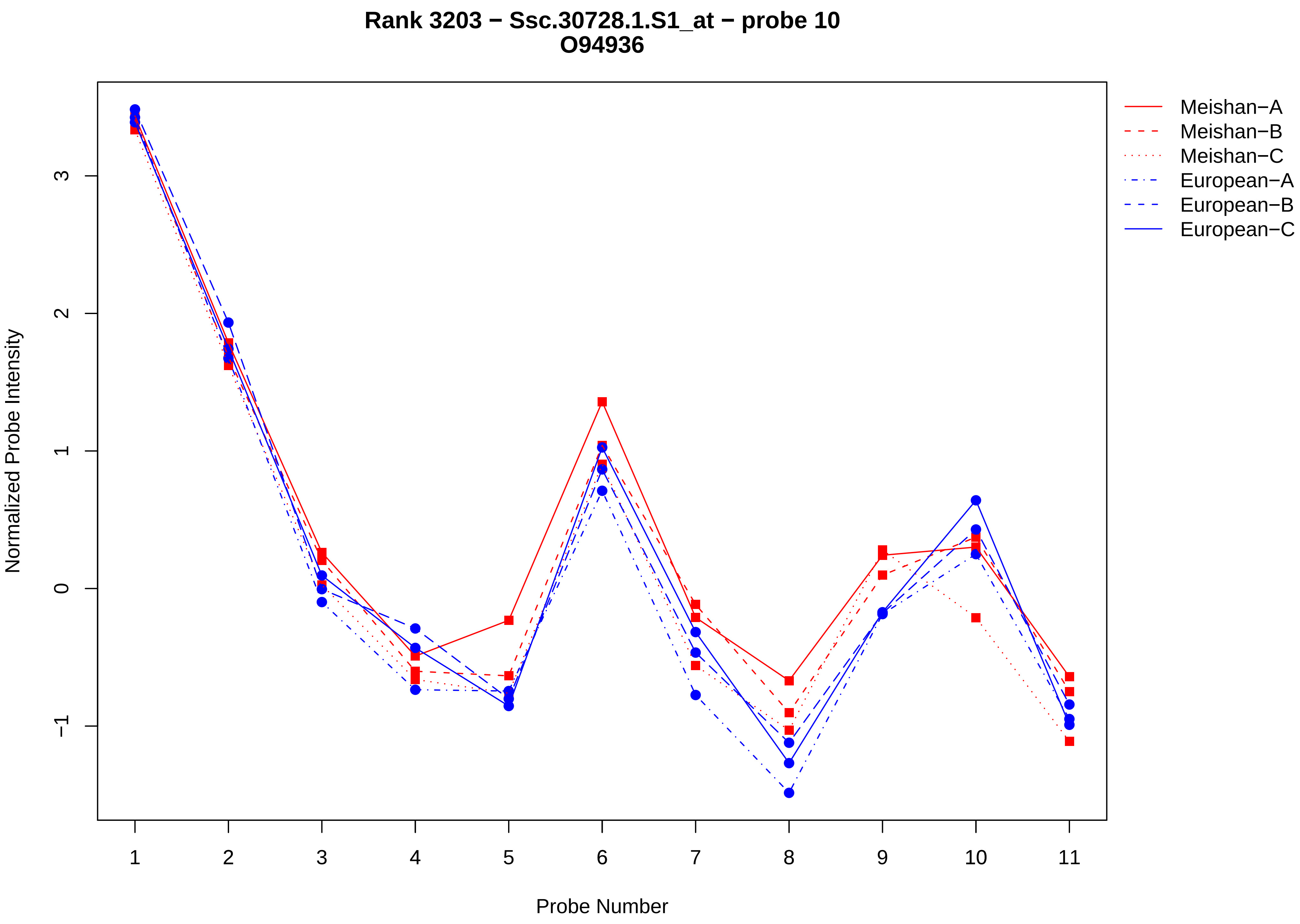

Rank 3203 − Ssc.30728.1.S1_at − probe 10
O94936
Normalized Probe Intensity
−1
0
1
2
3
1
2
3
4
5
6
7
8
9
10
11
Probe Number
Meishan−A
Meishan−B
Meishan−C
European−A
European−B
European−C

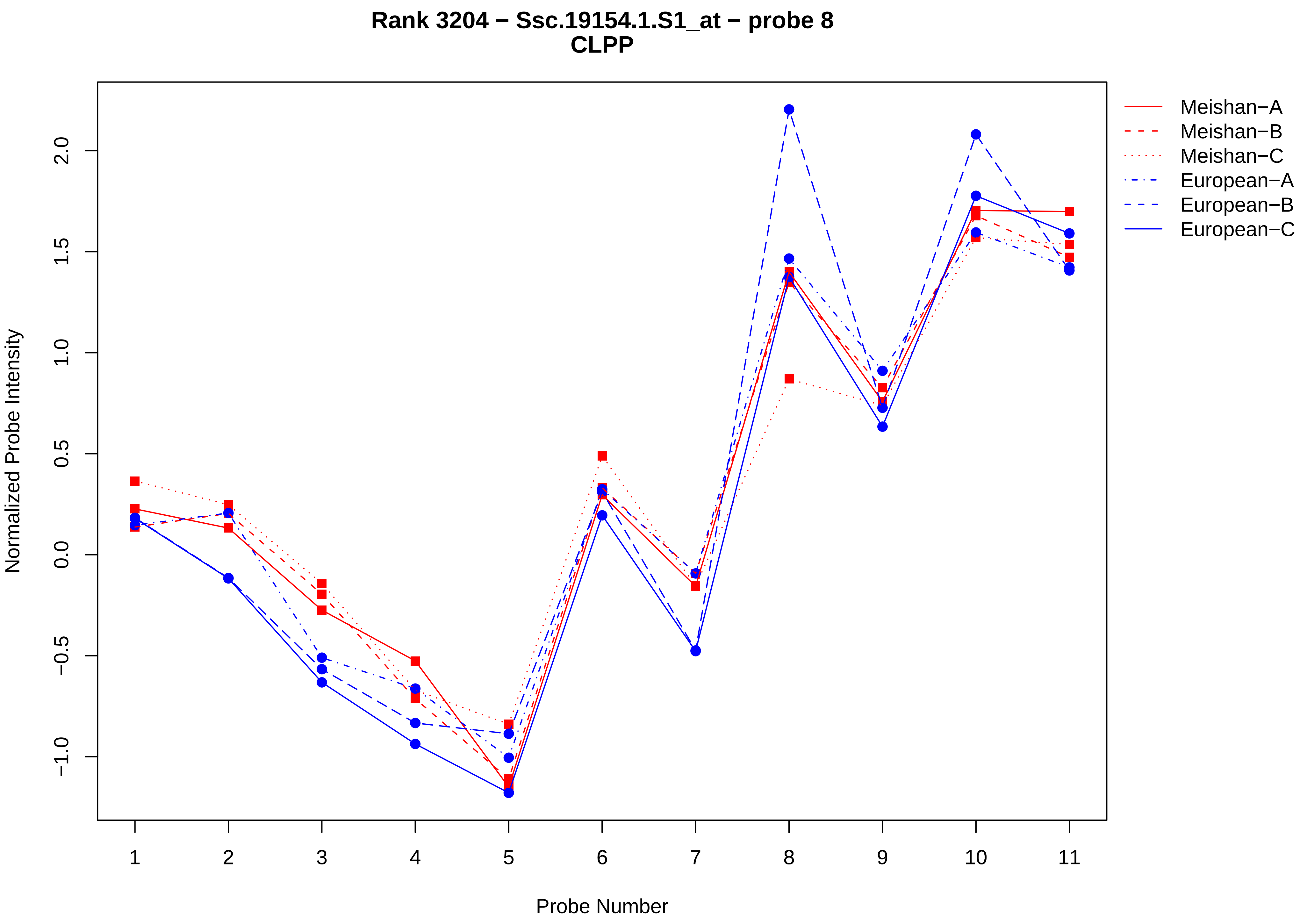
Rank 3204 − Ssc.19154.1.S1_at − probe 8
CLPP
Normalized Probe Intensity
−1.0
−0.5
0.0
0.5
1.0
1.5
2.0
Probe Number
1
2
3
4
5
6
7
8
9
10
11
Meishan−A
Meishan−B
Meishan−C
European−A
European−B
European−C

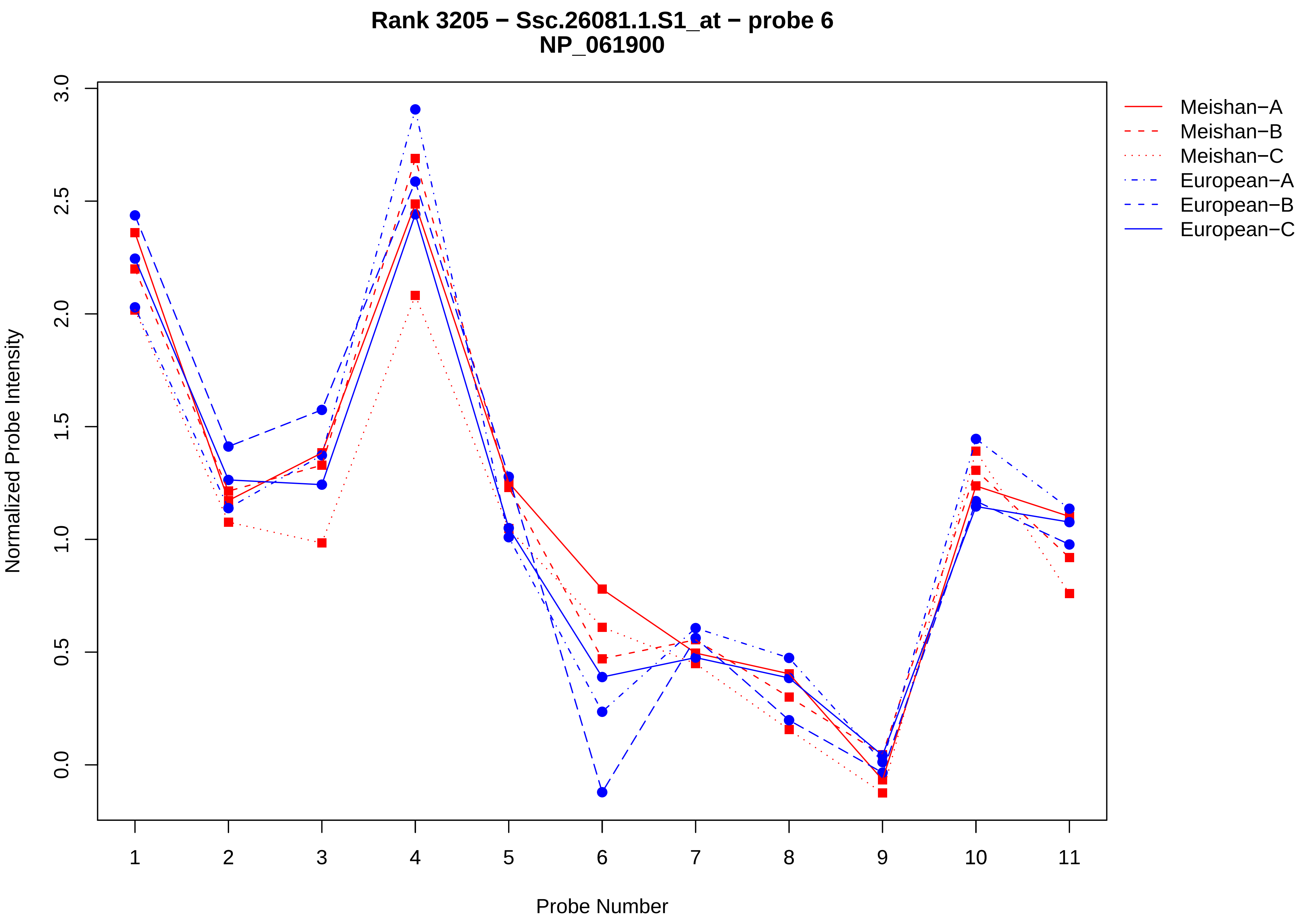

Rank 3205 − Ssc.26081.1.S1_at − probe 6
NP_061900
Normalized Probe Intensity
0.0
0.5
1.0
1.5
2.0
2.5
3.0
1
2
3
4
5
6
7
8
9
10
11
Probe Number
Meishan−A
Meishan−B
Meishan−C
European−A
European−B
European−C

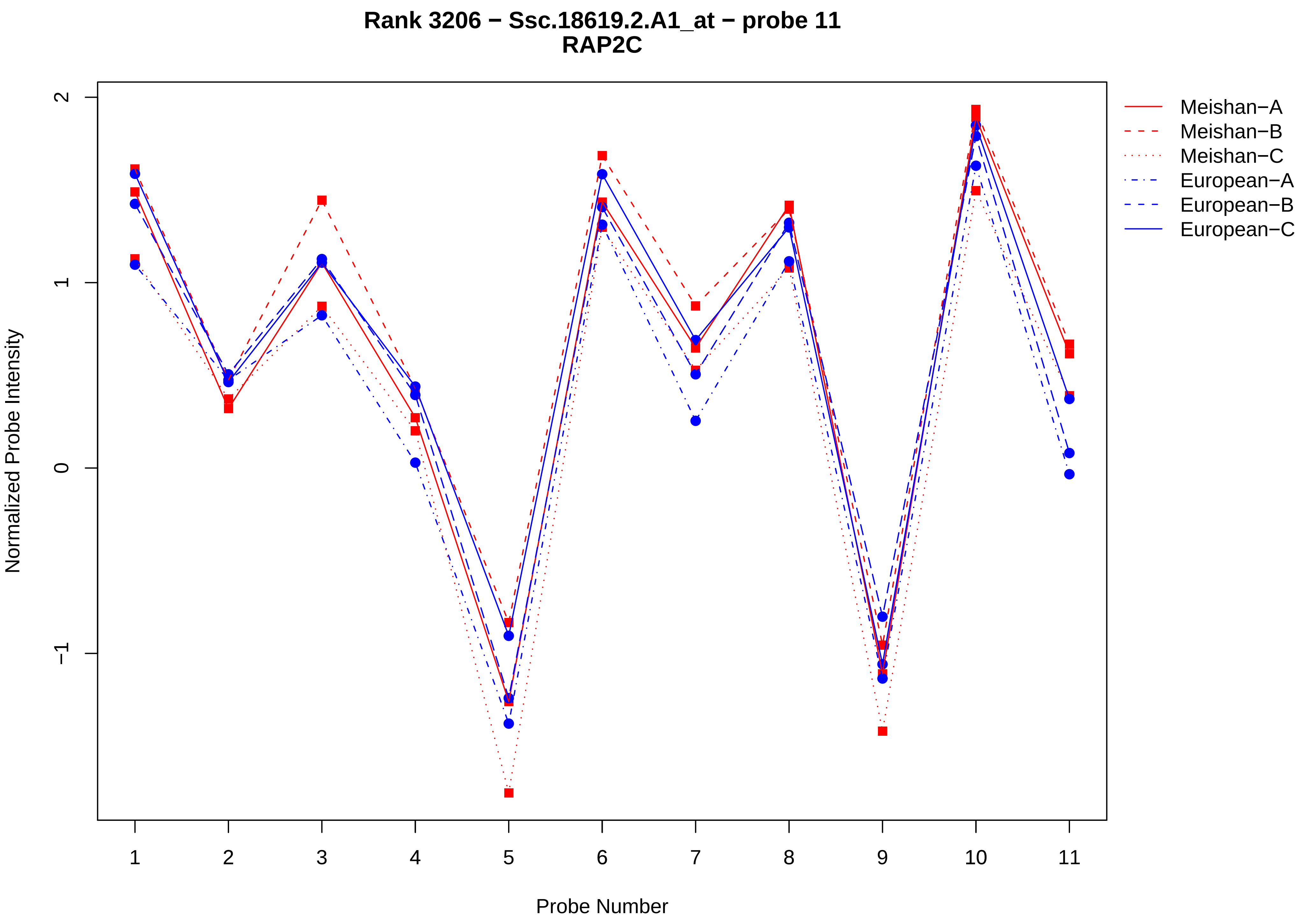

Rank 3206 − Ssc.18619.2.A1_at − probe 11
RAP2C
Normalized Probe Intensity
Probe Number
Meishan−A
Meishan−B
Meishan−C
European−A
European−B
European−C
2
1
0
−1
1
2
3
4
5
6
7
8
9
10
11

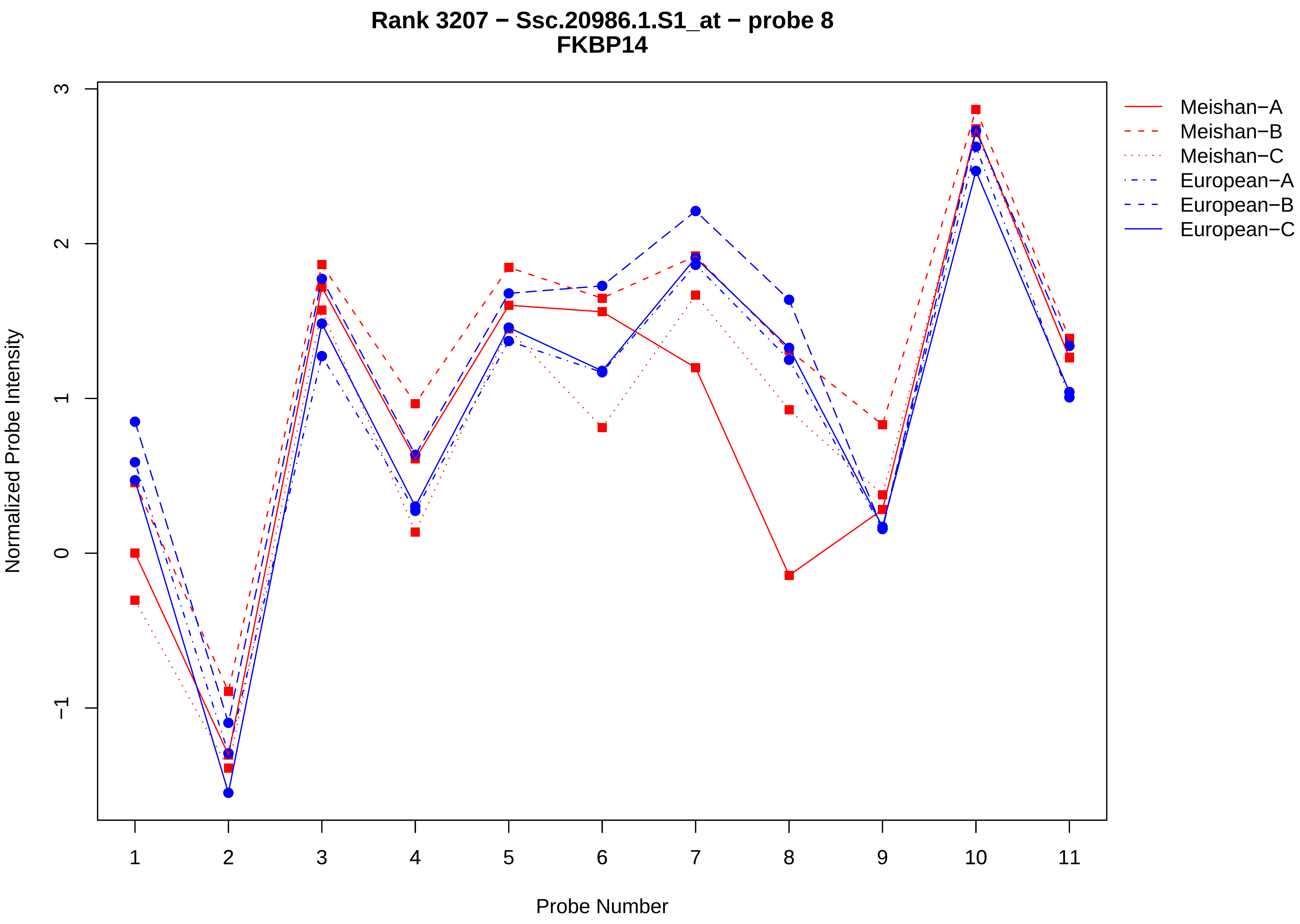
Rank 3207 − Ssc.20986.1.S1_at − probe 8
FKBP14
Normalized Probe Intensity
Probe Number
−1
0
1
2
3
1
2
3
4
5
6
7
8
9
10
11
Meishan−A
Meishan−B
Meishan−C
European−A
European−B
European−C

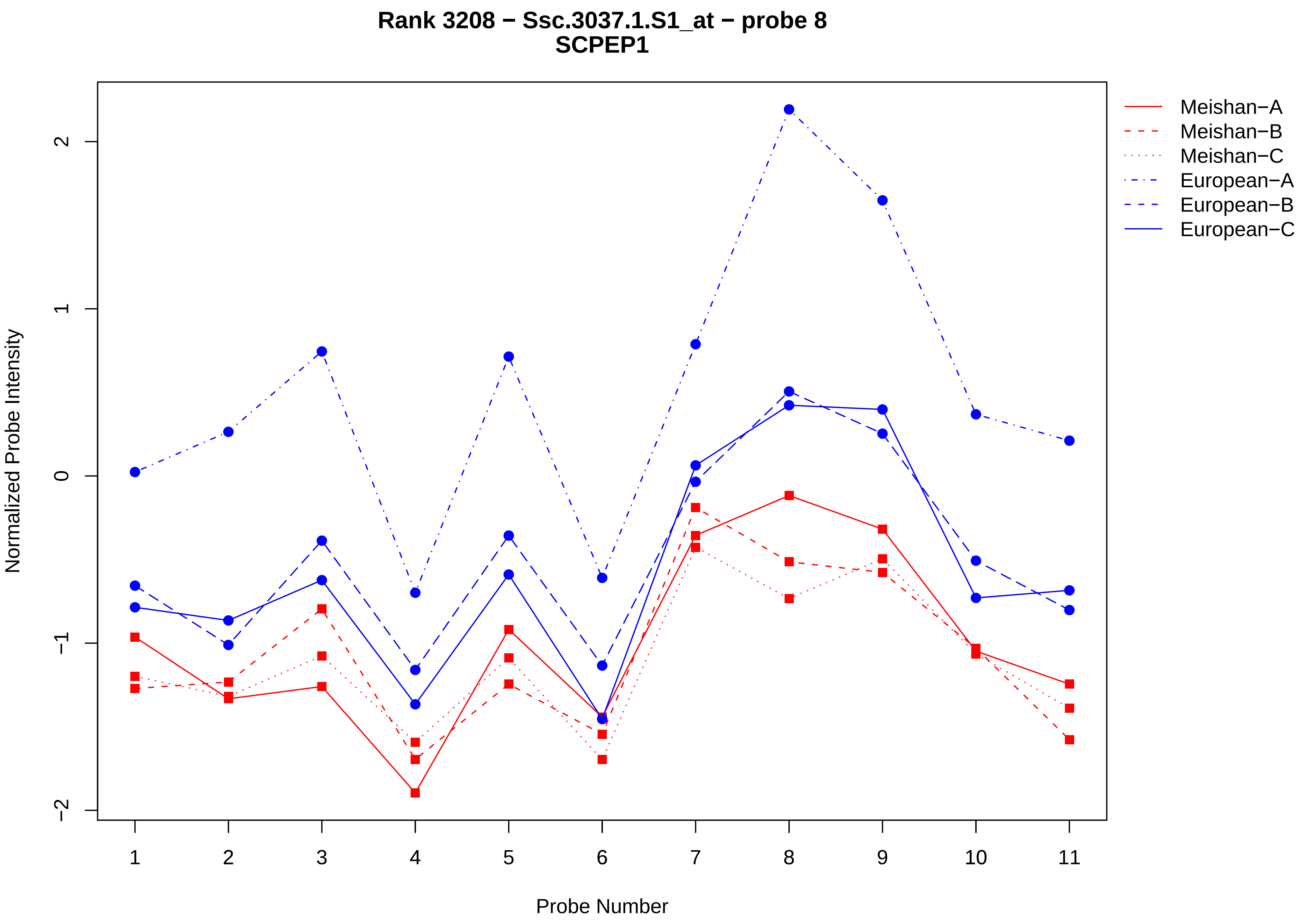

Rank 3208 − Ssc.3037.1.S1_at − probe 8
SCPEP1
Normalized Probe Intensity
Probe Number
Meishan−A
Meishan−B
Meishan−C
European−A
European−B
European−C
−2
−1
0
1
2
1
2
3
4
5
6
7
8
9
10
11

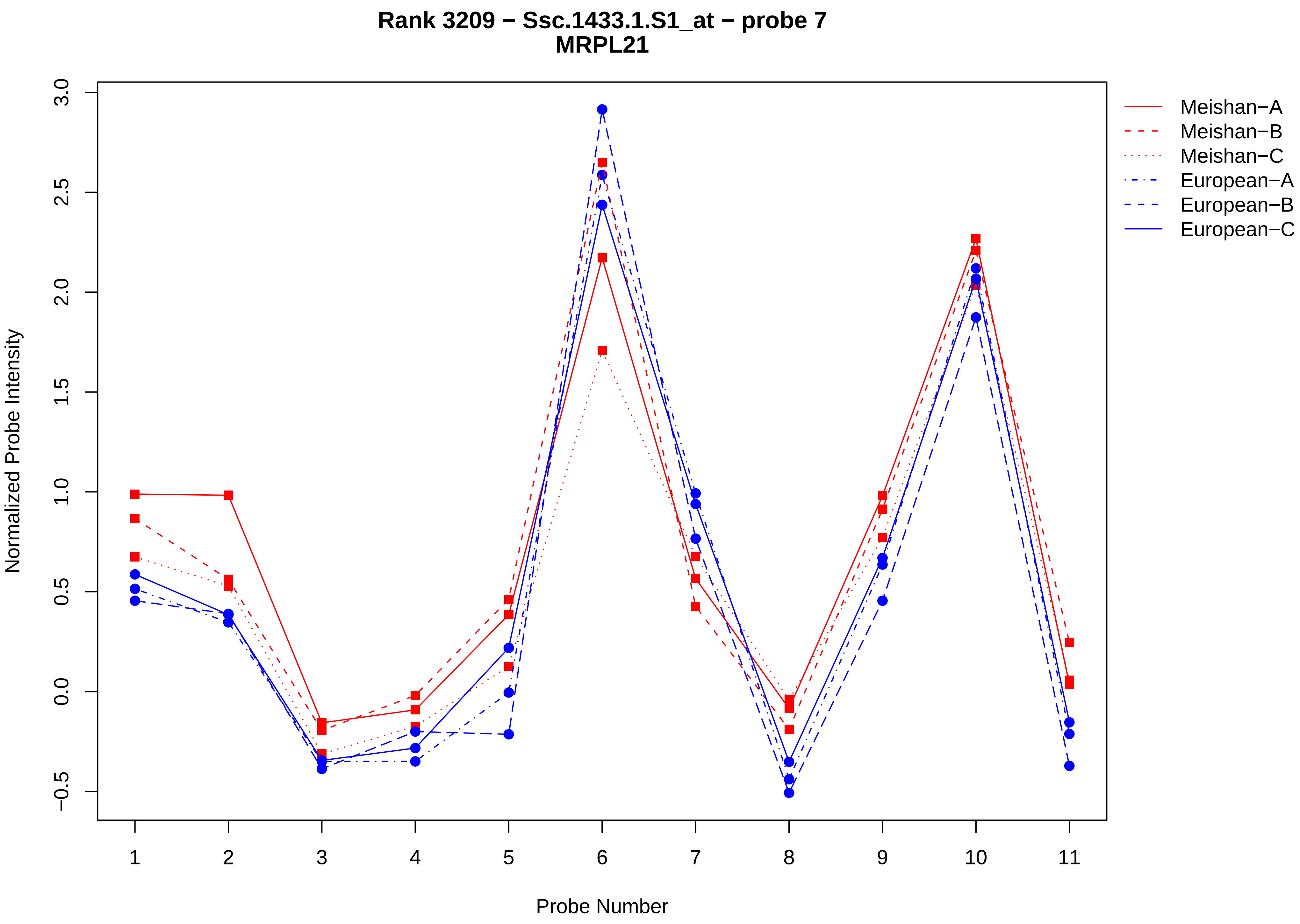

Rank 3209 − Ssc.1433.1.S1_at − probe 7
MRPL21
Normalized Probe Intensity
Probe Number
−0.5
0.0
0.5
1.0
1.5
2.0
2.5
3.0
1
2
3
4
5
6
7
8
9
10
11
Meishan−A
Meishan−B
Meishan−C
European−A
European−B
European−C

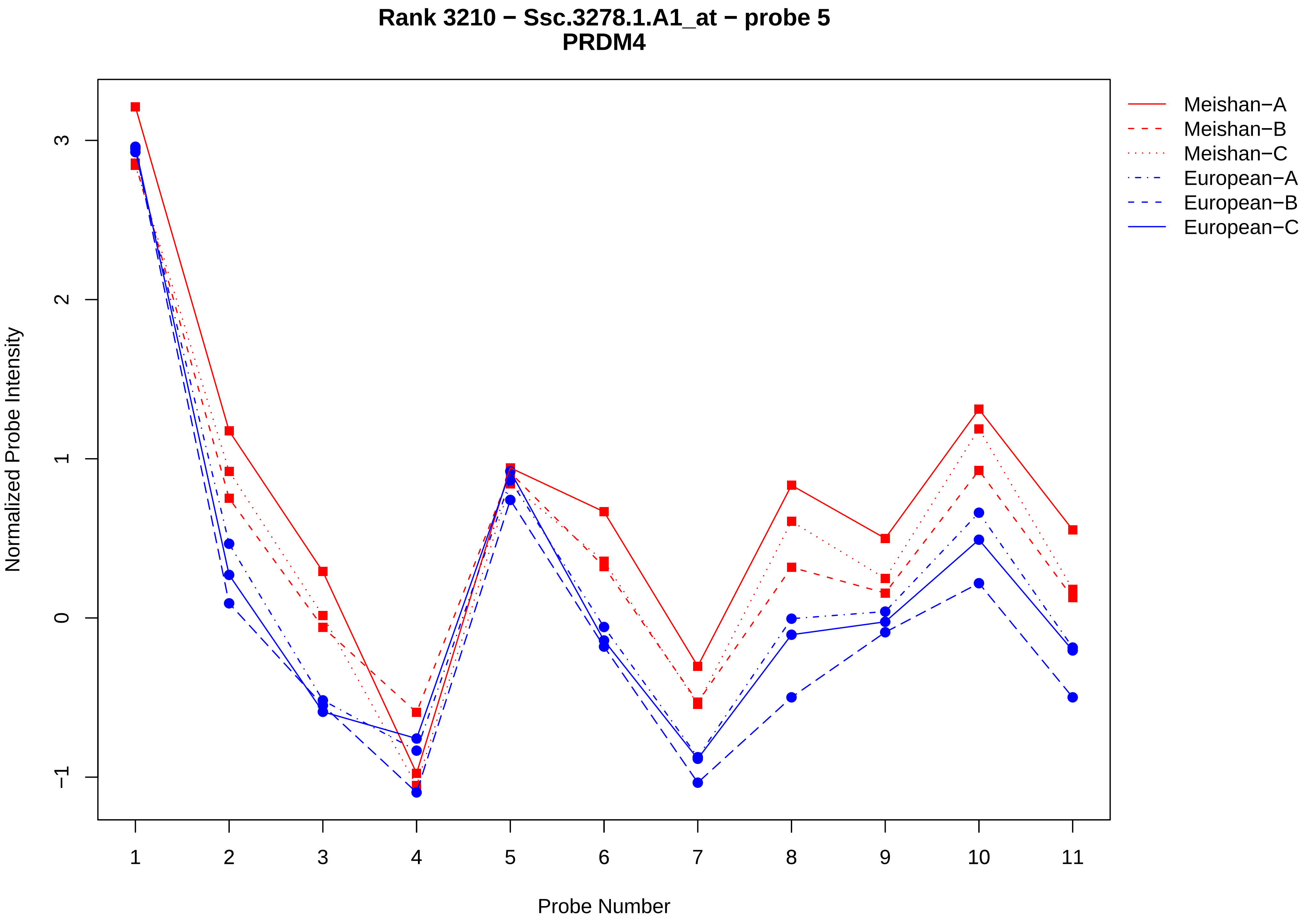

Rank 3210 − Ssc.3278.1.A1_at − probe 5
PRDM4
Normalized Probe Intensity
Probe Number
−1
0
1
2
3
1
2
3
4
5
6
7
8
9
10
11
Meishan−A
Meishan−B
Meishan−C
European−A
European−B
European−C

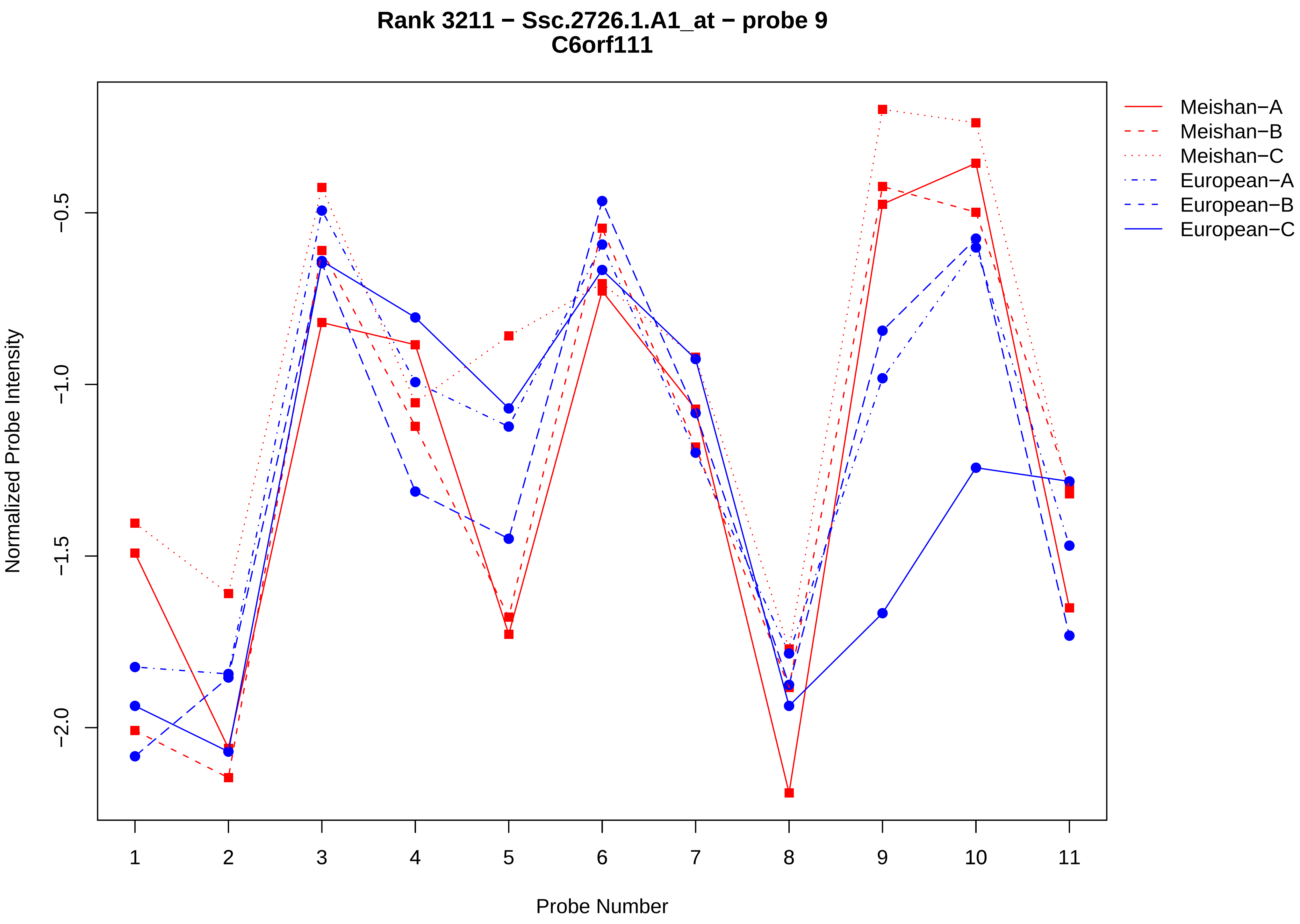

Rank 3211 − Ssc.2726.1.A1_at − probe 9
C6orf111
Normalized Probe Intensity
−0.5
−1.0
−1.5
−2.0
1
2
3
4
5
6
7
8
9
10
11
Probe Number
Meishan−A
Meishan−B
Meishan−C
European−A
European−B
European−C

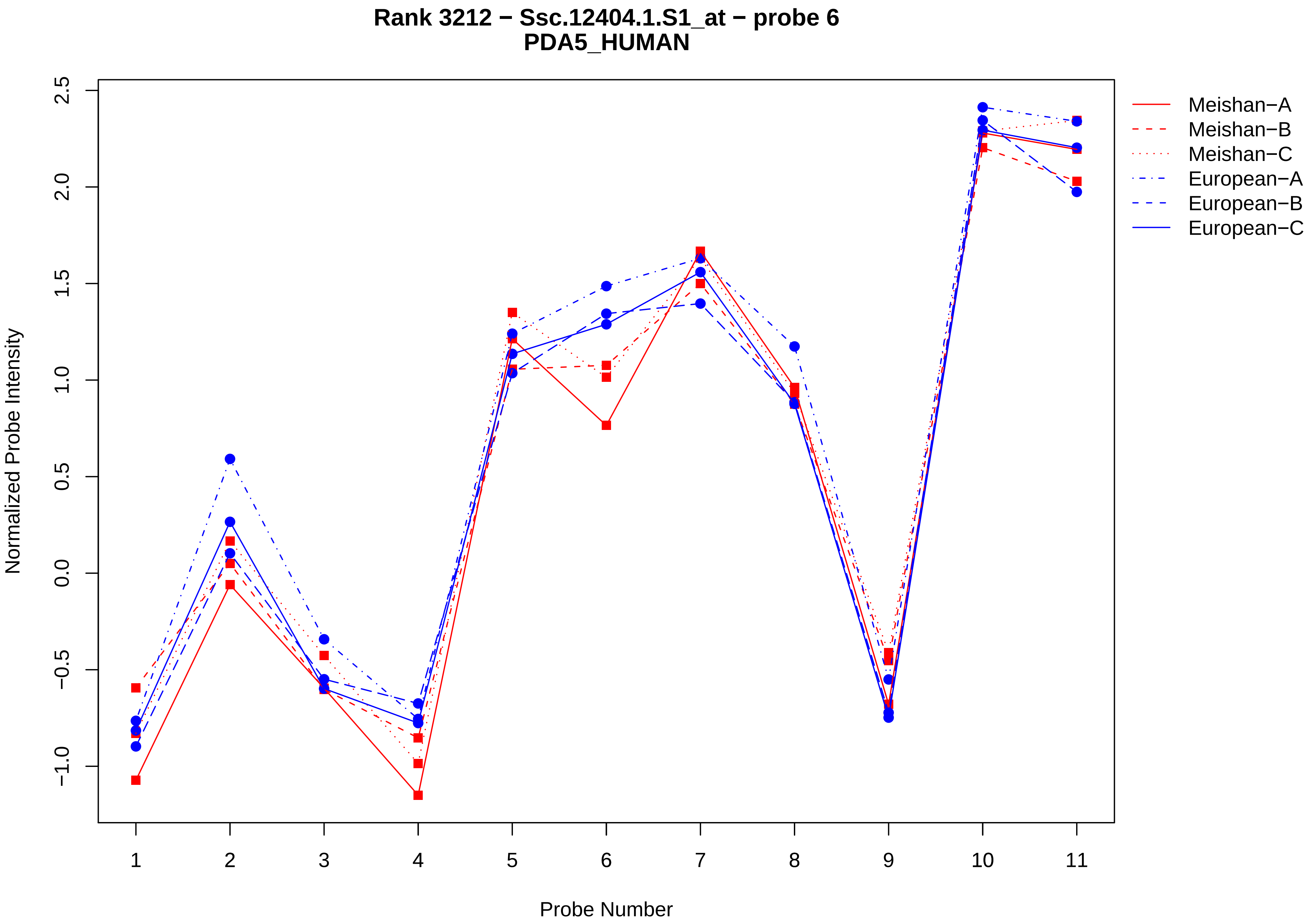

Rank 3212 − Ssc.12404.1.S1_at − probe 6
PDA5_HUMAN
Normalized Probe Intensity
Probe Number
Meishan−A
Meishan−B
Meishan−C
European−A
European−B
European−C

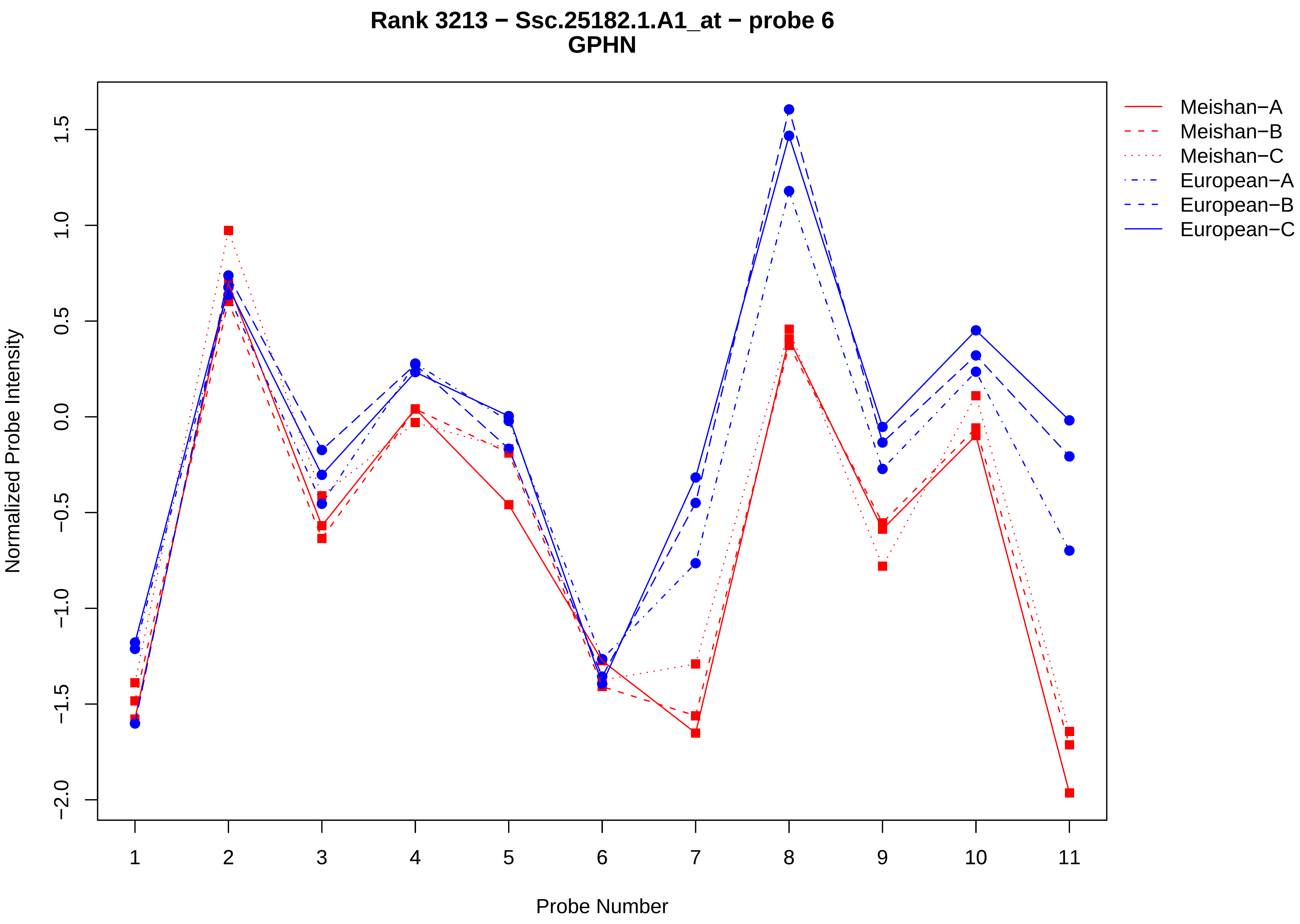

Rank 3213 − Ssc.25182.1.A1_at − probe 6
GPHN
Normalized Probe Intensity
Probe Number
Meishan−A
Meishan−B
Meishan−C
European−A
European−B
European−C

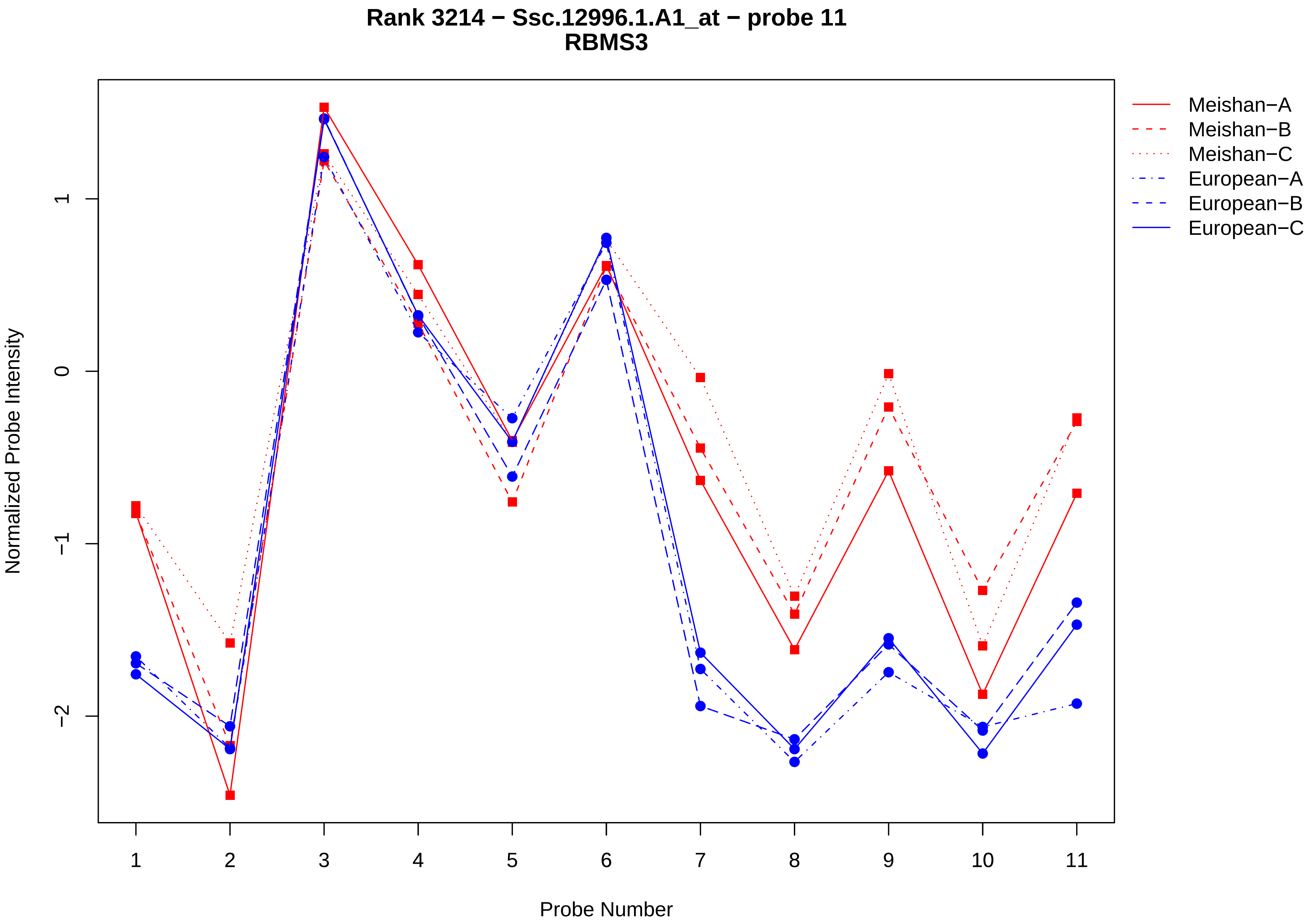

Rank 3214 − Ssc.12996.1.A1_at − probe 11
RBMS3
Normalized Probe Intensity
Probe Number
1
0
−1
−2
1
2
3
4
5
6
7
8
9
10
11
Meishan−A
Meishan−B
Meishan−C
European−A
European−B
European−C

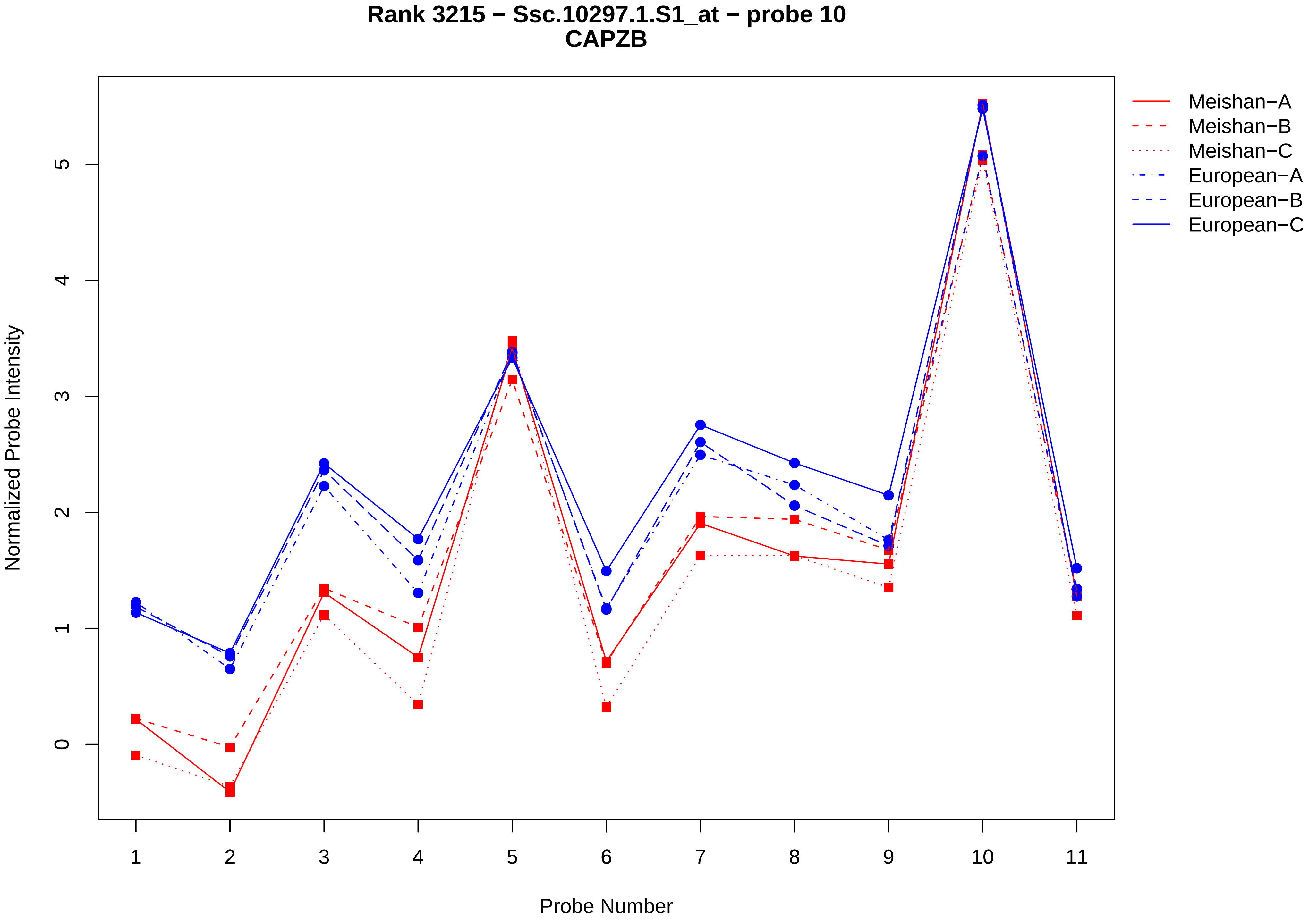
Rank 3215 − Ssc.10297.1.S1_at − probe 10
CAPZB
Normalized Probe Intensity
Probe Number
0
1
2
3
4
5
1
2
3
4
5
6
7
8
9
10
11
Meishan−A
Meishan−B
Meishan−C
European−A
European−B
European−C

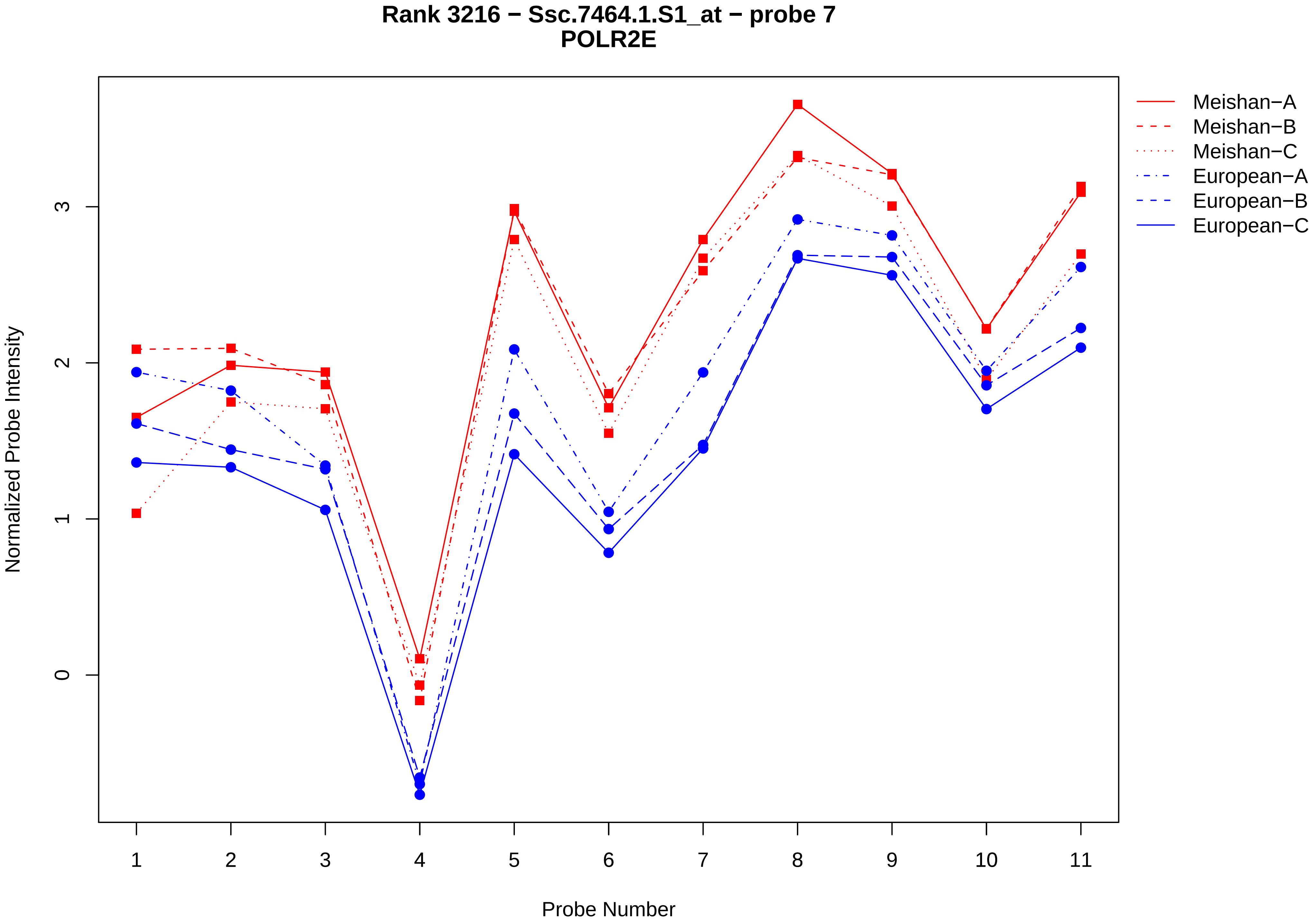

Rank 3216 − Ssc.7464.1.S1_at − probe 7
POLR2E
Normalized Probe Intensity
Probe Number
0
1
2
3
1
2
3
4
5
6
7
8
9
10
11
Meishan−A
Meishan−B
Meishan−C
European−A
European−B
European−C

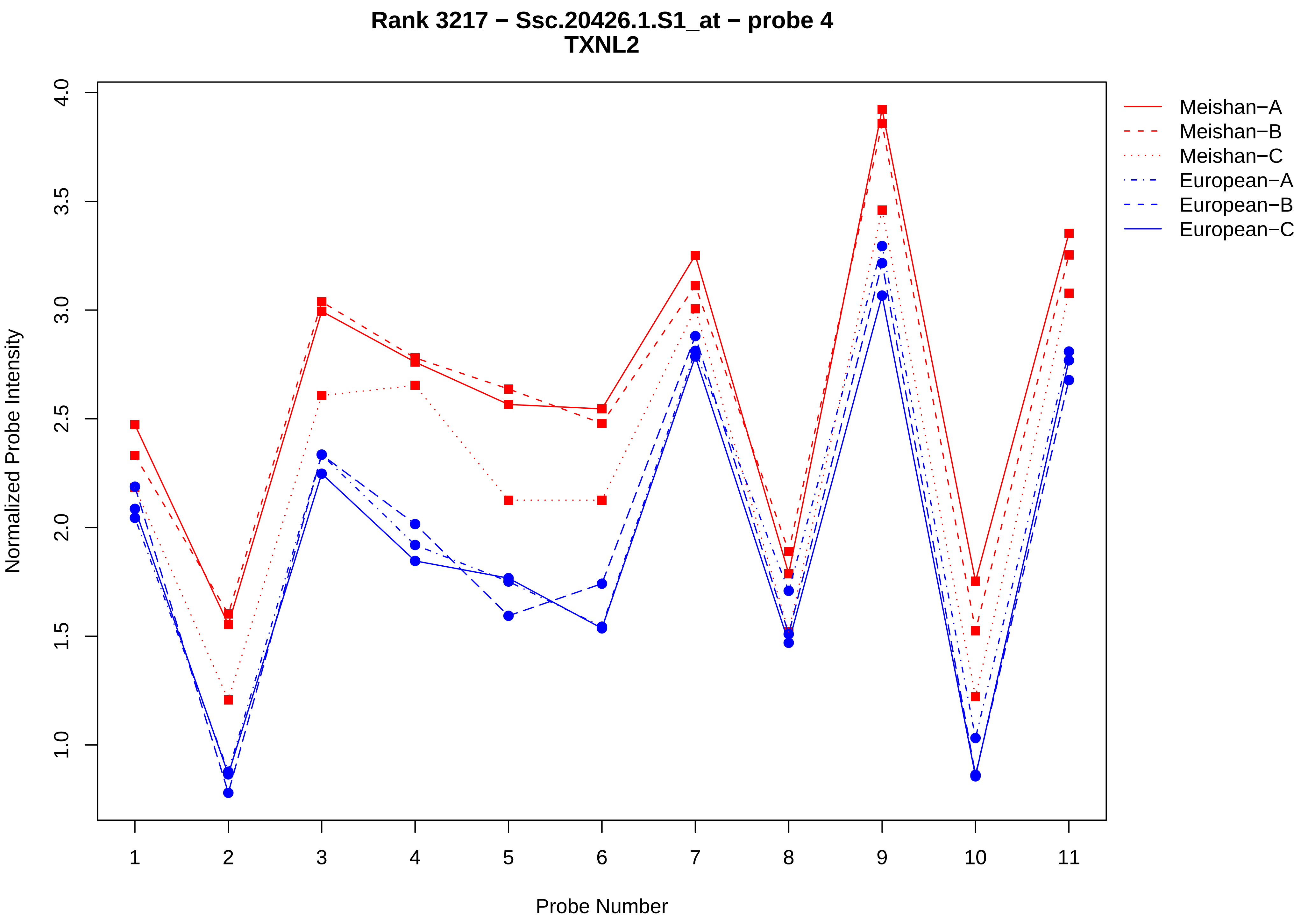

Rank 3217 − Ssc.20426.1.S1_at − probe 4
TXNL2
Normalized Probe Intensity
1.0
1.5
2.0
2.5
3.0
3.5
4.0
1
2
3
4
5
6
7
8
9
10
11
Probe Number
Meishan−A
Meishan−B
Meishan−C
European−A
European−B
European−C

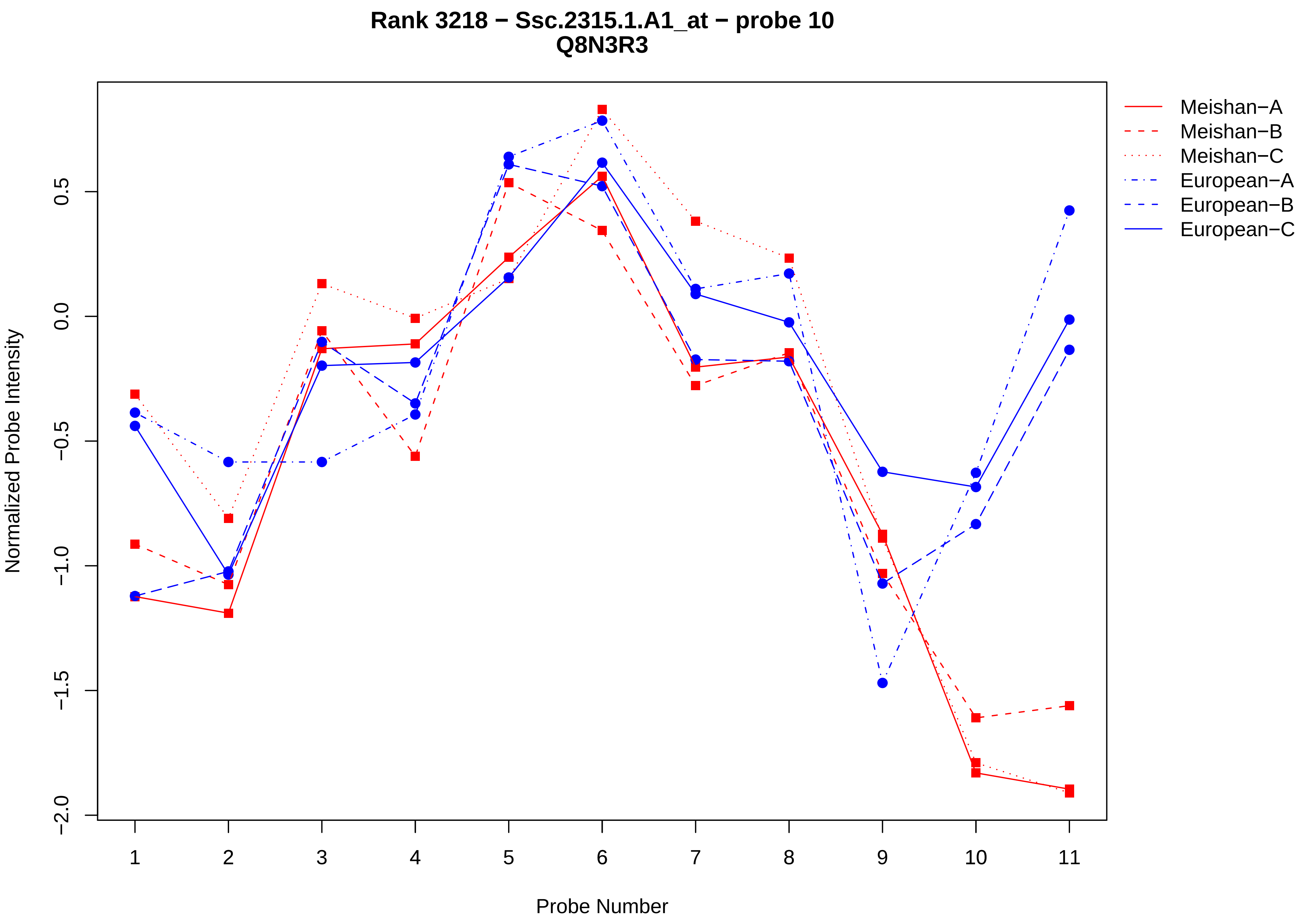

Rank 3218 − Ssc.2315.1.A1_at − probe 10
Q8N3R3
Normalized Probe Intensity
Probe Number
−2.0
−1.5
−1.0
−0.5
0.0
0.5
1
2
3
4
5
6
7
8
9
10
11
Meishan−A
Meishan−B
Meishan−C
European−A
European−B
European−C

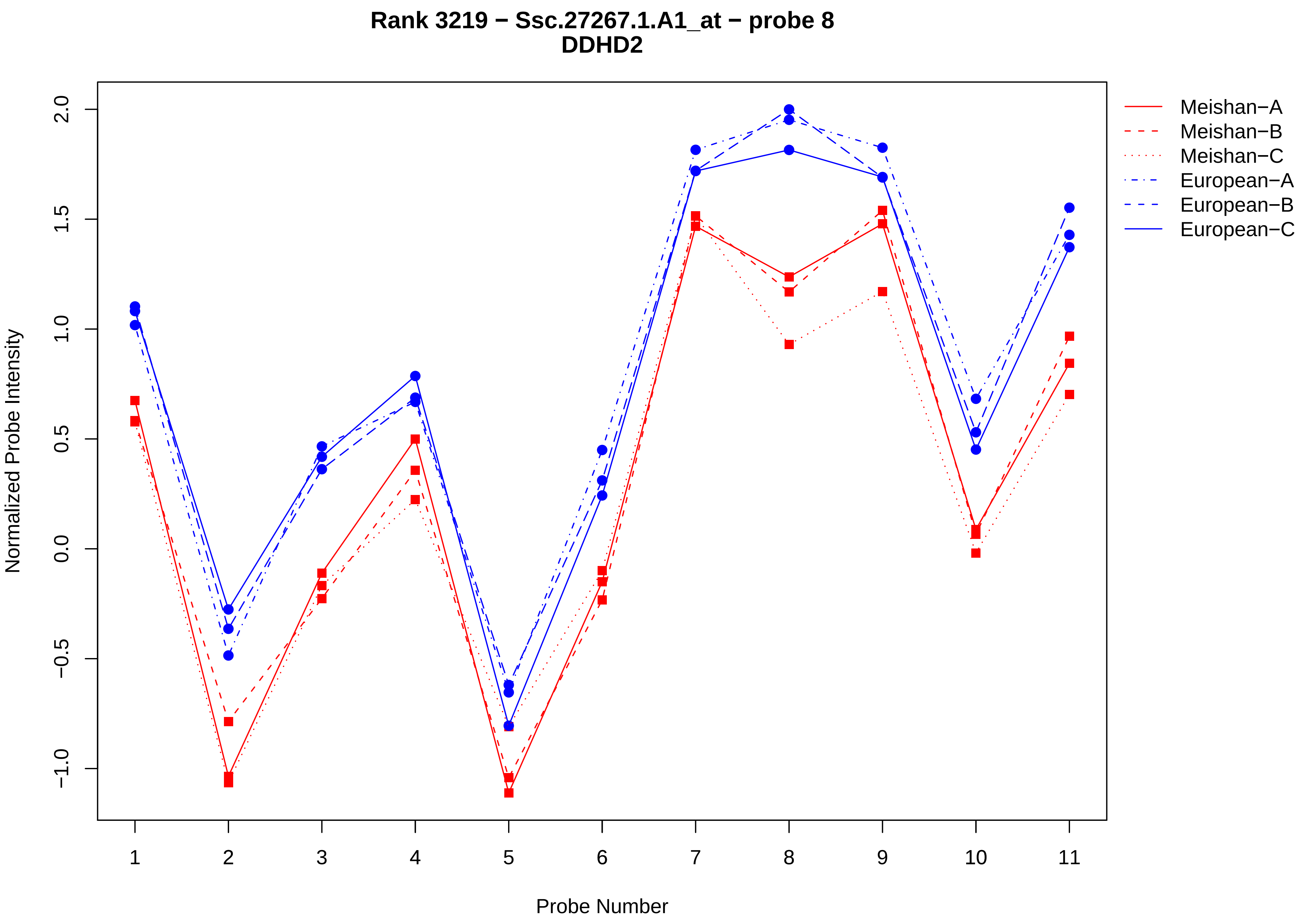

Rank 3219 − Ssc.27267.1.A1_at − probe 8
DDHD2
Normalized Probe Intensity
Probe Number
−1.0
−0.5
0.0
0.5
1.0
1.5
2.0
1
2
3
4
5
6
7
8
9
10
11
Meishan−A
Meishan−B
Meishan−C
European−A
European−B
European−C

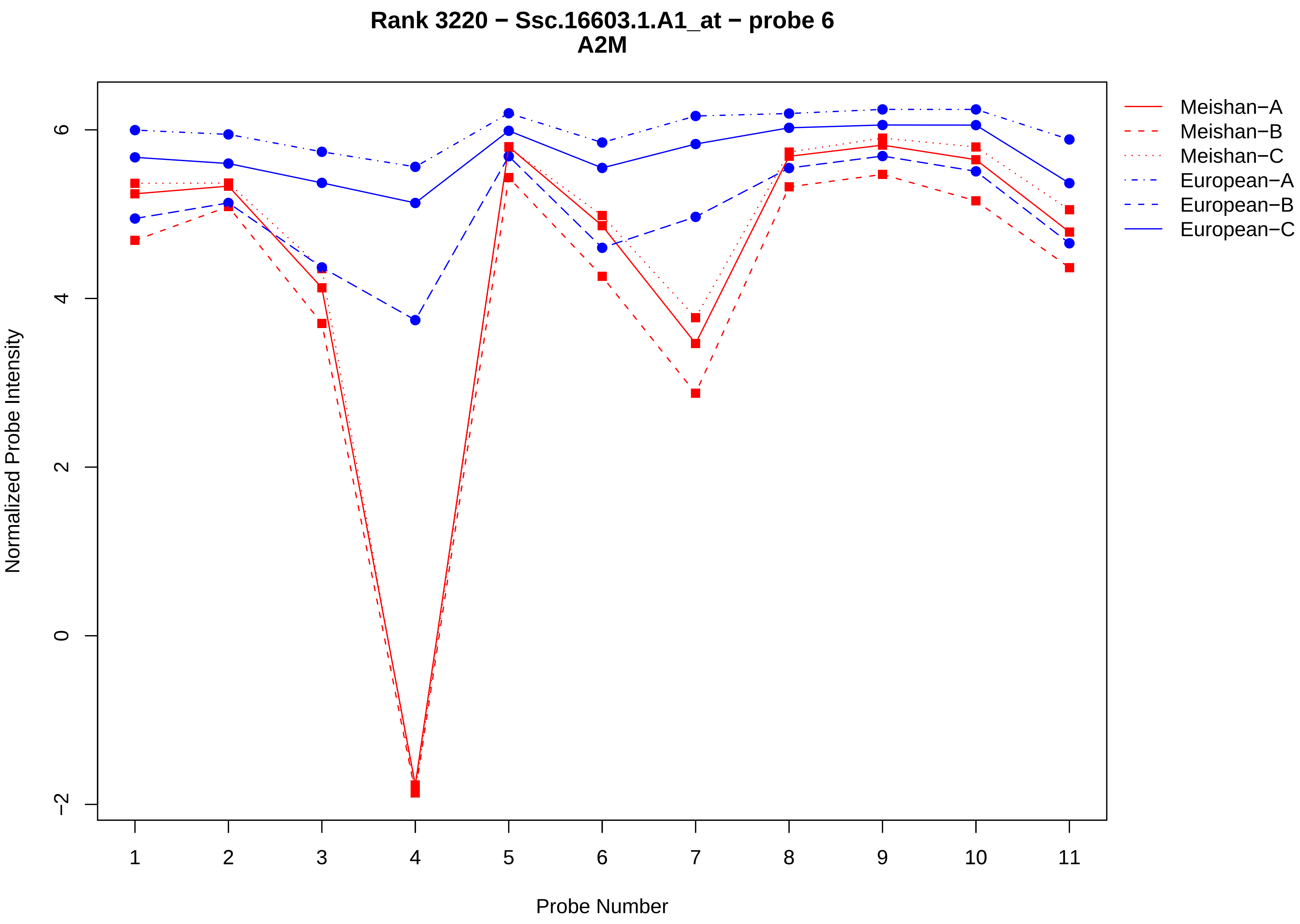

Rank 3220 − Ssc.16603.1.A1_at − probe 6
A2M
Normalized Probe Intensity
Probe Number
−2
0
2
4
6
1
2
3
4
5
6
7
8
9
10
11
Meishan−A
Meishan−B
Meishan−C
European−A
European−B
European−C

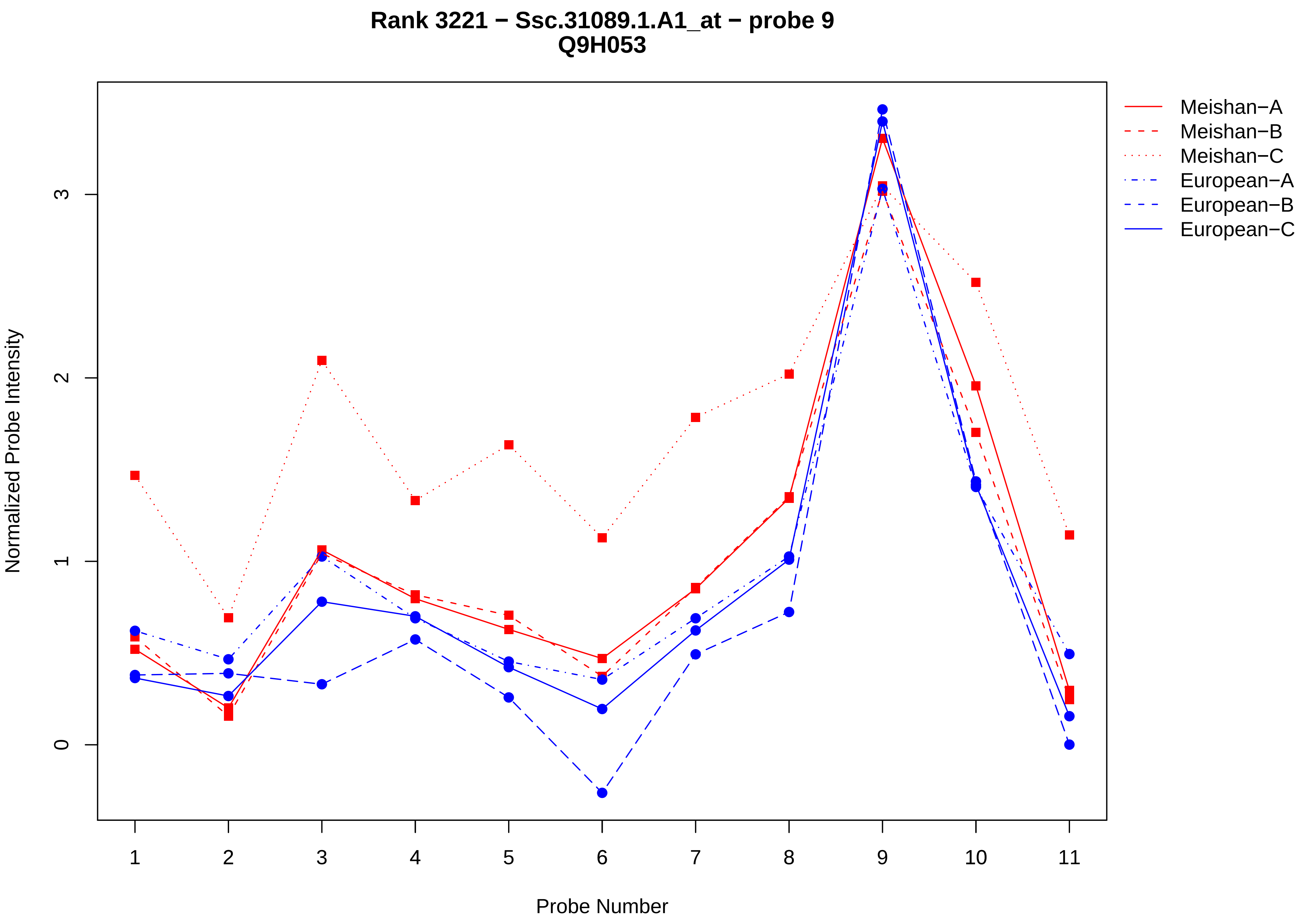

Rank 3221 − Ssc.31089.1.A1_at − probe 9
Q9H053
Normalized Probe Intensity
Probe Number
0
1
2
3
1
2
3
4
5
6
7
8
9
10
11
Meishan−A
Meishan−B
Meishan−C
European−A
European−B
European−C

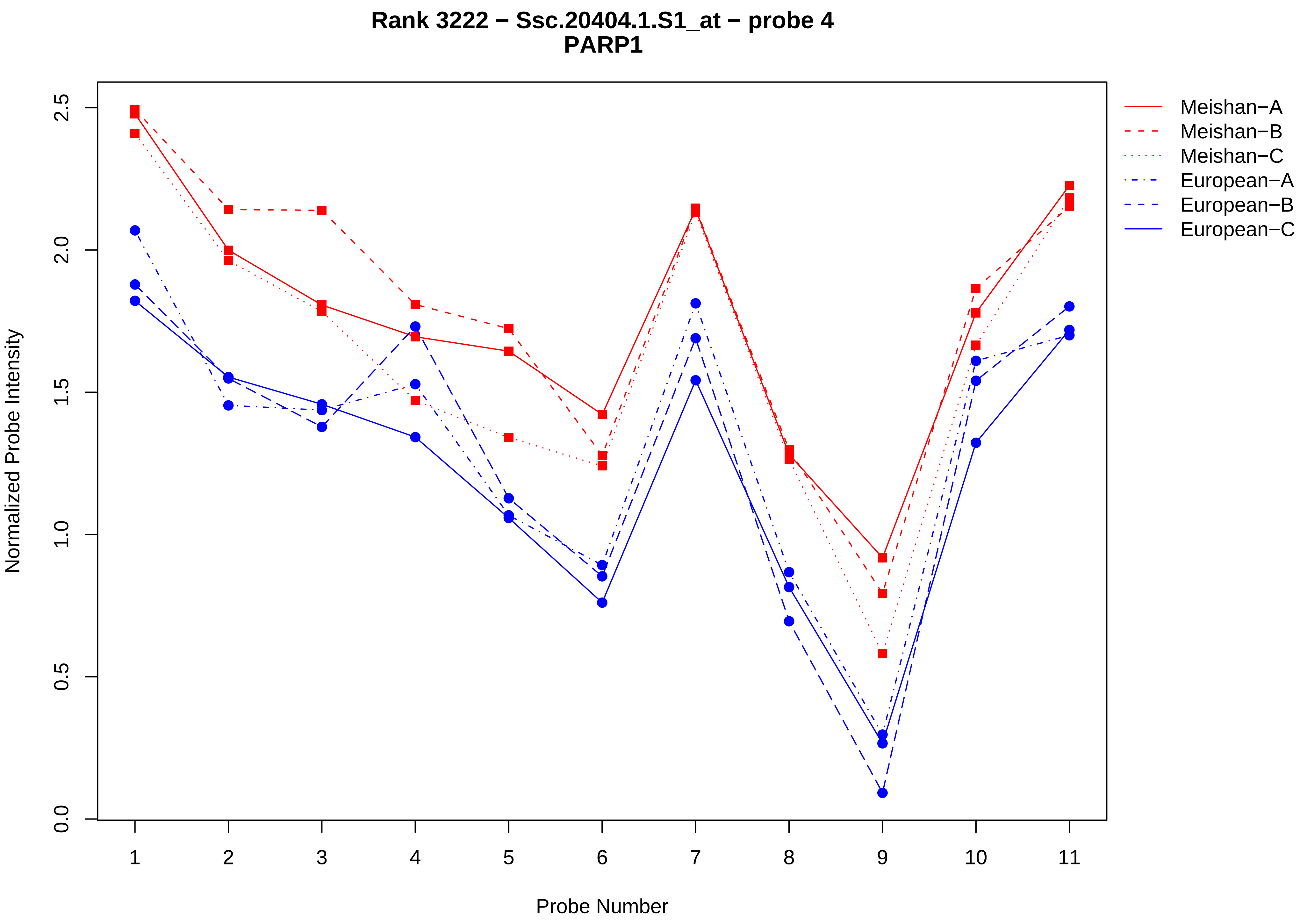

Rank 3222 − Ssc.20404.1.S1_at − probe 4
PARP1
Normalized Probe Intensity
0.0
0.5
1.0
1.5
2.0
2.5
Probe Number
1
2
3
4
5
6
7
8
9
10
11
Meishan−A
Meishan−B
Meishan−C
European−A
European−B
European−C

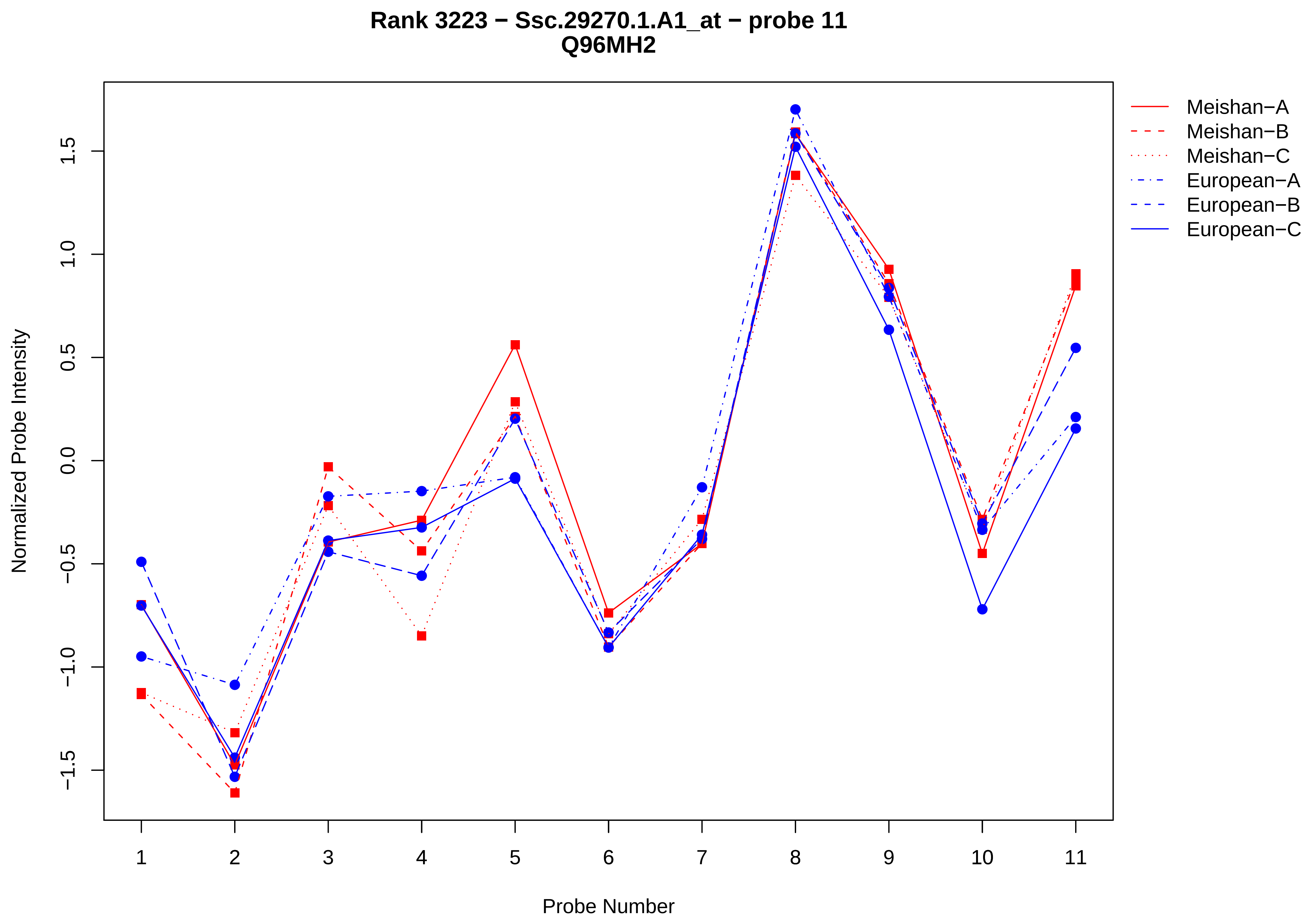
Rank 3223 − Ssc.29270.1.A1_at − probe 11
Q96MH2
Normalized Probe Intensity
Probe Number
1.5
1.0
0.5
0.0
−0.5
−1.0
−1.5
1
2
3
4
5
6
7
8
9
10
11
Meishan−A
Meishan−B
Meishan−C
European−A
European−B
European−C

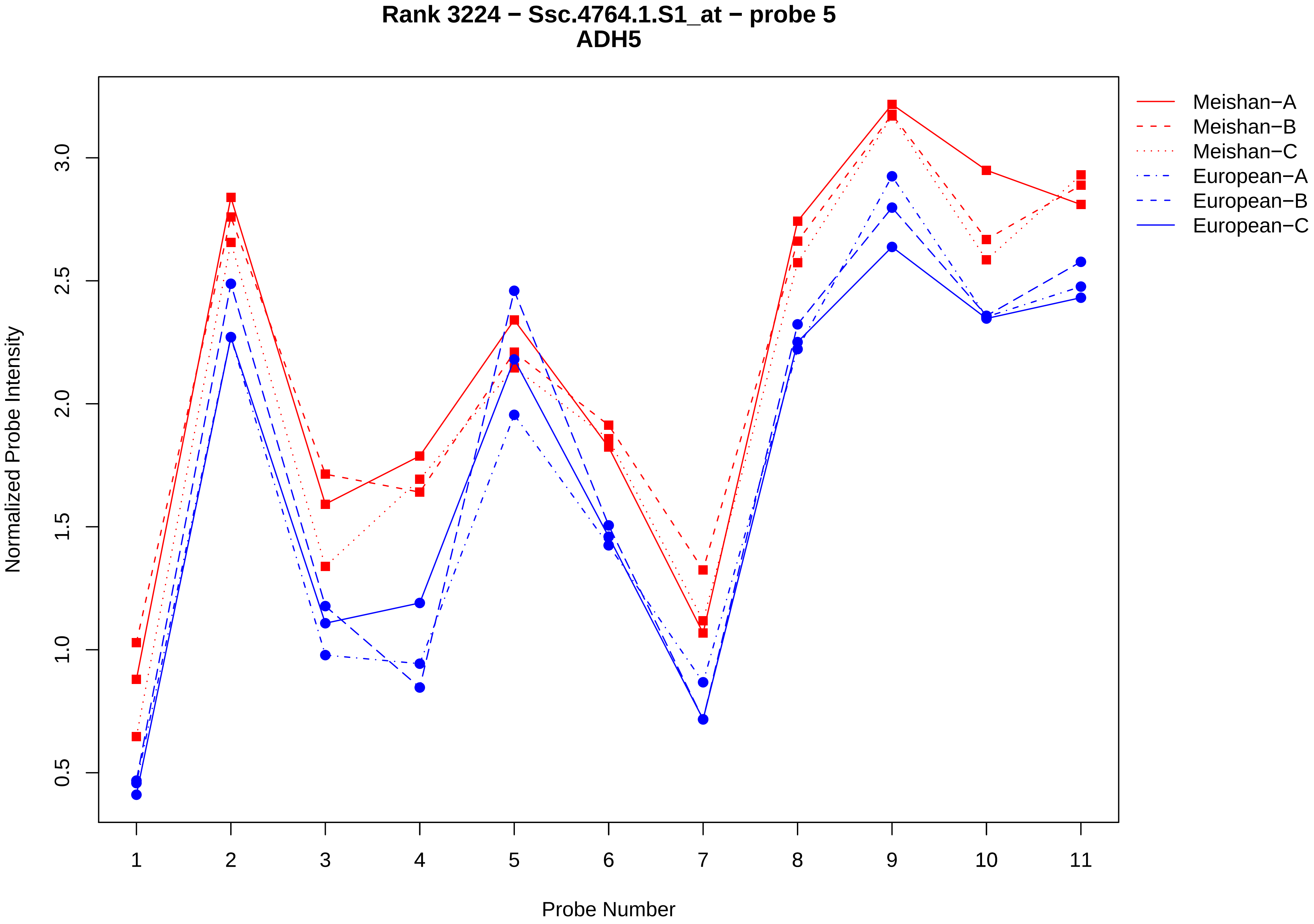

Rank 3224 − Ssc.4764.1.S1_at − probe 5
ADH5
Normalized Probe Intensity
Probe Number
0.5
1.0
1.5
2.0
2.5
3.0
1
2
3
4
5
6
7
8
9
10
11
Meishan−A
Meishan−B
Meishan−C
European−A
European−B
European−C

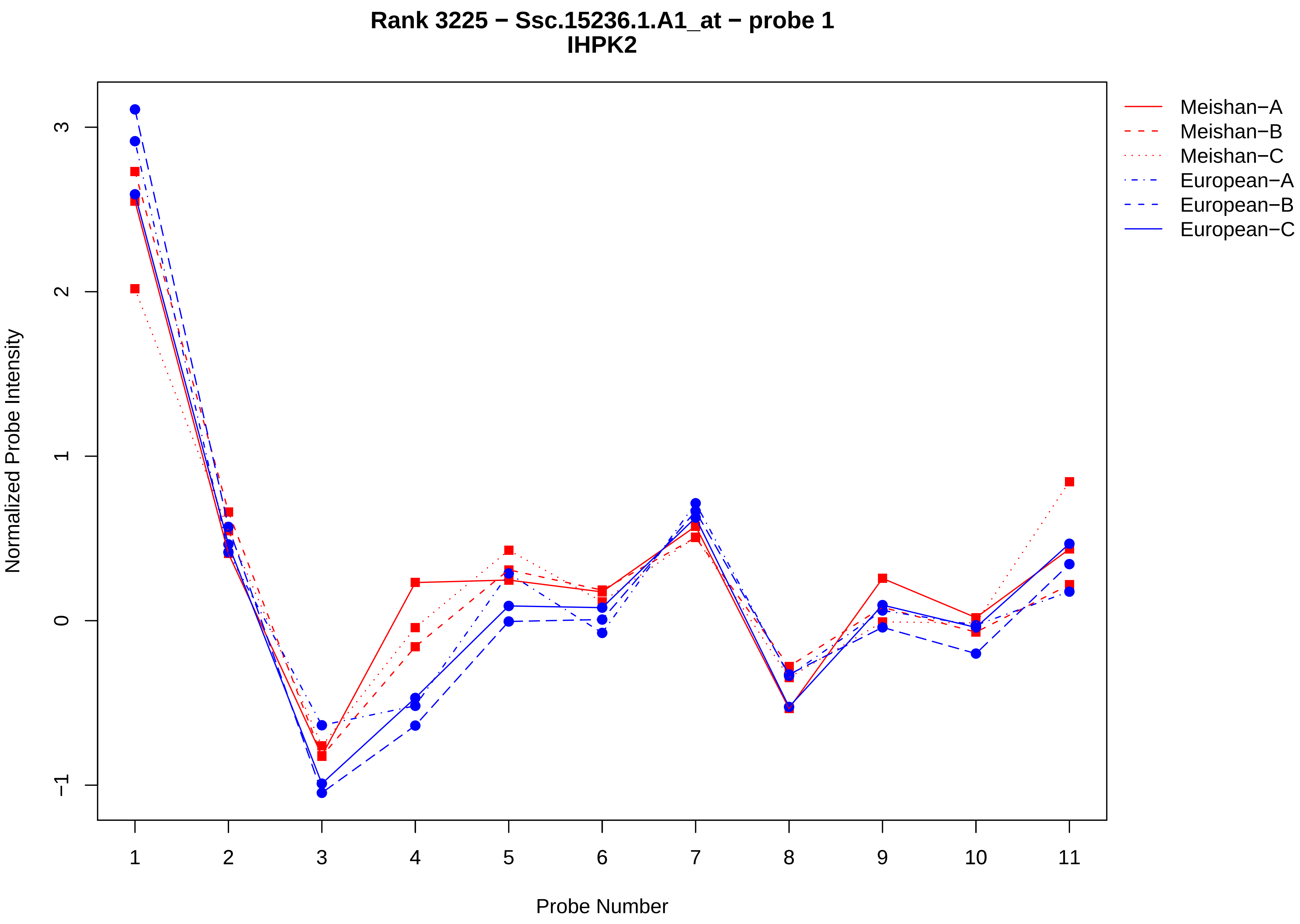

Rank 3225 − Ssc.15236.1.A1_at − probe 1
IHPK2
Normalized Probe Intensity
Probe Number
−1
0
1
2
3
1
2
3
4
5
6
7
8
9
10
11
Meishan−A
Meishan−B
Meishan−C
European−A
European−B
European−C

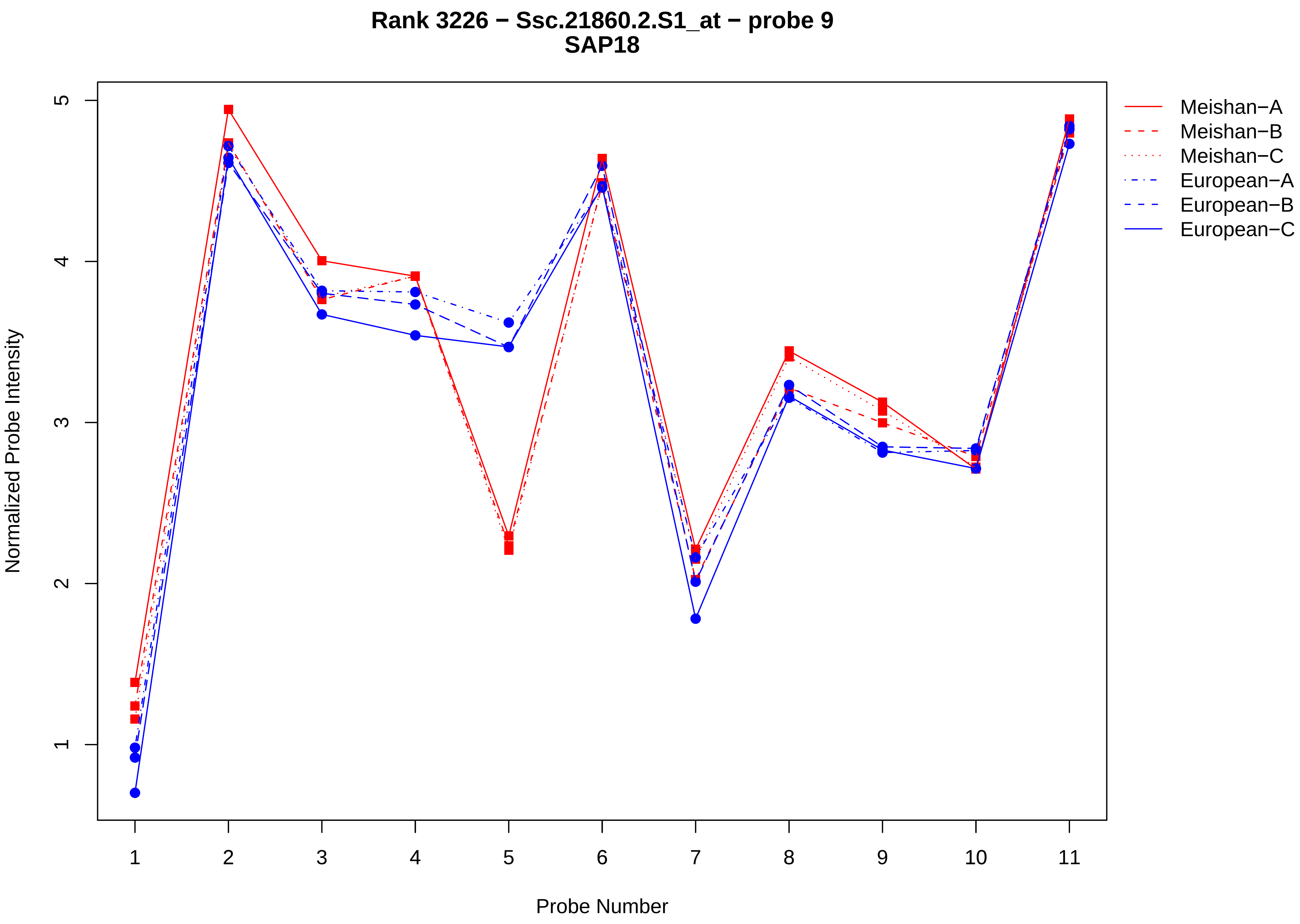
Rank 3226 − Ssc.21860.2.S1_at − probe 9
SAP18
Normalized Probe Intensity
Probe Number
1
2
3
4
5
1
2
3
4
5
6
7
8
9
10
11
Meishan−A
Meishan−B
Meishan−C
European−A
European−B
European−C

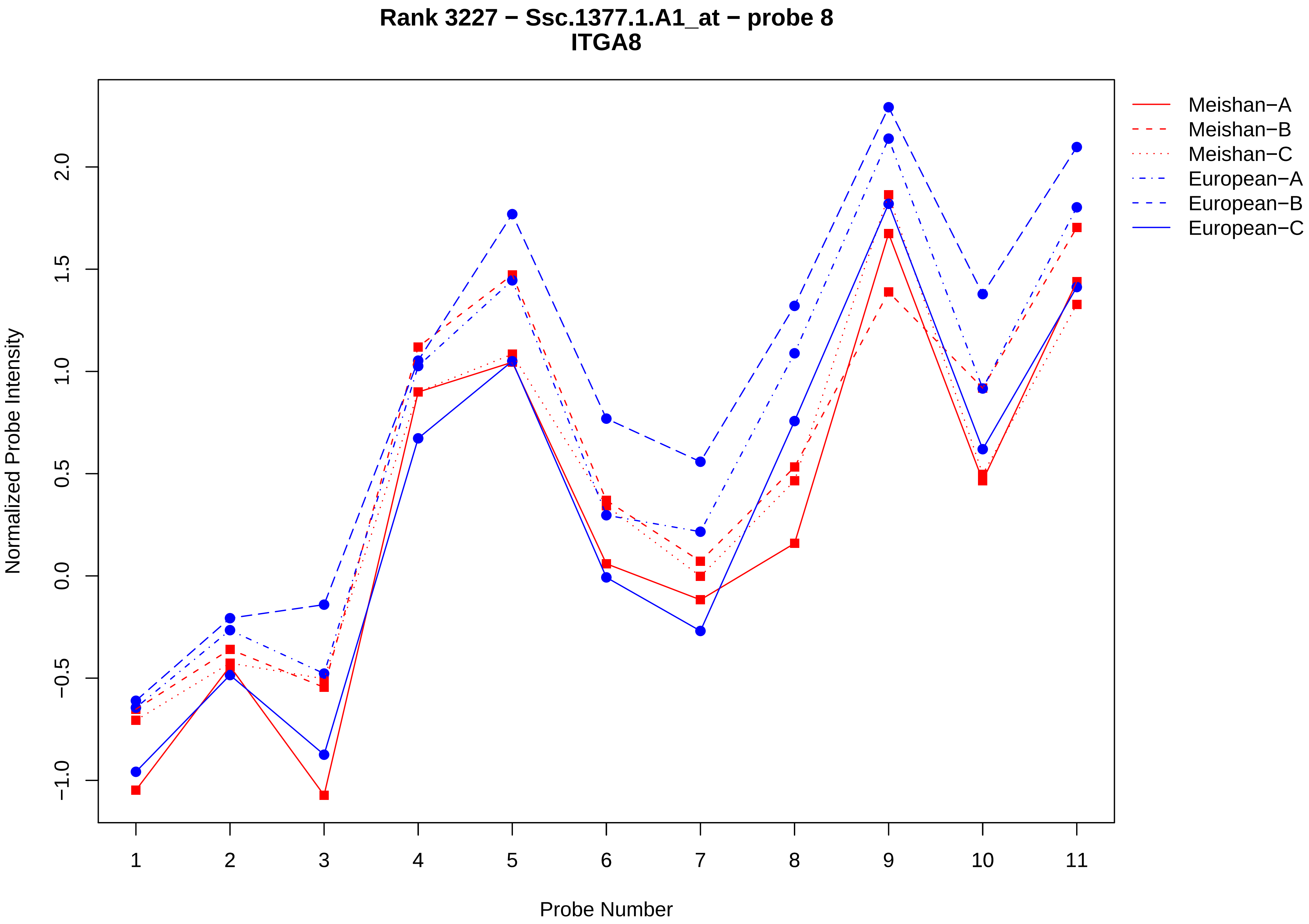

Rank 3227 − Ssc.1377.1.A1_at − probe 8
ITGA8
Normalized Probe Intensity
Probe Number
−1.0
−0.5
0.0
0.5
1.0
1.5
2.0
1
2
3
4
5
6
7
8
9
10
11
Meishan−A
Meishan−B
Meishan−C
European−A
European−B
European−C

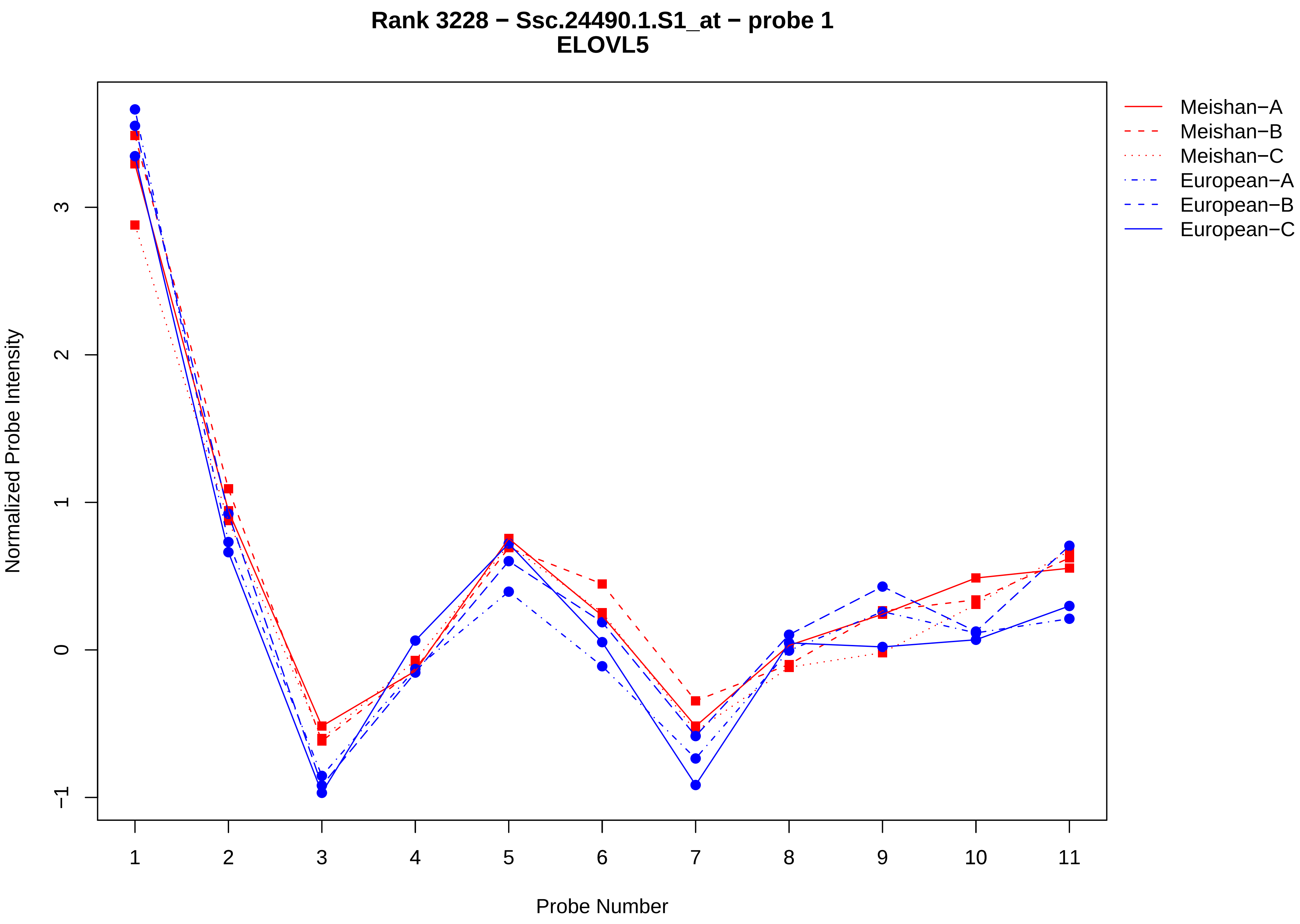

Rank 3228 − Ssc.24490.1.S1_at − probe 1
ELOVL5
Normalized Probe Intensity
Probe Number
−1
0
1
2
3
1
2
3
4
5
6
7
8
9
10
11
Meishan−A
Meishan−B
Meishan−C
European−A
European−B
European−C

Rank 3229 − Ssc.9373.1.A1_at − probe 6
Q9UPZ6

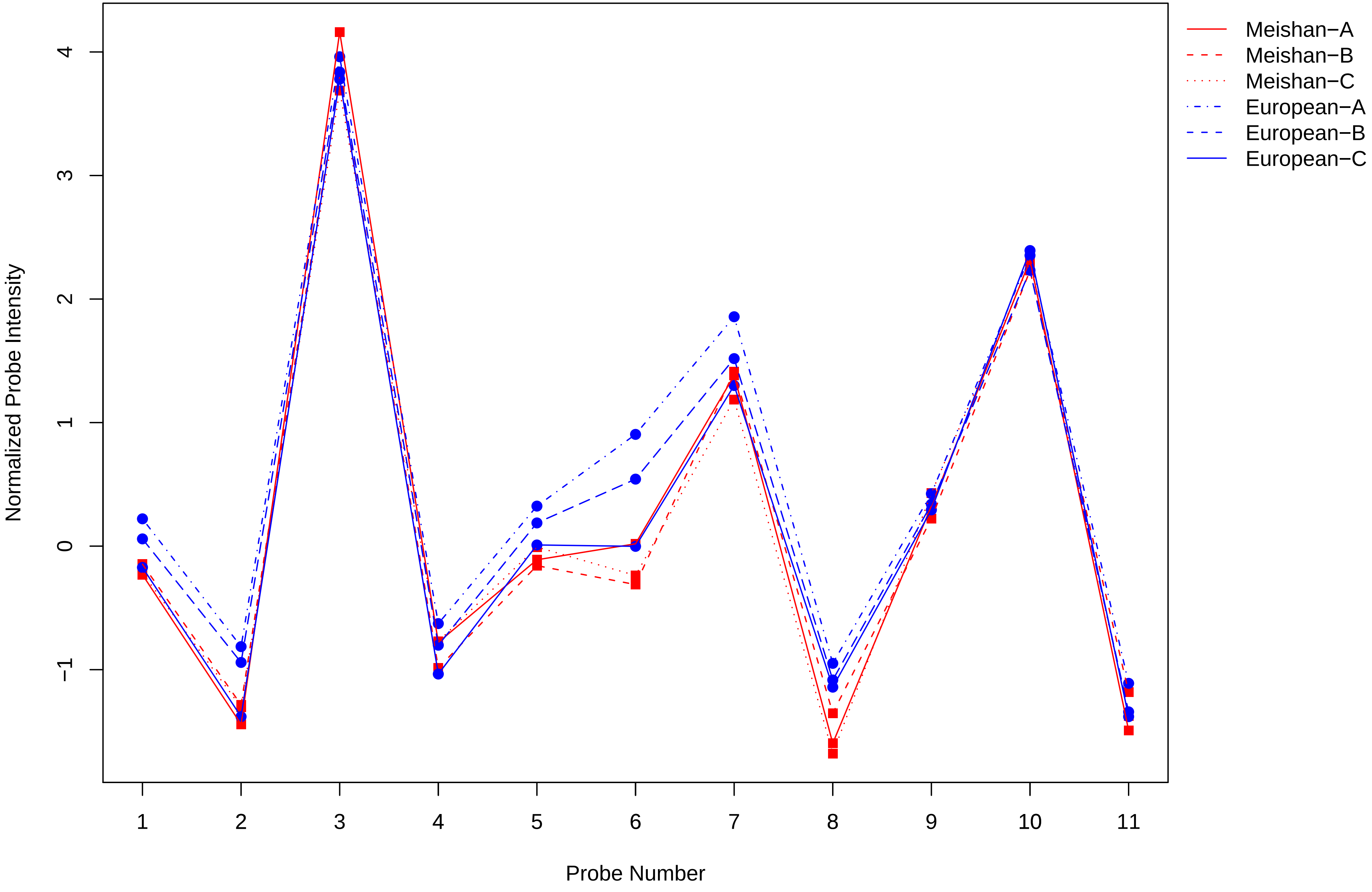

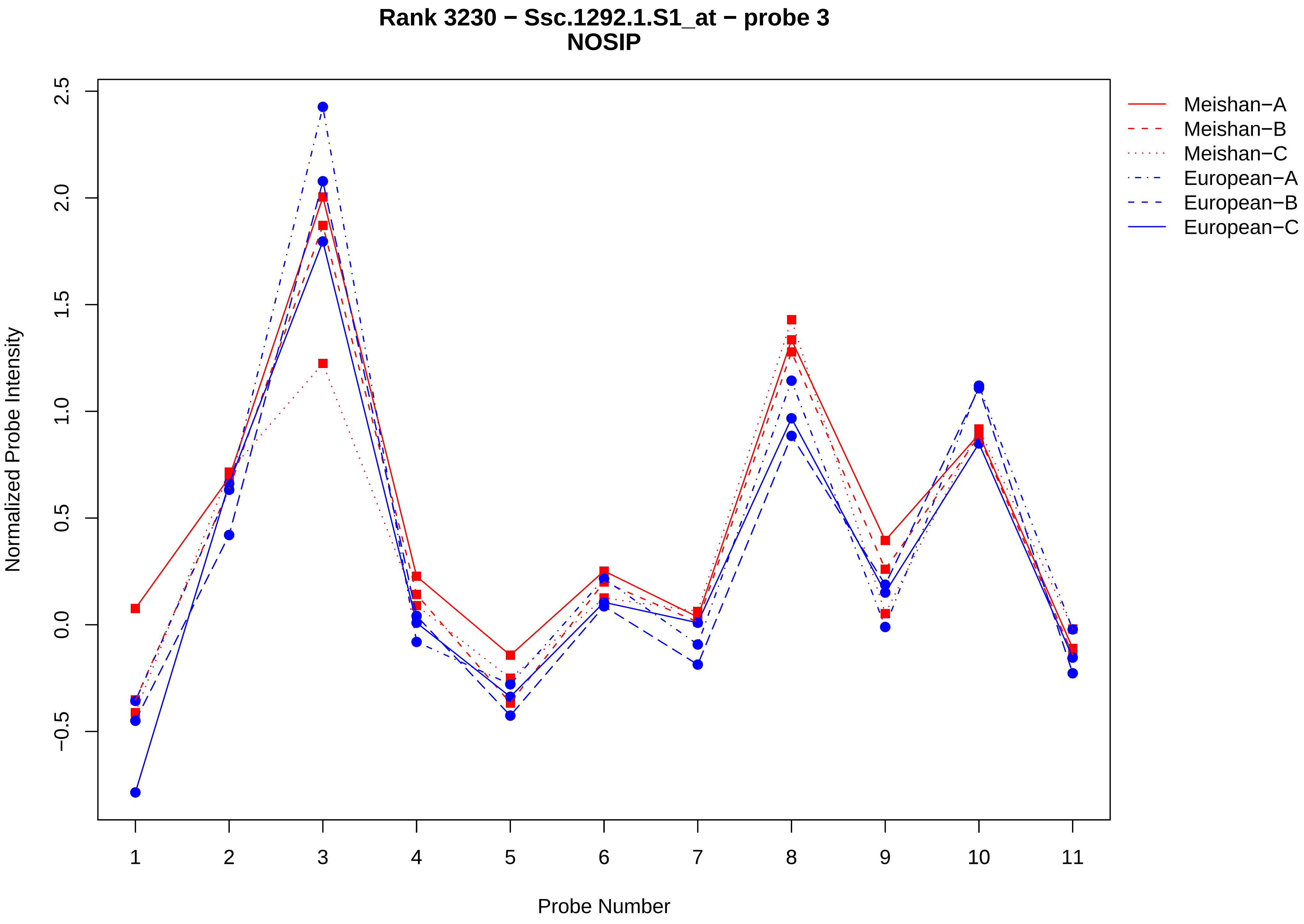

Rank 3230 − Ssc.1292.1.S1_at − probe 3
NOSIP
Normalized Probe Intensity
Probe Number
−0.5
0.0
0.5
1.0
1.5
2.0
2.5
1
2
3
4
5
6
7
8
9
10
11
Meishan−A
Meishan−B
Meishan−C
European−A
European−B
European−C

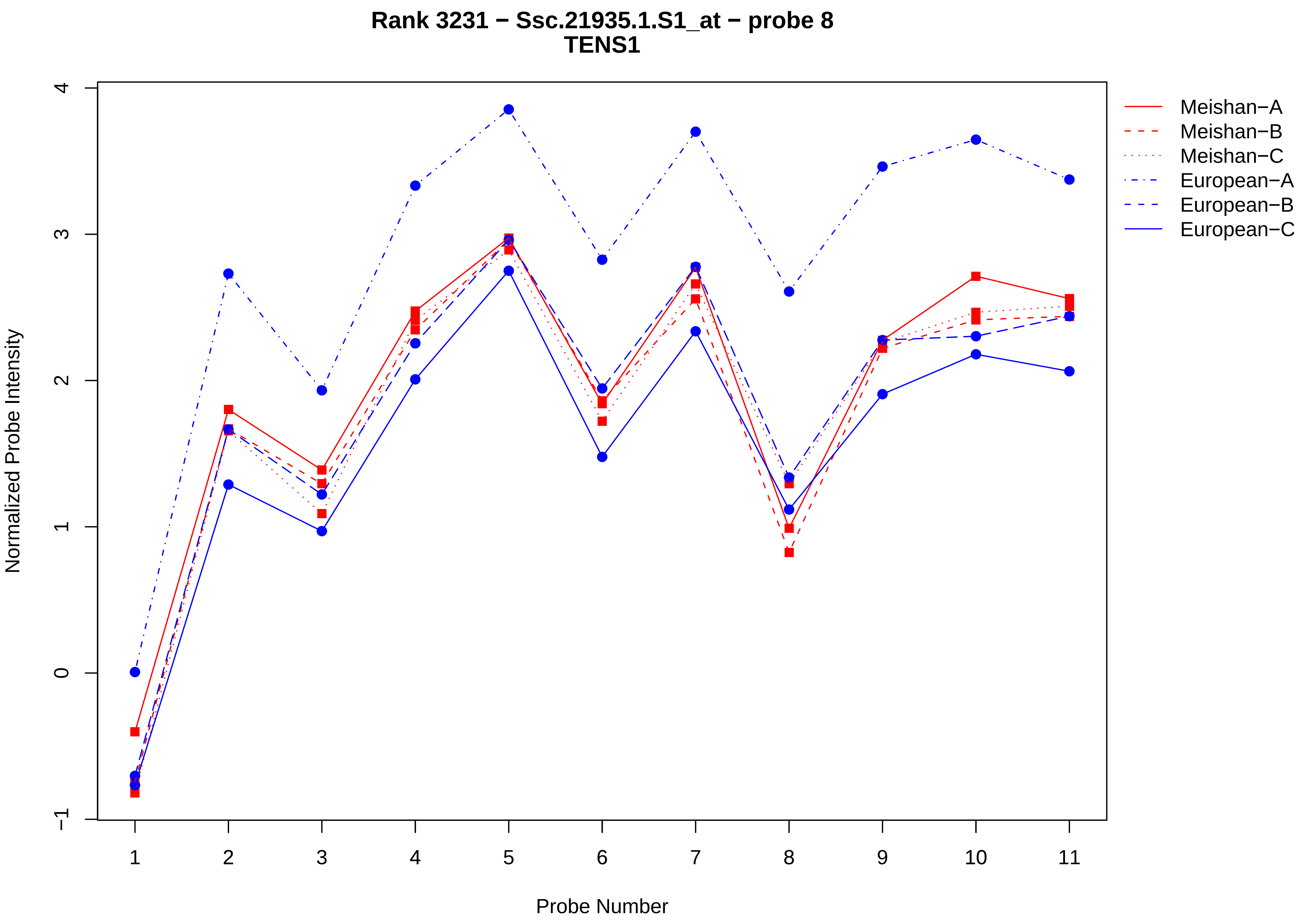

Rank 3231 − Ssc.21935.1.S1_at − probe 8
TENS1
Normalized Probe Intensity
Probe Number
−1
0
1
2
3
4
1
2
3
4
5
6
7
8
9
10
11
Meishan−A
Meishan−B
Meishan−C
European−A
European−B
European−C

Rank 3232 − Ssc.27189.1.A1_at − probe 11
Q5VSS2

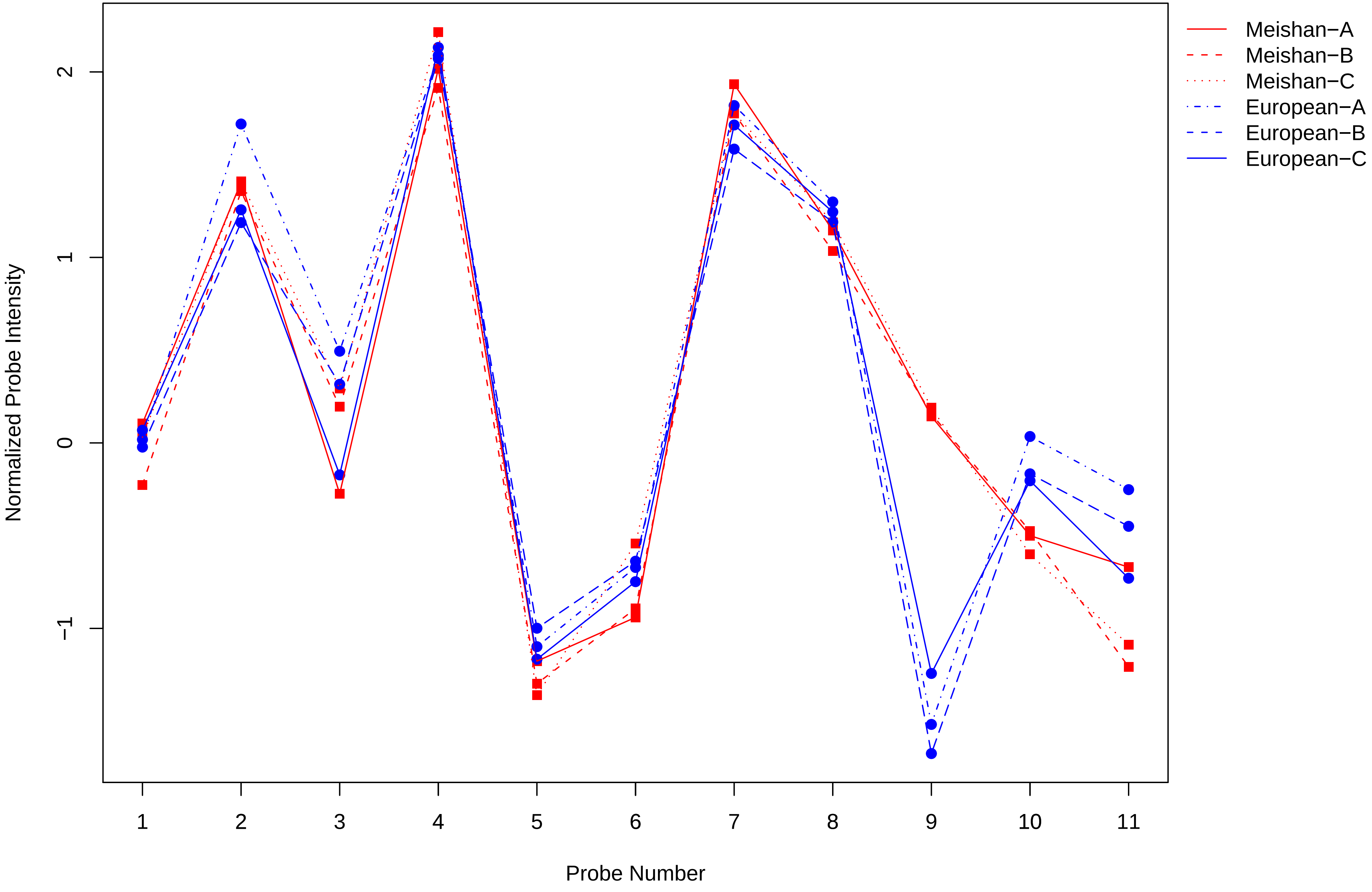

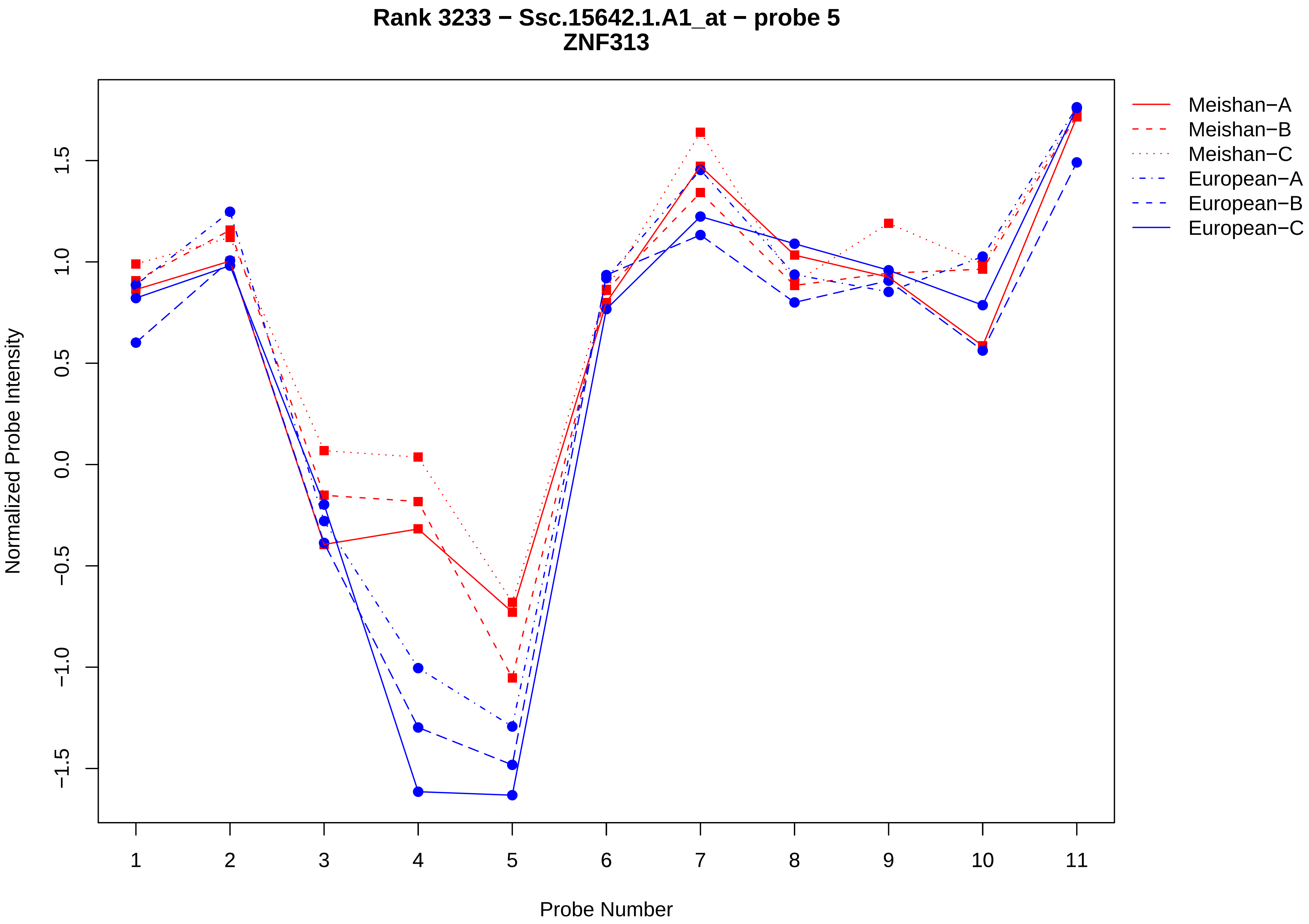

Rank 3233 − Ssc.15642.1.A1_at − probe 5
ZNF313
Normalized Probe Intensity
Probe Number
−1.5
−1.0
−0.5
0.0
0.5
1.0
1.5
1
2
3
4
5
6
7
8
9
10
11
Meishan−A
Meishan−B
Meishan−C
European−A
European−B
European−C

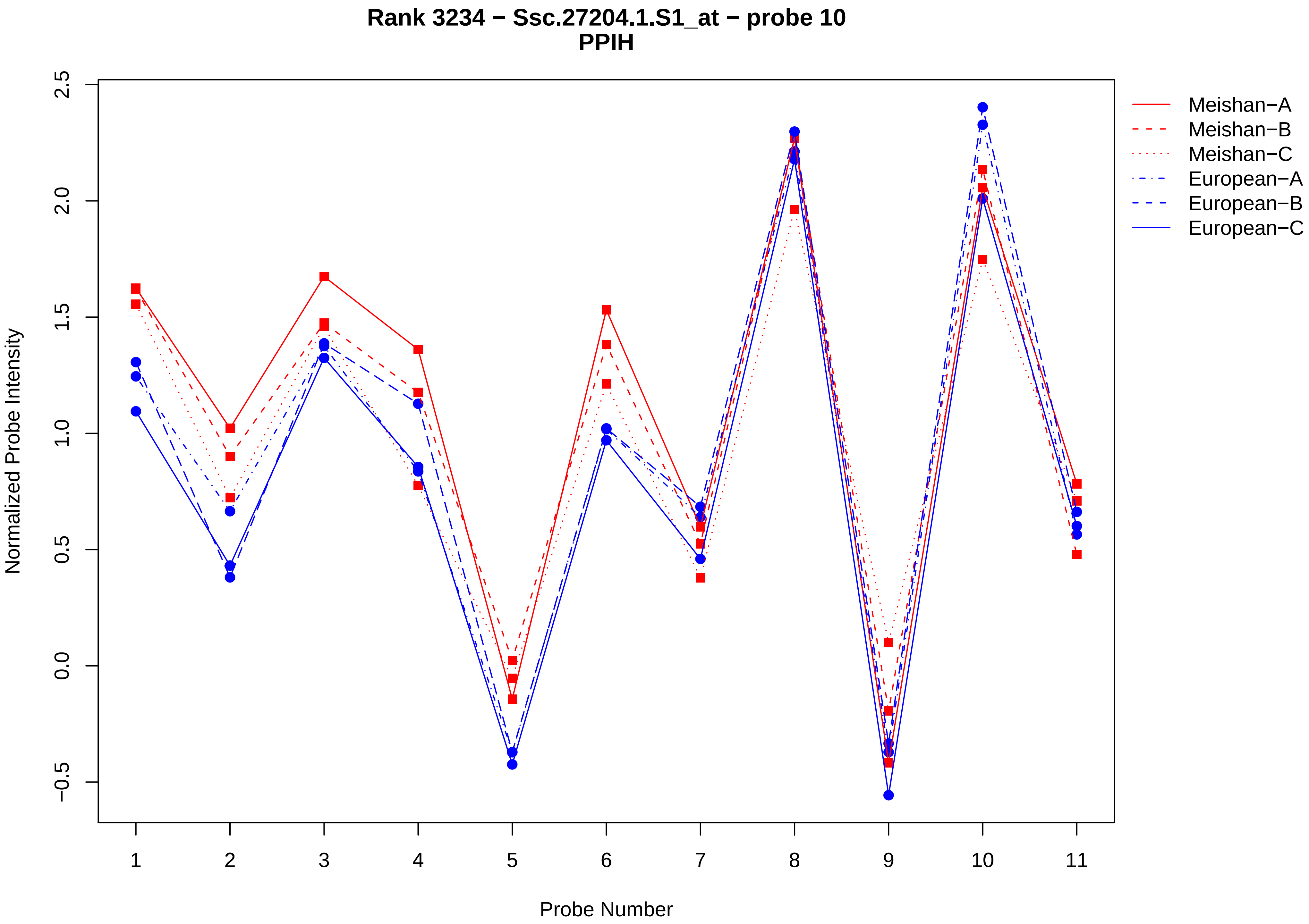

Rank 3234 − Ssc.27204.1.S1_at − probe 10
PPIH
Normalized Probe Intensity
Probe Number
−0.5
0.0
0.5
1.0
1.5
2.0
2.5
1
2
3
4
5
6
7
8
9
10
11
Meishan−A
Meishan−B
Meishan−C
European−A
European−B
European−C

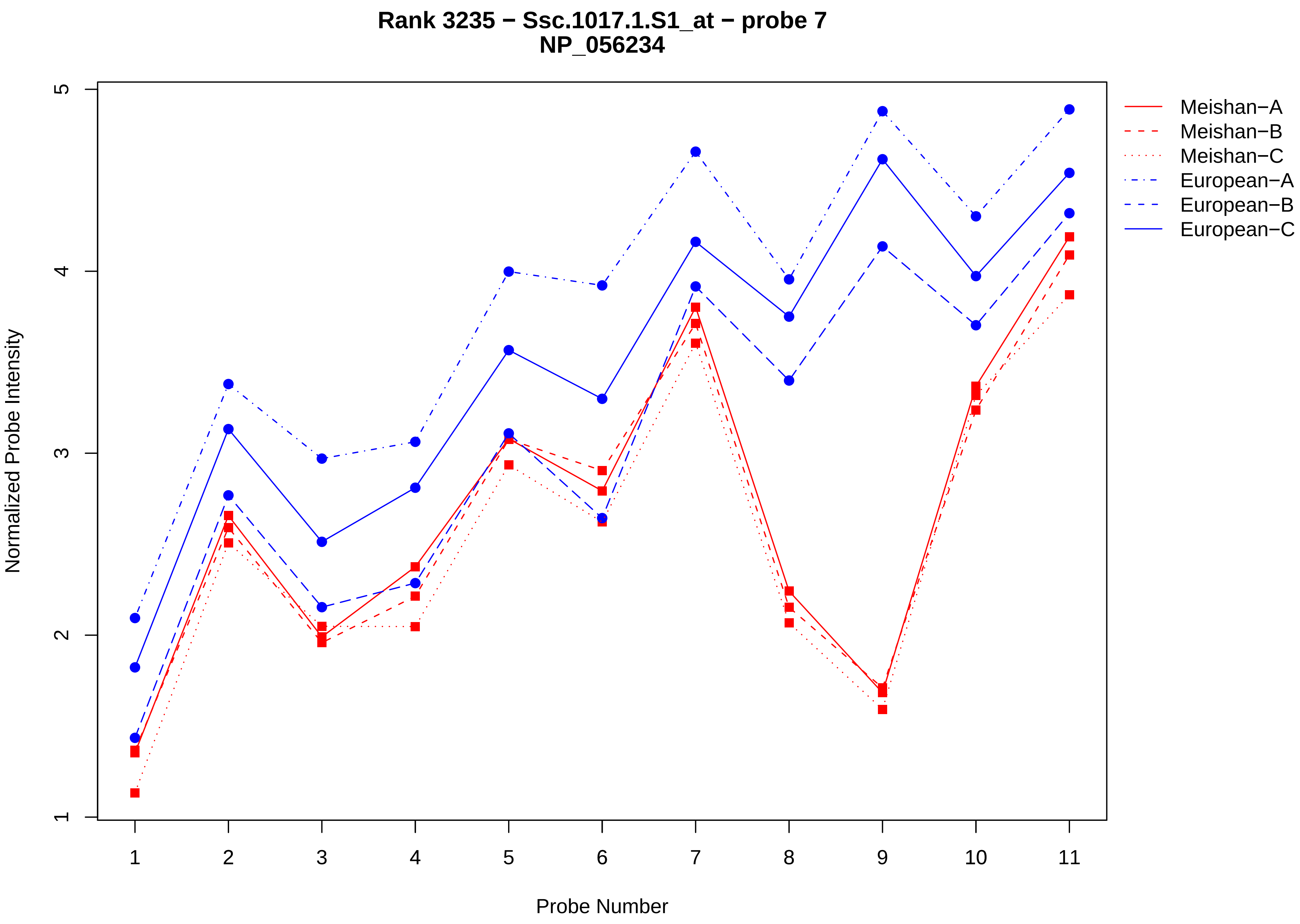

Rank 3235 − Ssc.1017.1.S1_at − probe 7
NP_056234
Normalized Probe Intensity
Probe Number
1
2
3
4
5
1
2
3
4
5
6
7
8
9
10
11
Meishan−A
Meishan−B
Meishan−C
European−A
European−B
European−C

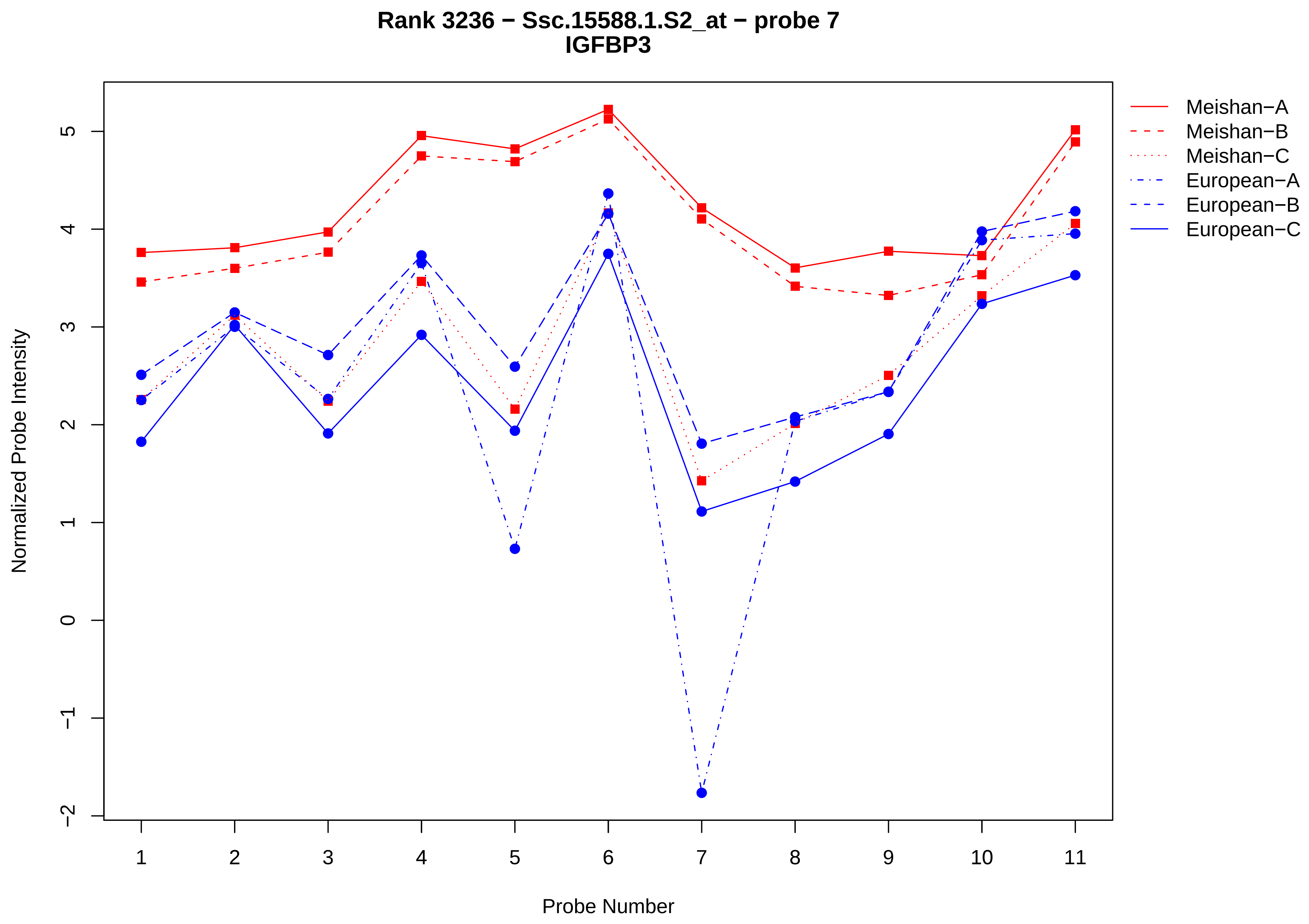
Rank 3236 − Ssc.15588.1.S2_at − probe 7
IGFBP3
Normalized Probe Intensity
Probe Number
−2
−1
0
1
2
3
4
5
1
2
3
4
5
6
7
8
9
10
11
Meishan−A
Meishan−B
Meishan−C
European−A
European−B
European−C

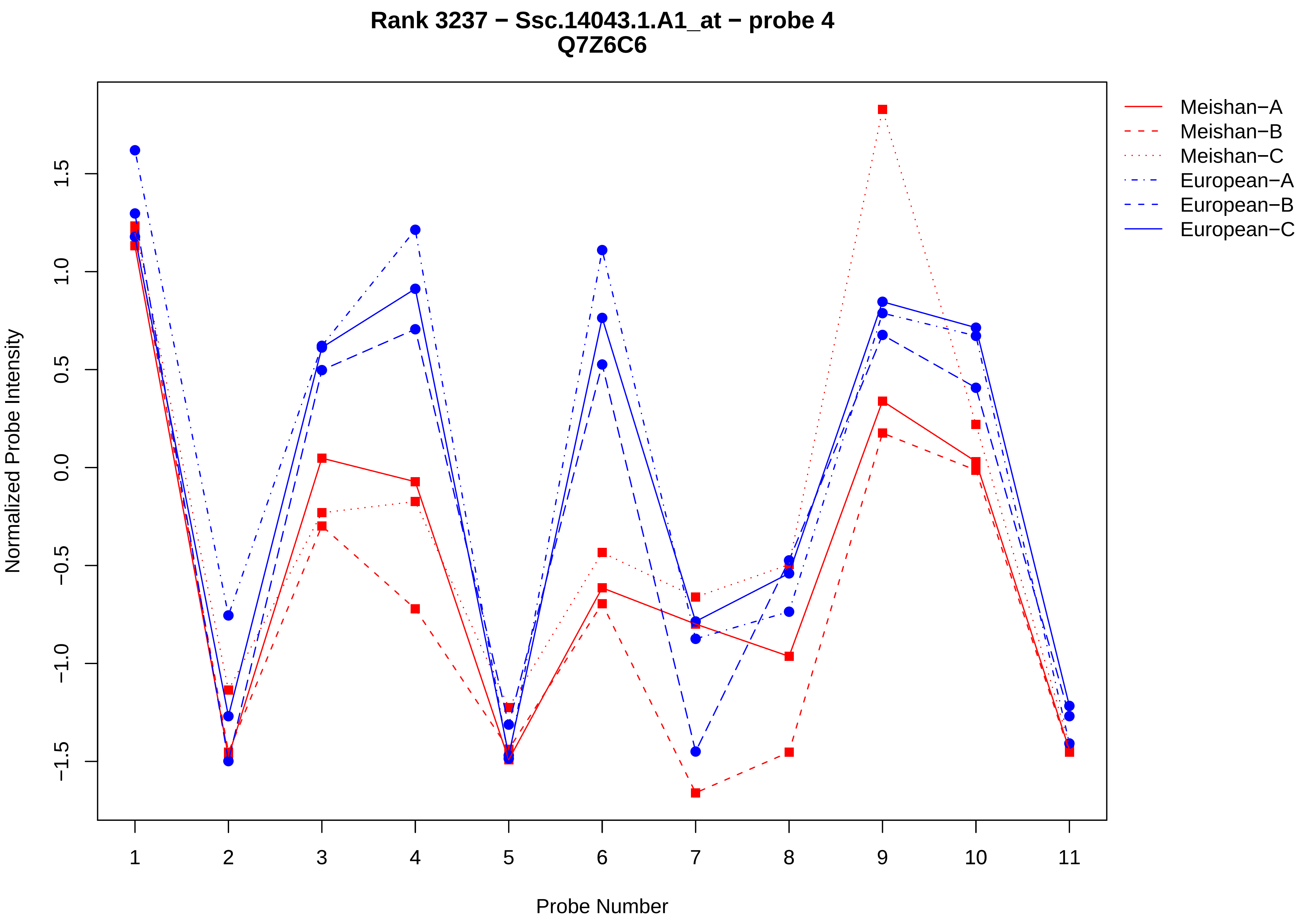
Rank 3237 − Ssc.14043.1.A1_at − probe 4
Q7Z6C6
Normalized Probe Intensity
Probe Number
−1.5
−1.0
−0.5
0.0
0.5
1.0
1.5
1
2
3
4
5
6
7
8
9
10
11
Meishan−A
Meishan−B
Meishan−C
European−A
European−B
European−C

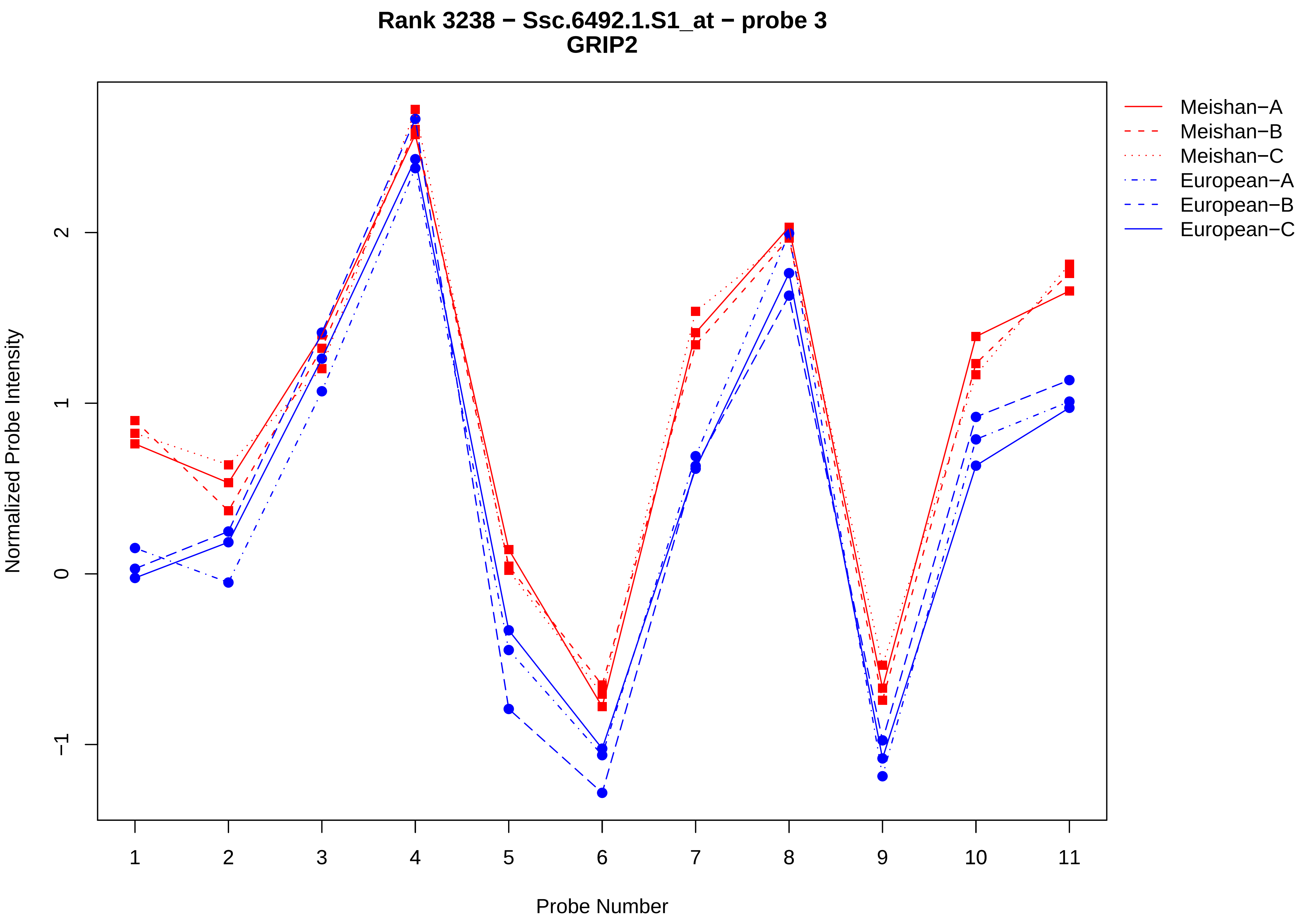

Rank 3238 − Ssc.6492.1.S1_at − probe 3
GRIP2
Normalized Probe Intensity
Probe Number
−1
0
1
2
1
2
3
4
5
6
7
8
9
10
11
Meishan−A
Meishan−B
Meishan−C
European−A
European−B
European−C

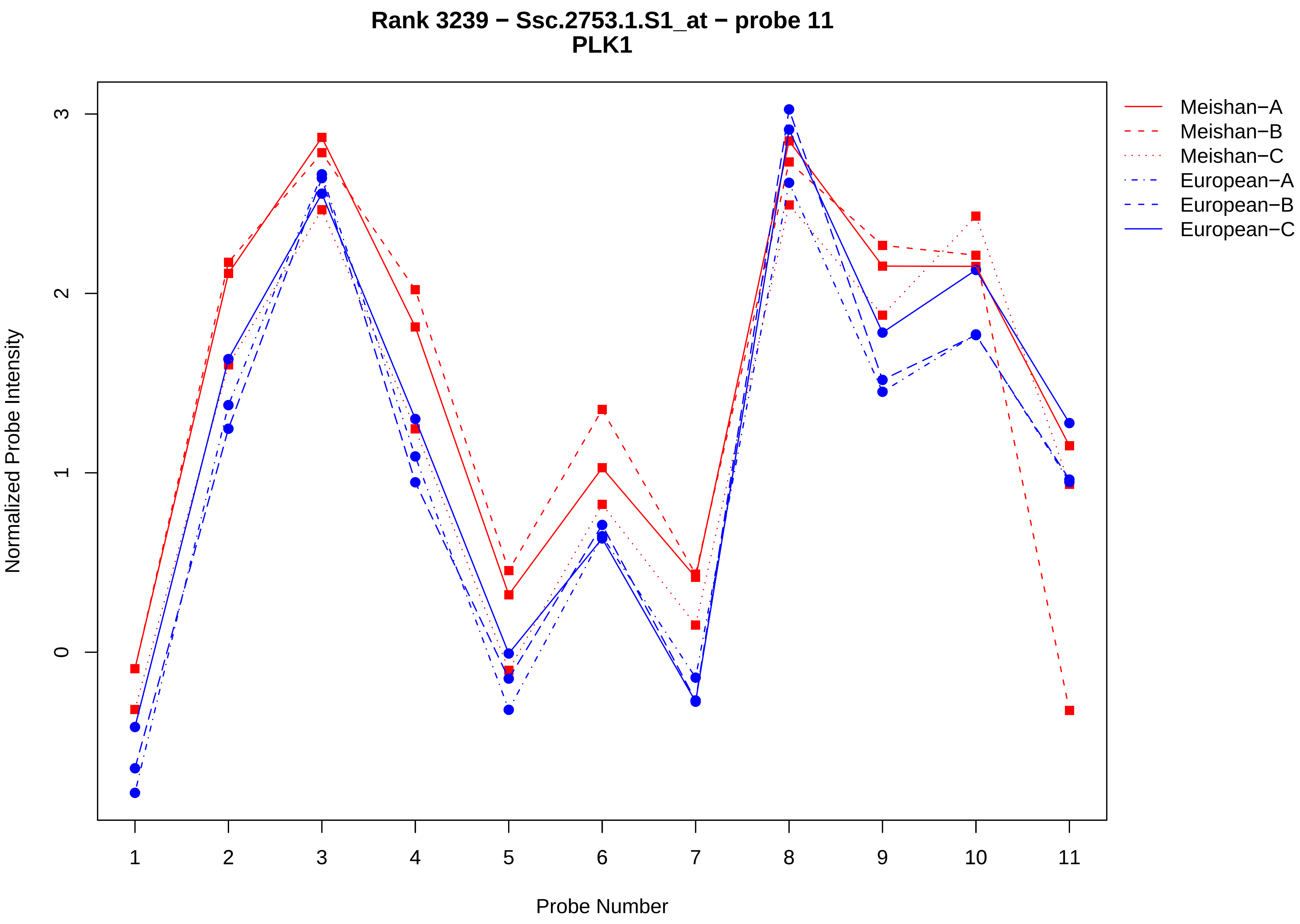

Rank 3239 − Ssc.2753.1.S1_at − probe 11
PLK1
Normalized Probe Intensity
Probe Number
0
1
2
3
1
2
3
4
5
6
7
8
9
10
11
Meishan−A
Meishan−B
Meishan−C
European−A
European−B
European−C

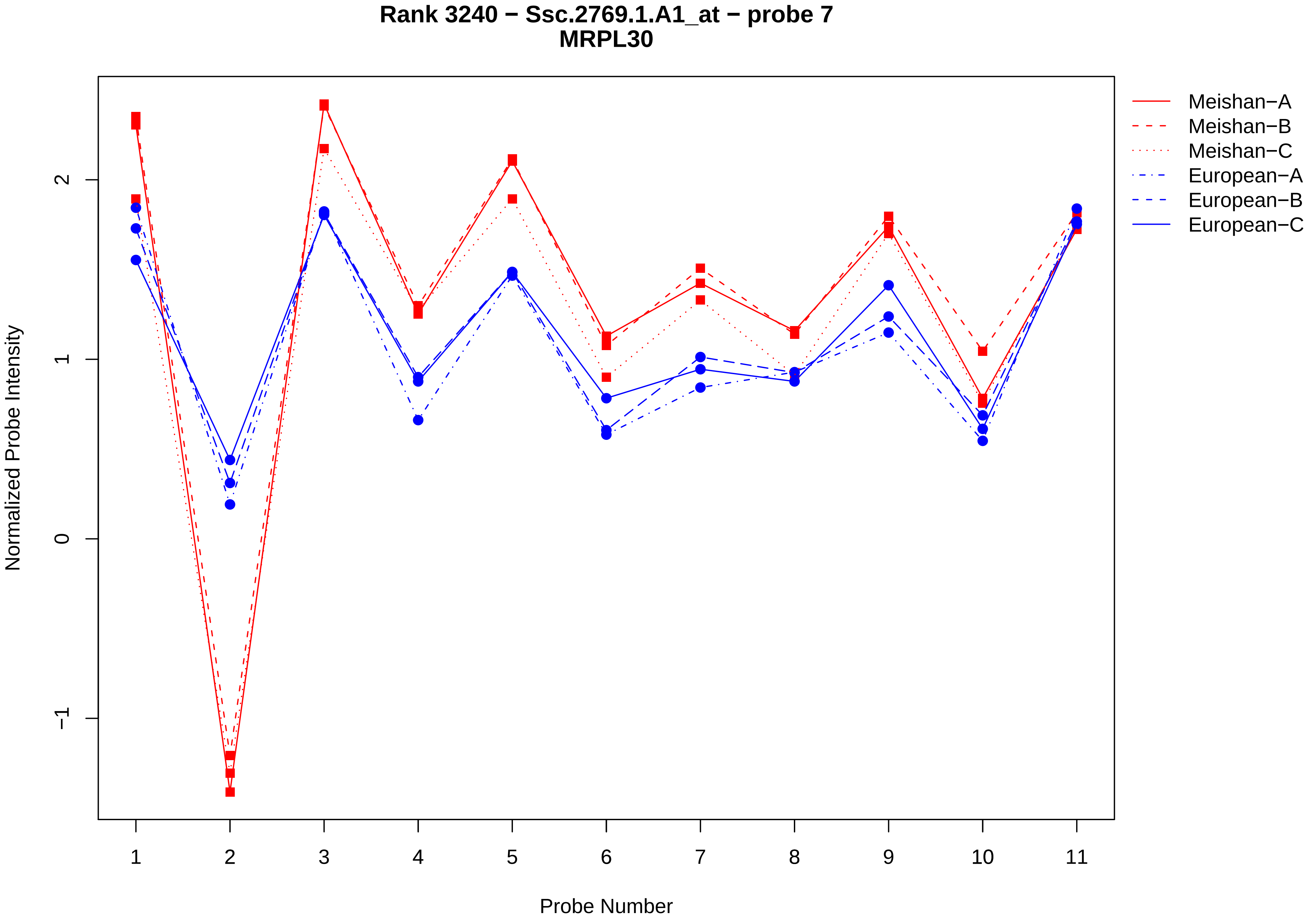
Rank 3240 − Ssc.2769.1.A1_at − probe 7
MRPL30
Normalized Probe Intensity
Probe Number
−1
0
1
2
1
2
3
4
5
6
7
8
9
10
11
Meishan−A
Meishan−B
Meishan−C
European−A
European−B
European−C

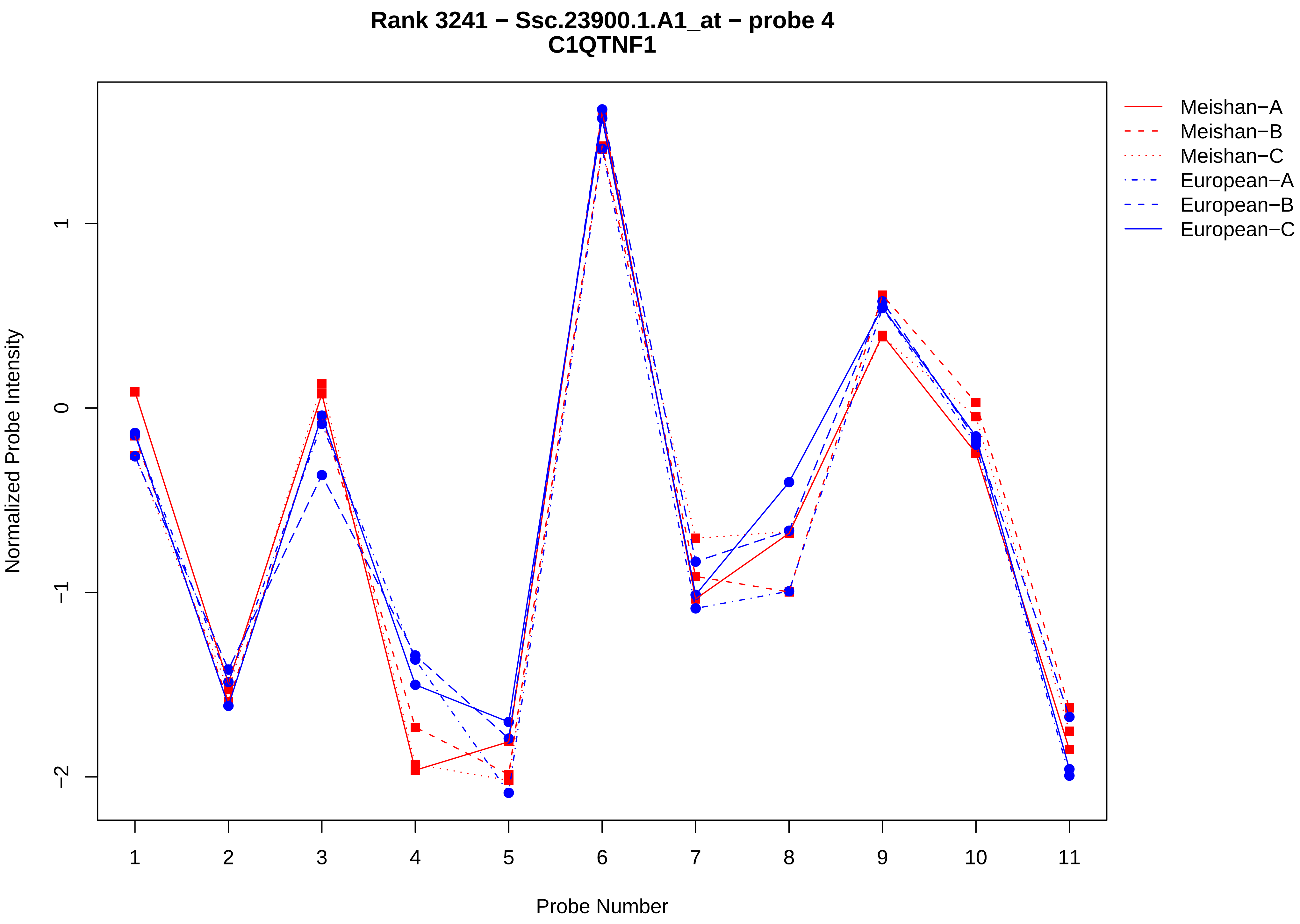

Rank 3241 − Ssc.23900.1.A1_at − probe 4
C1QTNF1
Normalized Probe Intensity
Probe Number
1
0
−1
−2
1
2
3
4
5
6
7
8
9
10
11
Meishan−A
Meishan−B
Meishan−C
European−A
European−B
European−C

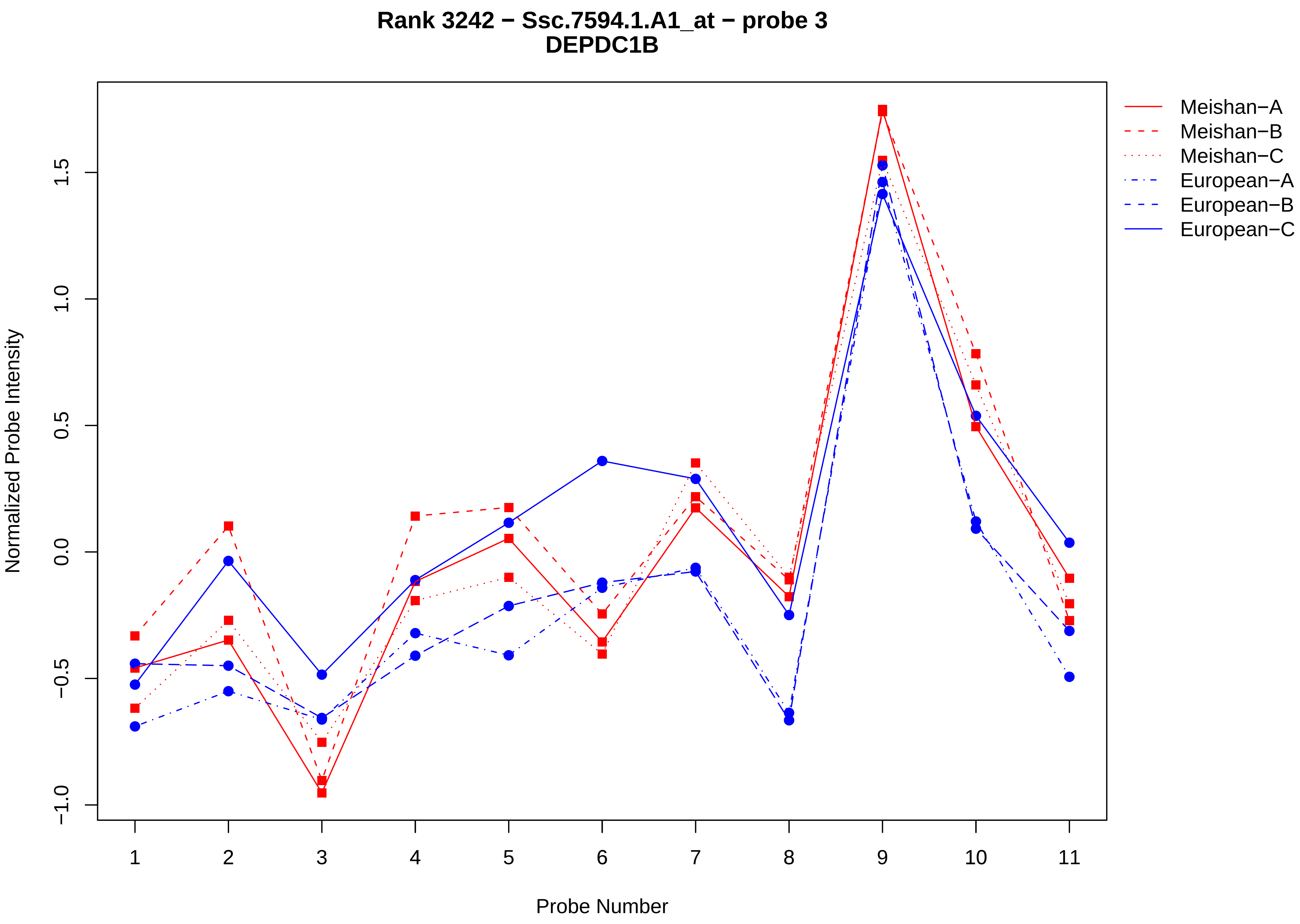
Rank 3242 − Ssc.7594.1.A1_at − probe 3
DEPDC1B
Normalized Probe Intensity
Probe Number
−1.0
−0.5
0.0
0.5
1.0
1.5
1
2
3
4
5
6
7
8
9
10
11
Meishan−A
Meishan−B
Meishan−C
European−A
European−B
European−C

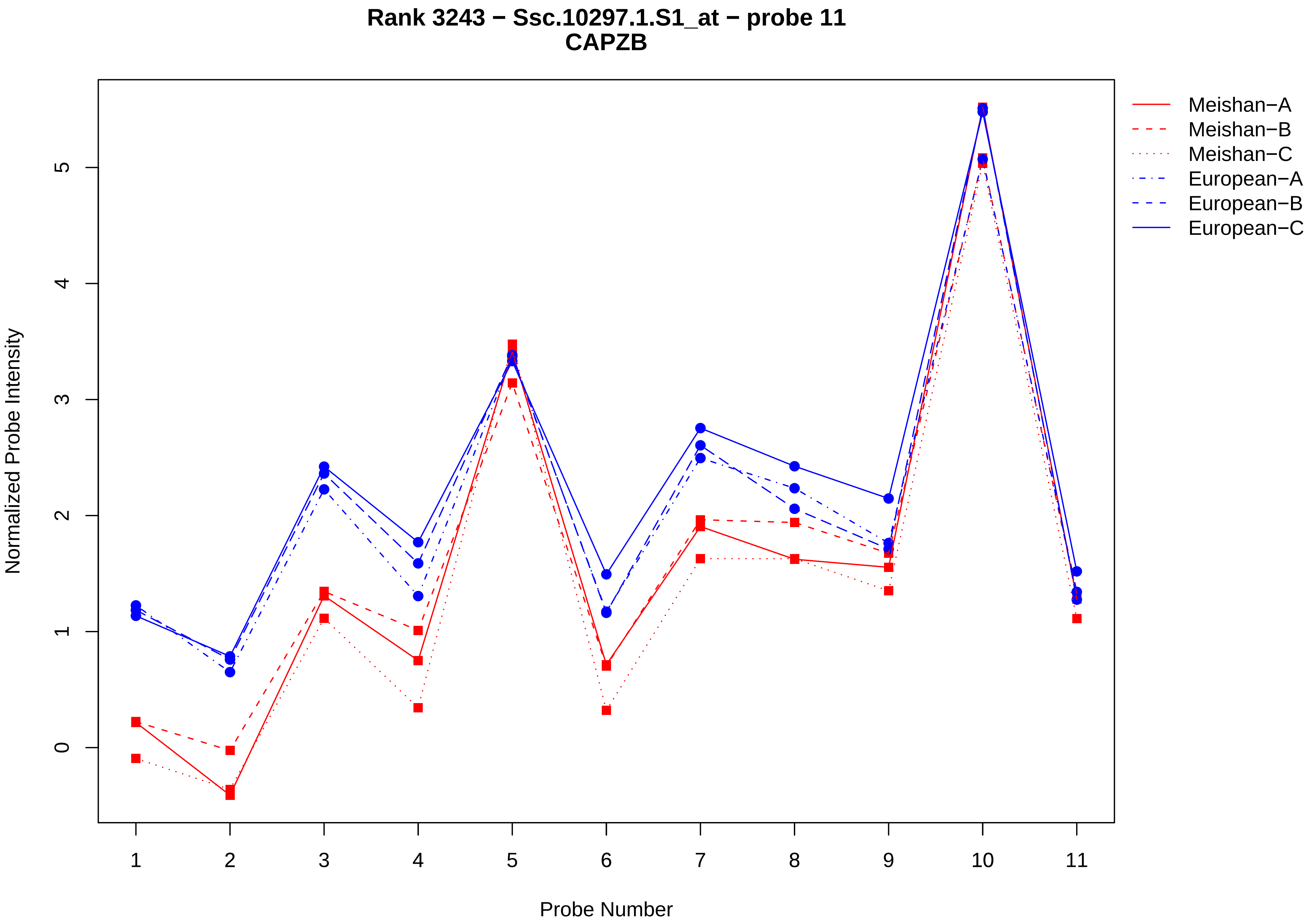

Rank 3243 − Ssc.10297.1.S1_at − probe 11
CAPZB
Normalized Probe Intensity
Probe Number
0
1
2
3
4
5
1
2
3
4
5
6
7
8
9
10
11
Meishan−A
Meishan−B
Meishan−C
European−A
European−B
European−C

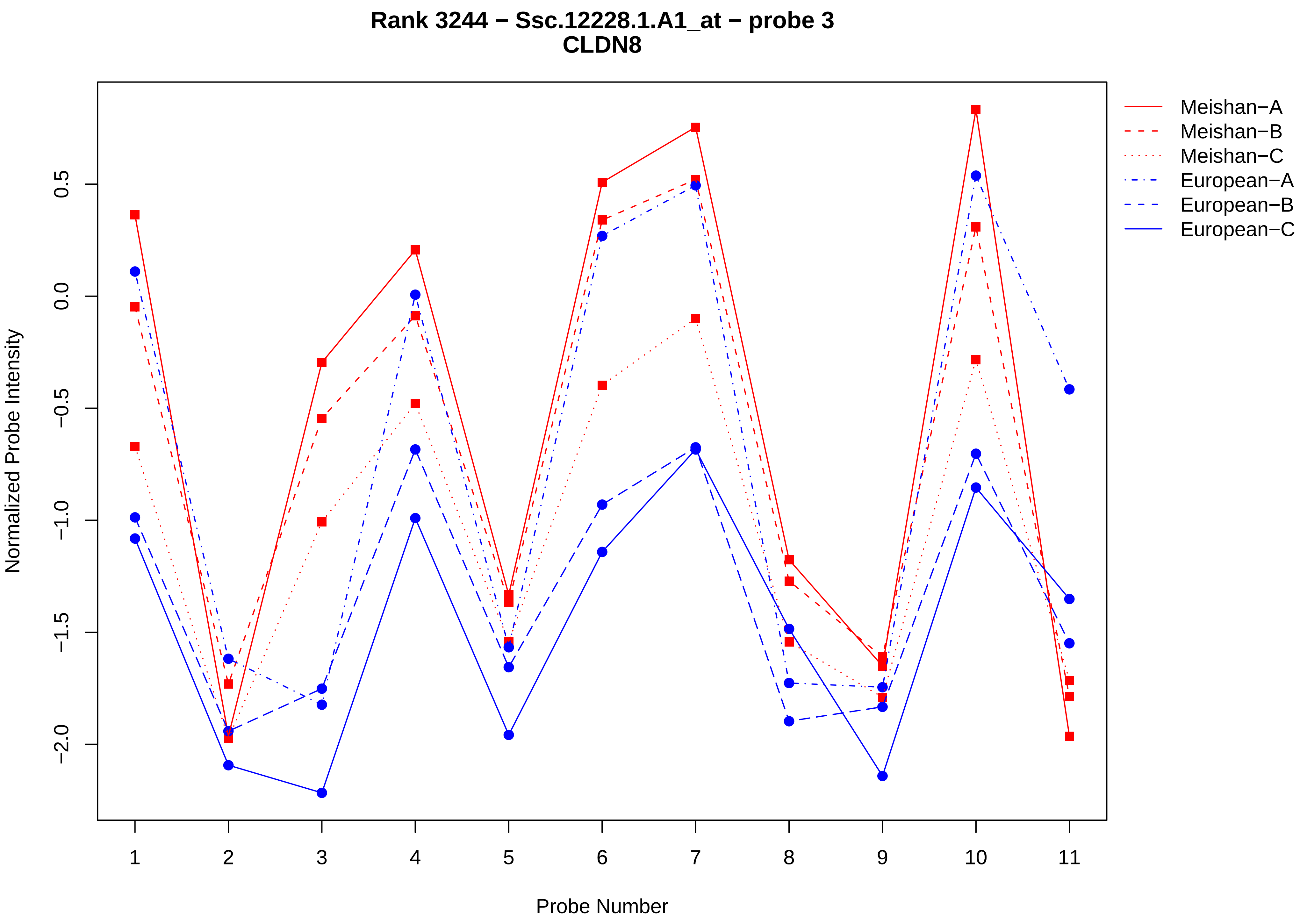

Rank 3244 − Ssc.12228.1.A1_at − probe 3
CLDN8
Normalized Probe Intensity
Probe Number
0.5
0.0
−0.5
−1.0
−1.5
−2.0
1
2
3
4
5
6
7
8
9
10
11
Meishan−A
Meishan−B
Meishan−C
European−A
European−B
European−C

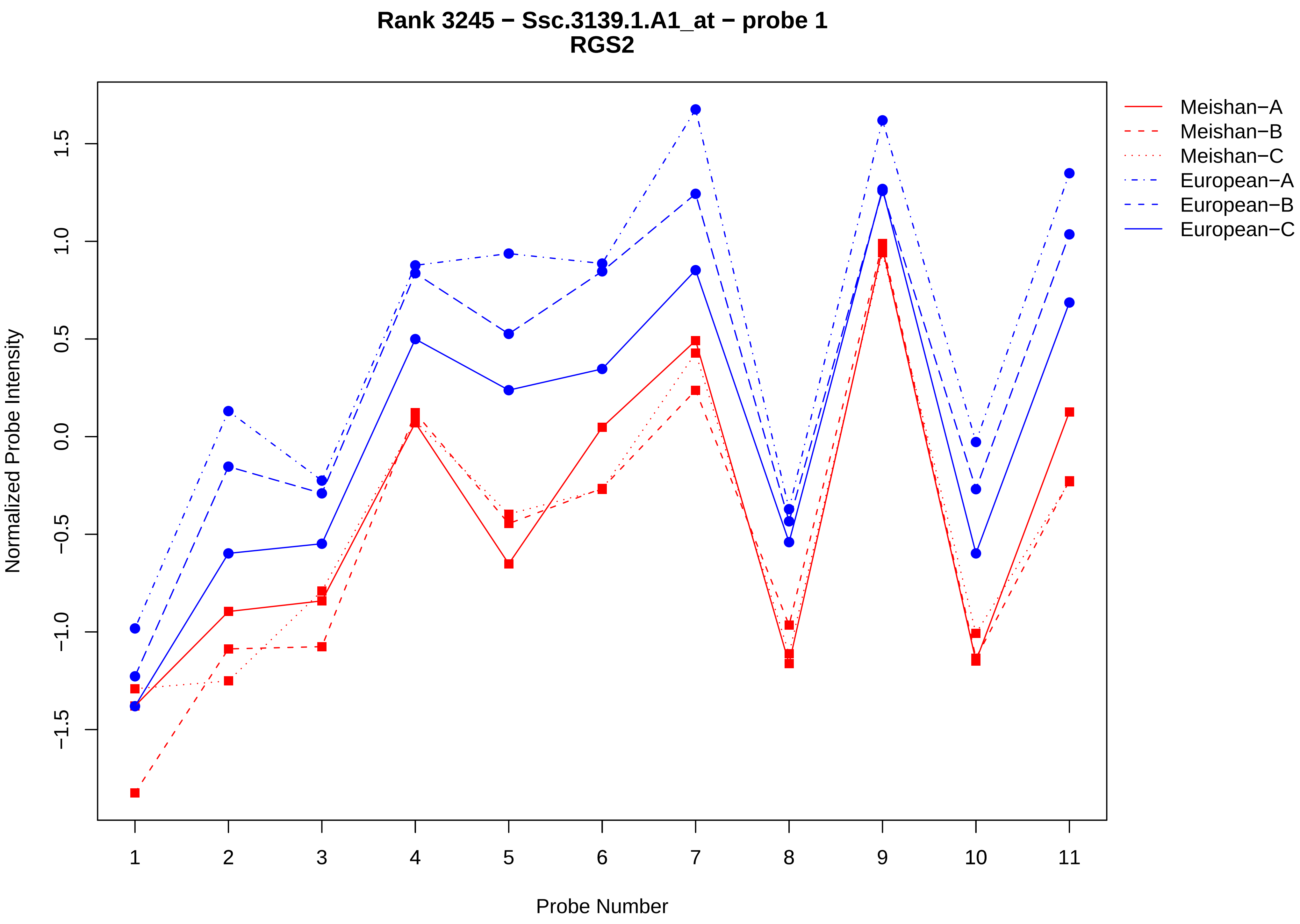

Rank 3245 − Ssc.3139.1.A1_at − probe 1
RGS2
Normalized Probe Intensity
−1.5
−1.0
−0.5
0.0
0.5
1.0
1.5
1
2
3
4
5
6
7
8
9
10
11
Probe Number
Meishan−A
Meishan−B
Meishan−C
European−A
European−B
European−C

Rank 3246 − Ssc.29390.1.A1_at − probe 3
TM7SF1

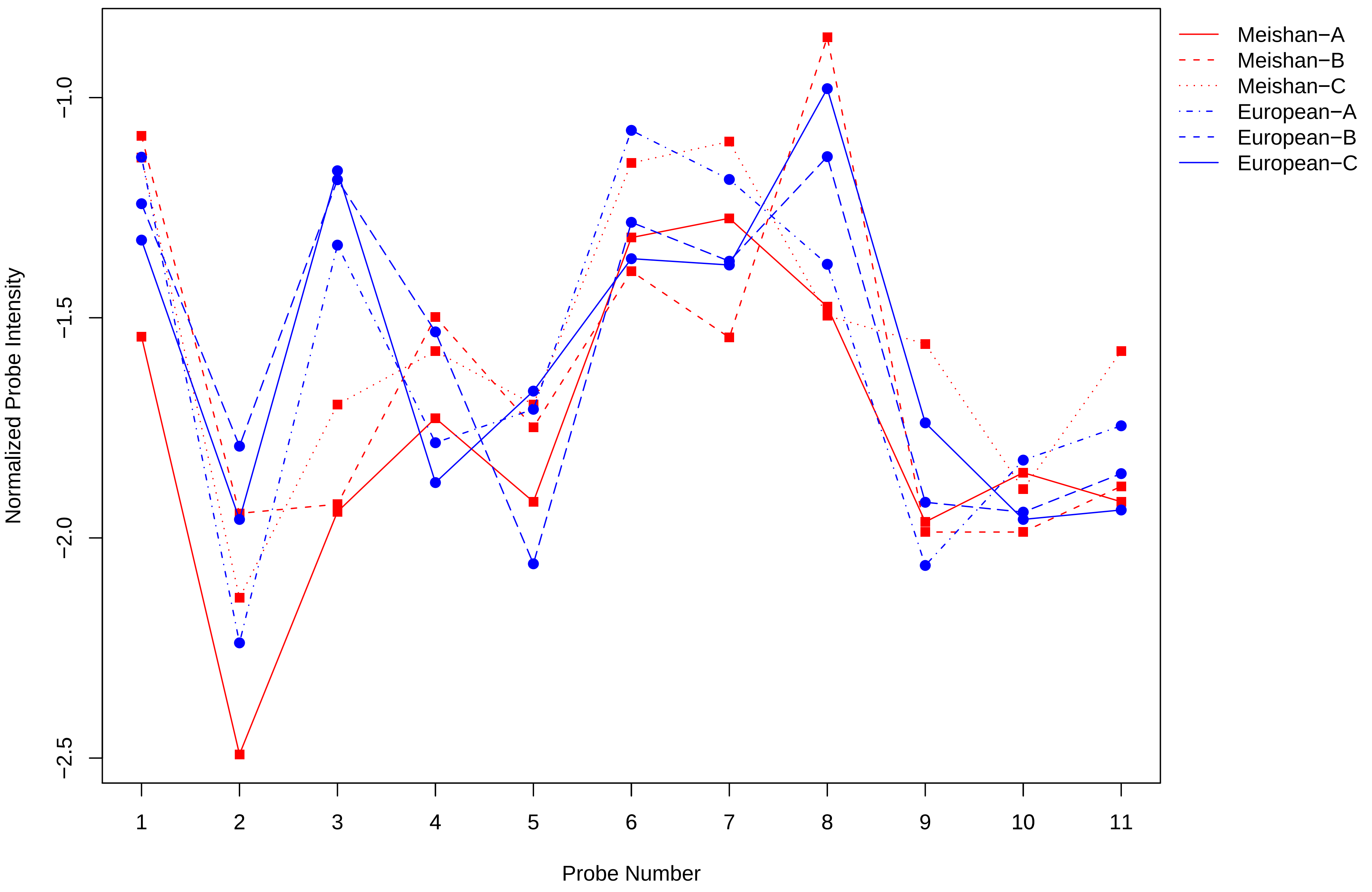

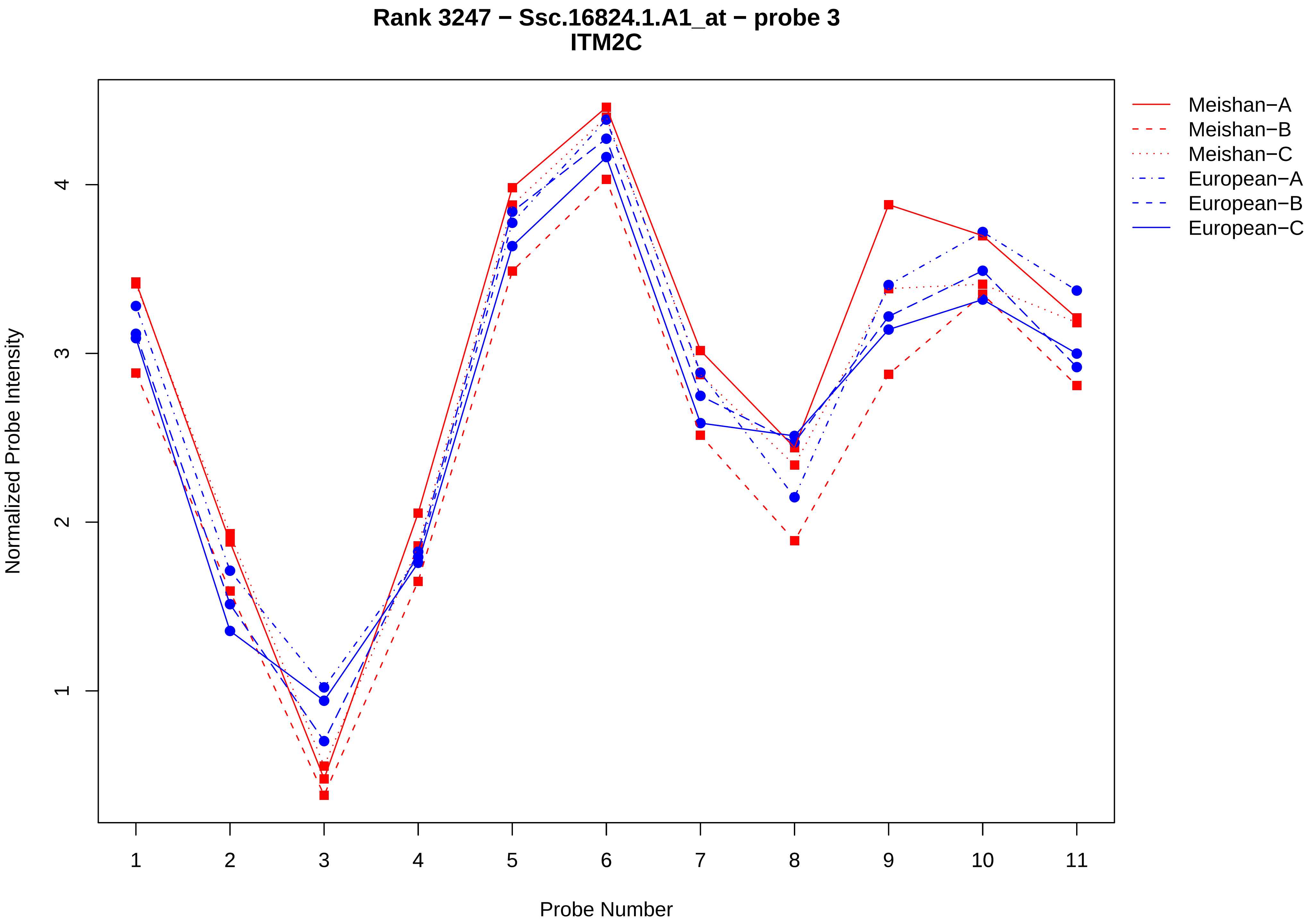

Rank 3247 − Ssc.16824.1.A1_at − probe 3
ITM2C
Normalized Probe Intensity
Probe Number
1
2
3
4
1
2
3
4
5
6
7
8
9
10
11
Meishan−A
Meishan−B
Meishan−C
European−A
European−B
European−C

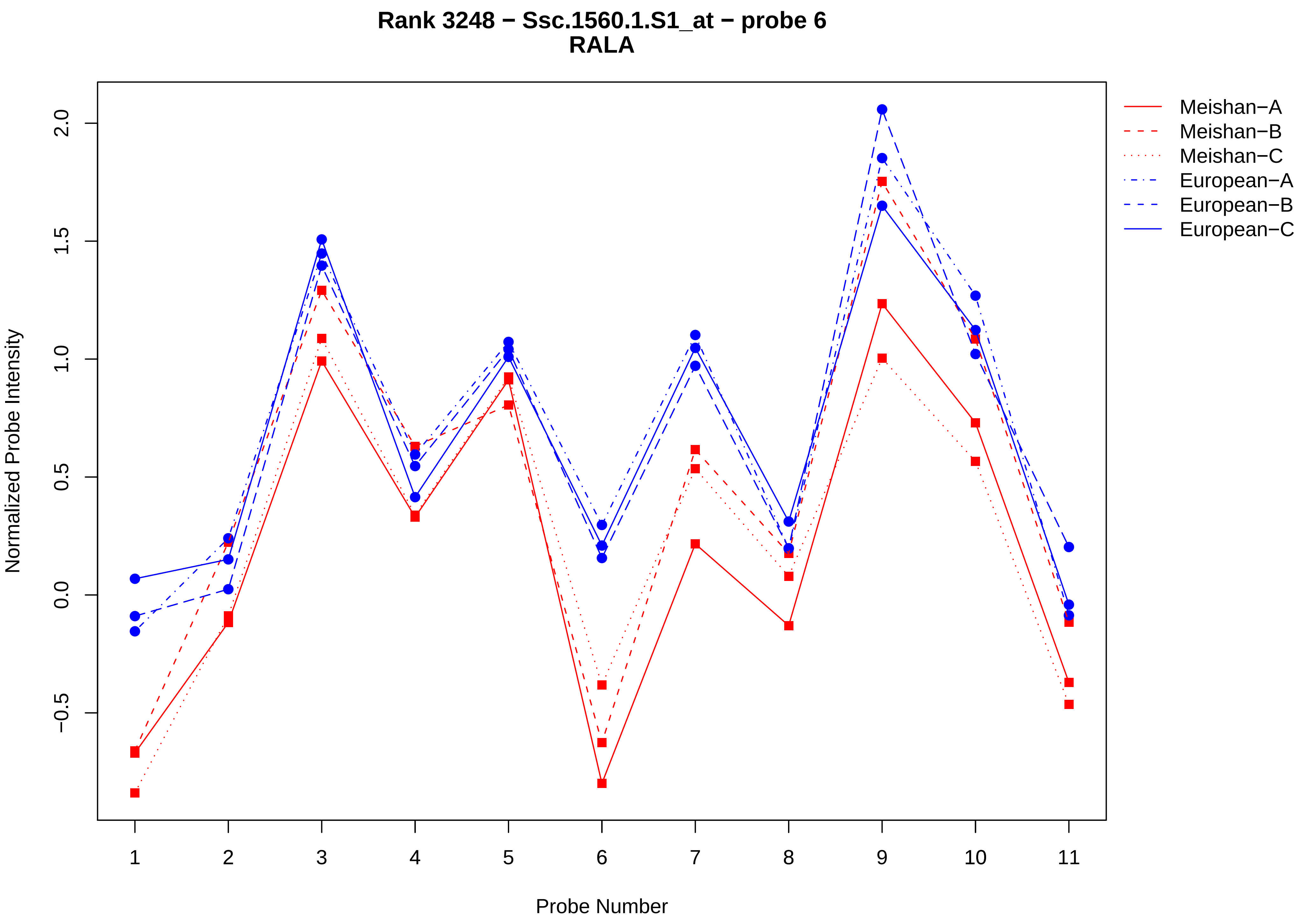
Rank 3248 − Ssc.1560.1.S1_at − probe 6
RALA
Normalized Probe Intensity
Probe Number
−0.5
0.0
0.5
1.0
1.5
2.0
1
2
3
4
5
6
7
8
9
10
11
Meishan−A
Meishan−B
Meishan−C
European−A
European−B
European−C

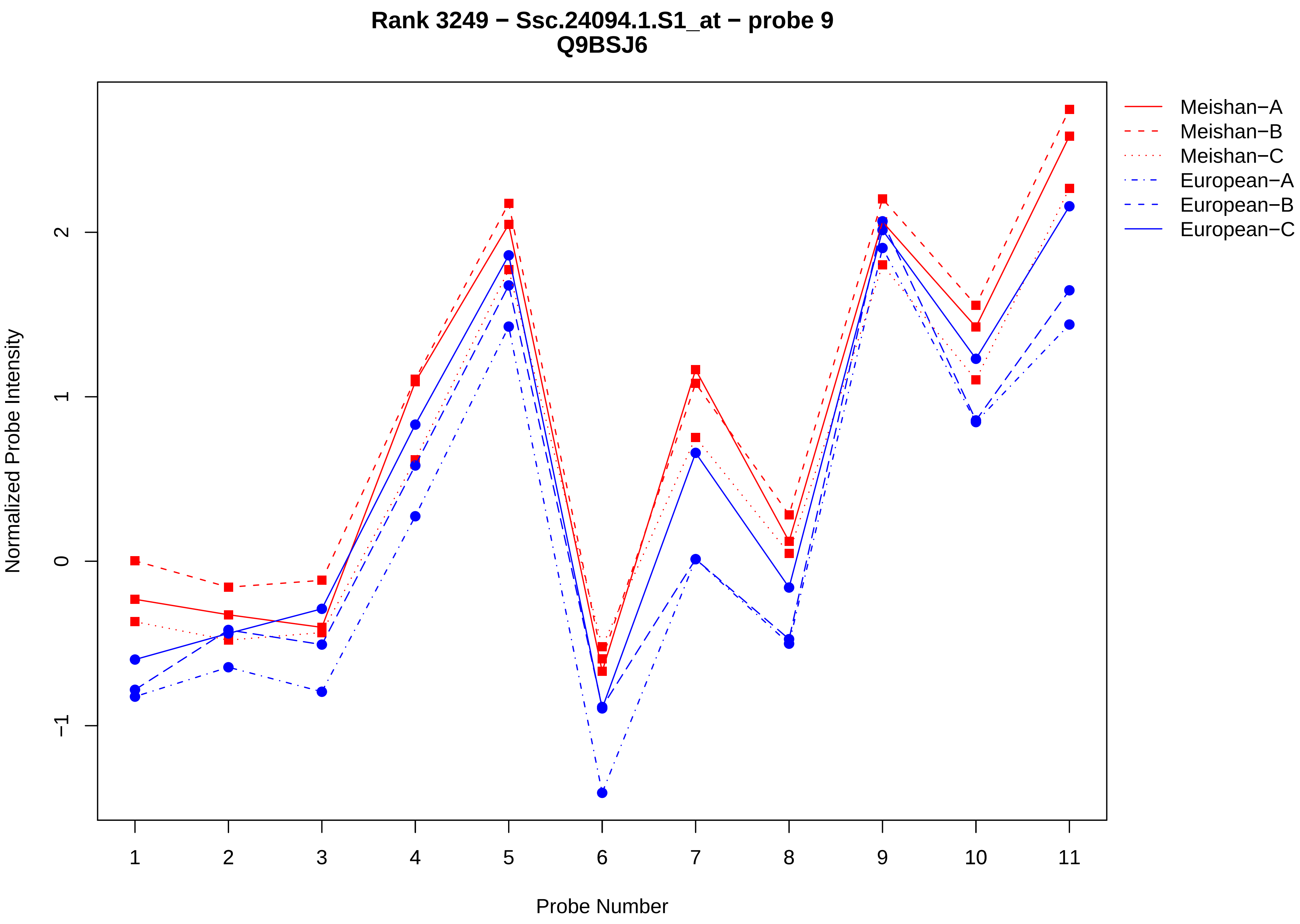

Rank 3249 − Ssc.24094.1.S1_at − probe 9
Q9BSJ6
Normalized Probe Intensity
Probe Number
−1
0
1
2
1
2
3
4
5
6
7
8
9
10
11
Meishan−A
Meishan−B
Meishan−C
European−A
European−B
European−C

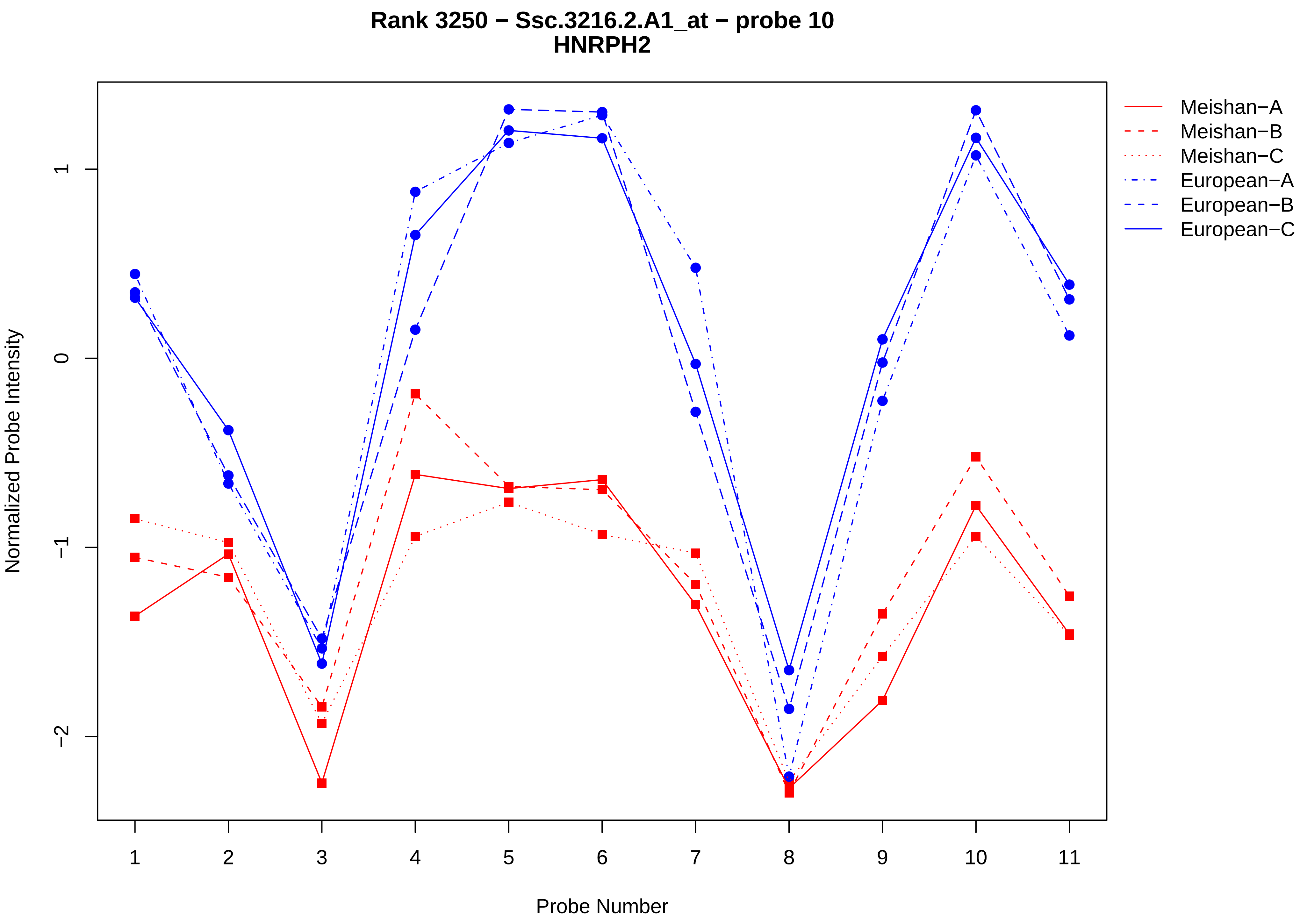

Rank 3250 − Ssc.3216.2.A1_at − probe 10
HNRPH2
Normalized Probe Intensity
Probe Number
Meishan−A
Meishan−B
Meishan−C
European−A
European−B
European−C
1
0
−1
−2
1
2
3
4
5
6
7
8
9
10
11

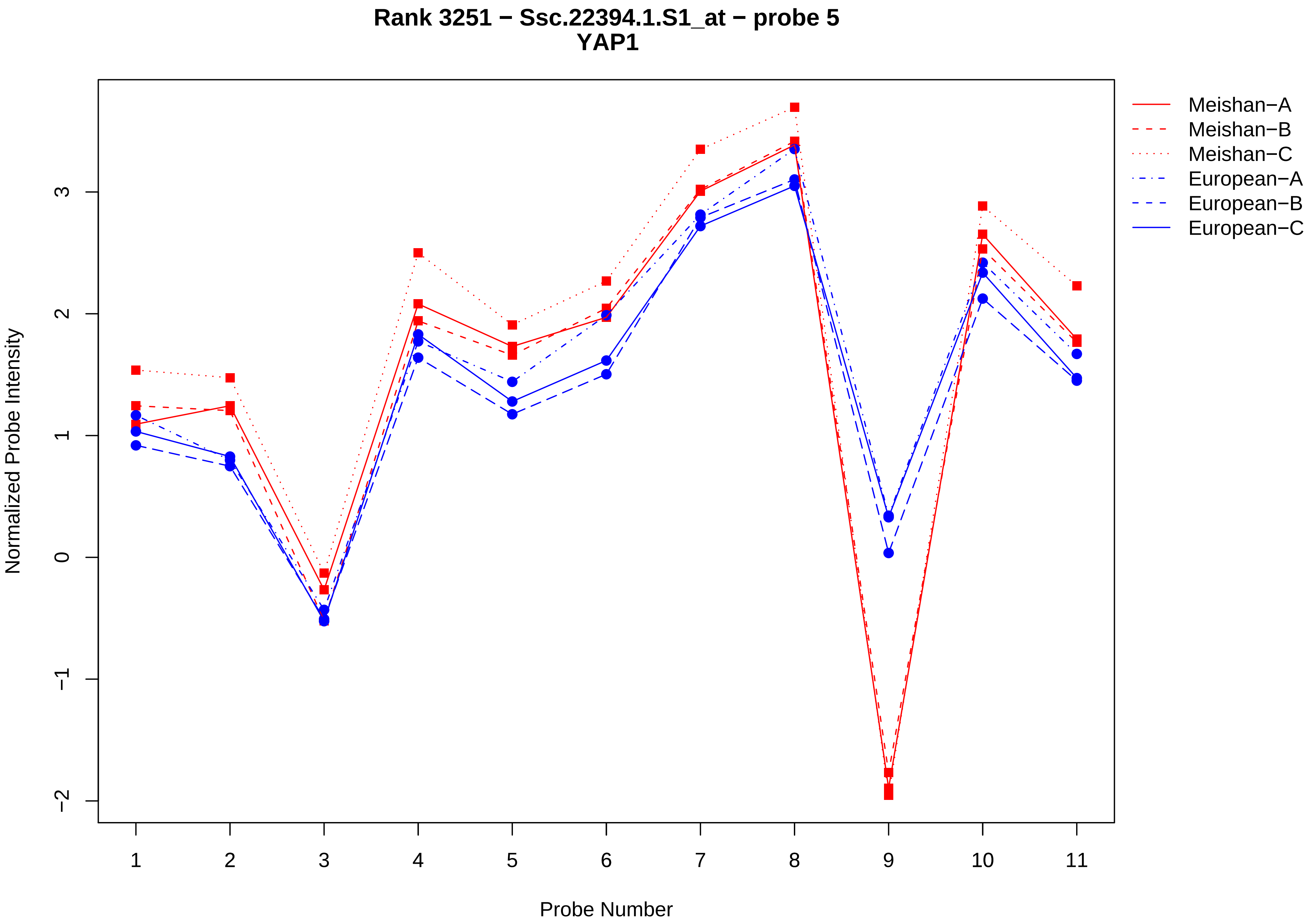

Rank 3251 − Ssc.22394.1.S1_at − probe 5
YAP1
Normalized Probe Intensity
Probe Number
−2
−1
0
1
2
3
1
2
3
4
5
6
7
8
9
10
11
Meishan−A
Meishan−B
Meishan−C
European−A
European−B
European−C

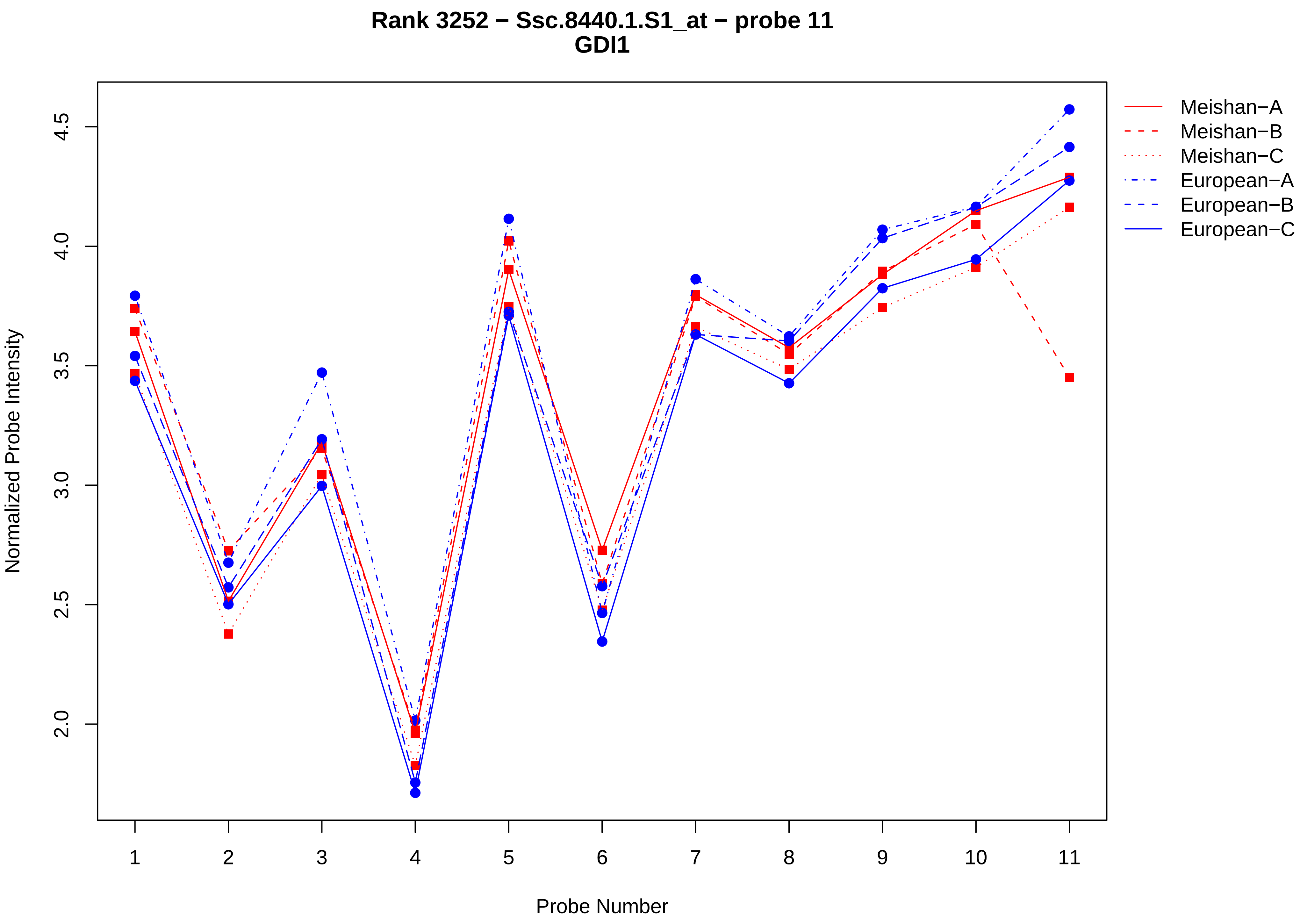

Rank 3252 − Ssc.8440.1.S1_at − probe 11
GDI1
Normalized Probe Intensity
Probe Number
2.0
2.5
3.0
3.5
4.0
4.5
1
2
3
4
5
6
7
8
9
10
11
Meishan−A
Meishan−B
Meishan−C
European−A
European−B
European−C

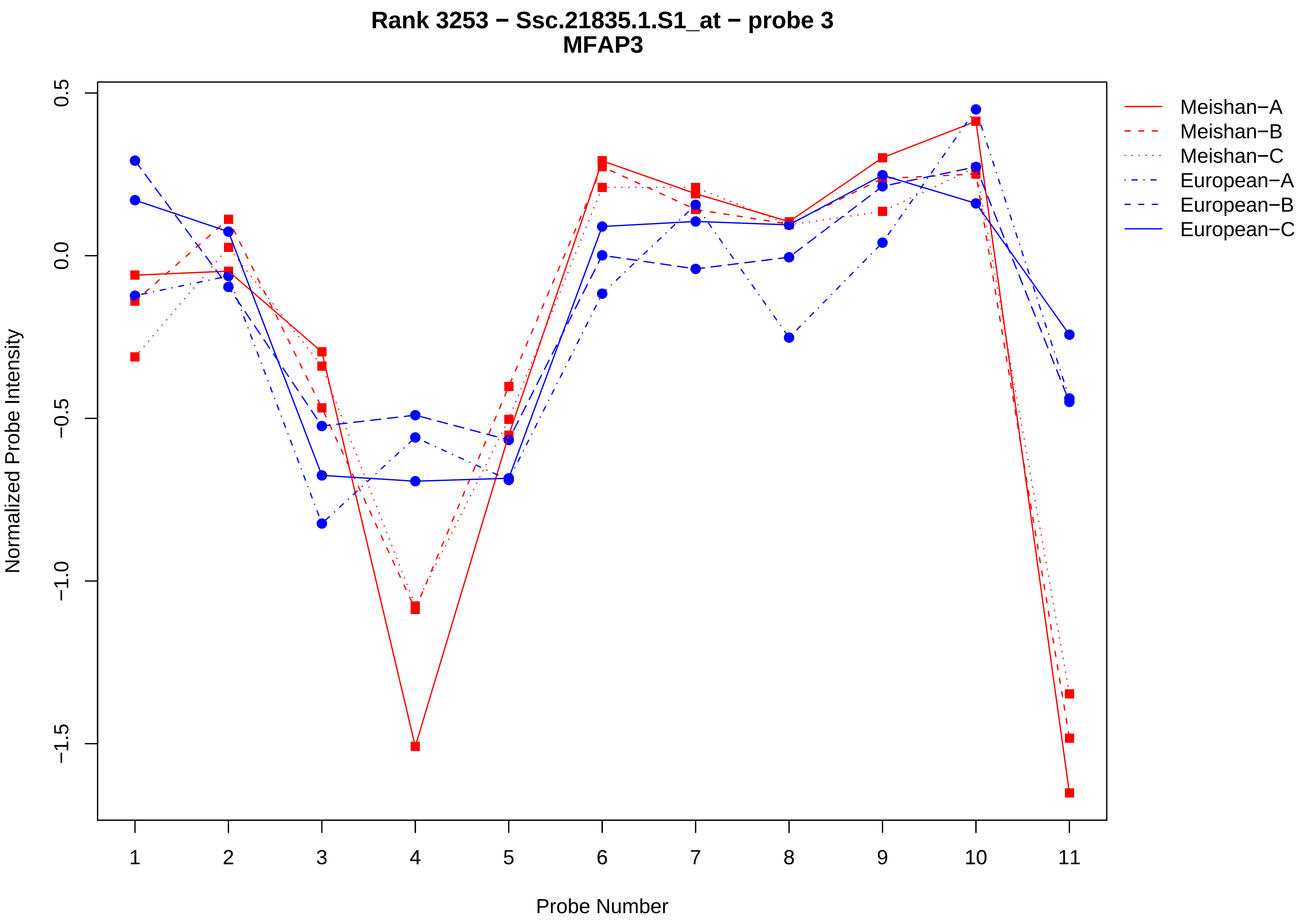

Rank 3253 − Ssc.21835.1.S1_at − probe 3
MFAP3
Normalized Probe Intensity
Probe Number
0.5
0.0
−0.5
−1.0
−1.5
1
2
3
4
5
6
7
8
9
10
11
Meishan−A
Meishan−B
Meishan−C
European−A
European−B
European−C

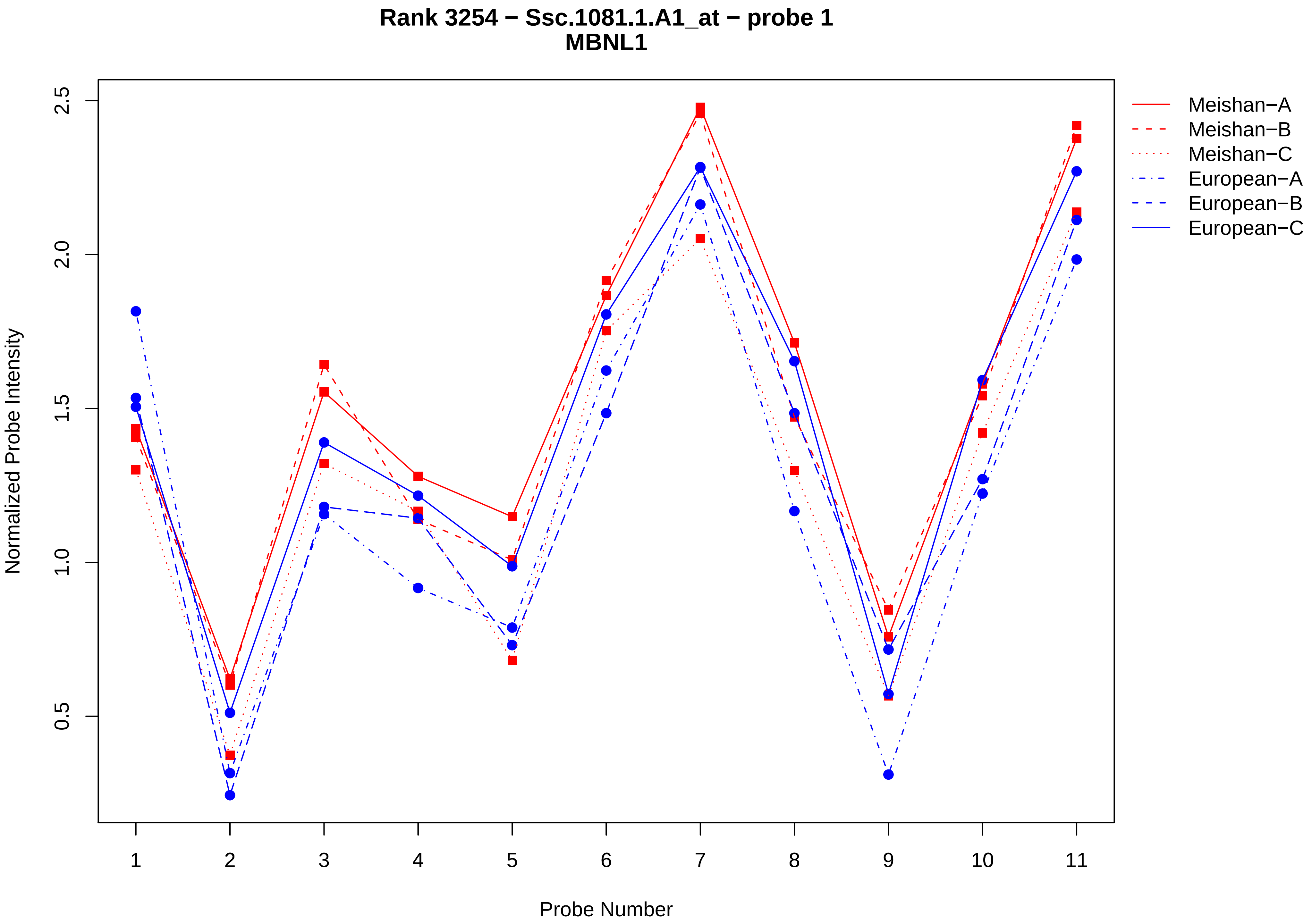

Rank 3254 − Ssc.1081.1.A1_at − probe 1
MBNL1
Normalized Probe Intensity
Probe Number
0.5
1.0
1.5
2.0
2.5
1
2
3
4
5
6
7
8
9
10
11
Meishan−A
Meishan−B
Meishan−C
European−A
European−B
European−C

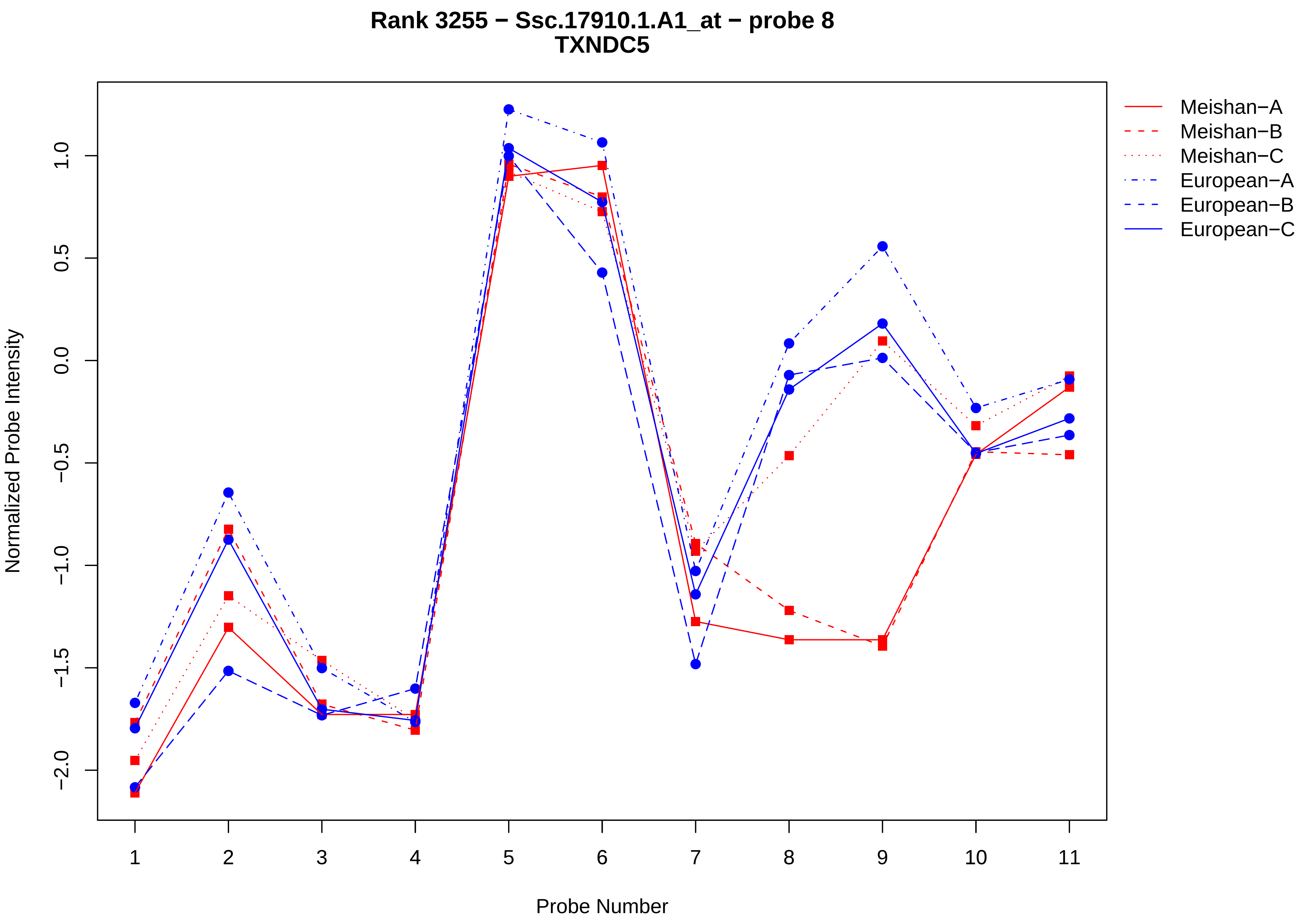

Rank 3255 − Ssc.17910.1.A1_at − probe 8
TXNDC5
Normalized Probe Intensity
Probe Number
−2.0
−1.5
−1.0
−0.5
0.0
0.5
1.0
1
2
3
4
5
6
7
8
9
10
11
Meishan−A
Meishan−B
Meishan−C
European−A
European−B
European−C

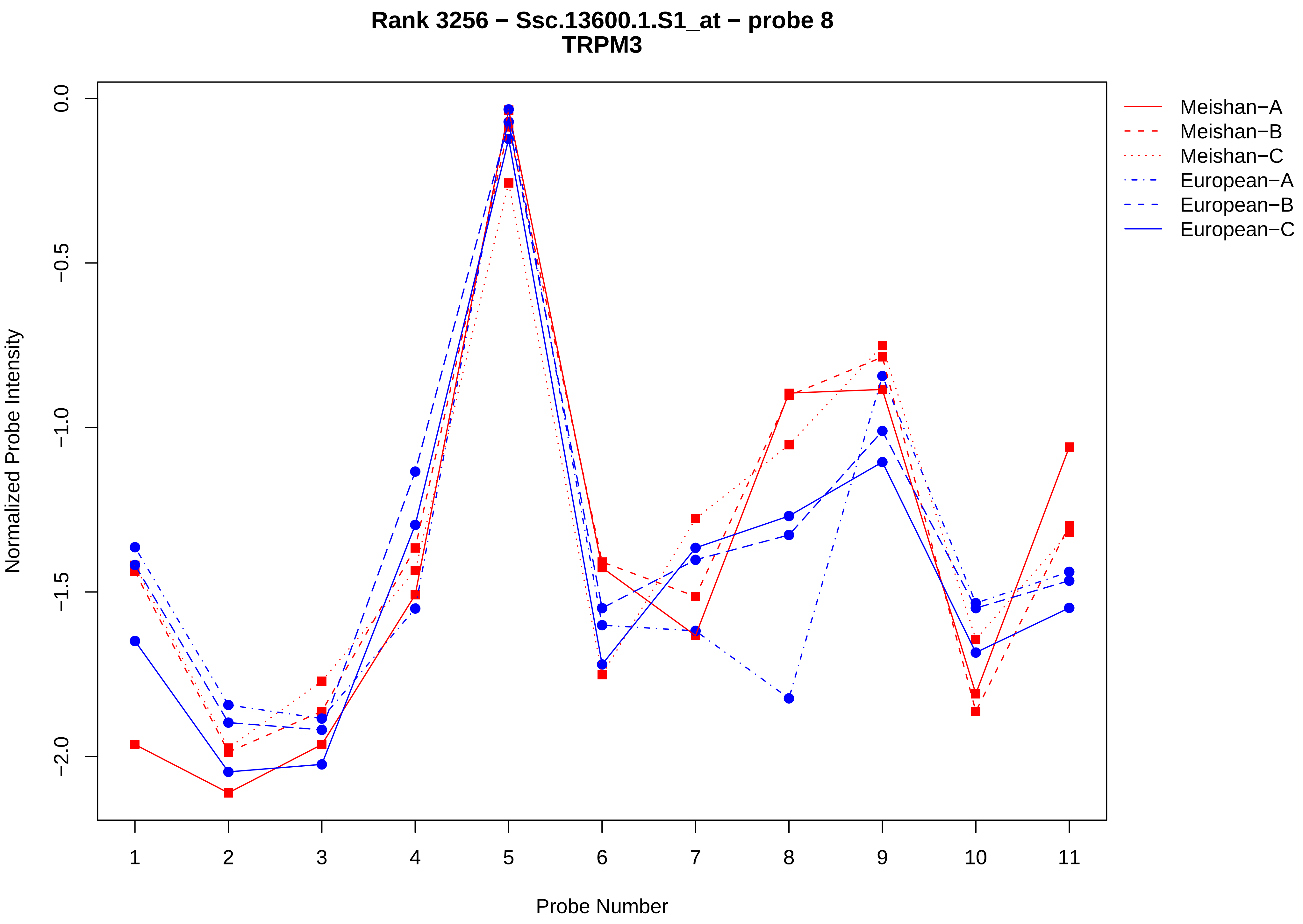

Rank 3256 − Ssc.13600.1.S1_at − probe 8
TRPM3
Normalized Probe Intensity
0.0
−0.5
−1.0
−1.5
−2.0
1
2
3
4
5
6
7
8
9
10
11
Probe Number
Meishan−A
Meishan−B
Meishan−C
European−A
European−B
European−C

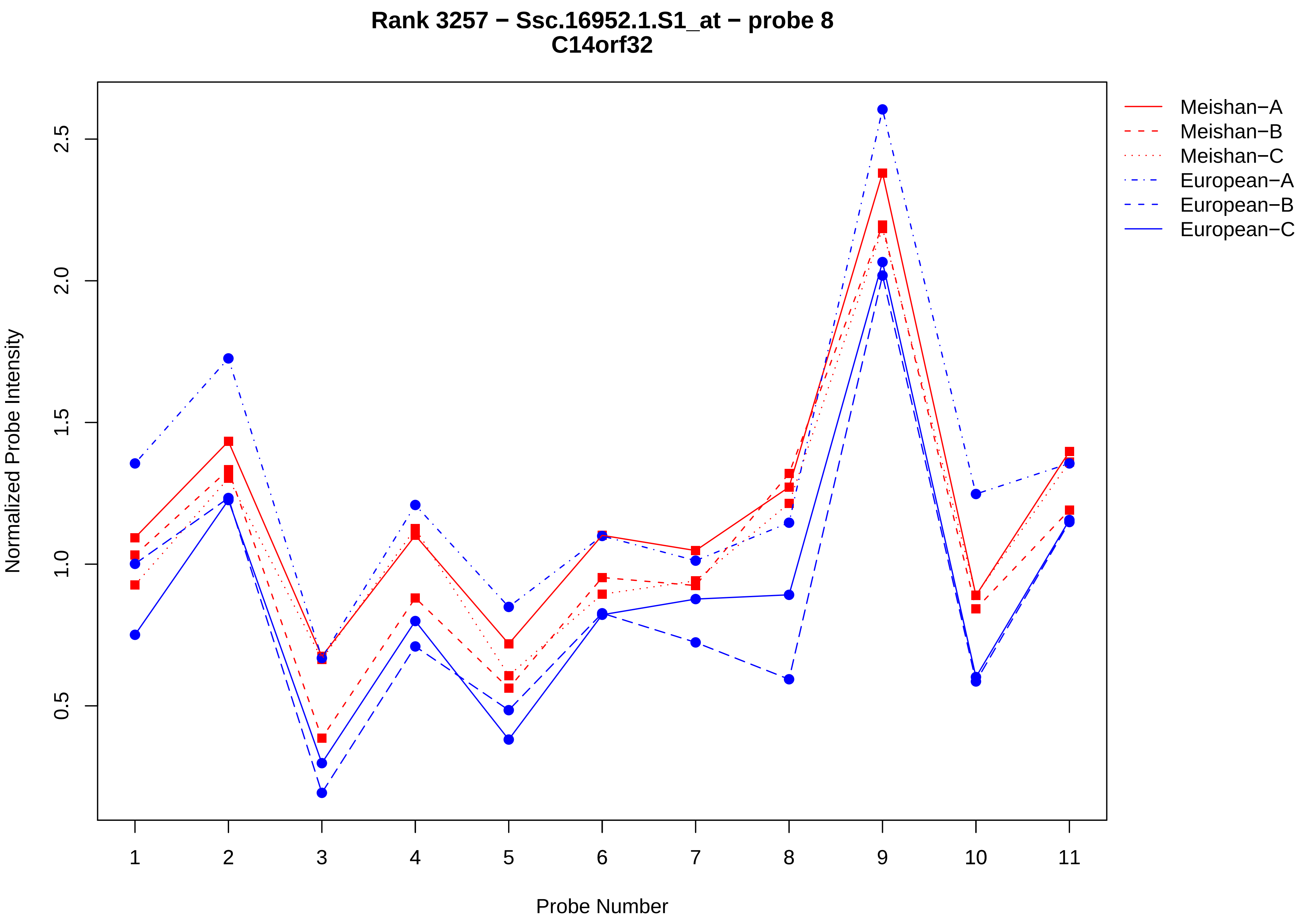

Rank 3257 − Ssc.16952.1.S1_at − probe 8
C14orf32
Normalized Probe Intensity
0.5
1.0
1.5
2.0
2.5
1
2
3
4
5
6
7
8
9
10
11
Probe Number
Meishan−A
Meishan−B
Meishan−C
European−A
European−B
European−C

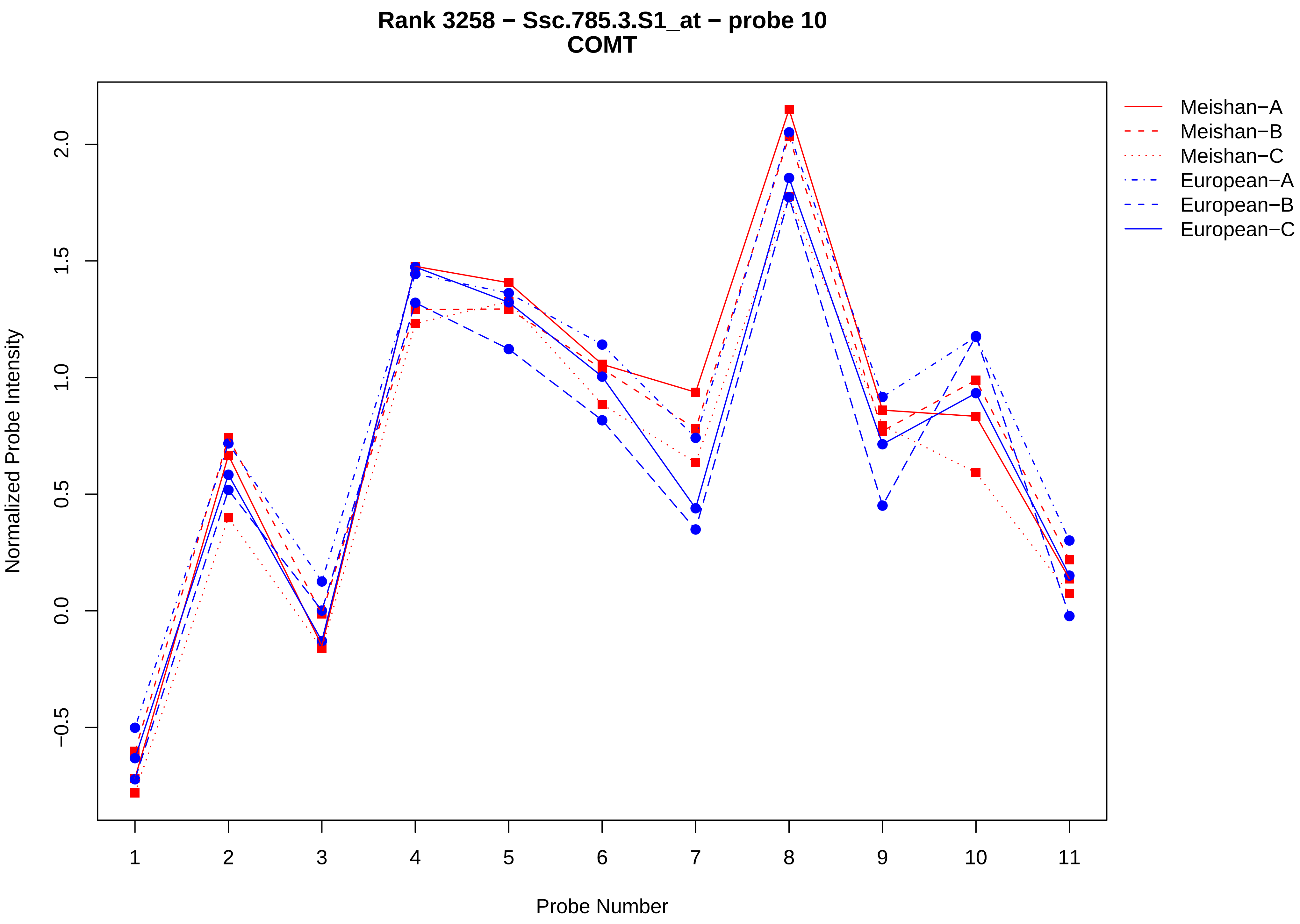

Rank 3258 − Ssc.785.3.S1_at − probe 10
COMT
Normalized Probe Intensity
Probe Number
Meishan−A
Meishan−B
Meishan−C
European−A
European−B
European−C
−0.5
0.0
0.5
1.0
1.5
2.0
1
2
3
4
5
6
7
8
9
10
11

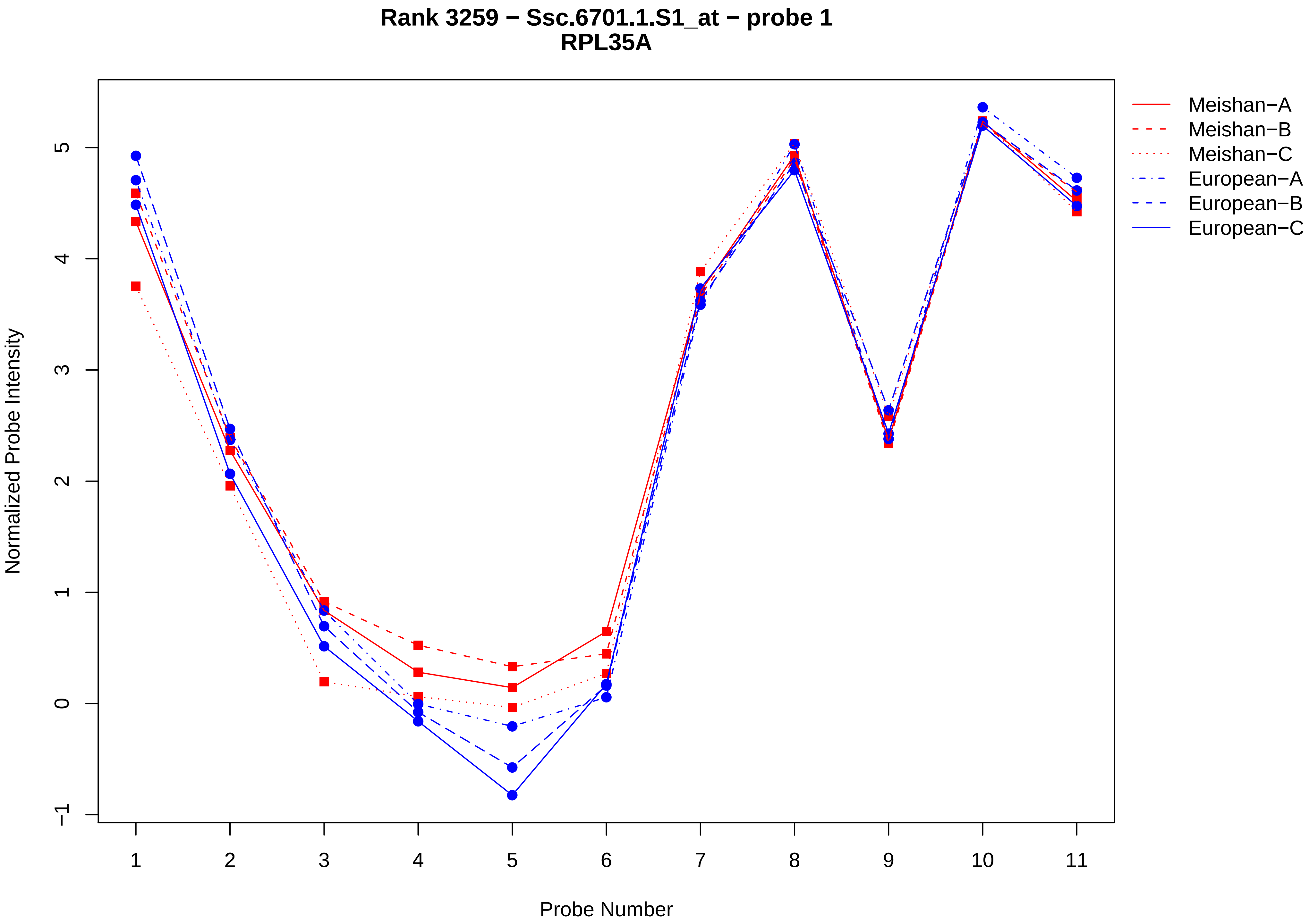

Rank 3259 − Ssc.6701.1.S1_at − probe 1
RPL35A
Normalized Probe Intensity
Probe Number
Meishan−A
Meishan−B
Meishan−C
European−A
European−B
European−C

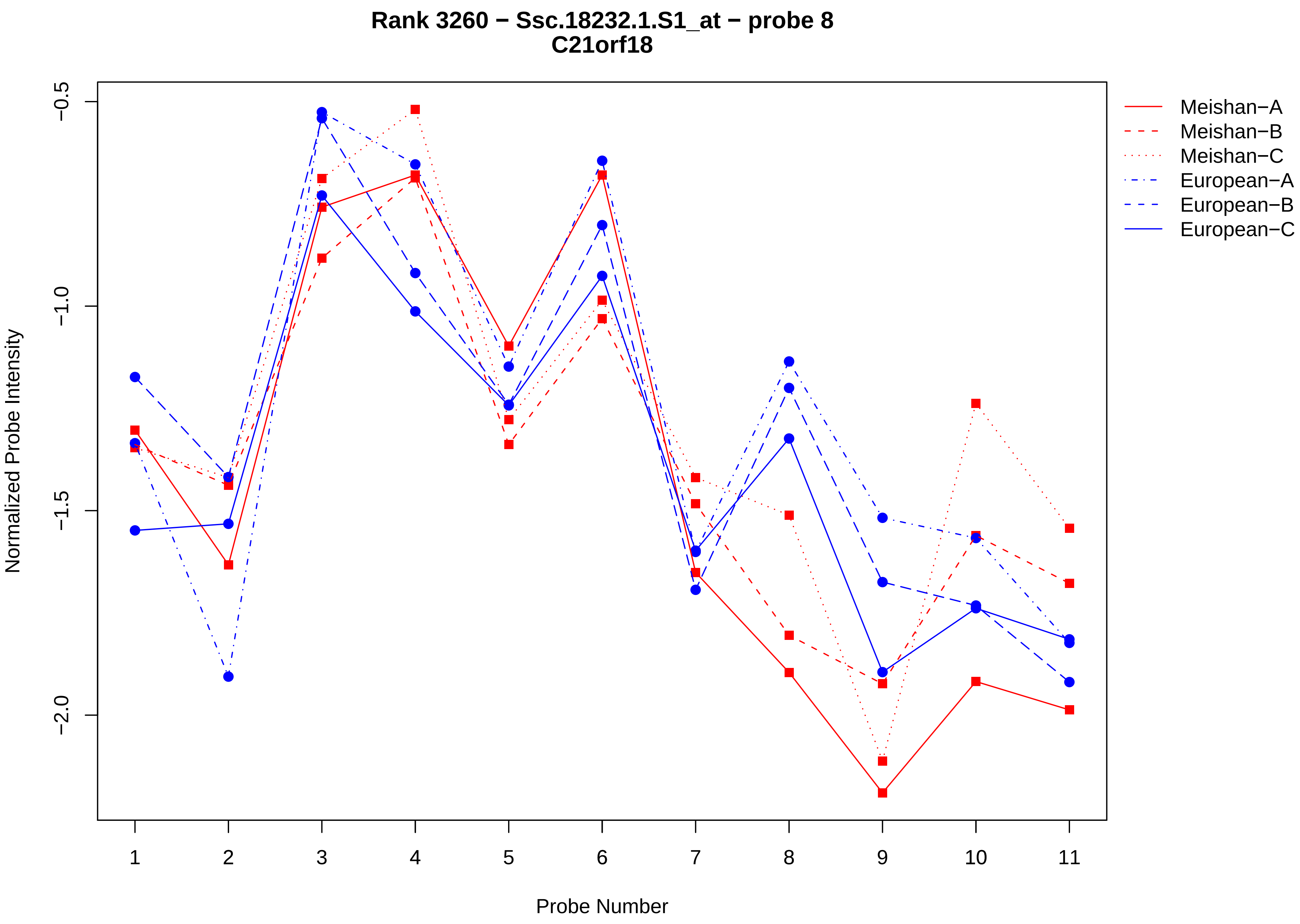

Rank 3260 − Ssc.18232.1.S1_at − probe 8
C21orf18
Normalized Probe Intensity
−0.5
−1.0
−1.5
−2.0
Probe Number
1
2
3
4
5
6
7
8
9
10
11
Meishan−A
Meishan−B
Meishan−C
European−A
European−B
European−C

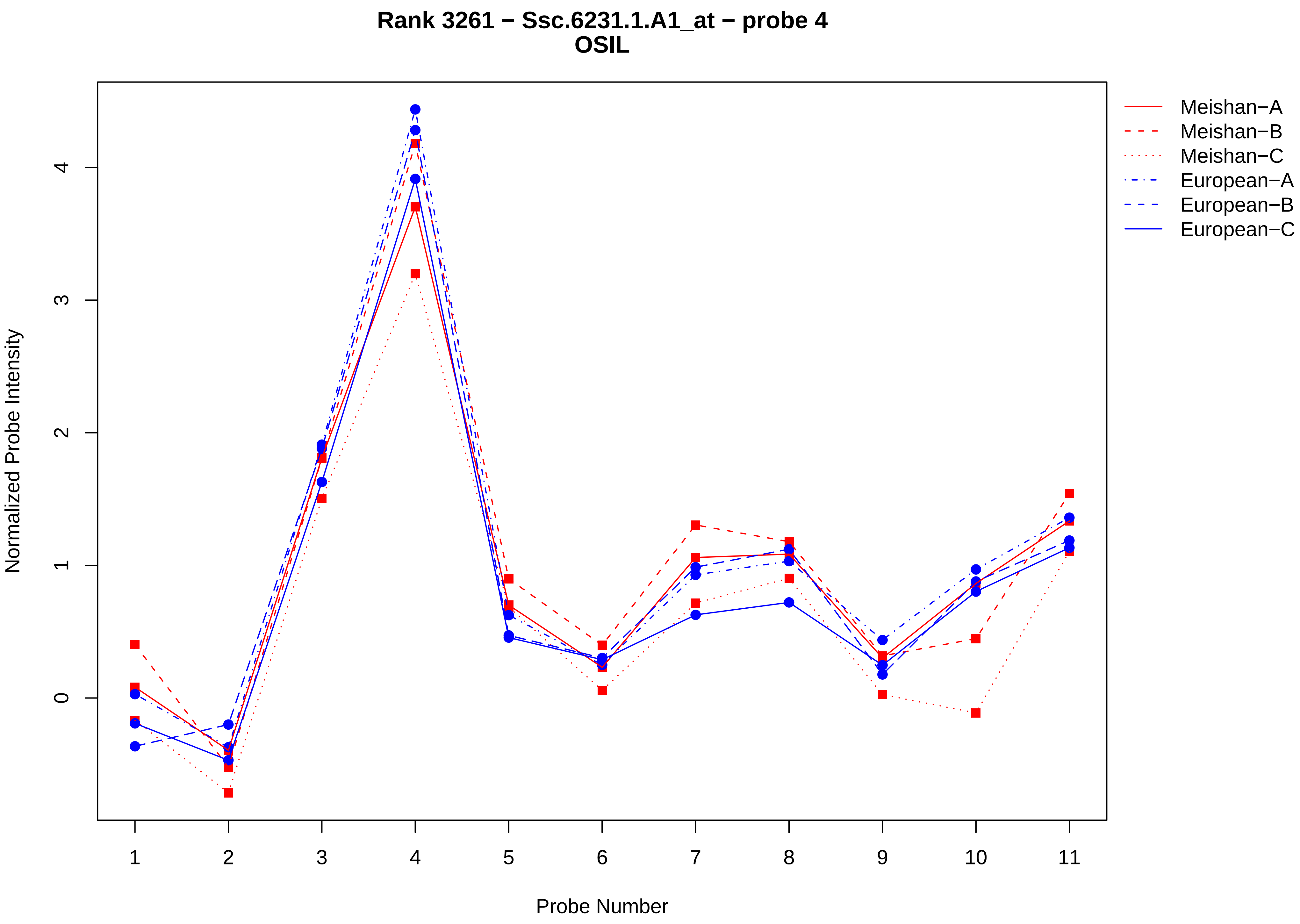

Rank 3261 − Ssc.6231.1.A1_at − probe 4
OSIL
Normalized Probe Intensity
Probe Number
0
1
2
3
4
1
2
3
4
5
6
7
8
9
10
11
Meishan−A
Meishan−B
Meishan−C
European−A
European−B
European−C

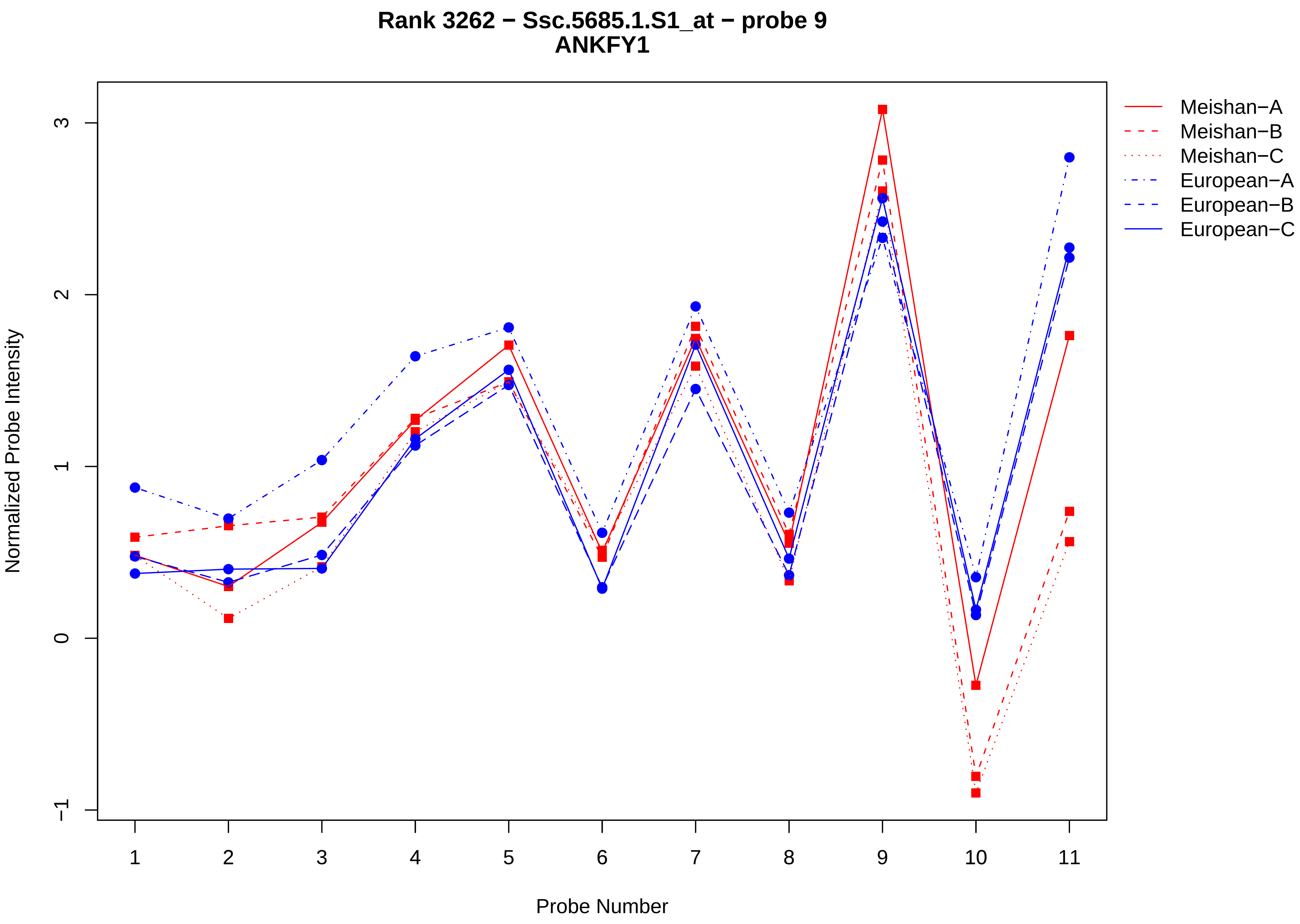

Rank 3262 − Ssc.5685.1.S1_at − probe 9
ANKFY1
Normalized Probe Intensity
Probe Number
−1
0
1
2
3
1
2
3
4
5
6
7
8
9
10
11
Meishan−A
Meishan−B
Meishan−C
European−A
European−B
European−C

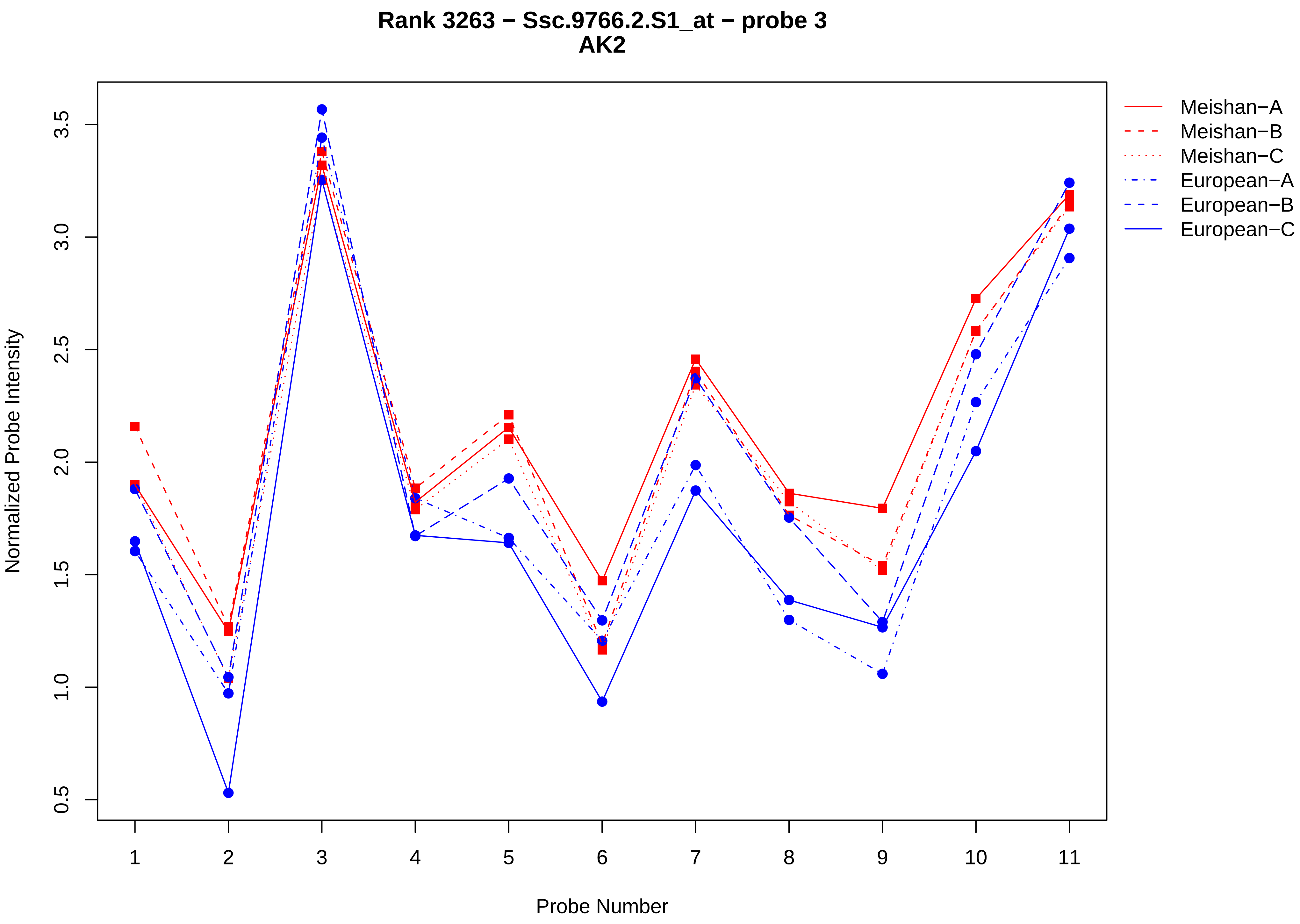
Rank 3263 − Ssc.9766.2.S1_at − probe 3
AK2
Normalized Probe Intensity
0.5
1.0
1.5
2.0
2.5
3.0
3.5
Probe Number
1
2
3
4
5
6
7
8
9
10
11
Meishan−A
Meishan−B
Meishan−C
European−A
European−B
European−C

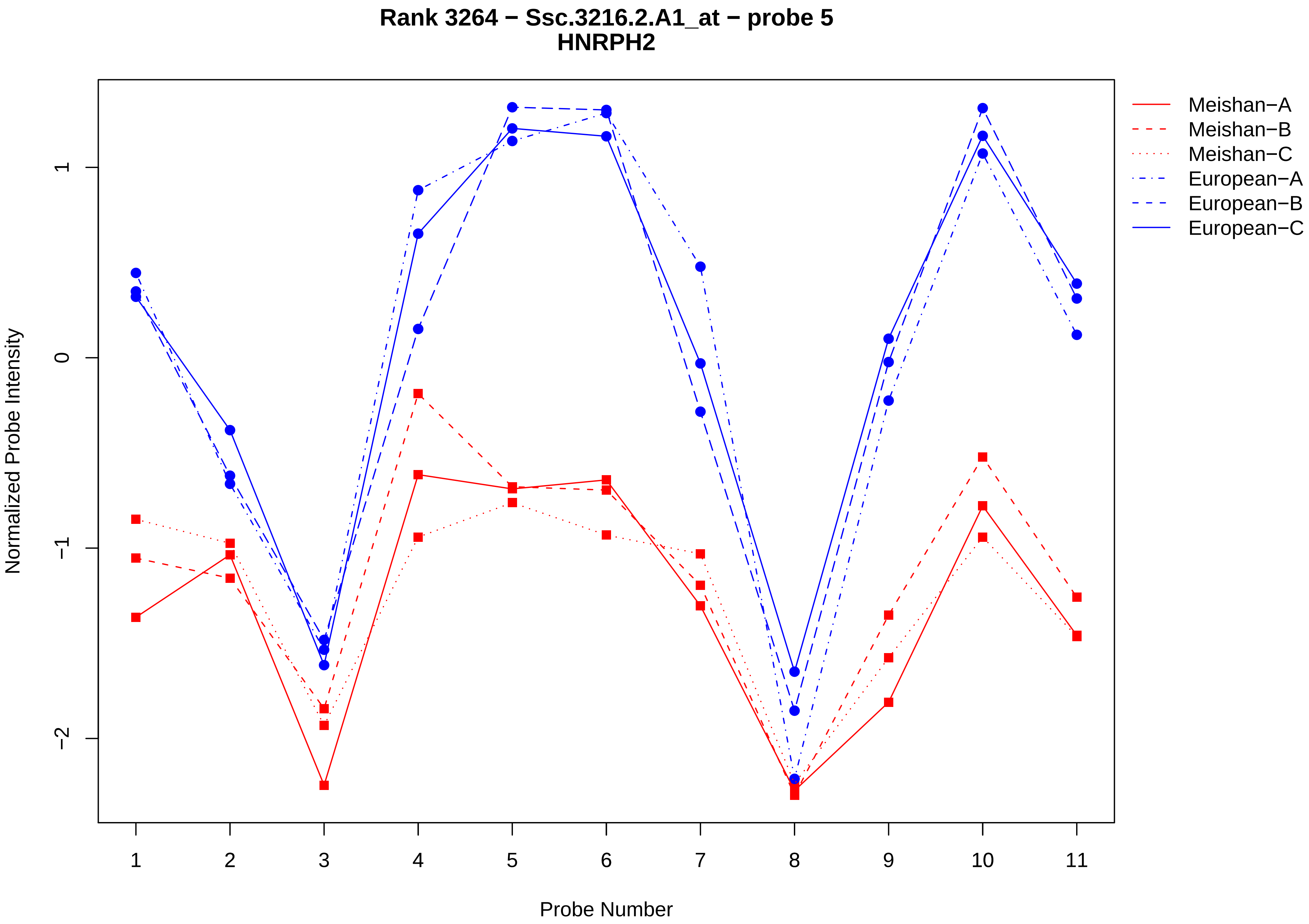

Rank 3264 − Ssc.3216.2.A1_at − probe 5
HNRPH2
Normalized Probe Intensity
Probe Number
1
0
−1
−2
1
2
3
4
5
6
7
8
9
10
11
Meishan−A
Meishan−B
Meishan−C
European−A
European−B
European−C

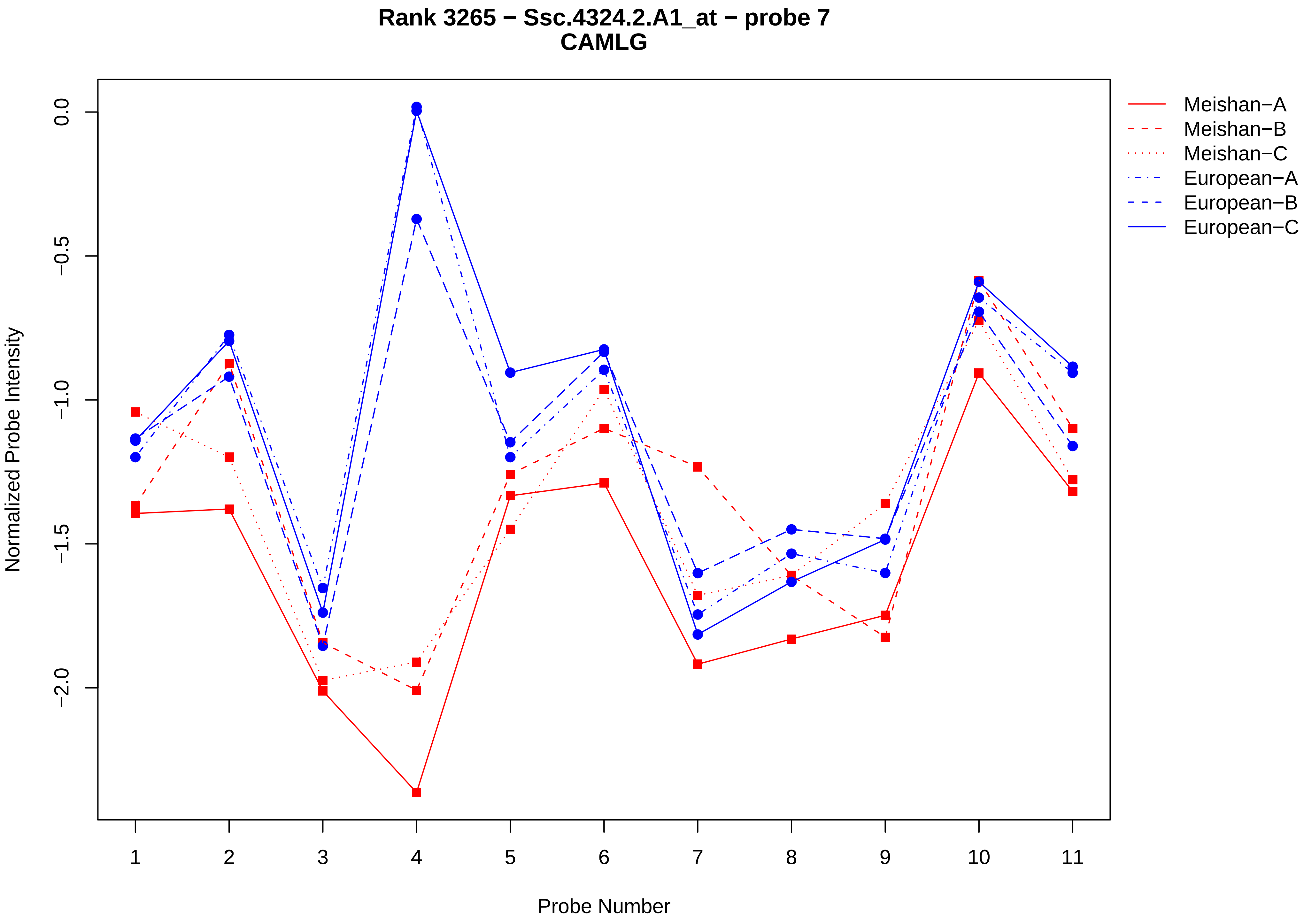

Rank 3265 − Ssc.4324.2.A1_at − probe 7
CAMLG
Normalized Probe Intensity
0.0
−0.5
−1.0
−1.5
−2.0
1
2
3
4
5
6
7
8
9
10
11
Probe Number
Meishan−A
Meishan−B
Meishan−C
European−A
European−B
European−C

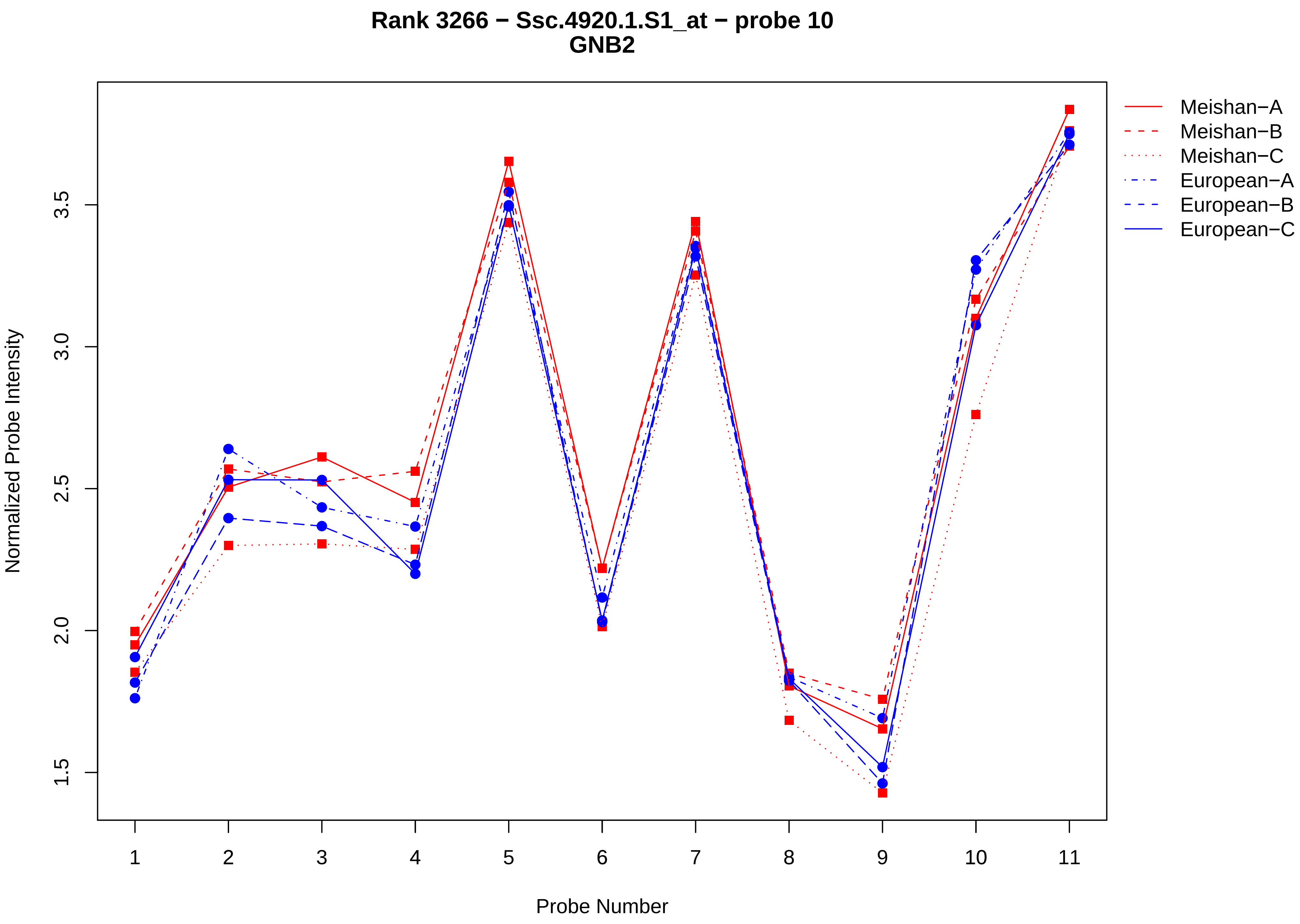

Rank 3266 − Ssc.4920.1.S1_at − probe 10
GNB2
Normalized Probe Intensity
Probe Number
1.5
2.0
2.5
3.0
3.5
1
2
3
4
5
6
7
8
9
10
11
Meishan−A
Meishan−B
Meishan−C
European−A
European−B
European−C

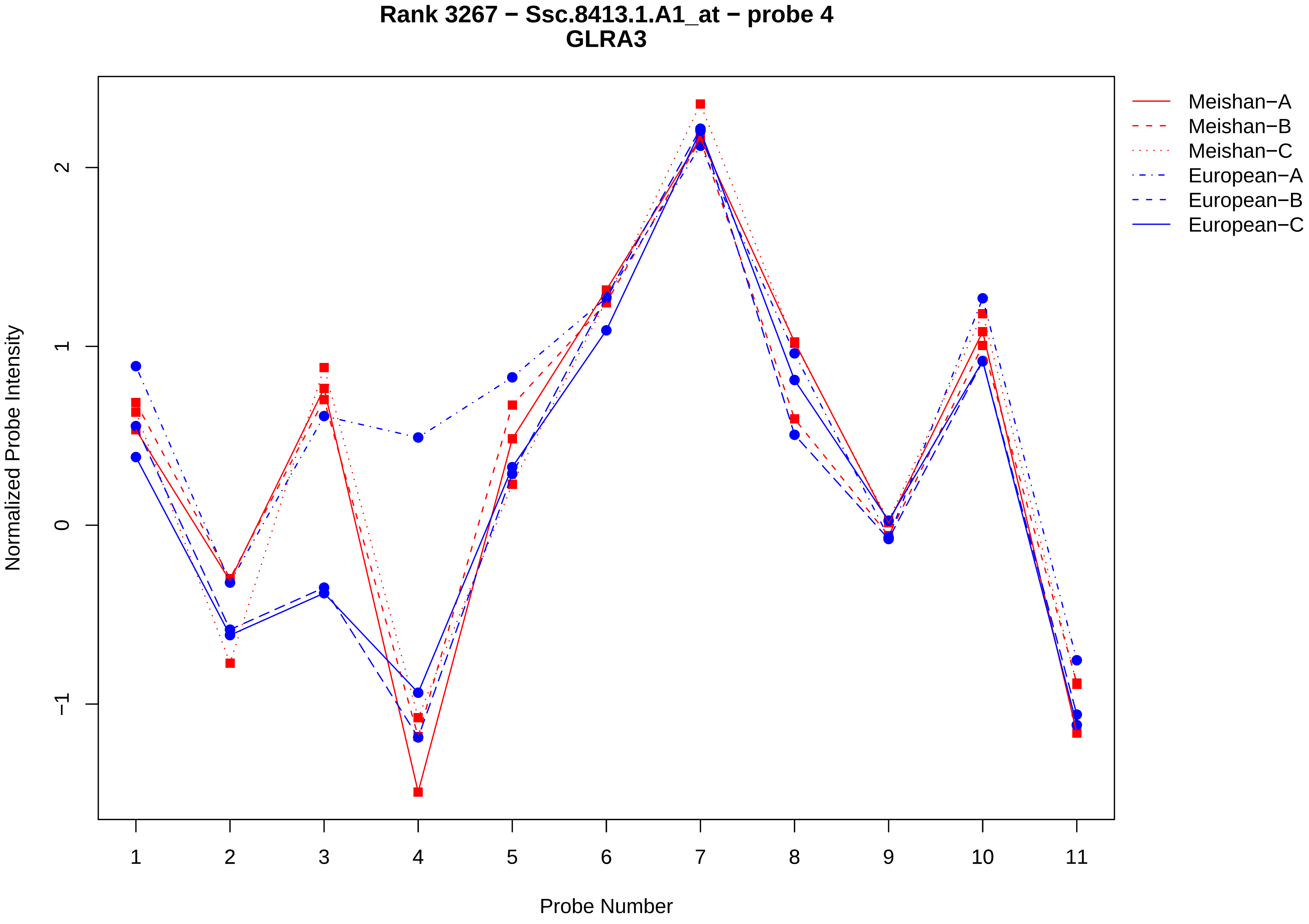

Rank 3267 − Ssc.8413.1.A1_at − probe 4
GLRA3
Normalized Probe Intensity
Probe Number
Meishan−A
Meishan−B
Meishan−C
European−A
European−B
European−C
−1
0
1
2
1
2
3
4
5
6
7
8
9
10
11

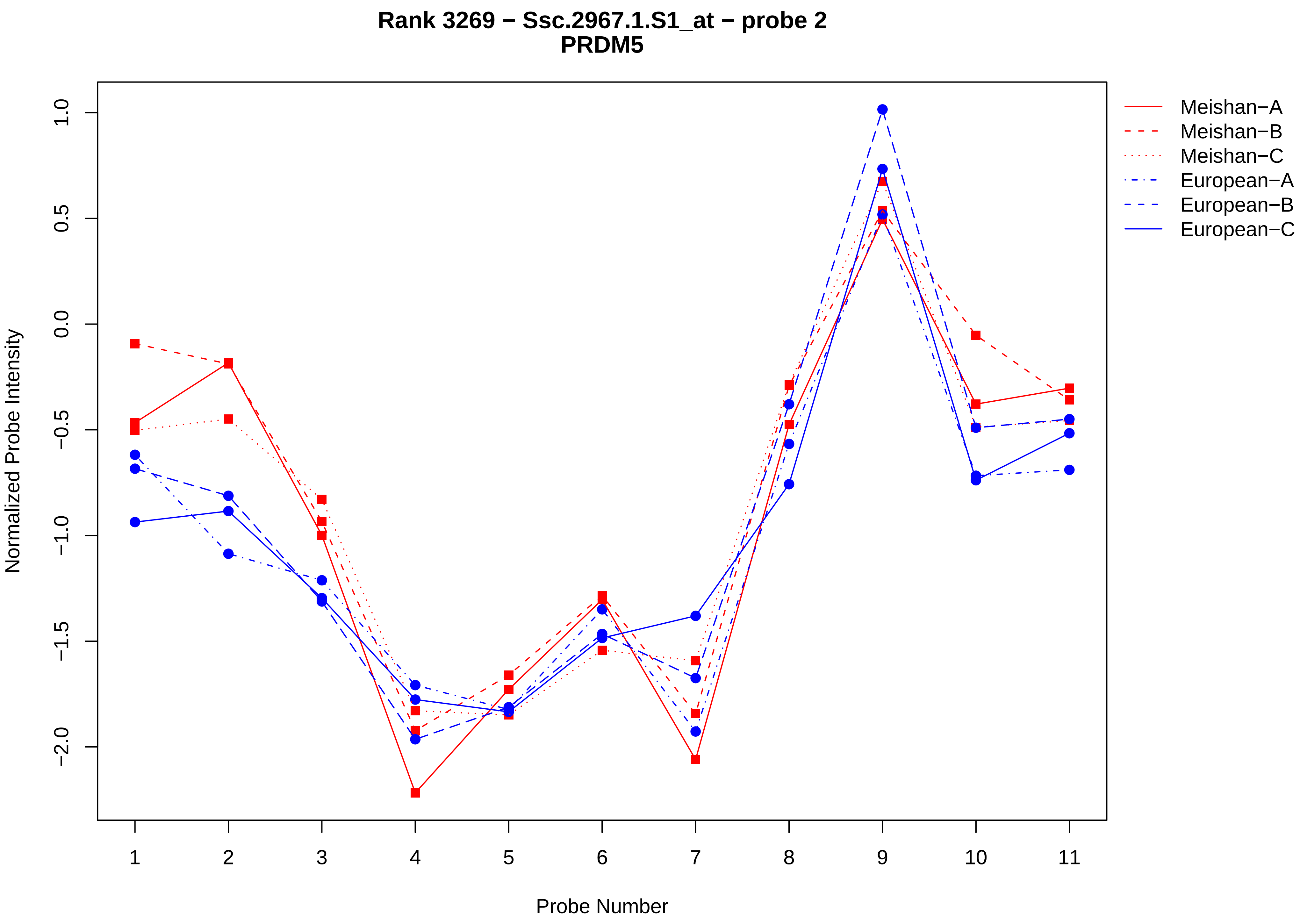

Rank 3269 − Ssc.2967.1.S1_at − probe 2
PRDM5
Normalized Probe Intensity
Probe Number
1.0
0.5
0.0
−0.5
−1.0
−1.5
−2.0
1
2
3
4
5
6
7
8
9
10
11
Meishan−A
Meishan−B
Meishan−C
European−A
European−B
European−C

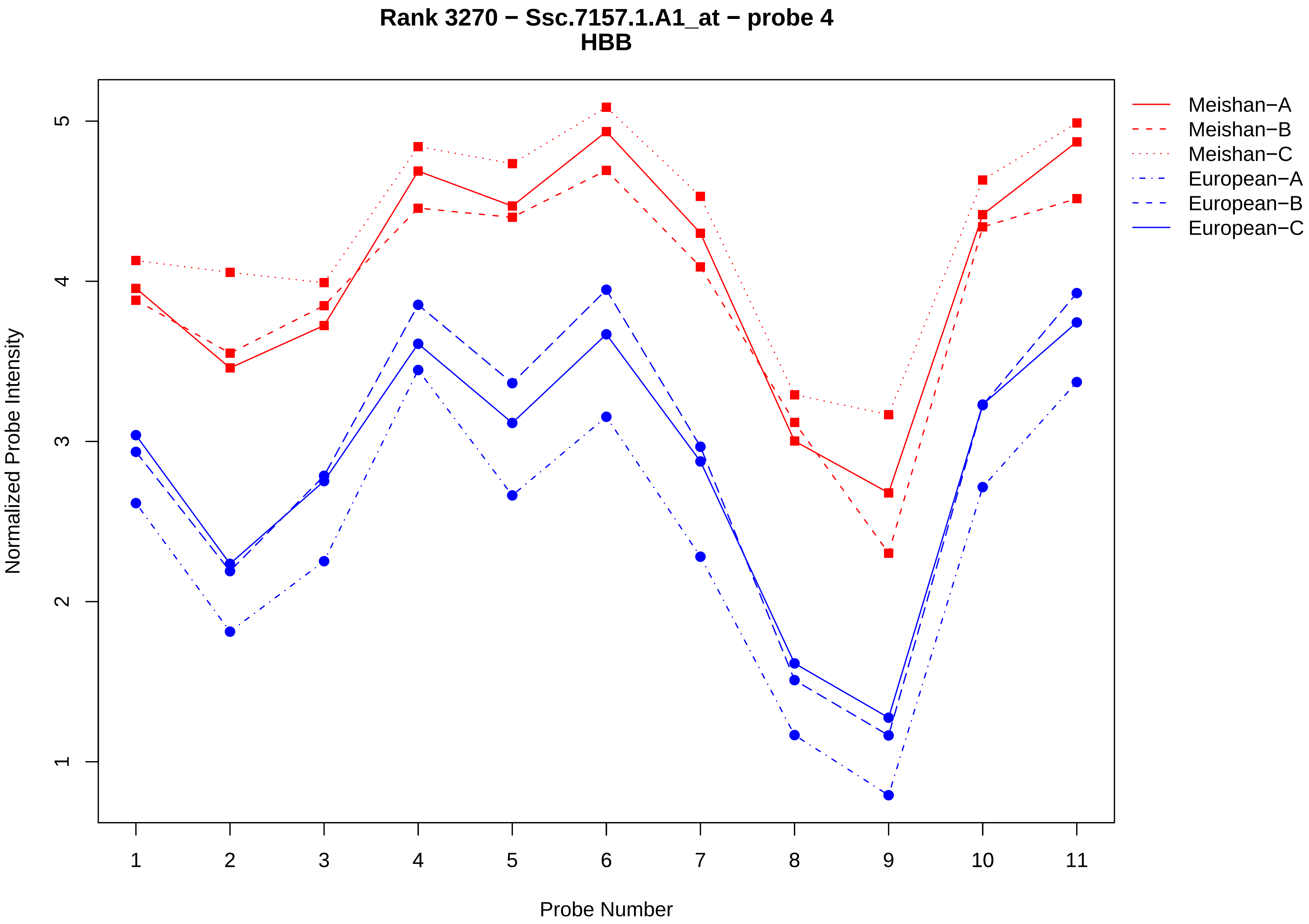
Rank 3270 − Ssc.7157.1.A1_at − probe 4
HBB
Normalized Probe Intensity
Probe Number
1
2
3
4
5
1
2
3
4
5
6
7
8
9
10
11
Meishan−A
Meishan−B
Meishan−C
European−A
European−B
European−C

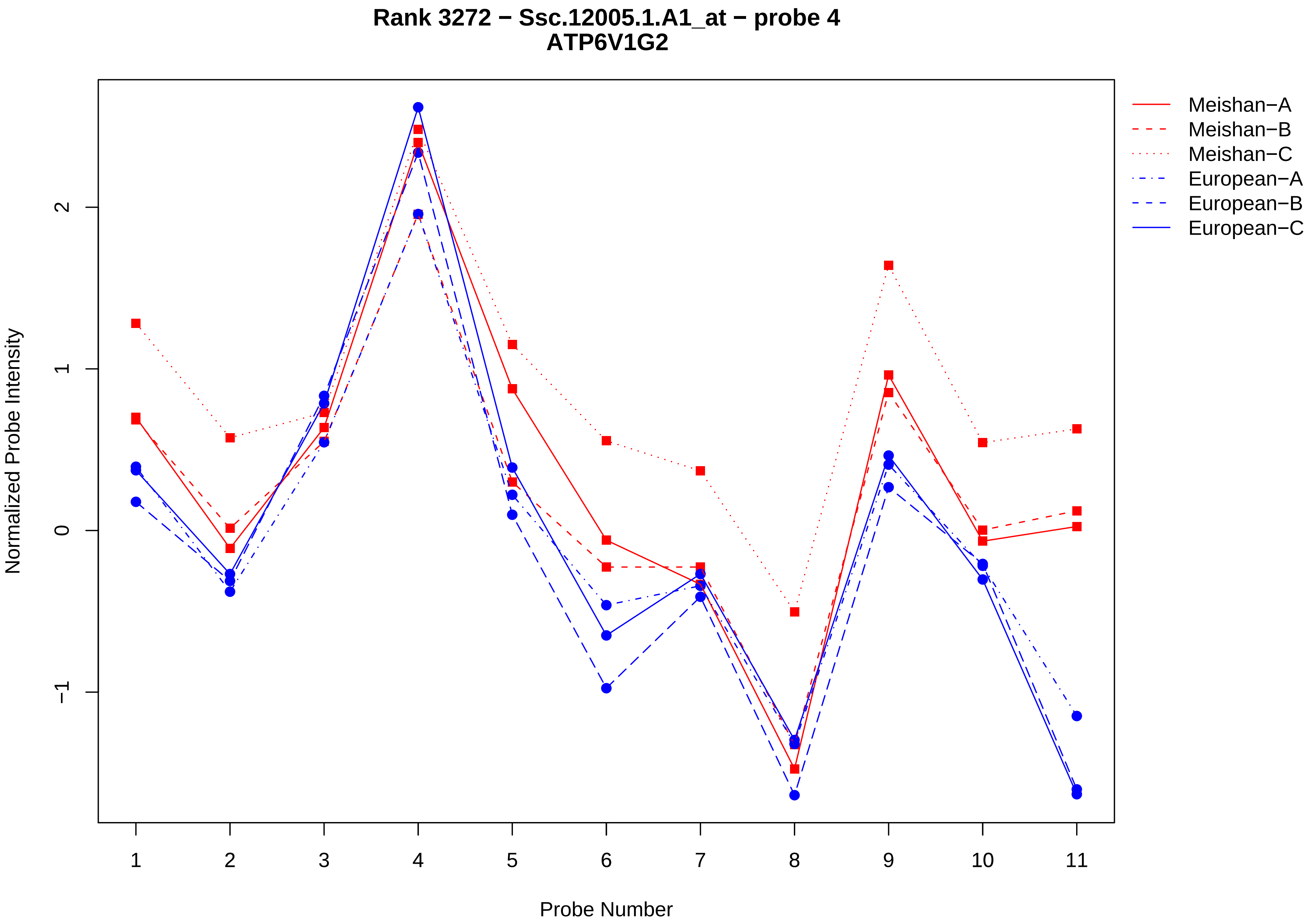
Rank 3272 − Ssc.12005.1.A1_at − probe 4
ATP6V1G2
Normalized Probe Intensity
Probe Number
Meishan−A
Meishan−B
Meishan−C
European−A
European−B
European−C
−1
0
1
2
1
2
3
4
5
6
7
8
9
10
11

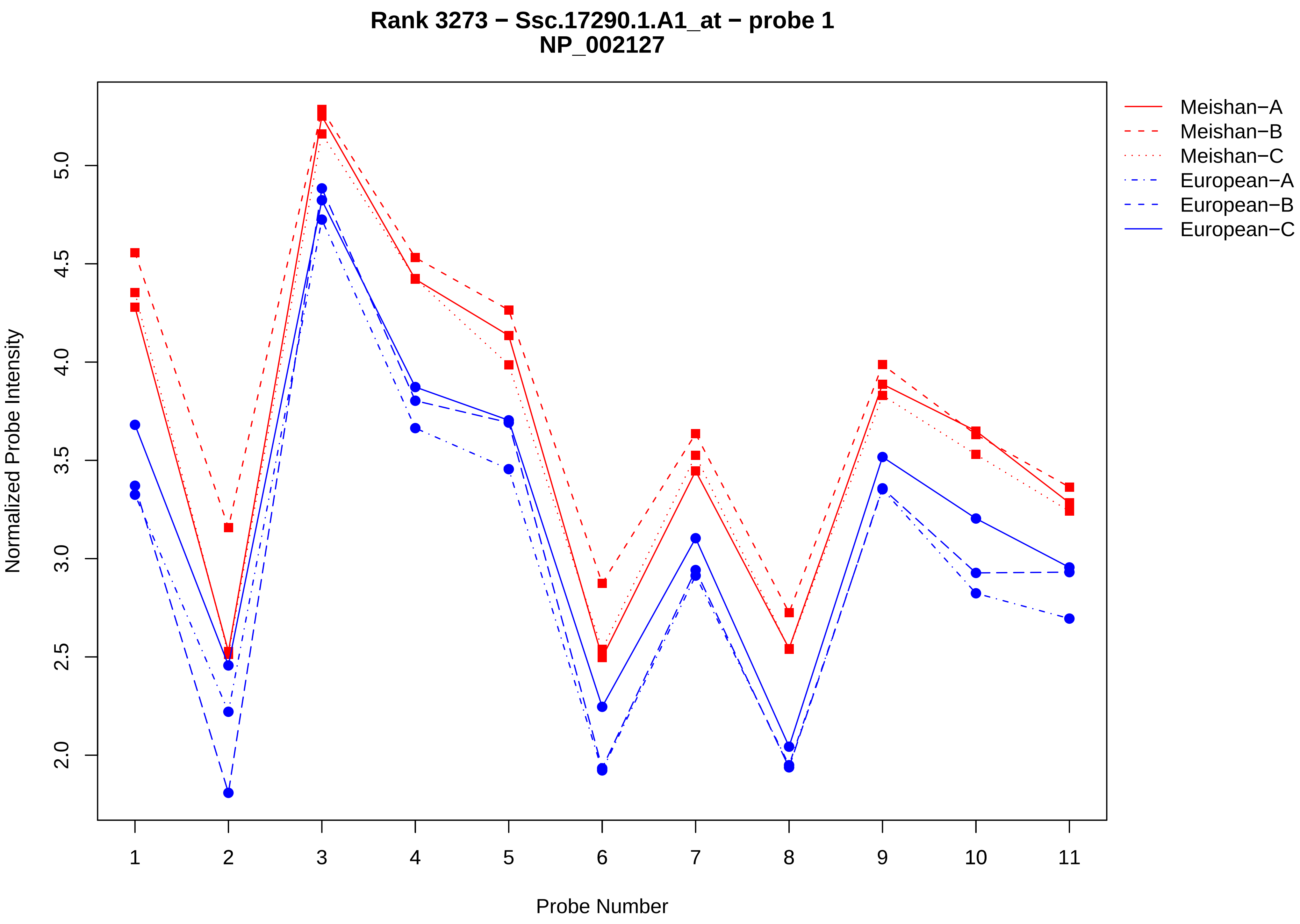
Rank 3273 − Ssc.17290.1.A1_at − probe 1
NP_002127
Normalized Probe Intensity
Probe Number
Meishan−A
Meishan−B
Meishan−C
European−A
European−B
European−C
2.0
2.5
3.0
3.5
4.0
4.5
5.0
1
2
3
4
5
6
7
8
9
10
11

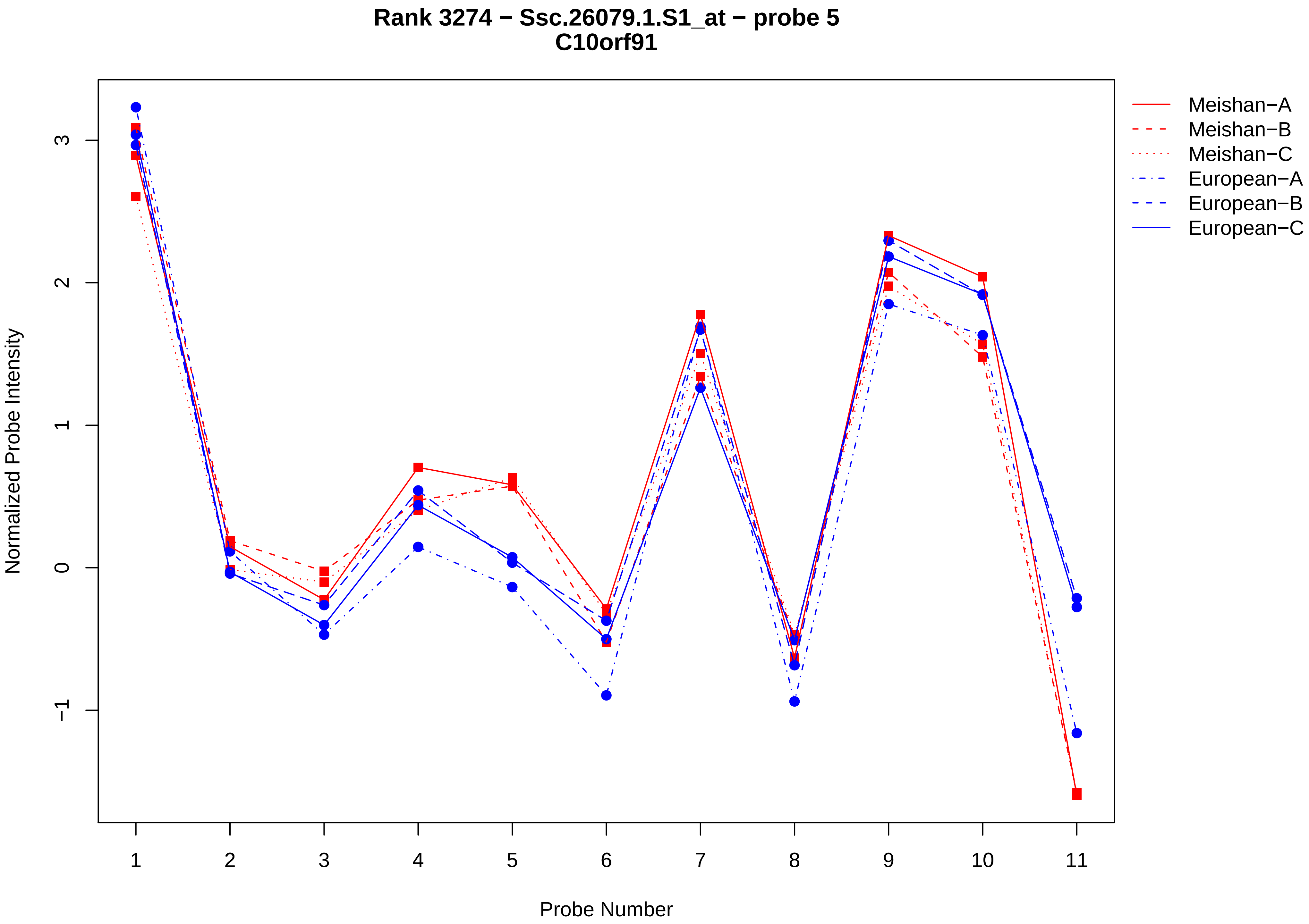

Rank 3274 − Ssc.26079.1.S1_at − probe 5
C10orf91
Normalized Probe Intensity
Probe Number
−1
0
1
2
3
1
2
3
4
5
6
7
8
9
10
11
Meishan−A
Meishan−B
Meishan−C
European−A
European−B
European−C

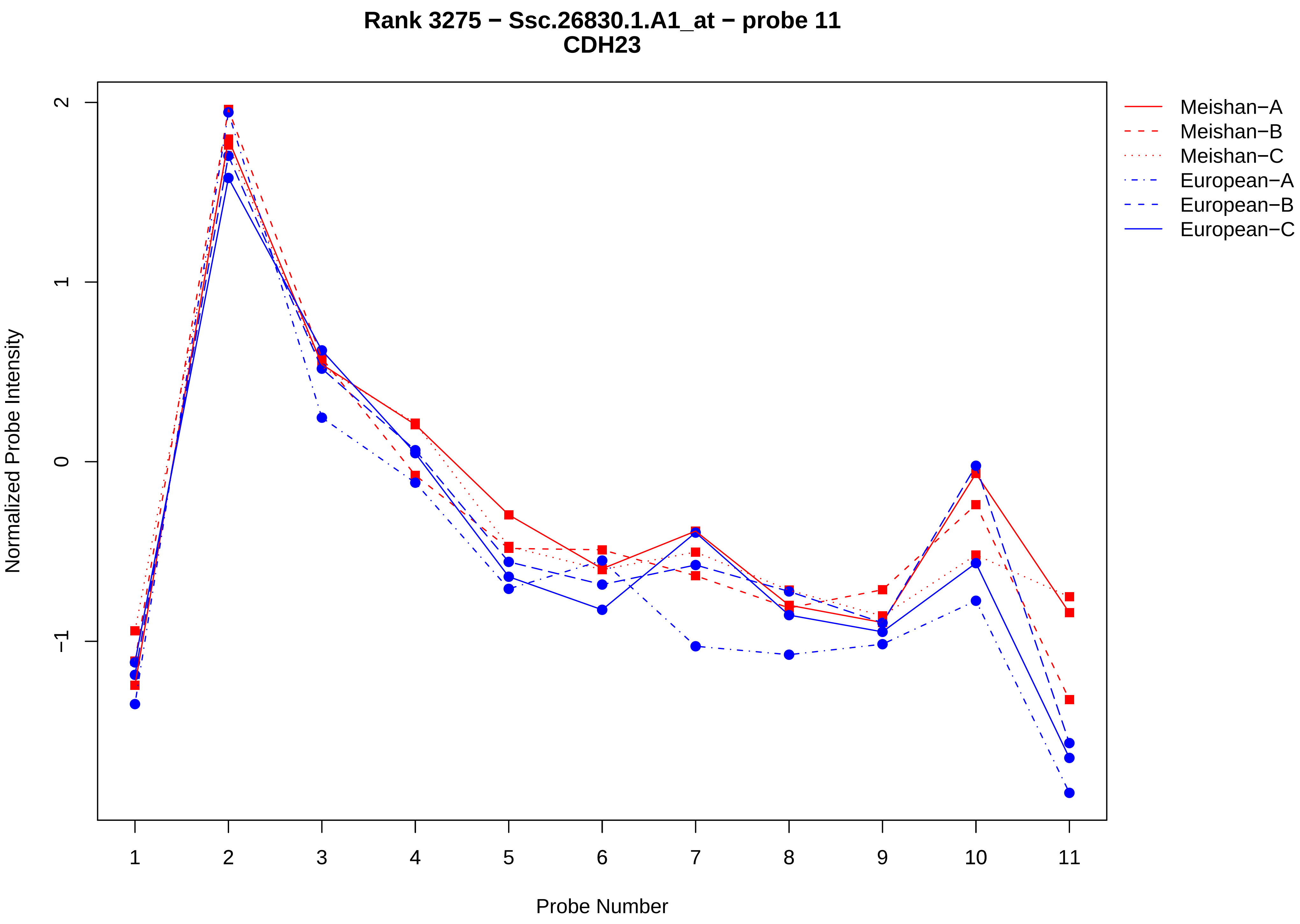

Rank 3275 − Ssc.26830.1.A1_at − probe 11
CDH23
Normalized Probe Intensity
Probe Number
2
1
0
−1
1
2
3
4
5
6
7
8
9
10
11
Meishan−A
Meishan−B
Meishan−C
European−A
European−B
European−C

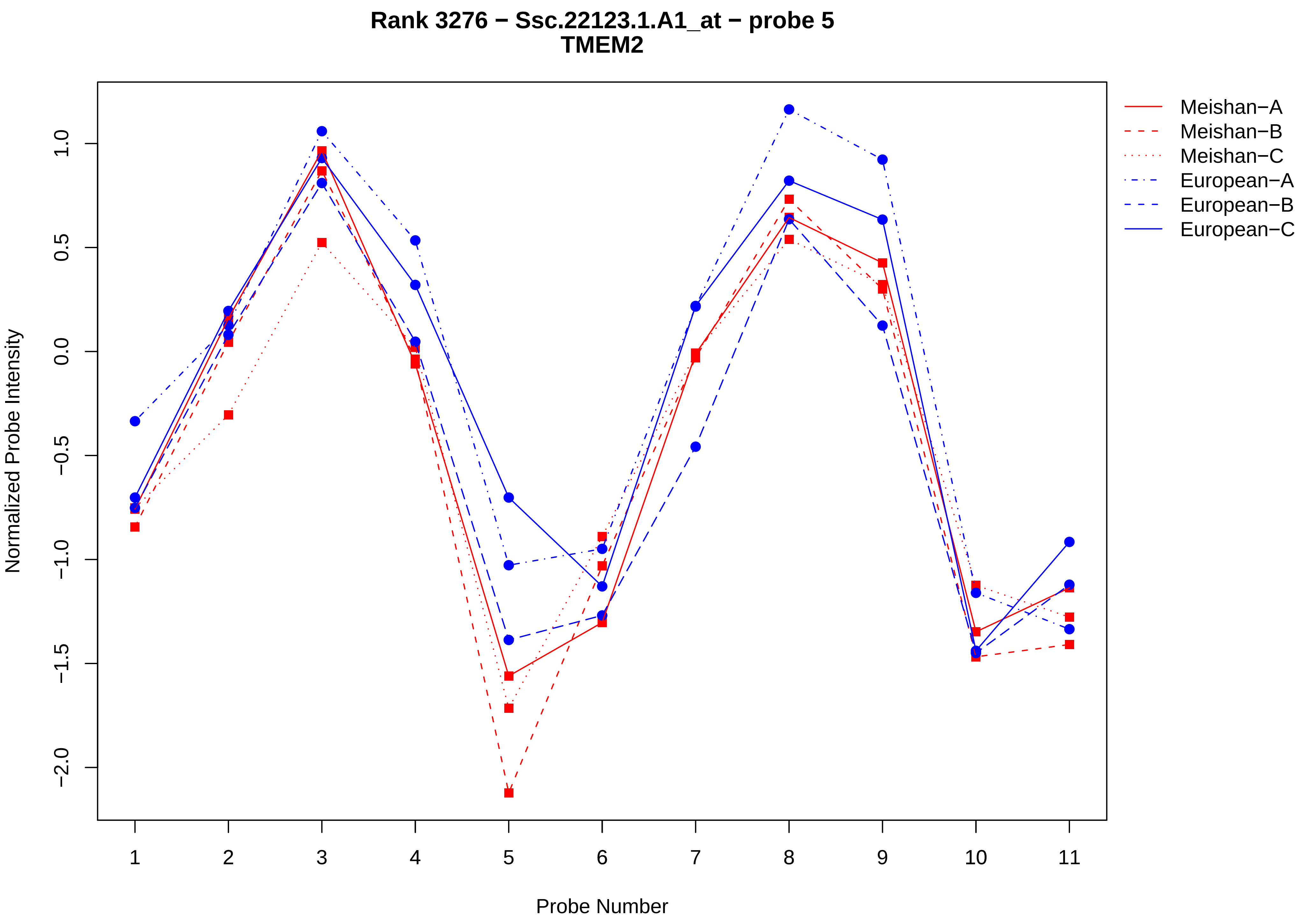

Rank 3276 − Ssc.22123.1.A1_at − probe 5
TMEM2
Normalized Probe Intensity
Probe Number
1.0
0.5
0.0
−0.5
−1.0
−1.5
−2.0
1
2
3
4
5
6
7
8
9
10
11
Meishan−A
Meishan−B
Meishan−C
European−A
European−B
European−C

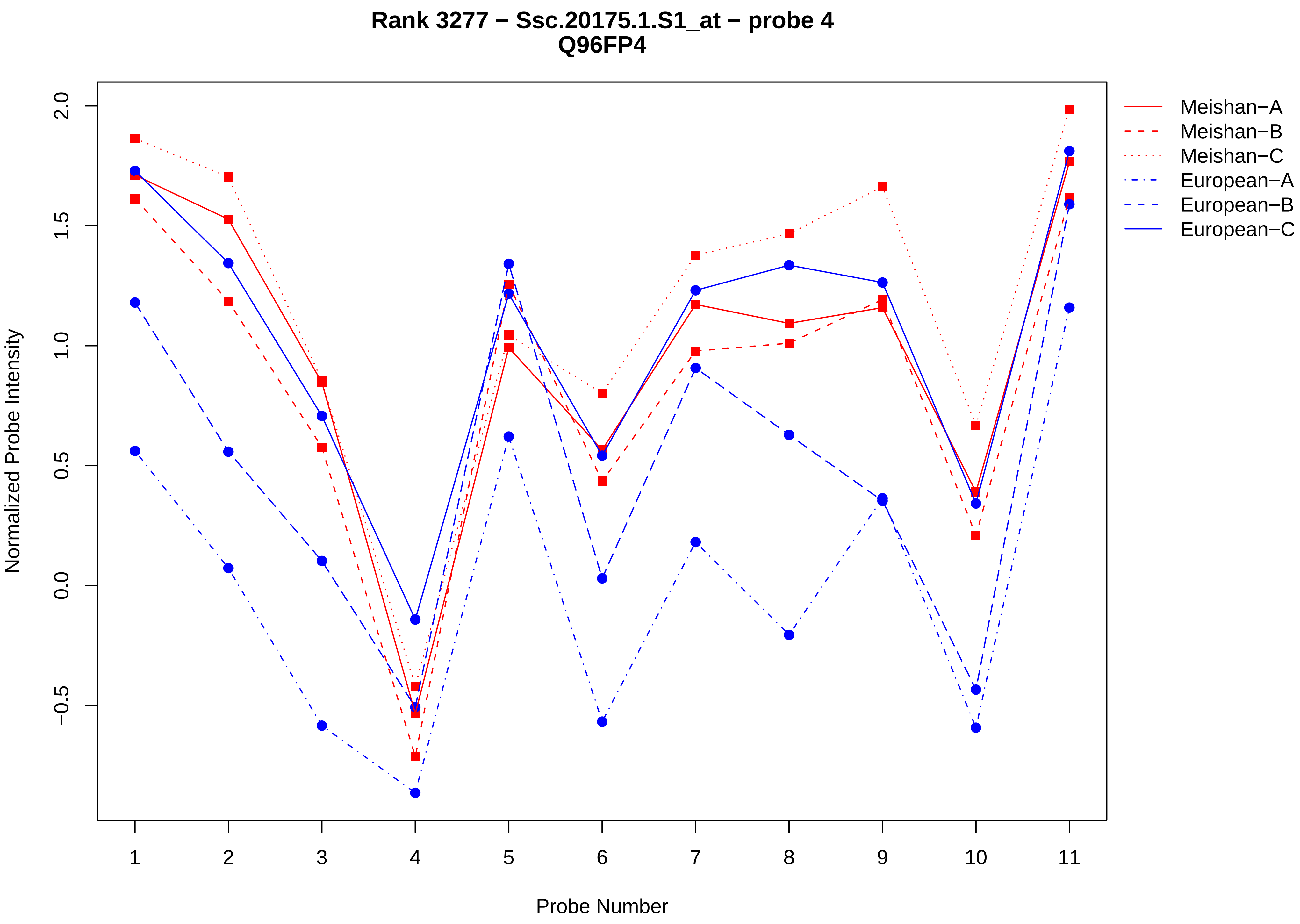
Rank 3277 − Ssc.20175.1.S1_at − probe 4
Q96FP4
Normalized Probe Intensity
Probe Number
Meishan−A
Meishan−B
Meishan−C
European−A
European−B
European−C
−0.5
0.0
0.5
1.0
1.5
2.0
1
2
3
4
5
6
7
8
9
10
11

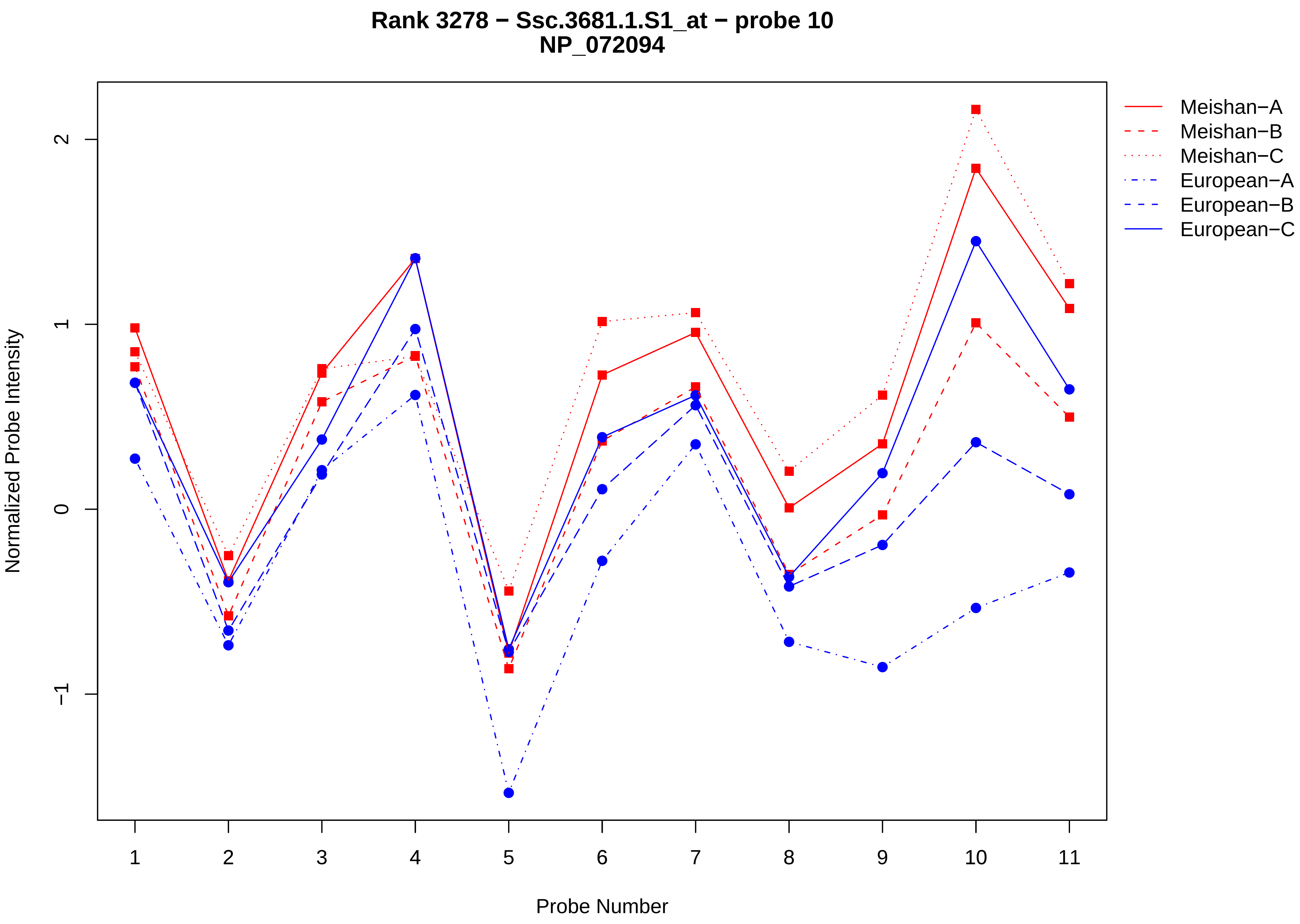

Rank 3278 − Ssc.3681.1.S1_at − probe 10
NP_072094
Normalized Probe Intensity
Probe Number
−1
0
1
2
1
2
3
4
5
6
7
8
9
10
11
Meishan−A
Meishan−B
Meishan−C
European−A
European−B
European−C

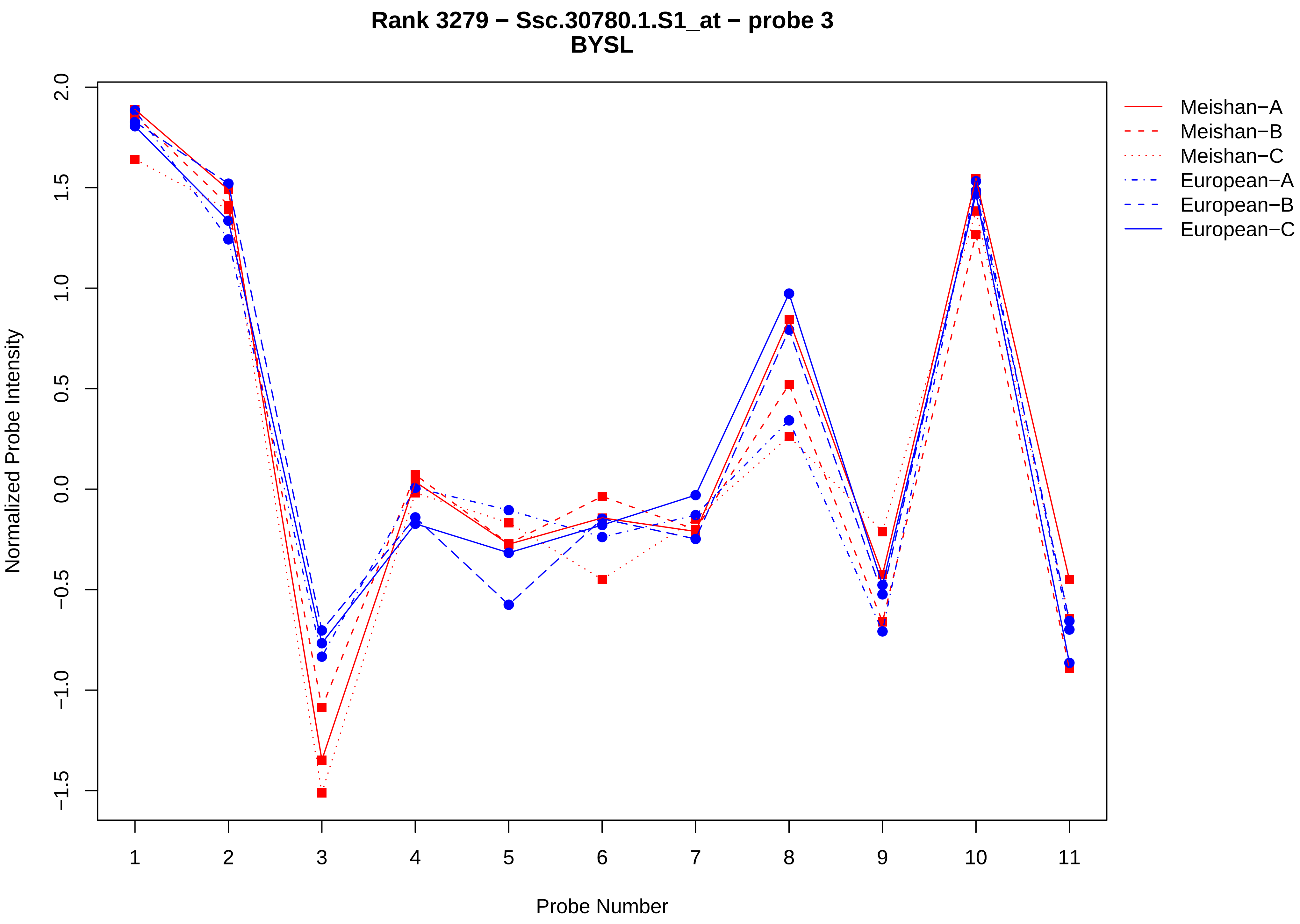

Rank 3279 − Ssc.30780.1.S1_at − probe 3
BYSL
Normalized Probe Intensity
Probe Number
−1.5
−1.0
−0.5
0.0
0.5
1.0
1.5
2.0
1
2
3
4
5
6
7
8
9
10
11
Meishan−A
Meishan−B
Meishan−C
European−A
European−B
European−C

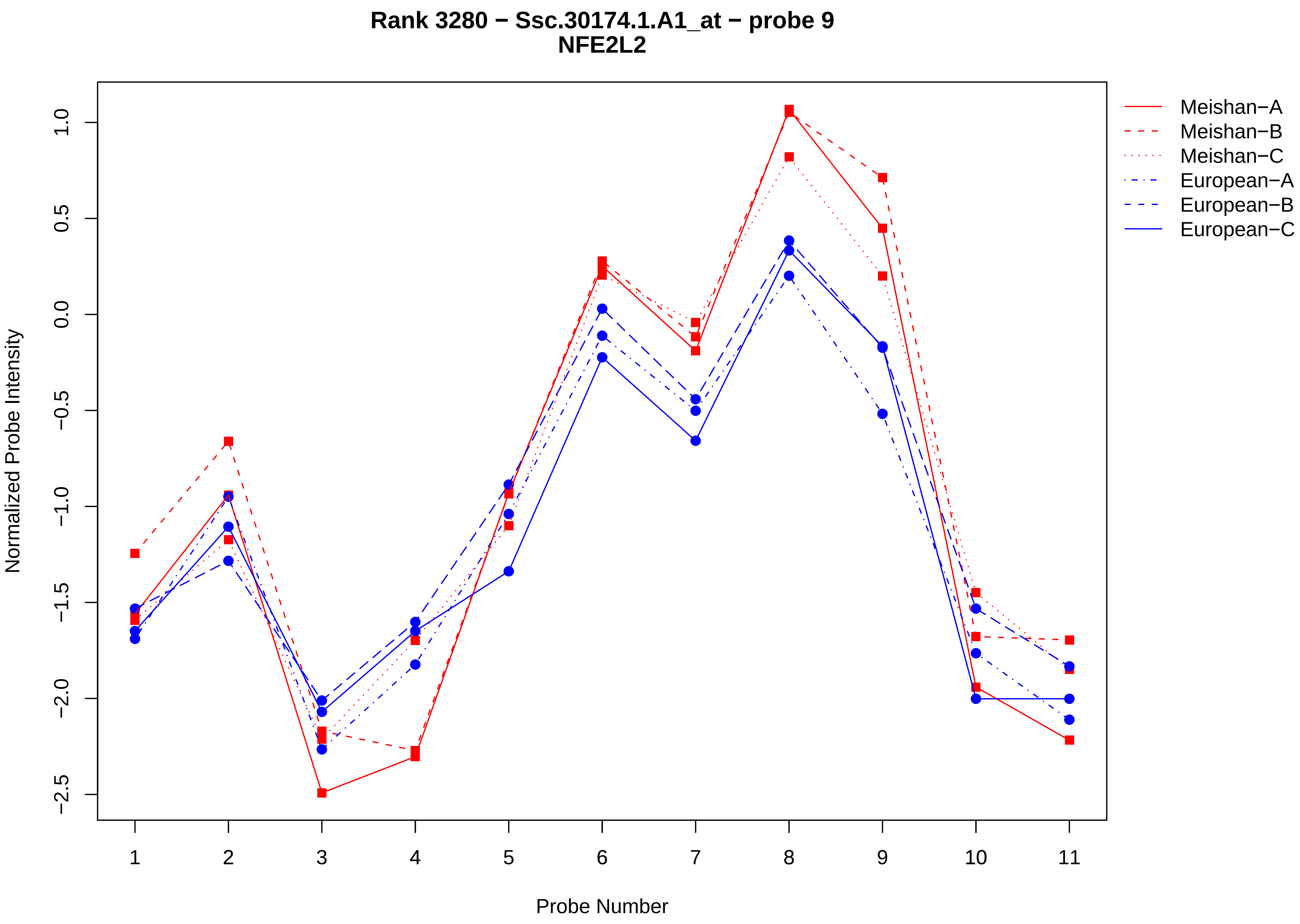

Rank 3280 − Ssc.30174.1.A1_at − probe 9
NFE2L2
Normalized Probe Intensity
Probe Number
1.0
0.5
0.0
−0.5
−1.0
−1.5
−2.0
−2.5
1
2
3
4
5
6
7
8
9
10
11
Meishan−A
Meishan−B
Meishan−C
European−A
European−B
European−C

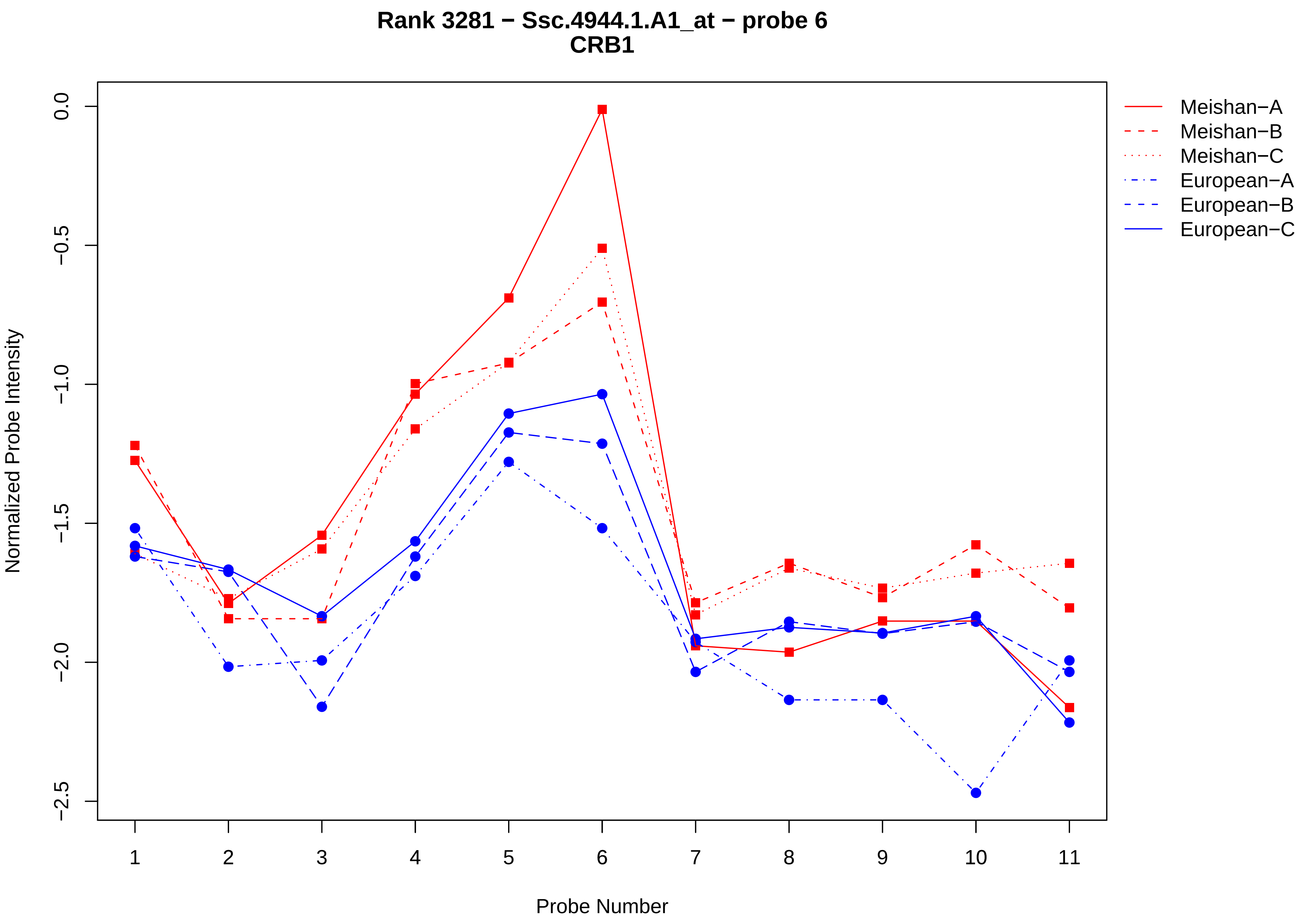
Rank 3281 − Ssc.4944.1.A1_at − probe 6
CRB1
Normalized Probe Intensity
0.0
−0.5
−1.0
−1.5
−2.0
−2.5
1
2
3
4
5
6
7
8
9
10
11
Probe Number
Meishan−A
Meishan−B
Meishan−C
European−A
European−B
European−C

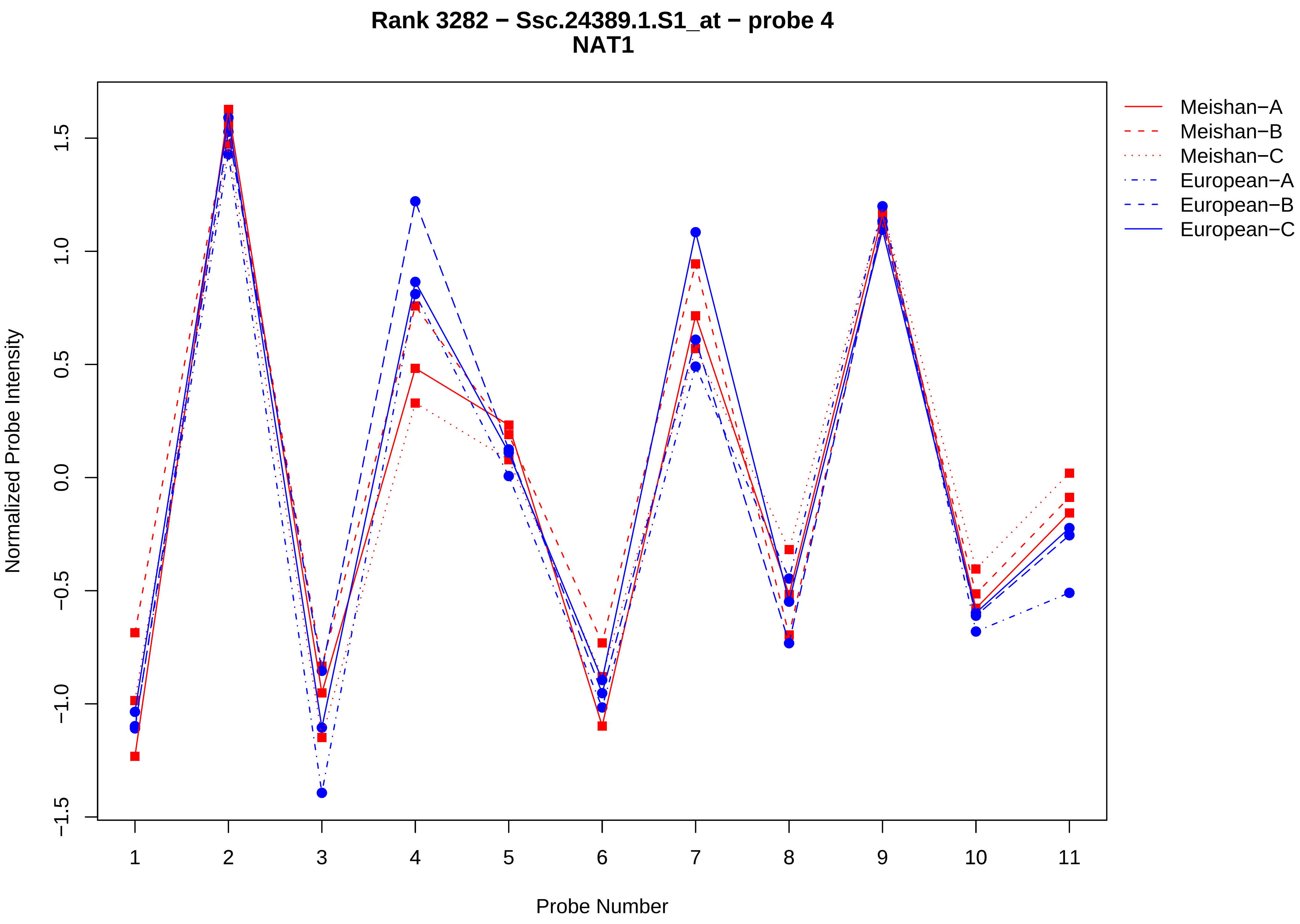

Rank 3282 − Ssc.24389.1.S1_at − probe 4
NAT1
Normalized Probe Intensity
Probe Number
−1.5
−1.0
−0.5
0.0
0.5
1.0
1.5
1
2
3
4
5
6
7
8
9
10
11
Meishan−A
Meishan−B
Meishan−C
European−A
European−B
European−C

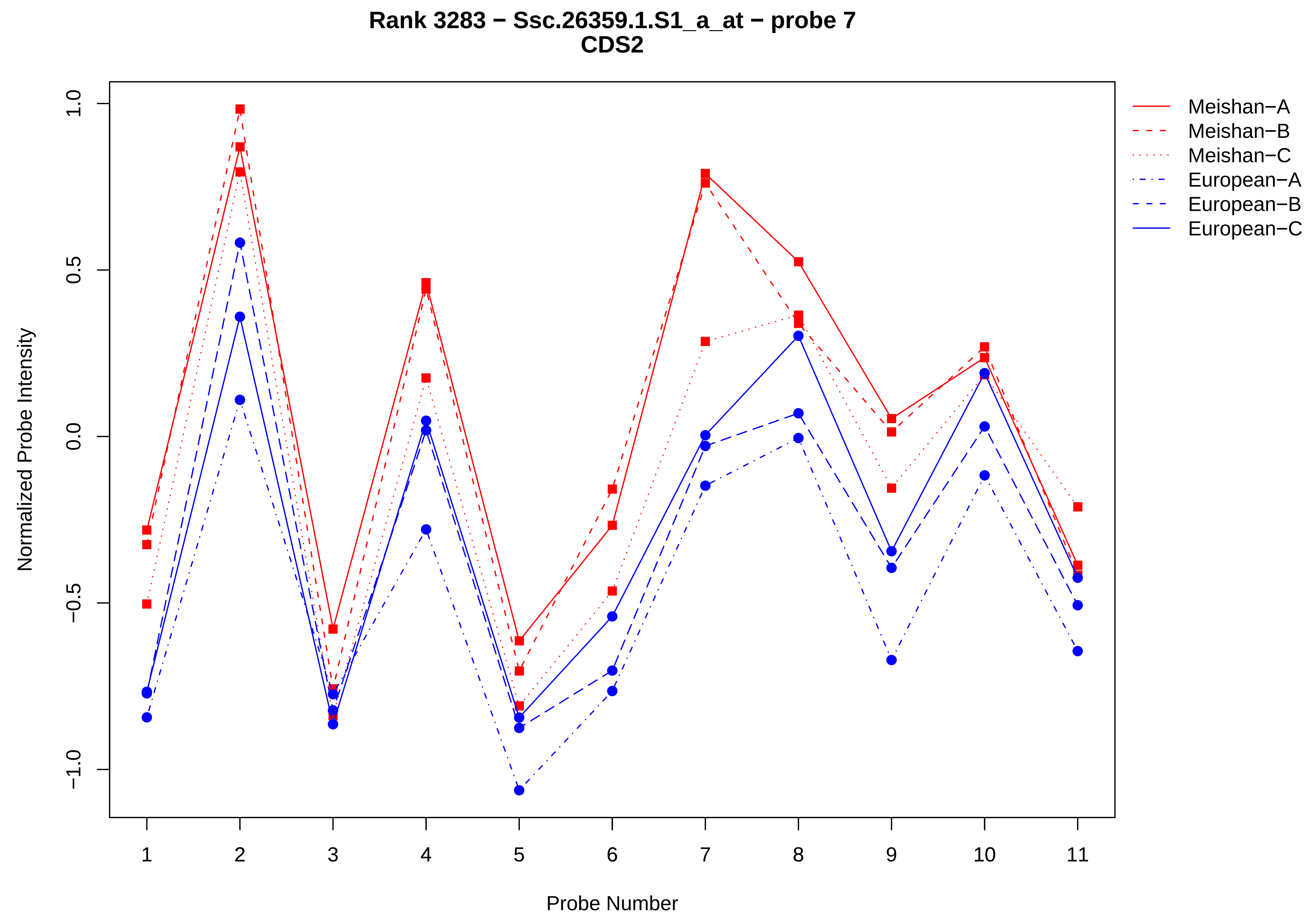
Rank 3283 − Ssc.26359.1.S1_a_at − probe 7
CDS2
Normalized Probe Intensity
Probe Number
1.0
0.5
0.0
−0.5
−1.0
1
2
3
4
5
6
7
8
9
10
11
Meishan−A
Meishan−B
Meishan−C
European−A
European−B
European−C

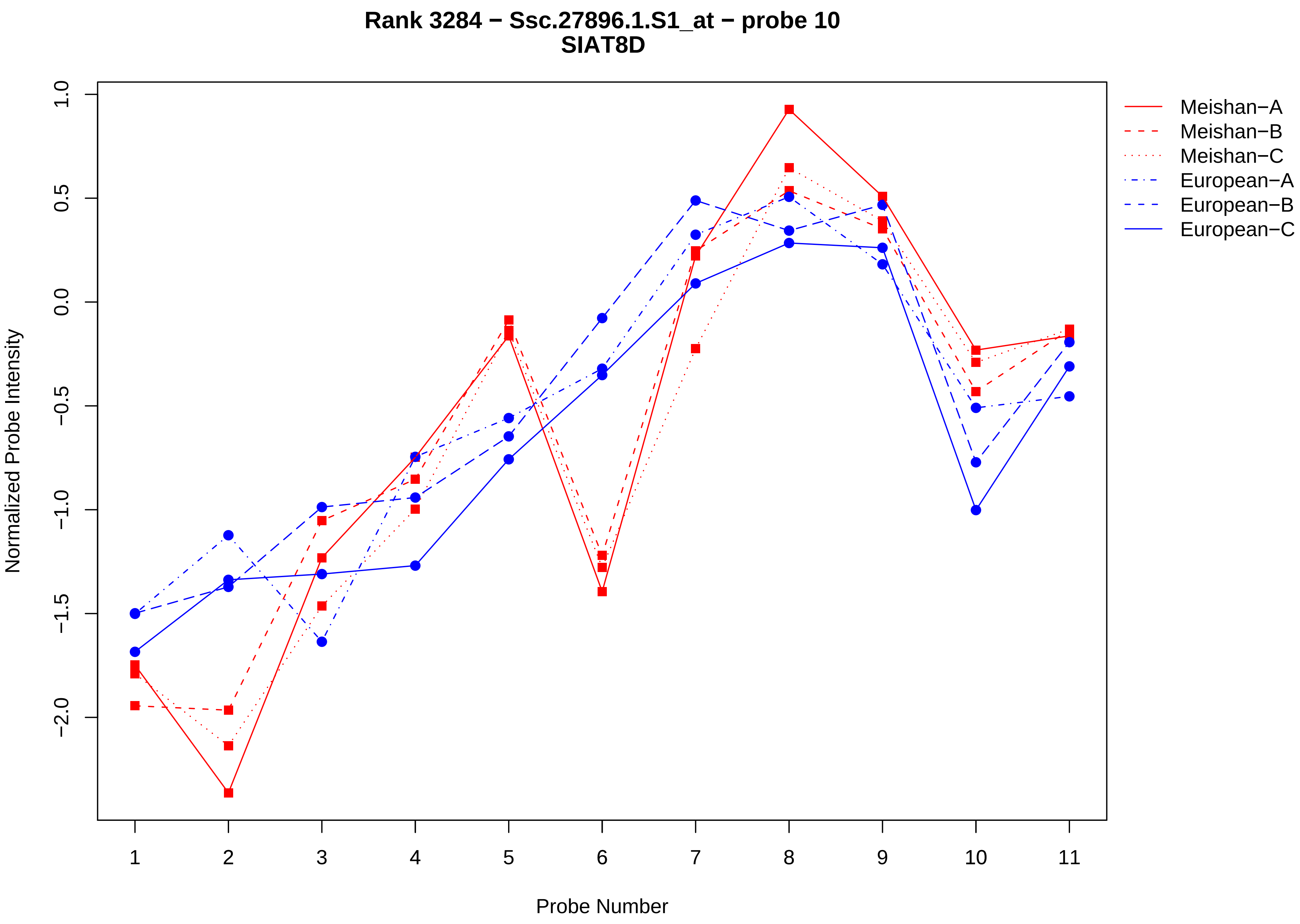

Rank 3284 − Ssc.27896.1.S1_at − probe 10
SIAT8D
Normalized Probe Intensity
Probe Number
1.0
0.5
0.0
−0.5
−1.0
−1.5
−2.0
1
2
3
4
5
6
7
8
9
10
11
Meishan−A
Meishan−B
Meishan−C
European−A
European−B
European−C

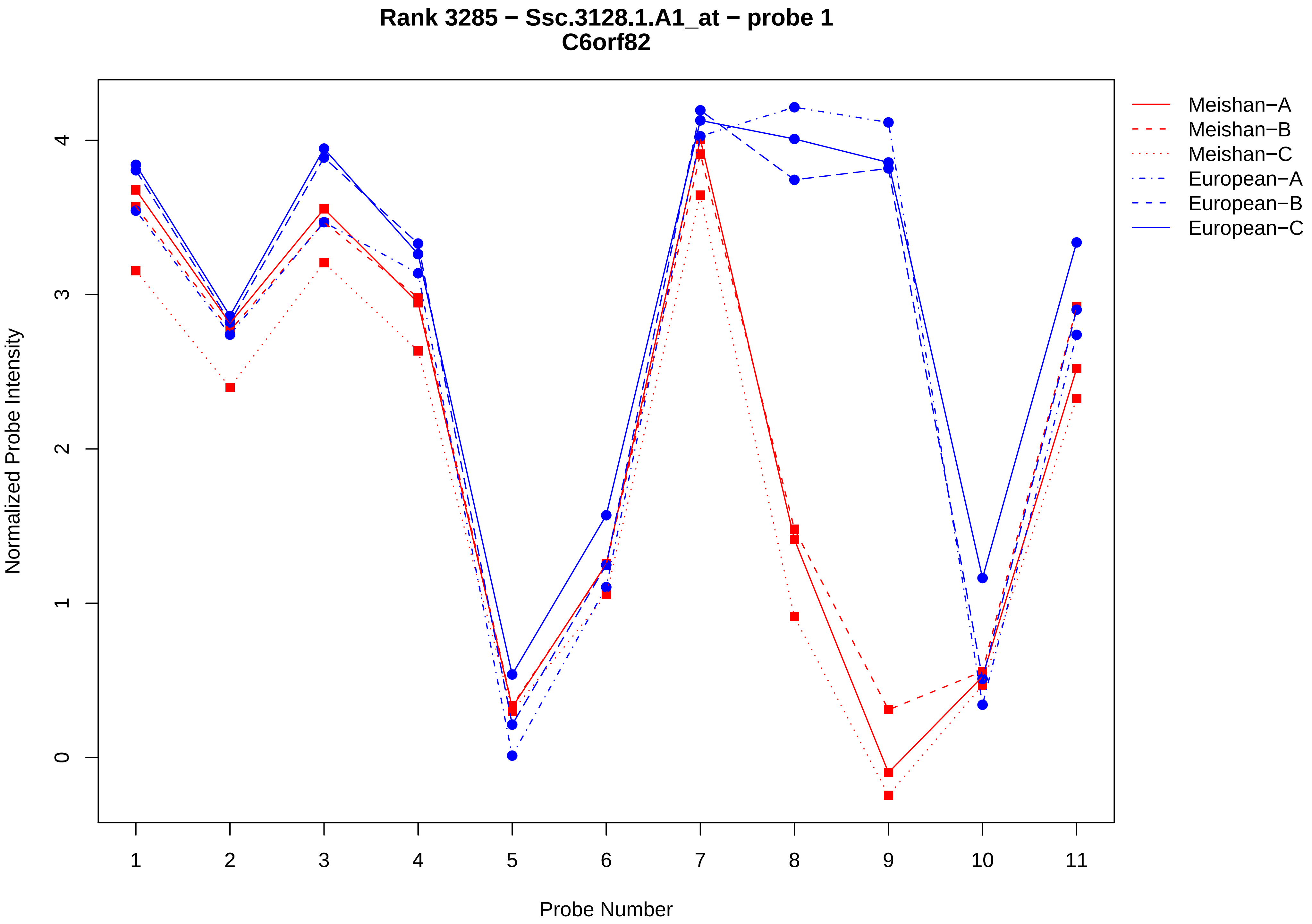
Rank 3285 − Ssc.3128.1.A1_at − probe 1
C6orf82
Normalized Probe Intensity
Probe Number
0
1
2
3
4
1
2
3
4
5
6
7
8
9
10
11
Meishan−A
Meishan−B
Meishan−C
European−A
European−B
European−C

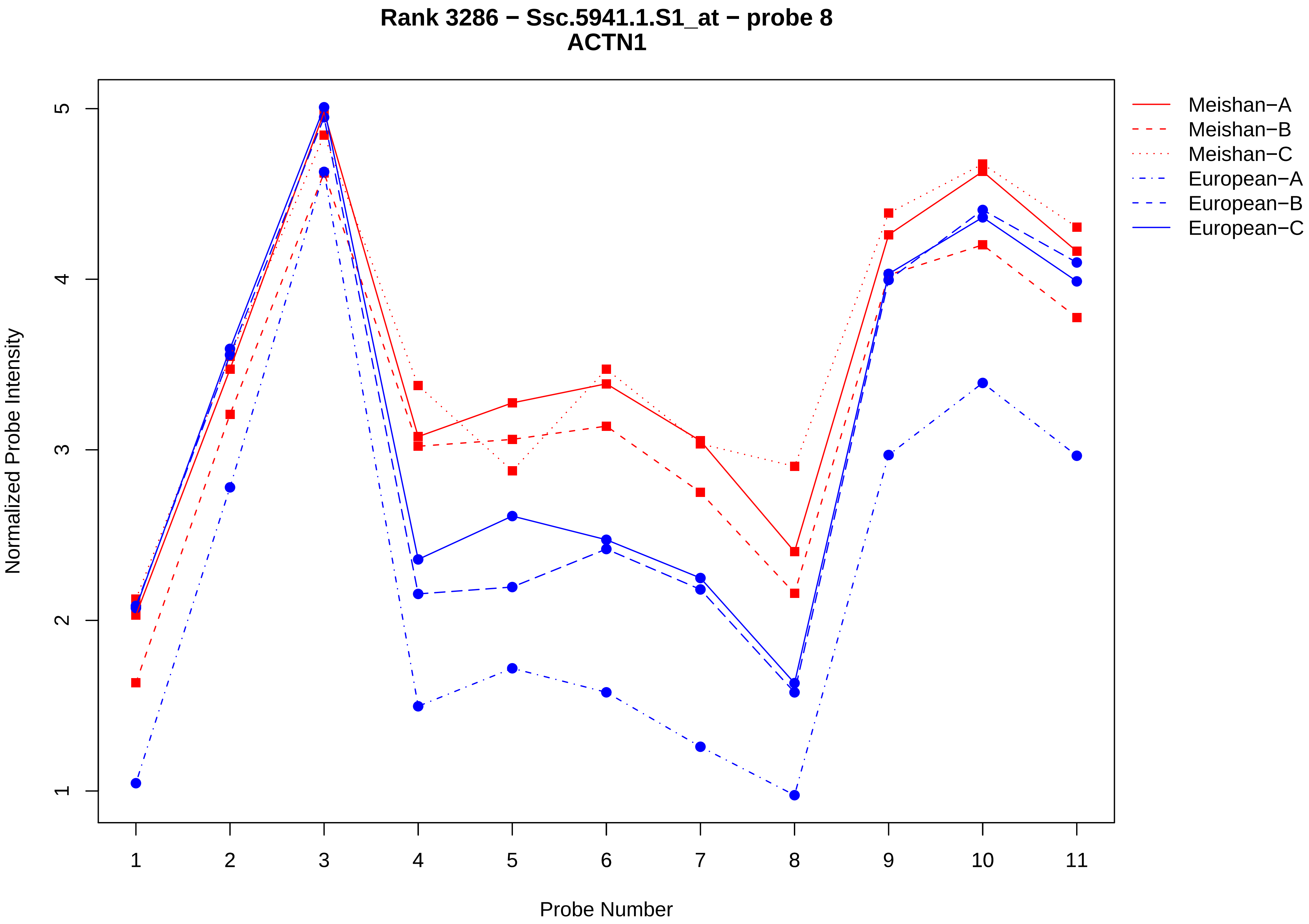

Rank 3286 − Ssc.5941.1.S1_at − probe 8
ACTN1
Normalized Probe Intensity
Probe Number
1
2
3
4
5
1
2
3
4
5
6
7
8
9
10
11
Meishan−A
Meishan−B
Meishan−C
European−A
European−B
European−C

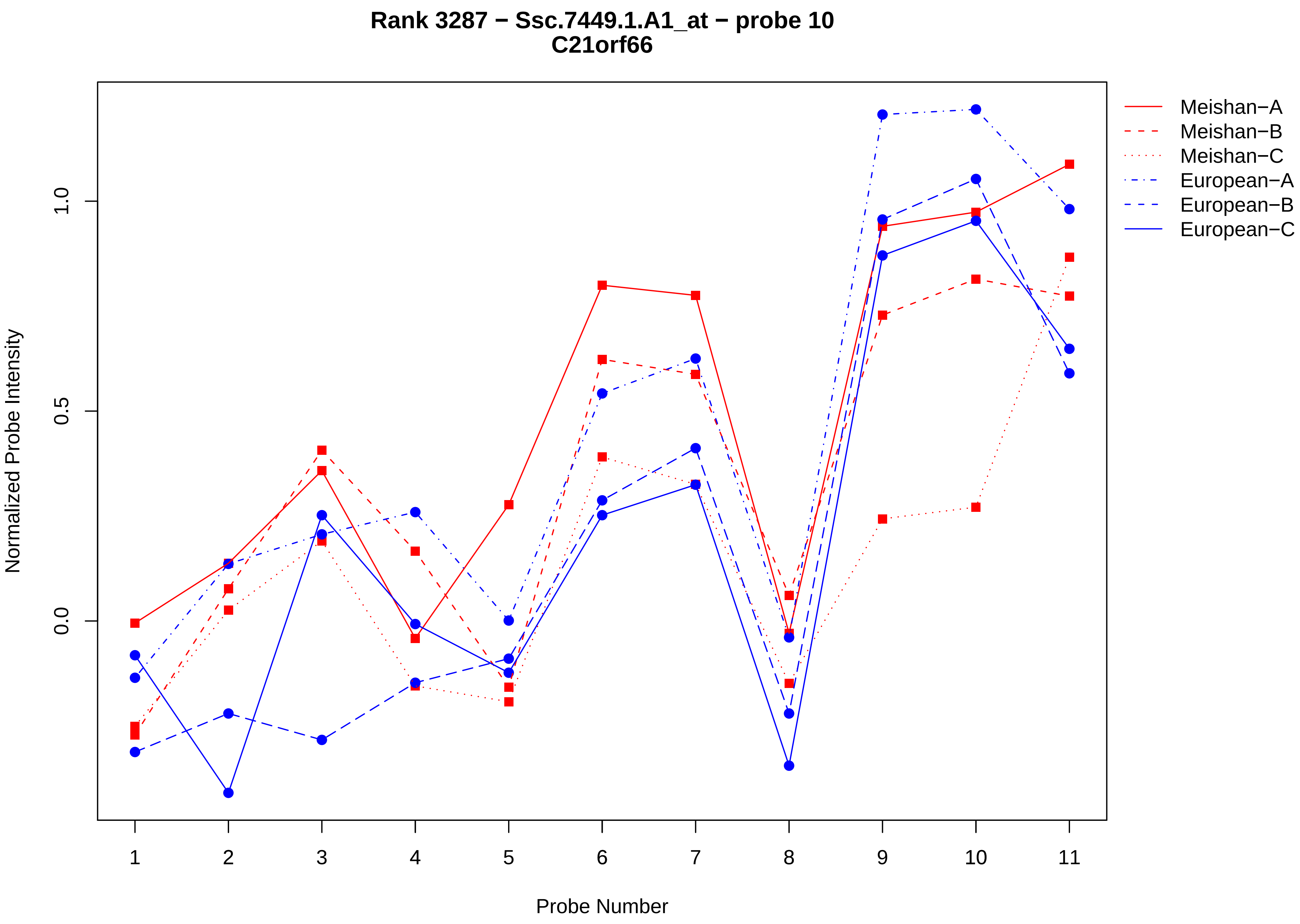

Rank 3287 − Ssc.7449.1.A1_at − probe 10
C21orf66
Normalized Probe Intensity
0.0
0.5
1.0
Probe Number
1
2
3
4
5
6
7
8
9
10
11
Meishan−A
Meishan−B
Meishan−C
European−A
European−B
European−C

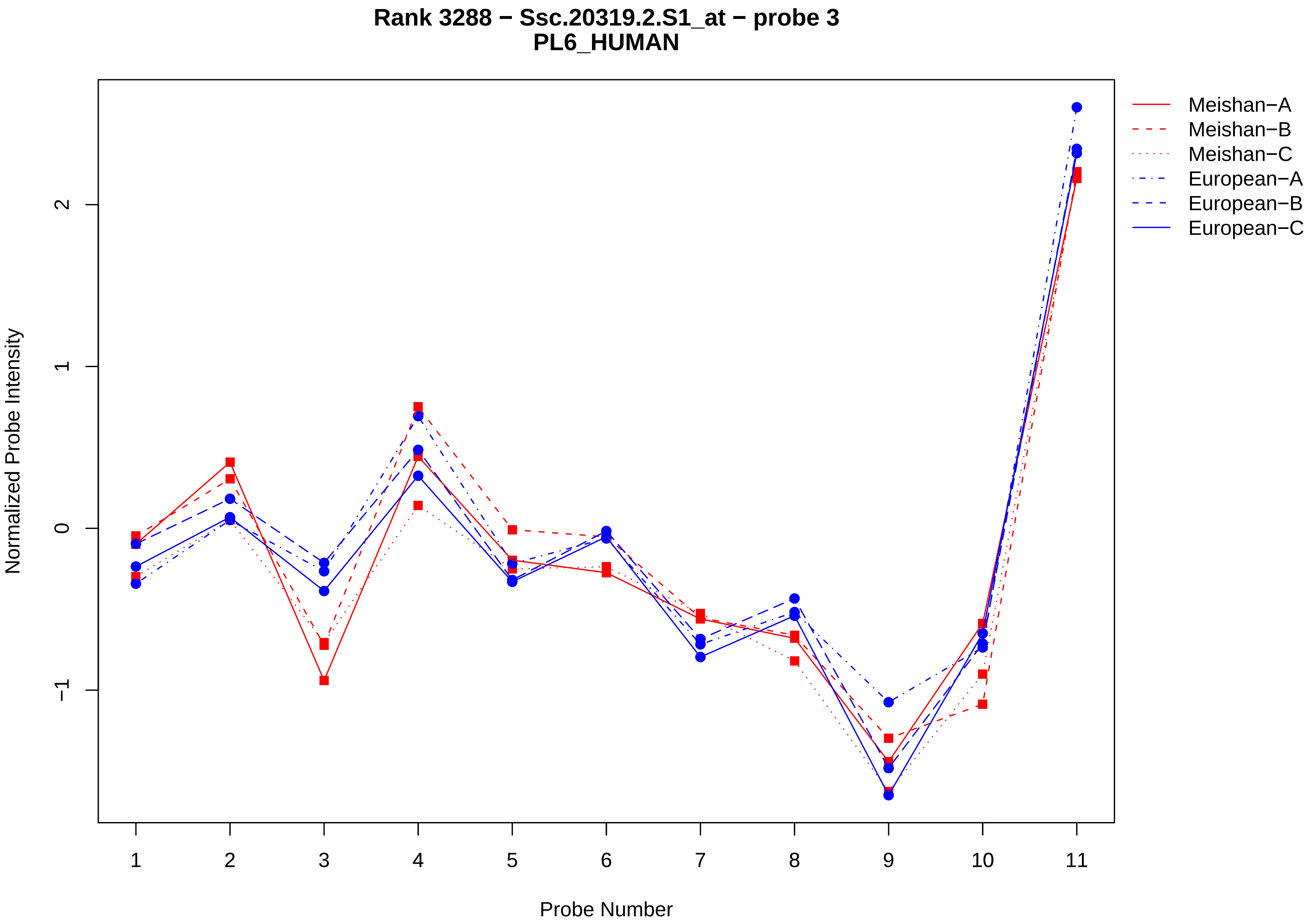

Rank 3288 − Ssc.20319.2.S1_at − probe 3
PL6_HUMAN
Normalized Probe Intensity
Probe Number
−1
0
1
2
1
2
3
4
5
6
7
8
9
10
11
Meishan−A
Meishan−B
Meishan−C
European−A
European−B
European−C

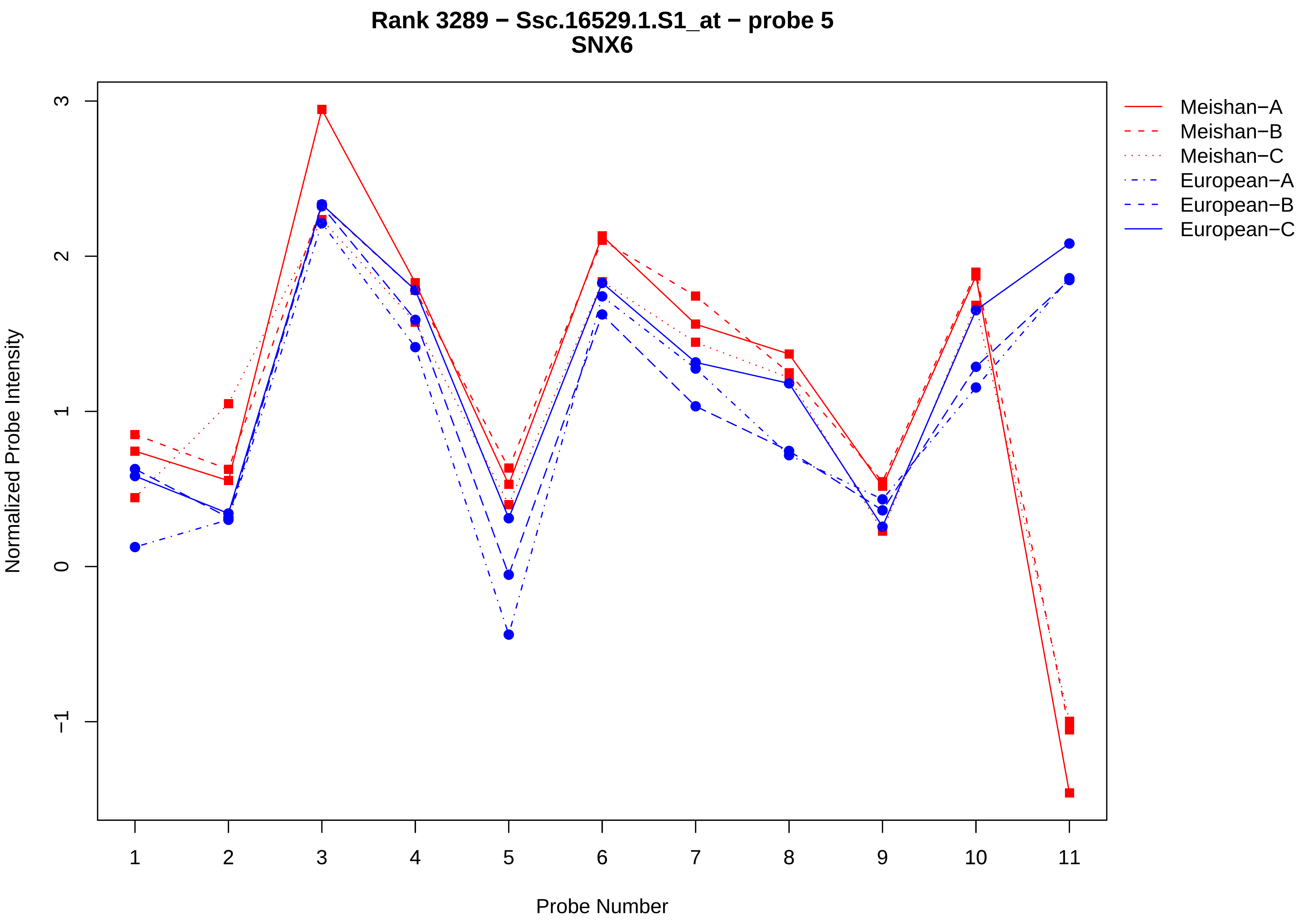

Rank 3289 − Ssc.16529.1.S1_at − probe 5
SNX6
Normalized Probe Intensity
Probe Number
−1
0
1
2
3
1
2
3
4
5
6
7
8
9
10
11
Meishan−A
Meishan−B
Meishan−C
European−A
European−B
European−C

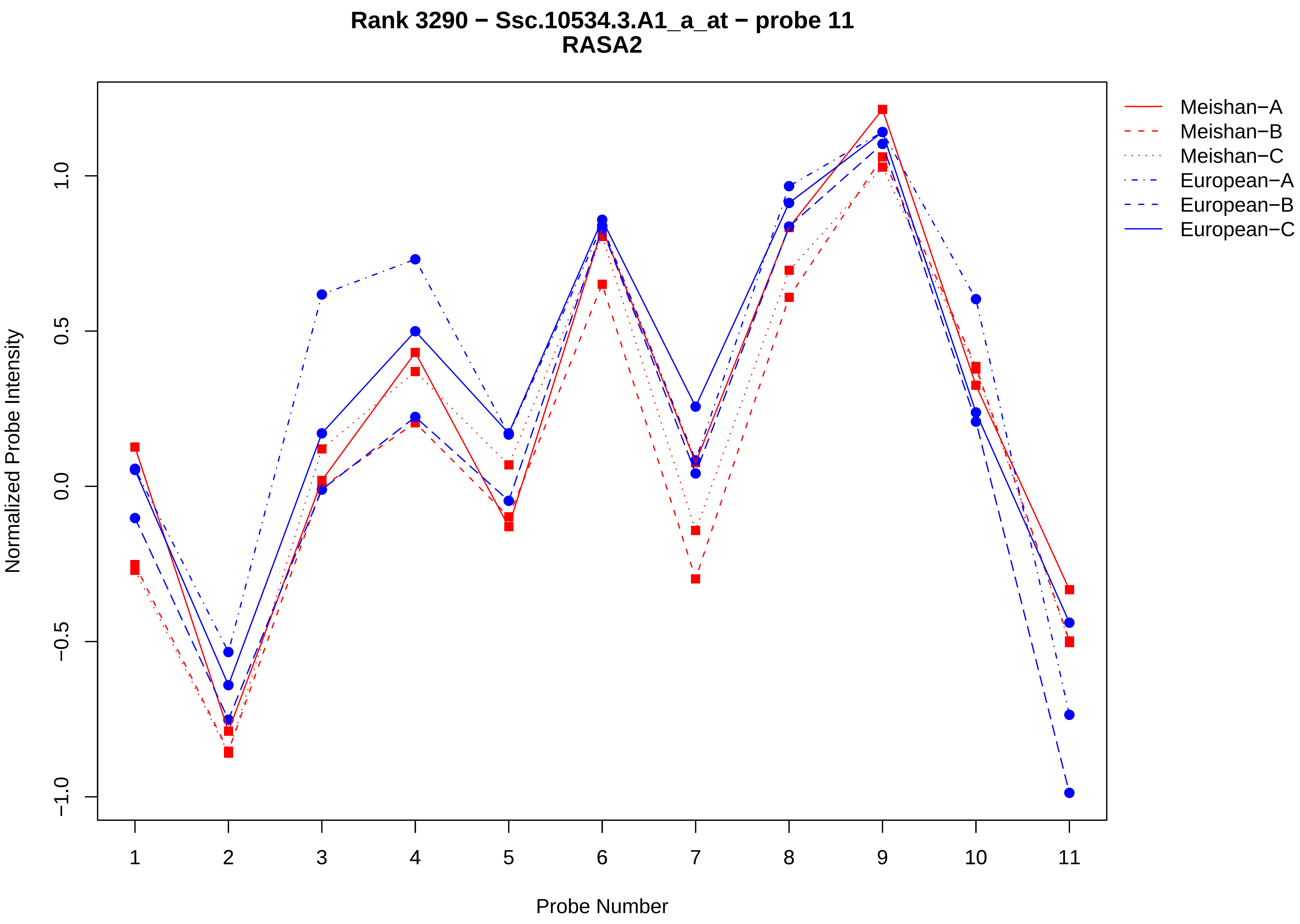

Rank 3290 − Ssc.10534.3.A1_a_at − probe 11
RASA2
Normalized Probe Intensity
Probe Number
1.0
0.5
0.0
−0.5
−1.0
1
2
3
4
5
6
7
8
9
10
11
Meishan−A
Meishan−B
Meishan−C
European−A
European−B
European−C

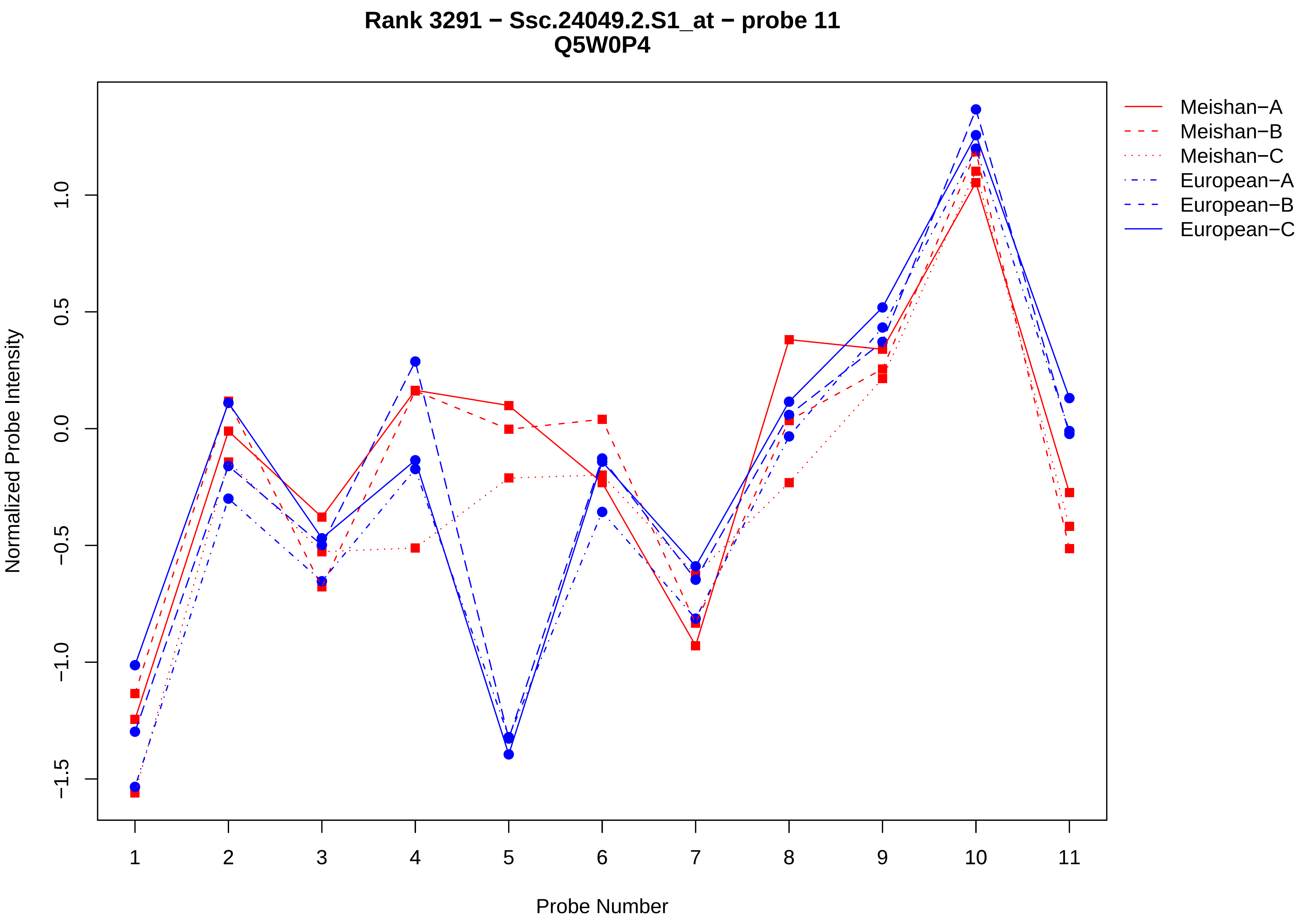
Rank 3291 − Ssc.24049.2.S1_at − probe 11
Q5W0P4
Normalized Probe Intensity
Probe Number
−1.5
−1.0
−0.5
0.0
0.5
1.0
1
2
3
4
5
6
7
8
9
10
11
Meishan−A
Meishan−B
Meishan−C
European−A
European−B
European−C

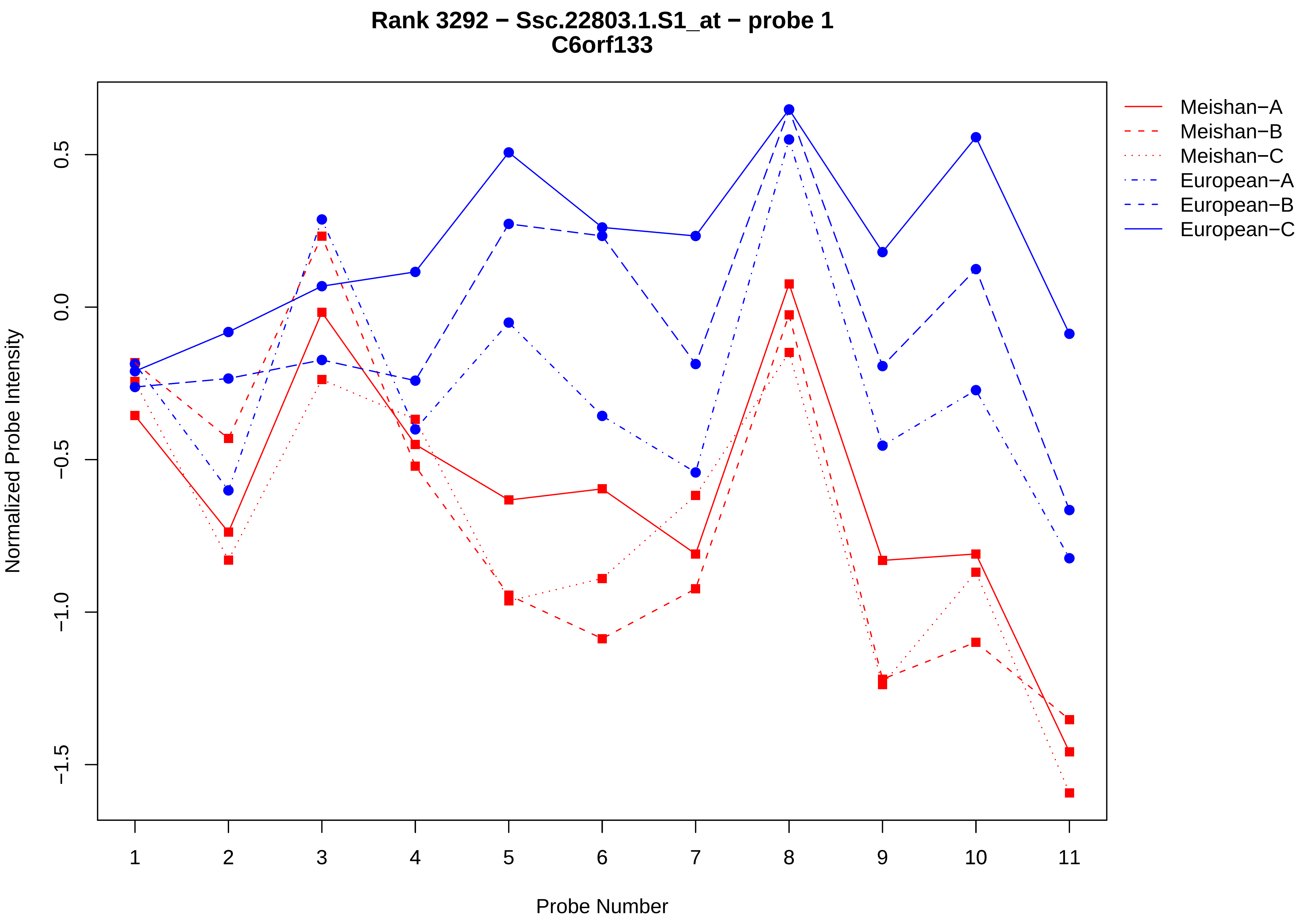

Rank 3292 − Ssc.22803.1.S1_at − probe 1
C6orf133
Normalized Probe Intensity
Probe Number
0.5
0.0
−0.5
−1.0
−1.5
1
2
3
4
5
6
7
8
9
10
11
Meishan−A
Meishan−B
Meishan−C
European−A
European−B
European−C

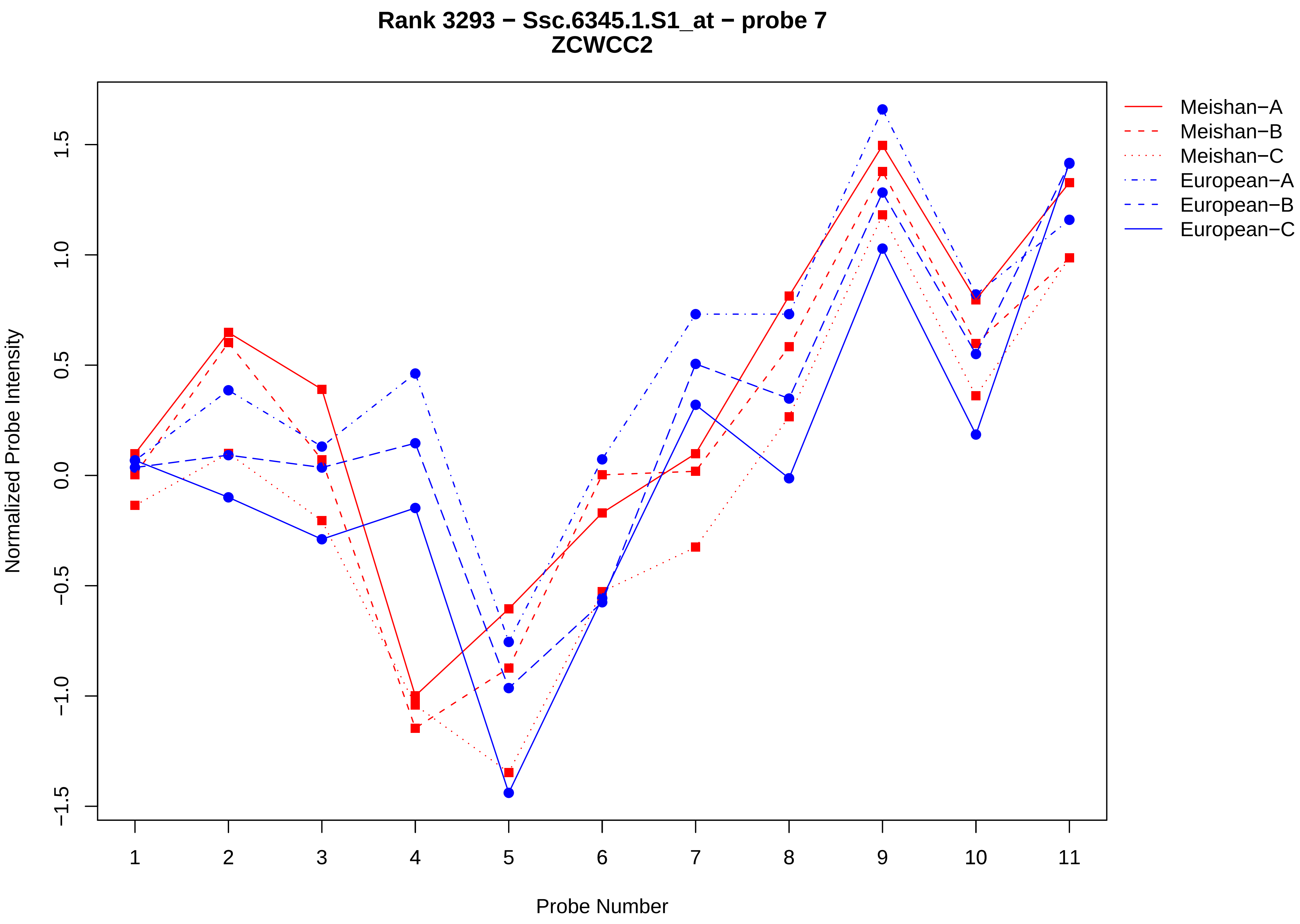

Rank 3293 − Ssc.6345.1.S1_at − probe 7
ZCWCC2
Normalized Probe Intensity
Probe Number
1.5
1.0
0.5
0.0
−0.5
−1.0
−1.5
1
2
3
4
5
6
7
8
9
10
11
Meishan−A
Meishan−B
Meishan−C
European−A
European−B
European−C

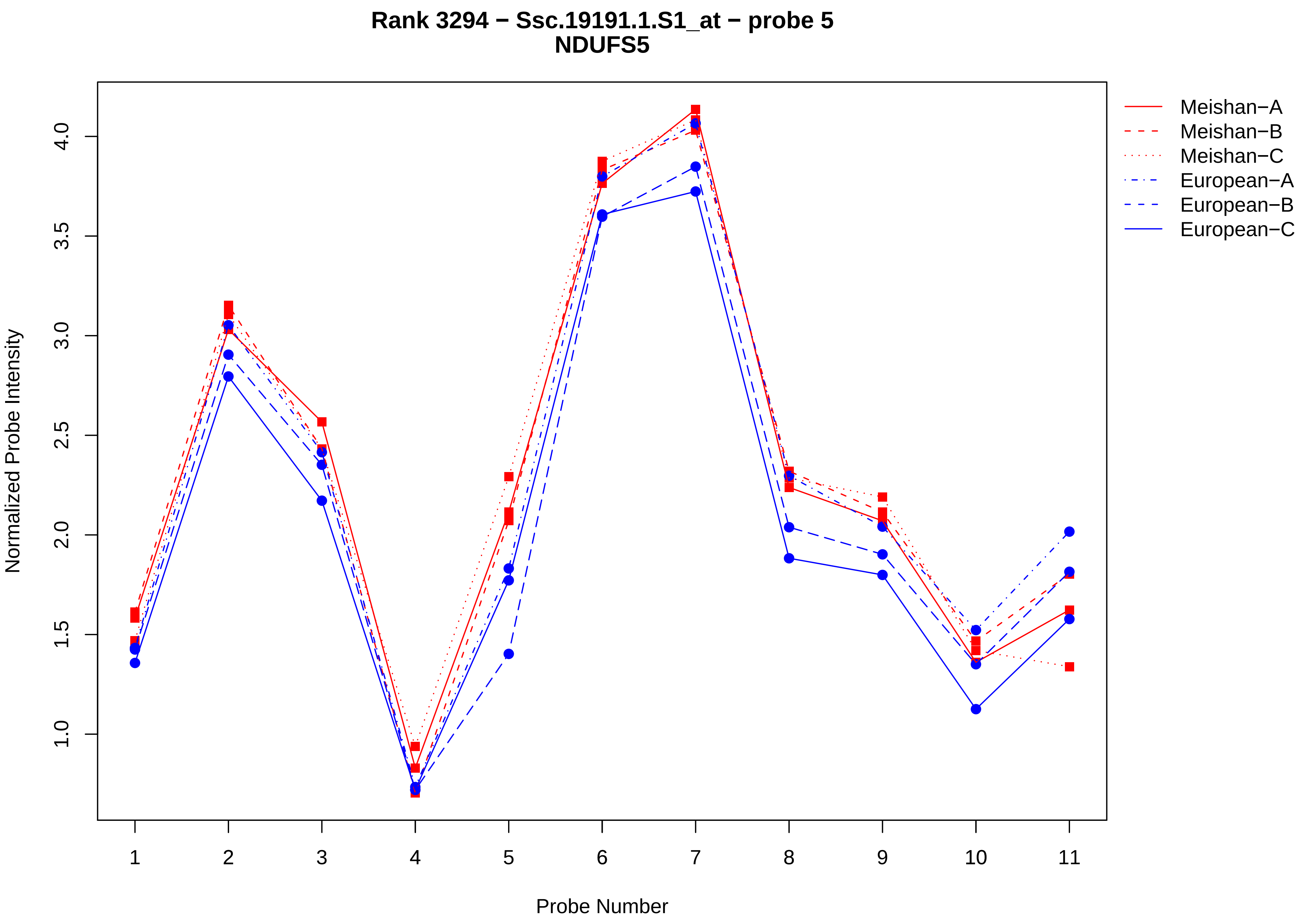

Rank 3294 − Ssc.19191.1.S1_at − probe 5
NDUFS5
Normalized Probe Intensity
Probe Number
Meishan−A
Meishan−B
Meishan−C
European−A
European−B
European−C
1.0
1.5
2.0
2.5
3.0
3.5
4.0
1
2
3
4
5
6
7
8
9
10
11

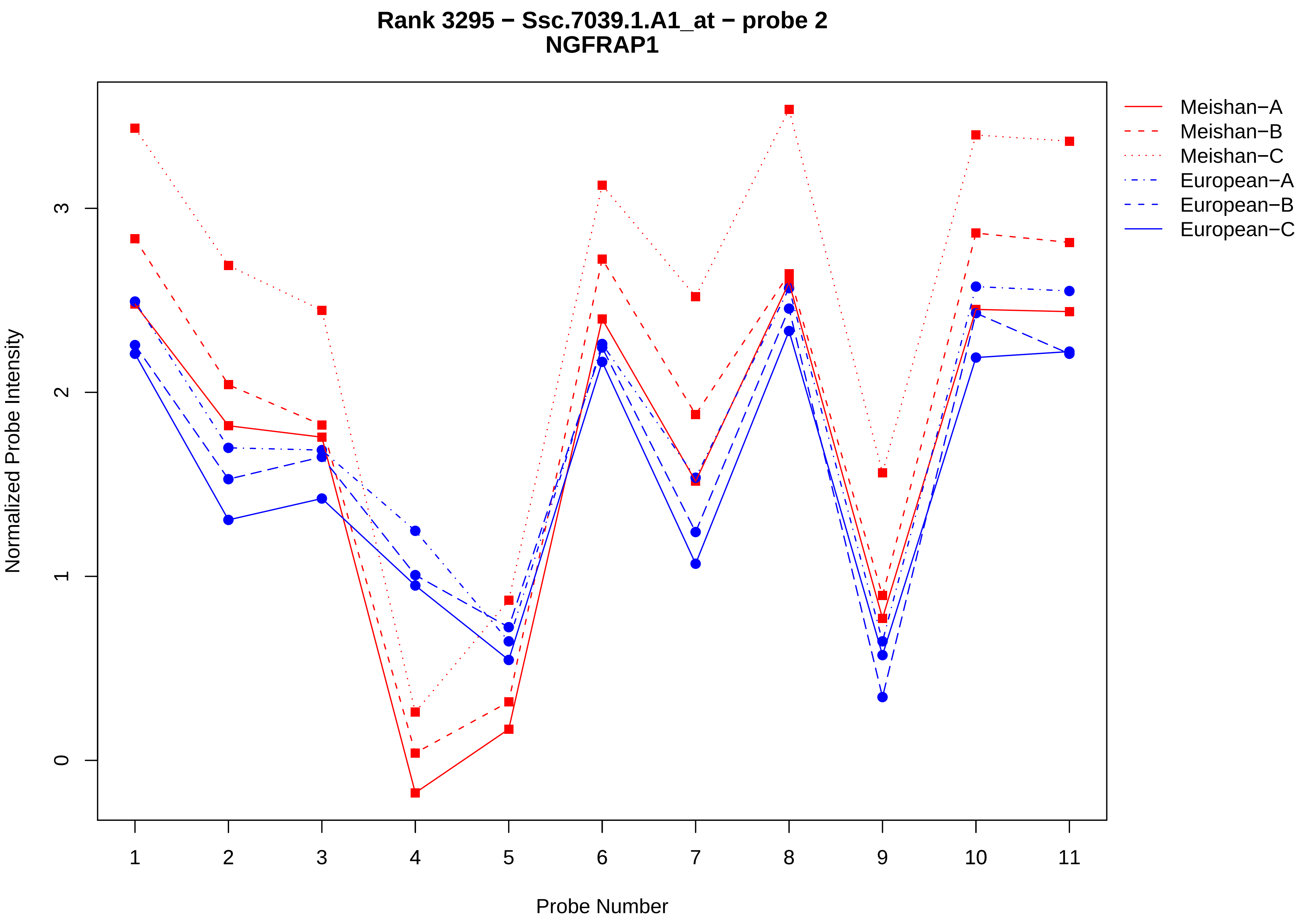

Rank 3295 − Ssc.7039.1.A1_at − probe 2
NGFRAP1
Normalized Probe Intensity
Probe Number
0
1
2
3
1
2
3
4
5
6
7
8
9
10
11
Meishan−A
Meishan−B
Meishan−C
European−A
European−B
European−C

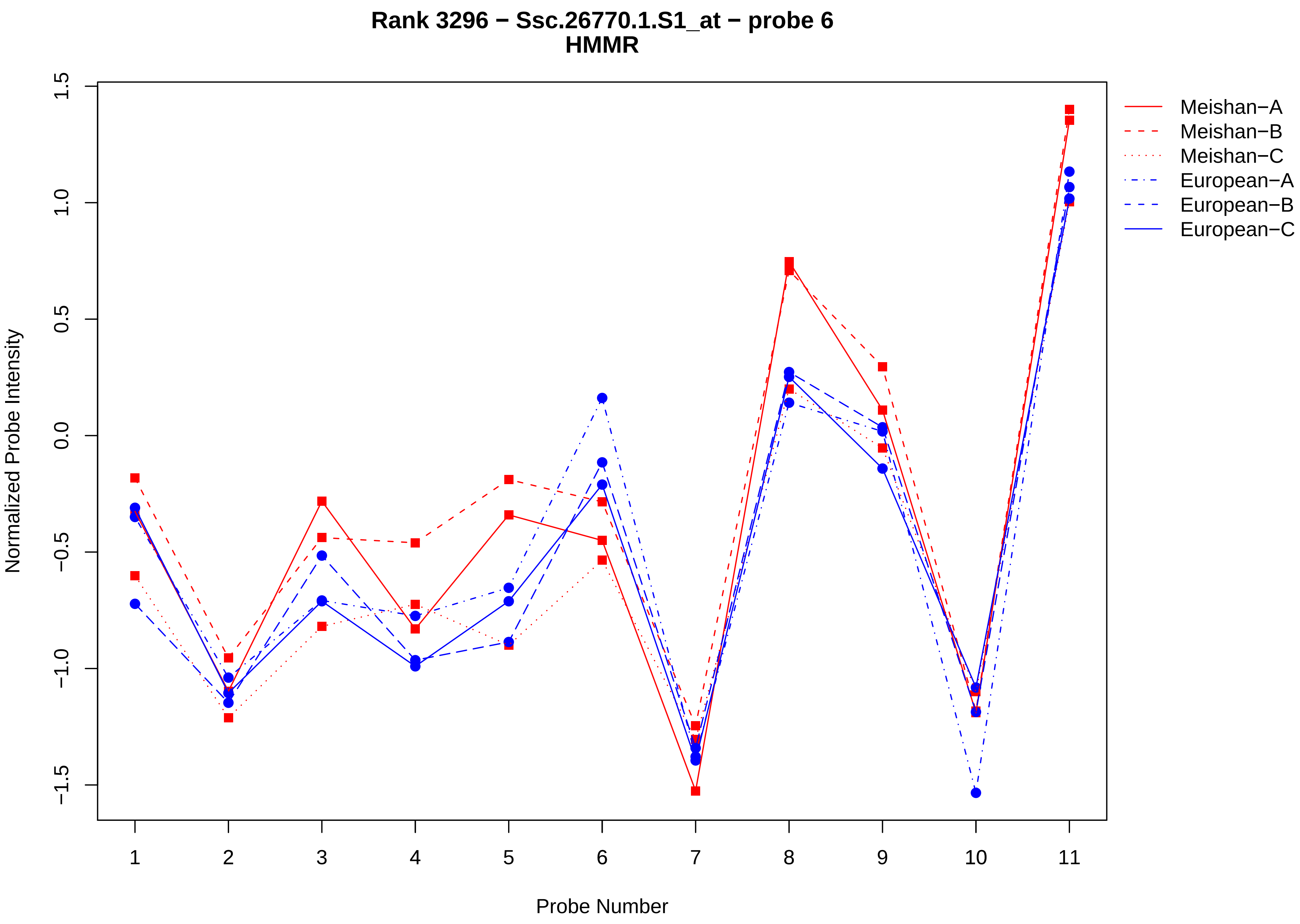

Rank 3296 − Ssc.26770.1.S1_at − probe 6
HMMR
Normalized Probe Intensity
Probe Number
Meishan−A
Meishan−B
Meishan−C
European−A
European−B
European−C
1.5
1.0
0.5
0.0
−0.5
−1.0
−1.5
1
2
3
4
5
6
7
8
9
10
11

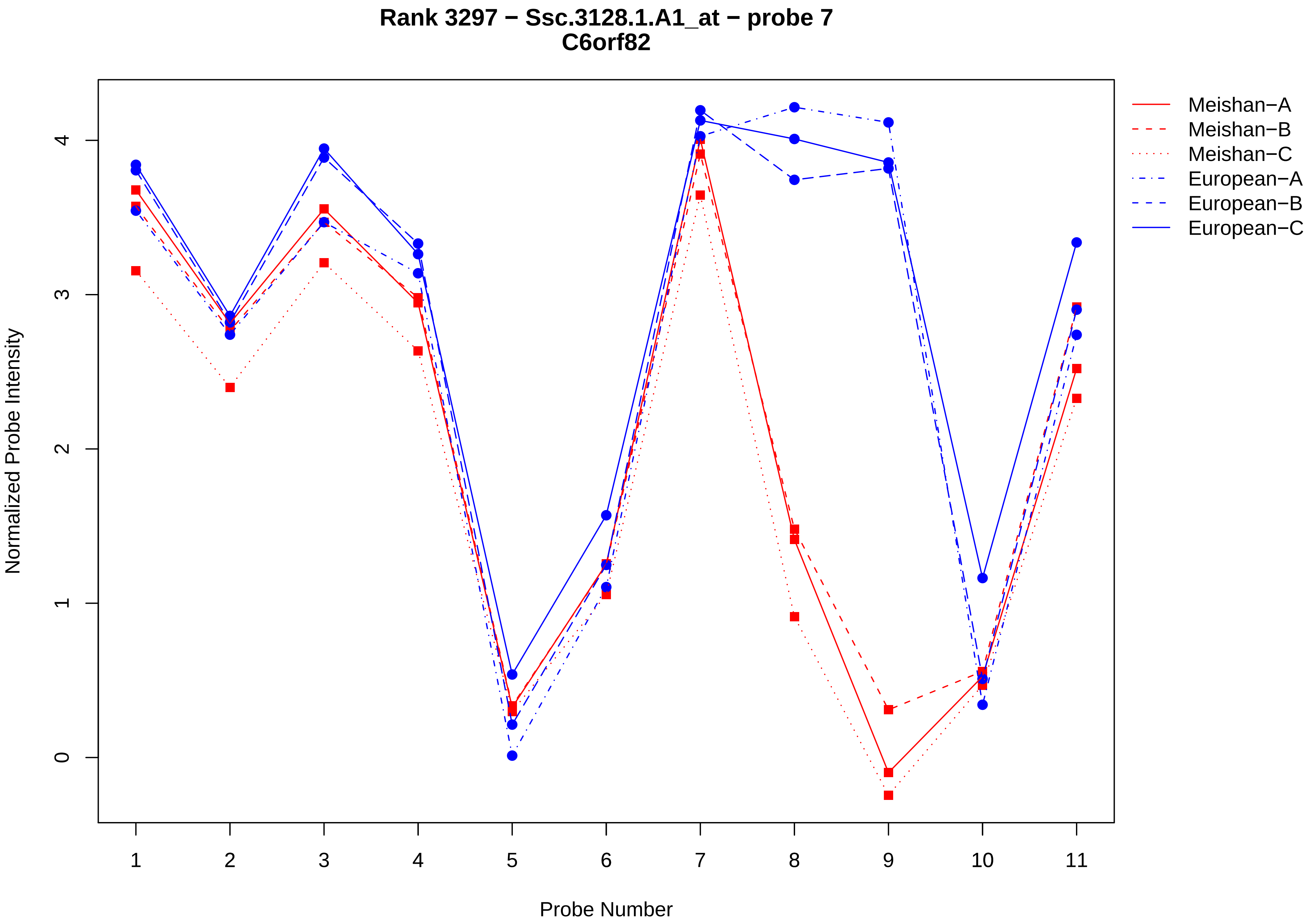

Rank 3297 − Ssc.3128.1.A1_at − probe 7
C6orf82
Normalized Probe Intensity
0
1
2
3
4
Probe Number
1
2
3
4
5
6
7
8
9
10
11
Meishan−A
Meishan−B
Meishan−C
European−A
European−B
European−C

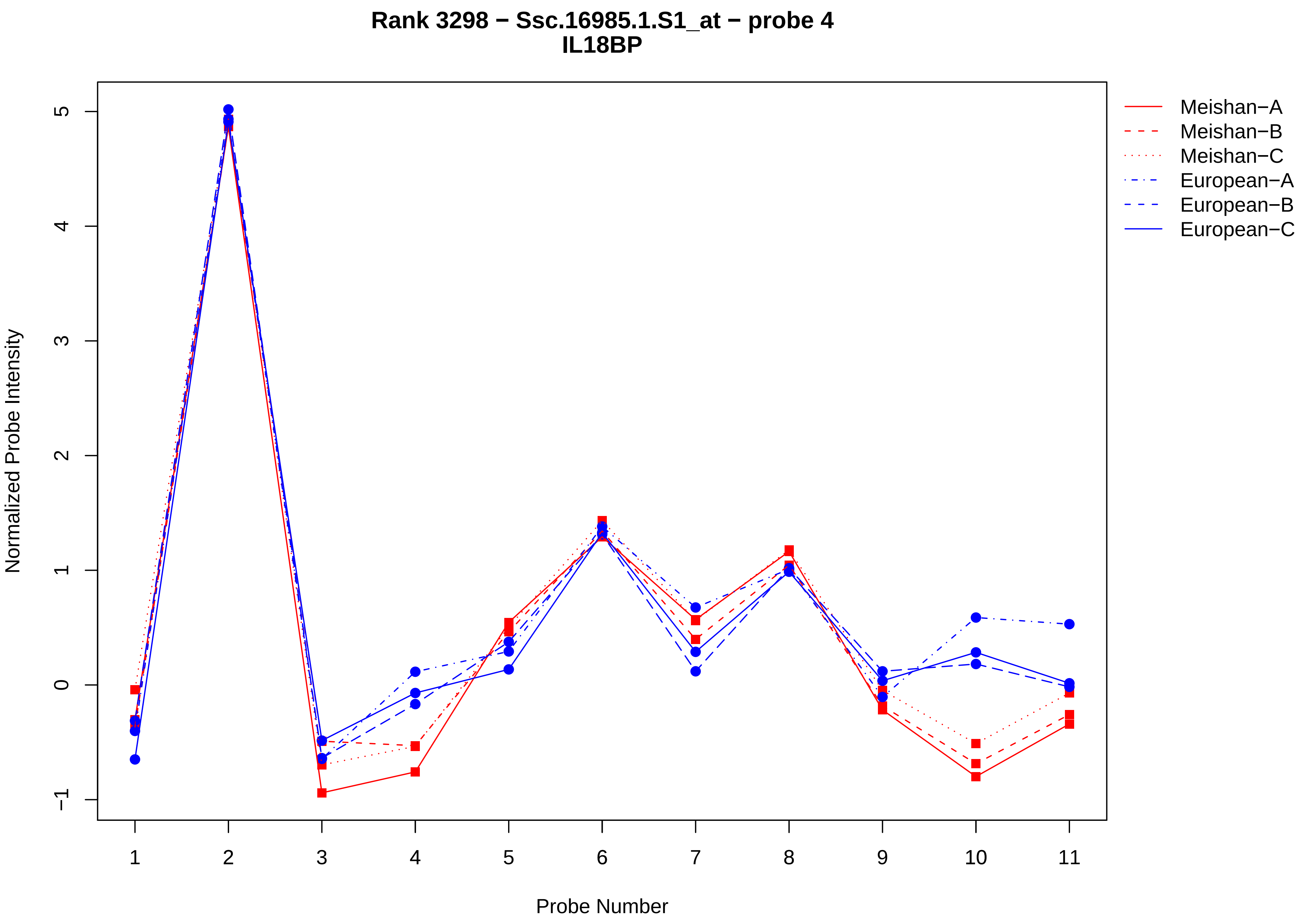

Rank 3298 − Ssc.16985.1.S1_at − probe 4
IL18BP
Normalized Probe Intensity
Probe Number
−1
0
1
2
3
4
5
1
2
3
4
5
6
7
8
9
10
11
Meishan−A
Meishan−B
Meishan−C
European−A
European−B
European−C

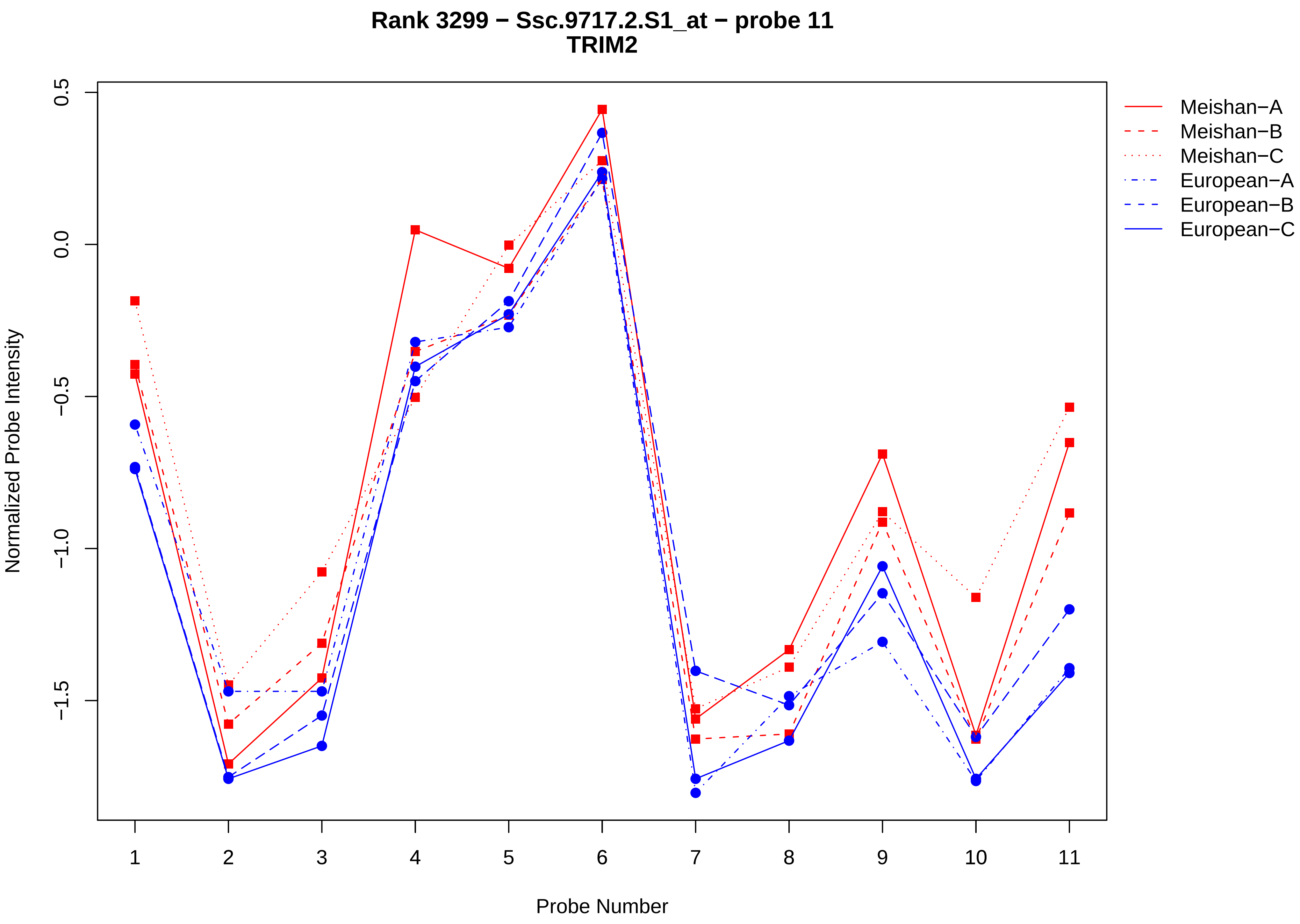
Rank 3299 − Ssc.9717.2.S1_at − probe 11
TRIM2
Normalized Probe Intensity
Probe Number
0.5
0.0
−0.5
−1.0
−1.5
1
2
3
4
5
6
7
8
9
10
11
Meishan−A
Meishan−B
Meishan−C
European−A
European−B
European−C

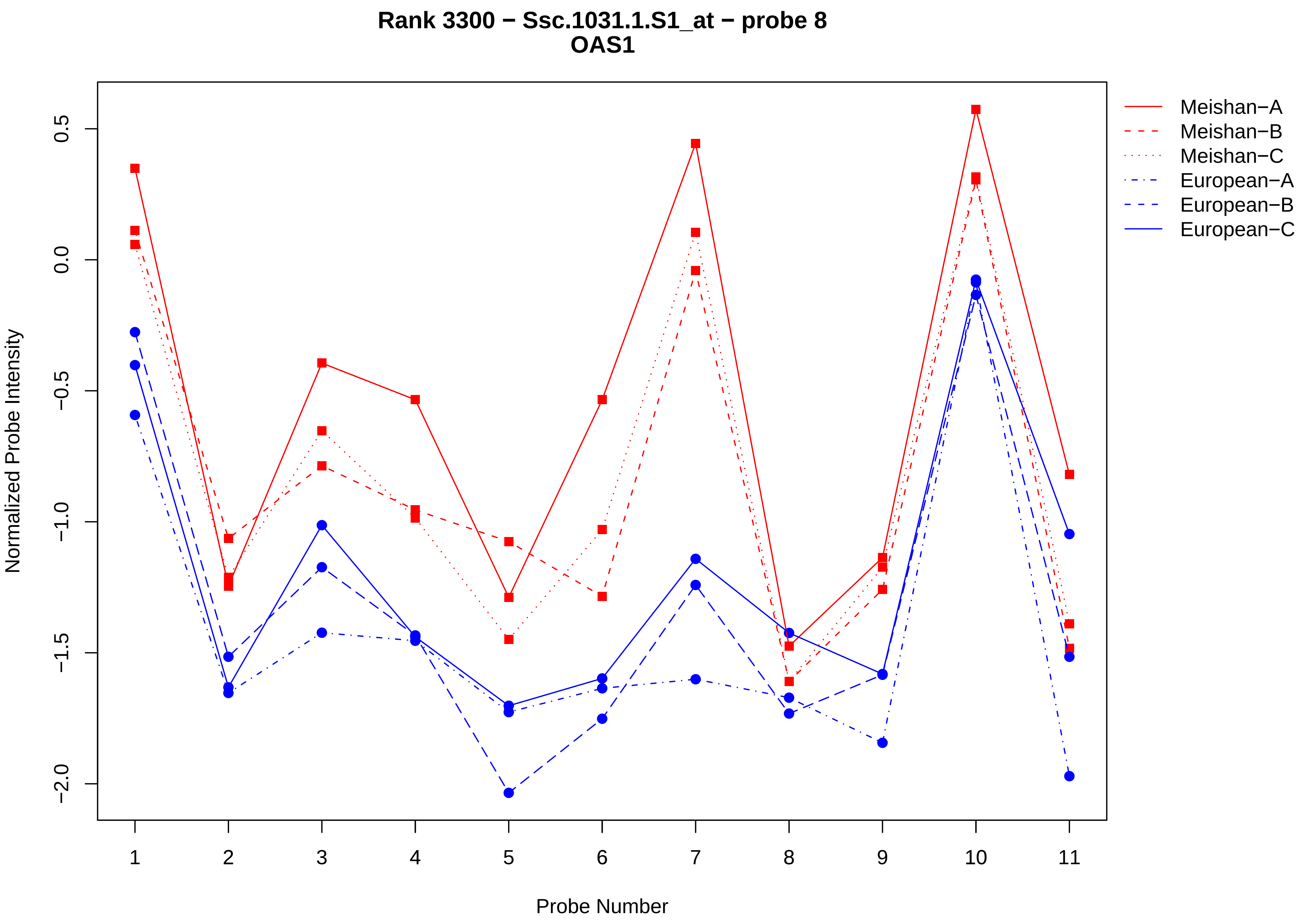

Rank 3300 − Ssc.1031.1.S1_at − probe 8
OAS1
Normalized Probe Intensity
Probe Number
0.5
0.0
−0.5
−1.0
−1.5
−2.0
1
2
3
4
5
6
7
8
9
10
11
Meishan−A
Meishan−B
Meishan−C
European−A
European−B
European−C

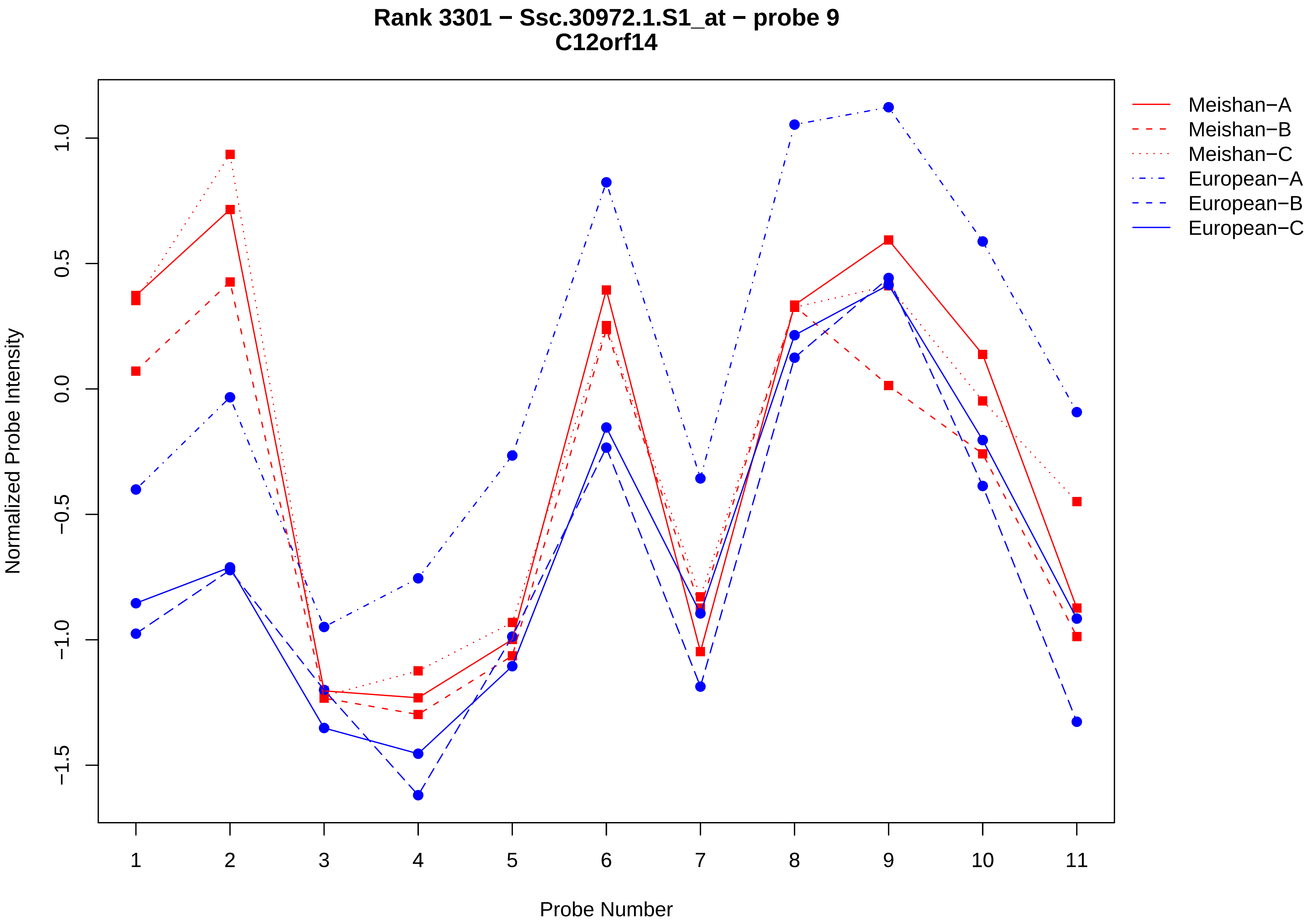

Rank 3301 − Ssc.30972.1.S1_at − probe 9
C12orf14
Normalized Probe Intensity
Probe Number
1.0
0.5
0.0
−0.5
−1.0
−1.5
1
2
3
4
5
6
7
8
9
10
11
Meishan−A
Meishan−B
Meishan−C
European−A
European−B
European−C

Rank 3302 − Ssc.11771.1.A1_at − probe 1
NP_055205

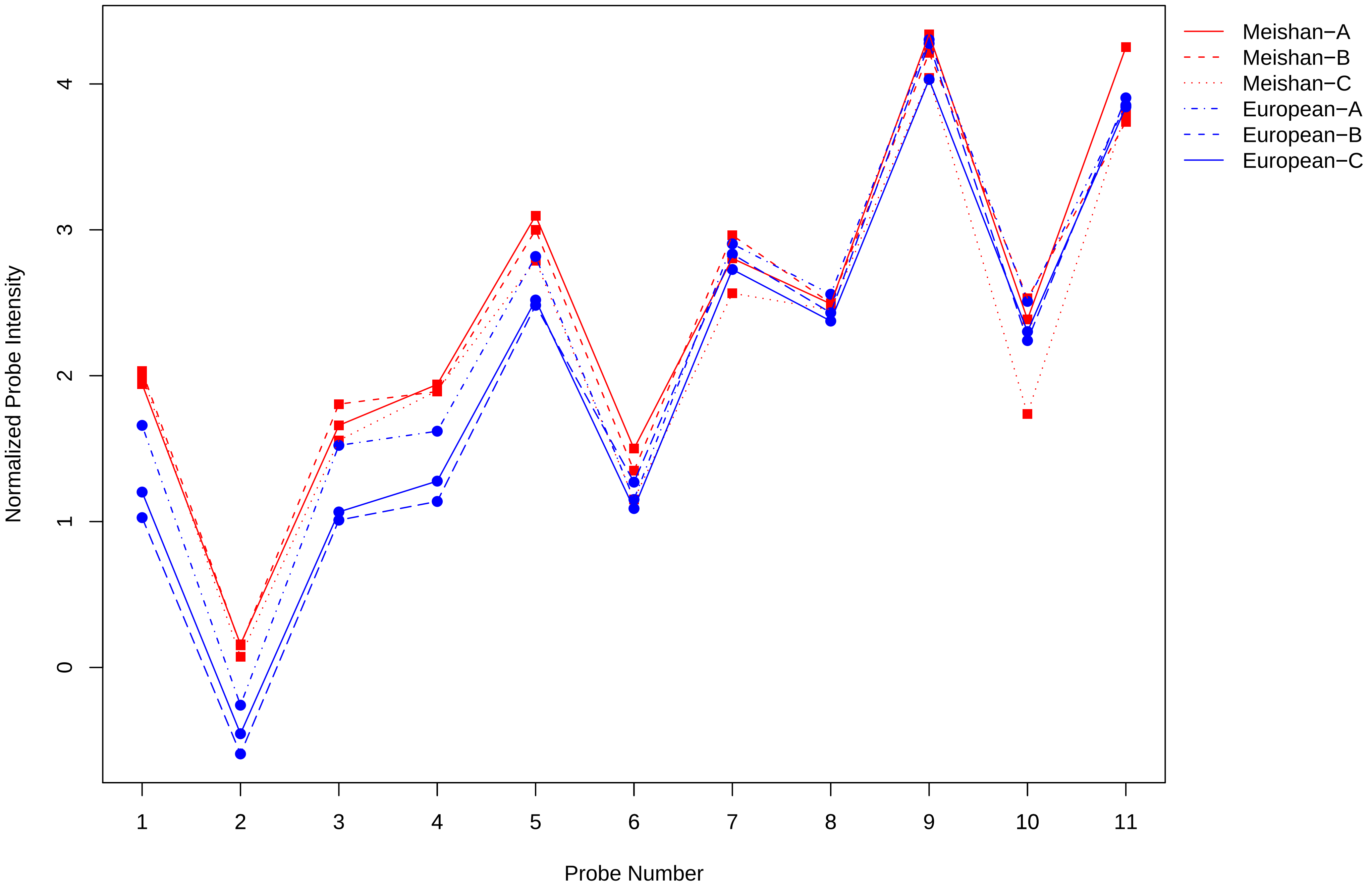

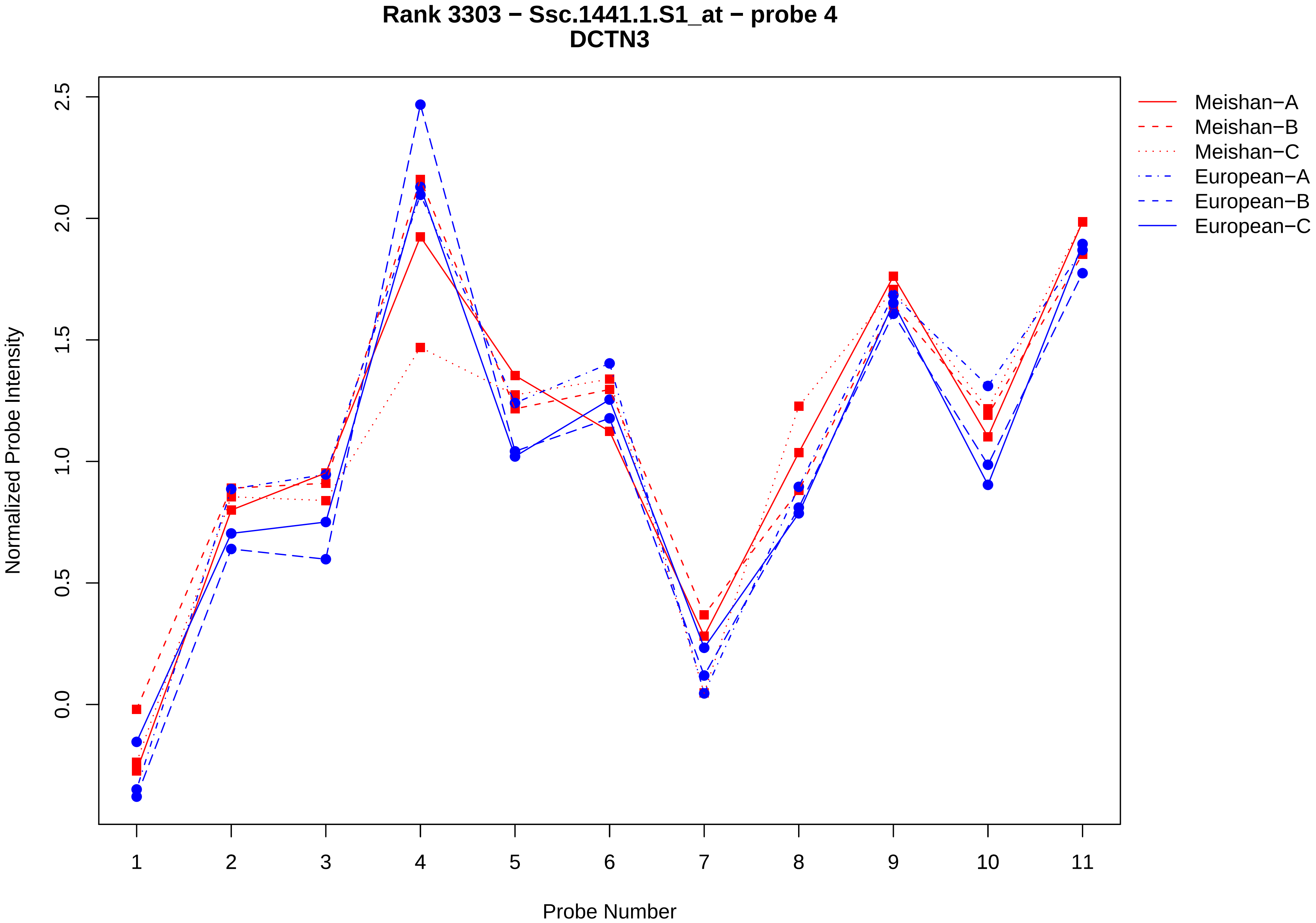

Rank 3303 − Ssc.1441.1.S1_at − probe 4
DCTN3
Normalized Probe Intensity
0.0
0.5
1.0
1.5
2.0
2.5
1
2
3
4
5
6
7
8
9
10
11
Probe Number
Meishan−A
Meishan−B
Meishan−C
European−A
European−B
European−C

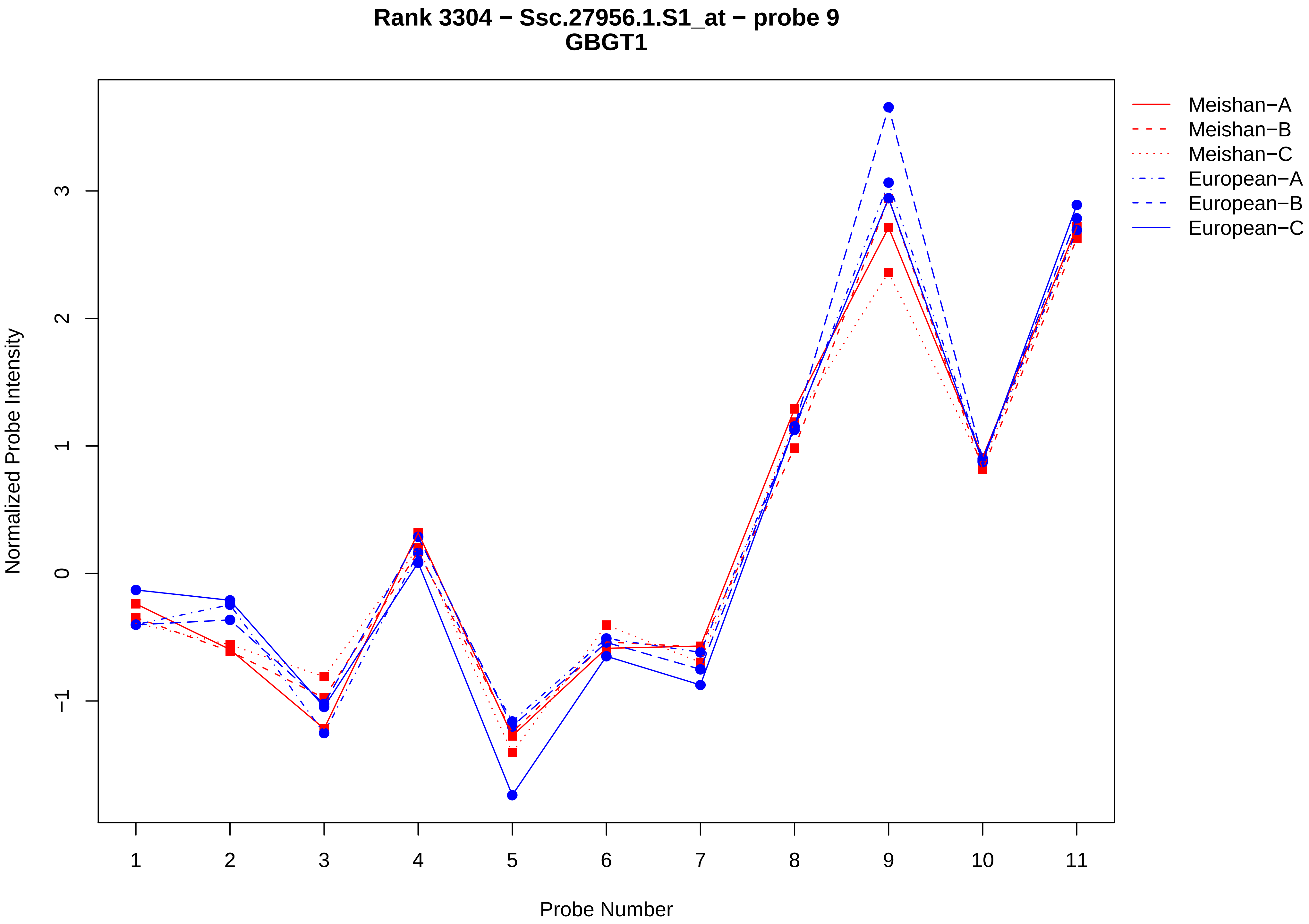

Rank 3304 − Ssc.27956.1.S1_at − probe 9
GBGT1
Normalized Probe Intensity
Probe Number
−1
0
1
2
3
1
2
3
4
5
6
7
8
9
10
11
Meishan−A
Meishan−B
Meishan−C
European−A
European−B
European−C

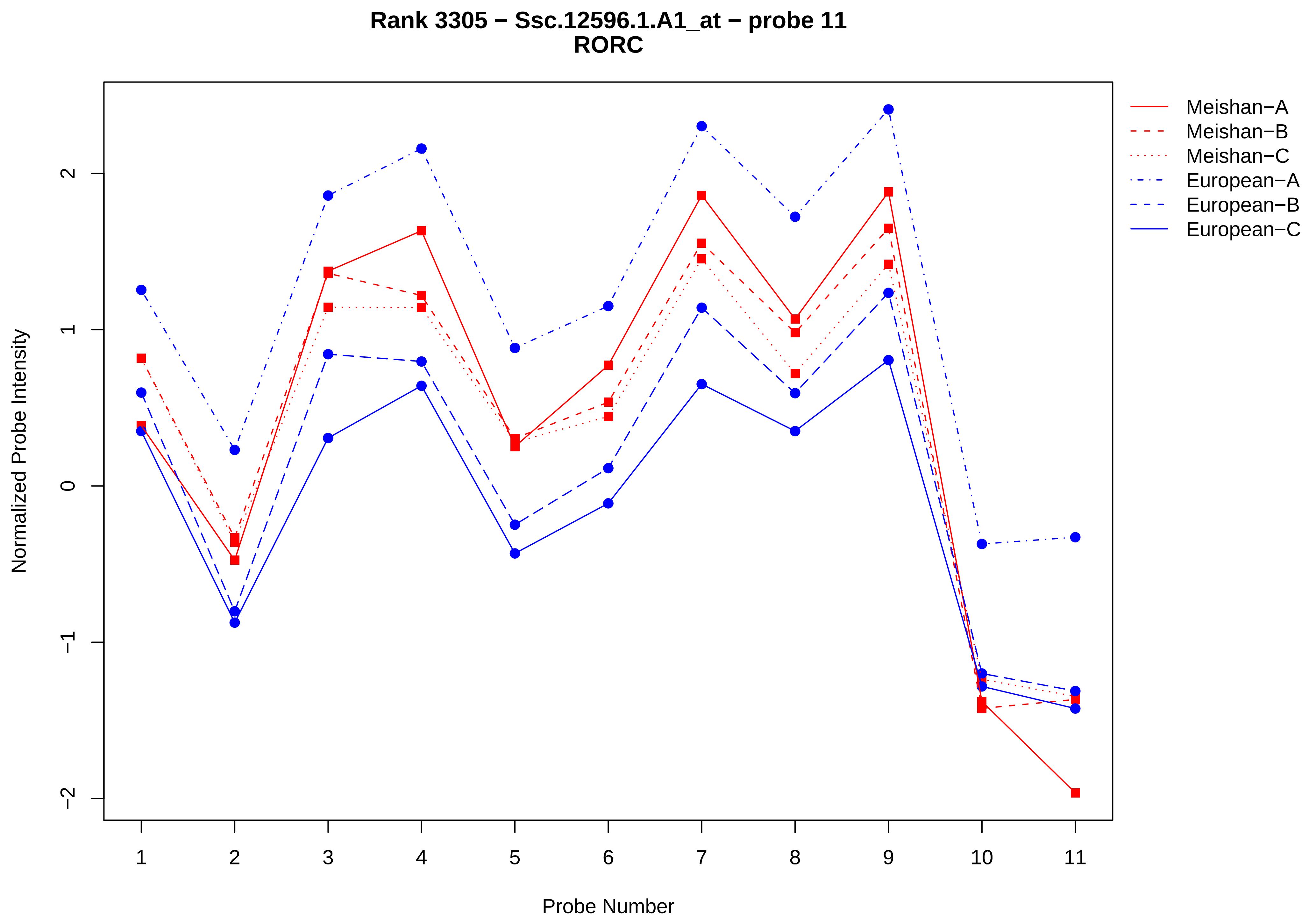

Rank 3305 − Ssc.12596.1.A1_at − probe 11
RORC
Normalized Probe Intensity
Probe Number
Meishan−A
Meishan−B
Meishan−C
European−A
European−B
European−C
−2
−1
0
1
2
1
2
3
4
5
6
7
8
9
10
11

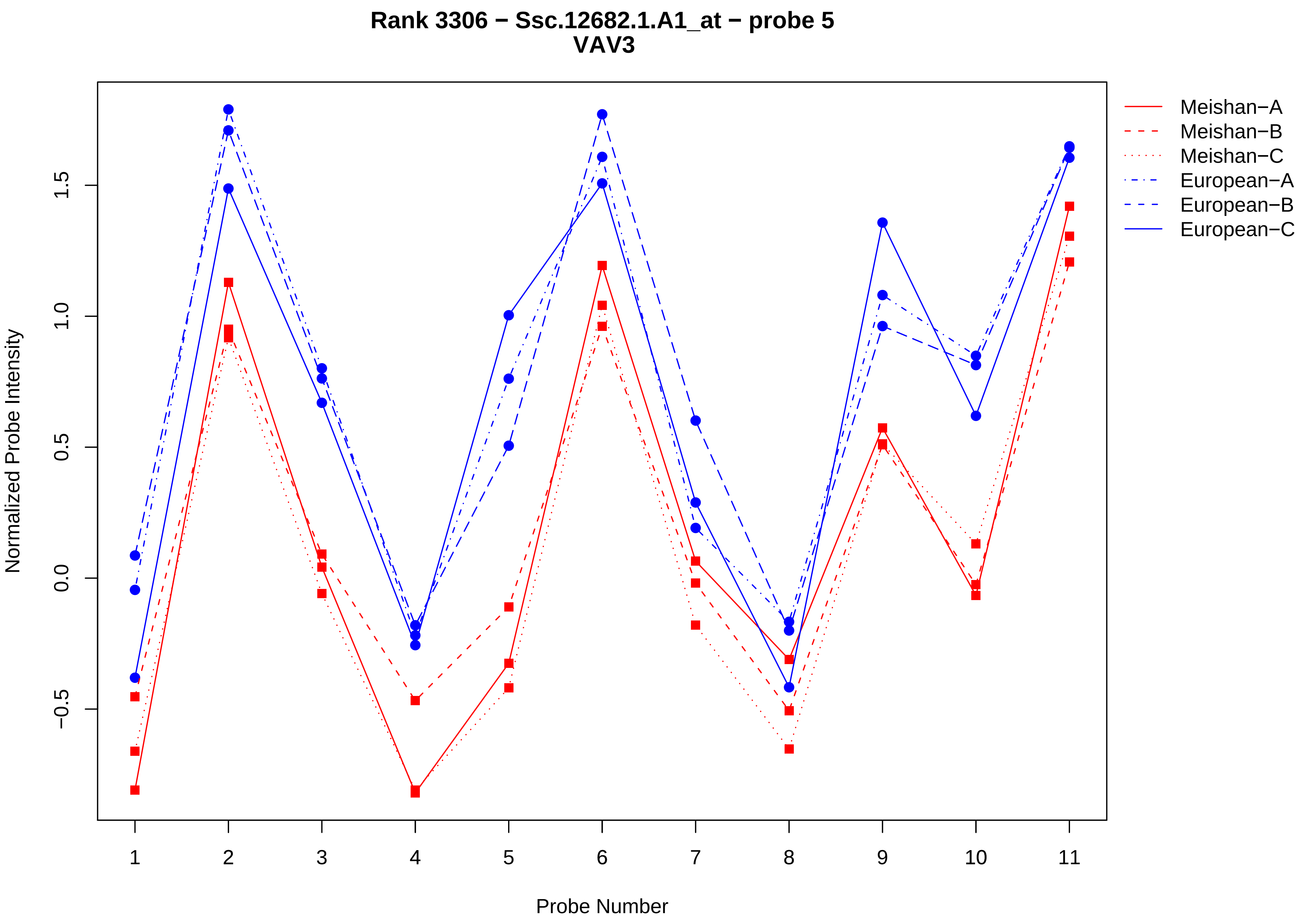

Rank 3306 − Ssc.12682.1.A1_at − probe 5
VAV3
Normalized Probe Intensity
Probe Number
−0.5
0.0
0.5
1.0
1.5
1
2
3
4
5
6
7
8
9
10
11
Meishan−A
Meishan−B
Meishan−C
European−A
European−B
European−C

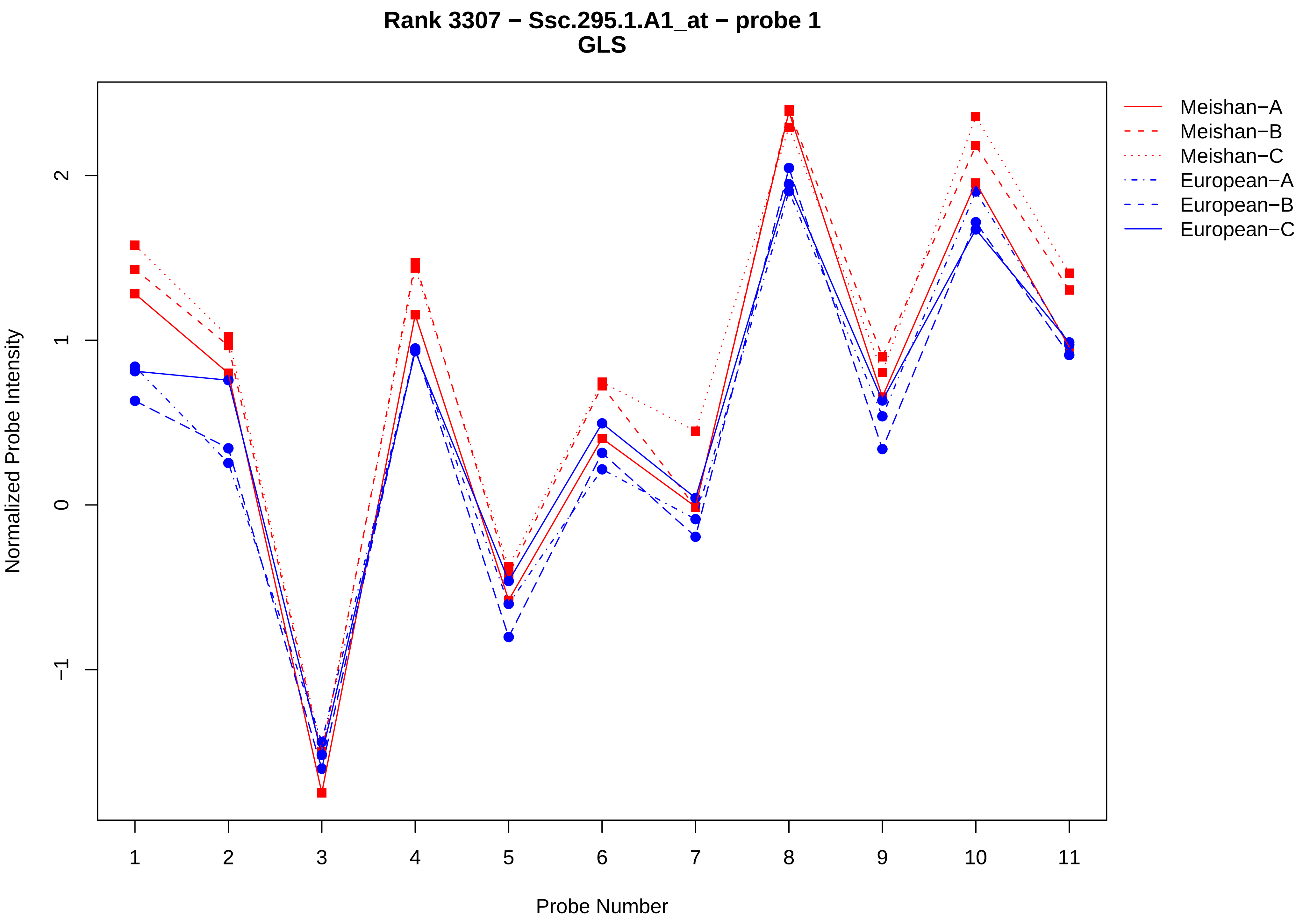

Rank 3307 − Ssc.295.1.A1_at − probe 1
GLS
Normalized Probe Intensity
−1
0
1
2
1
2
3
4
5
6
7
8
9
10
11
Probe Number
Meishan−A
Meishan−B
Meishan−C
European−A
European−B
European−C

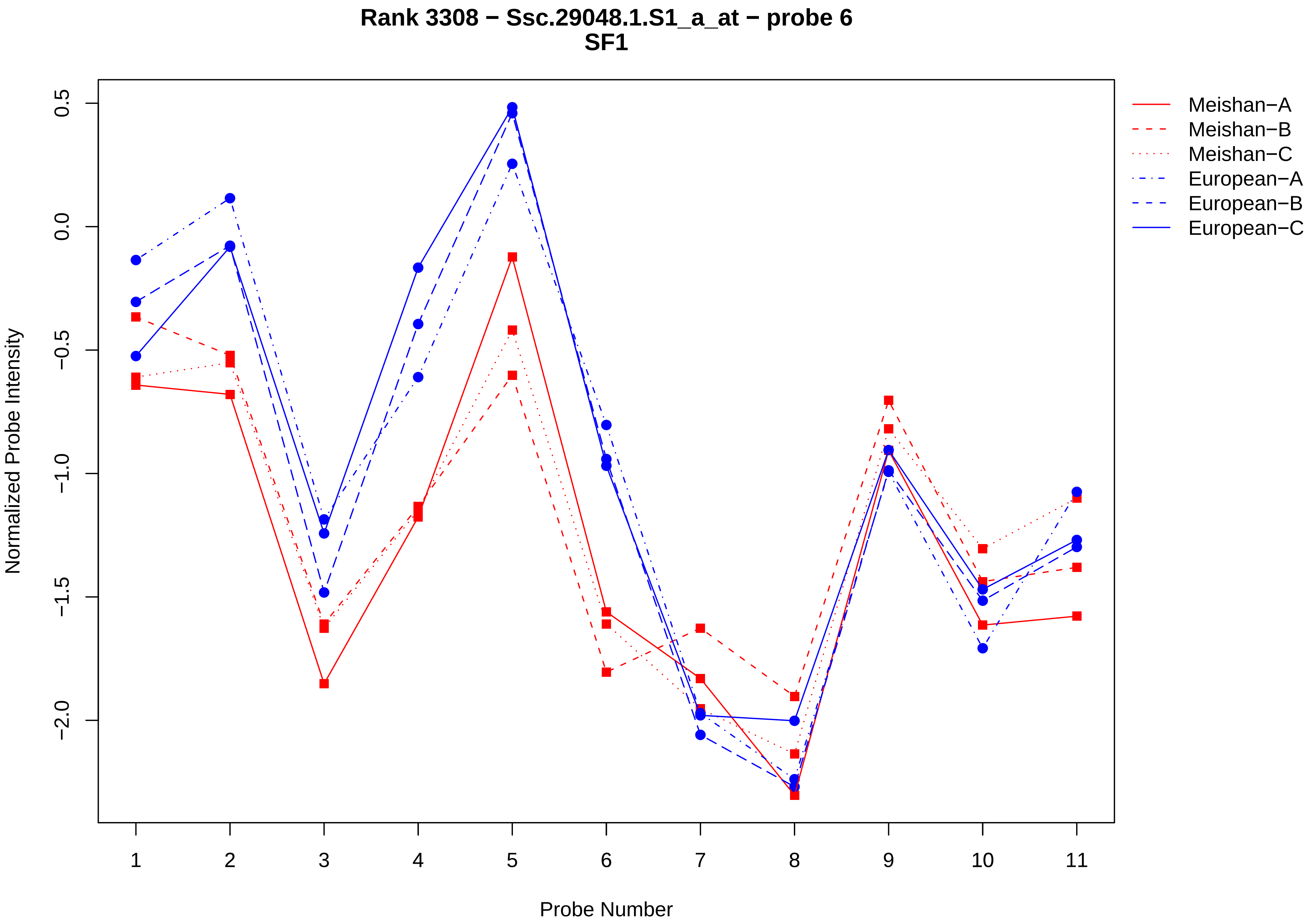

Rank 3308 − Ssc.29048.1.S1_a_at − probe 6
SF1
Normalized Probe Intensity
0.5
0.0
−0.5
−1.0
−1.5
−2.0
1
2
3
4
5
6
7
8
9
10
11
Probe Number
Meishan−A
Meishan−B
Meishan−C
European−A
European−B
European−C

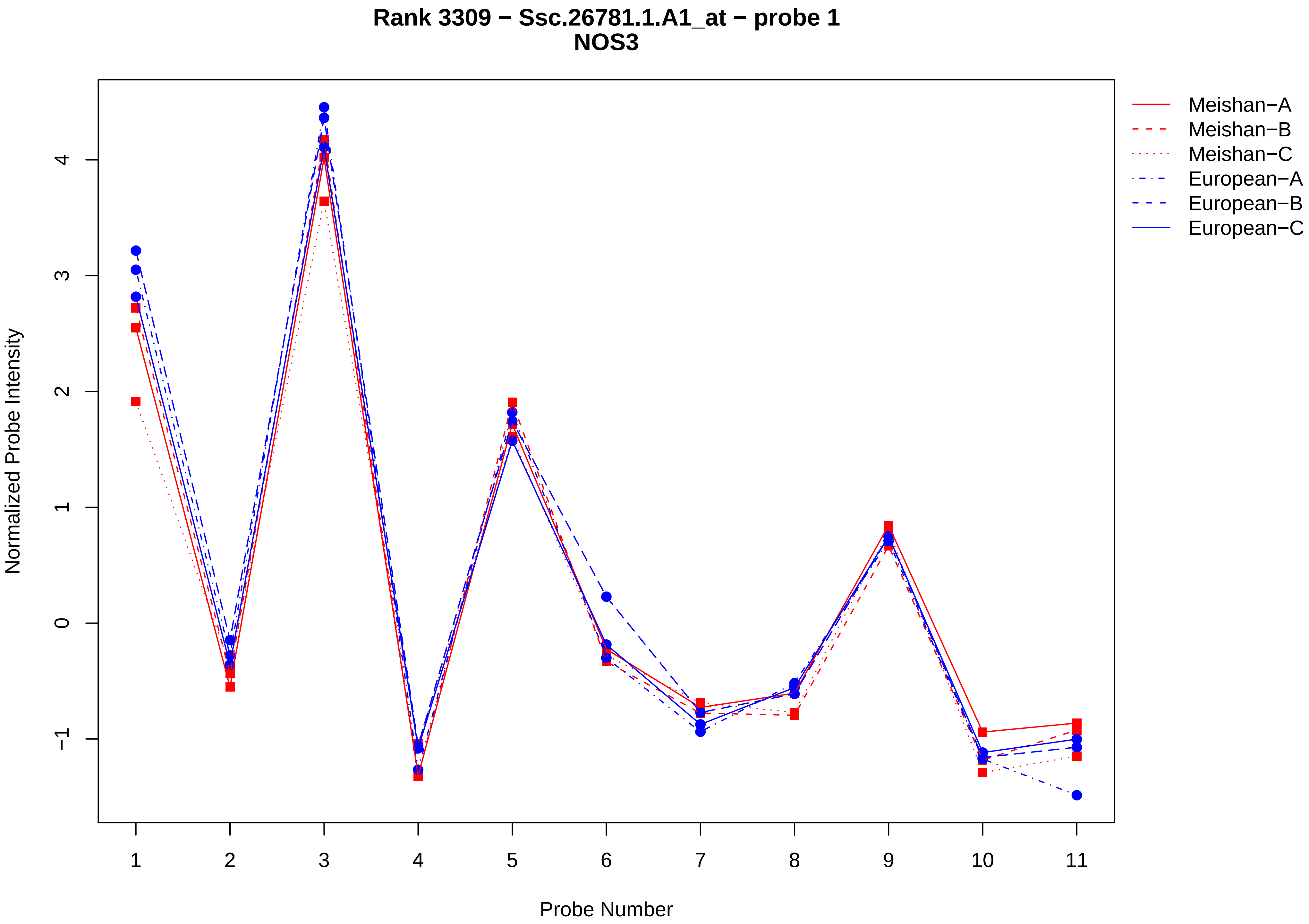
Rank 3309 − Ssc.26781.1.A1_at − probe 1
NOS3
Normalized Probe Intensity
Probe Number
−1
0
1
2
3
4
1
2
3
4
5
6
7
8
9
10
11
Meishan−A
Meishan−B
Meishan−C
European−A
European−B
European−C

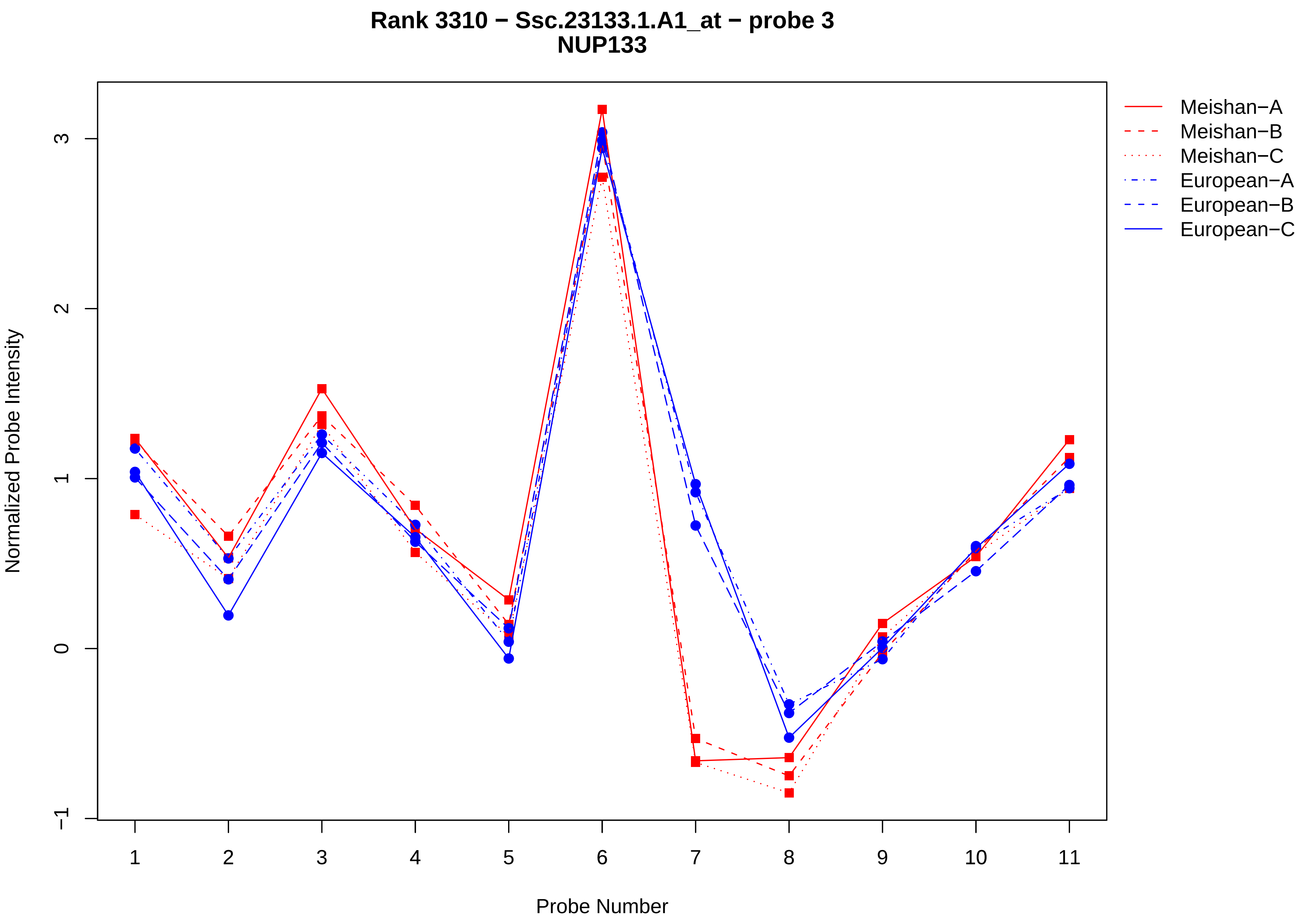

Rank 3310 − Ssc.23133.1.A1_at − probe 3
NUP133
Normalized Probe Intensity
Probe Number
−1
0
1
2
3
1
2
3
4
5
6
7
8
9
10
11
Meishan−A
Meishan−B
Meishan−C
European−A
European−B
European−C

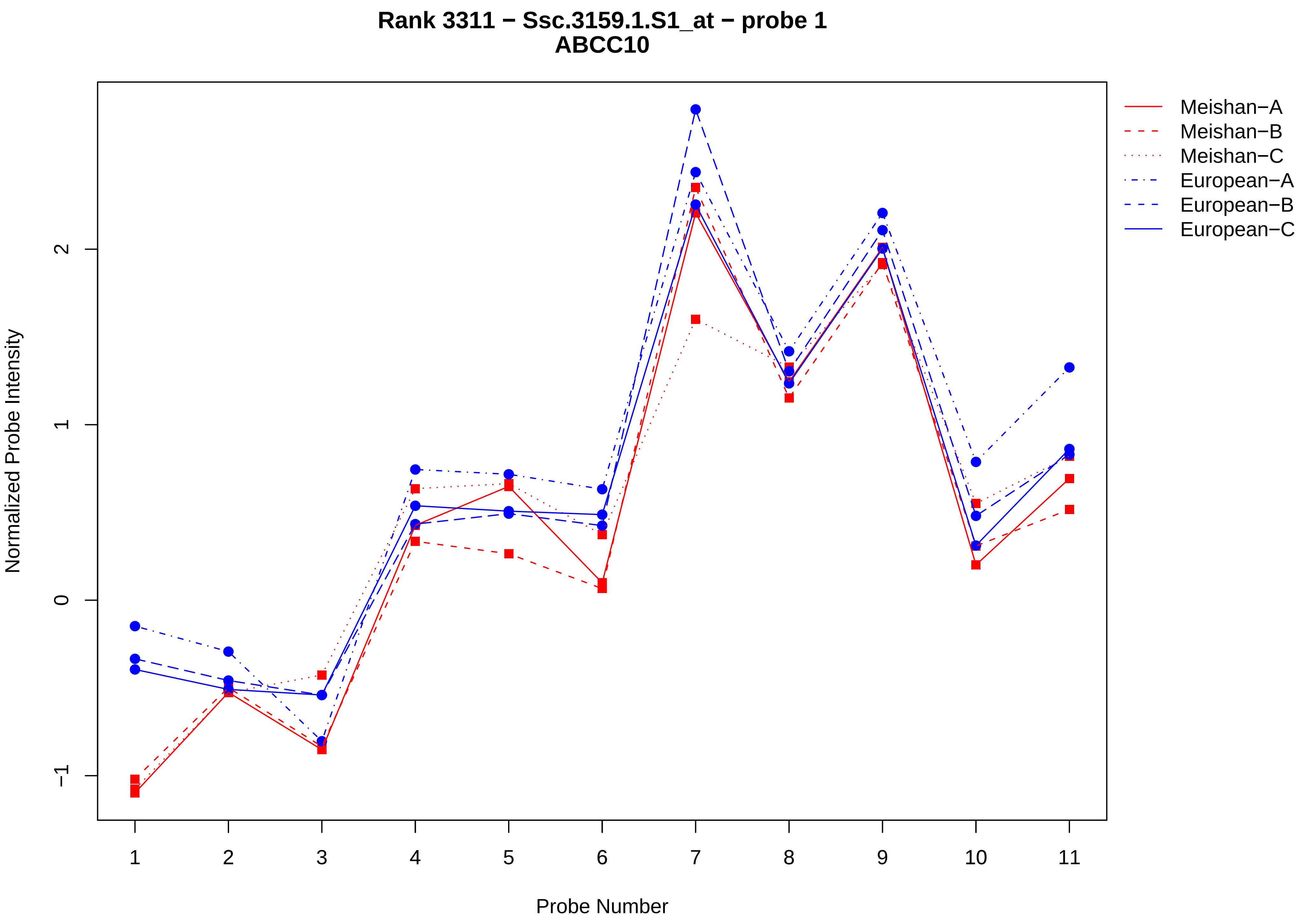

Rank 3311 − Ssc.3159.1.S1_at − probe 1
ABCC10
Normalized Probe Intensity
Probe Number
−1
0
1
2
1
2
3
4
5
6
7
8
9
10
11
Meishan−A
Meishan−B
Meishan−C
European−A
European−B
European−C

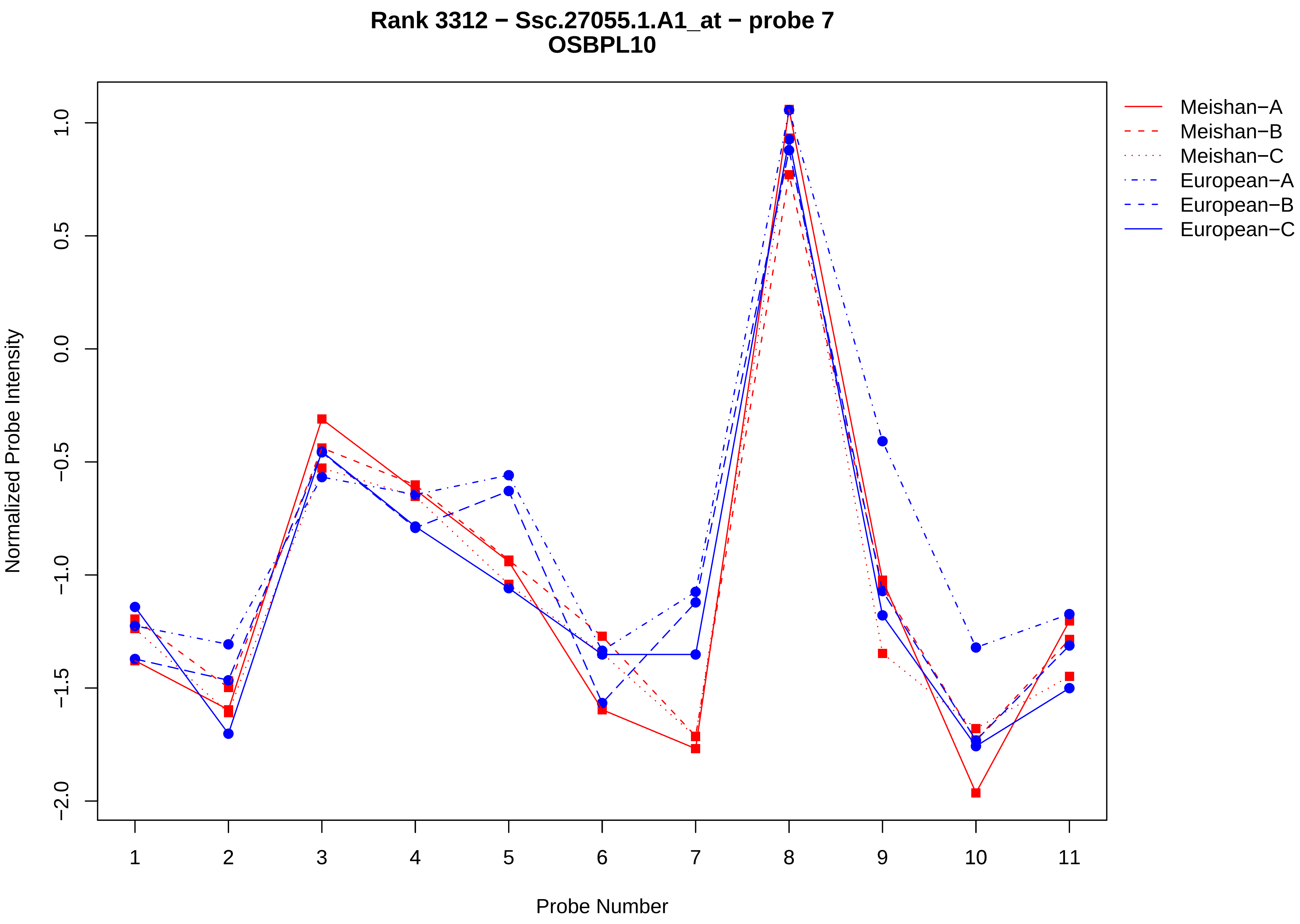

Rank 3312 − Ssc.27055.1.A1_at − probe 7
OSBPL10
Normalized Probe Intensity
Probe Number
1.0
0.5
0.0
−0.5
−1.0
−1.5
−2.0
1
2
3
4
5
6
7
8
9
10
11
Meishan−A
Meishan−B
Meishan−C
European−A
European−B
European−C

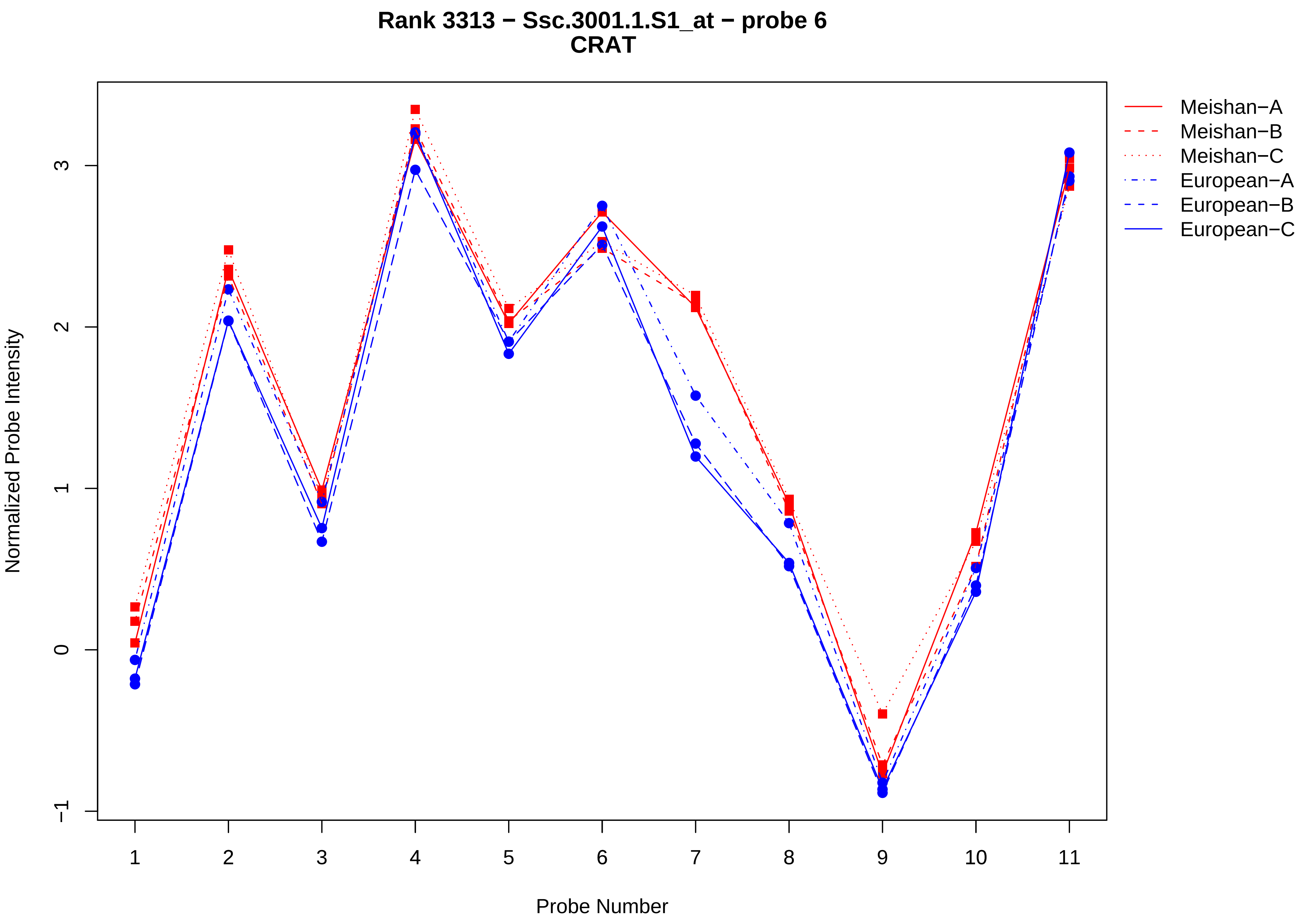

Rank 3313 − Ssc.3001.1.S1_at − probe 6
CRAT
Normalized Probe Intensity
Probe Number
Meishan−A
Meishan−B
Meishan−C
European−A
European−B
European−C
−1
0
1
2
3
1
2
3
4
5
6
7
8
9
10
11

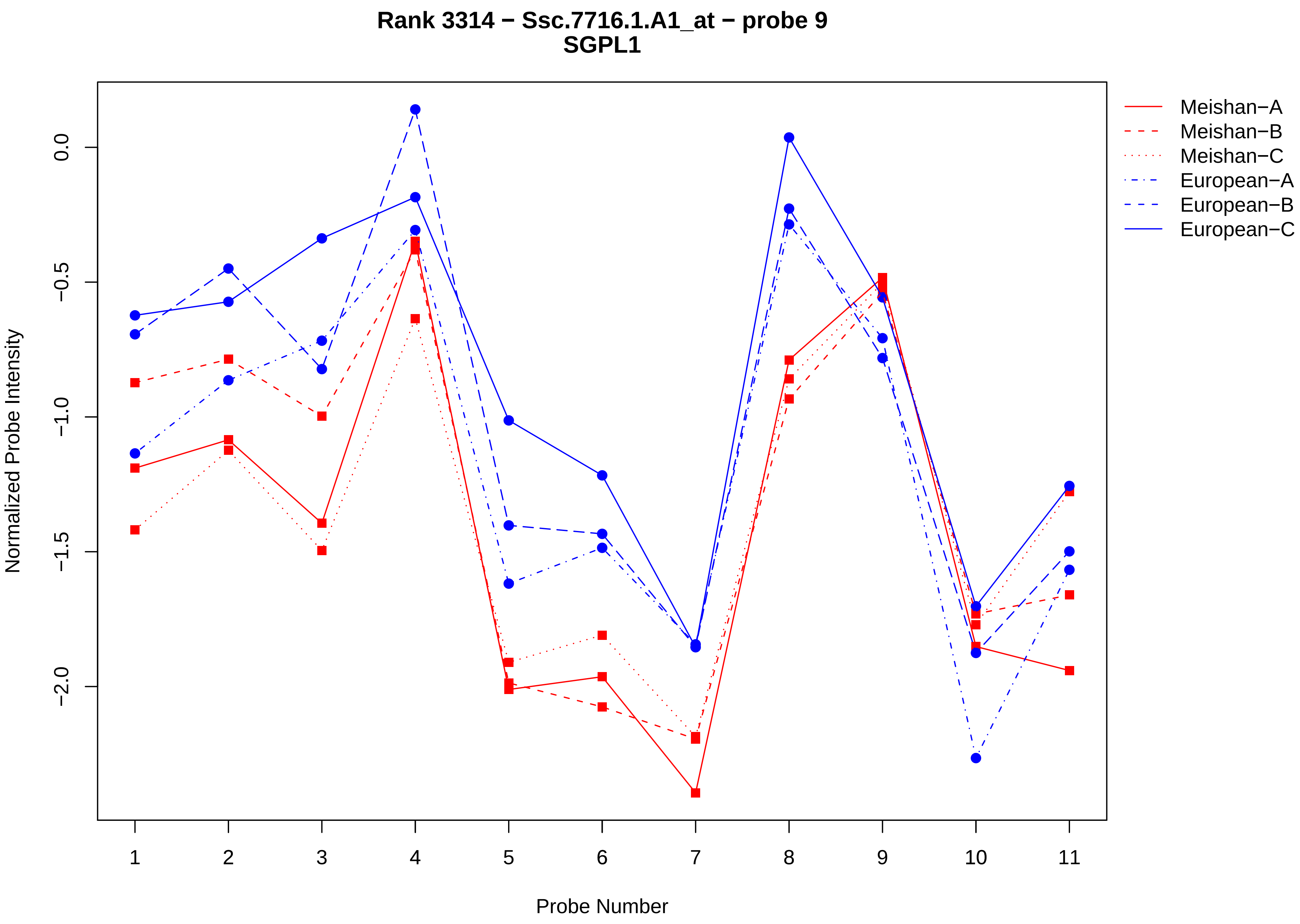
Rank 3314 − Ssc.7716.1.A1_at − probe 9
SGPL1
Normalized Probe Intensity
Probe Number
0.0
−0.5
−1.0
−1.5
−2.0
1
2
3
4
5
6
7
8
9
10
11
Meishan−A
Meishan−B
Meishan−C
European−A
European−B
European−C

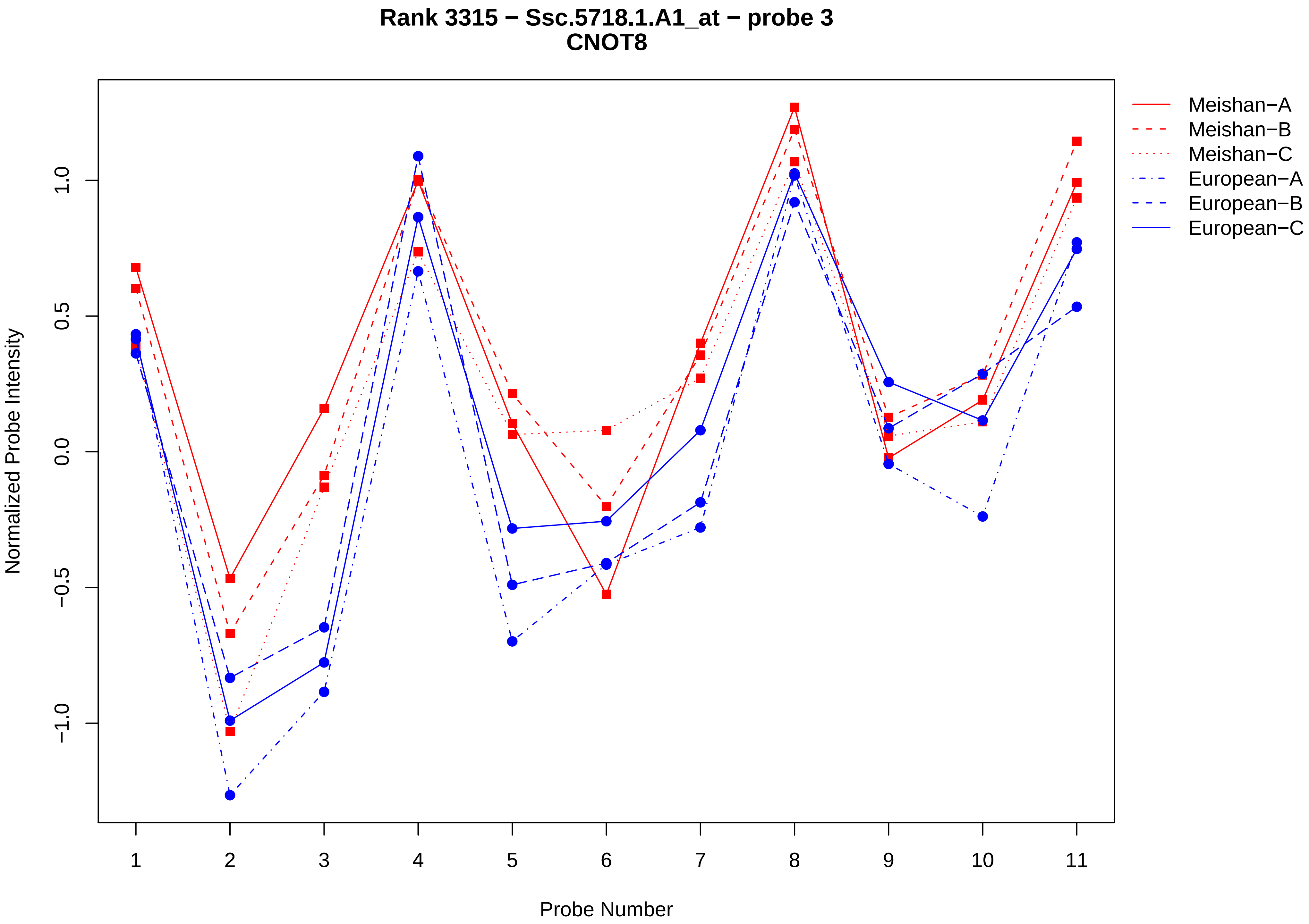

Rank 3315 − Ssc.5718.1.A1_at − probe 3
CNOT8
Normalized Probe Intensity
−1.0
−0.5
0.0
0.5
1.0
1
2
3
4
5
6
7
8
9
10
11
Probe Number
Meishan−A
Meishan−B
Meishan−C
European−A
European−B
European−C

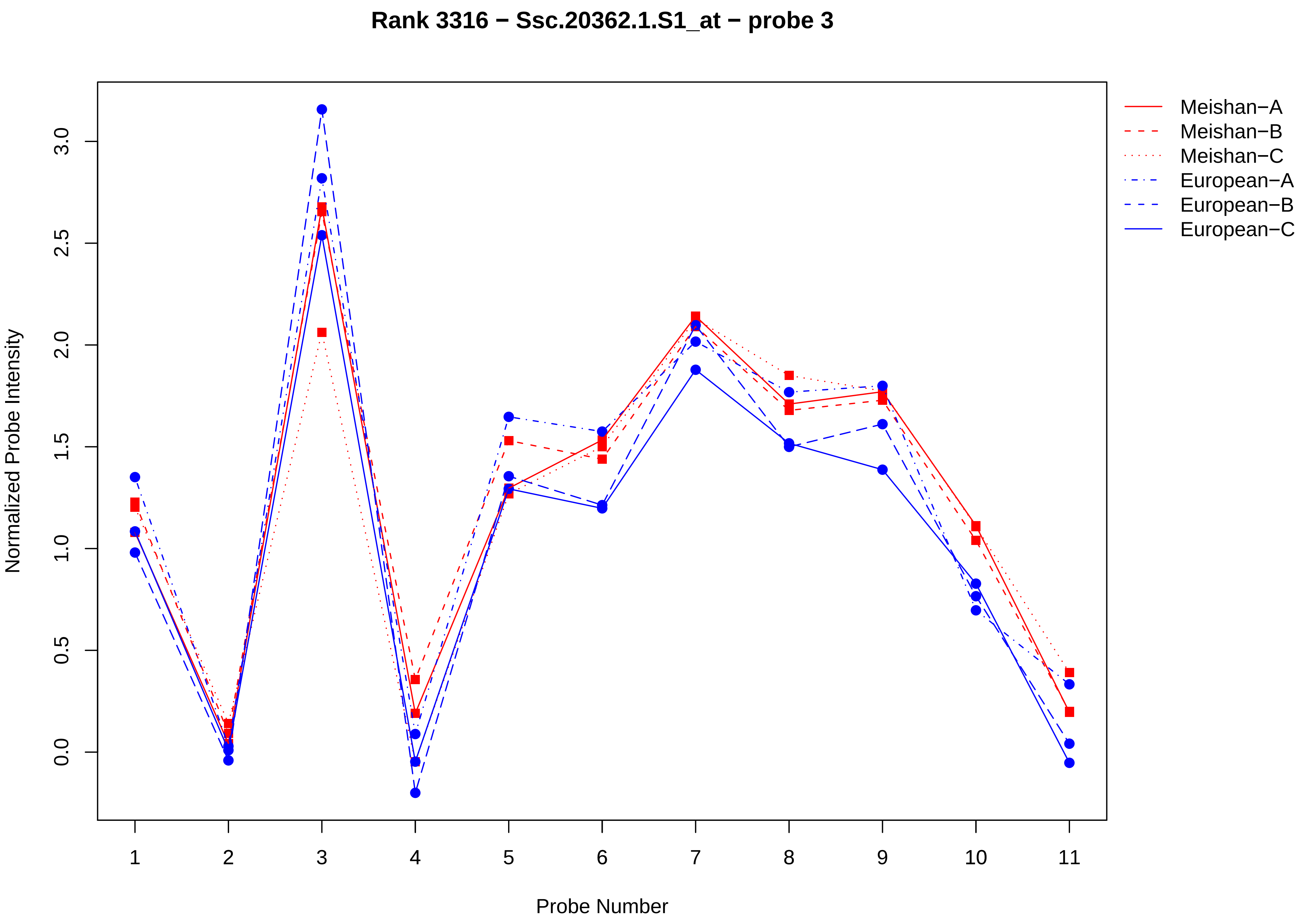

Rank 3316 − Ssc.20362.1.S1_at − probe 3
Normalized Probe Intensity
Probe Number
0.0
0.5
1.0
1.5
2.0
2.5
3.0
1
2
3
4
5
6
7
8
9
10
11
Meishan−A
Meishan−B
Meishan−C
European−A
European−B
European−C

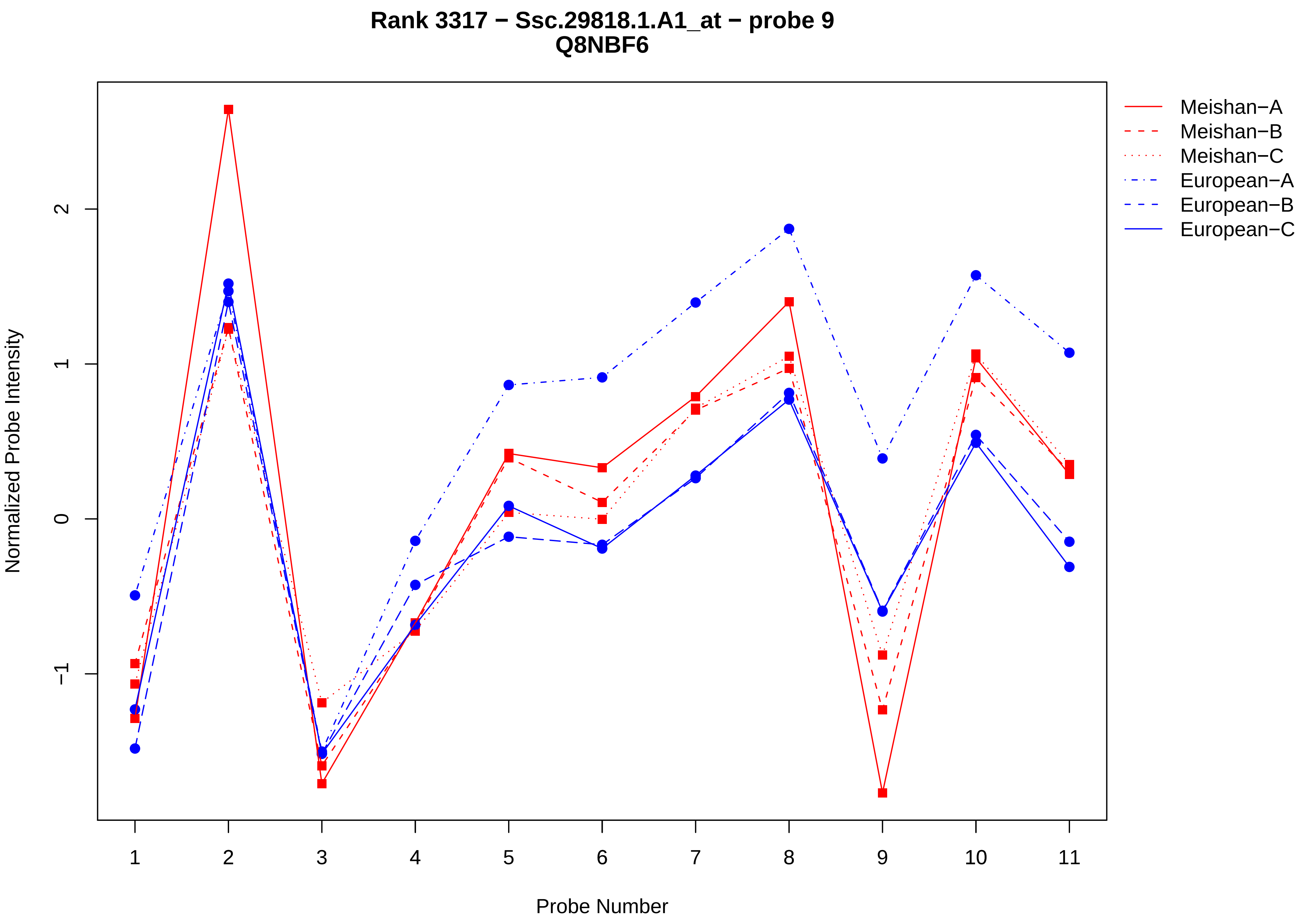
Rank 3317 − Ssc.29818.1.A1_at − probe 9
Q8NBF6
Normalized Probe Intensity
Probe Number
−1
0
1
2
1
2
3
4
5
6
7
8
9
10
11
Meishan−A
Meishan−B
Meishan−C
European−A
European−B
European−C

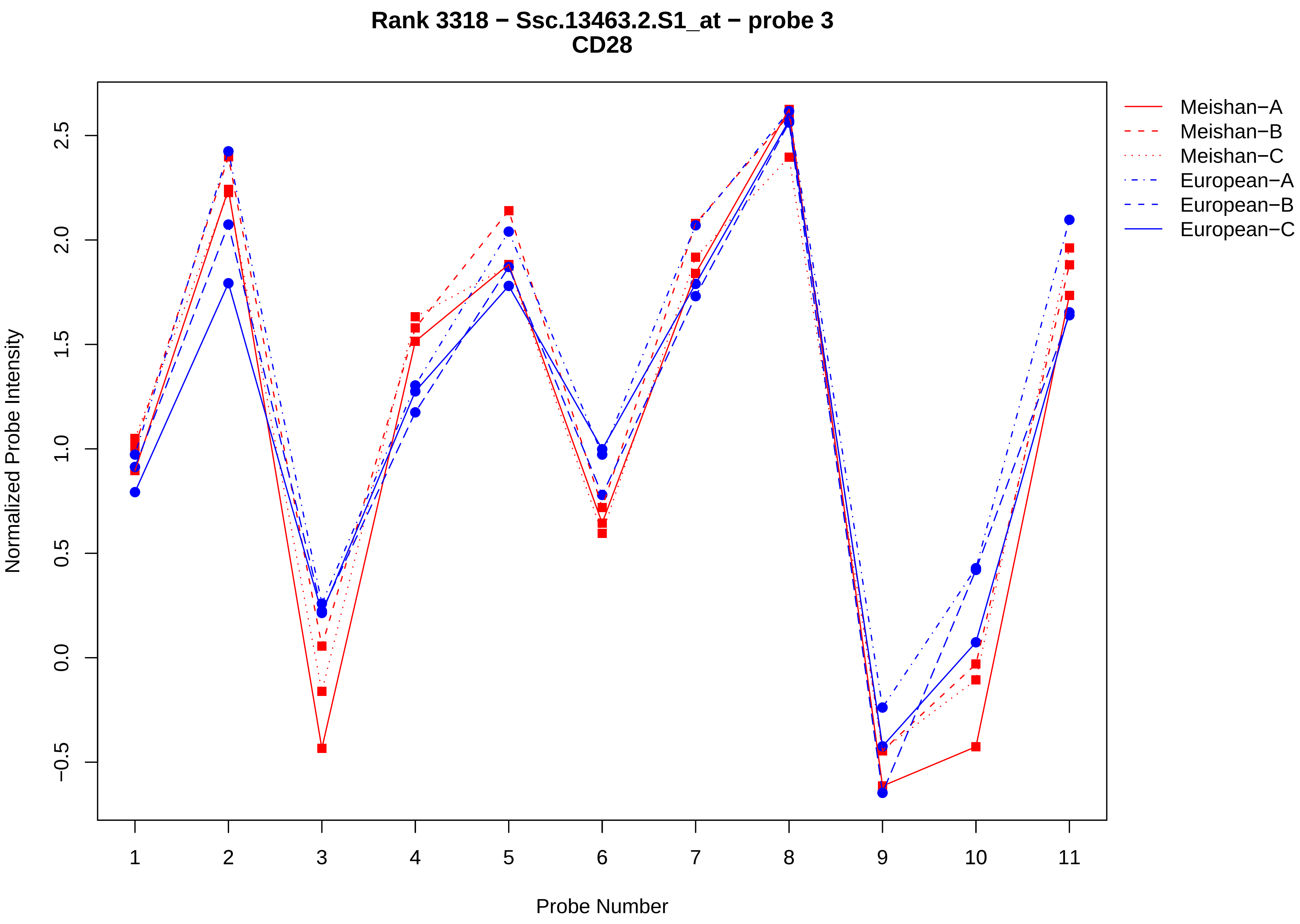

Rank 3318 − Ssc.13463.2.S1_at − probe 3
CD28
Normalized Probe Intensity
Probe Number
−0.5
0.0
0.5
1.0
1.5
2.0
2.5
1
2
3
4
5
6
7
8
9
10
11
Meishan−A
Meishan−B
Meishan−C
European−A
European−B
European−C

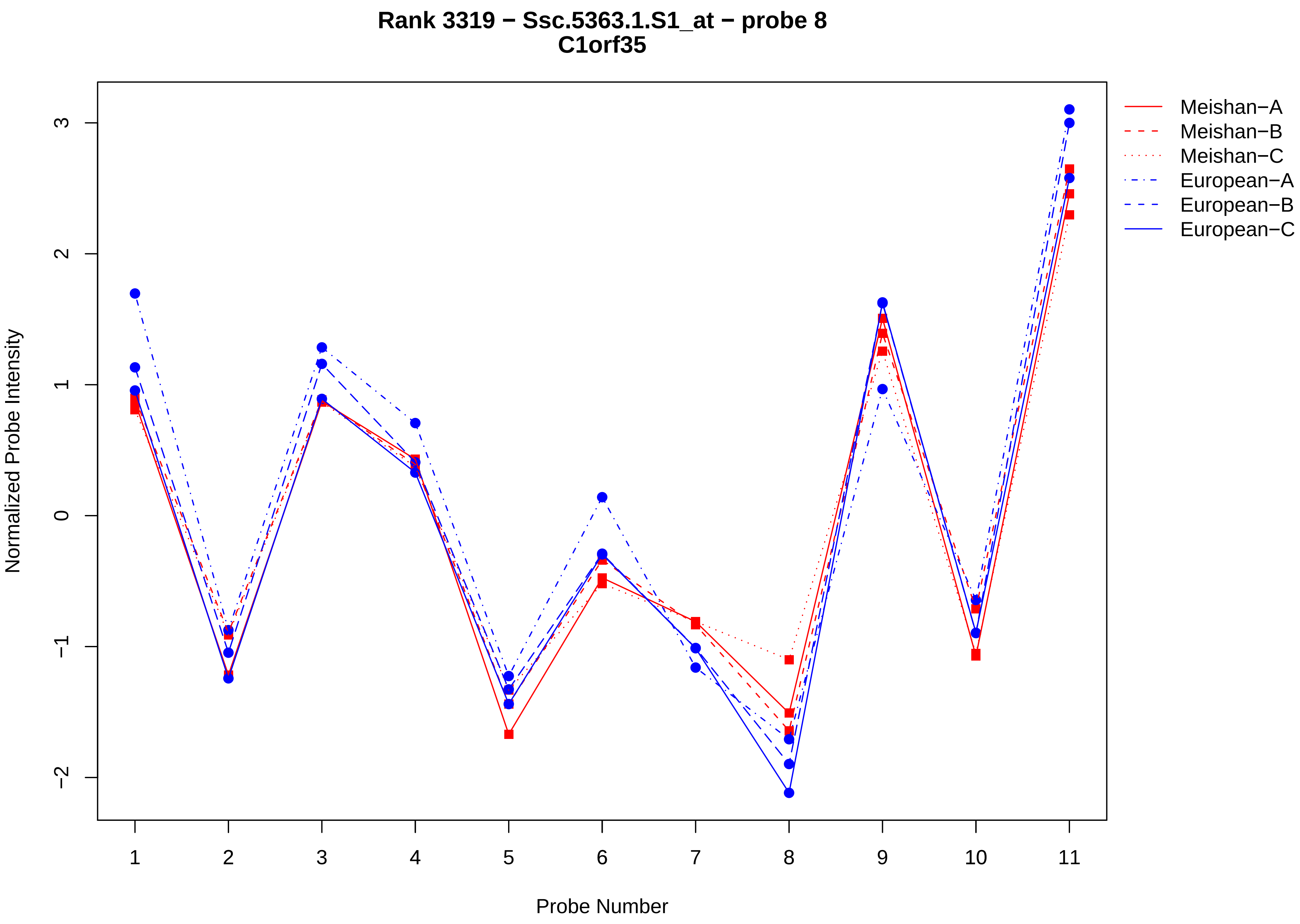

Rank 3319 − Ssc.5363.1.S1_at − probe 8
C1orf35
Normalized Probe Intensity
Probe Number
Meishan−A
Meishan−B
Meishan−C
European−A
European−B
European−C

Rank 3320 − Ssc.22097.1.A1_at − probe 7
EYA3

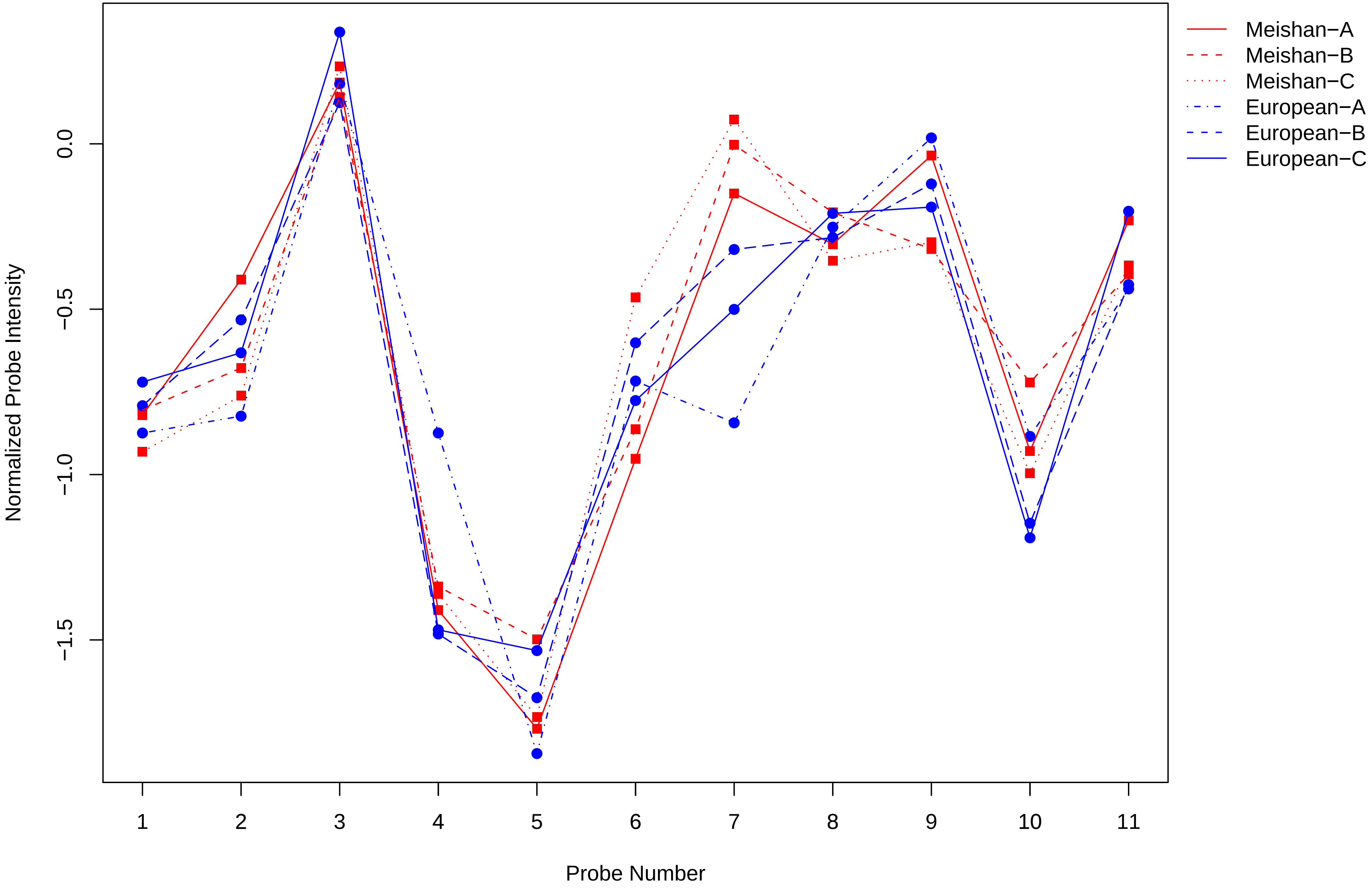

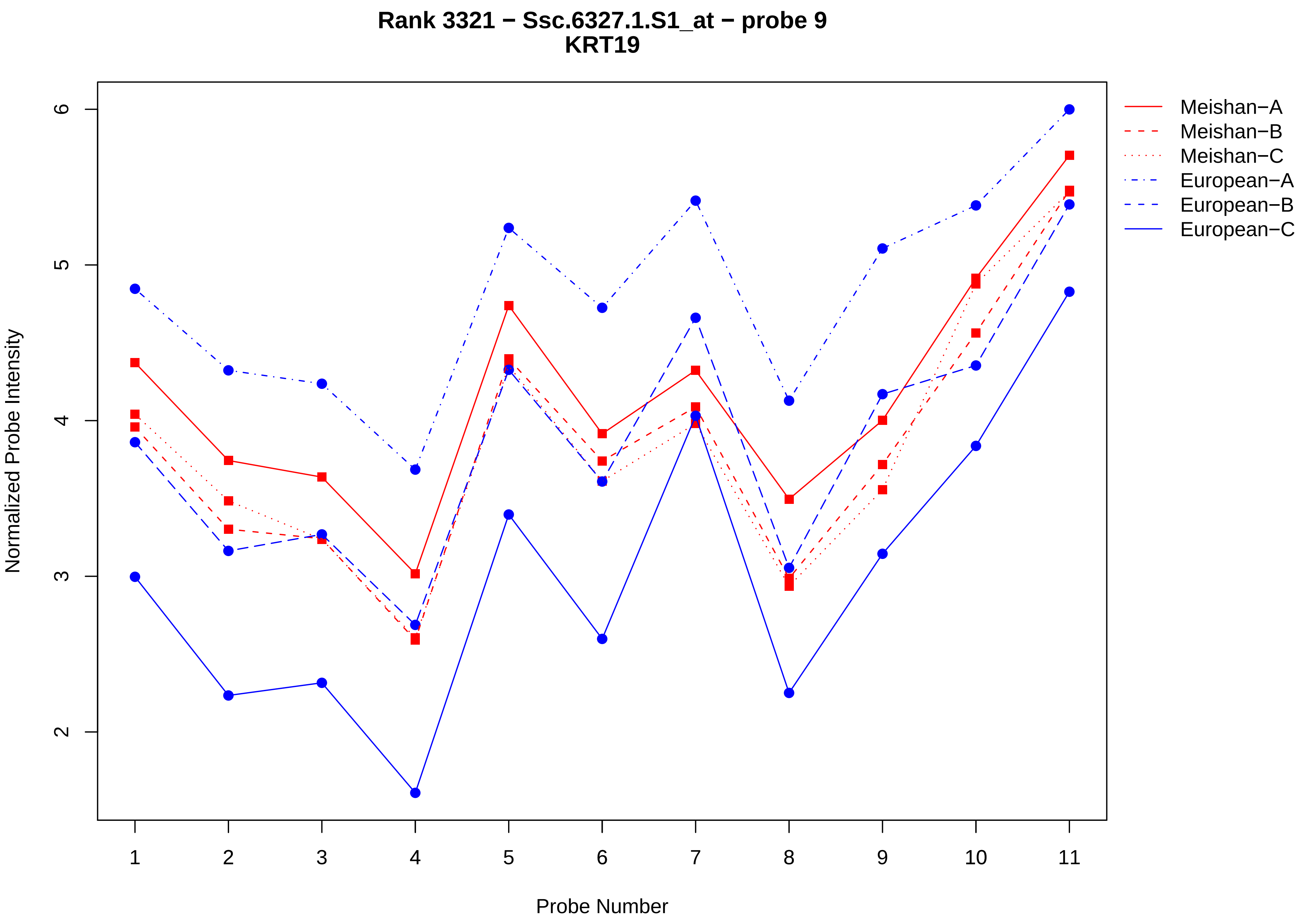
Rank 3321 − Ssc.6327.1.S1_at − probe 9
KRT19
Normalized Probe Intensity
Probe Number
2
3
4
5
6
1
2
3
4
5
6
7
8
9
10
11
Meishan−A
Meishan−B
Meishan−C
European−A
European−B
European−C

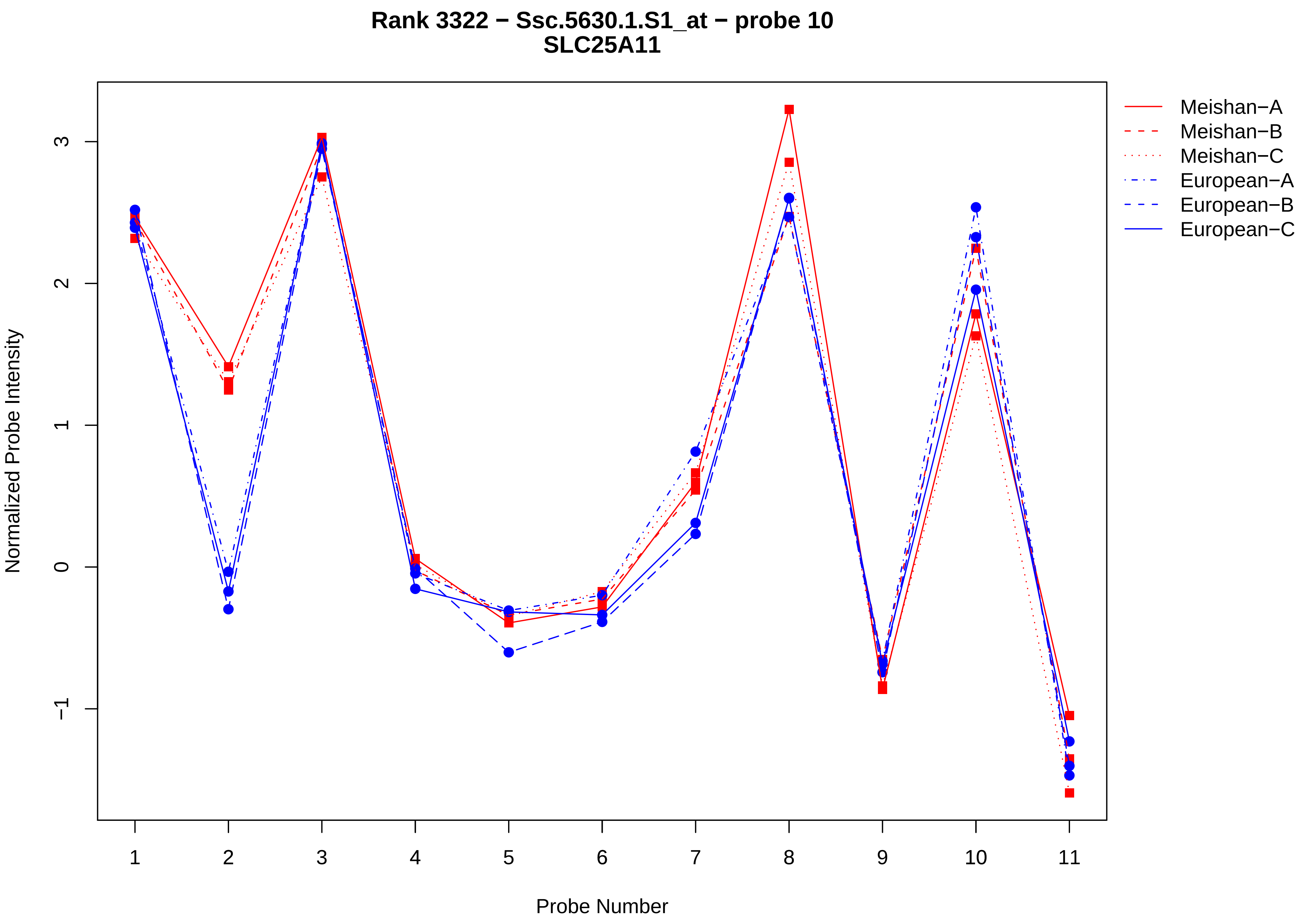
Rank 3322 − Ssc.5630.1.S1_at − probe 10
SLC25A11
Normalized Probe Intensity
Probe Number
−1
0
1
2
3
1
2
3
4
5
6
7
8
9
10
11
Meishan−A
Meishan−B
Meishan−C
European−A
European−B
European−C

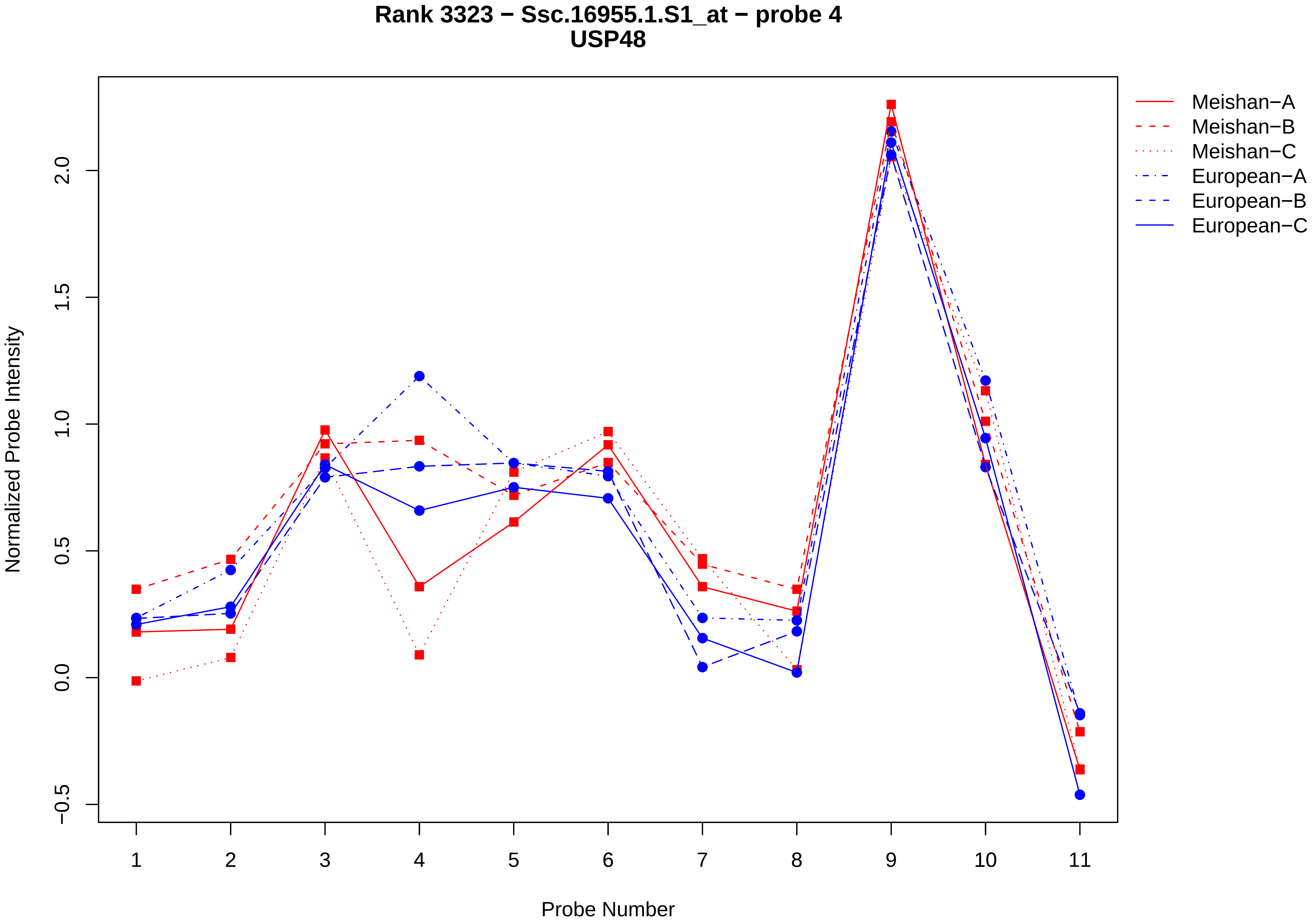
Rank 3323 − Ssc.16955.1.S1_at − probe 4
USP48
Normalized Probe Intensity
Probe Number
−0.5
0.0
0.5
1.0
1.5
2.0
1
2
3
4
5
6
7
8
9
10
11
Meishan−A
Meishan−B
Meishan−C
European−A
European−B
European−C

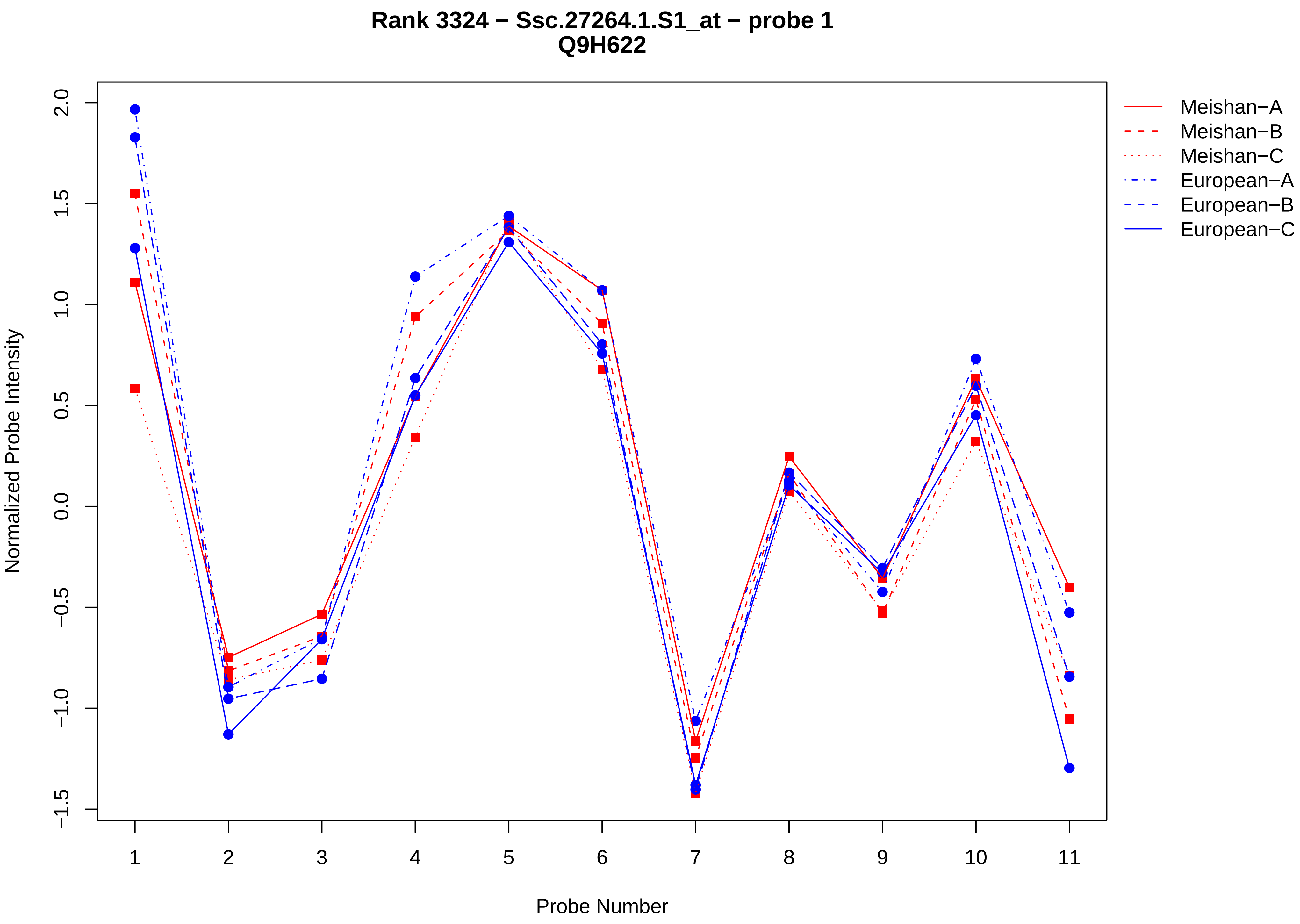

Rank 3324 − Ssc.27264.1.S1_at − probe 1
Q9H622
Normalized Probe Intensity
Probe Number
Meishan−A
Meishan−B
Meishan−C
European−A
European−B
European−C

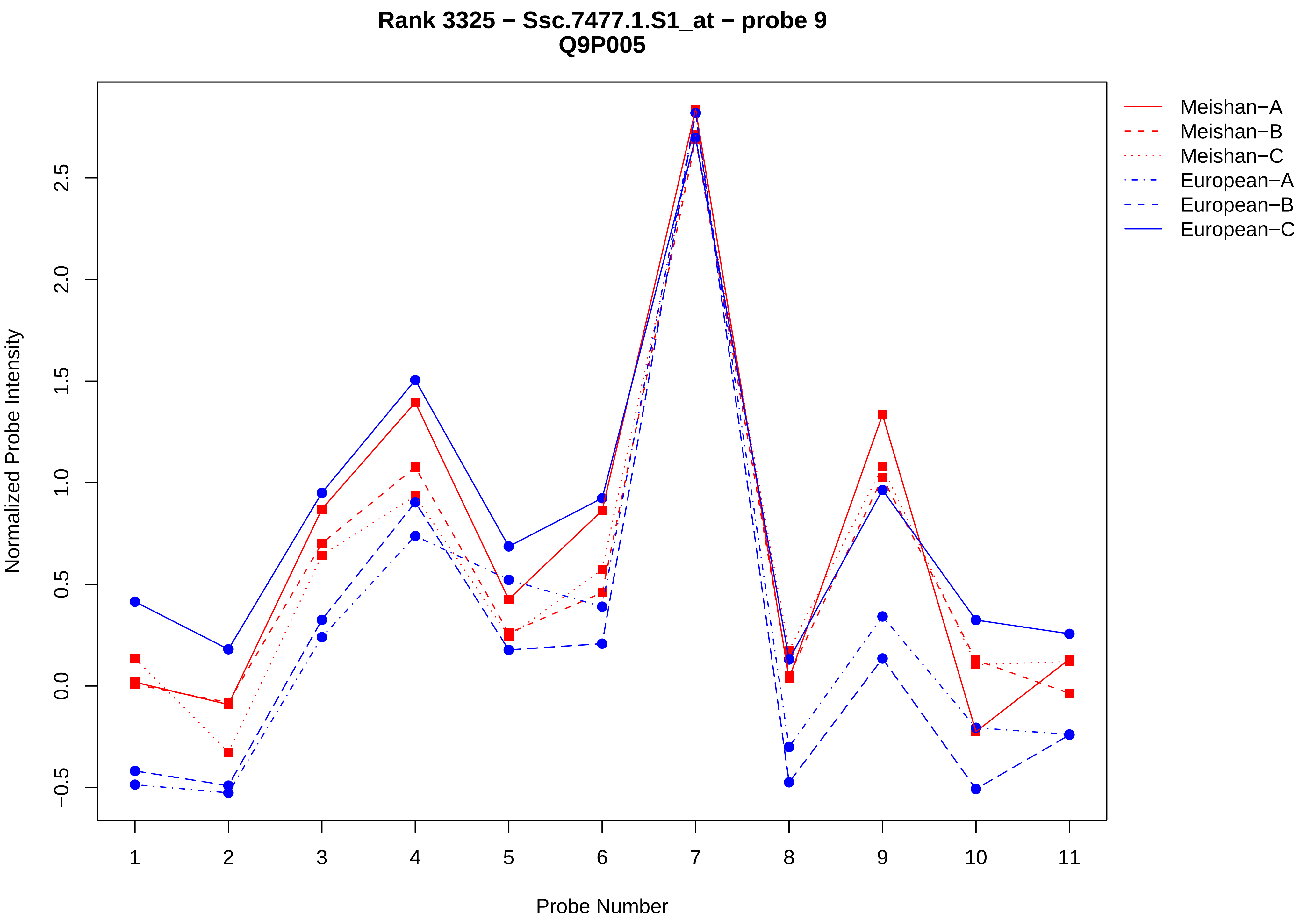

Rank 3325 − Ssc.7477.1.S1_at − probe 9
Q9P005
Normalized Probe Intensity
Probe Number
−0.5
0.0
0.5
1.0
1.5
2.0
2.5
1
2
3
4
5
6
7
8
9
10
11
Meishan−A
Meishan−B
Meishan−C
European−A
European−B
European−C

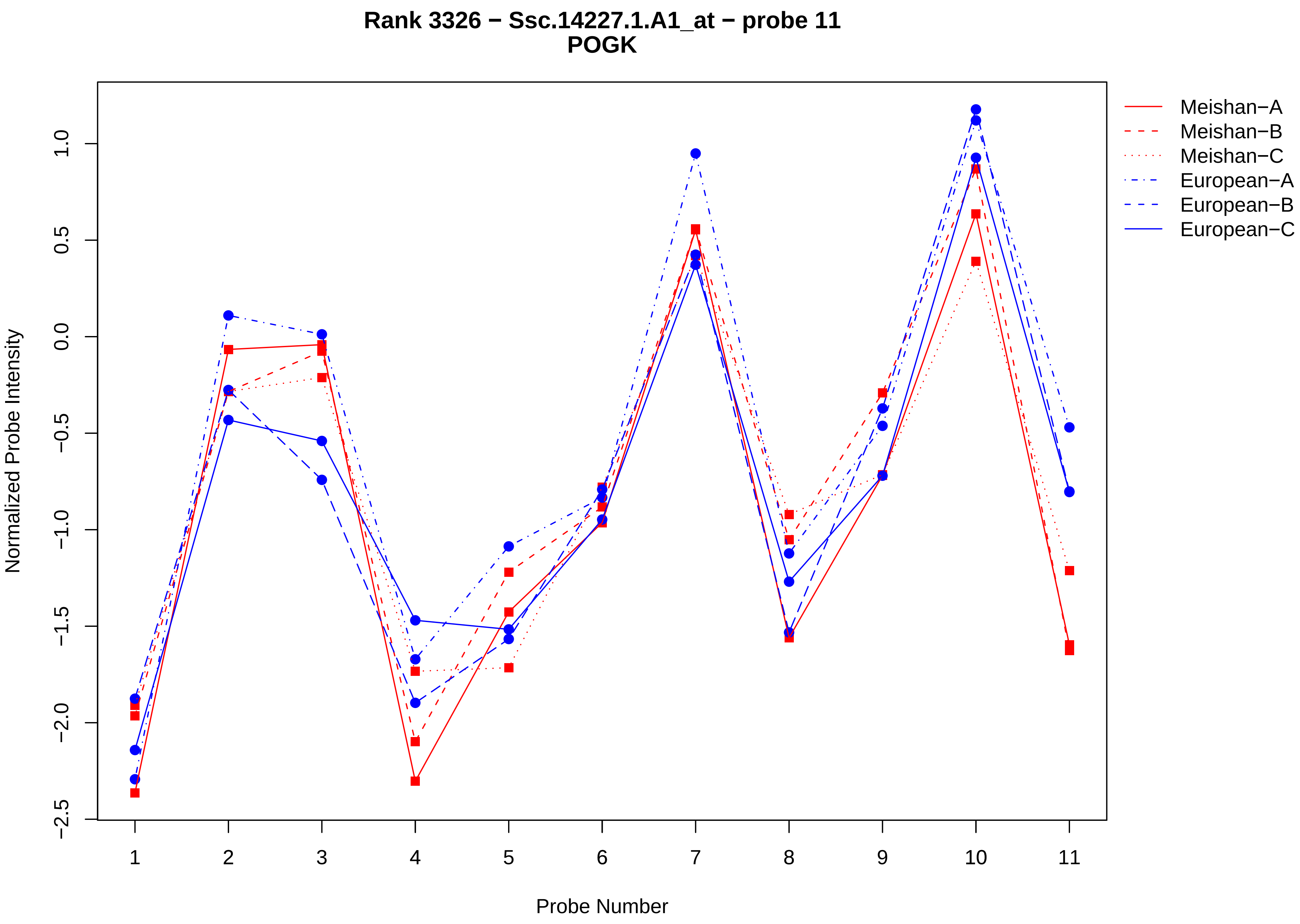
Rank 3326 − Ssc.14227.1.A1_at − probe 11
POGK
Normalized Probe Intensity
Probe Number
Meishan−A
Meishan−B
Meishan−C
European−A
European−B
European−C
1.0
0.5
0.0
−0.5
−1.0
−1.5
−2.0
−2.5
1
2
3
4
5
6
7
8
9
10
11

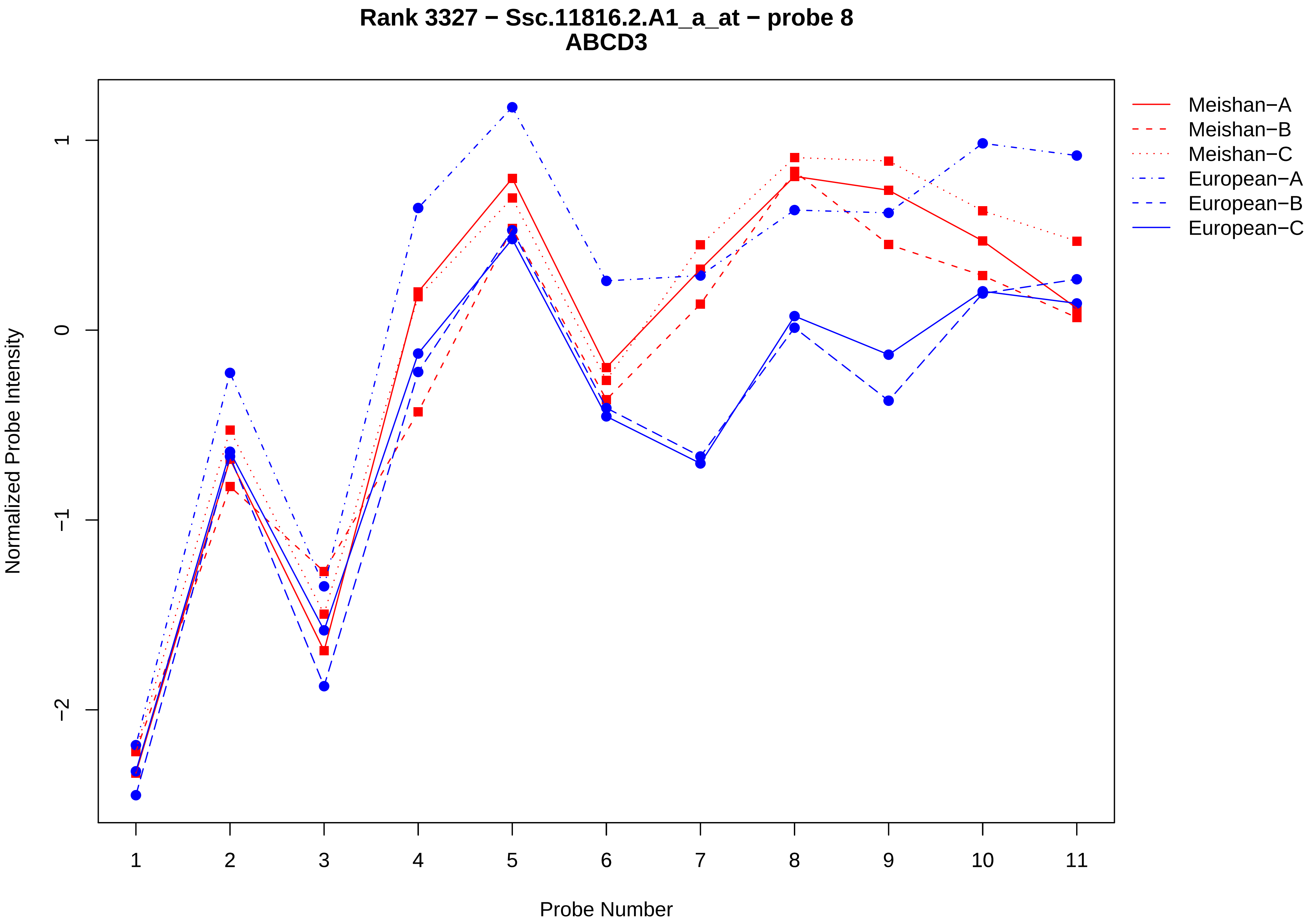

Rank 3327 − Ssc.11816.2.A1_a_at − probe 8
ABCD3
Normalized Probe Intensity
Probe Number
−2
−1
0
1
1
2
3
4
5
6
7
8
9
10
11
Meishan−A
Meishan−B
Meishan−C
European−A
European−B
European−C

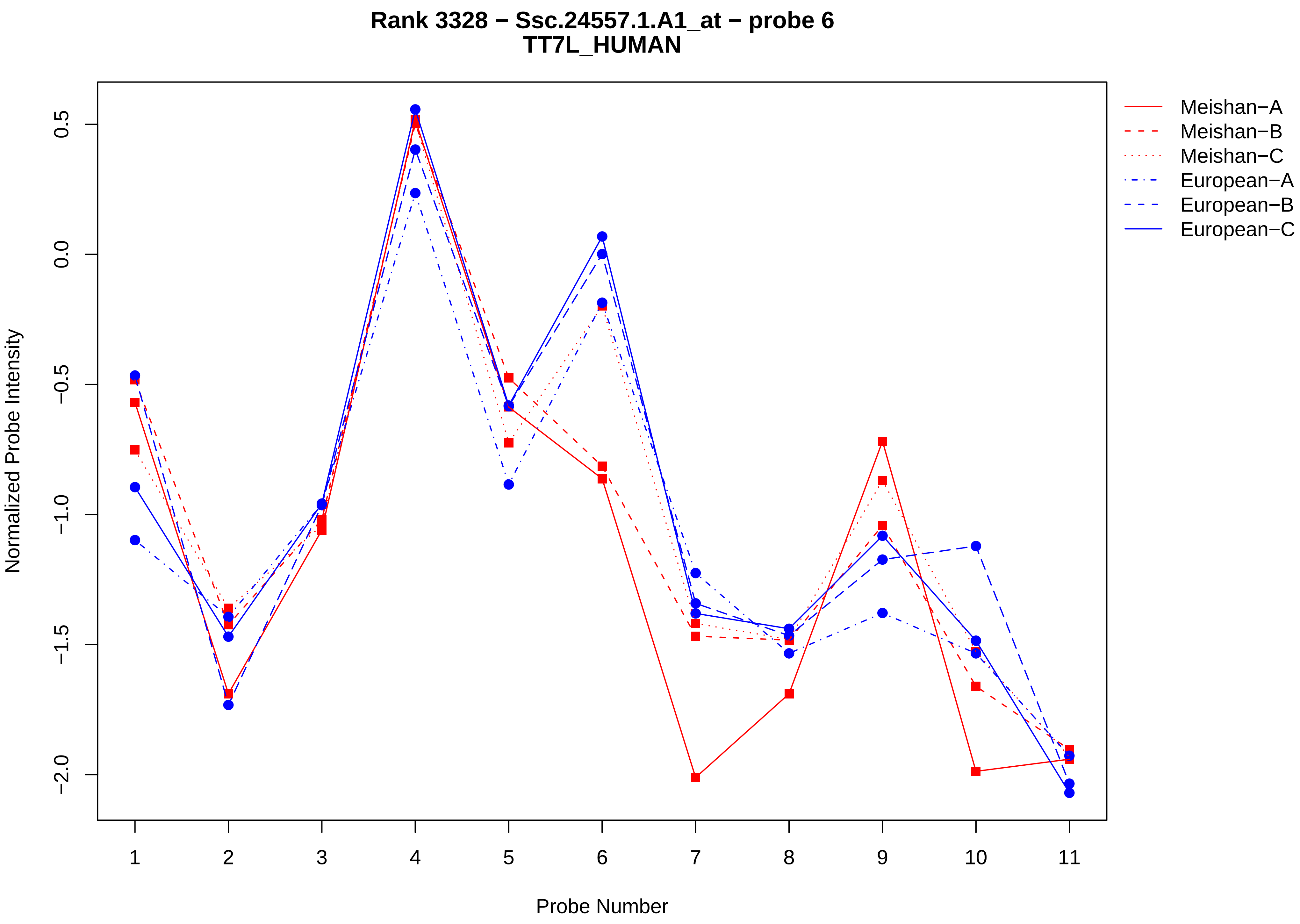

Rank 3328 − Ssc.24557.1.A1_at − probe 6
TT7L_HUMAN
Normalized Probe Intensity
Probe Number
0.5
0.0
−0.5
−1.0
−1.5
−2.0
1
2
3
4
5
6
7
8
9
10
11
Meishan−A
Meishan−B
Meishan−C
European−A
European−B
European−C

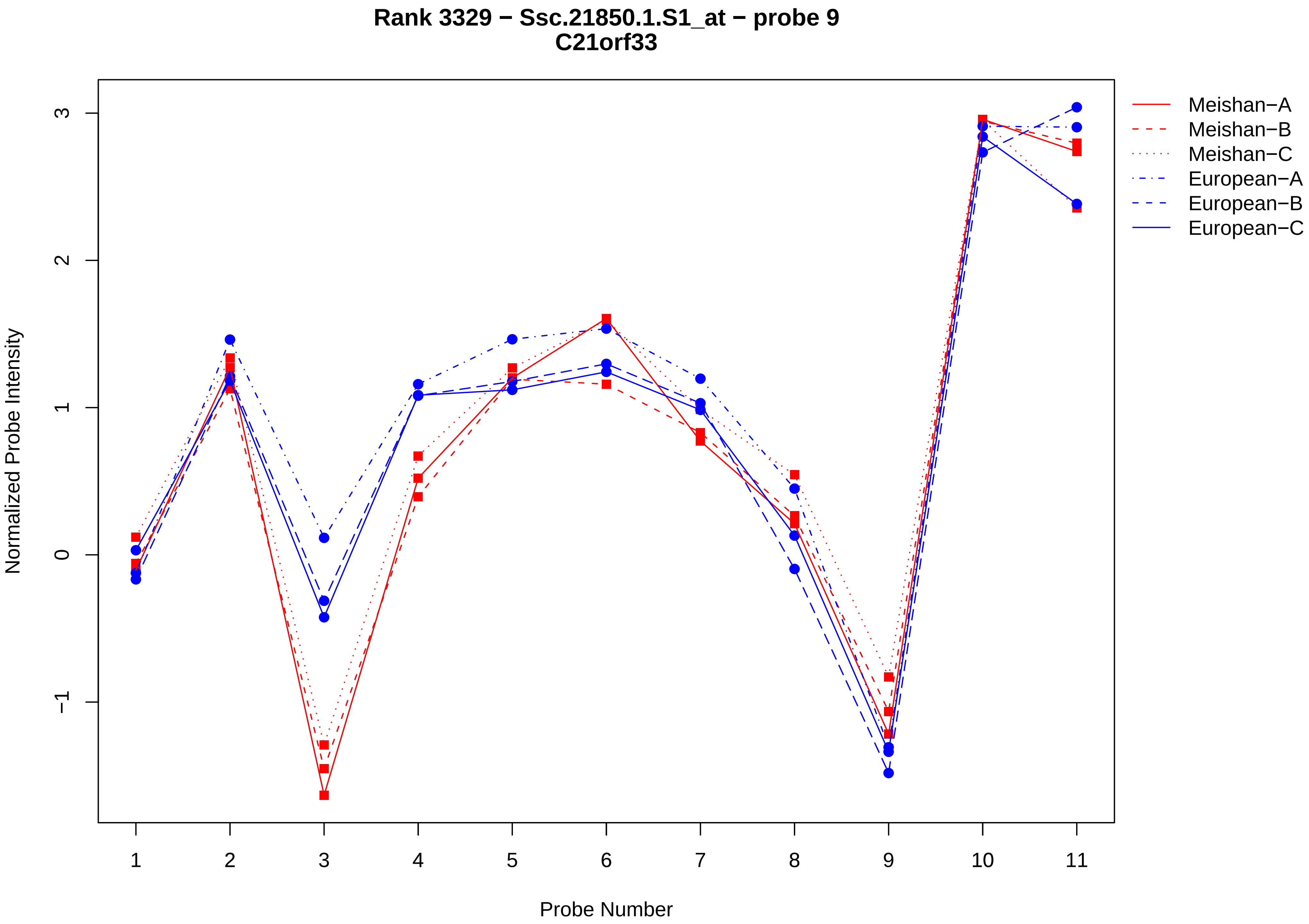

Rank 3329 − Ssc.21850.1.S1_at − probe 9
C21orf33
Normalized Probe Intensity
Probe Number
−1
0
1
2
3
1
2
3
4
5
6
7
8
9
10
11
Meishan−A
Meishan−B
Meishan−C
European−A
European−B
European−C

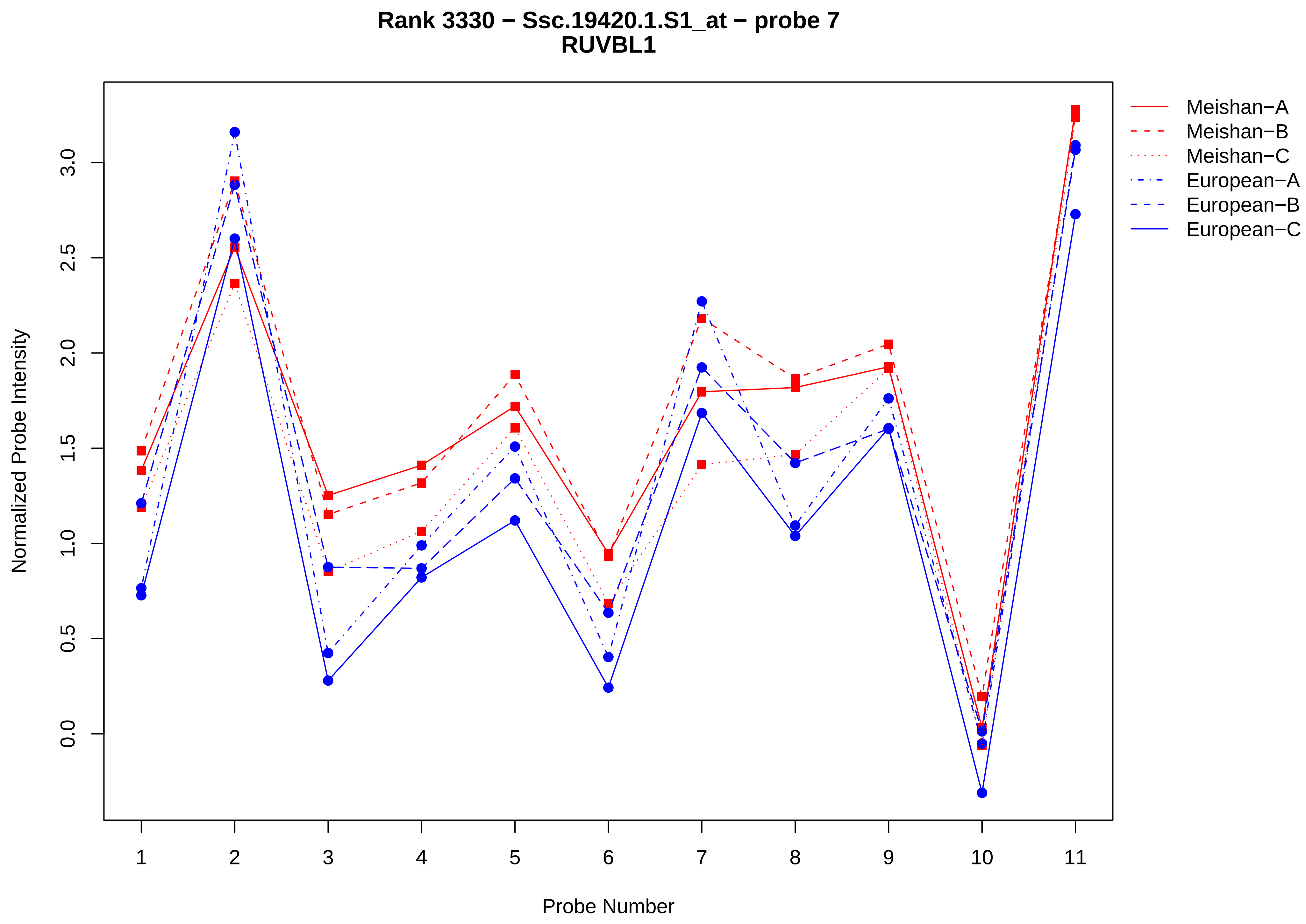

Rank 3330 − Ssc.19420.1.S1_at − probe 7
RUVBL1
Normalized Probe Intensity
Probe Number
Meishan−A
Meishan−B
Meishan−C
European−A
European−B
European−C
0.0
0.5
1.0
1.5
2.0
2.5
3.0
1
2
3
4
5
6
7
8
9
10
11

Rank 3331 − Ssc.30581.1.A1_at − probe 5
CDC14A

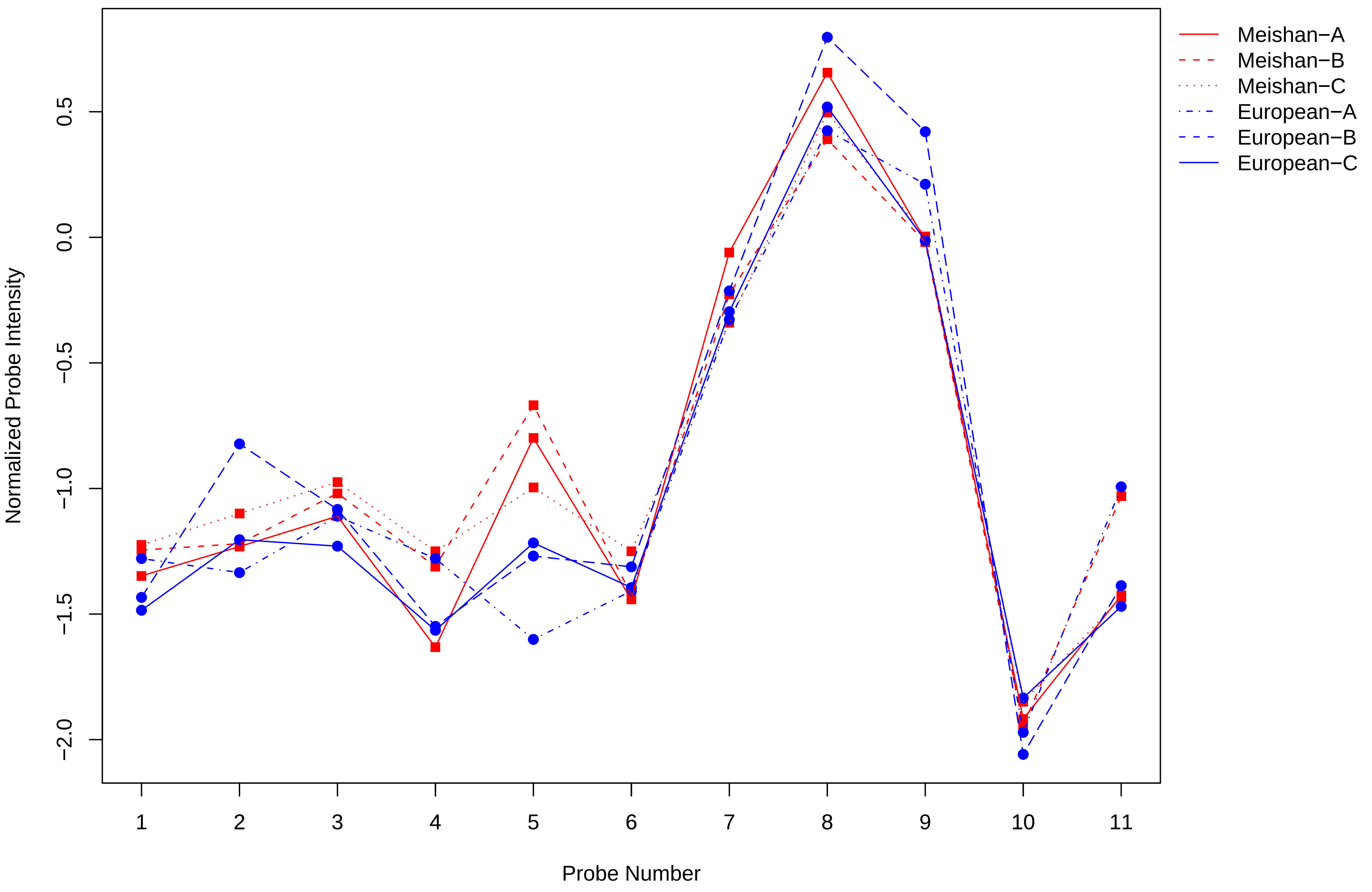

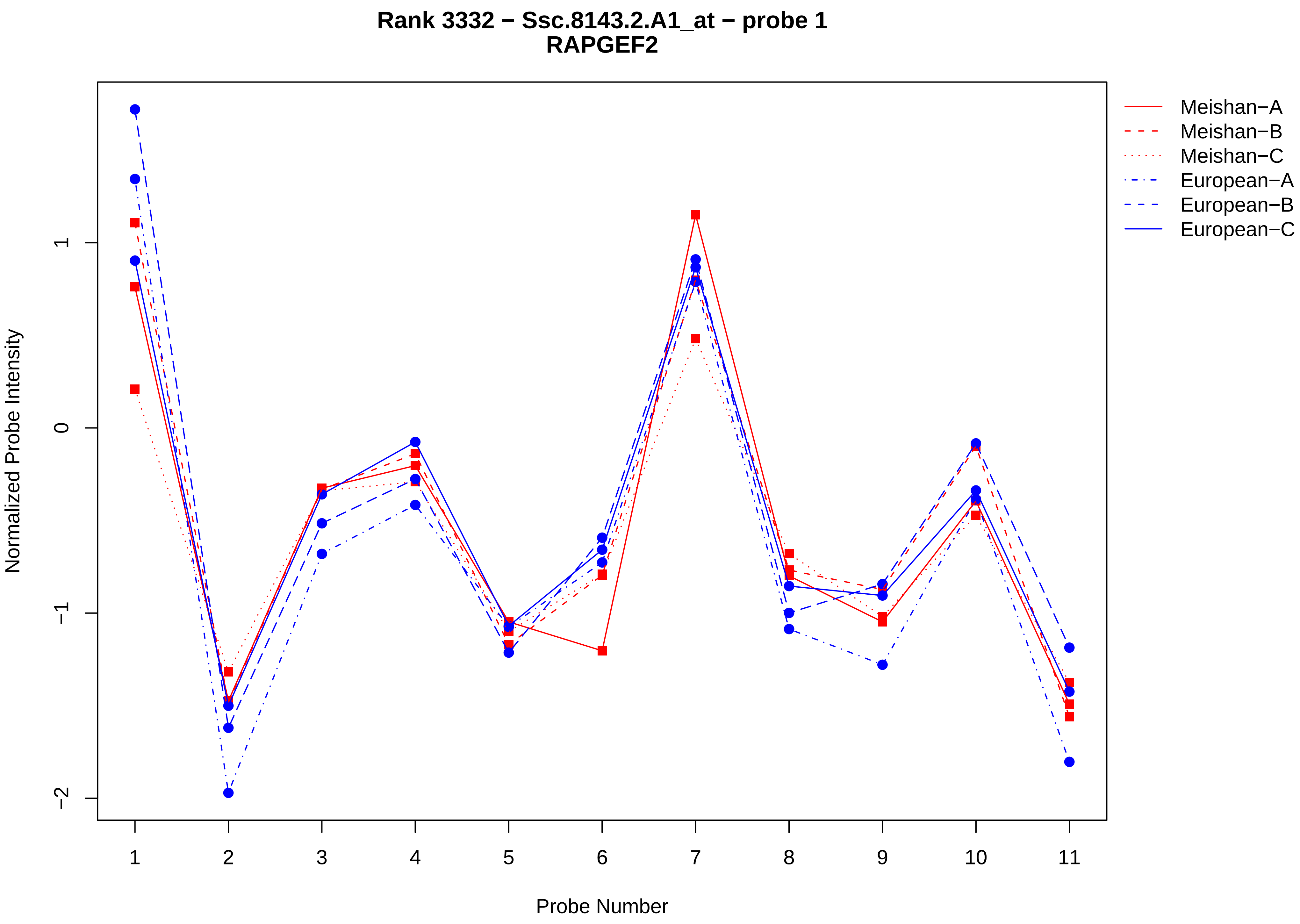

Rank 3332 − Ssc.8143.2.A1_at − probe 1
RAPGEF2
Normalized Probe Intensity
Probe Number
Meishan−A
Meishan−B
Meishan−C
European−A
European−B
European−C
1
0
−1
−2
1
2
3
4
5
6
7
8
9
10
11

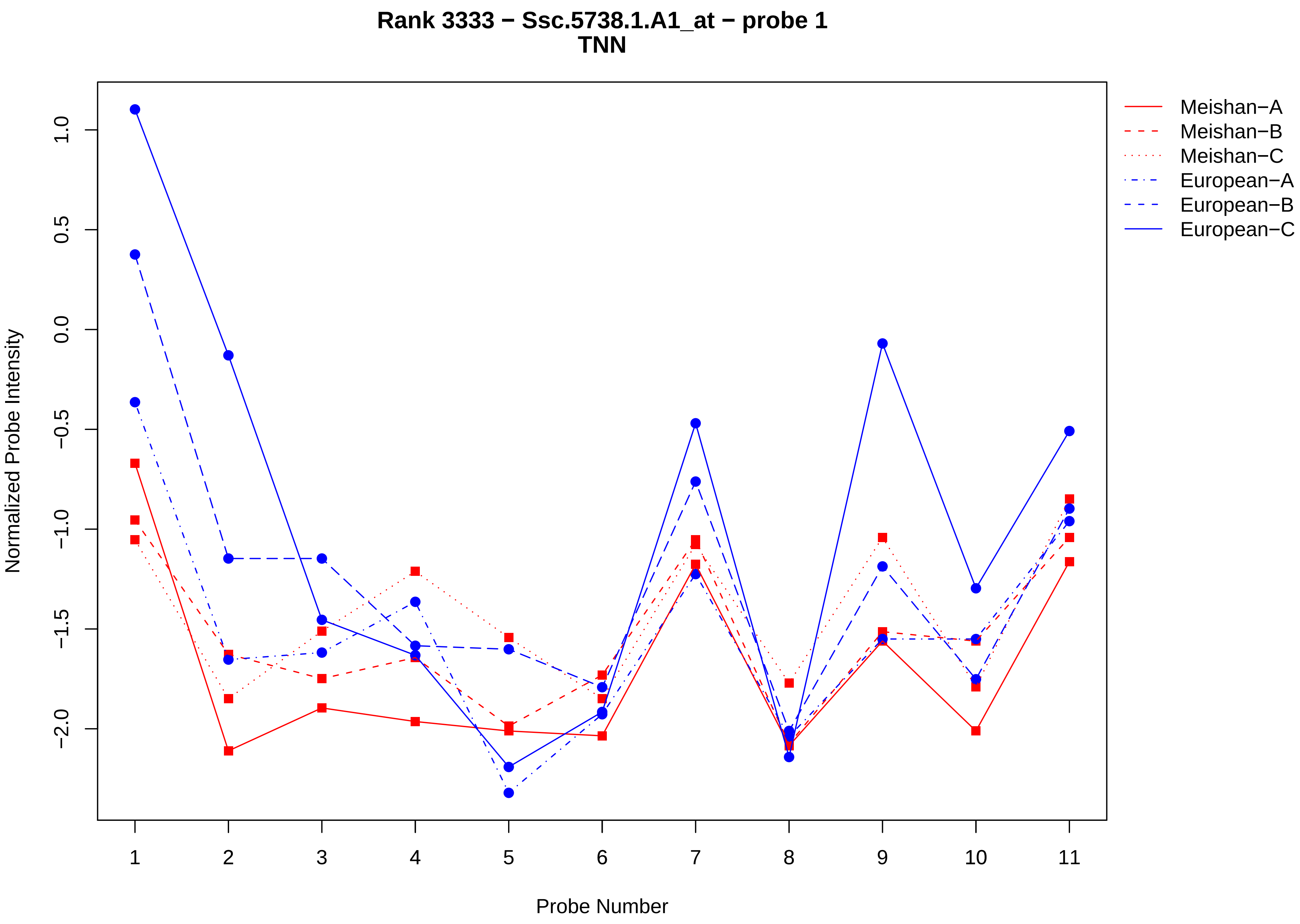

Rank 3333 − Ssc.5738.1.A1_at − probe 1
TNN
Normalized Probe Intensity
Probe Number
1.0
0.5
0.0
−0.5
−1.0
−1.5
−2.0
1
2
3
4
5
6
7
8
9
10
11
Meishan−A
Meishan−B
Meishan−C
European−A
European−B
European−C

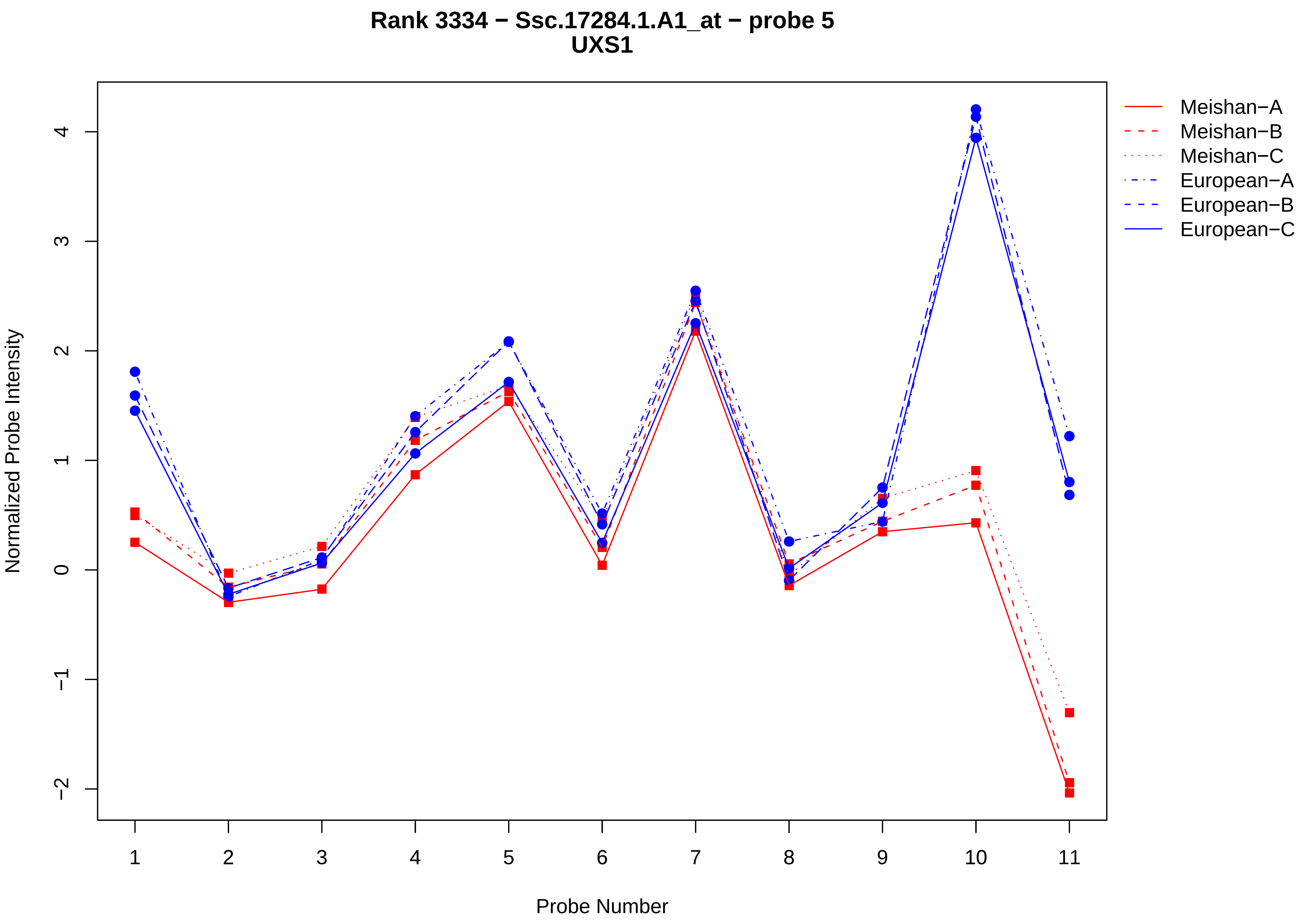

Rank 3334 − Ssc.17284.1.A1_at − probe 5
UXS1
Normalized Probe Intensity
Probe Number
−2
−1
0
1
2
3
4
1
2
3
4
5
6
7
8
9
10
11
Meishan−A
Meishan−B
Meishan−C
European−A
European−B
European−C

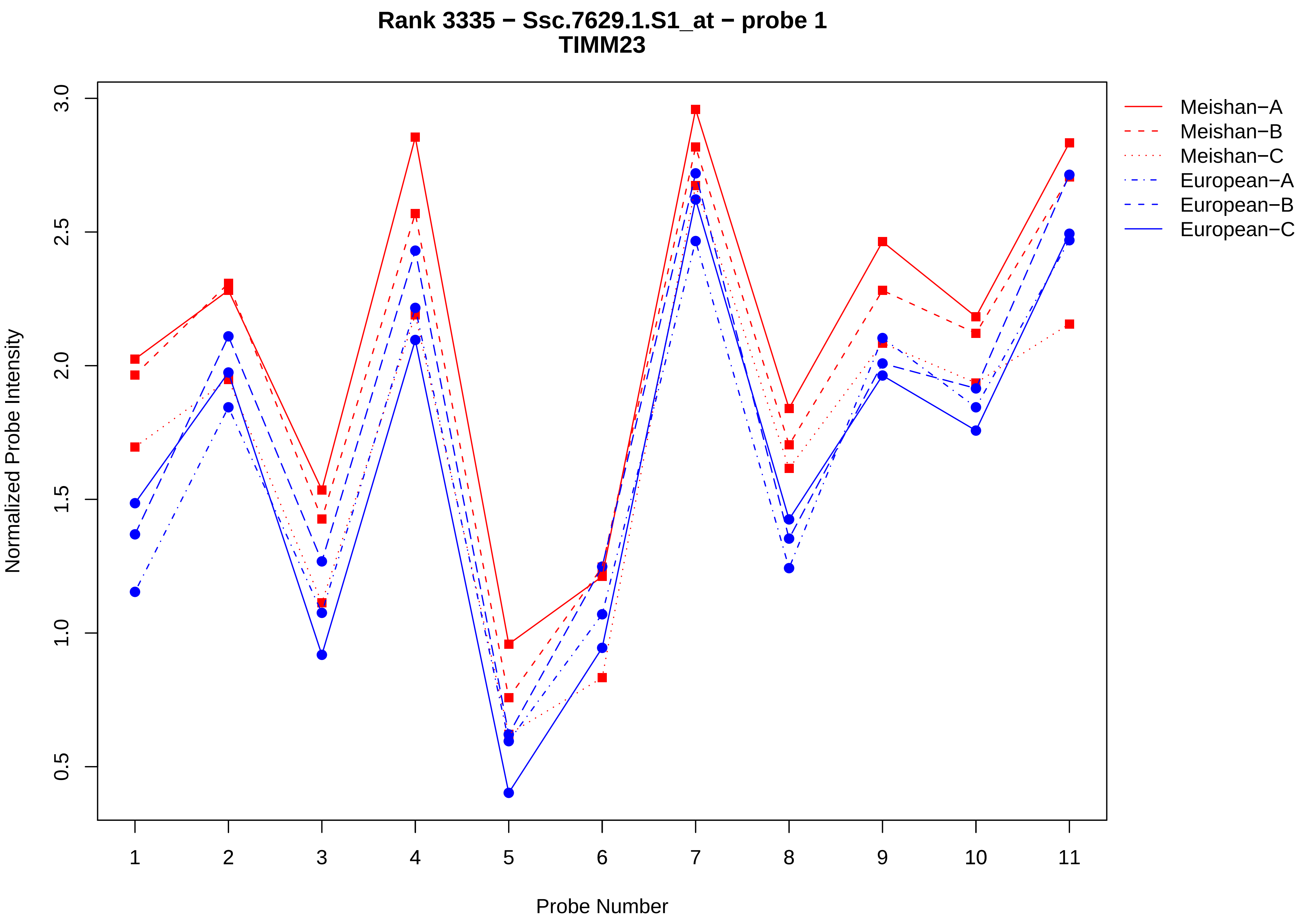

Rank 3335 − Ssc.7629.1.S1_at − probe 1
TIMM23
Normalized Probe Intensity
0.5
1.0
1.5
2.0
2.5
3.0
1
2
3
4
5
6
7
8
9
10
11
Probe Number
Meishan−A
Meishan−B
Meishan−C
European−A
European−B
European−C

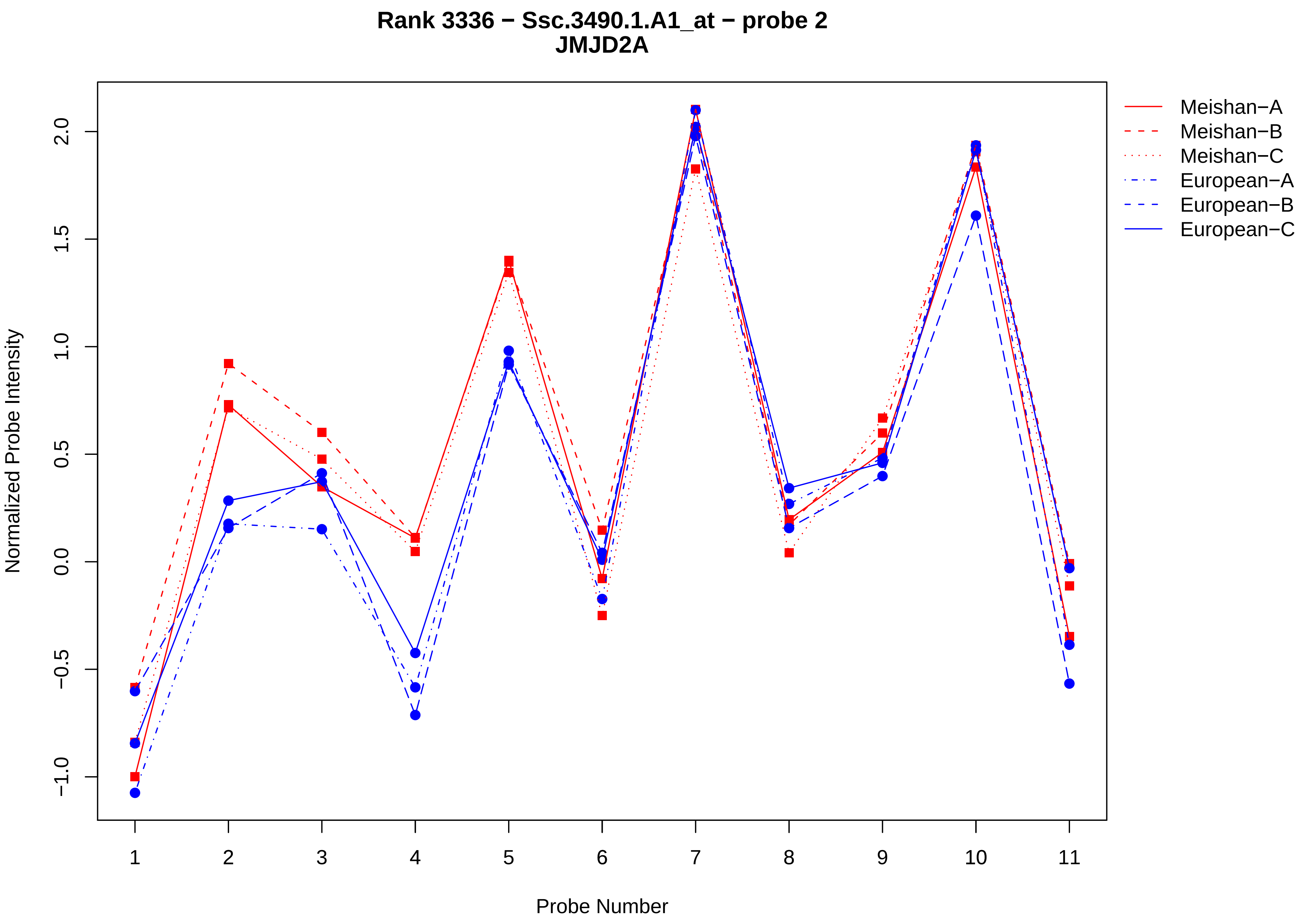

Rank 3336 − Ssc.3490.1.A1_at − probe 2
JMJD2A
Normalized Probe Intensity
Probe Number
−1.0
−0.5
0.0
0.5
1.0
1.5
2.0
1
2
3
4
5
6
7
8
9
10
11
Meishan−A
Meishan−B
Meishan−C
European−A
European−B
European−C

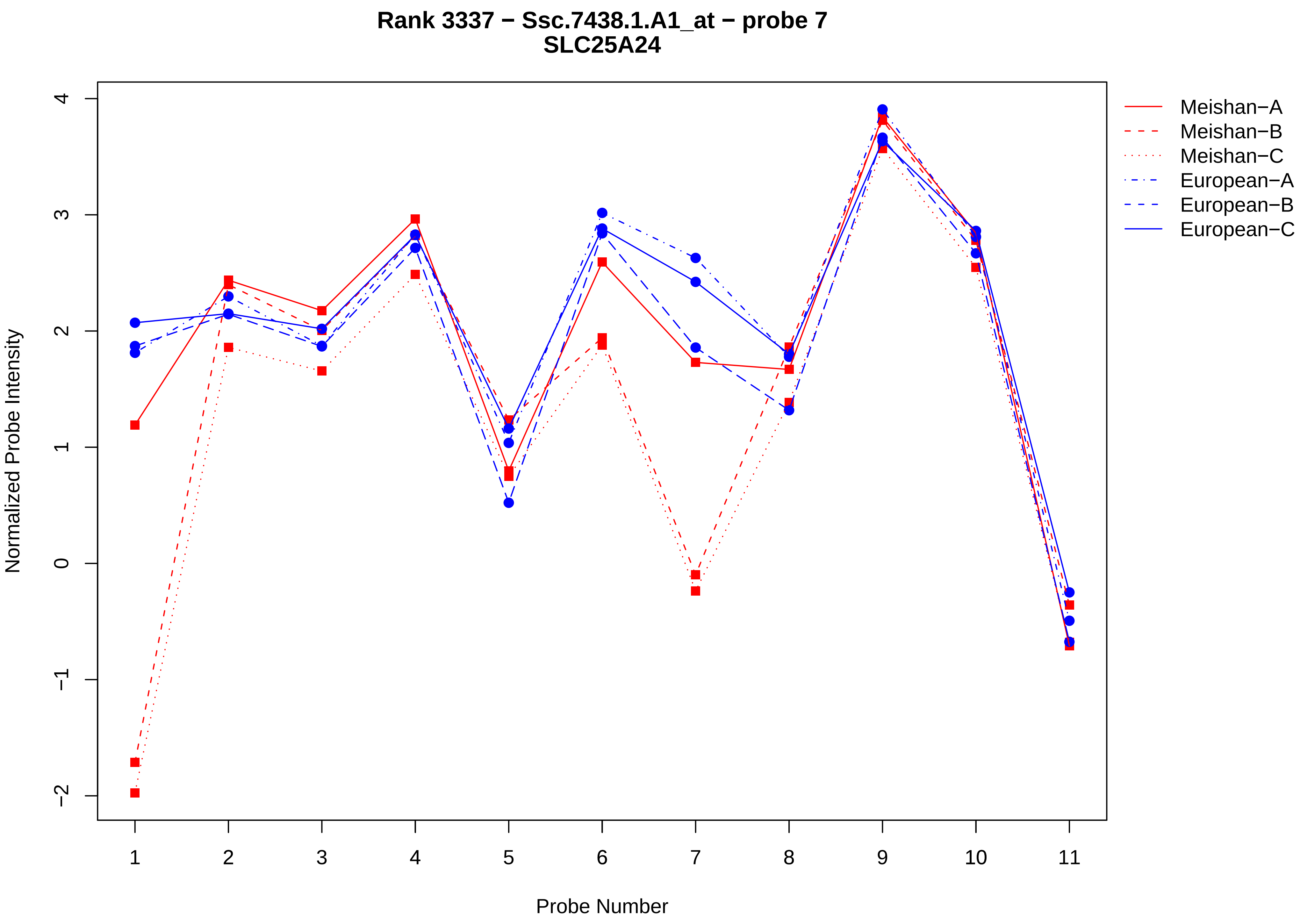

Rank 3337 − Ssc.7438.1.A1_at − probe 7
SLC25A24
Normalized Probe Intensity
Probe Number
Meishan−A
Meishan−B
Meishan−C
European−A
European−B
European−C

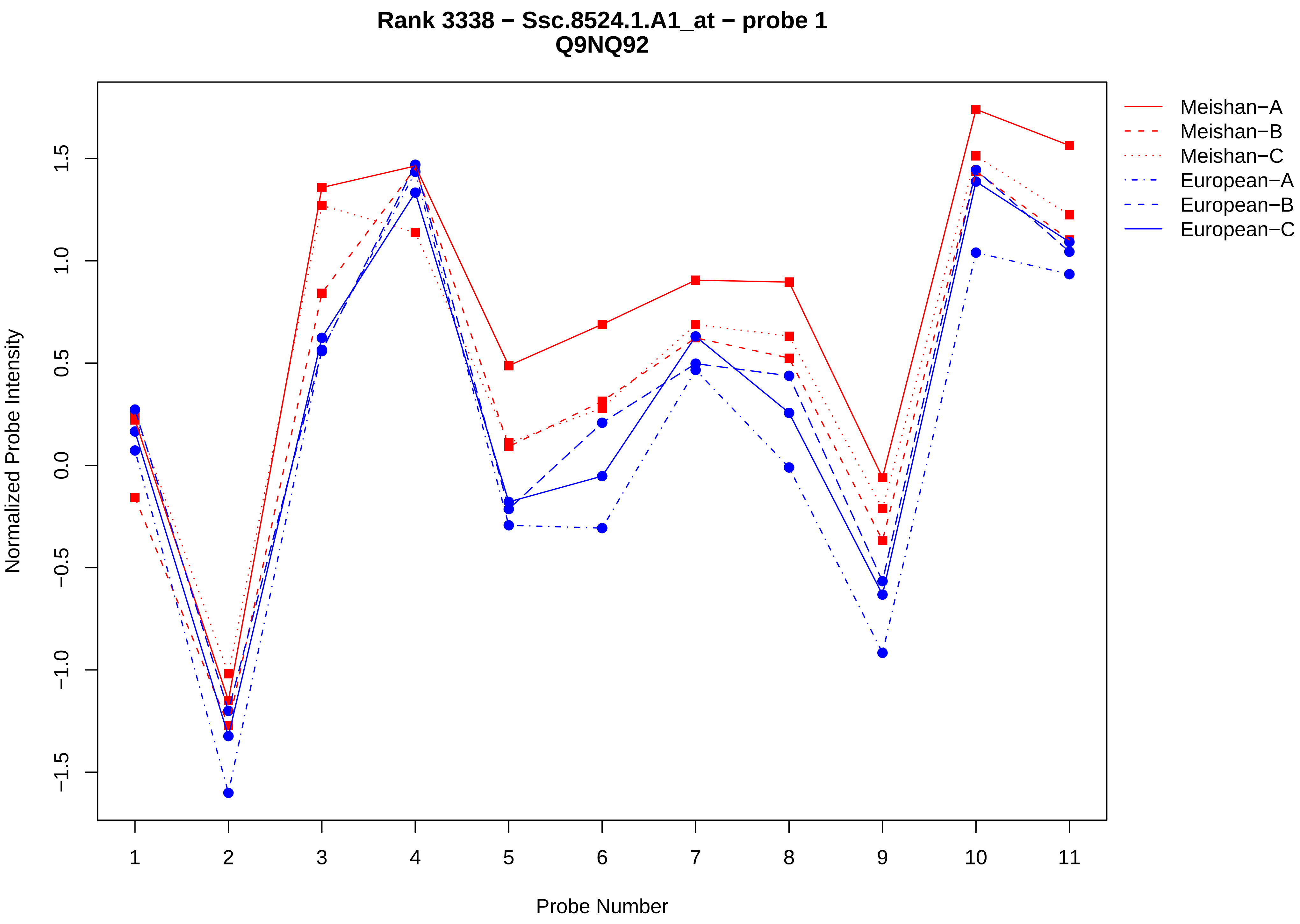

Rank 3338 − Ssc.8524.1.A1_at − probe 1
Q9NQ92
Normalized Probe Intensity
Probe Number
−1.5
−1.0
−0.5
0.0
0.5
1.0
1.5
1
2
3
4
5
6
7
8
9
10
11
Meishan−A
Meishan−B
Meishan−C
European−A
European−B
European−C

Rank 3339 − Ssc.11297.1.A1_at − probe 8
KCNK1

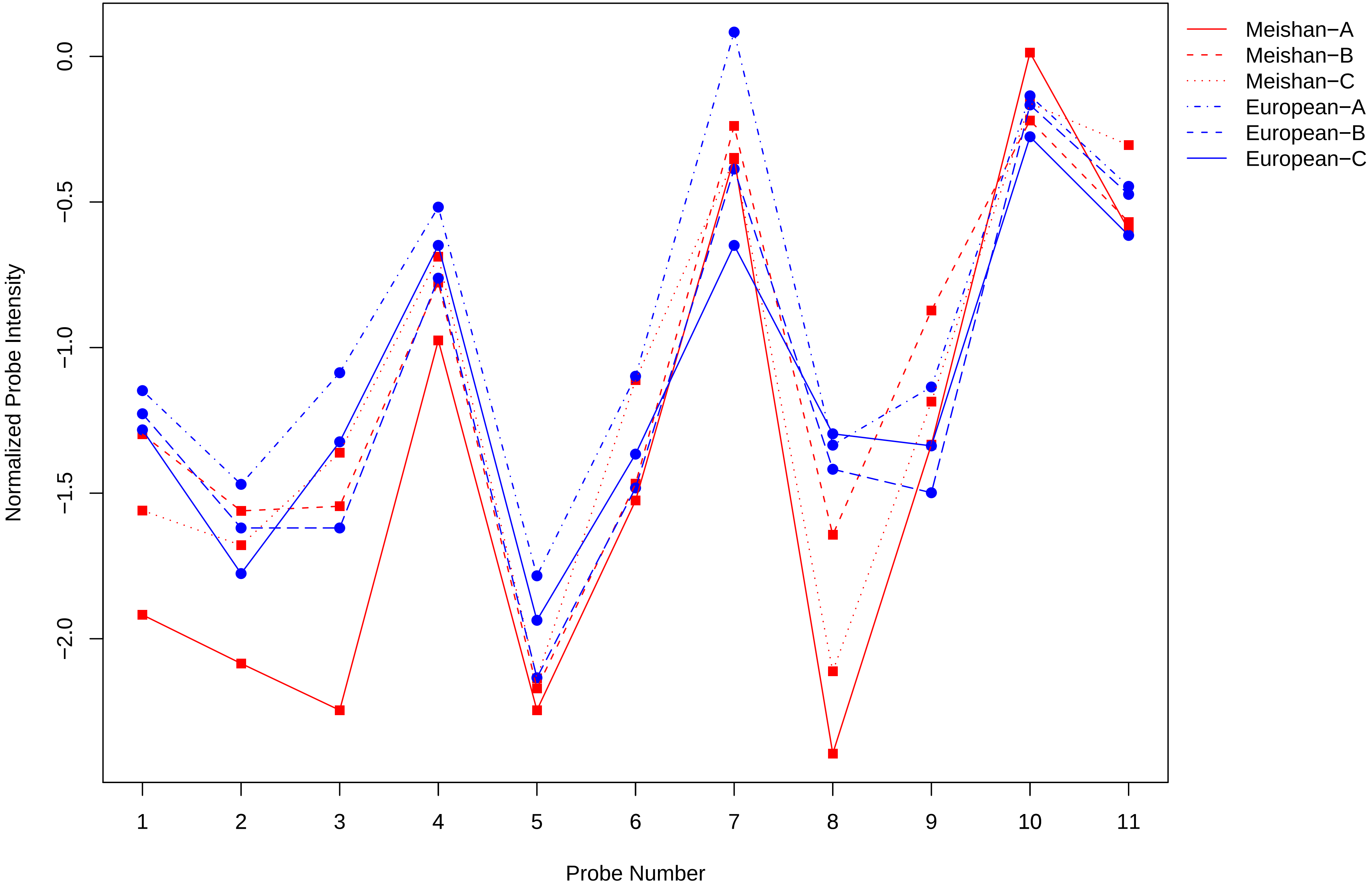

Rank 3340 − Ssc.1180.1.S1_at − probe 7
DNAJB2

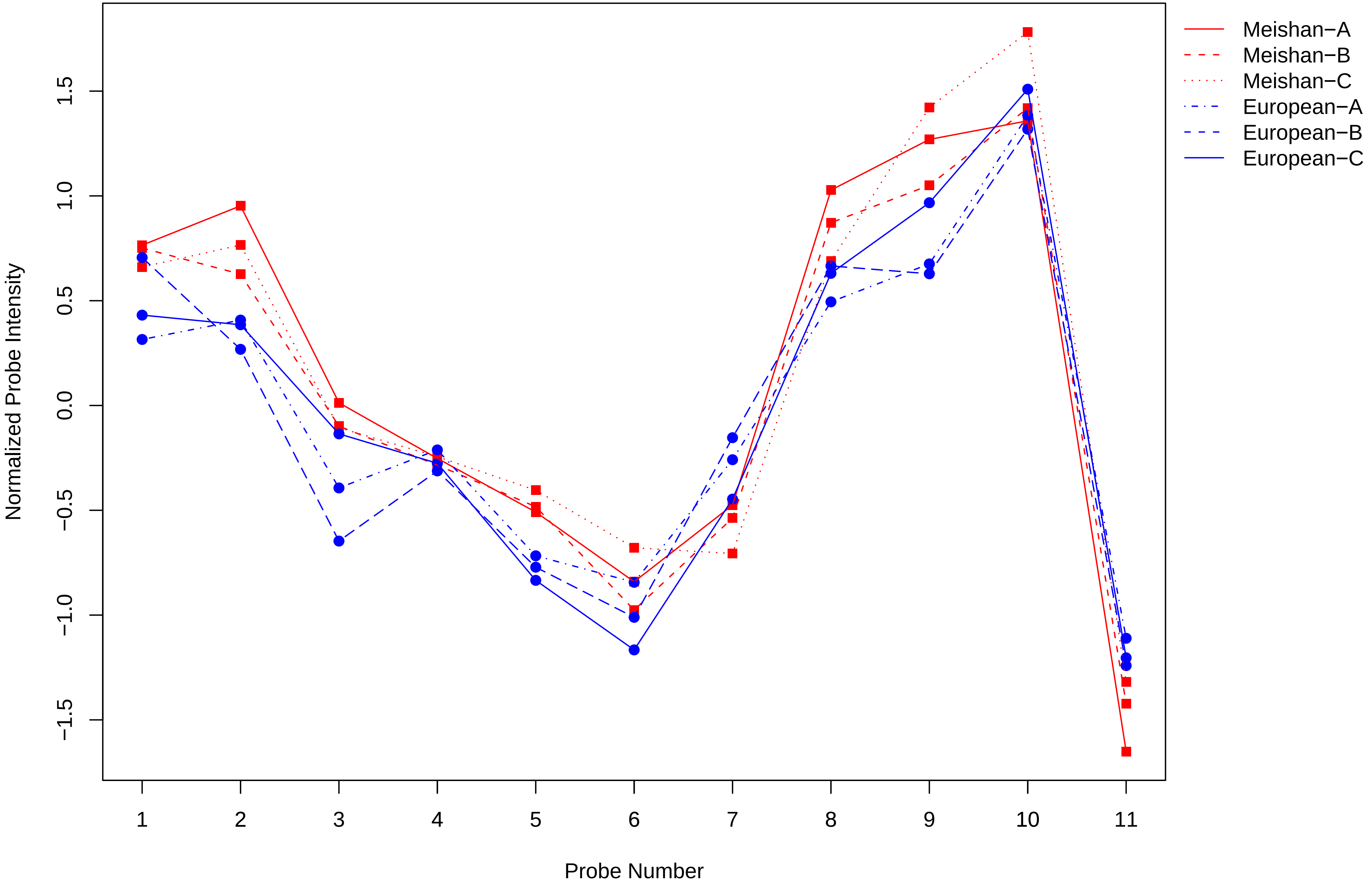

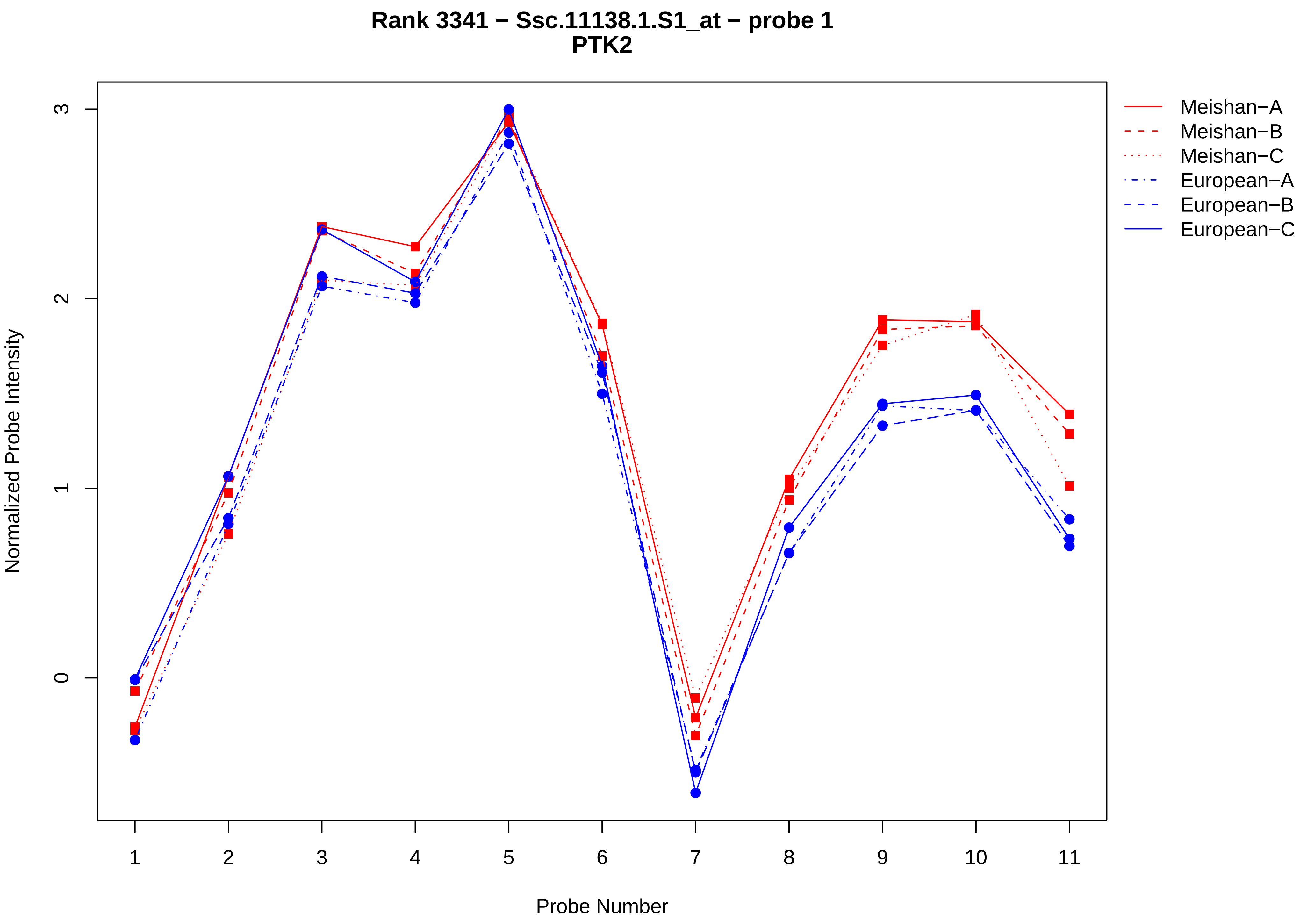

Rank 3341 − Ssc.11138.1.S1_at − probe 1
PTK2
Normalized Probe Intensity
Probe Number
0
1
2
3
1
2
3
4
5
6
7
8
9
10
11
Meishan−A
Meishan−B
Meishan−C
European−A
European−B
European−C

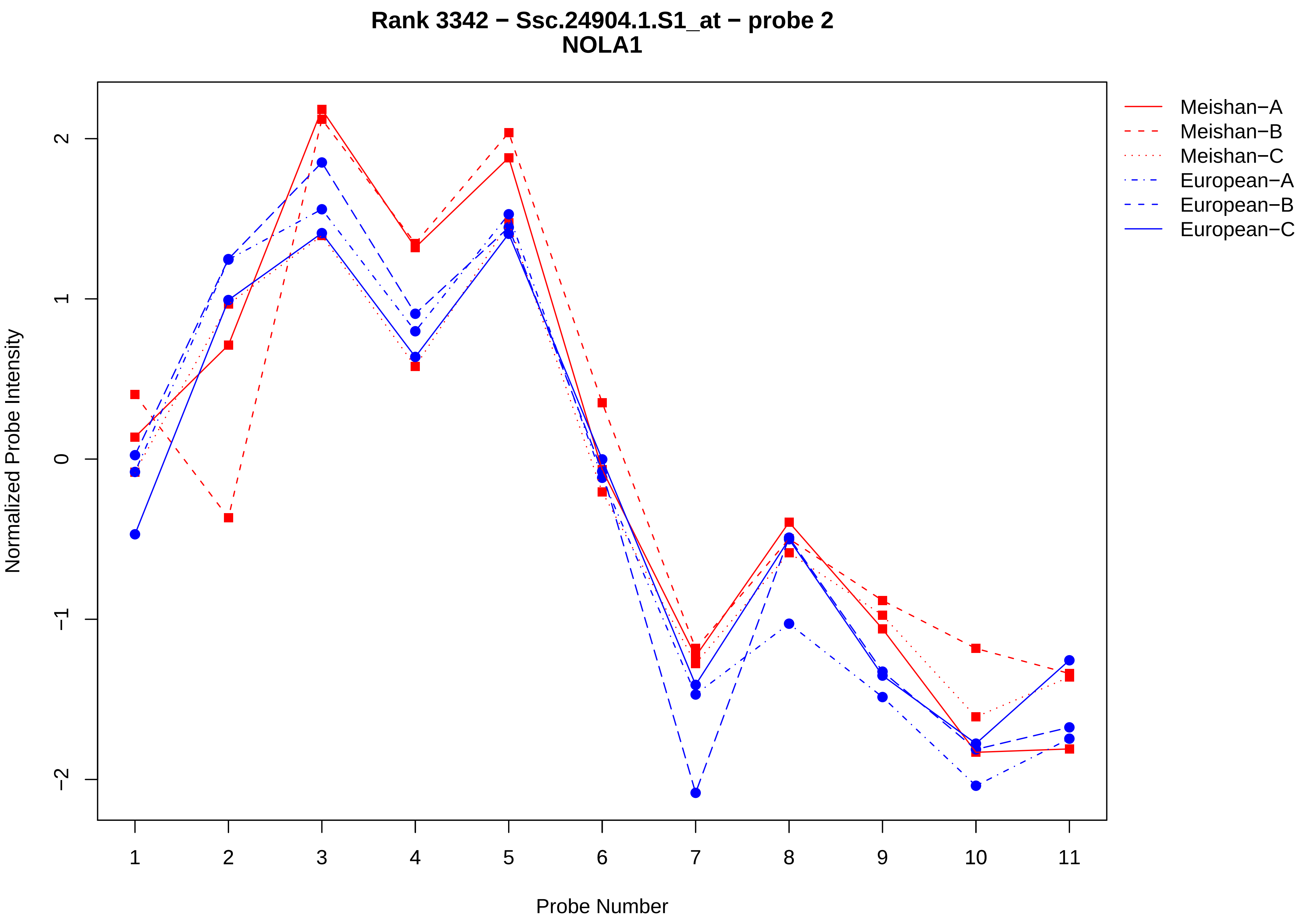
Rank 3342 − Ssc.24904.1.S1_at − probe 2
NOLA1
Normalized Probe Intensity
−2
−1
0
1
2
1
2
3
4
5
6
7
8
9
10
11
Probe Number
Meishan−A
Meishan−B
Meishan−C
European−A
European−B
European−C

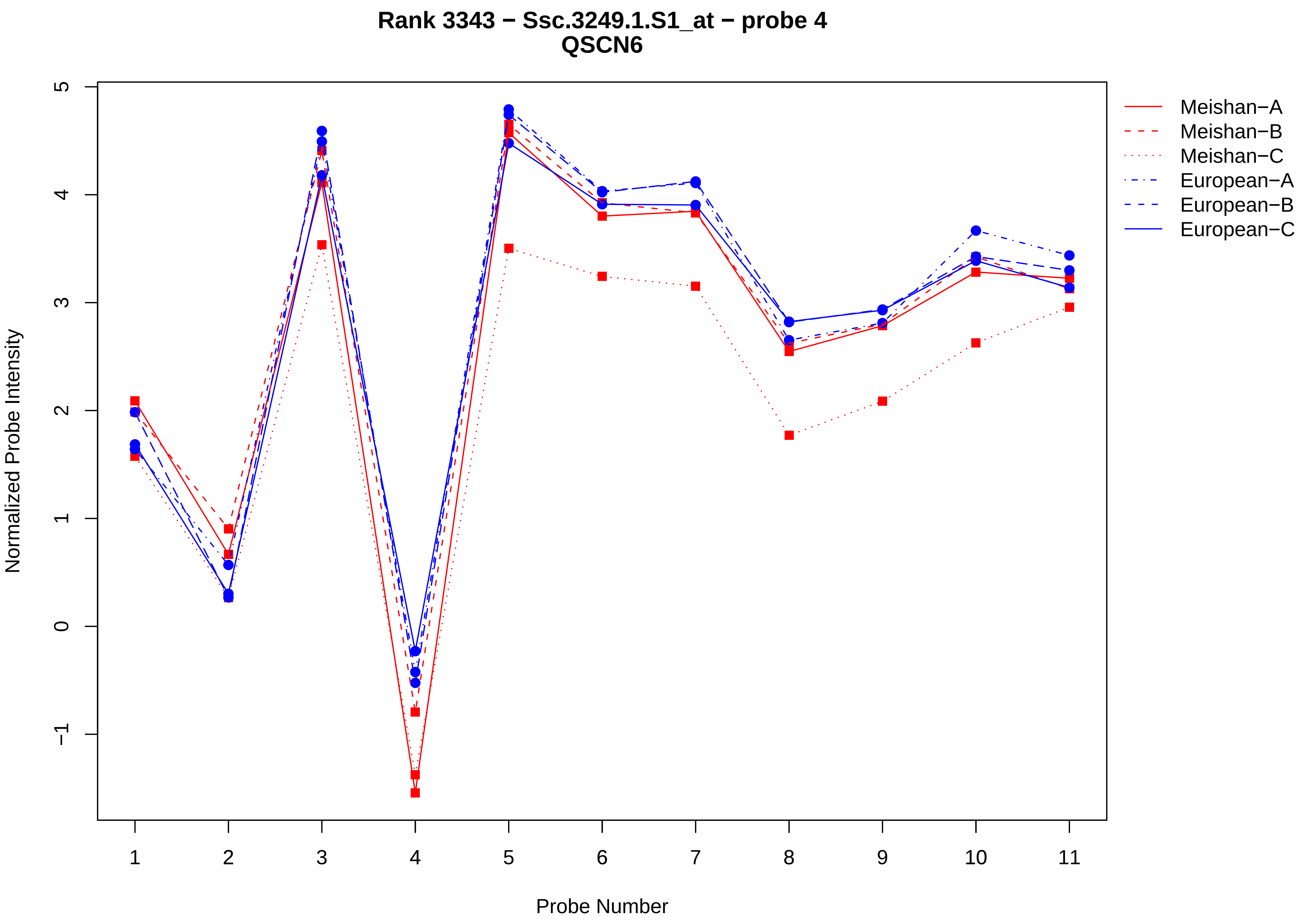

Rank 3343 − Ssc.3249.1.S1_at − probe 4
QSCN6
Normalized Probe Intensity
Probe Number
Meishan−A
Meishan−B
Meishan−C
European−A
European−B
European−C

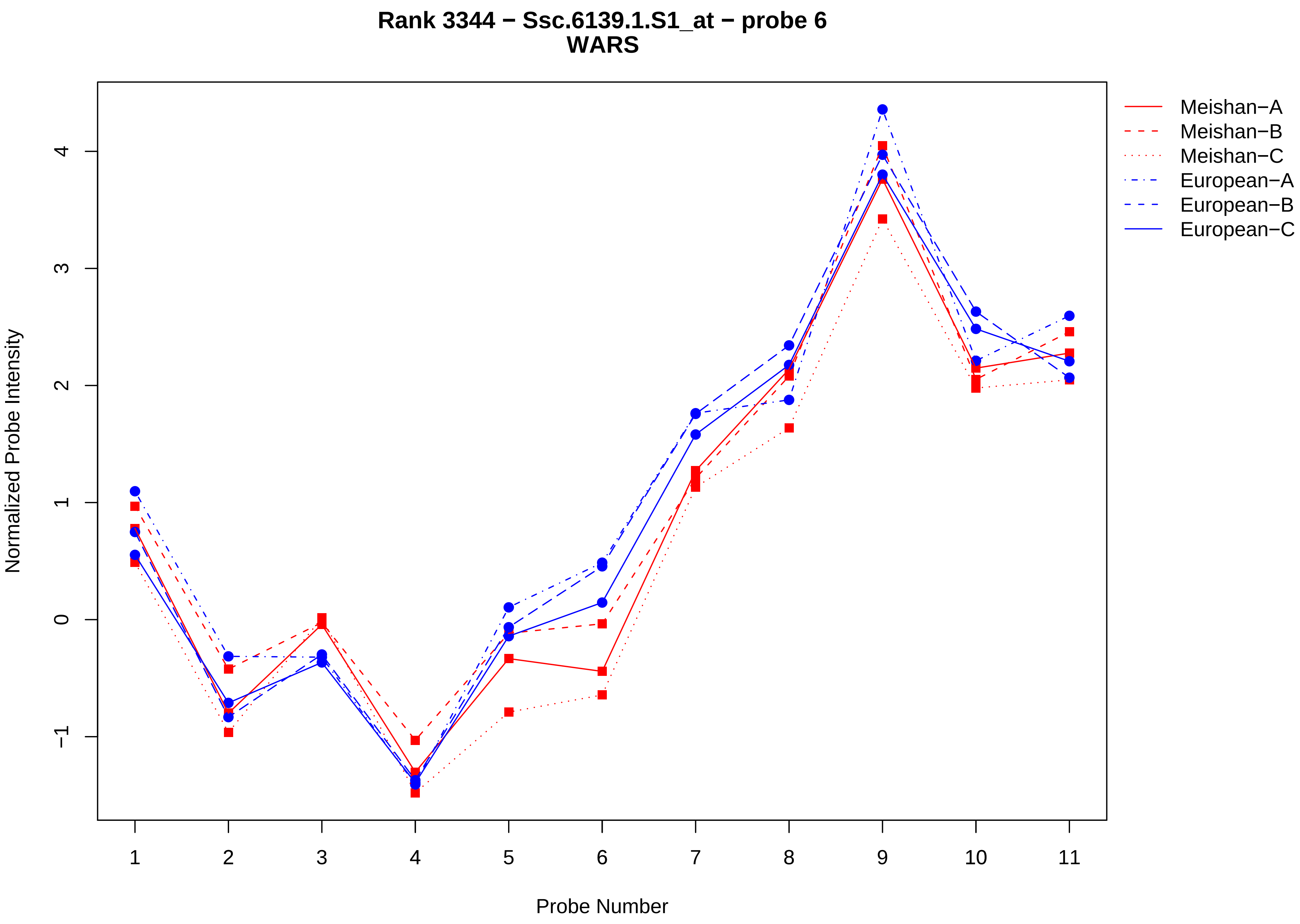

Rank 3344 − Ssc.6139.1.S1_at − probe 6
WARS
Normalized Probe Intensity
Probe Number
Meishan−A
Meishan−B
Meishan−C
European−A
European−B
European−C

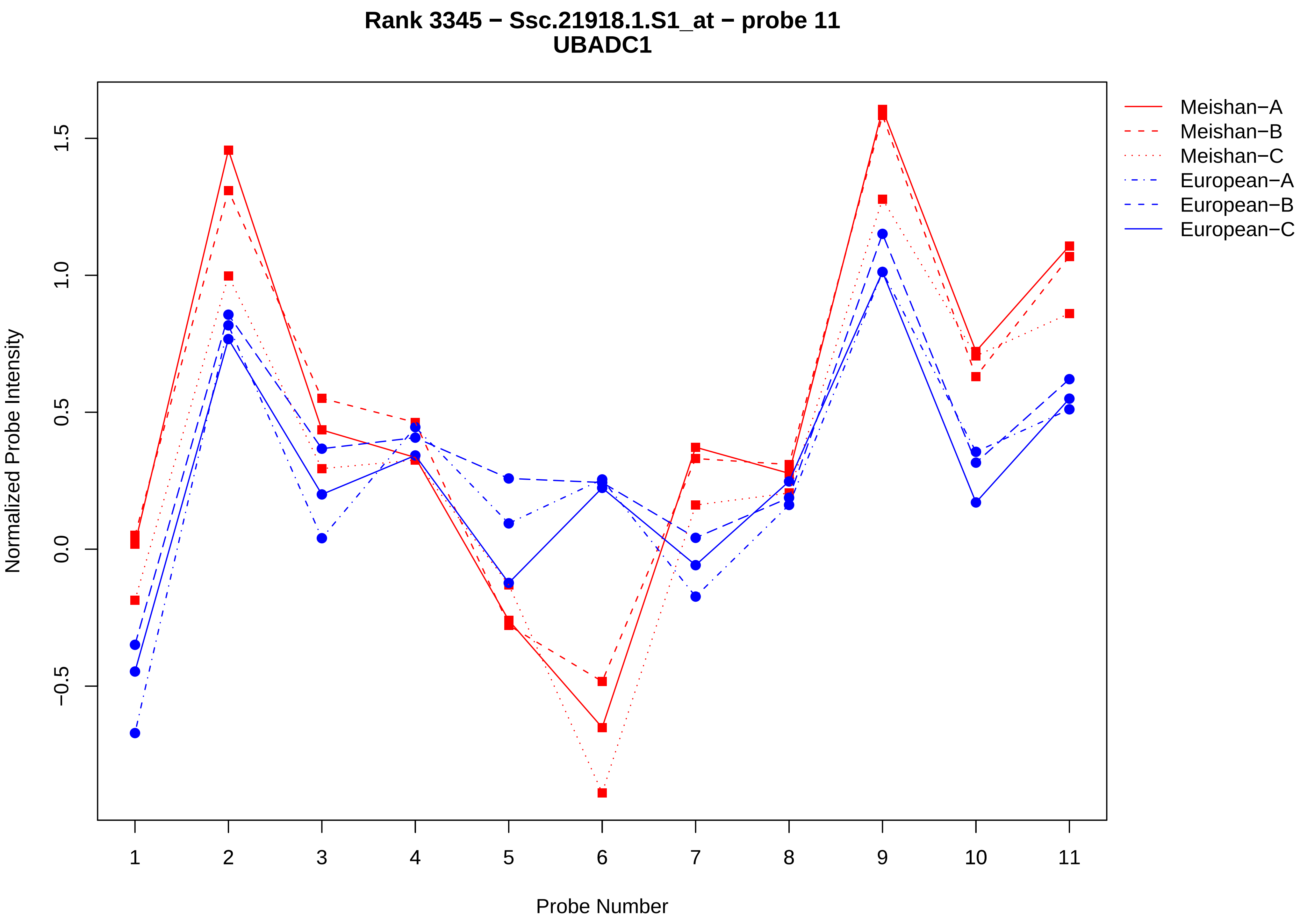
Rank 3345 − Ssc.21918.1.S1_at − probe 11
UBADC1
Normalized Probe Intensity
Probe Number
−0.5
0.0
0.5
1.0
1.5
1
2
3
4
5
6
7
8
9
10
11
Meishan−A
Meishan−B
Meishan−C
European−A
European−B
European−C

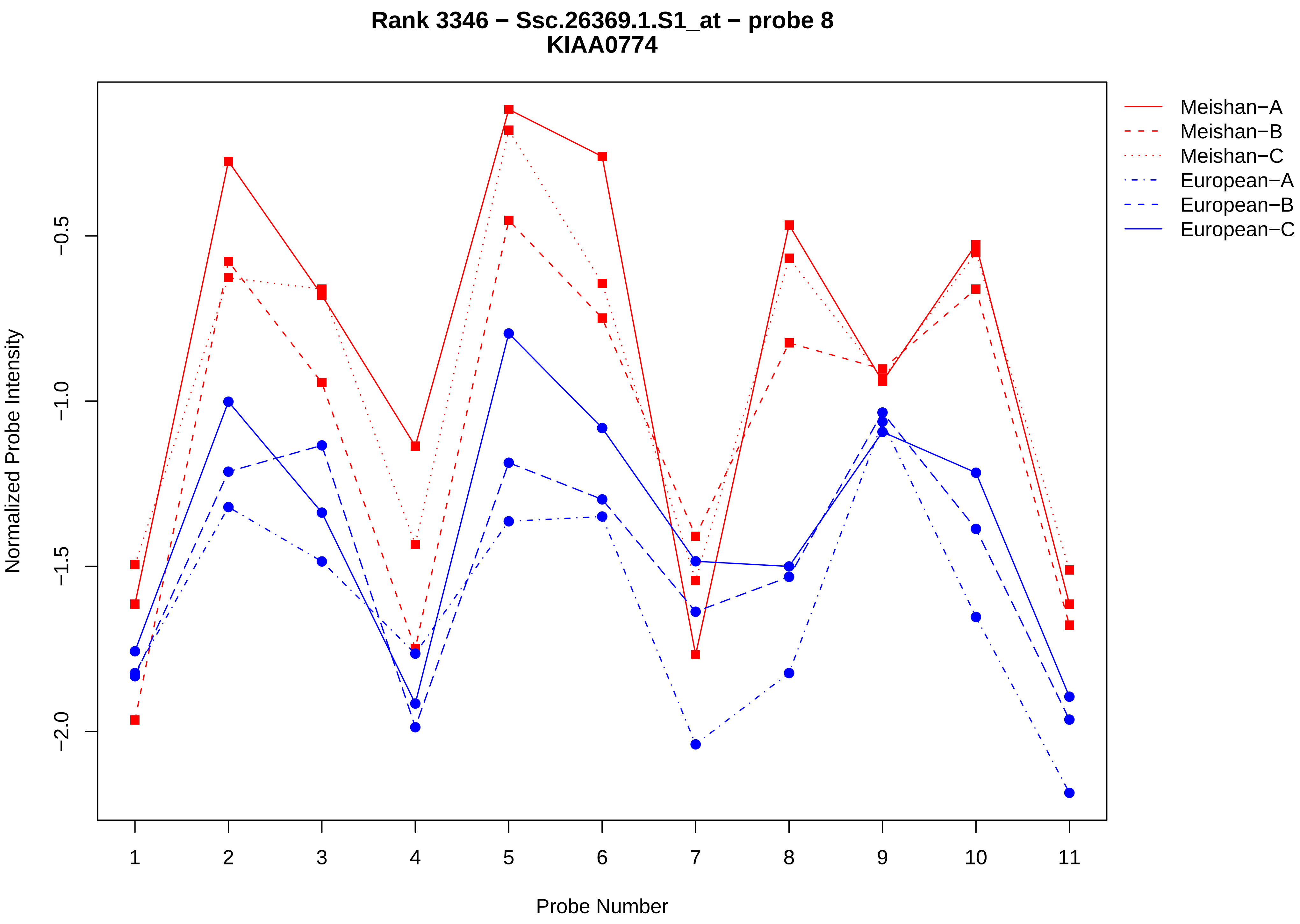
Rank 3346 − Ssc.26369.1.S1_at − probe 8
KIAA0774
Normalized Probe Intensity
−0.5
−1.0
−1.5
−2.0
1
2
3
4
5
6
7
8
9
10
11
Probe Number
Meishan−A
Meishan−B
Meishan−C
European−A
European−B
European−C

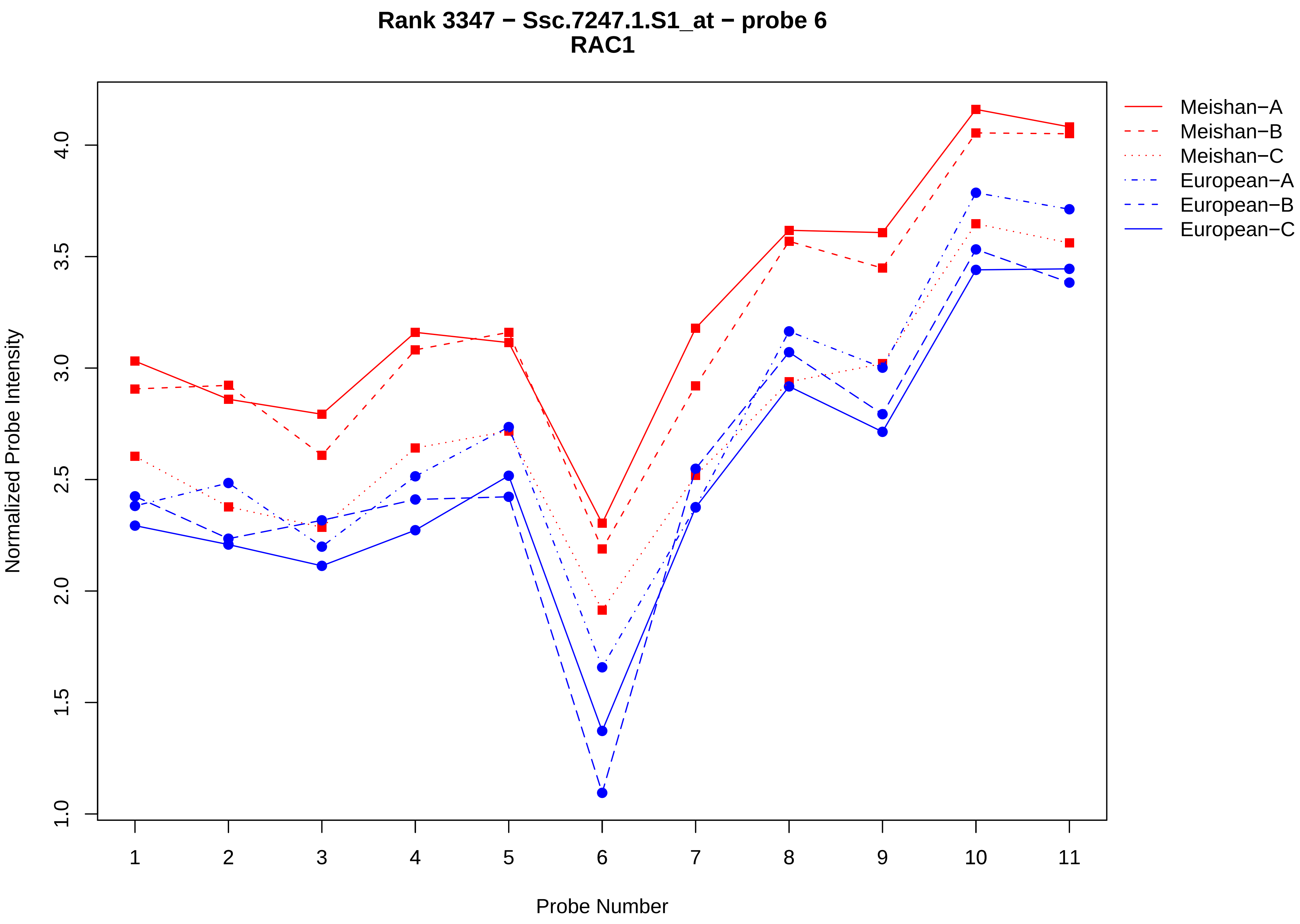

Rank 3347 − Ssc.7247.1.S1_at − probe 6
RAC1
Normalized Probe Intensity
Probe Number
1.0
1.5
2.0
2.5
3.0
3.5
4.0
1
2
3
4
5
6
7
8
9
10
11
Meishan−A
Meishan−B
Meishan−C
European−A
European−B
European−C

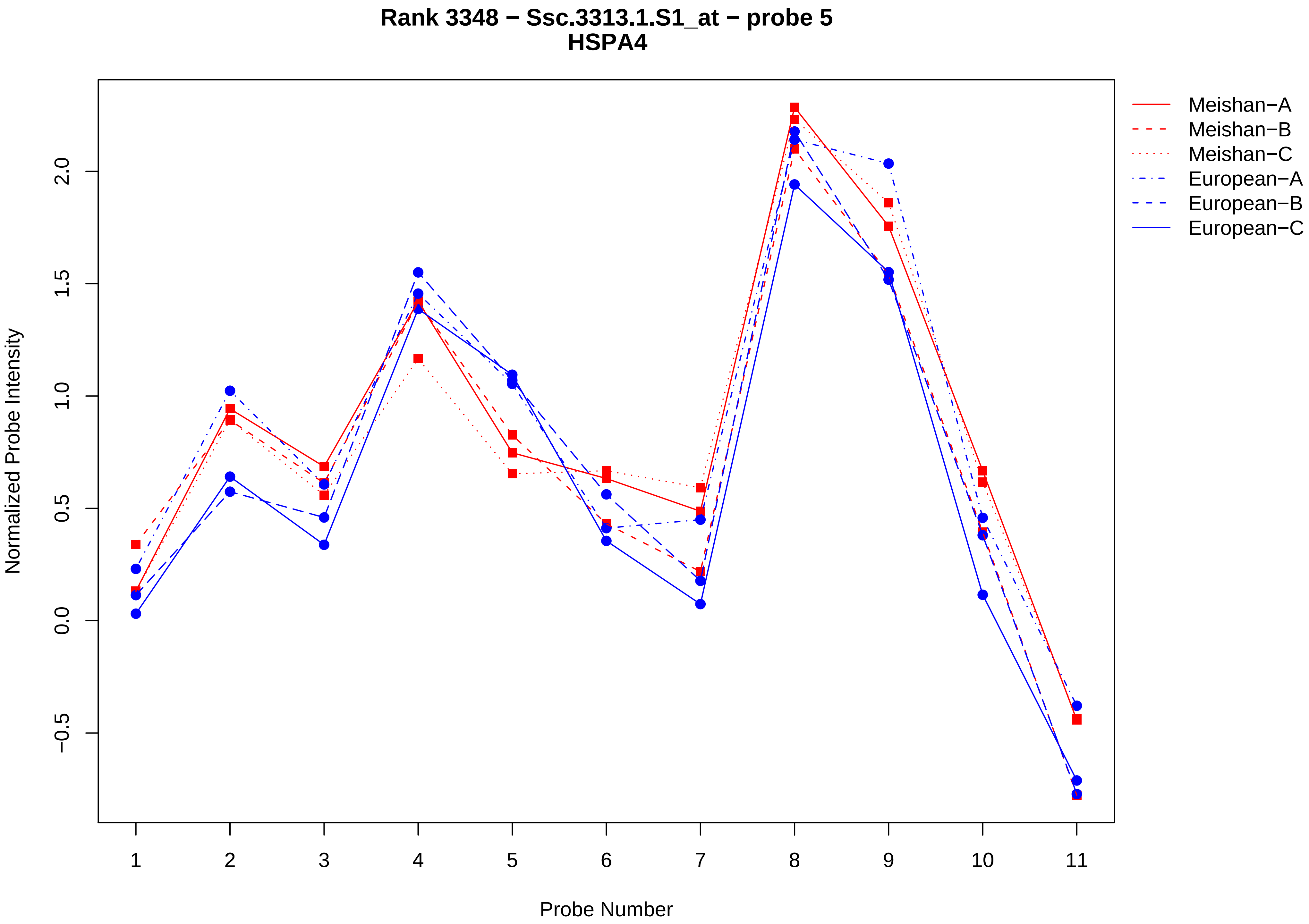

Rank 3348 − Ssc.3313.1.S1_at − probe 5
HSPA4
Normalized Probe Intensity
Probe Number
−0.5
0.0
0.5
1.0
1.5
2.0
1
2
3
4
5
6
7
8
9
10
11
Meishan−A
Meishan−B
Meishan−C
European−A
European−B
European−C

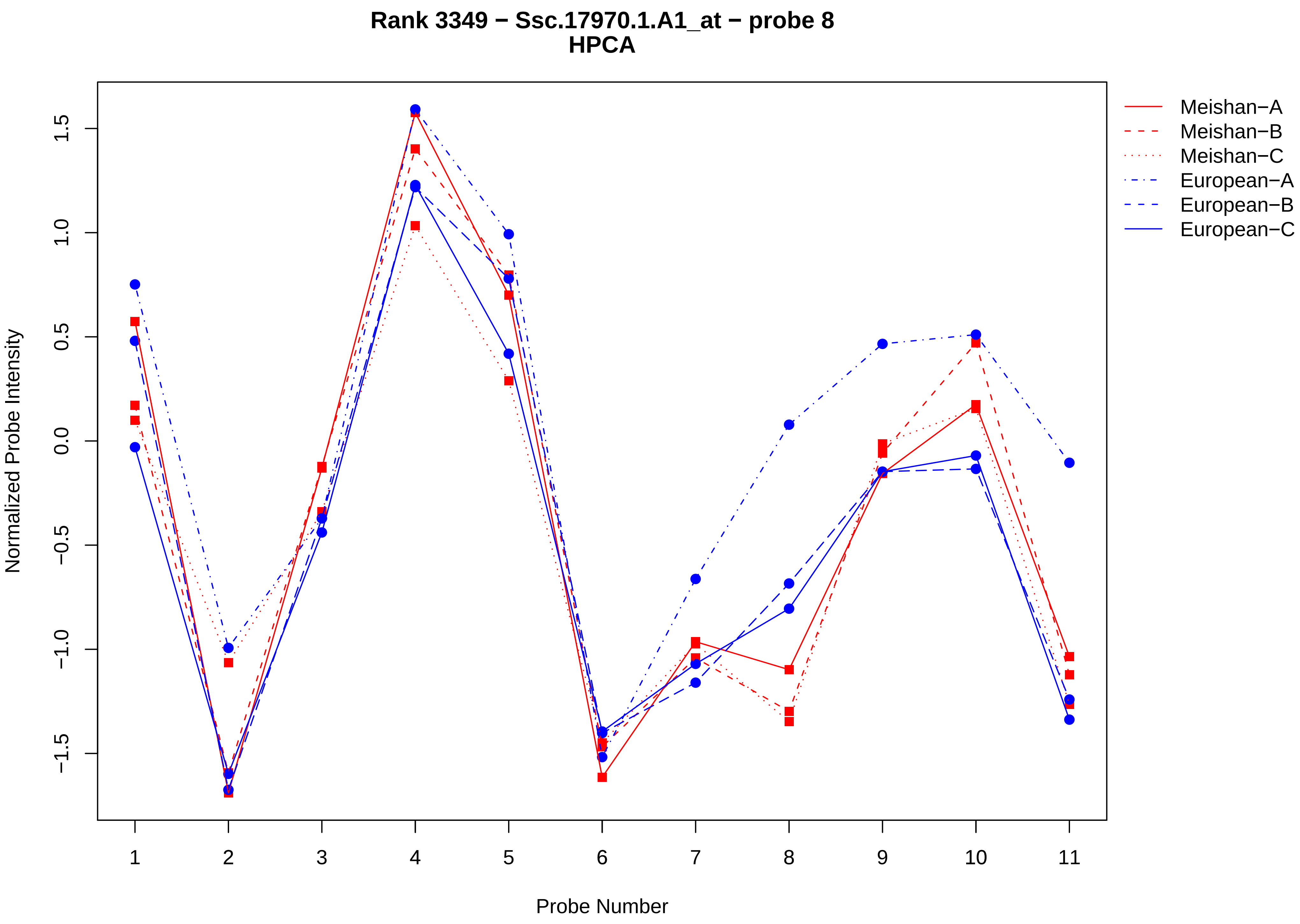

Rank 3349 − Ssc.17970.1.A1_at − probe 8
HPCA
Normalized Probe Intensity
Probe Number
1.5
1.0
0.5
0.0
−0.5
−1.0
−1.5
1
2
3
4
5
6
7
8
9
10
11
Meishan−A
Meishan−B
Meishan−C
European−A
European−B
European−C

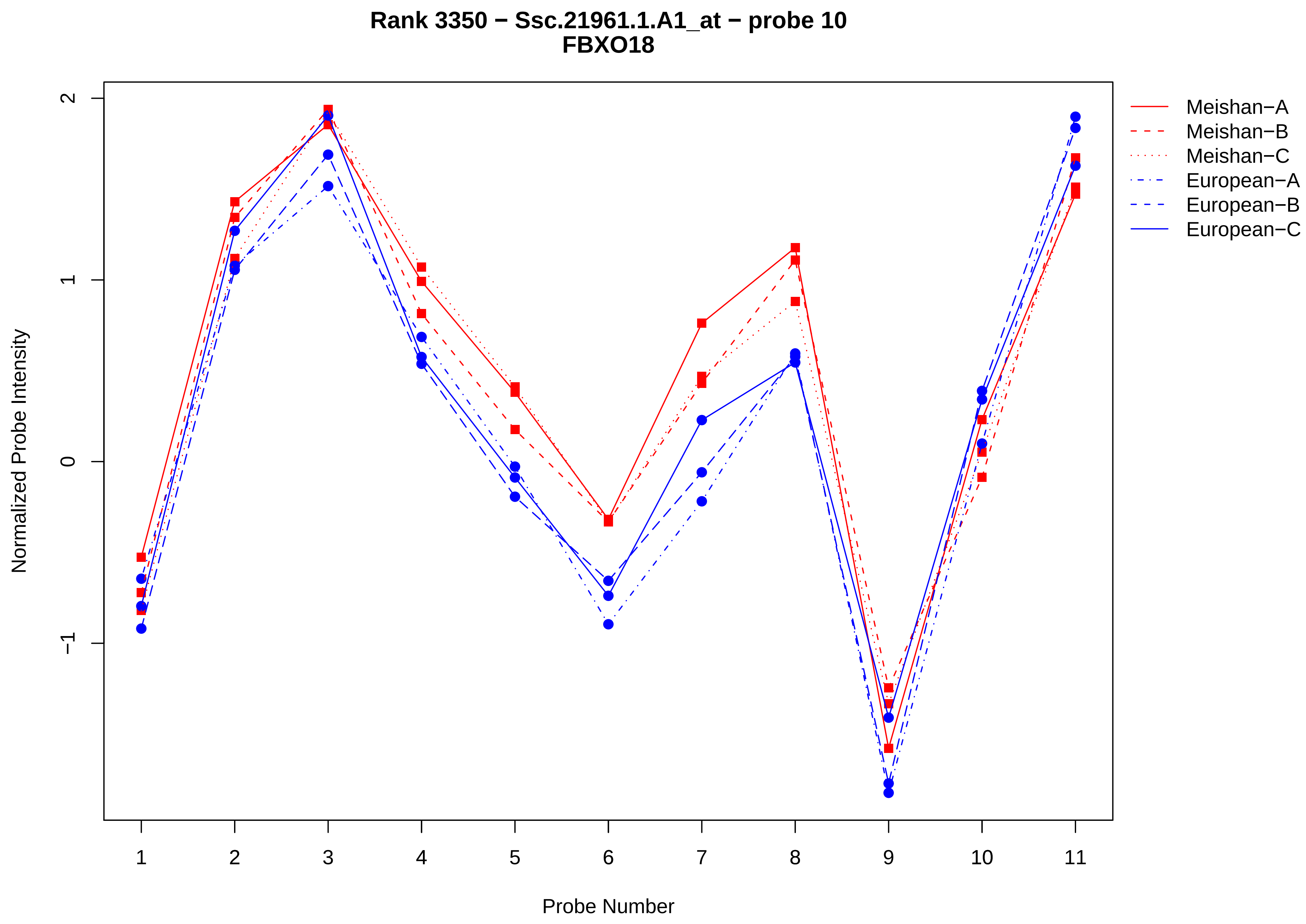

Rank 3350 − Ssc.21961.1.A1_at − probe 10
FBXO18
Normalized Probe Intensity
Probe Number
−1
0
1
2
1
2
3
4
5
6
7
8
9
10
11
Meishan−A
Meishan−B
Meishan−C
European−A
European−B
European−C

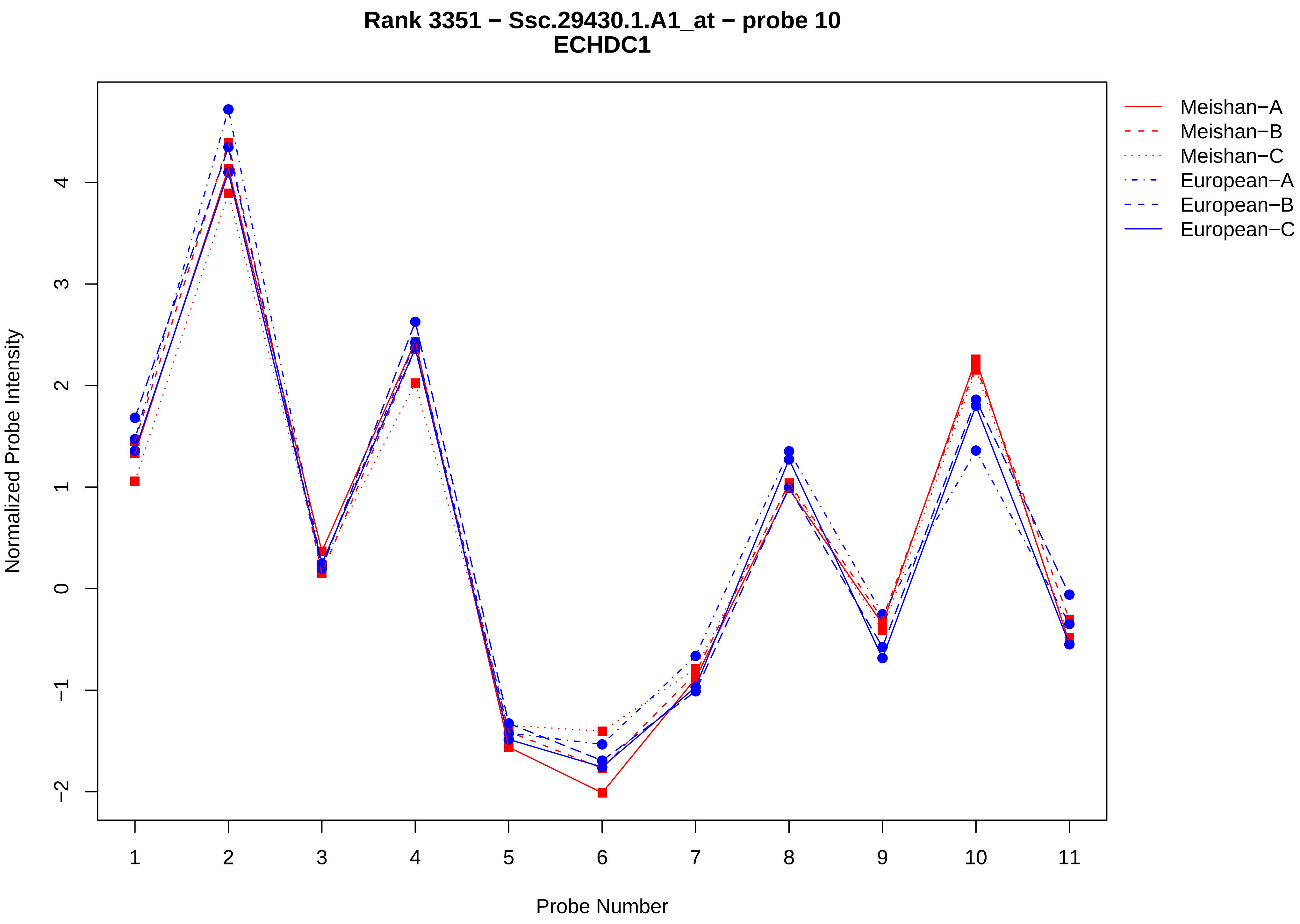

Rank 3351 − Ssc.29430.1.A1_at − probe 10
ECHDC1
Normalized Probe Intensity
Probe Number
Meishan−A
Meishan−B
Meishan−C
European−A
European−B
European−C

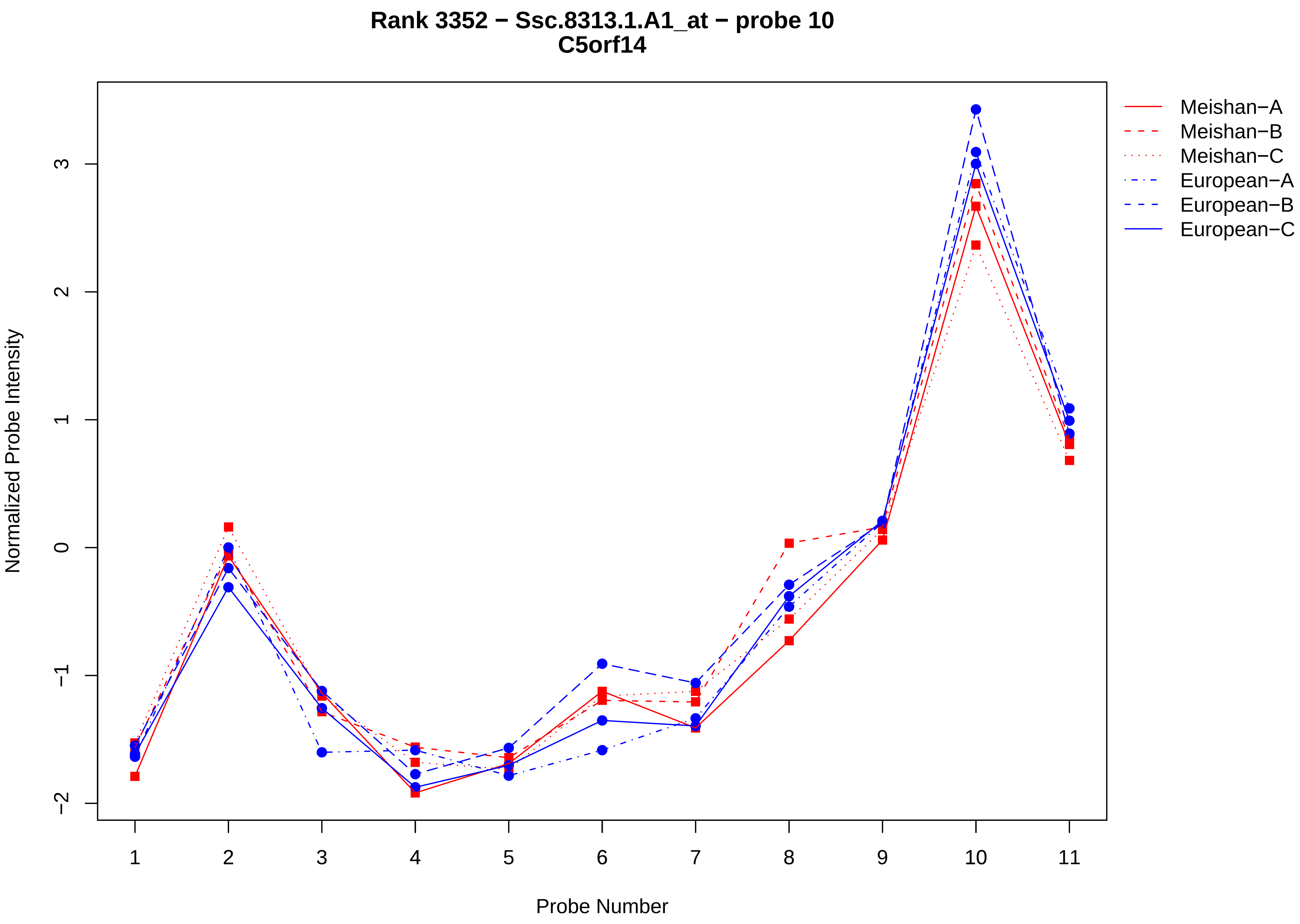

Rank 3352 − Ssc.8313.1.A1_at − probe 10
C5orf14
Normalized Probe Intensity
Probe Number
Meishan−A
Meishan−B
Meishan−C
European−A
European−B
European−C

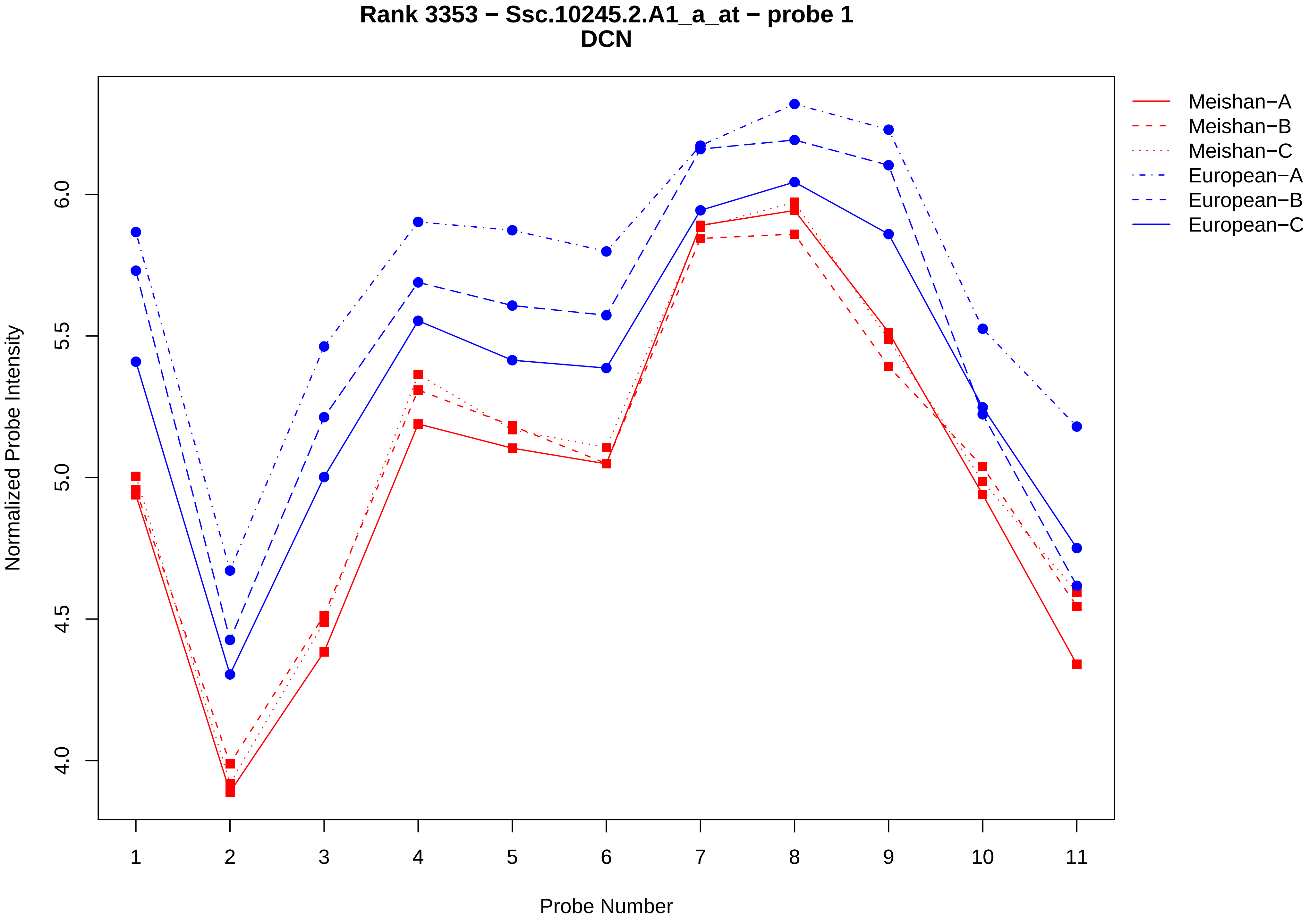

Rank 3353 − Ssc.10245.2.A1_a_at − probe 1
DCN
Normalized Probe Intensity
Probe Number
Meishan−A
Meishan−B
Meishan−C
European−A
European−B
European−C
4.0
4.5
5.0
5.5
6.0
1
2
3
4
5
6
7
8
9
10
11

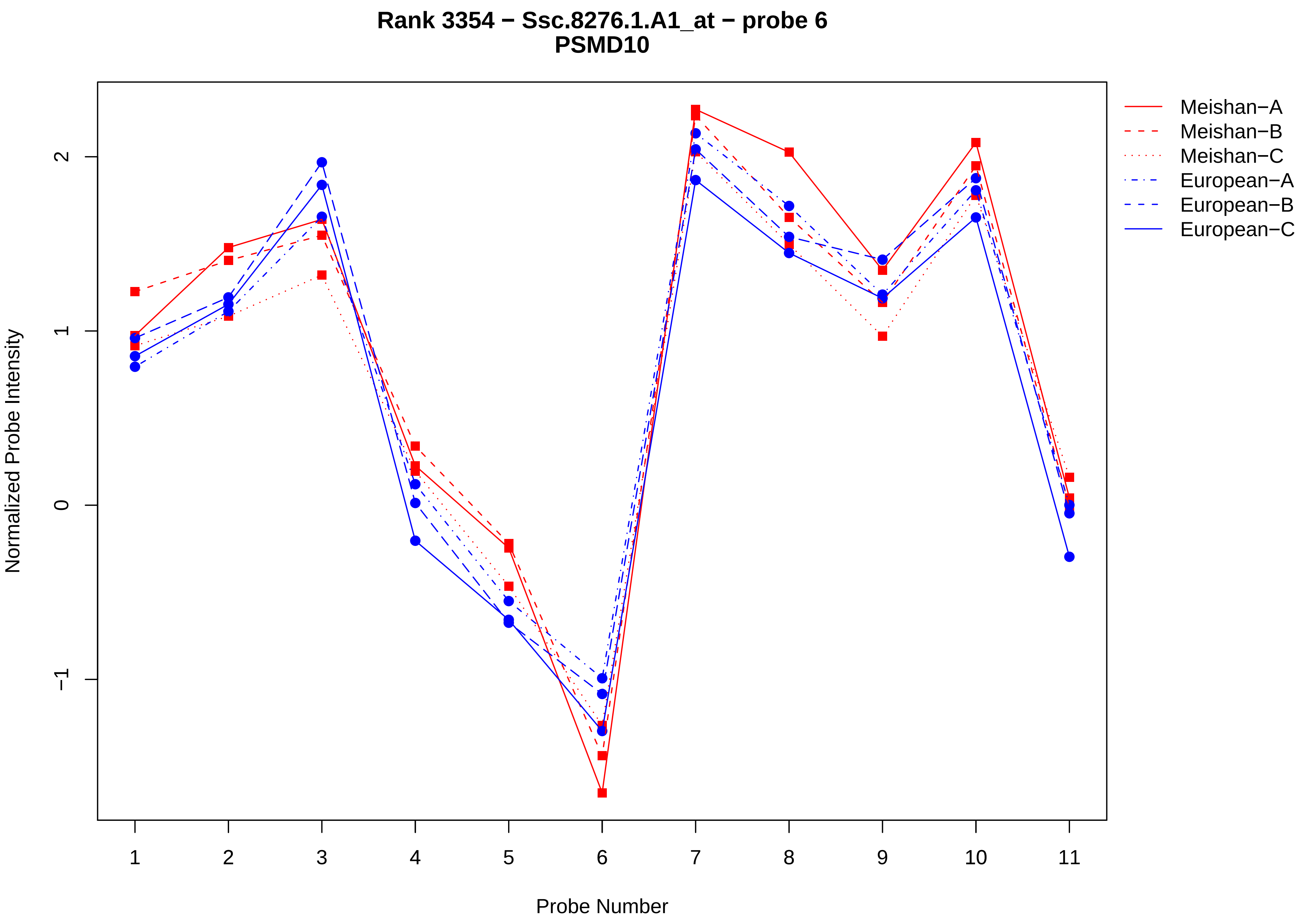
Rank 3354 − Ssc.8276.1.A1_at − probe 6
PSMD10
Normalized Probe Intensity
Probe Number
−1
0
1
2
1
2
3
4
5
6
7
8
9
10
11
Meishan−A
Meishan−B
Meishan−C
European−A
European−B
European−C

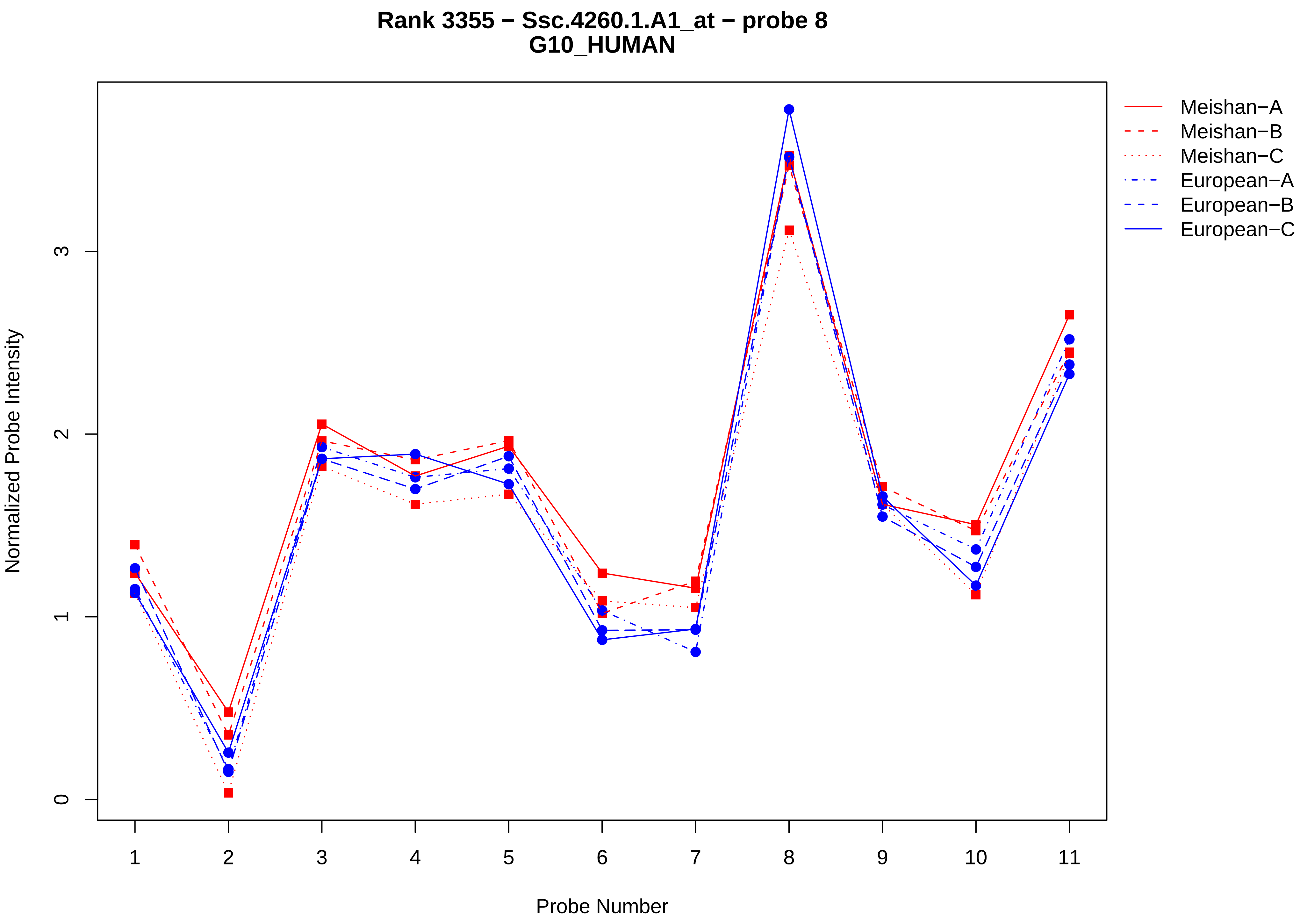

Rank 3355 − Ssc.4260.1.A1_at − probe 8
G10_HUMAN
Normalized Probe Intensity
0
1
2
3
Probe Number
1
2
3
4
5
6
7
8
9
10
11
Meishan−A
Meishan−B
Meishan−C
European−A
European−B
European−C

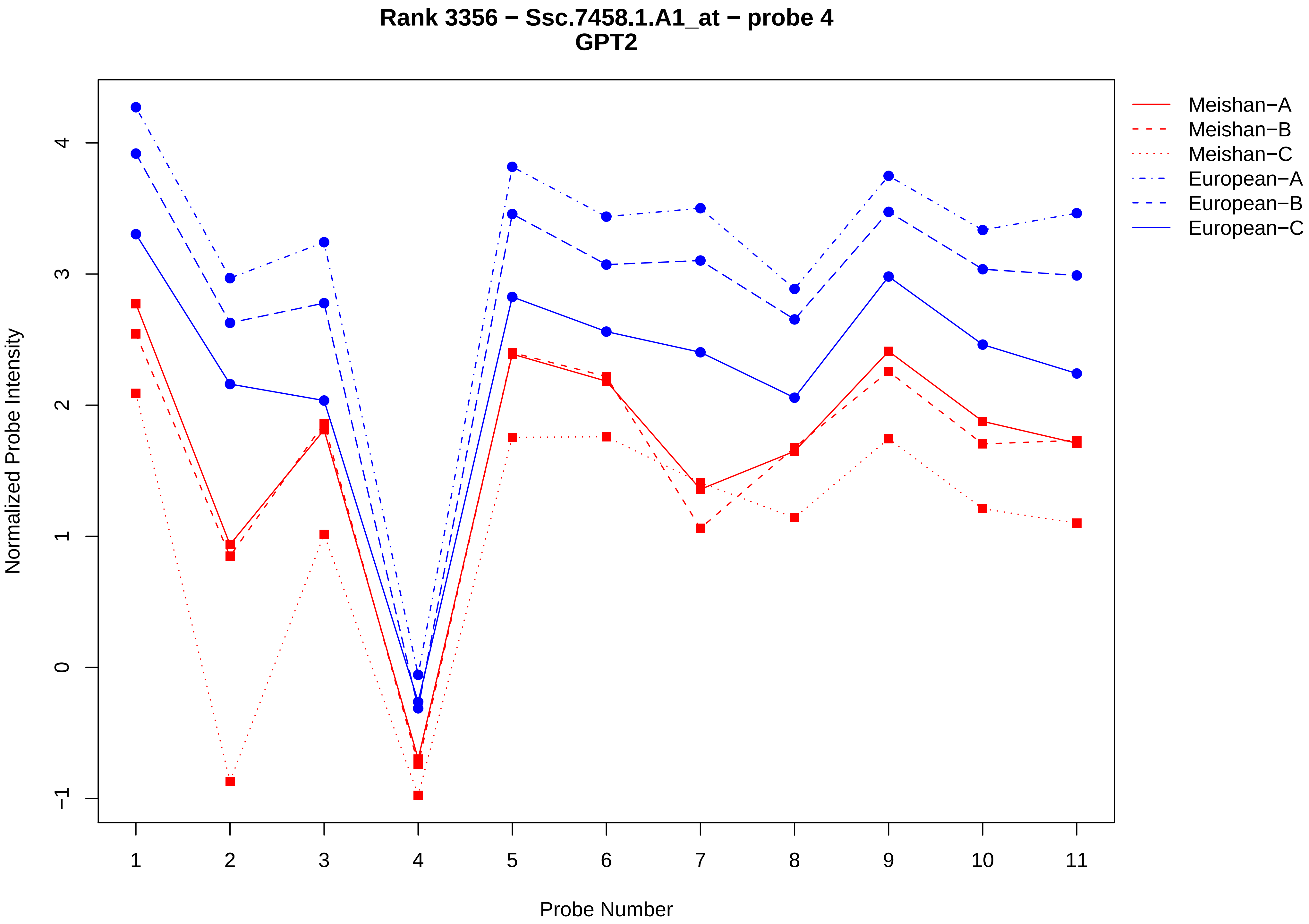

Rank 3356 − Ssc.7458.1.A1_at − probe 4
GPT2
Normalized Probe Intensity
Probe Number
−1
0
1
2
3
4
1
2
3
4
5
6
7
8
9
10
11
Meishan−A
Meishan−B
Meishan−C
European−A
European−B
European−C

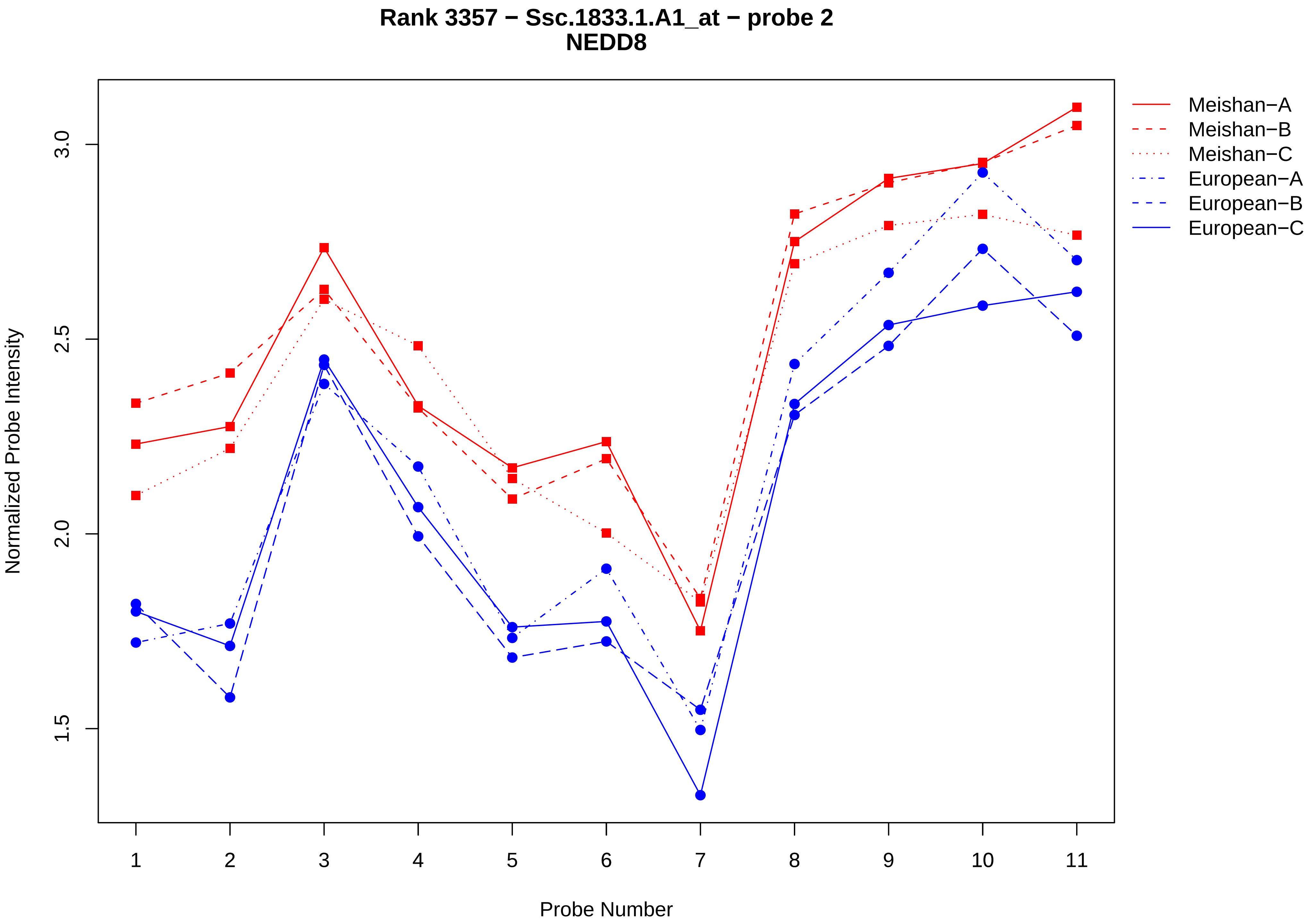
Rank 3357 − Ssc.1833.1.A1_at − probe 2
NEDD8
Normalized Probe Intensity
1.5
2.0
2.5
3.0
1
2
3
4
5
6
7
8
9
10
11
Probe Number
Meishan−A
Meishan−B
Meishan−C
European−A
European−B
European−C

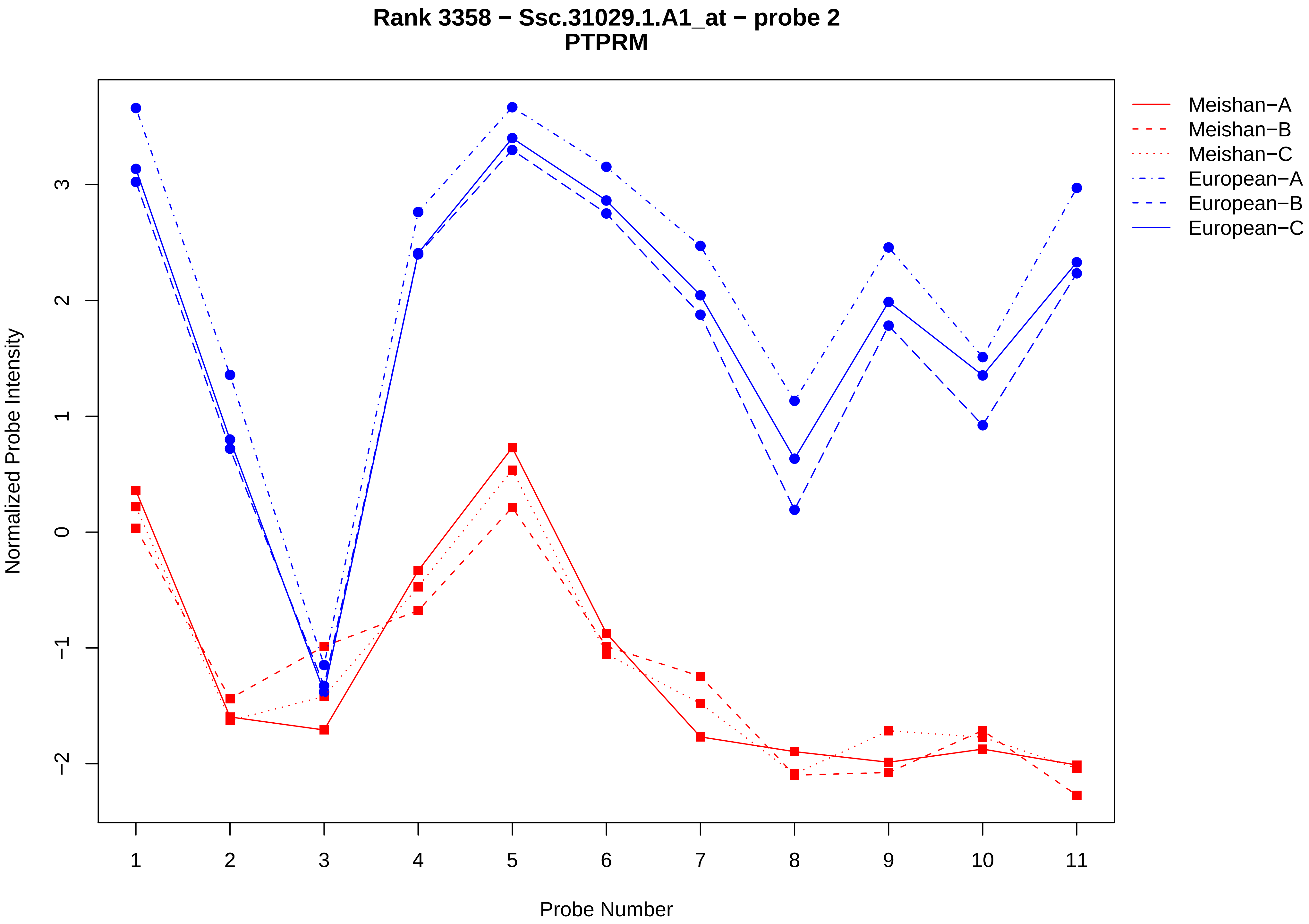

Rank 3358 − Ssc.31029.1.A1_at − probe 2
PTPRM
Normalized Probe Intensity
Probe Number
−2
−1
0
1
2
3
1
2
3
4
5
6
7
8
9
10
11
Meishan−A
Meishan−B
Meishan−C
European−A
European−B
European−C

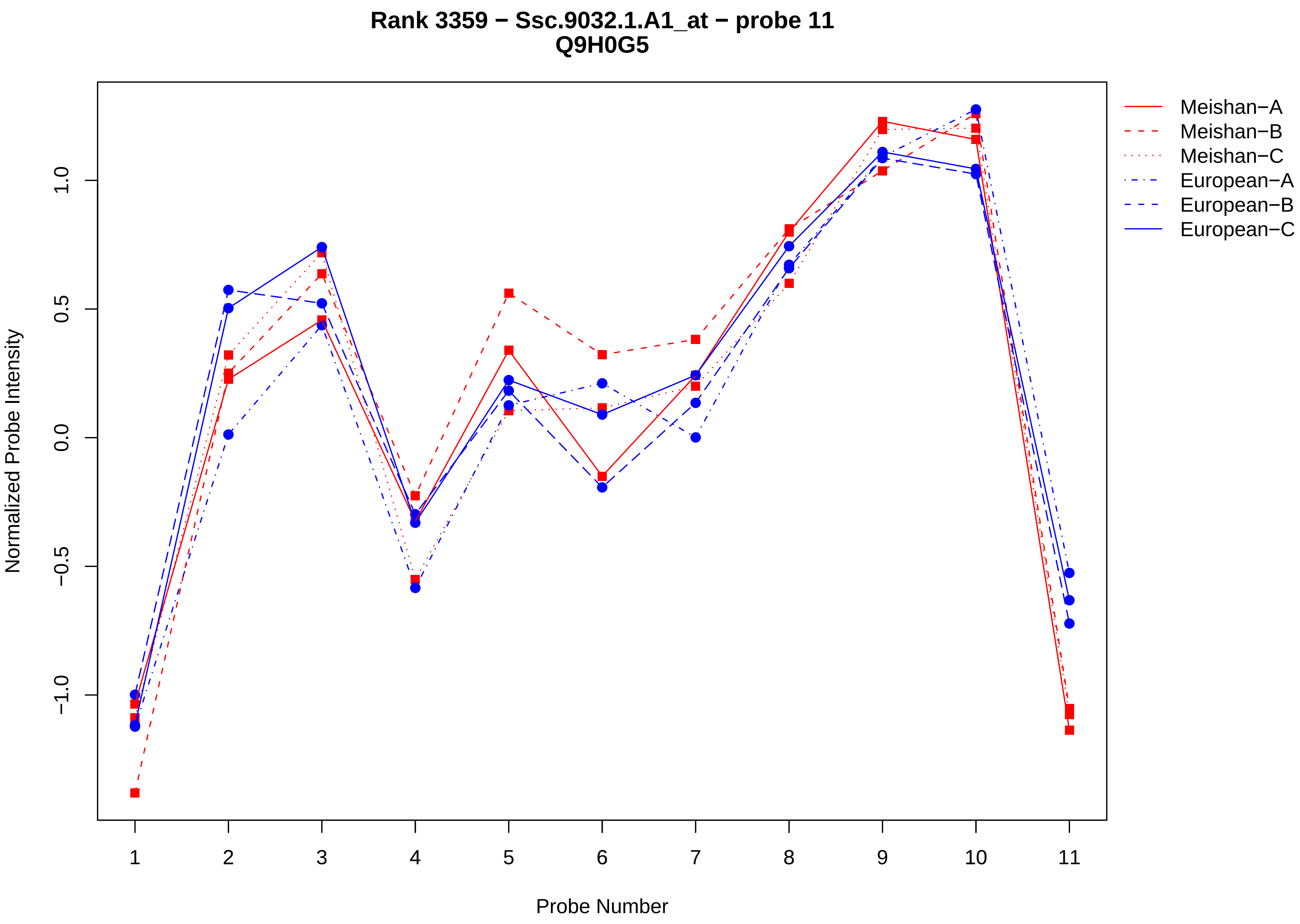

Rank 3359 − Ssc.9032.1.A1_at − probe 11
Q9H0G5
Normalized Probe Intensity
Probe Number
−1.0
−0.5
0.0
0.5
1.0
1
2
3
4
5
6
7
8
9
10
11
Meishan−A
Meishan−B
Meishan−C
European−A
European−B
European−C

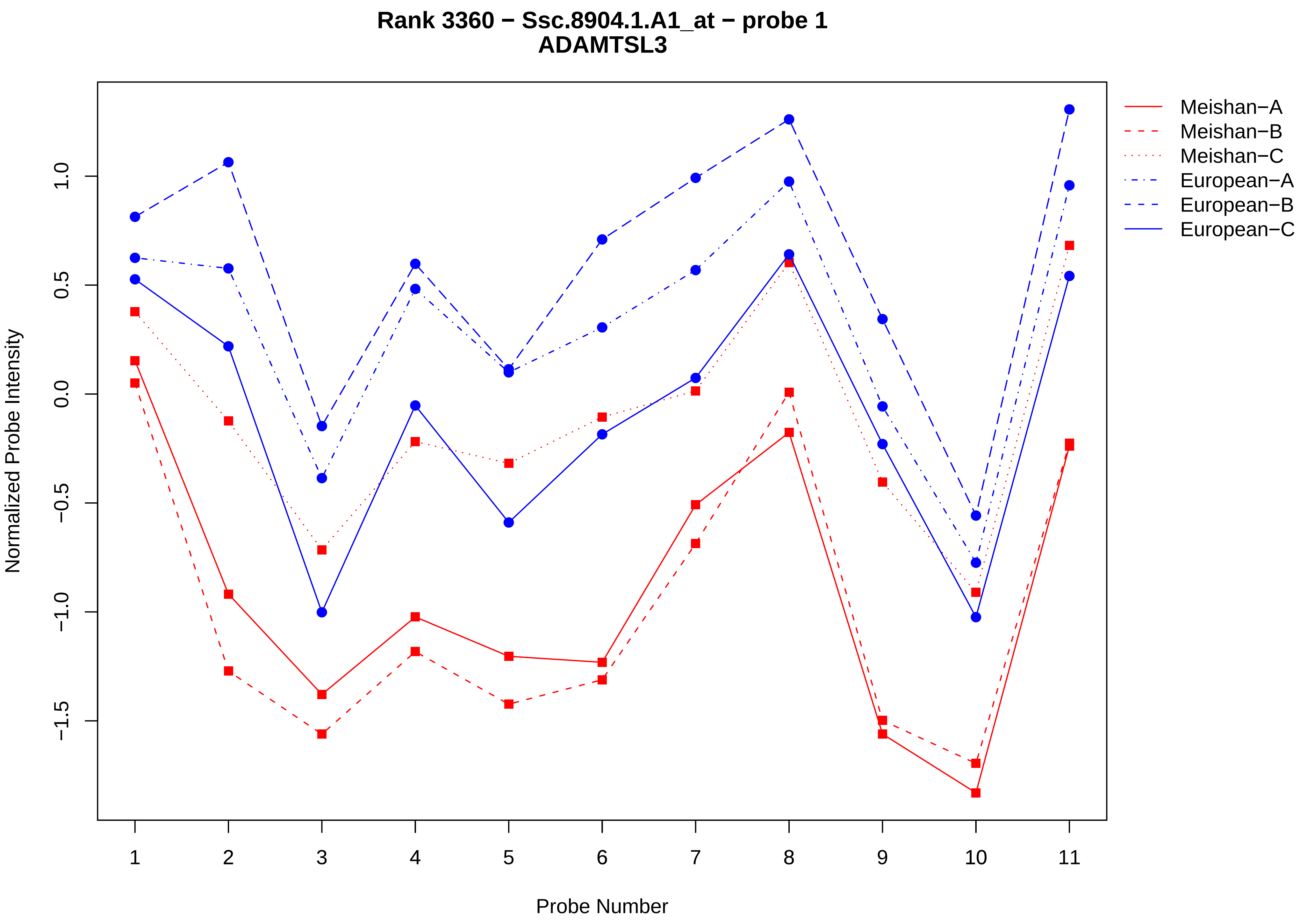
Rank 3360 − Ssc.8904.1.A1_at − probe 1
ADAMTSL3
Normalized Probe Intensity
Probe Number
Meishan−A
Meishan−B
Meishan−C
European−A
European−B
European−C
1.0
0.5
0.0
−0.5
−1.0
−1.5
1
2
3
4
5
6
7
8
9
10
11

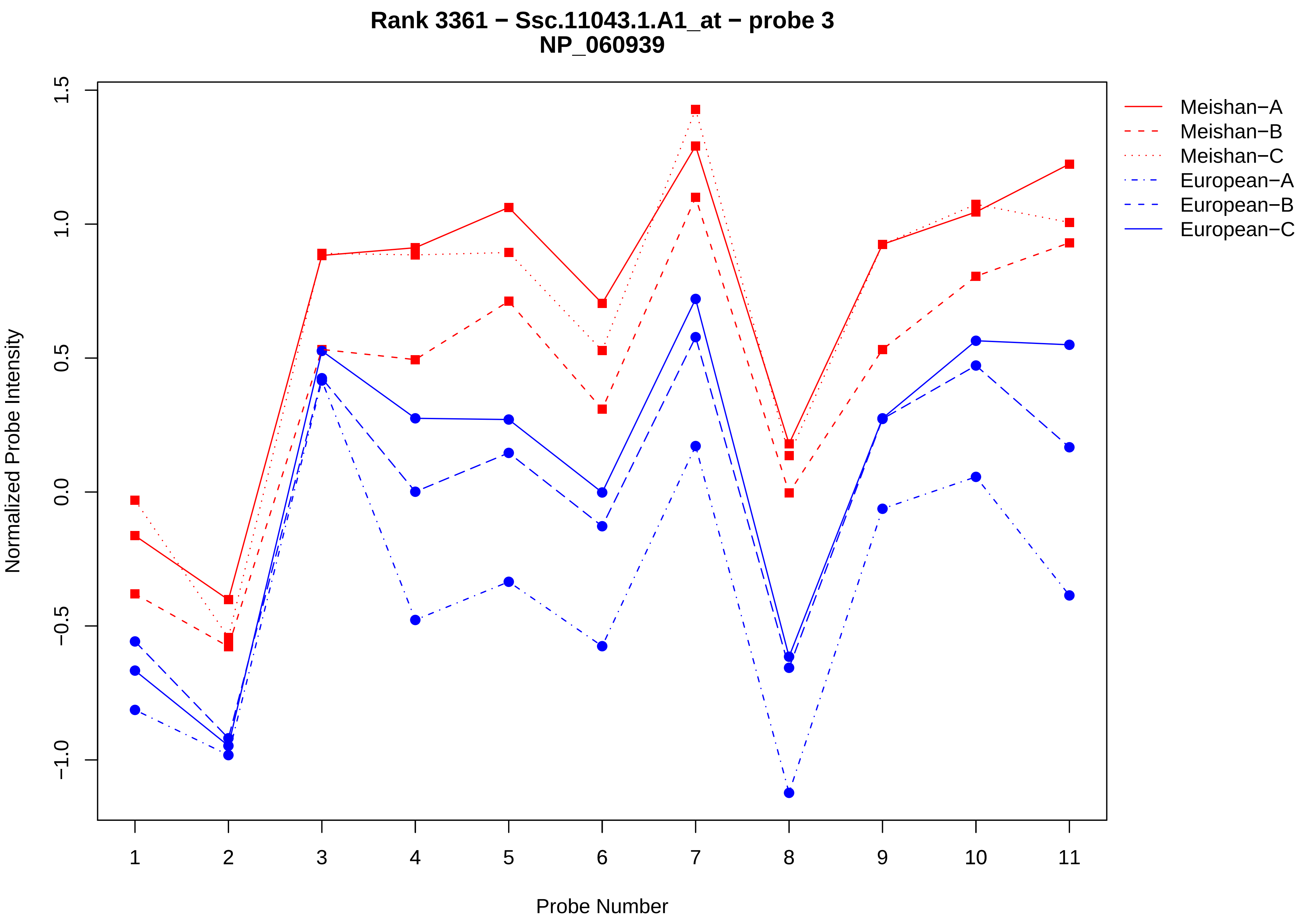

Rank 3361 − Ssc.11043.1.A1_at − probe 3
NP_060939
Normalized Probe Intensity
Probe Number
1.5
1.0
0.5
0.0
−0.5
−1.0
1
2
3
4
5
6
7
8
9
10
11
Meishan−A
Meishan−B
Meishan−C
European−A
European−B
European−C

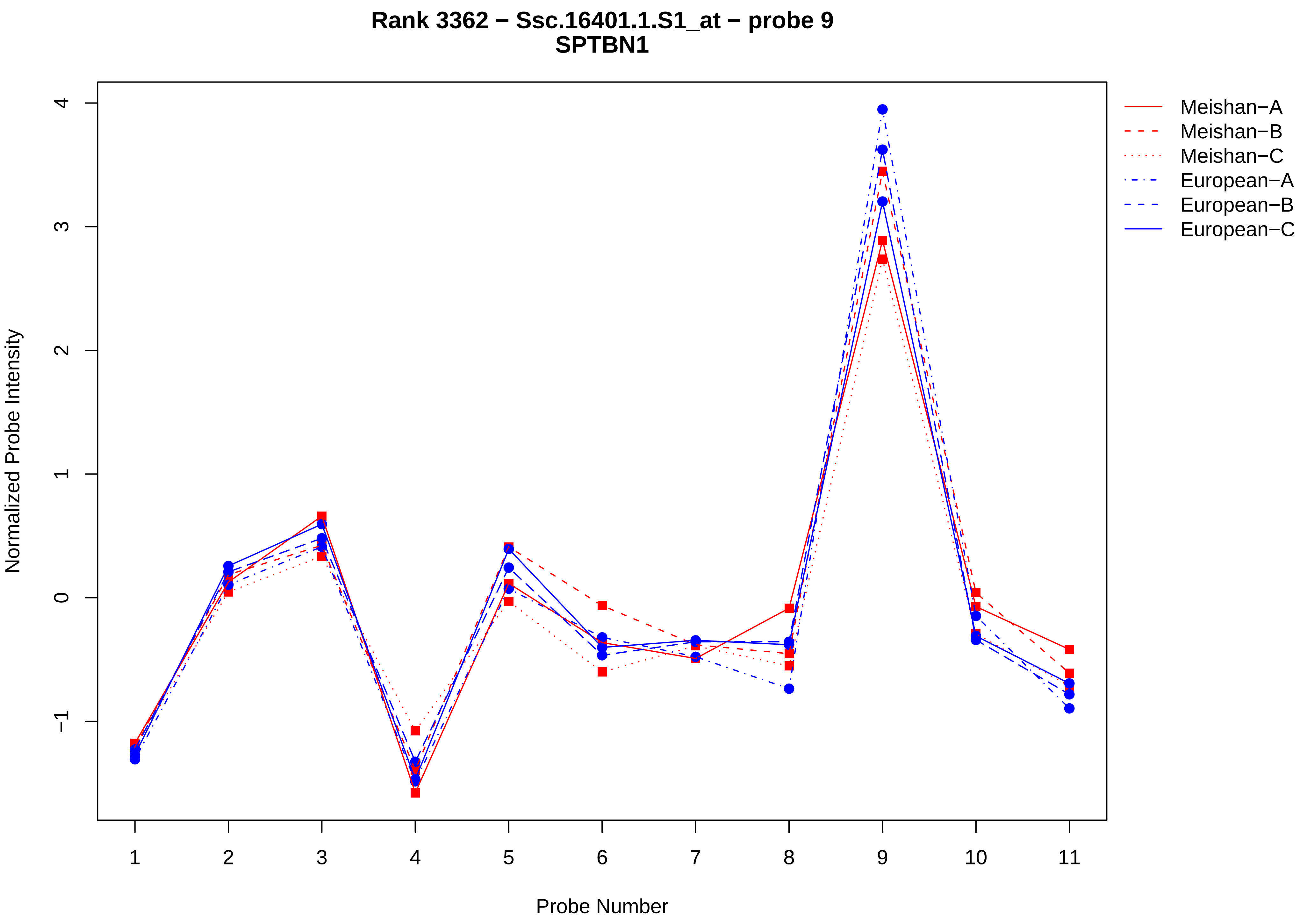

Rank 3362 − Ssc.16401.1.S1_at − probe 9
SPTBN1
Normalized Probe Intensity
Probe Number
Meishan−A
Meishan−B
Meishan−C
European−A
European−B
European−C
−1
0
1
2
3
4
1
2
3
4
5
6
7
8
9
10
11

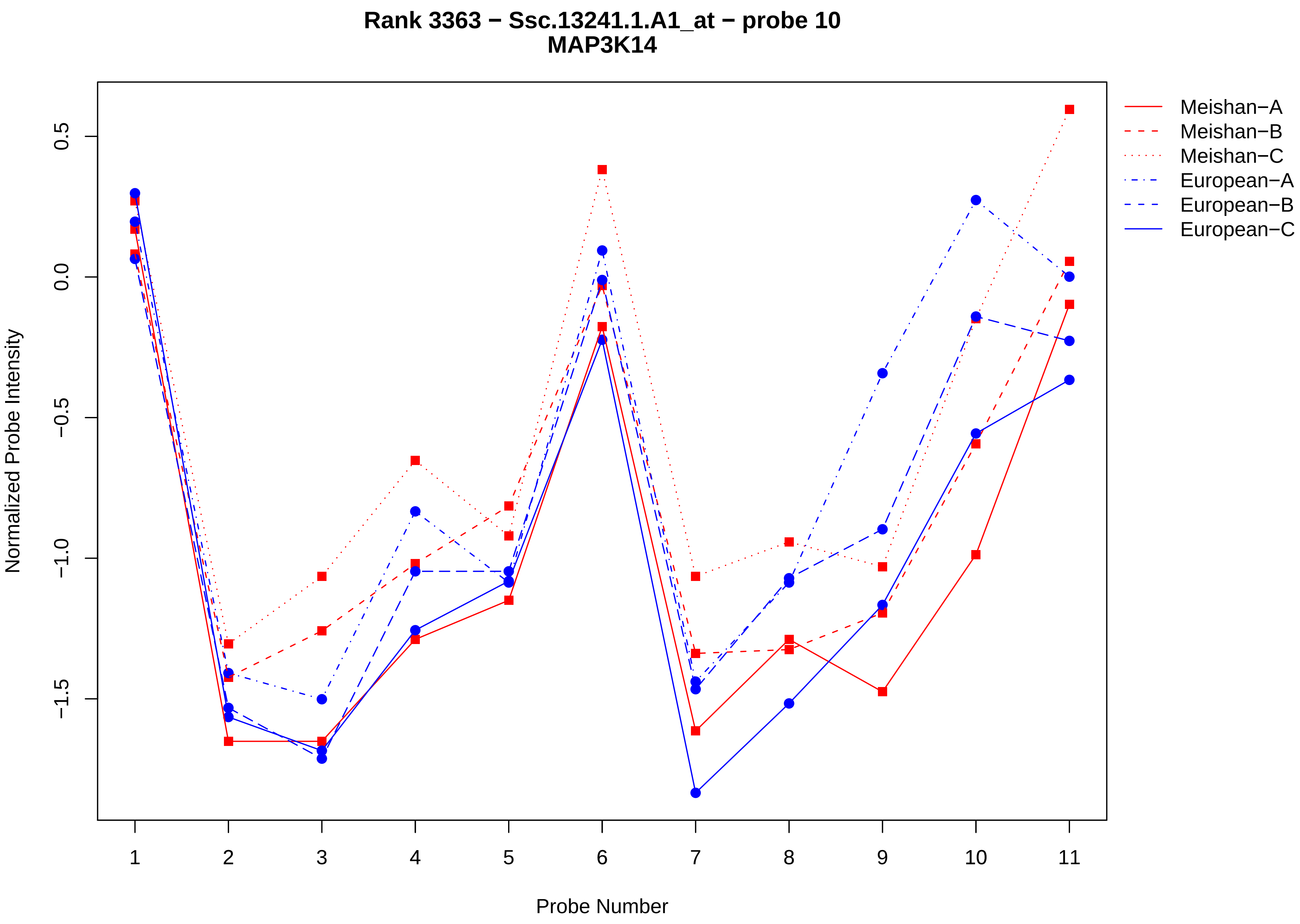

Rank 3363 − Ssc.13241.1.A1_at − probe 10
MAP3K14
Normalized Probe Intensity
Probe Number
0.5
0.0
−0.5
−1.0
−1.5
1
2
3
4
5
6
7
8
9
10
11
Meishan−A
Meishan−B
Meishan−C
European−A
European−B
European−C

Rank 3364 − Ssc.1758.2.S1_at − probe 6
TNIP1

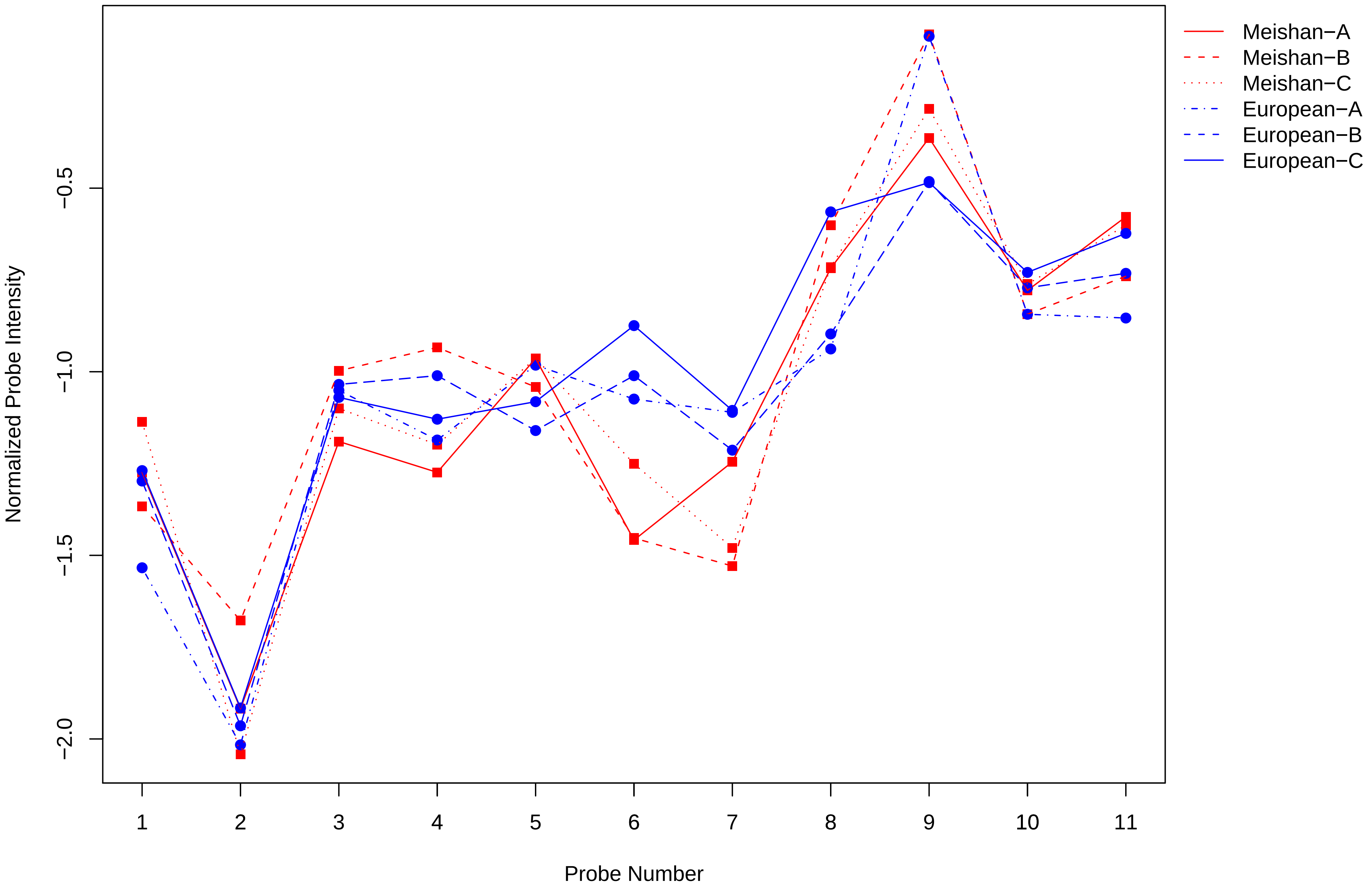

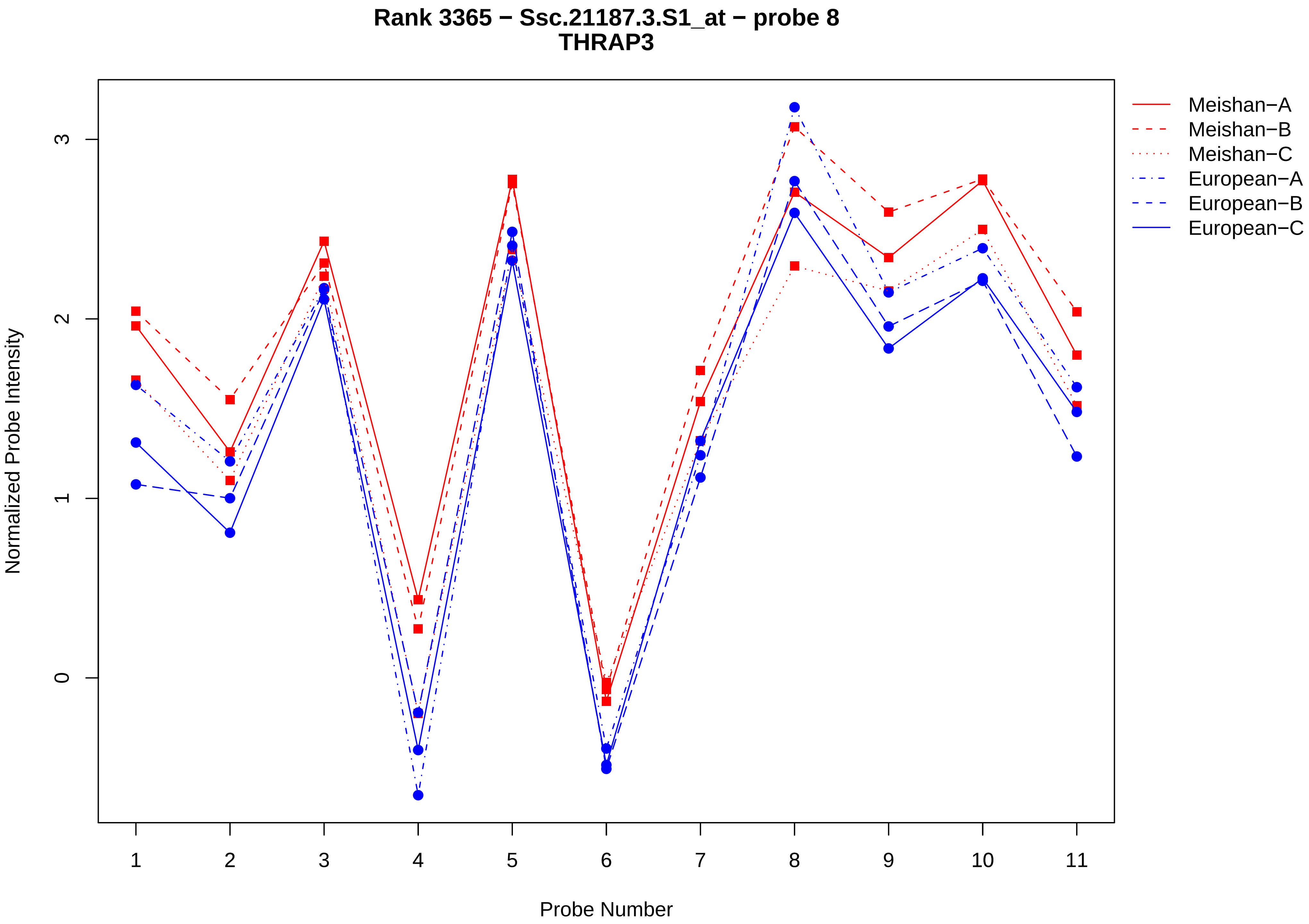

Rank 3365 − Ssc.21187.3.S1_at − probe 8
THRAP3
Normalized Probe Intensity
Probe Number
0
1
2
3
1
2
3
4
5
6
7
8
9
10
11
Meishan−A
Meishan−B
Meishan−C
European−A
European−B
European−C

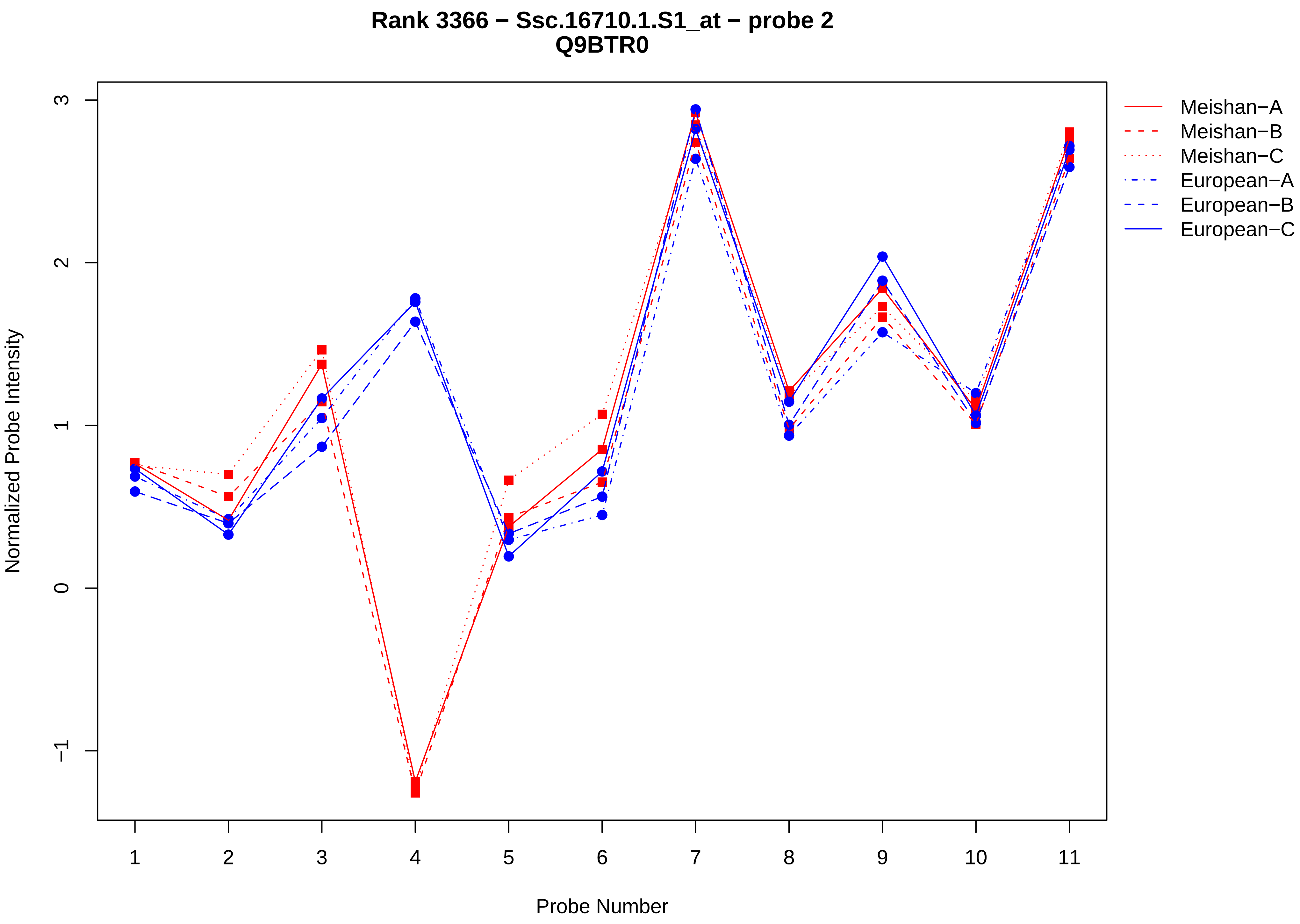

Rank 3366 − Ssc.16710.1.S1_at − probe 2
Q9BTR0
Normalized Probe Intensity
Probe Number
−1
0
1
2
3
1
2
3
4
5
6
7
8
9
10
11
Meishan−A
Meishan−B
Meishan−C
European−A
European−B
European−C

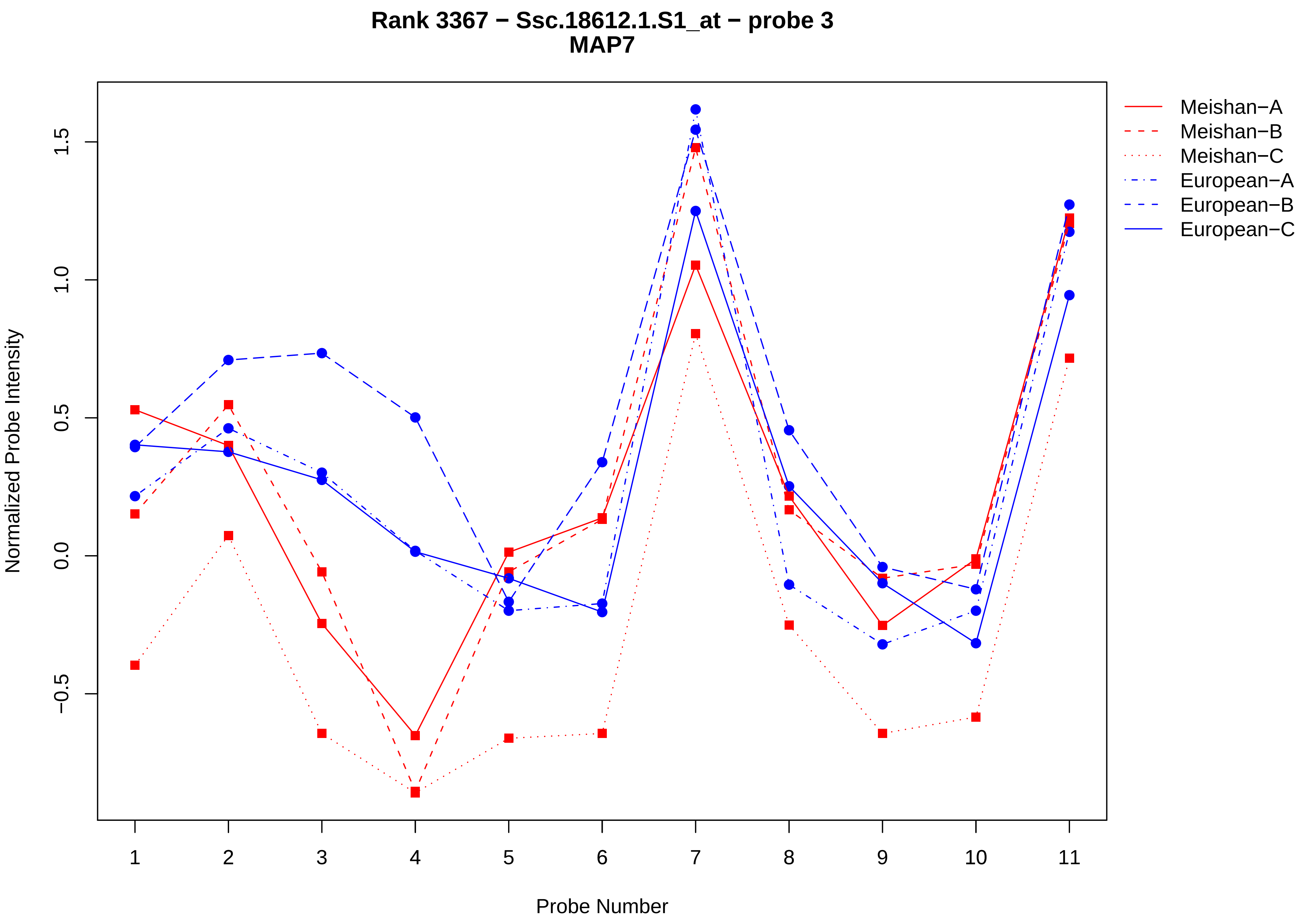

Rank 3367 − Ssc.18612.1.S1_at − probe 3
MAP7
Normalized Probe Intensity
Probe Number
−0.5
0.0
0.5
1.0
1.5
1
2
3
4
5
6
7
8
9
10
11
Meishan−A
Meishan−B
Meishan−C
European−A
European−B
European−C

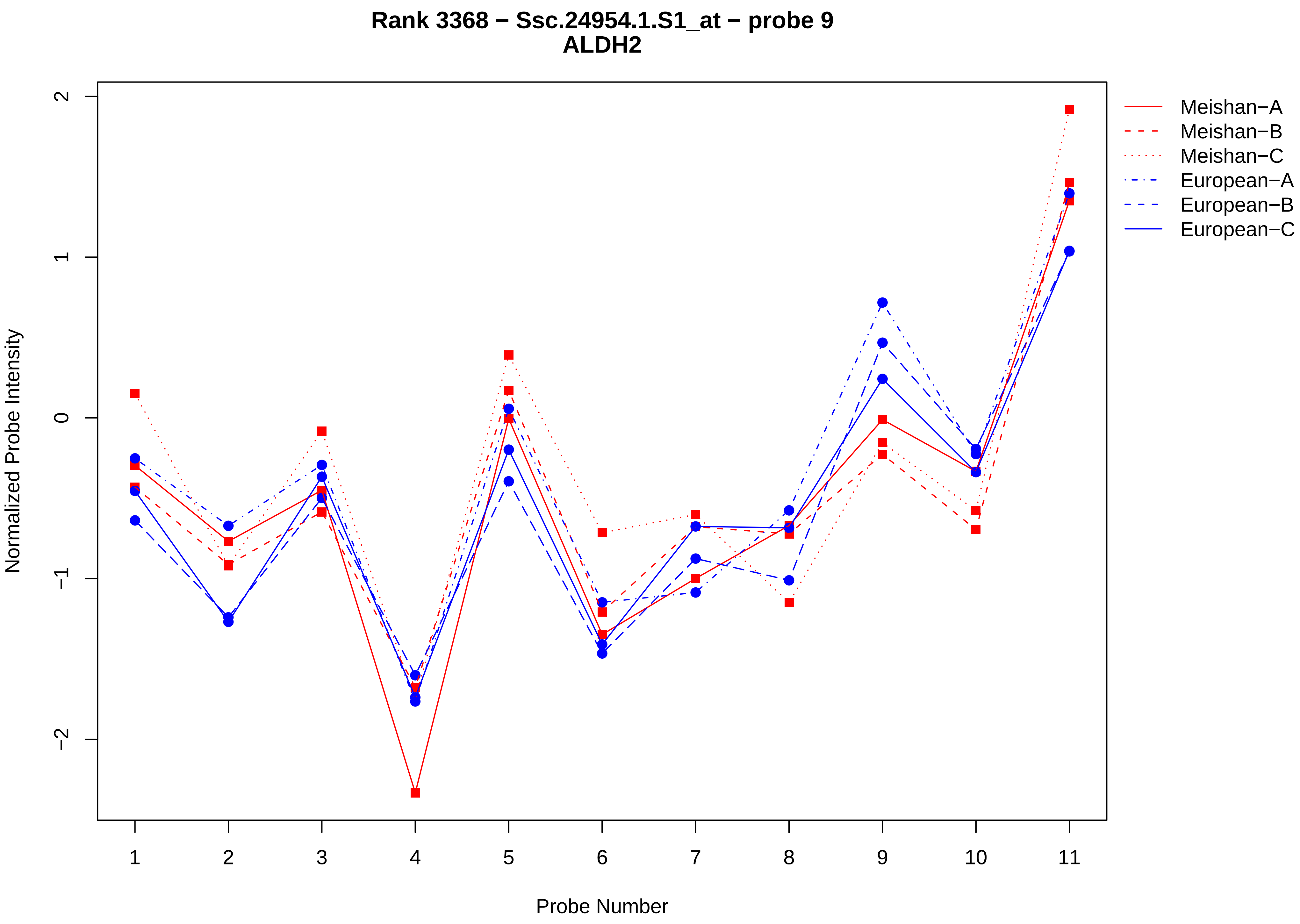

Rank 3368 − Ssc.24954.1.S1_at − probe 9
ALDH2
Normalized Probe Intensity
Probe Number
−2
−1
0
1
2
1
2
3
4
5
6
7
8
9
10
11
Meishan−A
Meishan−B
Meishan−C
European−A
European−B
European−C

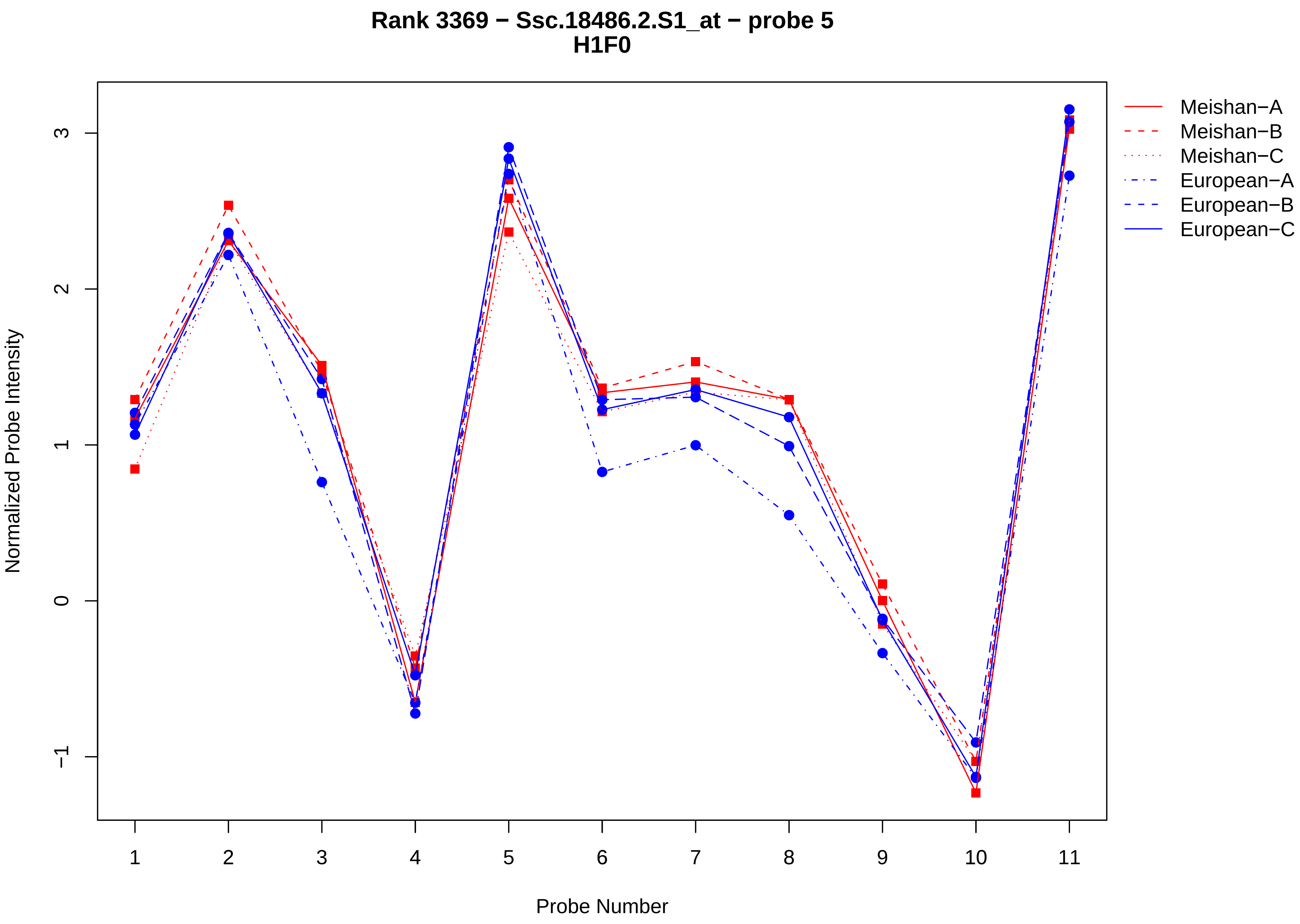

Rank 3369 − Ssc.18486.2.S1_at − probe 5
H1F0
Normalized Probe Intensity
Probe Number
−1
0
1
2
3
1
2
3
4
5
6
7
8
9
10
11
Meishan−A
Meishan−B
Meishan−C
European−A
European−B
European−C

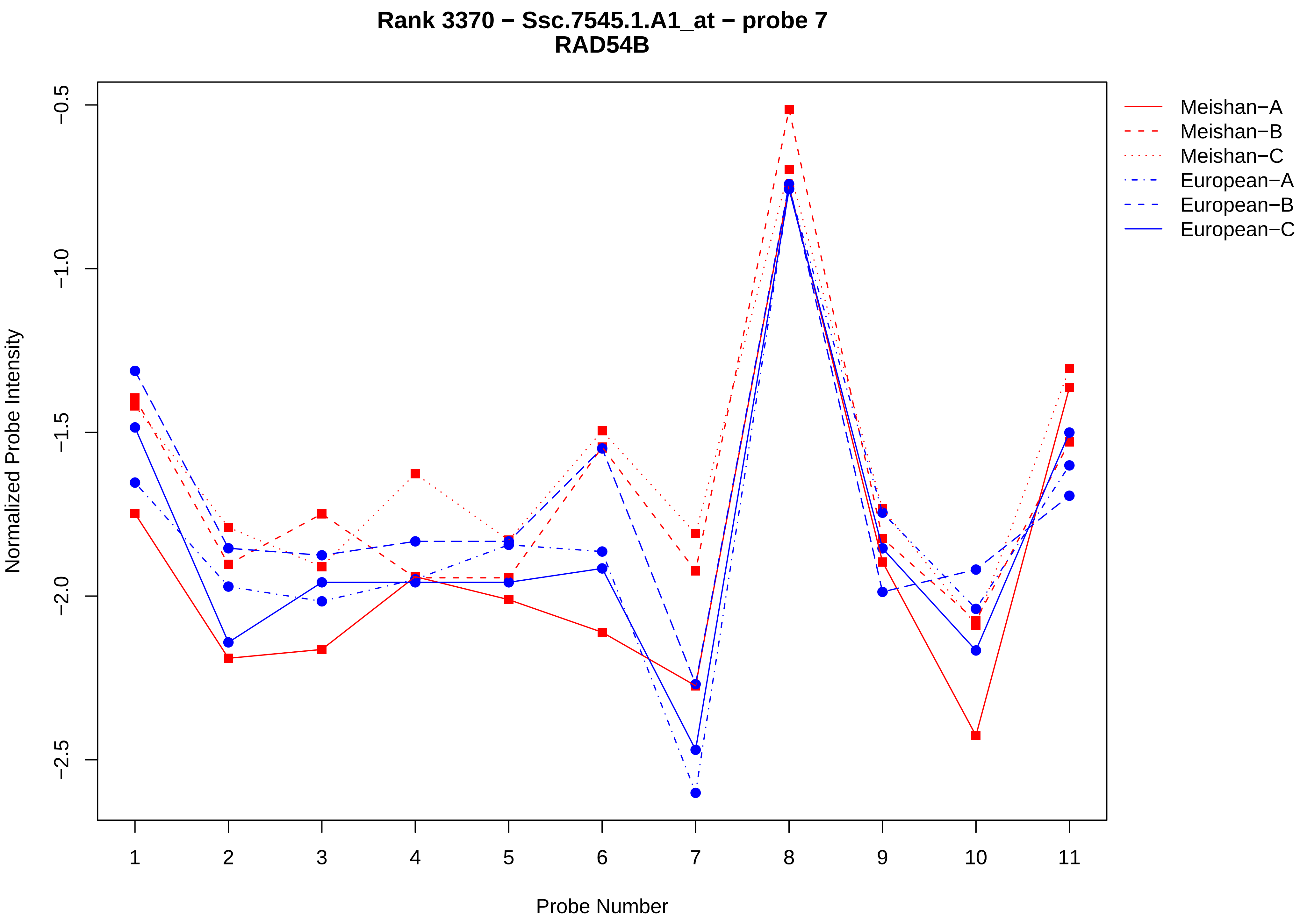
Rank 3370 − Ssc.7545.1.A1_at − probe 7
RAD54B
Normalized Probe Intensity
Probe Number
−0.5
−1.0
−1.5
−2.0
−2.5
1
2
3
4
5
6
7
8
9
10
11
Meishan−A
Meishan−B
Meishan−C
European−A
European−B
European−C

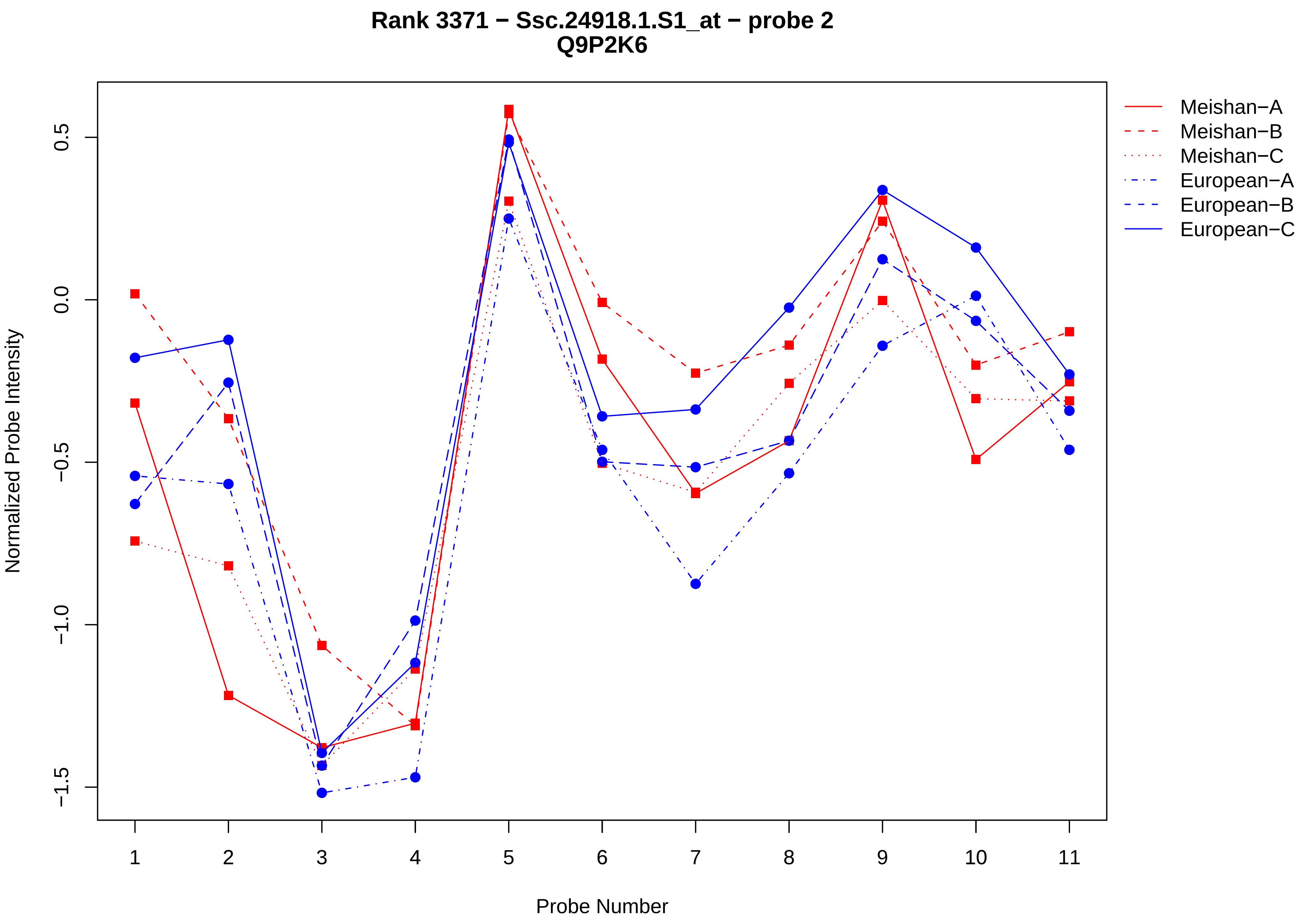

Rank 3371 − Ssc.24918.1.S1_at − probe 2
Q9P2K6
Normalized Probe Intensity
Probe Number
0.5
0.0
−0.5
−1.0
−1.5
1
2
3
4
5
6
7
8
9
10
11
Meishan−A
Meishan−B
Meishan−C
European−A
European−B
European−C

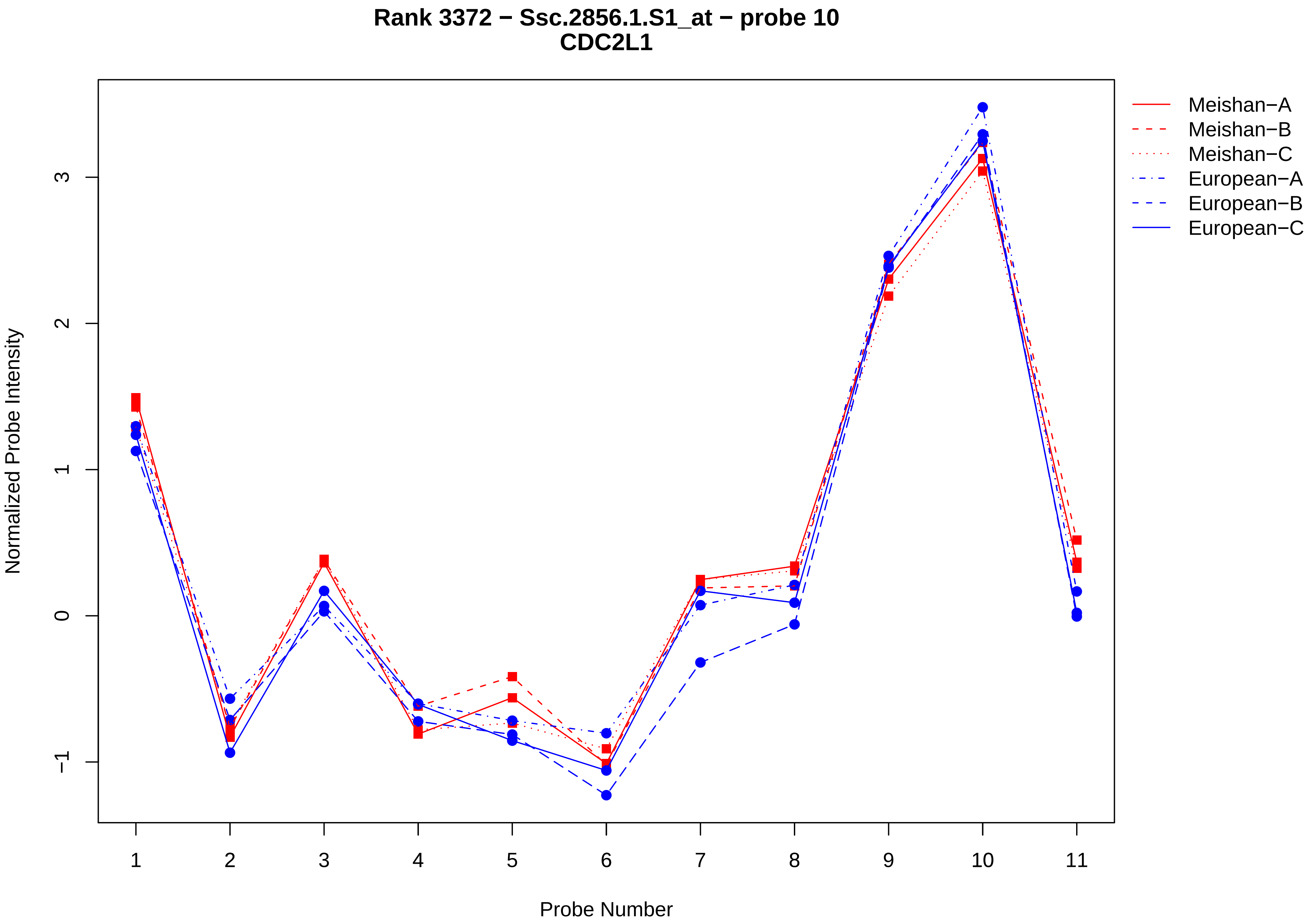

Rank 3372 − Ssc.2856.1.S1_at − probe 10
CDC2L1
Normalized Probe Intensity
Probe Number
−1
0
1
2
3
1
2
3
4
5
6
7
8
9
10
11
Meishan−A
Meishan−B
Meishan−C
European−A
European−B
European−C

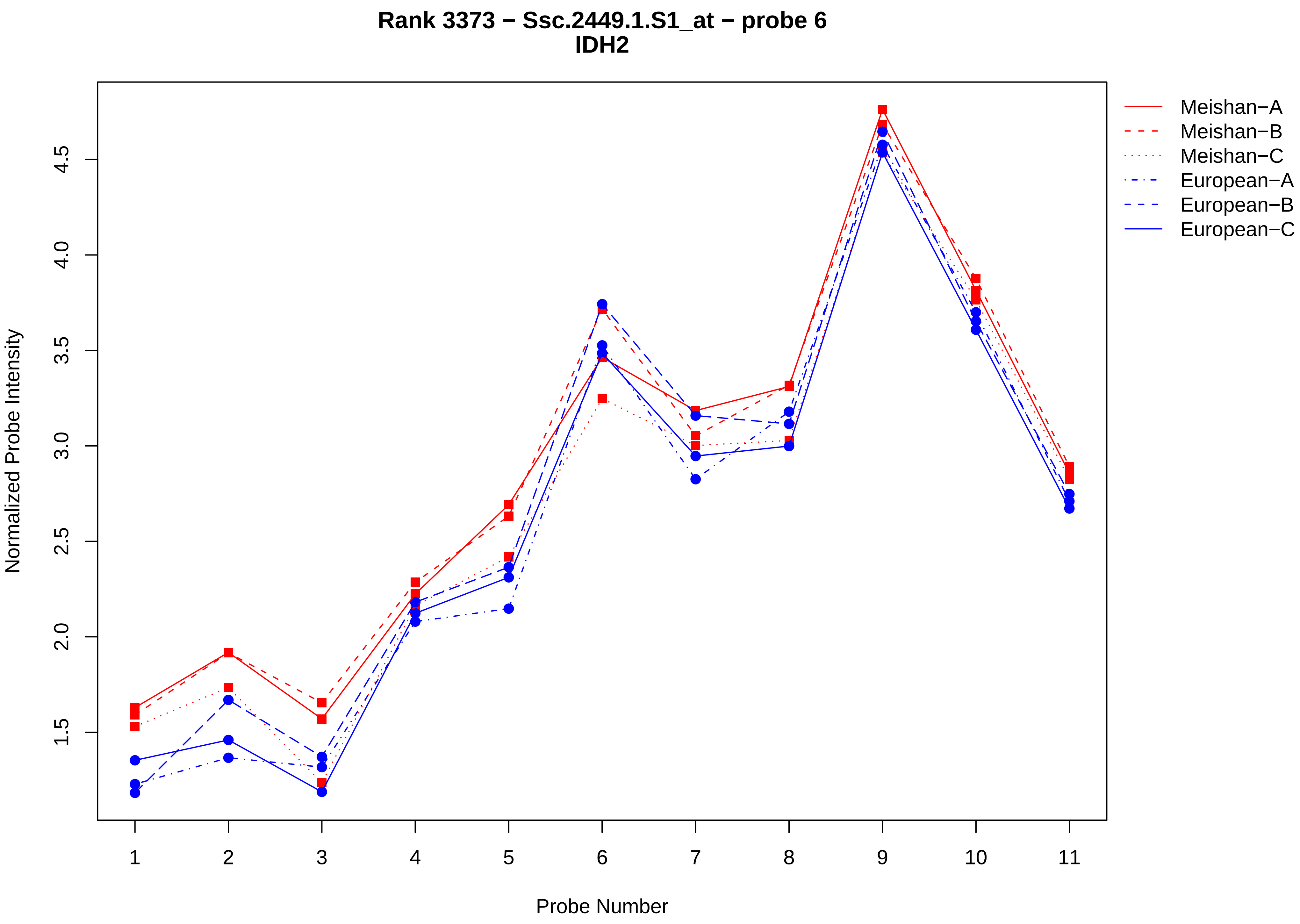

Rank 3373 − Ssc.2449.1.S1_at − probe 6
IDH2
Normalized Probe Intensity
Probe Number
1.5
2.0
2.5
3.0
3.5
4.0
4.5
1
2
3
4
5
6
7
8
9
10
11
Meishan−A
Meishan−B
Meishan−C
European−A
European−B
European−C

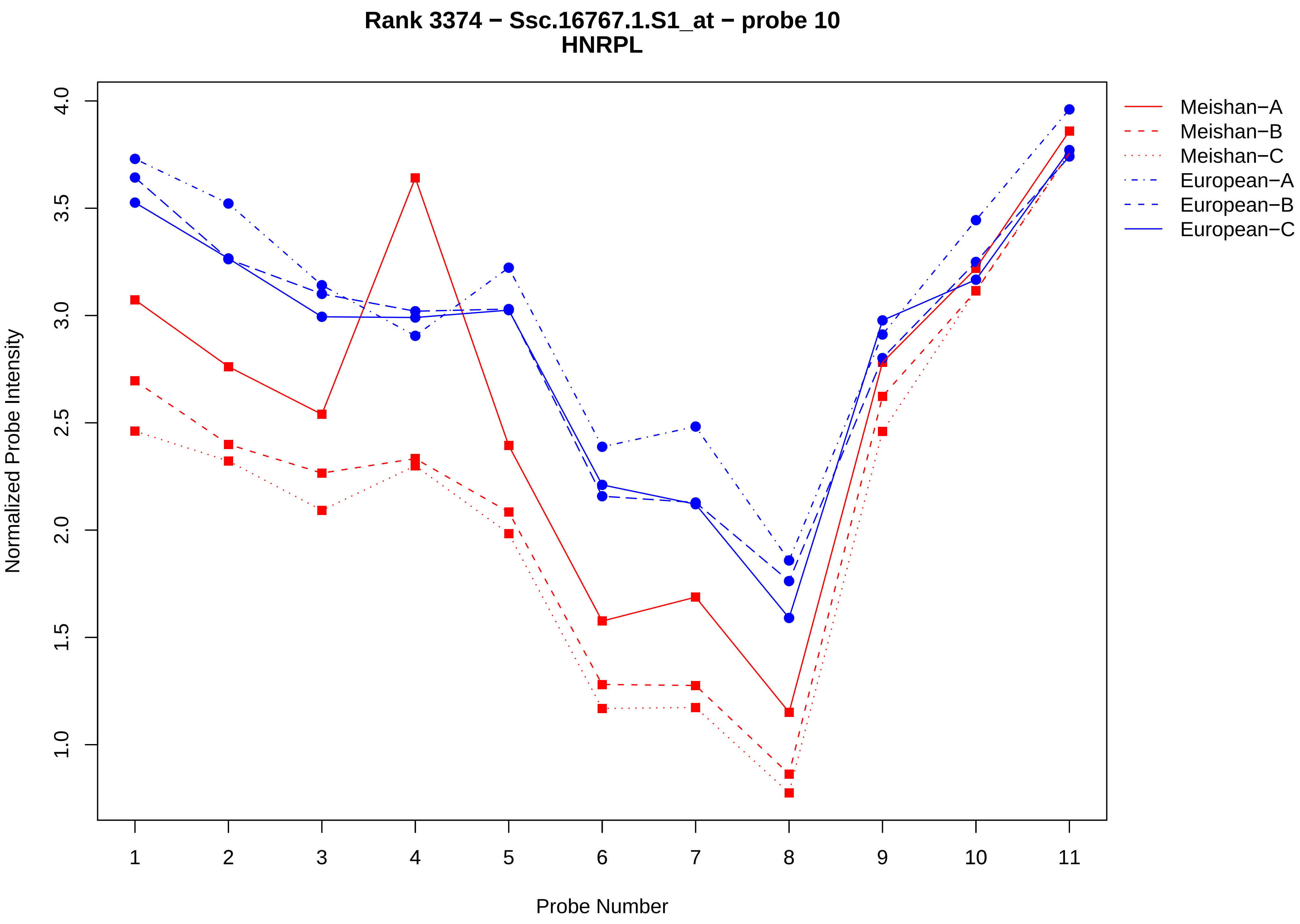
Rank 3374 − Ssc.16767.1.S1_at − probe 10
HNRPL
Normalized Probe Intensity
Probe Number
1.0
1.5
2.0
2.5
3.0
3.5
4.0
1
2
3
4
5
6
7
8
9
10
11
Meishan−A
Meishan−B
Meishan−C
European−A
European−B
European−C

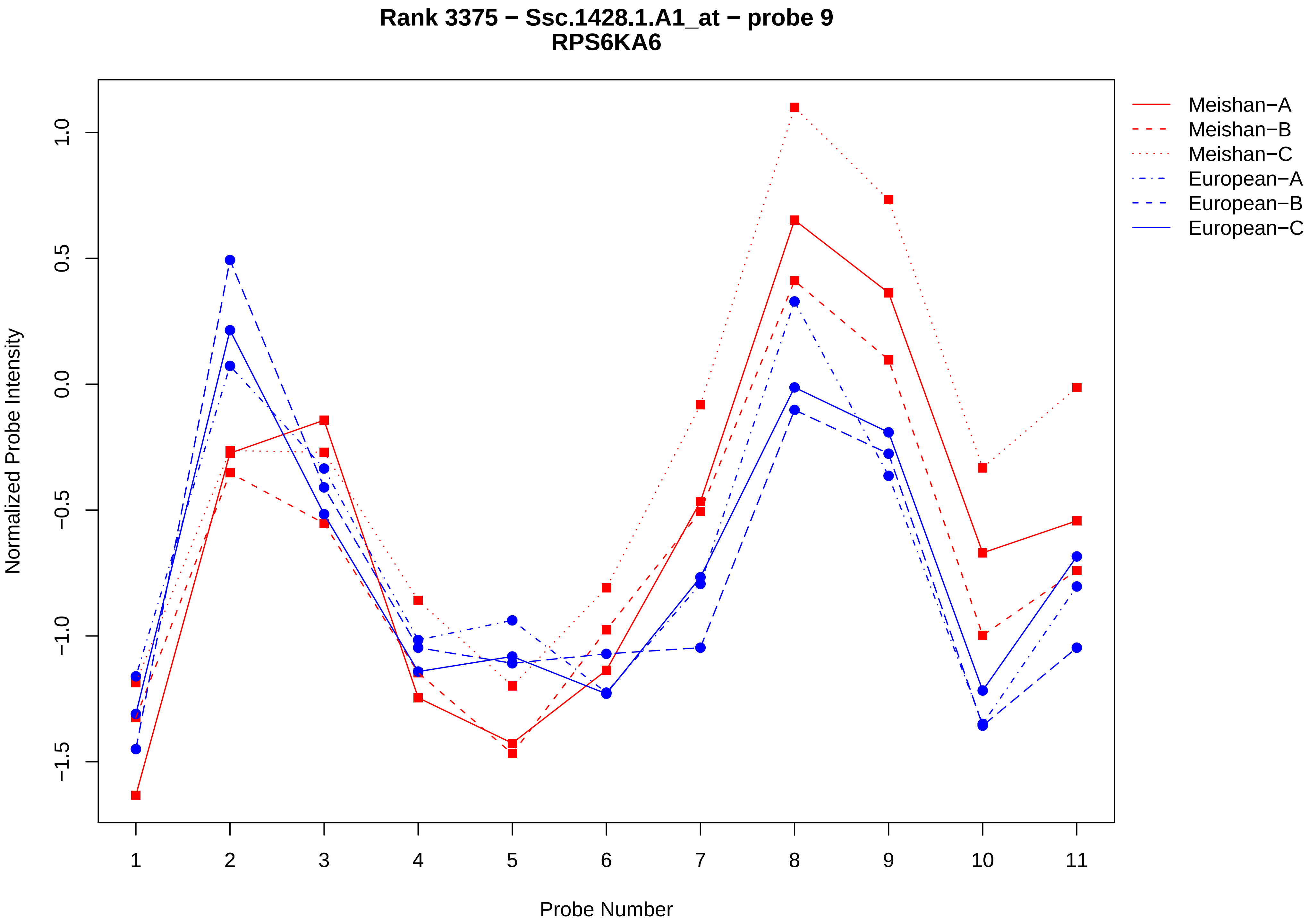

Rank 3375 − Ssc.1428.1.A1_at − probe 9
RPS6KA6
Normalized Probe Intensity
Probe Number
1.0
0.5
0.0
−0.5
−1.0
−1.5
1
2
3
4
5
6
7
8
9
10
11
Meishan−A
Meishan−B
Meishan−C
European−A
European−B
European−C

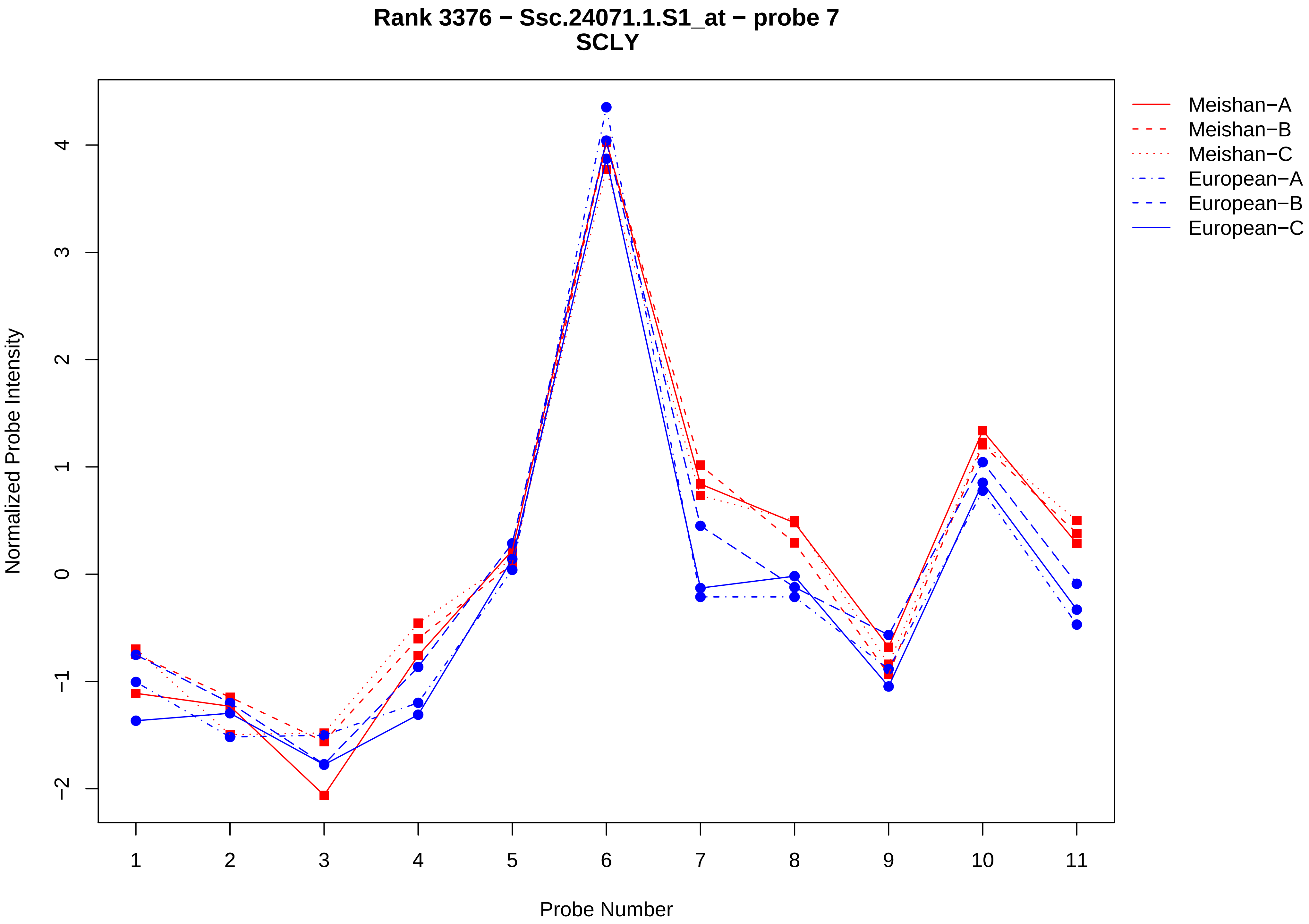

Rank 3376 − Ssc.24071.1.S1_at − probe 7
SCLY
Normalized Probe Intensity
Probe Number
−2
−1
0
1
2
3
4
1
2
3
4
5
6
7
8
9
10
11
Meishan−A
Meishan−B
Meishan−C
European−A
European−B
European−C

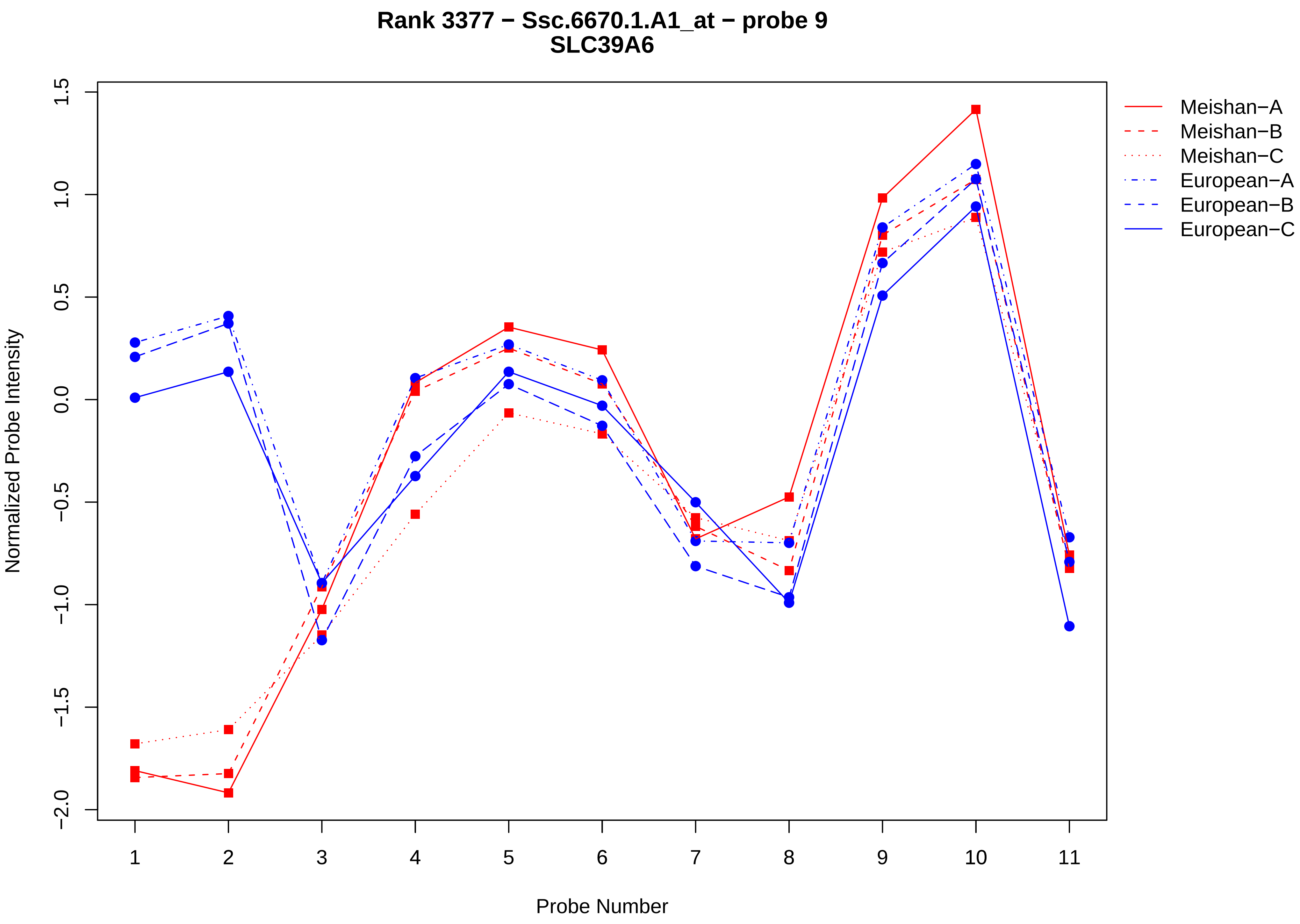

Rank 3377 − Ssc.6670.1.A1_at − probe 9
SLC39A6
Normalized Probe Intensity
Probe Number
1.5
1.0
0.5
0.0
−0.5
−1.0
−1.5
−2.0
1
2
3
4
5
6
7
8
9
10
11
Meishan−A
Meishan−B
Meishan−C
European−A
European−B
European−C

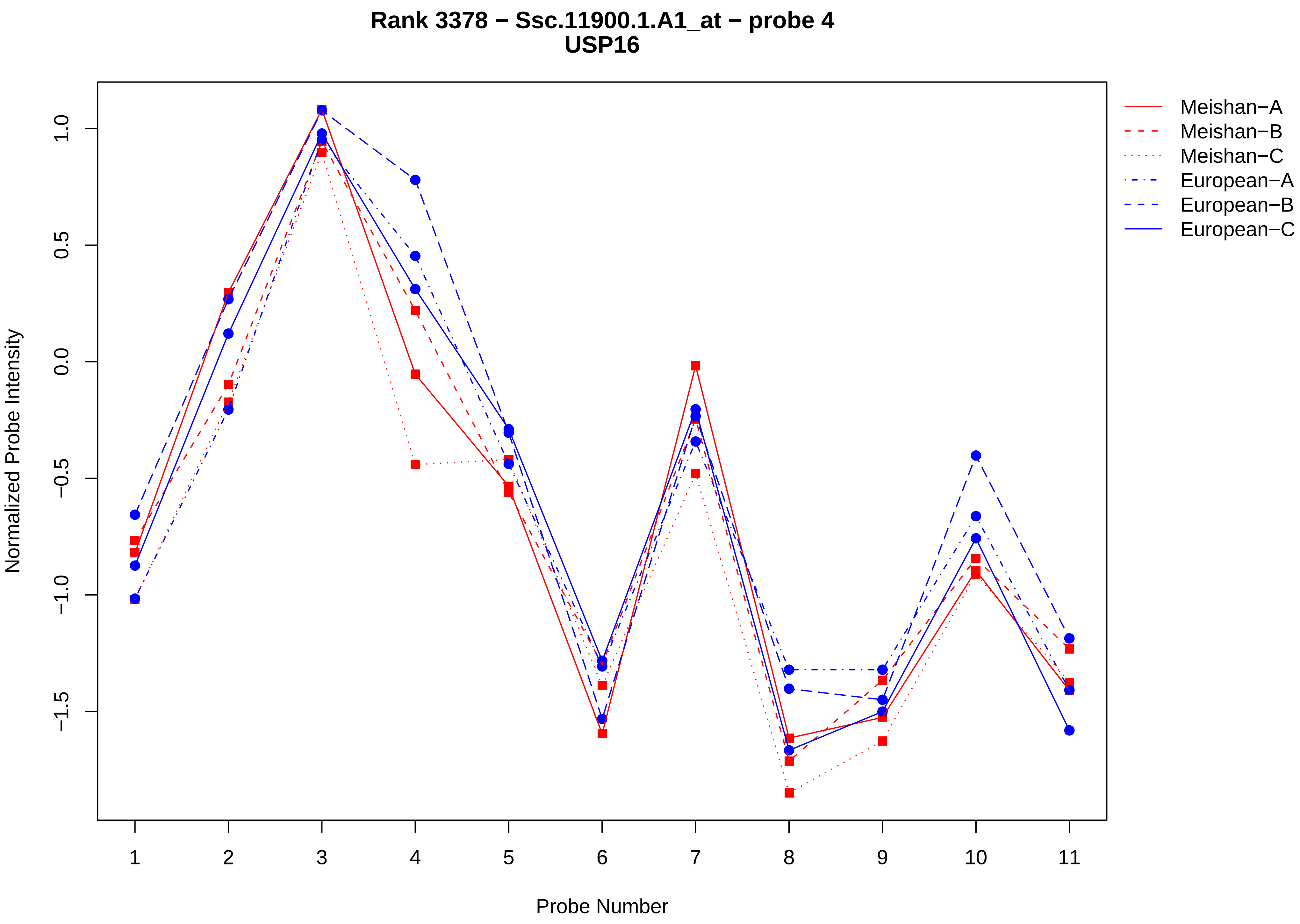

Rank 3378 − Ssc.11900.1.A1_at − probe 4
USP16
Normalized Probe Intensity
Probe Number
1.0
0.5
0.0
−0.5
−1.0
−1.5
1
2
3
4
5
6
7
8
9
10
11
Meishan−A
Meishan−B
Meishan−C
European−A
European−B
European−C

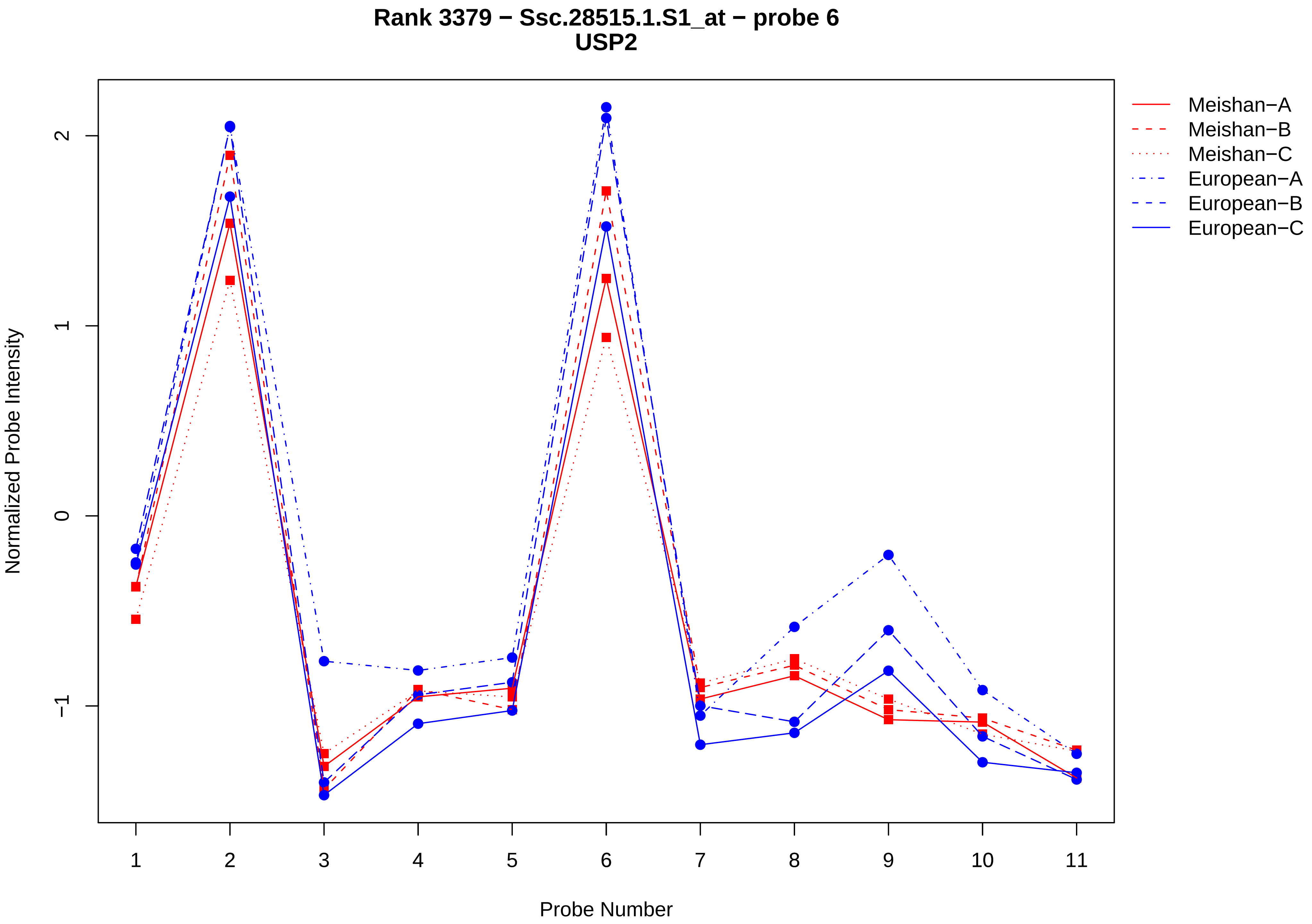
Rank 3379 − Ssc.28515.1.S1_at − probe 6
USP2
Normalized Probe Intensity
Probe Number
−1
0
1
2
1
2
3
4
5
6
7
8
9
10
11
Meishan−A
Meishan−B
Meishan−C
European−A
European−B
European−C

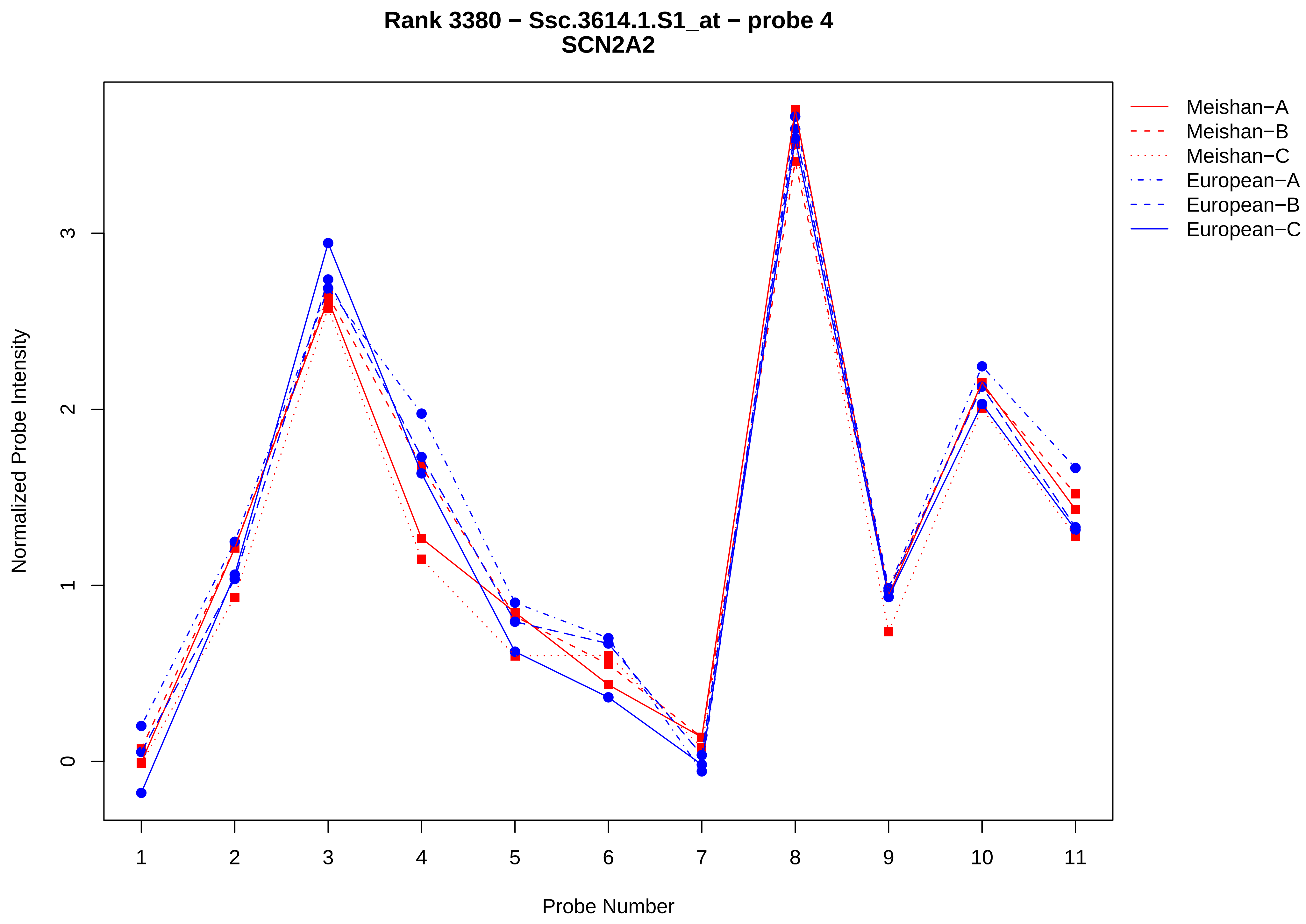

Rank 3380 − Ssc.3614.1.S1_at − probe 4
SCN2A2
Normalized Probe Intensity
Probe Number
0
1
2
3
1
2
3
4
5
6
7
8
9
10
11
Meishan−A
Meishan−B
Meishan−C
European−A
European−B
European−C

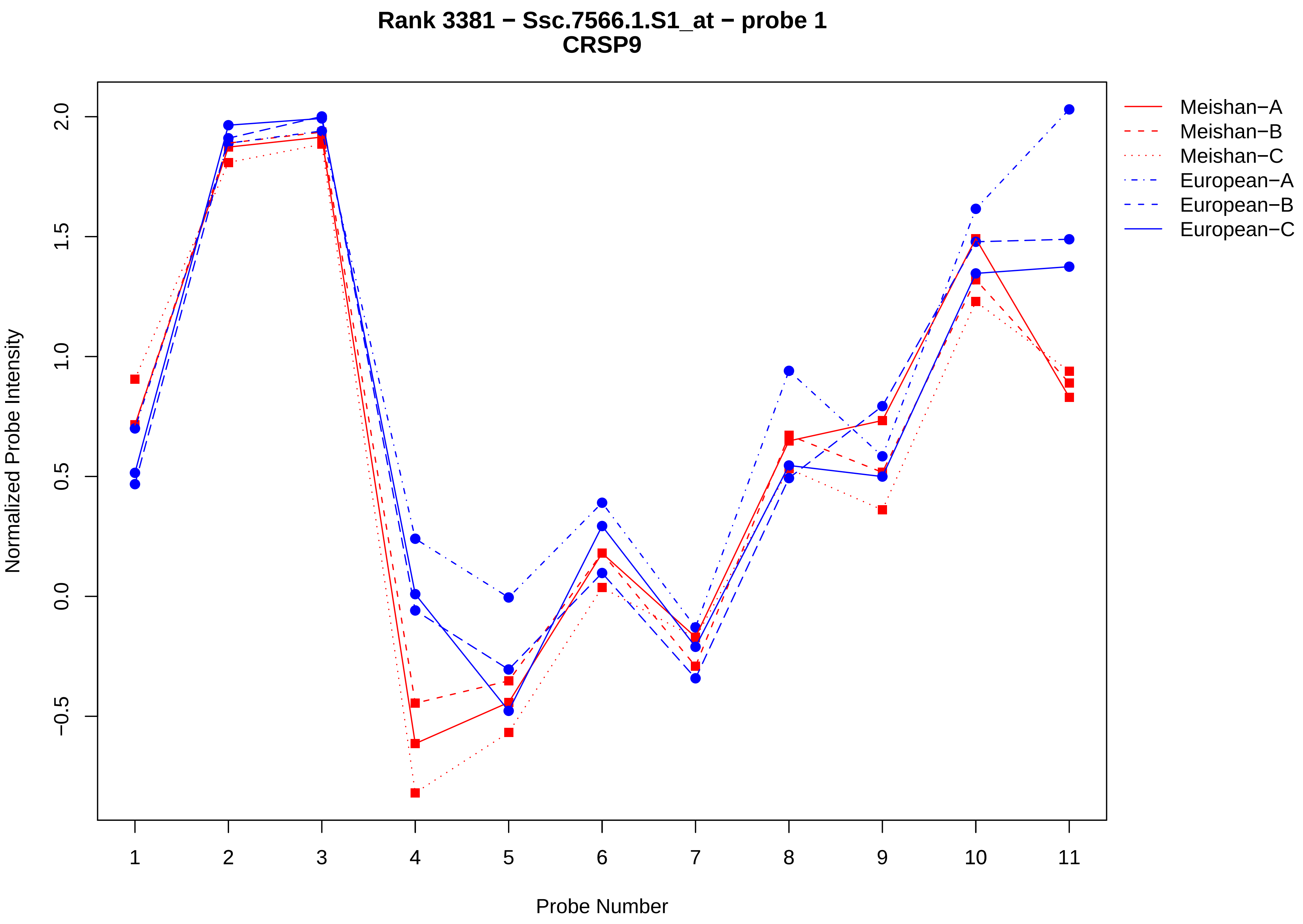

Rank 3381 − Ssc.7566.1.S1_at − probe 1
CRSP9
Normalized Probe Intensity
Probe Number
−0.5
0.0
0.5
1.0
1.5
2.0
1
2
3
4
5
6
7
8
9
10
11
Meishan−A
Meishan−B
Meishan−C
European−A
European−B
European−C

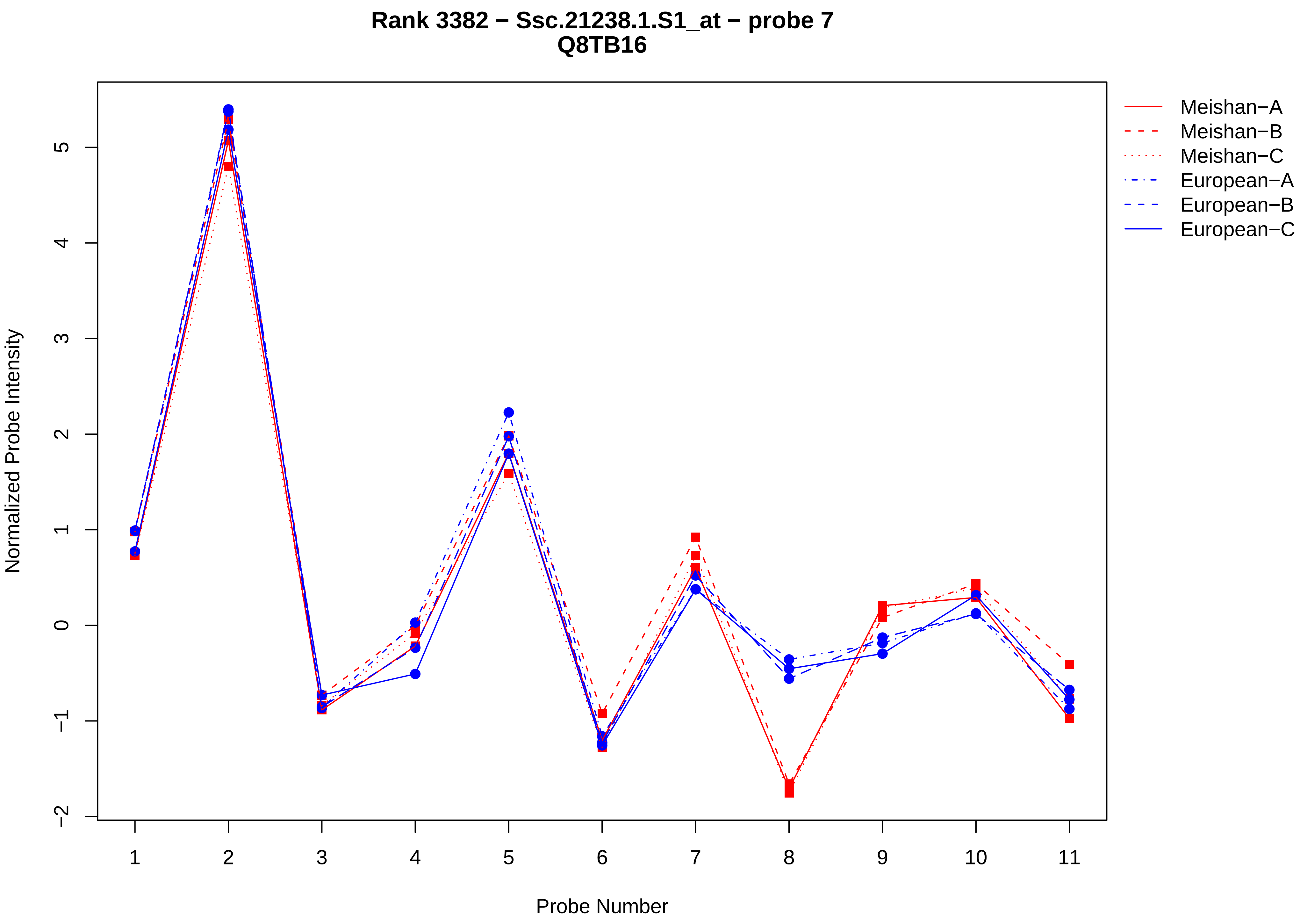

Rank 3382 − Ssc.21238.1.S1_at − probe 7
Q8TB16
Normalized Probe Intensity
Probe Number
Meishan−A
Meishan−B
Meishan−C
European−A
European−B
European−C

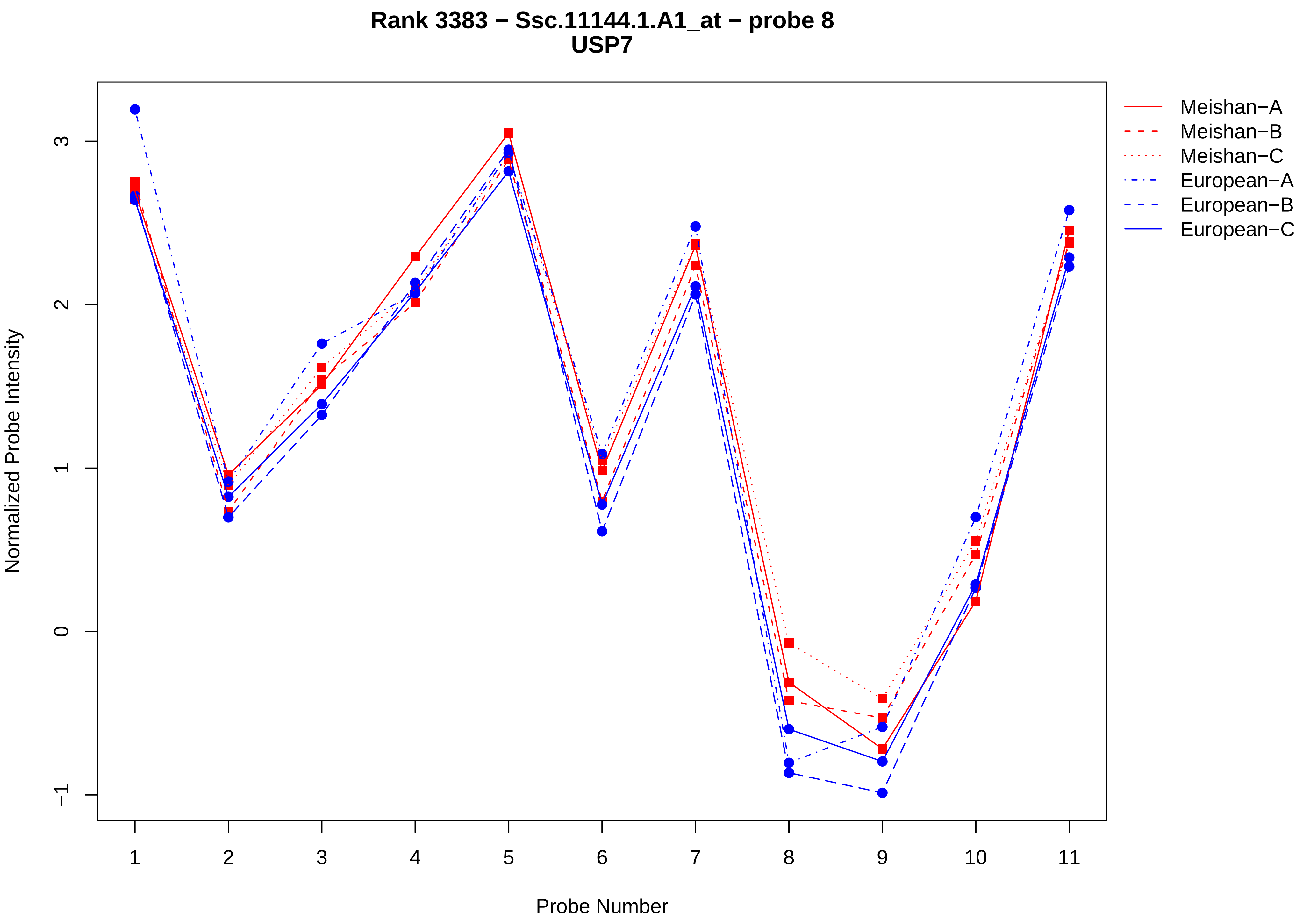

Rank 3383 − Ssc.11144.1.A1_at − probe 8
USP7
Normalized Probe Intensity
Probe Number
Meishan−A
Meishan−B
Meishan−C
European−A
European−B
European−C
−1
0
1
2
3
1
2
3
4
5
6
7
8
9
10
11

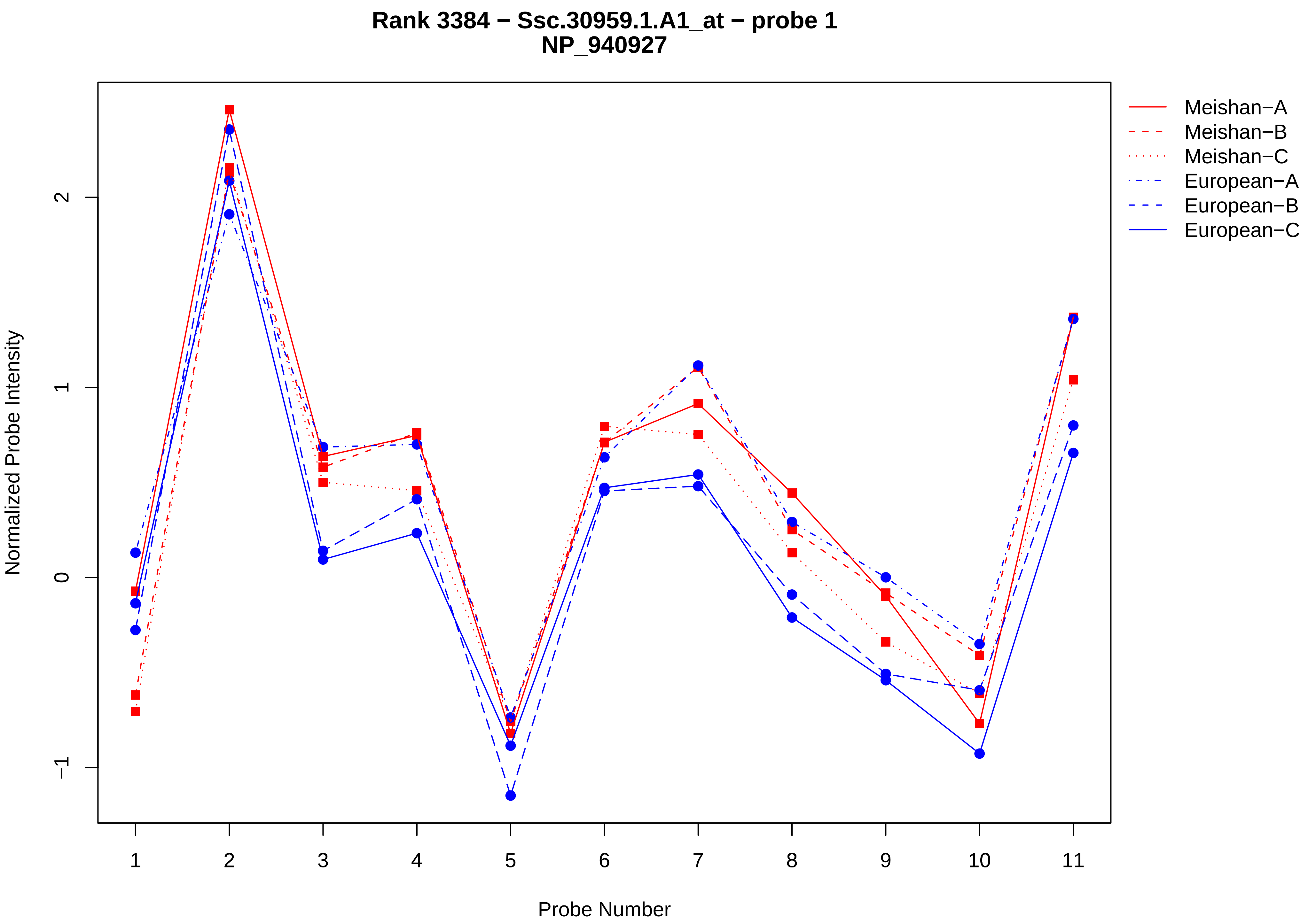
Rank 3384 − Ssc.30959.1.A1_at − probe 1
NP_940927
Normalized Probe Intensity
Probe Number
−1
0
1
2
1
2
3
4
5
6
7
8
9
10
11
Meishan−A
Meishan−B
Meishan−C
European−A
European−B
European−C

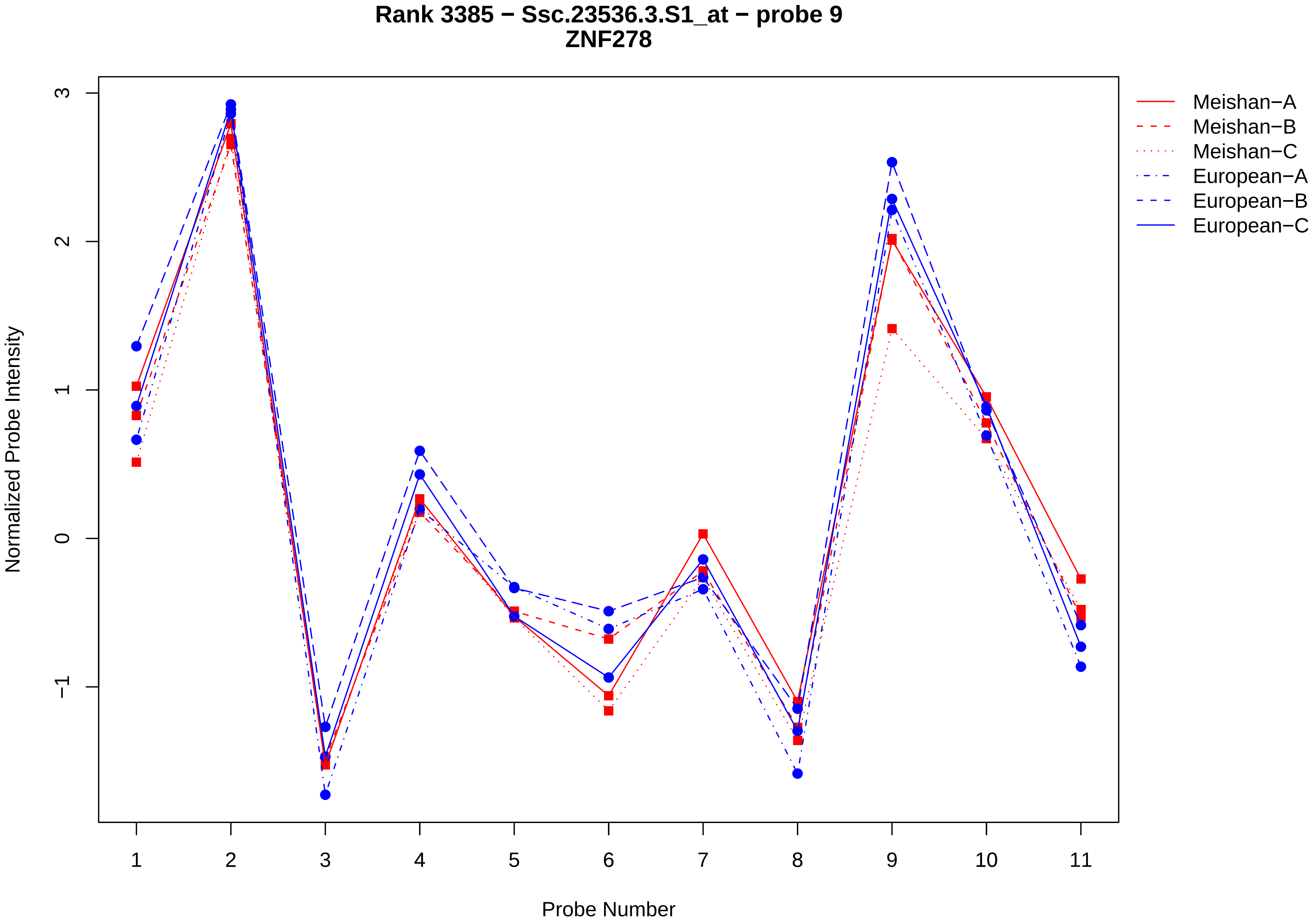

Rank 3385 − Ssc.23536.3.S1_at − probe 9
ZNF278
Normalized Probe Intensity
Probe Number
Meishan−A
Meishan−B
Meishan−C
European−A
European−B
European−C
−1
0
1
2
3
1
2
3
4
5
6
7
8
9
10
11

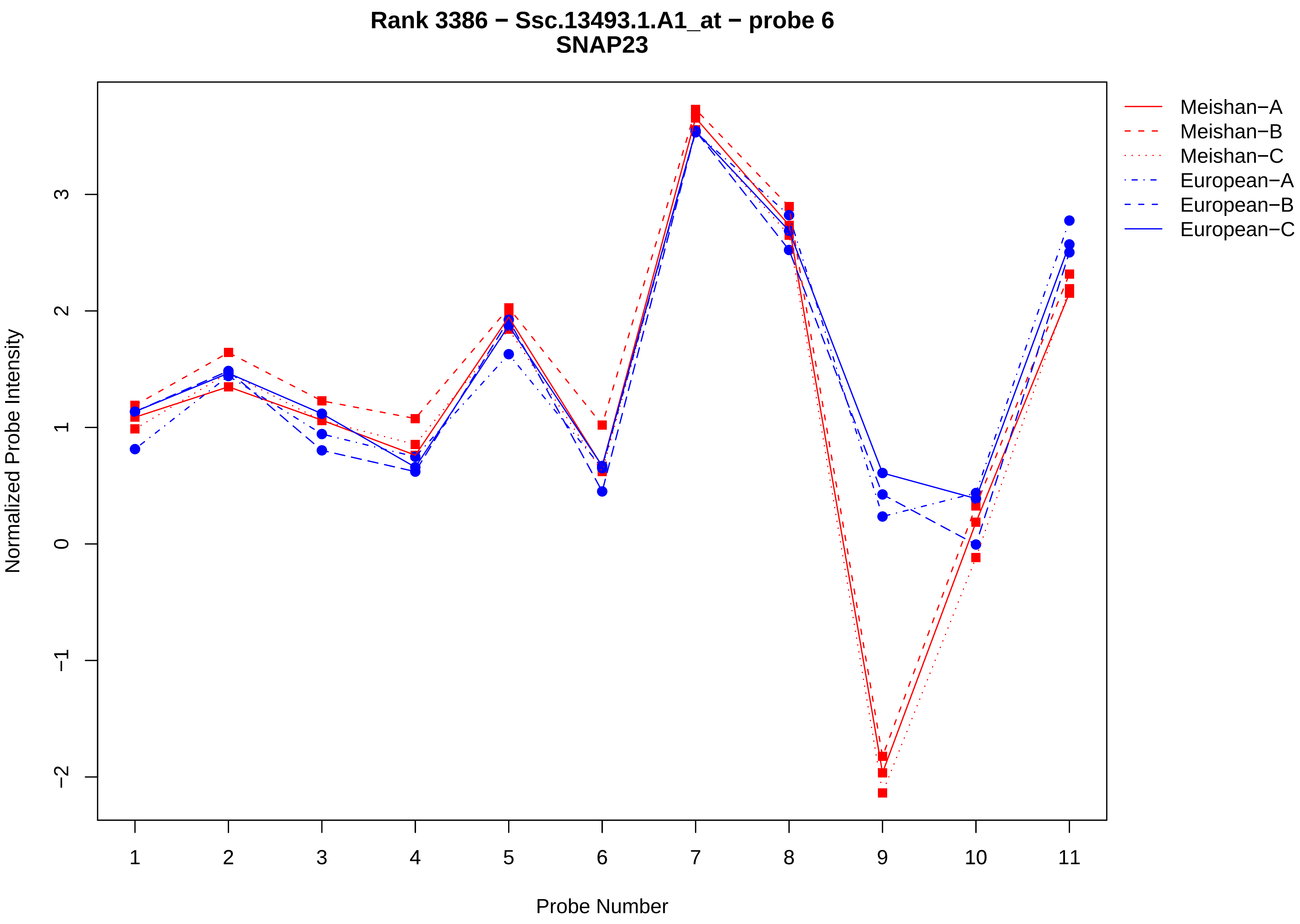

Rank 3386 − Ssc.13493.1.A1_at − probe 6
SNAP23
Normalized Probe Intensity
Probe Number
Meishan−A
Meishan−B
Meishan−C
European−A
European−B
European−C
−2
−1
0
1
2
3
1
2
3
4
5
6
7
8
9
10
11

Rank 3387 − Ssc.2191.1.S1_at − probe 6
APRT

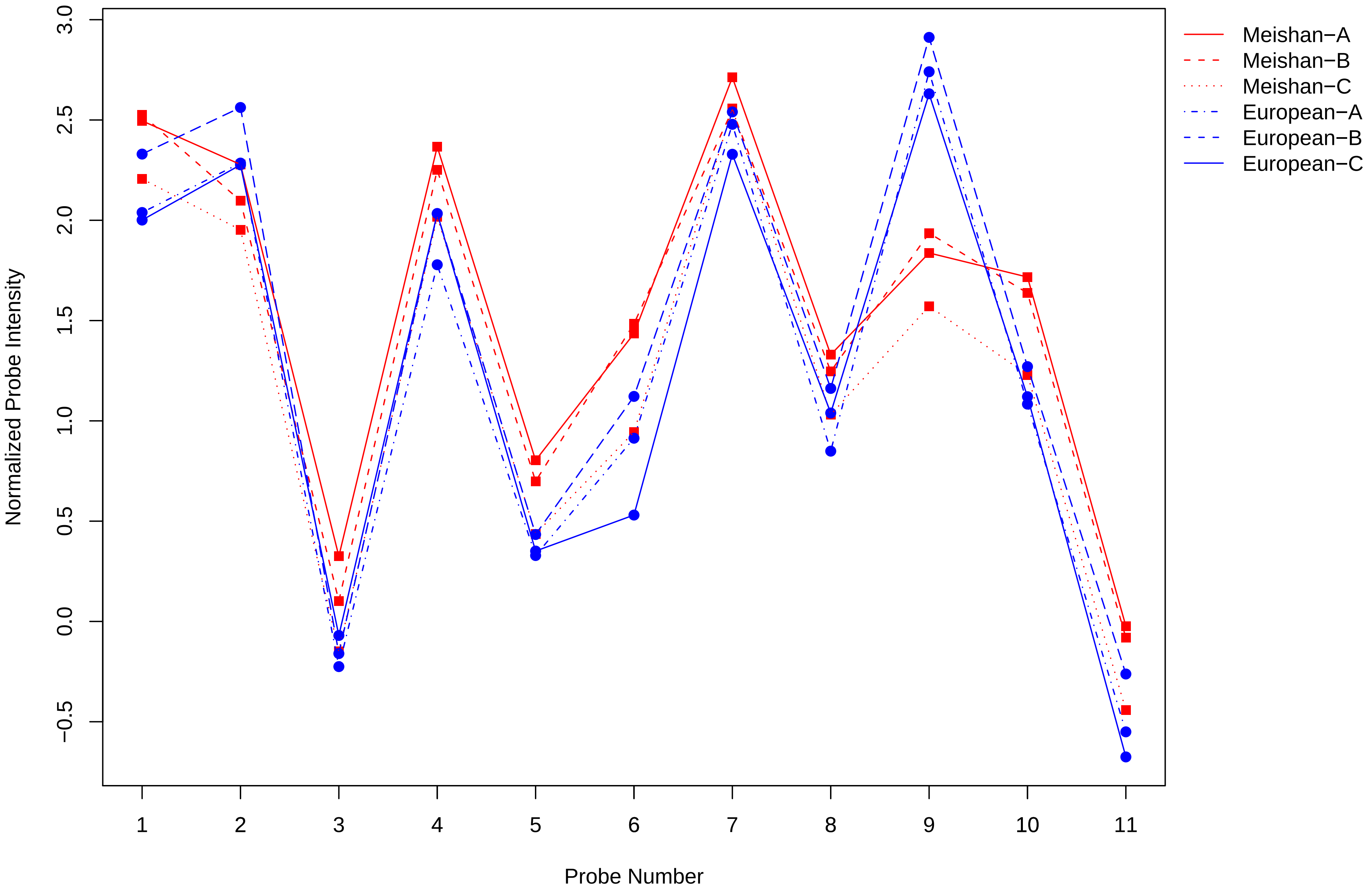

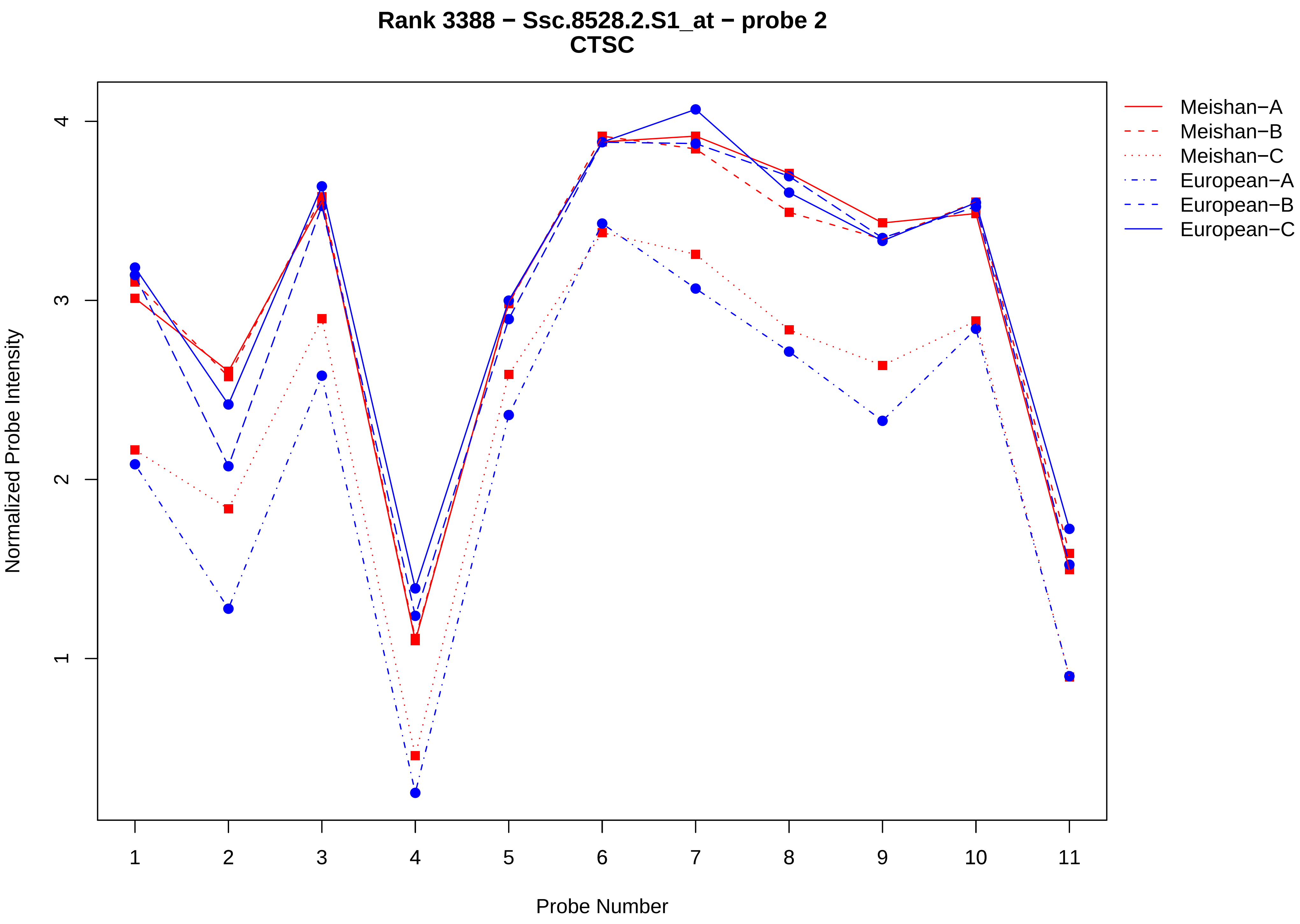

Rank 3388 − Ssc.8528.2.S1_at − probe 2
CTSC
Normalized Probe Intensity
Probe Number
1
2
3
4
1
2
3
4
5
6
7
8
9
10
11
Meishan−A
Meishan−B
Meishan−C
European−A
European−B
European−C

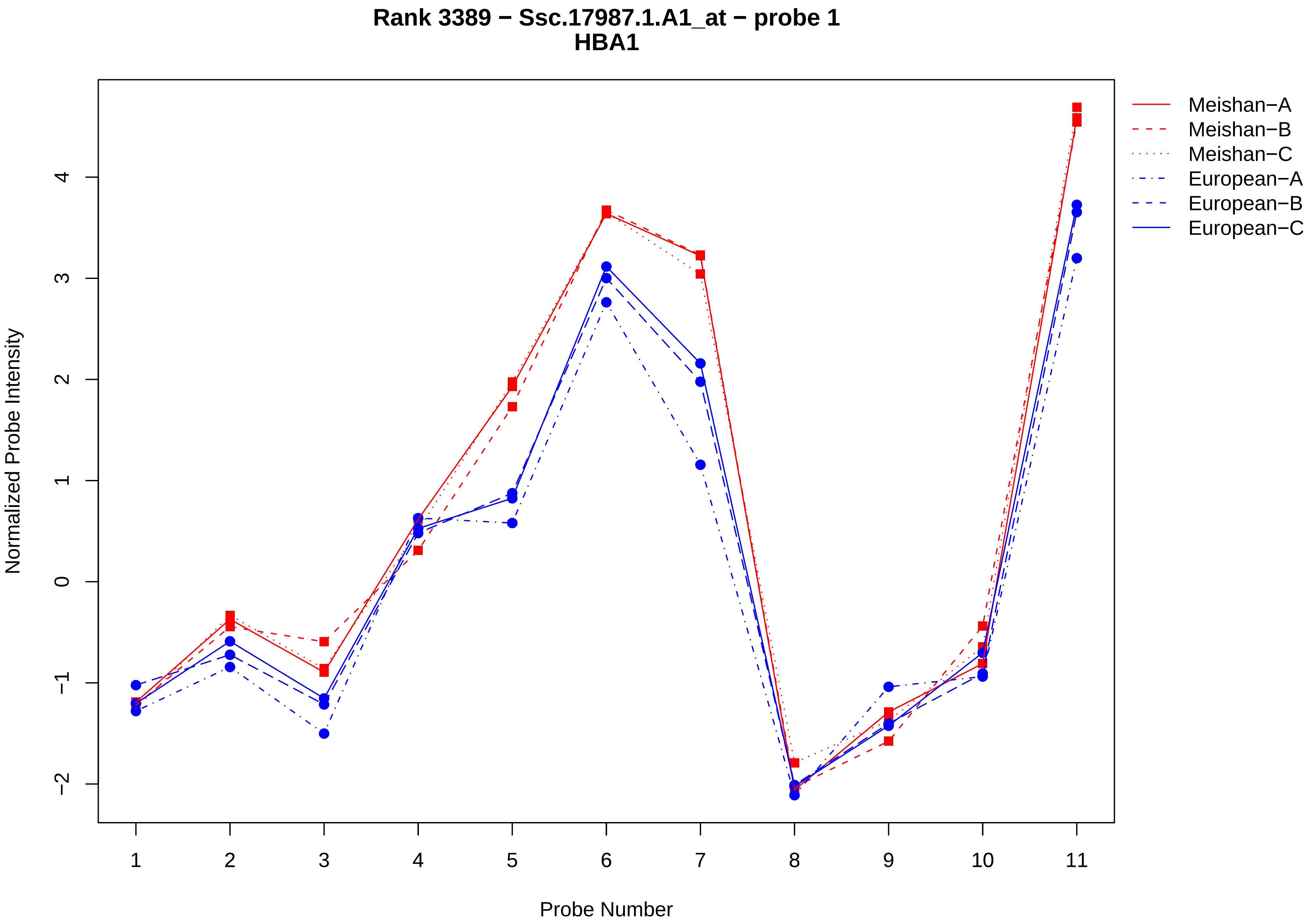

Rank 3389 − Ssc.17987.1.A1_at − probe 1
HBA1
Normalized Probe Intensity
Probe Number
−2
−1
0
1
2
3
4
1
2
3
4
5
6
7
8
9
10
11
Meishan−A
Meishan−B
Meishan−C
European−A
European−B
European−C

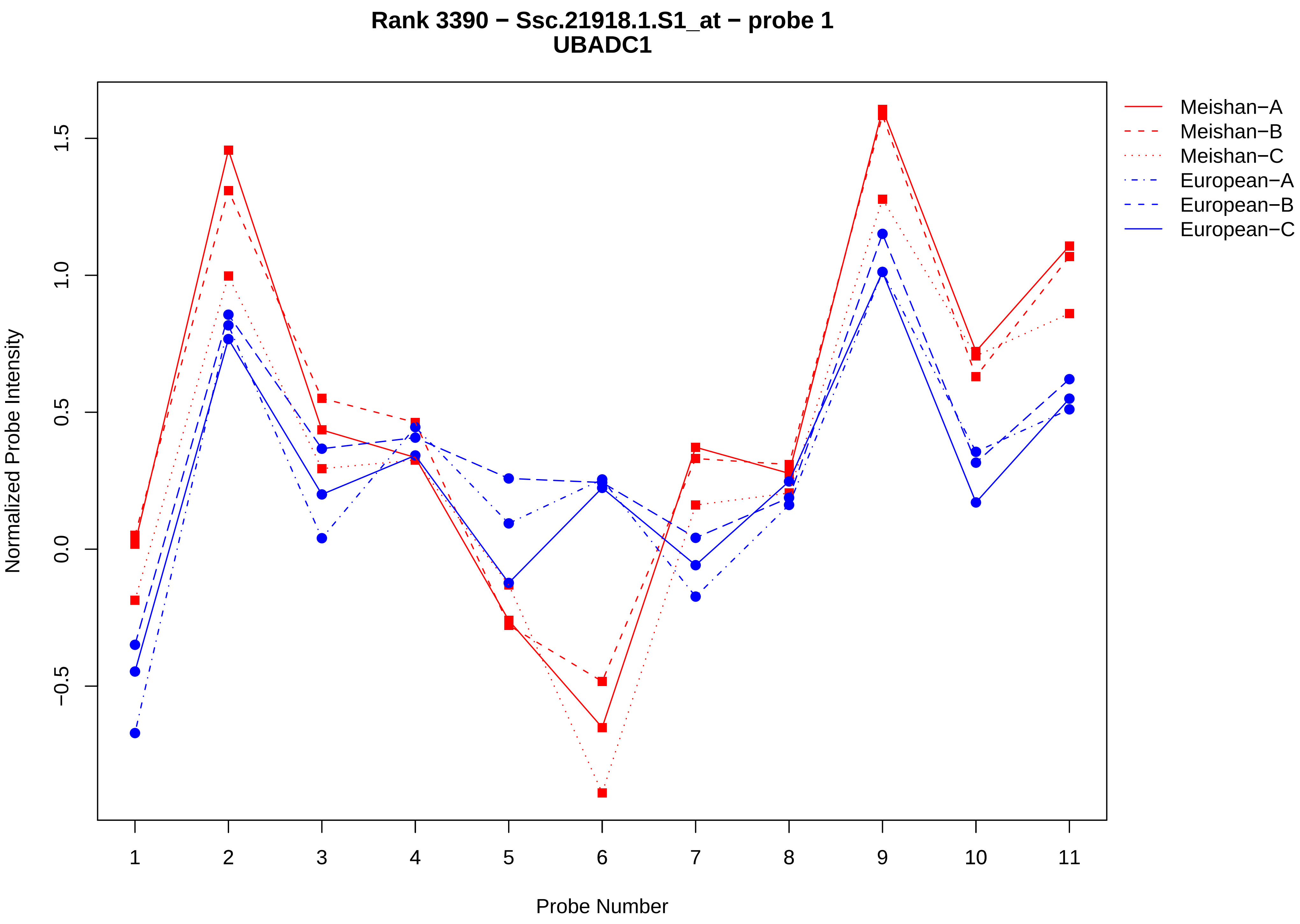

Rank 3390 − Ssc.21918.1.S1_at − probe 1
UBADC1
Normalized Probe Intensity
Probe Number
−0.5
0.0
0.5
1.0
1.5
1
2
3
4
5
6
7
8
9
10
11
Meishan−A
Meishan−B
Meishan−C
European−A
European−B
European−C

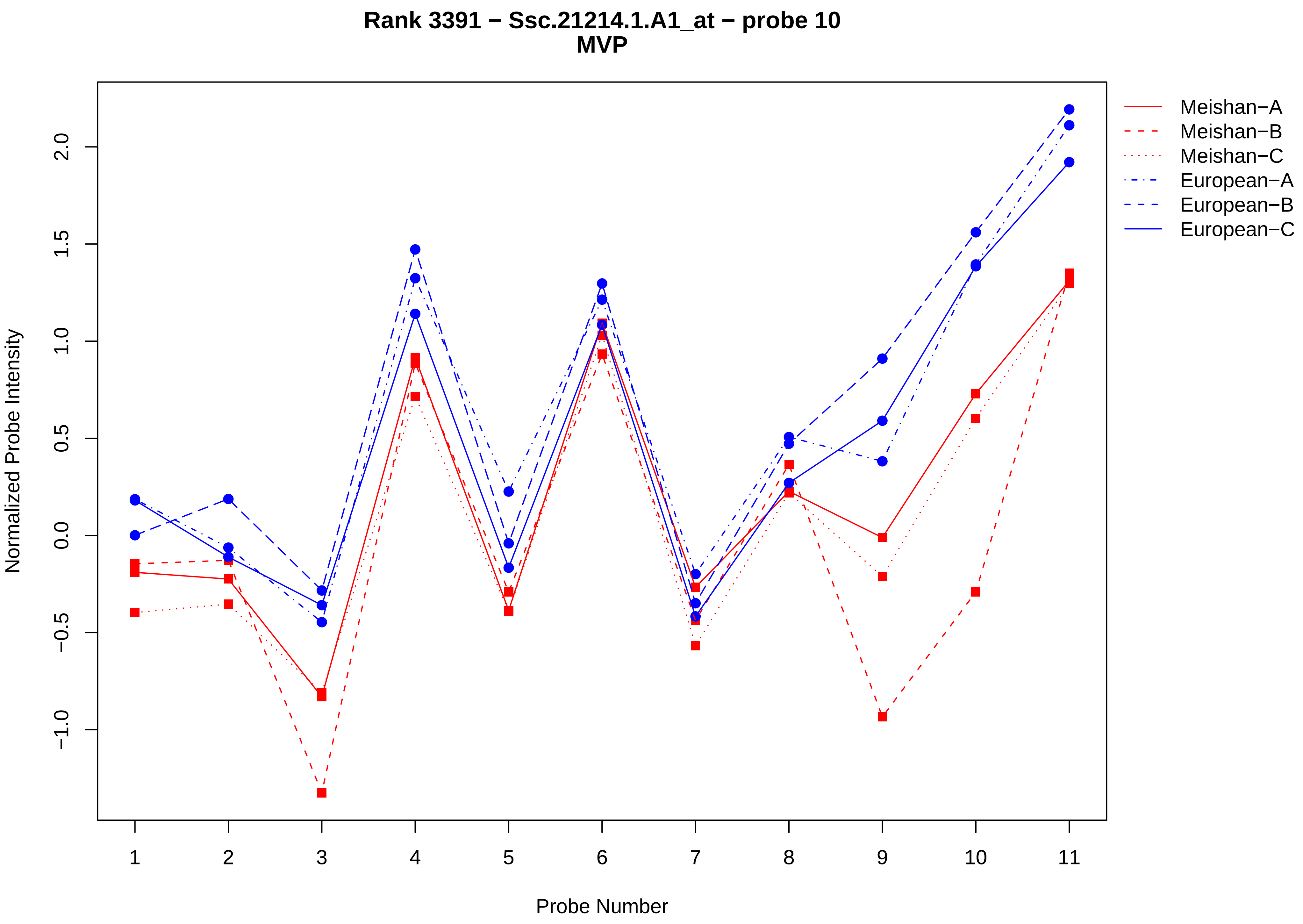

Rank 3391 − Ssc.21214.1.A1_at − probe 10
MVP
Normalized Probe Intensity
Probe Number
Meishan−A
Meishan−B
Meishan−C
European−A
European−B
European−C

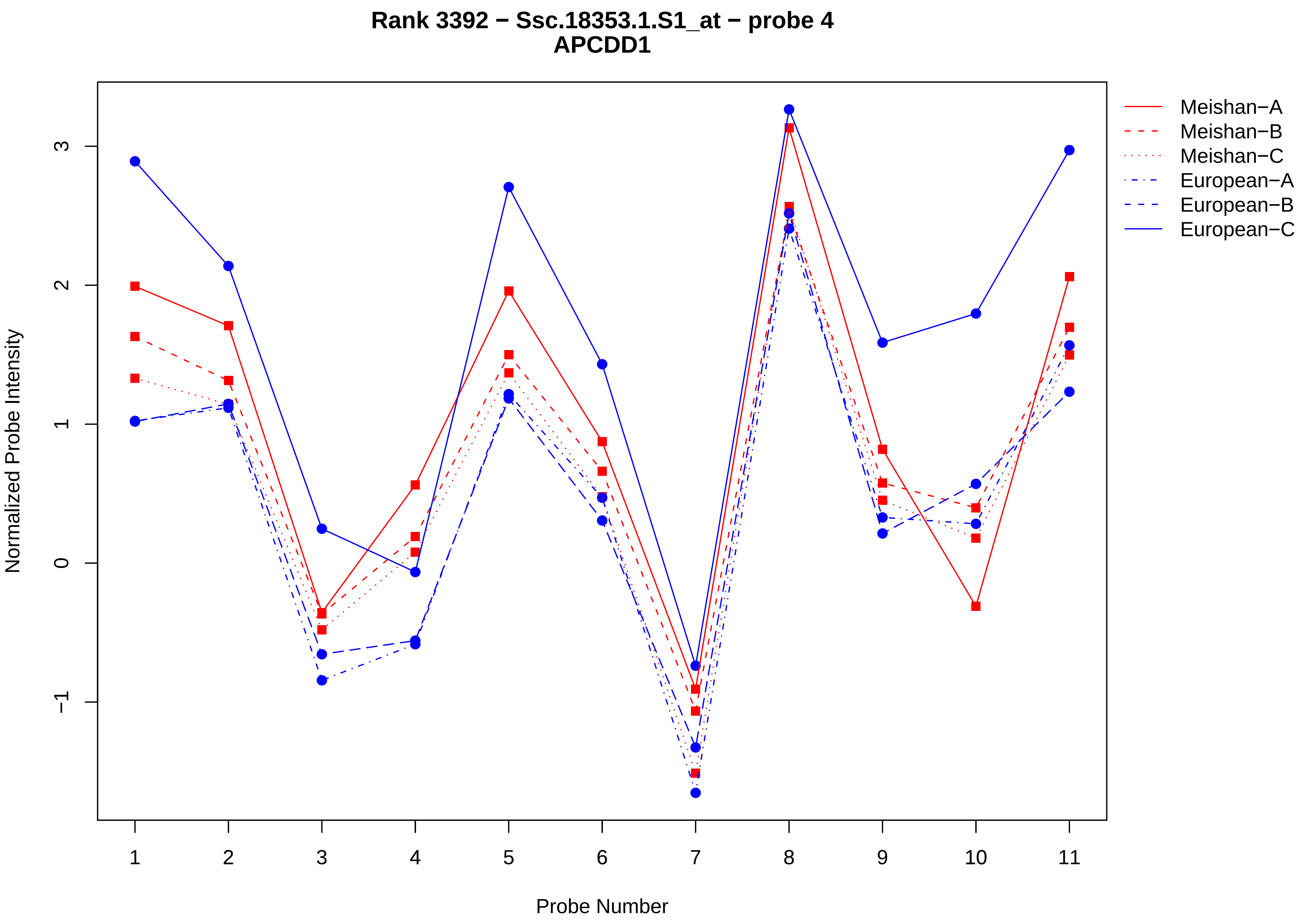
Rank 3392 − Ssc.18353.1.S1_at − probe 4
APCDD1
Normalized Probe Intensity
Probe Number
−1
0
1
2
3
1
2
3
4
5
6
7
8
9
10
11
Meishan−A
Meishan−B
Meishan−C
European−A
European−B
European−C

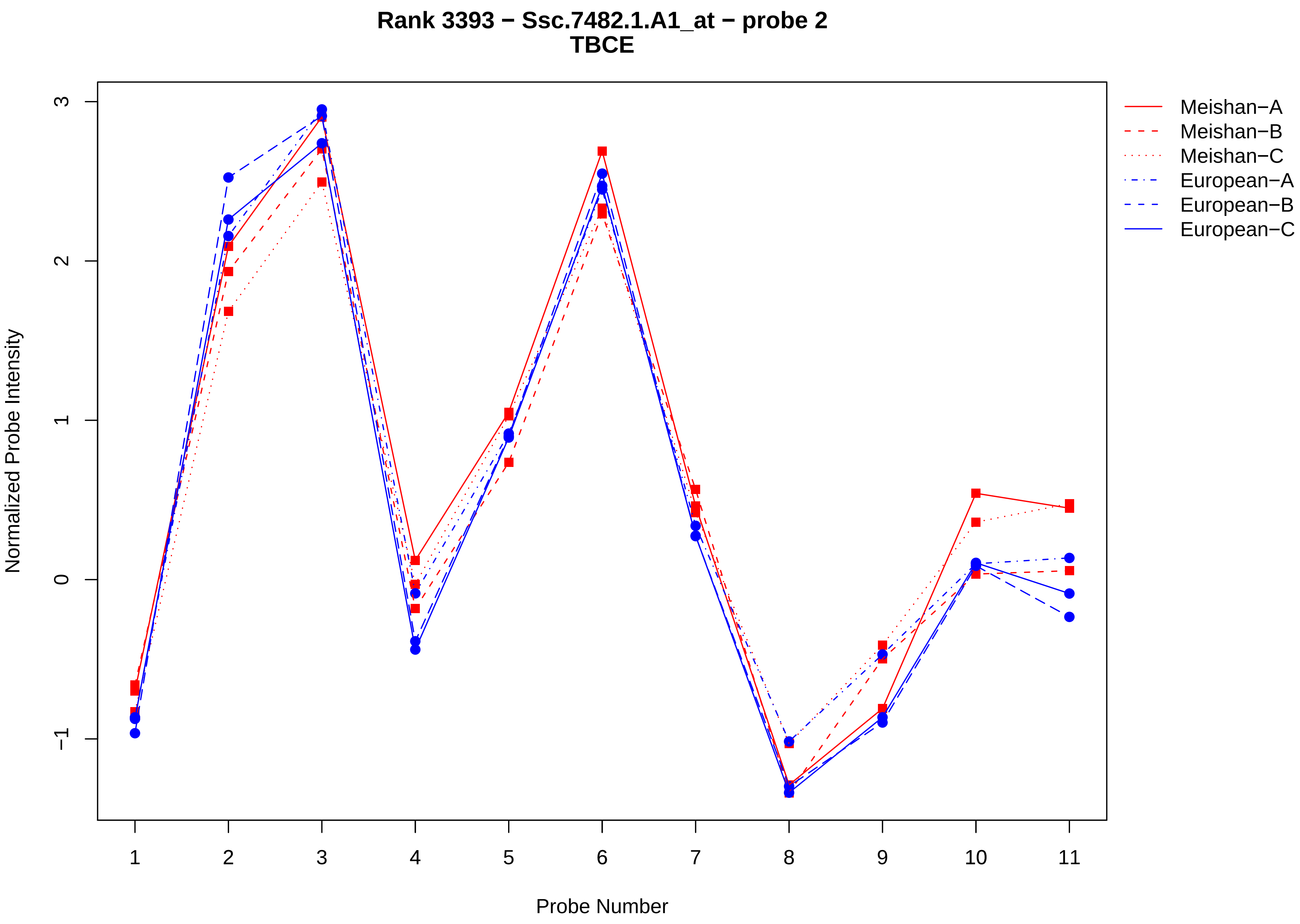

Rank 3393 − Ssc.7482.1.A1_at − probe 2
TBCE
Normalized Probe Intensity
Probe Number
Meishan−A
Meishan−B
Meishan−C
European−A
European−B
European−C

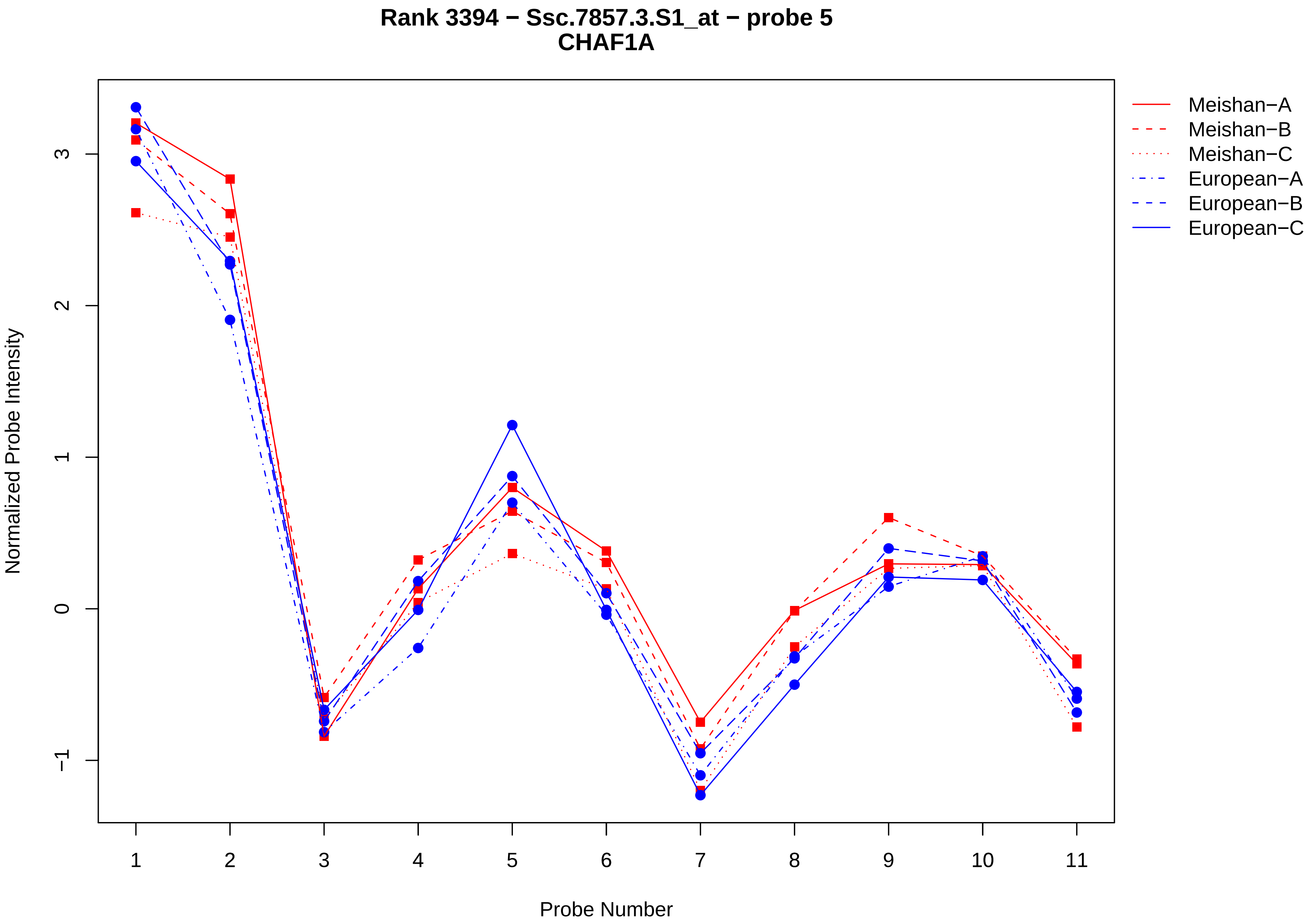

Rank 3394 − Ssc.7857.3.S1_at − probe 5
CHAF1A
Normalized Probe Intensity
Probe Number
−1
0
1
2
3
1
2
3
4
5
6
7
8
9
10
11
Meishan−A
Meishan−B
Meishan−C
European−A
European−B
European−C

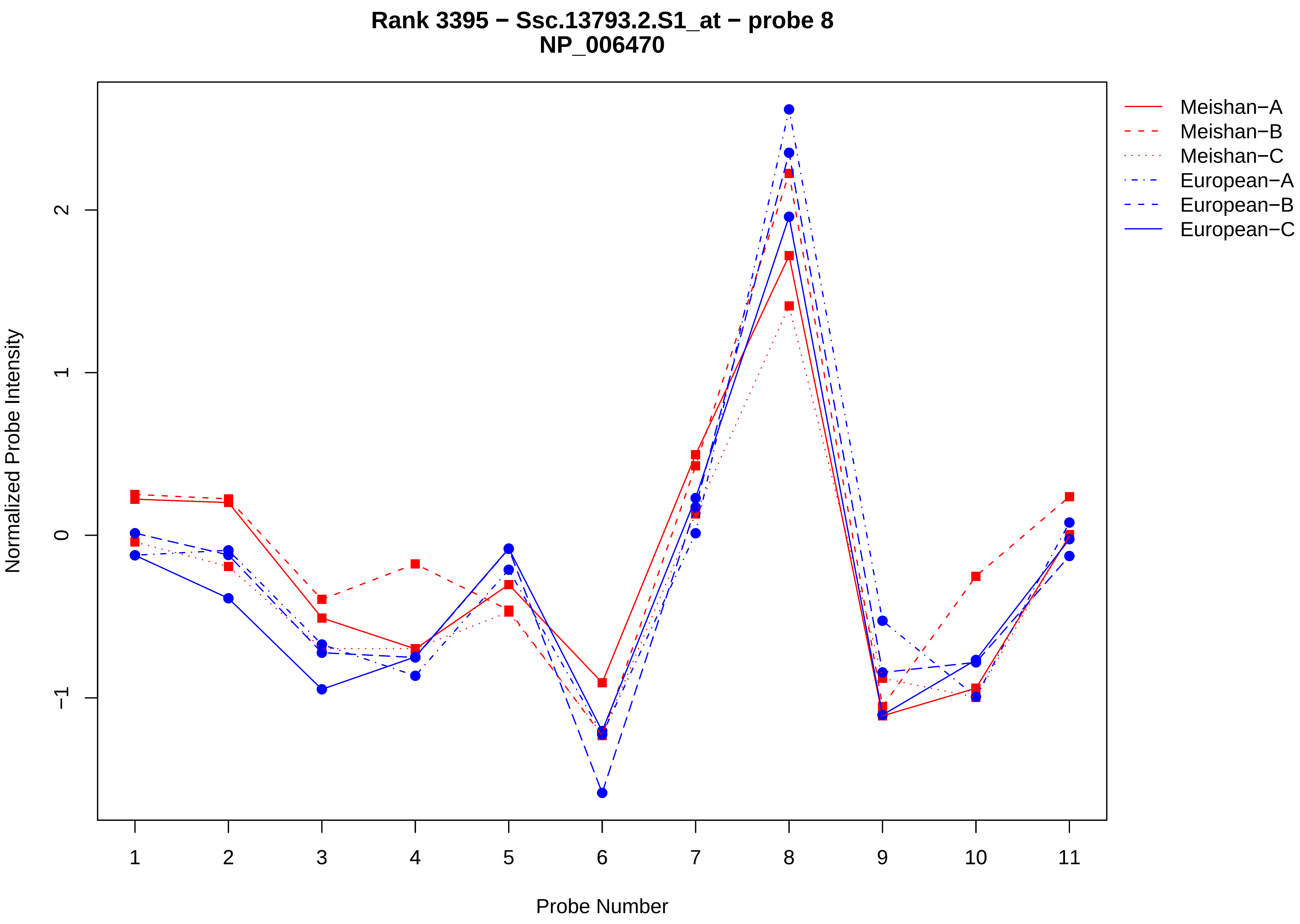

Rank 3395 − Ssc.13793.2.S1_at − probe 8
NP_006470
Normalized Probe Intensity
Probe Number
−1
0
1
2
1
2
3
4
5
6
7
8
9
10
11
Meishan−A
Meishan−B
Meishan−C
European−A
European−B
European−C

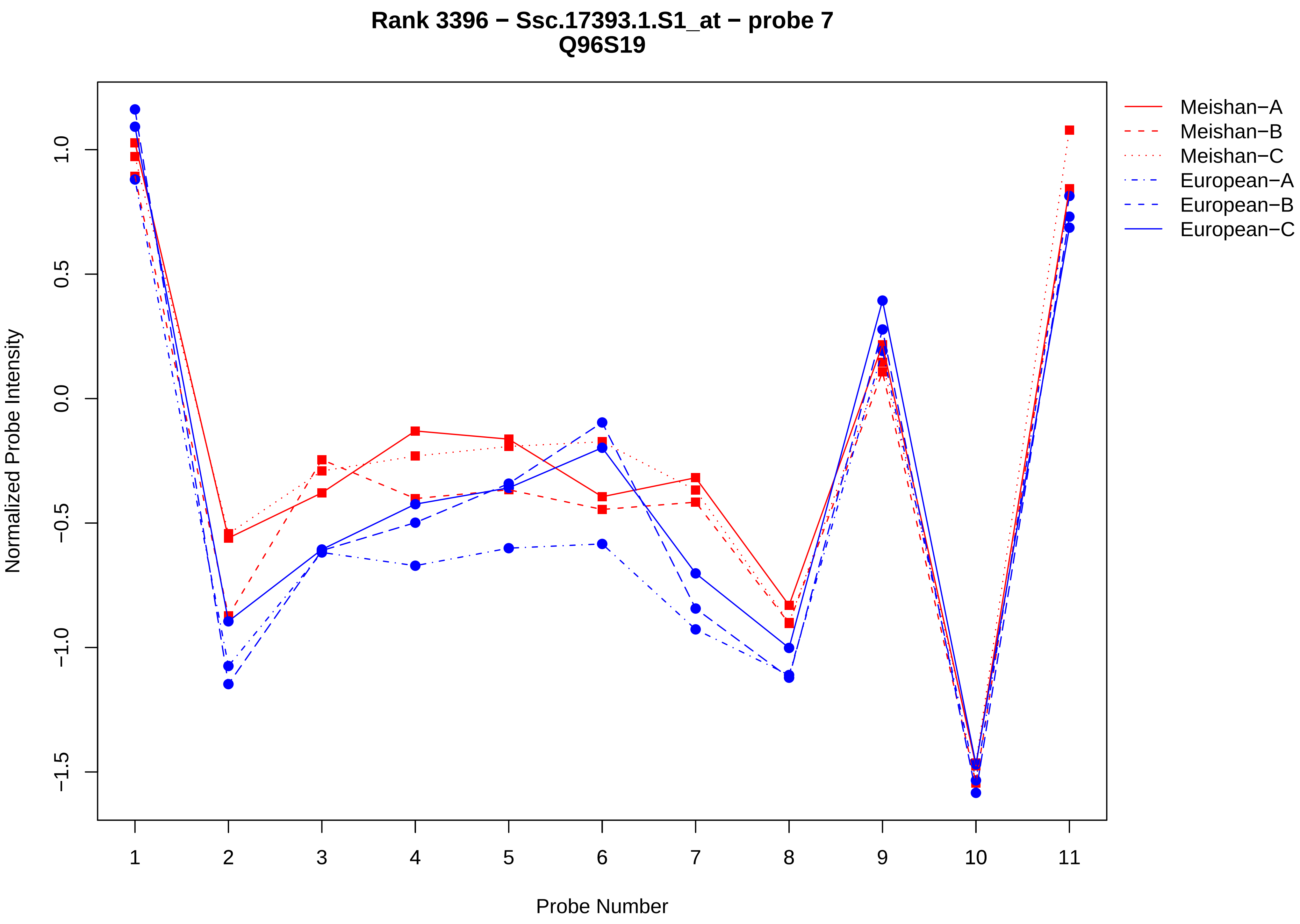

Rank 3396 − Ssc.17393.1.S1_at − probe 7
Q96S19
Normalized Probe Intensity
Probe Number
1.0
0.5
0.0
−0.5
−1.0
−1.5
1
2
3
4
5
6
7
8
9
10
11
Meishan−A
Meishan−B
Meishan−C
European−A
European−B
European−C

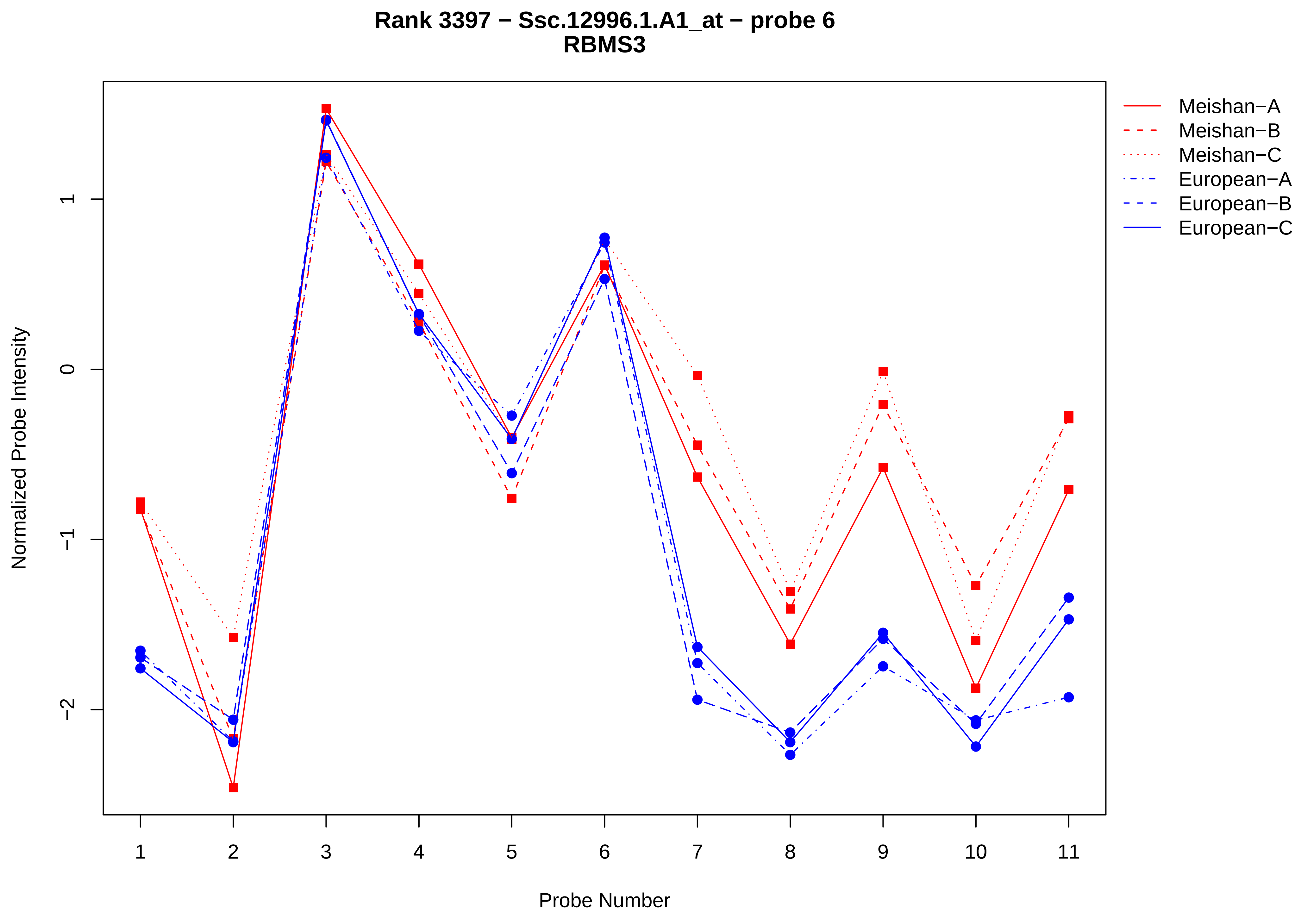
Rank 3397 − Ssc.12996.1.A1_at − probe 6
RBMS3
Normalized Probe Intensity
−2
−1
0
1
1
2
3
4
5
6
7
8
9
10
11
Probe Number
Meishan−A
Meishan−B
Meishan−C
European−A
European−B
European−C

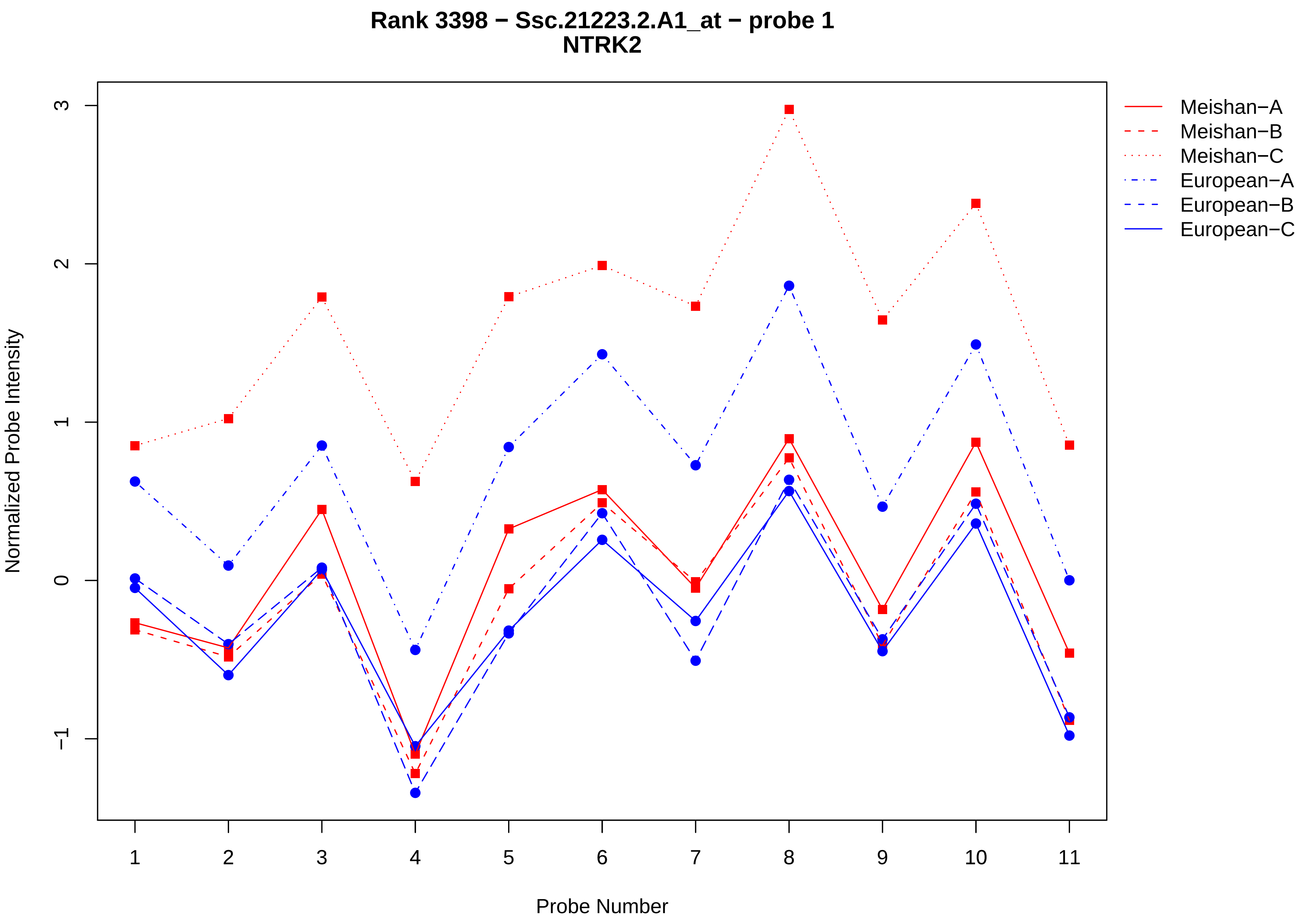

Rank 3398 − Ssc.21223.2.A1_at − probe 1
NTRK2
Normalized Probe Intensity
Probe Number
Meishan−A
Meishan−B
Meishan−C
European−A
European−B
European−C
−1
0
1
2
3
1
2
3
4
5
6
7
8
9
10
11

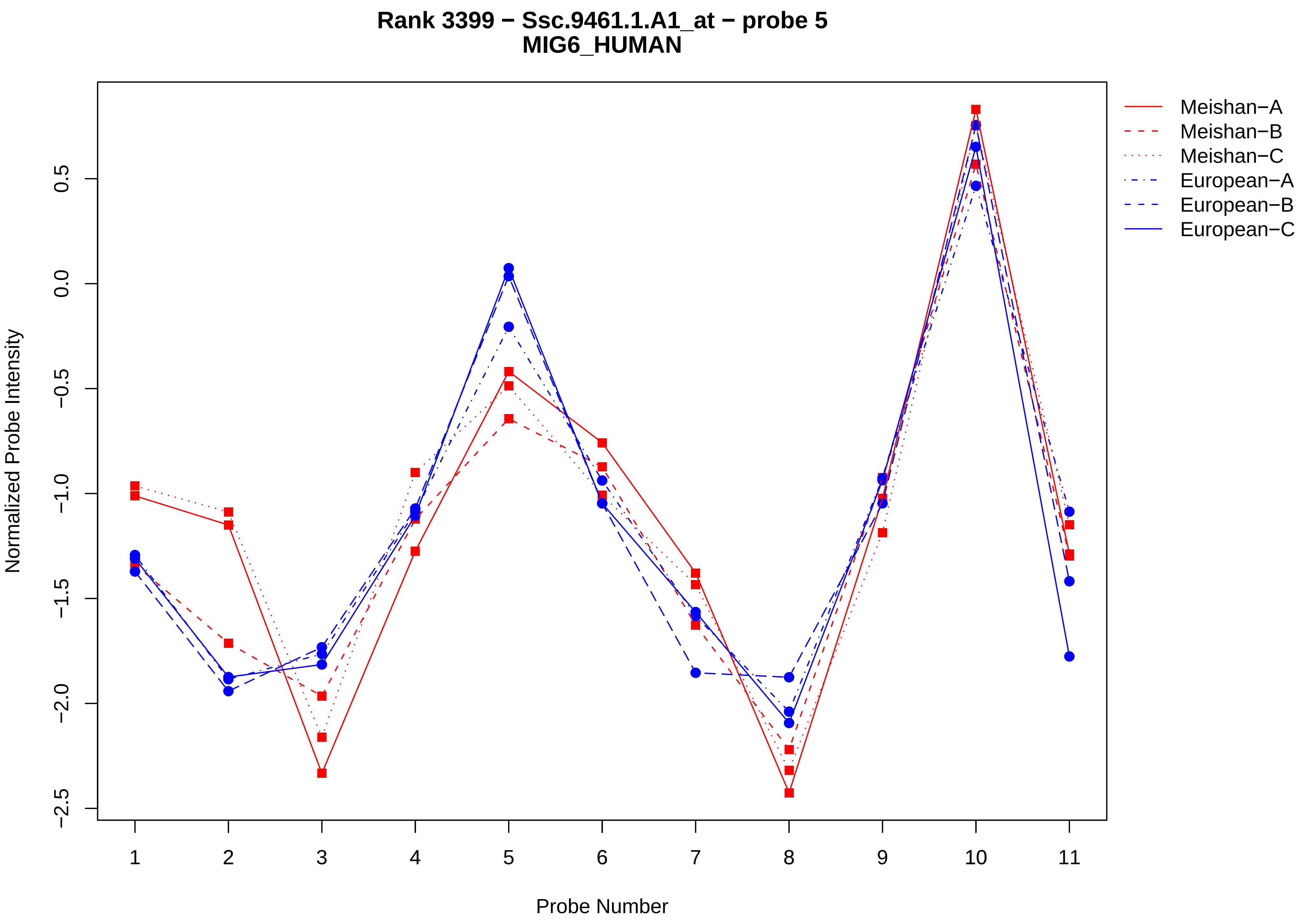

Rank 3399 − Ssc.9461.1.A1_at − probe 5
MIG6_HUMAN
Normalized Probe Intensity
Probe Number
Meishan−A
Meishan−B
Meishan−C
European−A
European−B
European−C

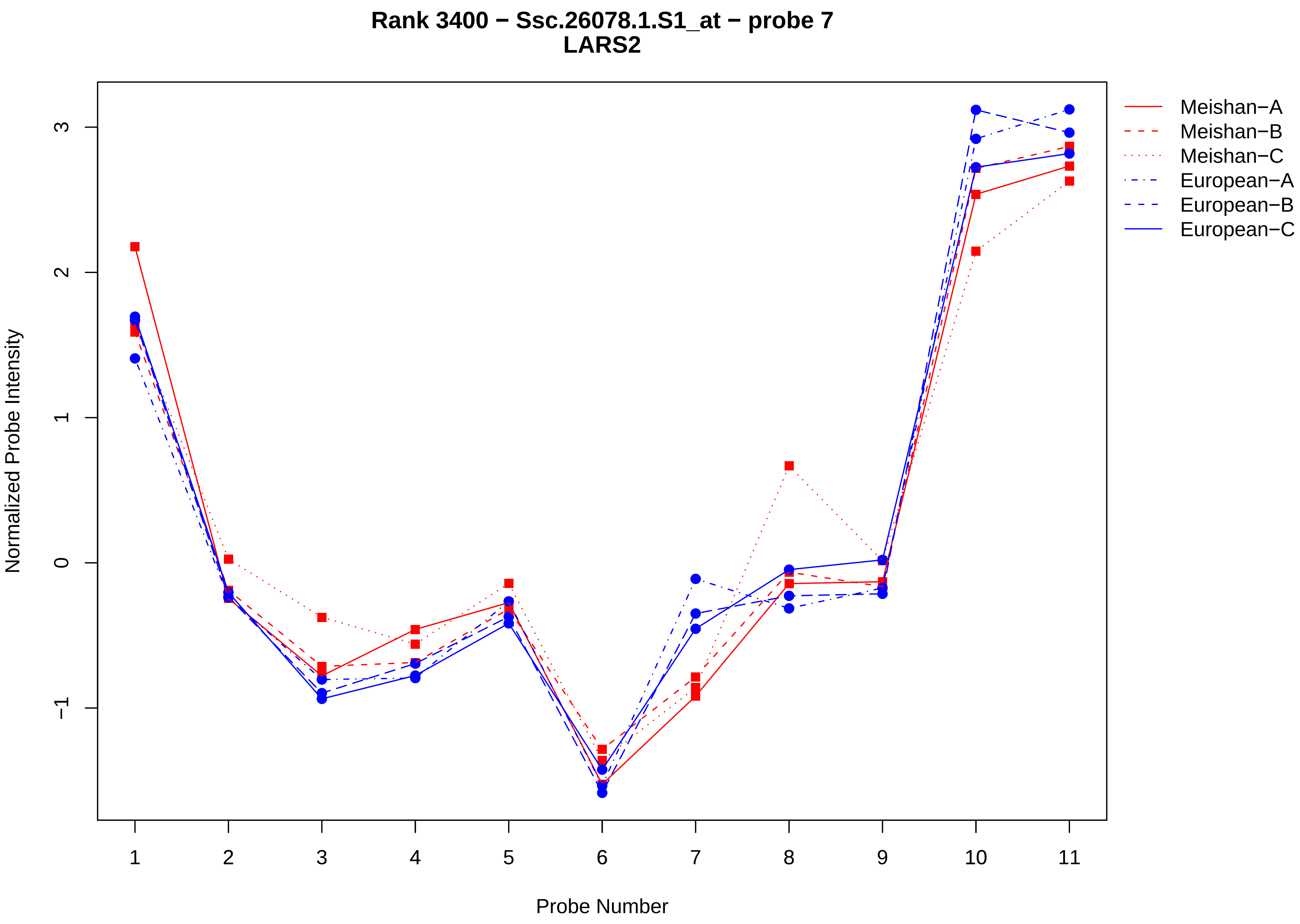

Rank 3400 − Ssc.26078.1.S1_at − probe 7
LARS2
Normalized Probe Intensity
Probe Number
−1
0
1
2
3
1
2
3
4
5
6
7
8
9
10
11
Meishan−A
Meishan−B
Meishan−C
European−A
European−B
European−C

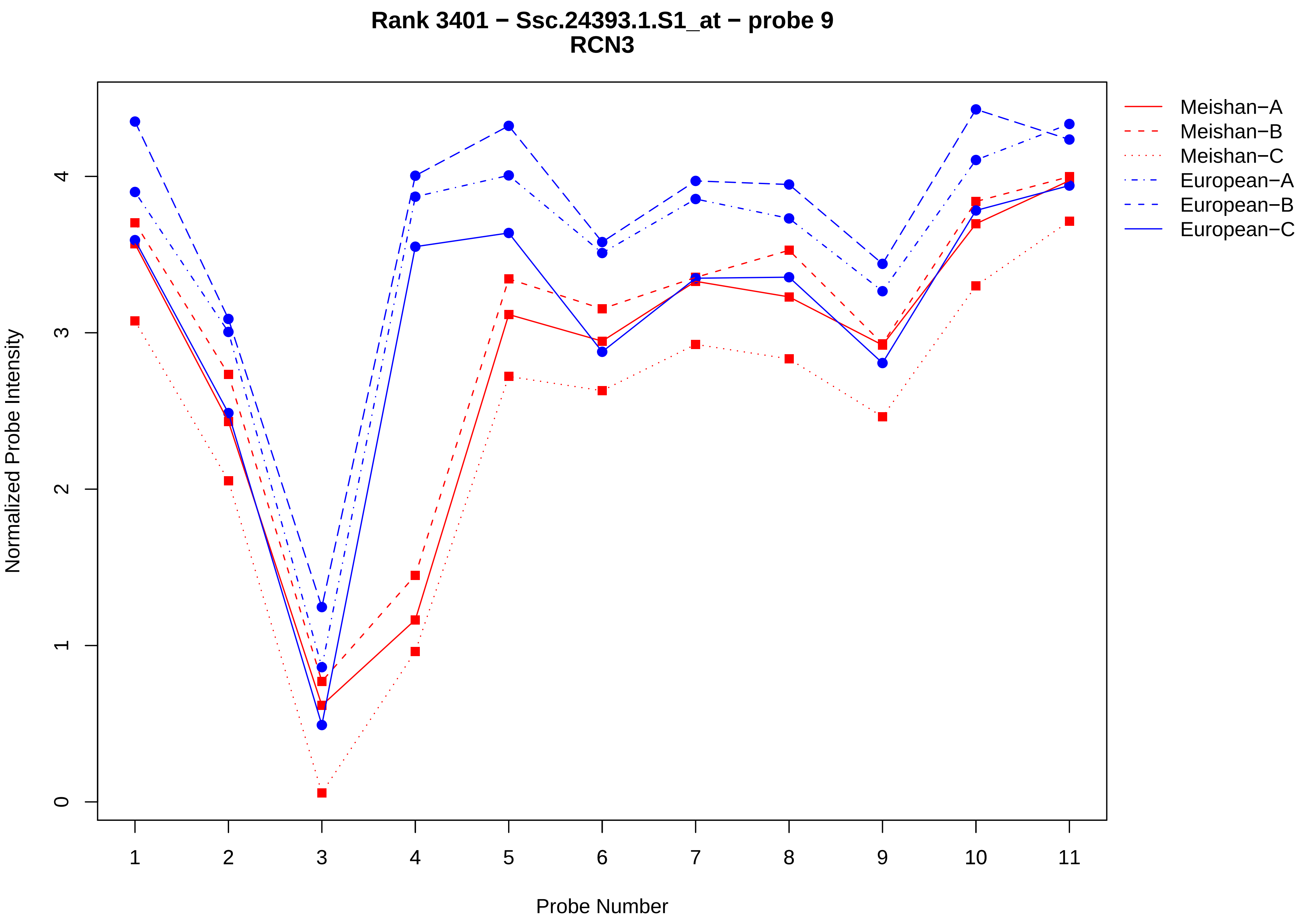
Rank 3401 − Ssc.24393.1.S1_at − probe 9
RCN3
Normalized Probe Intensity
Probe Number
0
1
2
3
4
1
2
3
4
5
6
7
8
9
10
11
Meishan−A
Meishan−B
Meishan−C
European−A
European−B
European−C

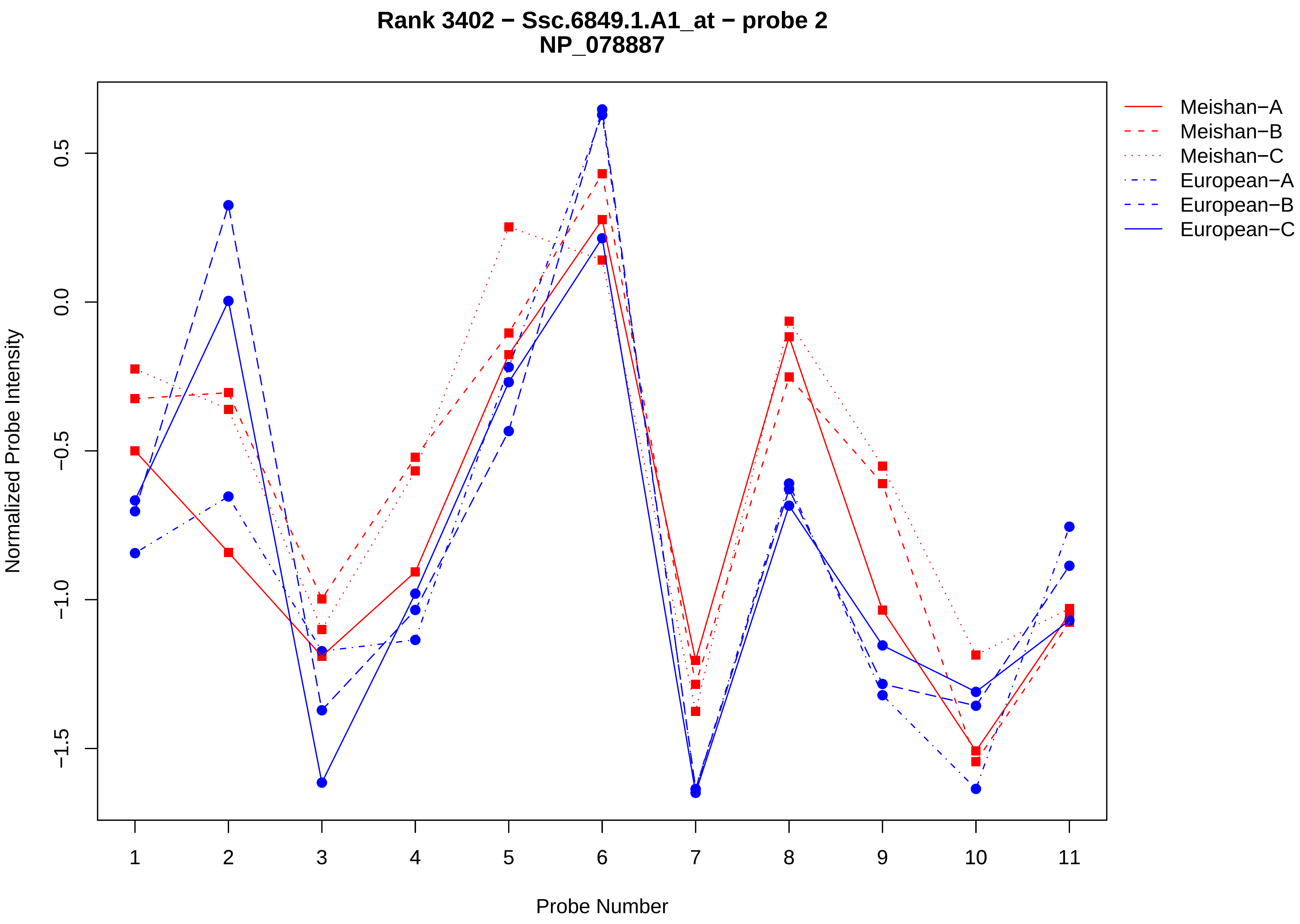

Rank 3402 − Ssc.6849.1.A1_at − probe 2
NP_078887
Normalized Probe Intensity
Probe Number
0.5
0.0
−0.5
−1.0
−1.5
1
2
3
4
5
6
7
8
9
10
11
Meishan−A
Meishan−B
Meishan−C
European−A
European−B
European−C

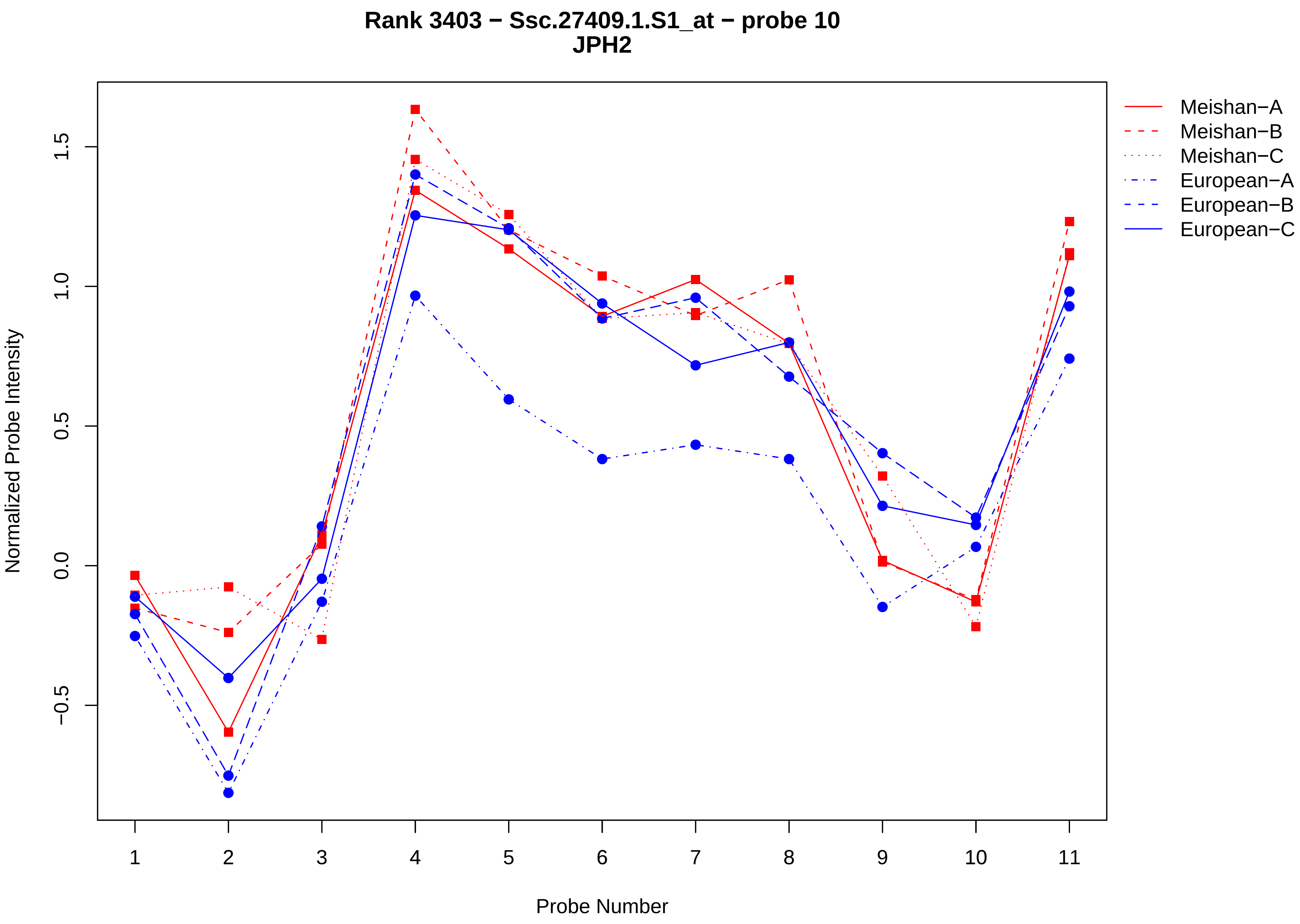

Rank 3403 − Ssc.27409.1.S1_at − probe 10
JPH2
Normalized Probe Intensity
Probe Number
−0.5
0.0
0.5
1.0
1.5
1
2
3
4
5
6
7
8
9
10
11
Meishan−A
Meishan−B
Meishan−C
European−A
European−B
European−C

Rank 3404 − Ssc.6579.1.S1_at − probe 11
NP_055412

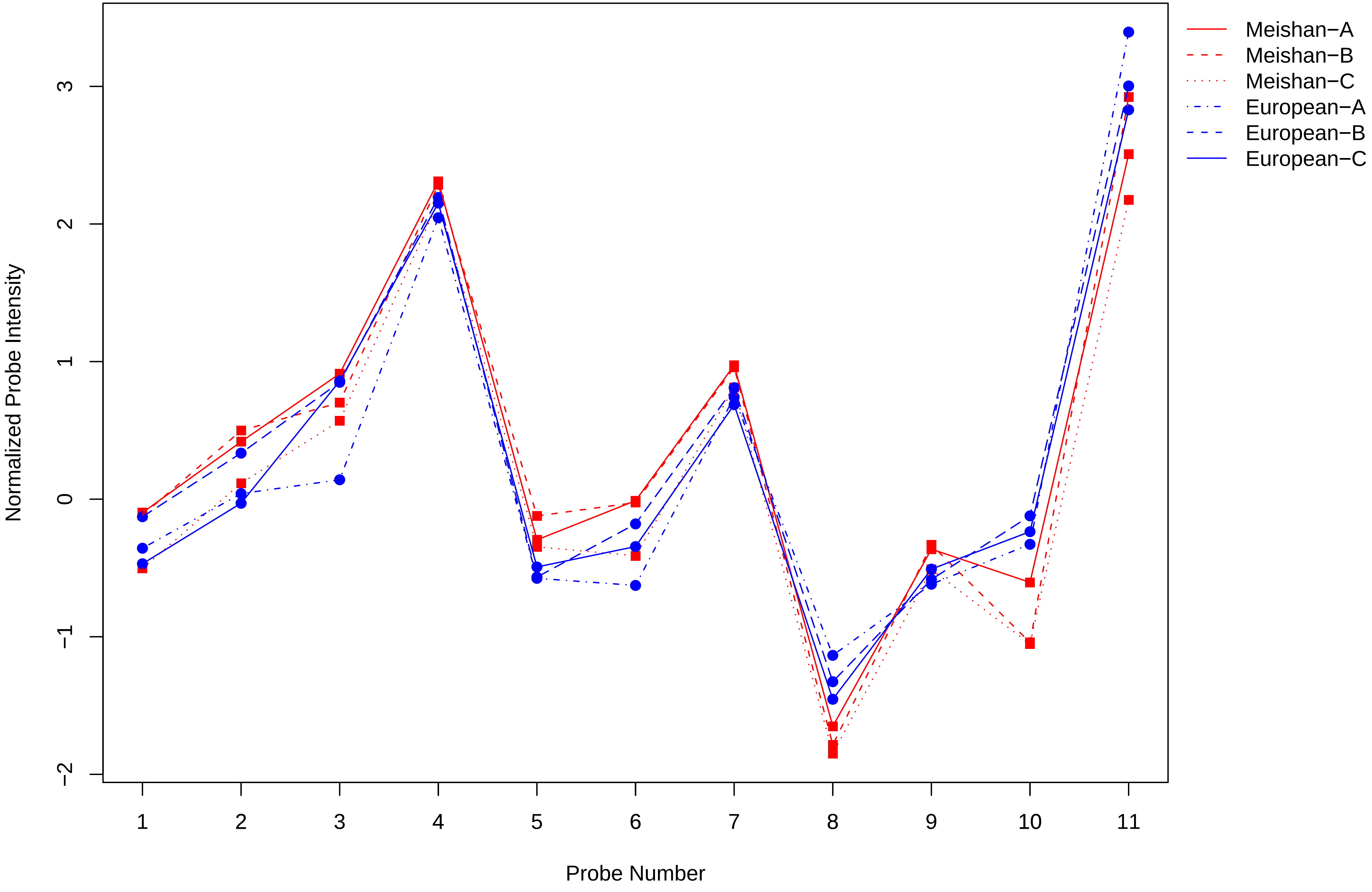

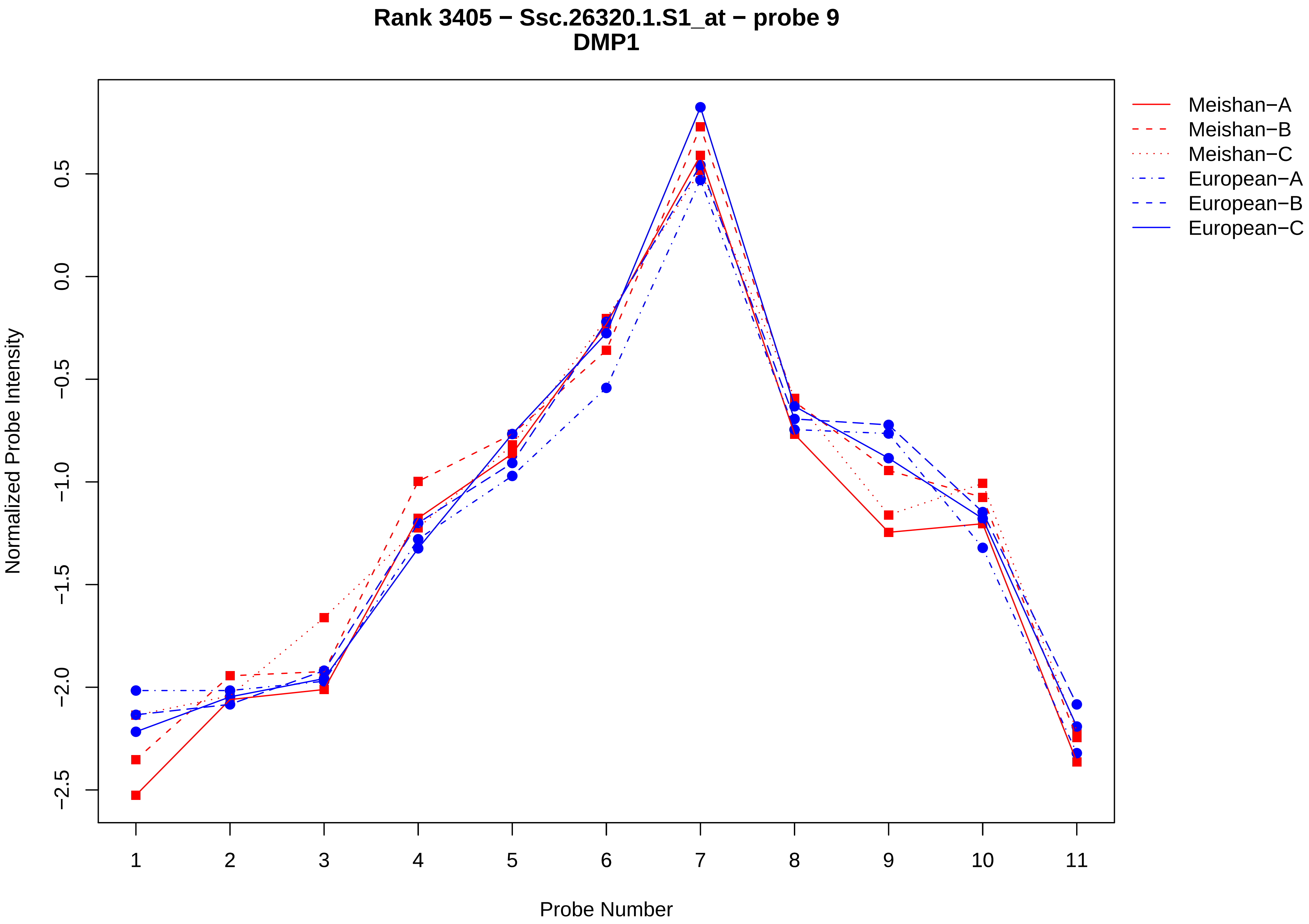

Rank 3405 − Ssc.26320.1.S1_at − probe 9
DMP1
Normalized Probe Intensity
Probe Number
0.5
0.0
−0.5
−1.0
−1.5
−2.0
−2.5
1
2
3
4
5
6
7
8
9
10
11
Meishan−A
Meishan−B
Meishan−C
European−A
European−B
European−C

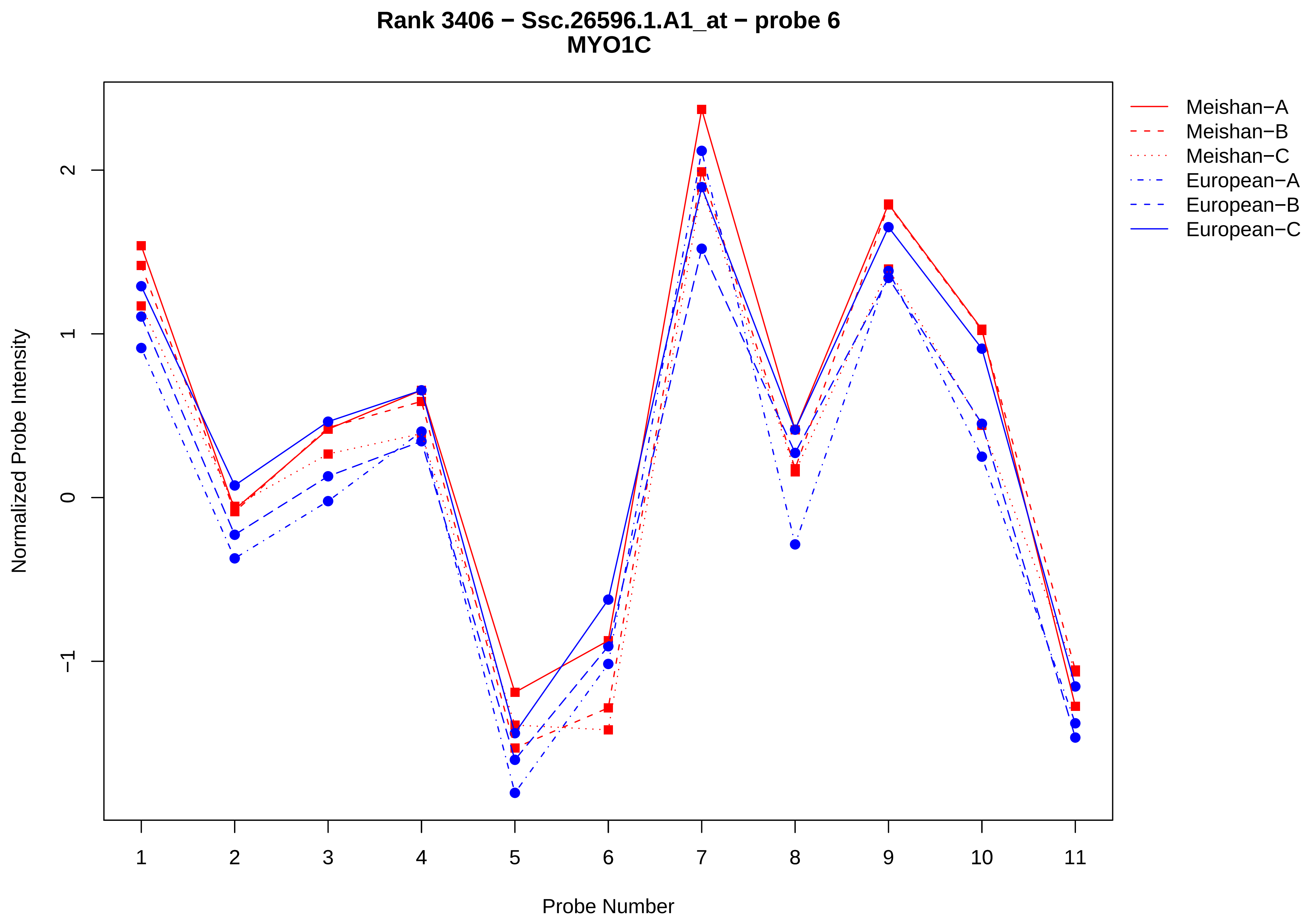

Rank 3406 − Ssc.26596.1.A1_at − probe 6
MYO1C
Normalized Probe Intensity
Probe Number
−1
0
1
2
1
2
3
4
5
6
7
8
9
10
11
Meishan−A
Meishan−B
Meishan−C
European−A
European−B
European−C

Rank 3407 − Ssc.12032.1.S1_at − probe 5
PLXNA4

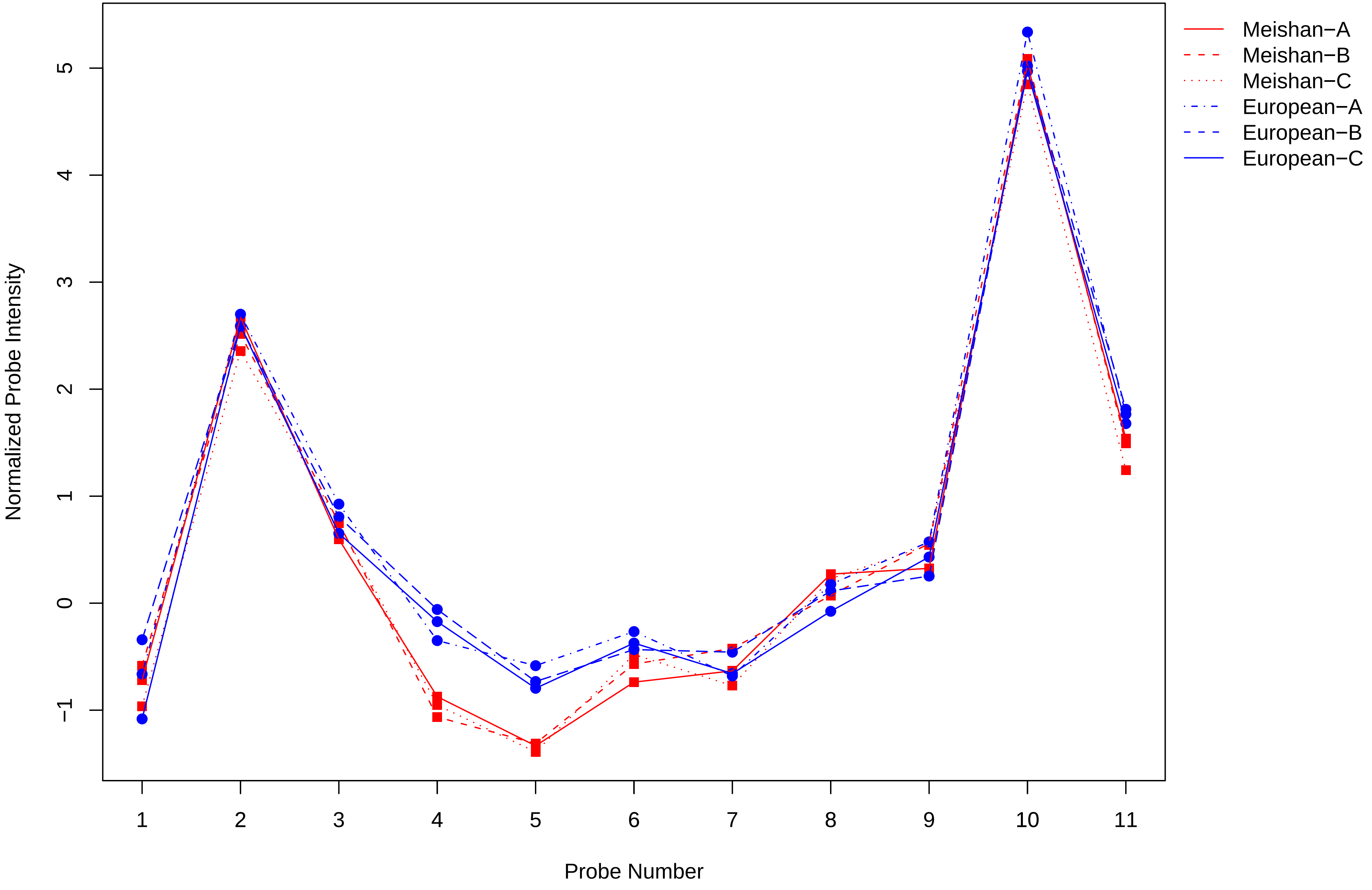

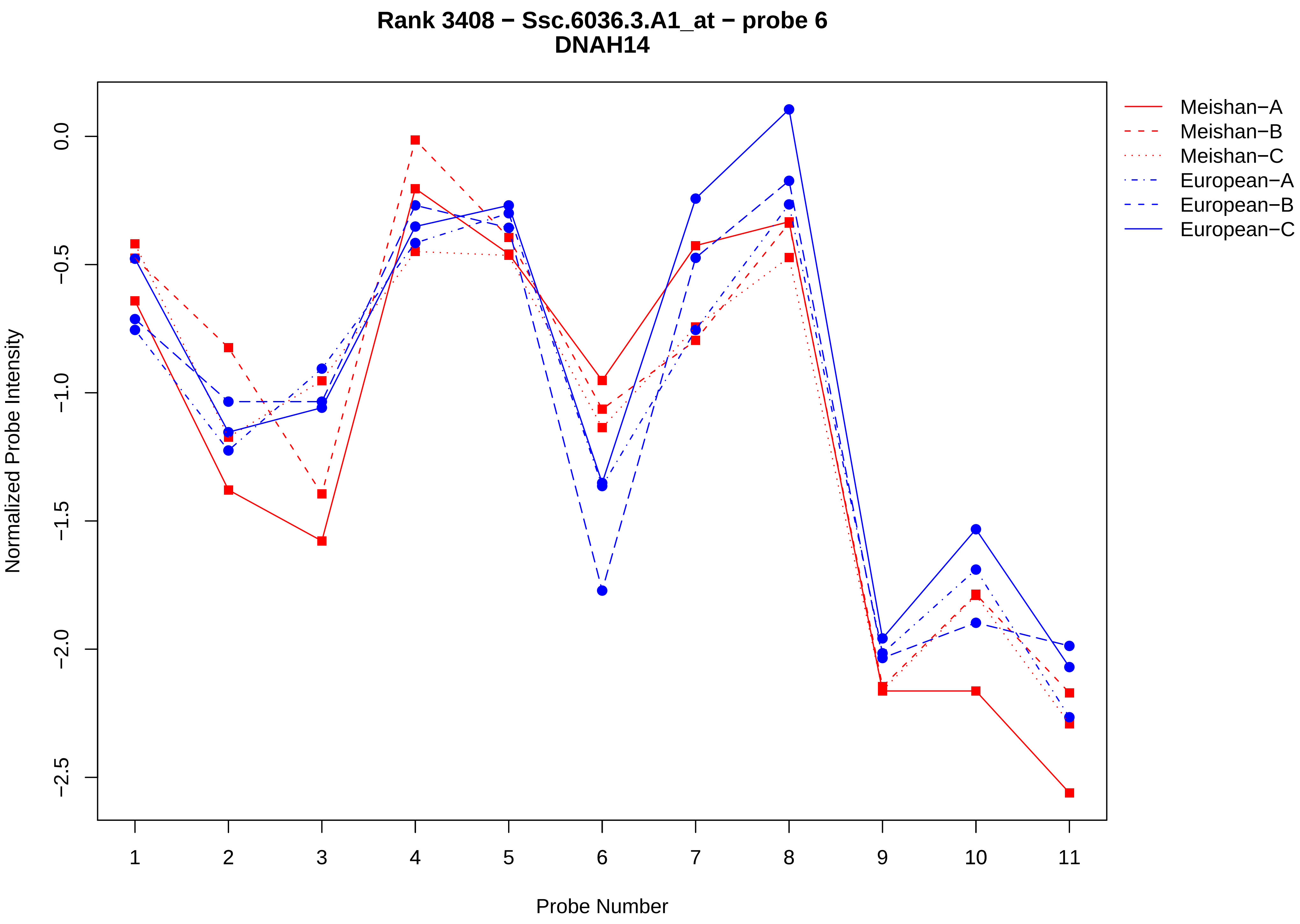

Rank 3408 − Ssc.6036.3.A1_at − probe 6
DNAH14
Normalized Probe Intensity
Probe Number
0.0
−0.5
−1.0
−1.5
−2.0
−2.5
1
2
3
4
5
6
7
8
9
10
11
Meishan−A
Meishan−B
Meishan−C
European−A
European−B
European−C

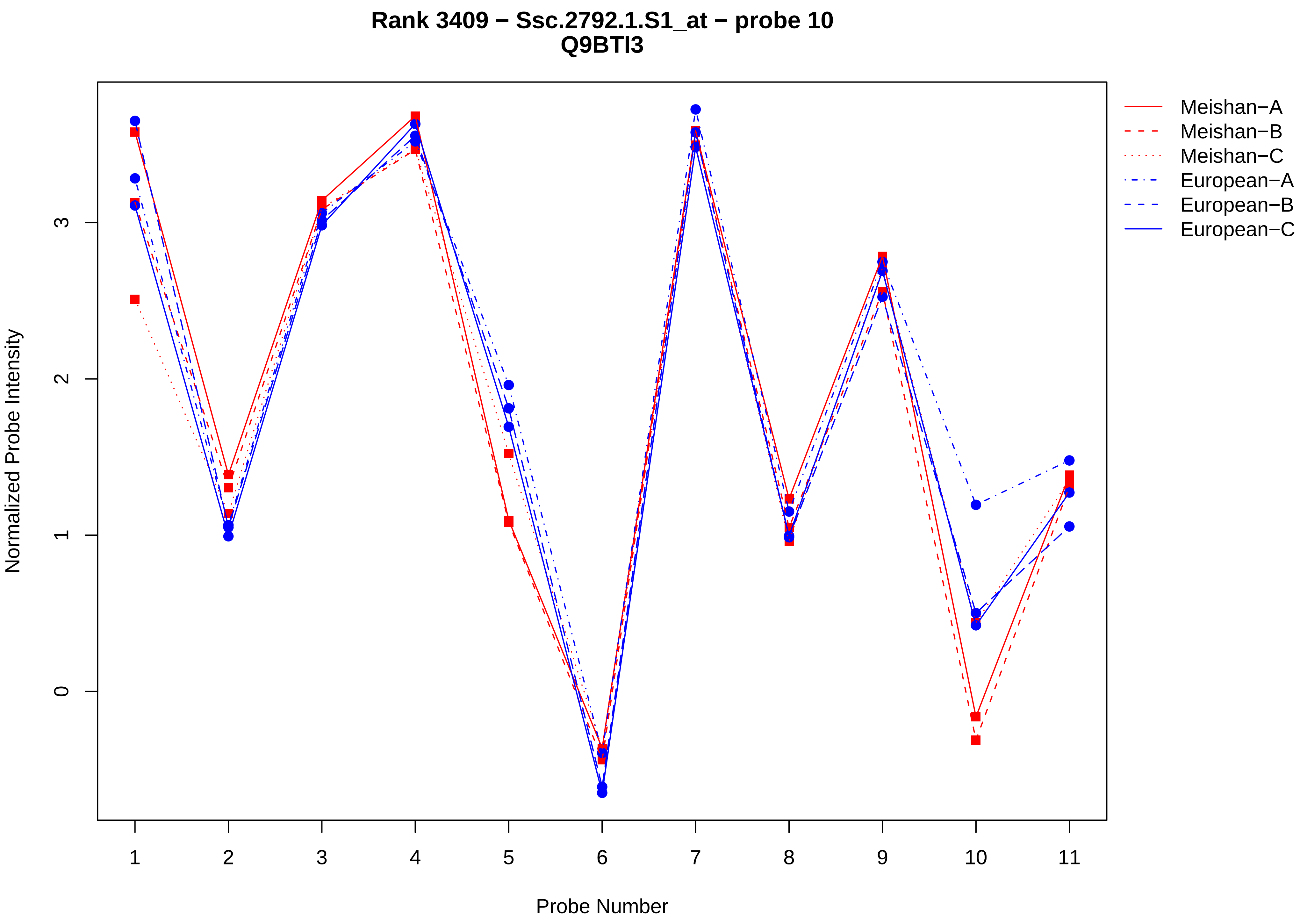

Rank 3409 − Ssc.2792.1.S1_at − probe 10
Q9BTI3
Normalized Probe Intensity
Probe Number
0
1
2
3
1
2
3
4
5
6
7
8
9
10
11
Meishan−A
Meishan−B
Meishan−C
European−A
European−B
European−C

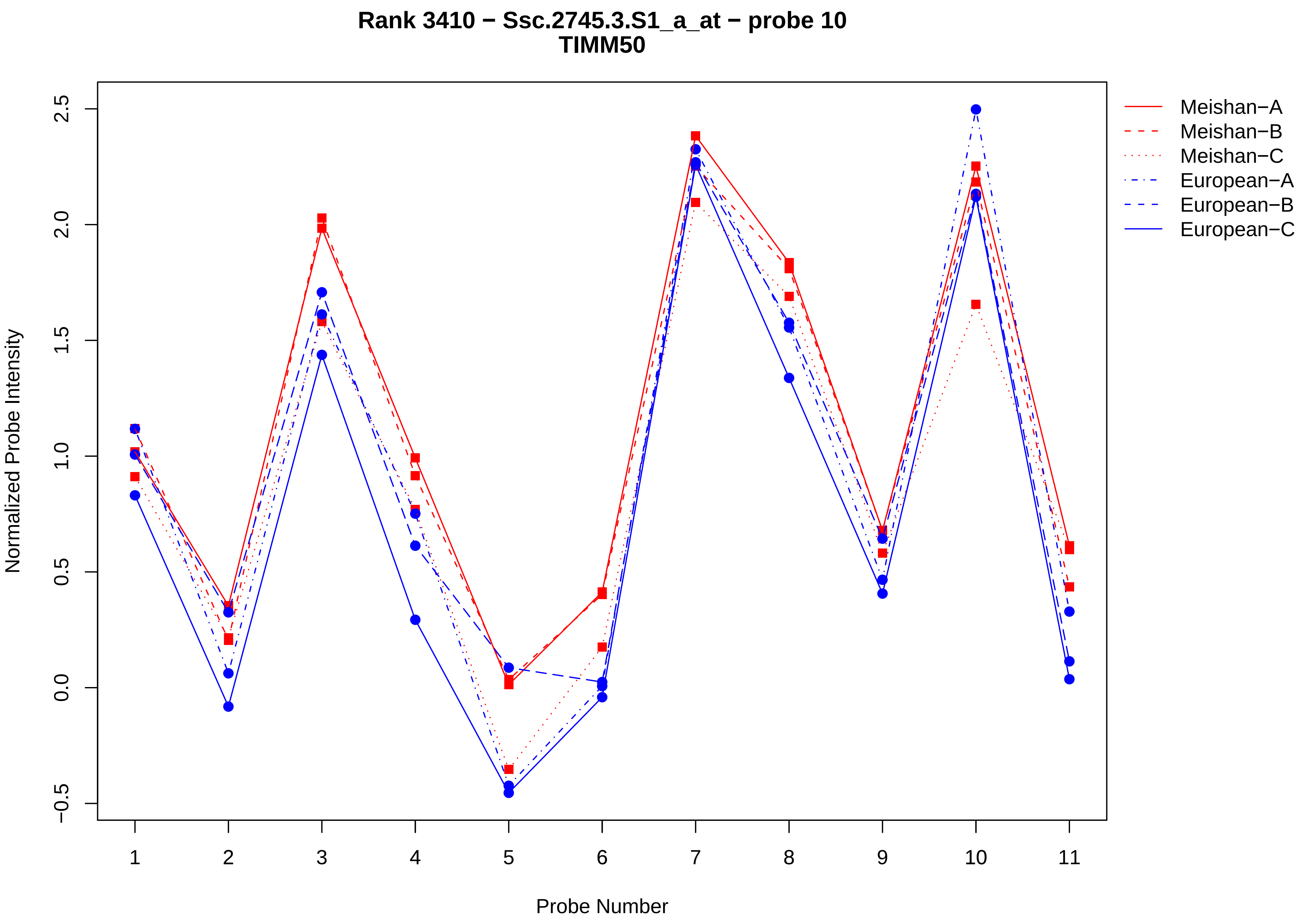

Rank 3410 − Ssc.2745.3.S1_a_at − probe 10
TIMM50
Normalized Probe Intensity
−0.5
0.0
0.5
1.0
1.5
2.0
2.5
Probe Number
1
2
3
4
5
6
7
8
9
10
11
Meishan−A
Meishan−B
Meishan−C
European−A
European−B
European−C

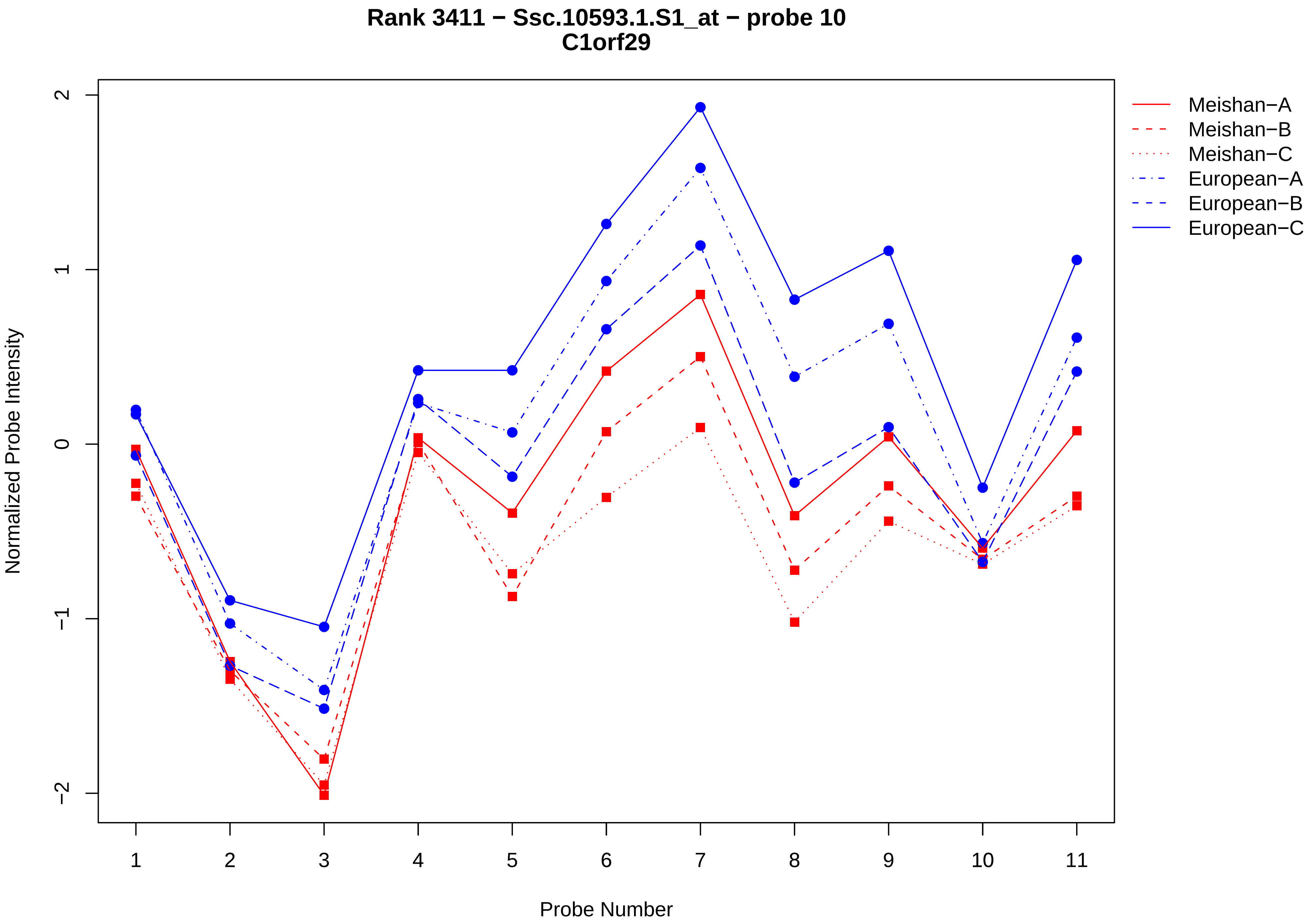

Rank 3411 − Ssc.10593.1.S1_at − probe 10
C1orf29
Normalized Probe Intensity
Probe Number
Meishan−A
Meishan−B
Meishan−C
European−A
European−B
European−C
2
1
0
−1
−2
1
2
3
4
5
6
7
8
9
10
11

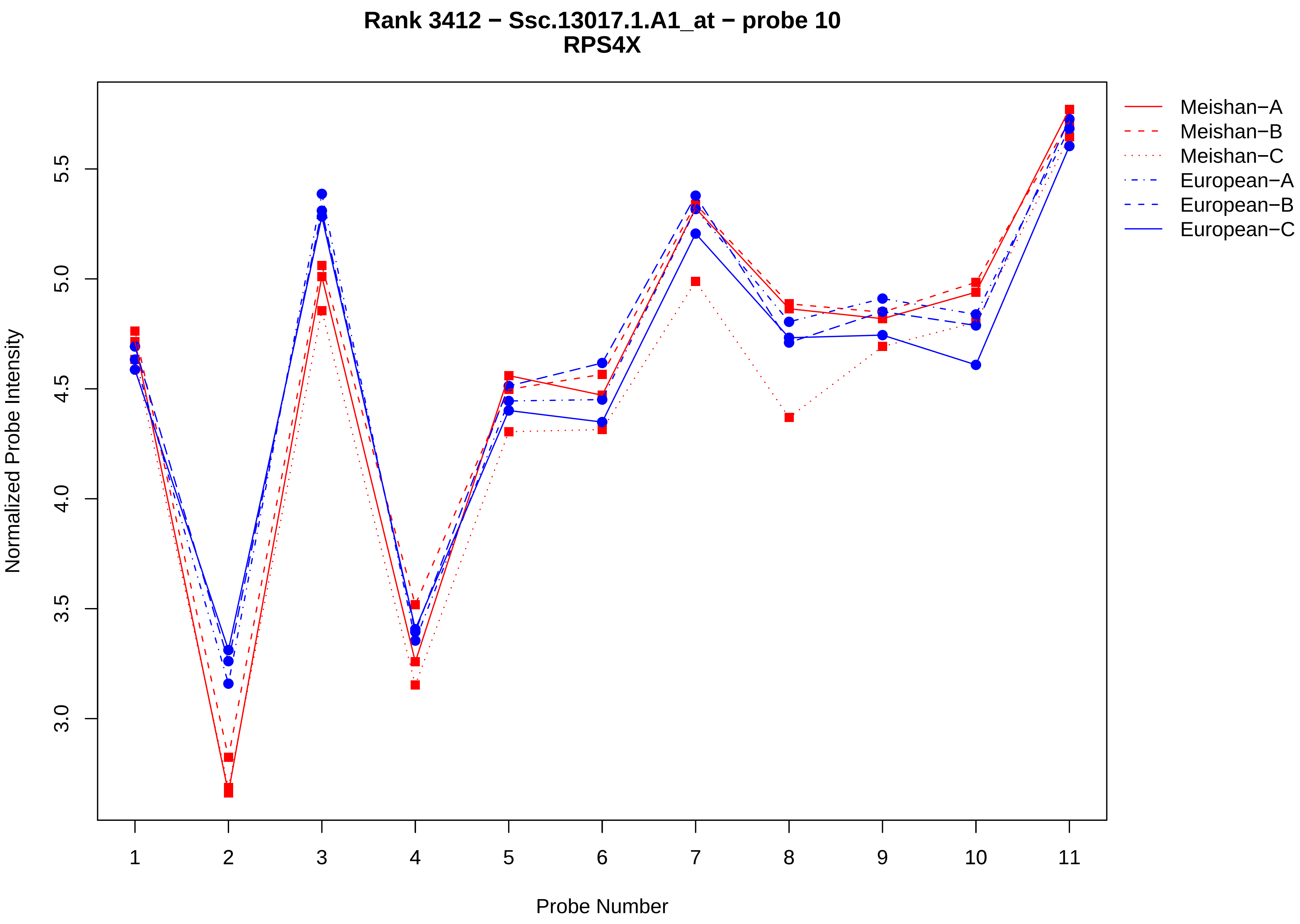
Rank 3412 − Ssc.13017.1.A1_at − probe 10
RPS4X
Normalized Probe Intensity
Probe Number
3.0
3.5
4.0
4.5
5.0
5.5
1
2
3
4
5
6
7
8
9
10
11
Meishan−A
Meishan−B
Meishan−C
European−A
European−B
European−C

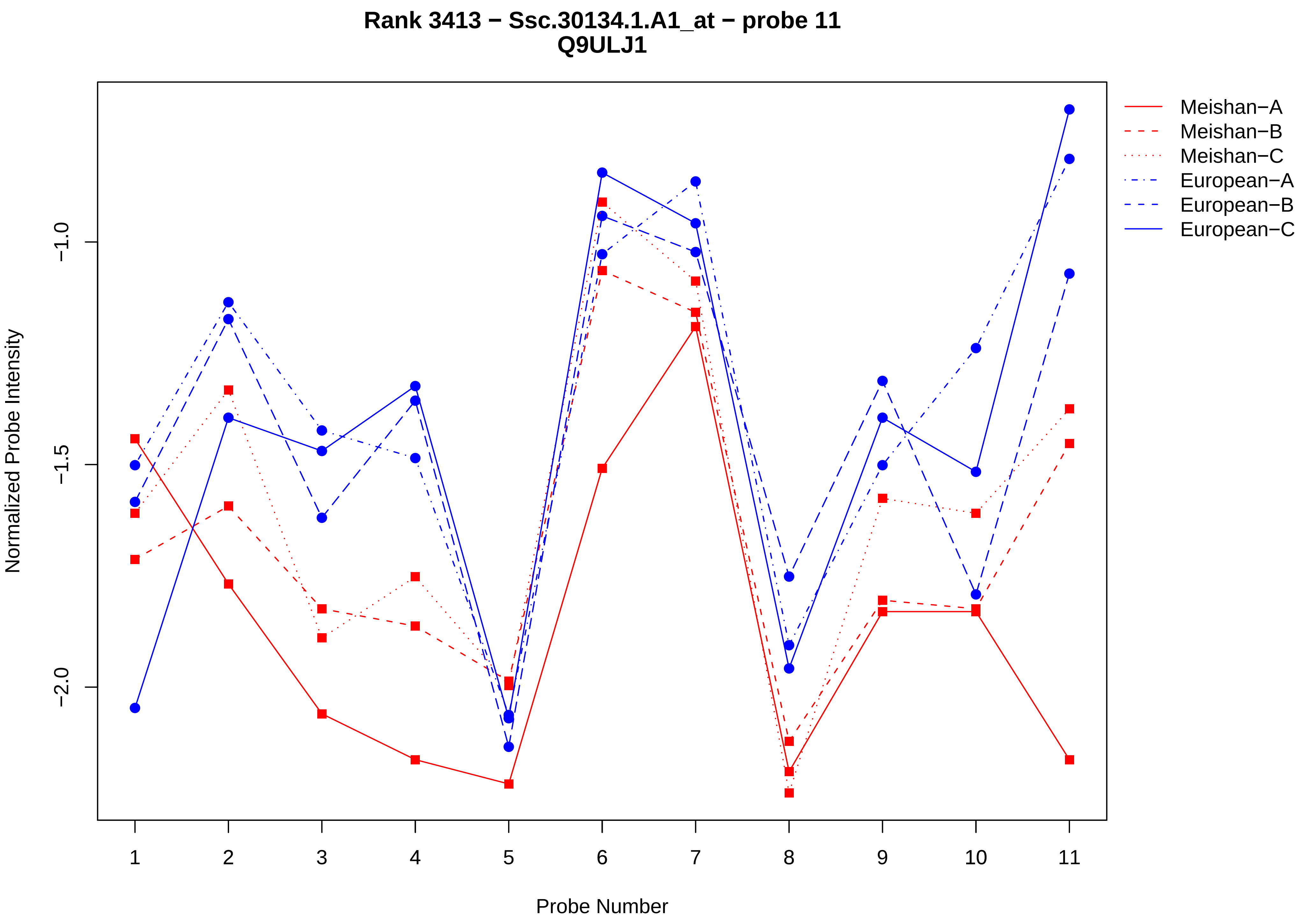

Rank 3413 − Ssc.30134.1.A1_at − probe 11
Q9ULJ1
Normalized Probe Intensity
−1.0
−1.5
−2.0
1
2
3
4
5
6
7
8
9
10
11
Probe Number
Meishan−A
Meishan−B
Meishan−C
European−A
European−B
European−C

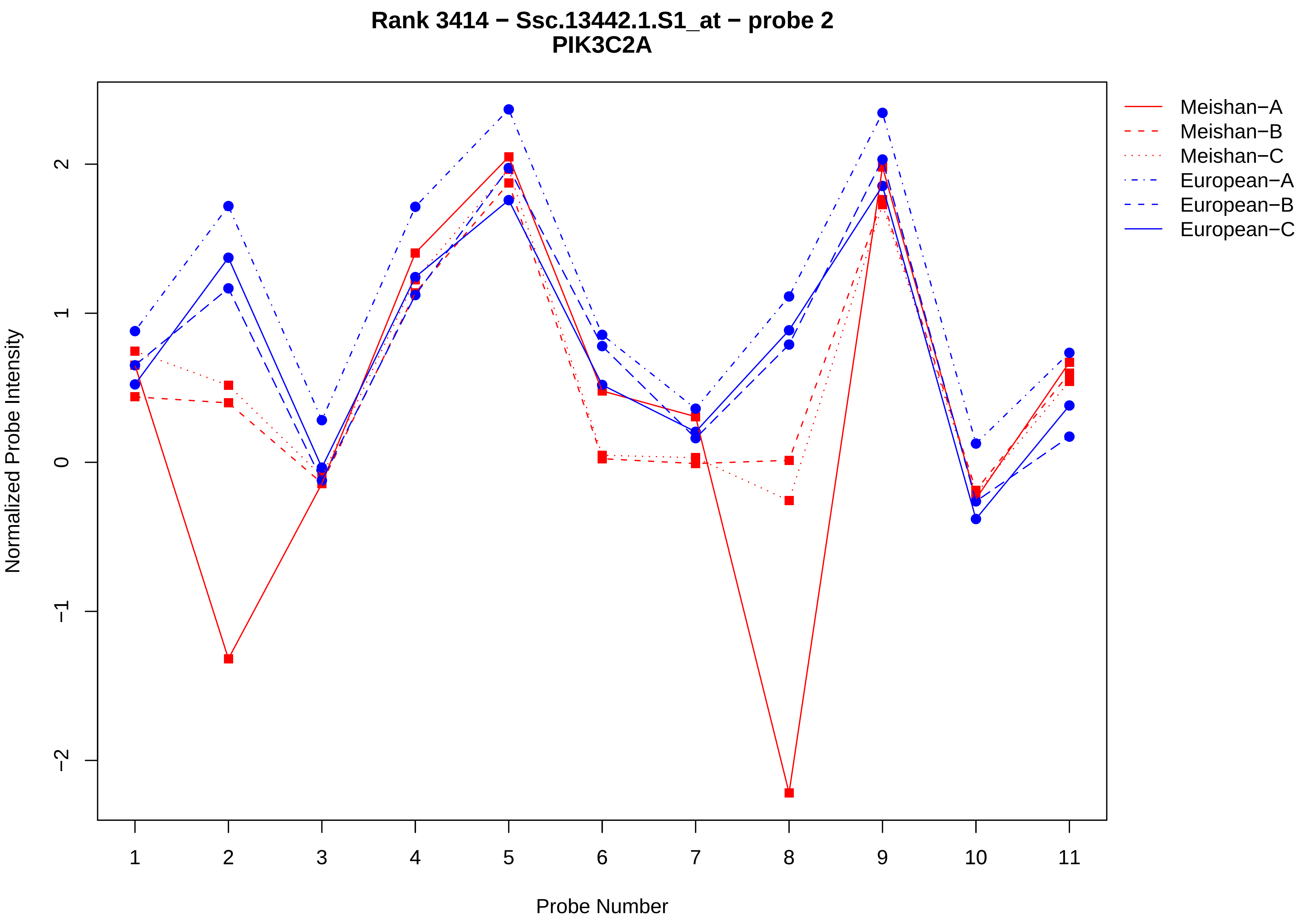

Rank 3414 − Ssc.13442.1.S1_at − probe 2
PIK3C2A
Normalized Probe Intensity
Probe Number
−2
−1
0
1
2
1
2
3
4
5
6
7
8
9
10
11
Meishan−A
Meishan−B
Meishan−C
European−A
European−B
European−C

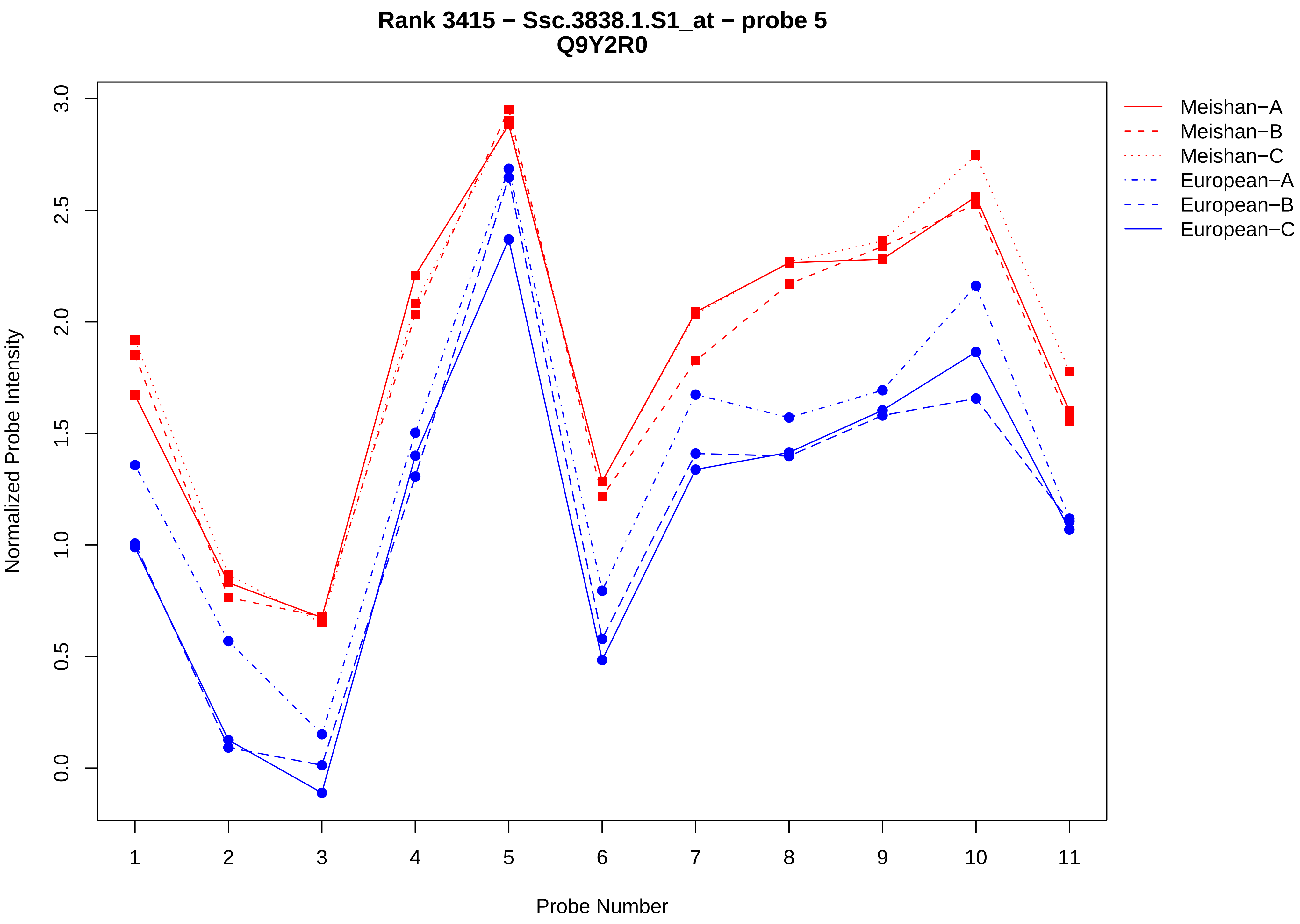
Rank 3415 − Ssc.3838.1.S1_at − probe 5
Q9Y2R0
Normalized Probe Intensity
Probe Number
Meishan−A
Meishan−B
Meishan−C
European−A
European−B
European−C
0.0
0.5
1.0
1.5
2.0
2.5
3.0
1
2
3
4
5
6
7
8
9
10
11

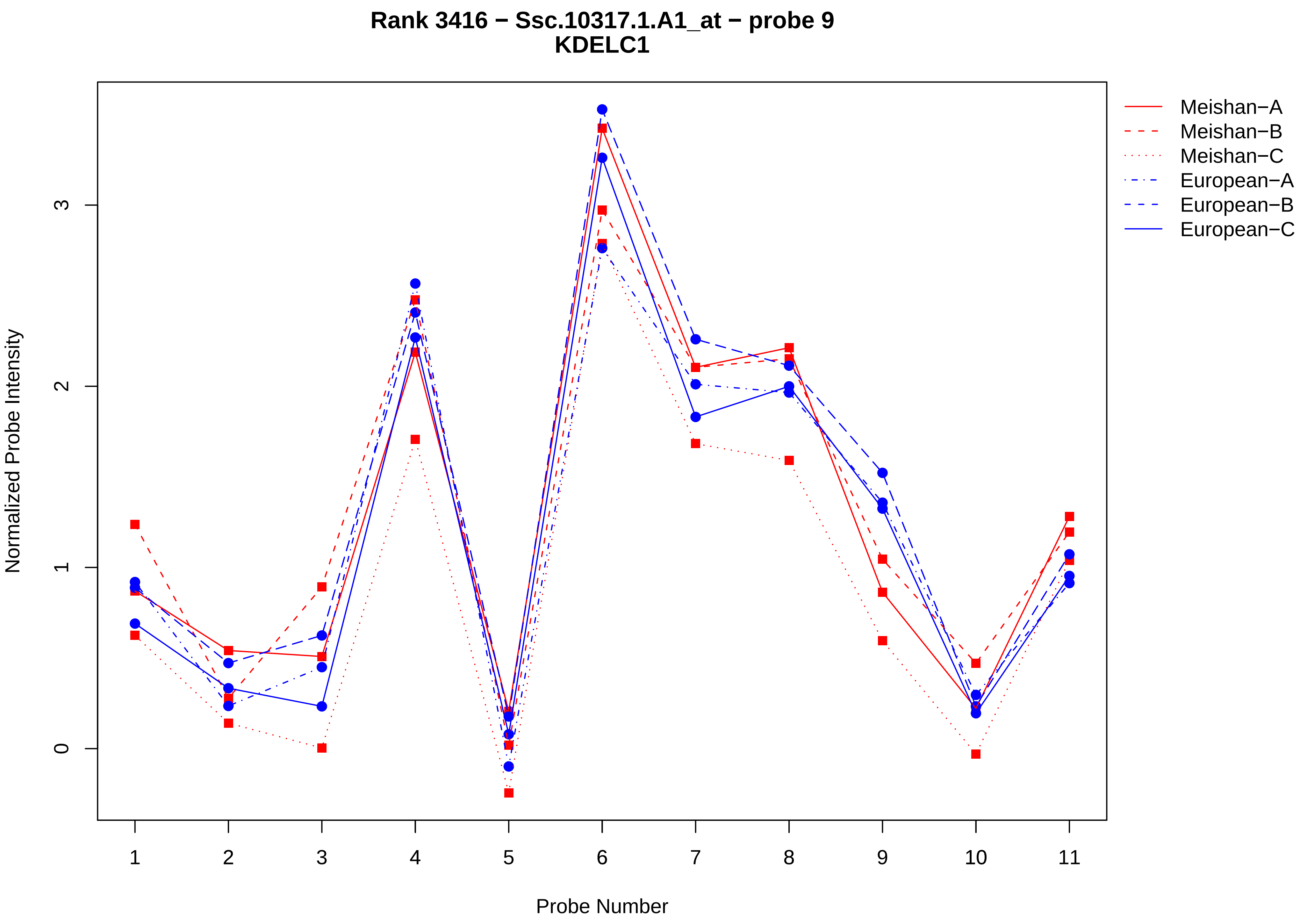

Rank 3416 − Ssc.10317.1.A1_at − probe 9
KDELC1
Normalized Probe Intensity
Probe Number
0
1
2
3
1
2
3
4
5
6
7
8
9
10
11
Meishan−A
Meishan−B
Meishan−C
European−A
European−B
European−C

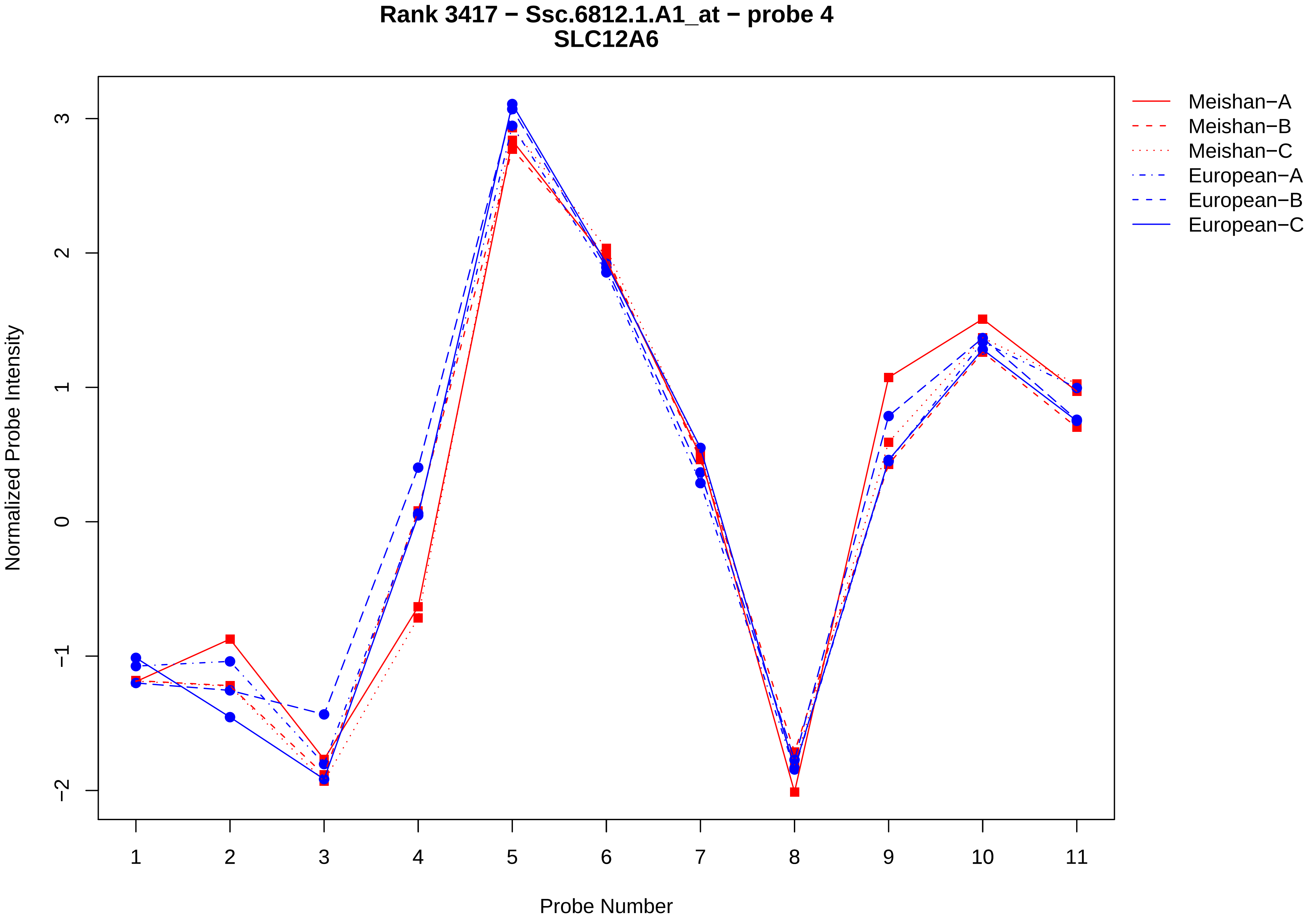

Rank 3417 − Ssc.6812.1.A1_at − probe 4
SLC12A6
Normalized Probe Intensity
Probe Number
−2
−1
0
1
2
3
1
2
3
4
5
6
7
8
9
10
11
Meishan−A
Meishan−B
Meishan−C
European−A
European−B
European−C

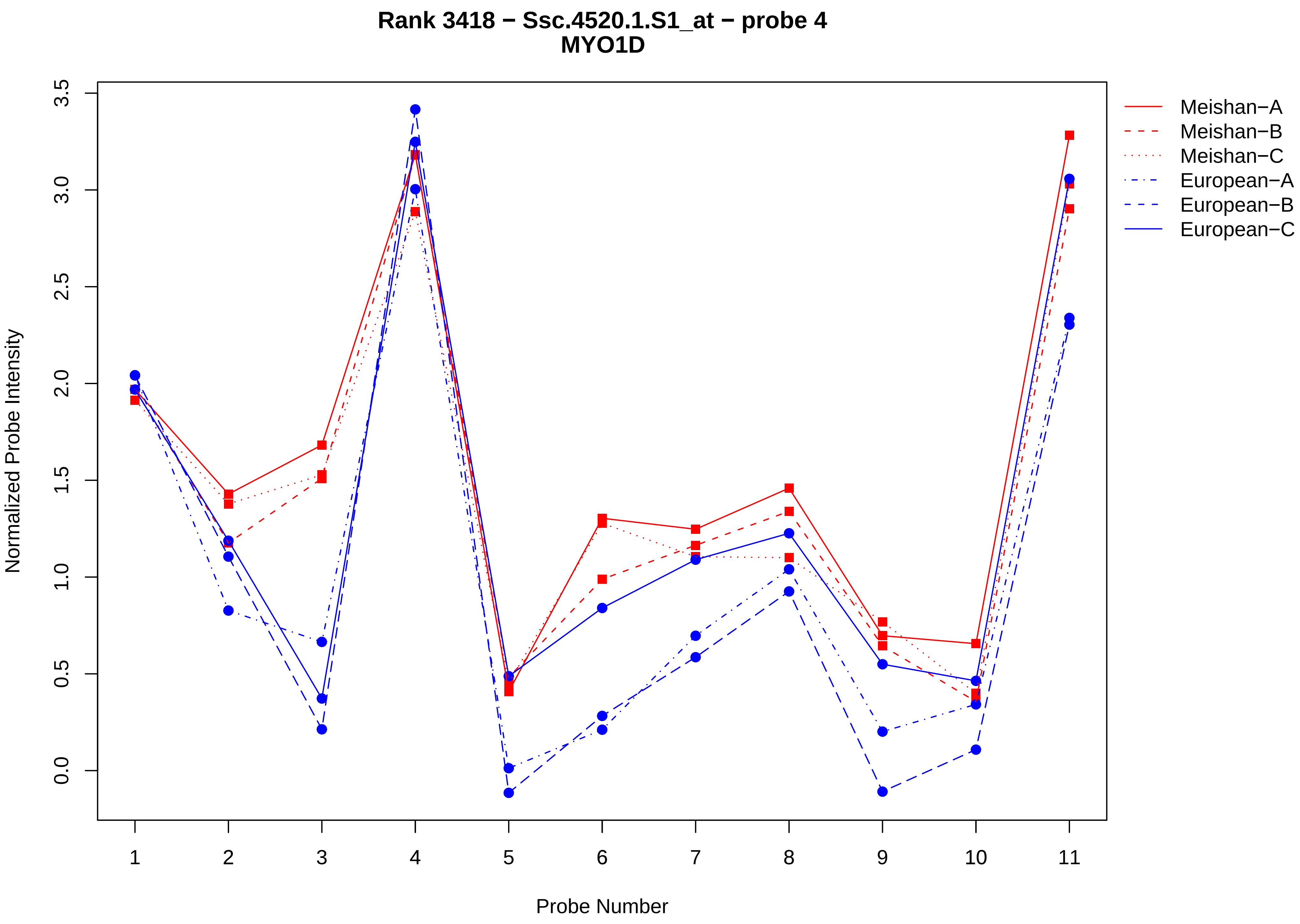

Rank 3418 − Ssc.4520.1.S1_at − probe 4
MYO1D
Normalized Probe Intensity
Probe Number
0.0
0.5
1.0
1.5
2.0
2.5
3.0
3.5
1
2
3
4
5
6
7
8
9
10
11
Meishan−A
Meishan−B
Meishan−C
European−A
European−B
European−C

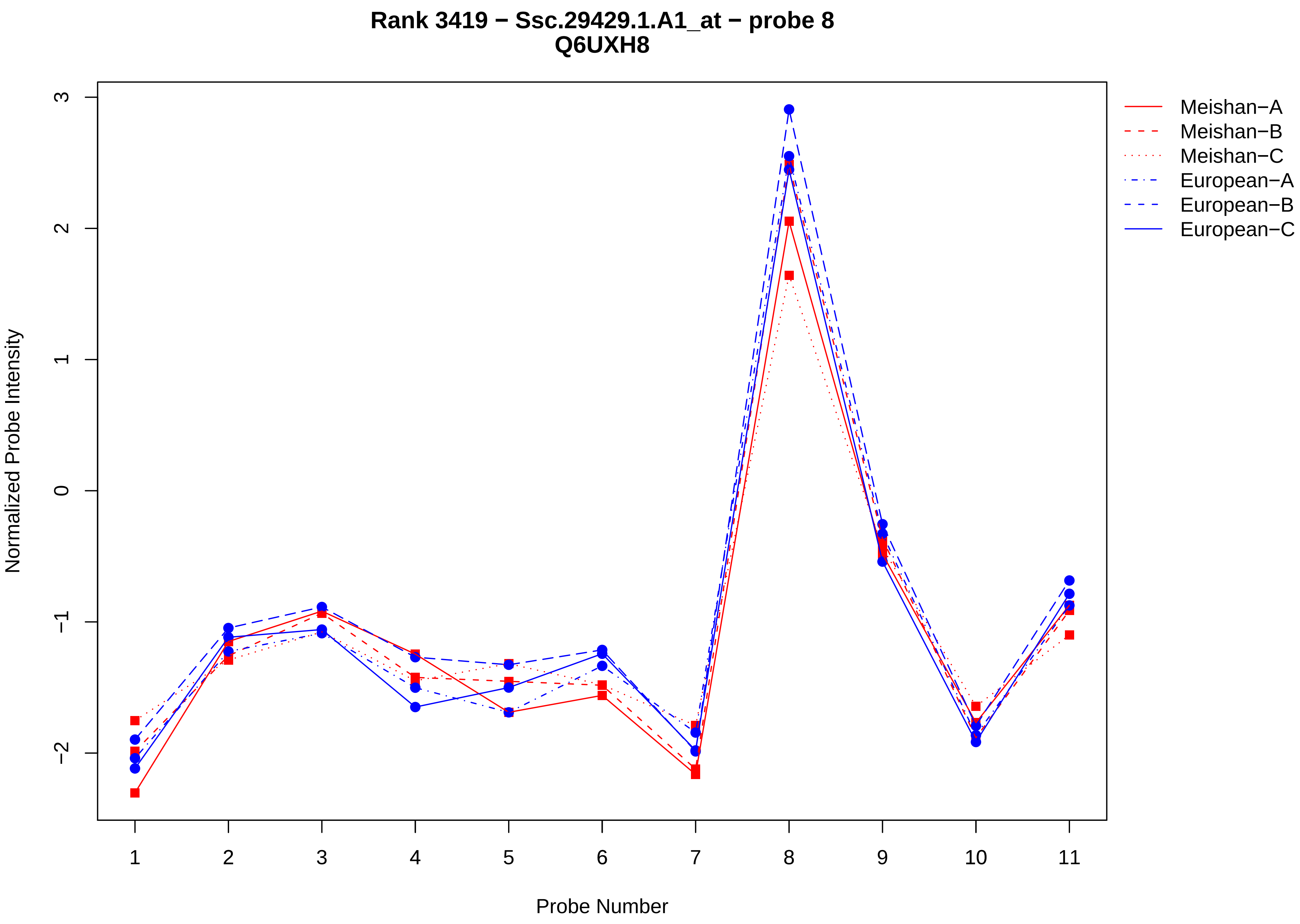

Rank 3419 − Ssc.29429.1.A1_at − probe 8
Q6UXH8
Normalized Probe Intensity
Probe Number
Meishan−A
Meishan−B
Meishan−C
European−A
European−B
European−C

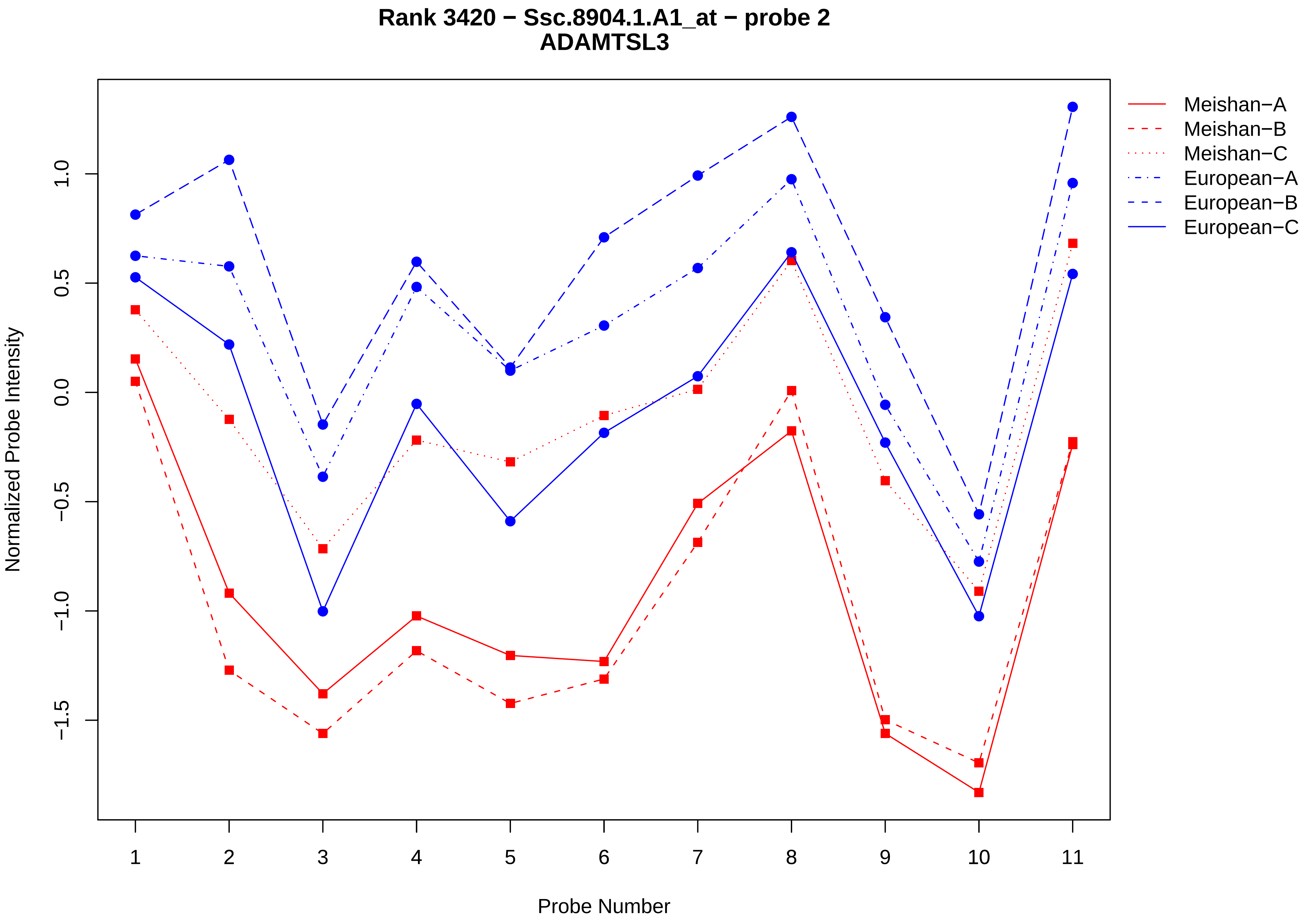

Rank 3420 − Ssc.8904.1.A1_at − probe 2
ADAMTSL3
Normalized Probe Intensity
Probe Number
Meishan−A
Meishan−B
Meishan−C
European−A
European−B
European−C
1.0
0.5
0.0
−0.5
−1.0
−1.5
1
2
3
4
5
6
7
8
9
10
11

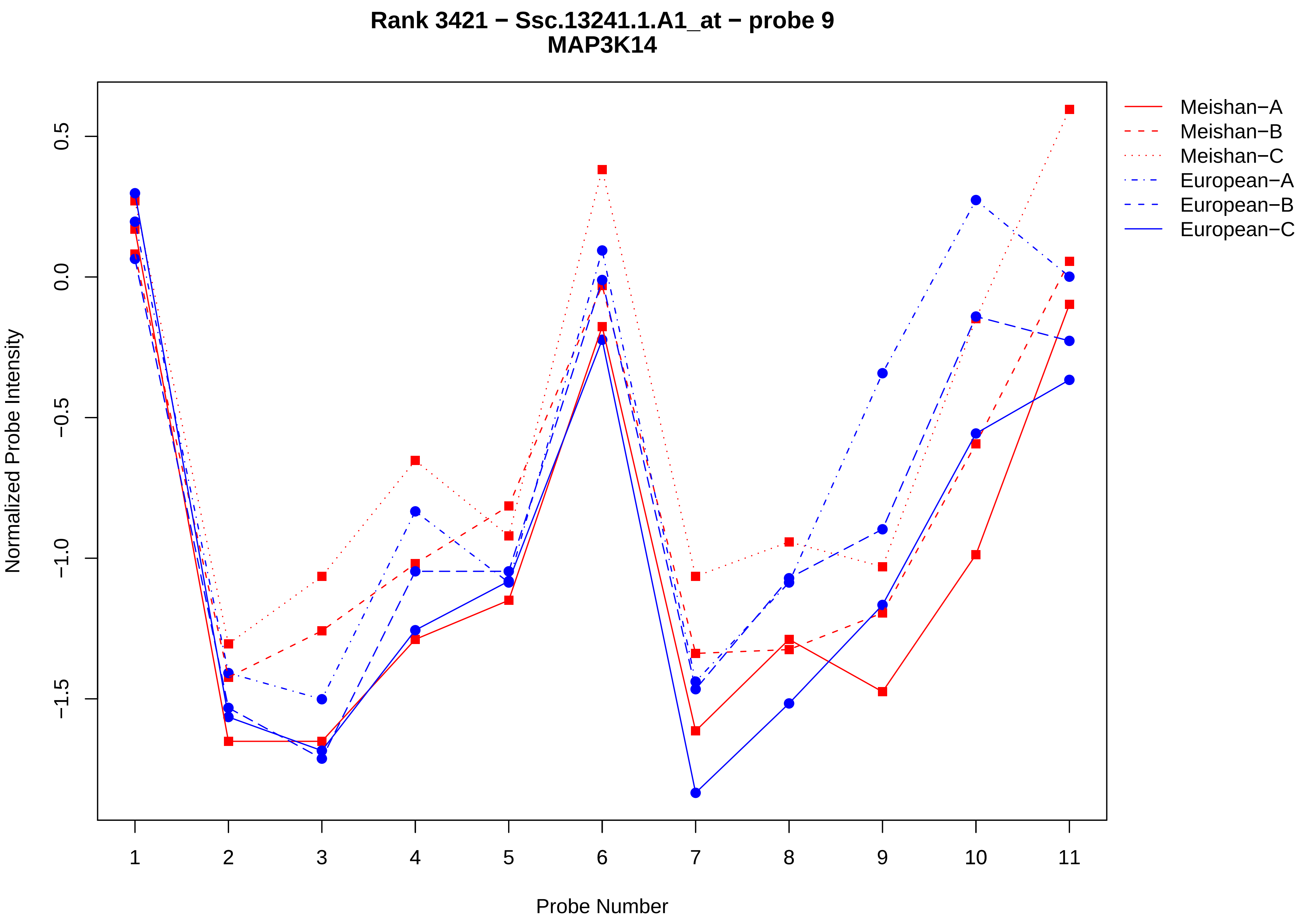

Rank 3421 − Ssc.13241.1.A1_at − probe 9
MAP3K14
Normalized Probe Intensity
Probe Number
0.5
0.0
−0.5
−1.0
−1.5
1
2
3
4
5
6
7
8
9
10
11
Meishan−A
Meishan−B
Meishan−C
European−A
European−B
European−C

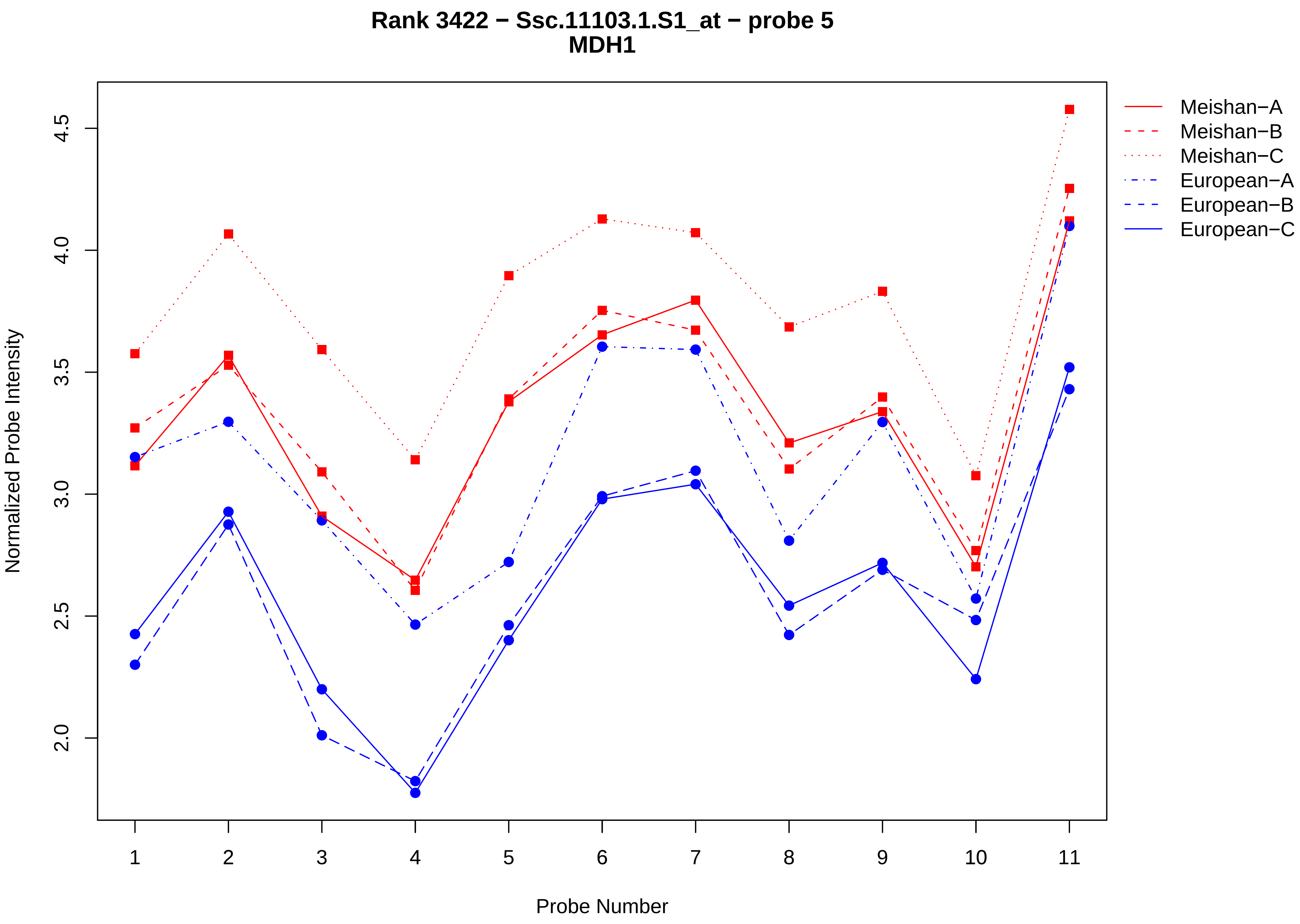

Rank 3422 − Ssc.11103.1.S1_at − probe 5
MDH1
Normalized Probe Intensity
Probe Number
2.0
2.5
3.0
3.5
4.0
4.5
1
2
3
4
5
6
7
8
9
10
11
Meishan−A
Meishan−B
Meishan−C
European−A
European−B
European−C

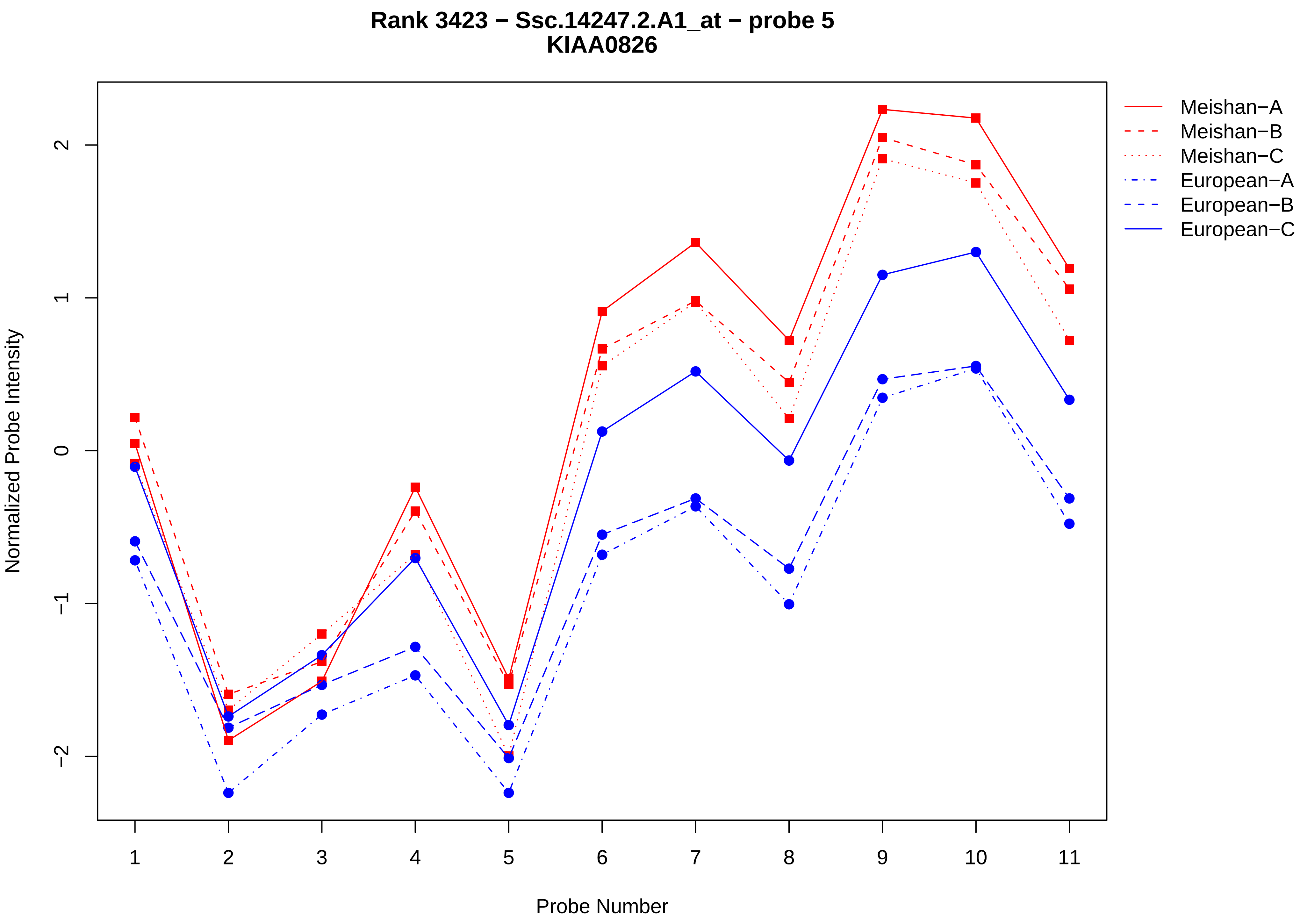
Rank 3423 − Ssc.14247.2.A1_at − probe 5
KIAA0826
Normalized Probe Intensity
Probe Number
Meishan−A
Meishan−B
Meishan−C
European−A
European−B
European−C
−2
−1
0
1
2
1
2
3
4
5
6
7
8
9
10
11

Rank 3424 − Ssc.19292.2.A1_at − probe 3
SIAT7F

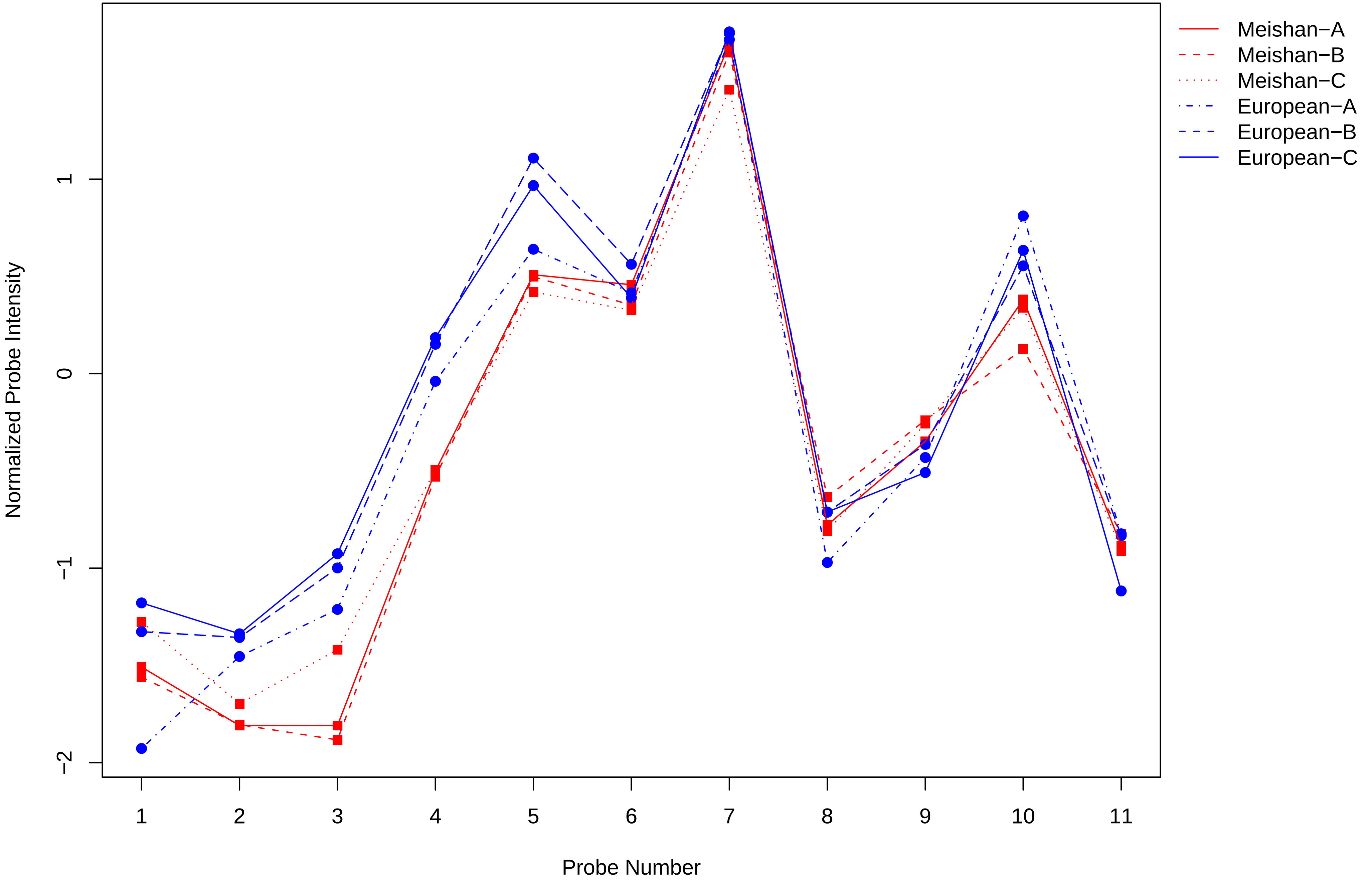

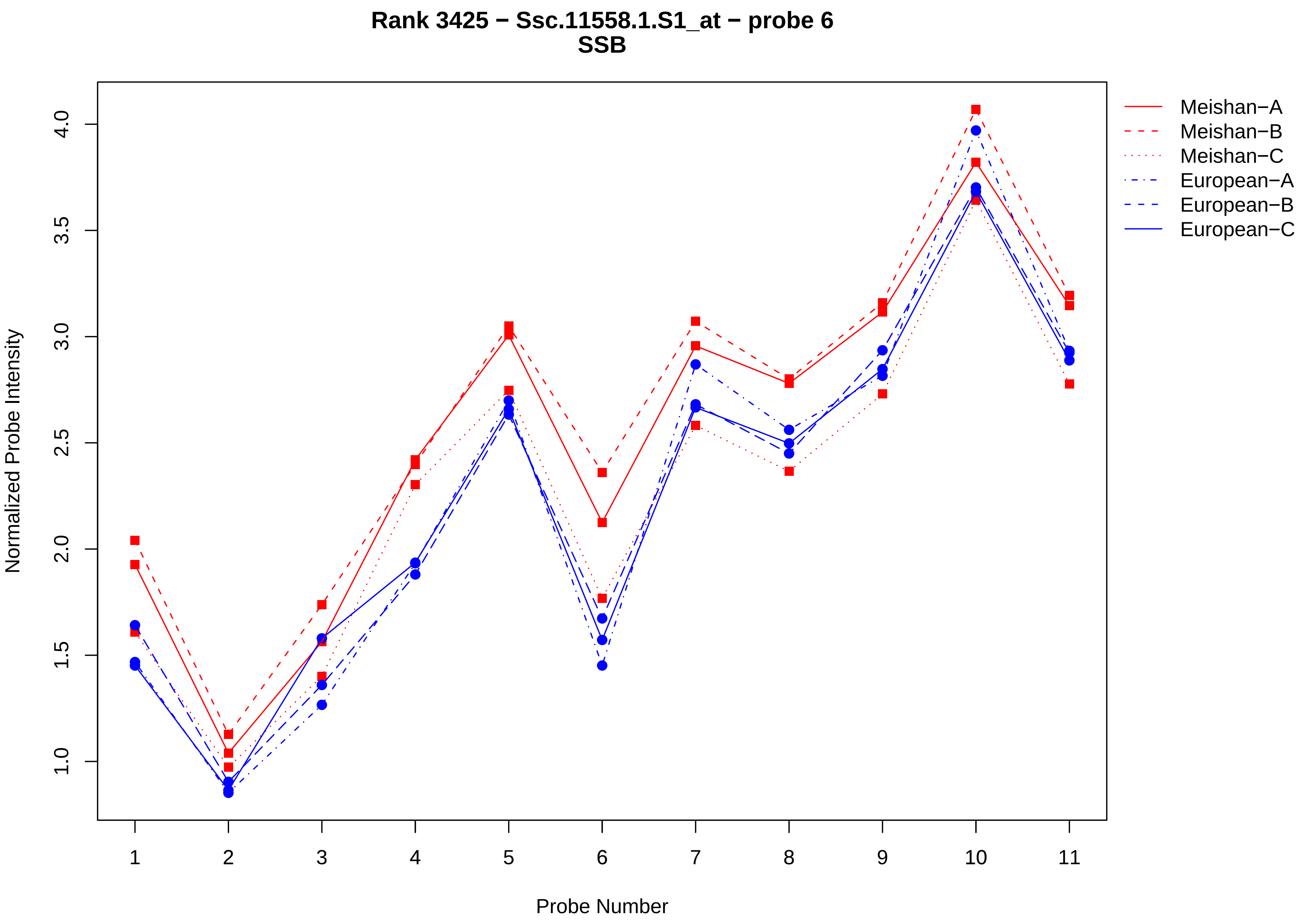

Rank 3425 − Ssc.11558.1.S1_at − probe 6
SSB
Normalized Probe Intensity
1.0
1.5
2.0
2.5
3.0
3.5
4.0
1
2
3
4
5
6
7
8
9
10
11
Probe Number
Meishan−A
Meishan−B
Meishan−C
European−A
European−B
European−C

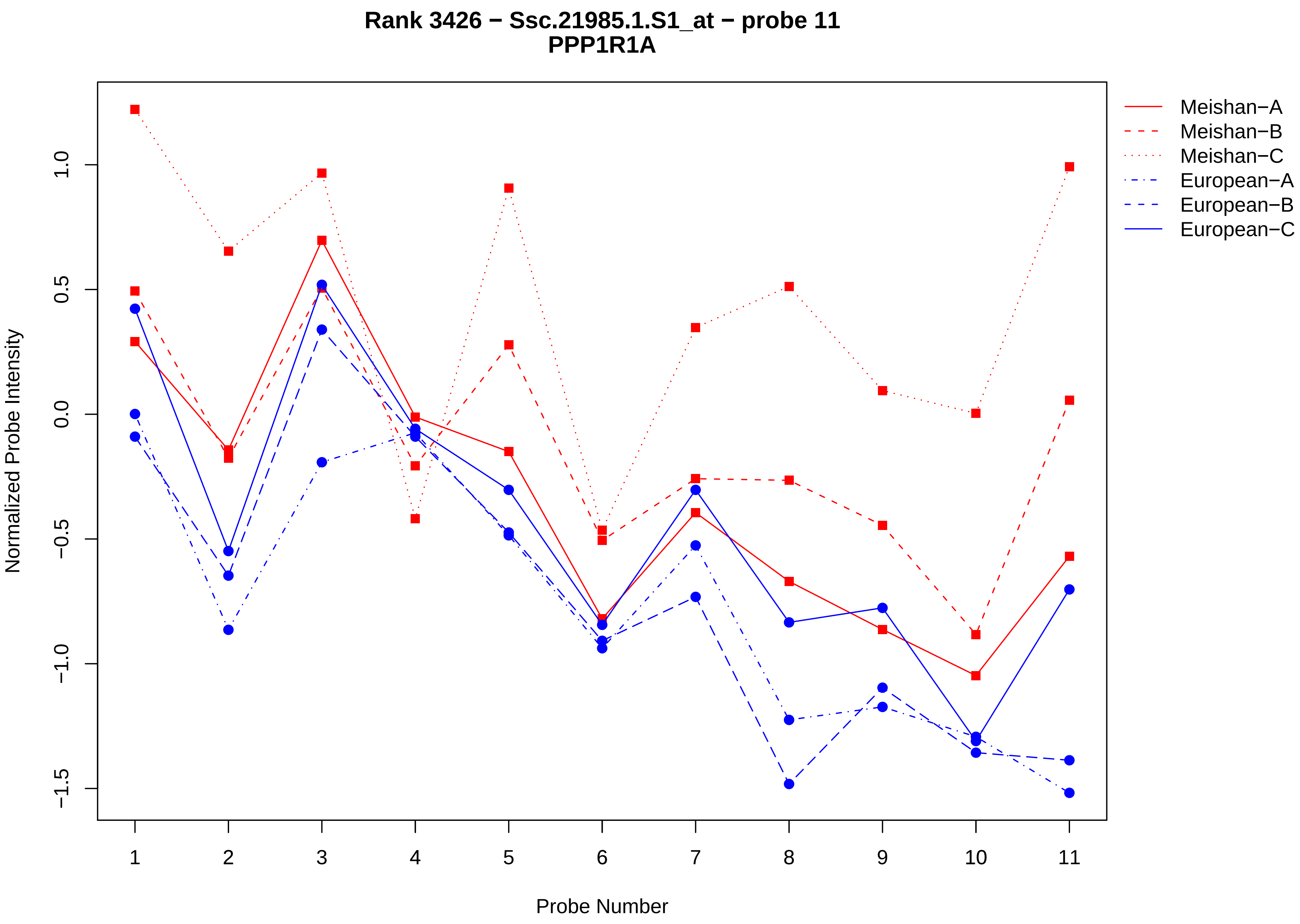

Rank 3426 − Ssc.21985.1.S1_at − probe 11
PPP1R1A
Normalized Probe Intensity
Probe Number
1.0
0.5
0.0
−0.5
−1.0
−1.5
1
2
3
4
5
6
7
8
9
10
11
Meishan−A
Meishan−B
Meishan−C
European−A
European−B
European−C

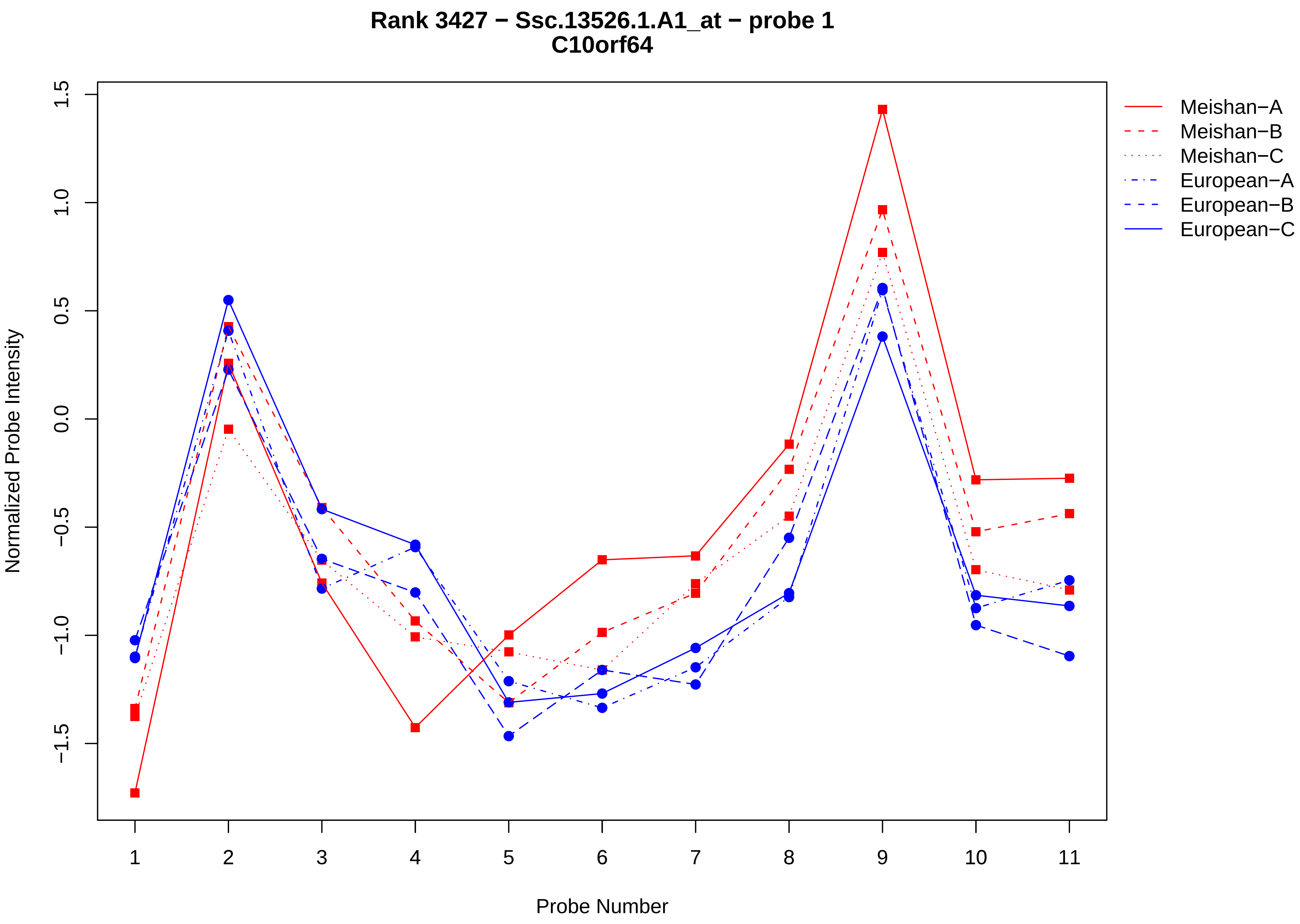

Rank 3427 − Ssc.13526.1.A1_at − probe 1
C10orf64
Normalized Probe Intensity
Probe Number
1.5
1.0
0.5
0.0
−0.5
−1.0
−1.5
1
2
3
4
5
6
7
8
9
10
11
Meishan−A
Meishan−B
Meishan−C
European−A
European−B
European−C

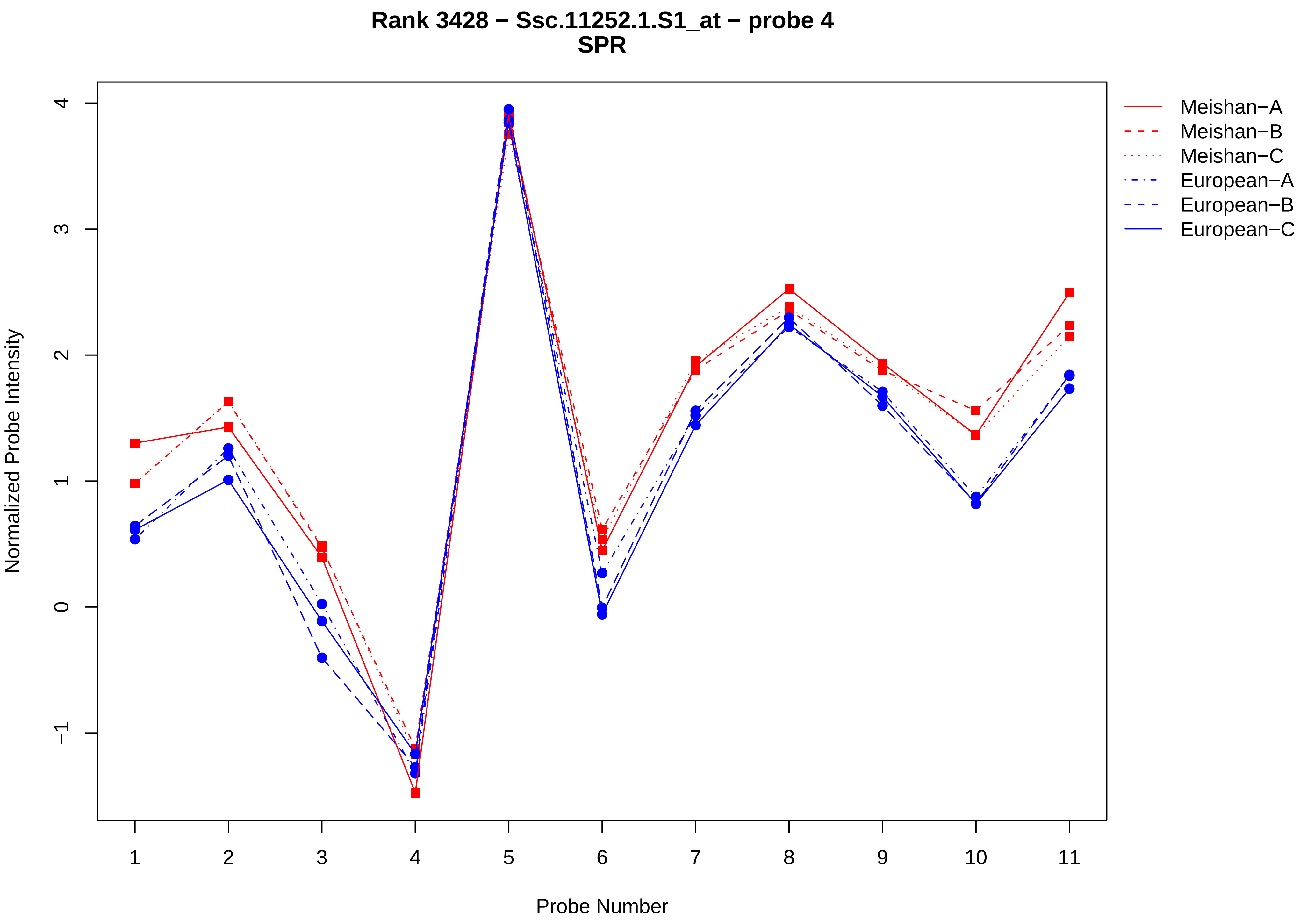
Rank 3428 − Ssc.11252.1.S1_at − probe 4
SPR
Normalized Probe Intensity
Probe Number
Meishan−A
Meishan−B
Meishan−C
European−A
European−B
European−C
−1
0
1
2
3
4
1
2
3
4
5
6
7
8
9
10
11

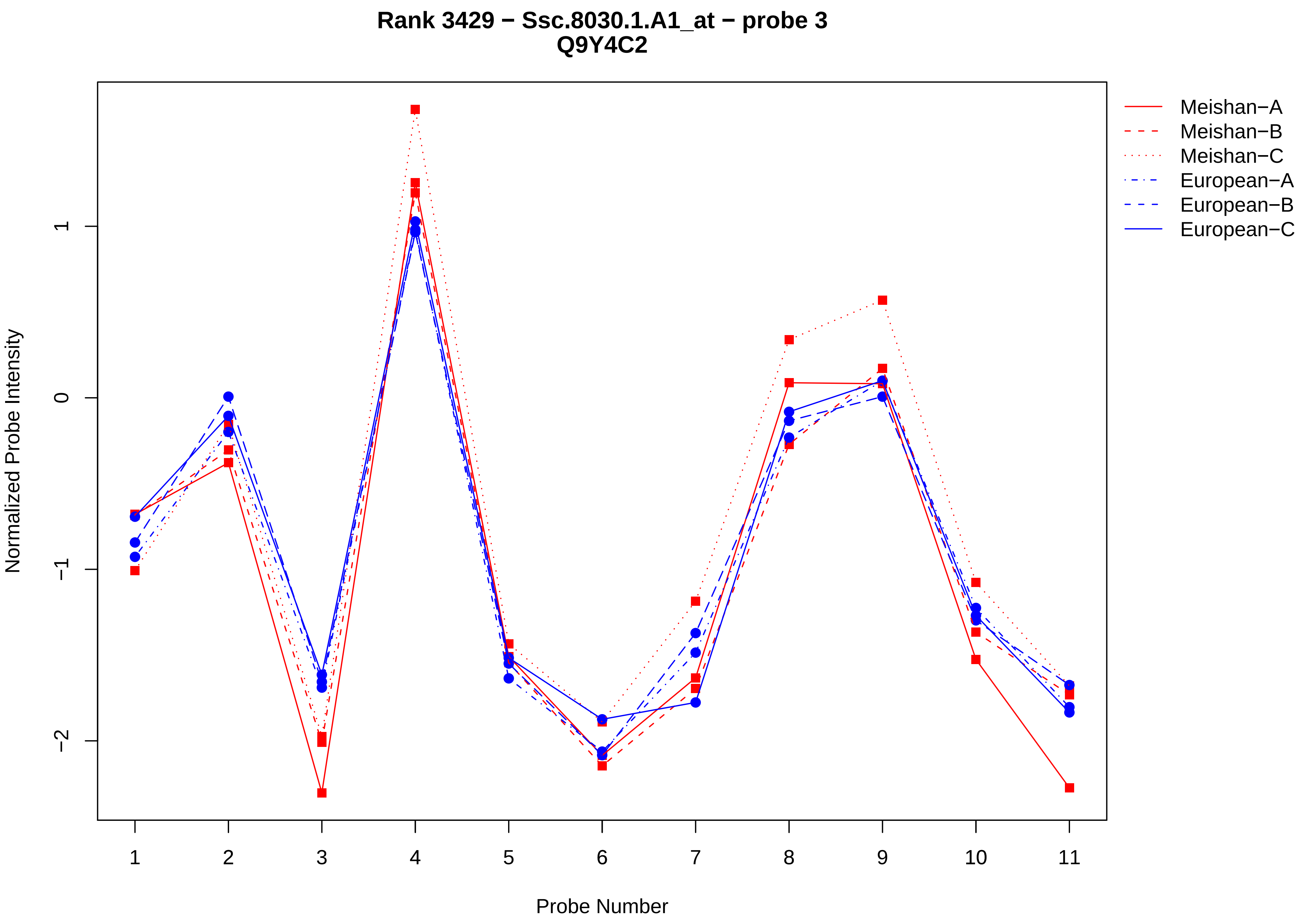
Rank 3429 − Ssc.8030.1.A1_at − probe 3
Q9Y4C2
Normalized Probe Intensity
Probe Number
Meishan−A
Meishan−B
Meishan−C
European−A
European−B
European−C
1
0
−1
−2
1
2
3
4
5
6
7
8
9
10
11

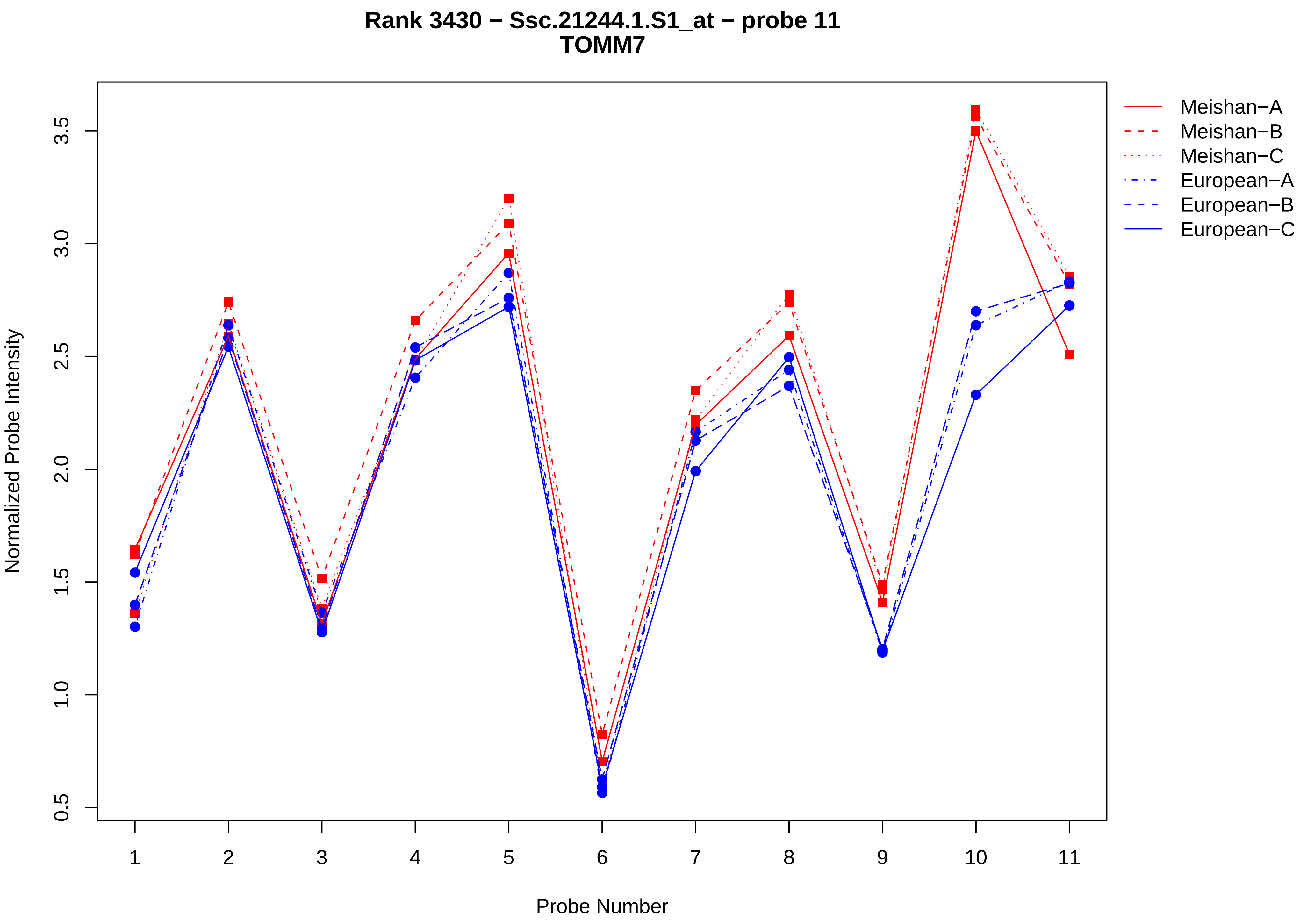

Rank 3430 − Ssc.21244.1.S1_at − probe 11
TOMM7
Normalized Probe Intensity
Probe Number
0.5
1.0
1.5
2.0
2.5
3.0
3.5
1
2
3
4
5
6
7
8
9
10
11
Meishan−A
Meishan−B
Meishan−C
European−A
European−B
European−C

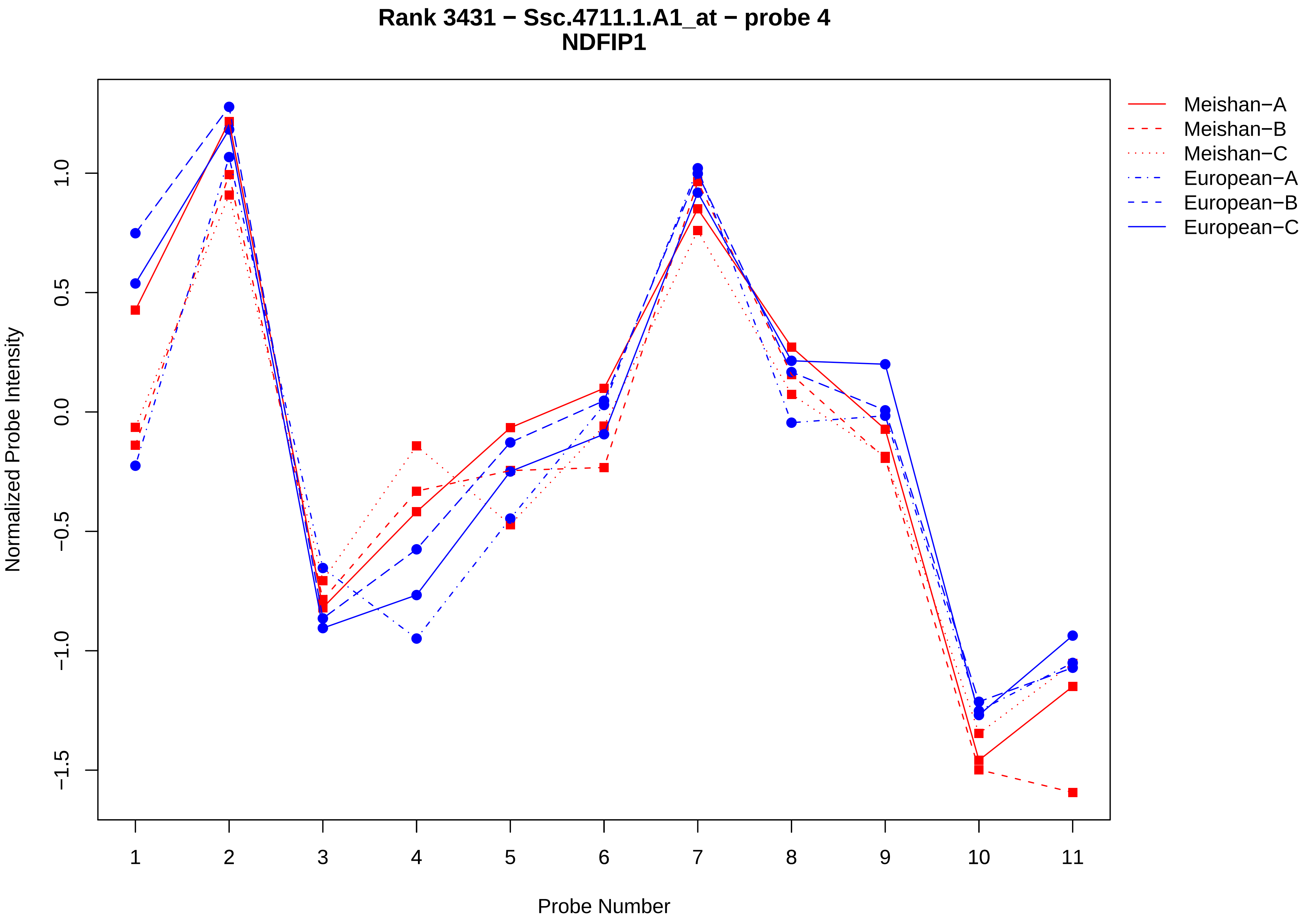

Rank 3431 − Ssc.4711.1.A1_at − probe 4
NDFIP1
Normalized Probe Intensity
Probe Number
1.0
0.5
0.0
−0.5
−1.0
−1.5
1
2
3
4
5
6
7
8
9
10
11
Meishan−A
Meishan−B
Meishan−C
European−A
European−B
European−C

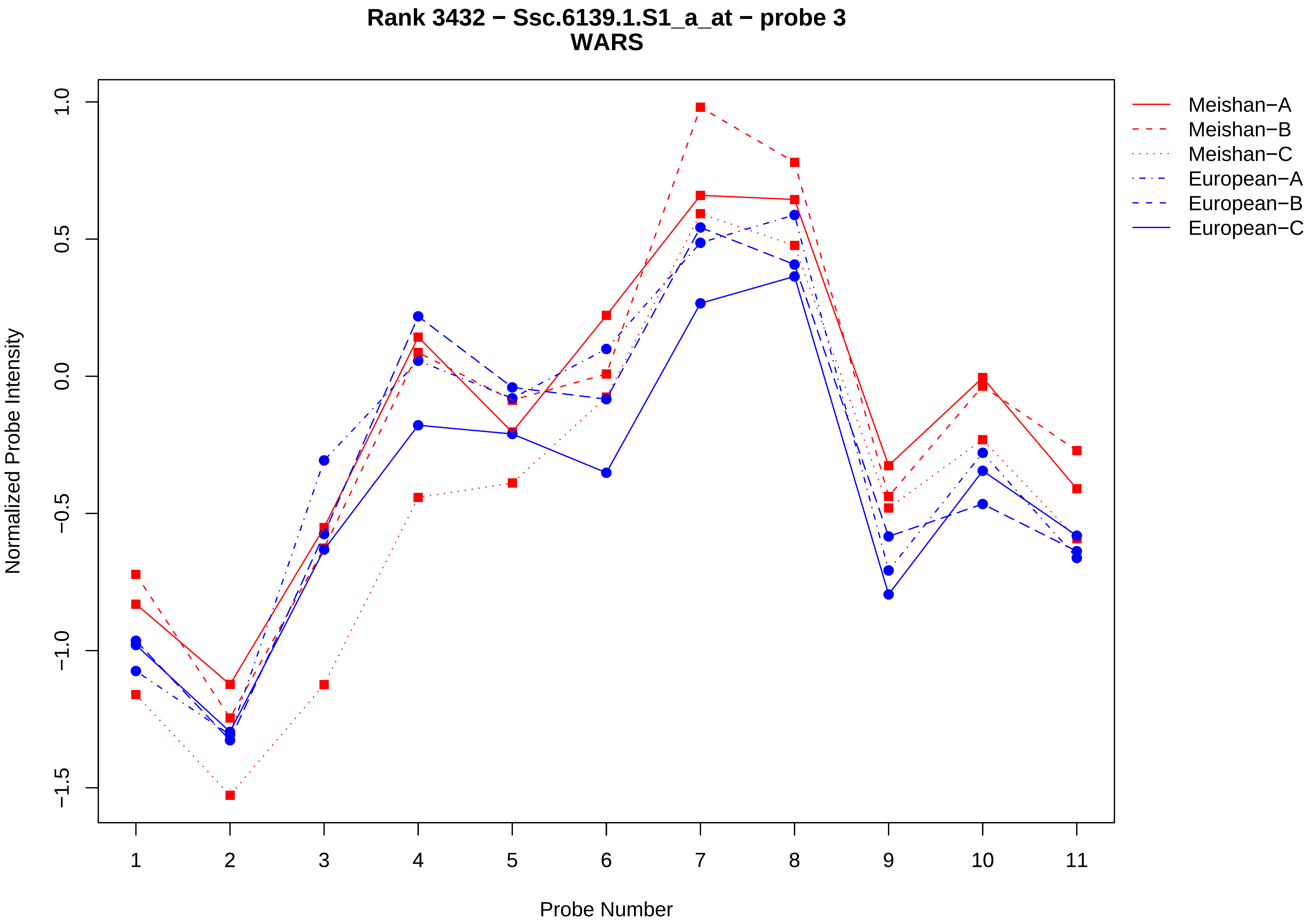

Rank 3432 − Ssc.6139.1.S1_a_at − probe 3
WARS
Normalized Probe Intensity
Probe Number
1.0
0.5
0.0
−0.5
−1.0
−1.5
1
2
3
4
5
6
7
8
9
10
11
Meishan−A
Meishan−B
Meishan−C
European−A
European−B
European−C

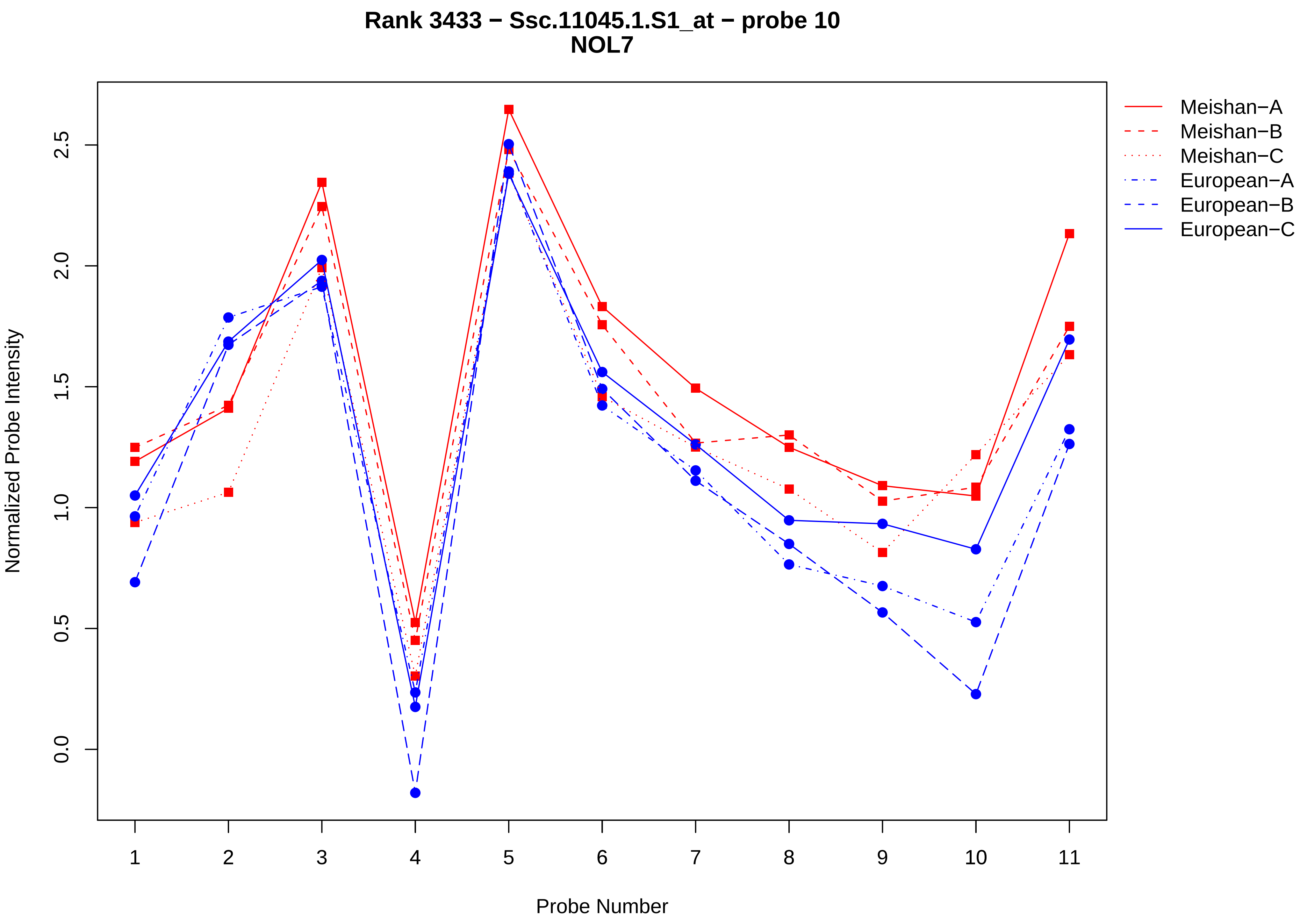

Rank 3433 − Ssc.11045.1.S1_at − probe 10
NOL7
Normalized Probe Intensity
Probe Number
0.0
0.5
1.0
1.5
2.0
2.5
1
2
3
4
5
6
7
8
9
10
11
Meishan−A
Meishan−B
Meishan−C
European−A
European−B
European−C

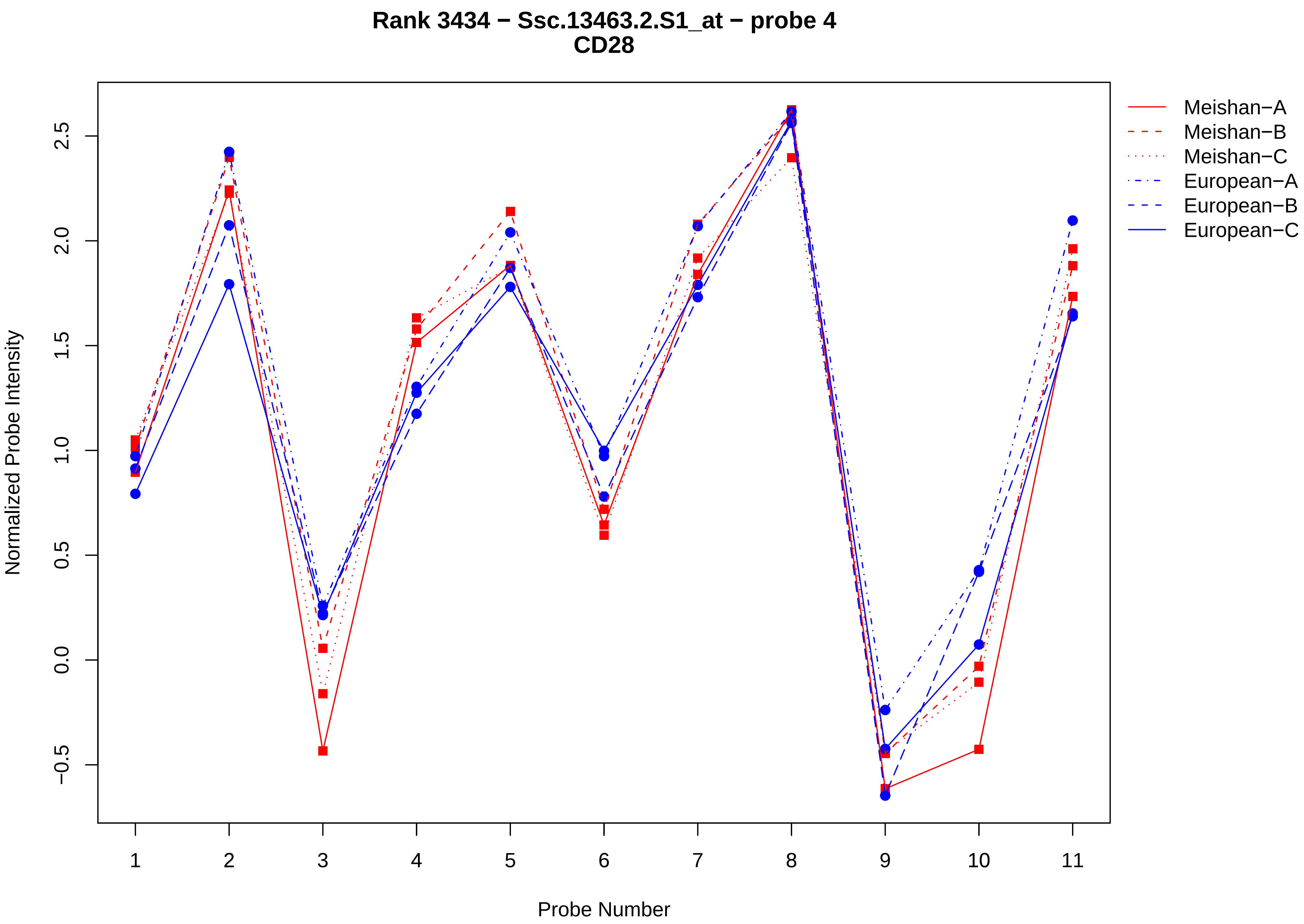

Rank 3434 − Ssc.13463.2.S1_at − probe 4
CD28
Normalized Probe Intensity
Probe Number
−0.5
0.0
0.5
1.0
1.5
2.0
2.5
1
2
3
4
5
6
7
8
9
10
11
Meishan−A
Meishan−B
Meishan−C
European−A
European−B
European−C

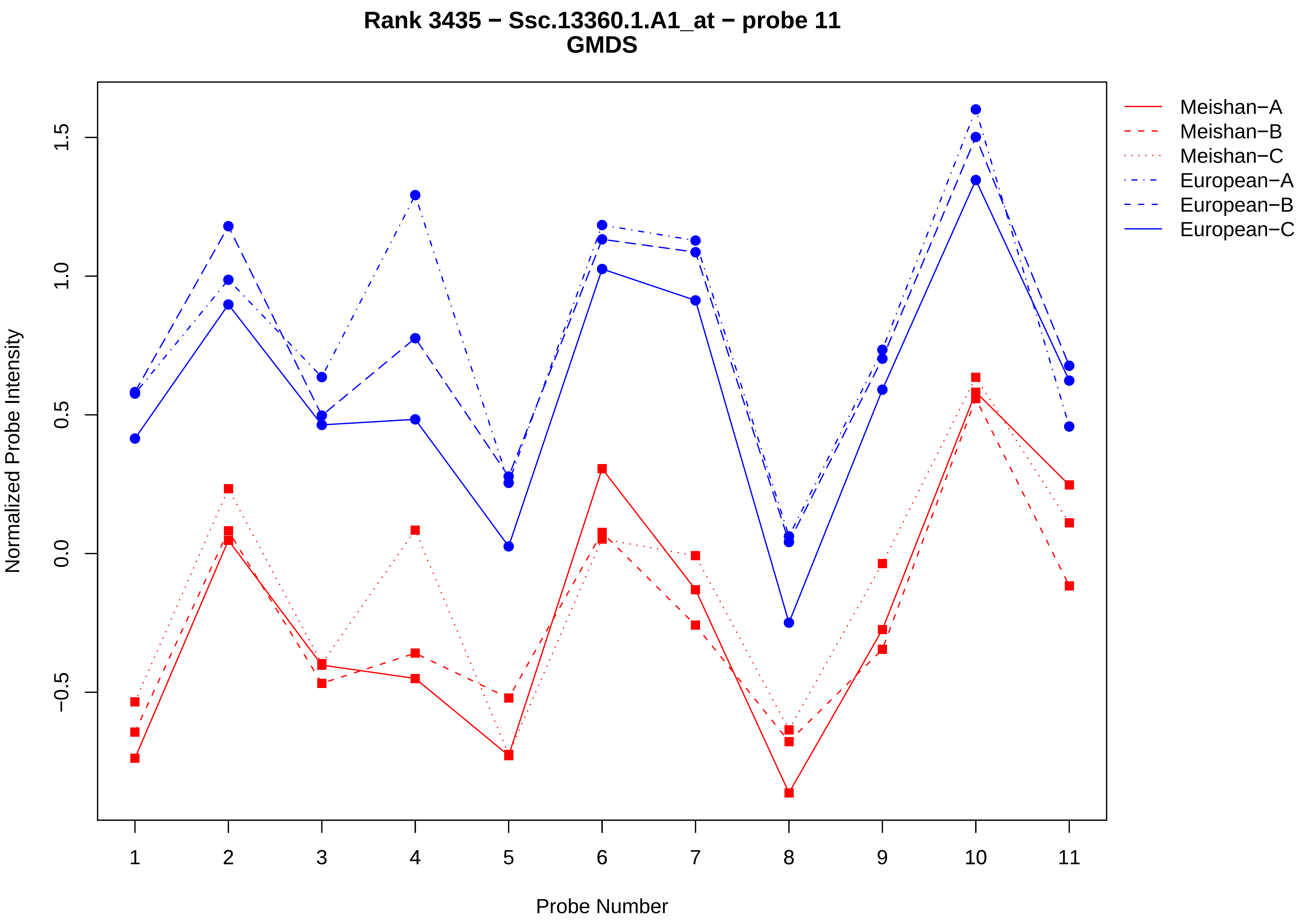

Rank 3435 − Ssc.13360.1.A1_at − probe 11
GMDS
Normalized Probe Intensity
Probe Number
−0.5
0.0
0.5
1.0
1.5
1
2
3
4
5
6
7
8
9
10
11
Meishan−A
Meishan−B
Meishan−C
European−A
European−B
European−C

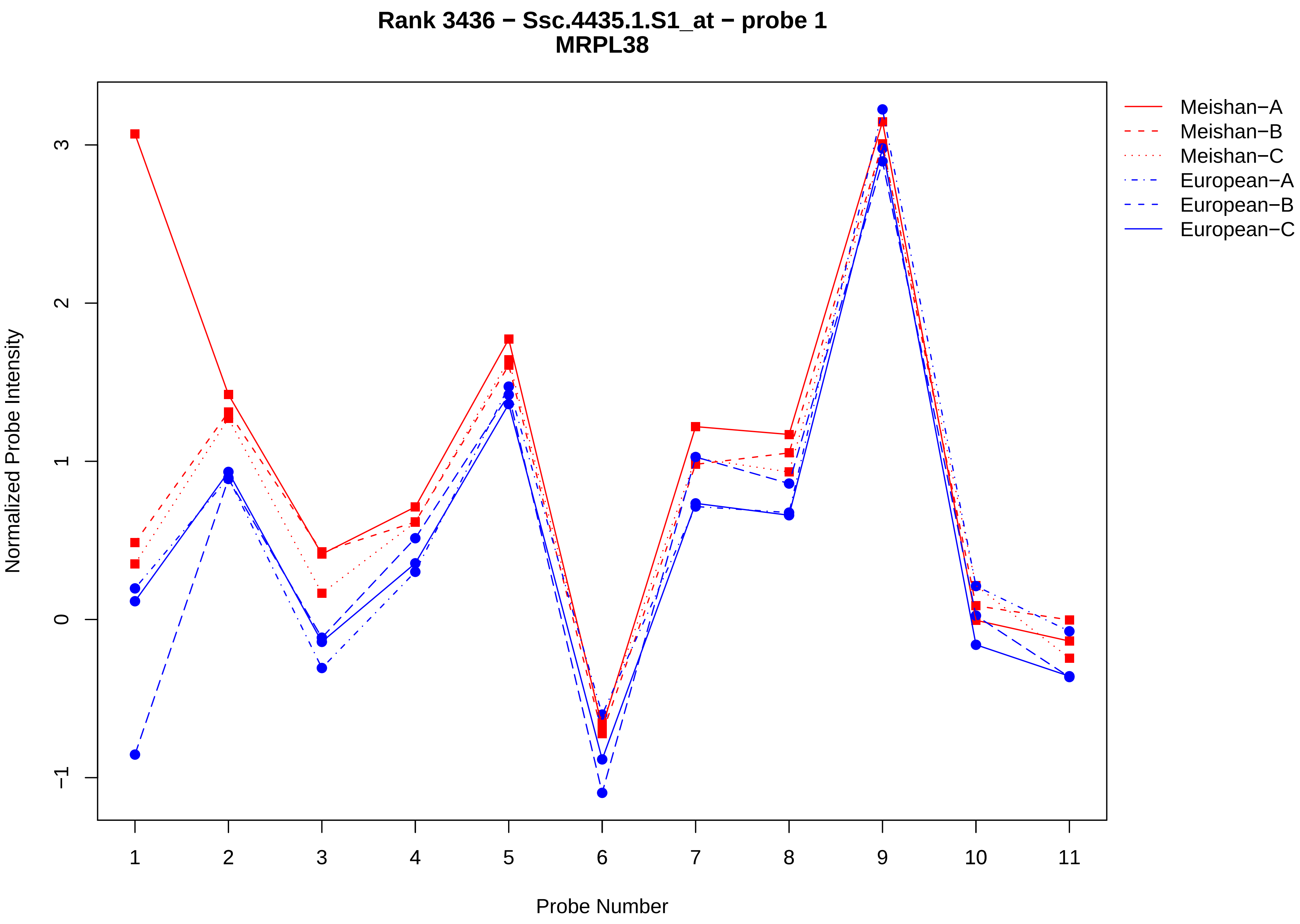
Rank 3436 − Ssc.4435.1.S1_at − probe 1
MRPL38
Normalized Probe Intensity
Probe Number
Meishan−A
Meishan−B
Meishan−C
European−A
European−B
European−C
−1
0
1
2
3
1
2
3
4
5
6
7
8
9
10
11

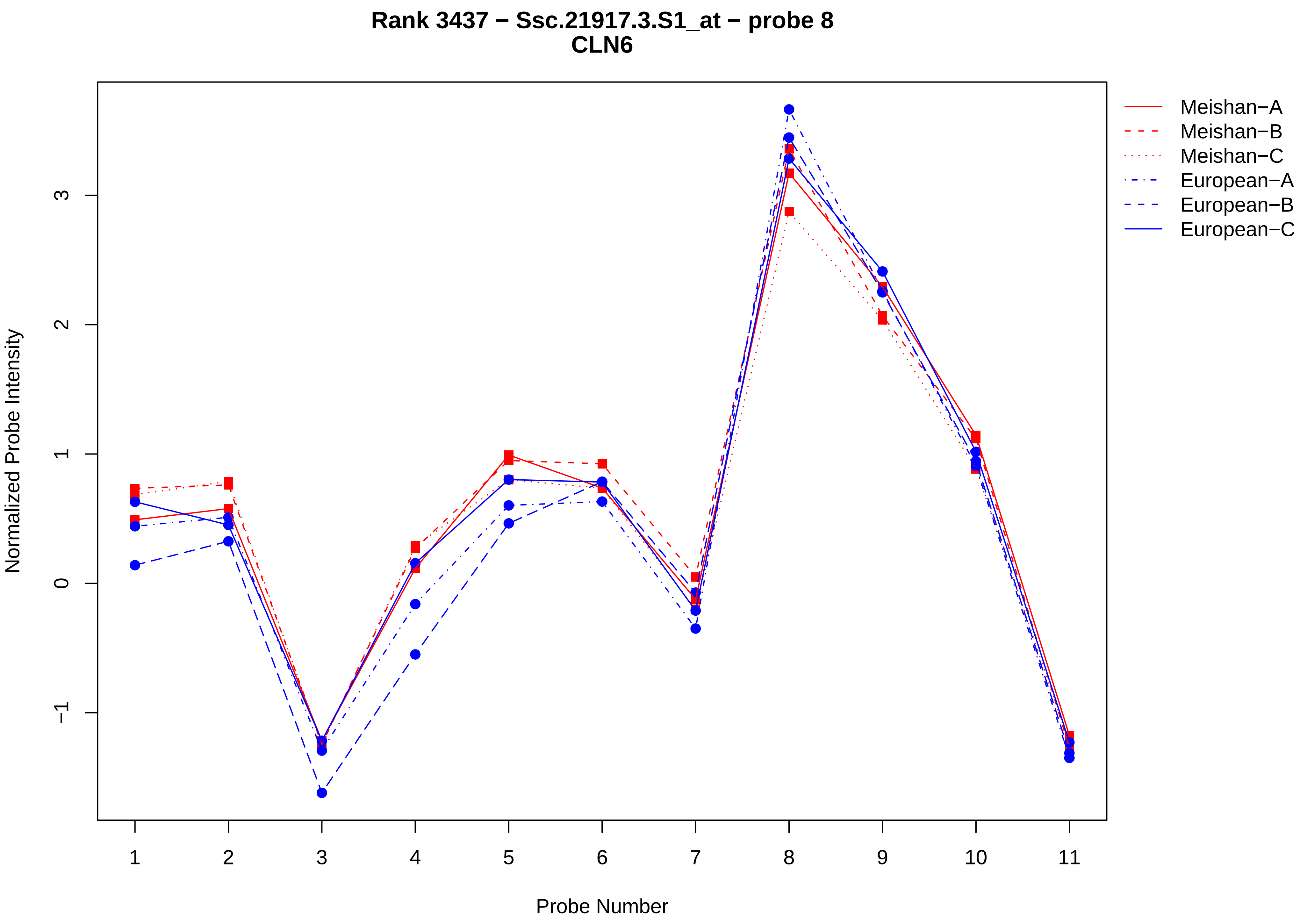

Rank 3437 − Ssc.21917.3.S1_at − probe 8
CLN6
Normalized Probe Intensity
Probe Number
Meishan−A
Meishan−B
Meishan−C
European−A
European−B
European−C

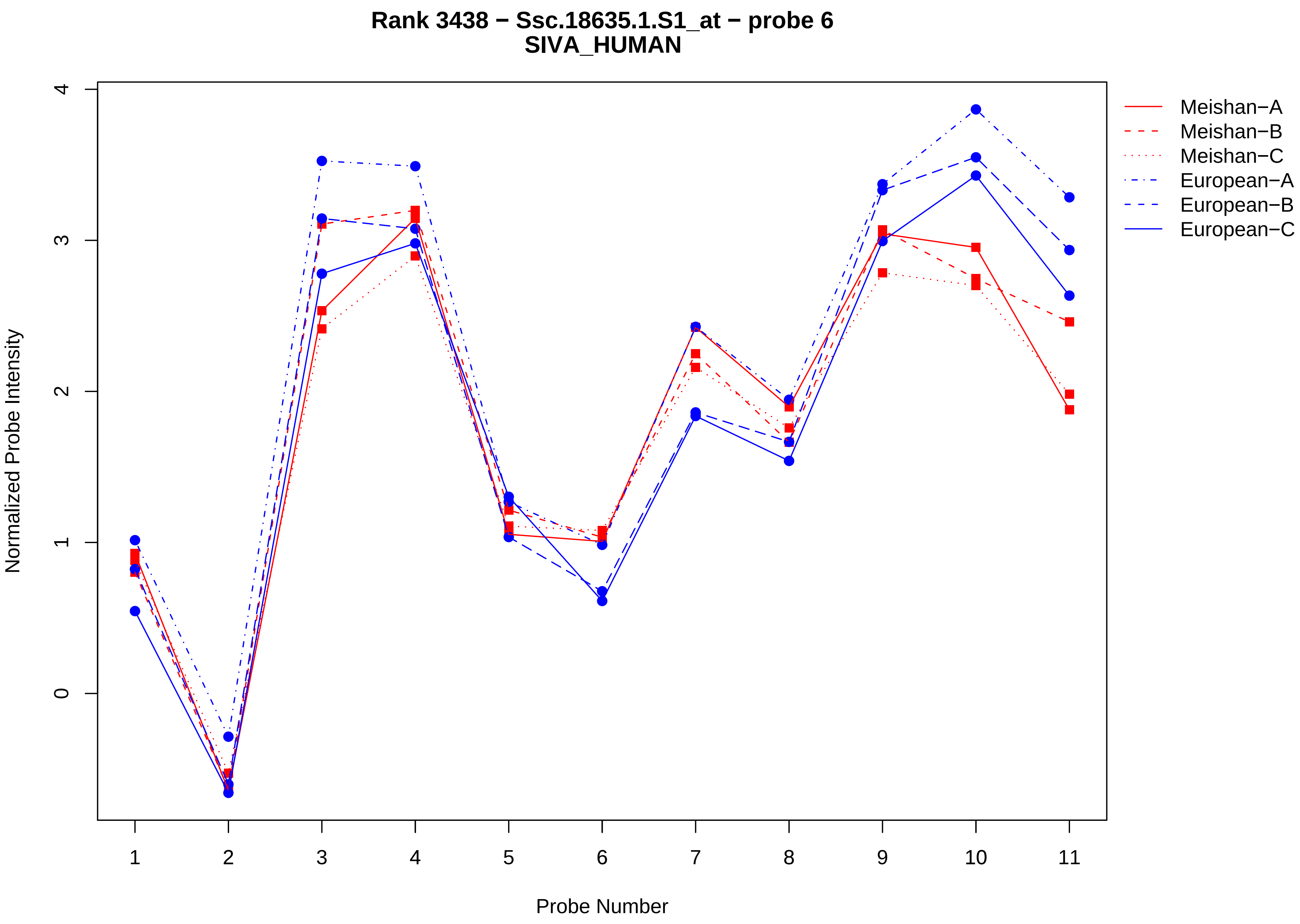

Rank 3438 − Ssc.18635.1.S1_at − probe 6
SIVA_HUMAN
Normalized Probe Intensity
Probe Number
0
1
2
3
4
1
2
3
4
5
6
7
8
9
10
11
Meishan−A
Meishan−B
Meishan−C
European−A
European−B
European−C

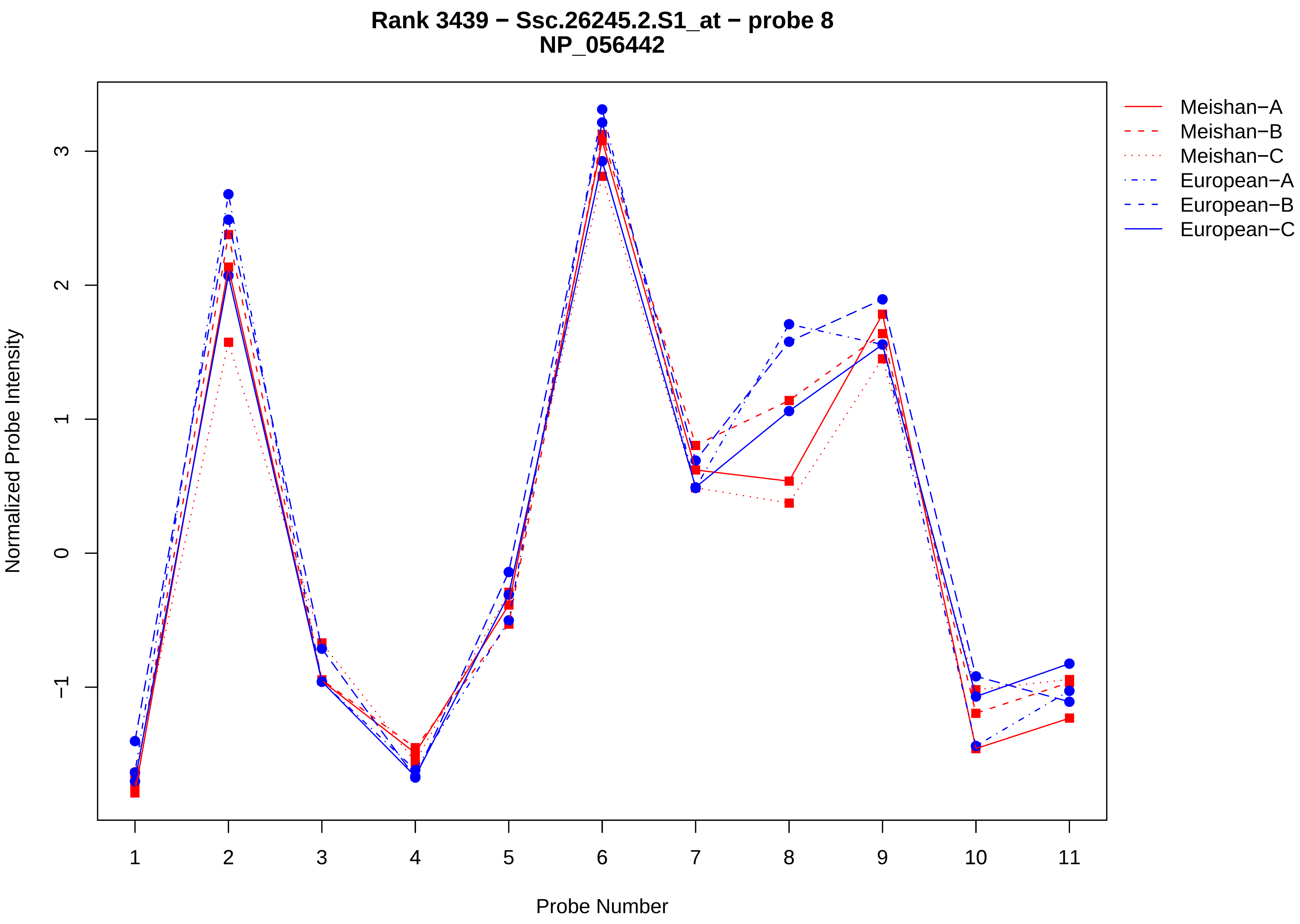

Rank 3439 − Ssc.26245.2.S1_at − probe 8
NP_056442
Normalized Probe Intensity
Probe Number
Meishan−A
Meishan−B
Meishan−C
European−A
European−B
European−C

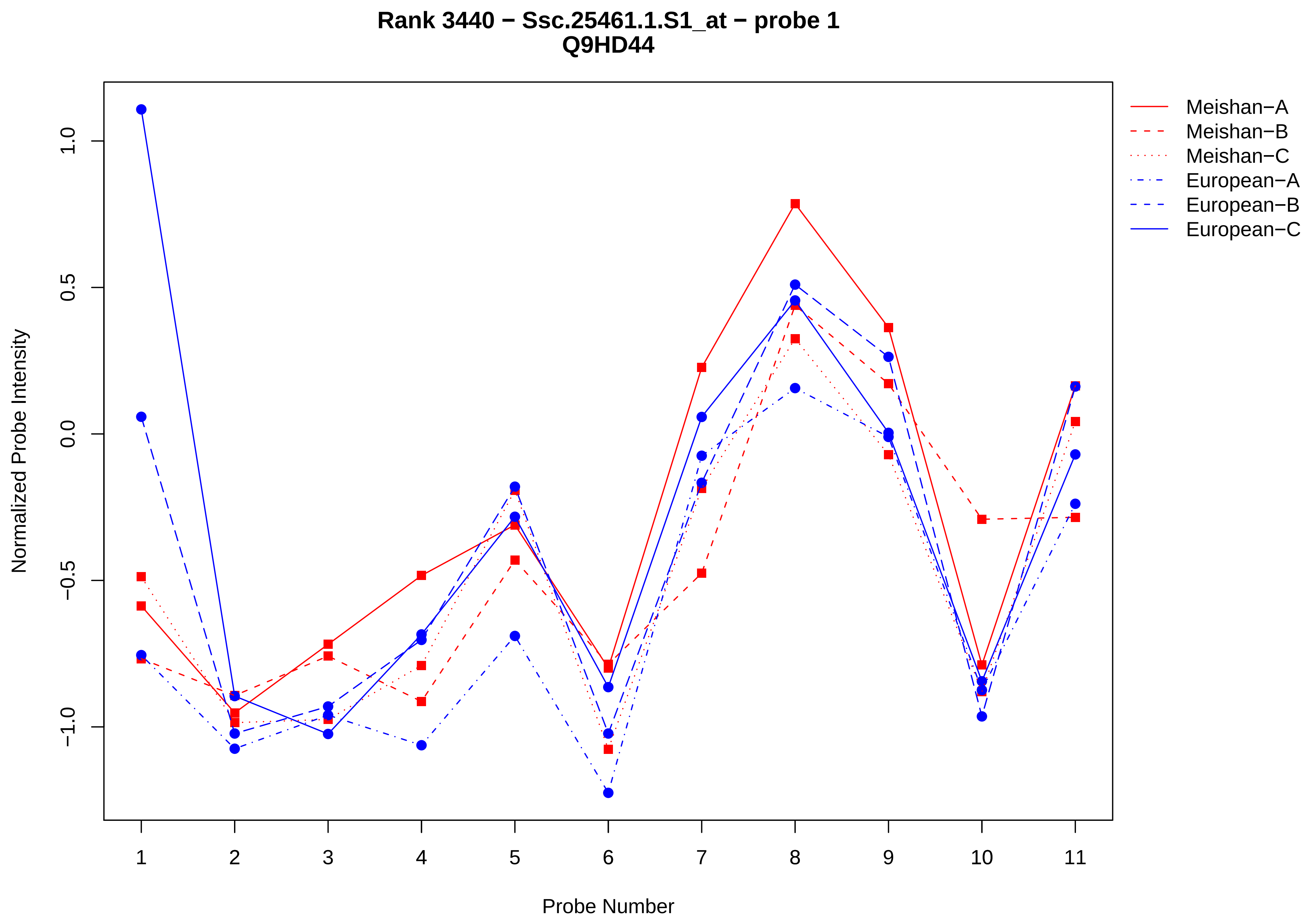

Rank 3440 − Ssc.25461.1.S1_at − probe 1
Q9HD44
Normalized Probe Intensity
Probe Number
Meishan−A
Meishan−B
Meishan−C
European−A
European−B
European−C
1.0
0.5
0.0
−0.5
−1.0
1
2
3
4
5
6
7
8
9
10
11

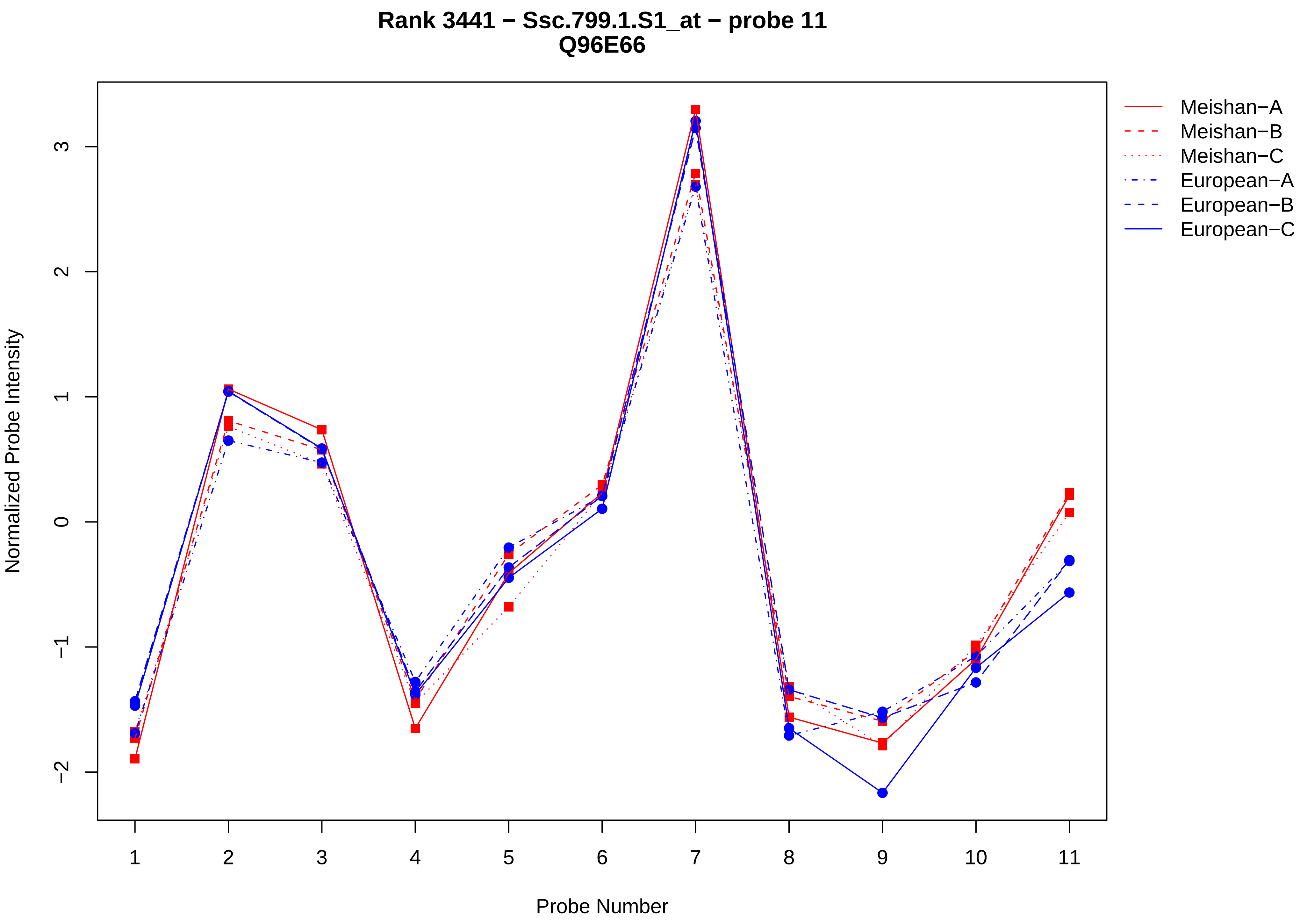

Rank 3441 − Ssc.799.1.S1_at − probe 11
Q96E66
Normalized Probe Intensity
Probe Number
−2
−1
0
1
2
3
1
2
3
4
5
6
7
8
9
10
11
Meishan−A
Meishan−B
Meishan−C
European−A
European−B
European−C

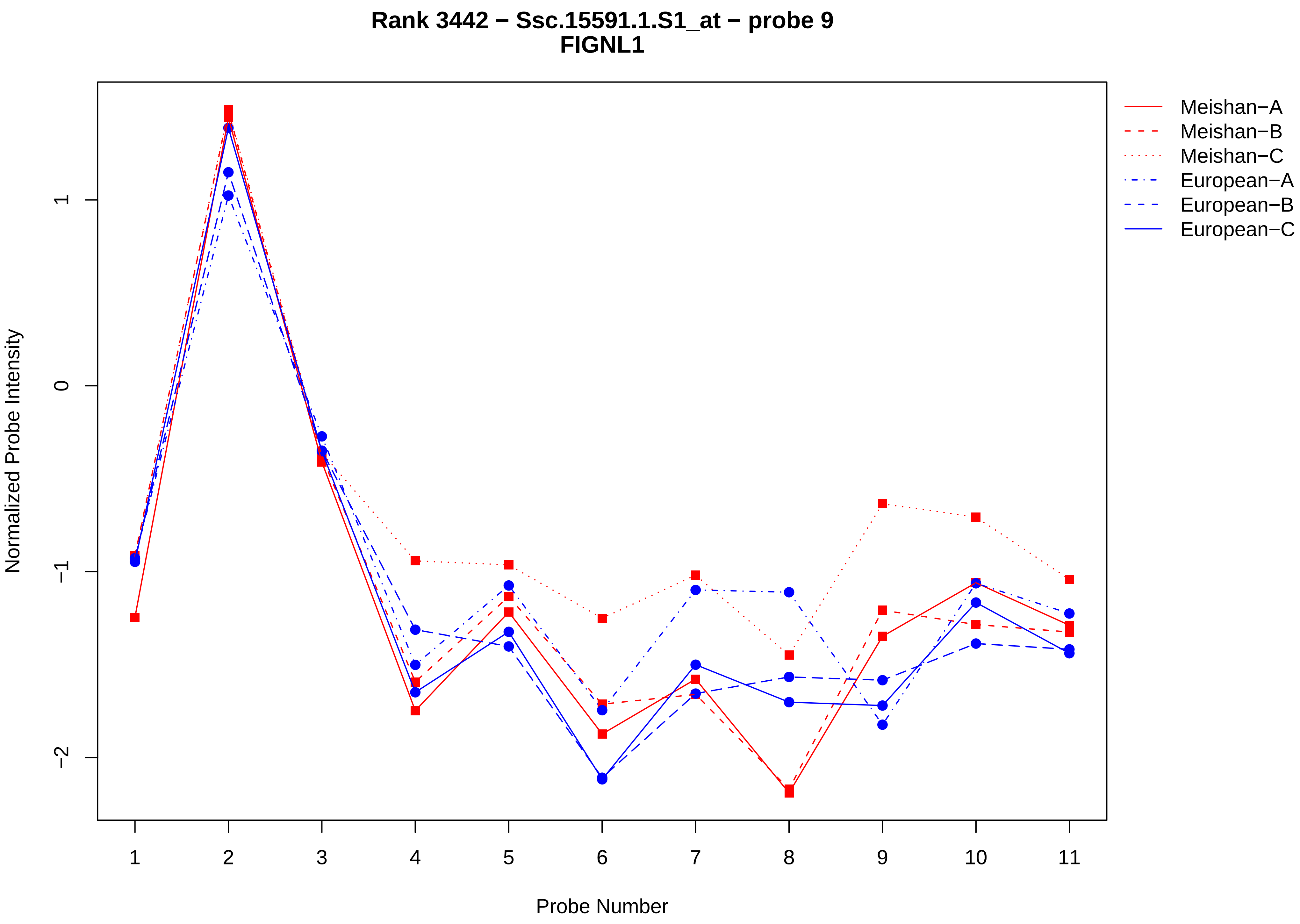

Rank 3442 − Ssc.15591.1.S1_at − probe 9
FIGNL1
Normalized Probe Intensity
1
0
−1
−2
1
2
3
4
5
6
7
8
9
10
11
Probe Number
Meishan−A
Meishan−B
Meishan−C
European−A
European−B
European−C

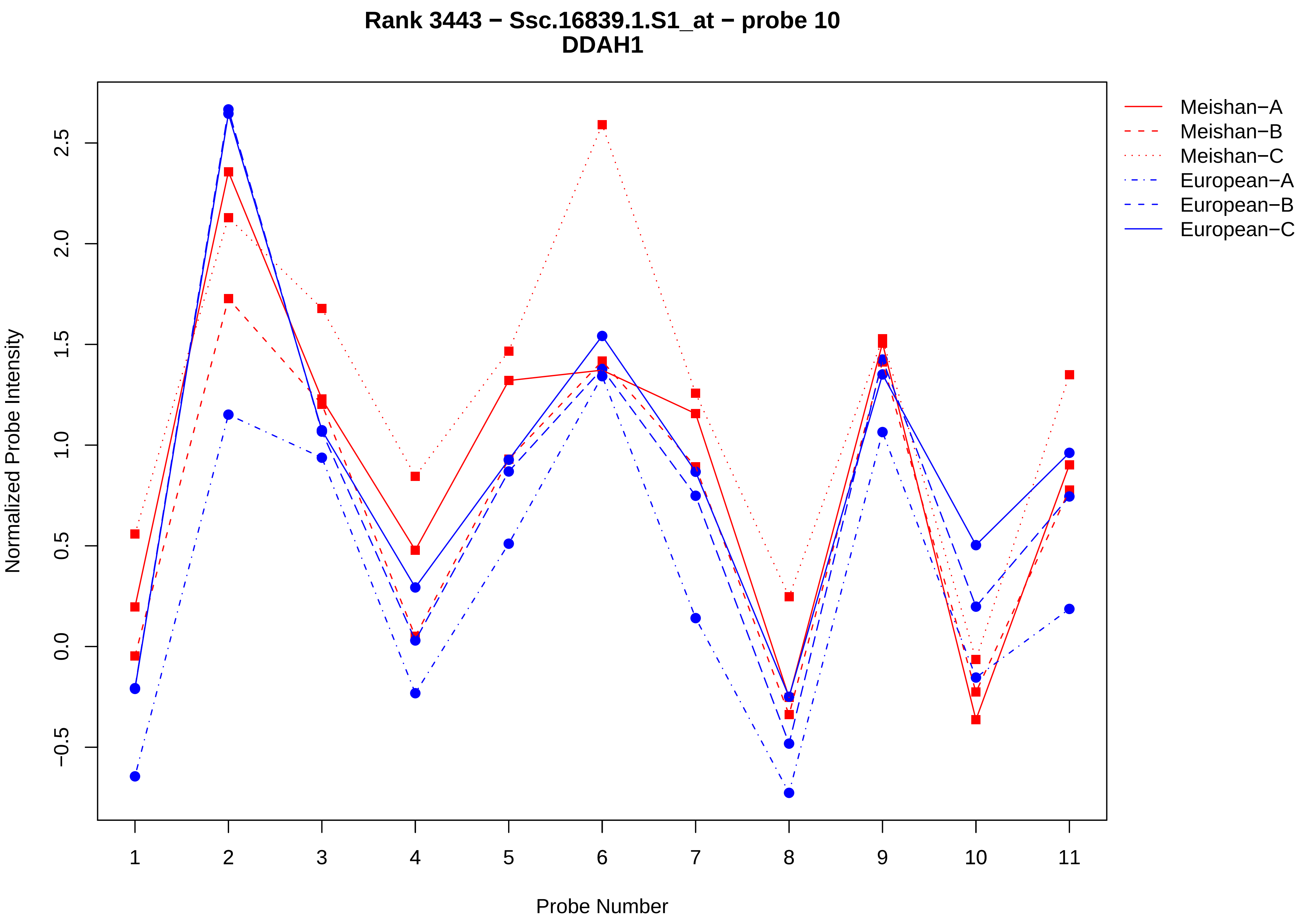

Rank 3443 − Ssc.16839.1.S1_at − probe 10
DDAH1
Normalized Probe Intensity
Probe Number
Meishan−A
Meishan−B
Meishan−C
European−A
European−B
European−C

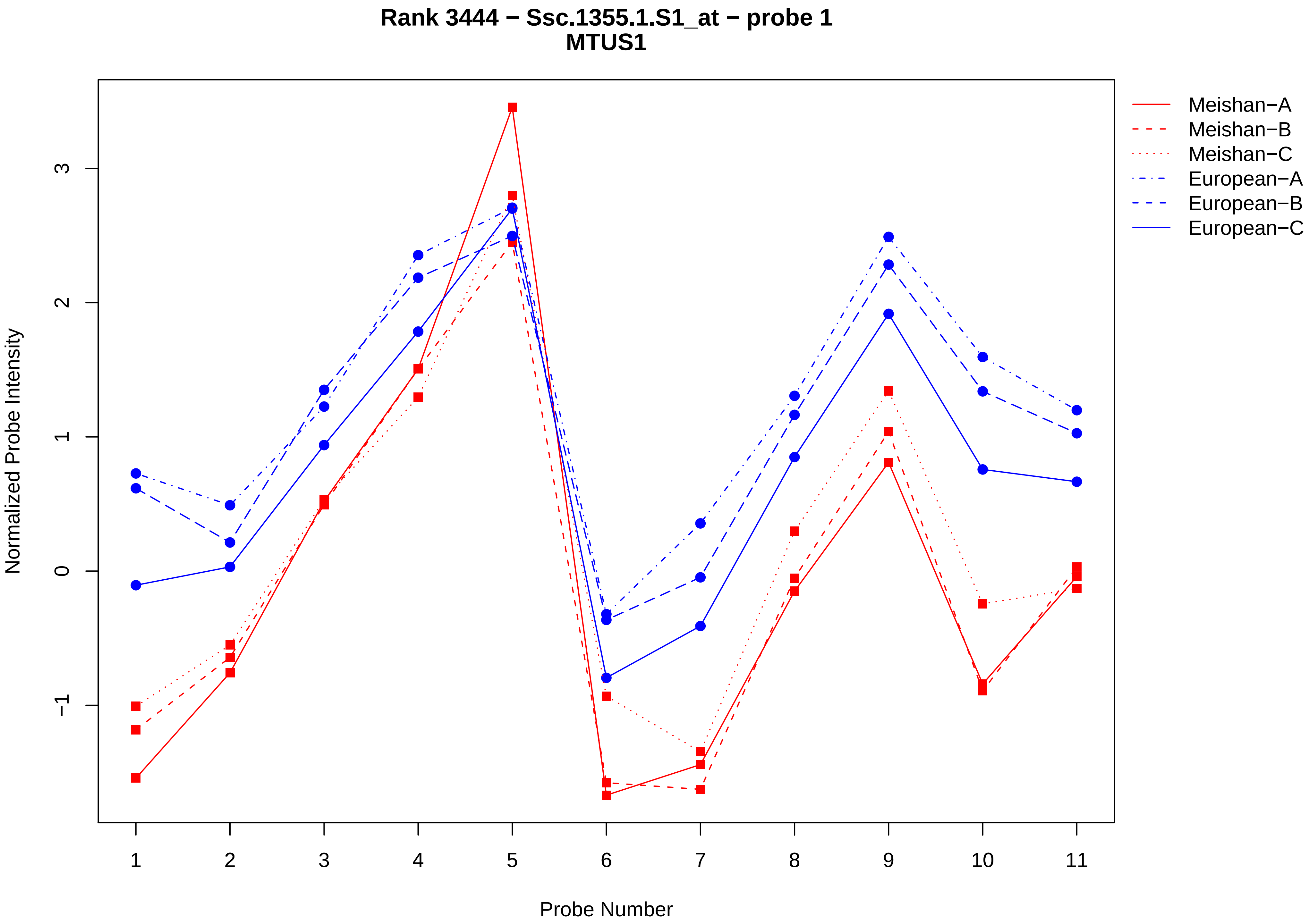

Rank 3444 − Ssc.1355.1.S1_at − probe 1
MTUS1
Normalized Probe Intensity
Probe Number
−1
0
1
2
3
1
2
3
4
5
6
7
8
9
10
11
Meishan−A
Meishan−B
Meishan−C
European−A
European−B
European−C

Rank 3445 − Ssc.28112.1.A1_at − probe 5
PPEF2

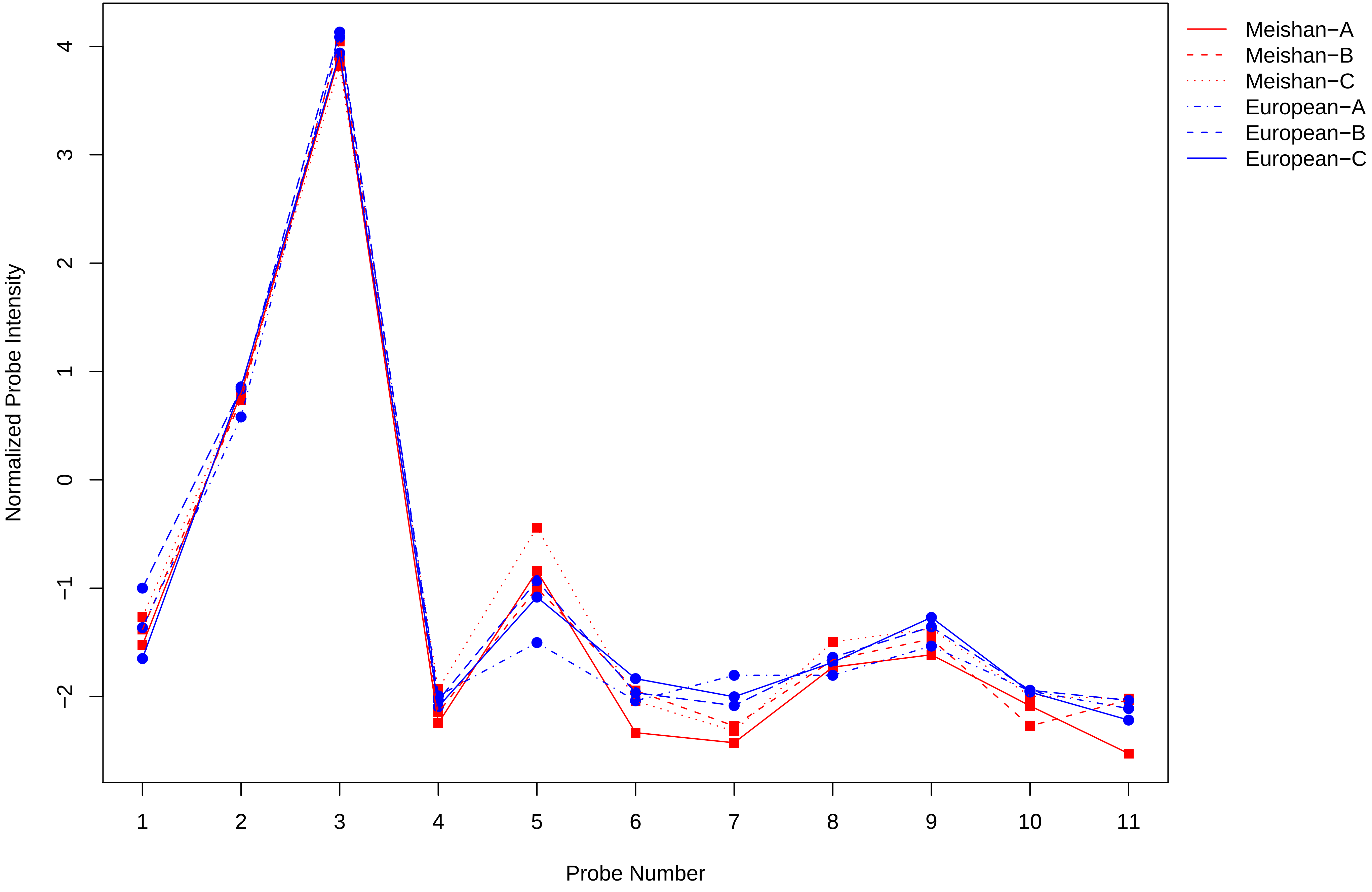

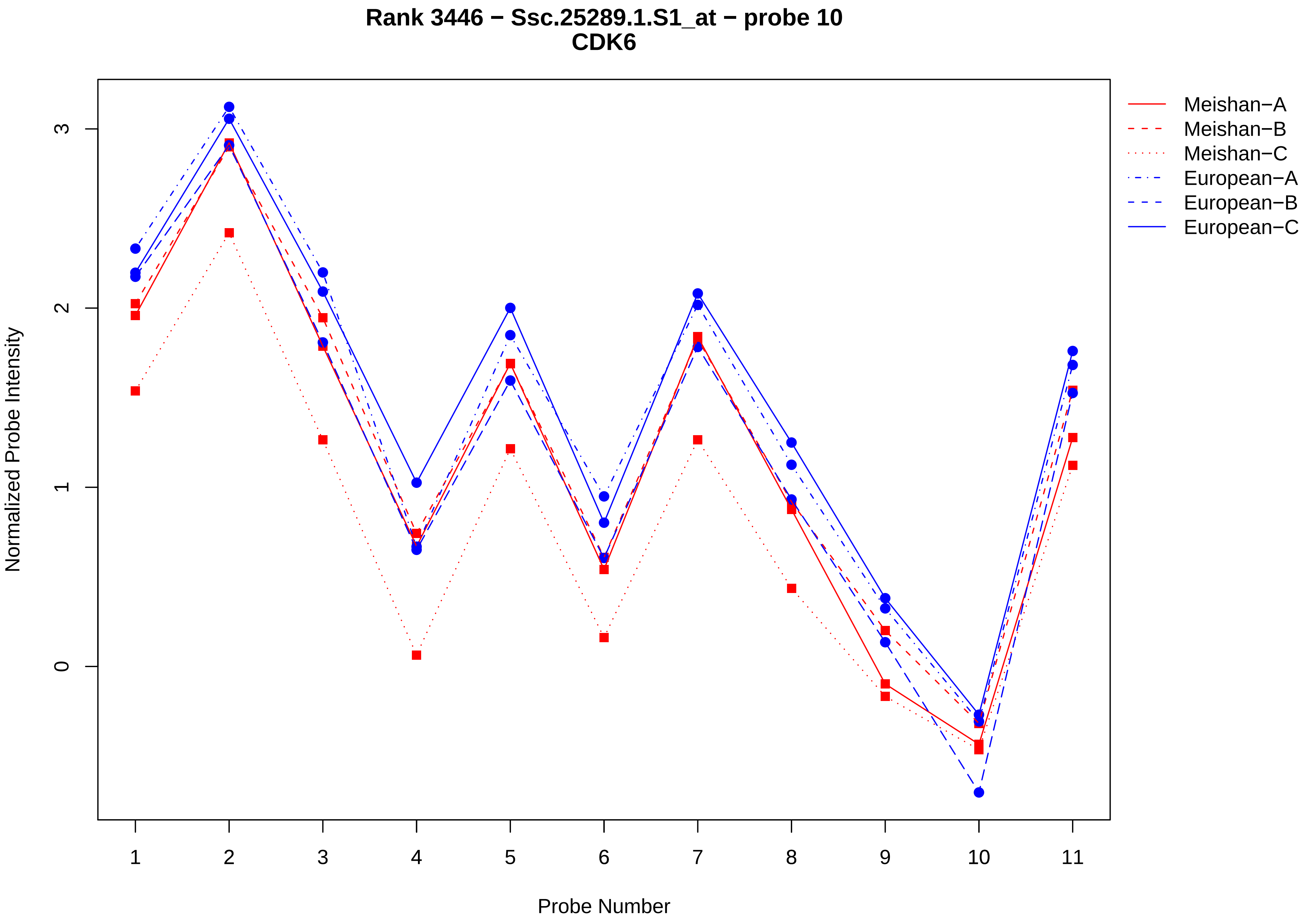

Rank 3446 − Ssc.25289.1.S1_at − probe 10
CDK6
Normalized Probe Intensity
Probe Number
0
1
2
3
1
2
3
4
5
6
7
8
9
10
11
Meishan−A
Meishan−B
Meishan−C
European−A
European−B
European−C

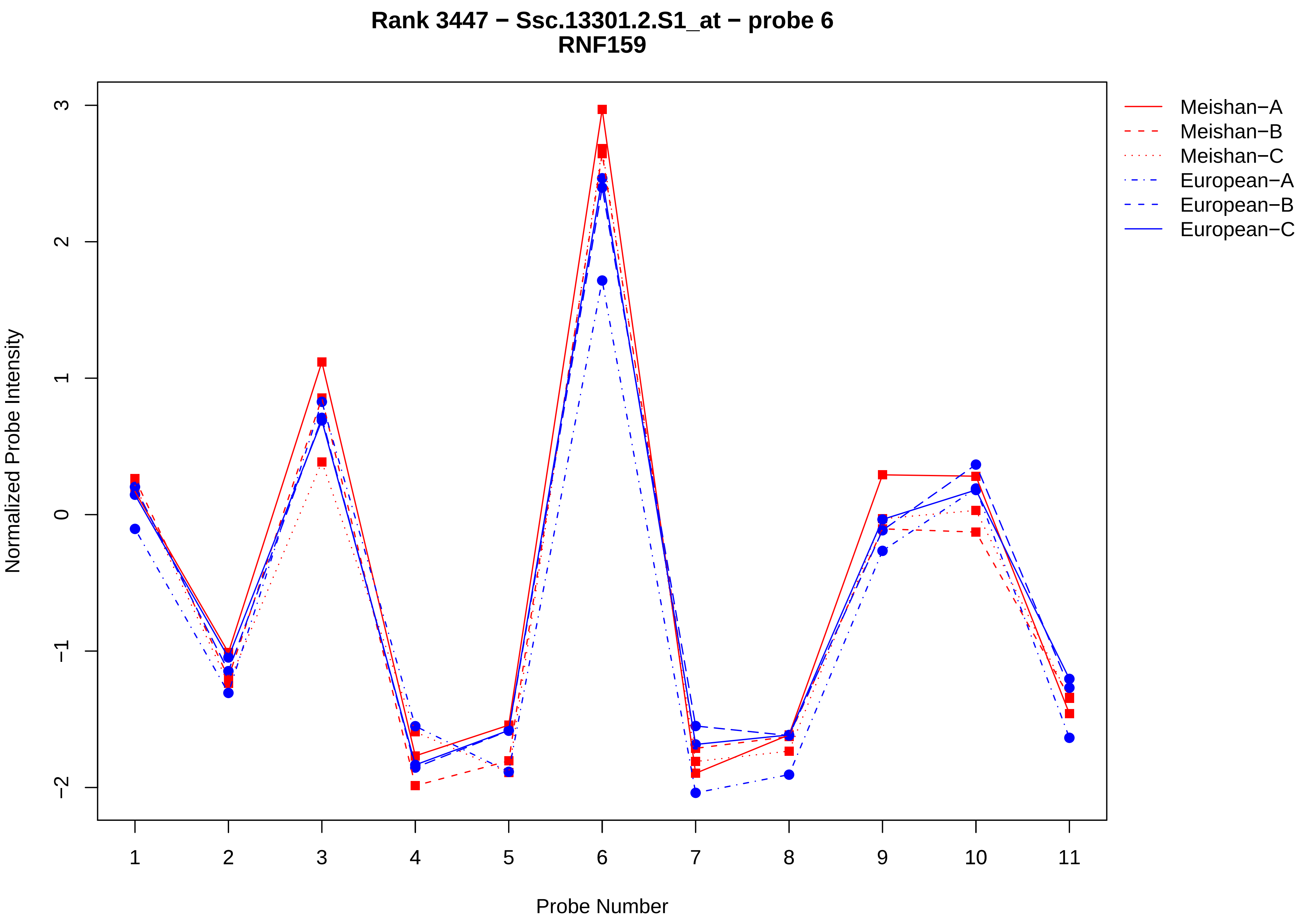

Rank 3447 − Ssc.13301.2.S1_at − probe 6
RNF159
Normalized Probe Intensity
Probe Number
Meishan−A
Meishan−B
Meishan−C
European−A
European−B
European−C
−2
−1
0
1
2
3
1
2
3
4
5
6
7
8
9
10
11

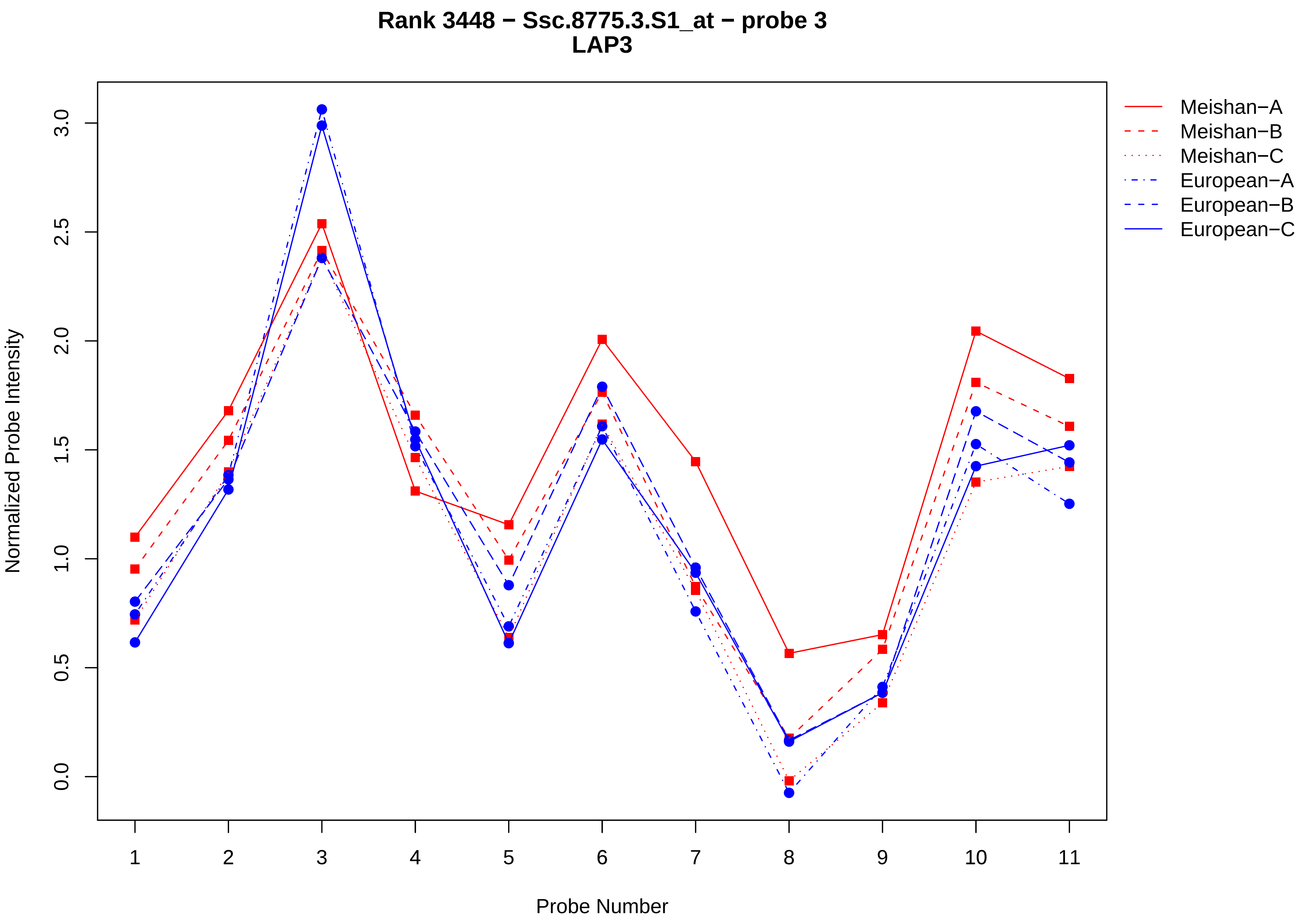
Rank 3448 − Ssc.8775.3.S1_at − probe 3
LAP3
Normalized Probe Intensity
Probe Number
0.0
0.5
1.0
1.5
2.0
2.5
3.0
1
2
3
4
5
6
7
8
9
10
11
Meishan−A
Meishan−B
Meishan−C
European−A
European−B
European−C

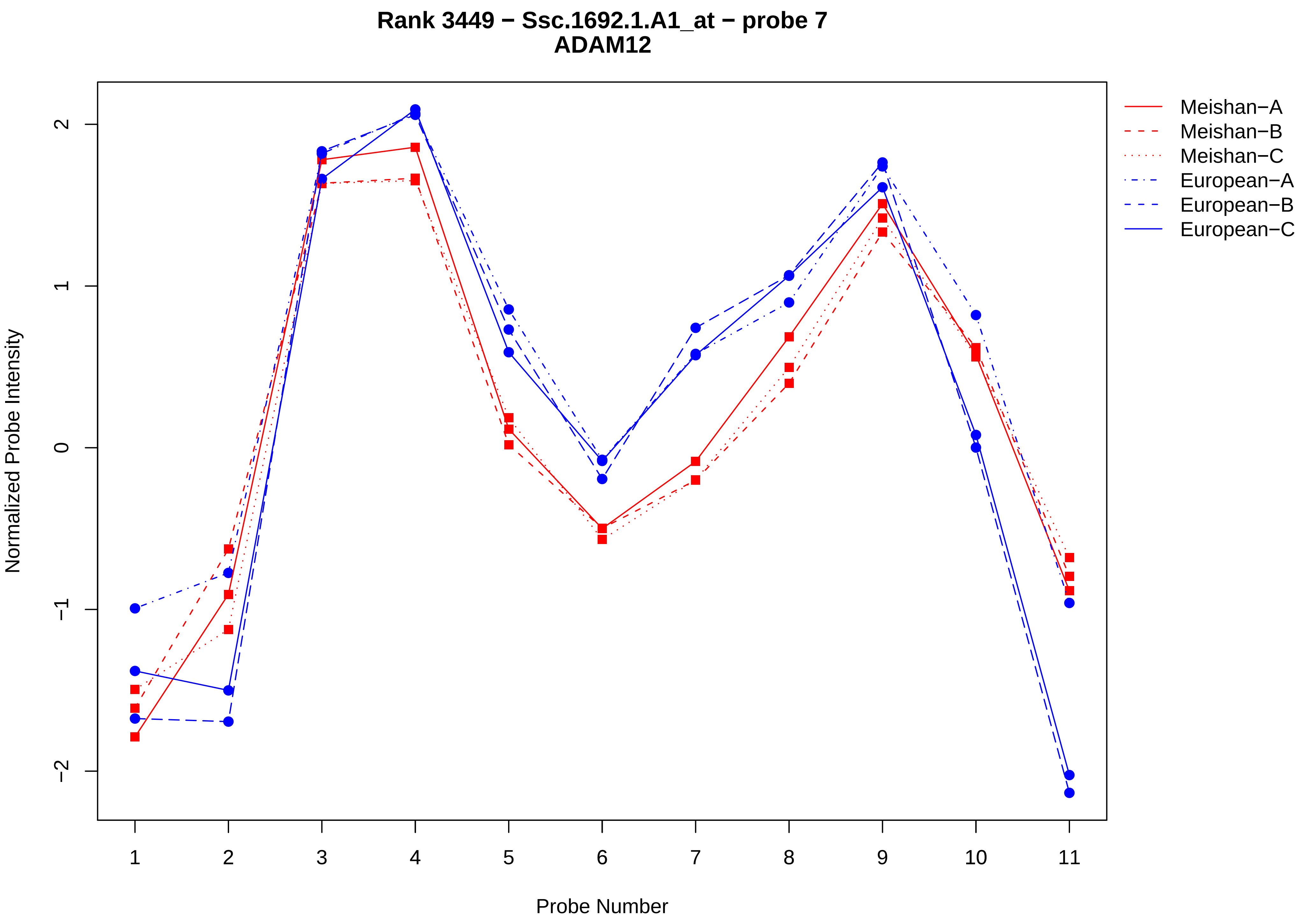

Rank 3449 − Ssc.1692.1.A1_at − probe 7
ADAM12
Normalized Probe Intensity
Probe Number
−2
−1
0
1
2
1
2
3
4
5
6
7
8
9
10
11
Meishan−A
Meishan−B
Meishan−C
European−A
European−B
European−C

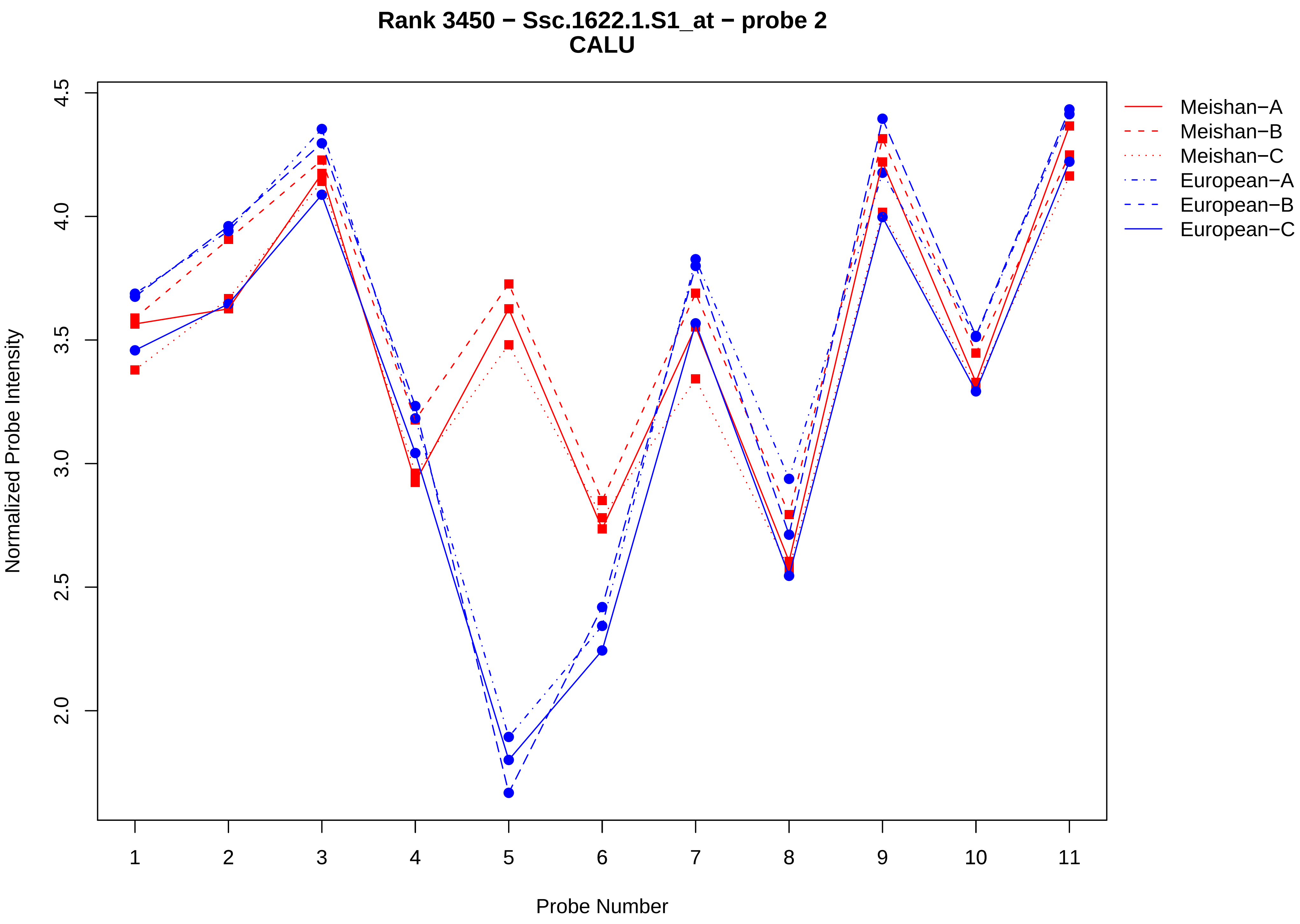

Rank 3450 − Ssc.1622.1.S1_at − probe 2
CALU
Normalized Probe Intensity
Probe Number
Meishan−A
Meishan−B
Meishan−C
European−A
European−B
European−C

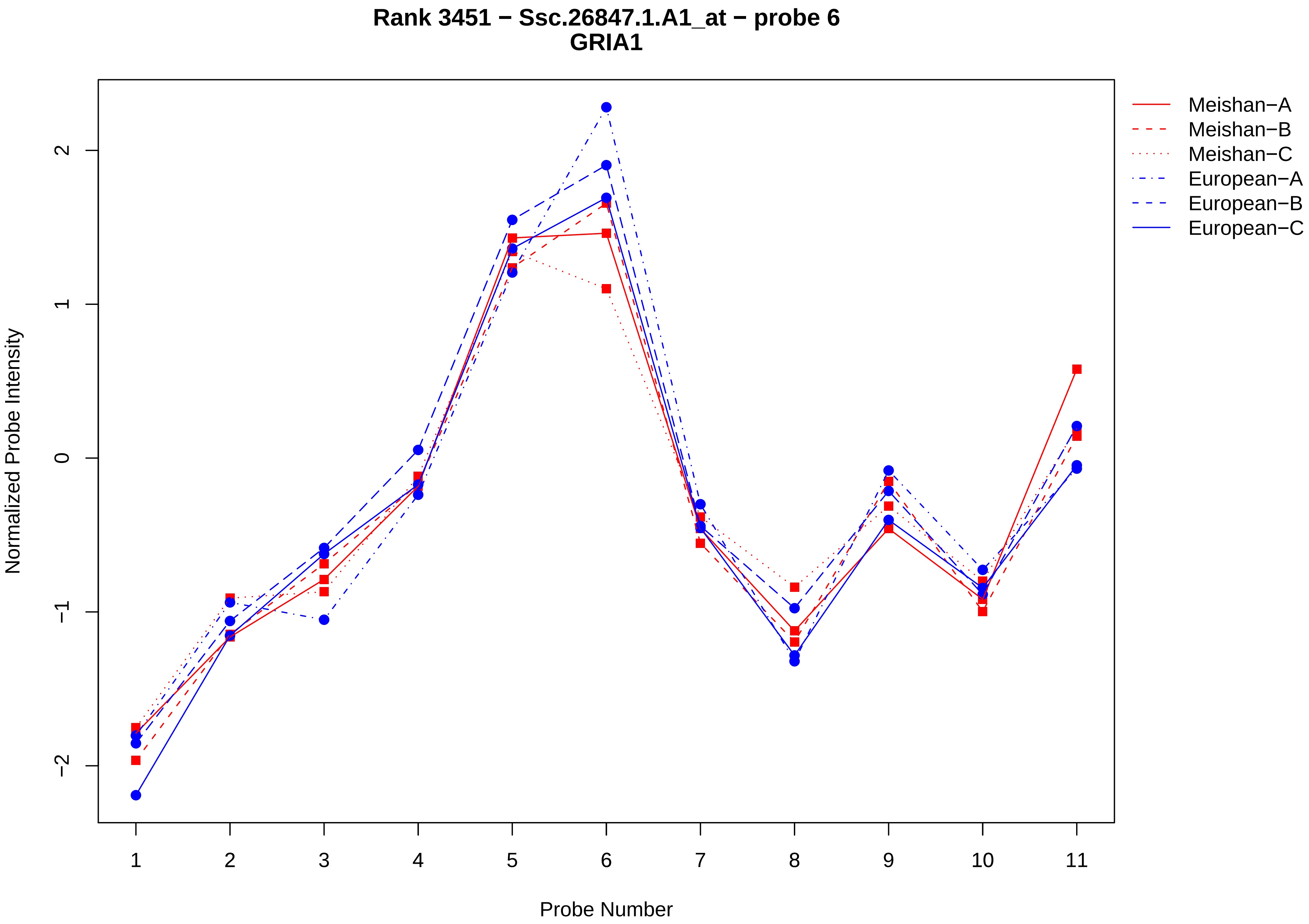

Rank 3451 − Ssc.26847.1.A1_at − probe 6
GRIA1
Normalized Probe Intensity
Probe Number
Meishan−A
Meishan−B
Meishan−C
European−A
European−B
European−C

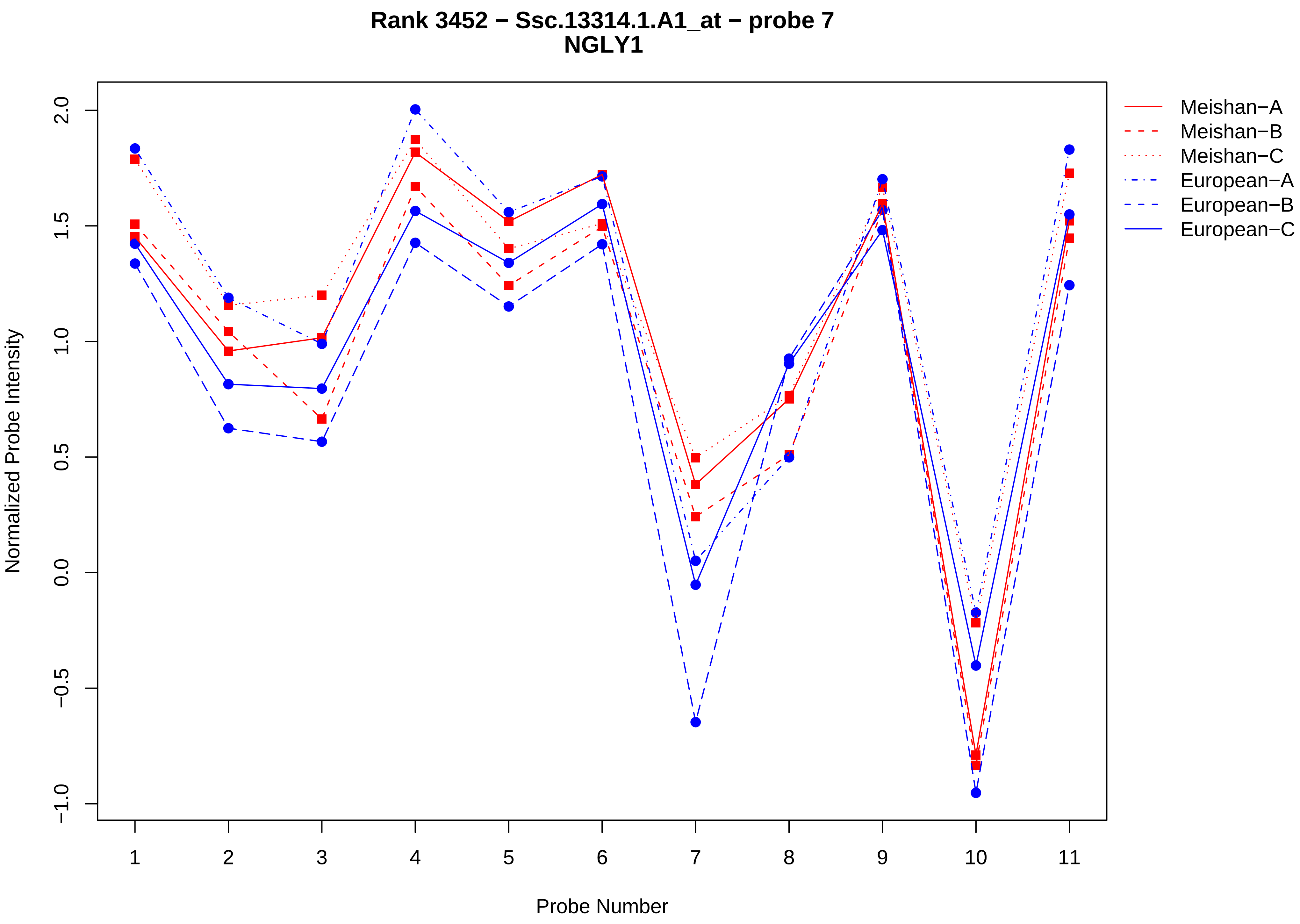
Rank 3452 − Ssc.13314.1.A1_at − probe 7
NGLY1
Normalized Probe Intensity
Probe Number
Meishan−A
Meishan−B
Meishan−C
European−A
European−B
European−C
−1.0
−0.5
0.0
0.5
1.0
1.5
2.0
1
2
3
4
5
6
7
8
9
10
11

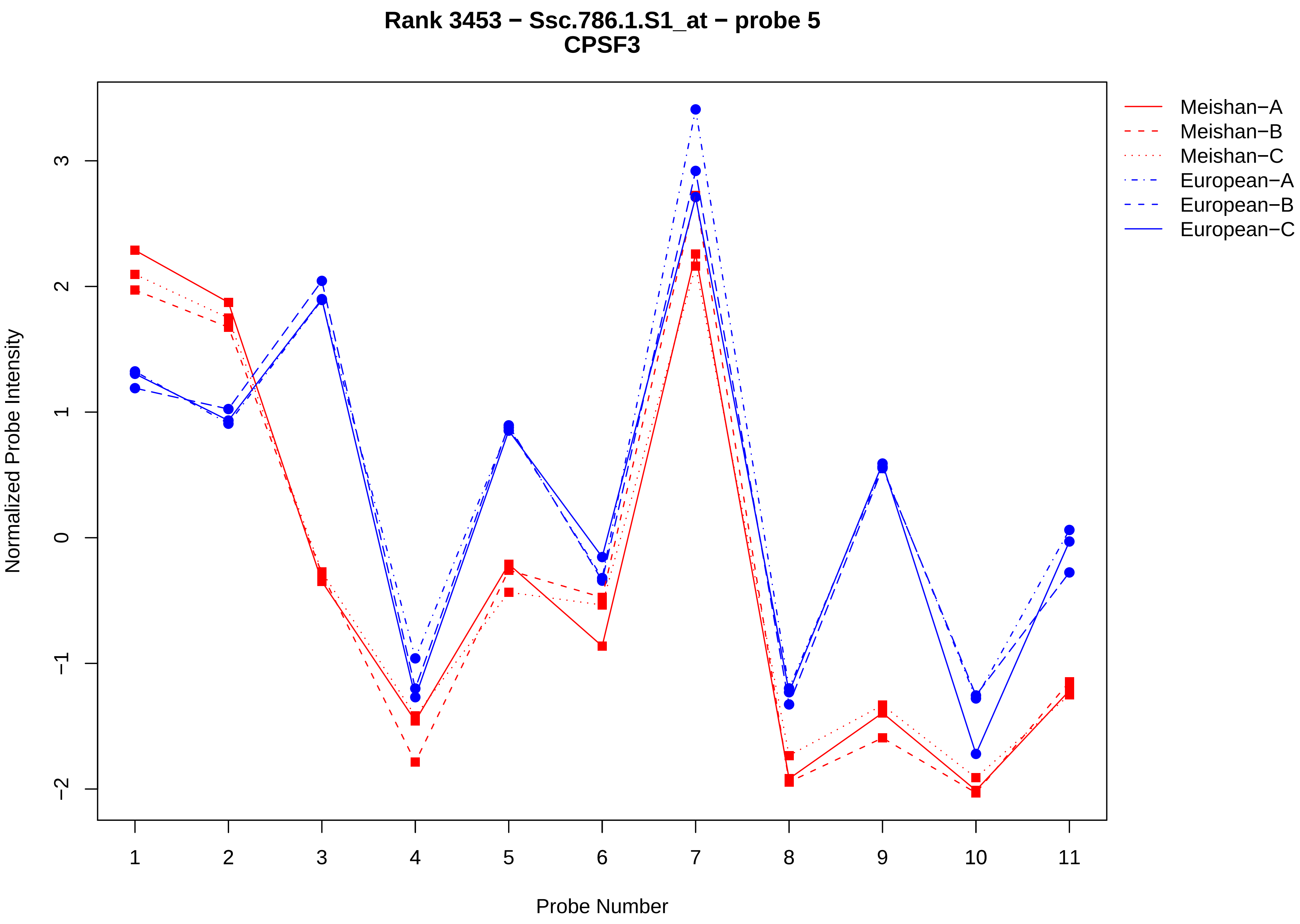

Rank 3453 − Ssc.786.1.S1_at − probe 5
CPSF3
Normalized Probe Intensity
Probe Number
Meishan−A
Meishan−B
Meishan−C
European−A
European−B
European−C

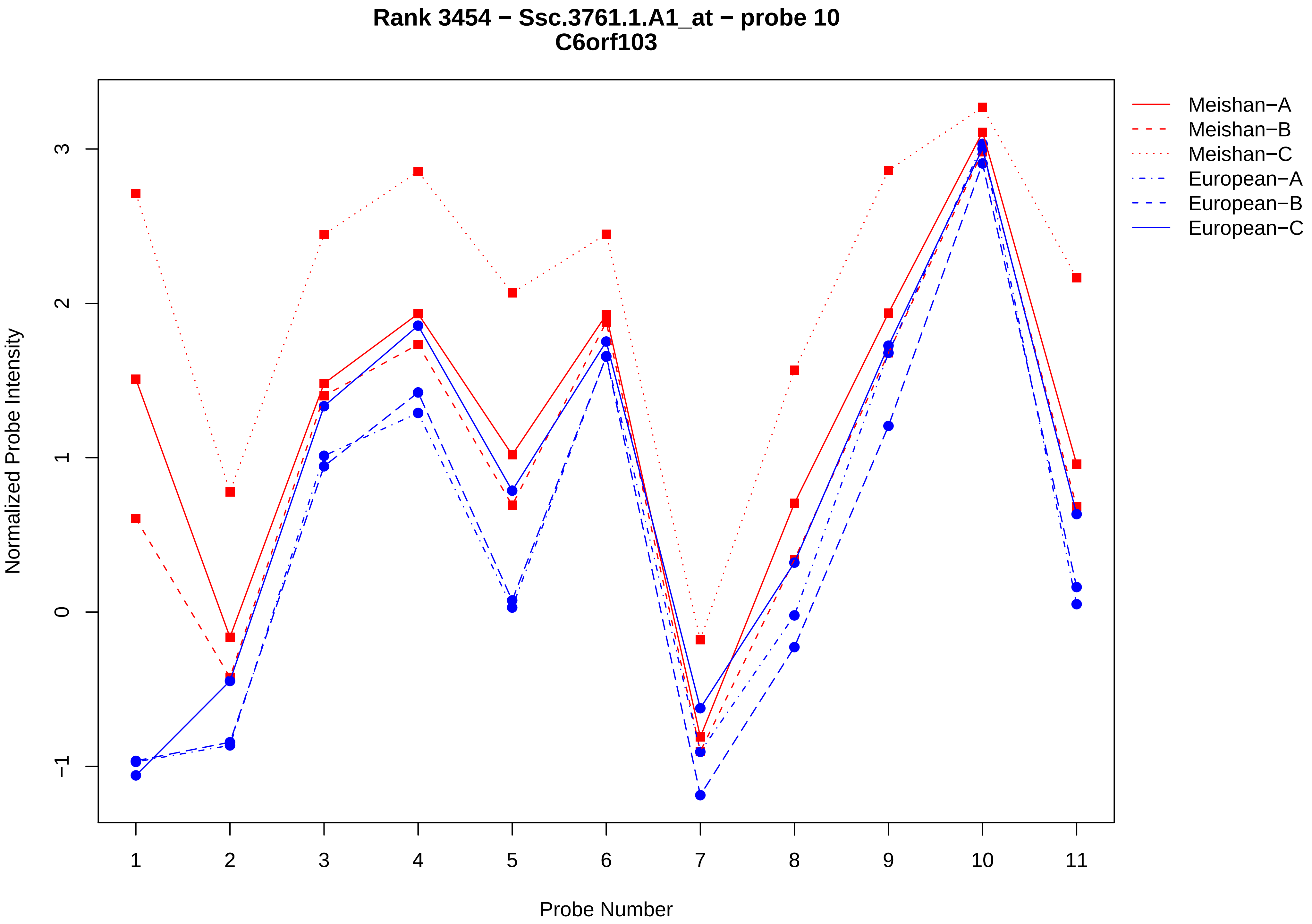
Rank 3454 − Ssc.3761.1.A1_at − probe 10
C6orf103
Normalized Probe Intensity
Probe Number
Meishan−A
Meishan−B
Meishan−C
European−A
European−B
European−C
−1
0
1
2
3
1
2
3
4
5
6
7
8
9
10
11

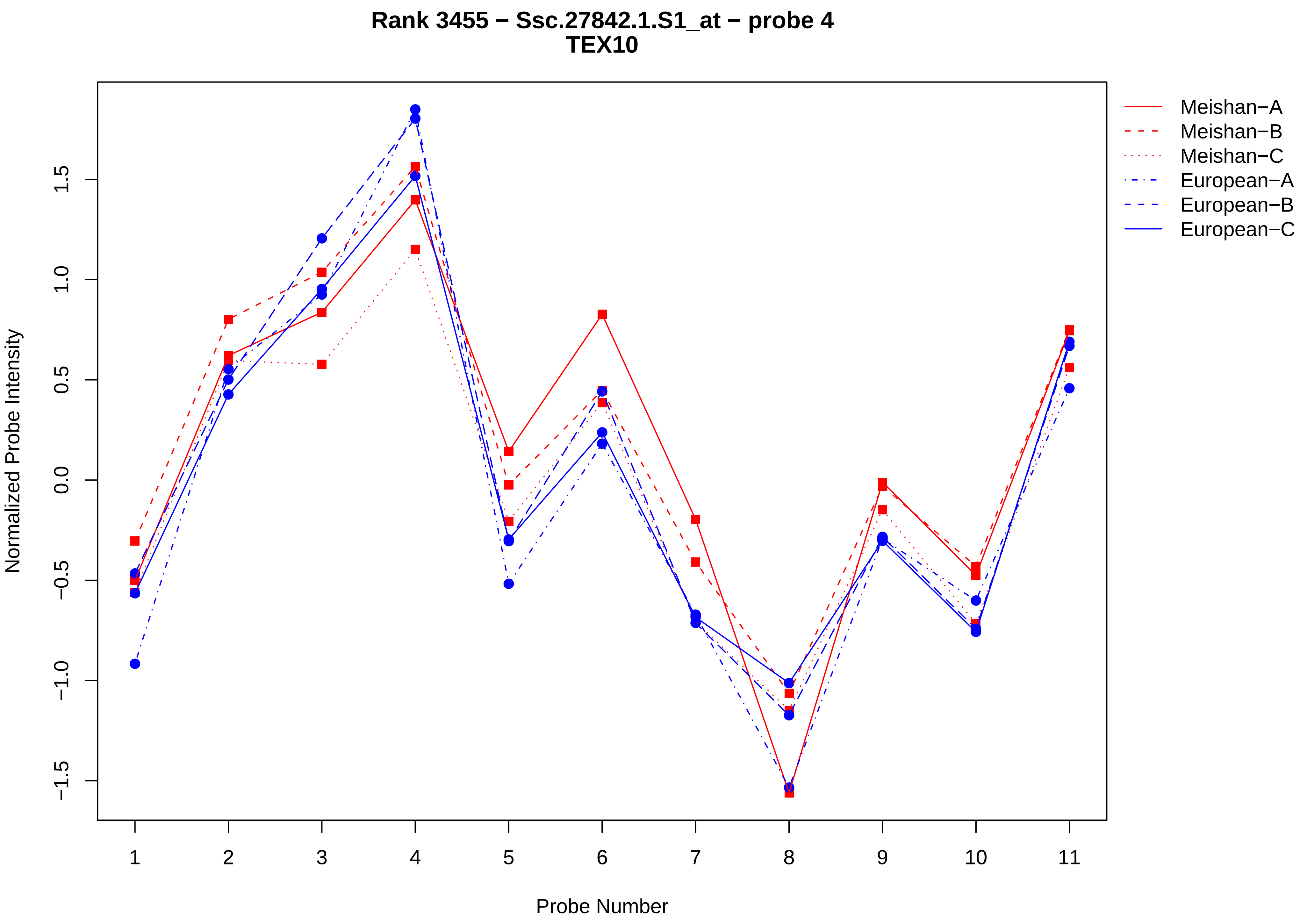
Rank 3455 − Ssc.27842.1.S1_at − probe 4
TEX10
Normalized Probe Intensity
Probe Number
Meishan−A
Meishan−B
Meishan−C
European−A
European−B
European−C
−1.5
−1.0
−0.5
0.0
0.5
1.0
1.5
1
2
3
4
5
6
7
8
9
10
11

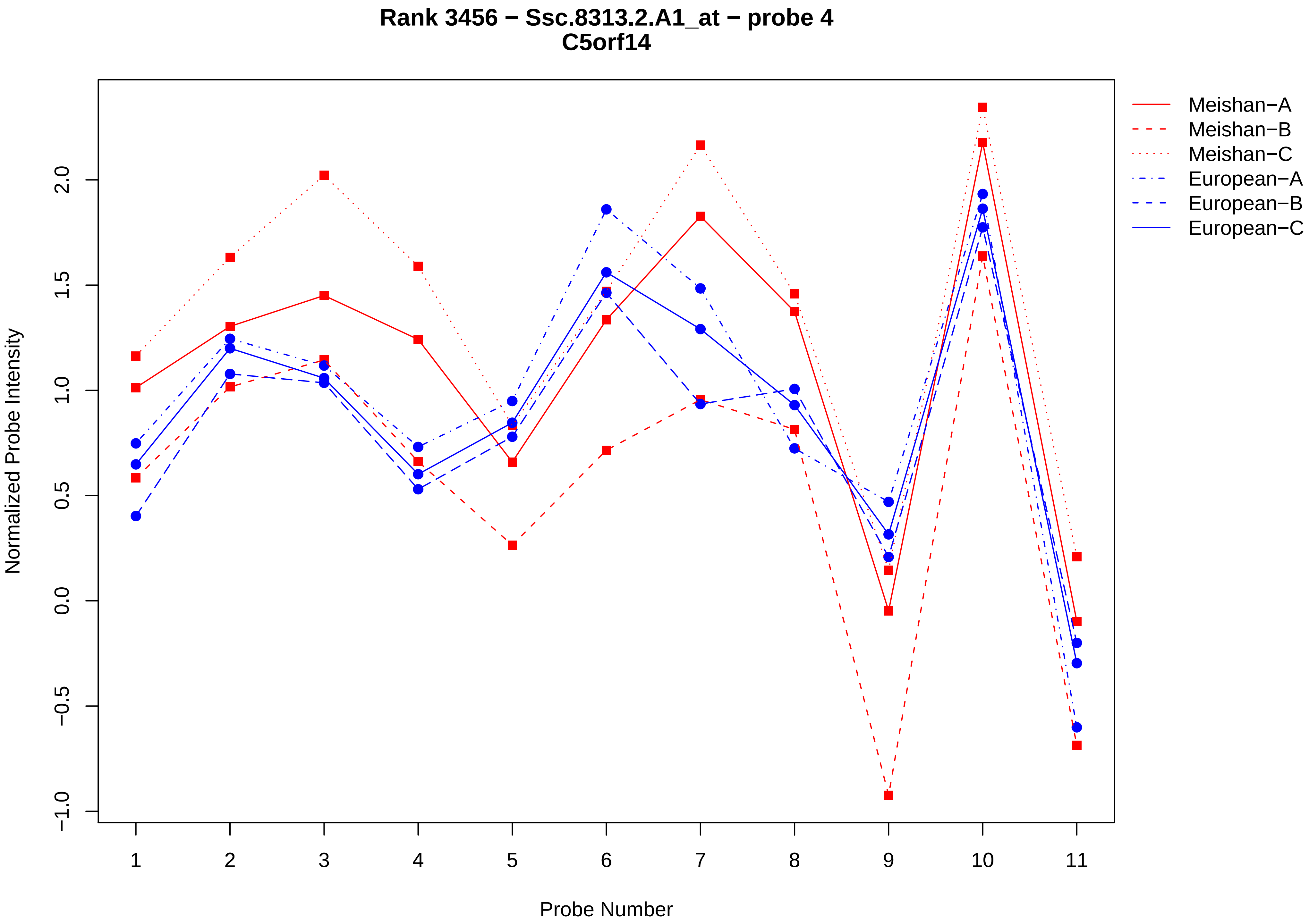

Rank 3456 − Ssc.8313.2.A1_at − probe 4
C5orf14
Normalized Probe Intensity
Probe Number
−1.0
−0.5
0.0
0.5
1.0
1.5
2.0
1
2
3
4
5
6
7
8
9
10
11
Meishan−A
Meishan−B
Meishan−C
European−A
European−B
European−C

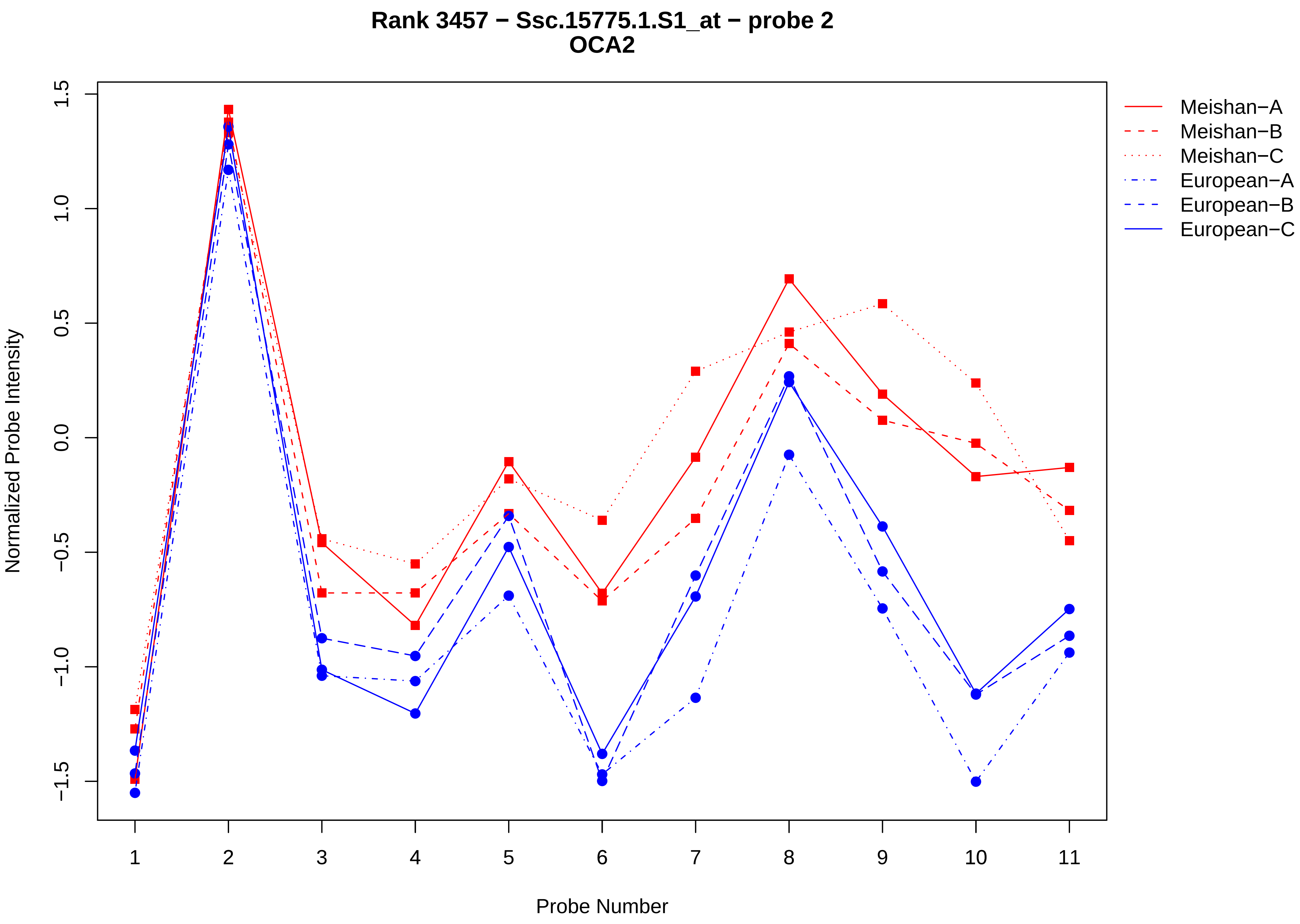

Rank 3457 − Ssc.15775.1.S1_at − probe 2
OCA2
Normalized Probe Intensity
Probe Number
1.5
1.0
0.5
0.0
−0.5
−1.0
−1.5
1
2
3
4
5
6
7
8
9
10
11
Meishan−A
Meishan−B
Meishan−C
European−A
European−B
European−C

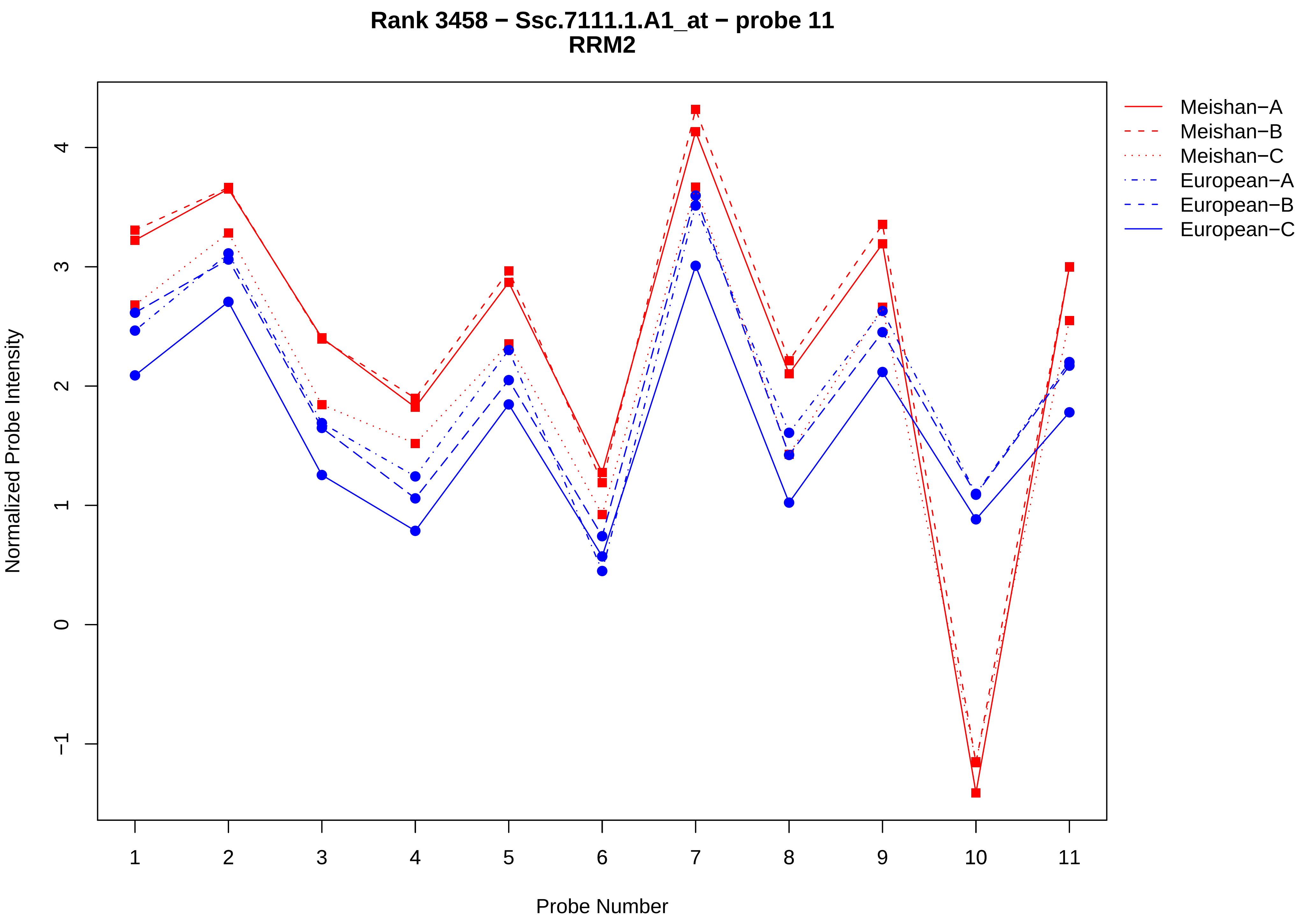

Rank 3458 − Ssc.7111.1.A1_at − probe 11
RRM2
Normalized Probe Intensity
Probe Number
−1
0
1
2
3
4
1
2
3
4
5
6
7
8
9
10
11
Meishan−A
Meishan−B
Meishan−C
European−A
European−B
European−C

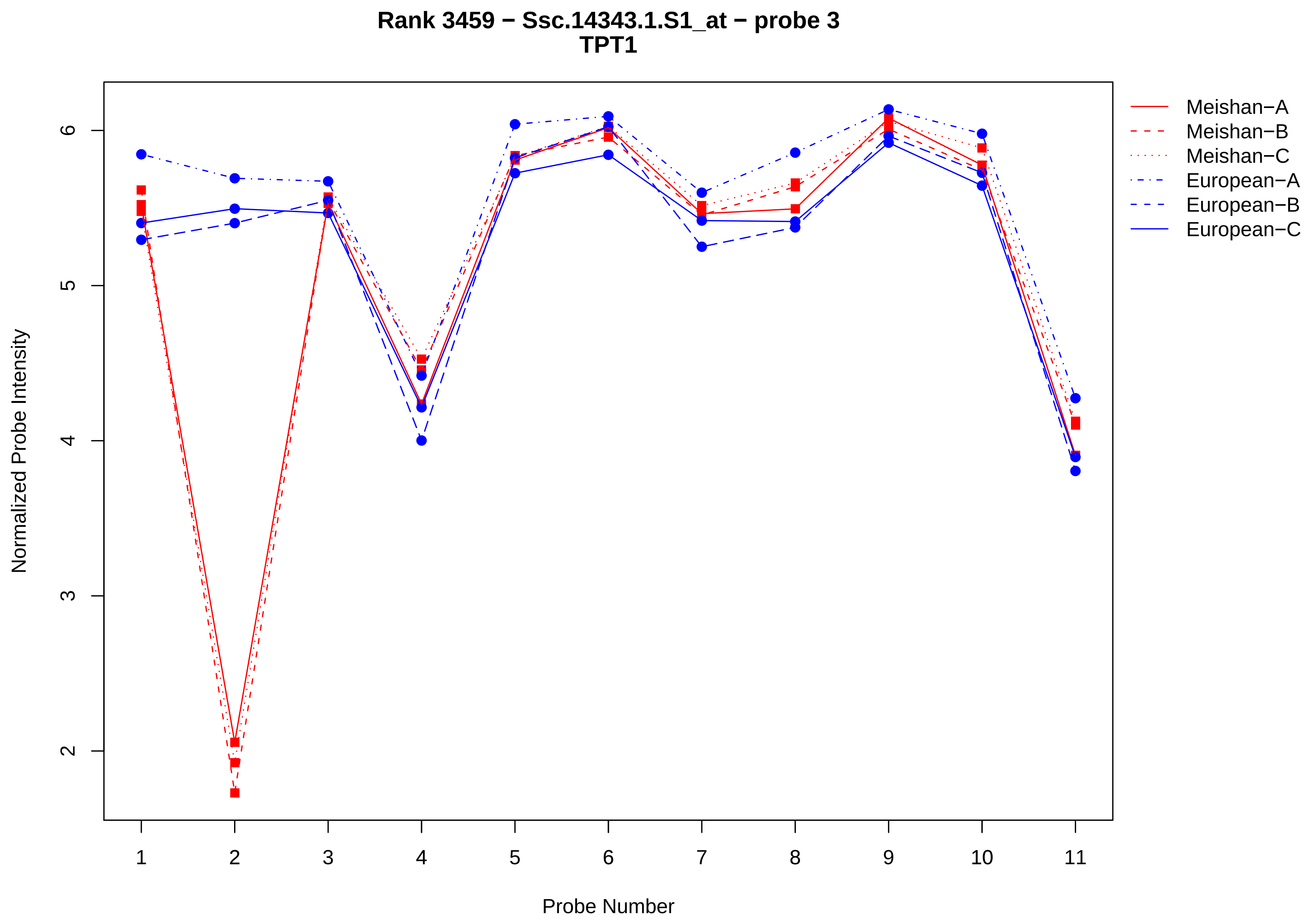

Rank 3459 − Ssc.14343.1.S1_at − probe 3
TPT1
Normalized Probe Intensity
Probe Number
Meishan−A
Meishan−B
Meishan−C
European−A
European−B
European−C
2
3
4
5
6
1
2
3
4
5
6
7
8
9
10
11

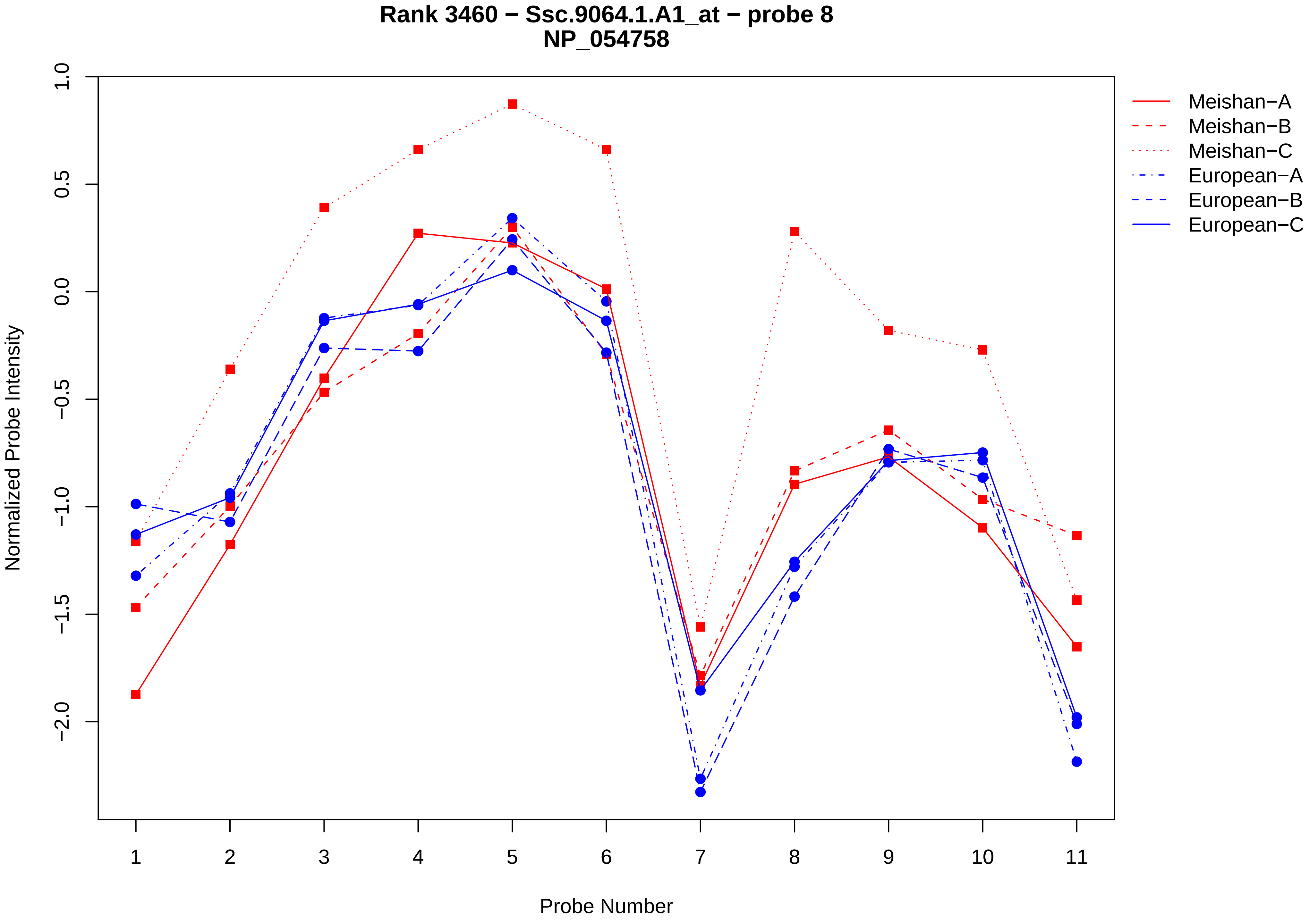

Rank 3460 − Ssc.9064.1.A1_at − probe 8
NP_054758
Normalized Probe Intensity
1.0
0.5
0.0
−0.5
−1.0
−1.5
−2.0
1
2
3
4
5
6
7
8
9
10
11
Probe Number
Meishan−A
Meishan−B
Meishan−C
European−A
European−B
European−C

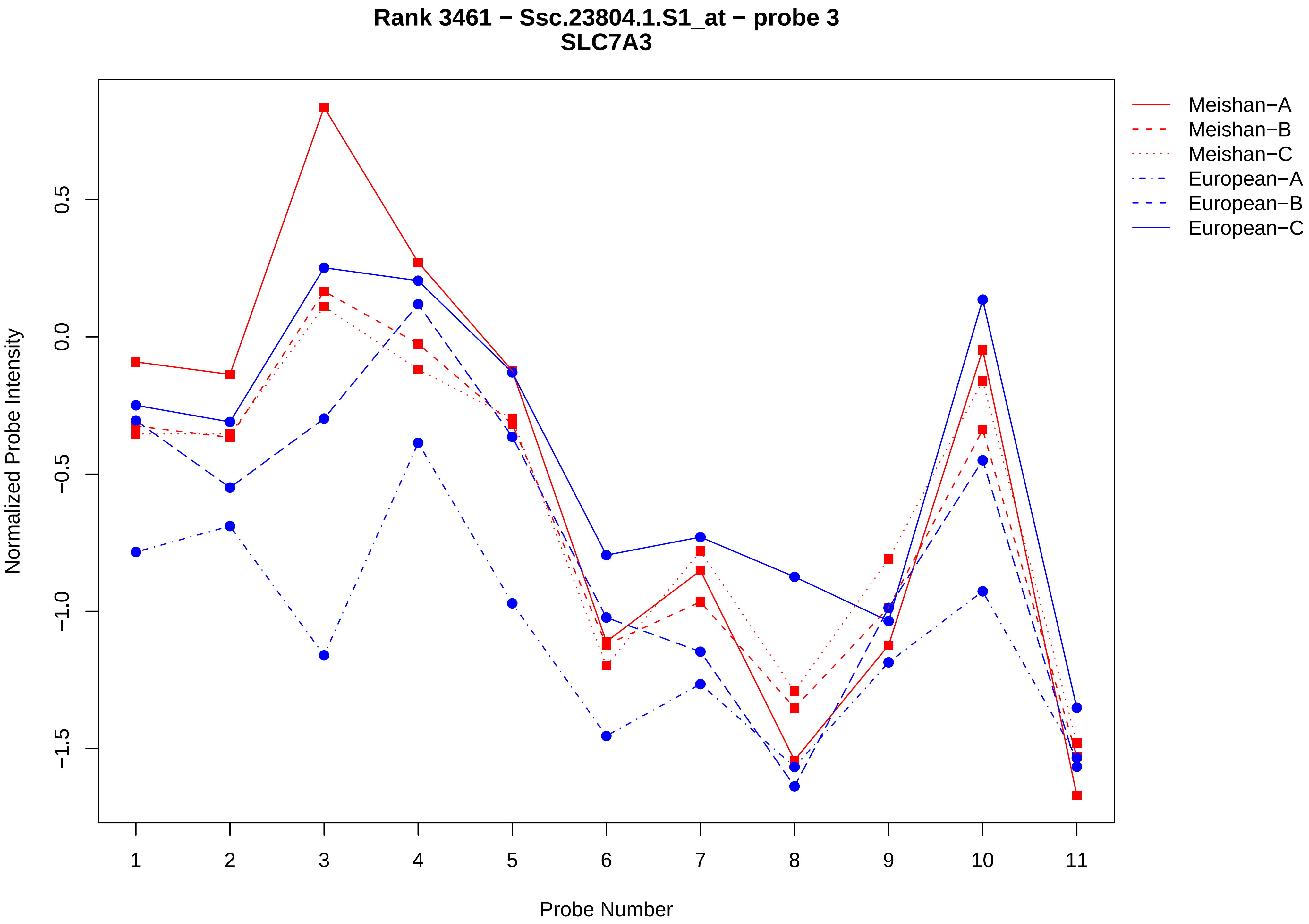

Rank 3461 − Ssc.23804.1.S1_at − probe 3
SLC7A3
Normalized Probe Intensity
Probe Number
0.5
0.0
−0.5
−1.0
−1.5
1
2
3
4
5
6
7
8
9
10
11
Meishan−A
Meishan−B
Meishan−C
European−A
European−B
European−C

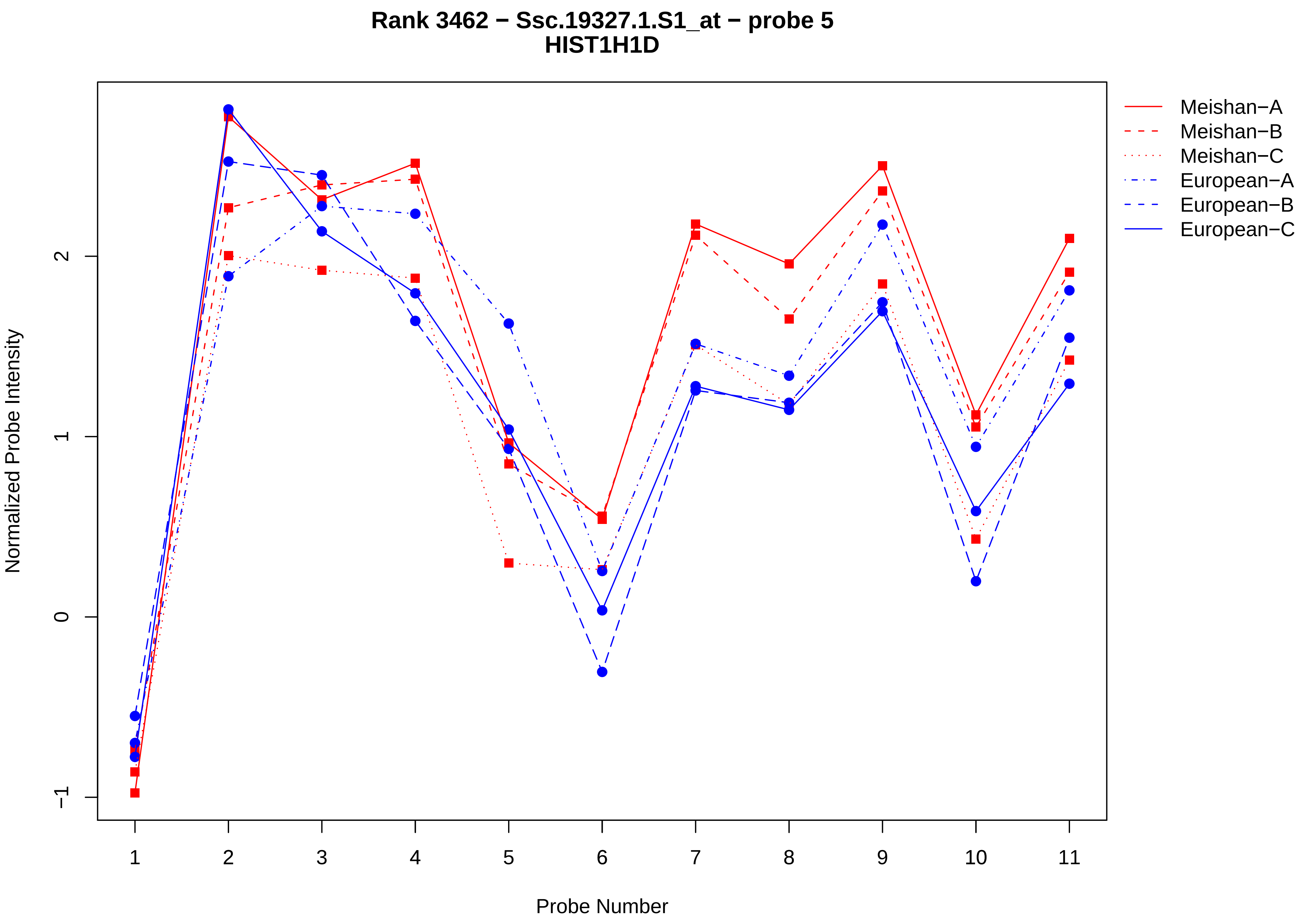

Rank 3462 − Ssc.19327.1.S1_at − probe 5
HIST1H1D
Normalized Probe Intensity
Probe Number
−1
0
1
2
1
2
3
4
5
6
7
8
9
10
11
Meishan−A
Meishan−B
Meishan−C
European−A
European−B
European−C

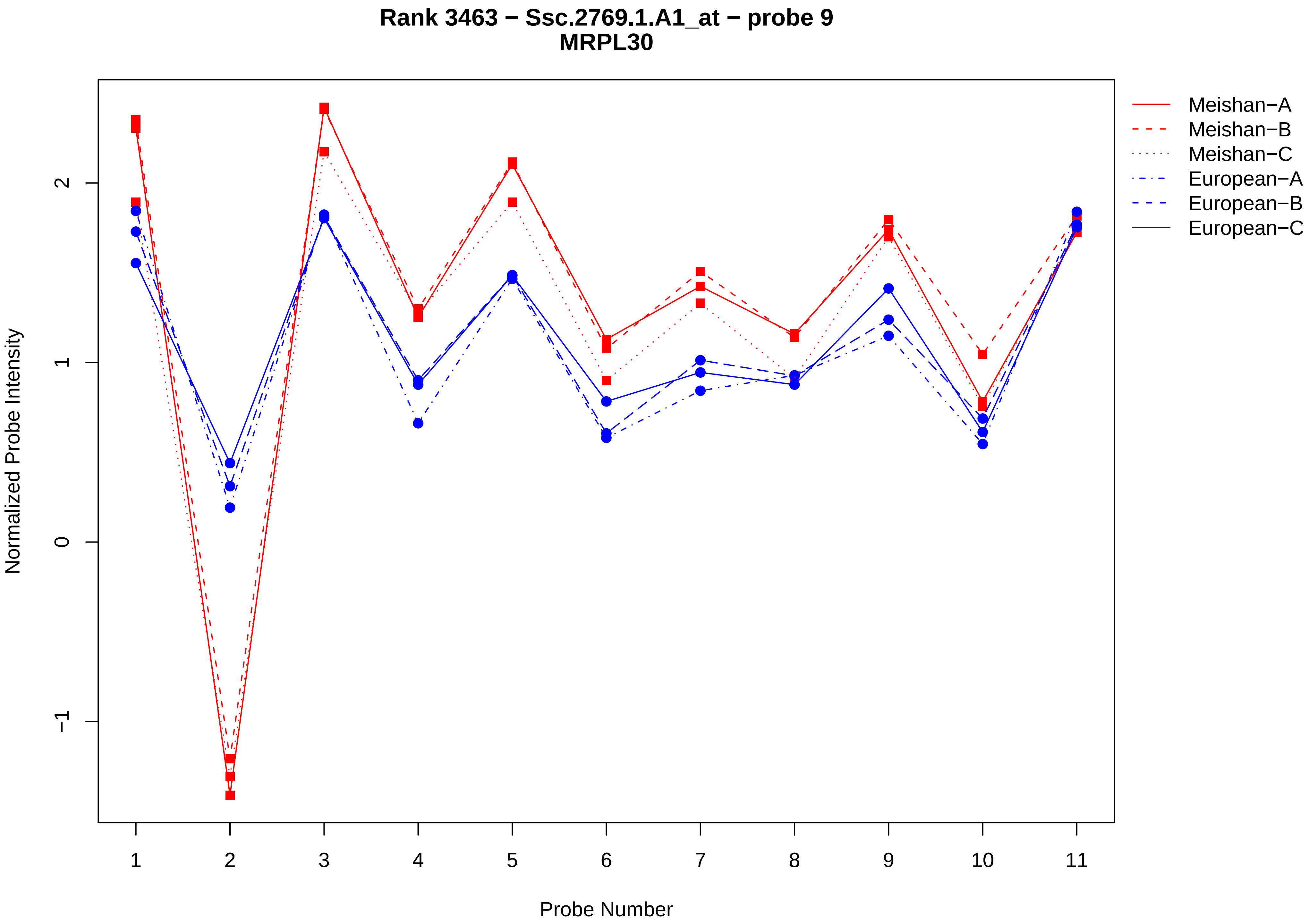

Rank 3463 − Ssc.2769.1.A1_at − probe 9
MRPL30
Normalized Probe Intensity
Probe Number
−1
0
1
2
1
2
3
4
5
6
7
8
9
10
11
Meishan−A
Meishan−B
Meishan−C
European−A
European−B
European−C

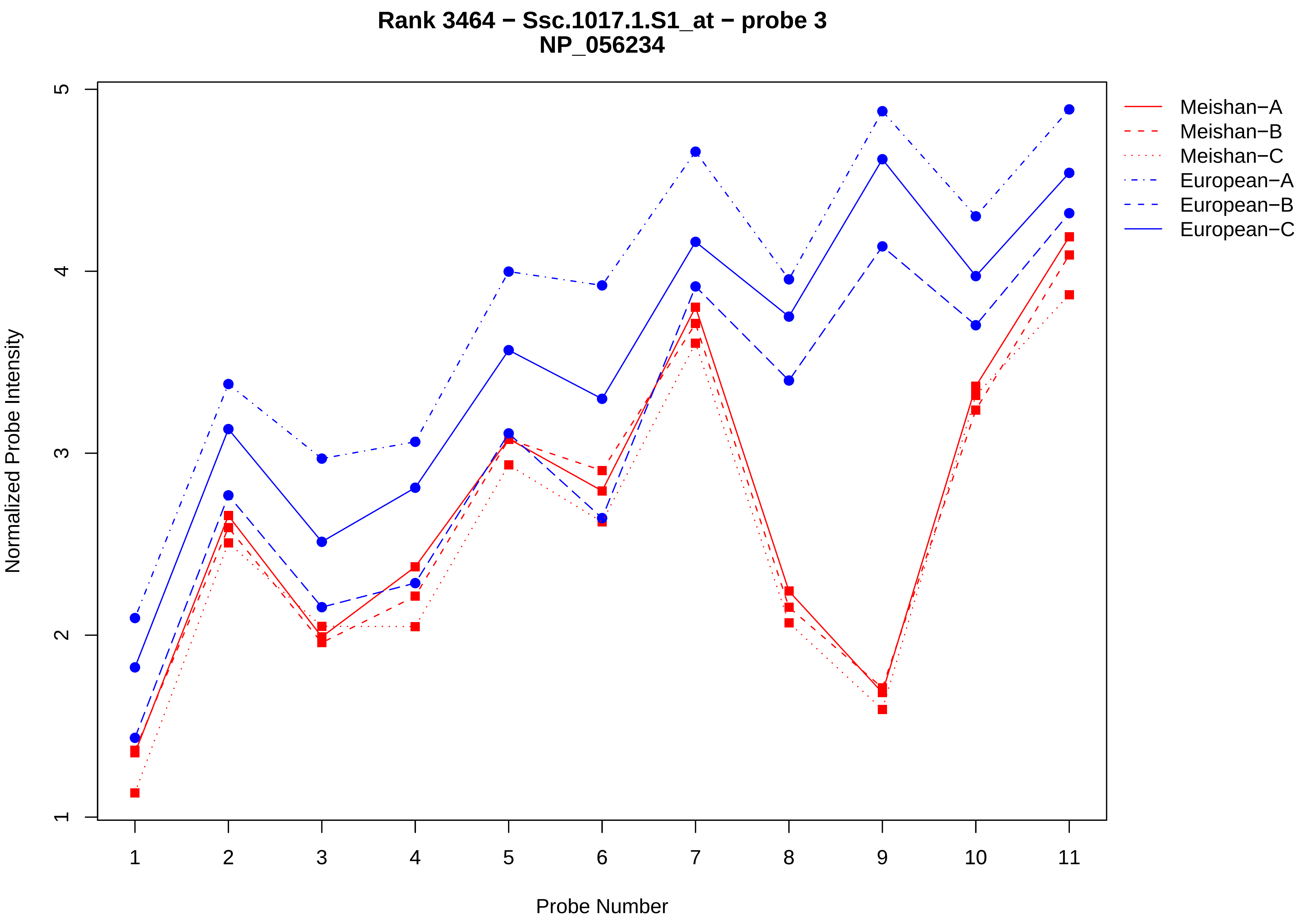

Rank 3464 − Ssc.1017.1.S1_at − probe 3
NP_056234
Normalized Probe Intensity
Probe Number
1
2
3
4
5
1
2
3
4
5
6
7
8
9
10
11
Meishan−A
Meishan−B
Meishan−C
European−A
European−B
European−C

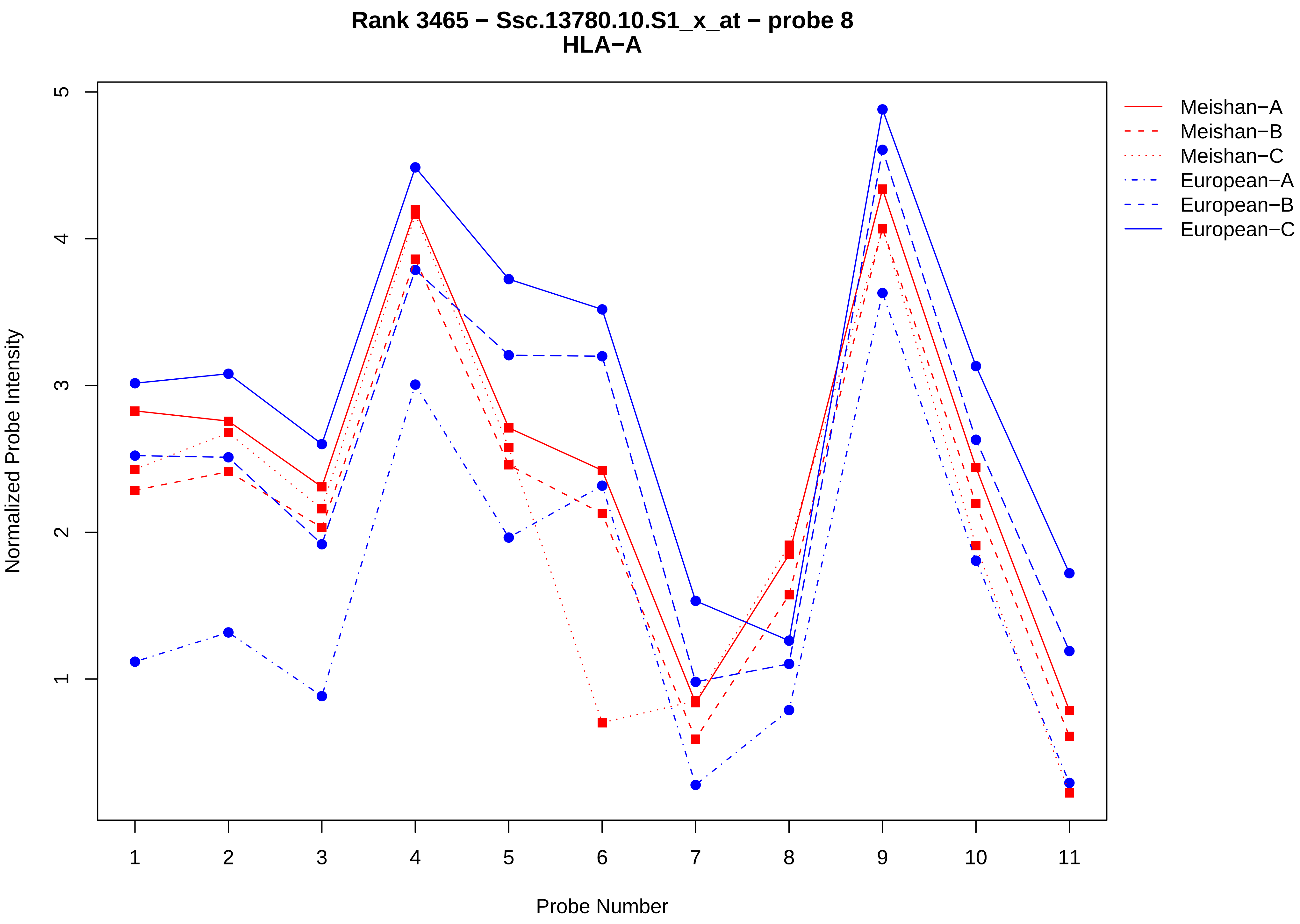

Rank 3465 − Ssc.13780.10.S1_x_at − probe 8
HLA−A
Normalized Probe Intensity
Probe Number
1
2
3
4
5
1
2
3
4
5
6
7
8
9
10
11
Meishan−A
Meishan−B
Meishan−C
European−A
European−B
European−C

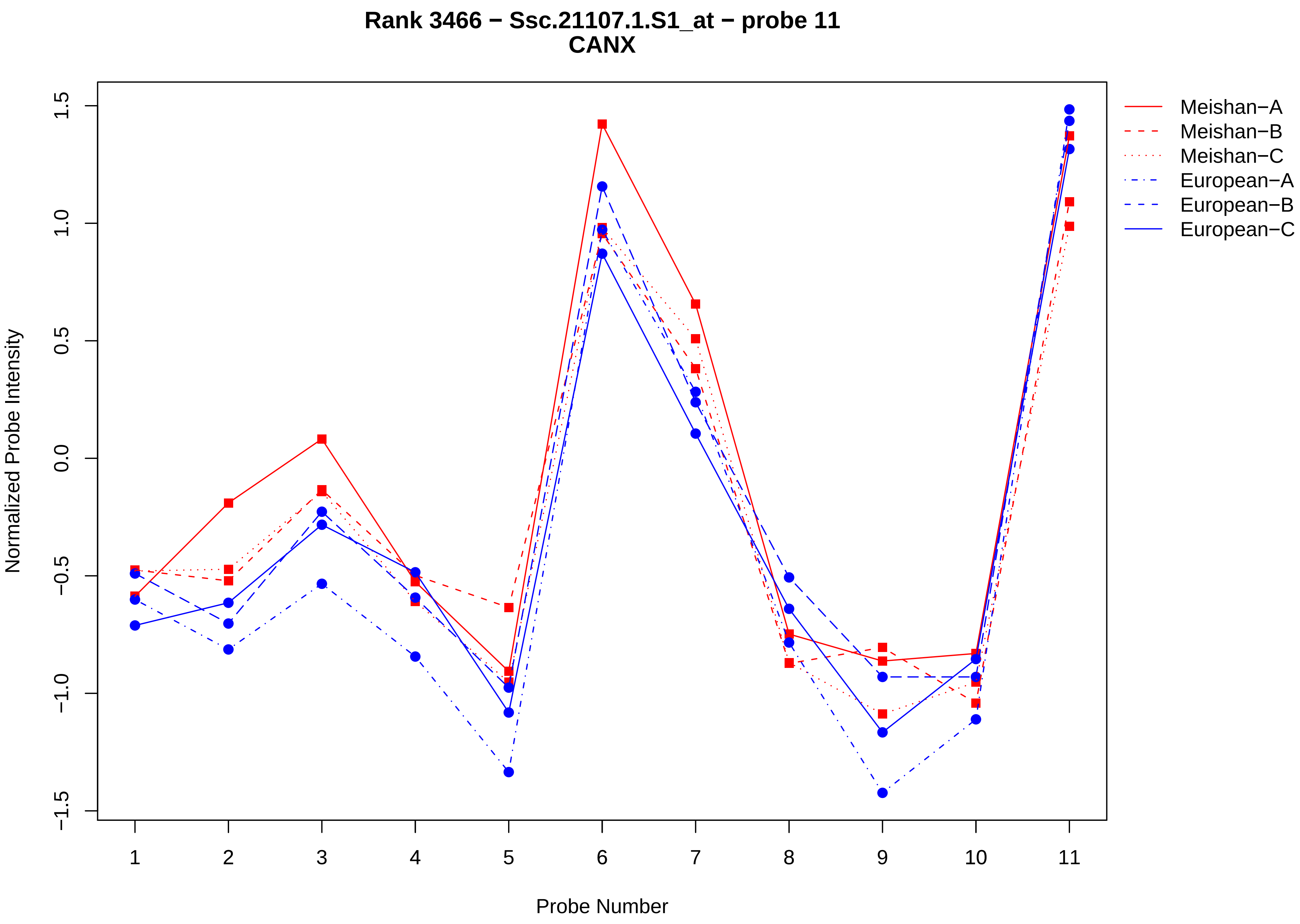

Rank 3466 − Ssc.21107.1.S1_at − probe 11
CANX
Normalized Probe Intensity
1.5
1.0
0.5
0.0
−0.5
−1.0
−1.5
1
2
3
4
5
6
7
8
9
10
11
Probe Number
Meishan−A
Meishan−B
Meishan−C
European−A
European−B
European−C

Rank 3467 − Ssc.24178.1.A1_at − probe 9
DISC1

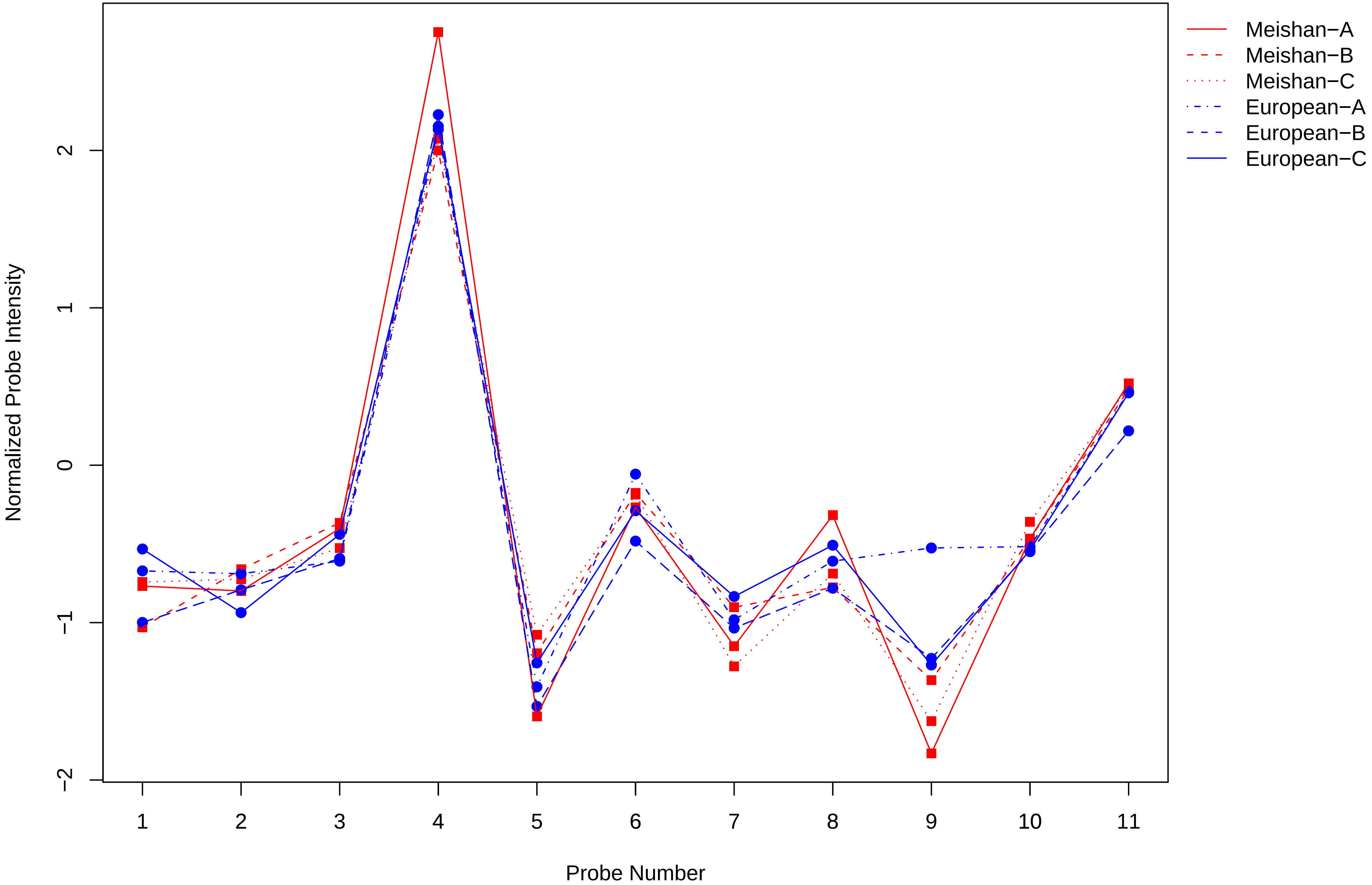

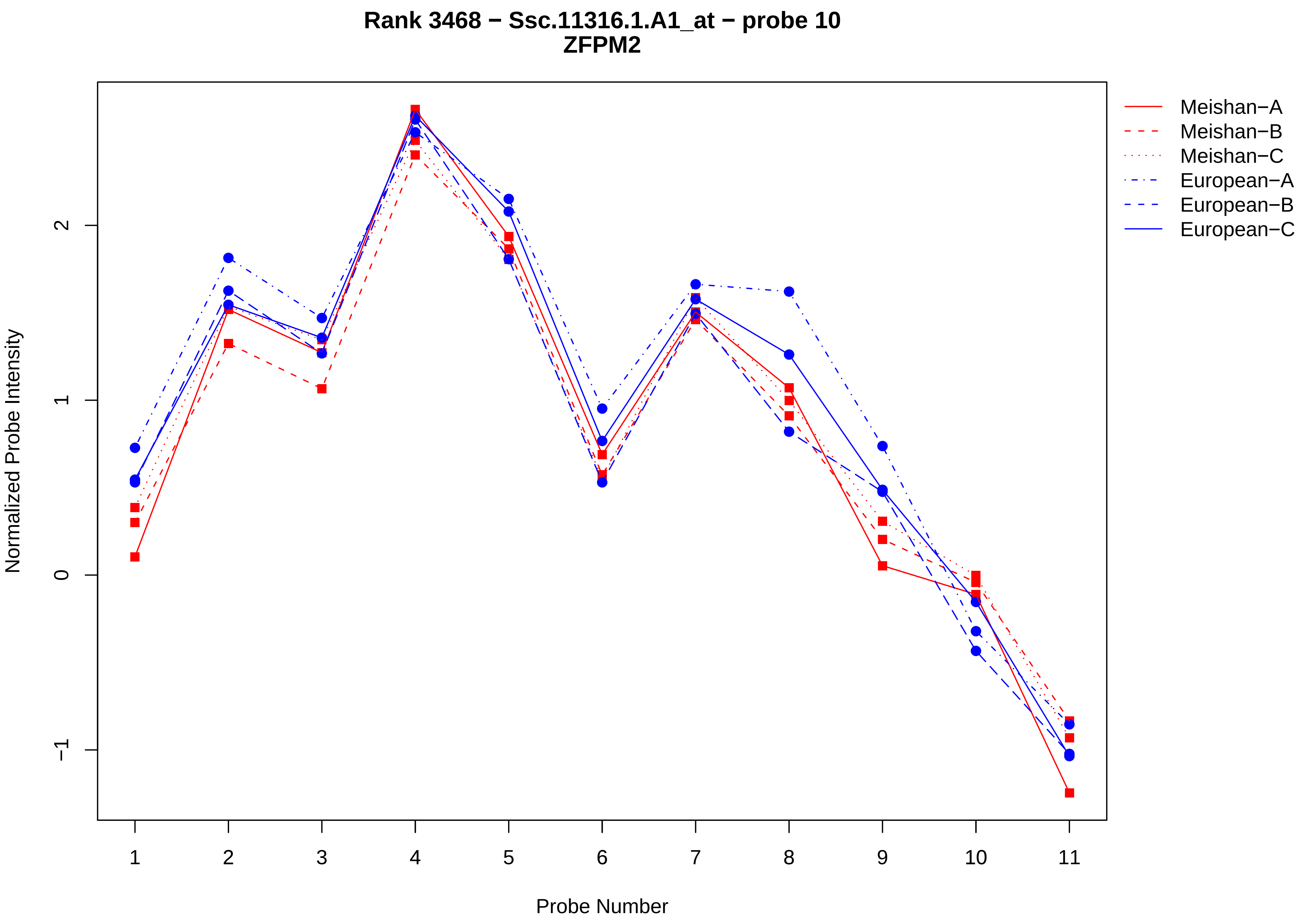

Rank 3468 − Ssc.11316.1.A1_at − probe 10
ZFPM2
Normalized Probe Intensity
Probe Number
Meishan−A
Meishan−B
Meishan−C
European−A
European−B
European−C
−1
0
1
2
1
2
3
4
5
6
7
8
9
10
11

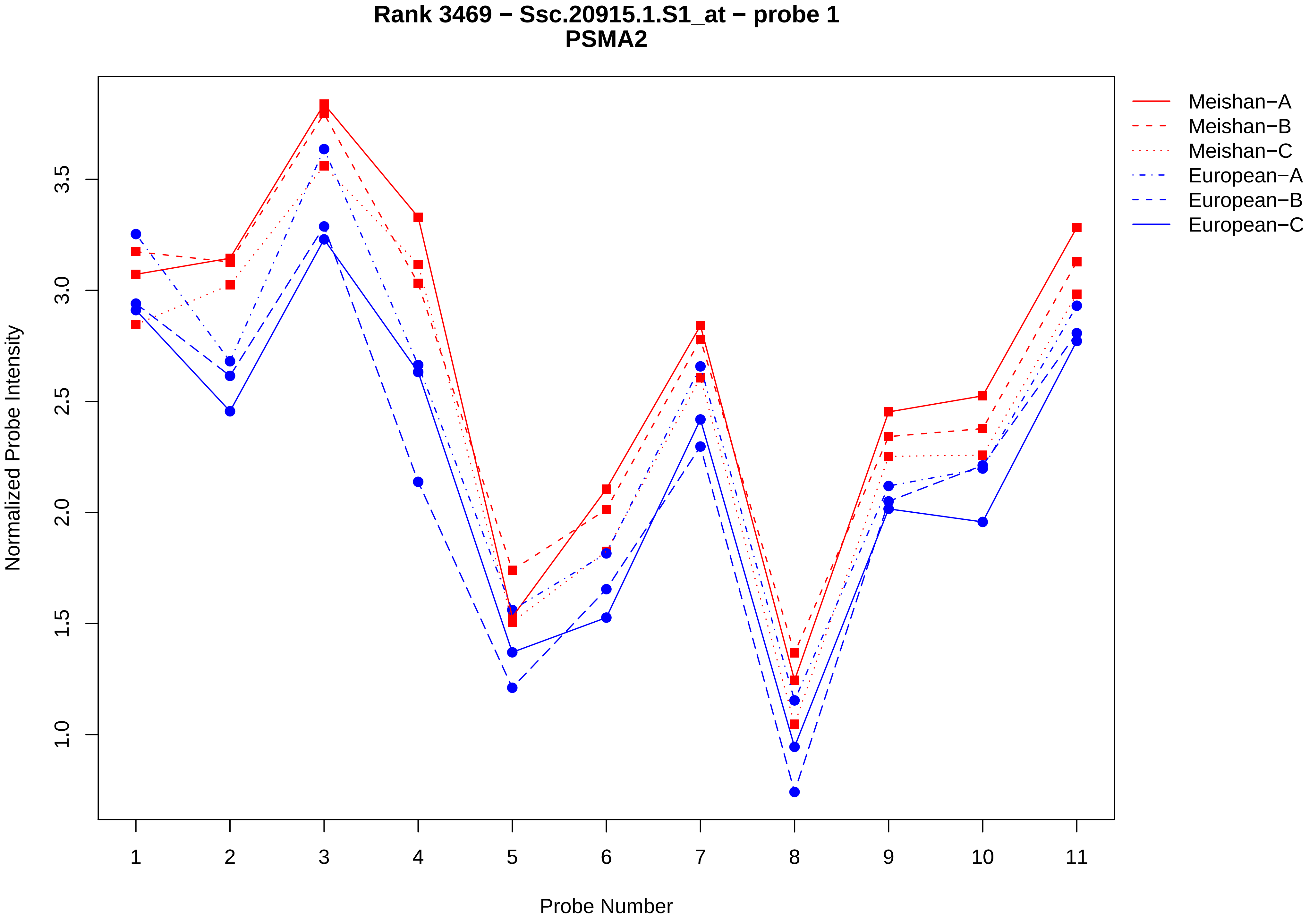

Rank 3469 − Ssc.20915.1.S1_at − probe 1
PSMA2
Normalized Probe Intensity
Probe Number
1.0
1.5
2.0
2.5
3.0
3.5
1
2
3
4
5
6
7
8
9
10
11
Meishan−A
Meishan−B
Meishan−C
European−A
European−B
European−C

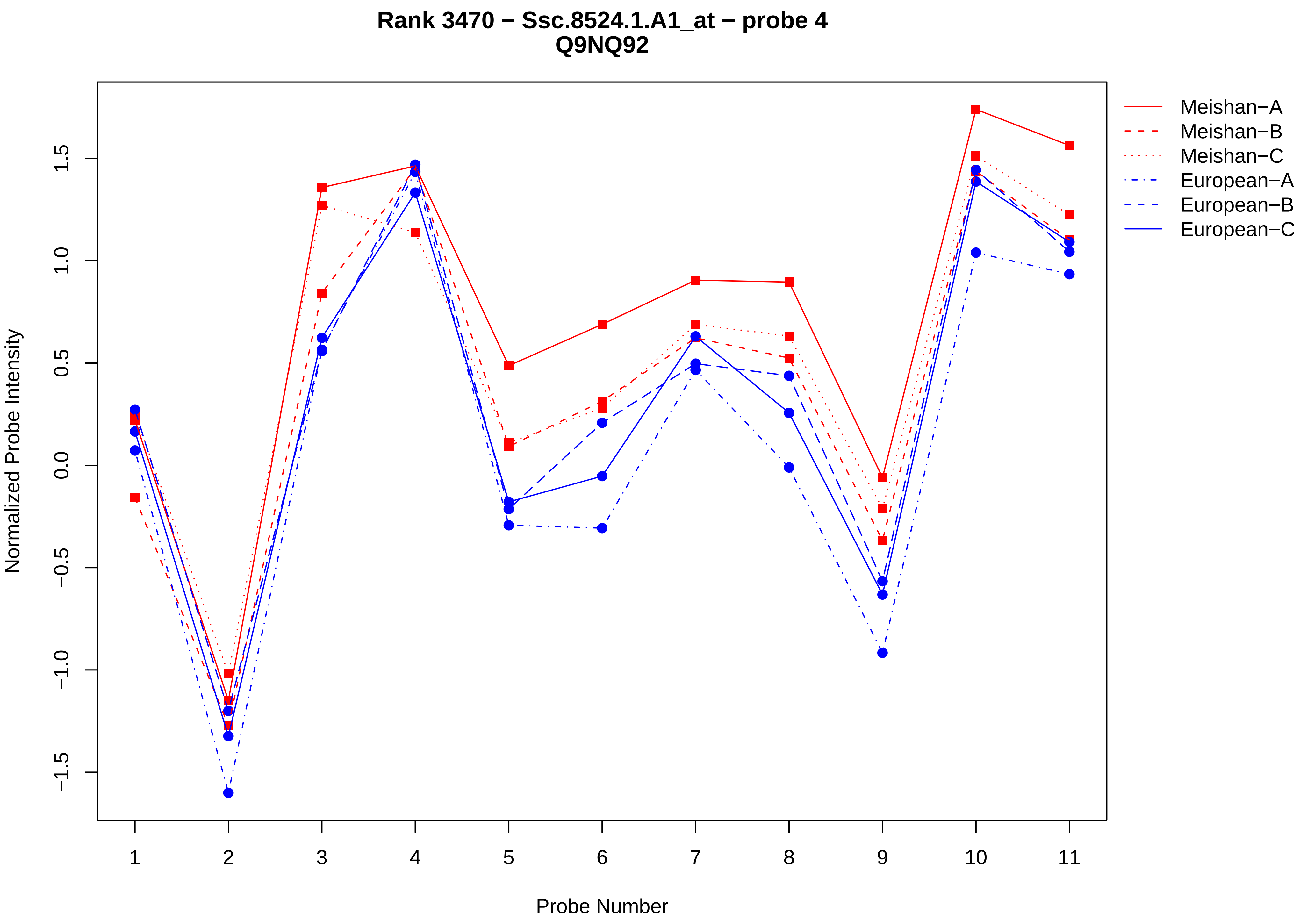

Rank 3470 − Ssc.8524.1.A1_at − probe 4
Q9NQ92
Normalized Probe Intensity
Probe Number
Meishan−A
Meishan−B
Meishan−C
European−A
European−B
European−C

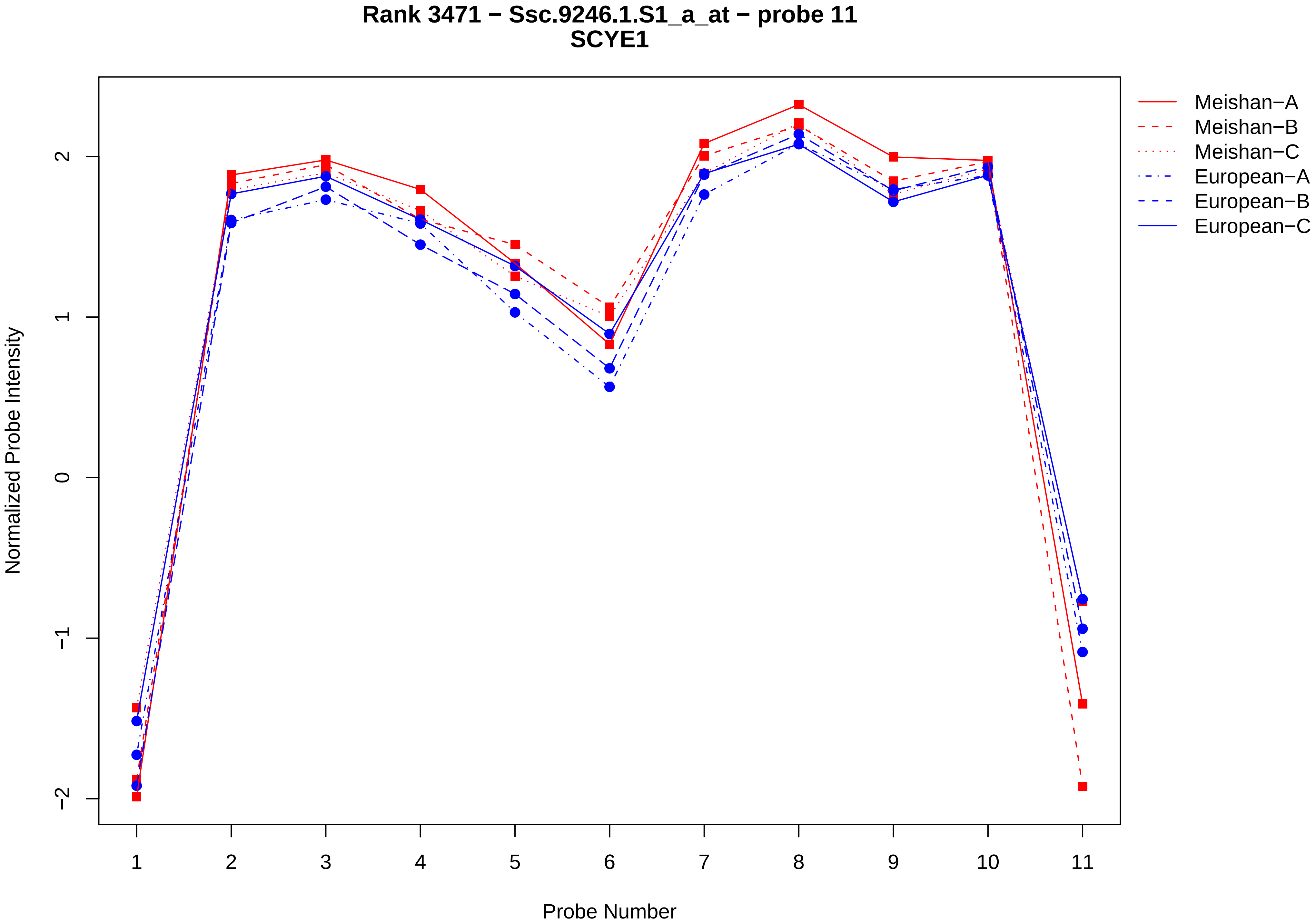

Rank 3471 − Ssc.9246.1.S1_a_at − probe 11
SCYE1
Normalized Probe Intensity
Probe Number
Meishan−A
Meishan−B
Meishan−C
European−A
European−B
European−C
−2
−1
0
1
2
1
2
3
4
5
6
7
8
9
10
11

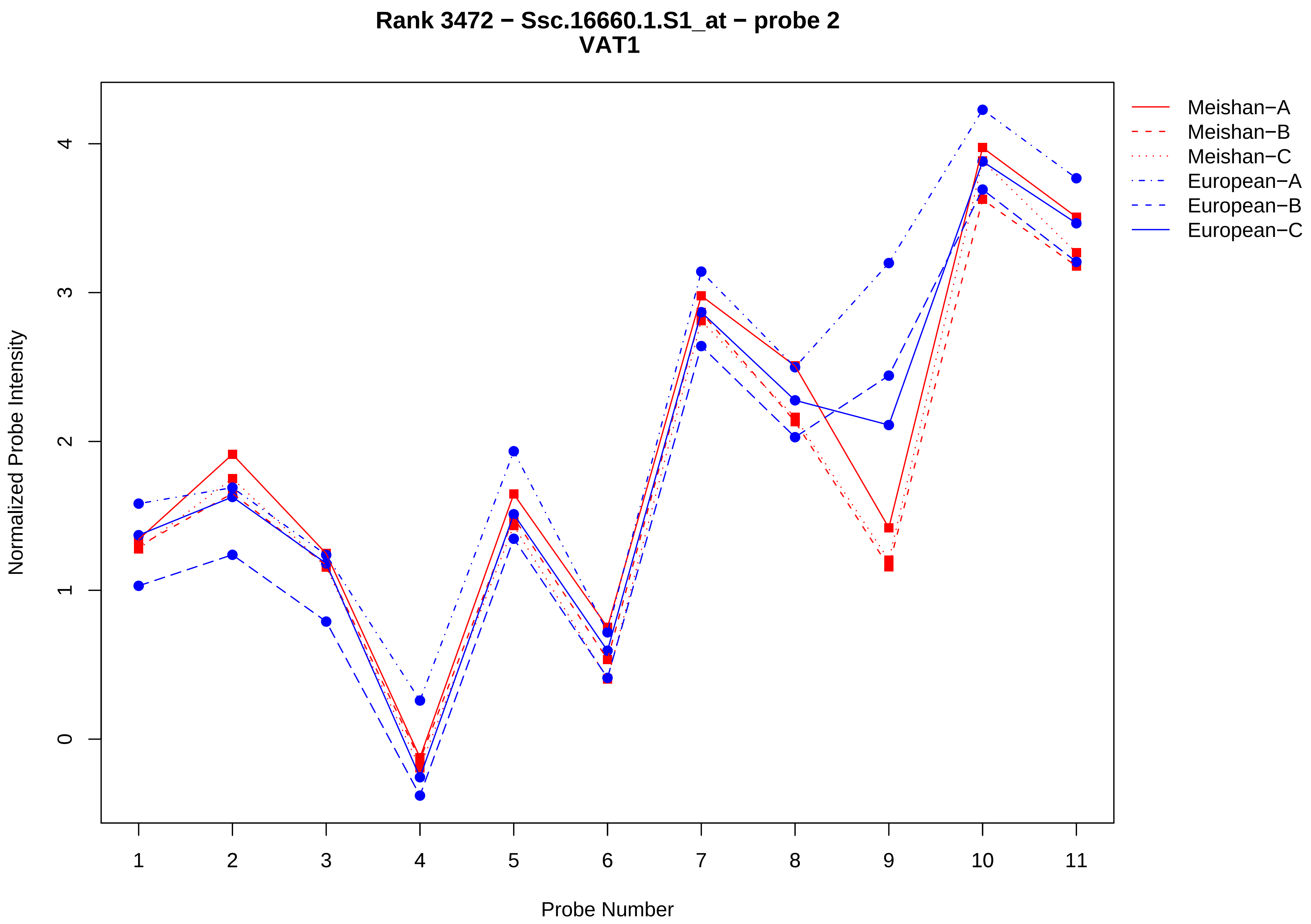

Rank 3472 − Ssc.16660.1.S1_at − probe 2
VAT1
Normalized Probe Intensity
Probe Number
0
1
2
3
4
1
2
3
4
5
6
7
8
9
10
11
Meishan−A
Meishan−B
Meishan−C
European−A
European−B
European−C

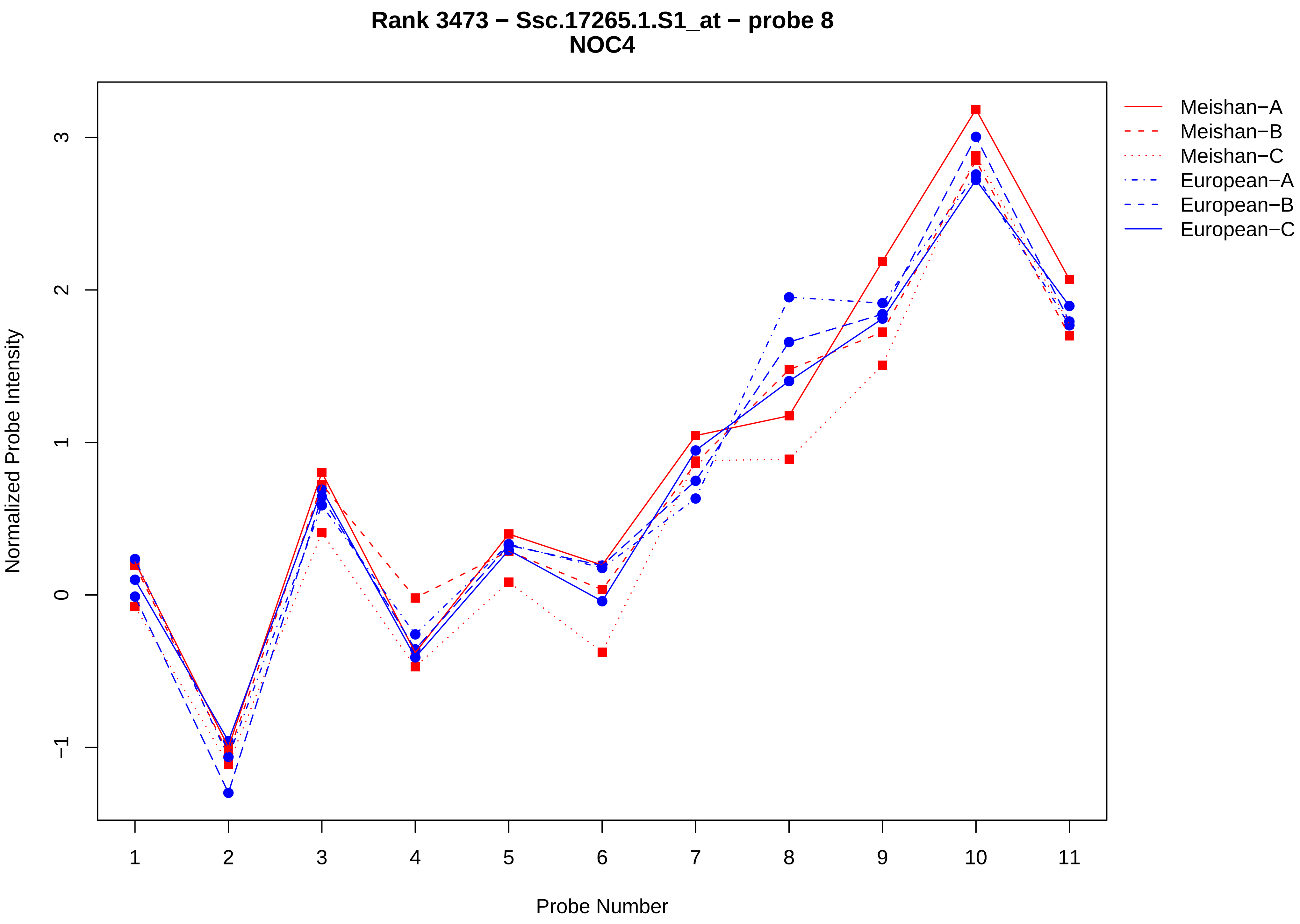

Rank 3473 − Ssc.17265.1.S1_at − probe 8
NOC4
Normalized Probe Intensity
Probe Number
−1
0
1
2
3
1
2
3
4
5
6
7
8
9
10
11
Meishan−A
Meishan−B
Meishan−C
European−A
European−B
European−C

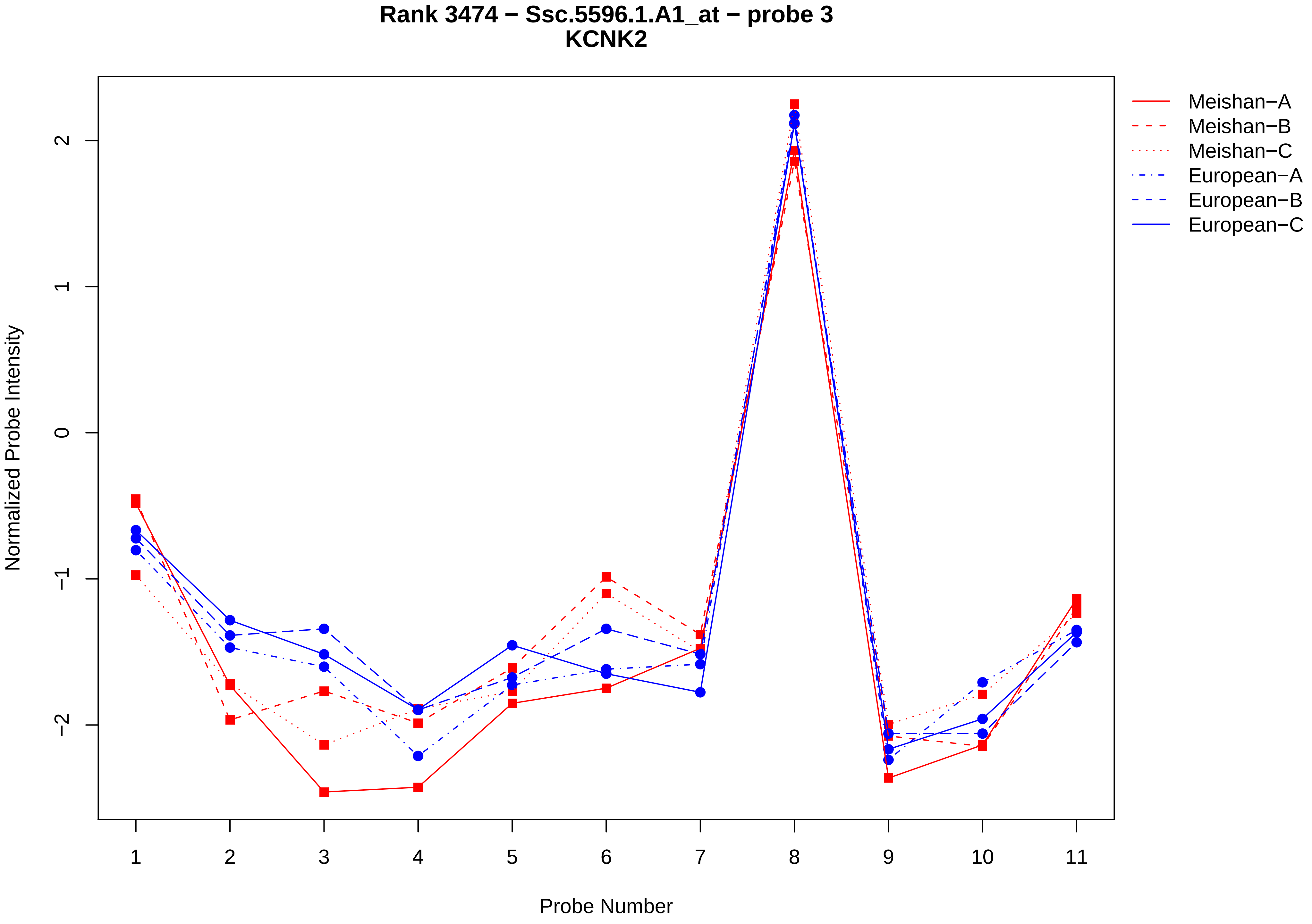

Rank 3474 − Ssc.5596.1.A1_at − probe 3
KCNK2
Normalized Probe Intensity
Probe Number
Meishan−A
Meishan−B
Meishan−C
European−A
European−B
European−C

Rank 3475 − Ssc.3035.1.S1_at − probe 9
CHTF18

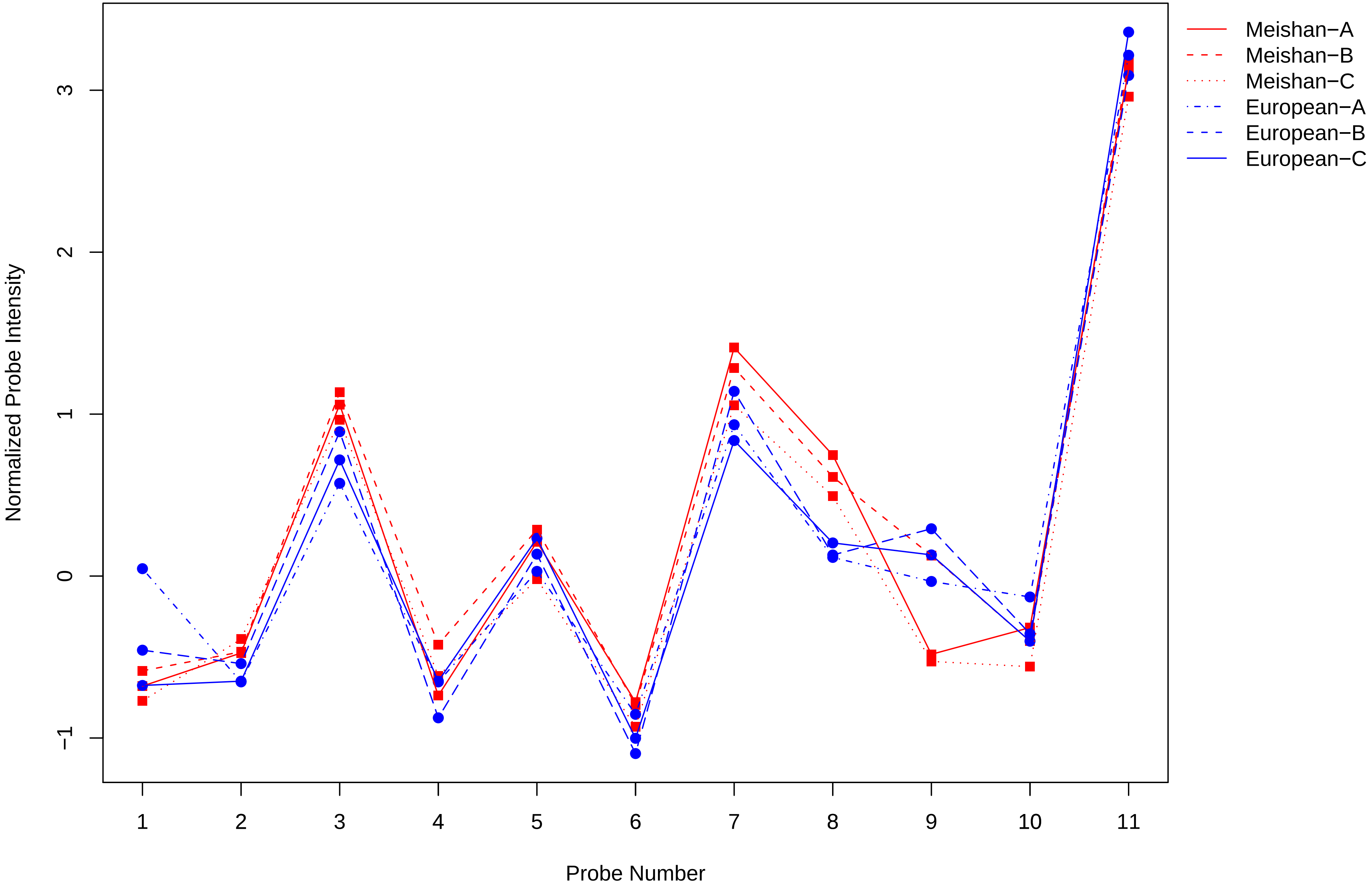

Rank 3476 − Ssc.15669.1.A1_at − probe 5
CSNK1D

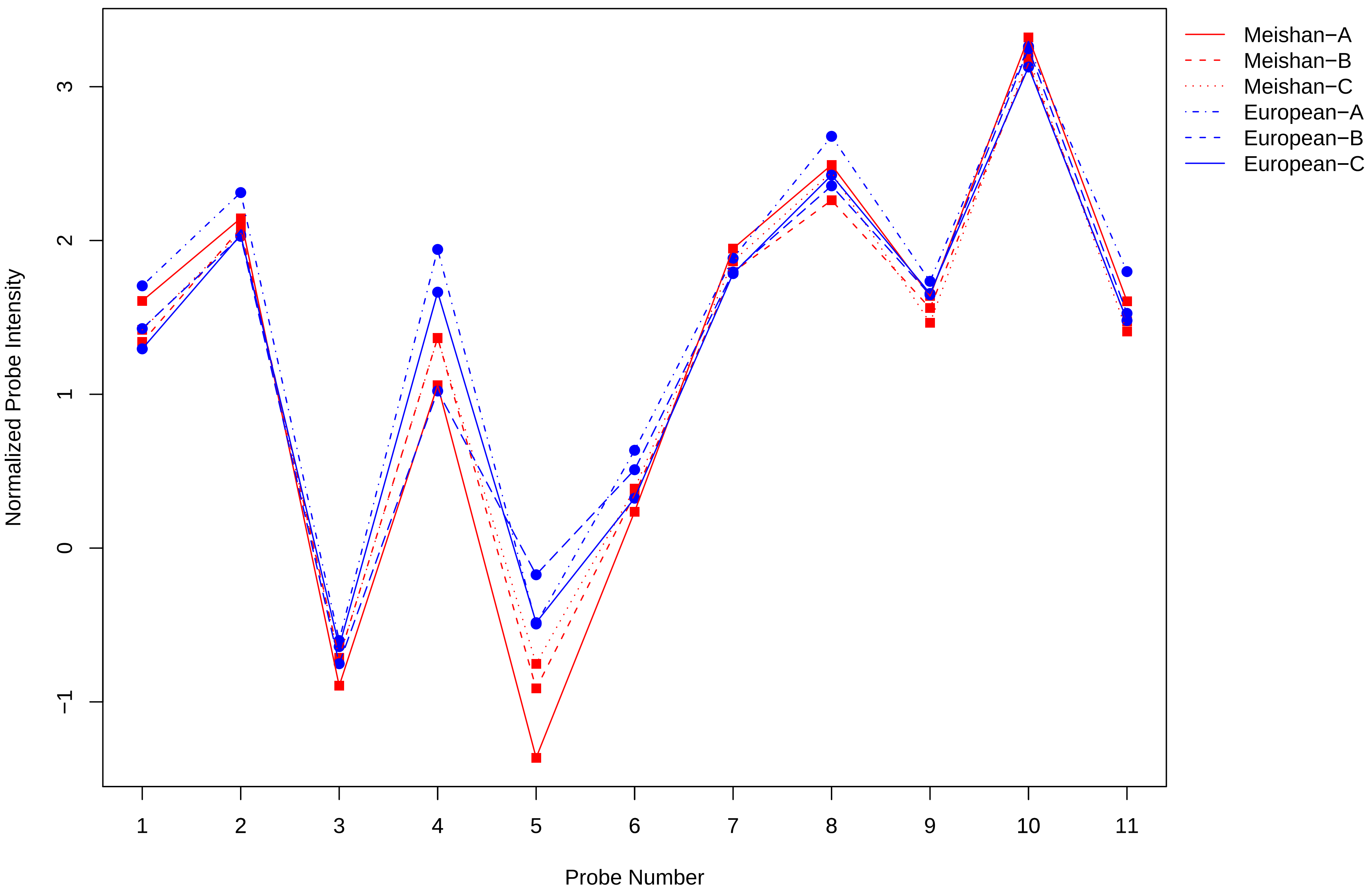

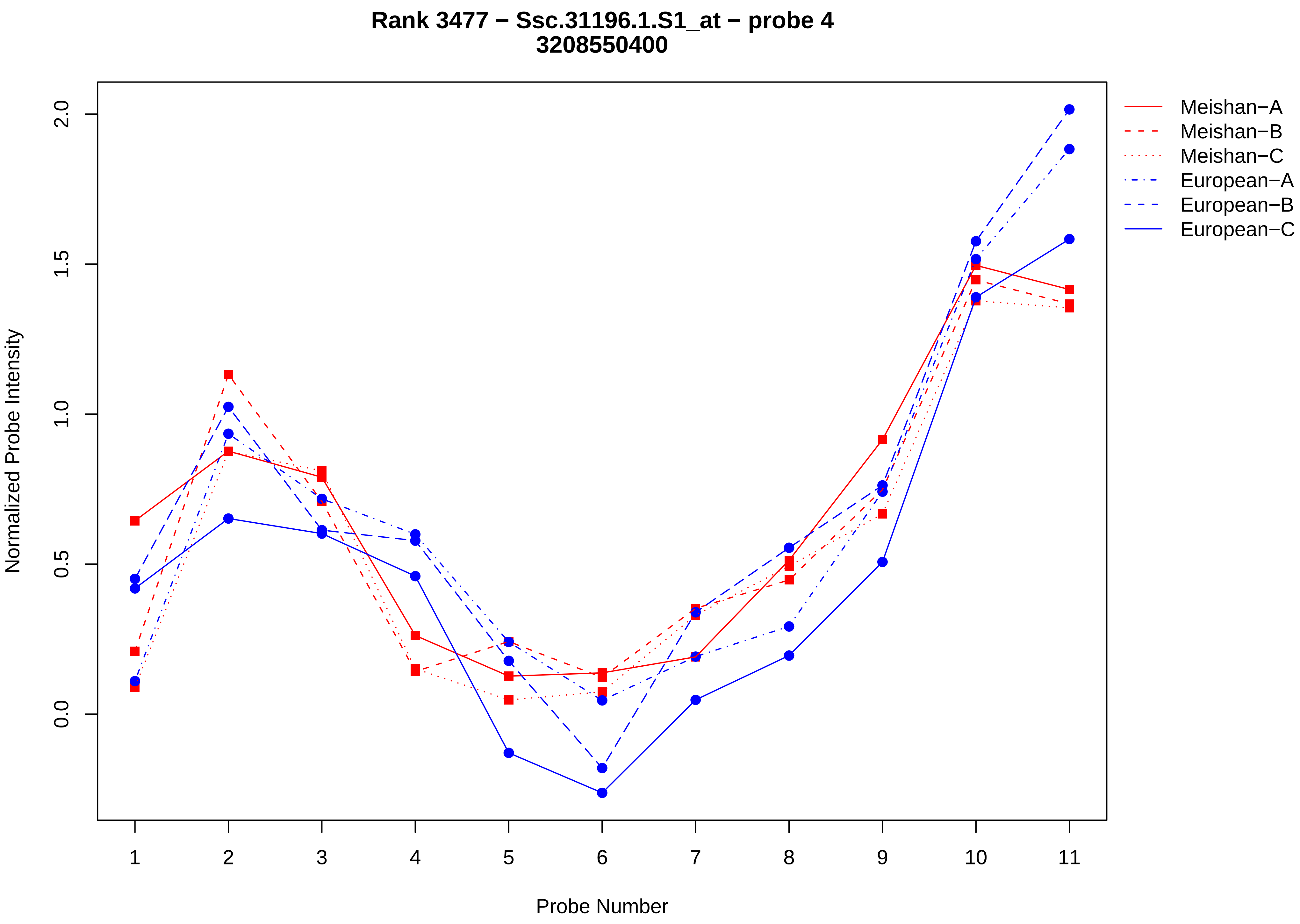

Rank 3477 − Ssc.31196.1.S1_at − probe 4
3208550400
Normalized Probe Intensity
0.0
0.5
1.0
1.5
2.0
1
2
3
4
5
6
7
8
9
10
11
Probe Number
Meishan−A
Meishan−B
Meishan−C
European−A
European−B
European−C

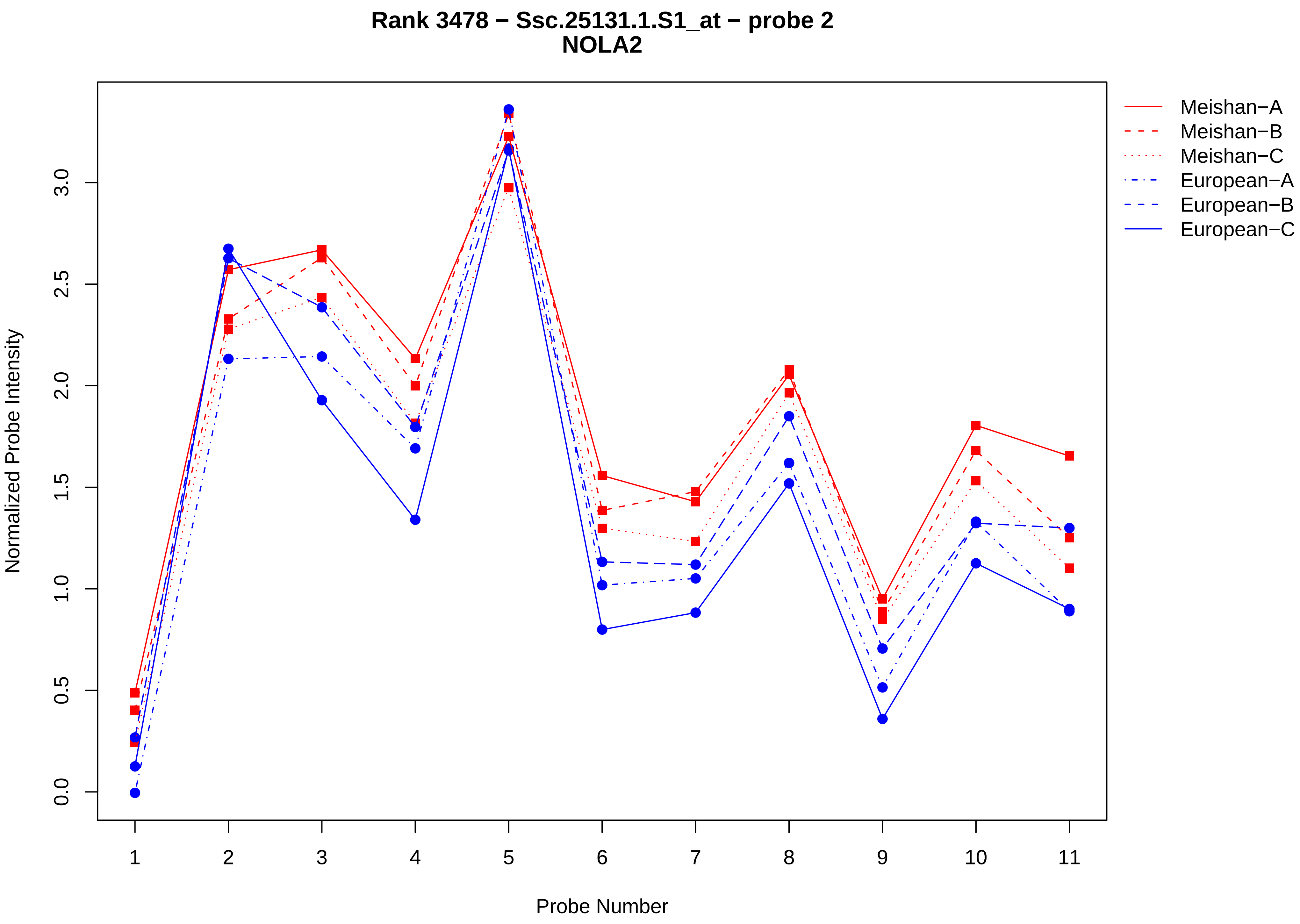

Rank 3478 − Ssc.25131.1.S1_at − probe 2
NOLA2
Normalized Probe Intensity
0.0
0.5
1.0
1.5
2.0
2.5
3.0
Probe Number
1
2
3
4
5
6
7
8
9
10
11
Meishan−A
Meishan−B
Meishan−C
European−A
European−B
European−C

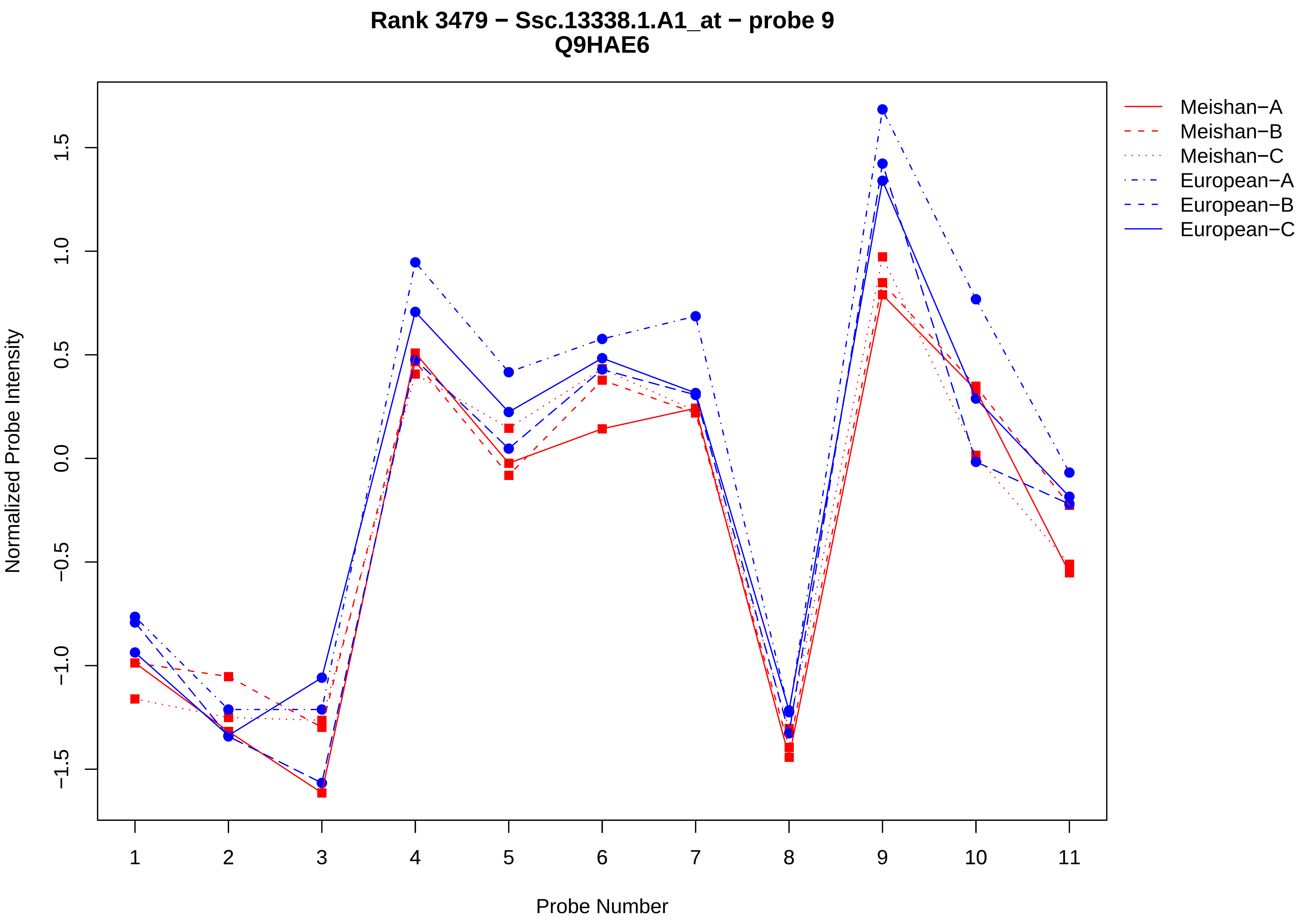
Rank 3479 − Ssc.13338.1.A1_at − probe 9
Q9HAE6
Normalized Probe Intensity
Probe Number
1.5
1.0
0.5
0.0
−0.5
−1.0
−1.5
1
2
3
4
5
6
7
8
9
10
11
Meishan−A
Meishan−B
Meishan−C
European−A
European−B
European−C

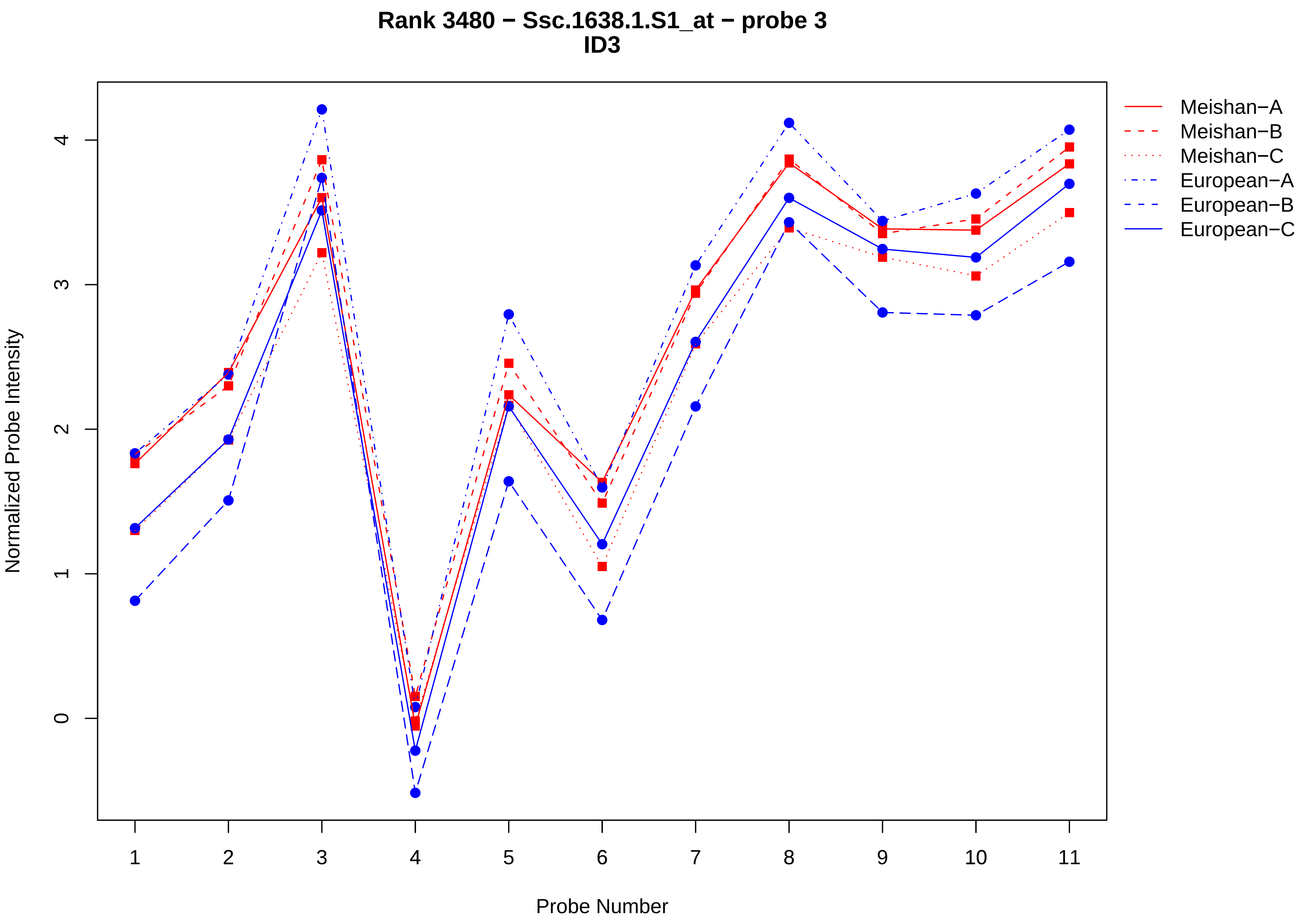

Rank 3480 − Ssc.1638.1.S1_at − probe 3
ID3
Normalized Probe Intensity
Probe Number
0
1
2
3
4
1
2
3
4
5
6
7
8
9
10
11
Meishan−A
Meishan−B
Meishan−C
European−A
European−B
European−C

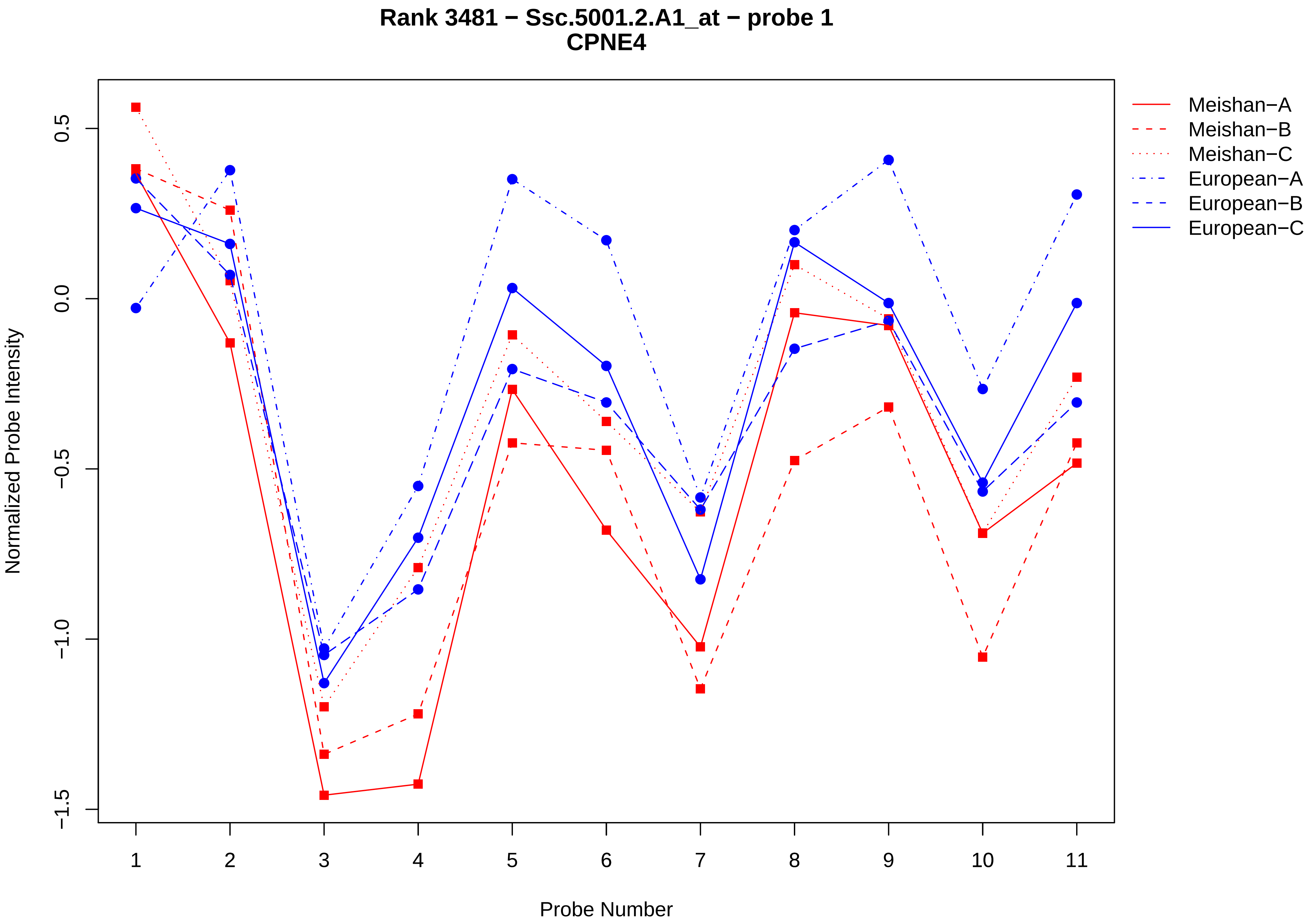

Rank 3481 − Ssc.5001.2.A1_at − probe 1
CPNE4
Normalized Probe Intensity
Probe Number
0.5
0.0
−0.5
−1.0
−1.5
1
2
3
4
5
6
7
8
9
10
11
Meishan−A
Meishan−B
Meishan−C
European−A
European−B
European−C

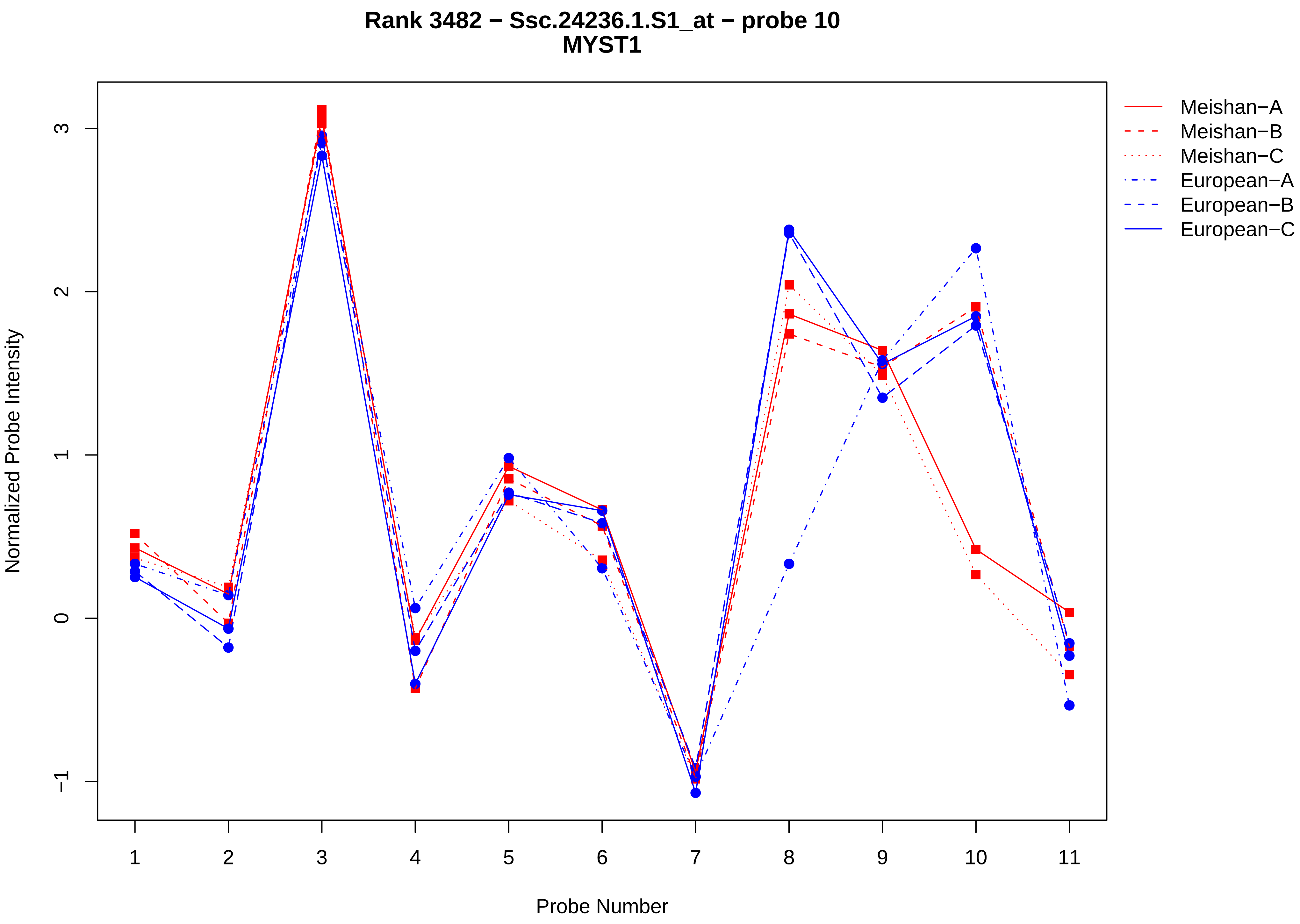

Rank 3482 − Ssc.24236.1.S1_at − probe 10
MYST1
Normalized Probe Intensity
Probe Number
Meishan−A
Meishan−B
Meishan−C
European−A
European−B
European−C

Rank 3483 − Ssc.6344.1.S1_at − probe 5
SRA1

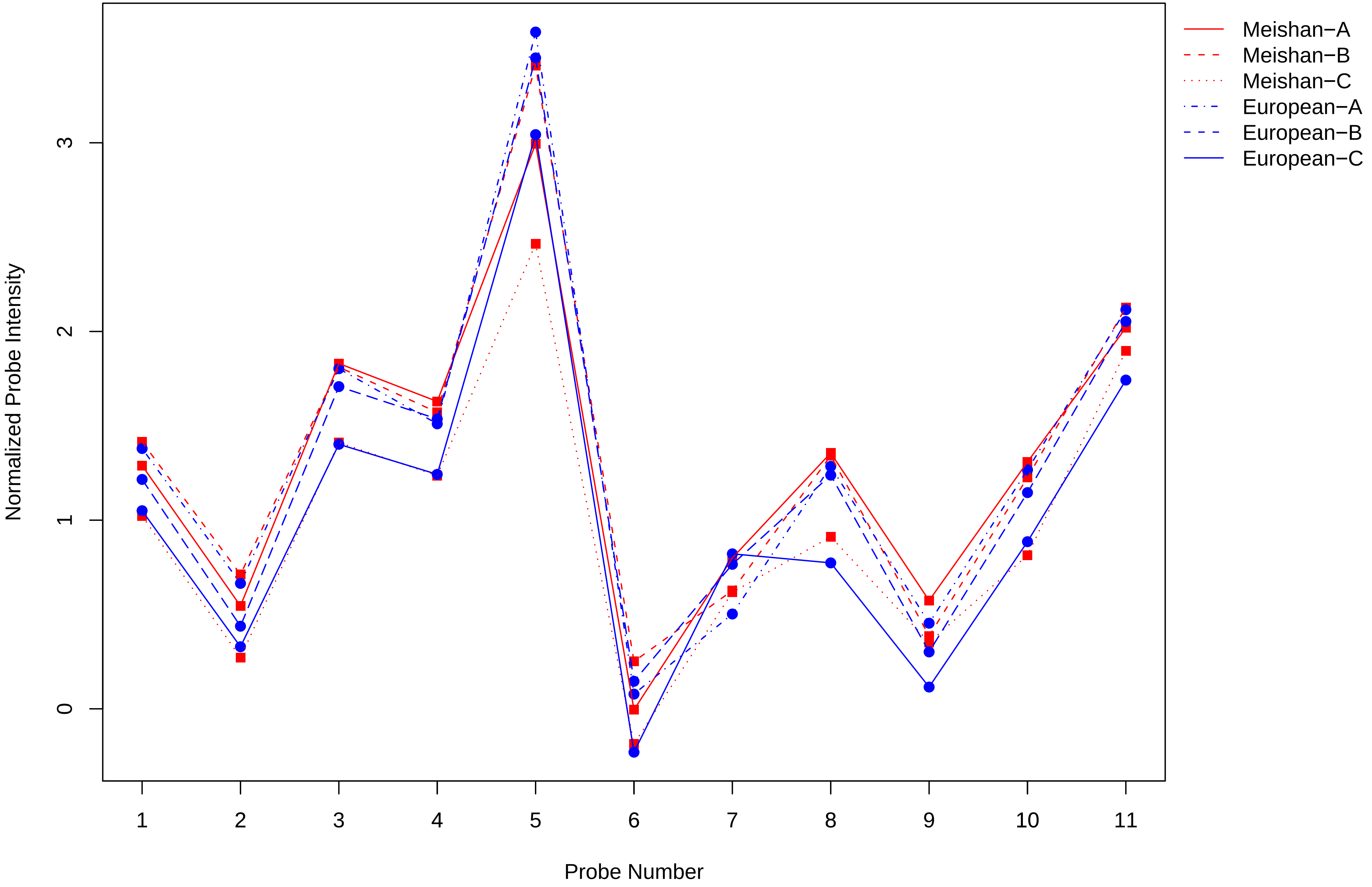

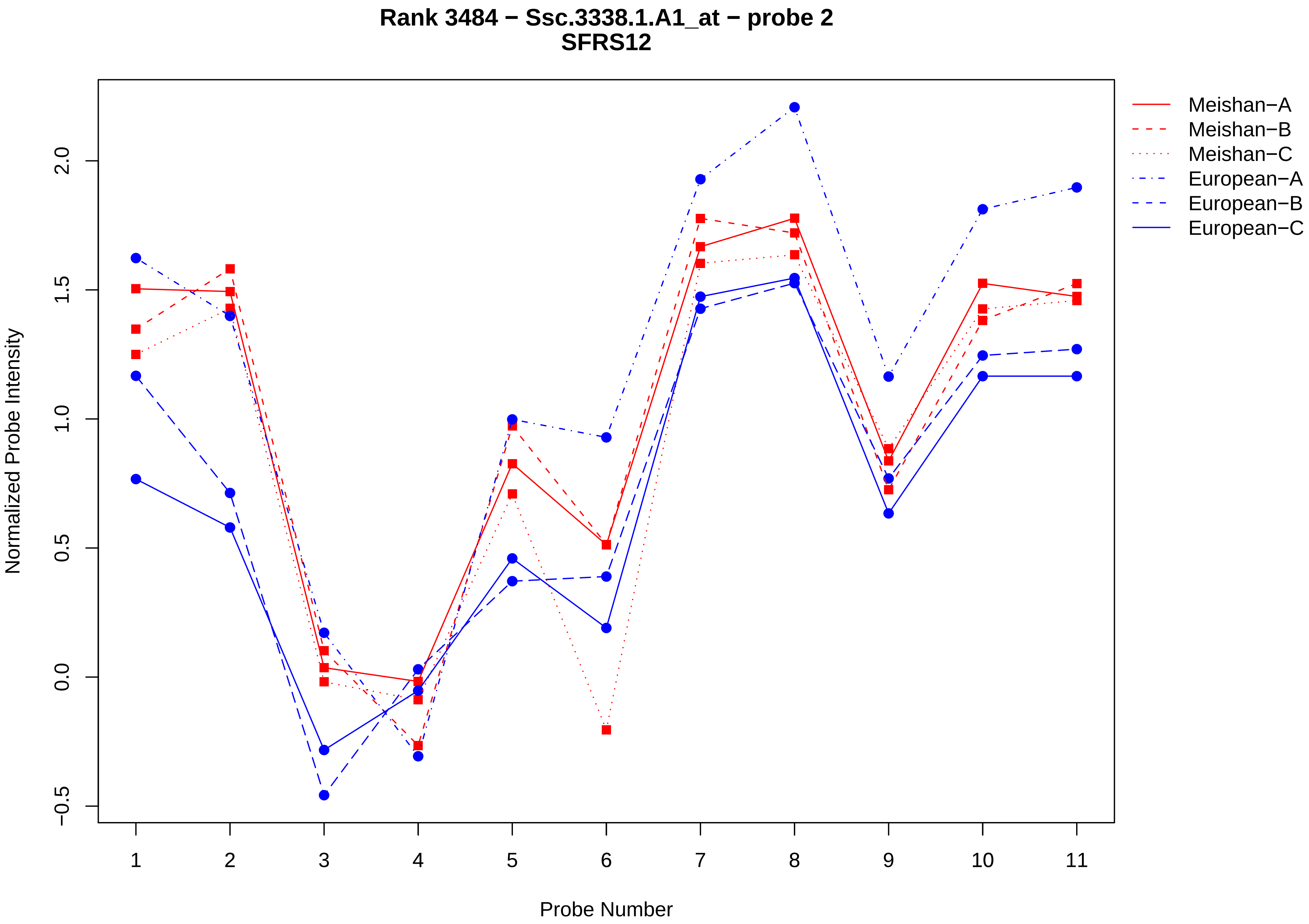

Rank 3484 − Ssc.3338.1.A1_at − probe 2
SFRS12
Normalized Probe Intensity
Probe Number
−0.5
0.0
0.5
1.0
1.5
2.0
1
2
3
4
5
6
7
8
9
10
11
Meishan−A
Meishan−B
Meishan−C
European−A
European−B
European−C

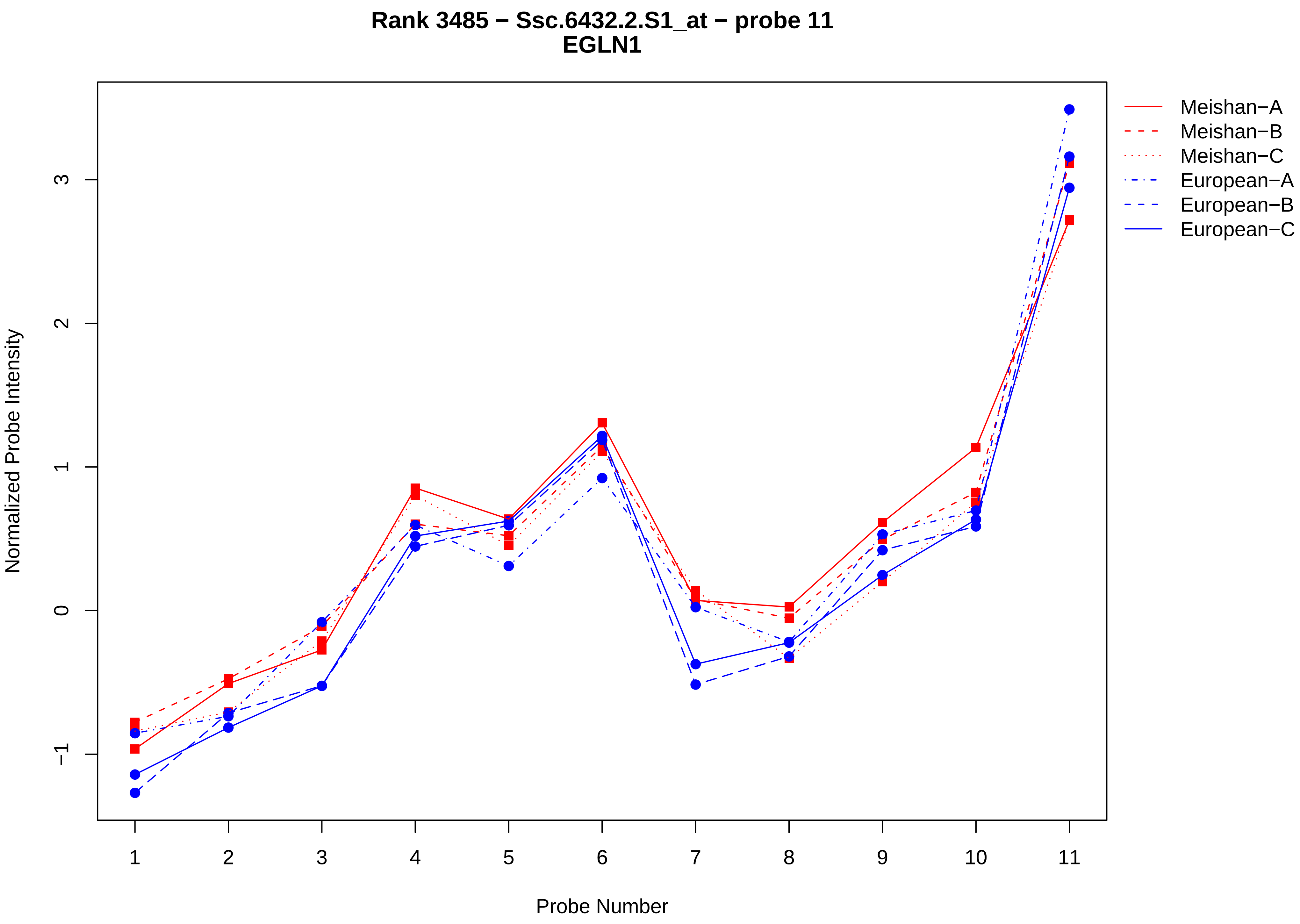

Rank 3485 − Ssc.6432.2.S1_at − probe 11
EGLN1
Normalized Probe Intensity
Probe Number
−1
0
1
2
3
1
2
3
4
5
6
7
8
9
10
11
Meishan−A
Meishan−B
Meishan−C
European−A
European−B
European−C

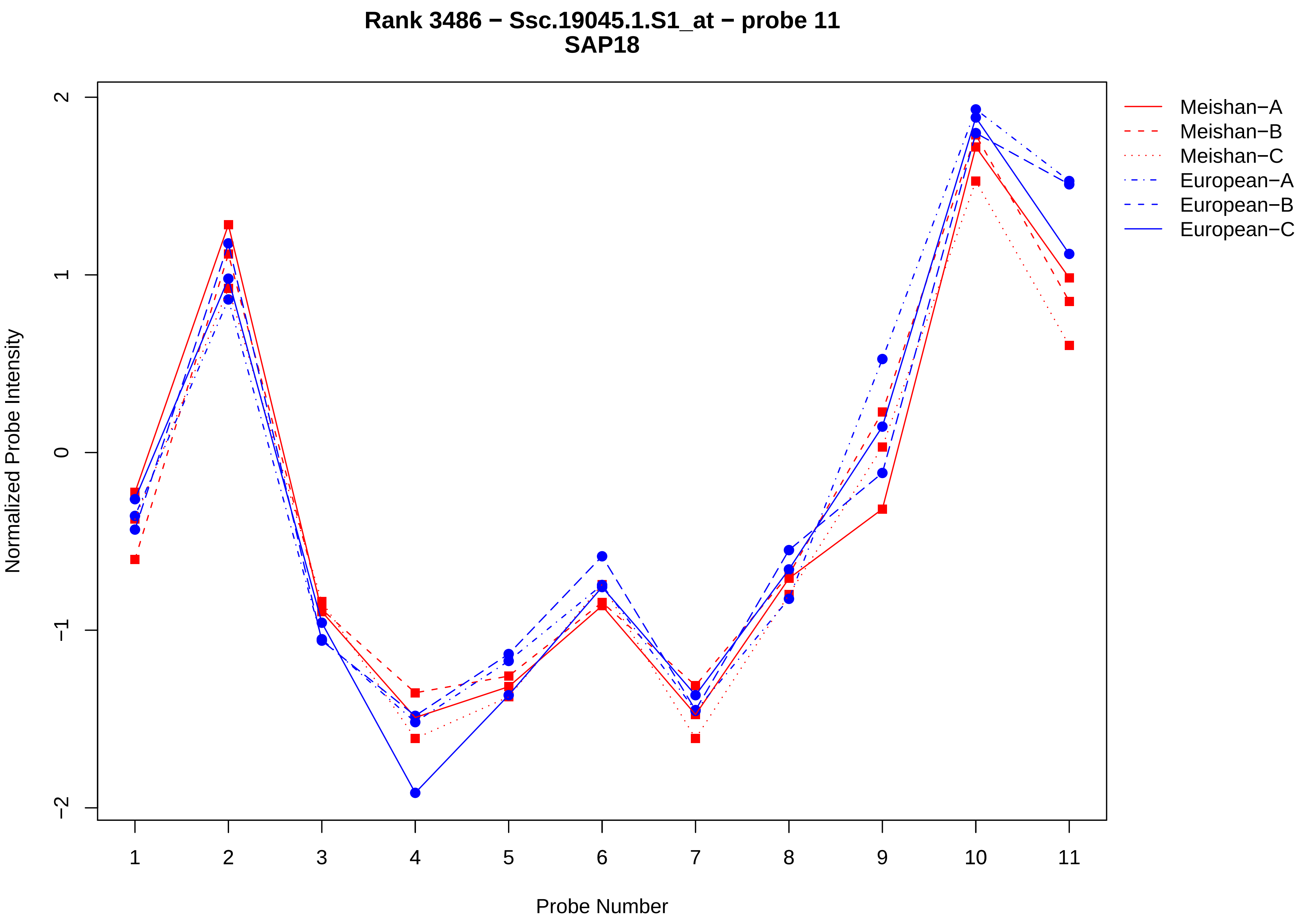

Rank 3486 − Ssc.19045.1.S1_at − probe 11
SAP18
Normalized Probe Intensity
Probe Number
Meishan−A
Meishan−B
Meishan−C
European−A
European−B
European−C

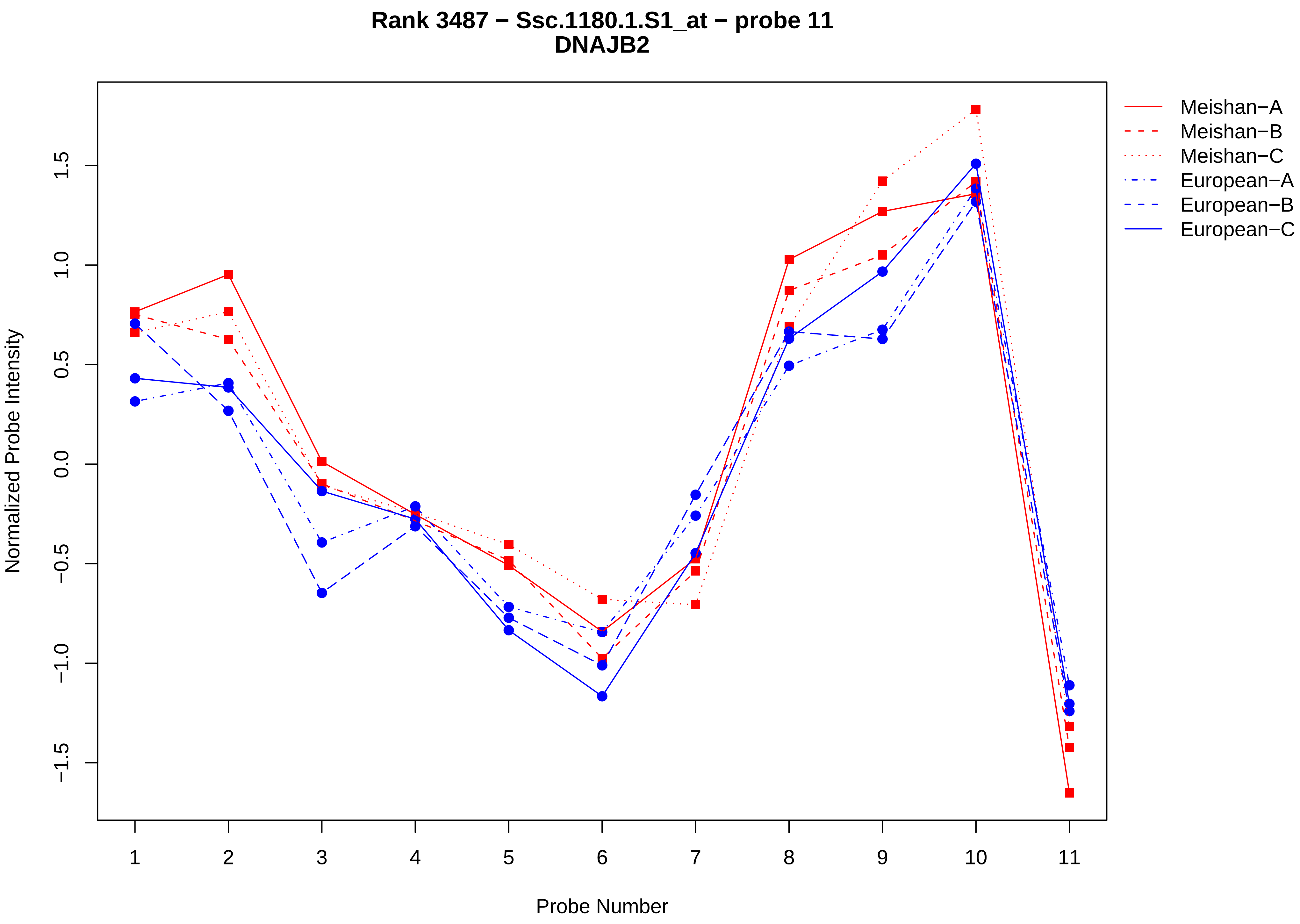

Rank 3487 − Ssc.1180.1.S1_at − probe 11
DNAJB2
Normalized Probe Intensity
Probe Number
Meishan−A
Meishan−B
Meishan−C
European−A
European−B
European−C
1.5
1.0
0.5
0.0
−0.5
−1.0
−1.5
1
2
3
4
5
6
7
8
9
10
11

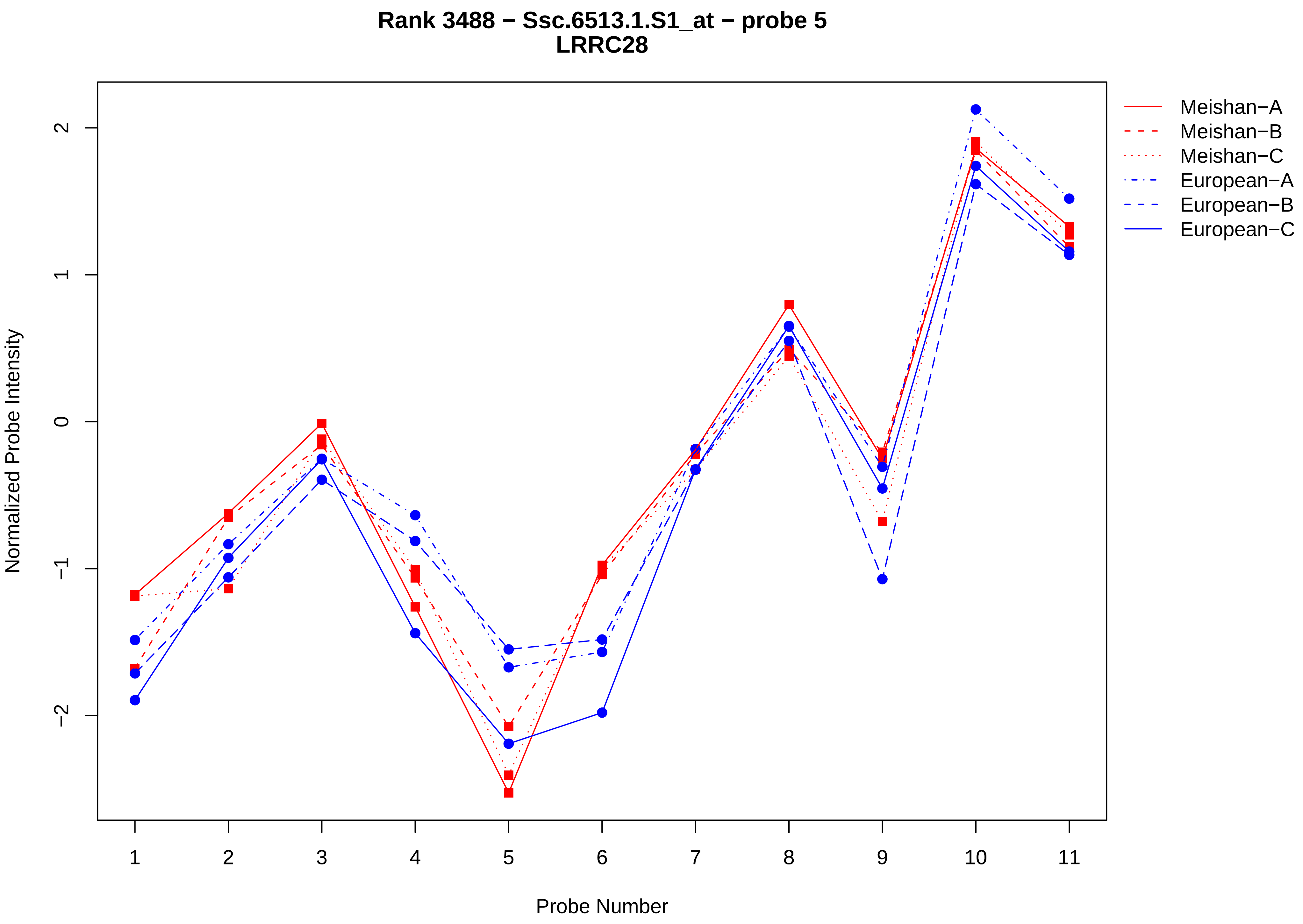

Rank 3488 − Ssc.6513.1.S1_at − probe 5
LRRC28
Normalized Probe Intensity
Probe Number
Meishan−A
Meishan−B
Meishan−C
European−A
European−B
European−C
2
1
0
−1
−2
1
2
3
4
5
6
7
8
9
10
11

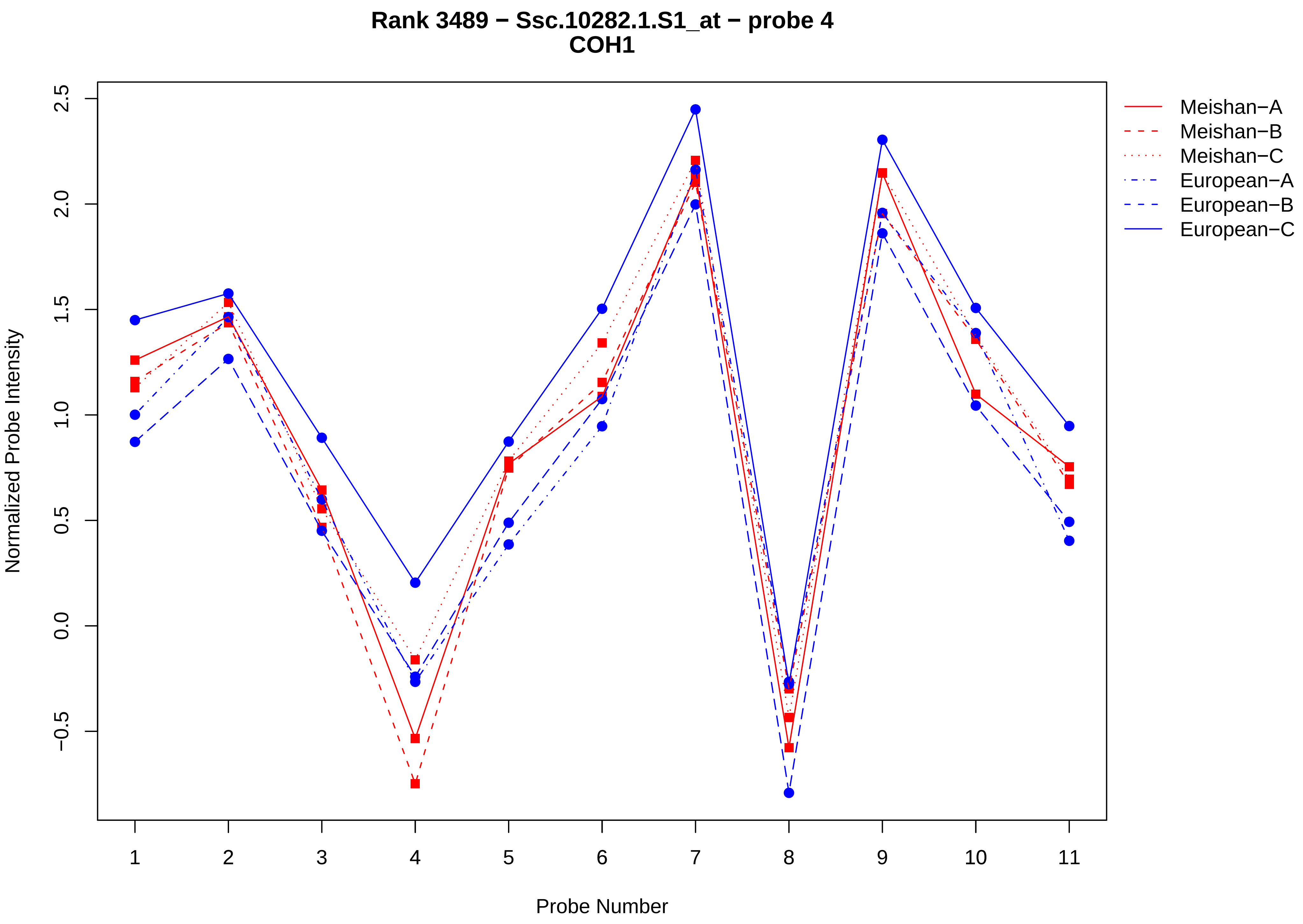

Rank 3489 − Ssc.10282.1.S1_at − probe 4
COH1
Normalized Probe Intensity
Probe Number
Meishan−A
Meishan−B
Meishan−C
European−A
European−B
European−C
−0.5
0.0
0.5
1.0
1.5
2.0
2.5
1
2
3
4
5
6
7
8
9
10
11

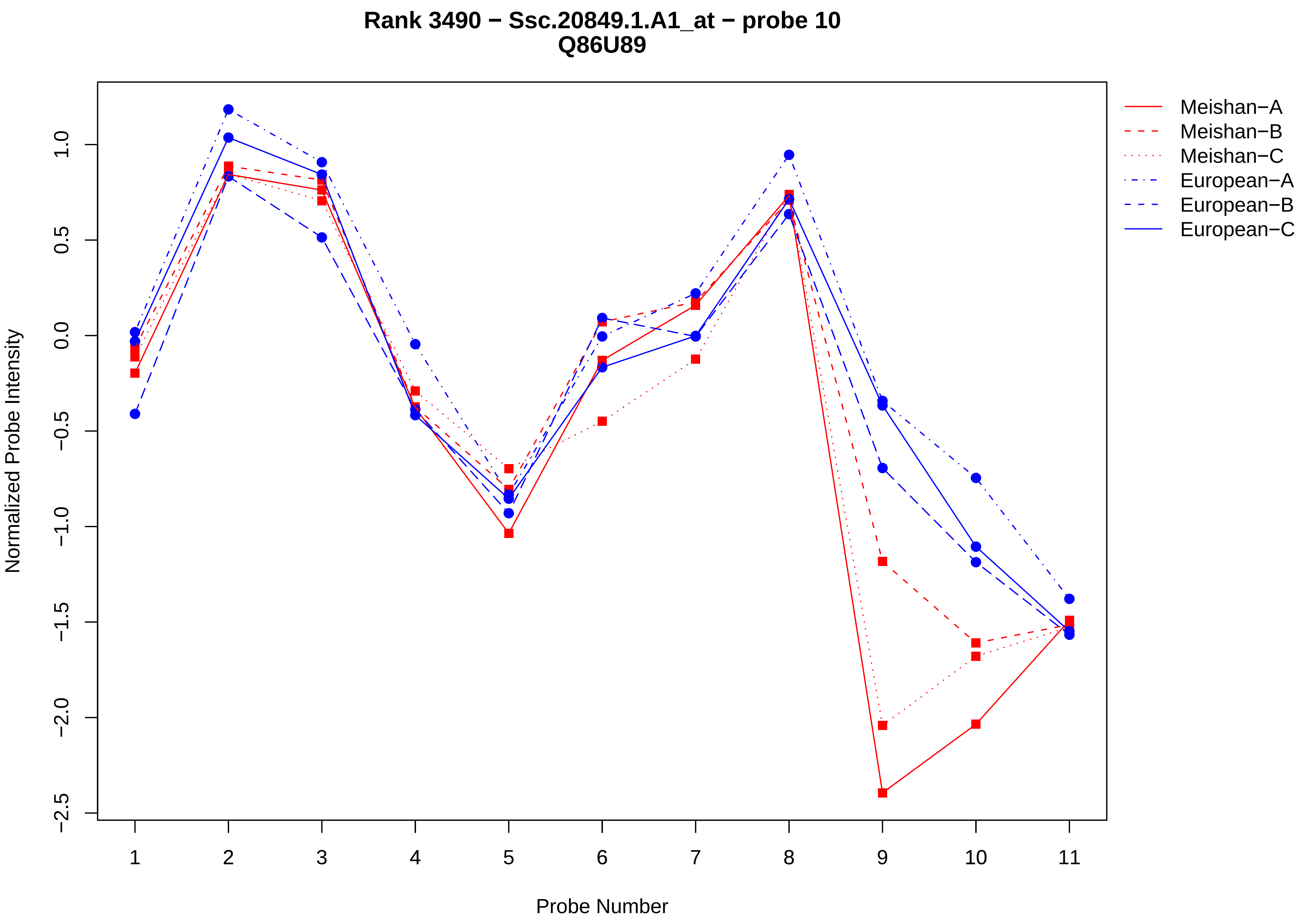

Rank 3490 − Ssc.20849.1.A1_at − probe 10
Q86U89
Normalized Probe Intensity
Probe Number
1.0
0.5
0.0
−0.5
−1.0
−1.5
−2.0
−2.5
1
2
3
4
5
6
7
8
9
10
11
Meishan−A
Meishan−B
Meishan−C
European−A
European−B
European−C

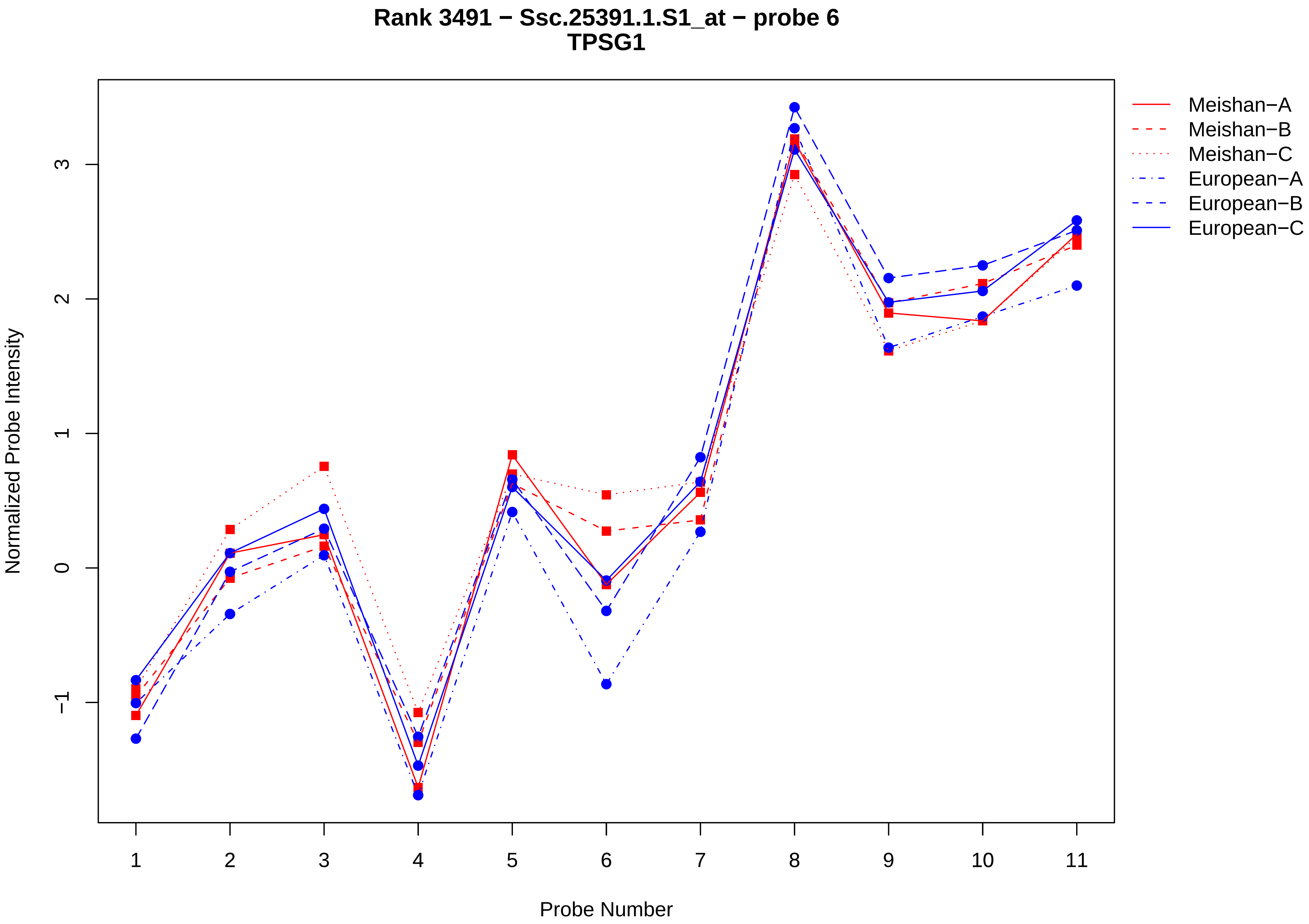

Rank 3491 − Ssc.25391.1.S1_at − probe 6
TPSG1
Normalized Probe Intensity
Probe Number
−1
0
1
2
3
1
2
3
4
5
6
7
8
9
10
11
Meishan−A
Meishan−B
Meishan−C
European−A
European−B
European−C

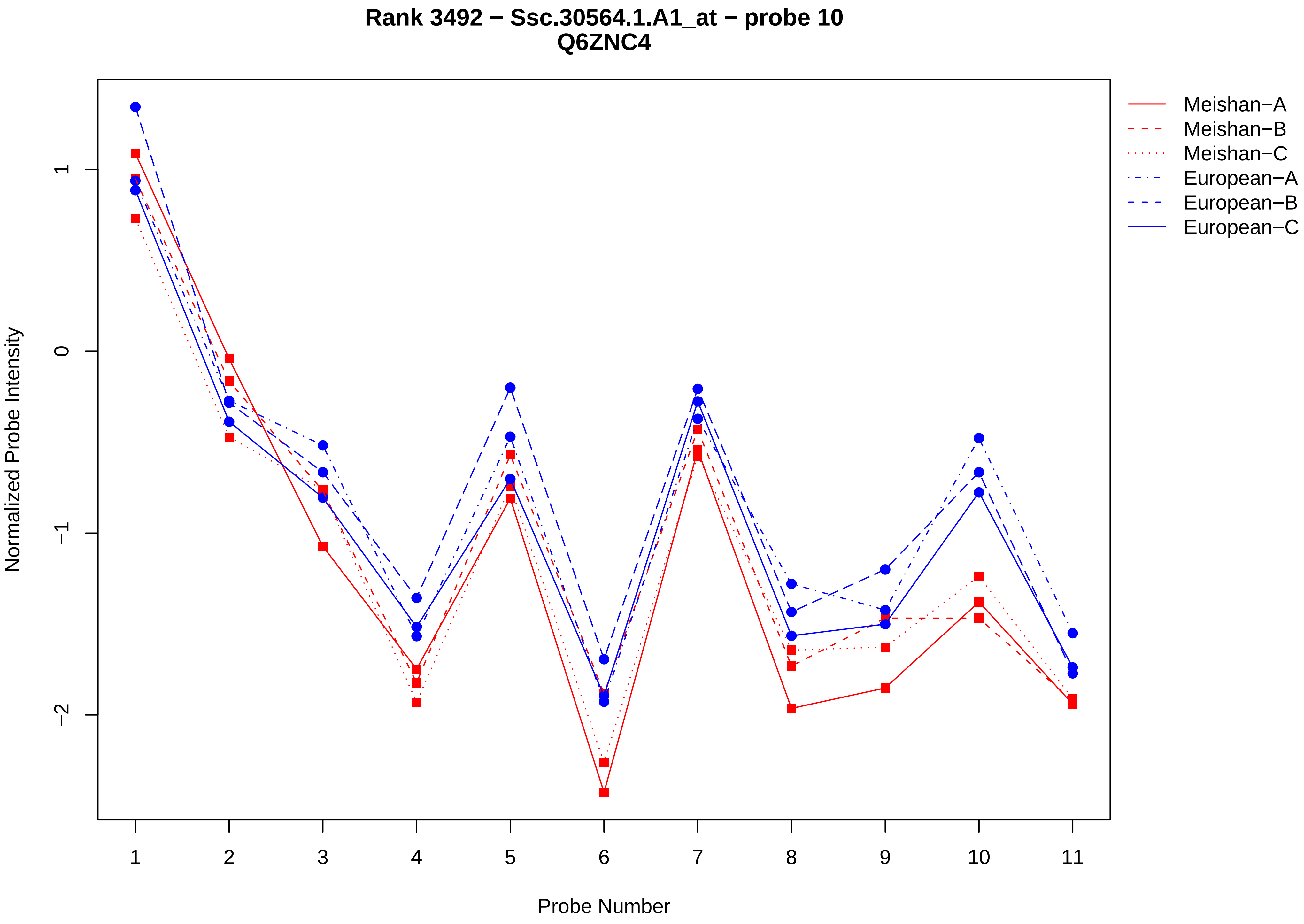

Rank 3492 − Ssc.30564.1.A1_at − probe 10
Q6ZNC4
Normalized Probe Intensity
Probe Number
1
0
−1
−2
1
2
3
4
5
6
7
8
9
10
11
Meishan−A
Meishan−B
Meishan−C
European−A
European−B
European−C

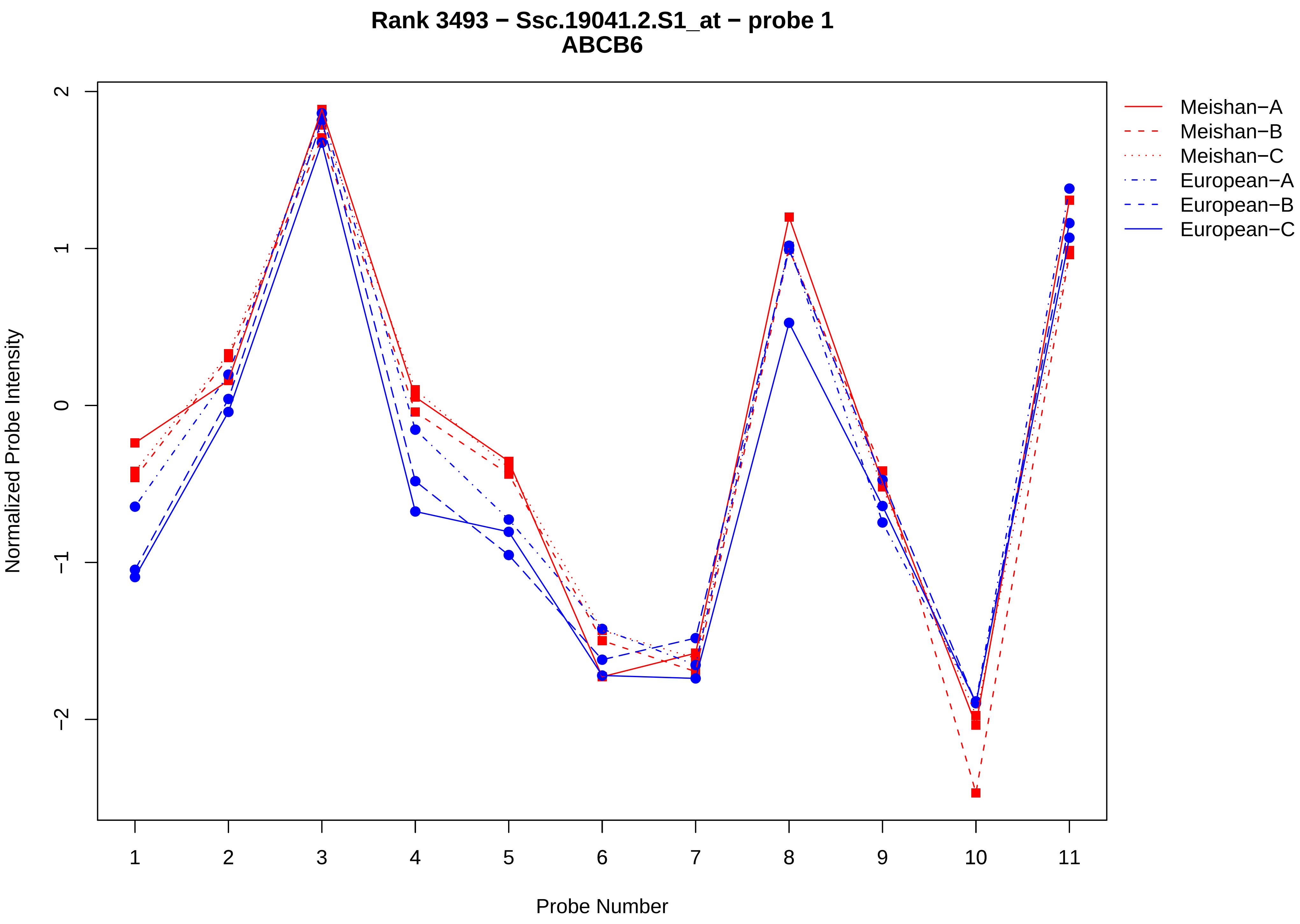
Rank 3493 − Ssc.19041.2.S1_at − probe 1
ABCB6
Normalized Probe Intensity
Probe Number
Meishan−A
Meishan−B
Meishan−C
European−A
European−B
European−C
2
1
0
−1
−2
1
2
3
4
5
6
7
8
9
10
11

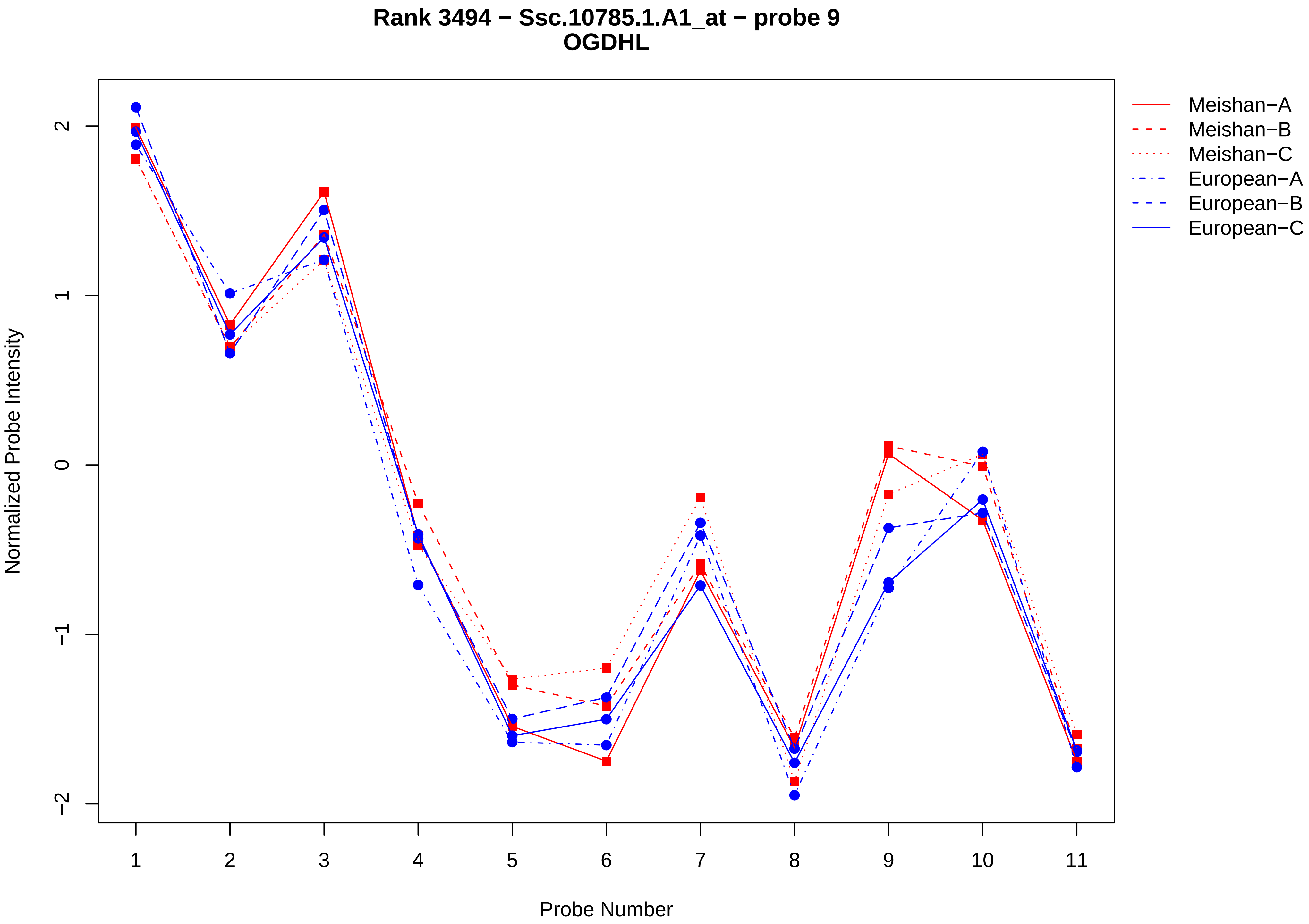

Rank 3494 − Ssc.10785.1.A1_at − probe 9
OGDHL
Normalized Probe Intensity
Probe Number
Meishan−A
Meishan−B
Meishan−C
European−A
European−B
European−C
2
1
0
−1
−2
1
2
3
4
5
6
7
8
9
10
11

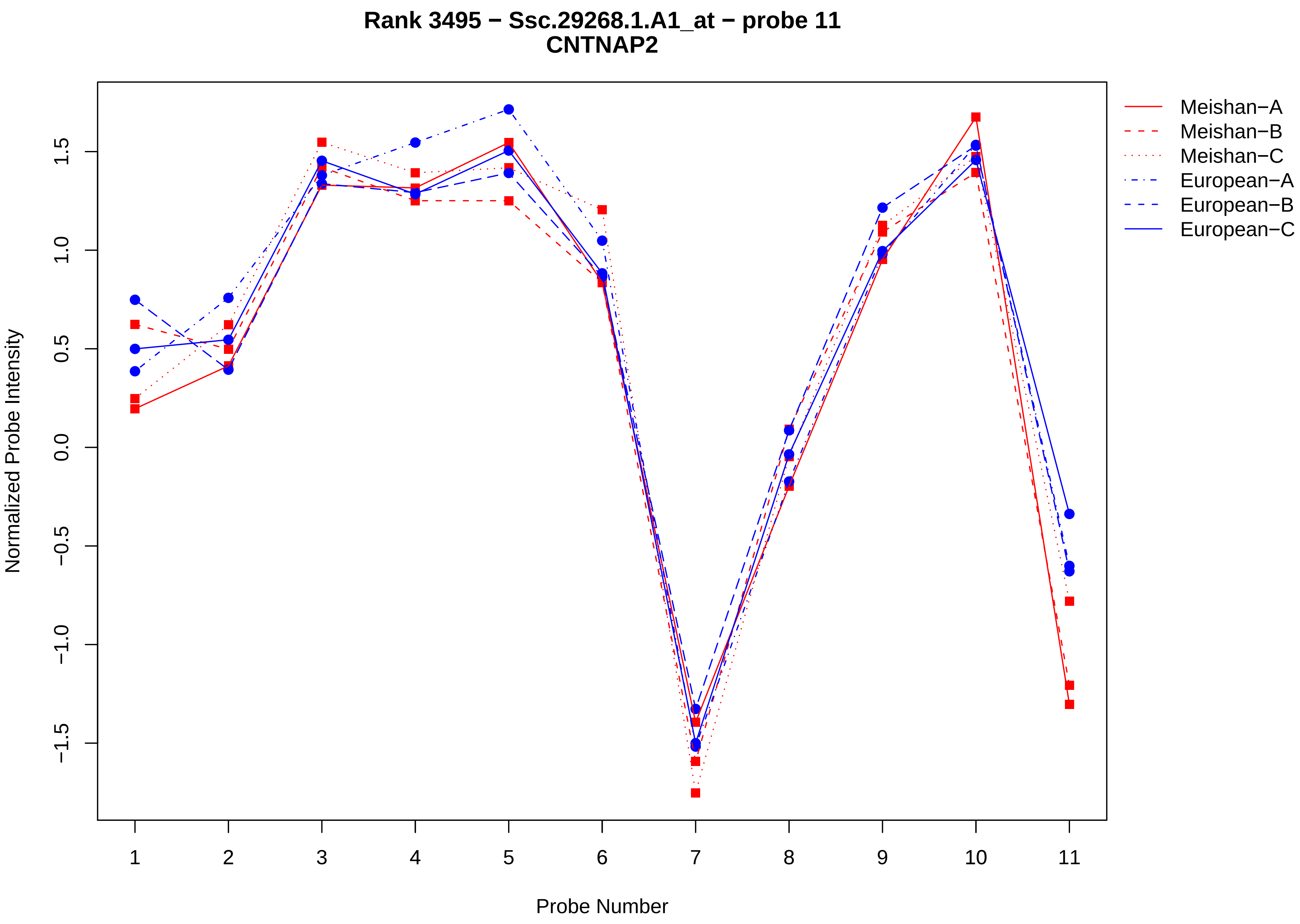

Rank 3495 − Ssc.29268.1.A1_at − probe 11
CNTNAP2
Normalized Probe Intensity
Probe Number
1.5
1.0
0.5
0.0
−0.5
−1.0
−1.5
1
2
3
4
5
6
7
8
9
10
11
Meishan−A
Meishan−B
Meishan−C
European−A
European−B
European−C

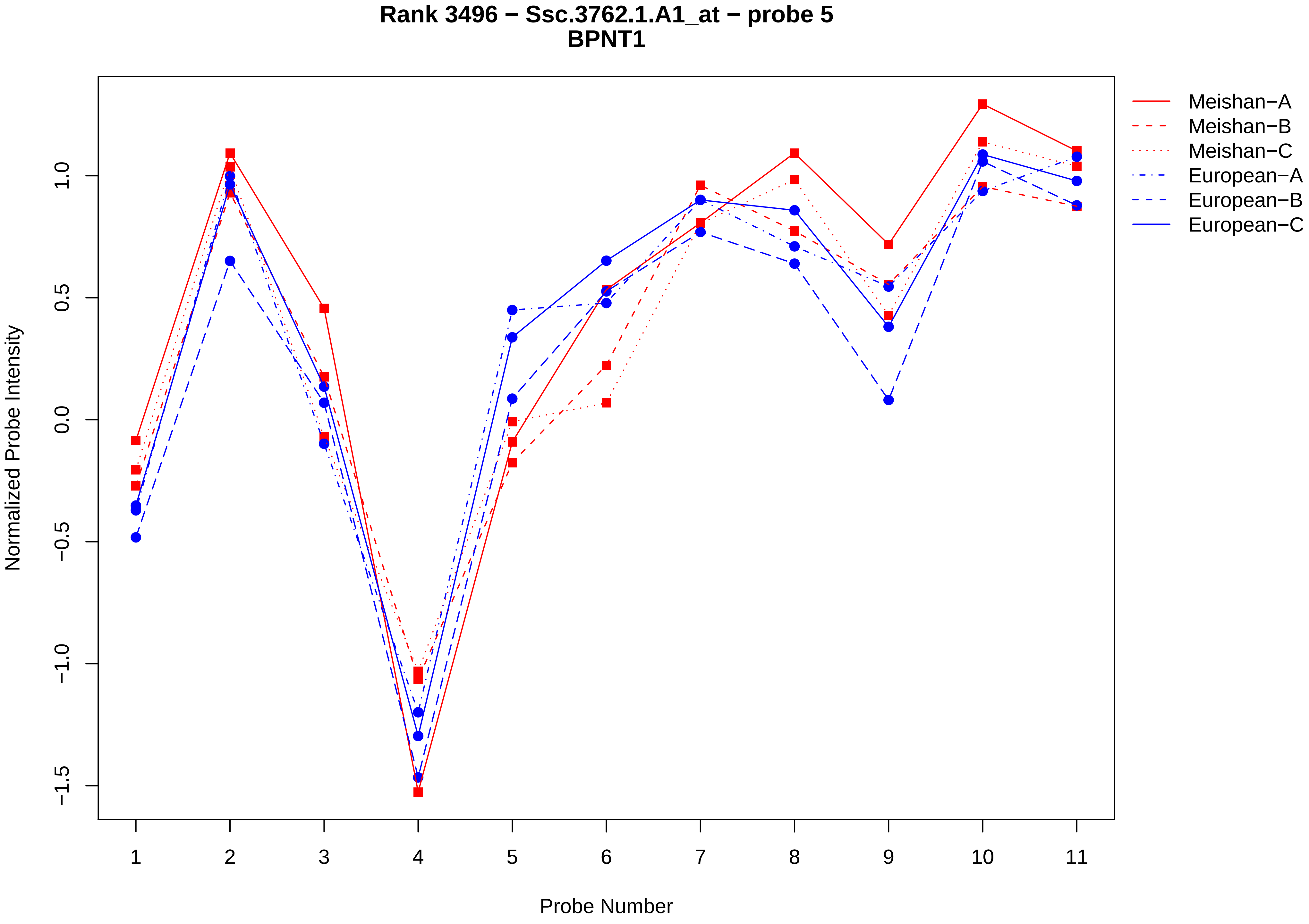
Rank 3496 − Ssc.3762.1.A1_at − probe 5
BPNT1
Normalized Probe Intensity
Probe Number
Meishan−A
Meishan−B
Meishan−C
European−A
European−B
European−C
−1.5
−1.0
−0.5
0.0
0.5
1.0
1
2
3
4
5
6
7
8
9
10
11

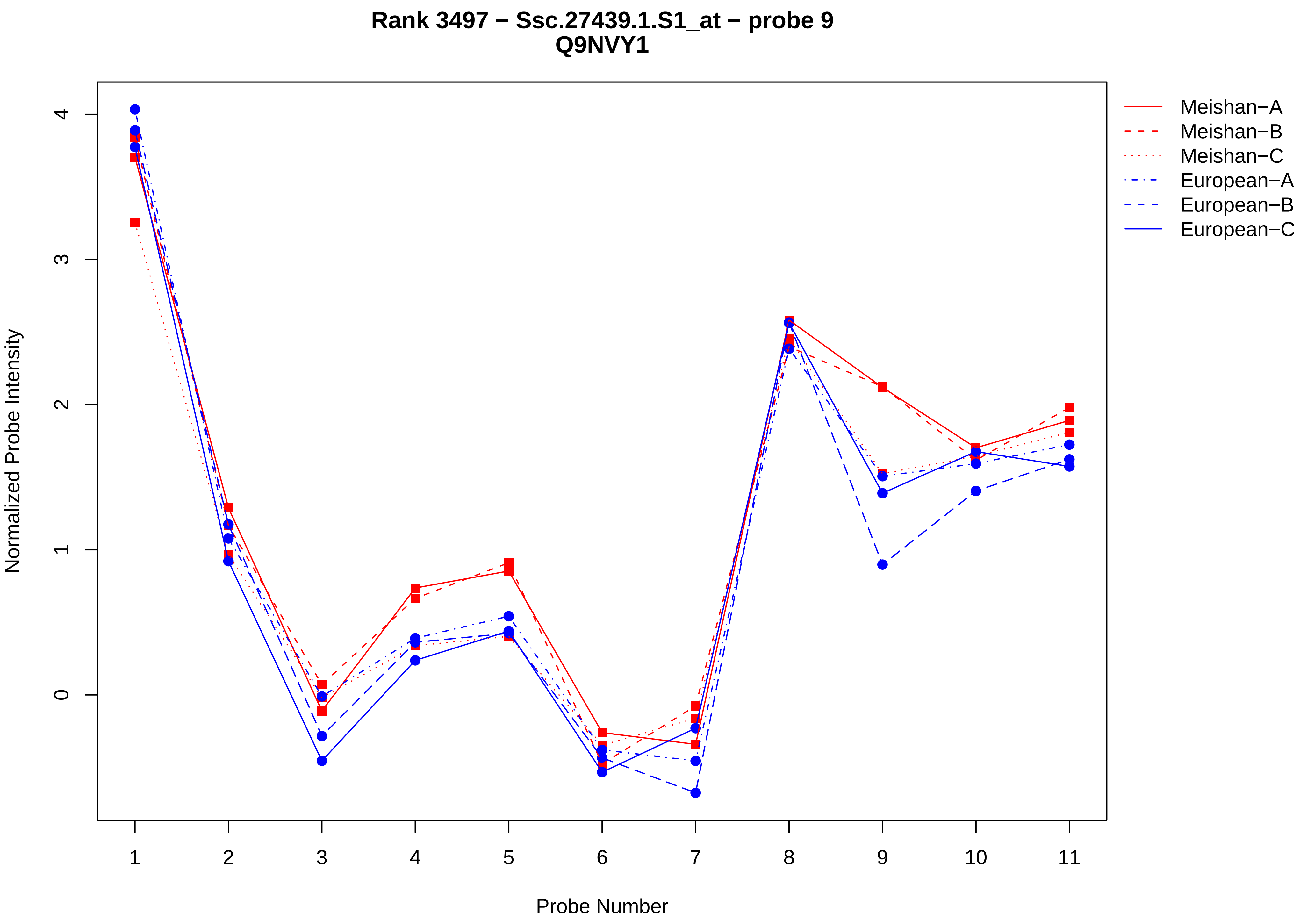

Rank 3497 − Ssc.27439.1.S1_at − probe 9
Q9NVY1
Normalized Probe Intensity
Probe Number
0
1
2
3
4
1
2
3
4
5
6
7
8
9
10
11
Meishan−A
Meishan−B
Meishan−C
European−A
European−B
European−C

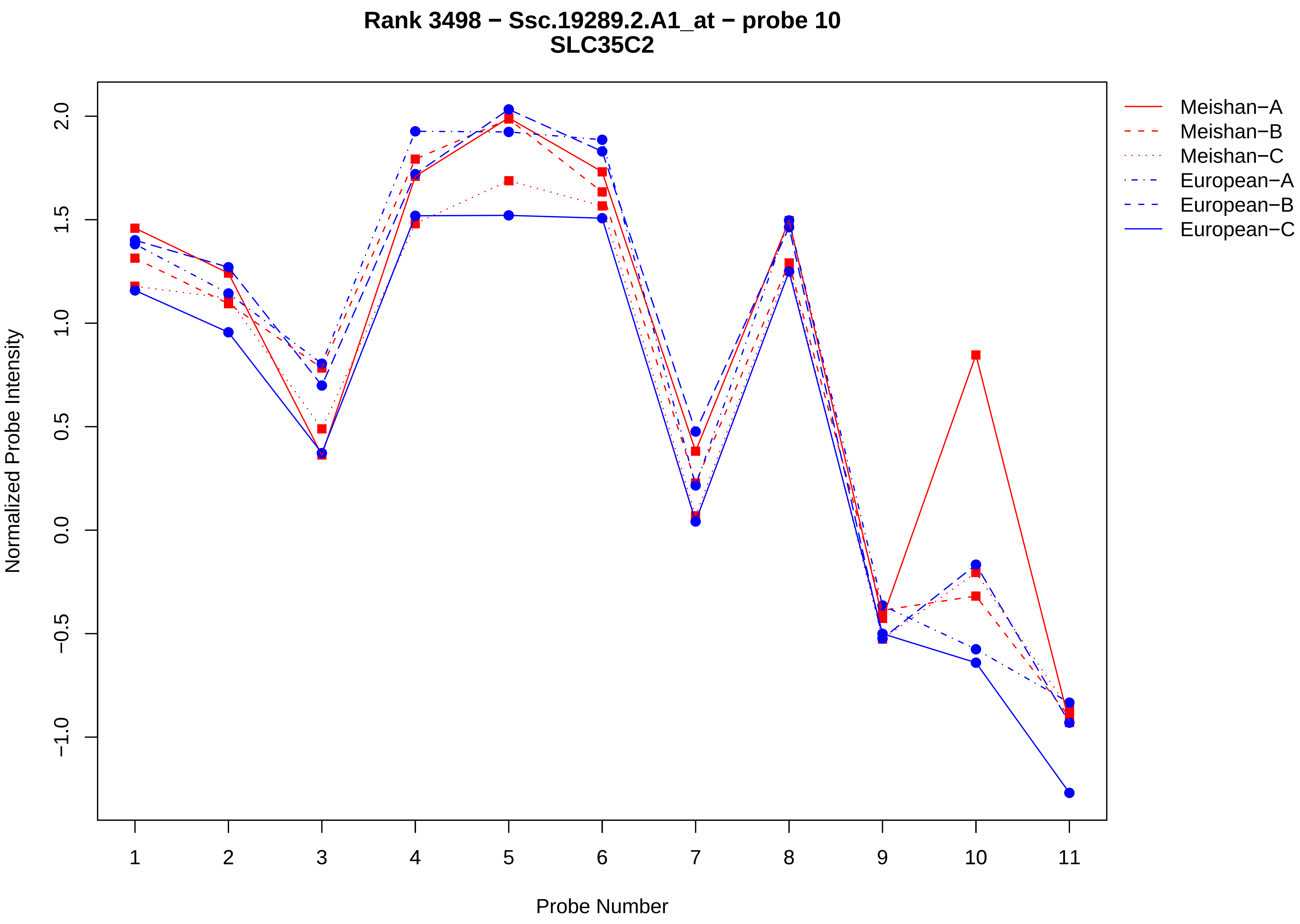

Rank 3498 − Ssc.19289.2.A1_at − probe 10
SLC35C2
Normalized Probe Intensity
Probe Number
−1.0
−0.5
0.0
0.5
1.0
1.5
2.0
1
2
3
4
5
6
7
8
9
10
11
Meishan−A
Meishan−B
Meishan−C
European−A
European−B
European−C

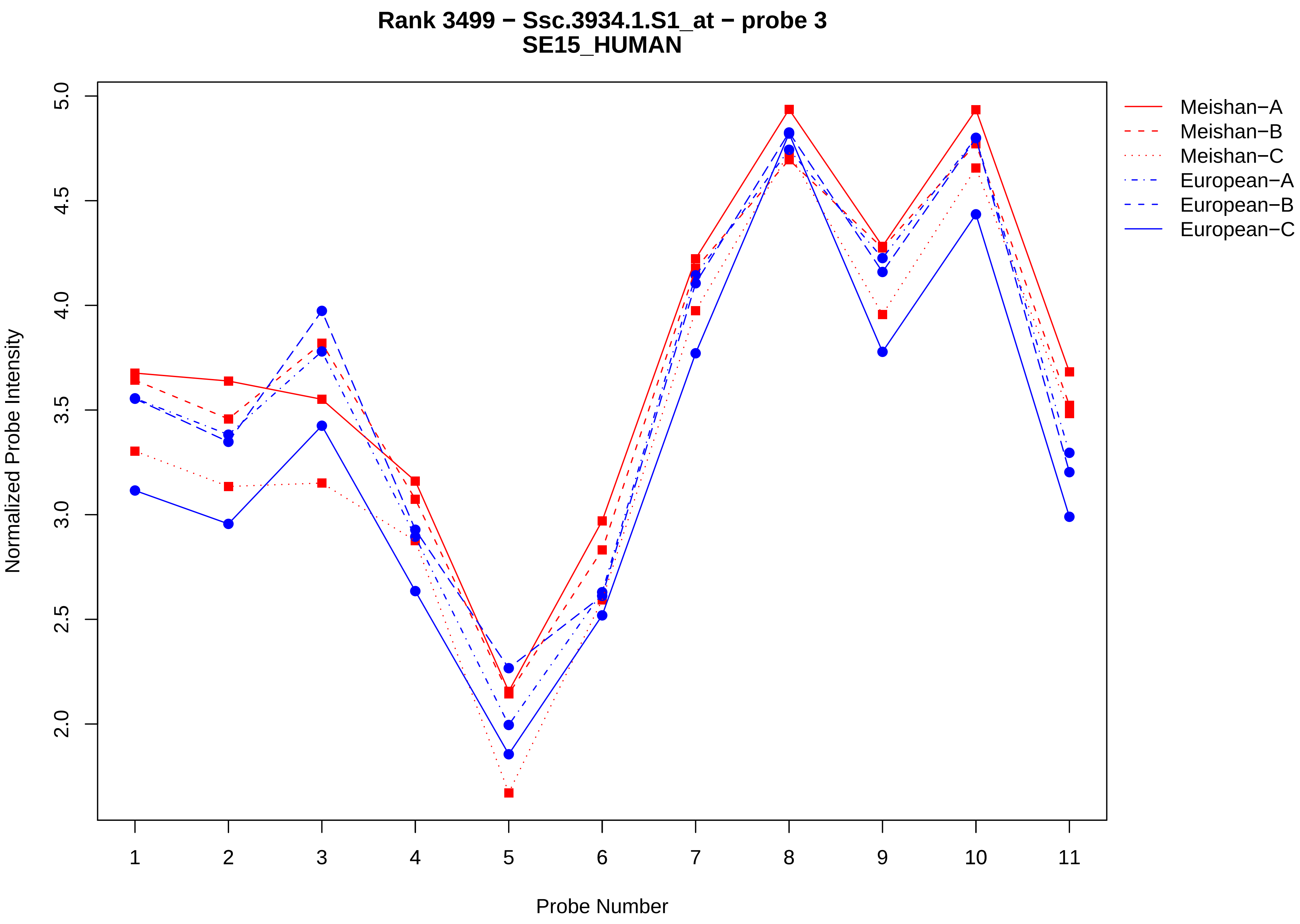

Rank 3499 − Ssc.3934.1.S1_at − probe 3
SE15_HUMAN
Normalized Probe Intensity
Probe Number
Meishan−A
Meishan−B
Meishan−C
European−A
European−B
European−C

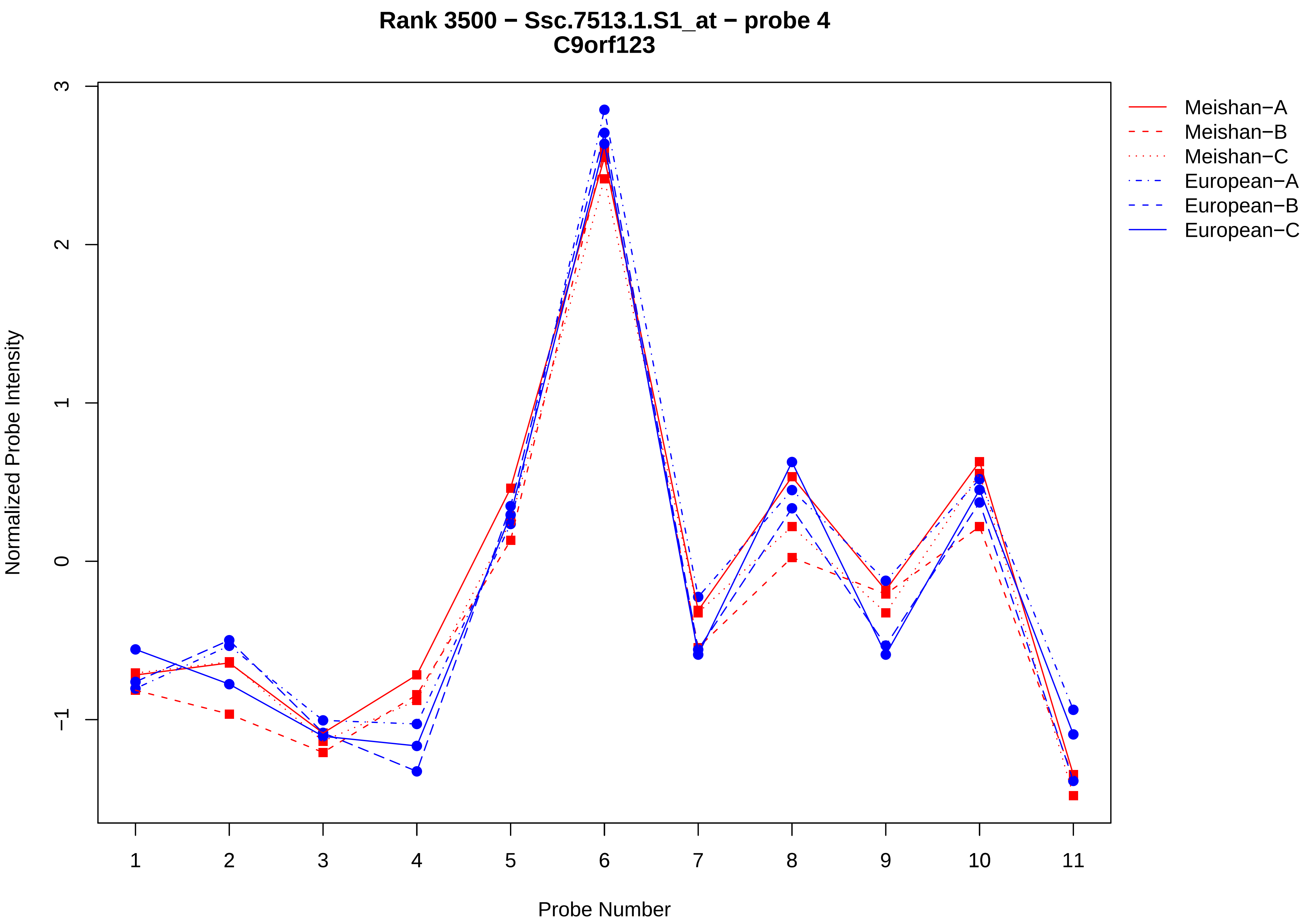
Rank 3500 − Ssc.7513.1.S1_at − probe 4
C9orf123
Normalized Probe Intensity
Probe Number
Meishan−A
Meishan−B
Meishan−C
European−A
European−B
European−C
3
2
1
0
−1
1
2
3
4
5
6
7
8
9
10
11

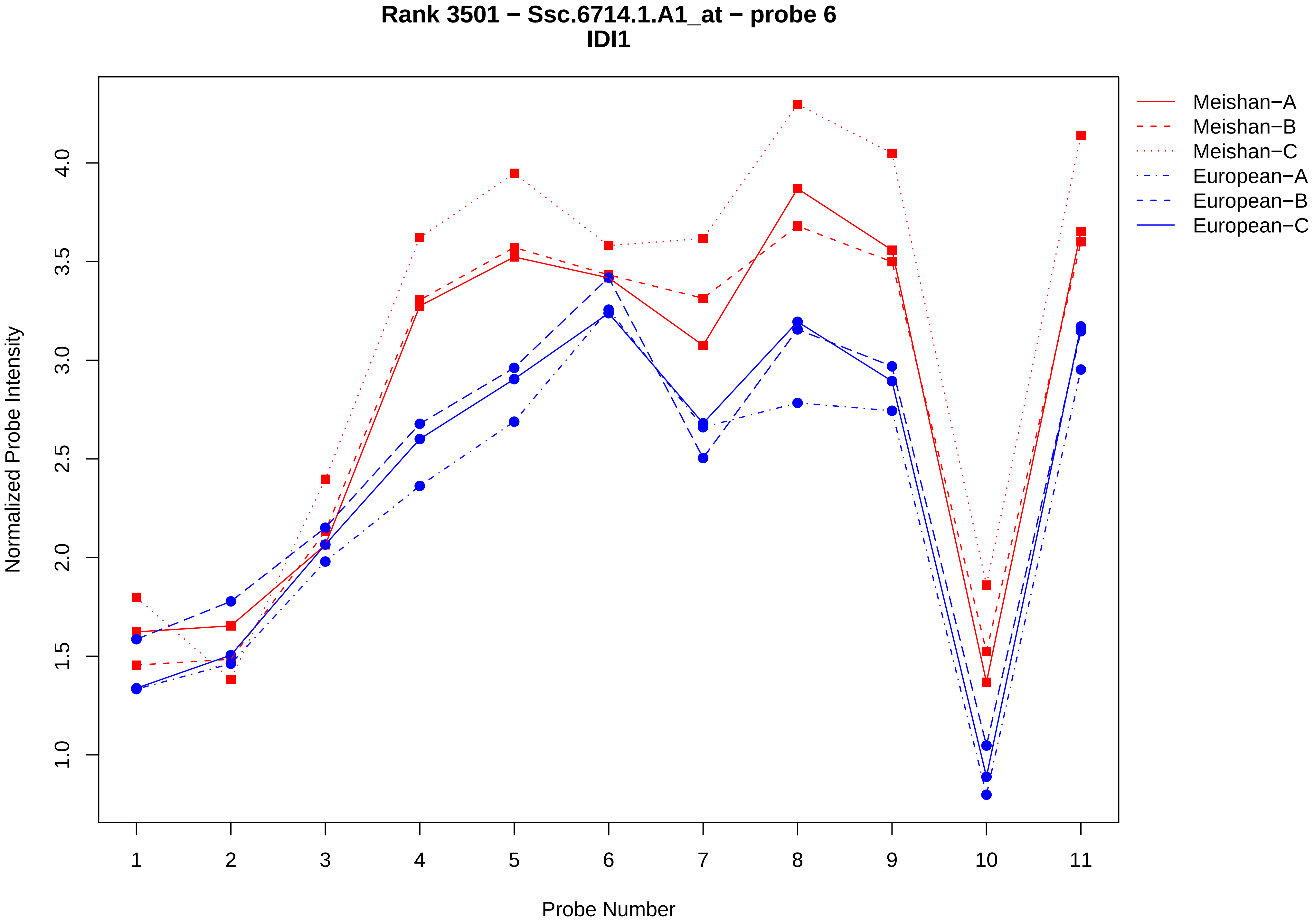

Rank 3501 − Ssc.6714.1.A1_at − probe 6
IDI1
Normalized Probe Intensity
Probe Number
1.0
1.5
2.0
2.5
3.0
3.5
4.0
1
2
3
4
5
6
7
8
9
10
11
Meishan−A
Meishan−B
Meishan−C
European−A
European−B
European−C

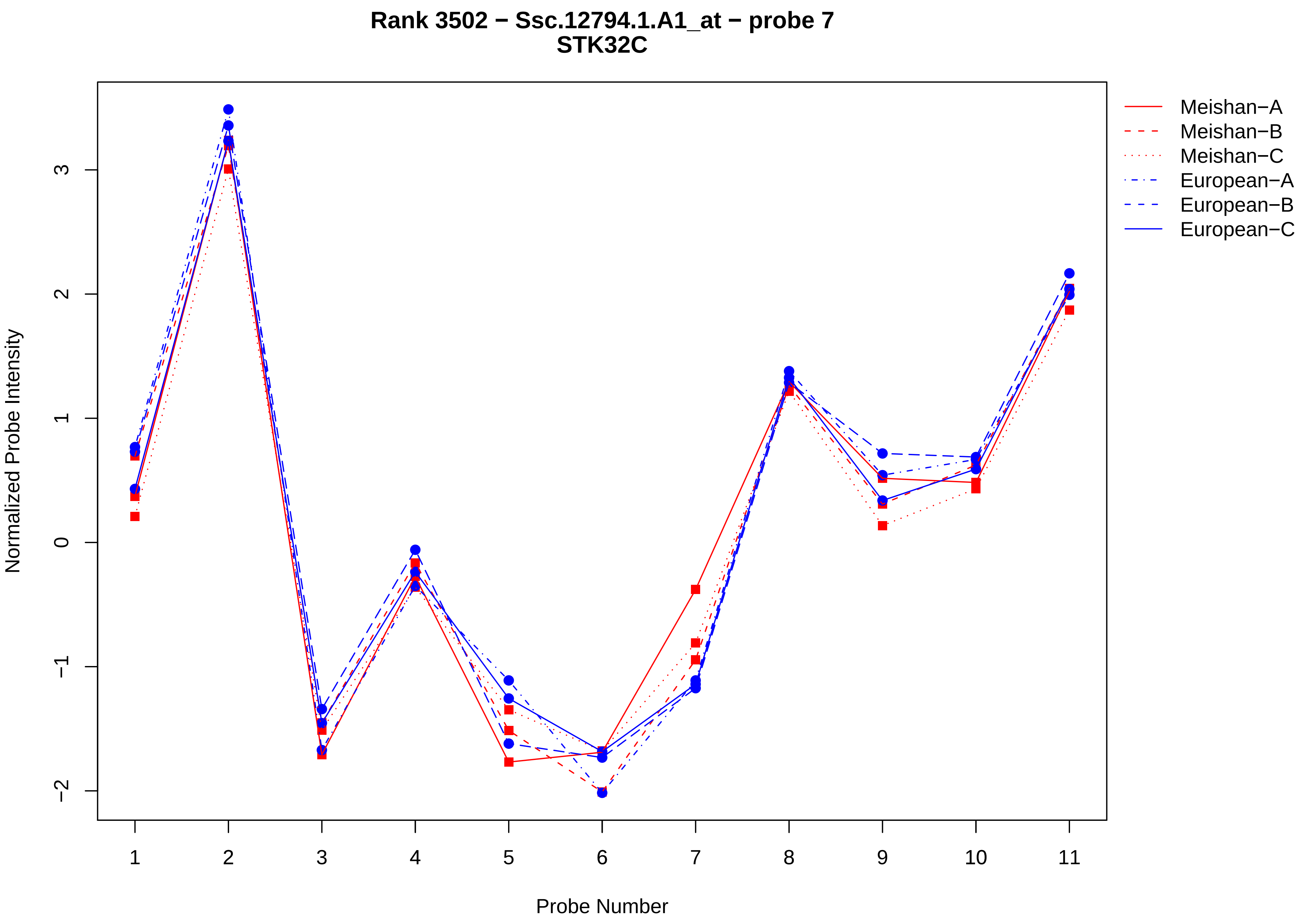

Rank 3502 − Ssc.12794.1.A1_at − probe 7
STK32C
Normalized Probe Intensity
Probe Number
Meishan−A
Meishan−B
Meishan−C
European−A
European−B
European−C
−2
−1
0
1
2
3
1
2
3
4
5
6
7
8
9
10
11

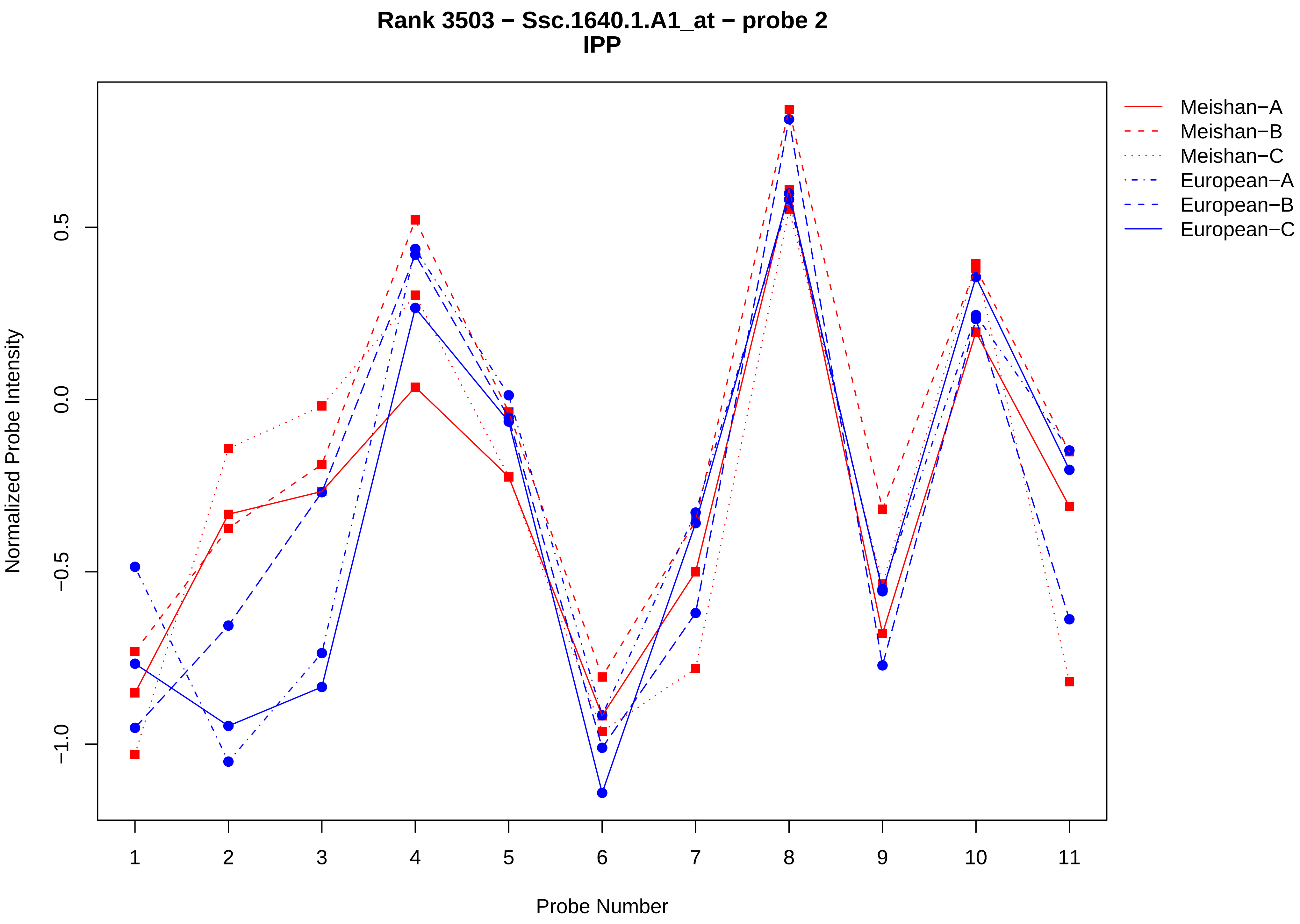

Rank 3503 − Ssc.1640.1.A1_at − probe 2
IPP
Normalized Probe Intensity
Probe Number
Meishan−A
Meishan−B
Meishan−C
European−A
European−B
European−C
0.5
0.0
−0.5
−1.0
1
2
3
4
5
6
7
8
9
10
11

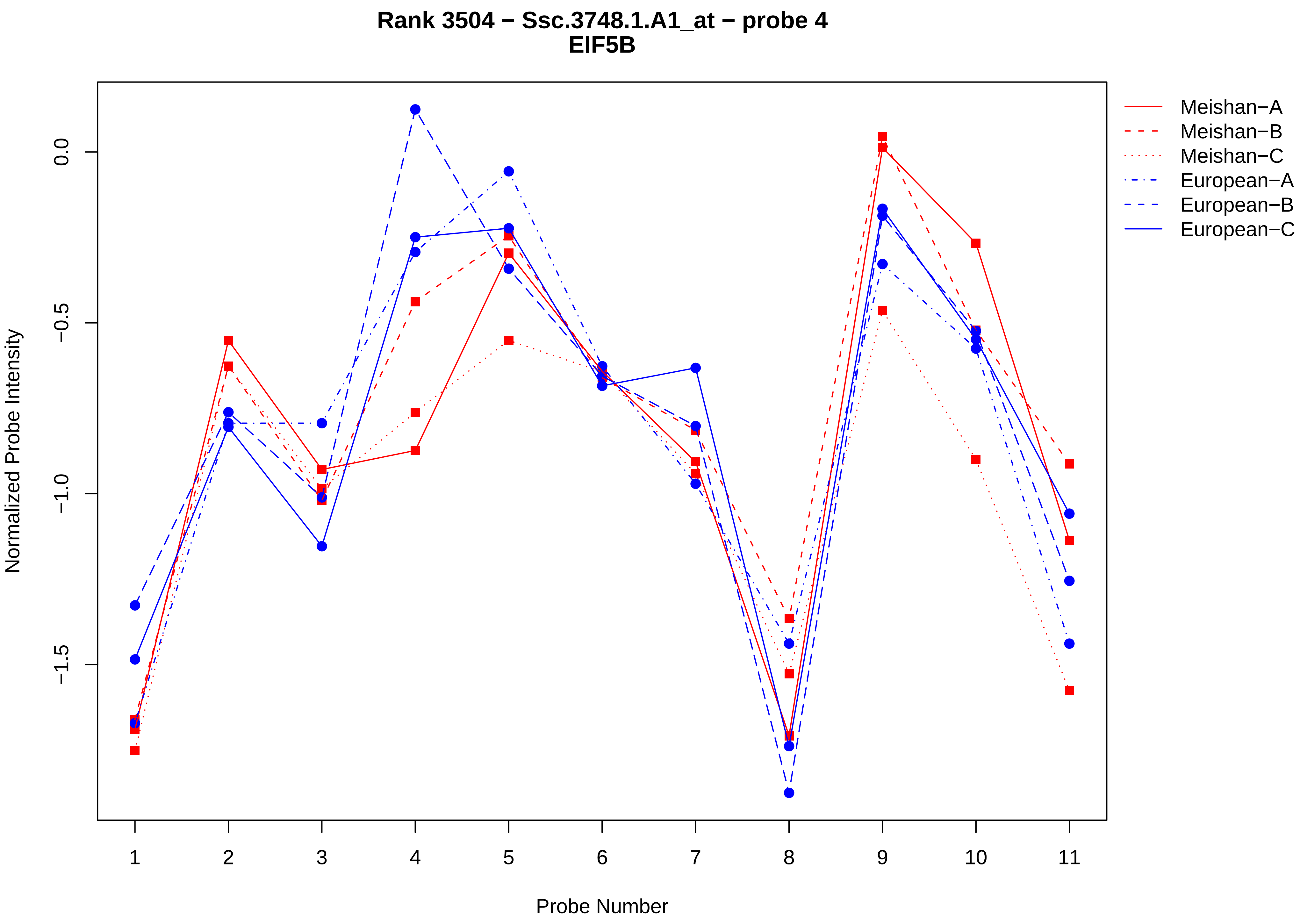

Rank 3504 − Ssc.3748.1.A1_at − probe 4
EIF5B
Normalized Probe Intensity
0.0
−0.5
−1.0
−1.5
Probe Number
1
2
3
4
5
6
7
8
9
10
11
Meishan−A
Meishan−B
Meishan−C
European−A
European−B
European−C

Rank 3505 − Ssc.7716.1.A1_at − probe 10
SGPL1

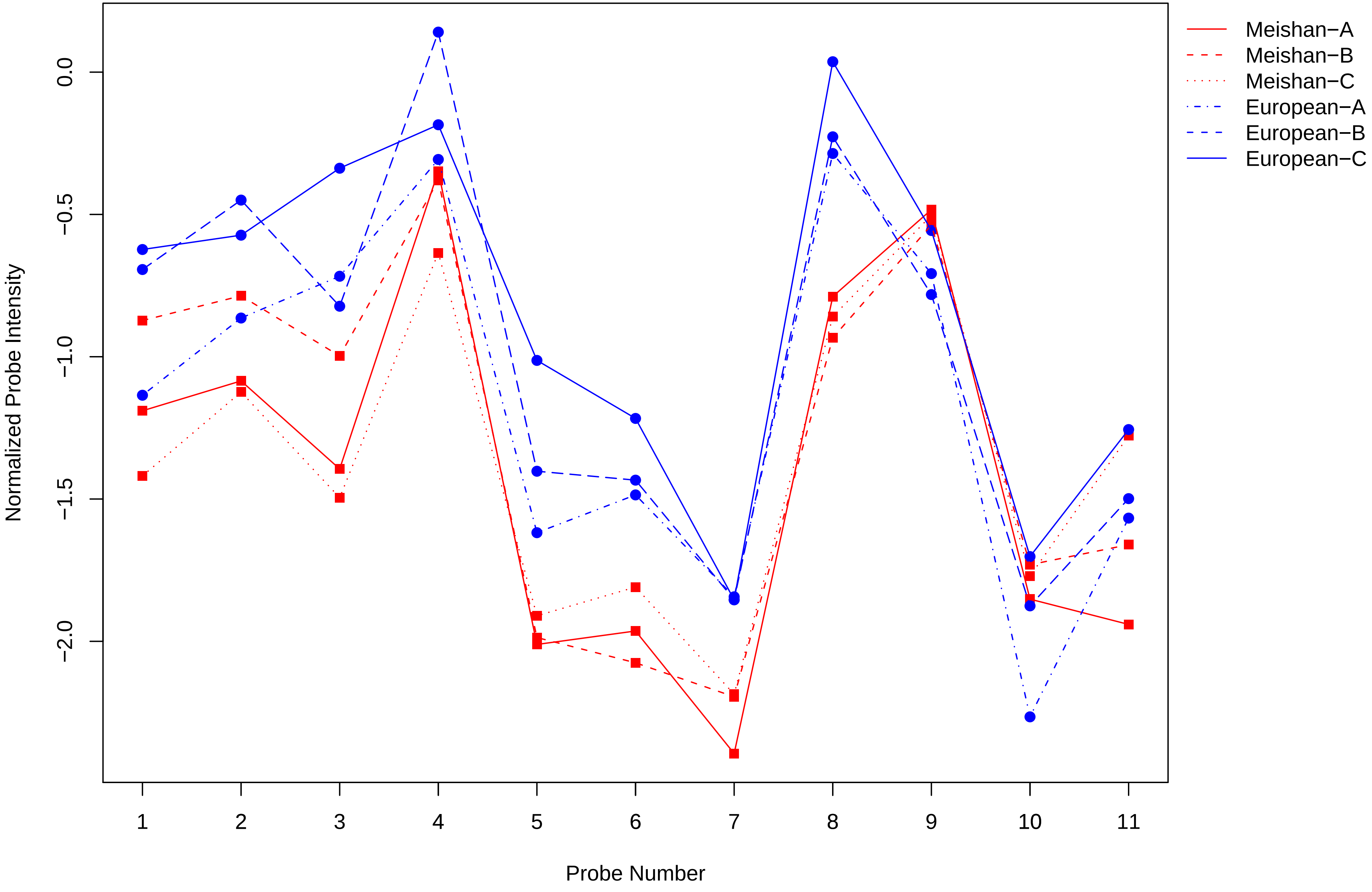

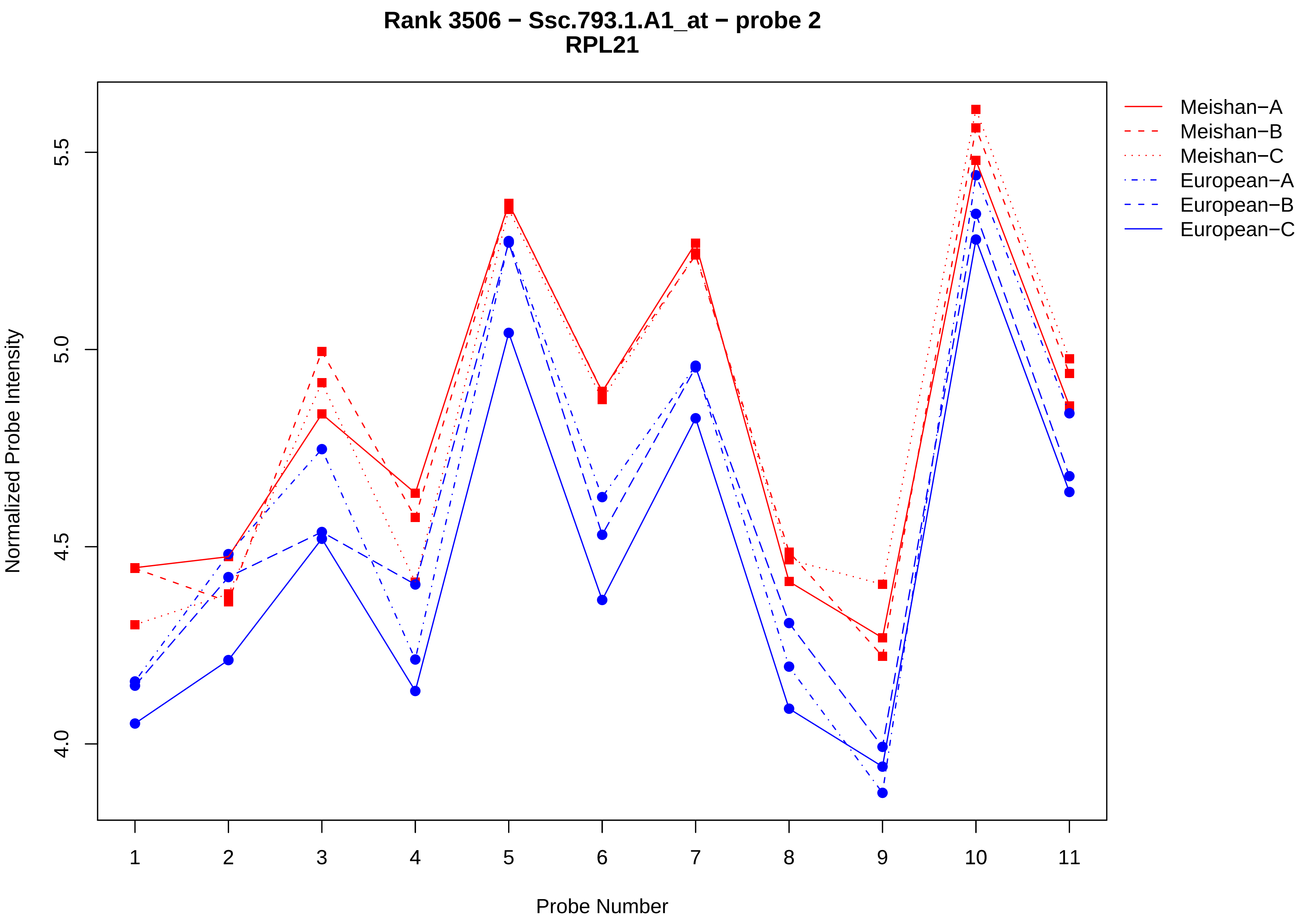

Rank 3506 − Ssc.793.1.A1_at − probe 2
RPL21
Normalized Probe Intensity
Probe Number
4.0
4.5
5.0
5.5
1
2
3
4
5
6
7
8
9
10
11
Meishan−A
Meishan−B
Meishan−C
European−A
European−B
European−C

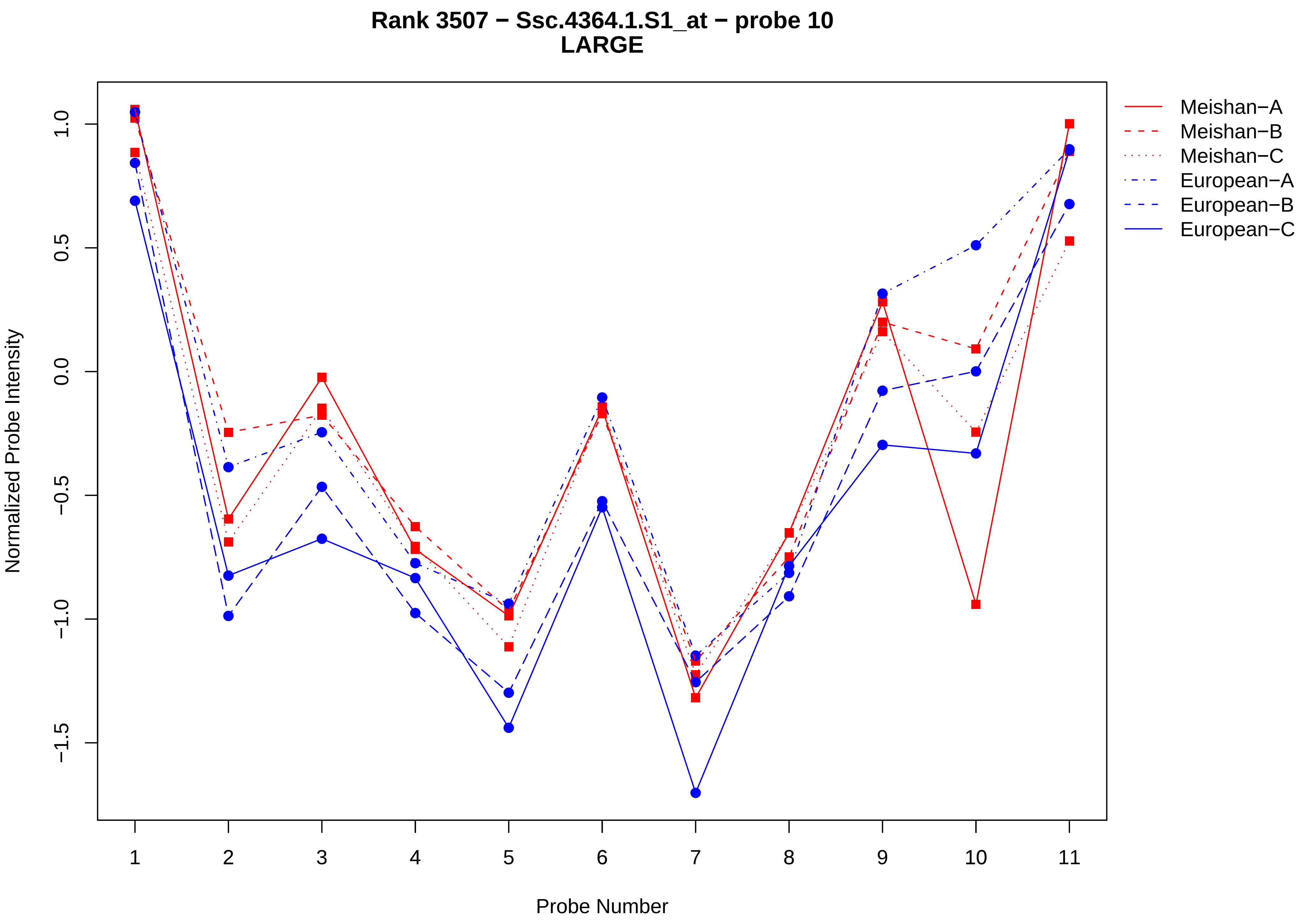

Rank 3507 − Ssc.4364.1.S1_at − probe 10
LARGE
Normalized Probe Intensity
1.0
0.5
0.0
−0.5
−1.0
−1.5
1
2
3
4
5
6
7
8
9
10
11
Probe Number
Meishan−A
Meishan−B
Meishan−C
European−A
European−B
European−C

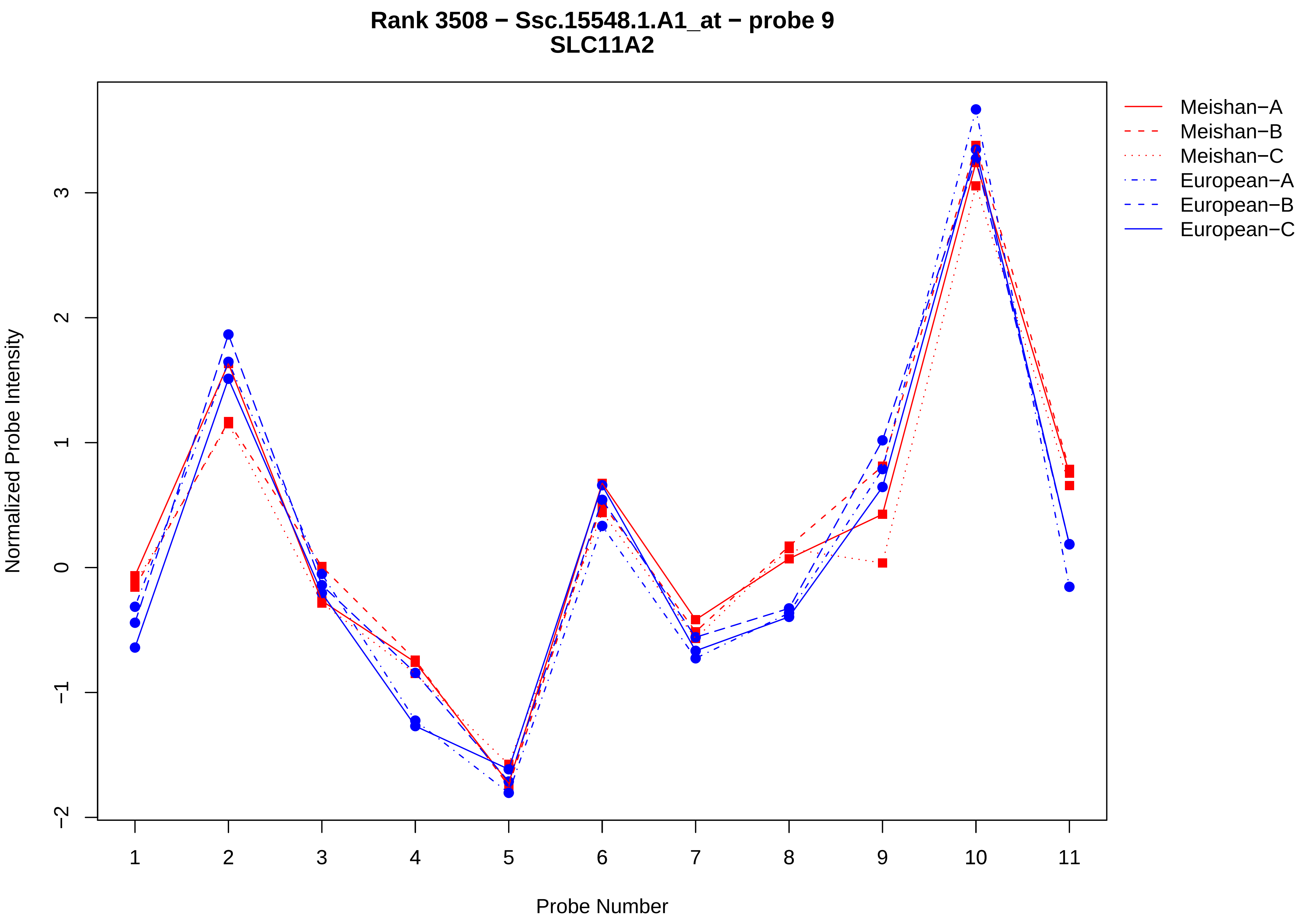
Rank 3508 − Ssc.15548.1.A1_at − probe 9
SLC11A2
Normalized Probe Intensity
Probe Number
Meishan−A
Meishan−B
Meishan−C
European−A
European−B
European−C
−2
−1
0
1
2
3
1
2
3
4
5
6
7
8
9
10
11

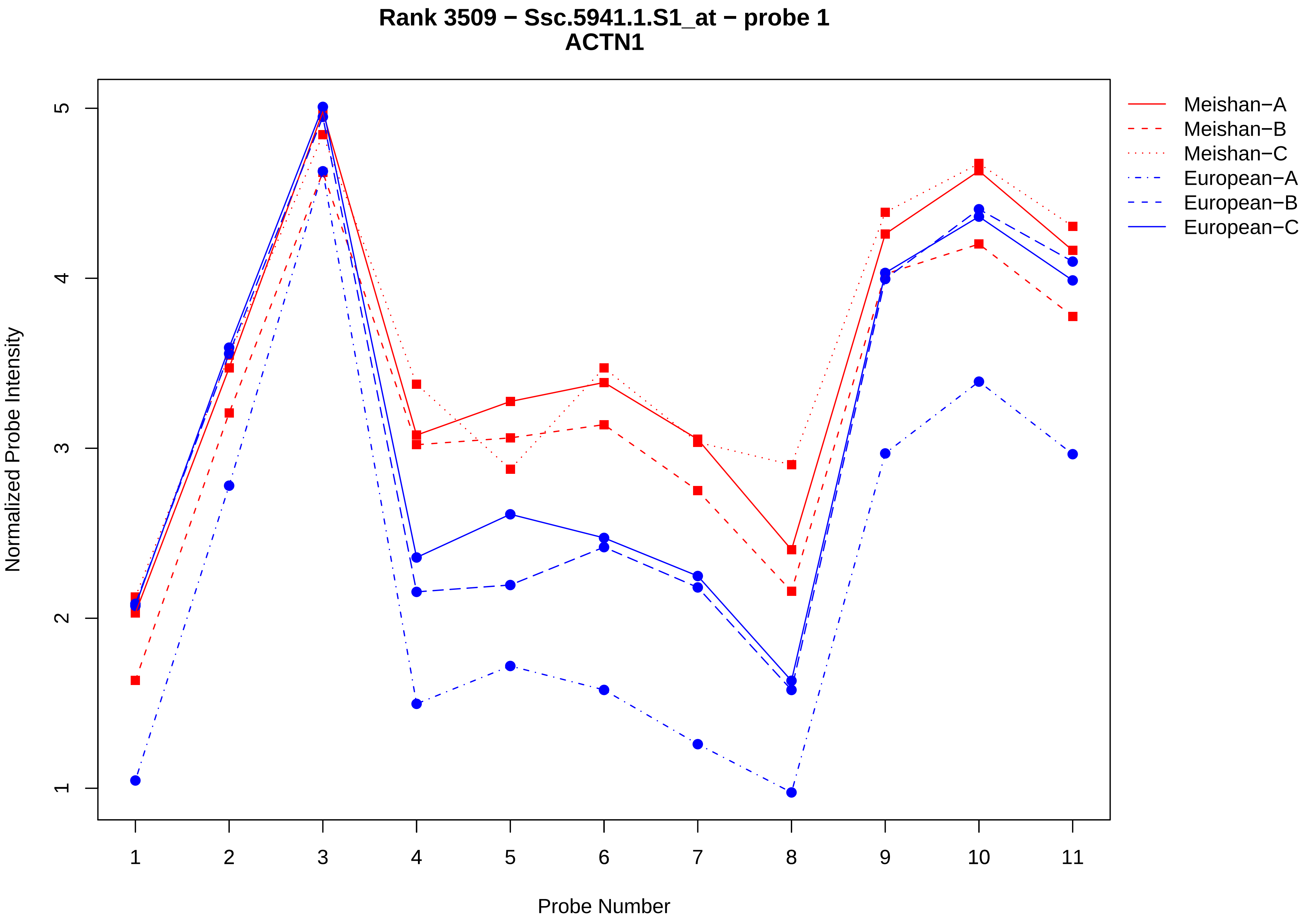

Rank 3509 − Ssc.5941.1.S1_at − probe 1
ACTN1
Normalized Probe Intensity
Probe Number
1
2
3
4
5
1
2
3
4
5
6
7
8
9
10
11
Meishan−A
Meishan−B
Meishan−C
European−A
European−B
European−C

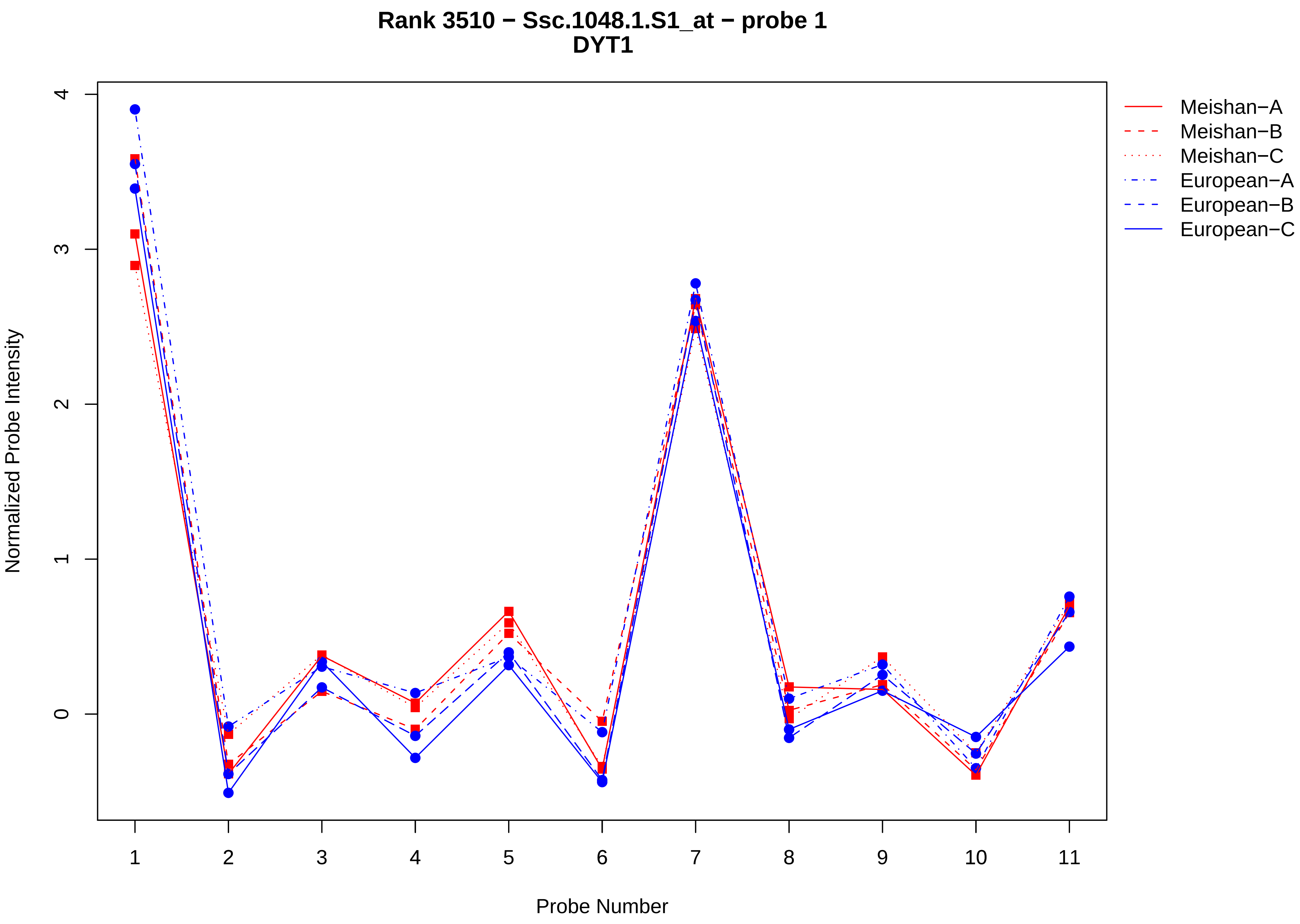

Rank 3510 − Ssc.1048.1.S1_at − probe 1
DYT1
Normalized Probe Intensity
Probe Number
0
1
2
3
4
1
2
3
4
5
6
7
8
9
10
11
Meishan−A
Meishan−B
Meishan−C
European−A
European−B
European−C

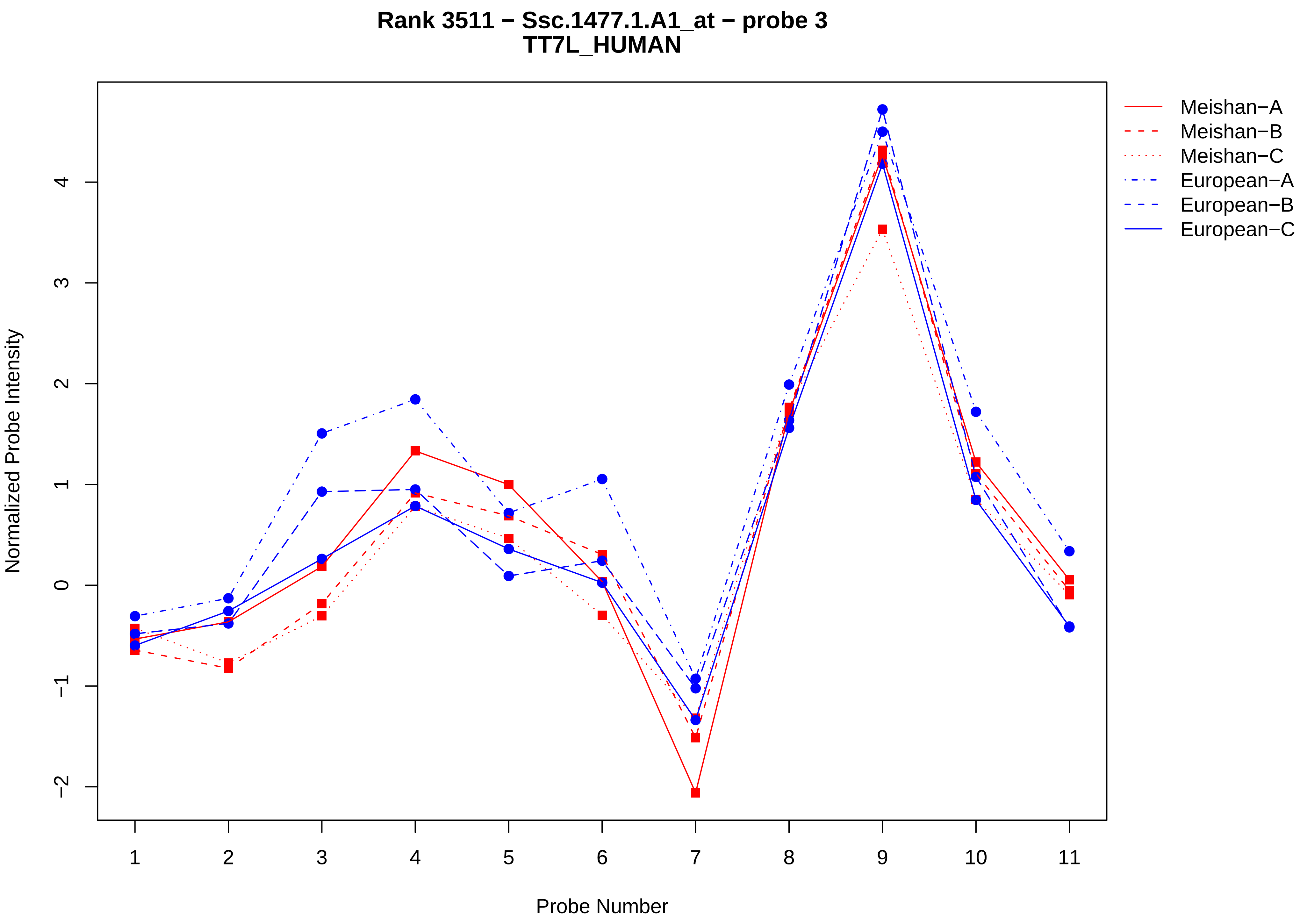

Rank 3511 − Ssc.1477.1.A1_at − probe 3
TT7L_HUMAN
Normalized Probe Intensity
Probe Number
−2
−1
0
1
2
3
4
1
2
3
4
5
6
7
8
9
10
11
Meishan−A
Meishan−B
Meishan−C
European−A
European−B
European−C

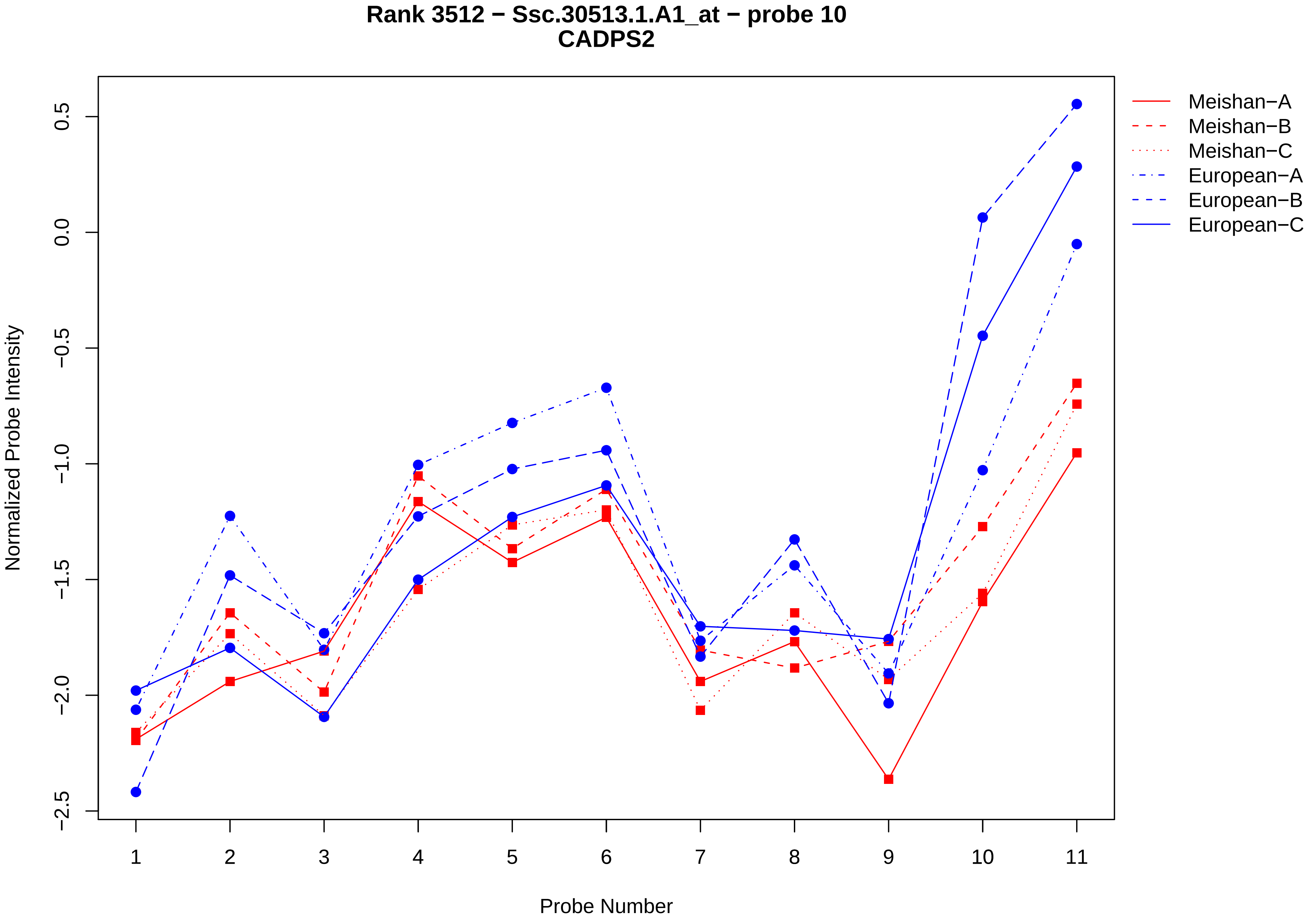

Rank 3512 − Ssc.30513.1.A1_at − probe 10
CADPS2
Normalized Probe Intensity
Probe Number
0.5
0.0
−0.5
−1.0
−1.5
−2.0
−2.5
1
2
3
4
5
6
7
8
9
10
11
Meishan−A
Meishan−B
Meishan−C
European−A
European−B
European−C

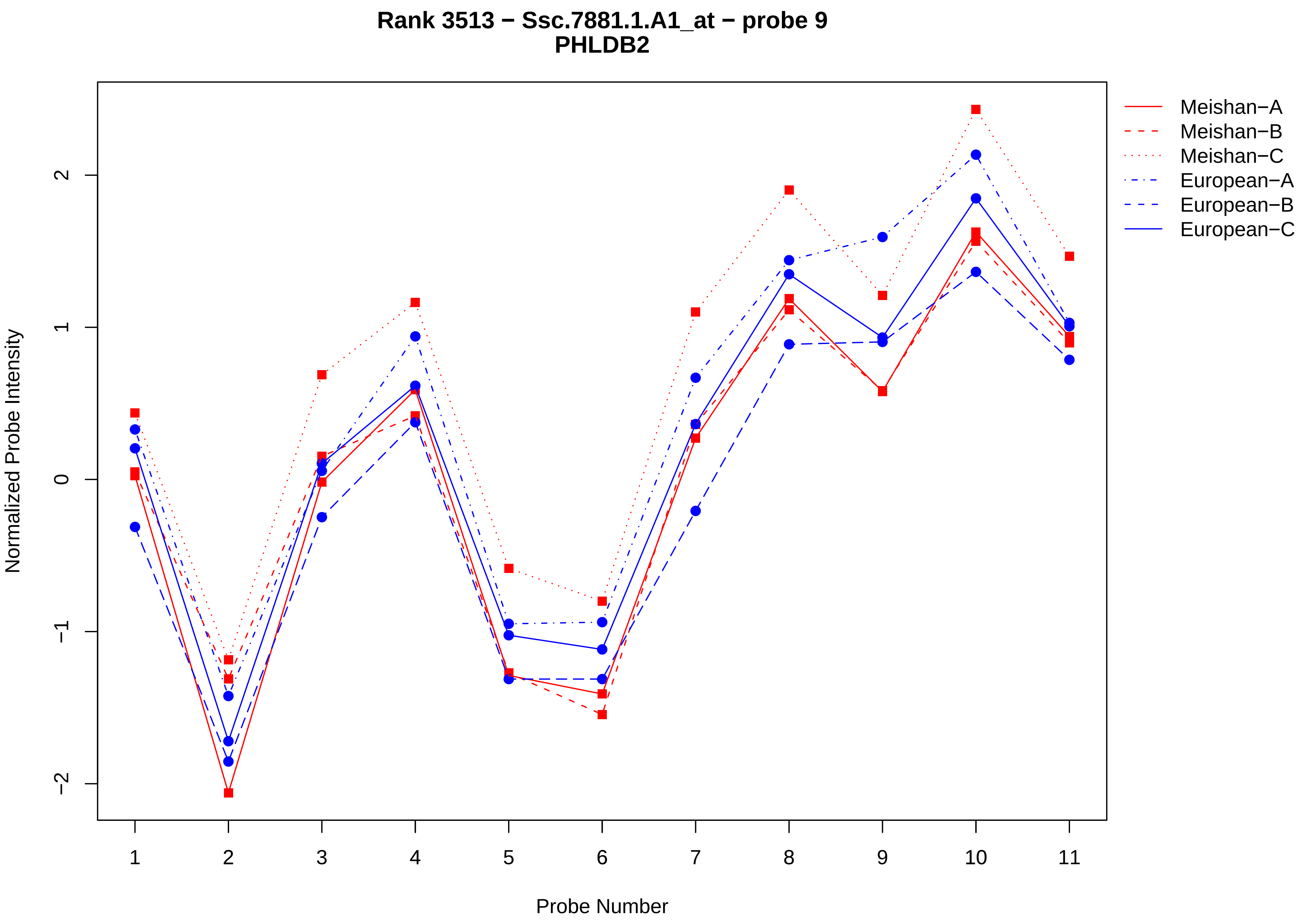

Rank 3513 − Ssc.7881.1.A1_at − probe 9
PHLDB2
Normalized Probe Intensity
Probe Number
Meishan−A
Meishan−B
Meishan−C
European−A
European−B
European−C

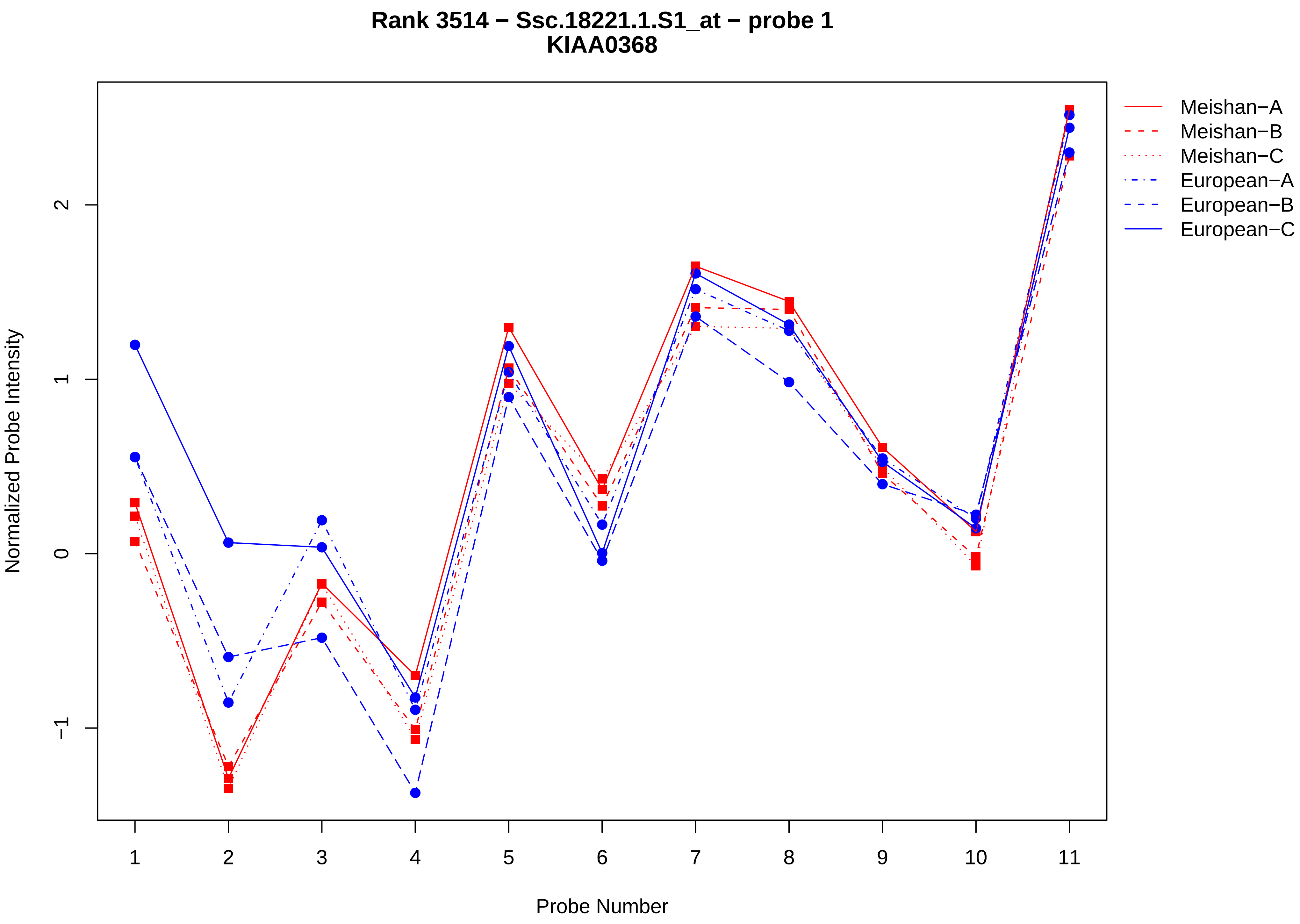
Rank 3514 − Ssc.18221.1.S1_at − probe 1
KIAA0368
Normalized Probe Intensity
Probe Number
−1
0
1
2
1
2
3
4
5
6
7
8
9
10
11
Meishan−A
Meishan−B
Meishan−C
European−A
European−B
European−C

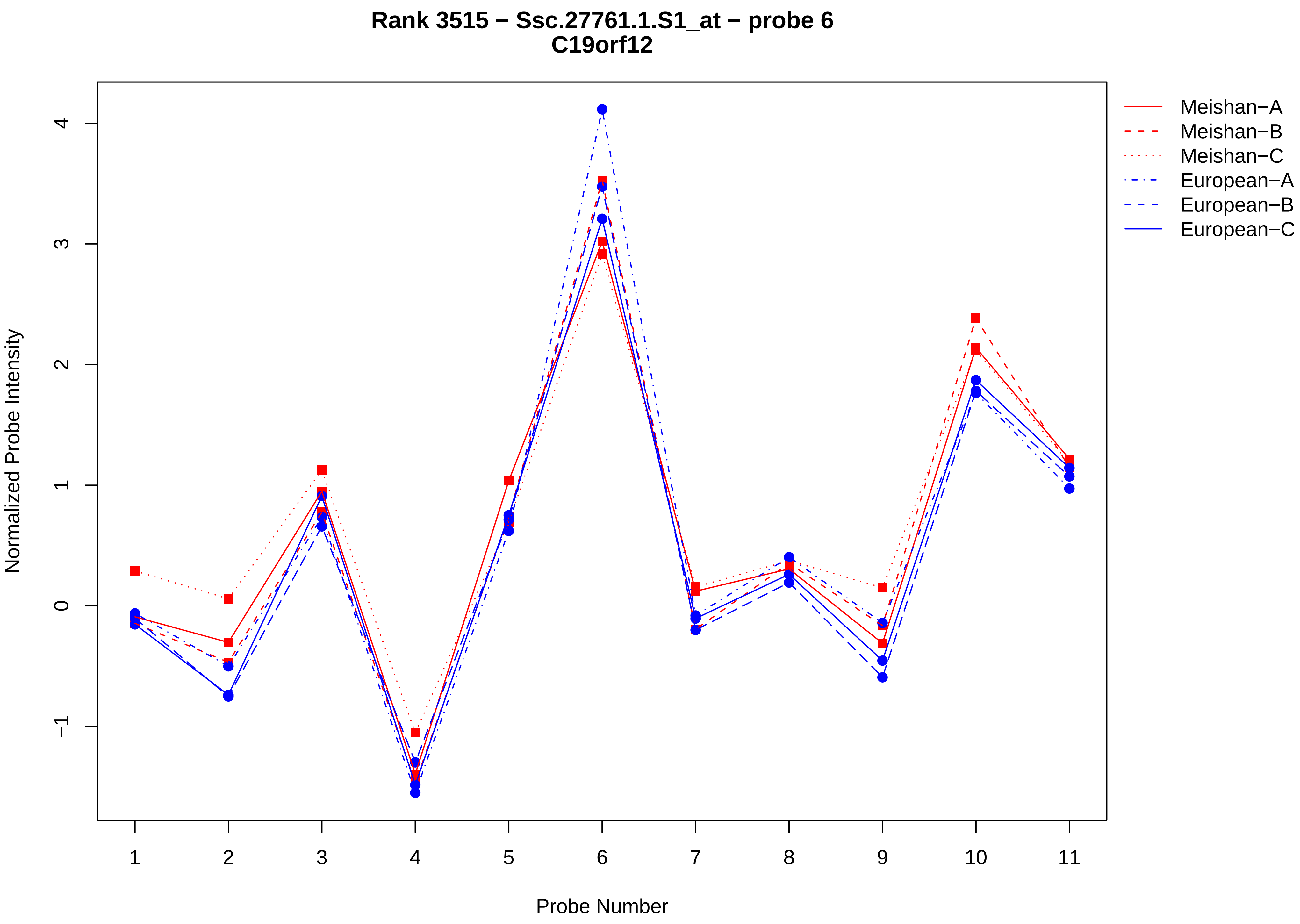

Rank 3515 − Ssc.27761.1.S1_at − probe 6
C19orf12
Normalized Probe Intensity
Probe Number
Meishan−A
Meishan−B
Meishan−C
European−A
European−B
European−C

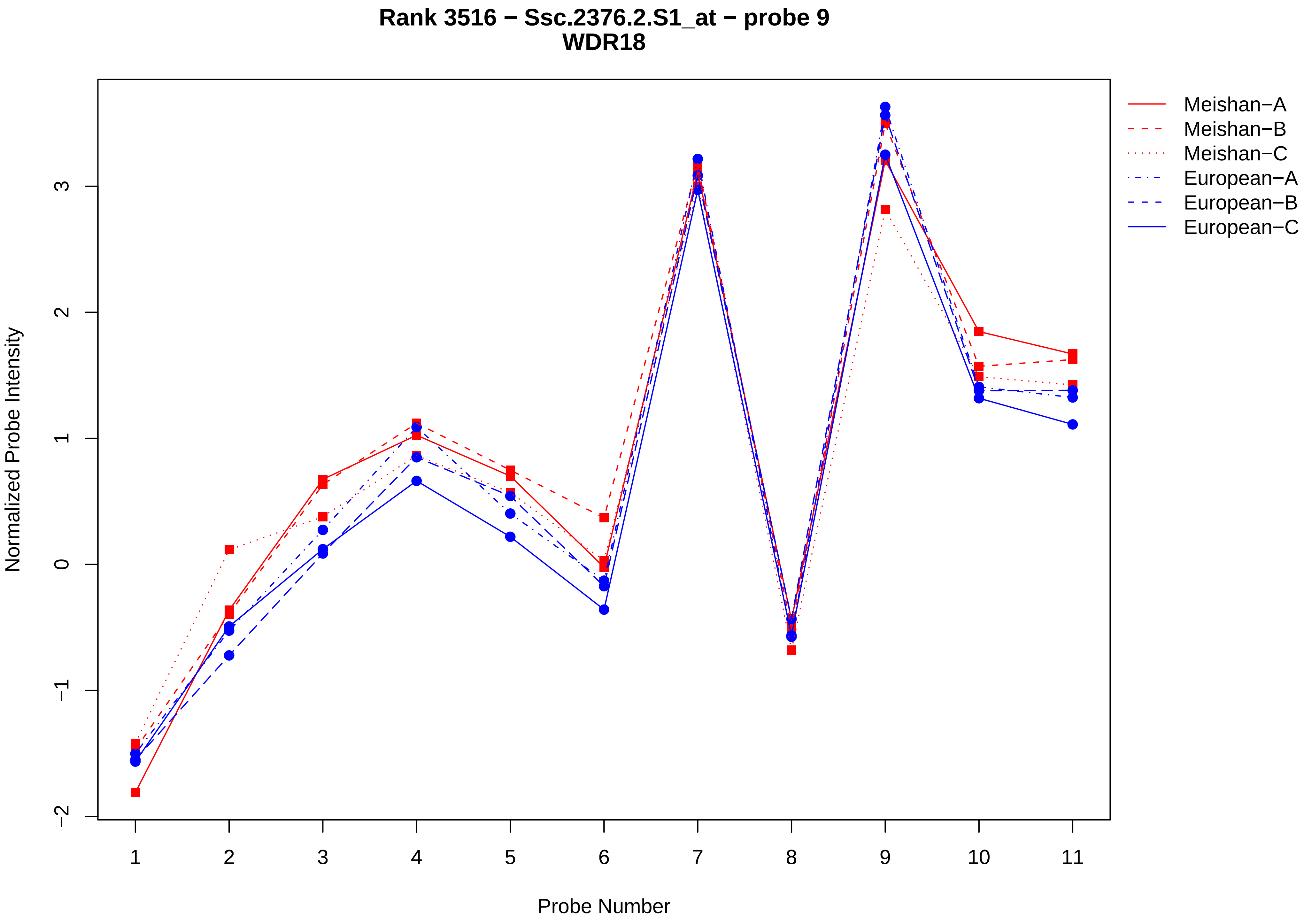

Rank 3516 − Ssc.2376.2.S1_at − probe 9
WDR18
Normalized Probe Intensity
Probe Number
−2
−1
0
1
2
3
1
2
3
4
5
6
7
8
9
10
11
Meishan−A
Meishan−B
Meishan−C
European−A
European−B
European−C

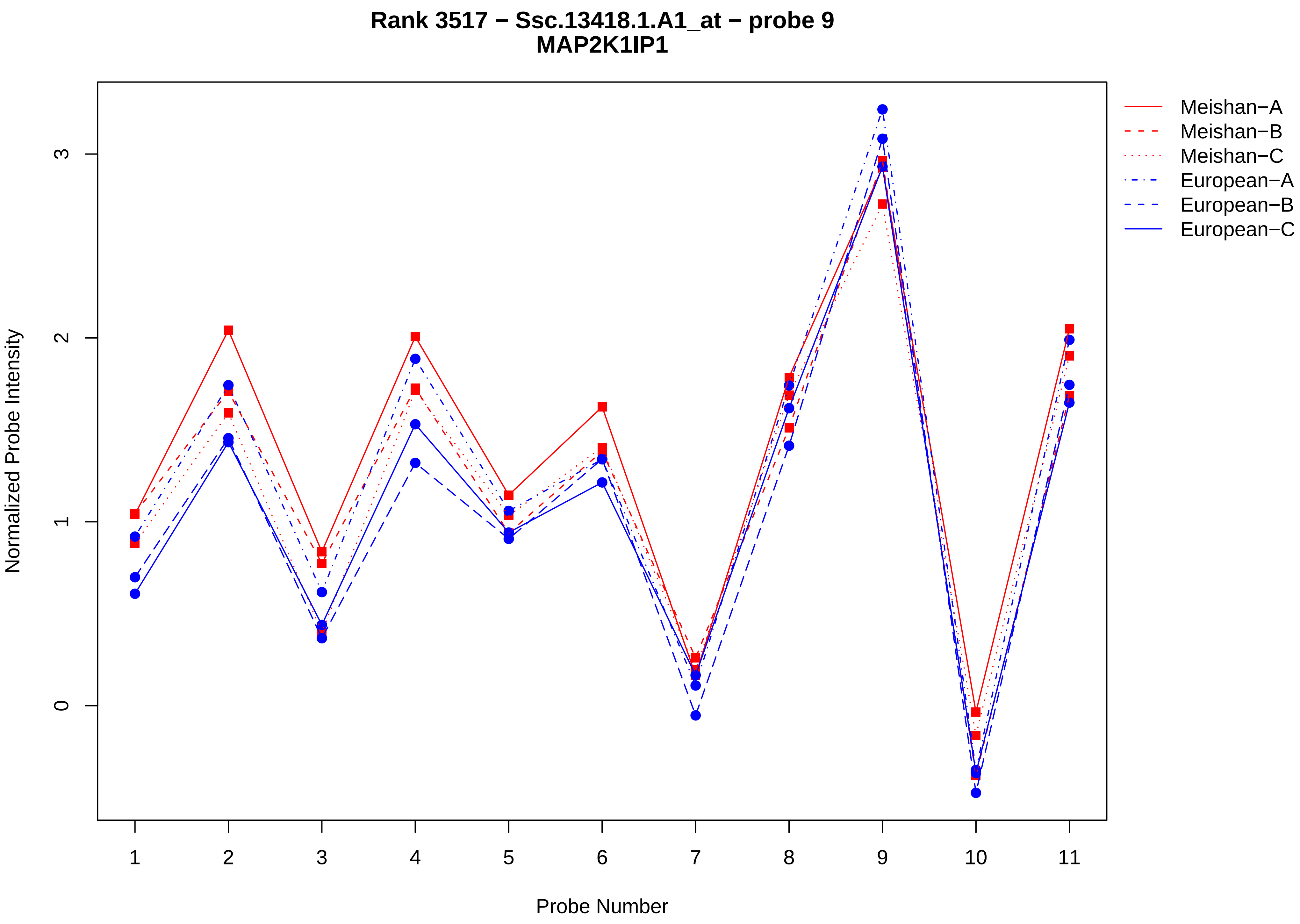

Rank 3517 − Ssc.13418.1.A1_at − probe 9
MAP2K1IP1
Normalized Probe Intensity
Probe Number
0
1
2
3
1
2
3
4
5
6
7
8
9
10
11
Meishan−A
Meishan−B
Meishan−C
European−A
European−B
European−C

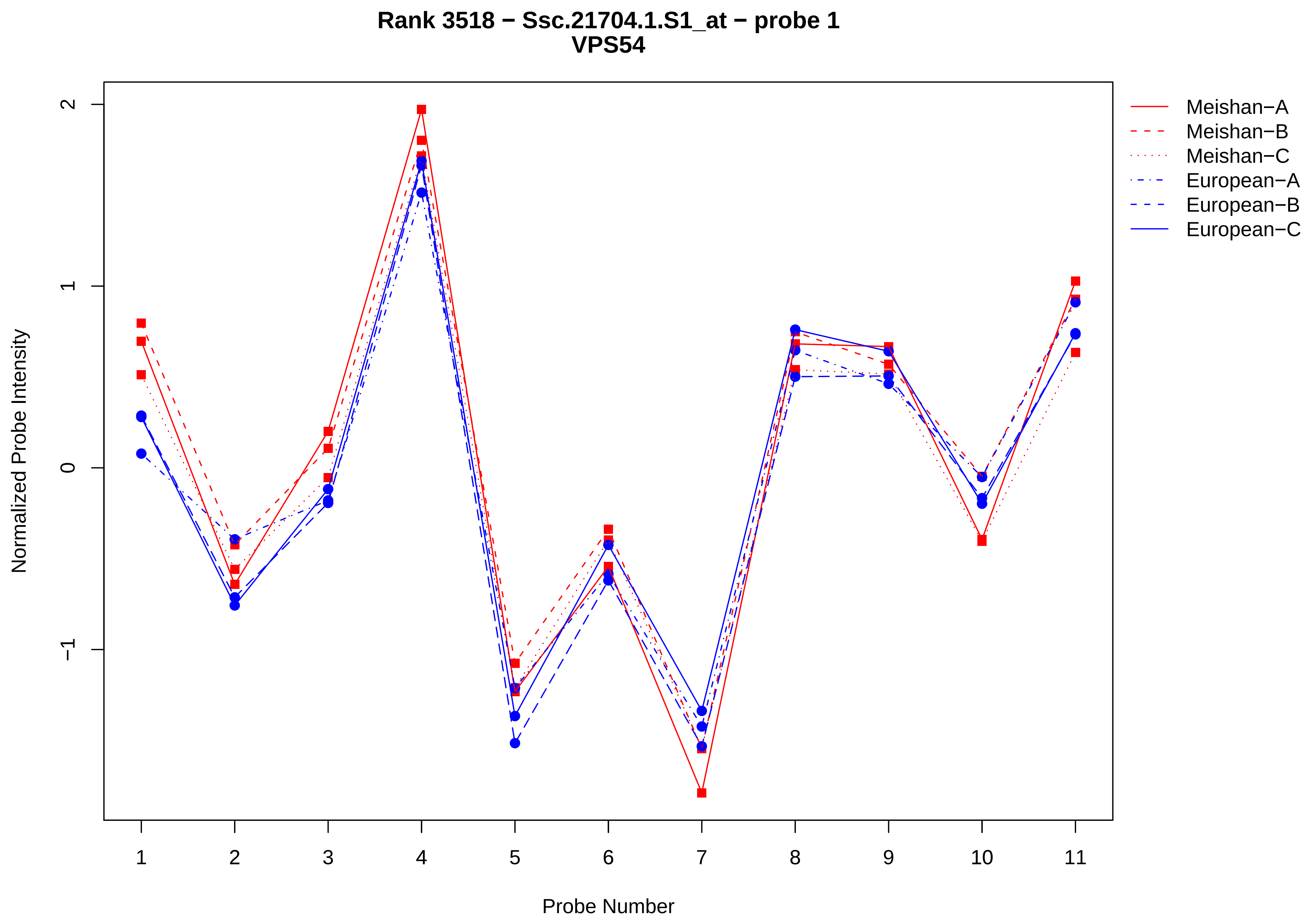

Rank 3518 − Ssc.21704.1.S1_at − probe 1
VPS54
Normalized Probe Intensity
Probe Number
−1
0
1
2
1
2
3
4
5
6
7
8
9
10
11
Meishan−A
Meishan−B
Meishan−C
European−A
European−B
European−C

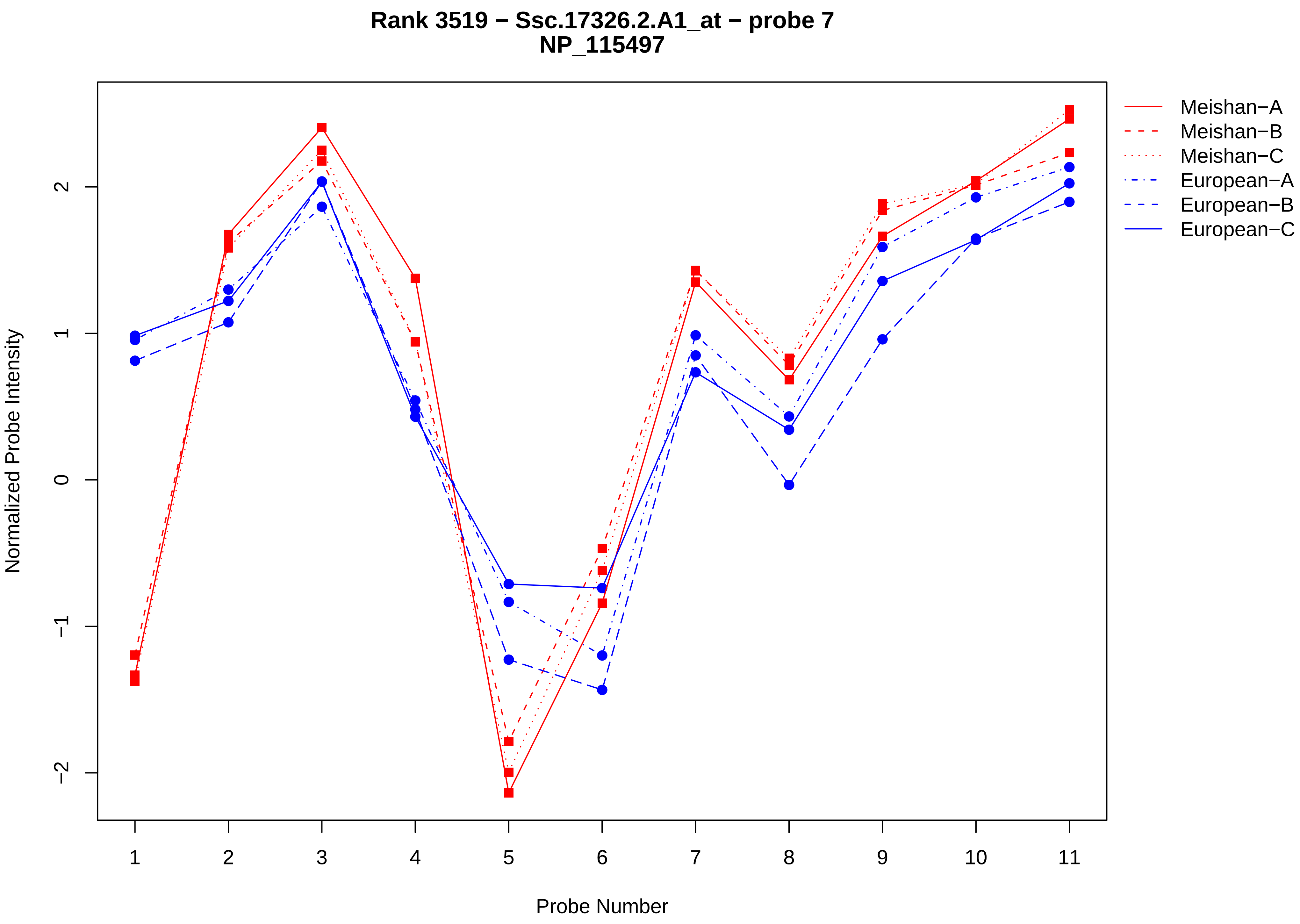

Rank 3519 − Ssc.17326.2.A1_at − probe 7
NP_115497
Normalized Probe Intensity
Probe Number
−2
−1
0
1
2
1
2
3
4
5
6
7
8
9
10
11
Meishan−A
Meishan−B
Meishan−C
European−A
European−B
European−C

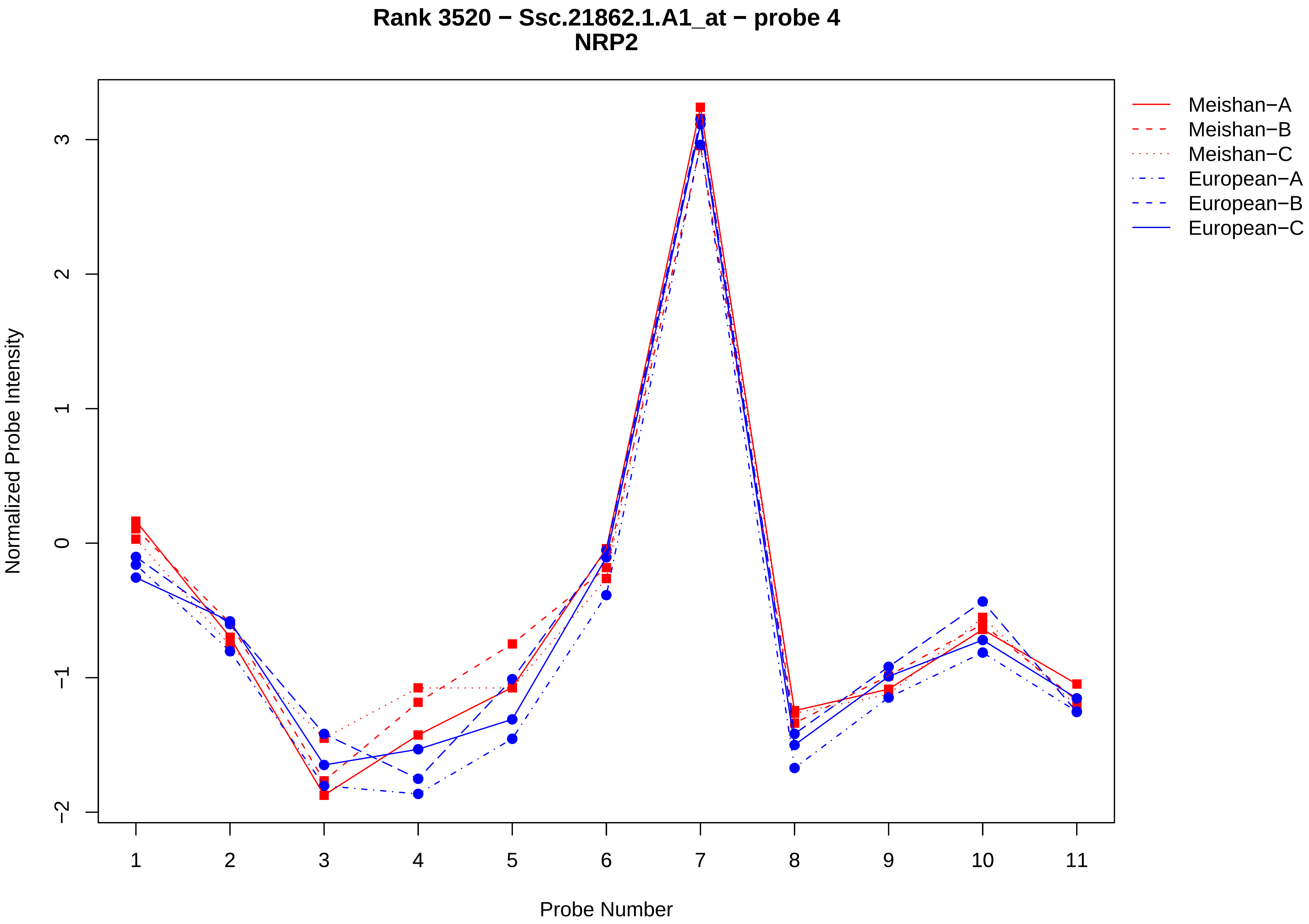

Rank 3520 − Ssc.21862.1.A1_at − probe 4
NRP2
Meishan−A
Meishan−B
Meishan−C
European−A
European−B
European−C
Normalized Probe Intensity
Probe Number
3
2
1
0
−1
−2
1
2
3
4
5
6
7
8
9
10
11

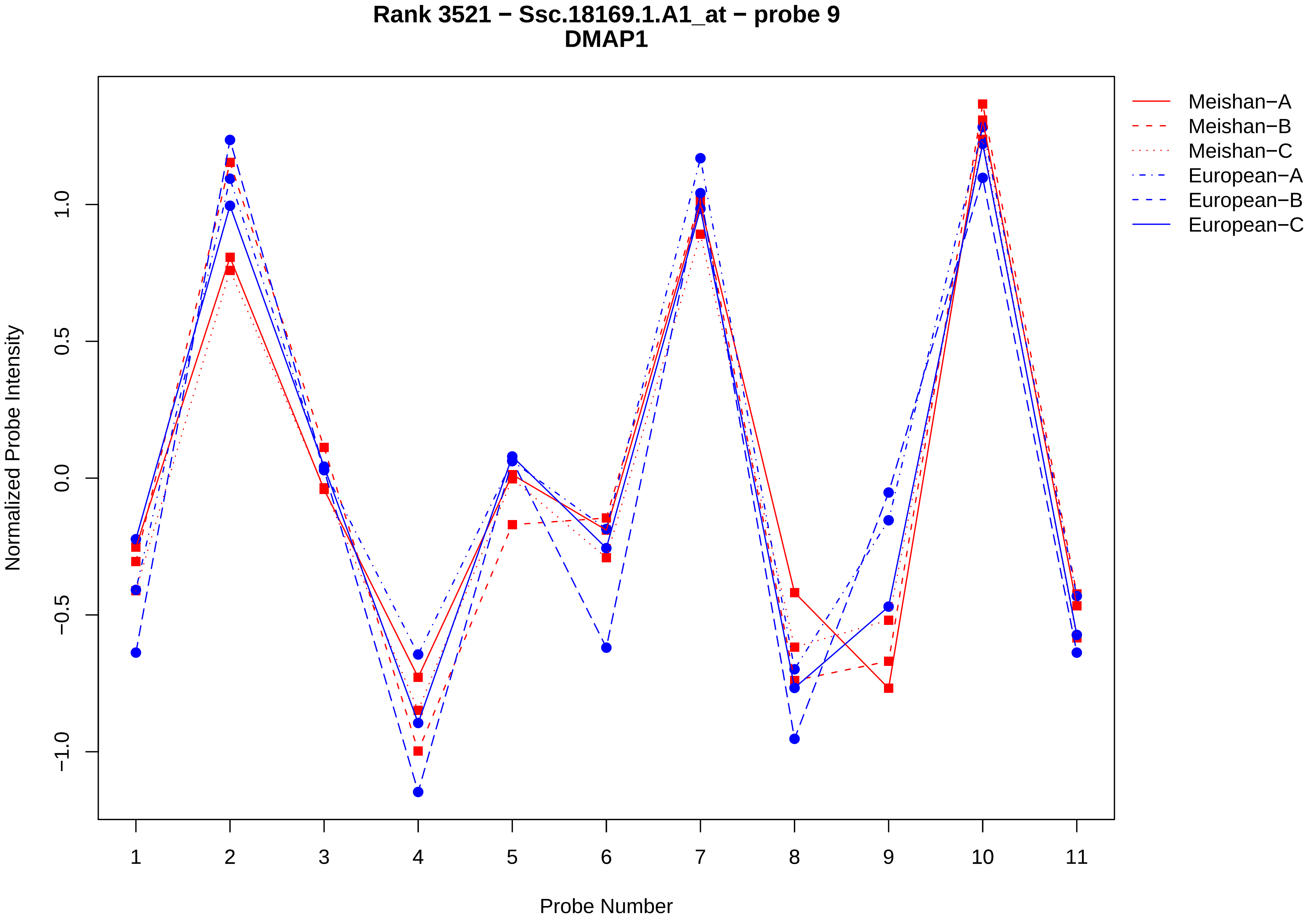

Rank 3521 − Ssc.18169.1.A1_at − probe 9
DMAP1
Normalized Probe Intensity
Probe Number
Meishan−A
Meishan−B
Meishan−C
European−A
European−B
European−C

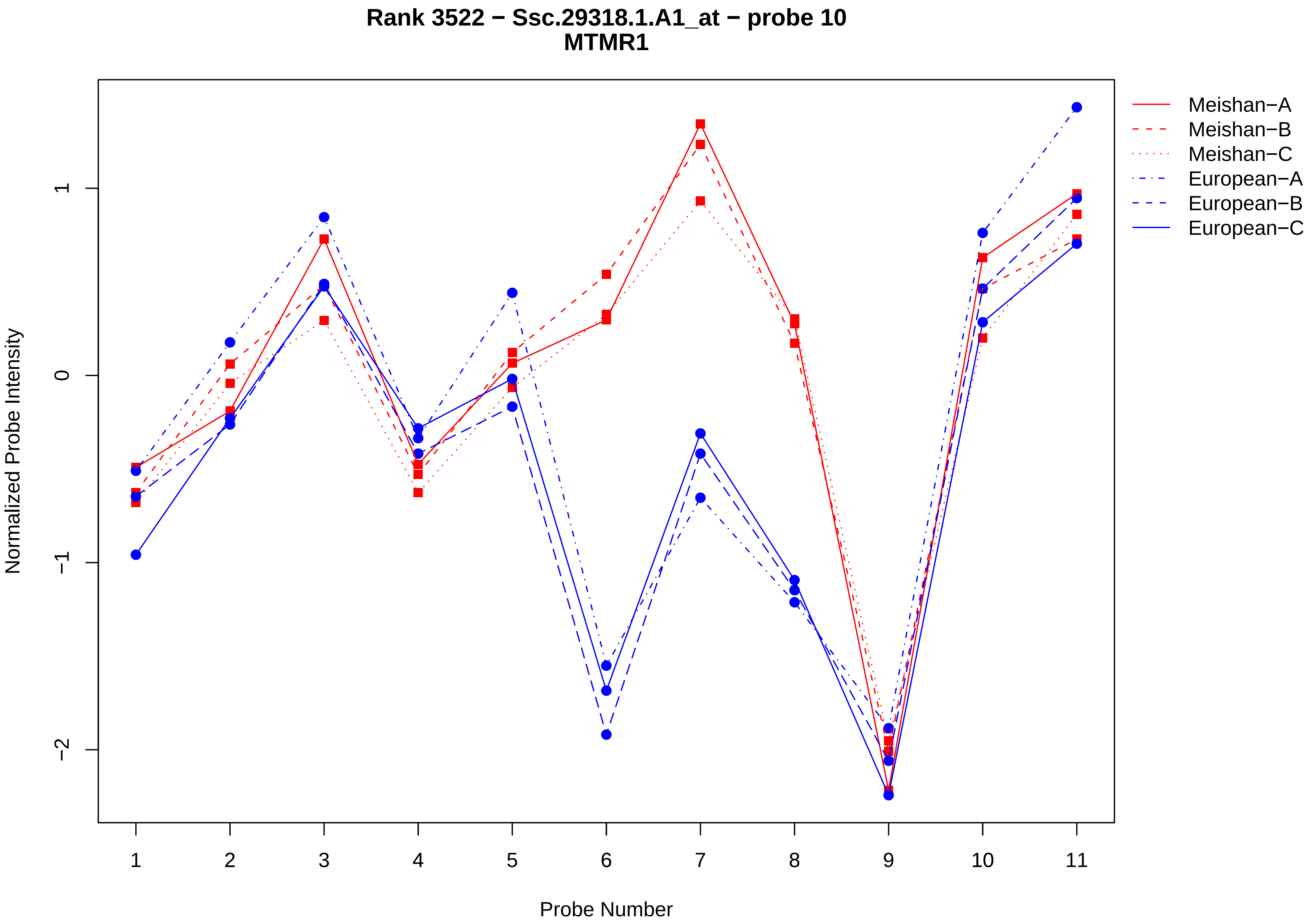

Rank 3522 − Ssc.29318.1.A1_at − probe 10
MTMR1
Normalized Probe Intensity
Probe Number
−2
−1
0
1
1
2
3
4
5
6
7
8
9
10
11
Meishan−A
Meishan−B
Meishan−C
European−A
European−B
European−C

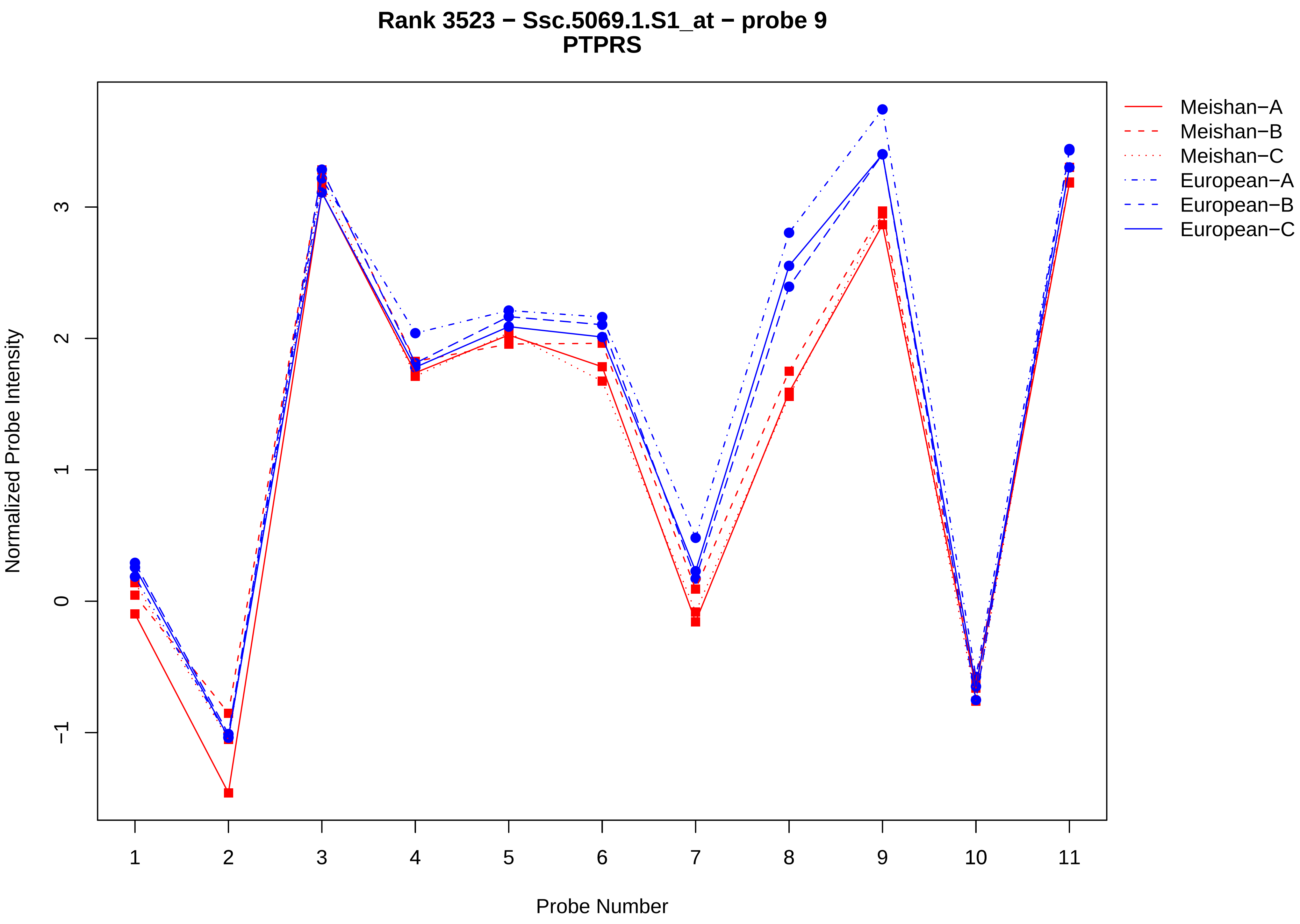

Rank 3523 − Ssc.5069.1.S1_at − probe 9
PTPRS
Normalized Probe Intensity
Probe Number
Meishan−A
Meishan−B
Meishan−C
European−A
European−B
European−C
−1
0
1
2
3
1
2
3
4
5
6
7
8
9
10
11

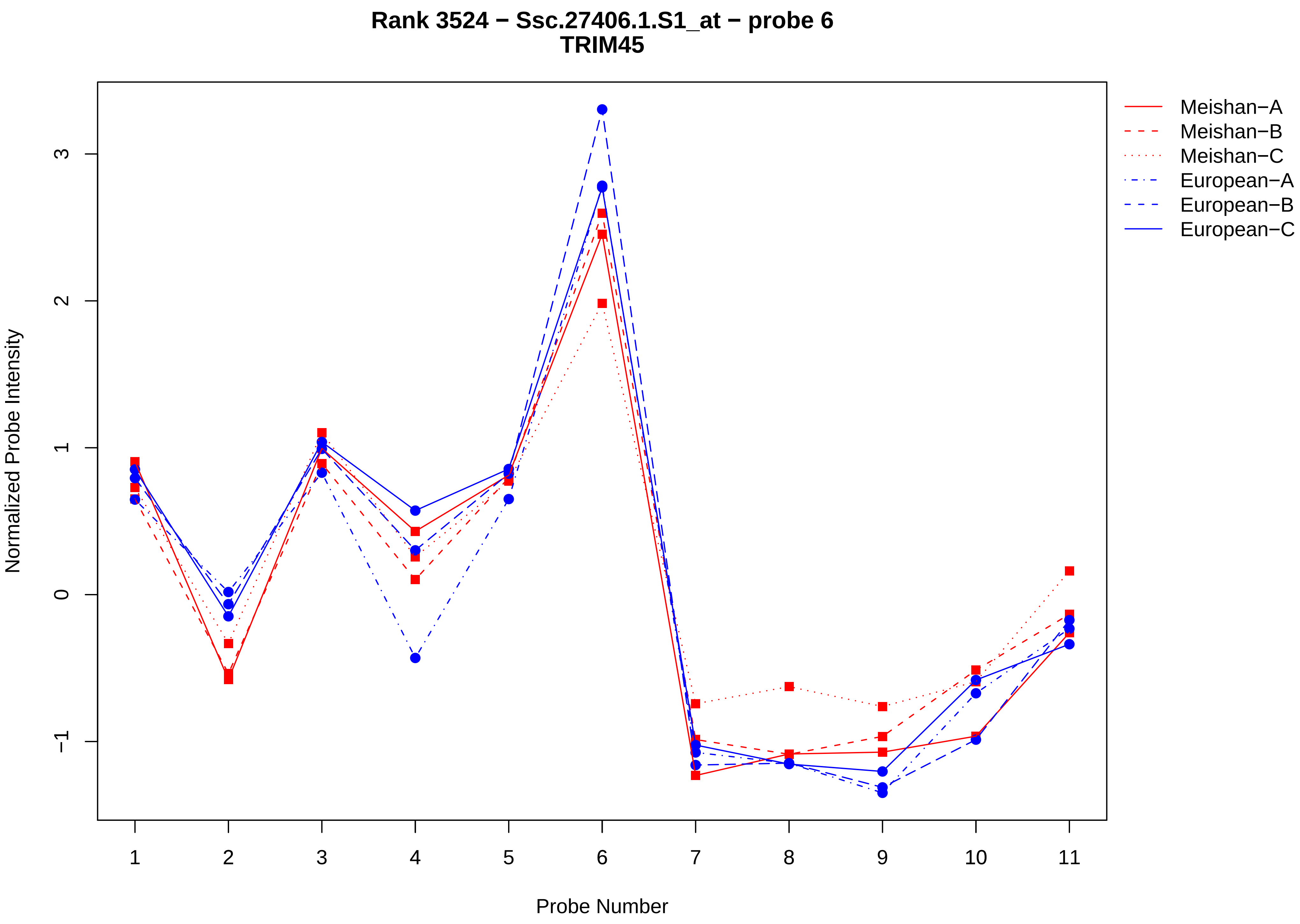
Rank 3524 − Ssc.27406.1.S1_at − probe 6
TRIM45
Normalized Probe Intensity
Probe Number
Meishan−A
Meishan−B
Meishan−C
European−A
European−B
European−C
−1
0
1
2
3
1
2
3
4
5
6
7
8
9
10
11

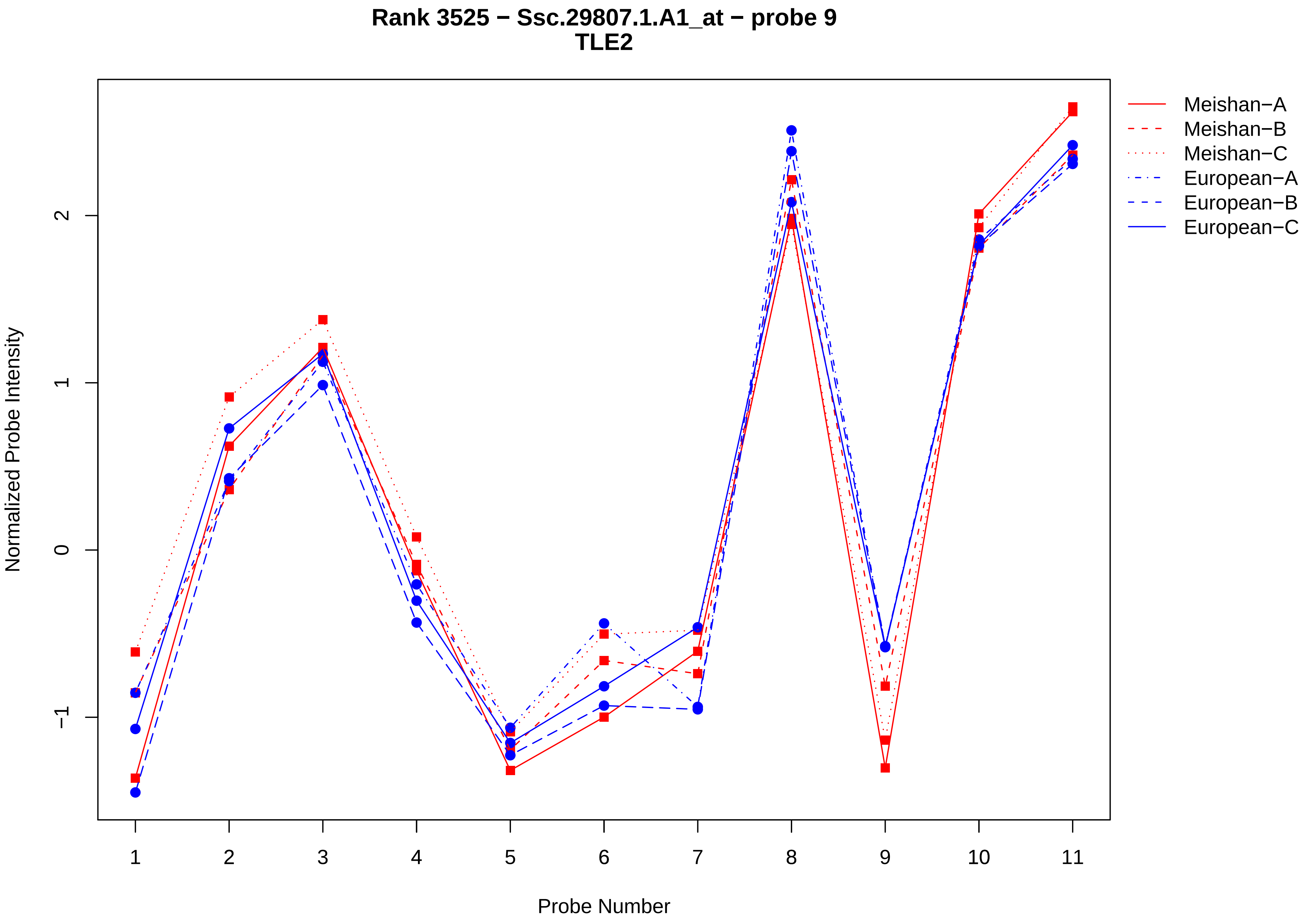
Rank 3525 − Ssc.29807.1.A1_at − probe 9
TLE2
Normalized Probe Intensity
Probe Number
Meishan−A
Meishan−B
Meishan−C
European−A
European−B
European−C
−1
0
1
2
1
2
3
4
5
6
7
8
9
10
11

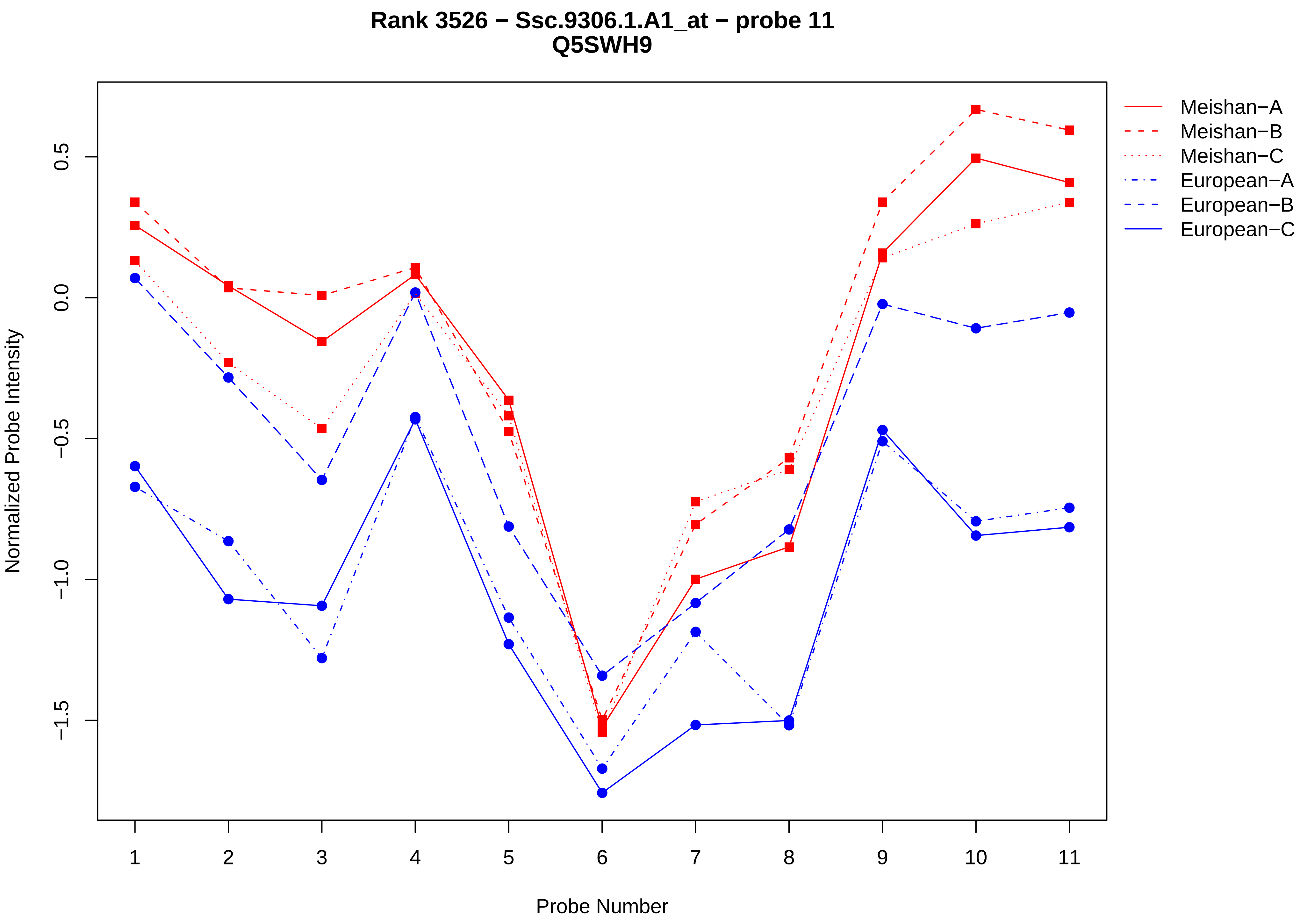

Rank 3526 − Ssc.9306.1.A1_at − probe 11
Q5SWH9
Normalized Probe Intensity
Probe Number
Meishan−A
Meishan−B
Meishan−C
European−A
European−B
European−C
0.5
0.0
−0.5
−1.0
−1.5
1
2
3
4
5
6
7
8
9
10
11

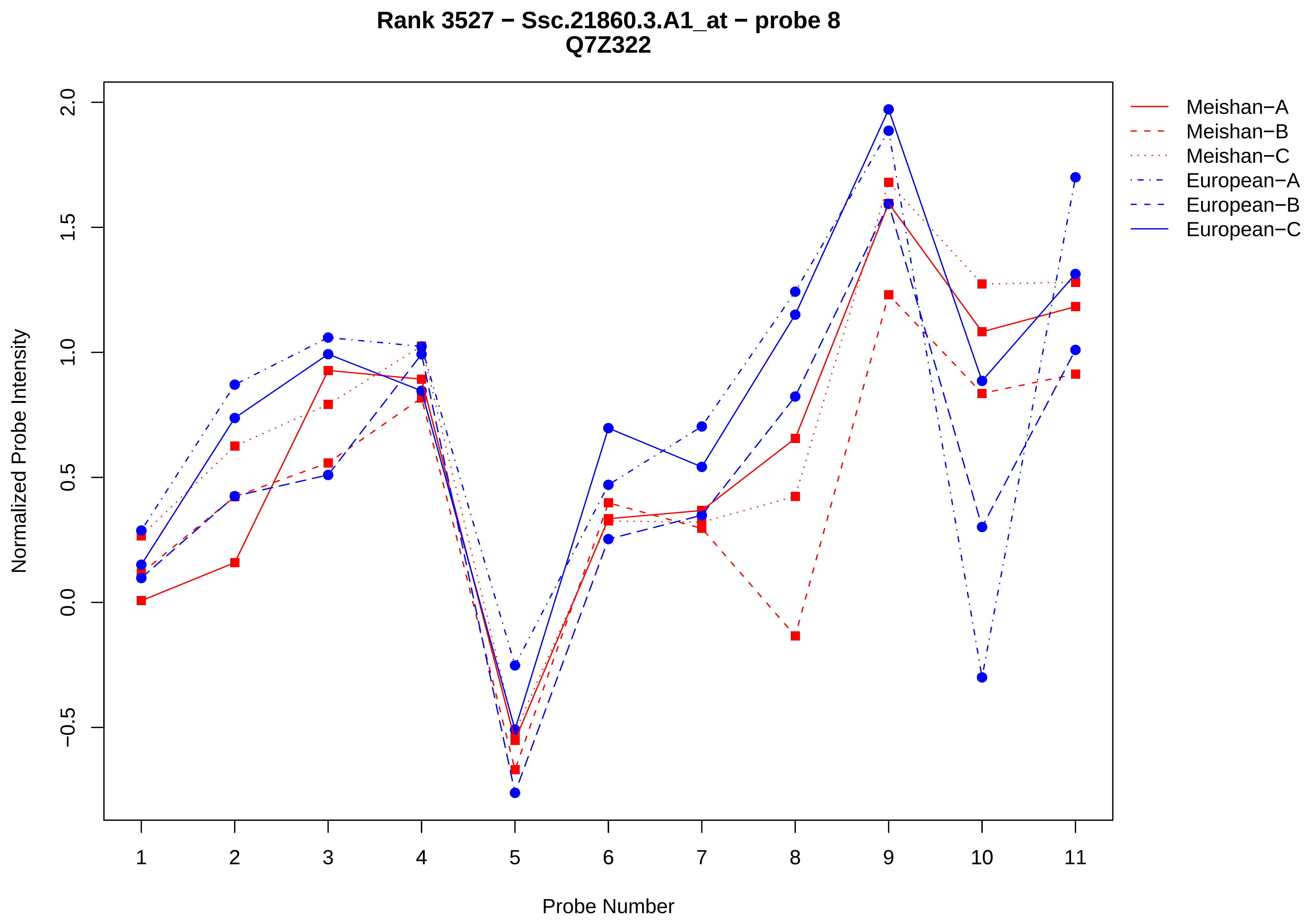

Rank 3527 − Ssc.21860.3.A1_at − probe 8
Q7Z322
Normalized Probe Intensity
Probe Number
Meishan−A
Meishan−B
Meishan−C
European−A
European−B
European−C
2.0
1.5
1.0
0.5
0.0
−0.5
1
2
3
4
5
6
7
8
9
10
11

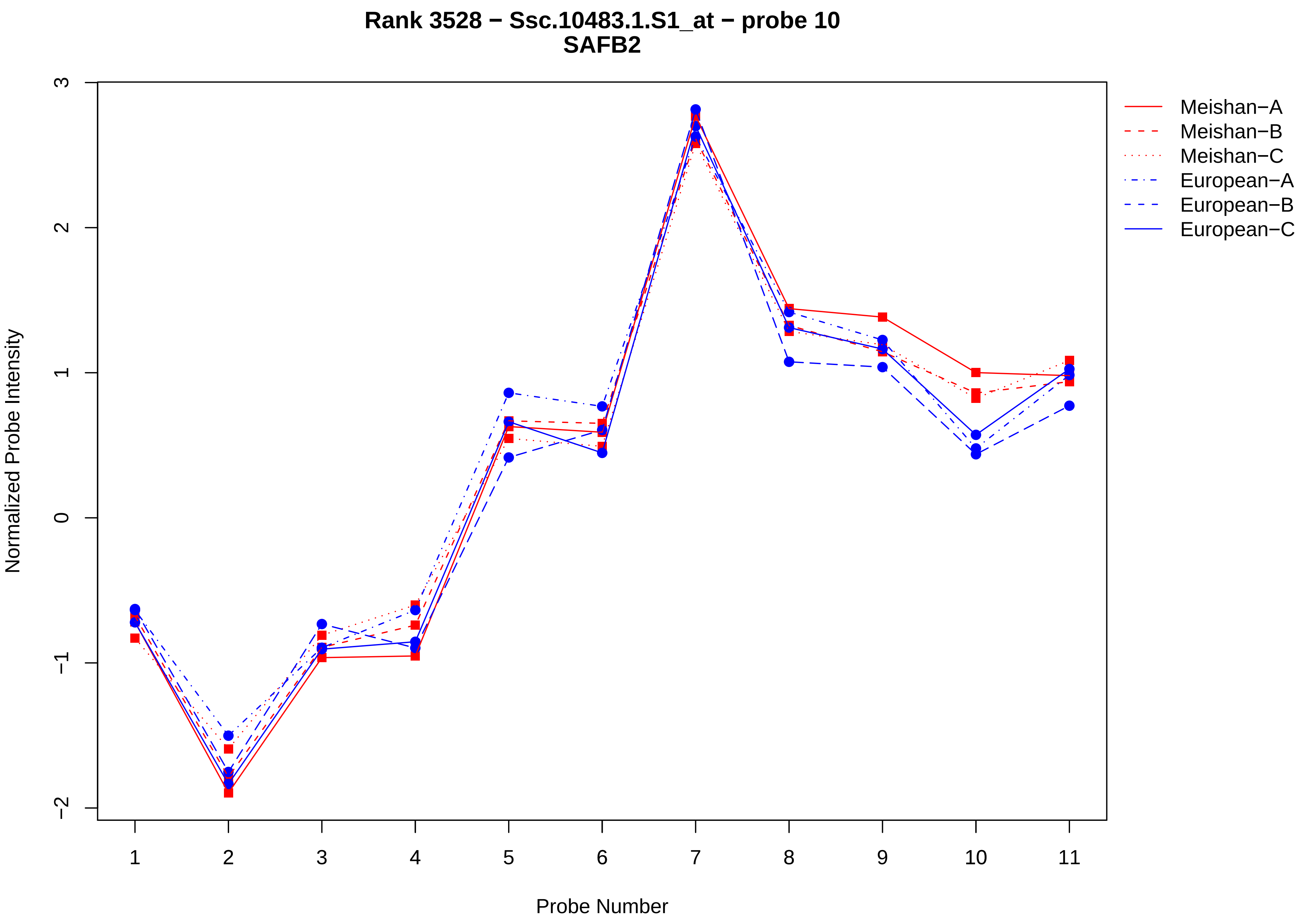
Rank 3528 − Ssc.10483.1.S1_at − probe 10
SAFB2
Normalized Probe Intensity
Probe Number
−2
−1
0
1
2
3
1
2
3
4
5
6
7
8
9
10
11
Meishan−A
Meishan−B
Meishan−C
European−A
European−B
European−C

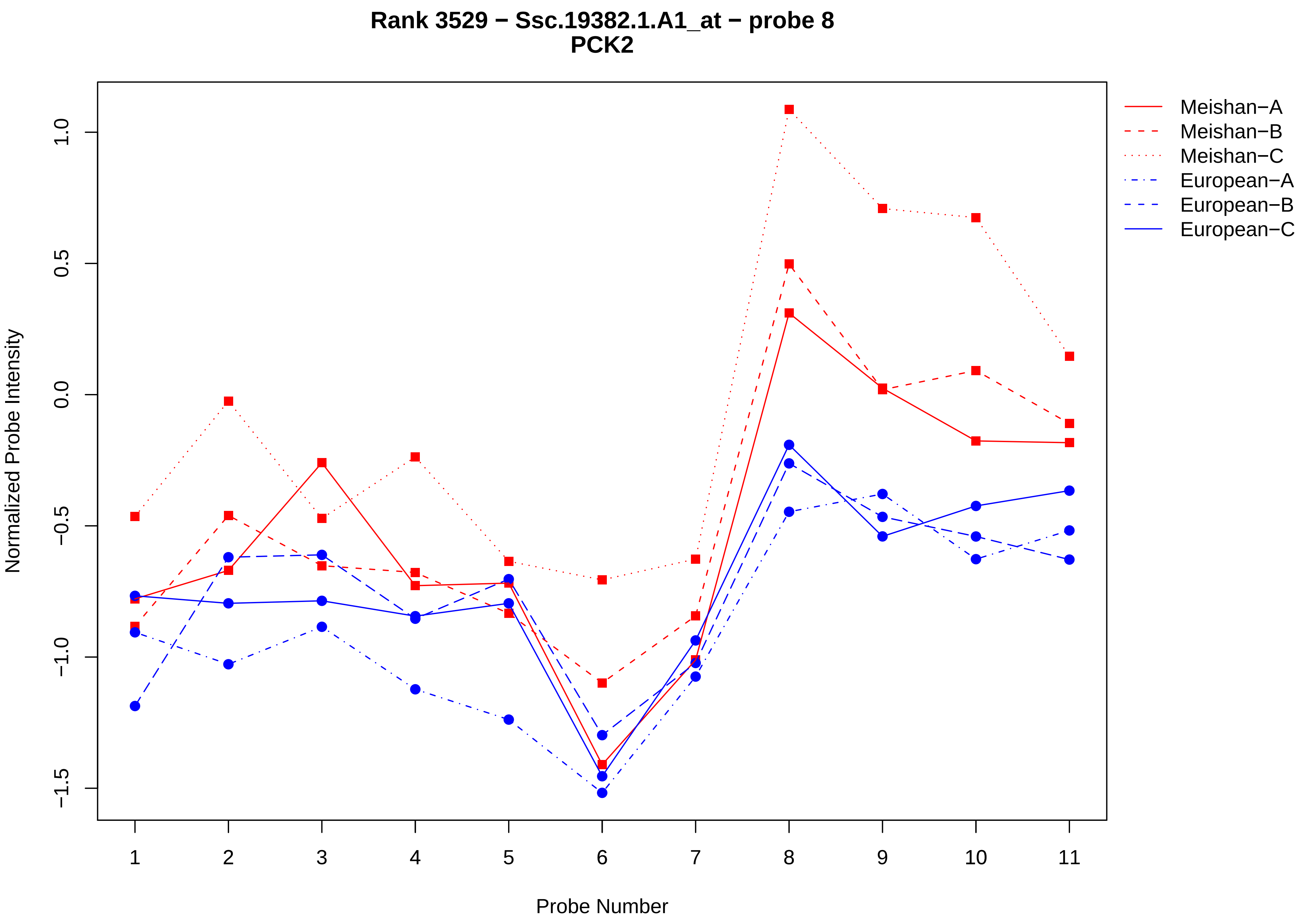

Rank 3529 − Ssc.19382.1.A1_at − probe 8
PCK2
Normalized Probe Intensity
1.0
0.5
0.0
−0.5
−1.0
−1.5
1
2
3
4
5
6
7
8
9
10
11
Probe Number
Meishan−A
Meishan−B
Meishan−C
European−A
European−B
European−C

Rank 3530 − Ssc.23468.2.S1_at − probe 11
PPAP2A

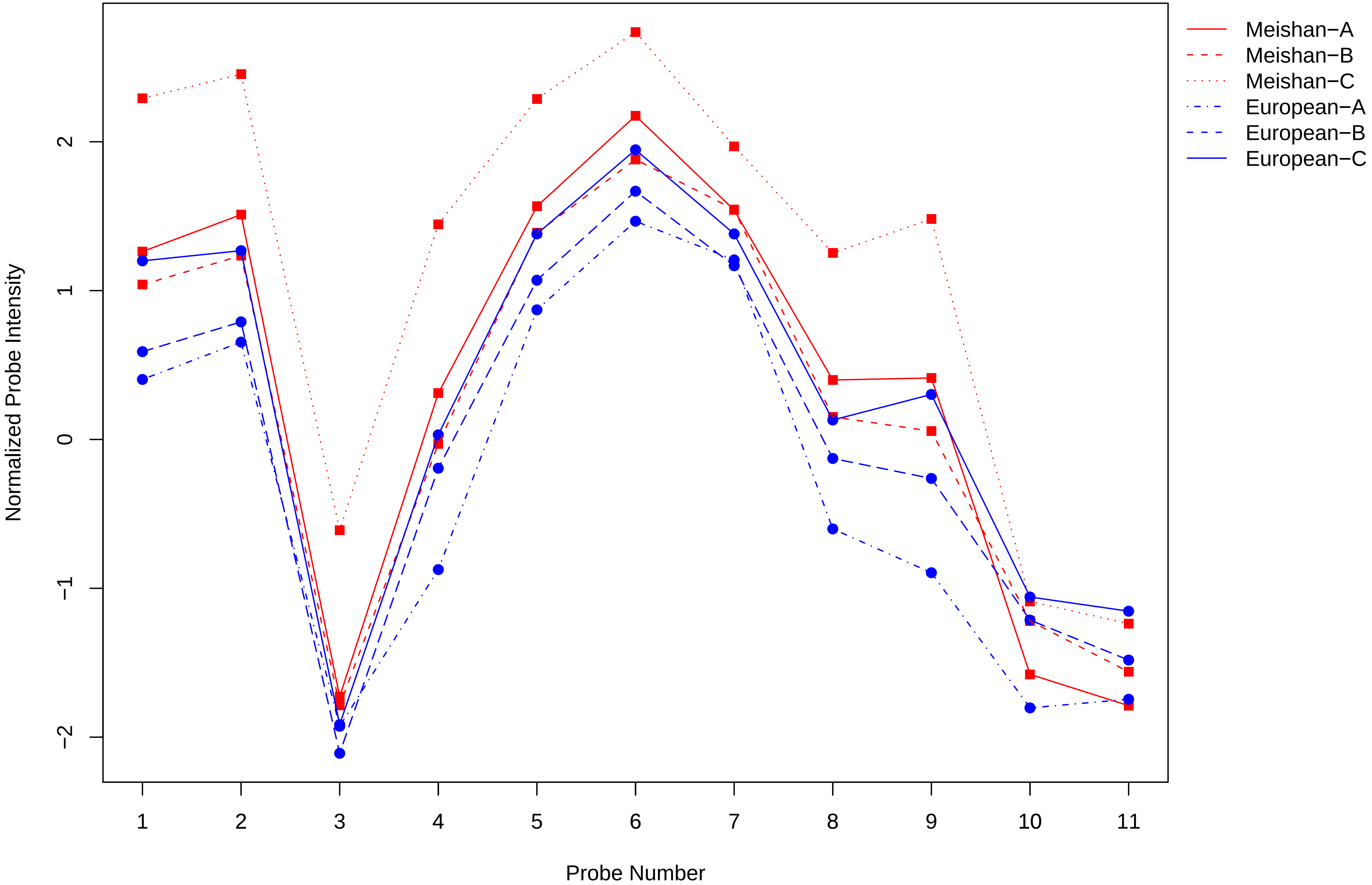

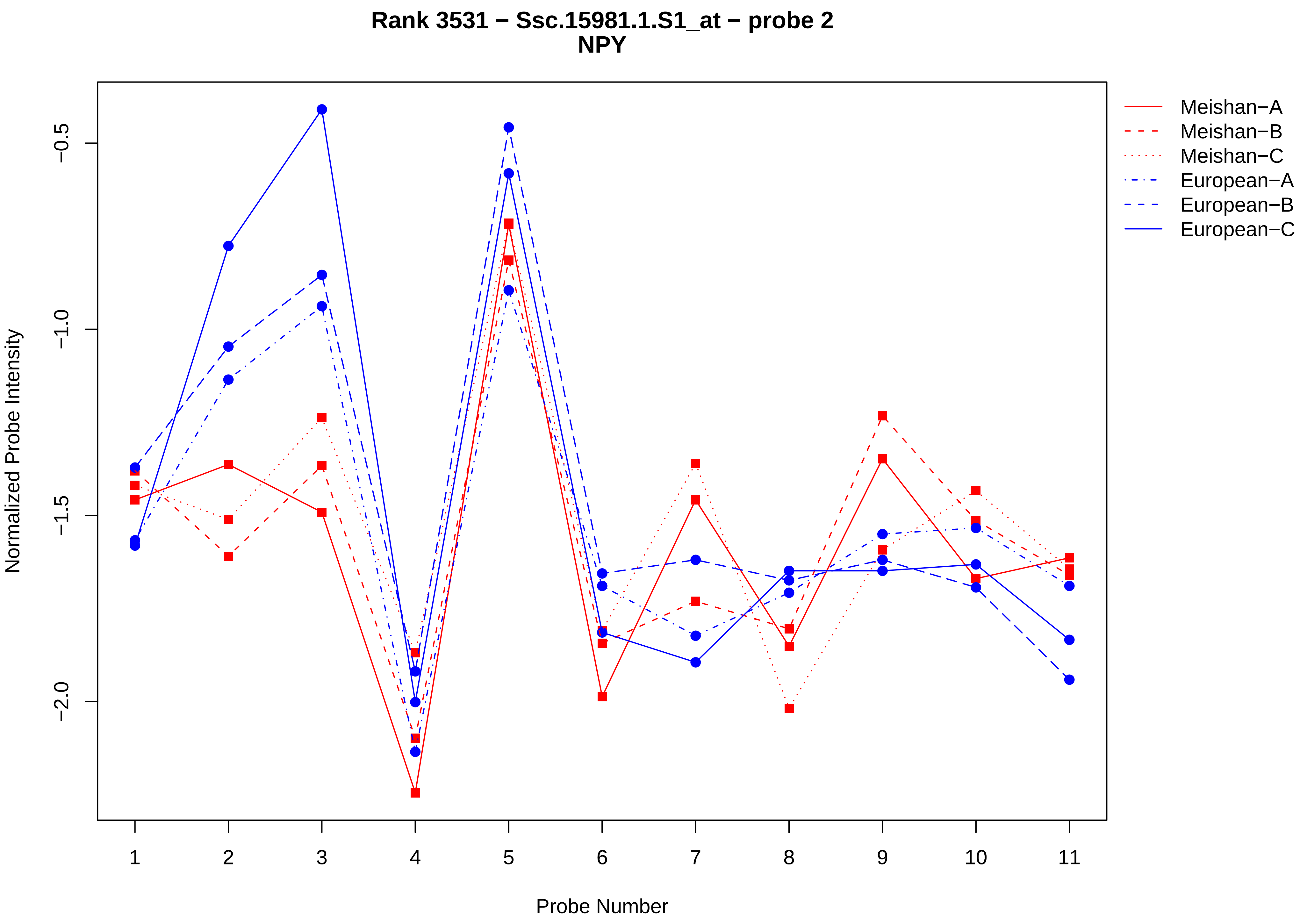

Rank 3531 − Ssc.15981.1.S1_at − probe 2
NPY
Normalized Probe Intensity
−0.5
−1.0
−1.5
−2.0
1
2
3
4
5
6
7
8
9
10
11
Probe Number
Meishan−A
Meishan−B
Meishan−C
European−A
European−B
European−C

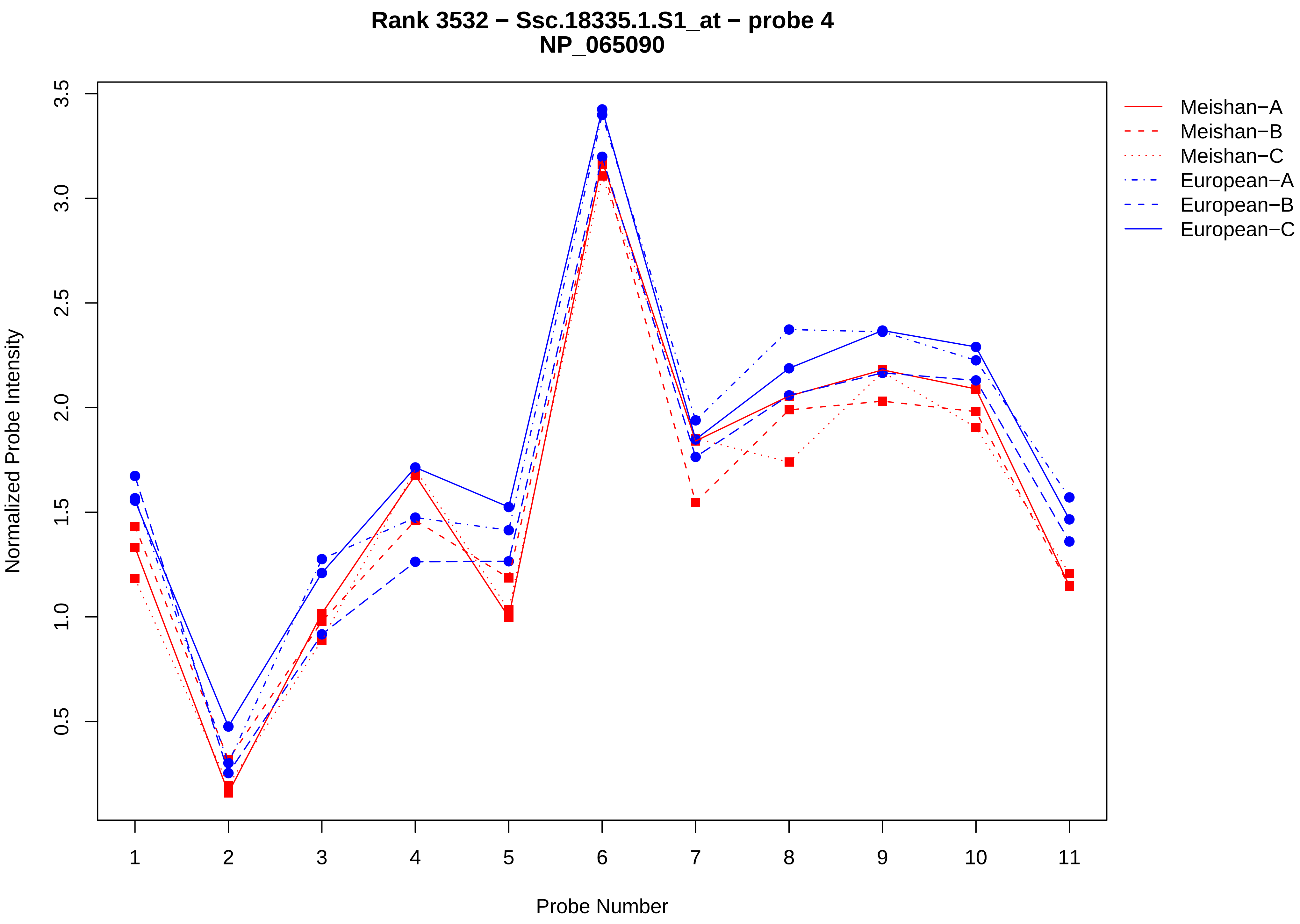

Rank 3532 − Ssc.18335.1.S1_at − probe 4
NP_065090
Normalized Probe Intensity
0.5
1.0
1.5
2.0
2.5
3.0
3.5
1
2
3
4
5
6
7
8
9
10
11
Probe Number
Meishan−A
Meishan−B
Meishan−C
European−A
European−B
European−C

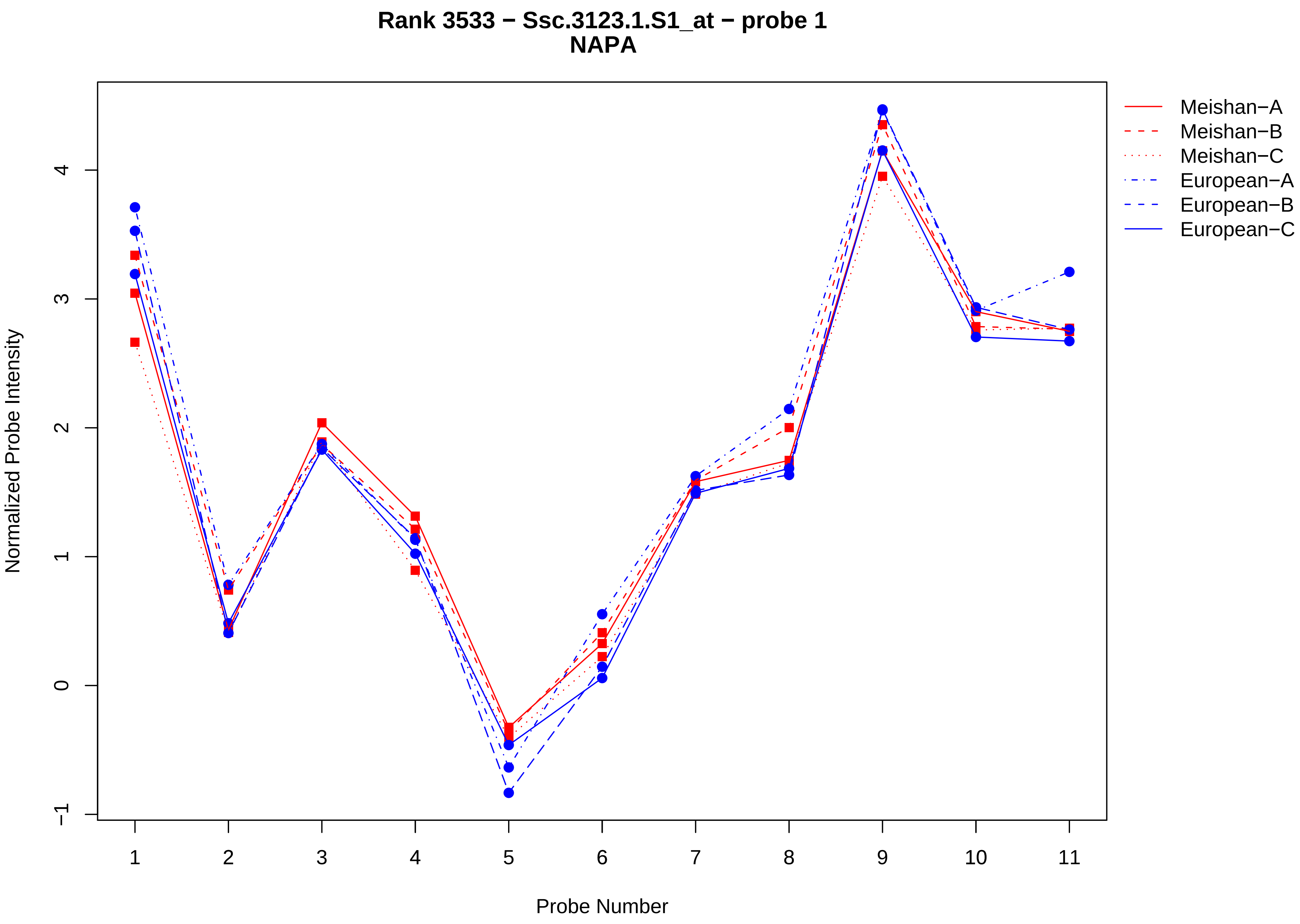
Rank 3533 − Ssc.3123.1.S1_at − probe 1
NAPA
Normalized Probe Intensity
Probe Number
−1
0
1
2
3
4
1
2
3
4
5
6
7
8
9
10
11
Meishan−A
Meishan−B
Meishan−C
European−A
European−B
European−C

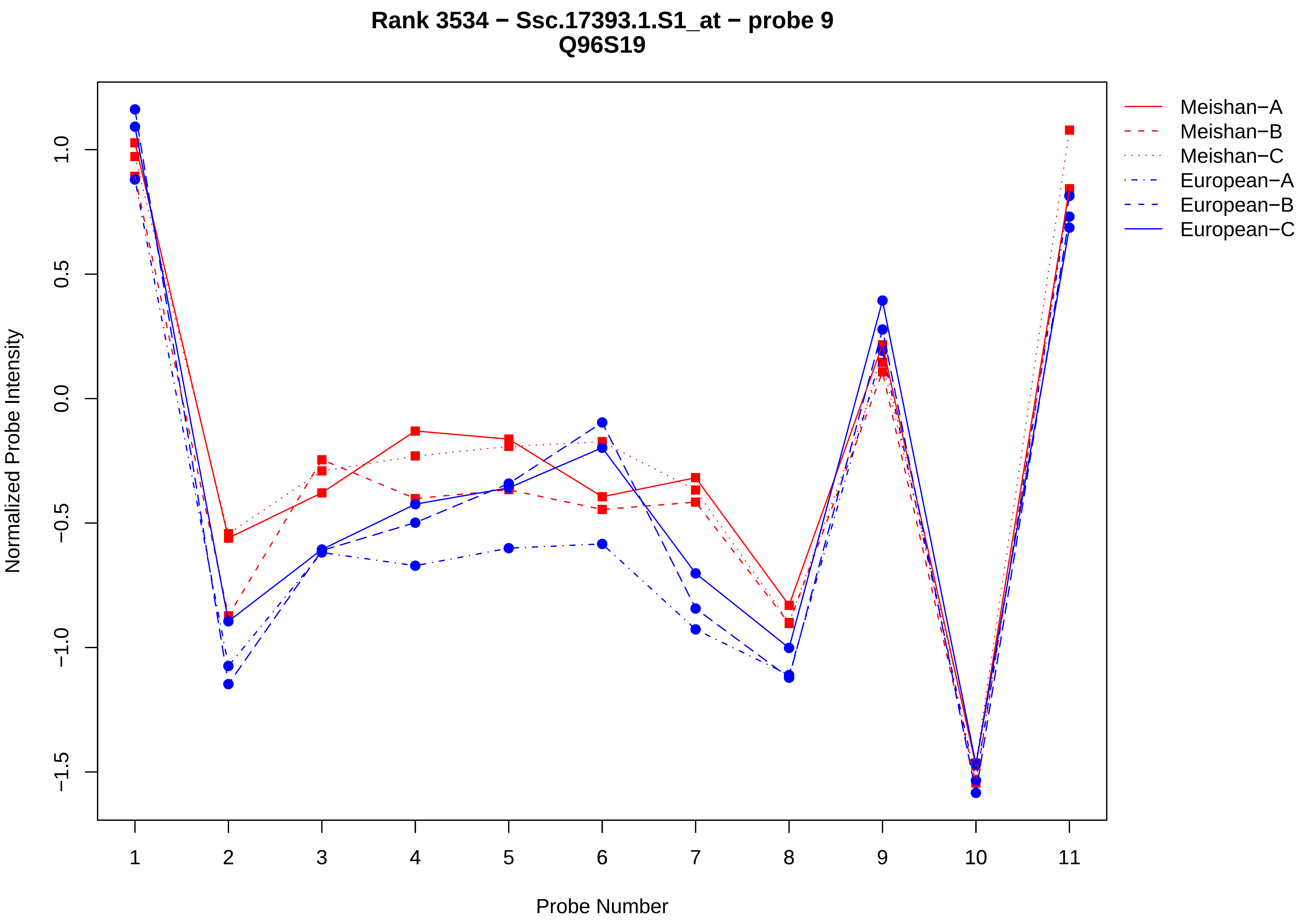
Rank 3534 − Ssc.17393.1.S1_at − probe 9
Q96S19
Normalized Probe Intensity
1.0
0.5
0.0
−0.5
−1.0
−1.5
Probe Number
1
2
3
4
5
6
7
8
9
10
11
Meishan−A
Meishan−B
Meishan−C
European−A
European−B
European−C

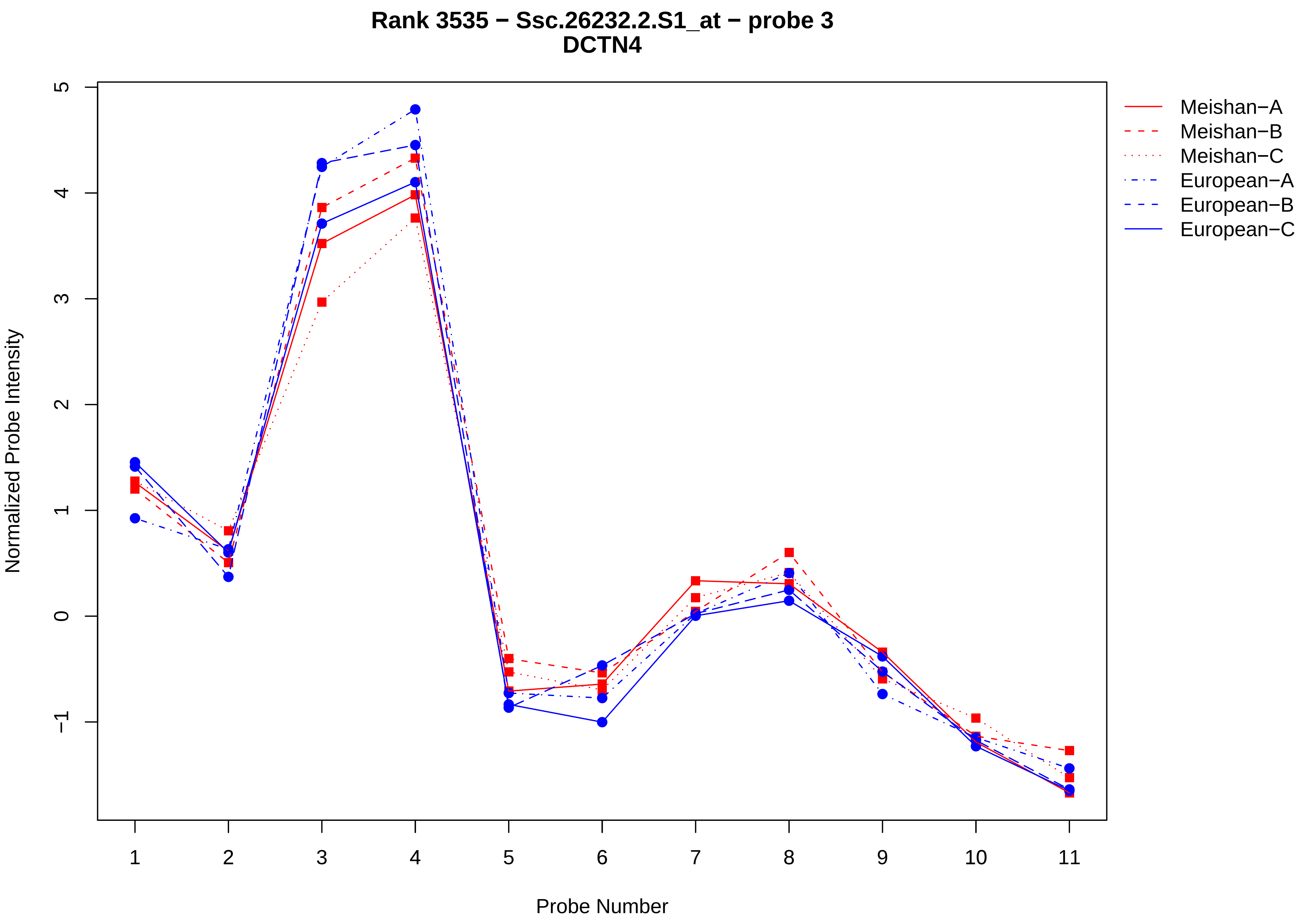

Rank 3535 − Ssc.26232.2.S1_at − probe 3
DCTN4
Normalized Probe Intensity
Probe Number
Meishan−A
Meishan−B
Meishan−C
European−A
European−B
European−C
−1
0
1
2
3
4
5
1
2
3
4
5
6
7
8
9
10
11

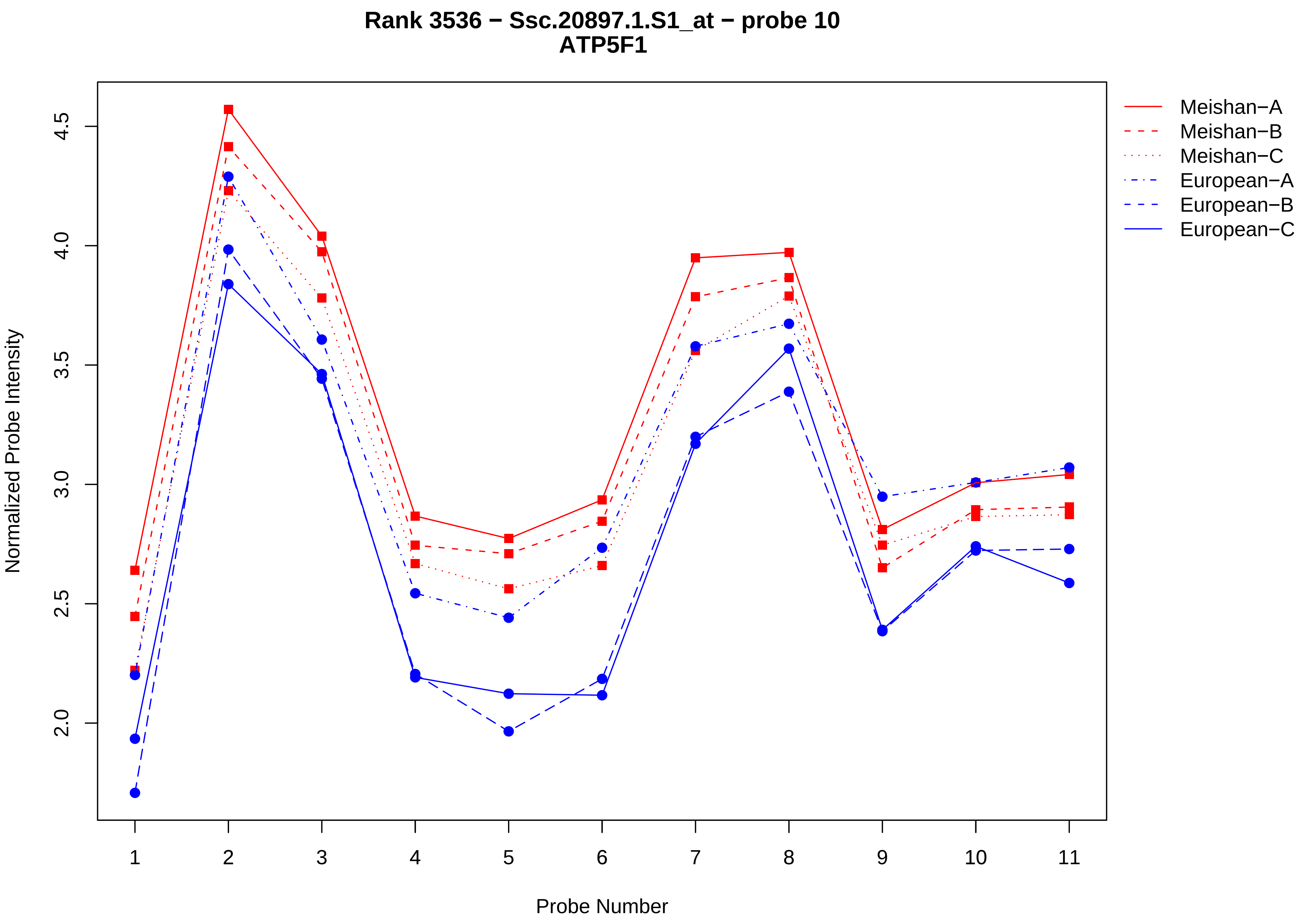

Rank 3536 − Ssc.20897.1.S1_at − probe 10
ATP5F1
Normalized Probe Intensity
Probe Number
Meishan−A
Meishan−B
Meishan−C
European−A
European−B
European−C

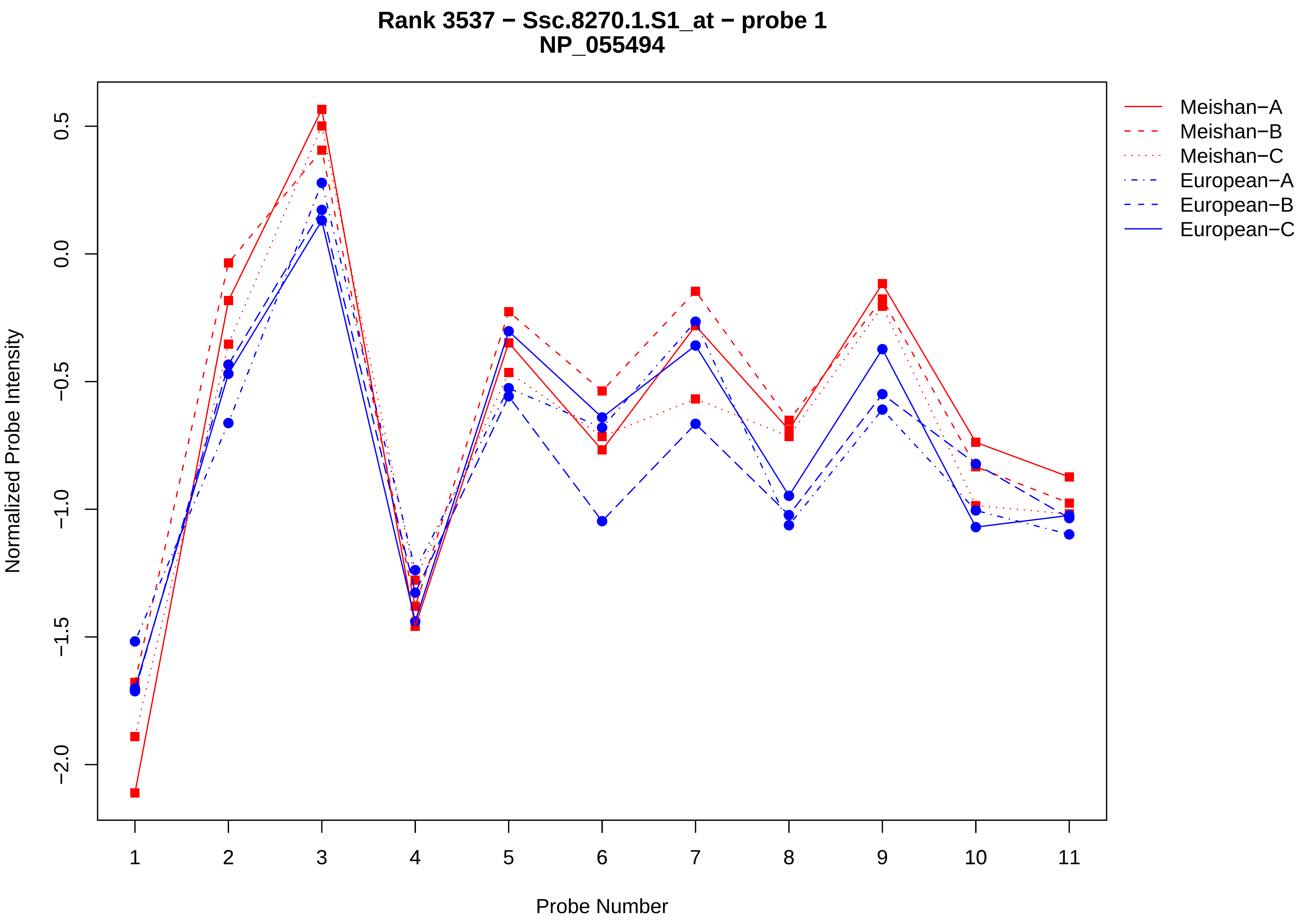

Rank 3537 − Ssc.8270.1.S1_at − probe 1
NP_055494
Normalized Probe Intensity
Probe Number
0.5
0.0
−0.5
−1.0
−1.5
−2.0
1
2
3
4
5
6
7
8
9
10
11
Meishan−A
Meishan−B
Meishan−C
European−A
European−B
European−C

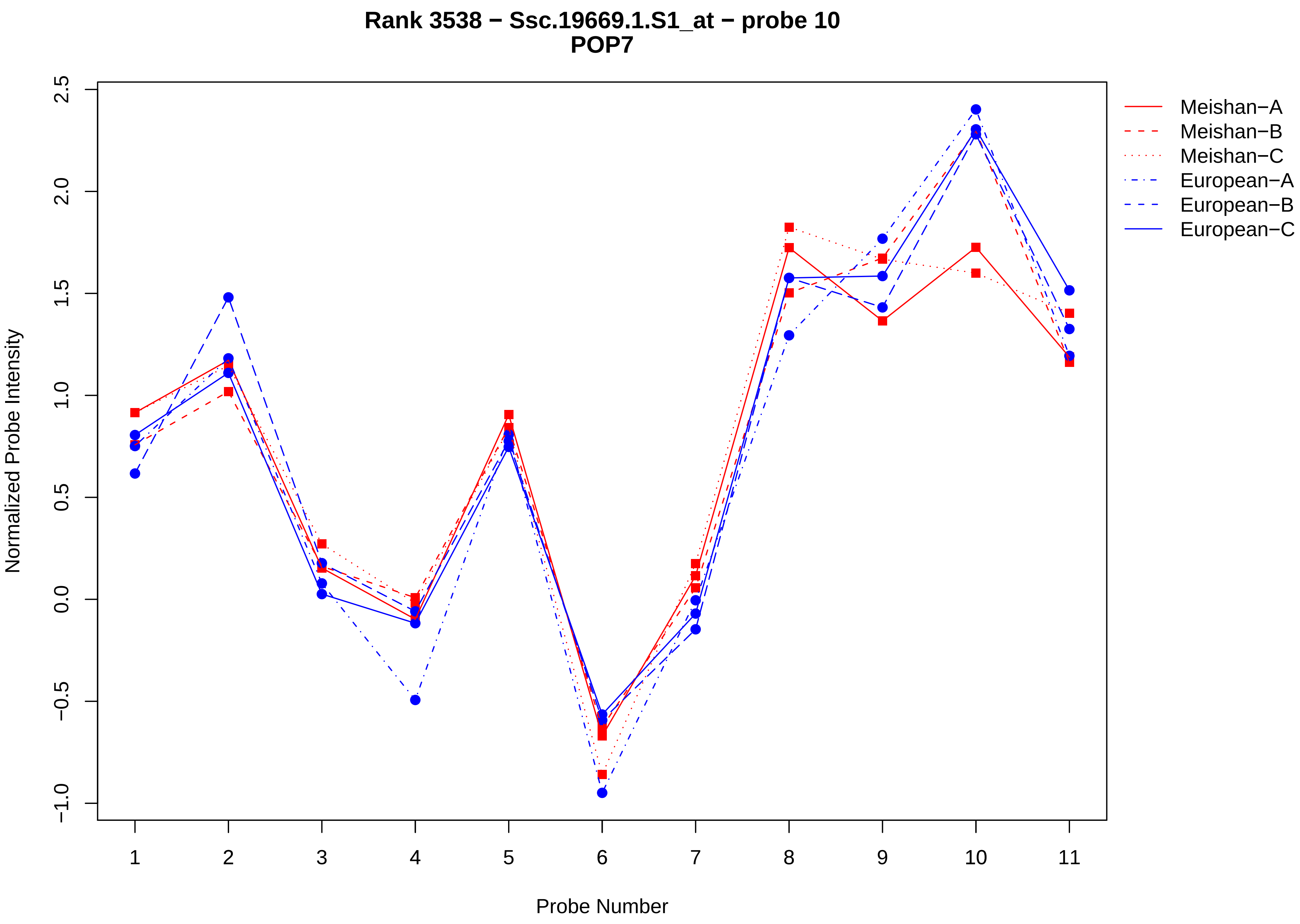

Rank 3538 − Ssc.19669.1.S1_at − probe 10
POP7
Normalized Probe Intensity
Probe Number
−1.0
−0.5
0.0
0.5
1.0
1.5
2.0
2.5
1
2
3
4
5
6
7
8
9
10
11
Meishan−A
Meishan−B
Meishan−C
European−A
European−B
European−C

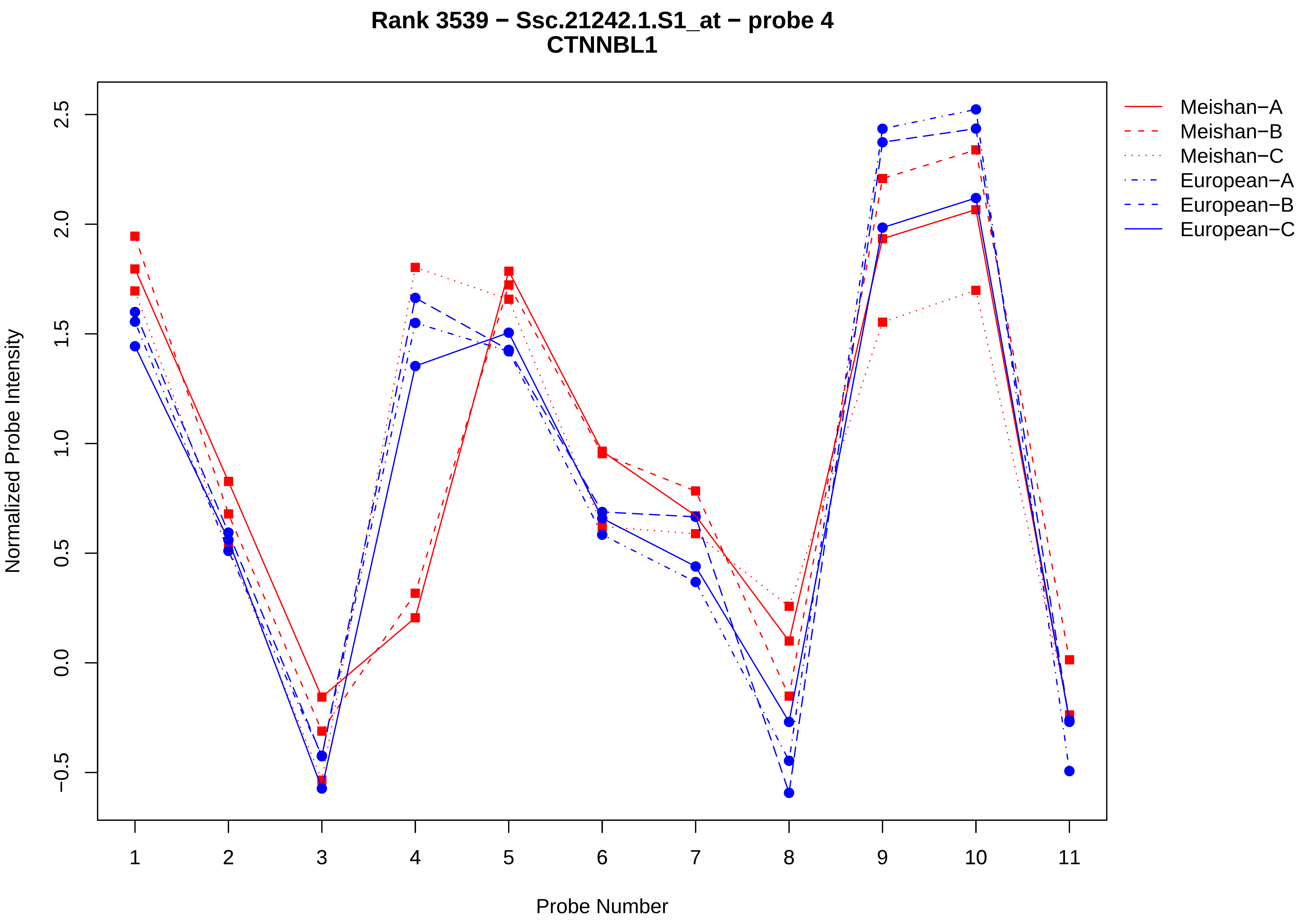
Rank 3539 − Ssc.21242.1.S1_at − probe 4
CTNNBL1
Normalized Probe Intensity
Probe Number
Meishan−A
Meishan−B
Meishan−C
European−A
European−B
European−C
−0.5
0.0
0.5
1.0
1.5
2.0
2.5
1
2
3
4
5
6
7
8
9
10
11

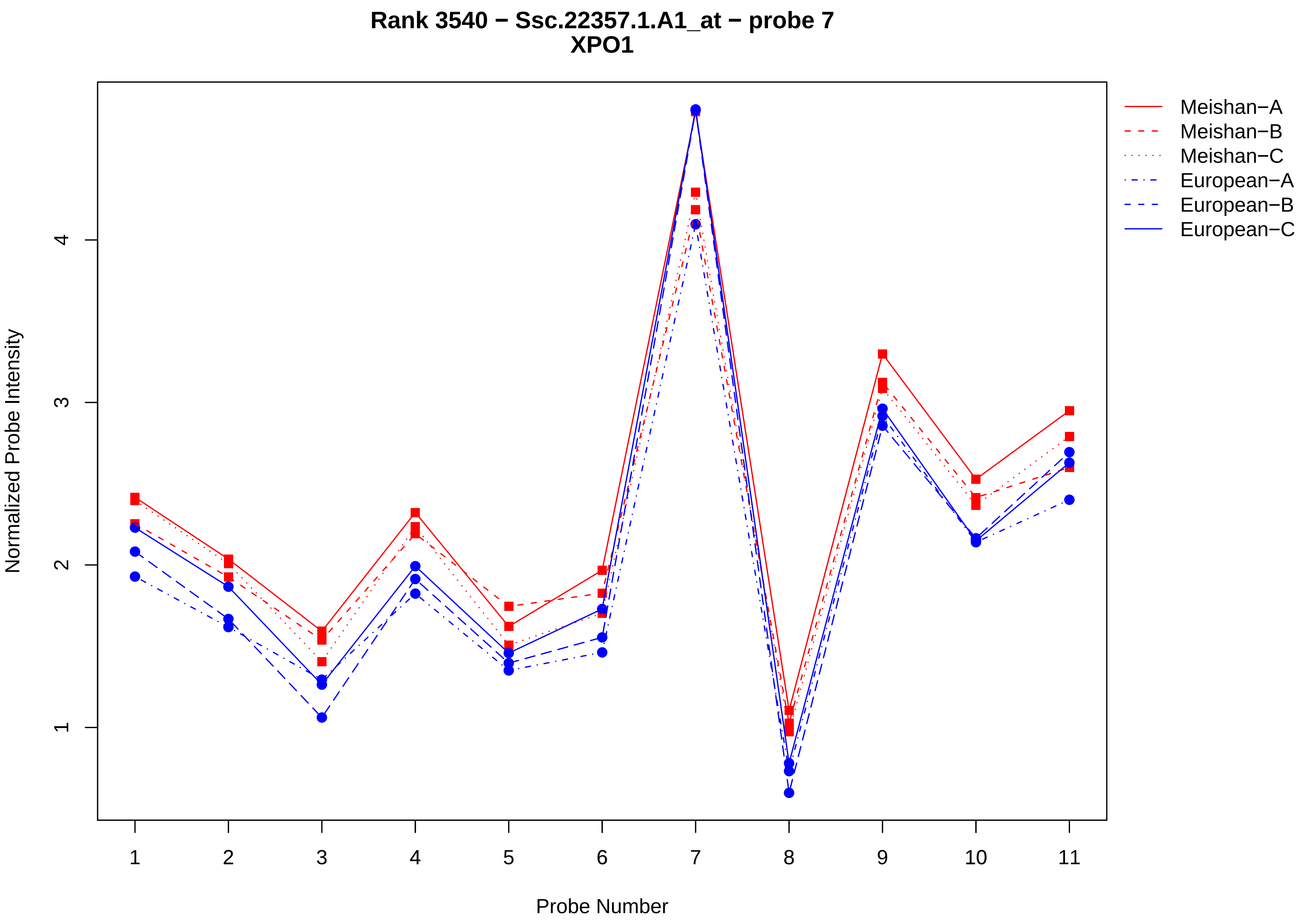

Rank 3540 − Ssc.22357.1.A1_at − probe 7
XPO1
Normalized Probe Intensity
Probe Number
1
2
3
4
1
2
3
4
5
6
7
8
9
10
11
Meishan−A
Meishan−B
Meishan−C
European−A
European−B
European−C

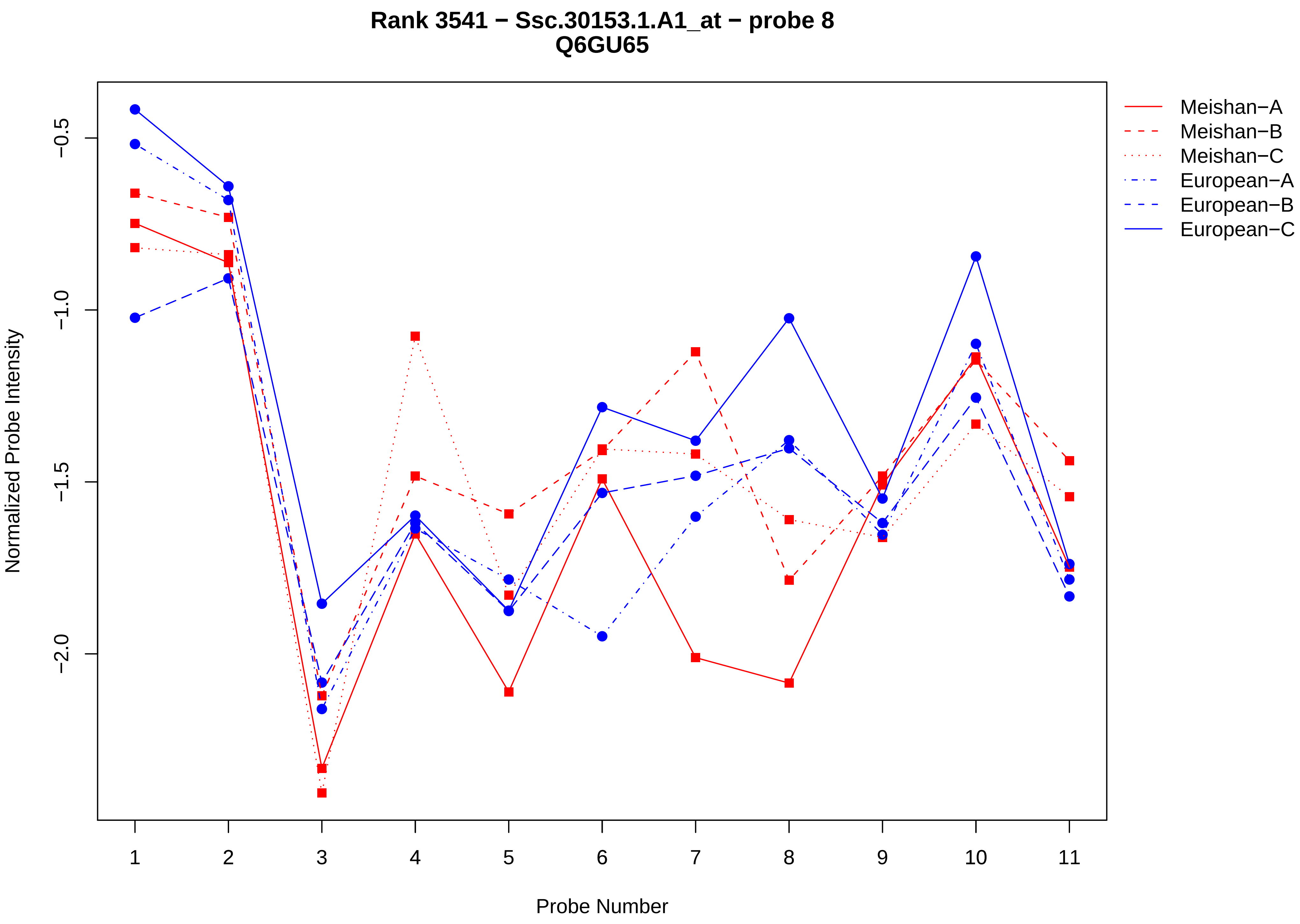

Rank 3541 − Ssc.30153.1.A1_at − probe 8
Q6GU65
Normalized Probe Intensity
−0.5
−1.0
−1.5
−2.0
Probe Number
1
2
3
4
5
6
7
8
9
10
11
Meishan−A
Meishan−B
Meishan−C
European−A
European−B
European−C

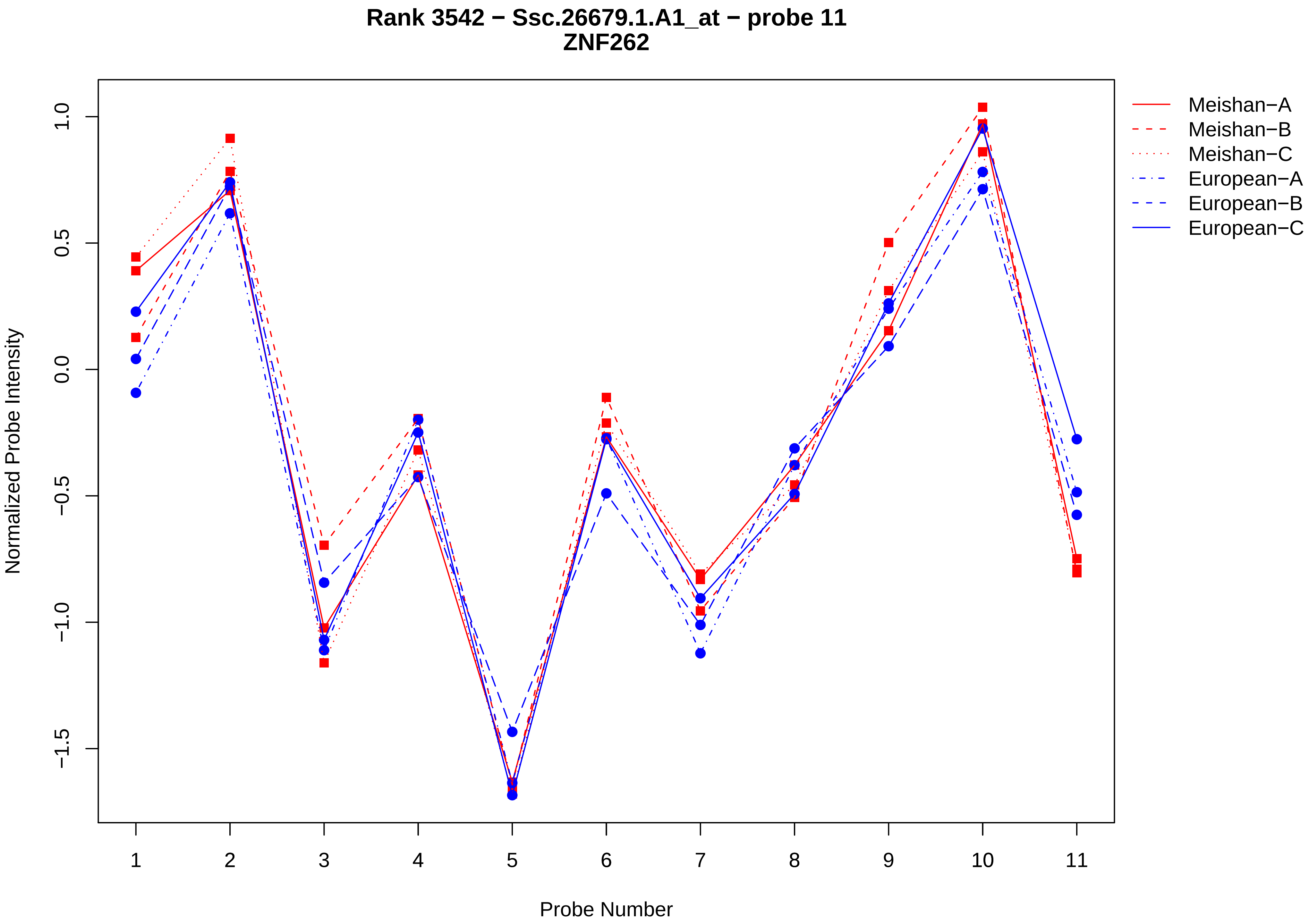

Rank 3542 − Ssc.26679.1.A1_at − probe 11
ZNF262
Normalized Probe Intensity
Probe Number
1.0
0.5
0.0
−0.5
−1.0
−1.5
1
2
3
4
5
6
7
8
9
10
11
Meishan−A
Meishan−B
Meishan−C
European−A
European−B
European−C

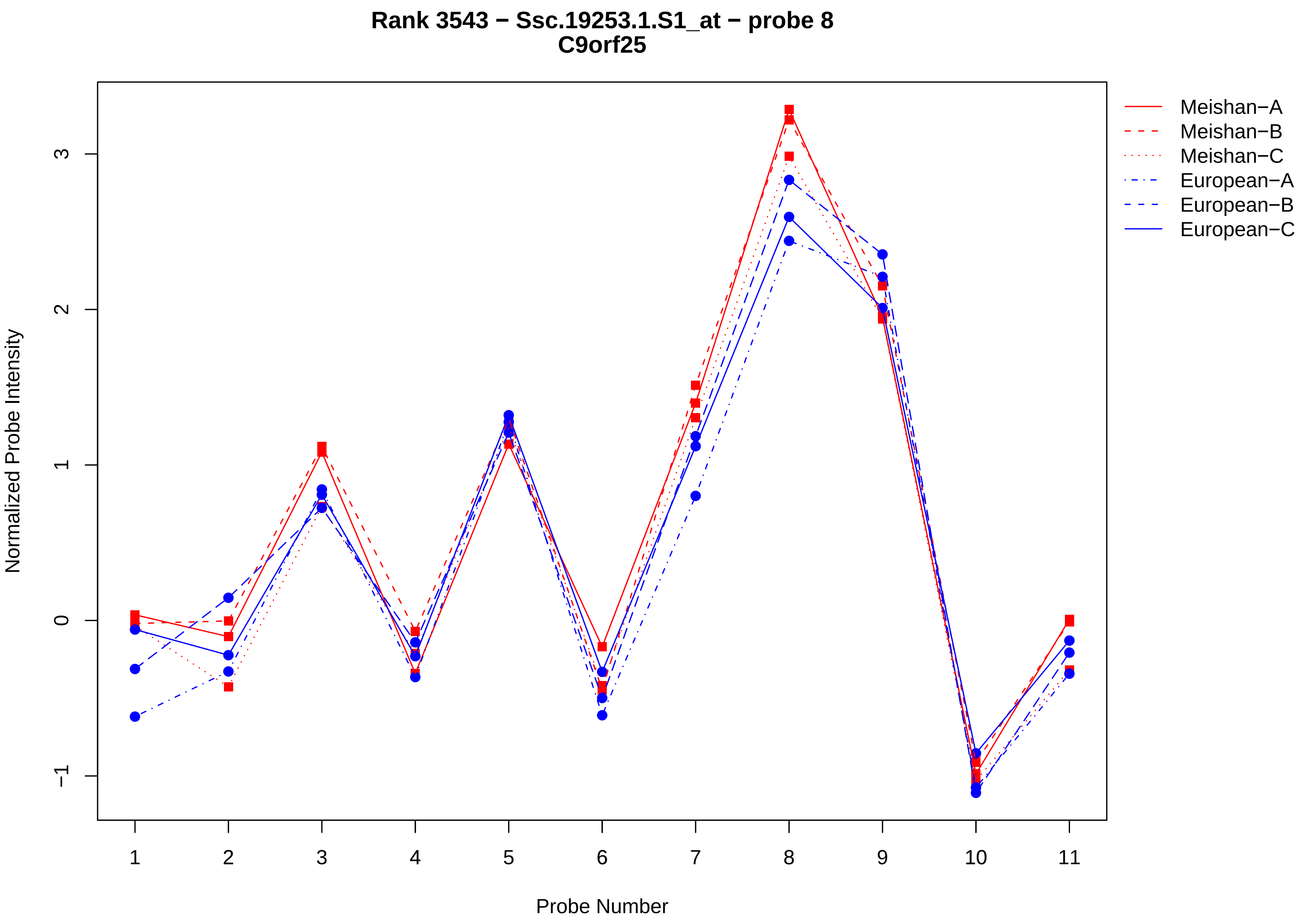
Rank 3543 − Ssc.19253.1.S1_at − probe 8
C9orf25
Normalized Probe Intensity
Probe Number
Meishan−A
Meishan−B
Meishan−C
European−A
European−B
European−C

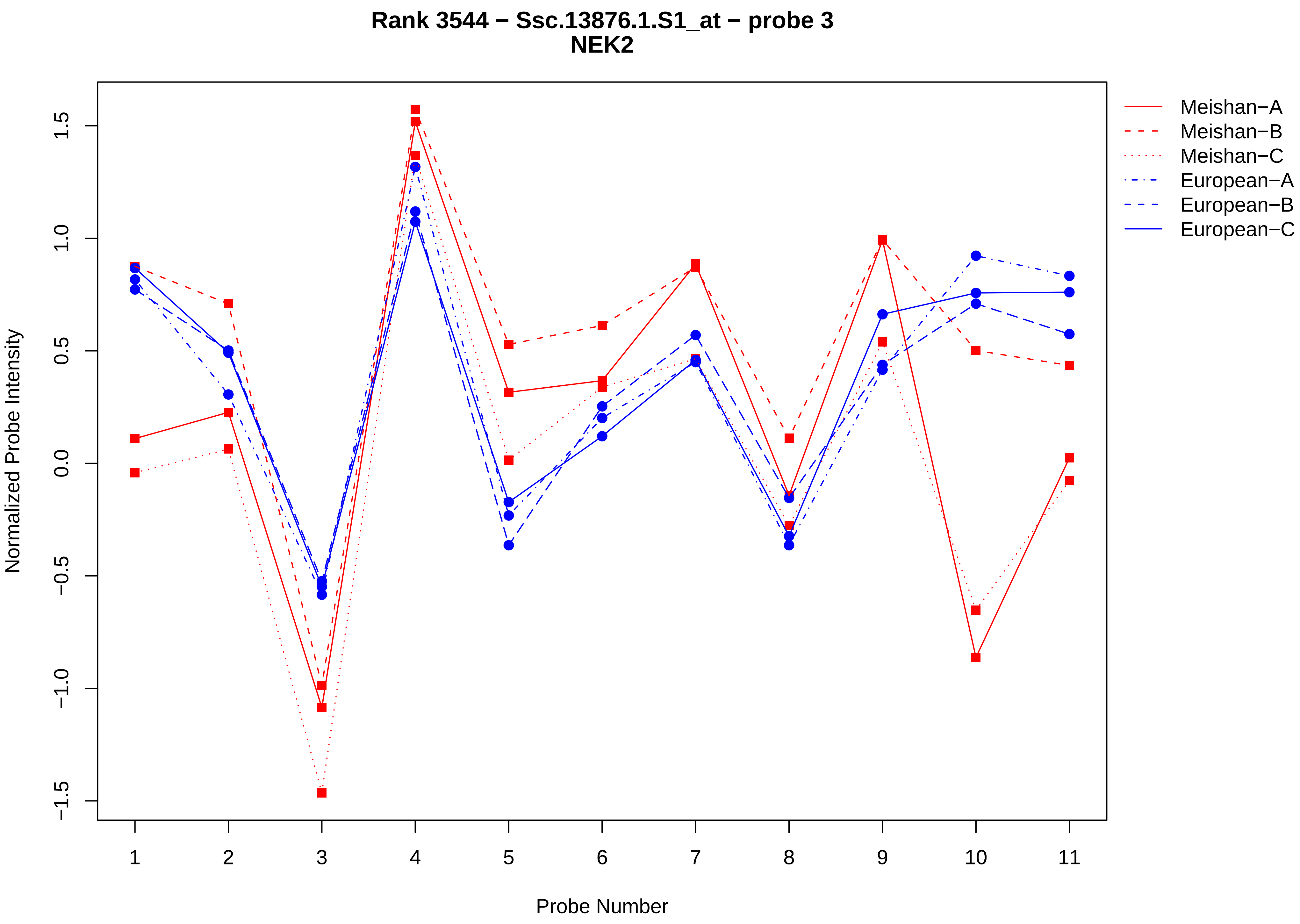

Rank 3544 − Ssc.13876.1.S1_at − probe 3
NEK2
Normalized Probe Intensity
Probe Number
−1.5
−1.0
−0.5
0.0
0.5
1.0
1.5
1
2
3
4
5
6
7
8
9
10
11
Meishan−A
Meishan−B
Meishan−C
European−A
European−B
European−C

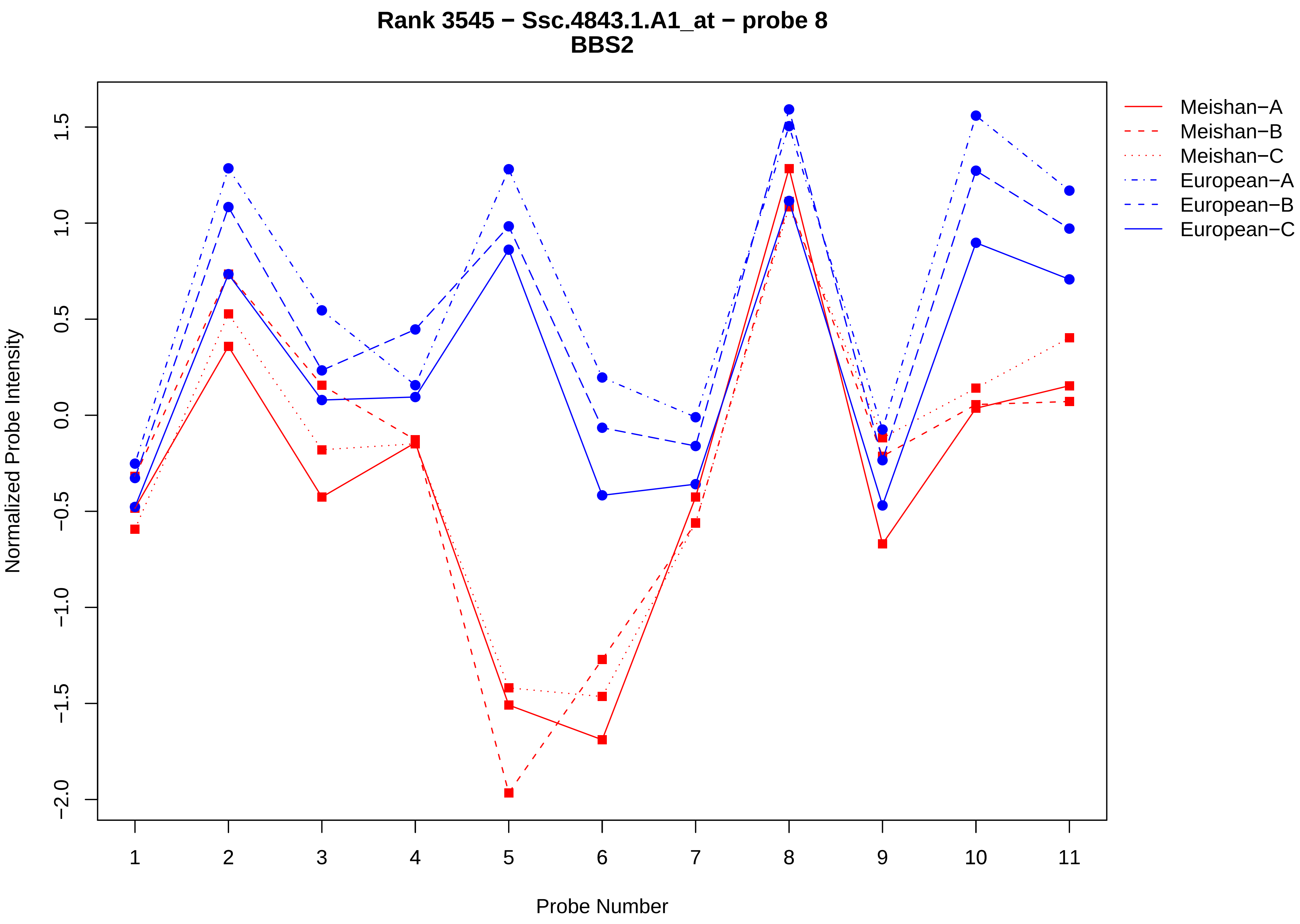

Rank 3545 − Ssc.4843.1.A1_at − probe 8
BBS2
Normalized Probe Intensity
Probe Number
Meishan−A
Meishan−B
Meishan−C
European−A
European−B
European−C

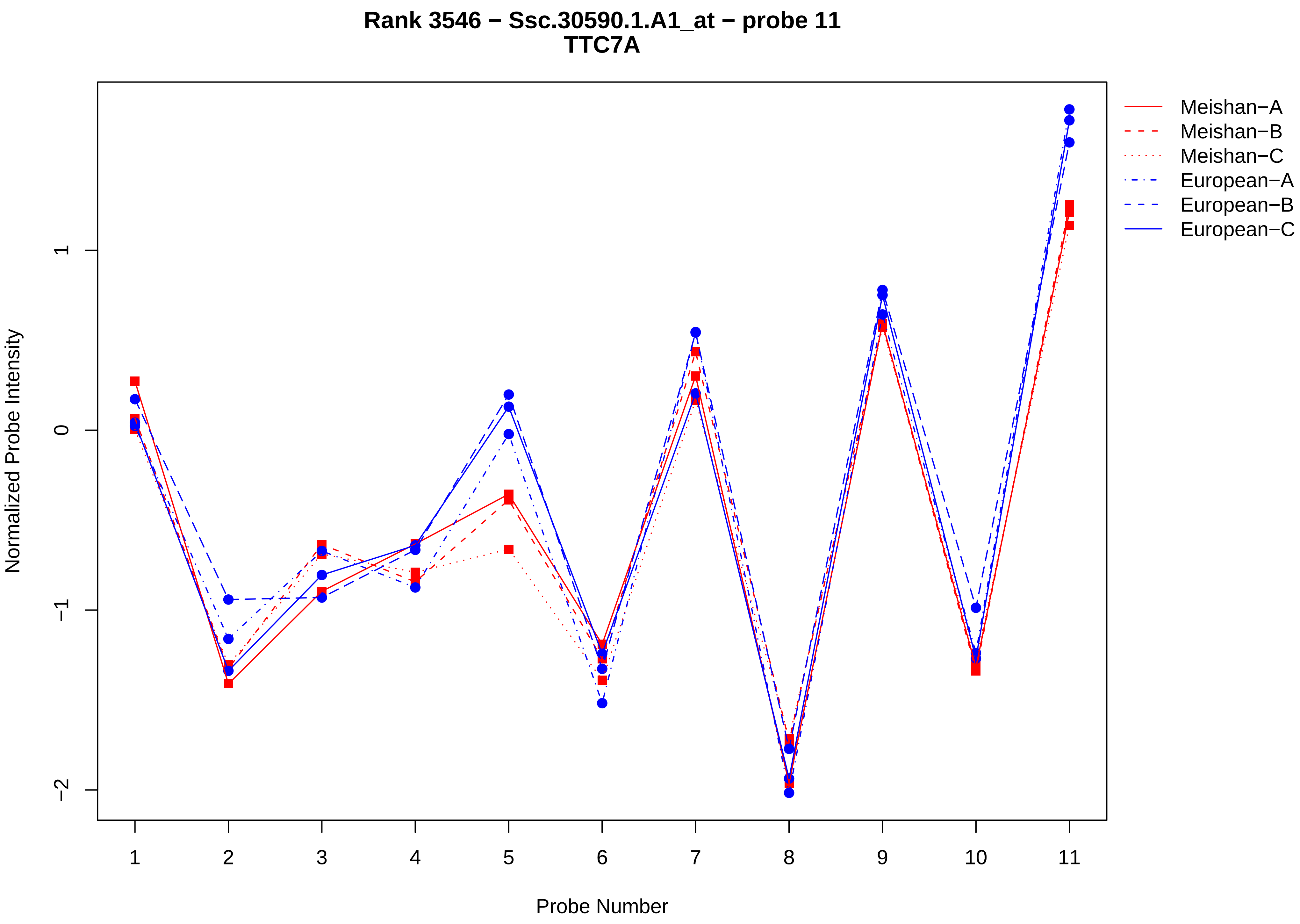

Rank 3546 − Ssc.30590.1.A1_at − probe 11
TTC7A
Normalized Probe Intensity
Probe Number
Meishan−A
Meishan−B
Meishan−C
European−A
European−B
European−C
−2
−1
0
1
1
2
3
4
5
6
7
8
9
10
11

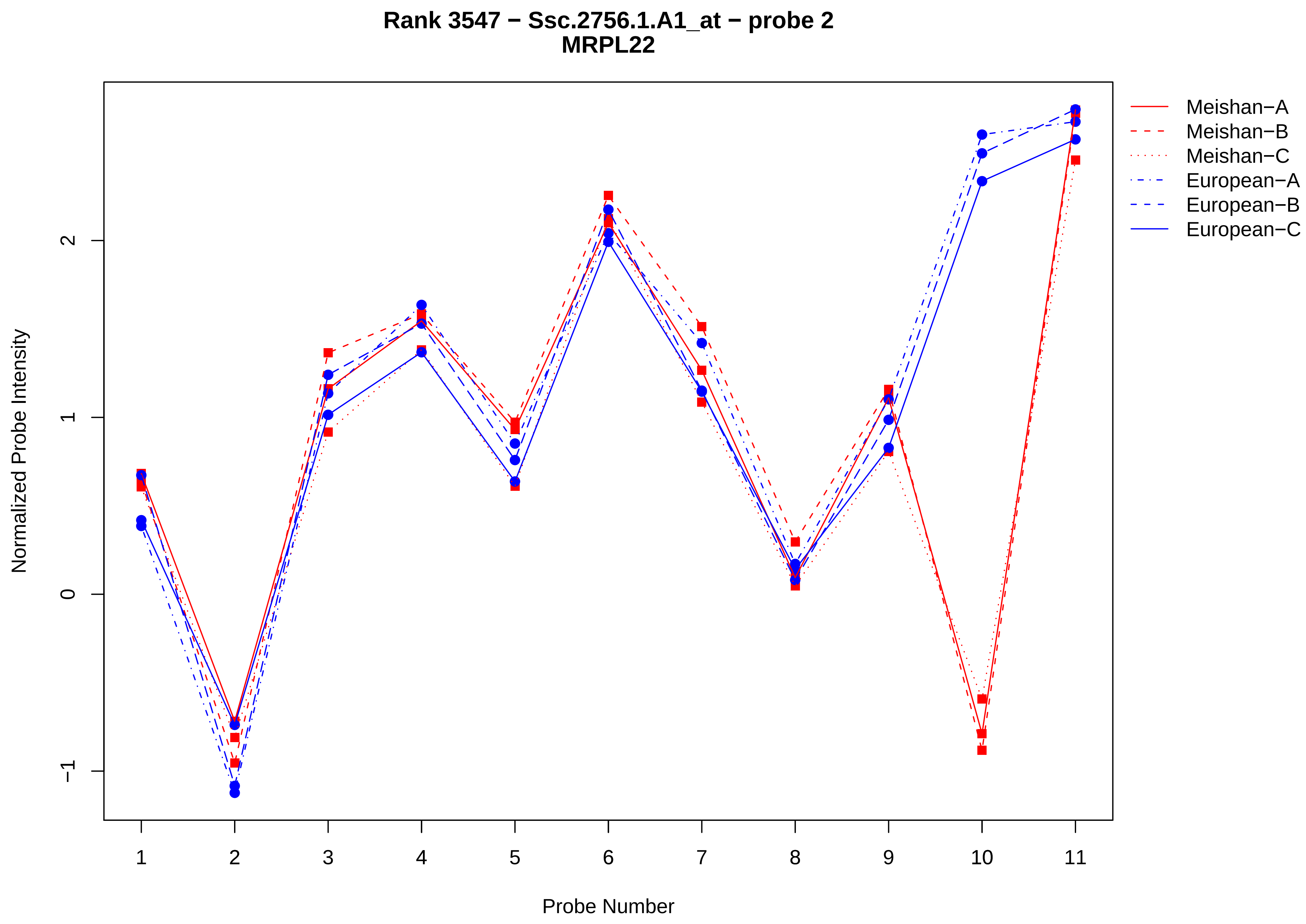

Rank 3547 − Ssc.2756.1.A1_at − probe 2
MRPL22
Normalized Probe Intensity
Probe Number
−1
0
1
2
1
2
3
4
5
6
7
8
9
10
11
Meishan−A
Meishan−B
Meishan−C
European−A
European−B
European−C

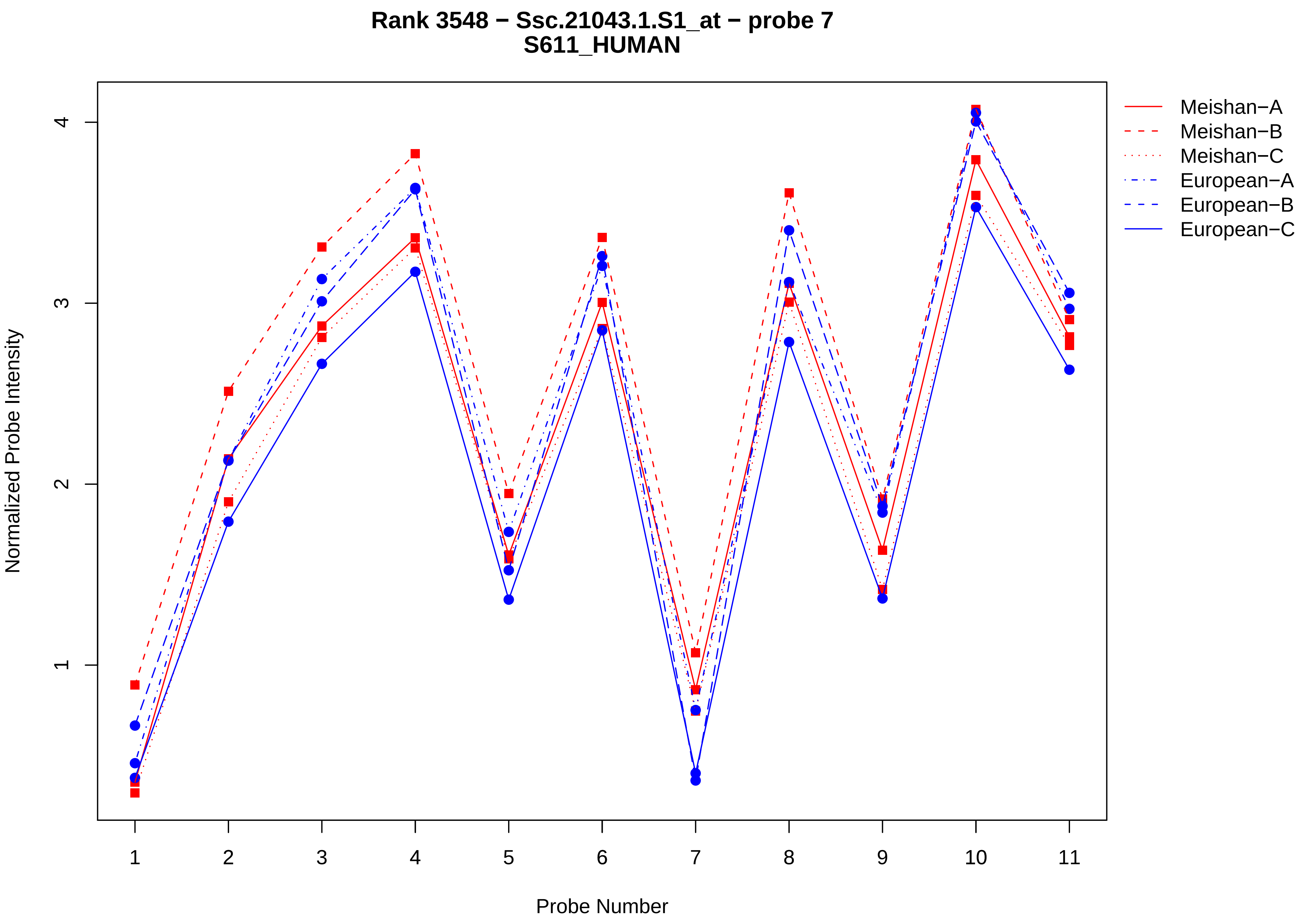

Rank 3548 − Ssc.21043.1.S1_at − probe 7
S611_HUMAN
Normalized Probe Intensity
Probe Number
1
2
3
4
1
2
3
4
5
6
7
8
9
10
11
Meishan−A
Meishan−B
Meishan−C
European−A
European−B
European−C

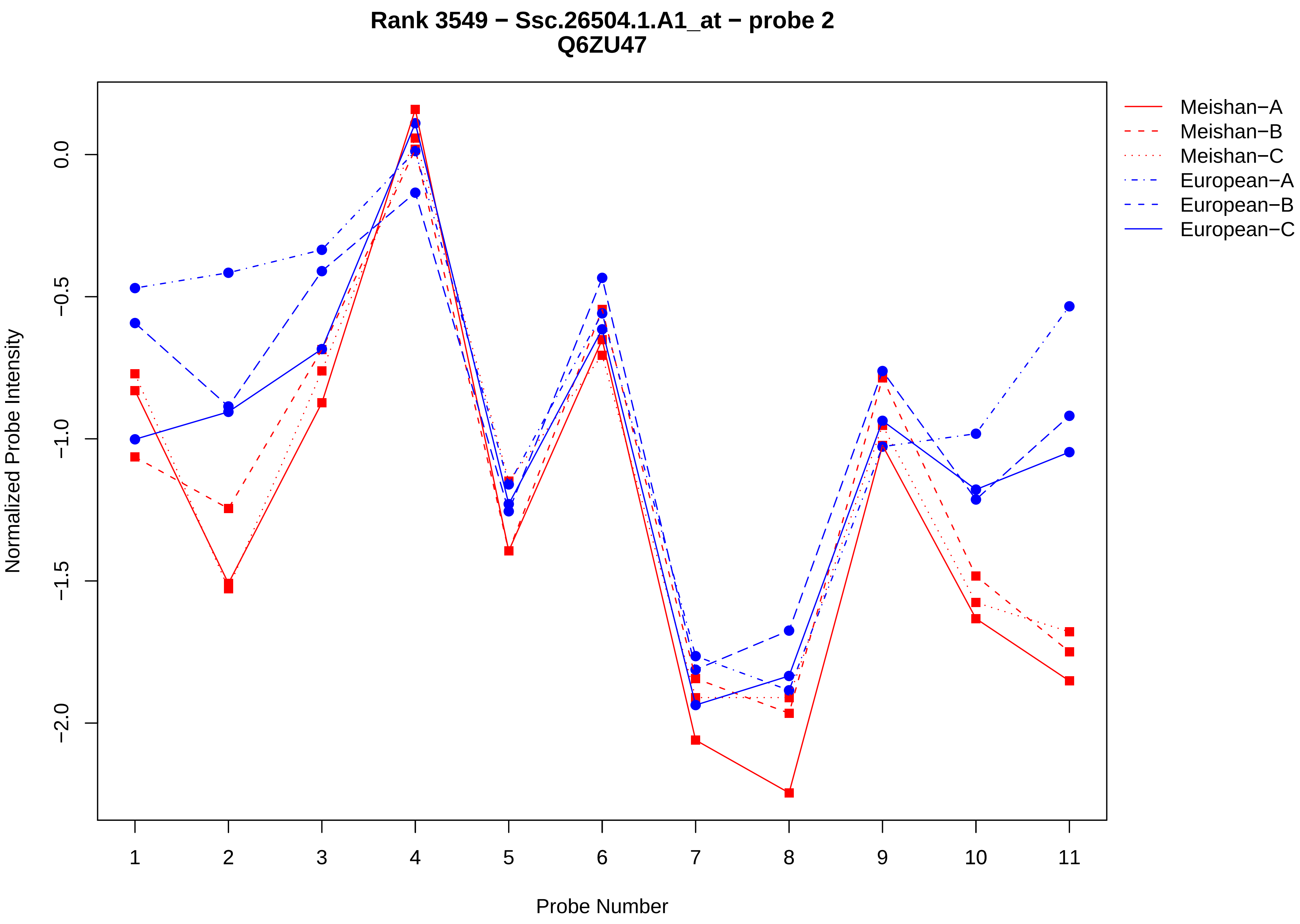

Rank 3549 − Ssc.26504.1.A1_at − probe 2
Q6ZU47
Normalized Probe Intensity
Probe Number
0.0
−0.5
−1.0
−1.5
−2.0
1
2
3
4
5
6
7
8
9
10
11
Meishan−A
Meishan−B
Meishan−C
European−A
European−B
European−C

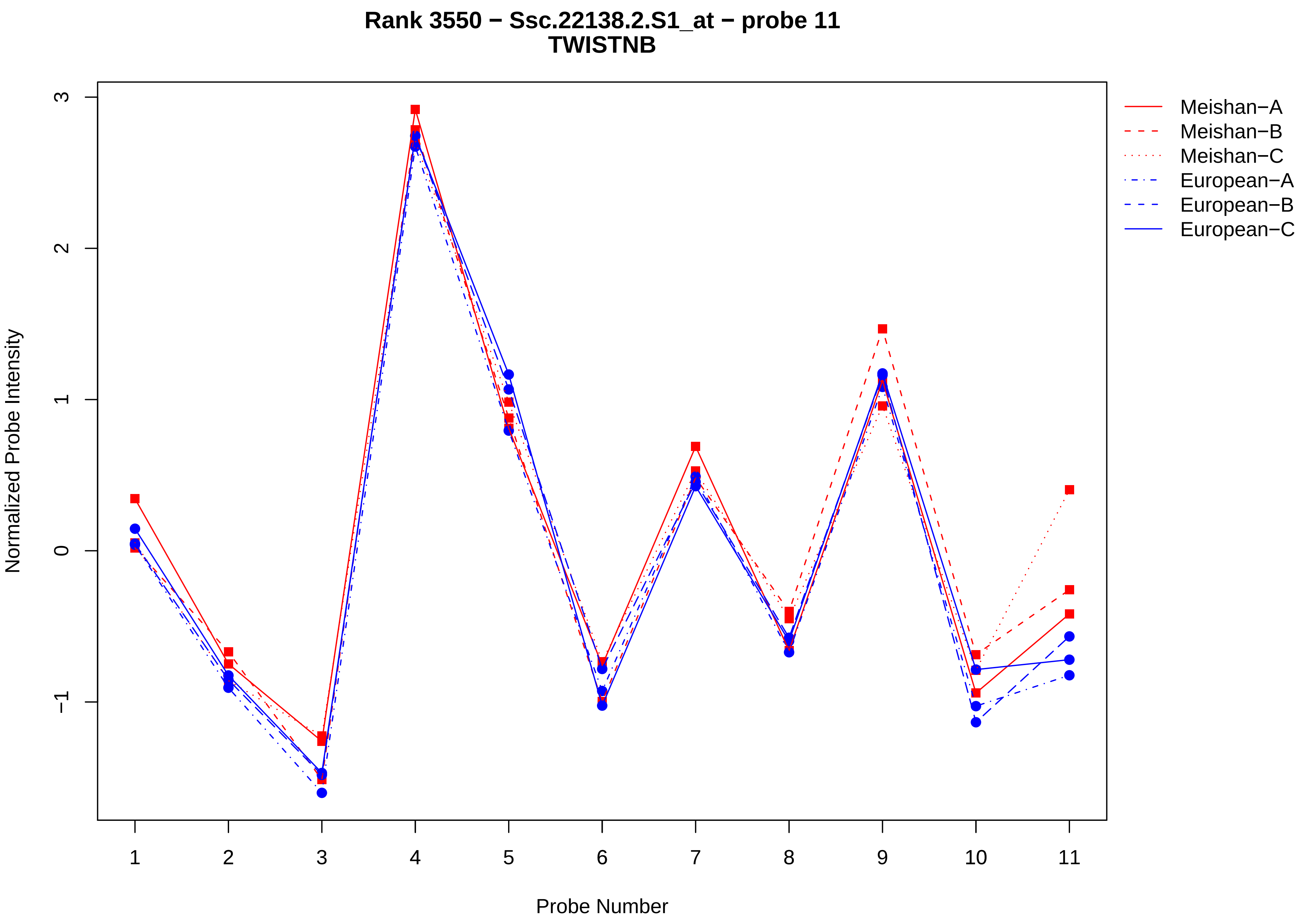

Rank 3550 − Ssc.22138.2.S1_at − probe 11
TWISTNB
Normalized Probe Intensity
Probe Number
Meishan−A
Meishan−B
Meishan−C
European−A
European−B
European−C
-1
0
1
2
3
1
2
3
4
5
6
7
8
9
10
11

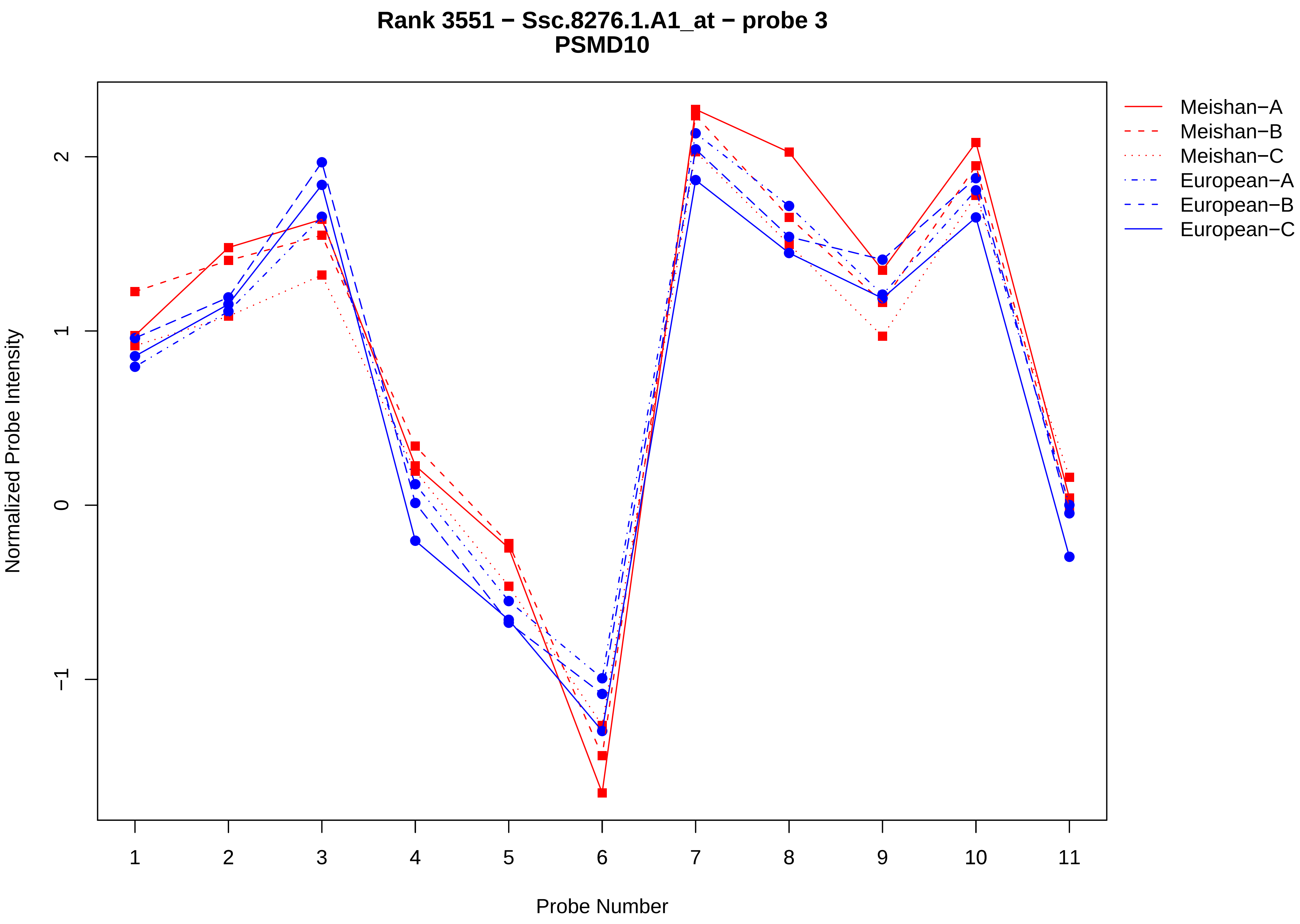

Rank 3551 − Ssc.8276.1.A1_at − probe 3
PSMD10
Normalized Probe Intensity
Probe Number
−1
0
1
2
1
2
3
4
5
6
7
8
9
10
11
Meishan−A
Meishan−B
Meishan−C
European−A
European−B
European−C

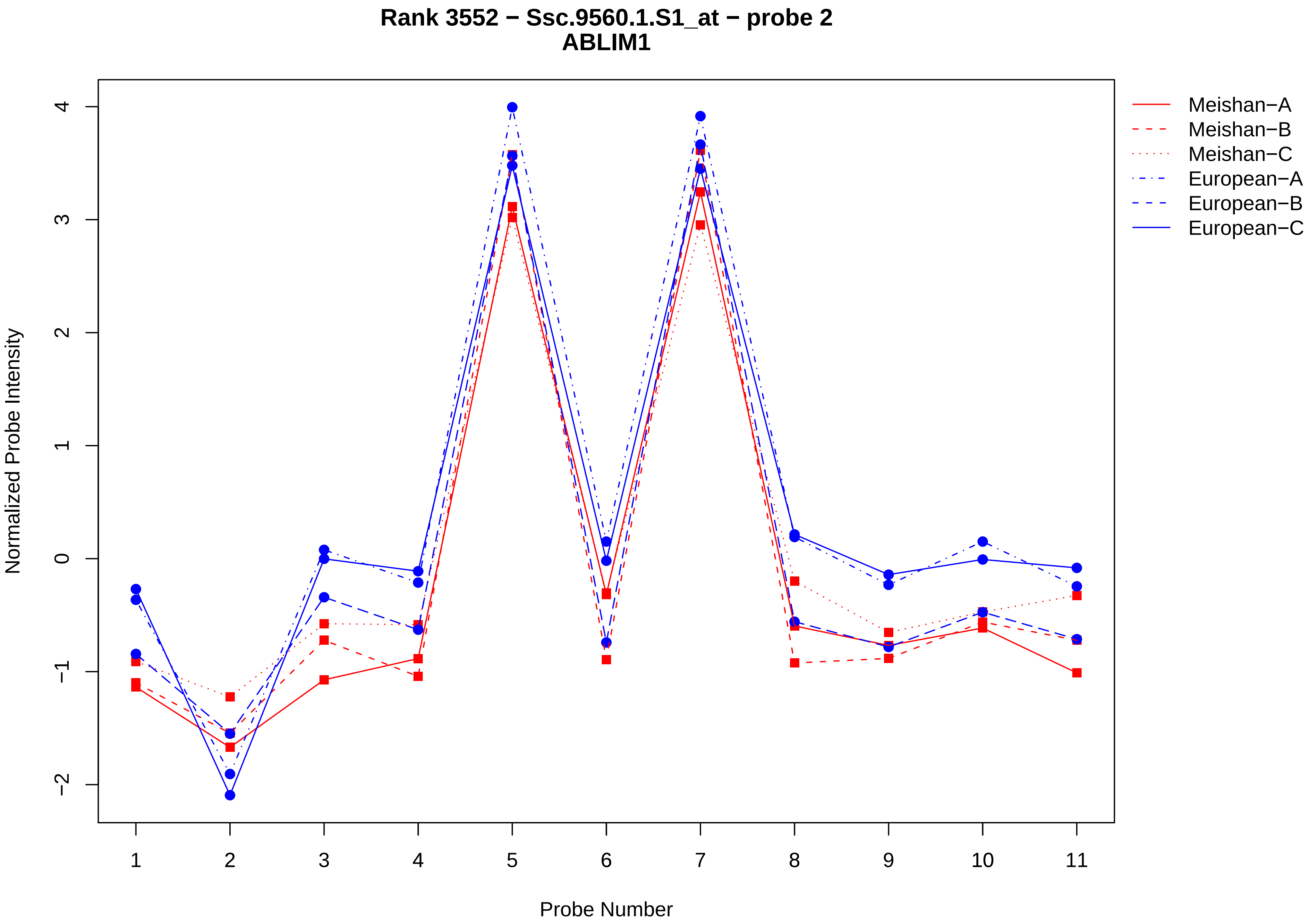
Rank 3552 − Ssc.9560.1.S1_at − probe 2
ABLIM1
Normalized Probe Intensity
Probe Number
Meishan−A
Meishan−B
Meishan−C
European−A
European−B
European−C
−2
−1
0
1
2
3
4
1
2
3
4
5
6
7
8
9
10
11

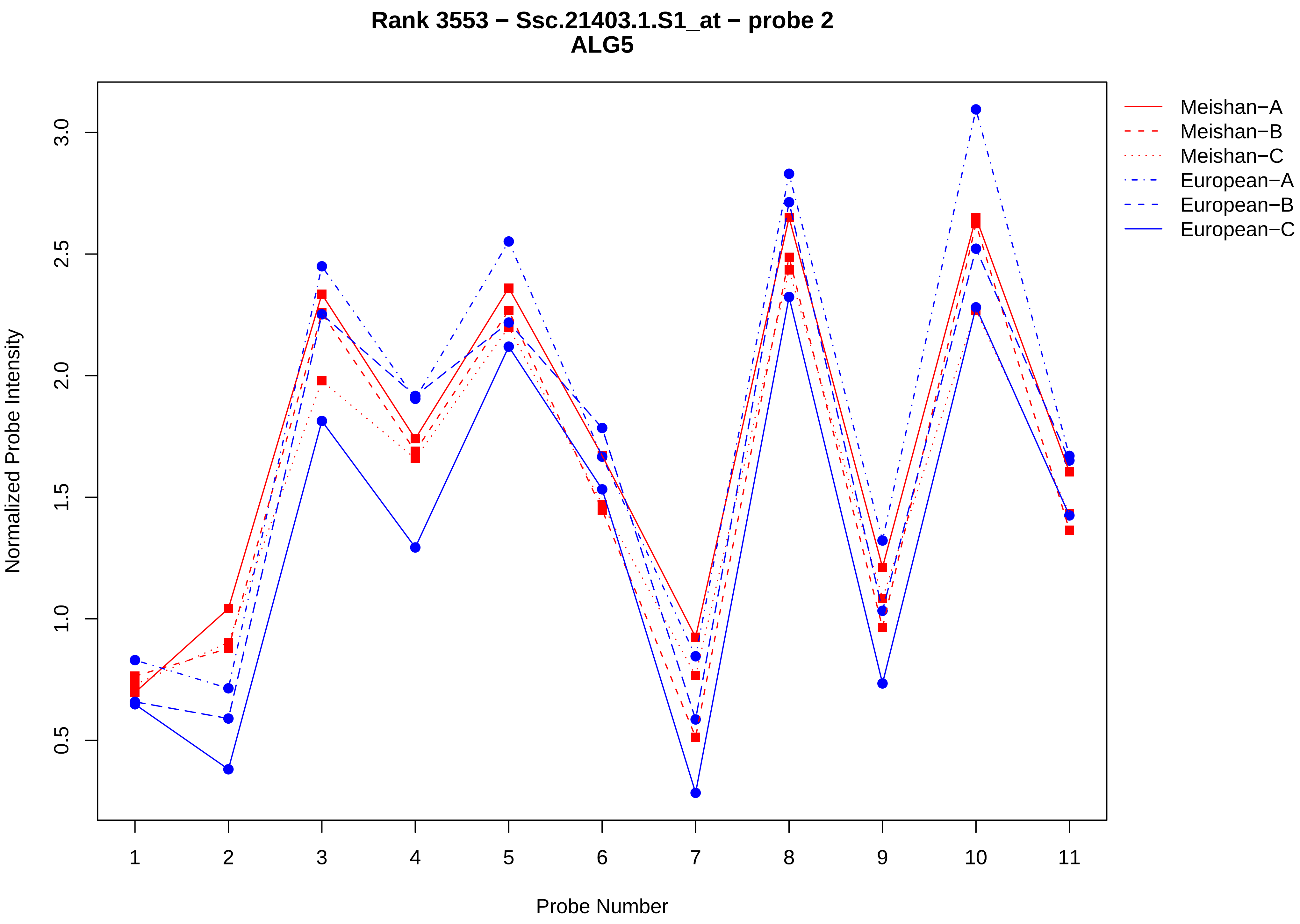

Rank 3553 − Ssc.21403.1.S1_at − probe 2
ALG5
Normalized Probe Intensity
Probe Number
0.5
1.0
1.5
2.0
2.5
3.0
1
2
3
4
5
6
7
8
9
10
11
Meishan−A
Meishan−B
Meishan−C
European−A
European−B
European−C

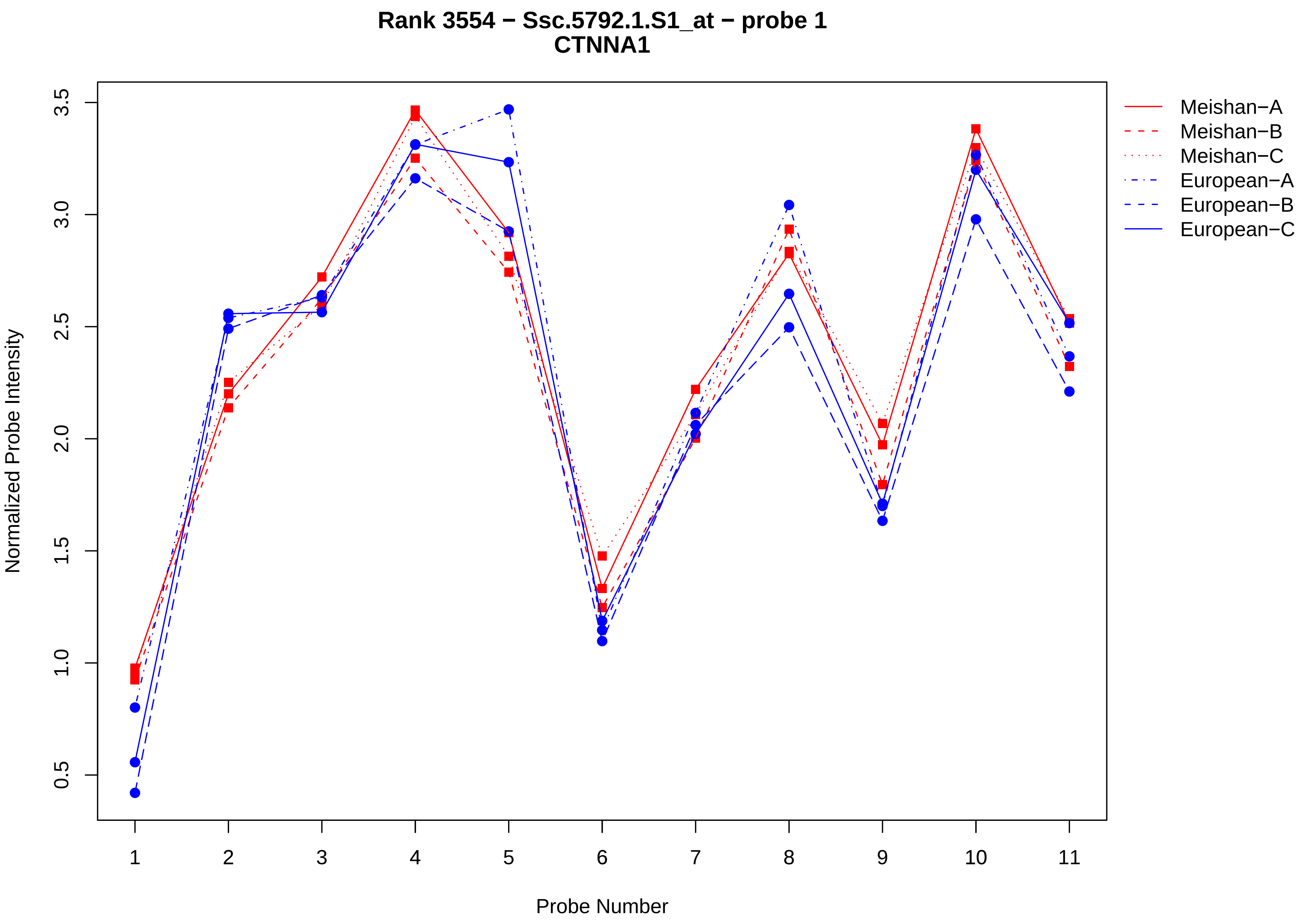

Rank 3554 − Ssc.5792.1.S1_at − probe 1
CTNNA1
Normalized Probe Intensity
Probe Number
0.5
1.0
1.5
2.0
2.5
3.0
3.5
1
2
3
4
5
6
7
8
9
10
11
Meishan−A
Meishan−B
Meishan−C
European−A
European−B
European−C

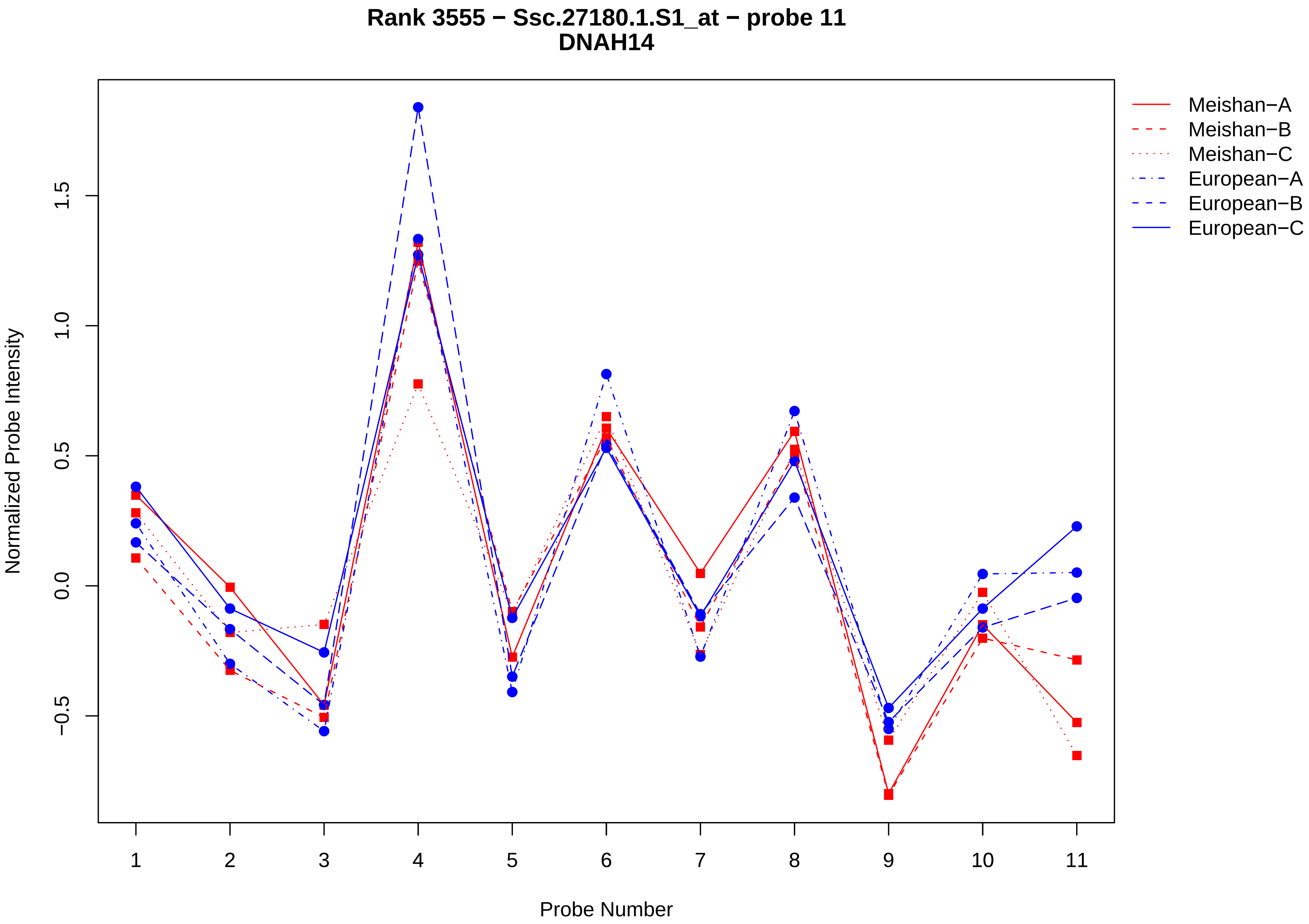

Rank 3555 − Ssc.27180.1.S1_at − probe 11
DNAH14
Normalized Probe Intensity
Probe Number
−0.5
0.0
0.5
1.0
1.5
1
2
3
4
5
6
7
8
9
10
11
Meishan−A
Meishan−B
Meishan−C
European−A
European−B
European−C

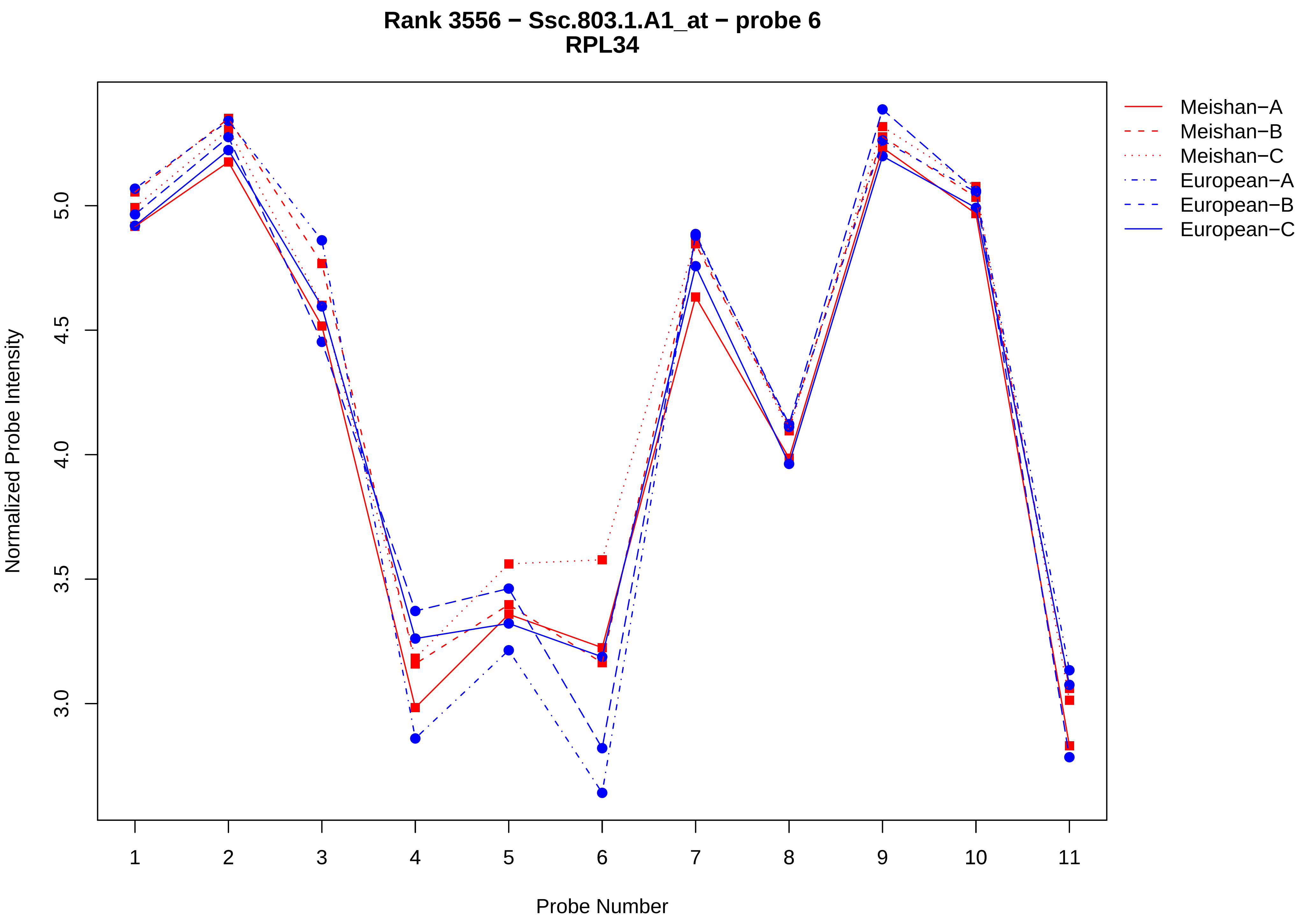

Rank 3556 − Ssc.803.1.A1_at − probe 6
RPL34
Normalized Probe Intensity
Probe Number
3.0
3.5
4.0
4.5
5.0
1
2
3
4
5
6
7
8
9
10
11
Meishan−A
Meishan−B
Meishan−C
European−A
European−B
European−C

Rank 3557 − Ssc.28652.1.A1_at − probe 5
PAQR3

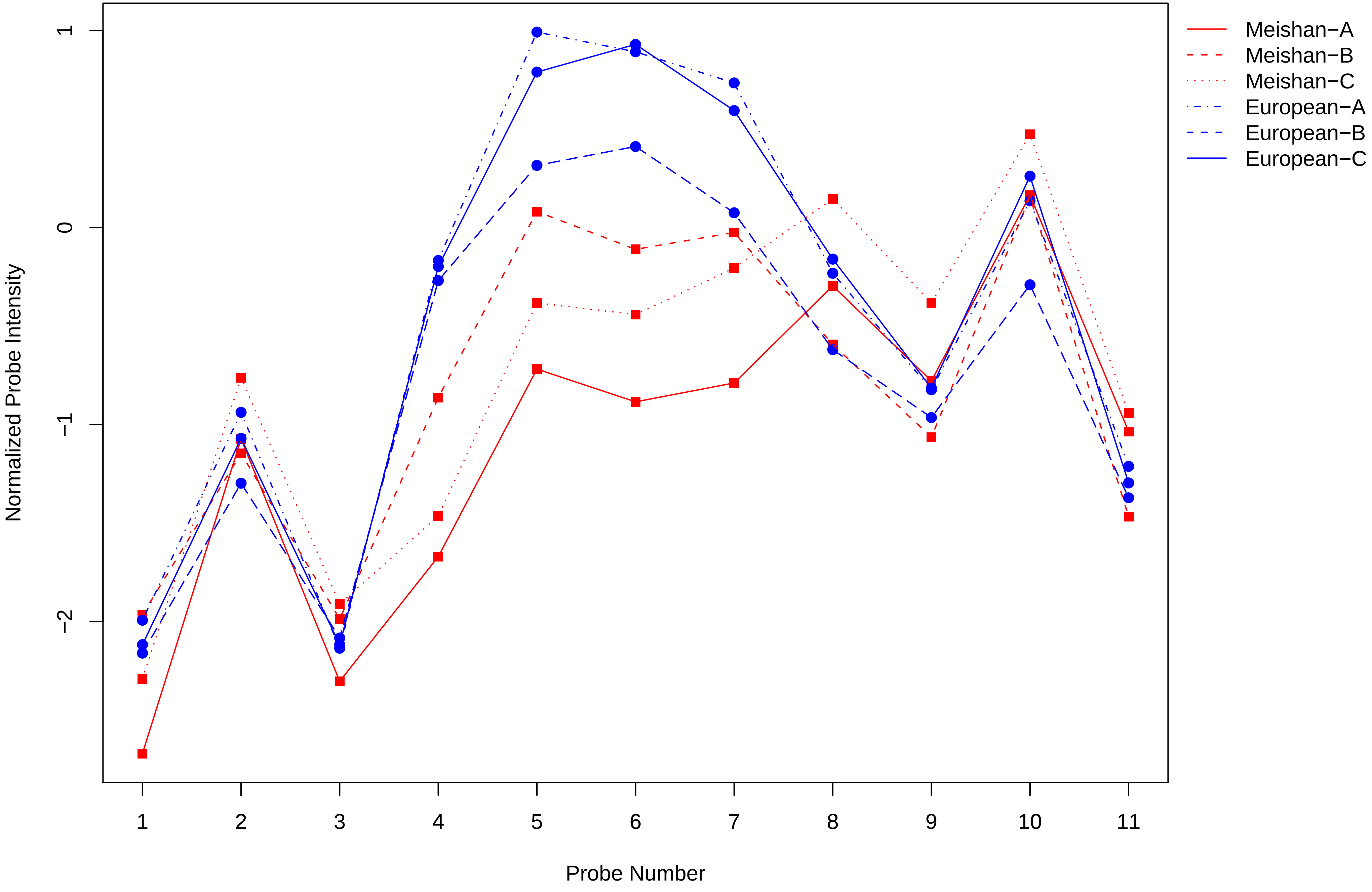

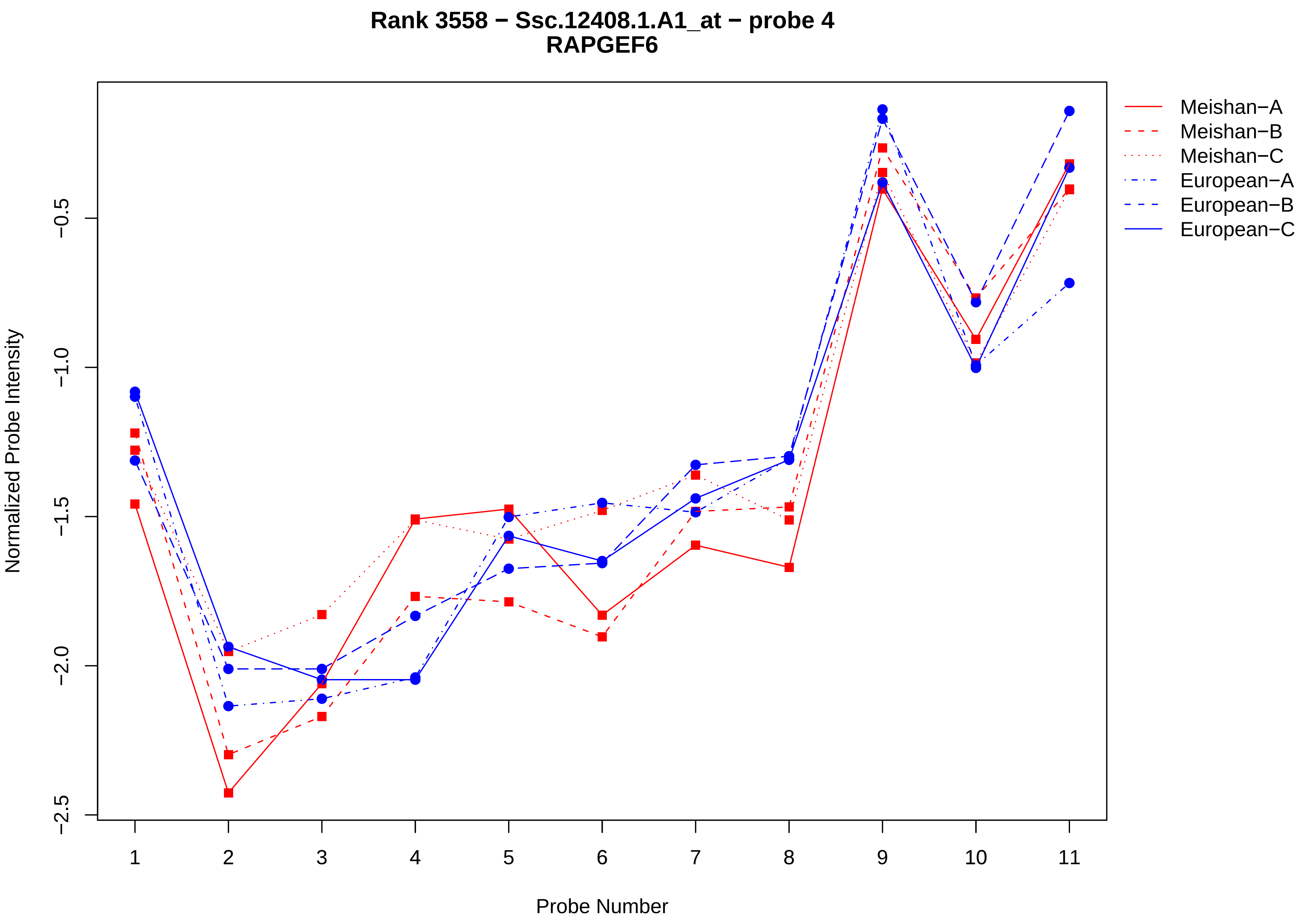

Rank 3558 − Ssc.12408.1.A1_at − probe 4
RAPGEF6
Normalized Probe Intensity
Probe Number
−0.5
−1.0
−1.5
−2.0
−2.5
1
2
3
4
5
6
7
8
9
10
11
Meishan−A
Meishan−B
Meishan−C
European−A
European−B
European−C

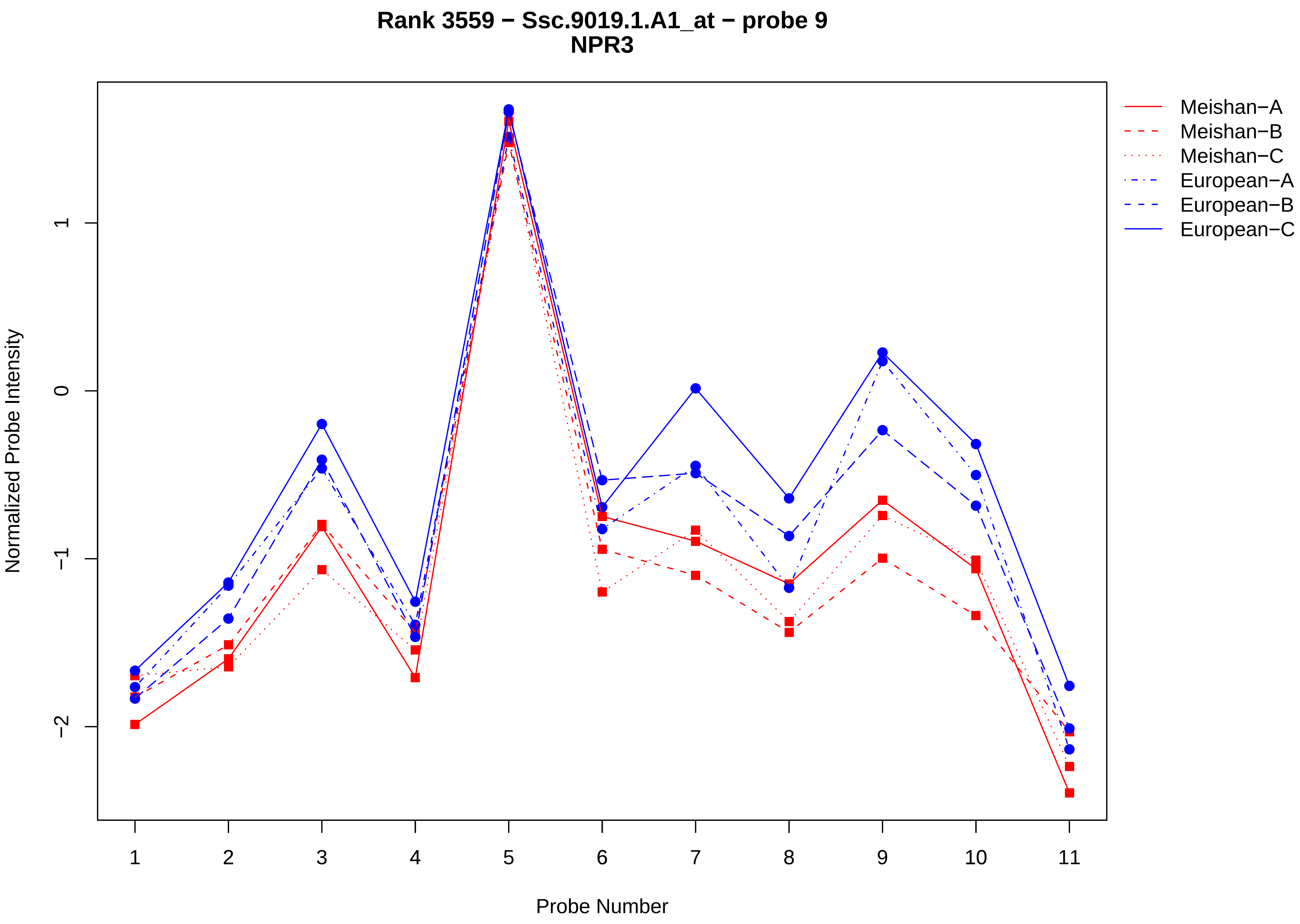

Rank 3559 − Ssc.9019.1.A1_at − probe 9
NPR3
Normalized Probe Intensity
Probe Number
1
0
−1
−2
1
2
3
4
5
6
7
8
9
10
11
Meishan−A
Meishan−B
Meishan−C
European−A
European−B
European−C

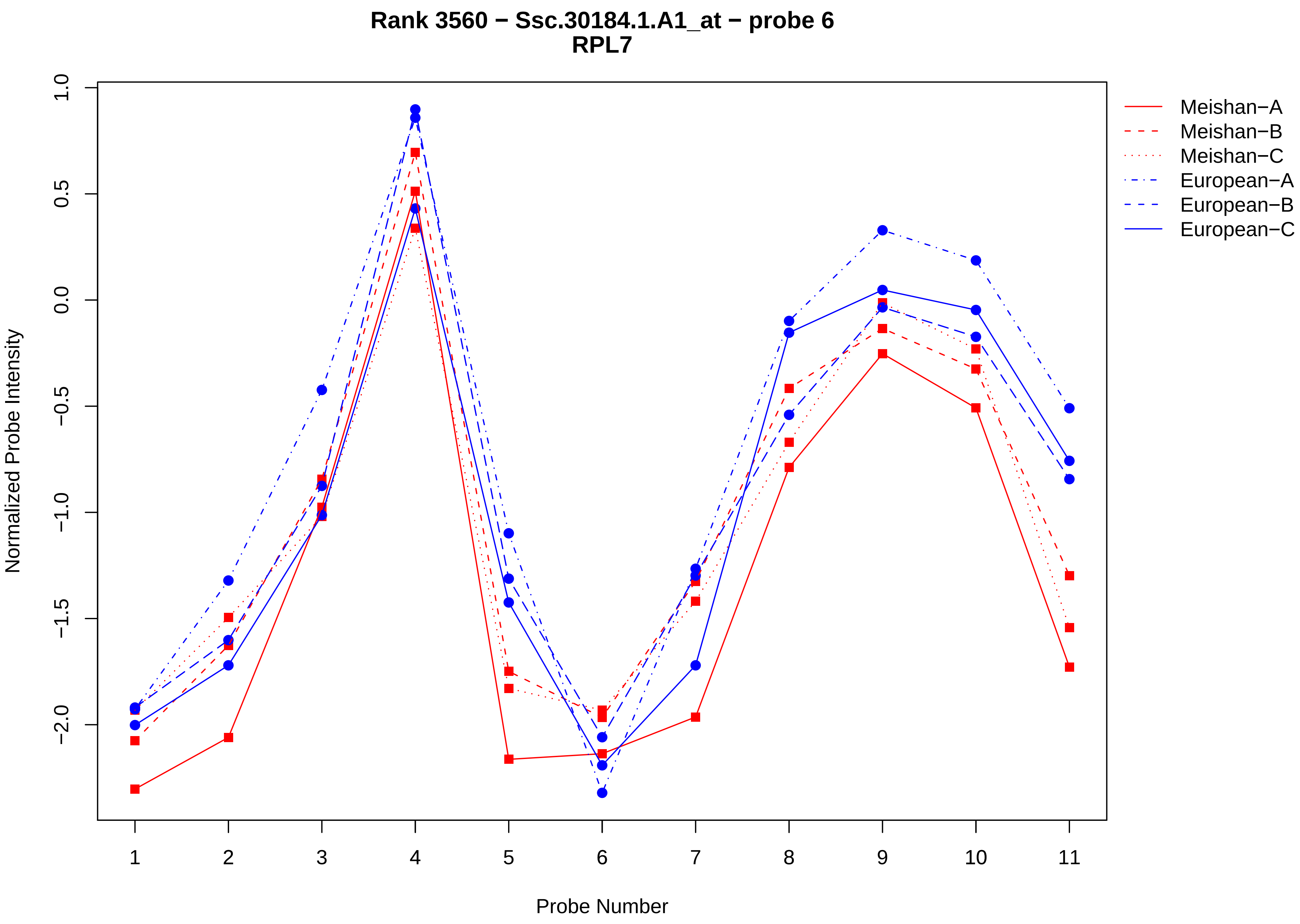

Rank 3560 − Ssc.30184.1.A1_at − probe 6
RPL7
Normalized Probe Intensity
Probe Number
1.0
0.5
0.0
−0.5
−1.0
−1.5
−2.0
1
2
3
4
5
6
7
8
9
10
11
Meishan−A
Meishan−B
Meishan−C
European−A
European−B
European−C

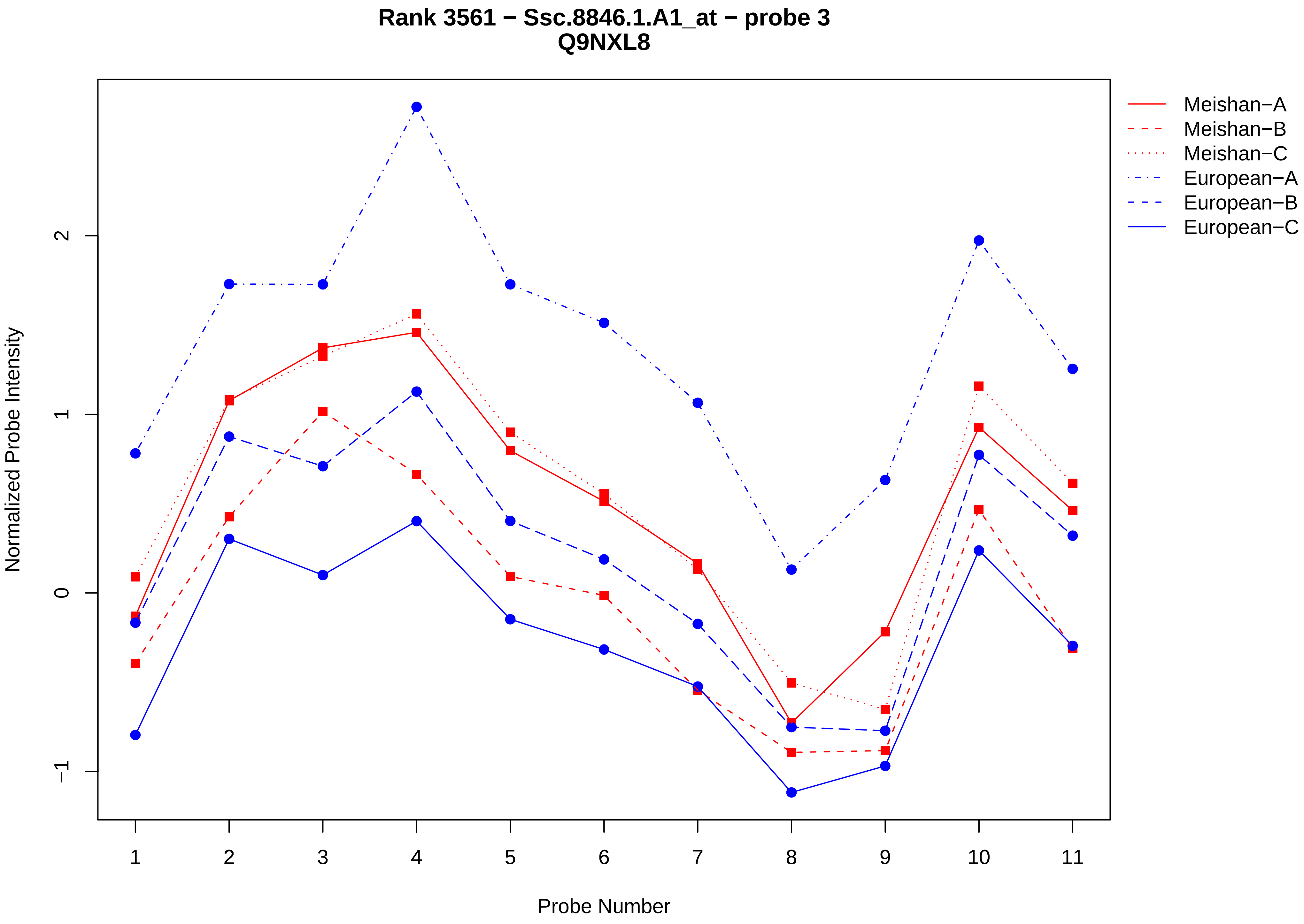

Rank 3561 − Ssc.8846.1.A1_at − probe 3
Q9NXL8
Normalized Probe Intensity
Probe Number
−1
0
1
2
1
2
3
4
5
6
7
8
9
10
11
Meishan−A
Meishan−B
Meishan−C
European−A
European−B
European−C

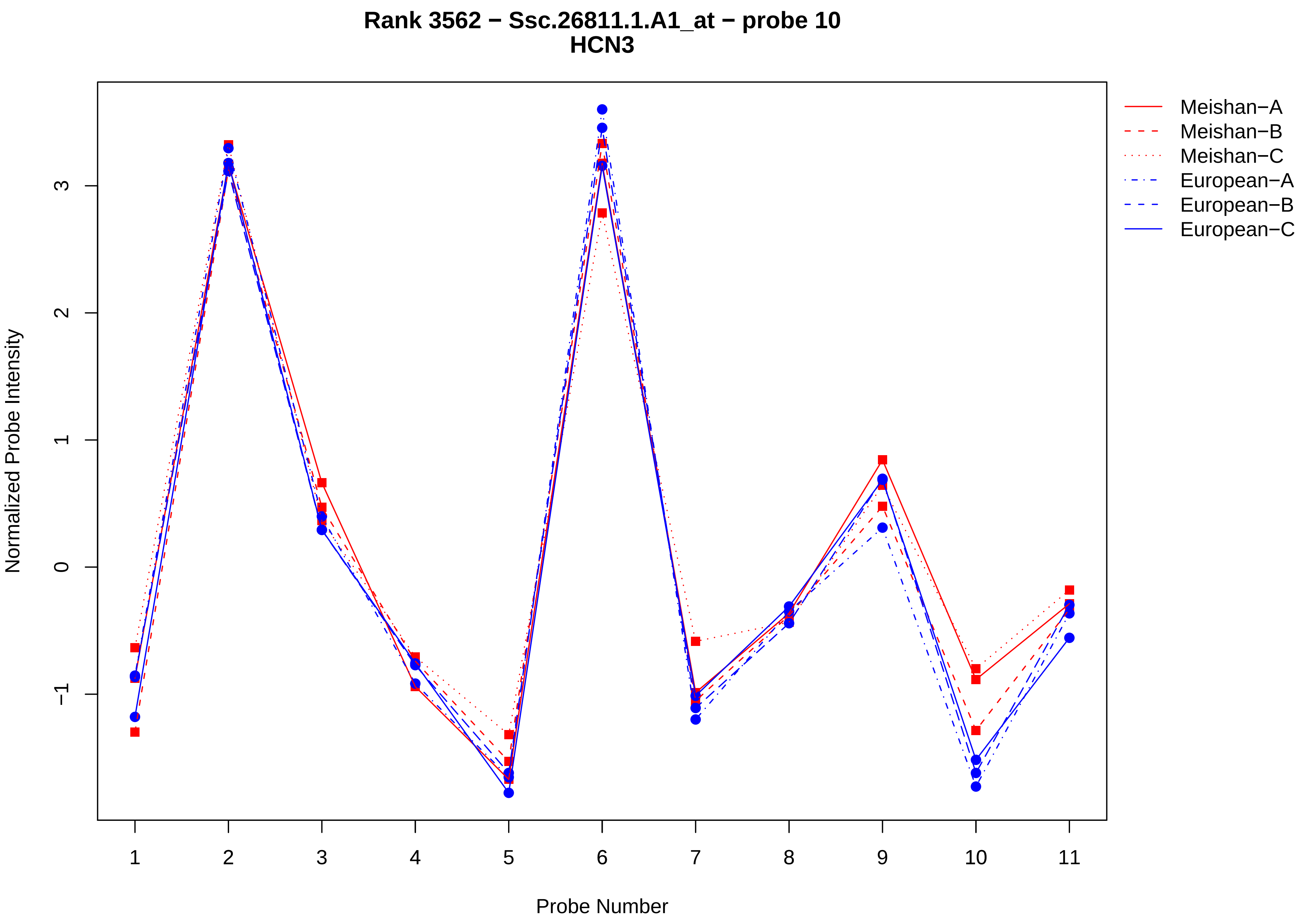

Rank 3562 − Ssc.26811.1.A1_at − probe 10
HCN3
Normalized Probe Intensity
Probe Number
Meishan−A
Meishan−B
Meishan−C
European−A
European−B
European−C
−1
0
1
2
3
1
2
3
4
5
6
7
8
9
10
11

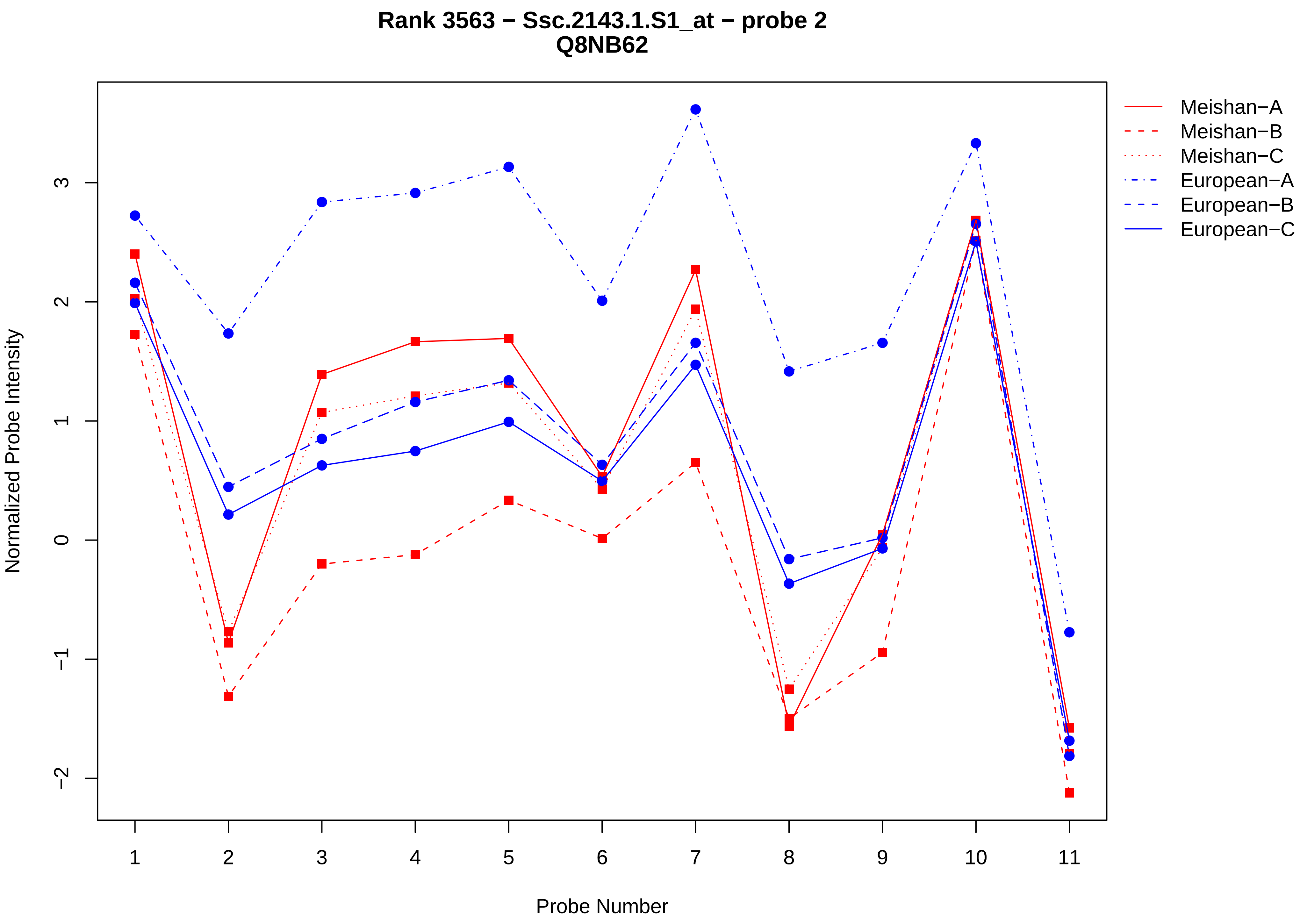
Rank 3563 − Ssc.2143.1.S1_at − probe 2
Q8NB62
Normalized Probe Intensity
Probe Number
Meishan−A
Meishan−B
Meishan−C
European−A
European−B
European−C

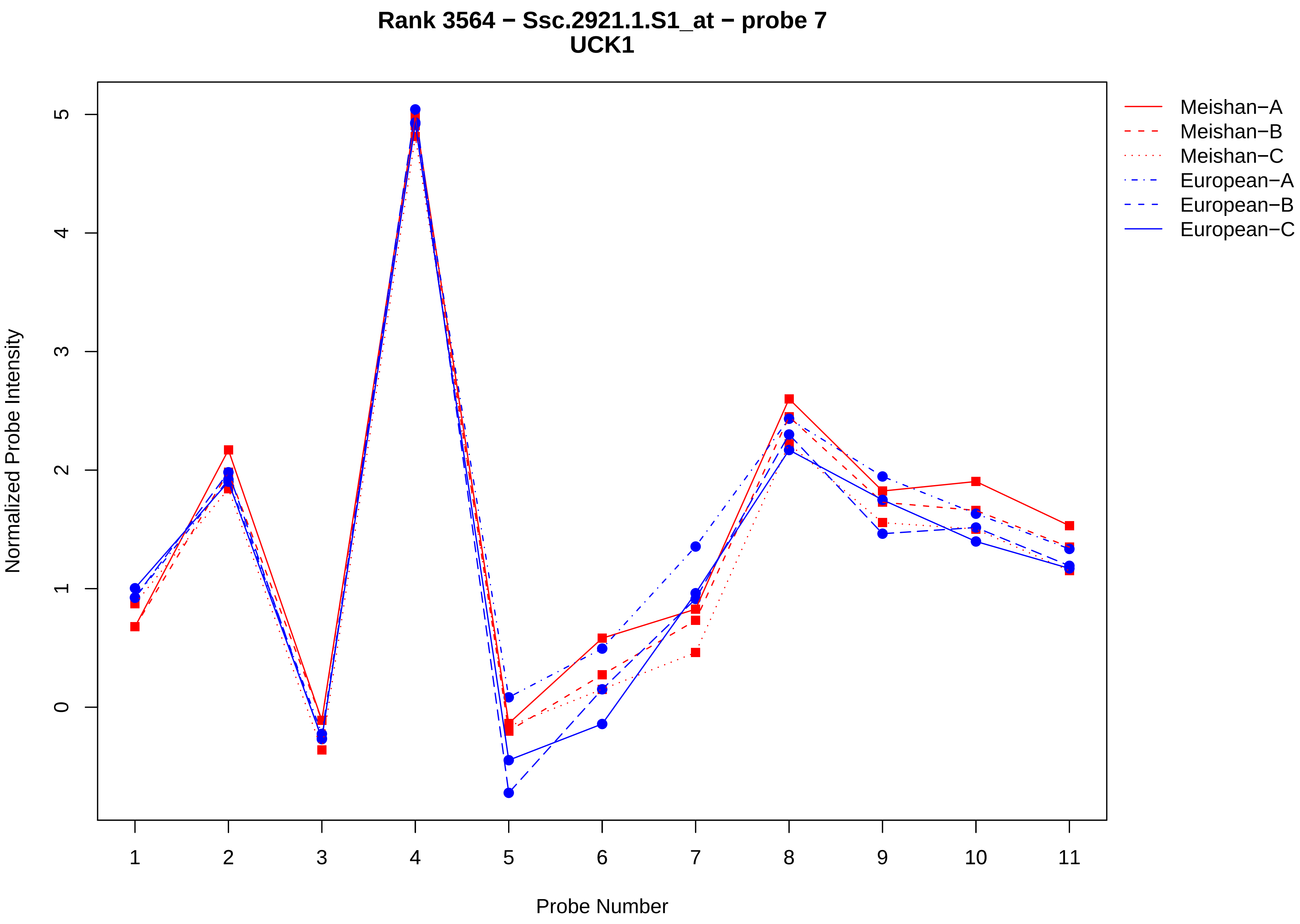
Rank 3564 − Ssc.2921.1.S1_at − probe 7
UCK1
Normalized Probe Intensity
Probe Number
0
1
2
3
4
5
1
2
3
4
5
6
7
8
9
10
11
Meishan−A
Meishan−B
Meishan−C
European−A
European−B
European−C

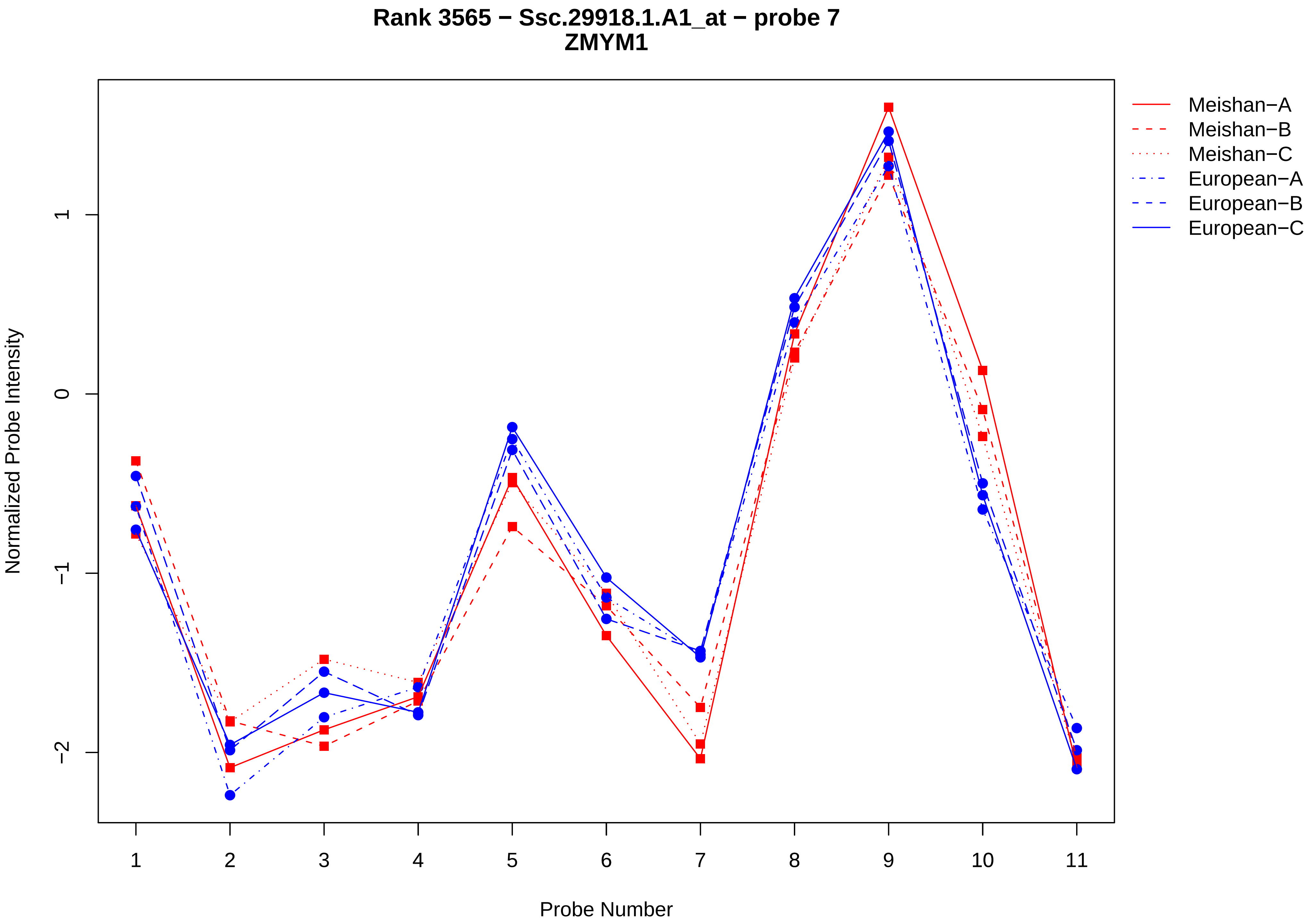

Rank 3565 − Ssc.29918.1.A1_at − probe 7
ZMYM1
Normalized Probe Intensity
Probe Number
1
0
−1
−2
1
2
3
4
5
6
7
8
9
10
11
Meishan−A
Meishan−B
Meishan−C
European−A
European−B
European−C

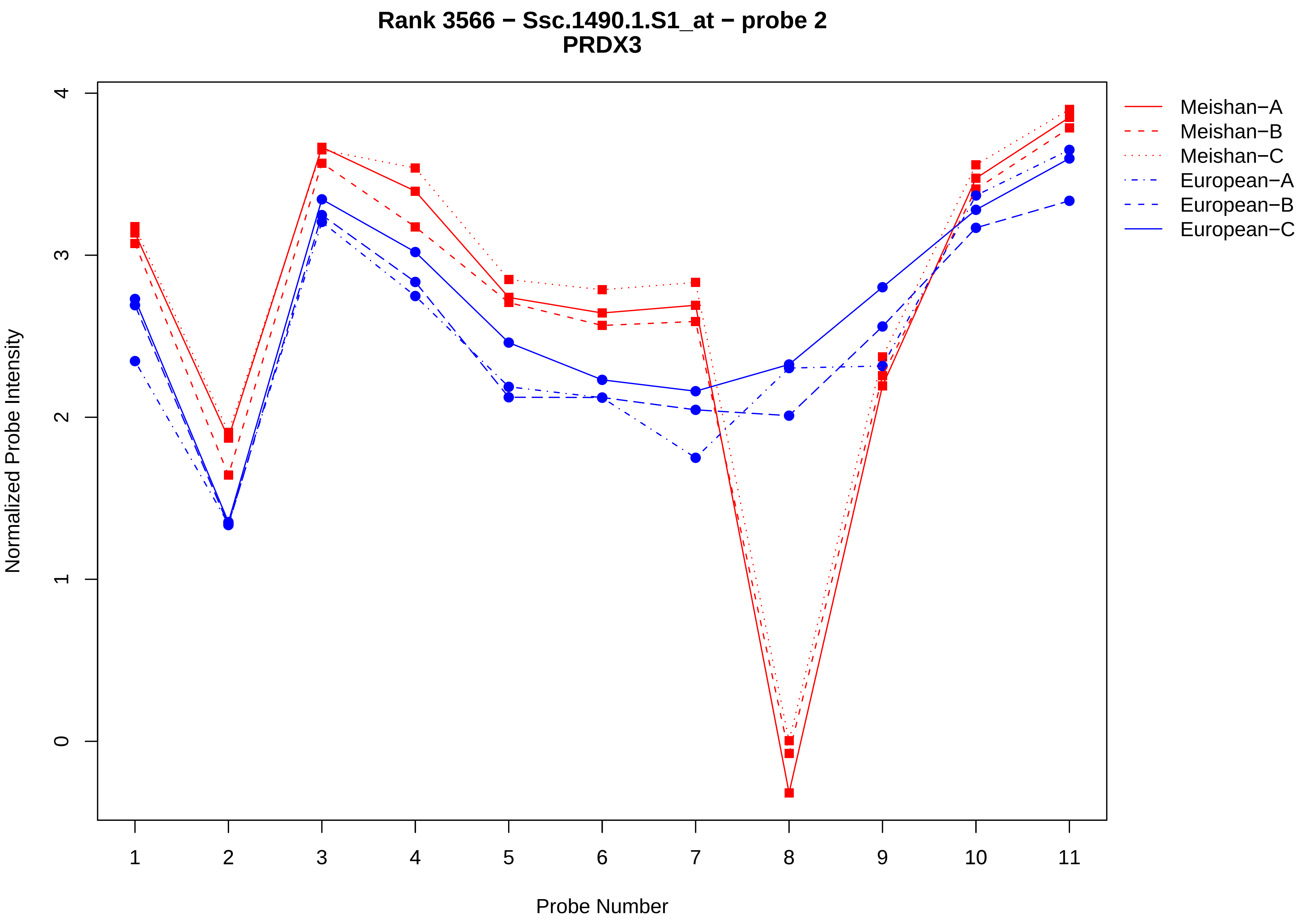
Rank 3566 − Ssc.1490.1.S1_at − probe 2
PRDX3
Normalized Probe Intensity
Probe Number
0
1
2
3
4
1
2
3
4
5
6
7
8
9
10
11
Meishan−A
Meishan−B
Meishan−C
European−A
European−B
European−C

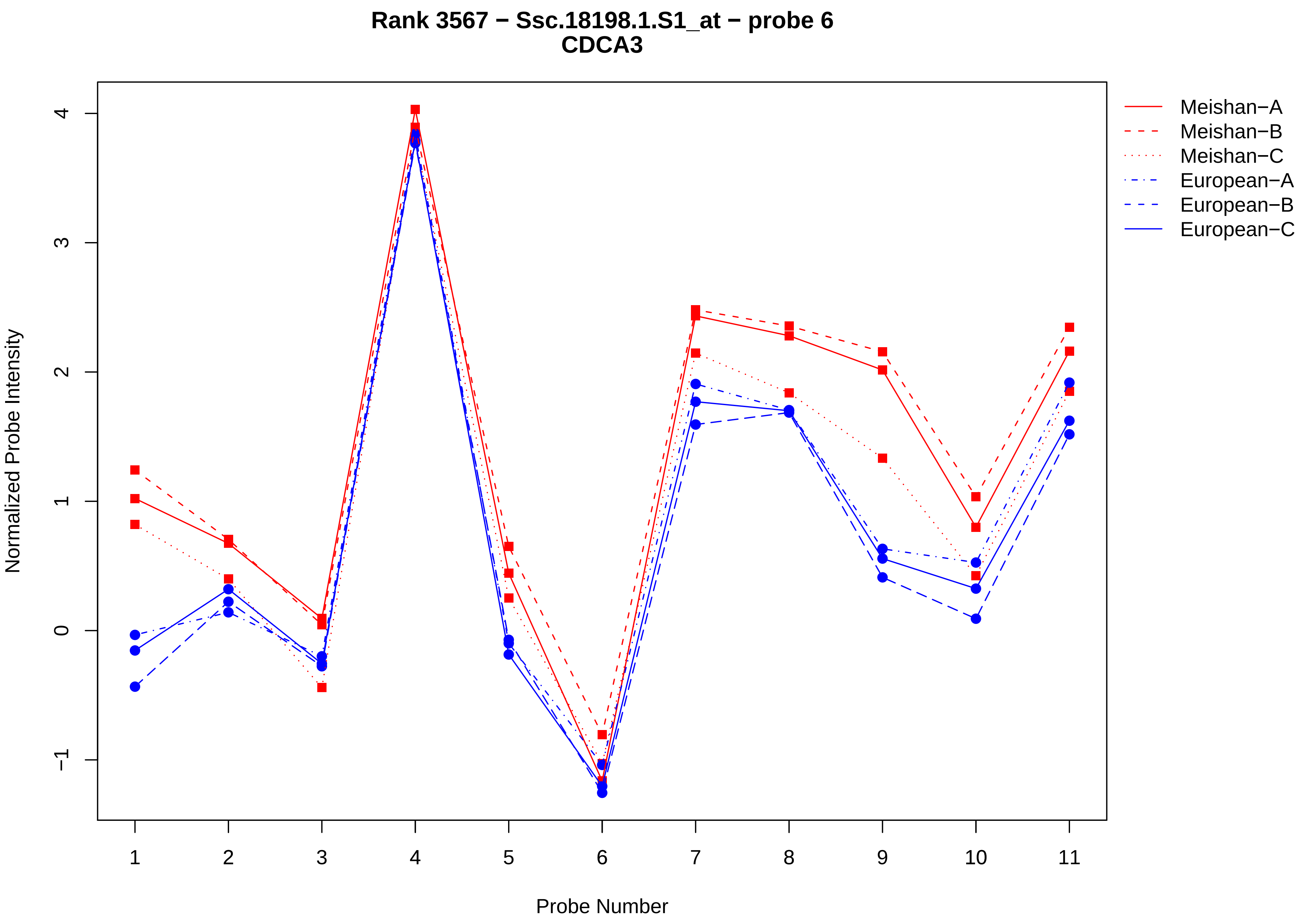

Rank 3567 − Ssc.18198.1.S1_at − probe 6
CDCA3
Normalized Probe Intensity
Probe Number
Meishan−A
Meishan−B
Meishan−C
European−A
European−B
European−C
−1
0
1
2
3
4
1
2
3
4
5
6
7
8
9
10
11

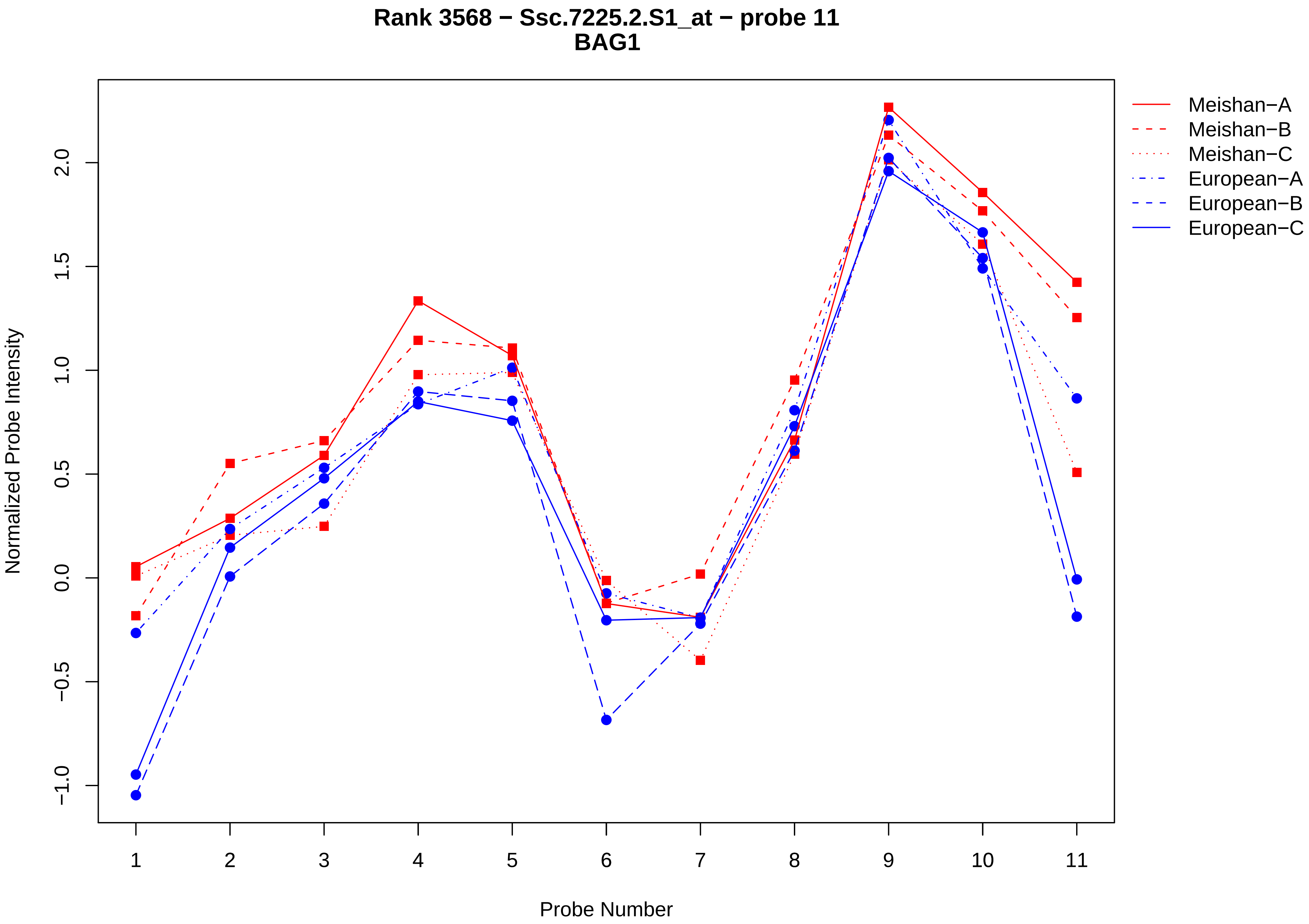

Rank 3568 − Ssc.7225.2.S1_at − probe 11
BAG1
Normalized Probe Intensity
Probe Number
Meishan−A
Meishan−B
Meishan−C
European−A
European−B
European−C
−1.0
−0.5
0.0
0.5
1.0
1.5
2.0
1
2
3
4
5
6
7
8
9
10
11

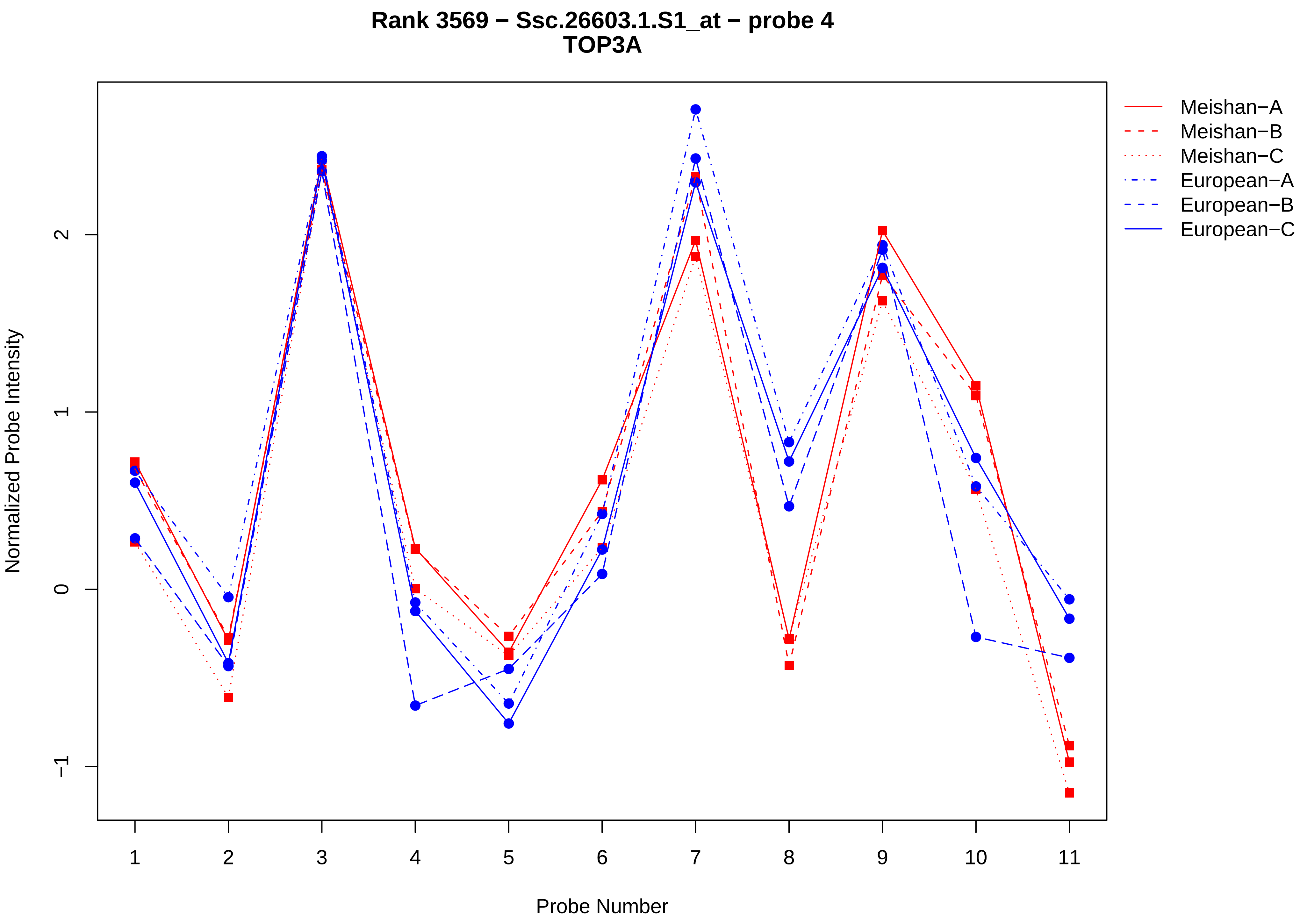

Rank 3569 − Ssc.26603.1.S1_at − probe 4
TOP3A
Normalized Probe Intensity
Probe Number
−1
0
1
2
1
2
3
4
5
6
7
8
9
10
11
Meishan−A
Meishan−B
Meishan−C
European−A
European−B
European−C

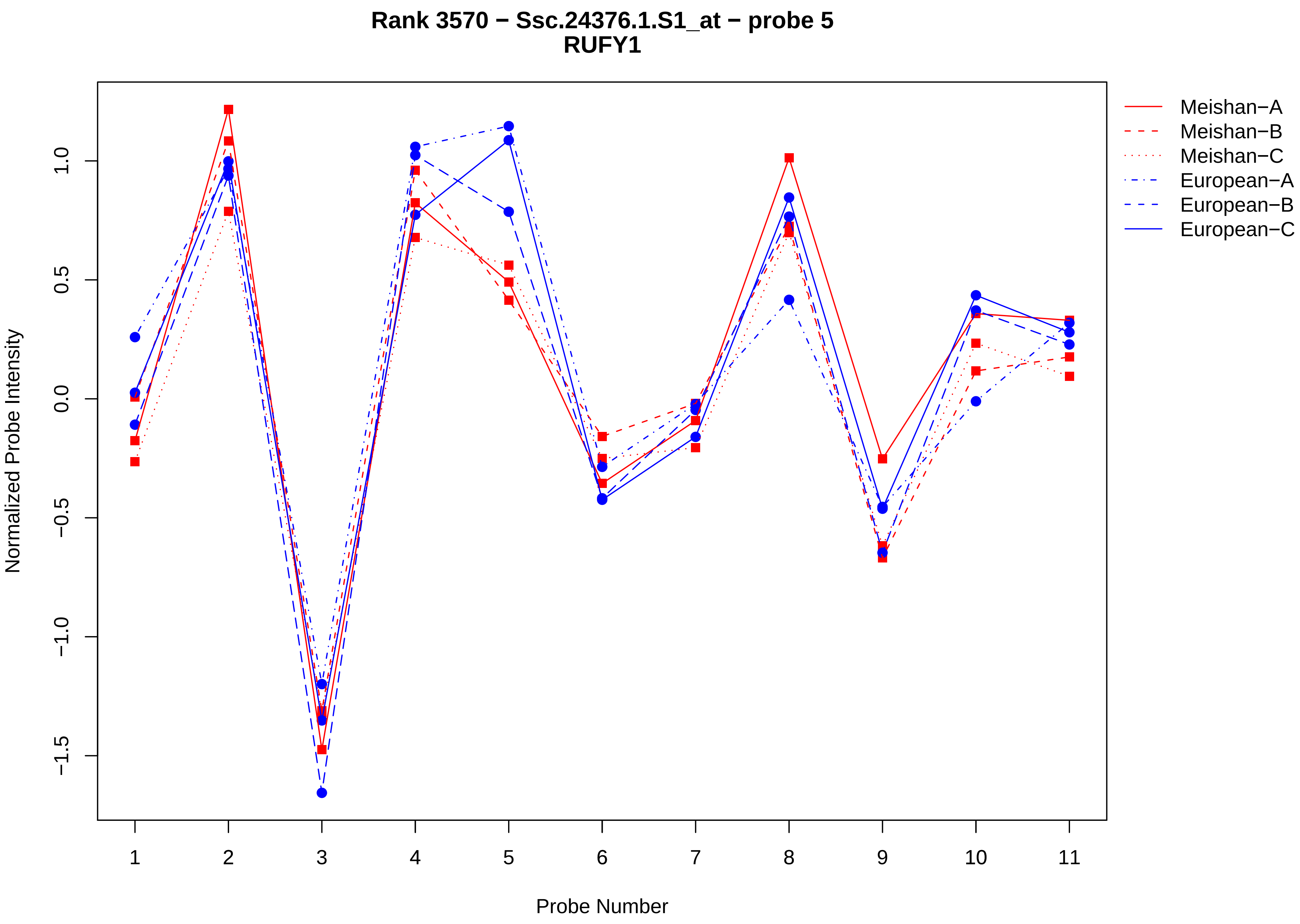

Rank 3570 − Ssc.24376.1.S1_at − probe 5
RUFY1
Normalized Probe Intensity
Probe Number
Meishan−A
Meishan−B
Meishan−C
European−A
European−B
European−C
1.0
0.5
0.0
−0.5
−1.0
−1.5
1
2
3
4
5
6
7
8
9
10
11

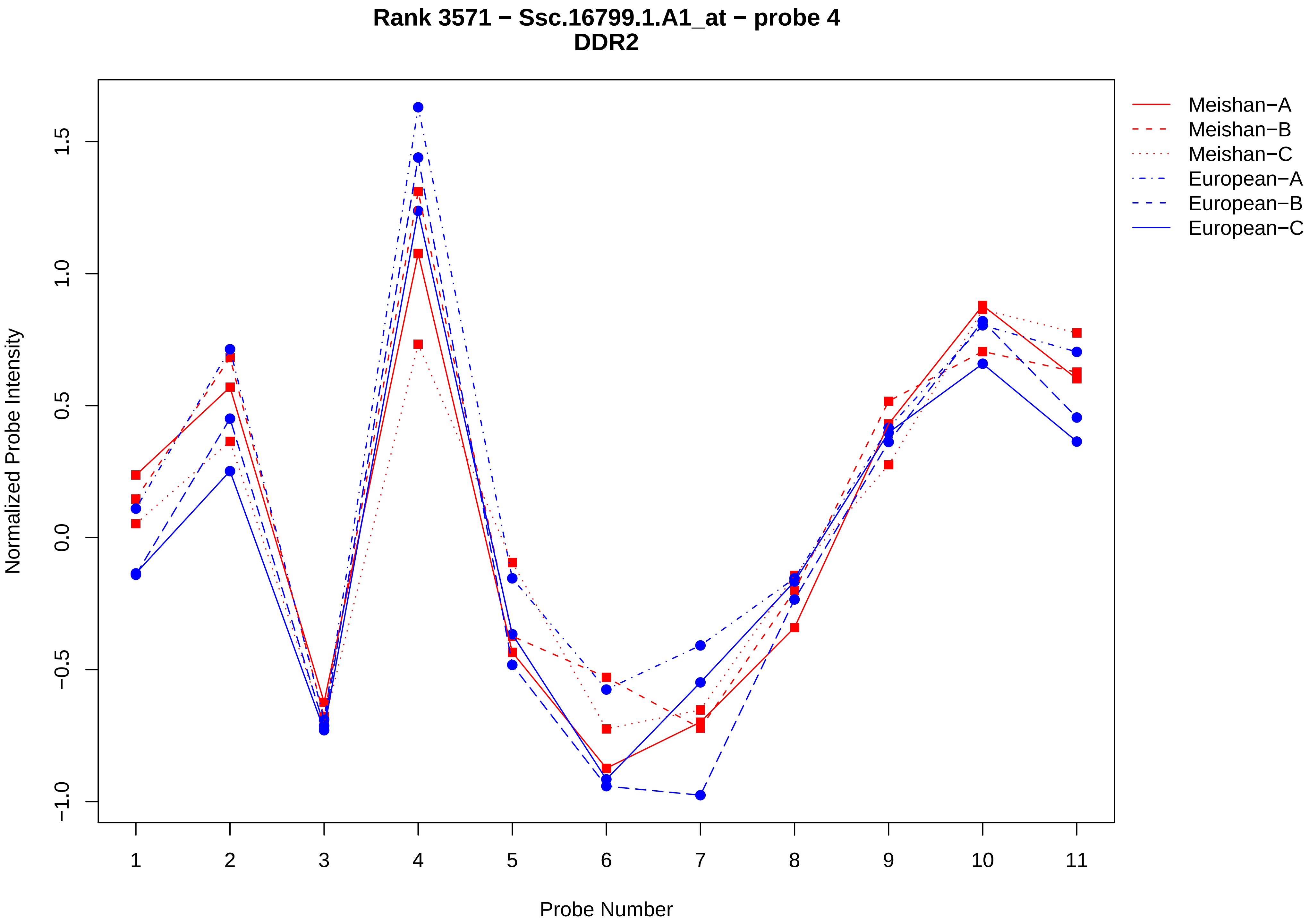

Rank 3571 − Ssc.16799.1.A1_at − probe 4
DDR2
Normalized Probe Intensity
Probe Number
−1.0
−0.5
0.0
0.5
1.0
1.5
1
2
3
4
5
6
7
8
9
10
11
Meishan−A
Meishan−B
Meishan−C
European−A
European−B
European−C

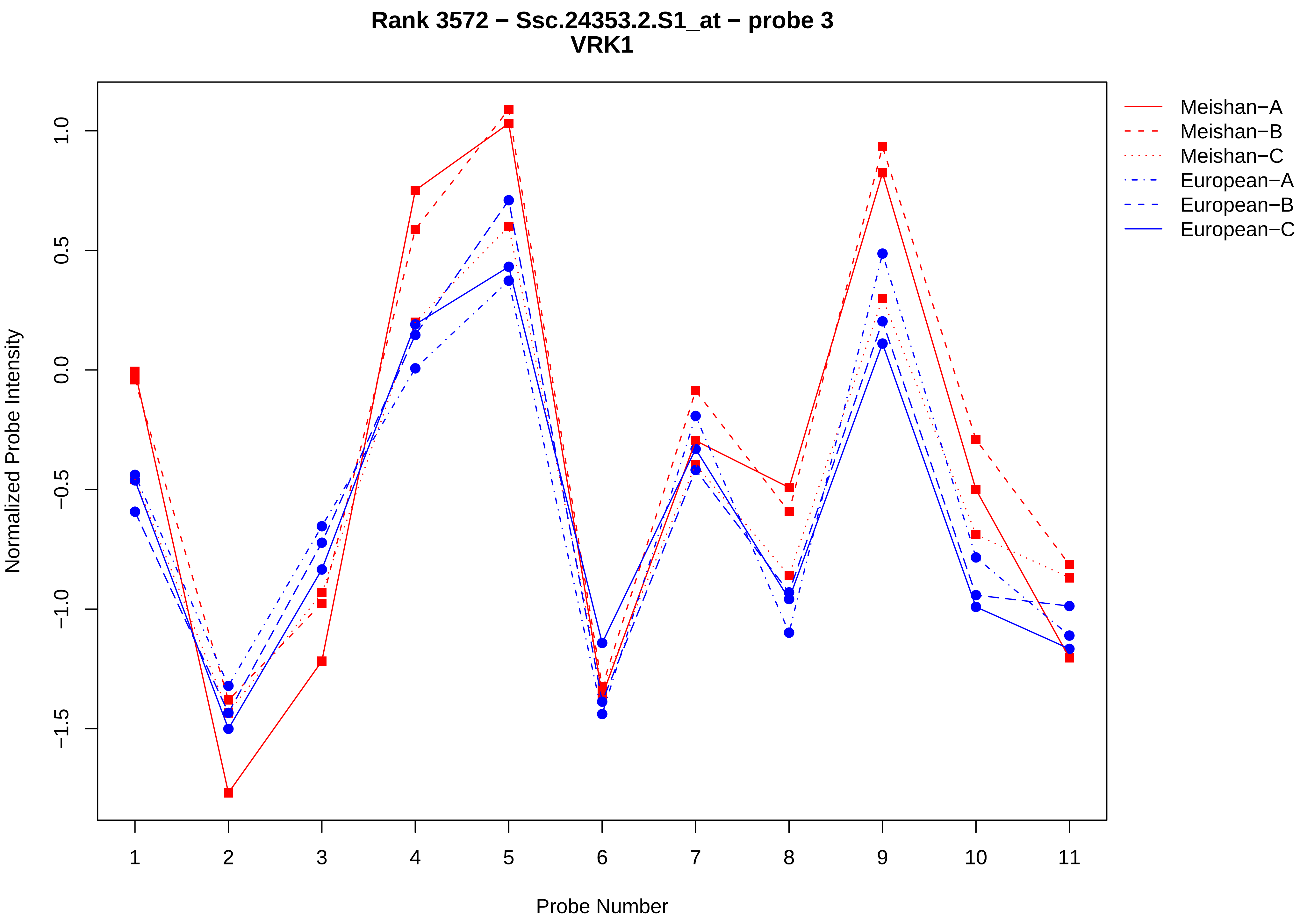

Rank 3572 − Ssc.24353.2.S1_at − probe 3
VRK1
Normalized Probe Intensity
Probe Number
1.0
0.5
0.0
−0.5
−1.0
−1.5
1
2
3
4
5
6
7
8
9
10
11
Meishan−A
Meishan−B
Meishan−C
European−A
European−B
European−C

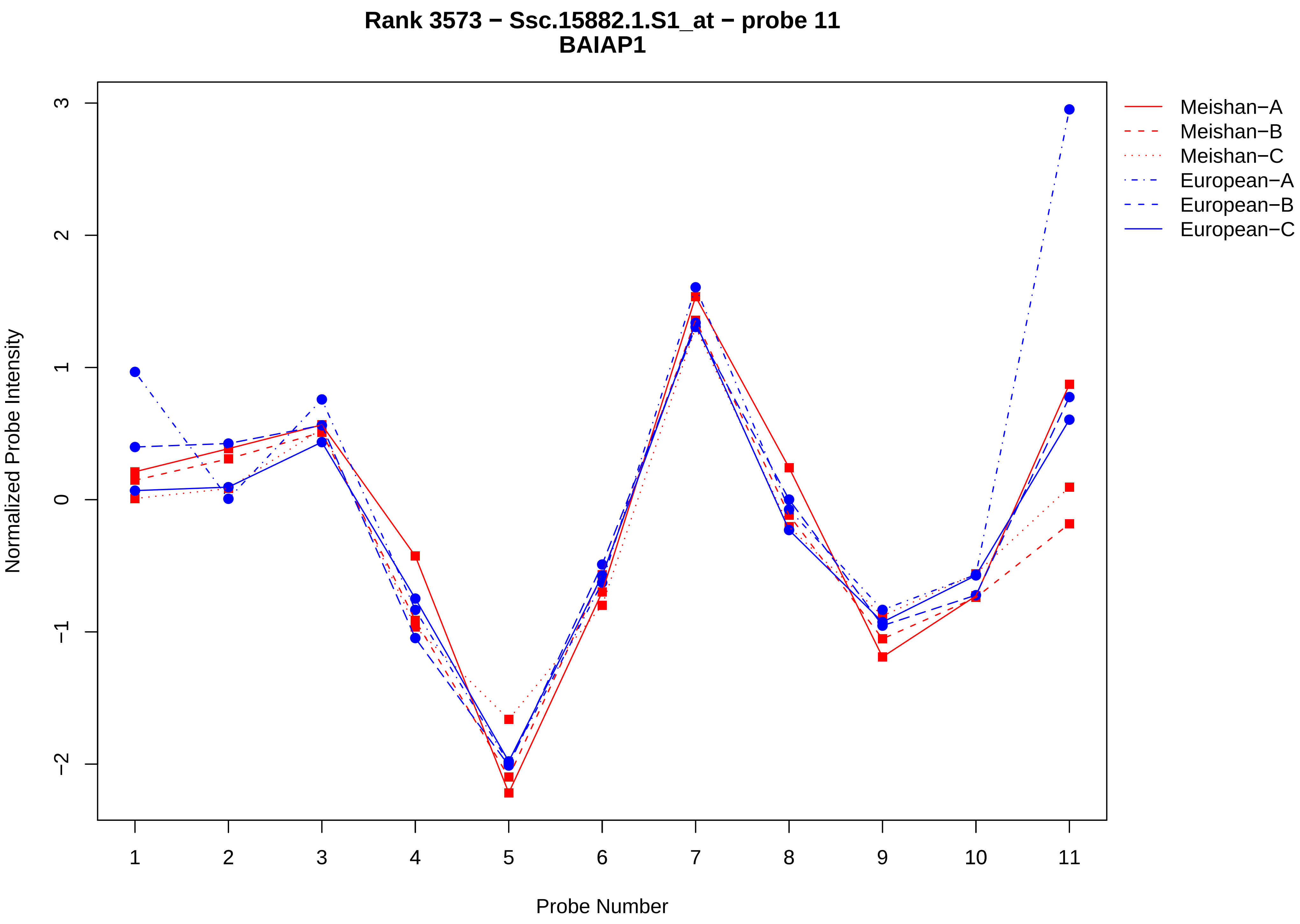

Rank 3573 − Ssc.15882.1.S1_at − probe 11
BAIAP1
Normalized Probe Intensity
Probe Number
Meishan−A
Meishan−B
Meishan−C
European−A
European−B
European−C

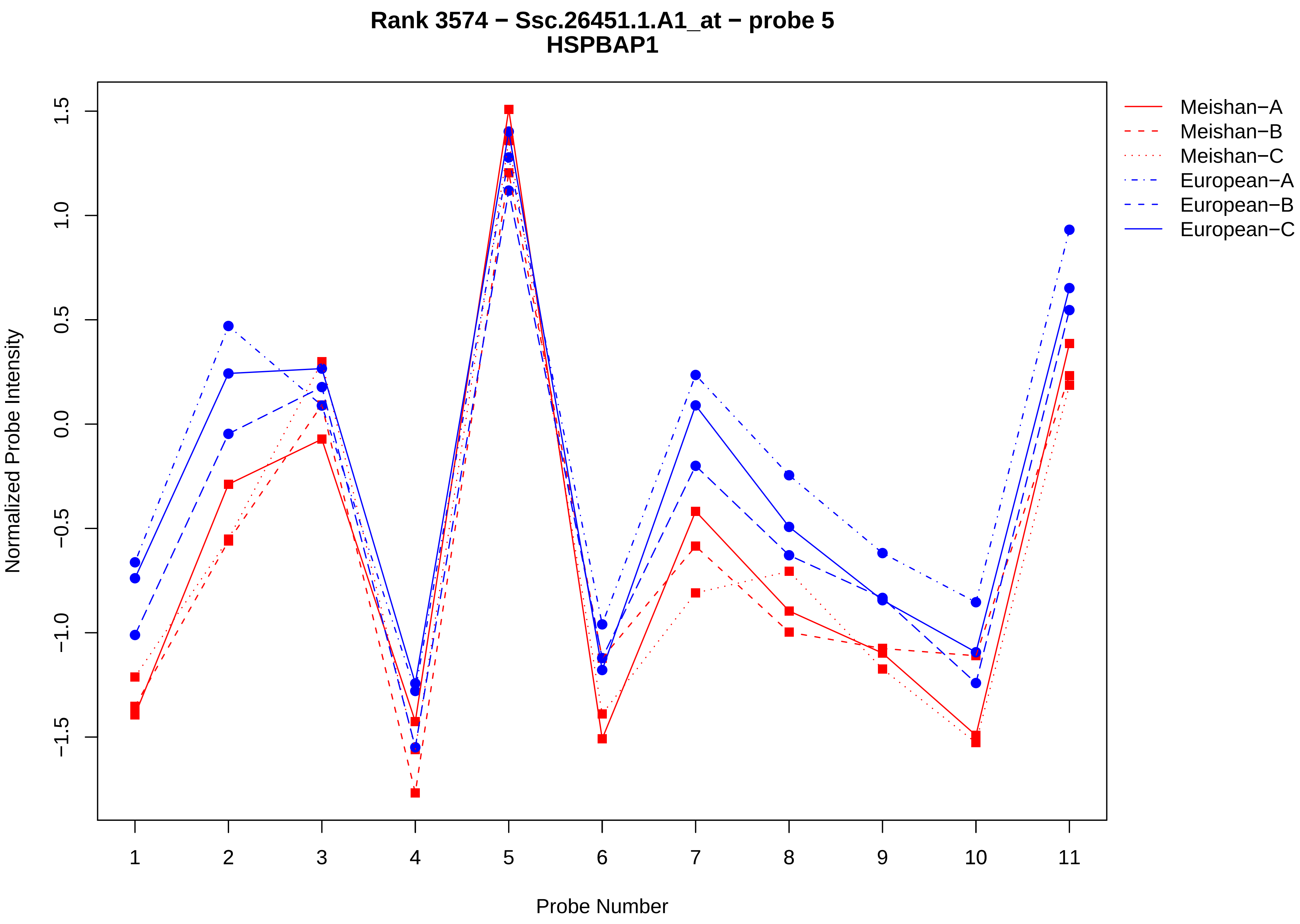

Rank 3574 − Ssc.26451.1.A1_at − probe 5
HSPBAP1
Normalized Probe Intensity
Probe Number
−1.5
−1.0
−0.5
0.0
0.5
1.0
1.5
1
2
3
4
5
6
7
8
9
10
11
Meishan−A
Meishan−B
Meishan−C
European−A
European−B
European−C

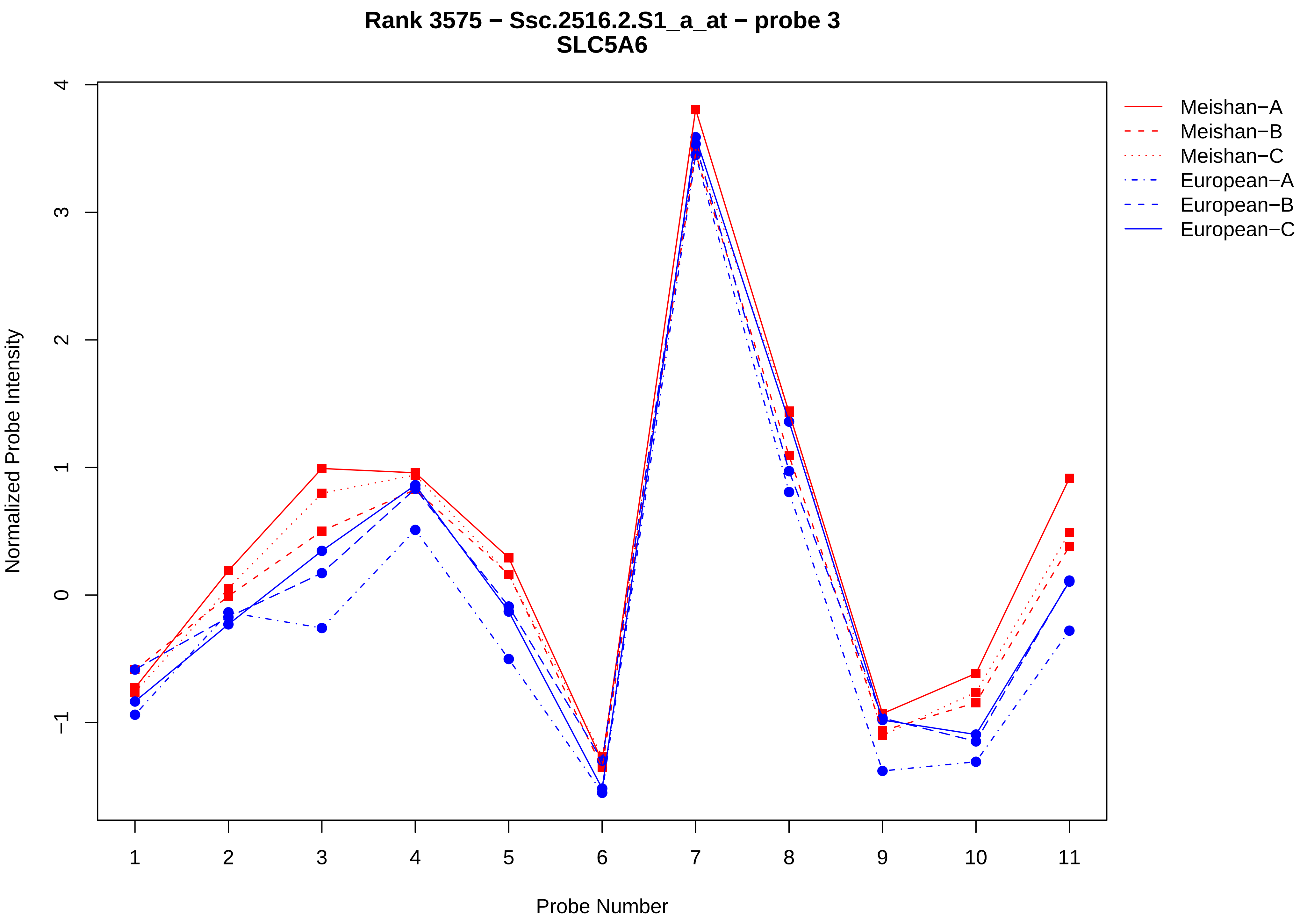

Rank 3575 − Ssc.2516.2.S1_a_at − probe 3
SLC5A6
Normalized Probe Intensity
Probe Number
−1
0
1
2
3
4
1
2
3
4
5
6
7
8
9
10
11
Meishan−A
Meishan−B
Meishan−C
European−A
European−B
European−C

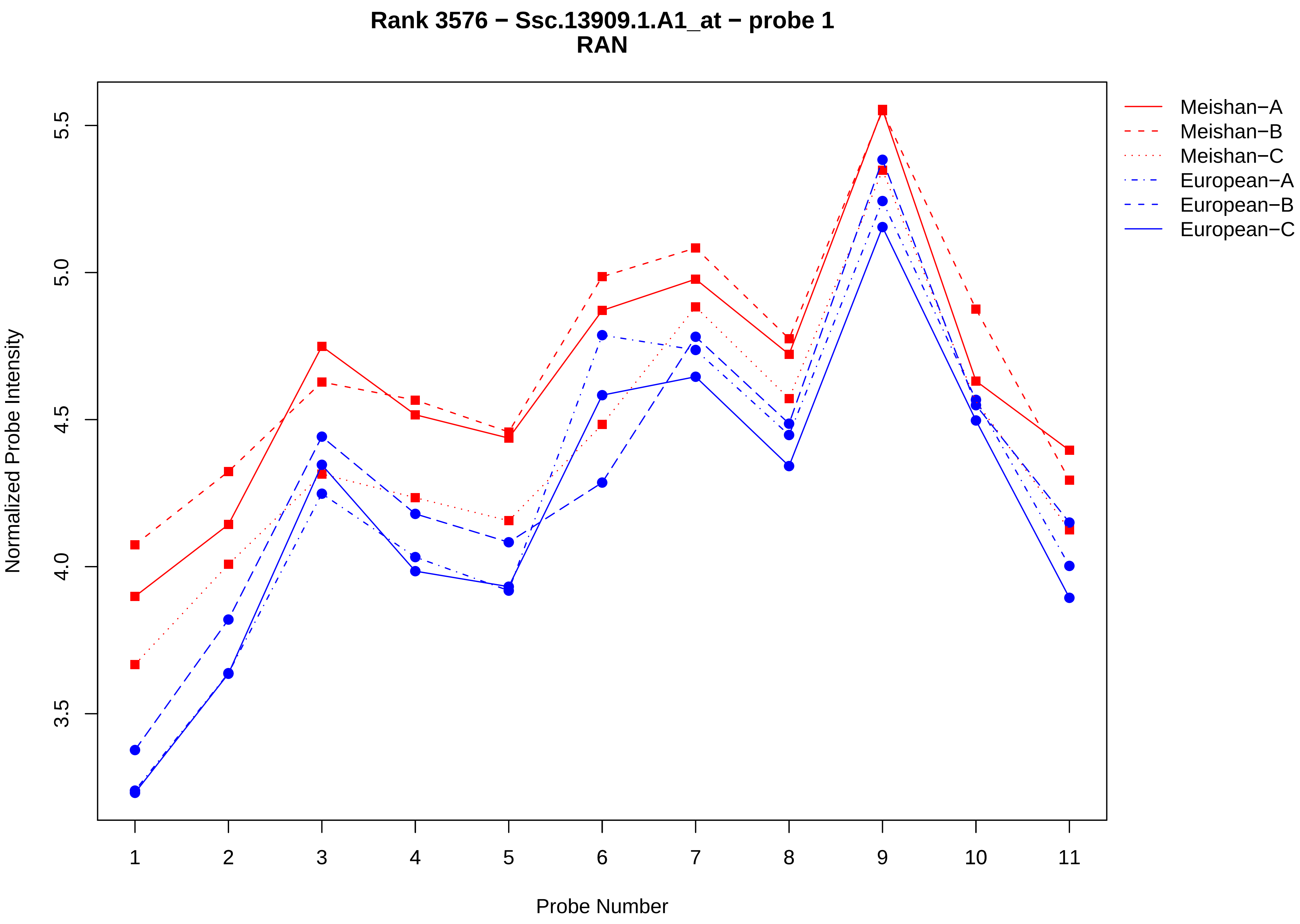
Rank 3576 − Ssc.13909.1.A1_at − probe 1
RAN
Normalized Probe Intensity
Probe Number
5.5
5.0
4.5
4.0
3.5
1
2
3
4
5
6
7
8
9
10
11
Meishan−A
Meishan−B
Meishan−C
European−A
European−B
European−C

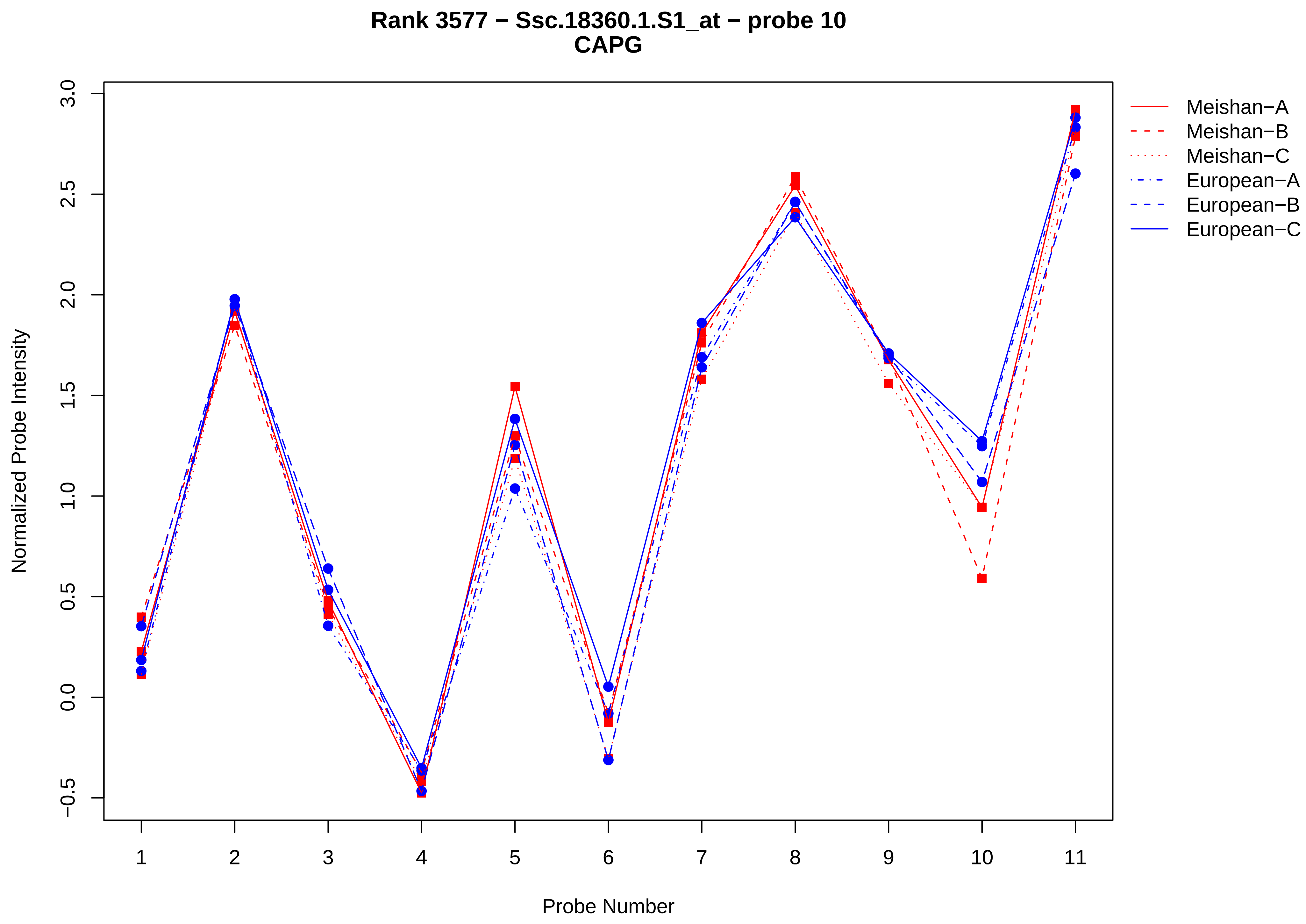

Rank 3577 − Ssc.18360.1.S1_at − probe 10
CAPG
Normalized Probe Intensity
Probe Number
Meishan−A
Meishan−B
Meishan−C
European−A
European−B
European−C

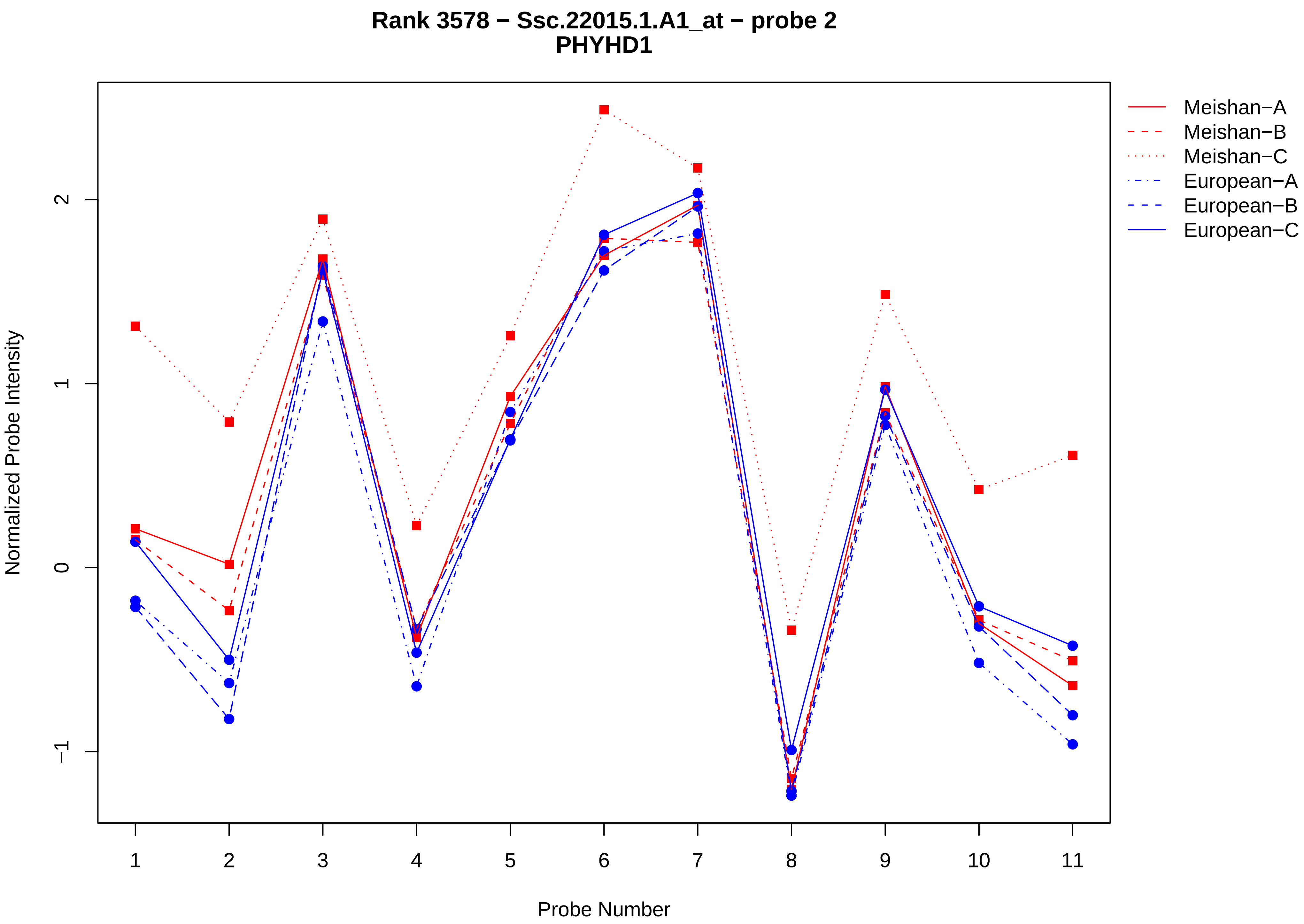

Rank 3578 − Ssc.22015.1.A1_at − probe 2
PHYHD1
Normalized Probe Intensity
Probe Number
Meishan−A
Meishan−B
Meishan−C
European−A
European−B
European−C

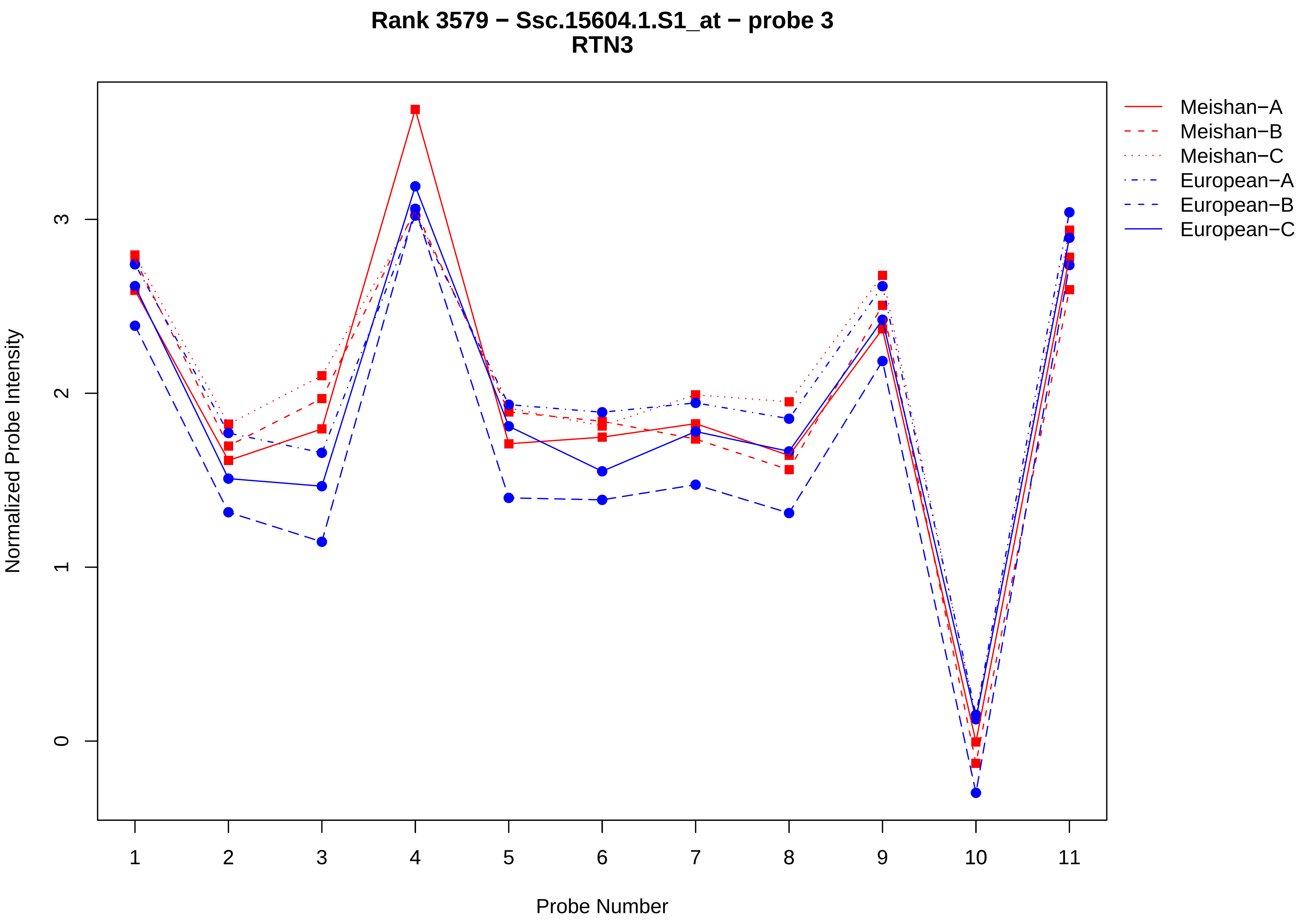

Rank 3579 − Ssc.15604.1.S1_at − probe 3
RTN3
Normalized Probe Intensity
Probe Number
0
1
2
3
1 2 3 4 5 6 7 8 9 10 11
Meishan−A
Meishan−B
Meishan−C
European−A
European−B
European−C

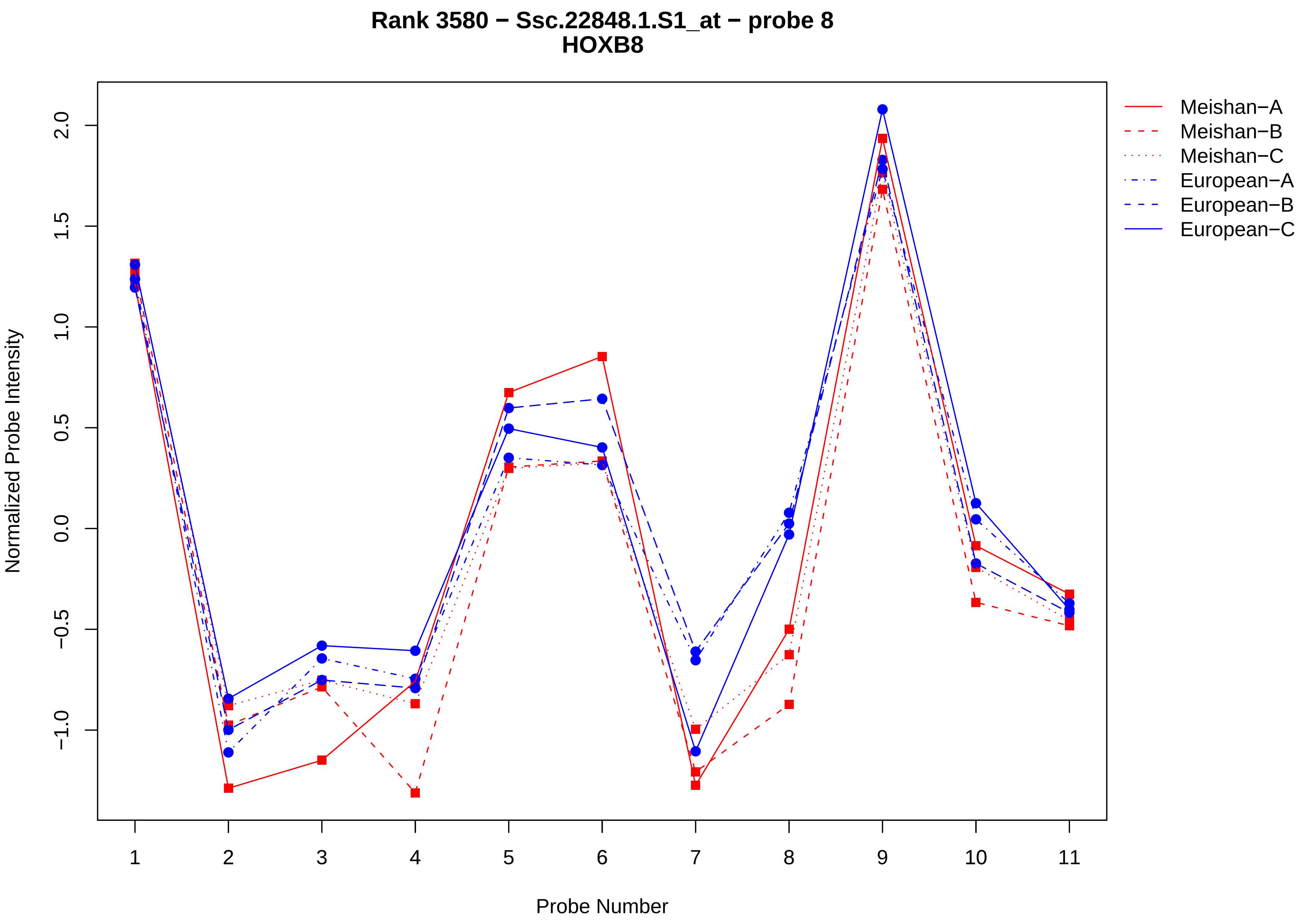

Rank 3580 − Ssc.22848.1.S1_at − probe 8
HOXB8
Normalized Probe Intensity
Probe Number
−1.0
−0.5
0.0
0.5
1.0
1.5
2.0
1
2
3
4
5
6
7
8
9
10
11
Meishan−A
Meishan−B
Meishan−C
European−A
European−B
European−C

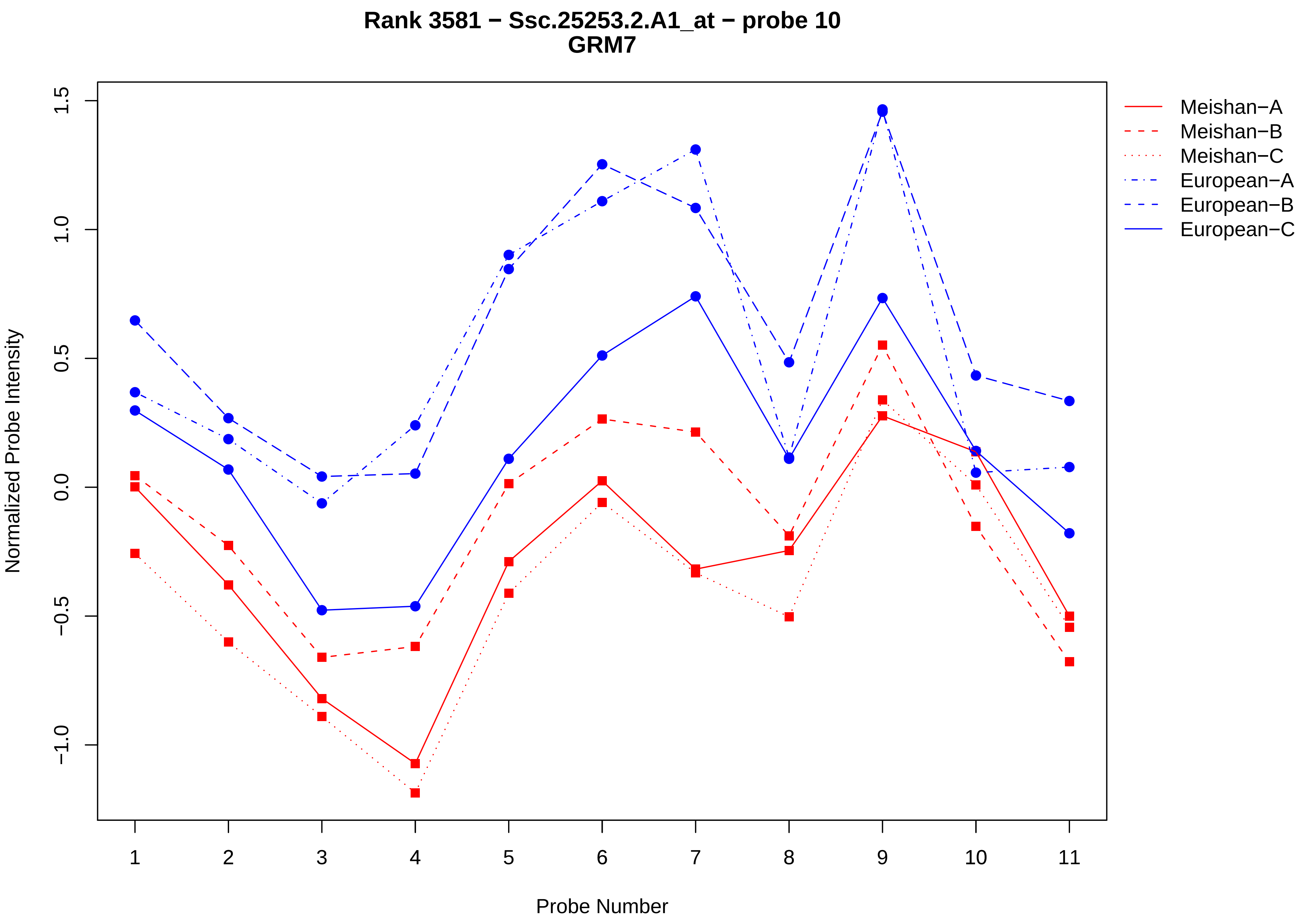
Rank 3581 − Ssc.25253.2.A1_at − probe 10
GRM7
Normalized Probe Intensity
Probe Number
1.5
1.0
0.5
0.0
−0.5
−1.0
1
2
3
4
5
6
7
8
9
10
11
Meishan−A
Meishan−B
Meishan−C
European−A
European−B
European−C

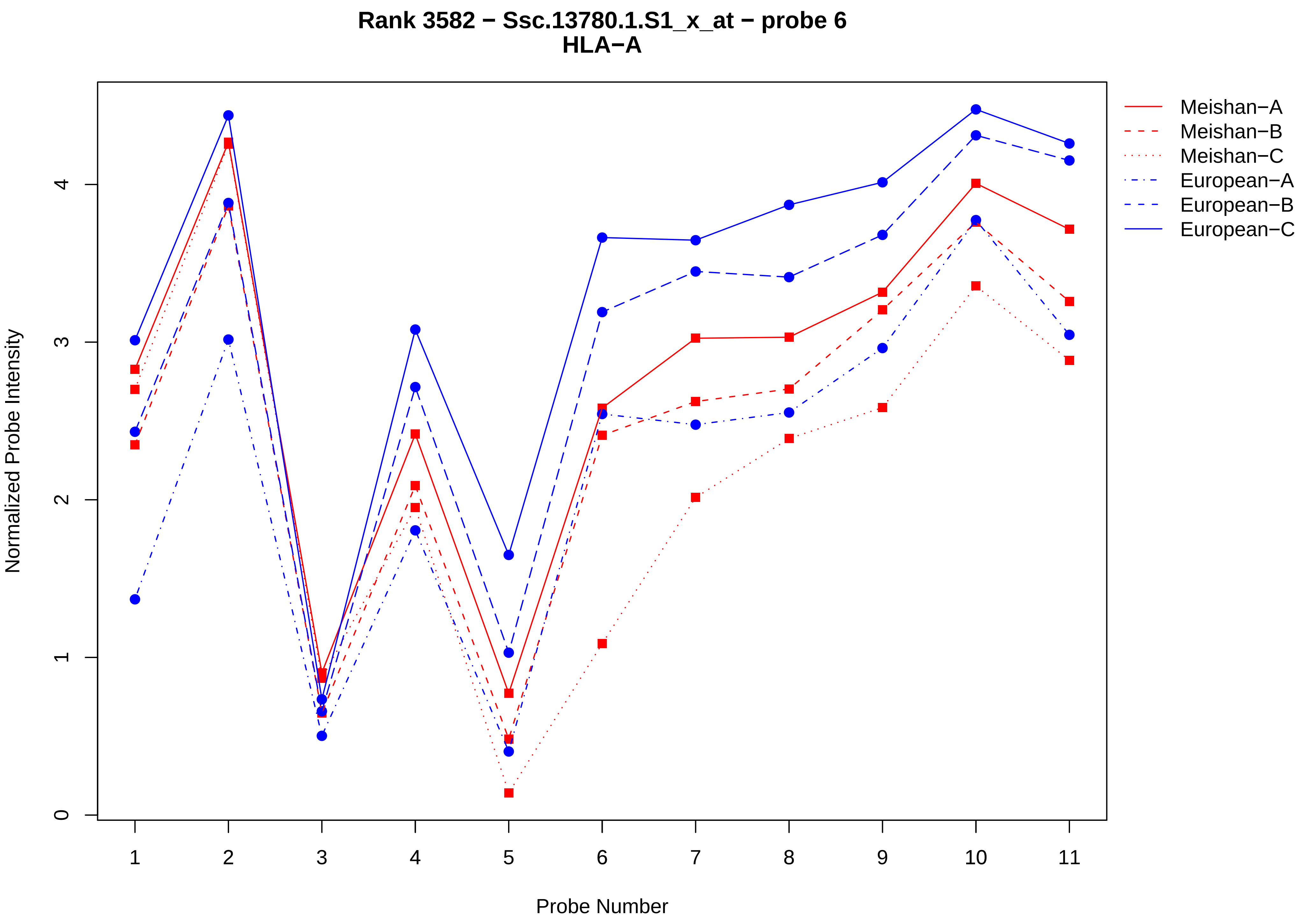

Rank 3582 − Ssc.13780.1.S1_x_at − probe 6
HLA−A
Normalized Probe Intensity
0
1
2
3
4
Probe Number
1
2
3
4
5
6
7
8
9
10
11
Meishan−A
Meishan−B
Meishan−C
European−A
European−B
European−C

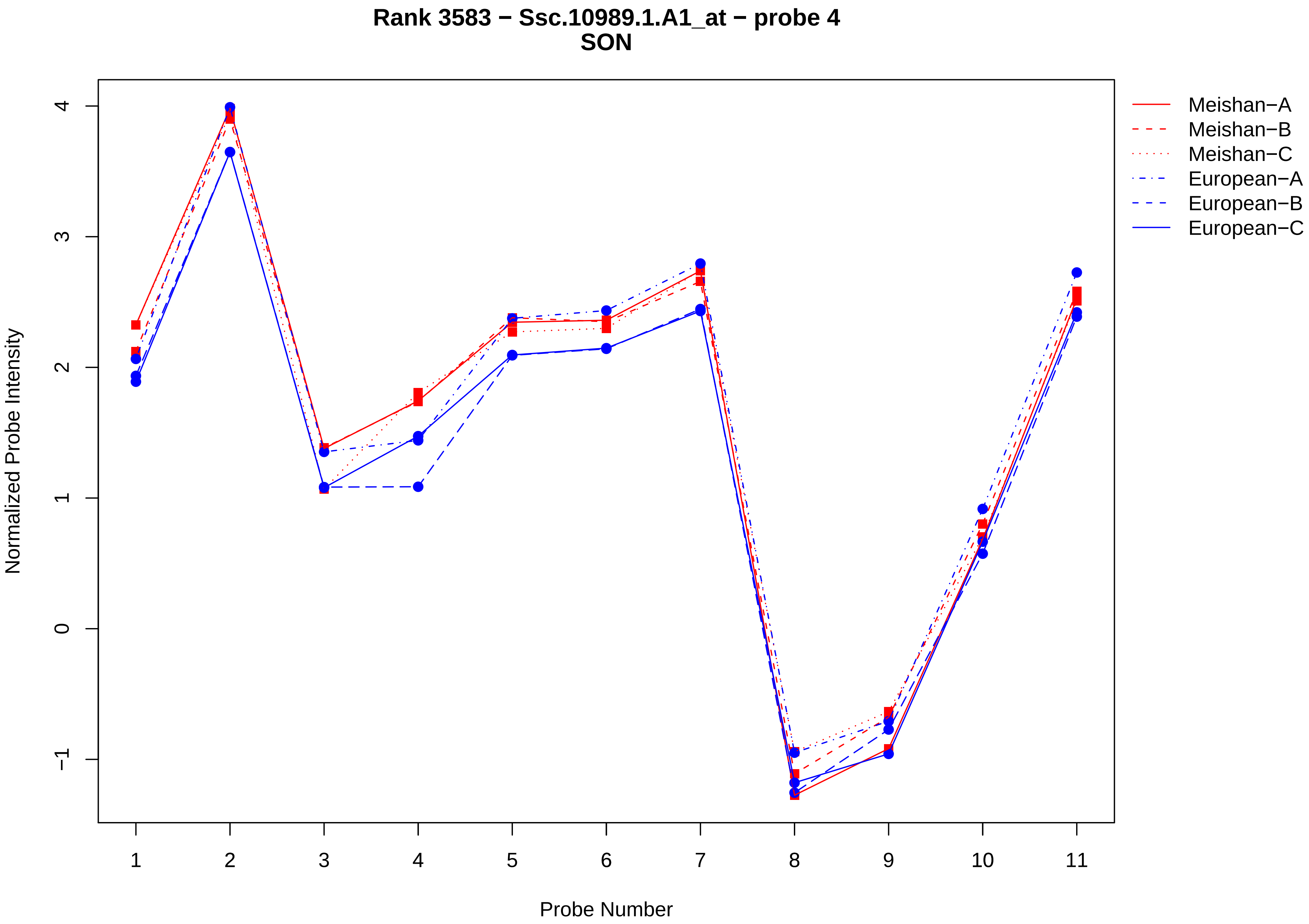

Rank 3583 − Ssc.10989.1.A1_at − probe 4
SON
Normalized Probe Intensity
Probe Number
Meishan−A
Meishan−B
Meishan−C
European−A
European−B
European−C
−1
0
1
2
3
4
1
2
3
4
5
6
7
8
9
10
11

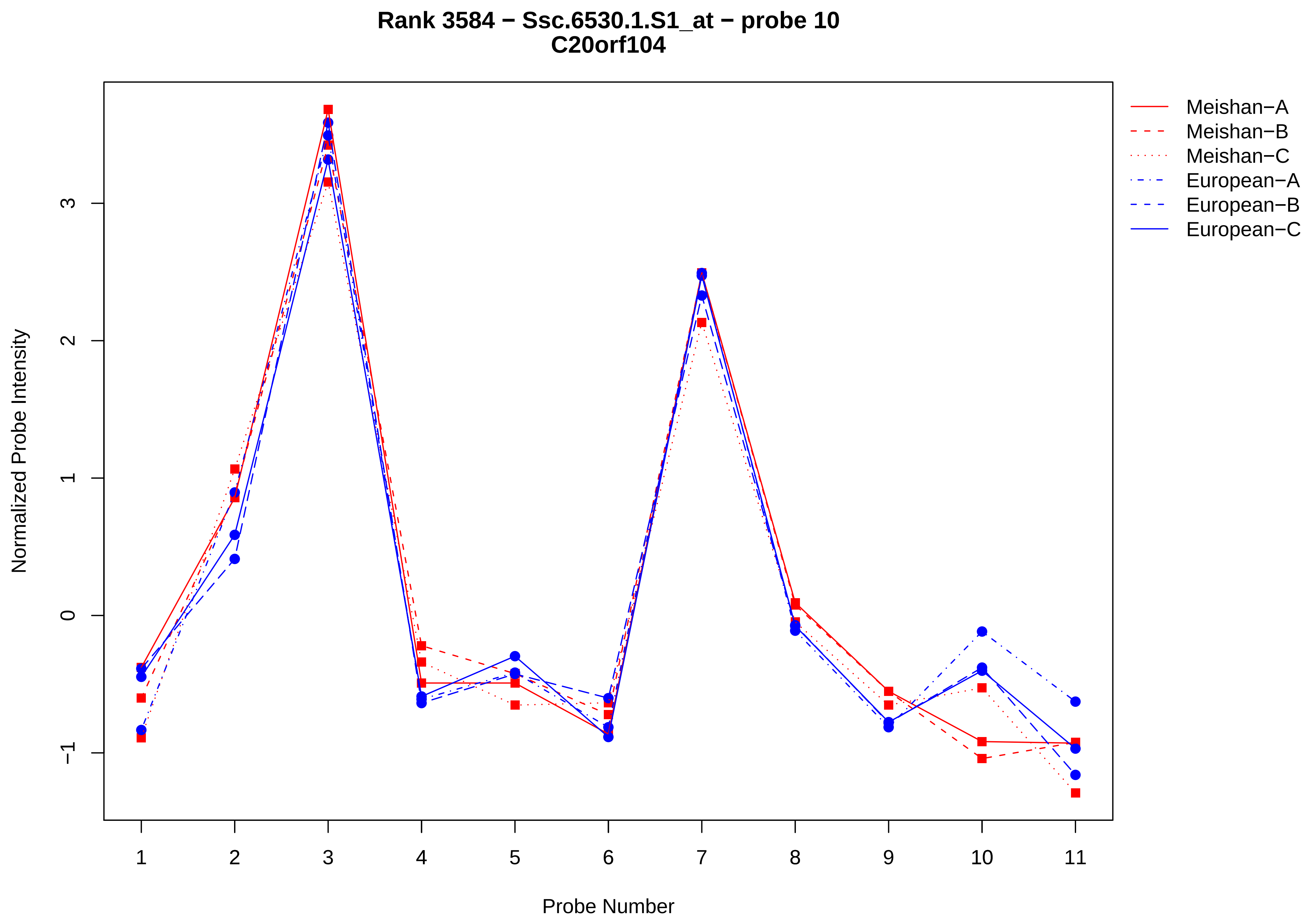

Rank 3584 − Ssc.6530.1.S1_at − probe 10
C20orf104
Normalized Probe Intensity
Probe Number
Meishan−A
Meishan−B
Meishan−C
European−A
European−B
European−C
−1
0
1
2
3
1
2
3
4
5
6
7
8
9
10
11

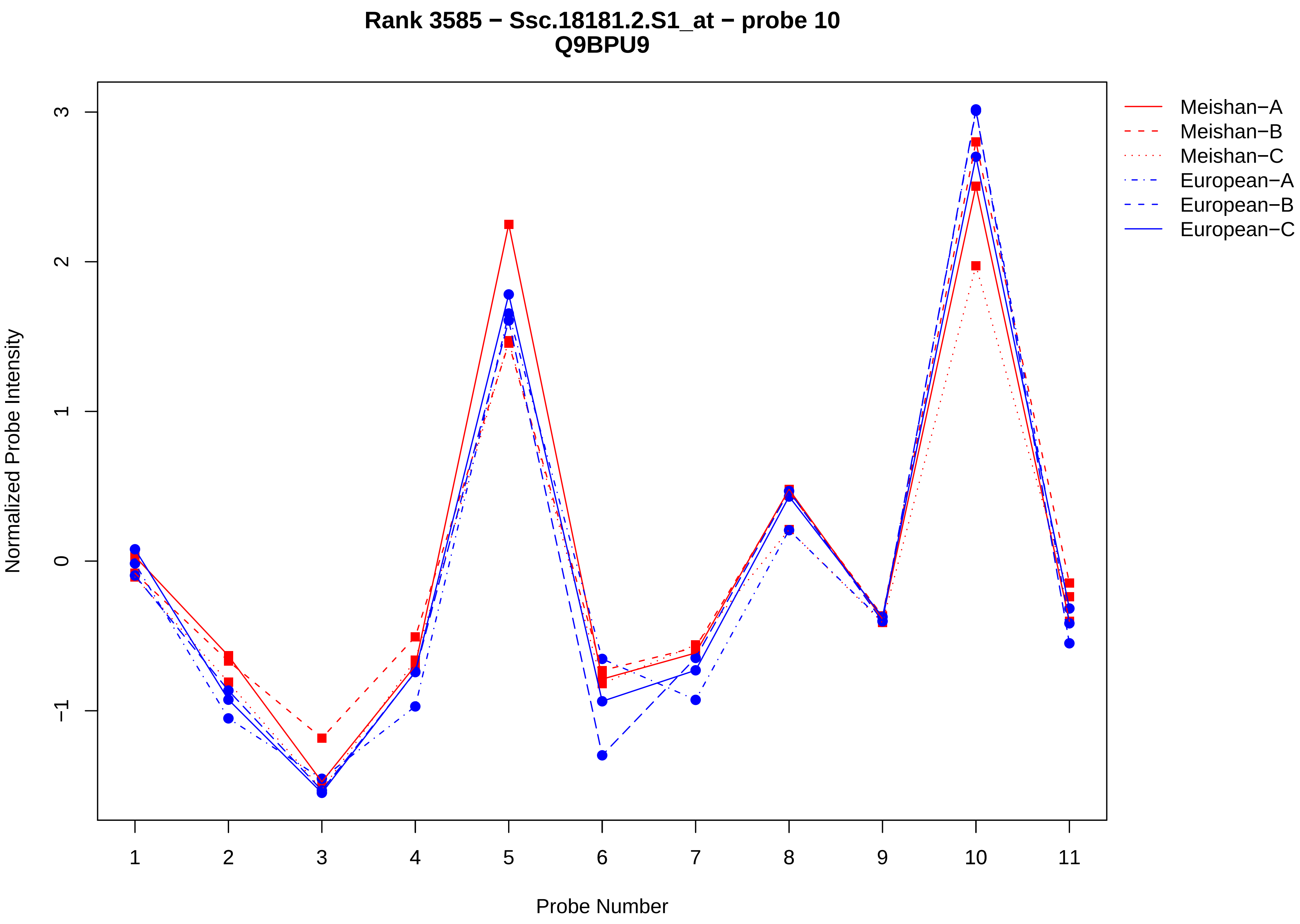

Rank 3585 − Ssc.18181.2.S1_at − probe 10
Q9BPU9
Normalized Probe Intensity
Probe Number
Meishan−A
Meishan−B
Meishan−C
European−A
European−B
European−C
−1
0
1
2
3
1
2
3
4
5
6
7
8
9
10
11

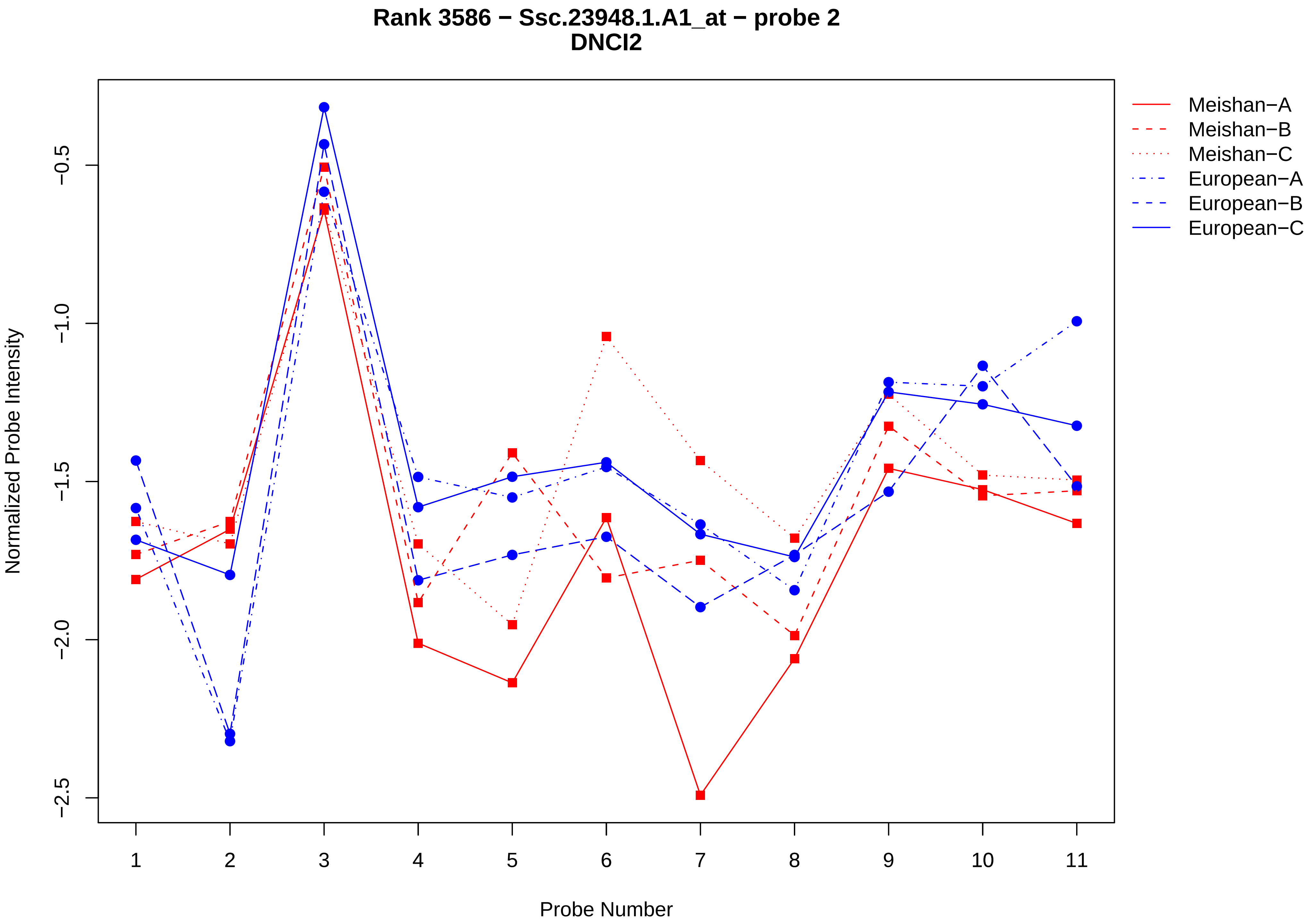

Rank 3586 − Ssc.23948.1.A1_at − probe 2
DNCI2
Normalized Probe Intensity
−0.5
−1.0
−1.5
−2.0
−2.5
1
2
3
4
5
6
7
8
9
10
11
Probe Number
Meishan−A
Meishan−B
Meishan−C
European−A
European−B
European−C

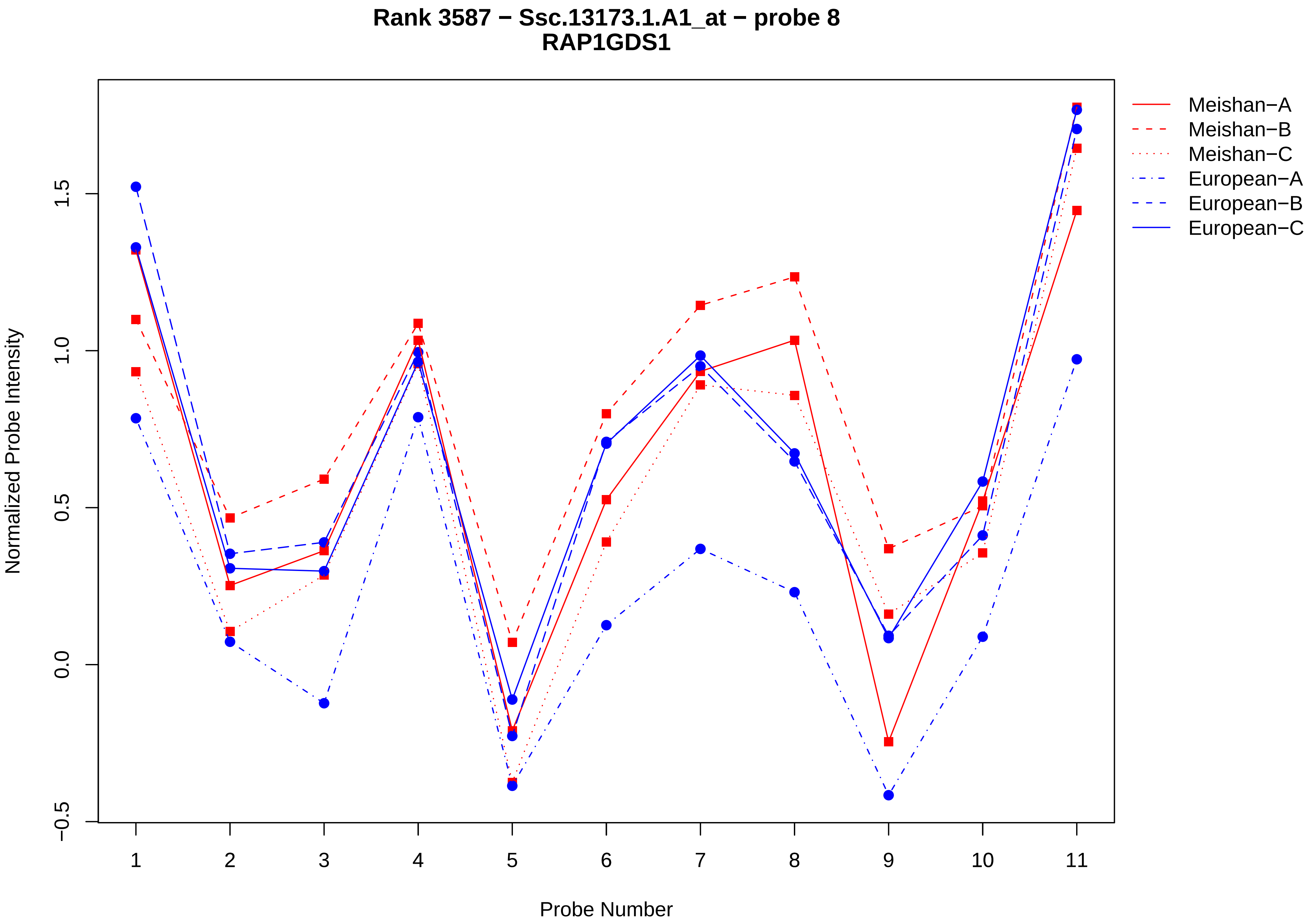

Rank 3587 − Ssc.13173.1.A1_at − probe 8
RAP1GDS1
Normalized Probe Intensity
Probe Number
−0.5
0.0
0.5
1.0
1.5
1
2
3
4
5
6
7
8
9
10
11
Meishan−A
Meishan−B
Meishan−C
European−A
European−B
European−C

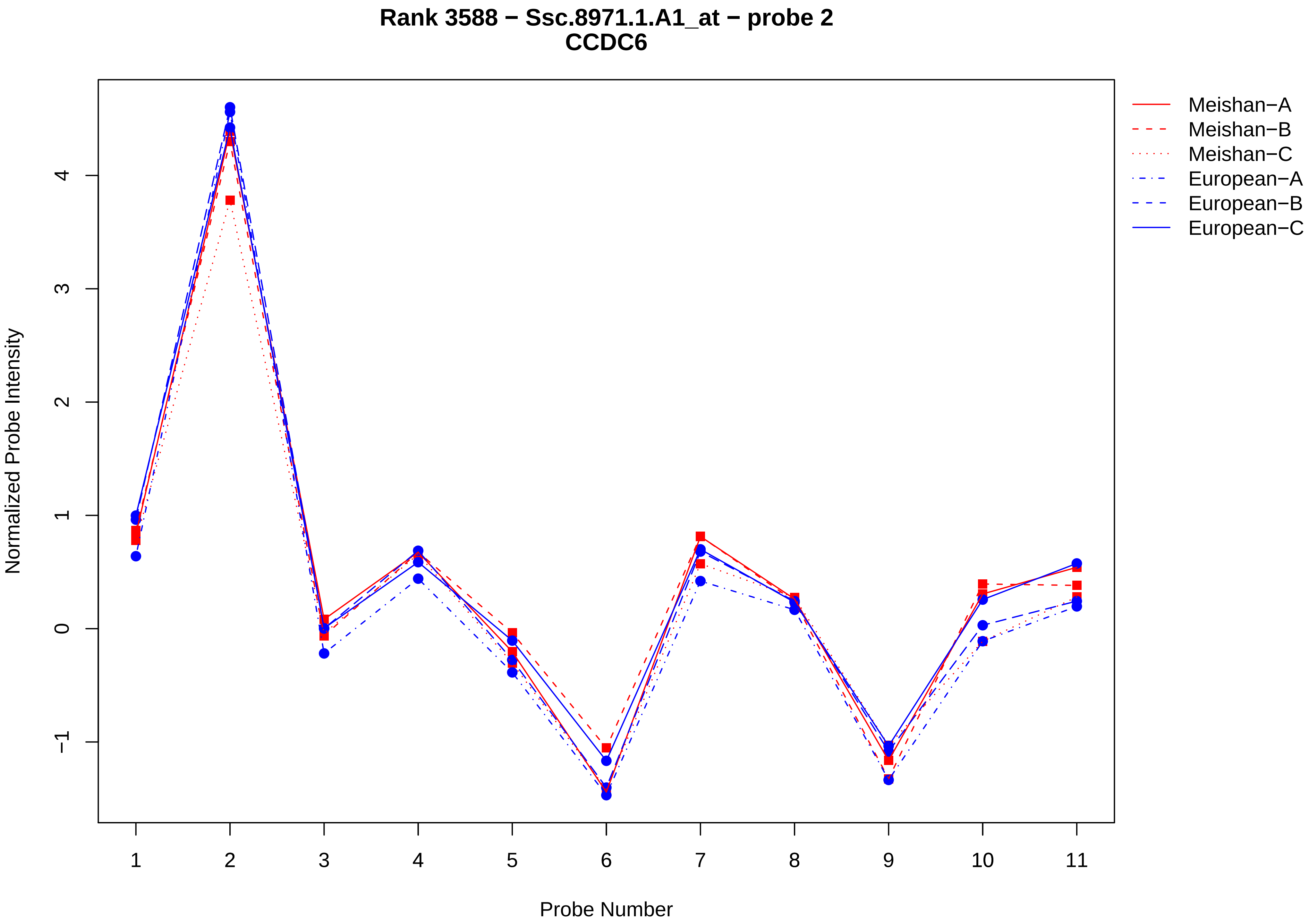

Rank 3588 − Ssc.8971.1.A1_at − probe 2
CCDC6
Normalized Probe Intensity
Probe Number
Meishan−A
Meishan−B
Meishan−C
European−A
European−B
European−C
−1
0
1
2
3
4
1
2
3
4
5
6
7
8
9
10
11

Rank 3589 − Ssc.1924.1.S1_at − probe 8
PRPSAP1

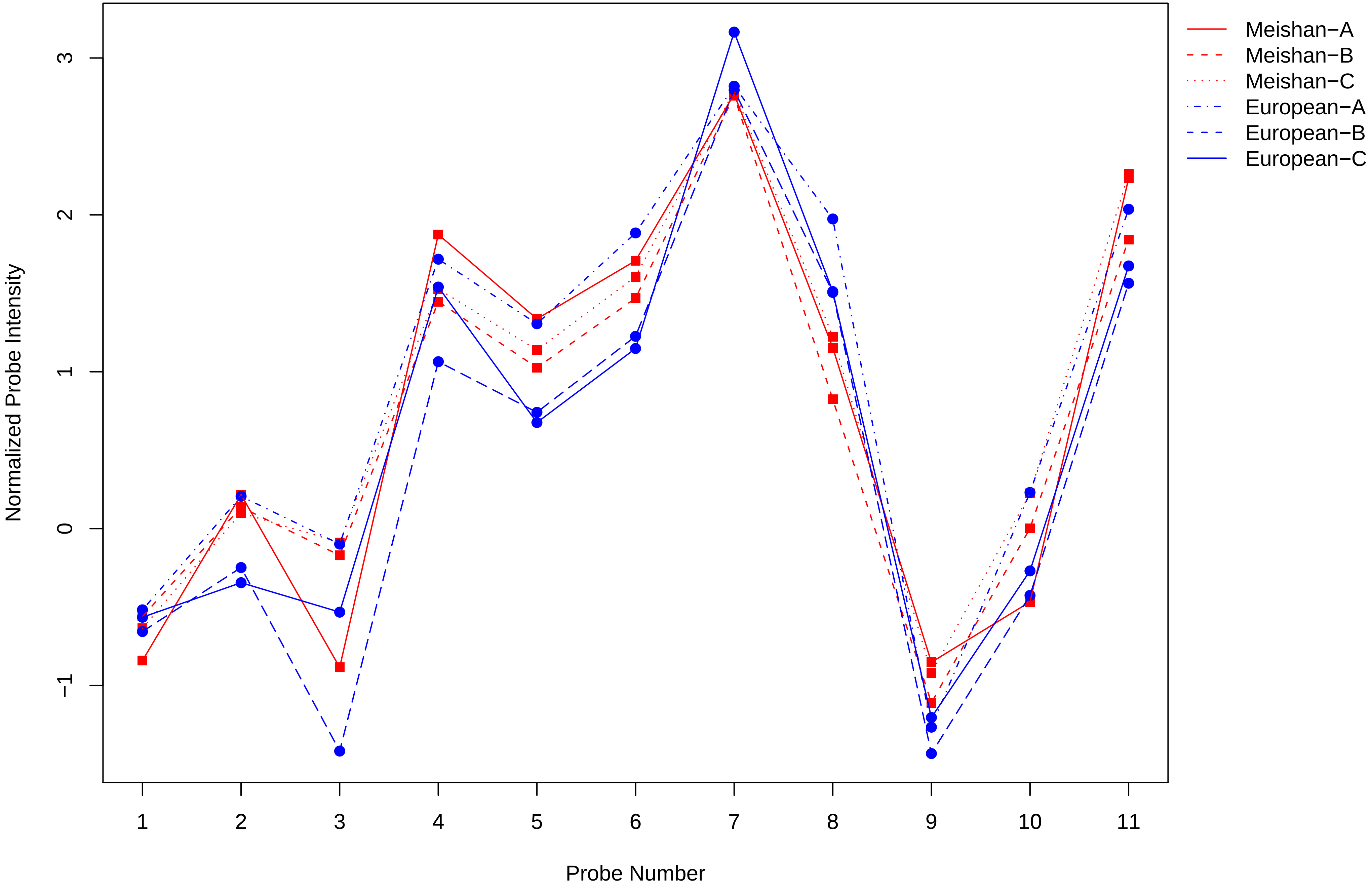

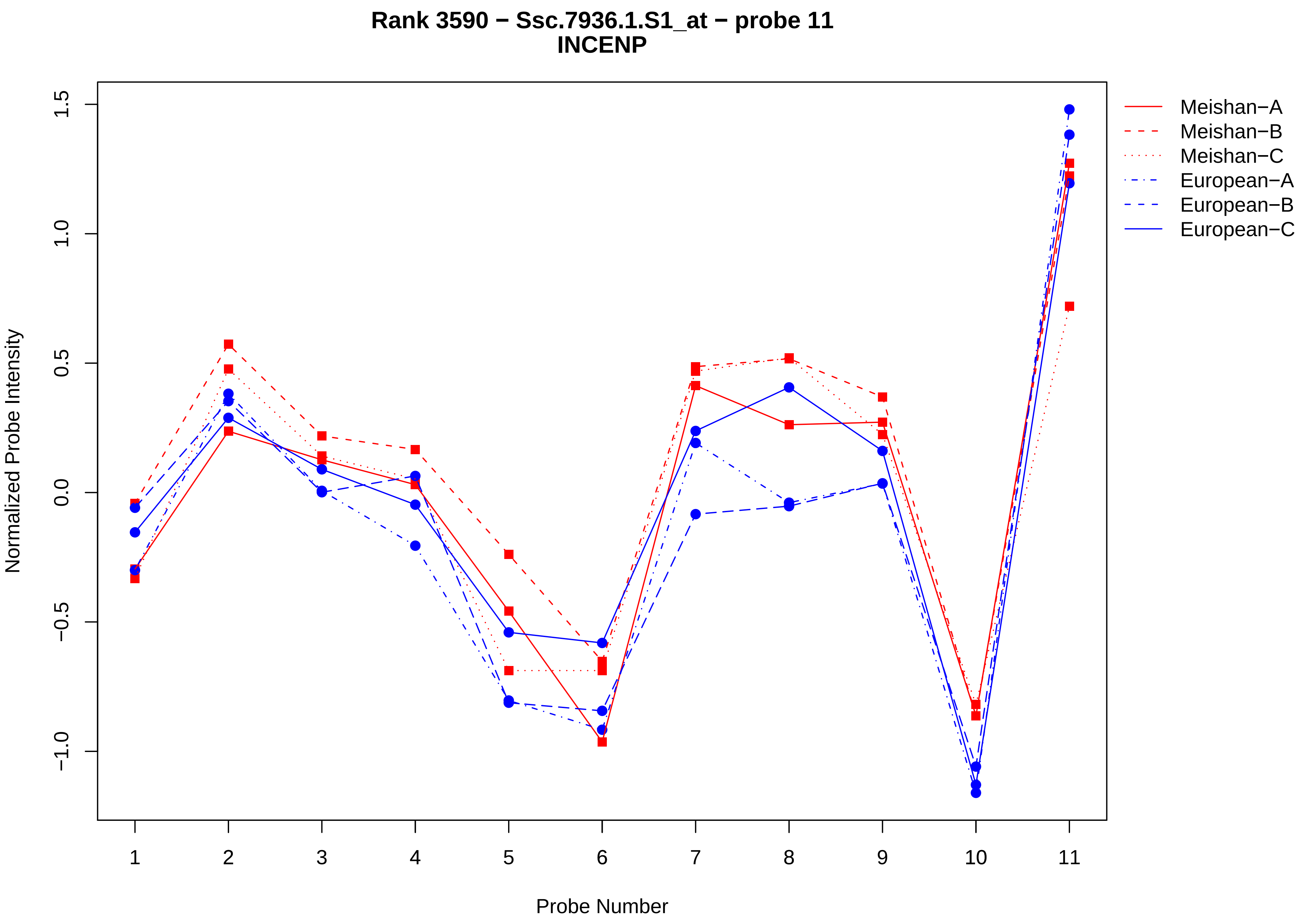
Rank 3590 − Ssc.7936.1.S1_at − probe 11
INCENP
Normalized Probe Intensity
Probe Number
Meishan−A
Meishan−B
Meishan−C
European−A
European−B
European−C
1.5
1.0
0.5
0.0
−0.5
−1.0
1
2
3
4
5
6
7
8
9
10
11

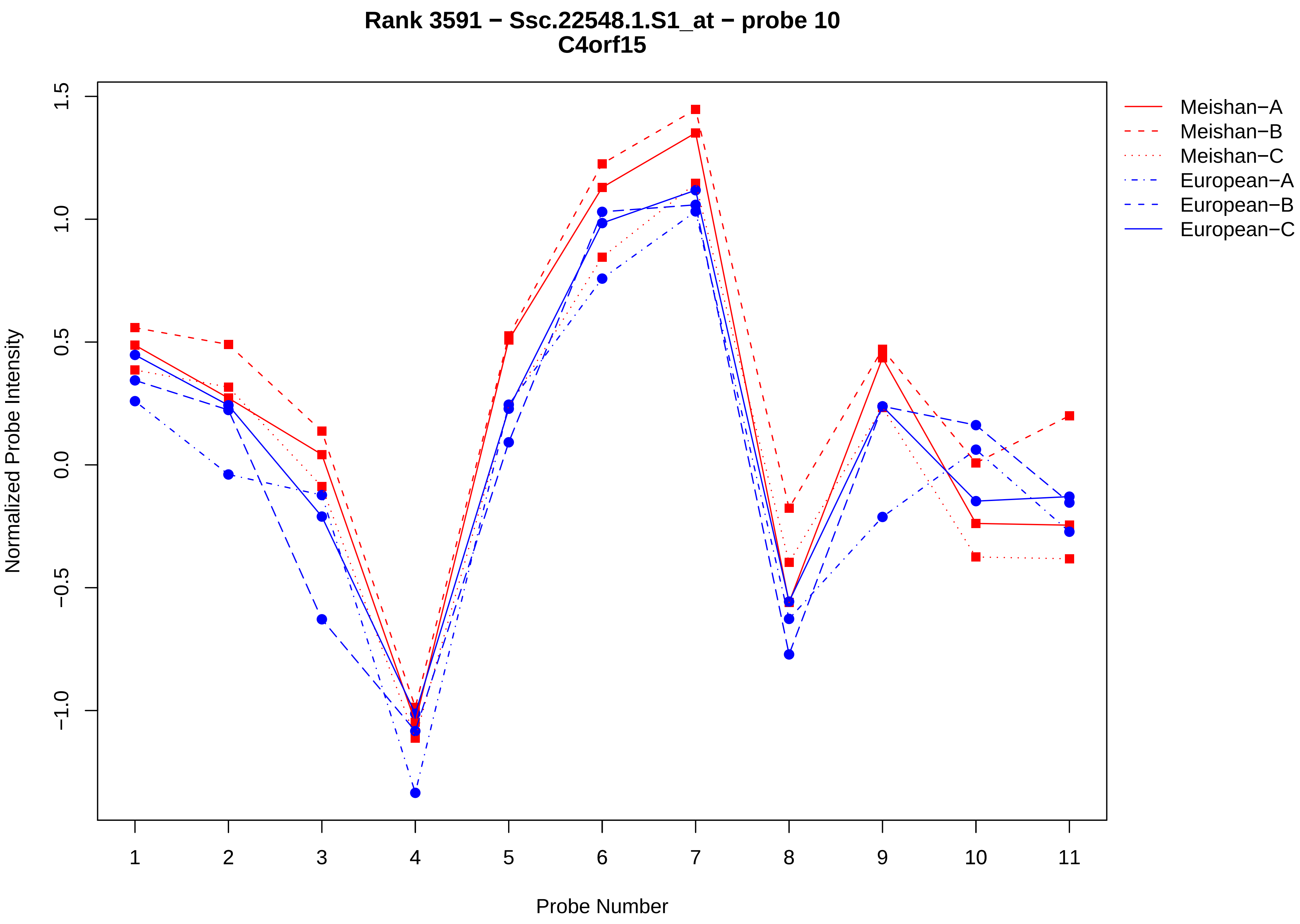
Rank 3591 − Ssc.22548.1.S1_at − probe 10
C4orf15
Normalized Probe Intensity
Probe Number
1.5
1.0
0.5
0.0
−0.5
−1.0
1
2
3
4
5
6
7
8
9
10
11
Meishan−A
Meishan−B
Meishan−C
European−A
European−B
European−C

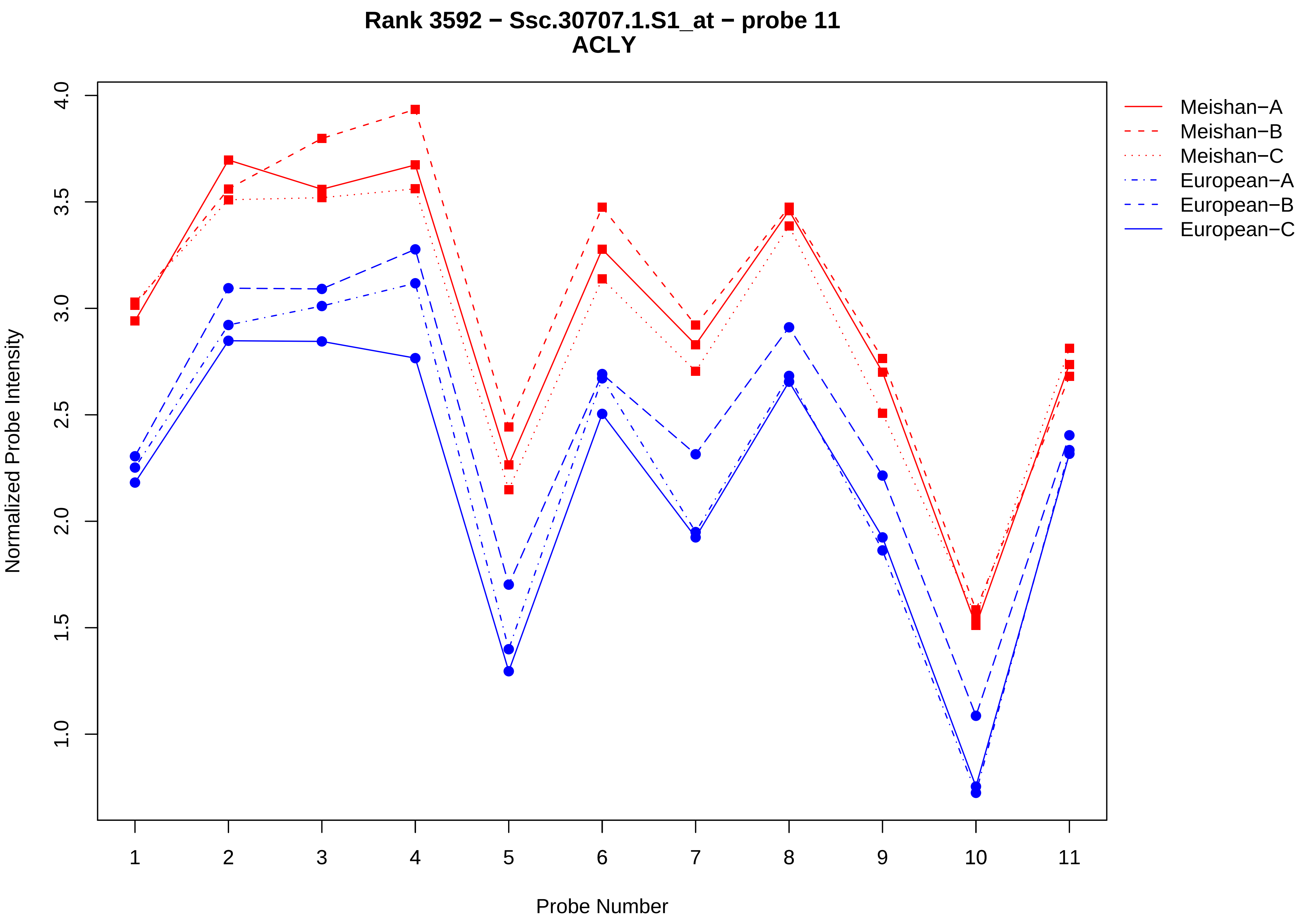

Rank 3592 − Ssc.30707.1.S1_at − probe 11
ACLY
Normalized Probe Intensity
Probe Number
1.0
1.5
2.0
2.5
3.0
3.5
4.0
1
2
3
4
5
6
7
8
9
10
11
Meishan−A
Meishan−B
Meishan−C
European−A
European−B
European−C

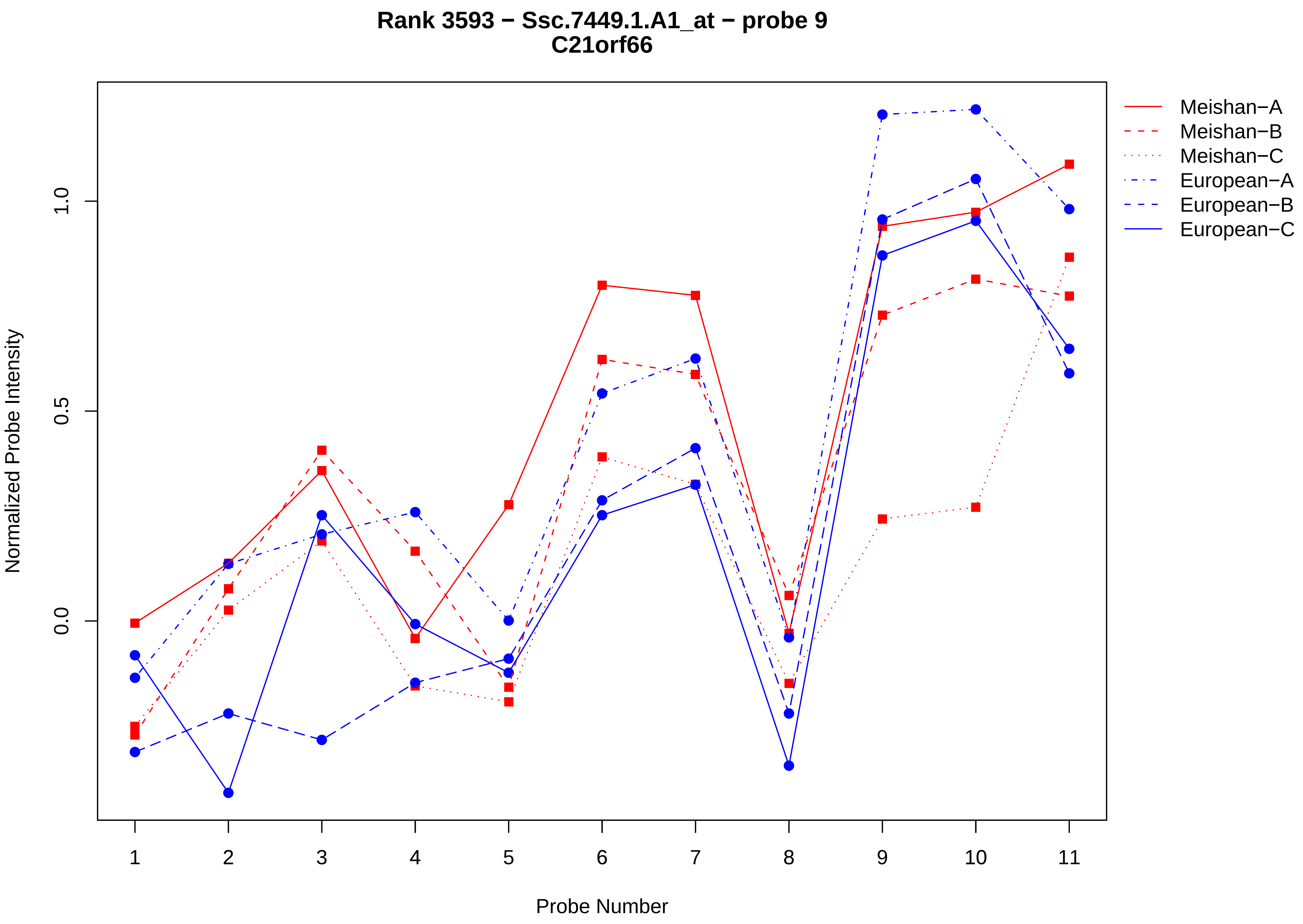

Rank 3593 − Ssc.7449.1.A1_at − probe 9
C21orf66
Normalized Probe Intensity
0.0
0.5
1.0
Probe Number
1
2
3
4
5
6
7
8
9
10
11
Meishan−A
Meishan−B
Meishan−C
European−A
European−B
European−C

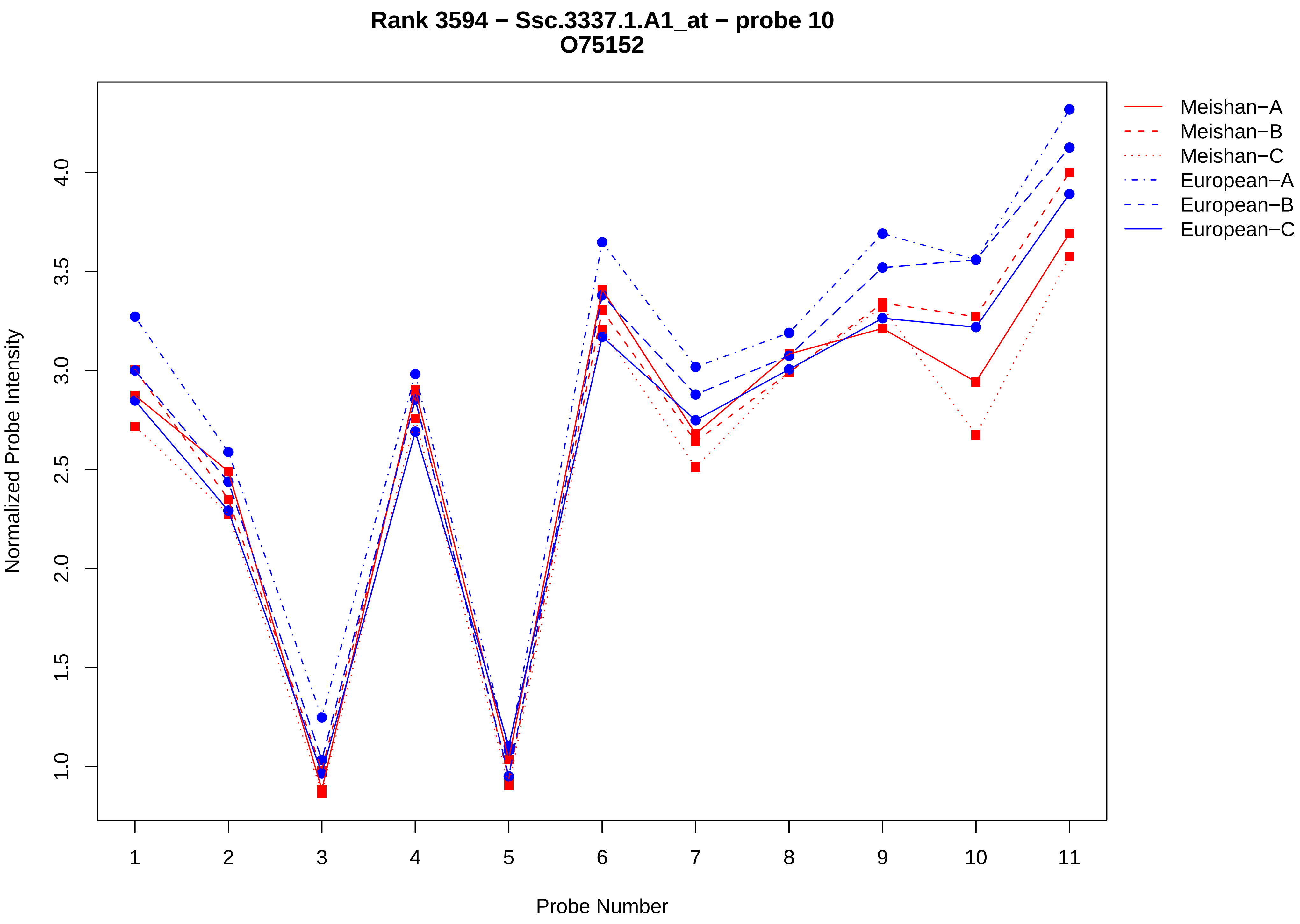

Rank 3594 − Ssc.3337.1.A1_at − probe 10
O75152
Normalized Probe Intensity
1.0
1.5
2.0
2.5
3.0
3.5
4.0
Probe Number
1
2
3
4
5
6
7
8
9
10
11
Meishan−A
Meishan−B
Meishan−C
European−A
European−B
European−C

Rank 3595 − Ssc.20163.1.S1_at − probe 2
C18orf22

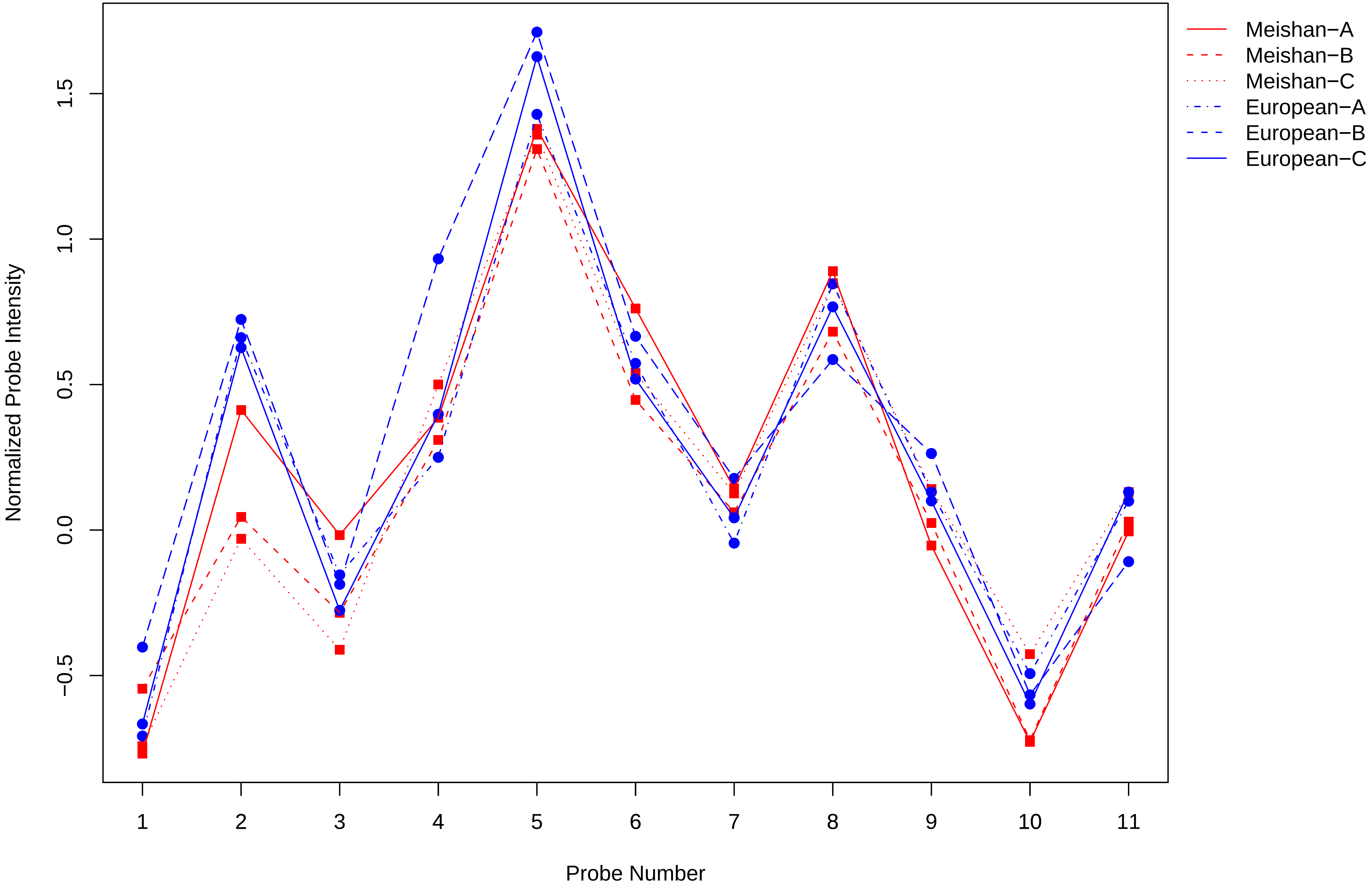

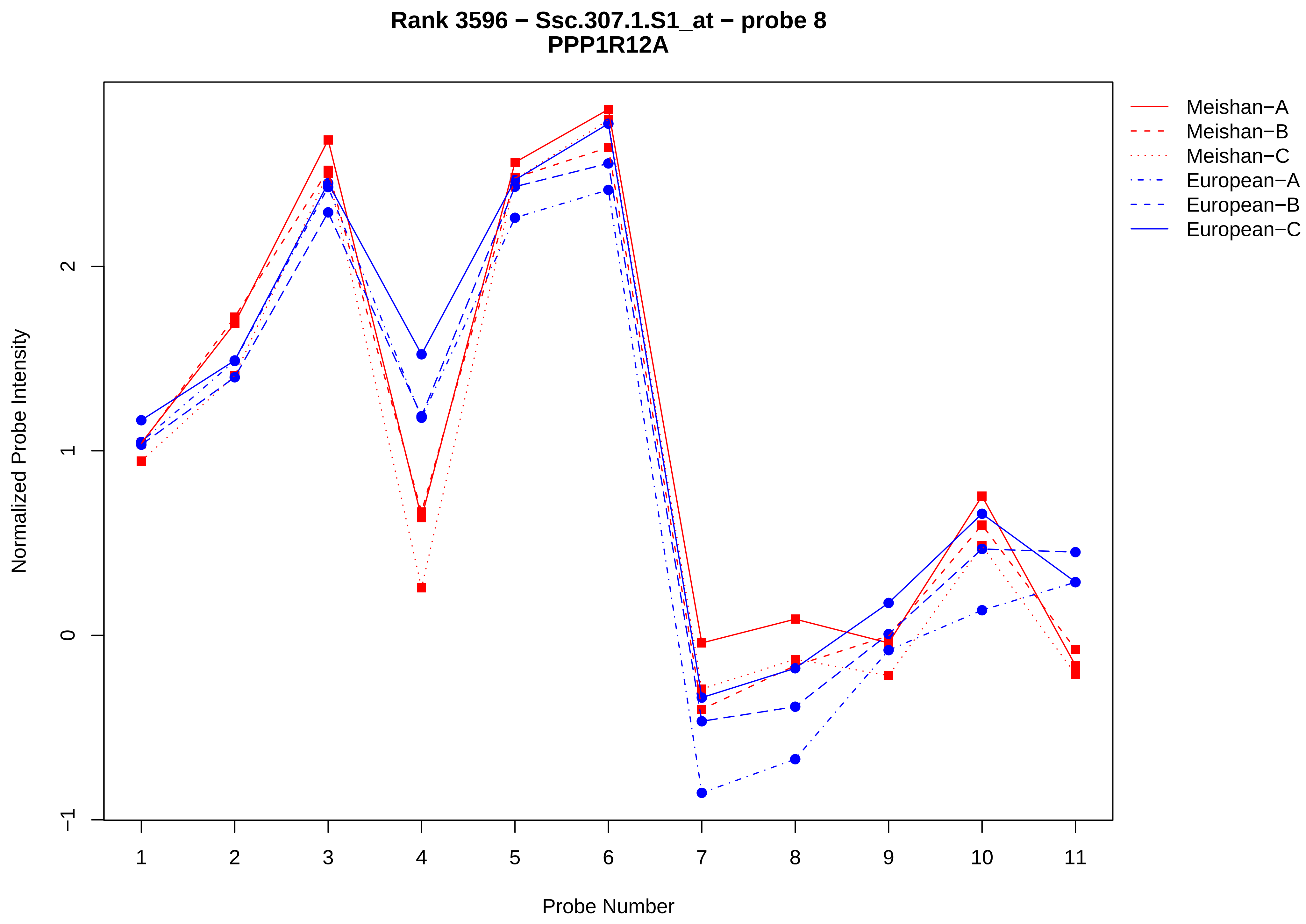

Rank 3596 − Ssc.307.1.S1_at − probe 8
PPP1R12A
Normalized Probe Intensity
Probe Number
−1
0
1
2
1
2
3
4
5
6
7
8
9
10
11
Meishan−A
Meishan−B
Meishan−C
European−A
European−B
European−C

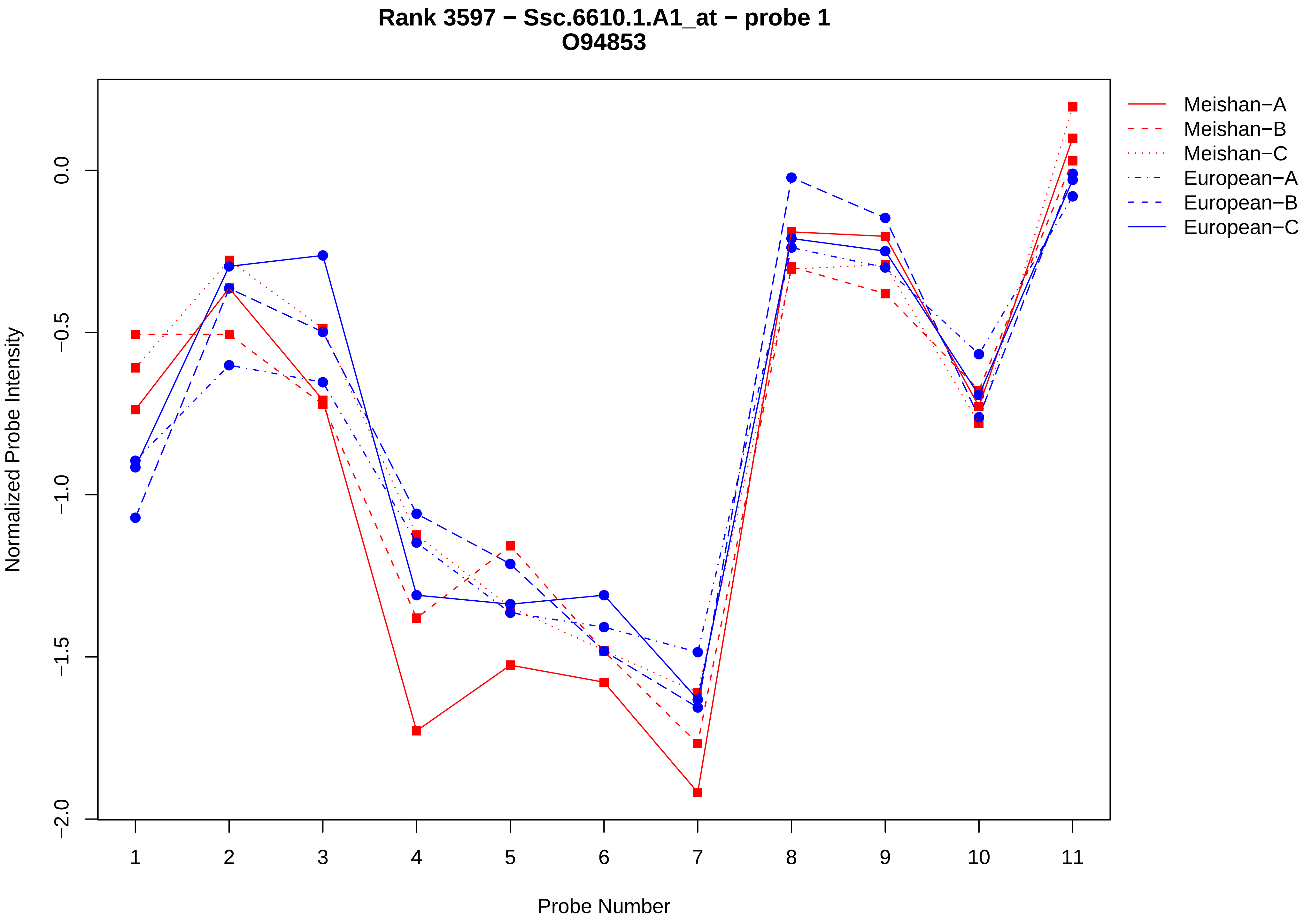

Rank 3597 − Ssc.6610.1.A1_at − probe 1
O94853
Normalized Probe Intensity
0.0
−0.5
−1.0
−1.5
−2.0
1
2
3
4
5
6
7
8
9
10
11
Probe Number
Meishan−A
Meishan−B
Meishan−C
European−A
European−B
European−C

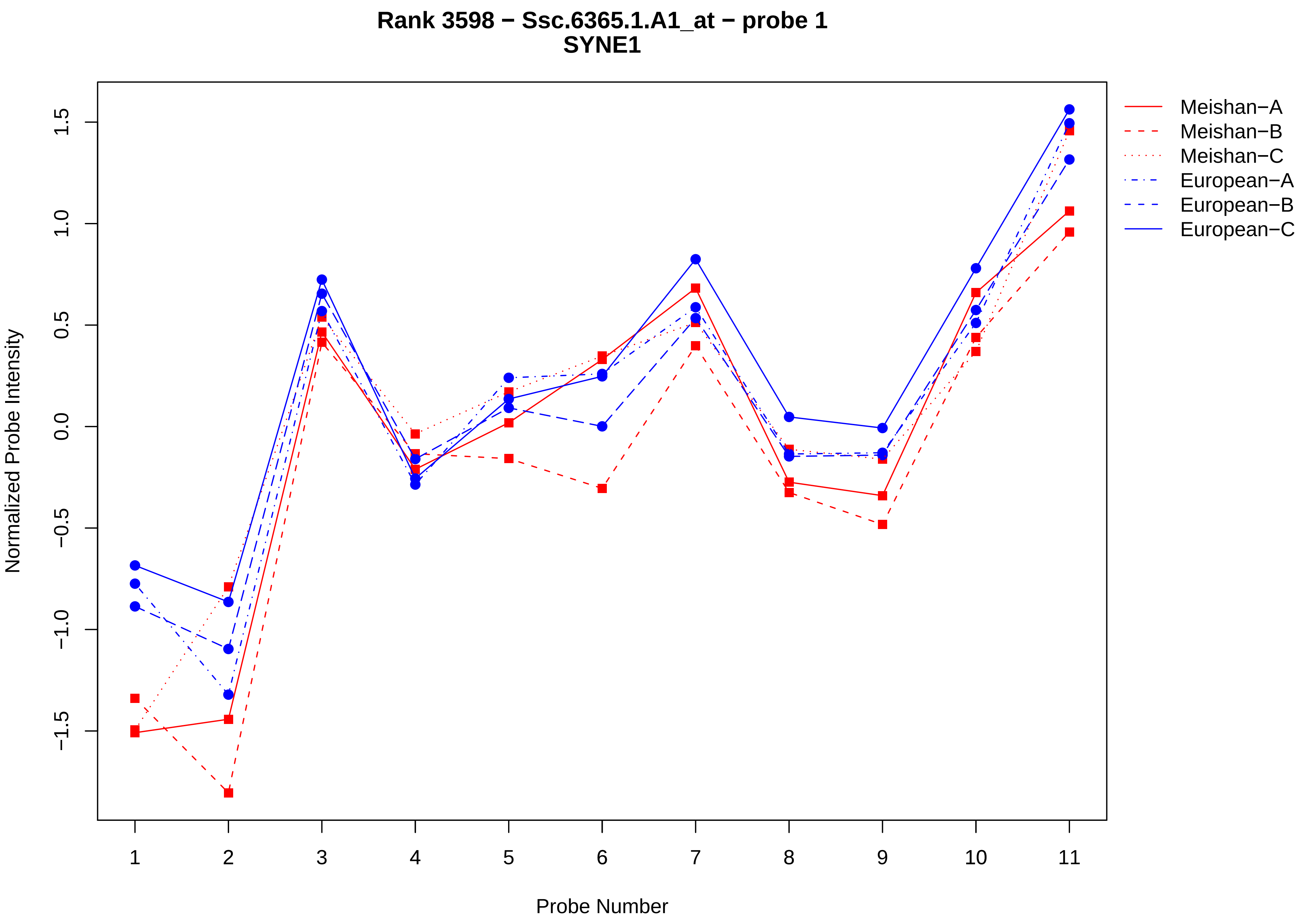
Rank 3598 − Ssc.6365.1.A1_at − probe 1
SYNE1
Normalized Probe Intensity
Probe Number
−1.5
−1.0
−0.5
0.0
0.5
1.0
1.5
1
2
3
4
5
6
7
8
9
10
11
Meishan−A
Meishan−B
Meishan−C
European−A
European−B
European−C

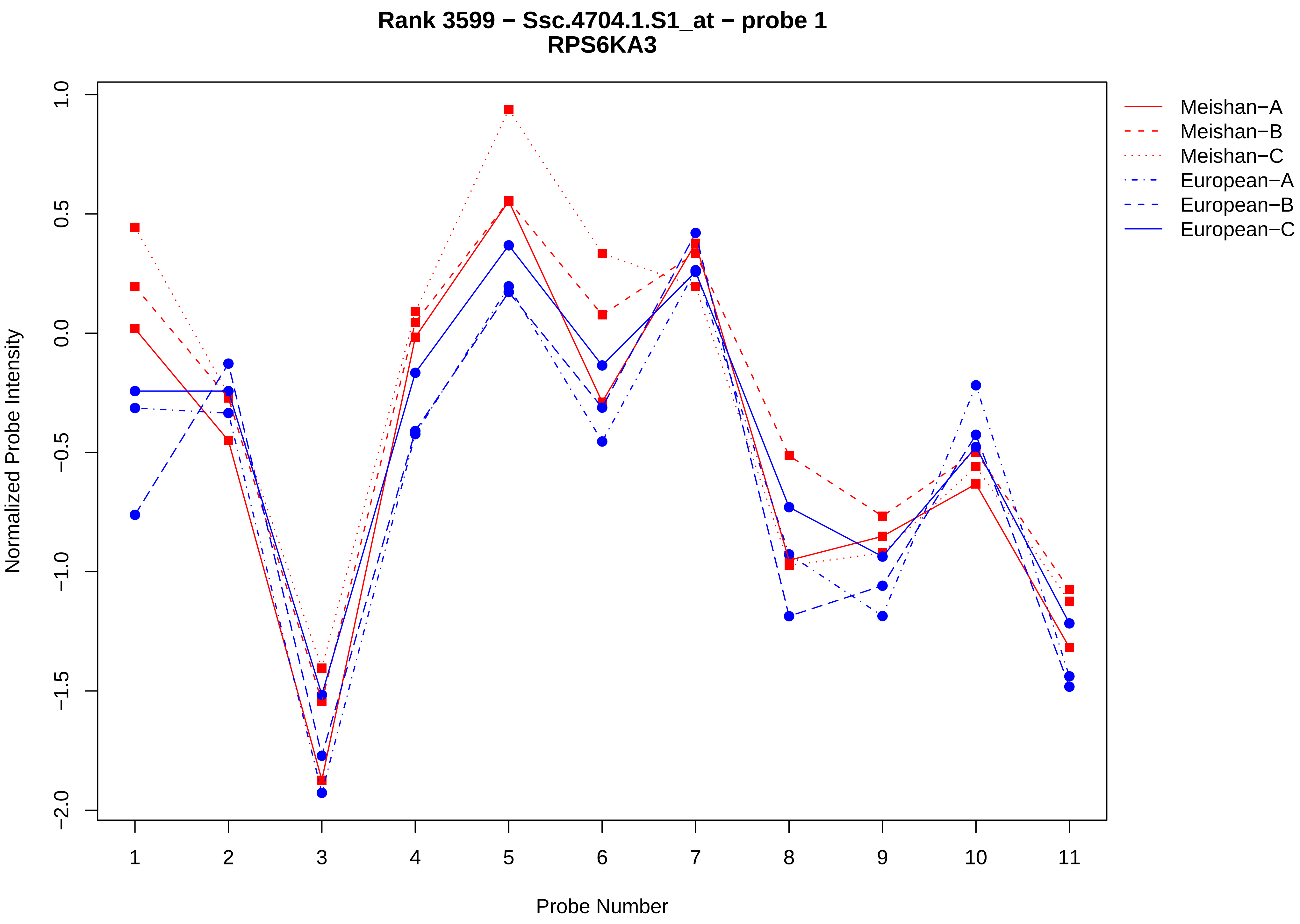

Rank 3599 − Ssc.4704.1.S1_at − probe 1
RPS6KA3
Normalized Probe Intensity
Probe Number
1.0
0.5
0.0
−0.5
−1.0
−1.5
−2.0
1
2
3
4
5
6
7
8
9
10
11
Meishan−A
Meishan−B
Meishan−C
European−A
European−B
European−C

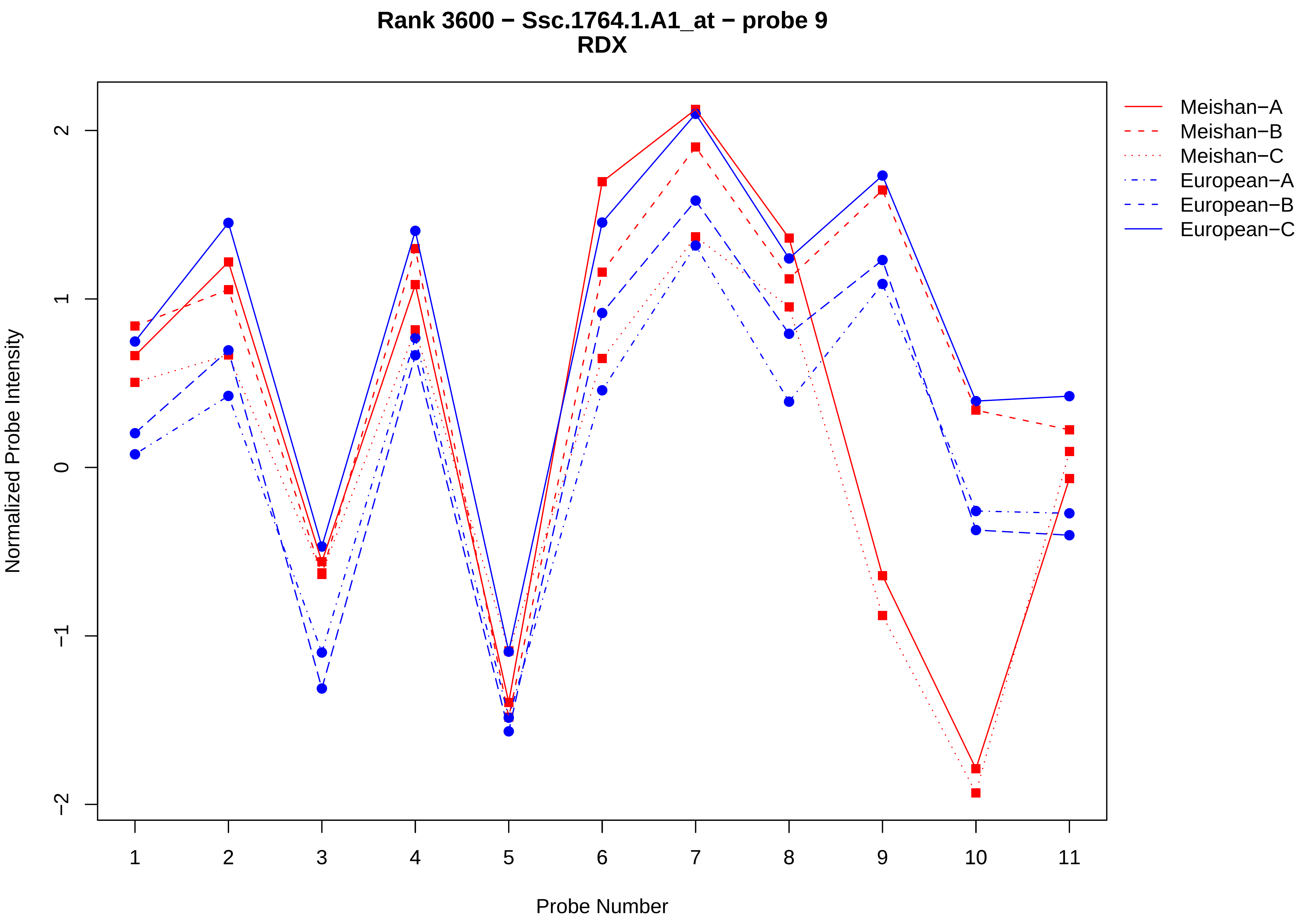

Rank 3600 − Ssc.1764.1.A1_at − probe 9
RDX
Normalized Probe Intensity
Probe Number
−2
−1
0
1
2
1
2
3
4
5
6
7
8
9
10
11
Meishan−A
Meishan−B
Meishan−C
European−A
European−B
European−C

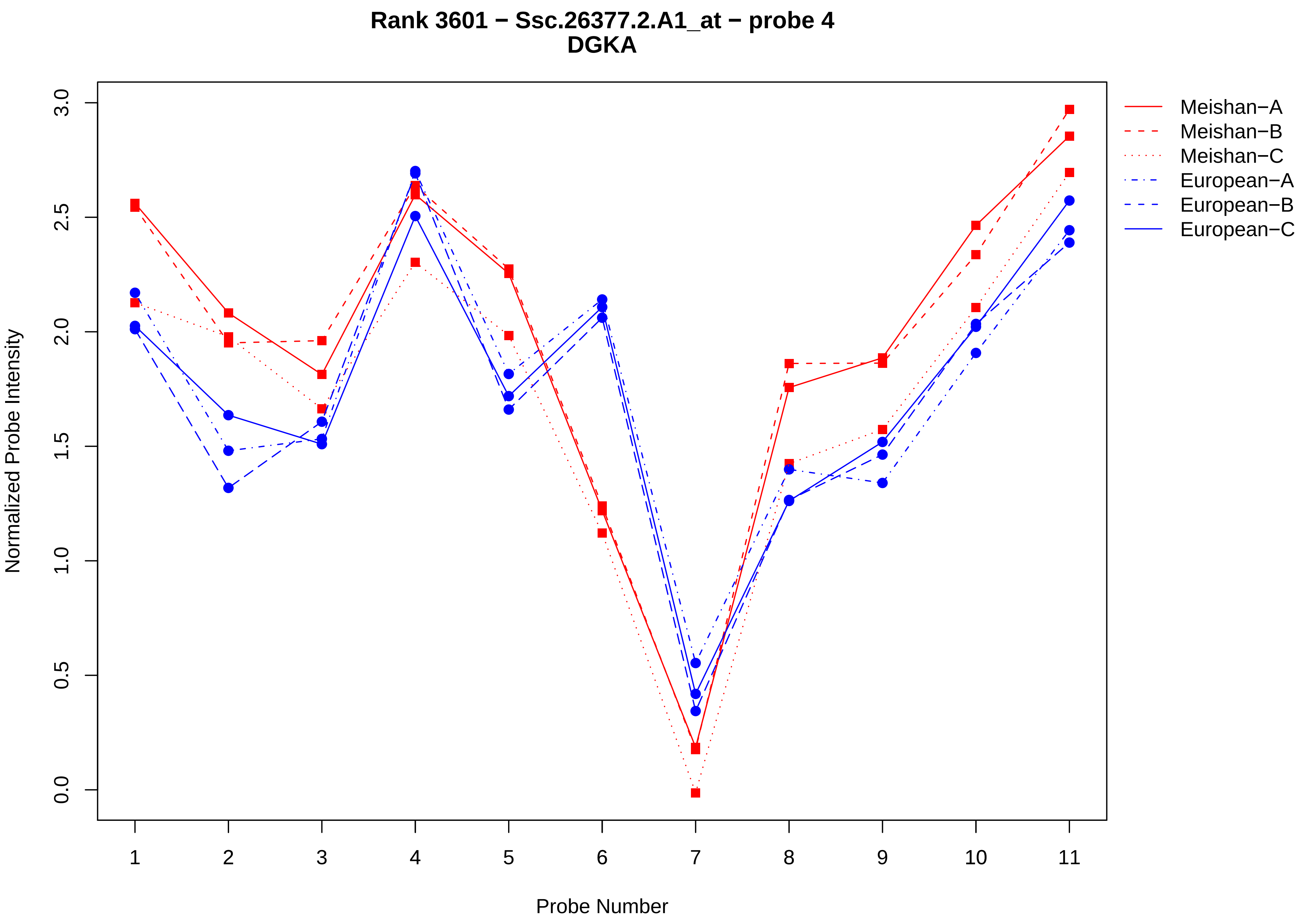
Rank 3601 − Ssc.26377.2.A1_at − probe 4
DGKA
Normalized Probe Intensity
0.0
0.5
1.0
1.5
2.0
2.5
3.0
1
2
3
4
5
6
7
8
9
10
11
Probe Number
Meishan−A
Meishan−B
Meishan−C
European−A
European−B
European−C

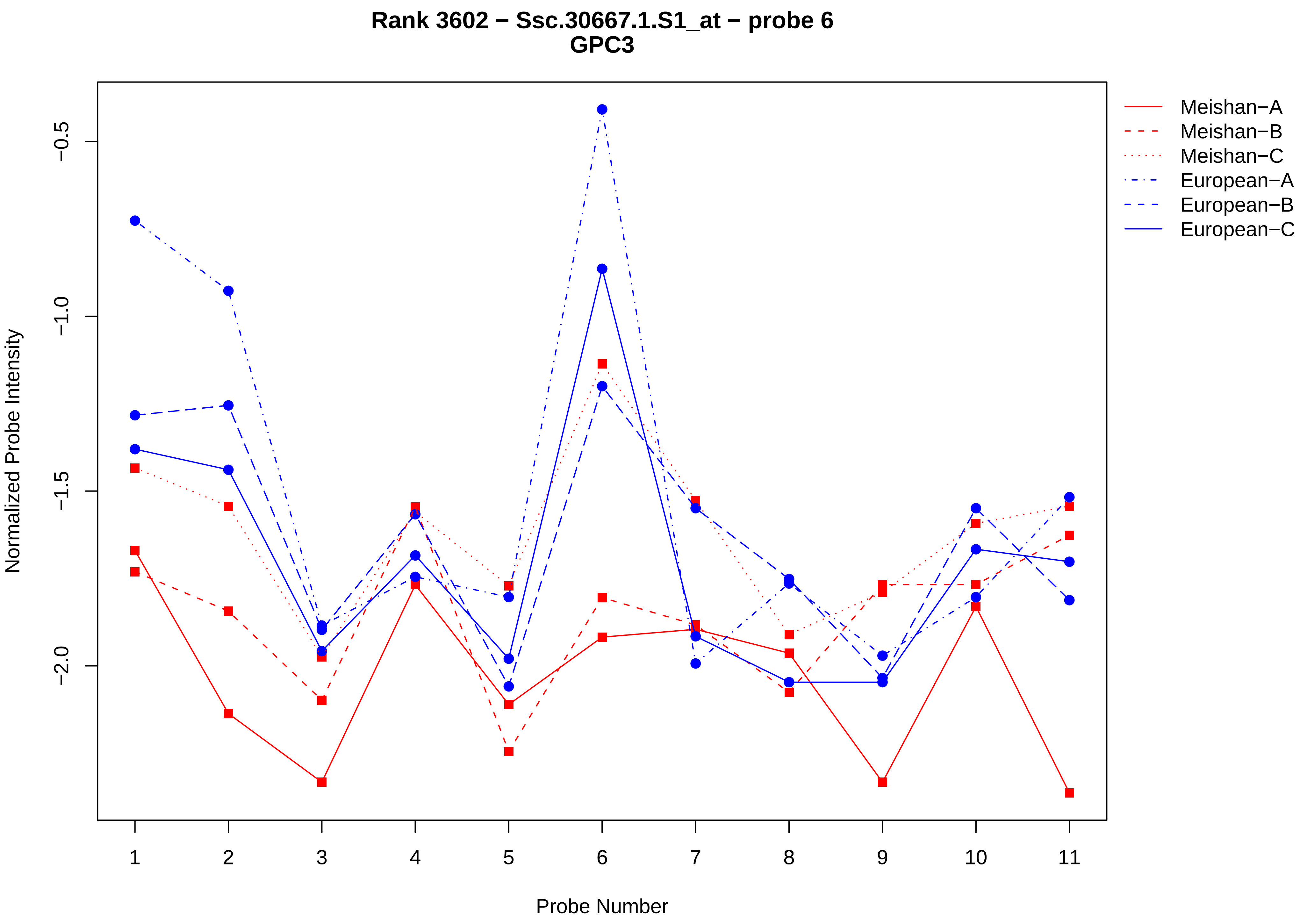

Rank 3602 − Ssc.30667.1.S1_at − probe 6
GPC3
Normalized Probe Intensity
−0.5
−1.0
−1.5
−2.0
1
2
3
4
5
6
7
8
9
10
11
Probe Number
Meishan−A
Meishan−B
Meishan−C
European−A
European−B
European−C

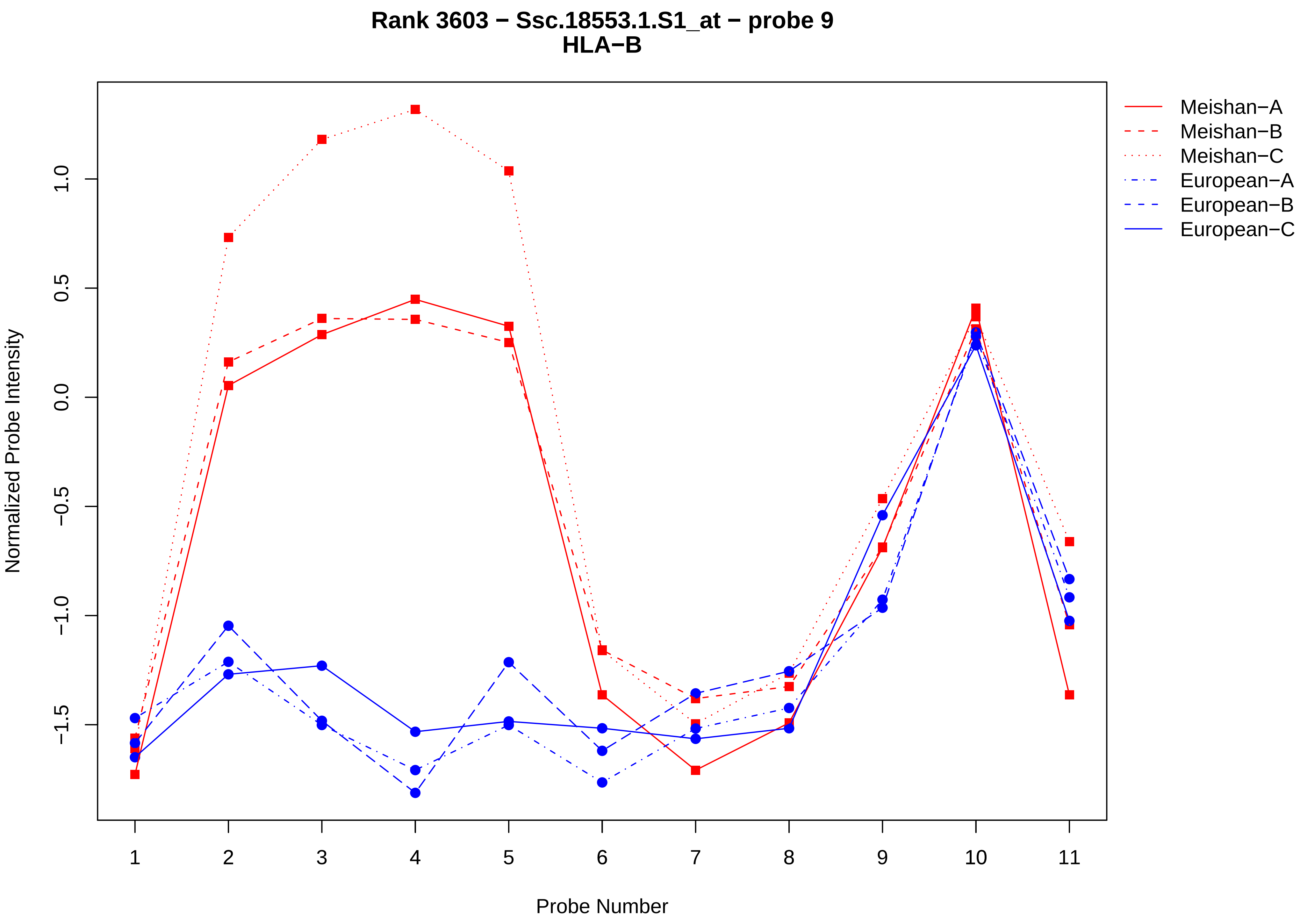

Rank 3603 − Ssc.18553.1.S1_at − probe 9
HLA−B
Normalized Probe Intensity
Probe Number
1.0
0.5
0.0
−0.5
−1.0
−1.5
1
2
3
4
5
6
7
8
9
10
11
Meishan−A
Meishan−B
Meishan−C
European−A
European−B
European−C

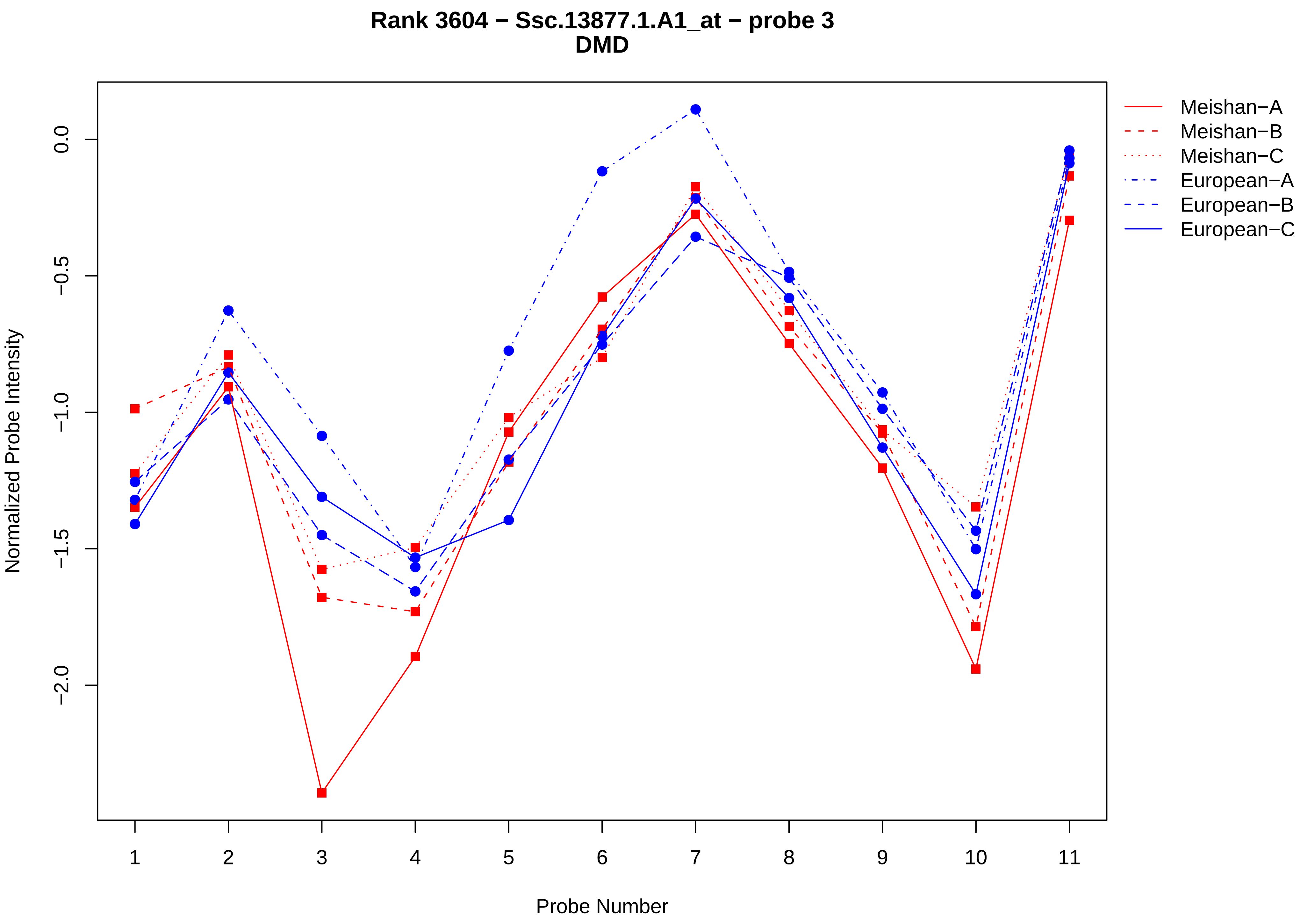
Rank 3604 − Ssc.13877.1.A1_at − probe 3
DMD
Normalized Probe Intensity
0.0
−0.5
−1.0
−1.5
−2.0
1
2
3
4
5
6
7
8
9
10
11
Probe Number
Meishan−A
Meishan−B
Meishan−C
European−A
European−B
European−C

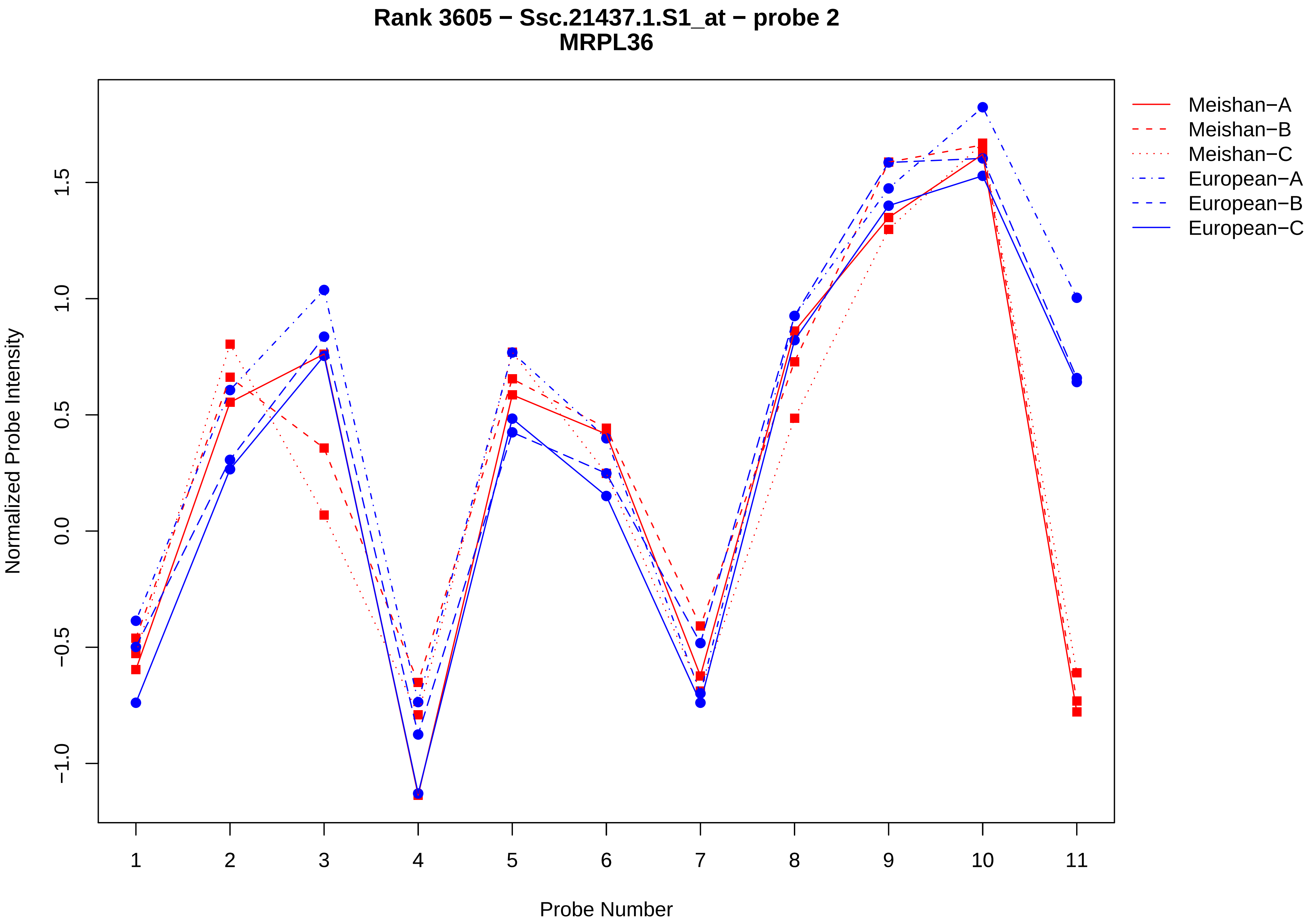

Rank 3605 − Ssc.21437.1.S1_at − probe 2
MRPL36
Normalized Probe Intensity
Probe Number
−1.0
−0.5
0.0
0.5
1.0
1.5
1
2
3
4
5
6
7
8
9
10
11
Meishan−A
Meishan−B
Meishan−C
European−A
European−B
European−C

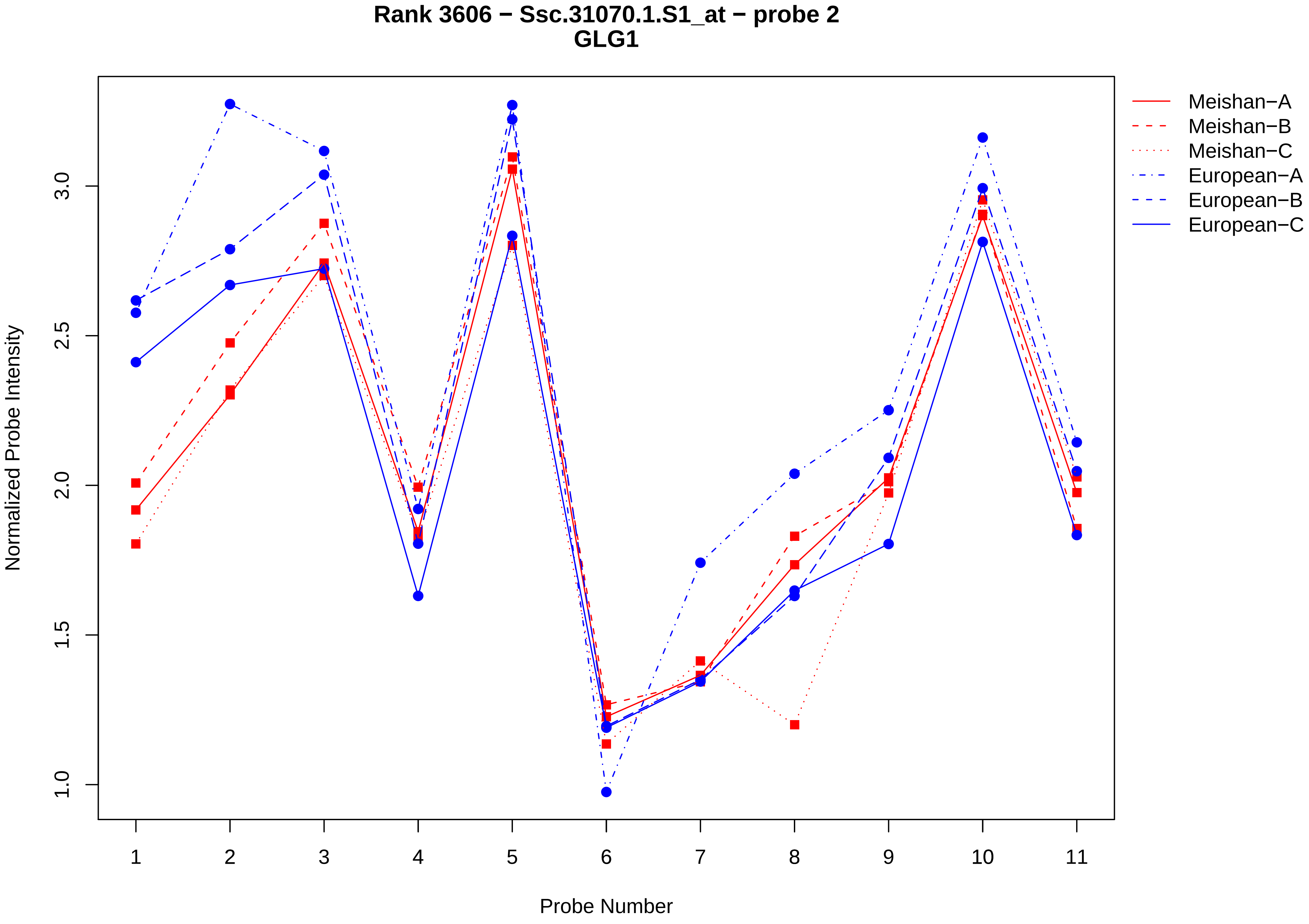

Rank 3606 − Ssc.31070.1.S1_at − probe 2
GLG1
Normalized Probe Intensity
Probe Number
1.0
1.5
2.0
2.5
3.0
1
2
3
4
5
6
7
8
9
10
11
Meishan−A
Meishan−B
Meishan−C
European−A
European−B
European−C

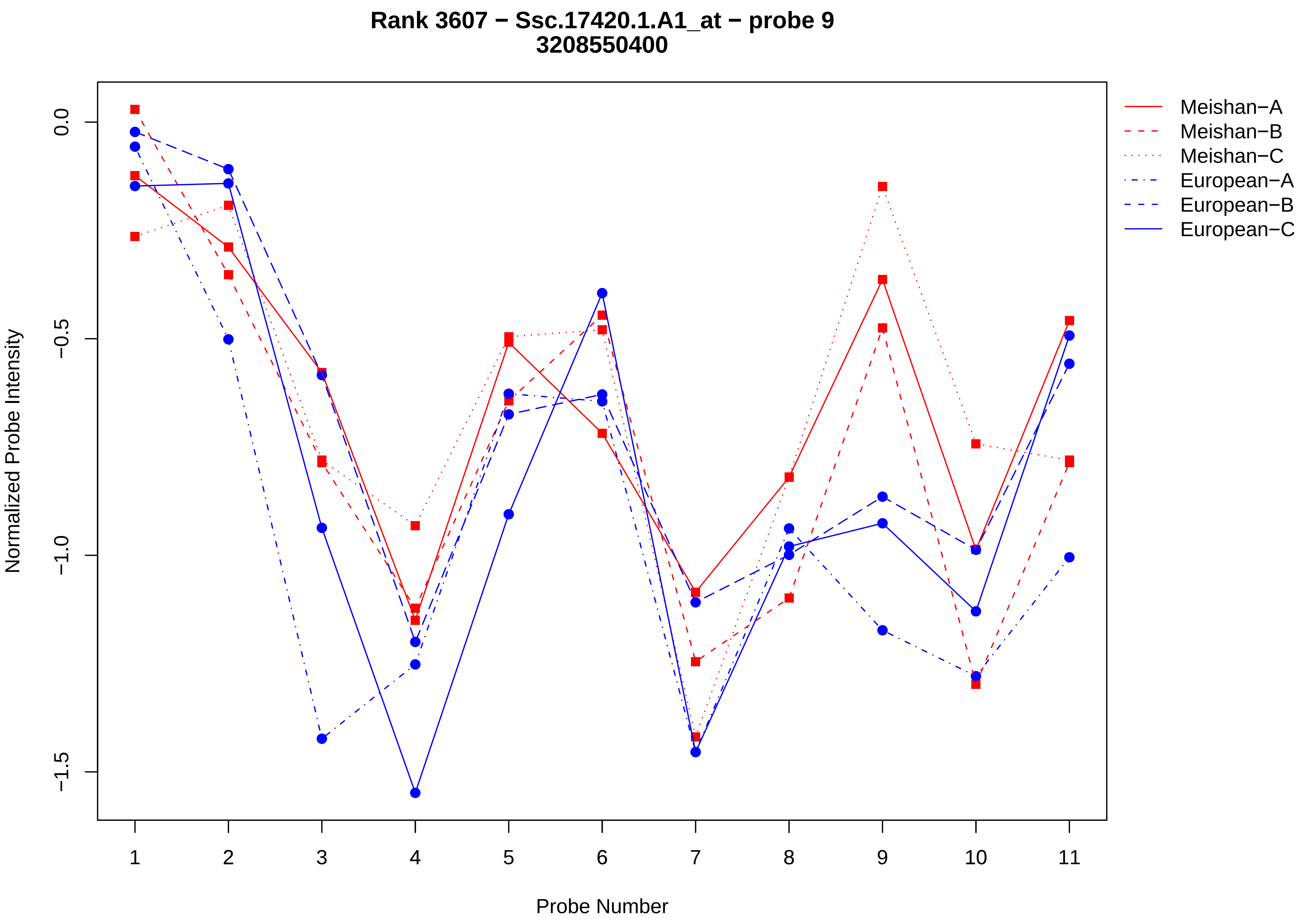

Rank 3607 − Ssc.17420.1.A1_at − probe 9
3208550400
Normalized Probe Intensity
Probe Number
0.0
−0.5
−1.0
−1.5
1
2
3
4
5
6
7
8
9
10
11
Meishan−A
Meishan−B
Meishan−C
European−A
European−B
European−C

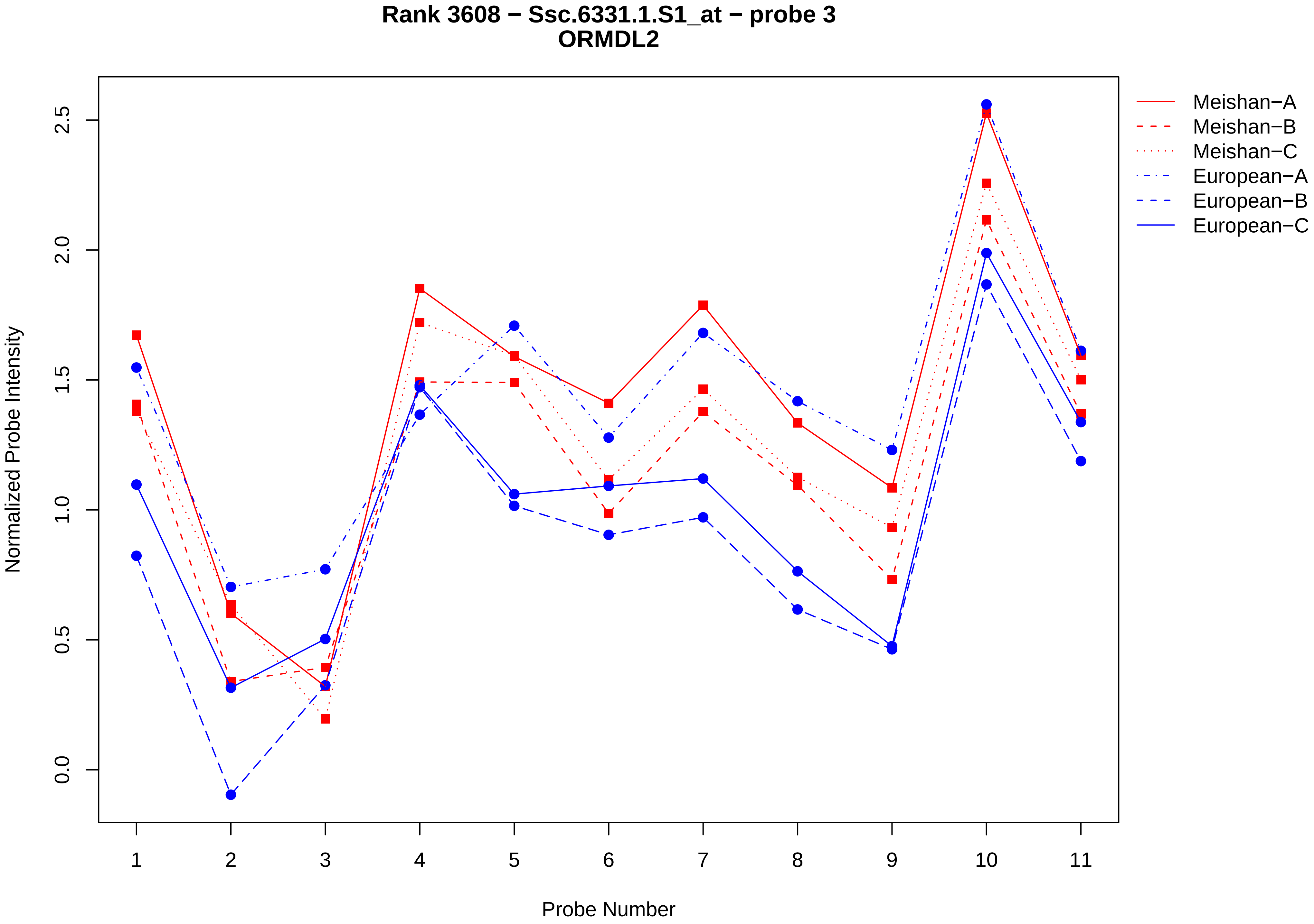

Rank 3608 − Ssc.6331.1.S1_at − probe 3
ORMDL2
Normalized Probe Intensity
0.0
0.5
1.0
1.5
2.0
2.5
1
2
3
4
5
6
7
8
9
10
11
Probe Number
Meishan−A
Meishan−B
Meishan−C
European−A
European−B
European−C

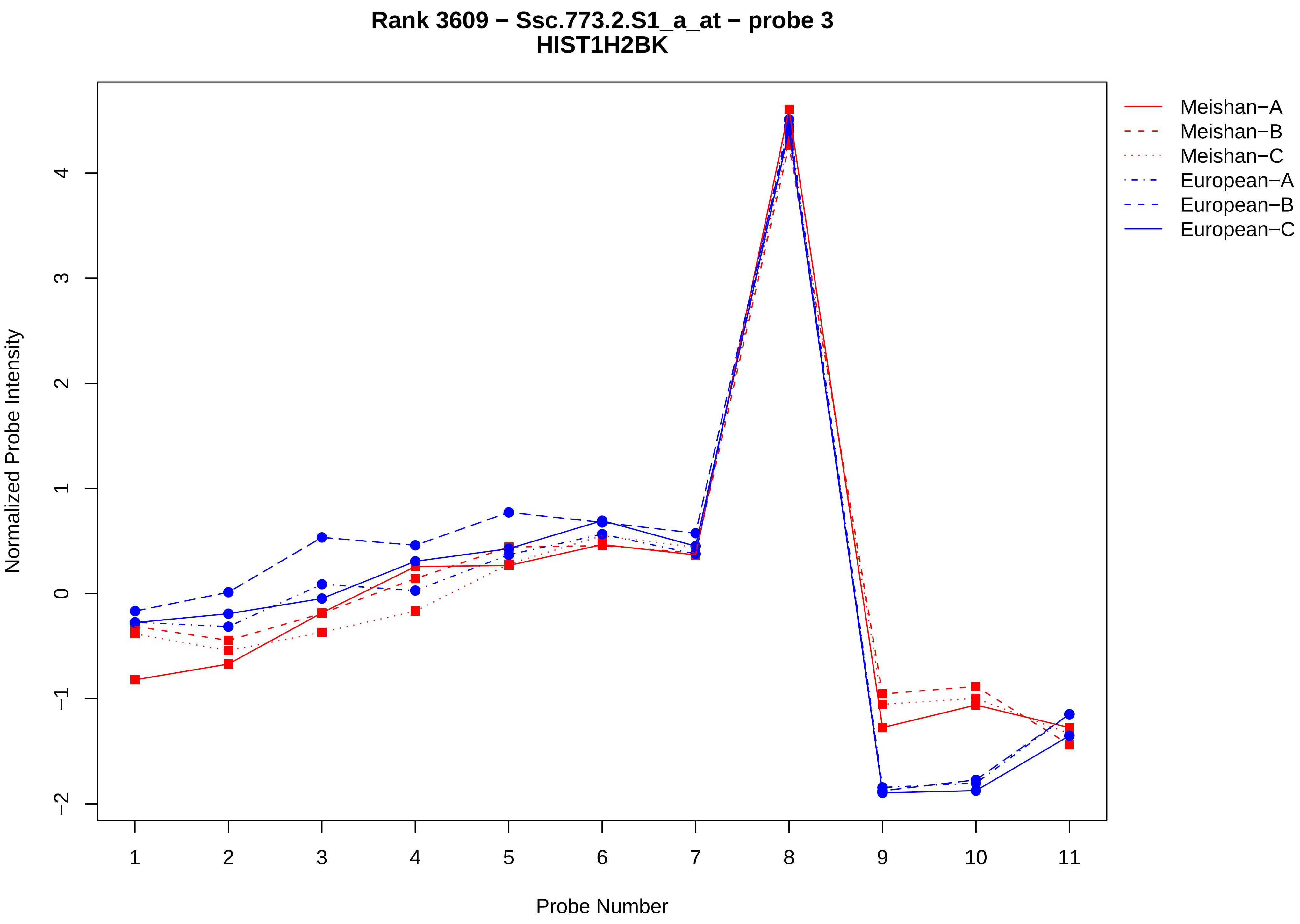
Rank 3609 − Ssc.773.2.S1_a_at − probe 3
HIST1H2BK
Normalized Probe Intensity
Probe Number
−2
−1
0
1
2
3
4
1
2
3
4
5
6
7
8
9
10
11
Meishan−A
Meishan−B
Meishan−C
European−A
European−B
European−C

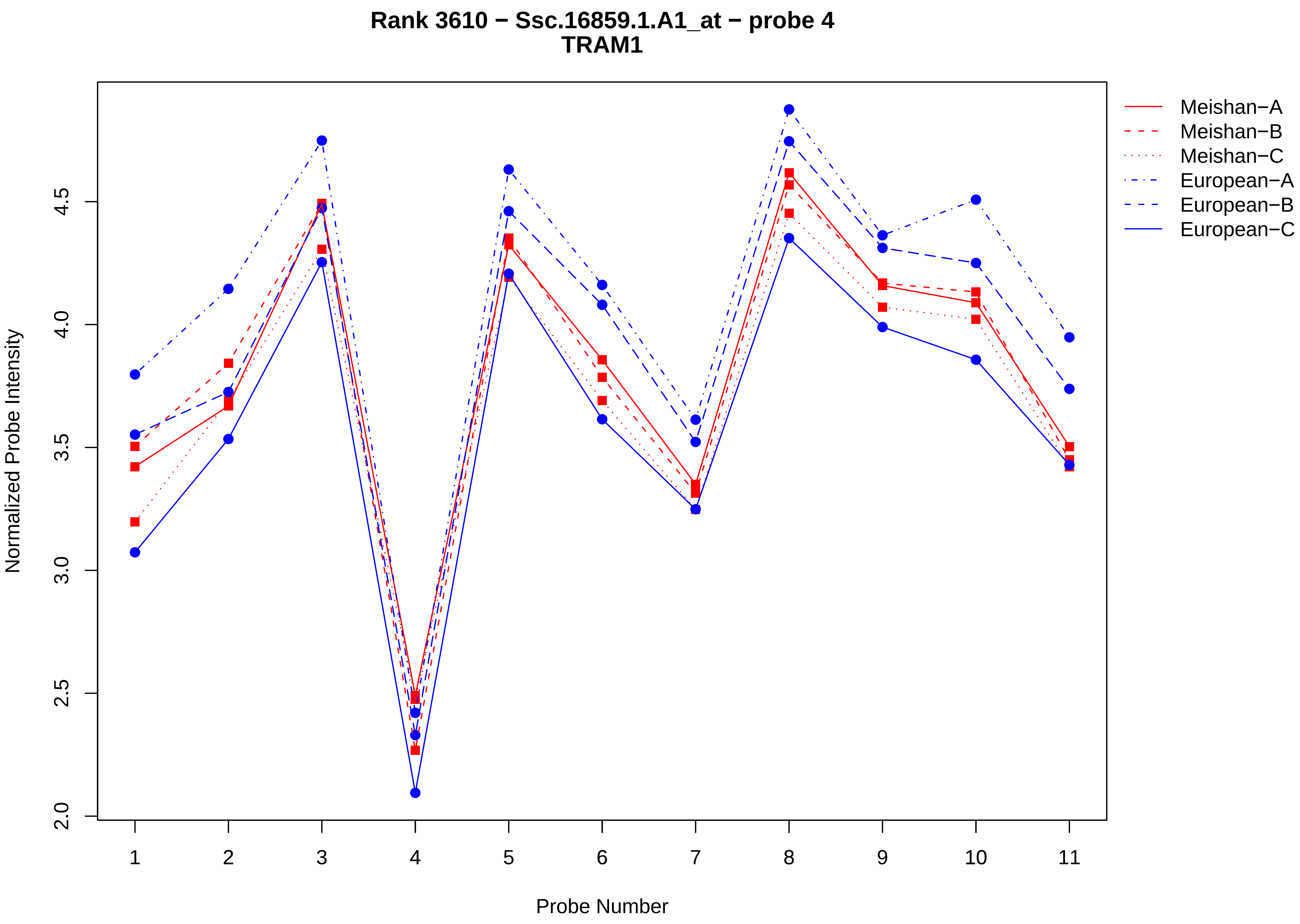

Rank 3610 − Ssc.16859.1.A1_at − probe 4
TRAM1
Normalized Probe Intensity
Probe Number
2.0
2.5
3.0
3.5
4.0
4.5
1
2
3
4
5
6
7
8
9
10
11
Meishan−A
Meishan−B
Meishan−C
European−A
European−B
European−C

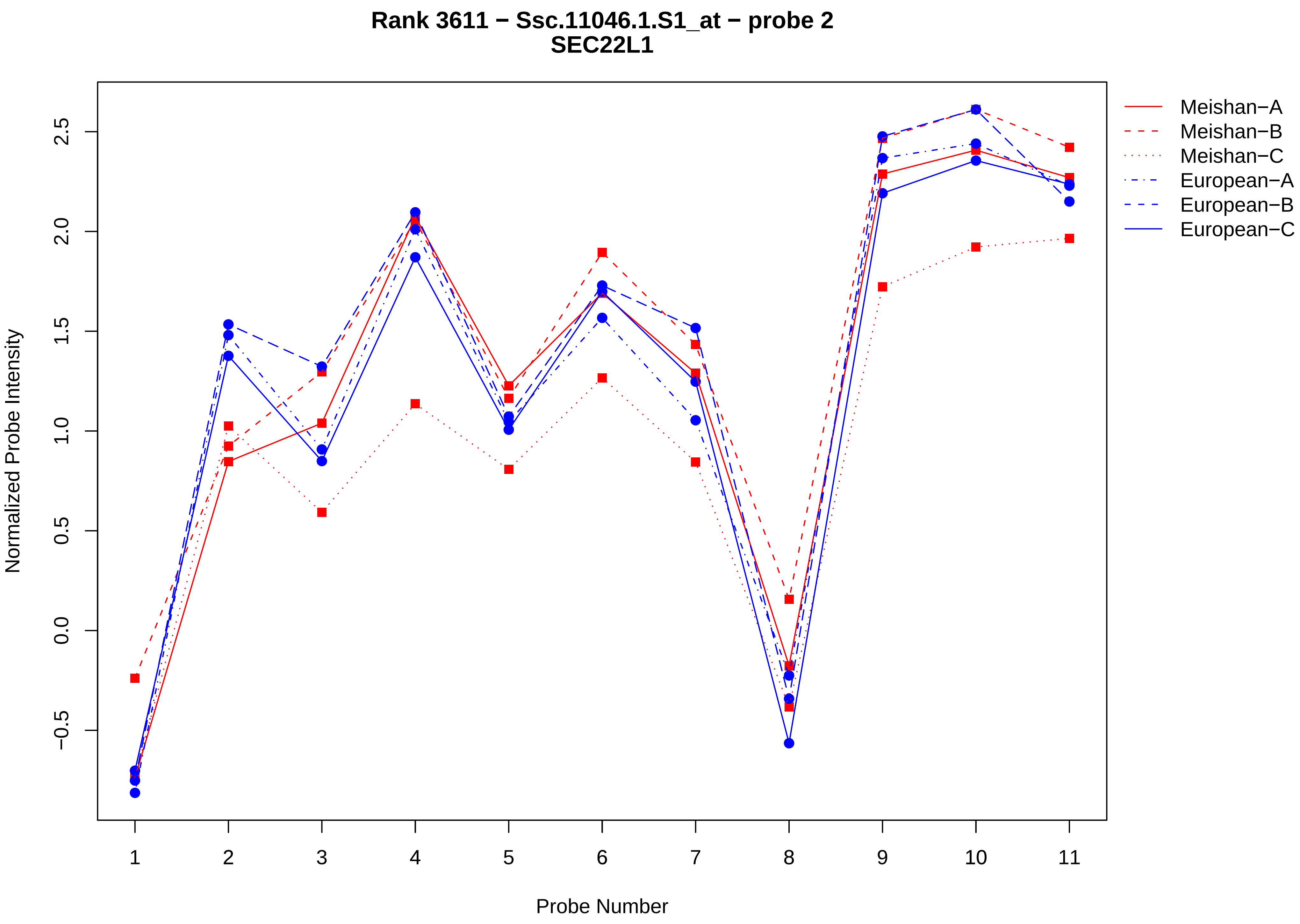
Rank 3611 − Ssc.11046.1.S1_at − probe 2
SEC22L1
Normalized Probe Intensity
Probe Number
−0.5
0.0
0.5
1.0
1.5
2.0
2.5
1
2
3
4
5
6
7
8
9
10
11
Meishan−A
Meishan−B
Meishan−C
European−A
European−B
European−C

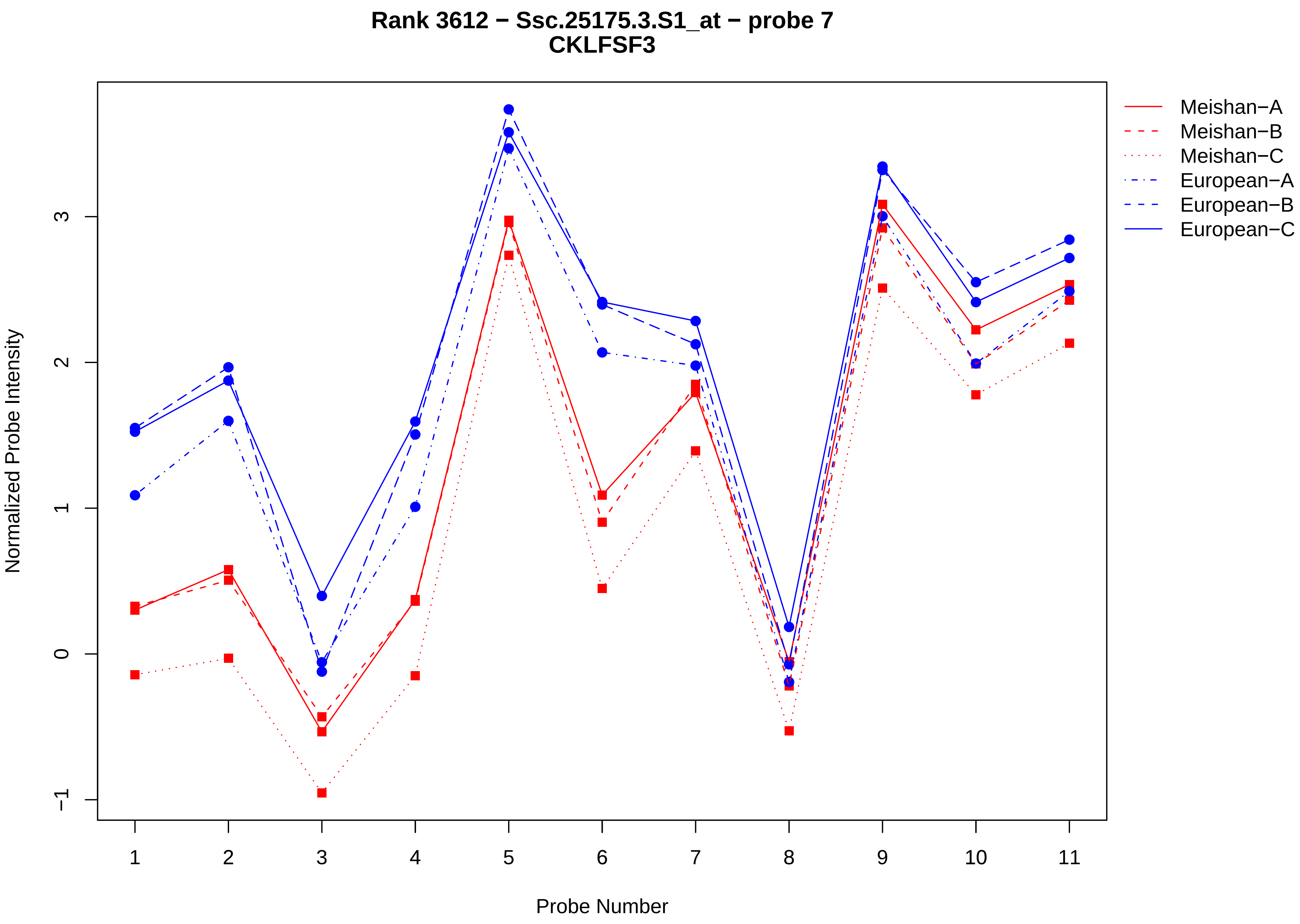

Rank 3612 − Ssc.25175.3.S1_at − probe 7
CKLFSF3
Normalized Probe Intensity
Probe Number
−1
0
1
2
3
1
2
3
4
5
6
7
8
9
10
11
Meishan−A
Meishan−B
Meishan−C
European−A
European−B
European−C

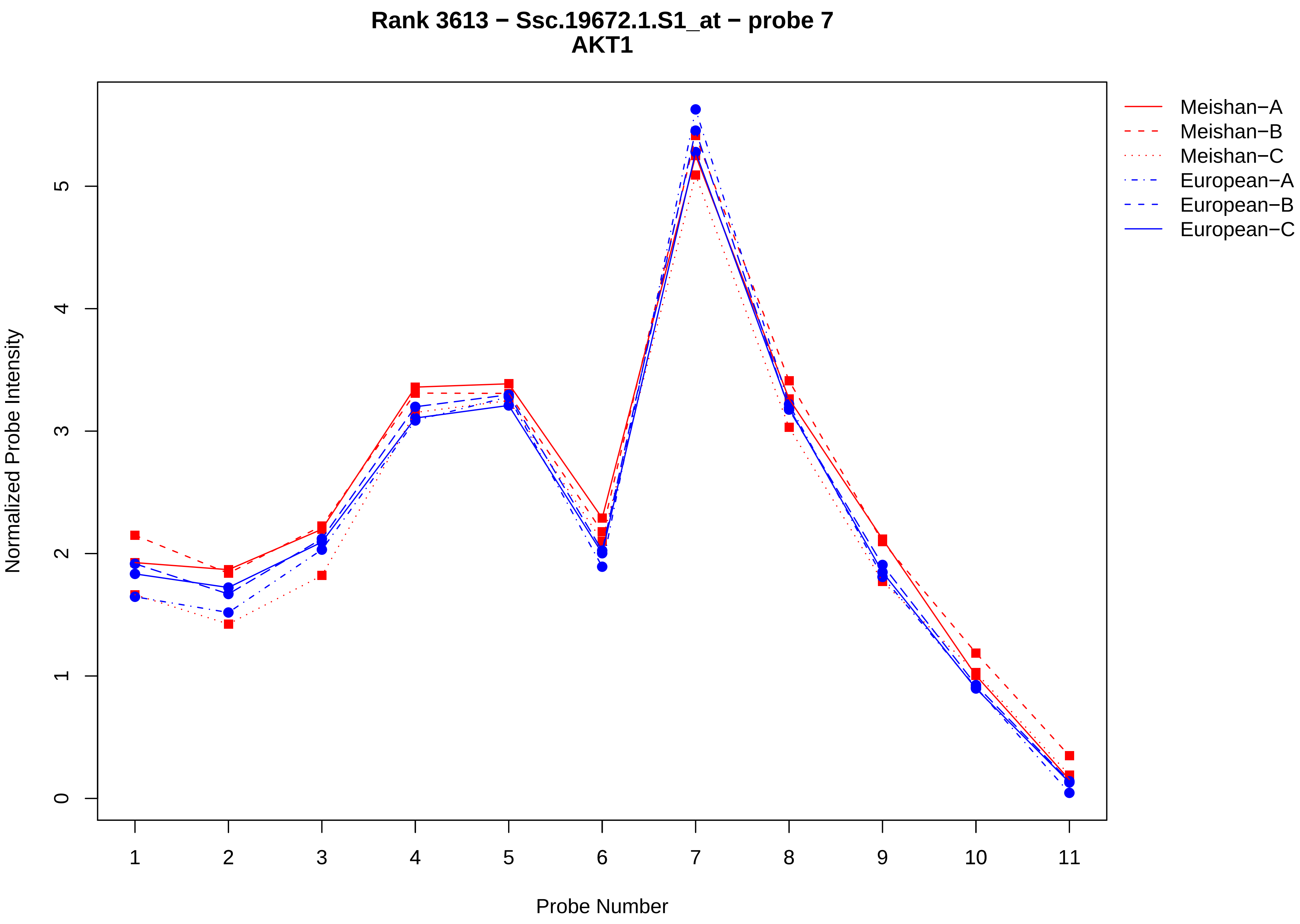

Rank 3613 − Ssc.19672.1.S1_at − probe 7
AKT1
Normalized Probe Intensity
Probe Number
0
1
2
3
4
5
1
2
3
4
5
6
7
8
9
10
11
Meishan−A
Meishan−B
Meishan−C
European−A
European−B
European−C

Rank 3614 − Ssc.19297.1.S1_at − probe 2
CLN2

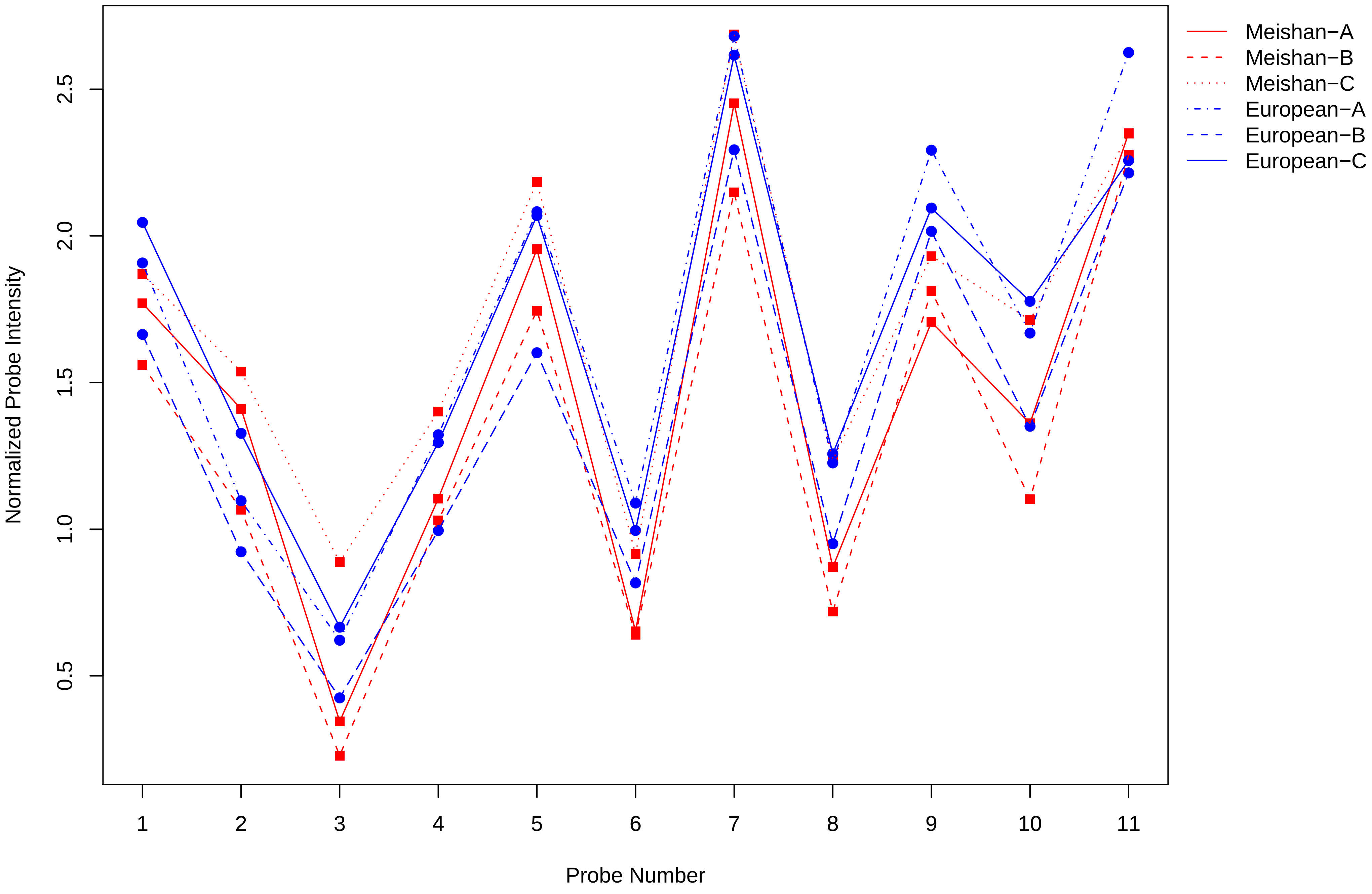

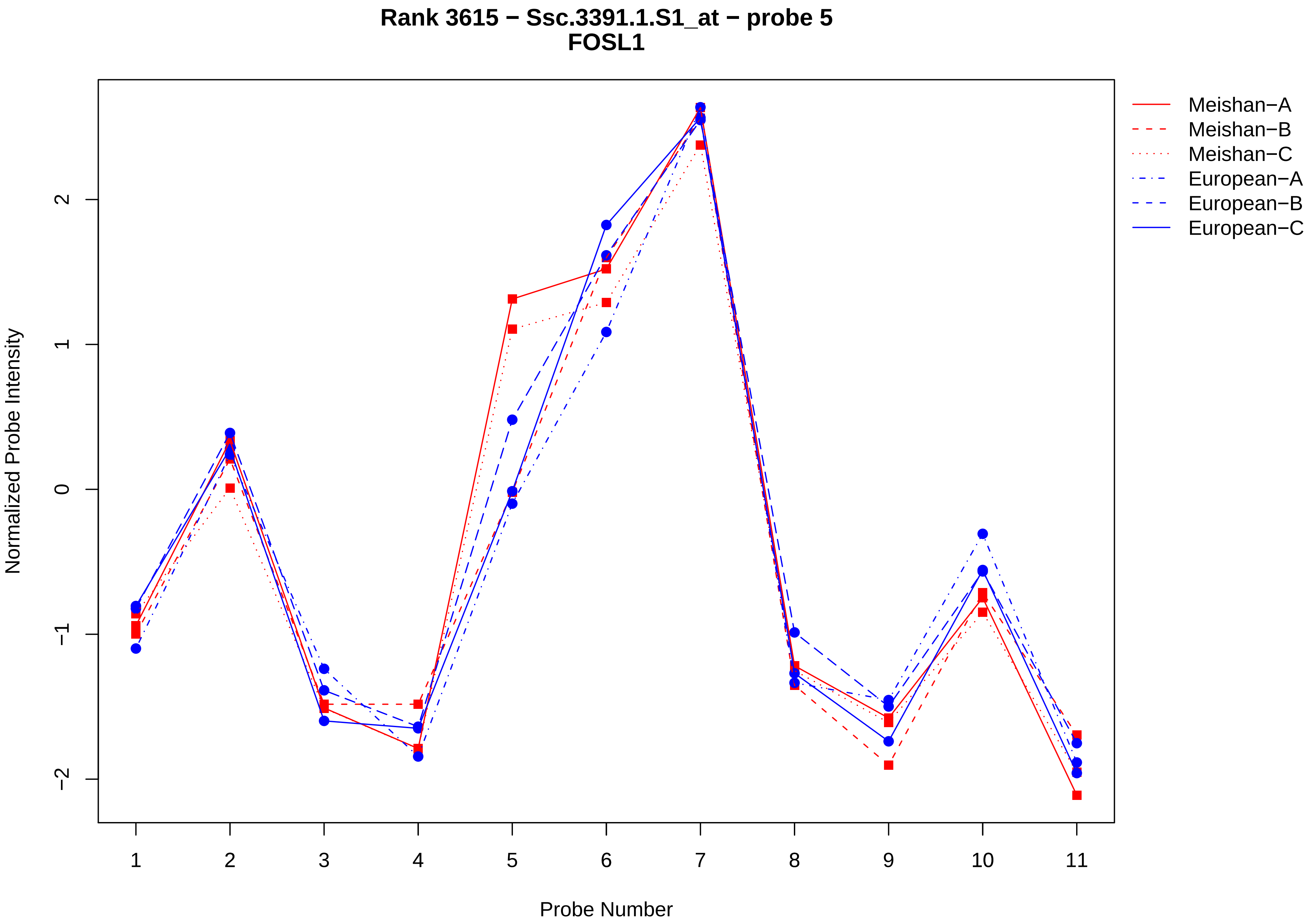

Rank 3615 − Ssc.3391.1.S1_at − probe 5
FOSL1
Normalized Probe Intensity
Probe Number
Meishan−A
Meishan−B
Meishan−C
European−A
European−B
European−C
−2
−1
0
1
2
1
2
3
4
5
6
7
8
9
10
11

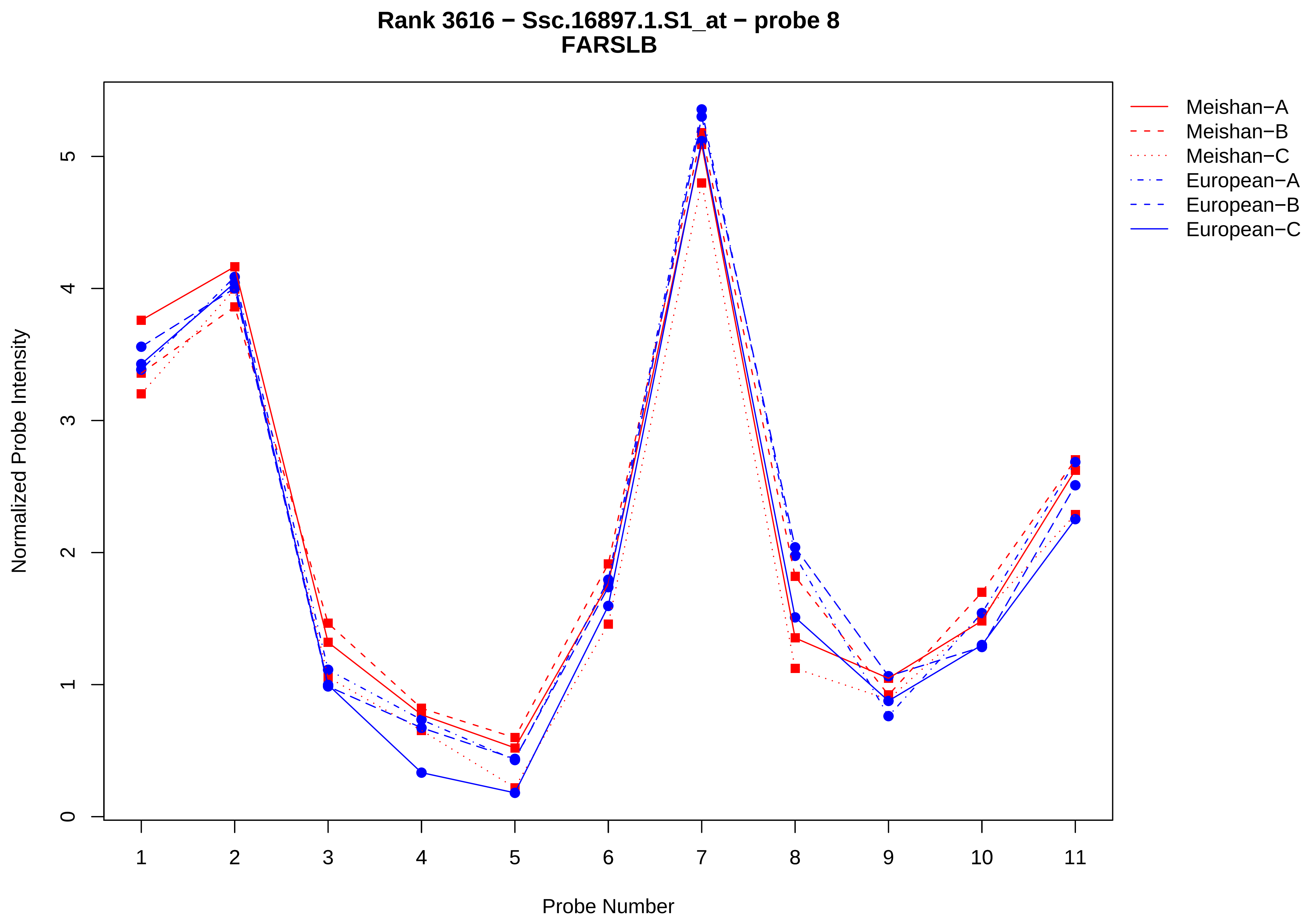

Rank 3616 − Ssc.16897.1.S1_at − probe 8
FARSLB
Normalized Probe Intensity
Probe Number
0
1
2
3
4
5
1
2
3
4
5
6
7
8
9
10
11
Meishan−A
Meishan−B
Meishan−C
European−A
European−B
European−C

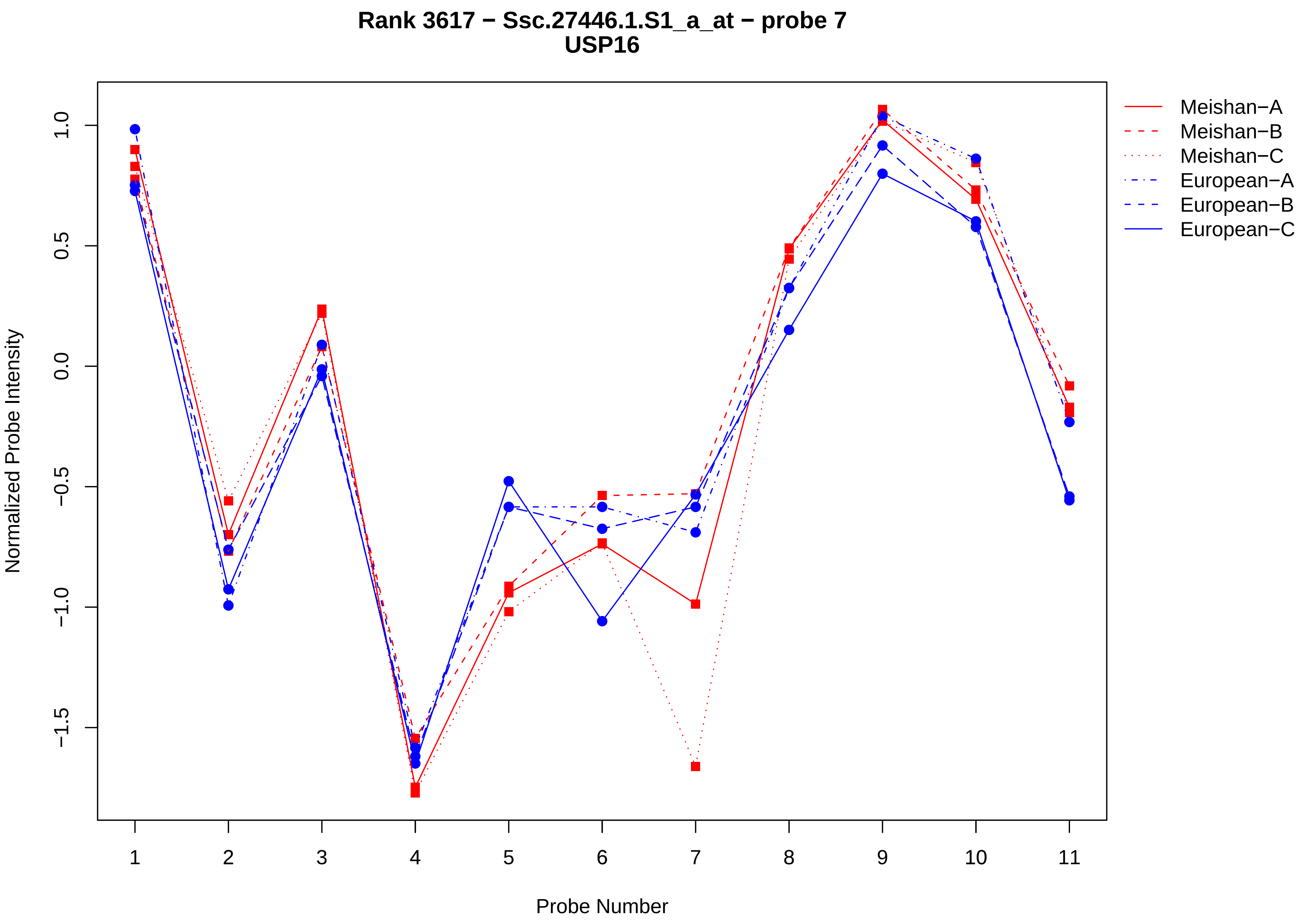
Rank 3617 − Ssc.27446.1.S1_a_at − probe 7
USP16
Normalized Probe Intensity
Probe Number
1.0
0.5
0.0
−0.5
−1.0
−1.5
1
2
3
4
5
6
7
8
9
10
11
Meishan−A
Meishan−B
Meishan−C
European−A
European−B
European−C

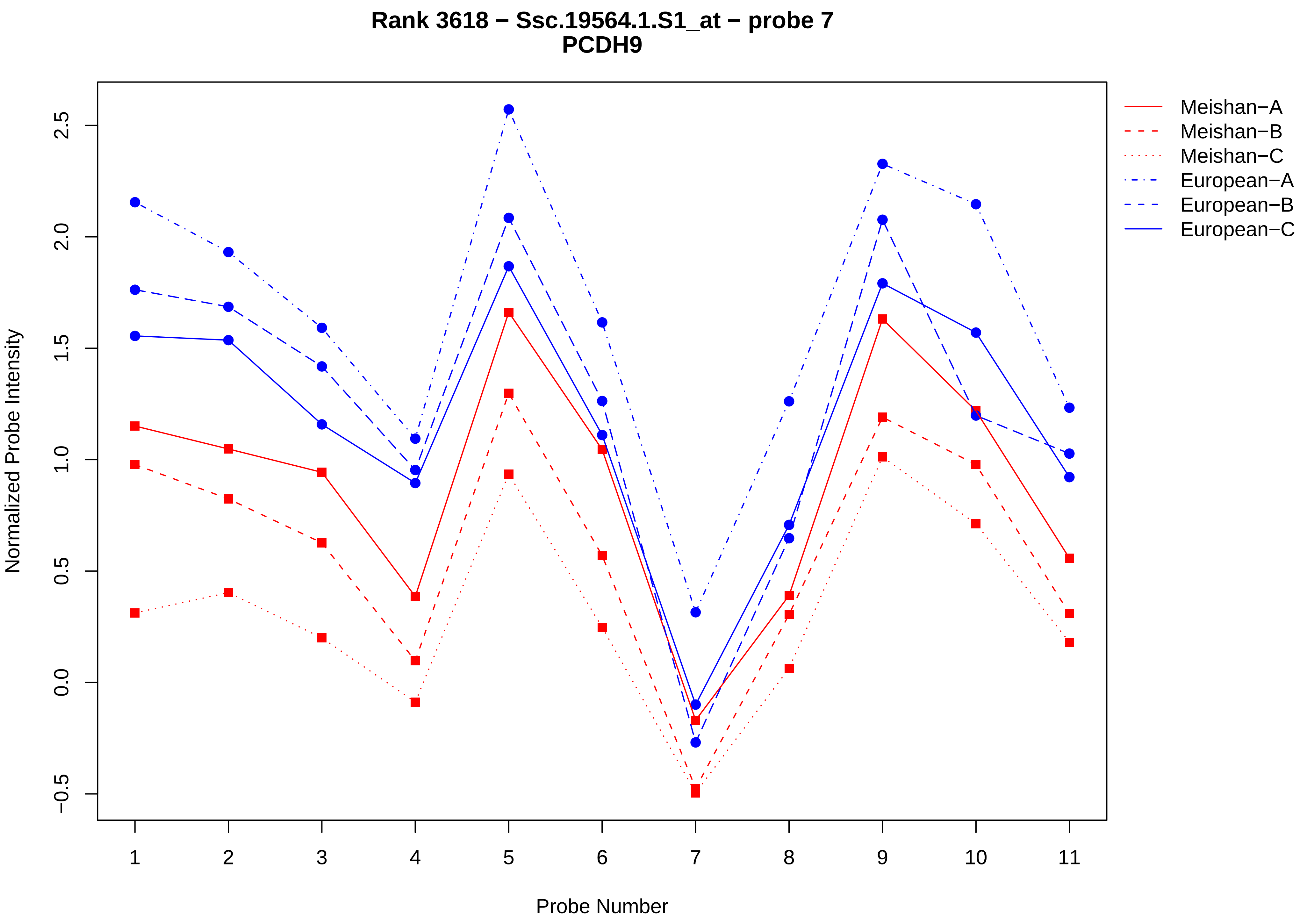

Rank 3618 − Ssc.19564.1.S1_at − probe 7
PCDH9
Normalized Probe Intensity
Probe Number
Meishan−A
Meishan−B
Meishan−C
European−A
European−B
European−C

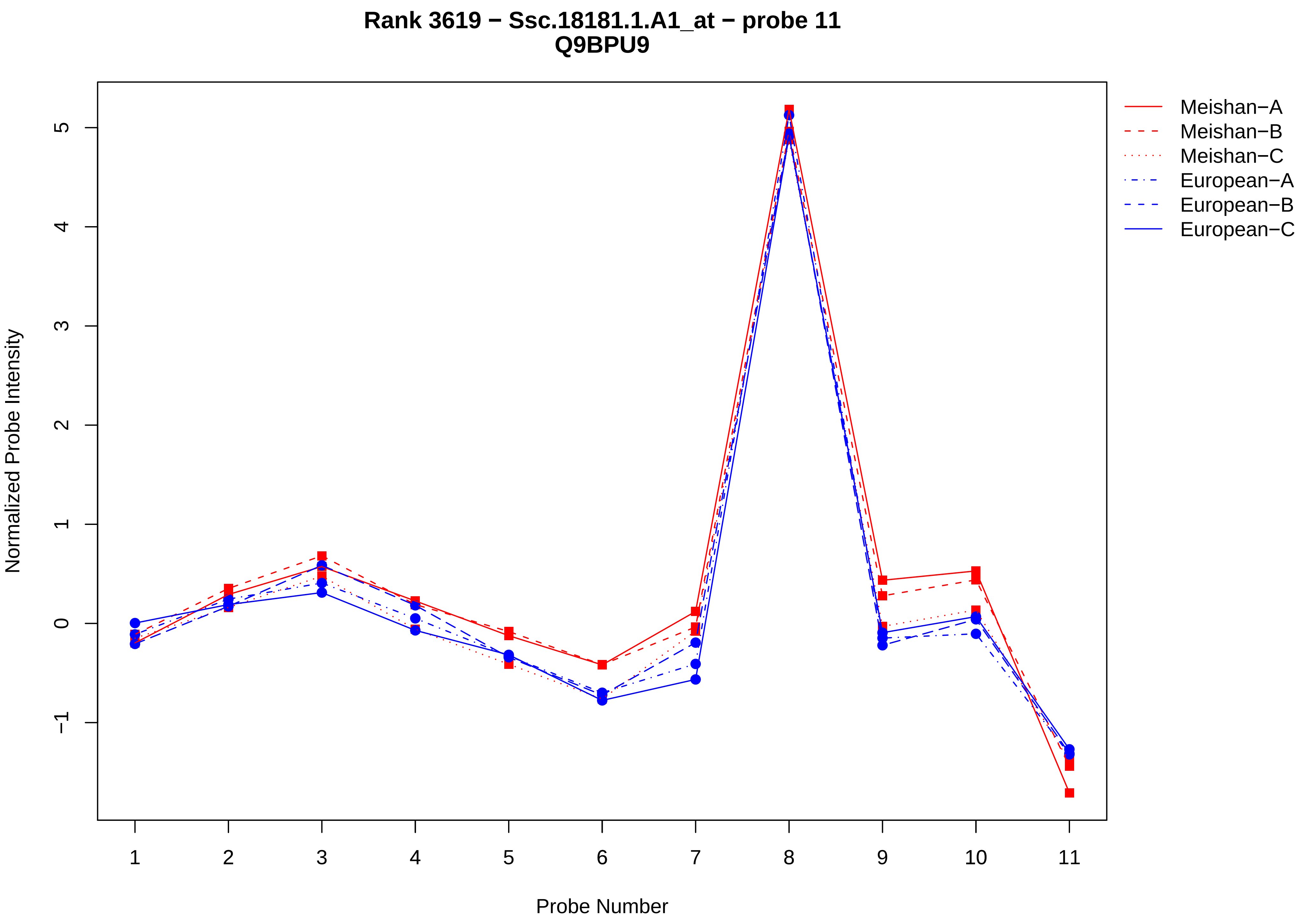
Rank 3619 − Ssc.18181.1.A1_at − probe 11
Q9BPU9
Normalized Probe Intensity
Probe Number
Meishan−A
Meishan−B
Meishan−C
European−A
European−B
European−C

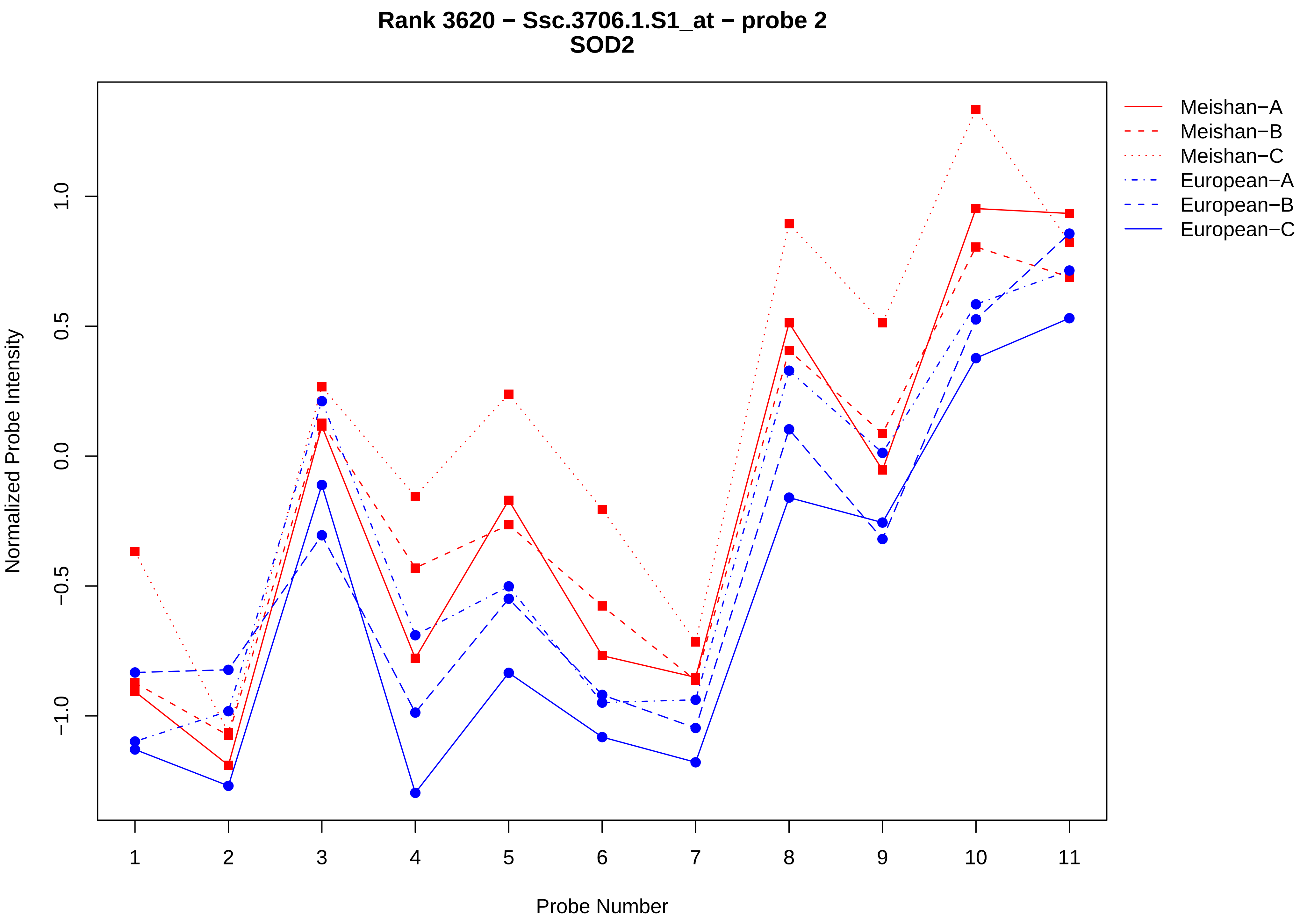

Rank 3620 − Ssc.3706.1.S1_at − probe 2
SOD2
Normalized Probe Intensity
Probe Number
1.0
0.5
0.0
−0.5
−1.0
1
2
3
4
5
6
7
8
9
10
11
Meishan−A
Meishan−B
Meishan−C
European−A
European−B
European−C

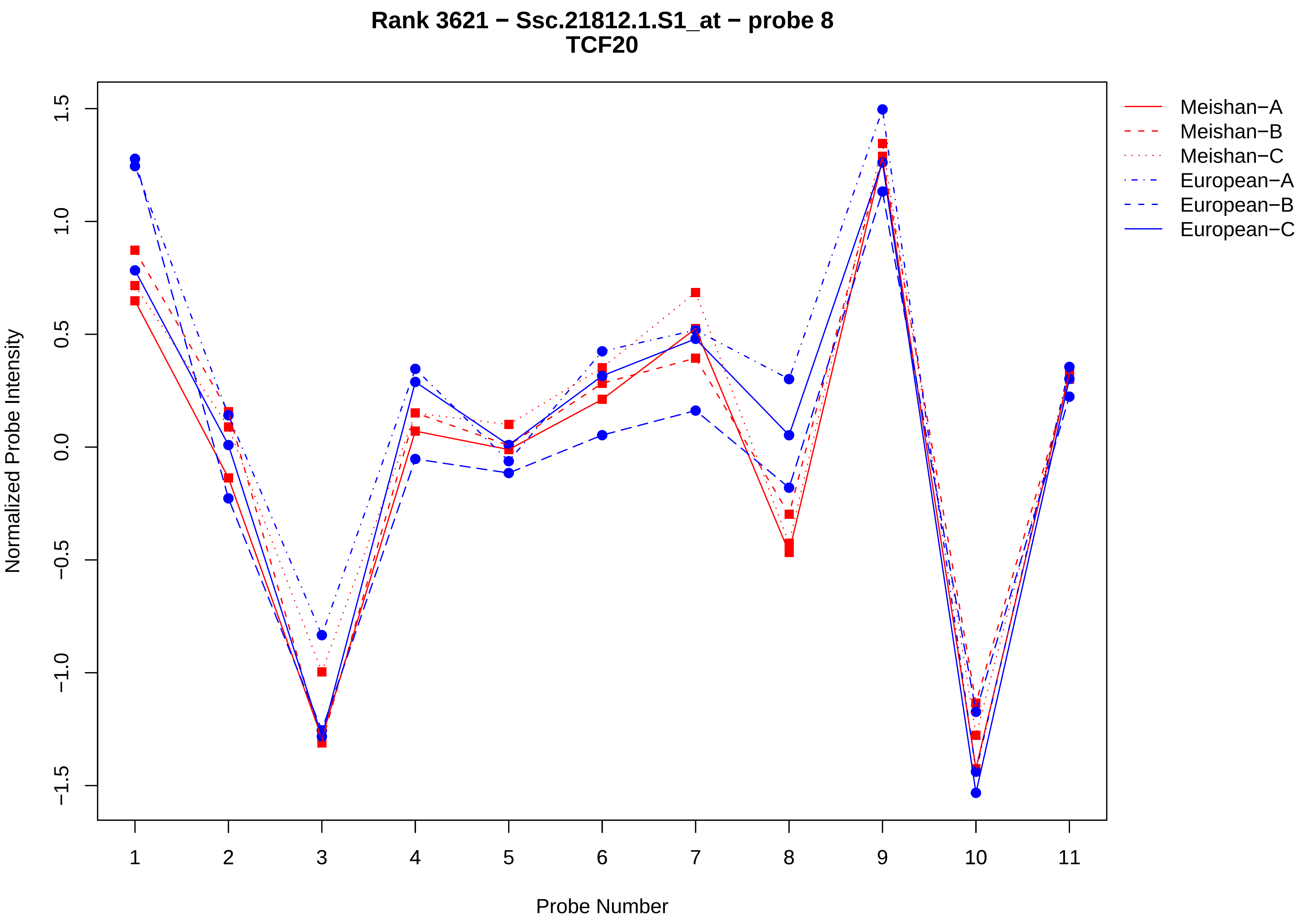

Rank 3621 − Ssc.21812.1.S1_at − probe 8
TCF20
Normalized Probe Intensity
Probe Number
Meishan−A
Meishan−B
Meishan−C
European−A
European−B
European−C
1.5
1.0
0.5
0.0
−0.5
−1.0
−1.5
1
2
3
4
5
6
7
8
9
10
11

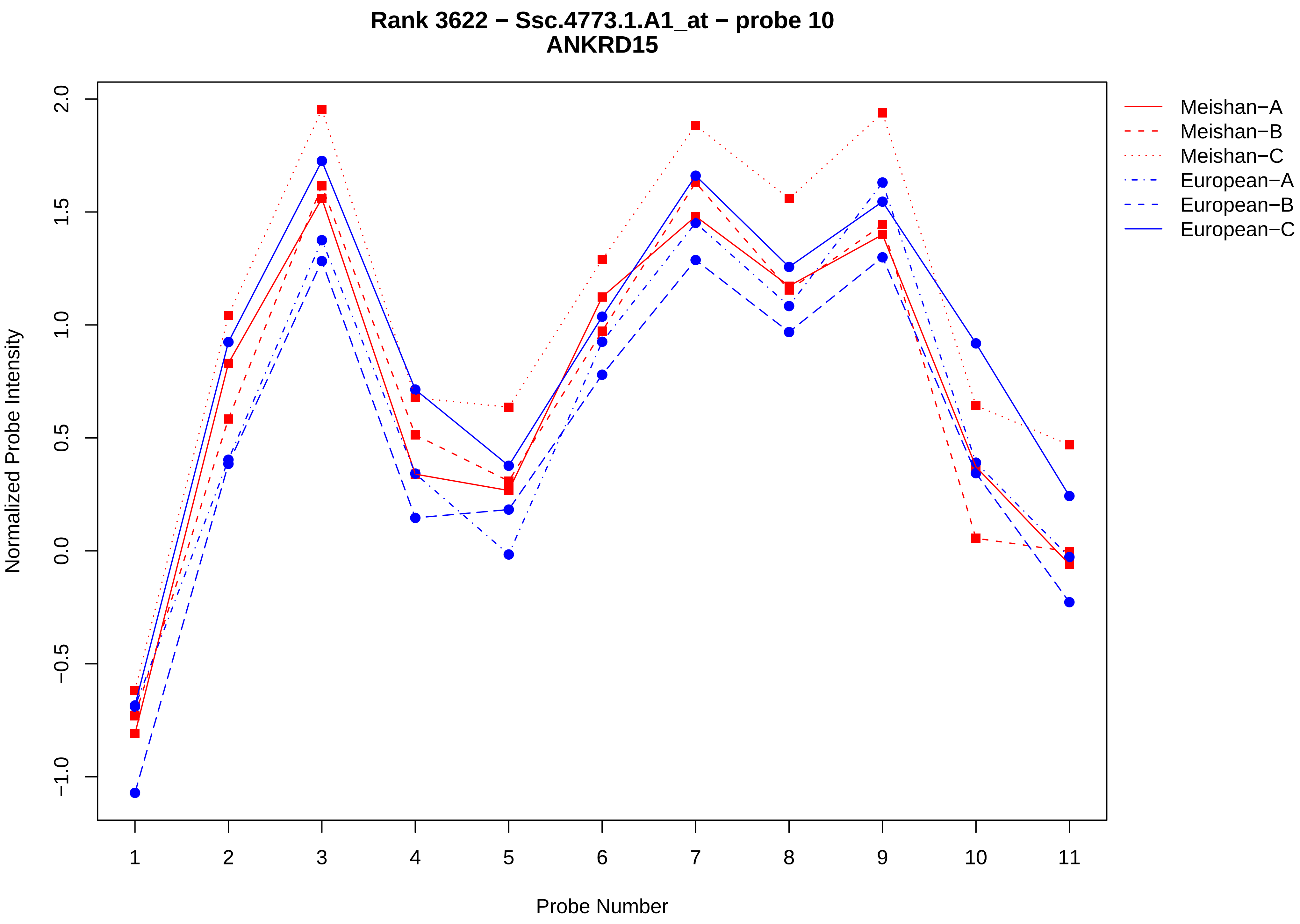

Rank 3622 − Ssc.4773.1.A1_at − probe 10
ANKRD15
Normalized Probe Intensity
Probe Number
−1.0
−0.5
0.0
0.5
1.0
1.5
2.0
1
2
3
4
5
6
7
8
9
10
11
Meishan−A
Meishan−B
Meishan−C
European−A
European−B
European−C

Rank 3623 − Ssc.11668.1.A1_at − probe 11
KPNA2

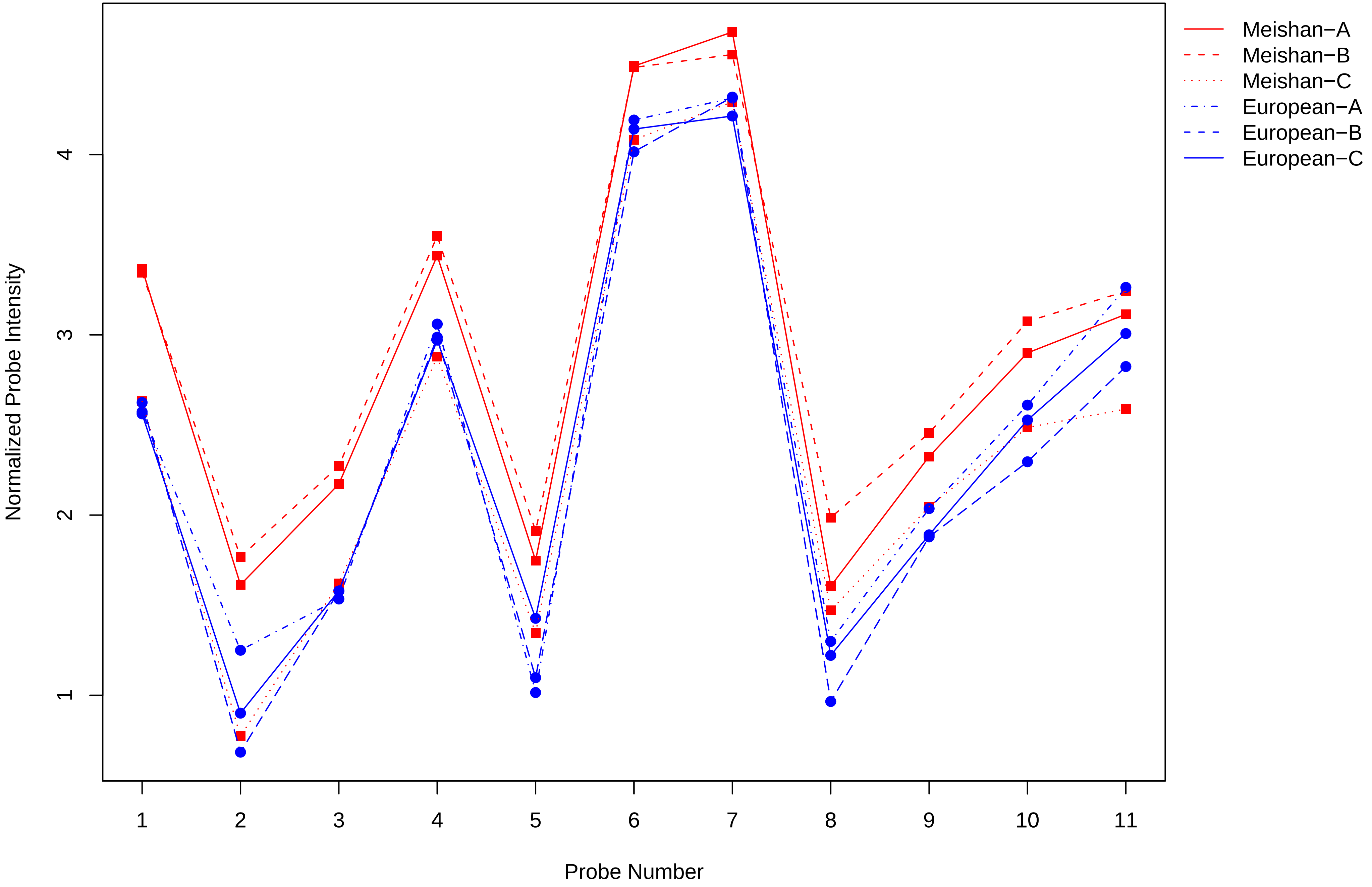

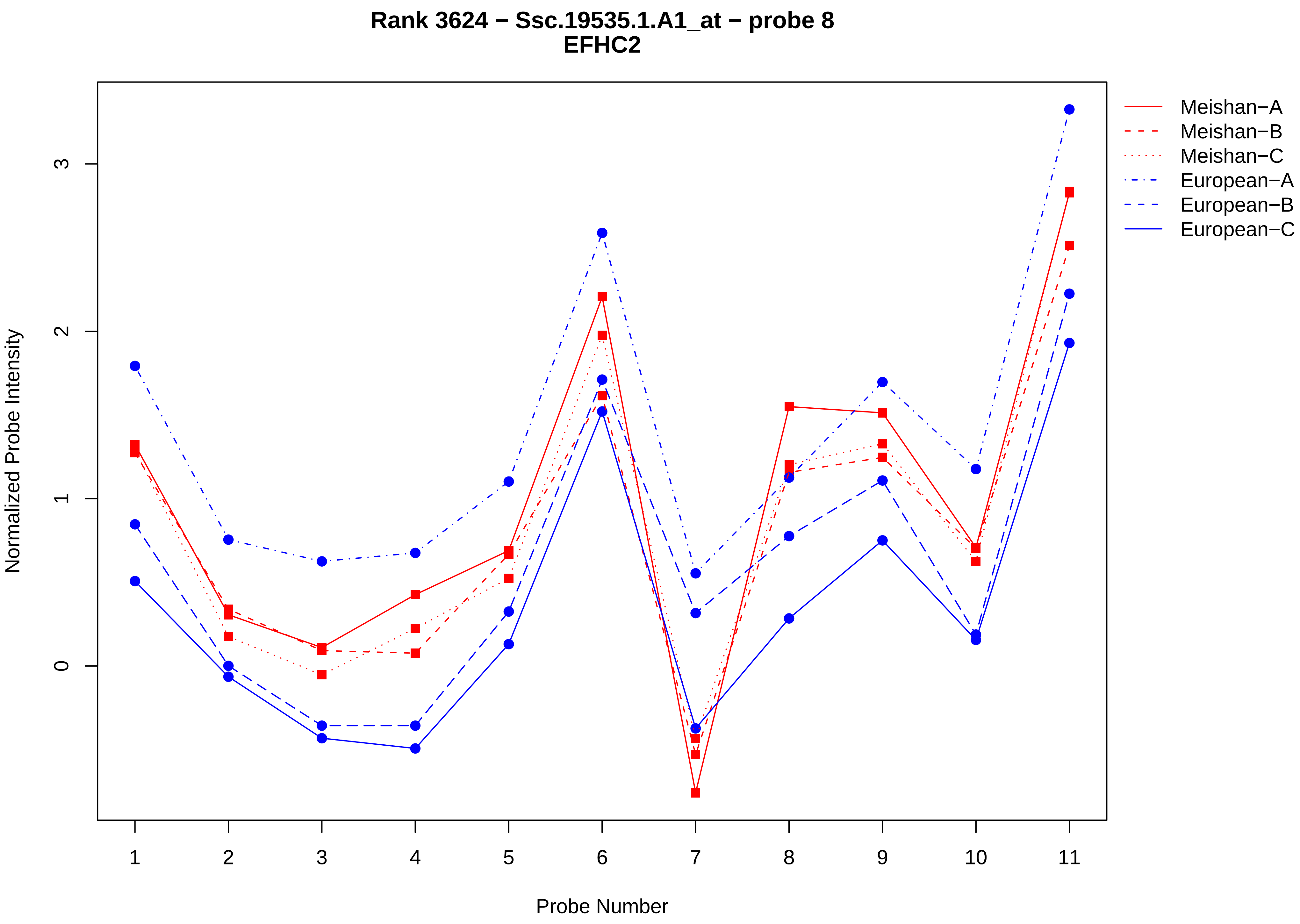

Rank 3624 − Ssc.19535.1.A1_at − probe 8
EFHC2
Normalized Probe Intensity
Probe Number
0
1
2
3
1
2
3
4
5
6
7
8
9
10
11
Meishan−A
Meishan−B
Meishan−C
European−A
European−B
European−C

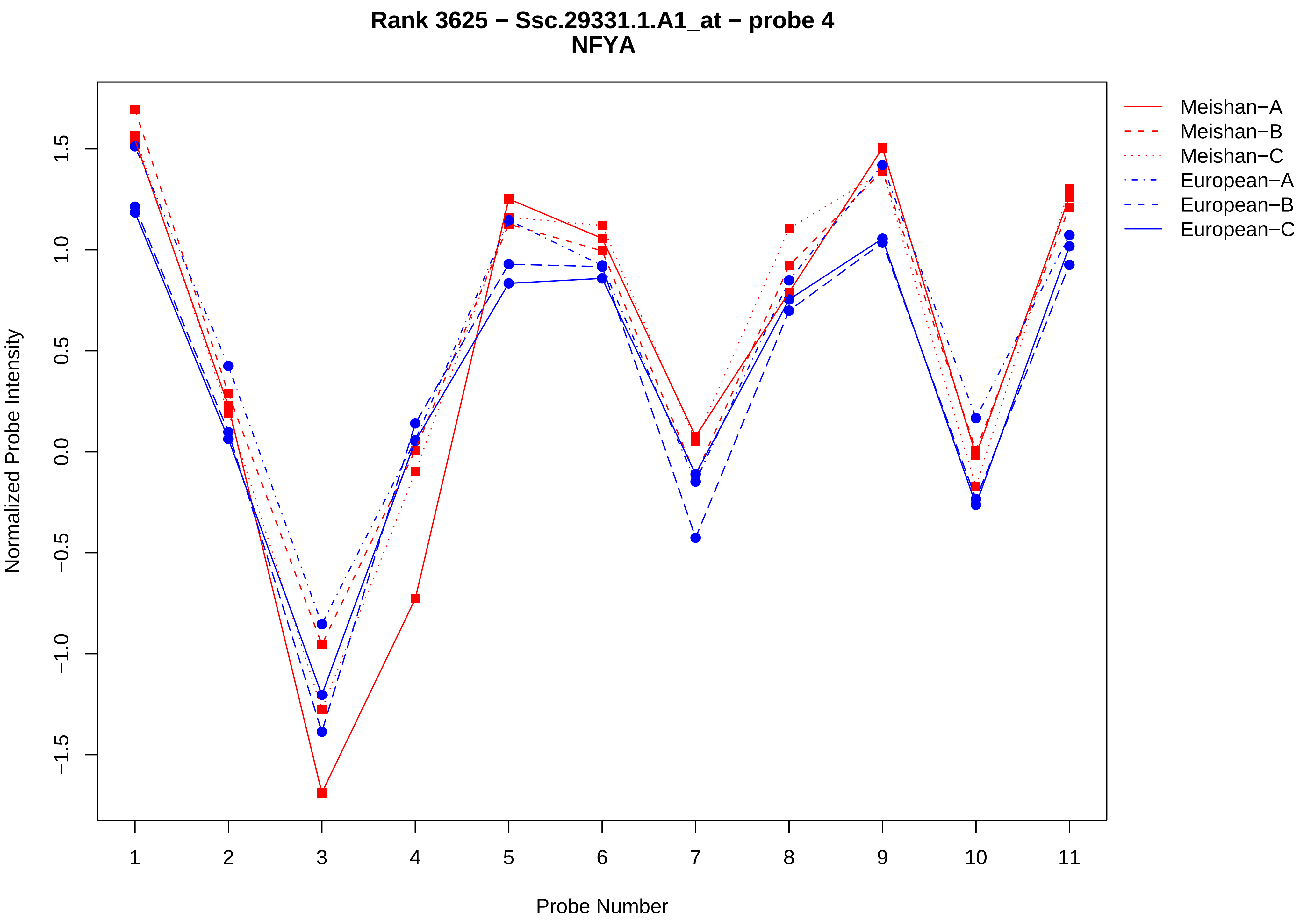

Rank 3625 − Ssc.29331.1.A1_at − probe 4
NFYA
Normalized Probe Intensity
Probe Number
1.5
1.0
0.5
0.0
−0.5
−1.0
−1.5
1
2
3
4
5
6
7
8
9
10
11
Meishan−A
Meishan−B
Meishan−C
European−A
European−B
European−C

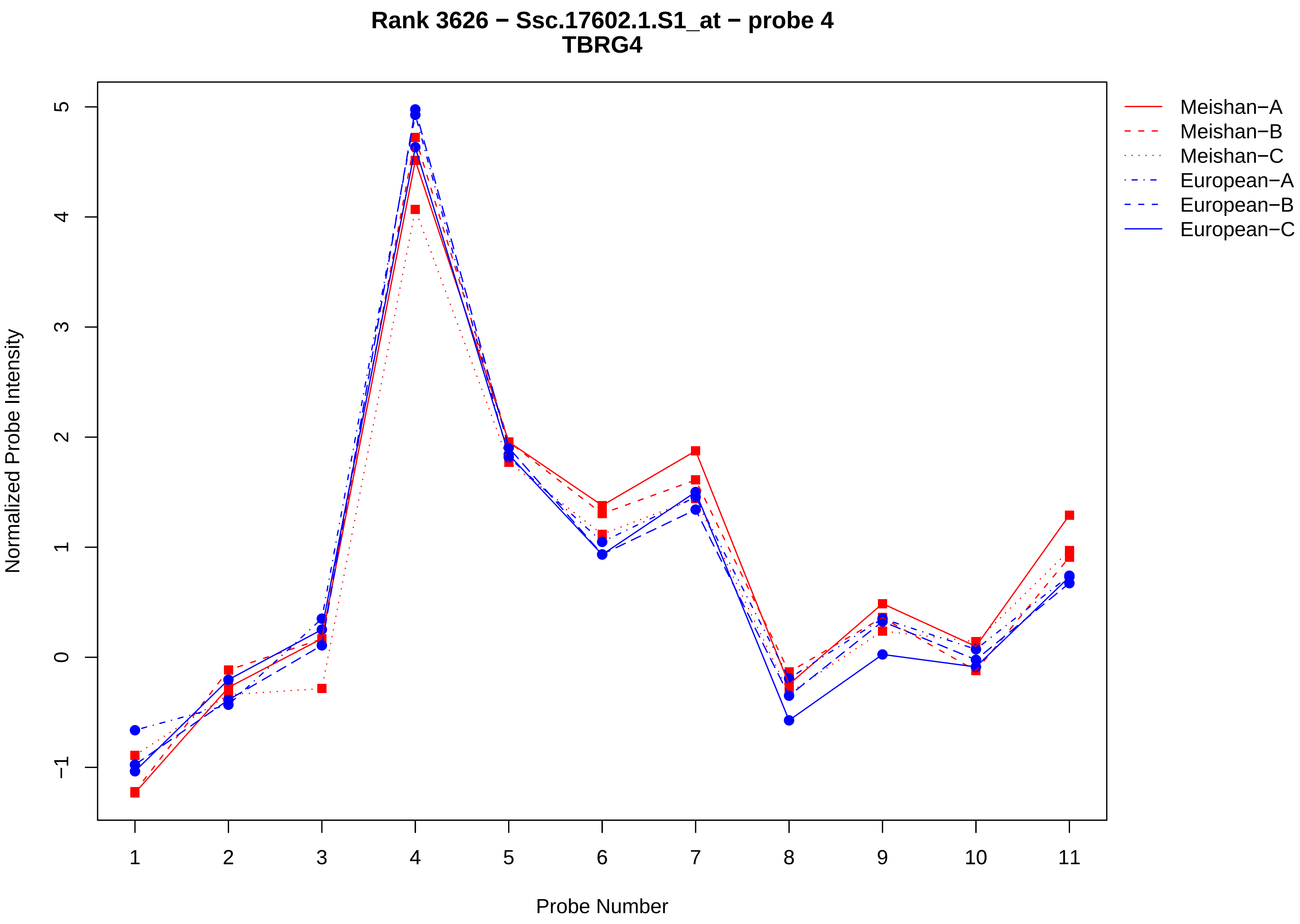

Rank 3626 − Ssc.17602.1.S1_at − probe 4
TBRG4
Normalized Probe Intensity
Probe Number
Meishan−A
Meishan−B
Meishan−C
European−A
European−B
European−C
−1
0
1
2
3
4
5
1
2
3
4
5
6
7
8
9
10
11

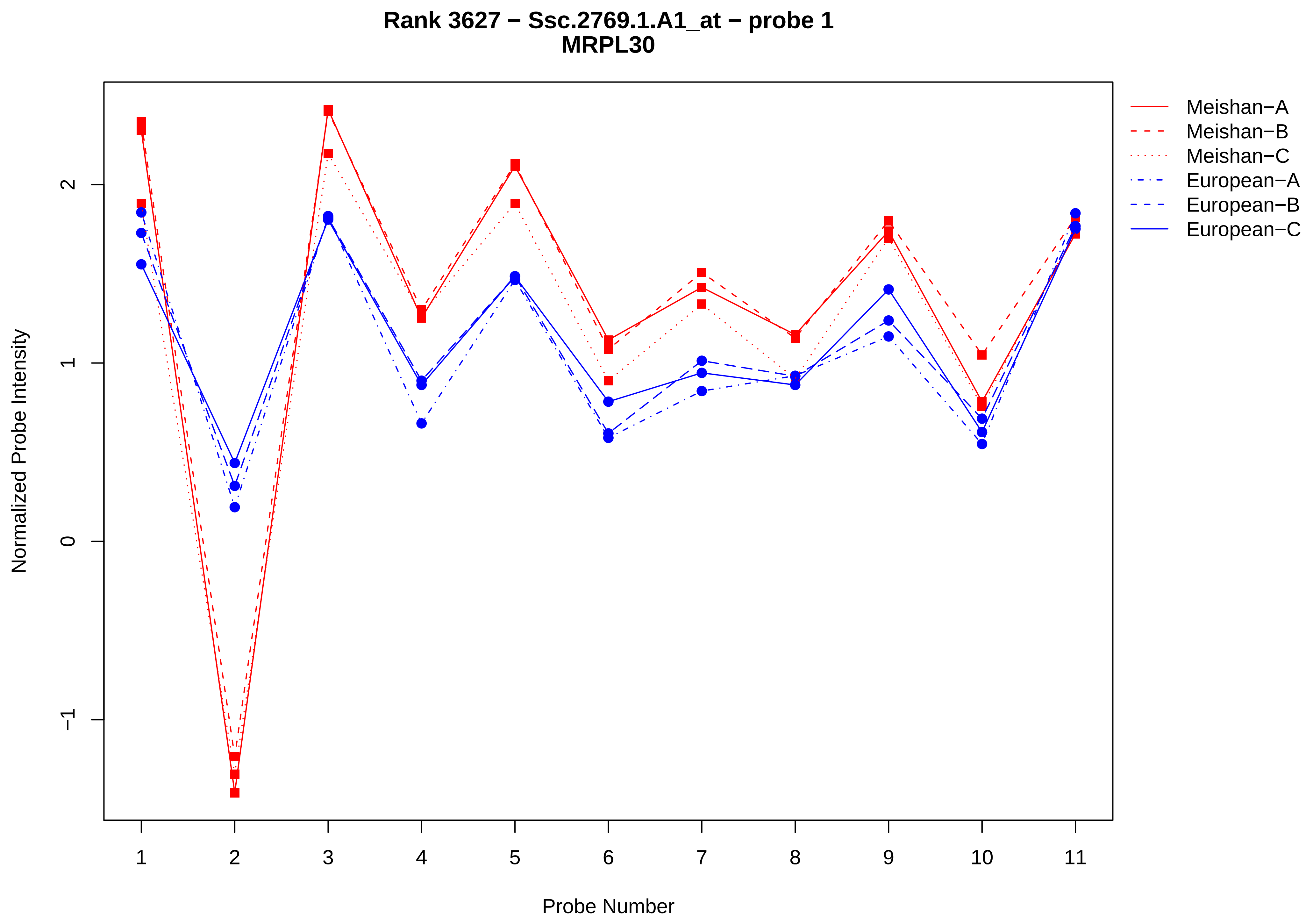

Rank 3627 − Ssc.2769.1.A1_at − probe 1
MRPL30
Normalized Probe Intensity
Probe Number
−1
0
1
2
1
2
3
4
5
6
7
8
9
10
11
Meishan−A
Meishan−B
Meishan−C
European−A
European−B
European−C

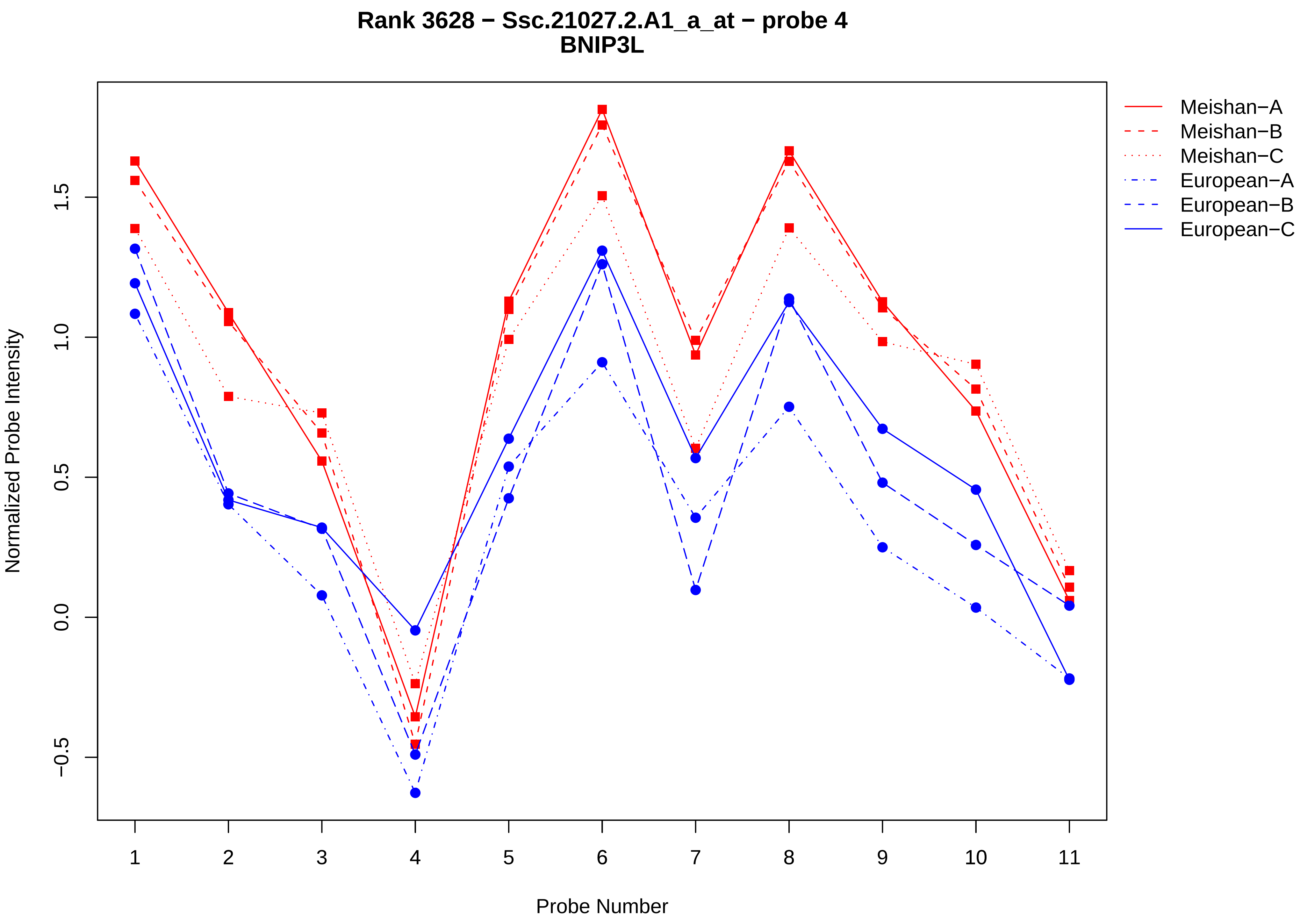

Rank 3628 − Ssc.21027.2.A1_a_at − probe 4
BNIP3L
Normalized Probe Intensity
Probe Number
−0.5
0.0
0.5
1.0
1.5
1
2
3
4
5
6
7
8
9
10
11
Meishan−A
Meishan−B
Meishan−C
European−A
European−B
European−C

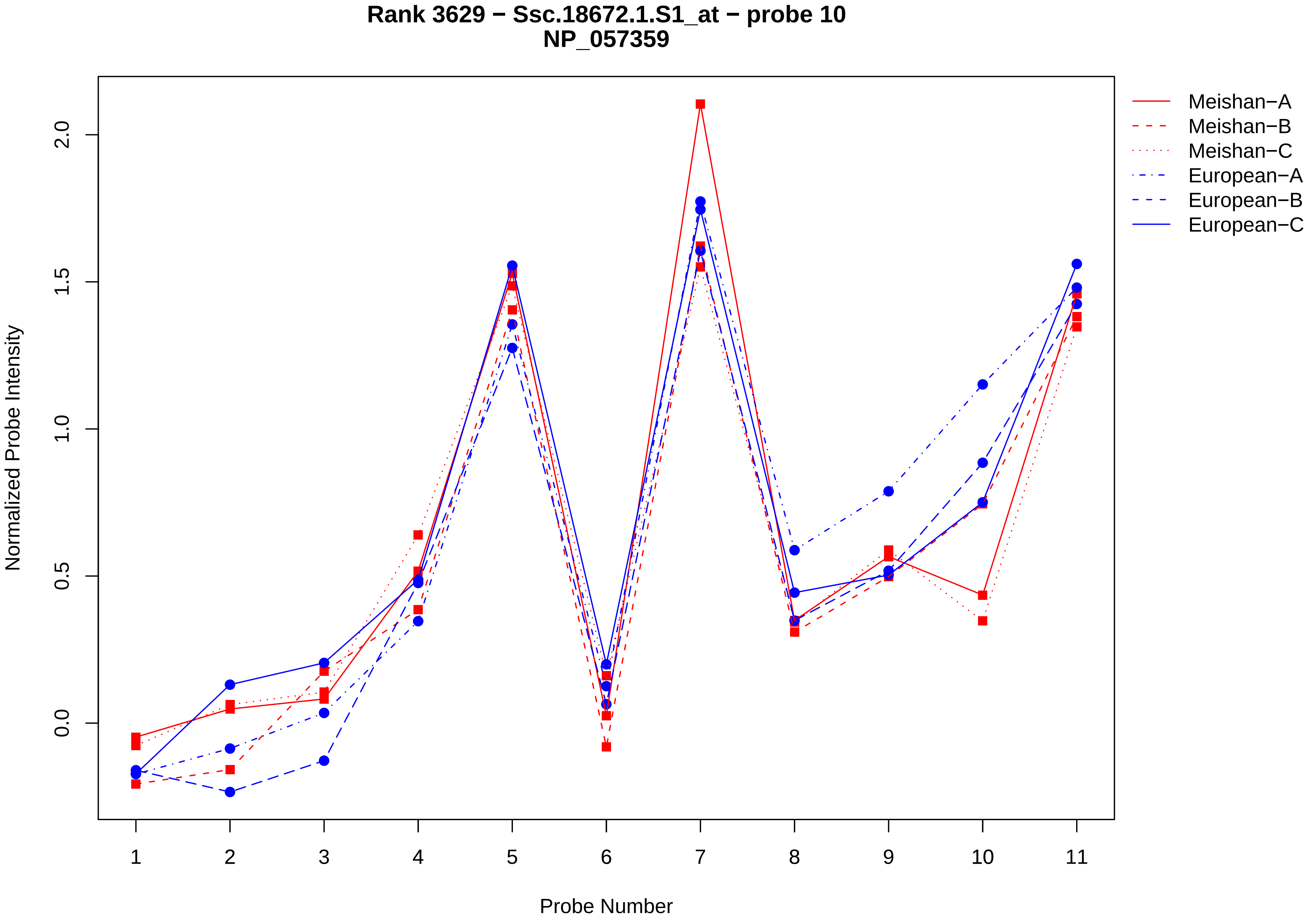

Rank 3629 − Ssc.18672.1.S1_at − probe 10
NP_057359
Normalized Probe Intensity
Probe Number
0.0
0.5
1.0
1.5
2.0
1
2
3
4
5
6
7
8
9
10
11
Meishan−A
Meishan−B
Meishan−C
European−A
European−B
European−C

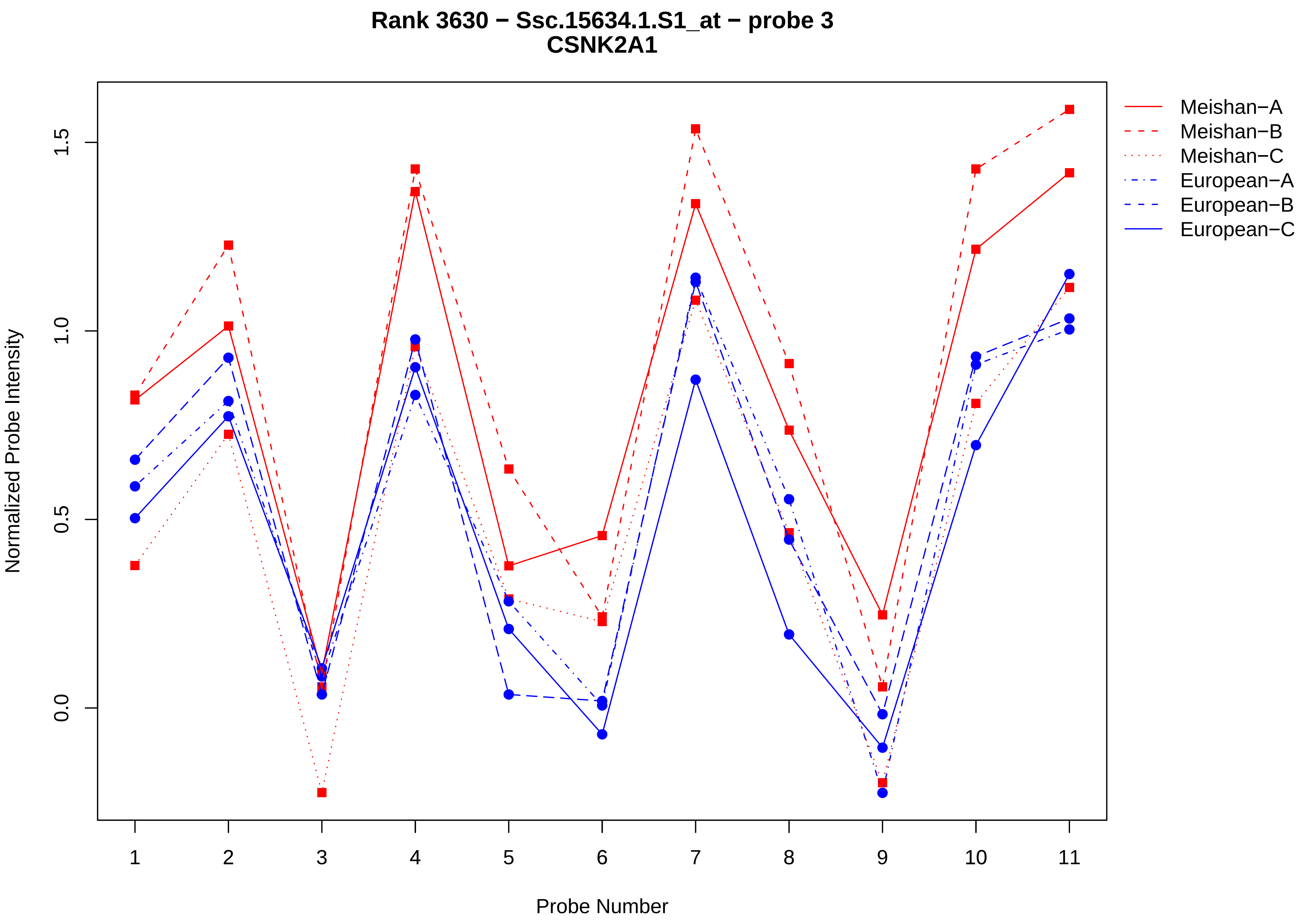

Rank 3630 − Ssc.15634.1.S1_at − probe 3
CSNK2A1
Normalized Probe Intensity
0.0
0.5
1.0
1.5
1
2
3
4
5
6
7
8
9
10
11
Probe Number
Meishan−A
Meishan−B
Meishan−C
European−A
European−B
European−C

Rank 3631 − Ssc.7455.1.A1_at − probe 4
AASDHPPT

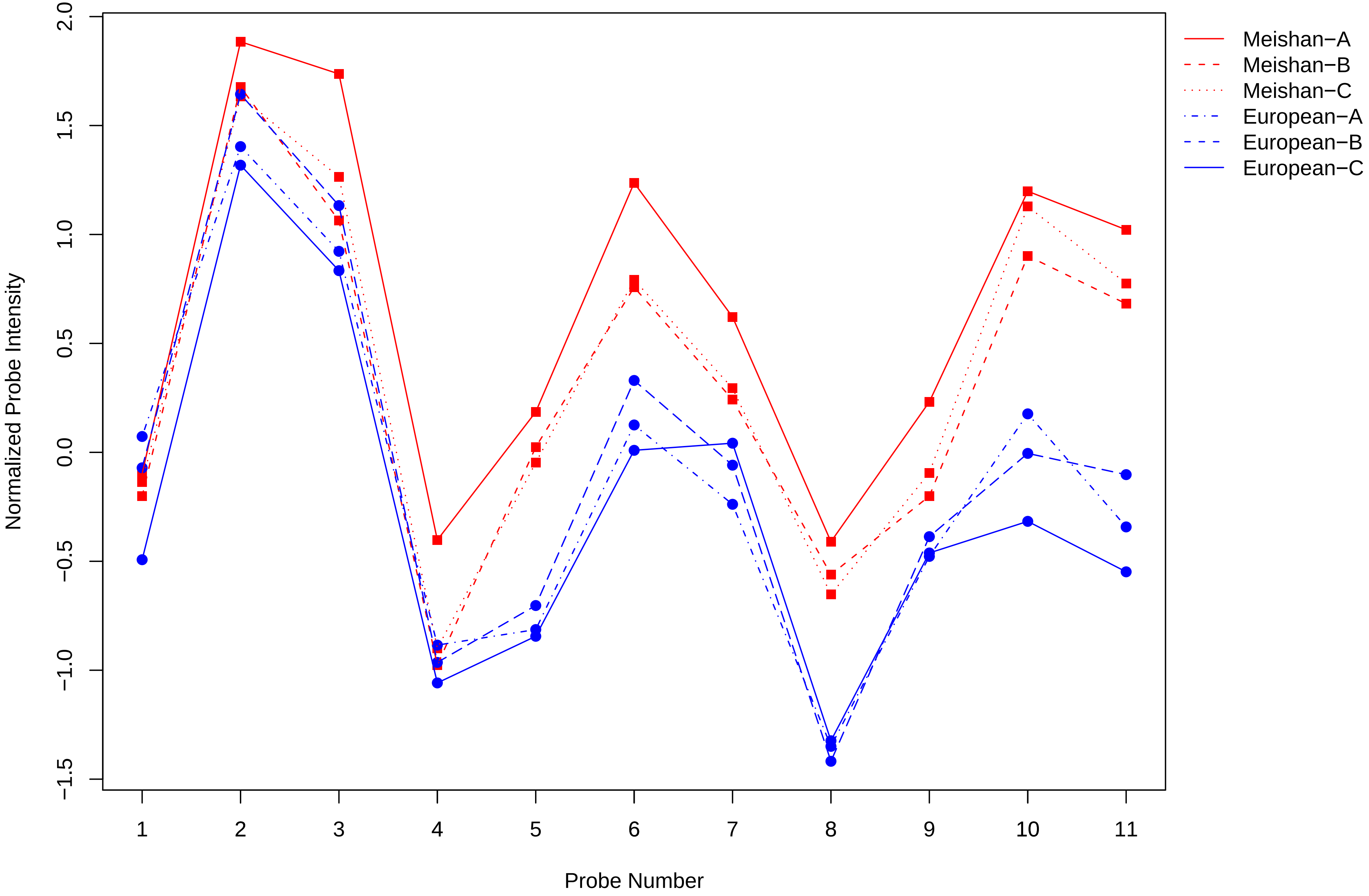

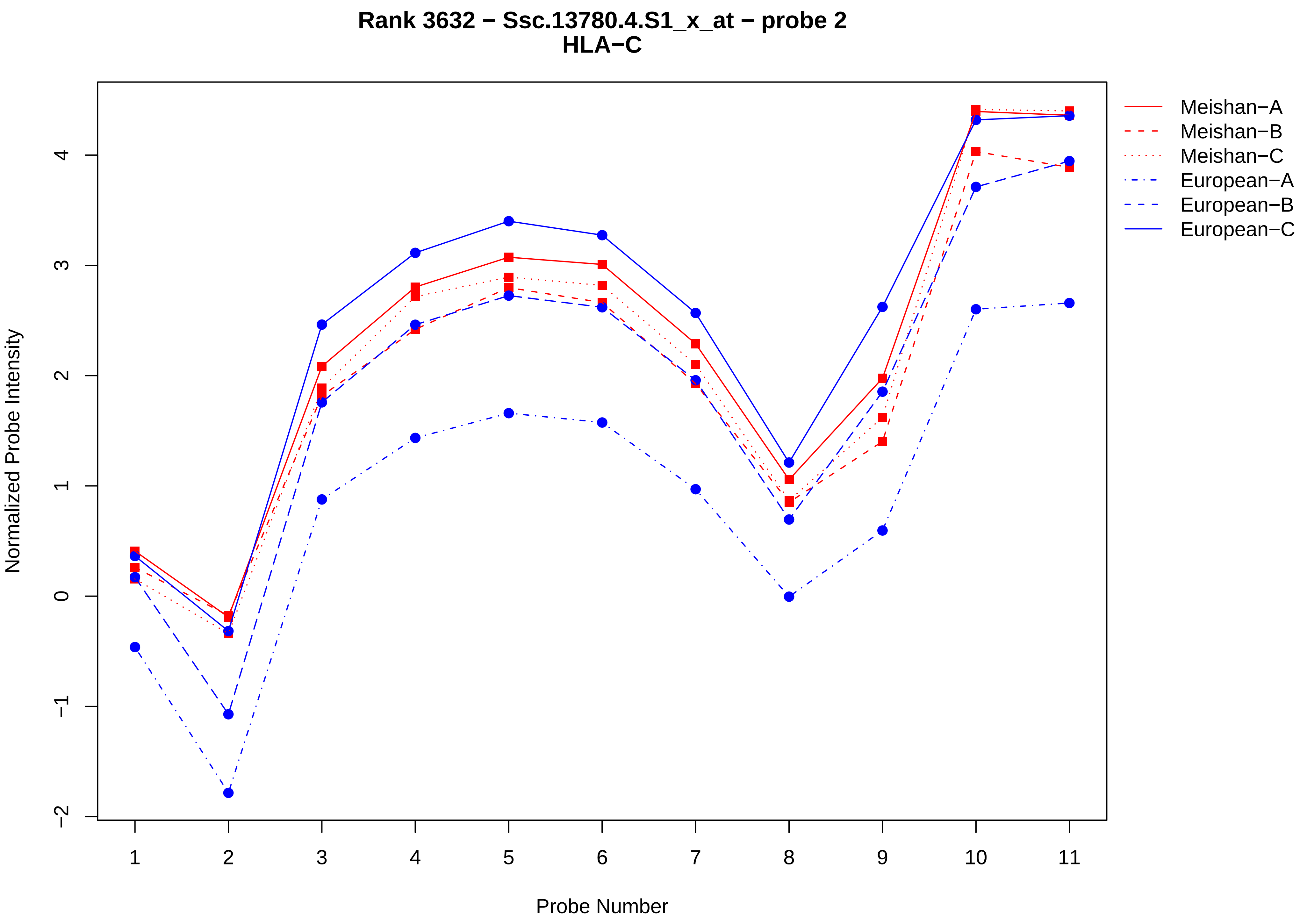

Rank 3632 − Ssc.13780.4.S1_x_at − probe 2
HLA−C
Normalized Probe Intensity
−2
−1
0
1
2
3
4
Probe Number
1
2
3
4
5
6
7
8
9
10
11
Meishan−A
Meishan−B
Meishan−C
European−A
European−B
European−C

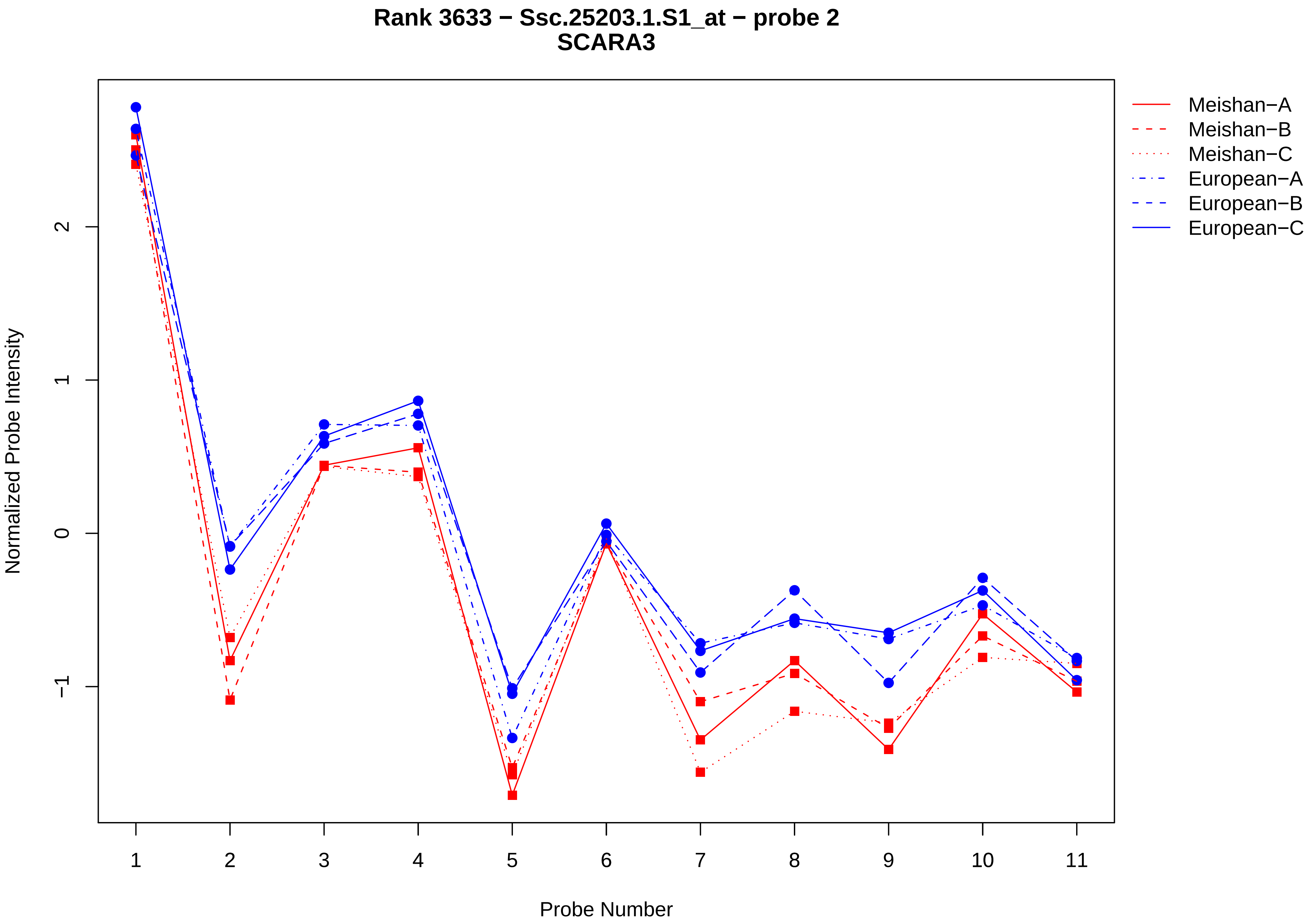
Rank 3633 − Ssc.25203.1.S1_at − probe 2
SCARA3
Normalized Probe Intensity
Probe Number
−1
0
1
2
1
2
3
4
5
6
7
8
9
10
11
Meishan−A
Meishan−B
Meishan−C
European−A
European−B
European−C

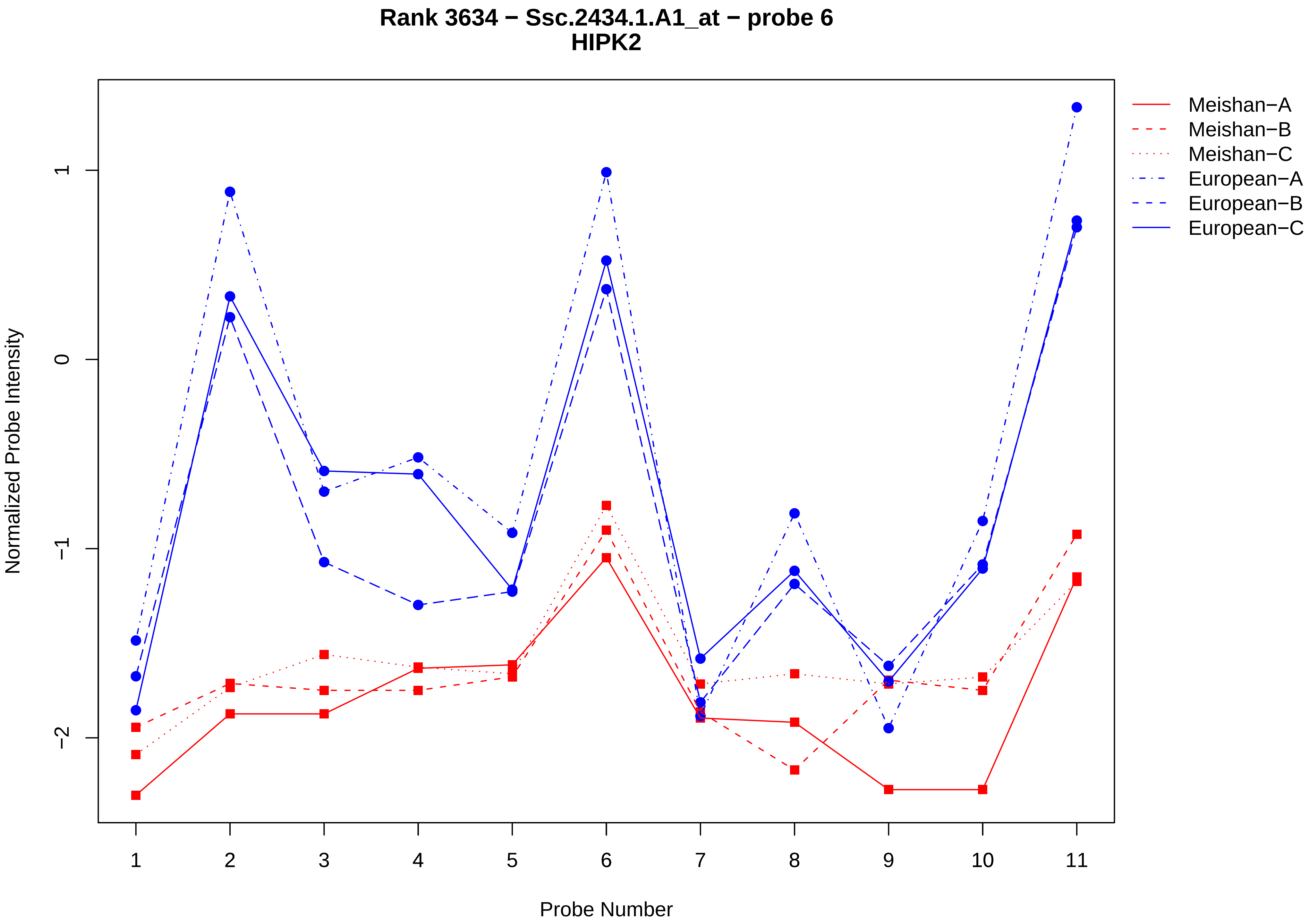

Rank 3634 − Ssc.2434.1.A1_at − probe 6
HIPK2
Normalized Probe Intensity
Probe Number
1
0
−1
−2
1
2
3
4
5
6
7
8
9
10
11
Meishan−A
Meishan−B
Meishan−C
European−A
European−B
European−C

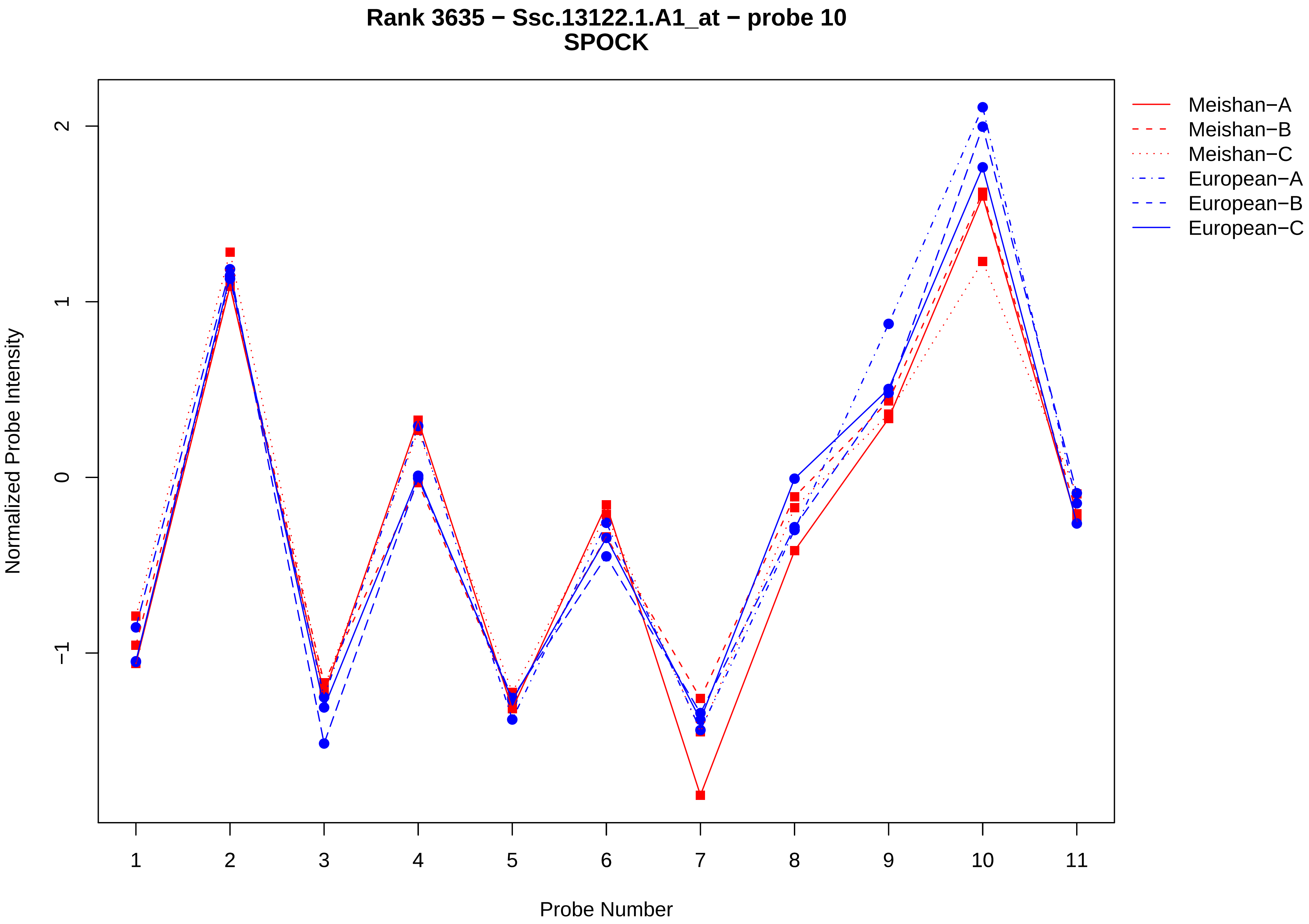

Rank 3635 − Ssc.13122.1.A1_at − probe 10
SPOCK
Normalized Probe Intensity
Probe Number
Meishan−A
Meishan−B
Meishan−C
European−A
European−B
European−C
−1
0
1
2
1
2
3
4
5
6
7
8
9
10
11

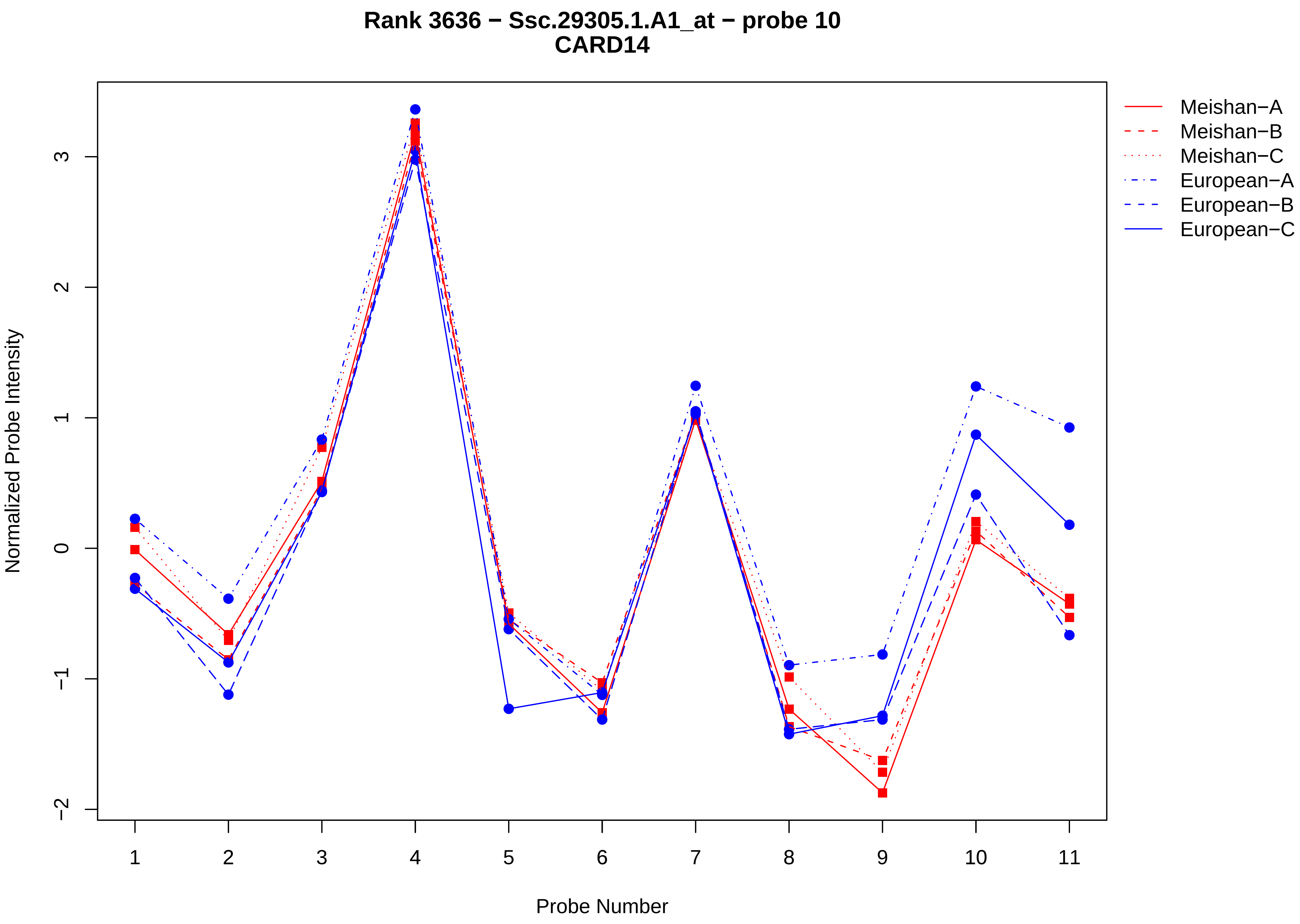

Rank 3636 − Ssc.29305.1.A1_at − probe 10
CARD14
Normalized Probe Intensity
Probe Number
−2
−1
0
1
2
3
1
2
3
4
5
6
7
8
9
10
11
Meishan−A
Meishan−B
Meishan−C
European−A
European−B
European−C

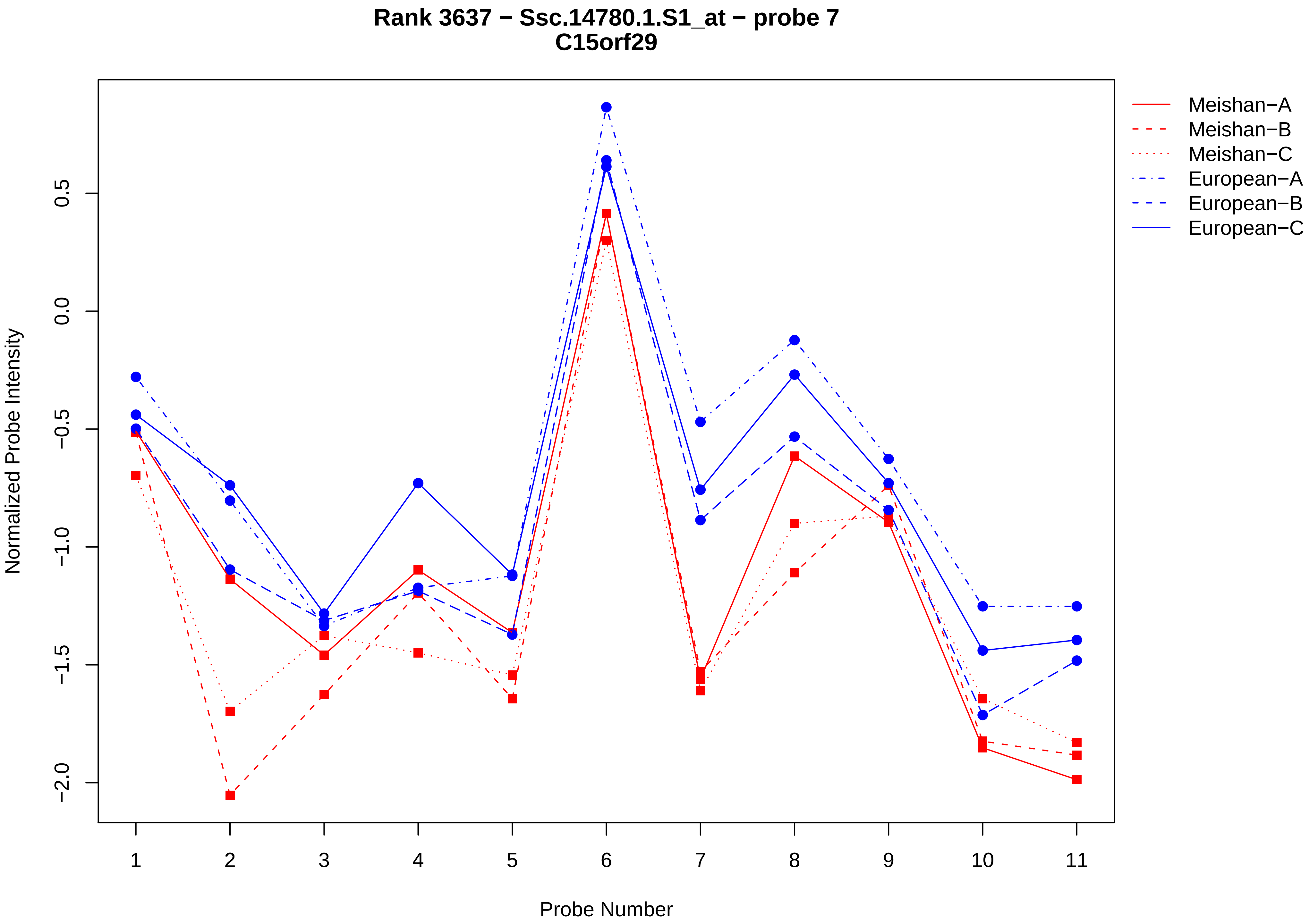

Rank 3637 − Ssc.14780.1.S1_at − probe 7
C15orf29
Normalized Probe Intensity
Probe Number
0.5
0.0
−0.5
−1.0
−1.5
−2.0
1
2
3
4
5
6
7
8
9
10
11
Meishan−A
Meishan−B
Meishan−C
European−A
European−B
European−C

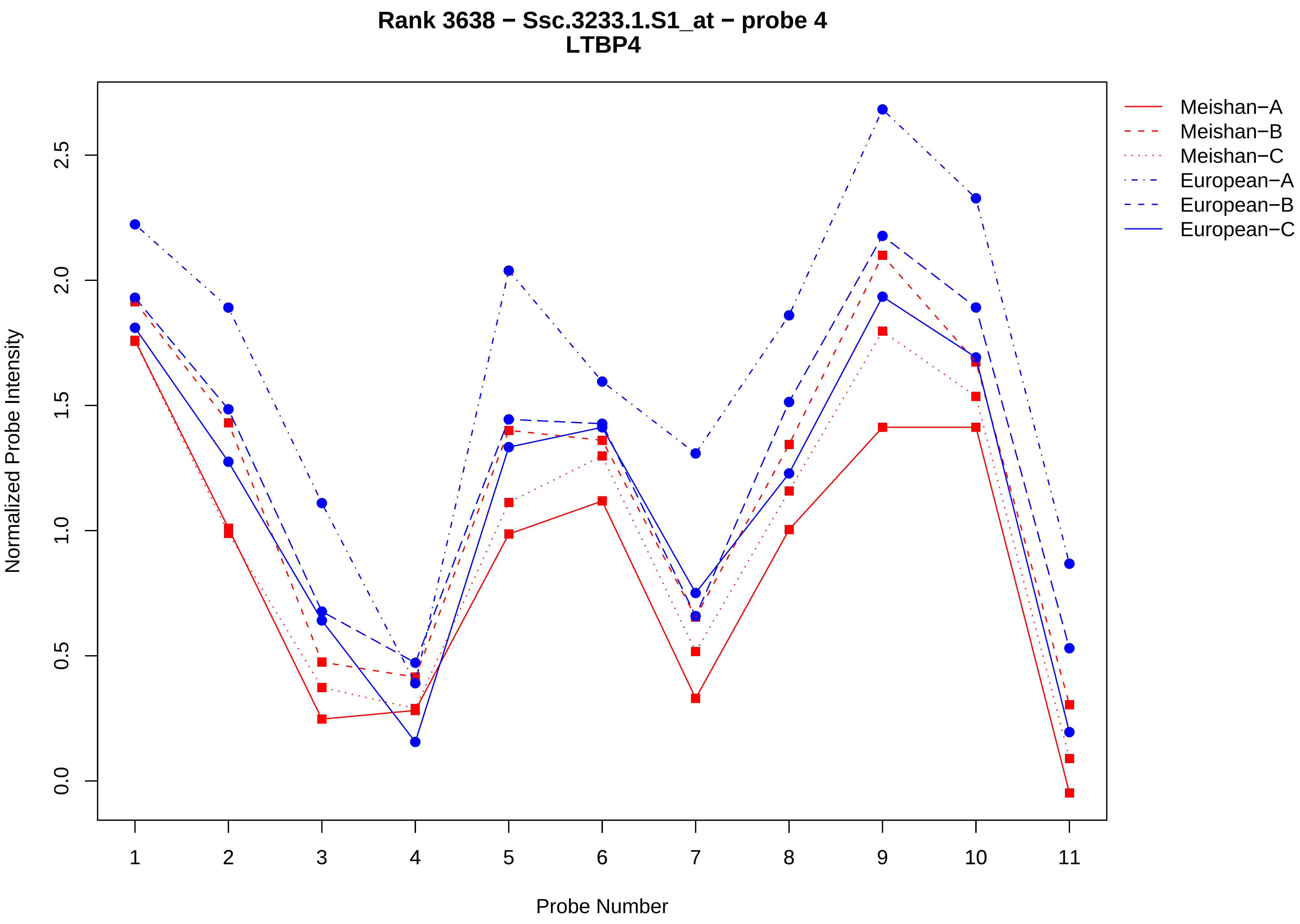

Rank 3638 − Ssc.3233.1.S1_at − probe 4
LTBP4
Normalized Probe Intensity
Probe Number
Meishan−A
Meishan−B
Meishan−C
European−A
European−B
European−C
0.0
0.5
1.0
1.5
2.0
2.5
1
2
3
4
5
6
7
8
9
10
11

Rank 3639 − Ssc.25042.1.A1_at − probe 9
FVR1_HUMAN

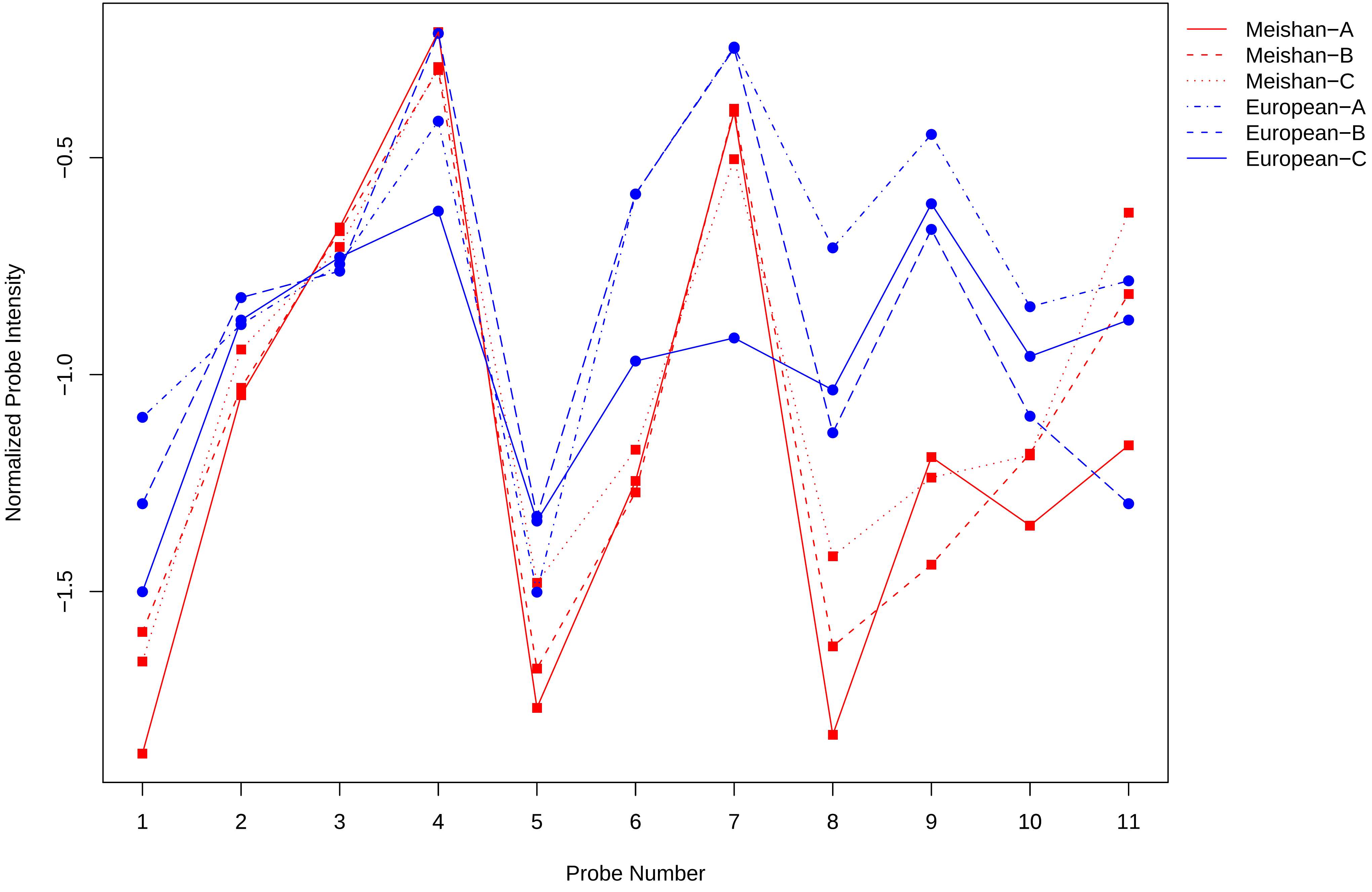

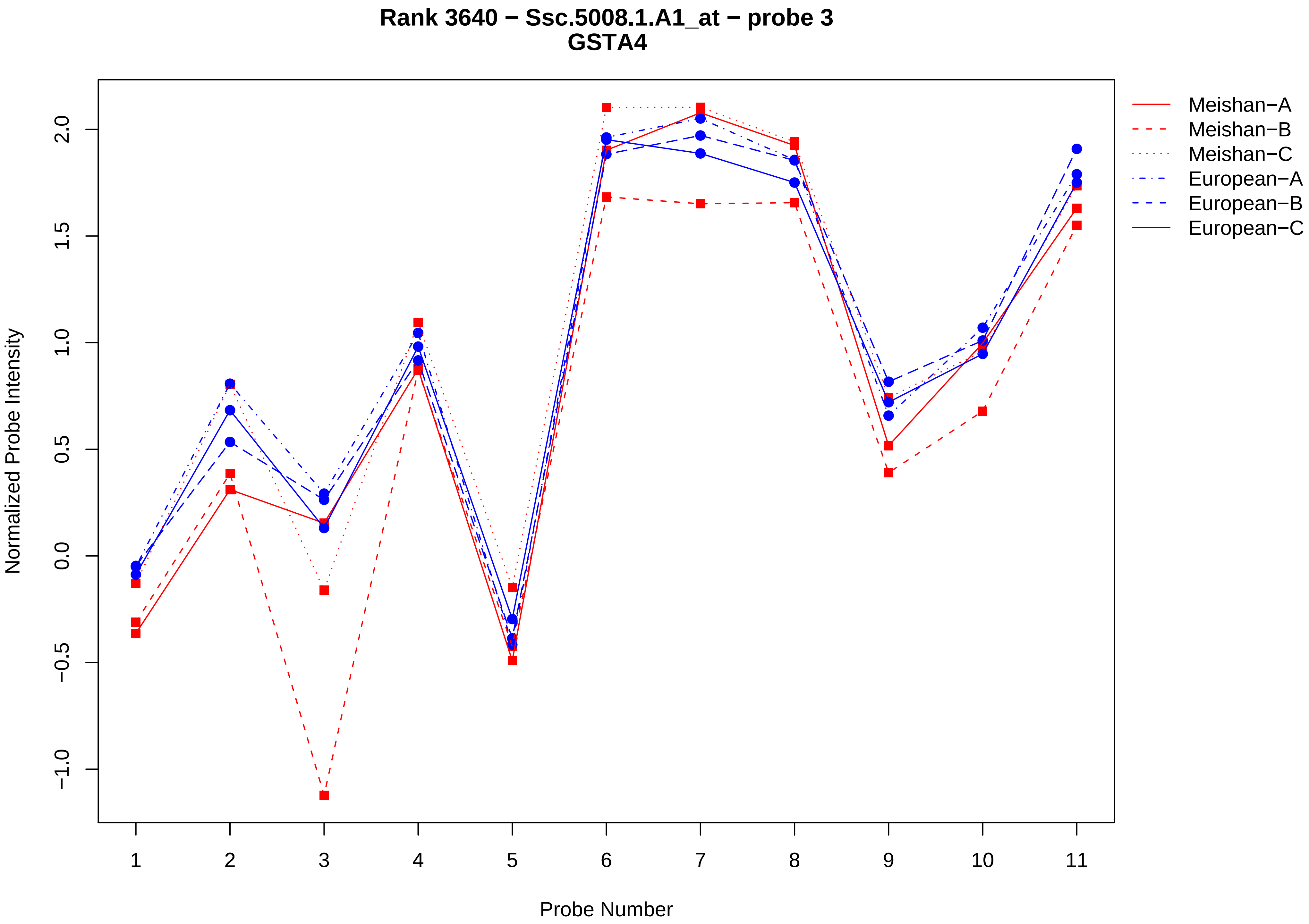

Rank 3640 − Ssc.5008.1.A1_at − probe 3
GSTA4
Normalized Probe Intensity
Probe Number
Meishan−A
Meishan−B
Meishan−C
European−A
European−B
European−C
2.0
1.5
1.0
0.5
0.0
−0.5
−1.0
1
2
3
4
5
6
7
8
9
10
11

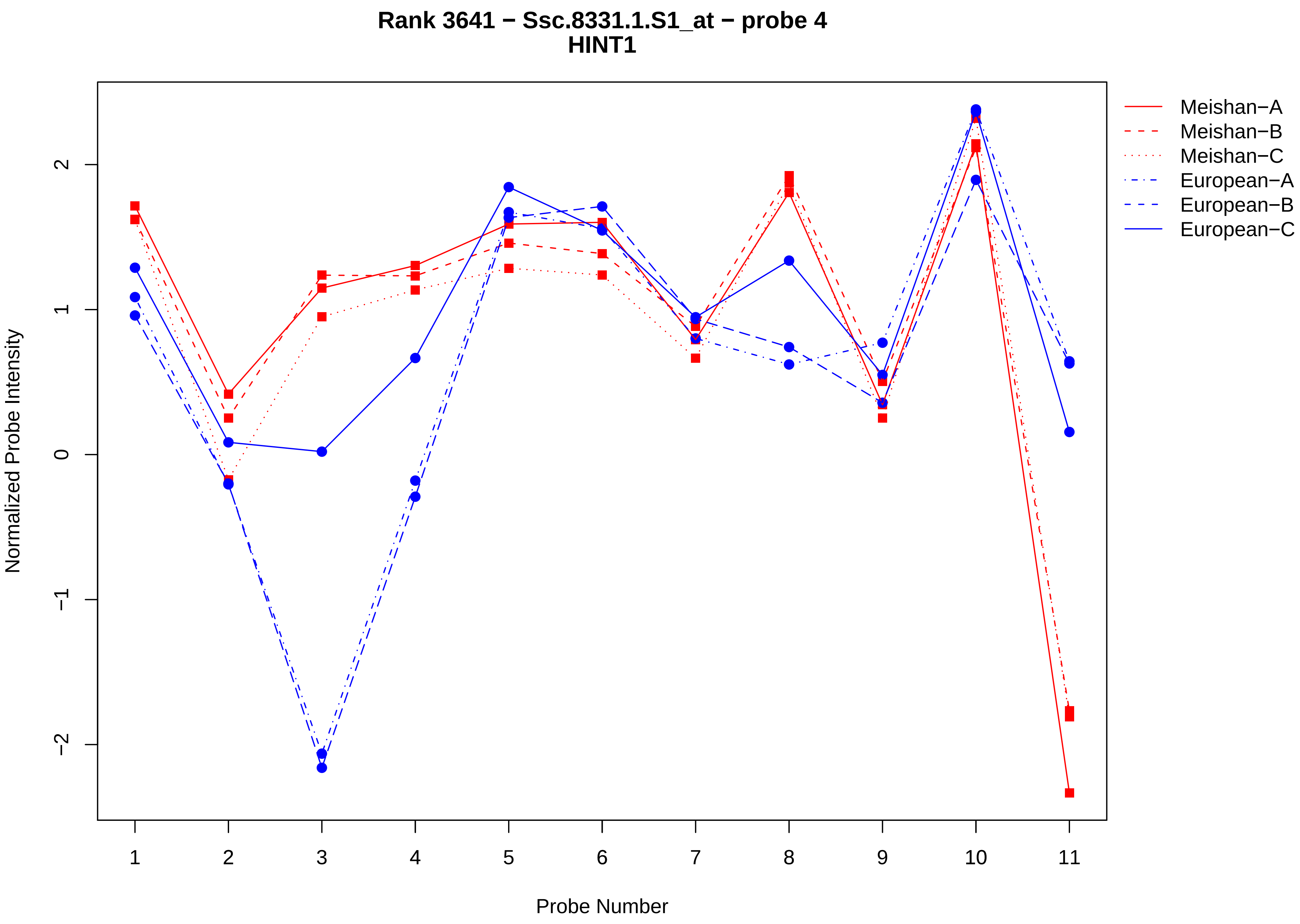

Rank 3641 − Ssc.8331.1.S1_at − probe 4
HINT1
Normalized Probe Intensity
Probe Number
−2
−1
0
1
2
1
2
3
4
5
6
7
8
9
10
11
Meishan−A
Meishan−B
Meishan−C
European−A
European−B
European−C

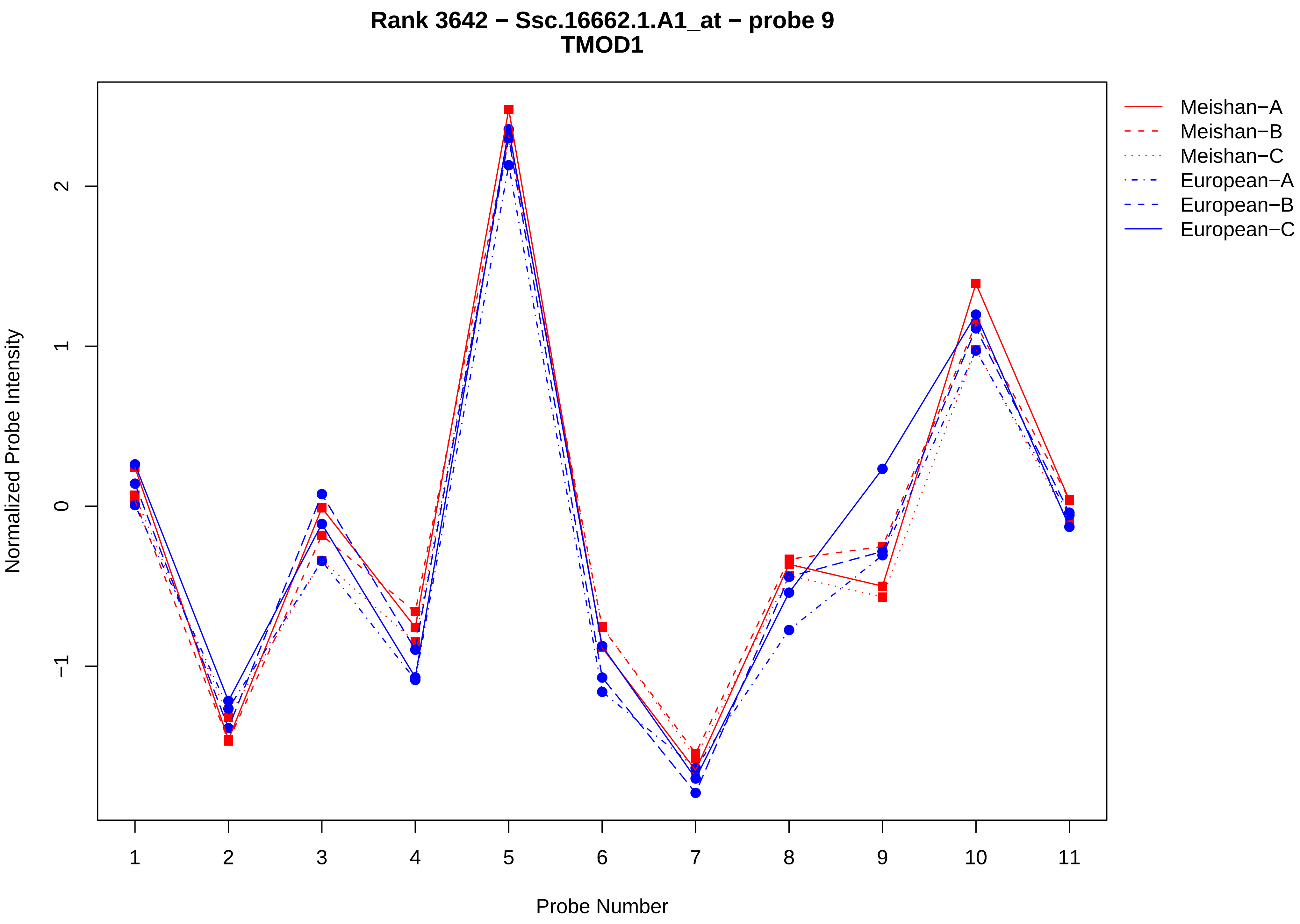

Rank 3642 − Ssc.16662.1.A1_at − probe 9
TMOD1
Normalized Probe Intensity
Probe Number
−1
0
1
2
1
2
3
4
5
6
7
8
9
10
11
Meishan−A
Meishan−B
Meishan−C
European−A
European−B
European−C

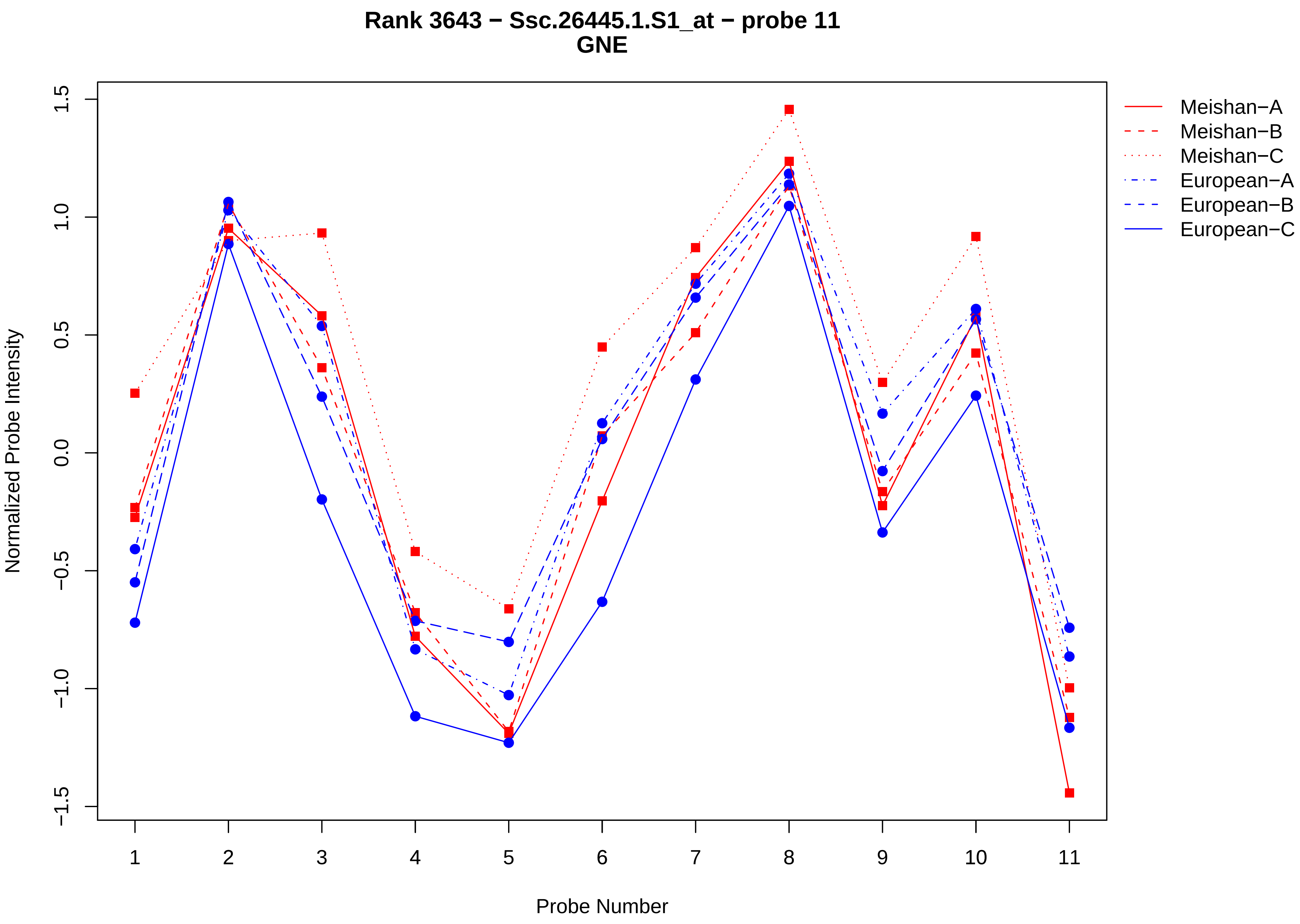
Rank 3643 − Ssc.26445.1.S1_at − probe 11
GNE
Normalized Probe Intensity
Probe Number
1.5
1.0
0.5
0.0
−0.5
−1.0
−1.5
1
2
3
4
5
6
7
8
9
10
11
Meishan−A
Meishan−B
Meishan−C
European−A
European−B
European−C

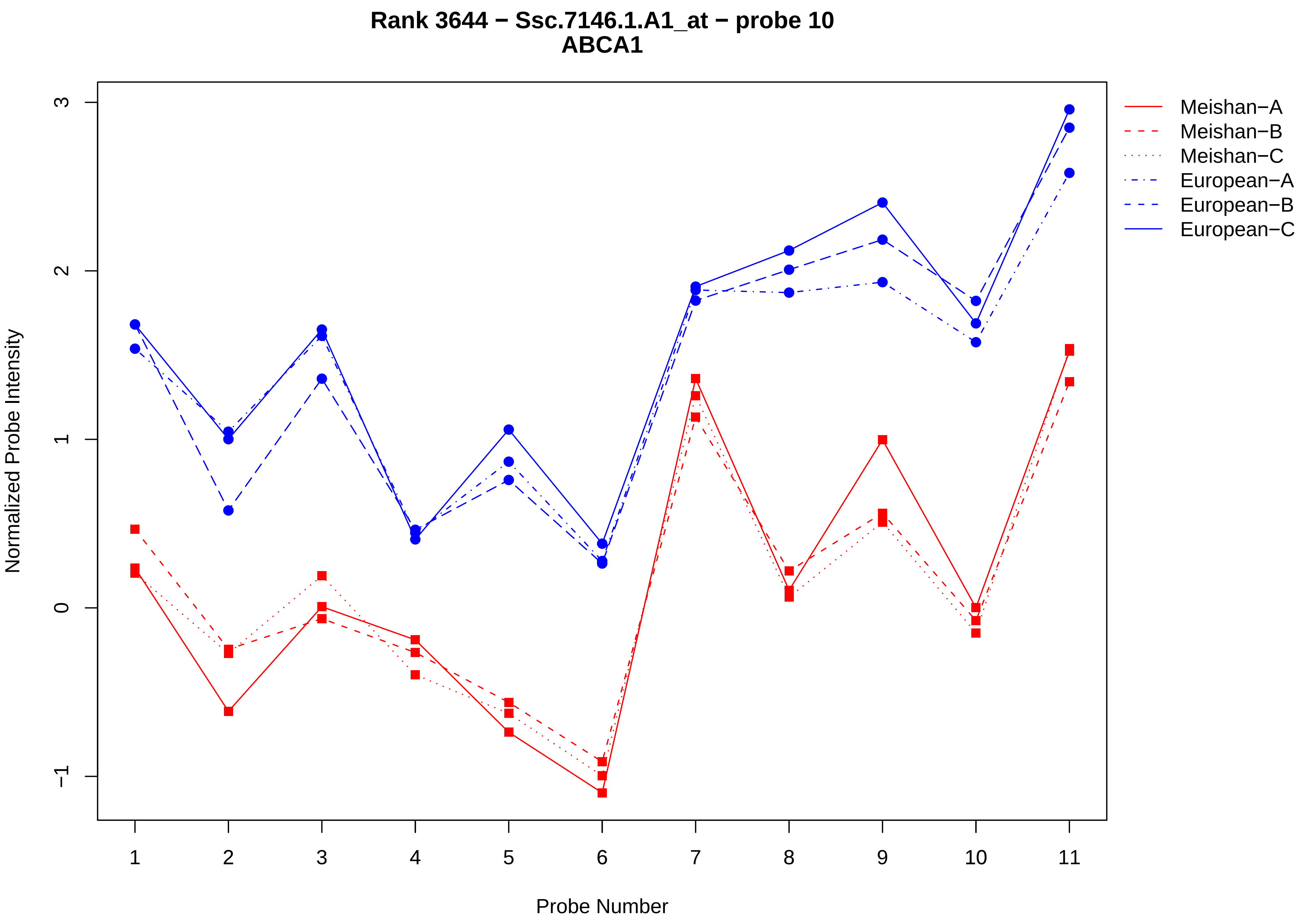

Rank 3644 − Ssc.7146.1.A1_at − probe 10
ABCA1
Normalized Probe Intensity
Probe Number
Meishan−A
Meishan−B
Meishan−C
European−A
European−B
European−C
−1
0
1
2
3
1
2
3
4
5
6
7
8
9
10
11

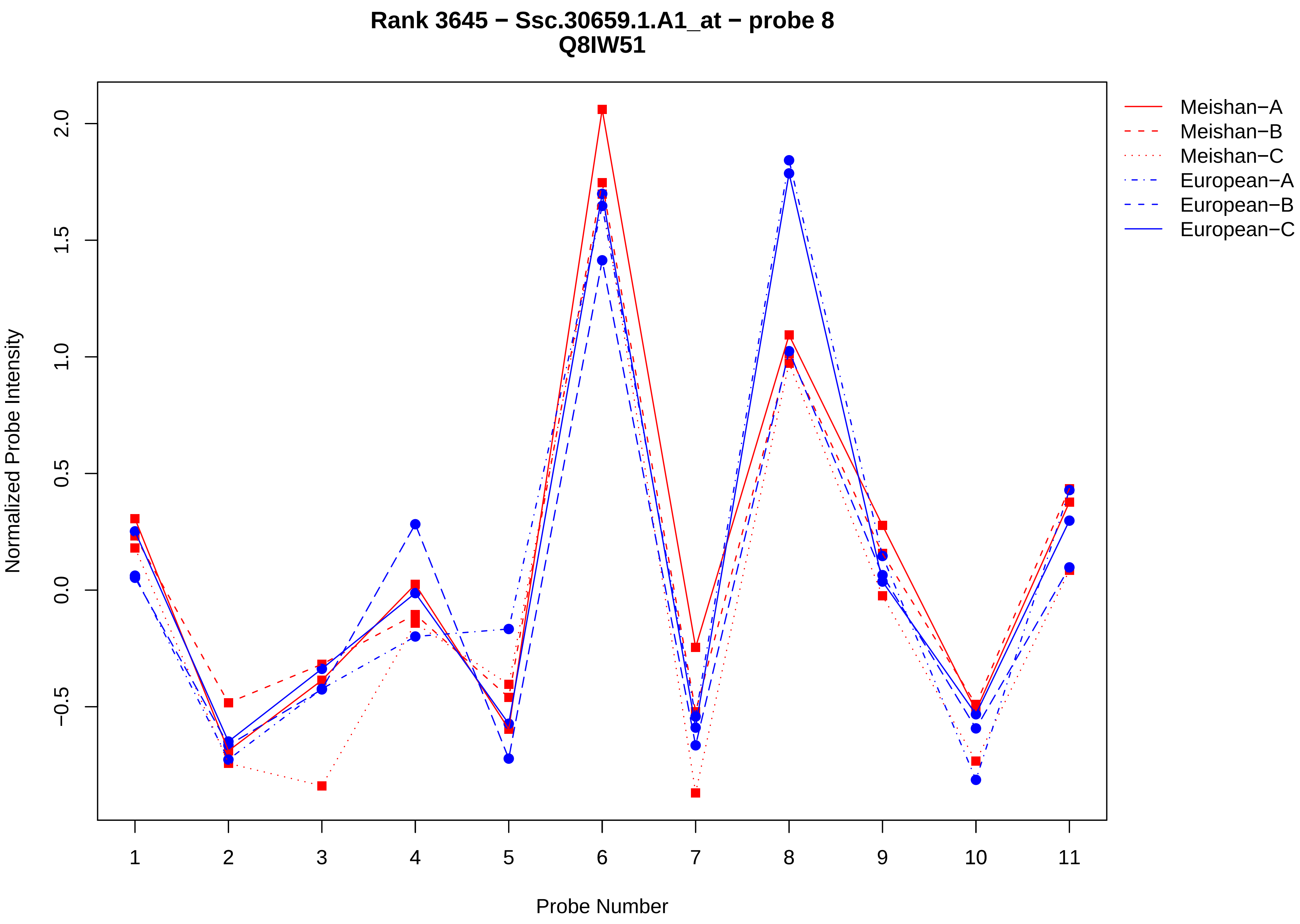
Rank 3645 − Ssc.30659.1.A1_at − probe 8
Q8IW51
Normalized Probe Intensity
Probe Number
Meishan−A
Meishan−B
Meishan−C
European−A
European−B
European−C
−0.5
0.0
0.5
1.0
1.5
2.0
1
2
3
4
5
6
7
8
9
10
11

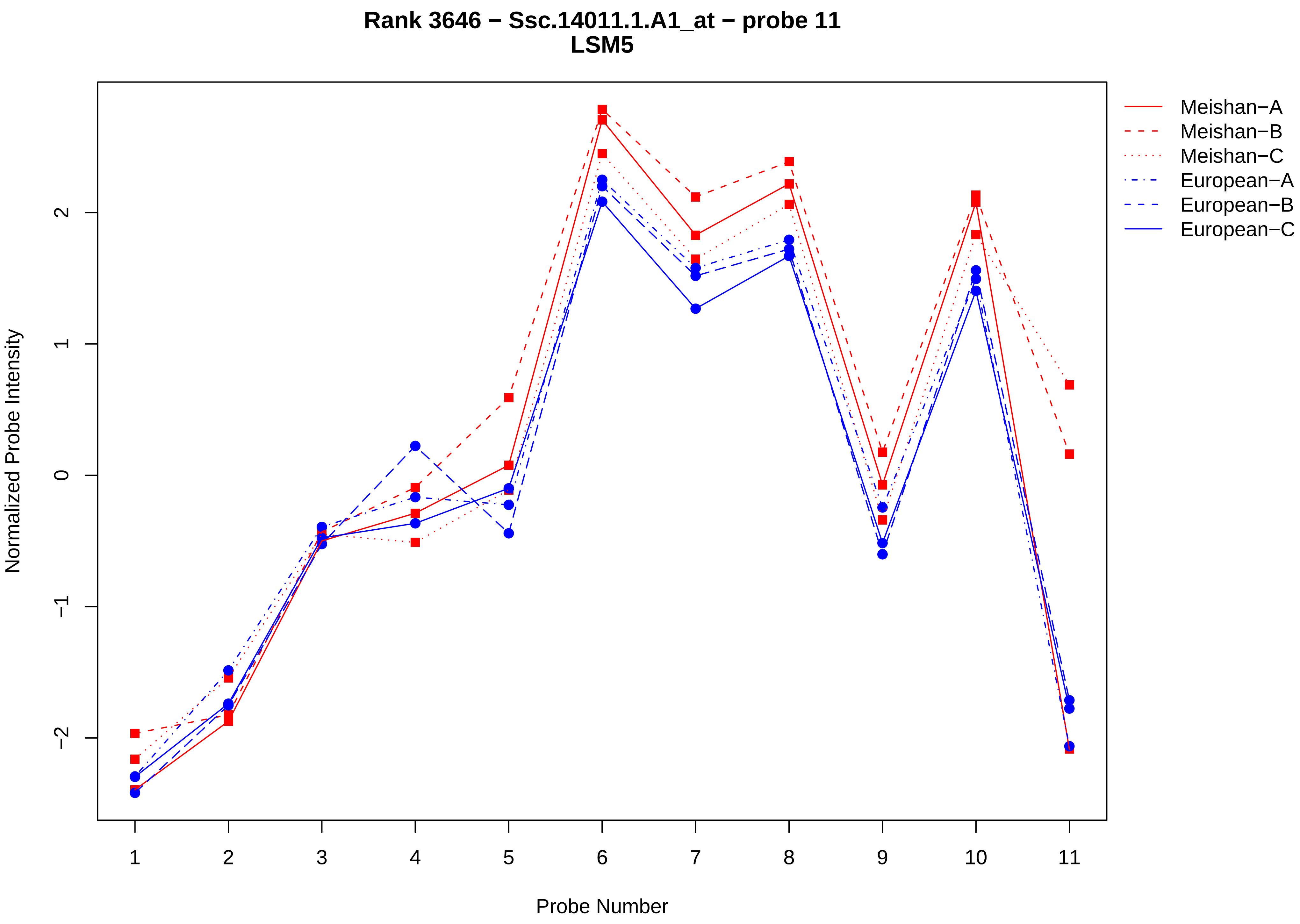
Rank 3646 − Ssc.14011.1.A1_at − probe 11
LSM5
Normalized Probe Intensity
Probe Number
−2
−1
0
1
2
1
2
3
4
5
6
7
8
9
10
11
Meishan−A
Meishan−B
Meishan−C
European−A
European−B
European−C

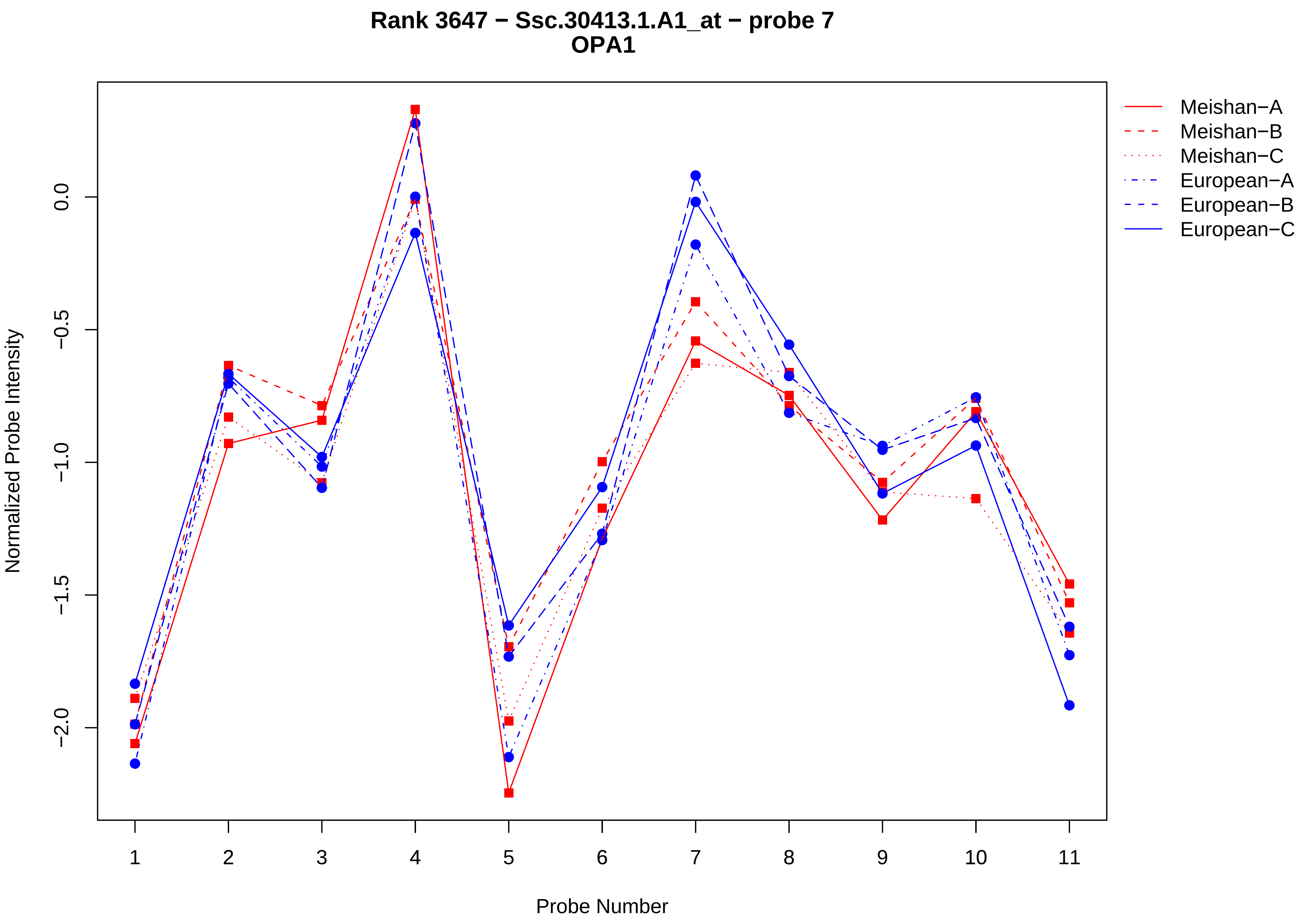

Rank 3647 − Ssc.30413.1.A1_at − probe 7
OPA1
Normalized Probe Intensity
Probe Number
0.0
−0.5
−1.0
−1.5
−2.0
1
2
3
4
5
6
7
8
9
10
11
Meishan−A
Meishan−B
Meishan−C
European−A
European−B
European−C

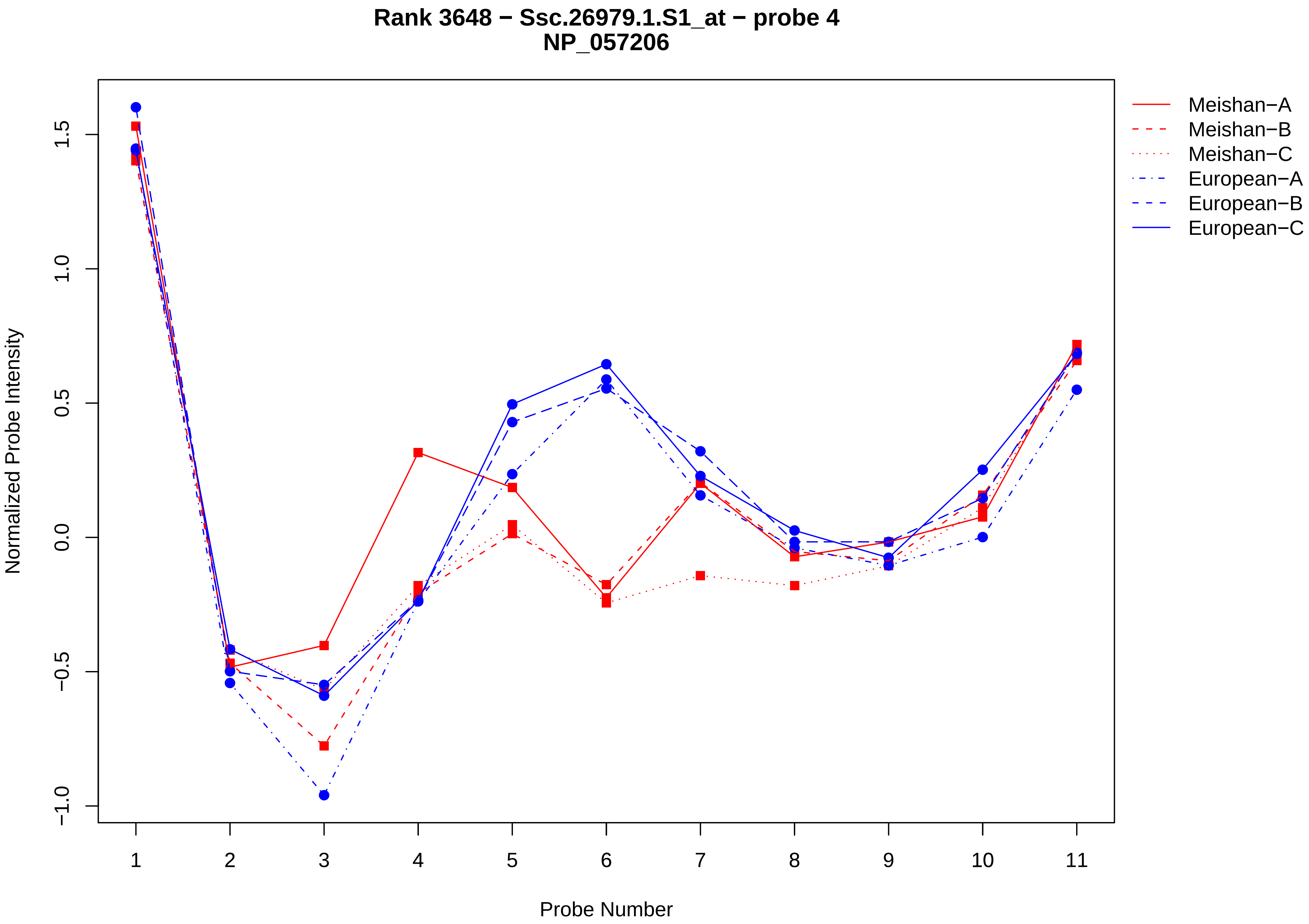
Rank 3648 − Ssc.26979.1.S1_at − probe 4
NP_057206
Normalized Probe Intensity
Probe Number
−1.0
−0.5
0.0
0.5
1.0
1.5
1
2
3
4
5
6
7
8
9
10
11
Meishan−A
Meishan−B
Meishan−C
European−A
European−B
European−C

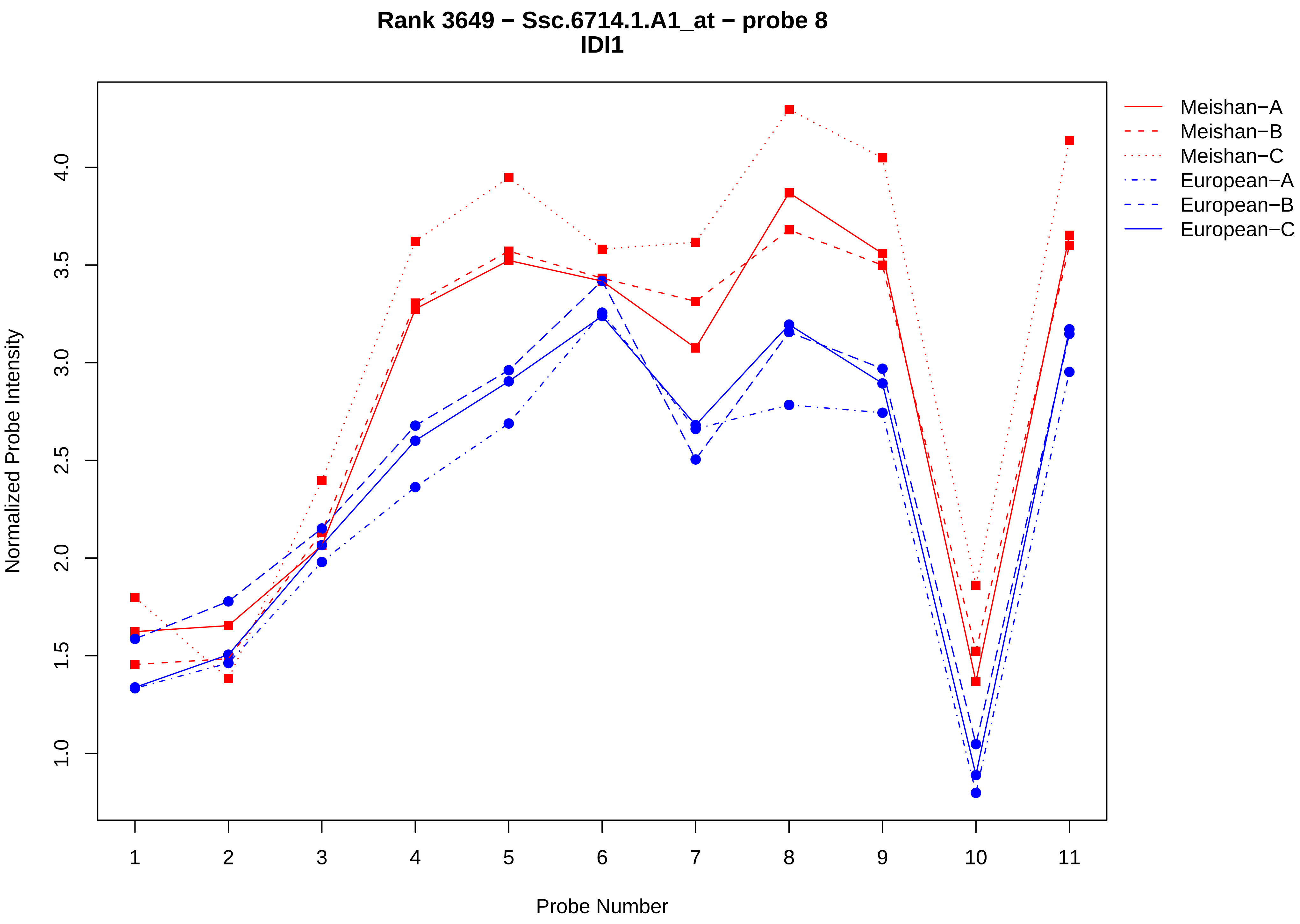

Rank 3649 − Ssc.6714.1.A1_at − probe 8
IDI1
Normalized Probe Intensity
Probe Number
Meishan−A
Meishan−B
Meishan−C
European−A
European−B
European−C

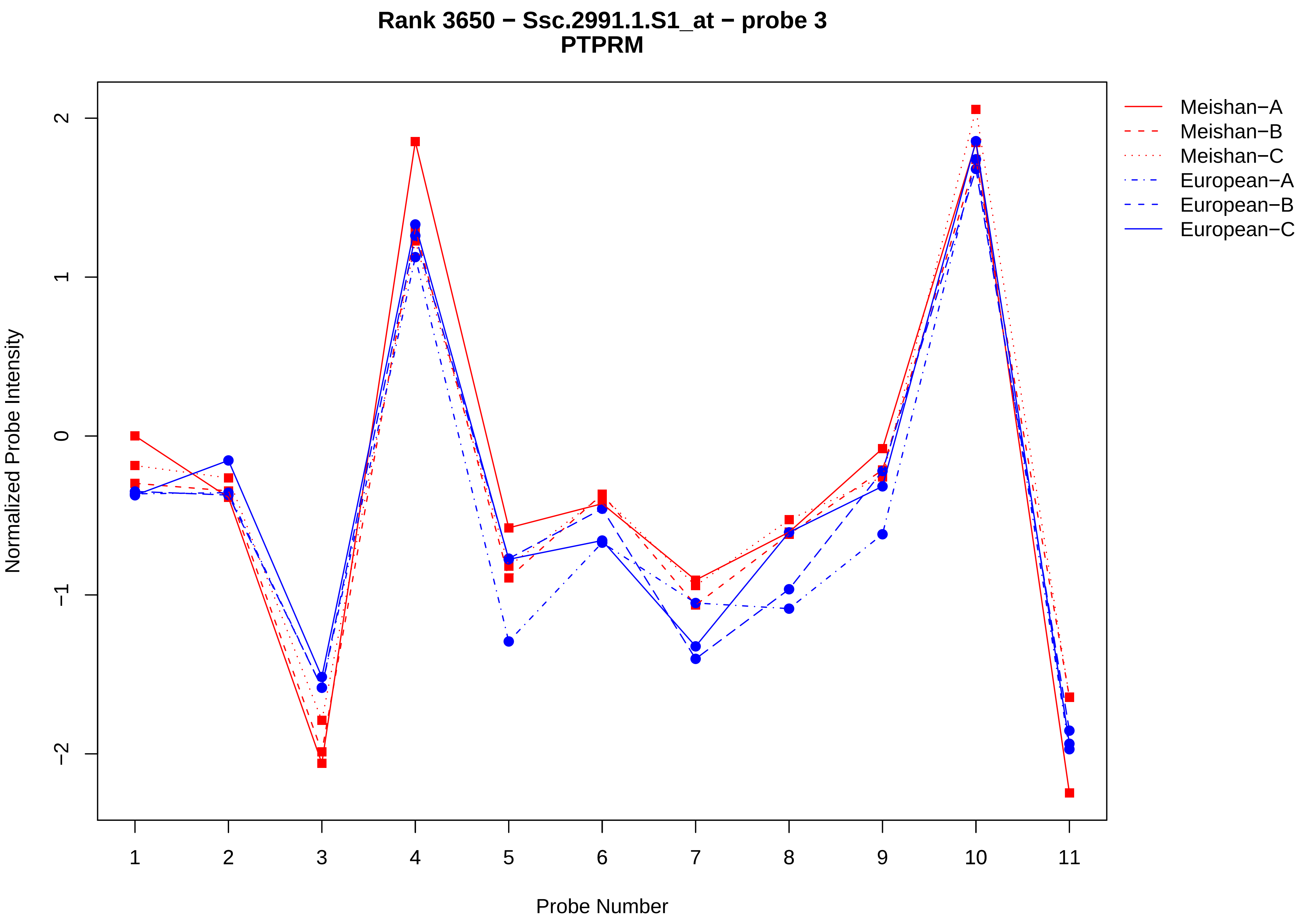

Rank 3650 − Ssc.2991.1.S1_at − probe 3
PTPRM
Normalized Probe Intensity
Probe Number
−2
−1
0
1
2
1
2
3
4
5
6
7
8
9
10
11
Meishan−A
Meishan−B
Meishan−C
European−A
European−B
European−C

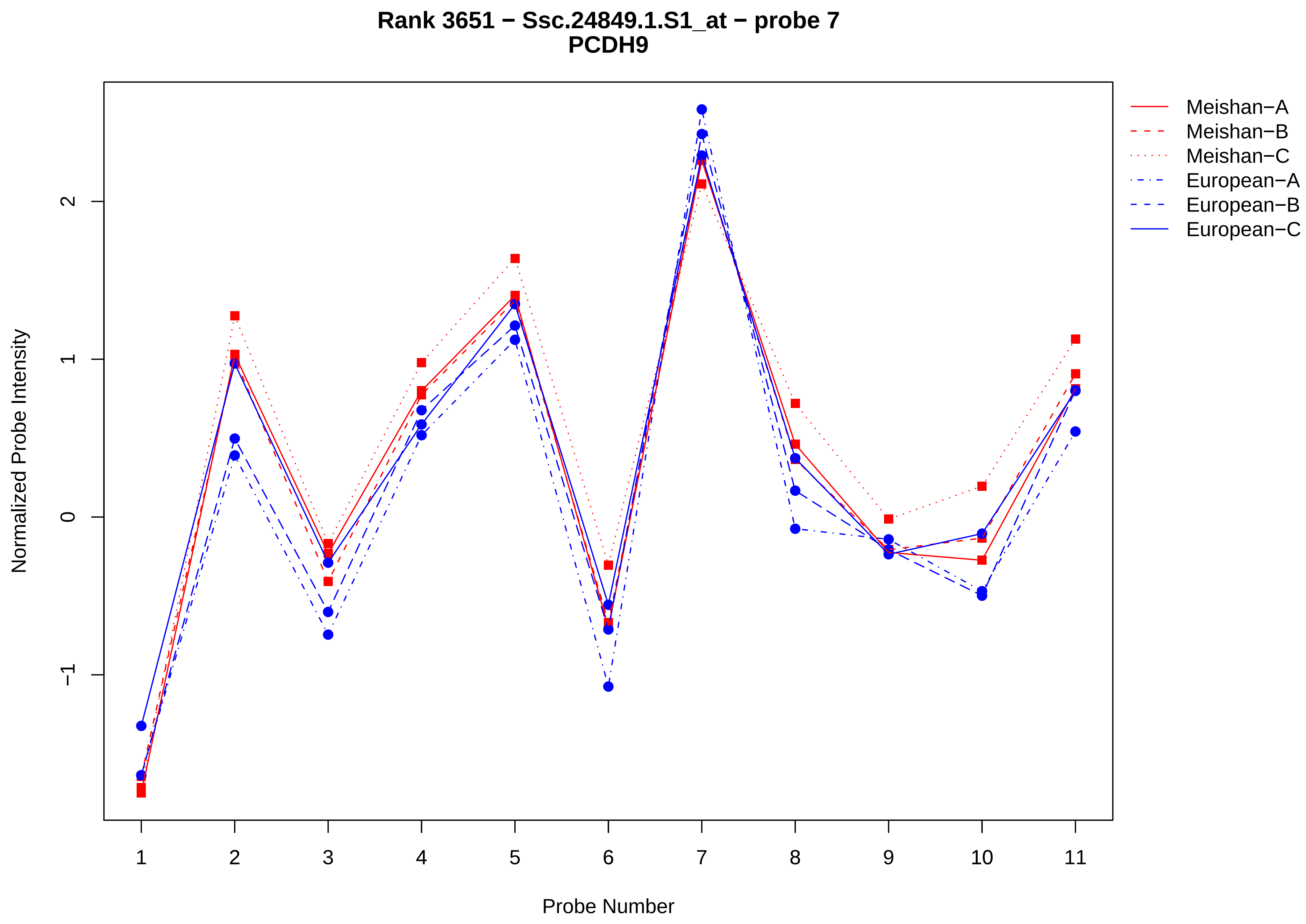

Rank 3651 − Ssc.24849.1.S1_at − probe 7
PCDH9
Normalized Probe Intensity
Probe Number
Meishan−A
Meishan−B
Meishan−C
European−A
European−B
European−C
−1
0
1
2
1
2
3
4
5
6
7
8
9
10
11

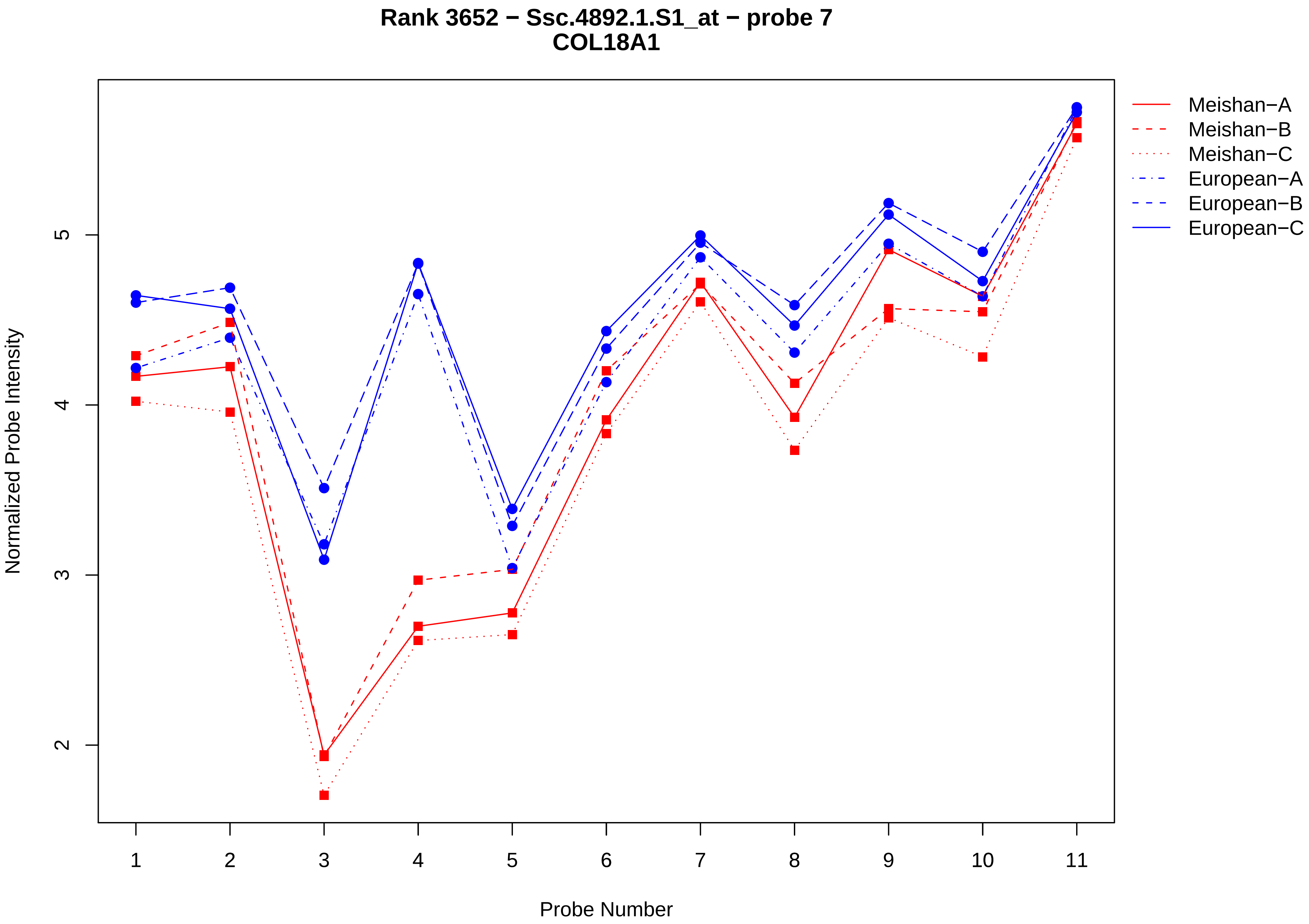

Rank 3652 − Ssc.4892.1.S1_at − probe 7
COL18A1
Normalized Probe Intensity
Probe Number
2
3
4
5
1
2
3
4
5
6
7
8
9
10
11
Meishan−A
Meishan−B
Meishan−C
European−A
European−B
European−C

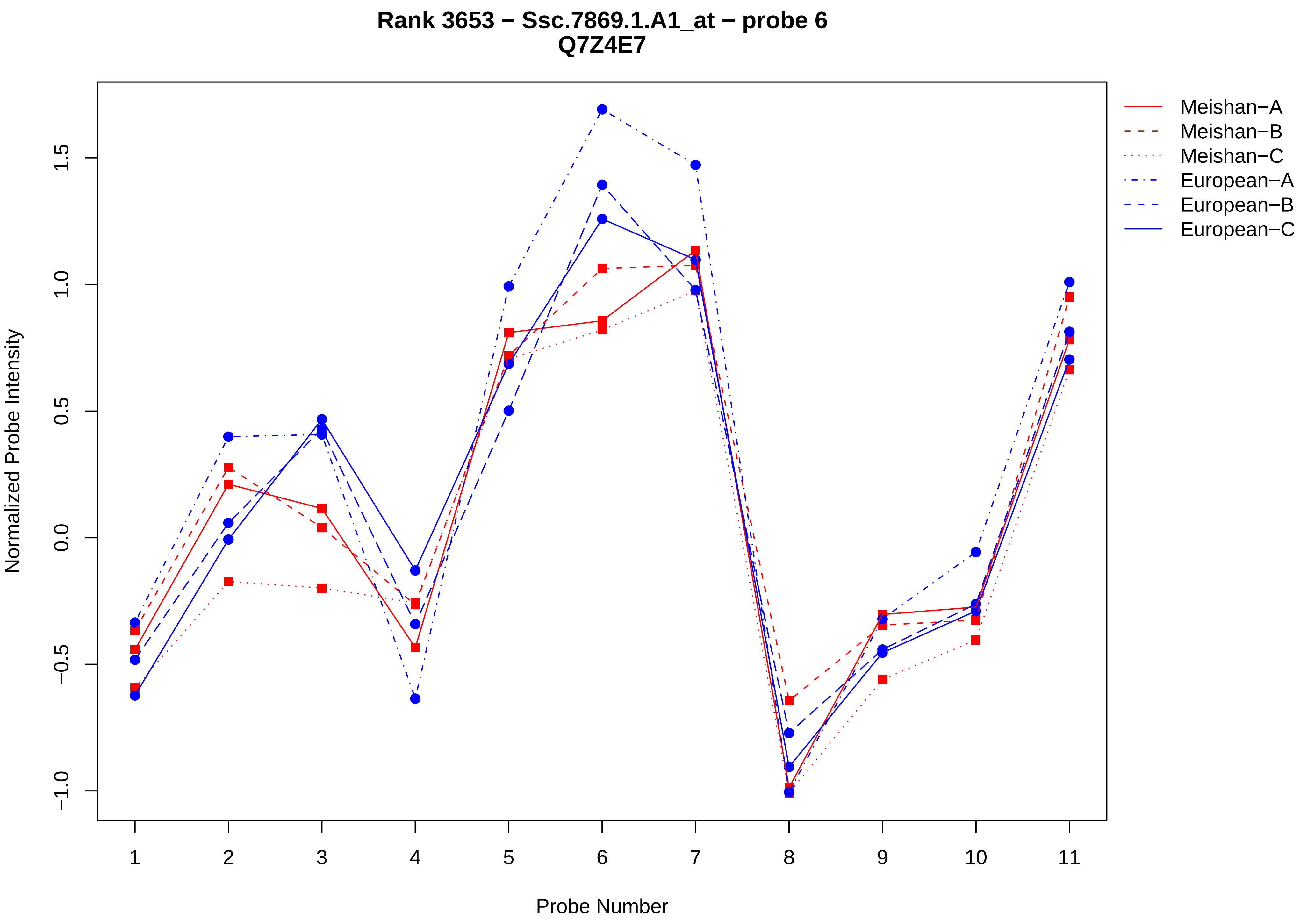

Rank 3653 − Ssc.7869.1.A1_at − probe 6
Q7Z4E7
Normalized Probe Intensity
Probe Number
Meishan−A
Meishan−B
Meishan−C
European−A
European−B
European−C

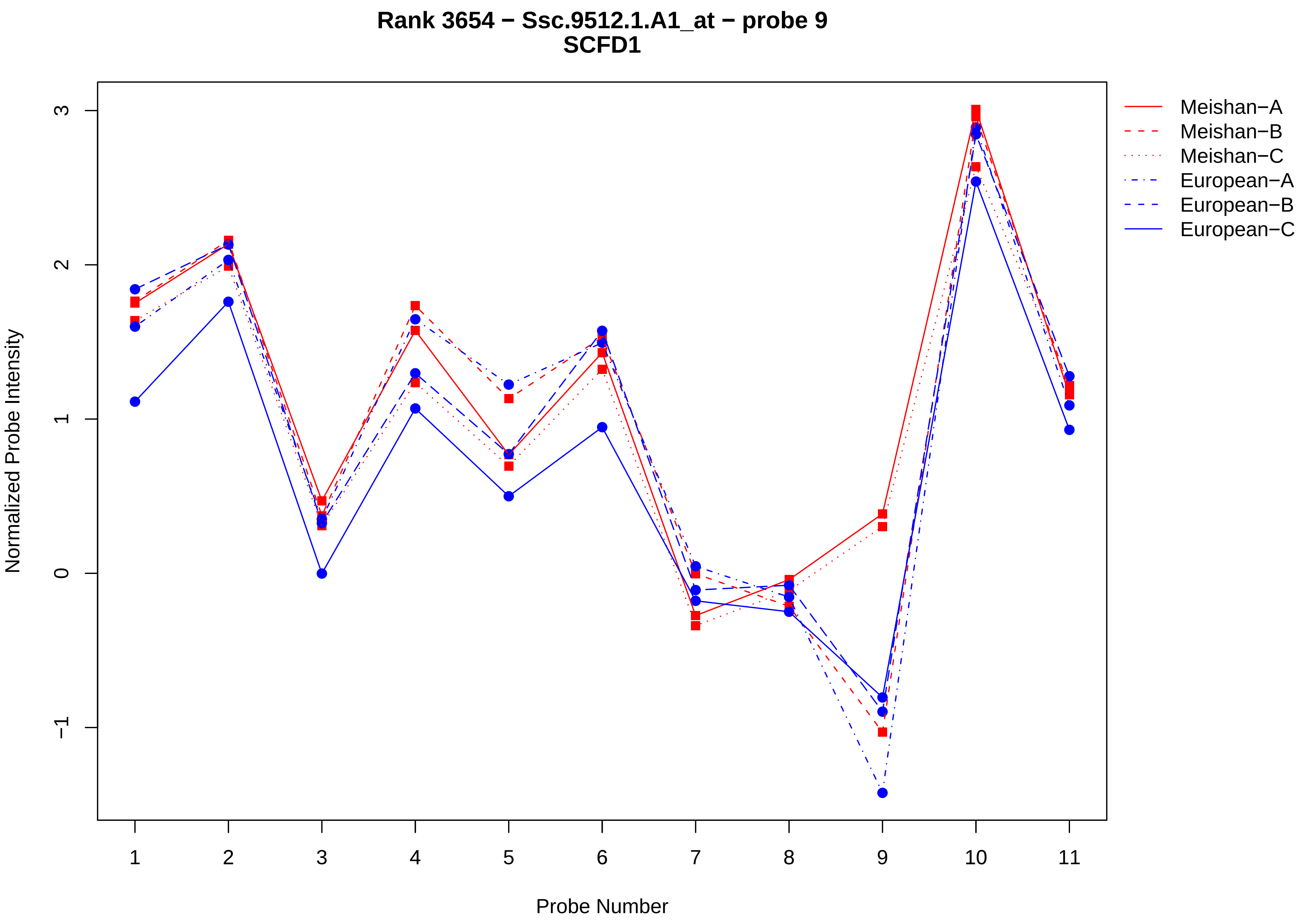

Rank 3654 − Ssc.9512.1.A1_at − probe 9
SCFD1
Normalized Probe Intensity
Probe Number
−1
0
1
2
3
1
2
3
4
5
6
7
8
9
10
11
Meishan−A
Meishan−B
Meishan−C
European−A
European−B
European−C

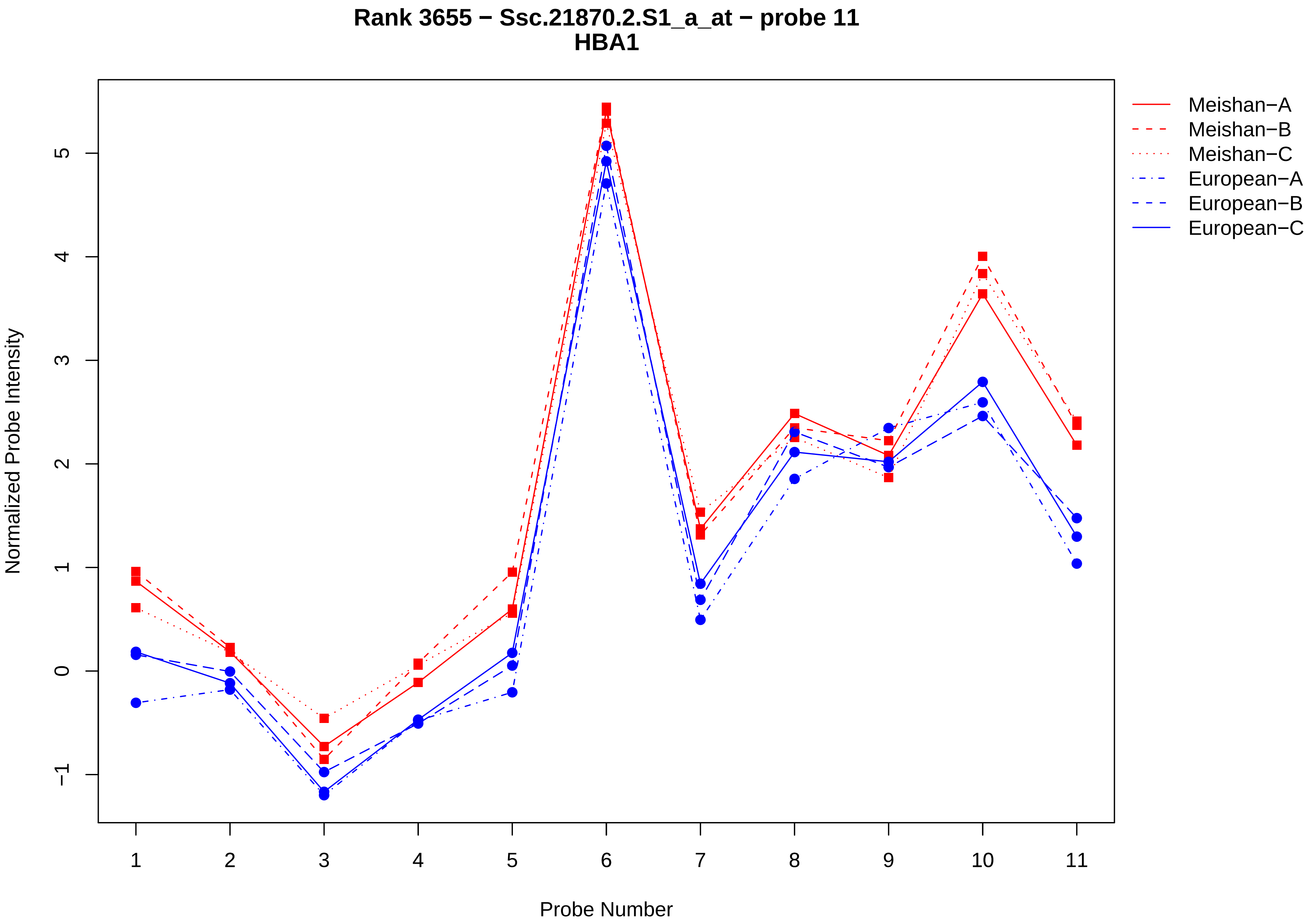

Rank 3655 − Ssc.21870.2.S1_a_at − probe 11
HBA1
Normalized Probe Intensity
Probe Number
Meishan−A
Meishan−B
Meishan−C
European−A
European−B
European−C
−1
0
1
2
3
4
5
1
2
3
4
5
6
7
8
9
10
11

Rank 3656 − Ssc.25882.1.S1_at − probe 4
SSPN

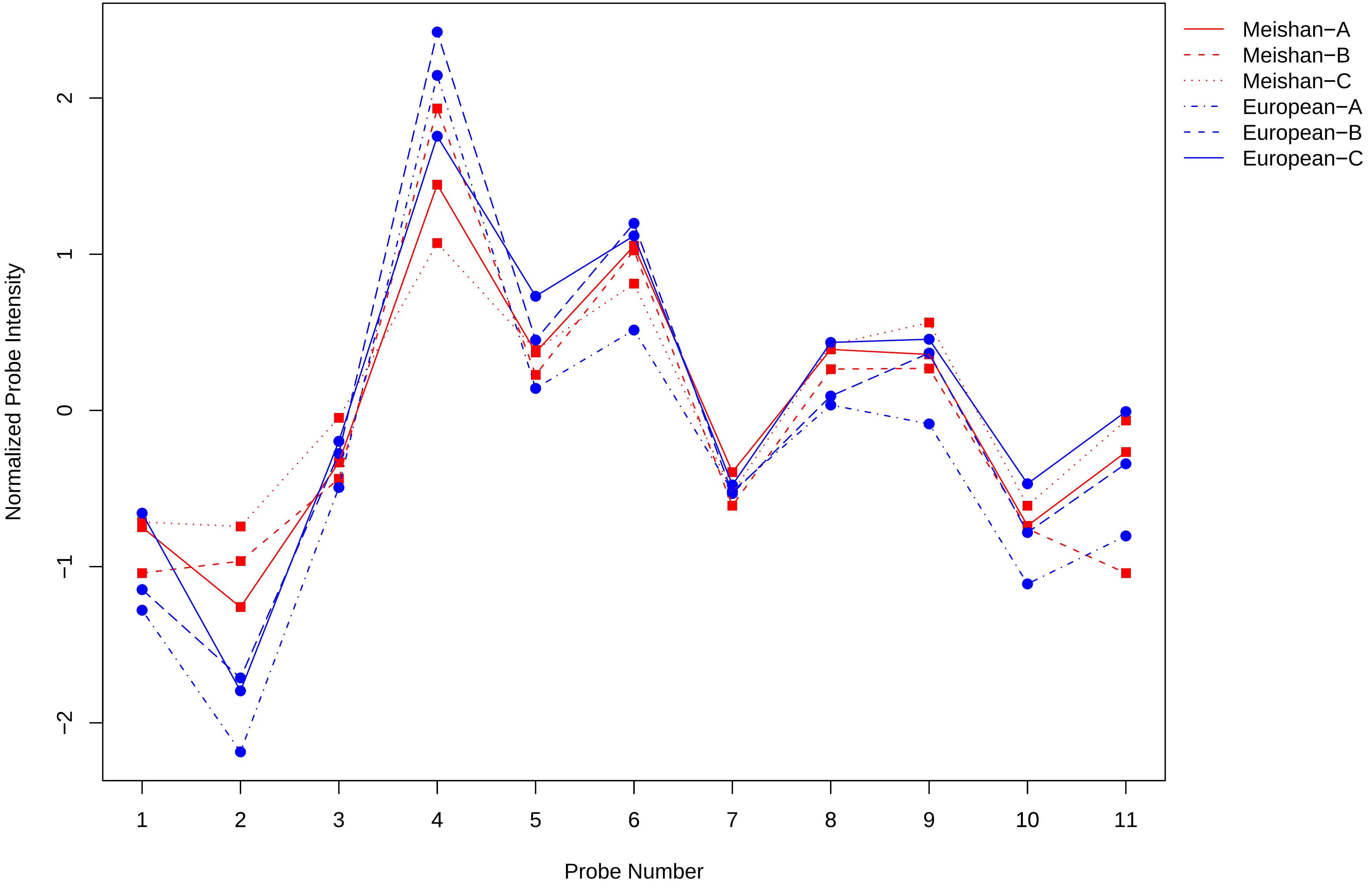

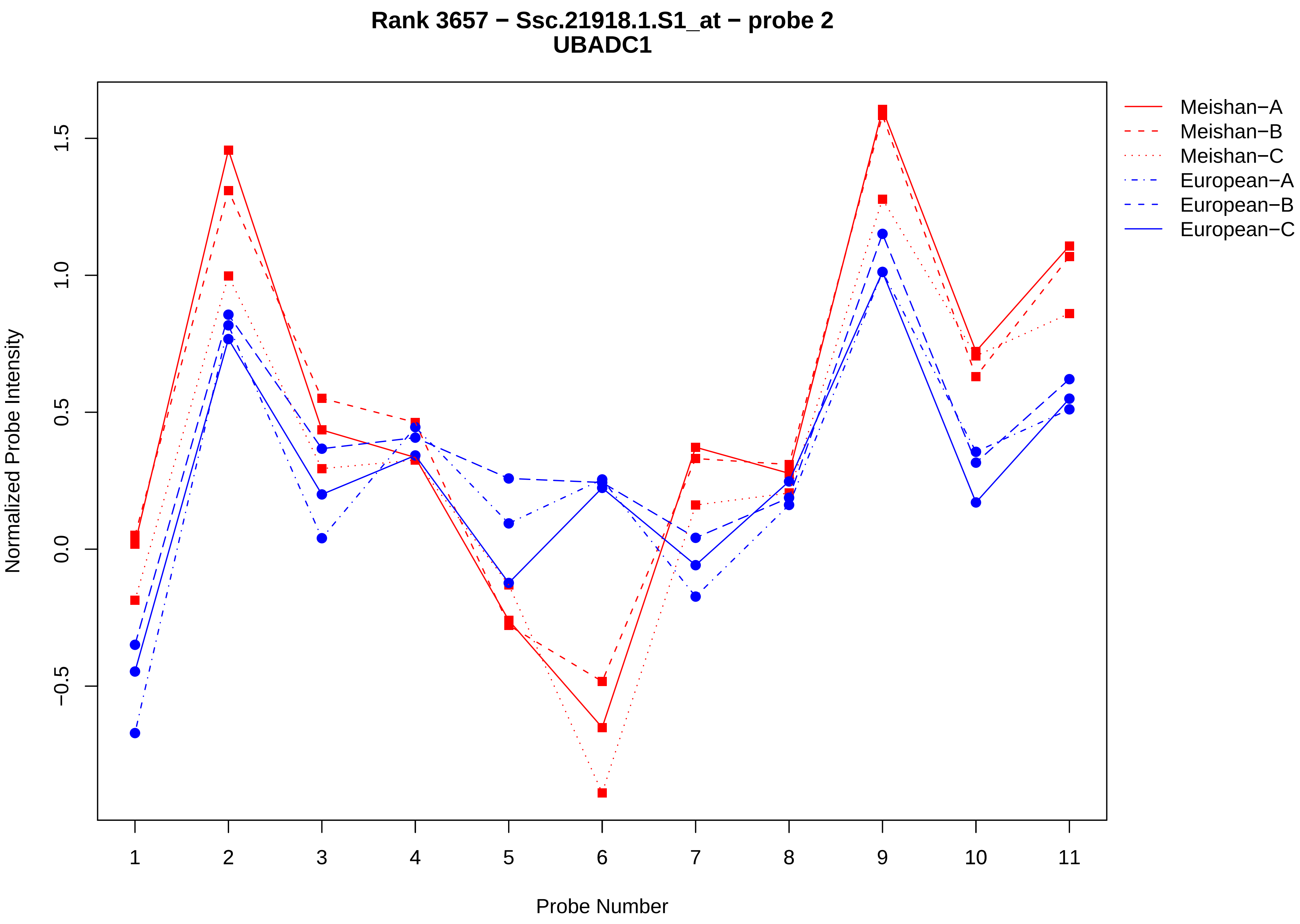

Rank 3657 − Ssc.21918.1.S1_at − probe 2
UBADC1
Normalized Probe Intensity
Probe Number
−0.5
0.0
0.5
1.0
1.5
1
2
3
4
5
6
7
8
9
10
11
Meishan−A
Meishan−B
Meishan−C
European−A
European−B
European−C

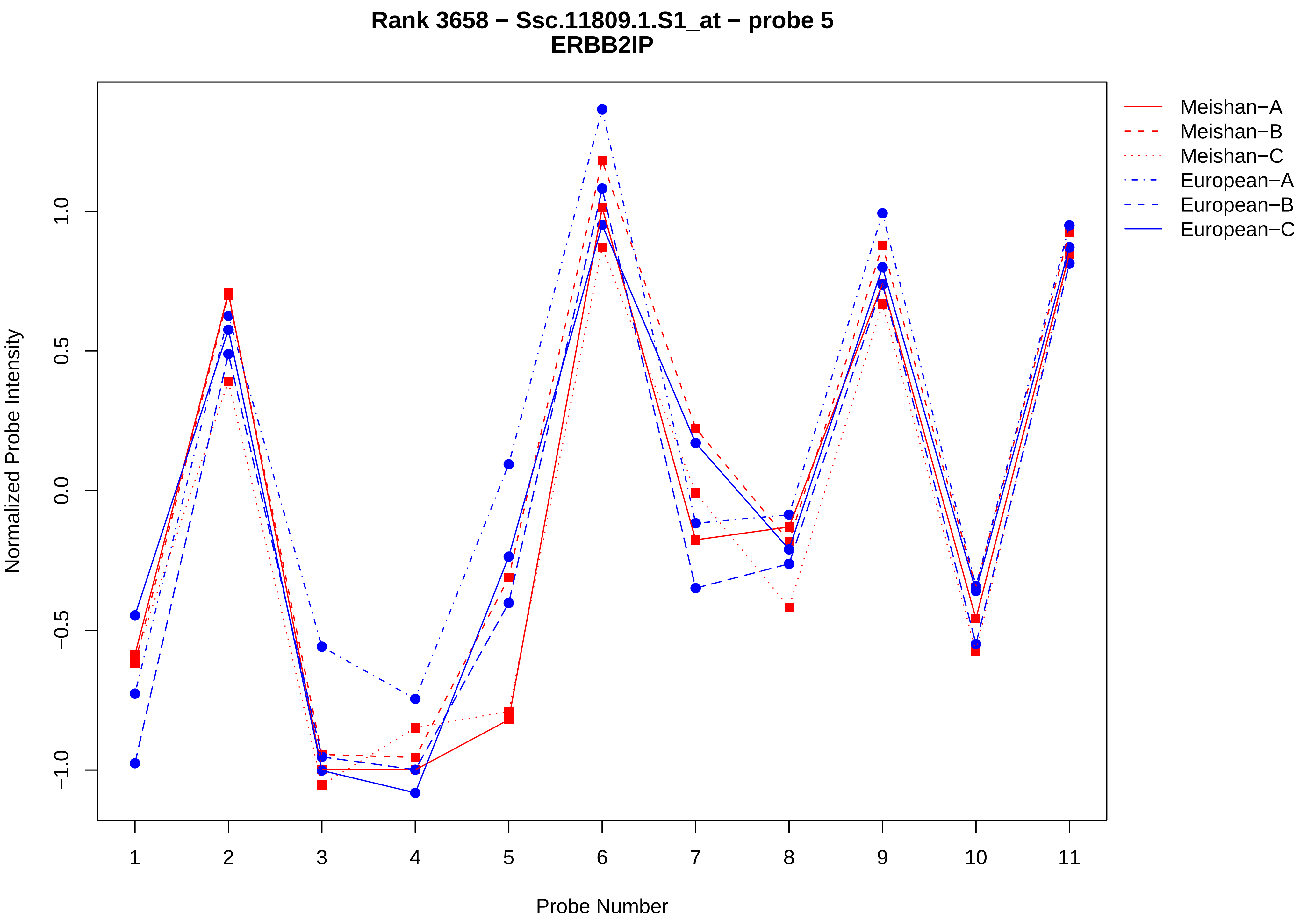

Rank 3658 − Ssc.11809.1.S1_at − probe 5
ERBB2IP
Normalized Probe Intensity
Probe Number
Meishan−A
Meishan−B
Meishan−C
European−A
European−B
European−C

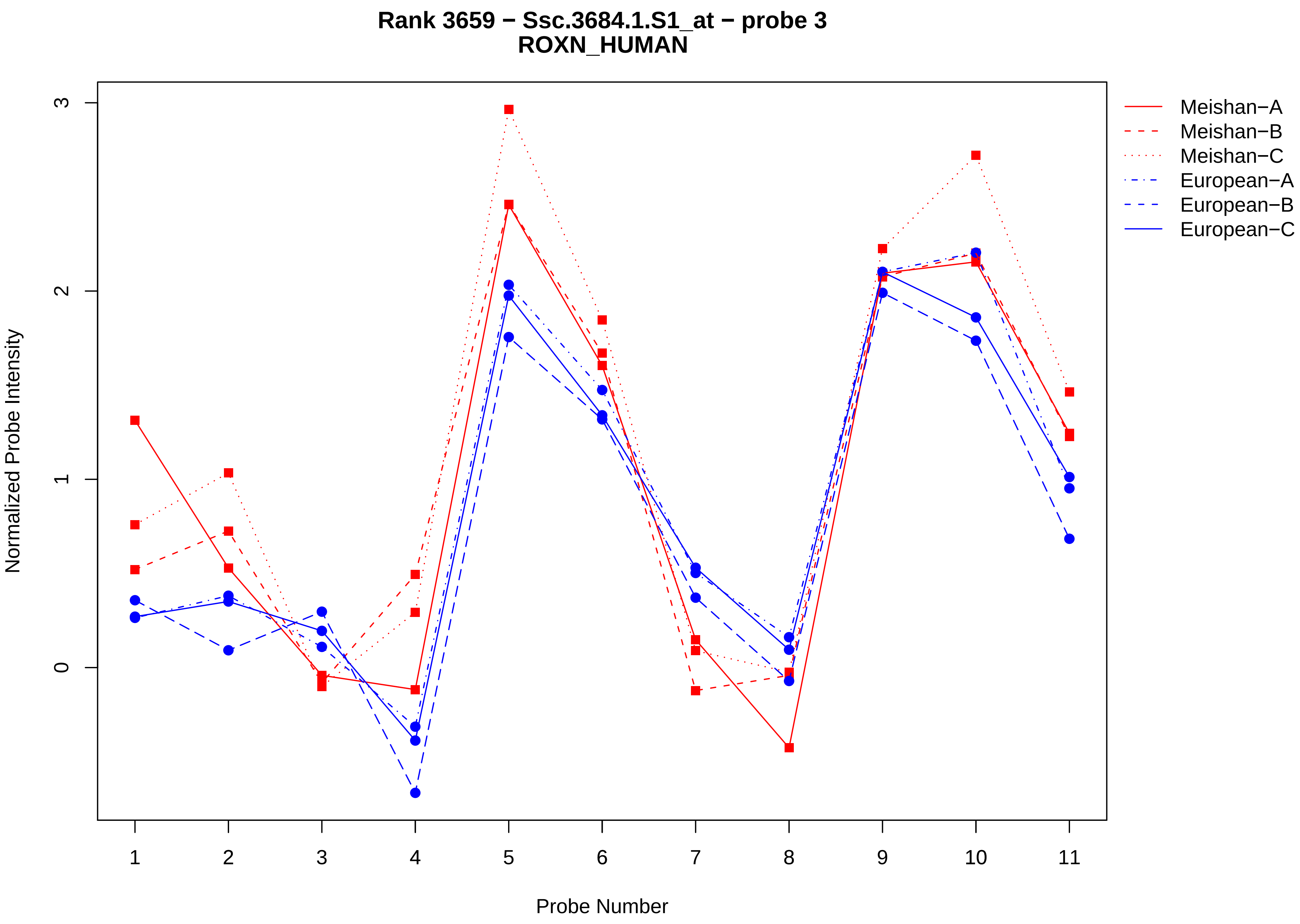

Rank 3659 − Ssc.3684.1.S1_at − probe 3
ROXN_HUMAN
Normalized Probe Intensity
Probe Number
0
1
2
3
1
2
3
4
5
6
7
8
9
10
11
Meishan−A
Meishan−B
Meishan−C
European−A
European−B
European−C

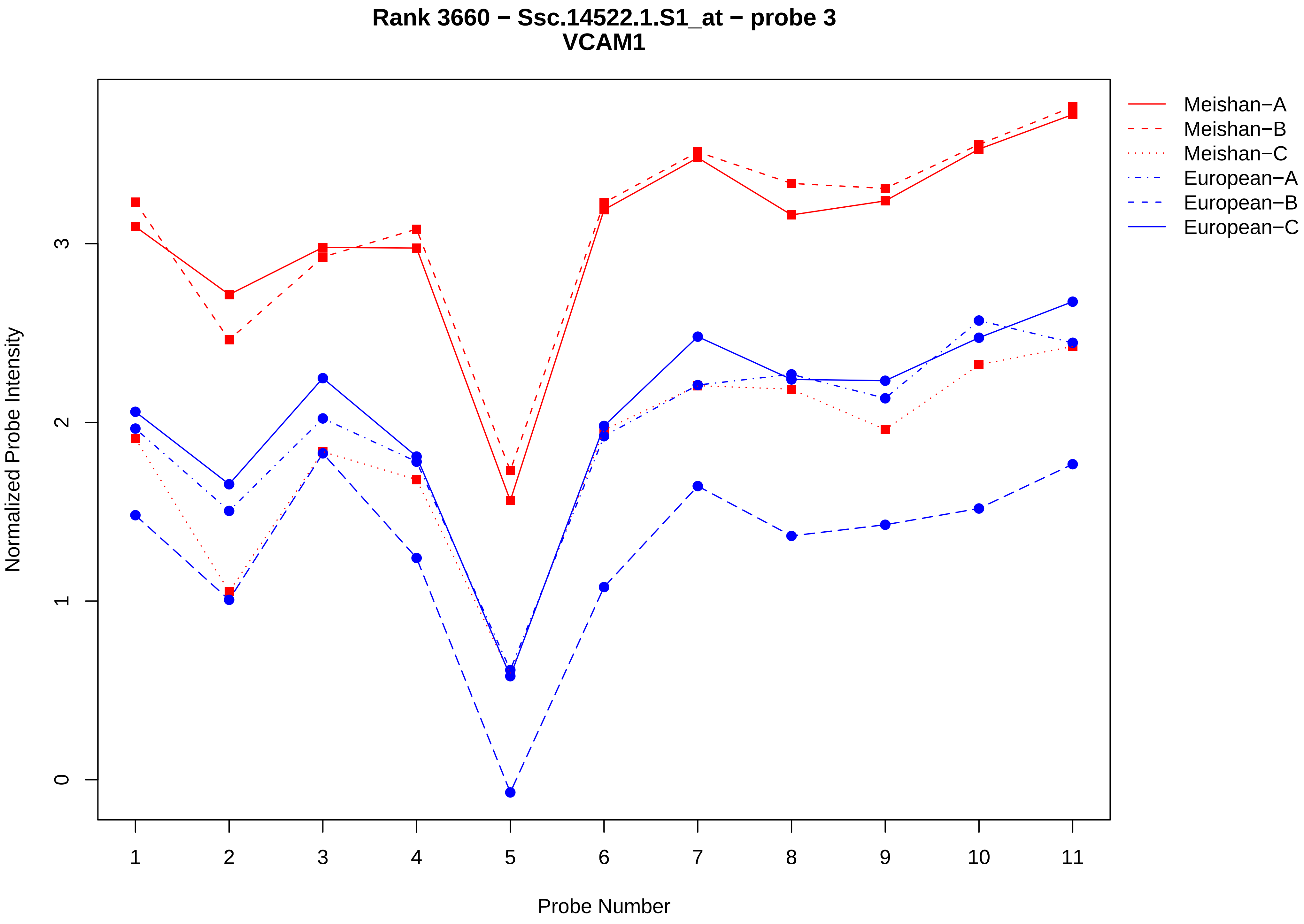

Rank 3660 − Ssc.14522.1.S1_at − probe 3
VCAM1
Normalized Probe Intensity
Probe Number
0
1
2
3
1
2
3
4
5
6
7
8
9
10
11
Meishan−A
Meishan−B
Meishan−C
European−A
European−B
European−C

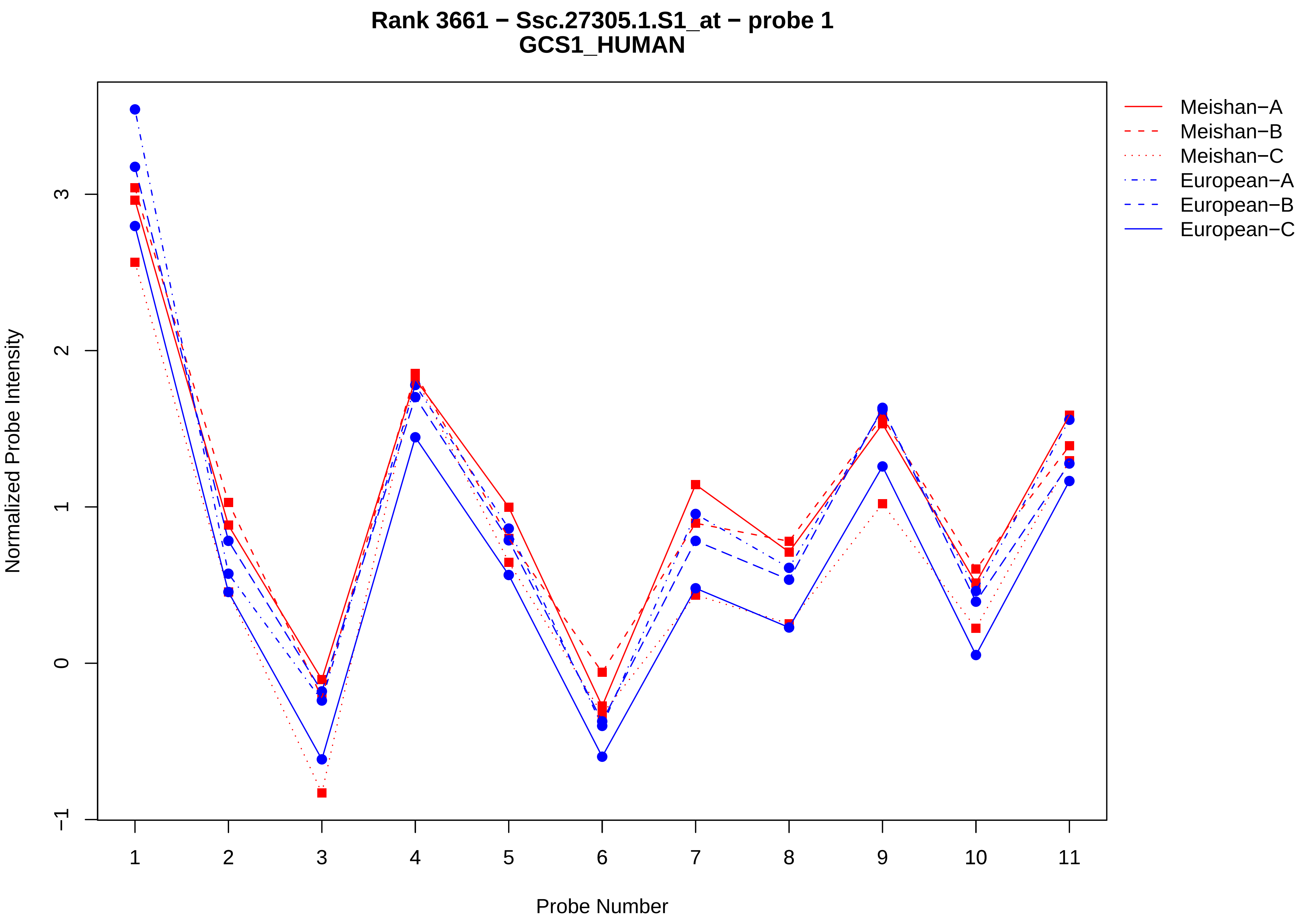

Rank 3661 − Ssc.27305.1.S1_at − probe 1
GCS1_HUMAN
Normalized Probe Intensity
Probe Number
Meishan−A
Meishan−B
Meishan−C
European−A
European−B
European−C
−1
0
1
2
3
1
2
3
4
5
6
7
8
9
10
11

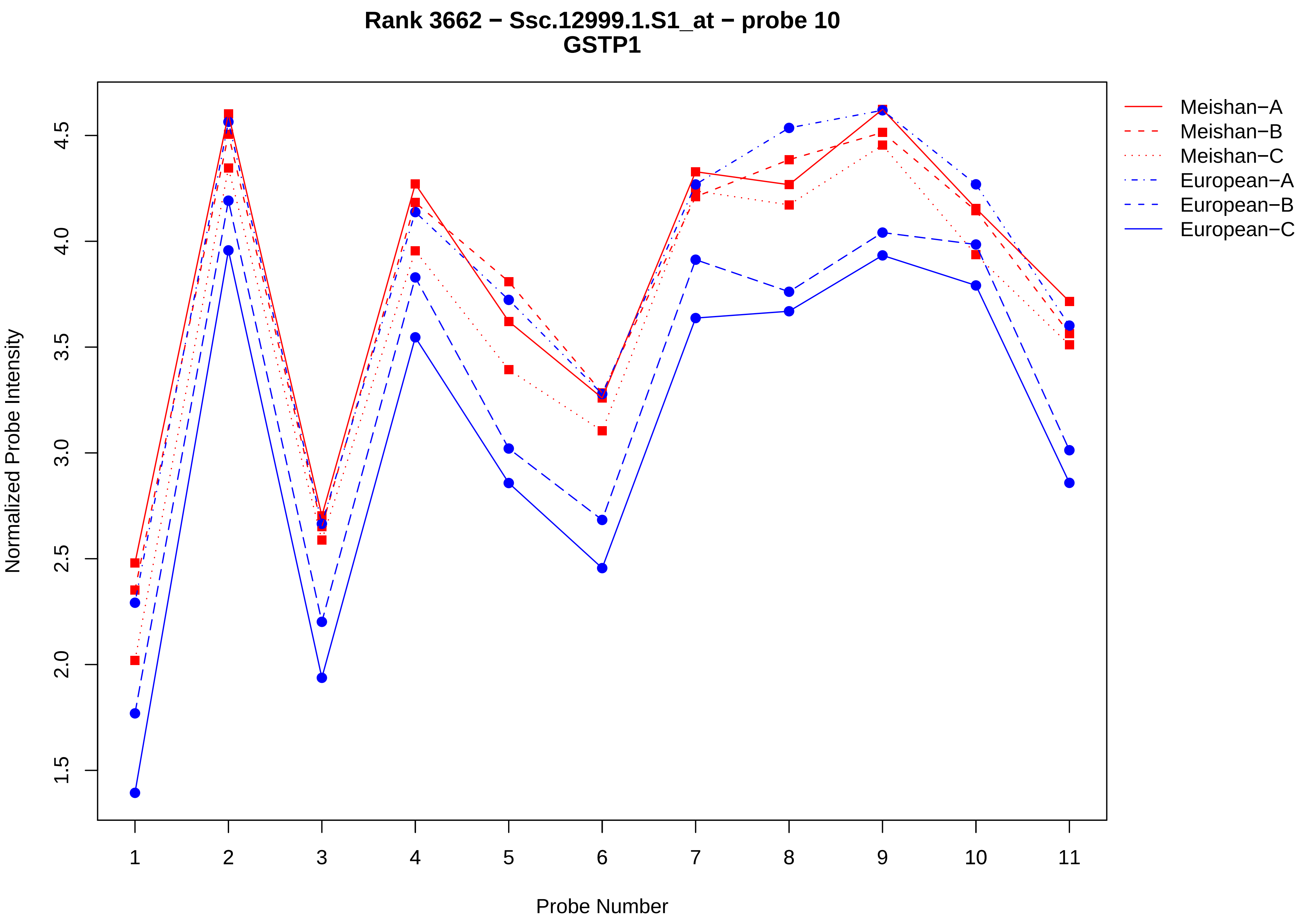

Rank 3662 − Ssc.12999.1.S1_at − probe 10
GSTP1
Normalized Probe Intensity
Probe Number
1.5
2.0
2.5
3.0
3.5
4.0
4.5
1
2
3
4
5
6
7
8
9
10
11
Meishan−A
Meishan−B
Meishan−C
European−A
European−B
European−C

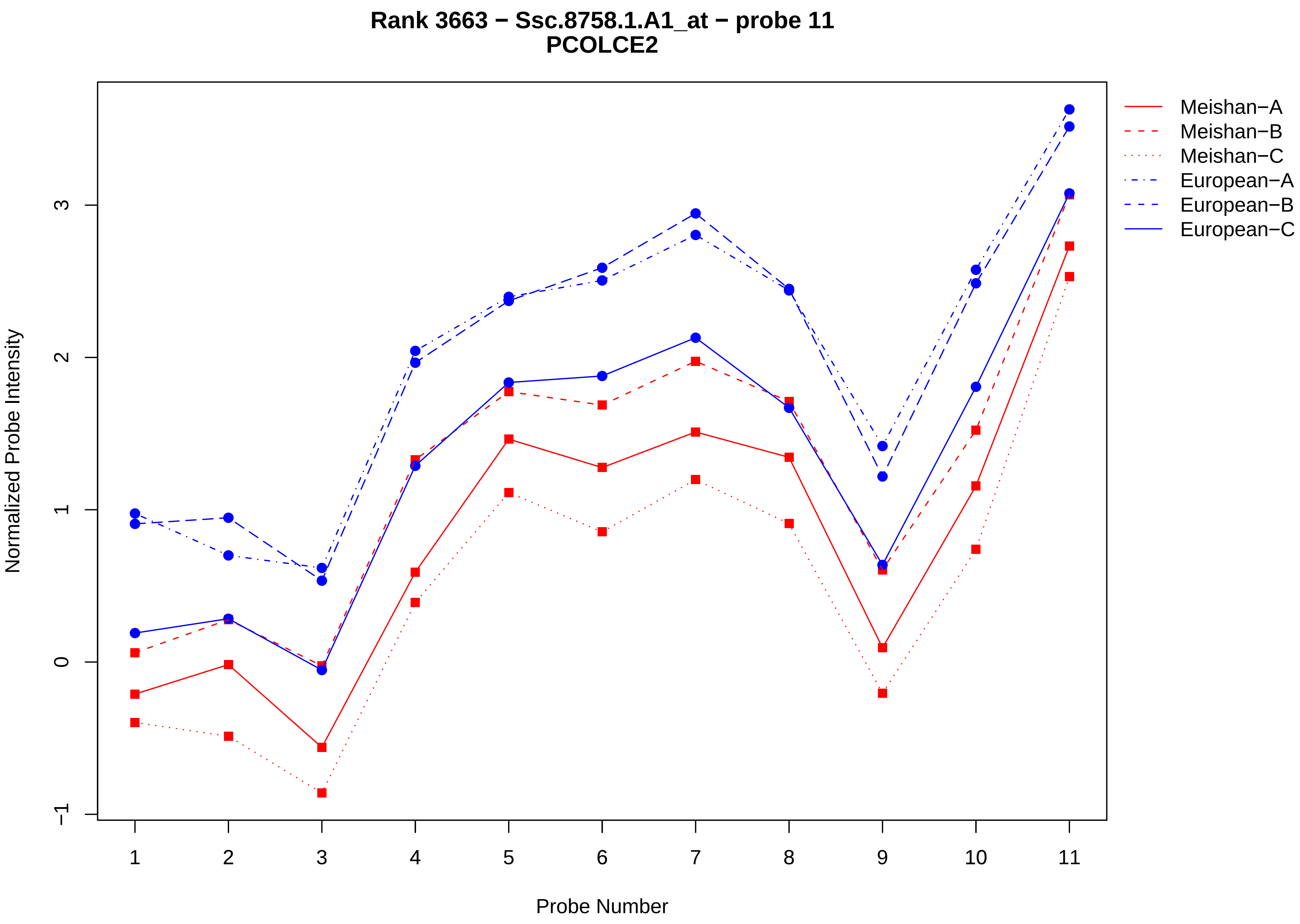

Rank 3663 − Ssc.8758.1.A1_at − probe 11
PCOLCE2
Normalized Probe Intensity
Probe Number
−1
0
1
2
3
1
2
3
4
5
6
7
8
9
10
11
Meishan−A
Meishan−B
Meishan−C
European−A
European−B
European−C

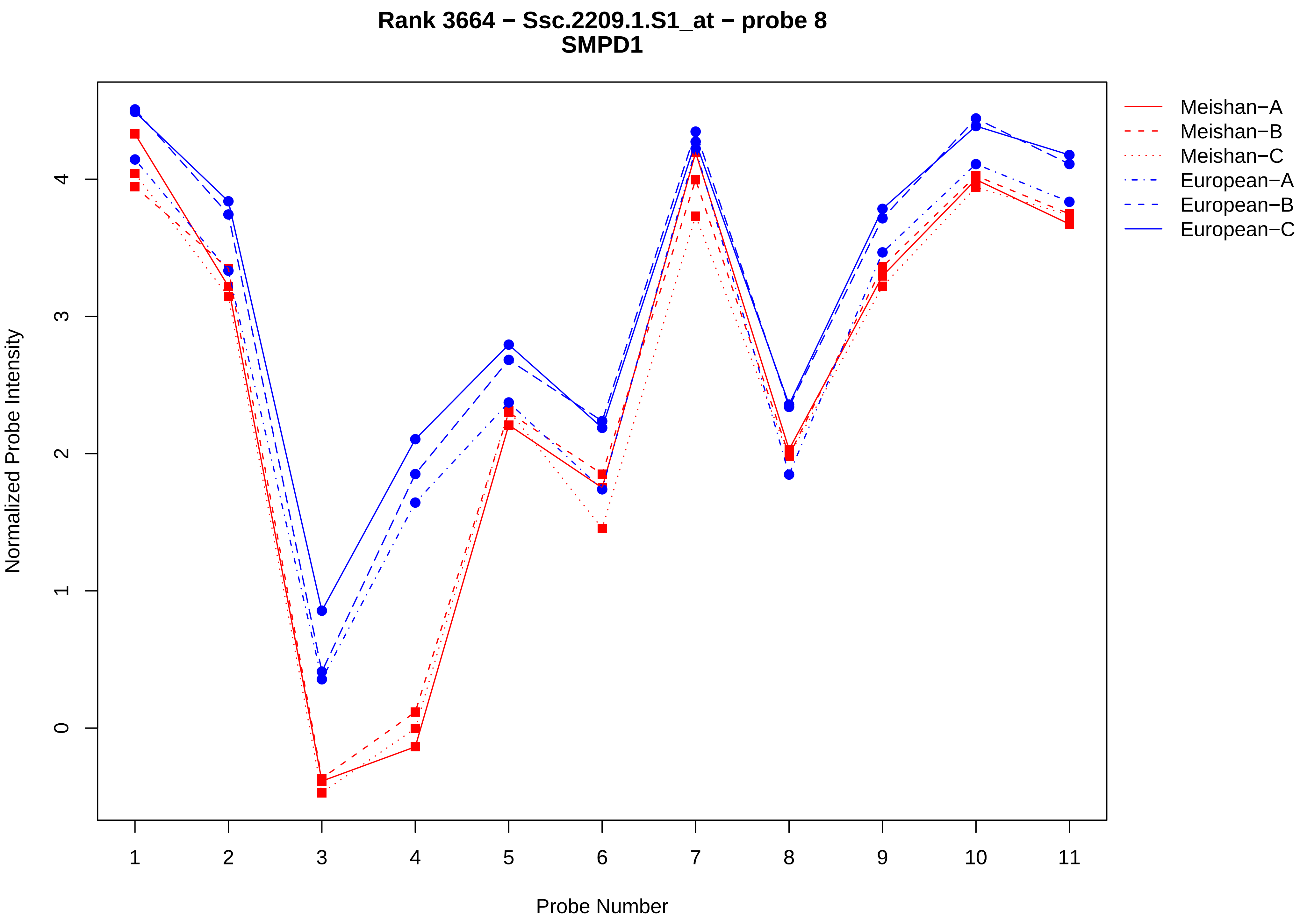

Rank 3664 − Ssc.2209.1.S1_at − probe 8
SMPD1
Normalized Probe Intensity
Probe Number
0
1
2
3
4
1
2
3
4
5
6
7
8
9
10
11
Meishan−A
Meishan−B
Meishan−C
European−A
European−B
European−C

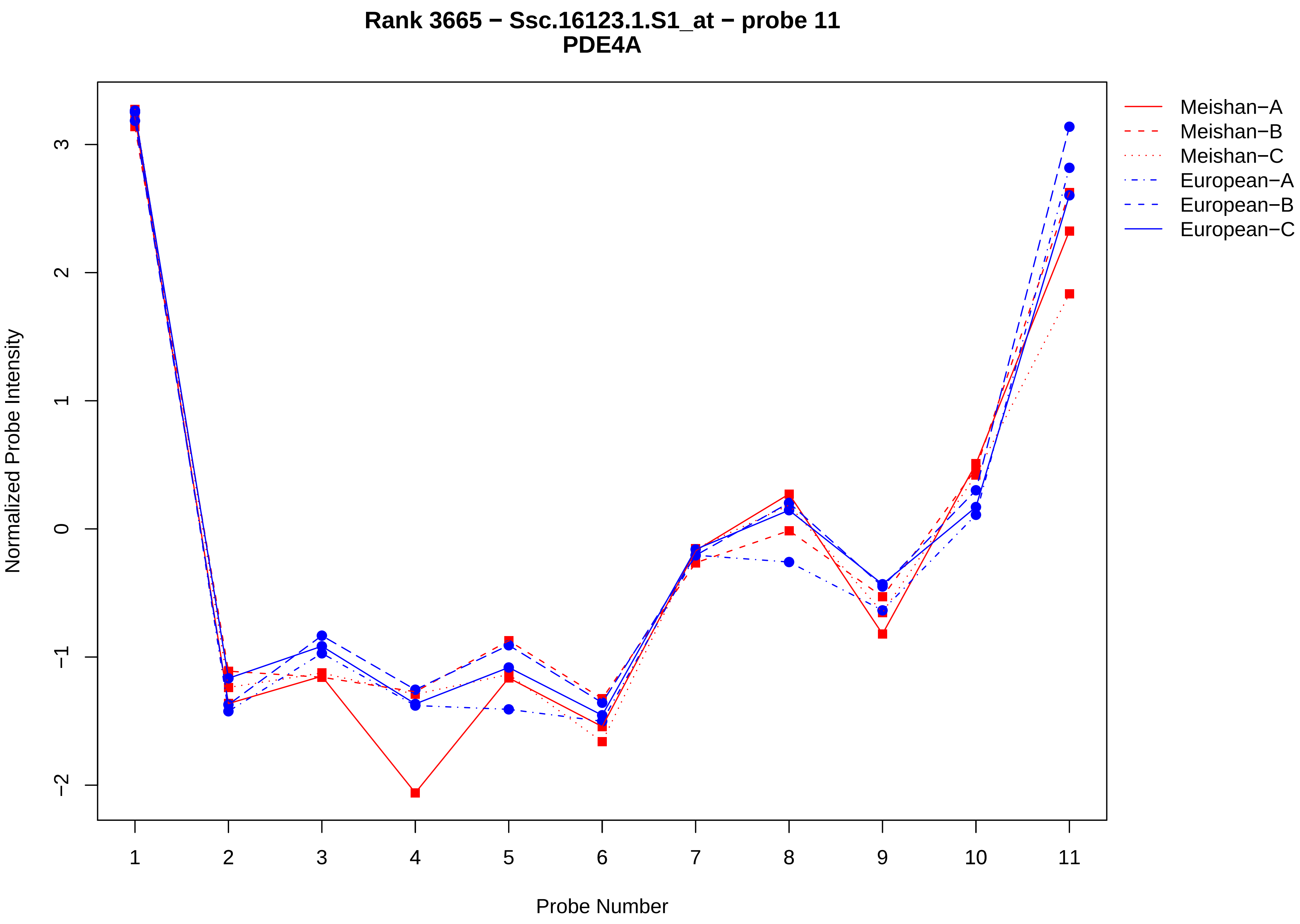
Rank 3665 − Ssc.16123.1.S1_at − probe 11
PDE4A
Normalized Probe Intensity
Probe Number
−2
−1
0
1
2
3
1
2
3
4
5
6
7
8
9
10
11
Meishan−A
Meishan−B
Meishan−C
European−A
European−B
European−C

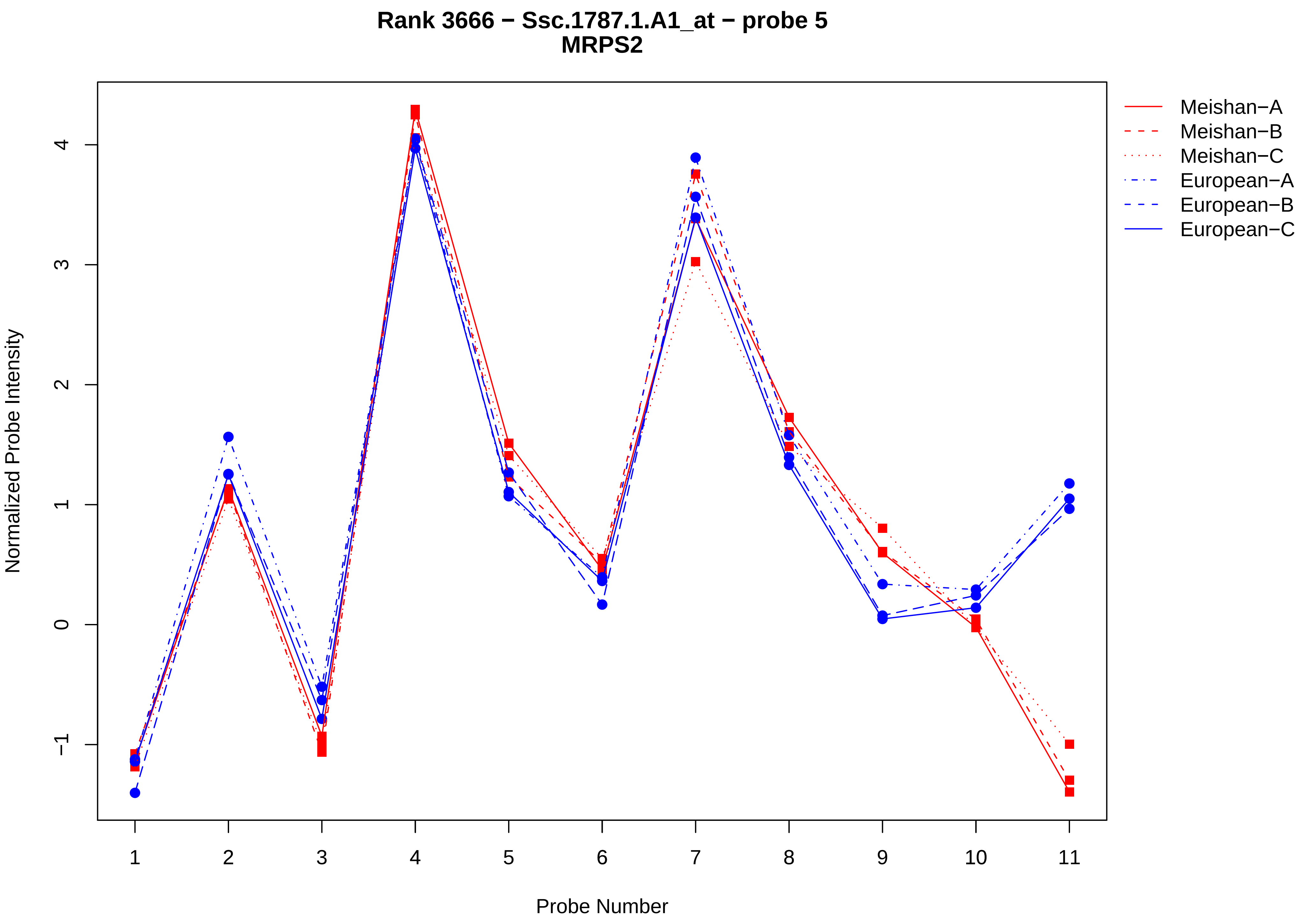
Rank 3666 − Ssc.1787.1.A1_at − probe 5
MRPS2
Normalized Probe Intensity
Probe Number
Meishan−A
Meishan−B
Meishan−C
European−A
European−B
European−C
−1
0
1
2
3
4
1
2
3
4
5
6
7
8
9
10
11

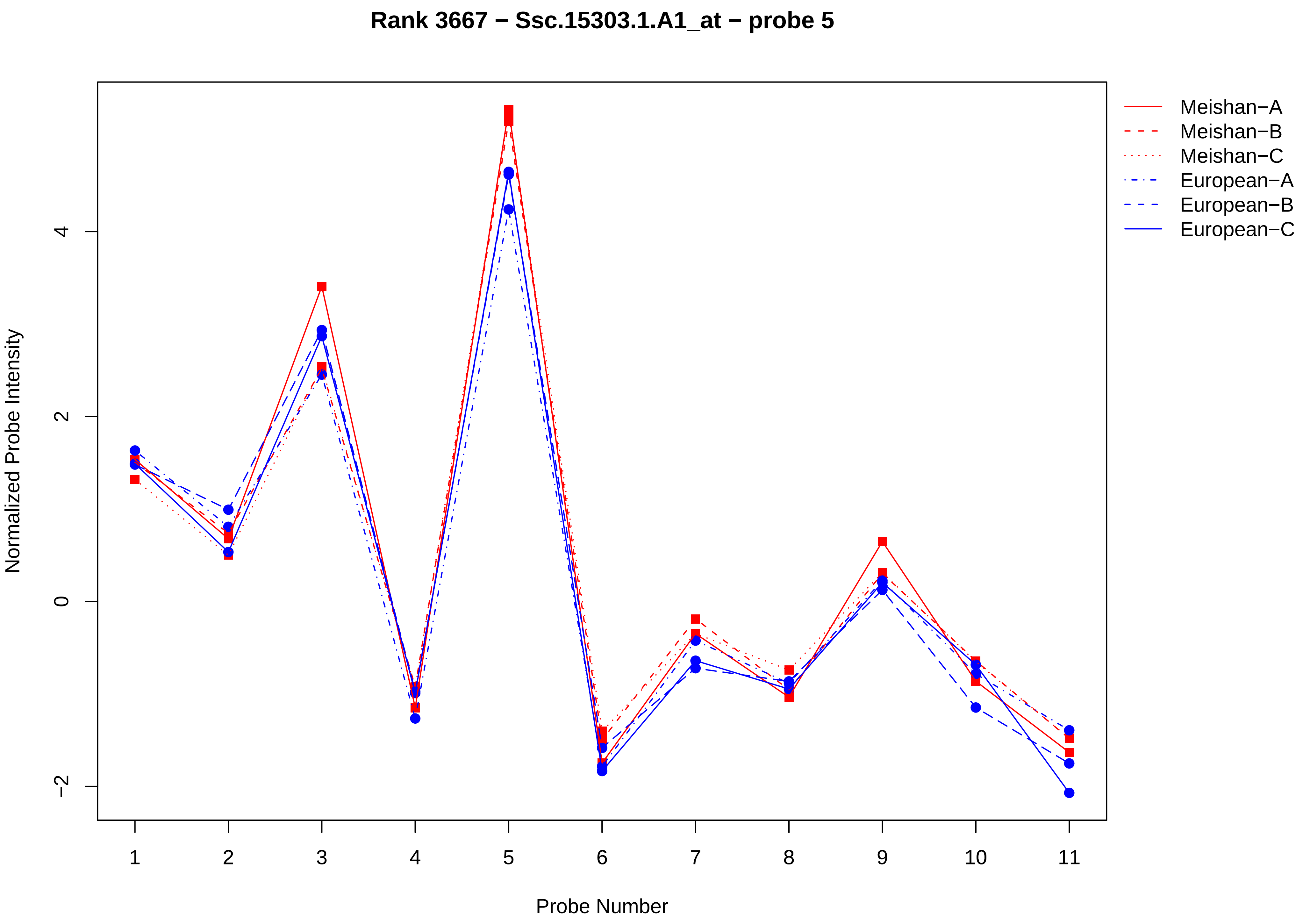

Rank 3667 − Ssc.15303.1.A1_at − probe 5
Normalized Probe Intensity
Probe Number
−2
0
2
4
1
2
3
4
5
6
7
8
9
10
11
Meishan−A
Meishan−B
Meishan−C
European−A
European−B
European−C

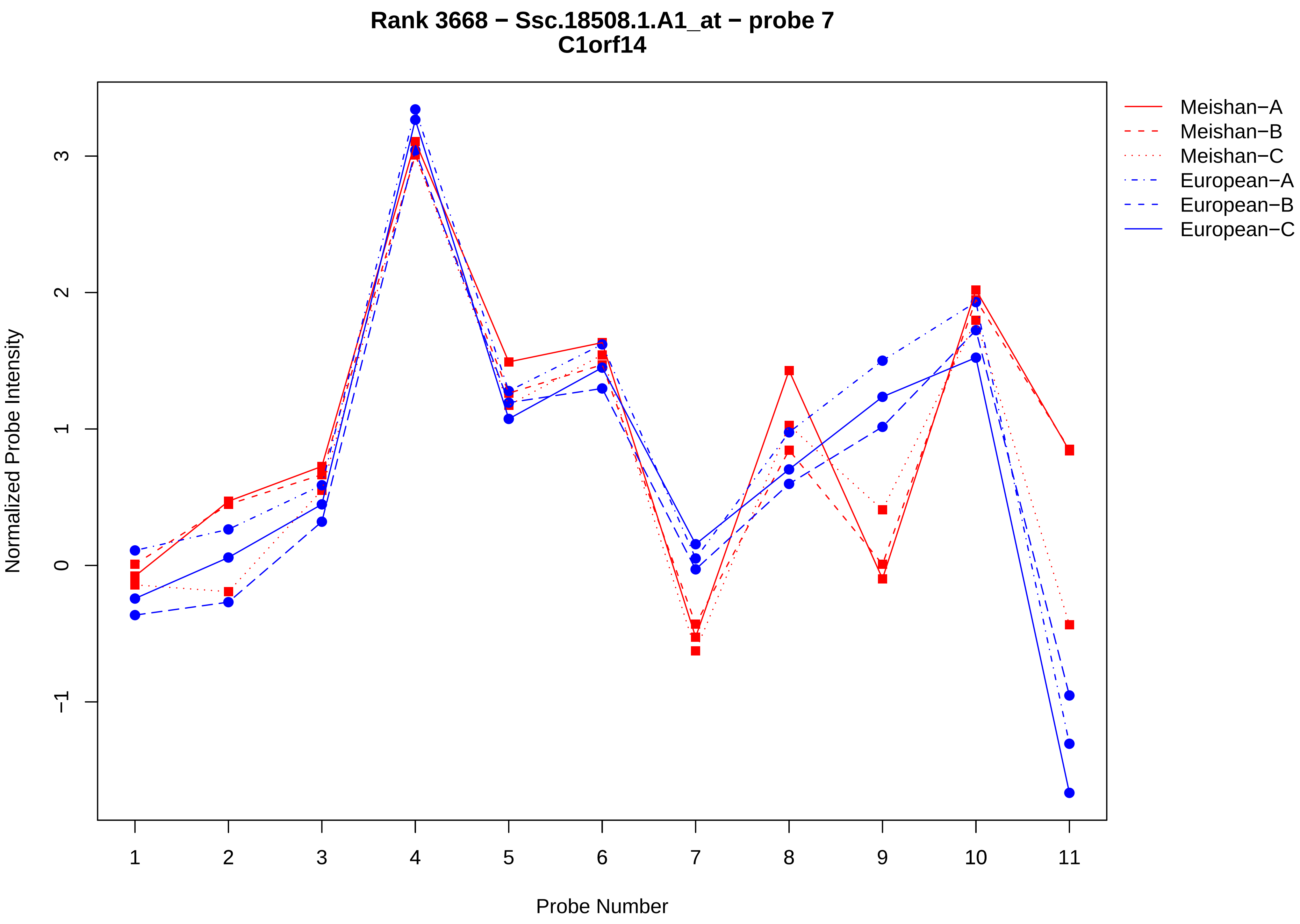

Rank 3668 − Ssc.18508.1.A1_at − probe 7
C1orf14
Normalized Probe Intensity
Probe Number
−1
0
1
2
3
1
2
3
4
5
6
7
8
9
10
11
Meishan−A
Meishan−B
Meishan−C
European−A
European−B
European−C

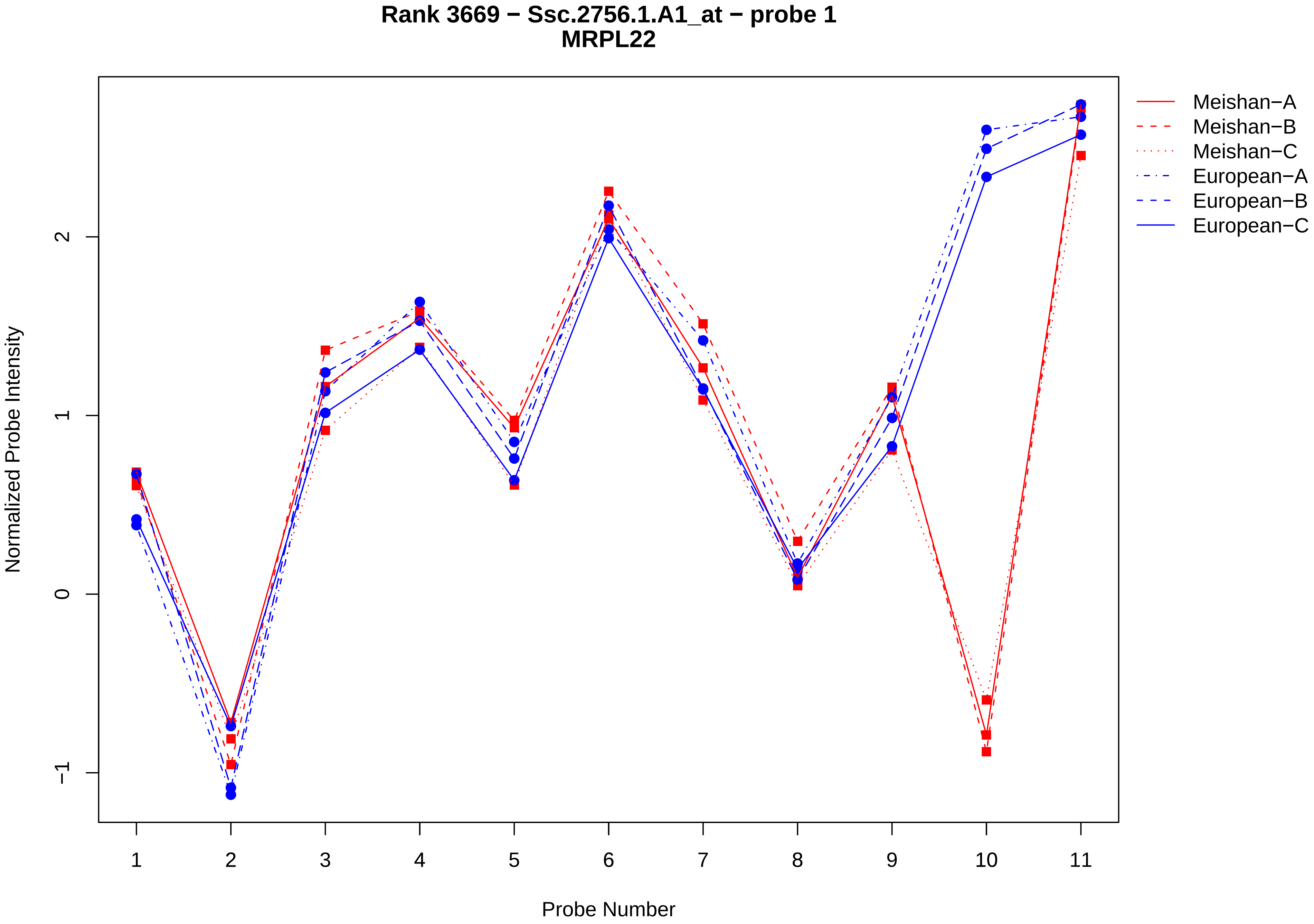
Rank 3669 − Ssc.2756.1.A1_at − probe 1
MRPL22
Normalized Probe Intensity
Probe Number
−1
0
1
2
1
2
3
4
5
6
7
8
9
10
11
Meishan−A
Meishan−B
Meishan−C
European−A
European−B
European−C

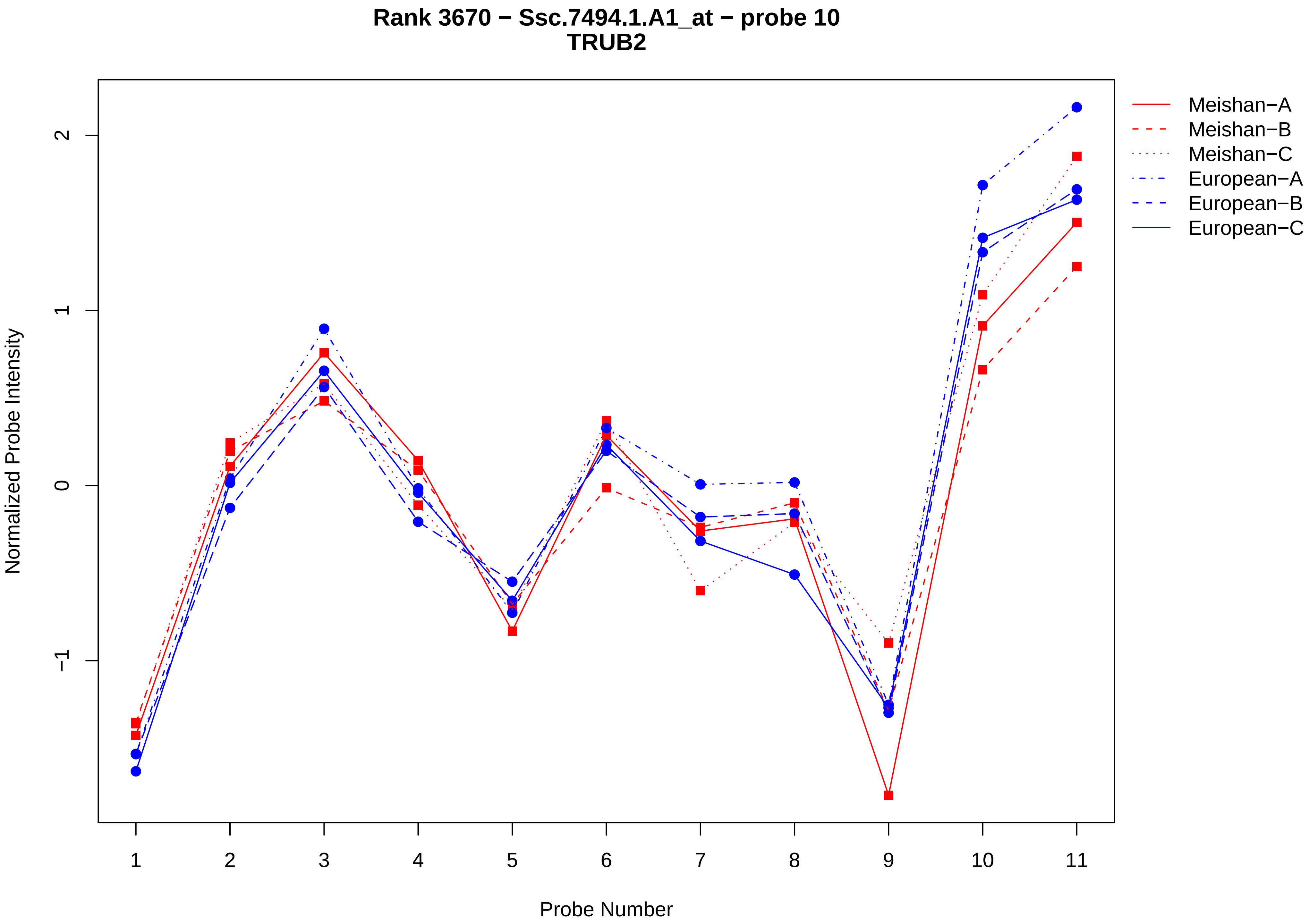

Rank 3670 − Ssc.7494.1.A1_at − probe 10
TRUB2
Normalized Probe Intensity
Probe Number
−1
0
1
2
1
2
3
4
5
6
7
8
9
10
11
Meishan−A
Meishan−B
Meishan−C
European−A
European−B
European−C

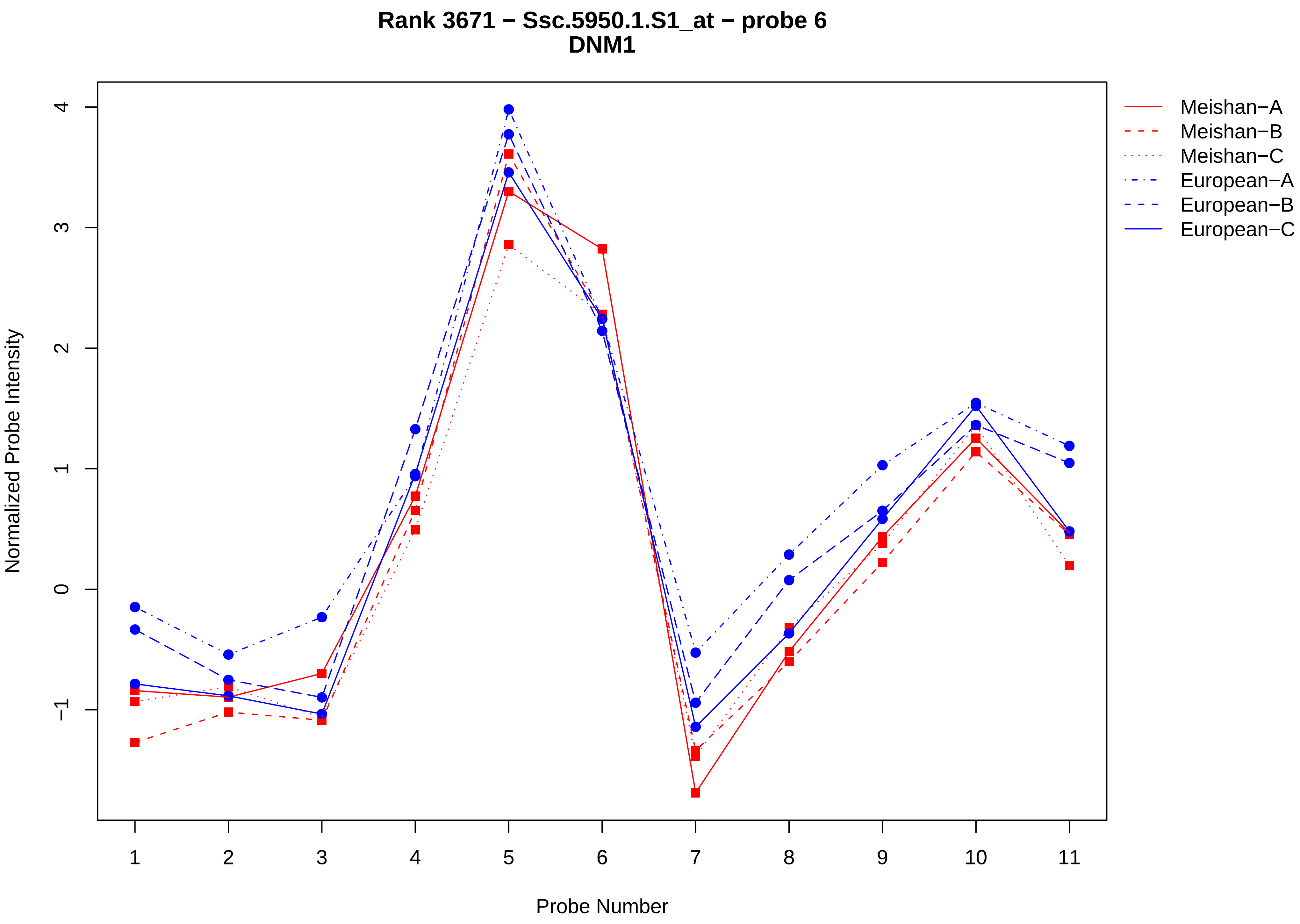

Rank 3671 − Ssc.5950.1.S1_at − probe 6
DNM1
Normalized Probe Intensity
Probe Number
−1
0
1
2
3
4
1
2
3
4
5
6
7
8
9
10
11
Meishan−A
Meishan−B
Meishan−C
European−A
European−B
European−C

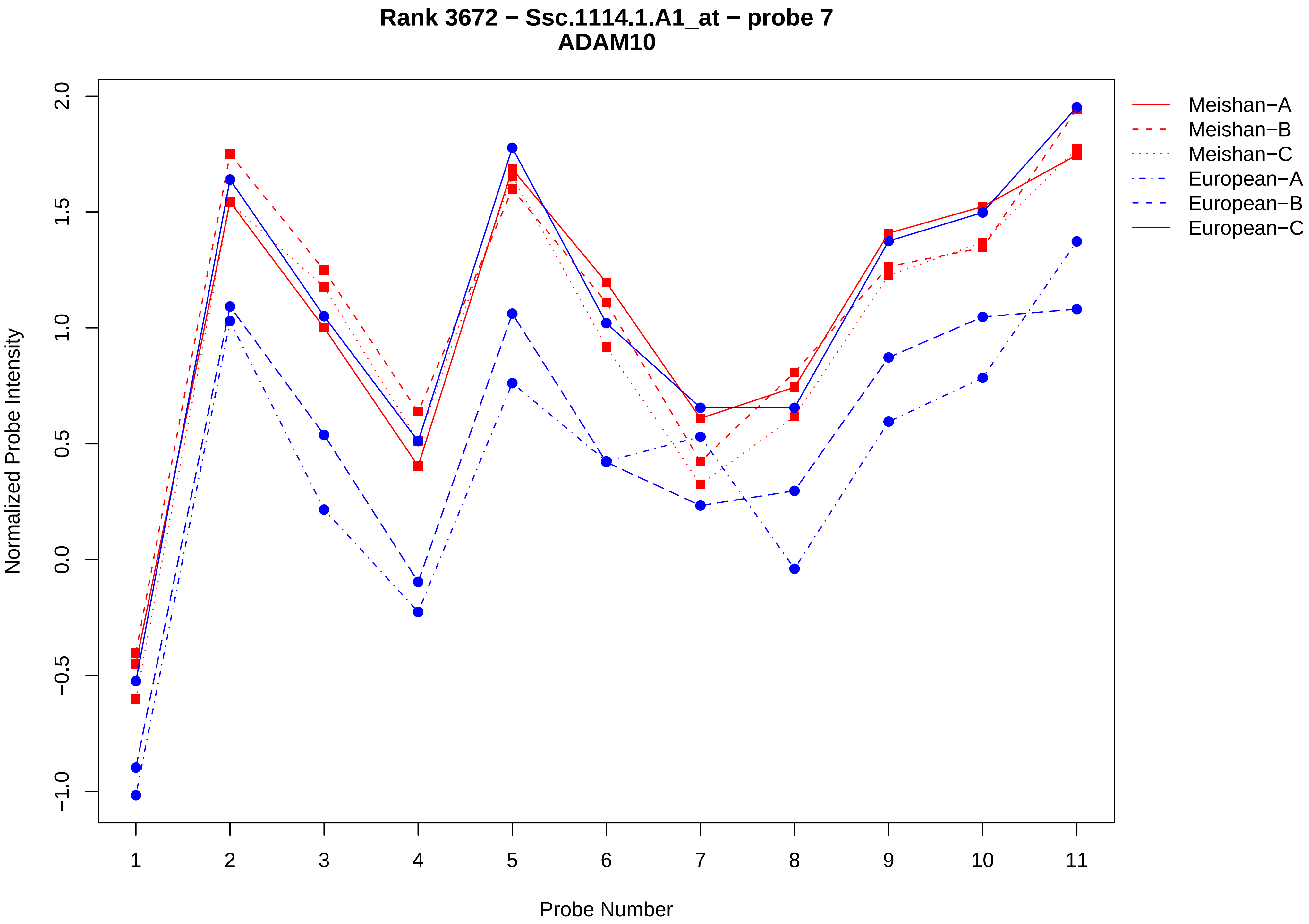
Rank 3672 − Ssc.1114.1.A1_at − probe 7
ADAM10
Normalized Probe Intensity
Probe Number
−1.0
−0.5
0.0
0.5
1.0
1.5
2.0
1
2
3
4
5
6
7
8
9
10
11
Meishan−A
Meishan−B
Meishan−C
European−A
European−B
European−C

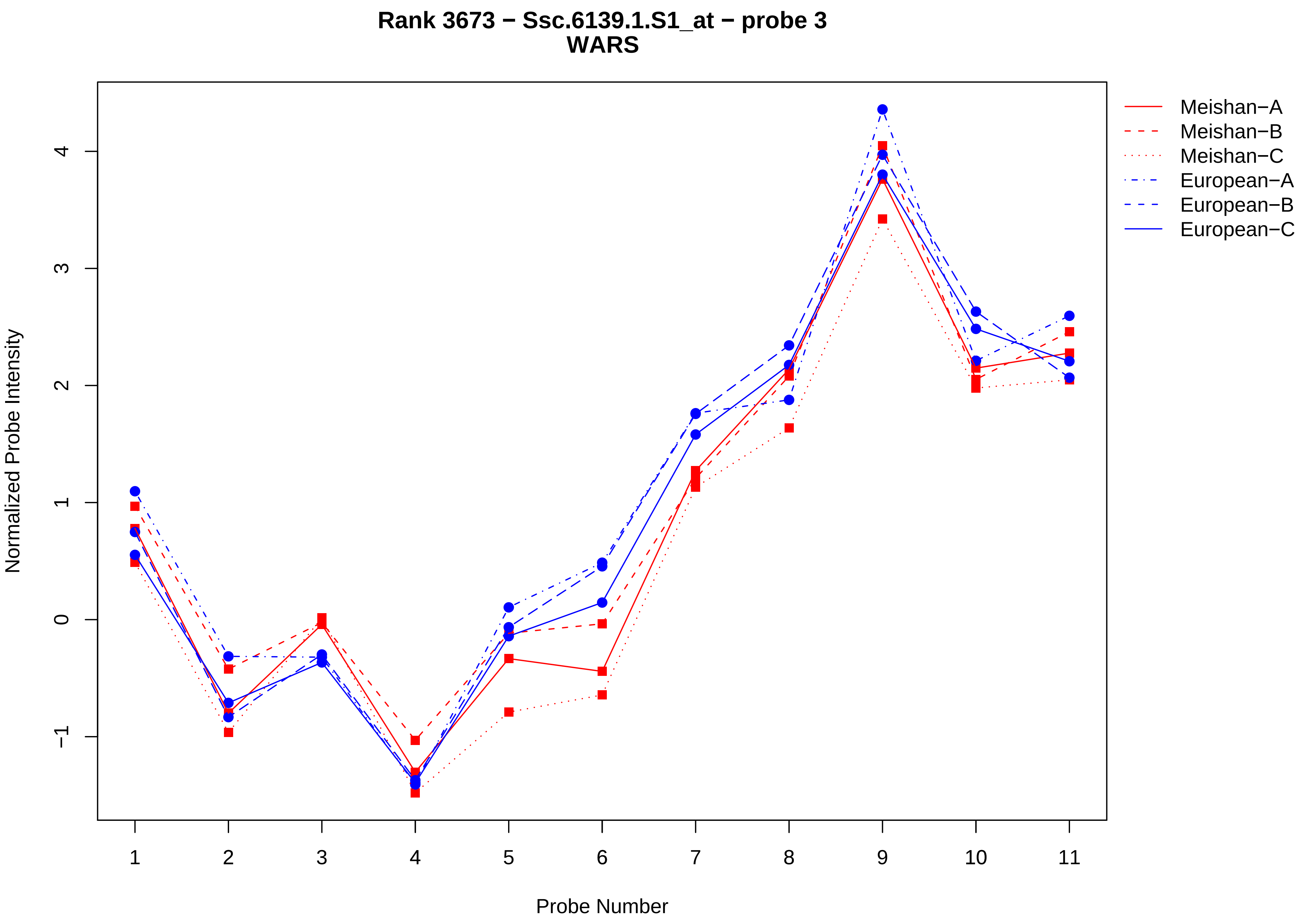

Rank 3673 − Ssc.6139.1.S1_at − probe 3
WARS
Normalized Probe Intensity
Probe Number
Meishan−A
Meishan−B
Meishan−C
European−A
European−B
European−C
−1
0
1
2
3
4
1
2
3
4
5
6
7
8
9
10
11

Rank 3674 − Ssc.21611.1.S1_at − probe 11
TRPC5

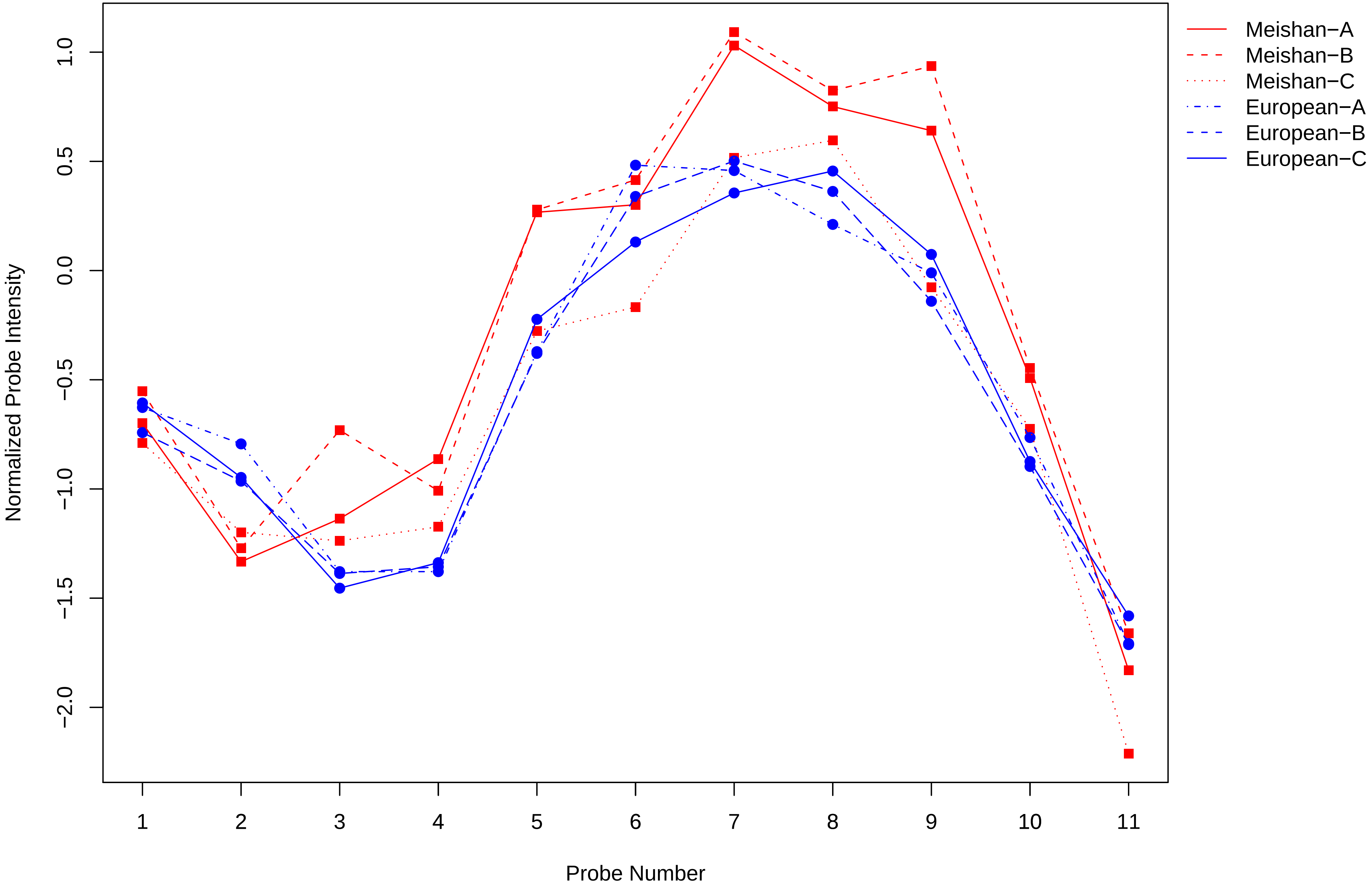

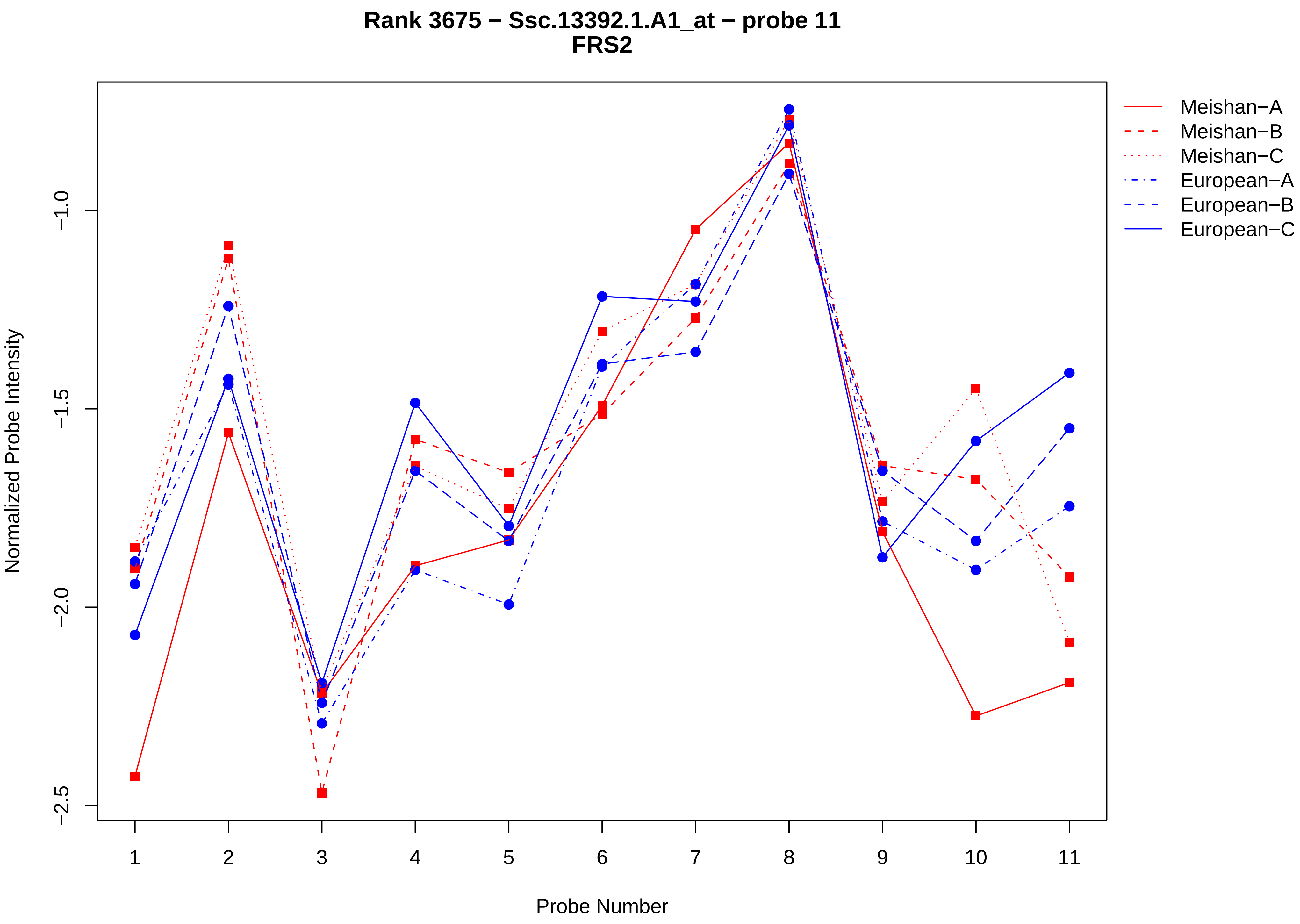

Rank 3675 − Ssc.13392.1.A1_at − probe 11
FRS2
Normalized Probe Intensity
−1.0
−1.5
−2.0
−2.5
Probe Number
1
2
3
4
5
6
7
8
9
10
11
Meishan−A
Meishan−B
Meishan−C
European−A
European−B
European−C

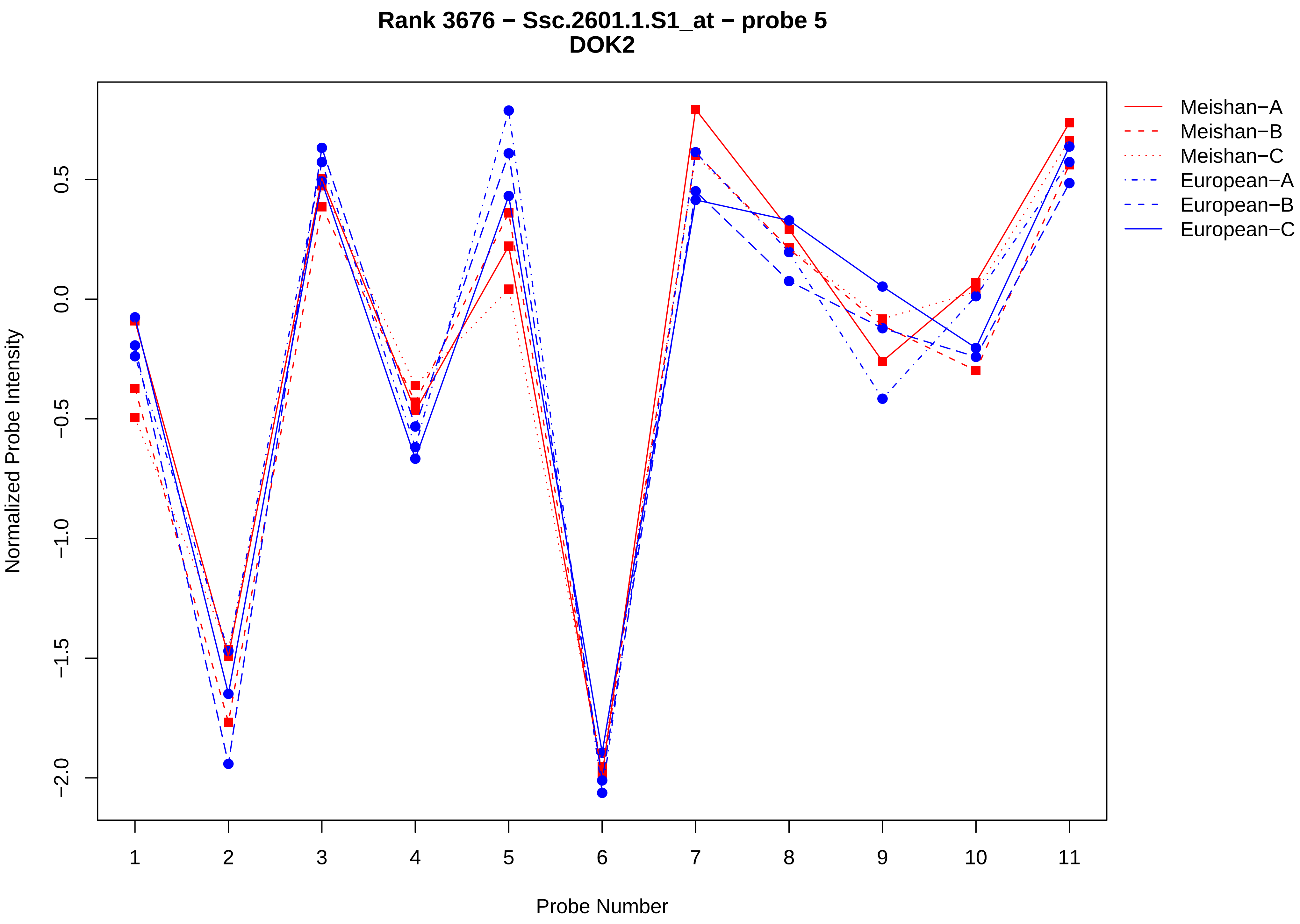

Rank 3676 − Ssc.2601.1.S1_at − probe 5
DOK2
Normalized Probe Intensity
Probe Number
−2.0
−1.5
−1.0
−0.5
0.0
0.5
1
2
3
4
5
6
7
8
9
10
11
Meishan−A
Meishan−B
Meishan−C
European−A
European−B
European−C

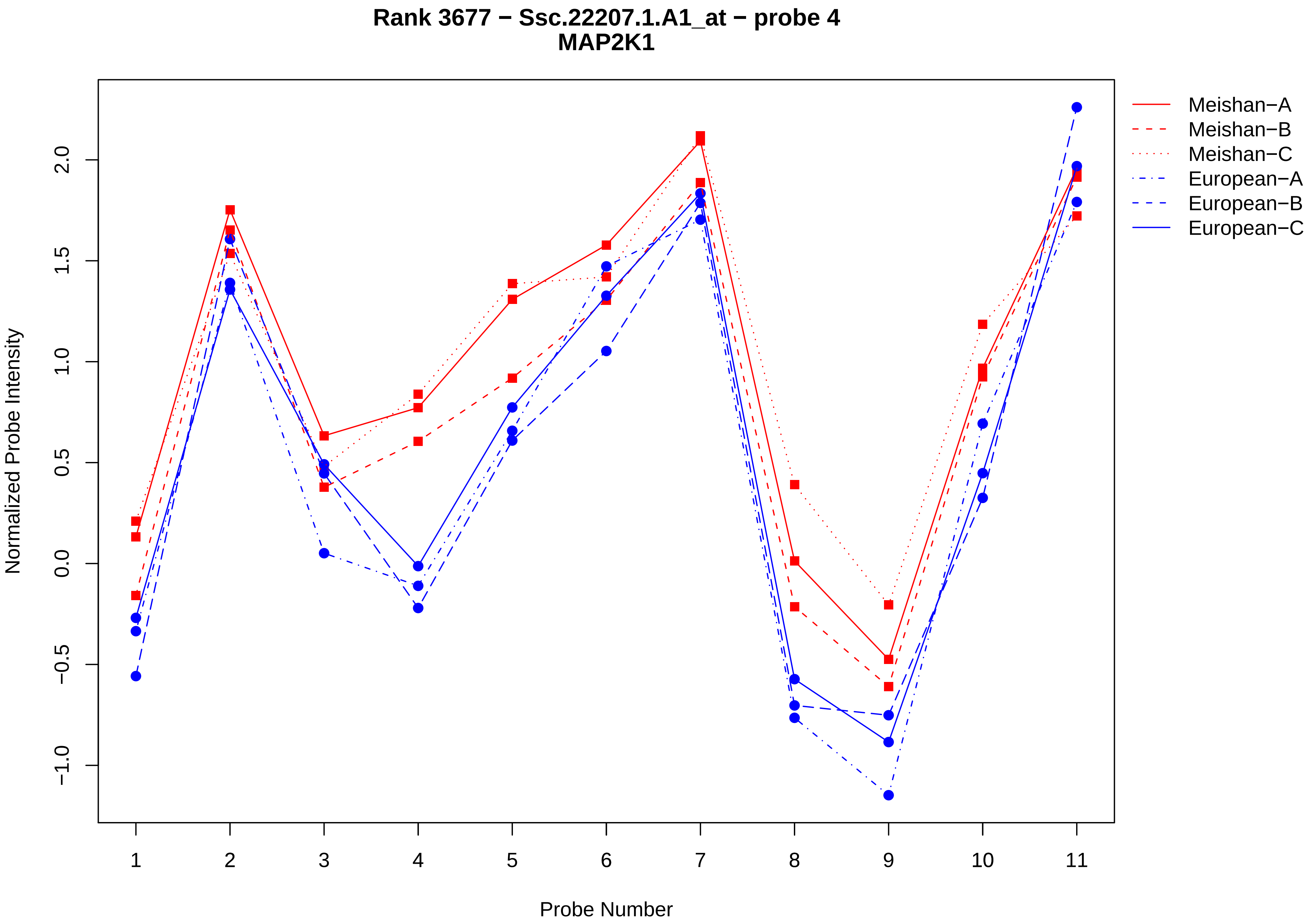

Rank 3677 − Ssc.22207.1.A1_at − probe 4
MAP2K1
Normalized Probe Intensity
Probe Number
−1.0
−0.5
0.0
0.5
1.0
1.5
2.0
1
2
3
4
5
6
7
8
9
10
11
Meishan−A
Meishan−B
Meishan−C
European−A
European−B
European−C

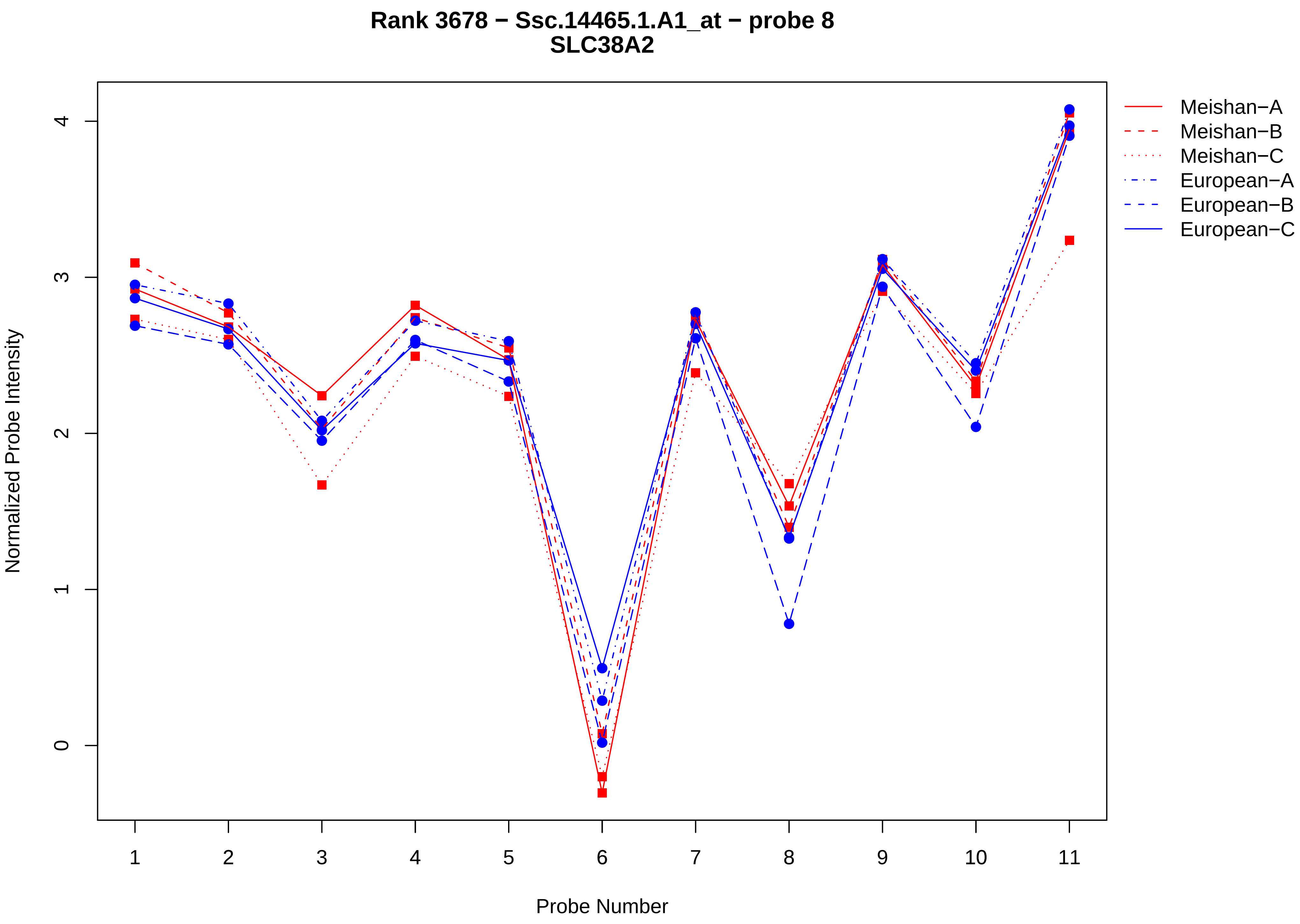

Rank 3678 − Ssc.14465.1.A1_at − probe 8
SLC38A2
Normalized Probe Intensity
Probe Number
0
1
2
3
4
1
2
3
4
5
6
7
8
9
10
11
Meishan−A
Meishan−B
Meishan−C
European−A
European−B
European−C

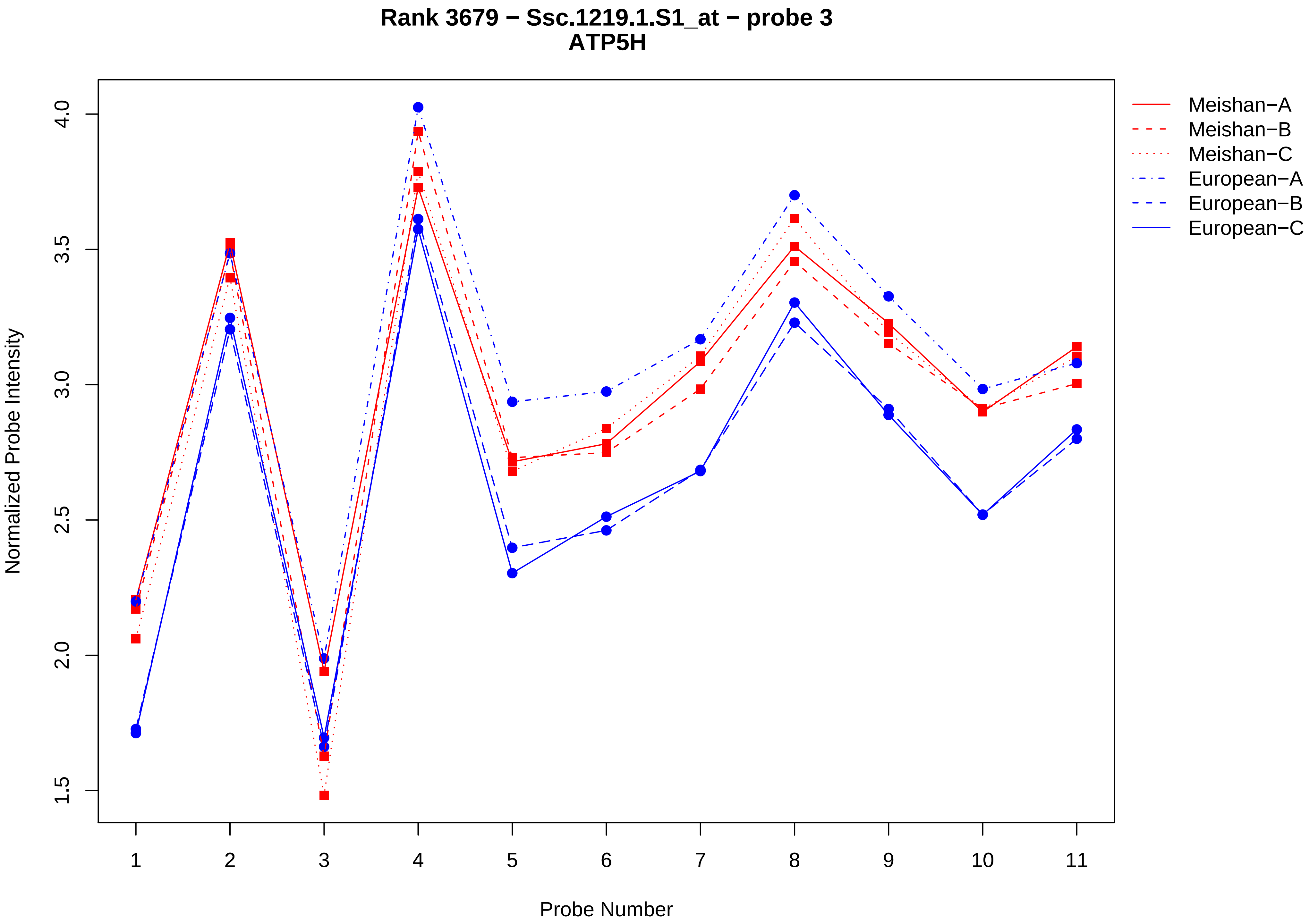

Rank 3679 − Ssc.1219.1.S1_at − probe 3
ATP5H
Normalized Probe Intensity
Probe Number
1.5
2.0
2.5
3.0
3.5
4.0
1
2
3
4
5
6
7
8
9
10
11
Meishan−A
Meishan−B
Meishan−C
European−A
European−B
European−C

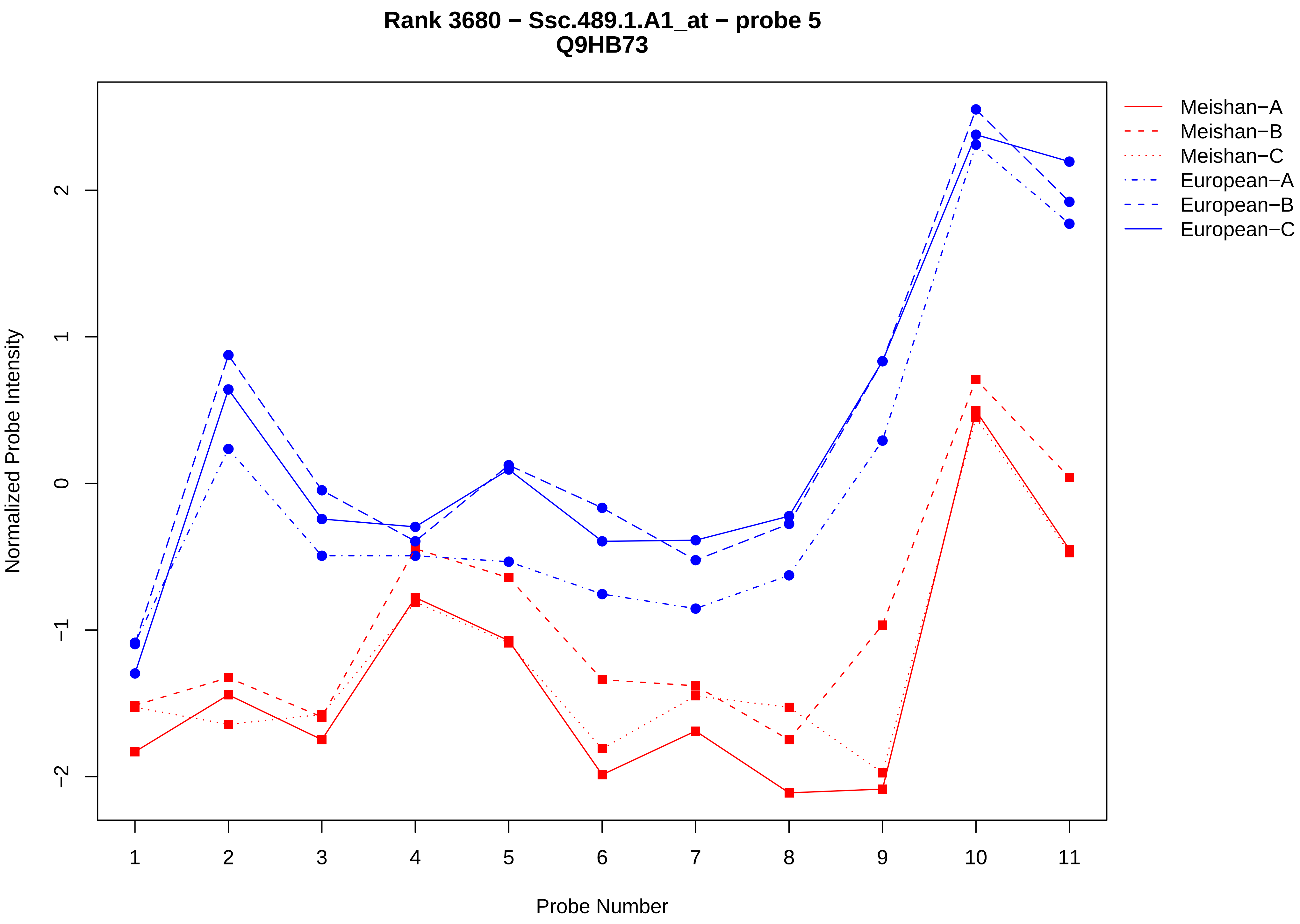

Rank 3680 − Ssc.489.1.A1_at − probe 5
Q9HB73
Normalized Probe Intensity
Probe Number
−2
−1
0
1
2
1
2
3
4
5
6
7
8
9
10
11
Meishan−A
Meishan−B
Meishan−C
European−A
European−B
European−C

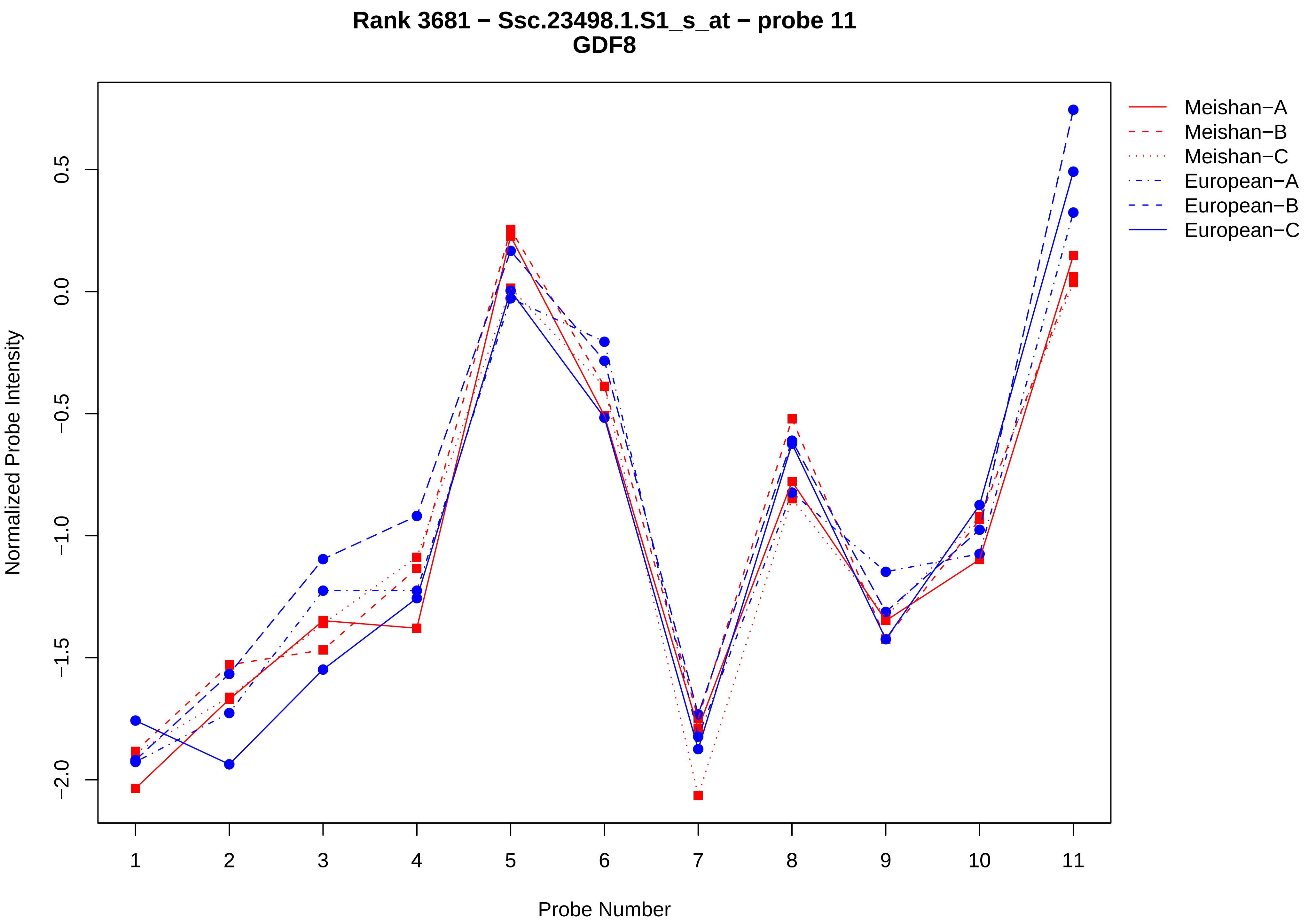

Rank 3681 − Ssc.23498.1.S1_s_at − probe 11
GDF8
Normalized Probe Intensity
Probe Number
0.5
0.0
−0.5
−1.0
−1.5
−2.0
1
2
3
4
5
6
7
8
9
10
11
Meishan−A
Meishan−B
Meishan−C
European−A
European−B
European−C

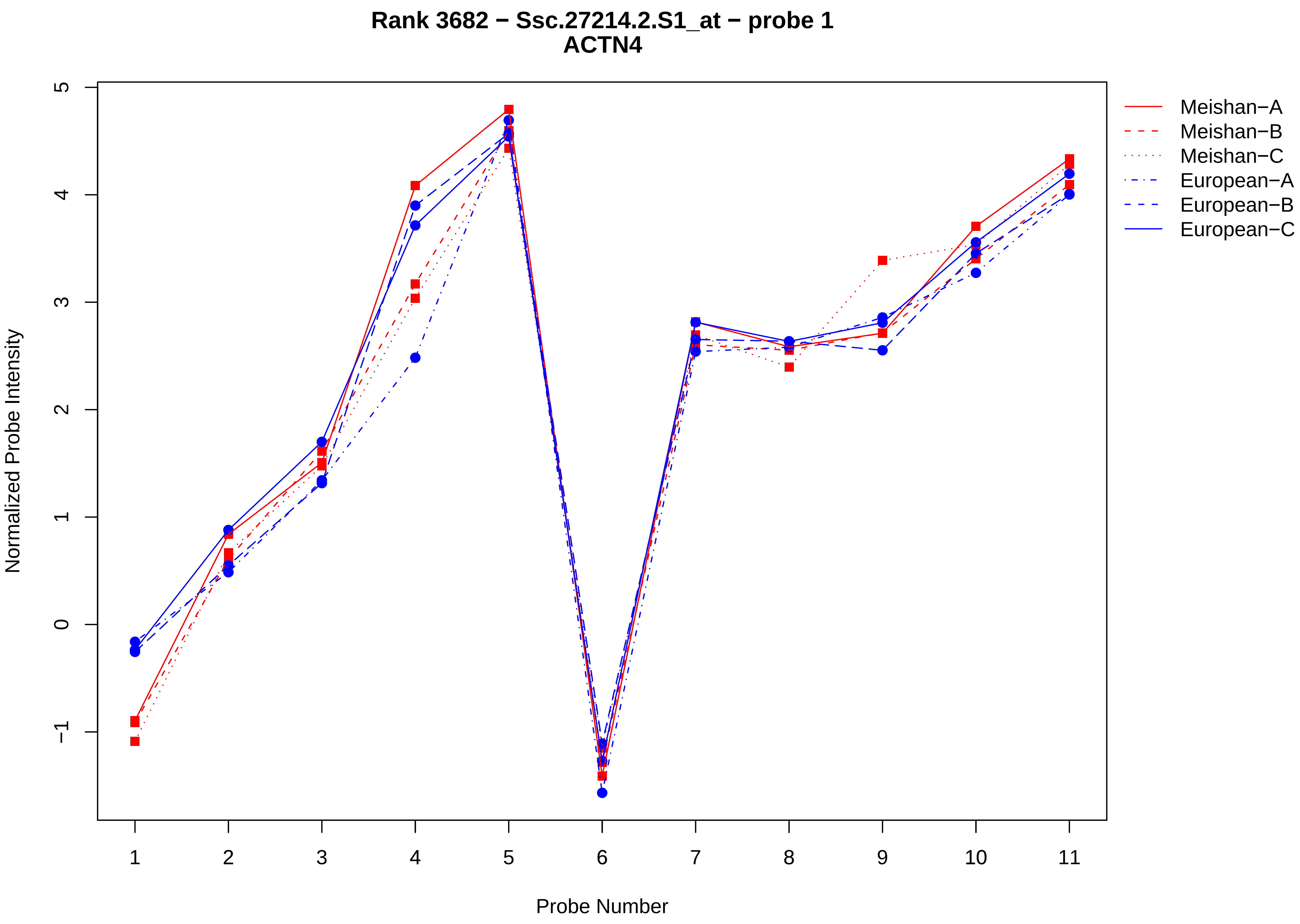

Rank 3682 − Ssc.27214.2.S1_at − probe 1
ACTN4
Normalized Probe Intensity
Probe Number
Meishan−A
Meishan−B
Meishan−C
European−A
European−B
European−C

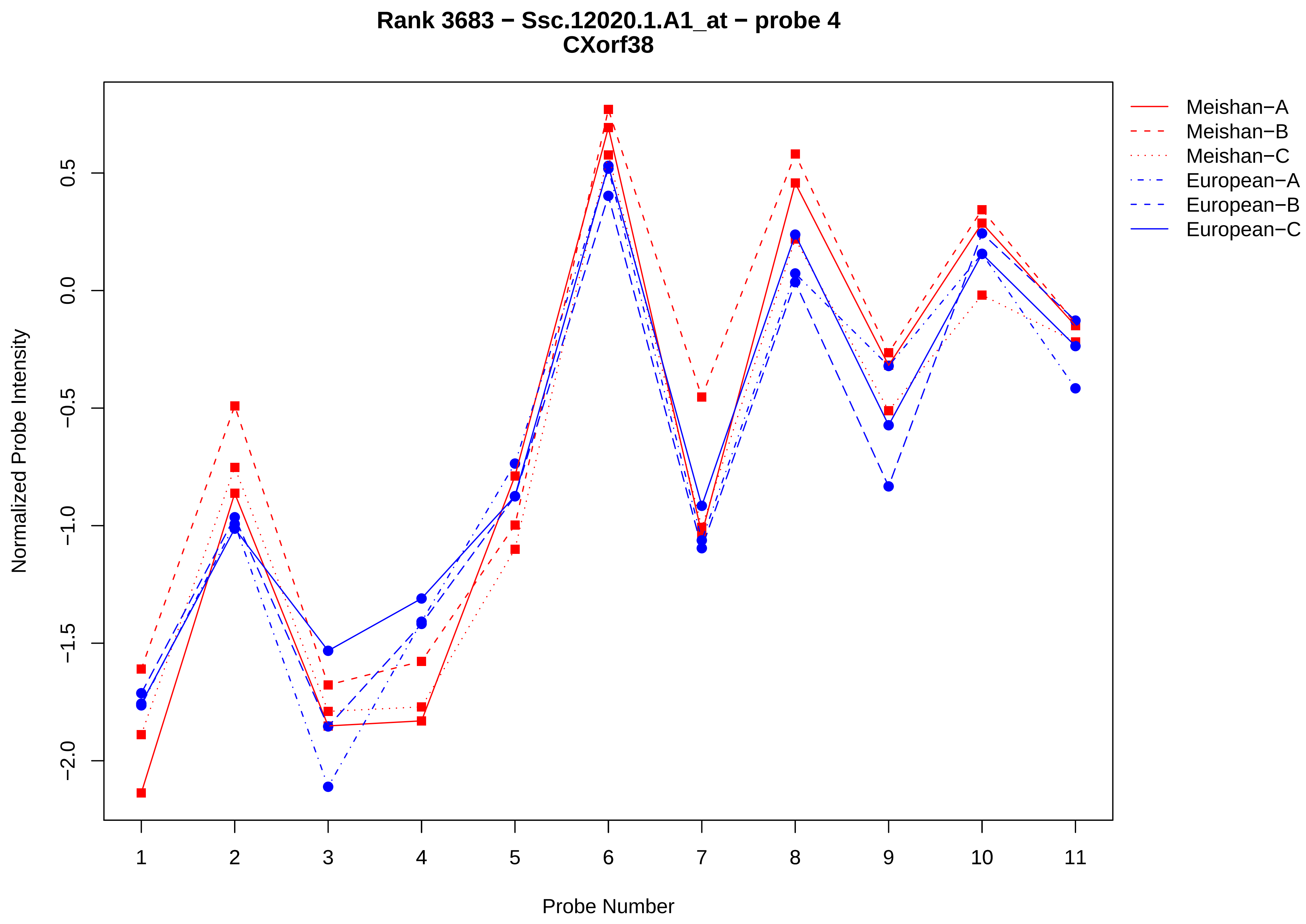

Rank 3683 − Ssc.12020.1.A1_at − probe 4
CXorf38
Normalized Probe Intensity
Probe Number
0.5
0.0
−0.5
−1.0
−1.5
−2.0
1
2
3
4
5
6
7
8
9
10
11
Meishan−A
Meishan−B
Meishan−C
European−A
European−B
European−C

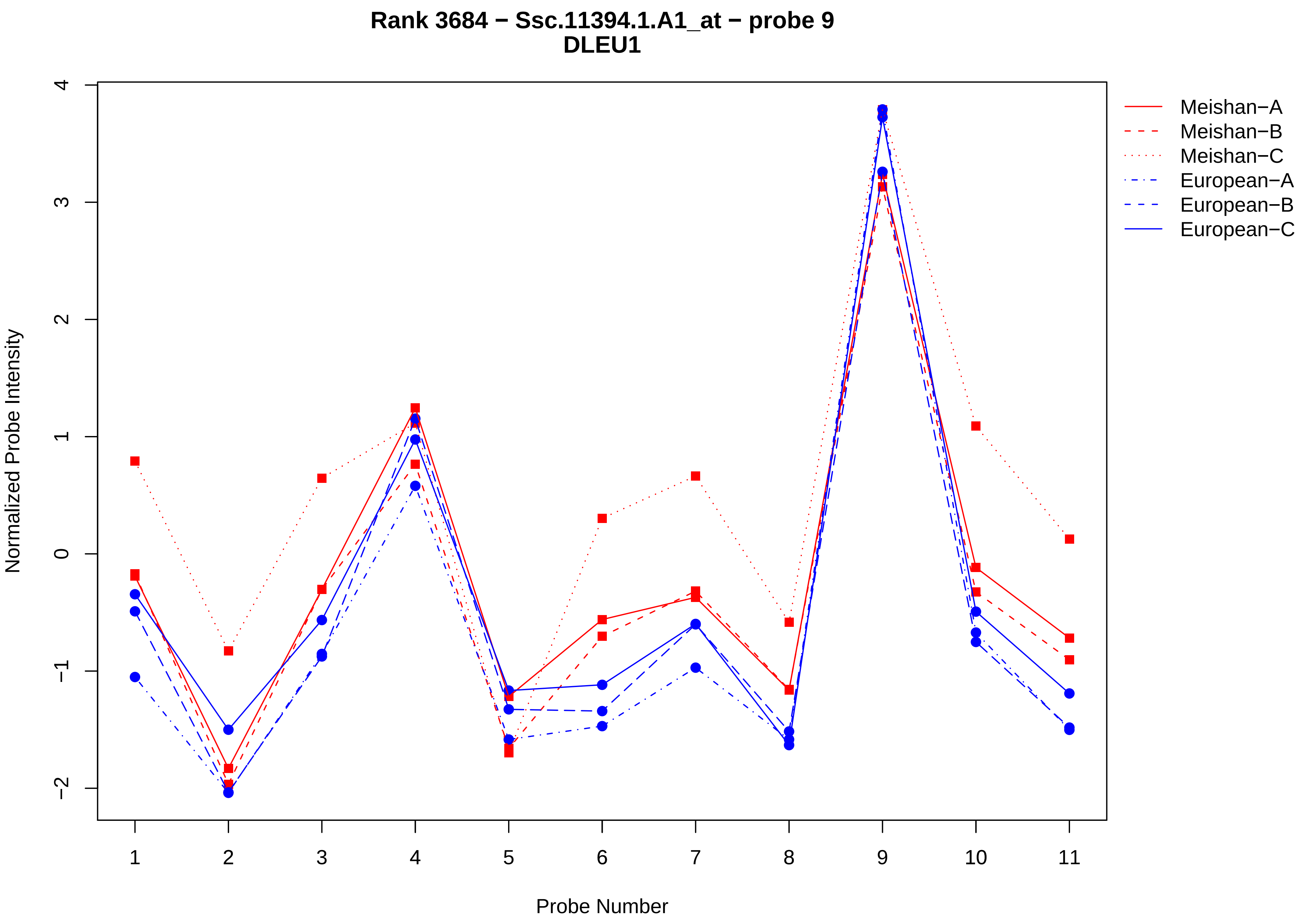

Rank 3684 − Ssc.11394.1.A1_at − probe 9
DLEU1
Normalized Probe Intensity
Probe Number
Meishan−A
Meishan−B
Meishan−C
European−A
European−B
European−C
−2
−1
0
1
2
3
4
1
2
3
4
5
6
7
8
9
10
11

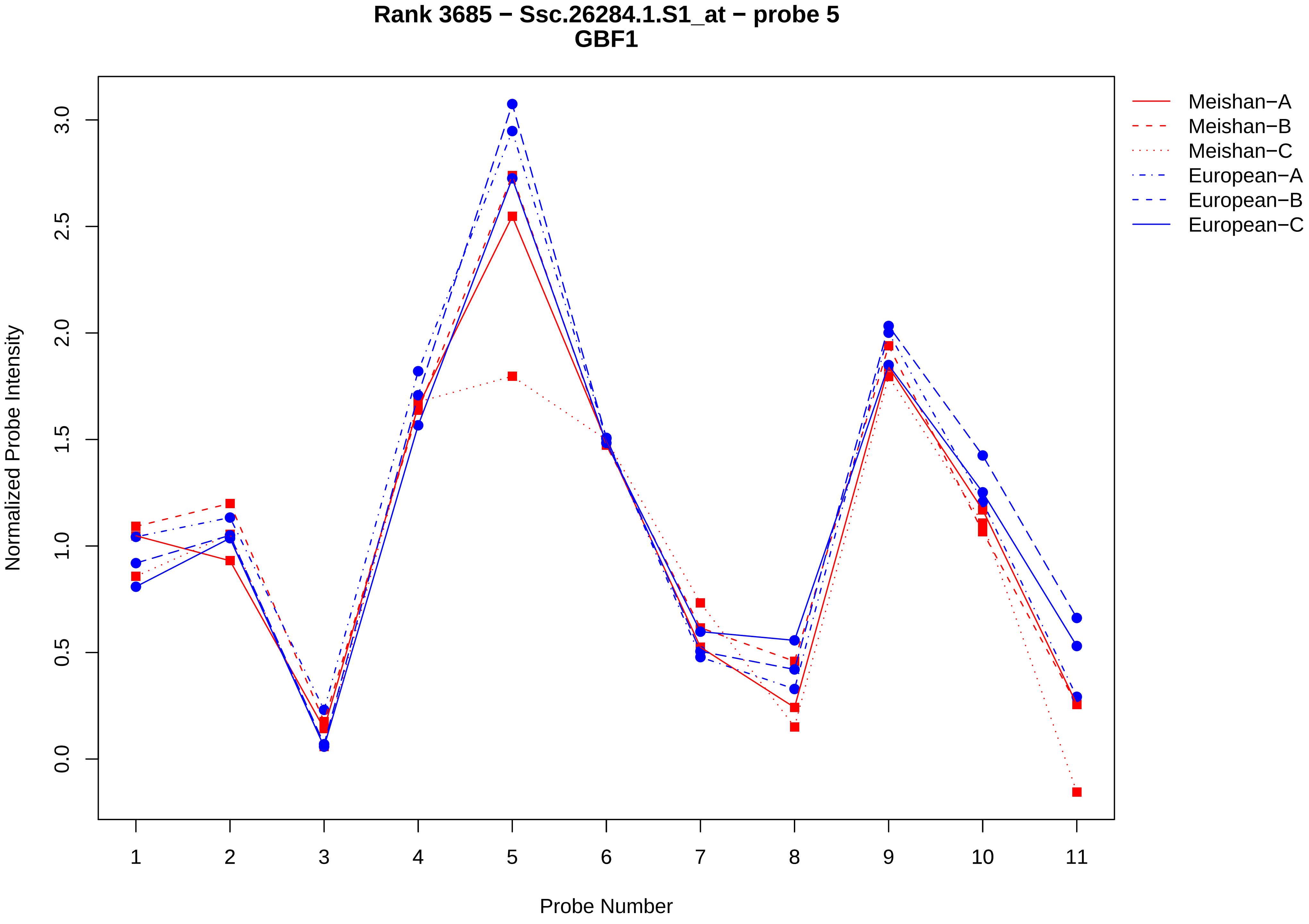

Rank 3685 − Ssc.26284.1.S1_at − probe 5
GBF1
Normalized Probe Intensity
0.0
0.5
1.0
1.5
2.0
2.5
3.0
1
2
3
4
5
6
7
8
9
10
11
Probe Number
Meishan−A
Meishan−B
Meishan−C
European−A
European−B
European−C

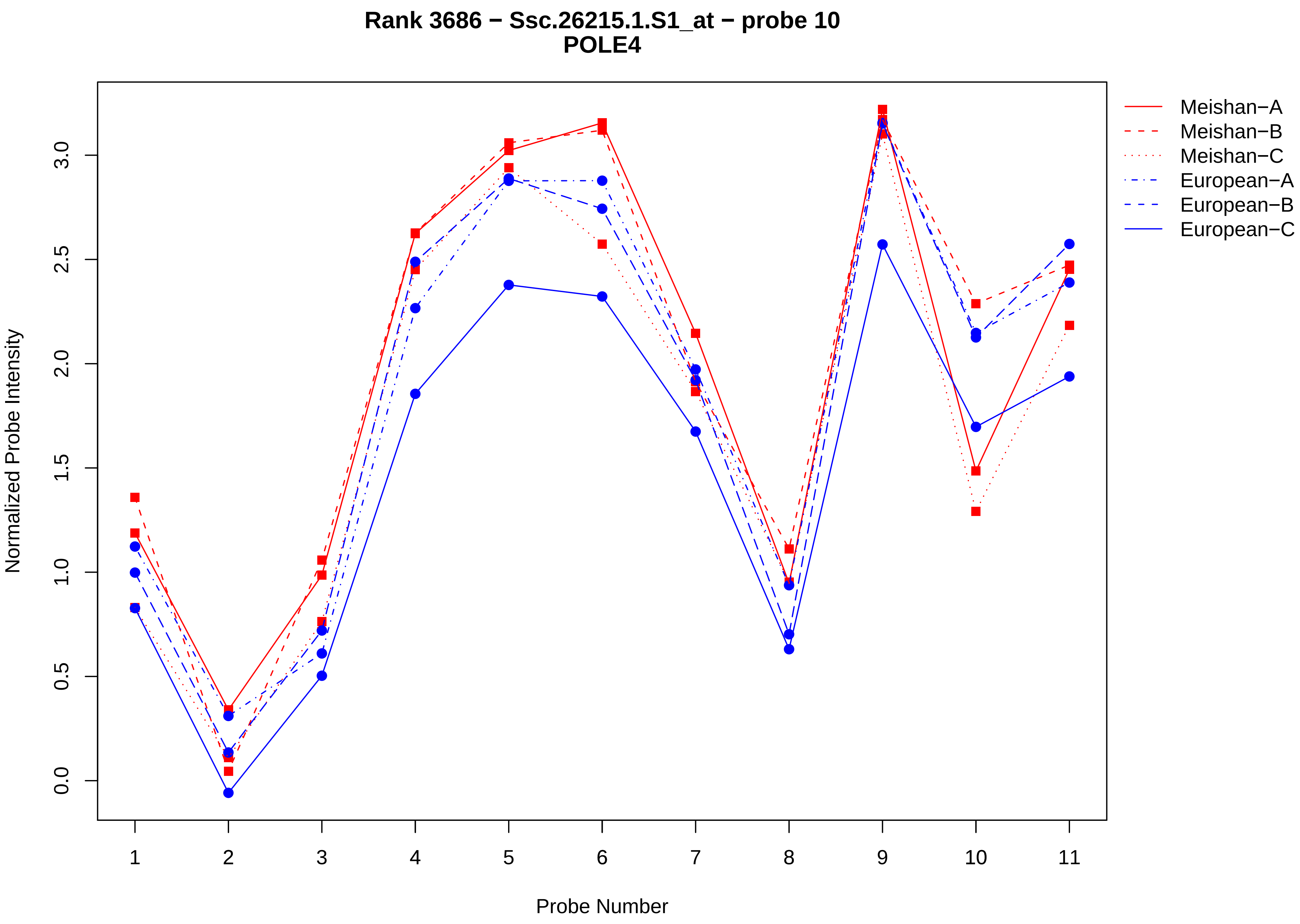

Rank 3686 − Ssc.26215.1.S1_at − probe 10
POLE4
Normalized Probe Intensity
0.0
0.5
1.0
1.5
2.0
2.5
3.0
1
2
3
4
5
6
7
8
9
10
11
Probe Number
Meishan−A
Meishan−B
Meishan−C
European−A
European−B
European−C

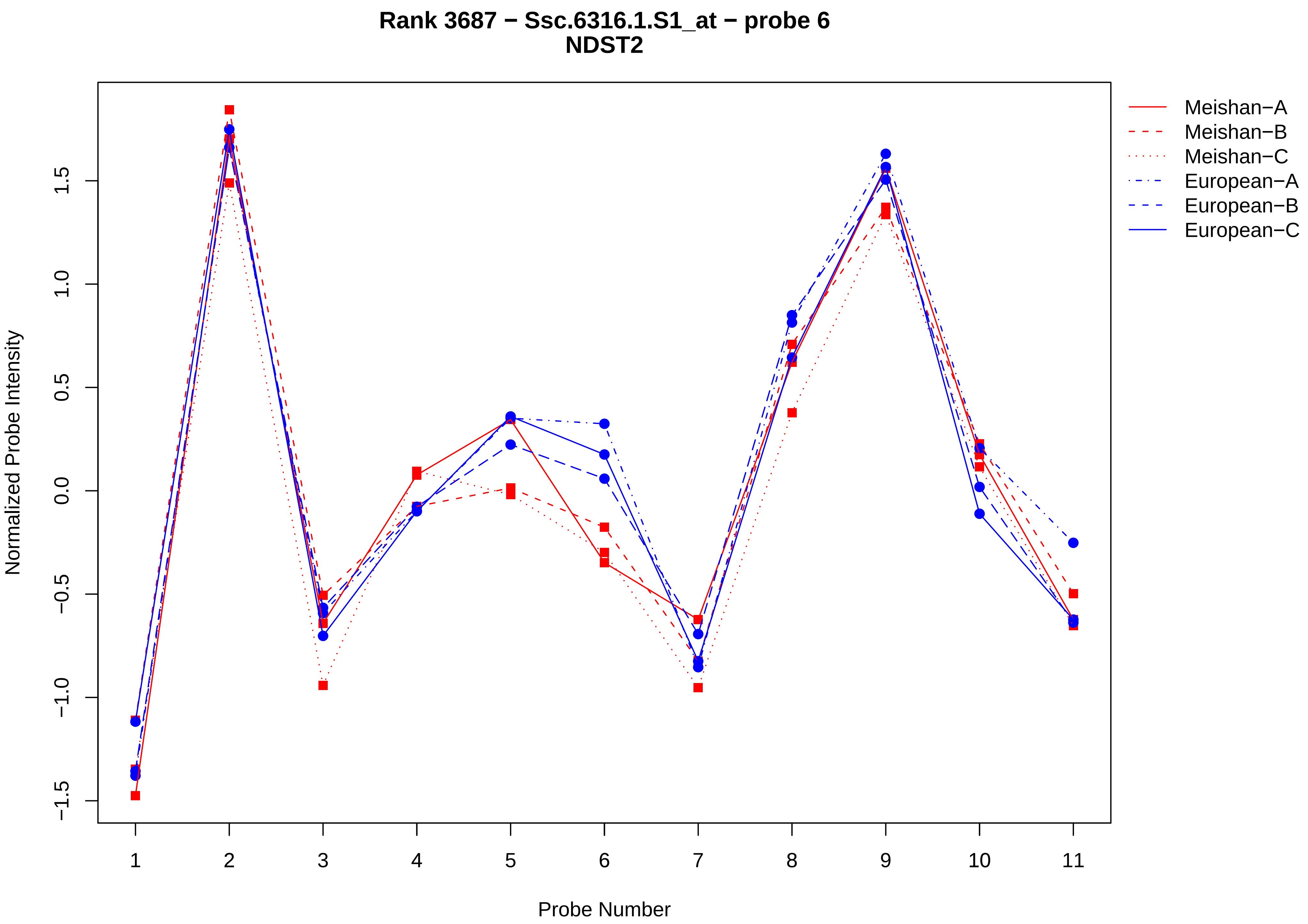

Rank 3687 − Ssc.6316.1.S1_at − probe 6
NDST2
Normalized Probe Intensity
Probe Number
Meishan−A
Meishan−B
Meishan−C
European−A
European−B
European−C

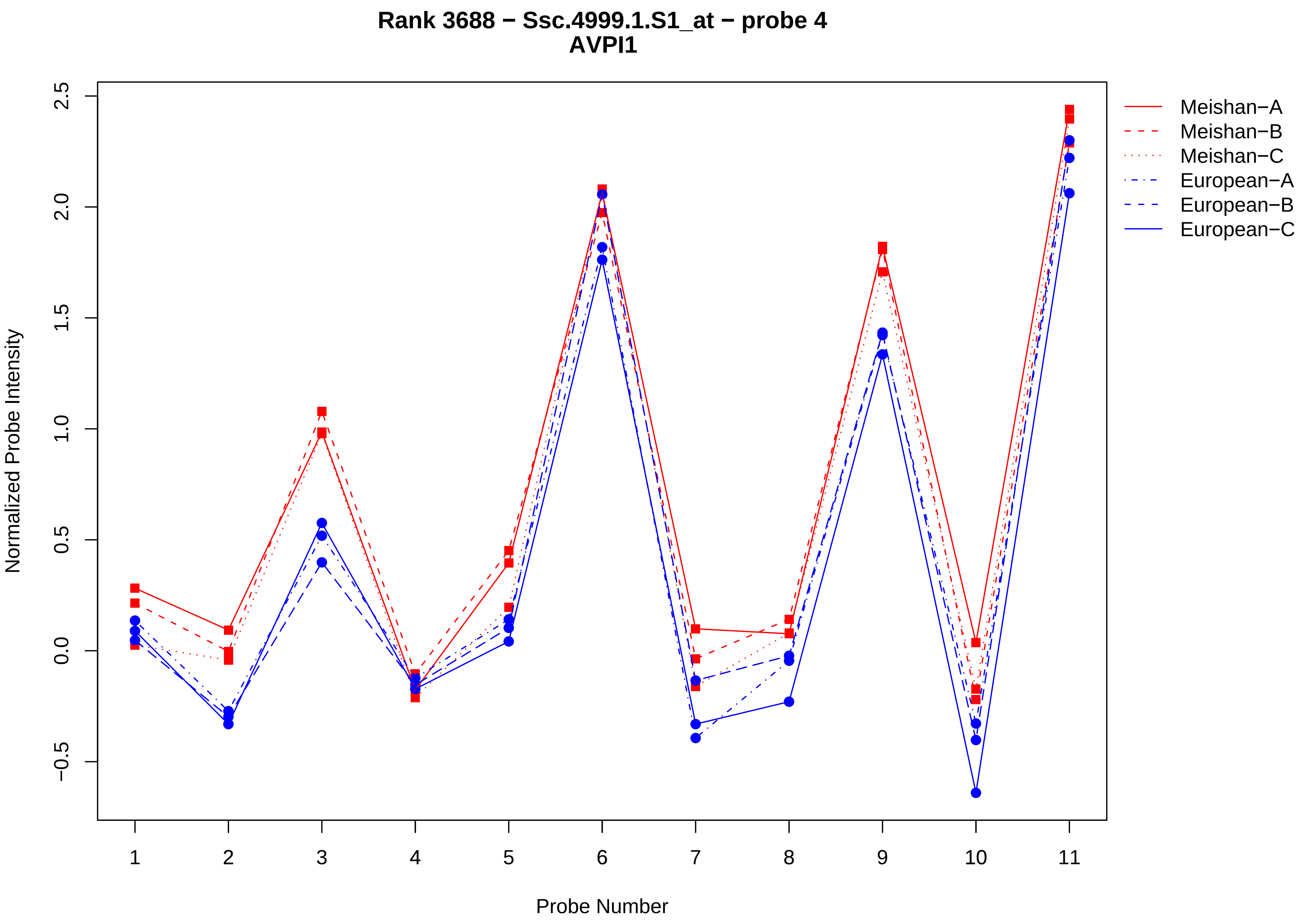
Rank 3688 − Ssc.4999.1.S1_at − probe 4
AVPI1
Normalized Probe Intensity
Probe Number
Meishan−A
Meishan−B
Meishan−C
European−A
European−B
European−C
−0.5
0.0
0.5
1.0
1.5
2.0
2.5
1
2
3
4
5
6
7
8
9
10
11

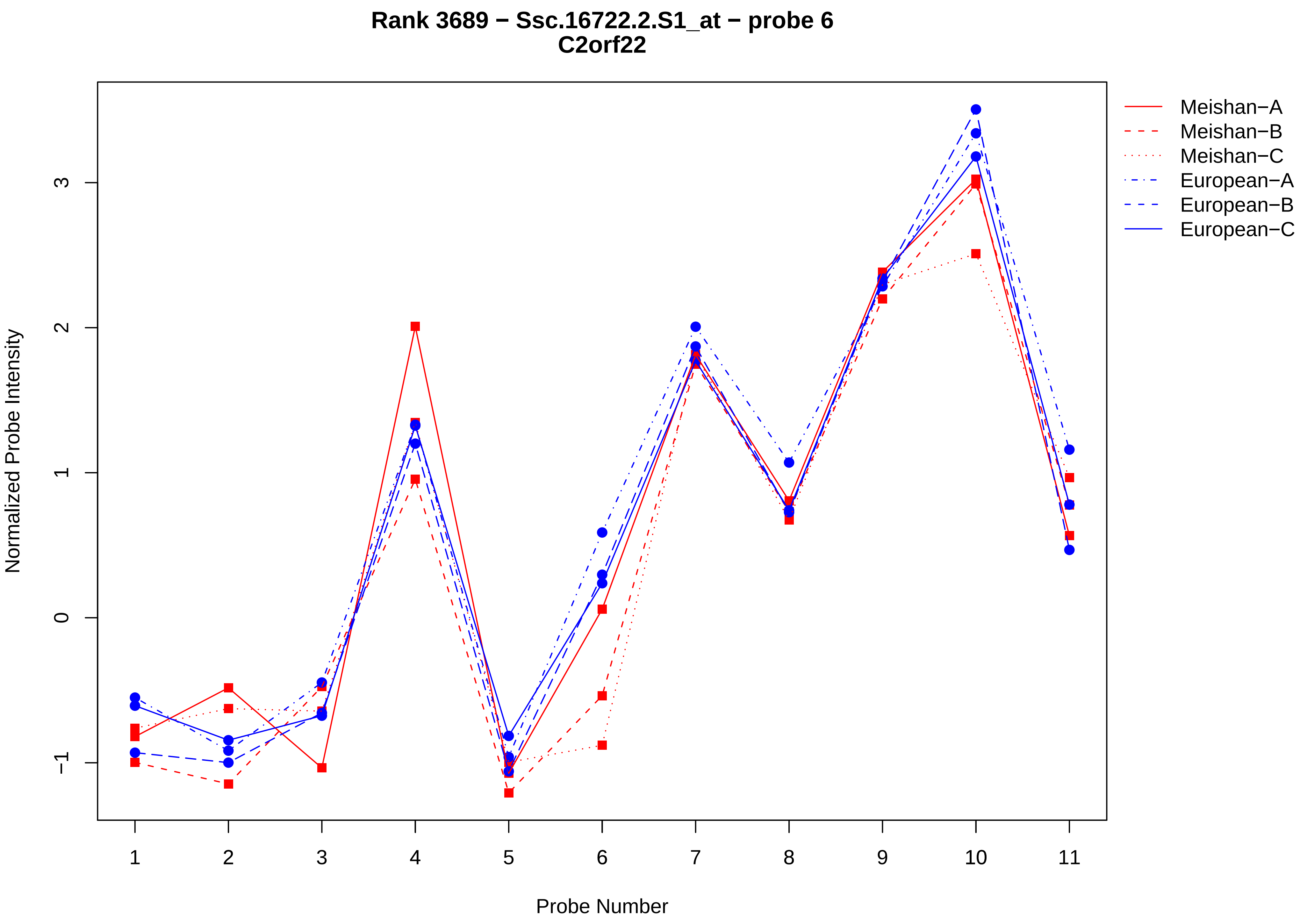

Rank 3689 − Ssc.16722.2.S1_at − probe 6
C2orf22
Normalized Probe Intensity
Probe Number
Meishan−A
Meishan−B
Meishan−C
European−A
European−B
European−C

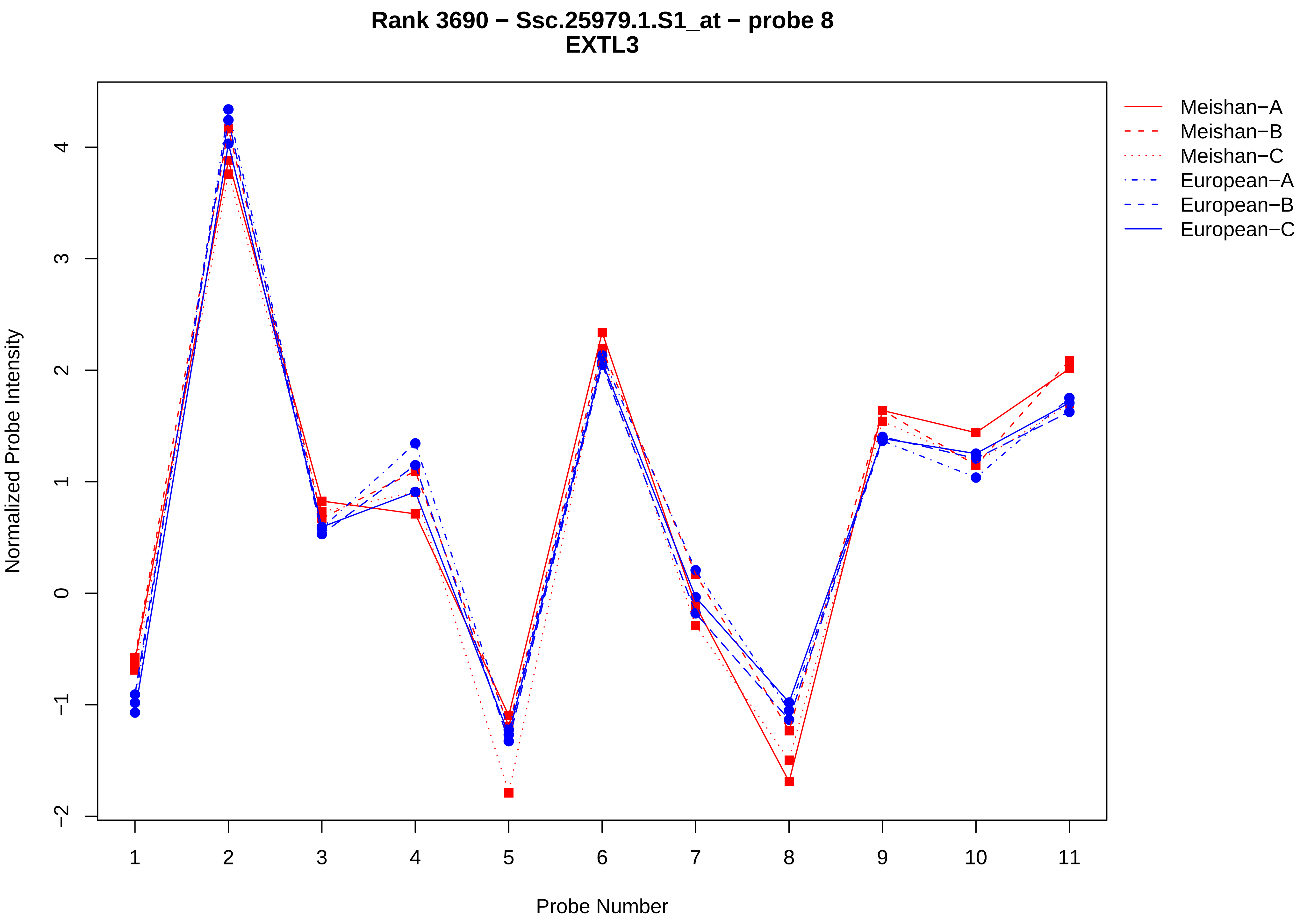

Rank 3690 − Ssc.25979.1.S1_at − probe 8
EXTL3
Normalized Probe Intensity
Probe Number
−2
−1
0
1
2
3
4
1
2
3
4
5
6
7
8
9
10
11
Meishan−A
Meishan−B
Meishan−C
European−A
European−B
European−C

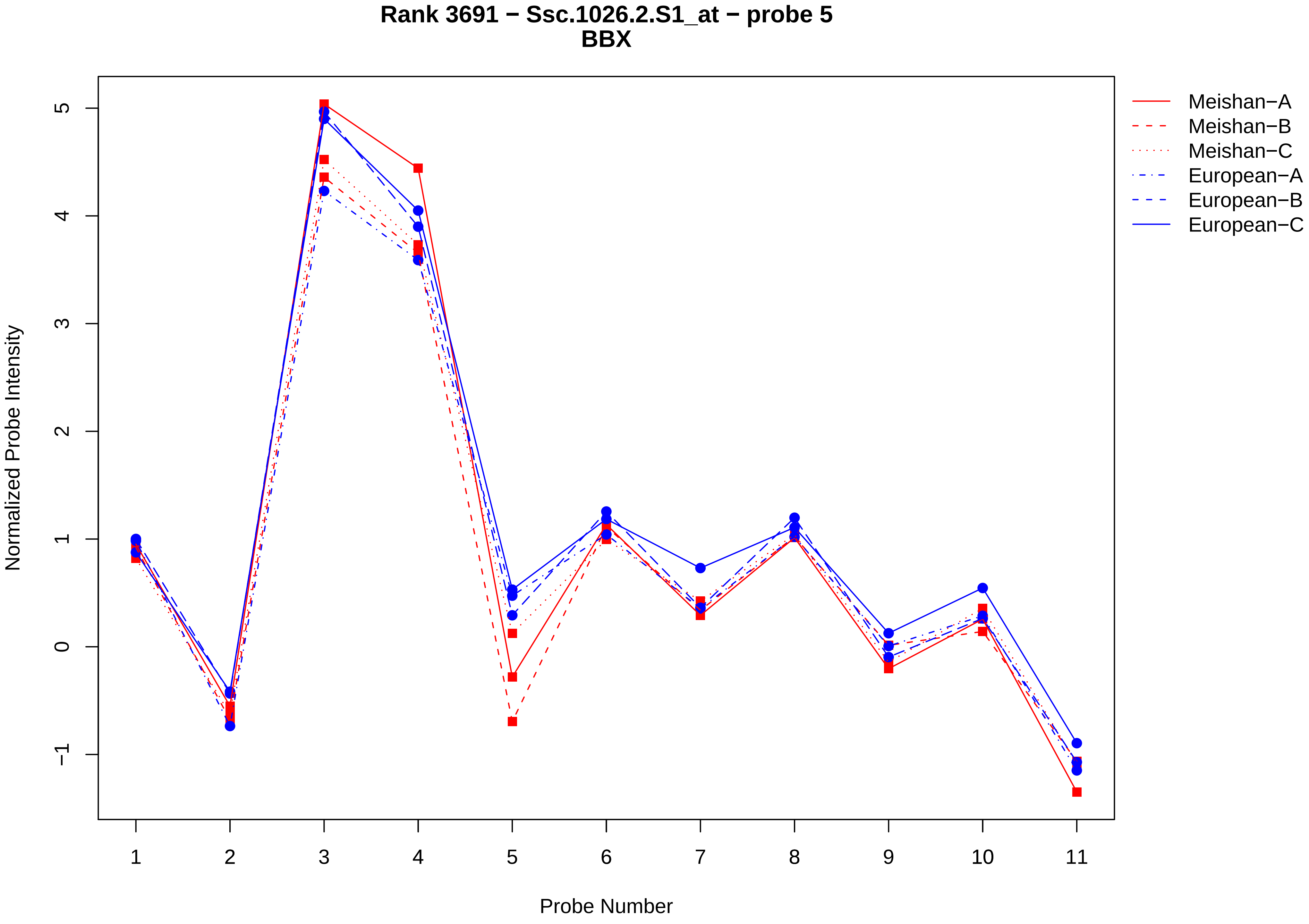

Rank 3691 − Ssc.1026.2.S1_at − probe 5
BBX
Normalized Probe Intensity
Probe Number
−1
0
1
2
3
4
5
1
2
3
4
5
6
7
8
9
10
11
Meishan−A
Meishan−B
Meishan−C
European−A
European−B
European−C

Rank 3692 − Ssc.7906.2.S1_at − probe 8
ARFGEF1

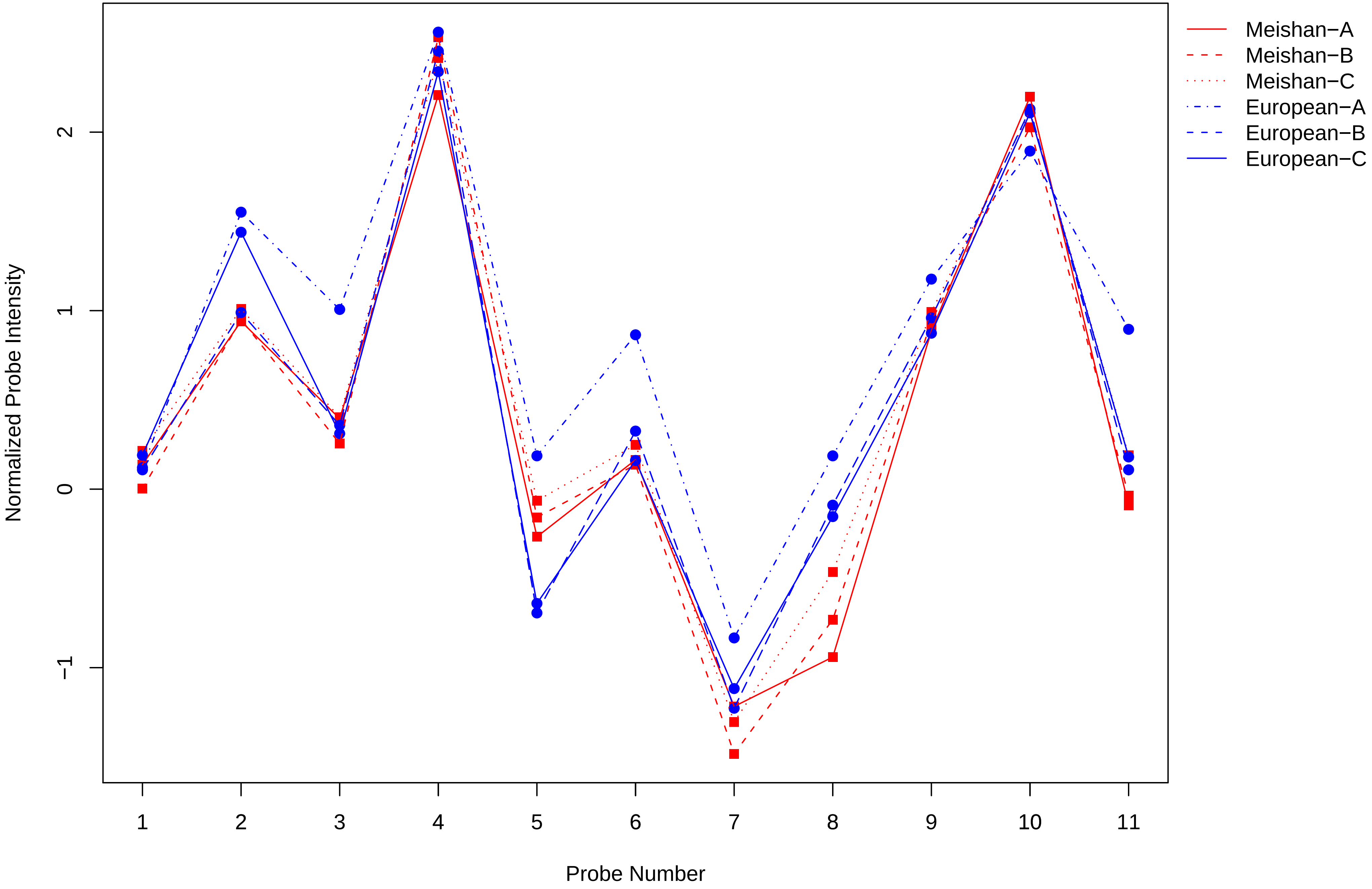

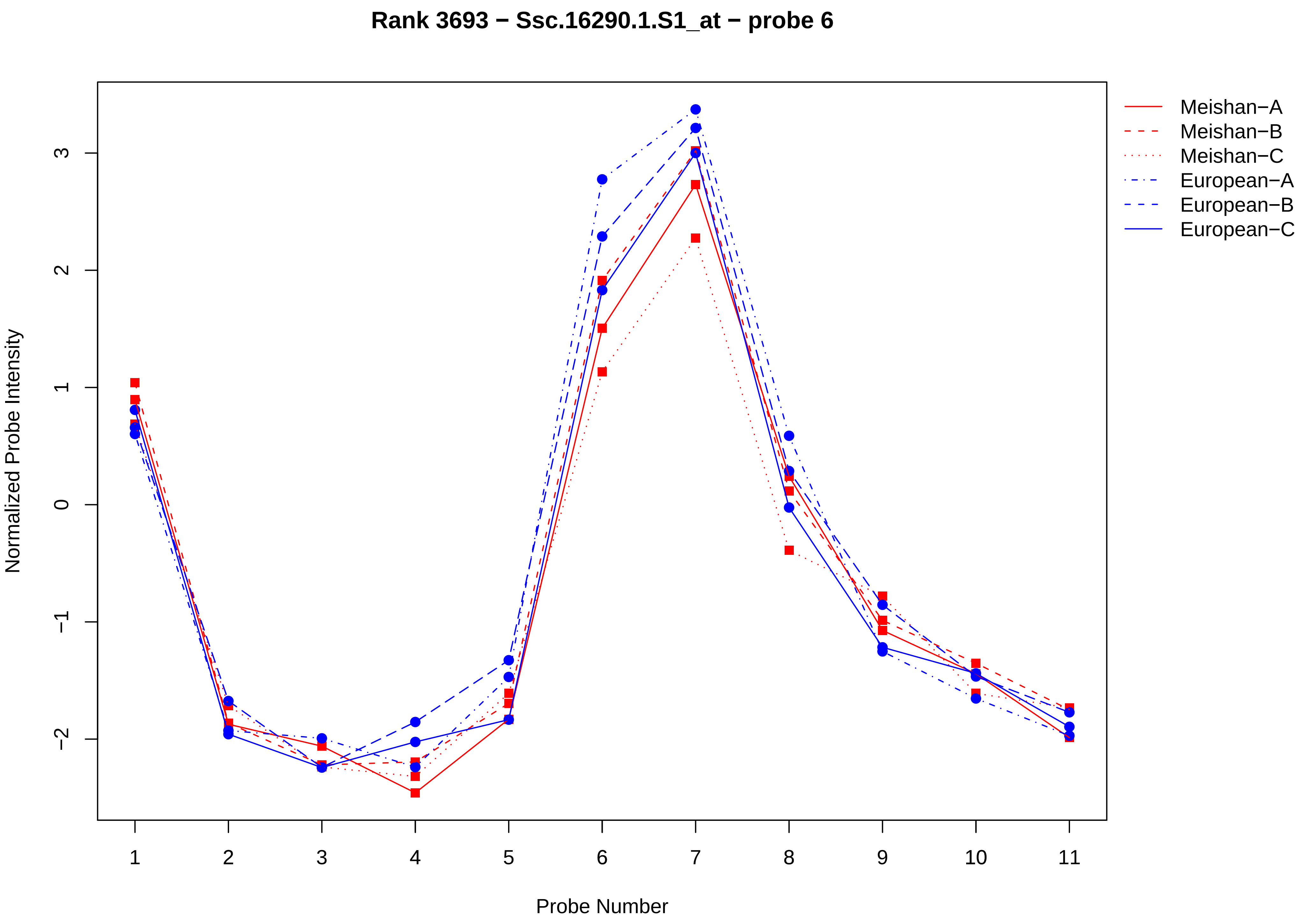

Rank 3693 − Ssc.16290.1.S1_at − probe 6
Normalized Probe Intensity
Probe Number
Meishan−A
Meishan−B
Meishan−C
European−A
European−B
European−C
−2
−1
0
1
2
3
1
2
3
4
5
6
7
8
9
10
11

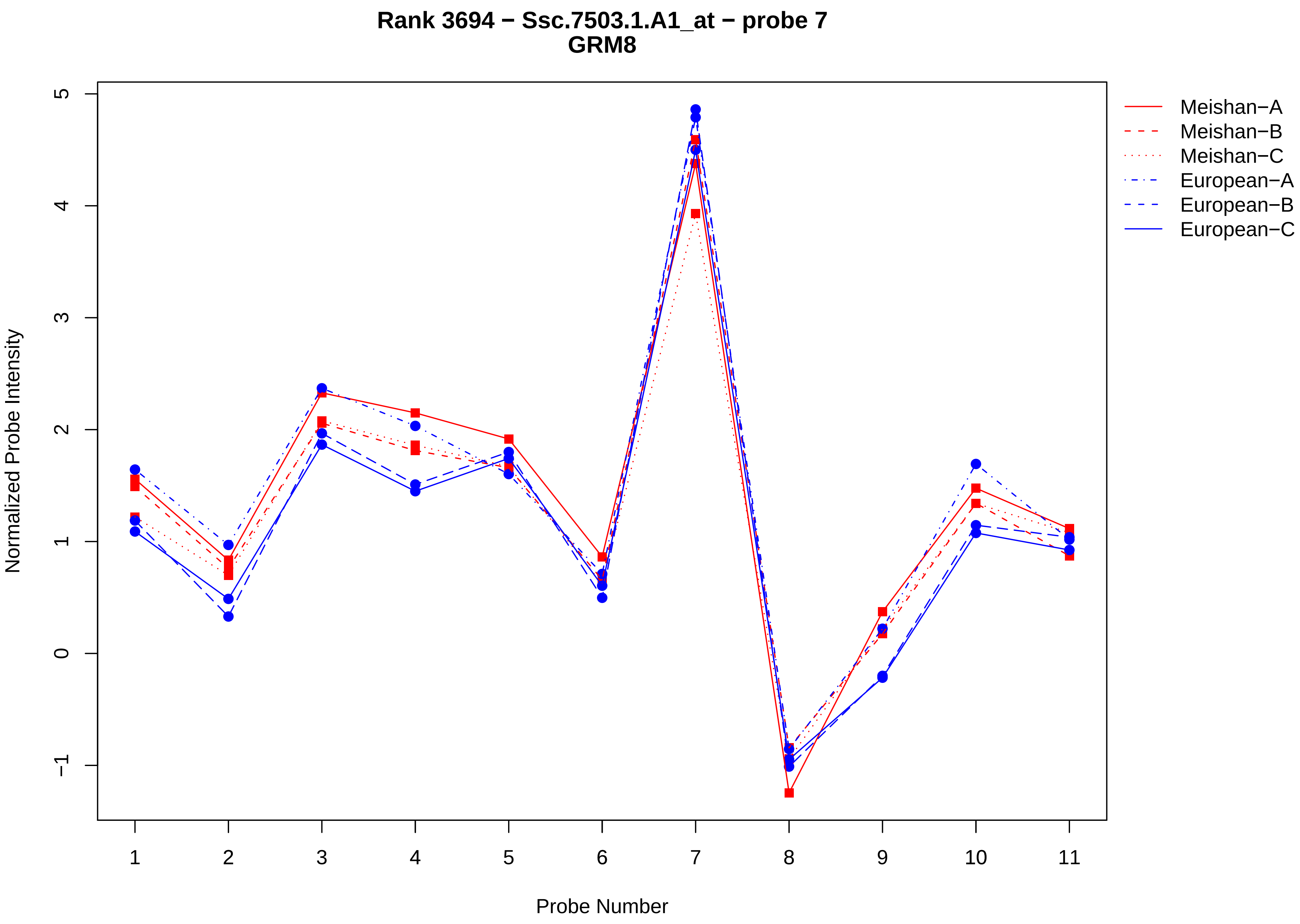

Rank 3694 − Ssc.7503.1.A1_at − probe 7
GRM8
Normalized Probe Intensity
Probe Number
−1
0
1
2
3
4
5
1
2
3
4
5
6
7
8
9
10
11
Meishan−A
Meishan−B
Meishan−C
European−A
European−B
European−C

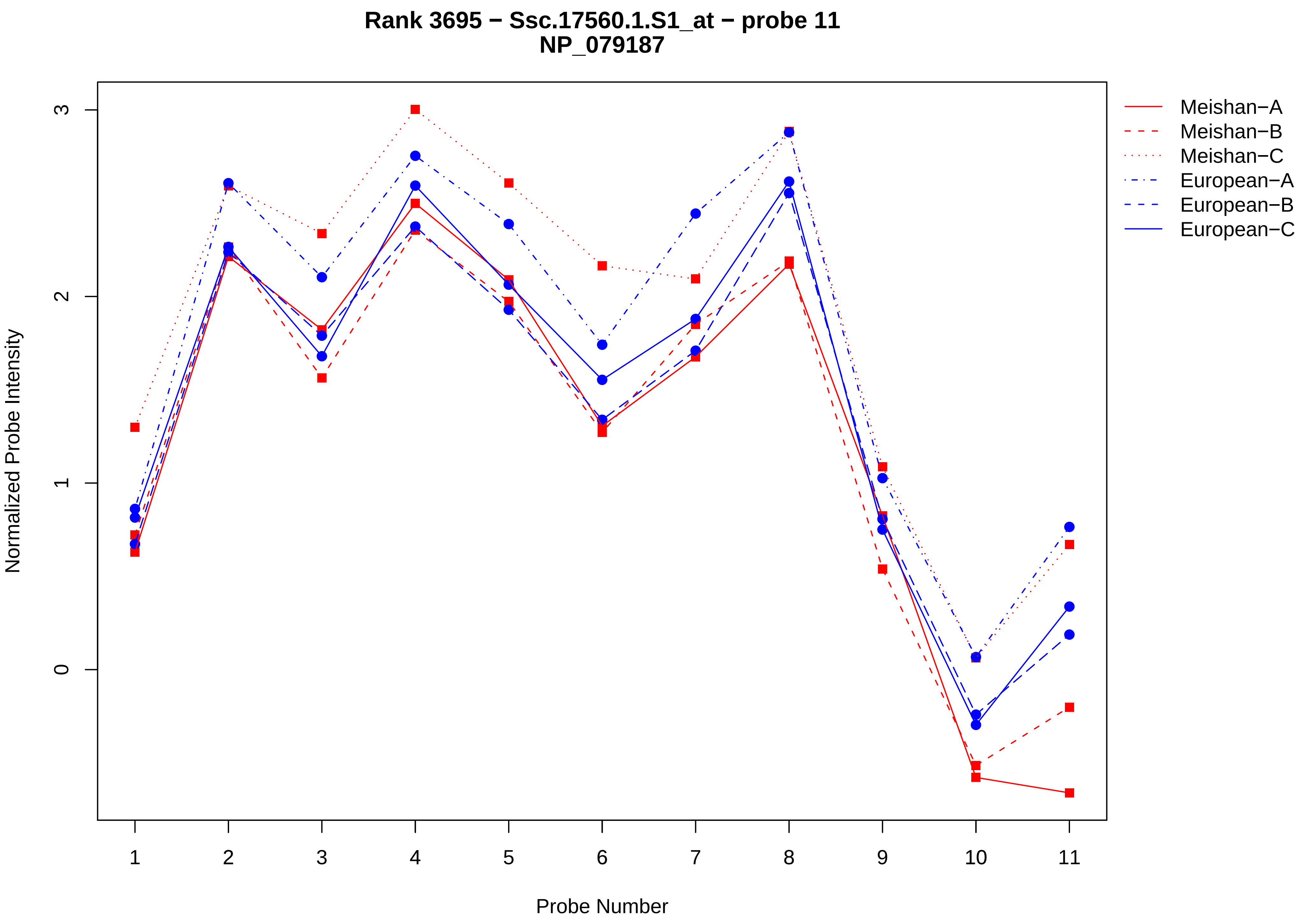

Rank 3695 − Ssc.17560.1.S1_at − probe 11
NP_079187
Normalized Probe Intensity
Probe Number
0
1
2
3
1
2
3
4
5
6
7
8
9
10
11
Meishan−A
Meishan−B
Meishan−C
European−A
European−B
European−C

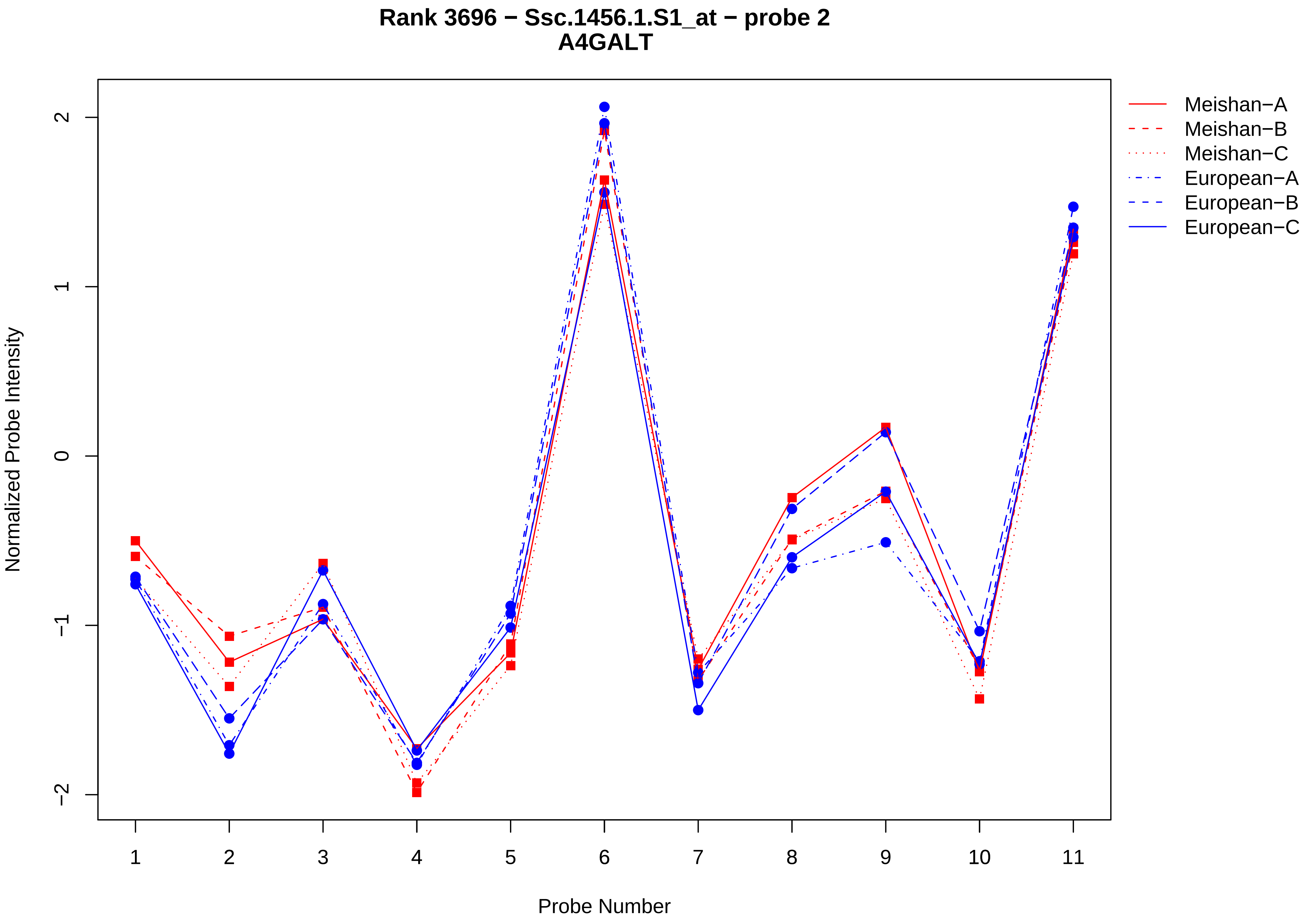
Rank 3696 − Ssc.1456.1.S1_at − probe 2
A4GALT
Normalized Probe Intensity
Probe Number
Meishan−A
Meishan−B
Meishan−C
European−A
European−B
European−C
−2
−1
0
1
2
1
2
3
4
5
6
7
8
9
10
11

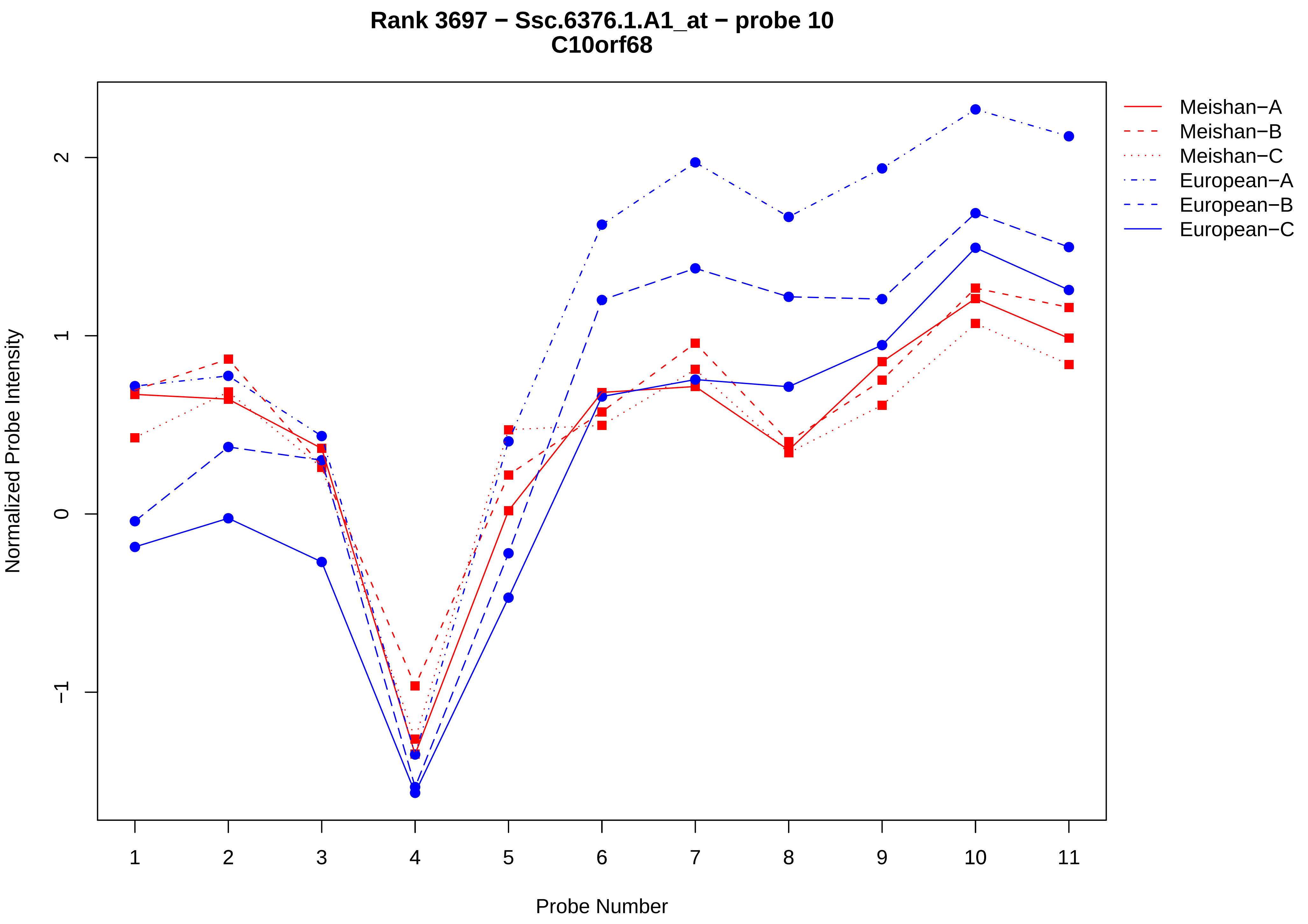

Rank 3697 − Ssc.6376.1.A1_at − probe 10
C10orf68
Normalized Probe Intensity
Probe Number
−1
0
1
2
1
2
3
4
5
6
7
8
9
10
11
Meishan−A
Meishan−B
Meishan−C
European−A
European−B
European−C

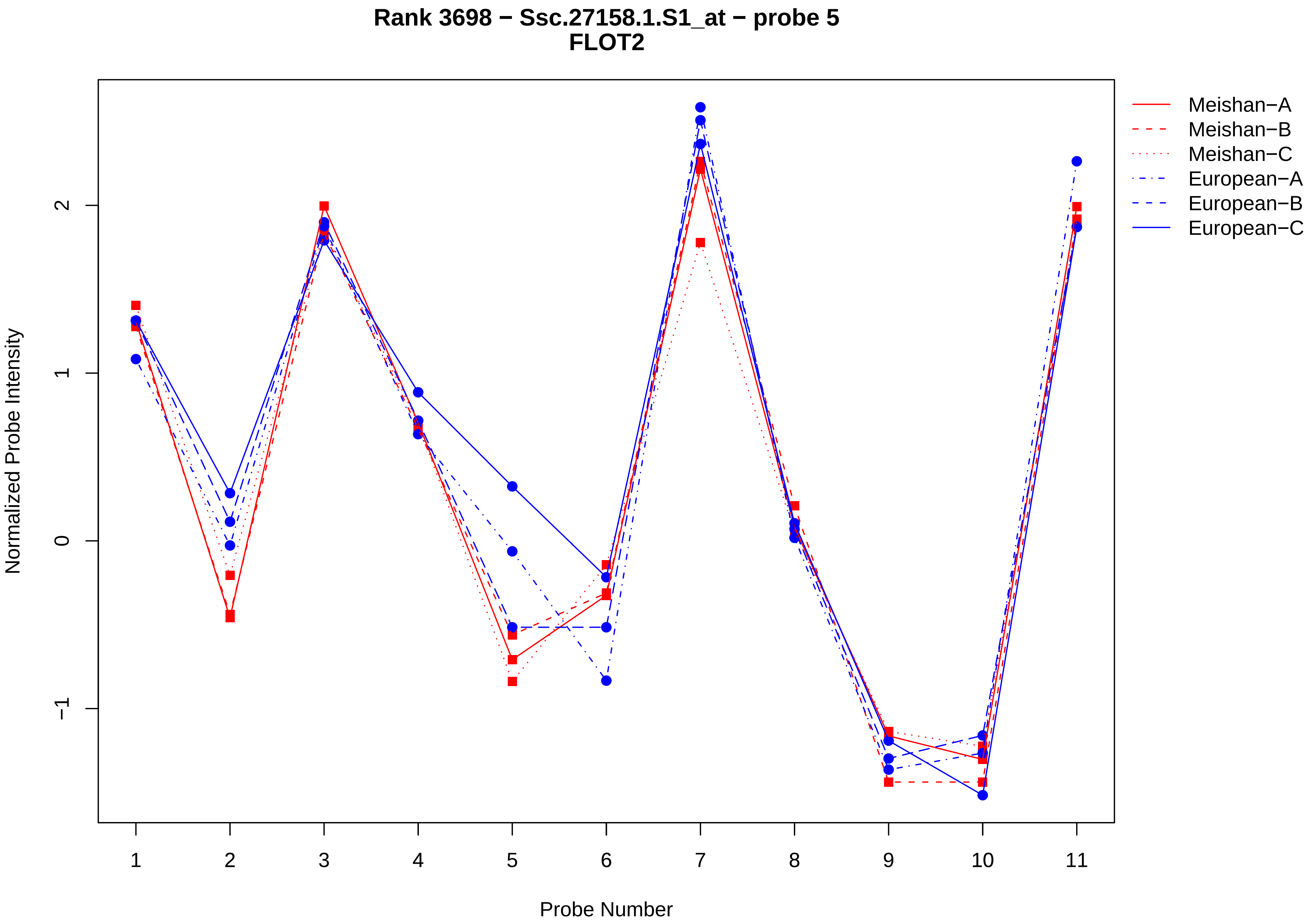

Rank 3698 − Ssc.27158.1.S1_at − probe 5
FLOT2
Normalized Probe Intensity
Probe Number
Meishan−A
Meishan−B
Meishan−C
European−A
European−B
European−C
−1
0
1
2
1
2
3
4
5
6
7
8
9
10
11

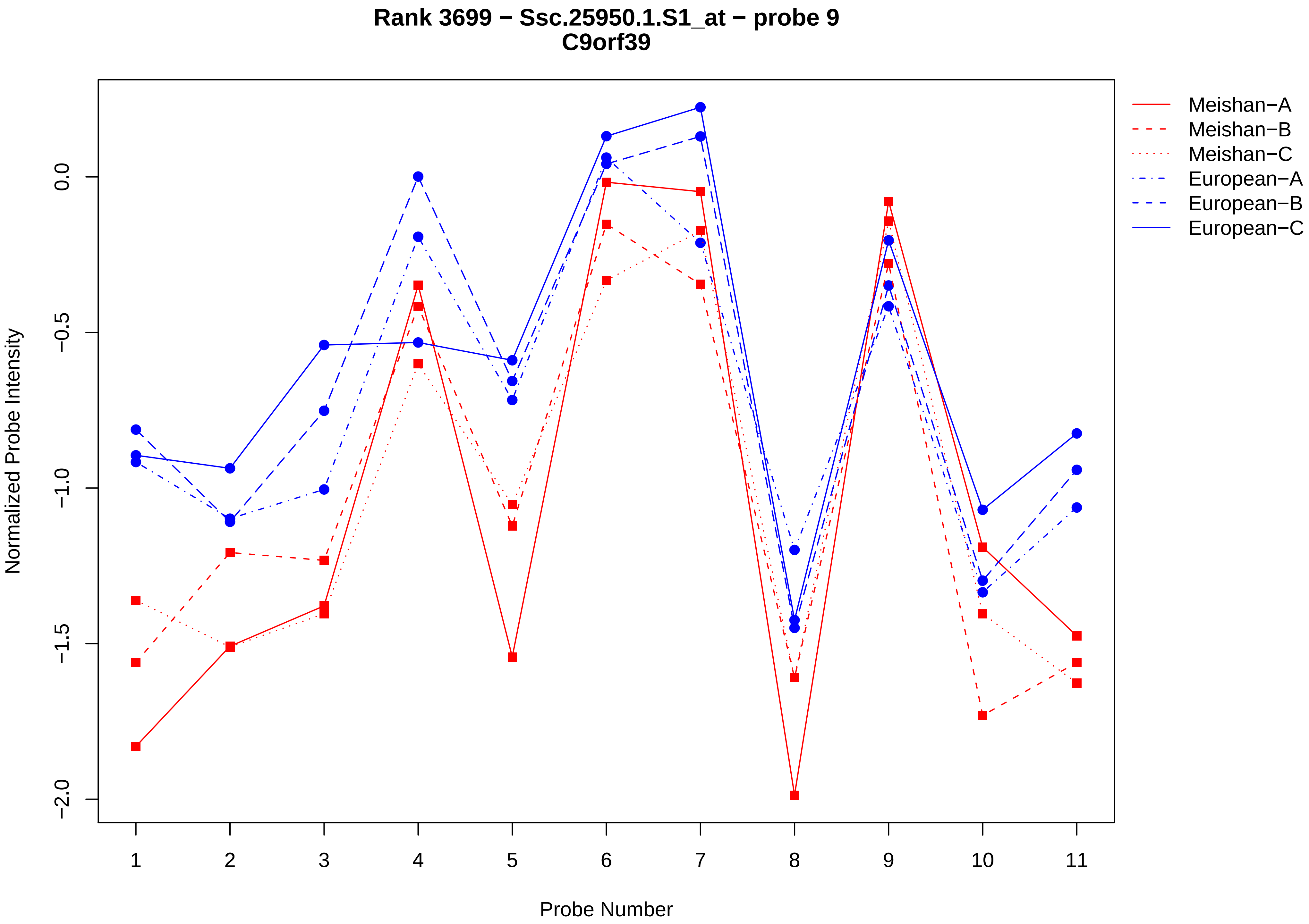

Rank 3699 − Ssc.25950.1.S1_at − probe 9
C9orf39
Normalized Probe Intensity
0.0
−0.5
−1.0
−1.5
−2.0
1
2
3
4
5
6
7
8
9
10
11
Probe Number
Meishan−A
Meishan−B
Meishan−C
European−A
European−B
European−C

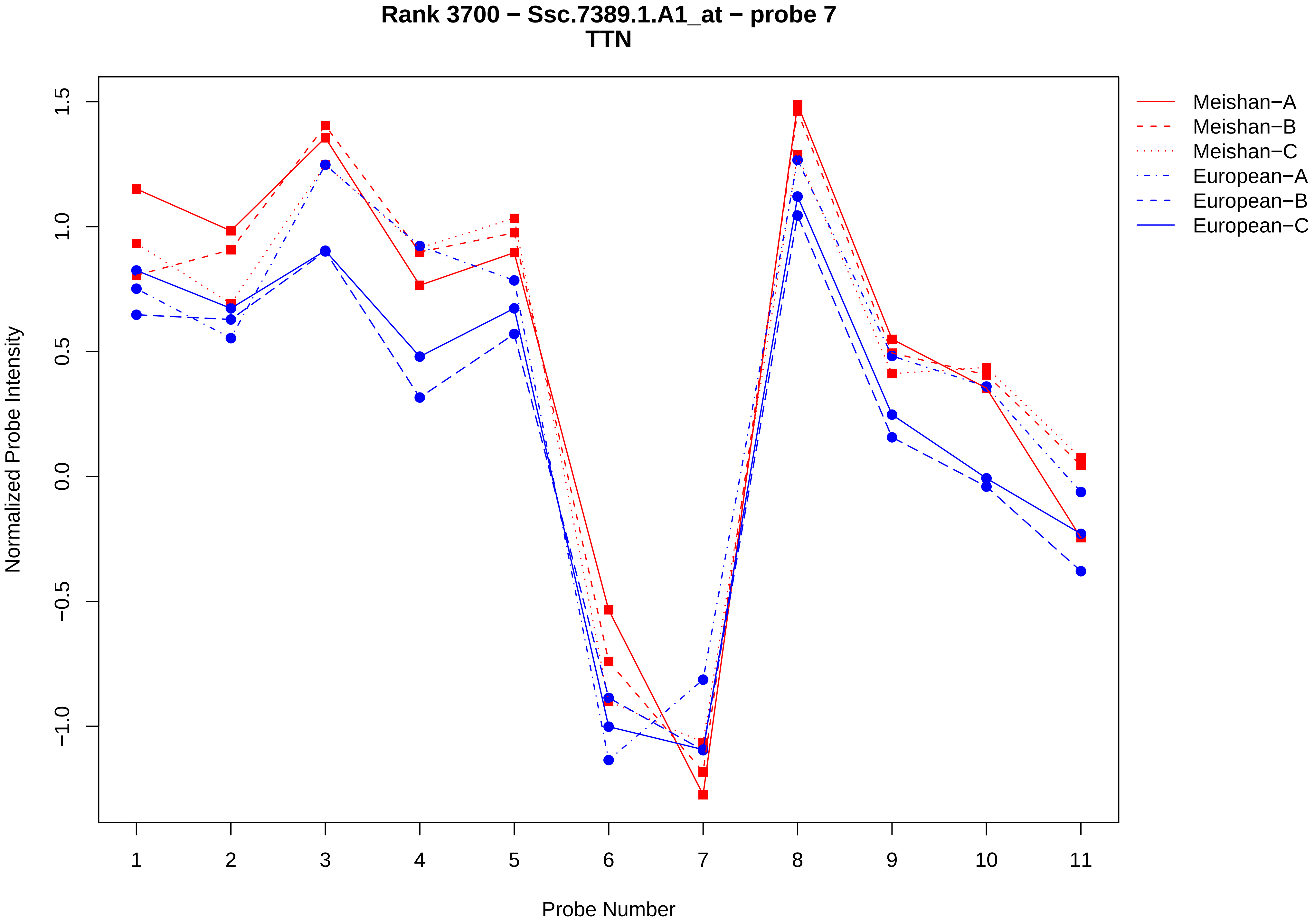

Rank 3700 − Ssc.7389.1.A1_at − probe 7
TTN
Normalized Probe Intensity
−1.0
−0.5
0.0
0.5
1.0
1.5
1
2
3
4
5
6
7
8
9
10
11
Probe Number
Meishan−A
Meishan−B
Meishan−C
European−A
European−B
European−C

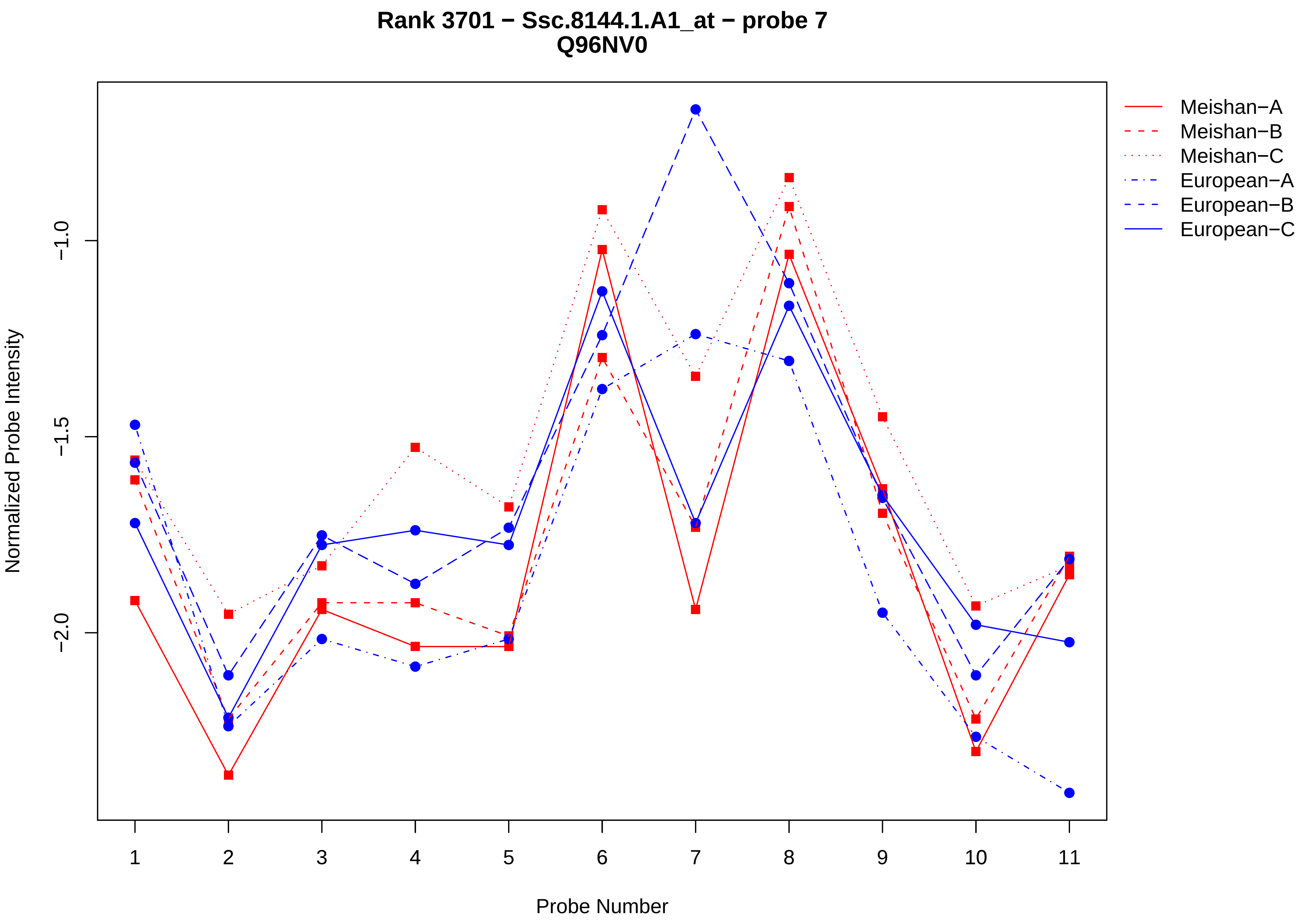
Rank 3701 − Ssc.8144.1.A1_at − probe 7
Q96NV0
Meishan−A
Meishan−B
Meishan−C
European−A
European−B
European−C
Normalized Probe Intensity
−1.0
−1.5
−2.0
1
2
3
4
5
6
7
8
9
10
11
Probe Number

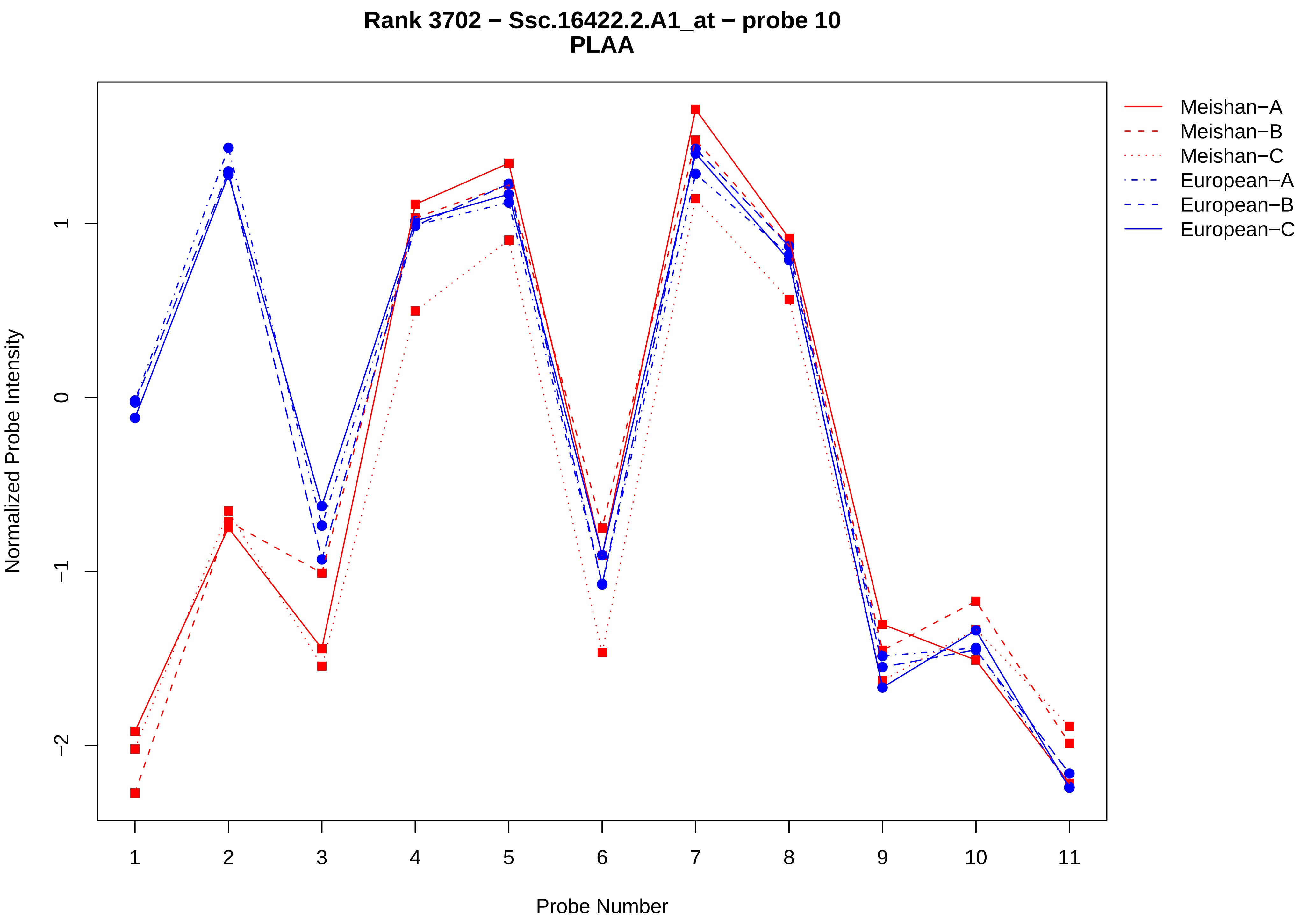

Rank 3702 − Ssc.16422.2.A1_at − probe 10
PLAA
Normalized Probe Intensity
Probe Number
Meishan−A
Meishan−B
Meishan−C
European−A
European−B
European−C
1
0
−1
−2
1
2
3
4
5
6
7
8
9
10
11

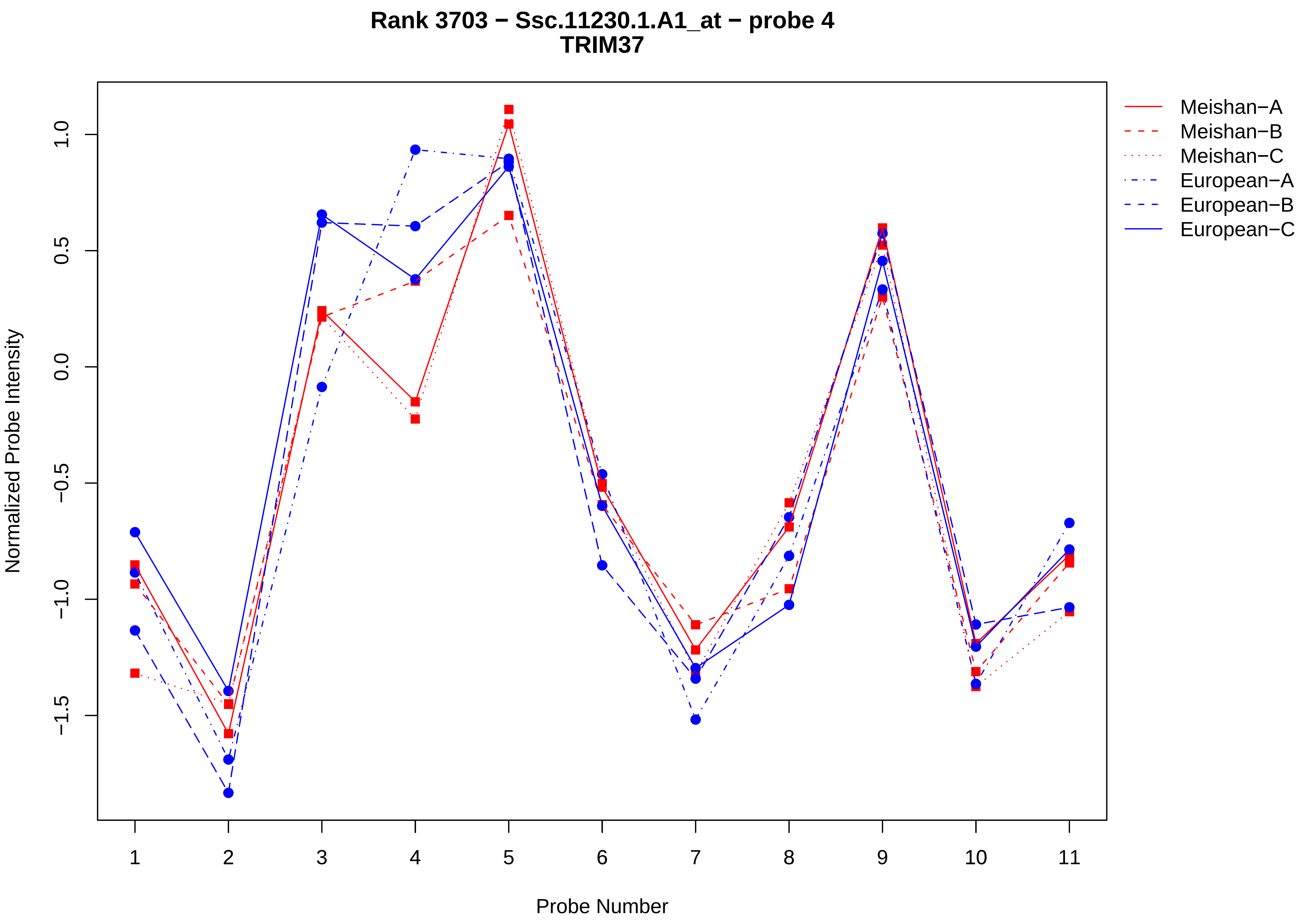

Rank 3703 − Ssc.11230.1.A1_at − probe 4
TRIM37
Normalized Probe Intensity
Probe Number
Meishan−A
Meishan−B
Meishan−C
European−A
European−B
European−C
1.0
0.5
0.0
−0.5
−1.0
−1.5
1
2
3
4
5
6
7
8
9
10
11

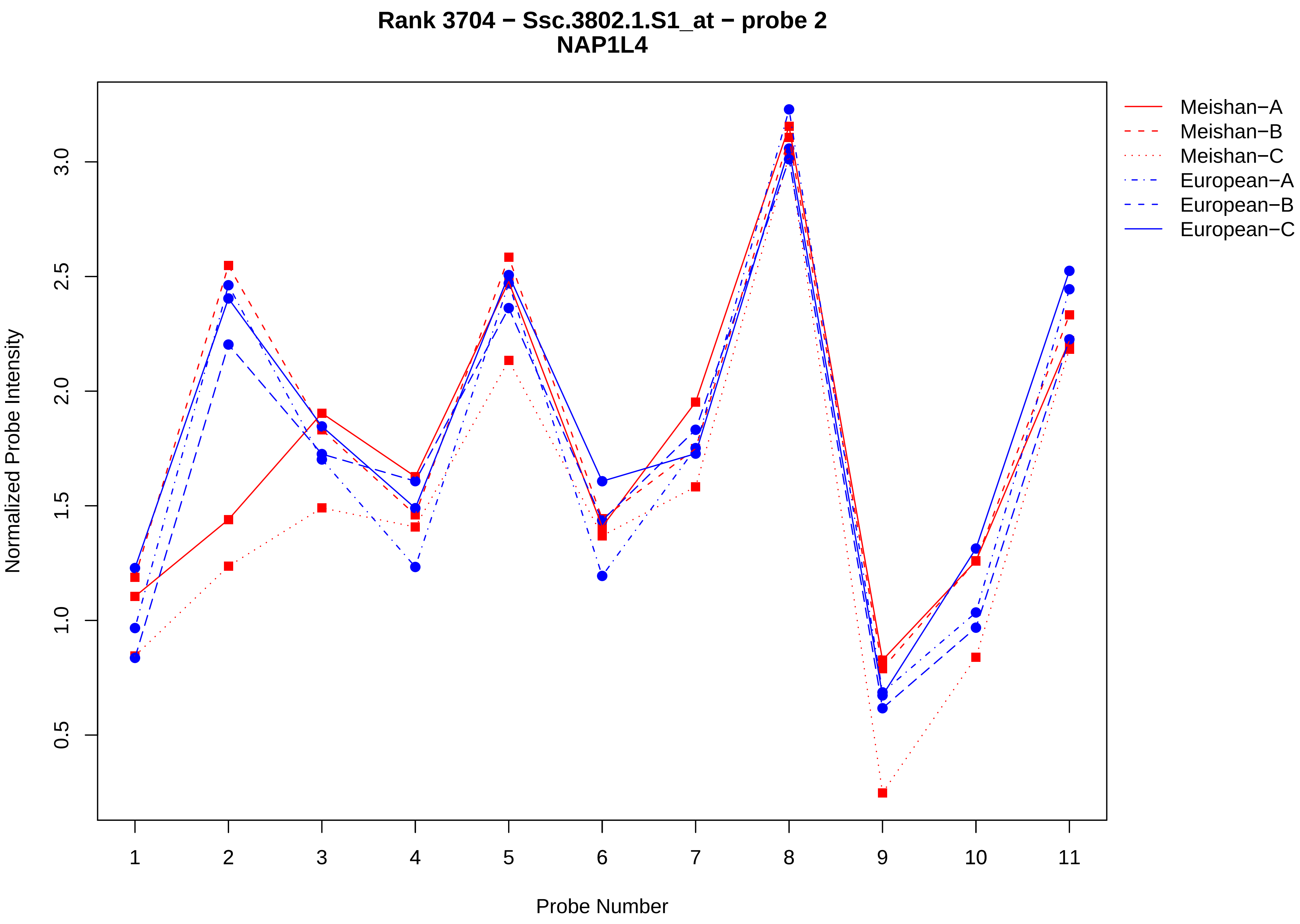

Rank 3704 − Ssc.3802.1.S1_at − probe 2
NAP1L4
Normalized Probe Intensity
Probe Number
0.5
1.0
1.5
2.0
2.5
3.0
1
2
3
4
5
6
7
8
9
10
11
Meishan−A
Meishan−B
Meishan−C
European−A
European−B
European−C

Rank 3705 − Ssc.6808.1.S1_at − probe 6
POLR2C

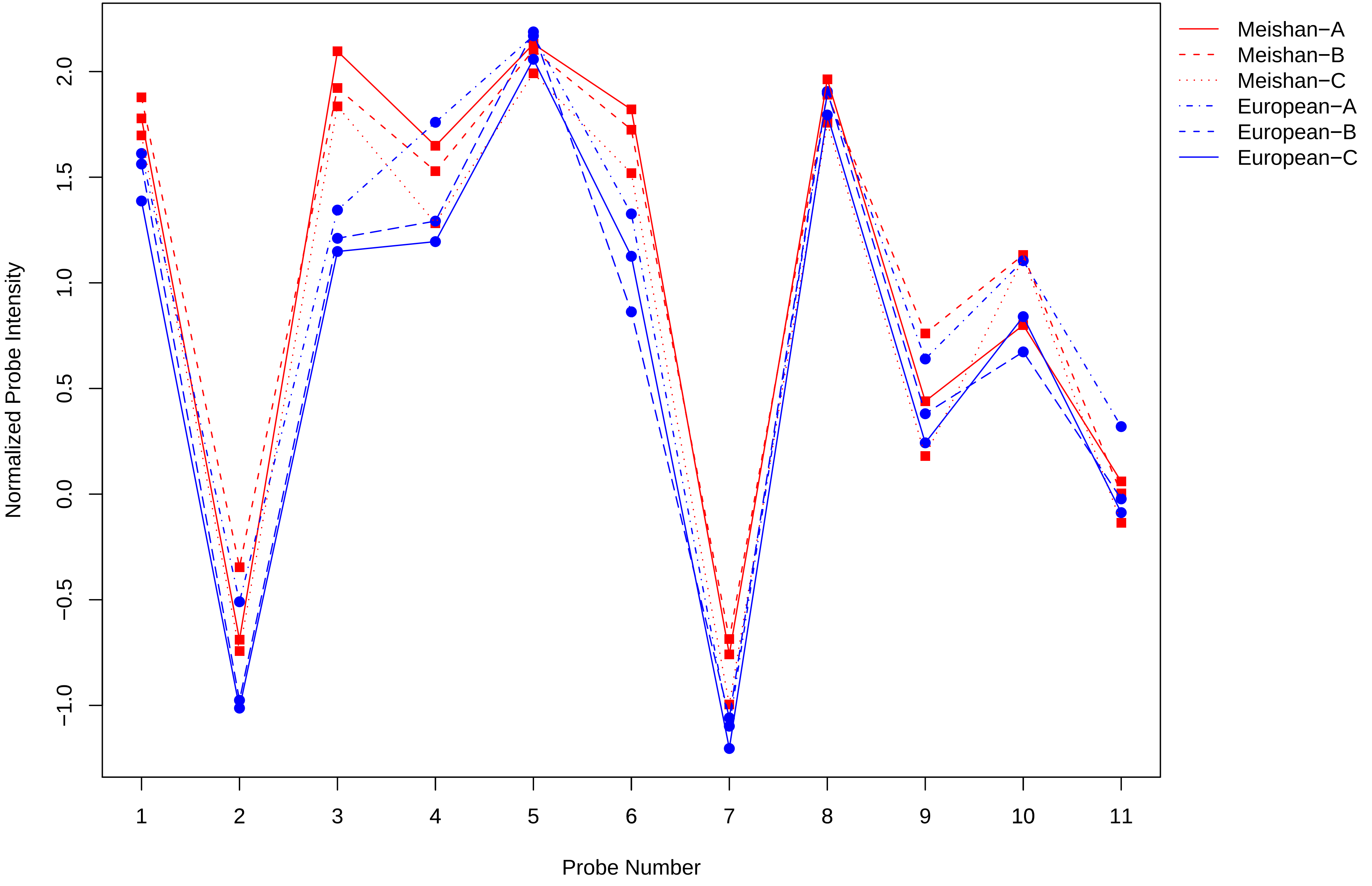

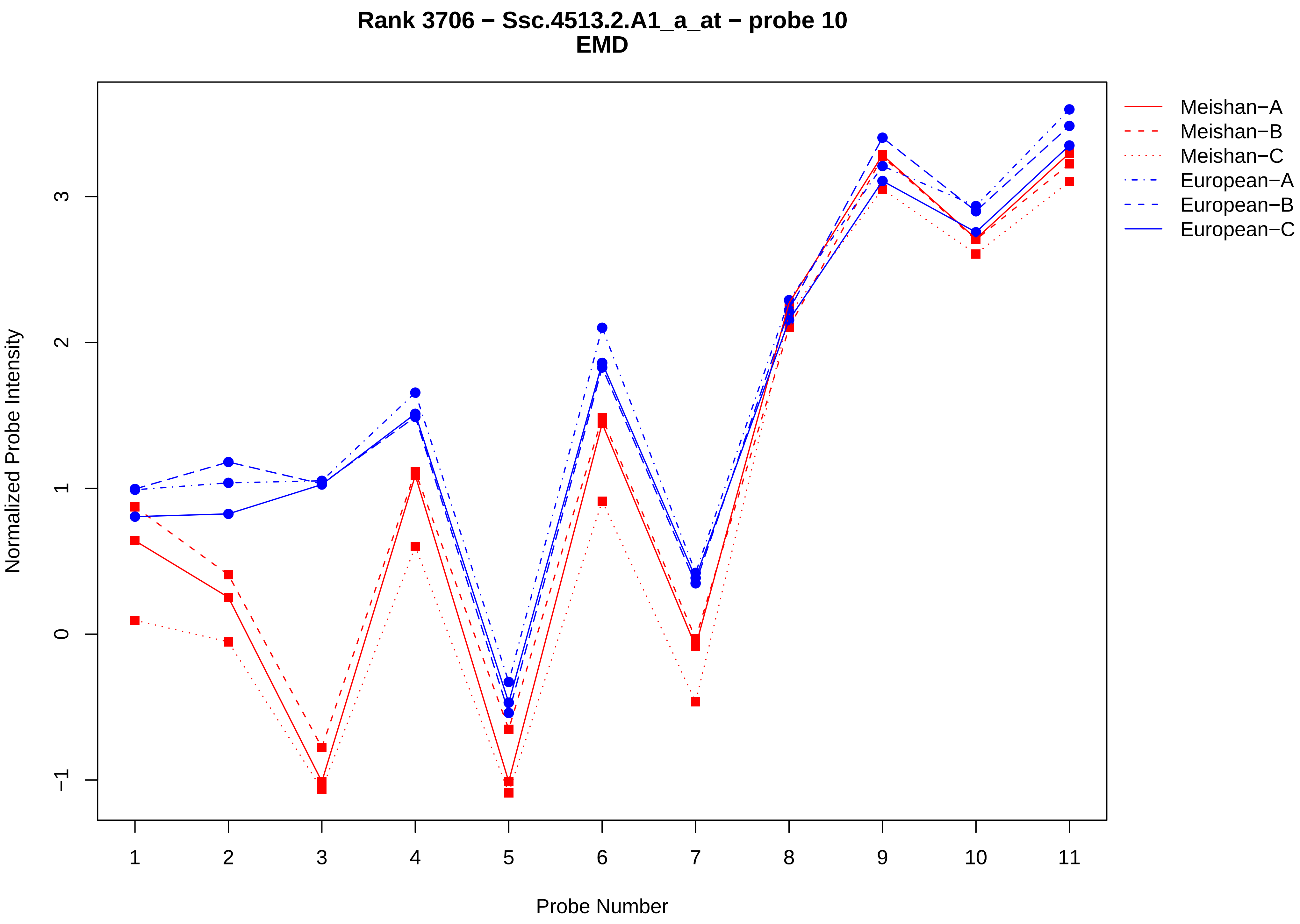
Rank 3706 − Ssc.4513.2.A1_a_at − probe 10
EMD
Normalized Probe Intensity
Probe Number
−1
0
1
2
3
1
2
3
4
5
6
7
8
9
10
11
Meishan−A
Meishan−B
Meishan−C
European−A
European−B
European−C

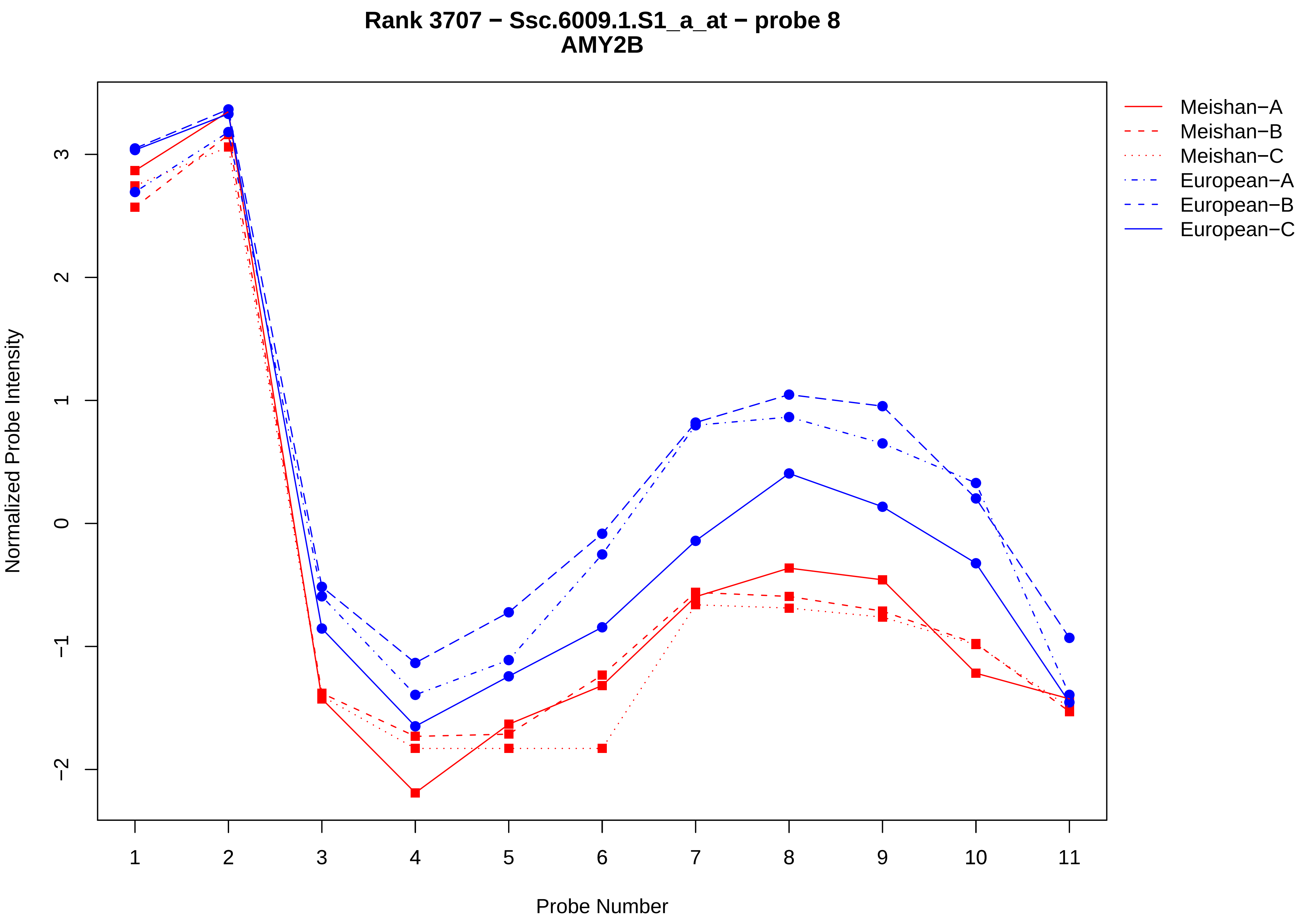
Rank 3707 − Ssc.6009.1.S1_a_at − probe 8
AMY2B
Normalized Probe Intensity
Probe Number
Meishan−A
Meishan−B
Meishan−C
European−A
European−B
European−C
−2
−1
0
1
2
3
1
2
3
4
5
6
7
8
9
10
11

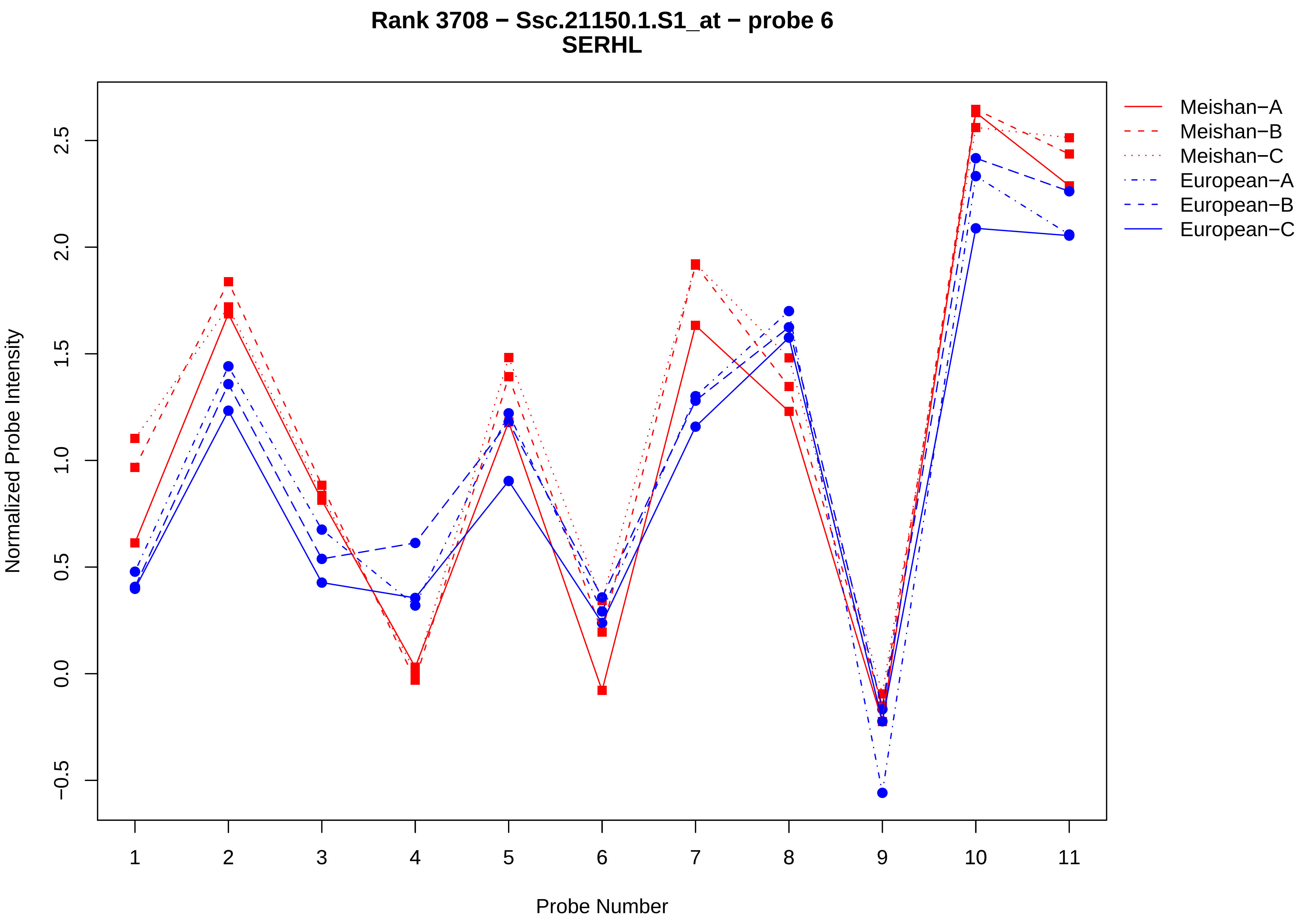
Rank 3708 − Ssc.21150.1.S1_at − probe 6
SERHL
Normalized Probe Intensity
Probe Number
Meishan−A
Meishan−B
Meishan−C
European−A
European−B
European−C
−0.5
0.0
0.5
1.0
1.5
2.0
2.5
1
2
3
4
5
6
7
8
9
10
11

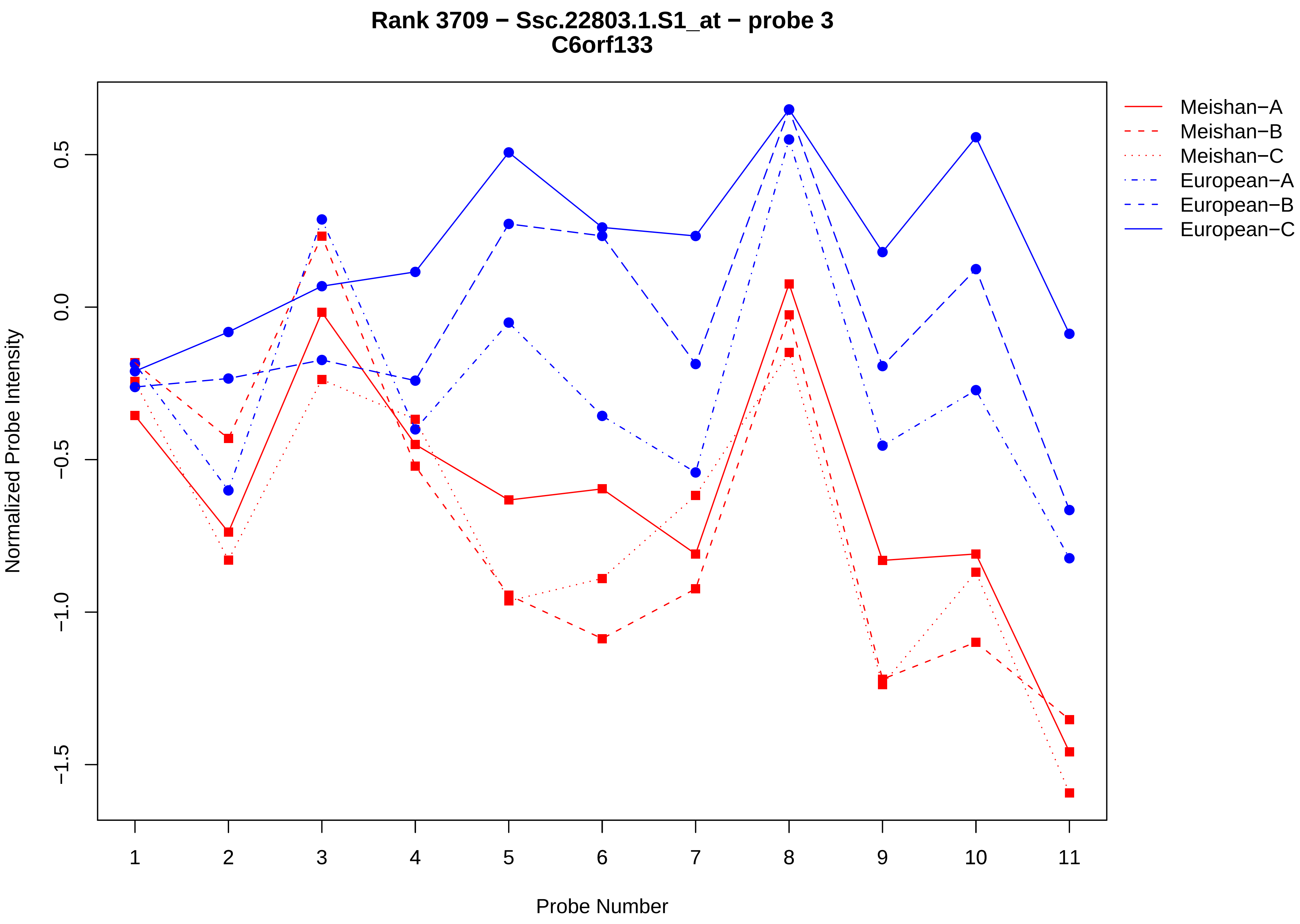
Rank 3709 − Ssc.22803.1.S1_at − probe 3
C6orf133
Normalized Probe Intensity
0.5
0.0
−0.5
−1.0
−1.5
1
2
3
4
5
6
7
8
9
10
11
Probe Number
Meishan−A
Meishan−B
Meishan−C
European−A
European−B
European−C

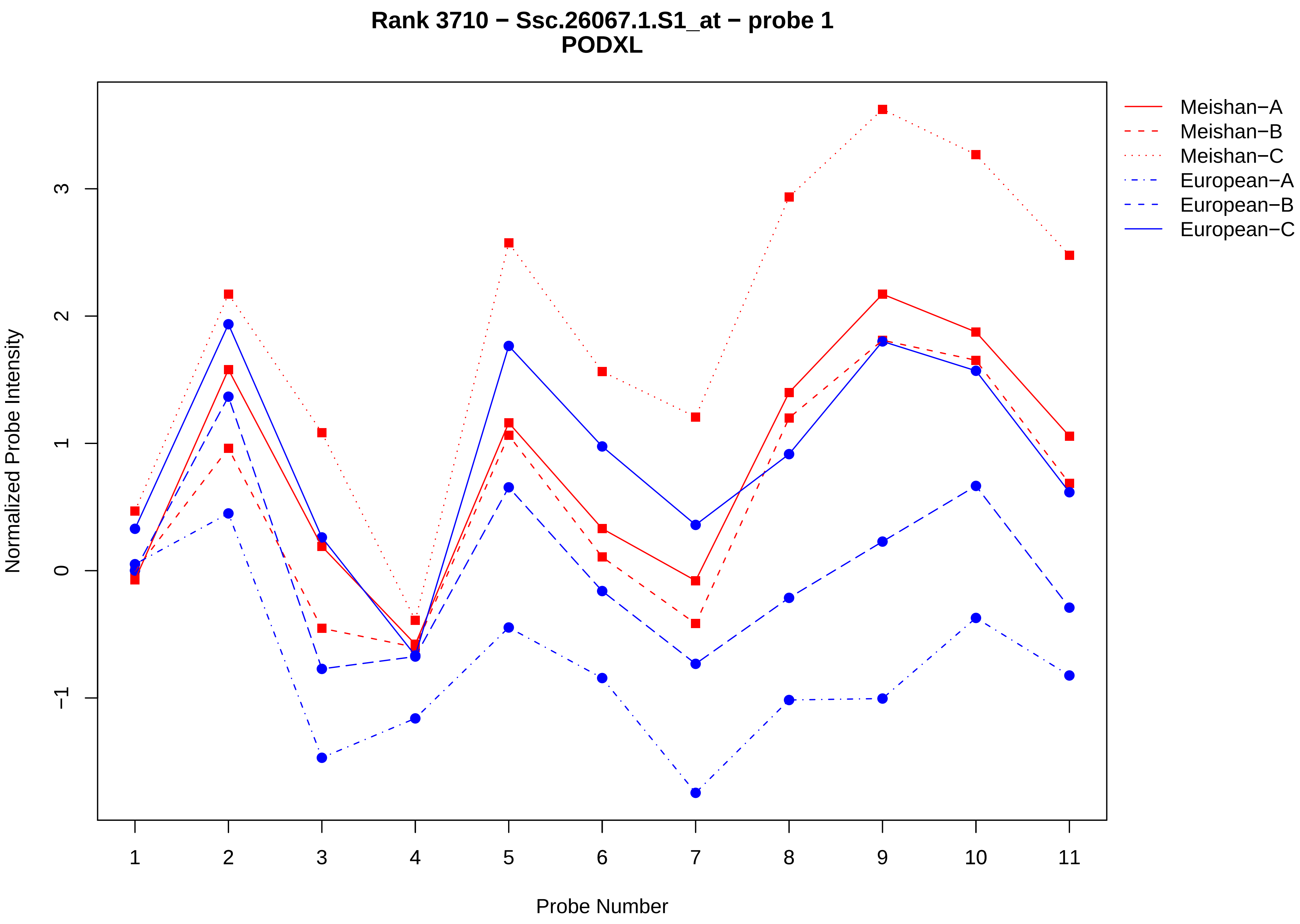
Rank 3710 − Ssc.26067.1.S1_at − probe 1
PODXL
Normalized Probe Intensity
Probe Number
Meishan−A
Meishan−B
Meishan−C
European−A
European−B
European−C
−1
0
1
2
3
1
2
3
4
5
6
7
8
9
10
11

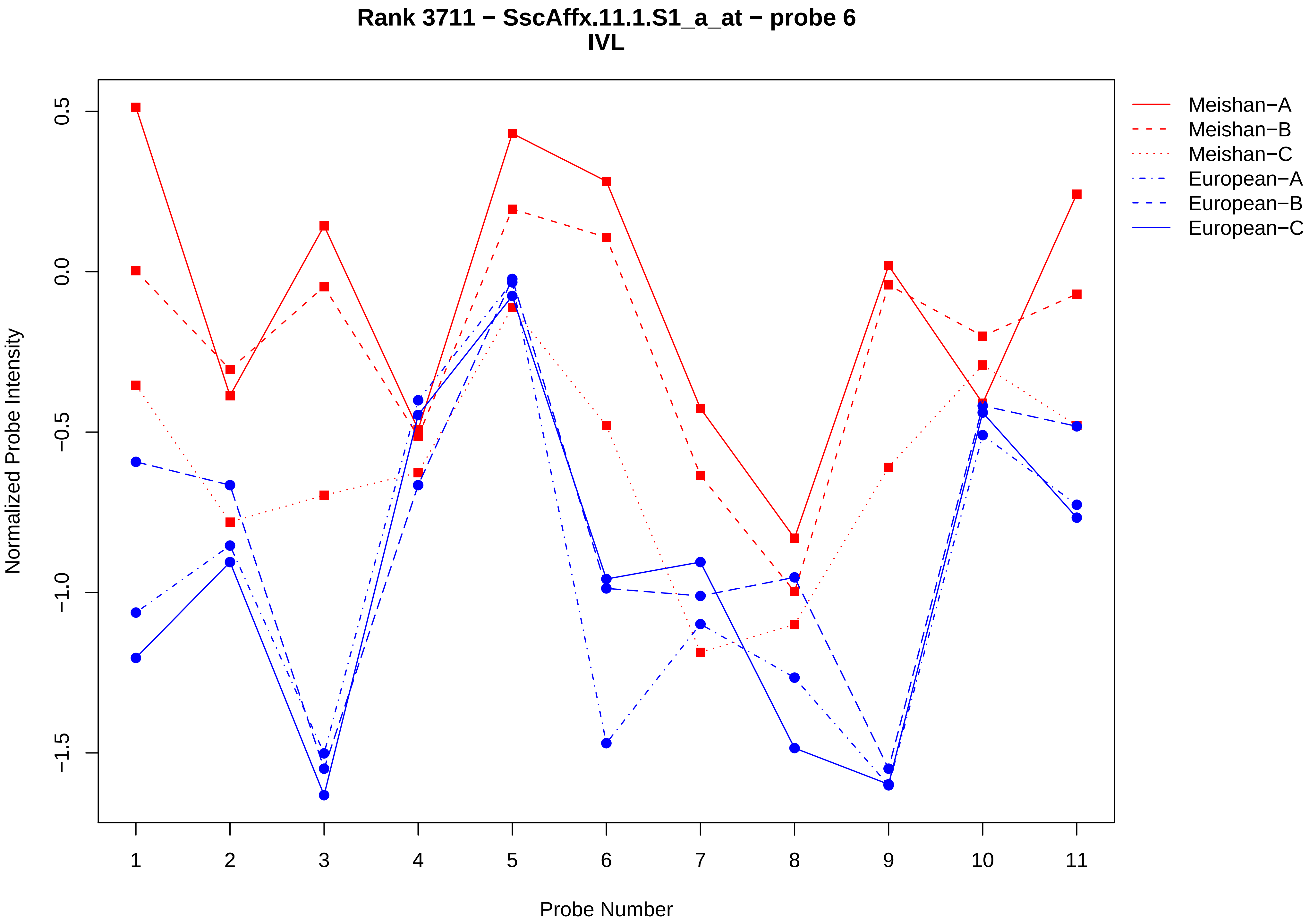

Rank 3711 − SscAffx.11.1.S1_a_at − probe 6
IVL
Normalized Probe Intensity
0.5
0.0
−0.5
−1.0
−1.5
1
2
3
4
5
6
7
8
9
10
11
Probe Number
Meishan−A
Meishan−B
Meishan−C
European−A
European−B
European−C

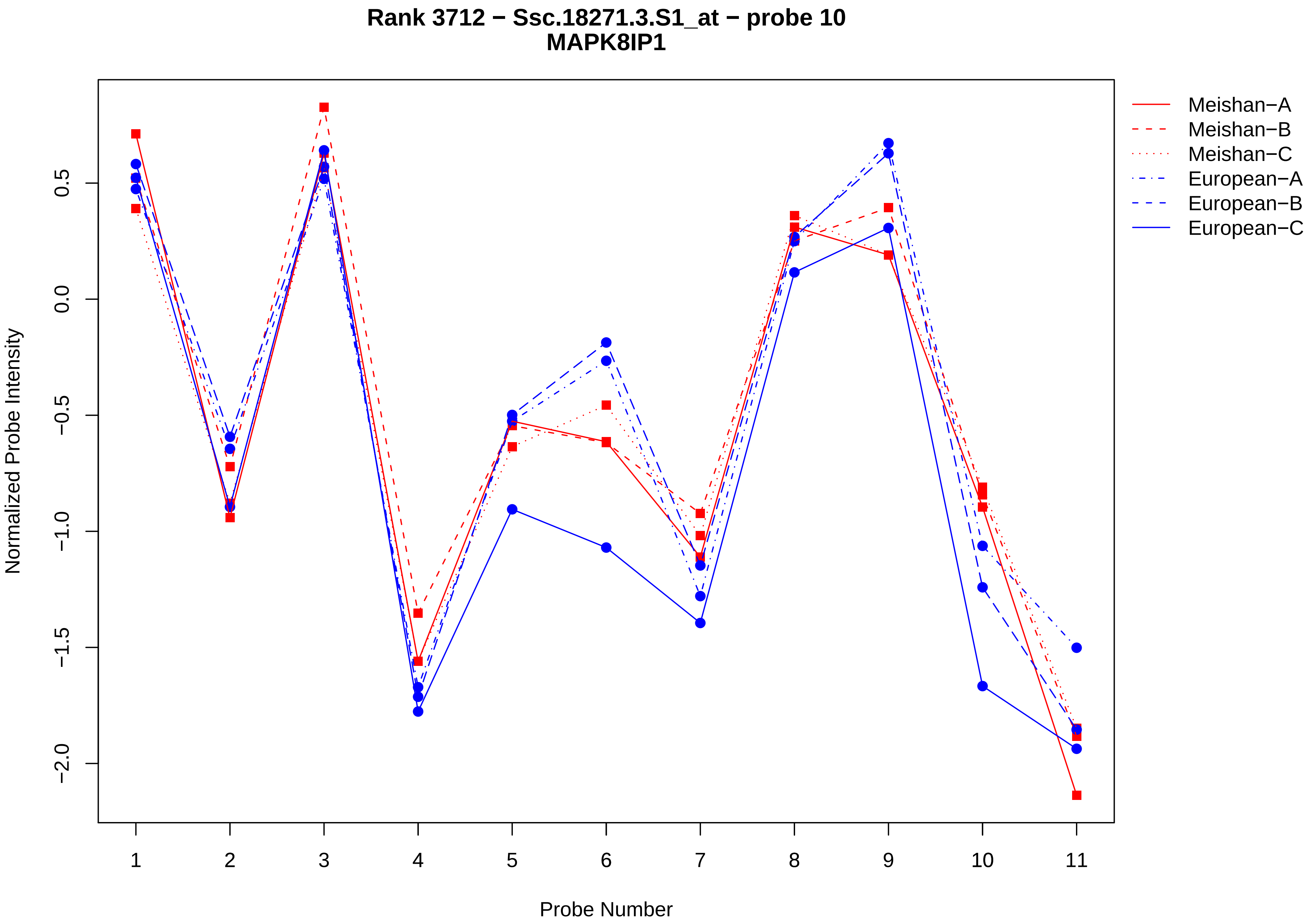

Rank 3712 − Ssc.18271.3.S1_at − probe 10
MAPK8IP1
Normalized Probe Intensity
Probe Number
−2.0
−1.5
−1.0
−0.5
0.0
0.5
1
2
3
4
5
6
7
8
9
10
11
Meishan−A
Meishan−B
Meishan−C
European−A
European−B
European−C

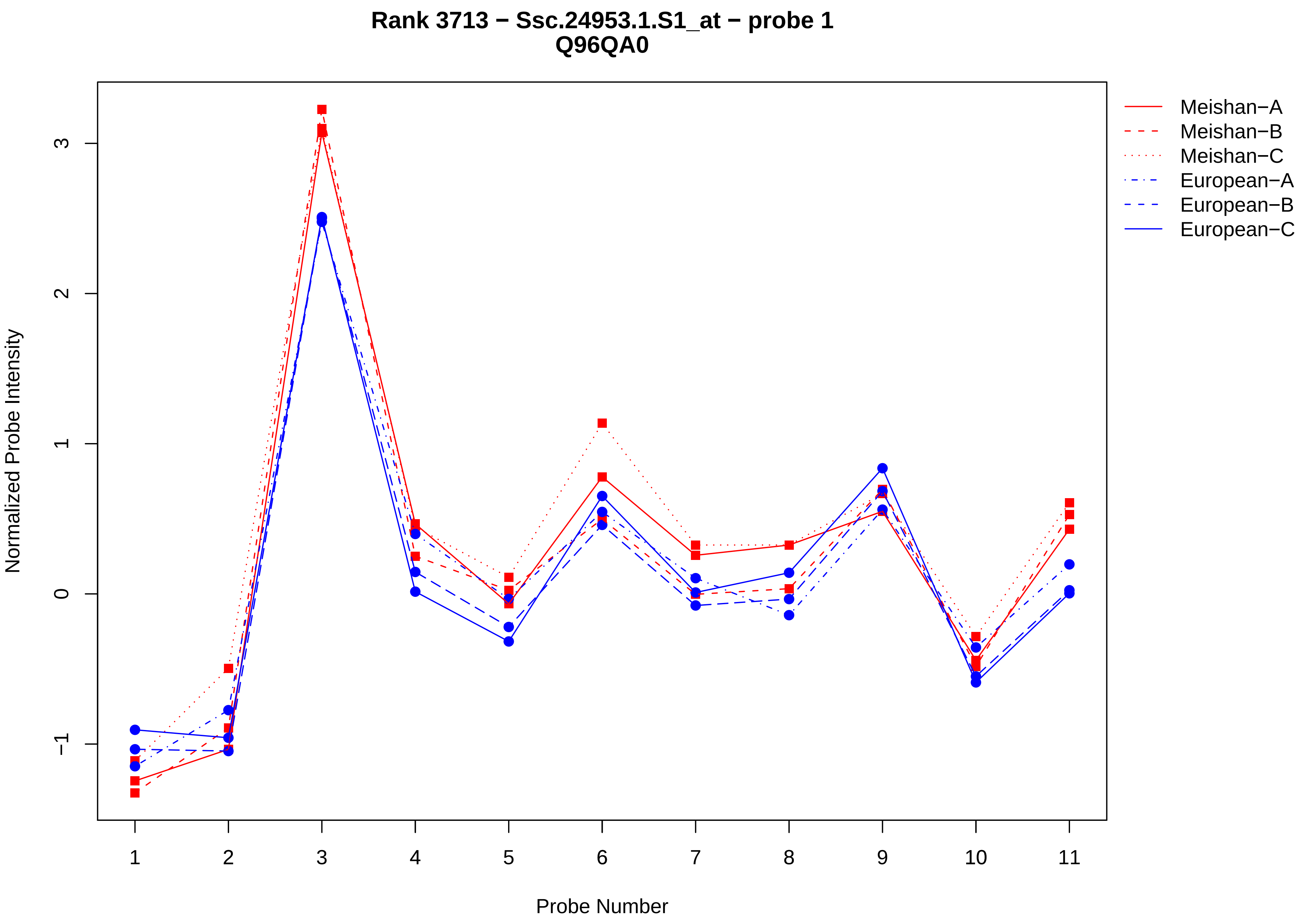

Rank 3713 − Ssc.24953.1.S1_at − probe 1
Q96QA0
Normalized Probe Intensity
Probe Number
Meishan−A
Meishan−B
Meishan−C
European−A
European−B
European−C
−1
0
1
2
3
1
2
3
4
5
6
7
8
9
10
11

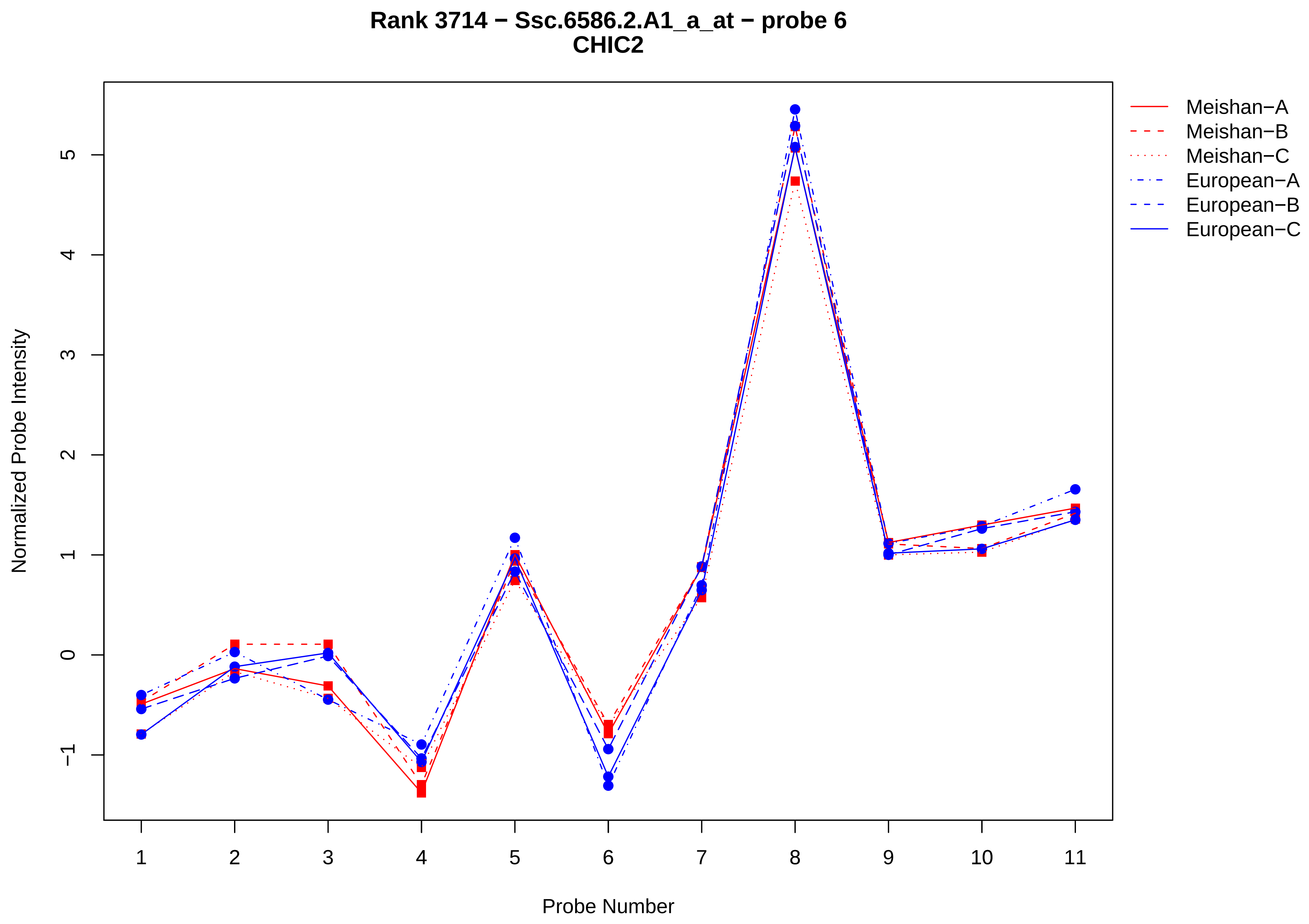

Rank 3714 − Ssc.6586.2.A1_a_at − probe 6
CHIC2
Normalized Probe Intensity
Probe Number
Meishan−A
Meishan−B
Meishan−C
European−A
European−B
European−C
−1
0
1
2
3
4
5
1
2
3
4
5
6
7
8
9
10
11

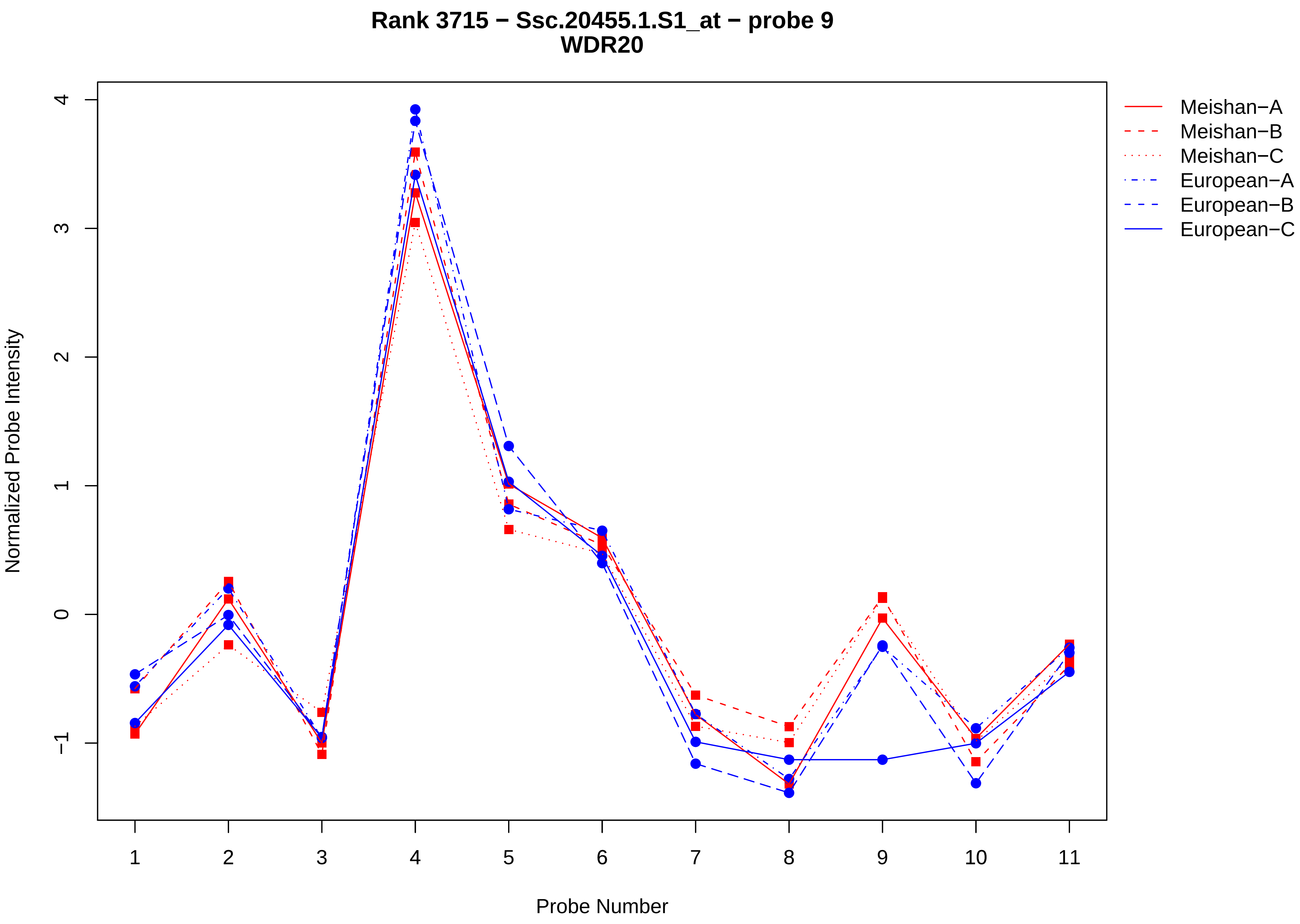

Rank 3715 − Ssc.20455.1.S1_at − probe 9
WDR20
Normalized Probe Intensity
Probe Number
Meishan−A
Meishan−B
Meishan−C
European−A
European−B
European−C

Rank 3716 − Ssc.14506.1.S1_at − probe 7
TOP2A

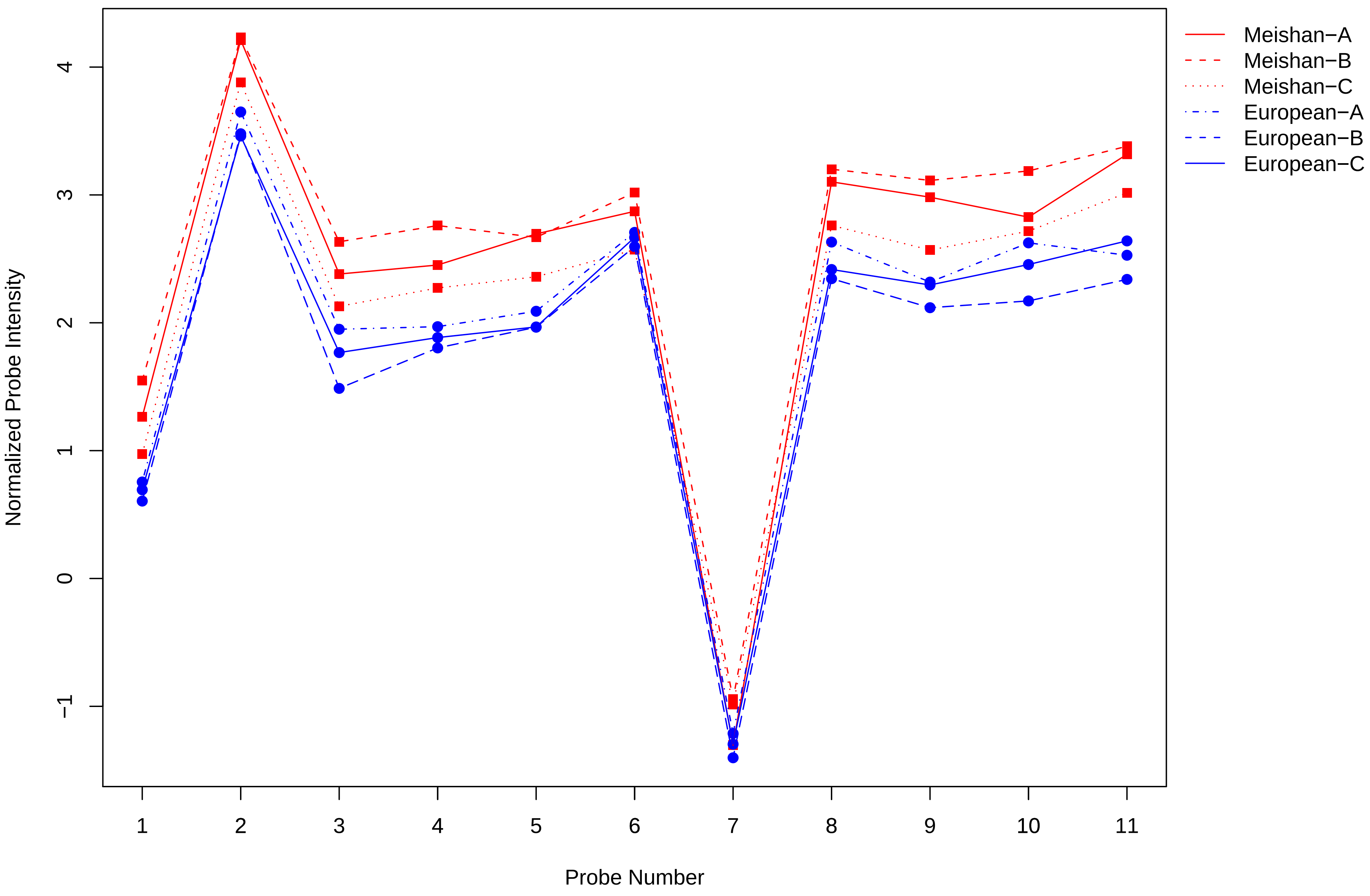

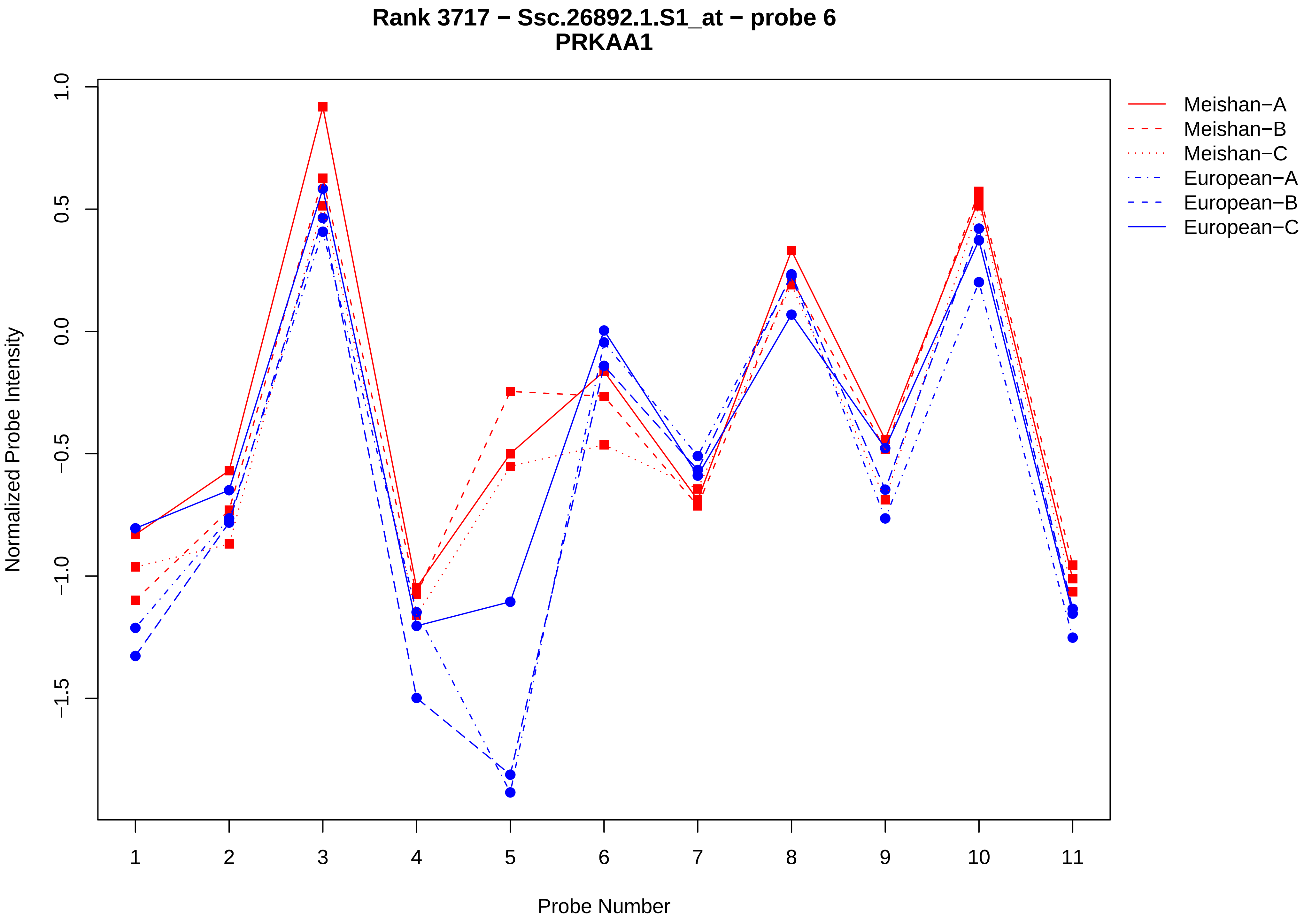

Rank 3717 − Ssc.26892.1.S1_at − probe 6
PRKAA1
Normalized Probe Intensity
1.0
0.5
0.0
−0.5
−1.0
−1.5
1
2
3
4
5
6
7
8
9
10
11
Probe Number
Meishan−A
Meishan−B
Meishan−C
European−A
European−B
European−C

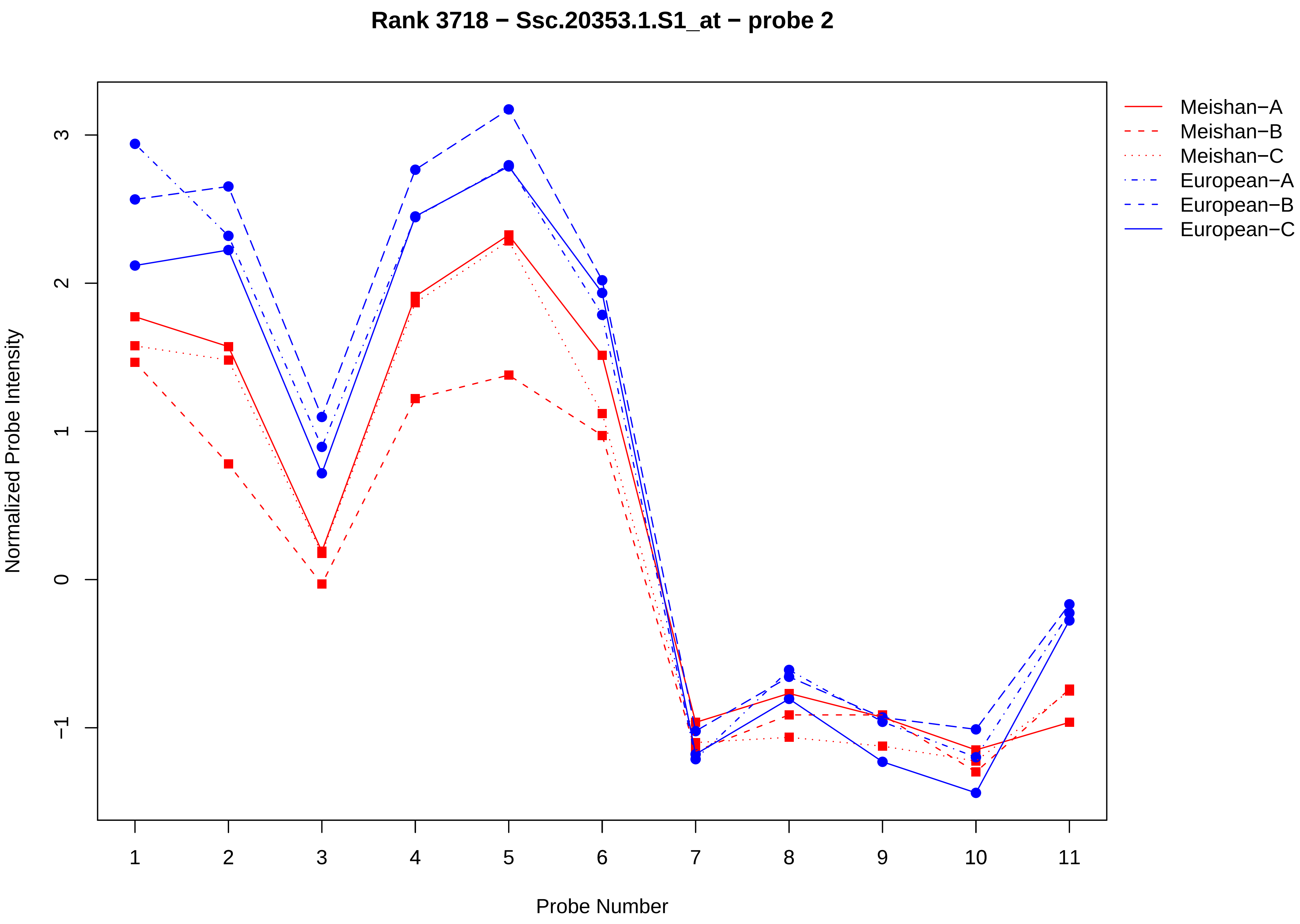

Rank 3718 − Ssc.20353.1.S1_at − probe 2
Normalized Probe Intensity
Probe Number
3
2
1
0
−1
1
2
3
4
5
6
7
8
9
10
11
Meishan−A
Meishan−B
Meishan−C
European−A
European−B
European−C

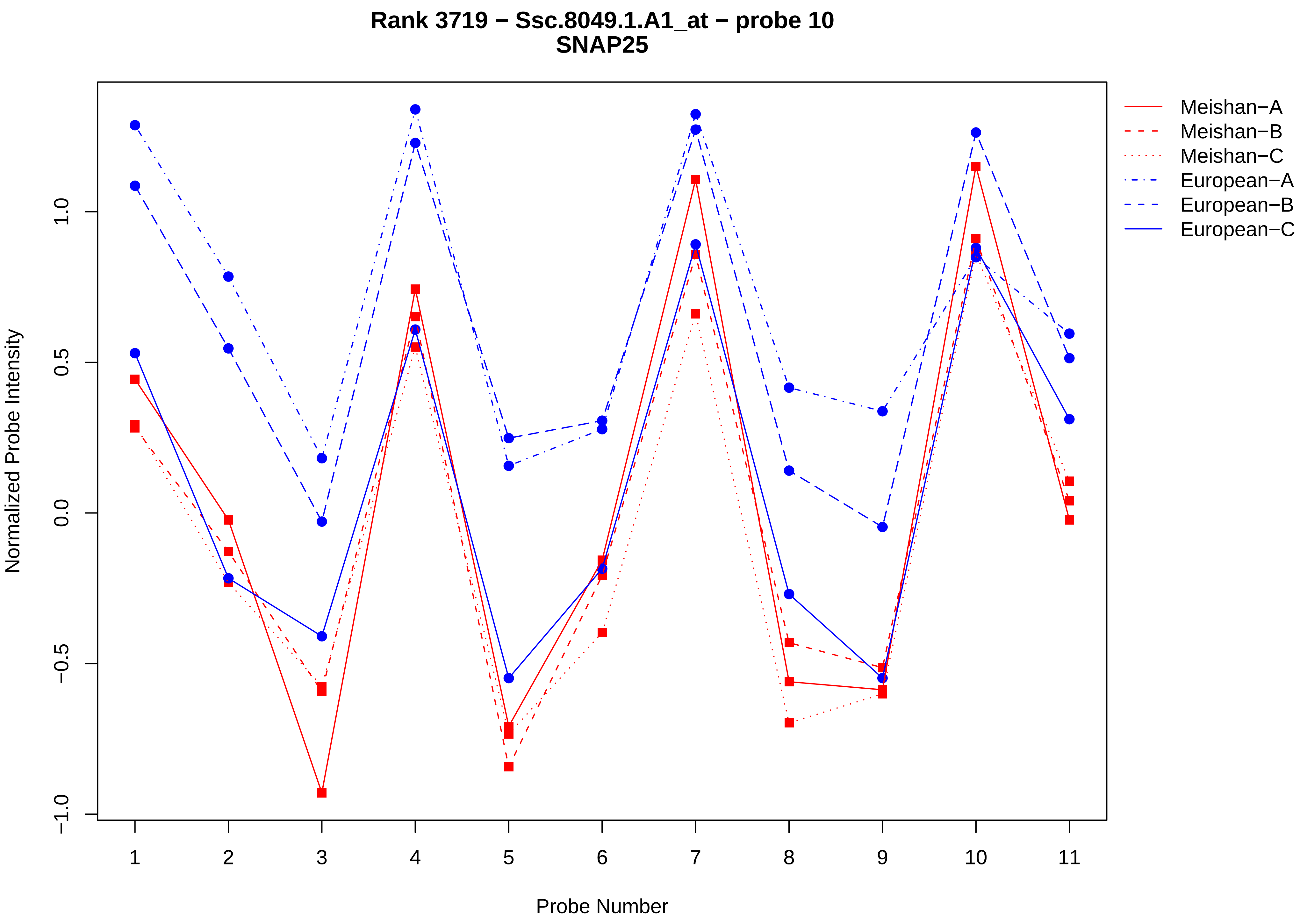

Rank 3719 − Ssc.8049.1.A1_at − probe 10
SNAP25
Normalized Probe Intensity
Probe Number
−1.0
−0.5
0.0
0.5
1.0
1
2
3
4
5
6
7
8
9
10
11
Meishan−A
Meishan−B
Meishan−C
European−A
European−B
European−C

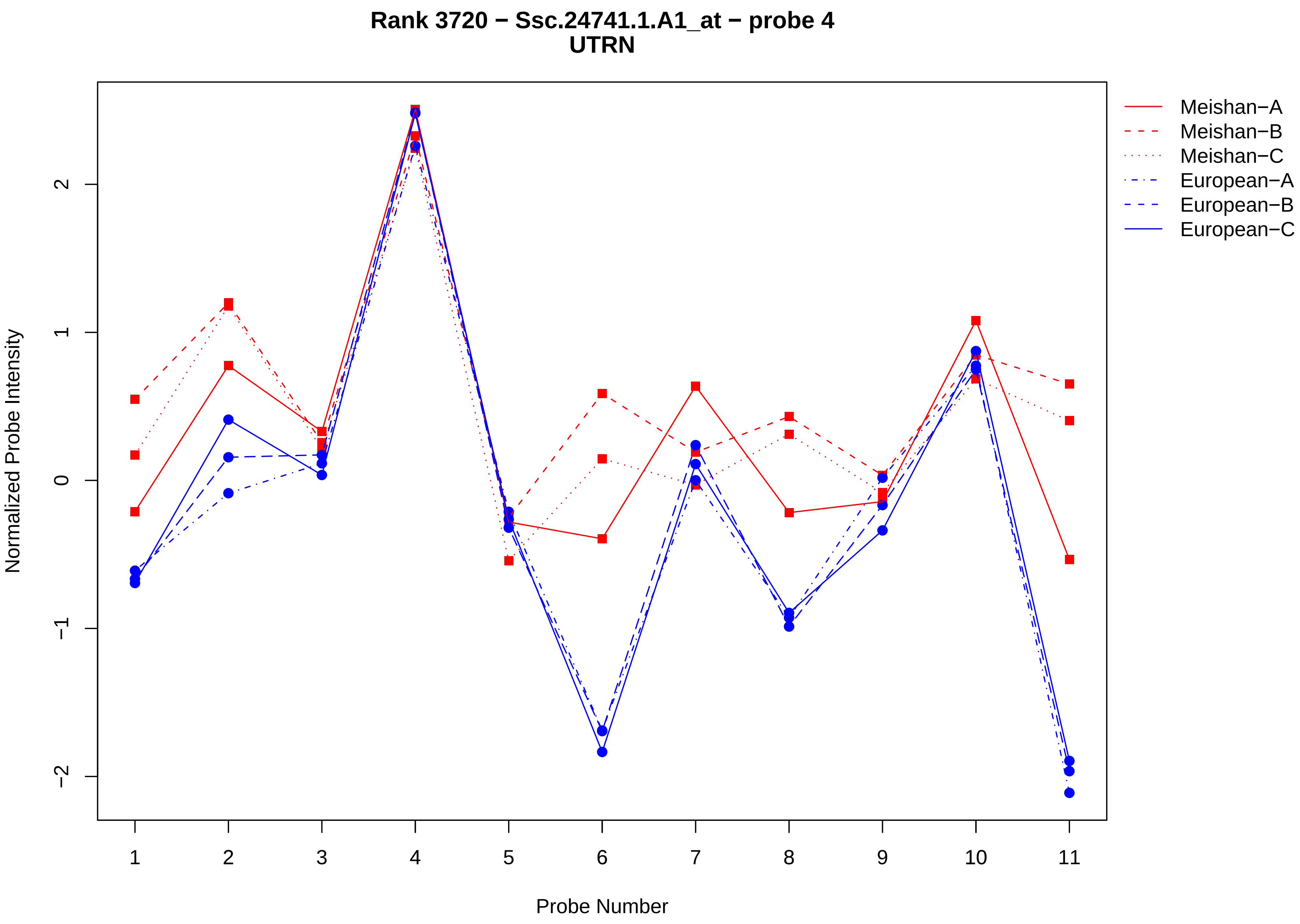

Rank 3720 − Ssc.24741.1.A1_at − probe 4
UTRN
Normalized Probe Intensity
Probe Number
−2
−1
0
1
2
1
2
3
4
5
6
7
8
9
10
11
Meishan−A
Meishan−B
Meishan−C
European−A
European−B
European−C

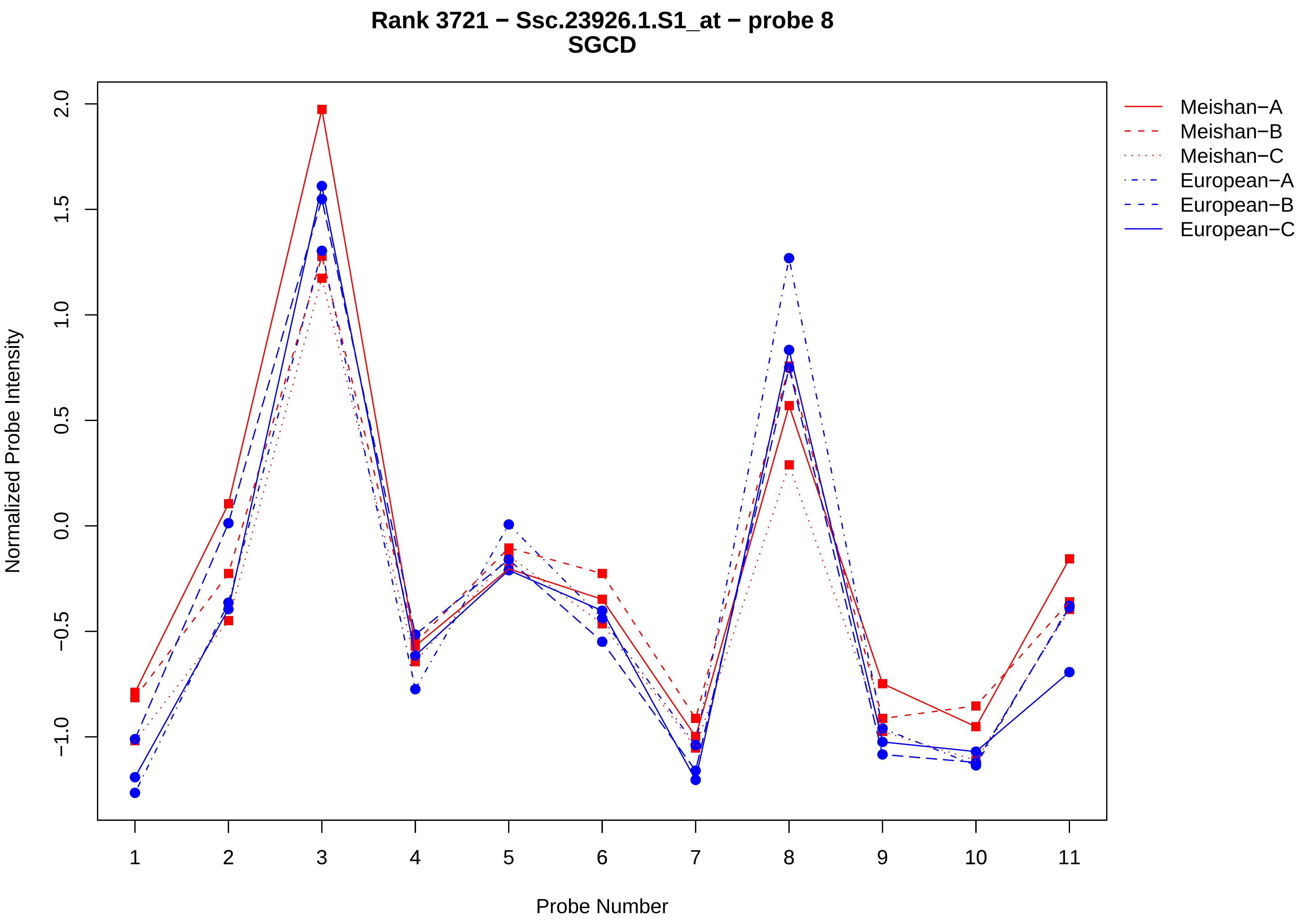

Rank 3721 − Ssc.23926.1.S1_at − probe 8
SGCD
Normalized Probe Intensity
Probe Number
Meishan−A
Meishan−B
Meishan−C
European−A
European−B
European−C
−1.0
−0.5
0.0
0.5
1.0
1.5
2.0
1
2
3
4
5
6
7
8
9
10
11

**Rank 3722 − Ssc.7928.1.A1_at − probe 3
STAM**

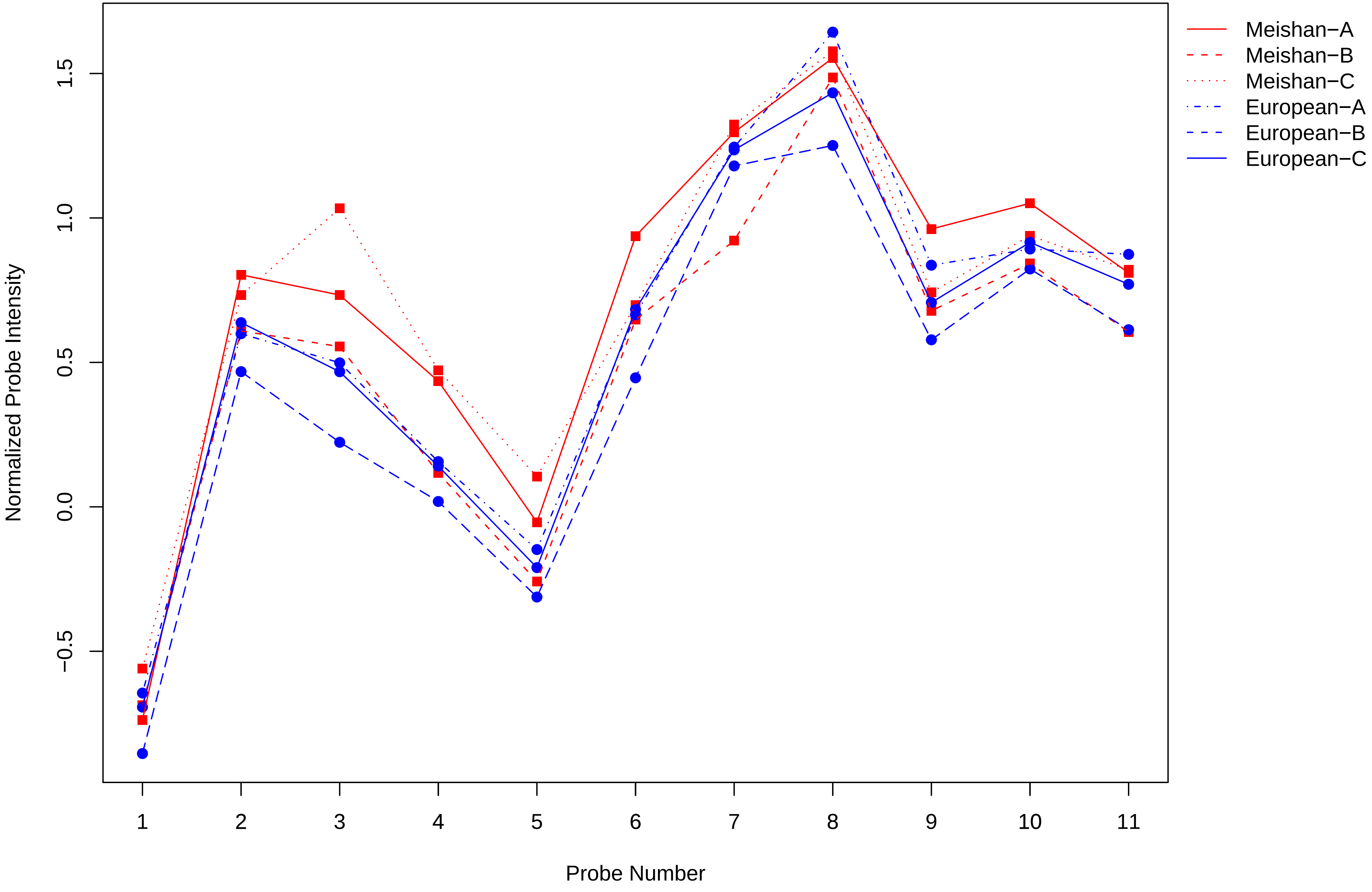

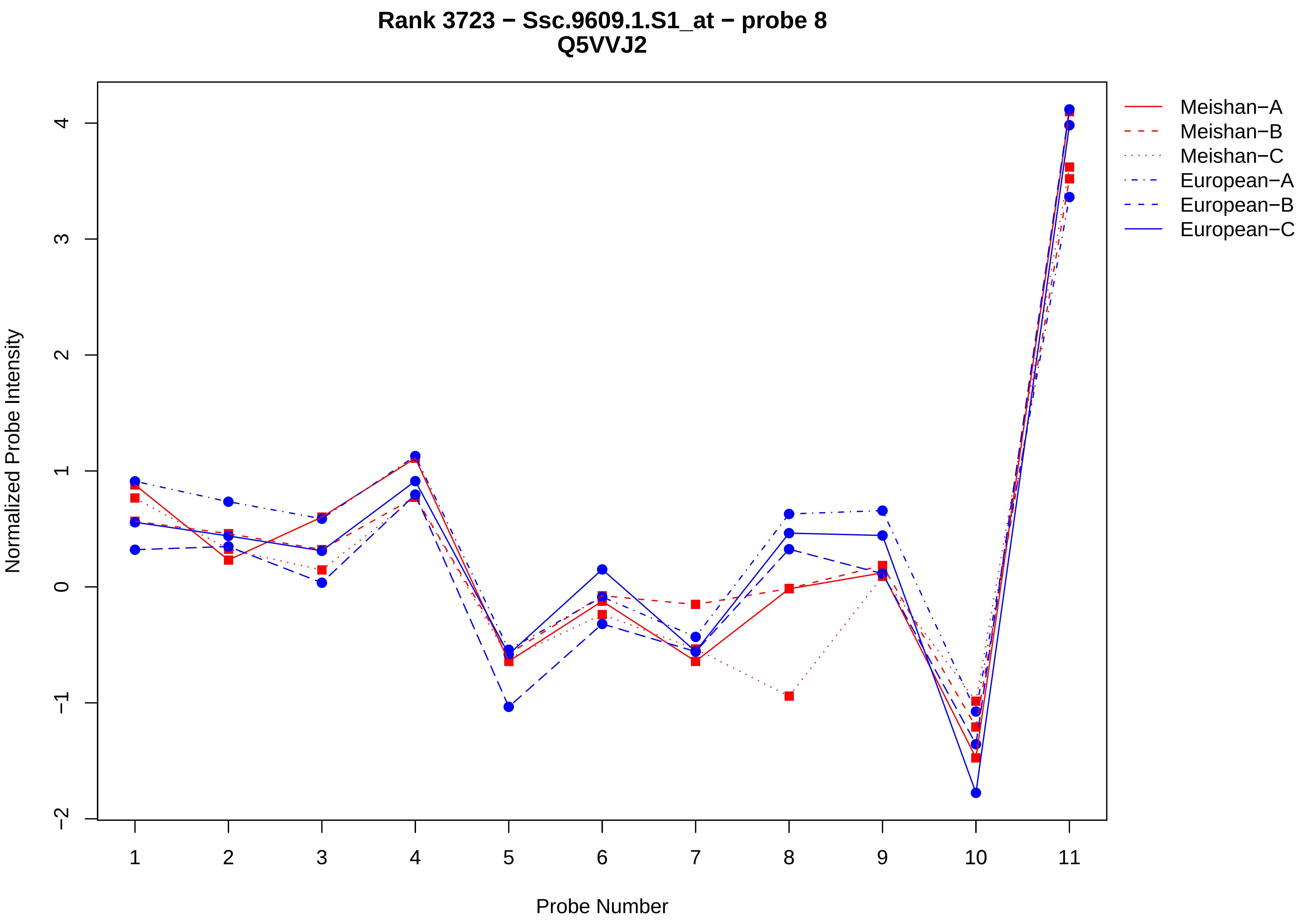

Rank 3723 − Ssc.9609.1.S1_at − probe 8
Q5VVJ2
Normalized Probe Intensity
Probe Number
−2
−1
0
1
2
3
4
1
2
3
4
5
6
7
8
9
10
11
Meishan−A
Meishan−B
Meishan−C
European−A
European−B
European−C

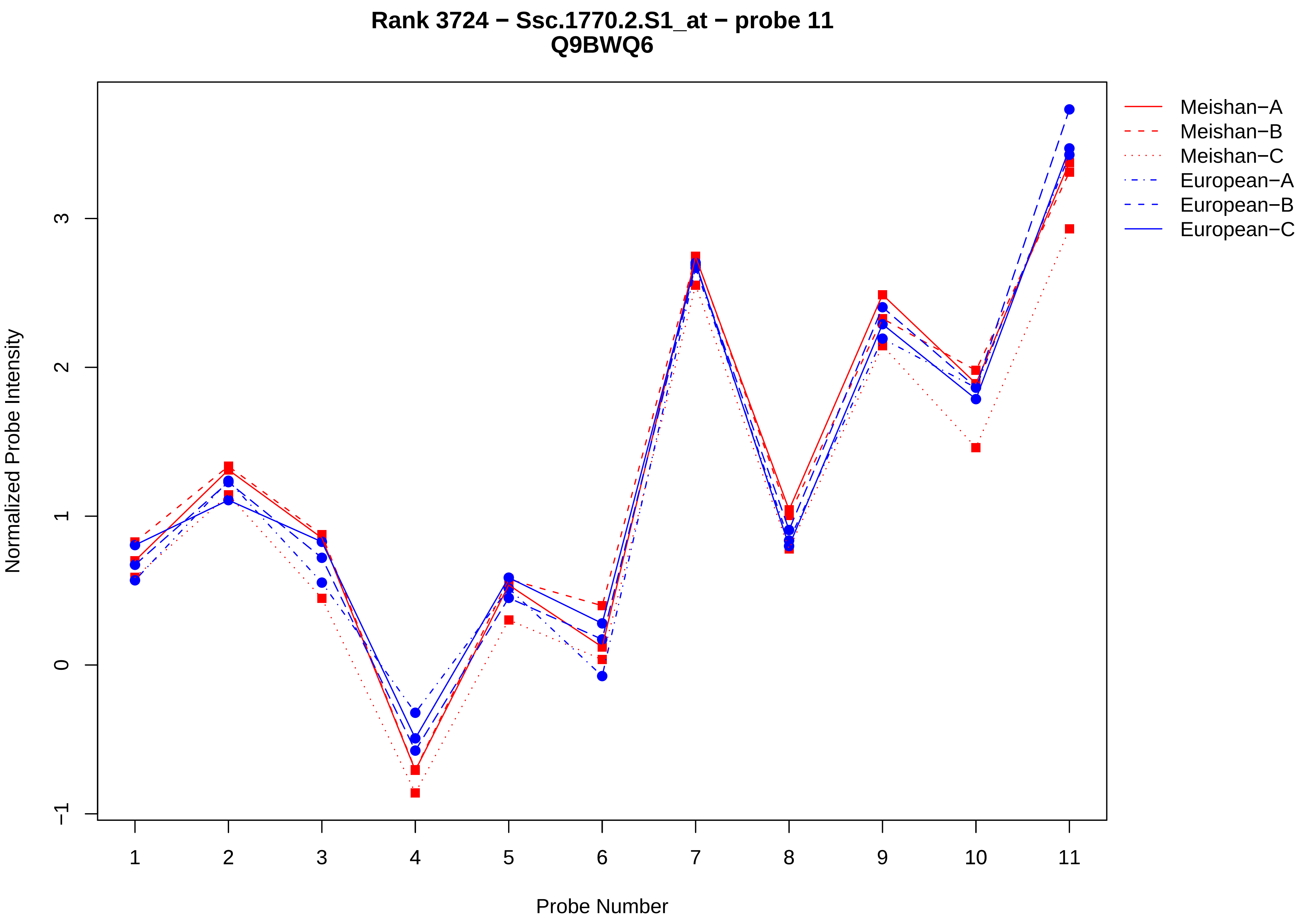

Rank 3724 − Ssc.1770.2.S1_at − probe 11
Q9BWQ6
Normalized Probe Intensity
Probe Number
−1
0
1
2
3
1
2
3
4
5
6
7
8
9
10
11
Meishan−A
Meishan−B
Meishan−C
European−A
European−B
European−C

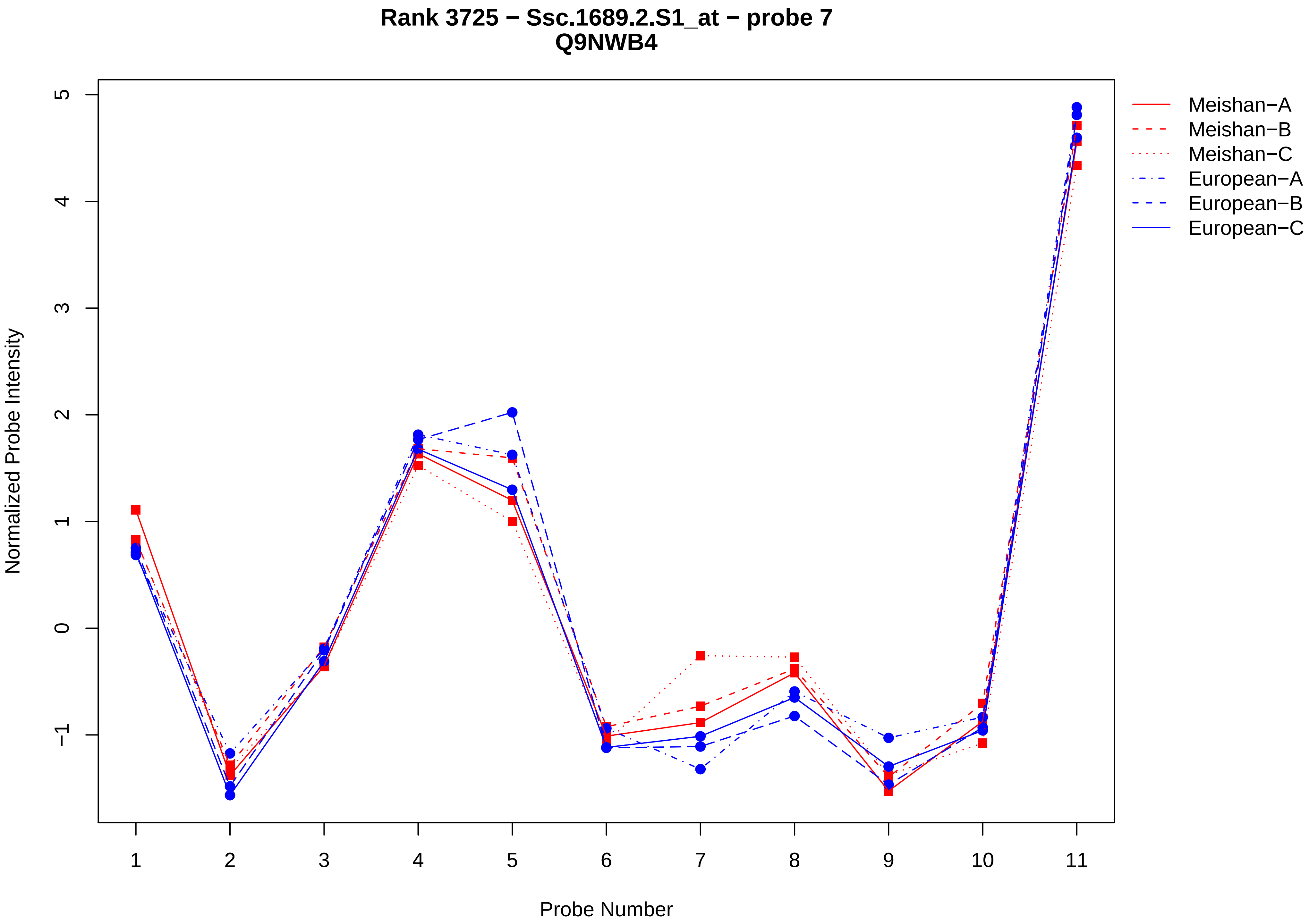

Rank 3725 − Ssc.1689.2.S1_at − probe 7
Q9NWB4
Normalized Probe Intensity
Probe Number
Meishan−A
Meishan−B
Meishan−C
European−A
European−B
European−C

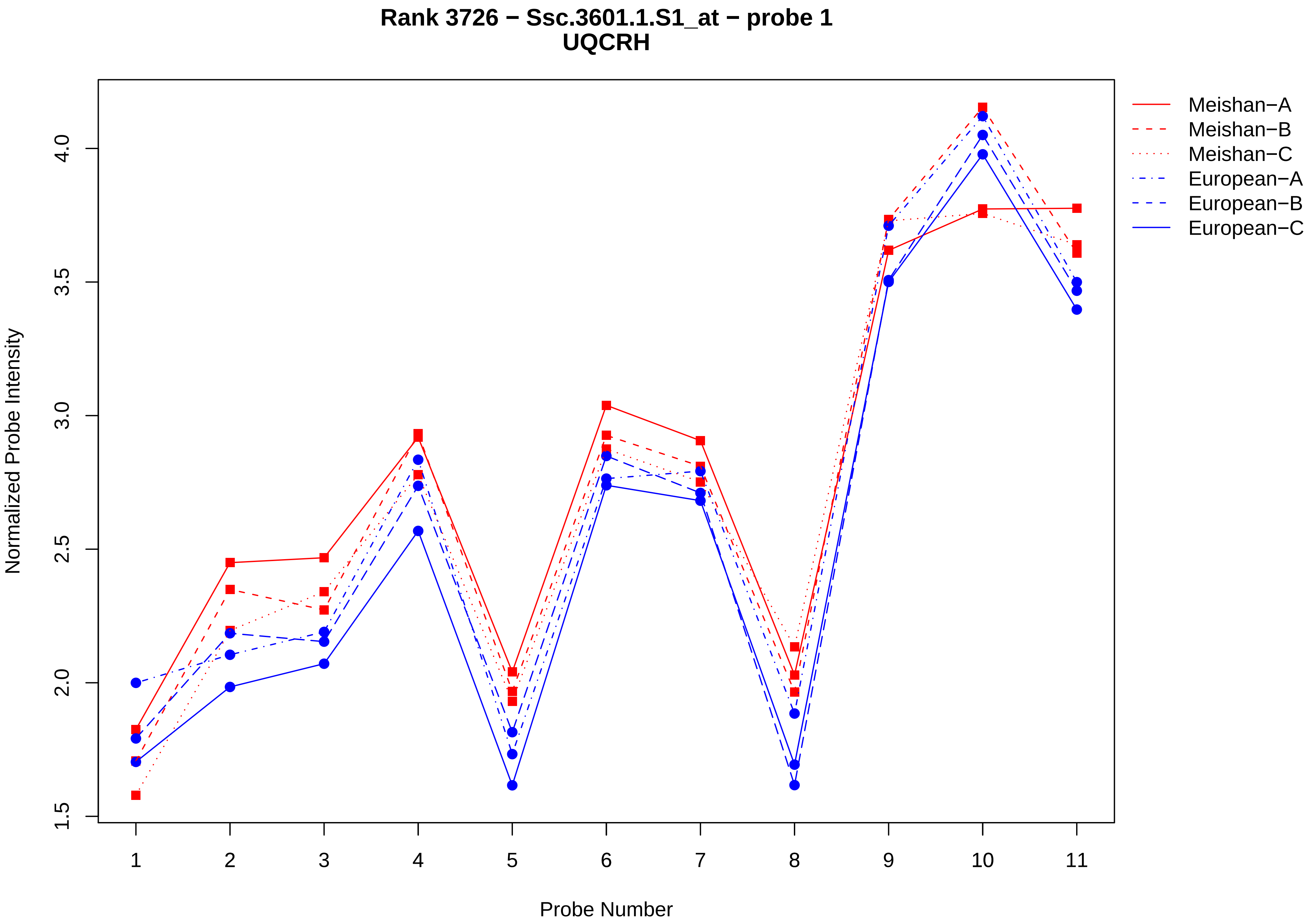

Rank 3726 − Ssc.3601.1.S1_at − probe 1
UQCRH
Normalized Probe Intensity
Probe Number
1.5
2.0
2.5
3.0
3.5
4.0
1
2
3
4
5
6
7
8
9
10
11
Meishan−A
Meishan−B
Meishan−C
European−A
European−B
European−C

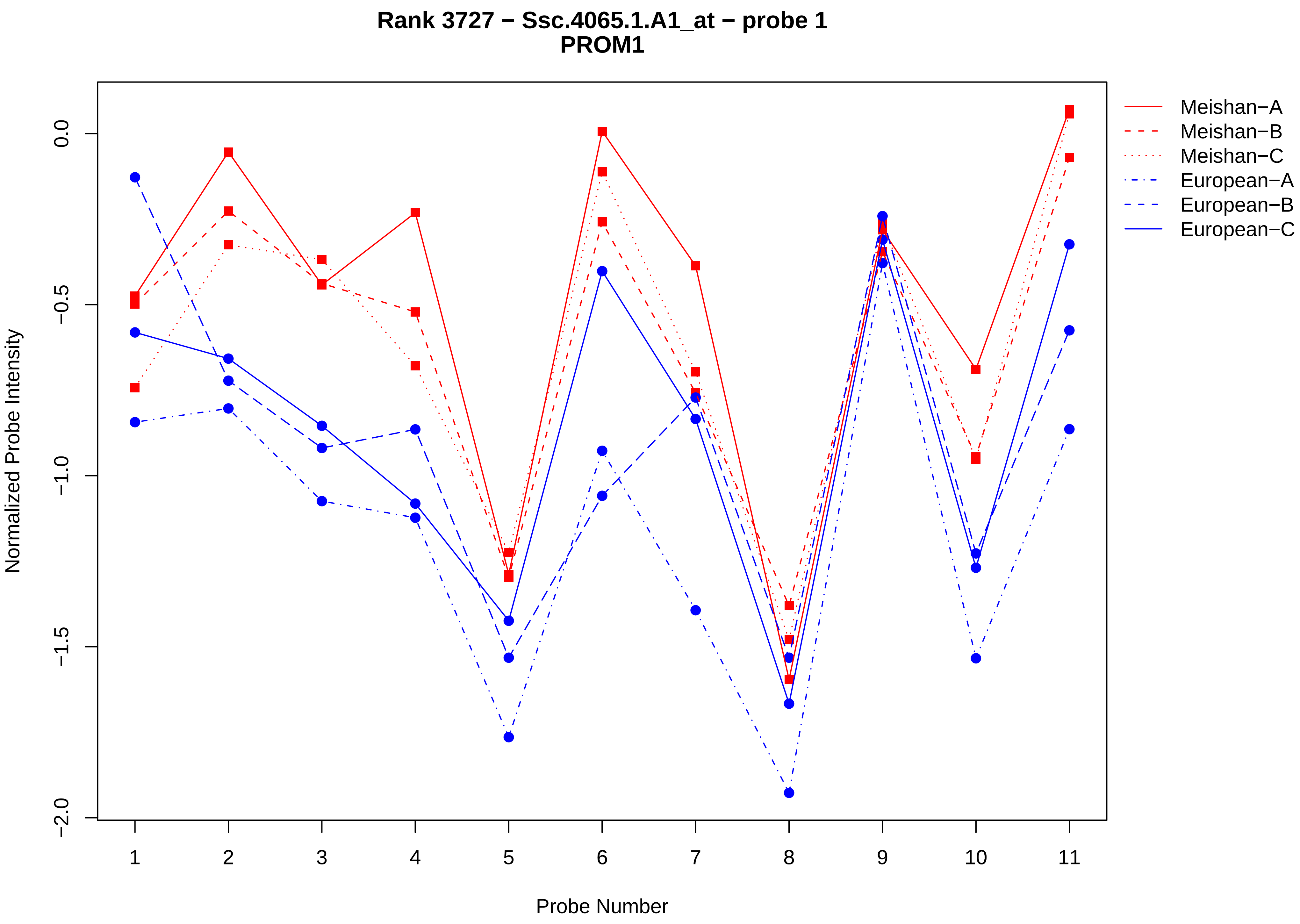
Rank 3727 − Ssc.4065.1.A1_at − probe 1
PROM1
Normalized Probe Intensity
Probe Number
0.0
−0.5
−1.0
−1.5
−2.0
1
2
3
4
5
6
7
8
9
10
11
Meishan−A
Meishan−B
Meishan−C
European−A
European−B
European−C

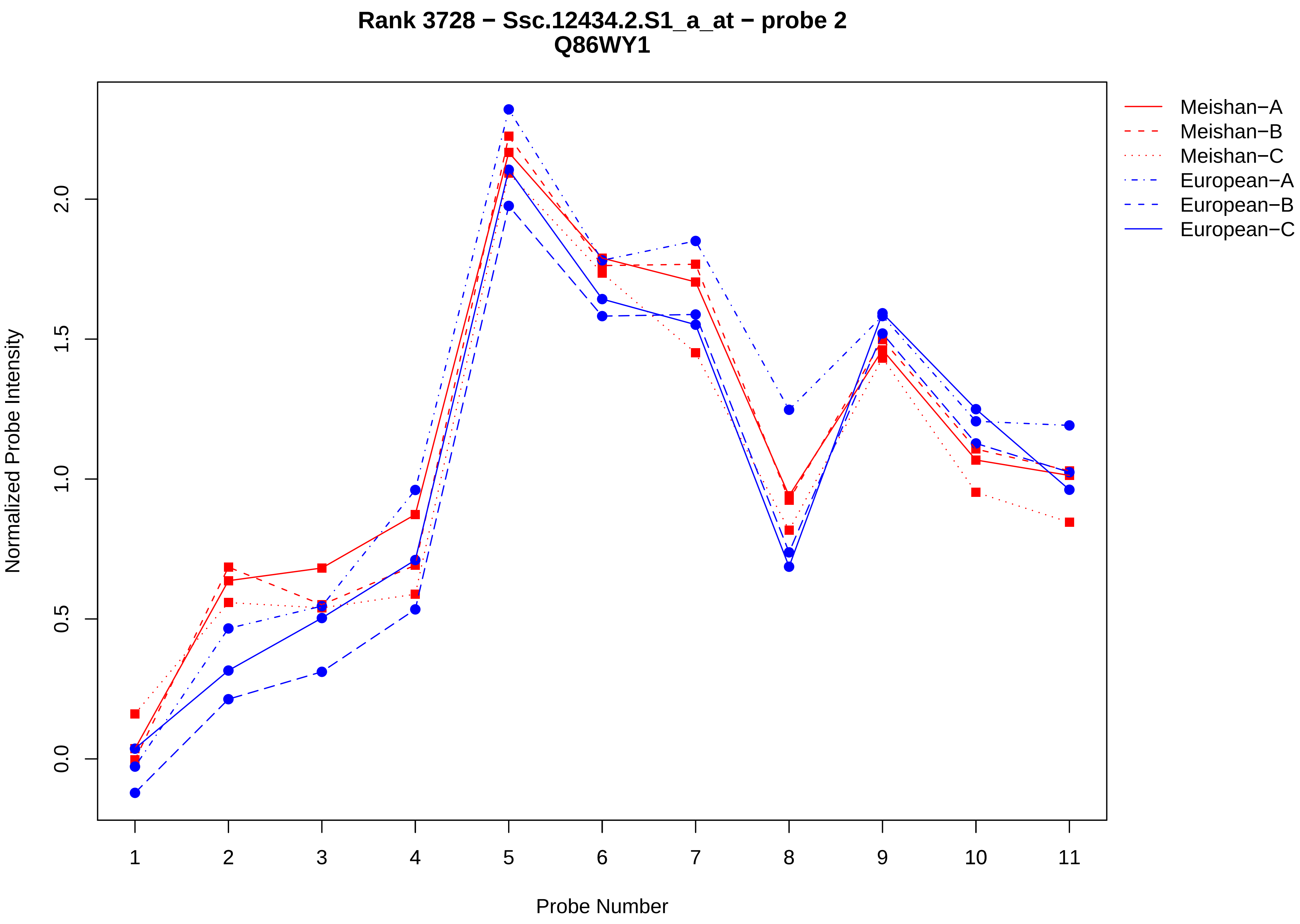

Rank 3728 − Ssc.12434.2.S1_a_at − probe 2
Q86WY1
Normalized Probe Intensity
0.0
0.5
1.0
1.5
2.0
1
2
3
4
5
6
7
8
9
10
11
Probe Number
Meishan−A
Meishan−B
Meishan−C
European−A
European−B
European−C

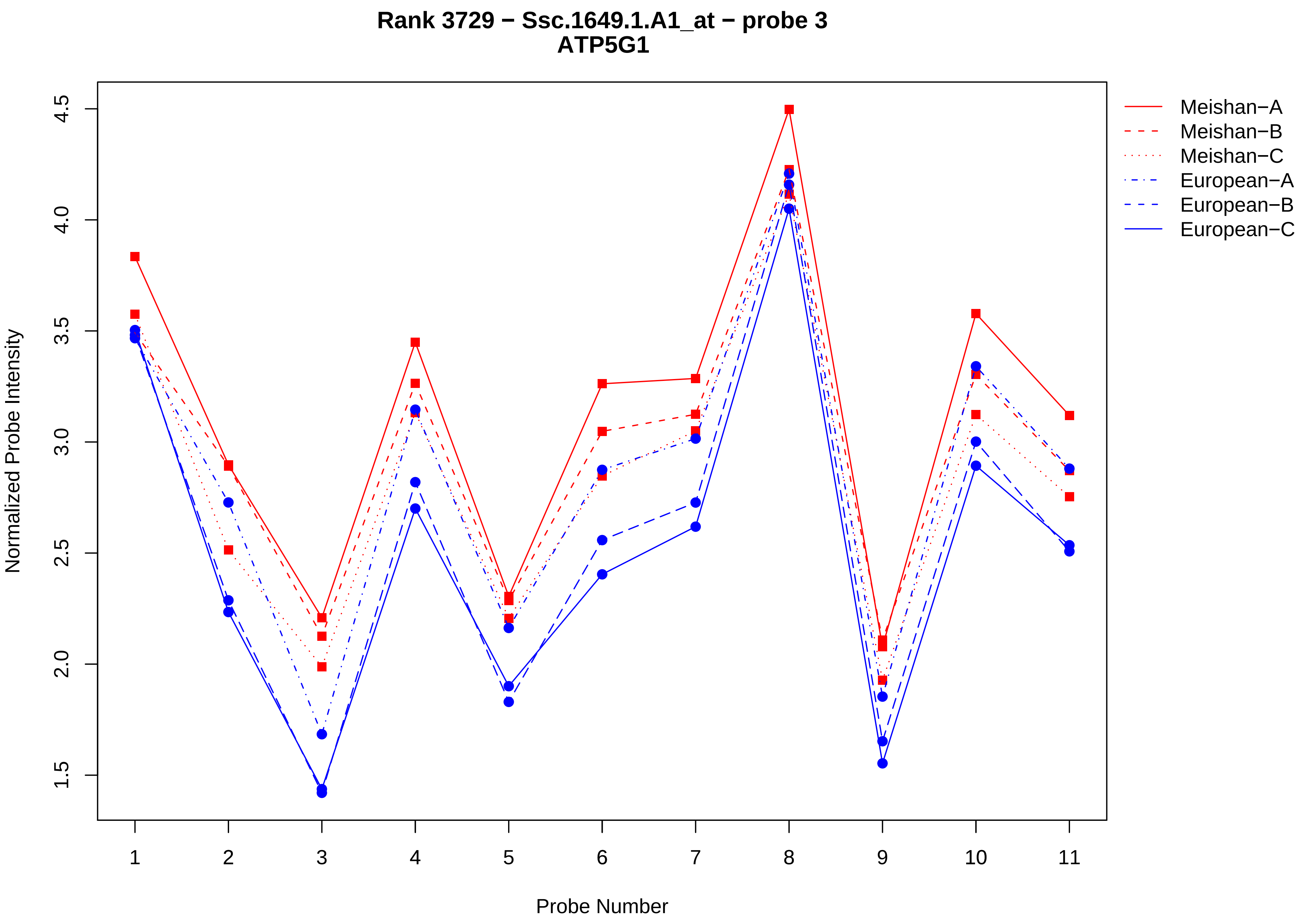
Rank 3729 − Ssc.1649.1.A1_at − probe 3
ATP5G1
Normalized Probe Intensity
Probe Number
1.5
2.0
2.5
3.0
3.5
4.0
4.5
1
2
3
4
5
6
7
8
9
10
11
Meishan−A
Meishan−B
Meishan−C
European−A
European−B
European−C

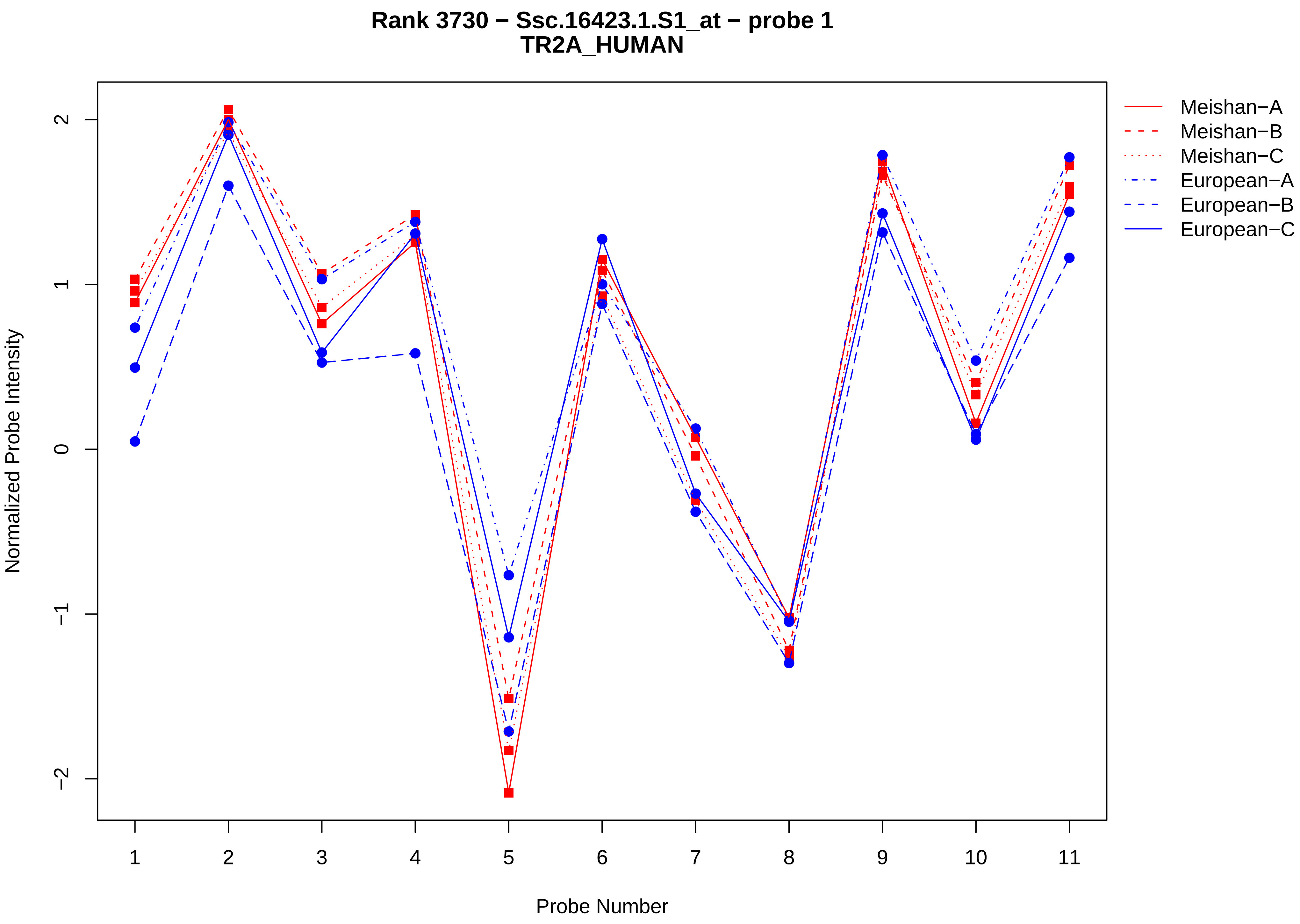

Rank 3730 − Ssc.16423.1.S1_at − probe 1
TR2A_HUMAN
Normalized Probe Intensity
Probe Number
Meishan−A
Meishan−B
Meishan−C
European−A
European−B
European−C
−2
−1
0
1
2
1
2
3
4
5
6
7
8
9
10
11

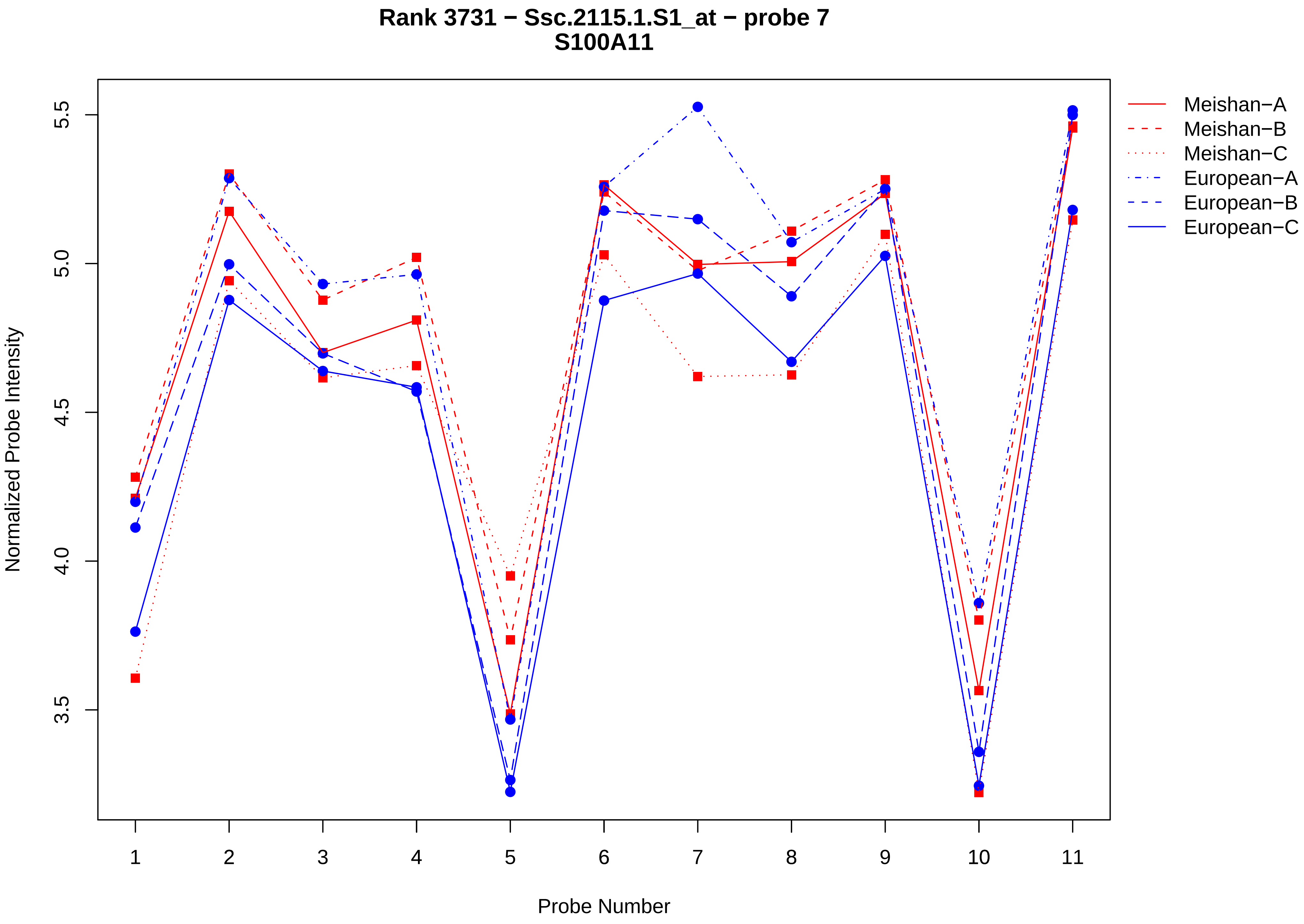

Rank 3731 − Ssc.2115.1.S1_at − probe 7
S100A11
Normalized Probe Intensity
Probe Number
3.5
4.0
4.5
5.0
5.5
1
2
3
4
5
6
7
8
9
10
11
Meishan−A
Meishan−B
Meishan−C
European−A
European−B
European−C

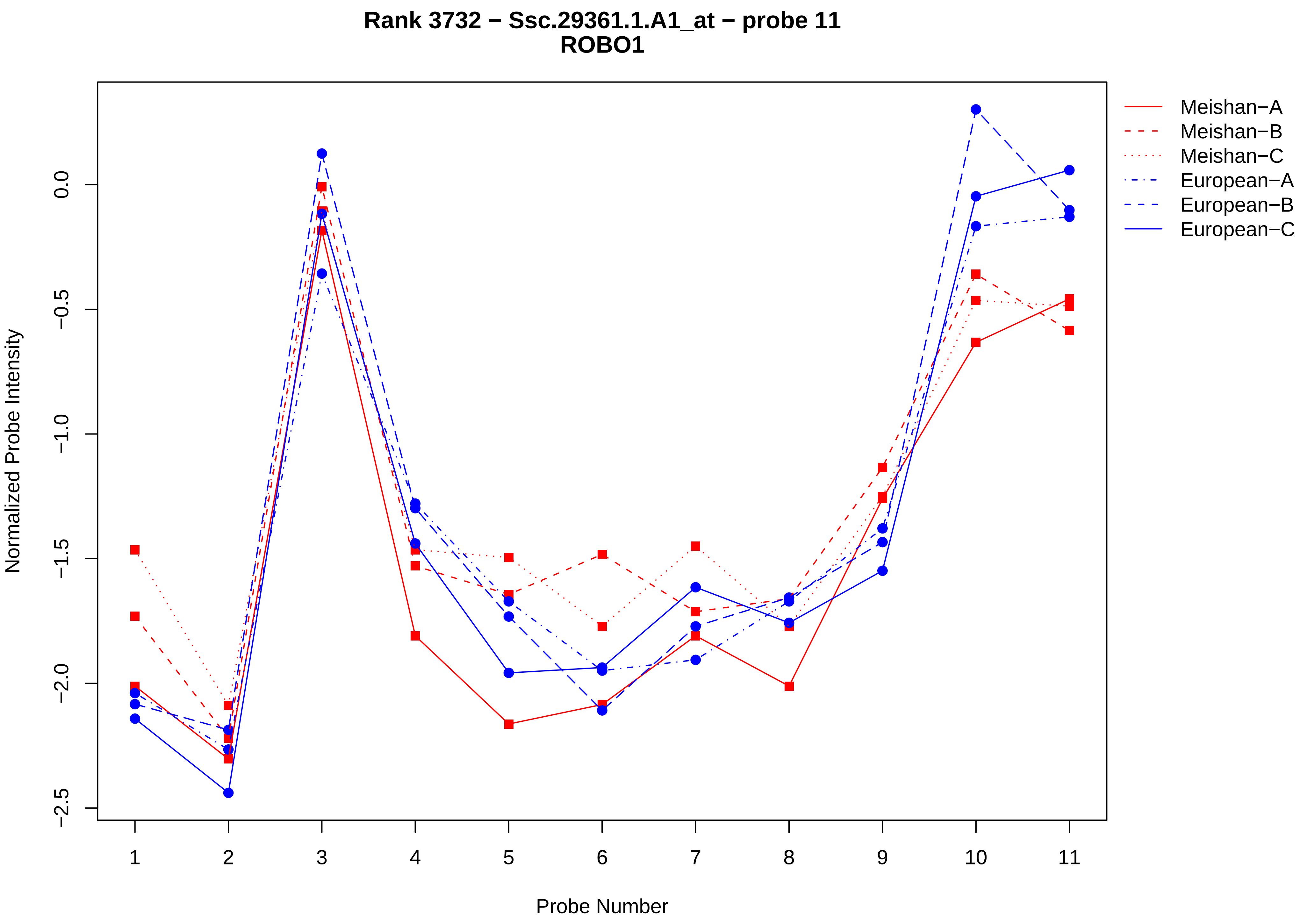
Rank 3732 − Ssc.29361.1.A1_at − probe 11
ROBO1
Normalized Probe Intensity
Probe Number
0.0
−0.5
−1.0
−1.5
−2.0
−2.5
1
2
3
4
5
6
7
8
9
10
11
Meishan−A
Meishan−B
Meishan−C
European−A
European−B
European−C

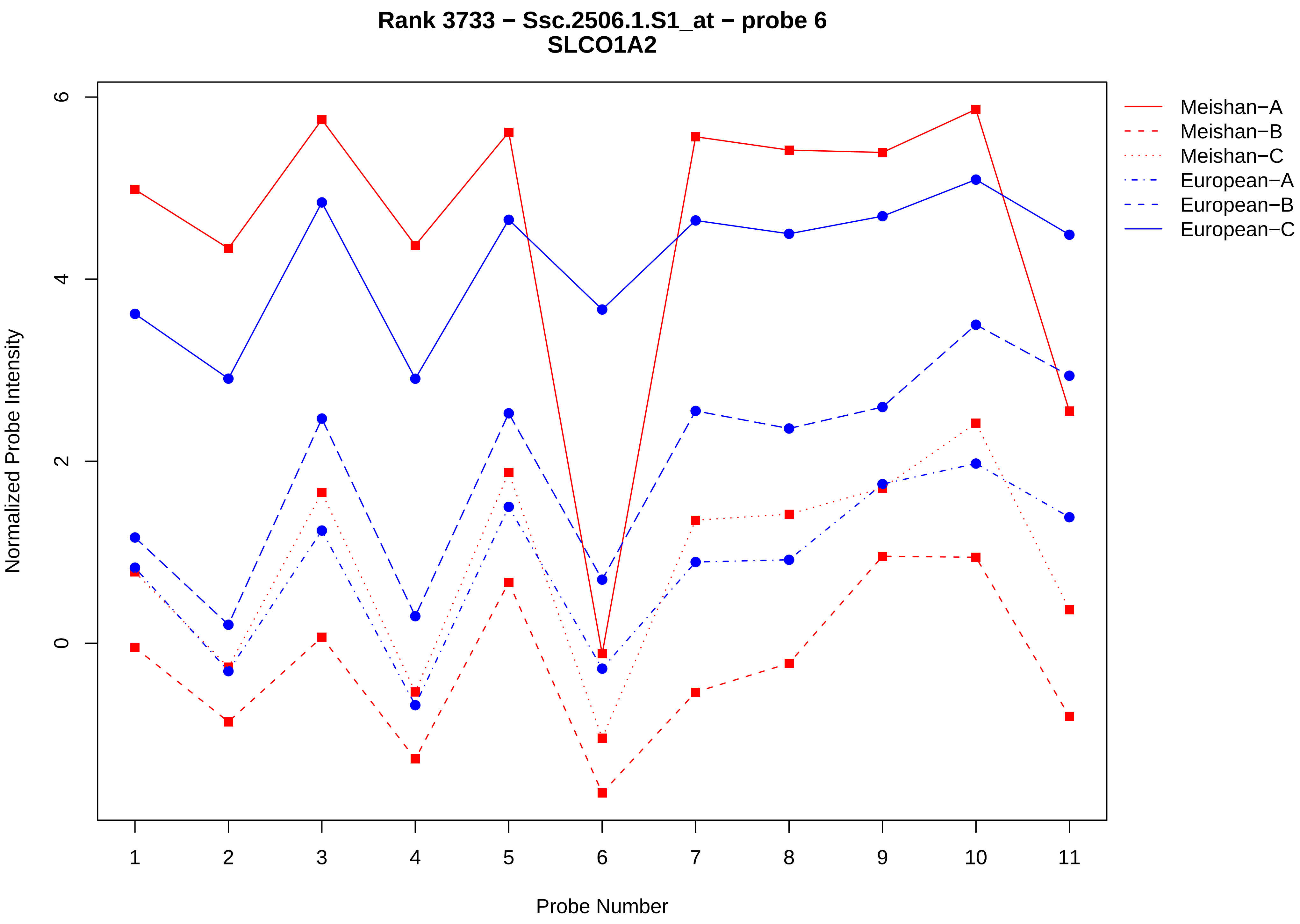
Rank 3733 − Ssc.2506.1.S1_at − probe 6
SLCO1A2
Normalized Probe Intensity
Probe Number
0
2
4
6
1
2
3
4
5
6
7
8
9
10
11
Meishan−A
Meishan−B
Meishan−C
European−A
European−B
European−C

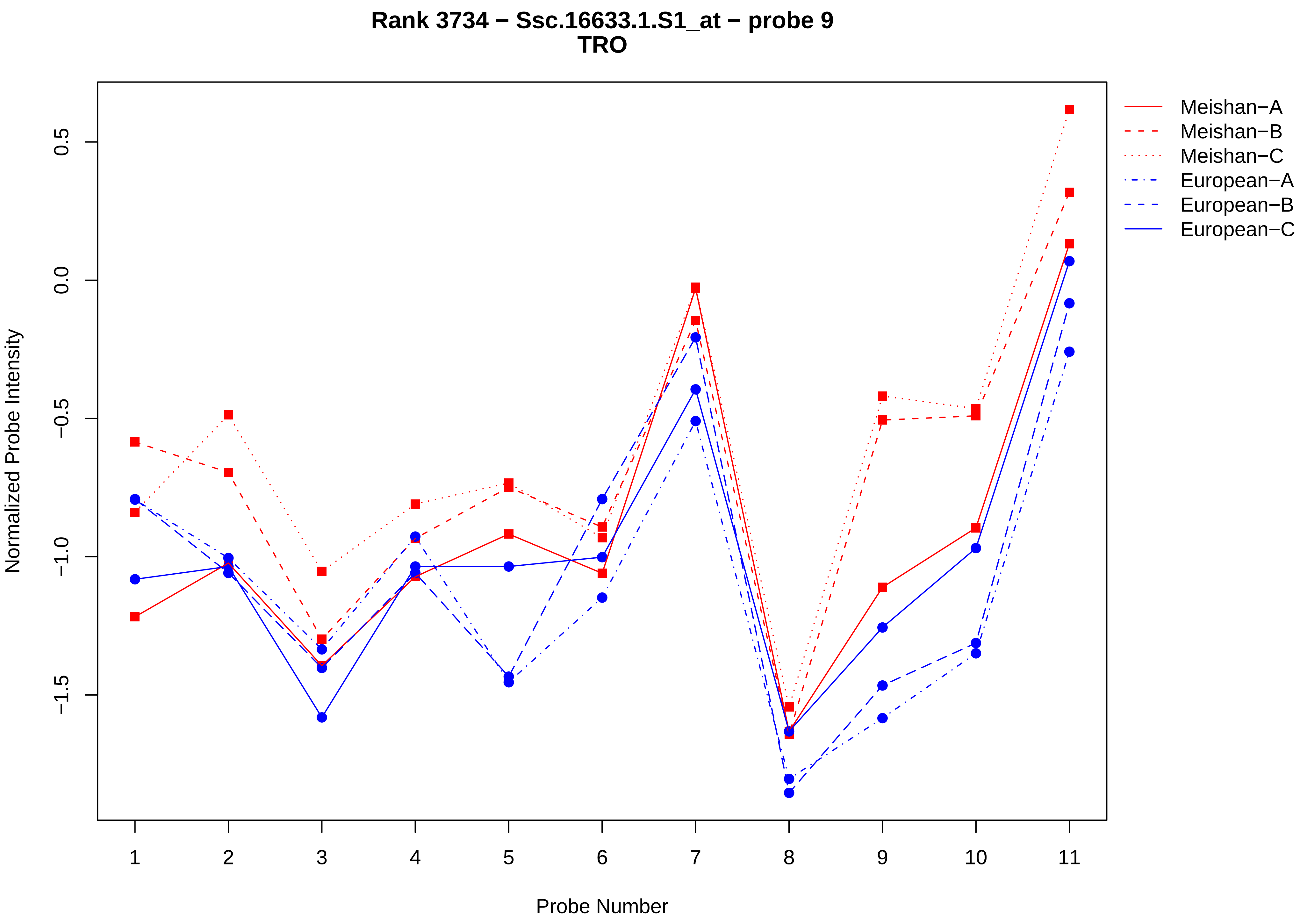
Rank 3734 − Ssc.16633.1.S1_at − probe 9
TRO
Normalized Probe Intensity
Probe Number
0.5
0.0
−0.5
−1.0
−1.5
1
2
3
4
5
6
7
8
9
10
11
Meishan−A
Meishan−B
Meishan−C
European−A
European−B
European−C

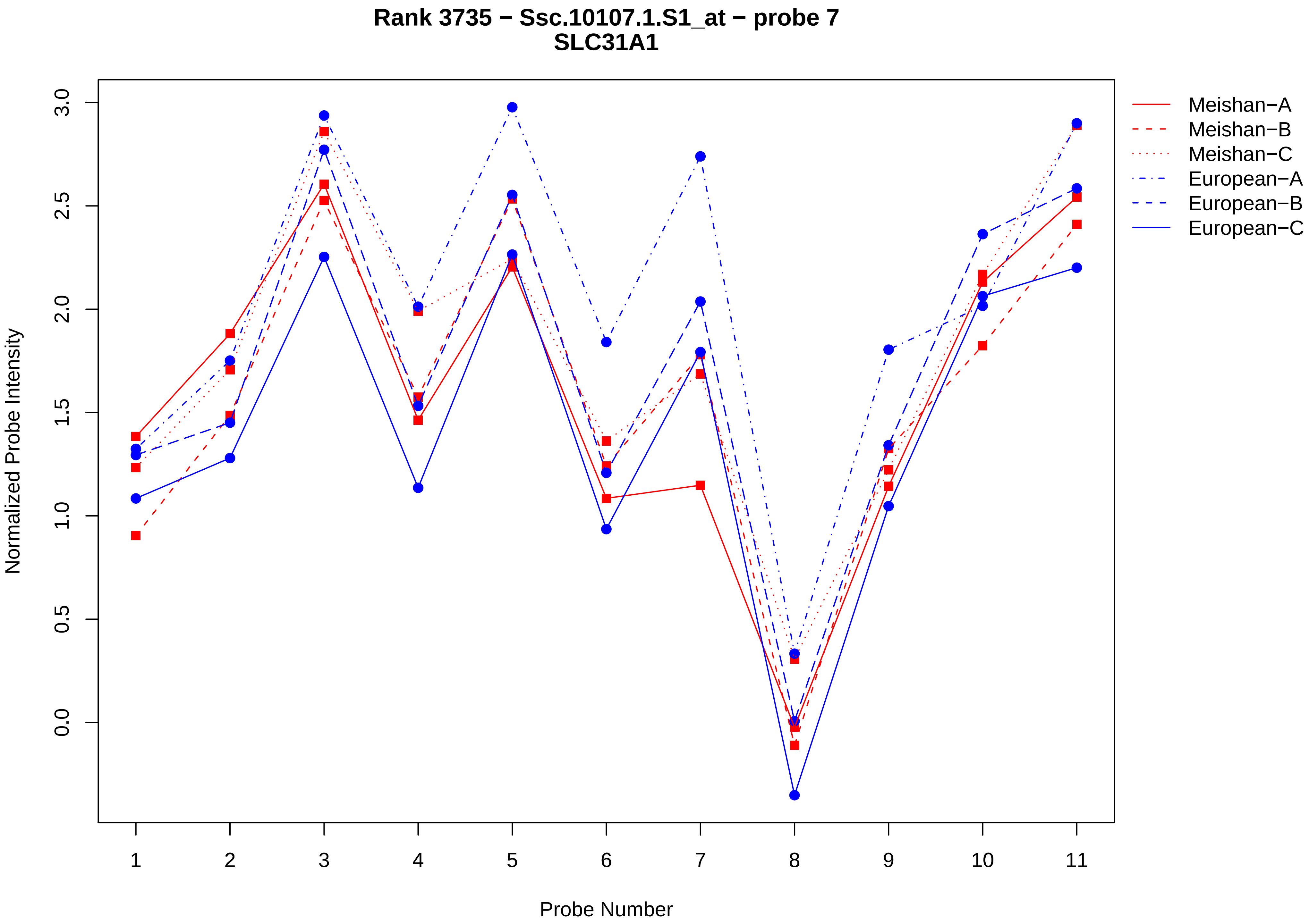

Rank 3735 − Ssc.10107.1.S1_at − probe 7
SLC31A1
Normalized Probe Intensity
Probe Number
0.0
0.5
1.0
1.5
2.0
2.5
3.0
1
2
3
4
5
6
7
8
9
10
11
Meishan−A
Meishan−B
Meishan−C
European−A
European−B
European−C

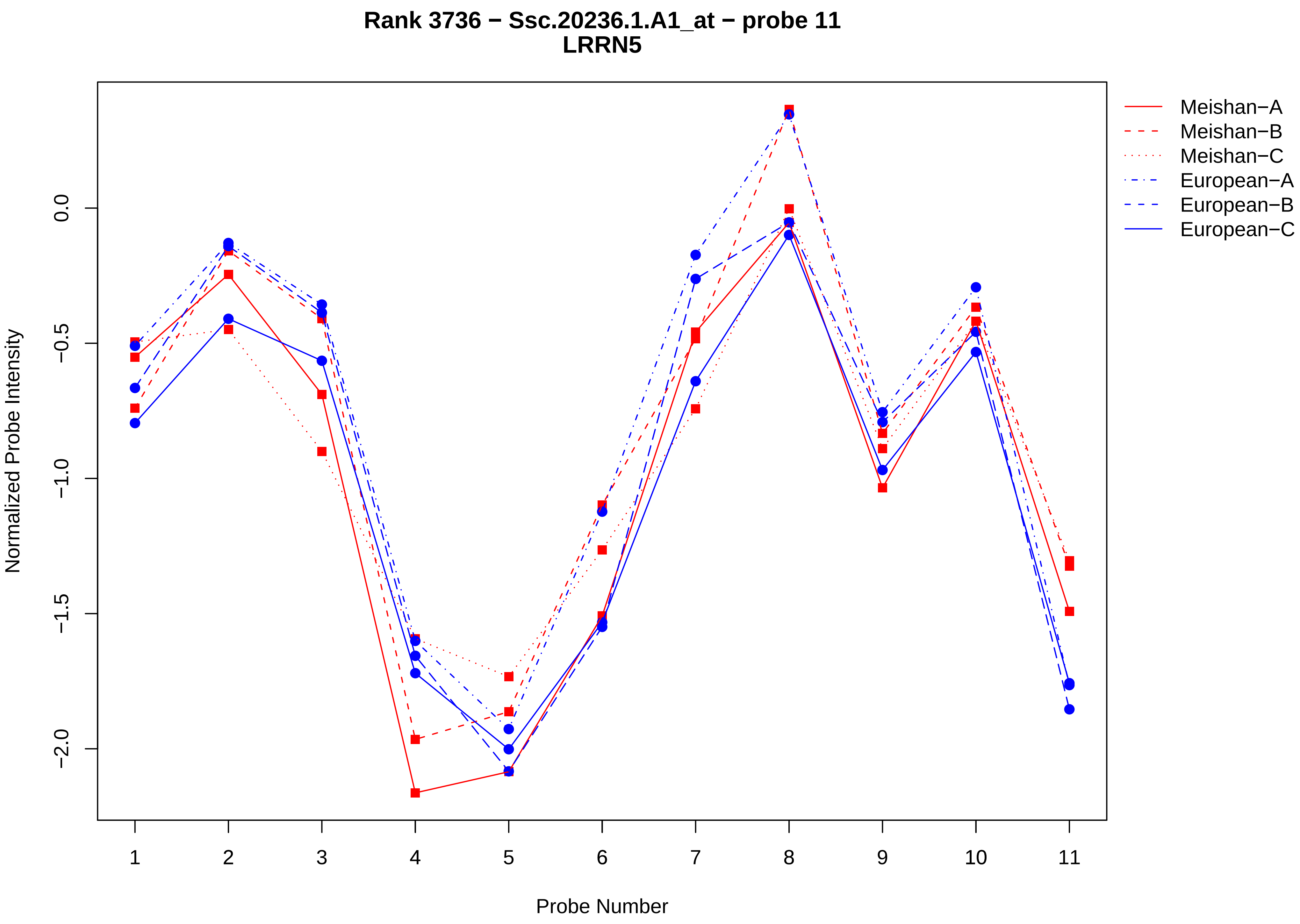

Rank 3736 − Ssc.20236.1.A1_at − probe 11
LRRN5
Normalized Probe Intensity
0.0
−0.5
−1.0
−1.5
−2.0
1
2
3
4
5
6
7
8
9
10
11
Probe Number
Meishan−A
Meishan−B
Meishan−C
European−A
European−B
European−C

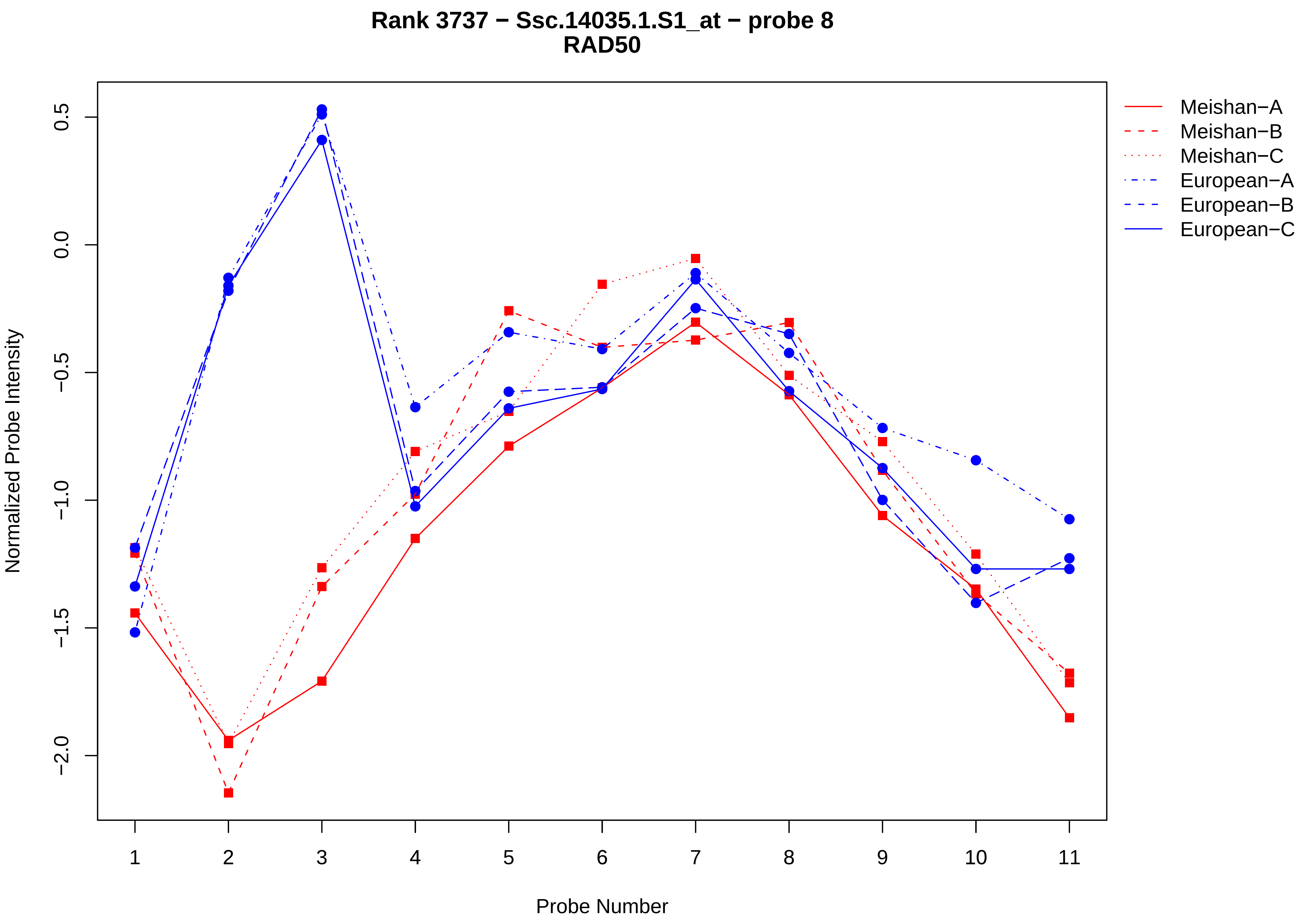
Rank 3737 − Ssc.14035.1.S1_at − probe 8
RAD50
Normalized Probe Intensity
Probe Number
0.5
0.0
−0.5
−1.0
−1.5
−2.0
1
2
3
4
5
6
7
8
9
10
11
Meishan−A
Meishan−B
Meishan−C
European−A
European−B
European−C

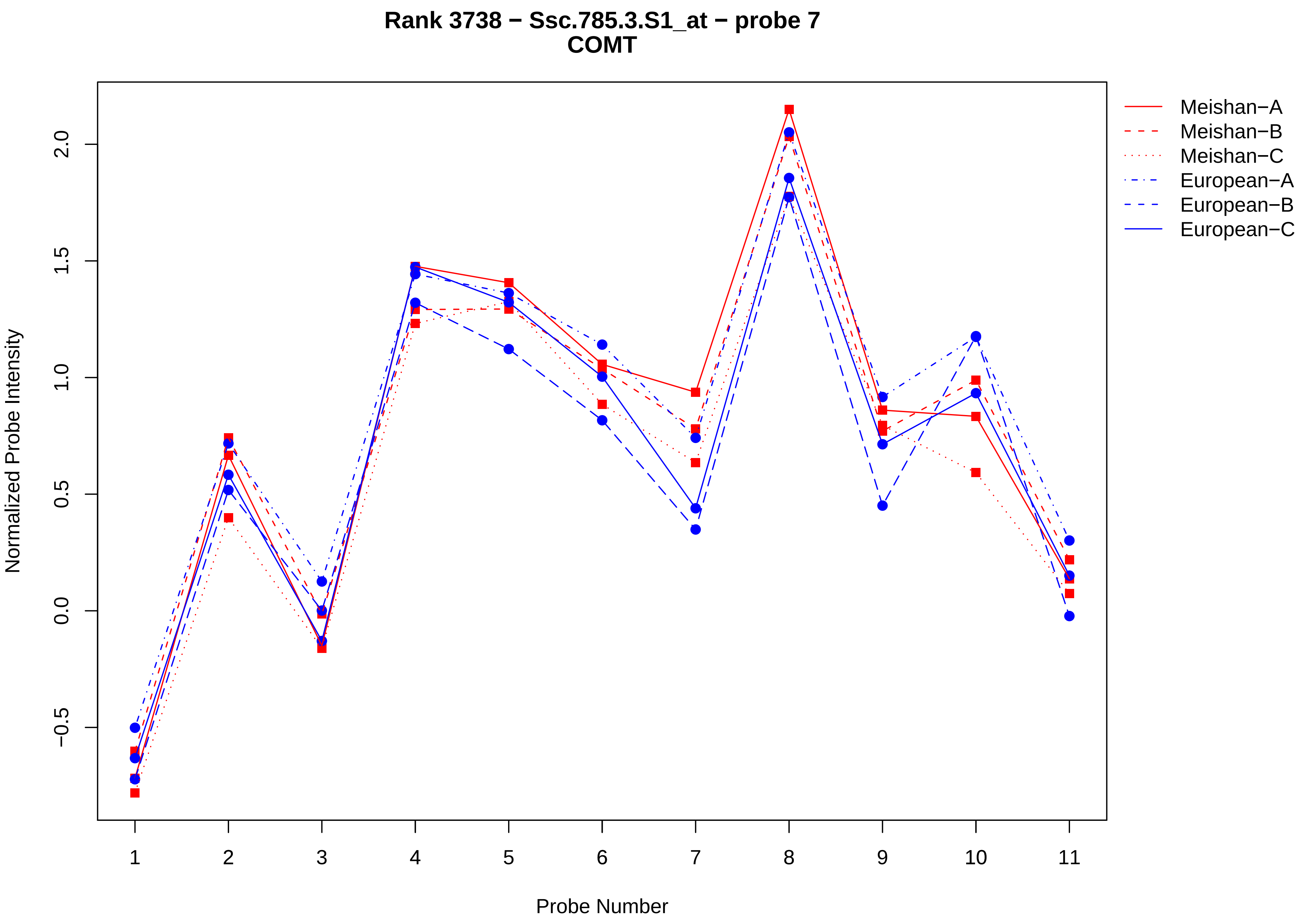

Rank 3738 − Ssc.785.3.S1_at − probe 7
COMT
Normalized Probe Intensity
Probe Number
−0.5
0.0
0.5
1.0
1.5
2.0
1
2
3
4
5
6
7
8
9
10
11
Meishan−A
Meishan−B
Meishan−C
European−A
European−B
European−C

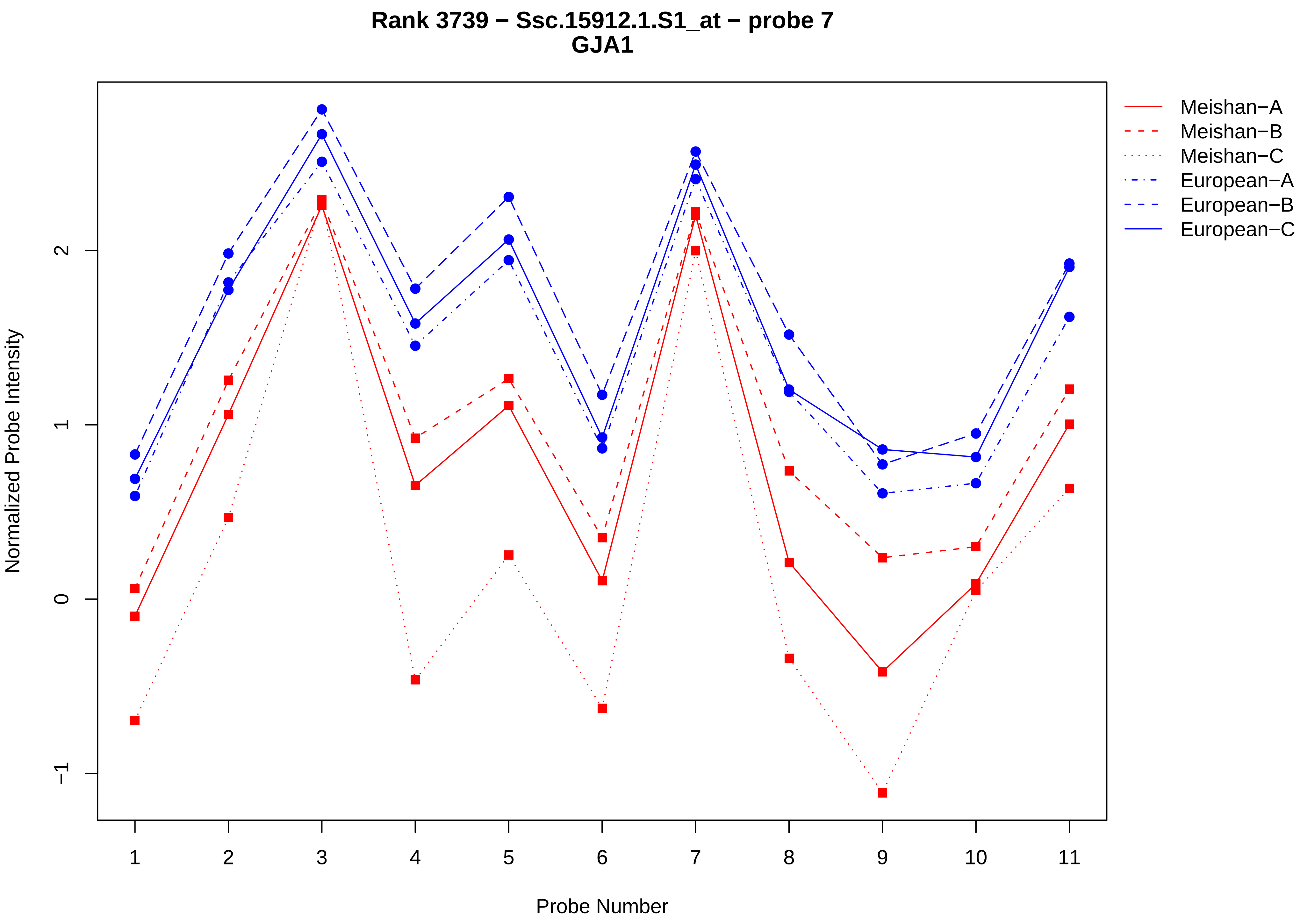

Rank 3739 − Ssc.15912.1.S1_at − probe 7
GJA1
Normalized Probe Intensity
Probe Number
−1
0
1
2
1
2
3
4
5
6
7
8
9
10
11
Meishan−A
Meishan−B
Meishan−C
European−A
European−B
European−C

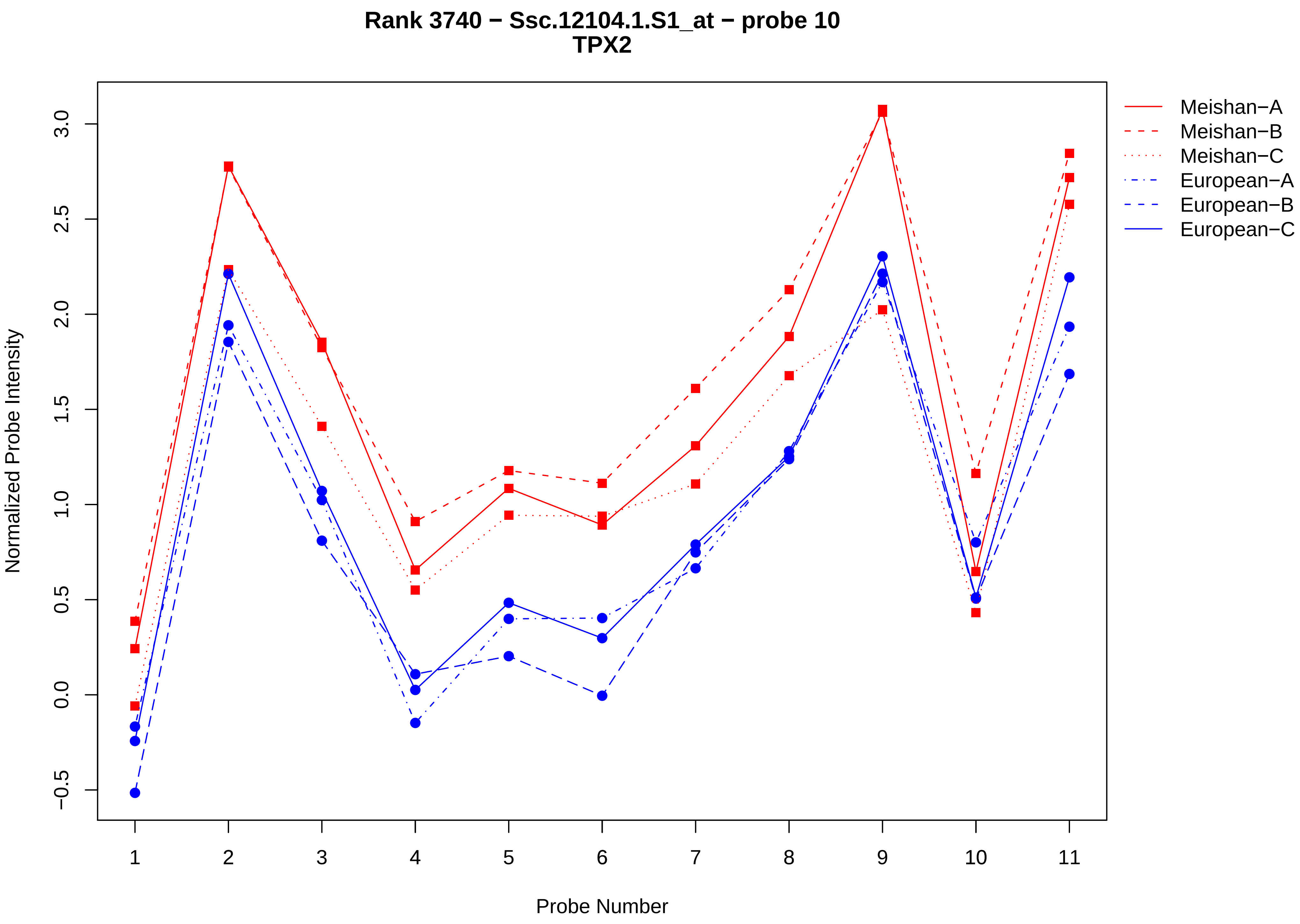

Rank 3740 − Ssc.12104.1.S1_at − probe 10
TPX2
Normalized Probe Intensity
Probe Number
Meishan−A
Meishan−B
Meishan−C
European−A
European−B
European−C
−0.5
0.0
0.5
1.0
1.5
2.0
2.5
3.0
1
2
3
4
5
6
7
8
9
10
11

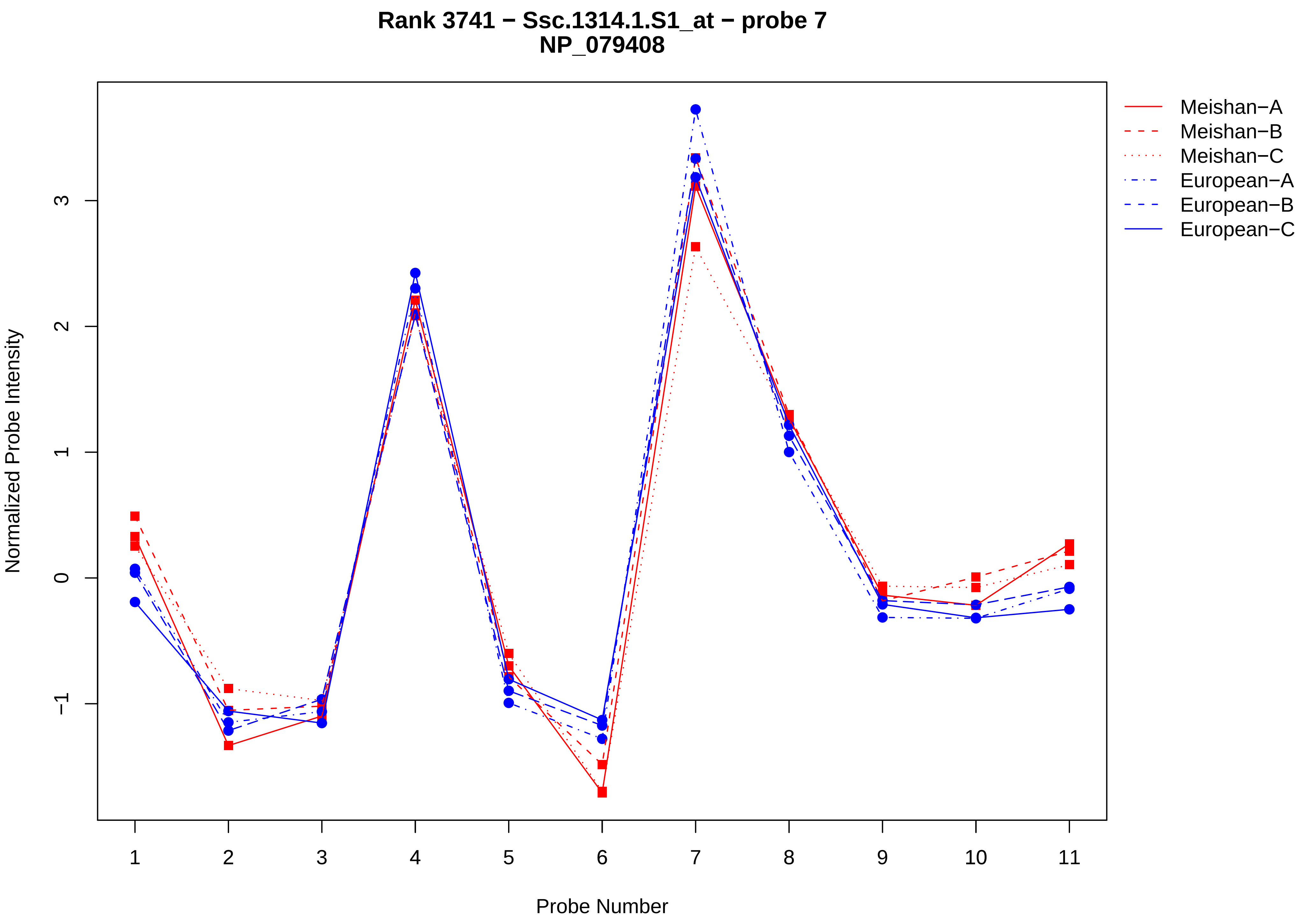

Rank 3741 − Ssc.1314.1.S1_at − probe 7
NP_079408
Normalized Probe Intensity
Probe Number
−1
0
1
2
3
1
2
3
4
5
6
7
8
9
10
11
Meishan−A
Meishan−B
Meishan−C
European−A
European−B
European−C

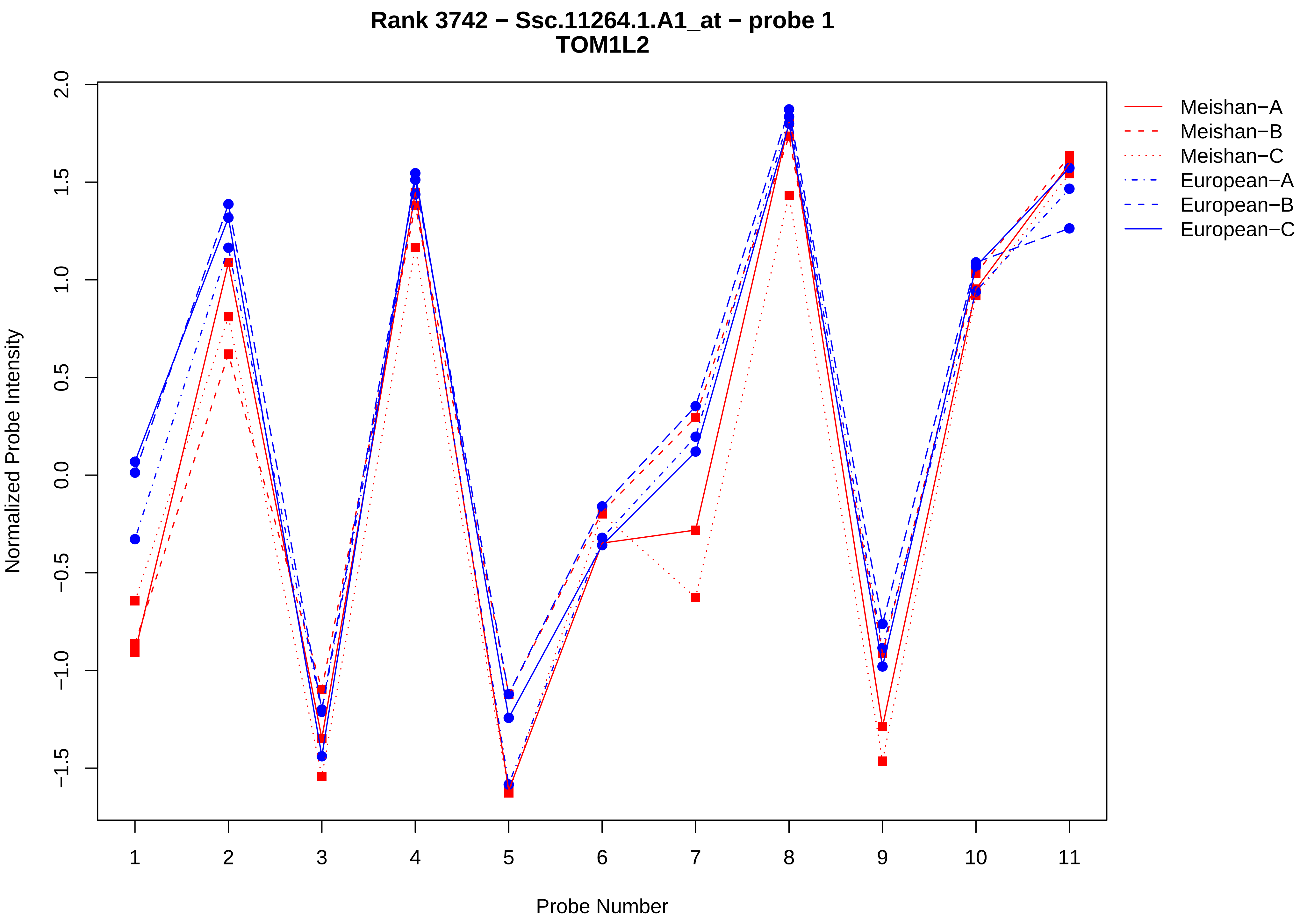

Rank 3742 − Ssc.11264.1.A1_at − probe 1
TOM1L2
Normalized Probe Intensity
Probe Number
Meishan−A
Meishan−B
Meishan−C
European−A
European−B
European−C
2.0
1.5
1.0
0.5
0.0
−0.5
−1.0
−1.5
1
2
3
4
5
6
7
8
9
10
11

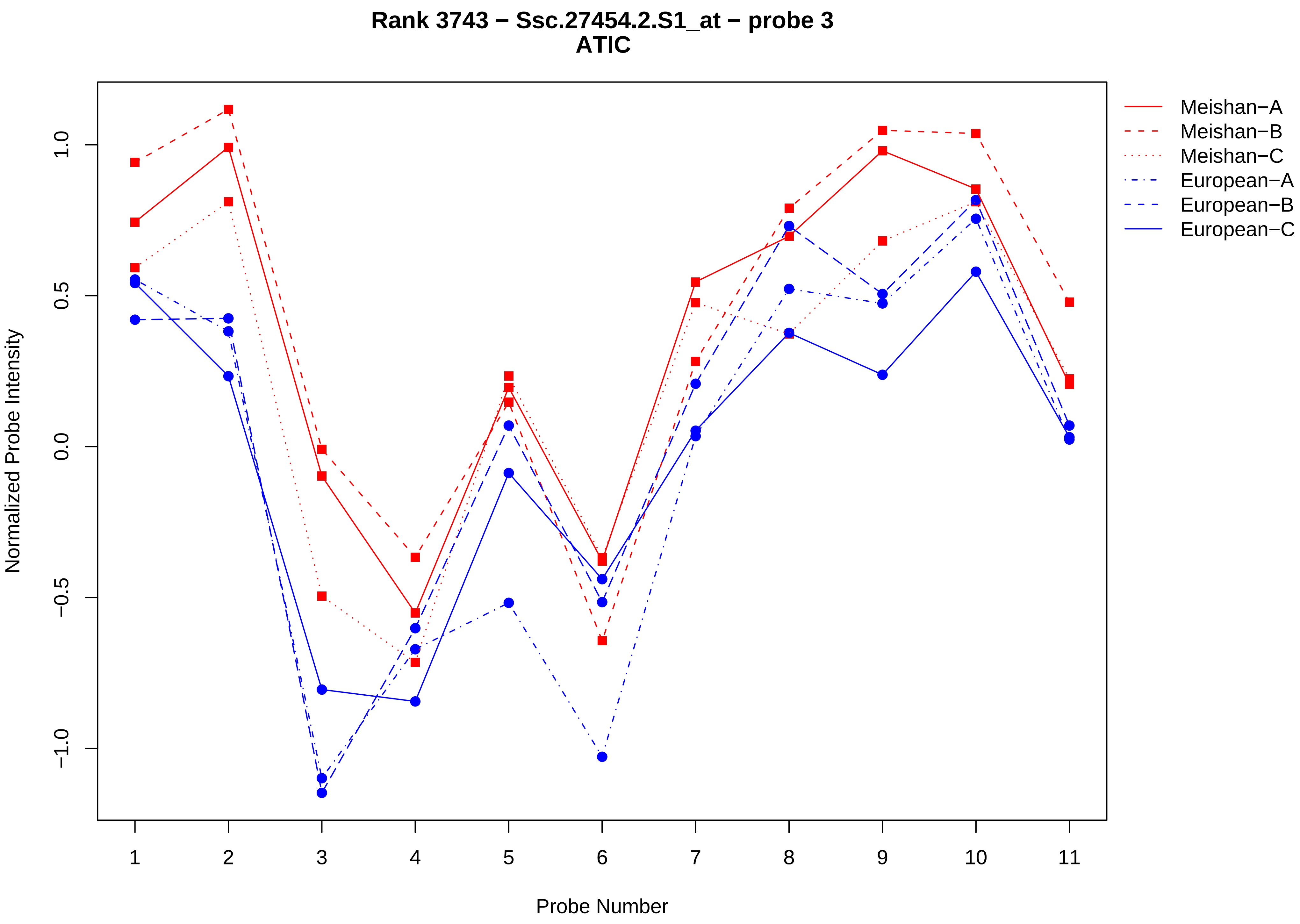

Rank 3743 − Ssc.27454.2.S1_at − probe 3
ATIC
Normalized Probe Intensity
Probe Number
1.0
0.5
0.0
−0.5
−1.0
1
2
3
4
5
6
7
8
9
10
11
Meishan−A
Meishan−B
Meishan−C
European−A
European−B
European−C

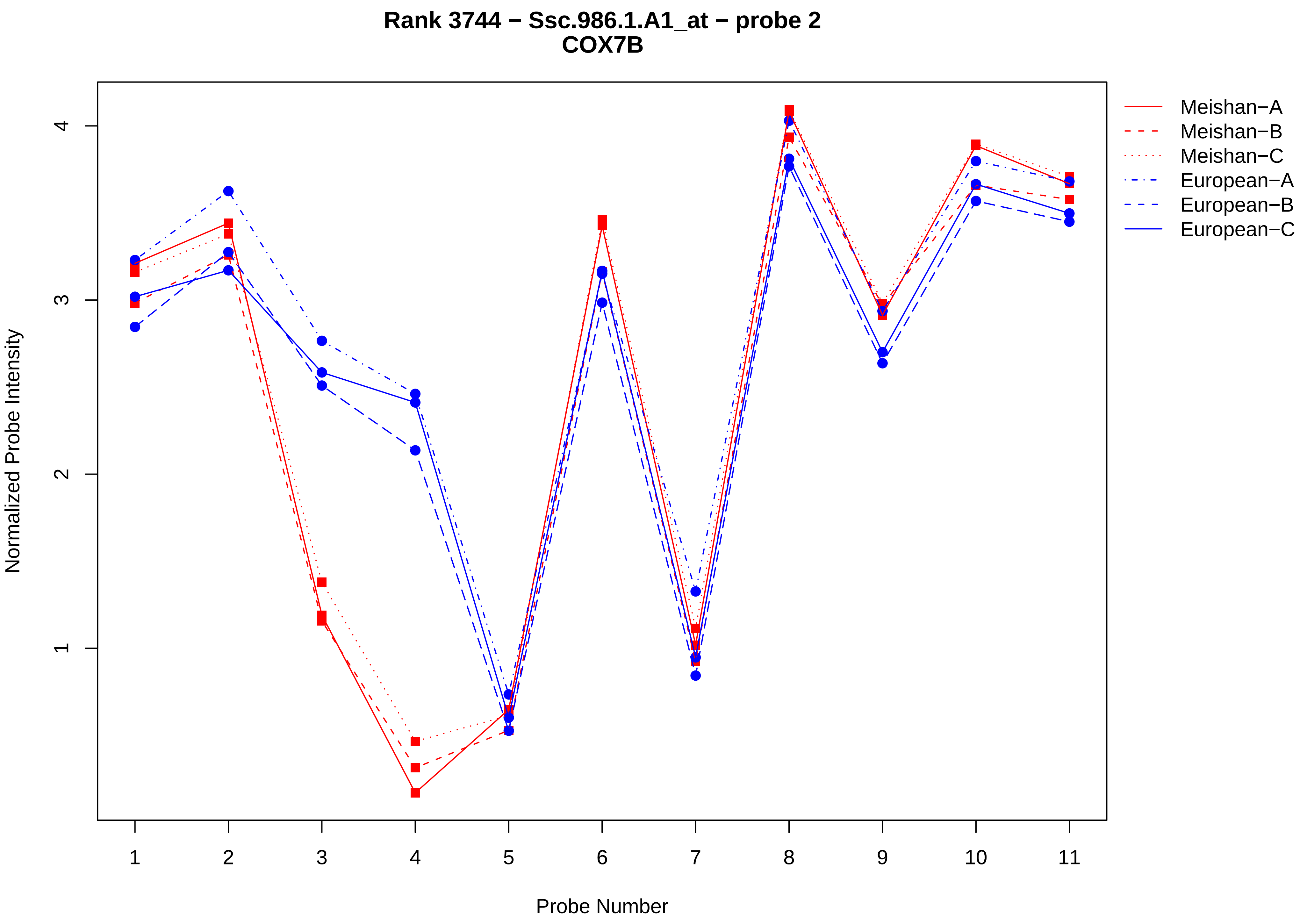

Rank 3744 − Ssc.986.1.A1_at − probe 2
COX7B
Normalized Probe Intensity
Probe Number
1
2
3
4
1
2
3
4
5
6
7
8
9
10
11
Meishan−A
Meishan−B
Meishan−C
European−A
European−B
European−C

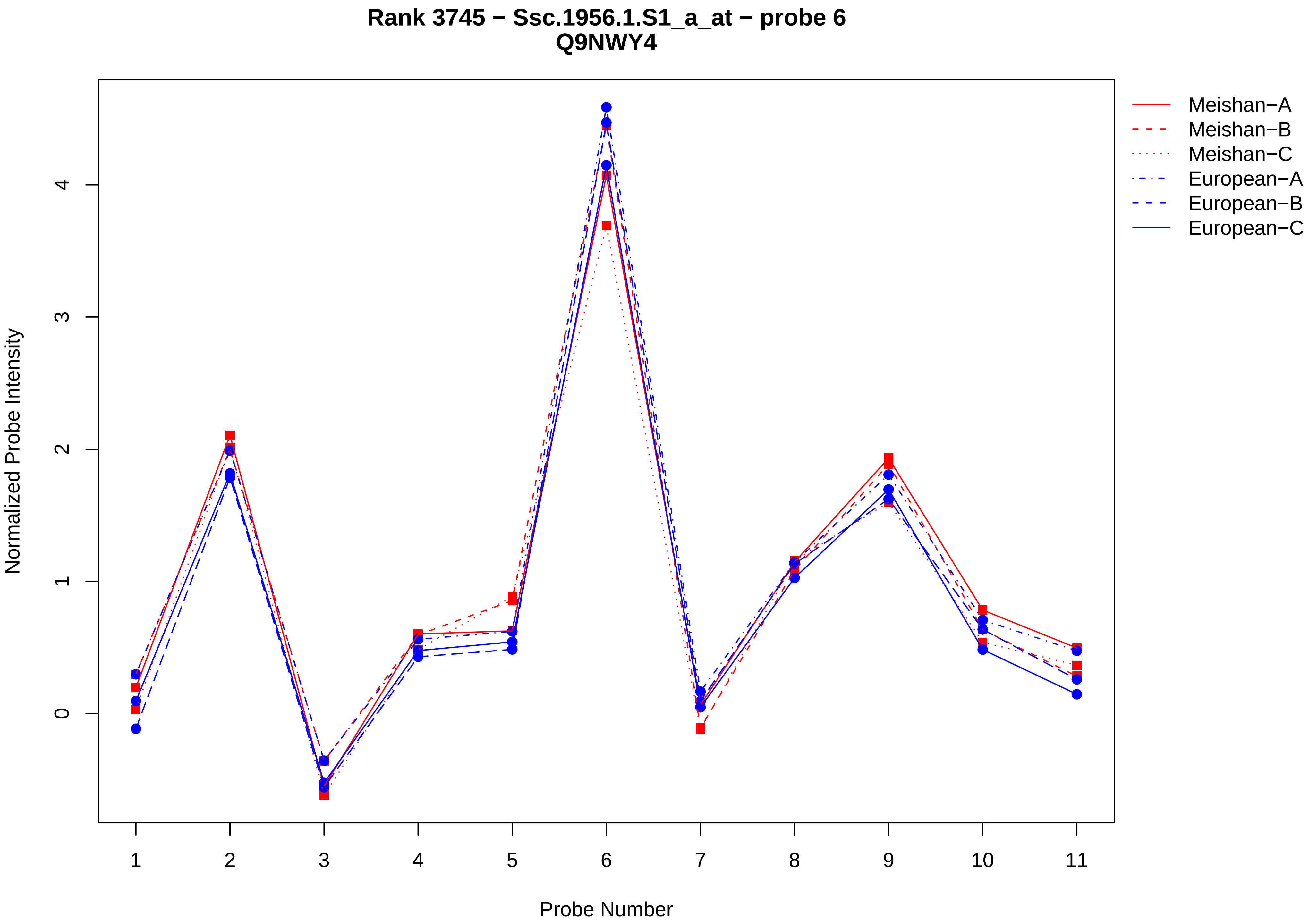

Rank 3745 − Ssc.1956.1.S1_a_at − probe 6
Q9NWY4
Normalized Probe Intensity
Probe Number
0
1
2
3
4
1
2
3
4
5
6
7
8
9
10
11
Meishan−A
Meishan−B
Meishan−C
European−A
European−B
European−C

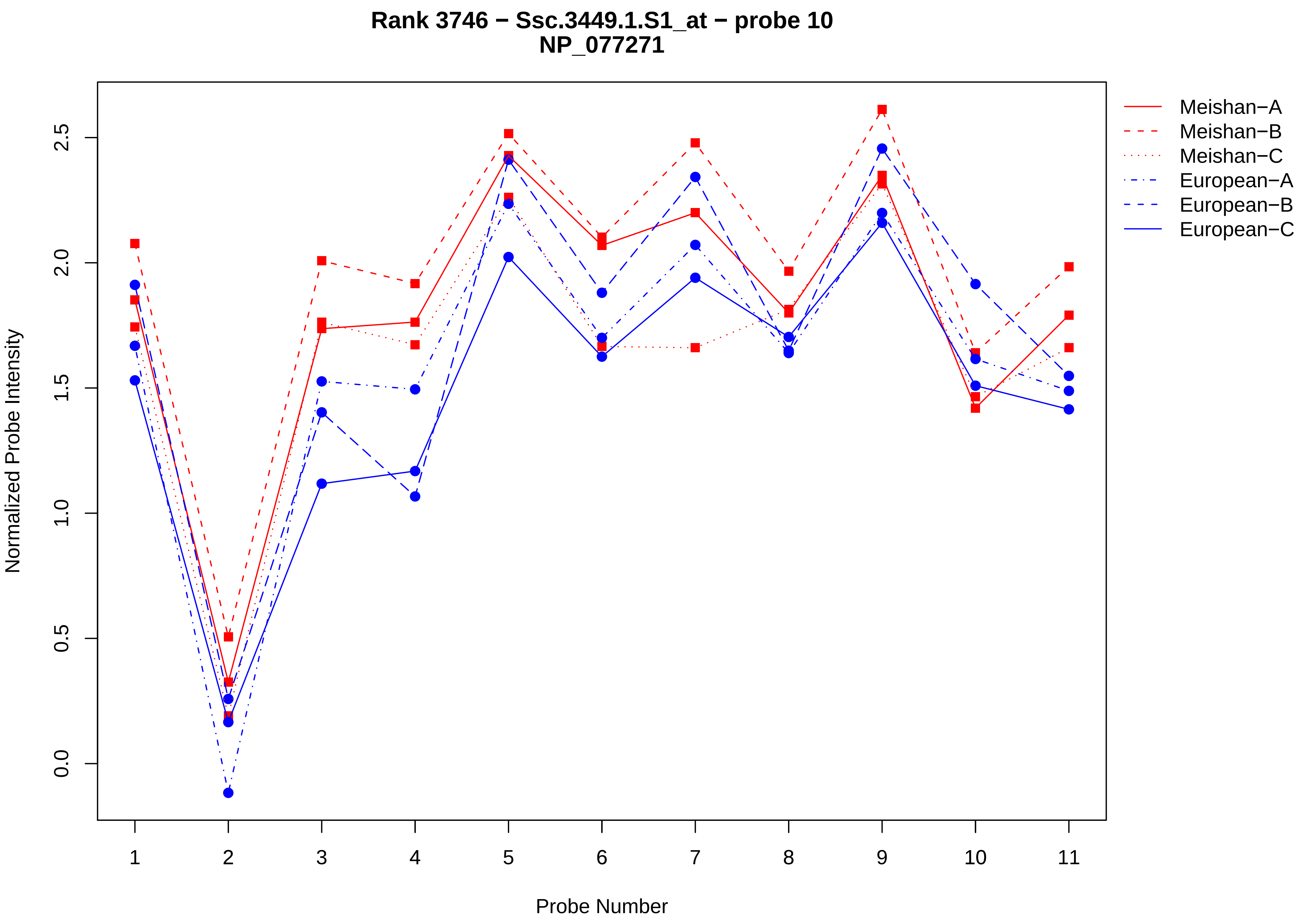

Rank 3746 − Ssc.3449.1.S1_at − probe 10
NP_077271
Normalized Probe Intensity
Probe Number
0.0
0.5
1.0
1.5
2.0
2.5
1
2
3
4
5
6
7
8
9
10
11
Meishan−A
Meishan−B
Meishan−C
European−A
European−B
European−C

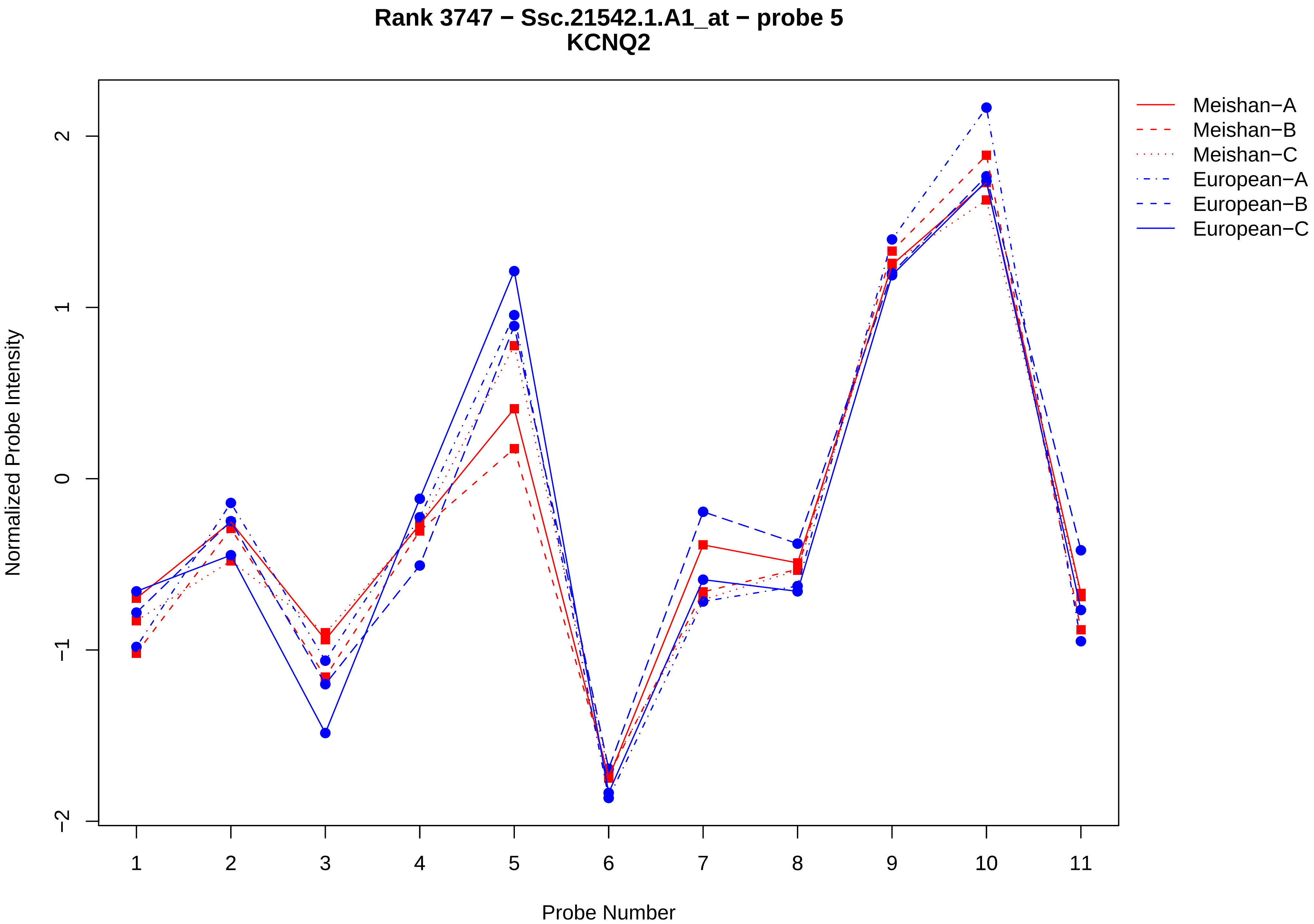

Rank 3747 − Ssc.21542.1.A1_at − probe 5
KCNQ2
Normalized Probe Intensity
Probe Number
−2
−1
0
1
2
1
2
3
4
5
6
7
8
9
10
11
Meishan−A
Meishan−B
Meishan−C
European−A
European−B
European−C

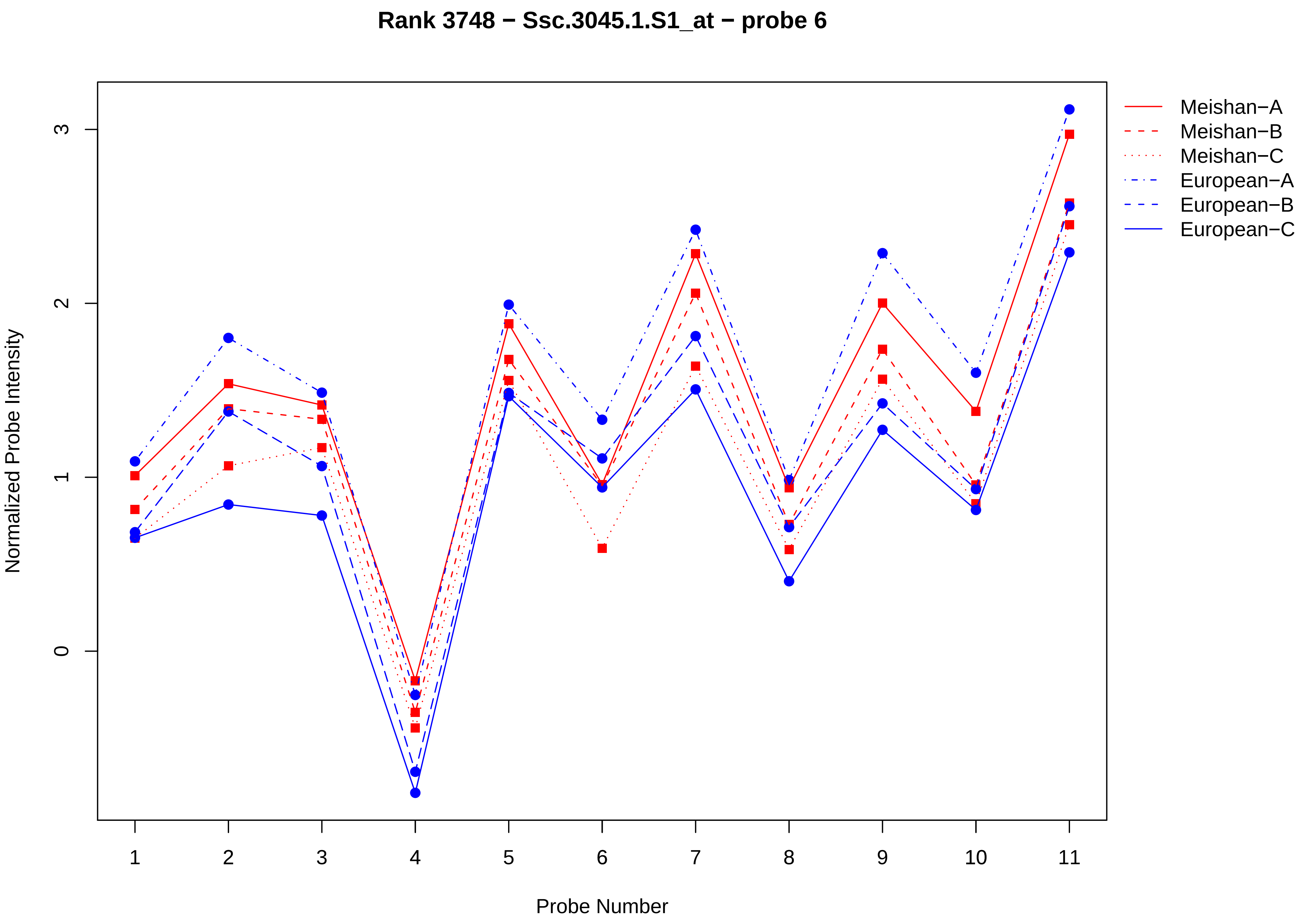

Rank 3748 − Ssc.3045.1.S1_at − probe 6
Normalized Probe Intensity
0
1
2
3
1
2
3
4
5
6
7
8
9
10
11
Probe Number
Meishan−A
Meishan−B
Meishan−C
European−A
European−B
European−C

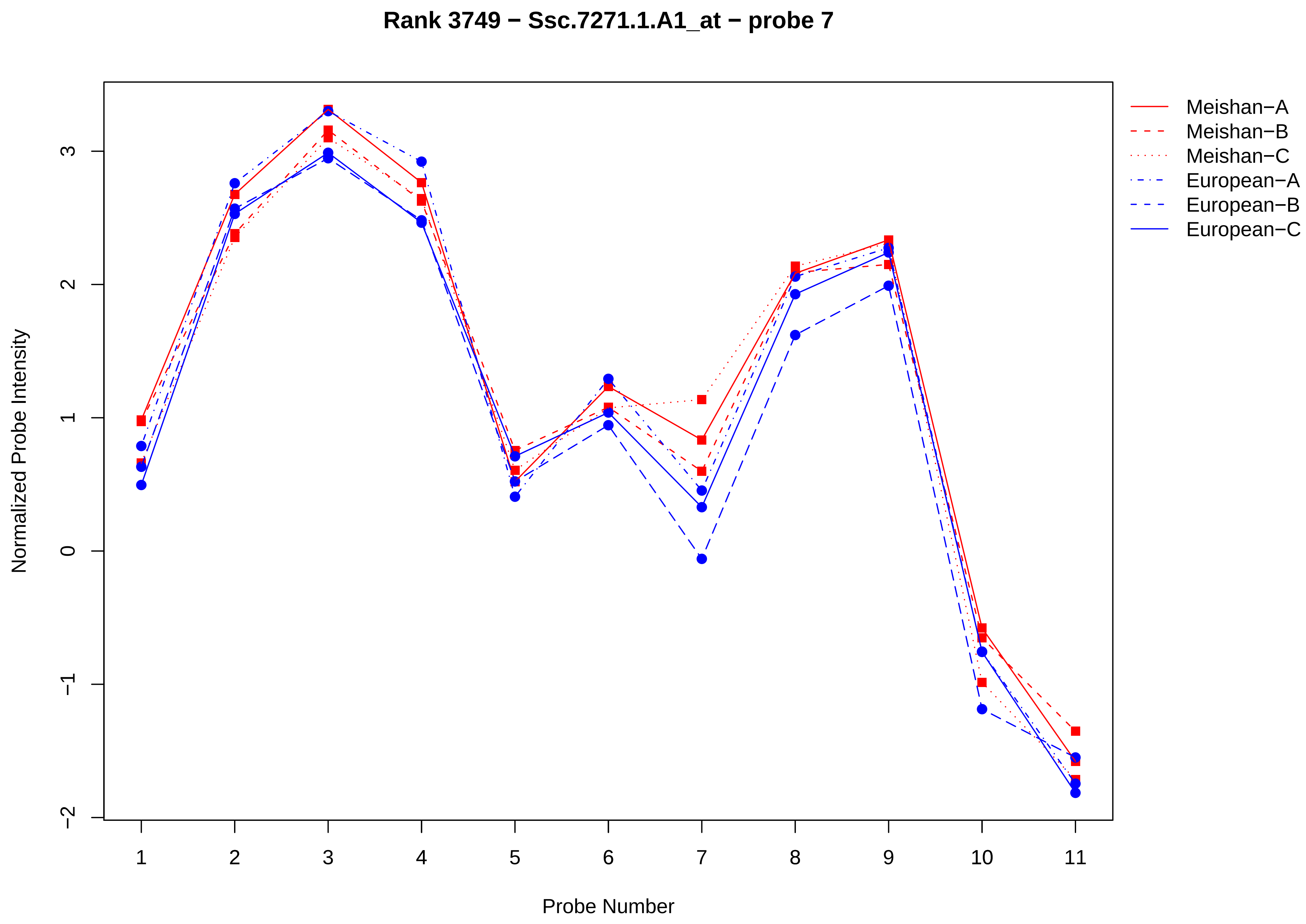

Rank 3749 − Ssc.7271.1.A1_at − probe 7
Normalized Probe Intensity
Probe Number
−2
−1
0
1
2
3
1
2
3
4
5
6
7
8
9
10
11
Meishan−A
Meishan−B
Meishan−C
European−A
European−B
European−C

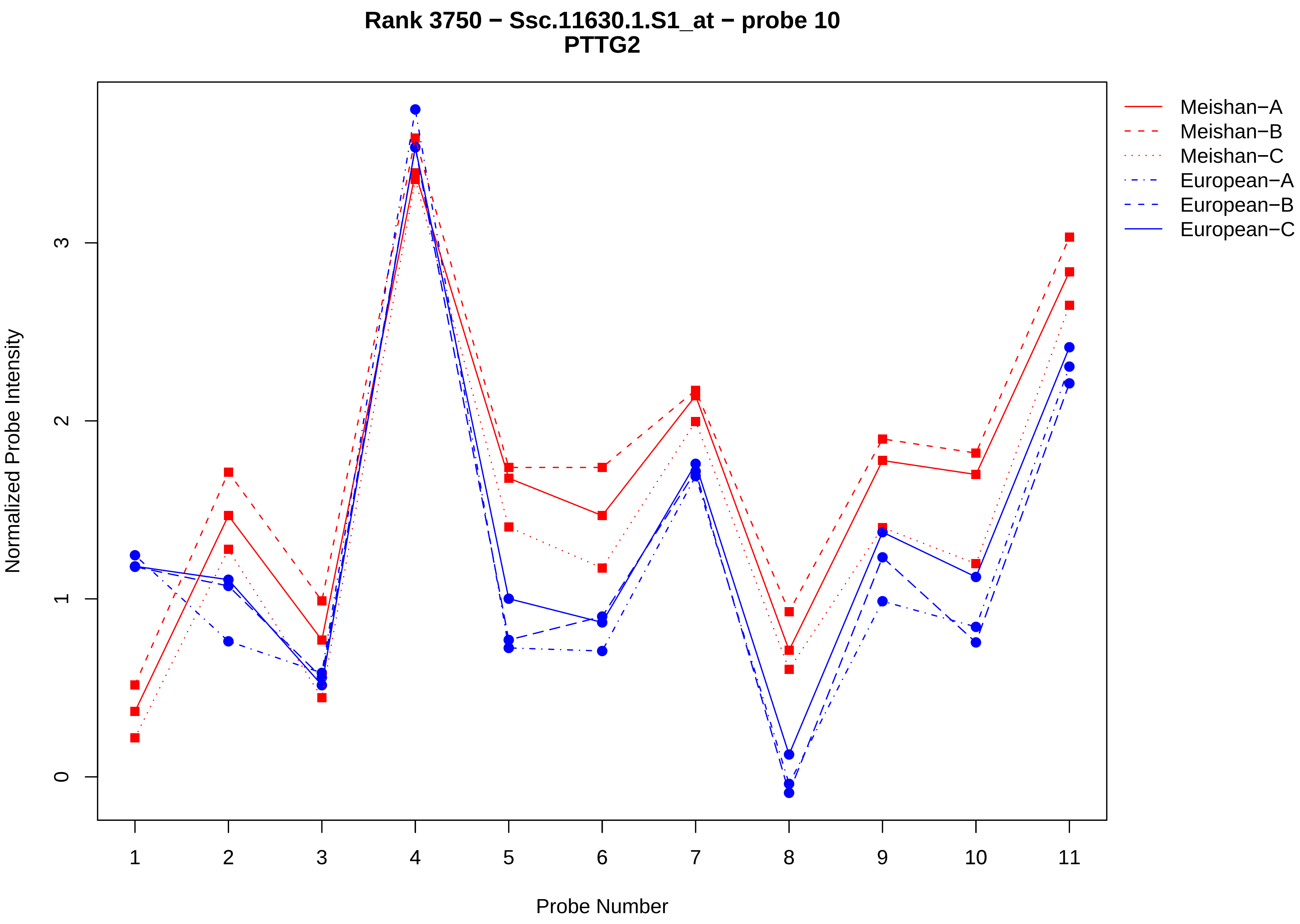

Rank 3750 − Ssc.11630.1.S1_at − probe 10
PTTG2
Normalized Probe Intensity
Probe Number
0
1
2
3
1
2
3
4
5
6
7
8
9
10
11
Meishan−A
Meishan−B
Meishan−C
European−A
European−B
European−C

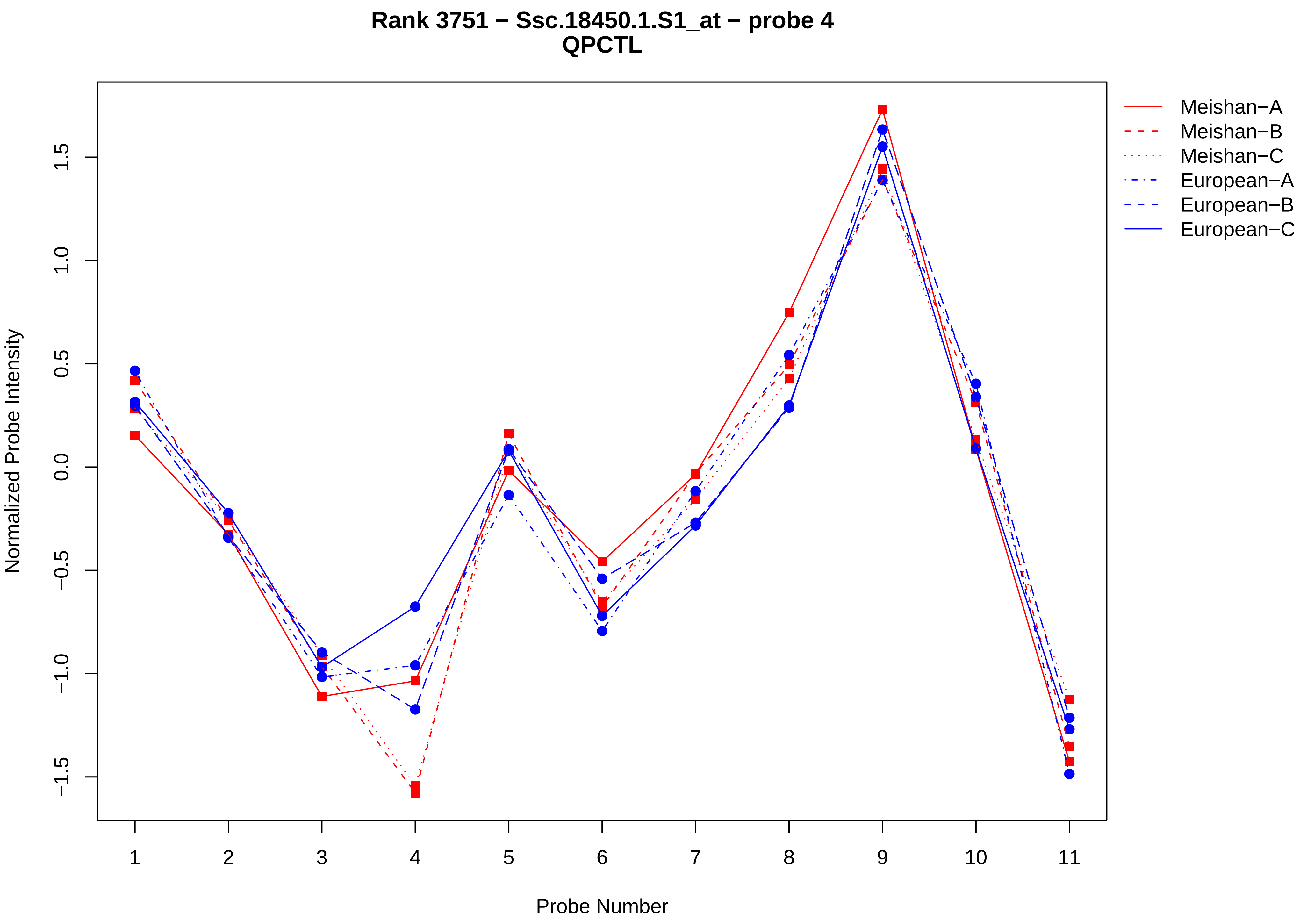

Rank 3751 − Ssc.18450.1.S1_at − probe 4
QPCTL
Normalized Probe Intensity
Probe Number
−1.5
−1.0
−0.5
0.0
0.5
1.0
1.5
1
2
3
4
5
6
7
8
9
10
11
Meishan−A
Meishan−B
Meishan−C
European−A
European−B
European−C

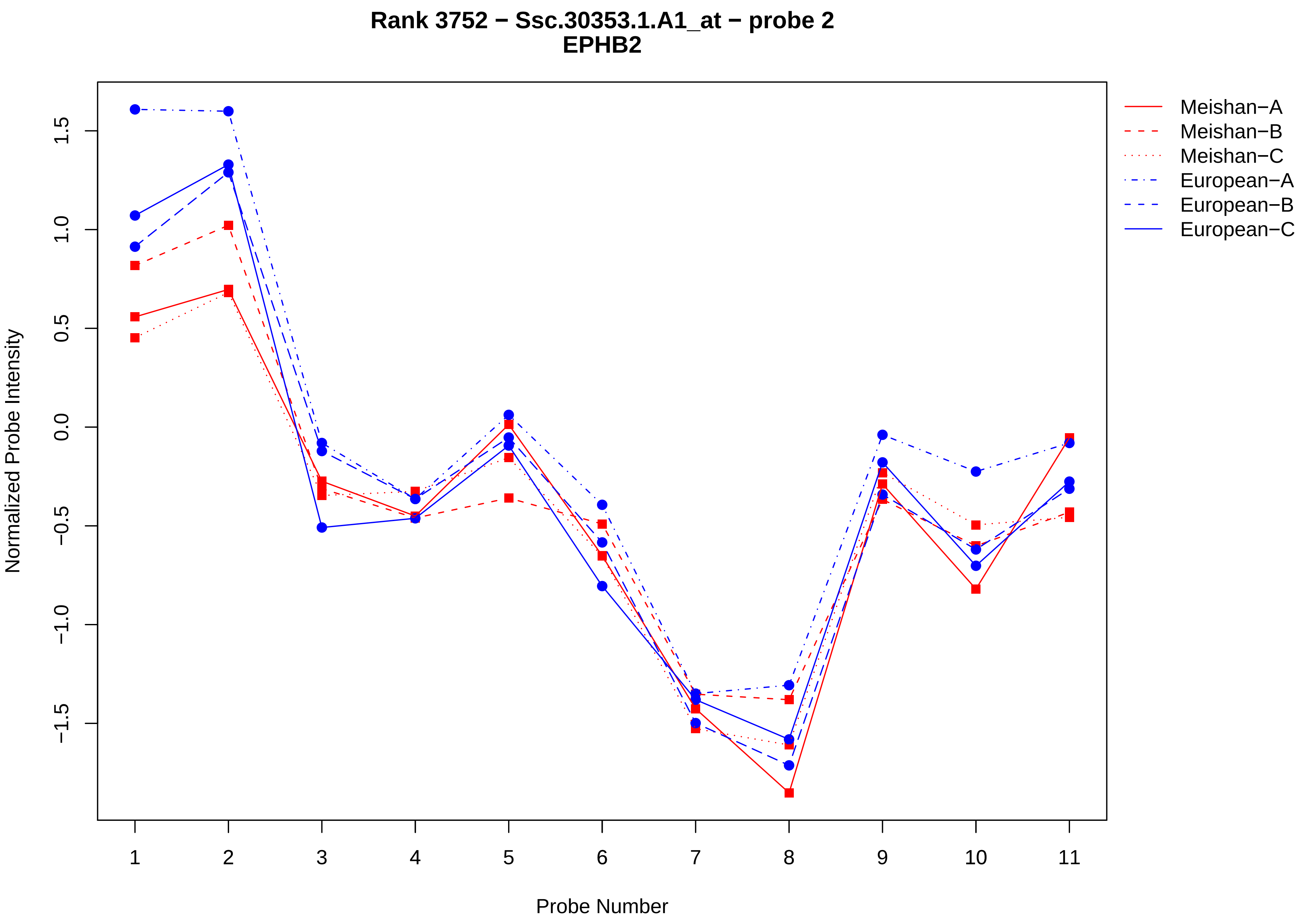
Rank 3752 − Ssc.30353.1.A1_at − probe 2
EPHB2
Normalized Probe Intensity
Probe Number
1.5
1.0
0.5
0.0
−0.5
−1.0
−1.5
1
2
3
4
5
6
7
8
9
10
11
Meishan−A
Meishan−B
Meishan−C
European−A
European−B
European−C

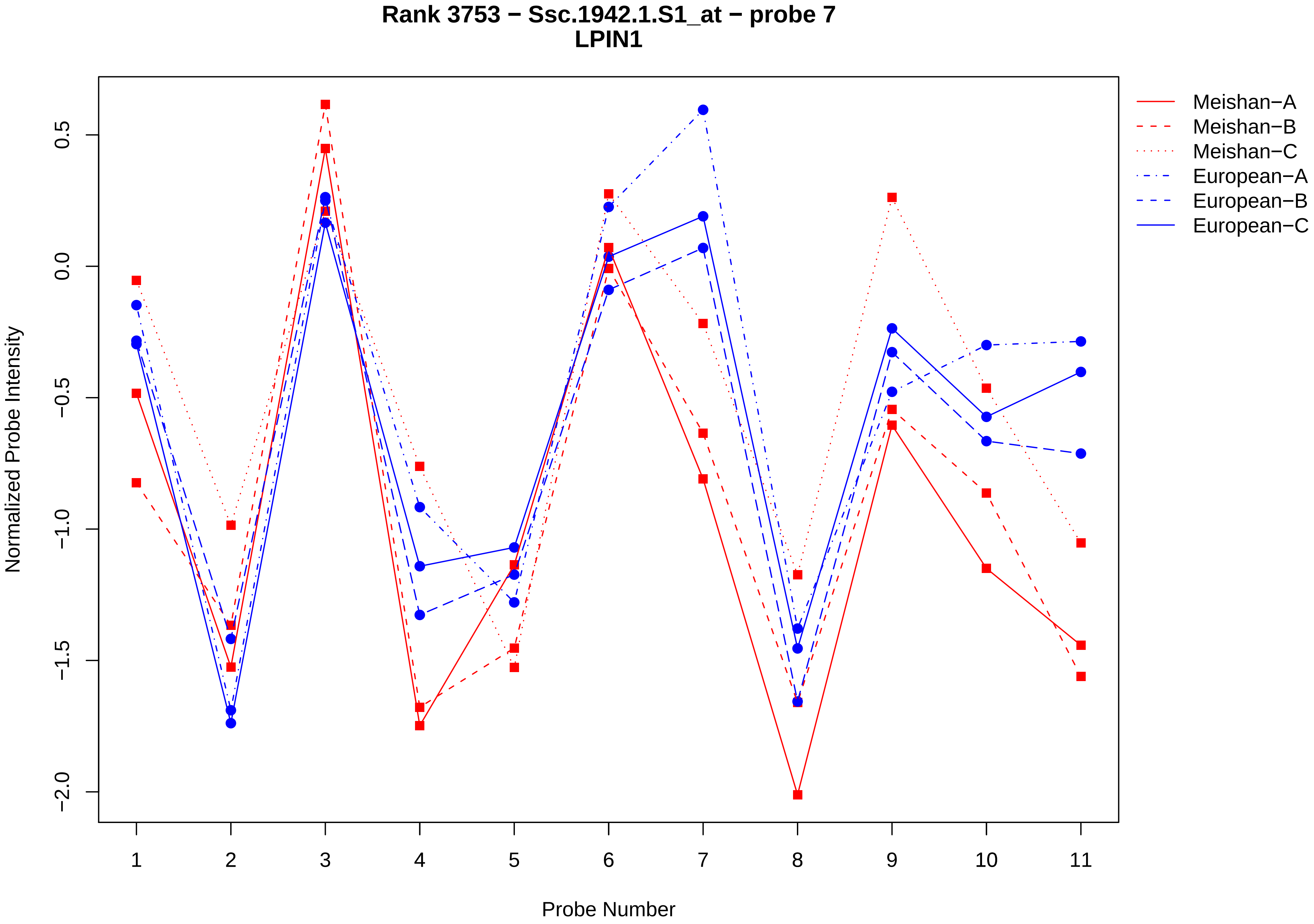

Rank 3753 − Ssc.1942.1.S1_at − probe 7
LPIN1
Normalized Probe Intensity
Probe Number
0.5
0.0
−0.5
−1.0
−1.5
−2.0
1
2
3
4
5
6
7
8
9
10
11
Meishan−A
Meishan−B
Meishan−C
European−A
European−B
European−C

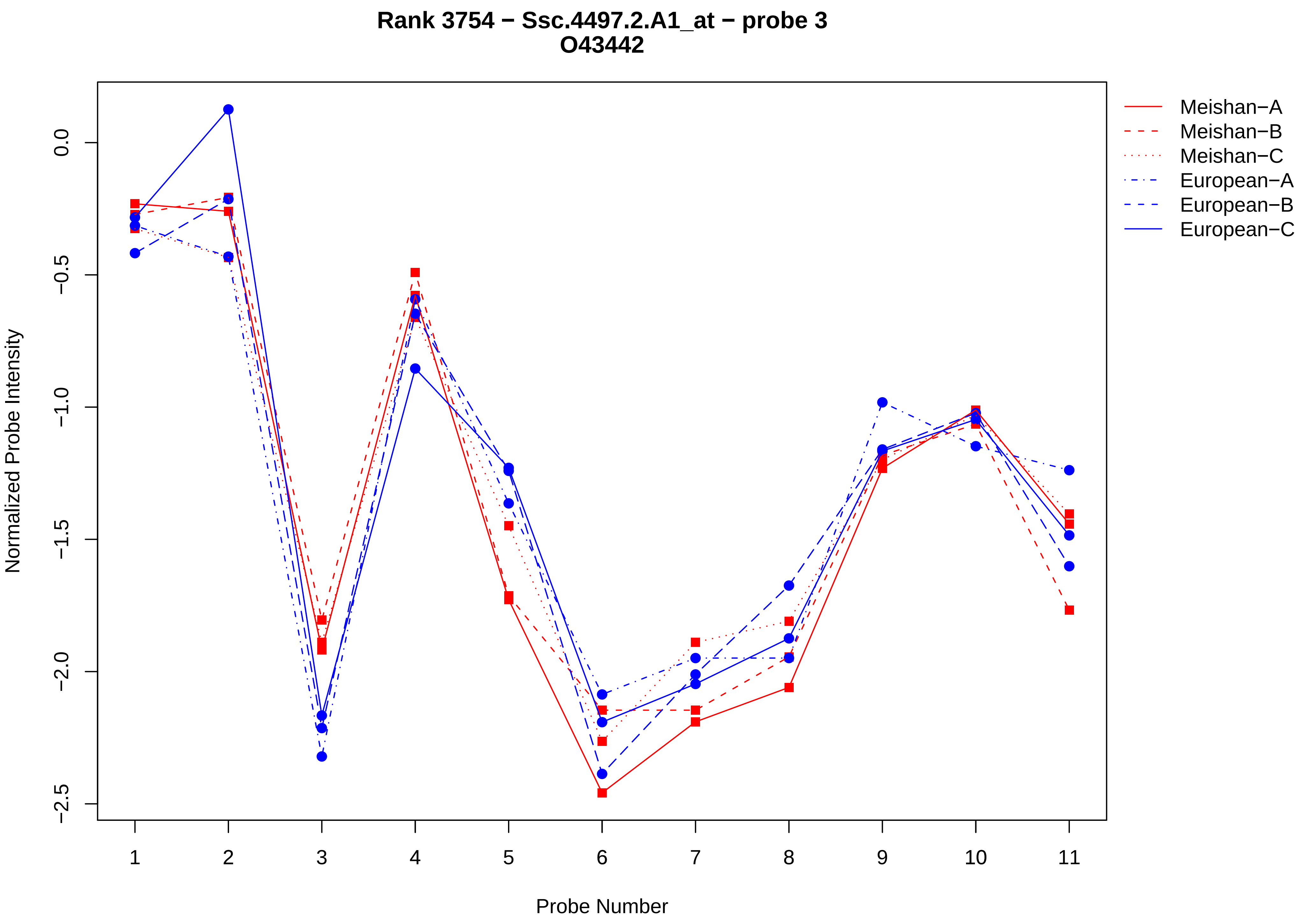

Rank 3754 − Ssc.4497.2.A1_at − probe 3
O43442
Normalized Probe Intensity
0.0
−0.5
−1.0
−1.5
−2.0
−2.5
1
2
3
4
5
6
7
8
9
10
11
Probe Number
Meishan−A
Meishan−B
Meishan−C
European−A
European−B
European−C

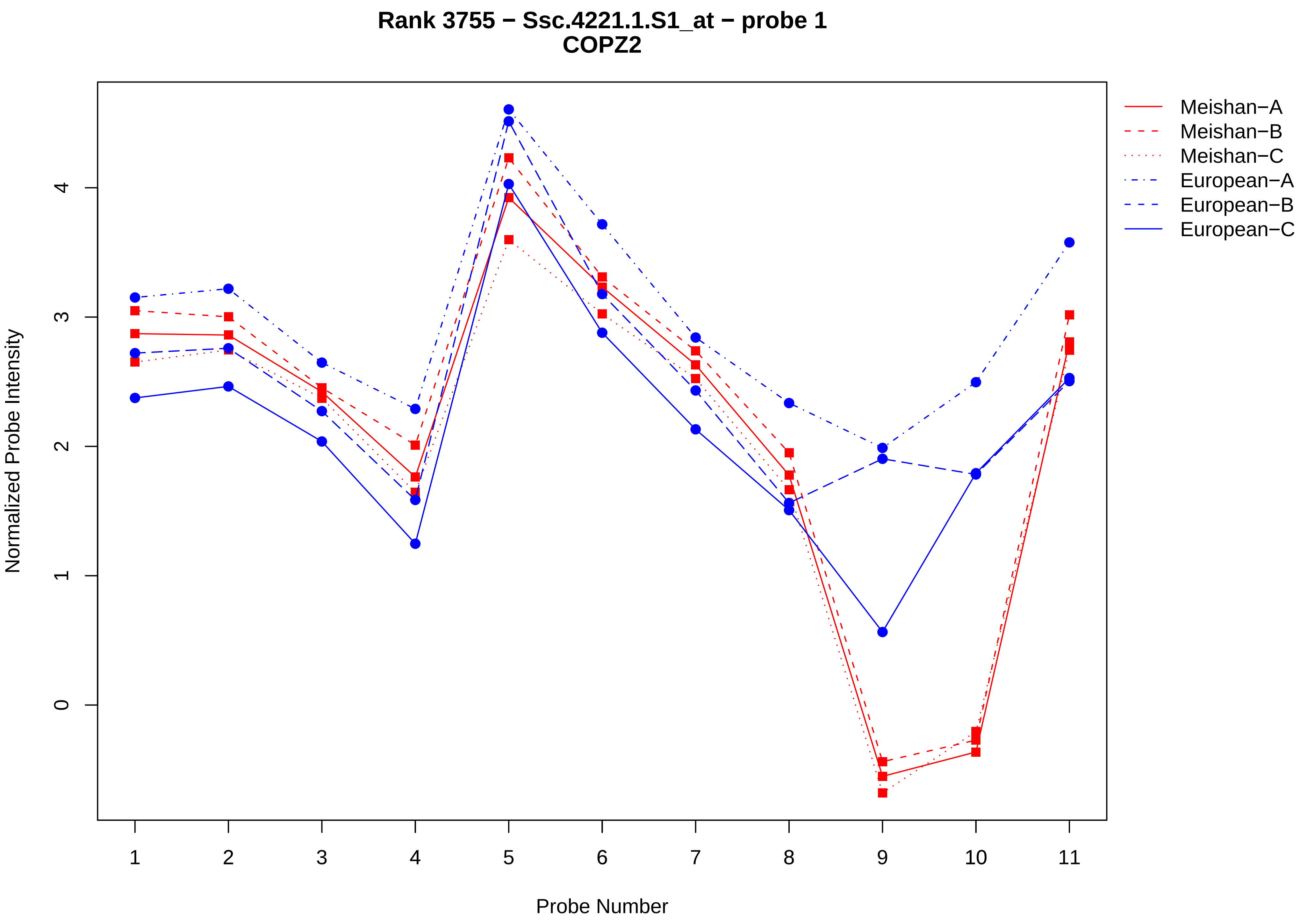

Rank 3755 − Ssc.4221.1.S1_at − probe 1
COPZ2
Normalized Probe Intensity
Probe Number
0
1
2
3
4
1
2
3
4
5
6
7
8
9
10
11
Meishan−A
Meishan−B
Meishan−C
European−A
European−B
European−C

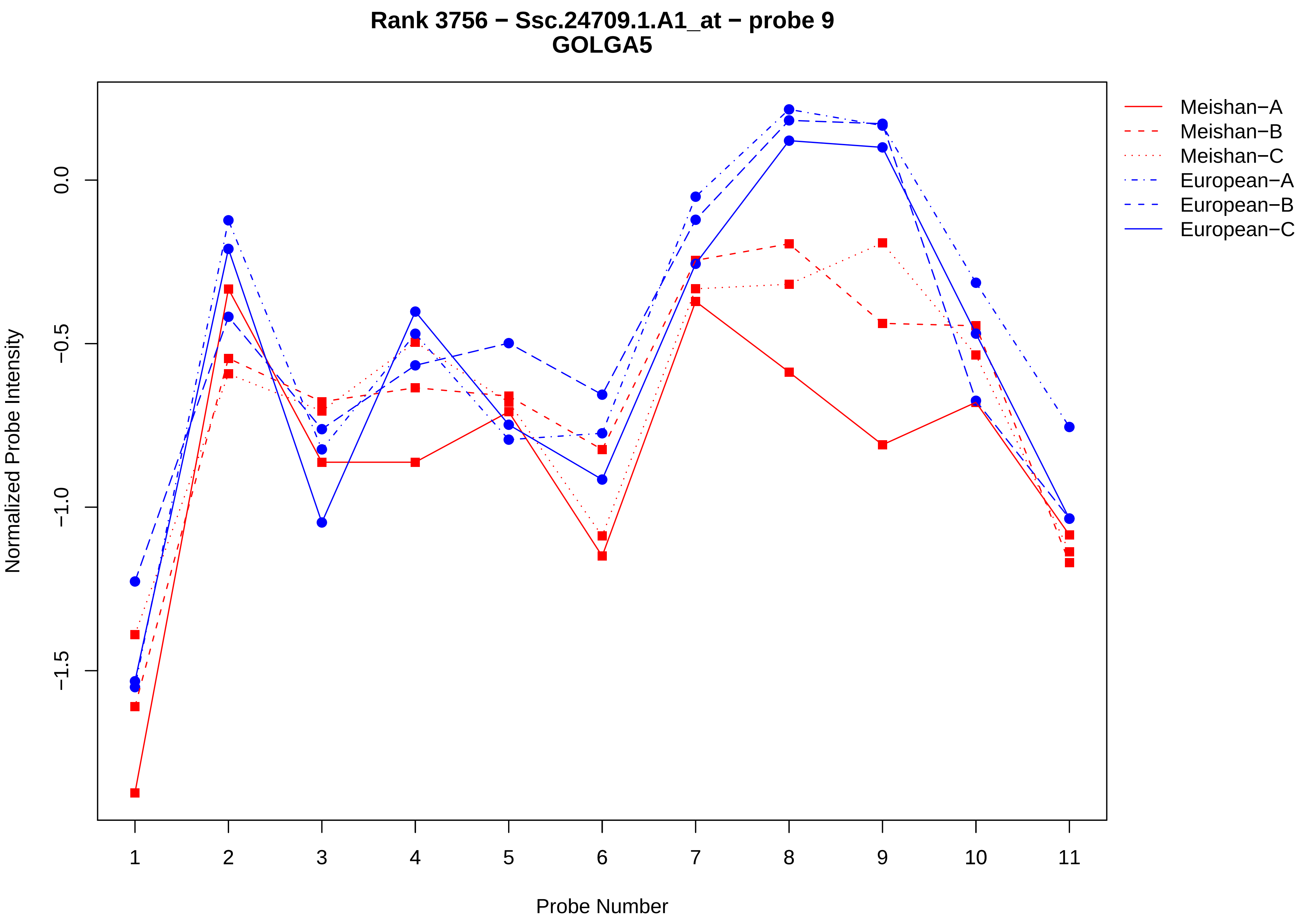

Rank 3756 − Ssc.24709.1.A1_at − probe 9
GOLGA5
Normalized Probe Intensity
Probe Number
0.0
−0.5
−1.0
−1.5
1
2
3
4
5
6
7
8
9
10
11
Meishan−A
Meishan−B
Meishan−C
European−A
European−B
European−C

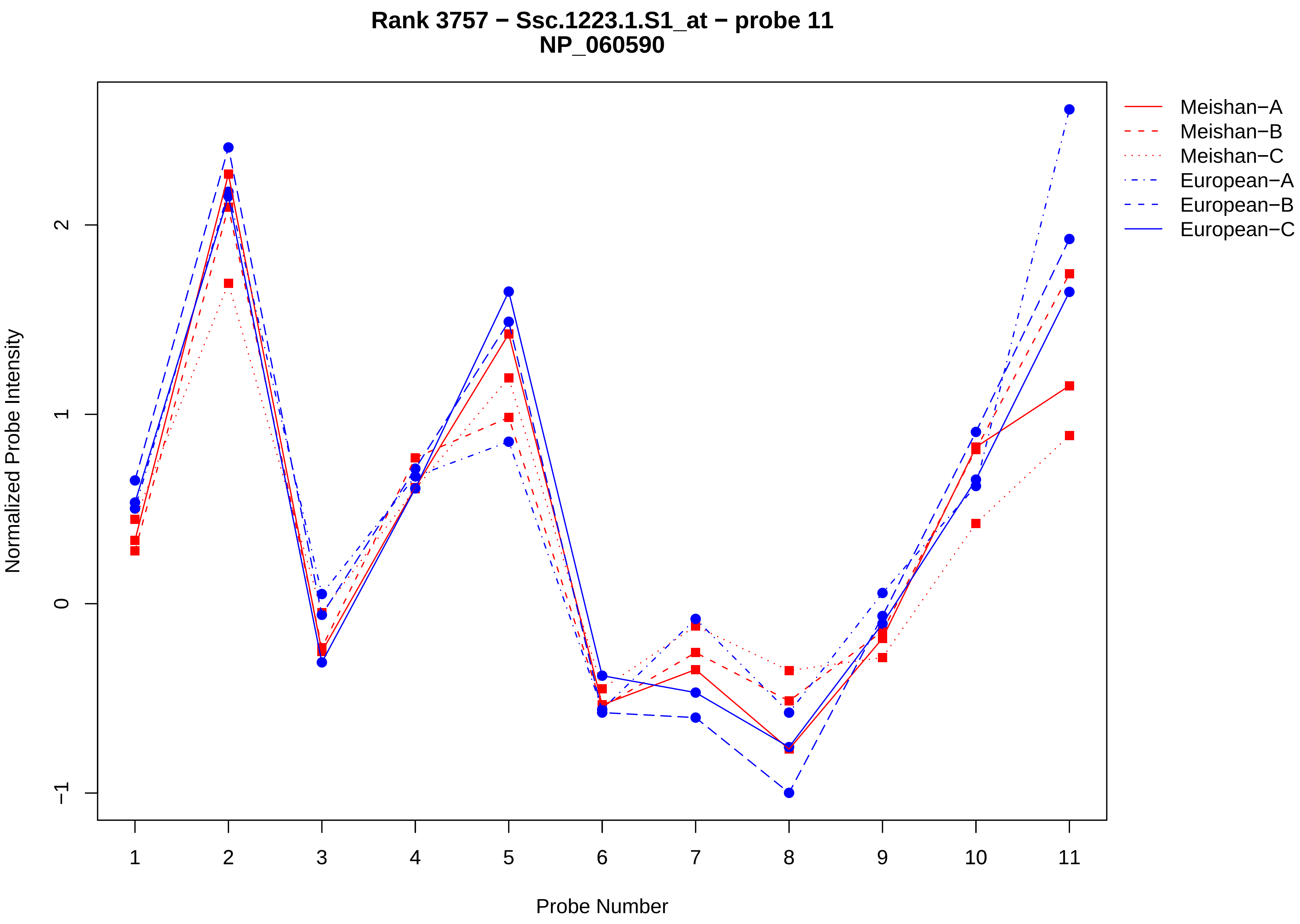

Rank 3757 − Ssc.1223.1.S1_at − probe 11
NP_060590
Normalized Probe Intensity
Probe Number
Meishan−A
Meishan−B
Meishan−C
European−A
European−B
European−C
−1
0
1
2
1
2
3
4
5
6
7
8
9
10
11

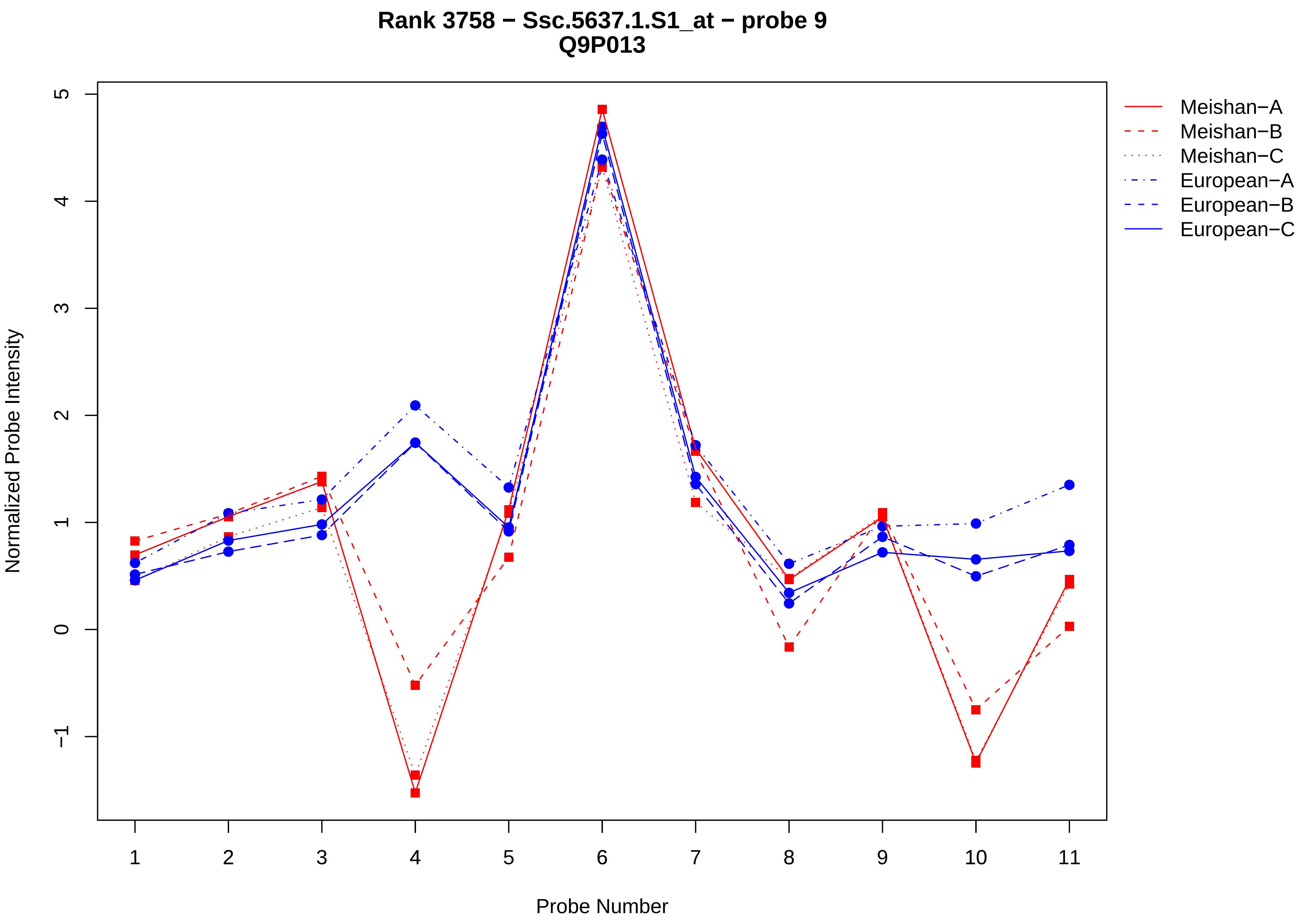

Rank 3758 − Ssc.5637.1.S1_at − probe 9
Q9P013
Normalized Probe Intensity
Probe Number
Meishan−A
Meishan−B
Meishan−C
European−A
European−B
European−C

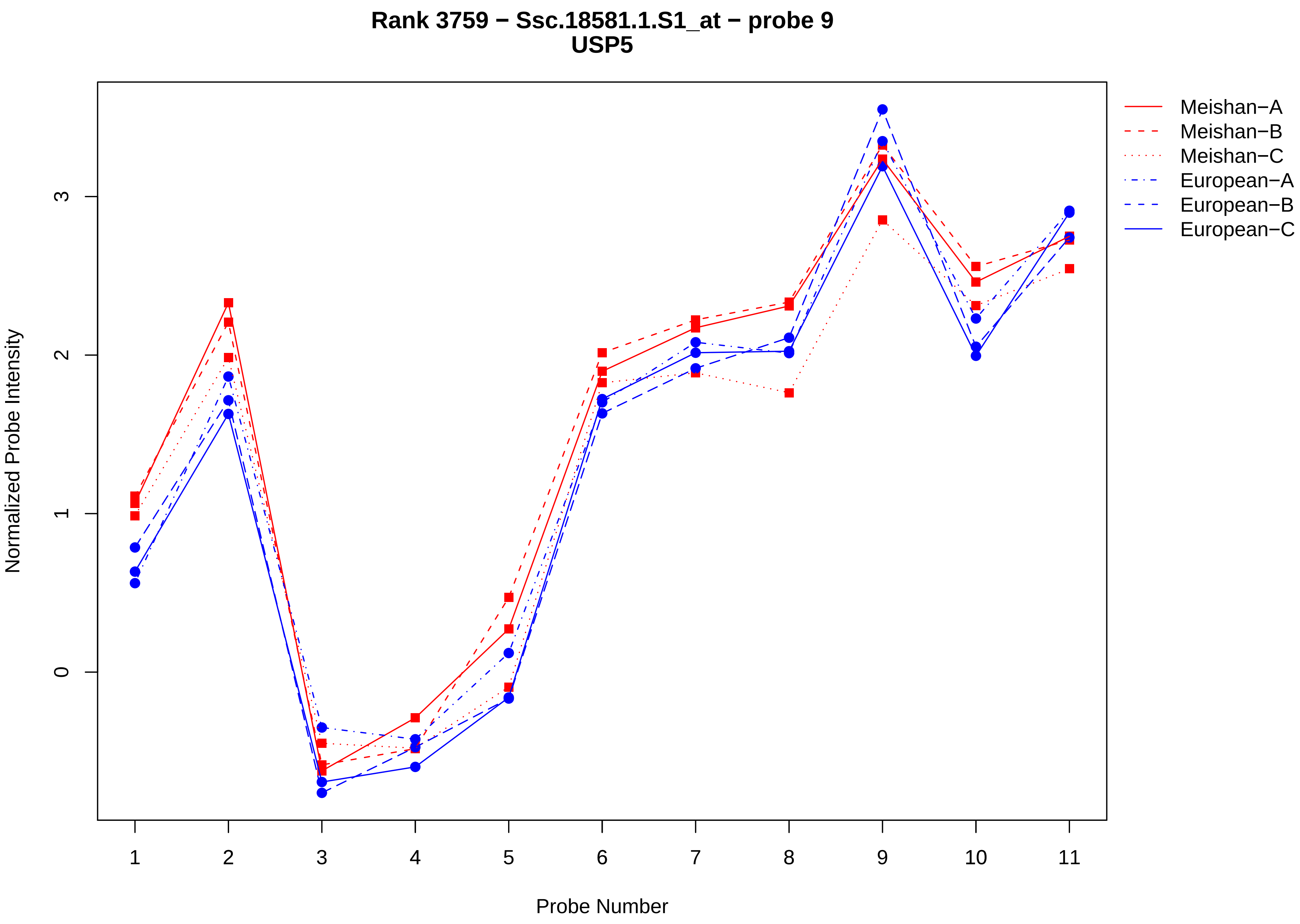

Rank 3759 − Ssc.18581.1.S1_at − probe 9
USP5
Normalized Probe Intensity
Probe Number
0
1
2
3
1
2
3
4
5
6
7
8
9
10
11
Meishan−A
Meishan−B
Meishan−C
European−A
European−B
European−C

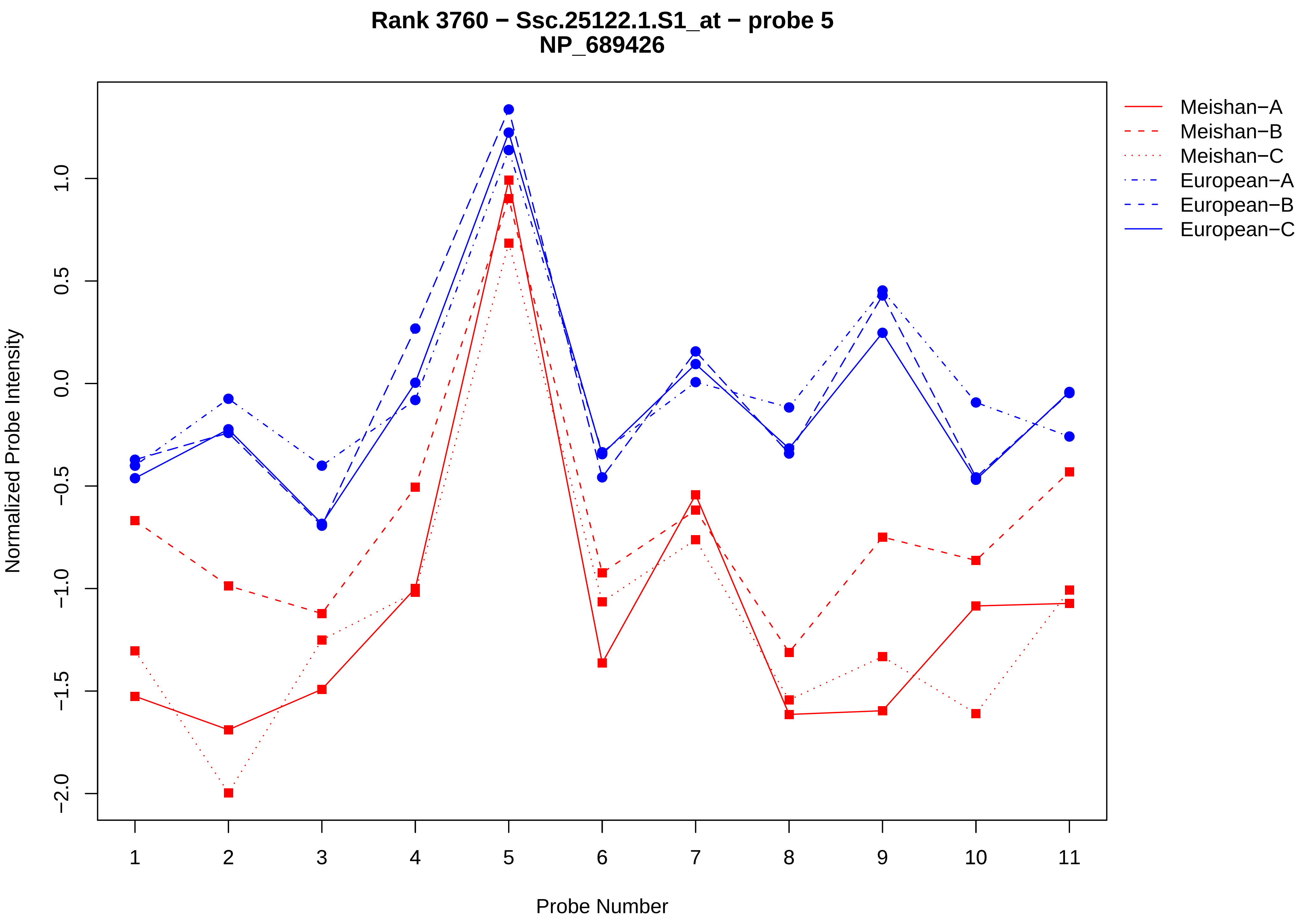

Rank 3760 − Ssc.25122.1.S1_at − probe 5
NP_689426
Normalized Probe Intensity
Probe Number
Meishan−A
Meishan−B
Meishan−C
European−A
European−B
European−C
1.0
0.5
0.0
−0.5
−1.0
−1.5
−2.0
1
2
3
4
5
6
7
8
9
10
11

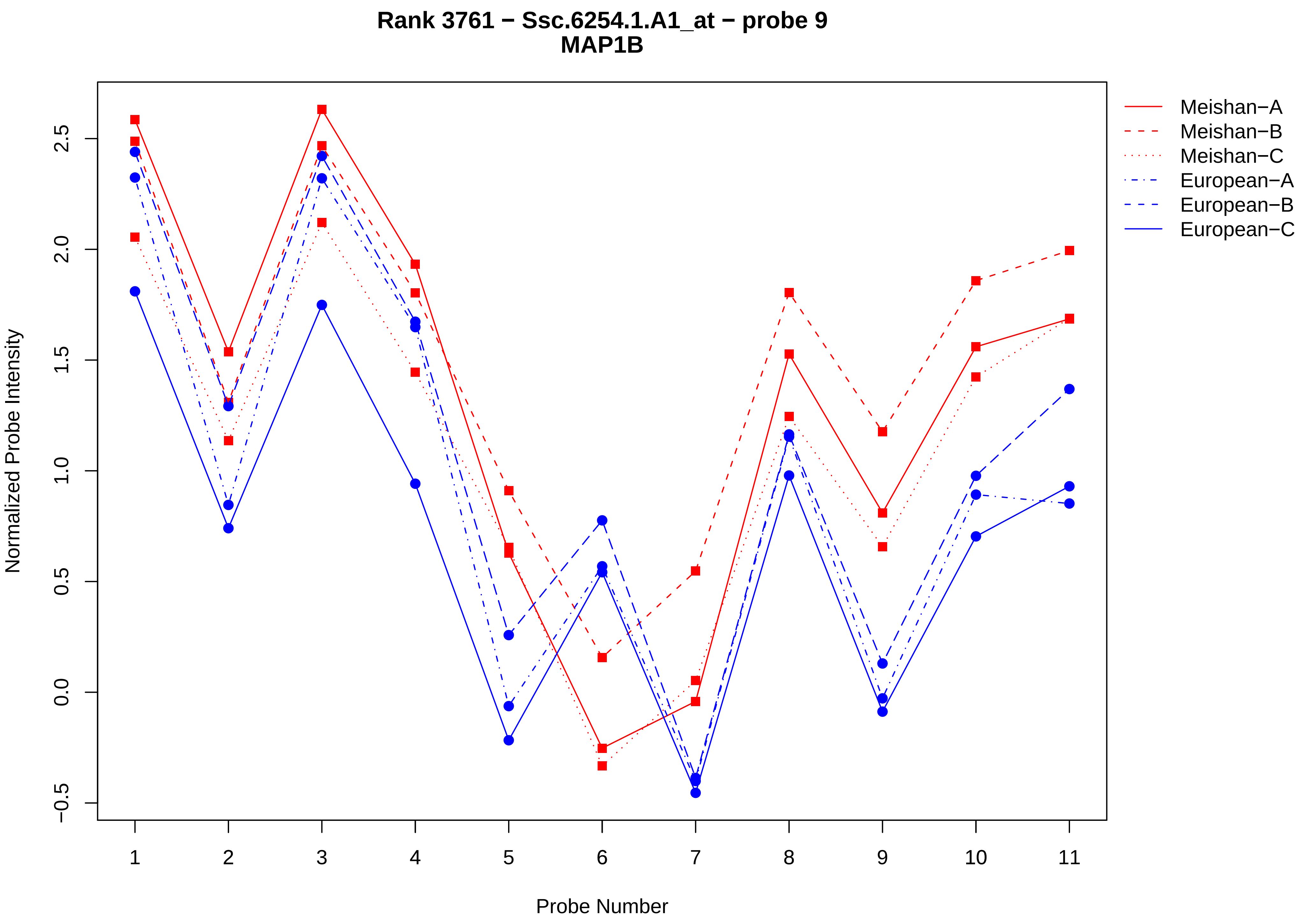

Rank 3761 − Ssc.6254.1.A1_at − probe 9
MAP1B
Normalized Probe Intensity
Probe Number
−0.5
0.0
0.5
1.0
1.5
2.0
2.5
1
2
3
4
5
6
7
8
9
10
11
Meishan−A
Meishan−B
Meishan−C
European−A
European−B
European−C

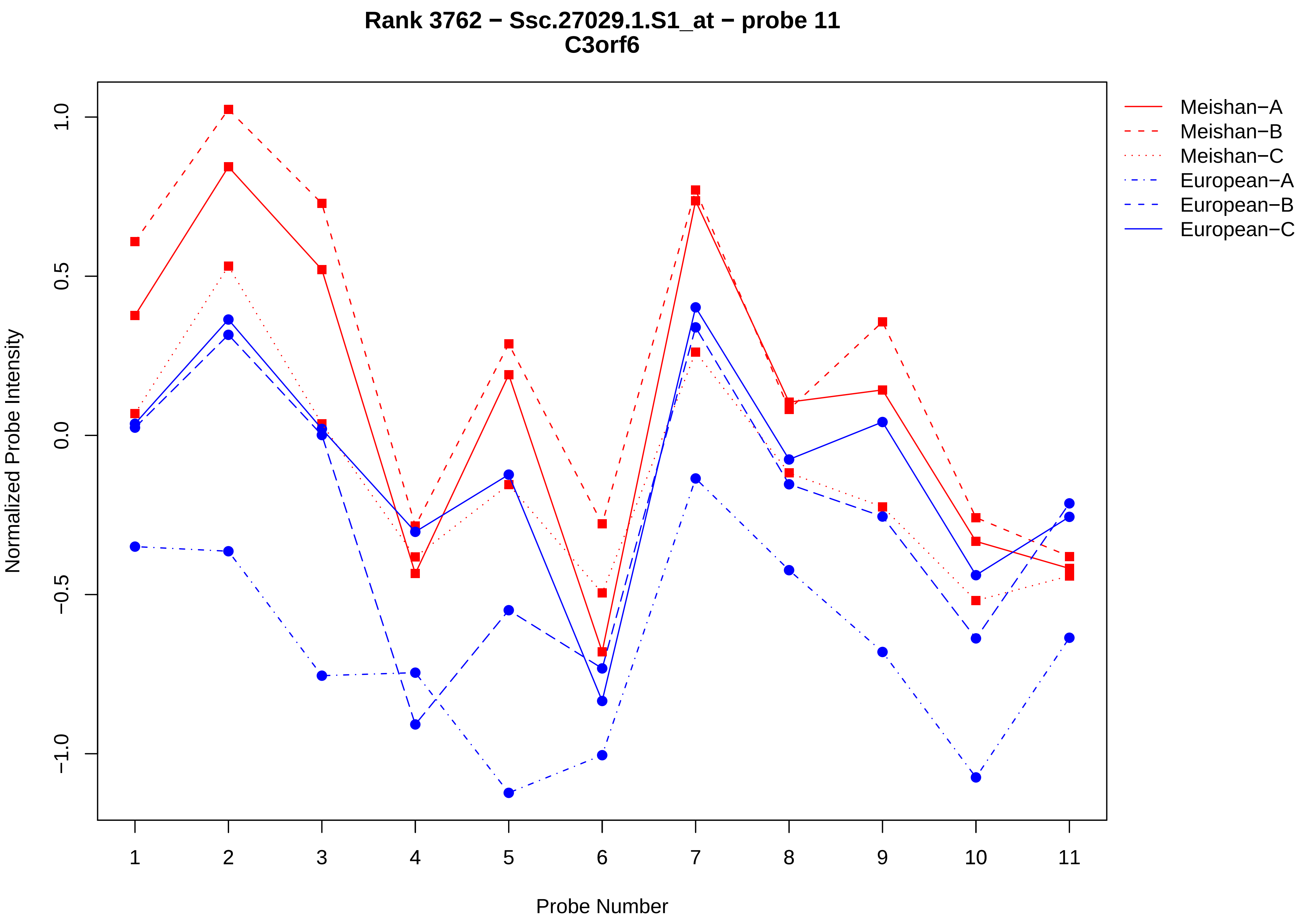

Rank 3762 − Ssc.27029.1.S1_at − probe 11
C3orf6
Normalized Probe Intensity
Probe Number
1.0
0.5
0.0
−0.5
−1.0
1
2
3
4
5
6
7
8
9
10
11
Meishan−A
Meishan−B
Meishan−C
European−A
European−B
European−C

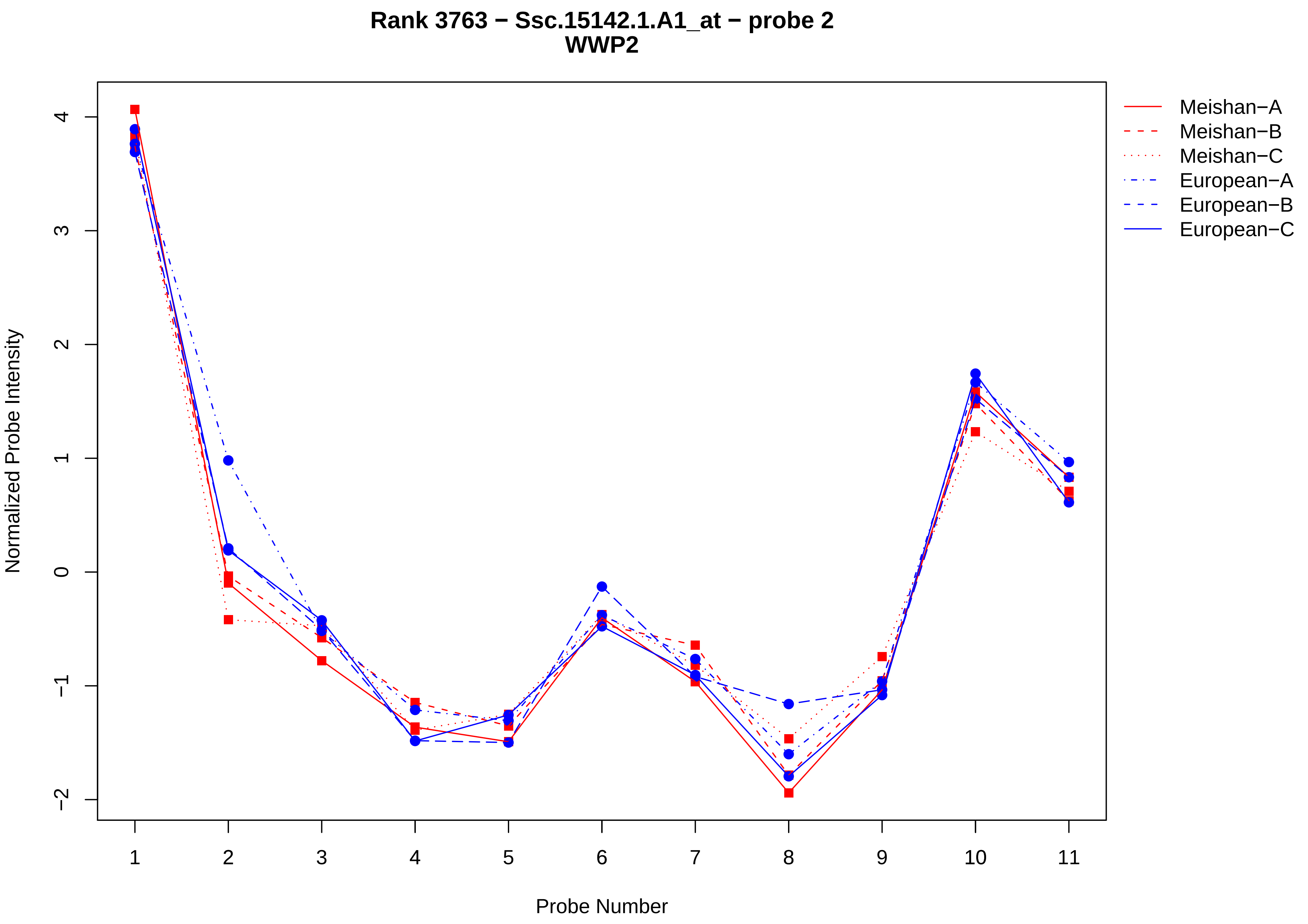

Rank 3763 − Ssc.15142.1.A1_at − probe 2
WWP2
Normalized Probe Intensity
Probe Number
−2
−1
0
1
2
3
4
1
2
3
4
5
6
7
8
9
10
11
Meishan−A
Meishan−B
Meishan−C
European−A
European−B
European−C

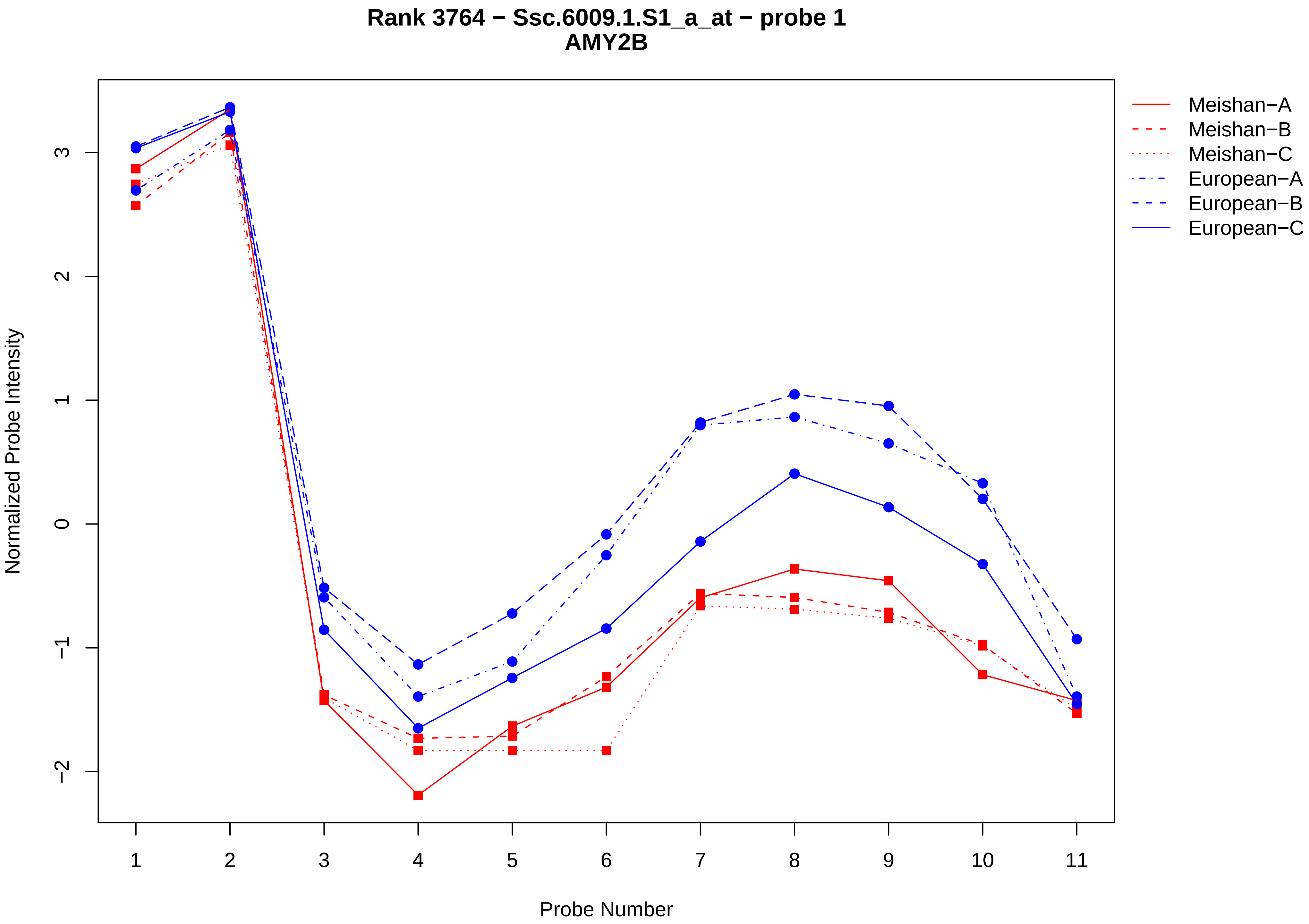
Rank 3764 − Ssc.6009.1.S1_a_at − probe 1
AMY2B
Normalized Probe Intensity
Probe Number
−2
−1
0
1
2
3
1
2
3
4
5
6
7
8
9
10
11
Meishan−A
Meishan−B
Meishan−C
European−A
European−B
European−C

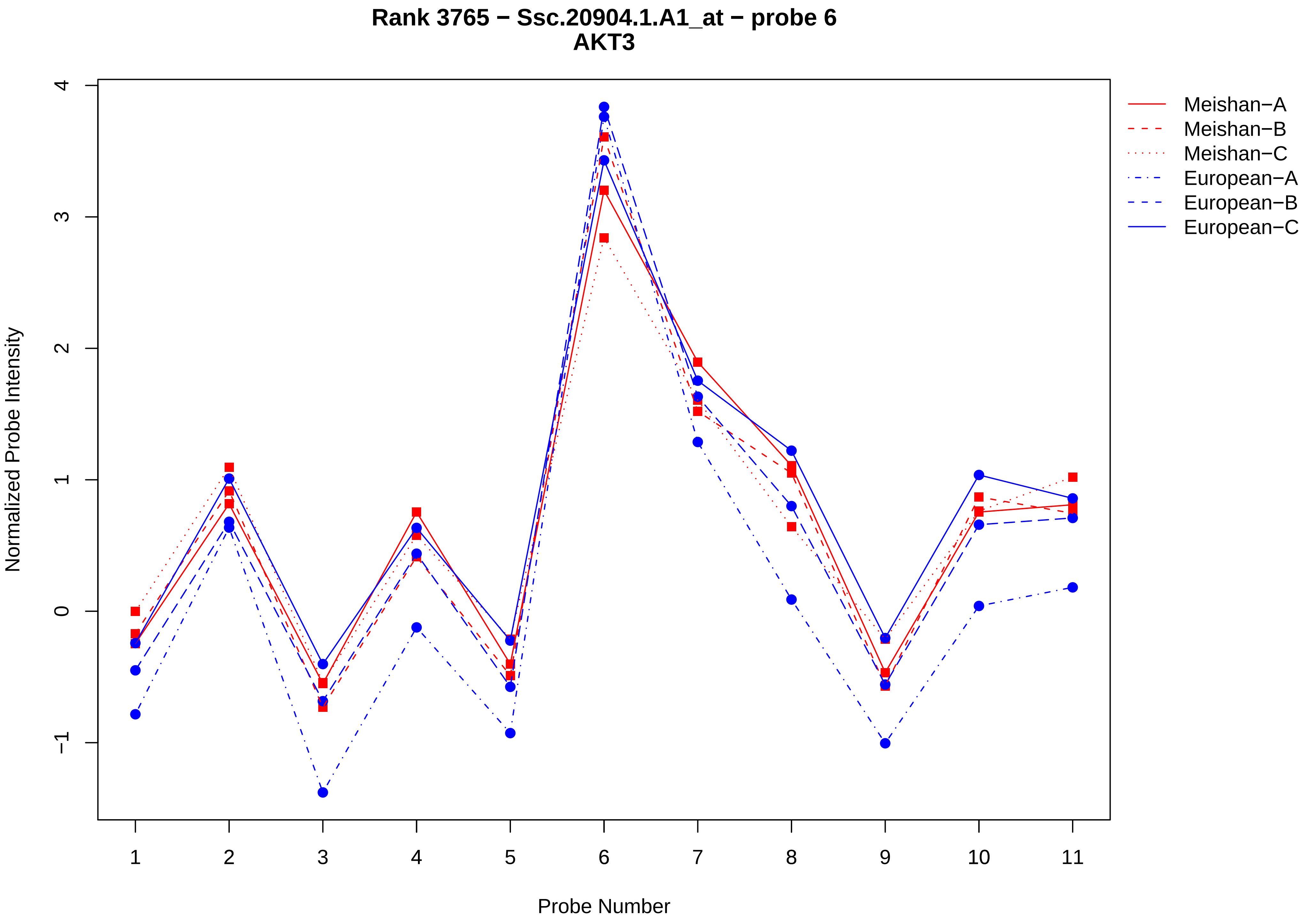

Rank 3765 − Ssc.20904.1.A1_at − probe 6
AKT3
Normalized Probe Intensity
Probe Number
Meishan−A
Meishan−B
Meishan−C
European−A
European−B
European−C

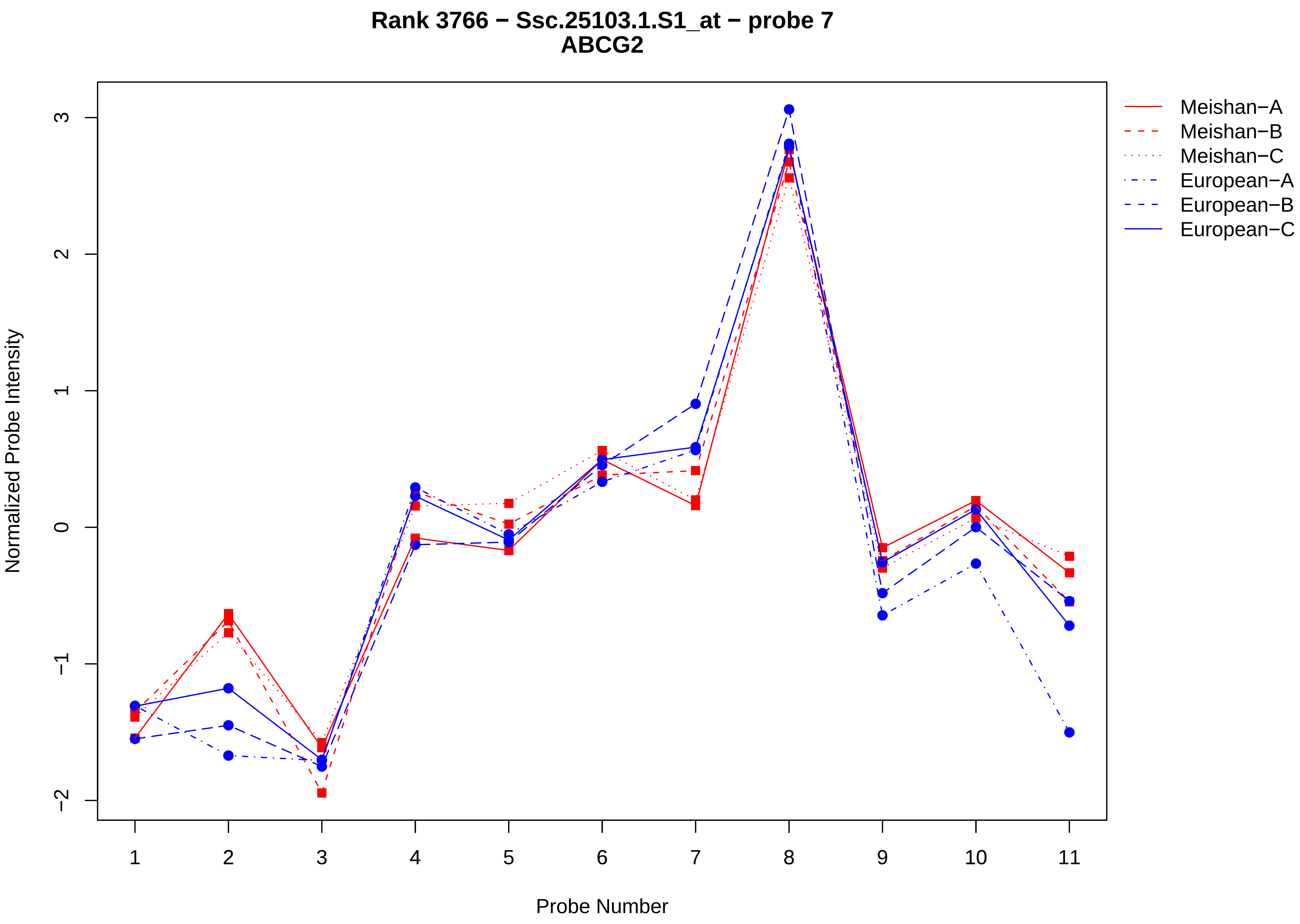

Rank 3766 − Ssc.25103.1.S1_at − probe 7
ABCG2
Normalized Probe Intensity
Probe Number
Meishan−A
Meishan−B
Meishan−C
European−A
European−B
European−C

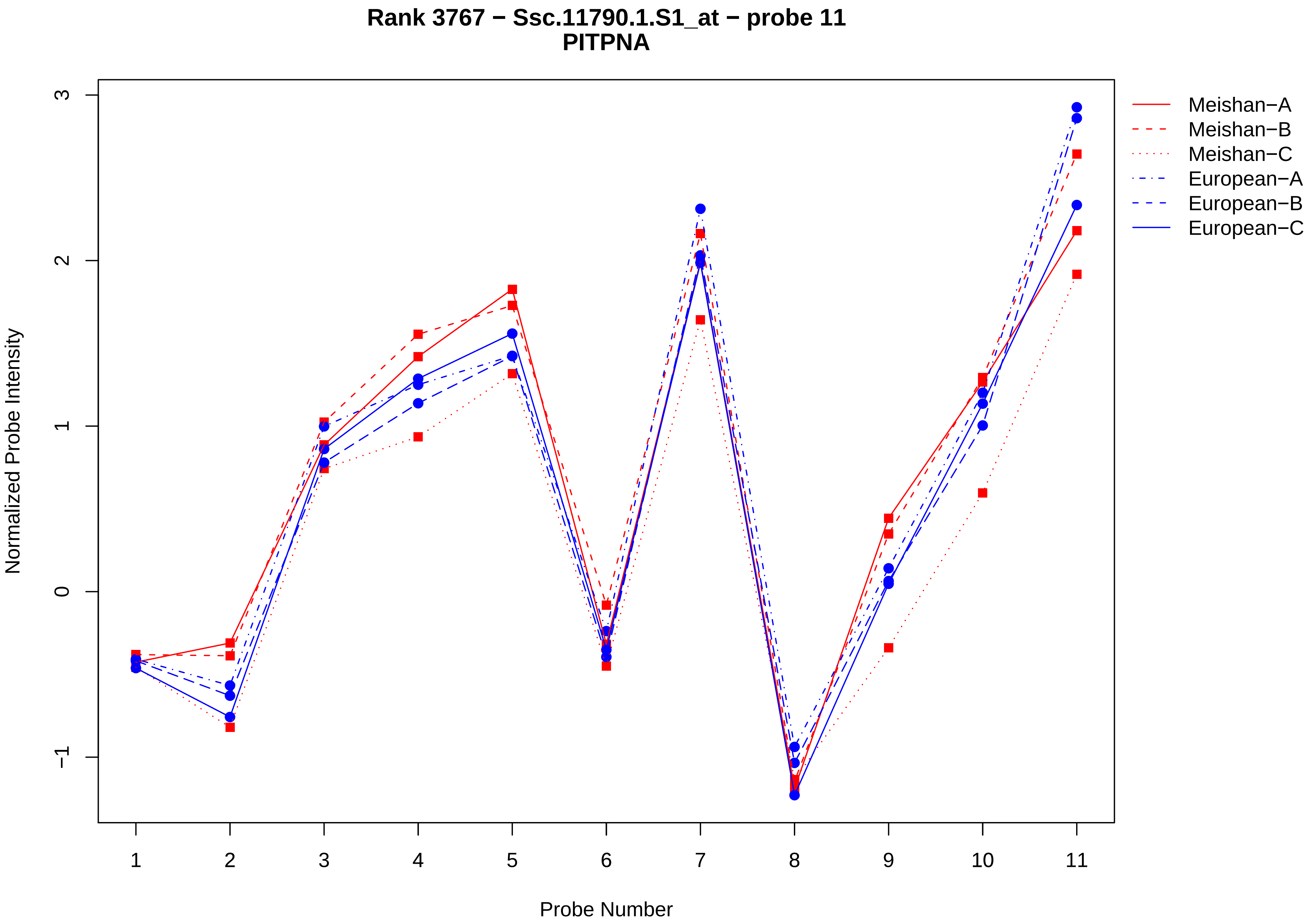

Rank 3767 − Ssc.11790.1.S1_at − probe 11
PITPNA
Normalized Probe Intensity
Probe Number
Meishan−A
Meishan−B
Meishan−C
European−A
European−B
European−C

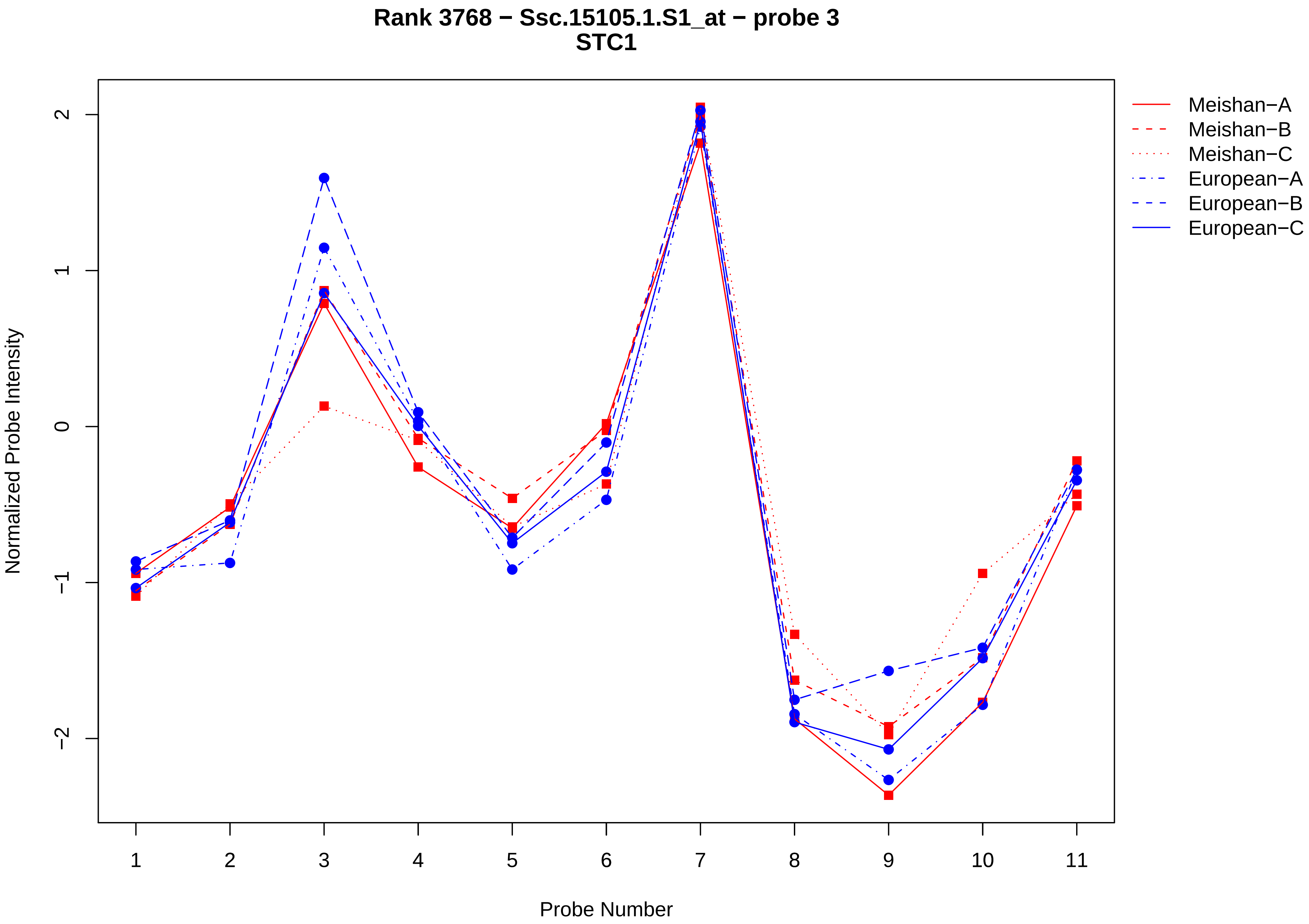
Rank 3768 − Ssc.15105.1.S1_at − probe 3
STC1
Normalized Probe Intensity
Probe Number
Meishan−A
Meishan−B
Meishan−C
European−A
European−B
European−C
−2
−1
0
1
2
1
2
3
4
5
6
7
8
9
10
11

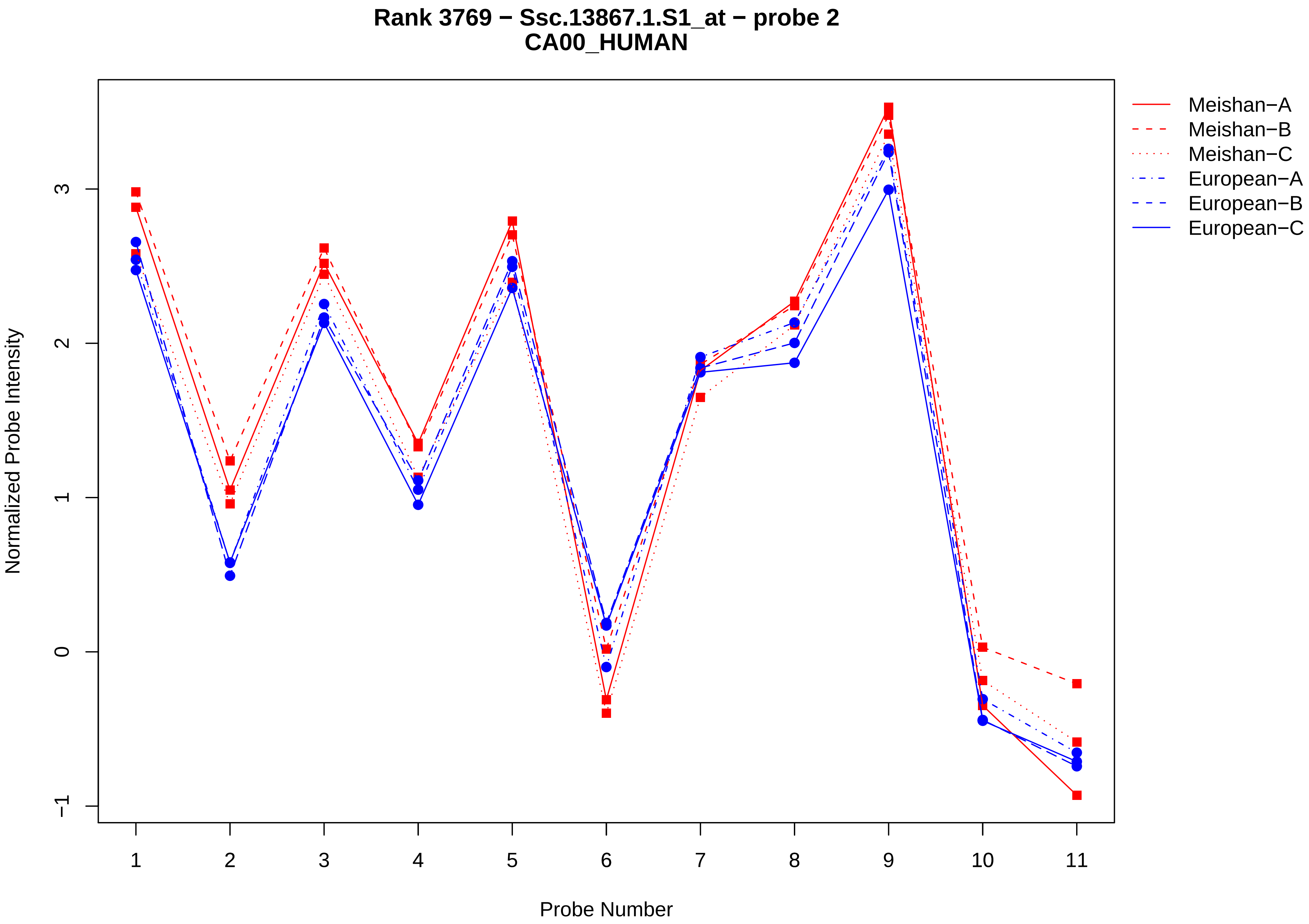

Rank 3769 − Ssc.13867.1.S1_at − probe 2
CA00_HUMAN
Normalized Probe Intensity
Probe Number
−1
0
1
2
3
1
2
3
4
5
6
7
8
9
10
11
Meishan−A
Meishan−B
Meishan−C
European−A
European−B
European−C

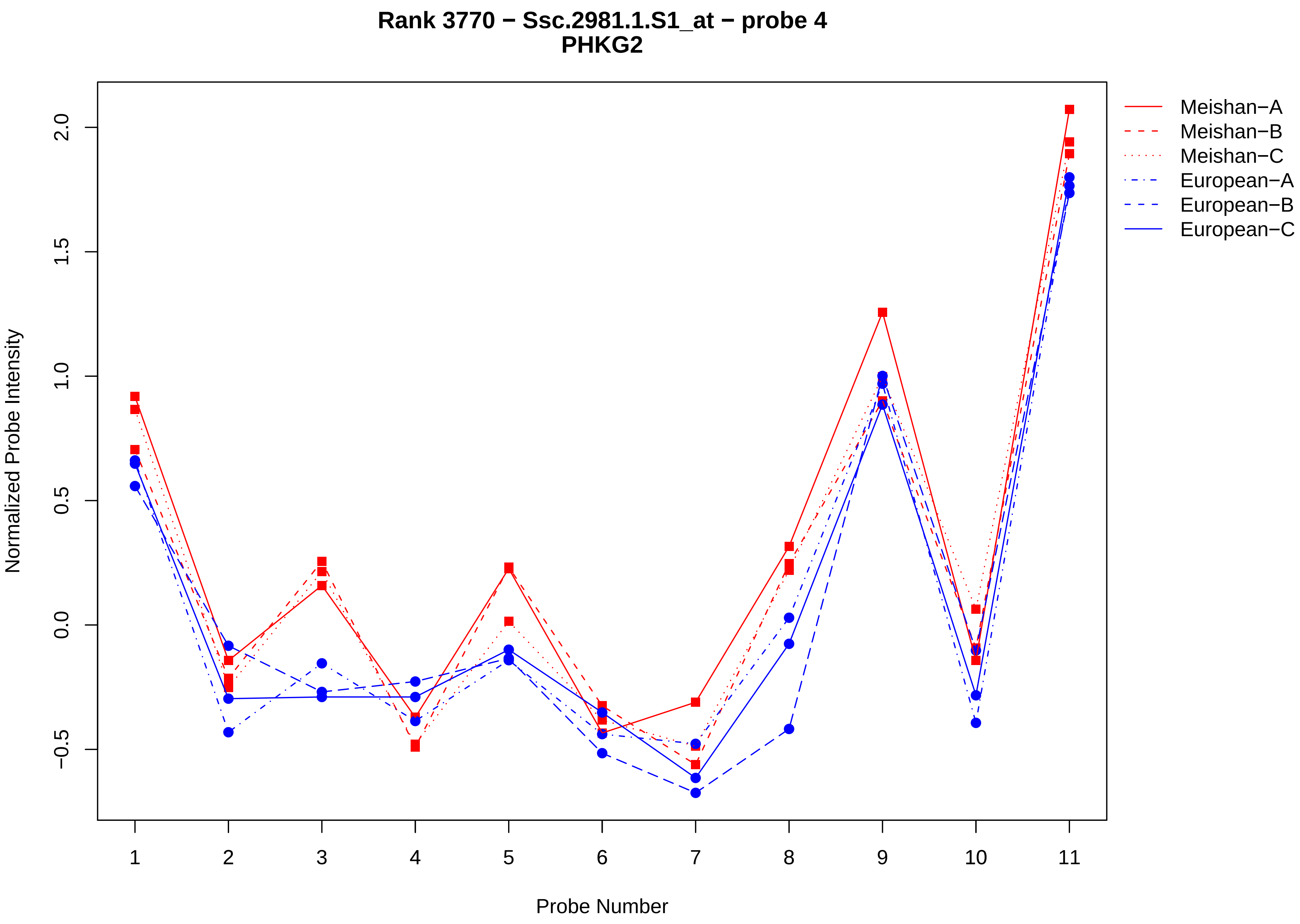
Rank 3770 − Ssc.2981.1.S1_at − probe 4
PHKG2
Normalized Probe Intensity
Probe Number
−0.5
0.0
0.5
1.0
1.5
2.0
1
2
3
4
5
6
7
8
9
10
11
Meishan−A
Meishan−B
Meishan−C
European−A
European−B
European−C

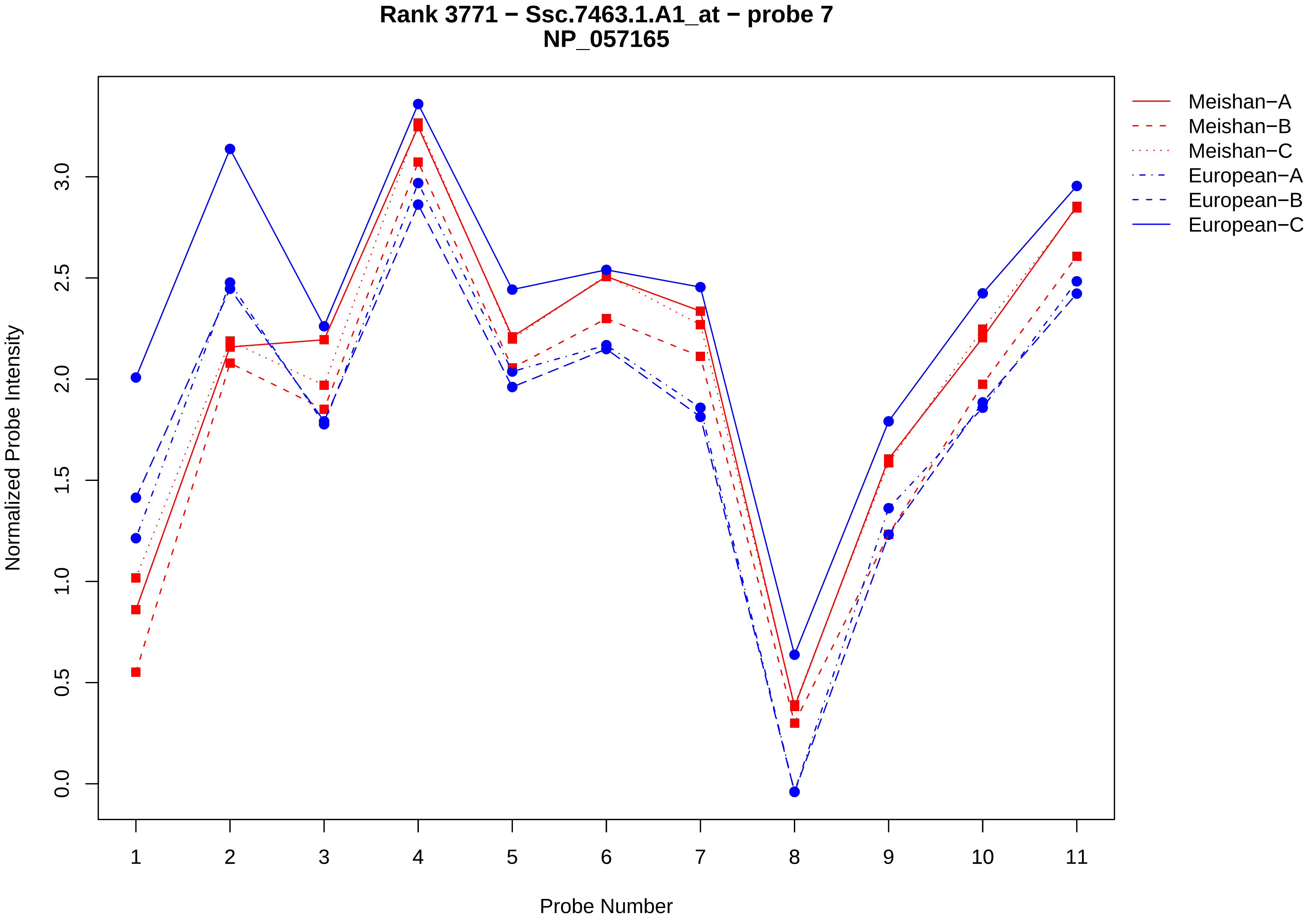
Rank 3771 − Ssc.7463.1.A1_at − probe 7
NP_057165
Normalized Probe Intensity
Probe Number
0.0
0.5
1.0
1.5
2.0
2.5
3.0
1
2
3
4
5
6
7
8
9
10
11
Meishan−A
Meishan−B
Meishan−C
European−A
European−B
European−C

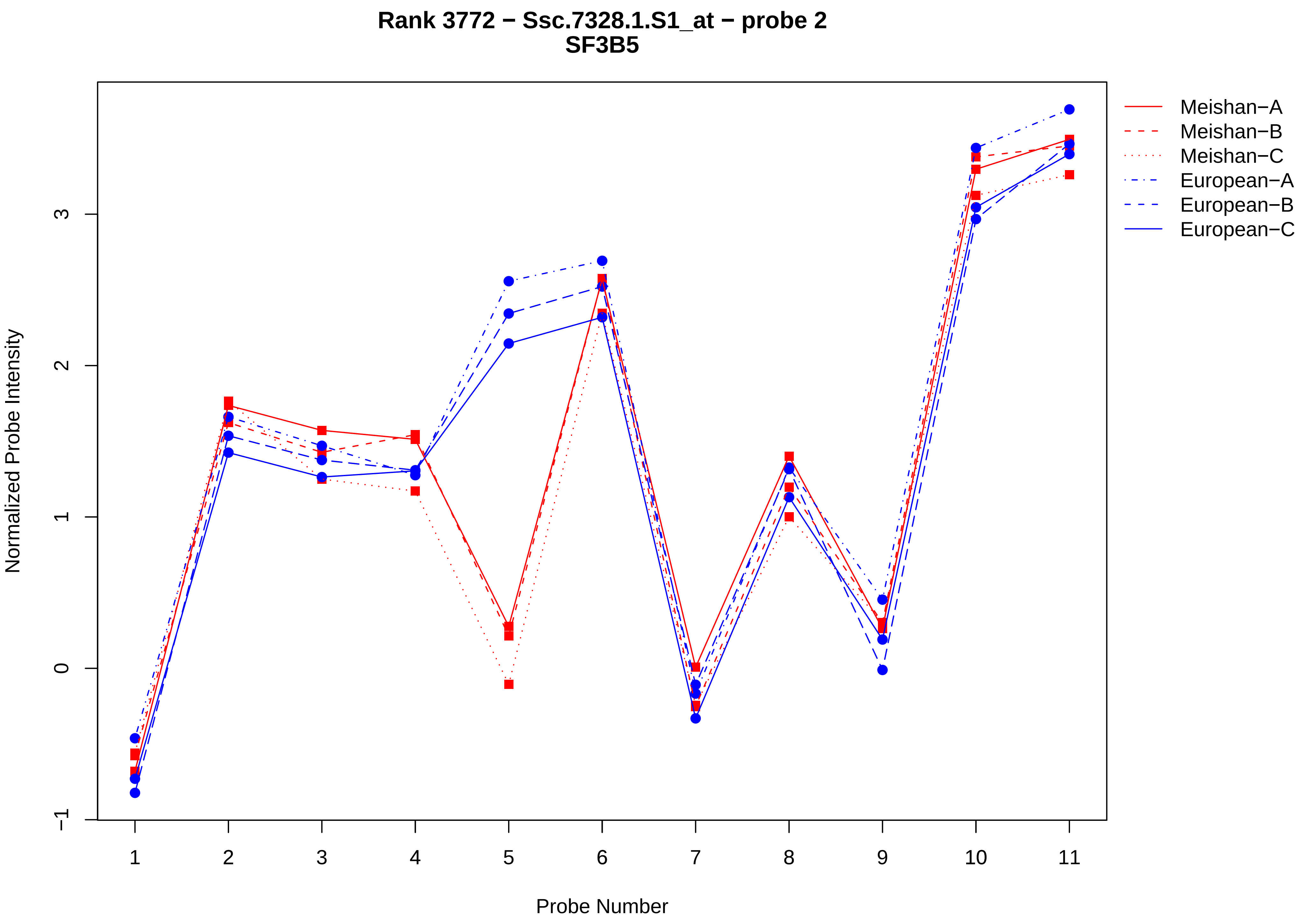
Rank 3772 − Ssc.7328.1.S1_at − probe 2
SF3B5
Normalized Probe Intensity
Probe Number
−1
0
1
2
3
1
2
3
4
5
6
7
8
9
10
11
Meishan−A
Meishan−B
Meishan−C
European−A
European−B
European−C

Rank 3773 − Ssc.30037.1.A1_at − probe 6
PIP5K1A

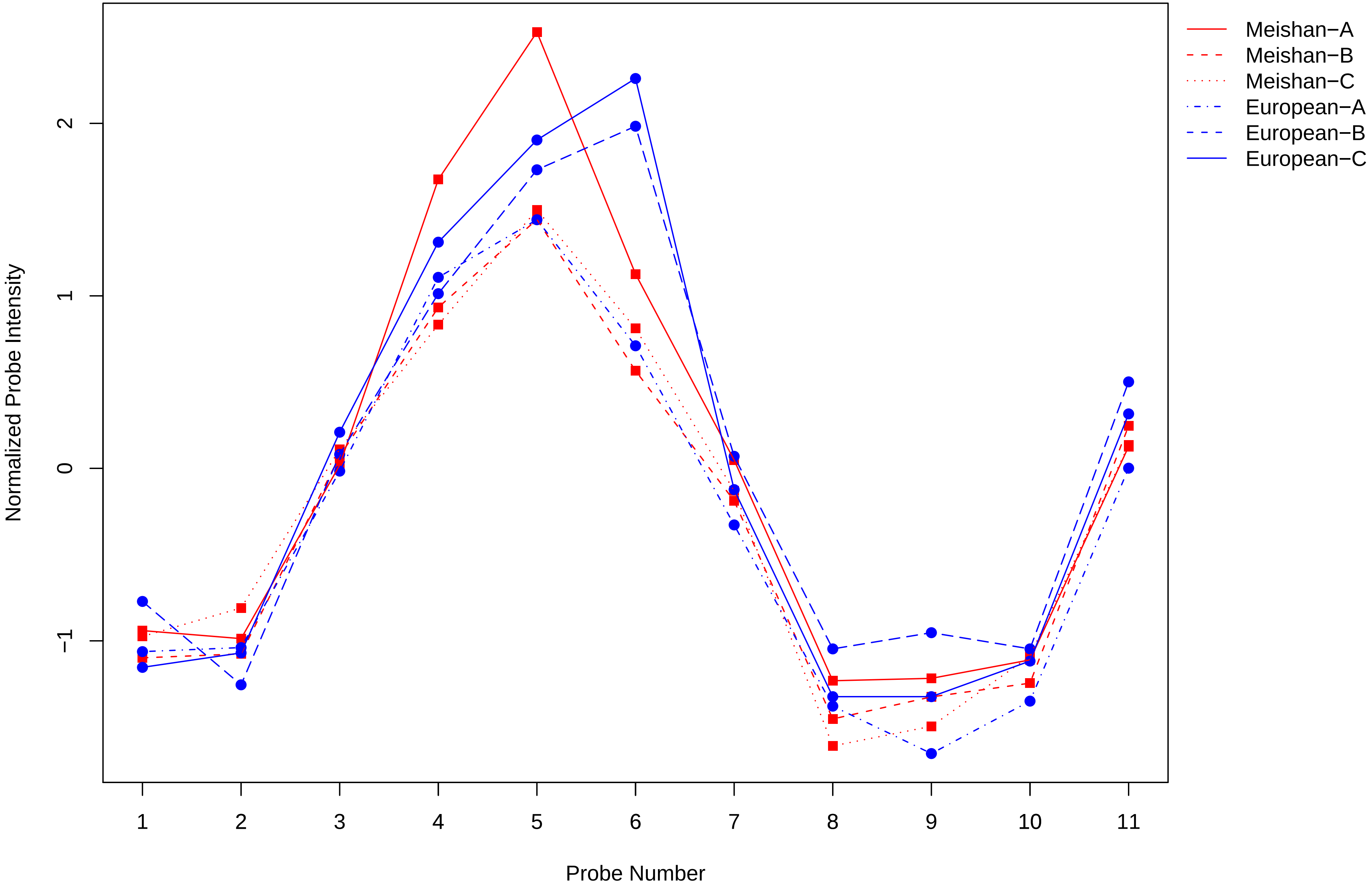

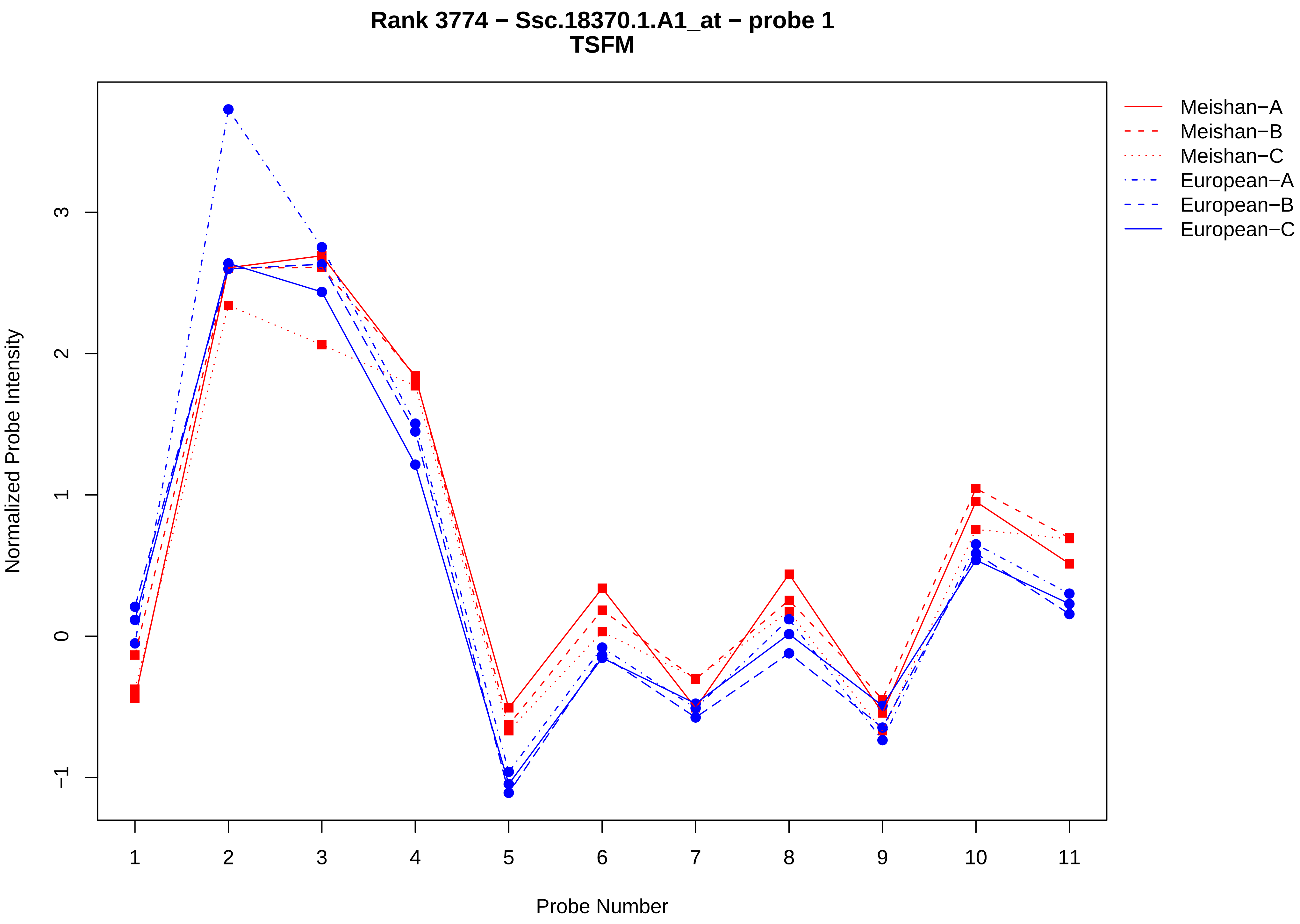

Rank 3774 − Ssc.18370.1.A1_at − probe 1
TSFM
Normalized Probe Intensity
−1
0
1
2
3
1
2
3
4
5
6
7
8
9
10
11
Probe Number
Meishan−A
Meishan−B
Meishan−C
European−A
European−B
European−C

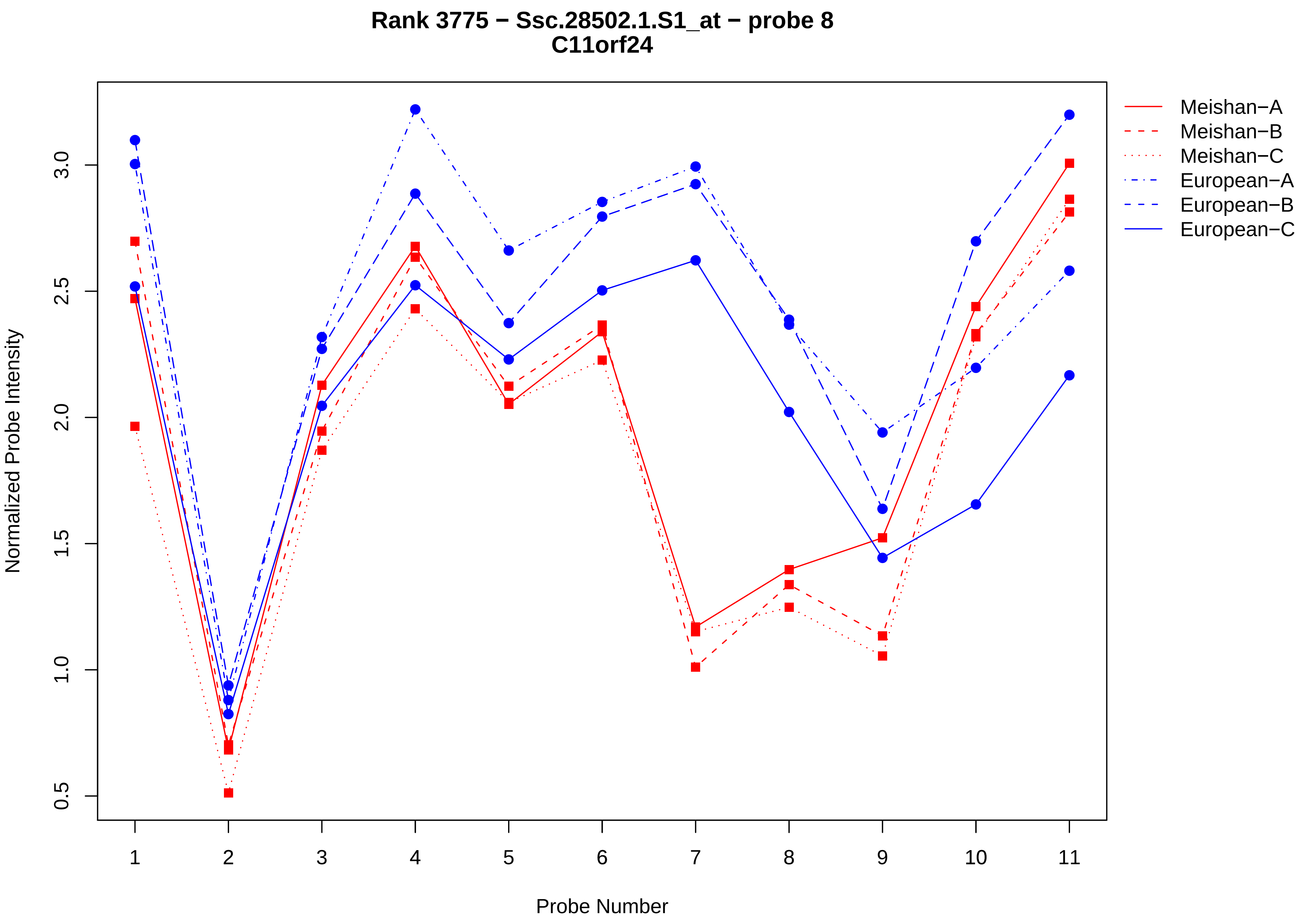
Rank 3775 − Ssc.28502.1.S1_at − probe 8
C11orf24
Normalized Probe Intensity
Probe Number
0.5
1.0
1.5
2.0
2.5
3.0
1
2
3
4
5
6
7
8
9
10
11
Meishan−A
Meishan−B
Meishan−C
European−A
European−B
European−C

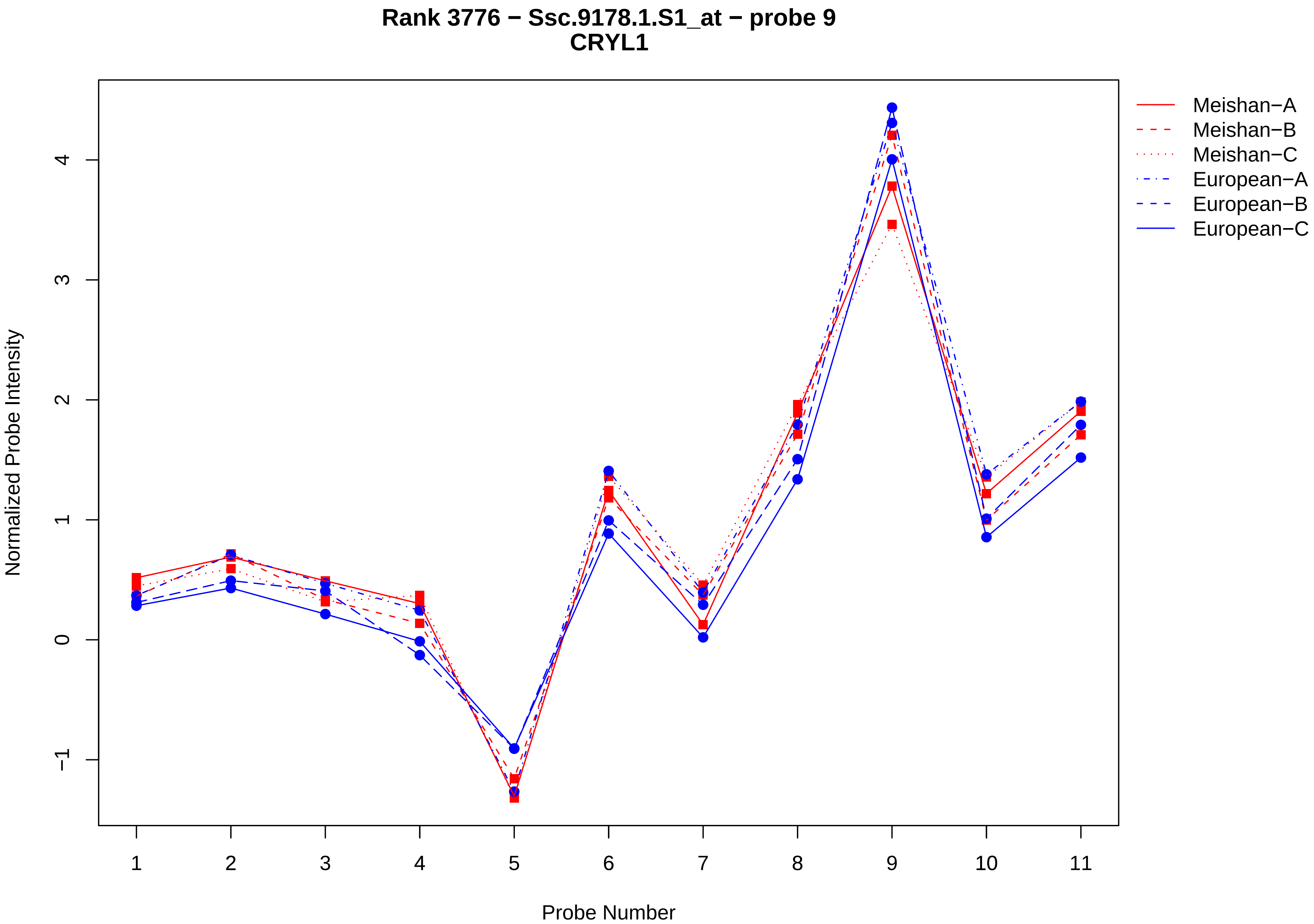

Rank 3776 − Ssc.9178.1.S1_at − probe 9
CRYL1
Normalized Probe Intensity
Probe Number
Meishan−A
Meishan−B
Meishan−C
European−A
European−B
European−C
−1
0
1
2
3
4
1
2
3
4
5
6
7
8
9
10
11

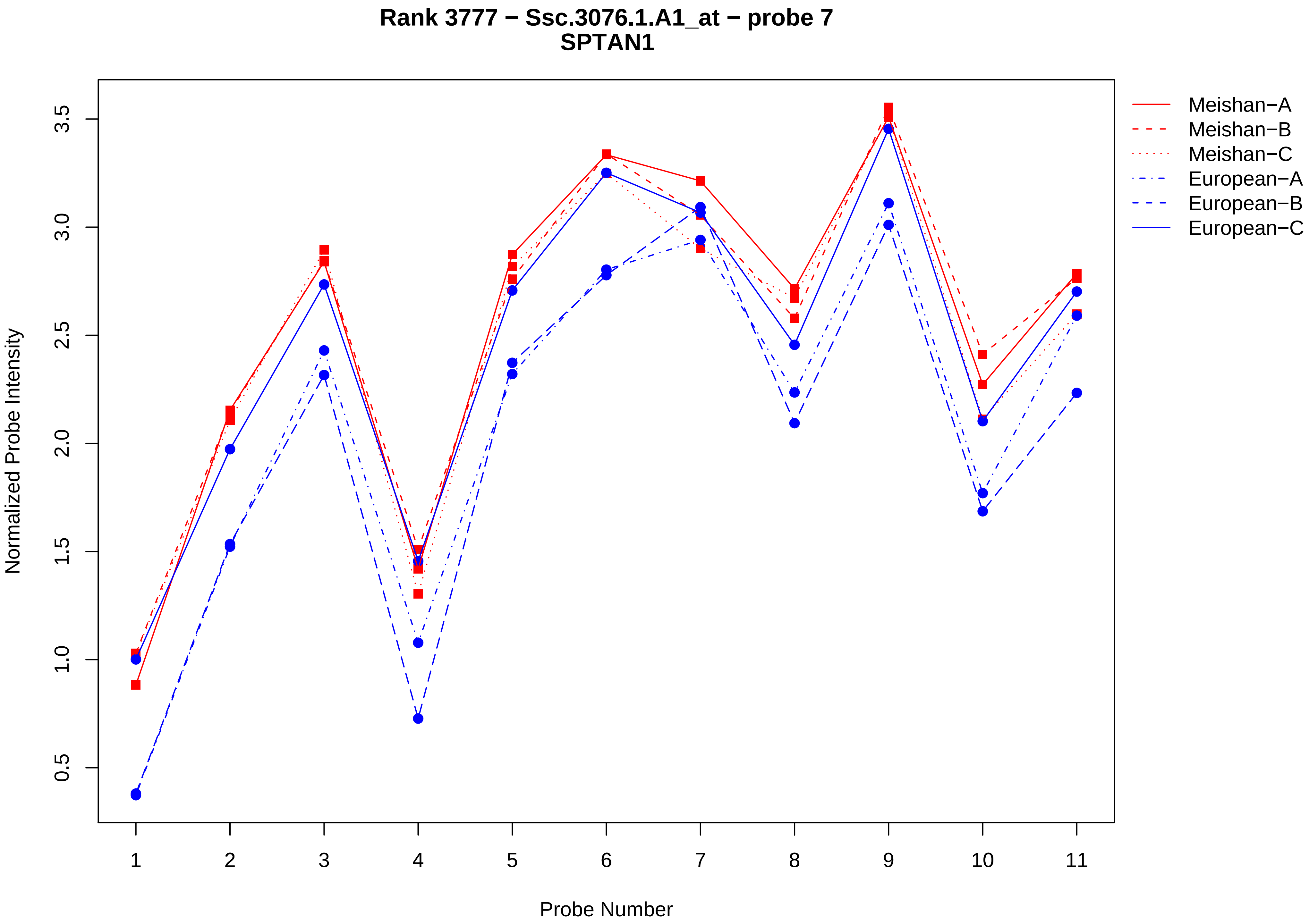

Rank 3777 − Ssc.3076.1.A1_at − probe 7
SPTAN1
Normalized Probe Intensity
Probe Number
0.5
1.0
1.5
2.0
2.5
3.0
3.5
1
2
3
4
5
6
7
8
9
10
11
Meishan−A
Meishan−B
Meishan−C
European−A
European−B
European−C

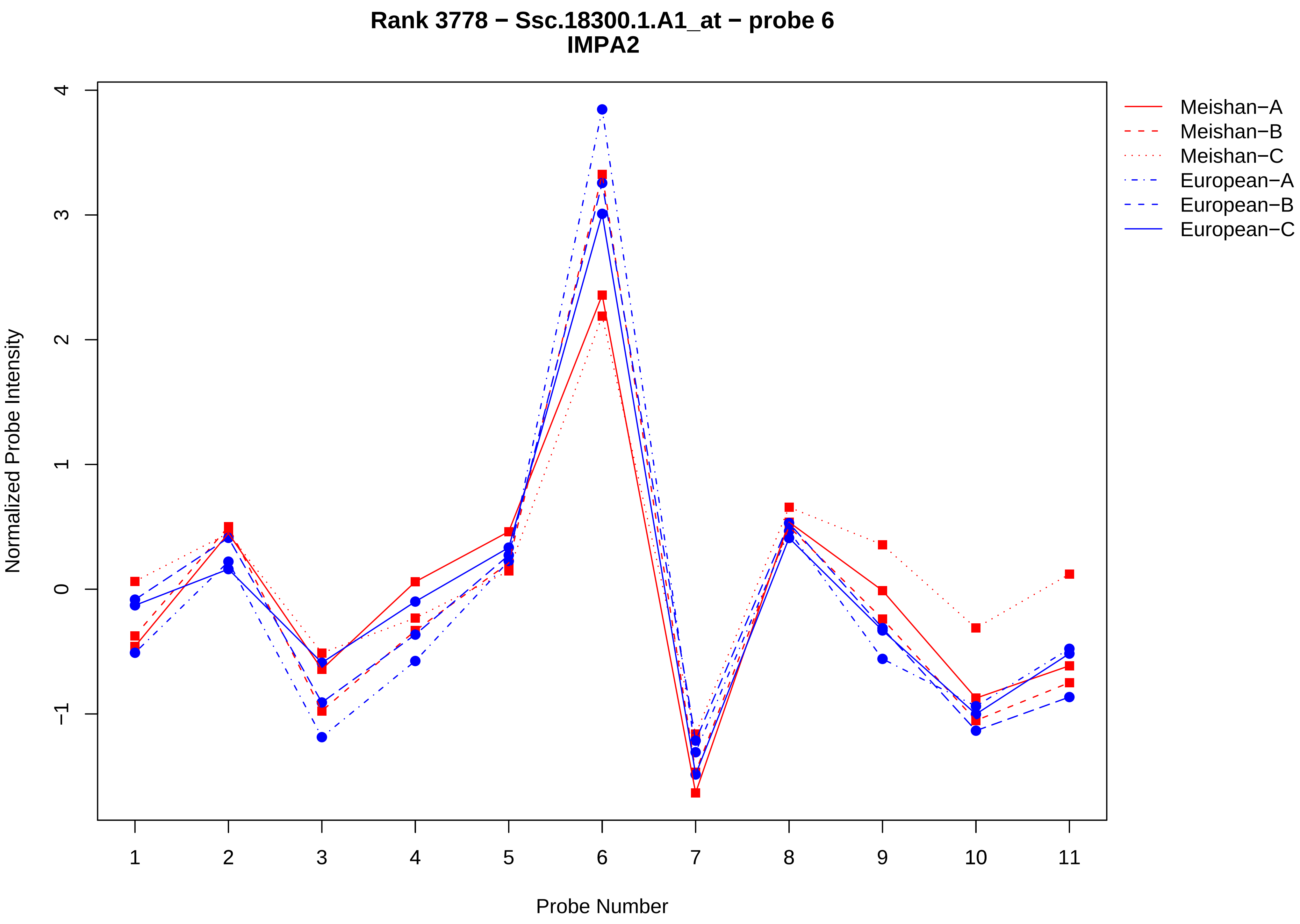

Rank 3778 − Ssc.18300.1.A1_at − probe 6
IMPA2
Normalized Probe Intensity
Probe Number
Meishan−A
Meishan−B
Meishan−C
European−A
European−B
European−C

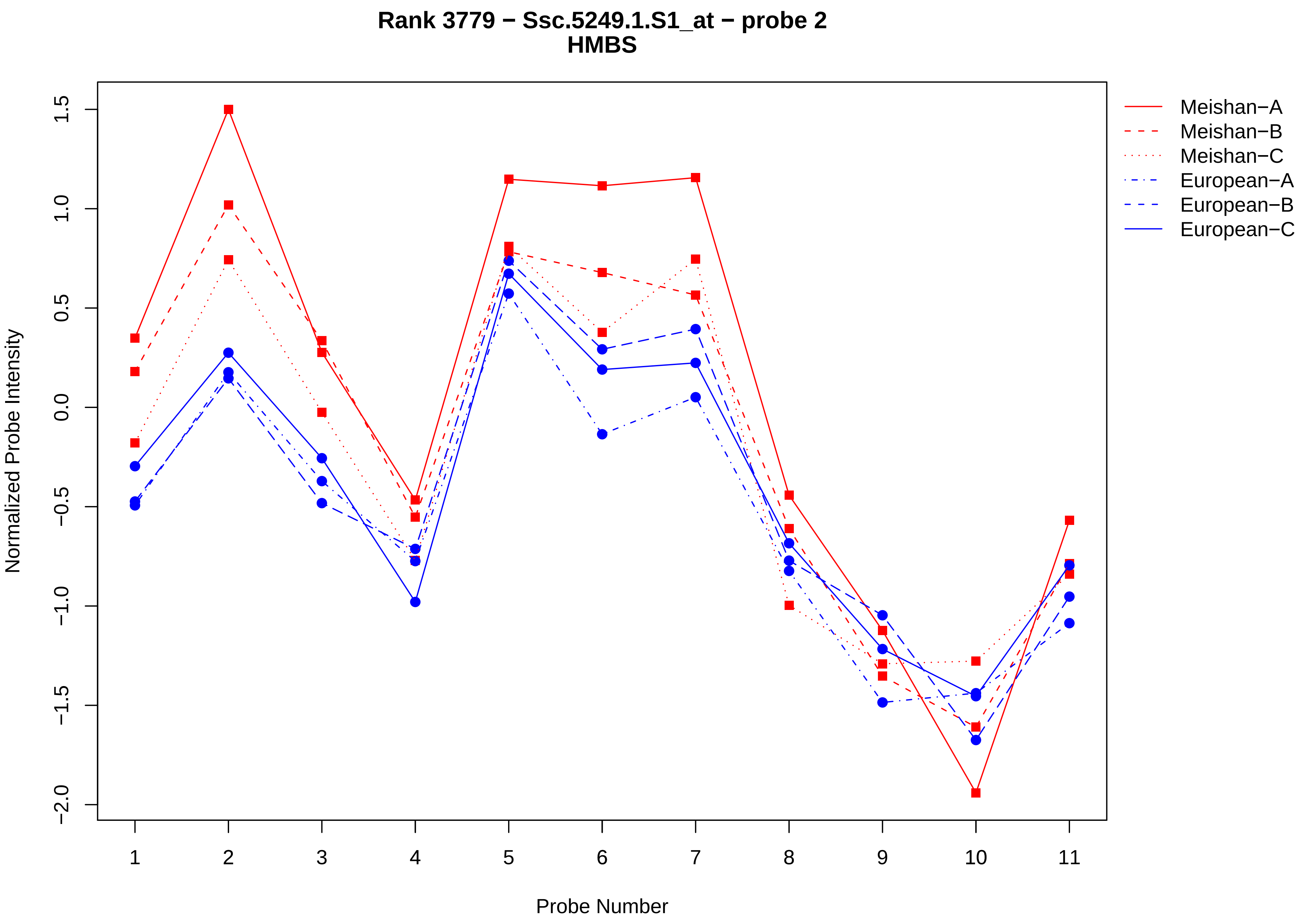

Rank 3779 − Ssc.5249.1.S1_at − probe 2
HMBS
Normalized Probe Intensity
Probe Number
1.5
1.0
0.5
0.0
−0.5
−1.0
−1.5
−2.0
1
2
3
4
5
6
7
8
9
10
11
Meishan−A
Meishan−B
Meishan−C
European−A
European−B
European−C

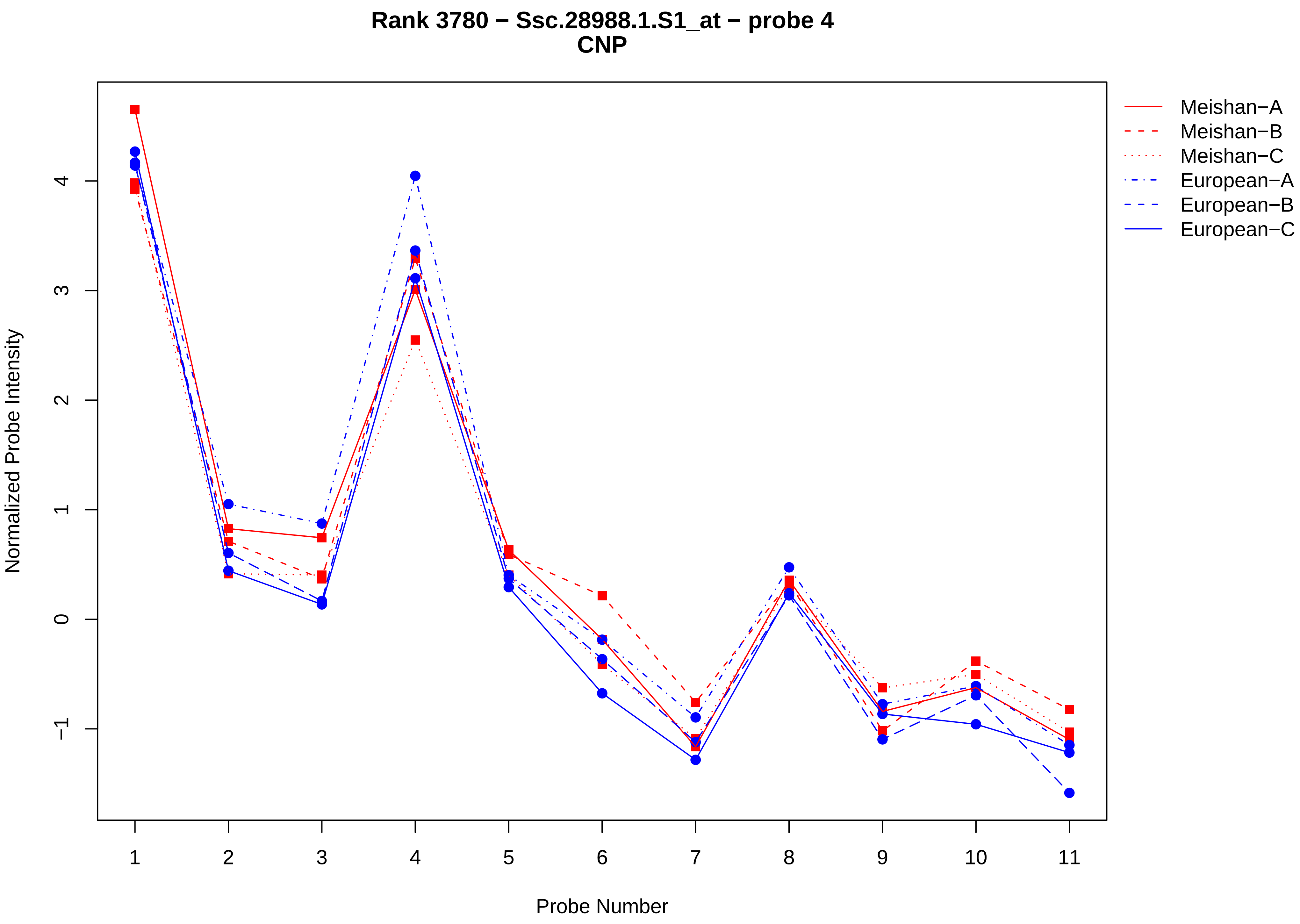

Rank 3780 − Ssc.28988.1.S1_at − probe 4
CNP
Normalized Probe Intensity
Probe Number
−1
0
1
2
3
4
1
2
3
4
5
6
7
8
9
10
11
Meishan−A
Meishan−B
Meishan−C
European−A
European−B
European−C

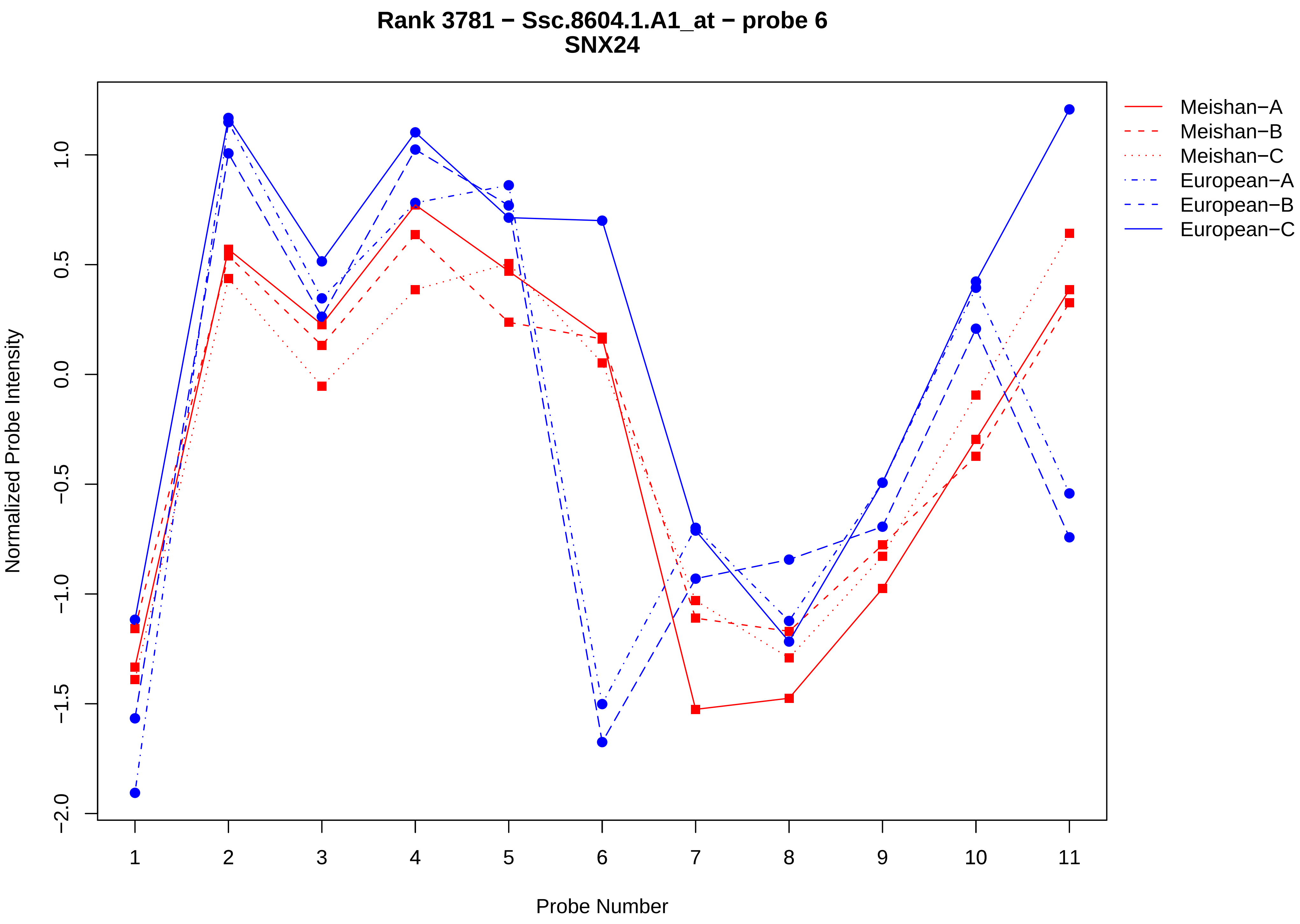

Rank 3781 − Ssc.8604.1.A1_at − probe 6
SNX24
Normalized Probe Intensity
−2.0
−1.5
−1.0
−0.5
0.0
0.5
1.0
1
2
3
4
5
6
7
8
9
10
11
Probe Number
Meishan−A
Meishan−B
Meishan−C
European−A
European−B
European−C

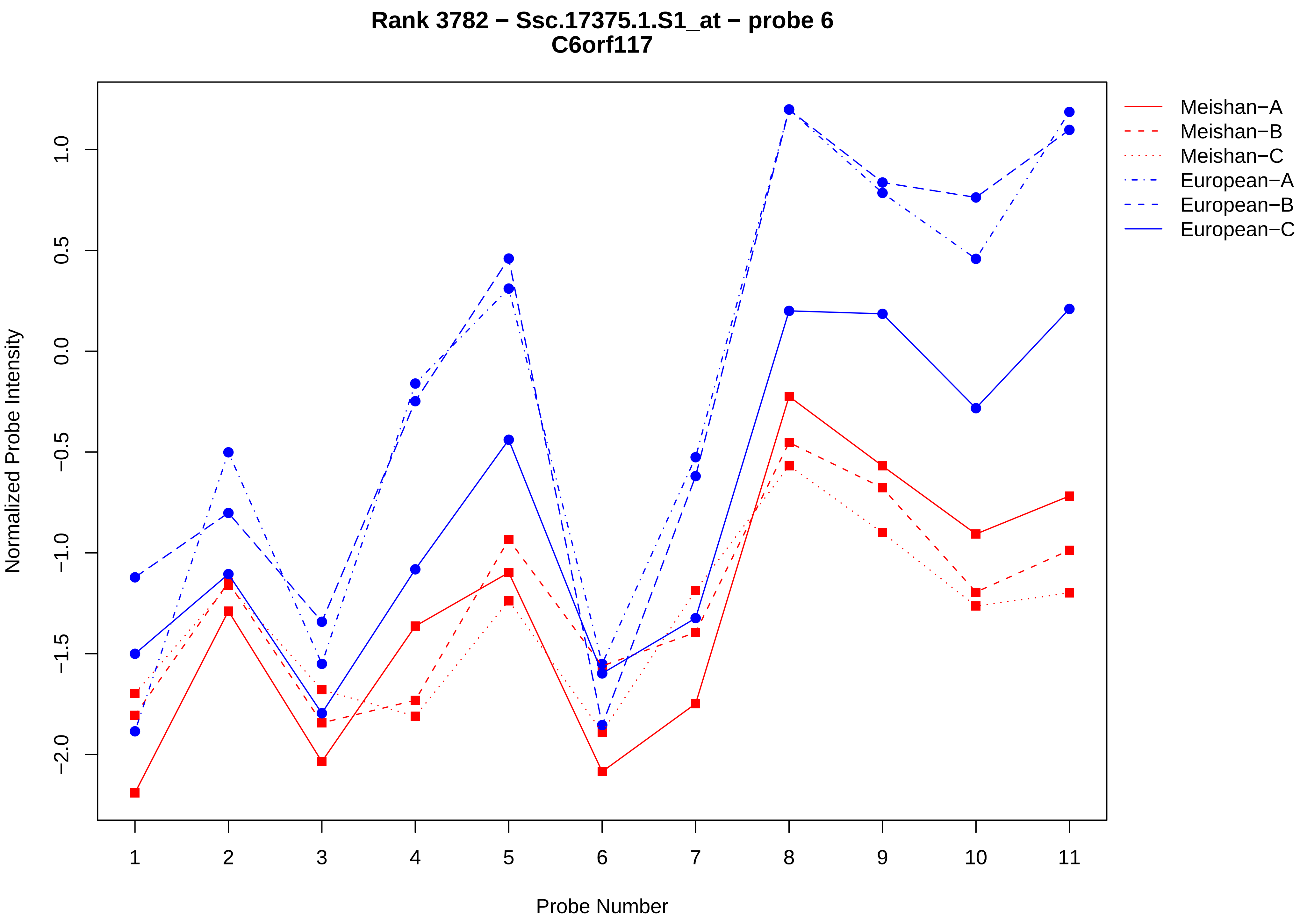
Rank 3782 − Ssc.17375.1.S1_at − probe 6
C6orf117
Normalized Probe Intensity
Probe Number
1.0
0.5
0.0
−0.5
−1.0
−1.5
−2.0
1
2
3
4
5
6
7
8
9
10
11
Meishan−A
Meishan−B
Meishan−C
European−A
European−B
European−C

Rank 3783 − Ssc.6009.1.S1_at − probe 11
AMY2B

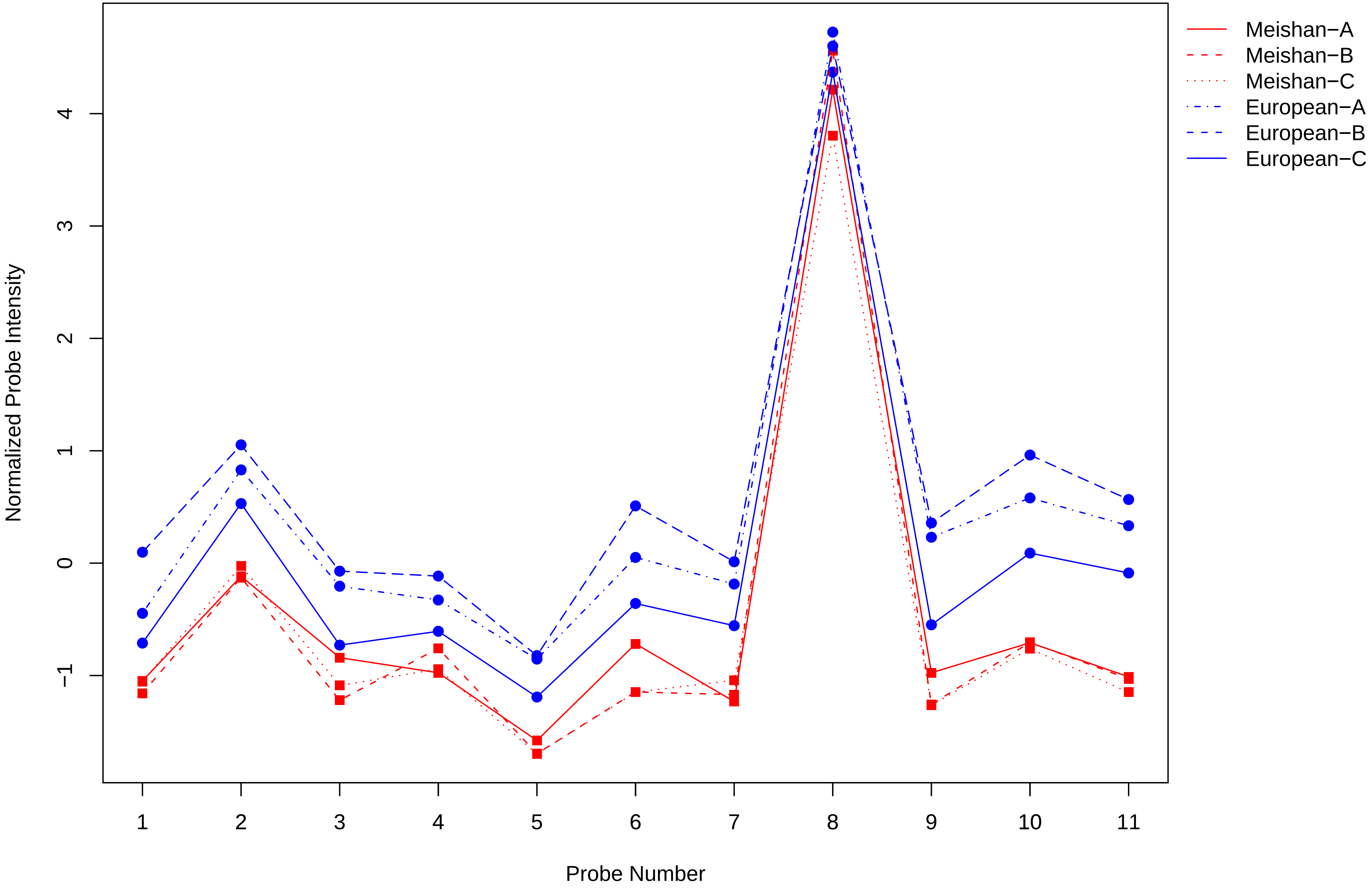

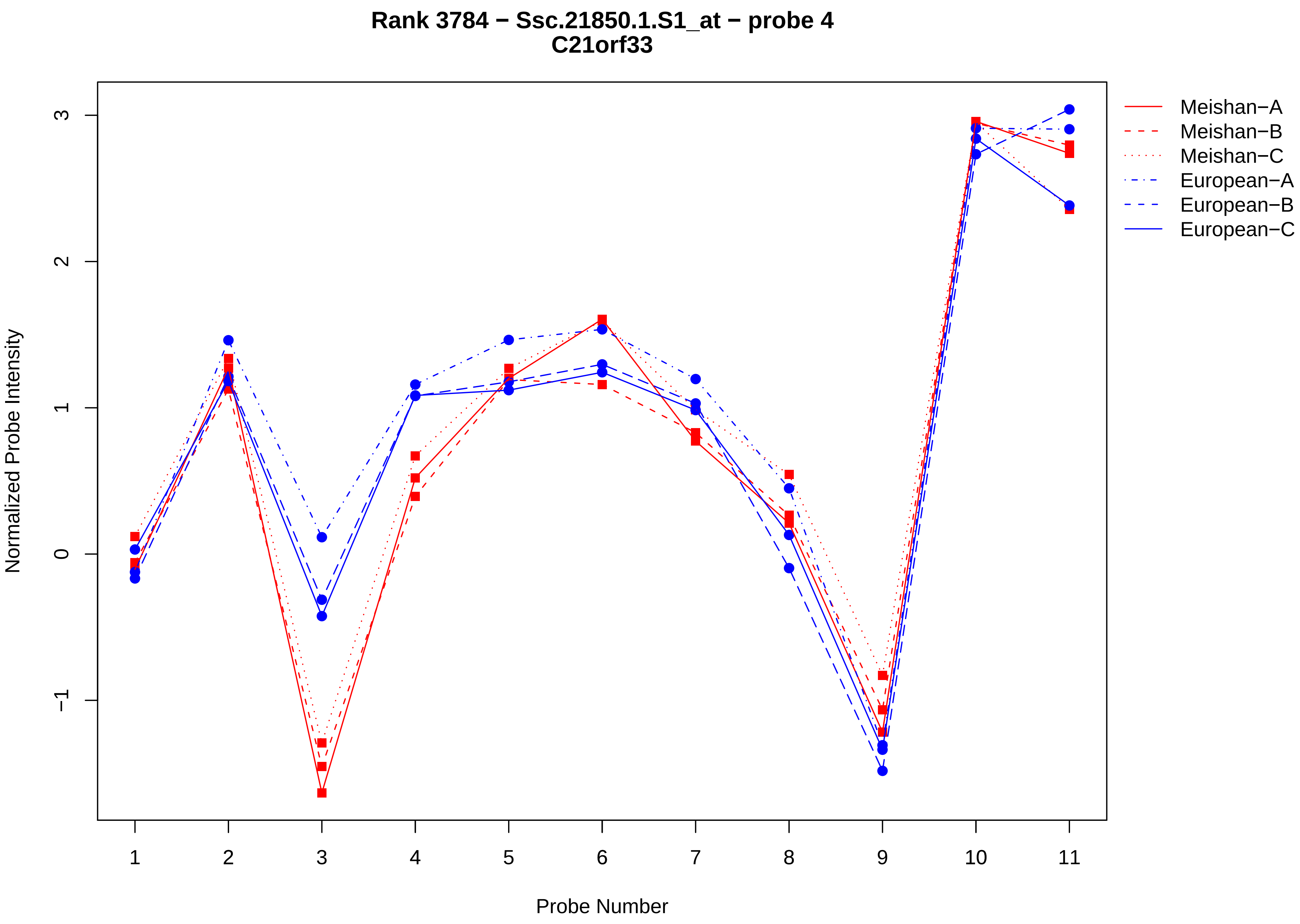

Rank 3784 − Ssc.21850.1.S1_at − probe 4
C21orf33
Normalized Probe Intensity
Probe Number
−1
0
1
2
3
1
2
3
4
5
6
7
8
9
10
11
Meishan−A
Meishan−B
Meishan−C
European−A
European−B
European−C

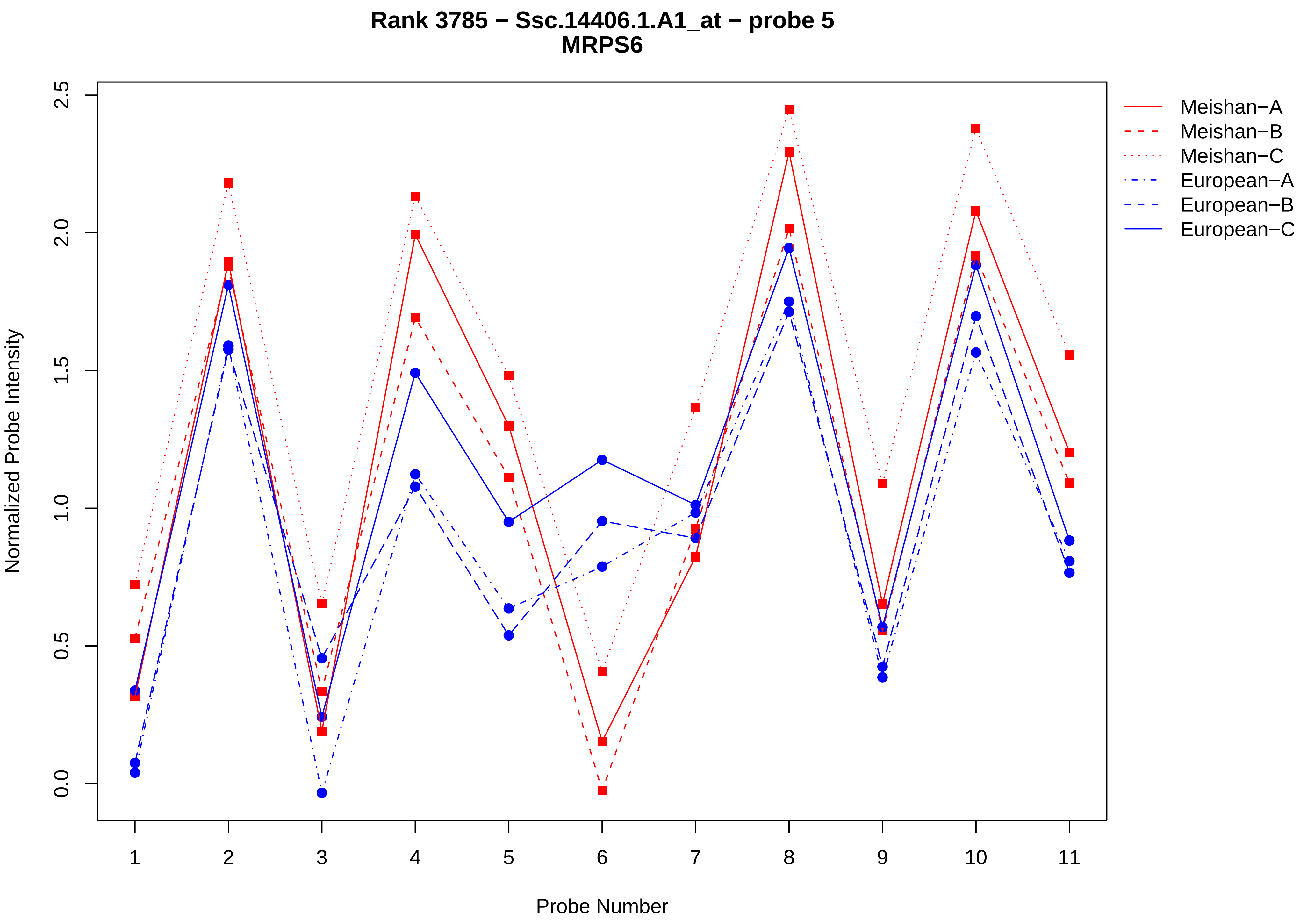

Rank 3785 − Ssc.14406.1.A1_at − probe 5
MRPS6
Normalized Probe Intensity
0.0
0.5
1.0
1.5
2.0
2.5
1
2
3
4
5
6
7
8
9
10
11
Probe Number
Meishan−A
Meishan−B
Meishan−C
European−A
European−B
European−C

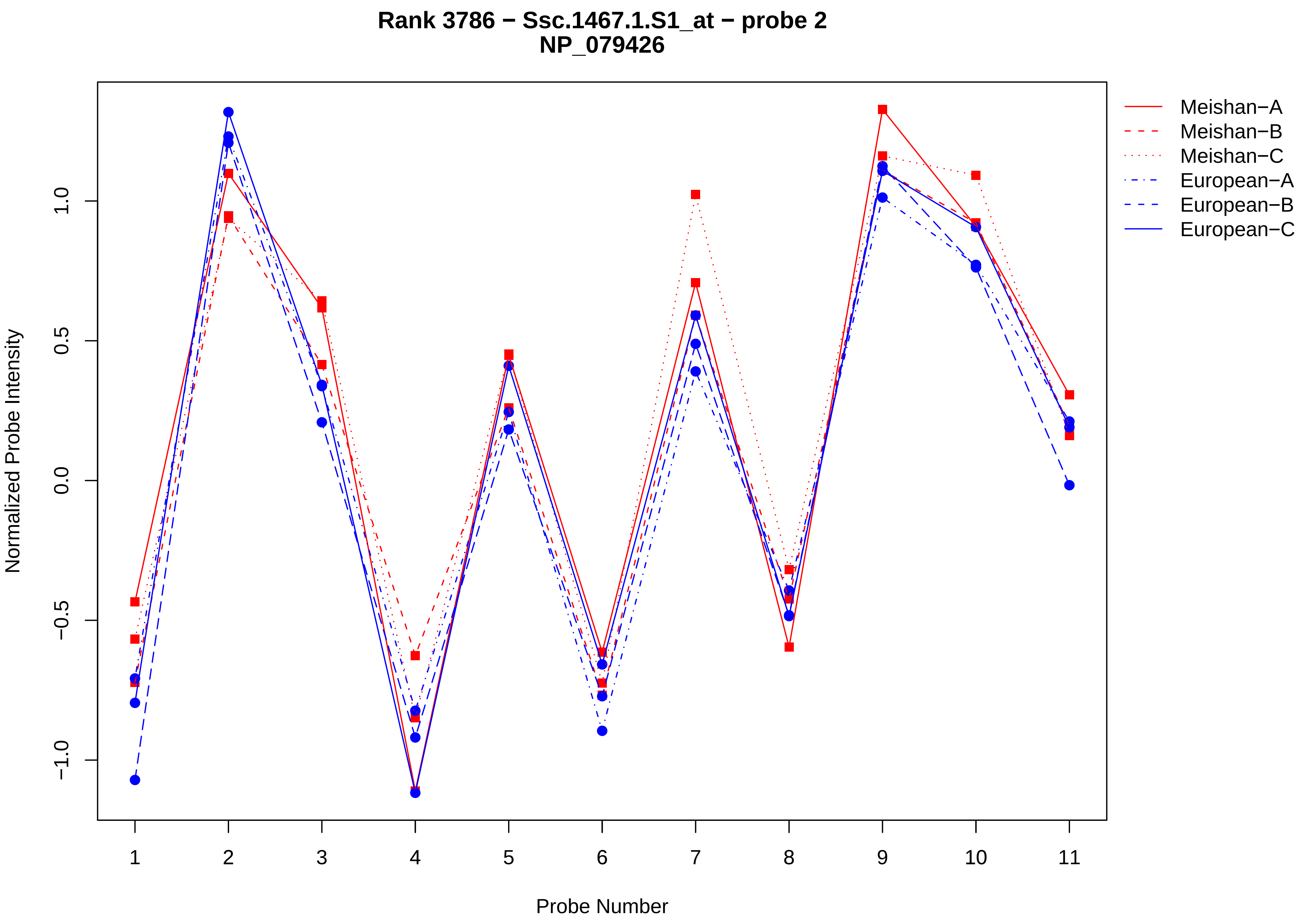

Rank 3786 − Ssc.1467.1.S1_at − probe 2
NP_079426
Normalized Probe Intensity
Probe Number
−1.0
−0.5
0.0
0.5
1.0
1
2
3
4
5
6
7
8
9
10
11
Meishan−A
Meishan−B
Meishan−C
European−A
European−B
European−C

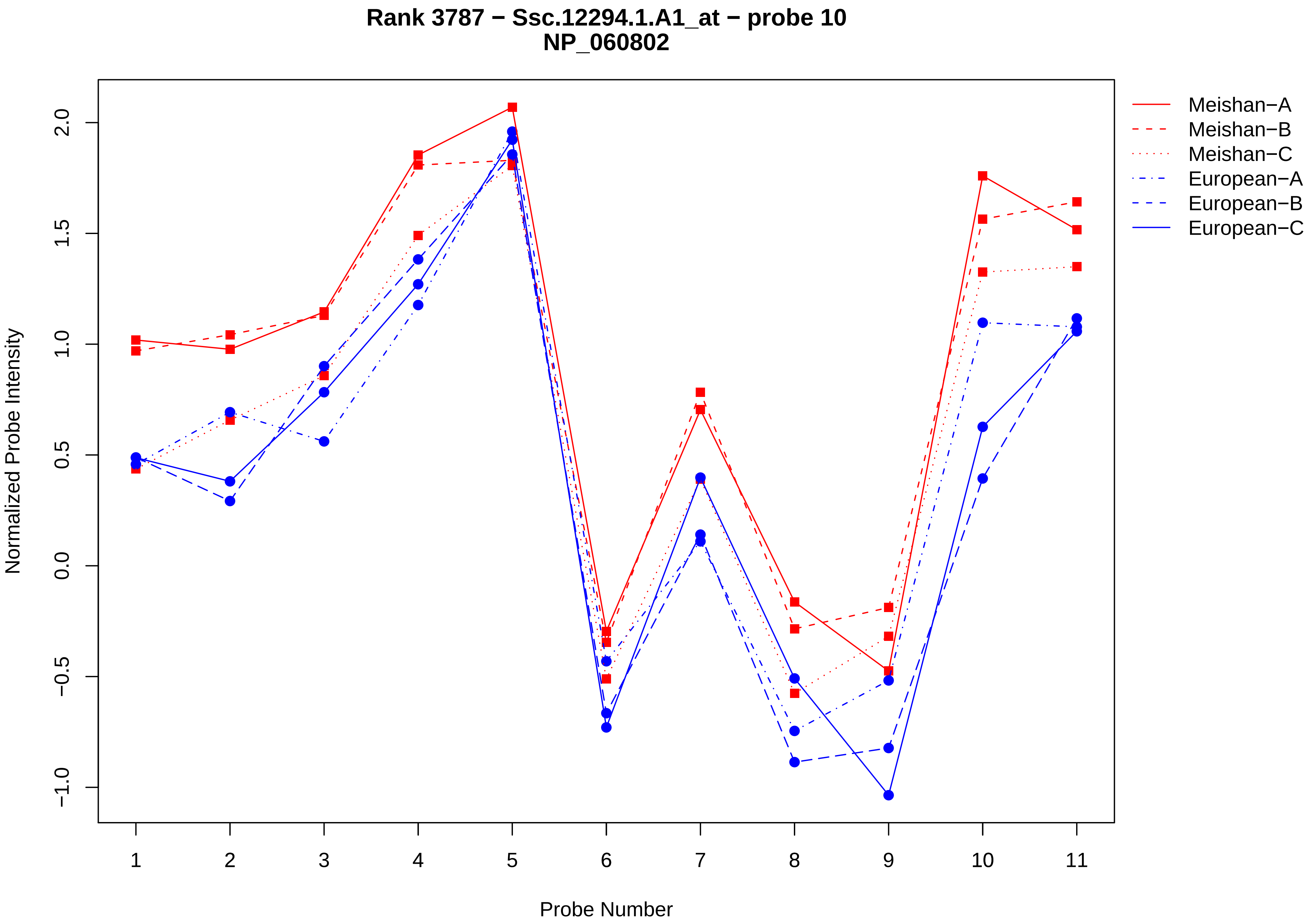

Rank 3787 − Ssc.12294.1.A1_at − probe 10
NP_060802
Normalized Probe Intensity
Probe Number
Meishan−A
Meishan−B
Meishan−C
European−A
European−B
European−C
−1.0
−0.5
0.0
0.5
1.0
1.5
2.0
1
2
3
4
5
6
7
8
9
10
11

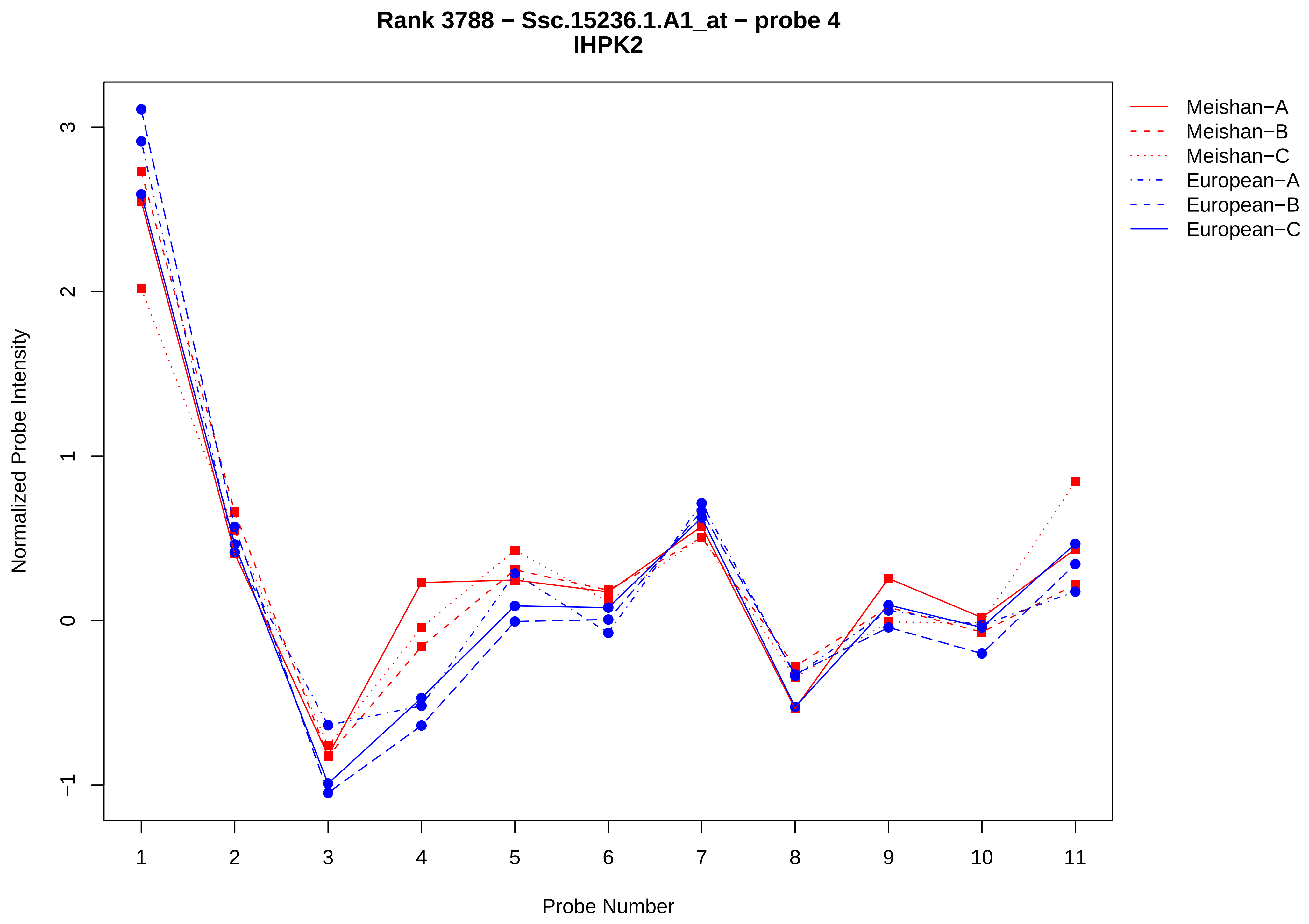

Rank 3788 − Ssc.15236.1.A1_at − probe 4
IHPK2
Normalized Probe Intensity
Probe Number
−1
0
1
2
3
1
2
3
4
5
6
7
8
9
10
11
Meishan−A
Meishan−B
Meishan−C
European−A
European−B
European−C

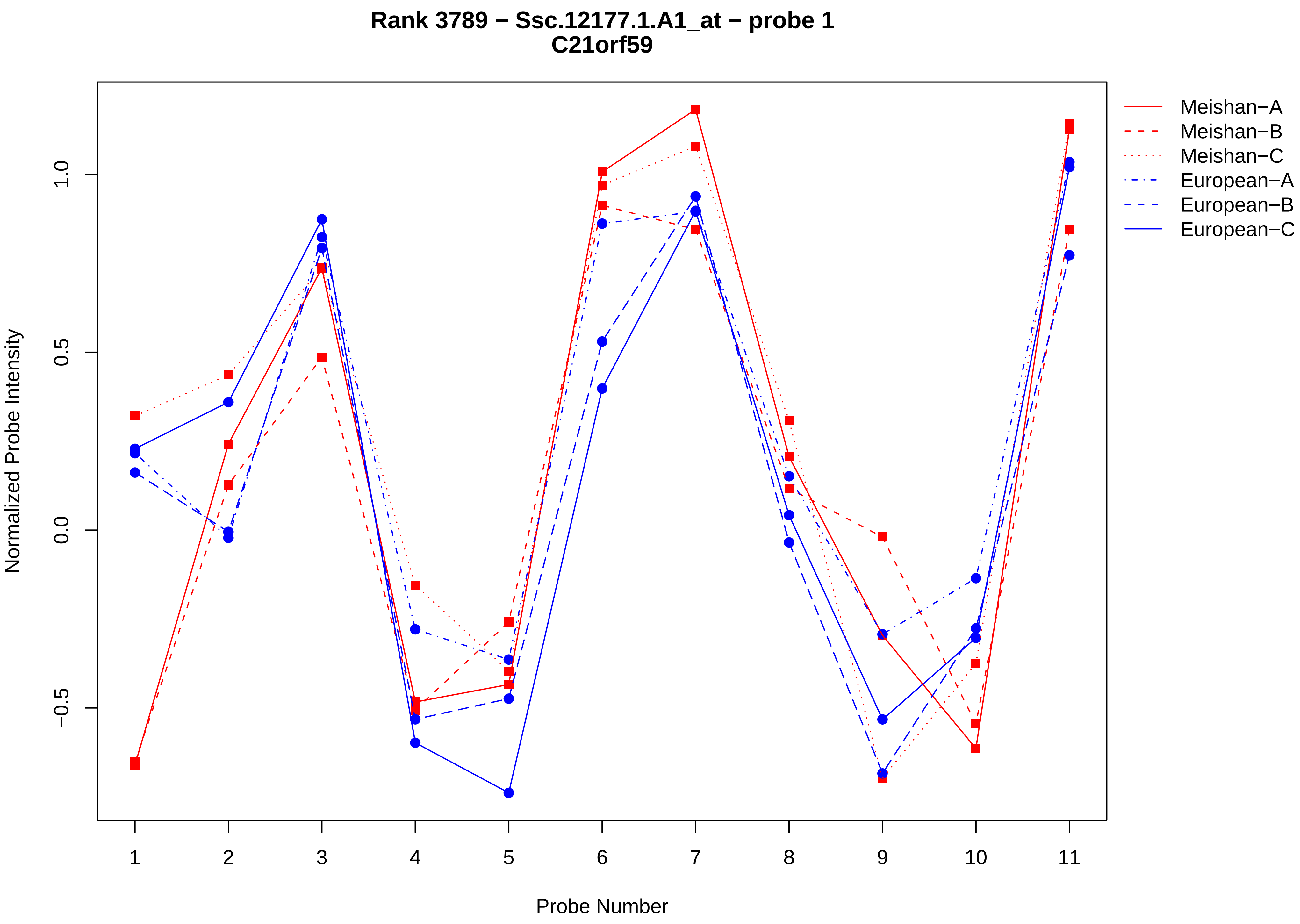

Rank 3789 − Ssc.12177.1.A1_at − probe 1
C21orf59
Normalized Probe Intensity
Probe Number
−0.5
0.0
0.5
1.0
1
2
3
4
5
6
7
8
9
10
11
Meishan−A
Meishan−B
Meishan−C
European−A
European−B
European−C

Rank 3790 − Ssc.26265.1.S1_at − probe 7
GRK6

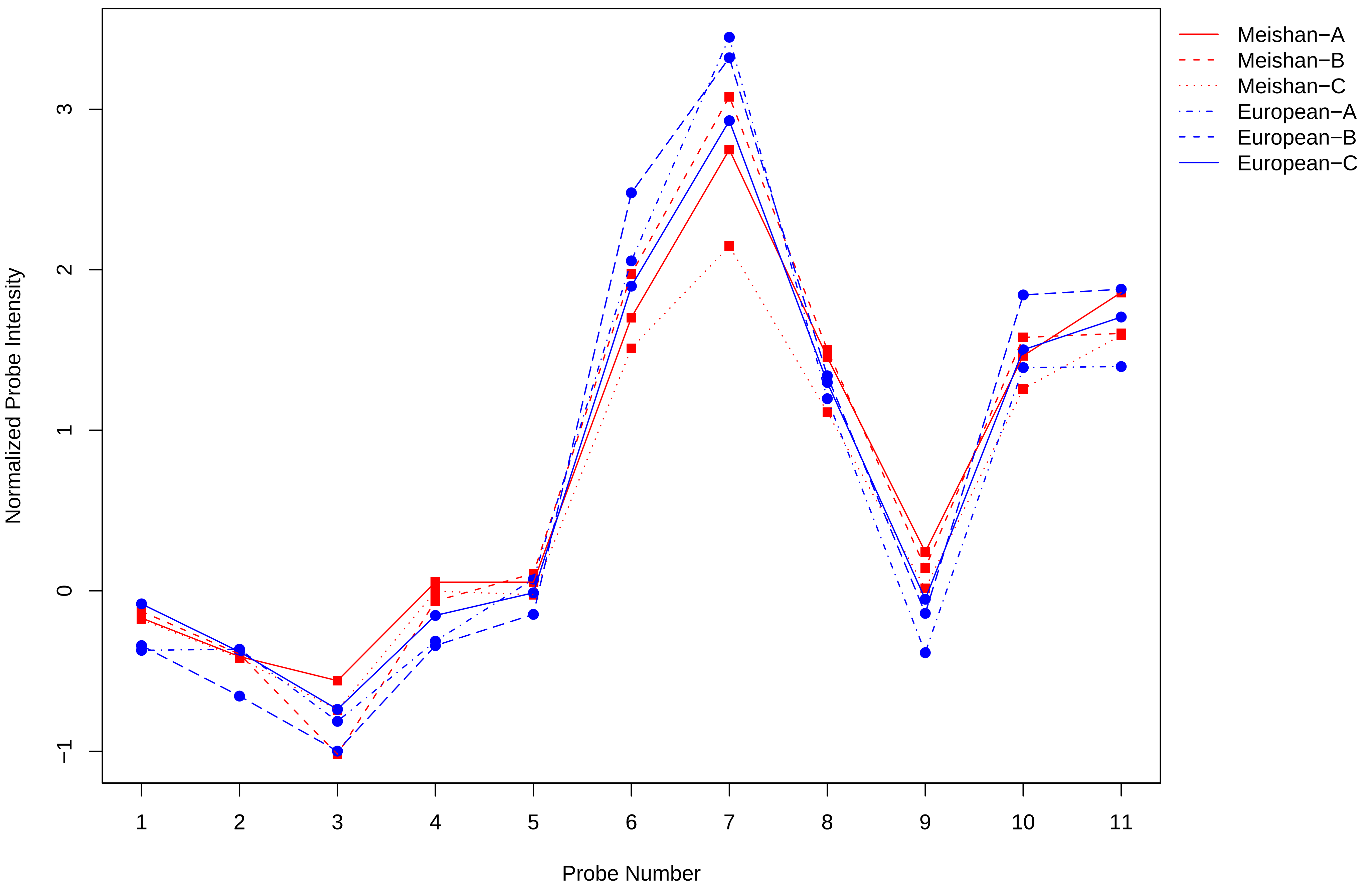

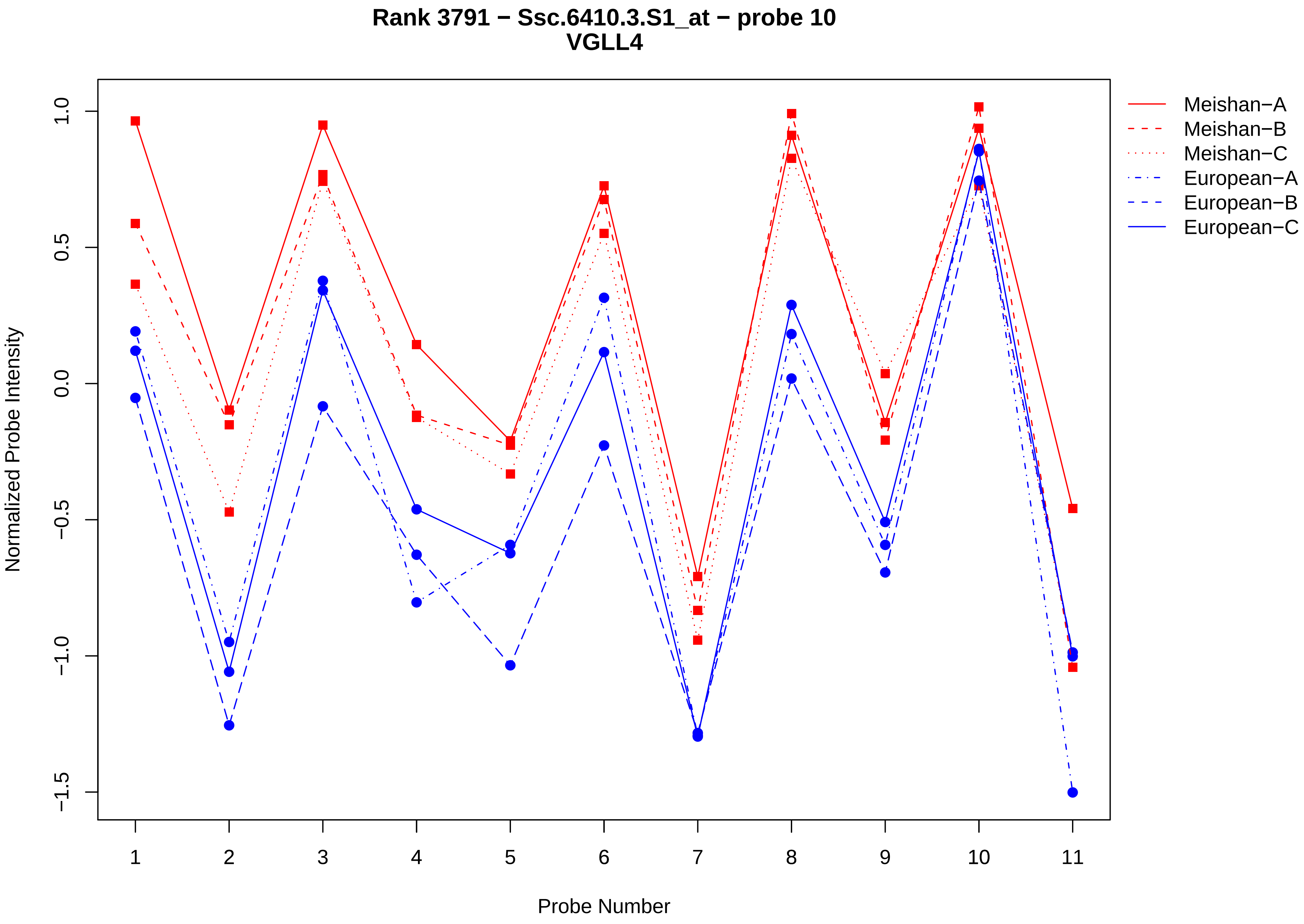

Rank 3791 − Ssc.6410.3.S1_at − probe 10
VGLL4
Normalized Probe Intensity
Probe Number
1.0
0.5
0.0
−0.5
−1.0
−1.5
1
2
3
4
5
6
7
8
9
10
11
Meishan−A
Meishan−B
Meishan−C
European−A
European−B
European−C

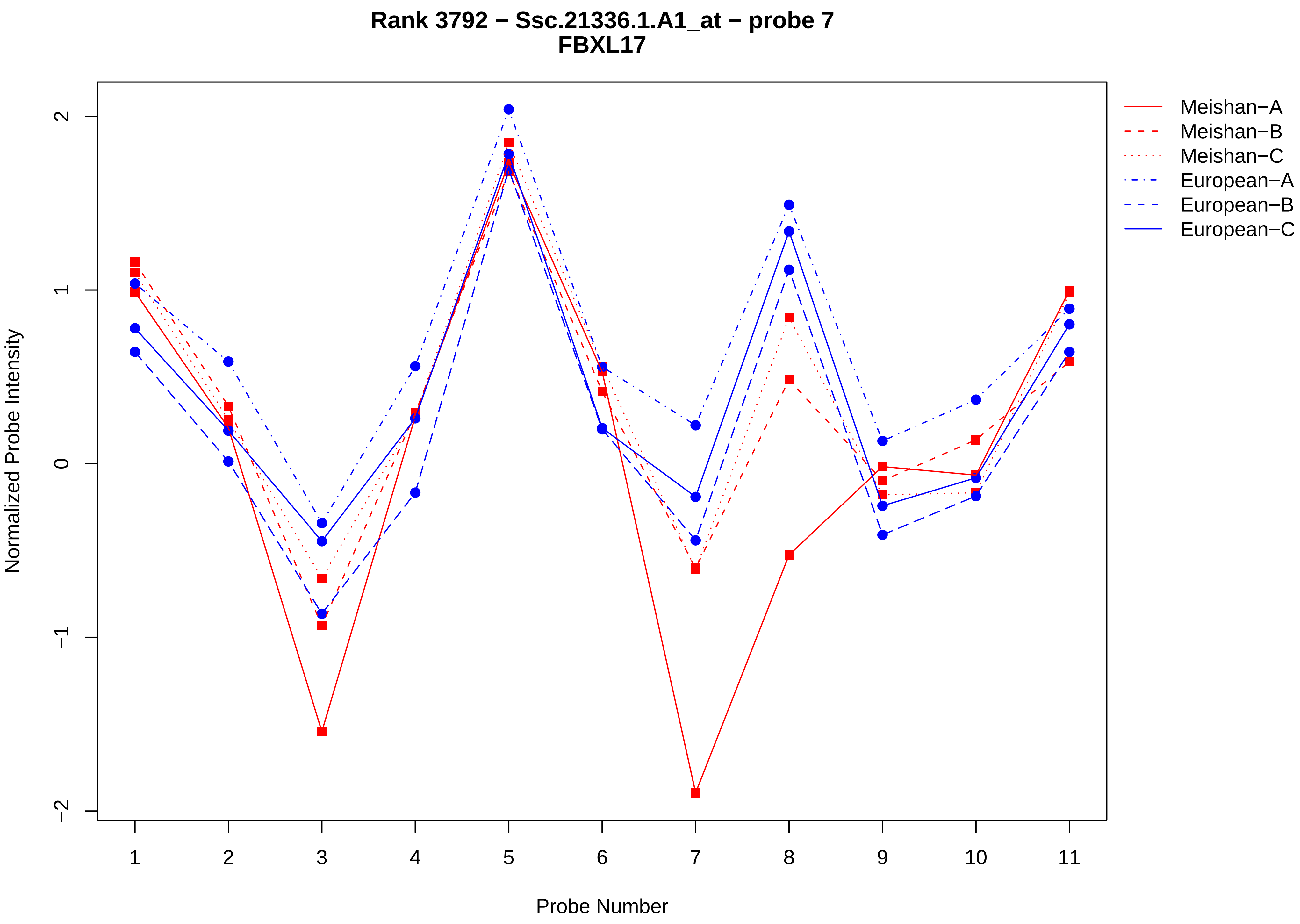

Rank 3792 − Ssc.21336.1.A1_at − probe 7
FBXL17
Normalized Probe Intensity
Probe Number
−2
−1
0
1
2
1
2
3
4
5
6
7
8
9
10
11
Meishan−A
Meishan−B
Meishan−C
European−A
European−B
European−C

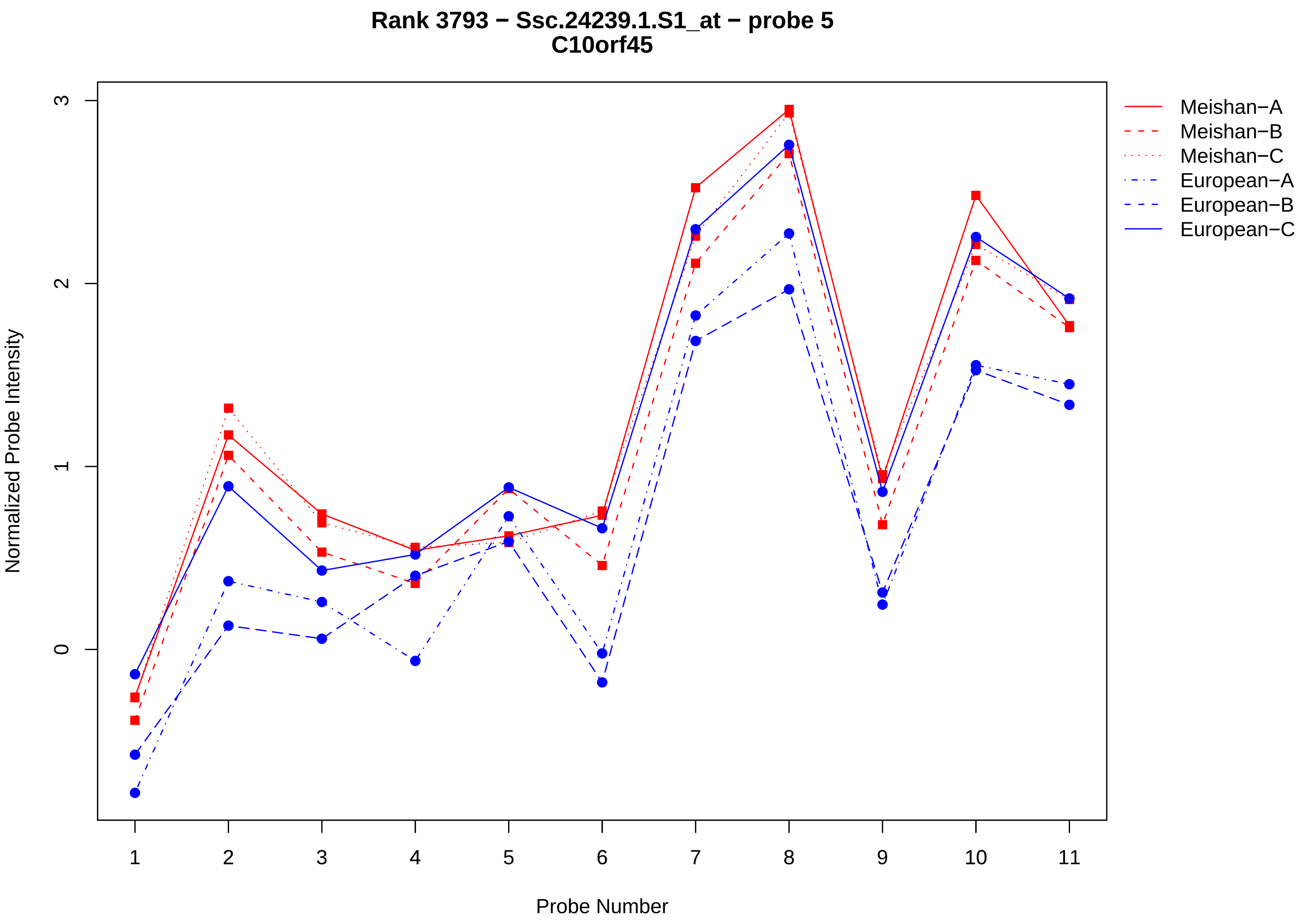

Rank 3793 − Ssc.24239.1.S1_at − probe 5
C10orf45
Normalized Probe Intensity
Probe Number
0
1
2
3
1
2
3
4
5
6
7
8
9
10
11
Meishan−A
Meishan−B
Meishan−C
European−A
European−B
European−C

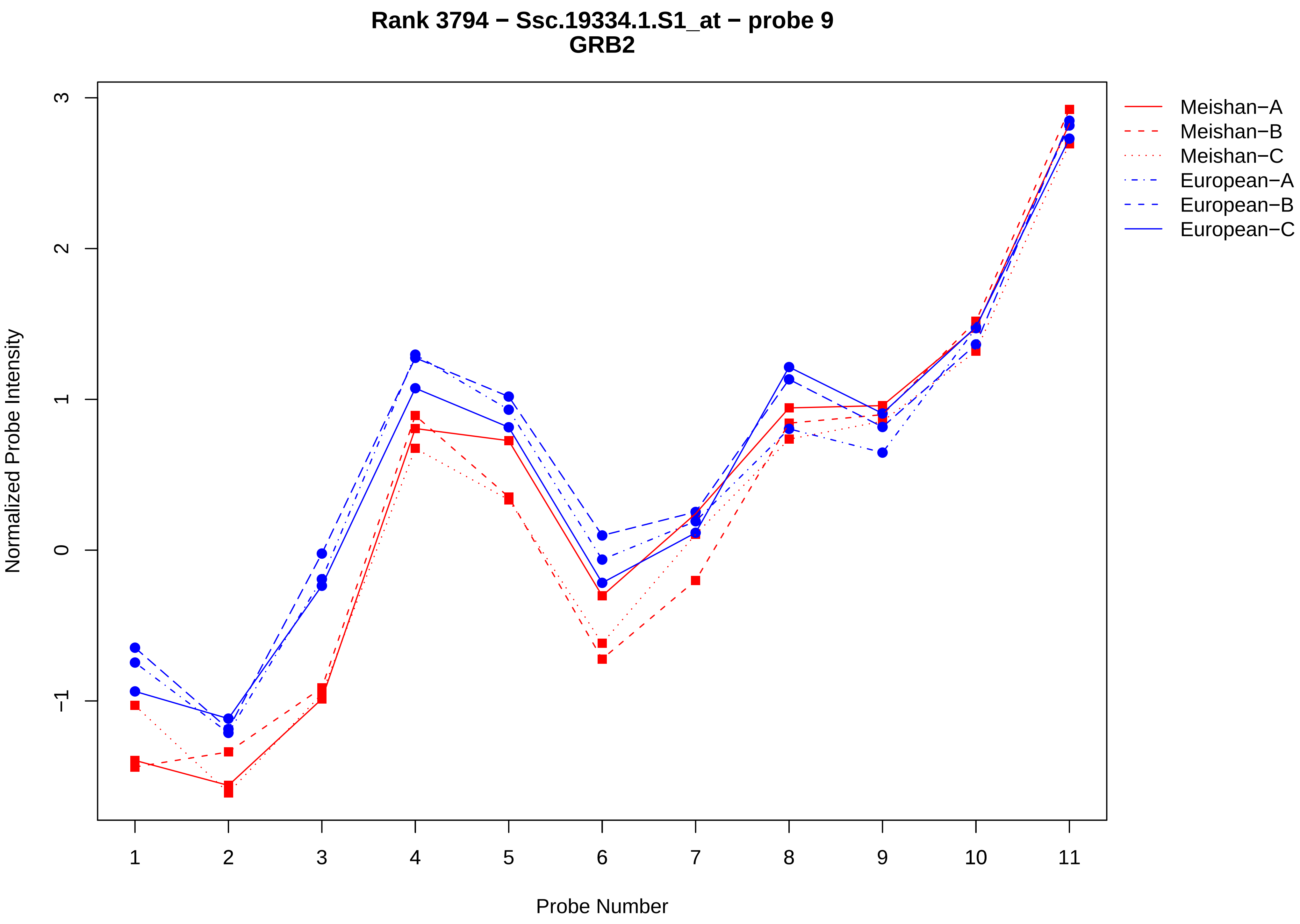

Rank 3794 − Ssc.19334.1.S1_at − probe 9
GRB2
Normalized Probe Intensity
Probe Number
Meishan−A
Meishan−B
Meishan−C
European−A
European−B
European−C

Rank 3795 − Ssc.8288.1.A1_at − probe 4
Q8TE36

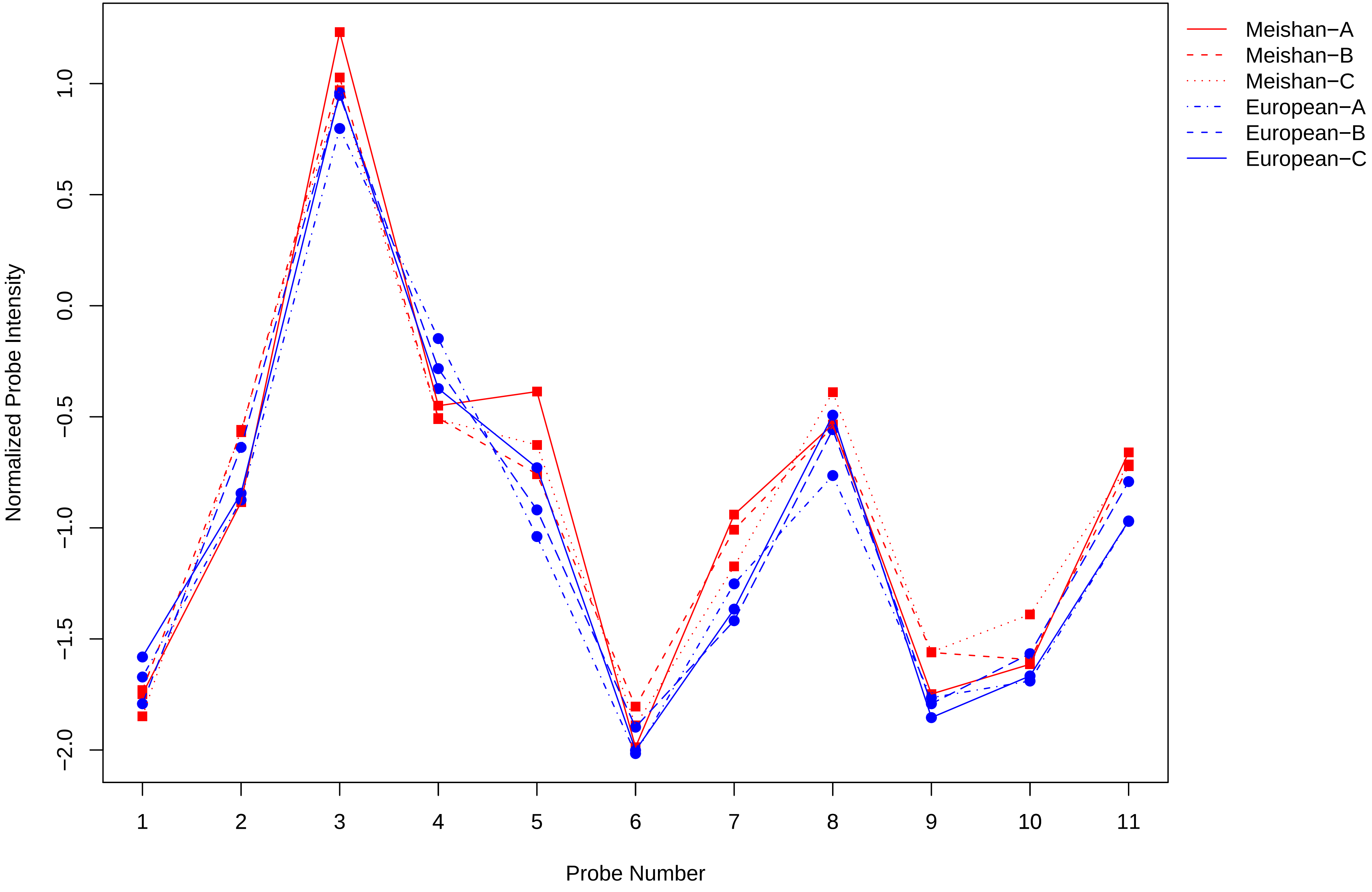

Rank 3796 − Ssc.2048.1.S1_at − probe 11
TIMM17A

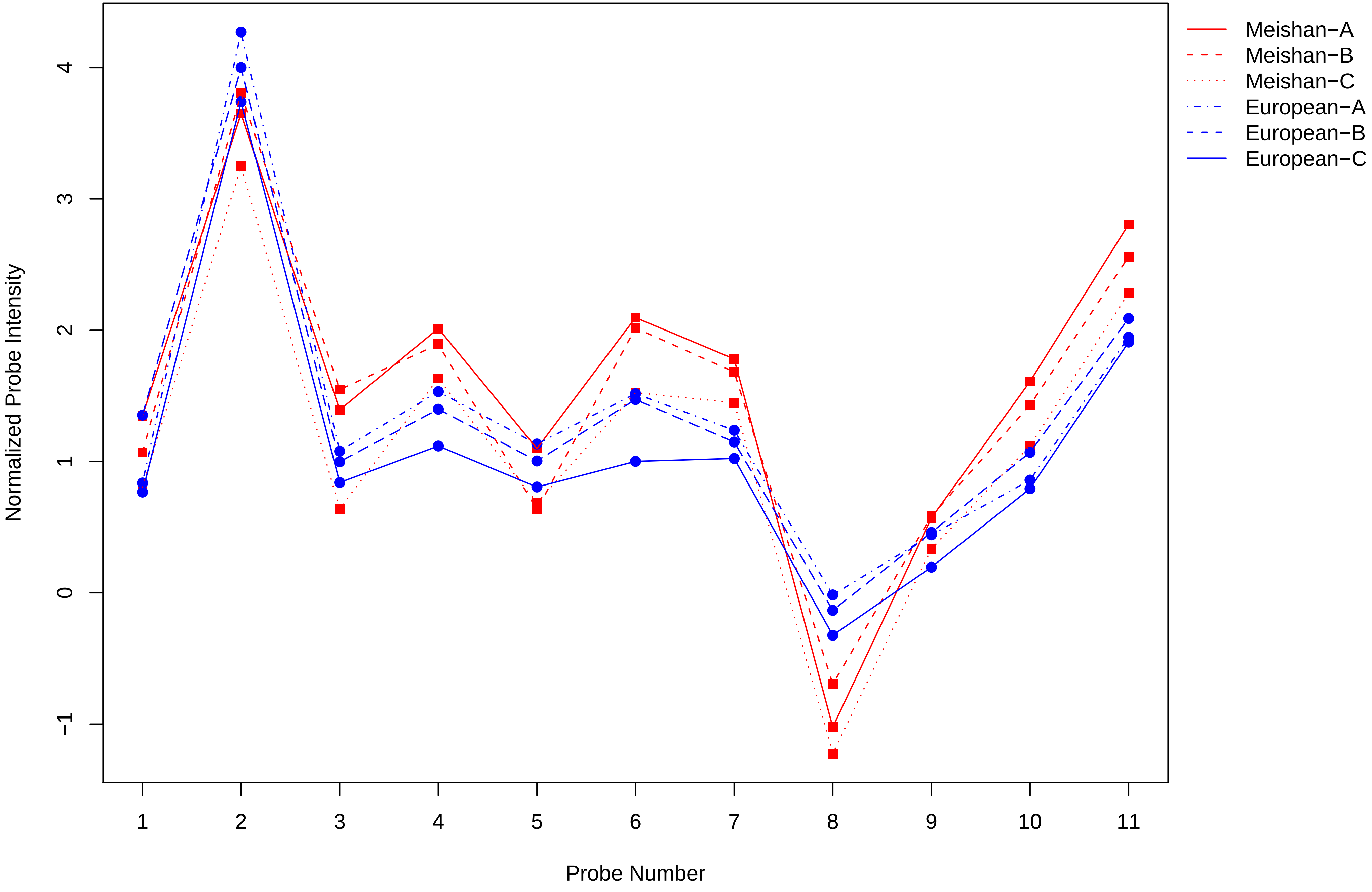

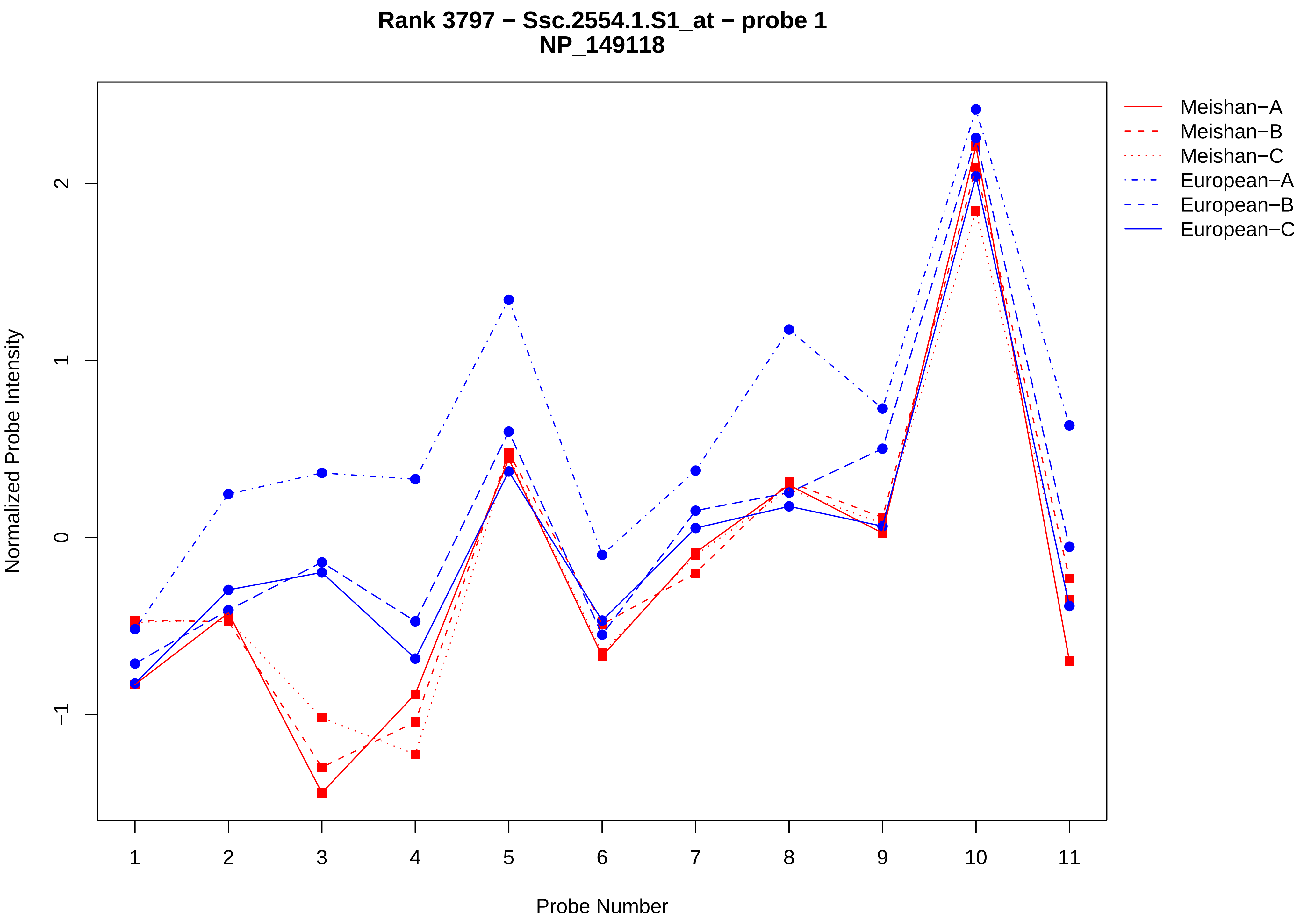

Rank 3797 − Ssc.2554.1.S1_at − probe 1
NP_149118
Meishan−A
Meishan−B
Meishan−C
European−A
European−B
European−C
Normalized Probe Intensity
Probe Number
−1
0
1
2
1
2
3
4
5
6
7
8
9
10
11

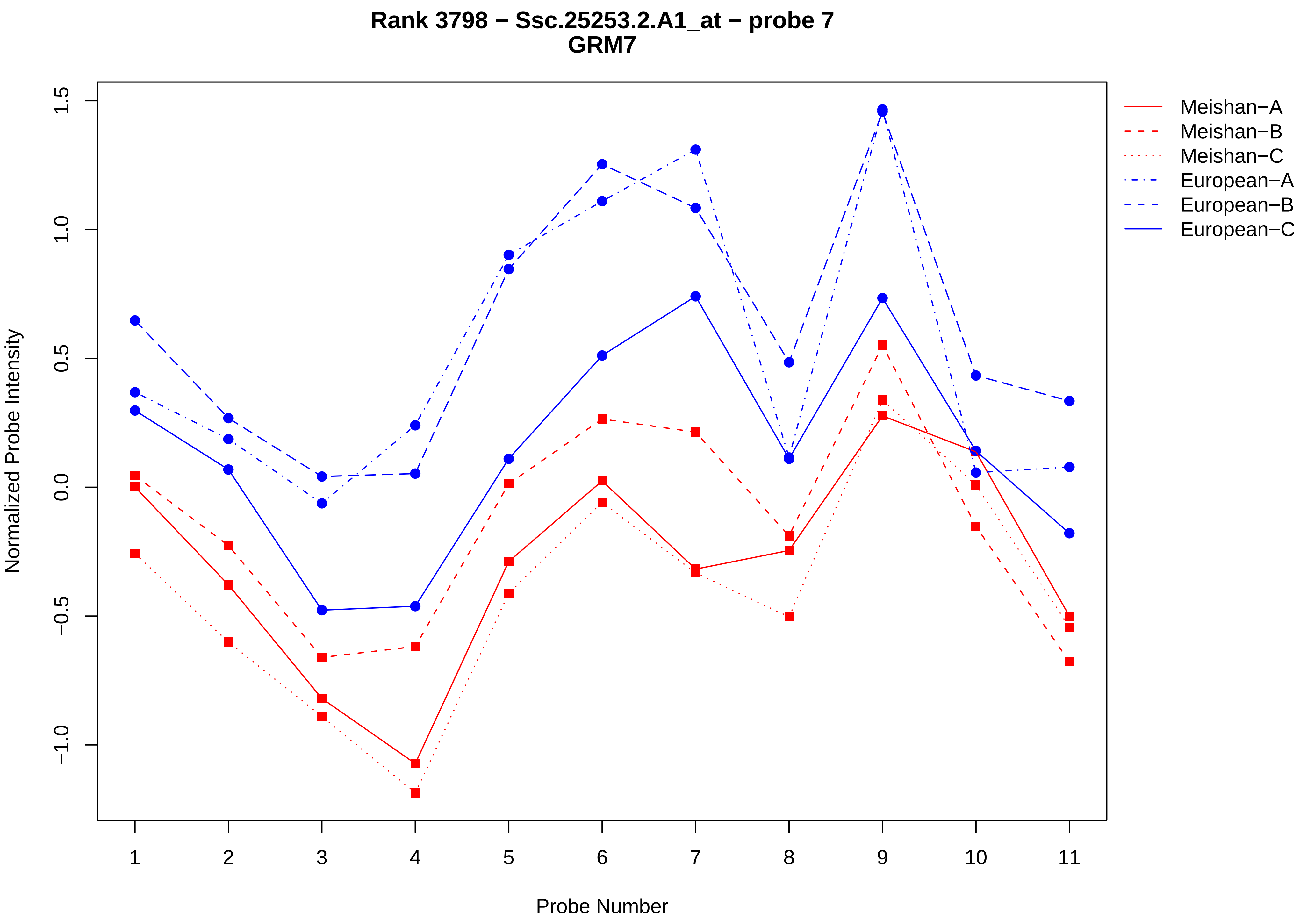
Rank 3798 − Ssc.25253.2.A1_at − probe 7
GRM7
Normalized Probe Intensity
Probe Number
−1.0
−0.5
0.0
0.5
1.0
1.5
1
2
3
4
5
6
7
8
9
10
11
Meishan−A
Meishan−B
Meishan−C
European−A
European−B
European−C

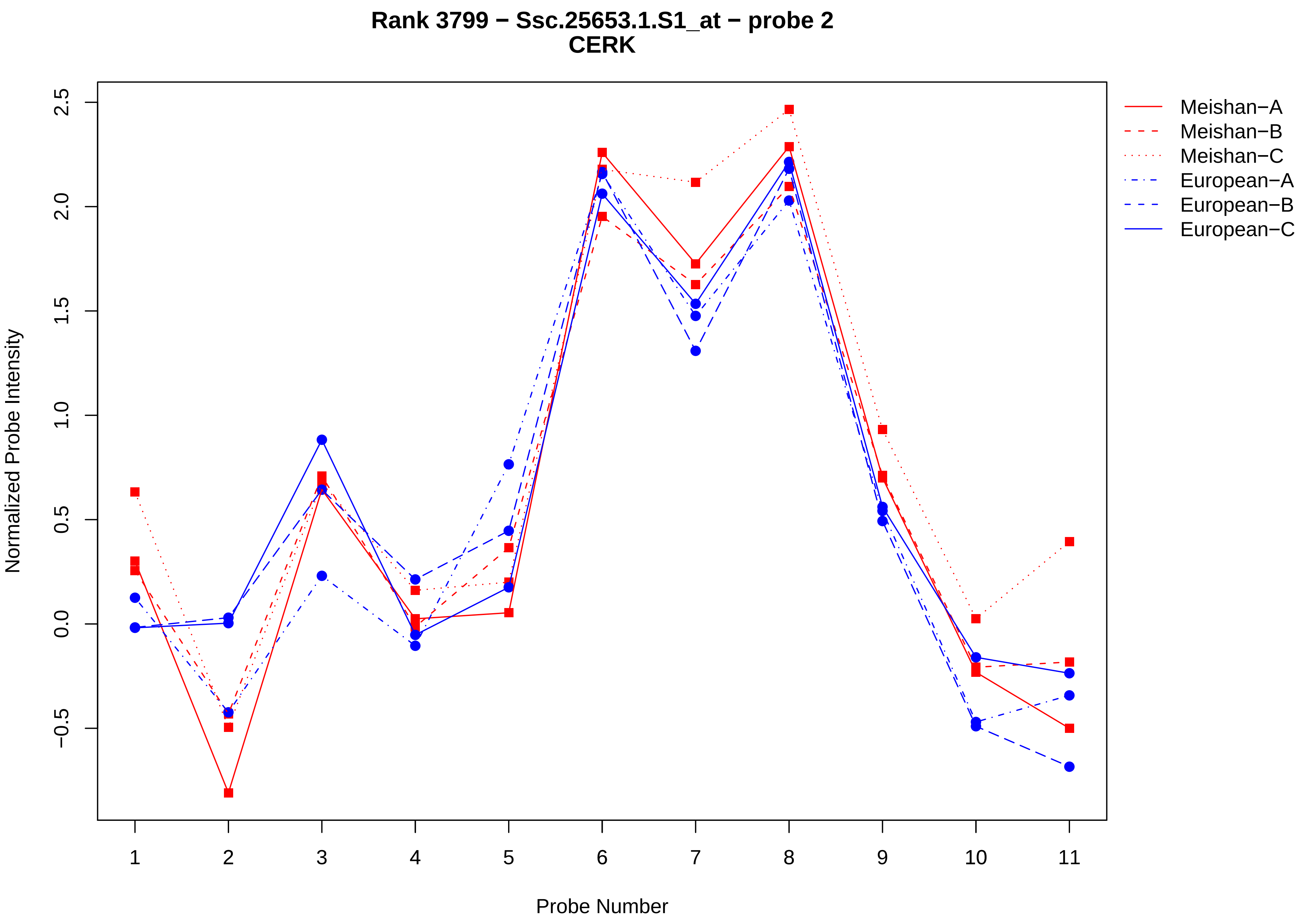
Rank 3799 − Ssc.25653.1.S1_at − probe 2
CERK
Normalized Probe Intensity
Probe Number
Meishan−A
Meishan−B
Meishan−C
European−A
European−B
European−C
2.5
2.0
1.5
1.0
0.5
0.0
−0.5
1
2
3
4
5
6
7
8
9
10
11

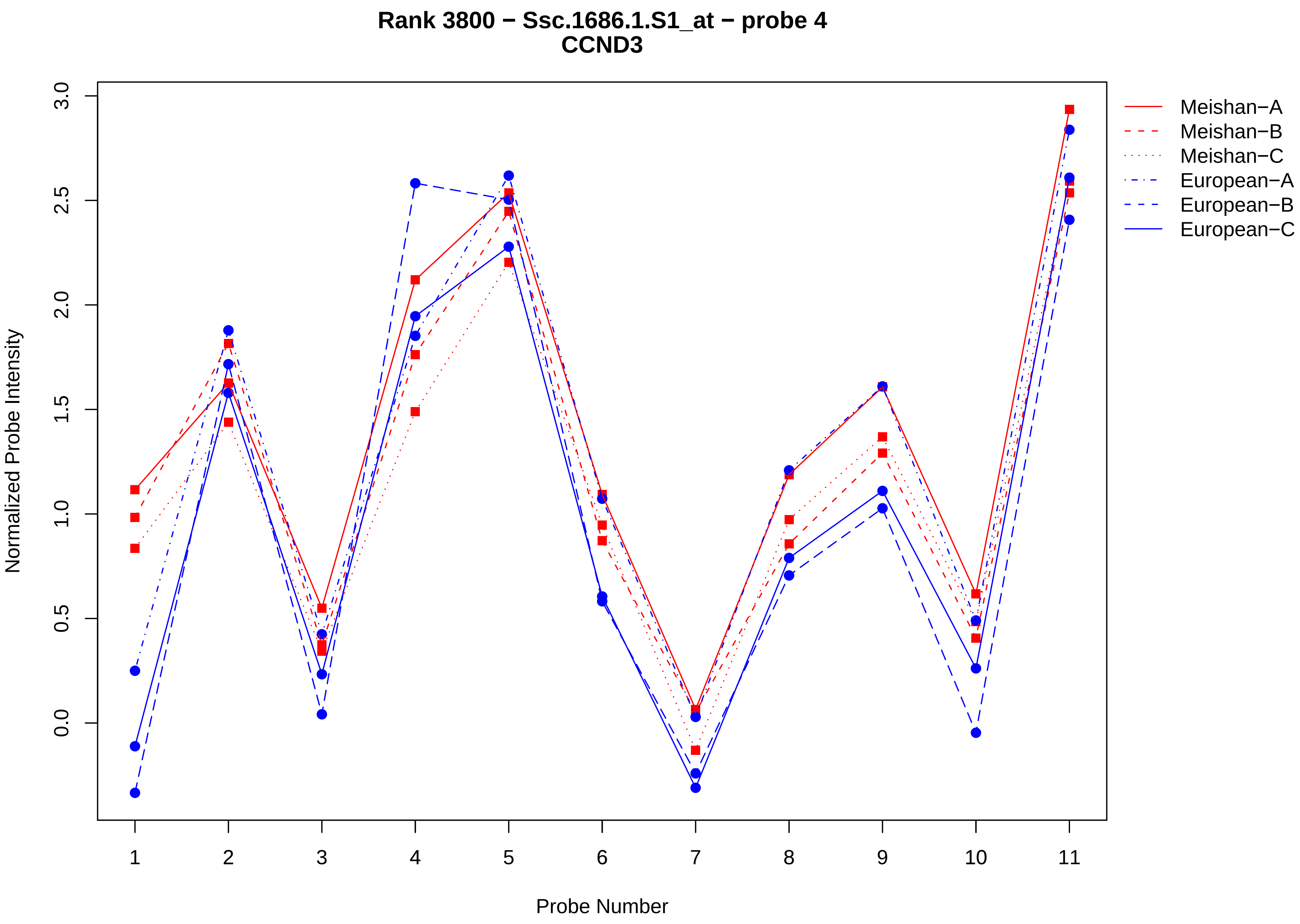

Rank 3800 − Ssc.1686.1.S1_at − probe 4
CCND3
Normalized Probe Intensity
0.0
0.5
1.0
1.5
2.0
2.5
3.0
1
2
3
4
5
6
7
8
9
10
11
Probe Number
Meishan−A
Meishan−B
Meishan−C
European−A
European−B
European−C

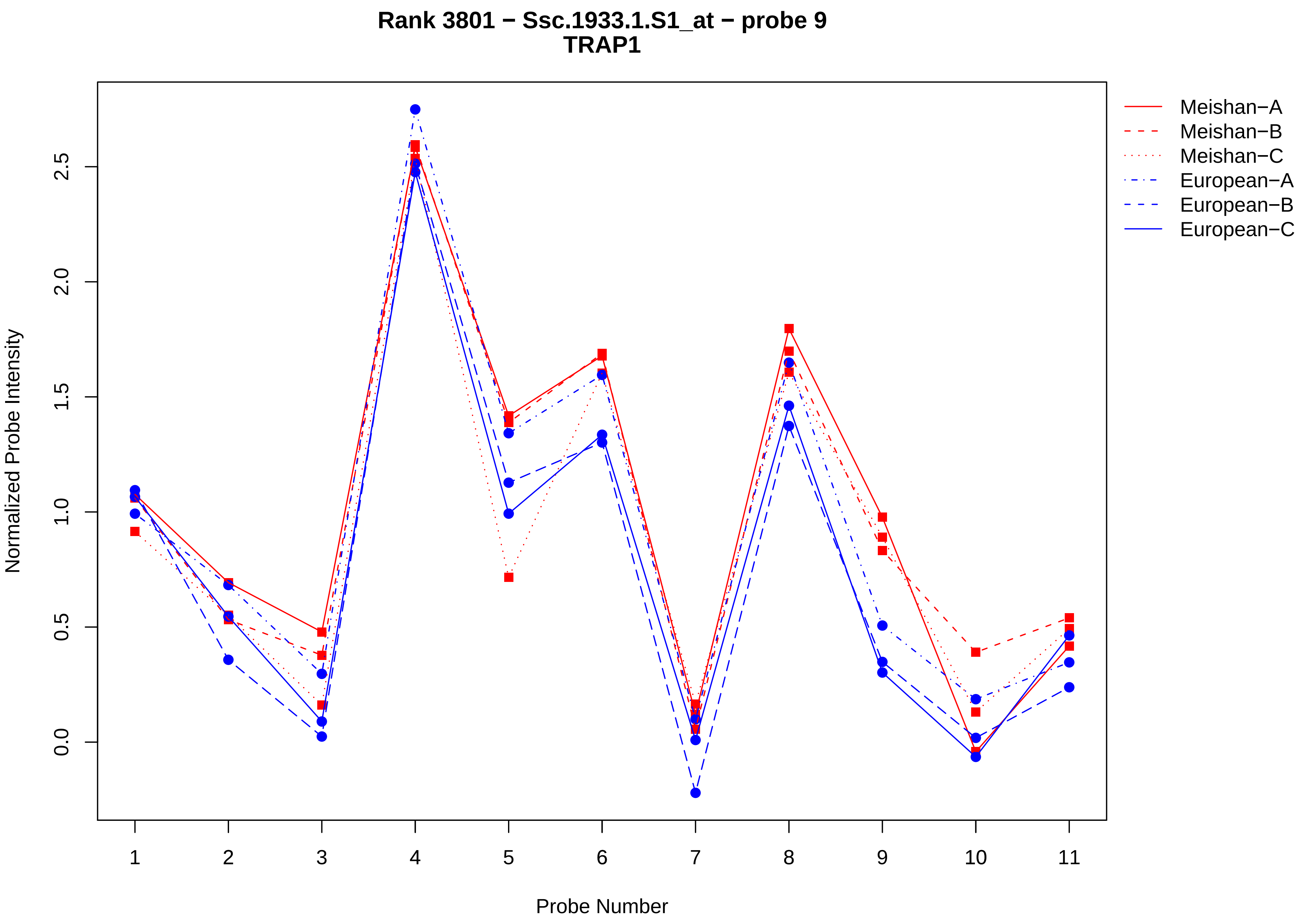

Rank 3801 − Ssc.1933.1.S1_at − probe 9
TRAP1
Normalized Probe Intensity
0.0
0.5
1.0
1.5
2.0
2.5
1
2
3
4
5
6
7
8
9
10
11
Probe Number
Meishan−A
Meishan−B
Meishan−C
European−A
European−B
European−C

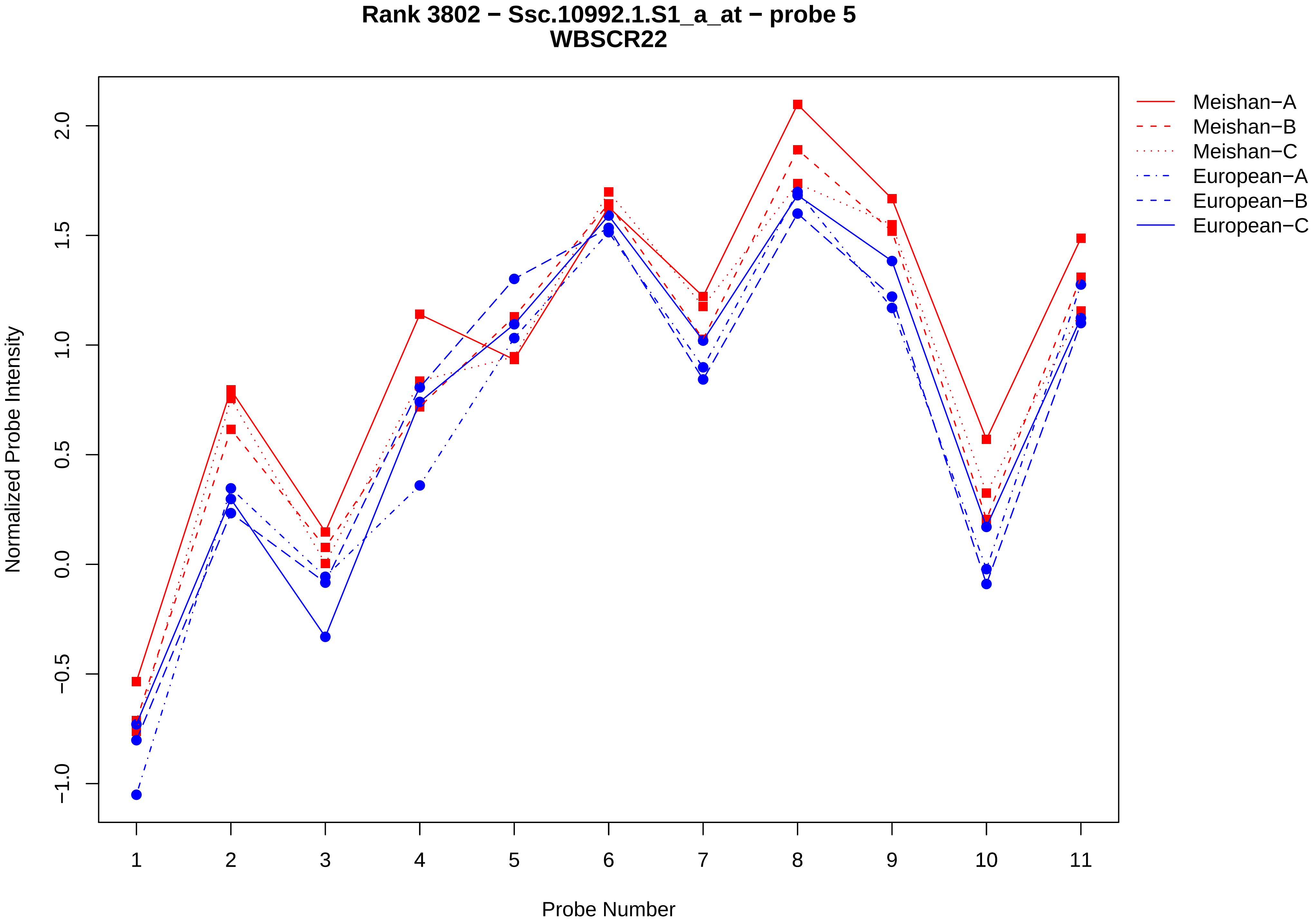

Rank 3802 − Ssc.10992.1.S1_a_at − probe 5
WBSCR22
Normalized Probe Intensity
Probe Number
−1.0
−0.5
0.0
0.5
1.0
1.5
2.0
1
2
3
4
5
6
7
8
9
10
11
Meishan−A
Meishan−B
Meishan−C
European−A
European−B
European−C

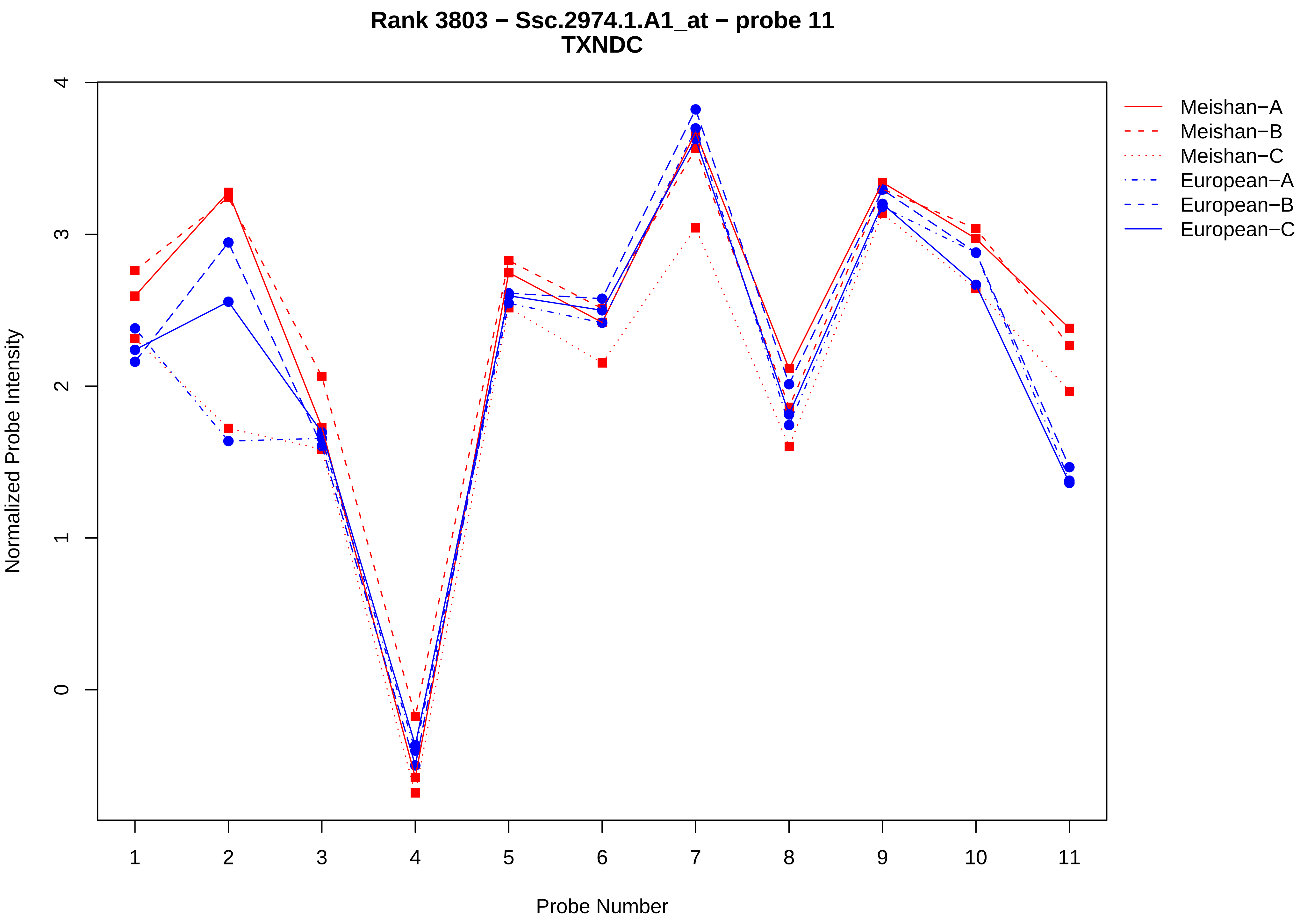

Rank 3803 − Ssc.2974.1.A1_at − probe 11
TXNDC
Normalized Probe Intensity
Probe Number
0
1
2
3
4
1
2
3
4
5
6
7
8
9
10
11
Meishan−A
Meishan−B
Meishan−C
European−A
European−B
European−C

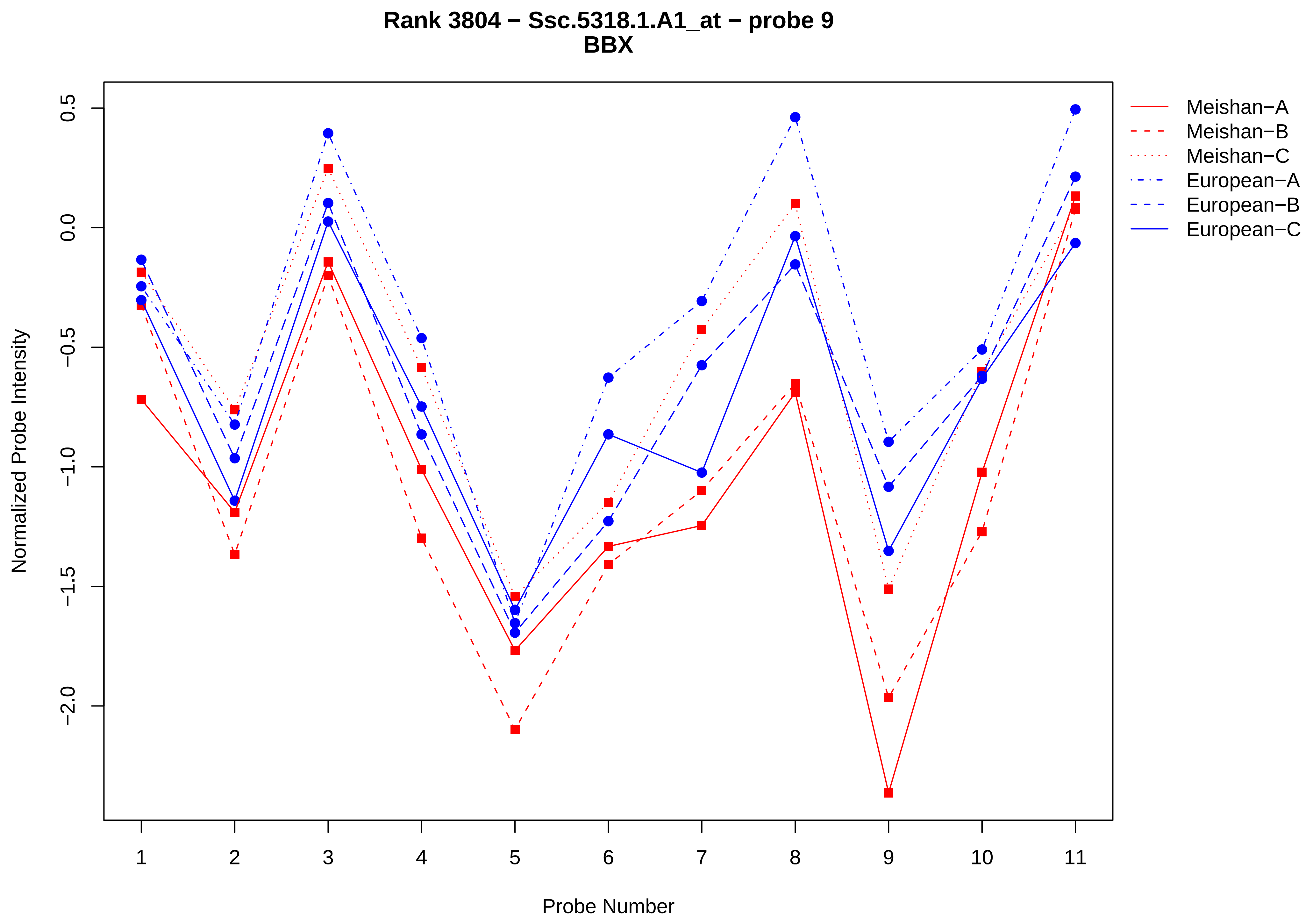

Rank 3804 − Ssc.5318.1.A1_at − probe 9
BBX
Normalized Probe Intensity
Probe Number
0.5
0.0
−0.5
−1.0
−1.5
−2.0
1
2
3
4
5
6
7
8
9
10
11
Meishan−A
Meishan−B
Meishan−C
European−A
European−B
European−C

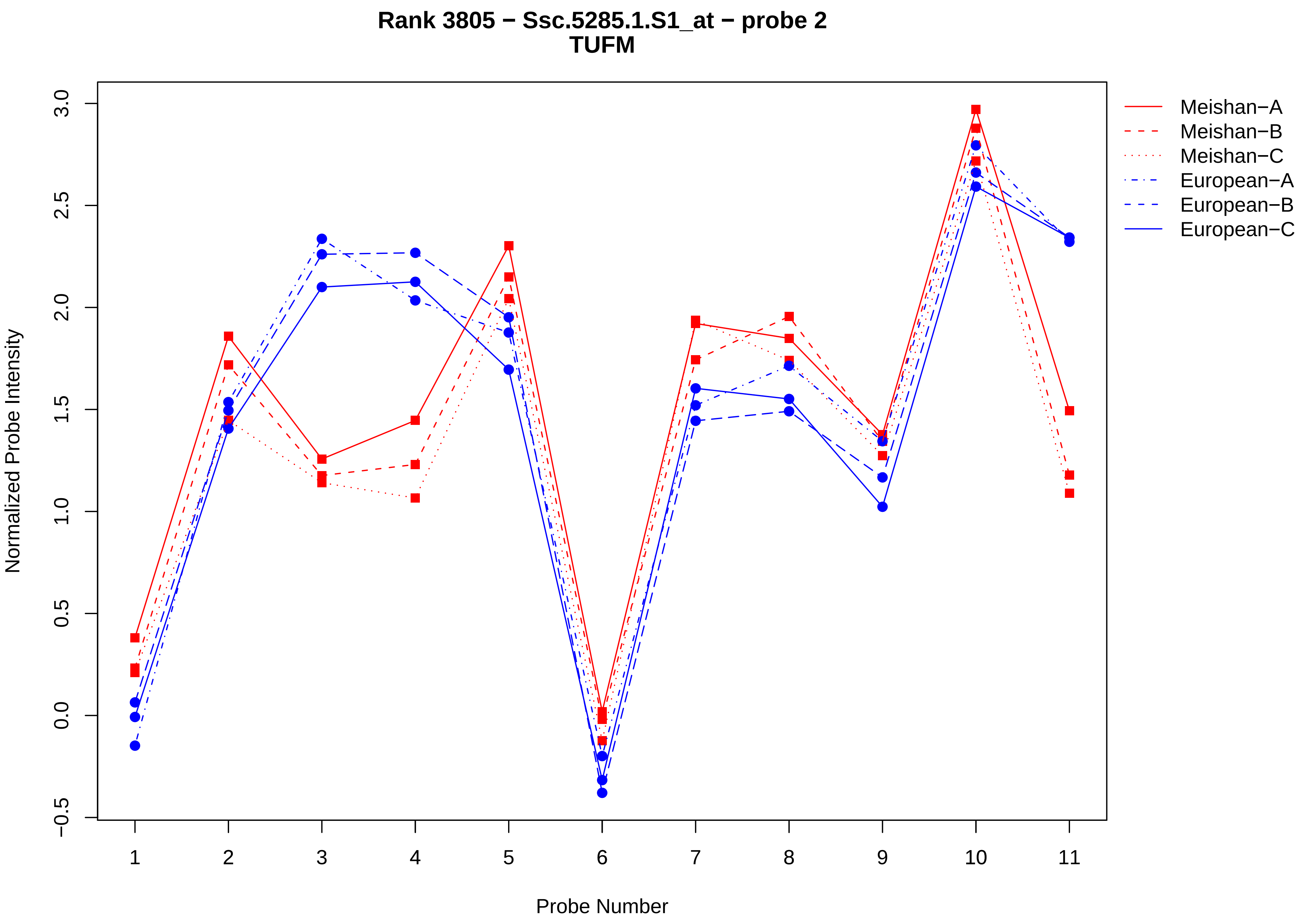

Rank 3805 − Ssc.5285.1.S1_at − probe 2
TUFM
Normalized Probe Intensity
Probe Number
Meishan−A
Meishan−B
Meishan−C
European−A
European−B
European−C

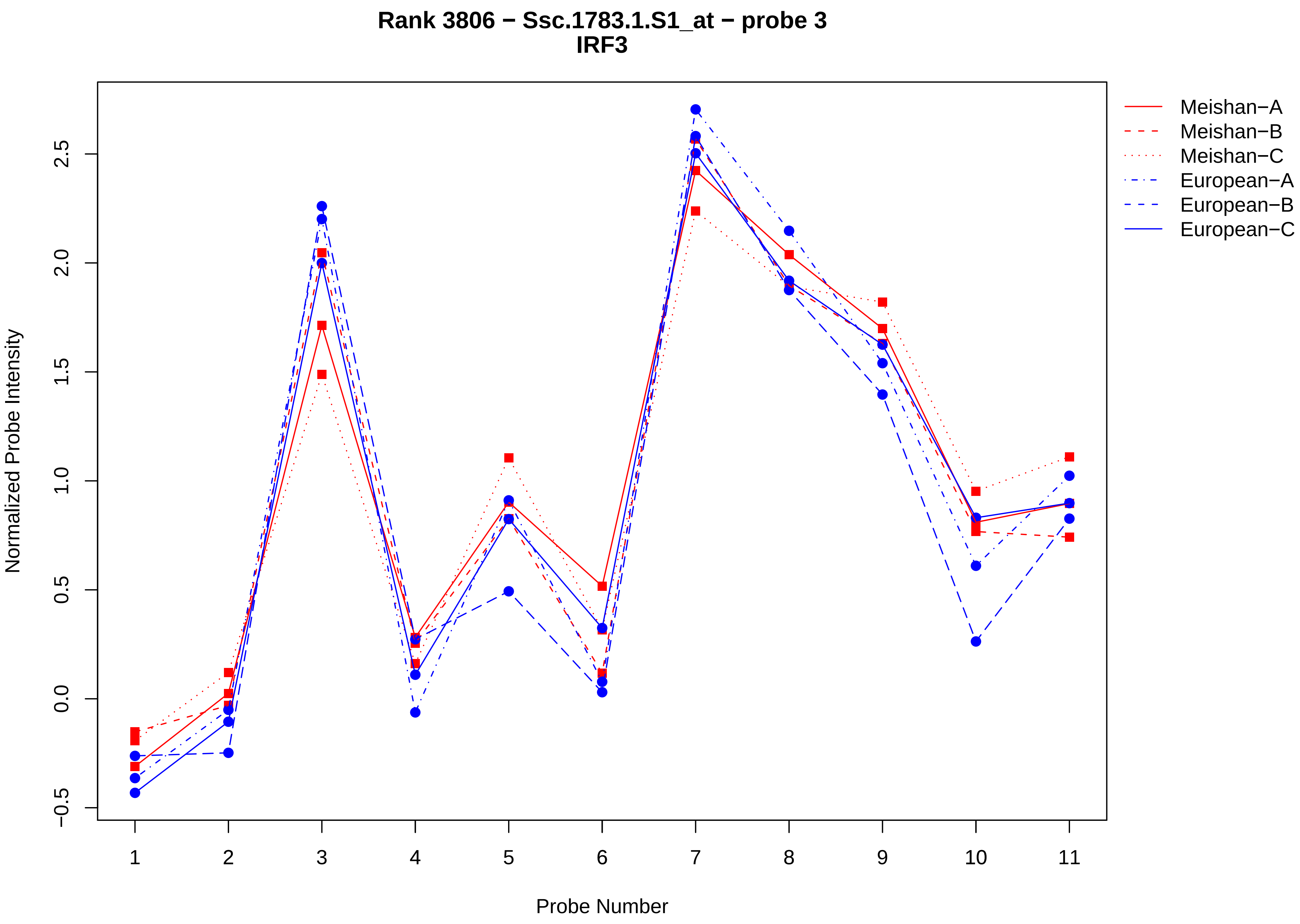

Rank 3806 − Ssc.1783.1.S1_at − probe 3
IRF3
Normalized Probe Intensity
Probe Number
−0.5
0.0
0.5
1.0
1.5
2.0
2.5
1
2
3
4
5
6
7
8
9
10
11
Meishan−A
Meishan−B
Meishan−C
European−A
European−B
European−C

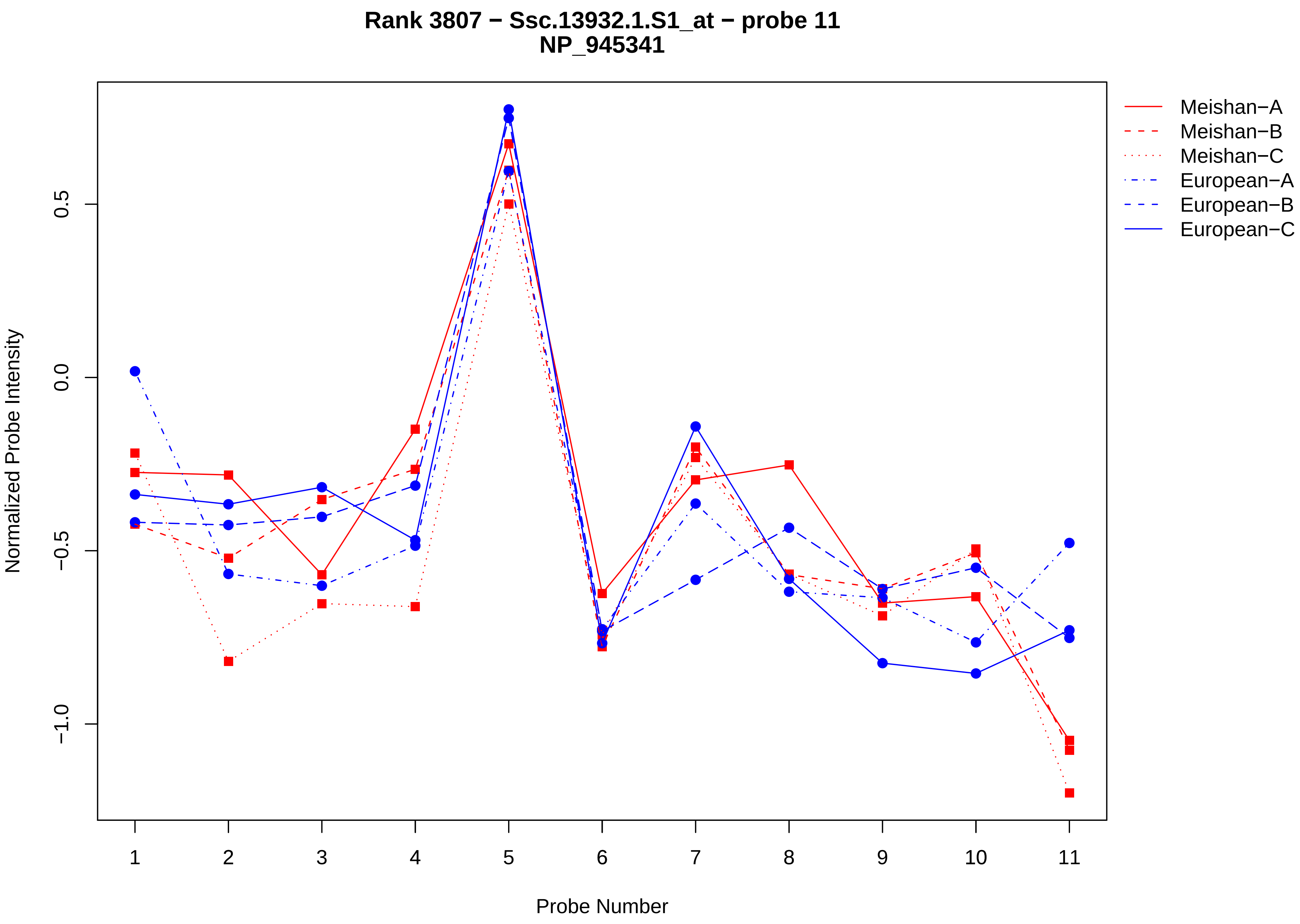
Rank 3807 − Ssc.13932.1.S1_at − probe 11
NP_945341
Normalized Probe Intensity
Probe Number
0.5
0.0
−0.5
−1.0
1
2
3
4
5
6
7
8
9
10
11
Meishan−A
Meishan−B
Meishan−C
European−A
European−B
European−C

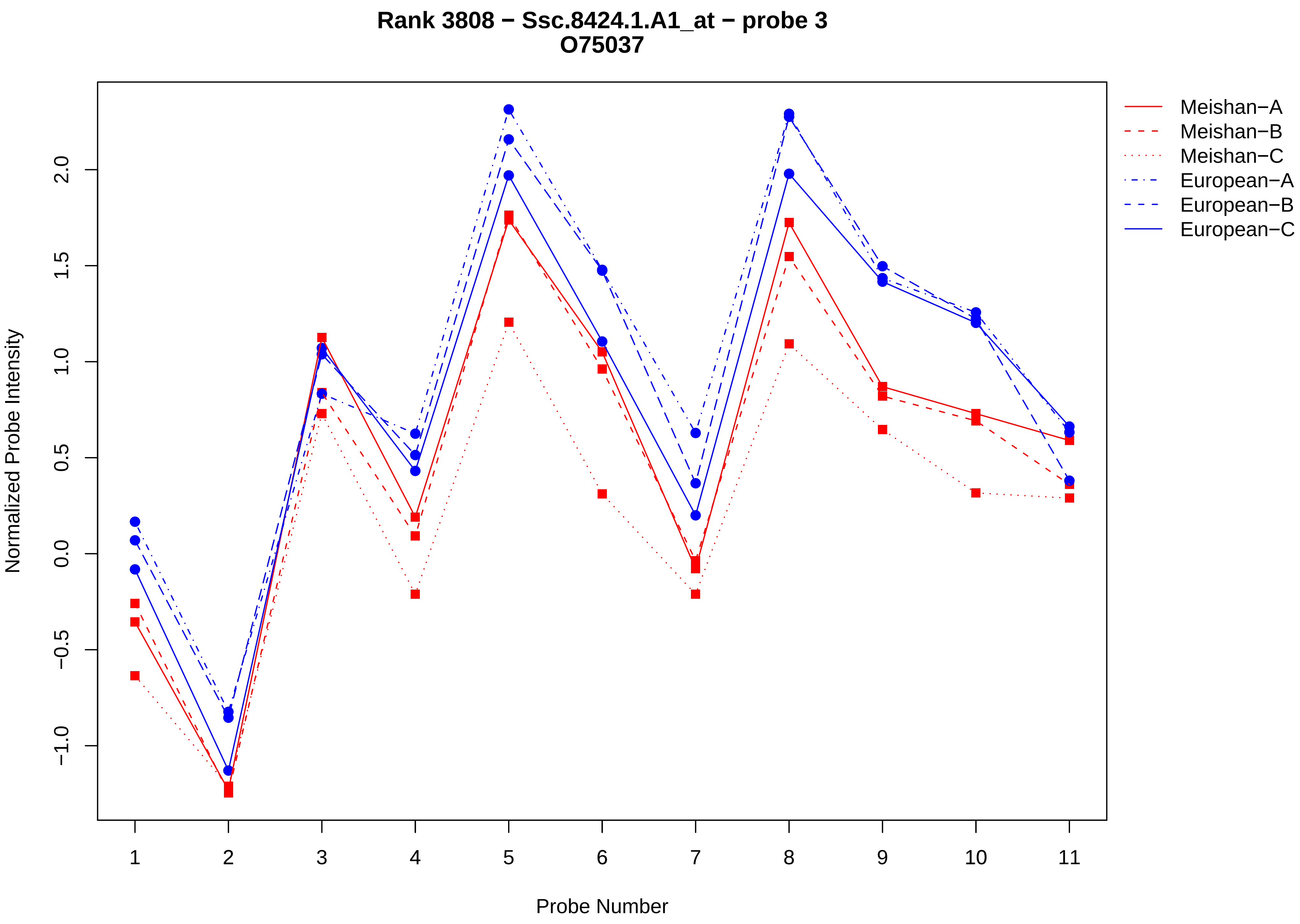

Rank 3808 − Ssc.8424.1.A1_at − probe 3
O75037
Normalized Probe Intensity
Probe Number
−1.0
−0.5
0.0
0.5
1.0
1.5
2.0
1
2
3
4
5
6
7
8
9
10
11
Meishan−A
Meishan−B
Meishan−C
European−A
European−B
European−C

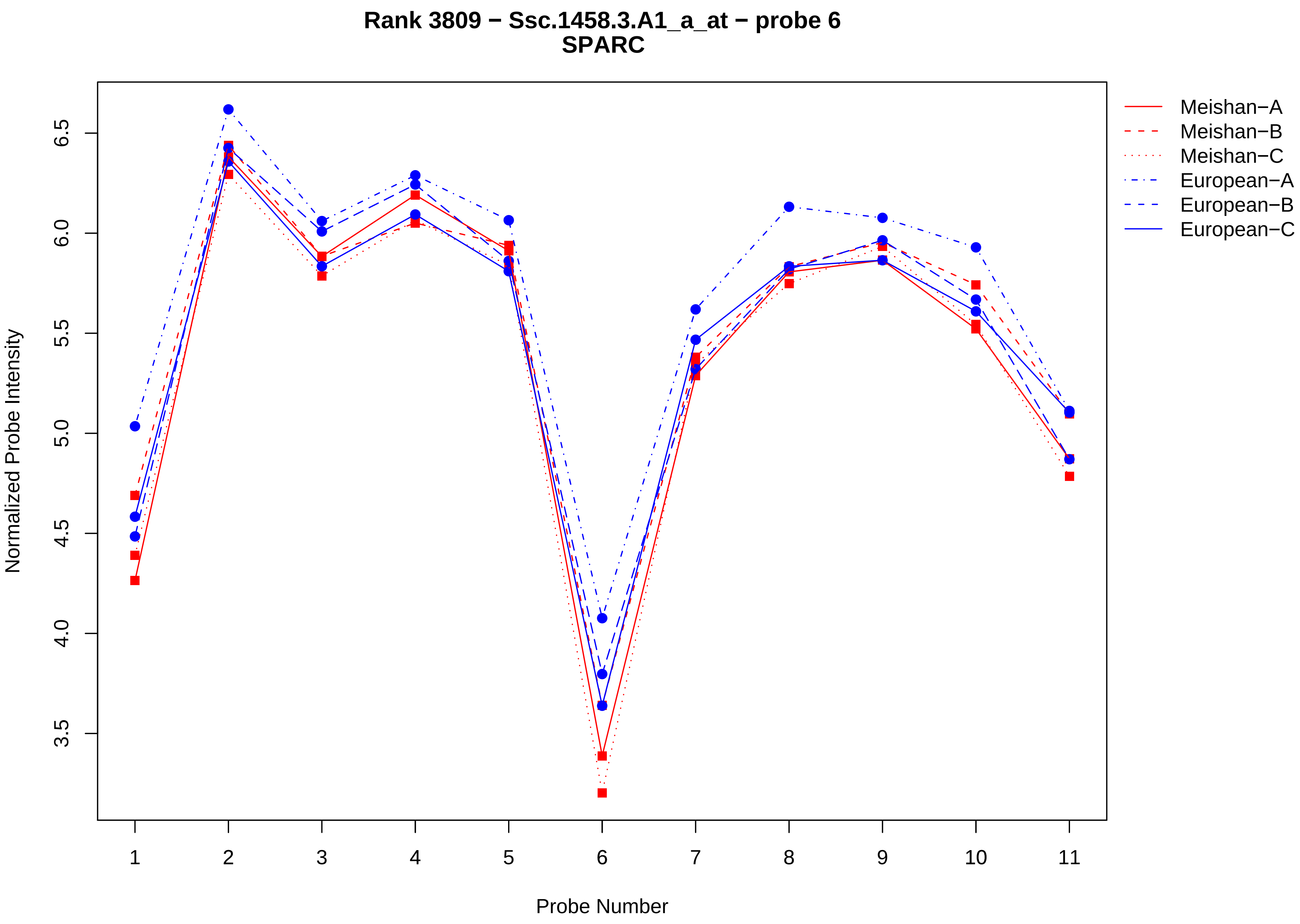

Rank 3809 − Ssc.1458.3.A1_a_at − probe 6
SPARC
Normalized Probe Intensity
Probe Number
Meishan−A
Meishan−B
Meishan−C
European−A
European−B
European−C
3.5
4.0
4.5
5.0
5.5
6.0
6.5
1
2
3
4
5
6
7
8
9
10
11

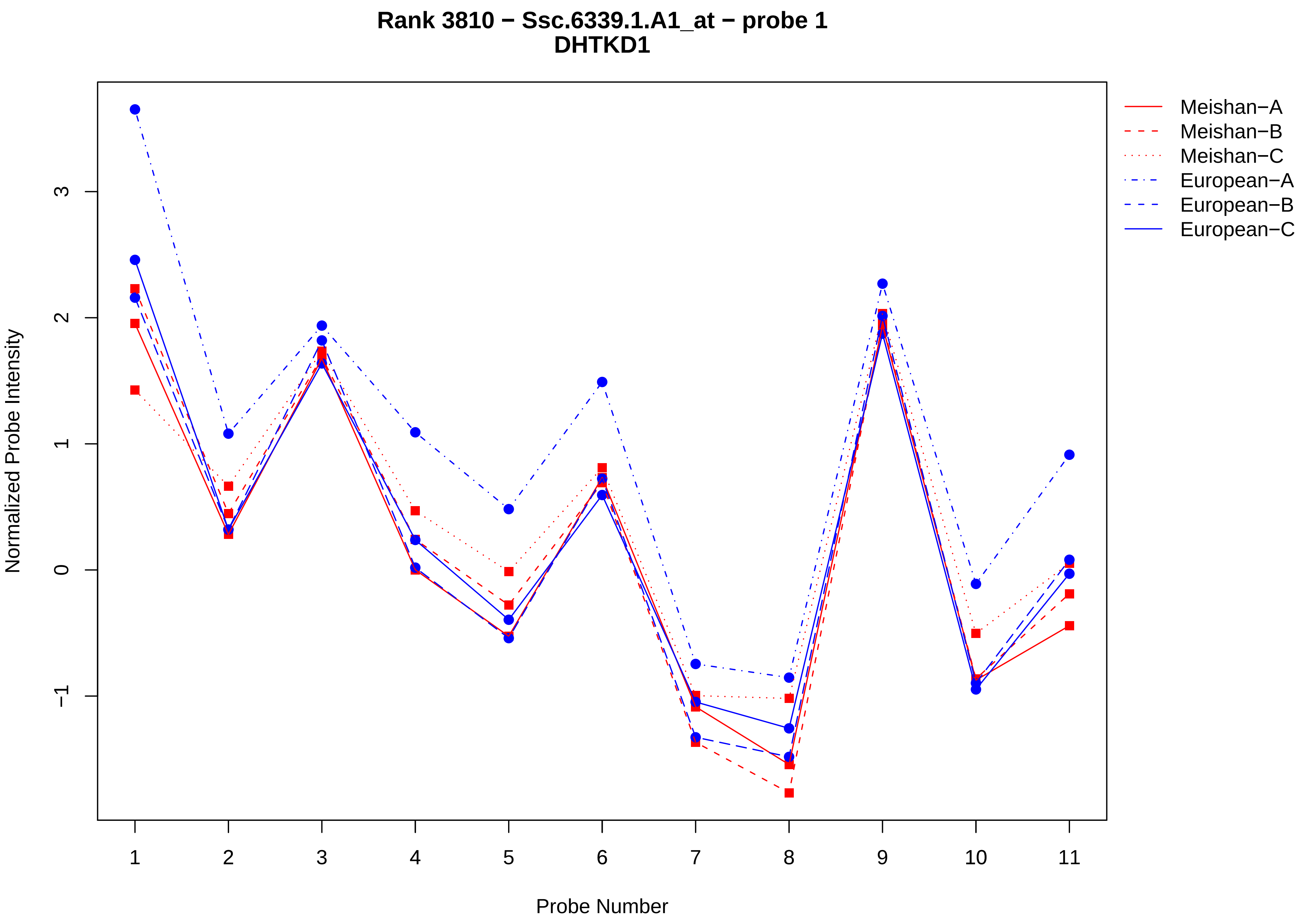

Rank 3810 − Ssc.6339.1.A1_at − probe 1
DHTKD1
Normalized Probe Intensity
Probe Number
−1
0
1
2
3
1
2
3
4
5
6
7
8
9
10
11
Meishan−A
Meishan−B
Meishan−C
European−A
European−B
European−C

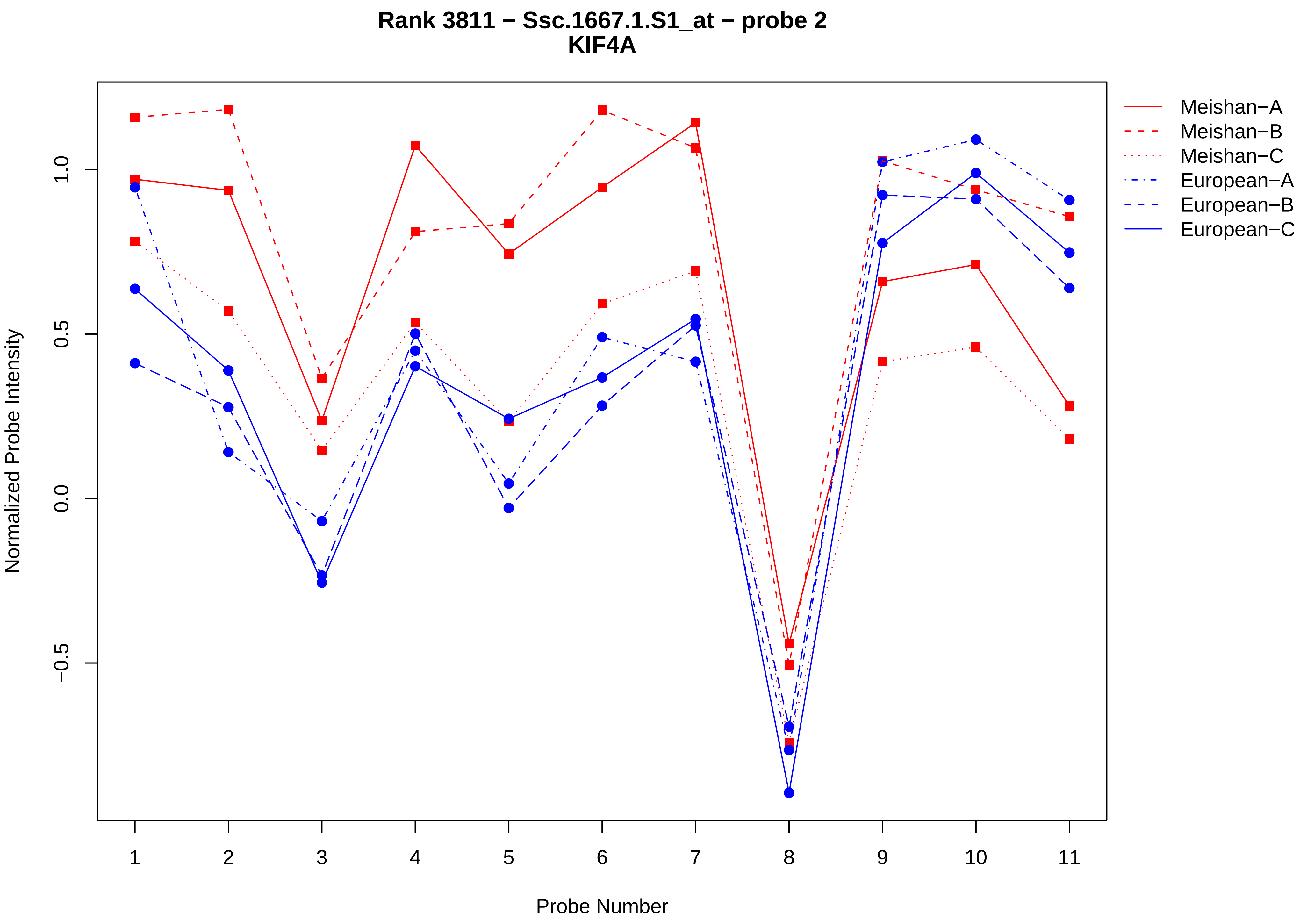
Rank 3811 − Ssc.1667.1.S1_at − probe 2
KIF4A
Normalized Probe Intensity
−0.5
0.0
0.5
1.0
1
2
3
4
5
6
7
8
9
10
11
Probe Number
Meishan−A
Meishan−B
Meishan−C
European−A
European−B
European−C

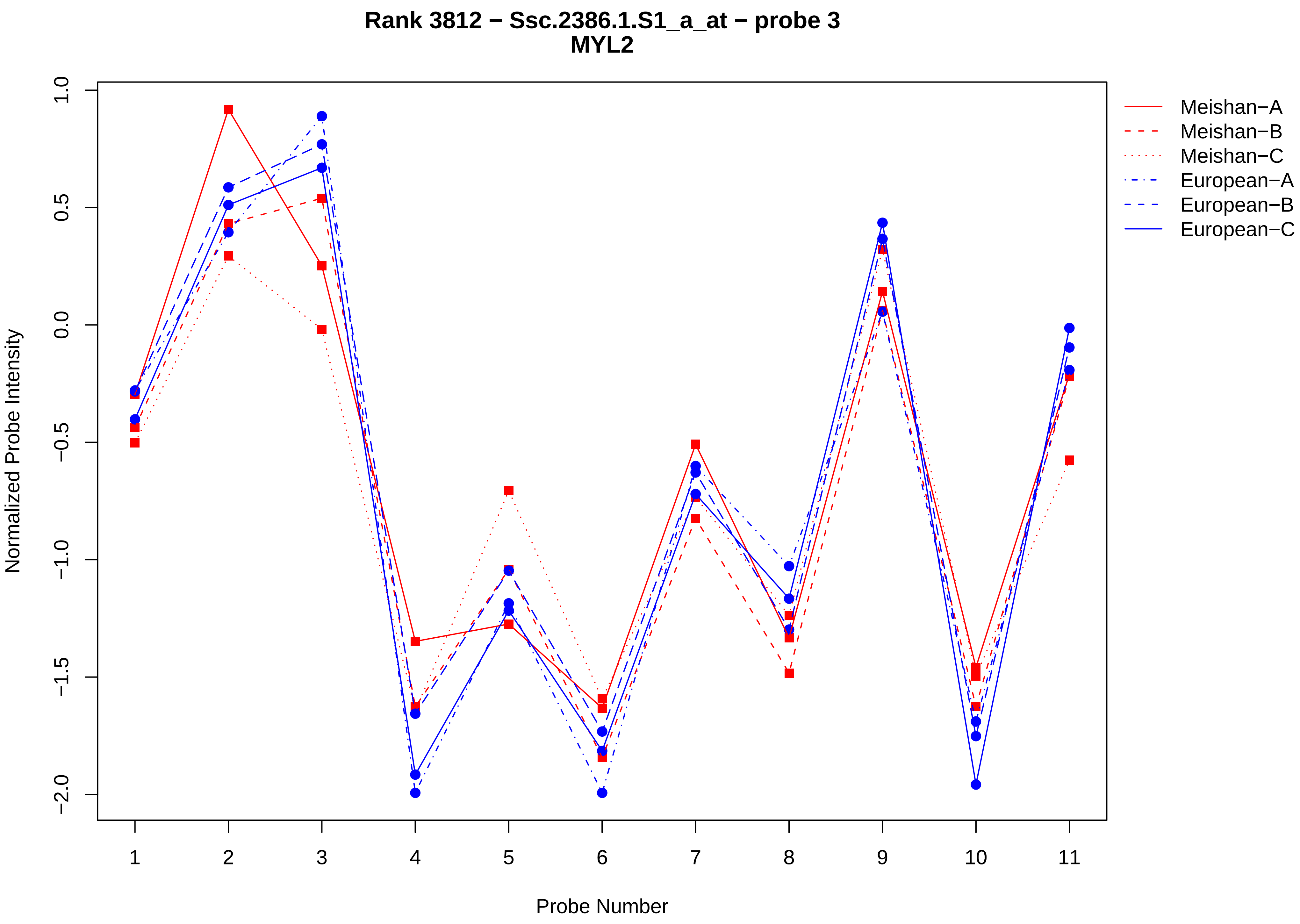

Rank 3812 − Ssc.2386.1.S1_a_at − probe 3
MYL2
Normalized Probe Intensity
Probe Number
1.0
0.5
0.0
−0.5
−1.0
−1.5
−2.0
1
2
3
4
5
6
7
8
9
10
11
Meishan−A
Meishan−B
Meishan−C
European−A
European−B
European−C

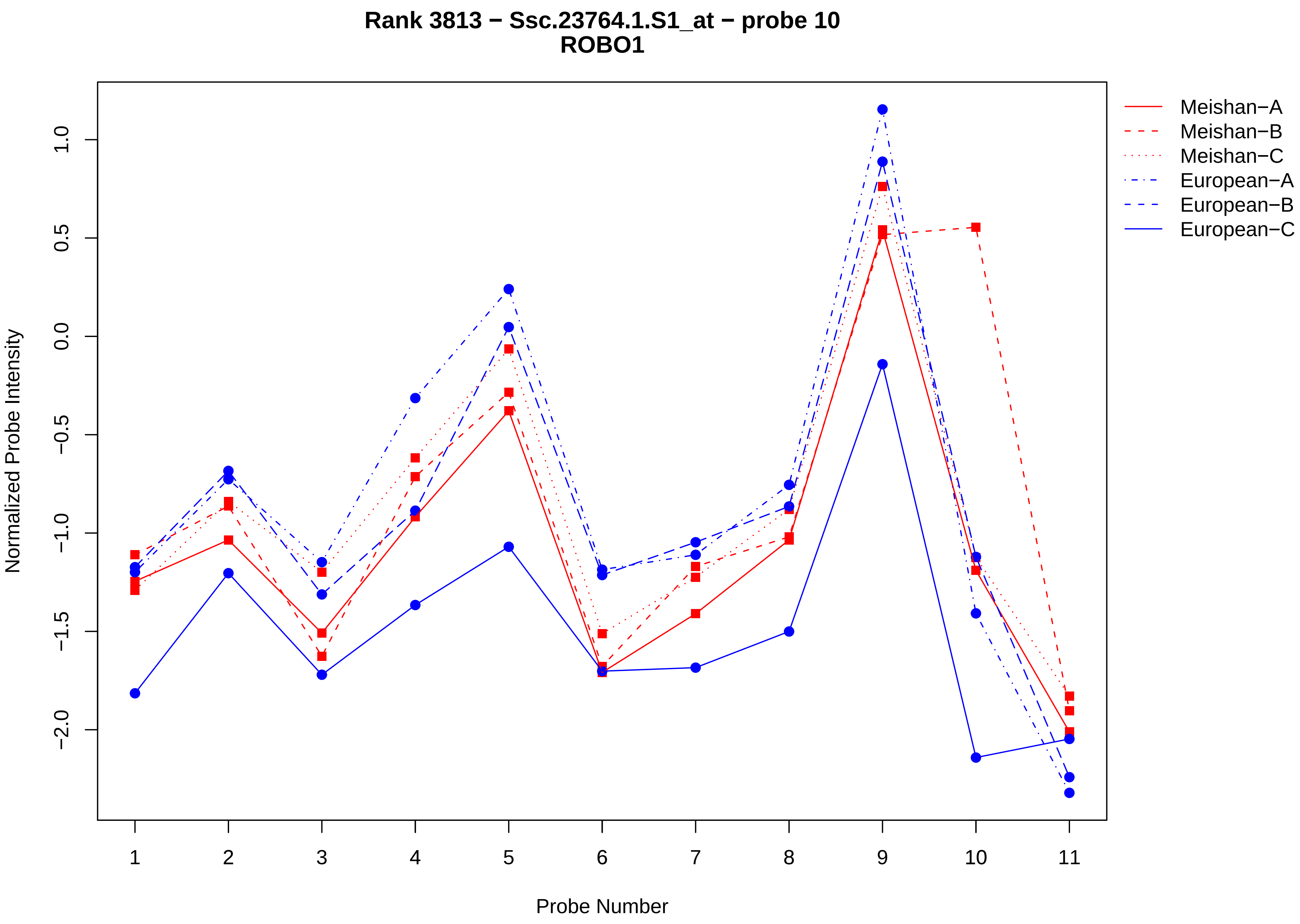

Rank 3813 − Ssc.23764.1.S1_at − probe 10
ROBO1
Normalized Probe Intensity
1.0
0.5
0.0
−0.5
−1.0
−1.5
−2.0
1
2
3
4
5
6
7
8
9
10
11
Probe Number
Meishan−A
Meishan−B
Meishan−C
European−A
European−B
European−C

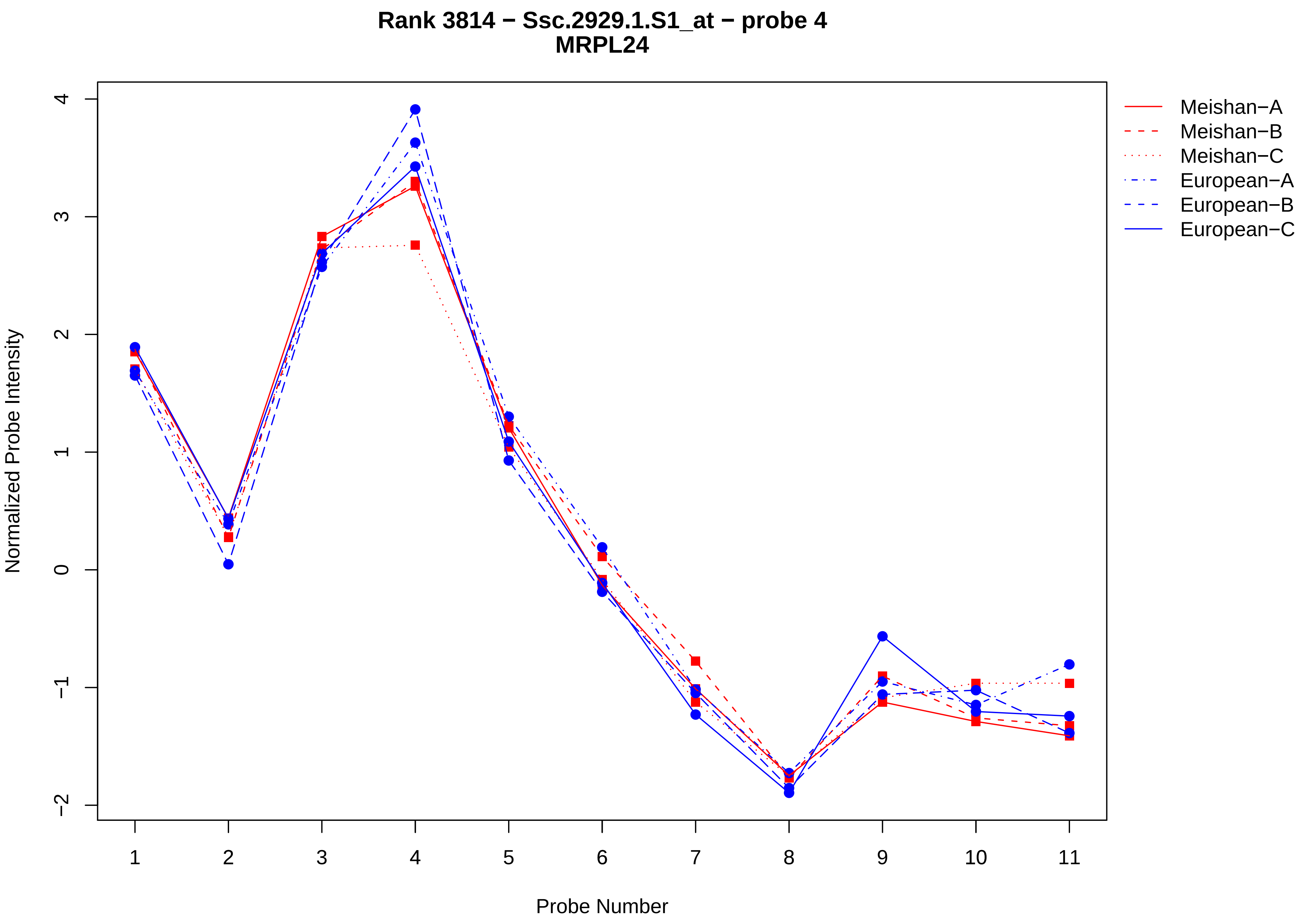

Rank 3814 − Ssc.2929.1.S1_at − probe 4
MRPL24
Normalized Probe Intensity
Probe Number
−2
−1
0
1
2
3
4
1
2
3
4
5
6
7
8
9
10
11
Meishan−A
Meishan−B
Meishan−C
European−A
European−B
European−C

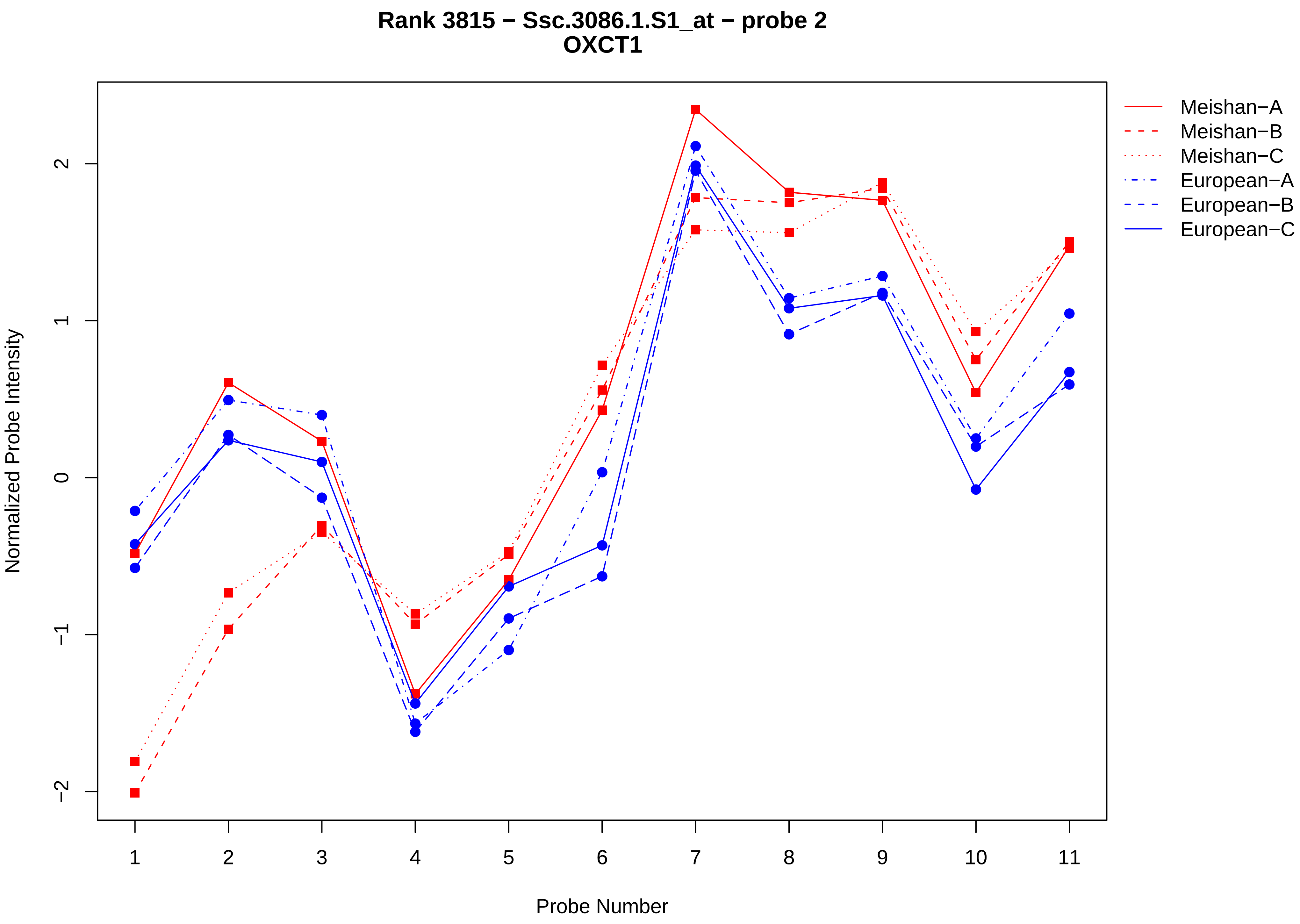

Rank 3815 − Ssc.3086.1.S1_at − probe 2
OXCT1
Normalized Probe Intensity
Probe Number
−2
−1
0
1
2
1
2
3
4
5
6
7
8
9
10
11
Meishan−A
Meishan−B
Meishan−C
European−A
European−B
European−C

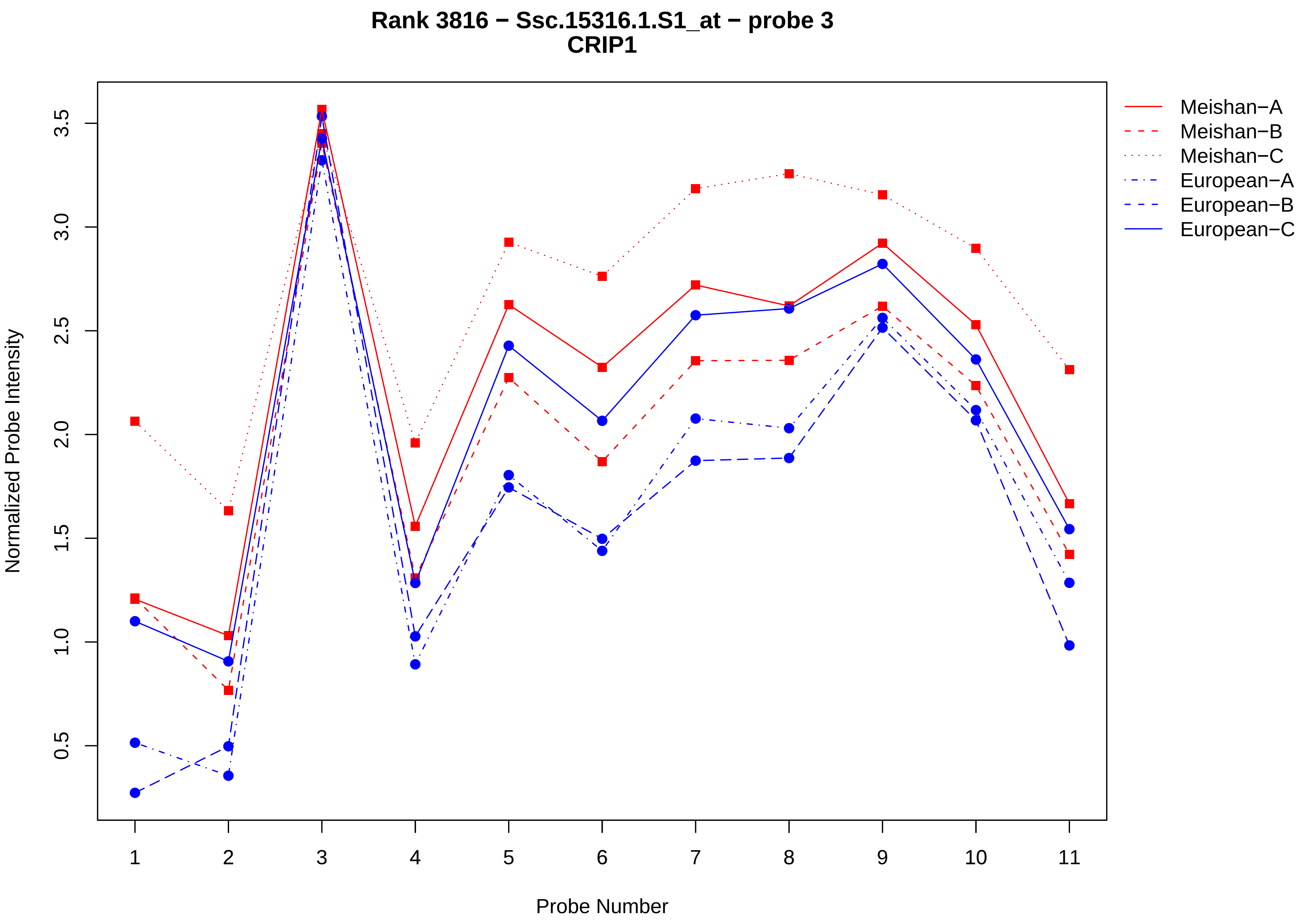
Rank 3816 − Ssc.15316.1.S1_at − probe 3
CRIP1
Normalized Probe Intensity
Probe Number
0.5
1.0
1.5
2.0
2.5
3.0
3.5
1
2
3
4
5
6
7
8
9
10
11
Meishan−A
Meishan−B
Meishan−C
European−A
European−B
European−C

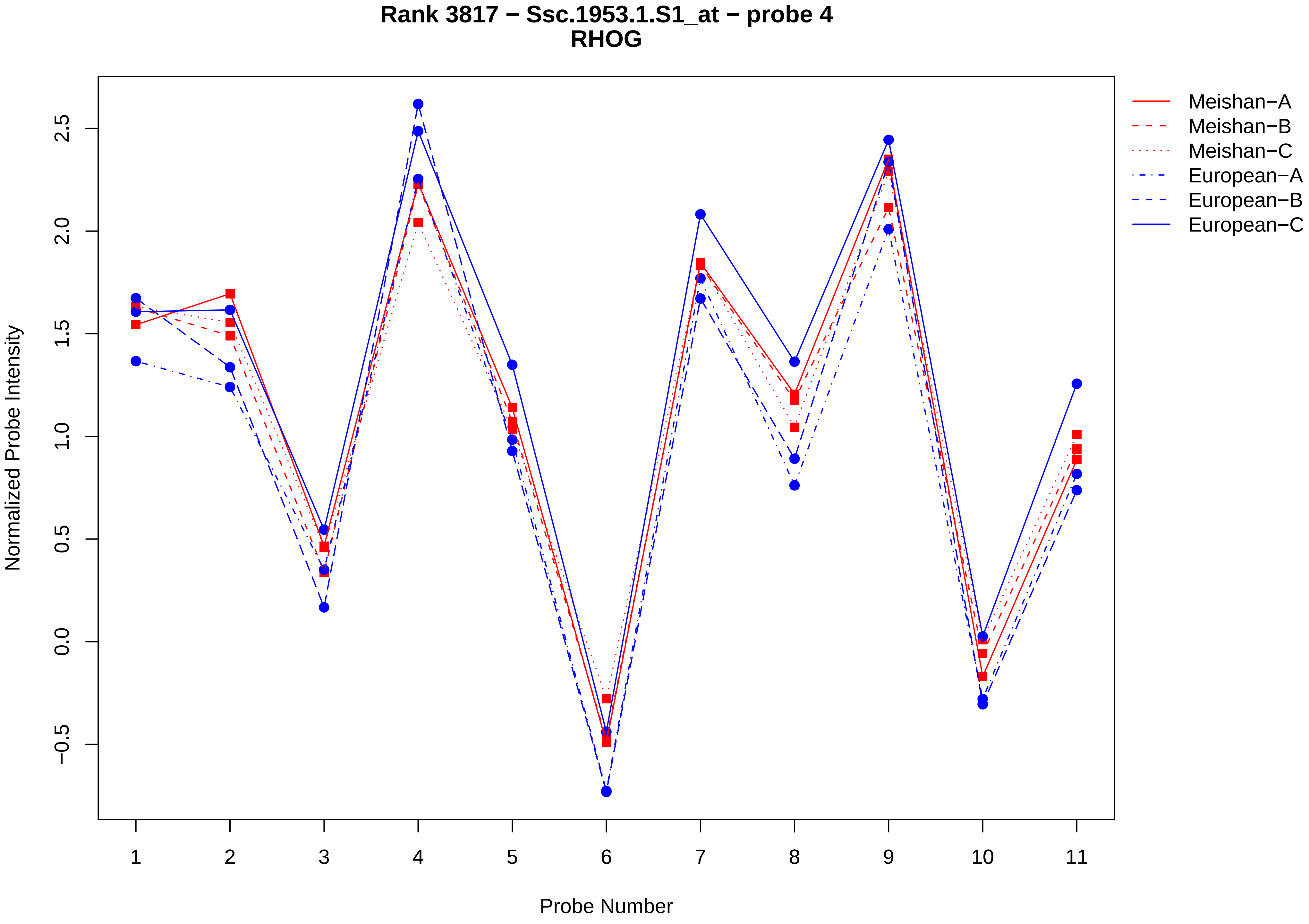

Rank 3817 − Ssc.1953.1.S1_at − probe 4
RHOG
Normalized Probe Intensity
−0.5
0.0
0.5
1.0
1.5
2.0
2.5
1
2
3
4
5
6
7
8
9
10
11
Probe Number
Meishan−A
Meishan−B
Meishan−C
European−A
European−B
European−C

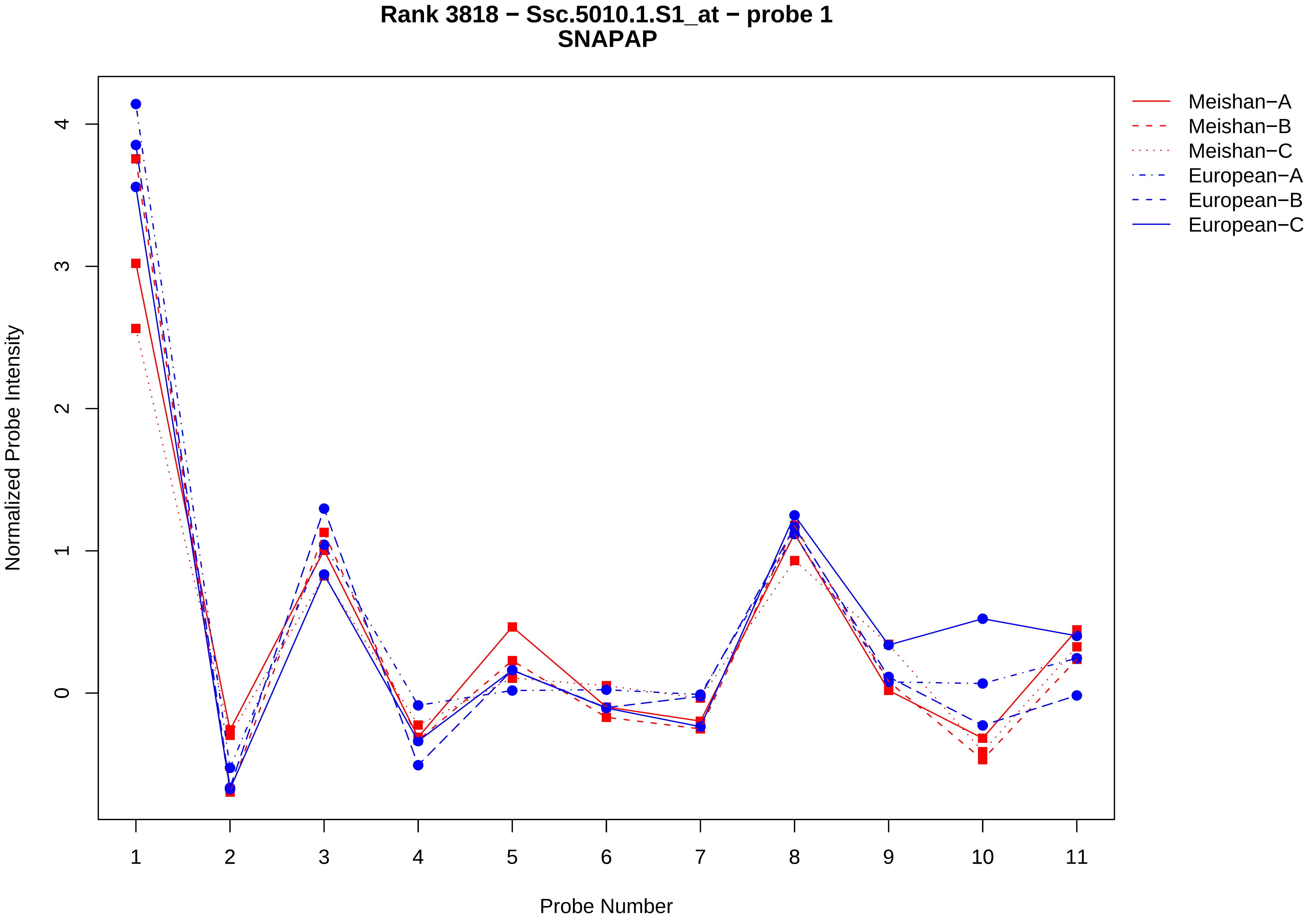

Rank 3818 − Ssc.5010.1.S1_at − probe 1
SNAPAP
Normalized Probe Intensity
Probe Number
0
1
2
3
4
1
2
3
4
5
6
7
8
9
10
11
Meishan−A
Meishan−B
Meishan−C
European−A
European−B
European−C

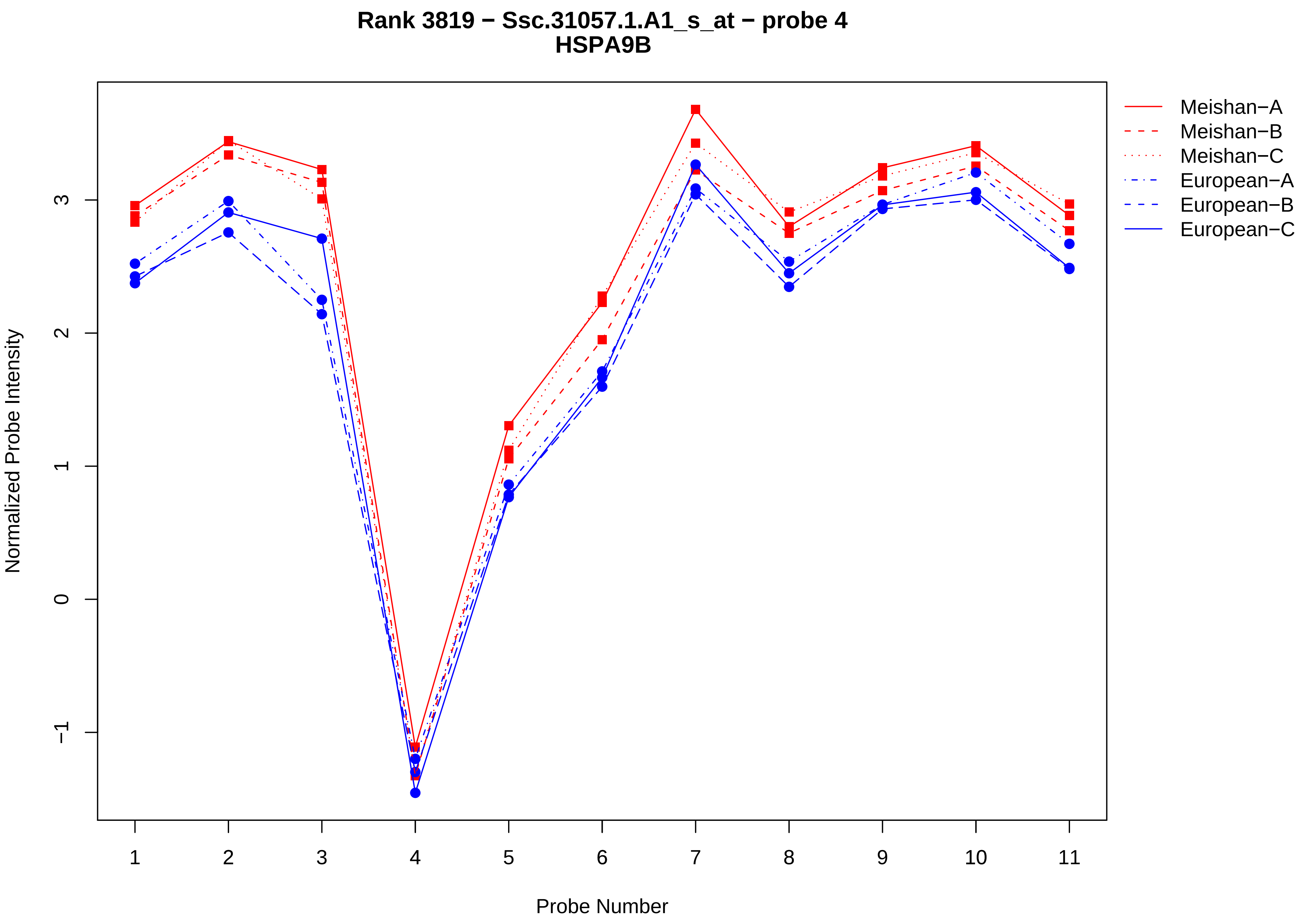

Rank 3819 − Ssc.31057.1.A1_s_at − probe 4
HSPA9B
Normalized Probe Intensity
Probe Number
−1
0
1
2
3
1
2
3
4
5
6
7
8
9
10
11
Meishan−A
Meishan−B
Meishan−C
European−A
European−B
European−C

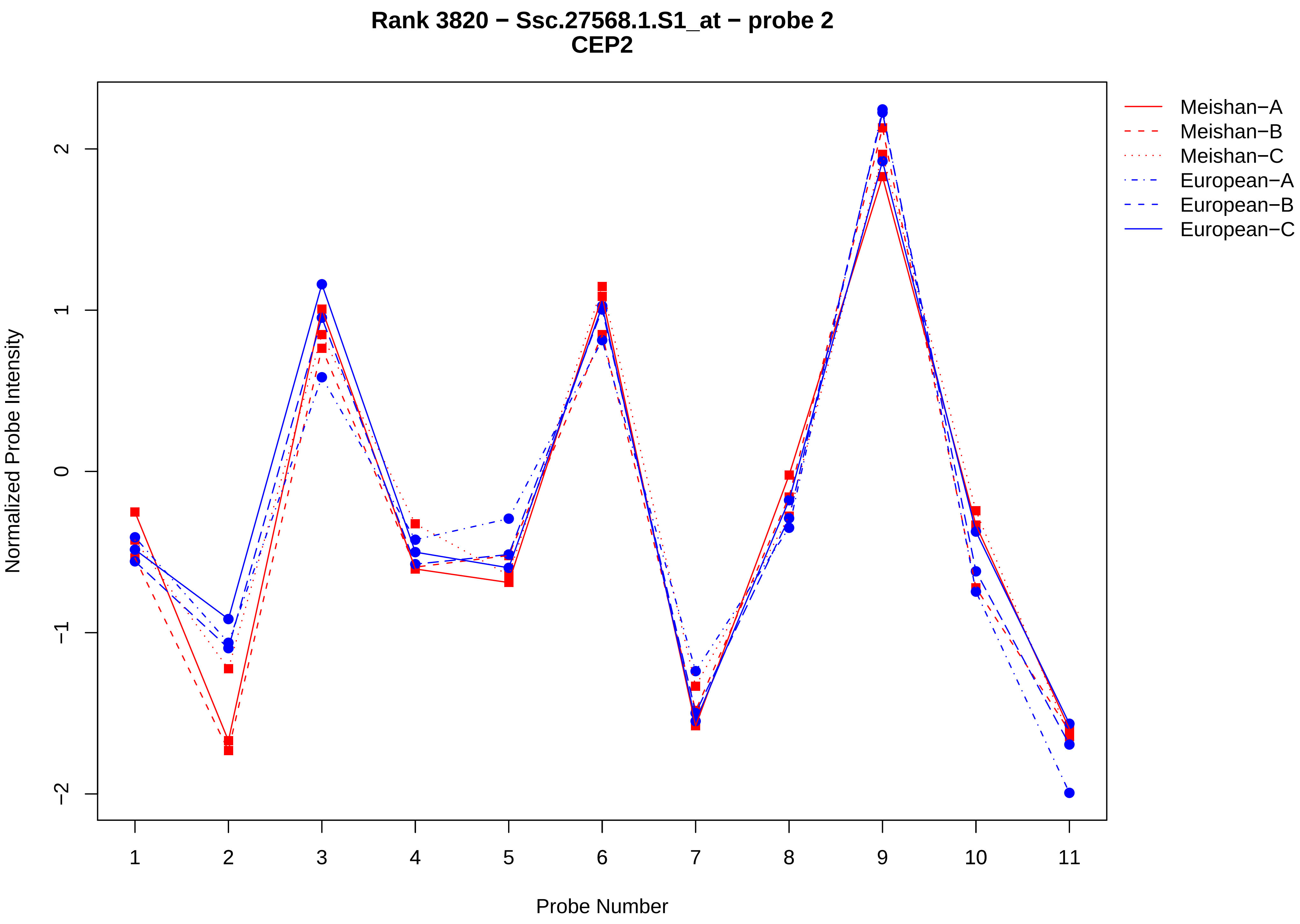
Rank 3820 − Ssc.27568.1.S1_at − probe 2
CEP2
Normalized Probe Intensity
Probe Number
−2
−1
0
1
2
1
2
3
4
5
6
7
8
9
10
11
Meishan−A
Meishan−B
Meishan−C
European−A
European−B
European−C

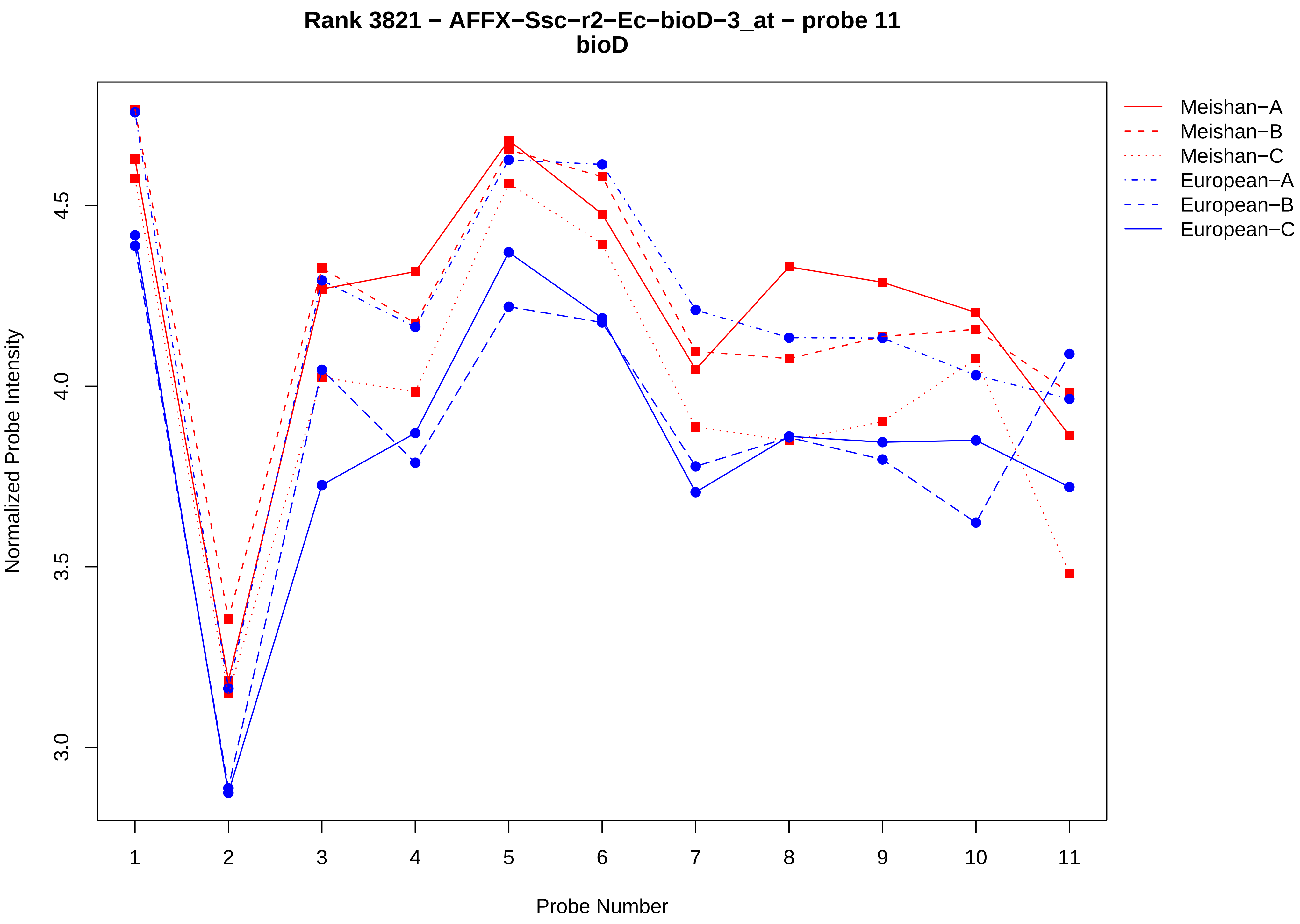
Rank 3821 − AFFX−Ssc−r2−Ec−bioD−3_at − probe 11
bioD
Normalized Probe Intensity
3.0
3.5
4.0
4.5
1
2
3
4
5
6
7
8
9
10
11
Probe Number
Meishan−A
Meishan−B
Meishan−C
European−A
European−B
European−C

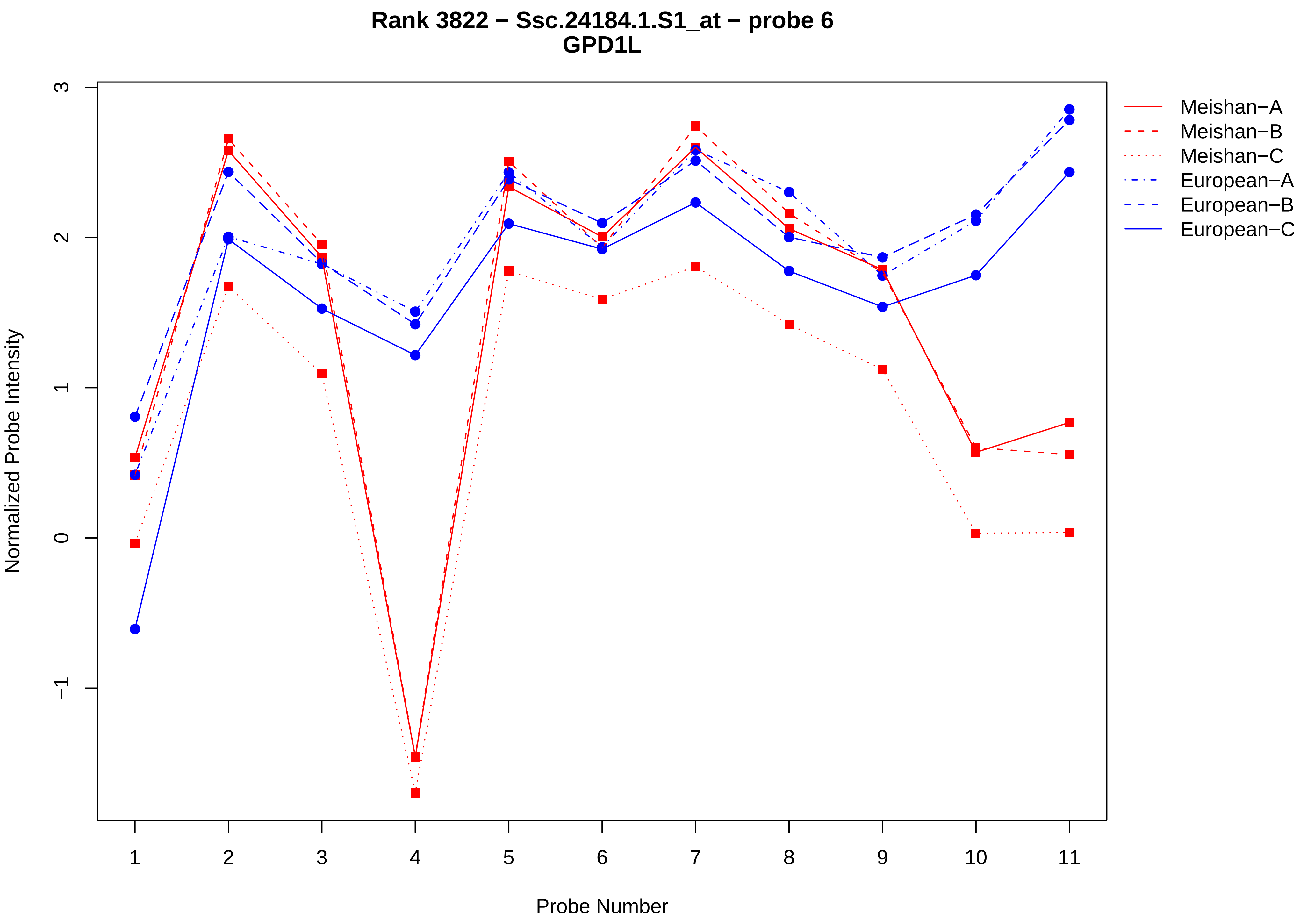

Rank 3822 − Ssc.24184.1.S1_at − probe 6
GPD1L
Normalized Probe Intensity
Probe Number
−1
0
1
2
3
1
2
3
4
5
6
7
8
9
10
11
Meishan−A
Meishan−B
Meishan−C
European−A
European−B
European−C

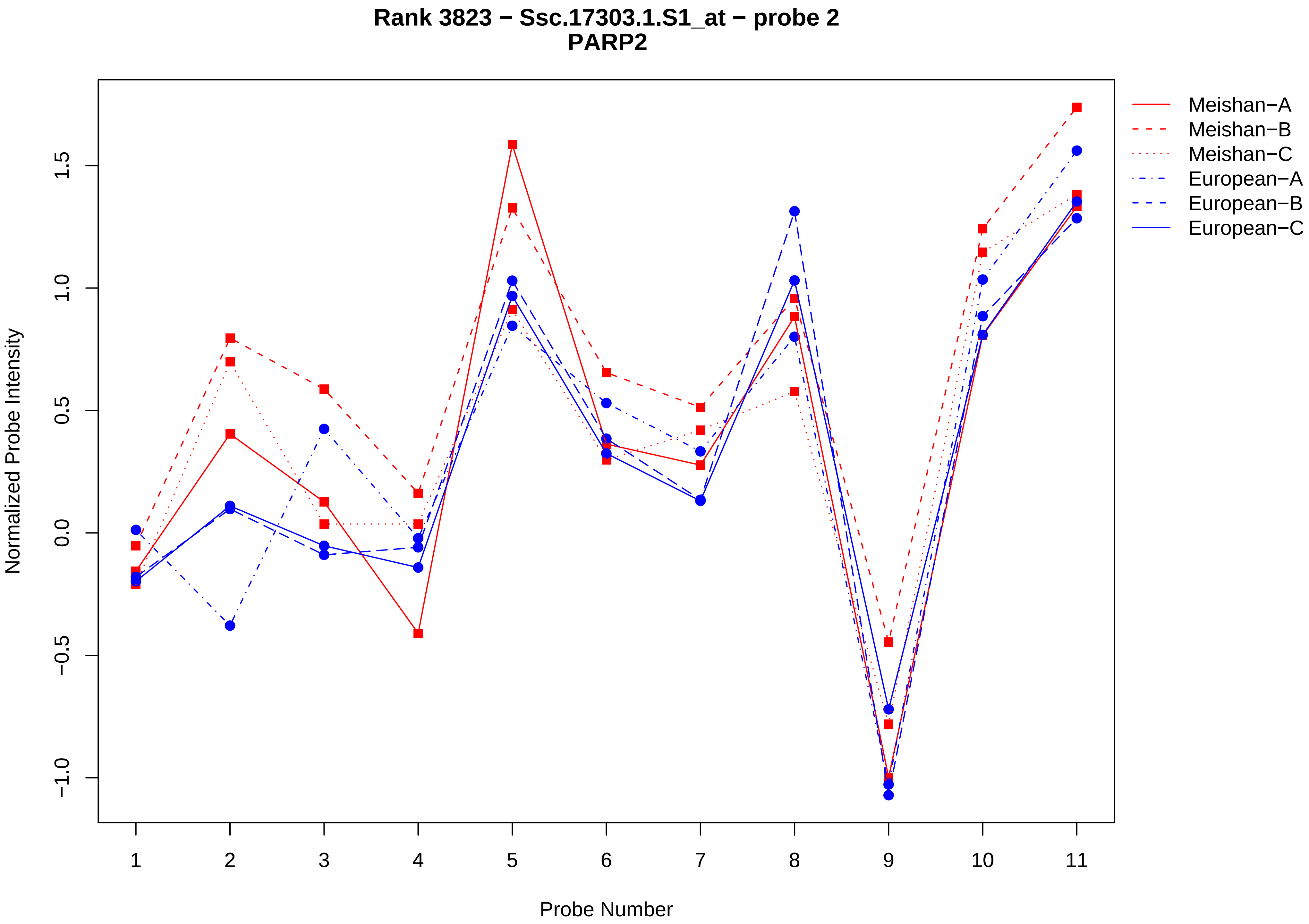

Rank 3823 − Ssc.17303.1.S1_at − probe 2
PARP2
Normalized Probe Intensity
Probe Number
Meishan−A
Meishan−B
Meishan−C
European−A
European−B
European−C
−1.0
−0.5
0.0
0.5
1.0
1.5
1
2
3
4
5
6
7
8
9
10
11

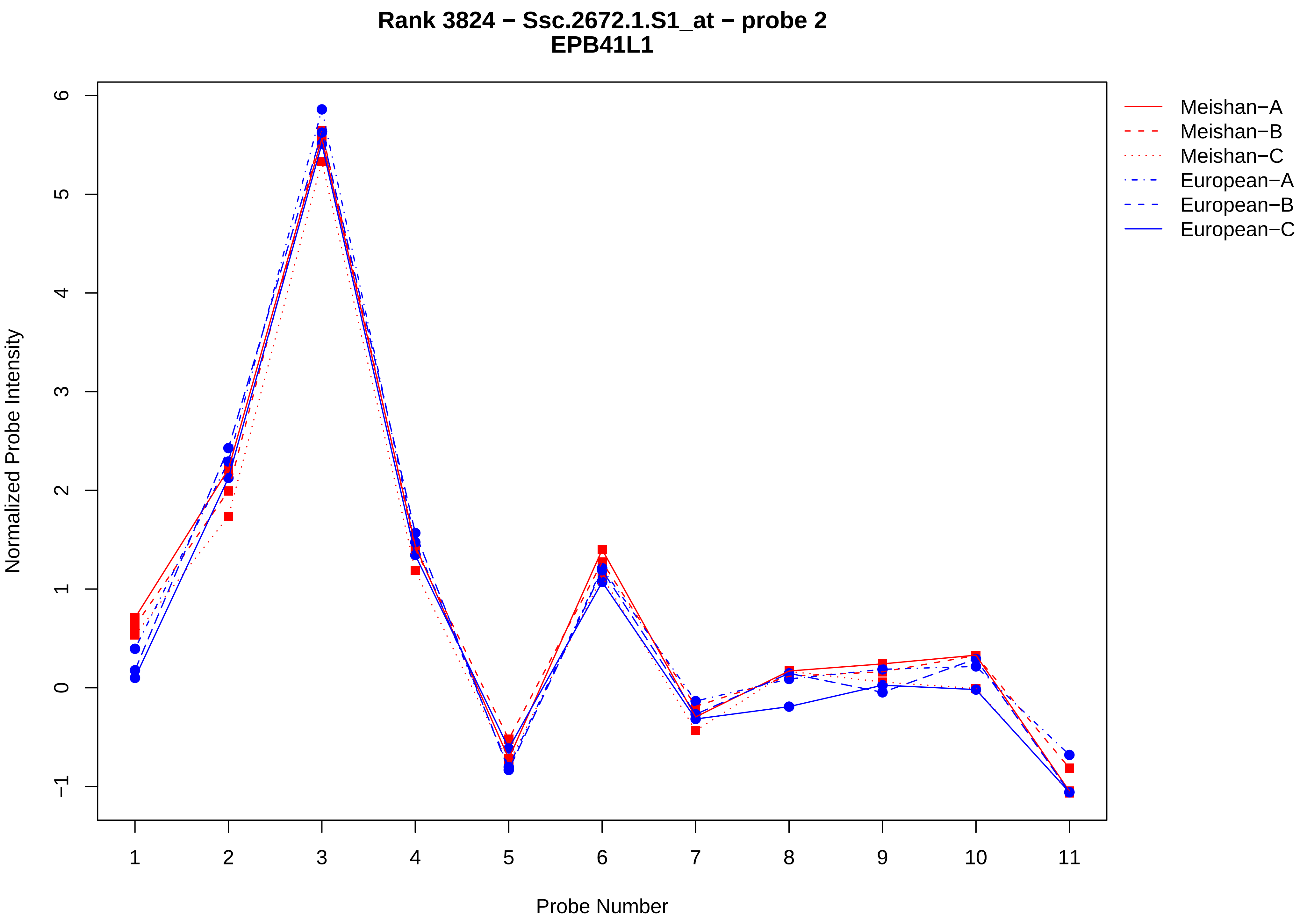

Rank 3824 − Ssc.2672.1.S1_at − probe 2
EPB41L1
Normalized Probe Intensity
Probe Number
−1
0
1
2
3
4
5
6
1
2
3
4
5
6
7
8
9
10
11
Meishan−A
Meishan−B
Meishan−C
European−A
European−B
European−C

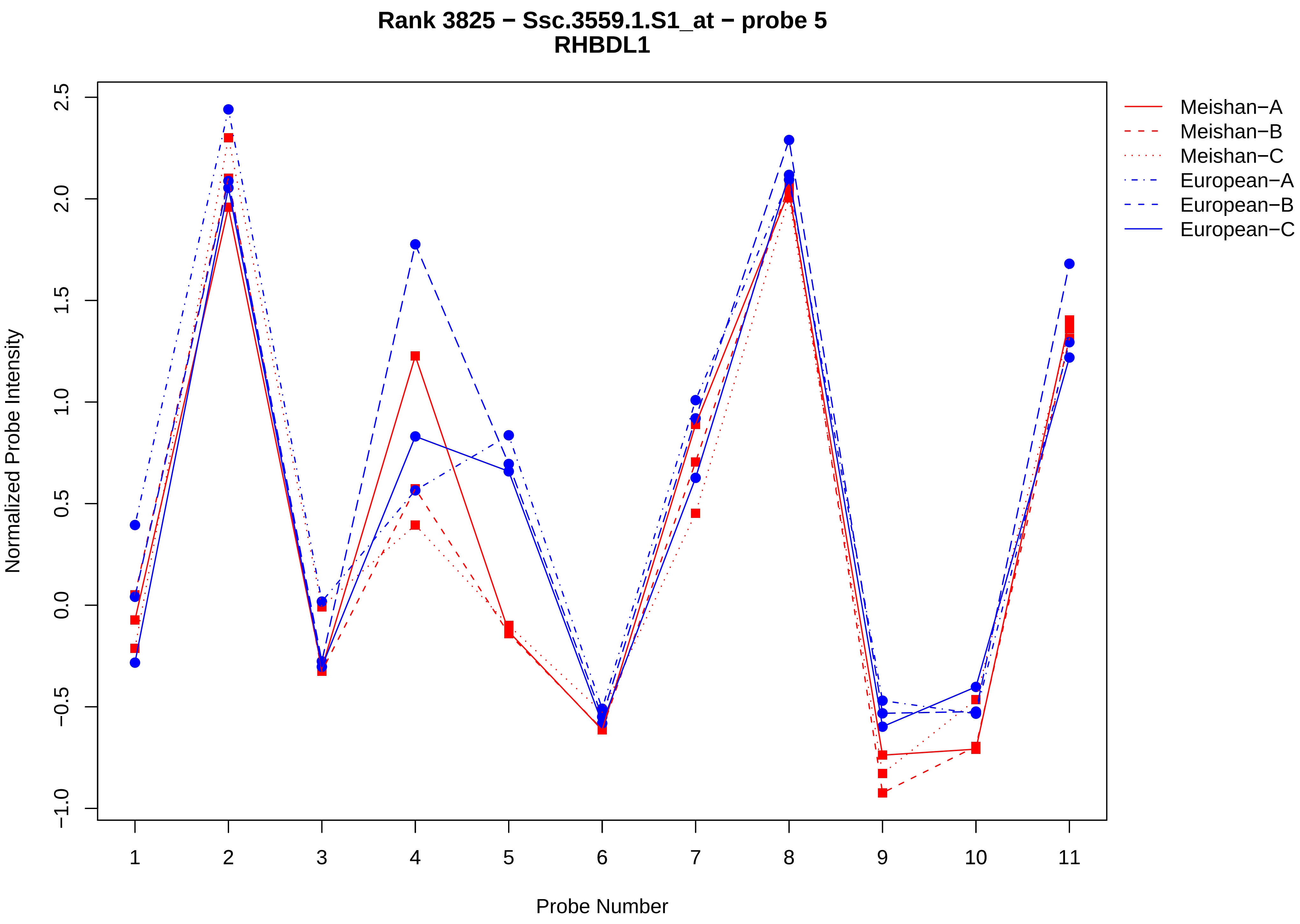

Rank 3825 − Ssc.3559.1.S1_at − probe 5
RHBDL1
Normalized Probe Intensity
Probe Number
−1.0
−0.5
0.0
0.5
1.0
1.5
2.0
2.5
1
2
3
4
5
6
7
8
9
10
11
Meishan−A
Meishan−B
Meishan−C
European−A
European−B
European−C

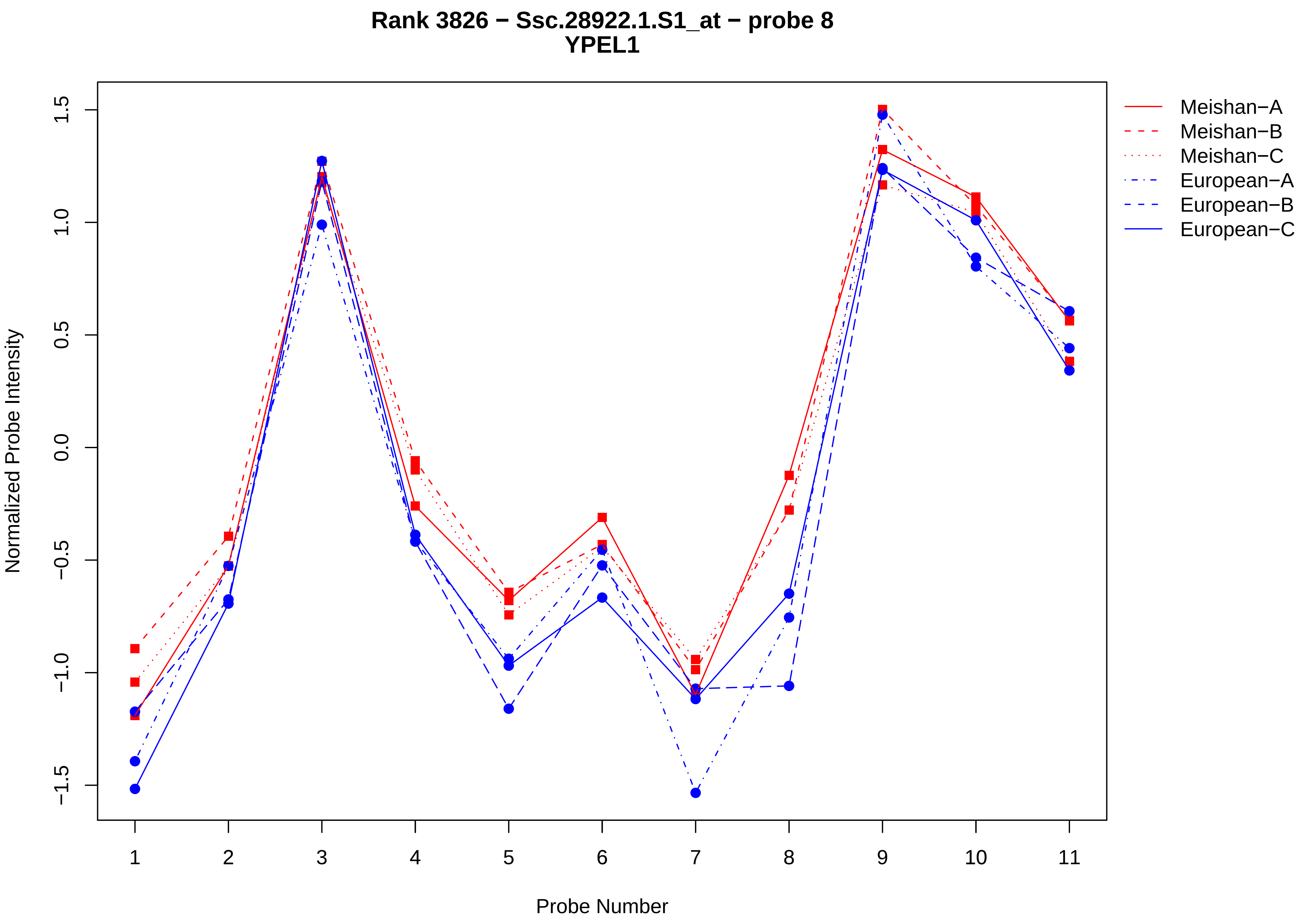

Rank 3826 − Ssc.28922.1.S1_at − probe 8
YPEL1
Normalized Probe Intensity
Probe Number
Meishan−A
Meishan−B
Meishan−C
European−A
European−B
European−C
1.5
1.0
0.5
0.0
−0.5
−1.0
−1.5
1
2
3
4
5
6
7
8
9
10
11

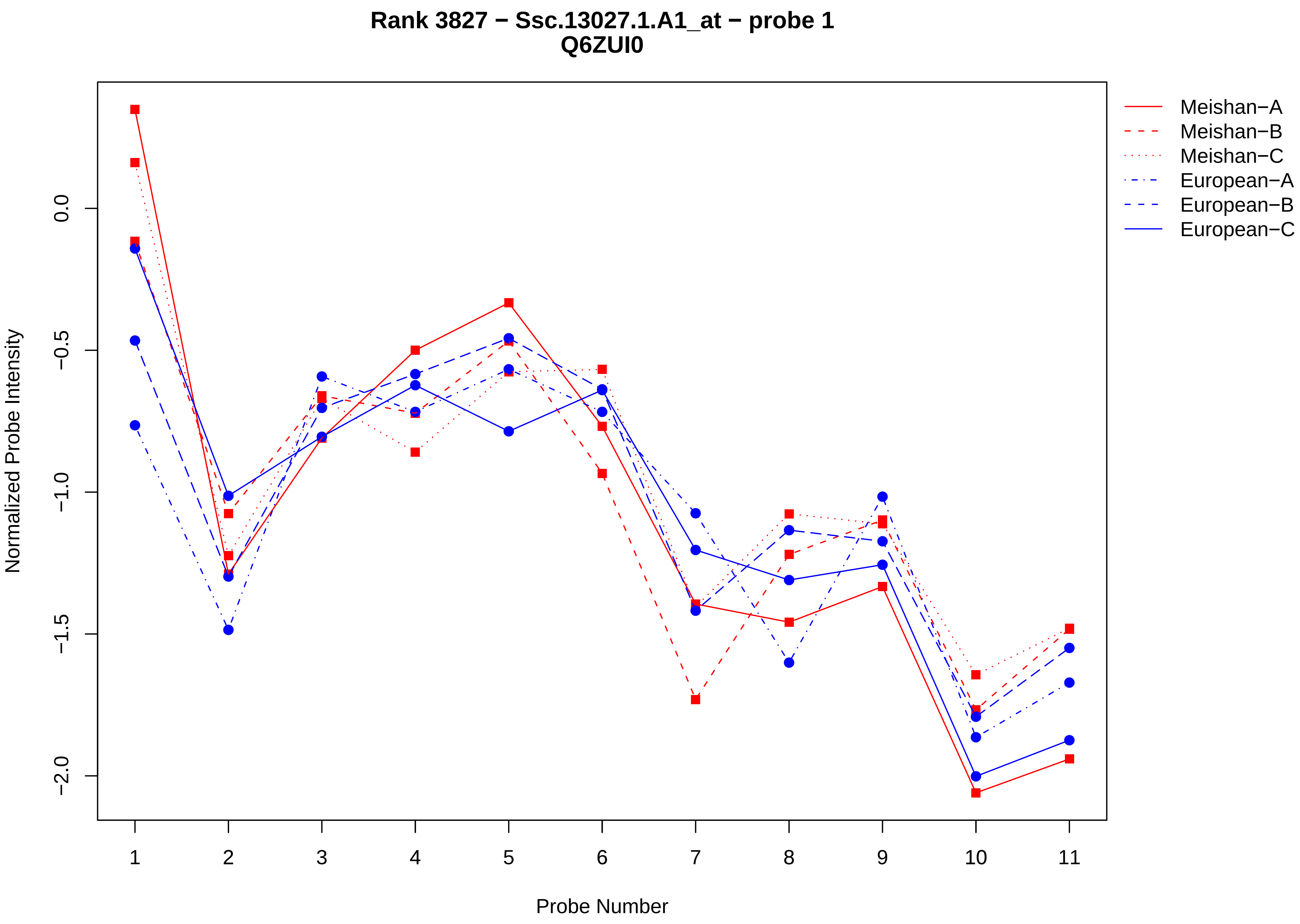

Rank 3827 − Ssc.13027.1.A1_at − probe 1
Q6ZUI0
Normalized Probe Intensity
Probe Number
0.0
−0.5
−1.0
−1.5
−2.0
1
2
3
4
5
6
7
8
9
10
11
Meishan−A
Meishan−B
Meishan−C
European−A
European−B
European−C

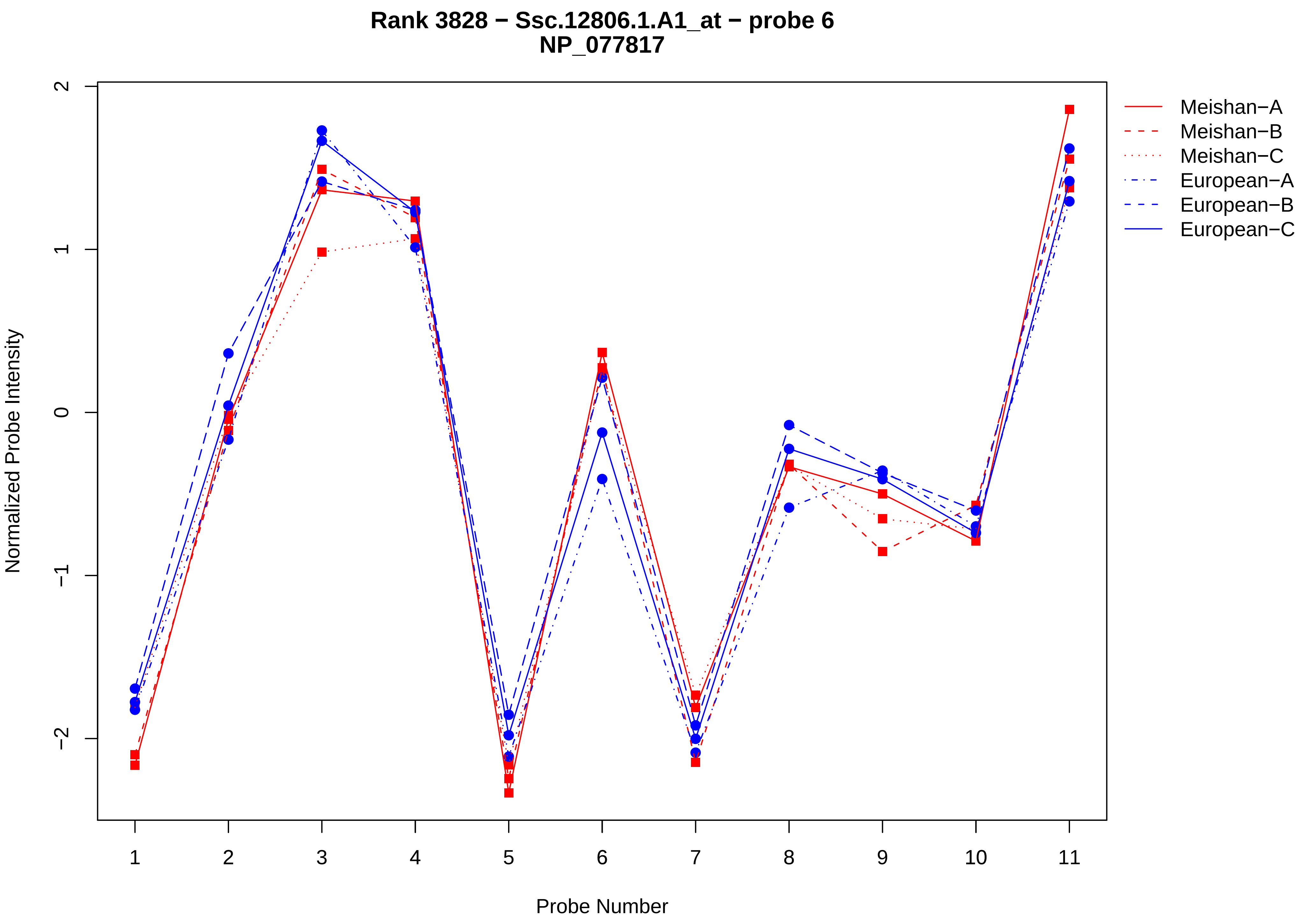

Rank 3828 − Ssc.12806.1.A1_at − probe 6
NP_077817
Normalized Probe Intensity
Probe Number
−2
−1
0
1
2
1
2
3
4
5
6
7
8
9
10
11
Meishan−A
Meishan−B
Meishan−C
European−A
European−B
European−C

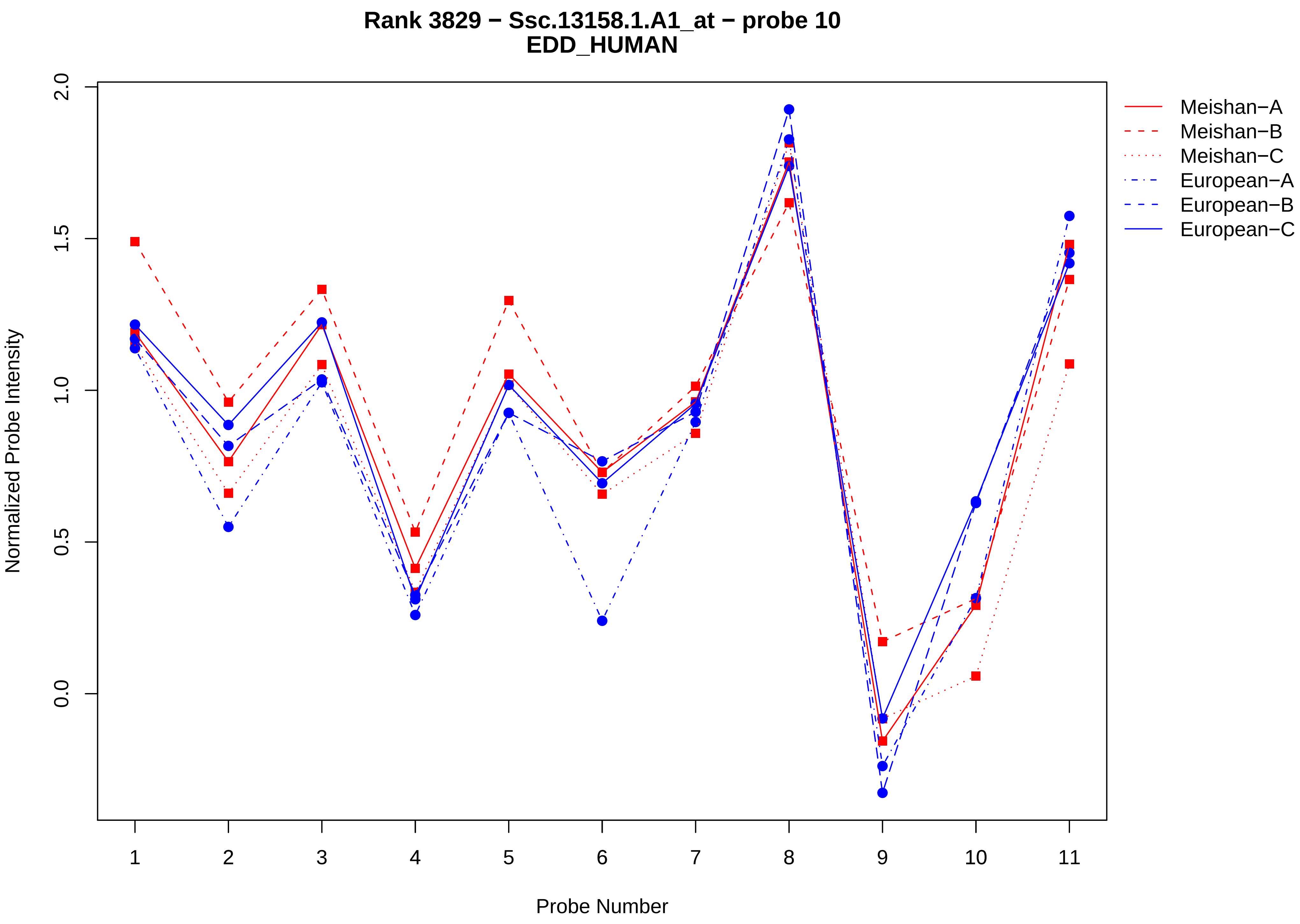

Rank 3829 − Ssc.13158.1.A1_at − probe 10
EDD_HUMAN
Normalized Probe Intensity
Probe Number
0.0
0.5
1.0
1.5
2.0
1
2
3
4
5
6
7
8
9
10
11
Meishan−A
Meishan−B
Meishan−C
European−A
European−B
European−C

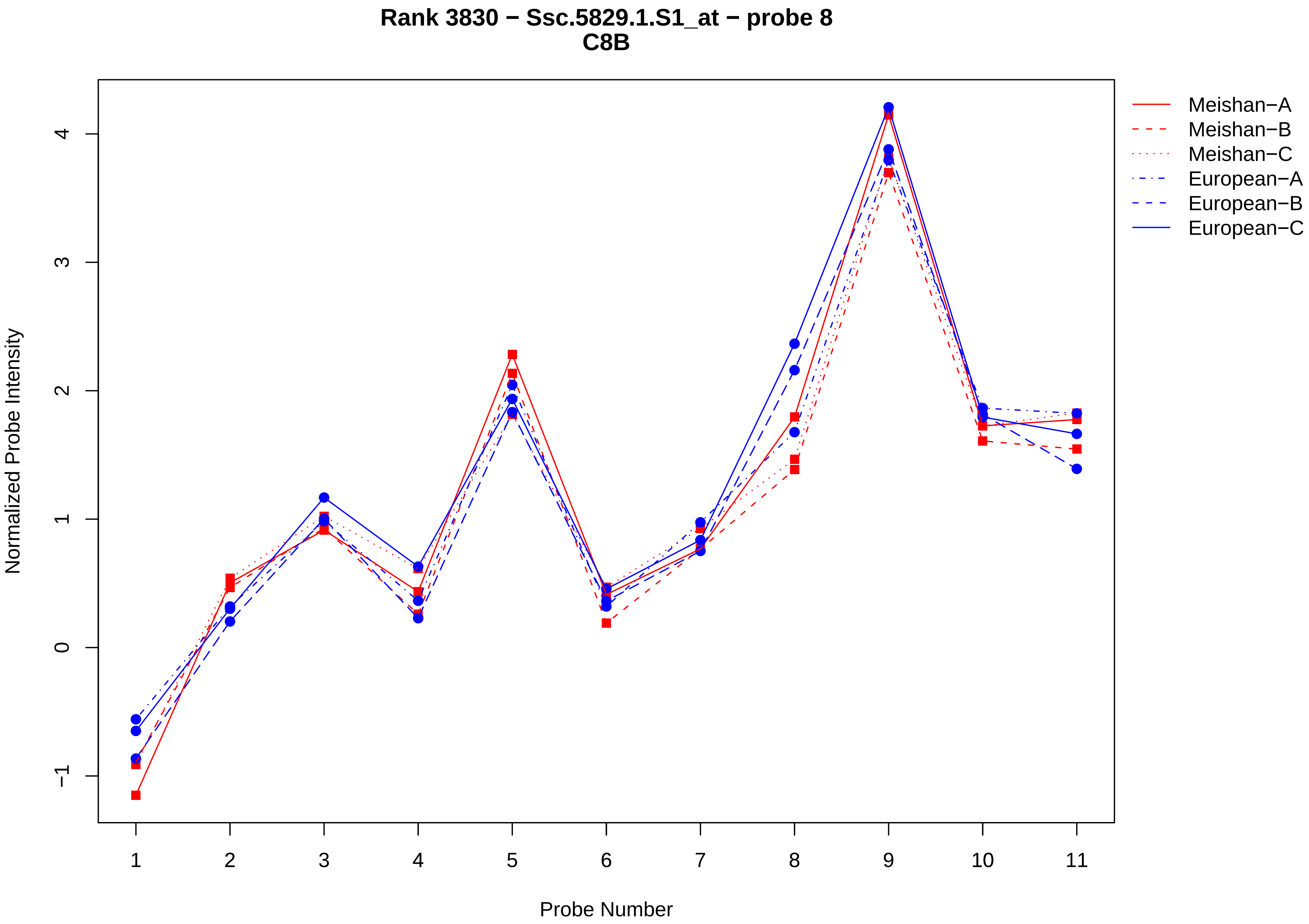
Rank 3830 − Ssc.5829.1.S1_at − probe 8
C8B
Normalized Probe Intensity
Probe Number
−1
0
1
2
3
4
1
2
3
4
5
6
7
8
9
10
11
Meishan−A
Meishan−B
Meishan−C
European−A
European−B
European−C

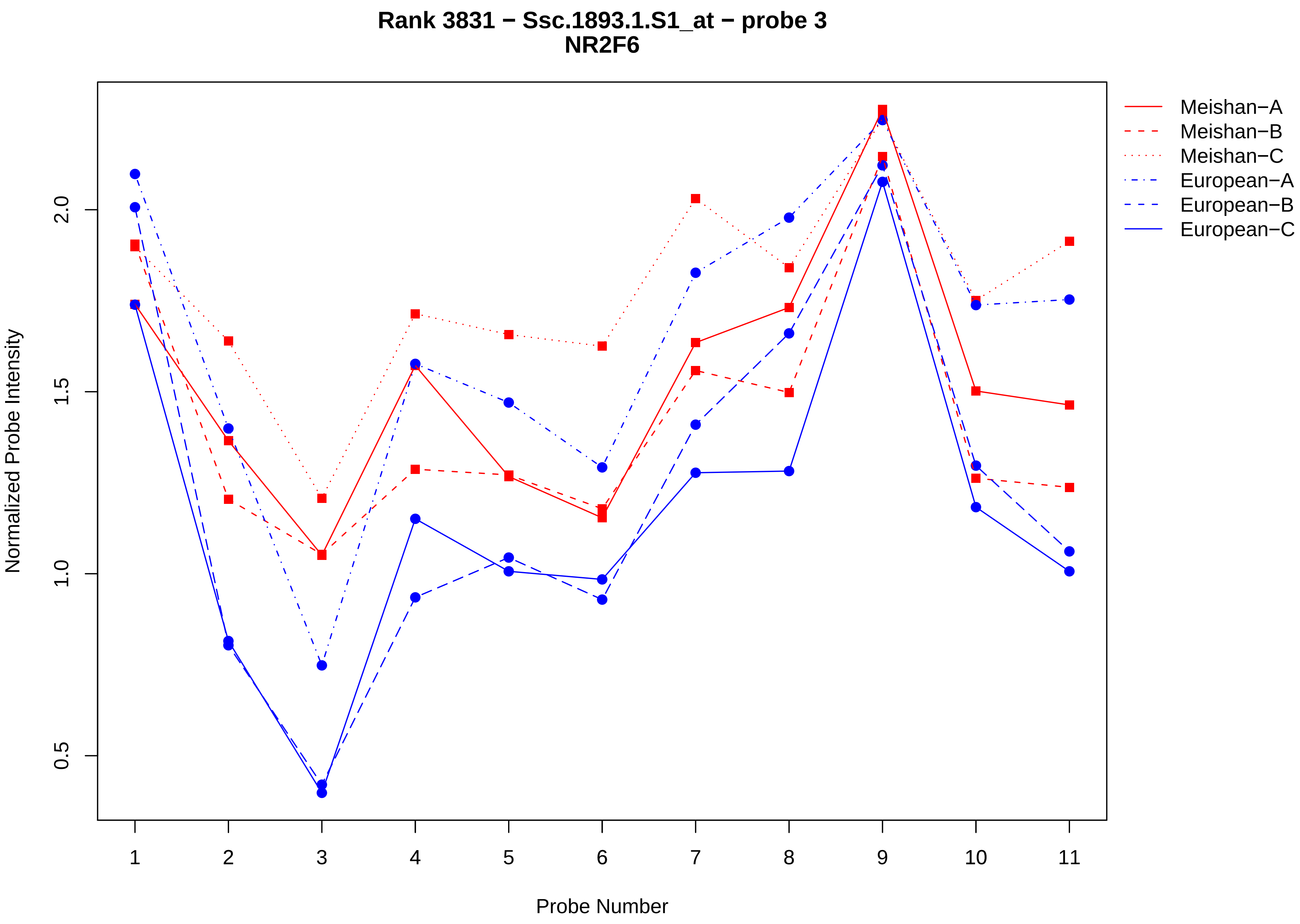
Rank 3831 − Ssc.1893.1.S1_at − probe 3
NR2F6
Normalized Probe Intensity
0.5
1.0
1.5
2.0
1 2 3 4 5 6 7 8 9 10 11
Probe Number
Meishan−A
Meishan−B
Meishan−C
European−A
European−B
European−C

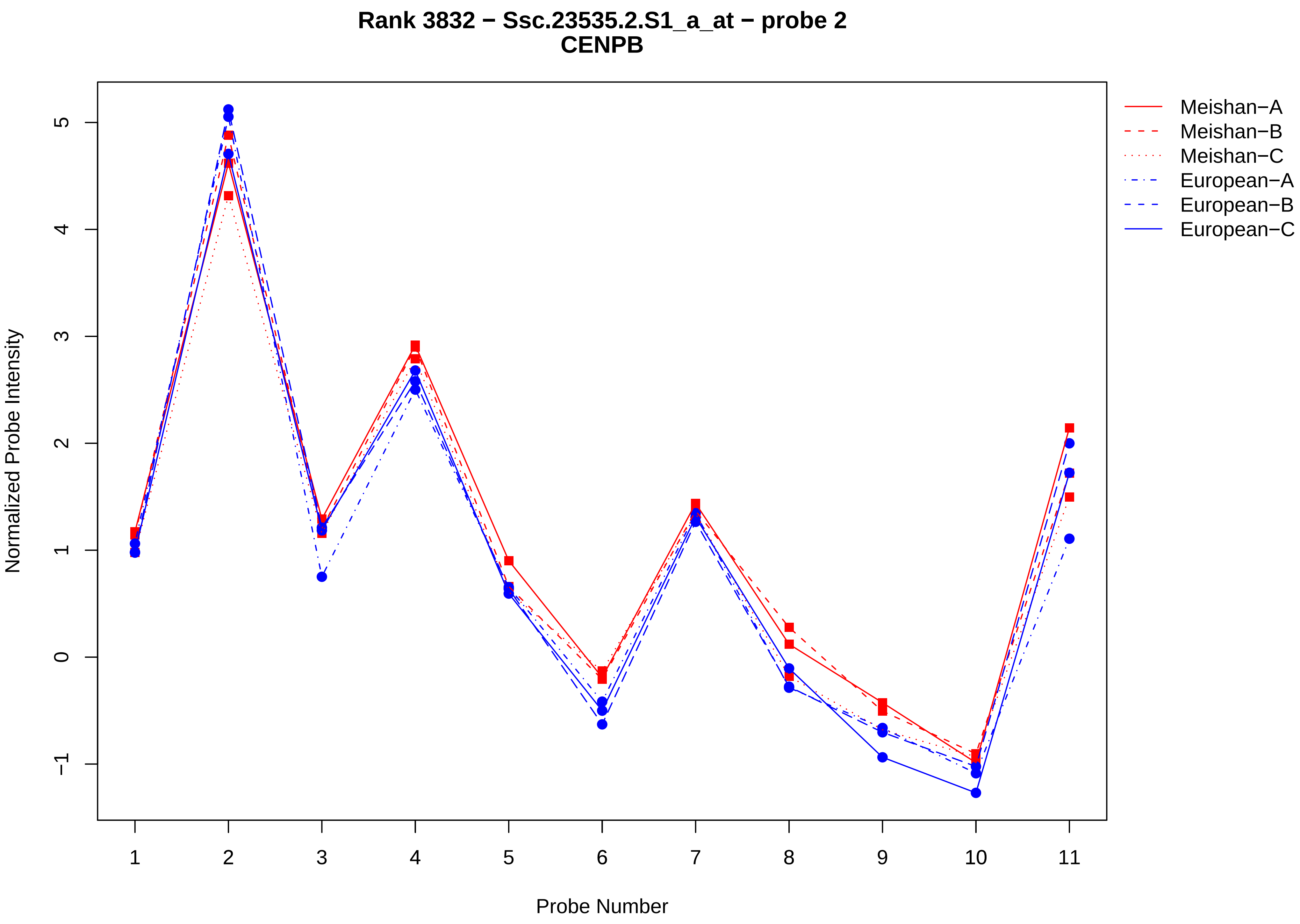

Rank 3832 − Ssc.23535.2.S1_a_at − probe 2
CENPB
Normalized Probe Intensity
Probe Number
Meishan−A
Meishan−B
Meishan−C
European−A
European−B
European−C

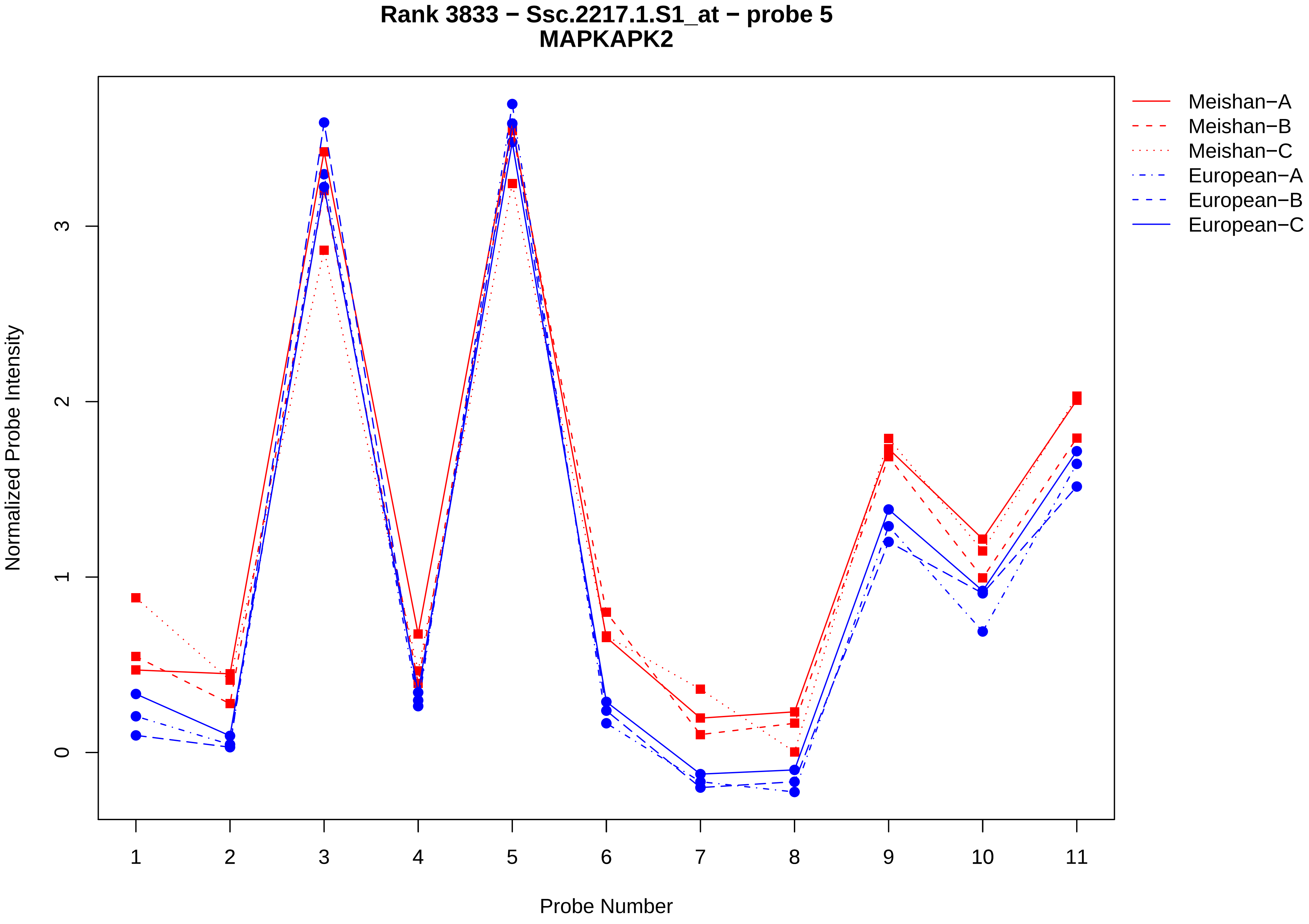

Rank 3833 − Ssc.2217.1.S1_at − probe 5
MAPKAPK2
Normalized Probe Intensity
Probe Number
0
1
2
3
1
2
3
4
5
6
7
8
9
10
11
Meishan−A
Meishan−B
Meishan−C
European−A
European−B
European−C

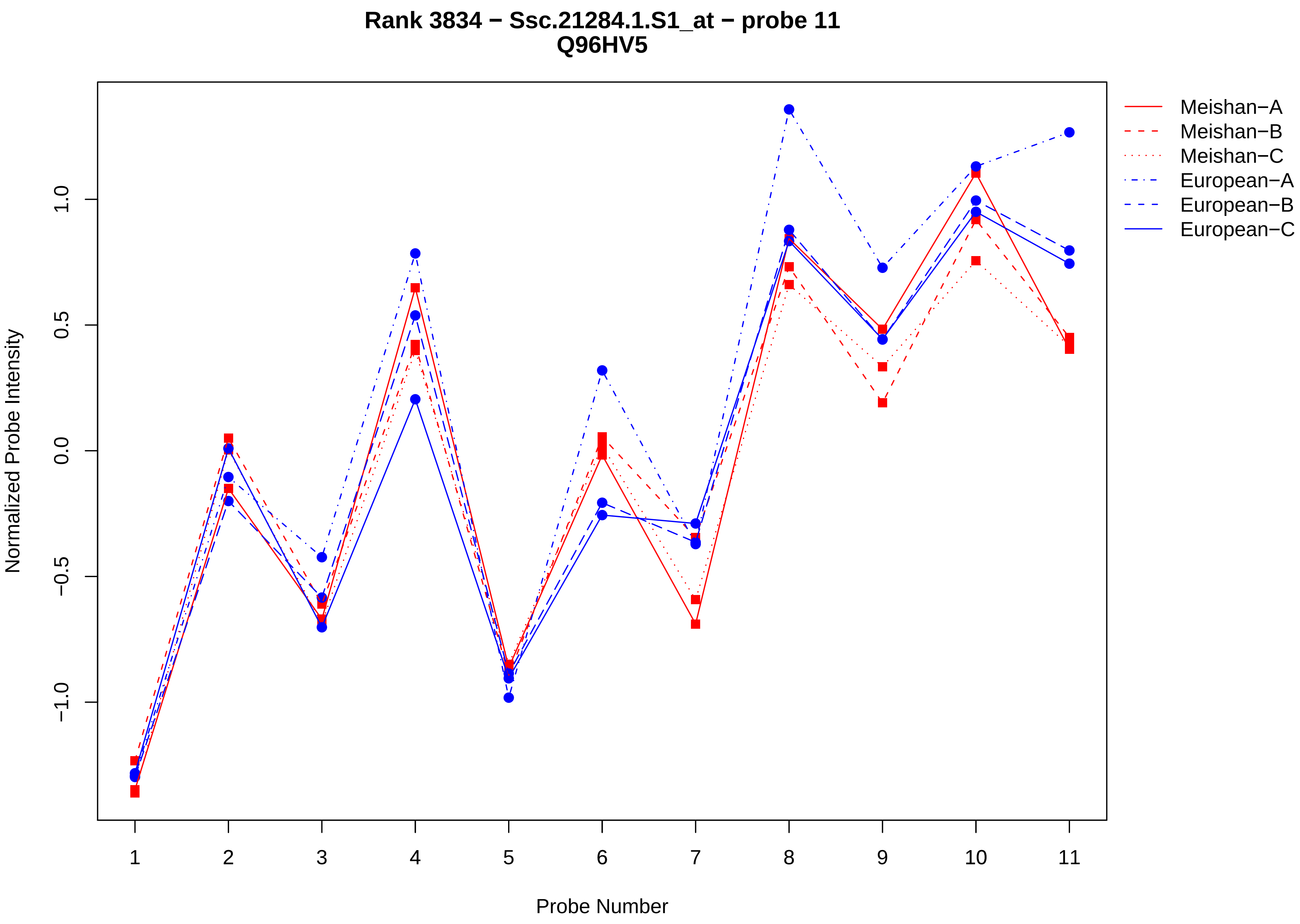

Rank 3834 − Ssc.21284.1.S1_at − probe 11
Q96HV5
Normalized Probe Intensity
−1.0
−0.5
0.0
0.5
1.0
1
2
3
4
5
6
7
8
9
10
11
Probe Number
Meishan−A
Meishan−B
Meishan−C
European−A
European−B
European−C

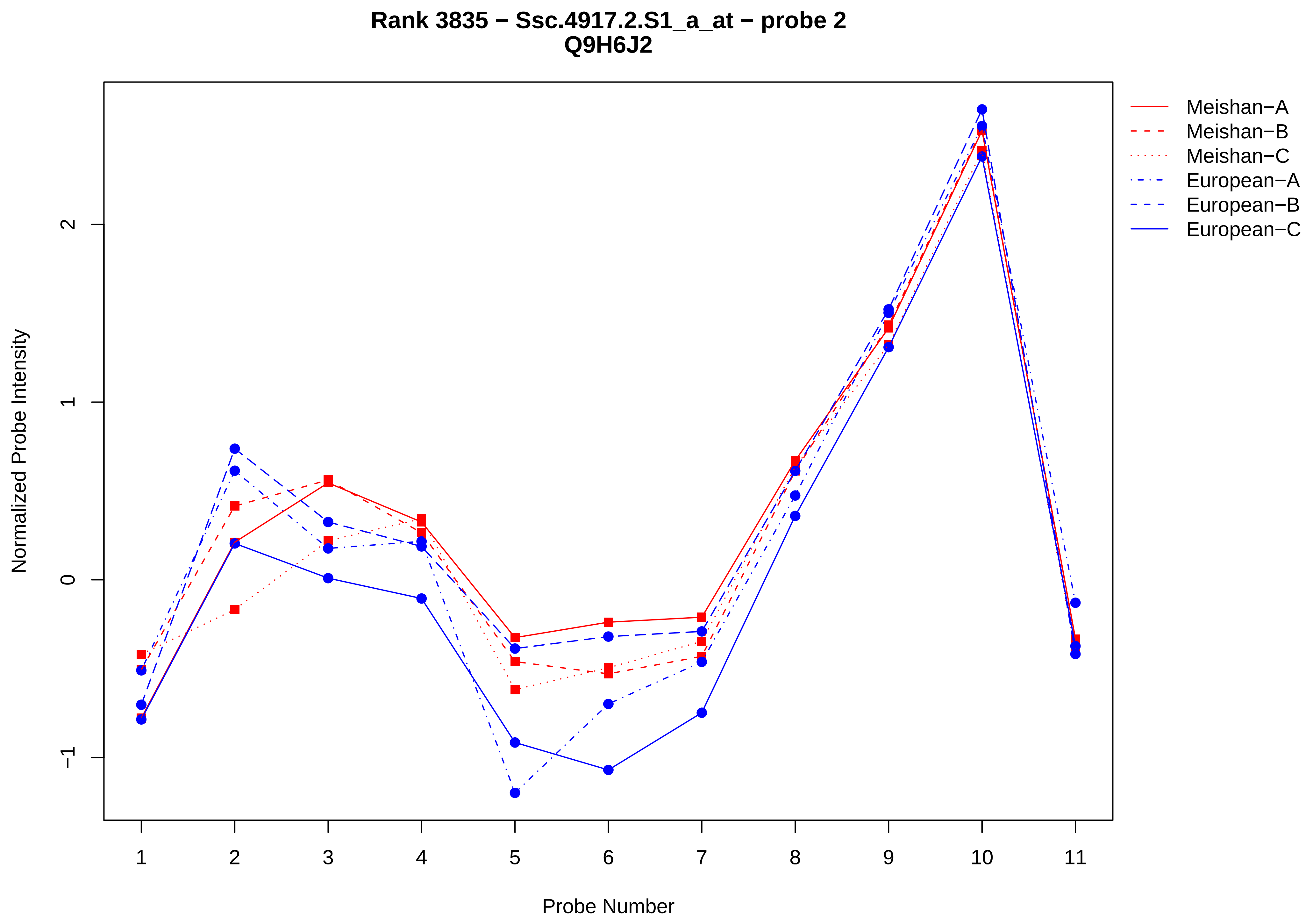

Rank 3835 − Ssc.4917.2.S1_a_at − probe 2
Q9H6J2
Normalized Probe Intensity
Probe Number
−1
0
1
2
1
2
3
4
5
6
7
8
9
10
11
Meishan−A
Meishan−B
Meishan−C
European−A
European−B
European−C

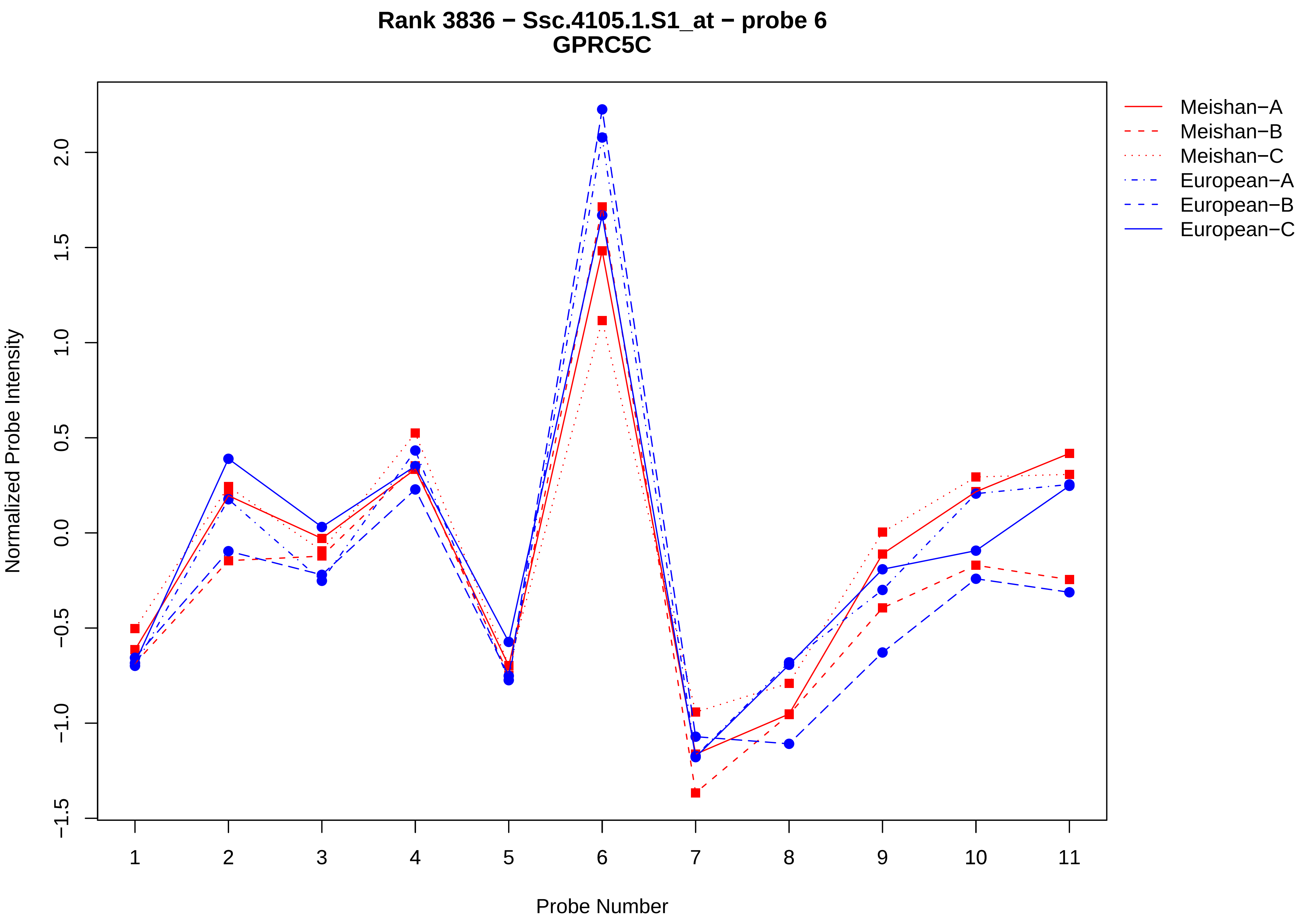

Rank 3836 − Ssc.4105.1.S1_at − probe 6
GPRC5C
Normalized Probe Intensity
Probe Number
Meishan−A
Meishan−B
Meishan−C
European−A
European−B
European−C
−1.5
−1.0
−0.5
0.0
0.5
1.0
1.5
2.0
1
2
3
4
5
6
7
8
9
10
11

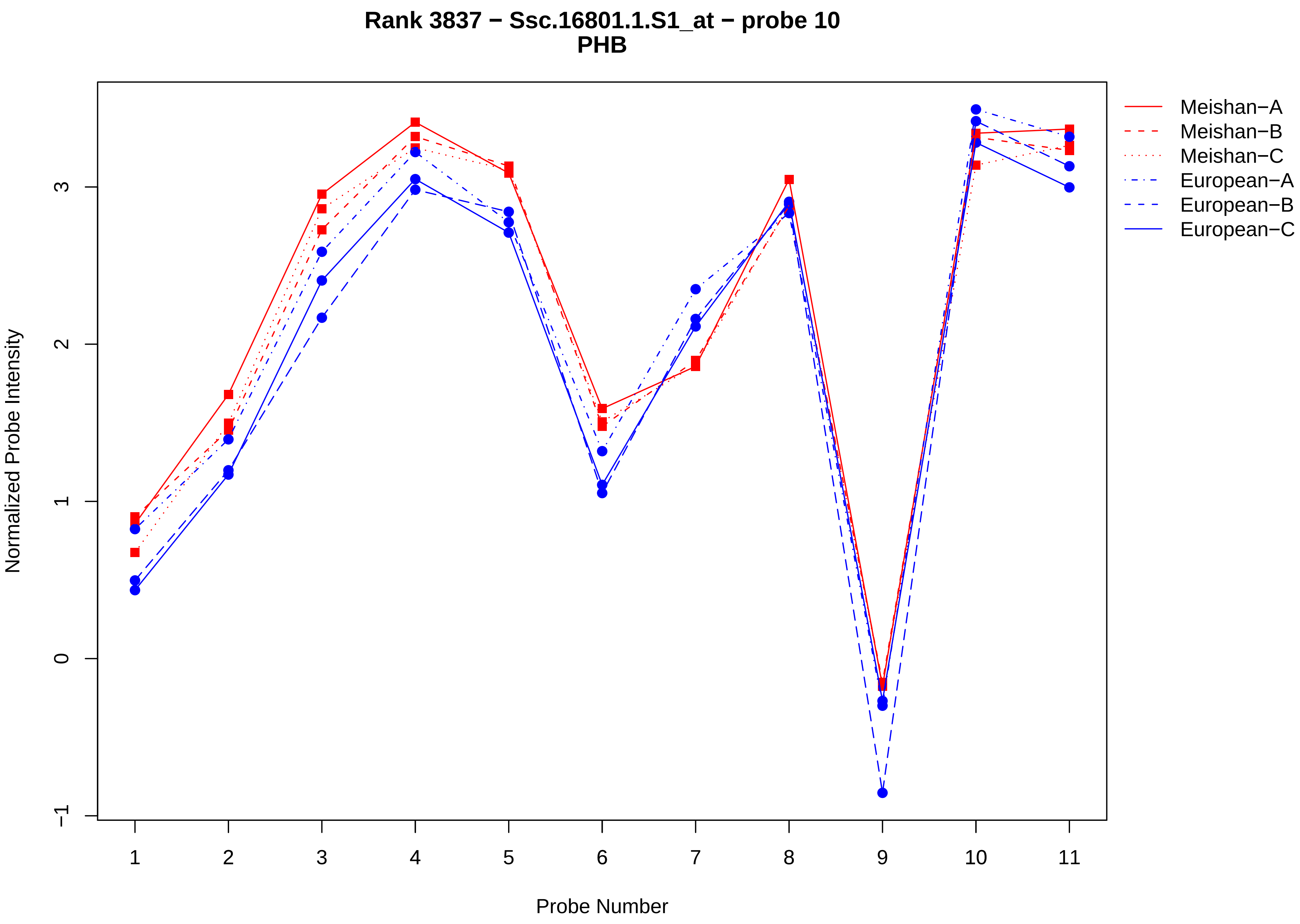

Rank 3837 − Ssc.16801.1.S1_at − probe 10
PHB
Normalized Probe Intensity
Probe Number
−1
0
1
2
3
1
2
3
4
5
6
7
8
9
10
11
Meishan−A
Meishan−B
Meishan−C
European−A
European−B
European−C

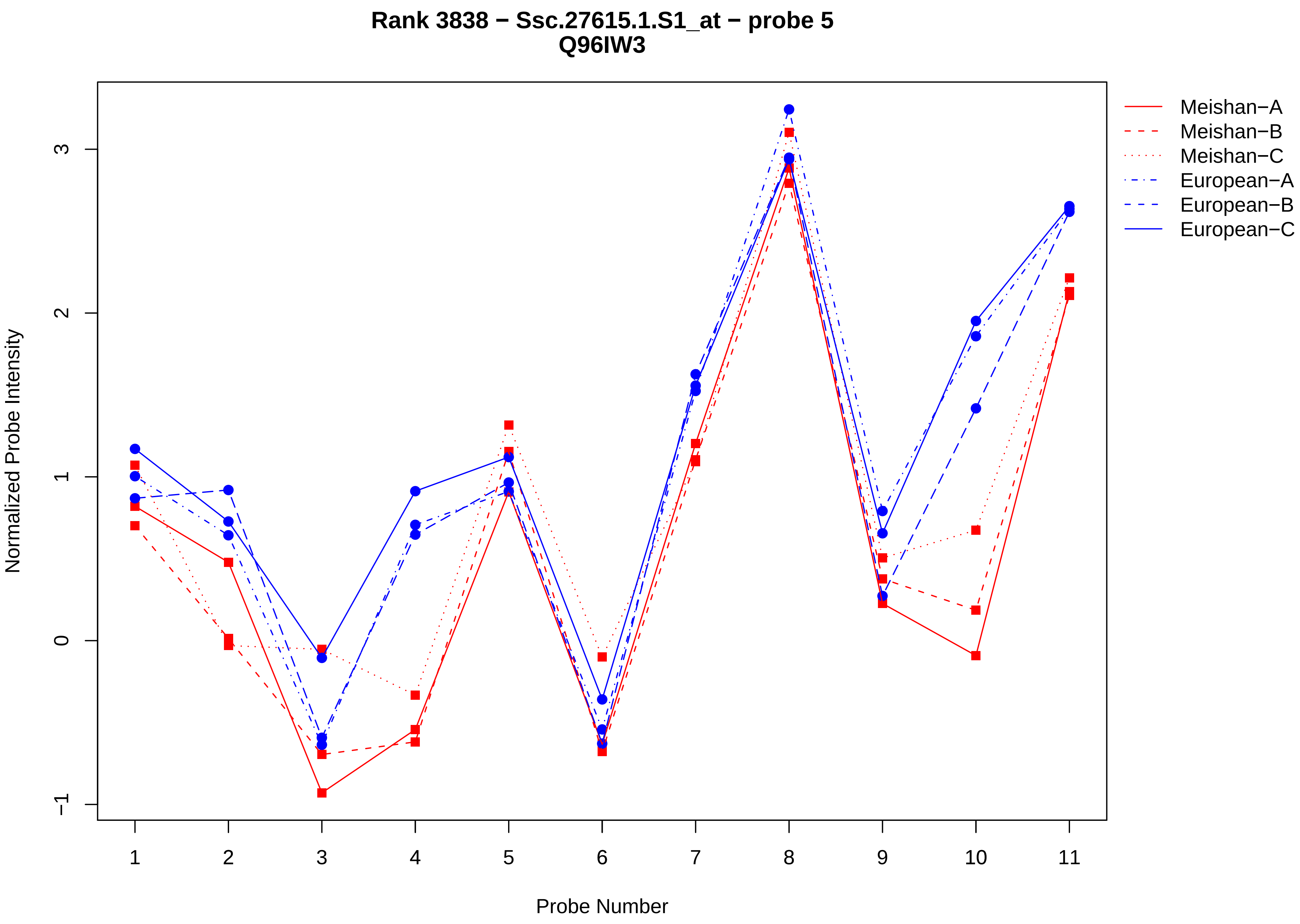
Rank 3838 − Ssc.27615.1.S1_at − probe 5
Q96IW3
Normalized Probe Intensity
Probe Number
Meishan−A
Meishan−B
Meishan−C
European−A
European−B
European−C

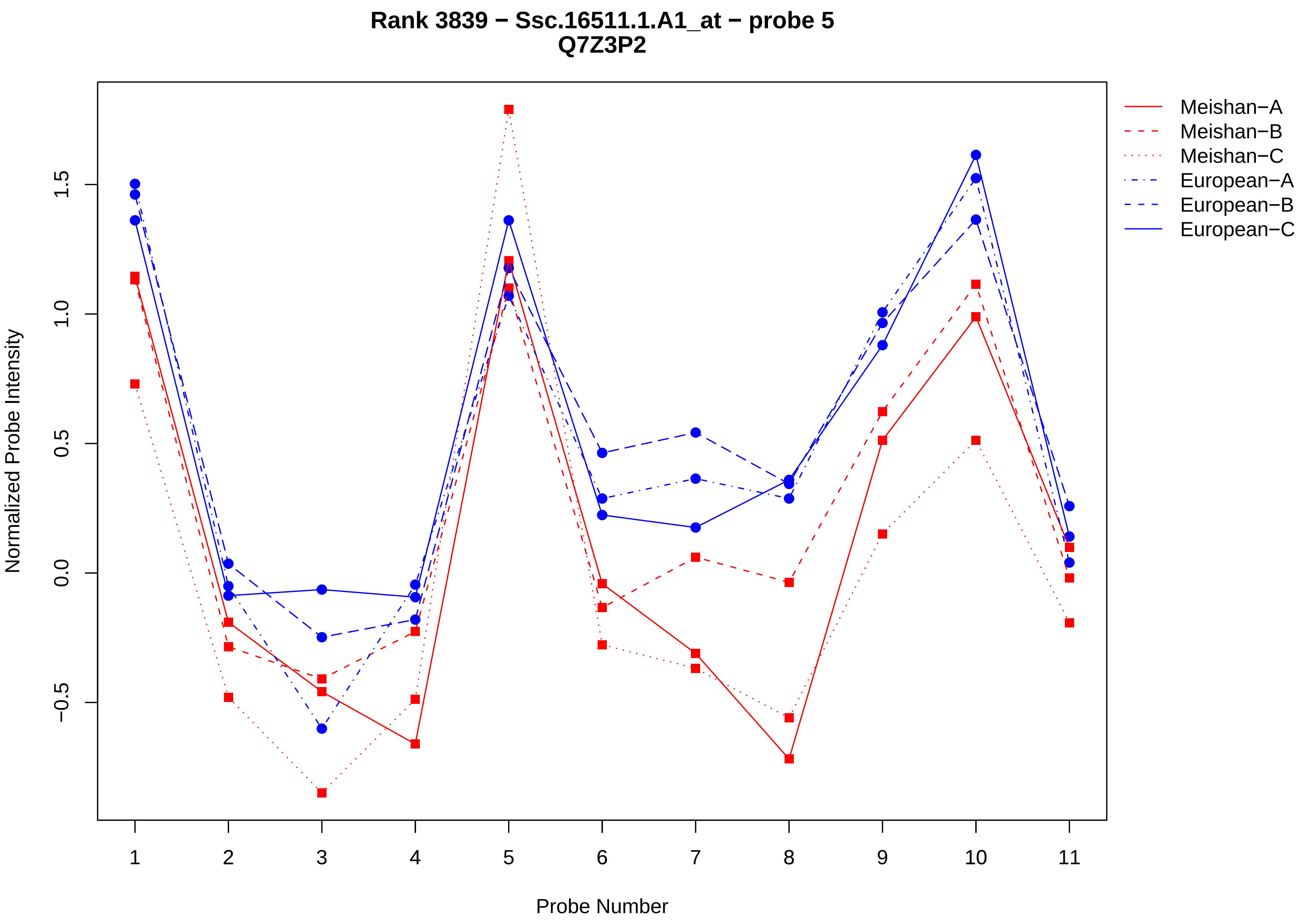

Rank 3839 − Ssc.16511.1.A1_at − probe 5
Q7Z3P2
Normalized Probe Intensity
Probe Number
−0.5
0.0
0.5
1.0
1.5
1
2
3
4
5
6
7
8
9
10
11
Meishan−A
Meishan−B
Meishan−C
European−A
European−B
European−C

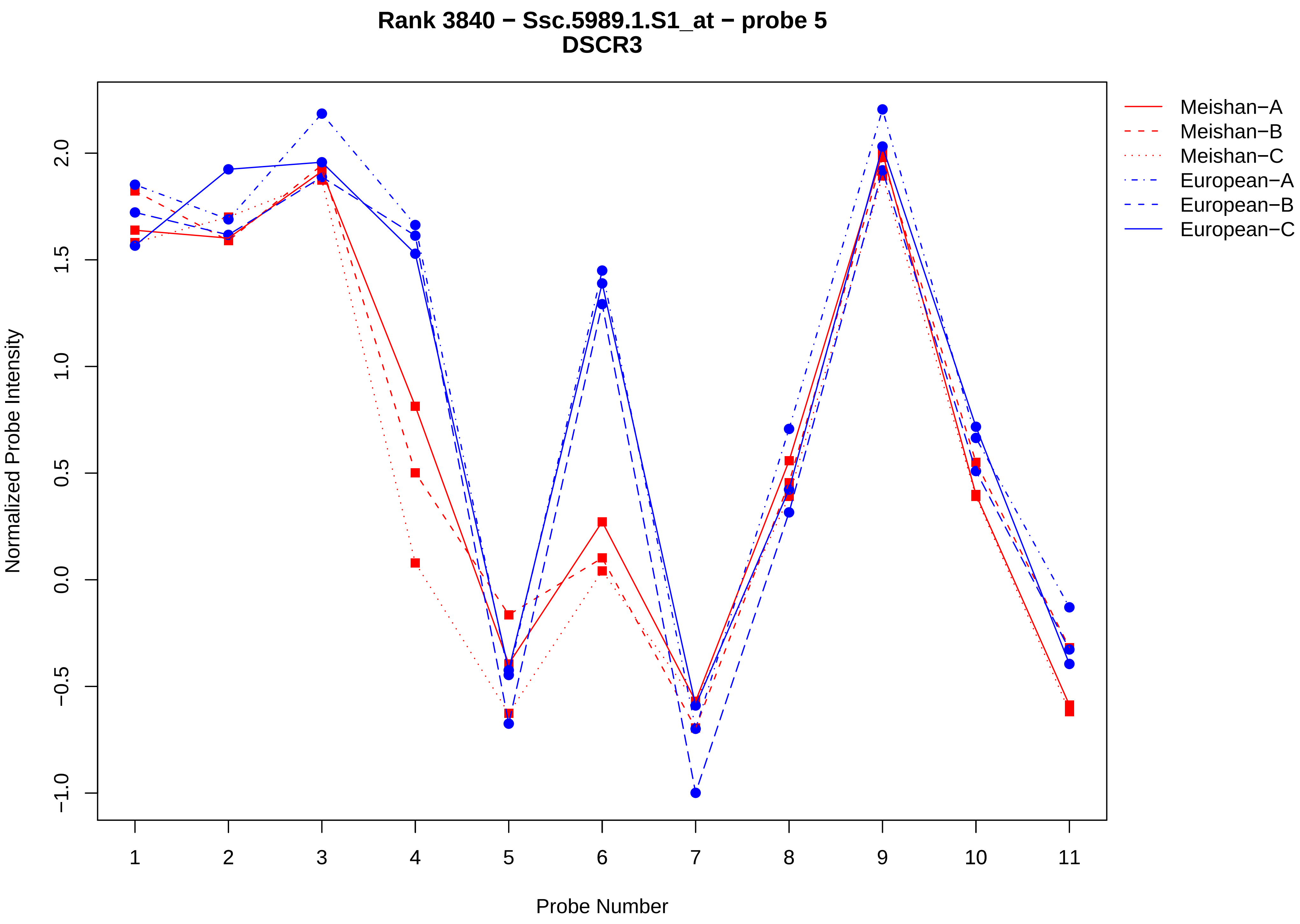

Rank 3840 − Ssc.5989.1.S1_at − probe 5
DSCR3
Normalized Probe Intensity
Probe Number
−1.0
−0.5
0.0
0.5
1.0
1.5
2.0
1
2
3
4
5
6
7
8
9
10
11
Meishan−A
Meishan−B
Meishan−C
European−A
European−B
European−C

Rank 3841 − Ssc.27410.1.S1_at − probe 4
MYCN

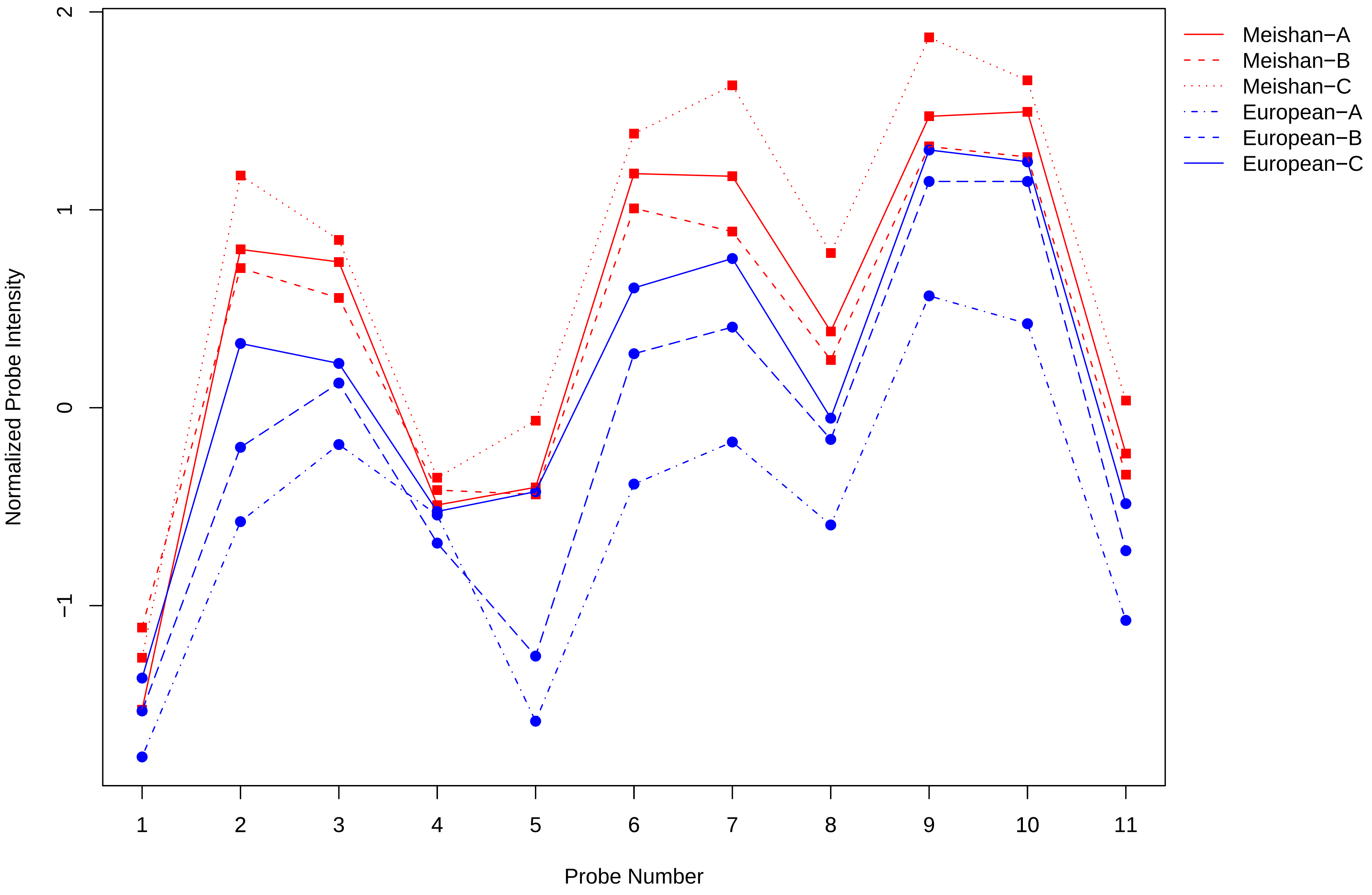

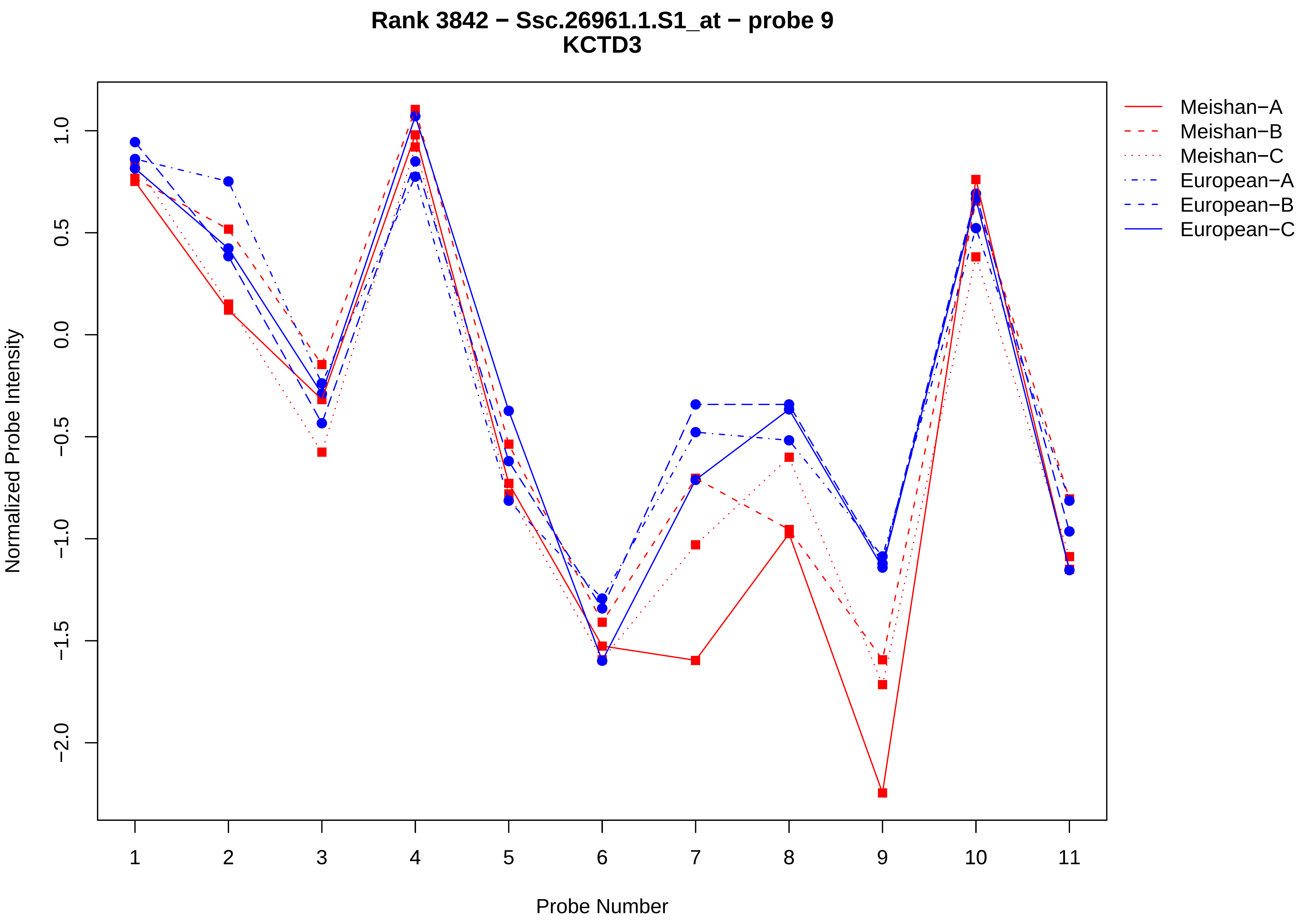

Rank 3842 − Ssc.26961.1.S1_at − probe 9
KCTD3
Normalized Probe Intensity
Probe Number
1.0
0.5
0.0
−0.5
−1.0
−1.5
−2.0
1
2
3
4
5
6
7
8
9
10
11
Meishan−A
Meishan−B
Meishan−C
European−A
European−B
European−C

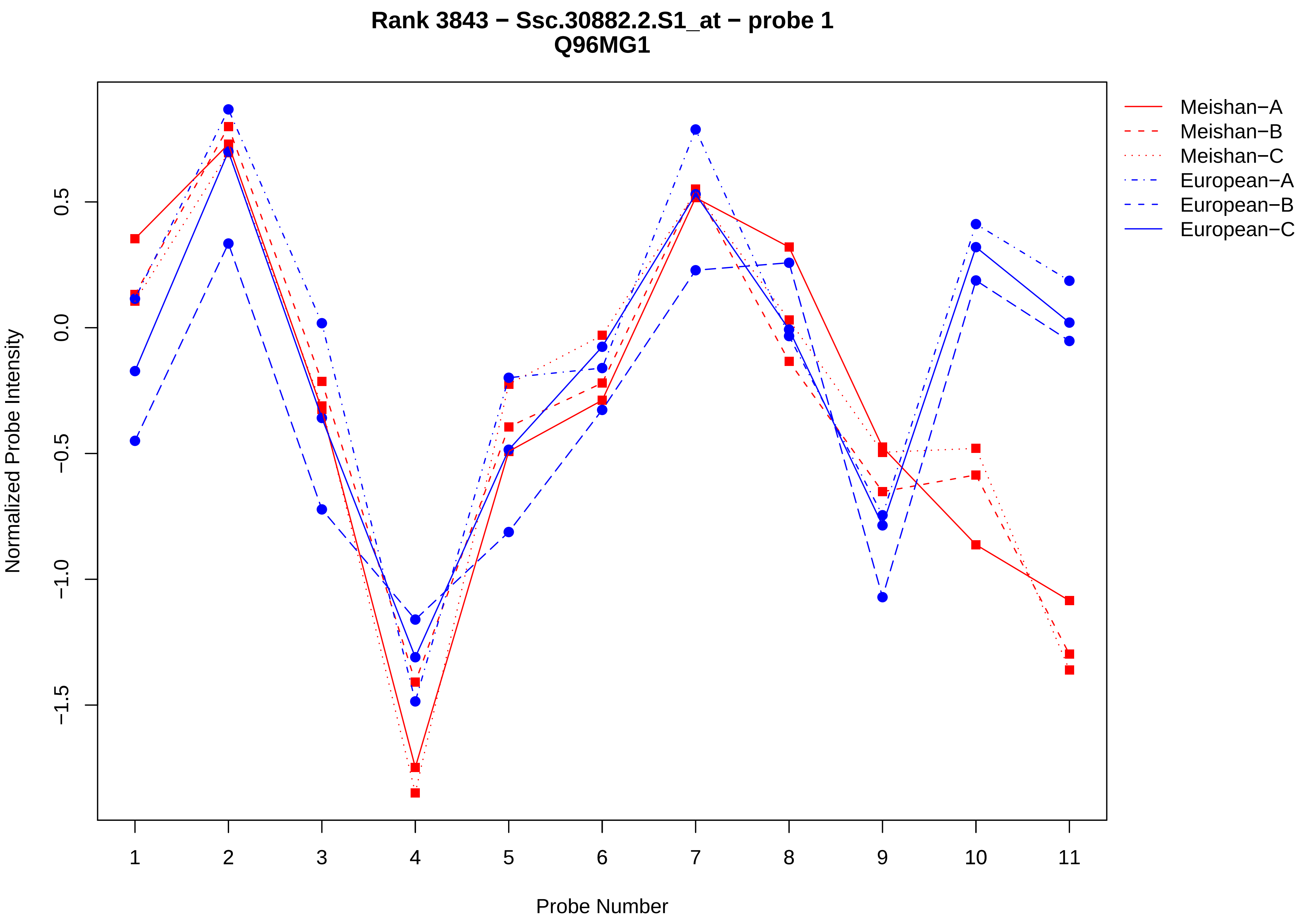

Rank 3843 − Ssc.30882.2.S1_at − probe 1
Q96MG1
Normalized Probe Intensity
Probe Number
0.5
0.0
−0.5
−1.0
−1.5
1
2
3
4
5
6
7
8
9
10
11
Meishan−A
Meishan−B
Meishan−C
European−A
European−B
European−C

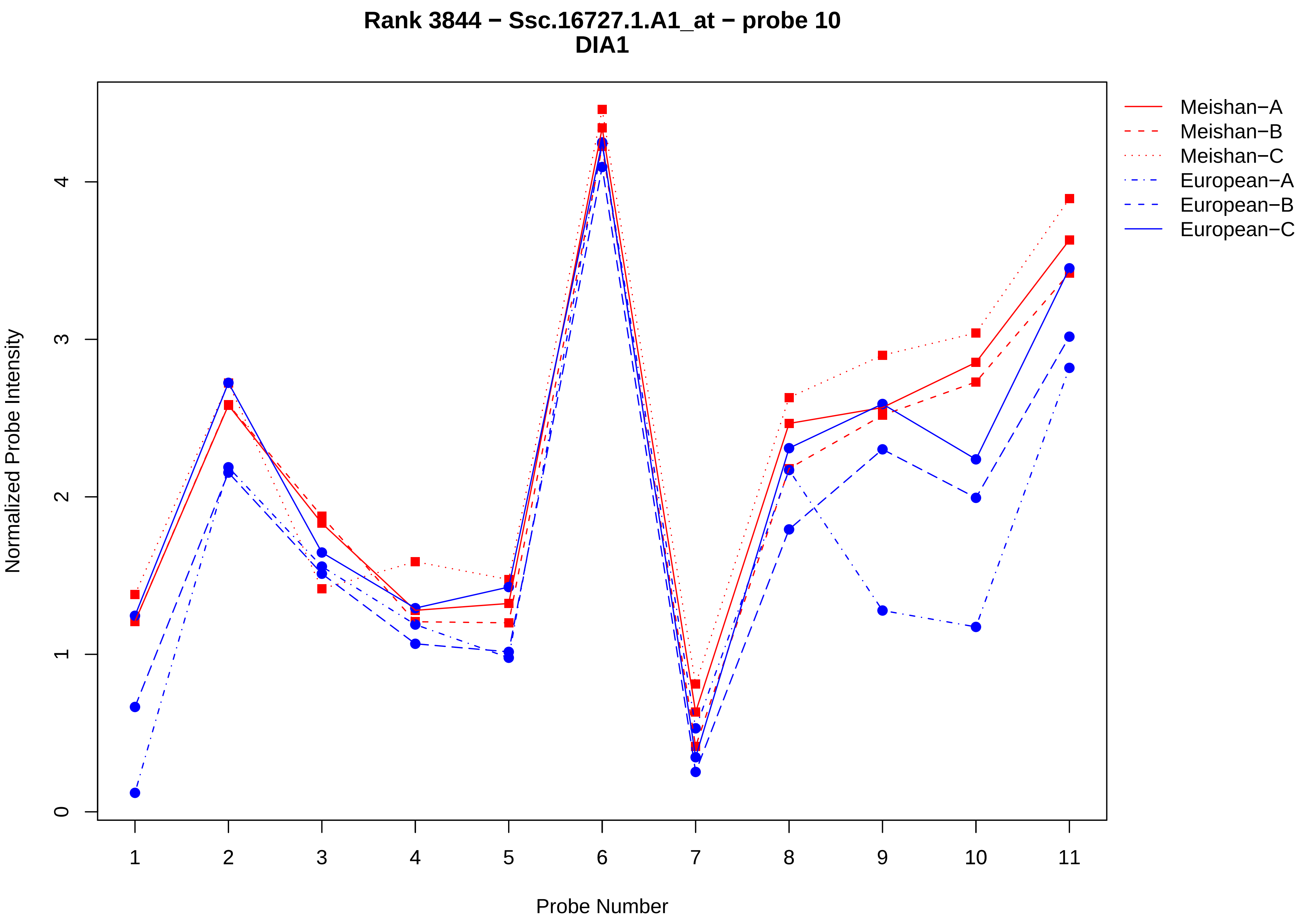

Rank 3844 − Ssc.16727.1.A1_at − probe 10
DIA1
Normalized Probe Intensity
Probe Number
0
1
2
3
4
1
2
3
4
5
6
7
8
9
10
11
Meishan−A
Meishan−B
Meishan−C
European−A
European−B
European−C

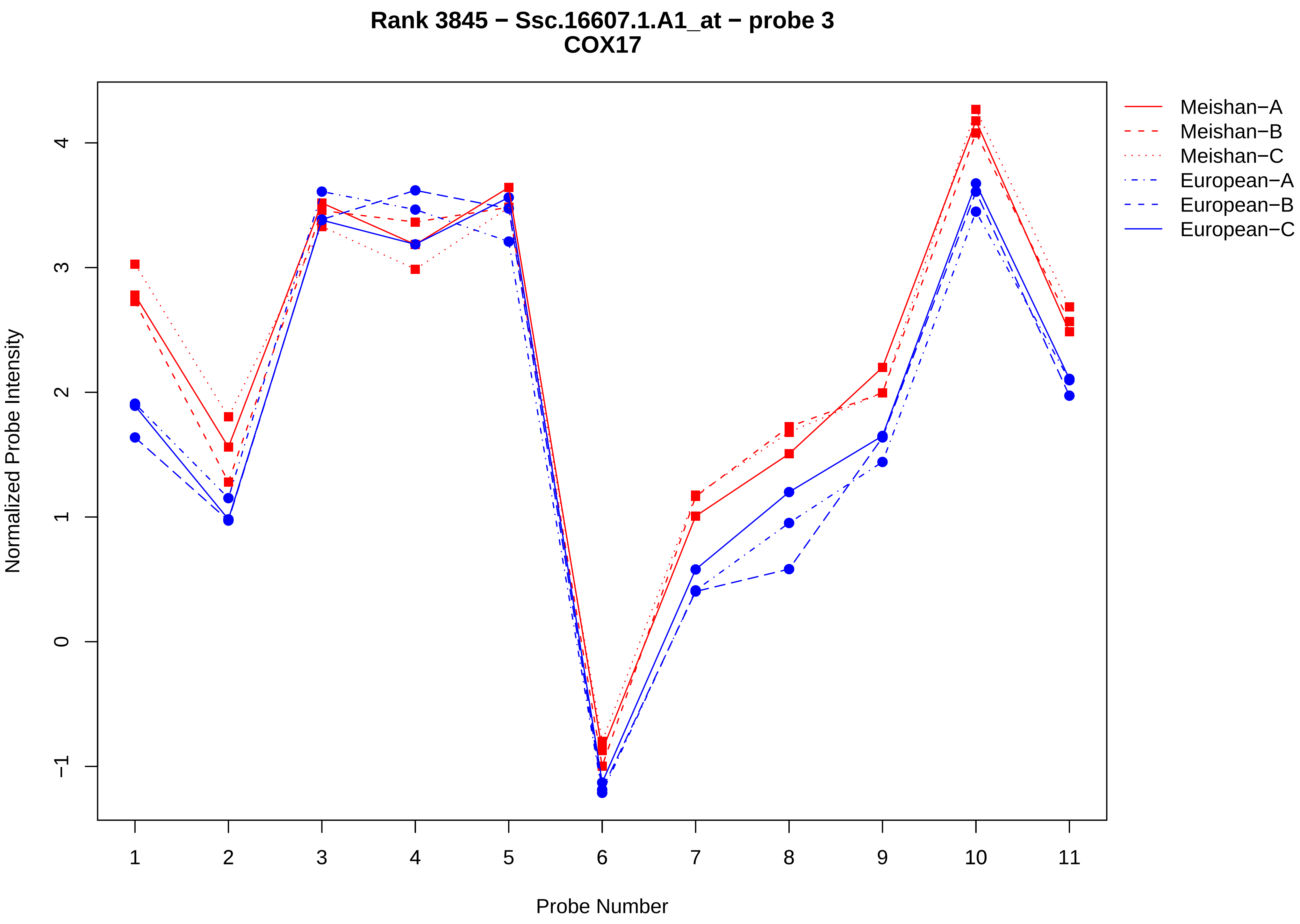

Rank 3845 − Ssc.16607.1.A1_at − probe 3
COX17
Normalized Probe Intensity
Probe Number
Meishan−A
Meishan−B
Meishan−C
European−A
European−B
European−C

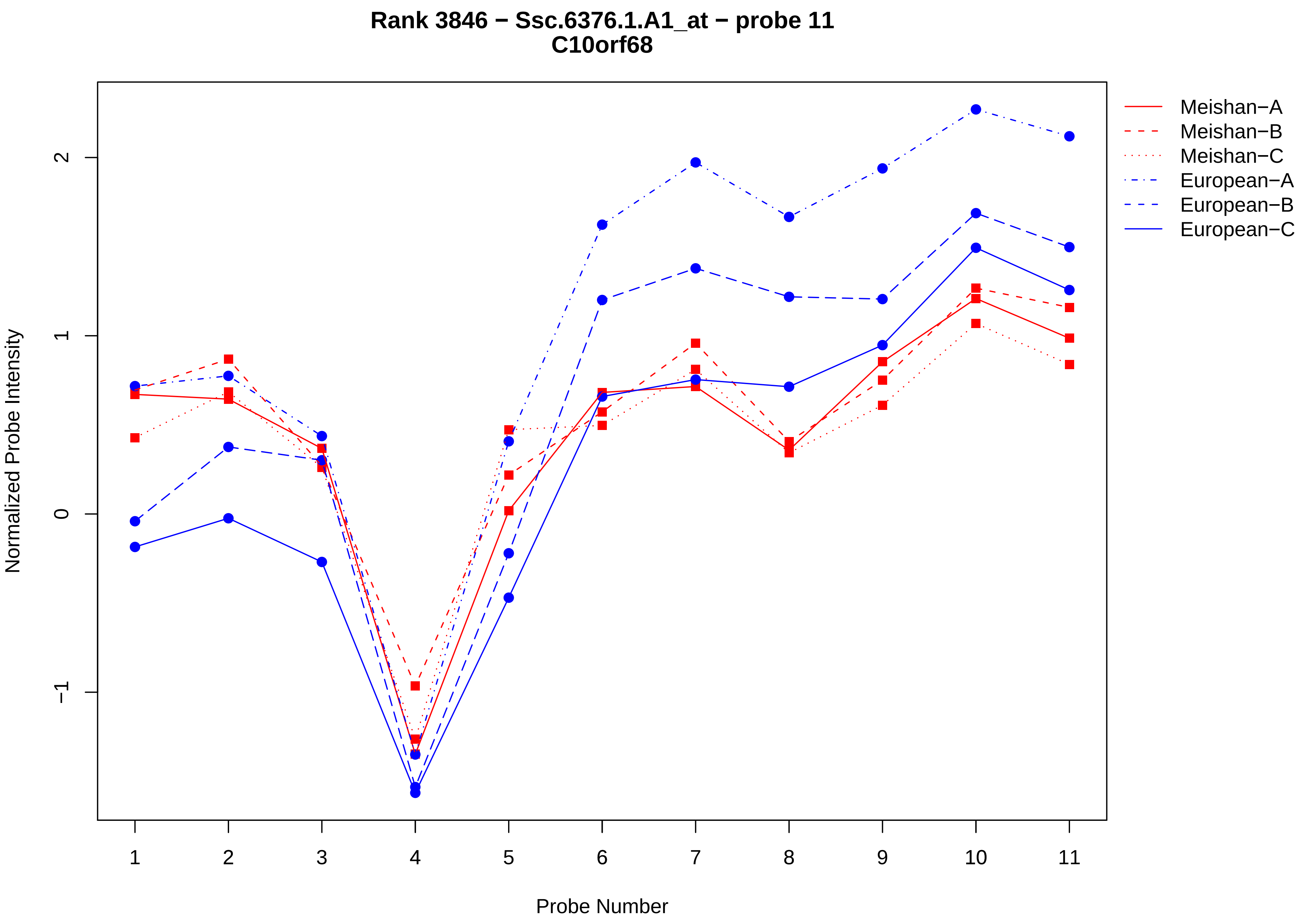

Rank 3846 − Ssc.6376.1.A1_at − probe 11
C10orf68
Normalized Probe Intensity
Probe Number
Meishan−A
Meishan−B
Meishan−C
European−A
European−B
European−C

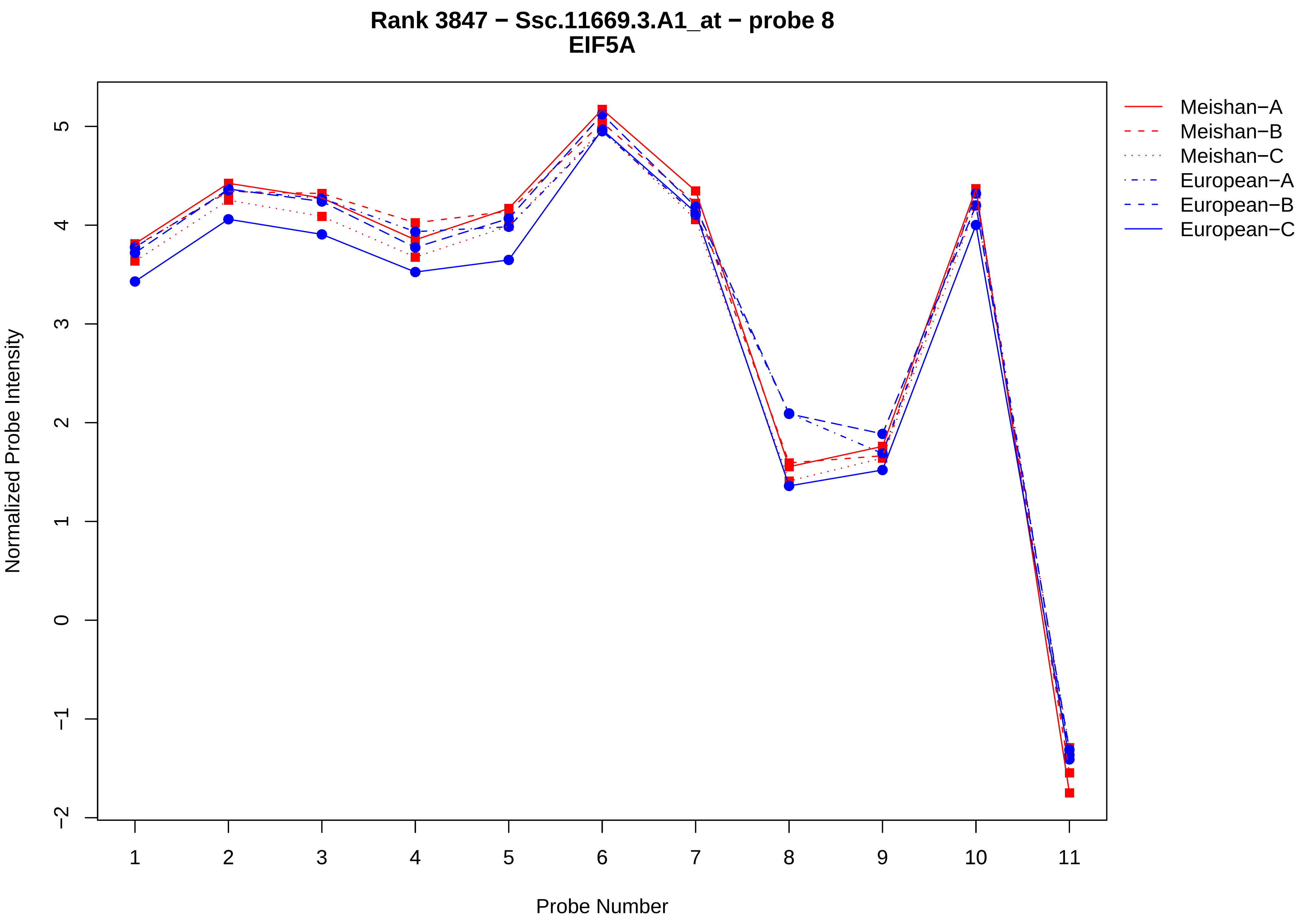

Rank 3847 − Ssc.11669.3.A1_at − probe 8
EIF5A
Normalized Probe Intensity
Probe Number
Meishan−A
Meishan−B
Meishan−C
European−A
European−B
European−C

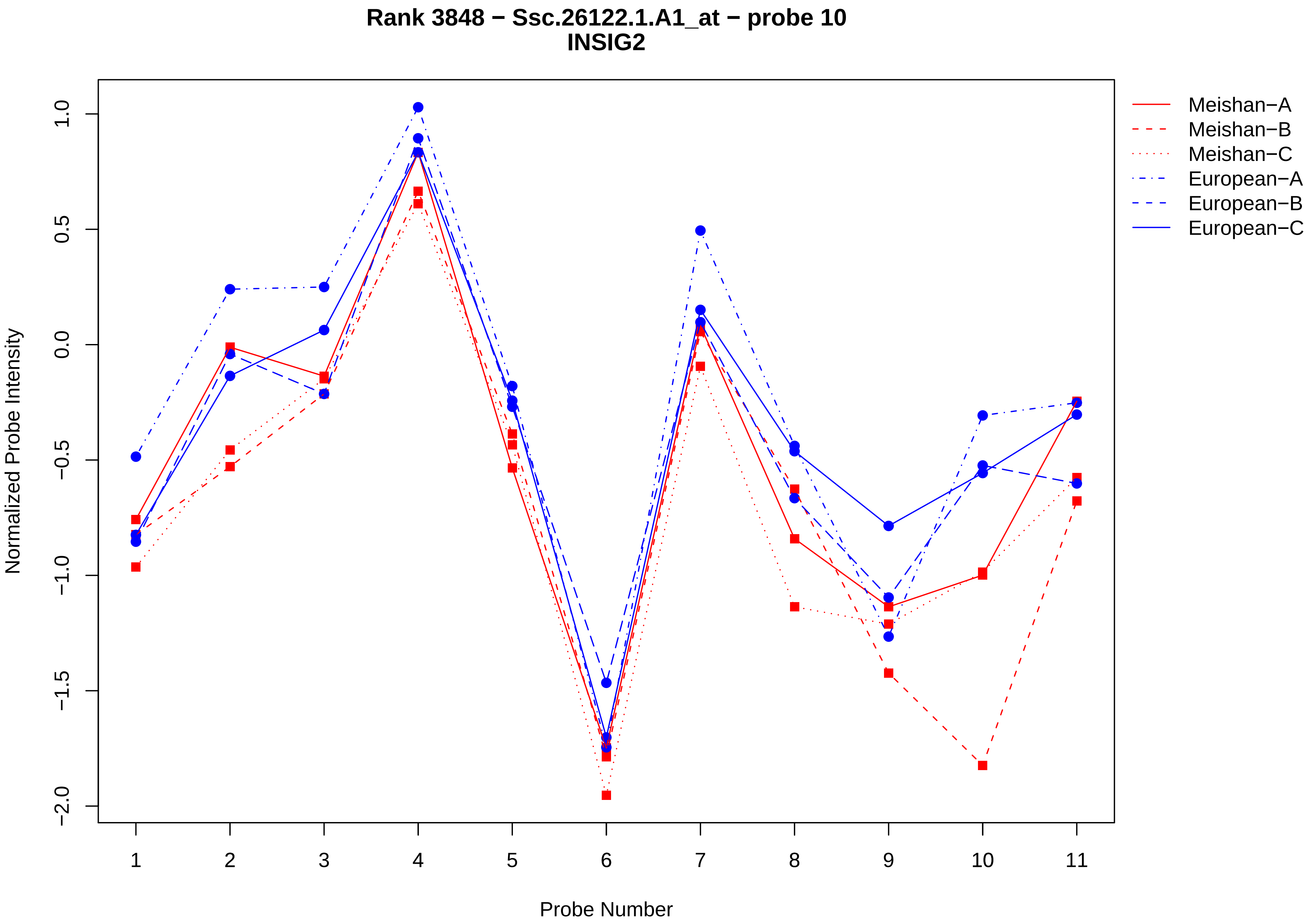
Rank 3848 − Ssc.26122.1.A1_at − probe 10
INSIG2
Normalized Probe Intensity
Probe Number
1.0
0.5
0.0
−0.5
−1.0
−1.5
−2.0
1
2
3
4
5
6
7
8
9
10
11
Meishan−A
Meishan−B
Meishan−C
European−A
European−B
European−C

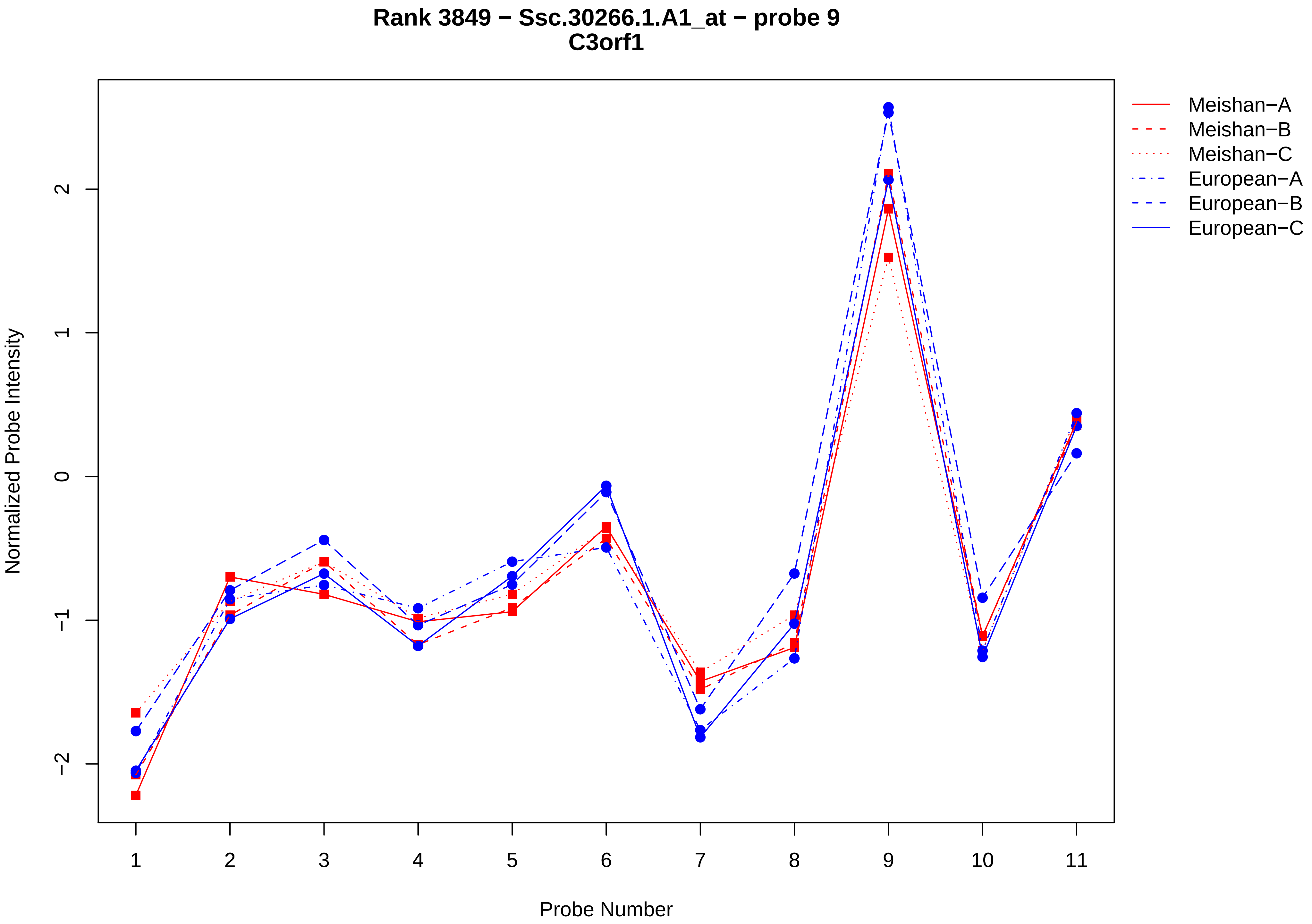

Rank 3849 − Ssc.30266.1.A1_at − probe 9
C3orf1
Normalized Probe Intensity
Probe Number
−2
−1
0
1
2
1
2
3
4
5
6
7
8
9
10
11
Meishan−A
Meishan−B
Meishan−C
European−A
European−B
European−C

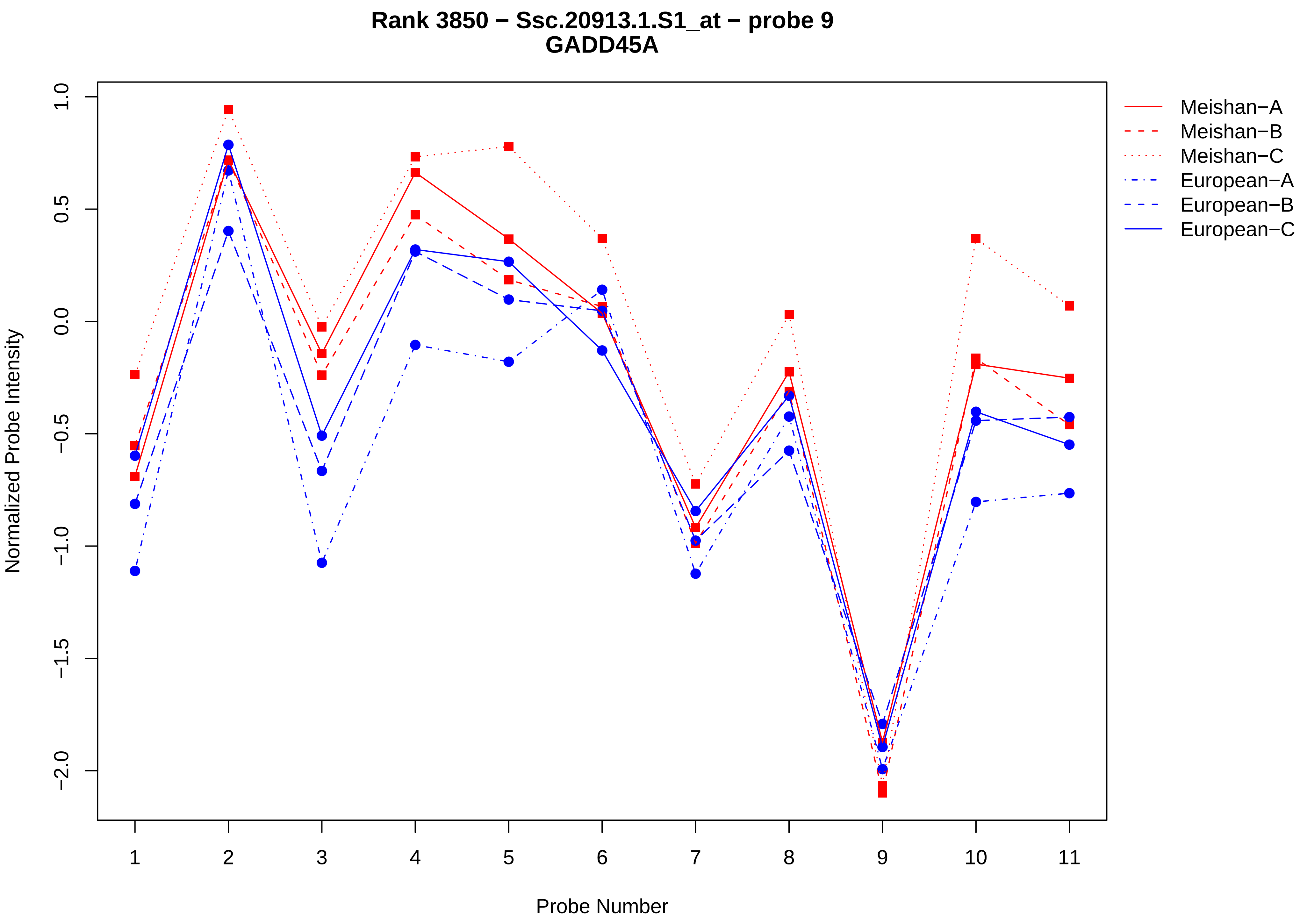

Rank 3850 − Ssc.20913.1.S1_at − probe 9
GADD45A
Normalized Probe Intensity
Probe Number
1.0
0.5
0.0
−0.5
−1.0
−1.5
−2.0
1
2
3
4
5
6
7
8
9
10
11
Meishan−A
Meishan−B
Meishan−C
European−A
European−B
European−C

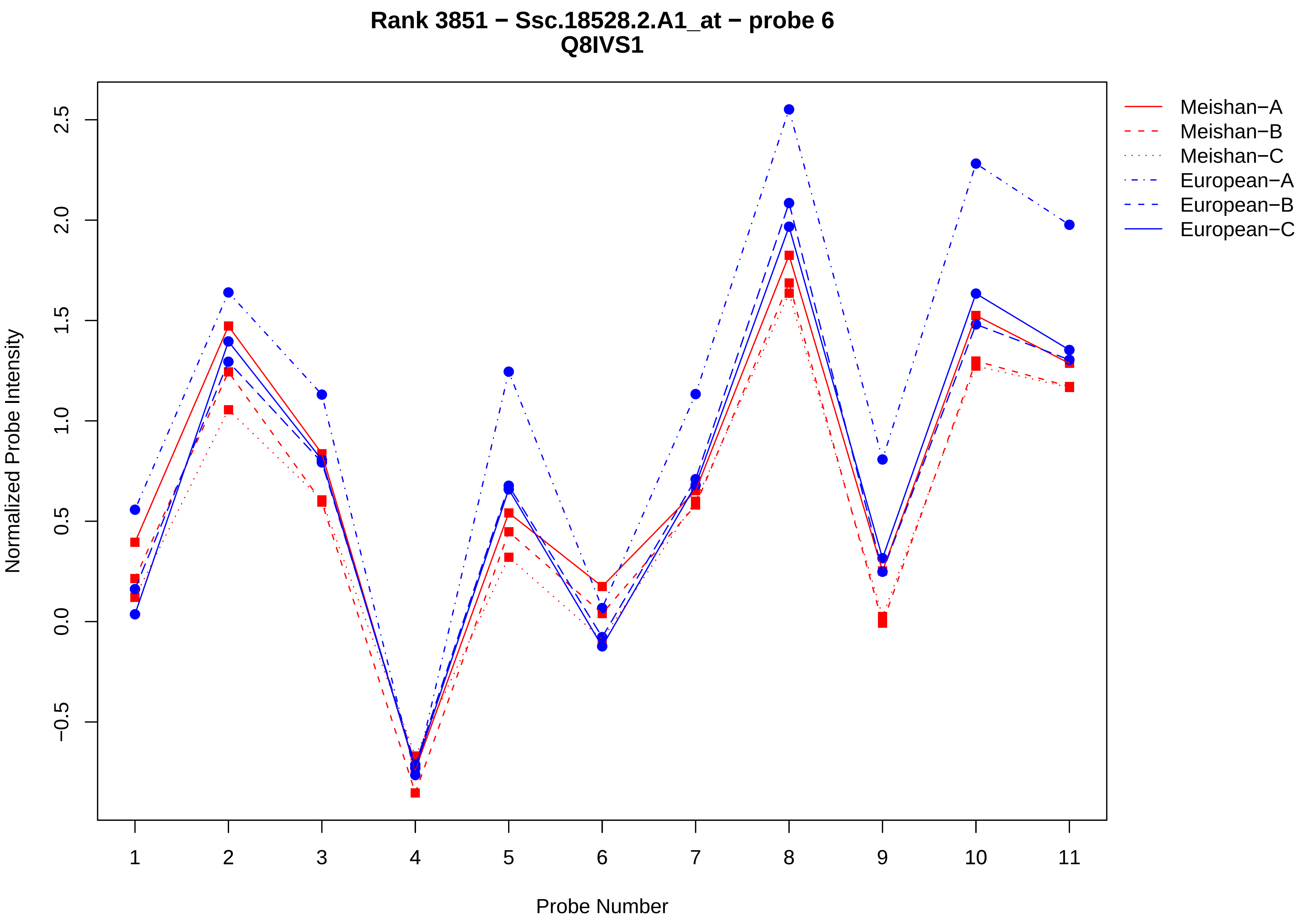

Rank 3851 − Ssc.18528.2.A1_at − probe 6
Q8IVS1
Normalized Probe Intensity
Probe Number
−0.5
0.0
0.5
1.0
1.5
2.0
2.5
1
2
3
4
5
6
7
8
9
10
11
Meishan−A
Meishan−B
Meishan−C
European−A
European−B
European−C

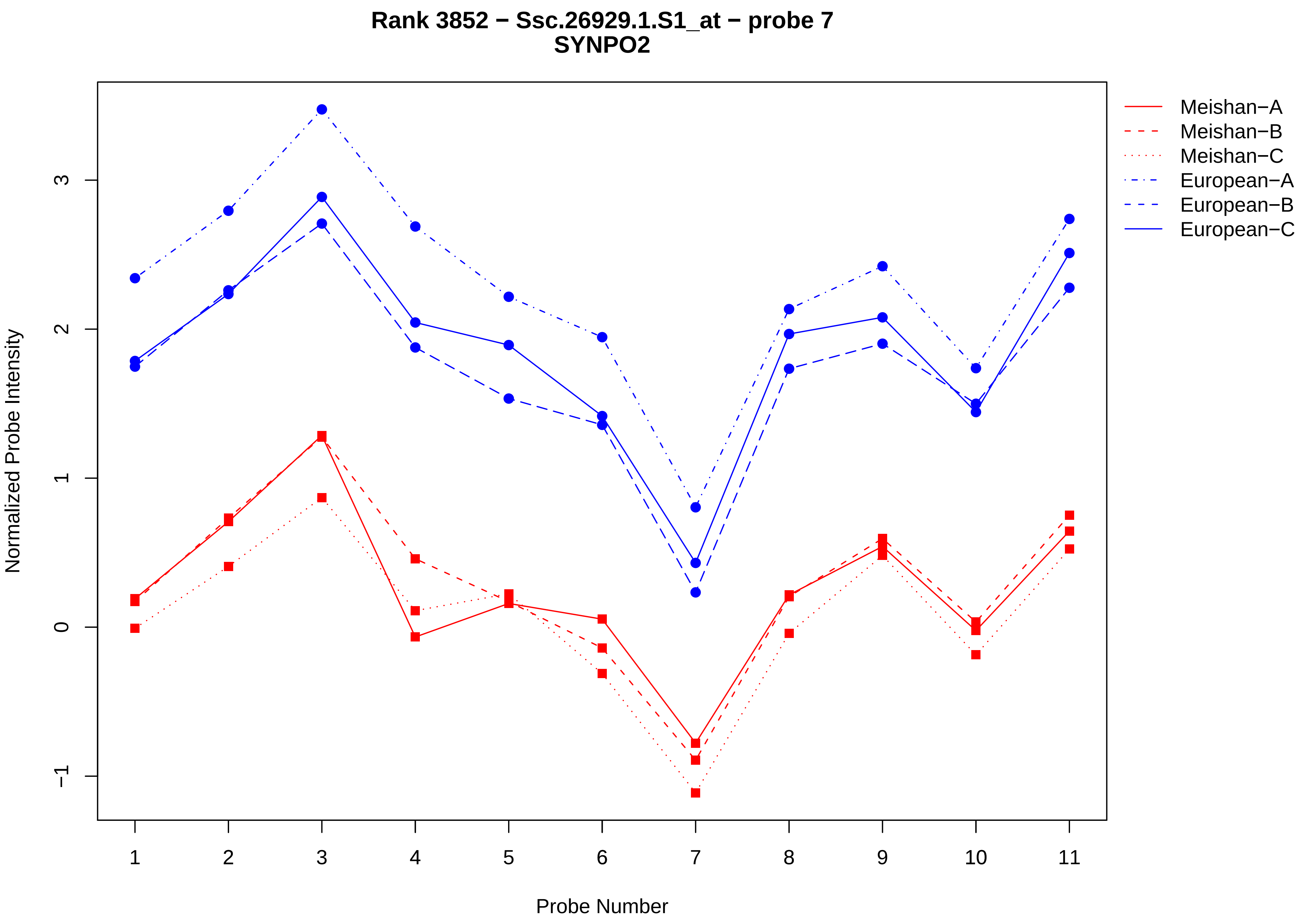

Rank 3852 − Ssc.26929.1.S1_at − probe 7
SYNPO2
Normalized Probe Intensity
Probe Number
Meishan−A
Meishan−B
Meishan−C
European−A
European−B
European−C
−1
0
1
2
3
1
2
3
4
5
6
7
8
9
10
11

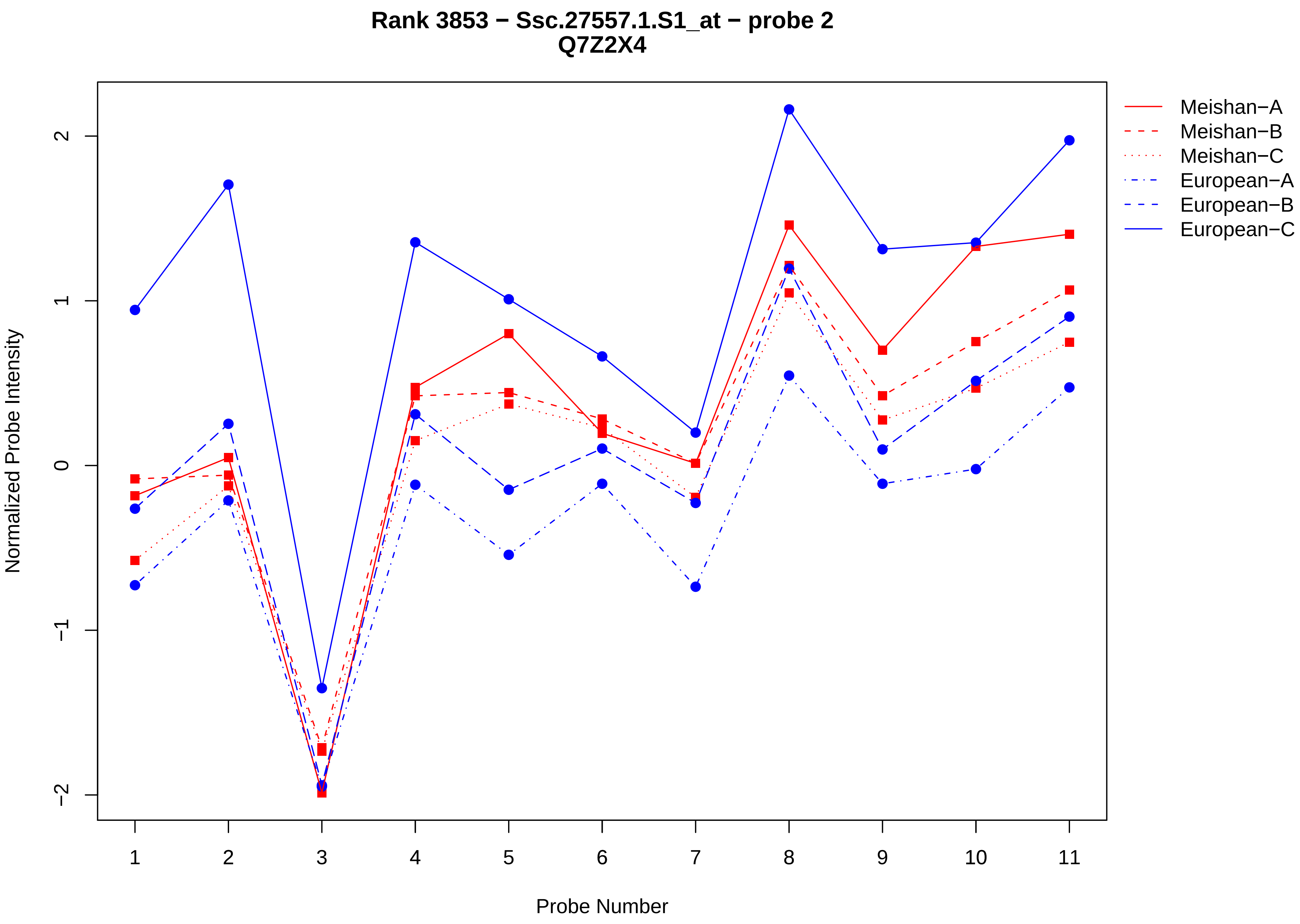

Rank 3853 − Ssc.27557.1.S1_at − probe 2
Q7Z2X4
Normalized Probe Intensity
Probe Number
Meishan−A
Meishan−B
Meishan−C
European−A
European−B
European−C
−2
−1
0
1
2
1
2
3
4
5
6
7
8
9
10
11

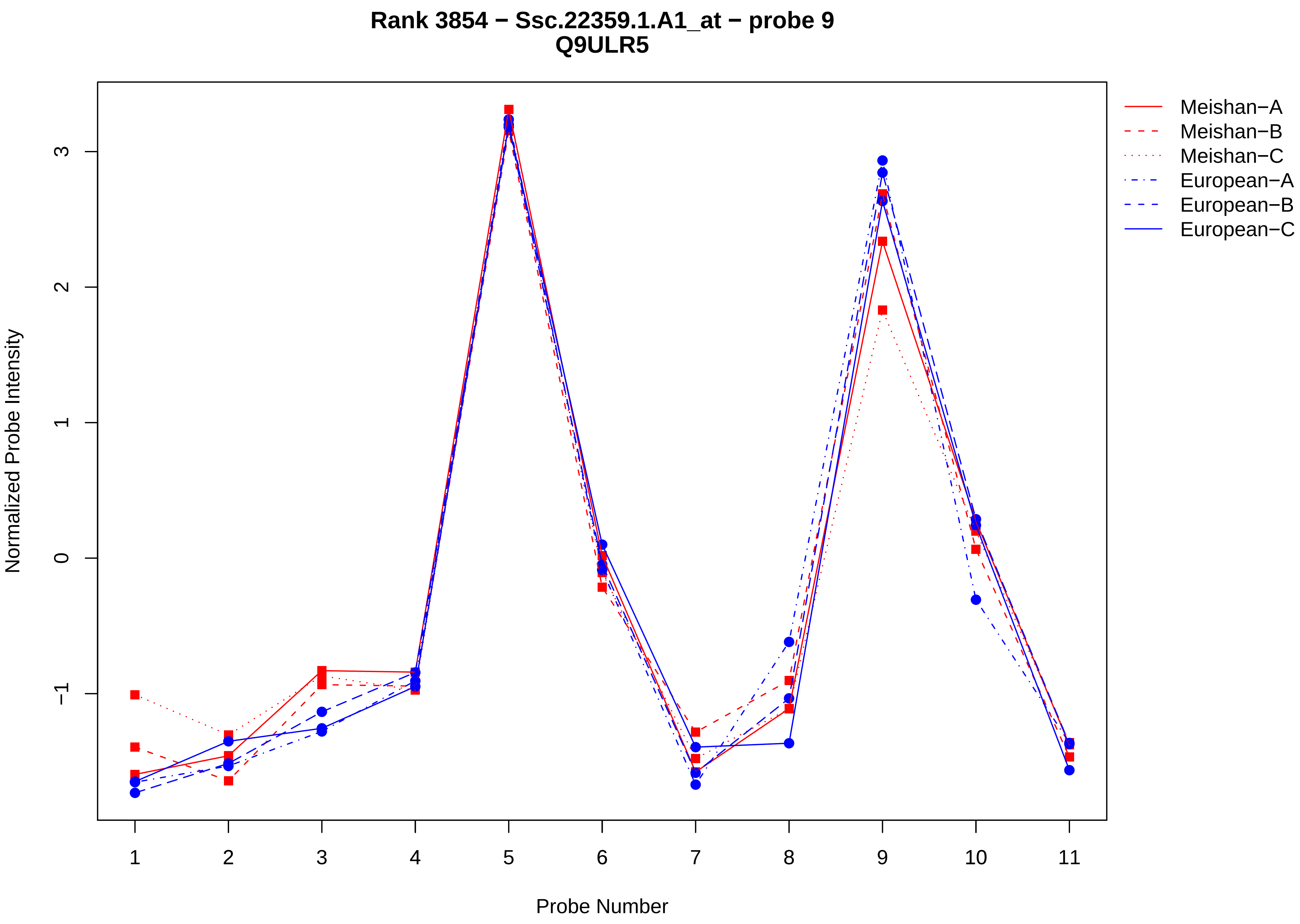

Rank 3854 − Ssc.22359.1.A1_at − probe 9
Q9ULR5
Normalized Probe Intensity
Probe Number
Meishan−A
Meishan−B
Meishan−C
European−A
European−B
European−C
−1
0
1
2
3
1
2
3
4
5
6
7
8
9
10
11

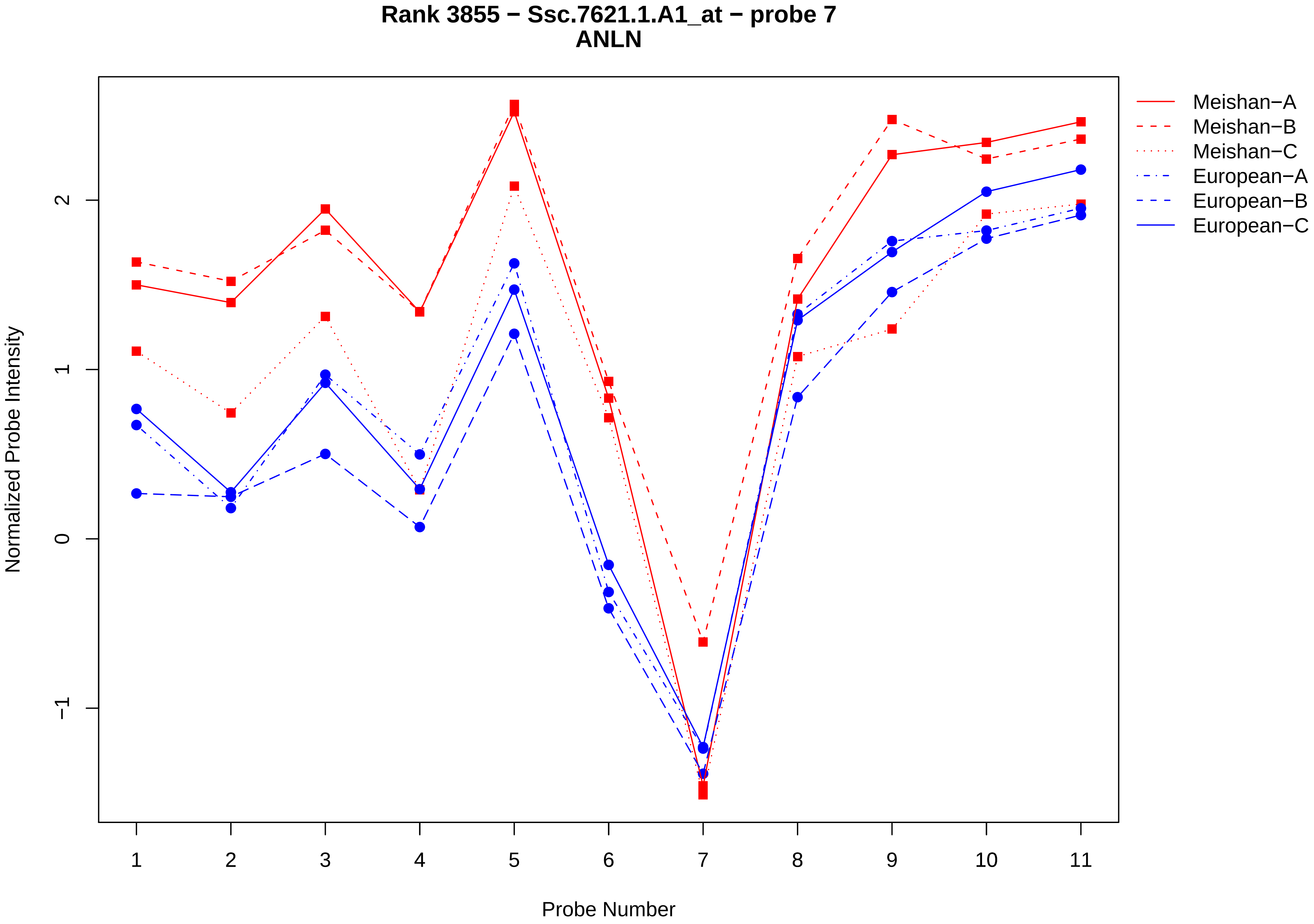

Rank 3855 − Ssc.7621.1.A1_at − probe 7
ANLN
Normalized Probe Intensity
Probe Number
−1
0
1
2
1
2
3
4
5
6
7
8
9
10
11
Meishan−A
Meishan−B
Meishan−C
European−A
European−B
European−C

Rank 3856 − Ssc.5638.1.S1_at − probe 9
FKBP4

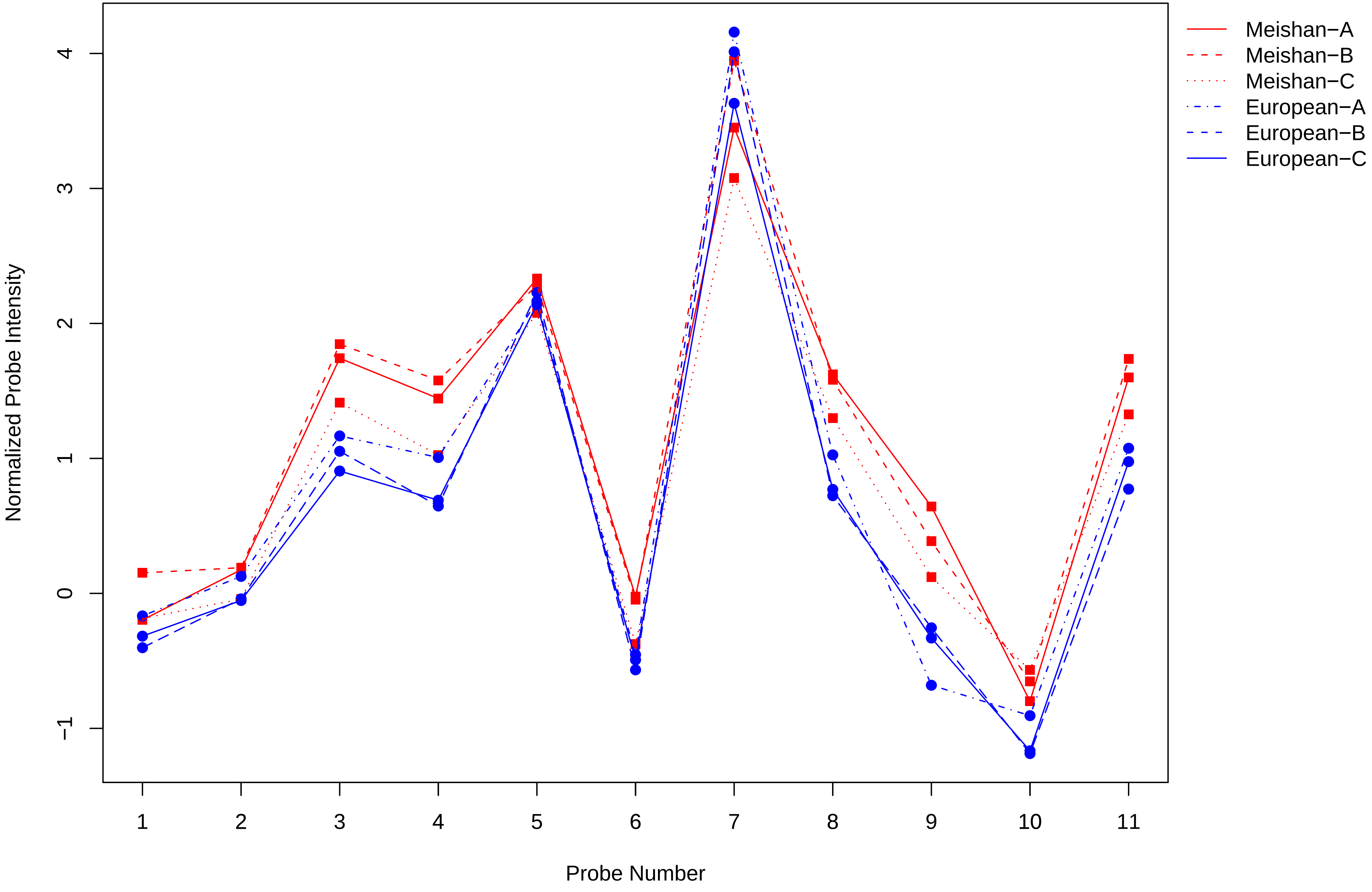

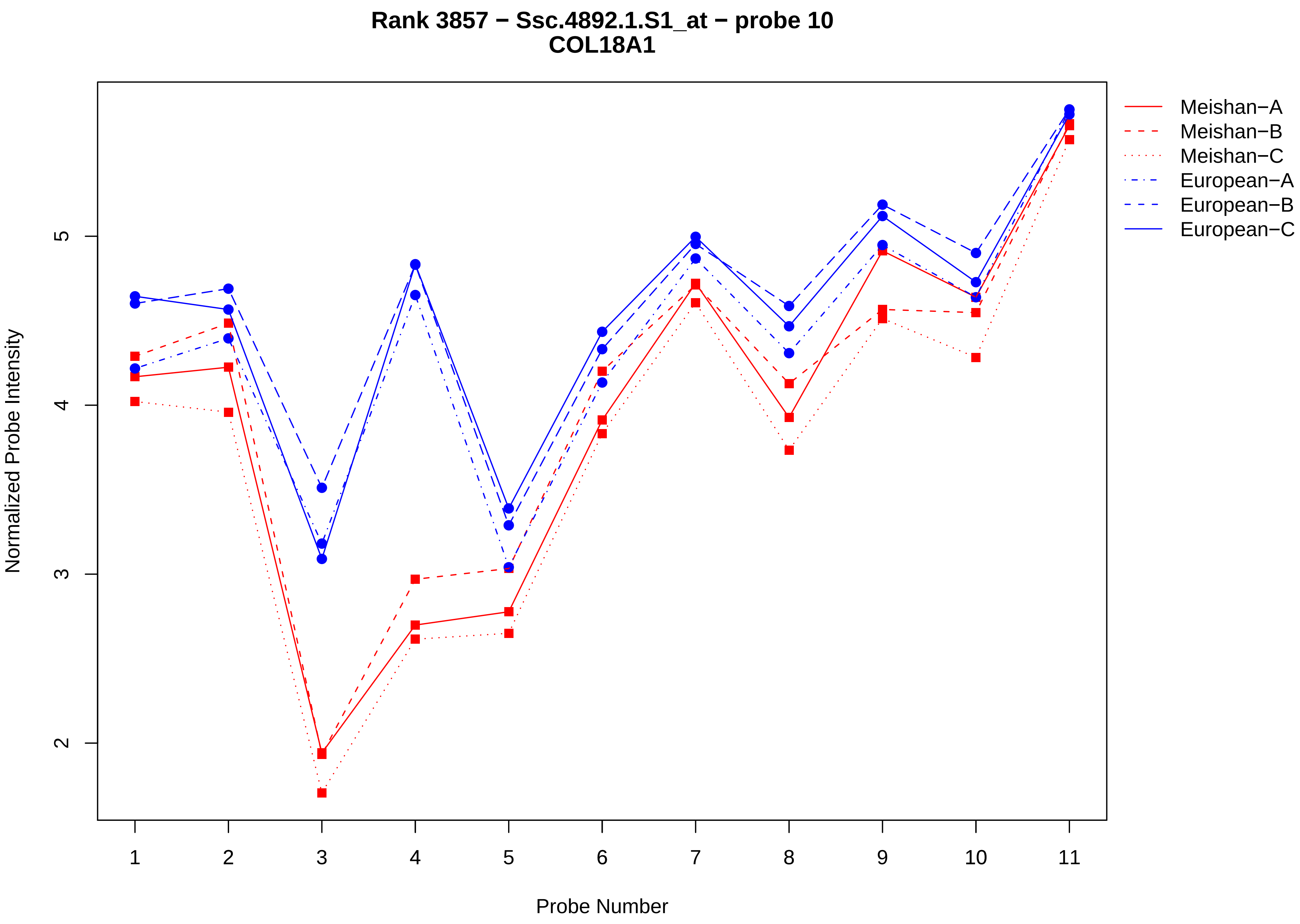
Rank 3857 − Ssc.4892.1.S1_at − probe 10
COL18A1
Normalized Probe Intensity
Probe Number
2
3
4
5
1
2
3
4
5
6
7
8
9
10
11
Meishan−A
Meishan−B
Meishan−C
European−A
European−B
European−C

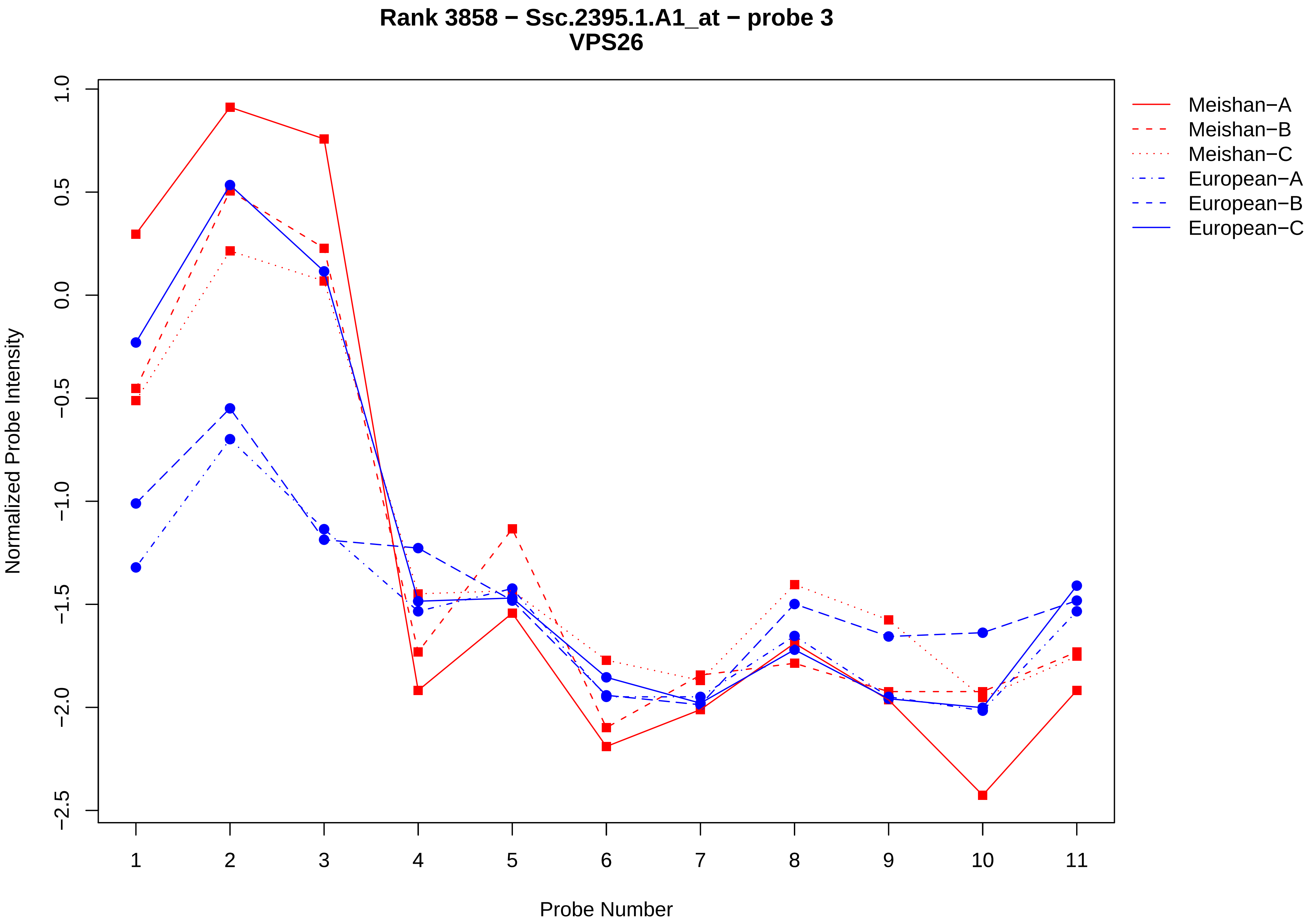

Rank 3858 − Ssc.2395.1.A1_at − probe 3
VPS26
Normalized Probe Intensity
Probe Number
1.0
0.5
0.0
−0.5
−1.0
−1.5
−2.0
−2.5
1
2
3
4
5
6
7
8
9
10
11
Meishan−A
Meishan−B
Meishan−C
European−A
European−B
European−C

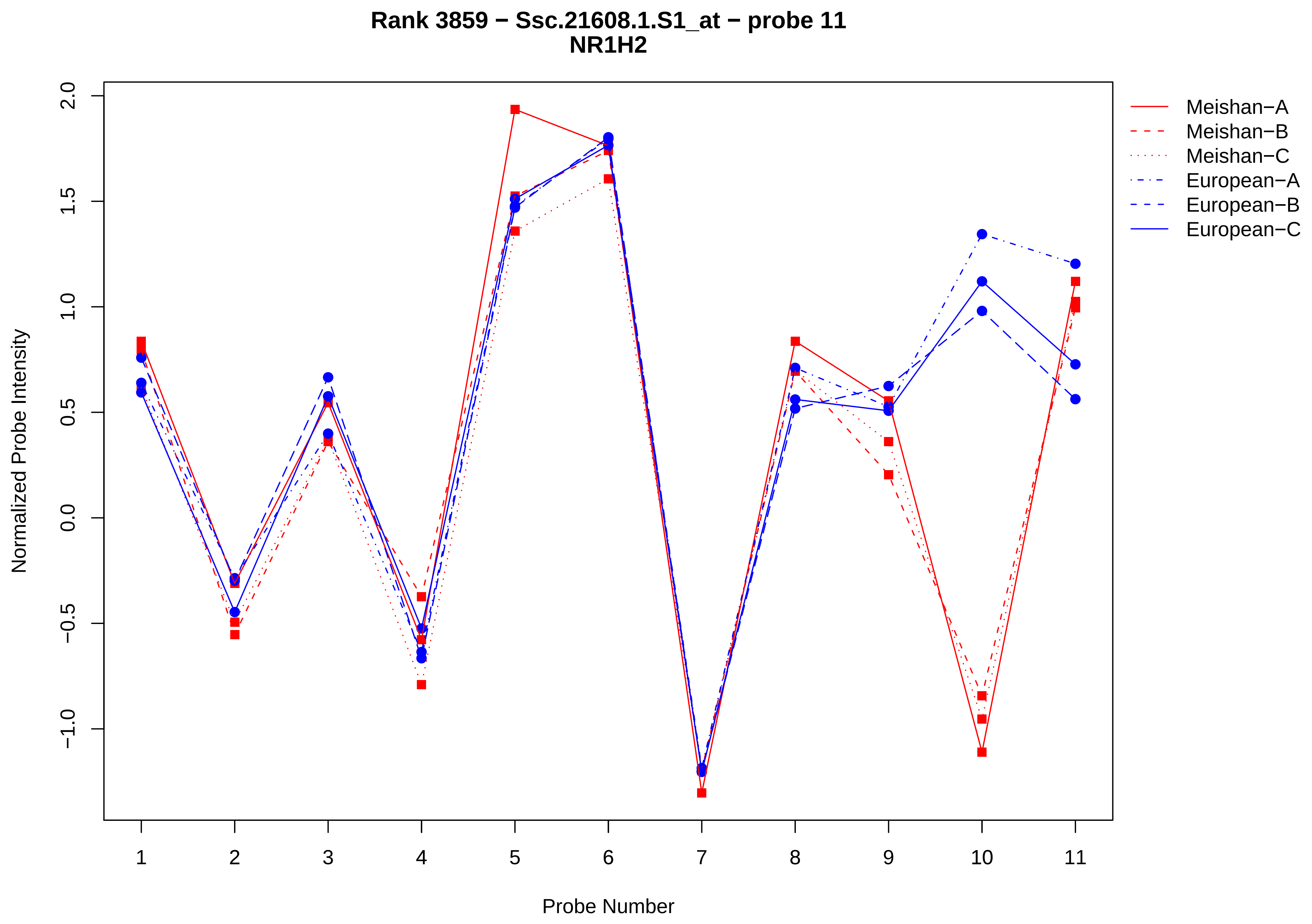

Rank 3859 − Ssc.21608.1.S1_at − probe 11
NR1H2
Normalized Probe Intensity
Probe Number
Meishan−A
Meishan−B
Meishan−C
European−A
European−B
European−C
2.0
1.5
1.0
0.5
0.0
−0.5
−1.0
1
2
3
4
5
6
7
8
9
10
11

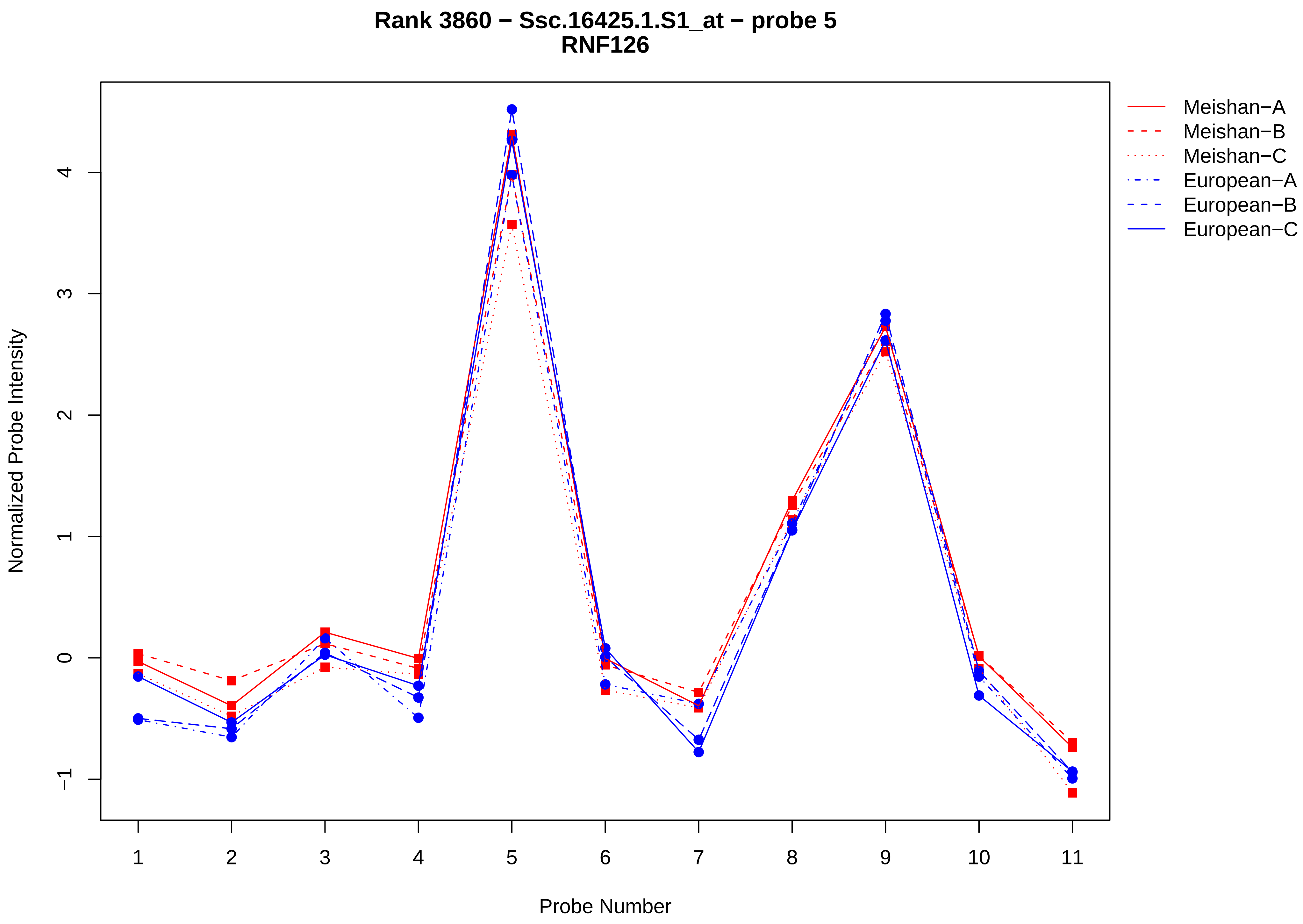
Rank 3860 − Ssc.16425.1.S1_at − probe 5
RNF126
Normalized Probe Intensity
Probe Number
Meishan−A
Meishan−B
Meishan−C
European−A
European−B
European−C
−1
0
1
2
3
4
1
2
3
4
5
6
7
8
9
10
11

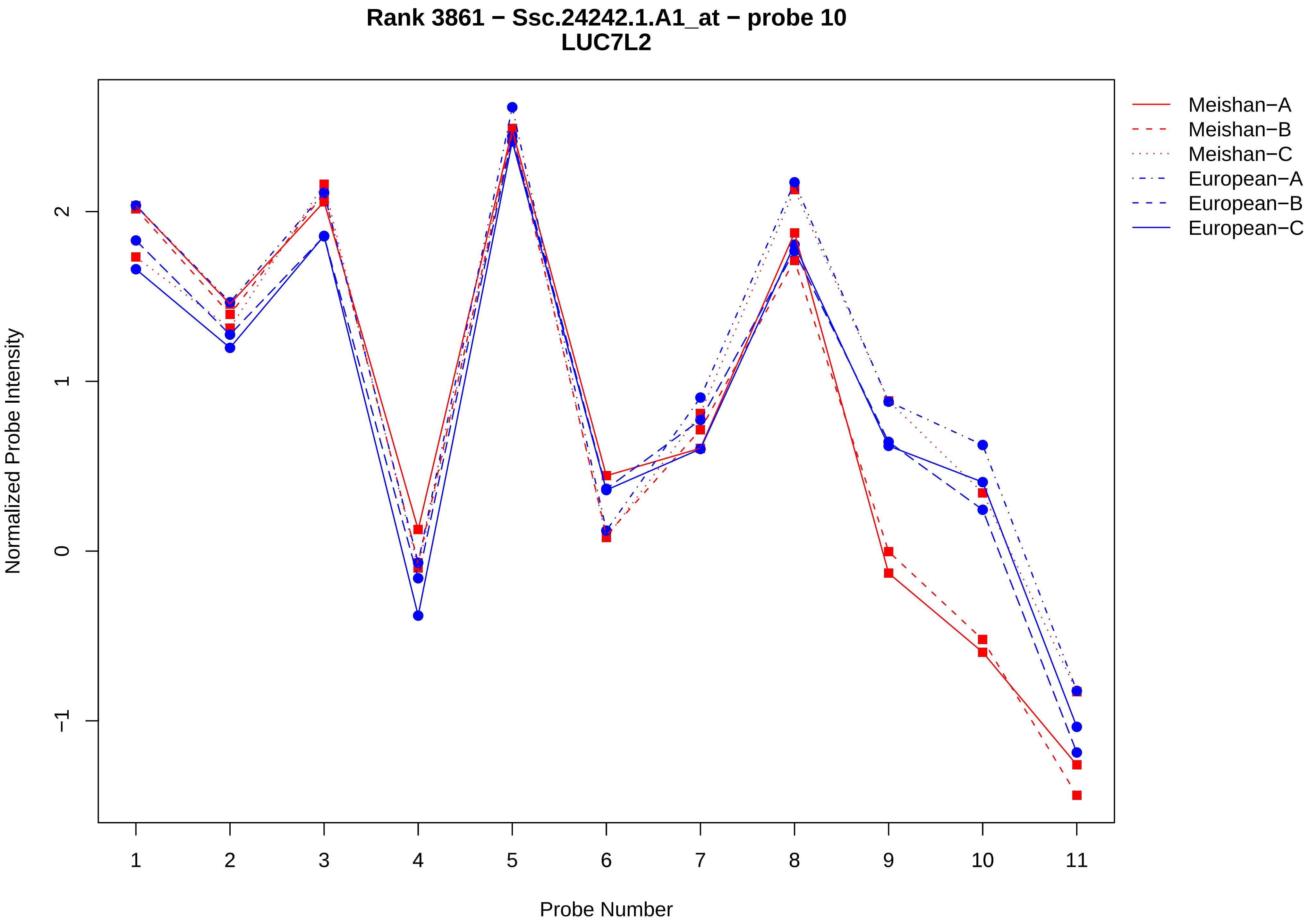

Rank 3861 − Ssc.24242.1.A1_at − probe 10
LUC7L2
Normalized Probe Intensity
Probe Number
−1
0
1
2
1
2
3
4
5
6
7
8
9
10
11
Meishan−A
Meishan−B
Meishan−C
European−A
European−B
European−C

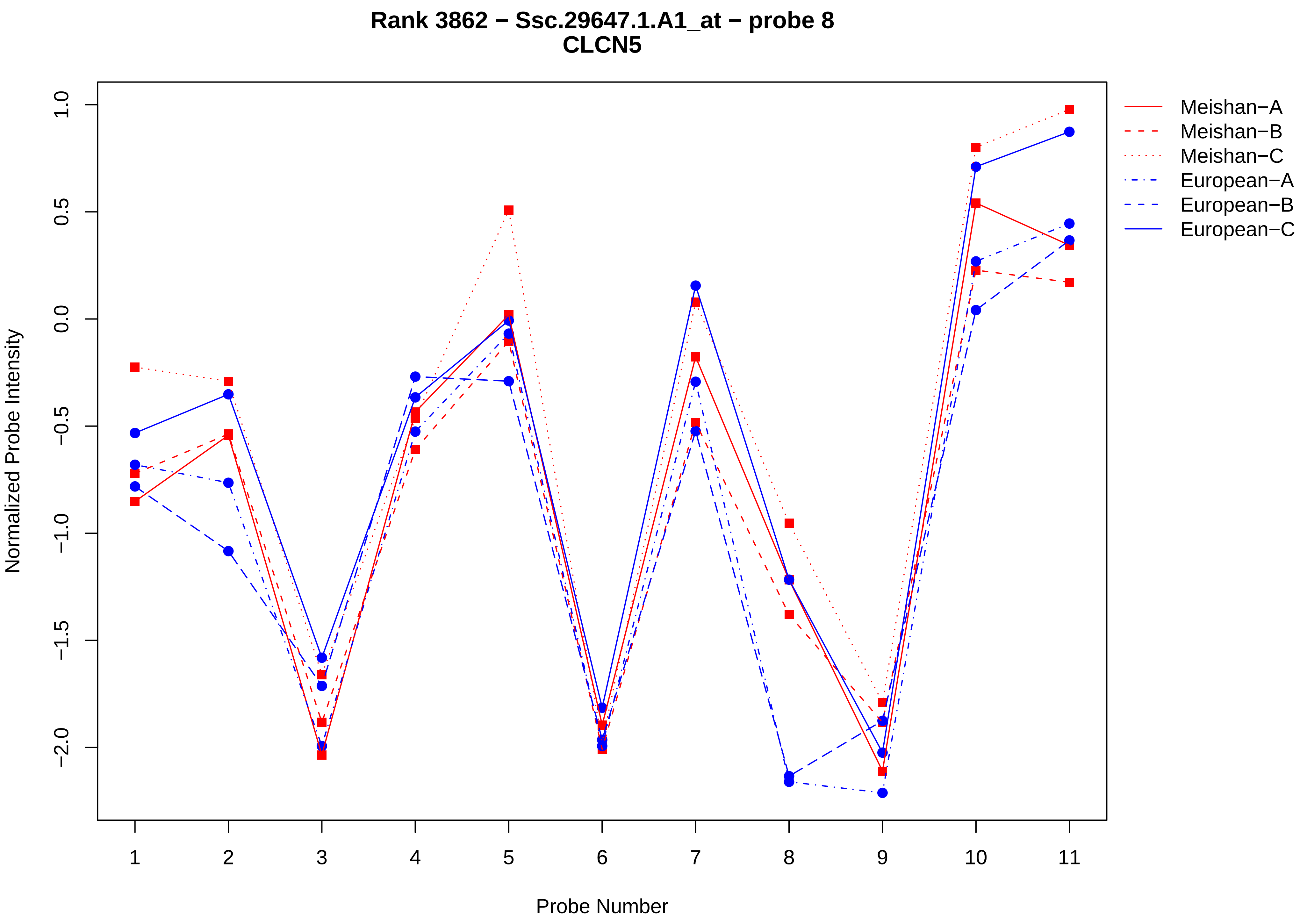

Rank 3862 − Ssc.29647.1.A1_at − probe 8
CLCN5
Normalized Probe Intensity
Probe Number
1.0
0.5
0.0
−0.5
−1.0
−1.5
−2.0
1
2
3
4
5
6
7
8
9
10
11
Meishan−A
Meishan−B
Meishan−C
European−A
European−B
European−C

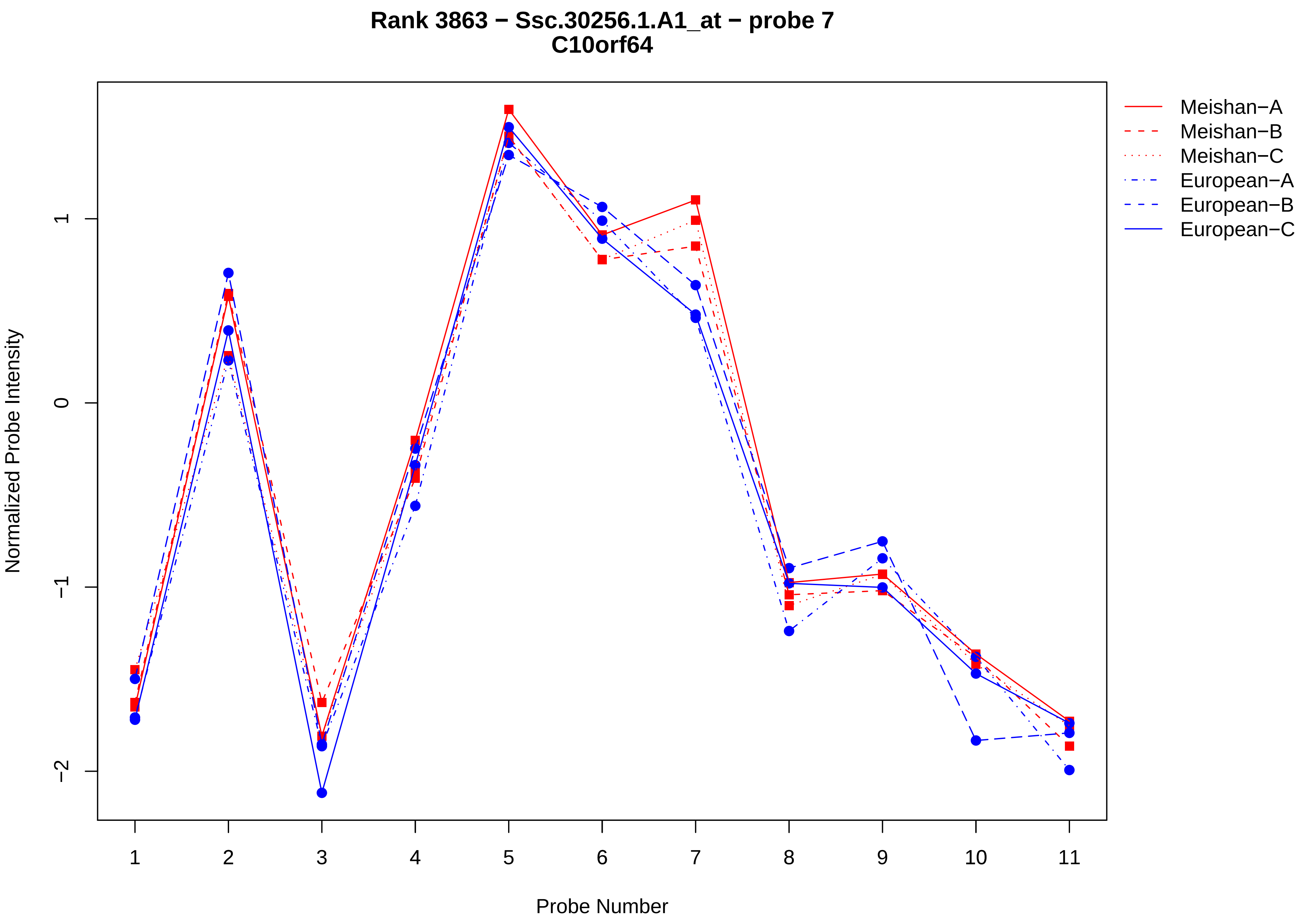
Rank 3863 − Ssc.30256.1.A1_at − probe 7
C10orf64
Normalized Probe Intensity
Probe Number
Meishan−A
Meishan−B
Meishan−C
European−A
European−B
European−C
−2
−1
0
1
1
2
3
4
5
6
7
8
9
10
11

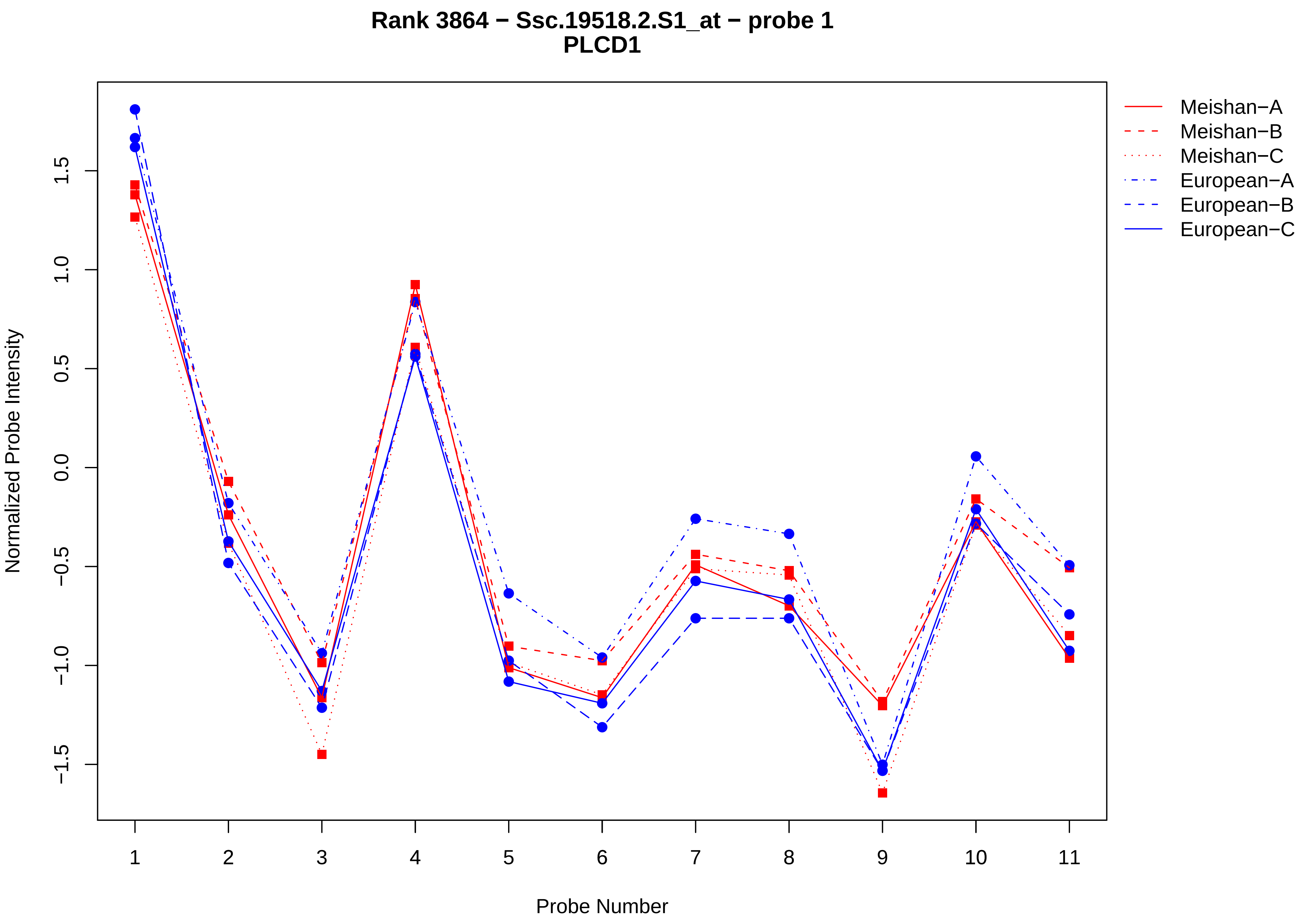

Rank 3864 − Ssc.19518.2.S1_at − probe 1
PLCD1
Normalized Probe Intensity
Probe Number
Meishan−A
Meishan−B
Meishan−C
European−A
European−B
European−C
−1.5
−1.0
−0.5
0.0
0.5
1.0
1.5
1
2
3
4
5
6
7
8
9
10
11

Rank 3865 − Ssc.2709.1.S1_at − probe 10
ERO1L

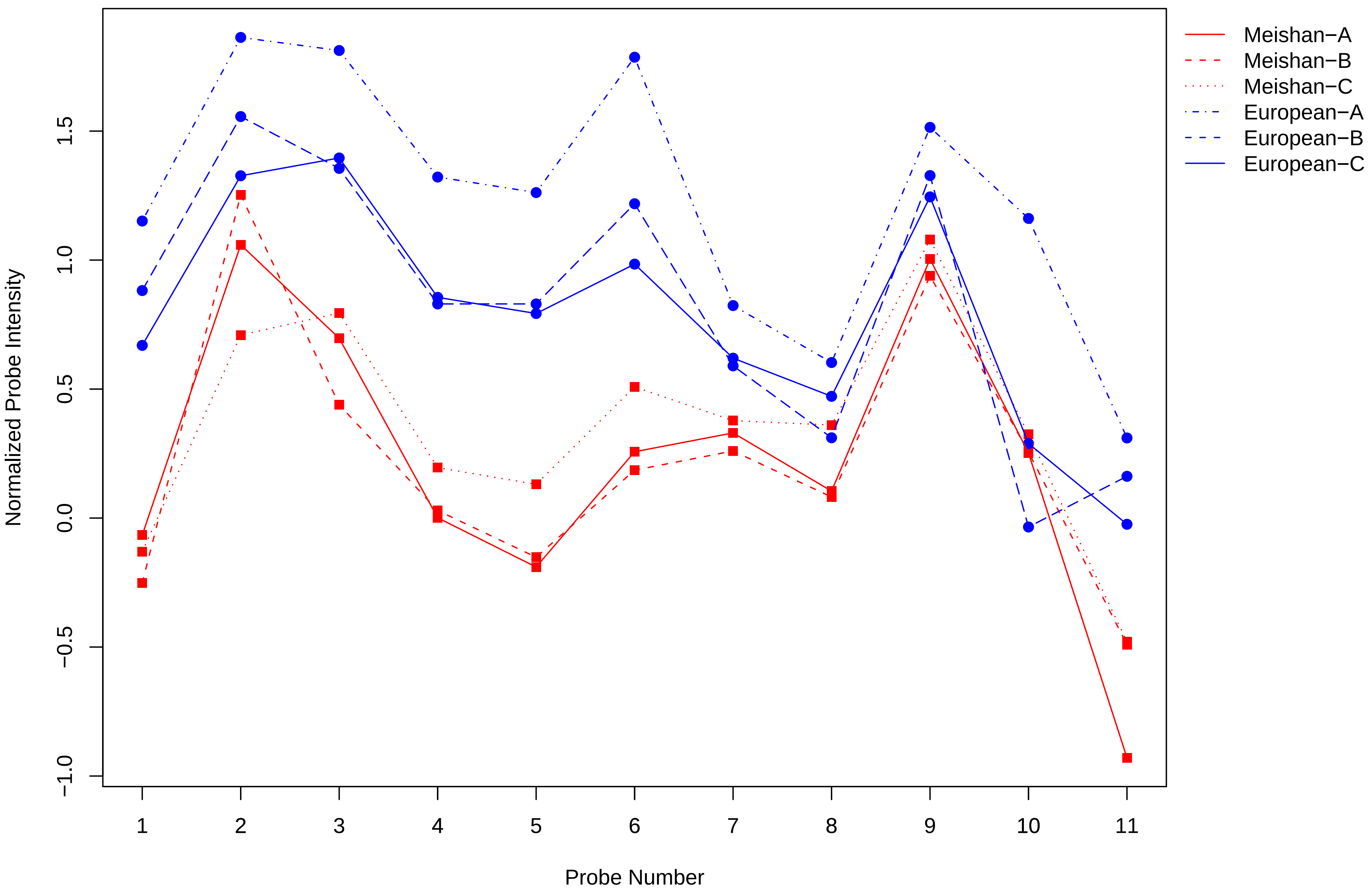

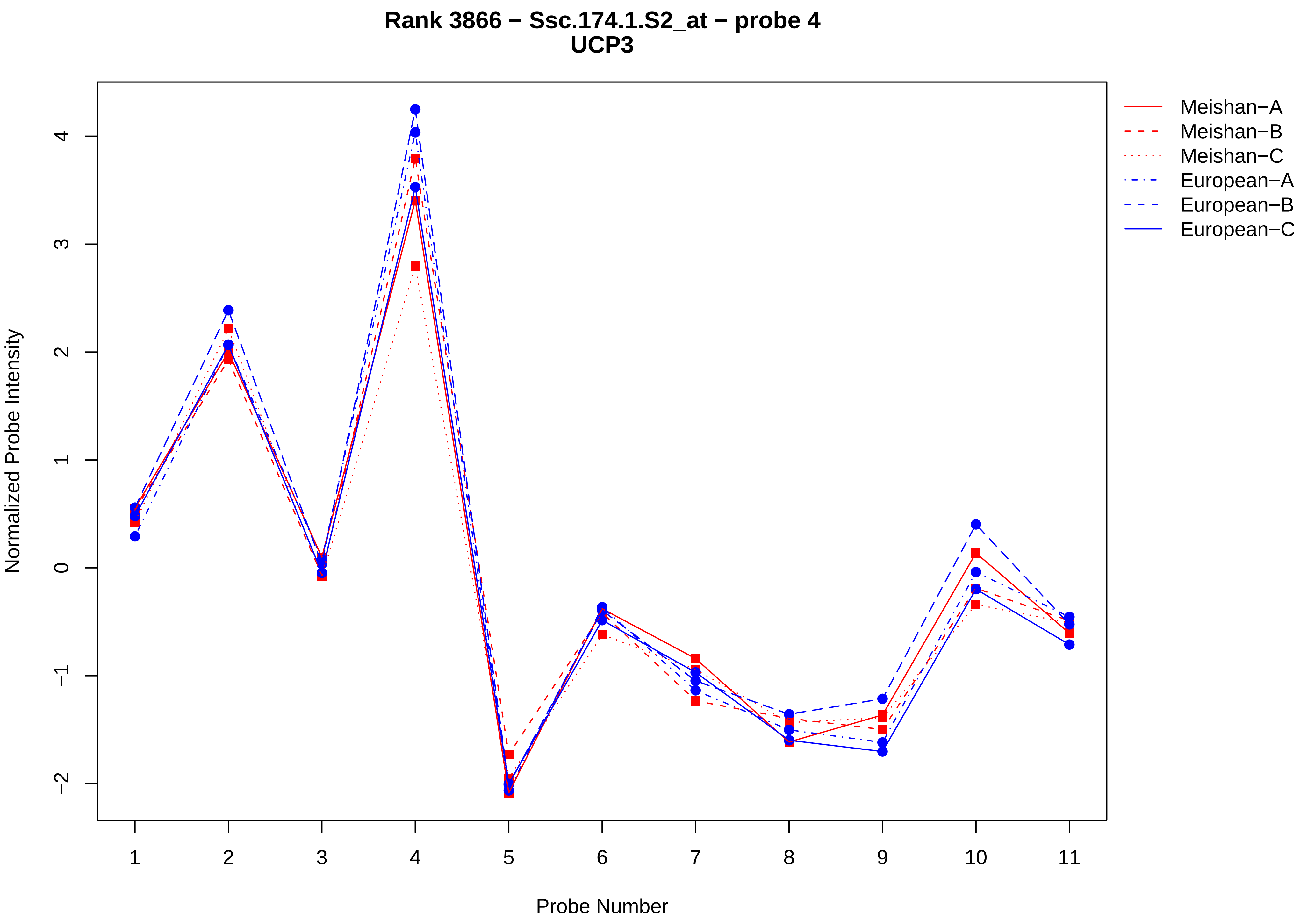

Rank 3866 − Ssc.174.1.S2_at − probe 4
UCP3
Normalized Probe Intensity
Probe Number
−2
−1
0
1
2
3
4
1
2
3
4
5
6
7
8
9
10
11
Meishan−A
Meishan−B
Meishan−C
European−A
European−B
European−C

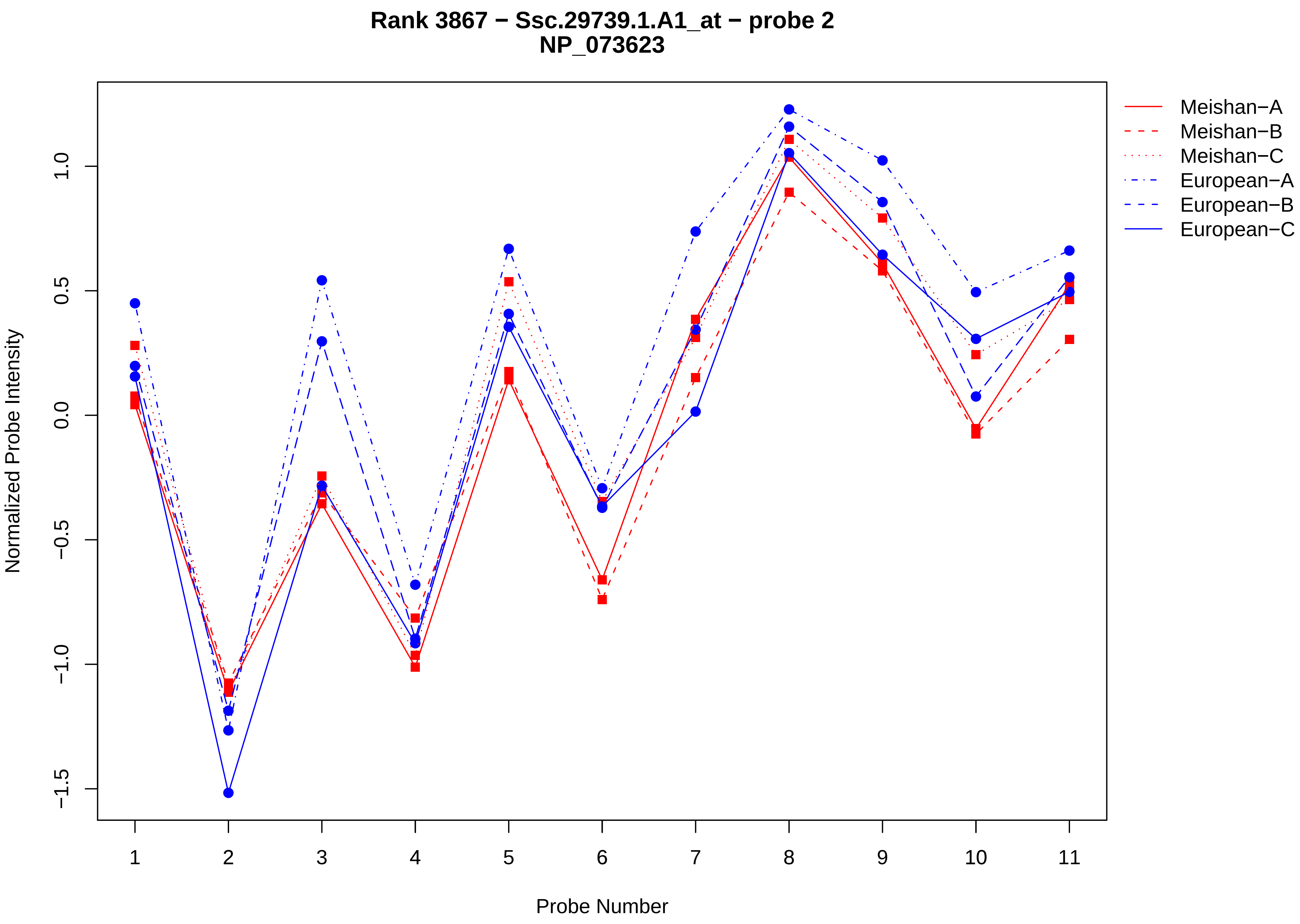
Rank 3867 − Ssc.29739.1.A1_at − probe 2
NP_073623
Normalized Probe Intensity
Probe Number
−1.5
−1.0
−0.5
0.0
0.5
1.0
1
2
3
4
5
6
7
8
9
10
11
Meishan−A
Meishan−B
Meishan−C
European−A
European−B
European−C

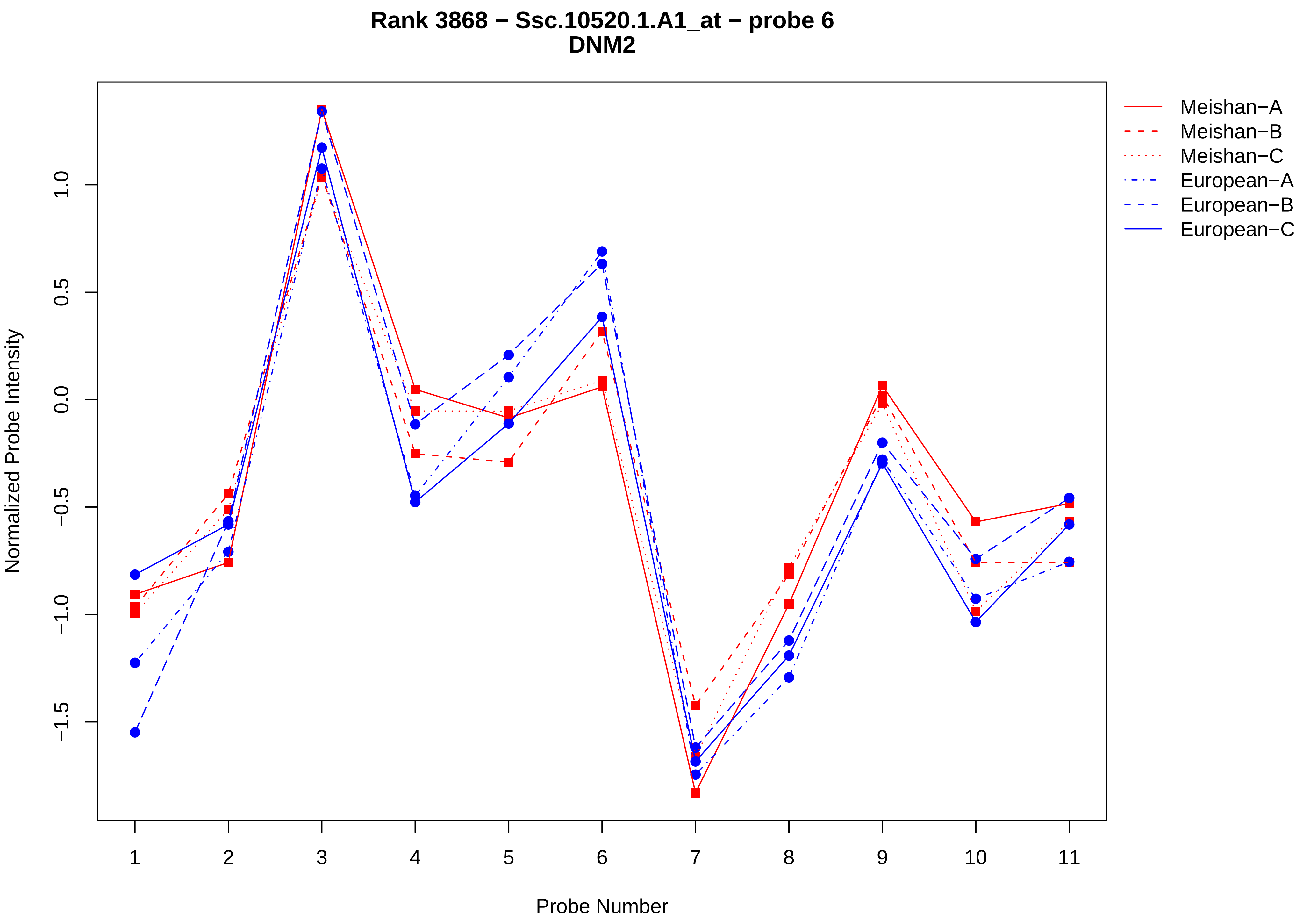

Rank 3868 − Ssc.10520.1.A1_at − probe 6
DNM2
Normalized Probe Intensity
Probe Number
1.0
0.5
0.0
−0.5
−1.0
−1.5
1
2
3
4
5
6
7
8
9
10
11
Meishan−A
Meishan−B
Meishan−C
European−A
European−B
European−C

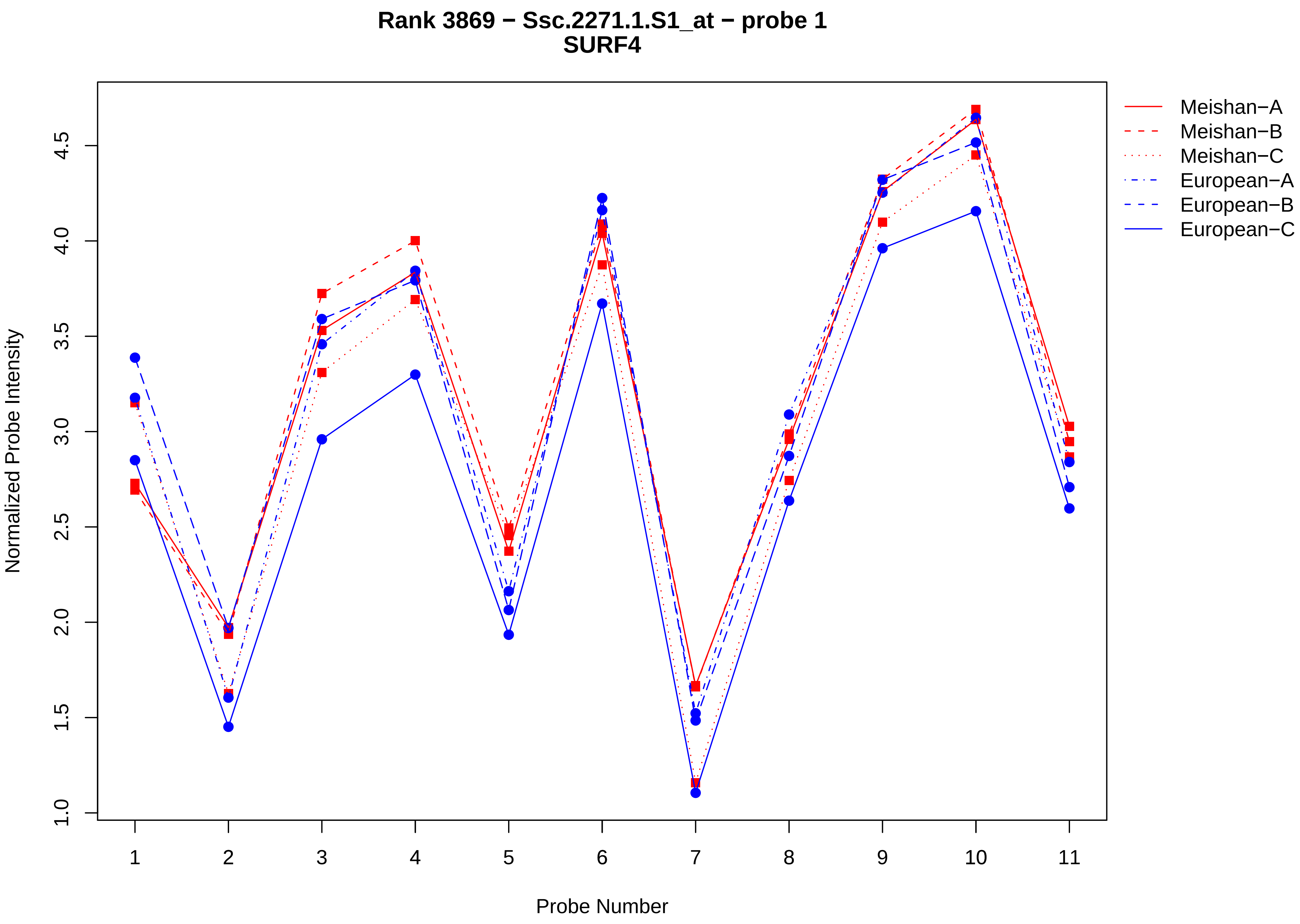

Rank 3869 − Ssc.2271.1.S1_at − probe 1
SURF4
Normalized Probe Intensity
1.0
1.5
2.0
2.5
3.0
3.5
4.0
4.5
Probe Number
1
2
3
4
5
6
7
8
9
10
11
Meishan−A
Meishan−B
Meishan−C
European−A
European−B
European−C

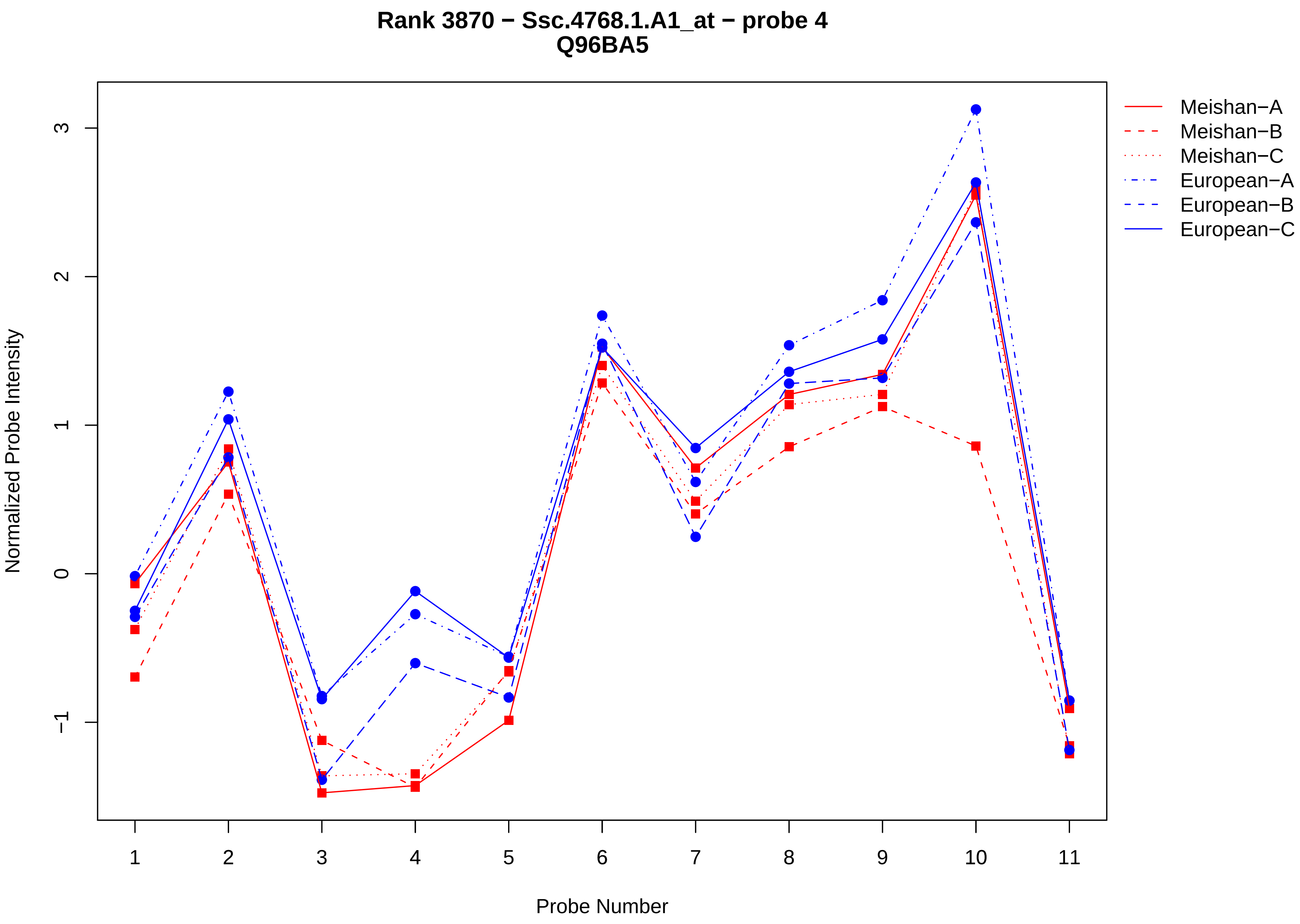
Rank 3870 − Ssc.4768.1.A1_at − probe 4
Q96BA5
Normalized Probe Intensity
Probe Number
Meishan−A
Meishan−B
Meishan−C
European−A
European−B
European−C
−1
0
1
2
3
1
2
3
4
5
6
7
8
9
10
11

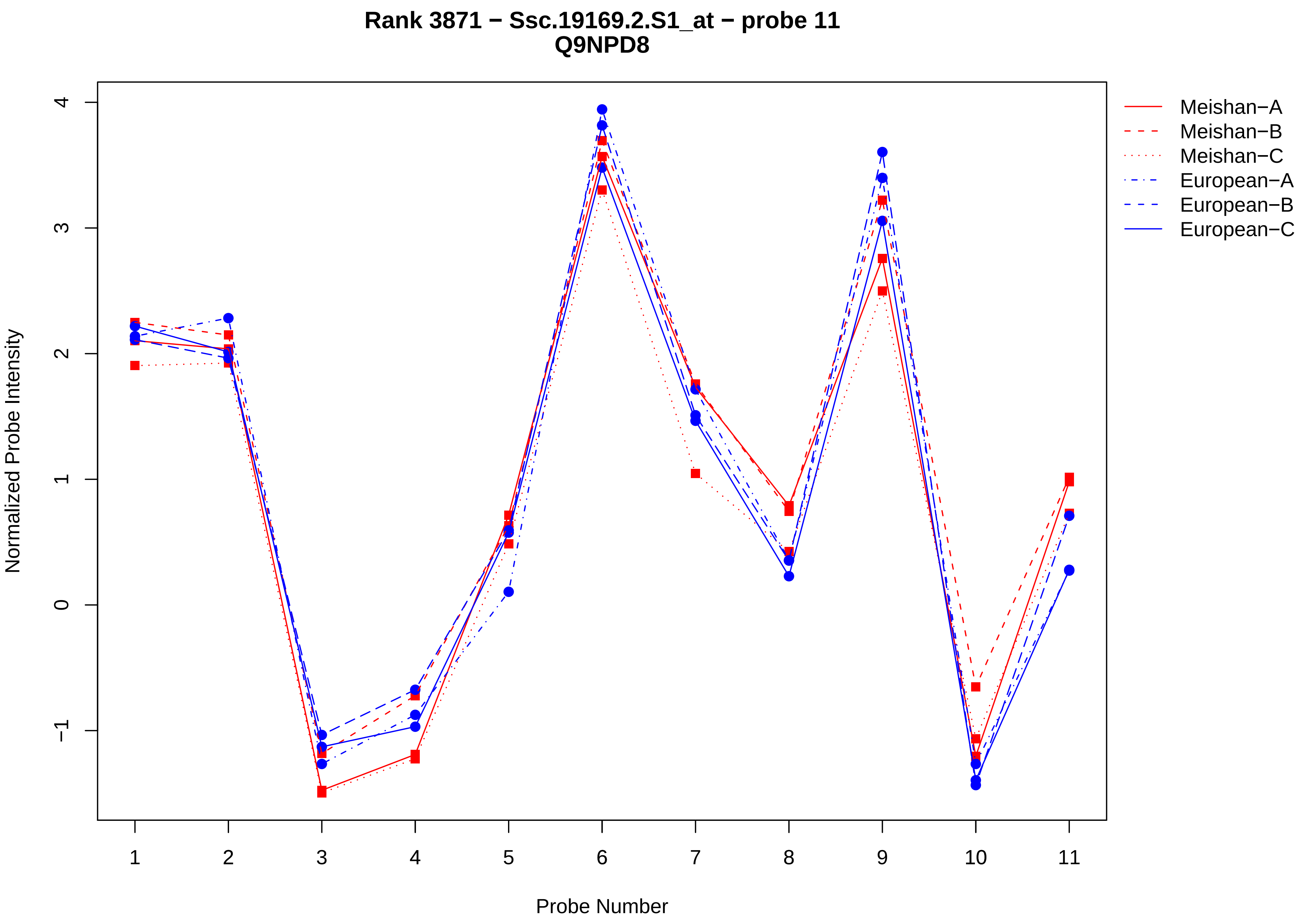
Rank 3871 − Ssc.19169.2.S1_at − probe 11
Q9NPD8
Normalized Probe Intensity
Probe Number
−1
0
1
2
3
4
1
2
3
4
5
6
7
8
9
10
11
Meishan−A
Meishan−B
Meishan−C
European−A
European−B
European−C

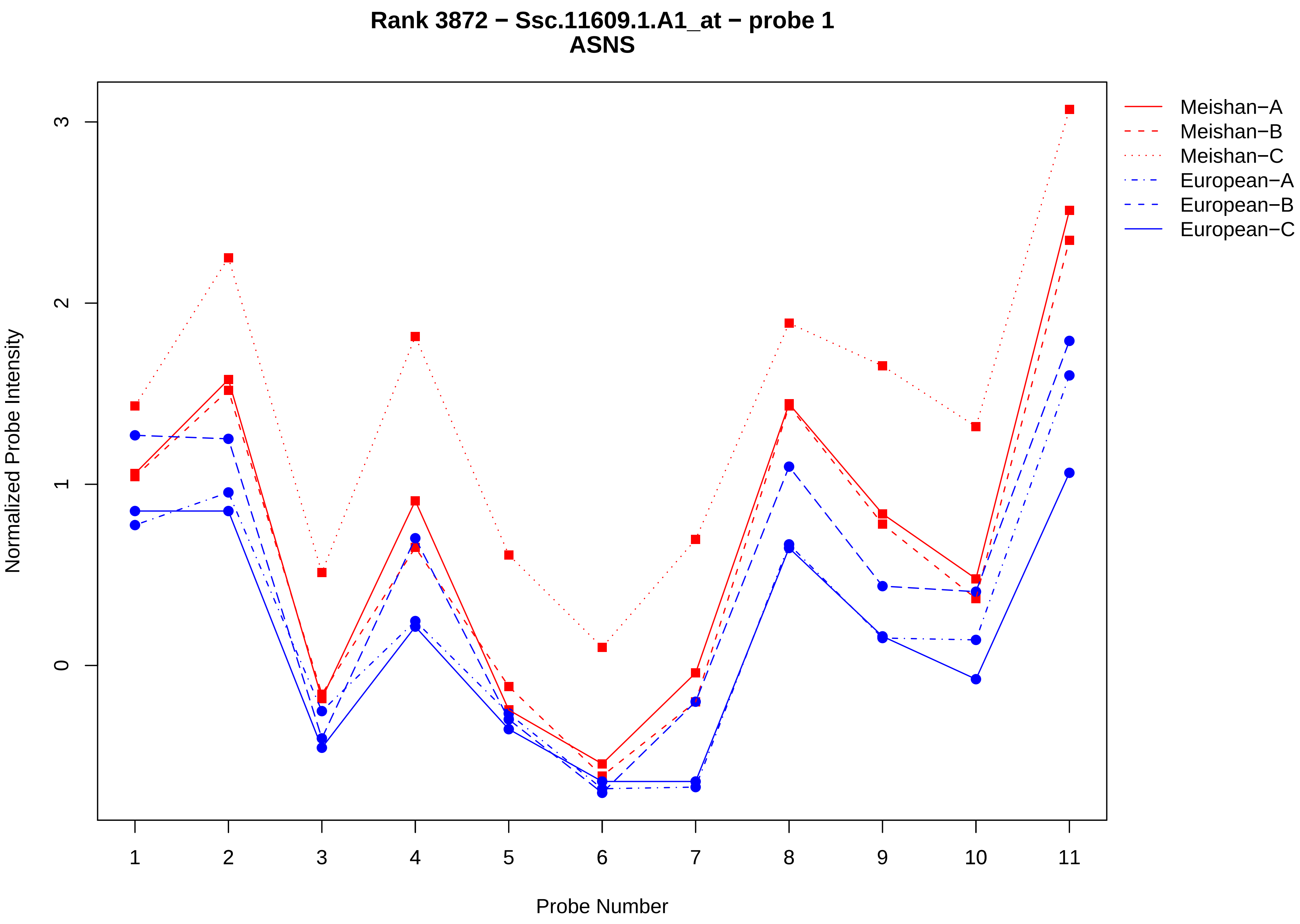
Rank 3872 − Ssc.11609.1.A1_at − probe 1
ASNS
Normalized Probe Intensity
Probe Number
0
1
2
3
1
2
3
4
5
6
7
8
9
10
11
Meishan−A
Meishan−B
Meishan−C
European−A
European−B
European−C

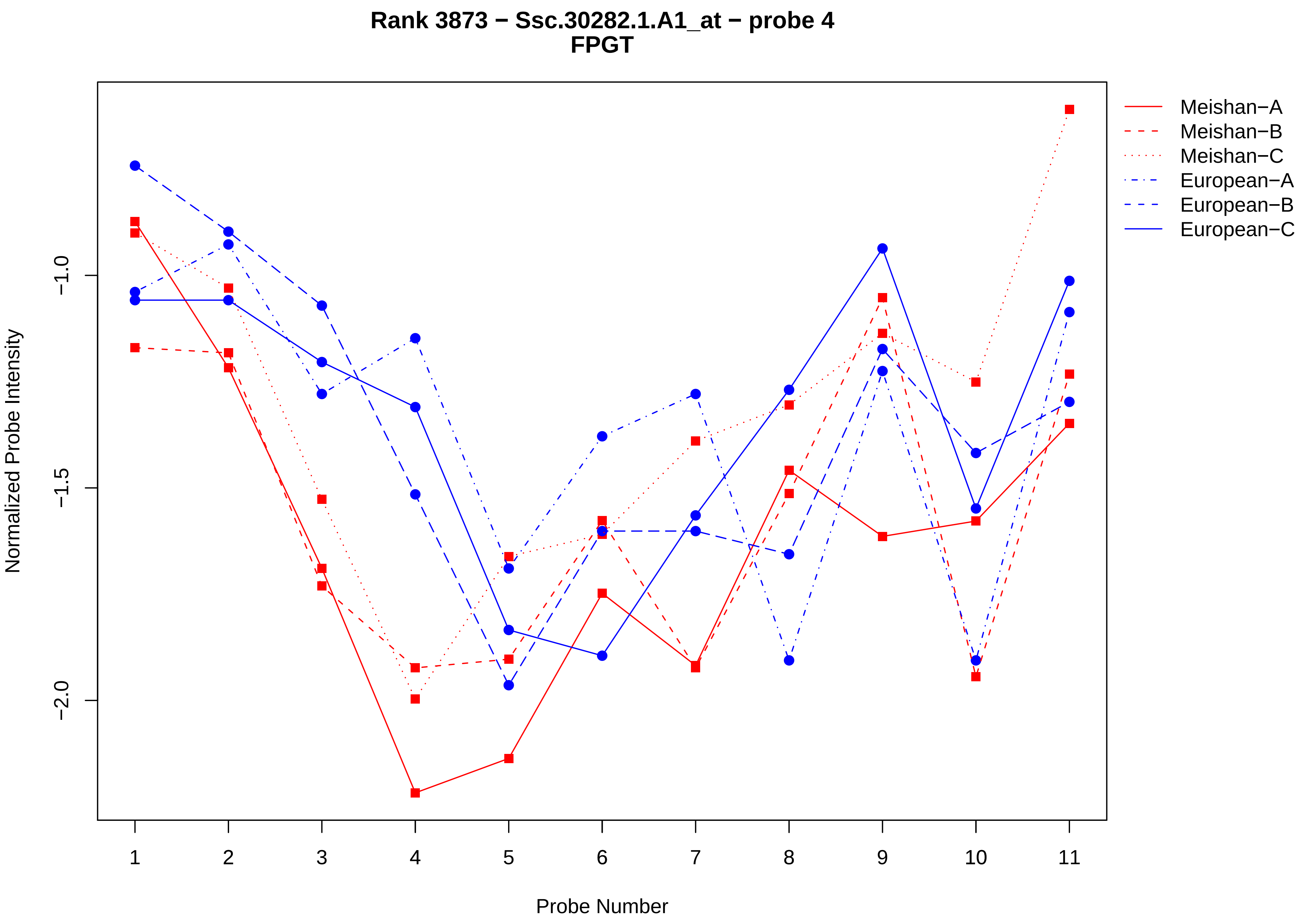

Rank 3873 − Ssc.30282.1.A1_at − probe 4
FPGT
Normalized Probe Intensity
−1.0
−1.5
−2.0
Probe Number
1
2
3
4
5
6
7
8
9
10
11
Meishan−A
Meishan−B
Meishan−C
European−A
European−B
European−C

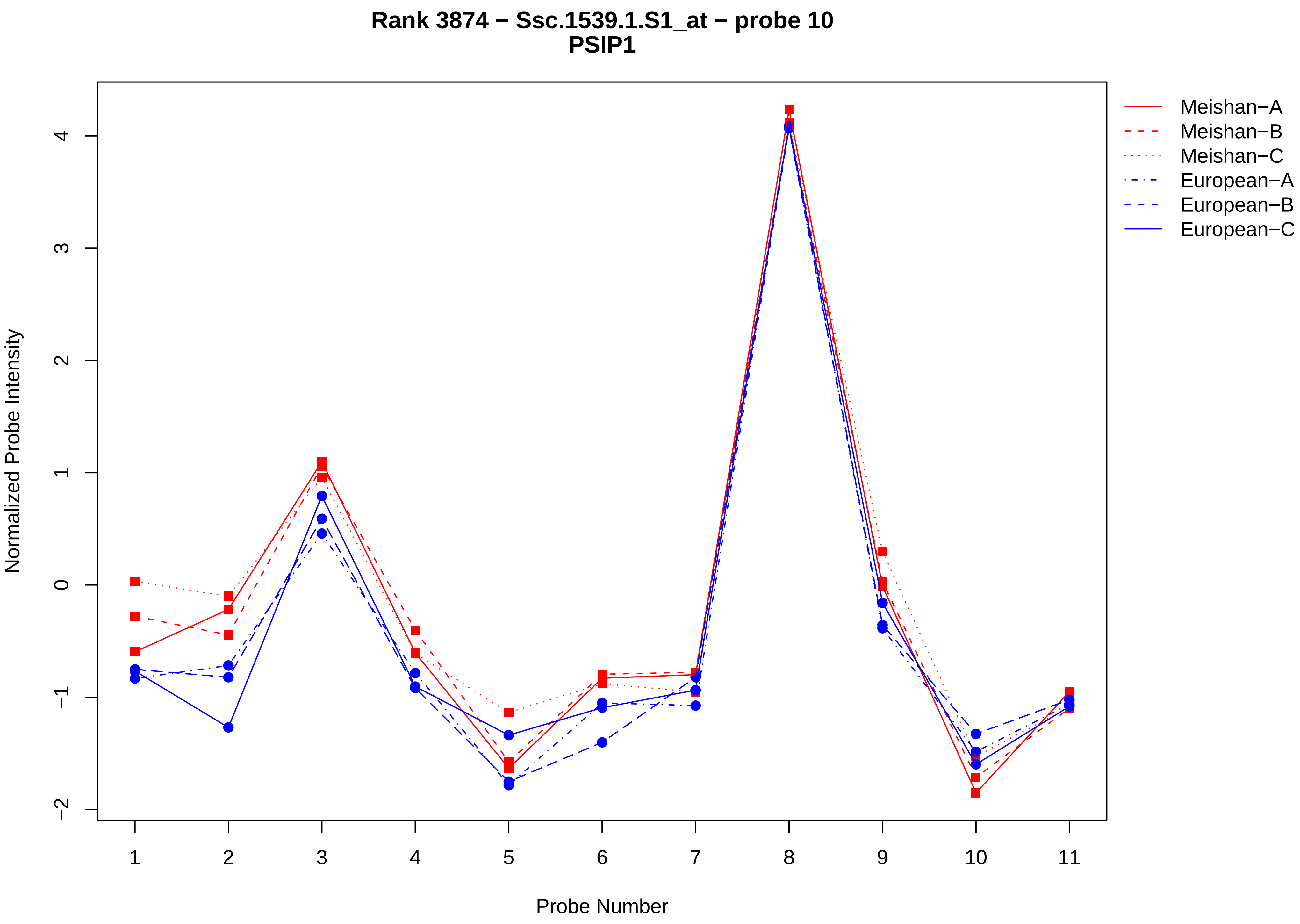

Rank 3874 − Ssc.1539.1.S1_at − probe 10
PSIP1
Normalized Probe Intensity
Probe Number
Meishan−A
Meishan−B
Meishan−C
European−A
European−B
European−C

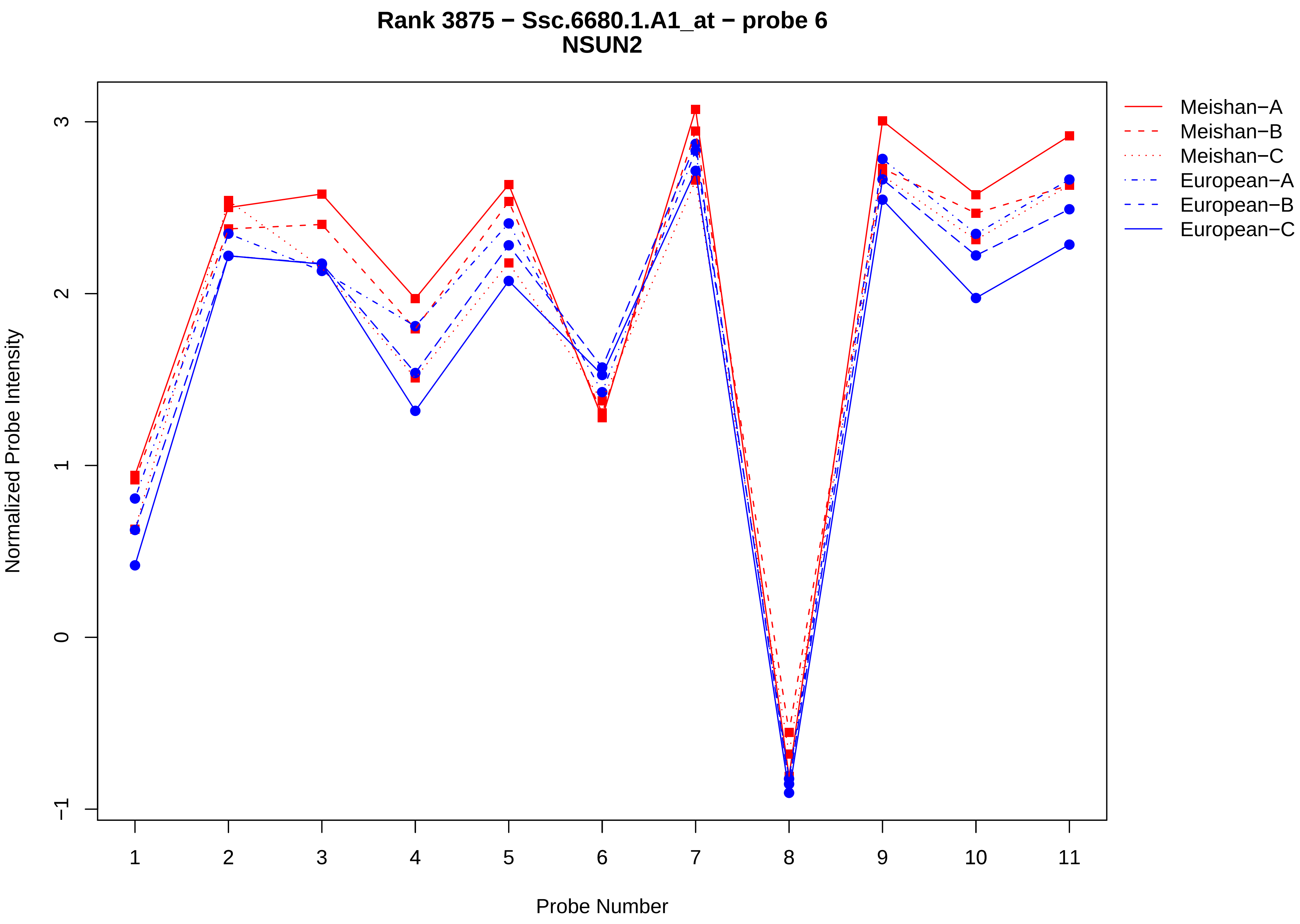
Rank 3875 − Ssc.6680.1.A1_at − probe 6
NSUN2
Normalized Probe Intensity
Probe Number
Meishan−A
Meishan−B
Meishan−C
European−A
European−B
European−C

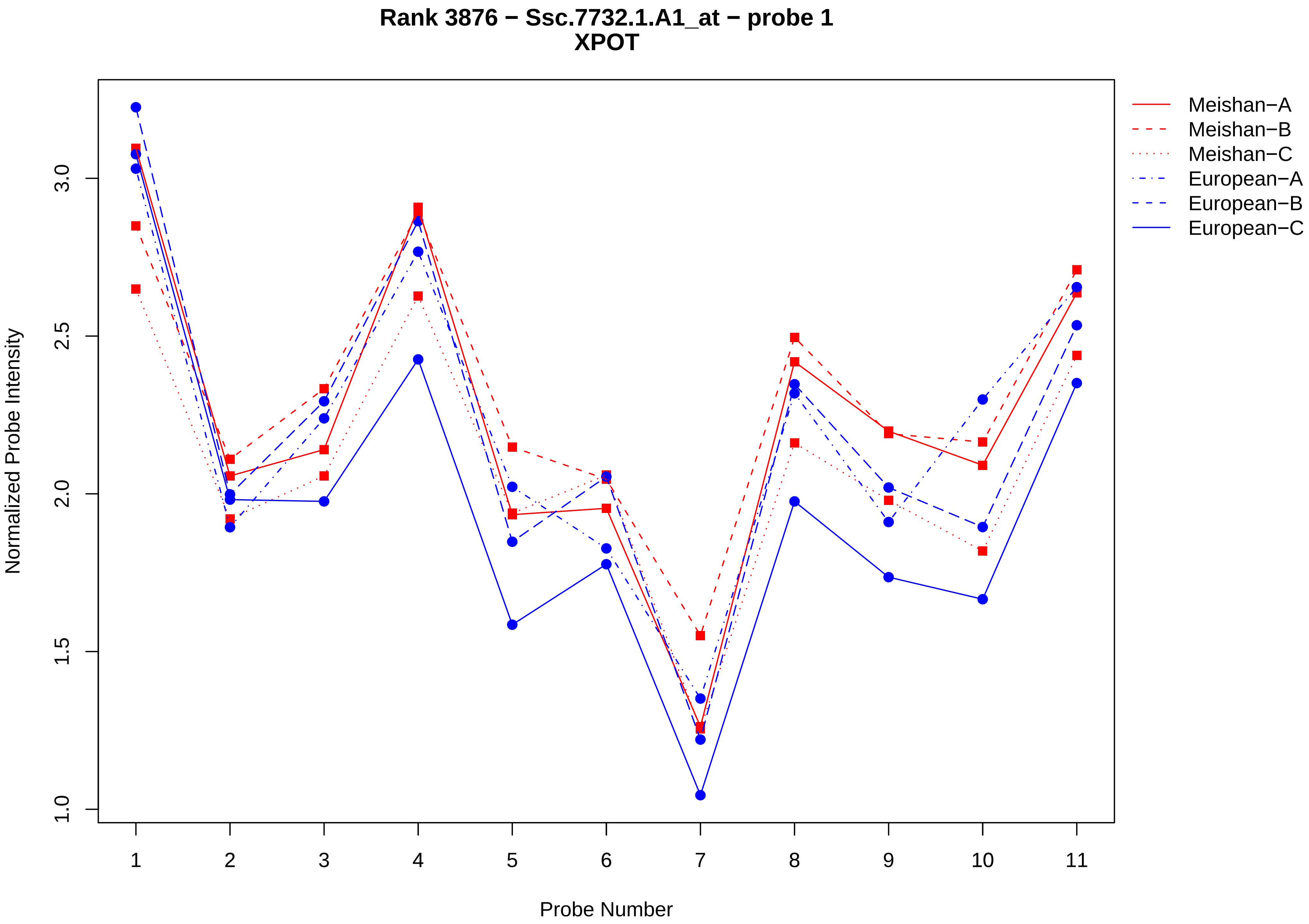

Rank 3876 − Ssc.7732.1.A1_at − probe 1
XPOT
Normalized Probe Intensity
Probe Number
1.0
1.5
2.0
2.5
3.0
1
2
3
4
5
6
7
8
9
10
11
Meishan−A
Meishan−B
Meishan−C
European−A
European−B
European−C

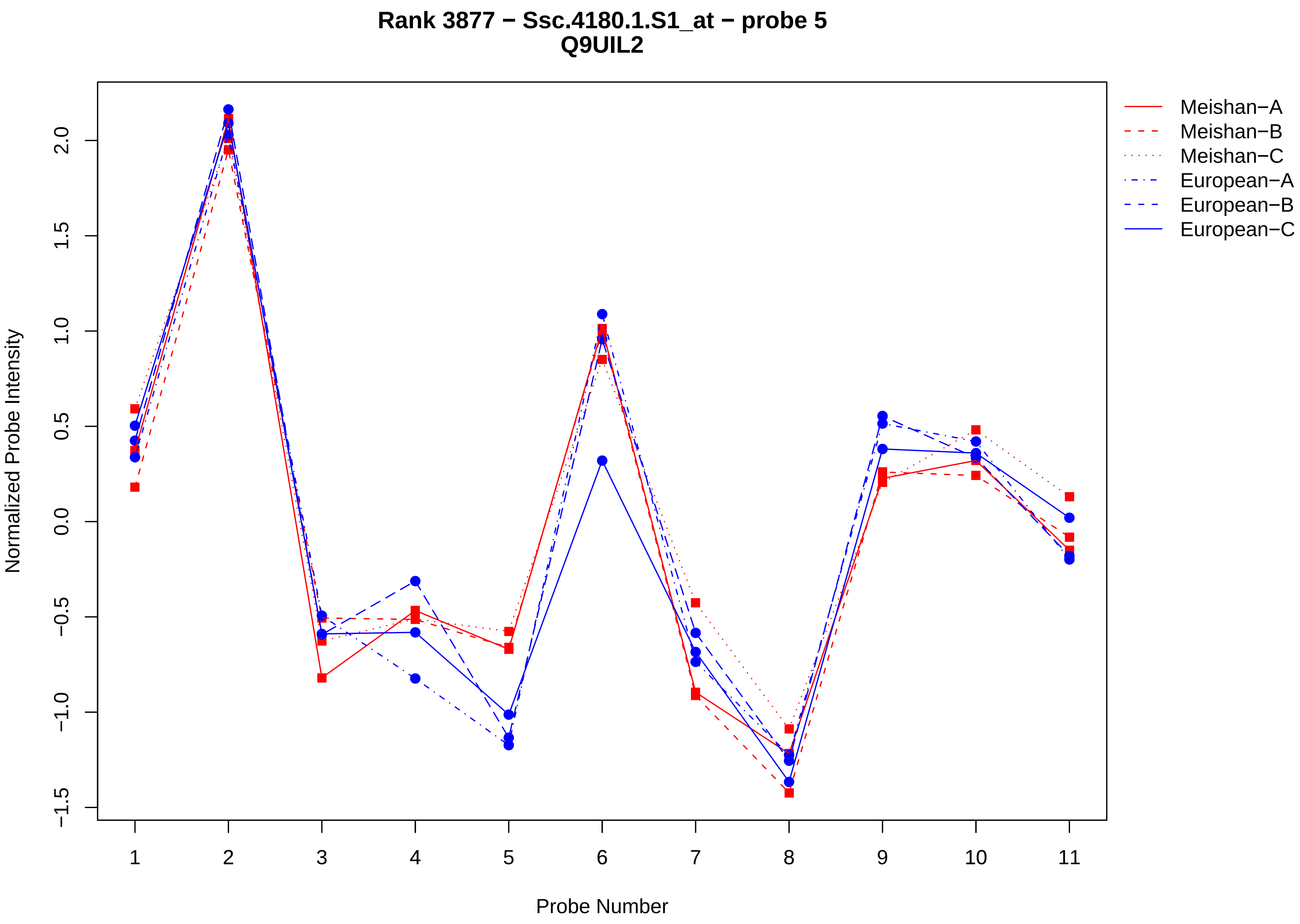
Rank 3877 − Ssc.4180.1.S1_at − probe 5
Q9UIL2
Normalized Probe Intensity
Probe Number
Meishan−A
Meishan−B
Meishan−C
European−A
European−B
European−C
−1.5
−1.0
−0.5
0.0
0.5
1.0
1.5
2.0
1
2
3
4
5
6
7
8
9
10
11

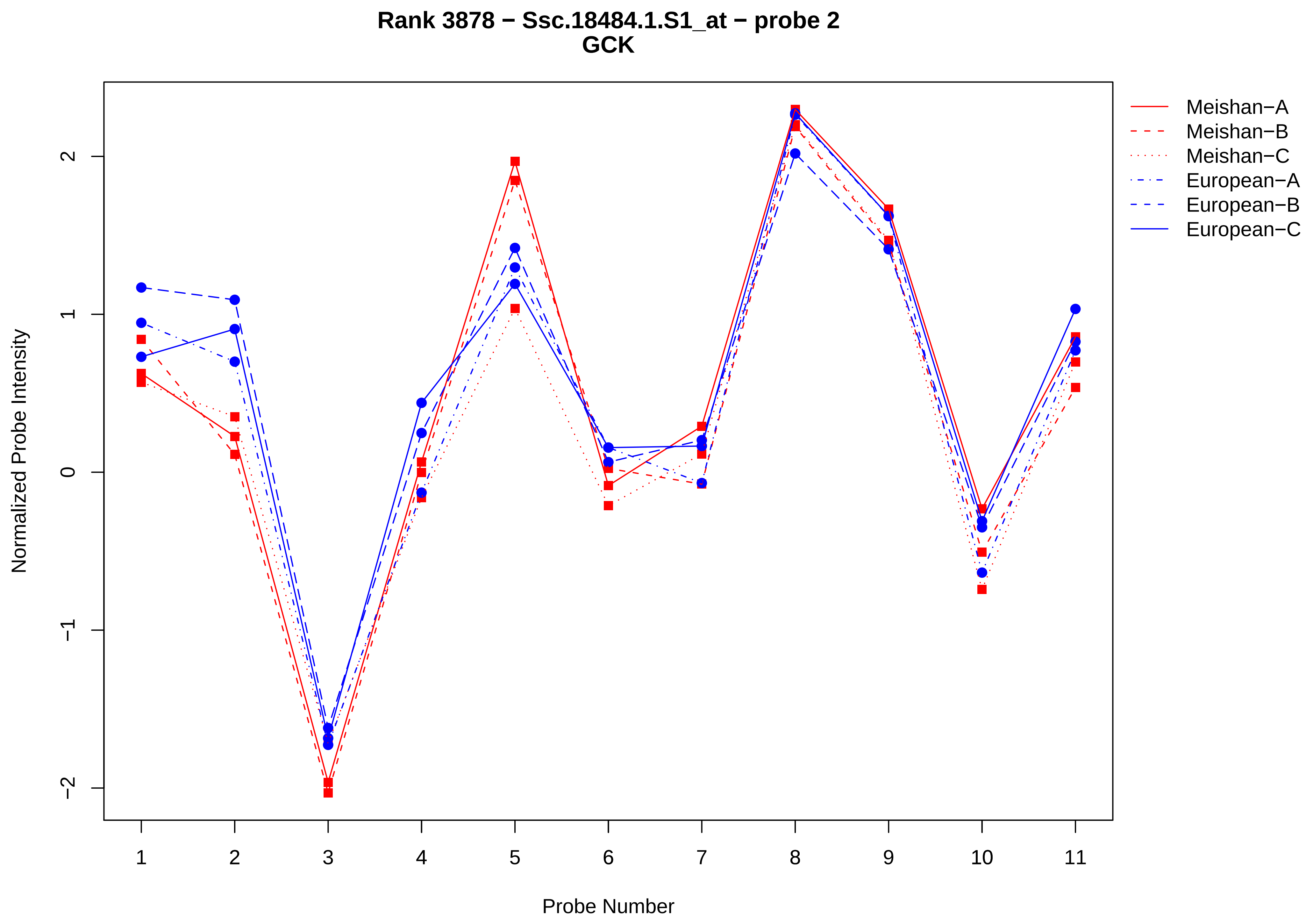

Rank 3878 − Ssc.18484.1.S1_at − probe 2
GCK
Normalized Probe Intensity
Probe Number
−2
−1
0
1
2
1
2
3
4
5
6
7
8
9
10
11
Meishan−A
Meishan−B
Meishan−C
European−A
European−B
European−C

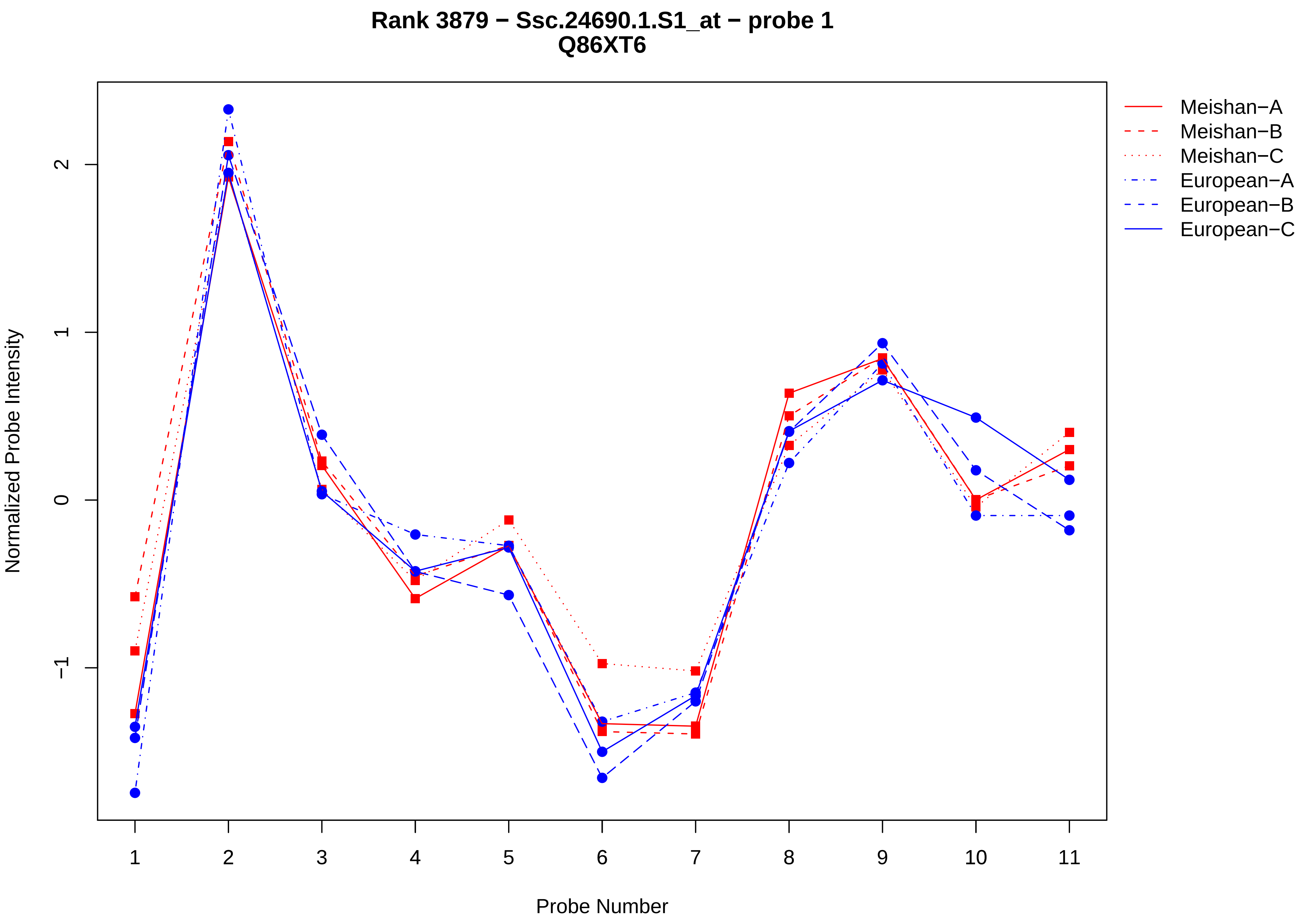

Rank 3879 − Ssc.24690.1.S1_at − probe 1
Q86XT6
Normalized Probe Intensity
Probe Number
Meishan−A
Meishan−B
Meishan−C
European−A
European−B
European−C
−1
0
1
2
1
2
3
4
5
6
7
8
9
10
11

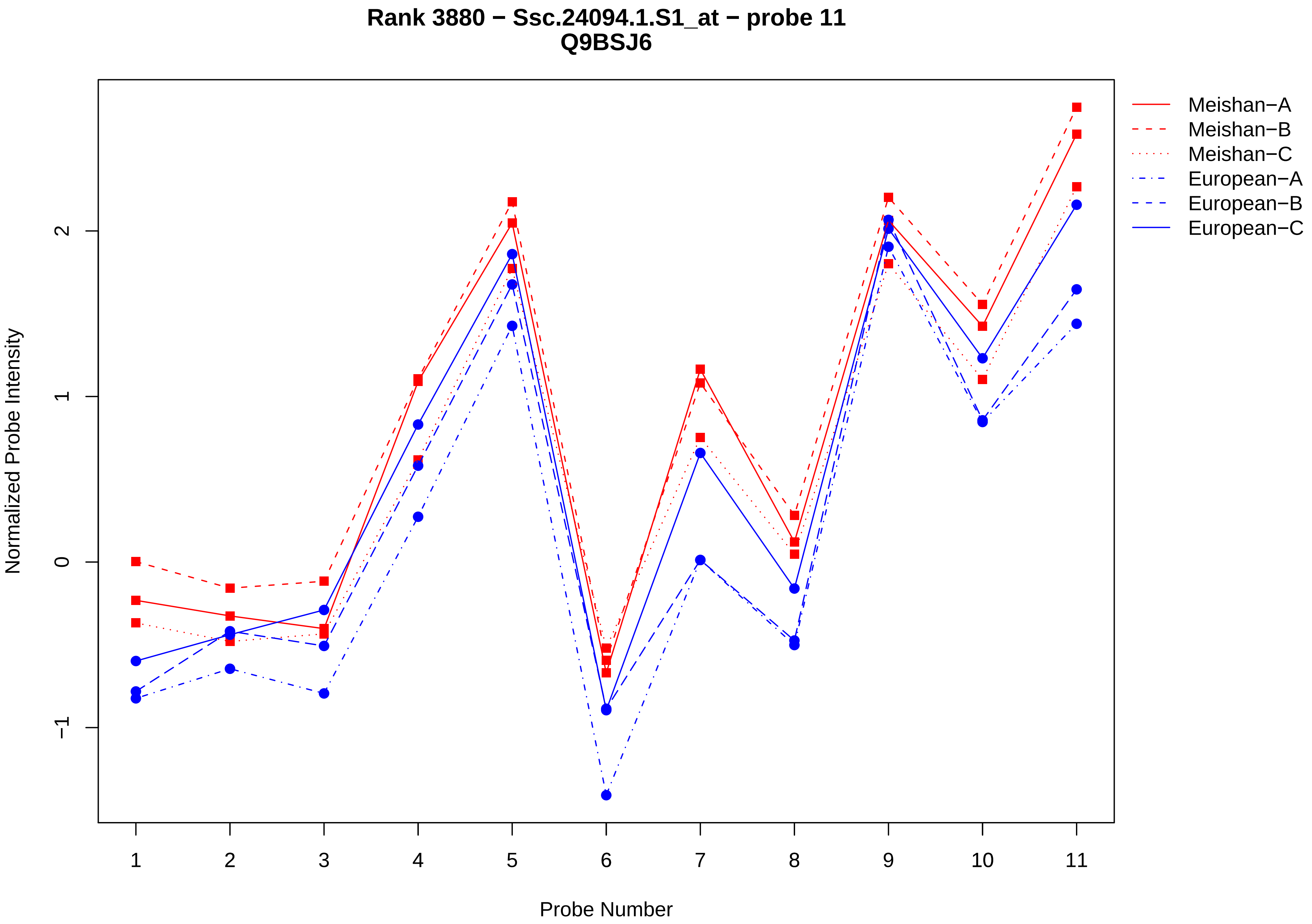

Rank 3880 − Ssc.24094.1.S1_at − probe 11
Q9BSJ6
Normalized Probe Intensity
Probe Number
−1
0
1
2
1
2
3
4
5
6
7
8
9
10
11
Meishan−A
Meishan−B
Meishan−C
European−A
European−B
European−C

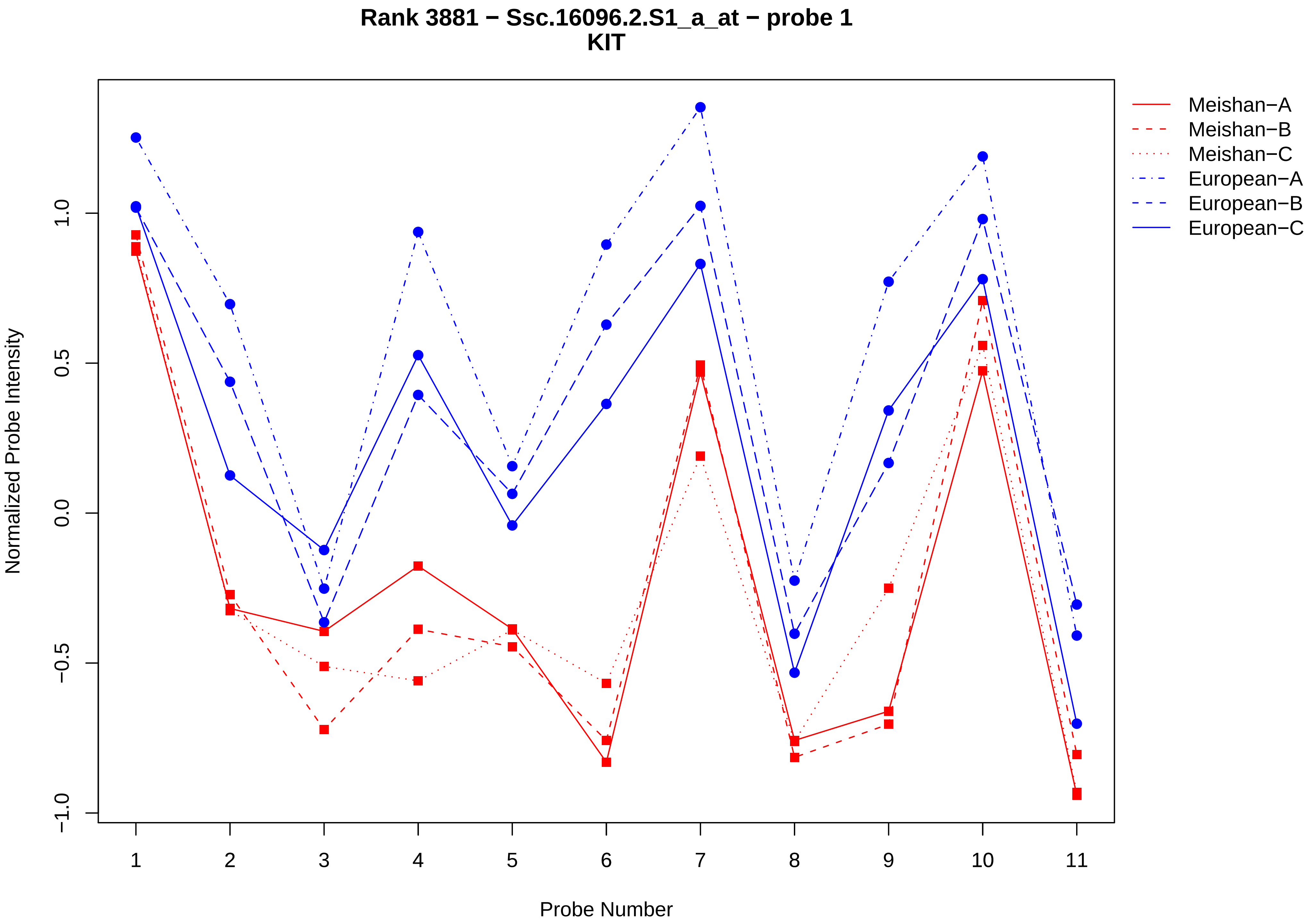

Rank 3881 − Ssc.16096.2.S1_a_at − probe 1
KIT
Normalized Probe Intensity
Probe Number
−1.0
−0.5
0.0
0.5
1.0
1
2
3
4
5
6
7
8
9
10
11
Meishan−A
Meishan−B
Meishan−C
European−A
European−B
European−C

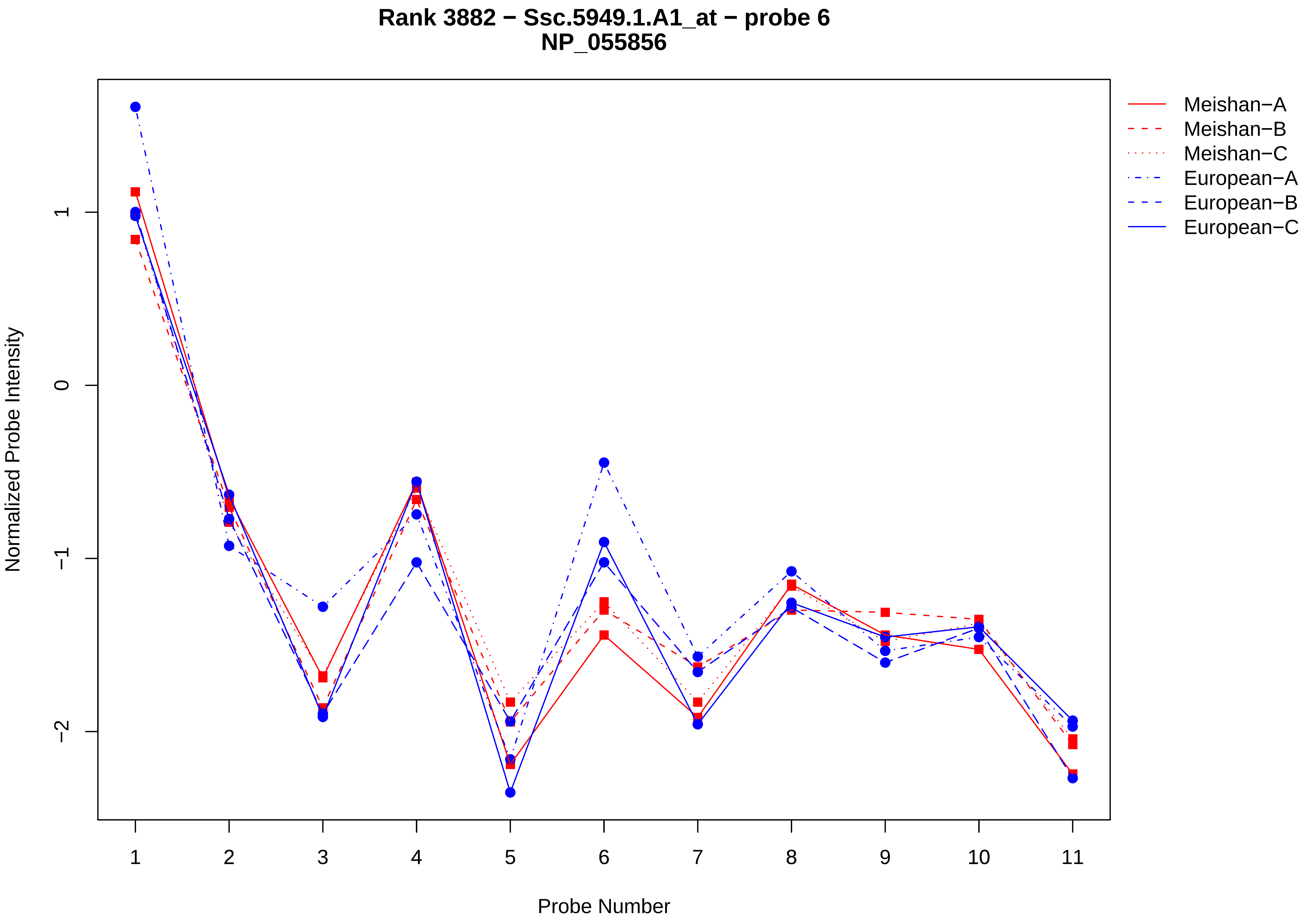
Rank 3882 − Ssc.5949.1.A1_at − probe 6
NP_055856
Normalized Probe Intensity
1
0
−1
−2
1
2
3
4
5
6
7
8
9
10
11
Probe Number
Meishan−A
Meishan−B
Meishan−C
European−A
European−B
European−C

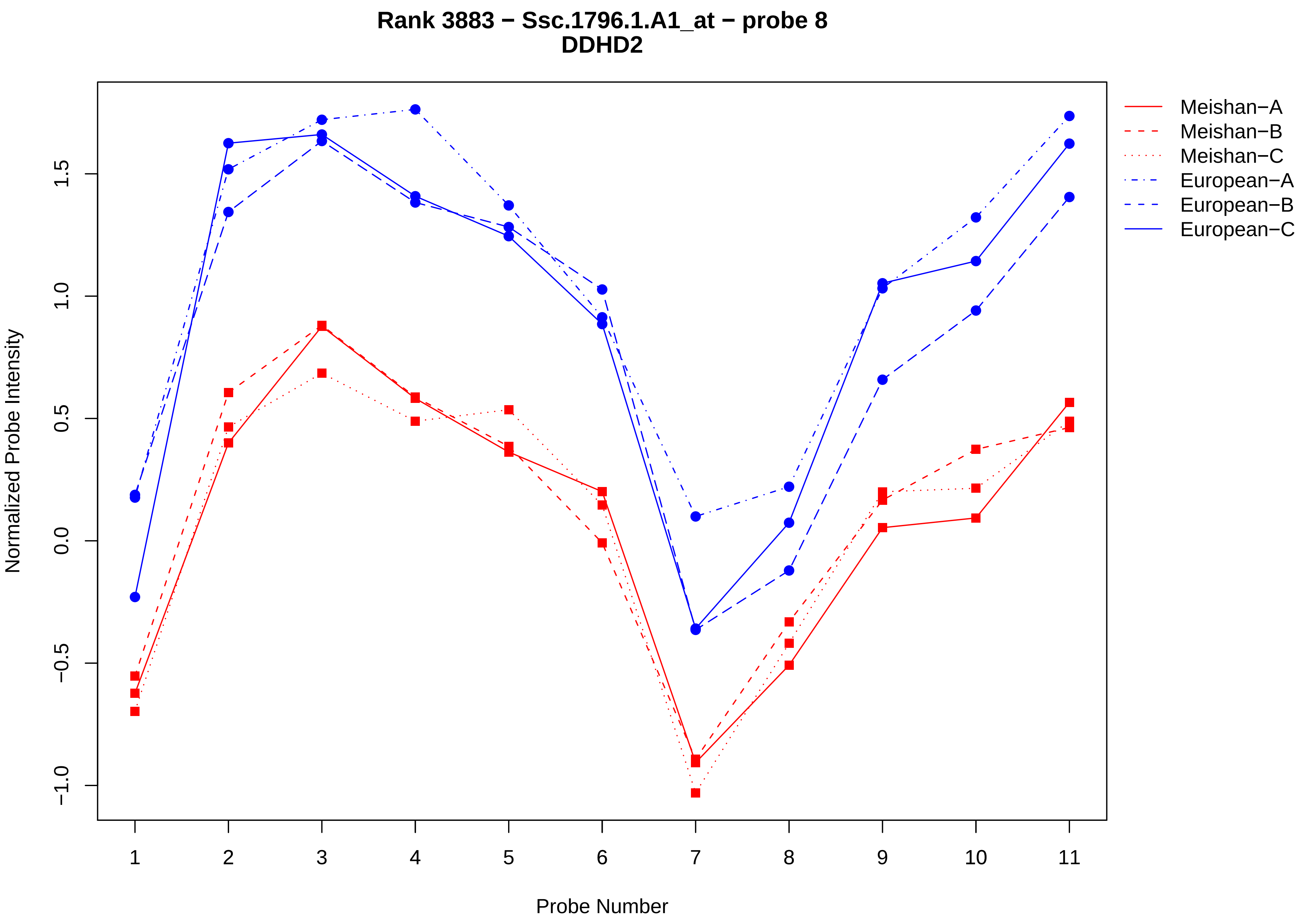

Rank 3883 − Ssc.1796.1.A1_at − probe 8
DDHD2
Normalized Probe Intensity
Probe Number
−1.0
−0.5
0.0
0.5
1.0
1.5
1
2
3
4
5
6
7
8
9
10
11
Meishan−A
Meishan−B
Meishan−C
European−A
European−B
European−C

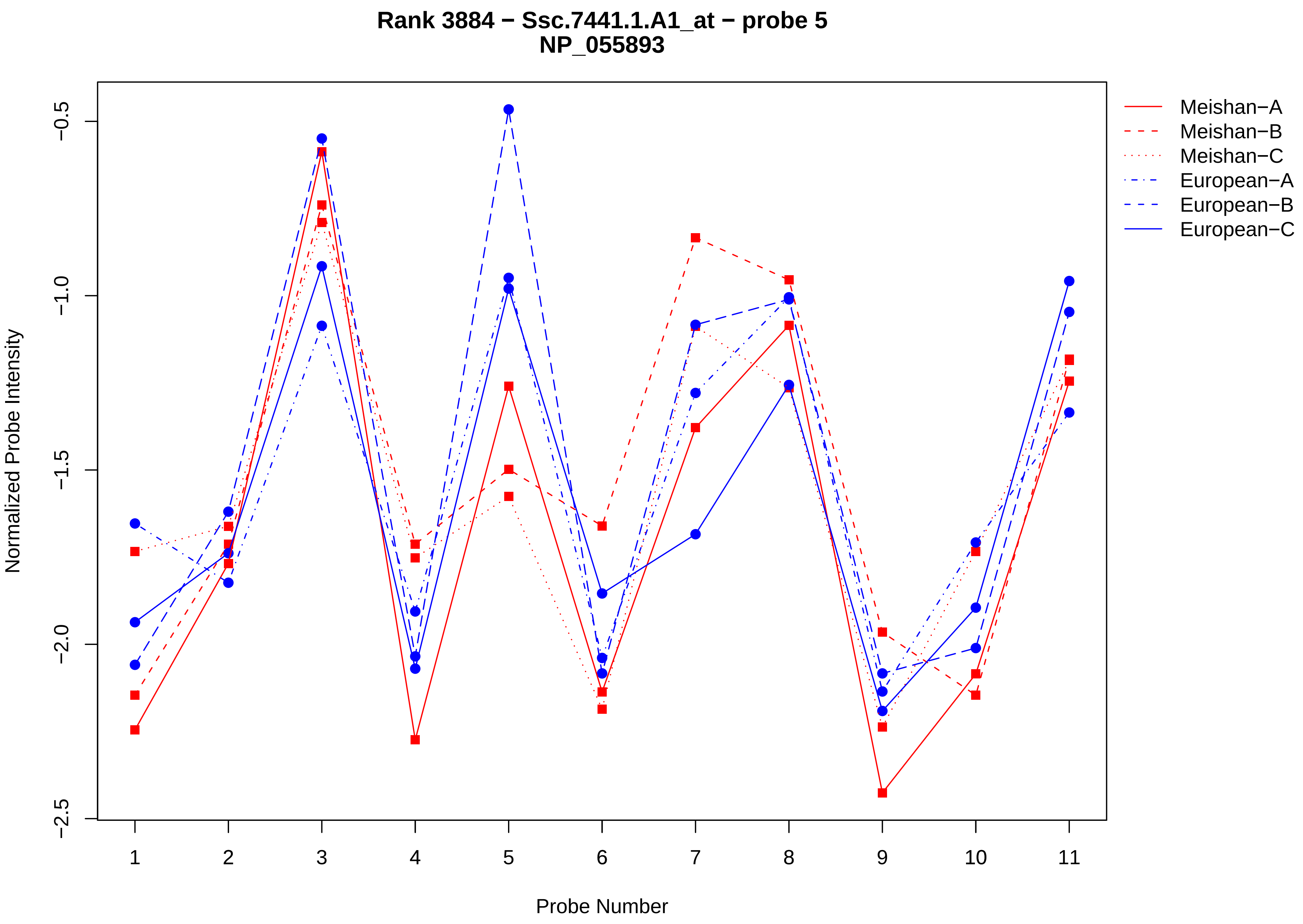

Rank 3884 − Ssc.7441.1.A1_at − probe 5
NP_055893
Normalized Probe Intensity
−0.5
−1.0
−1.5
−2.0
−2.5
1
2
3
4
5
6
7
8
9
10
11
Probe Number
Meishan−A
Meishan−B
Meishan−C
European−A
European−B
European−C

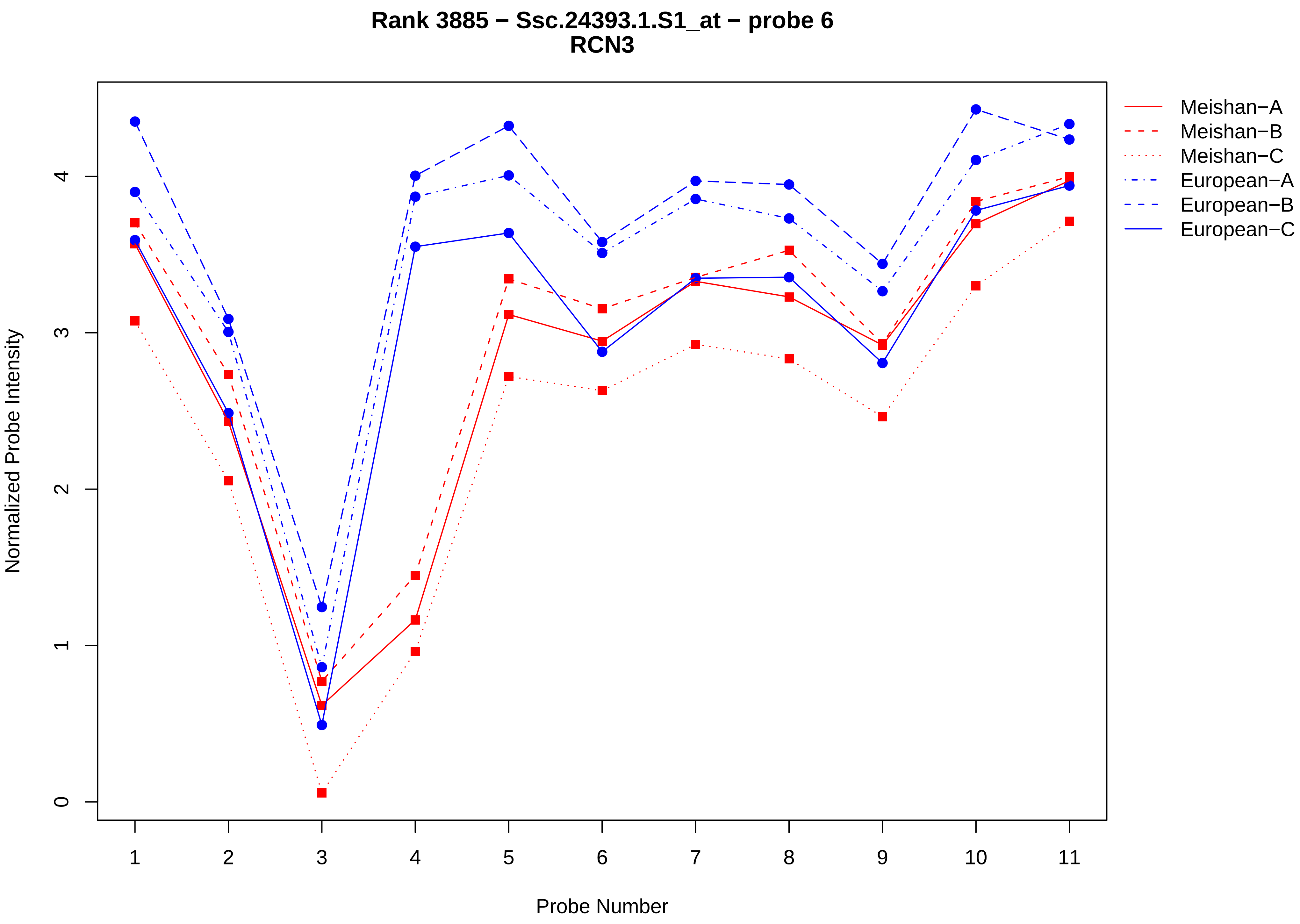

Rank 3885 − Ssc.24393.1.S1_at − probe 6
RCN3
Normalized Probe Intensity
Probe Number
0
1
2
3
4
1
2
3
4
5
6
7
8
9
10
11
Meishan−A
Meishan−B
Meishan−C
European−A
European−B
European−C

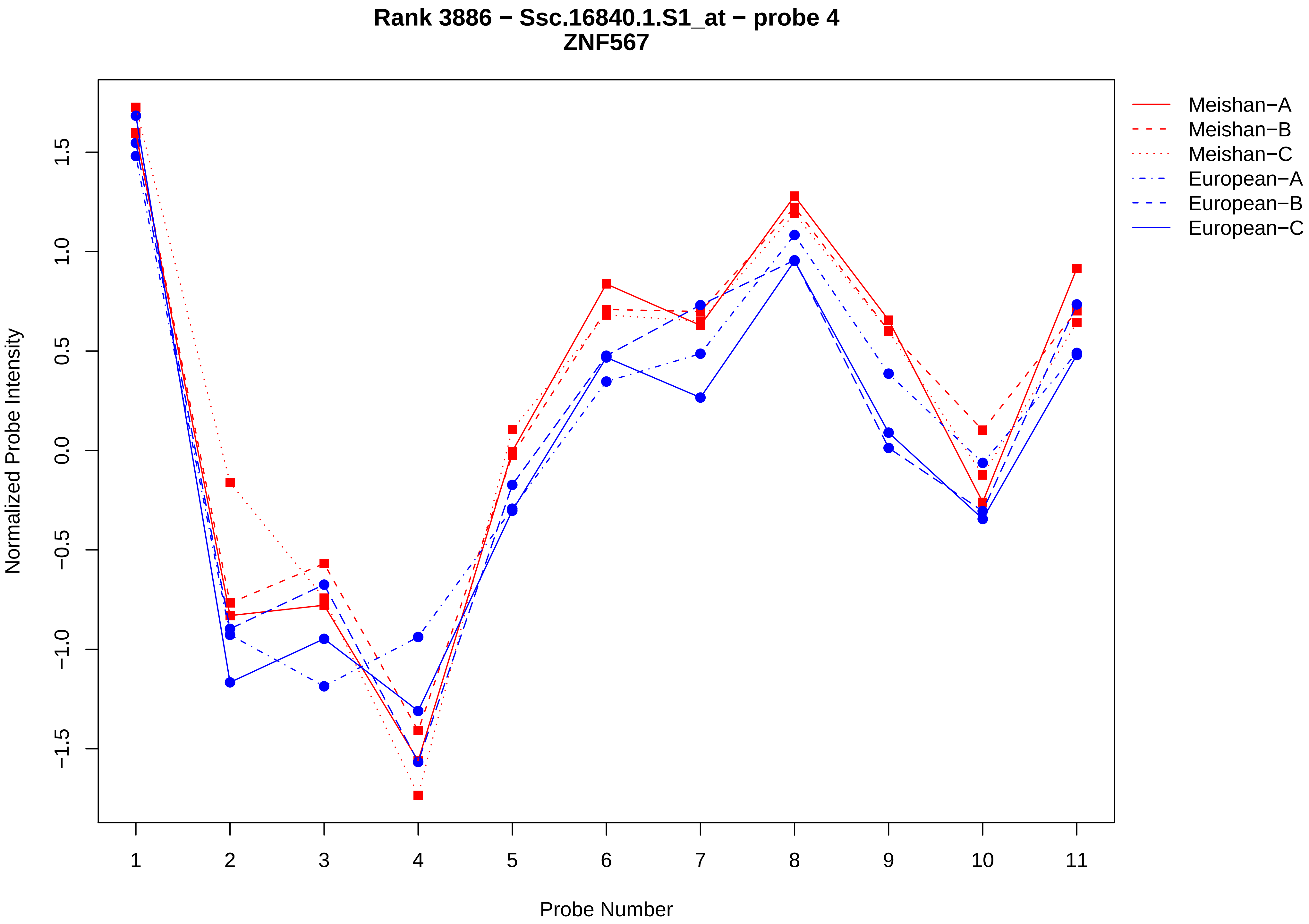

Rank 3886 − Ssc.16840.1.S1_at − probe 4
ZNF567
Normalized Probe Intensity
Probe Number
1.5
1.0
0.5
0.0
−0.5
−1.0
−1.5
1
2
3
4
5
6
7
8
9
10
11
Meishan−A
Meishan−B
Meishan−C
European−A
European−B
European−C

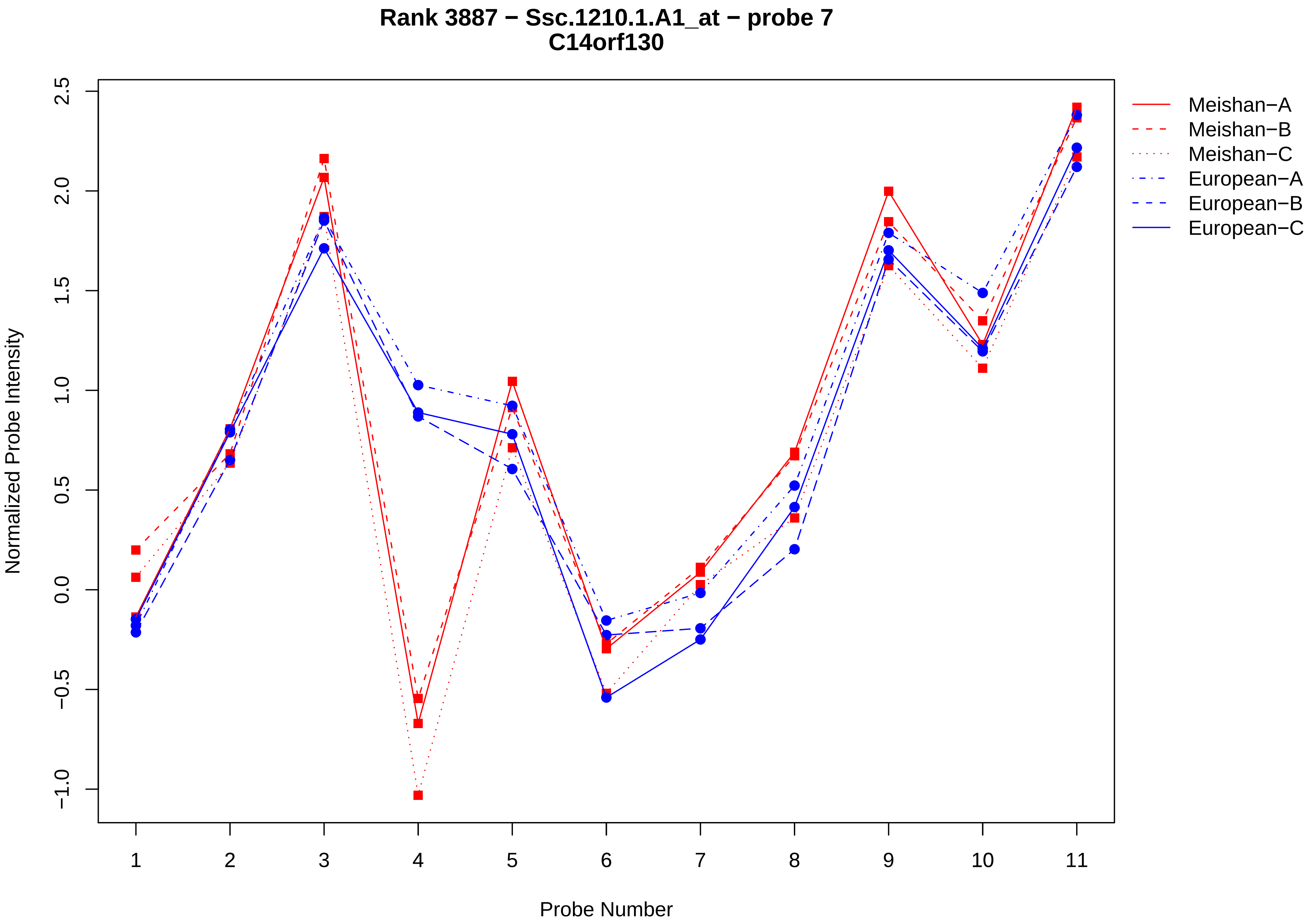

Rank 3887 − Ssc.1210.1.A1_at − probe 7
C14orf130
Normalized Probe Intensity
Probe Number
−1.0
−0.5
0.0
0.5
1.0
1.5
2.0
2.5
1
2
3
4
5
6
7
8
9
10
11
Meishan−A
Meishan−B
Meishan−C
European−A
European−B
European−C

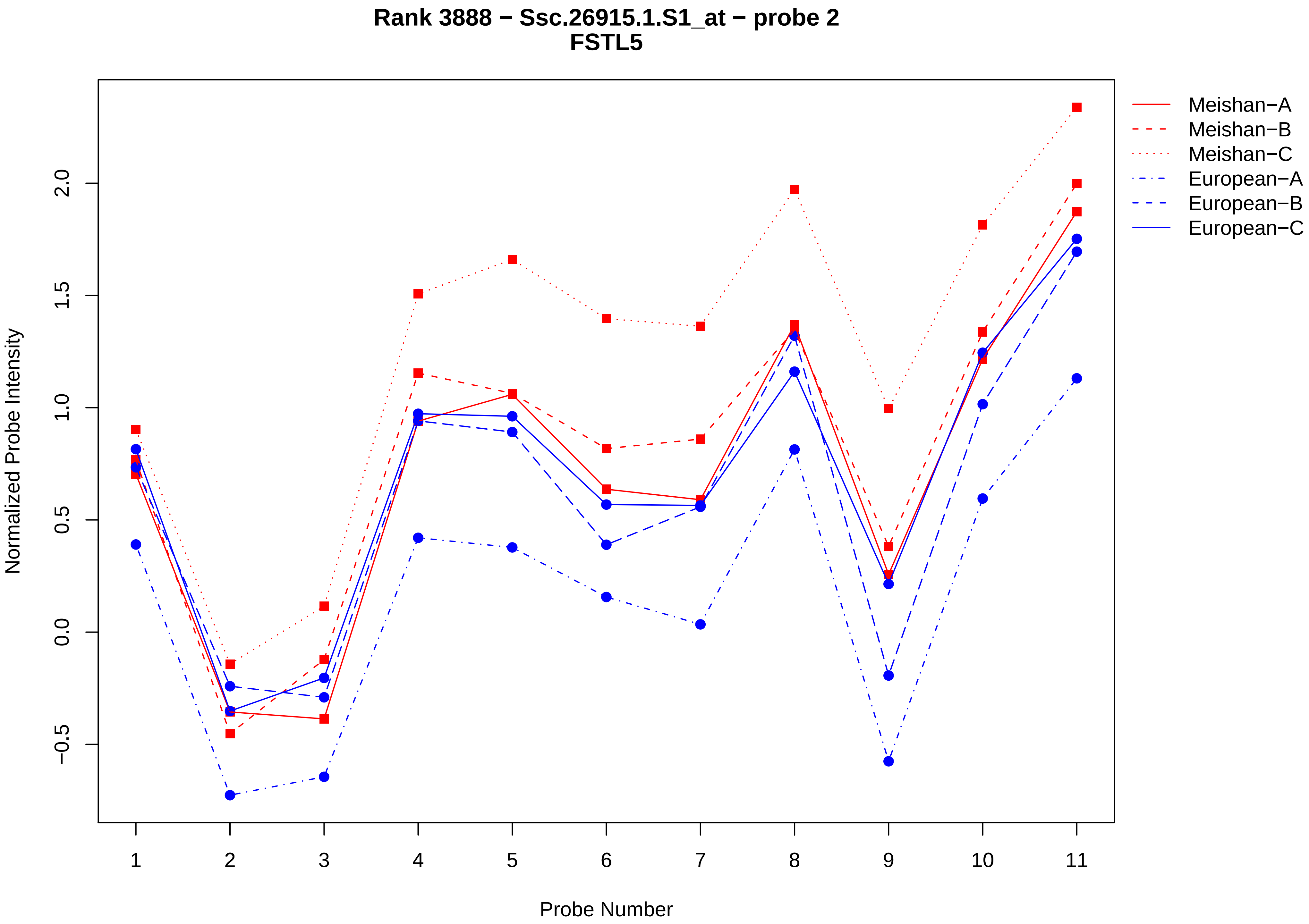

Rank 3888 − Ssc.26915.1.S1_at − probe 2
FSTL5
Normalized Probe Intensity
Probe Number
−0.5
0.0
0.5
1.0
1.5
2.0
1
2
3
4
5
6
7
8
9
10
11
Meishan−A
Meishan−B
Meishan−C
European−A
European−B
European−C

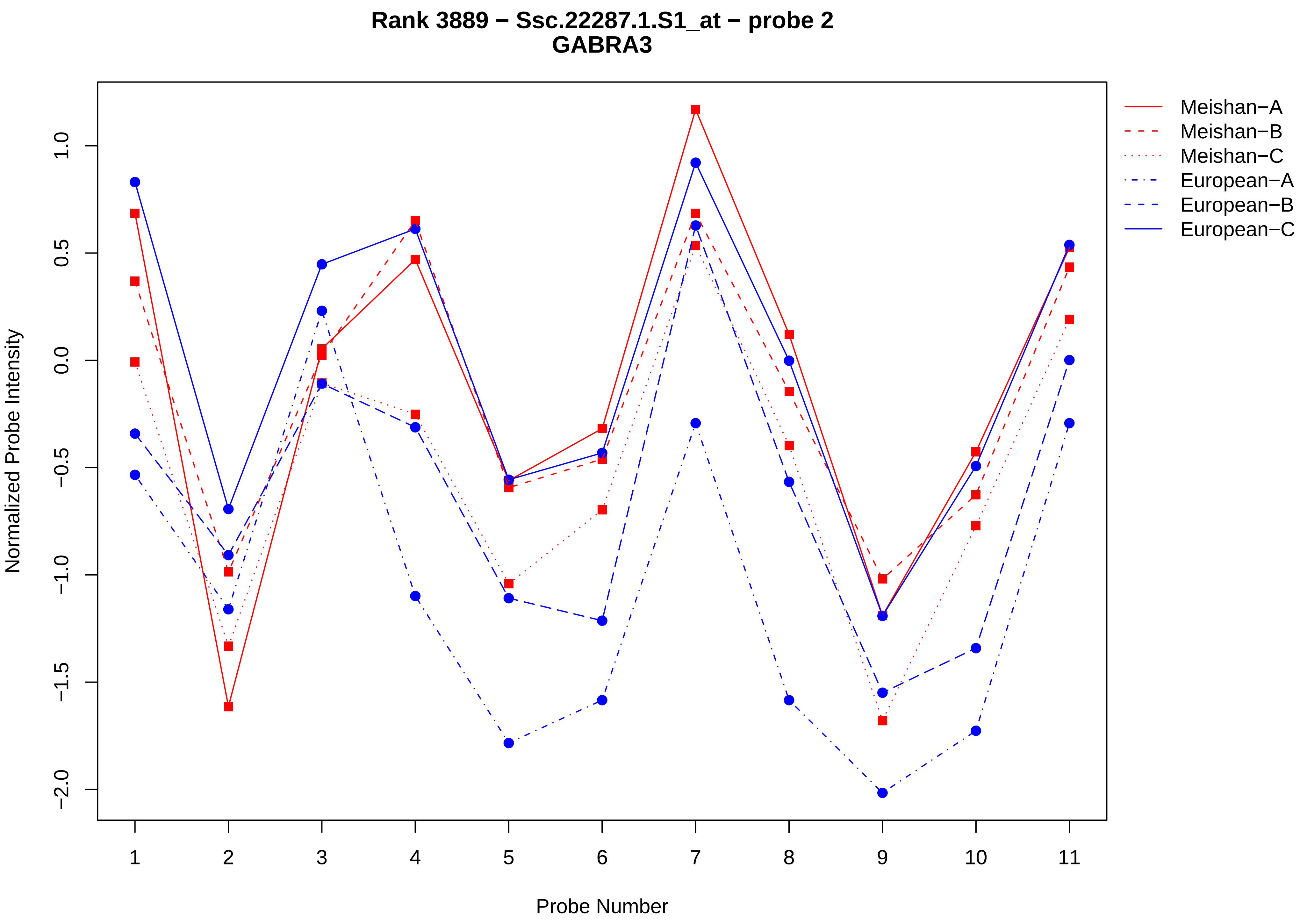

Rank 3889 − Ssc.22287.1.S1_at − probe 2
GABRA3
Normalized Probe Intensity
Probe Number
1.0
0.5
0.0
−0.5
−1.0
−1.5
−2.0
1
2
3
4
5
6
7
8
9
10
11
Meishan−A
Meishan−B
Meishan−C
European−A
European−B
European−C

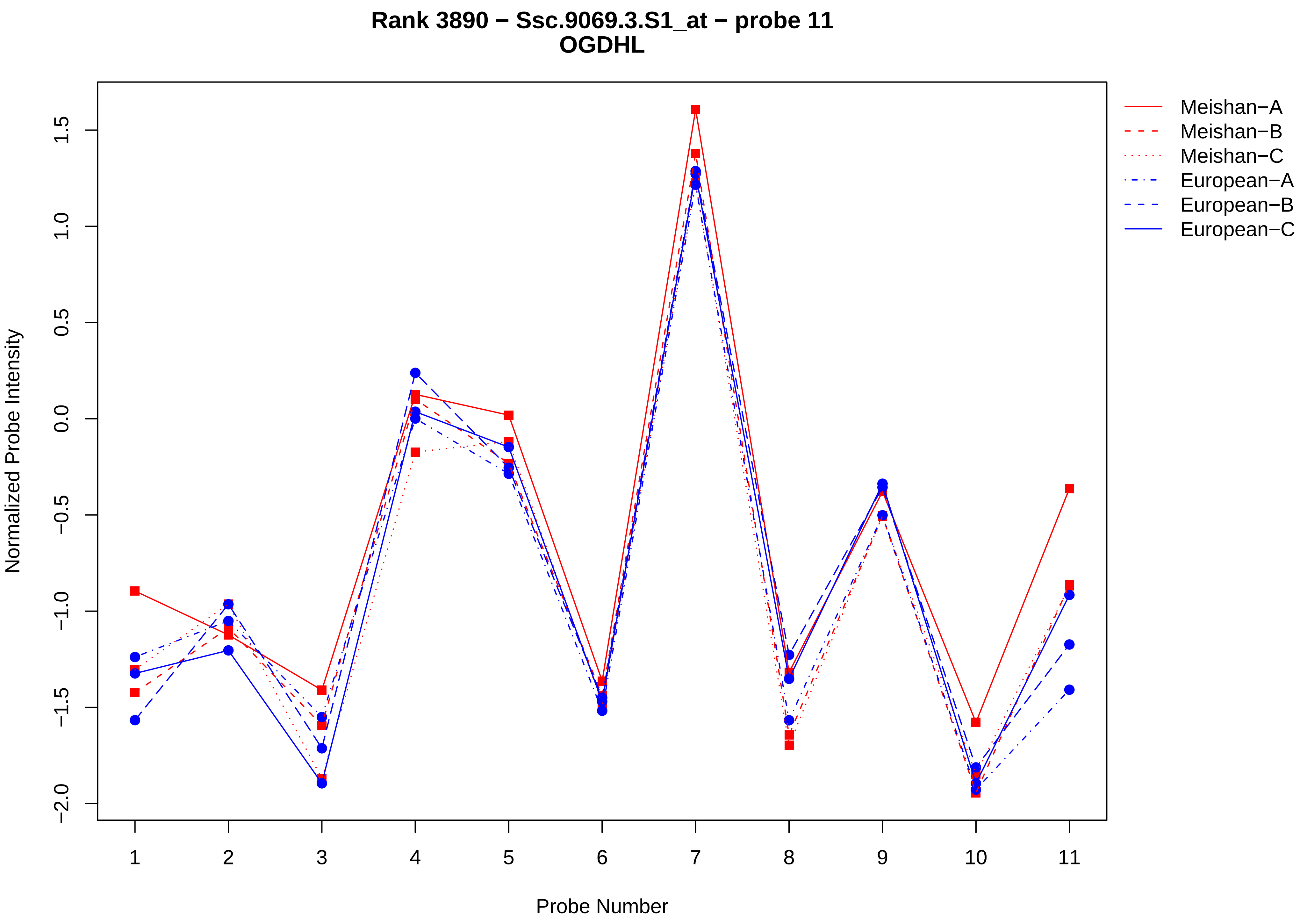

Rank 3890 − Ssc.9069.3.S1_at − probe 11
OGDHL
Normalized Probe Intensity
Probe Number
−2.0
−1.5
−1.0
−0.5
0.0
0.5
1.0
1.5
1
2
3
4
5
6
7
8
9
10
11
Meishan−A
Meishan−B
Meishan−C
European−A
European−B
European−C

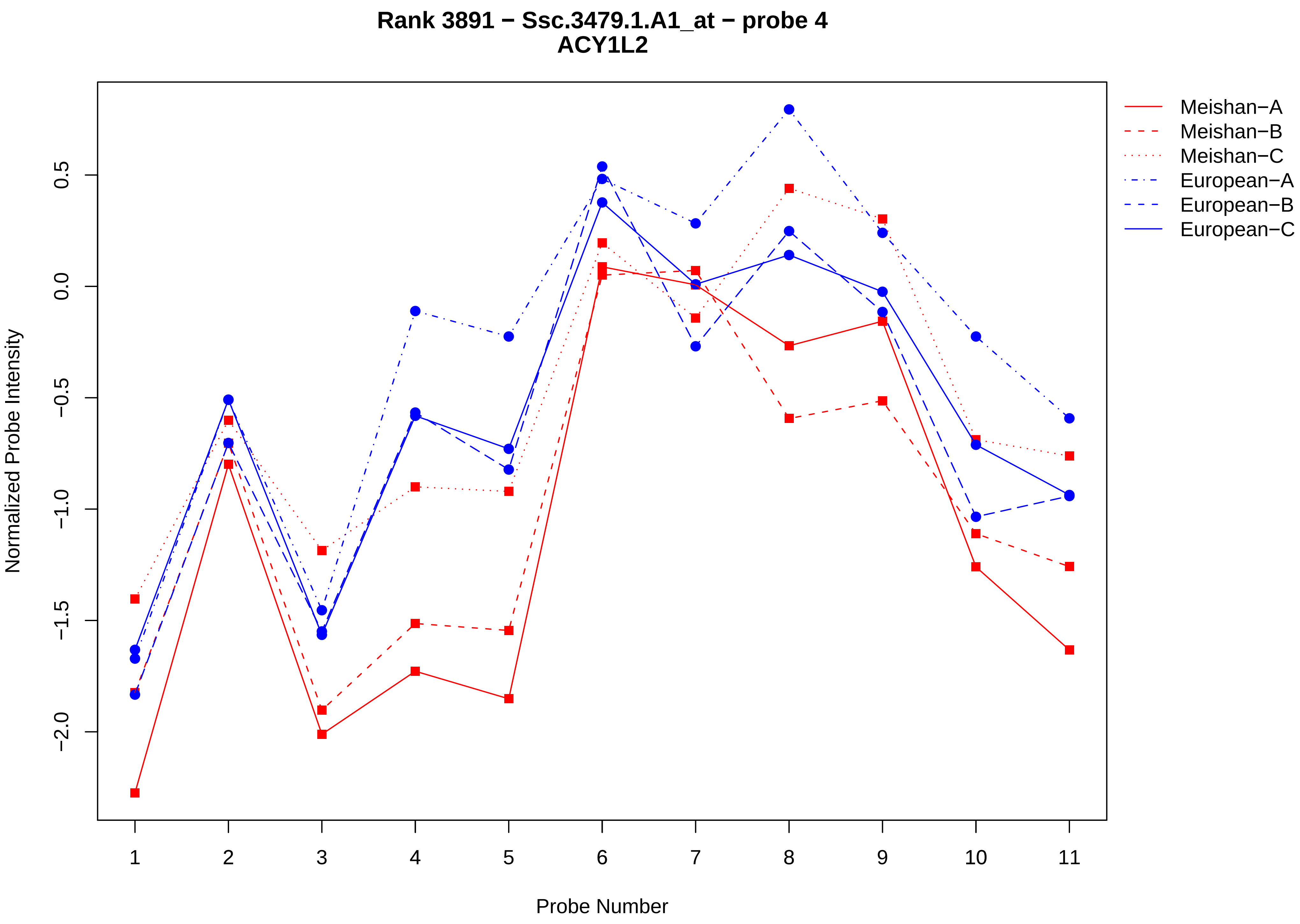

Rank 3891 − Ssc.3479.1.A1_at − probe 4
ACY1L2
Normalized Probe Intensity
Probe Number
0.5
0.0
−0.5
−1.0
−1.5
−2.0
1
2
3
4
5
6
7
8
9
10
11
Meishan−A
Meishan−B
Meishan−C
European−A
European−B
European−C

Rank 3892 − Ssc.4682.1.A1_at − probe 11
DDX18

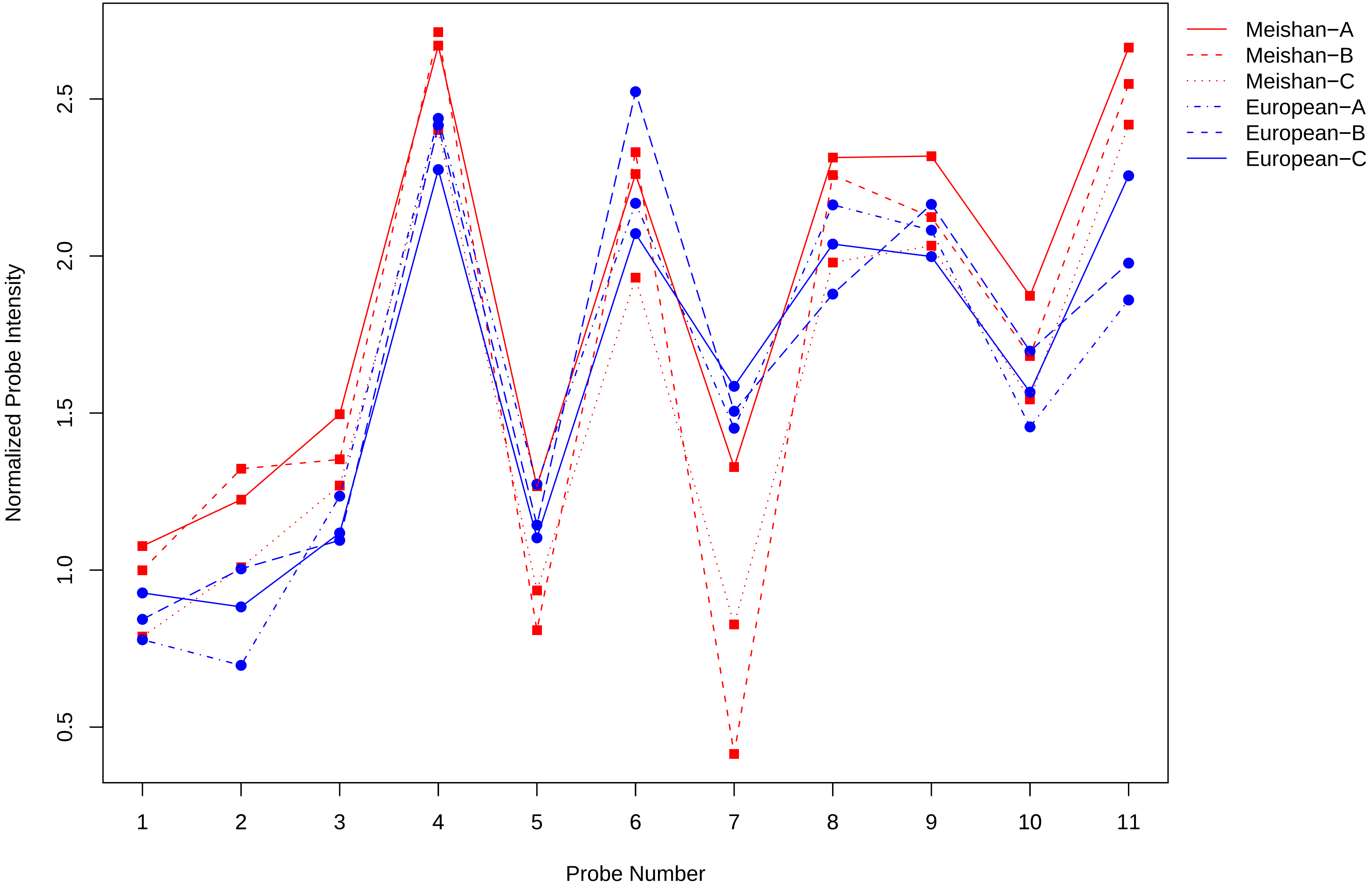

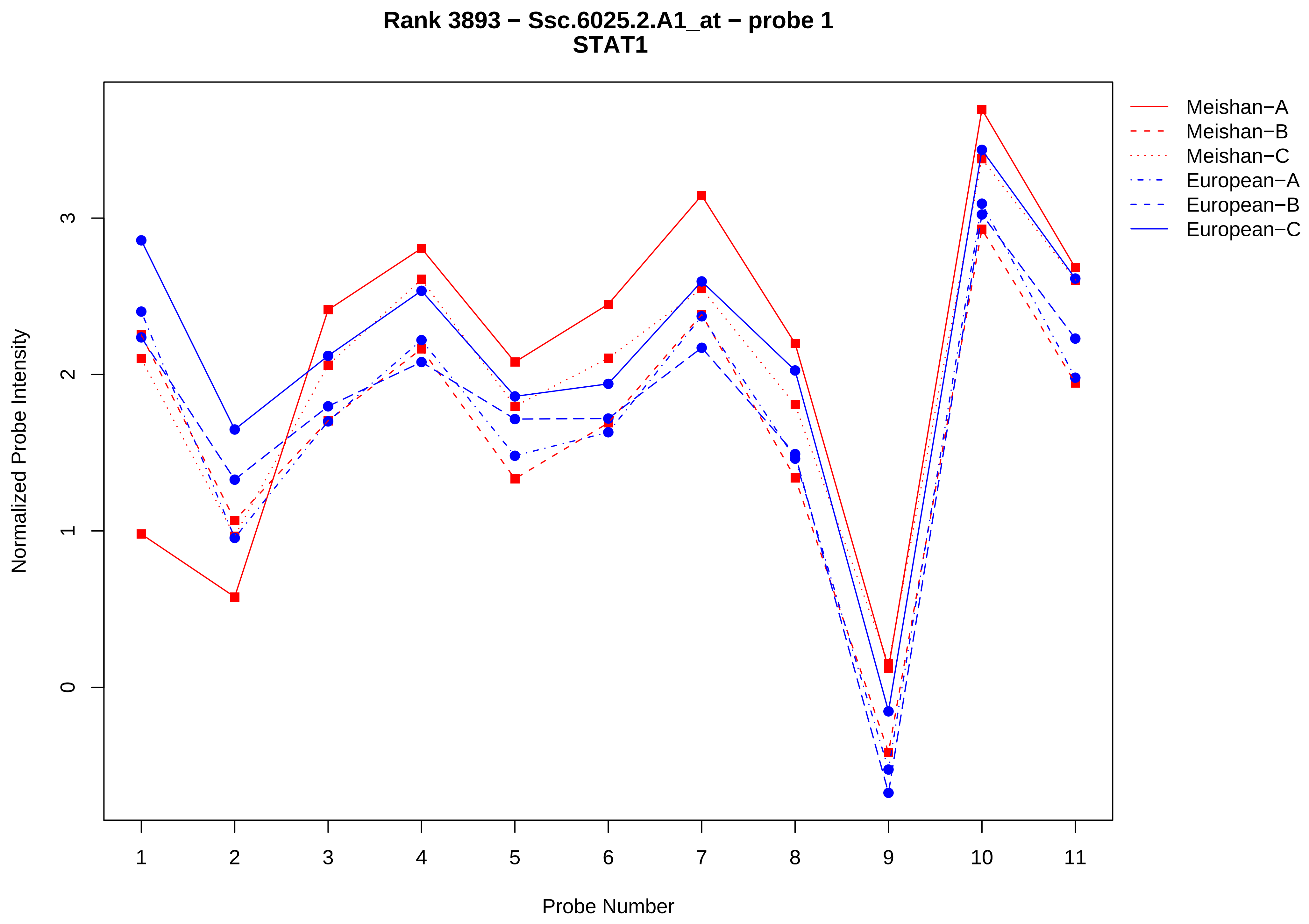
Rank 3893 − Ssc.6025.2.A1_at − probe 1
STAT1
Normalized Probe Intensity
Probe Number
0
1
2
3
1
2
3
4
5
6
7
8
9
10
11
Meishan−A
Meishan−B
Meishan−C
European−A
European−B
European−C

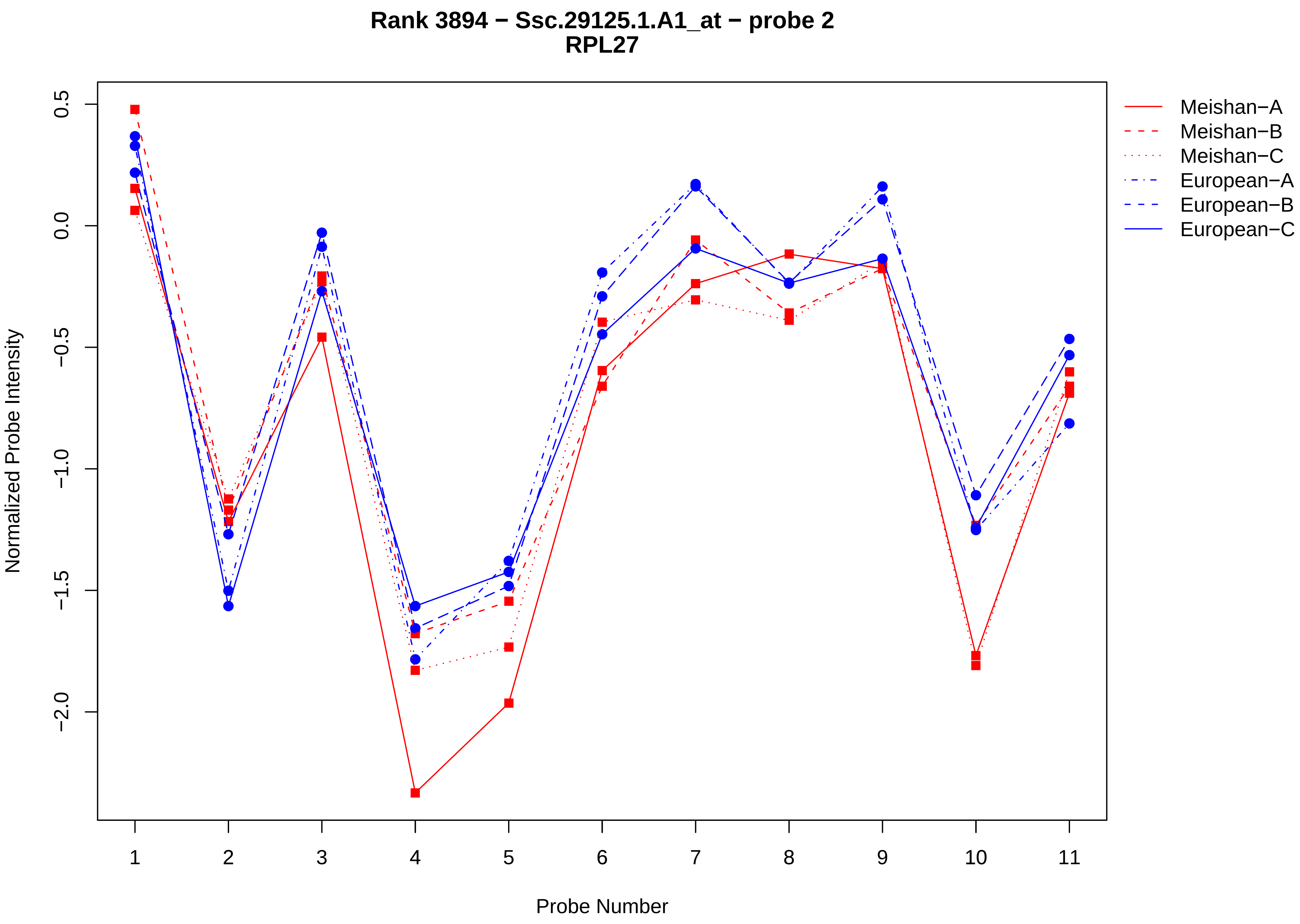

Rank 3894 − Ssc.29125.1.A1_at − probe 2
RPL27
Normalized Probe Intensity
Probe Number
0.5
0.0
−0.5
−1.0
−1.5
−2.0
1
2
3
4
5
6
7
8
9
10
11
Meishan−A
Meishan−B
Meishan−C
European−A
European−B
European−C

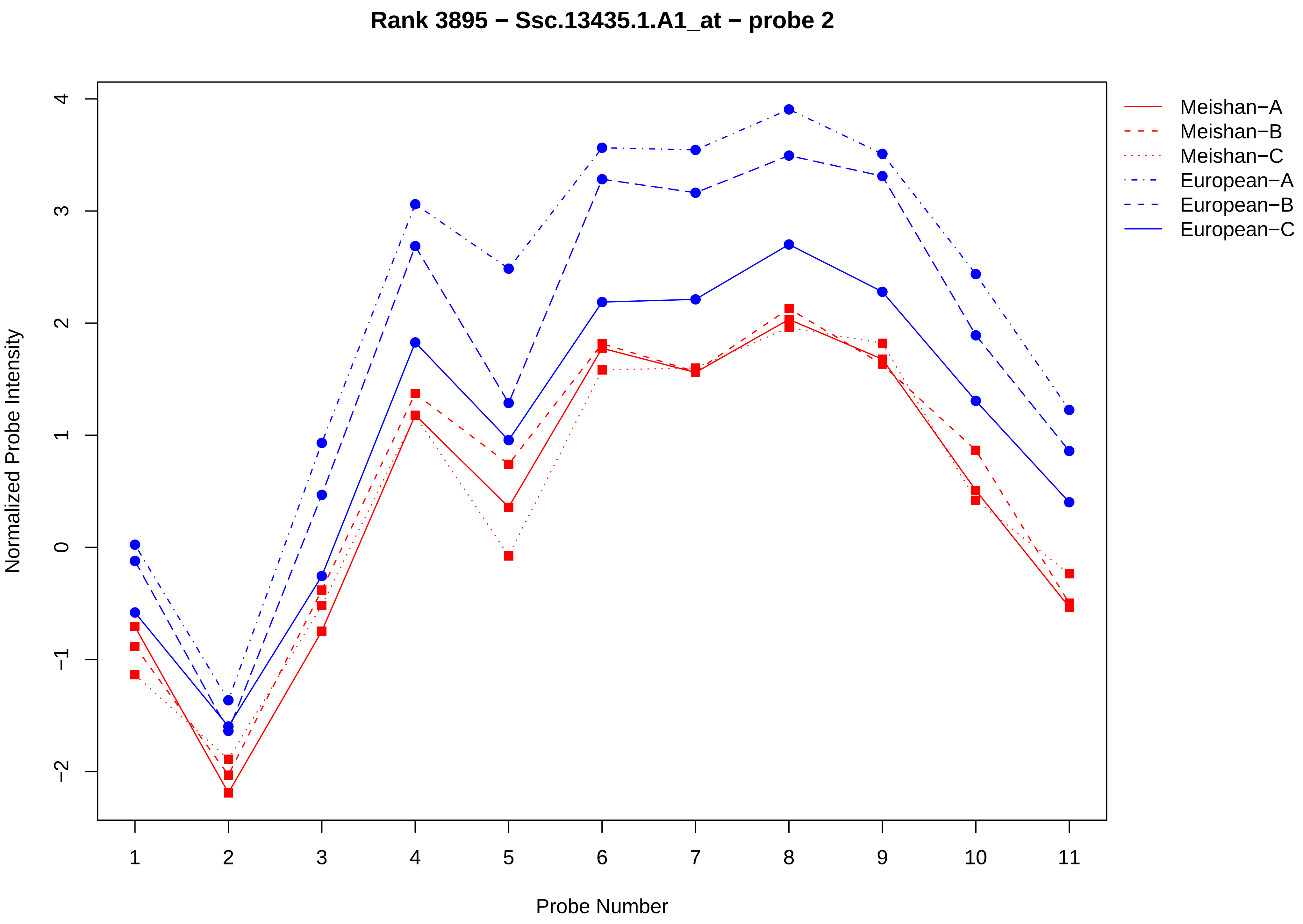

Rank 3895 − Ssc.13435.1.A1_at − probe 2
Normalized Probe Intensity
Probe Number
Meishan−A
Meishan−B
Meishan−C
European−A
European−B
European−C

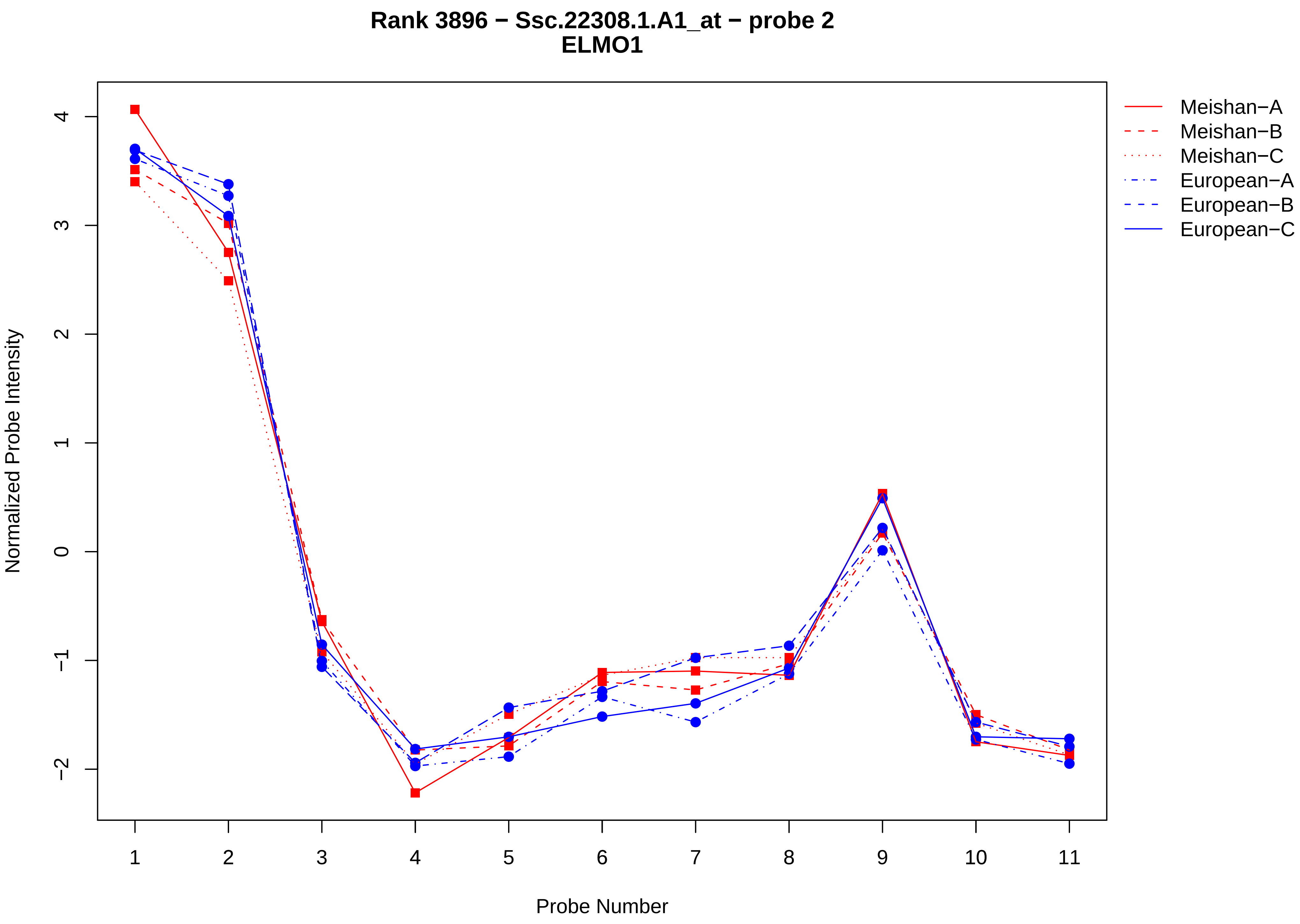

Rank 3896 − Ssc.22308.1.A1_at − probe 2
ELMO1
Normalized Probe Intensity
Probe Number
Meishan−A
Meishan−B
Meishan−C
European−A
European−B
European−C

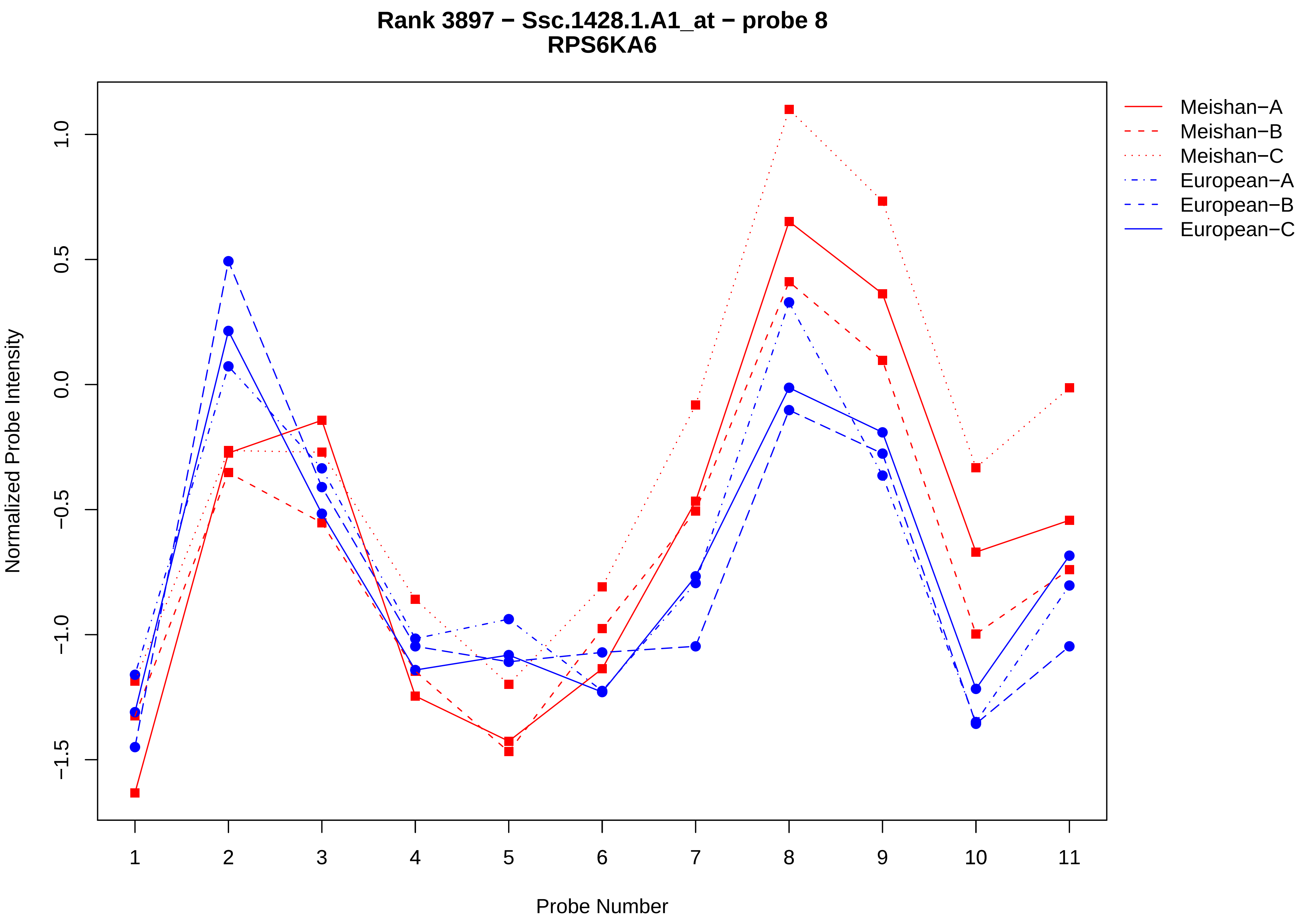

Rank 3897 − Ssc.1428.1.A1_at − probe 8
RPS6KA6
Normalized Probe Intensity
Probe Number
Meishan−A
Meishan−B
Meishan−C
European−A
European−B
European−C
1.0
0.5
0.0
−0.5
−1.0
−1.5
1
2
3
4
5
6
7
8
9
10
11

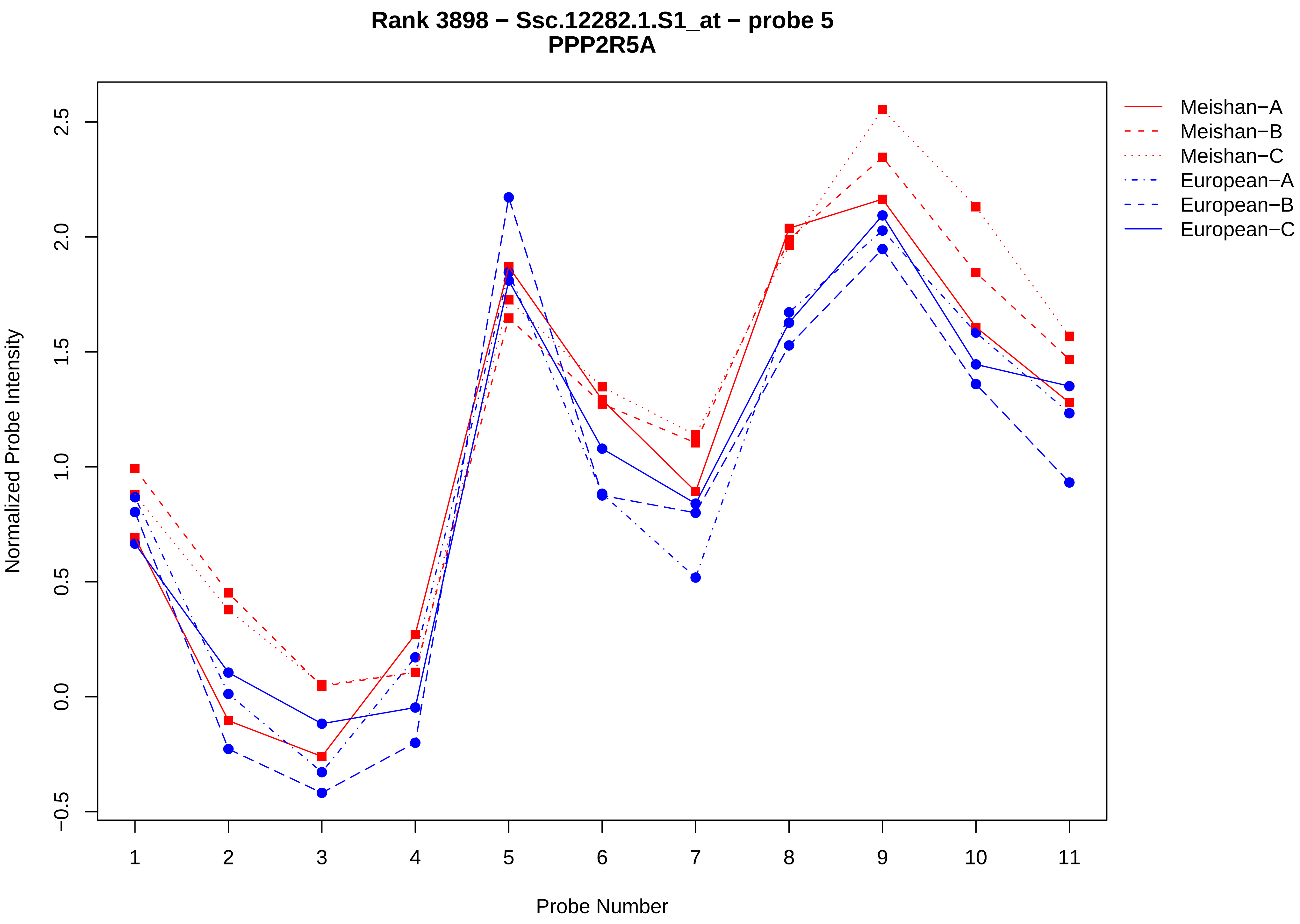

Rank 3898 − Ssc.12282.1.S1_at − probe 5
PPP2R5A
Normalized Probe Intensity
Probe Number
−0.5
0.0
0.5
1.0
1.5
2.0
2.5
1
2
3
4
5
6
7
8
9
10
11
Meishan−A
Meishan−B
Meishan−C
European−A
European−B
European−C

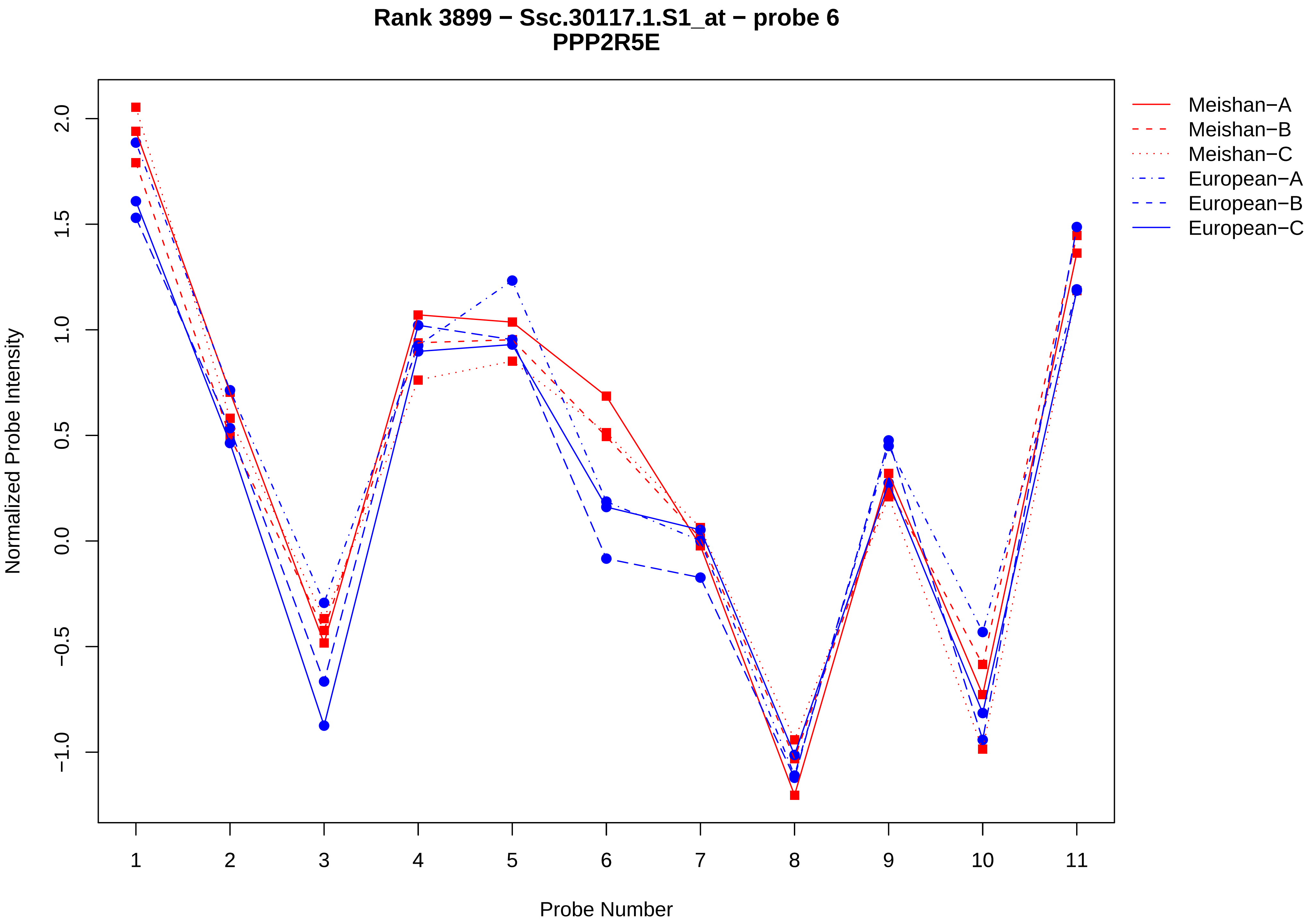
Rank 3899 − Ssc.30117.1.S1_at − probe 6
PPP2R5E
Normalized Probe Intensity
Probe Number
Meishan−A
Meishan−B
Meishan−C
European−A
European−B
European−C
2.0
1.5
1.0
0.5
0.0
−0.5
−1.0
1
2
3
4
5
6
7
8
9
10
11

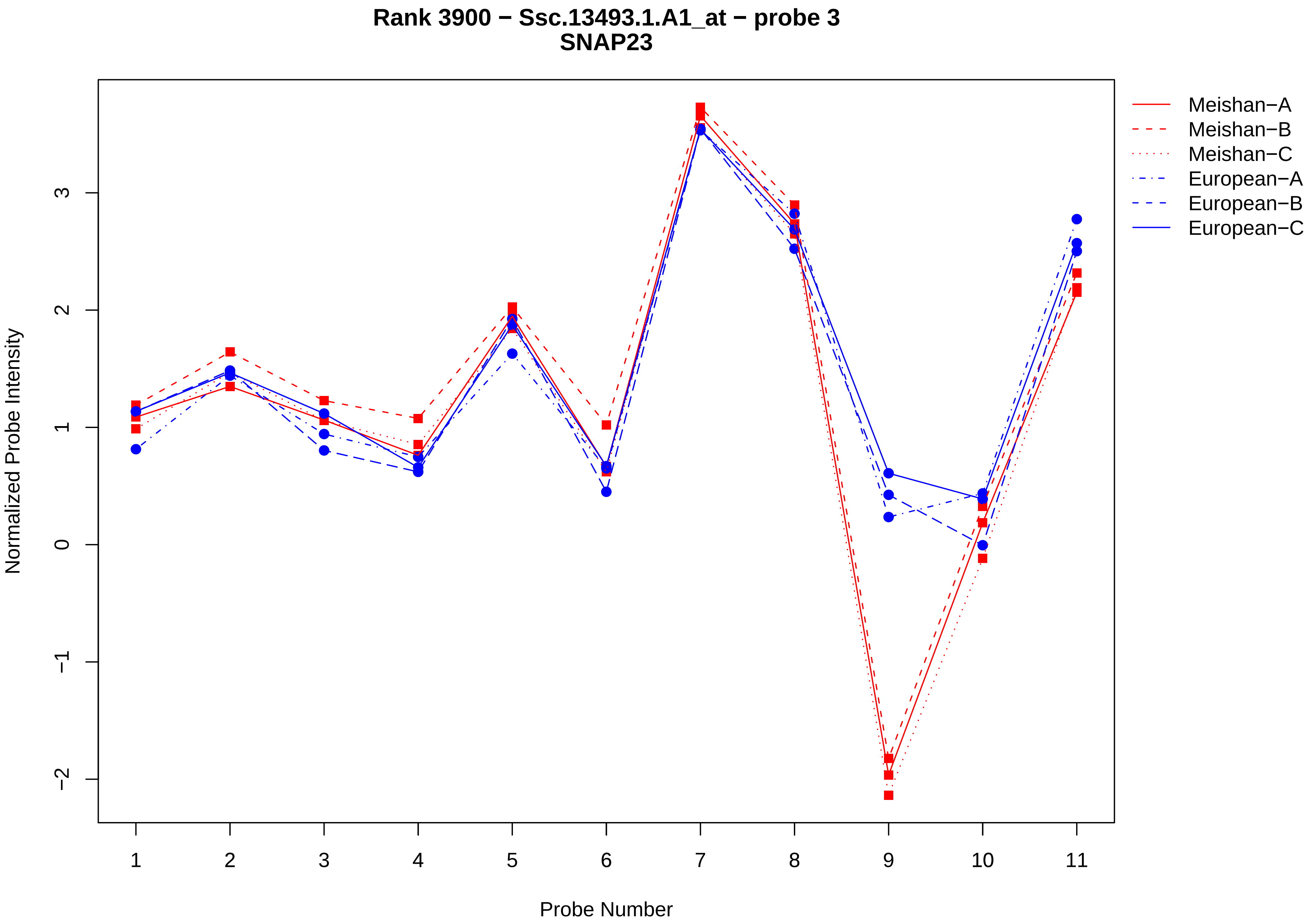

Rank 3900 − Ssc.13493.1.A1_at − probe 3
SNAP23
Normalized Probe Intensity
Probe Number
−2
−1
0
1
2
3
1
2
3
4
5
6
7
8
9
10
11
Meishan−A
Meishan−B
Meishan−C
European−A
European−B
European−C

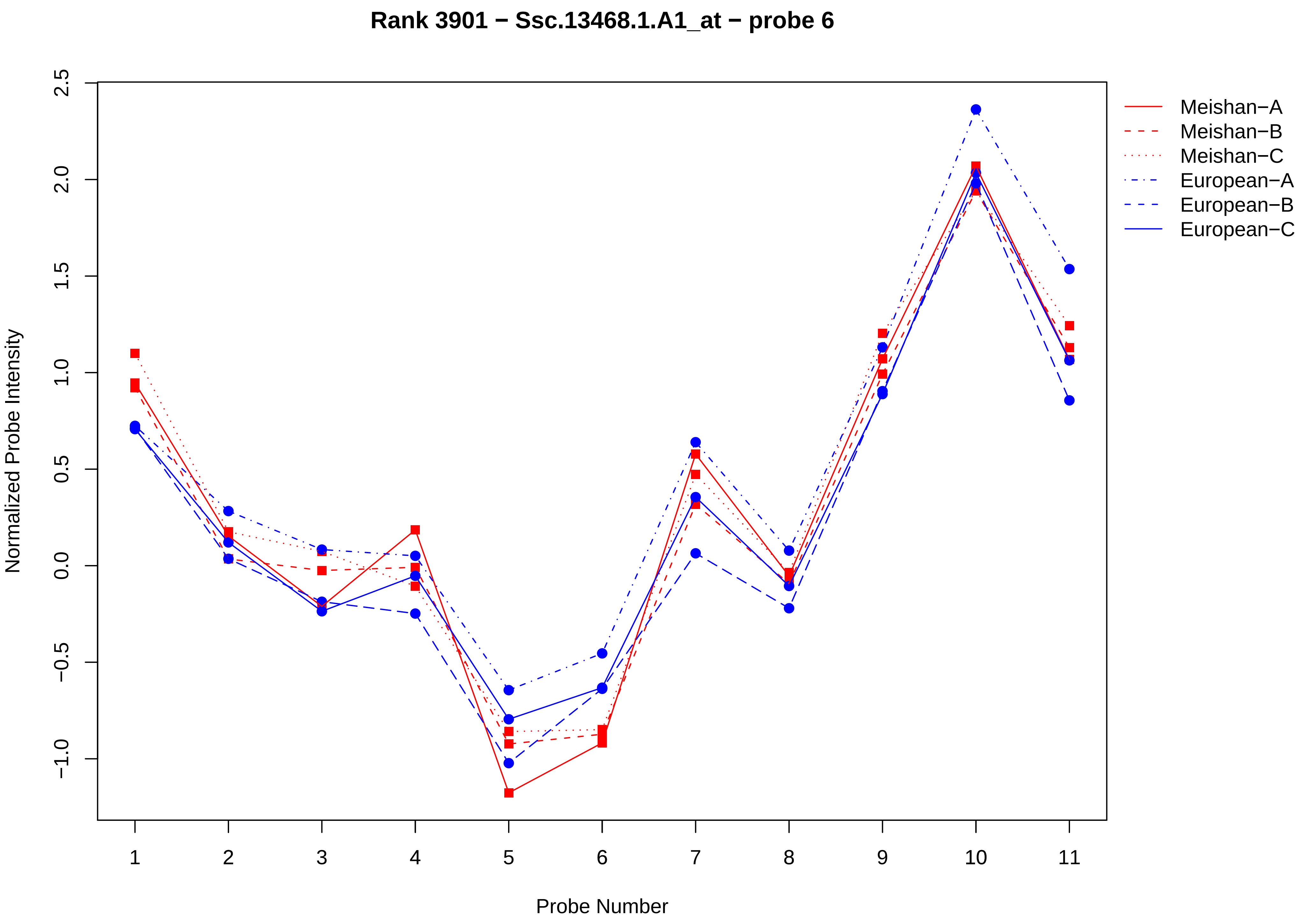
Rank 3901 − Ssc.13468.1.A1_at − probe 6
Normalized Probe Intensity
Probe Number
−1.0
−0.5
0.0
0.5
1.0
1.5
2.0
2.5
1
2
3
4
5
6
7
8
9
10
11
Meishan−A
Meishan−B
Meishan−C
European−A
European−B
European−C

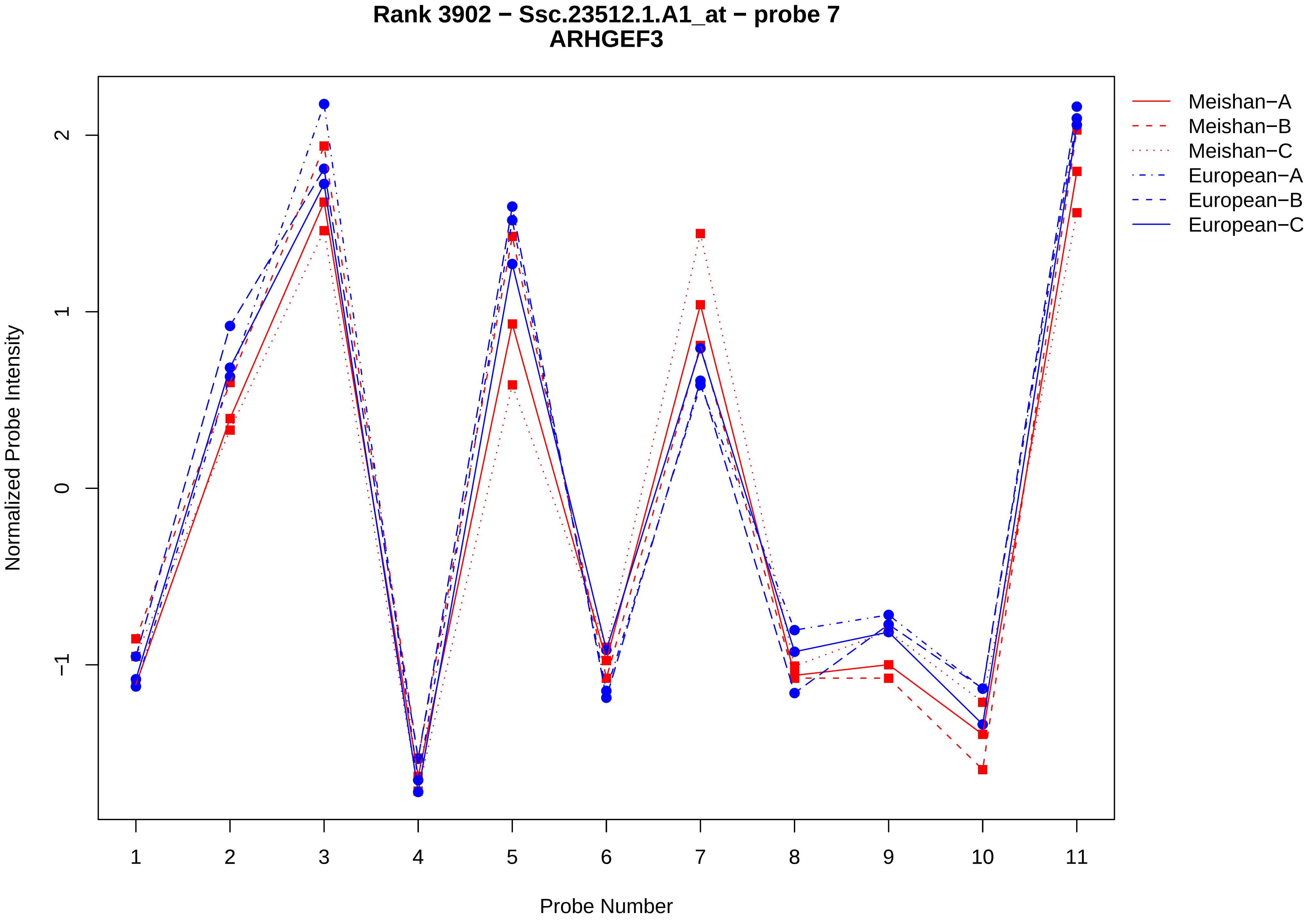

Rank 3902 − Ssc.23512.1.A1_at − probe 7
ARHGEF3
Normalized Probe Intensity
Probe Number
−1
0
1
2
1
2
3
4
5
6
7
8
9
10
11
Meishan−A
Meishan−B
Meishan−C
European−A
European−B
European−C

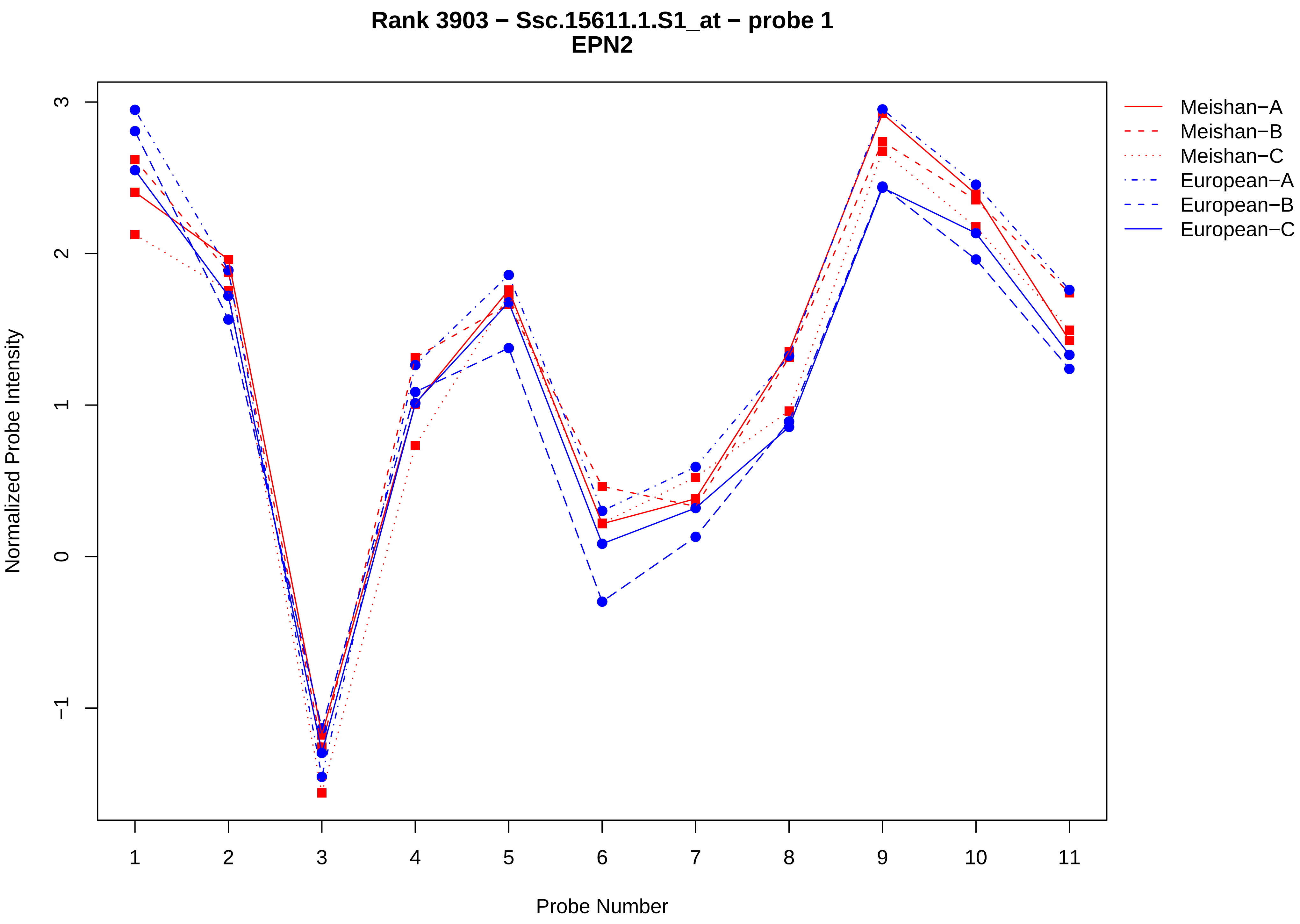

Rank 3903 − Ssc.15611.1.S1_at − probe 1
EPN2
Normalized Probe Intensity
Probe Number
−1
0
1
2
3
1
2
3
4
5
6
7
8
9
10
11
Meishan−A
Meishan−B
Meishan−C
European−A
European−B
European−C

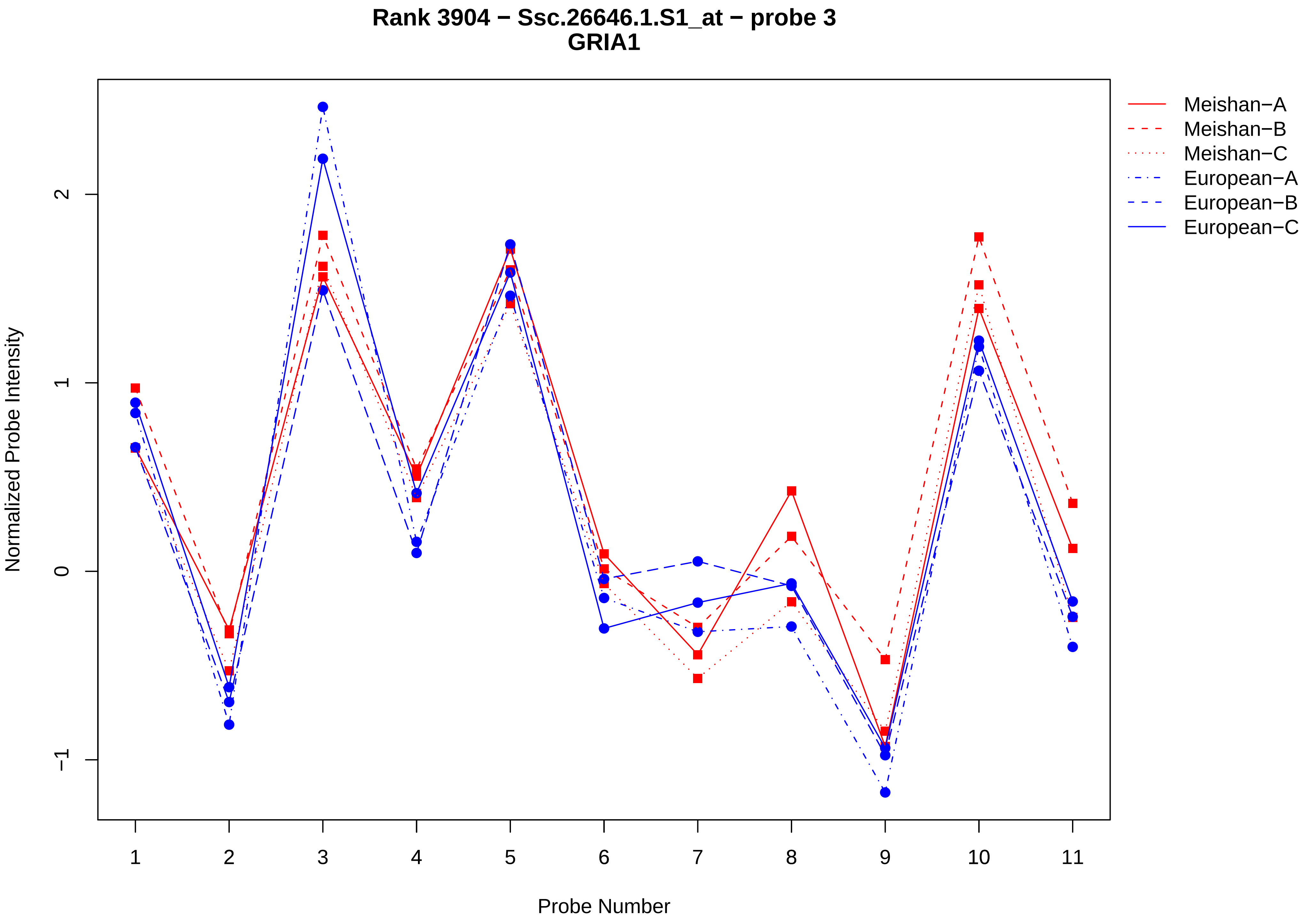
Rank 3904 − Ssc.26646.1.S1_at − probe 3
GRIA1
Normalized Probe Intensity
Probe Number
−1
0
1
2
1
2
3
4
5
6
7
8
9
10
11
Meishan−A
Meishan−B
Meishan−C
European−A
European−B
European−C

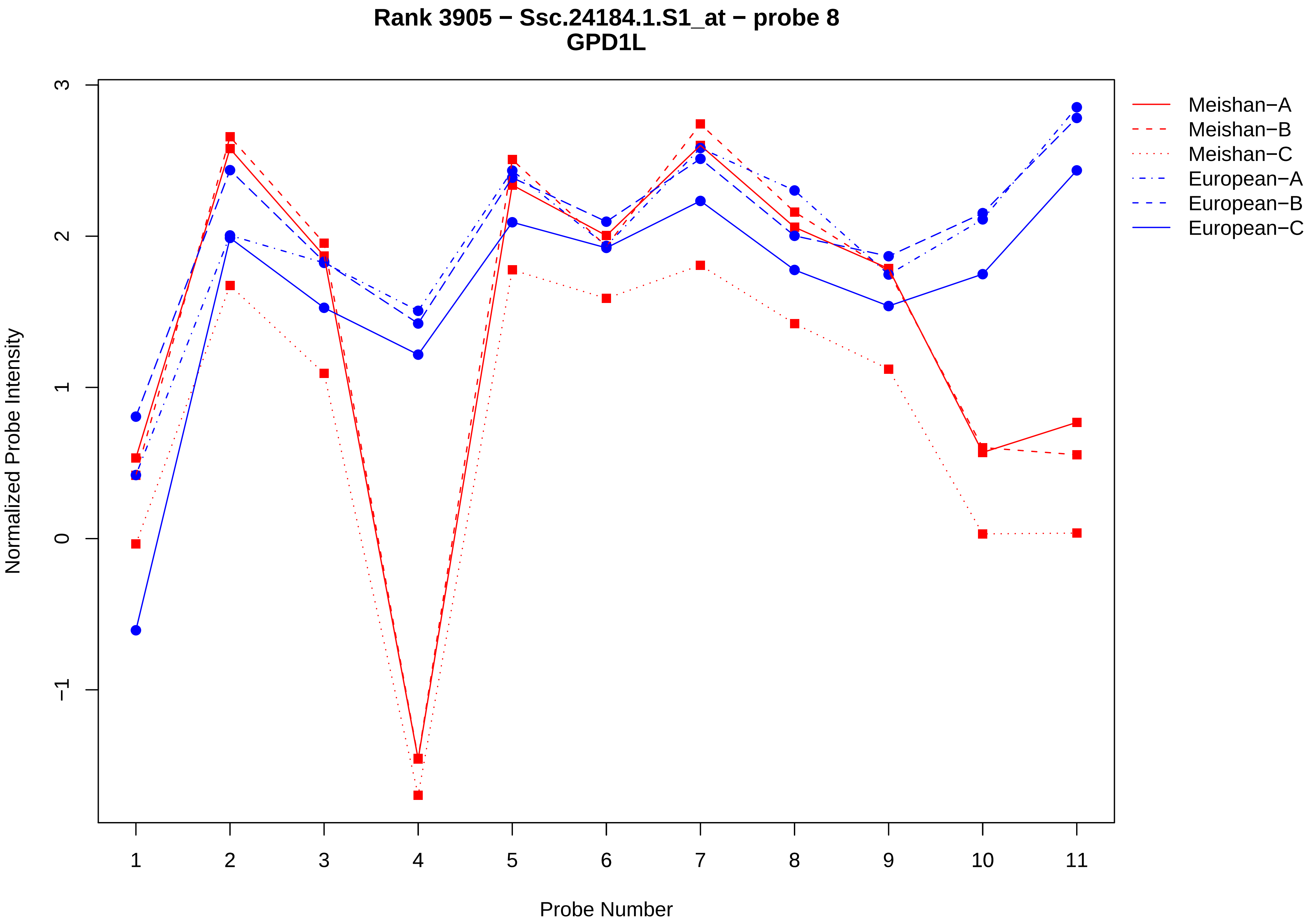

Rank 3905 − Ssc.24184.1.S1_at − probe 8
GPD1L
Normalized Probe Intensity
Probe Number
−1
0
1
2
3
1
2
3
4
5
6
7
8
9
10
11
Meishan−A
Meishan−B
Meishan−C
European−A
European−B
European−C

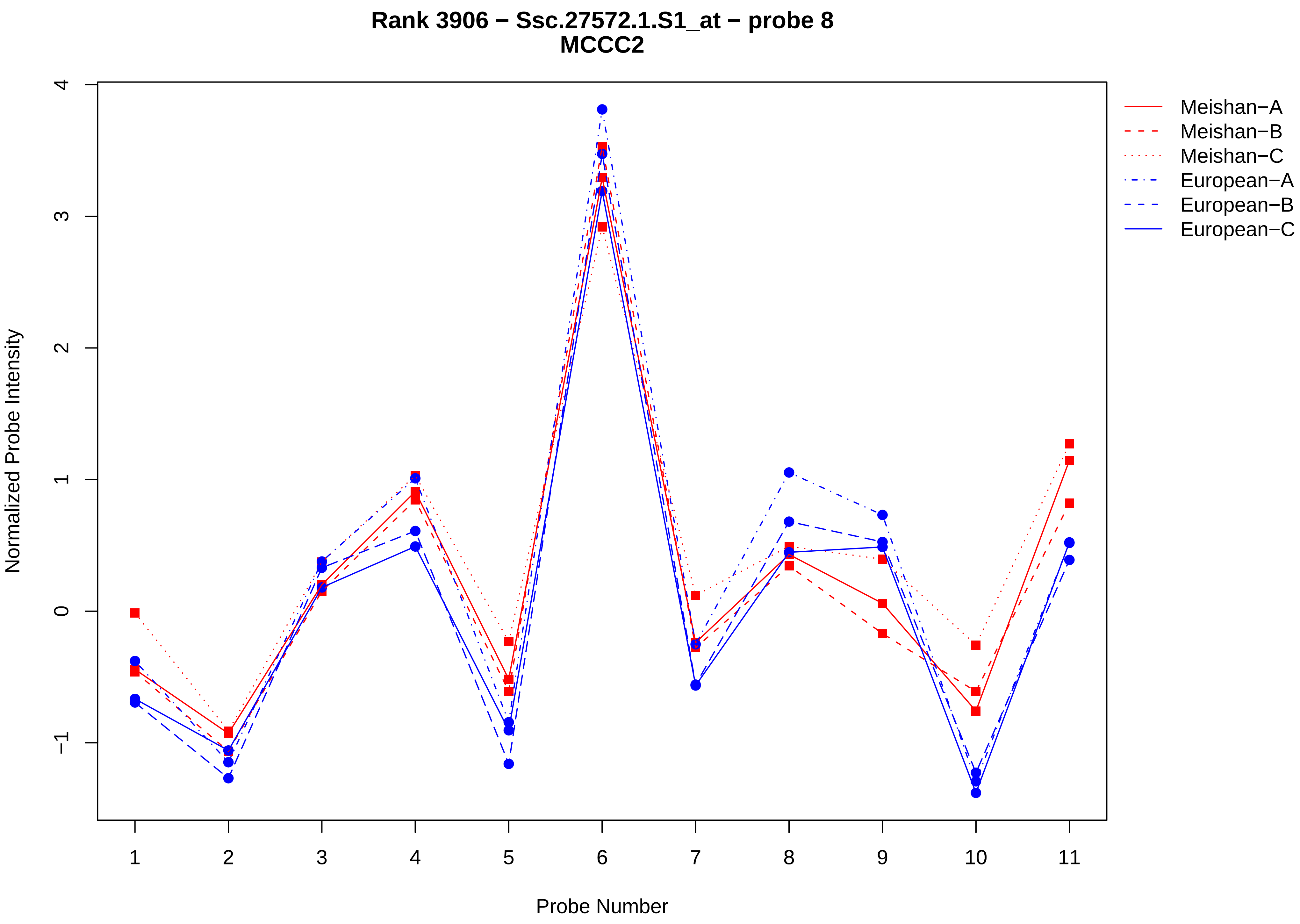

Rank 3906 − Ssc.27572.1.S1_at − probe 8
MCCC2
Normalized Probe Intensity
Probe Number
−1
0
1
2
3
4
1
2
3
4
5
6
7
8
9
10
11
Meishan−A
Meishan−B
Meishan−C
European−A
European−B
European−C

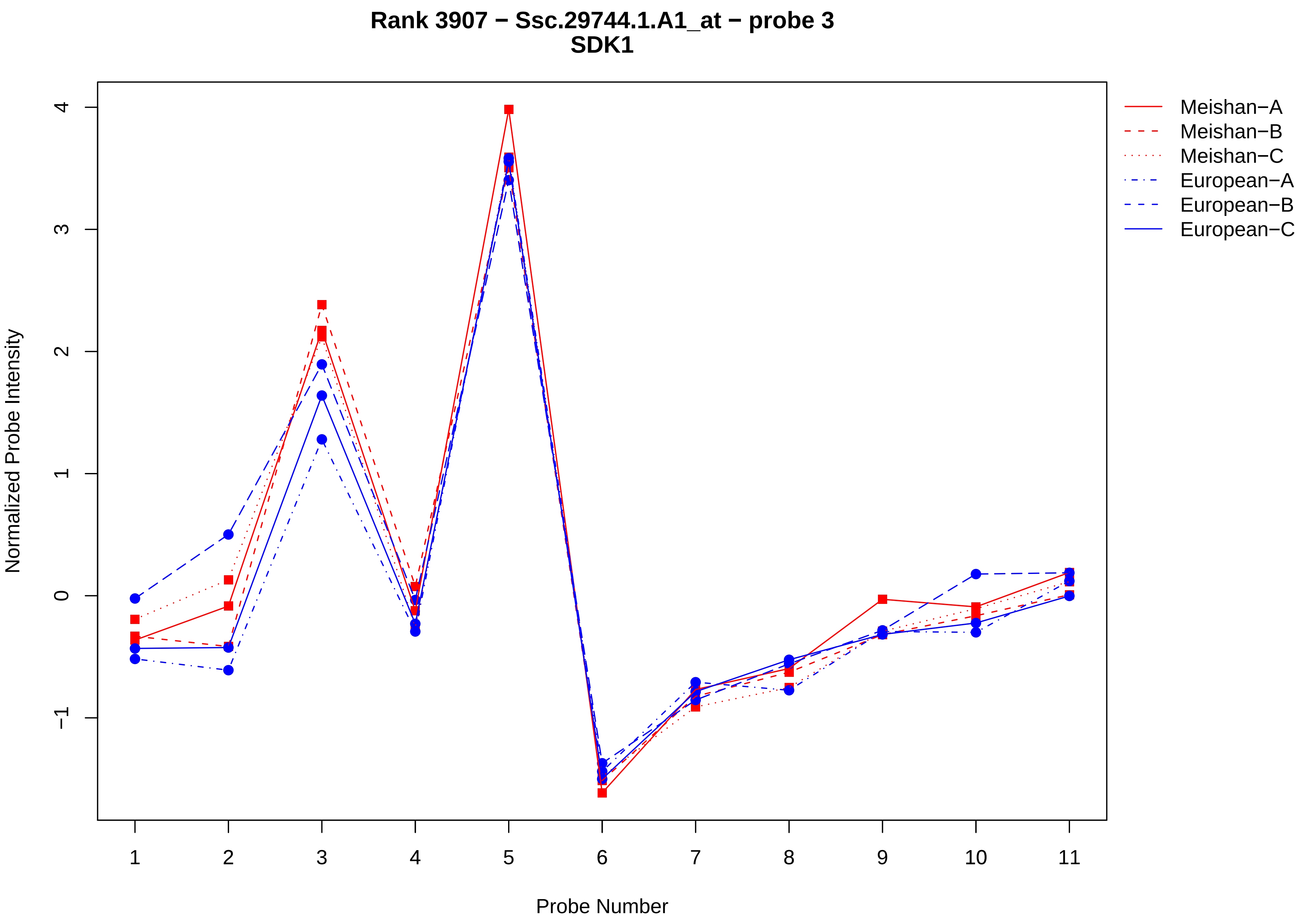

Rank 3907 − Ssc.29744.1.A1_at − probe 3
SDK1
Normalized Probe Intensity
Probe Number
−1
0
1
2
3
4
1
2
3
4
5
6
7
8
9
10
11
Meishan−A
Meishan−B
Meishan−C
European−A
European−B
European−C

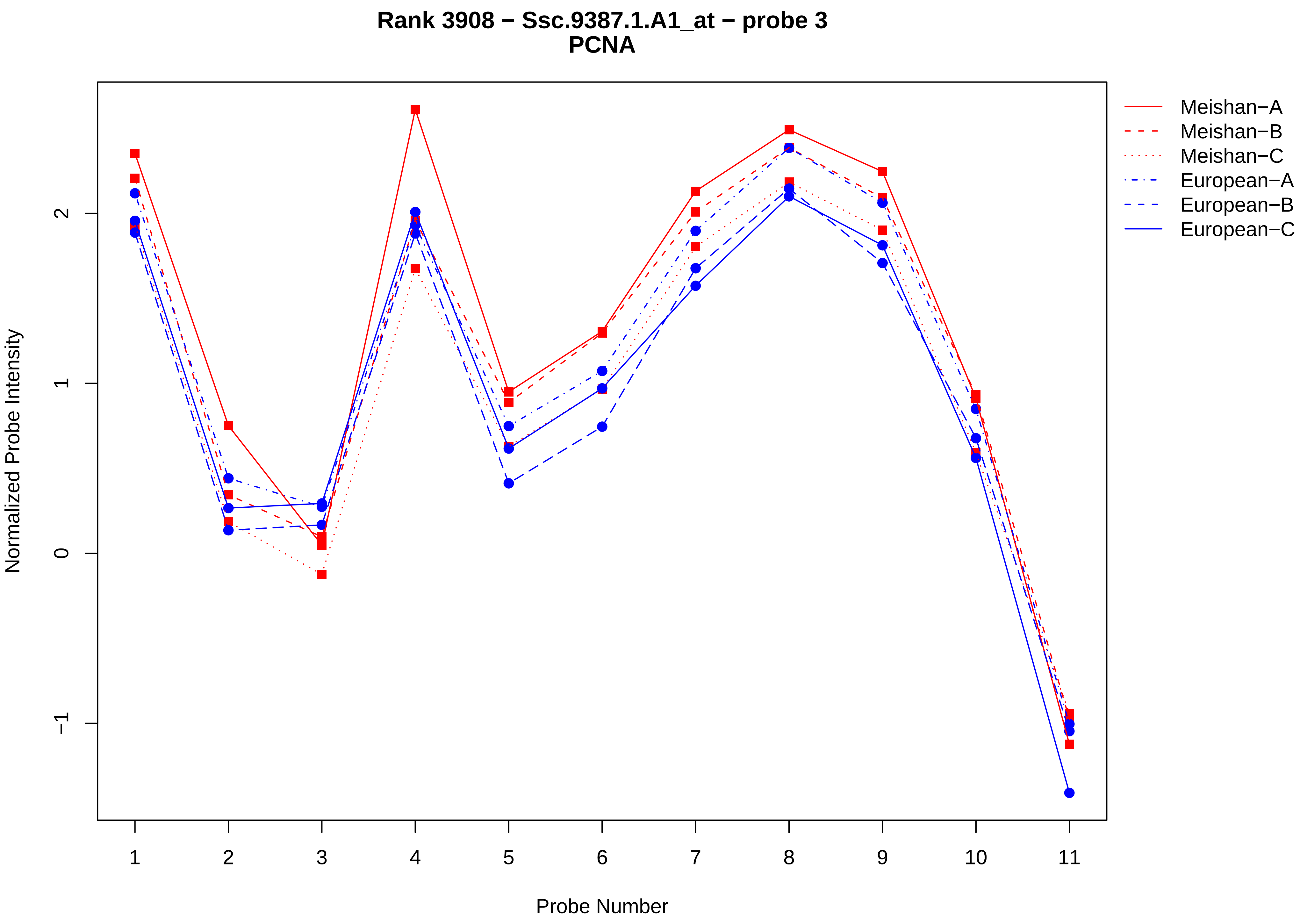

Rank 3908 − Ssc.9387.1.A1_at − probe 3
PCNA
Meishan−A
Meishan−B
Meishan−C
European−A
European−B
European−C
Normalized Probe Intensity
Probe Number
−1
0
1
2
1
2
3
4
5
6
7
8
9
10
11

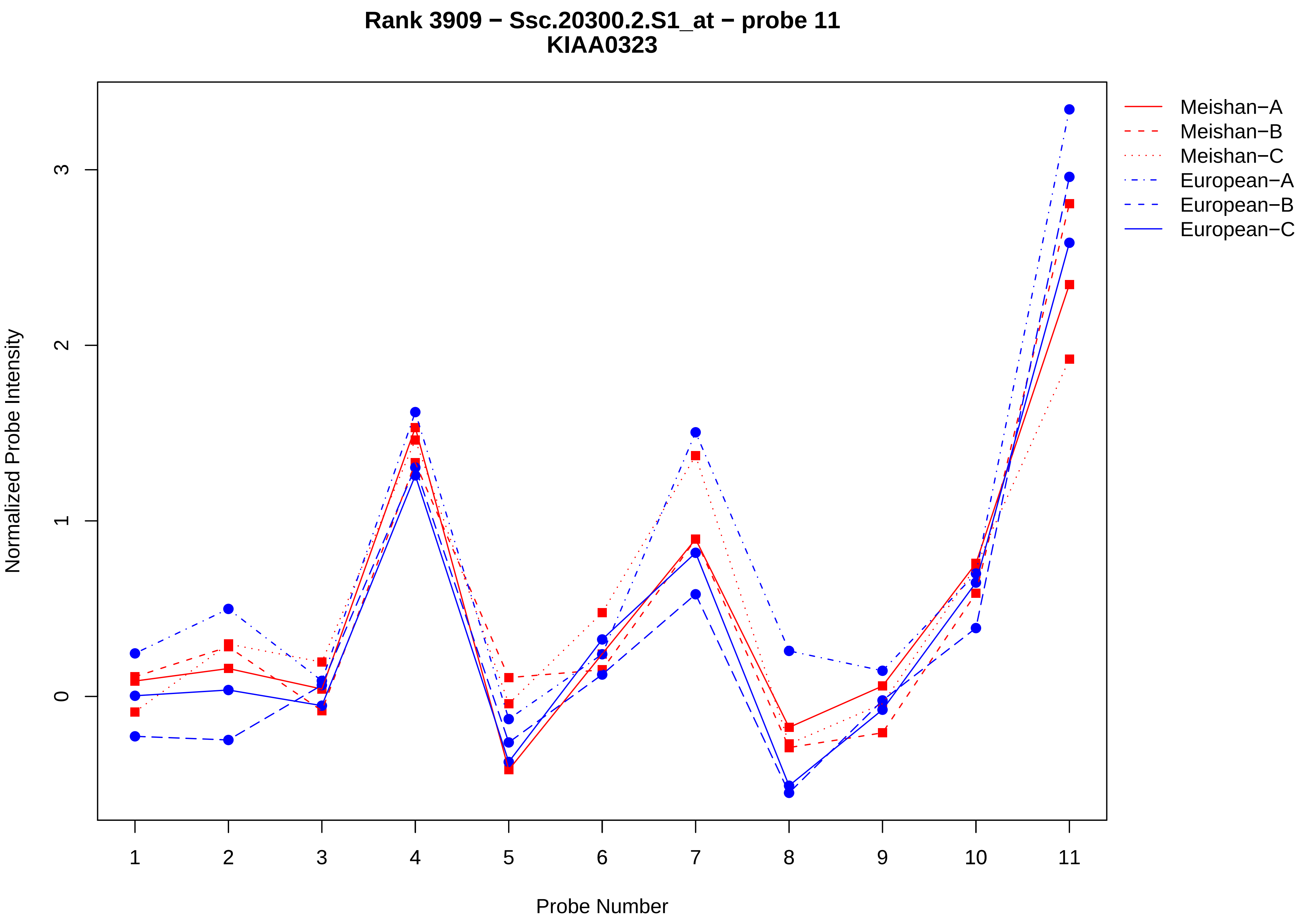

Rank 3909 − Ssc.20300.2.S1_at − probe 11
KIAA0323
Normalized Probe Intensity
Probe Number
0
1
2
3
1
2
3
4
5
6
7
8
9
10
11
Meishan−A
Meishan−B
Meishan−C
European−A
European−B
European−C

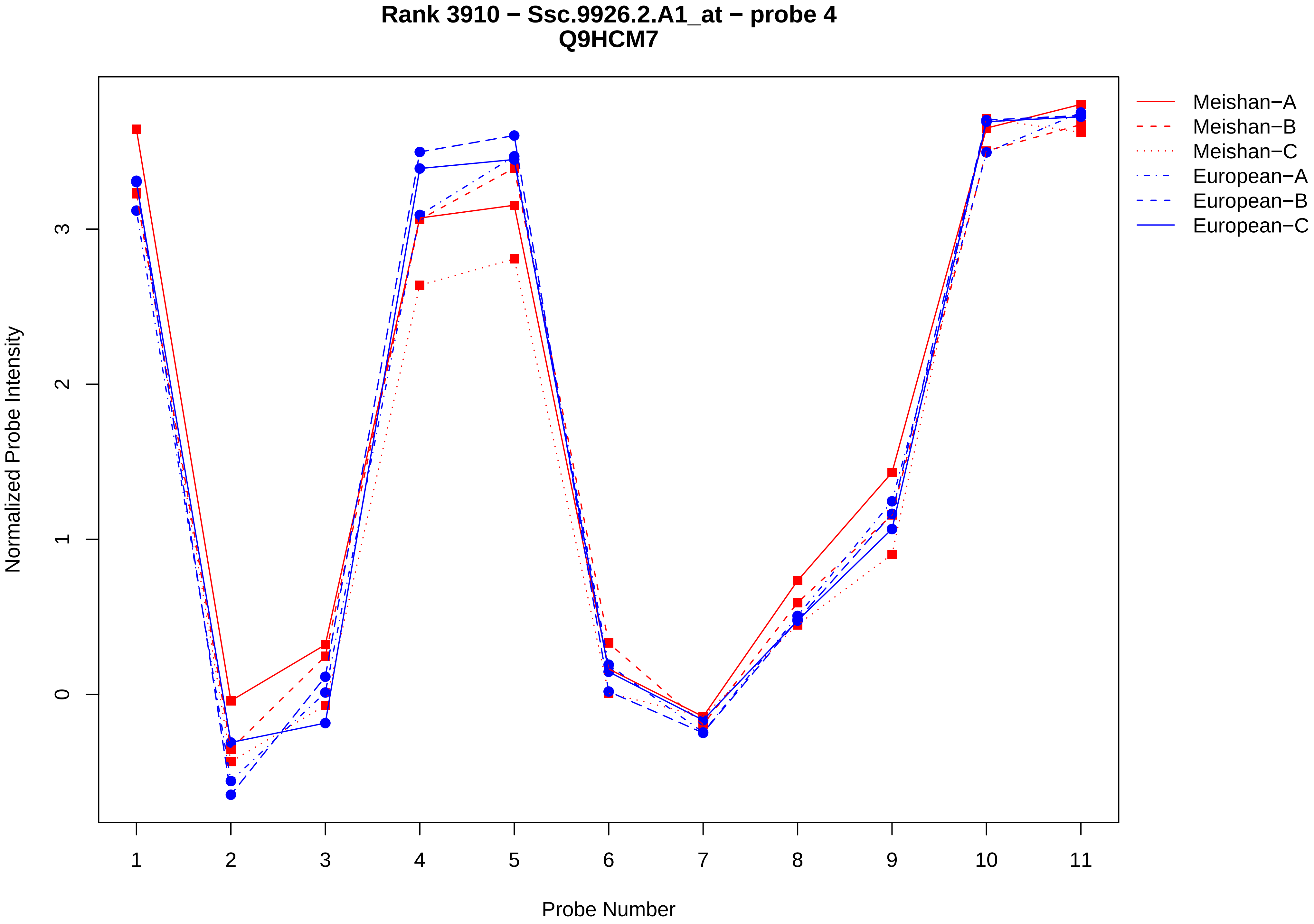

Rank 3910 − Ssc.9926.2.A1_at − probe 4
Q9HCM7
Normalized Probe Intensity
Probe Number
0
1
2
3
1
2
3
4
5
6
7
8
9
10
11
Meishan−A
Meishan−B
Meishan−C
European−A
European−B
European−C

Rank 3911 − Ssc.23296.1.S1_at − probe 8
NP_787050

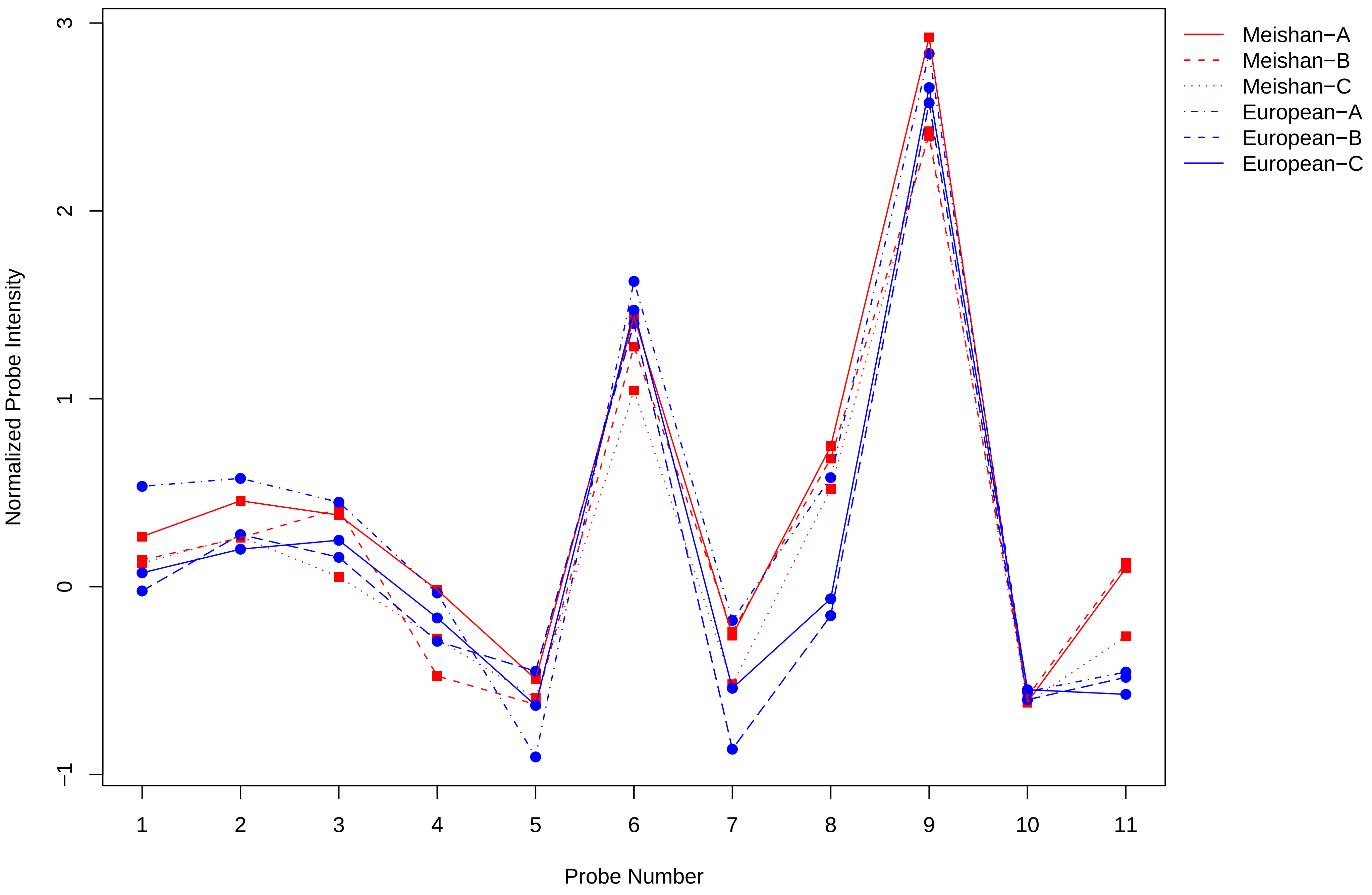

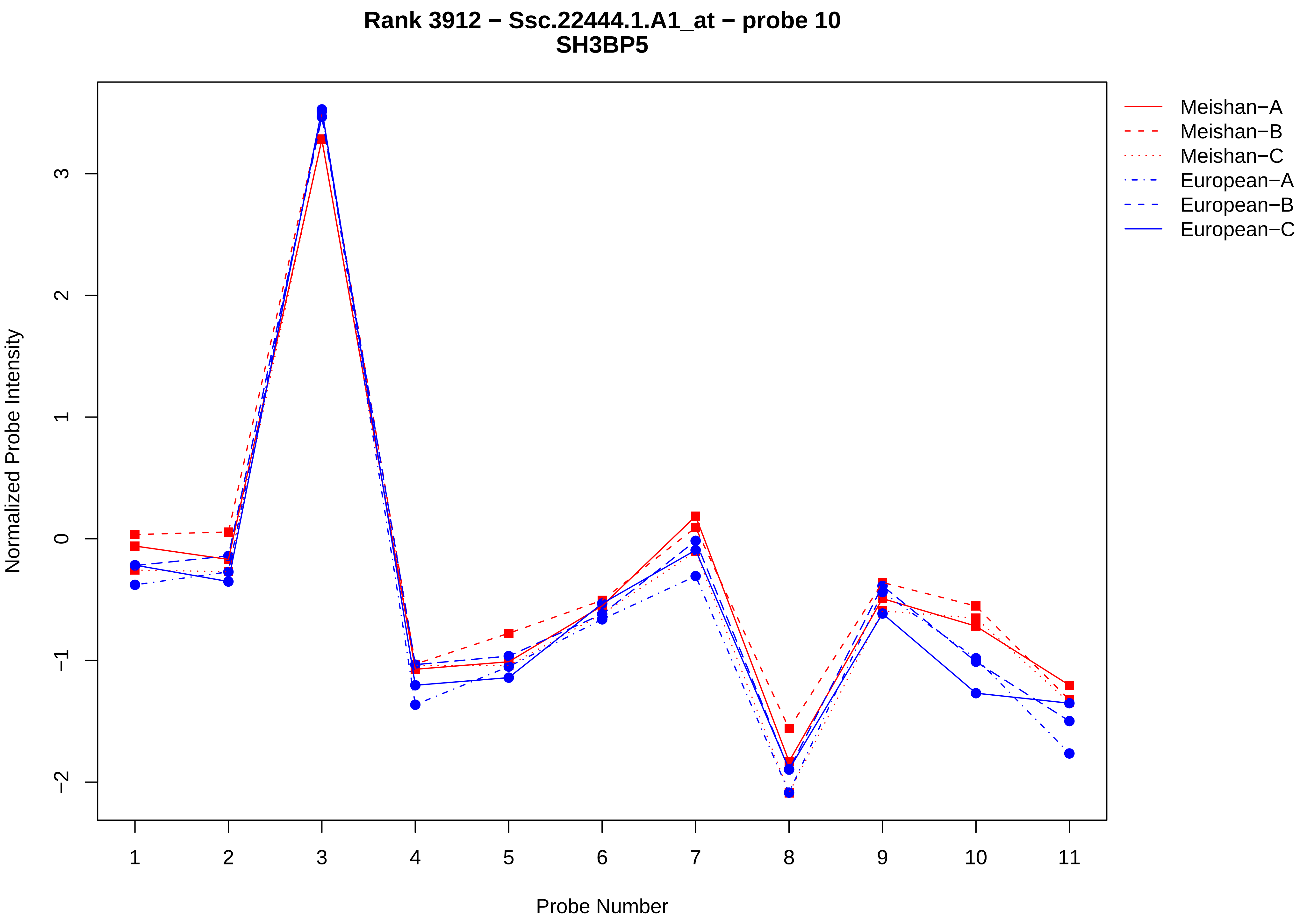
Rank 3912 − Ssc.22444.1.A1_at − probe 10
SH3BP5
Normalized Probe Intensity
Probe Number
3
2
1
0
−1
−2
1
2
3
4
5
6
7
8
9
10
11
Meishan−A
Meishan−B
Meishan−C
European−A
European−B
European−C

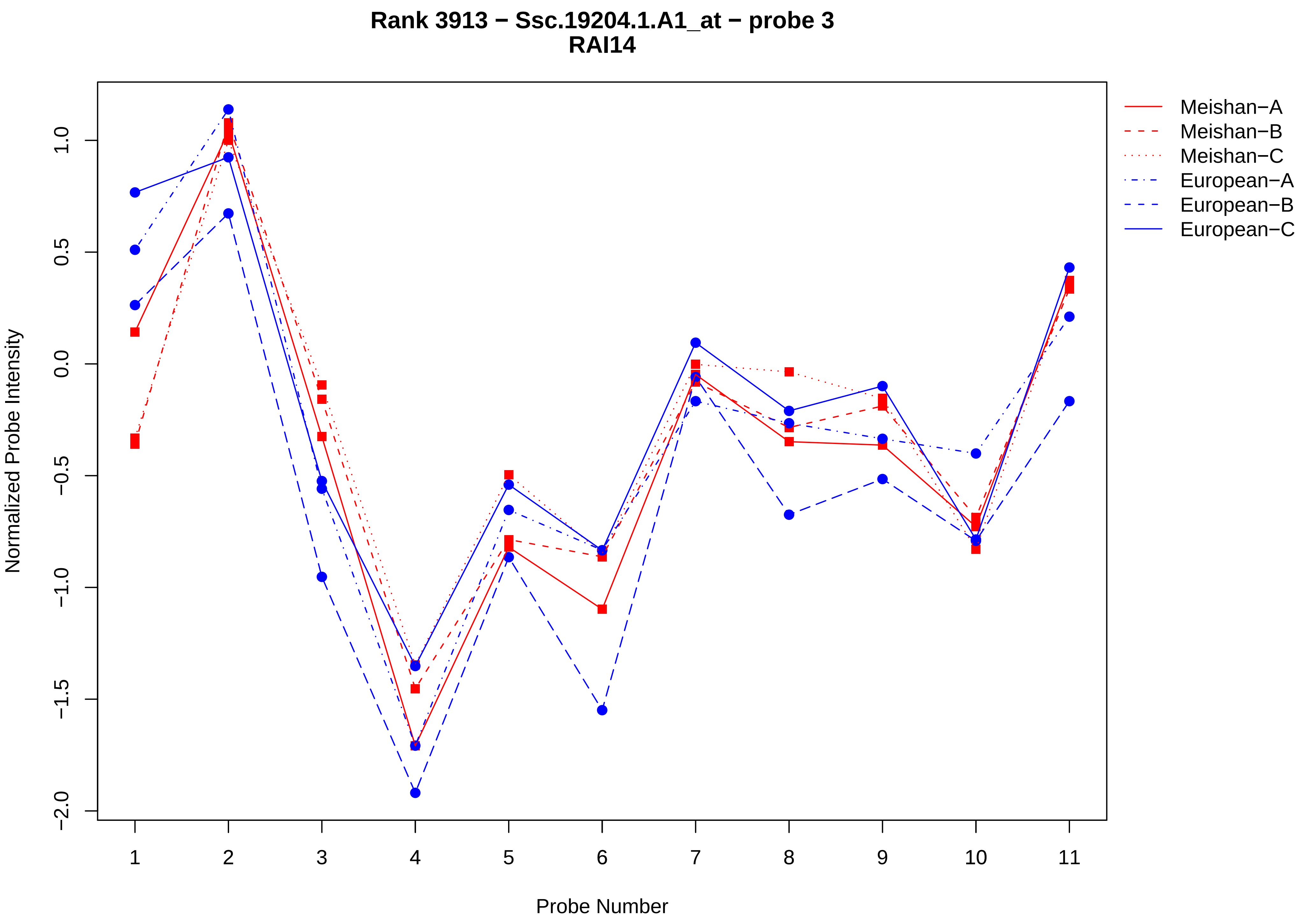
Rank 3913 − Ssc.19204.1.A1_at − probe 3
RAI14
Normalized Probe Intensity
Probe Number
Meishan−A
Meishan−B
Meishan−C
European−A
European−B
European−C
1.0
0.5
0.0
−0.5
−1.0
−1.5
−2.0
1
2
3
4
5
6
7
8
9
10
11

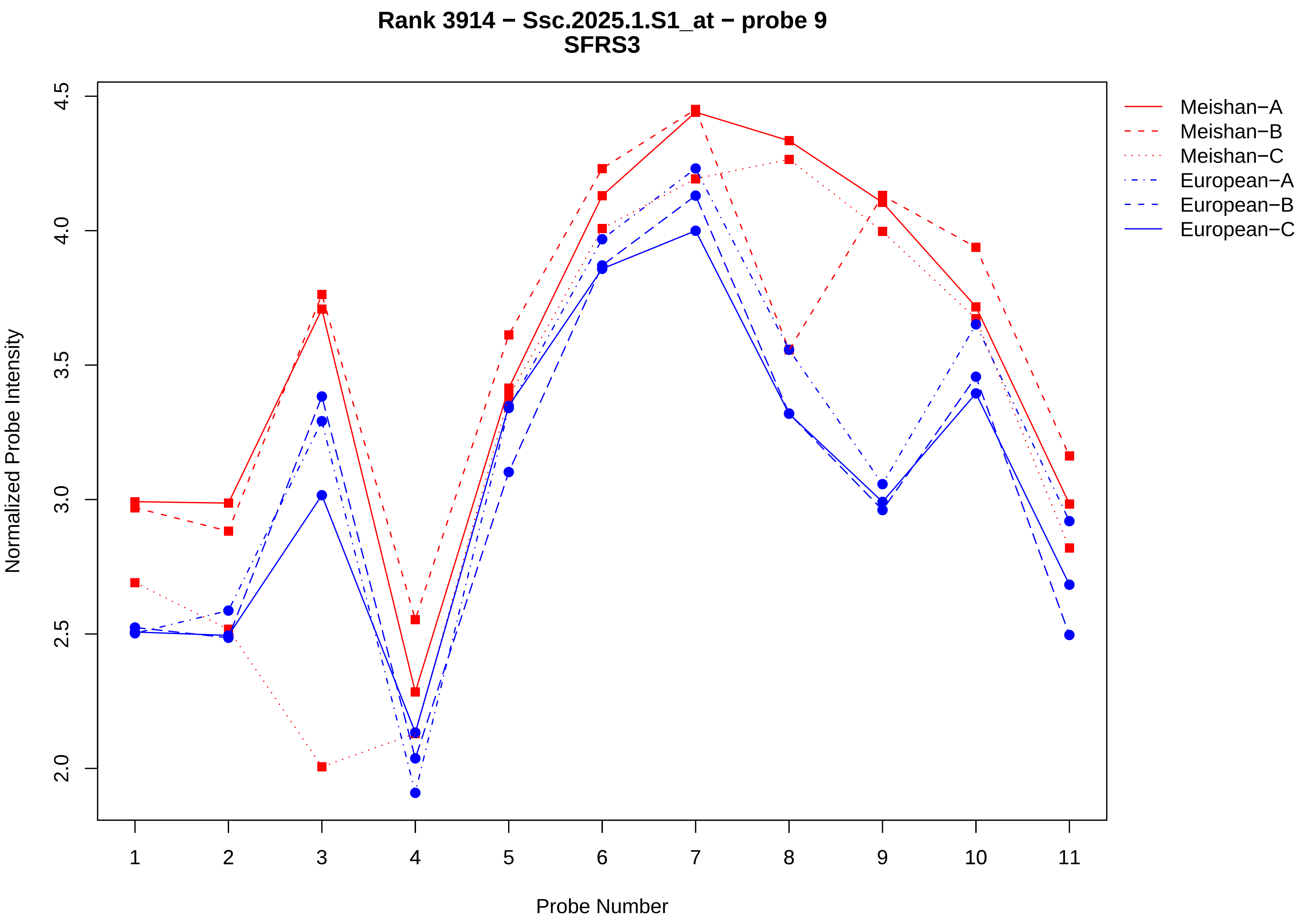

Rank 3914 − Ssc.2025.1.S1_at − probe 9
SFRS3
Normalized Probe Intensity
Probe Number
2.0
2.5
3.0
3.5
4.0
4.5
1
2
3
4
5
6
7
8
9
10
11
Meishan−A
Meishan−B
Meishan−C
European−A
European−B
European−C

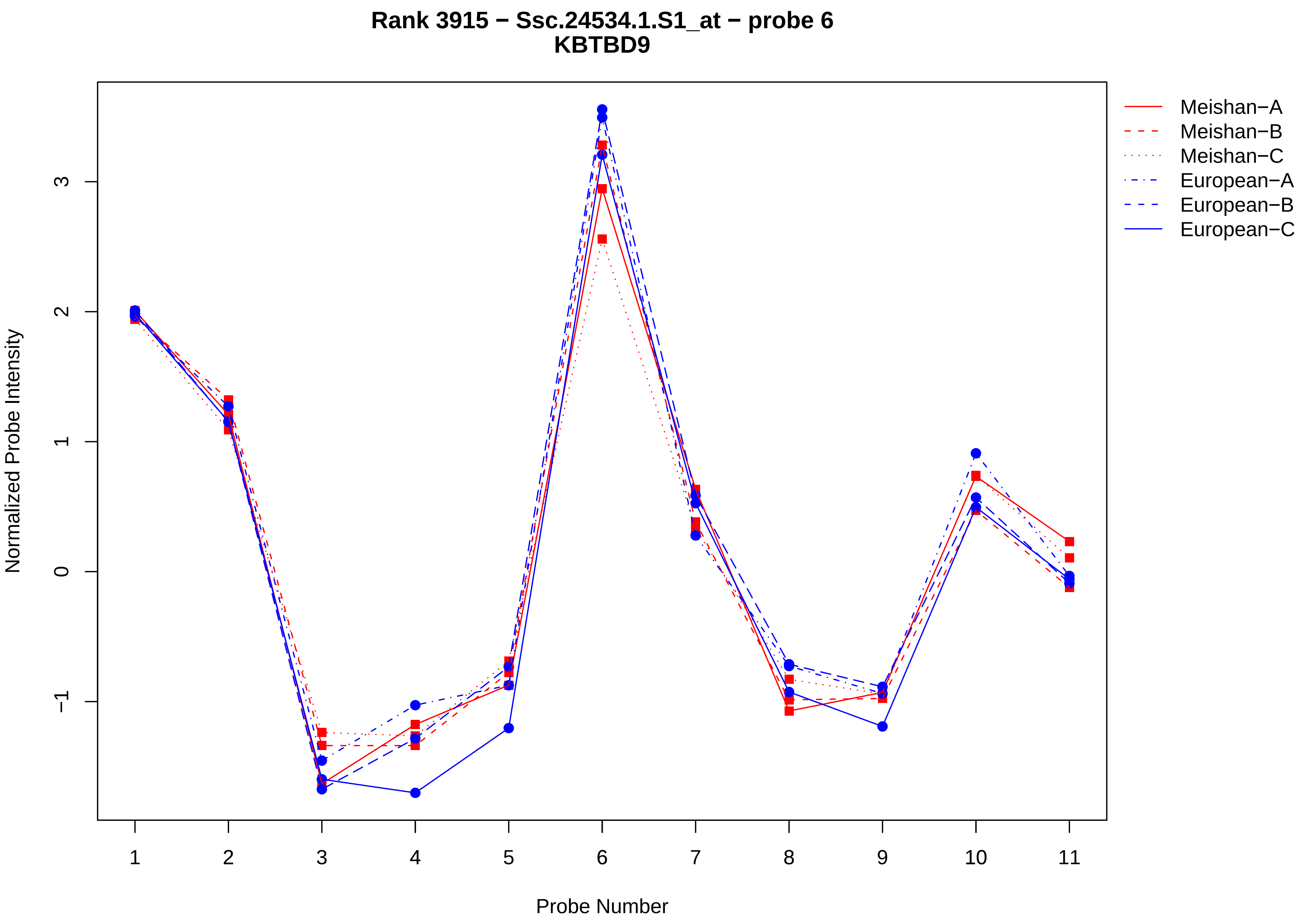
Rank 3915 − Ssc.24534.1.S1_at − probe 6
KBTBD9
Normalized Probe Intensity
Probe Number
Meishan−A
Meishan−B
Meishan−C
European−A
European−B
European−C
−1
0
1
2
3
1
2
3
4
5
6
7
8
9
10
11

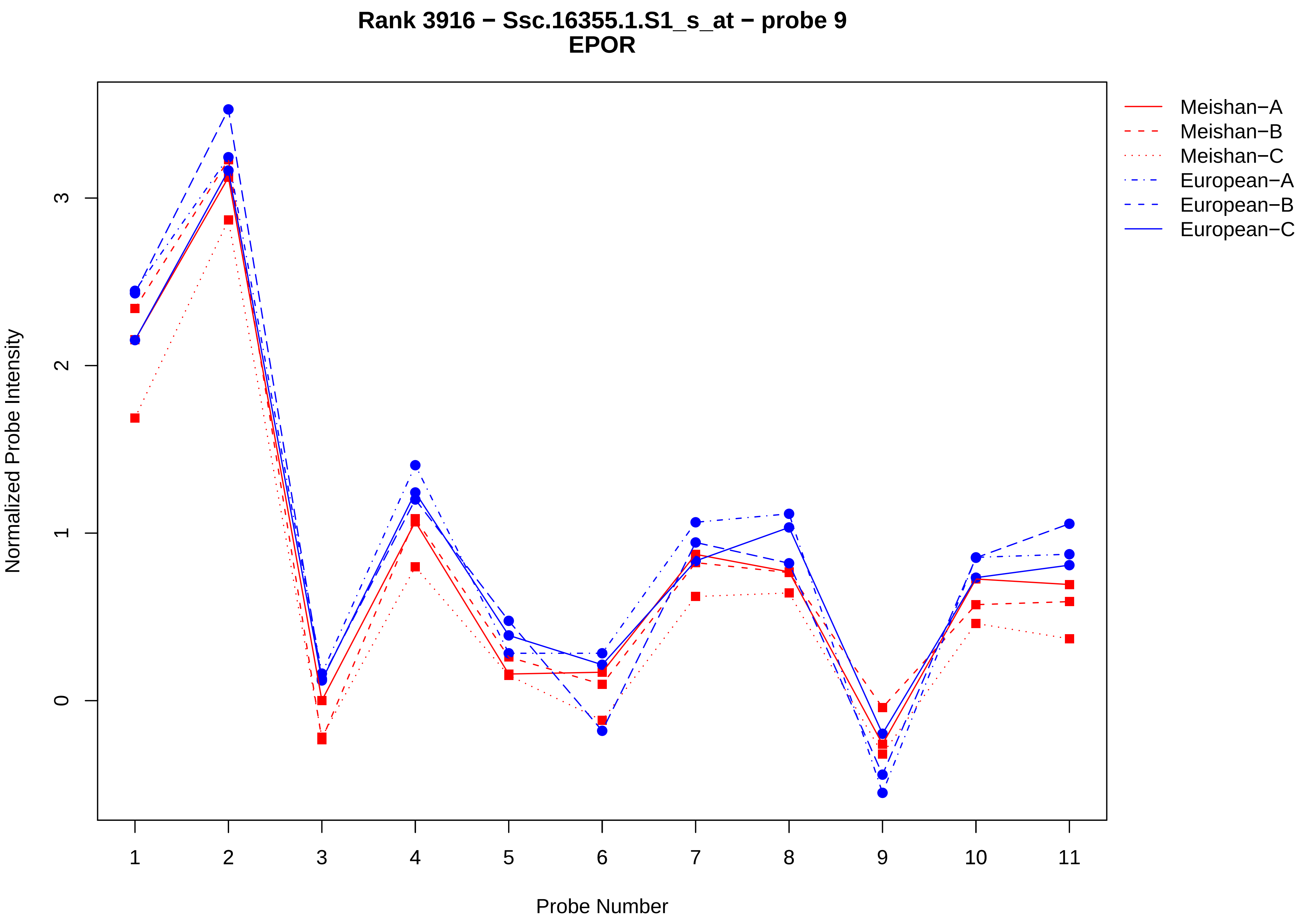

Rank 3916 − Ssc.16355.1.S1_s_at − probe 9
EPOR
Normalized Probe Intensity
Probe Number
0
1
2
3
1
2
3
4
5
6
7
8
9
10
11
Meishan−A
Meishan−B
Meishan−C
European−A
European−B
European−C

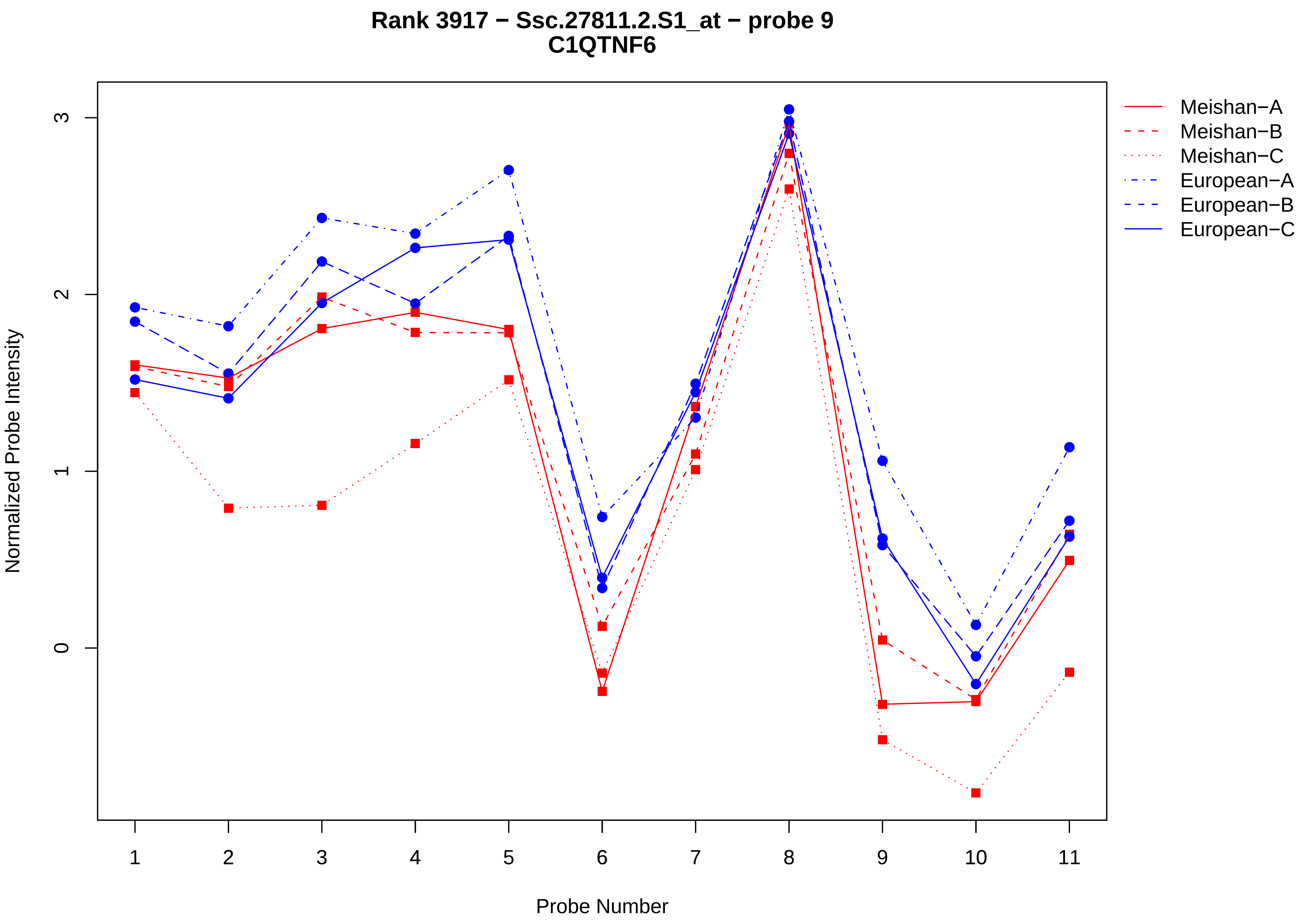
Rank 3917 − Ssc.27811.2.S1_at − probe 9
C1QTNF6
Normalized Probe Intensity
Probe Number
Meishan−A
Meishan−B
Meishan−C
European−A
European−B
European−C

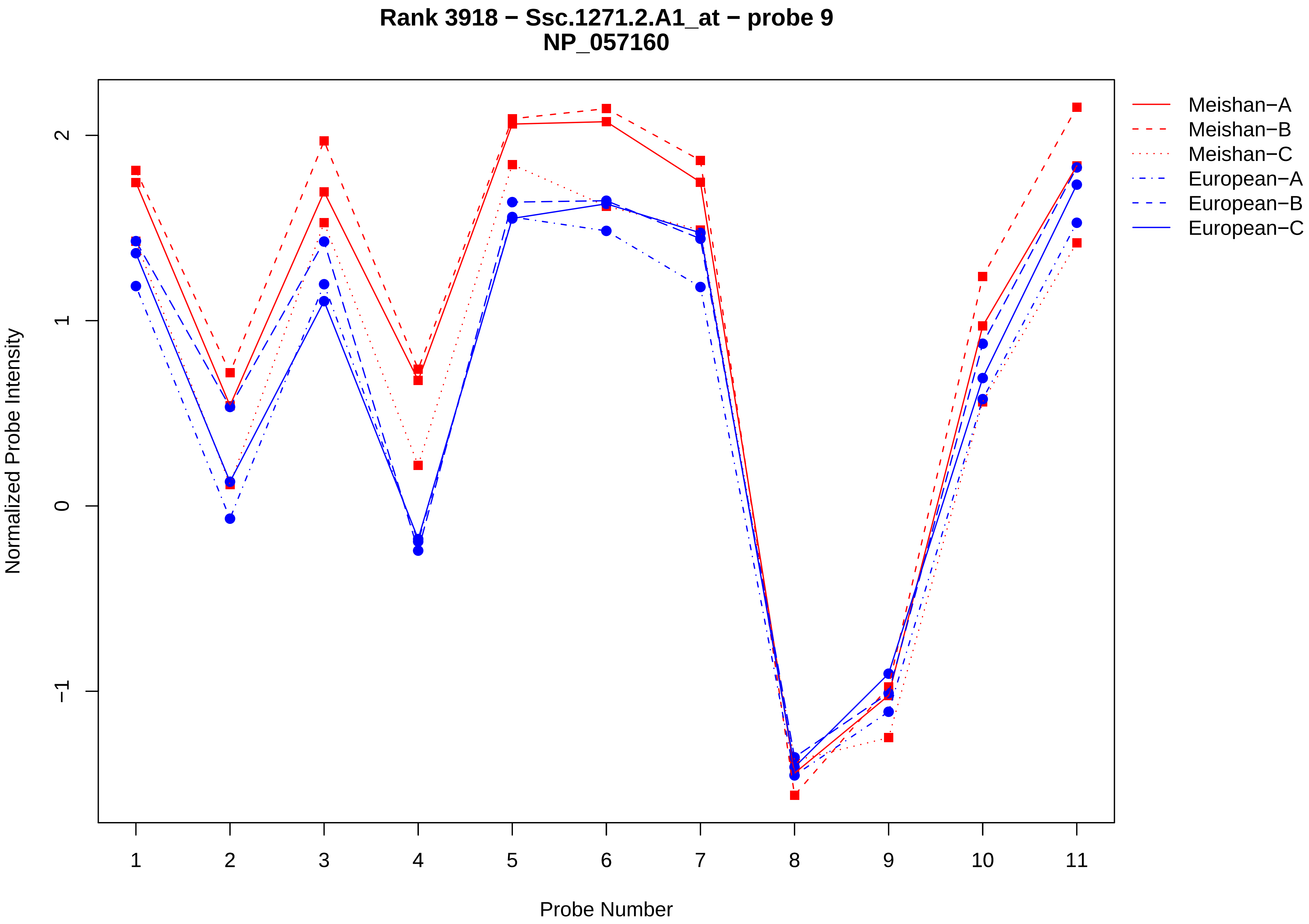
Rank 3918 − Ssc.1271.2.A1_at − probe 9
NP_057160
Normalized Probe Intensity
Probe Number
−1
0
1
2
1
2
3
4
5
6
7
8
9
10
11
Meishan−A
Meishan−B
Meishan−C
European−A
European−B
European−C

Rank 3919 − Ssc.15711.1.S1_at − probe 7
PRAF2

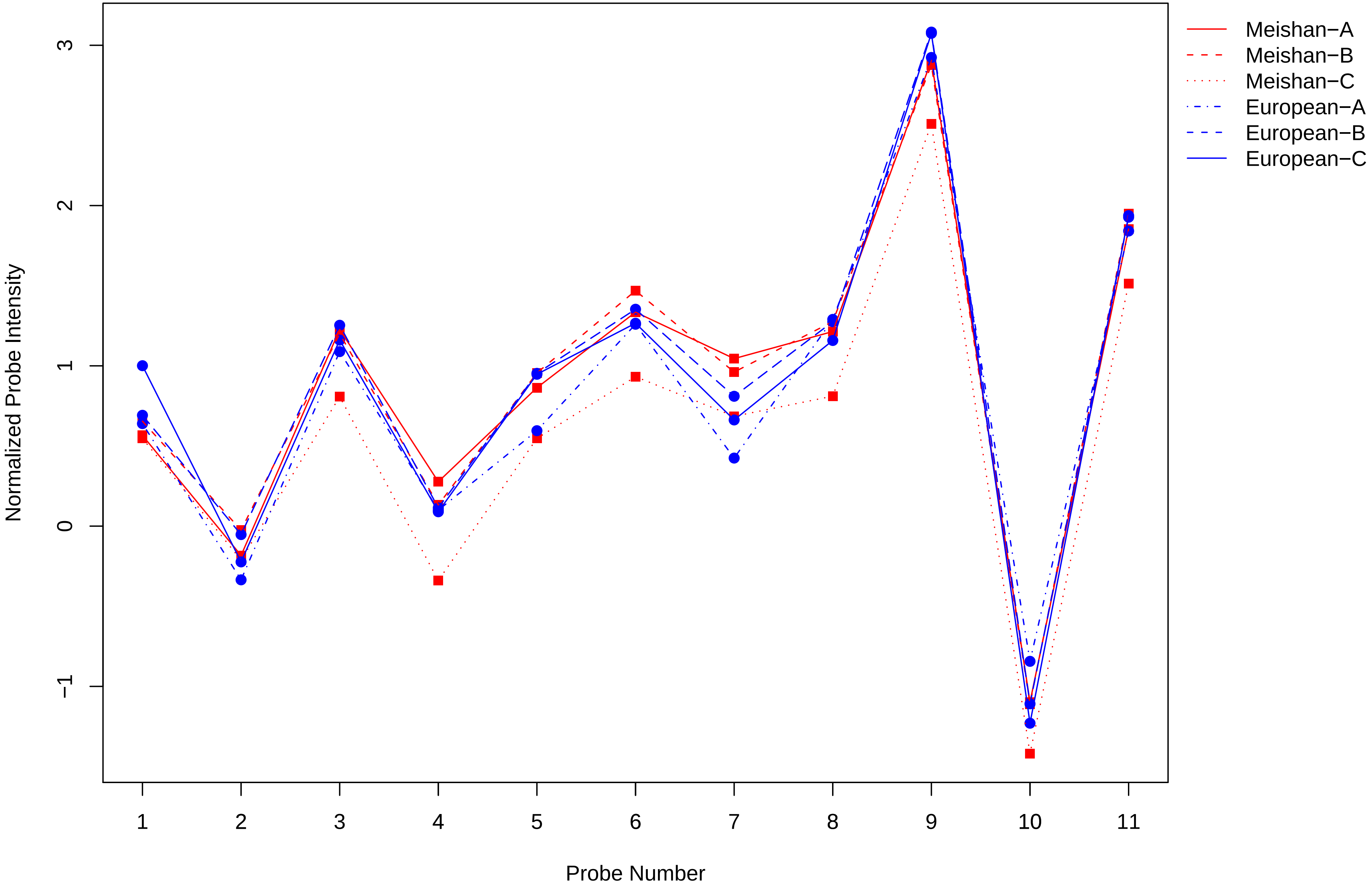

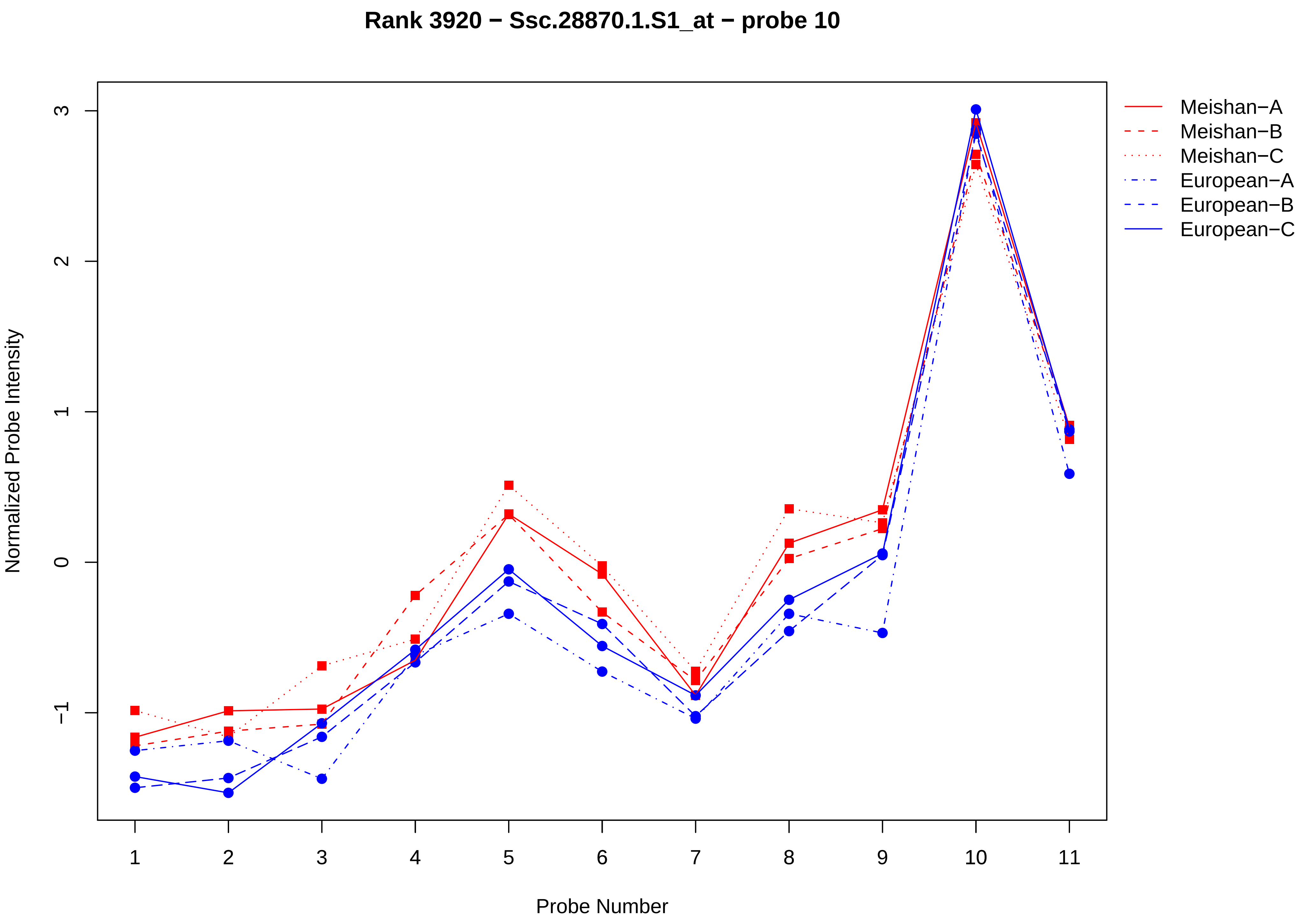

Rank 3920 − Ssc.28870.1.S1_at − probe 10
Normalized Probe Intensity
Probe Number
−1
0
1
2
3
1
2
3
4
5
6
7
8
9
10
11
Meishan−A
Meishan−B
Meishan−C
European−A
European−B
European−C

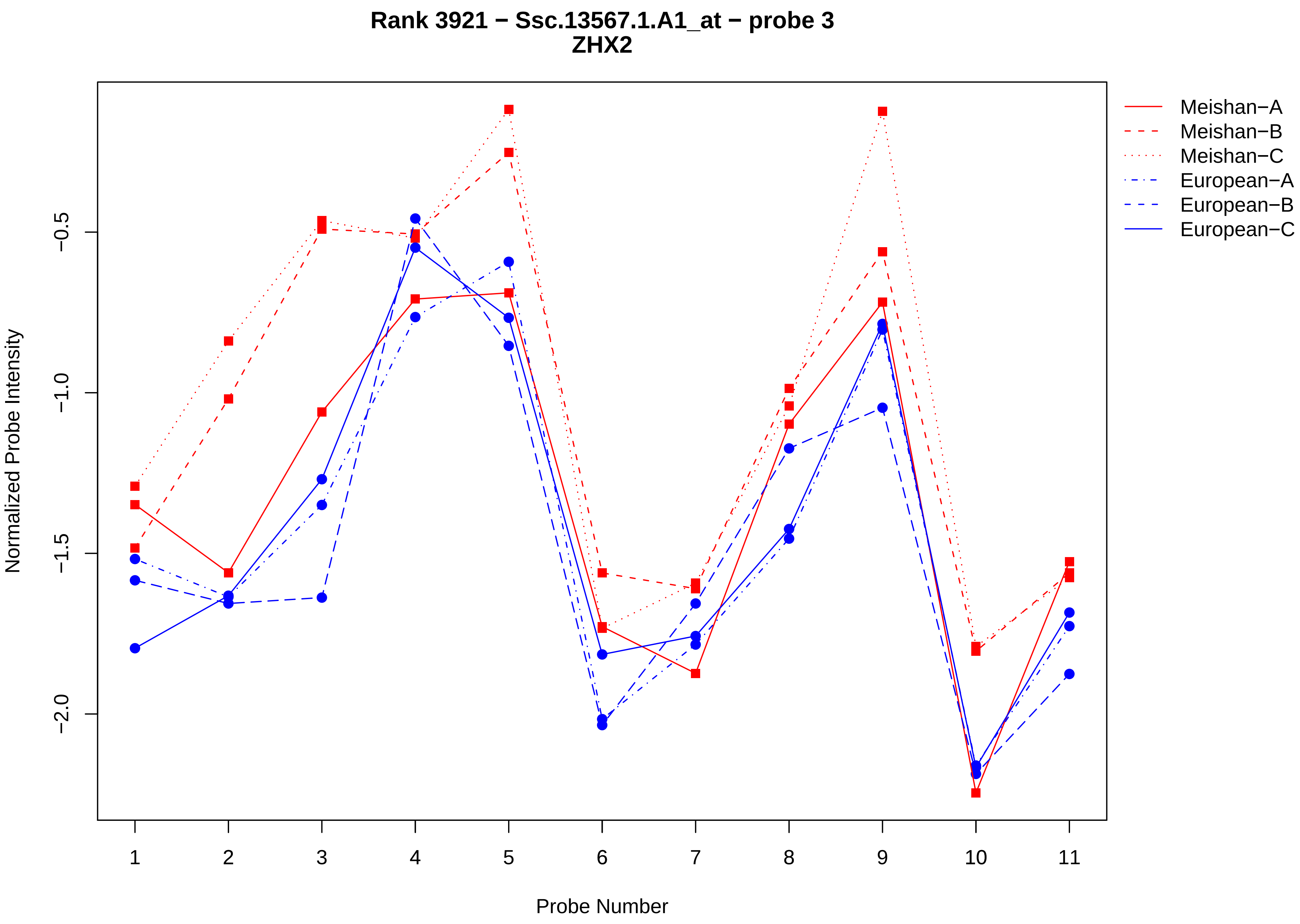

Rank 3921 − Ssc.13567.1.A1_at − probe 3
ZHX2
Normalized Probe Intensity
−0.5
−1.0
−1.5
−2.0
Probe Number
1
2
3
4
5
6
7
8
9
10
11
Meishan−A
Meishan−B
Meishan−C
European−A
European−B
European−C

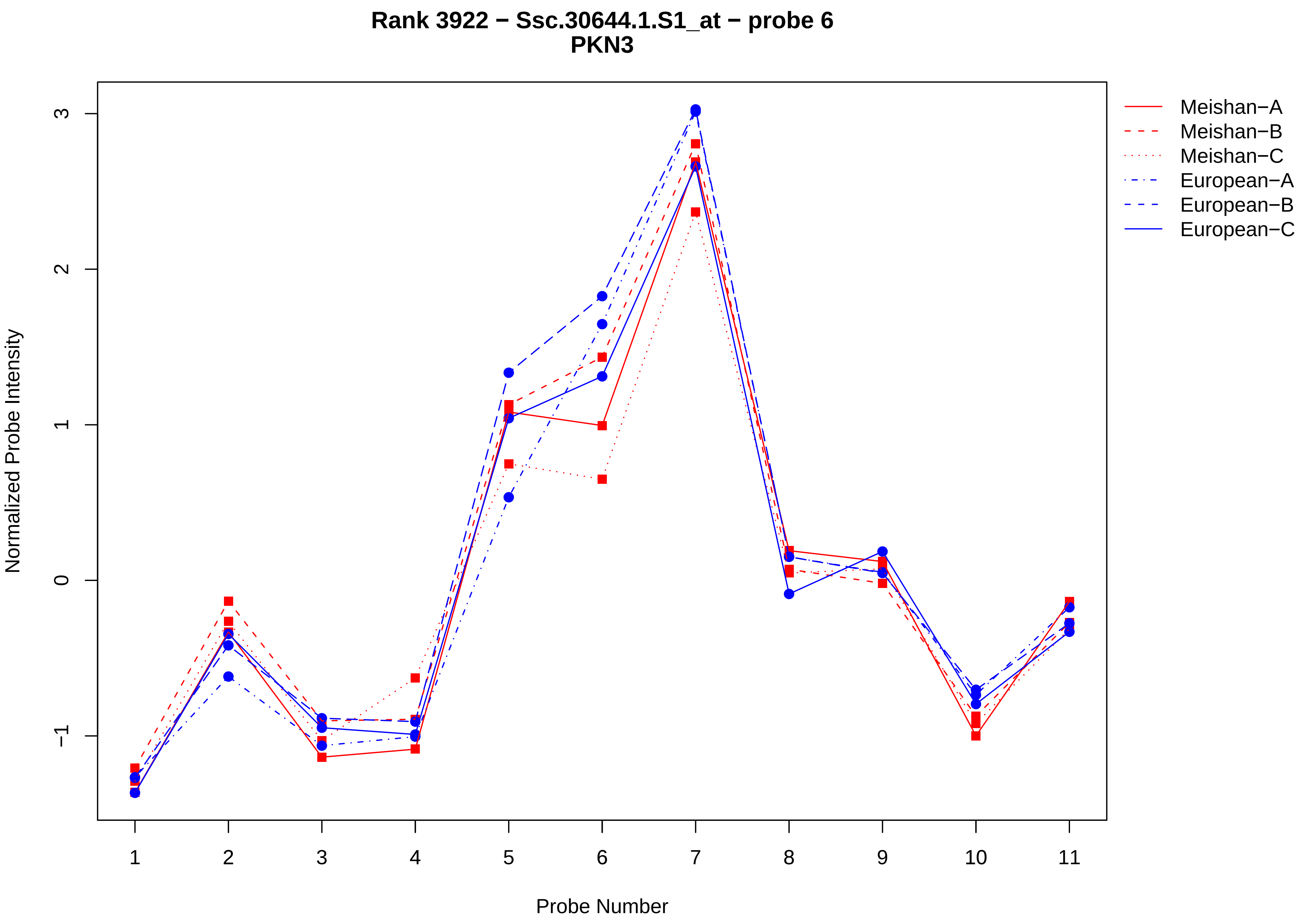

Rank 3922 − Ssc.30644.1.S1_at − probe 6
PKN3
Normalized Probe Intensity
Probe Number
Meishan−A
Meishan−B
Meishan−C
European−A
European−B
European−C

Rank 3923 − Ssc.26282.1.S1_a_at − probe 1
CMYA4

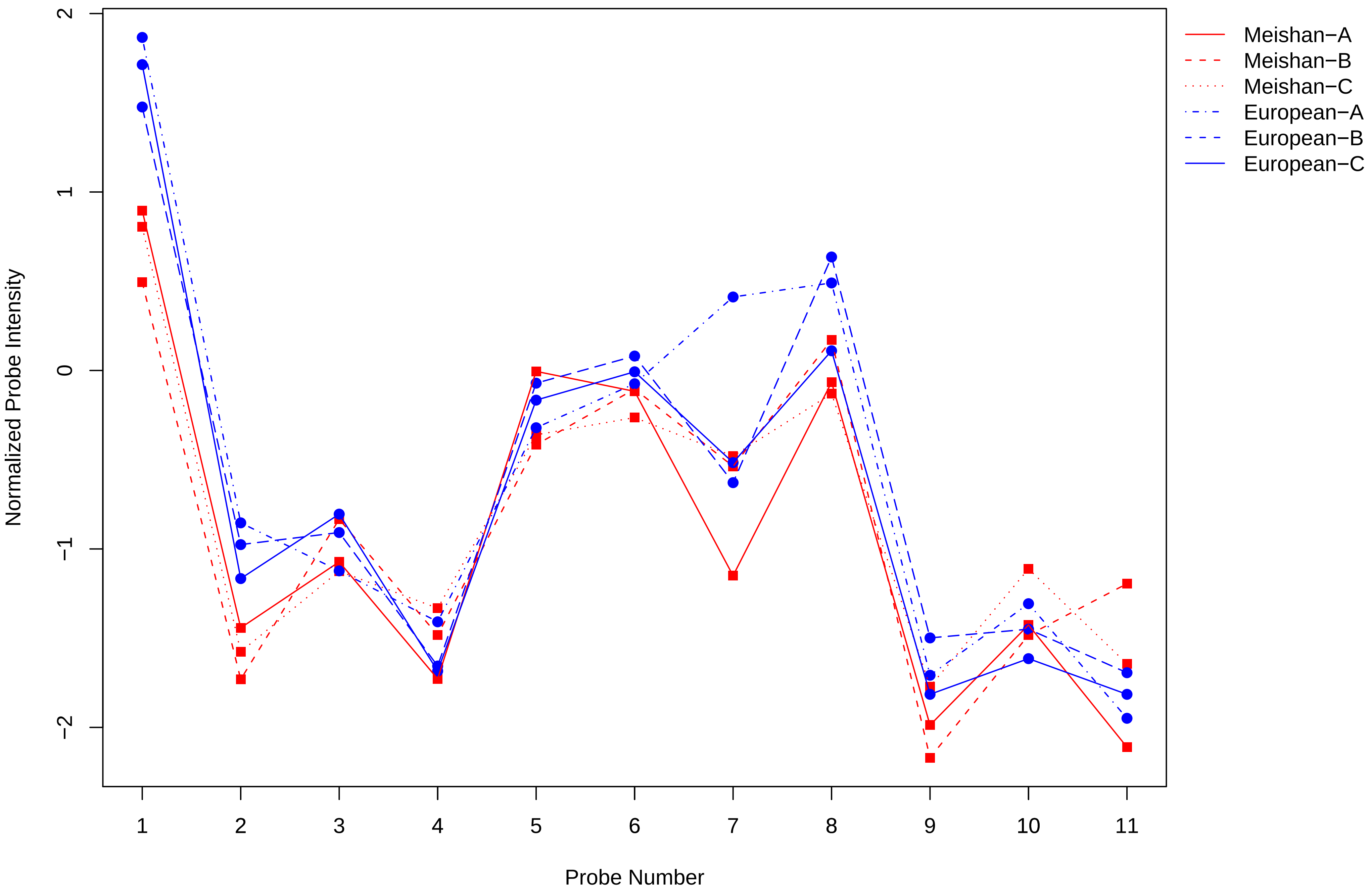

Rank 3924 − Ssc.13196.1.A1_at − probe 1
TAF2

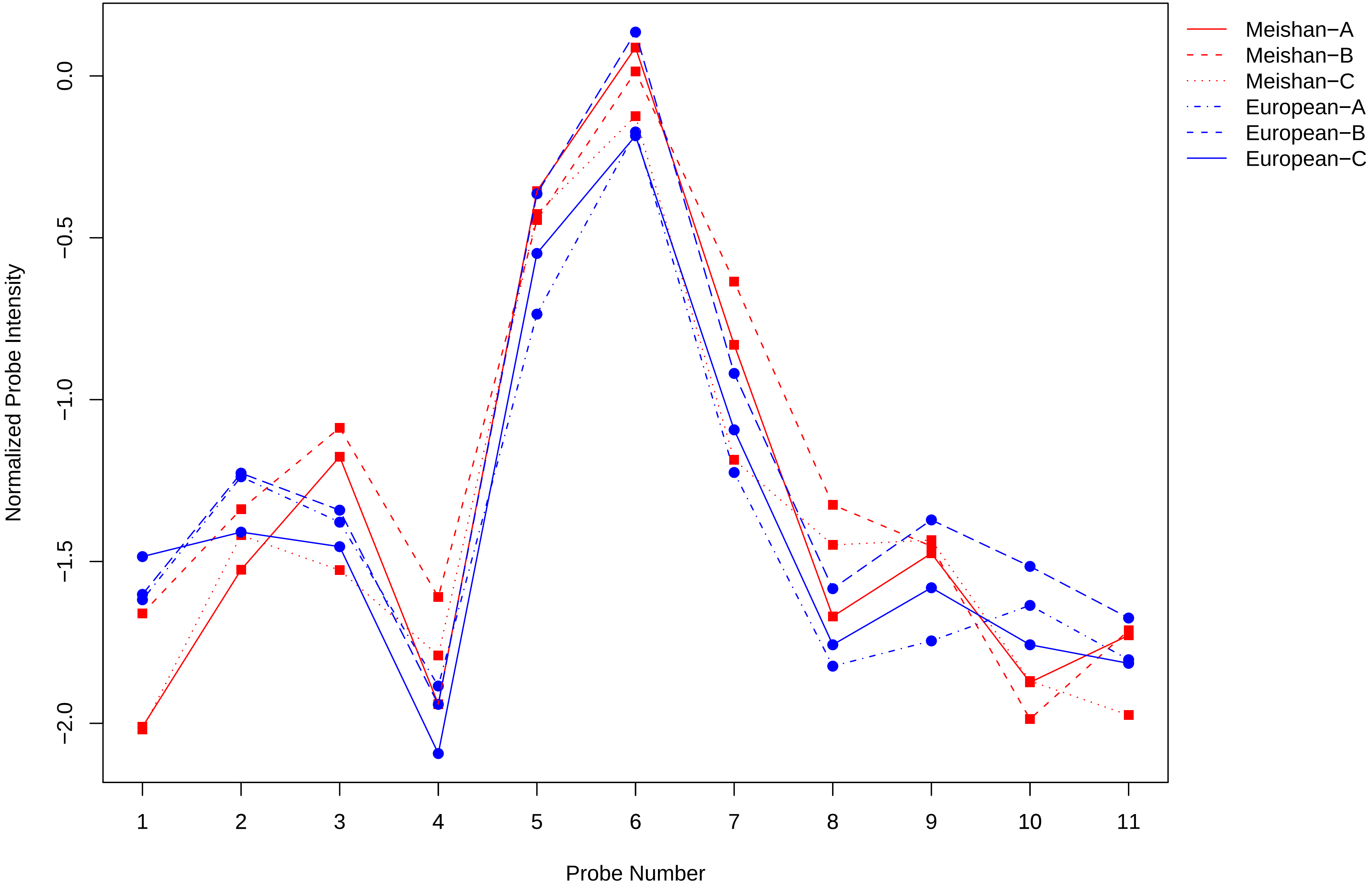

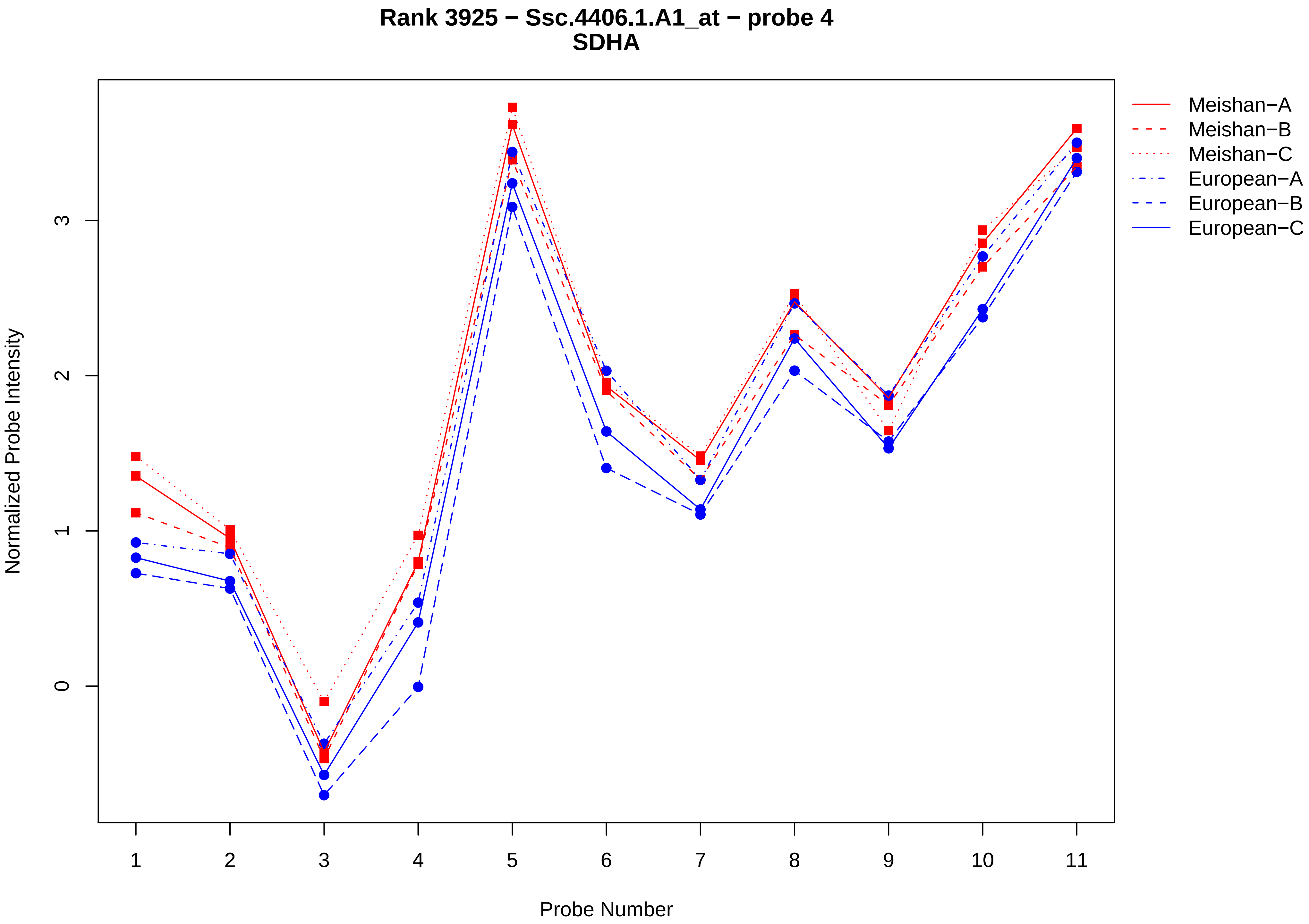

Rank 3925 − Ssc.4406.1.A1_at − probe 4
SDHA
Normalized Probe Intensity
Probe Number
0
1
2
3
1
2
3
4
5
6
7
8
9
10
11
Meishan−A
Meishan−B
Meishan−C
European−A
European−B
European−C

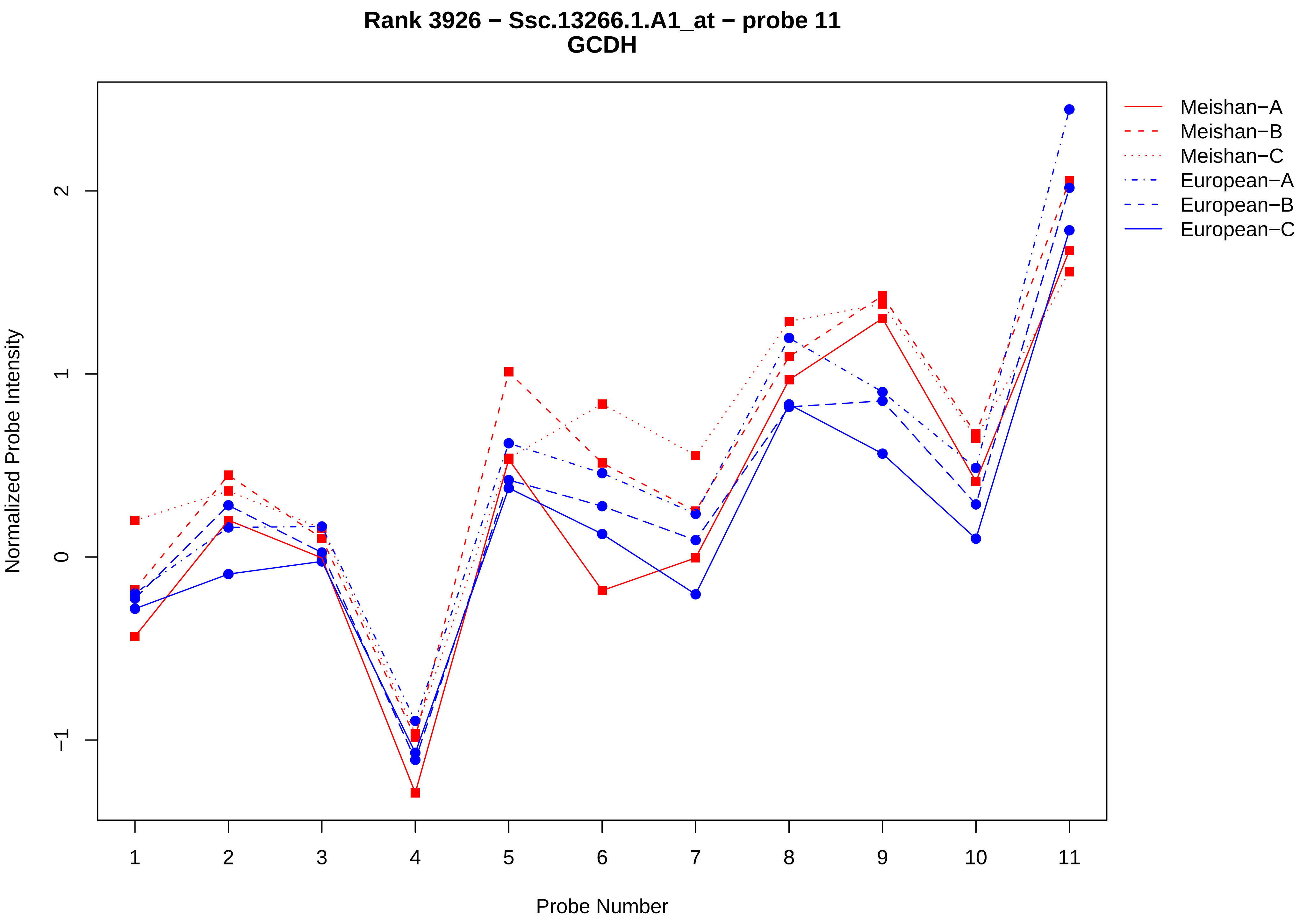
Rank 3926 − Ssc.13266.1.A1_at − probe 11
GCDH
Normalized Probe Intensity
Probe Number
−1
0
1
2
1
2
3
4
5
6
7
8
9
10
11
Meishan−A
Meishan−B
Meishan−C
European−A
European−B
European−C

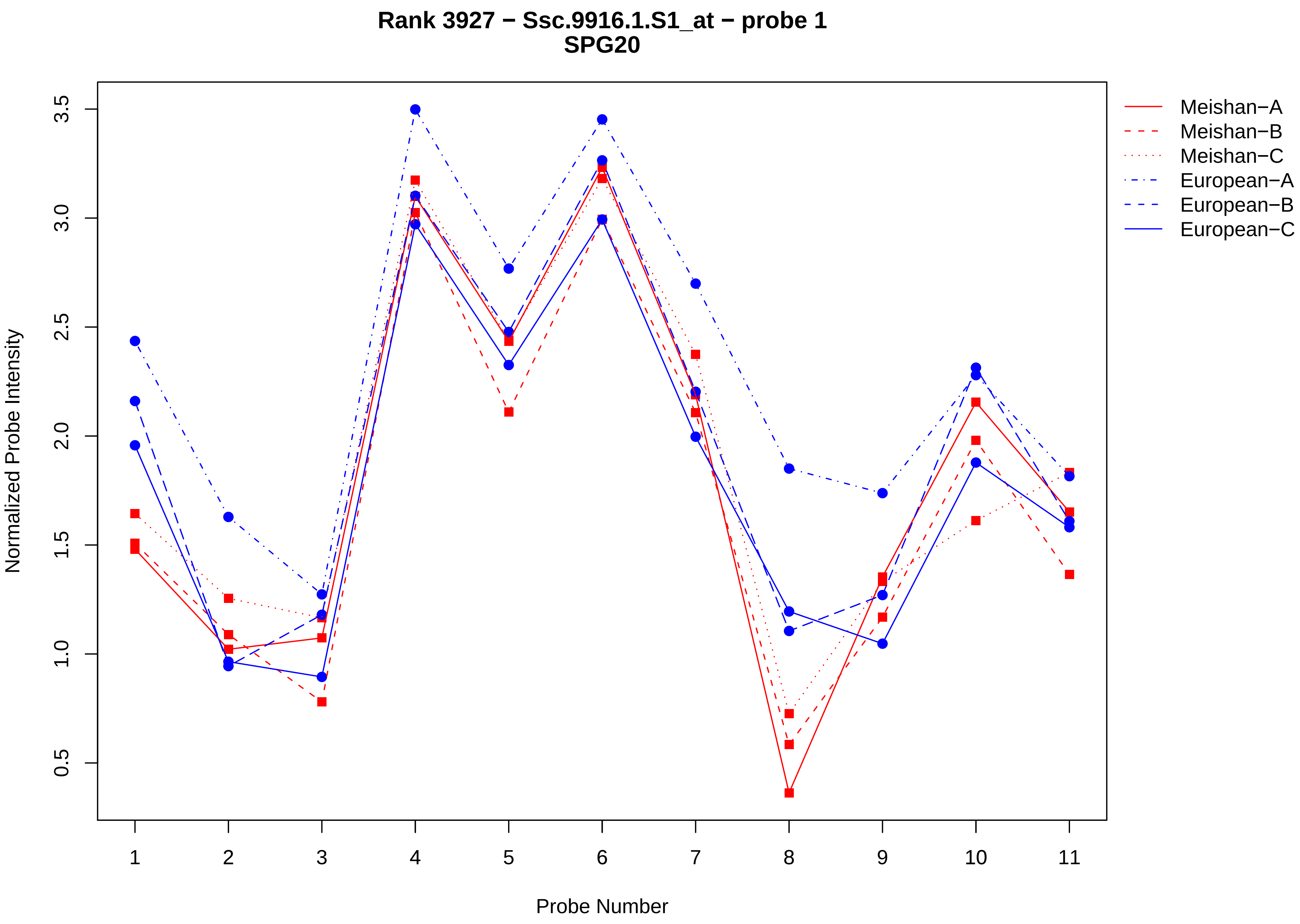

Rank 3927 − Ssc.9916.1.S1_at − probe 1
SPG20
Normalized Probe Intensity
Probe Number
0.5
1.0
1.5
2.0
2.5
3.0
3.5
1
2
3
4
5
6
7
8
9
10
11
Meishan−A
Meishan−B
Meishan−C
European−A
European−B
European−C

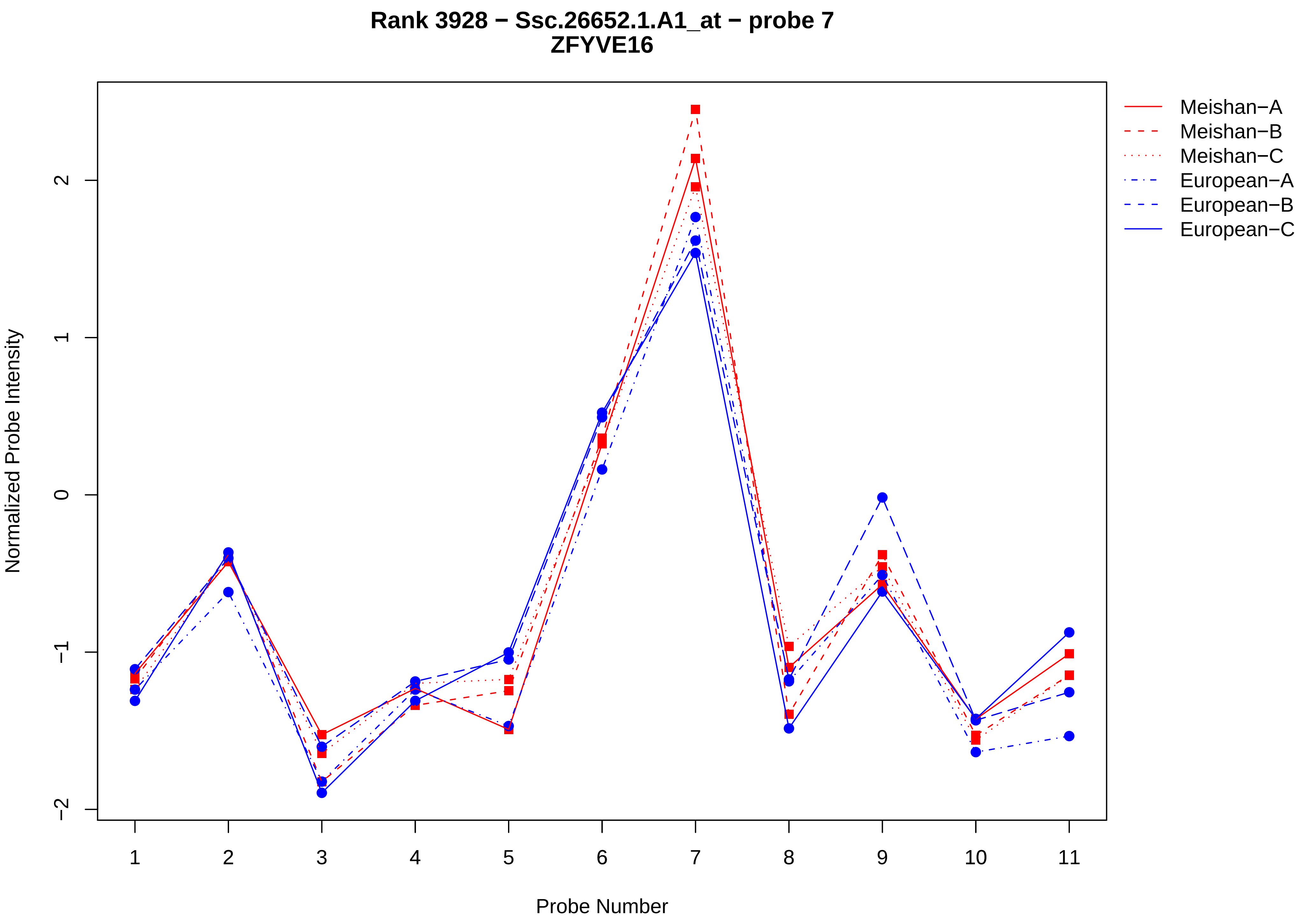

Rank 3928 − Ssc.26652.1.A1_at − probe 7
ZFYVE16
Normalized Probe Intensity
Probe Number
Meishan−A
Meishan−B
Meishan−C
European−A
European−B
European−C
−2
−1
0
1
2
1
2
3
4
5
6
7
8
9
10
11

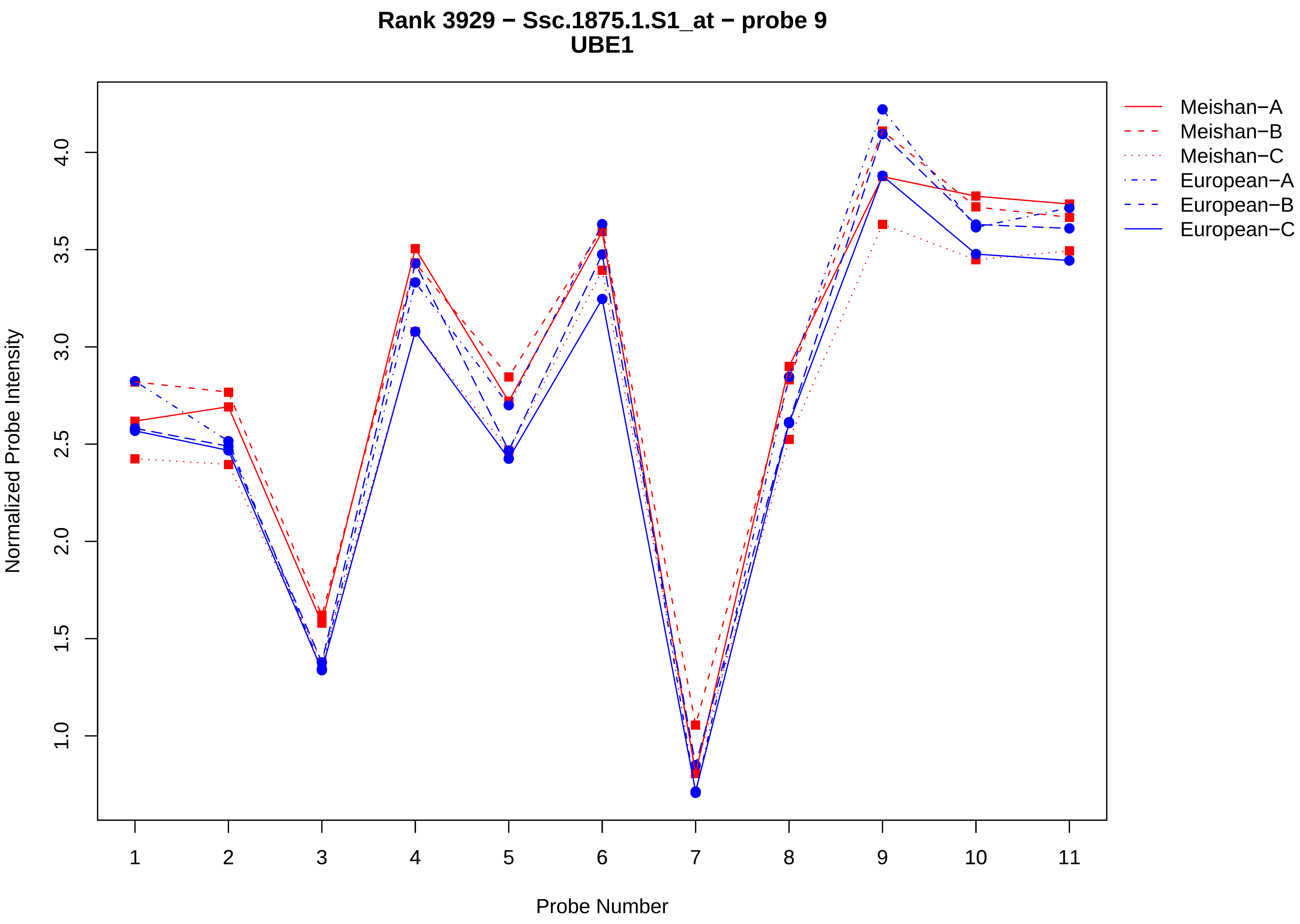

Rank 3929 − Ssc.1875.1.S1_at − probe 9
UBE1
Normalized Probe Intensity
Probe Number
1.0
1.5
2.0
2.5
3.0
3.5
4.0
1
2
3
4
5
6
7
8
9
10
11
Meishan−A
Meishan−B
Meishan−C
European−A
European−B
European−C

Rank 3930 − Ssc.27997.1.A1_at − probe 1
PML

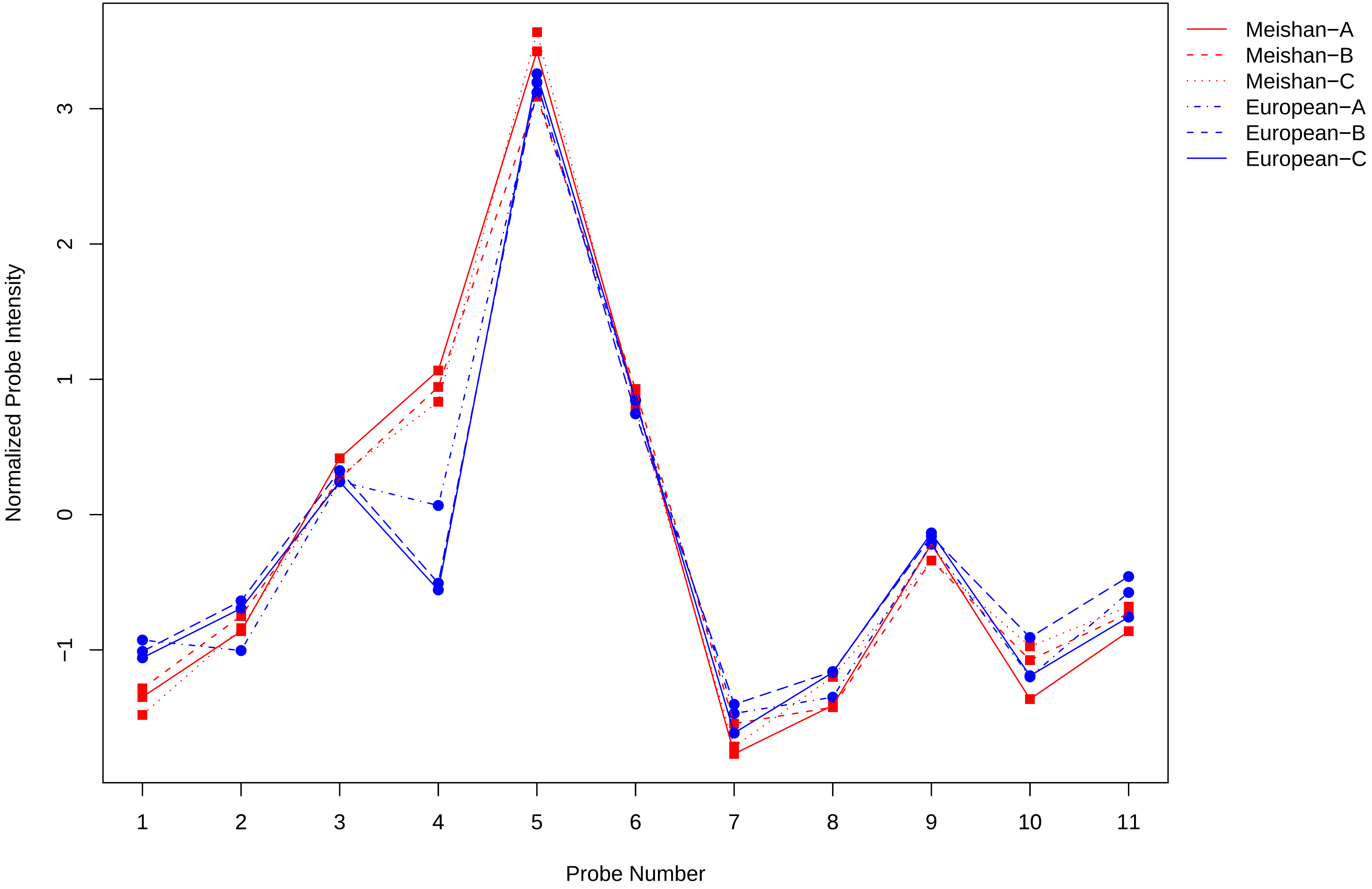

Rank 3931 − Ssc.18642.1.S1_at − probe 1
ITGB1BP2

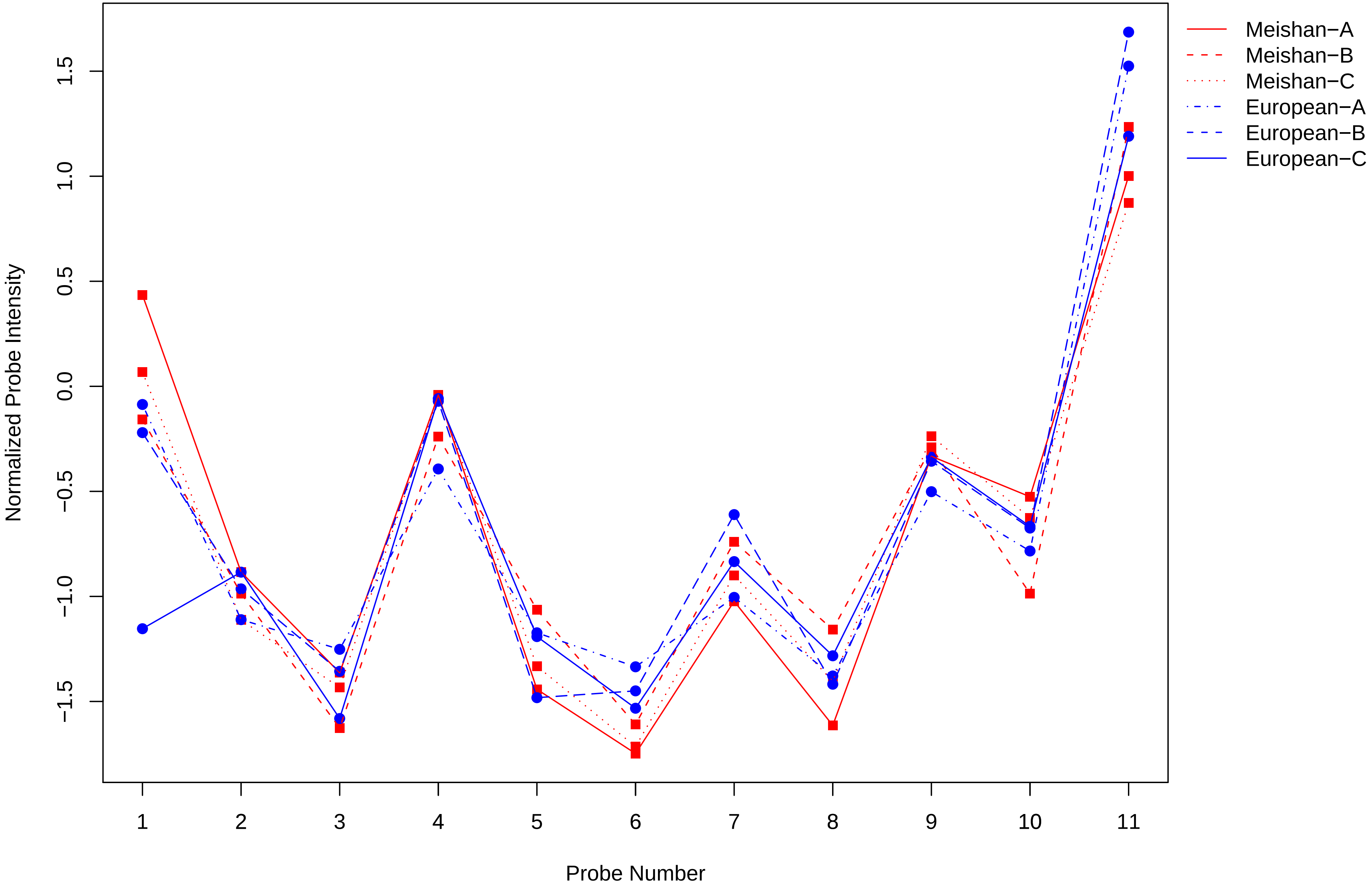

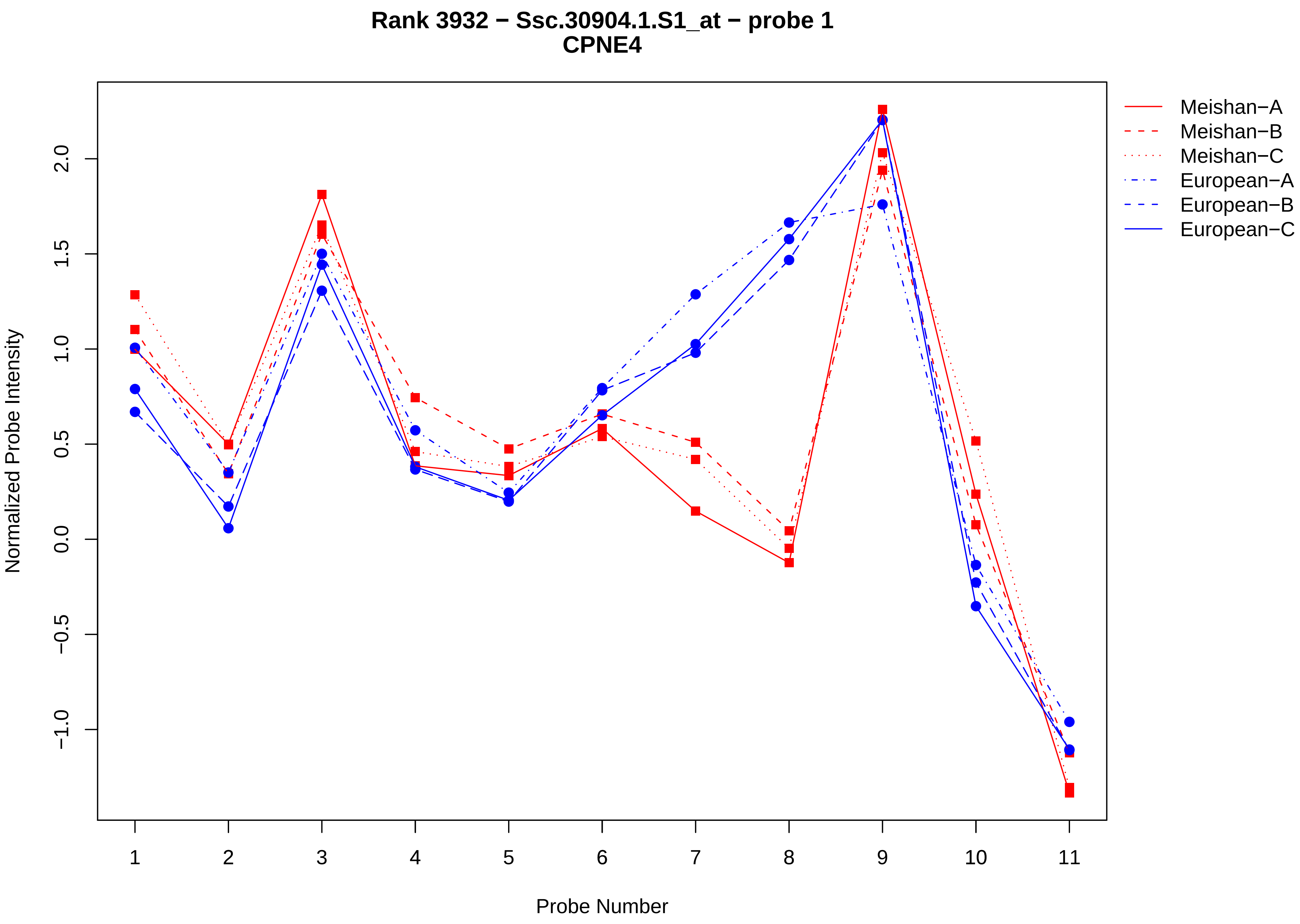
Rank 3932 − Ssc.30904.1.S1_at − probe 1
CPNE4
Normalized Probe Intensity
Probe Number
−1.0
−0.5
0.0
0.5
1.0
1.5
2.0
1
2
3
4
5
6
7
8
9
10
11
Meishan−A
Meishan−B
Meishan−C
European−A
European−B
European−C

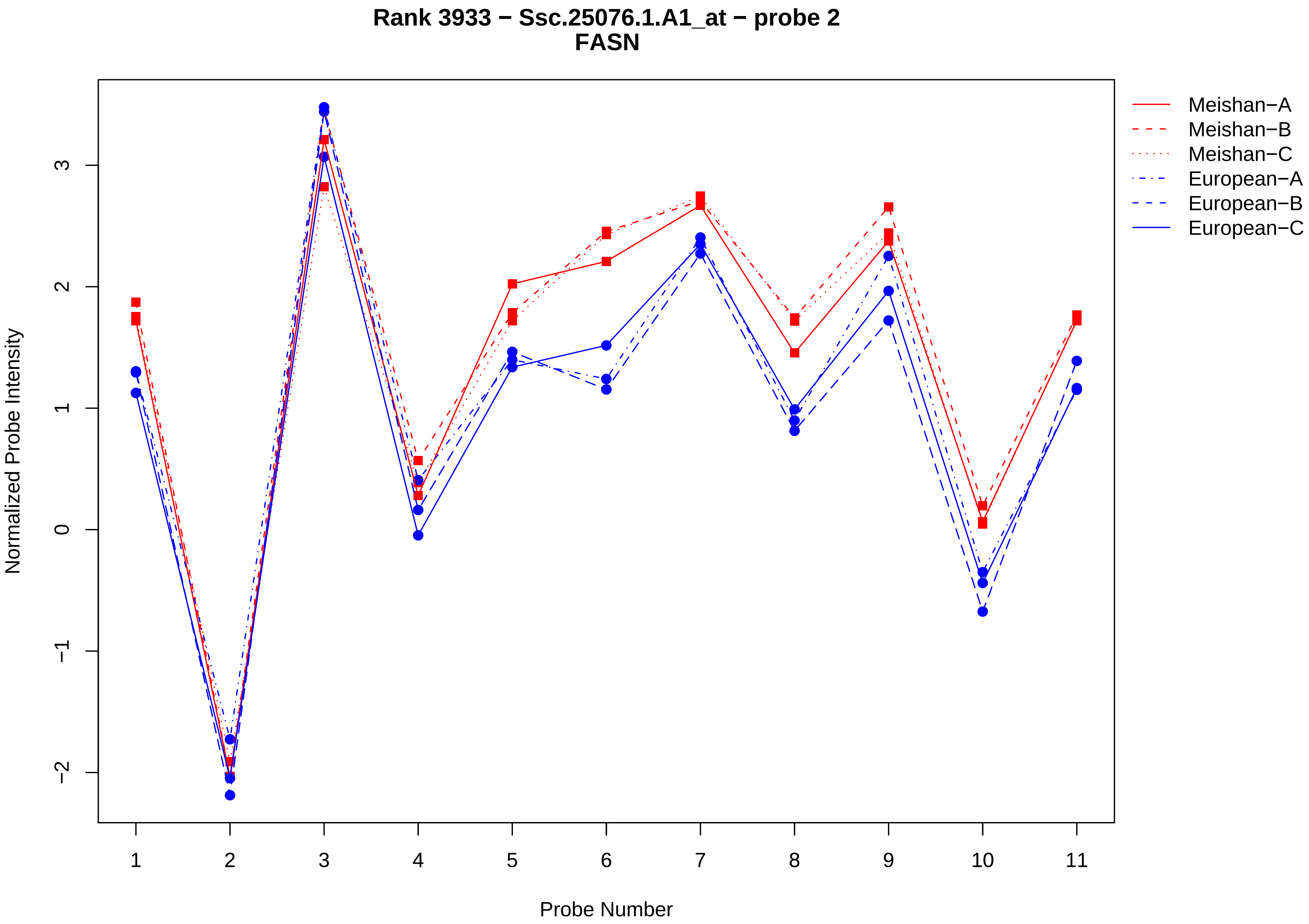

Rank 3933 − Ssc.25076.1.A1_at − probe 2
FASN
Normalized Probe Intensity
Probe Number
−2
−1
0
1
2
3
1
2
3
4
5
6
7
8
9
10
11
Meishan−A
Meishan−B
Meishan−C
European−A
European−B
European−C

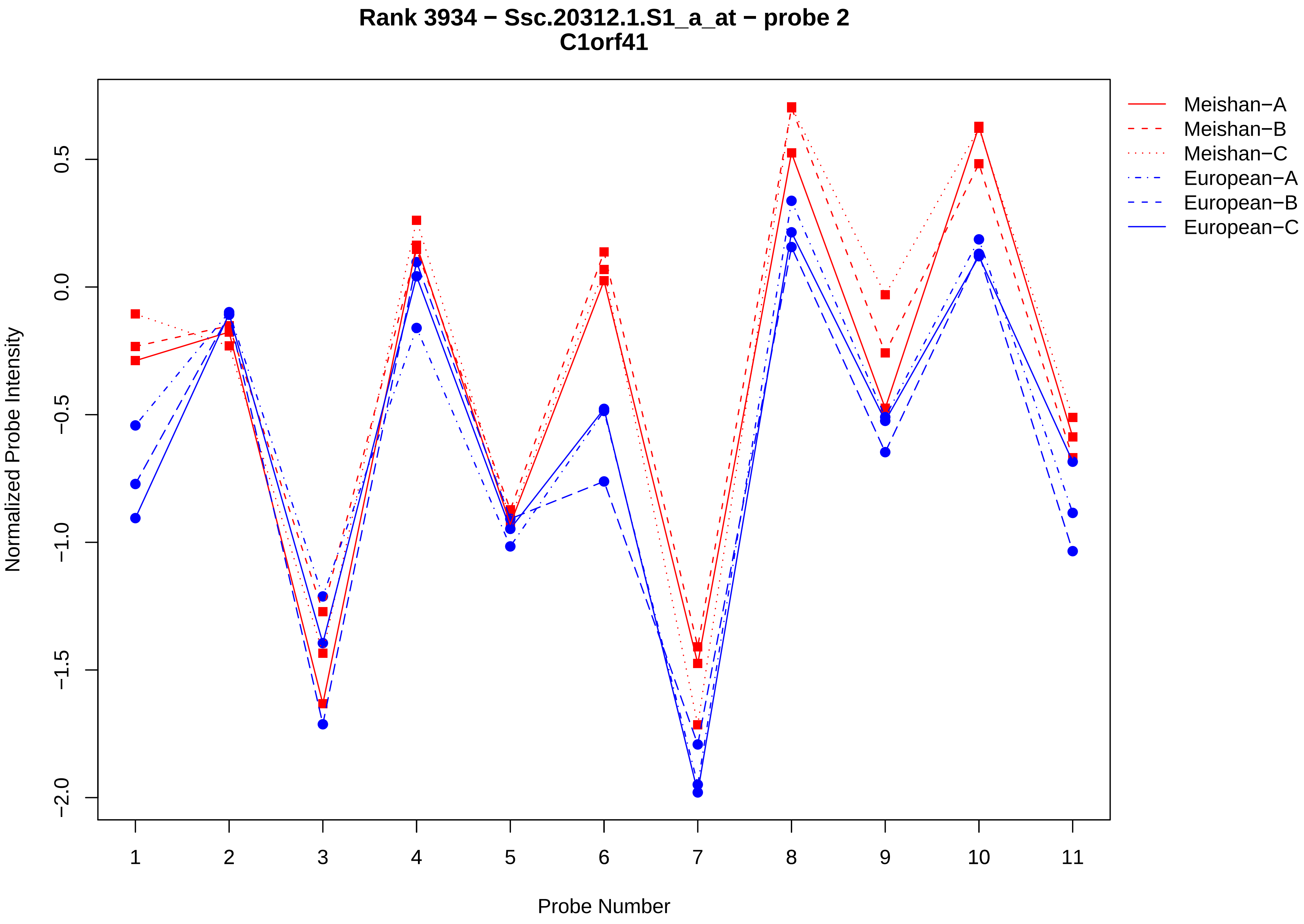

Rank 3934 − Ssc.20312.1.S1_a_at − probe 2
C1orf41
Normalized Probe Intensity
Probe Number
Meishan−A
Meishan−B
Meishan−C
European−A
European−B
European−C
0.5
0.0
−0.5
−1.0
−1.5
−2.0
1
2
3
4
5
6
7
8
9
10
11

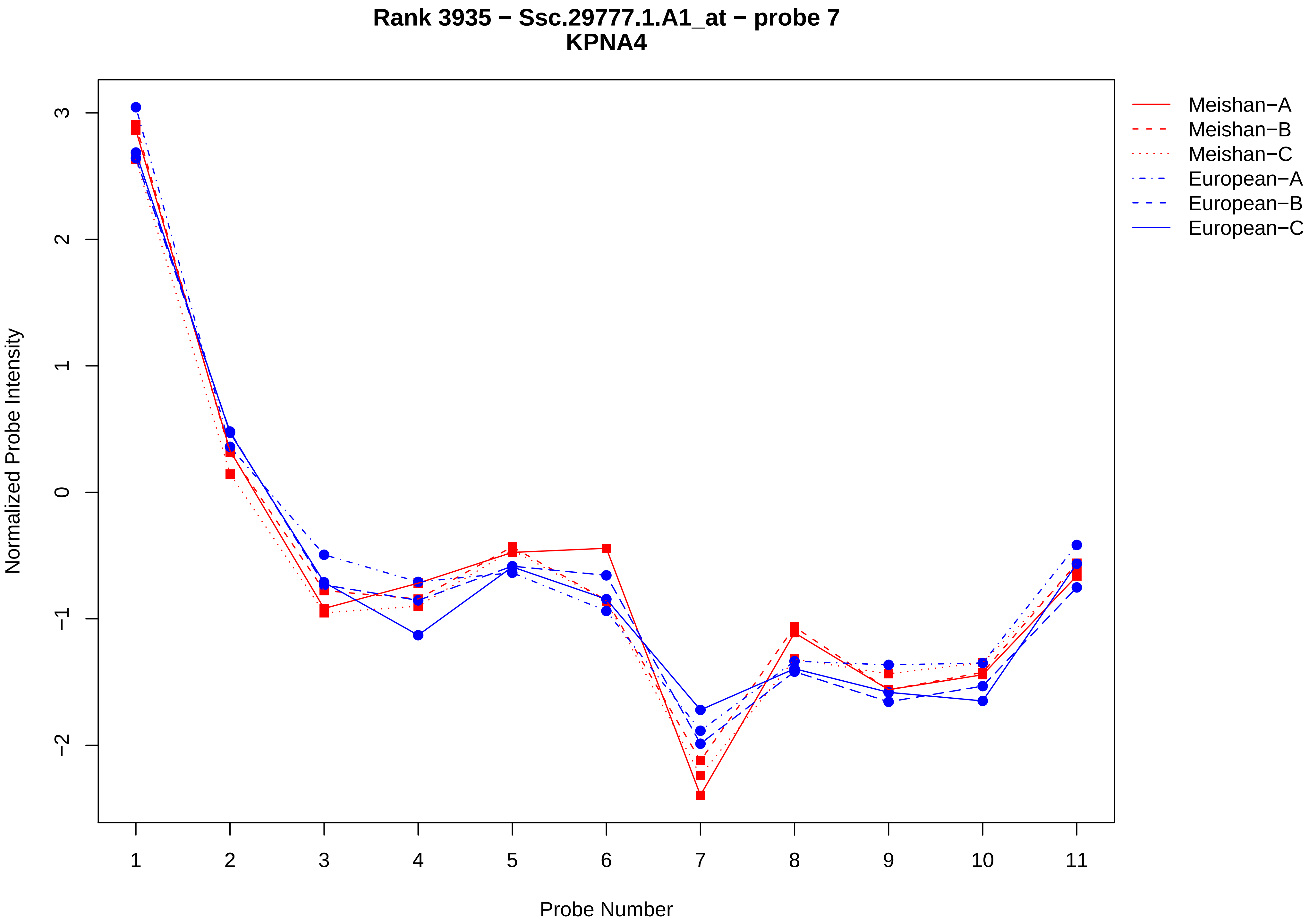

Rank 3935 − Ssc.29777.1.A1_at − probe 7
KPNA4
Normalized Probe Intensity
Probe Number
3
2
1
0
−1
−2
1
2
3
4
5
6
7
8
9
10
11
Meishan−A
Meishan−B
Meishan−C
European−A
European−B
European−C

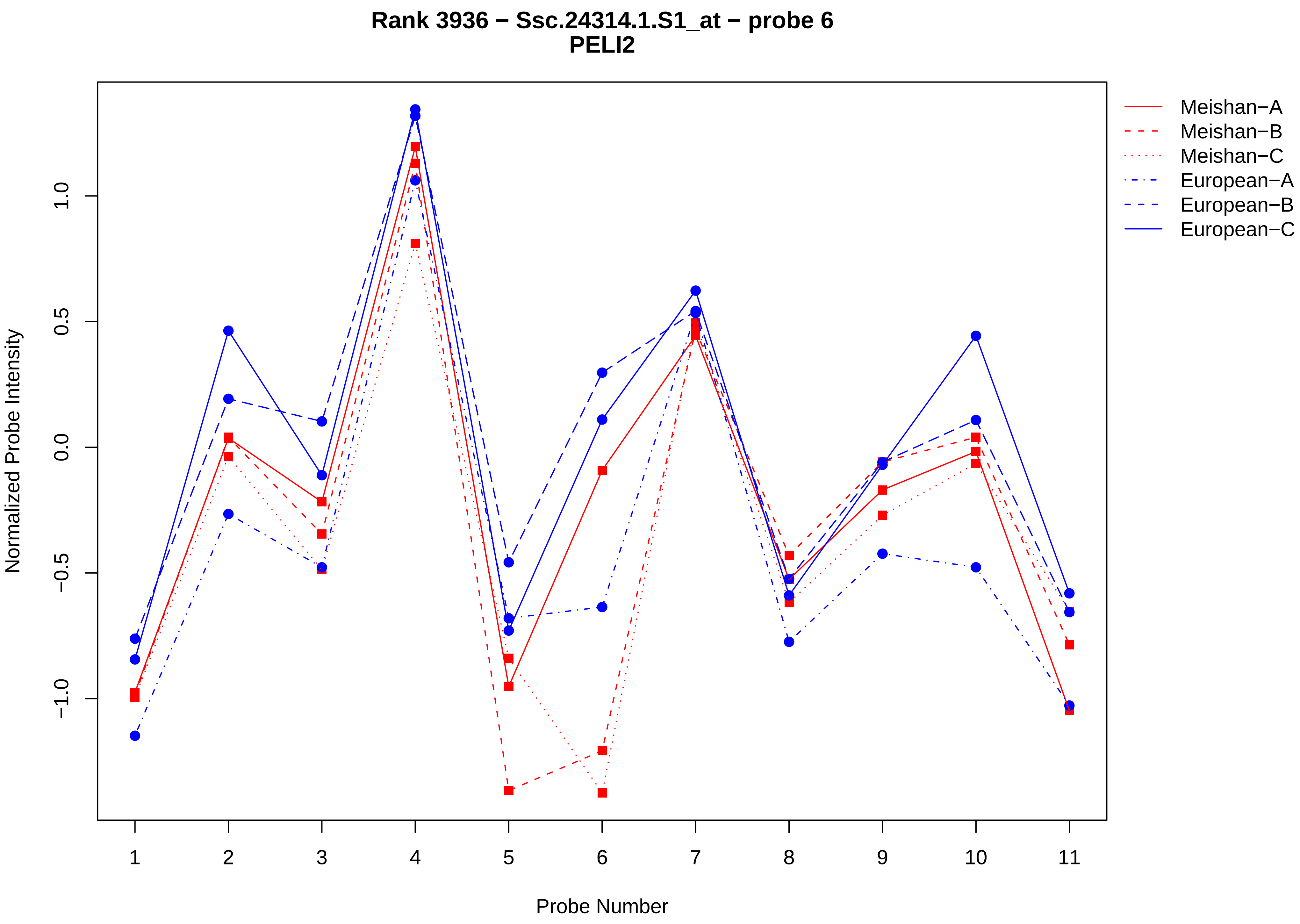
Rank 3936 − Ssc.24314.1.S1_at − probe 6
PELI2
Normalized Probe Intensity
Probe Number
−1.0
−0.5
0.0
0.5
1.0
1
2
3
4
5
6
7
8
9
10
11
Meishan−A
Meishan−B
Meishan−C
European−A
European−B
European−C

Rank 3937 − Ssc.29940.1.A1_at − probe 9
SMAP1

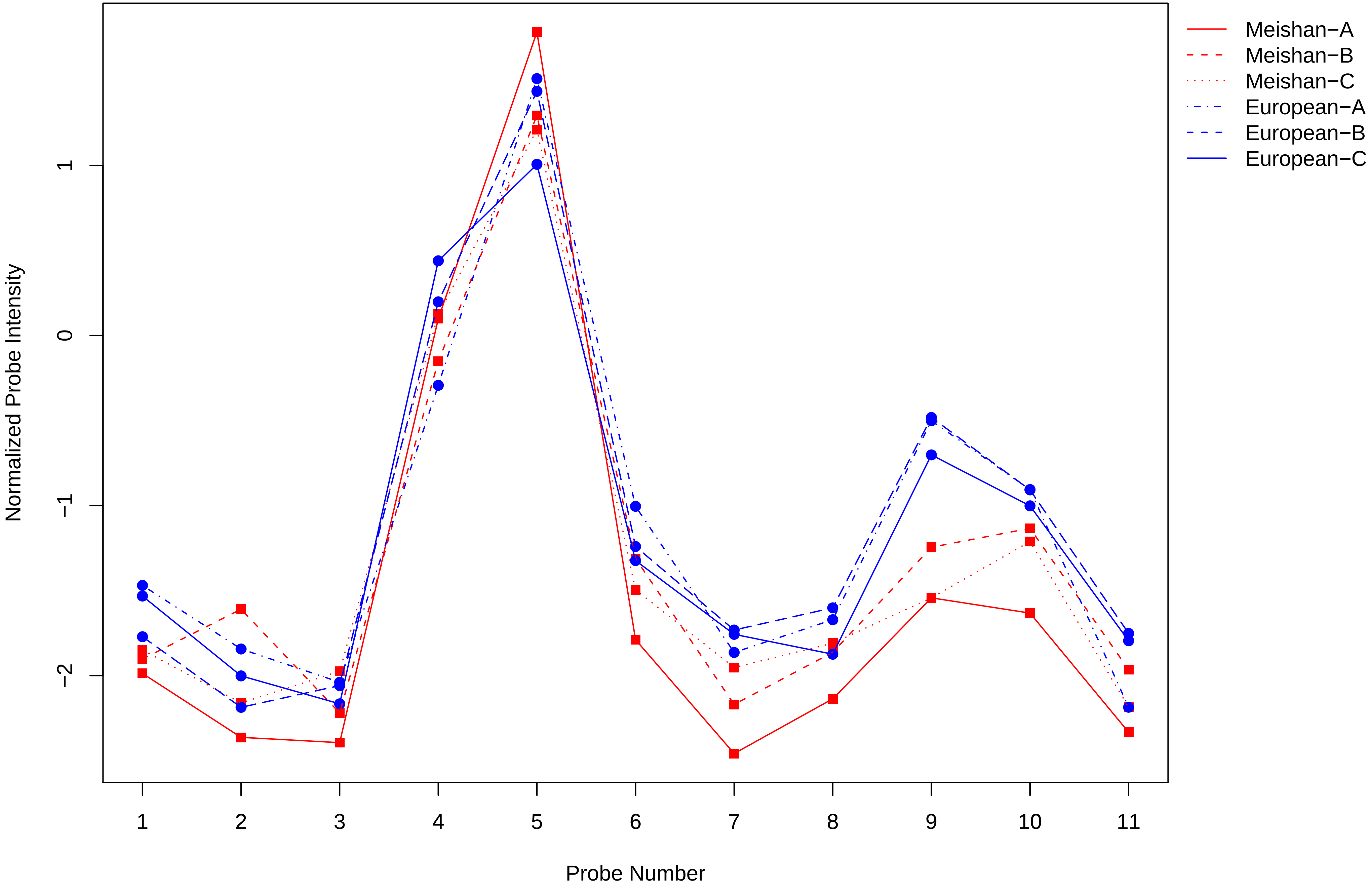

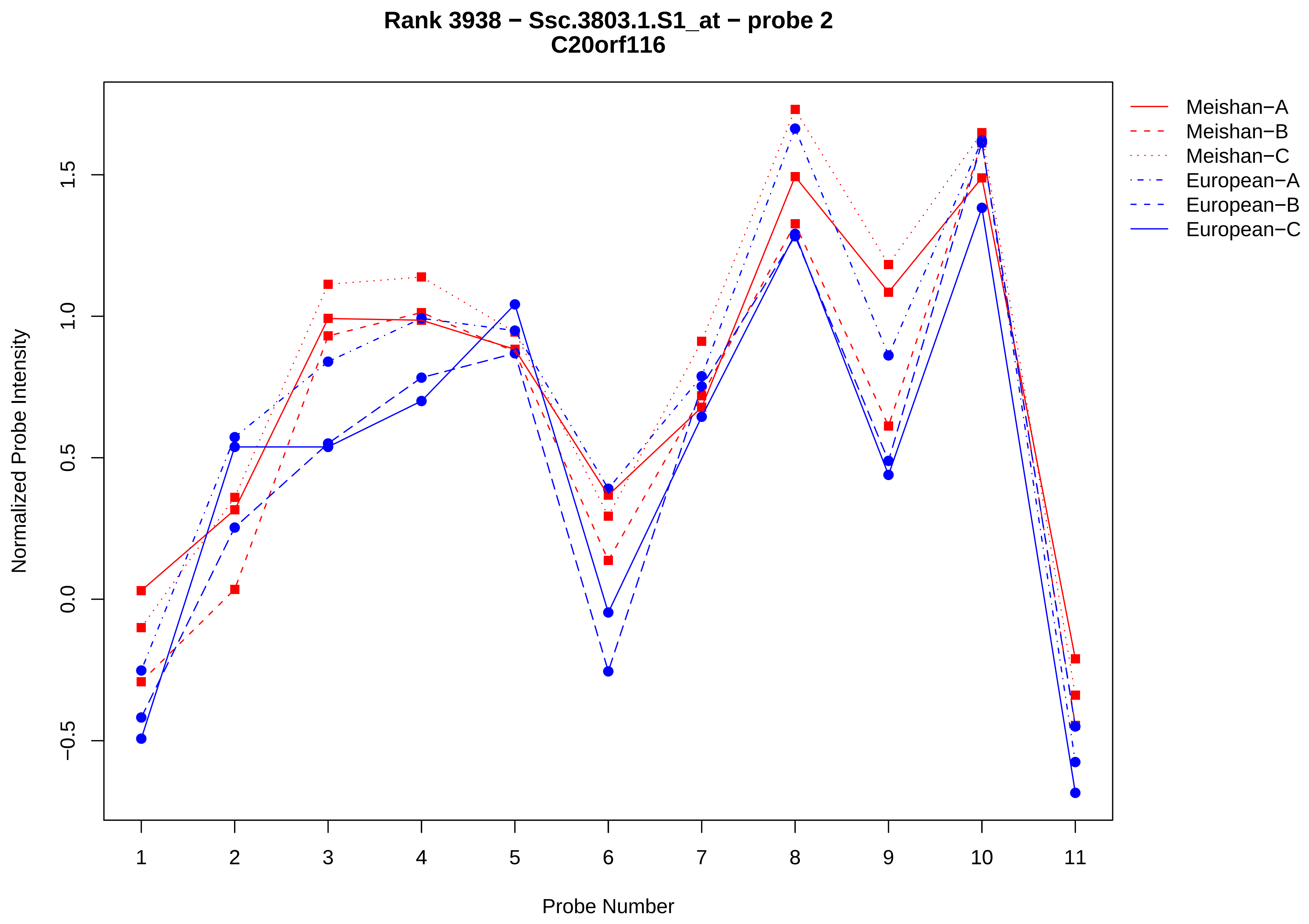

Rank 3938 − Ssc.3803.1.S1_at − probe 2
C20orf116
Meishan−A
Meishan−B
Meishan−C
European−A
European−B
European−C
Normalized Probe Intensity
Probe Number
−0.5
0.0
0.5
1.0
1.5
1
2
3
4
5
6
7
8
9
10
11

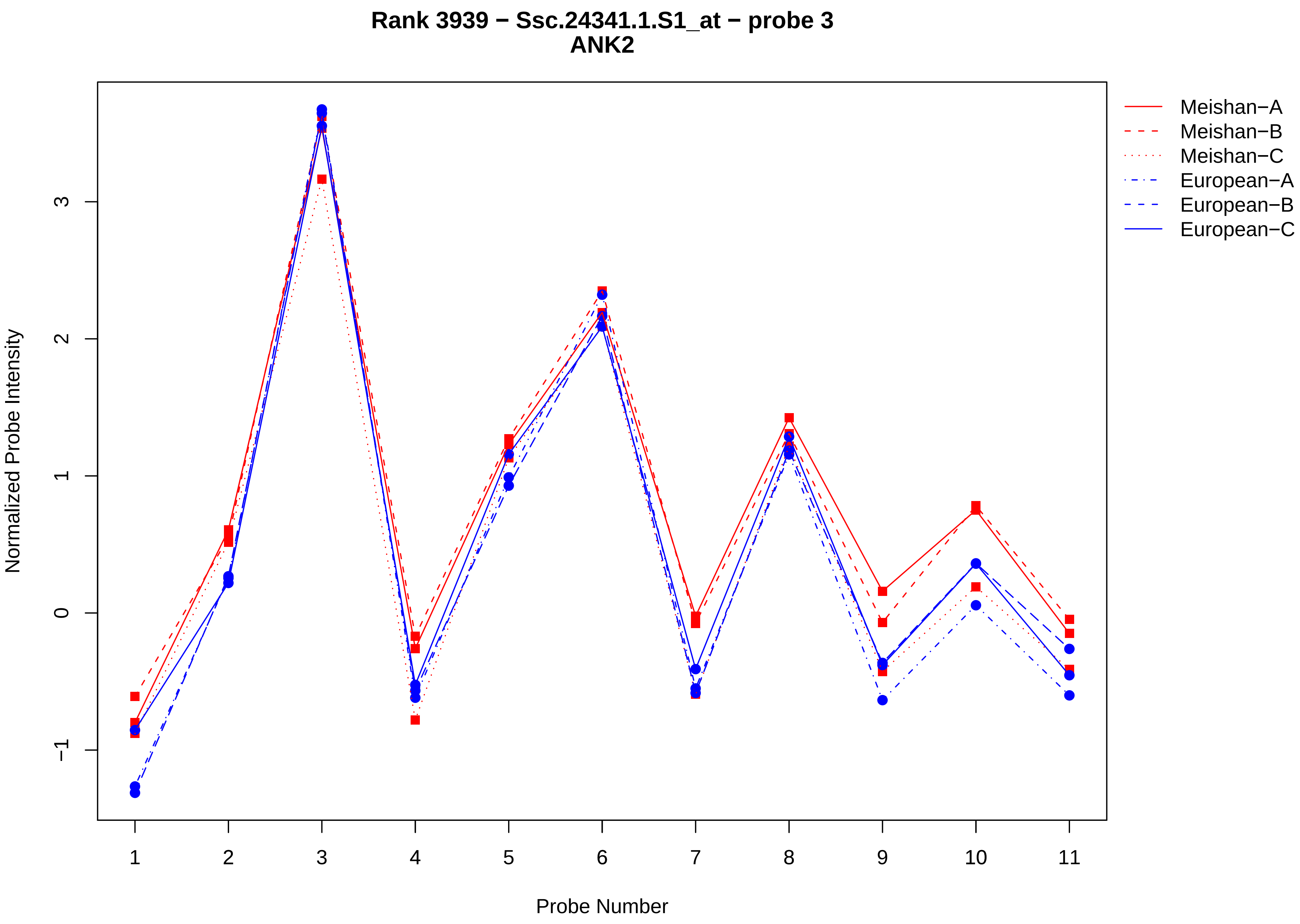

Rank 3939 − Ssc.24341.1.S1_at − probe 3
ANK2
Normalized Probe Intensity
Probe Number
−1
0
1
2
3
1
2
3
4
5
6
7
8
9
10
11
Meishan−A
Meishan−B
Meishan−C
European−A
European−B
European−C

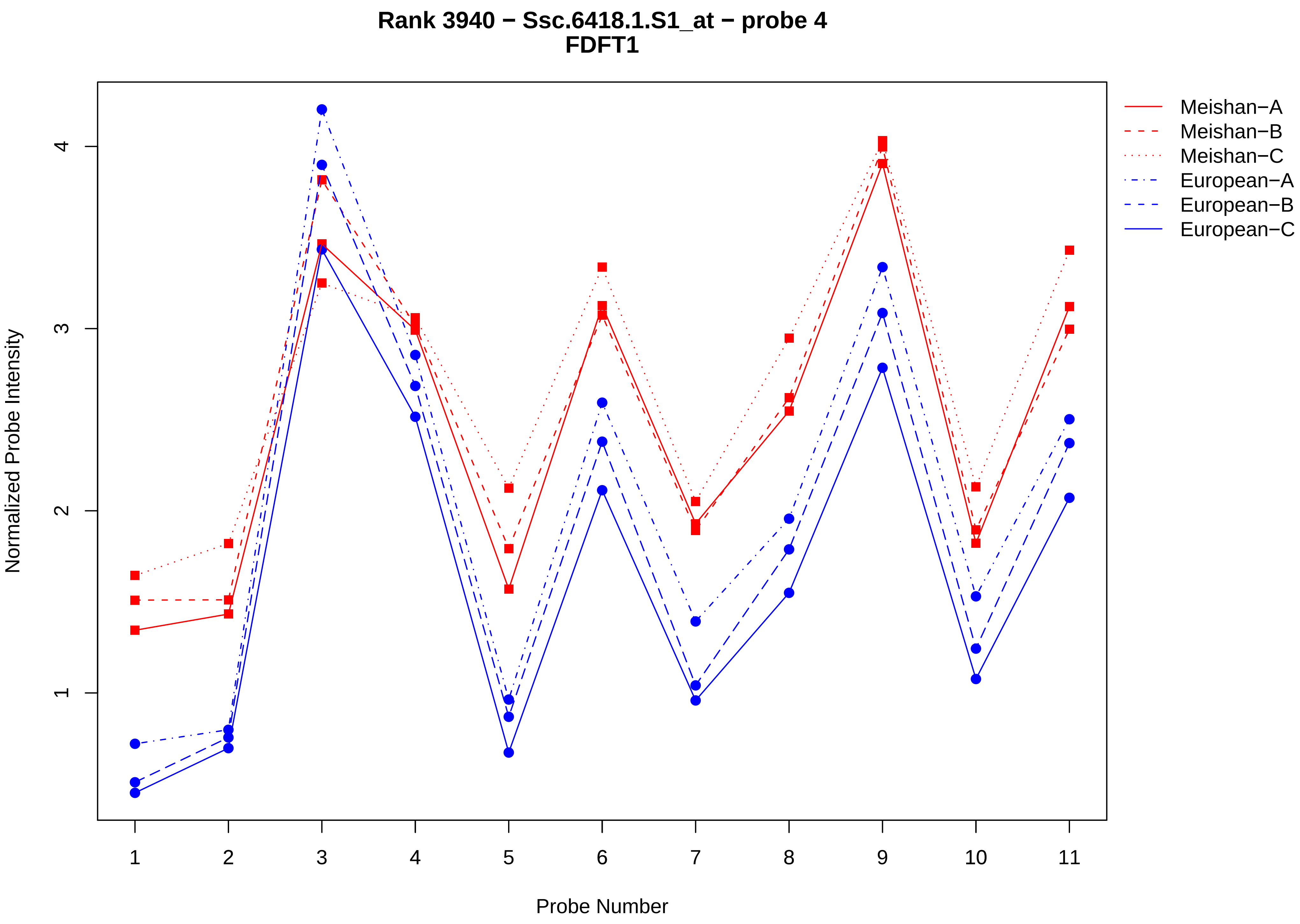

Rank 3940 − Ssc.6418.1.S1_at − probe 4
FDFT1
Normalized Probe Intensity
Probe Number
1
2
3
4
5
6
7
8
9
10
11
Meishan−A
Meishan−B
Meishan−C
European−A
European−B
European−C

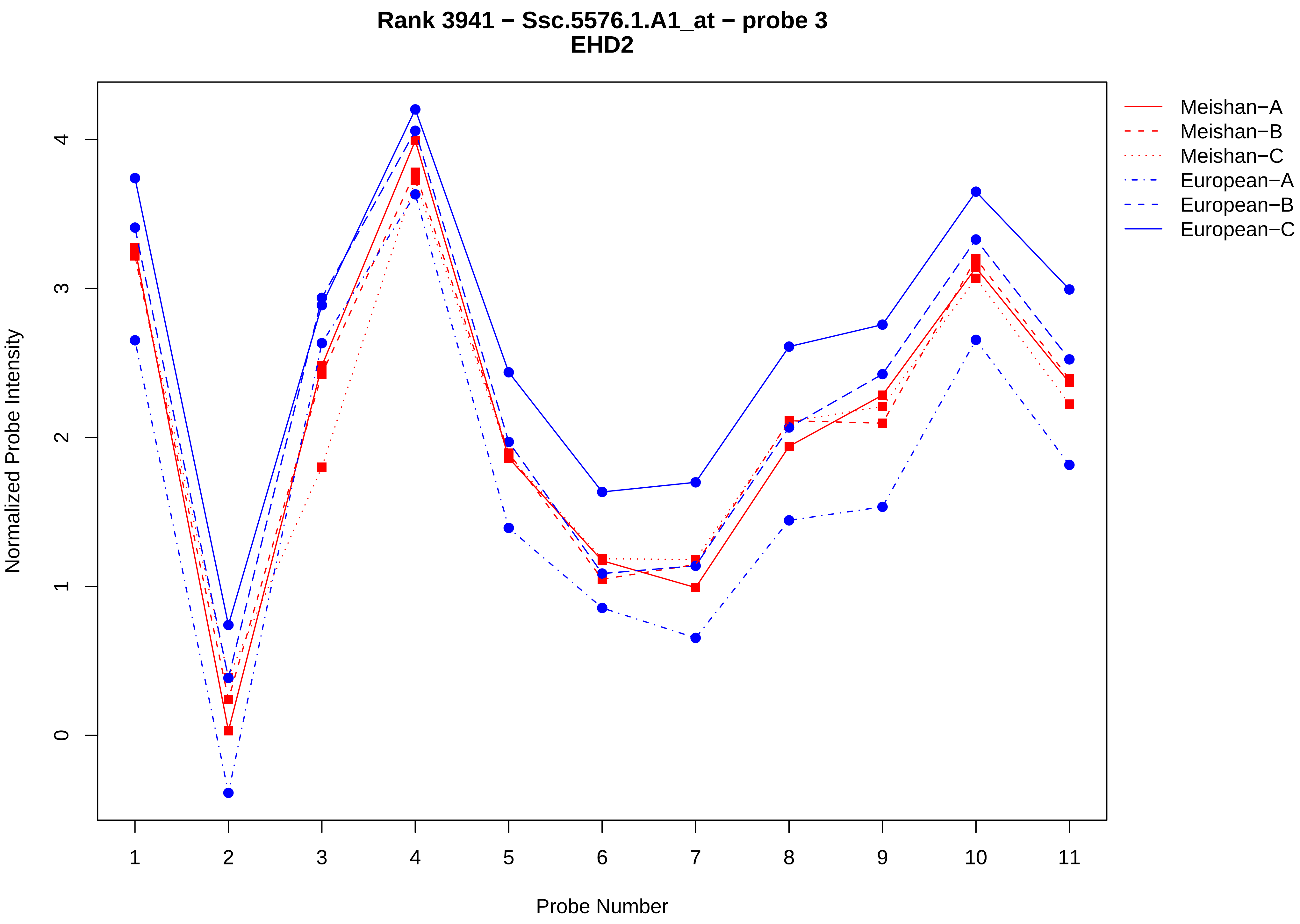
Rank 3941 − Ssc.5576.1.A1_at − probe 3
EHD2
Normalized Probe Intensity
Probe Number
0
1
2
3
4
1
2
3
4
5
6
7
8
9
10
11
Meishan−A
Meishan−B
Meishan−C
European−A
European−B
European−C

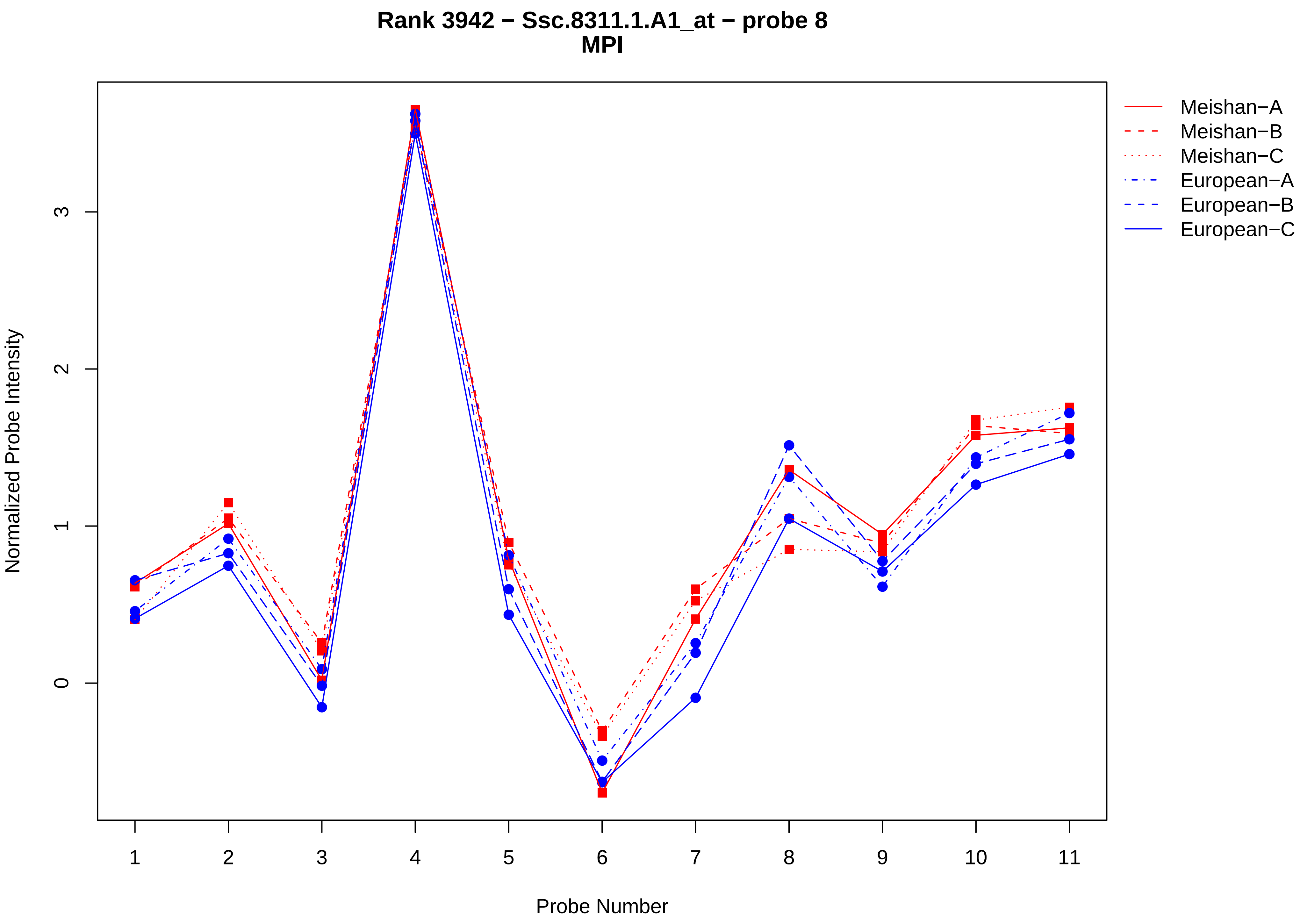

Rank 3942 − Ssc.8311.1.A1_at − probe 8
MPI
Normalized Probe Intensity
Probe Number
0
1
2
3
1
2
3
4
5
6
7
8
9
10
11
Meishan−A
Meishan−B
Meishan−C
European−A
European−B
European−C

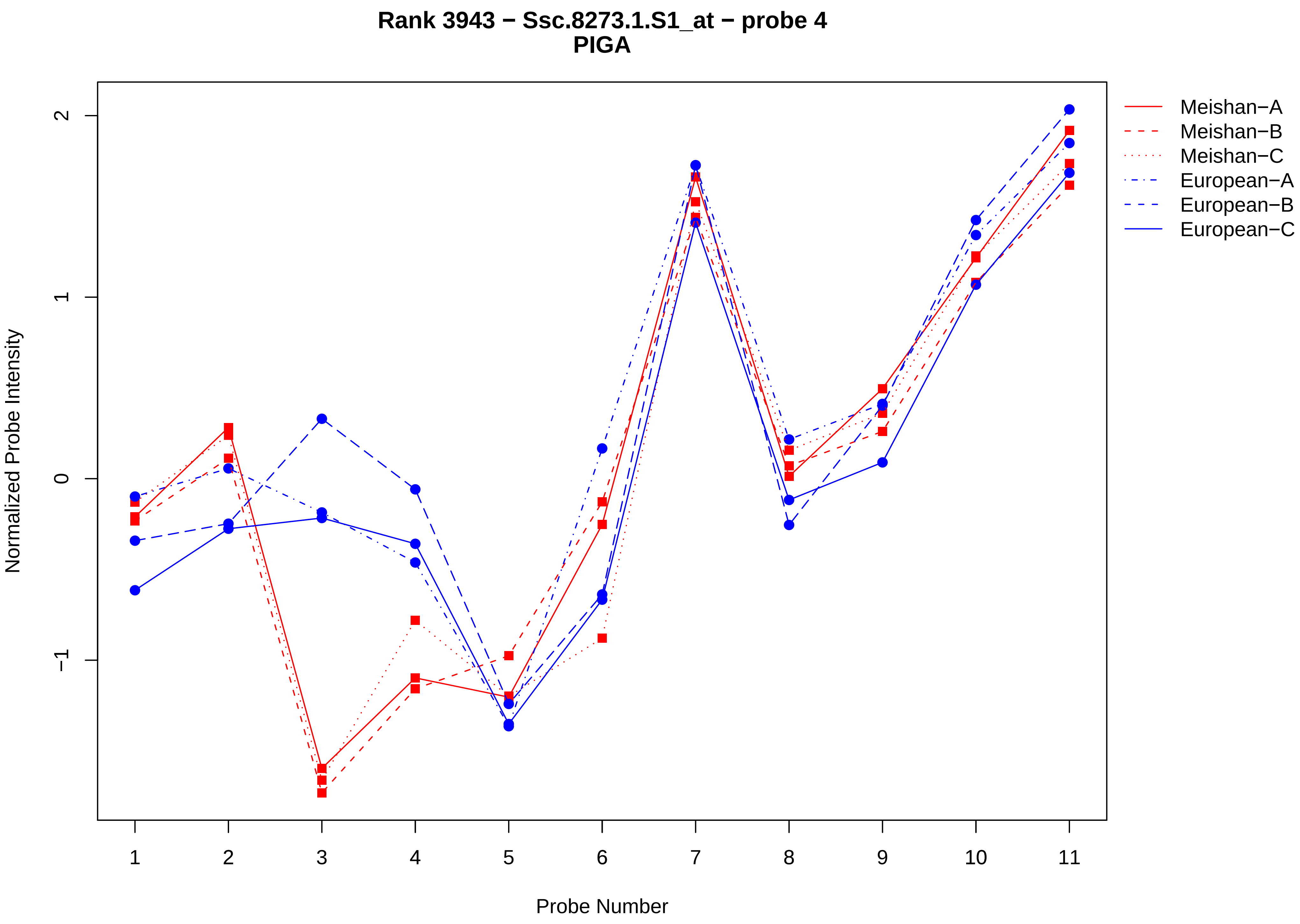

Rank 3943 − Ssc.8273.1.S1_at − probe 4
PIGA
Normalized Probe Intensity
Probe Number
−1
0
1
2
1
2
3
4
5
6
7
8
9
10
11
Meishan−A
Meishan−B
Meishan−C
European−A
European−B
European−C

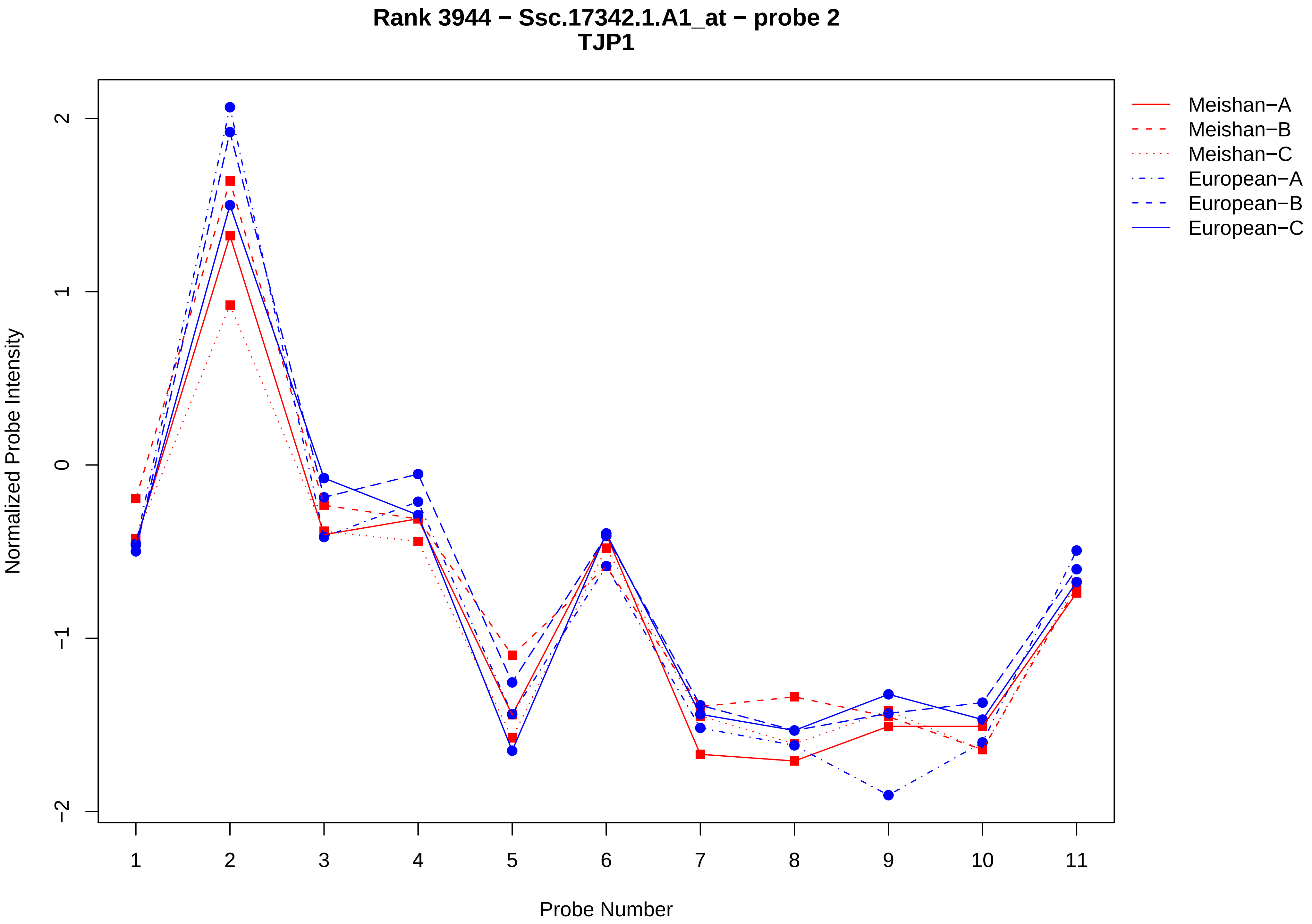

Rank 3944 − Ssc.17342.1.A1_at − probe 2
TJP1
Normalized Probe Intensity
Probe Number
Meishan−A
Meishan−B
Meishan−C
European−A
European−B
European−C
−2
−1
0
1
2
1
2
3
4
5
6
7
8
9
10
11

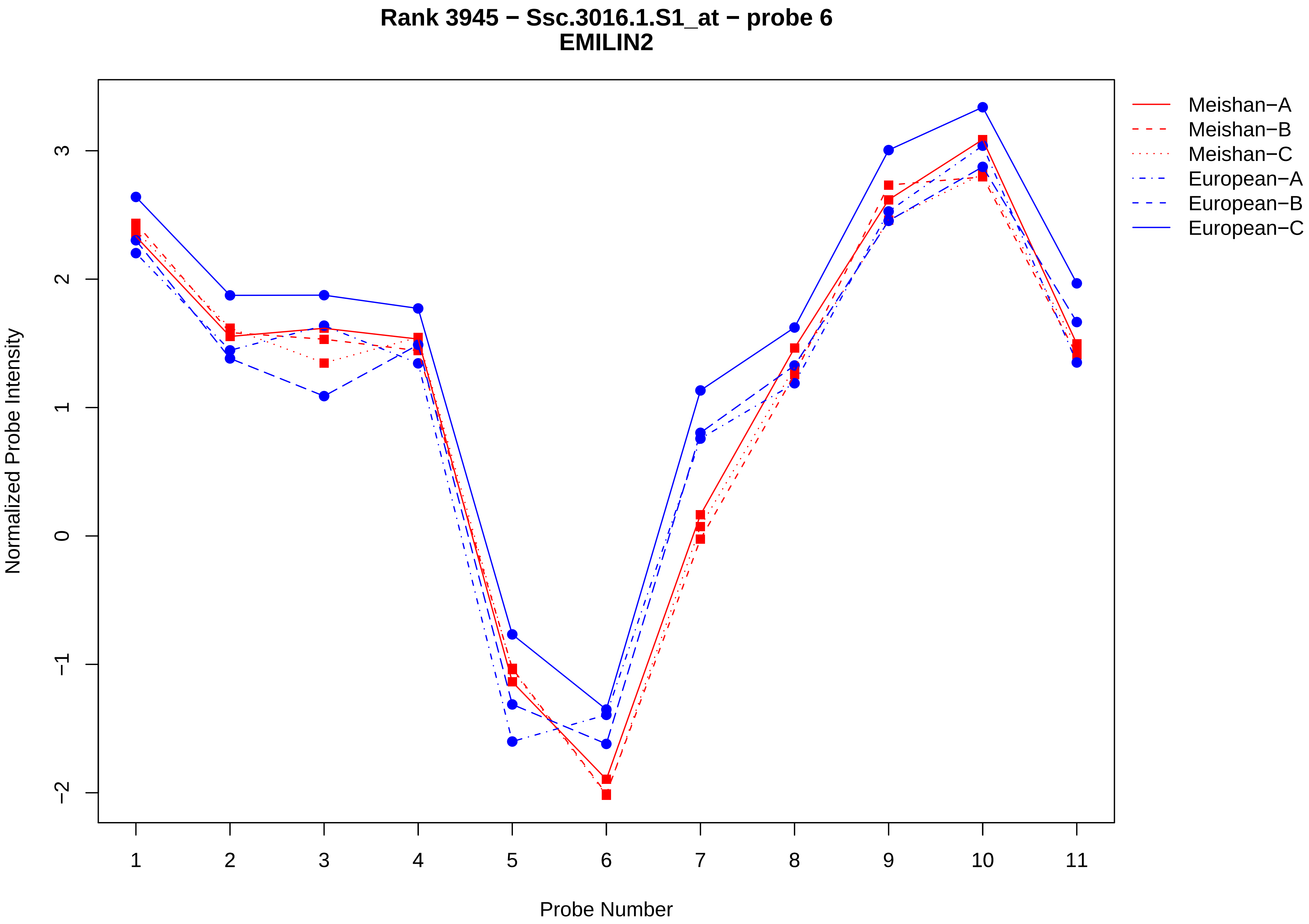

Rank 3945 − Ssc.3016.1.S1_at − probe 6
EMILIN2
Normalized Probe Intensity
Probe Number
−2
−1
0
1
2
3
1
2
3
4
5
6
7
8
9
10
11
Meishan−A
Meishan−B
Meishan−C
European−A
European−B
European−C

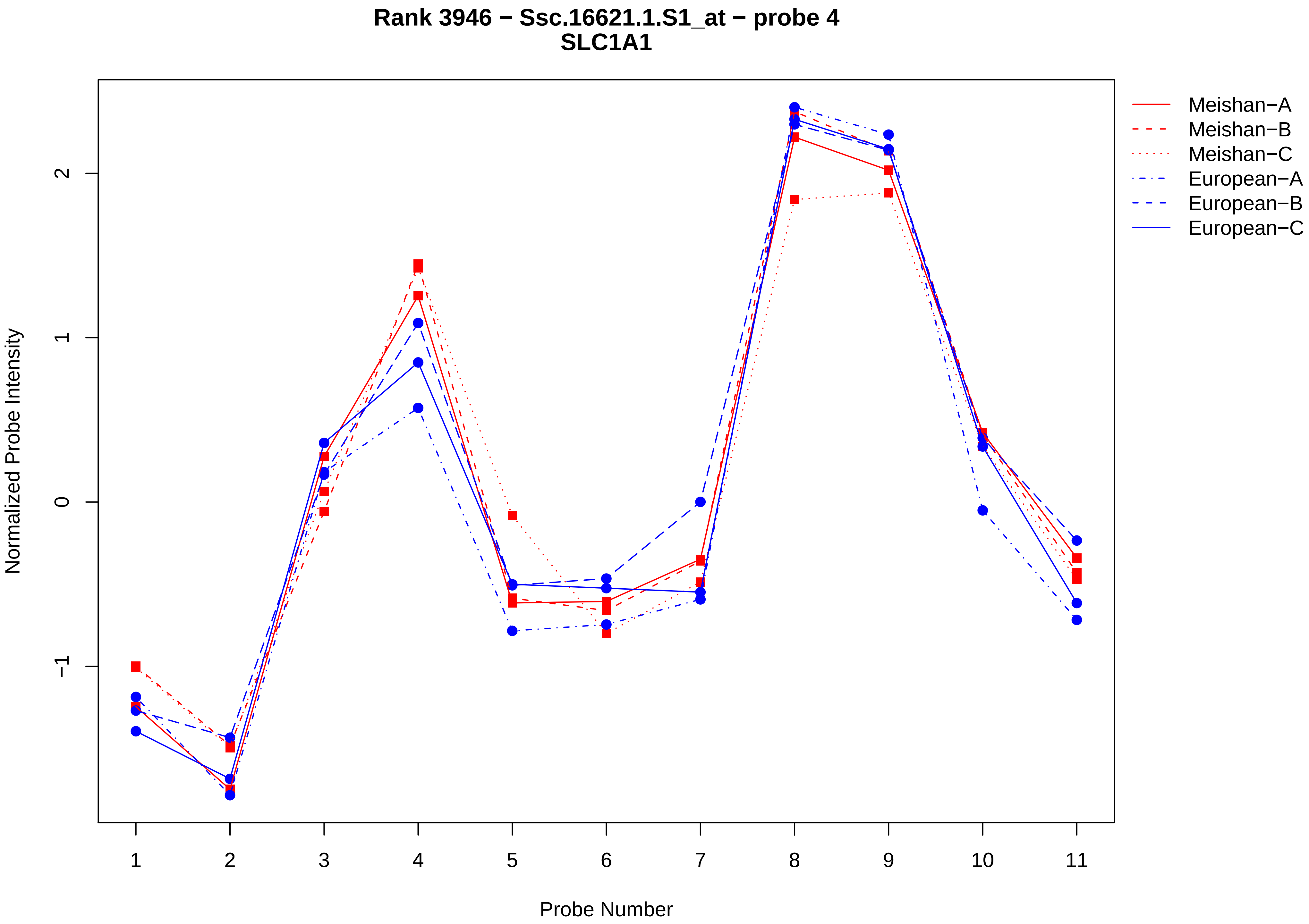

Rank 3946 − Ssc.16621.1.S1_at − probe 4
SLC1A1
Normalized Probe Intensity
Probe Number
−1
0
1
2
1
2
3
4
5
6
7
8
9
10
11
Meishan−A
Meishan−B
Meishan−C
European−A
European−B
European−C

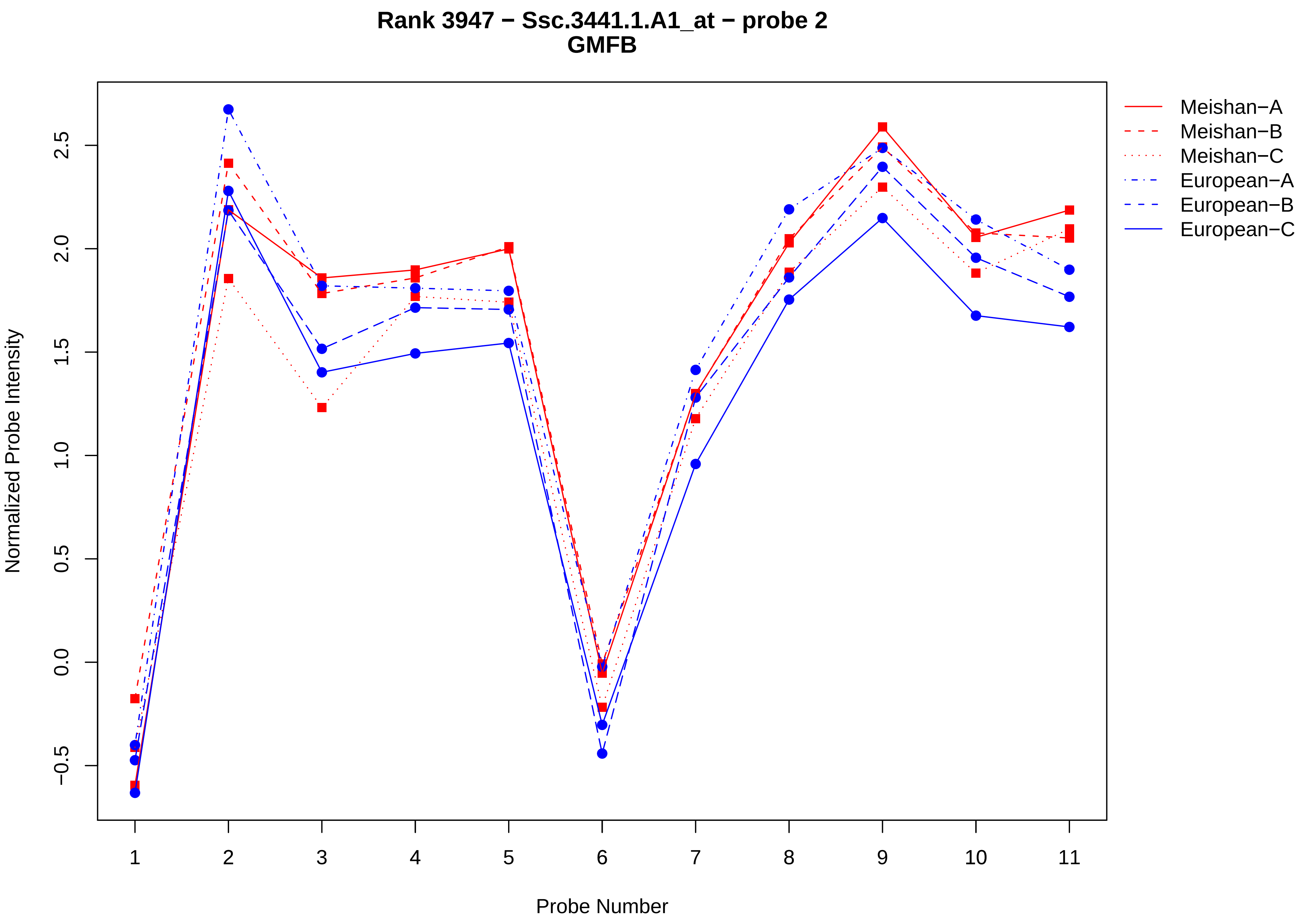
Rank 3947 − Ssc.3441.1.A1_at − probe 2
GMFB
Normalized Probe Intensity
Probe Number
−0.5
0.0
0.5
1.0
1.5
2.0
2.5
1
2
3
4
5
6
7
8
9
10
11
Meishan−A
Meishan−B
Meishan−C
European−A
European−B
European−C

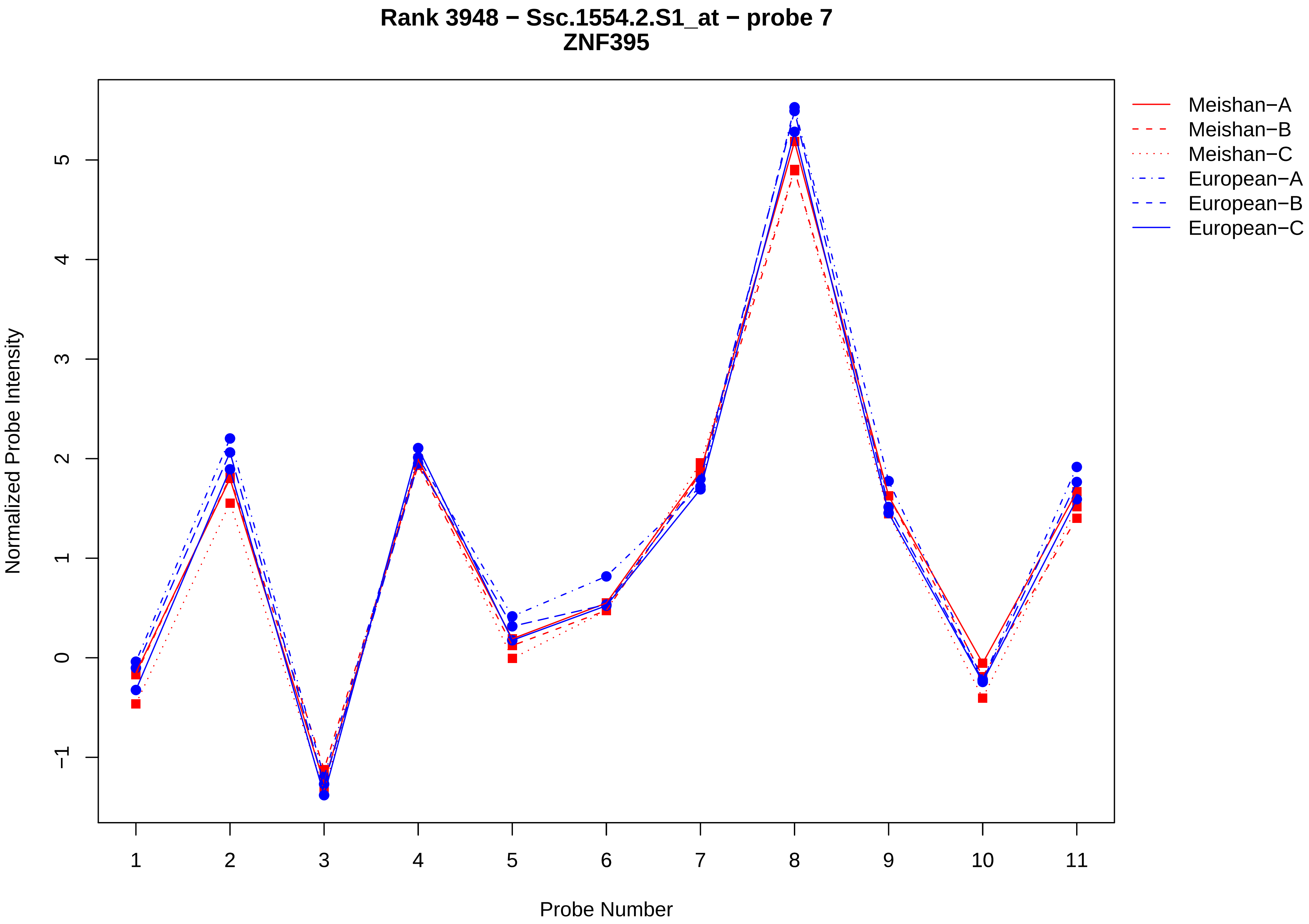

Rank 3948 − Ssc.1554.2.S1_at − probe 7
ZNF395
Normalized Probe Intensity
Probe Number
−1
0
1
2
3
4
5
1
2
3
4
5
6
7
8
9
10
11
Meishan−A
Meishan−B
Meishan−C
European−A
European−B
European−C

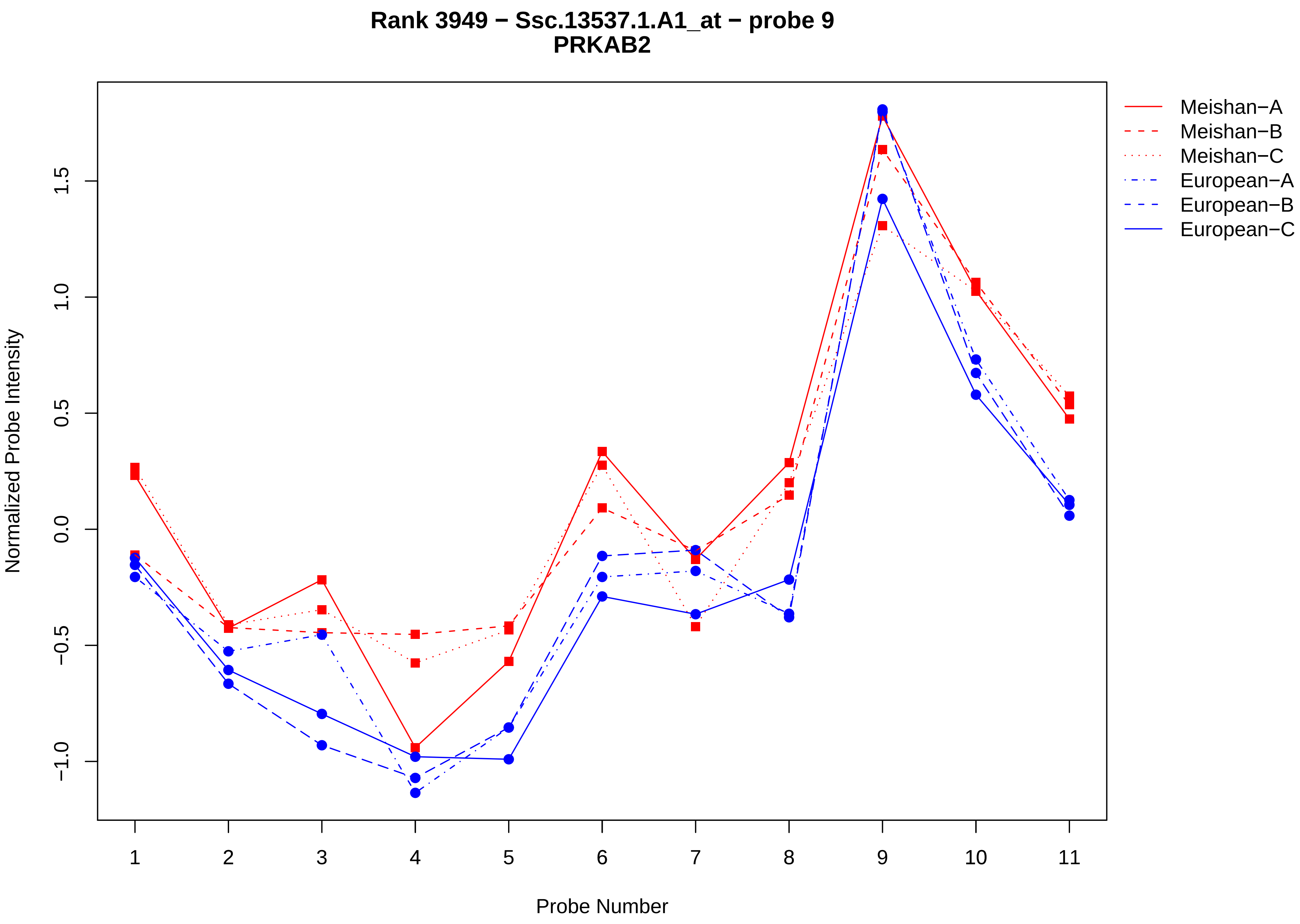

Rank 3949 − Ssc.13537.1.A1_at − probe 9
PRKAB2
Normalized Probe Intensity
Probe Number
−1.0
−0.5
0.0
0.5
1.0
1.5
1
2
3
4
5
6
7
8
9
10
11
Meishan−A
Meishan−B
Meishan−C
European−A
European−B
European−C

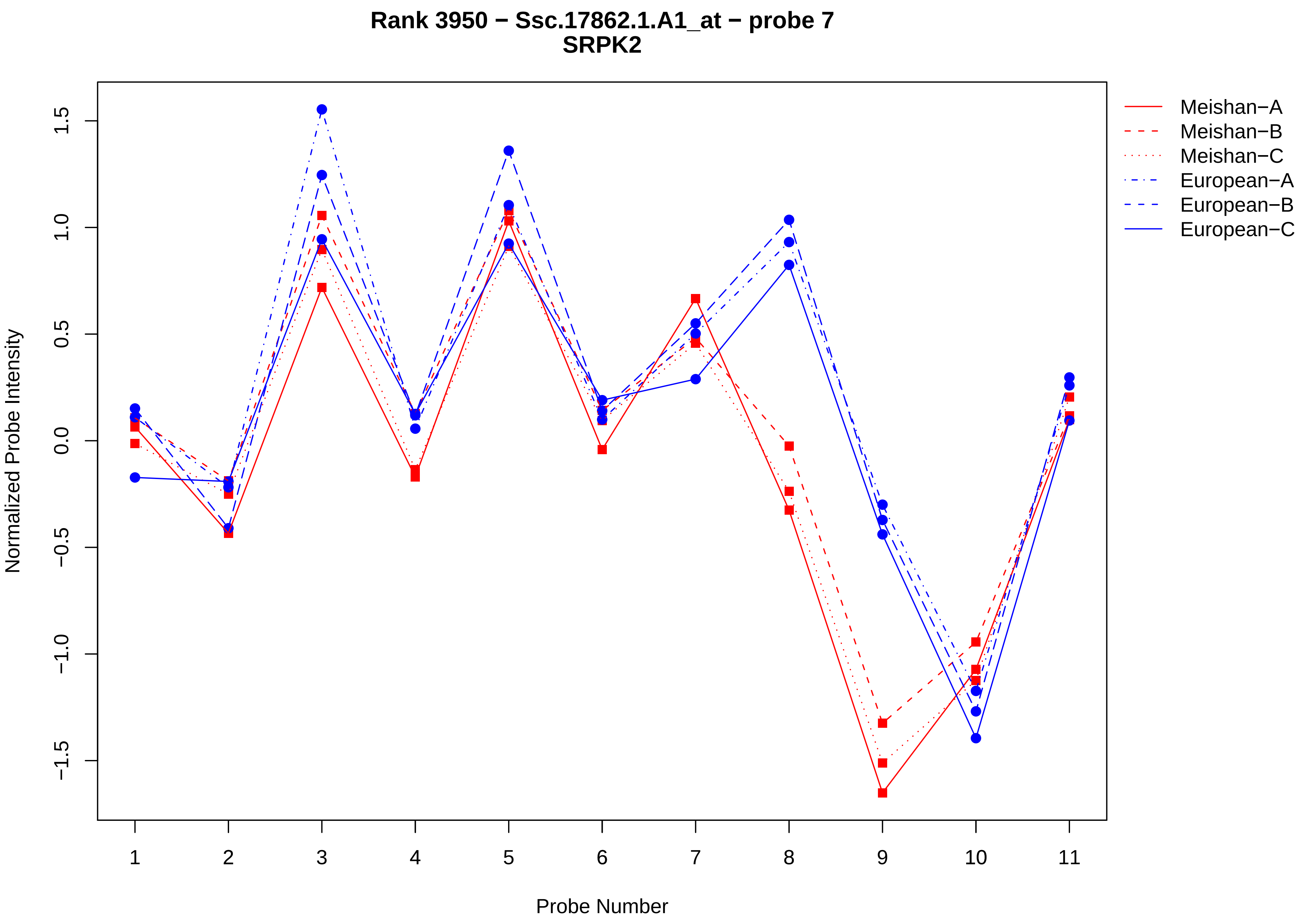

Rank 3950 − Ssc.17862.1.A1_at − probe 7
SRPK2
Normalized Probe Intensity
Probe Number
1.5
1.0
0.5
0.0
−0.5
−1.0
−1.5
1
2
3
4
5
6
7
8
9
10
11
Meishan−A
Meishan−B
Meishan−C
European−A
European−B
European−C

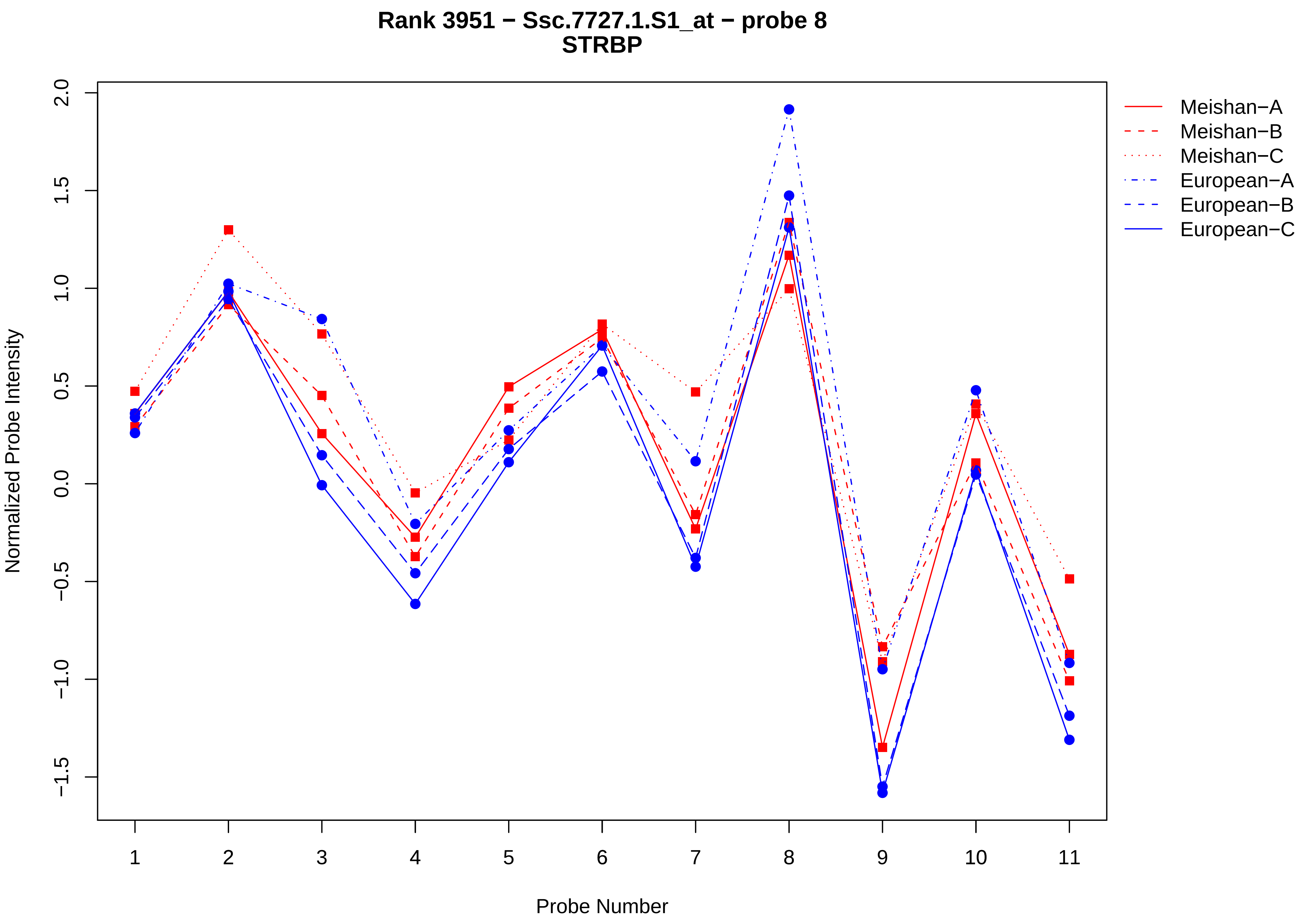

Rank 3951 − Ssc.7727.1.S1_at − probe 8
STRBP
Normalized Probe Intensity
Probe Number
2.0
1.5
1.0
0.5
0.0
−0.5
−1.0
−1.5
1
2
3
4
5
6
7
8
9
10
11
Meishan−A
Meishan−B
Meishan−C
European−A
European−B
European−C

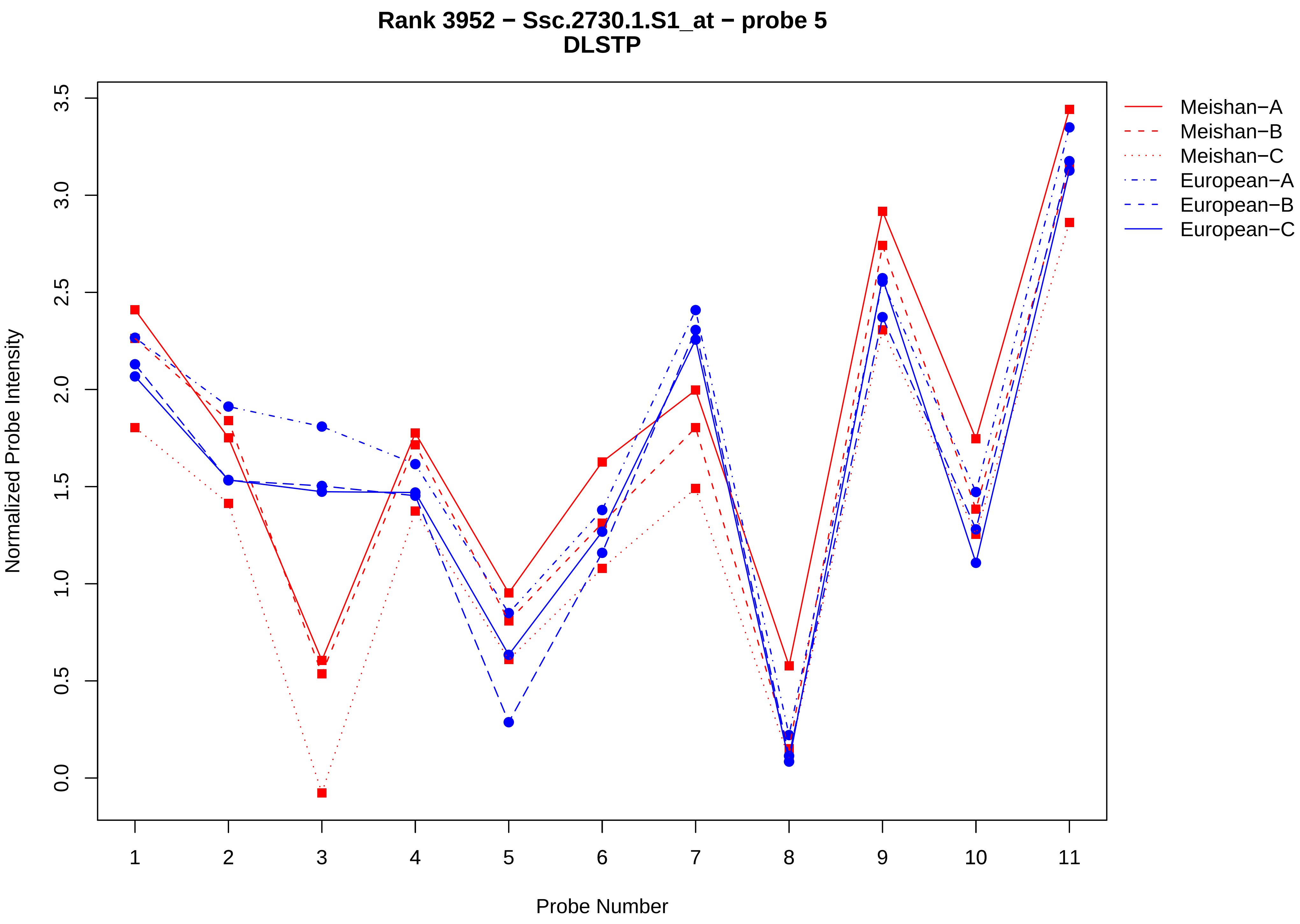

Rank 3952 − Ssc.2730.1.S1_at − probe 5
DLSTP
Normalized Probe Intensity
Probe Number
0.0
0.5
1.0
1.5
2.0
2.5
3.0
3.5
1 2 3 4 5 6 7 8 9 10 11
Meishan−A
Meishan−B
Meishan−C
European−A
European−B
European−C

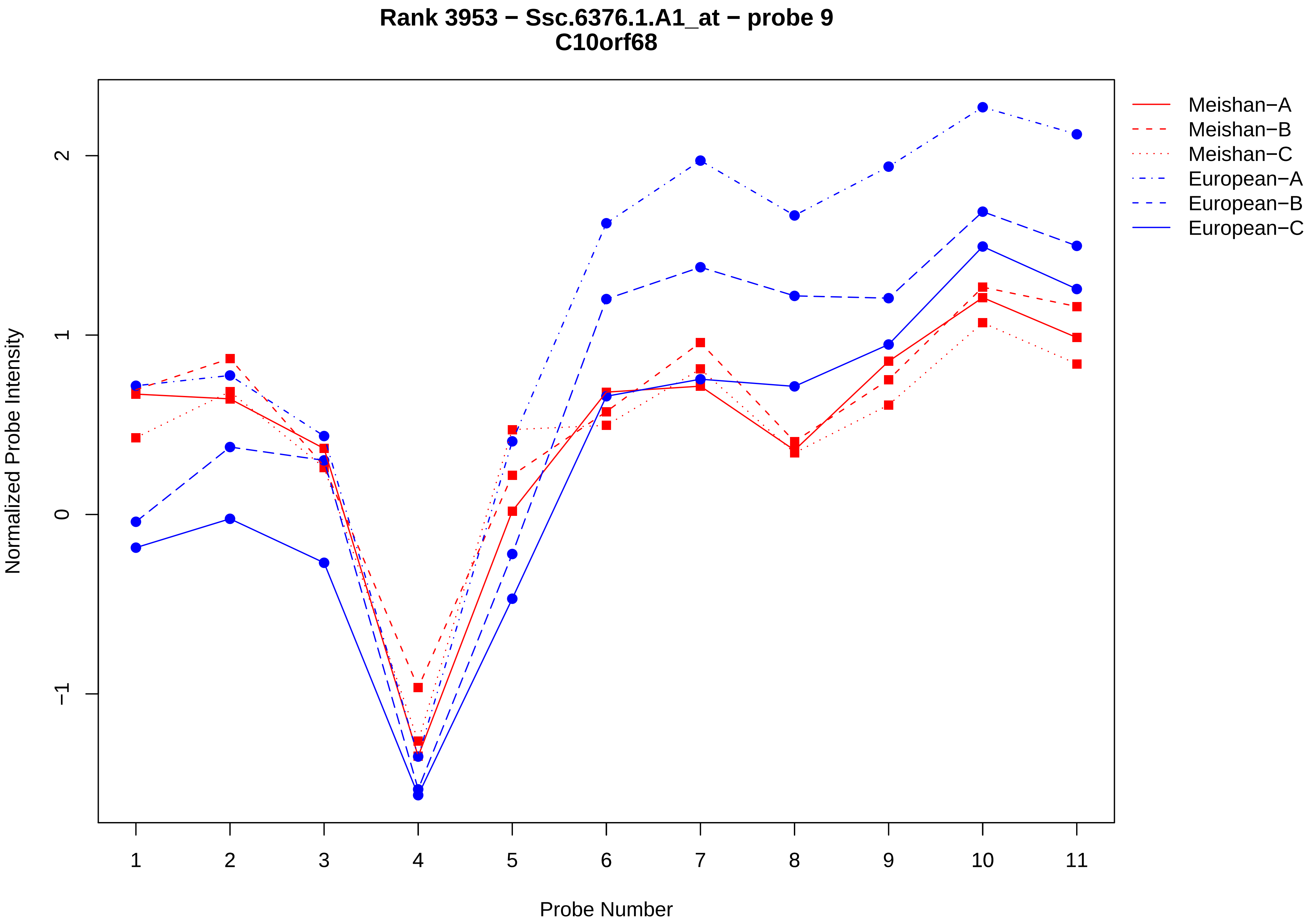
Rank 3953 − Ssc.6376.1.A1_at − probe 9
C10orf68
Normalized Probe Intensity
Probe Number
−1
0
1
2
1
2
3
4
5
6
7
8
9
10
11
Meishan−A
Meishan−B
Meishan−C
European−A
European−B
European−C

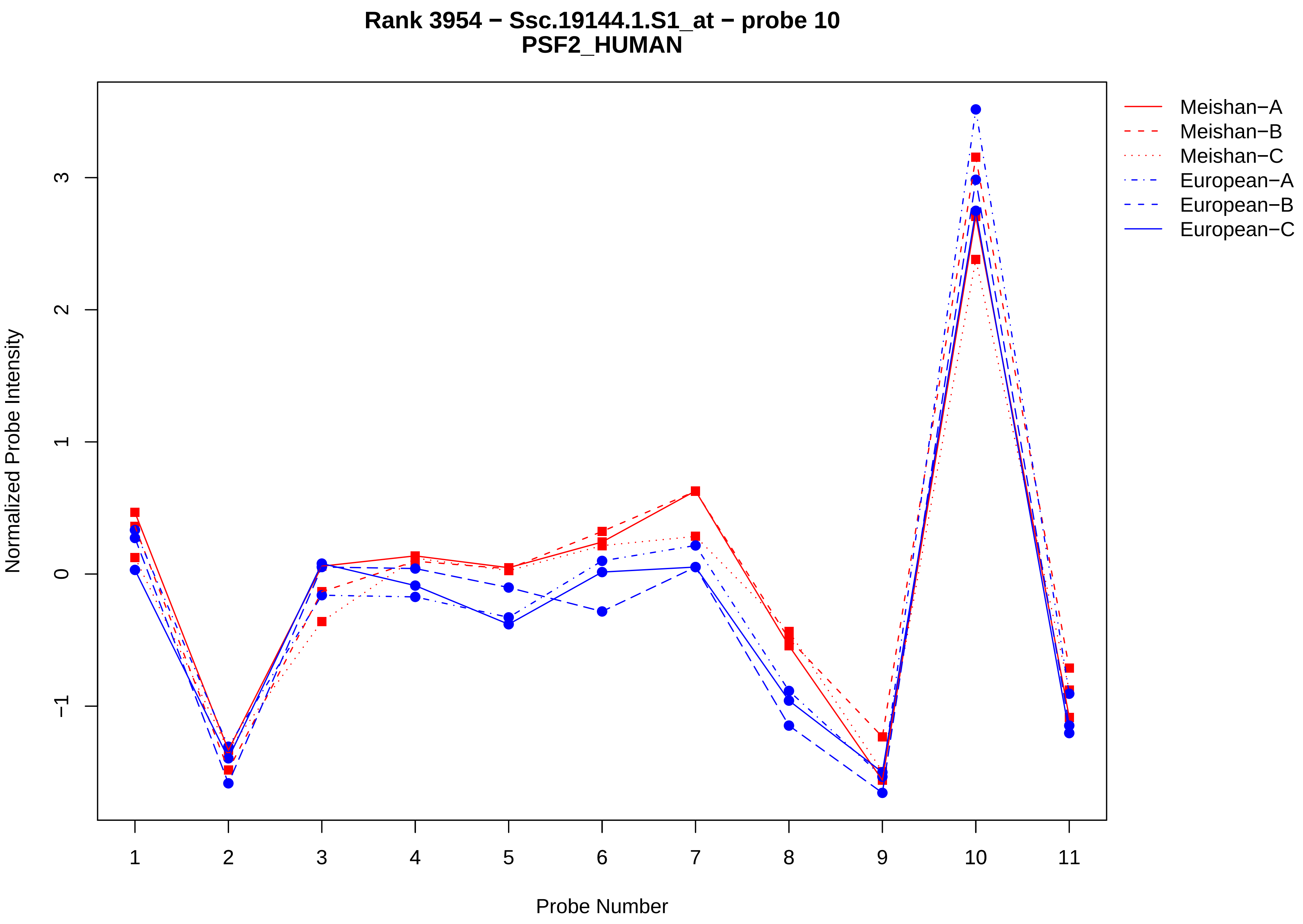
Rank 3954 − Ssc.19144.1.S1_at − probe 10
PSF2_HUMAN
Normalized Probe Intensity
Probe Number
Meishan−A
Meishan−B
Meishan−C
European−A
European−B
European−C
−1
0
1
2
3
1
2
3
4
5
6
7
8
9
10
11

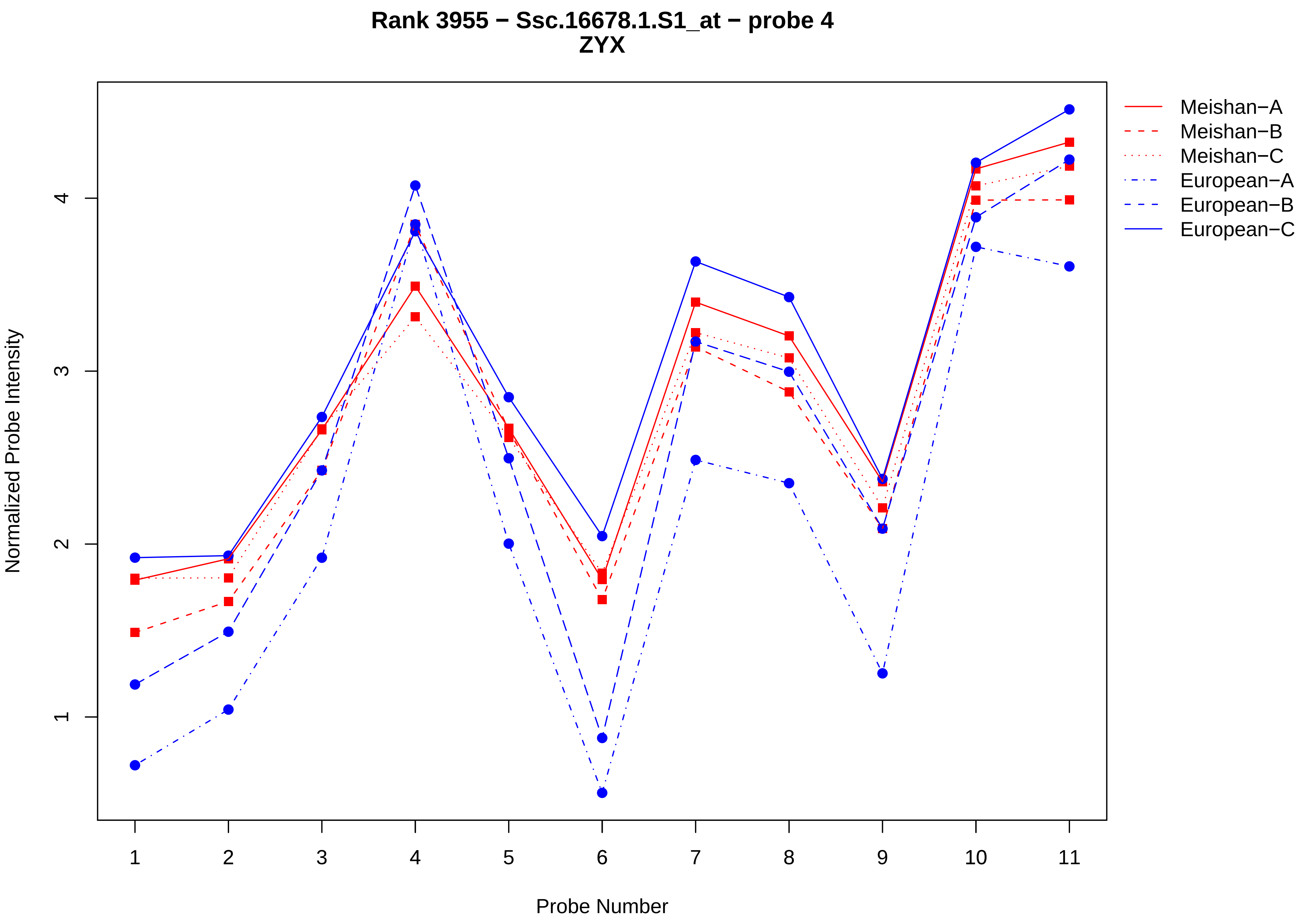

Rank 3955 − Ssc.16678.1.S1_at − probe 4
ZYX
Normalized Probe Intensity
Probe Number
1
2
3
4
5
6
7
8
9
10
11
Meishan−A
Meishan−B
Meishan−C
European−A
European−B
European−C

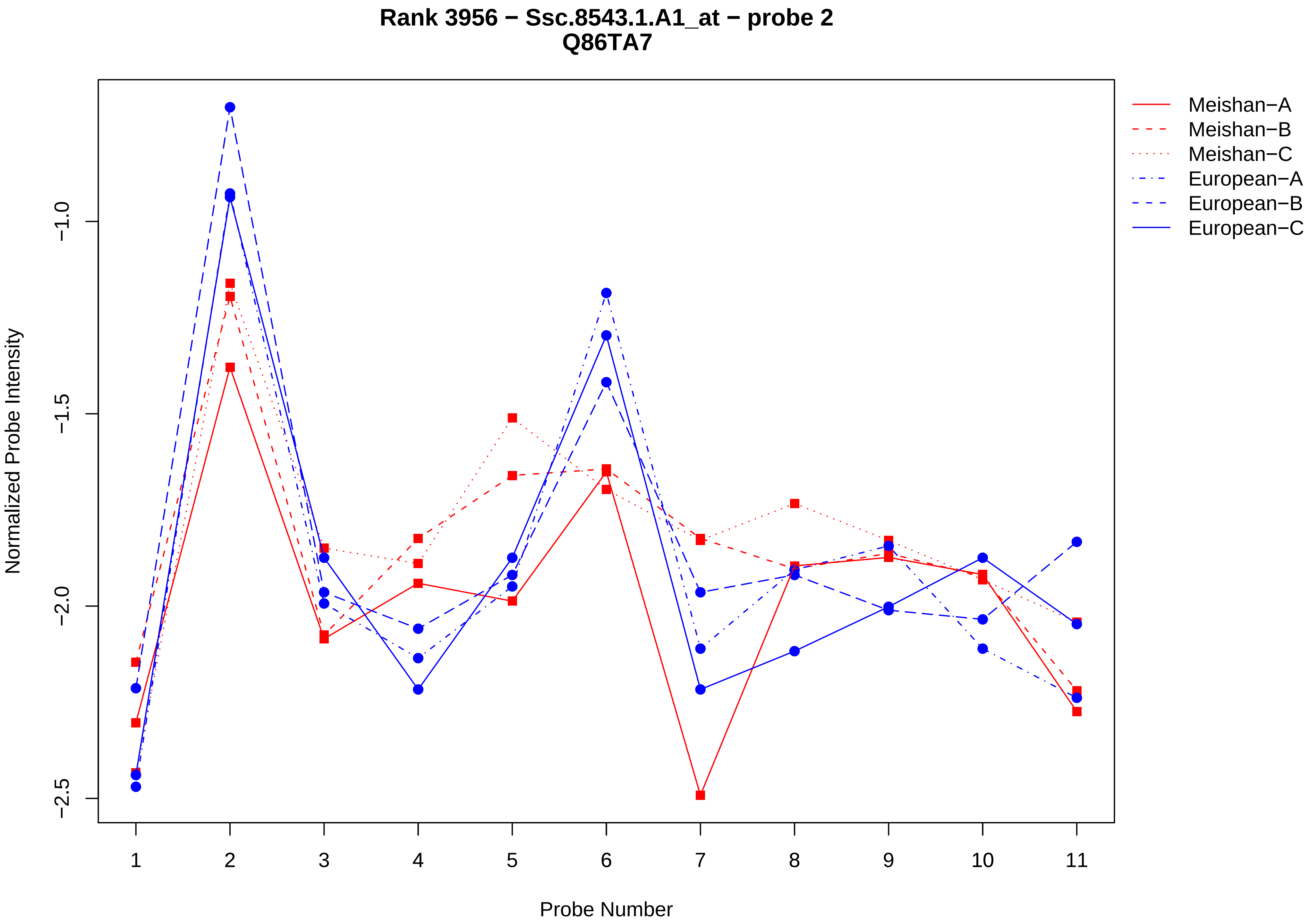

Rank 3956 − Ssc.8543.1.A1_at − probe 2
Q86TA7
Normalized Probe Intensity
−1.0
−1.5
−2.0
−2.5
1
2
3
4
5
6
7
8
9
10
11
Probe Number
Meishan−A
Meishan−B
Meishan−C
European−A
European−B
European−C

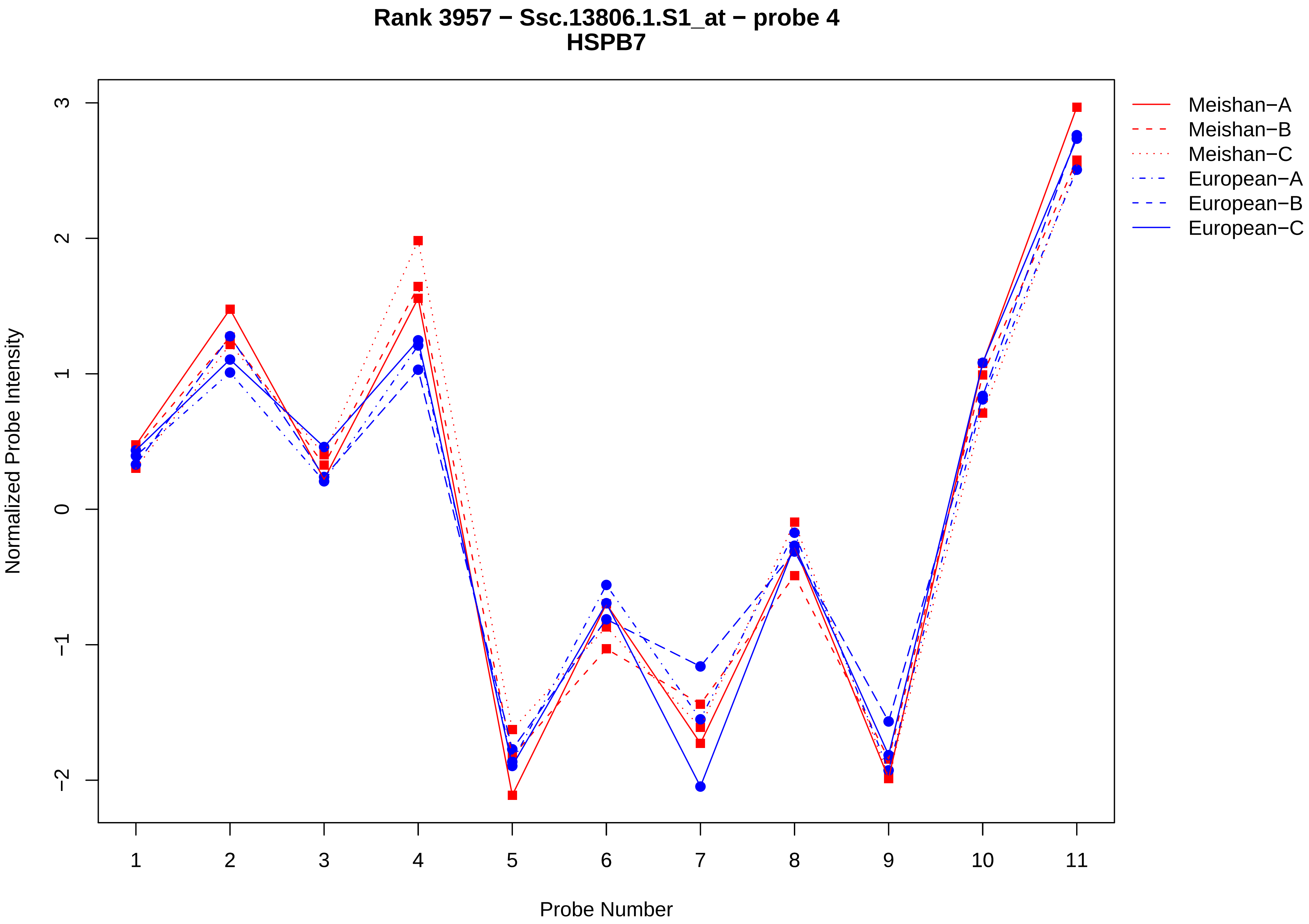

Rank 3957 − Ssc.13806.1.S1_at − probe 4
HSPB7
Normalized Probe Intensity
Probe Number
−2
−1
0
1
2
3
1
2
3
4
5
6
7
8
9
10
11
Meishan−A
Meishan−B
Meishan−C
European−A
European−B
European−C

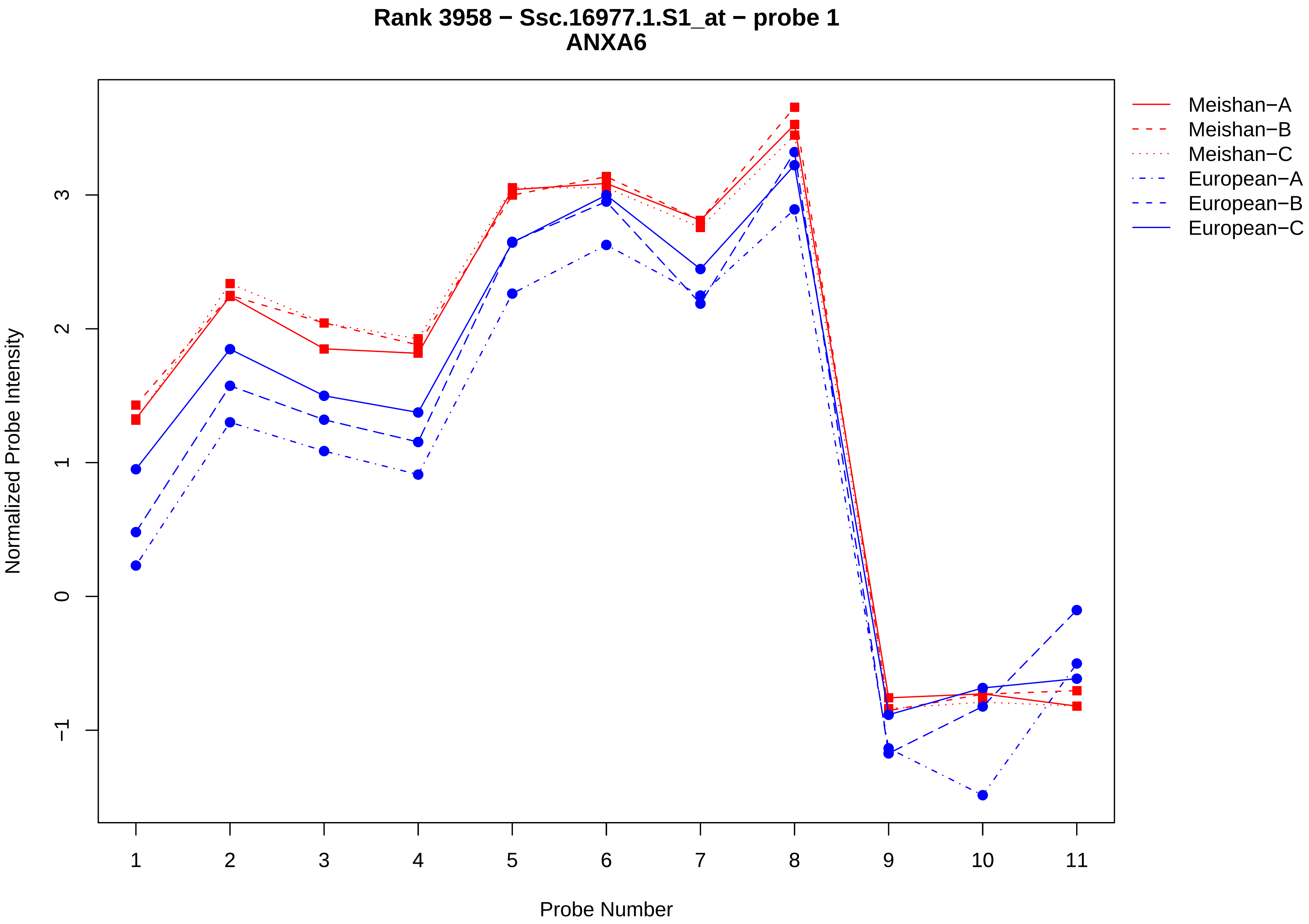

Rank 3958 − Ssc.16977.1.S1_at − probe 1
ANXA6
Normalized Probe Intensity
Probe Number
−1
0
1
2
3
1
2
3
4
5
6
7
8
9
10
11
Meishan−A
Meishan−B
Meishan−C
European−A
European−B
European−C

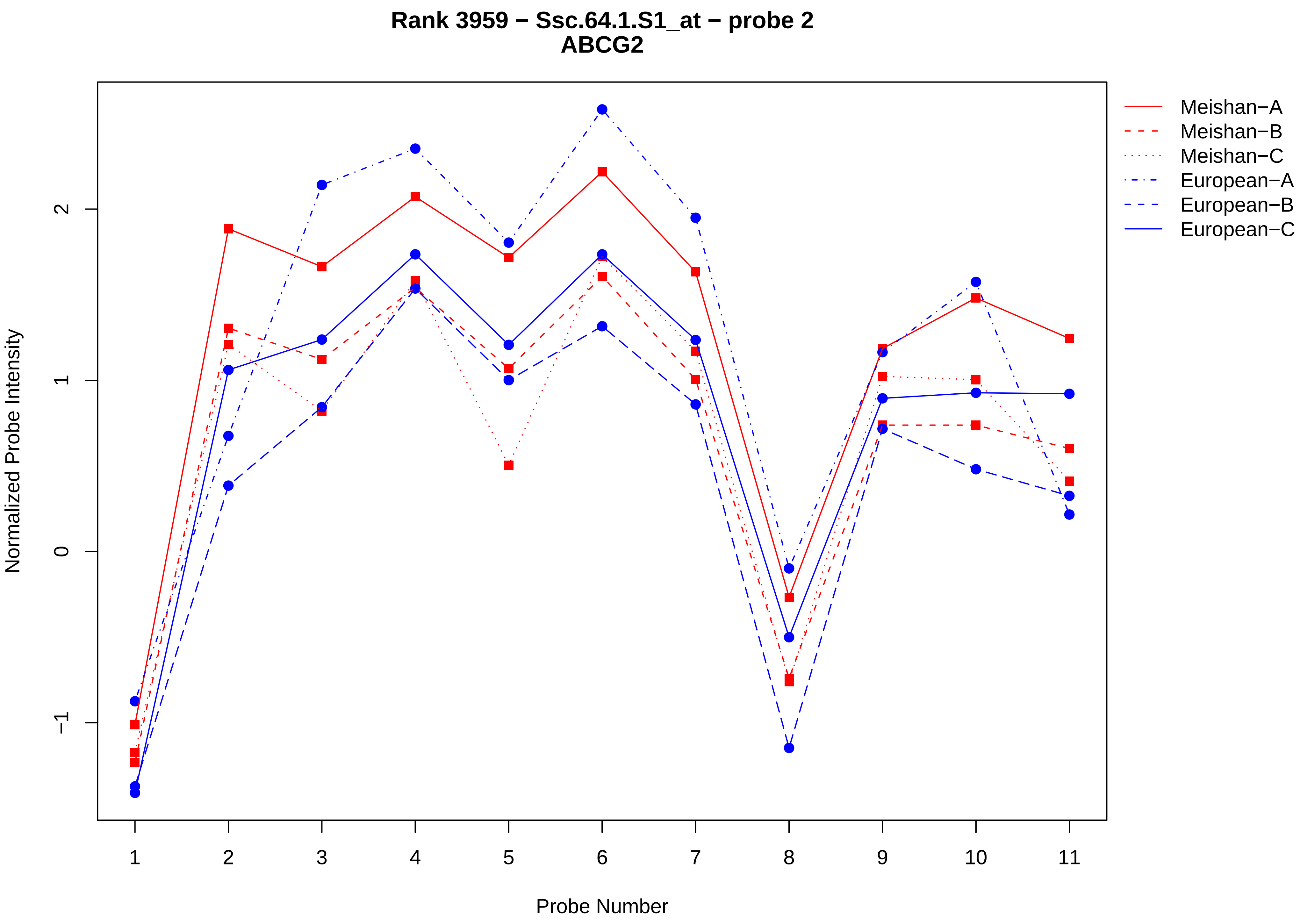

Rank 3959 − Ssc.64.1.S1_at − probe 2
ABCG2
Normalized Probe Intensity
Probe Number
−1
0
1
2
1
2
3
4
5
6
7
8
9
10
11
Meishan−A
Meishan−B
Meishan−C
European−A
European−B
European−C

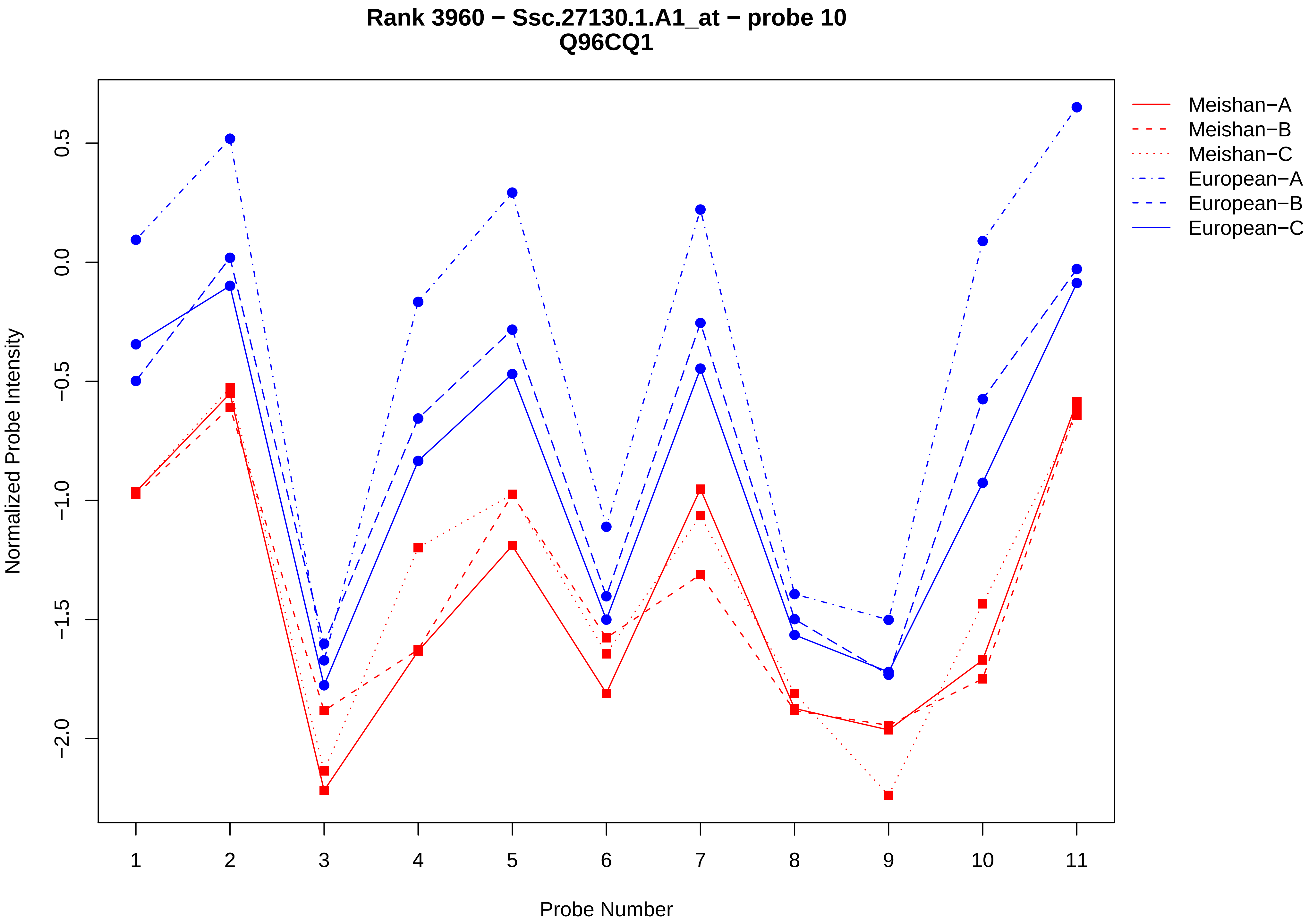

Rank 3960 − Ssc.27130.1.A1_at − probe 10
Q96CQ1
Normalized Probe Intensity
Probe Number
0.5
0.0
−0.5
−1.0
−1.5
−2.0
1
2
3
4
5
6
7
8
9
10
11
Meishan−A
Meishan−B
Meishan−C
European−A
European−B
European−C

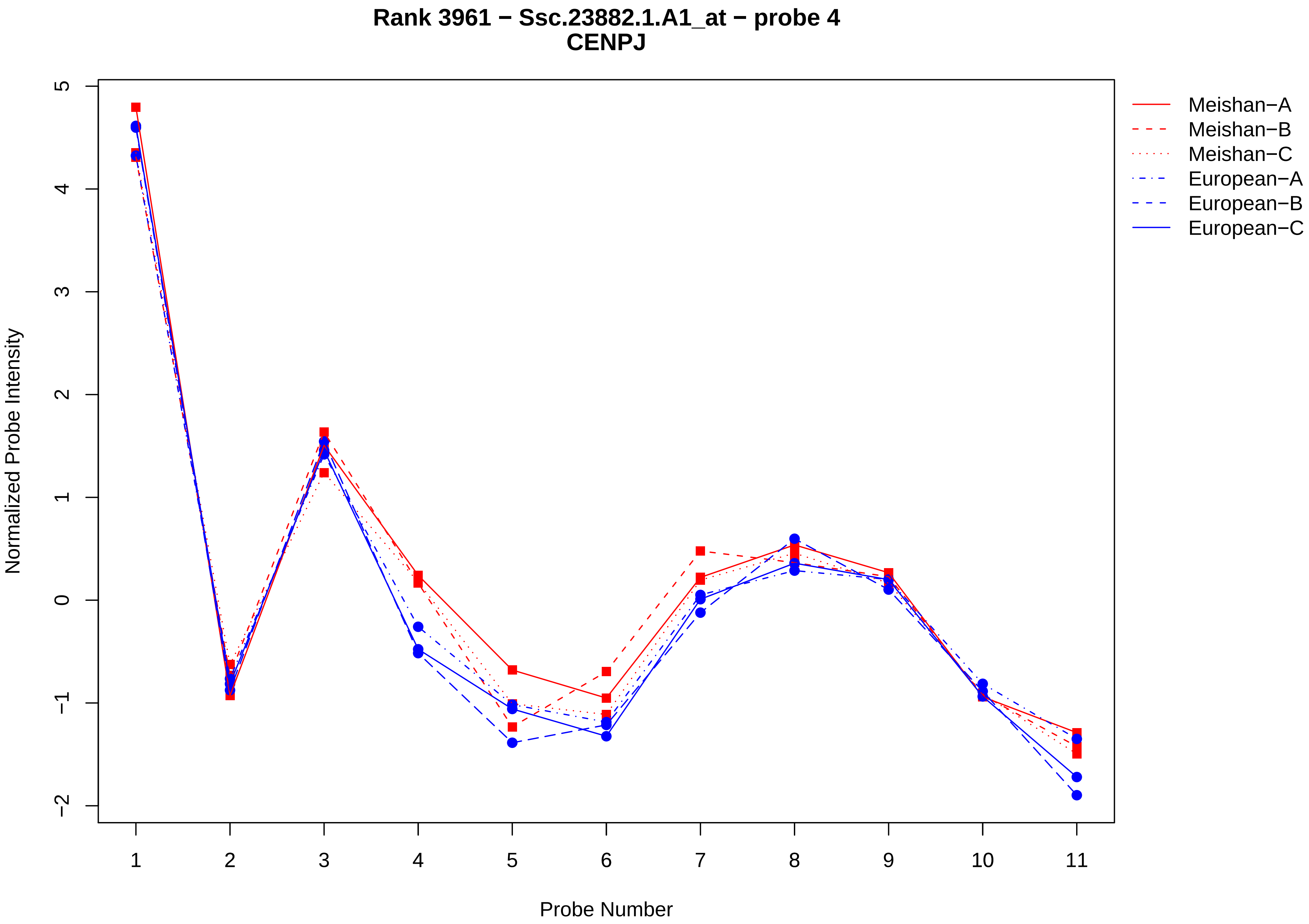

Rank 3961 − Ssc.23882.1.A1_at − probe 4
CENPJ
Normalized Probe Intensity
Probe Number
Meishan−A
Meishan−B
Meishan−C
European−A
European−B
European−C
−2
−1
0
1
2
3
4
5
1
2
3
4
5
6
7
8
9
10
11

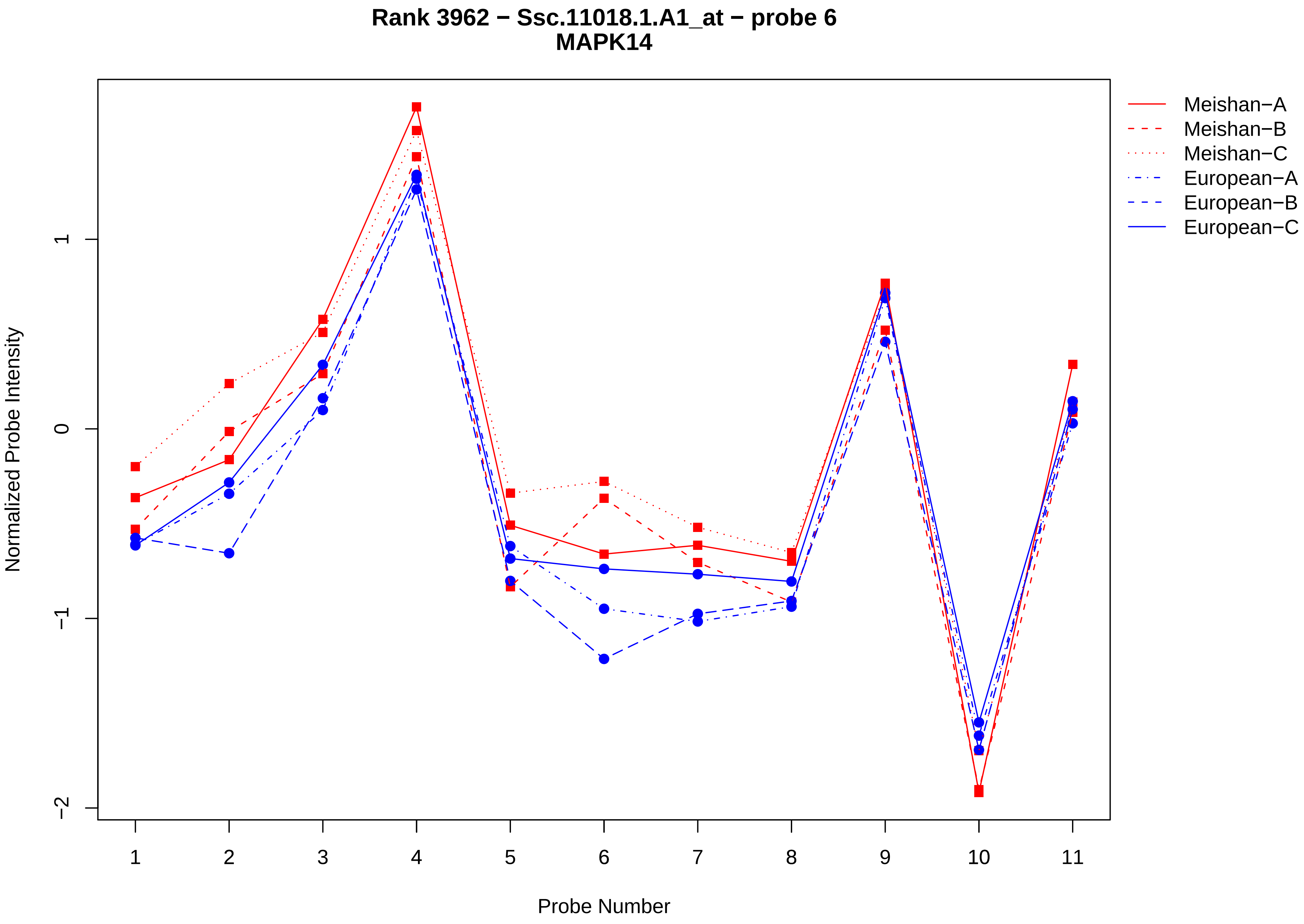

Rank 3962 − Ssc.11018.1.A1_at − probe 6
MAPK14
Normalized Probe Intensity
Probe Number
1
0
−1
−2
1
2
3
4
5
6
7
8
9
10
11
Meishan−A
Meishan−B
Meishan−C
European−A
European−B
European−C

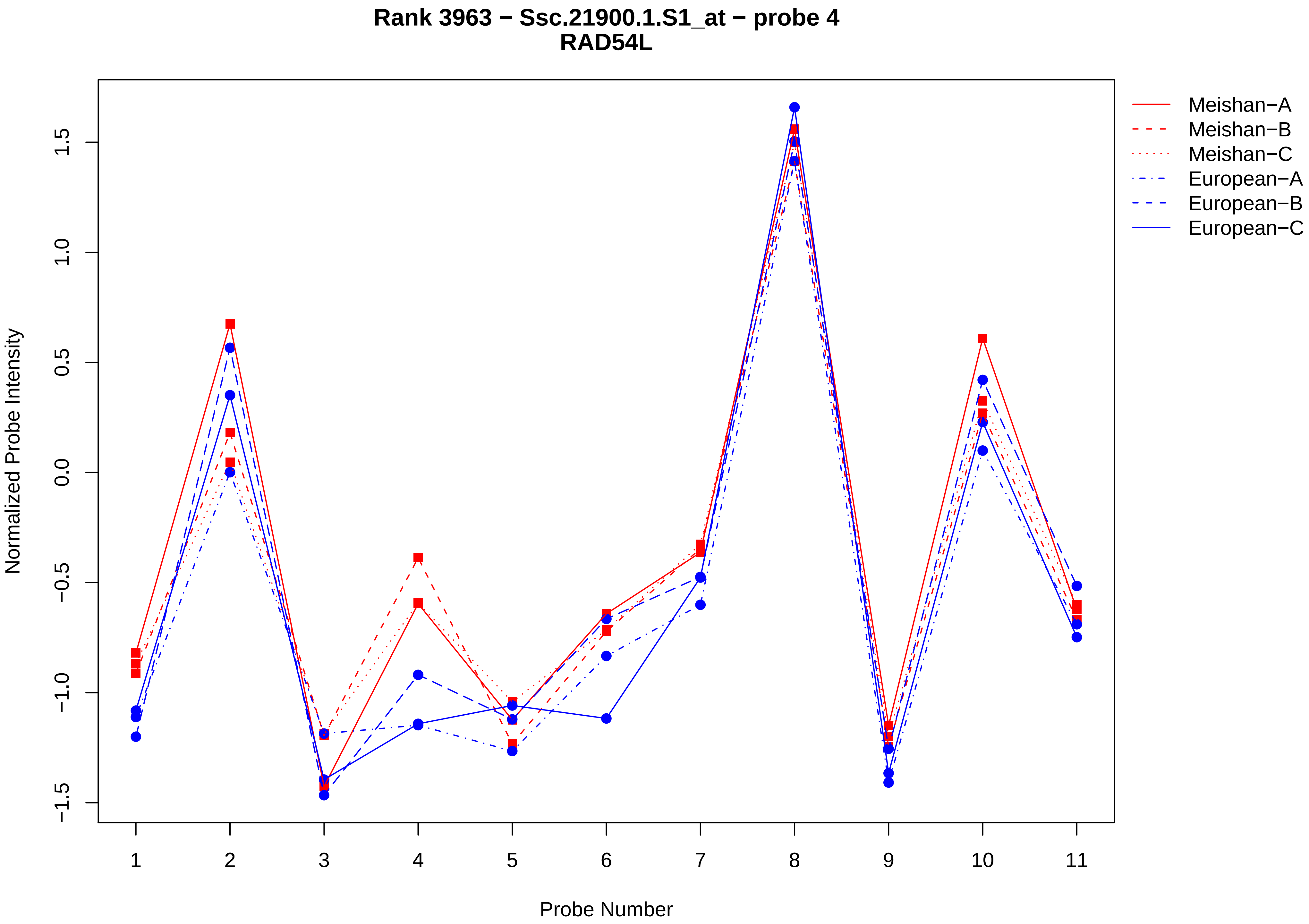

Rank 3963 − Ssc.21900.1.S1_at − probe 4
RAD54L
Normalized Probe Intensity
Probe Number
−1.5
−1.0
−0.5
0.0
0.5
1.0
1.5
1
2
3
4
5
6
7
8
9
10
11
Meishan−A
Meishan−B
Meishan−C
European−A
European−B
European−C

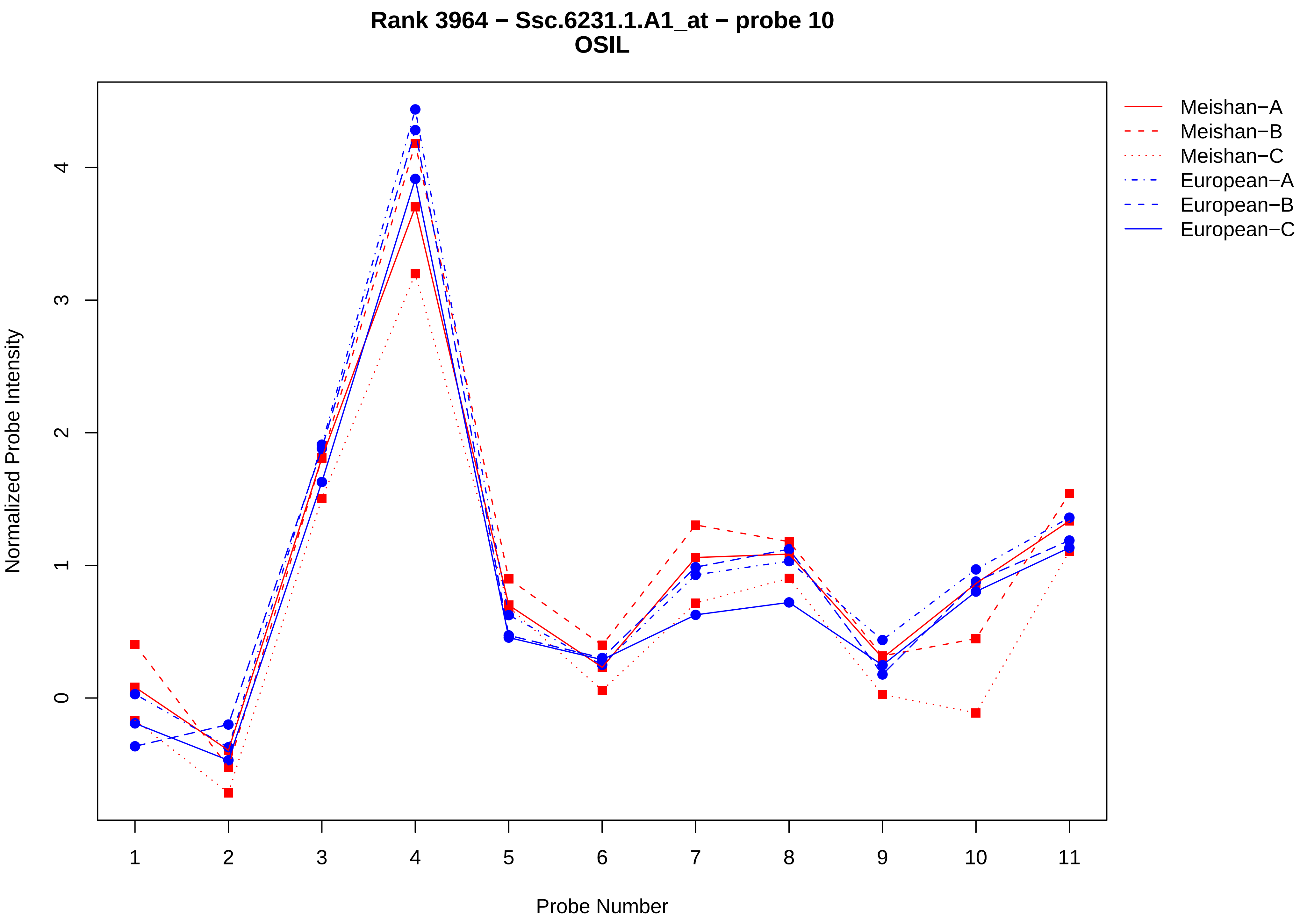

Rank 3964 − Ssc.6231.1.A1_at − probe 10
OSIL
Normalized Probe Intensity
Probe Number
Meishan−A
Meishan−B
Meishan−C
European−A
European−B
European−C
0
1
2
3
4
1
2
3
4
5
6
7
8
9
10
11

Rank 3965 − Ssc.7067.1.A1_at − probe 8
TM4SF6

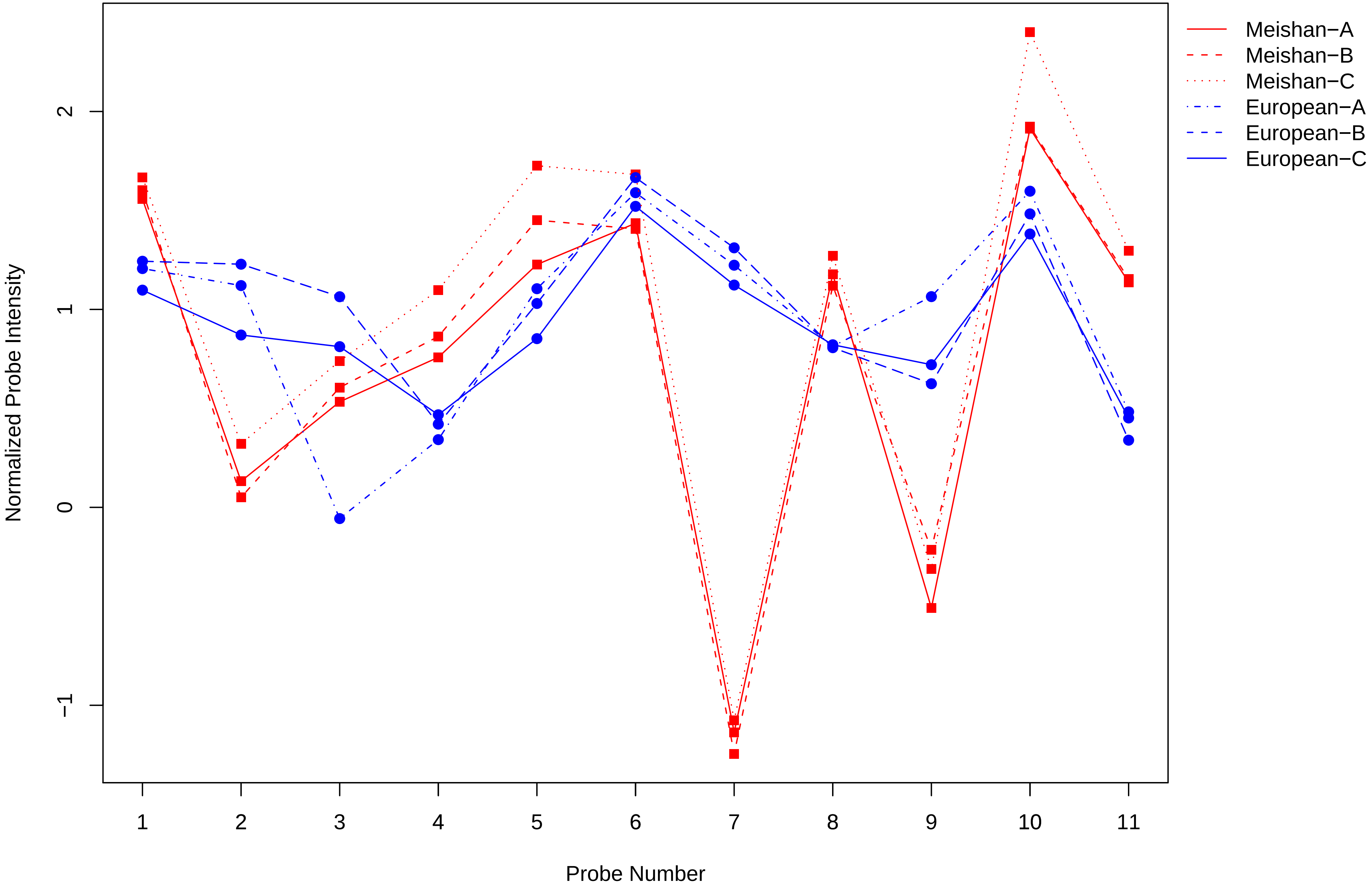

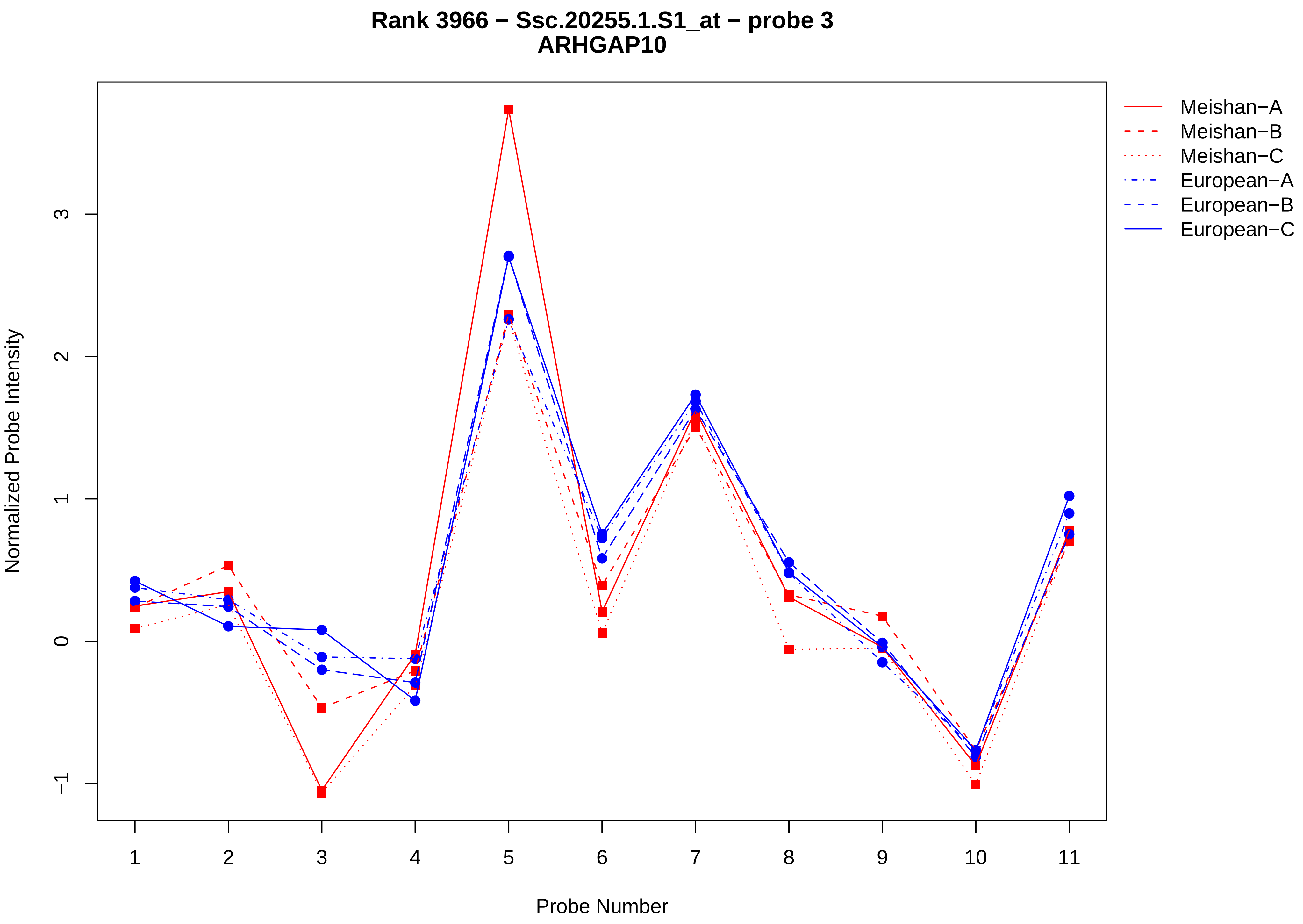
Rank 3966 − Ssc.20255.1.S1_at − probe 3
ARHGAP10
Normalized Probe Intensity
Probe Number
−1
0
1
2
3
1
2
3
4
5
6
7
8
9
10
11
Meishan−A
Meishan−B
Meishan−C
European−A
European−B
European−C

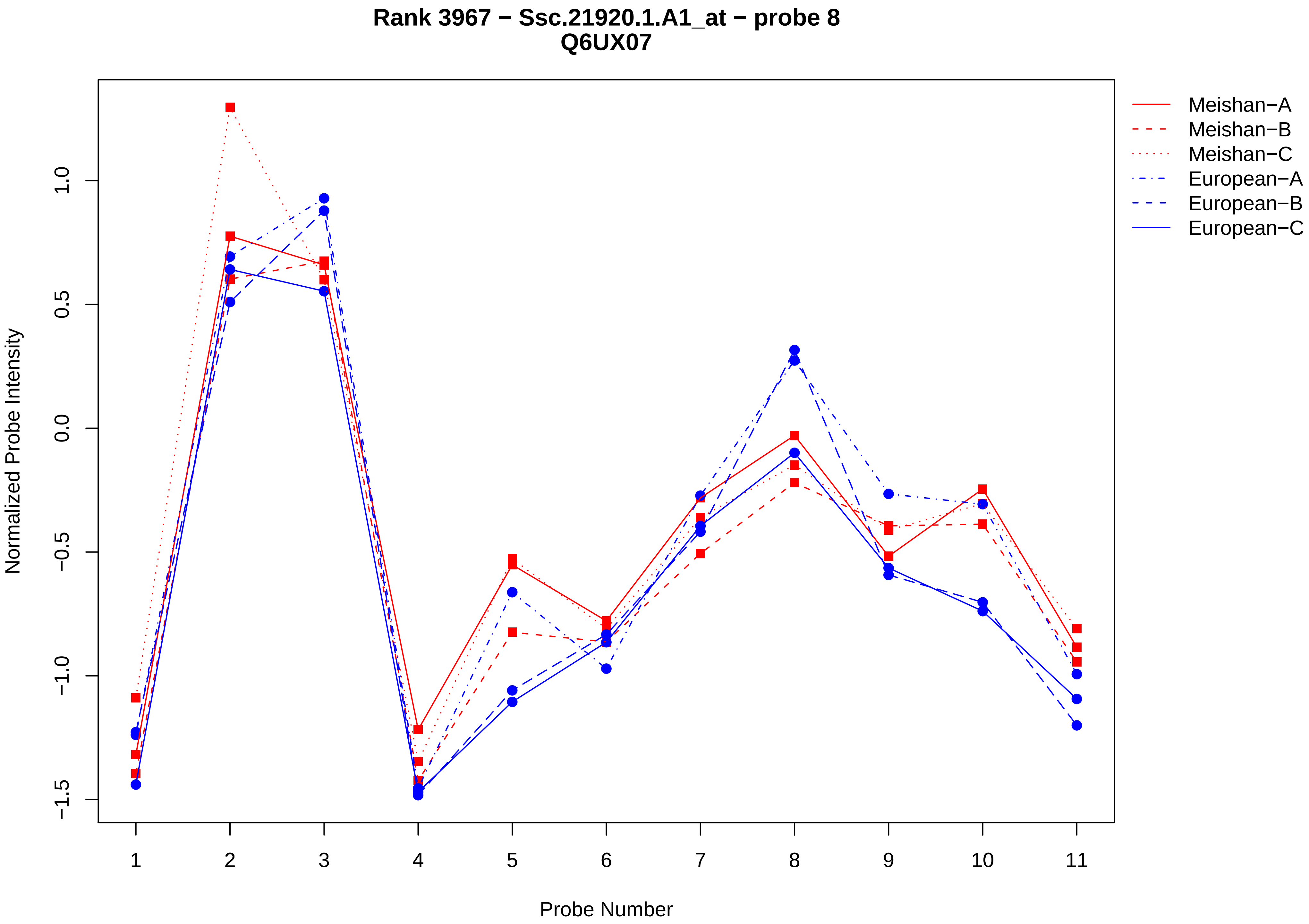

Rank 3967 − Ssc.21920.1.A1_at − probe 8
Q6UX07
Normalized Probe Intensity
Probe Number
Meishan−A
Meishan−B
Meishan−C
European−A
European−B
European−C
−1.5
−1.0
−0.5
0.0
0.5
1.0
1
2
3
4
5
6
7
8
9
10
11

Rank 3968 − Ssc.27439.1.S1_at − probe 1
Q9NVY1

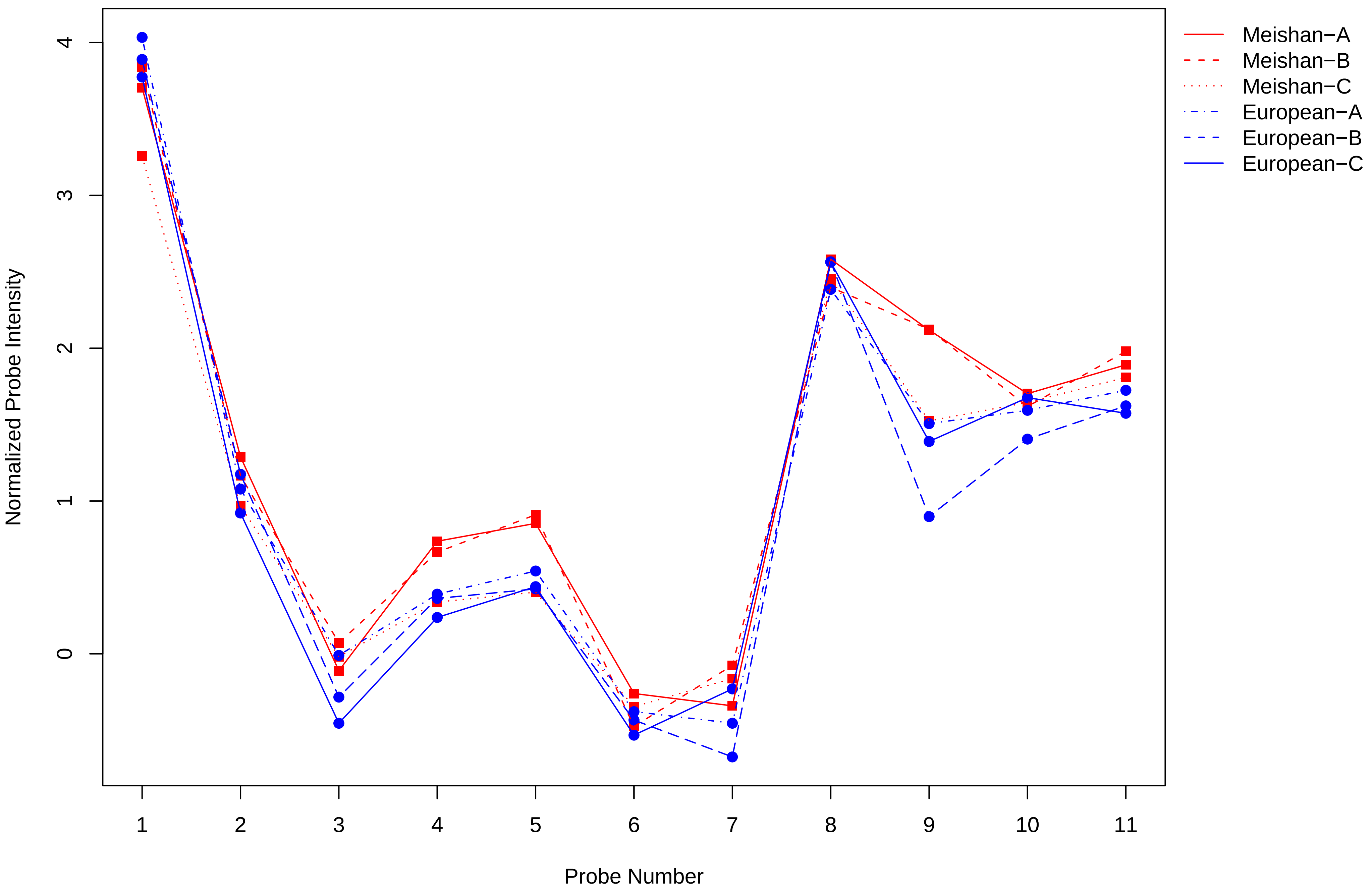

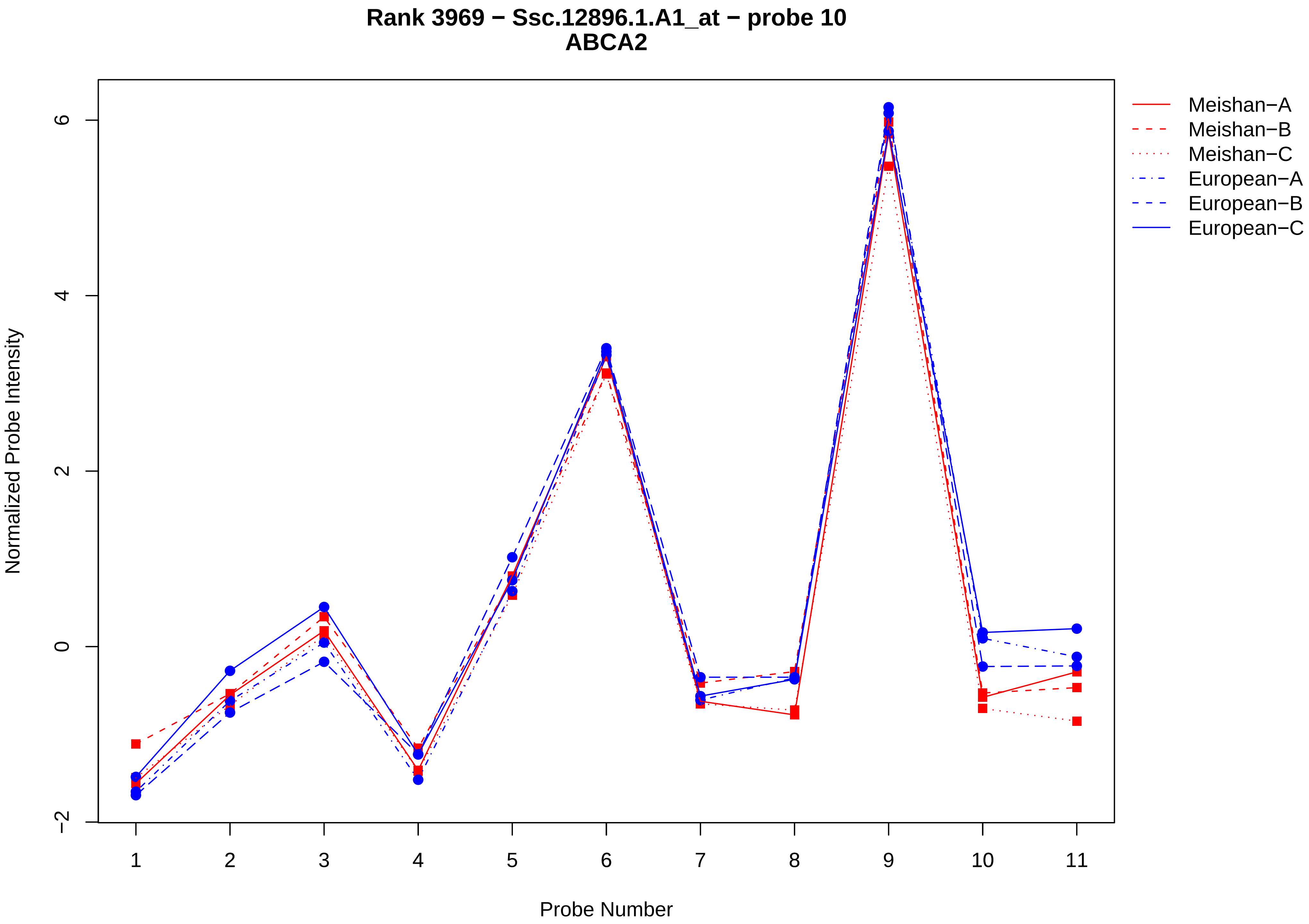

Rank 3969 − Ssc.12896.1.A1_at − probe 10
ABCA2
Normalized Probe Intensity
Probe Number
−2
0
2
4
6
1
2
3
4
5
6
7
8
9
10
11
Meishan−A
Meishan−B
Meishan−C
European−A
European−B
European−C

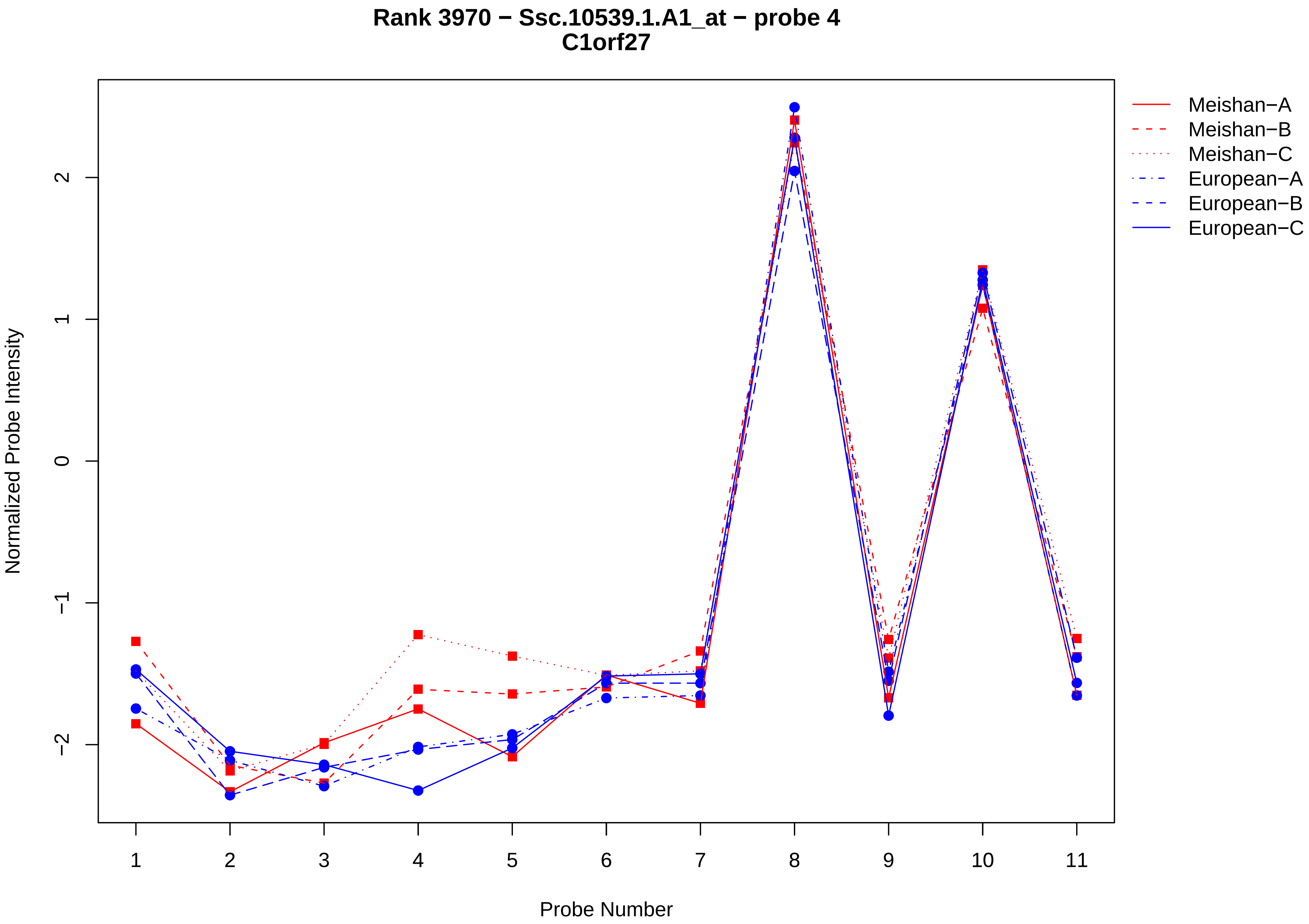

Rank 3970 − Ssc.10539.1.A1_at − probe 4
C1orf27
Normalized Probe Intensity
Probe Number
Meishan−A
Meishan−B
Meishan−C
European−A
European−B
European−C
2
1
0
−1
−2
1
2
3
4
5
6
7
8
9
10
11

Rank 3971 − Ssc.24045.1.S1_at − probe 11
NP_612404

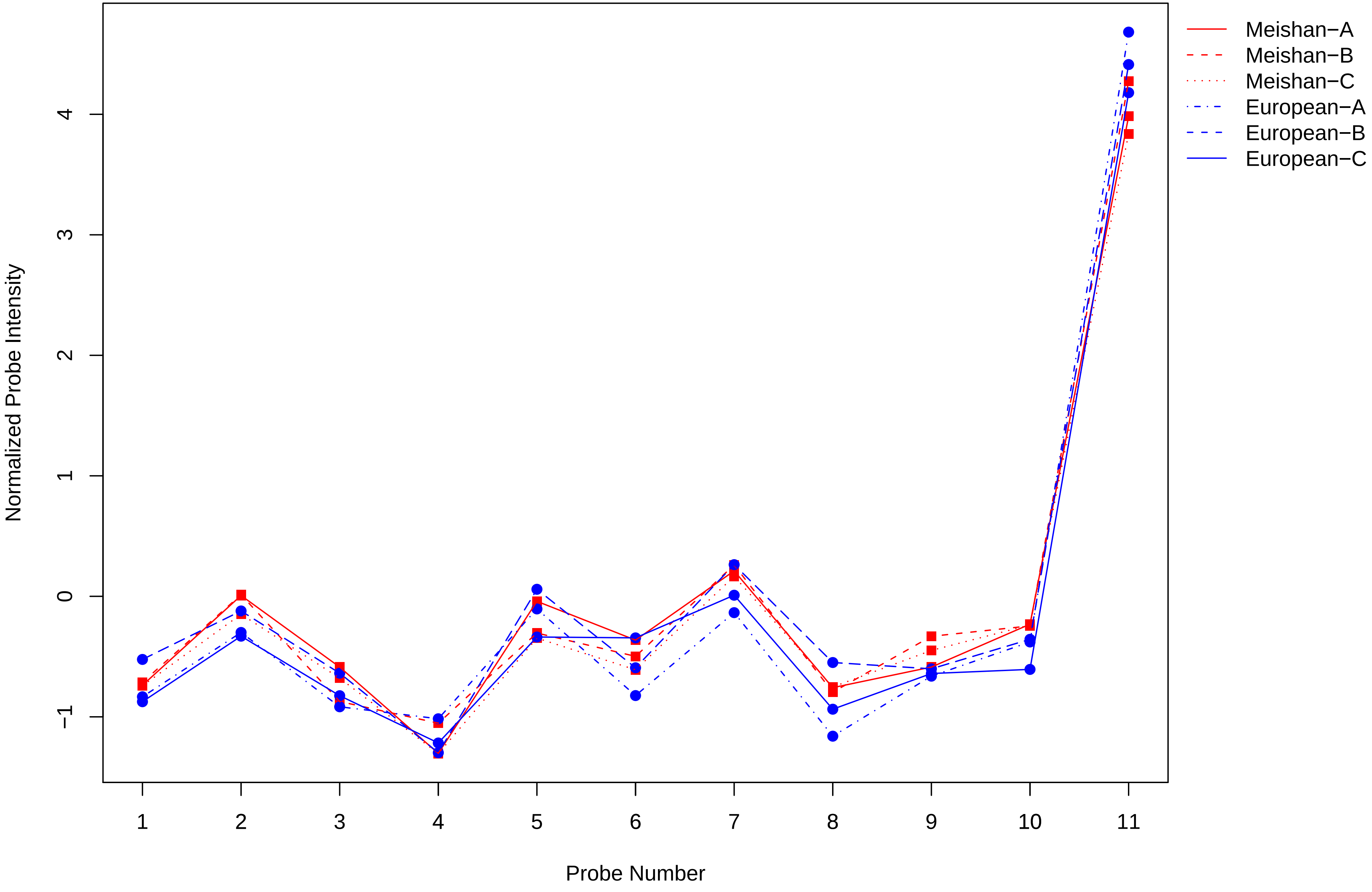

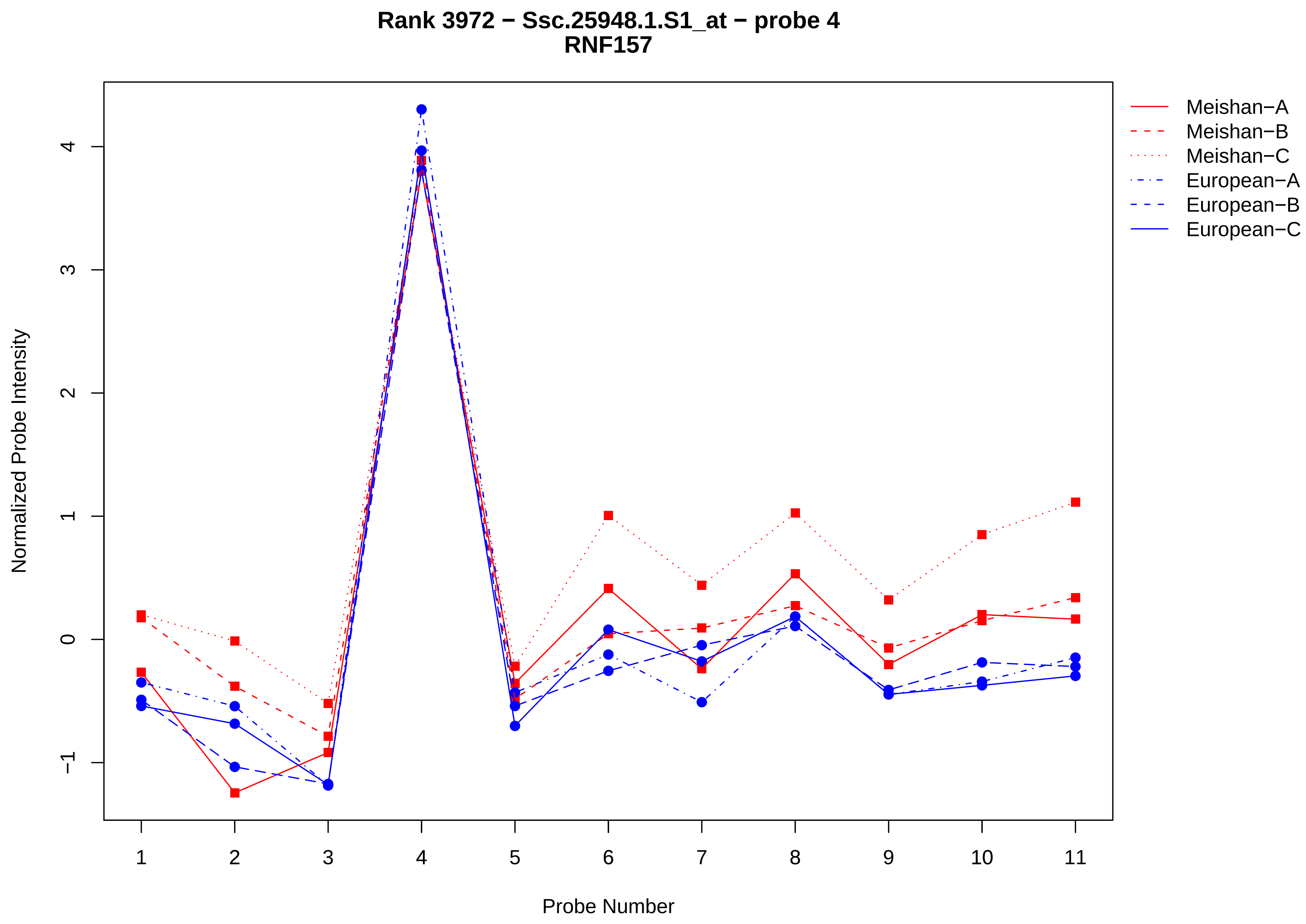

Rank 3972 − Ssc.25948.1.S1_at − probe 4
RNF157
Normalized Probe Intensity
Probe Number
−1
0
1
2
3
4
1
2
3
4
5
6
7
8
9
10
11
Meishan−A
Meishan−B
Meishan−C
European−A
European−B
European−C

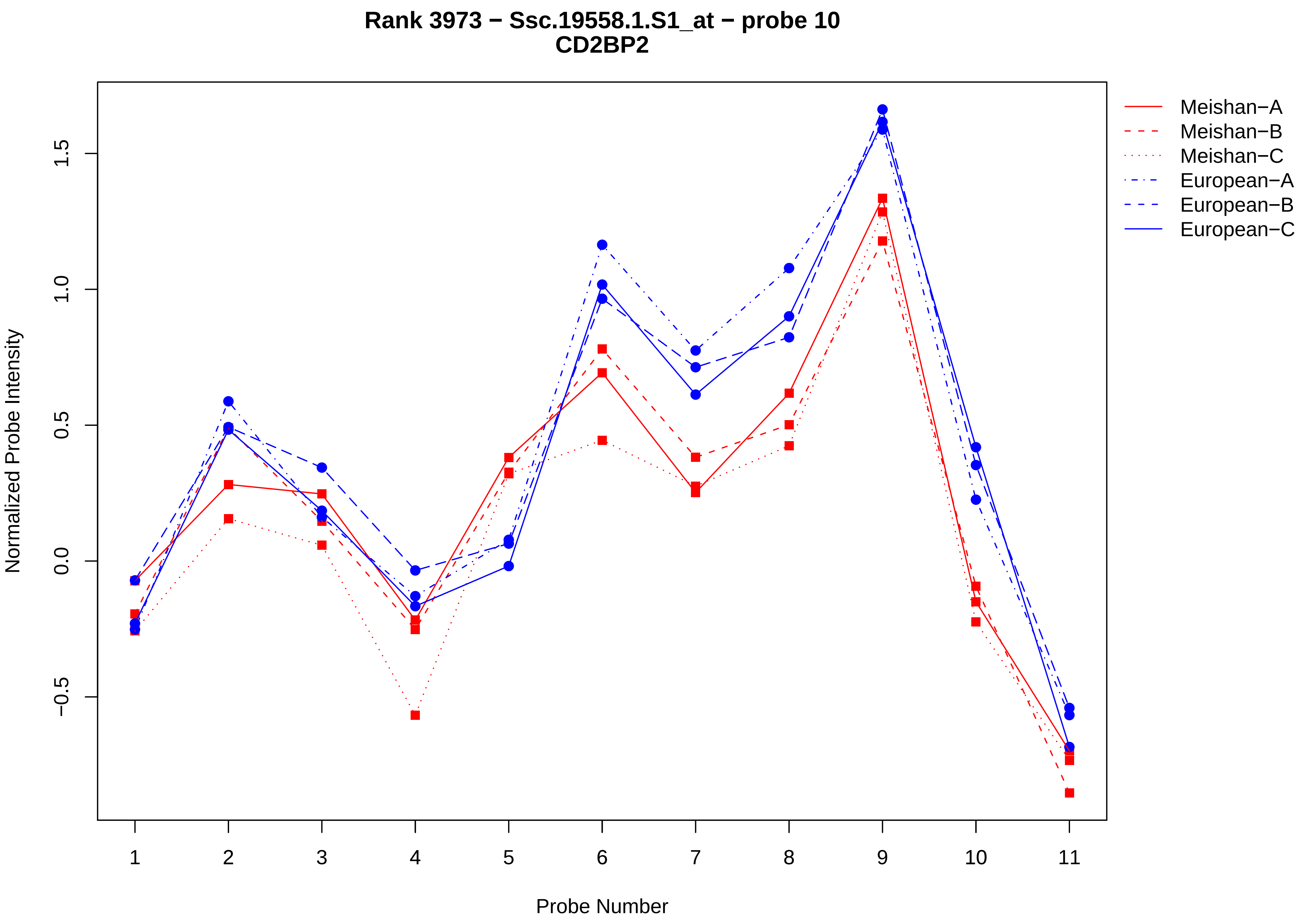
Rank 3973 − Ssc.19558.1.S1_at − probe 10
CD2BP2
Normalized Probe Intensity
Probe Number
−0.5
0.0
0.5
1.0
1.5
1
2
3
4
5
6
7
8
9
10
11
Meishan−A
Meishan−B
Meishan−C
European−A
European−B
European−C

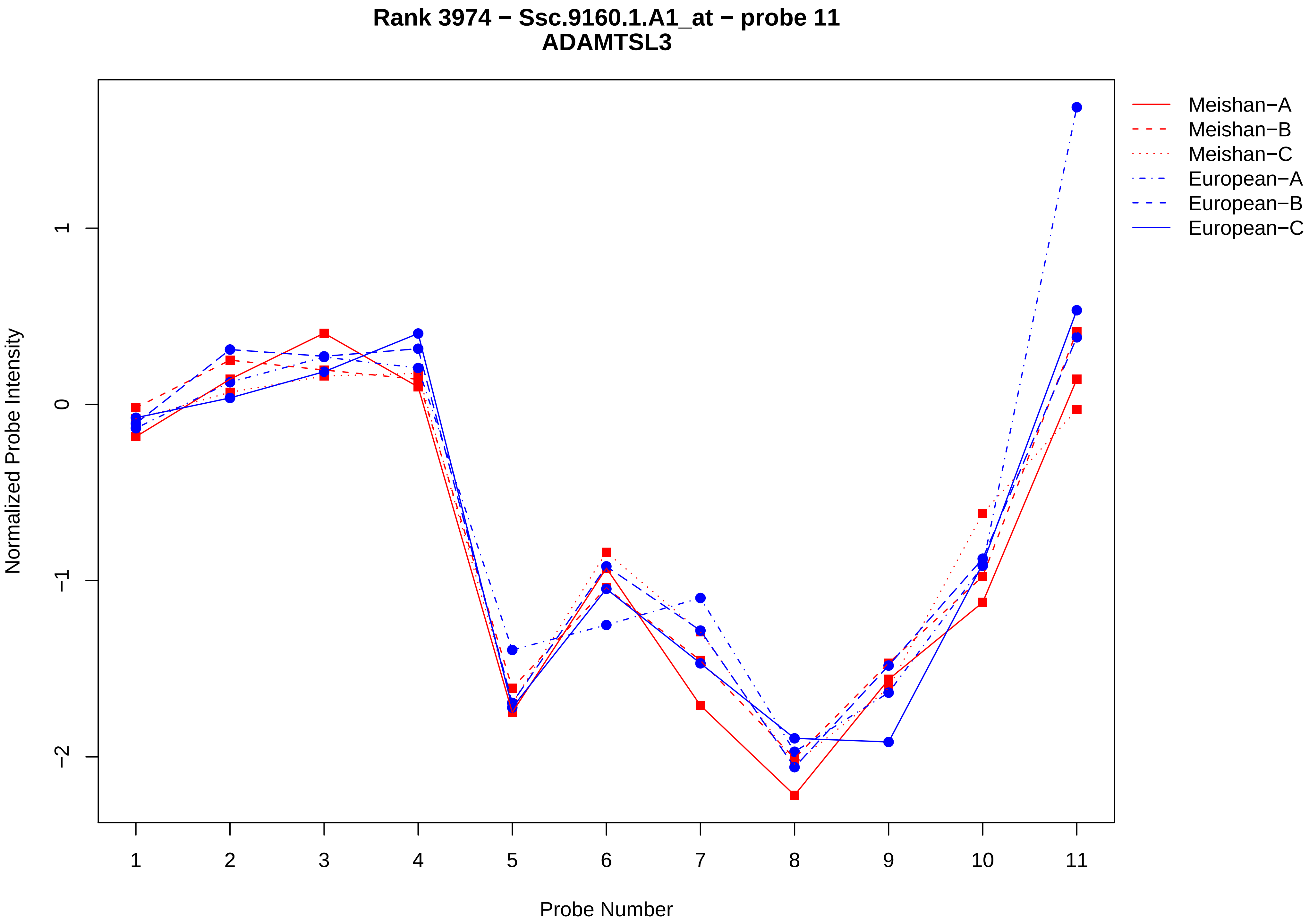

Rank 3974 − Ssc.9160.1.A1_at − probe 11
ADAMTSL3
Normalized Probe Intensity
Probe Number
Meishan−A
Meishan−B
Meishan−C
European−A
European−B
European−C

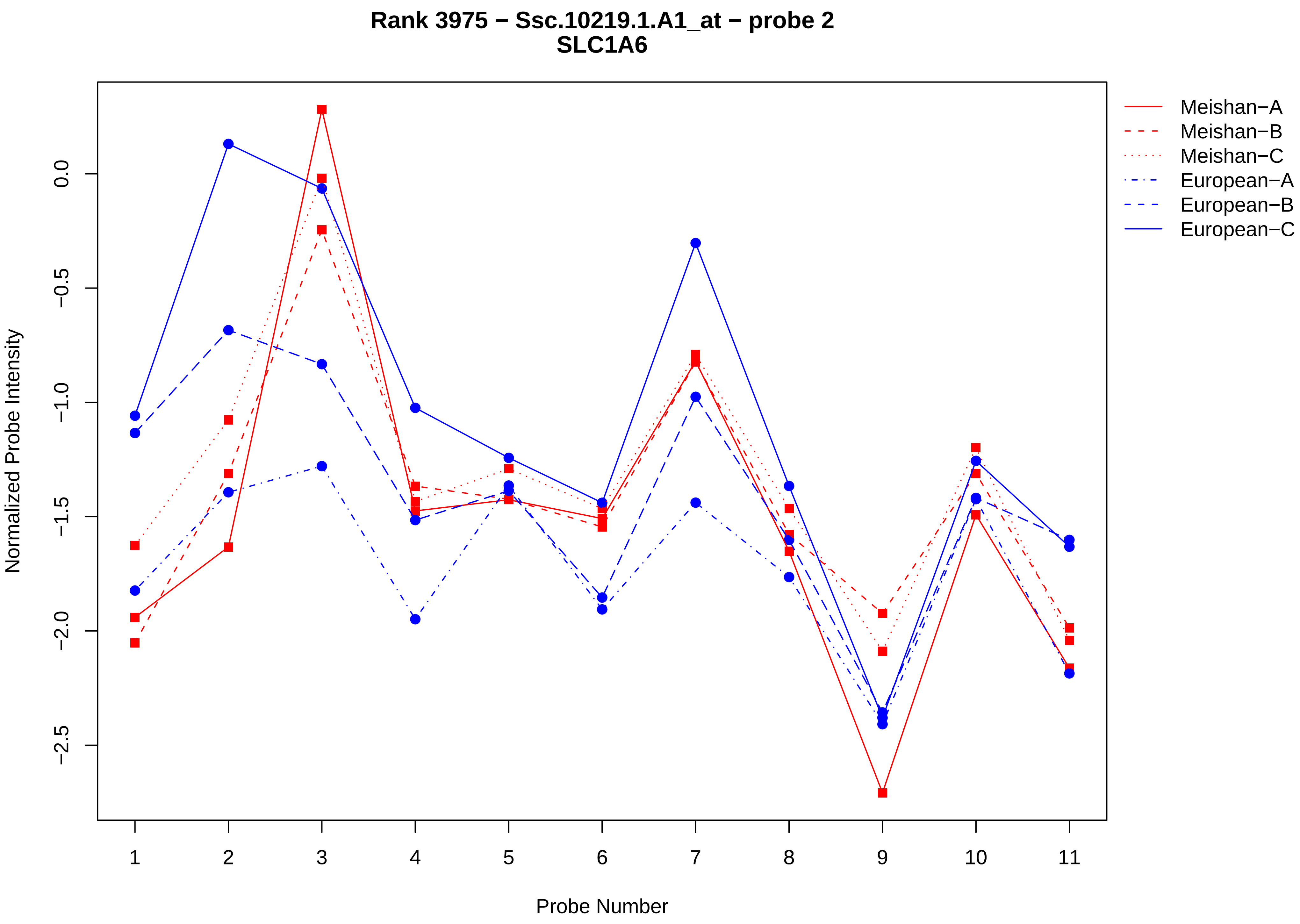

Rank 3975 − Ssc.10219.1.A1_at − probe 2
SLC1A6
Normalized Probe Intensity
Probe Number
0.0
−0.5
−1.0
−1.5
−2.0
−2.5
1
2
3
4
5
6
7
8
9
10
11
Meishan−A
Meishan−B
Meishan−C
European−A
European−B
European−C

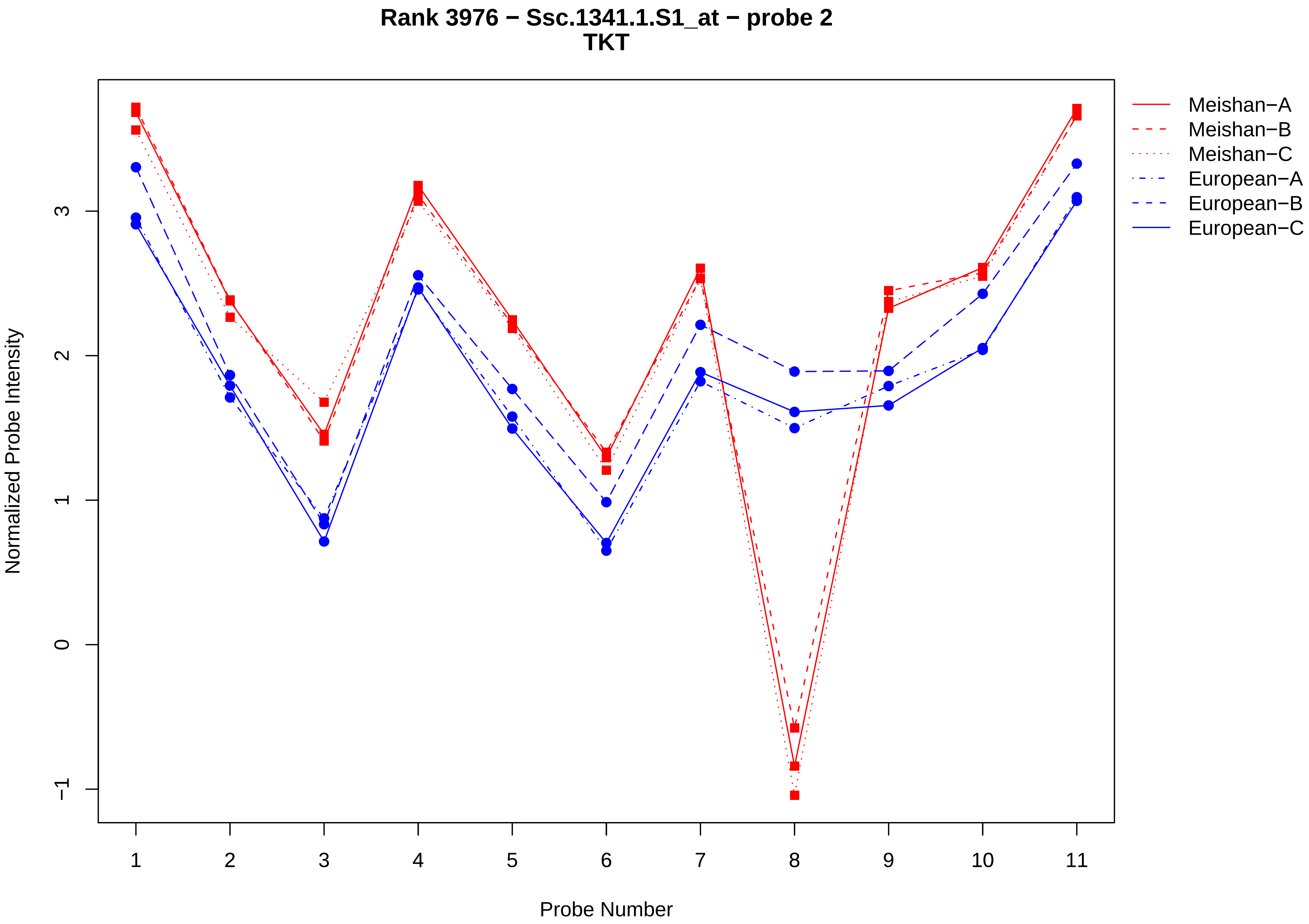
Rank 3976 − Ssc.1341.1.S1_at − probe 2
TKT
Normalized Probe Intensity
Probe Number
−1
0
1
2
3
1
2
3
4
5
6
7
8
9
10
11
Meishan−A
Meishan−B
Meishan−C
European−A
European−B
European−C

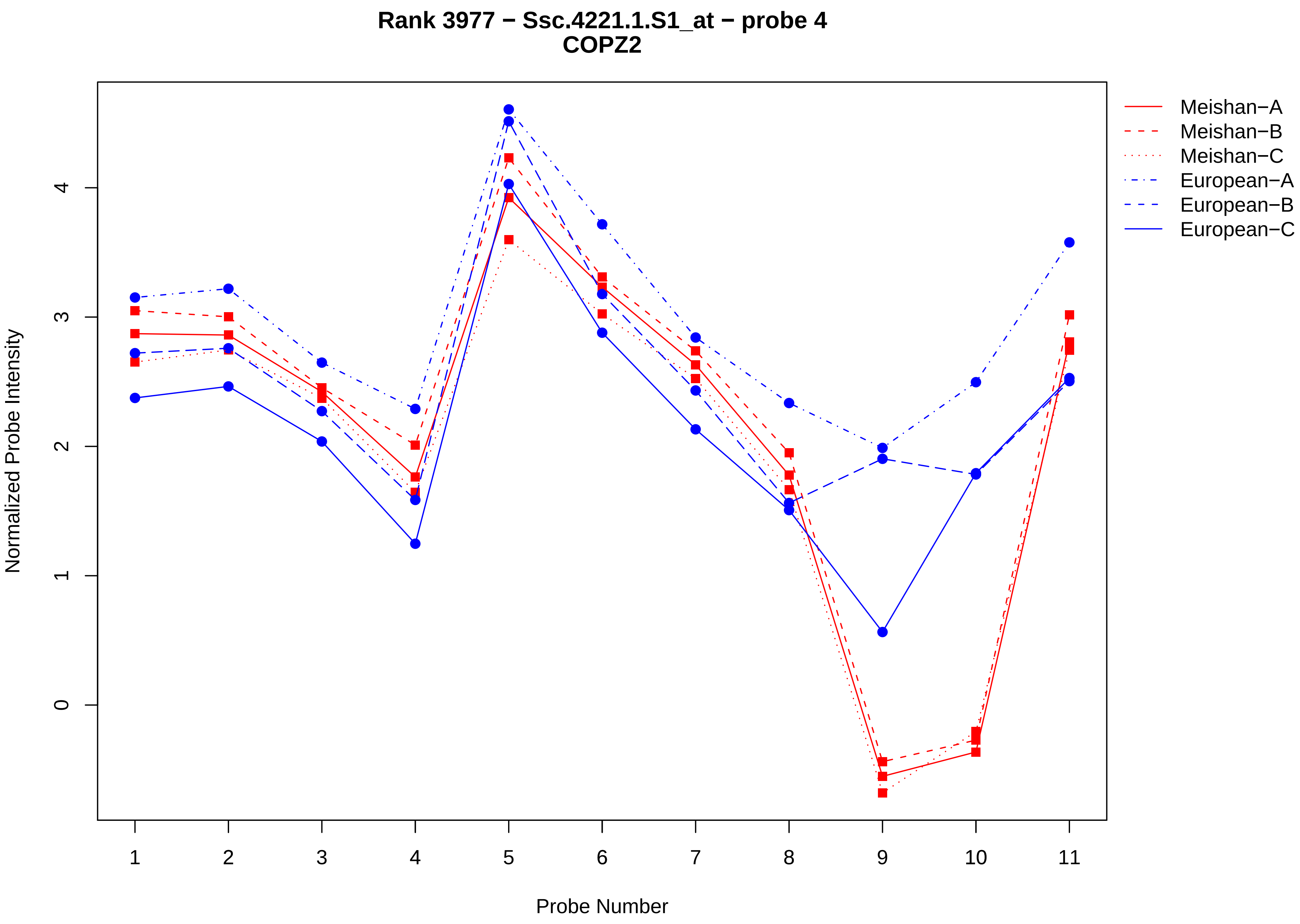

Rank 3977 − Ssc.4221.1.S1_at − probe 4
COPZ2
Normalized Probe Intensity
Probe Number
0
1
2
3
4
1
2
3
4
5
6
7
8
9
10
11
Meishan−A
Meishan−B
Meishan−C
European−A
European−B
European−C

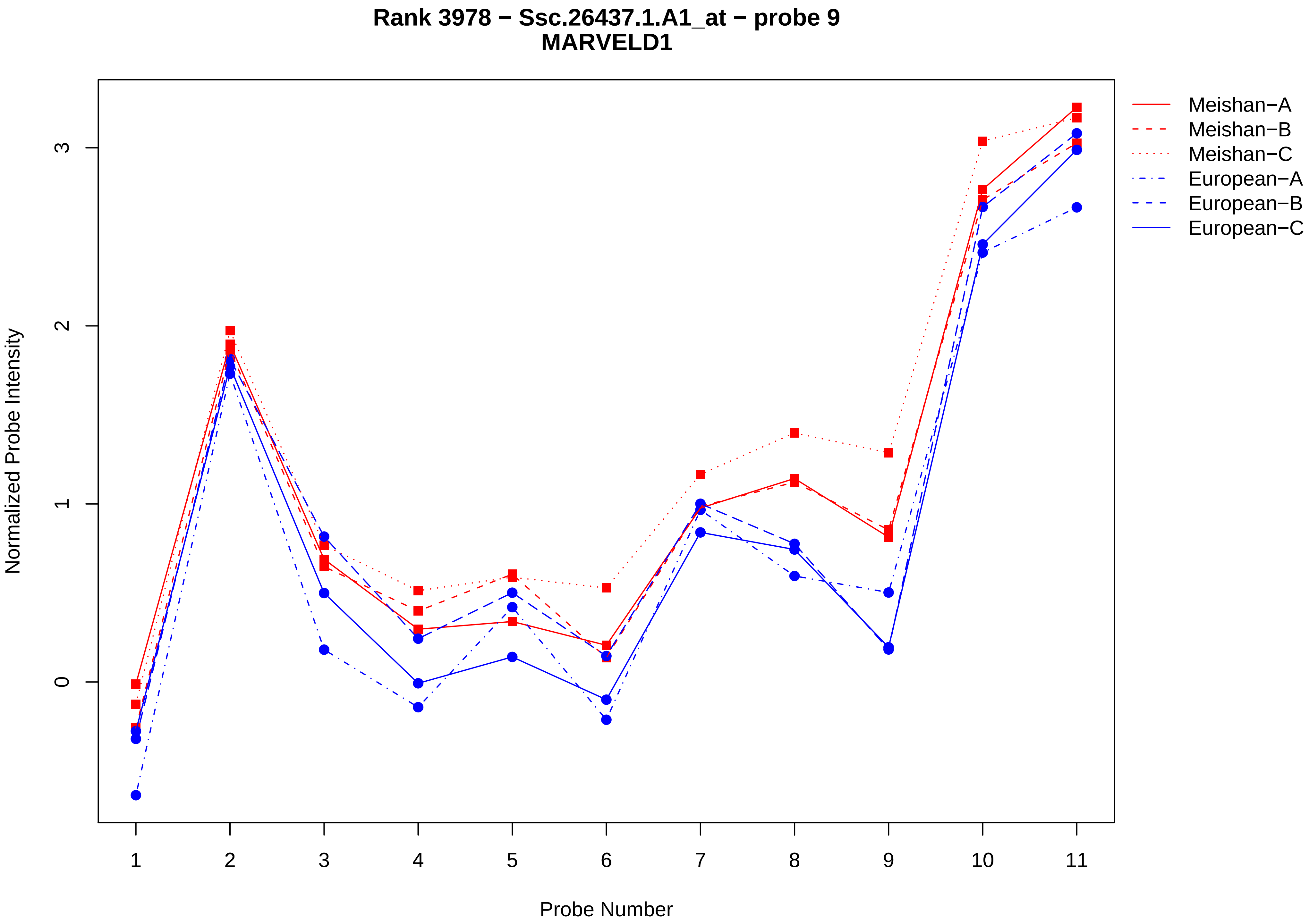

Rank 3978 − Ssc.26437.1.A1_at − probe 9
MARVELD1
Normalized Probe Intensity
0
1
2
3
Probe Number
1
2
3
4
5
6
7
8
9
10
11
Meishan−A
Meishan−B
Meishan−C
European−A
European−B
European−C

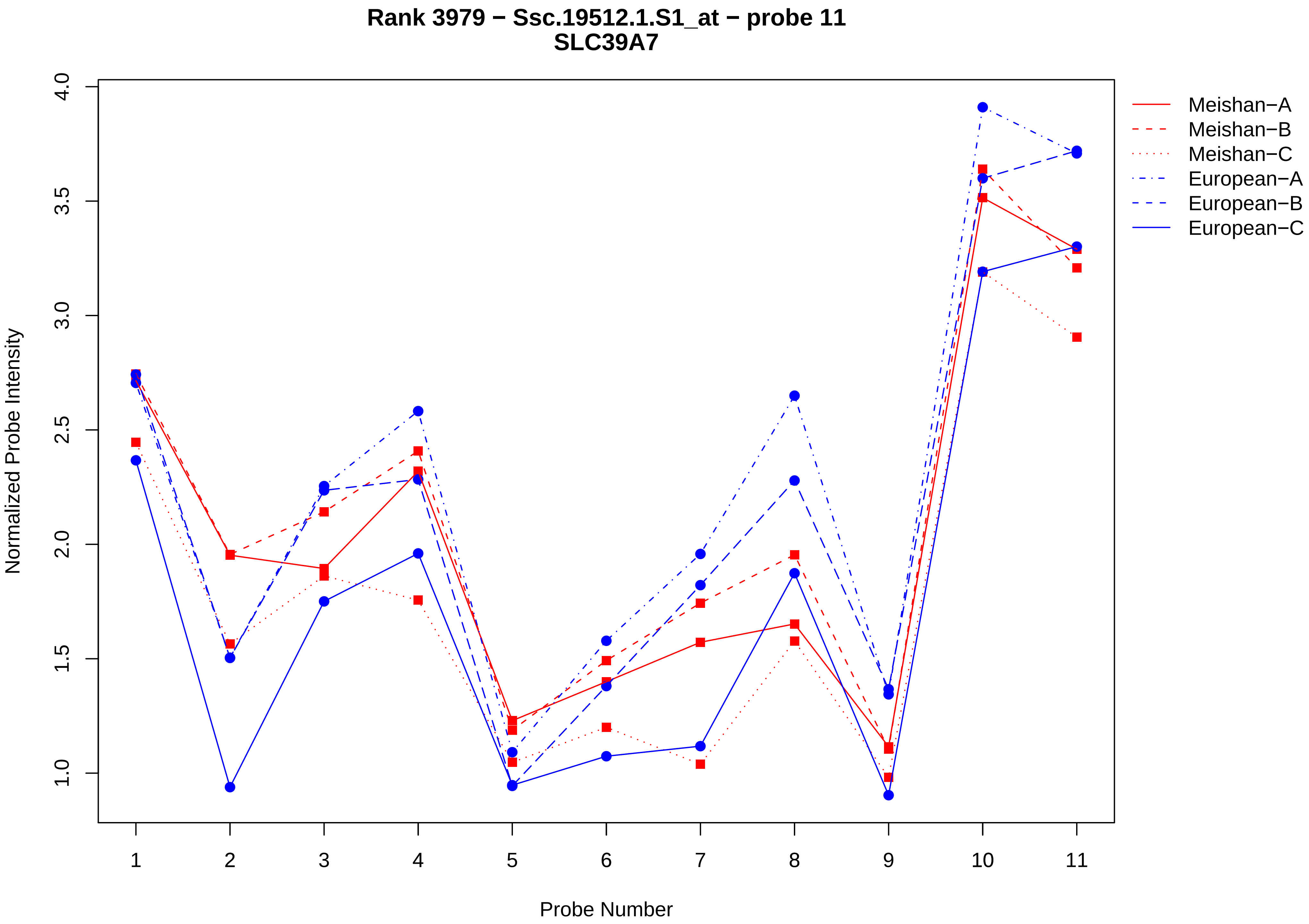

Rank 3979 − Ssc.19512.1.S1_at − probe 11
SLC39A7
Normalized Probe Intensity
Probe Number
Meishan−A
Meishan−B
Meishan−C
European−A
European−B
European−C
1.0
1.5
2.0
2.5
3.0
3.5
4.0
1
2
3
4
5
6
7
8
9
10
11

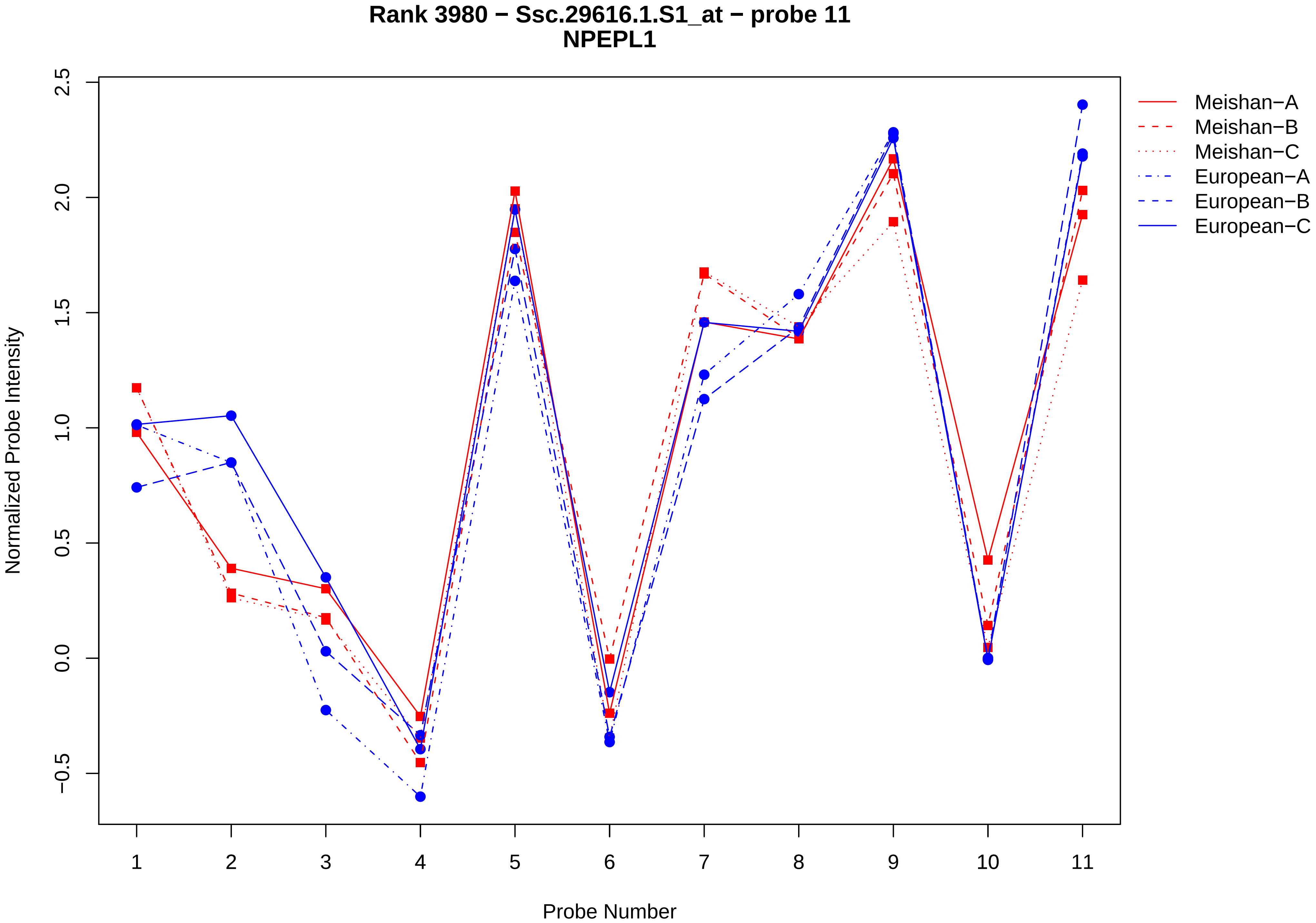

Rank 3980 − Ssc.29616.1.S1_at − probe 11
NPEPL1
Normalized Probe Intensity
Probe Number
Meishan−A
Meishan−B
Meishan−C
European−A
European−B
European−C
−0.5
0.0
0.5
1.0
1.5
2.0
2.5
1
2
3
4
5
6
7
8
9
10
11

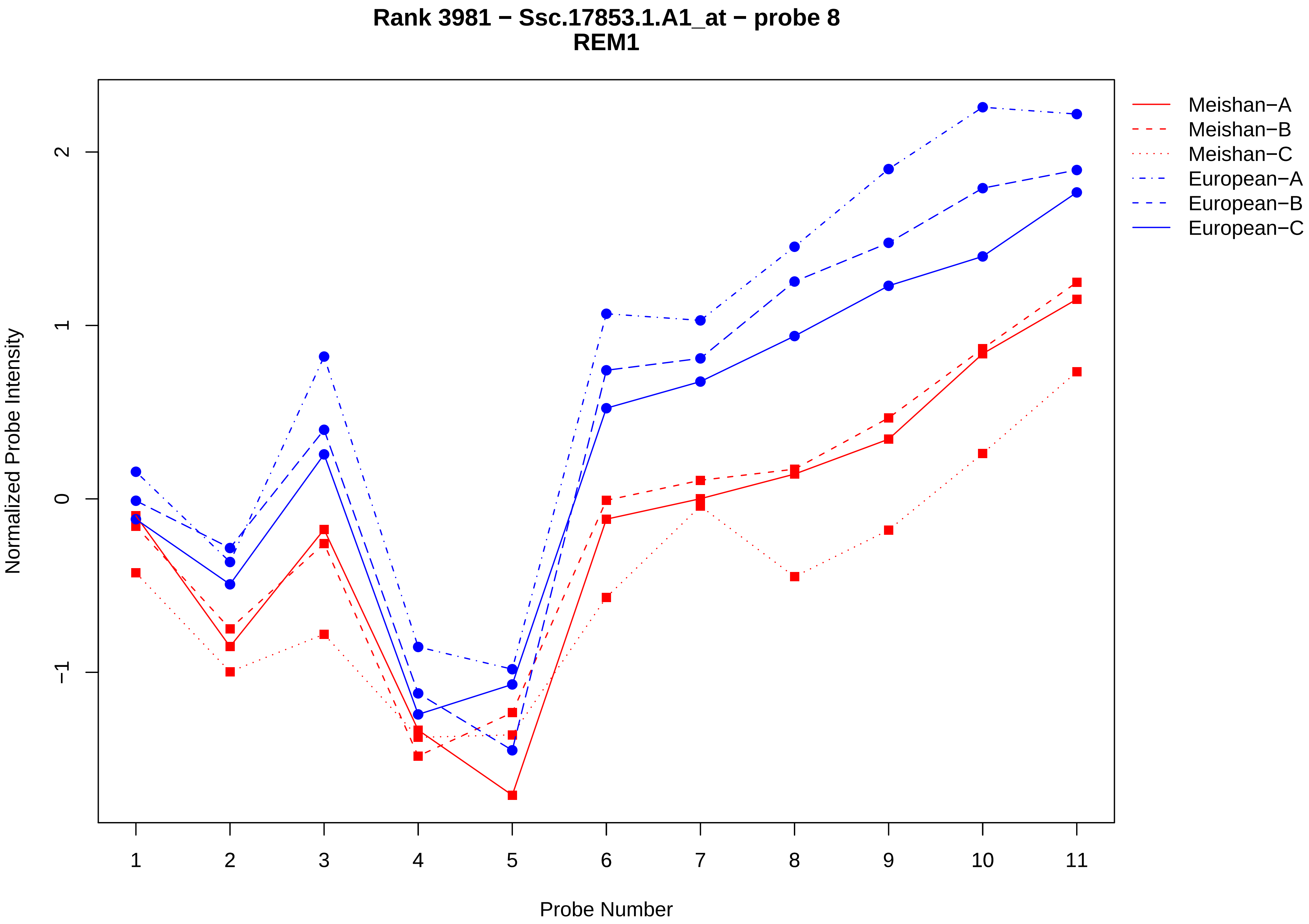

Rank 3981 − Ssc.17853.1.A1_at − probe 8
REM1
Normalized Probe Intensity
Probe Number
−1
0
1
2
1
2
3
4
5
6
7
8
9
10
11
Meishan−A
Meishan−B
Meishan−C
European−A
European−B
European−C

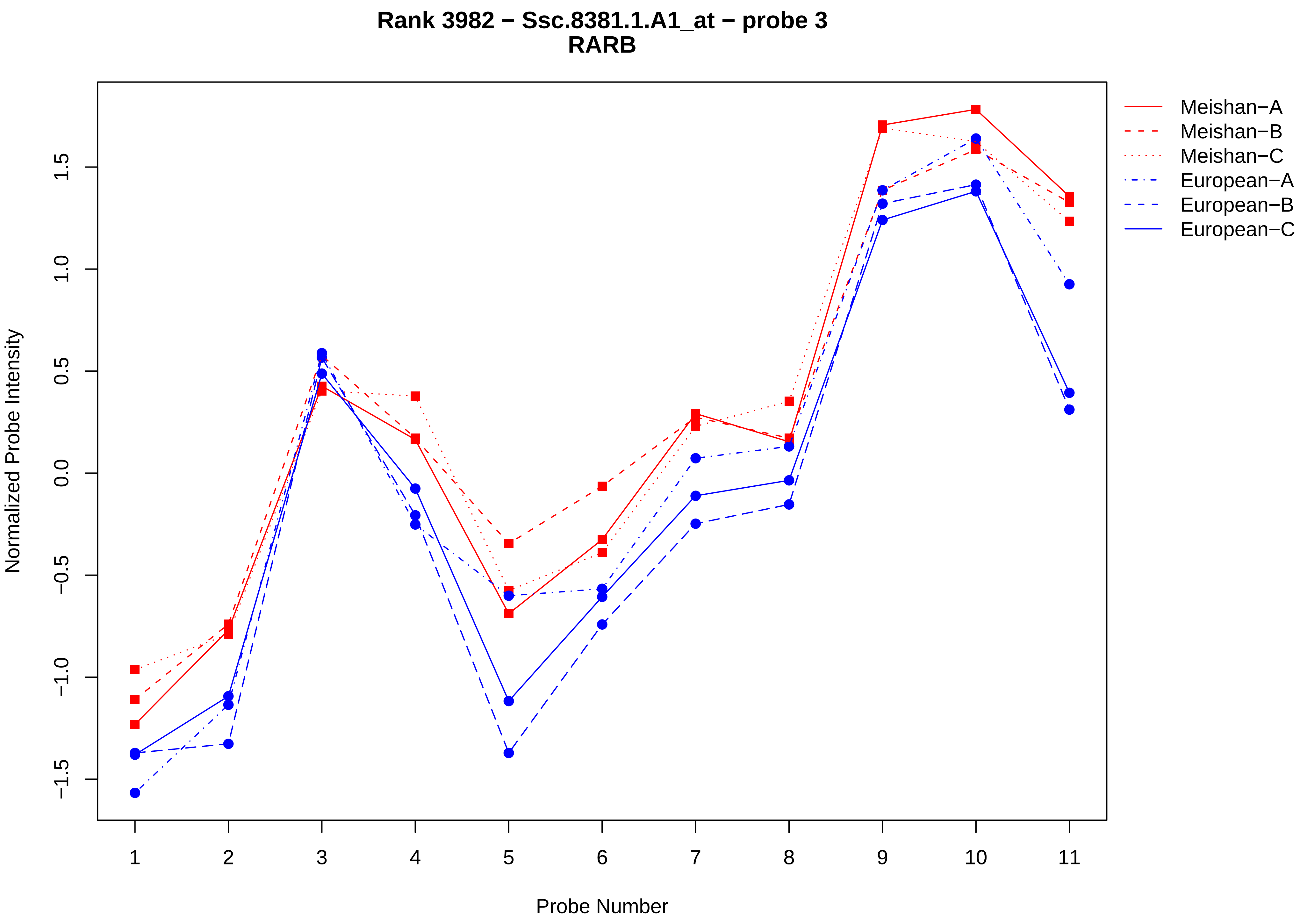

Rank 3982 − Ssc.8381.1.A1_at − probe 3
RARB
Normalized Probe Intensity
Probe Number
−1.5
−1.0
−0.5
0.0
0.5
1.0
1.5
1
2
3
4
5
6
7
8
9
10
11
Meishan−A
Meishan−B
Meishan−C
European−A
European−B
European−C

Rank 3983 − Ssc.6139.2.S1_at − probe 7
WARS

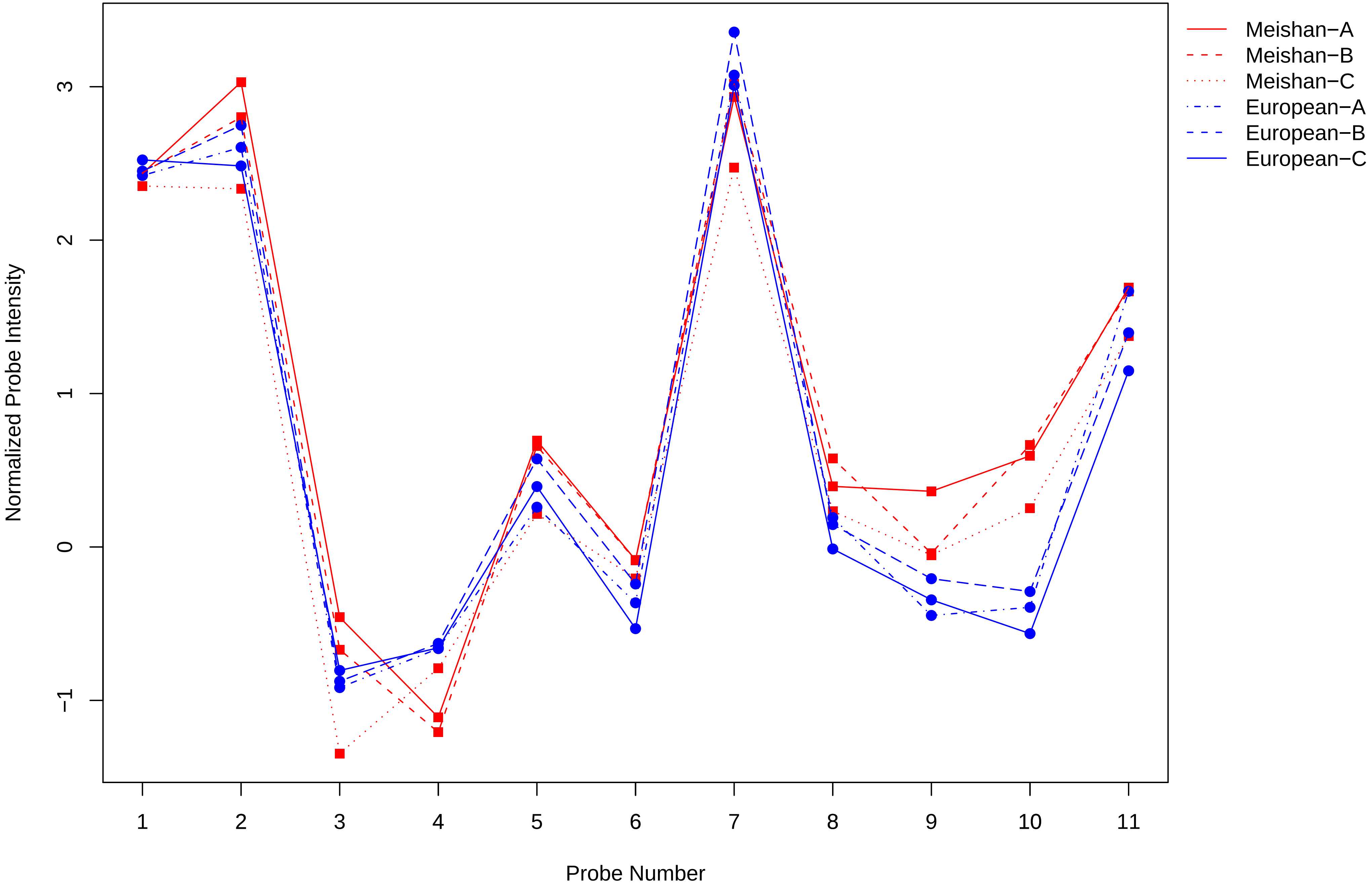

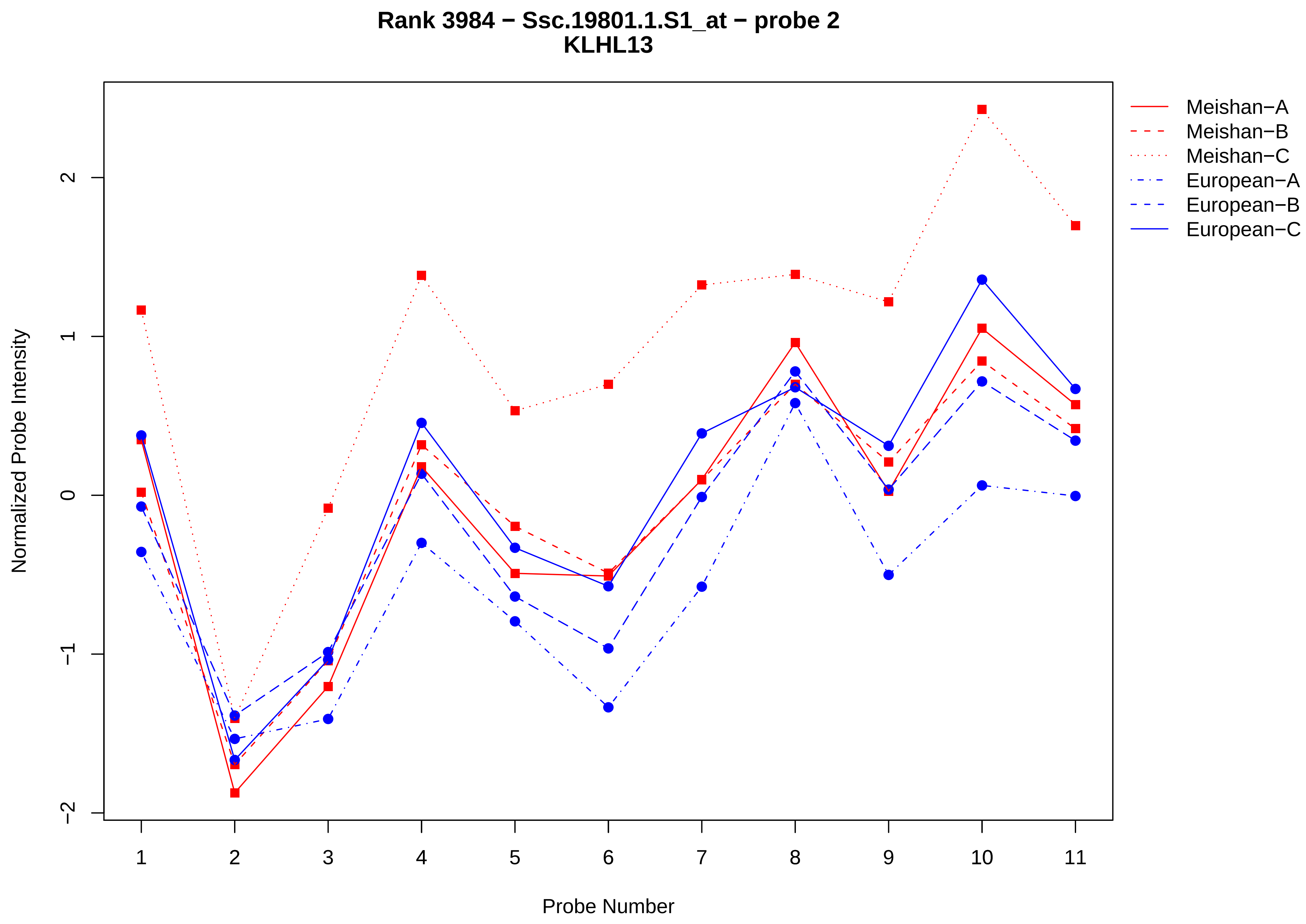
Rank 3984 − Ssc.19801.1.S1_at − probe 2
KLHL13
Normalized Probe Intensity
Probe Number
−2
−1
0
1
2
1
2
3
4
5
6
7
8
9
10
11
Meishan−A
Meishan−B
Meishan−C
European−A
European−B
European−C

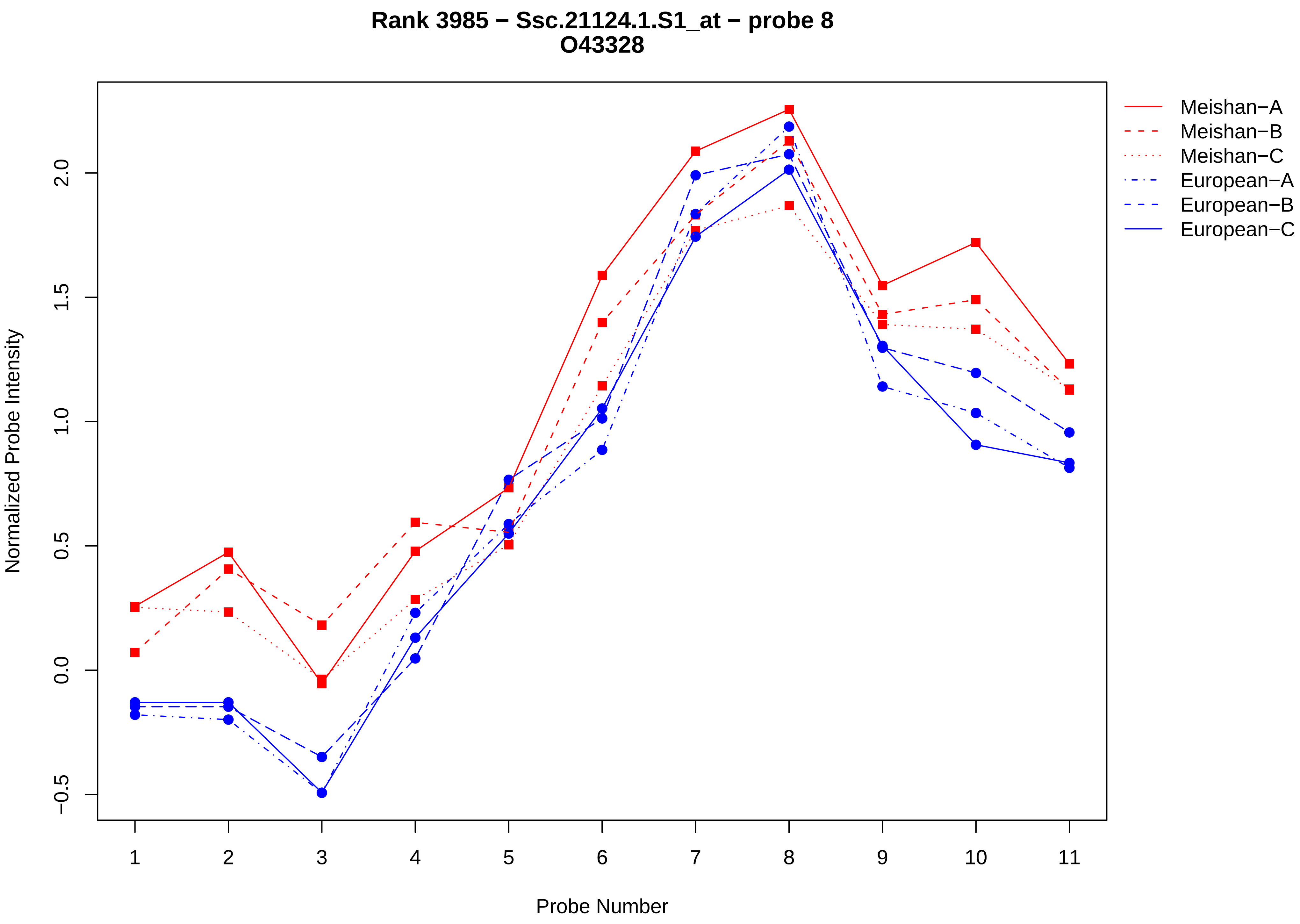
Rank 3985 − Ssc.21124.1.S1_at − probe 8
O43328
Normalized Probe Intensity
−0.5
0.0
0.5
1.0
1.5
2.0
1
2
3
4
5
6
7
8
9
10
11
Probe Number
Meishan−A
Meishan−B
Meishan−C
European−A
European−B
European−C

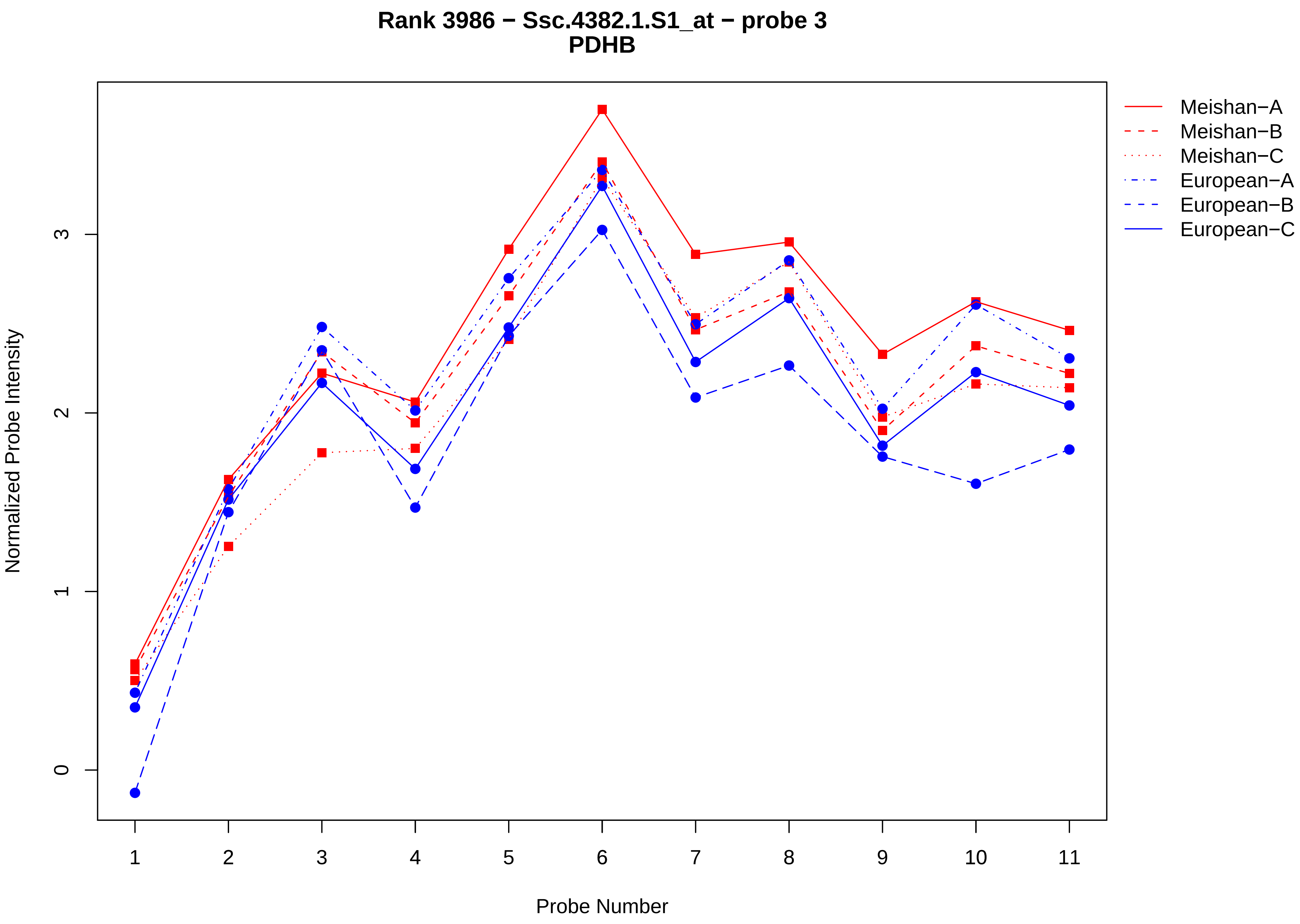

Rank 3986 − Ssc.4382.1.S1_at − probe 3
PDHB
Normalized Probe Intensity
Probe Number
0
1
2
3
1
2
3
4
5
6
7
8
9
10
11
Meishan−A
Meishan−B
Meishan−C
European−A
European−B
European−C

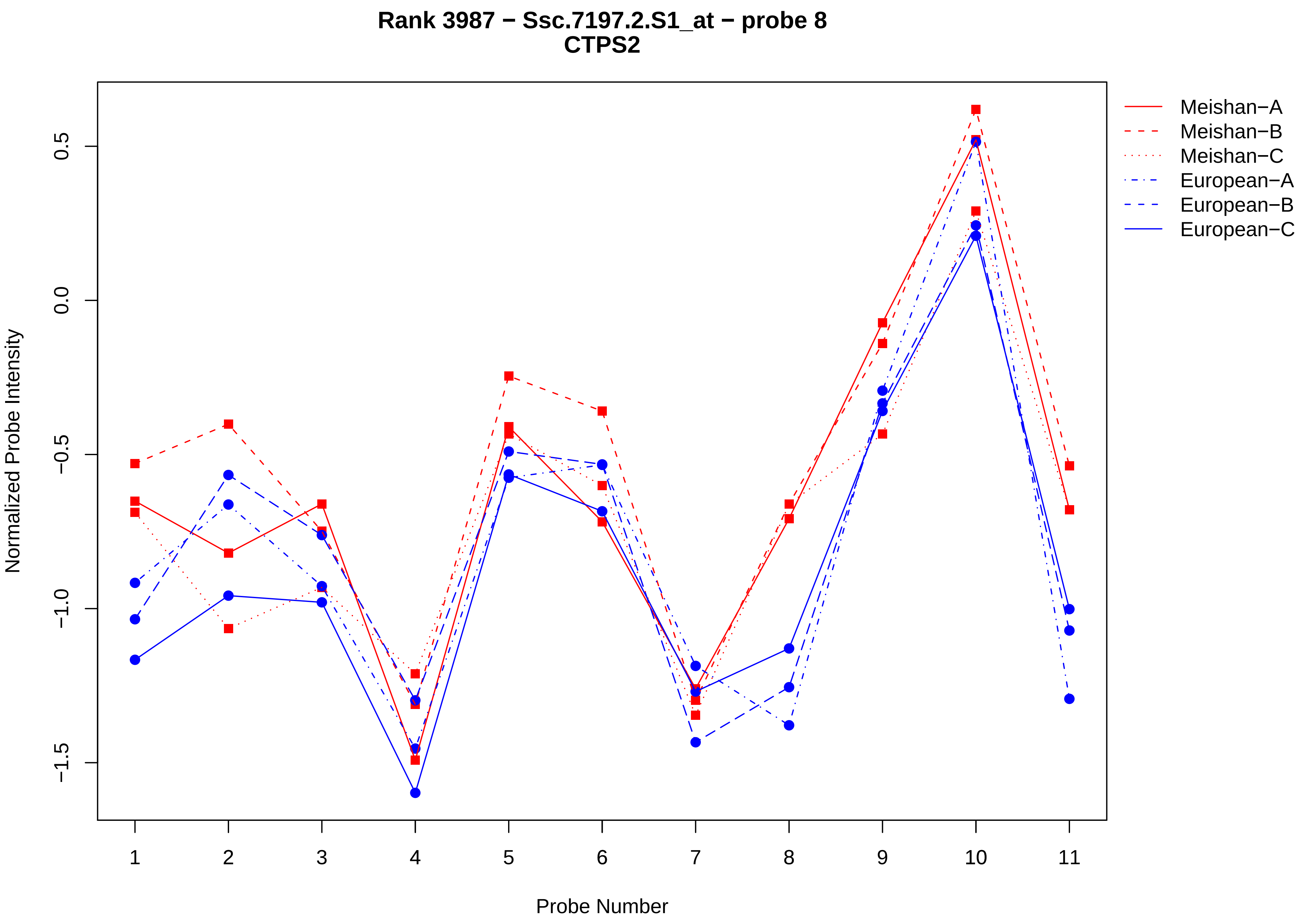

Rank 3987 − Ssc.7197.2.S1_at − probe 8
CTPS2
Normalized Probe Intensity
Probe Number
0.5
0.0
−0.5
−1.0
−1.5
1
2
3
4
5
6
7
8
9
10
11
Meishan−A
Meishan−B
Meishan−C
European−A
European−B
European−C

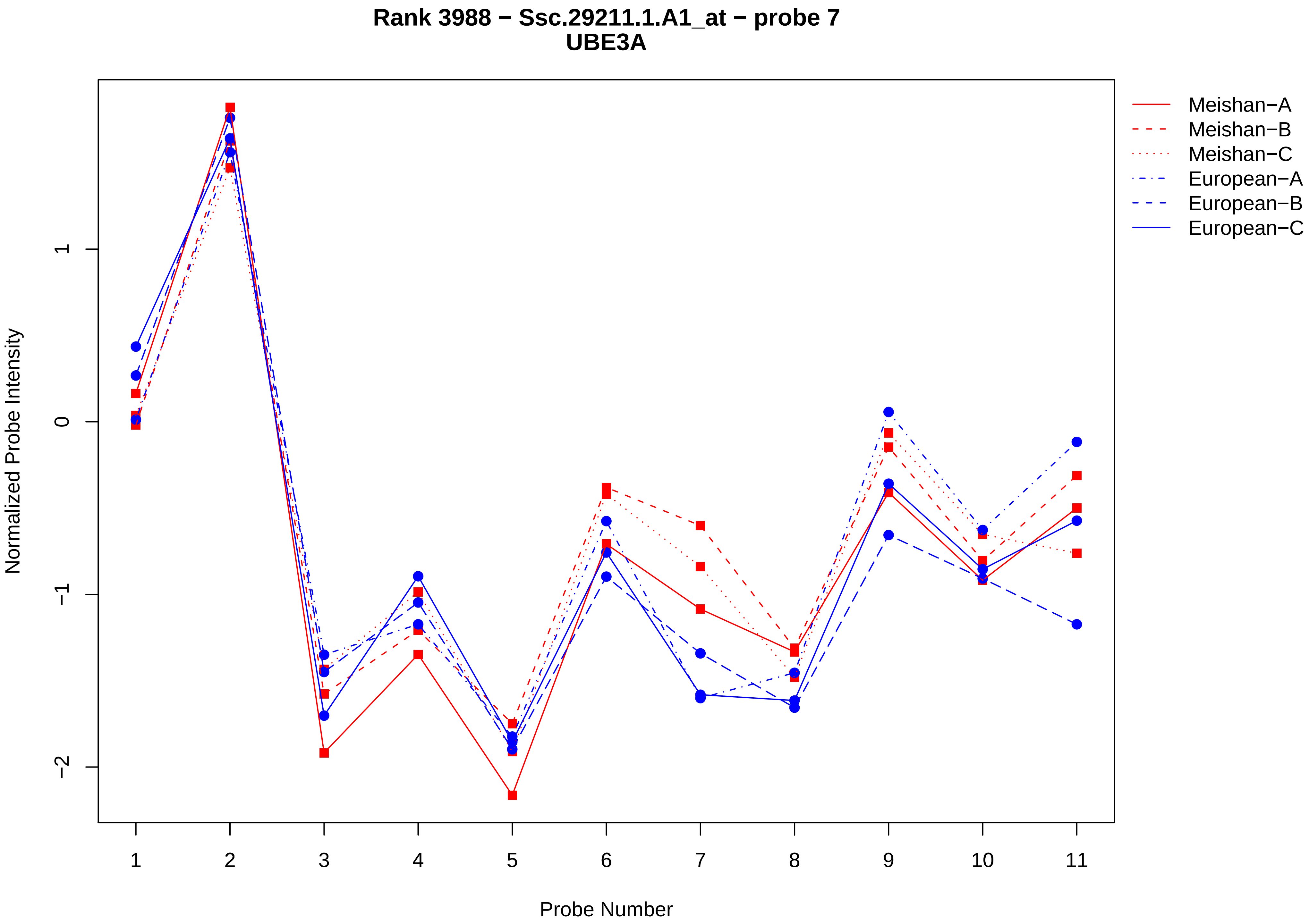
Rank 3988 − Ssc.29211.1.A1_at − probe 7
UBE3A
Normalized Probe Intensity
Probe Number
−2
−1
0
1
1
2
3
4
5
6
7
8
9
10
11
Meishan−A
Meishan−B
Meishan−C
European−A
European−B
European−C

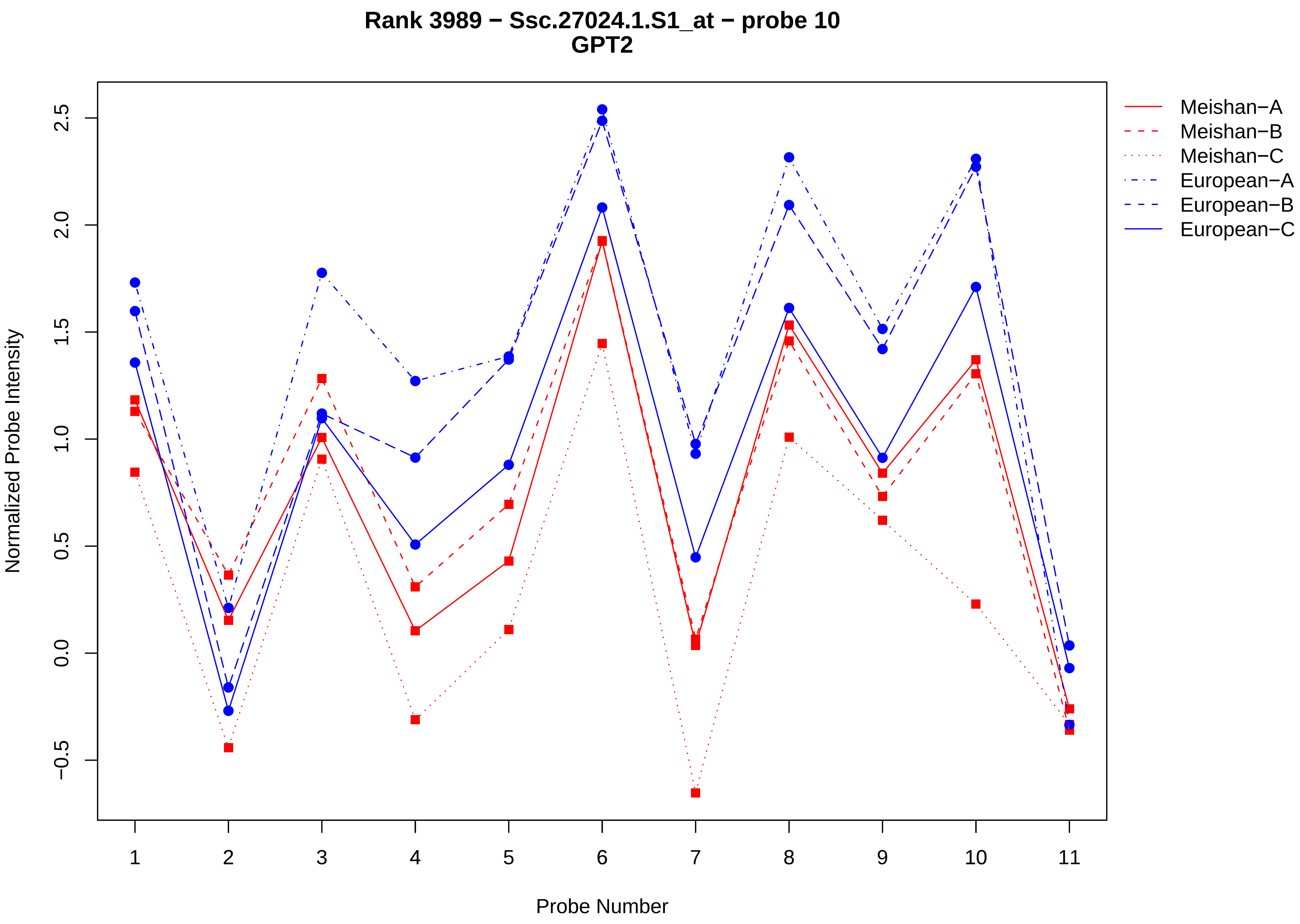
Rank 3989 − Ssc.27024.1.S1_at − probe 10
GPT2
Normalized Probe Intensity
Probe Number
−0.5
0.0
0.5
1.0
1.5
2.0
2.5
1
2
3
4
5
6
7
8
9
10
11
Meishan−A
Meishan−B
Meishan−C
European−A
European−B
European−C

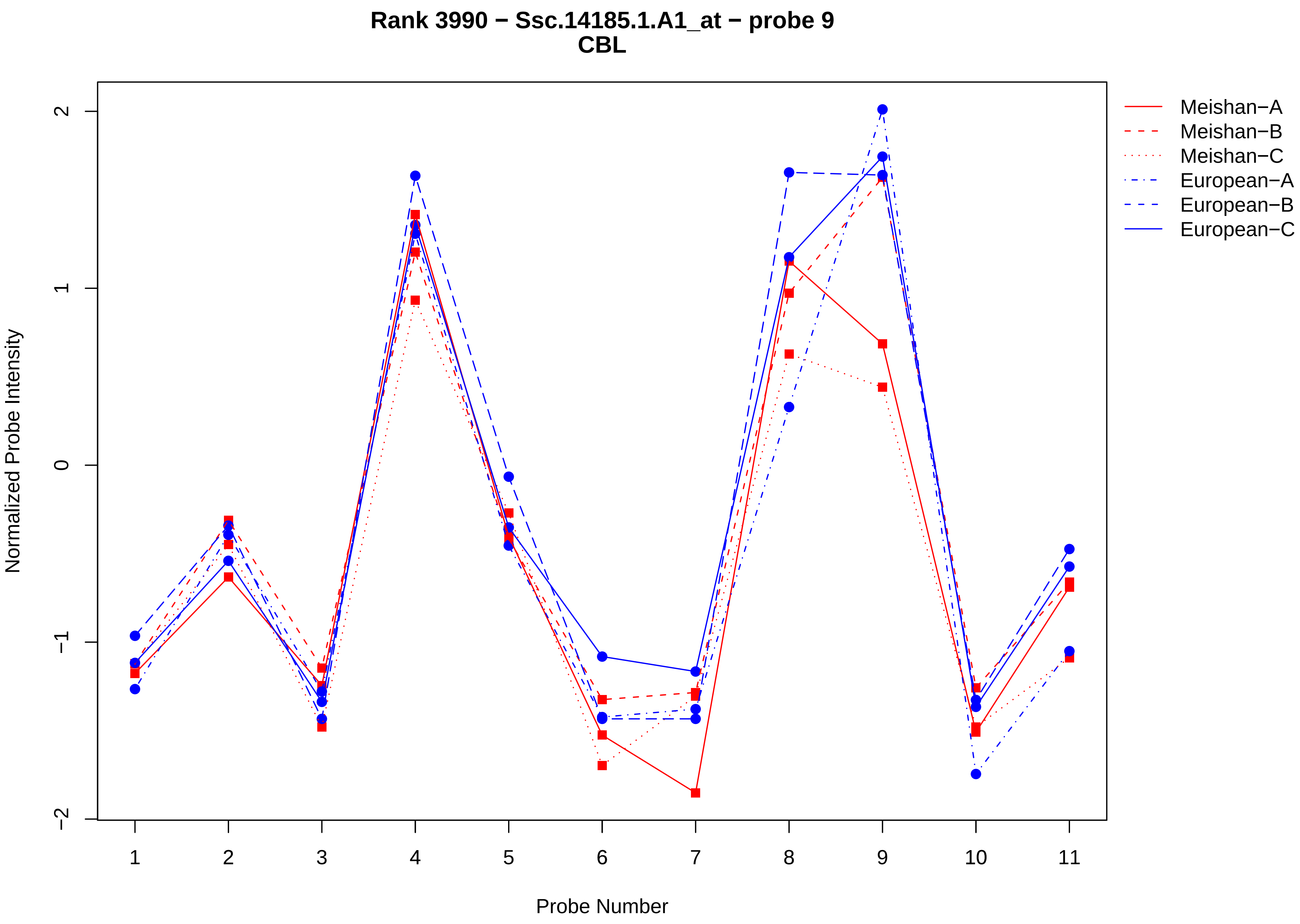

Rank 3990 − Ssc.14185.1.A1_at − probe 9
CBL
Normalized Probe Intensity
Probe Number
Meishan−A
Meishan−B
Meishan−C
European−A
European−B
European−C

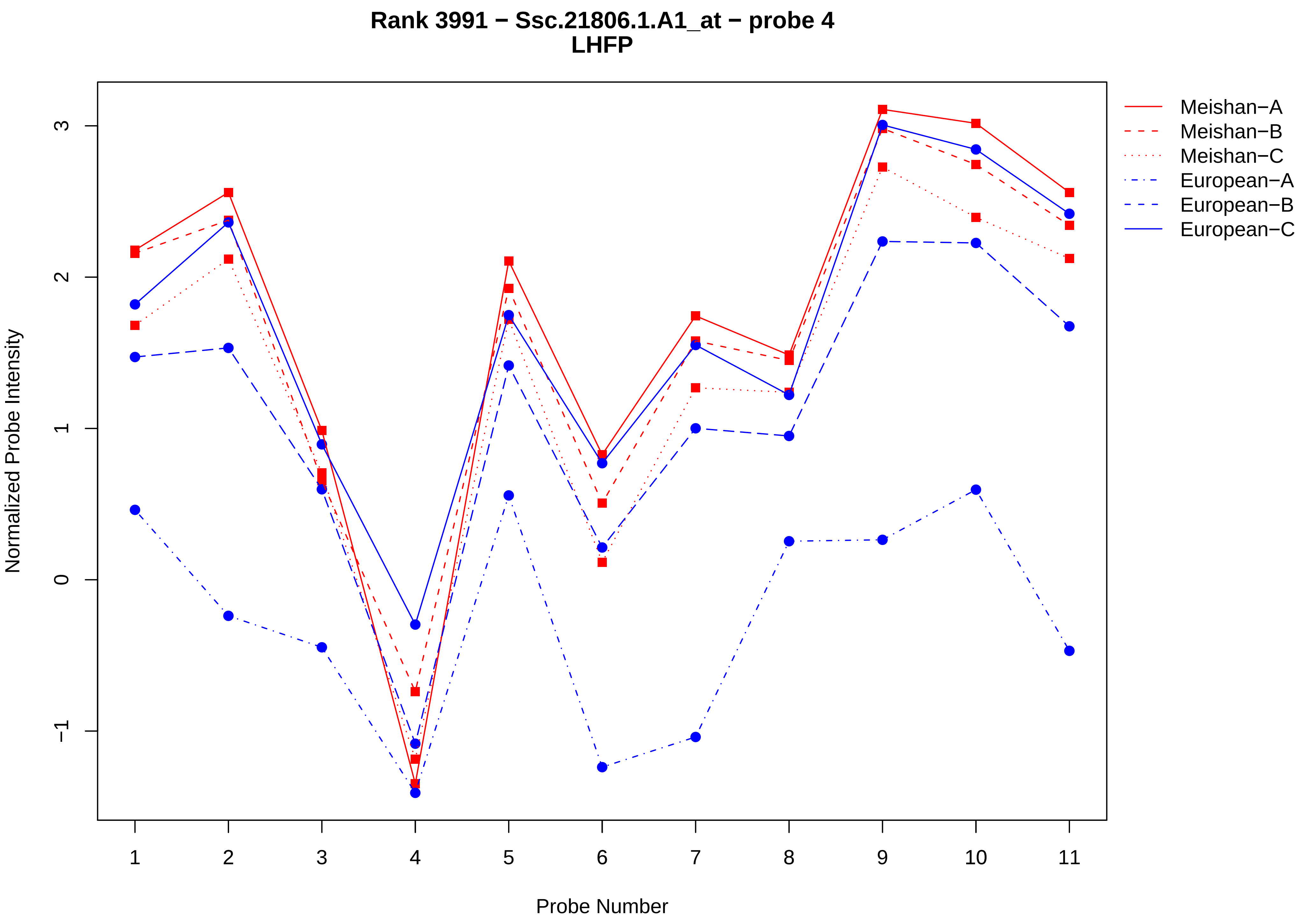

Rank 3991 − Ssc.21806.1.A1_at − probe 4
LHFP
Normalized Probe Intensity
Probe Number
Meishan−A
Meishan−B
Meishan−C
European−A
European−B
European−C

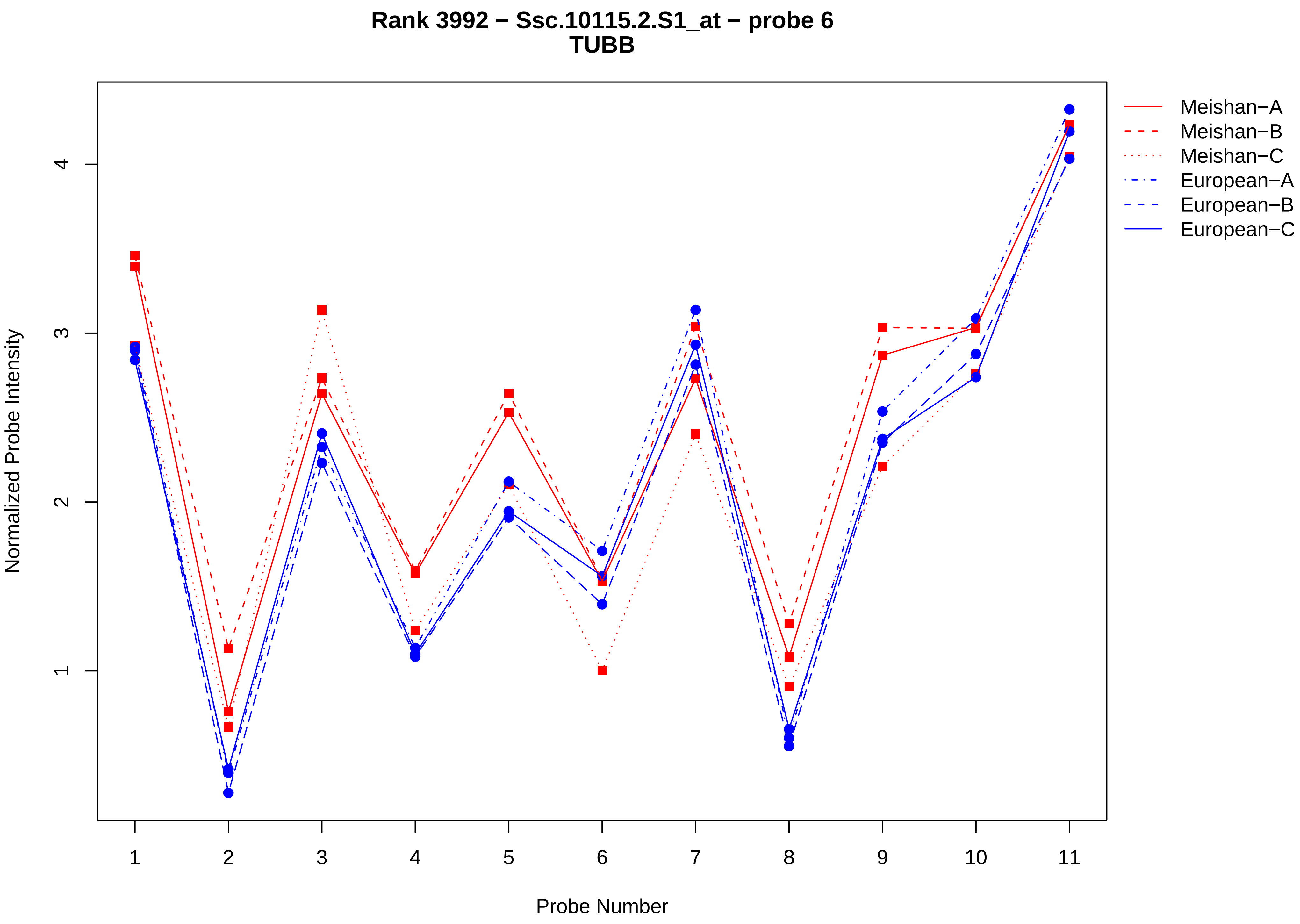

Rank 3992 − Ssc.10115.2.S1_at − probe 6
TUBB
Normalized Probe Intensity
Probe Number
1
2
3
4
1
2
3
4
5
6
7
8
9
10
11
Meishan−A
Meishan−B
Meishan−C
European−A
European−B
European−C

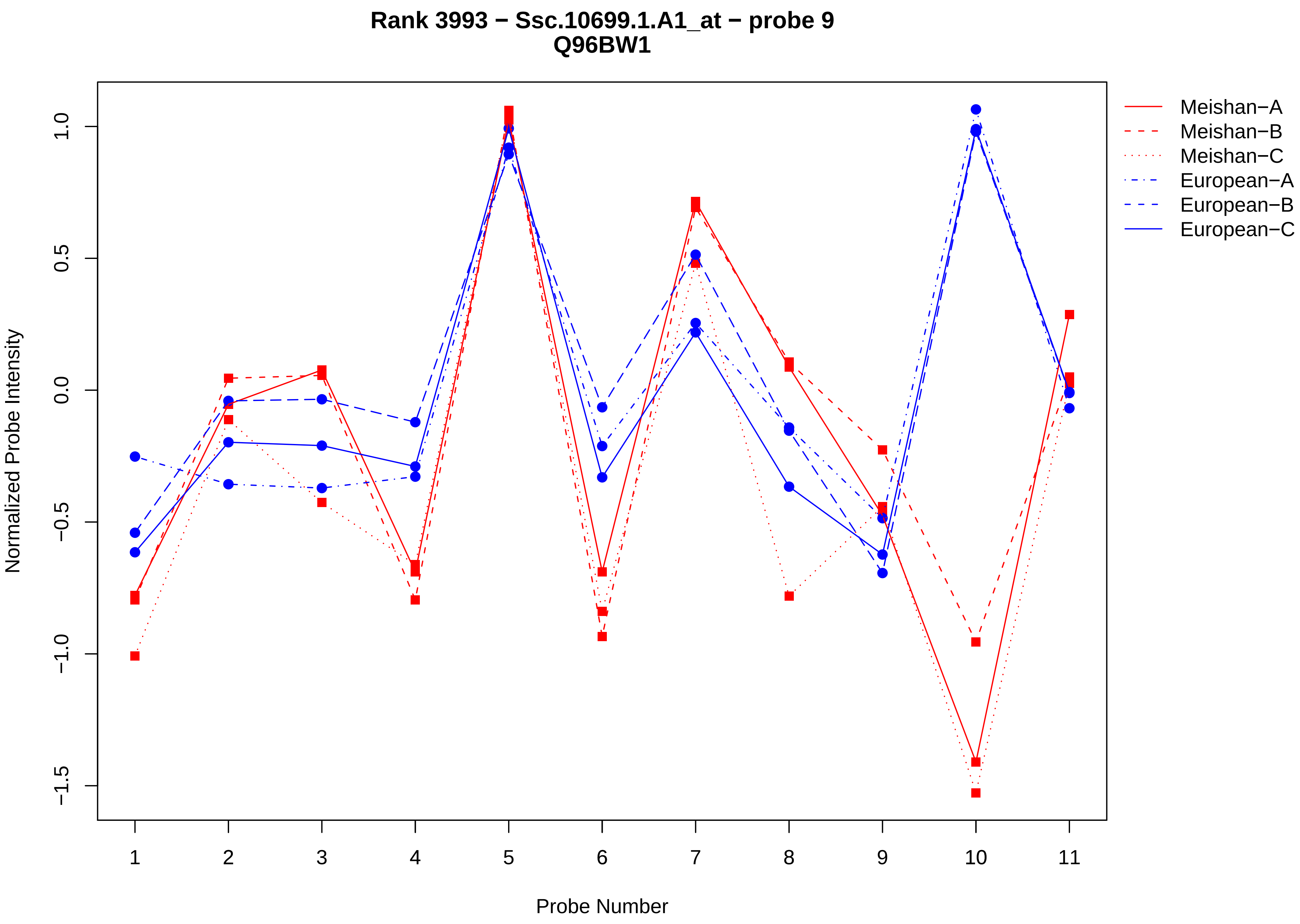

Rank 3993 − Ssc.10699.1.A1_at − probe 9
Q96BW1
Normalized Probe Intensity
Probe Number
Meishan−A
Meishan−B
Meishan−C
European−A
European−B
European−C
1.0
0.5
0.0
−0.5
−1.0
−1.5
1
2
3
4
5
6
7
8
9
10
11

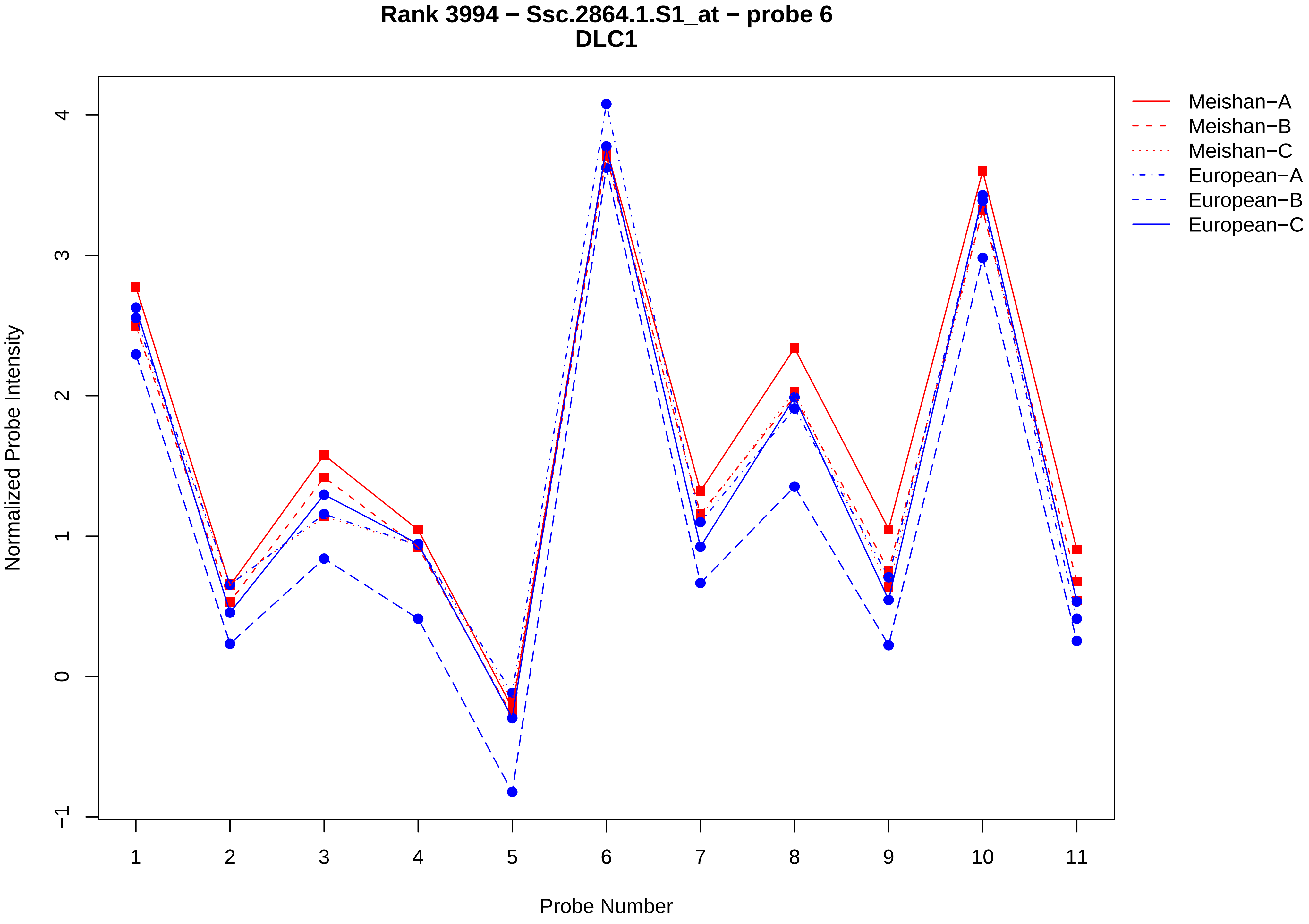

Rank 3994 − Ssc.2864.1.S1_at − probe 6
DLC1
Normalized Probe Intensity
Probe Number
−1
0
1
2
3
4
1
2
3
4
5
6
7
8
9
10
11
Meishan−A
Meishan−B
Meishan−C
European−A
European−B
European−C

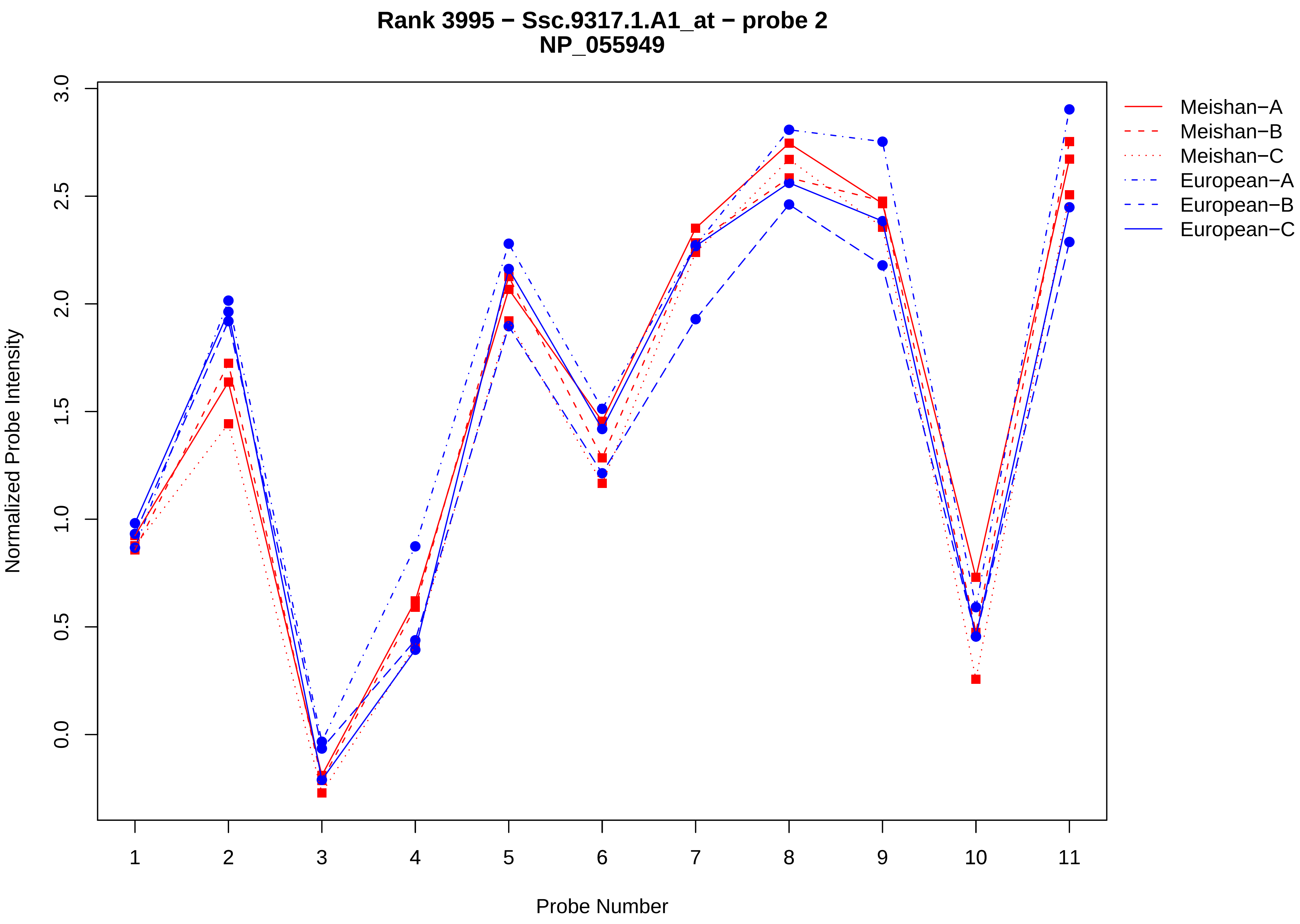

Rank 3995 − Ssc.9317.1.A1_at − probe 2
NP_055949
Normalized Probe Intensity
Probe Number
0.0
0.5
1.0
1.5
2.0
2.5
3.0
1
2
3
4
5
6
7
8
9
10
11
Meishan−A
Meishan−B
Meishan−C
European−A
European−B
European−C

Rank 3996 − Ssc.15114.1.S1_at − probe 9
DDEF1

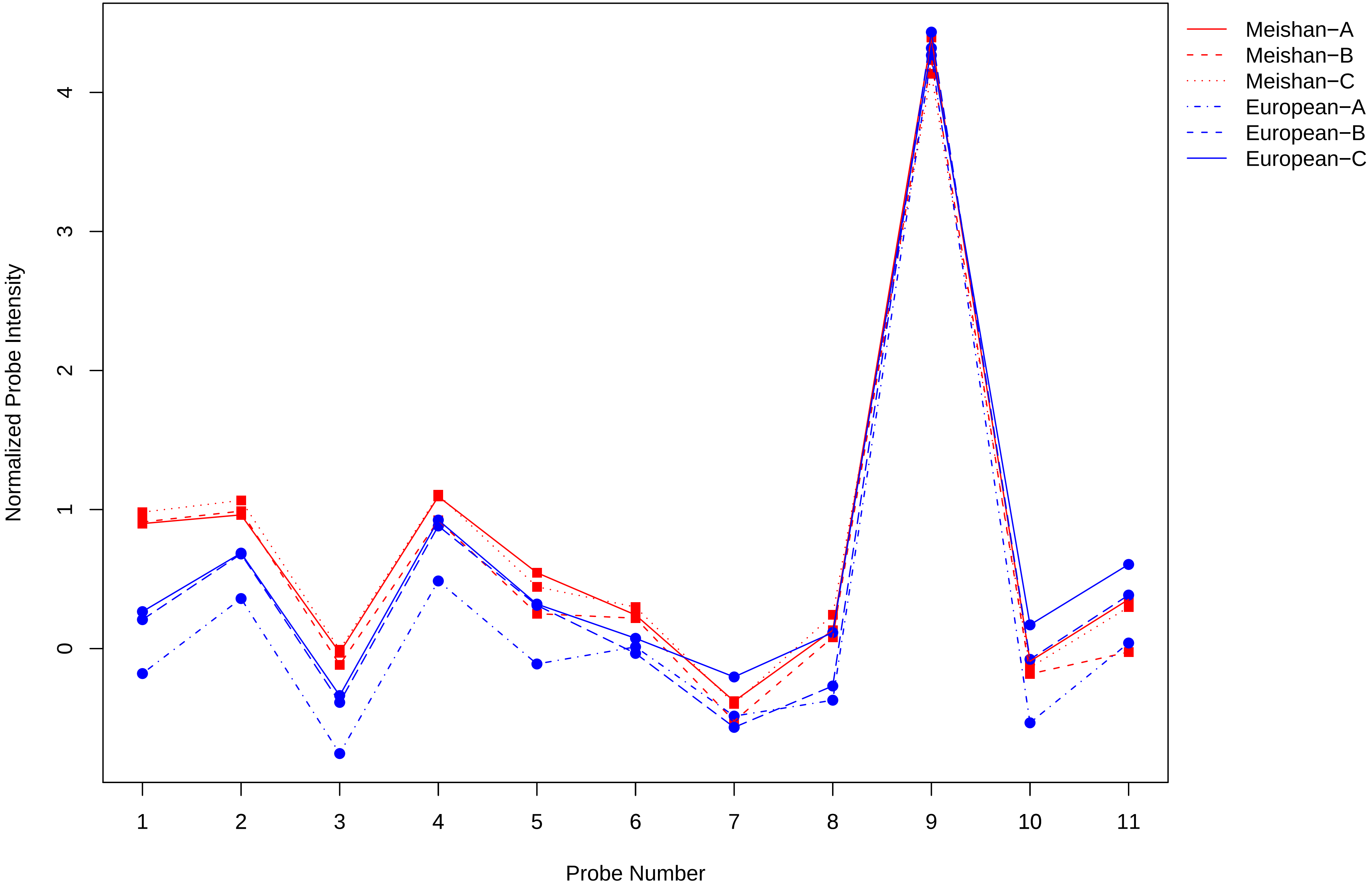

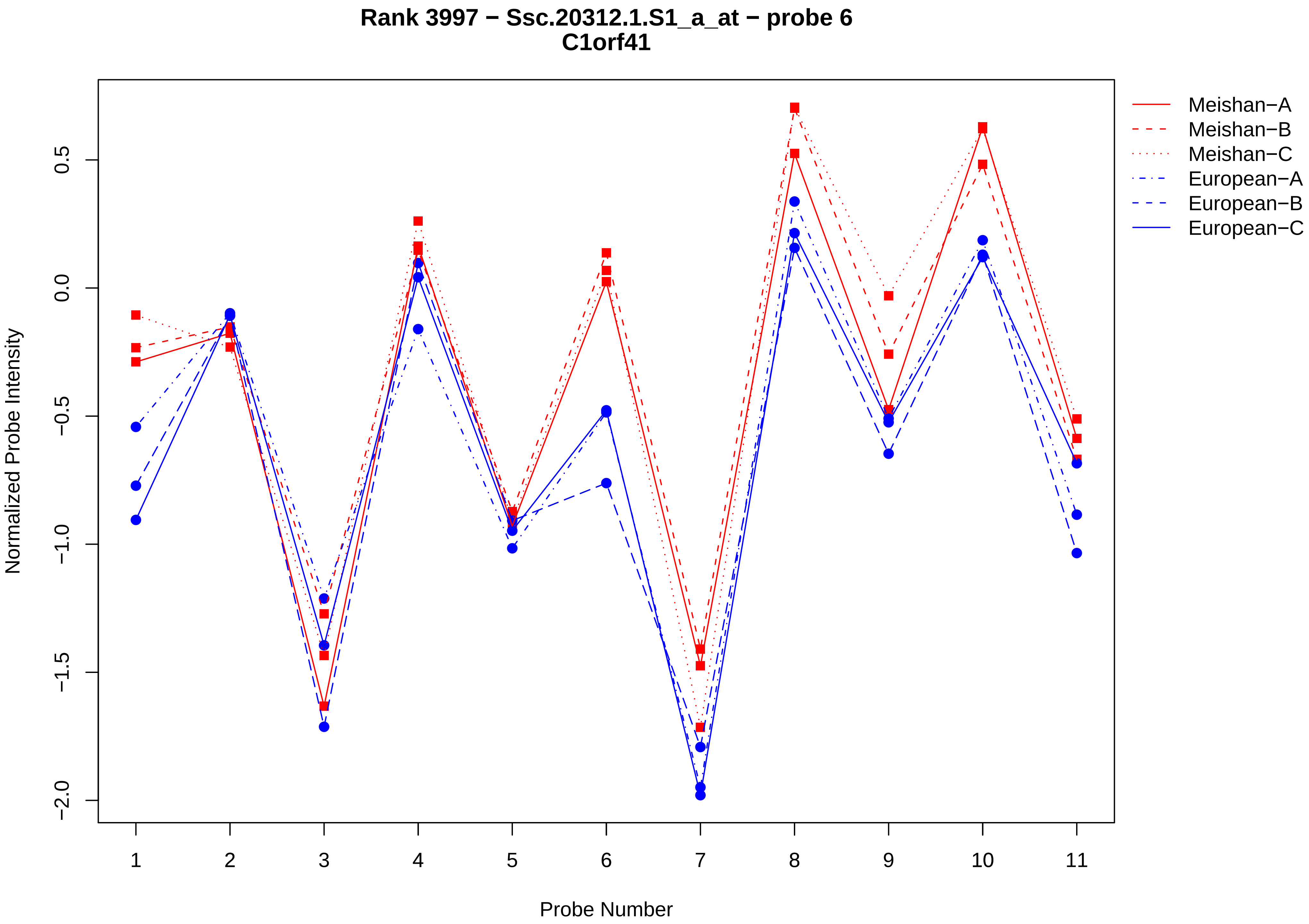

Rank 3997 − Ssc.20312.1.S1_a_at − probe 6
C1orf41
Normalized Probe Intensity
Probe Number
0.5
0.0
−0.5
−1.0
−1.5
−2.0
1
2
3
4
5
6
7
8
9
10
11
Meishan−A
Meishan−B
Meishan−C
European−A
European−B
European−C

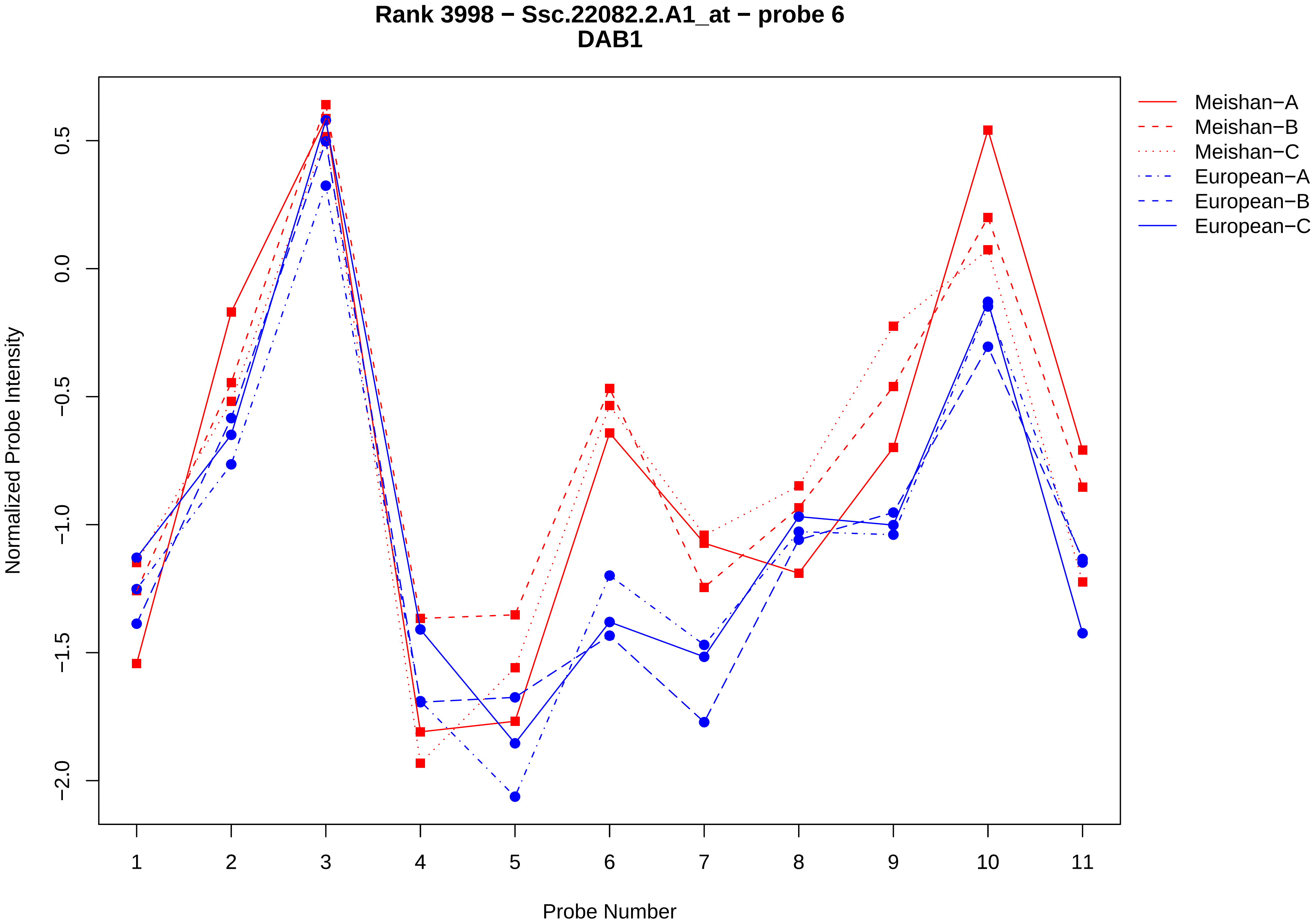

Rank 3998 − Ssc.22082.2.A1_at − probe 6
DAB1
Normalized Probe Intensity
Probe Number
Meishan−A
Meishan−B
Meishan−C
European−A
European−B
European−C
0.5
0.0
−0.5
−1.0
−1.5
−2.0
1
2
3
4
5
6
7
8
9
10
11

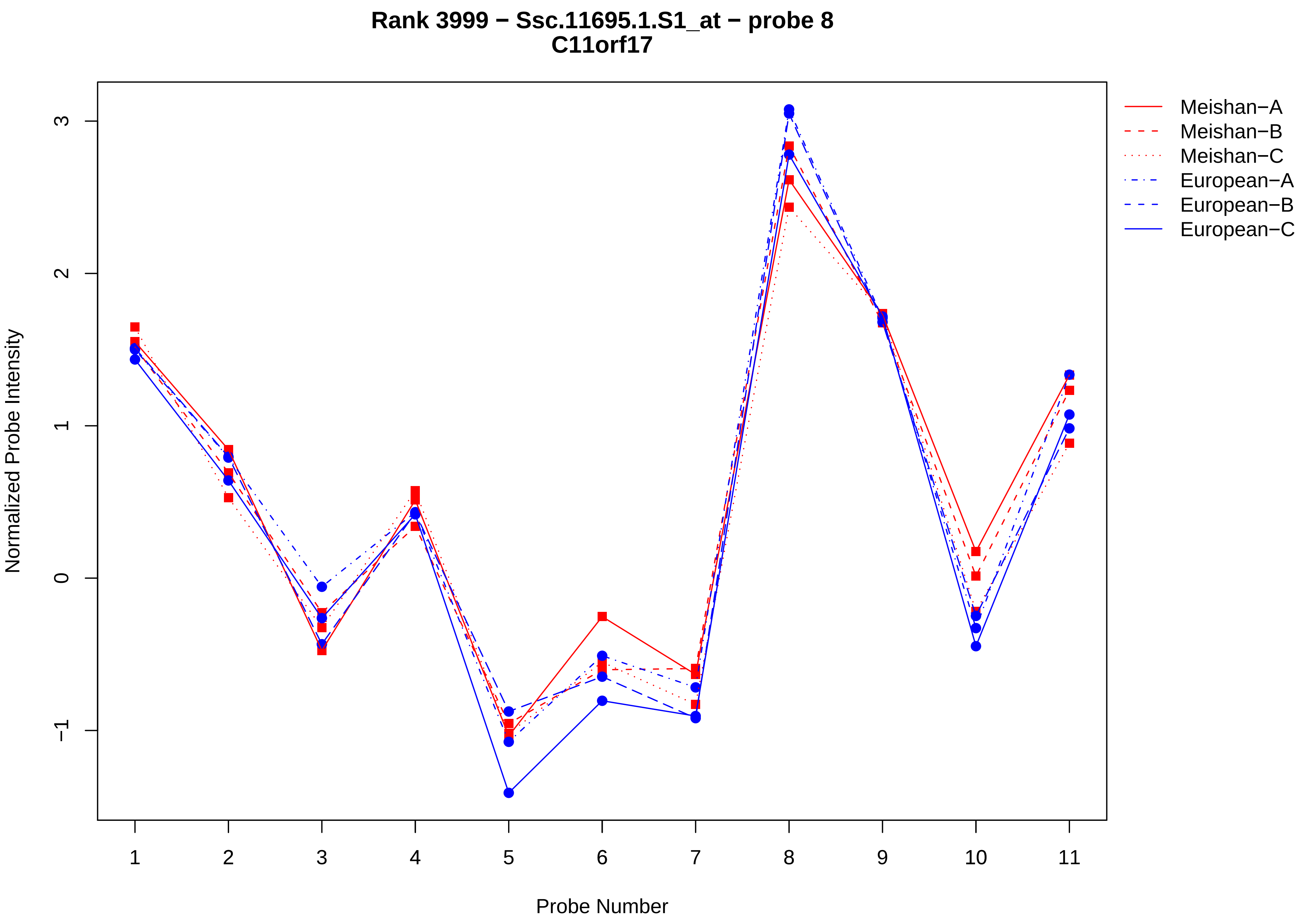

Rank 3999 − Ssc.11695.1.S1_at − probe 8
C11orf17
Normalized Probe Intensity
Probe Number
Meishan−A
Meishan−B
Meishan−C
European−A
European−B
European−C
−1
0
1
2
3
1
2
3
4
5
6
7
8
9
10
11

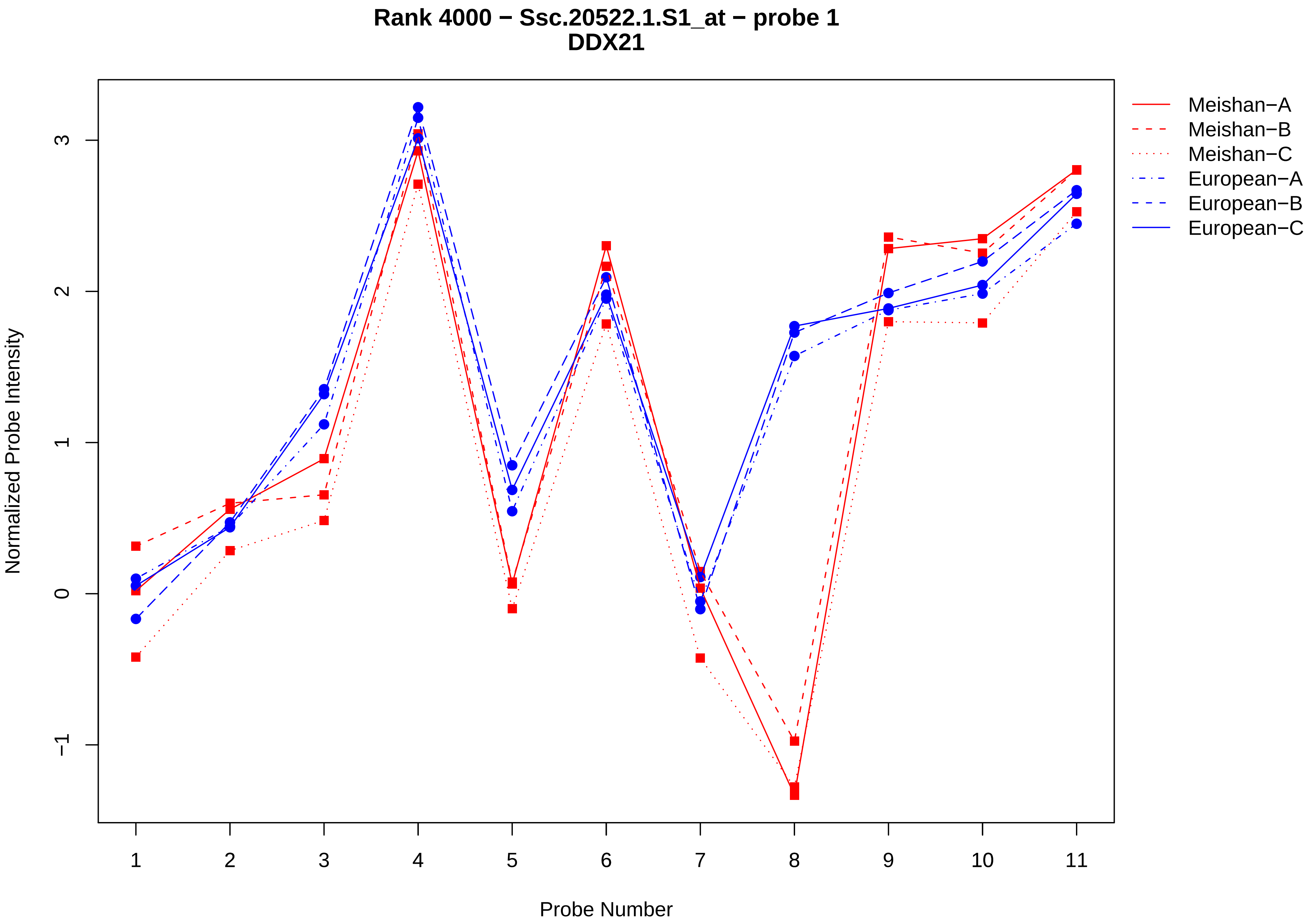

Rank 4000 − Ssc.20522.1.S1_at − probe 1
DDX21
Normalized Probe Intensity
Probe Number
Meishan−A
Meishan−B
Meishan−C
European−A
European−B
European−C

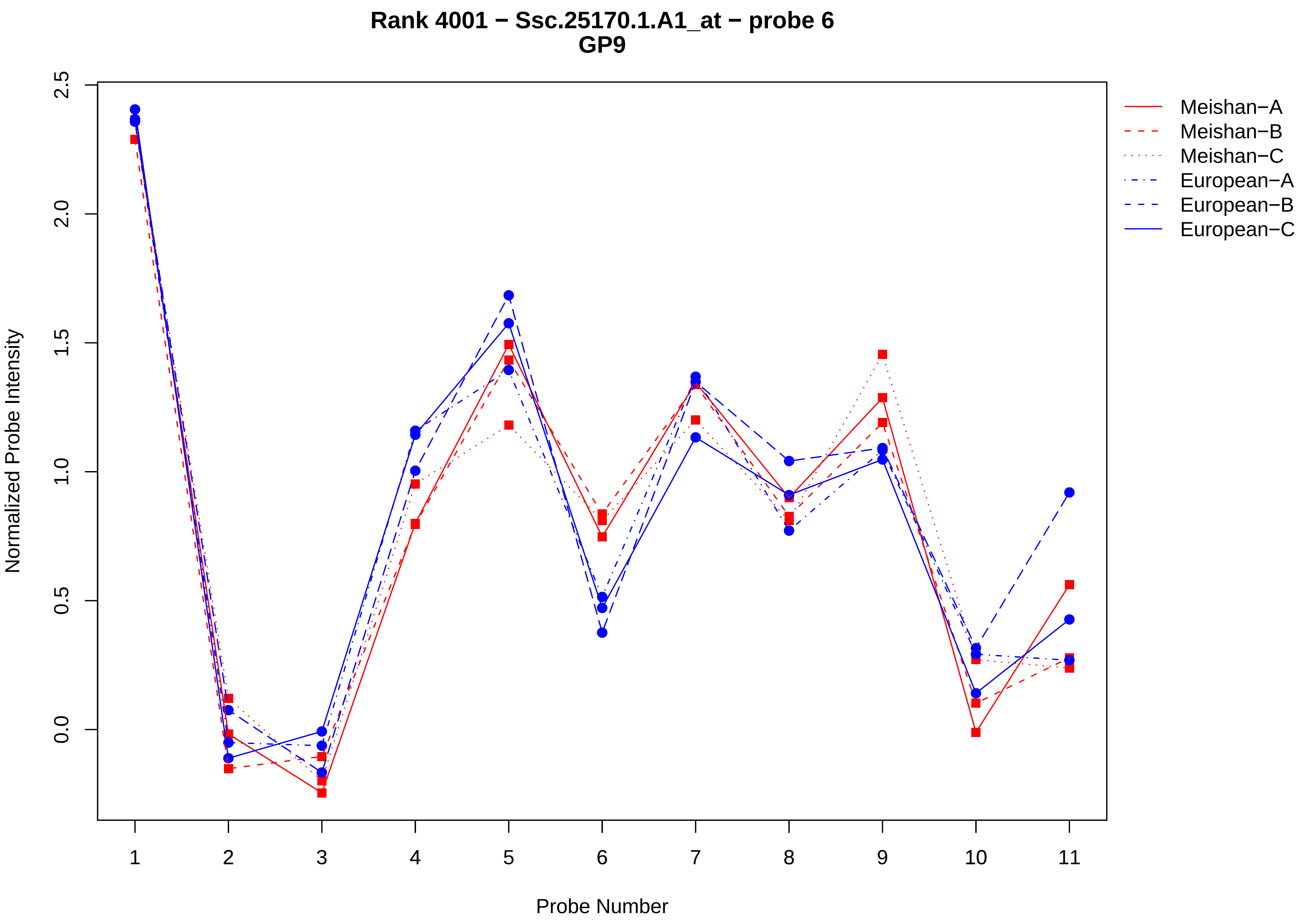
Rank 4001 − Ssc.25170.1.A1_at − probe 6
GP9
Normalized Probe Intensity
Probe Number
0.0
0.5
1.0
1.5
2.0
2.5
1
2
3
4
5
6
7
8
9
10
11
Meishan−A
Meishan−B
Meishan−C
European−A
European−B
European−C

Rank 4002 − Ssc.22067.1.A1_at − probe 1
SLC16A3

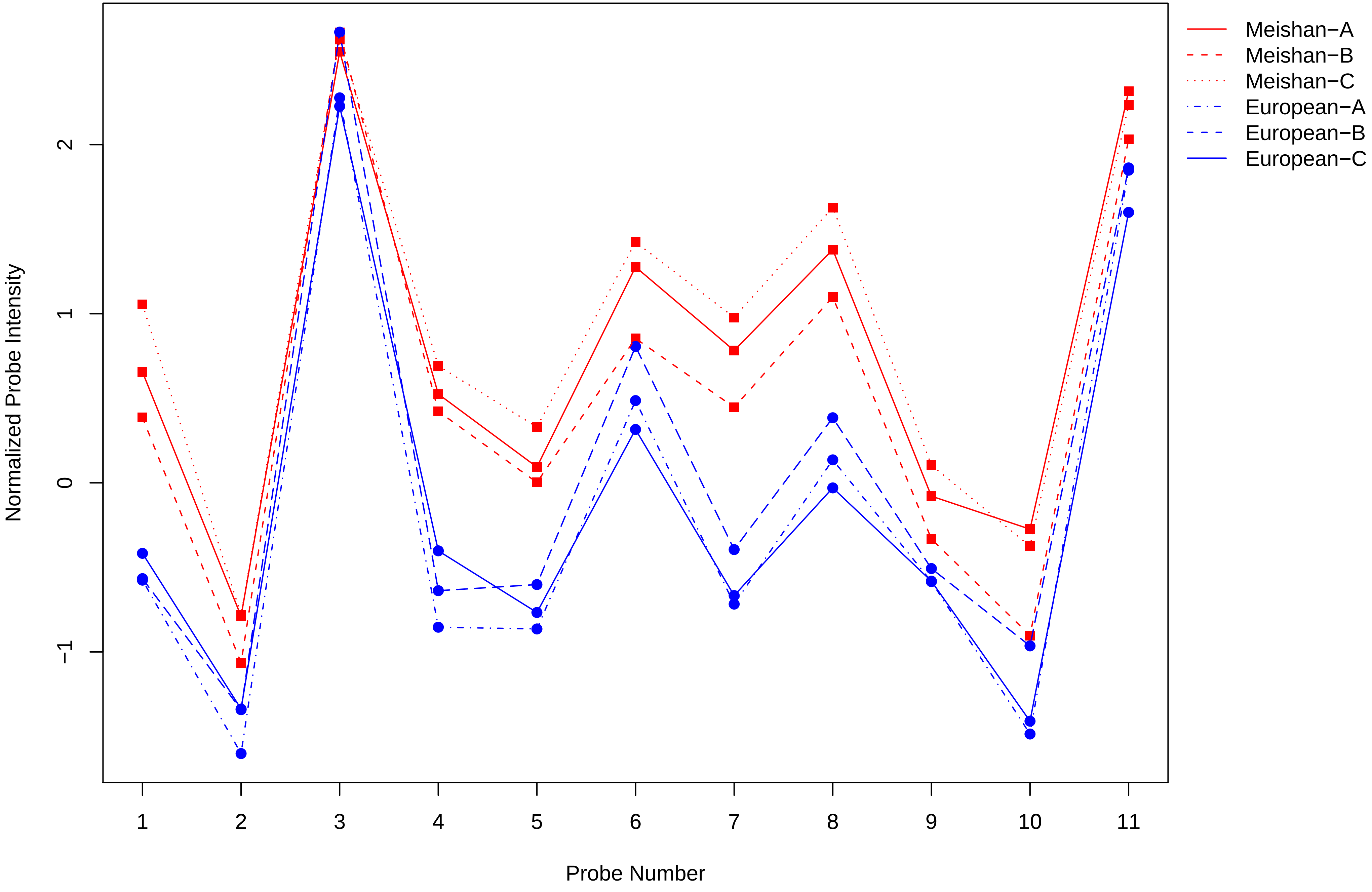

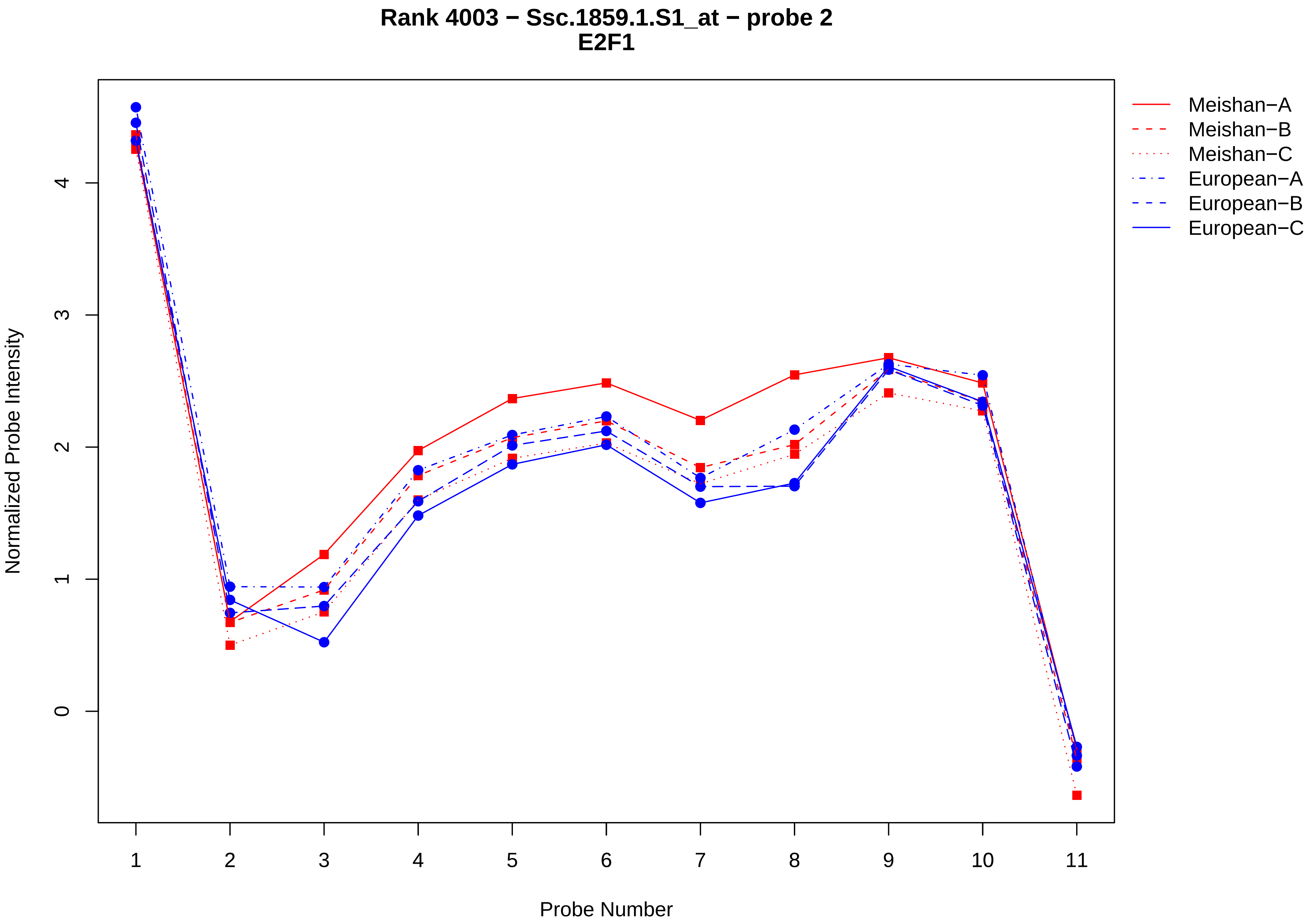

Rank 4003 − Ssc.1859.1.S1_at − probe 2
E2F1
Normalized Probe Intensity
Probe Number
0
1
2
3
4
1
2
3
4
5
6
7
8
9
10
11
Meishan−A
Meishan−B
Meishan−C
European−A
European−B
European−C

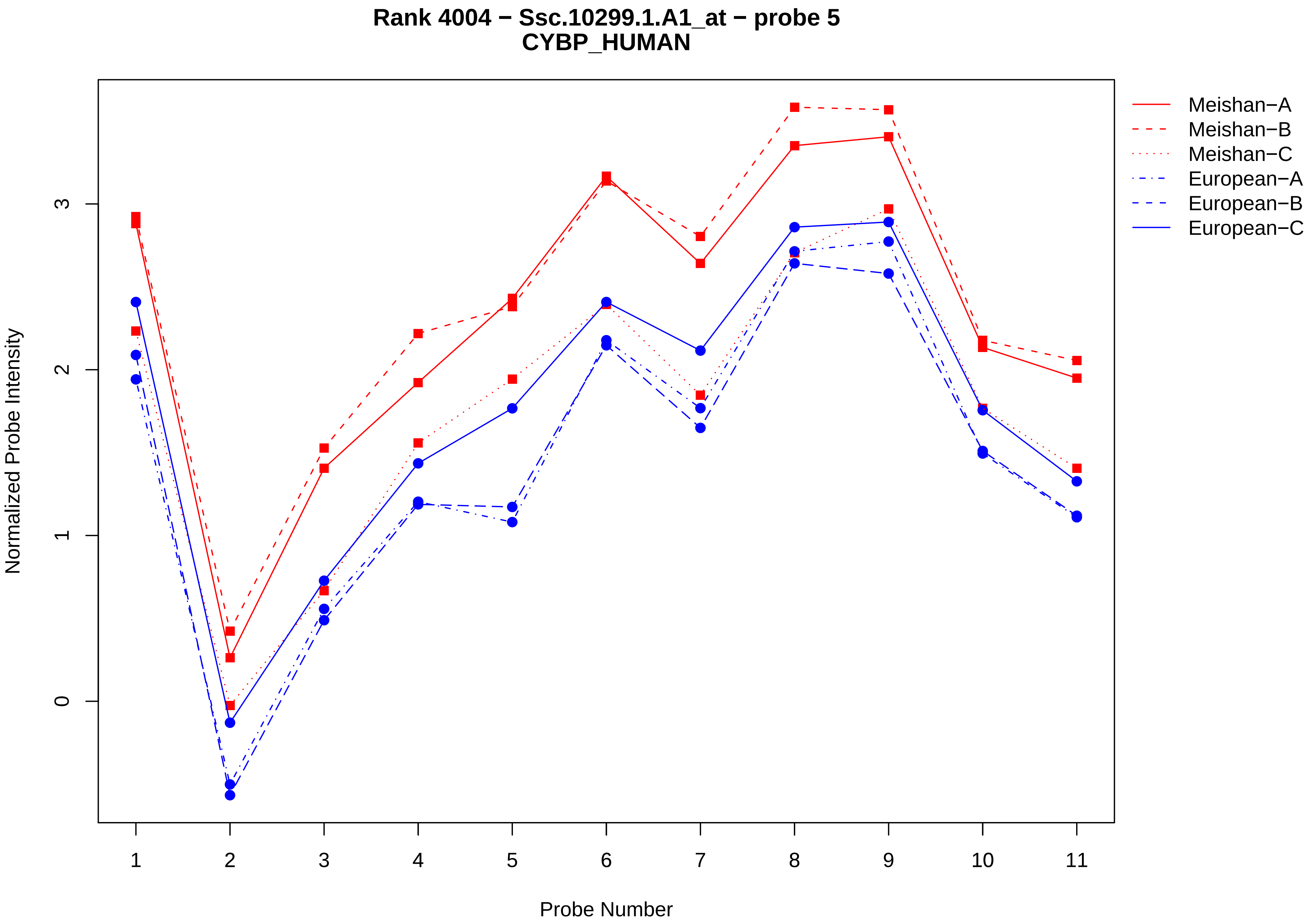

Rank 4004 − Ssc.10299.1.A1_at − probe 5
CYBP_HUMAN
Normalized Probe Intensity
Probe Number
0
1
2
3
1
2
3
4
5
6
7
8
9
10
11
Meishan−A
Meishan−B
Meishan−C
European−A
European−B
European−C

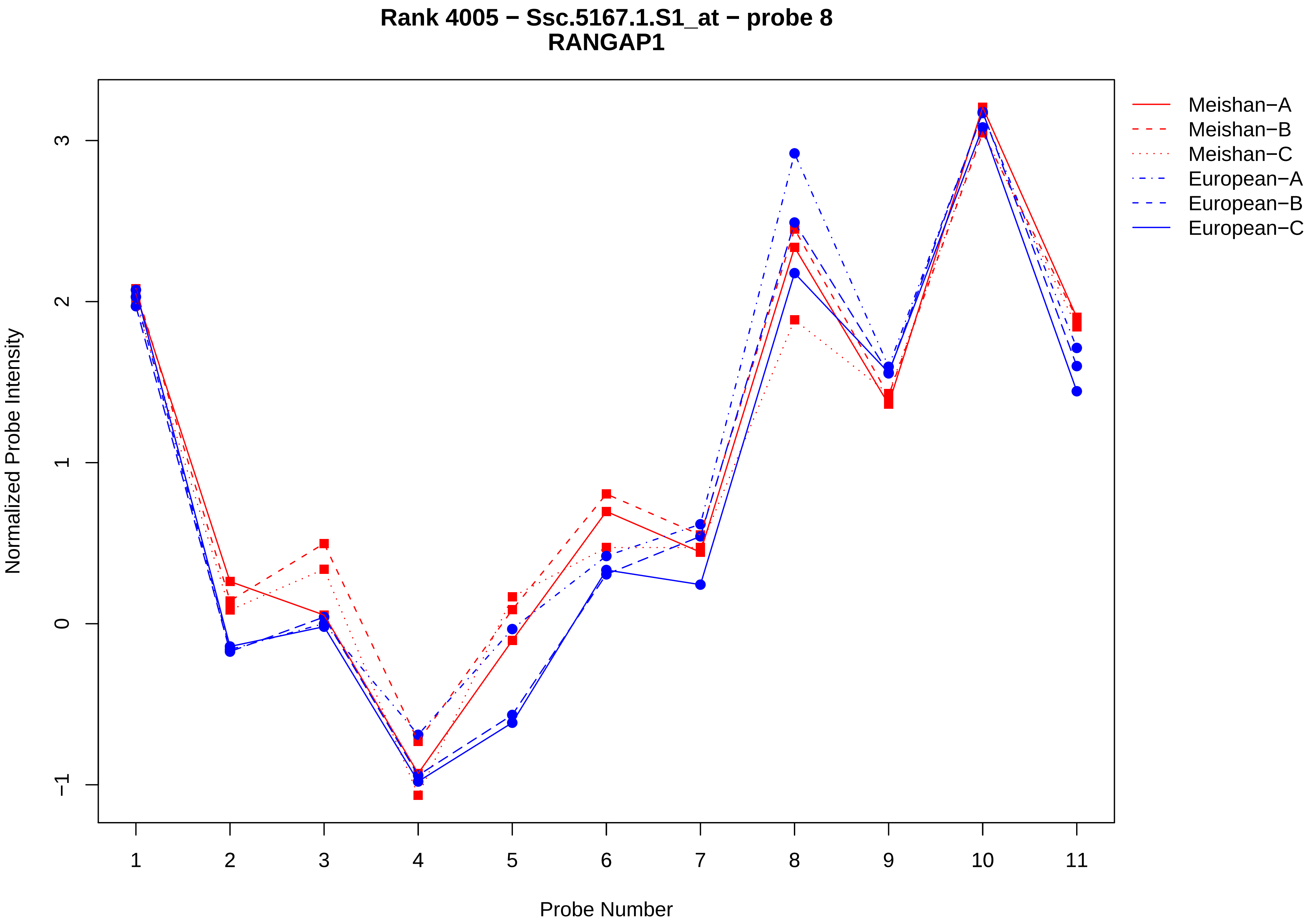

Rank 4005 − Ssc.5167.1.S1_at − probe 8
RANGAP1
Normalized Probe Intensity
Probe Number
−1
0
1
2
3
1
2
3
4
5
6
7
8
9
10
11
Meishan−A
Meishan−B
Meishan−C
European−A
European−B
European−C

Rank 4006 − Ssc.7890.1.S1_at − probe 2
Q9H7Z1

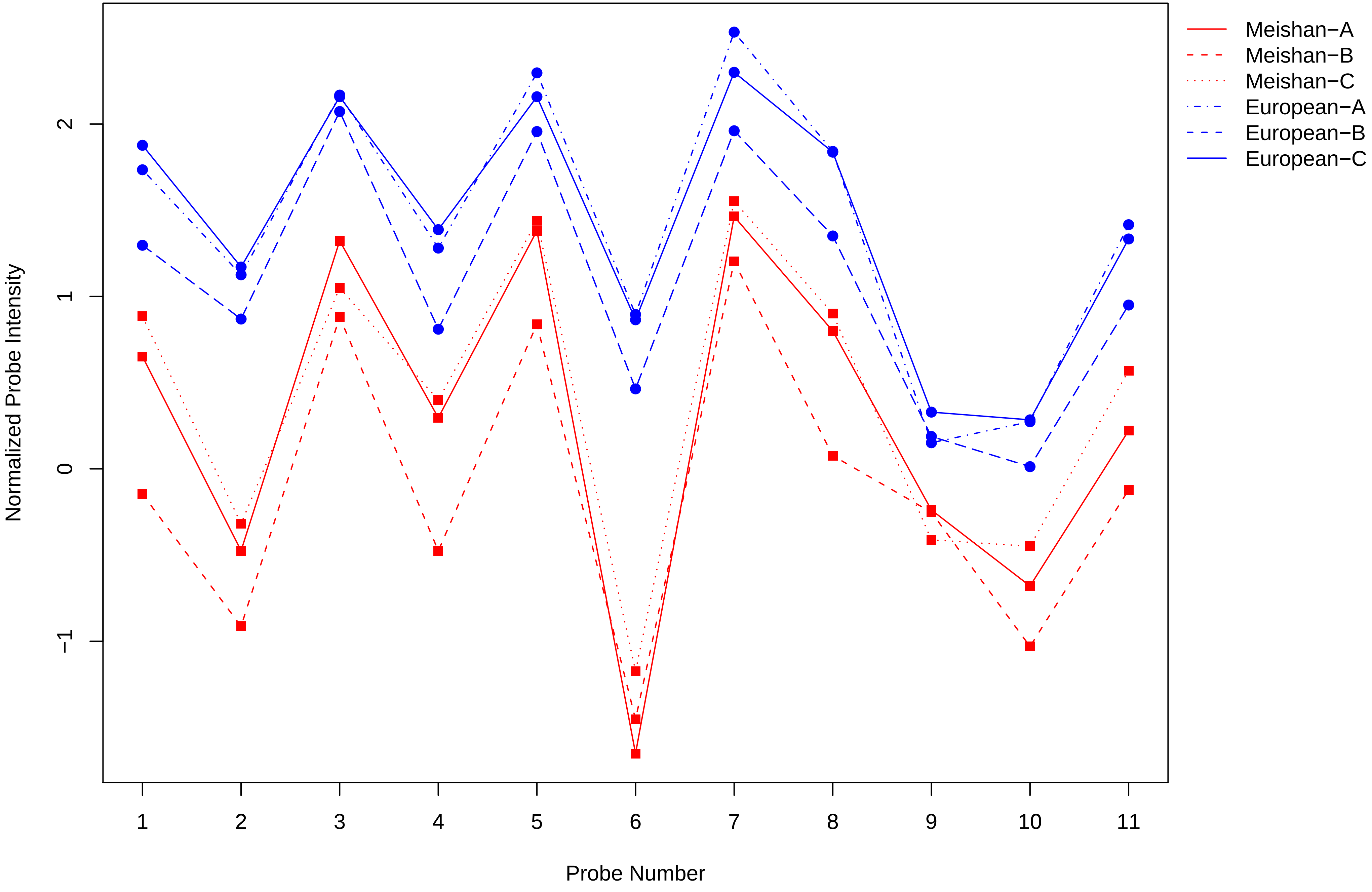

Rank 4007 − Ssc.20238.2.S1_a_at − probe 11
GNPDA1

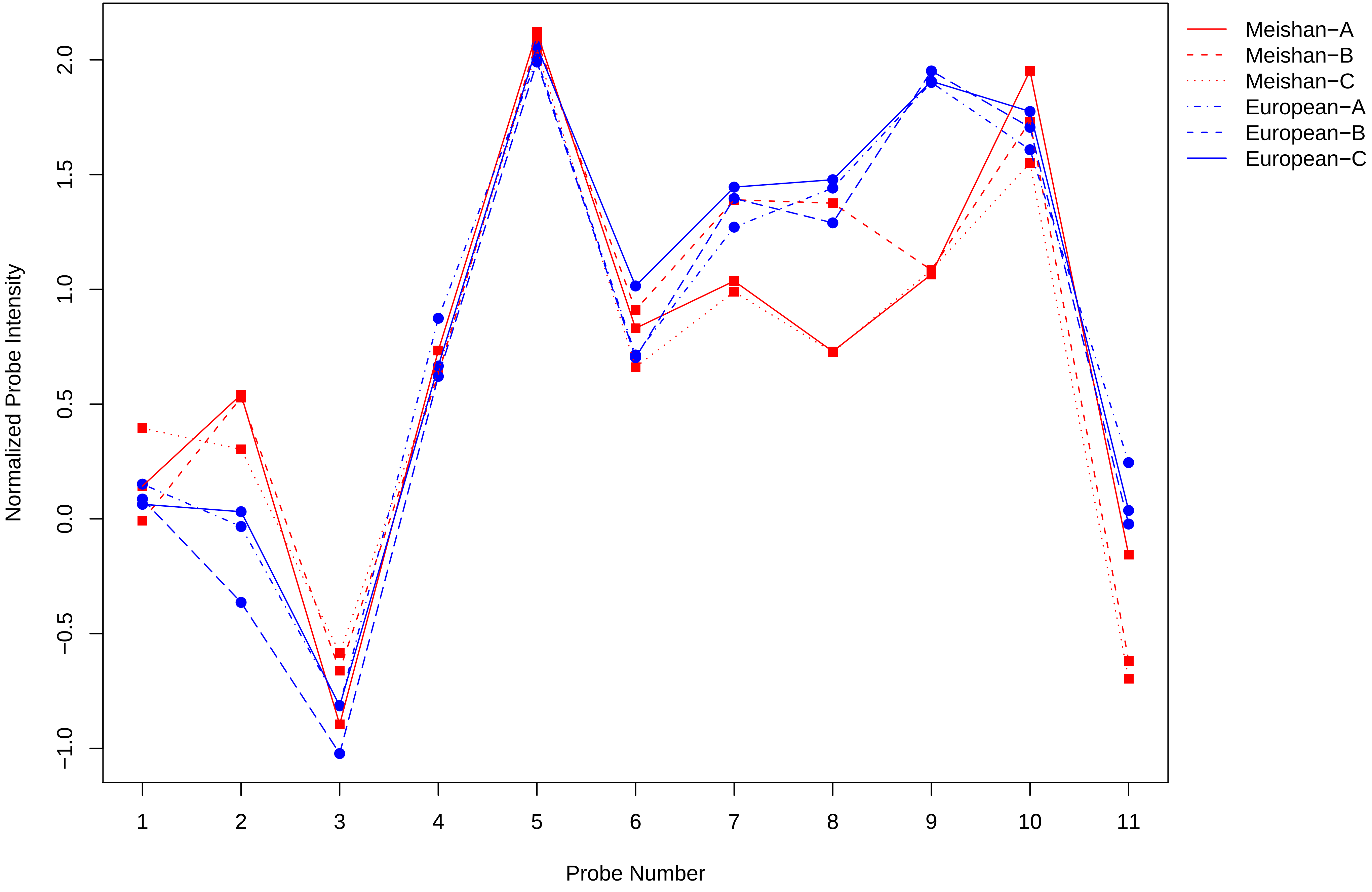

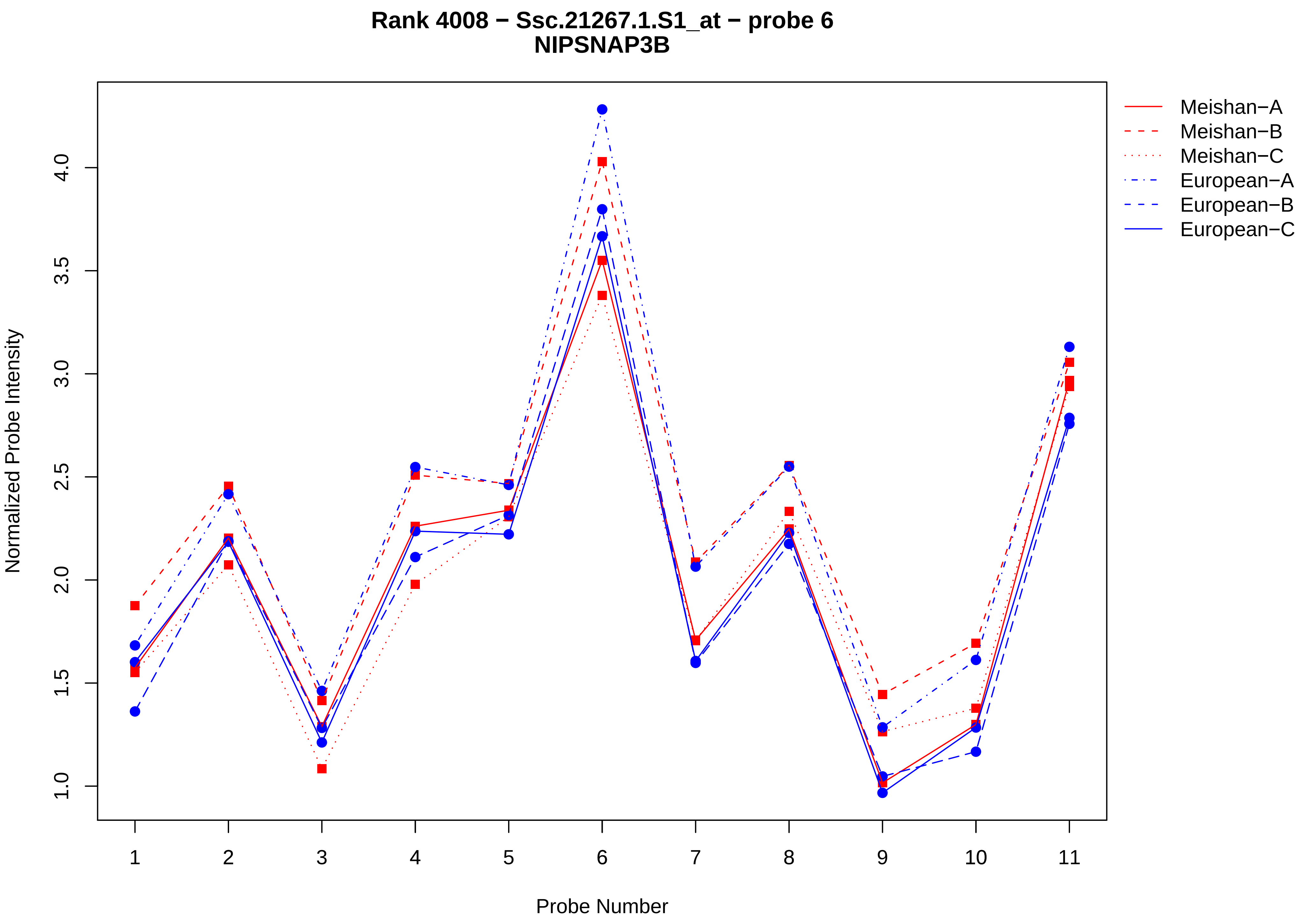

Rank 4008 − Ssc.21267.1.S1_at − probe 6
NIPSNAP3B
Normalized Probe Intensity
Probe Number
1.0
1.5
2.0
2.5
3.0
3.5
4.0
1
2
3
4
5
6
7
8
9
10
11
Meishan−A
Meishan−B
Meishan−C
European−A
European−B
European−C

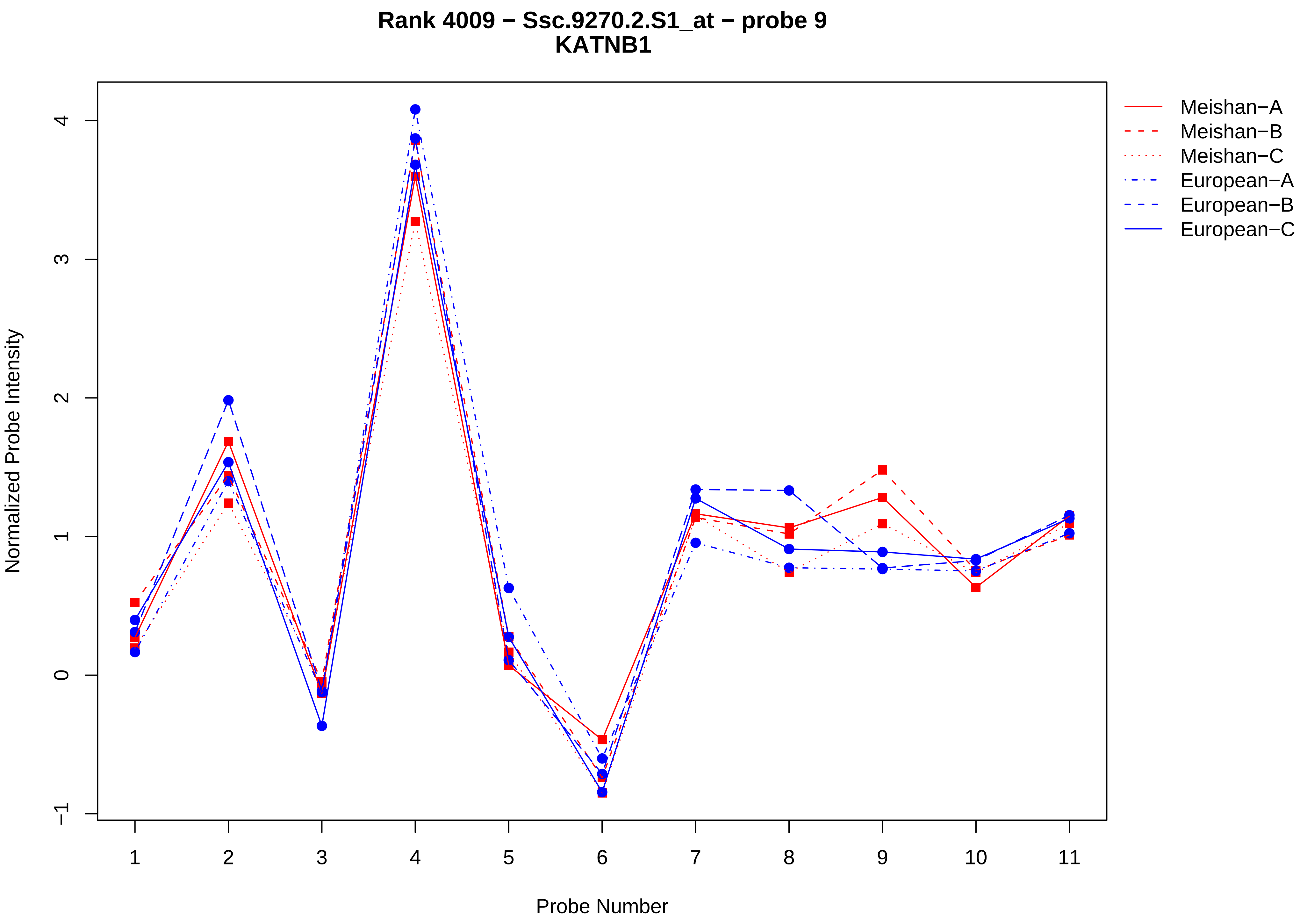
Rank 4009 − Ssc.9270.2.S1_at − probe 9
KATNB1
Normalized Probe Intensity
Probe Number
−1
0
1
2
3
4
1
2
3
4
5
6
7
8
9
10
11
Meishan−A
Meishan−B
Meishan−C
European−A
European−B
European−C

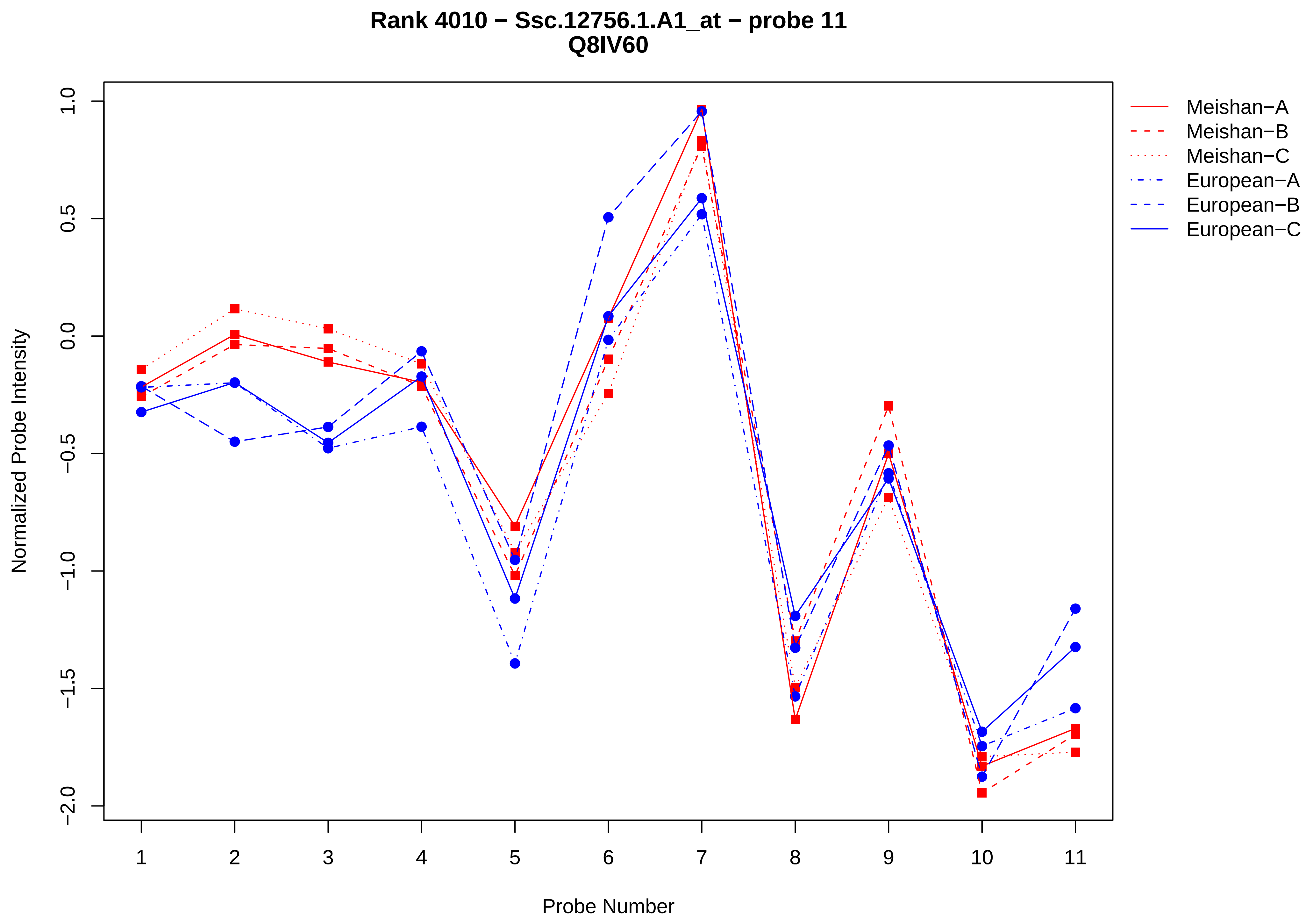

Rank 4010 − Ssc.12756.1.A1_at − probe 11
Q8IV60
Normalized Probe Intensity
Probe Number
1.0
0.5
0.0
−0.5
−1.0
−1.5
−2.0
1
2
3
4
5
6
7
8
9
10
11
Meishan−A
Meishan−B
Meishan−C
European−A
European−B
European−C

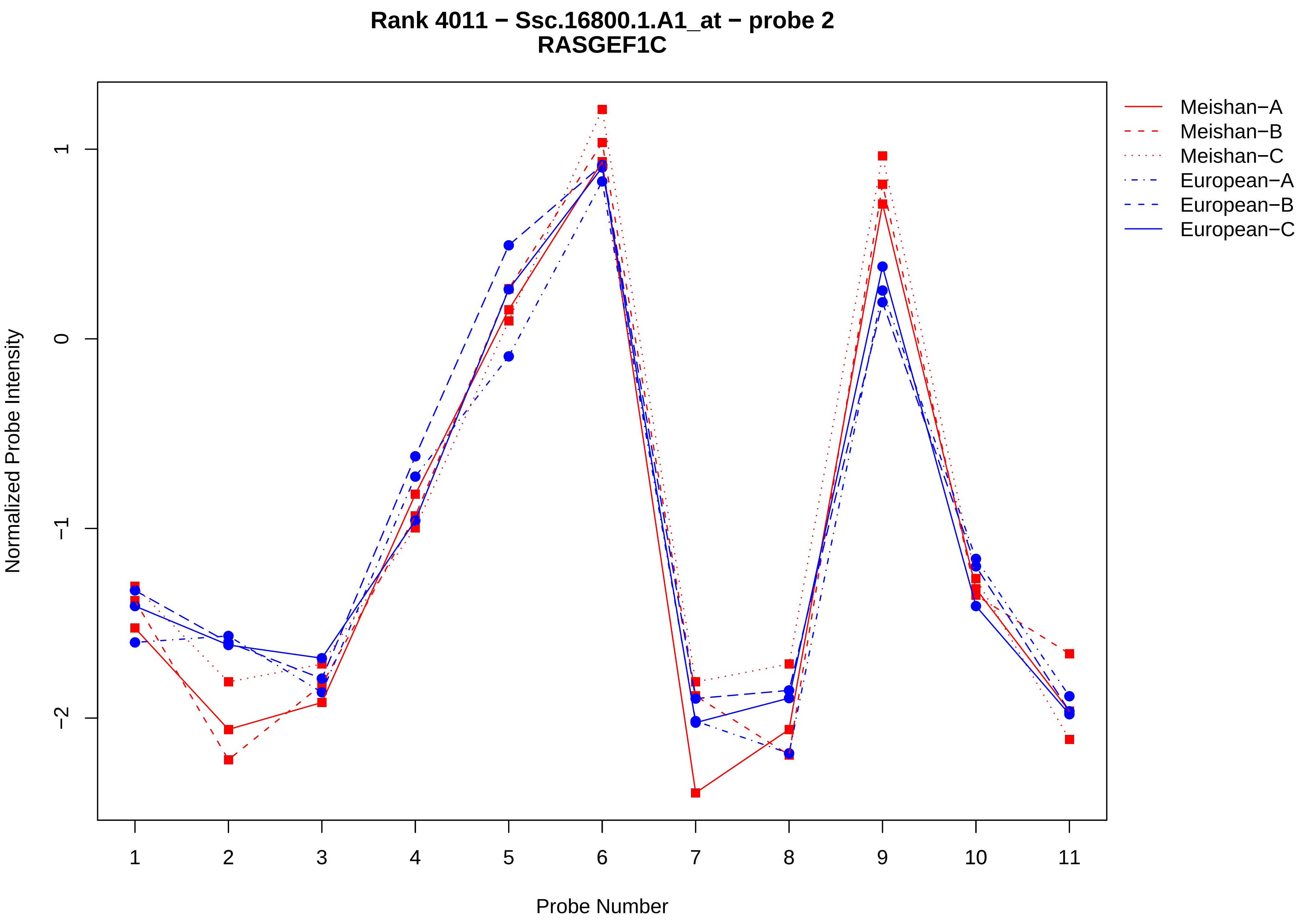

Rank 4011 − Ssc.16800.1.A1_at − probe 2
RASGEF1C
Normalized Probe Intensity
Probe Number
1
0
−1
−2
1
2
3
4
5
6
7
8
9
10
11
Meishan−A
Meishan−B
Meishan−C
European−A
European−B
European−C

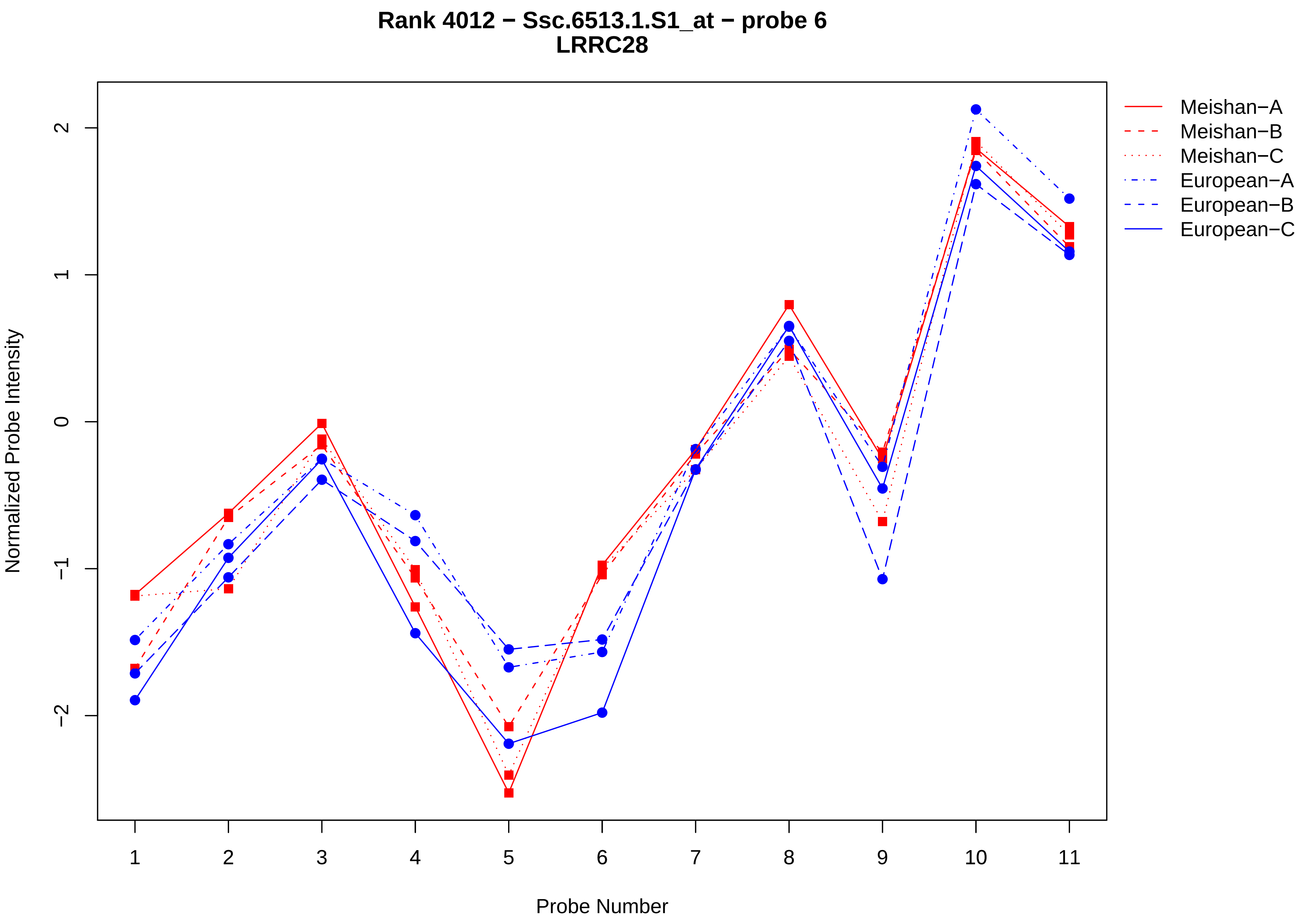

Rank 4012 − Ssc.6513.1.S1_at − probe 6
LRRC28
Normalized Probe Intensity
Probe Number
Meishan−A
Meishan−B
Meishan−C
European−A
European−B
European−C

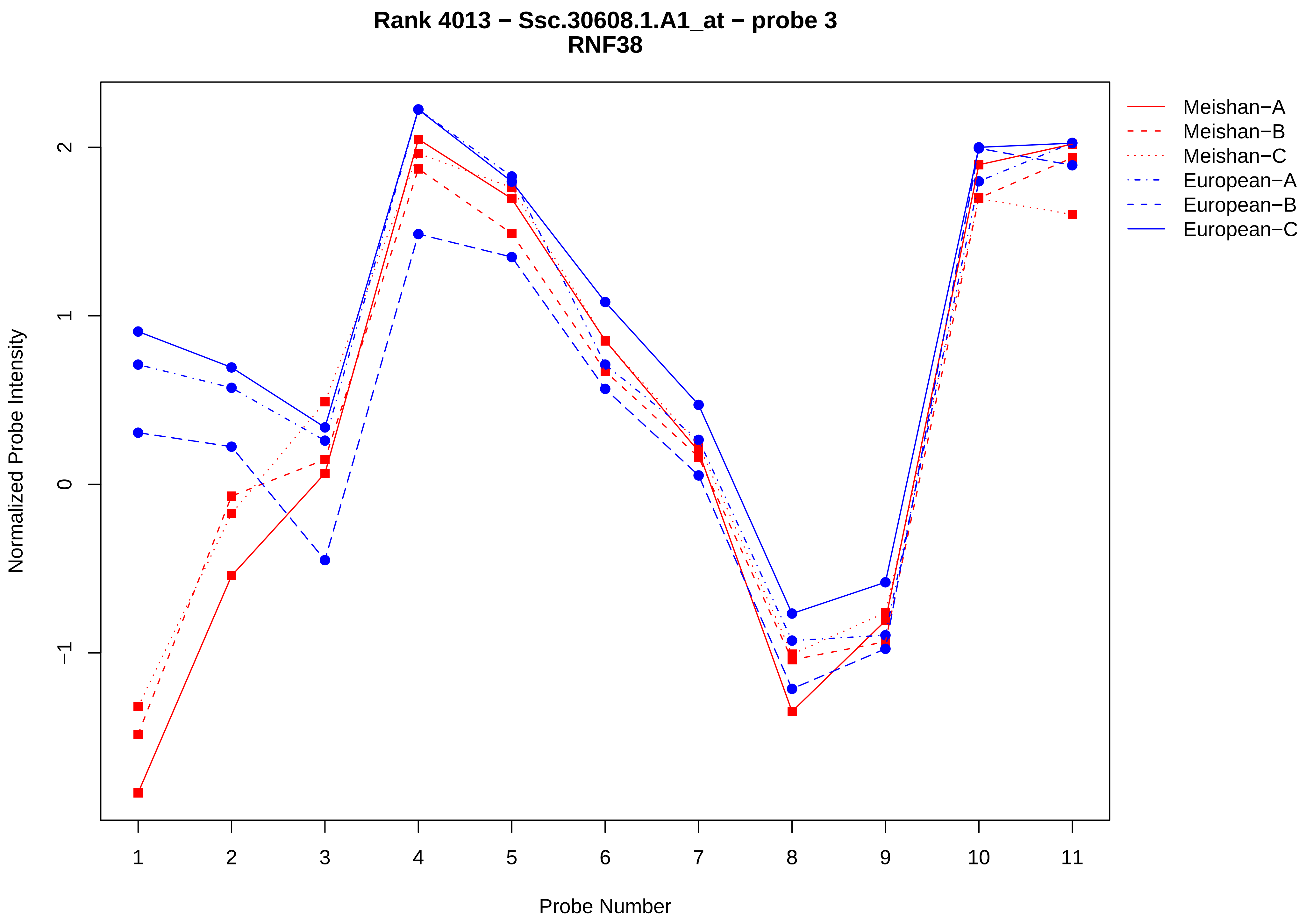

Rank 4013 − Ssc.30608.1.A1_at − probe 3
RNF38
Normalized Probe Intensity
Probe Number
Meishan−A
Meishan−B
Meishan−C
European−A
European−B
European−C

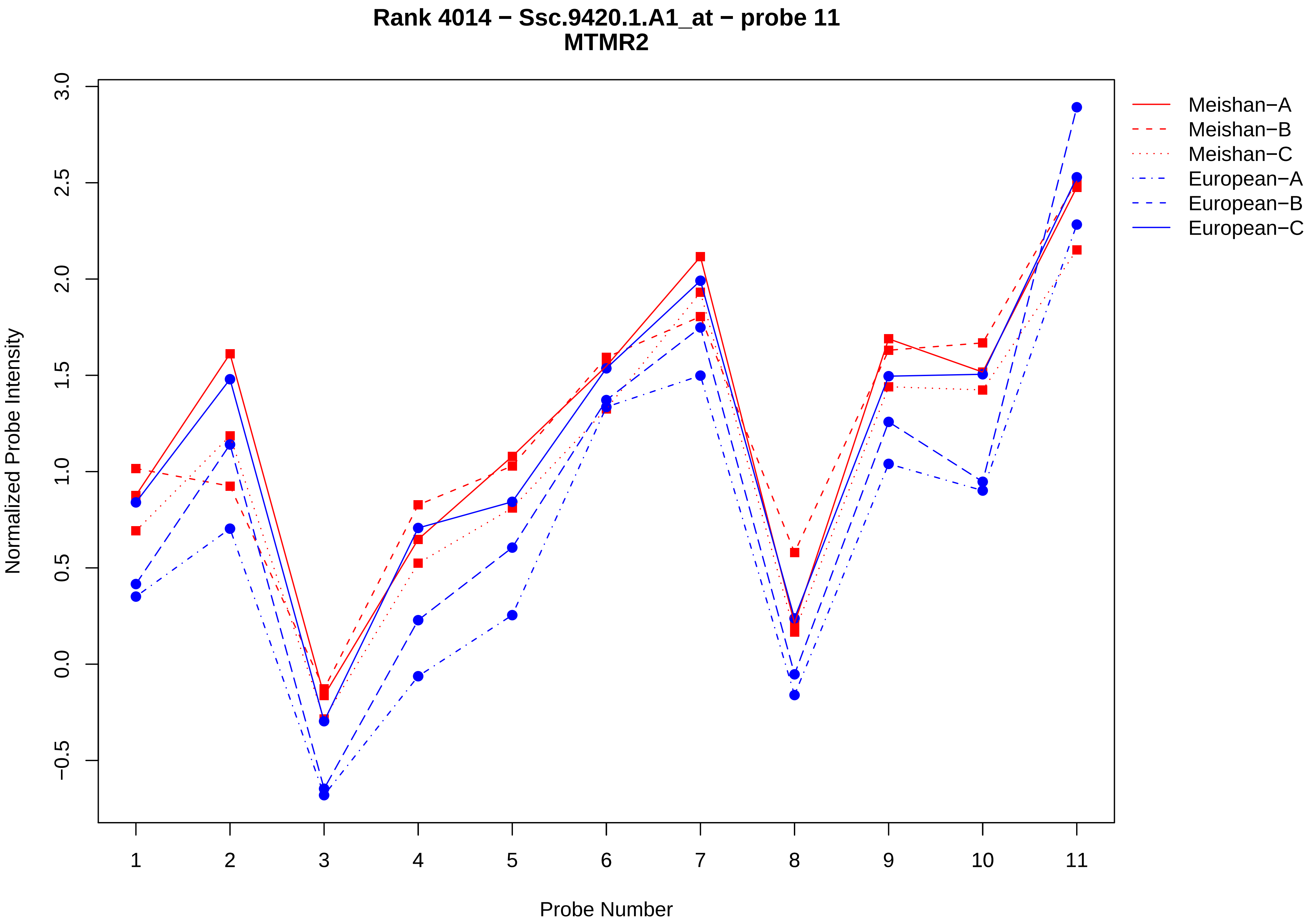

Rank 4014 − Ssc.9420.1.A1_at − probe 11
MTMR2
Normalized Probe Intensity
−0.5
0.0
0.5
1.0
1.5
2.0
2.5
3.0
1
2
3
4
5
6
7
8
9
10
11
Probe Number
Meishan−A
Meishan−B
Meishan−C
European−A
European−B
European−C

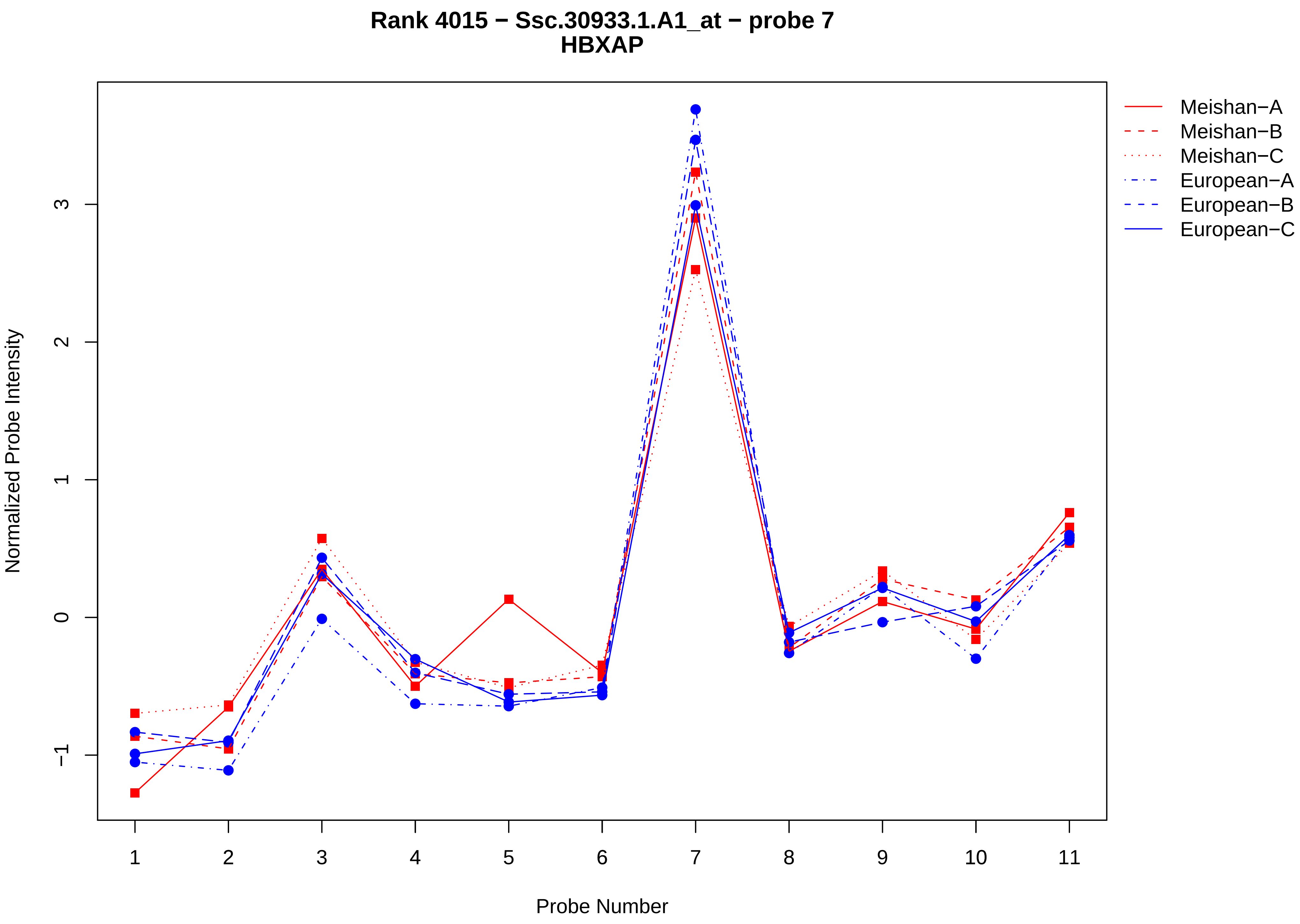
Rank 4015 − Ssc.30933.1.A1_at − probe 7
HBXAP
Normalized Probe Intensity
Probe Number
−1
0
1
2
3
1
2
3
4
5
6
7
8
9
10
11
Meishan−A
Meishan−B
Meishan−C
European−A
European−B
European−C

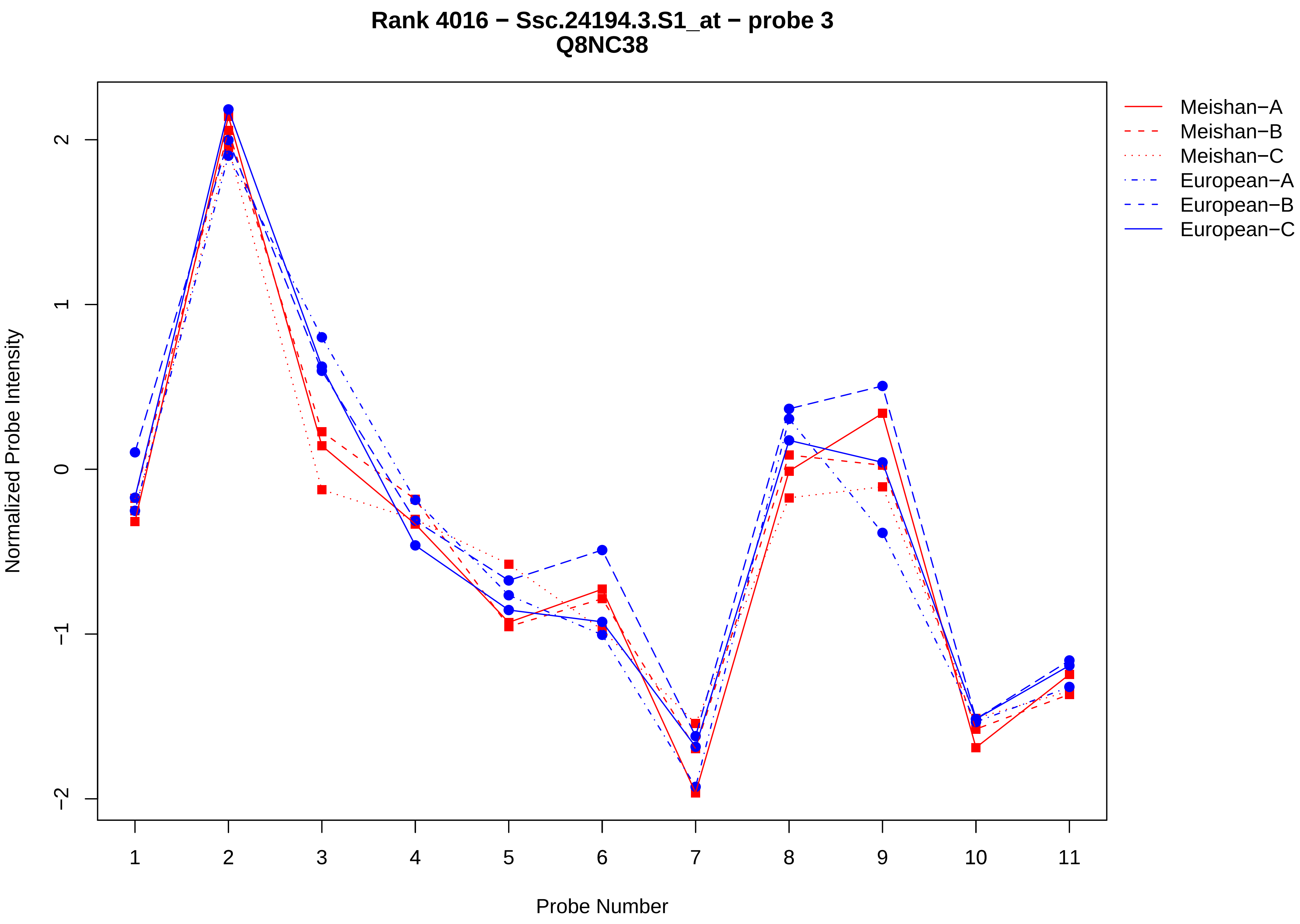
Rank 4016 − Ssc.24194.3.S1_at − probe 3
Q8NC38
Normalized Probe Intensity
Probe Number
−2
−1
0
1
2
1
2
3
4
5
6
7
8
9
10
11
Meishan−A
Meishan−B
Meishan−C
European−A
European−B
European−C

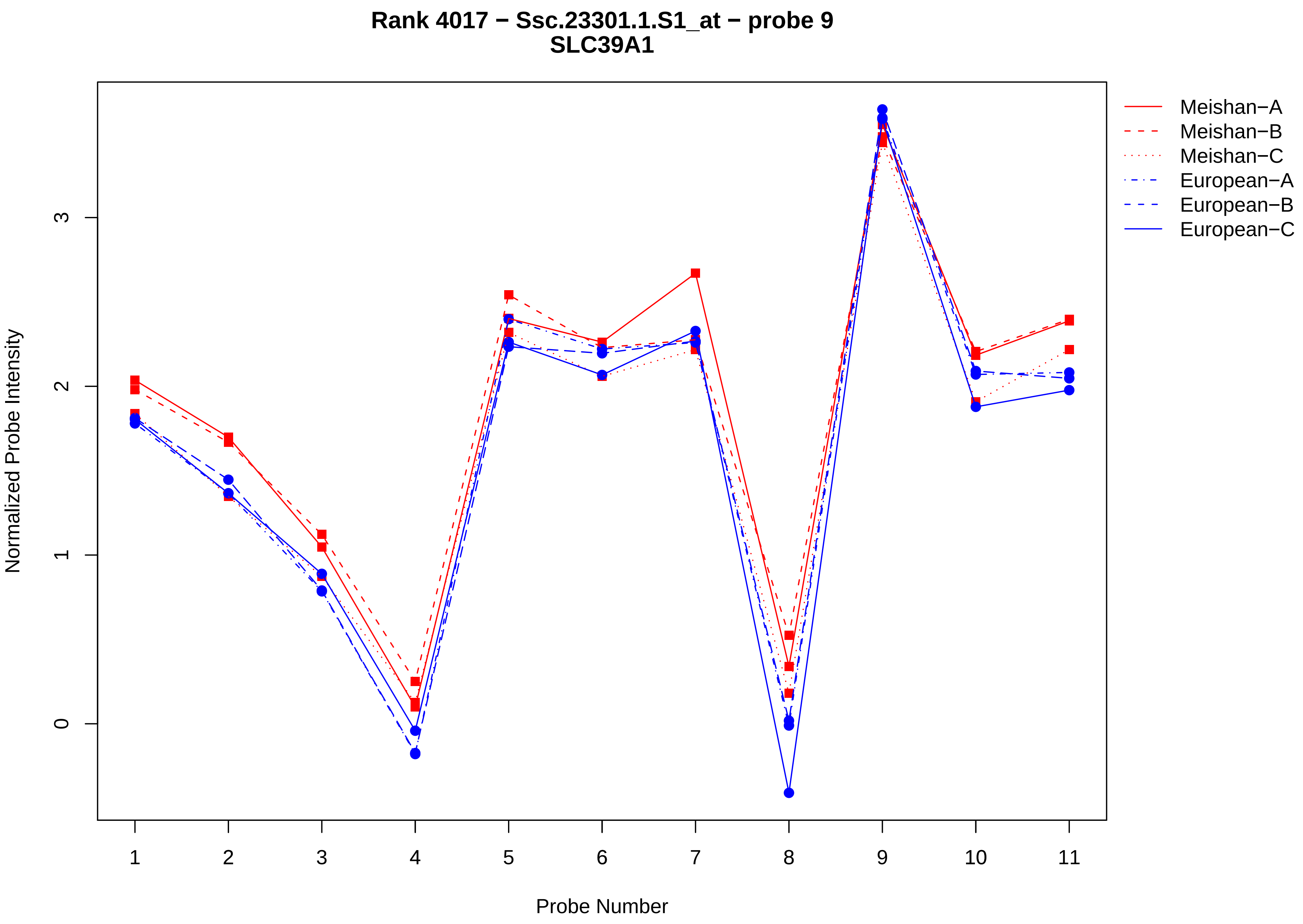
Rank 4017 − Ssc.23301.1.S1_at − probe 9
SLC39A1
Normalized Probe Intensity
Probe Number
0
1
2
3
1
2
3
4
5
6
7
8
9
10
11
Meishan−A
Meishan−B
Meishan−C
European−A
European−B
European−C

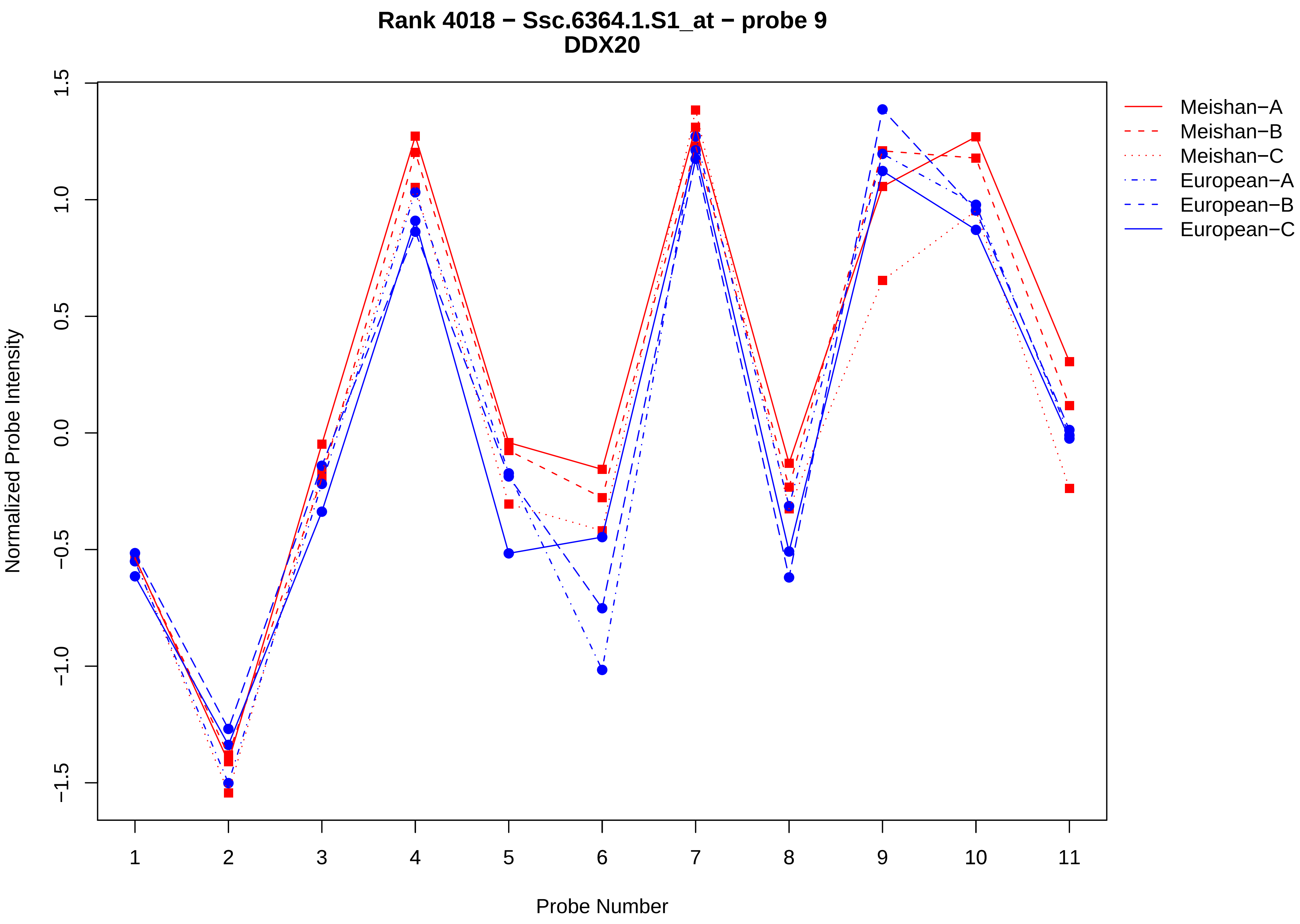

Rank 4018 − Ssc.6364.1.S1_at − probe 9
DDX20
Normalized Probe Intensity
Probe Number
1.5
1.0
0.5
0.0
−0.5
−1.0
−1.5
1
2
3
4
5
6
7
8
9
10
11
Meishan−A
Meishan−B
Meishan−C
European−A
European−B
European−C

Rank 4019 − Ssc.1461.1.S1_at − probe 2
COX7C

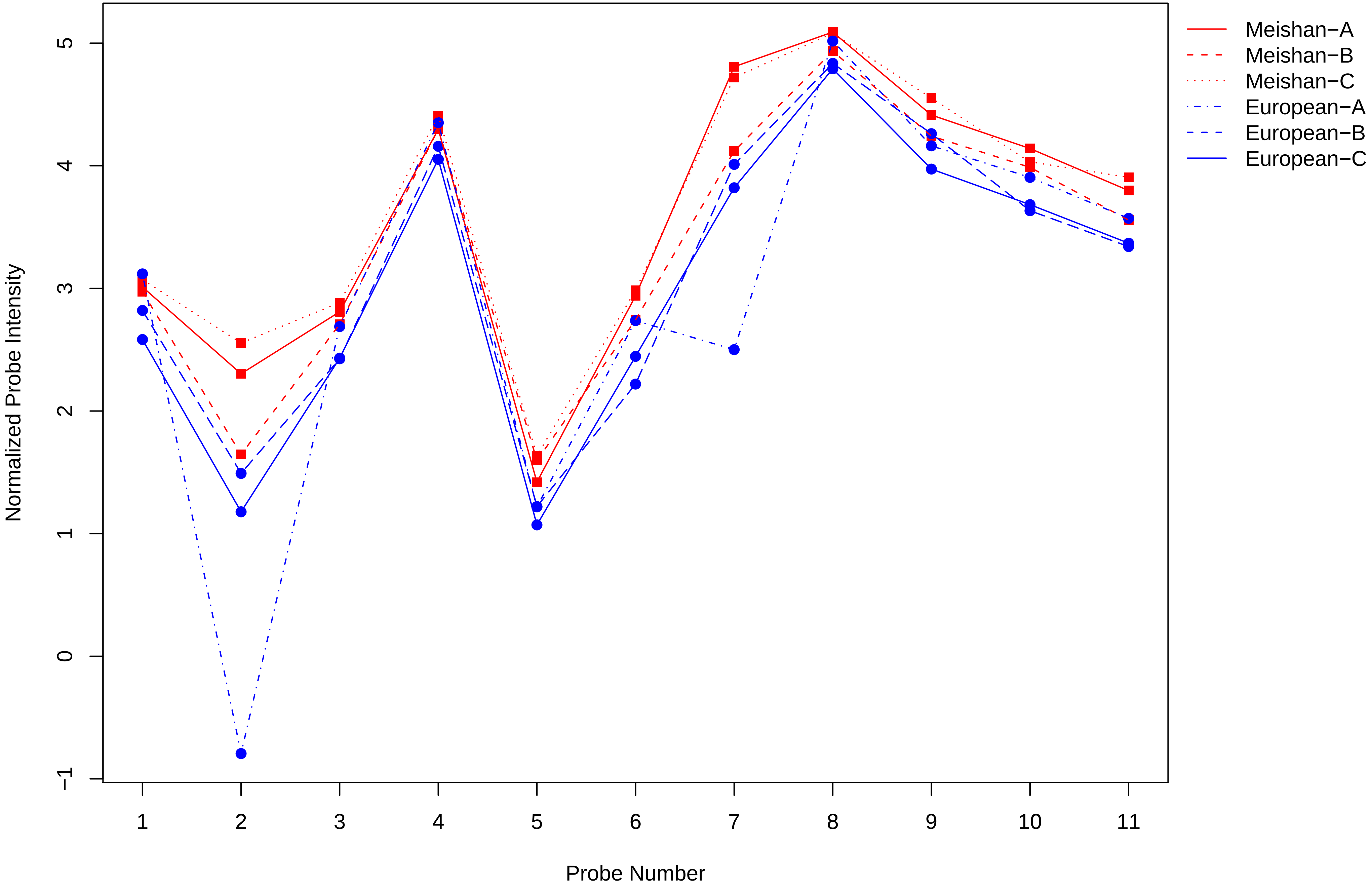

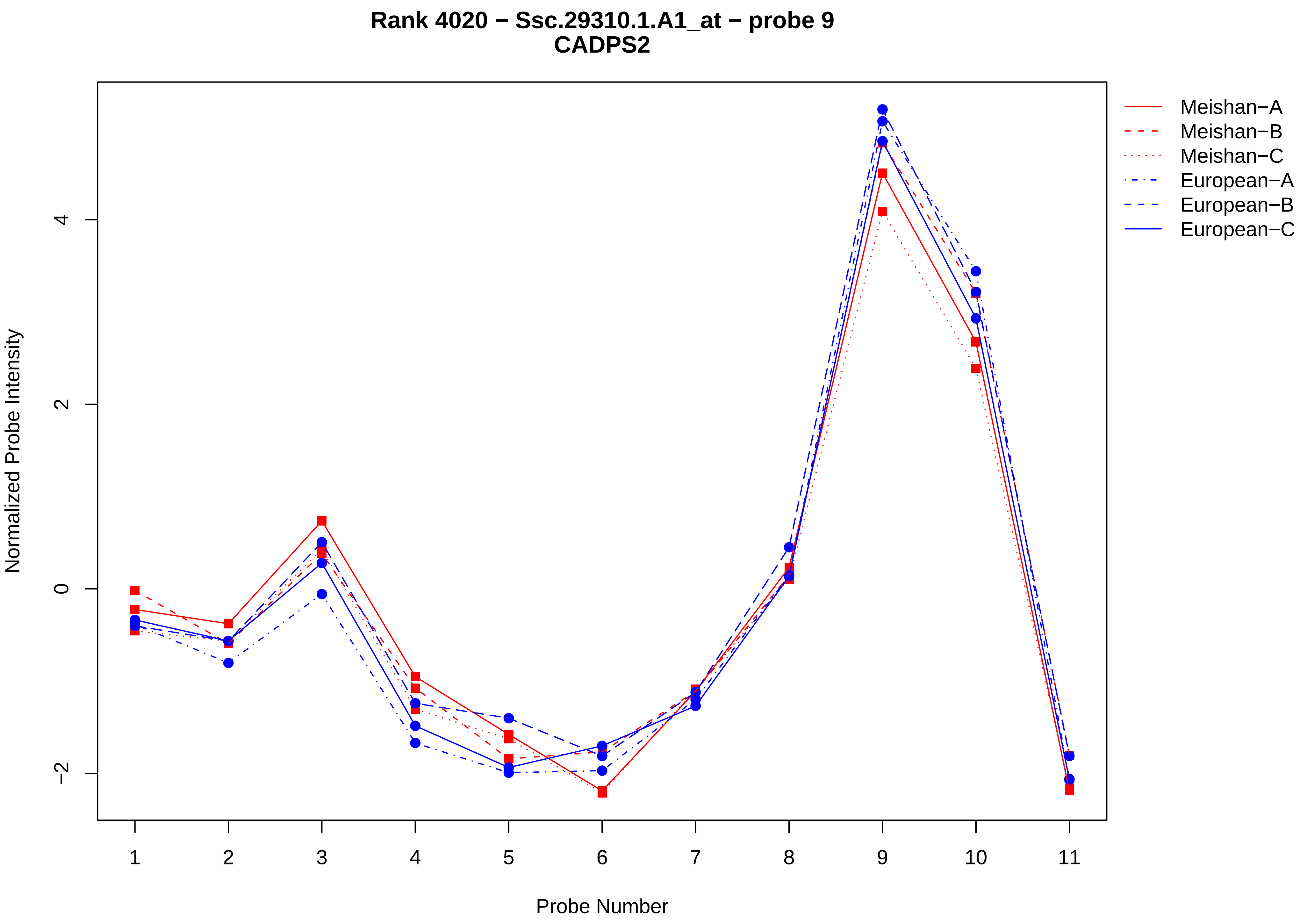

Rank 4020 − Ssc.29310.1.A1_at − probe 9
CADPS2
Normalized Probe Intensity
Probe Number
−2
0
2
4
1
2
3
4
5
6
7
8
9
10
11
Meishan−A
Meishan−B
Meishan−C
European−A
European−B
European−C

Rank 4021 − Ssc.26050.1.S1_at − probe 10
C5orf19

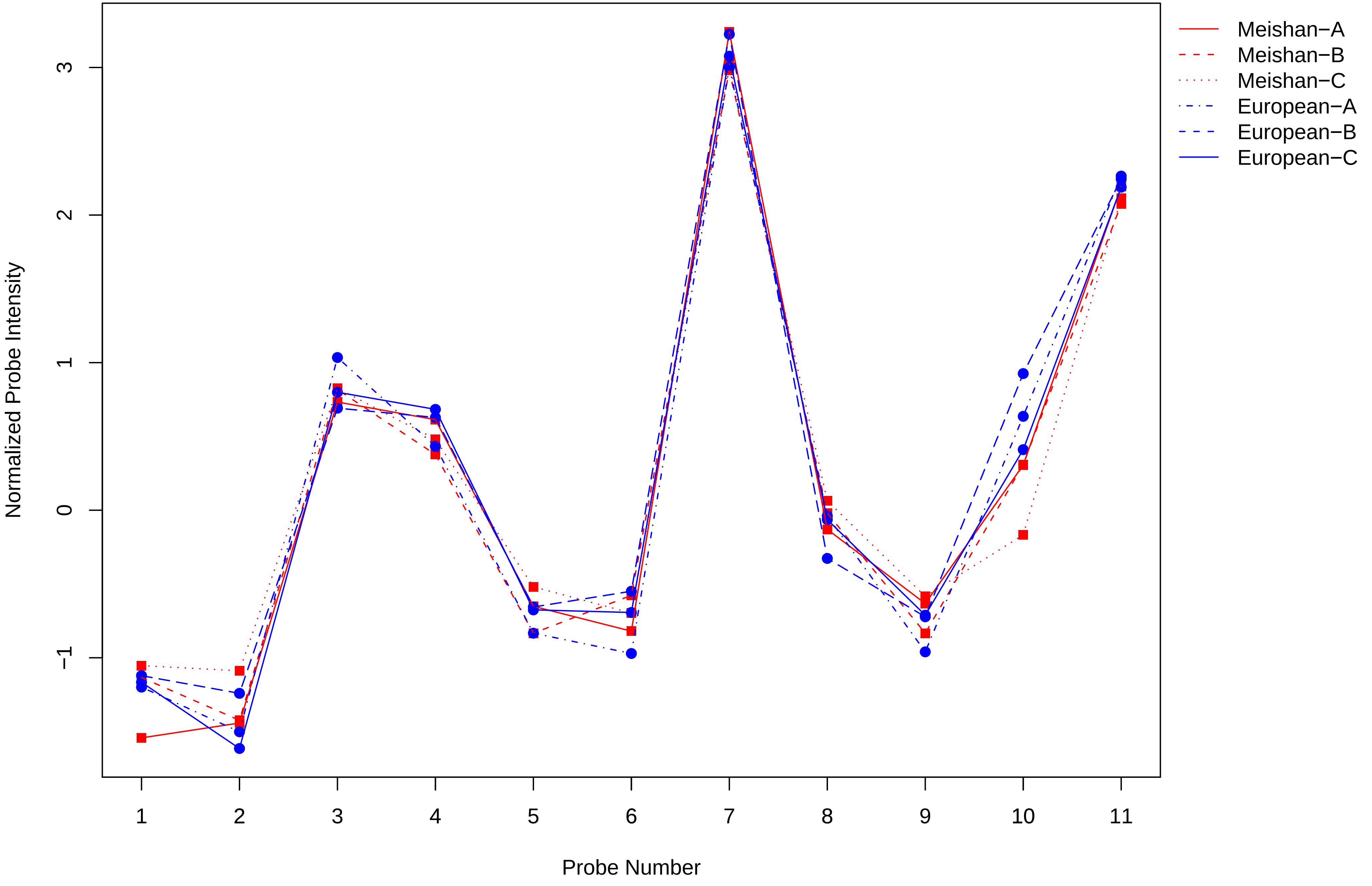

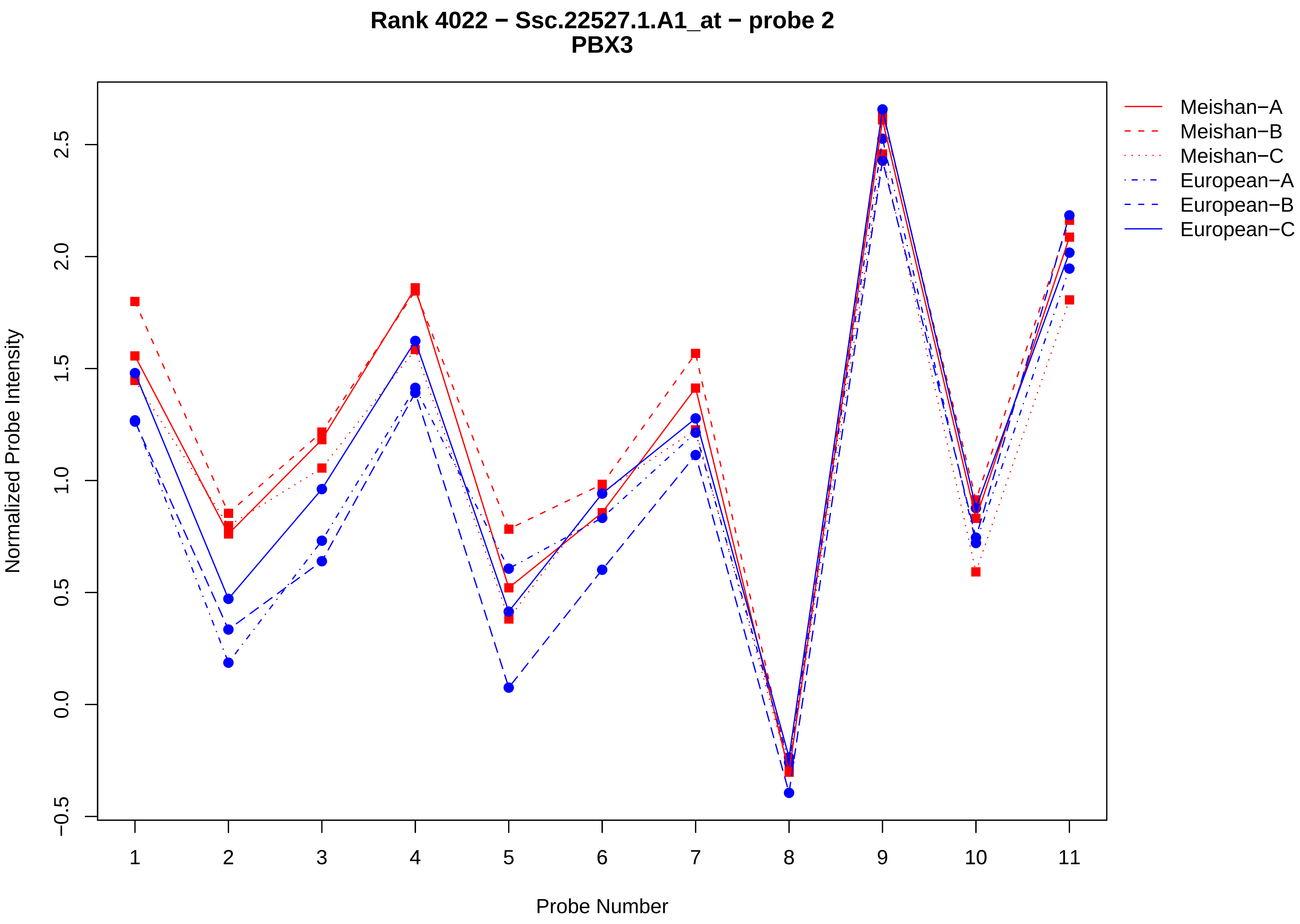

Rank 4022 − Ssc.22527.1.A1_at − probe 2
PBX3
Normalized Probe Intensity
Probe Number
−0.5
0.0
0.5
1.0
1.5
2.0
2.5
1
2
3
4
5
6
7
8
9
10
11
Meishan−A
Meishan−B
Meishan−C
European−A
European−B
European−C

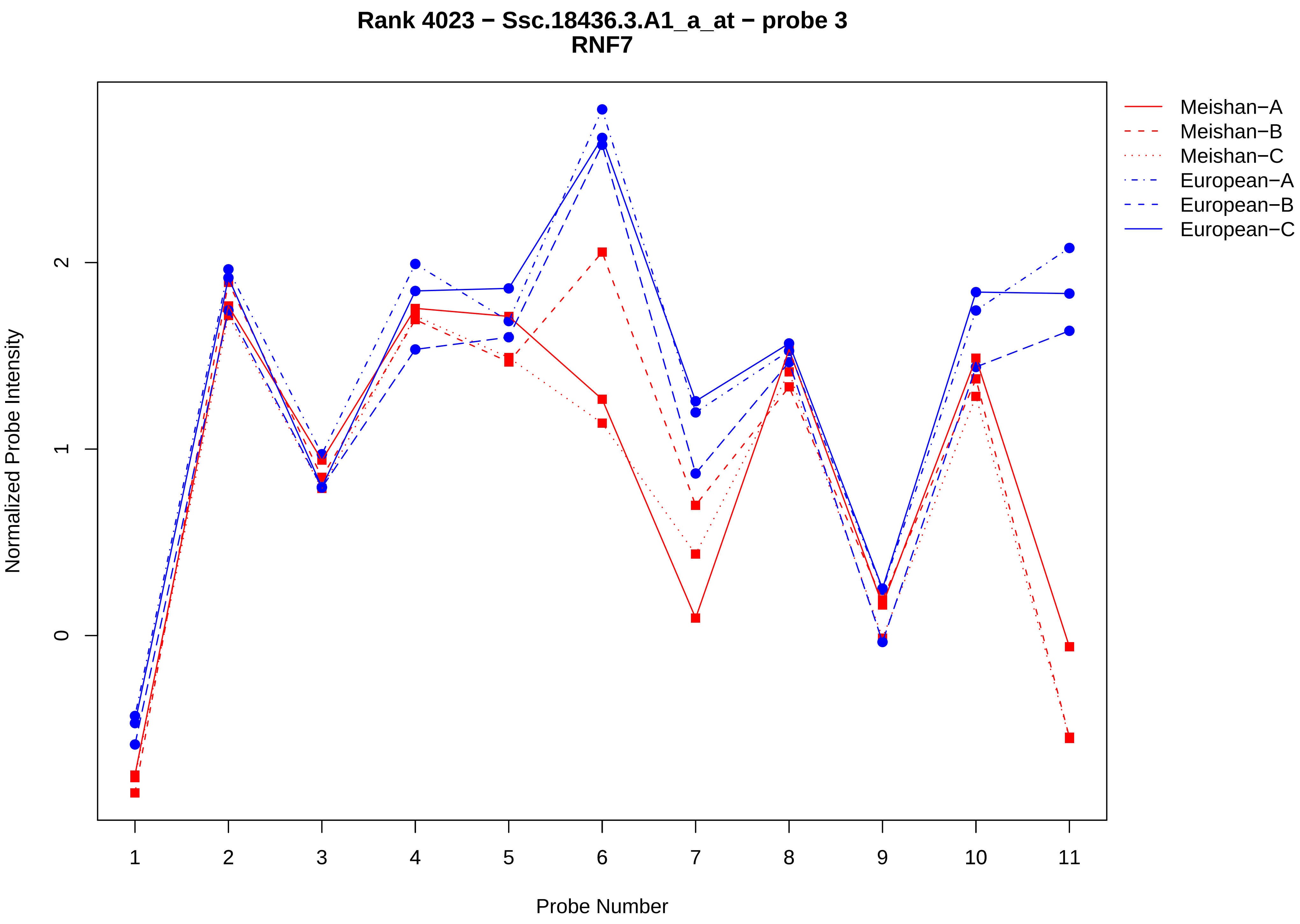
Rank 4023 − Ssc.18436.3.A1_a_at − probe 3
RNF7
Normalized Probe Intensity
0
1
2
1
2
3
4
5
6
7
8
9
10
11
Probe Number
Meishan−A
Meishan−B
Meishan−C
European−A
European−B
European−C

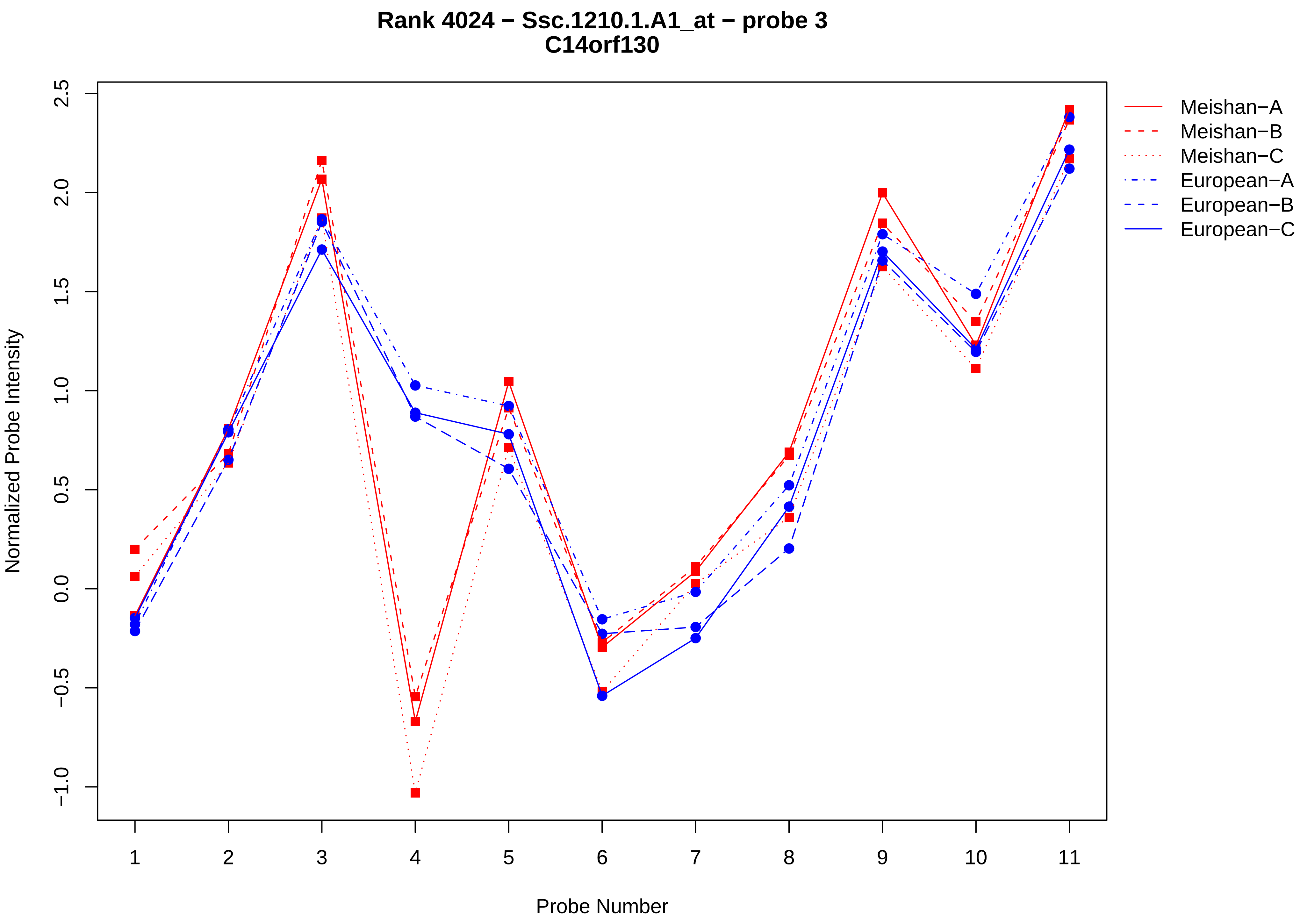

Rank 4024 − Ssc.1210.1.A1_at − probe 3
C14orf130
Normalized Probe Intensity
Probe Number
Meishan−A
Meishan−B
Meishan−C
European−A
European−B
European−C
2.5
2.0
1.5
1.0
0.5
0.0
−0.5
−1.0
1
2
3
4
5
6
7
8
9
10
11

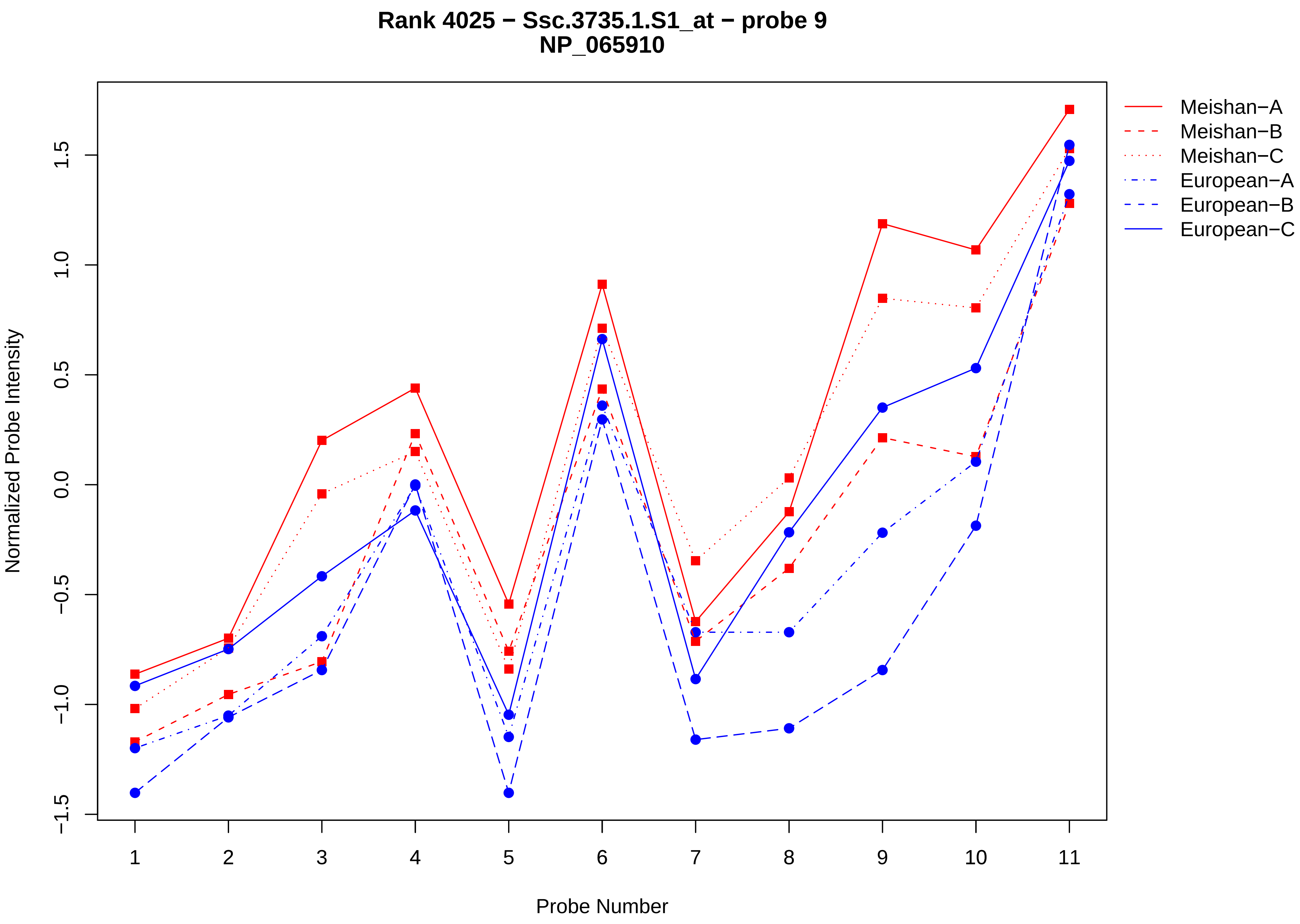
Rank 4025 − Ssc.3735.1.S1_at − probe 9
NP_065910
Normalized Probe Intensity
Probe Number
−1.5
−1.0
−0.5
0.0
0.5
1.0
1.5
1
2
3
4
5
6
7
8
9
10
11
Meishan−A
Meishan−B
Meishan−C
European−A
European−B
European−C

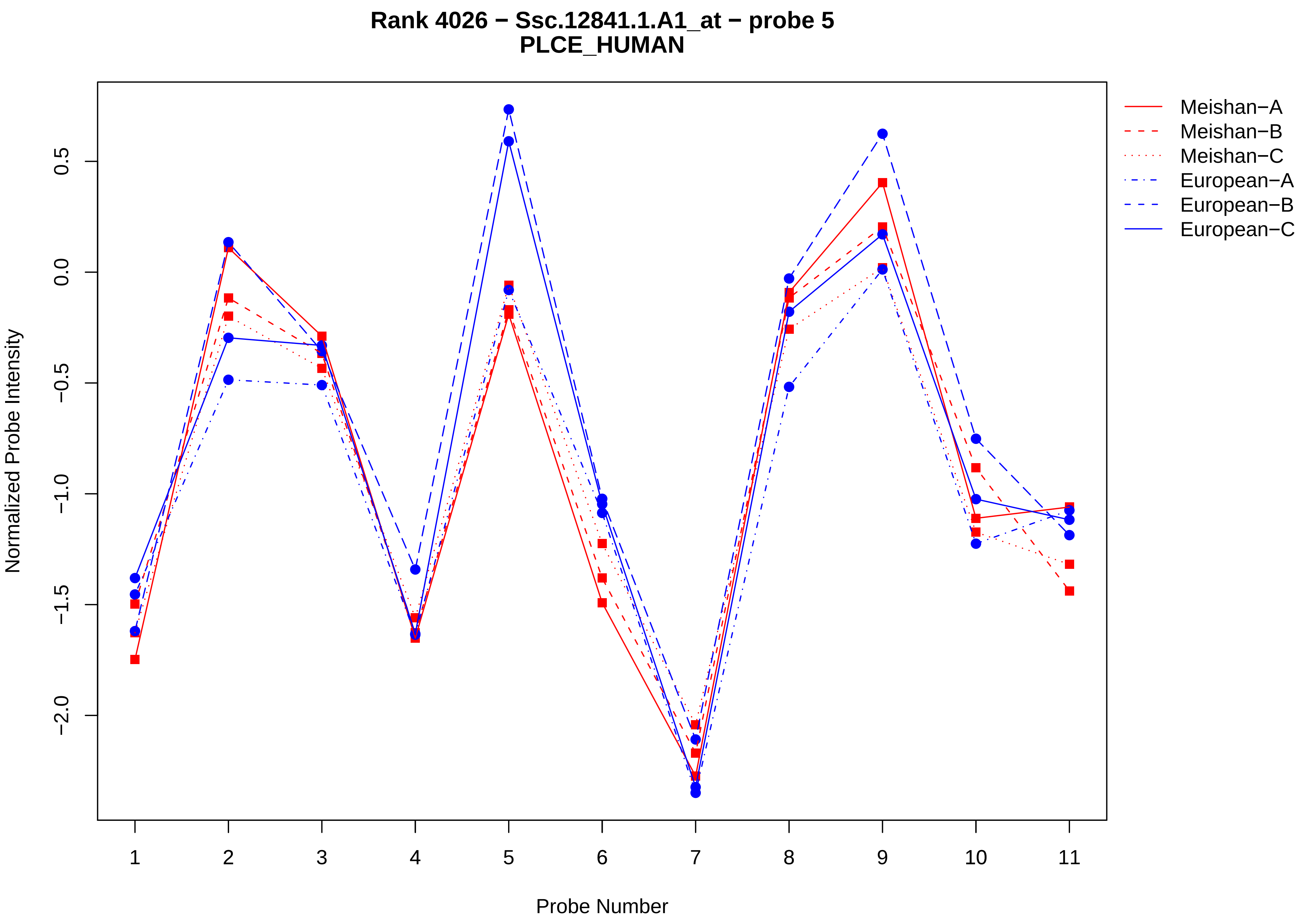

Rank 4026 − Ssc.12841.1.A1_at − probe 5
PLCE_HUMAN
Normalized Probe Intensity
Probe Number
0.5
0.0
−0.5
−1.0
−1.5
−2.0
1
2
3
4
5
6
7
8
9
10
11
Meishan−A
Meishan−B
Meishan−C
European−A
European−B
European−C

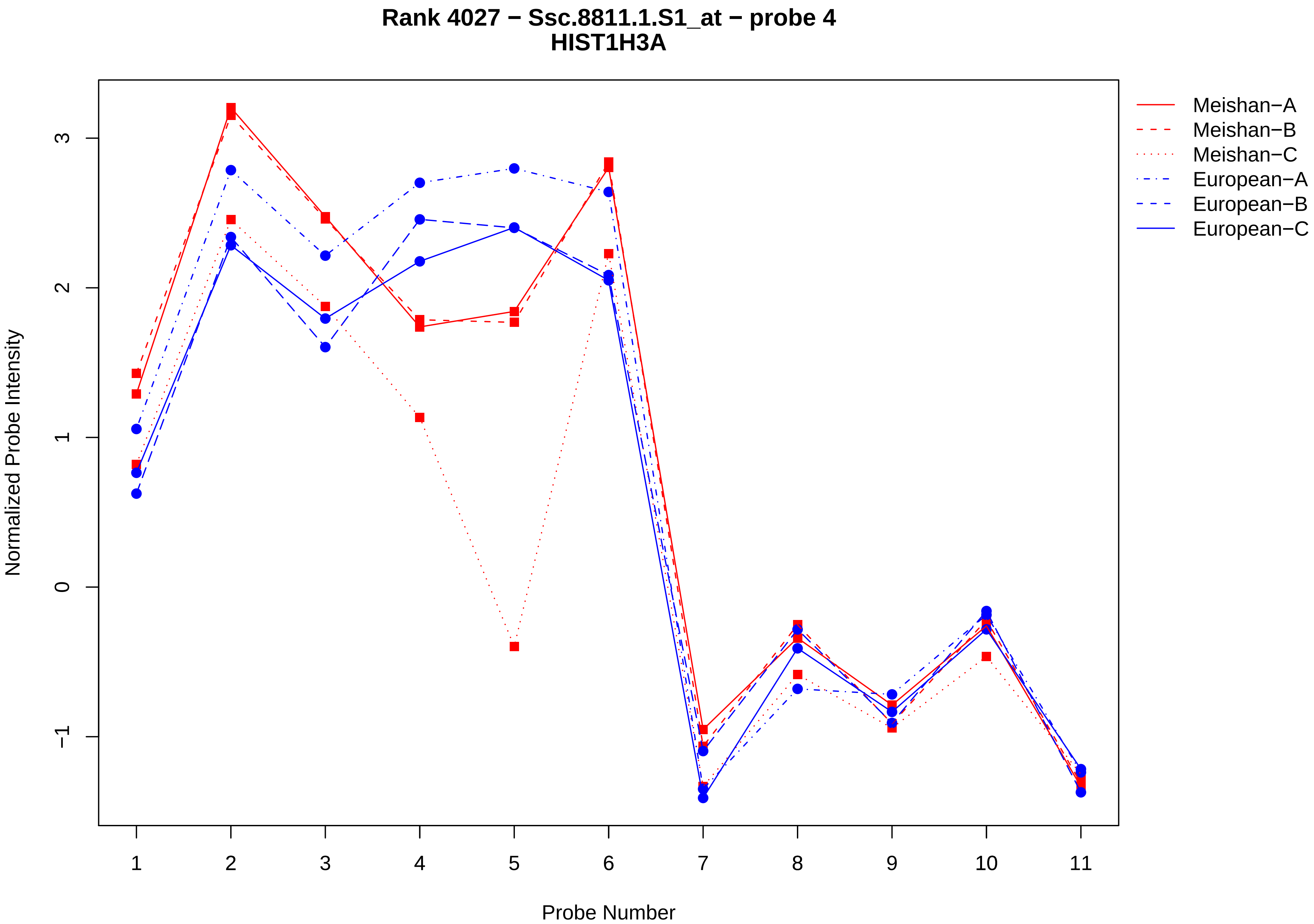

Rank 4027 − Ssc.8811.1.S1_at − probe 4
HIST1H3A
Normalized Probe Intensity
Probe Number
−1
0
1
2
3
1
2
3
4
5
6
7
8
9
10
11
Meishan−A
Meishan−B
Meishan−C
European−A
European−B
European−C

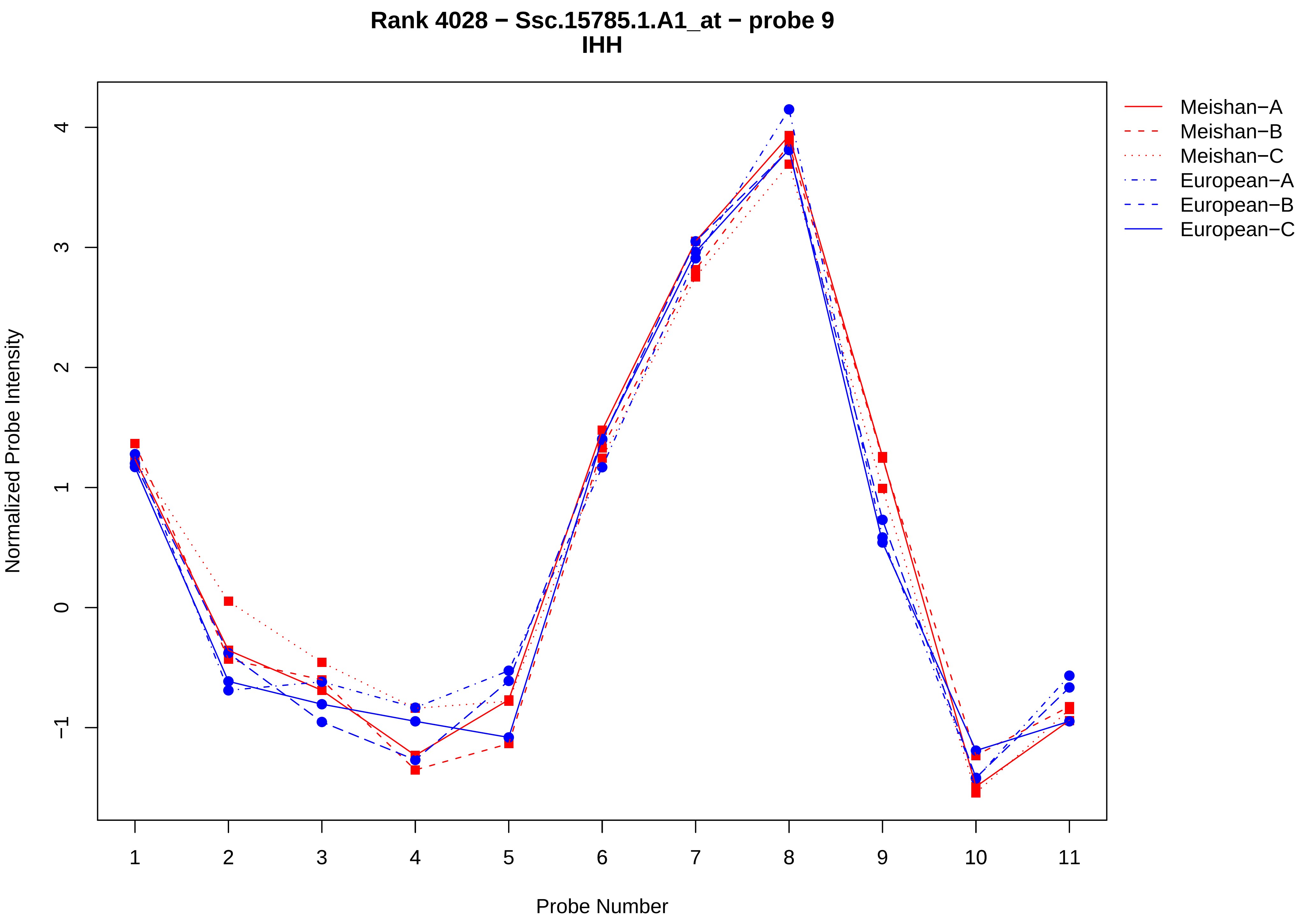

Rank 4028 − Ssc.15785.1.A1_at − probe 9
IHH
Normalized Probe Intensity
Probe Number
Meishan−A
Meishan−B
Meishan−C
European−A
European−B
European−C
−1
0
1
2
3
4
1
2
3
4
5
6
7
8
9
10
11

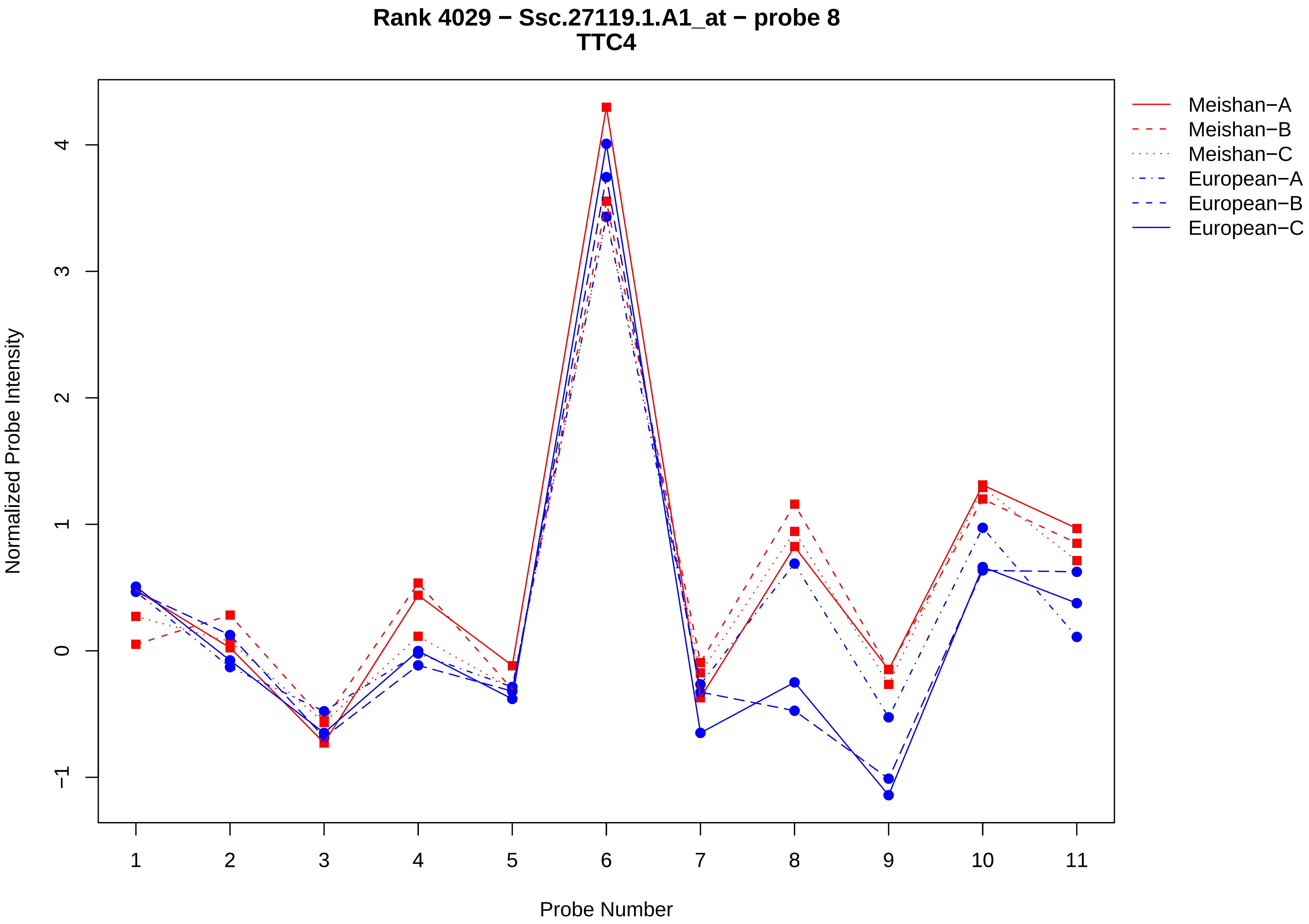

Rank 4029 − Ssc.27119.1.A1_at − probe 8
TTC4
Normalized Probe Intensity
Probe Number
Meishan−A
Meishan−B
Meishan−C
European−A
European−B
European−C
−1
0
1
2
3
4
1
2
3
4
5
6
7
8
9
10
11

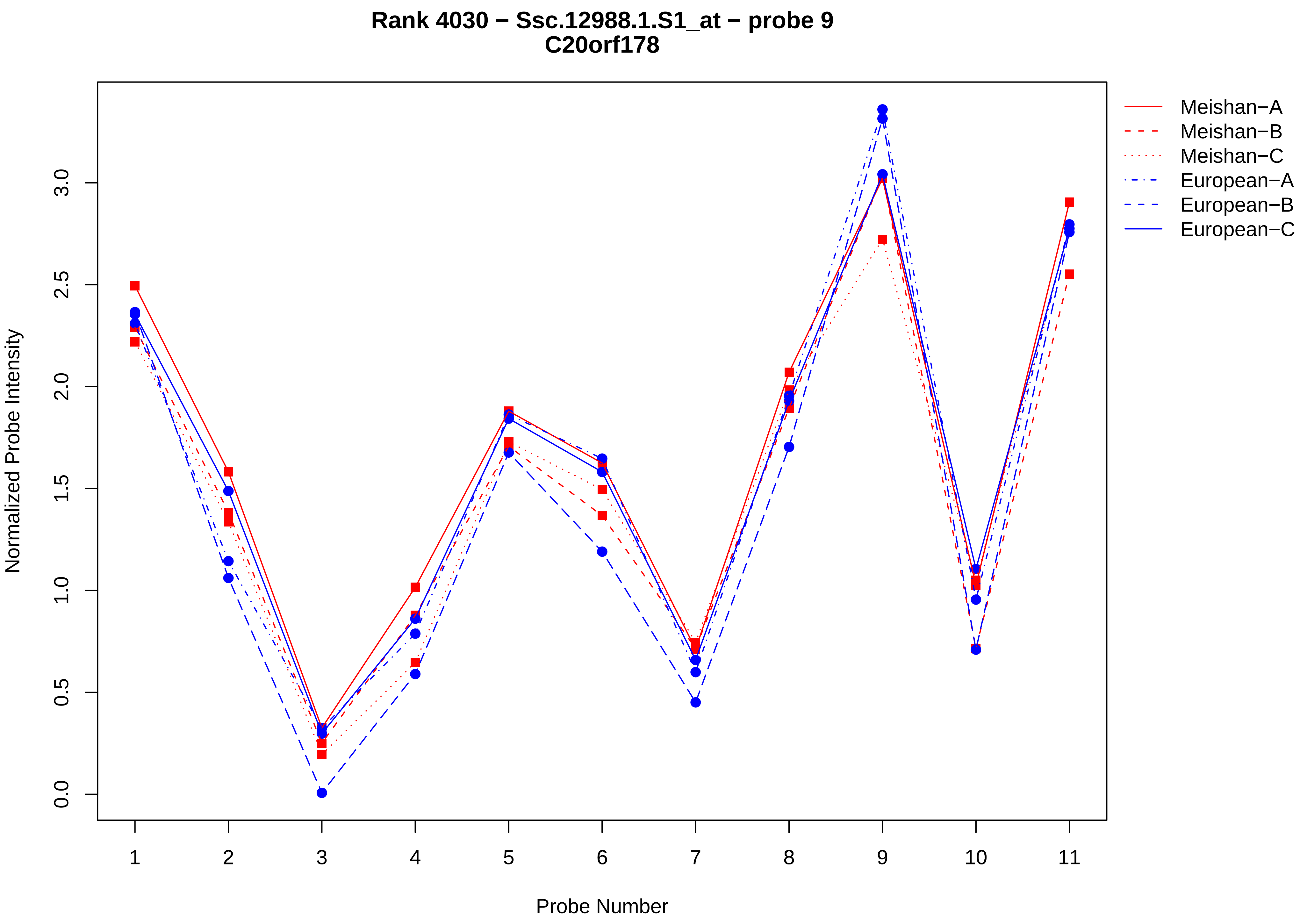

Rank 4030 − Ssc.12988.1.S1_at − probe 9
C20orf178
Normalized Probe Intensity
Probe Number
0.0
0.5
1.0
1.5
2.0
2.5
3.0
1
2
3
4
5
6
7
8
9
10
11
Meishan−A
Meishan−B
Meishan−C
European−A
European−B
European−C

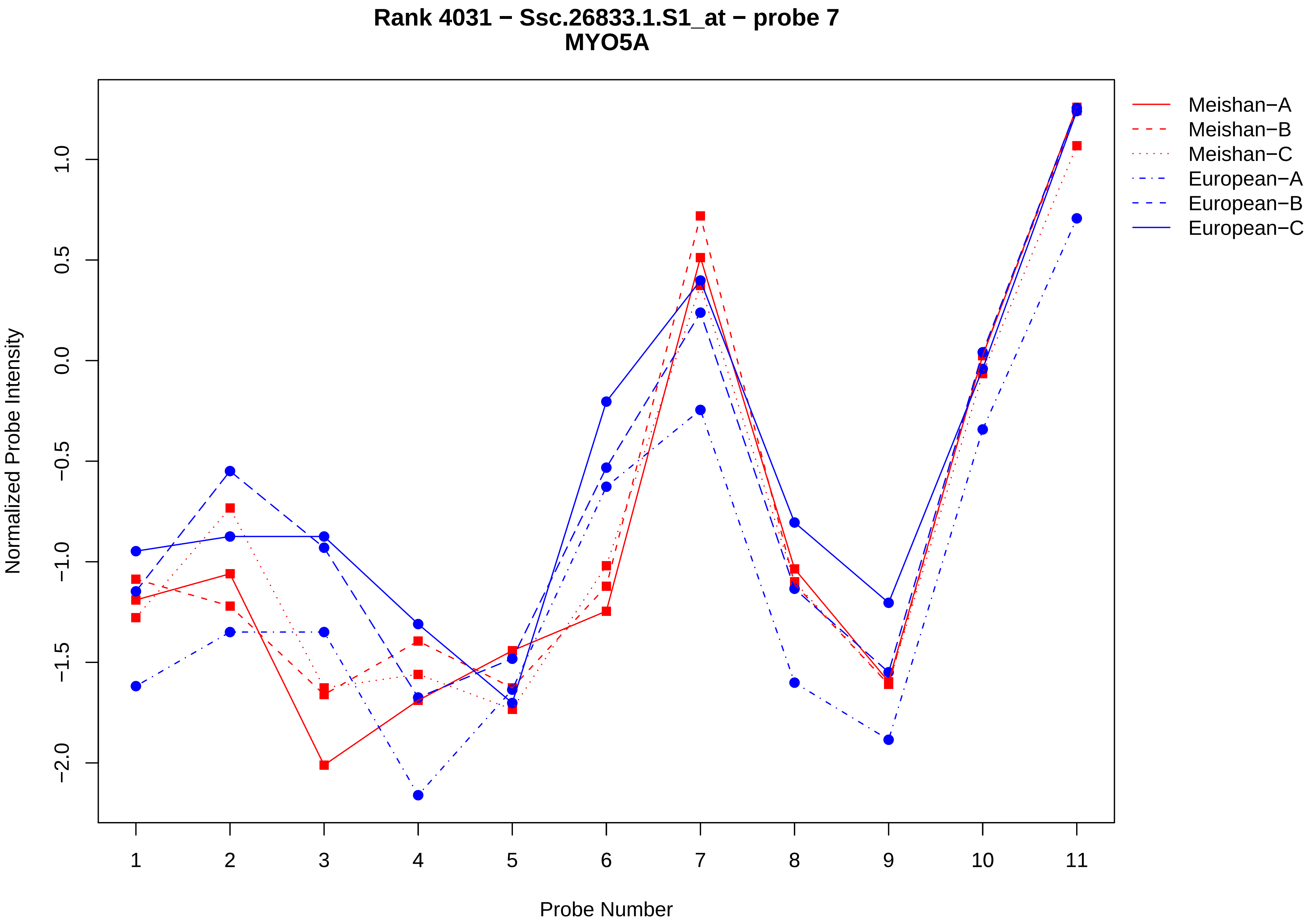

Rank 4031 − Ssc.26833.1.S1_at − probe 7
MYO5A
Normalized Probe Intensity
Probe Number
Meishan−A
Meishan−B
Meishan−C
European−A
European−B
European−C
1.0
0.5
0.0
−0.5
−1.0
−1.5
−2.0
1
2
3
4
5
6
7
8
9
10
11

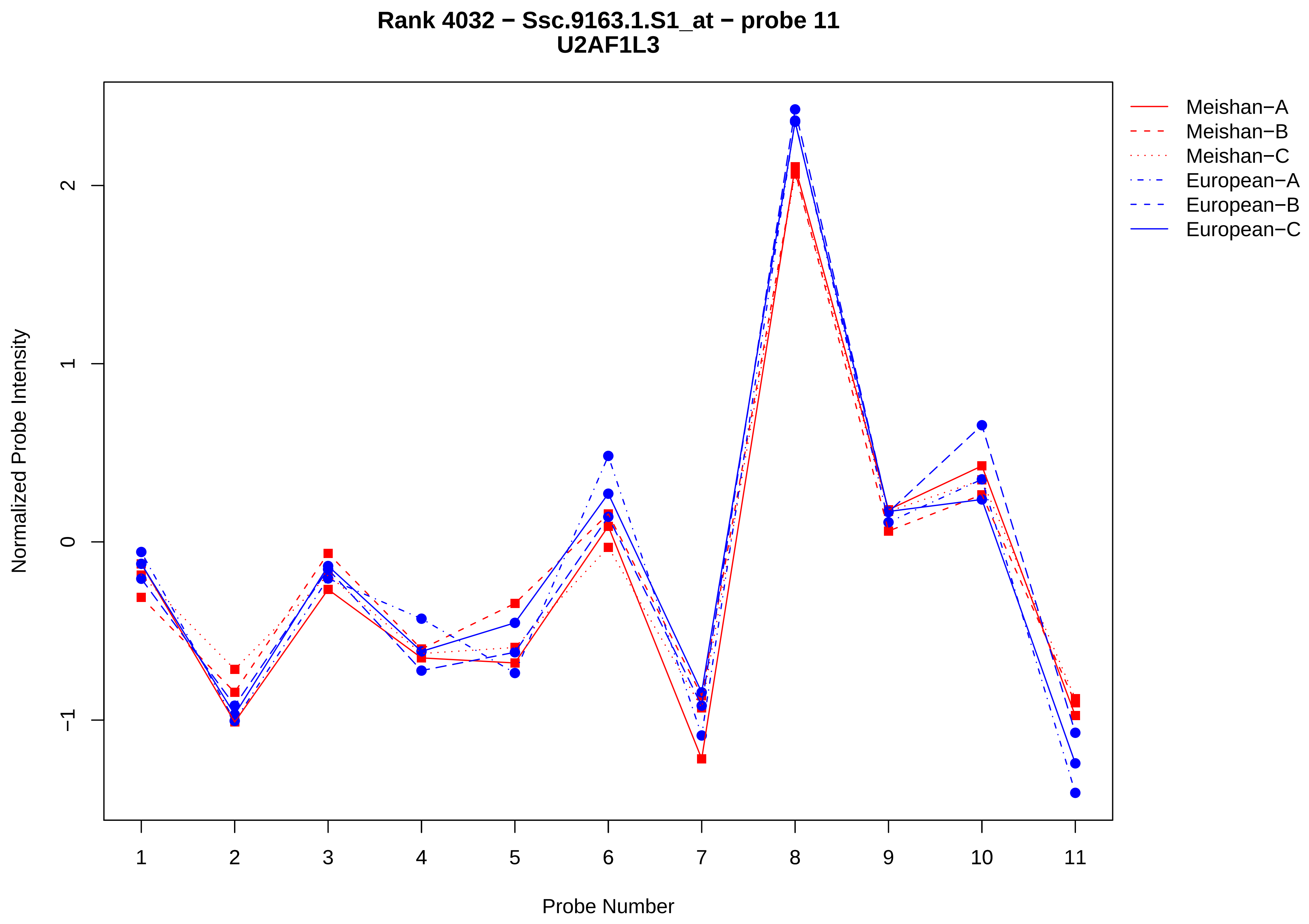

Rank 4032 − Ssc.9163.1.S1_at − probe 11
U2AF1L3
Normalized Probe Intensity
Probe Number
Meishan−A
Meishan−B
Meishan−C
European−A
European−B
European−C

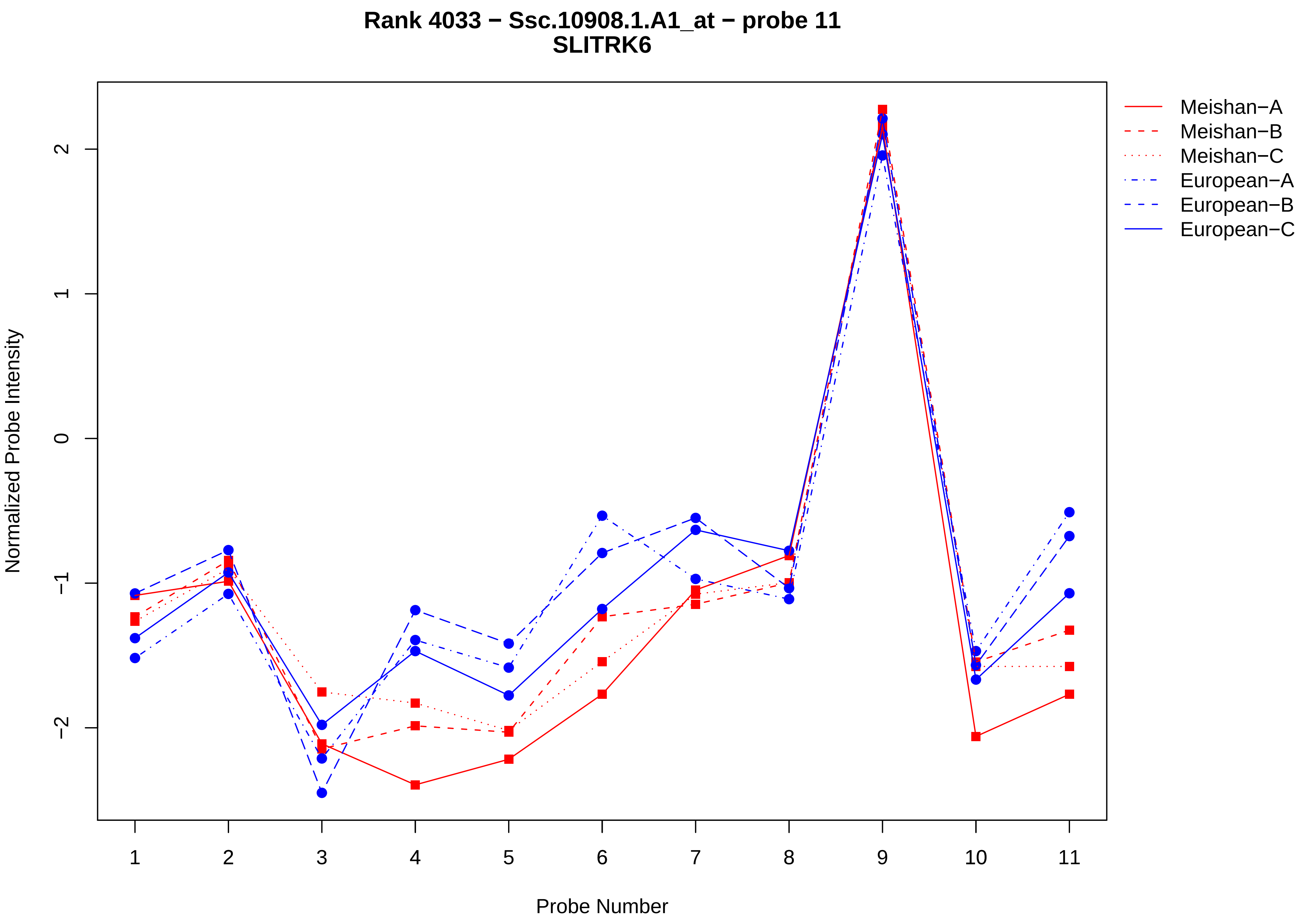

Rank 4033 − Ssc.10908.1.A1_at − probe 11
SLITRK6
Normalized Probe Intensity
Probe Number
Meishan−A
Meishan−B
Meishan−C
European−A
European−B
European−C
−2
−1
0
1
2
1
2
3
4
5
6
7
8
9
10
11

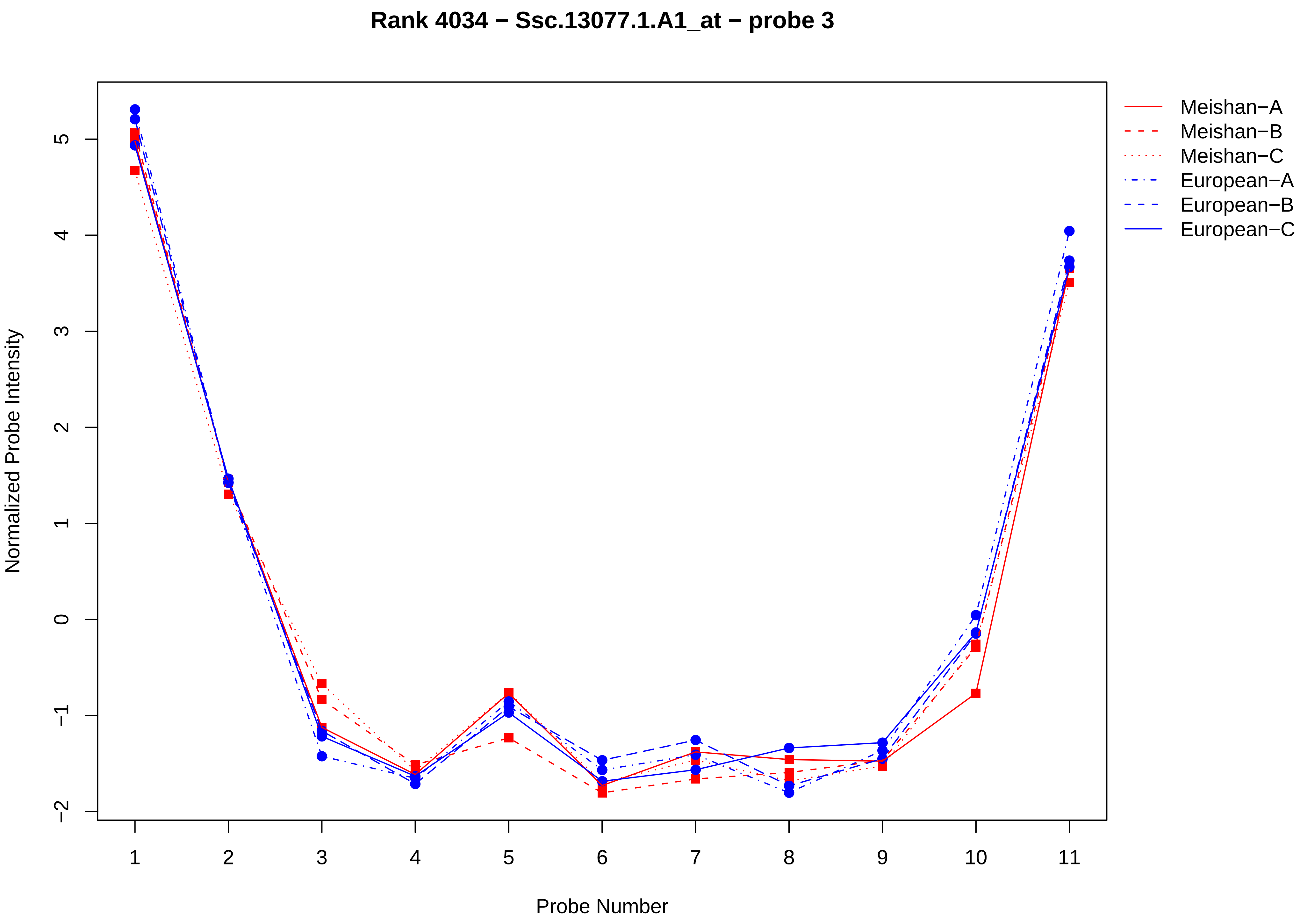

Rank 4034 − Ssc.13077.1.A1_at − probe 3
Normalized Probe Intensity
Probe Number
−2
−1
0
1
2
3
4
5
1
2
3
4
5
6
7
8
9
10
11
Meishan−A
Meishan−B
Meishan−C
European−A
European−B
European−C

Rank 4035 − Ssc.4228.1.S1_at − probe 9
NP_056352

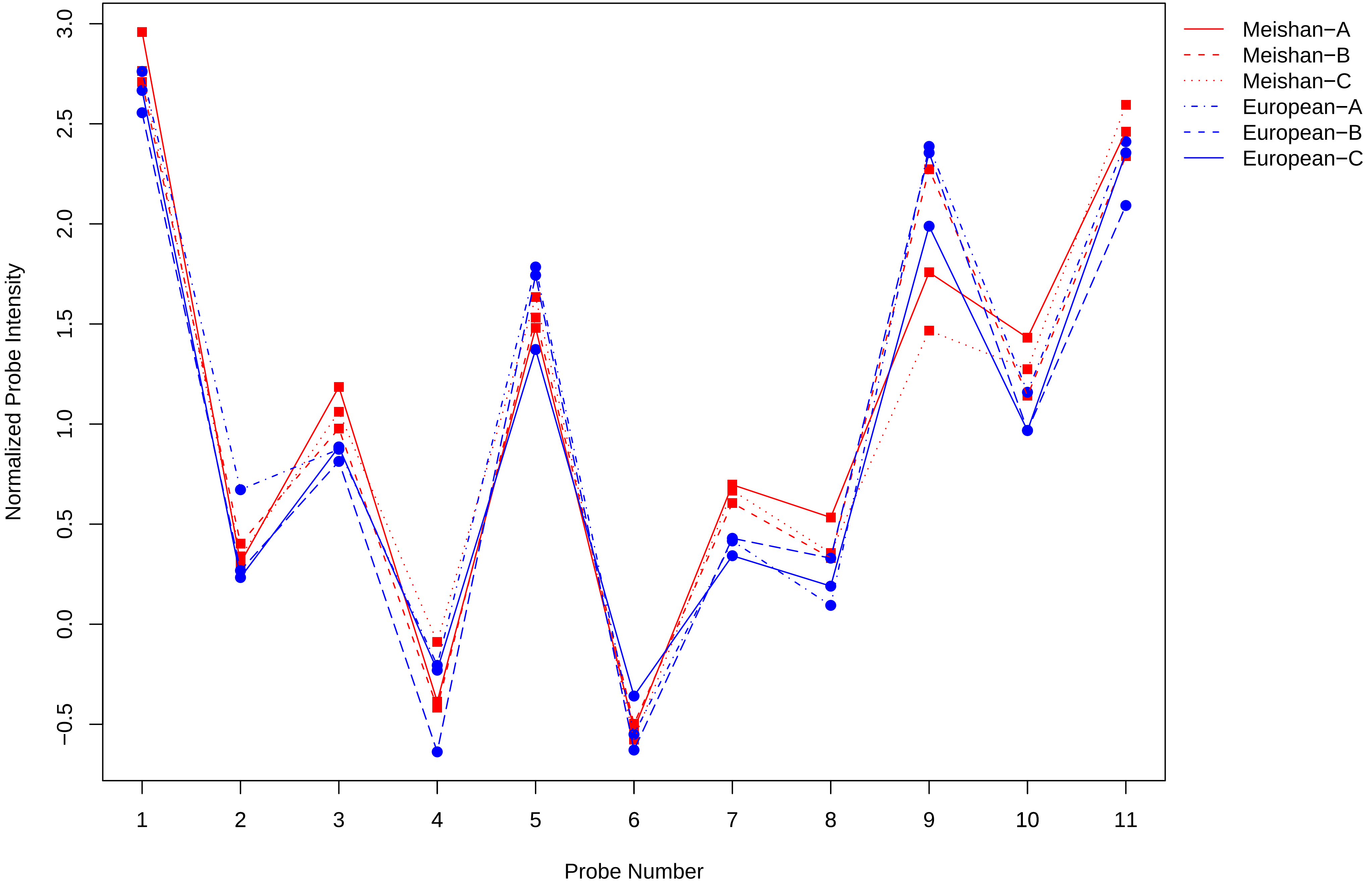

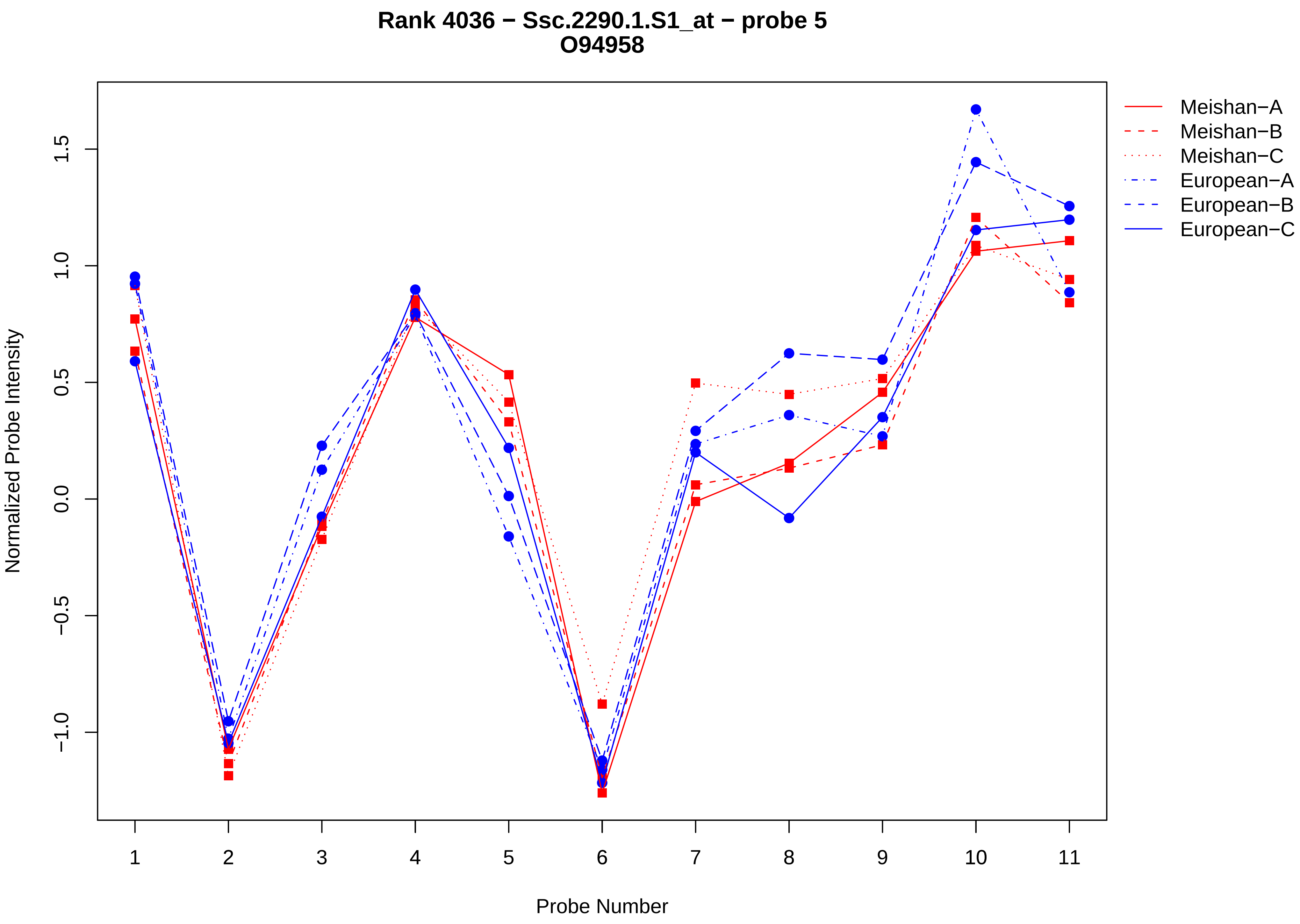

Rank 4036 − Ssc.2290.1.S1_at − probe 5
O94958
Normalized Probe Intensity
Probe Number
−1.0
−0.5
0.0
0.5
1.0
1.5
1
2
3
4
5
6
7
8
9
10
11
Meishan−A
Meishan−B
Meishan−C
European−A
European−B
European−C

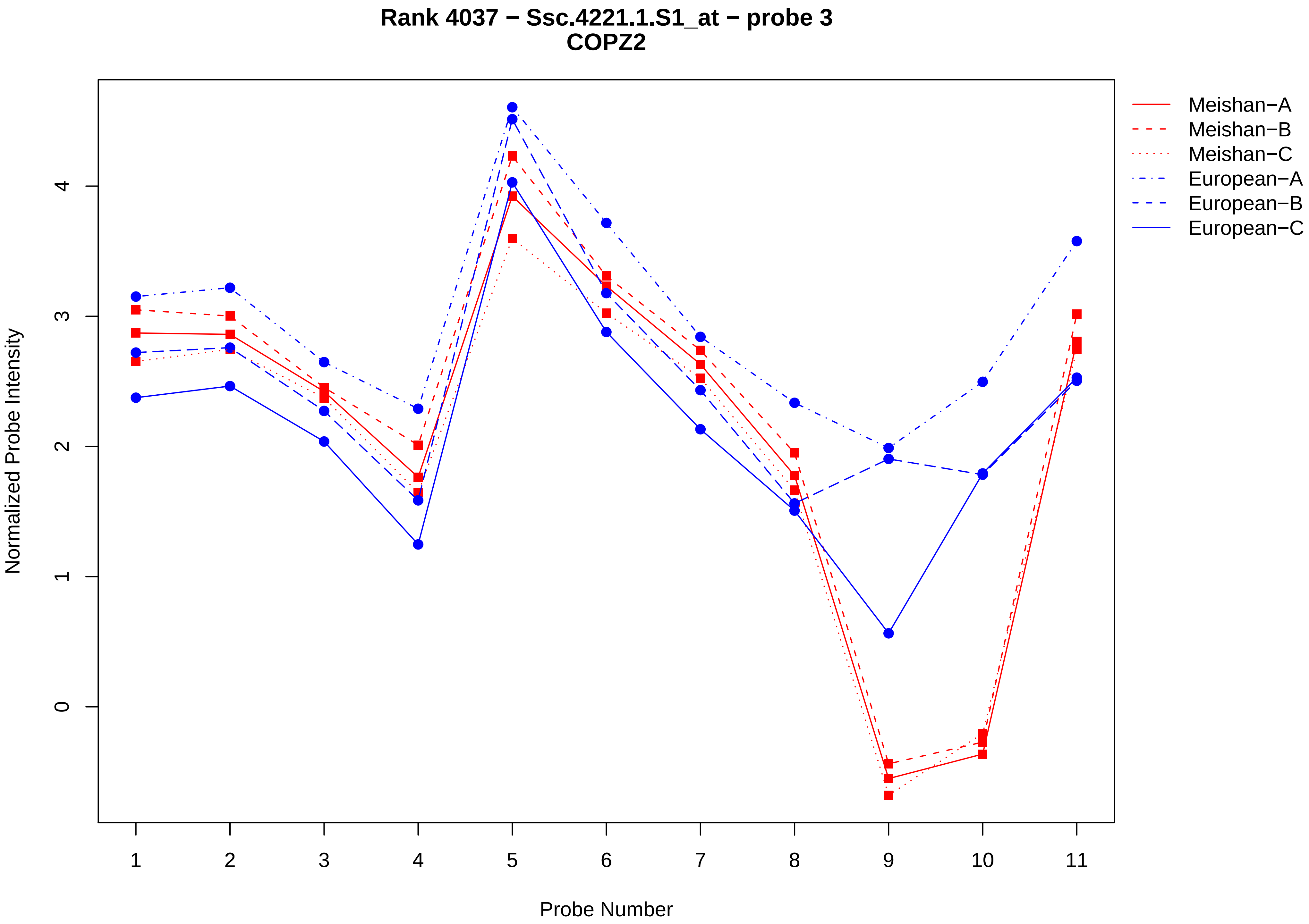
Rank 4037 − Ssc.4221.1.S1_at − probe 3
COPZ2
Normalized Probe Intensity
0
1
2
3
4
1
2
3
4
5
6
7
8
9
10
11
Probe Number
Meishan−A
Meishan−B
Meishan−C
European−A
European−B
European−C

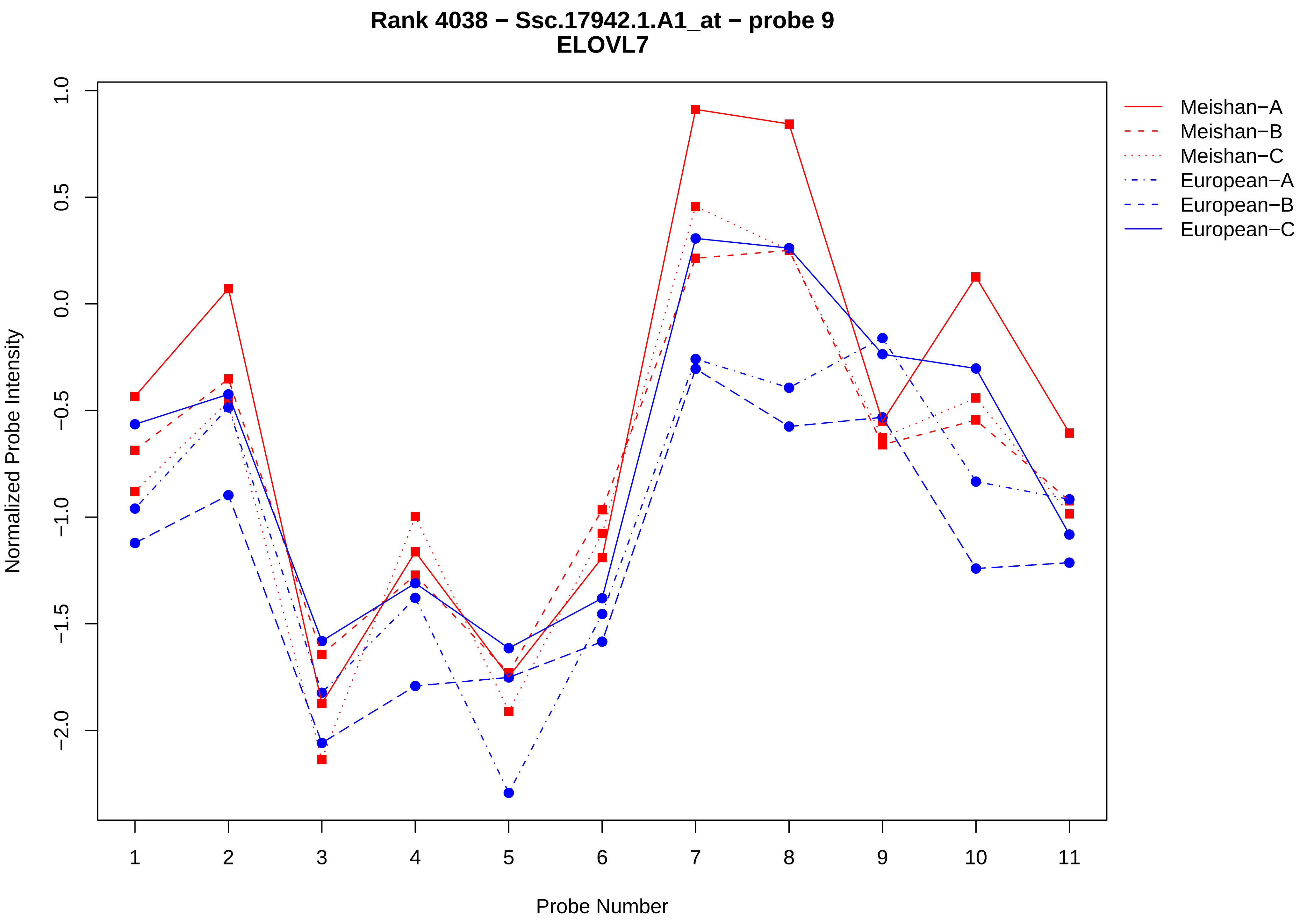

Rank 4038 − Ssc.17942.1.A1_at − probe 9
ELOVL7
Normalized Probe Intensity
Probe Number
1.0
0.5
0.0
−0.5
−1.0
−1.5
−2.0
1
2
3
4
5
6
7
8
9
10
11
Meishan−A
Meishan−B
Meishan−C
European−A
European−B
European−C

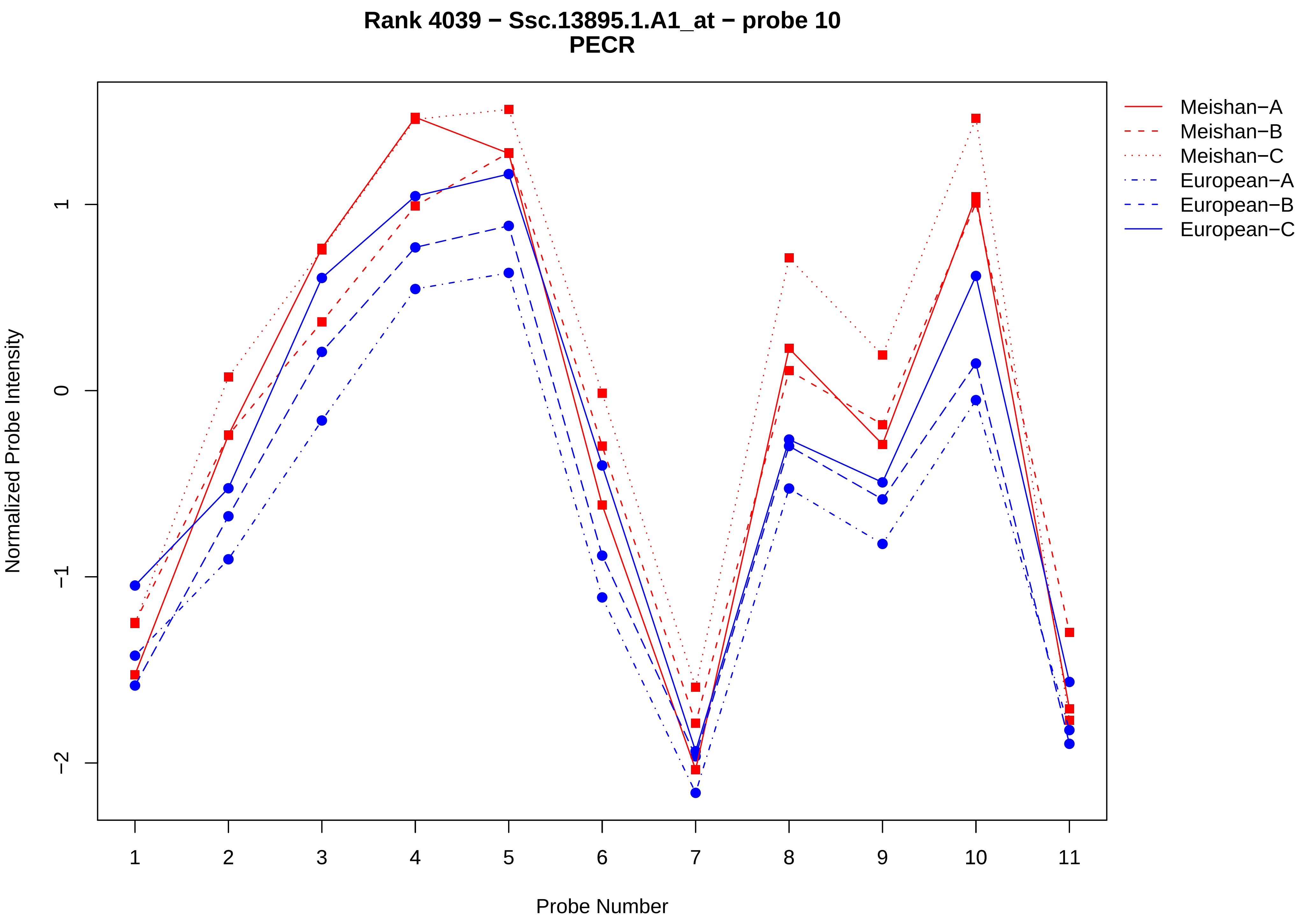

Rank 4039 − Ssc.13895.1.A1_at − probe 10
PECR
Normalized Probe Intensity
Probe Number
−2
−1
0
1
1
2
3
4
5
6
7
8
9
10
11
Meishan−A
Meishan−B
Meishan−C
European−A
European−B
European−C

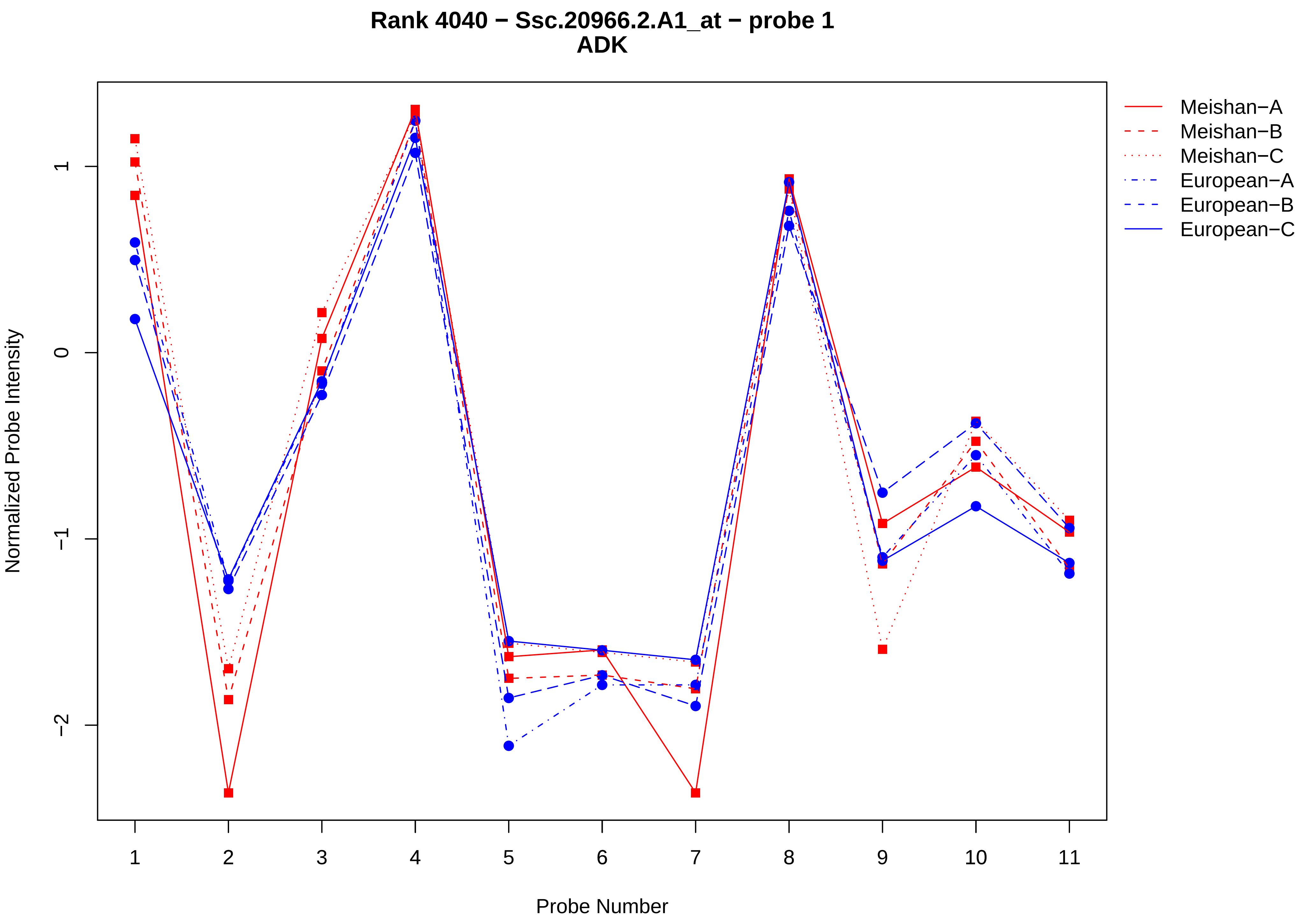

Rank 4040 − Ssc.20966.2.A1_at − probe 1
ADK
Normalized Probe Intensity
Probe Number
Meishan−A
Meishan−B
Meishan−C
European−A
European−B
European−C
1
0
−1
−2
1
2
3
4
5
6
7
8
9
10
11

Rank 4041 − Ssc.14490.1.S1_at − probe 9
LTB4DH

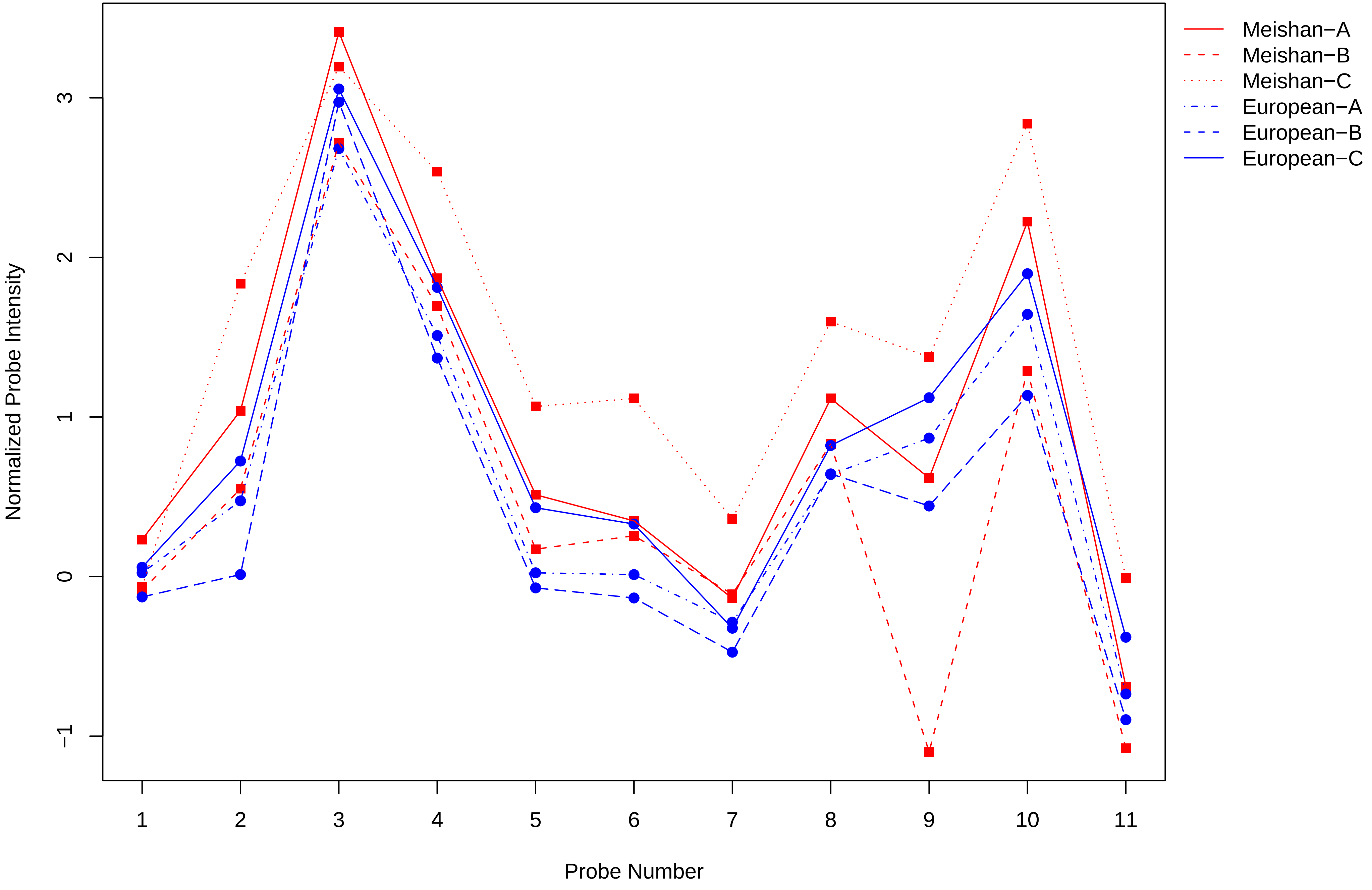

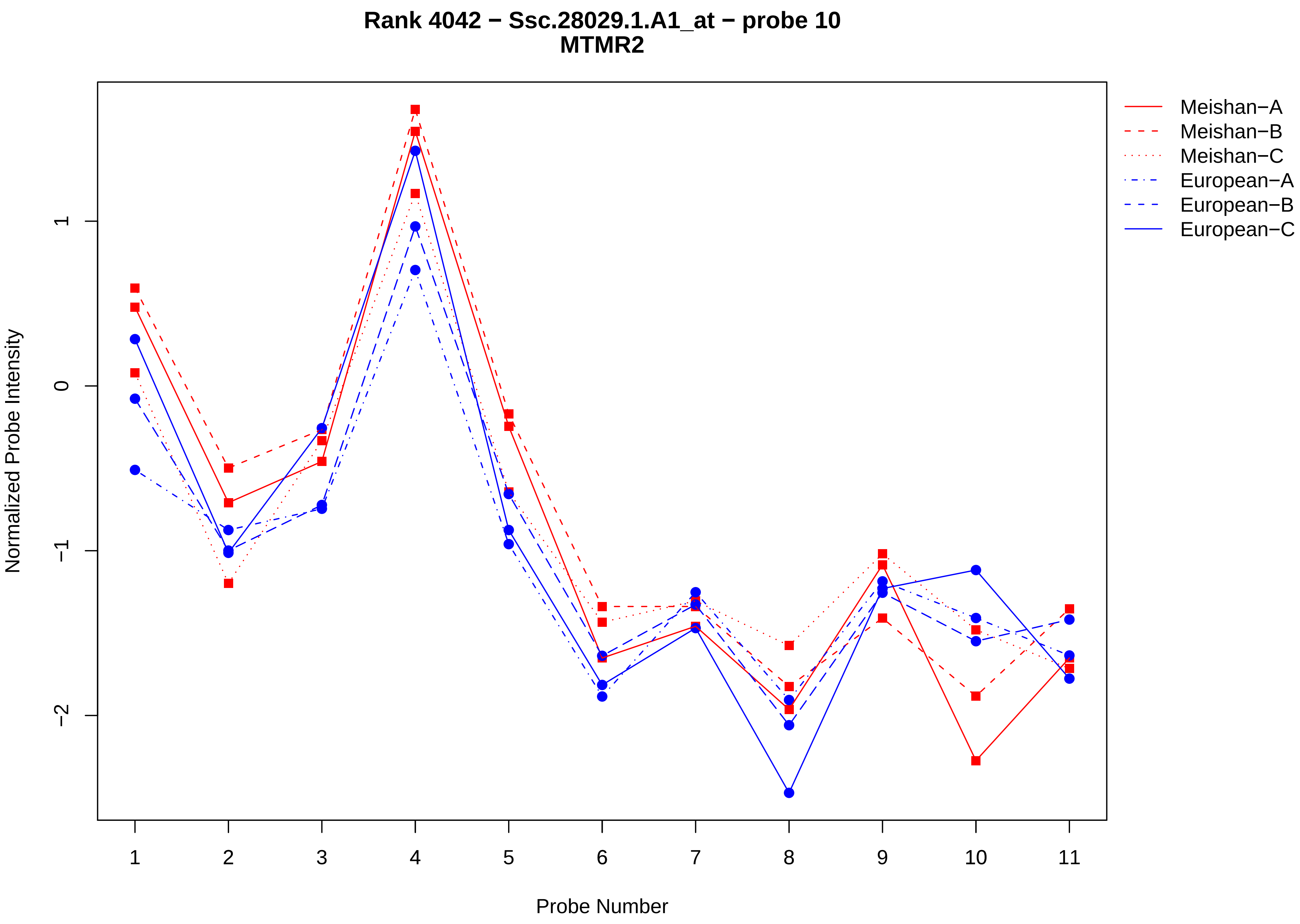

Rank 4042 − Ssc.28029.1.A1_at − probe 10
MTMR2
Meishan−A
Meishan−B
Meishan−C
European−A
European−B
European−C
Normalized Probe Intensity
Probe Number
1
0
−1
−2
1
2
3
4
5
6
7
8
9
10
11

Rank 4043 − Ssc.10245.2.A1_a_at − probe 8
DCN

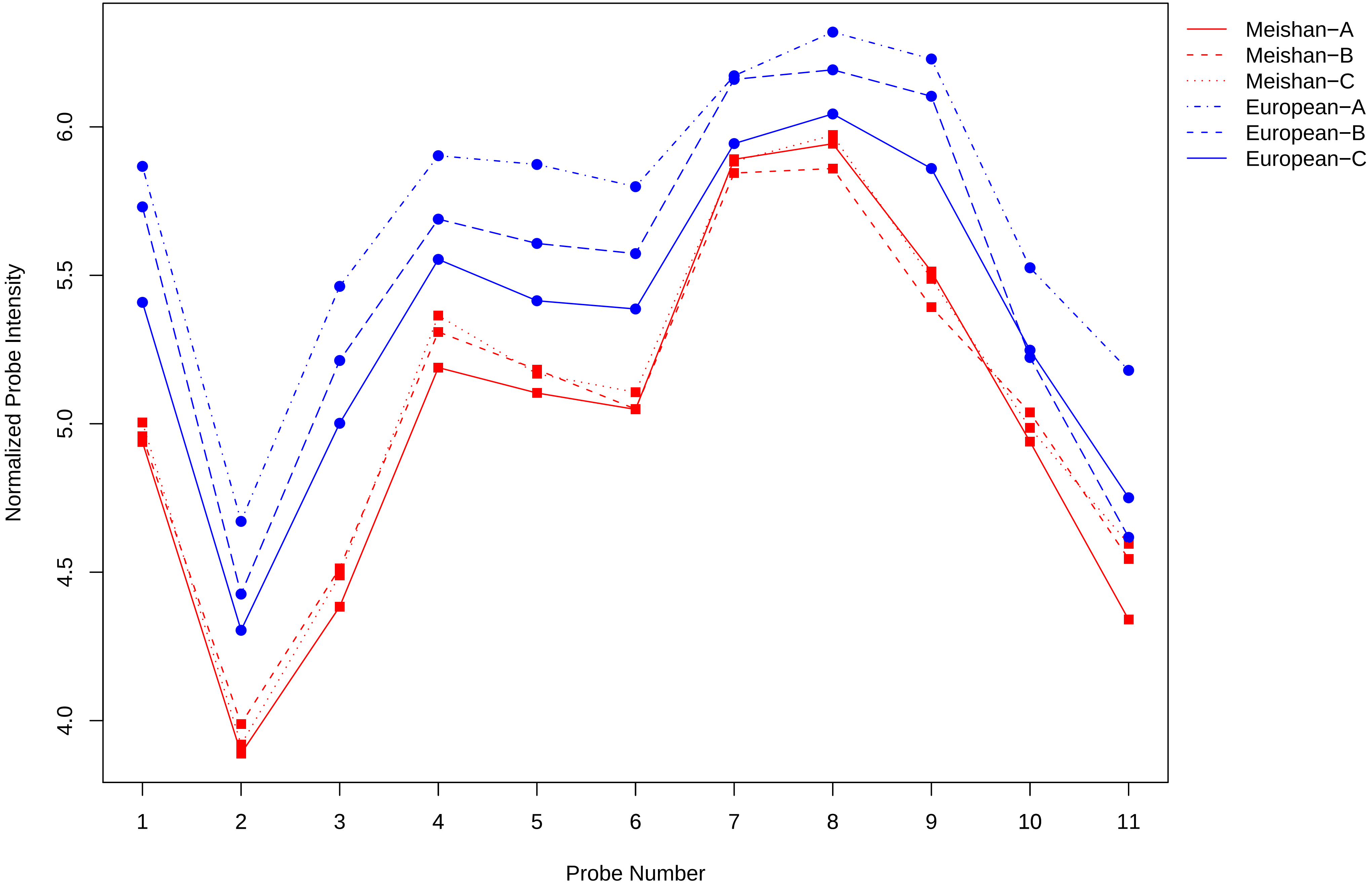

Rank 4044 − Ssc.7568.1.A1_at − probe 10
CCL28

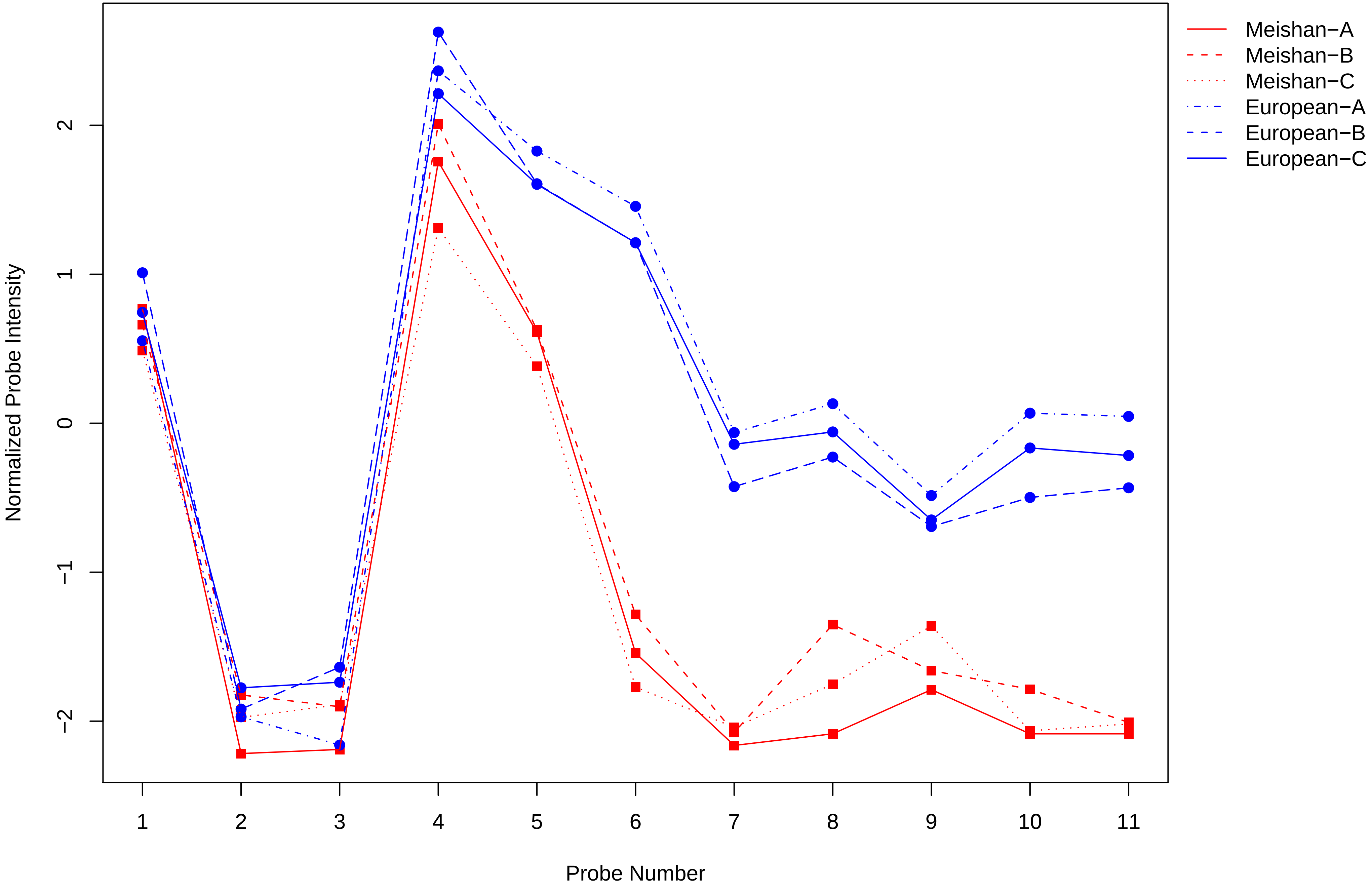

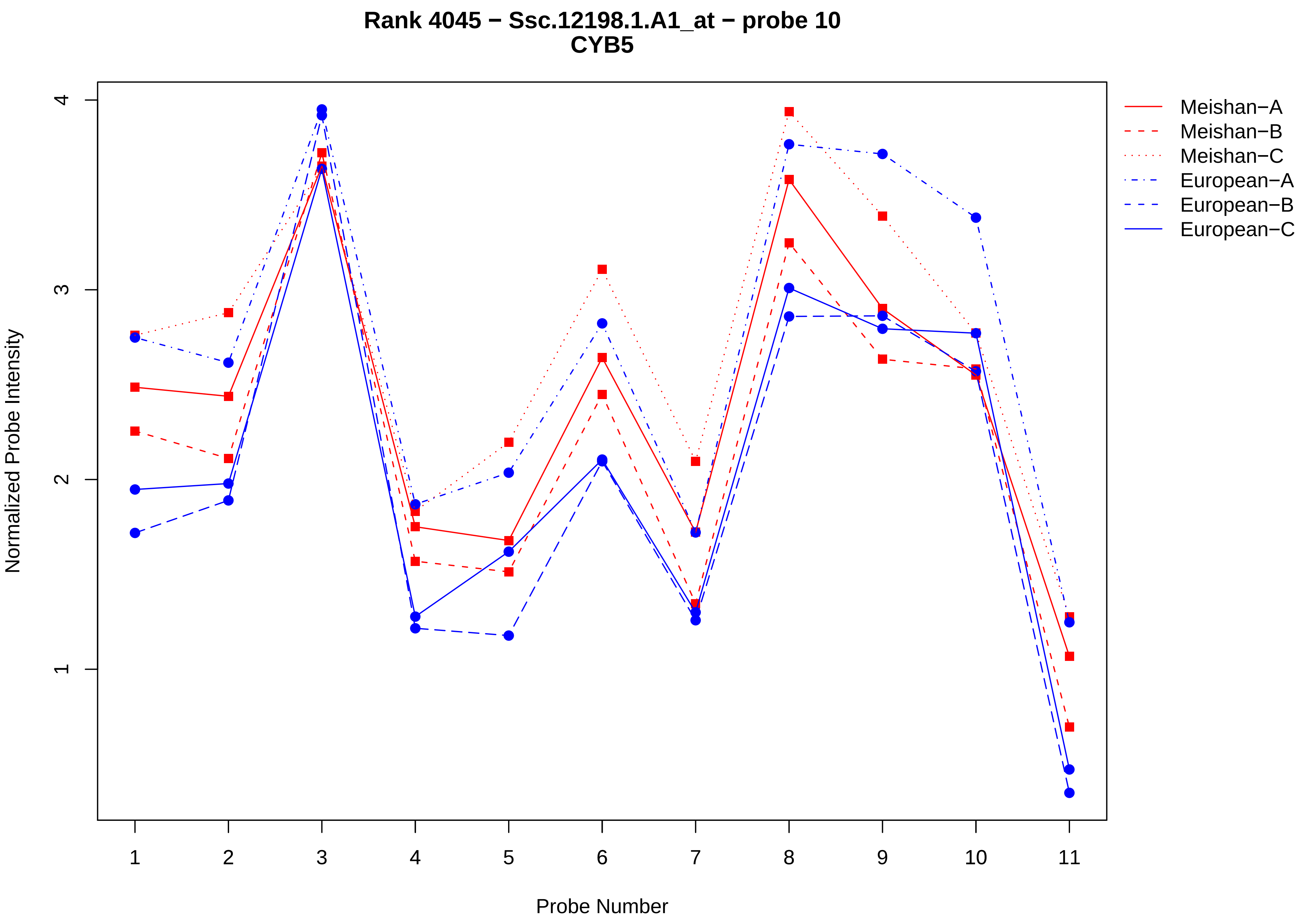

Rank 4045 − Ssc.12198.1.A1_at − probe 10
CYB5
Normalized Probe Intensity
Probe Number
1
2
3
4
1
2
3
4
5
6
7
8
9
10
11
Meishan−A
Meishan−B
Meishan−C
European−A
European−B
European−C

Rank 4046 − Ssc.9975.1.A1_at − probe 3
TGFB2

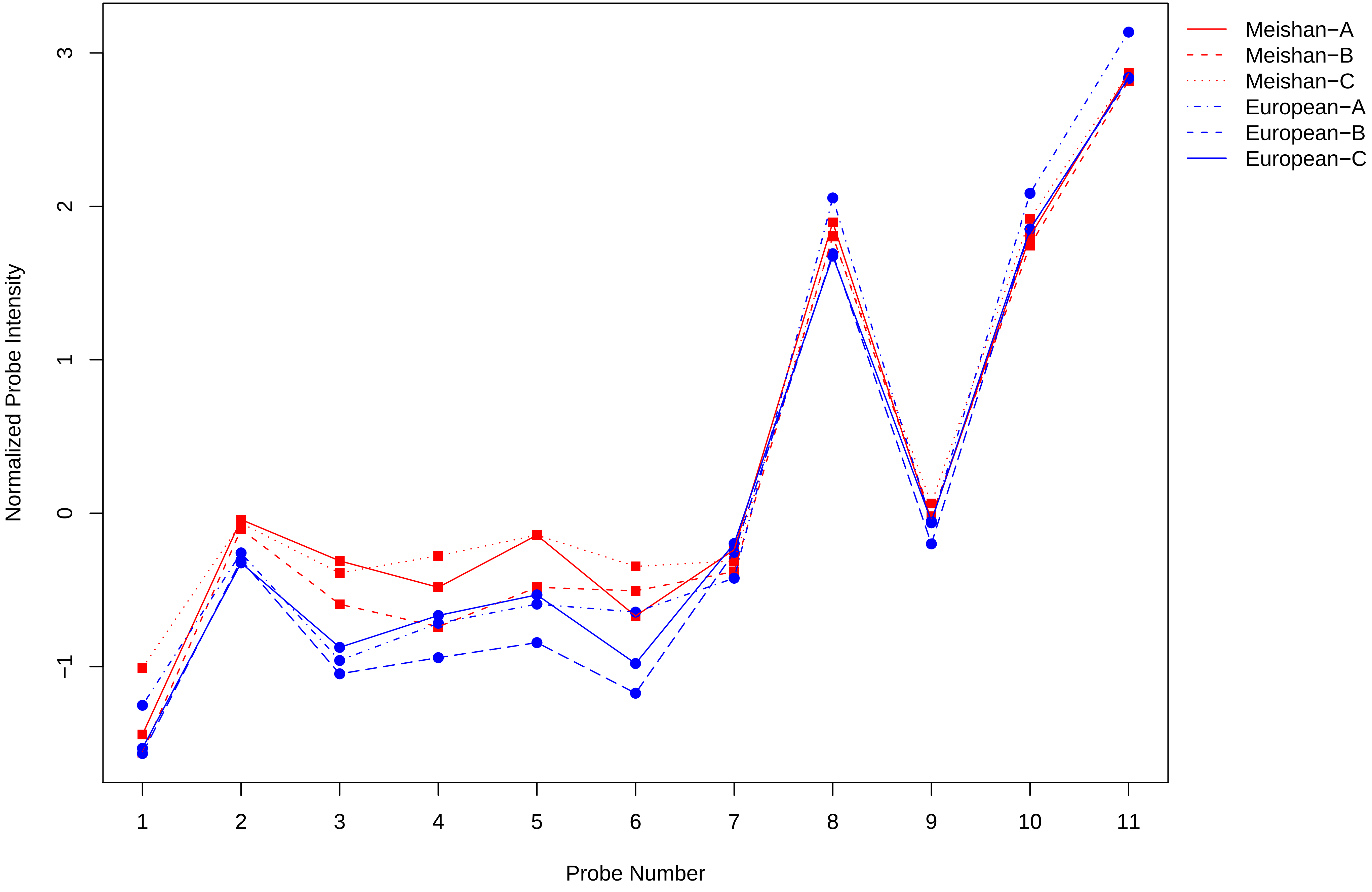

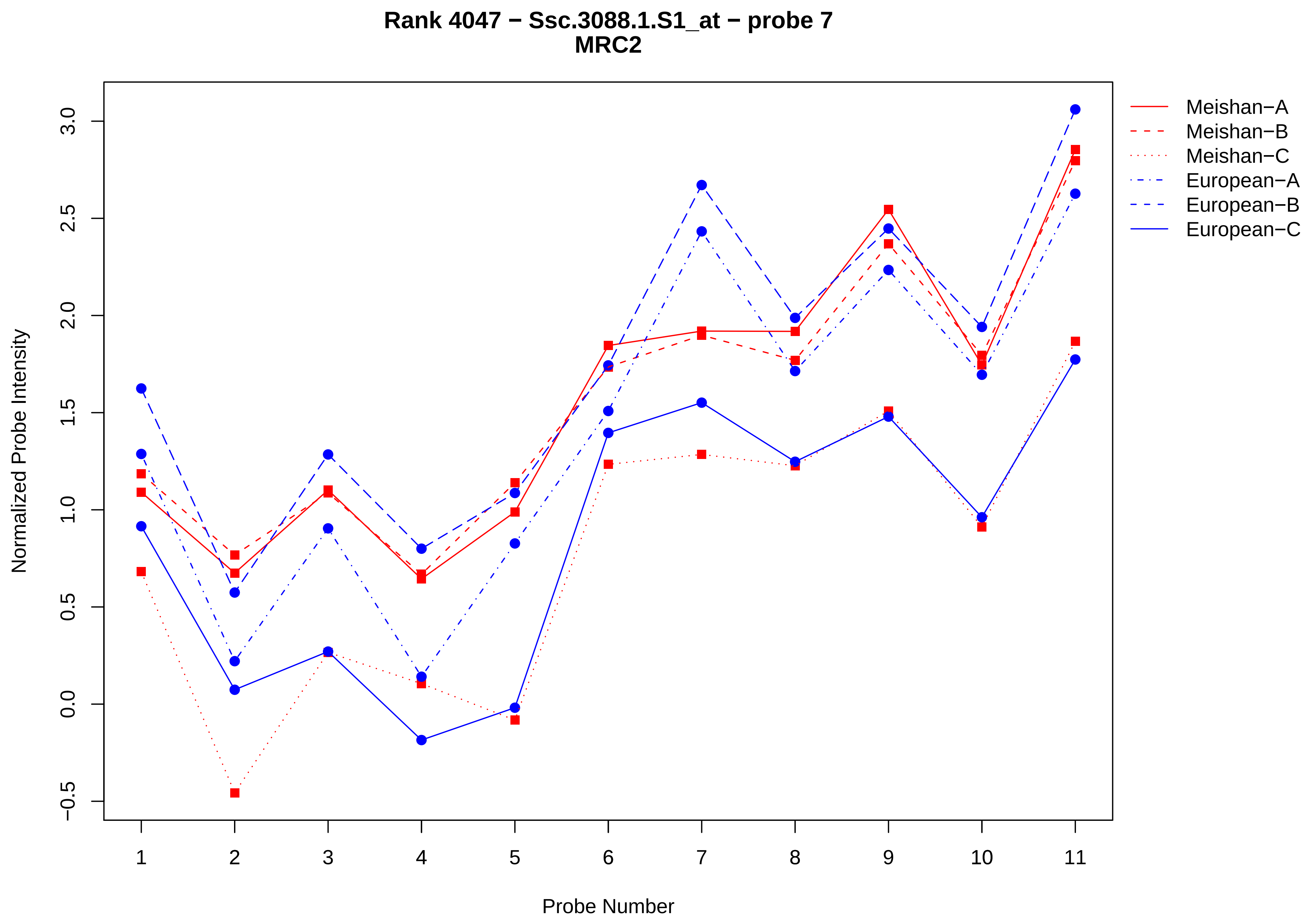
Rank 4047 − Ssc.3088.1.S1_at − probe 7
MRC2
Normalized Probe Intensity
Probe Number
−0.5
0.0
0.5
1.0
1.5
2.0
2.5
3.0
1
2
3
4
5
6
7
8
9
10
11
Meishan−A
Meishan−B
Meishan−C
European−A
European−B
European−C

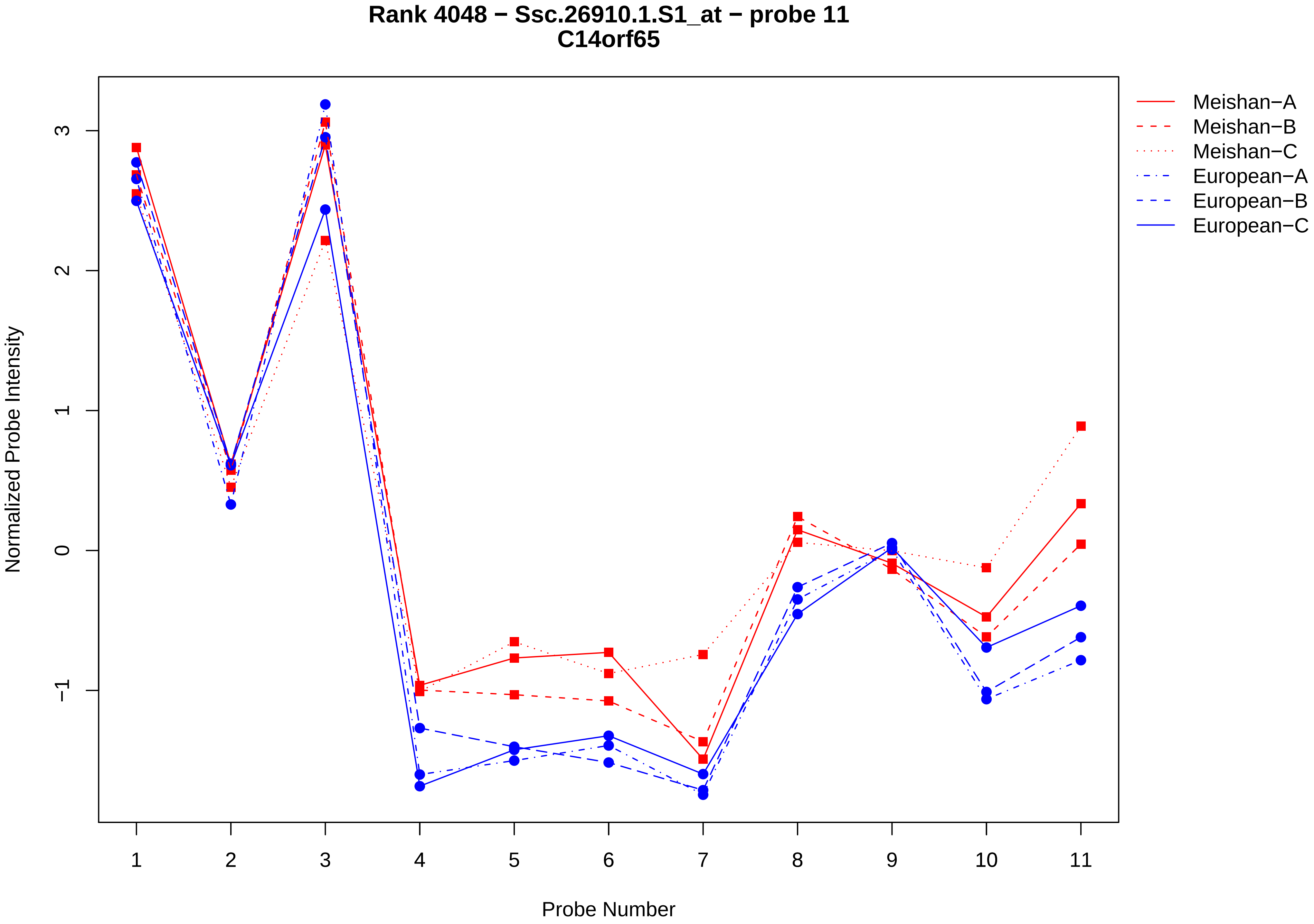
Rank 4048 − Ssc.26910.1.S1_at − probe 11
C14orf65
Normalized Probe Intensity
Probe Number
−1
0
1
2
3
1
2
3
4
5
6
7
8
9
10
11
Meishan−A
Meishan−B
Meishan−C
European−A
European−B
European−C

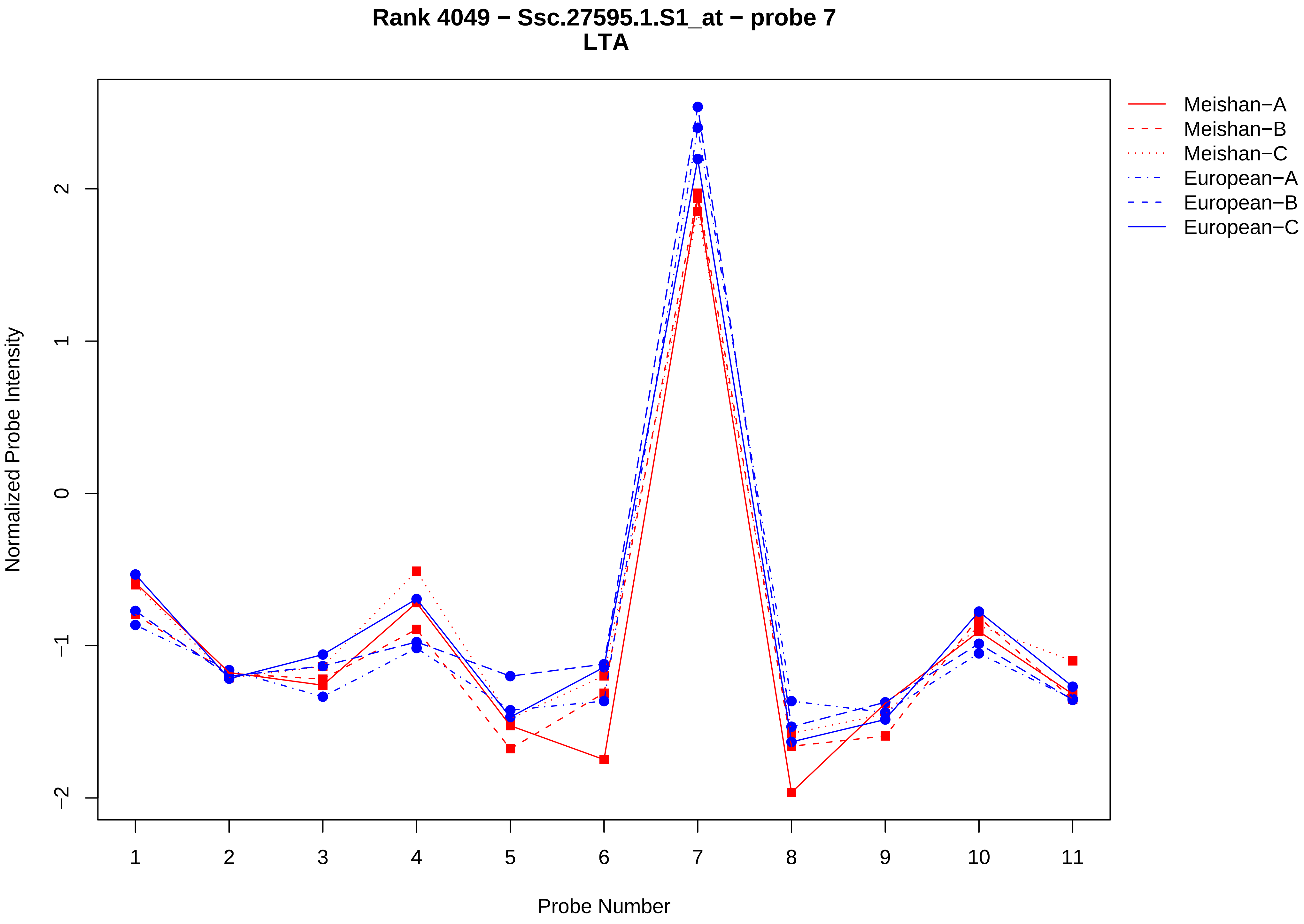

Rank 4049 − Ssc.27595.1.S1_at − probe 7
LTA
Normalized Probe Intensity
Probe Number
−2
−1
0
1
2
1
2
3
4
5
6
7
8
9
10
11
Meishan−A
Meishan−B
Meishan−C
European−A
European−B
European−C

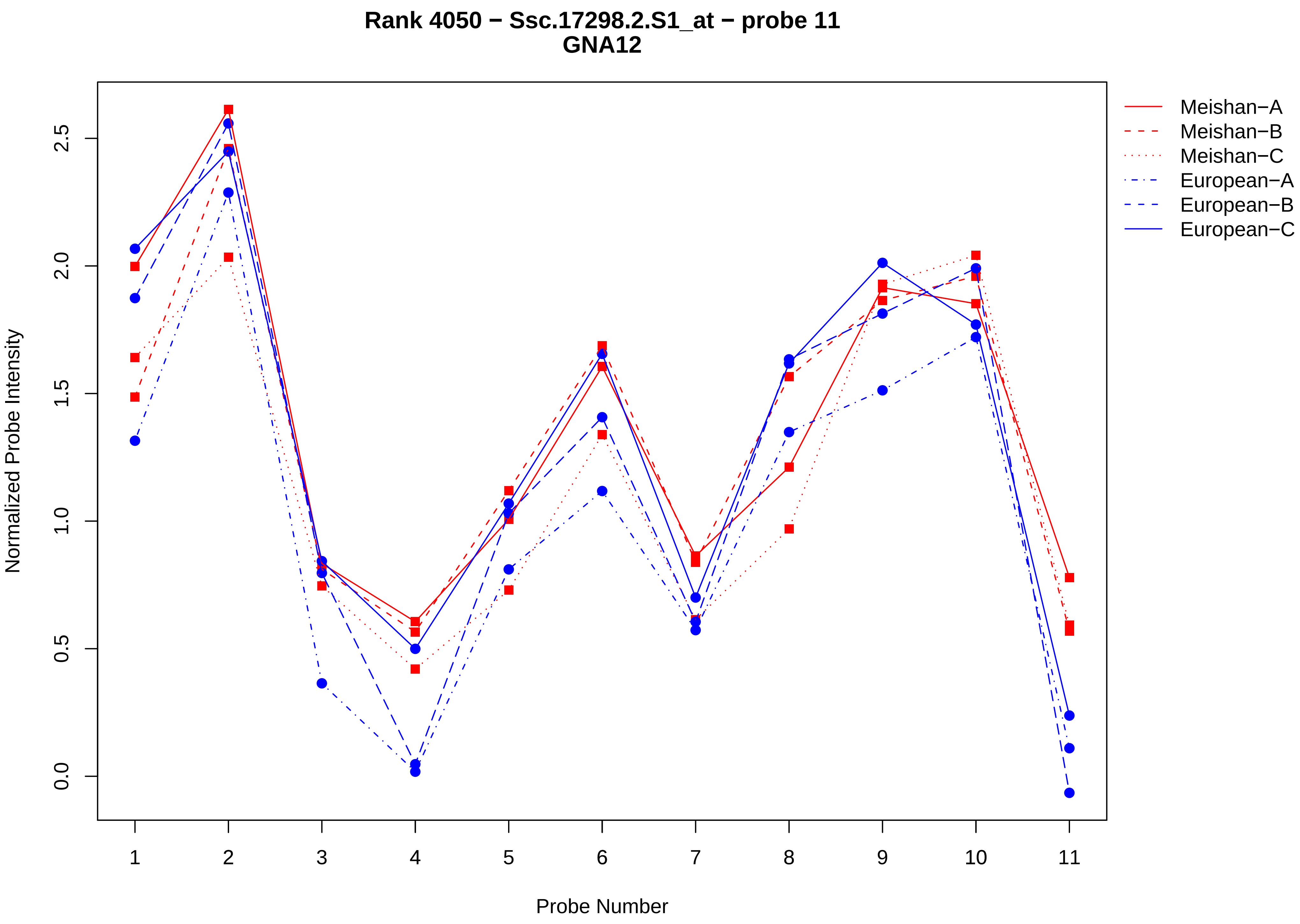

Rank 4050 − Ssc.17298.2.S1_at − probe 11
GNA12
Normalized Probe Intensity
Probe Number
0.0
0.5
1.0
1.5
2.0
2.5
1
2
3
4
5
6
7
8
9
10
11
Meishan−A
Meishan−B
Meishan−C
European−A
European−B
European−C

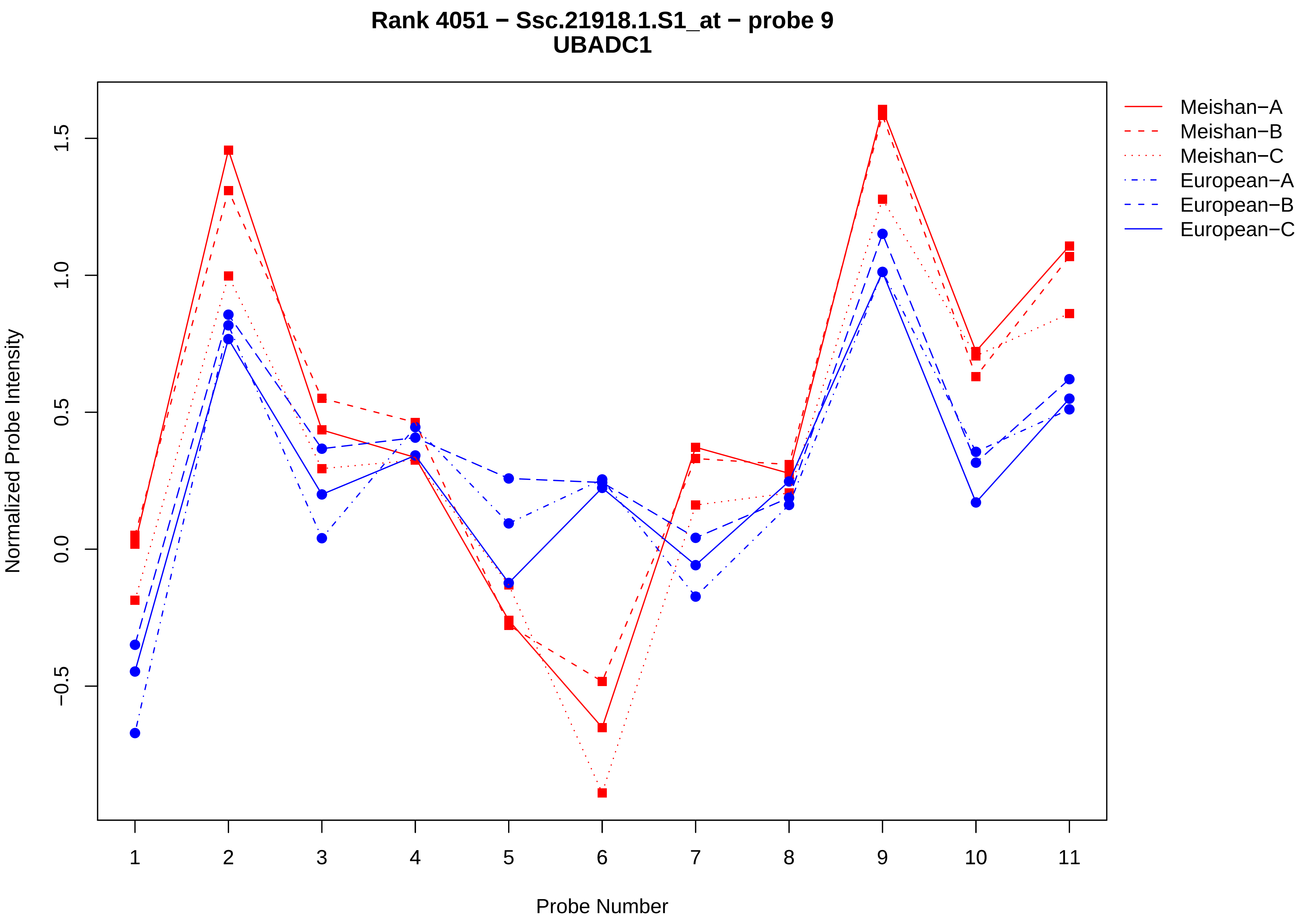

Rank 4051 − Ssc.21918.1.S1_at − probe 9
UBADC1
Normalized Probe Intensity
Probe Number
−0.5
0.0
0.5
1.0
1.5
1
2
3
4
5
6
7
8
9
10
11
Meishan−A
Meishan−B
Meishan−C
European−A
European−B
European−C

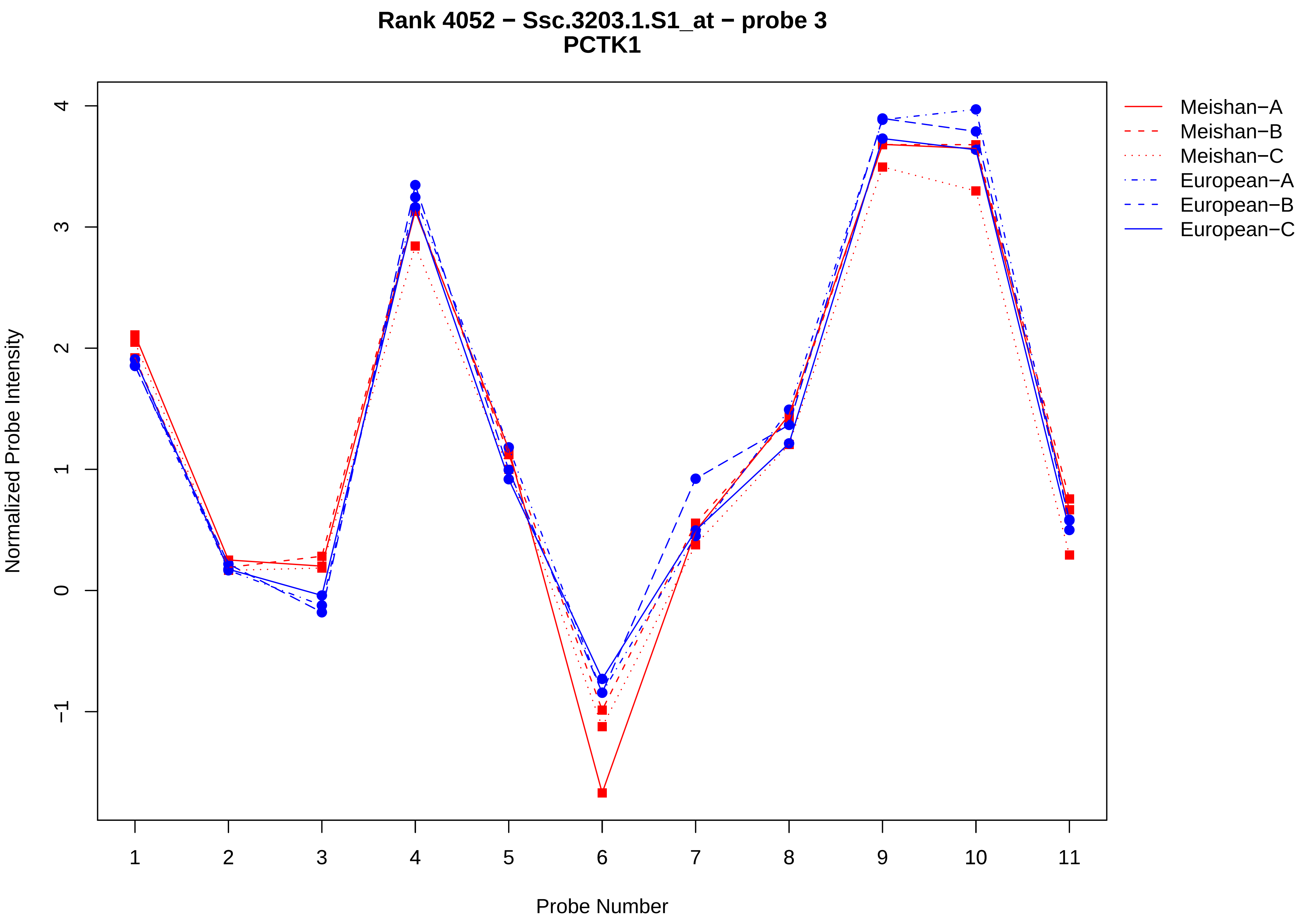
Rank 4052 − Ssc.3203.1.S1_at − probe 3
PCTK1
Normalized Probe Intensity
Probe Number
−1
0
1
2
3
4
1
2
3
4
5
6
7
8
9
10
11
Meishan−A
Meishan−B
Meishan−C
European−A
European−B
European−C

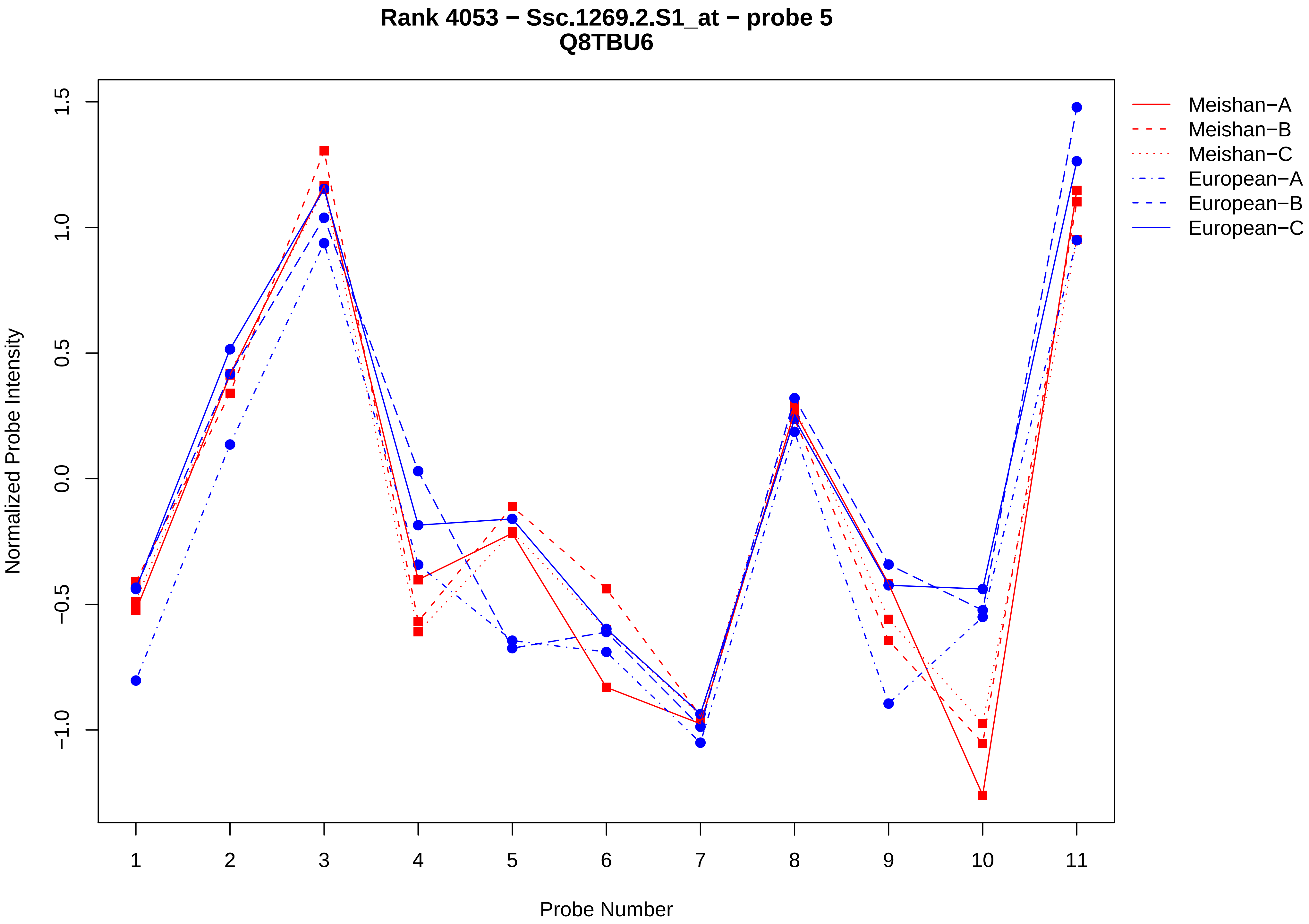

Rank 4053 − Ssc.1269.2.S1_at − probe 5
Q8TBU6
Normalized Probe Intensity
Probe Number
Meishan−A
Meishan−B
Meishan−C
European−A
European−B
European−C
1.5
1.0
0.5
0.0
−0.5
−1.0
1
2
3
4
5
6
7
8
9
10
11

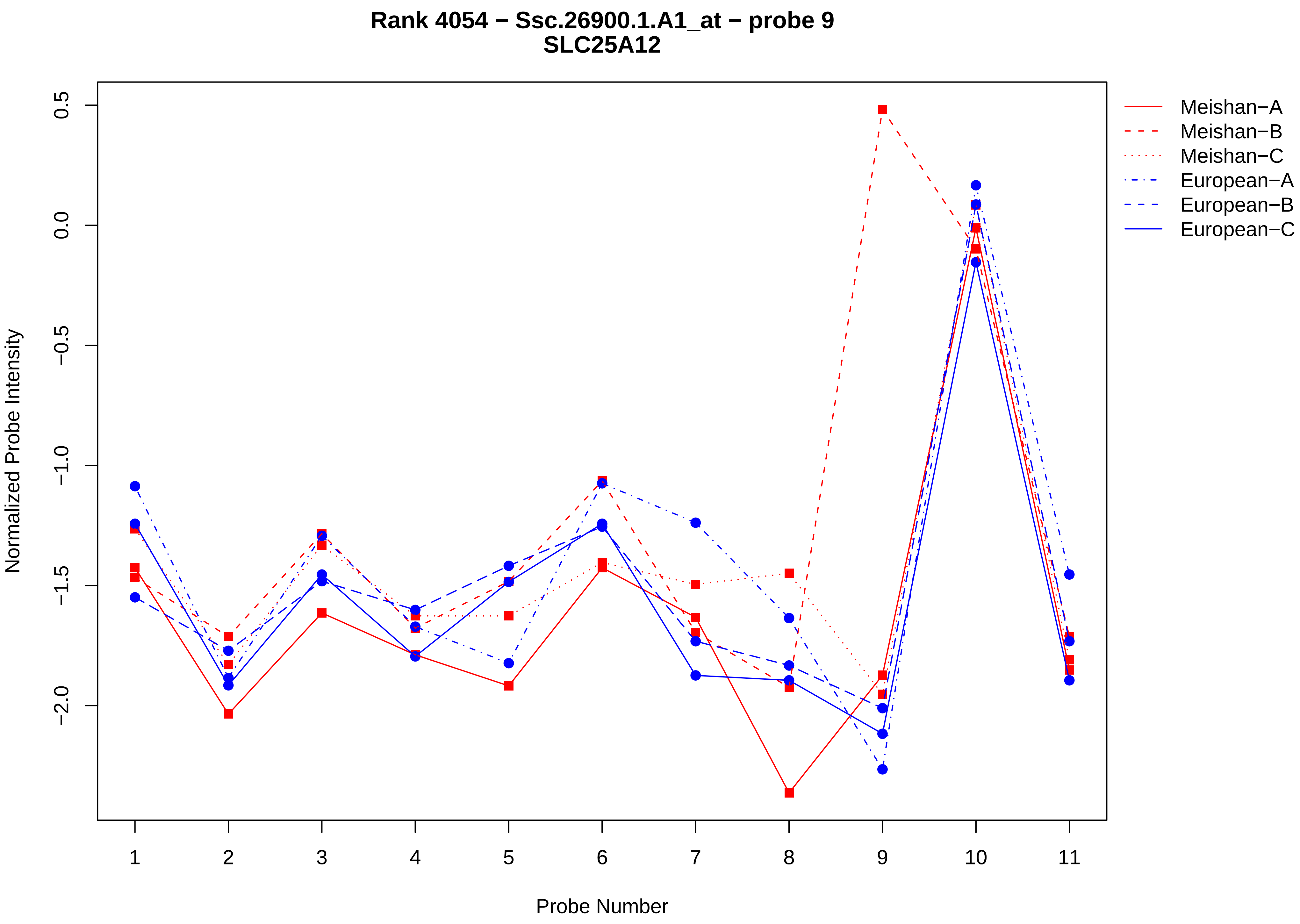

Rank 4054 − Ssc.26900.1.A1_at − probe 9
SLC25A12
Normalized Probe Intensity
Probe Number
0.5
0.0
−0.5
−1.0
−1.5
−2.0
1
2
3
4
5
6
7
8
9
10
11
Meishan−A
Meishan−B
Meishan−C
European−A
European−B
European−C

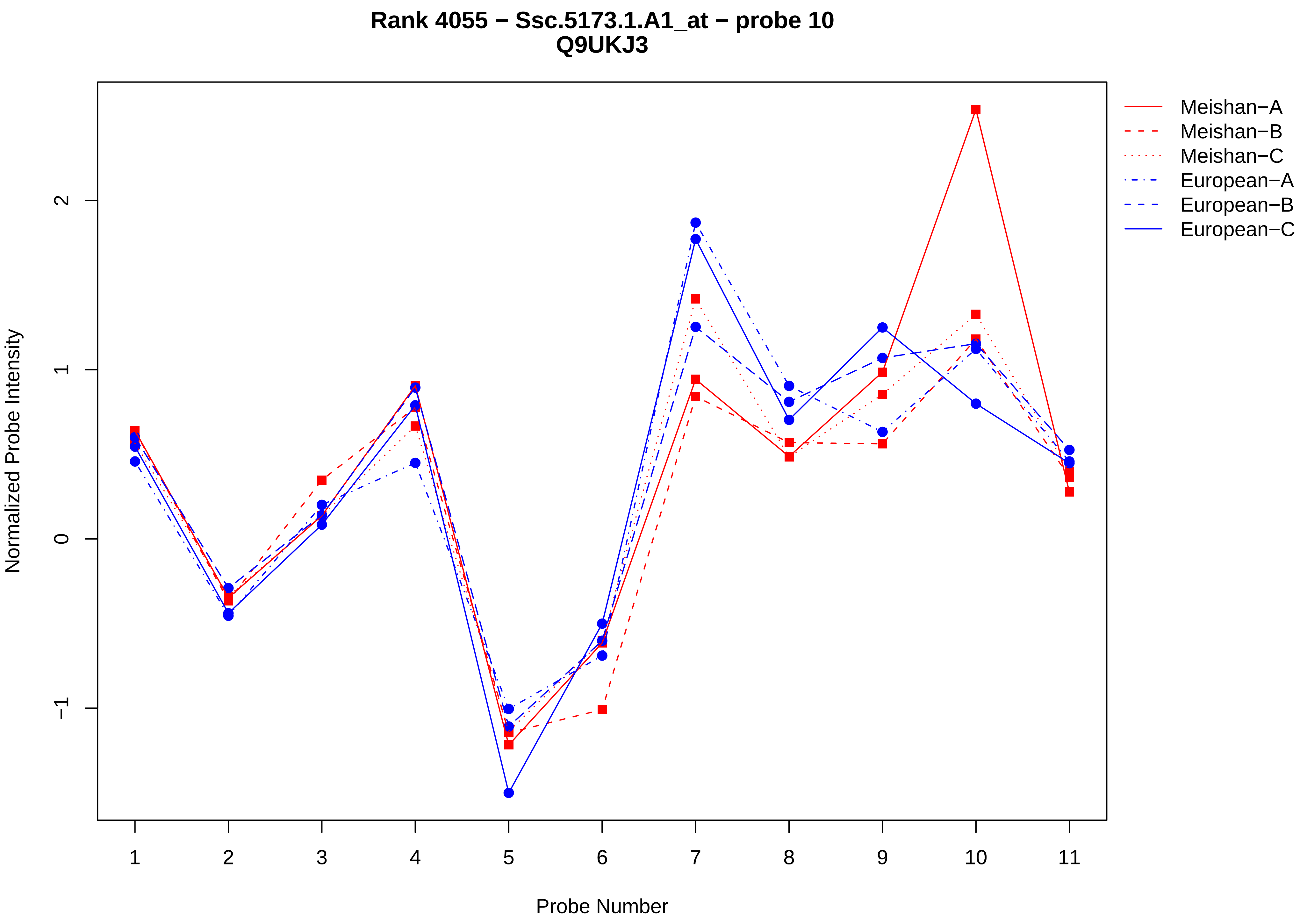

Rank 4055 − Ssc.5173.1.A1_at − probe 10
Q9UKJ3
Normalized Probe Intensity
Probe Number
−1
0
1
2
1
2
3
4
5
6
7
8
9
10
11
Meishan−A
Meishan−B
Meishan−C
European−A
European−B
European−C

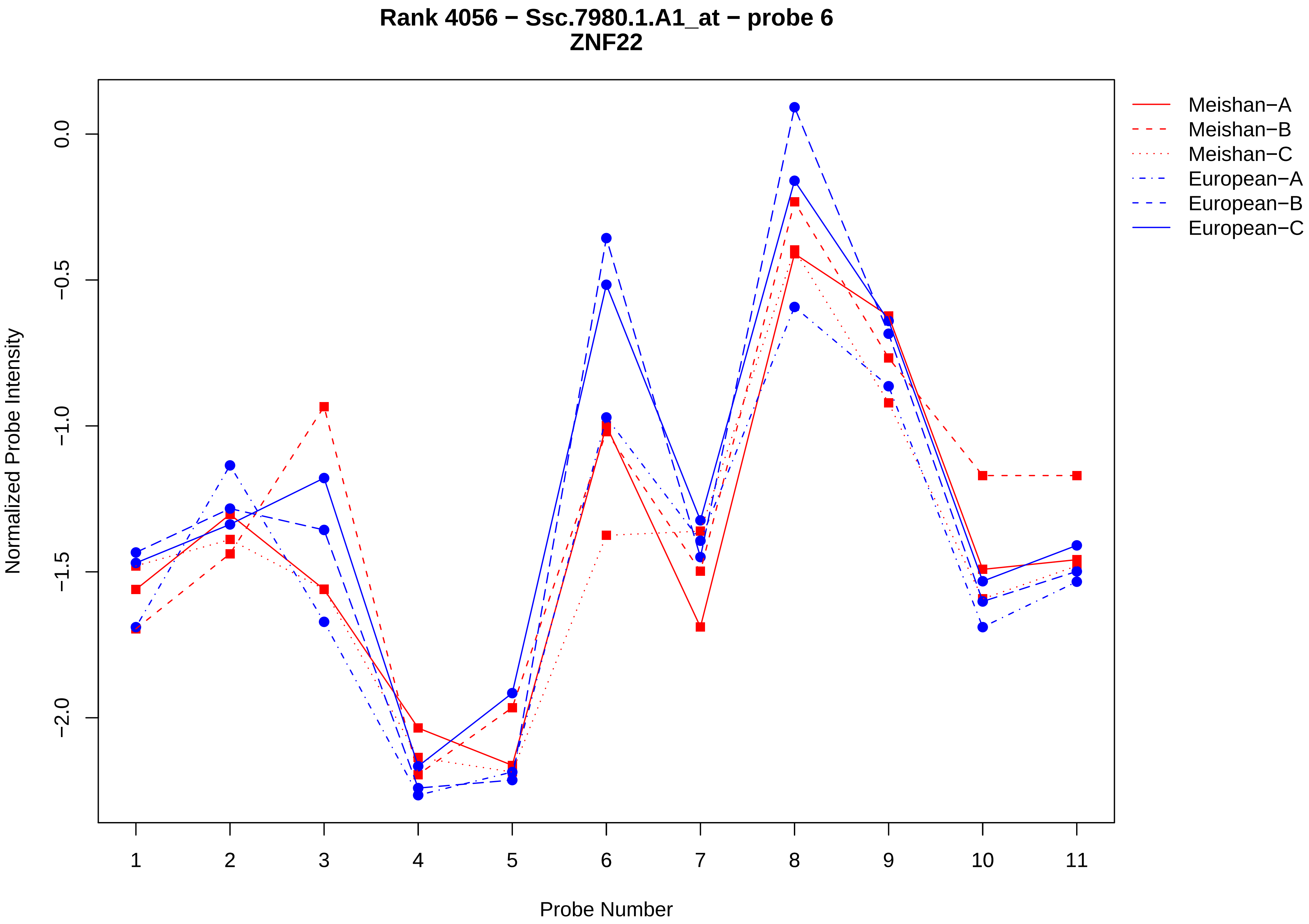

Rank 4056 − Ssc.7980.1.A1_at − probe 6
ZNF22
Normalized Probe Intensity
Probe Number
0.0
−0.5
−1.0
−1.5
−2.0
1
2
3
4
5
6
7
8
9
10
11
Meishan−A
Meishan−B
Meishan−C
European−A
European−B
European−C

Rank 4057 − Ssc.17853.1.A1_at − probe 4
REM1

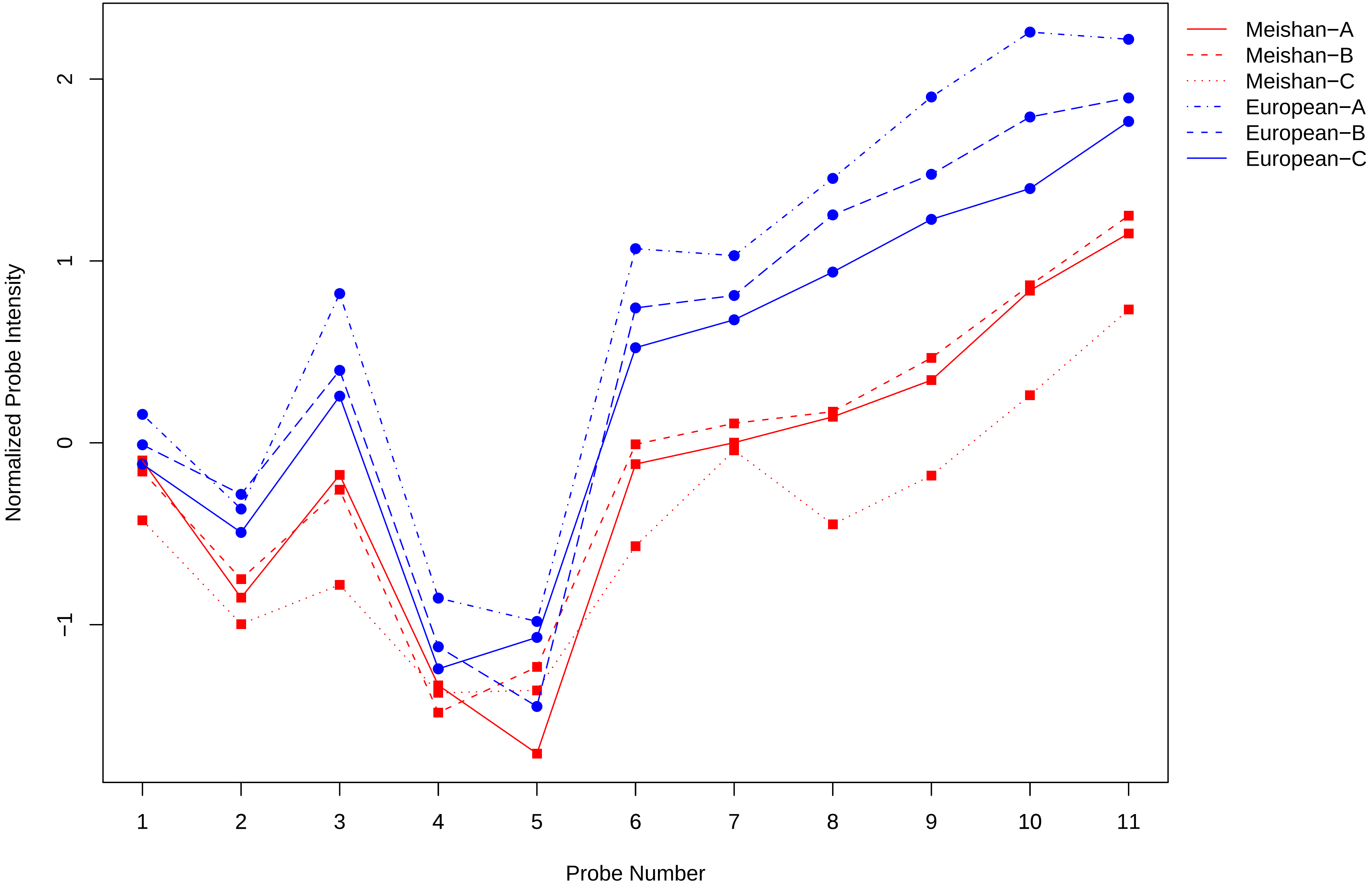

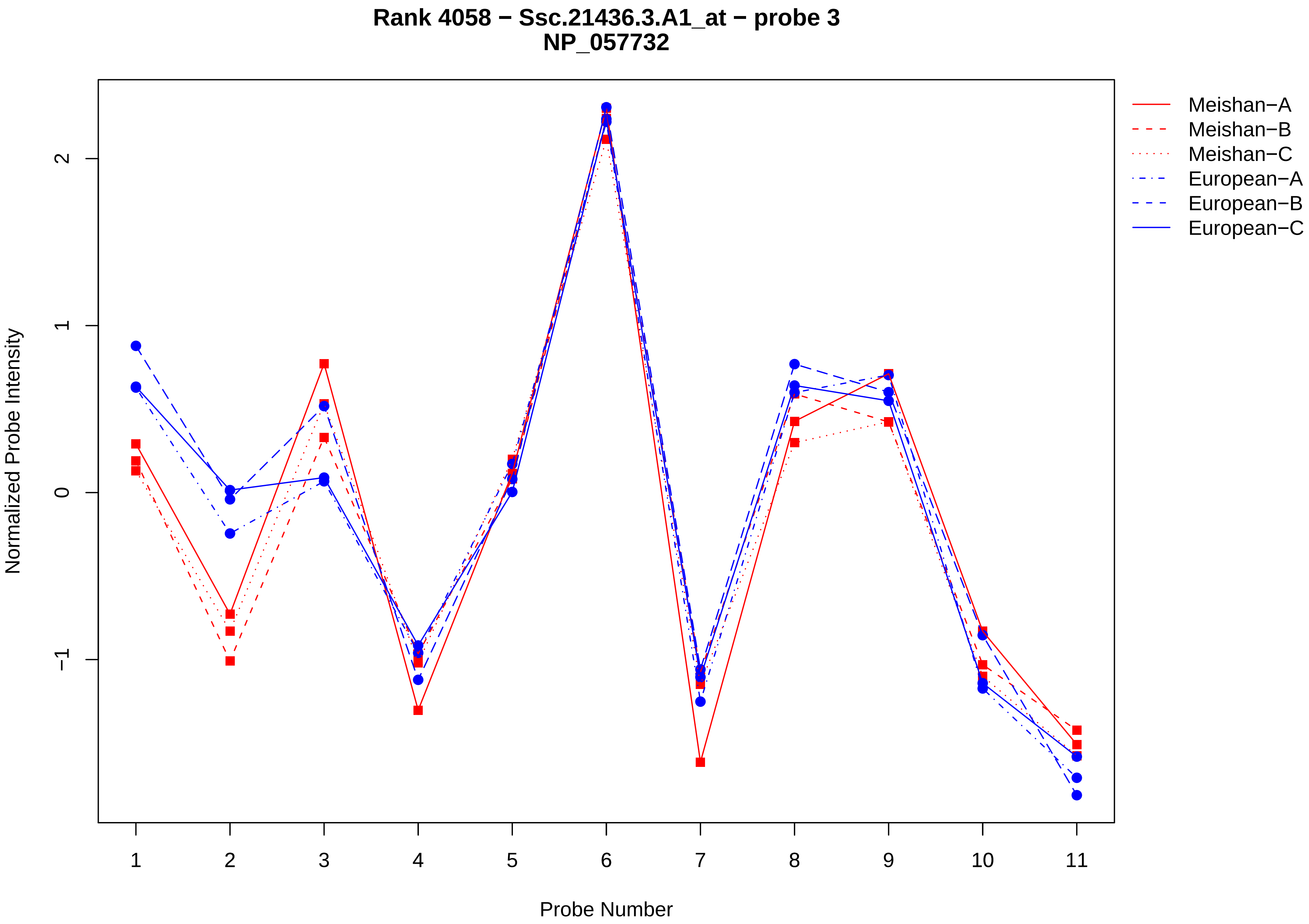

Rank 4058 − Ssc.21436.3.A1_at − probe 3
NP_057732
Normalized Probe Intensity
Probe Number
−1
0
1
2
1
2
3
4
5
6
7
8
9
10
11
Meishan−A
Meishan−B
Meishan−C
European−A
European−B
European−C

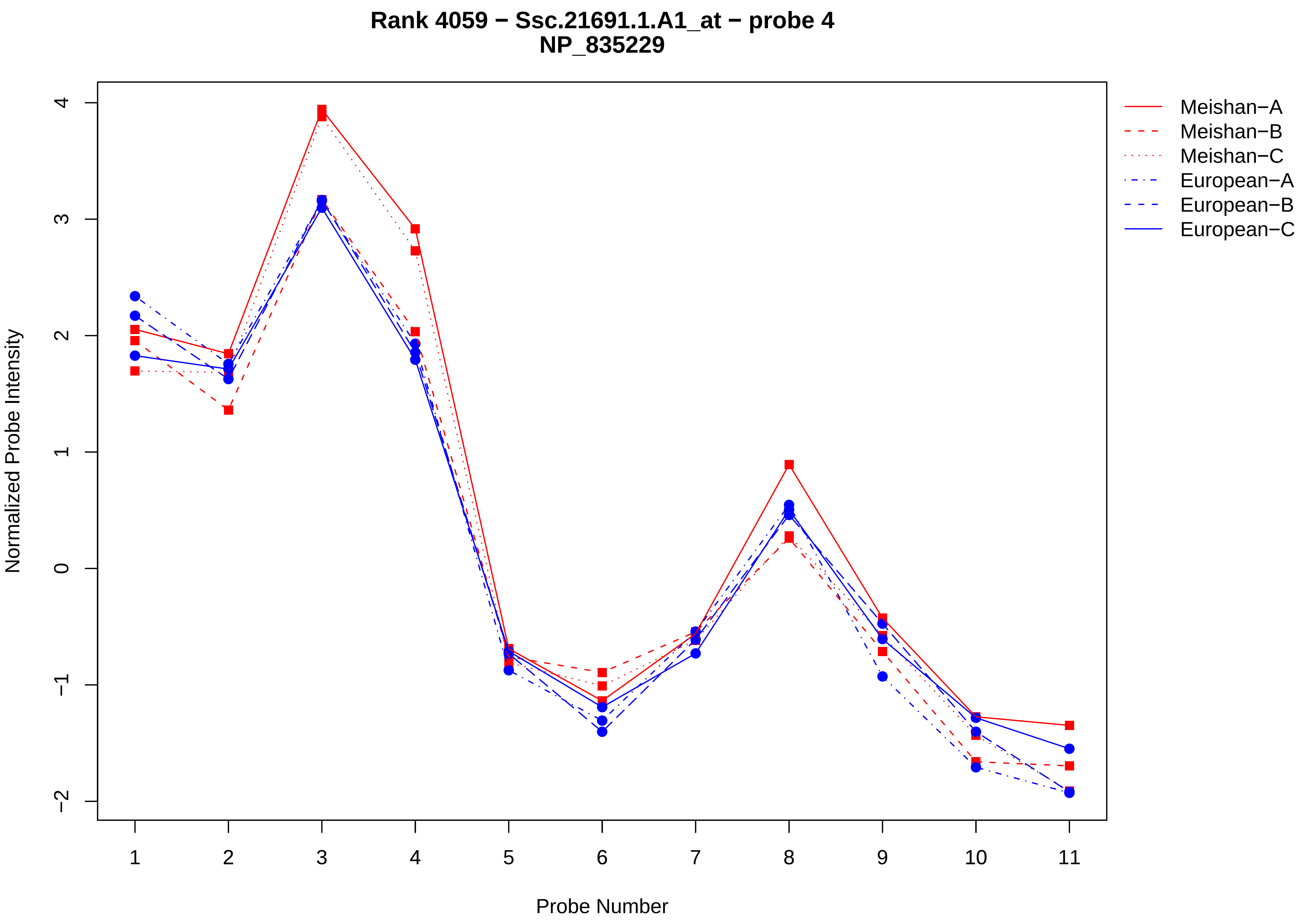
Rank 4059 − Ssc.21691.1.A1_at − probe 4
NP_835229
Normalized Probe Intensity
Probe Number
Meishan−A
Meishan−B
Meishan−C
European−A
European−B
European−C
−2
−1
0
1
2
3
4
1
2
3
4
5
6
7
8
9
10
11

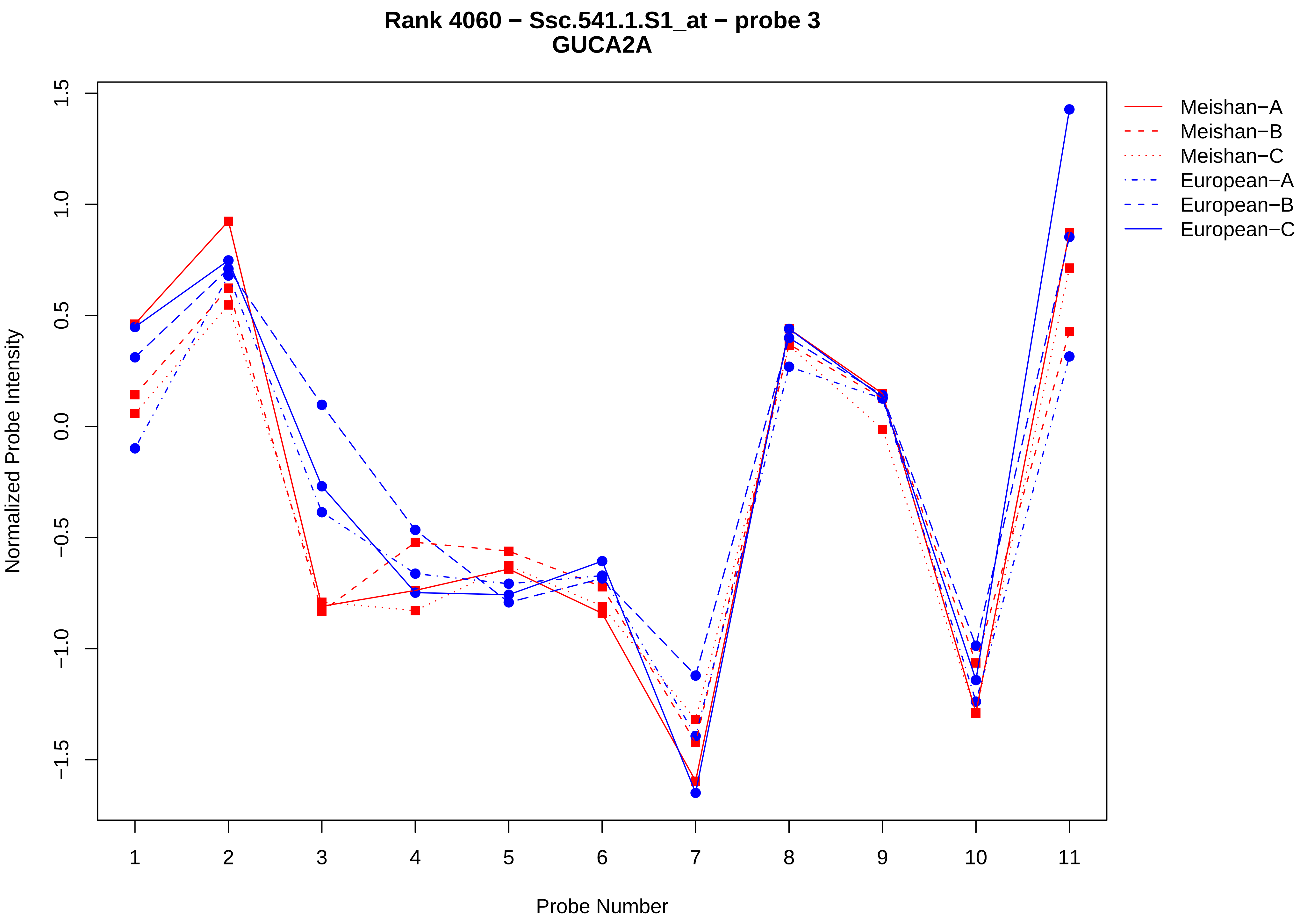

Rank 4060 − Ssc.541.1.S1_at − probe 3
GUCA2A
Normalized Probe Intensity
Probe Number
1.5
1.0
0.5
0.0
−0.5
−1.0
−1.5
1
2
3
4
5
6
7
8
9
10
11
Meishan−A
Meishan−B
Meishan−C
European−A
European−B
European−C

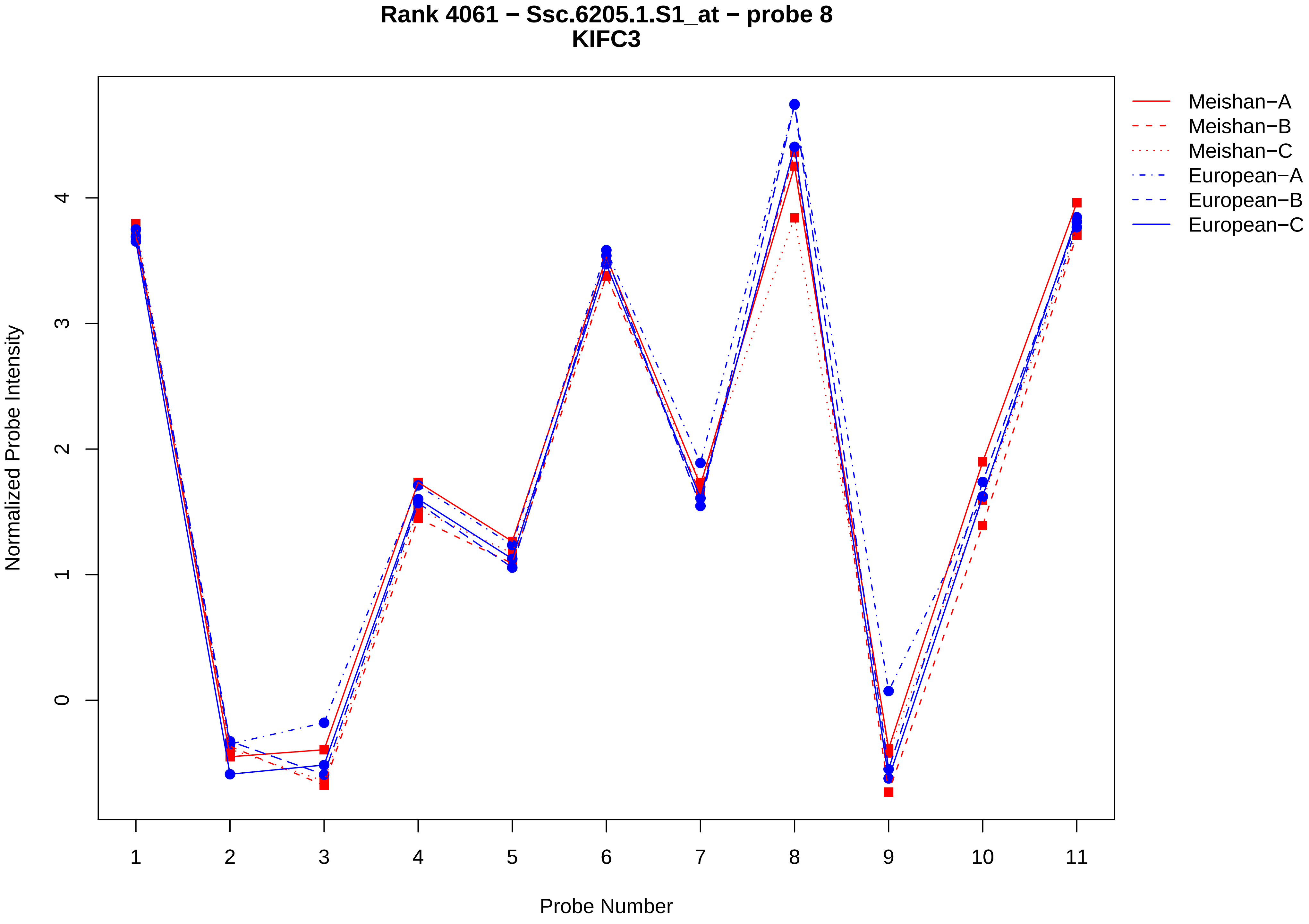

Rank 4061 − Ssc.6205.1.S1_at − probe 8
KIFC3
Normalized Probe Intensity
Probe Number
Meishan−A
Meishan−B
Meishan−C
European−A
European−B
European−C
0
1
2
3
4
1
2
3
4
5
6
7
8
9
10
11

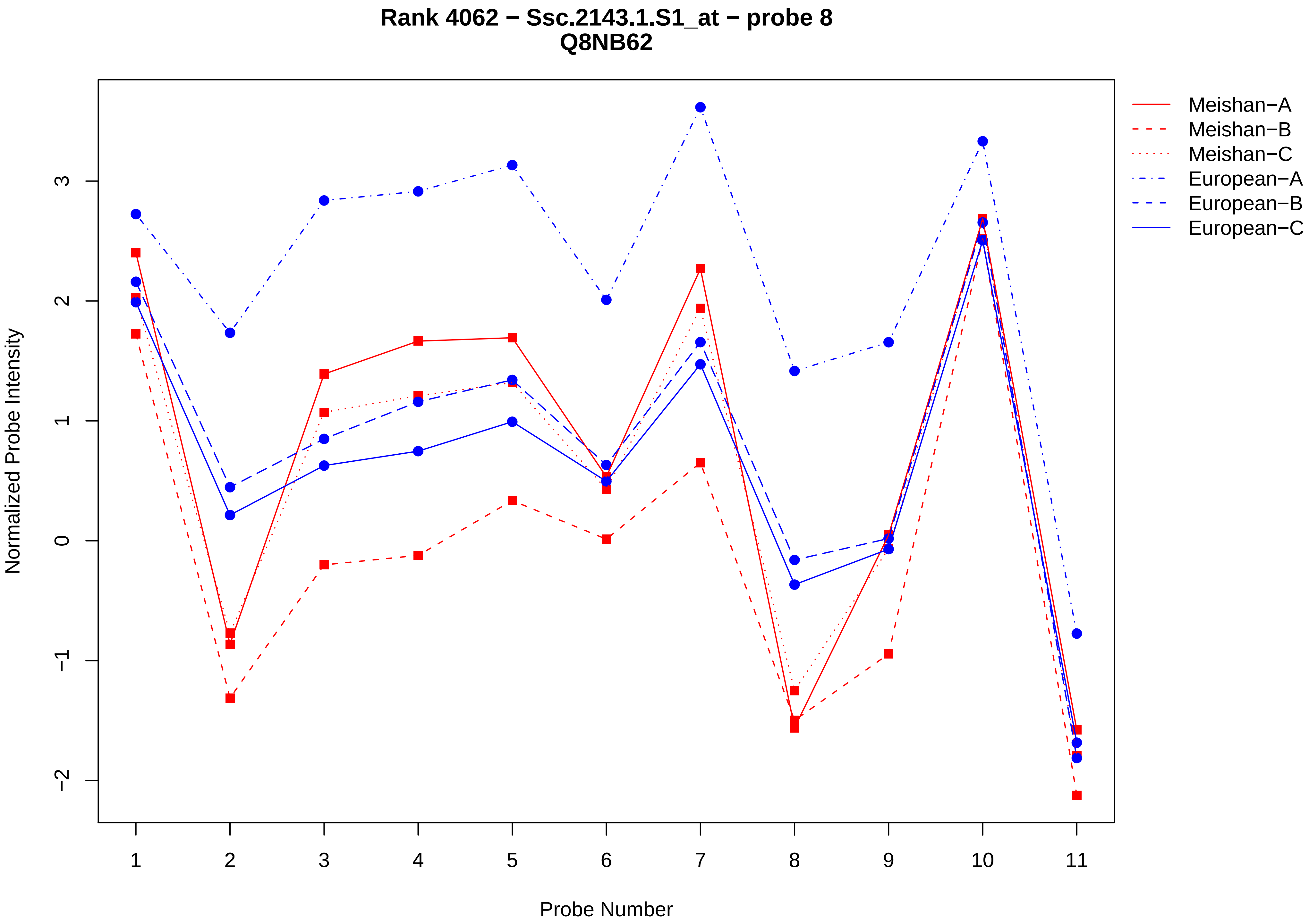
Rank 4062 − Ssc.2143.1.S1_at − probe 8
Q8NB62
Normalized Probe Intensity
Probe Number
Meishan−A
Meishan−B
Meishan−C
European−A
European−B
European−C
−2
−1
0
1
2
3
1
2
3
4
5
6
7
8
9
10
11

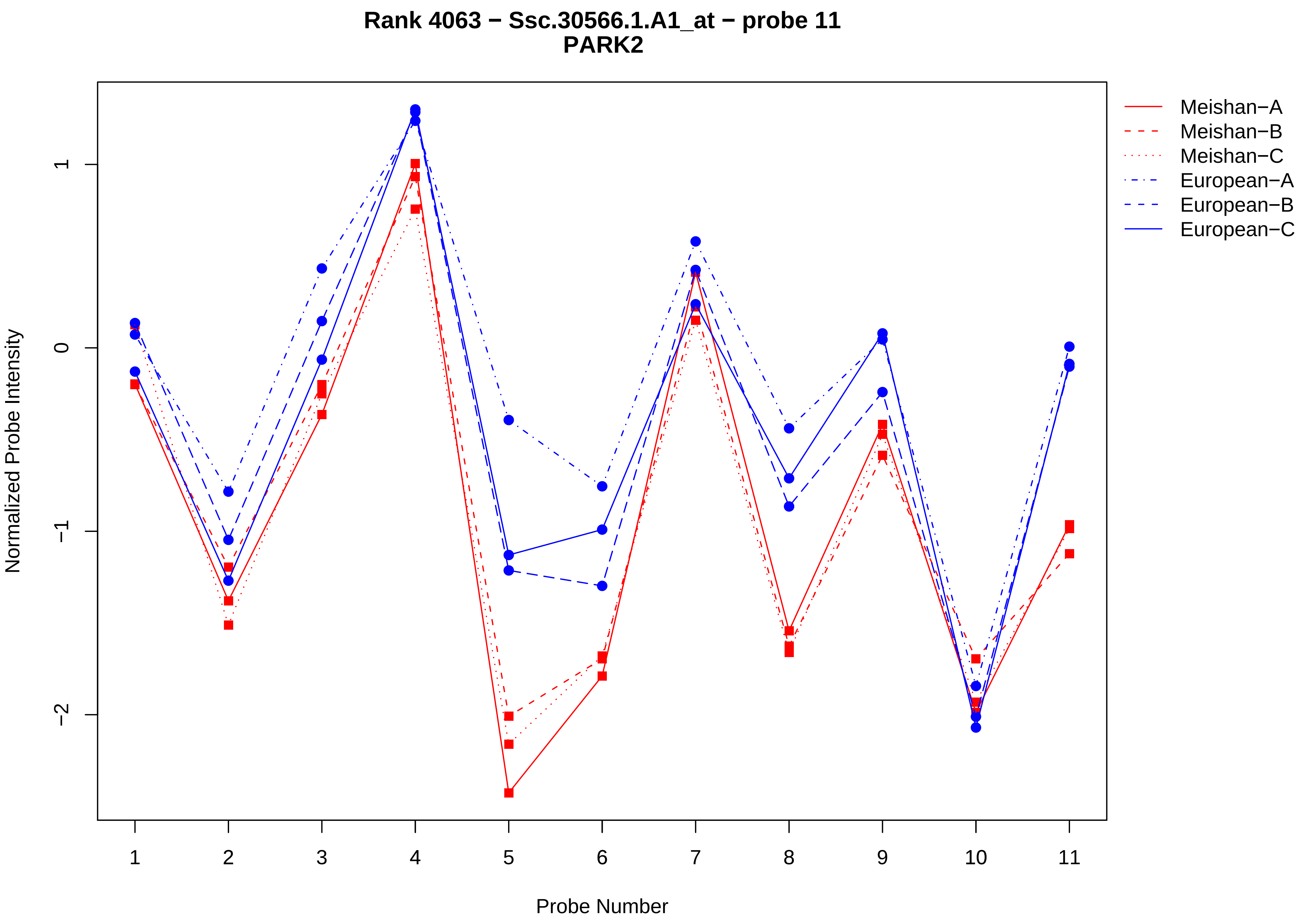

Rank 4063 − Ssc.30566.1.A1_at − probe 11
PARK2
Normalized Probe Intensity
Probe Number
Meishan−A
Meishan−B
Meishan−C
European−A
European−B
European−C
1
0
−1
−2
1
2
3
4
5
6
7
8
9
10
11

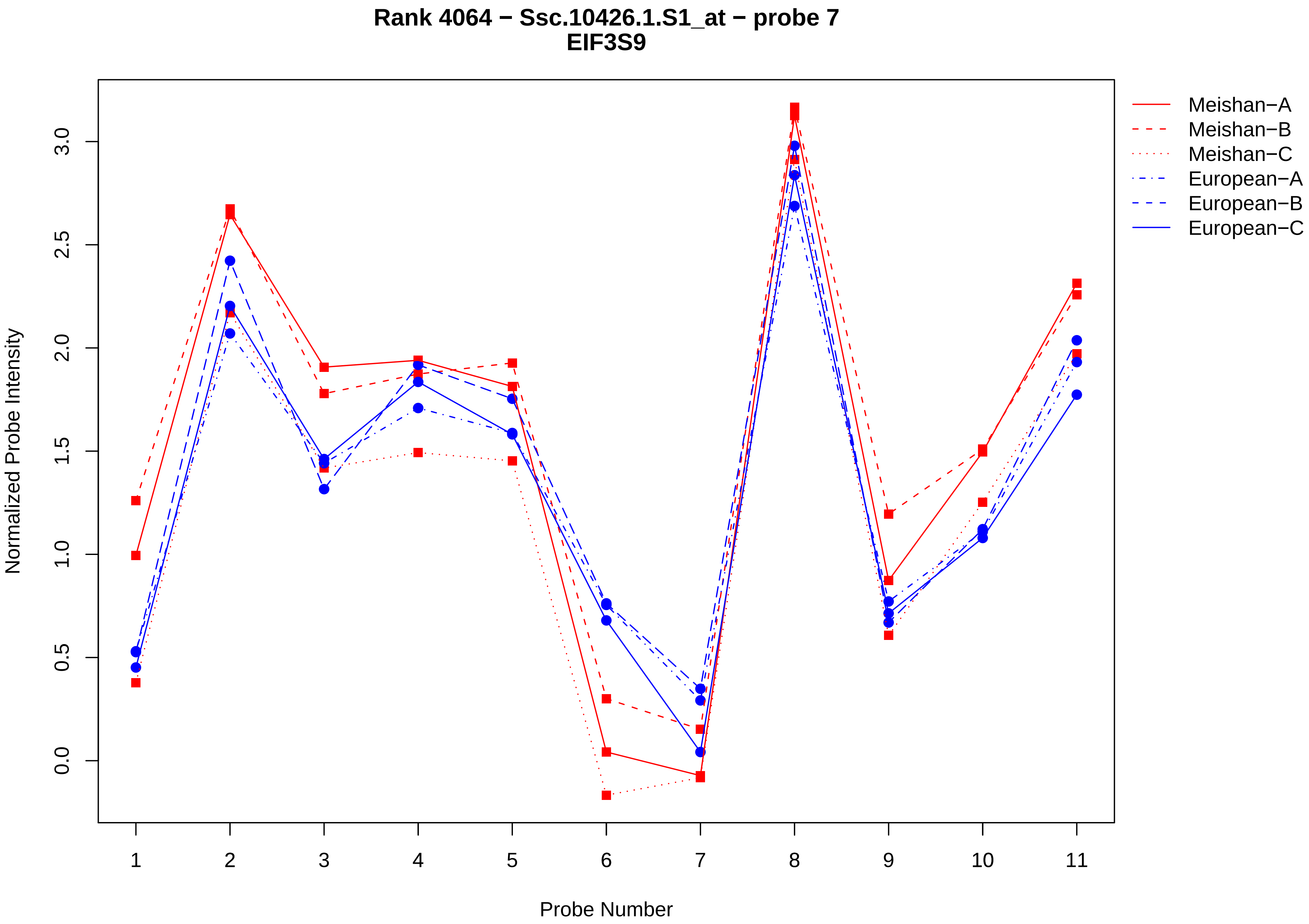

Rank 4064 − Ssc.10426.1.S1_at − probe 7
EIF3S9
Normalized Probe Intensity
Probe Number
0.0
0.5
1.0
1.5
2.0
2.5
3.0
1
2
3
4
5
6
7
8
9
10
11
Meishan−A
Meishan−B
Meishan−C
European−A
European−B
European−C

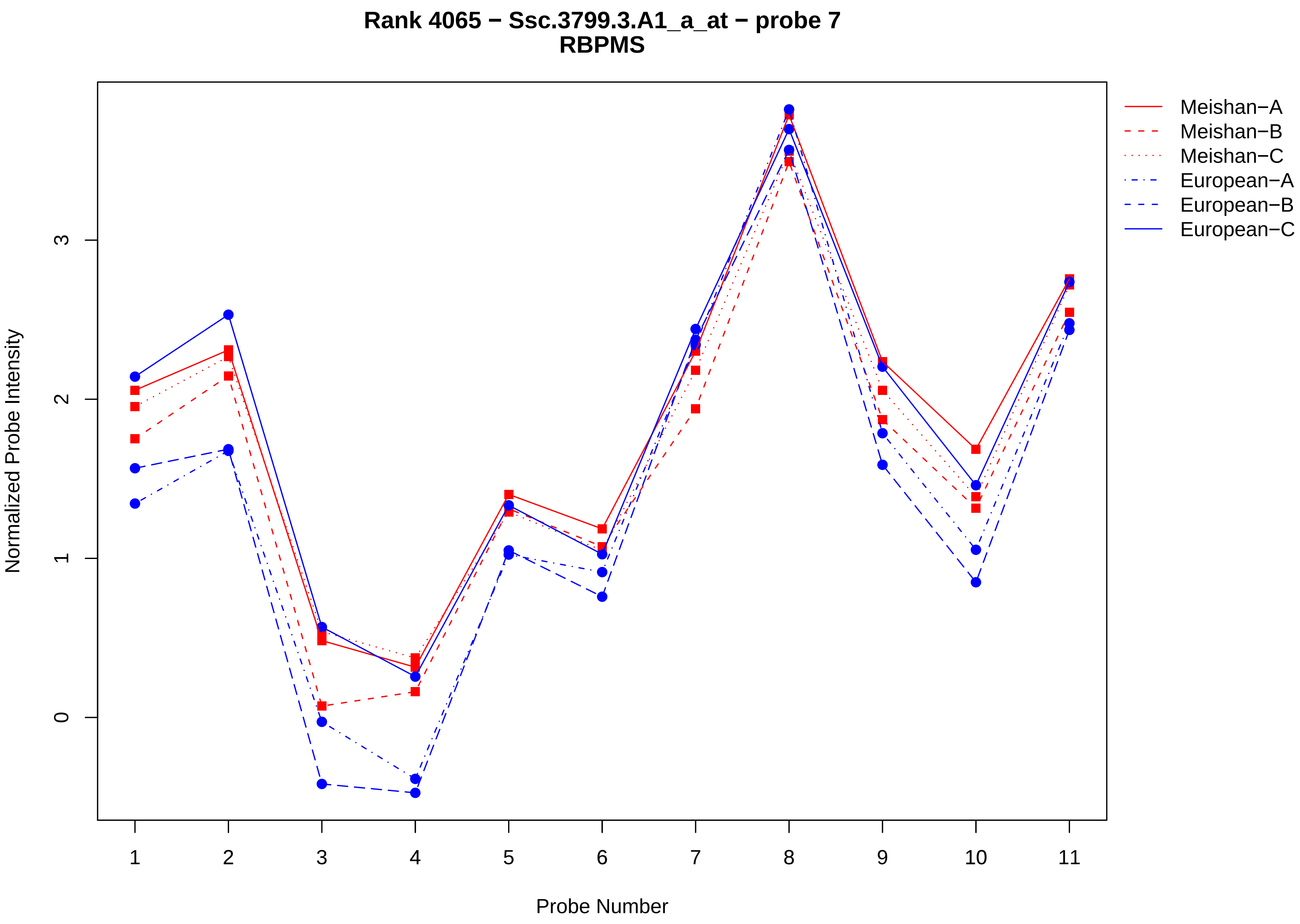

Rank 4065 − Ssc.3799.3.A1_a_at − probe 7
RBPMS
Normalized Probe Intensity
Probe Number
0
1
2
3
1
2
3
4
5
6
7
8
9
10
11
Meishan−A
Meishan−B
Meishan−C
European−A
European−B
European−C

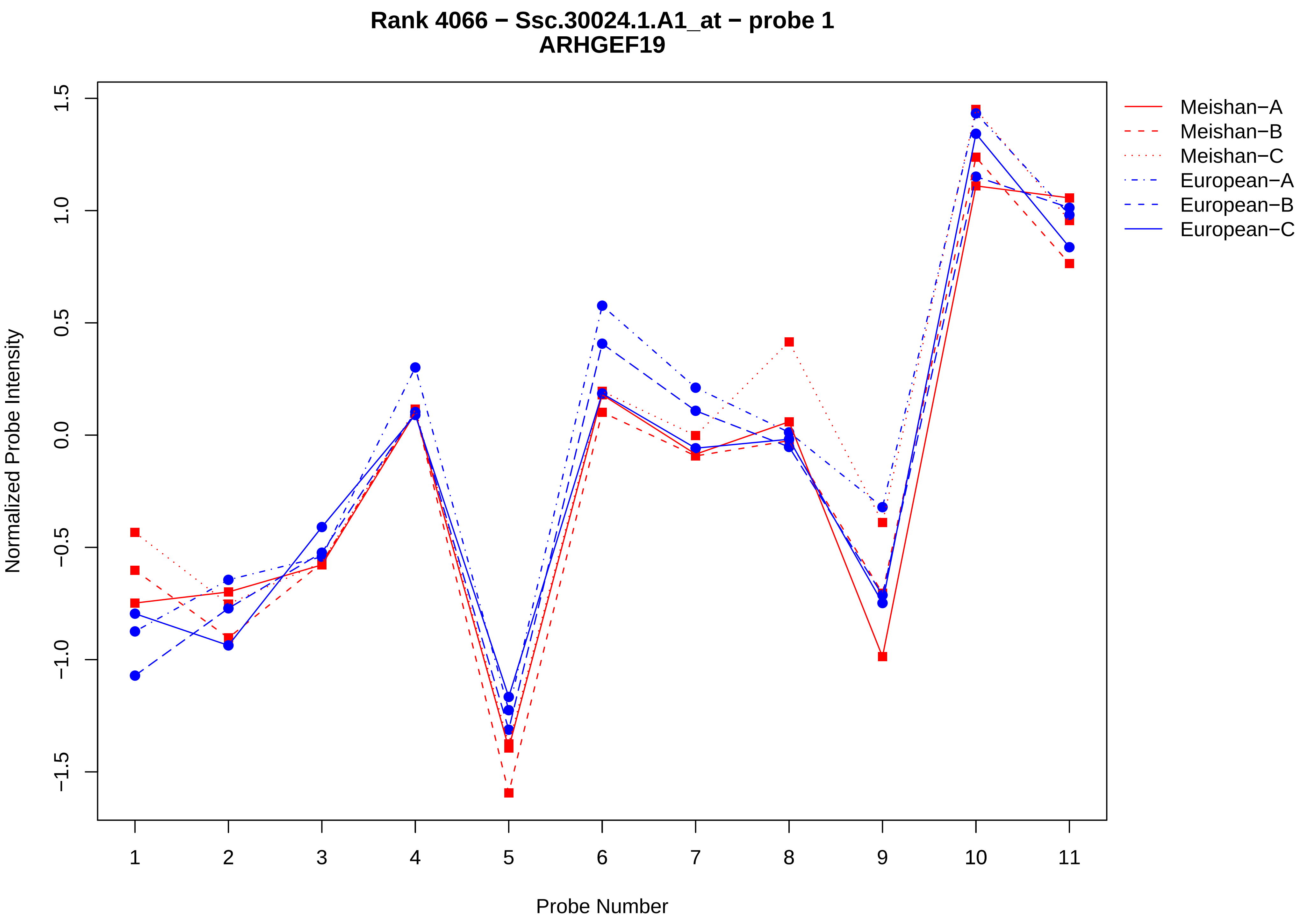

Rank 4066 − Ssc.30024.1.A1_at − probe 1
ARHGEF19
Normalized Probe Intensity
Probe Number
1.5
1.0
0.5
0.0
−0.5
−1.0
−1.5
1
2
3
4
5
6
7
8
9
10
11
Meishan−A
Meishan−B
Meishan−C
European−A
European−B
European−C

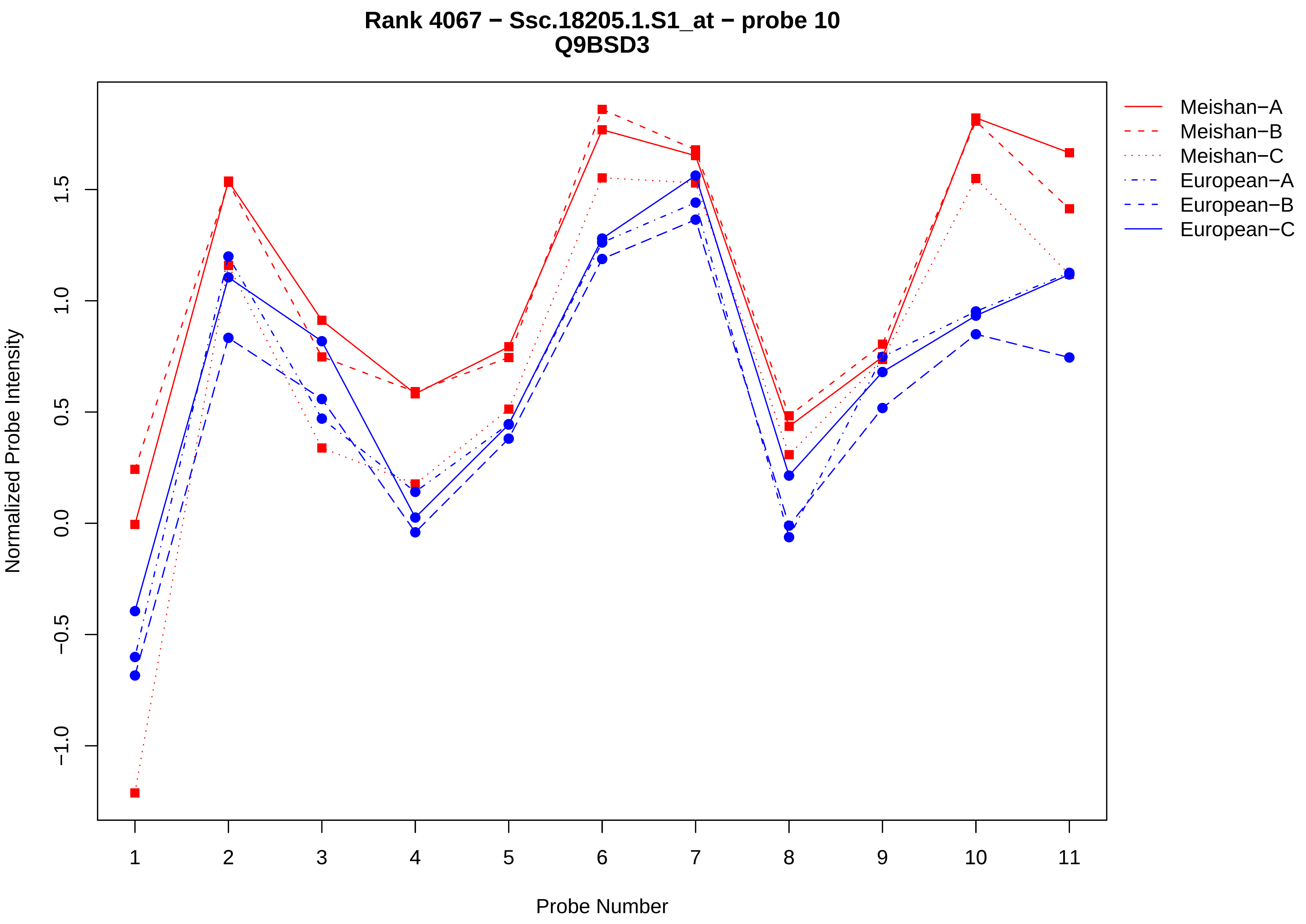

Rank 4067 − Ssc.18205.1.S1_at − probe 10
Q9BSD3
Normalized Probe Intensity
Probe Number
Meishan−A
Meishan−B
Meishan−C
European−A
European−B
European−C
−1.0
−0.5
0.0
0.5
1.0
1.5
1
2
3
4
5
6
7
8
9
10
11

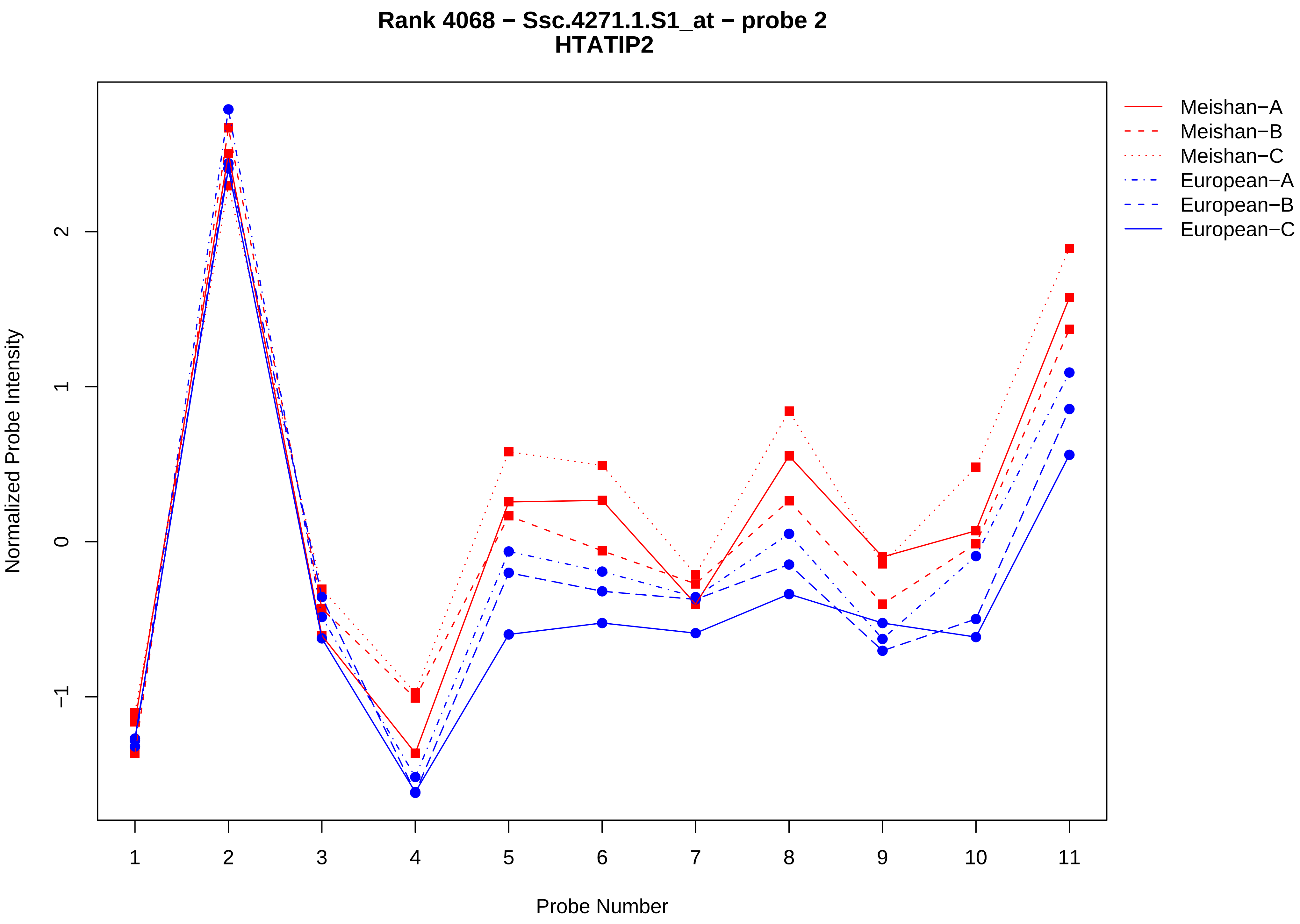

Rank 4068 − Ssc.4271.1.S1_at − probe 2
HTATIP2
Normalized Probe Intensity
Probe Number
−1
0
1
2
1
2
3
4
5
6
7
8
9
10
11
Meishan−A
Meishan−B
Meishan−C
European−A
European−B
European−C

Rank 4069 − Ssc.21280.2.A1_at − probe 5
CDC27

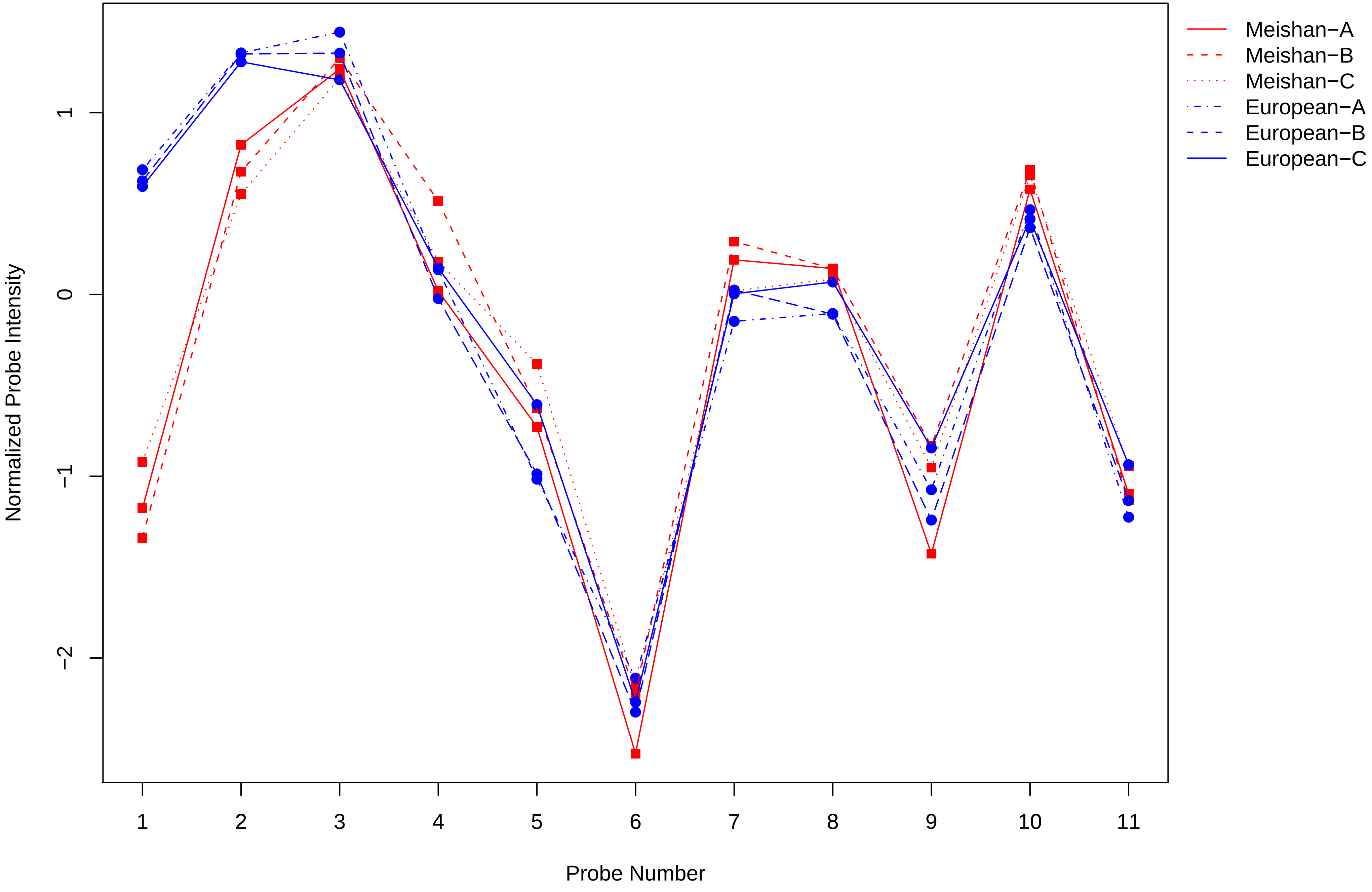

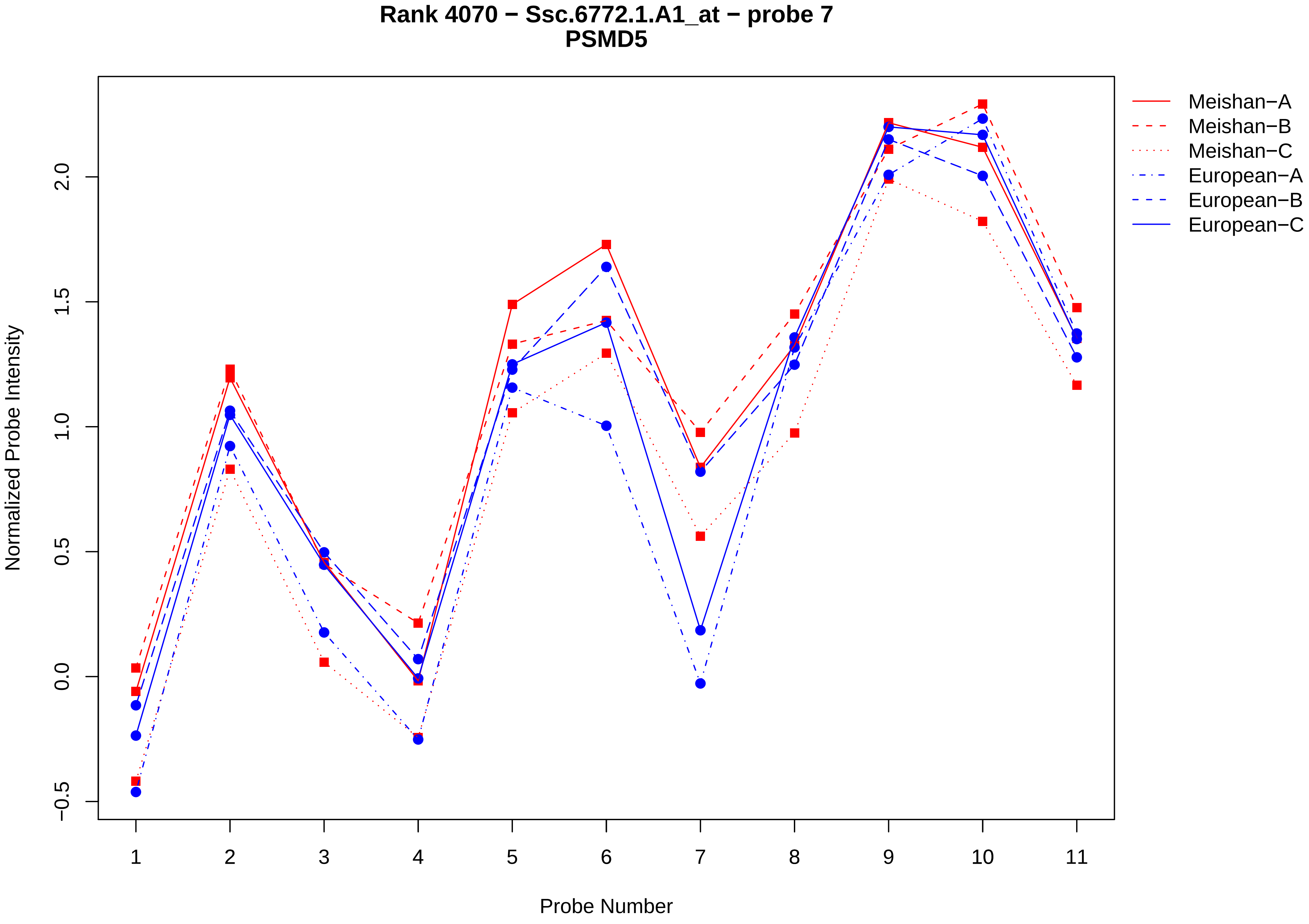

Rank 4070 − Ssc.6772.1.A1_at − probe 7
PSMD5
Normalized Probe Intensity
Probe Number
−0.5
0.0
0.5
1.0
1.5
2.0
1
2
3
4
5
6
7
8
9
10
11
Meishan−A
Meishan−B
Meishan−C
European−A
European−B
European−C

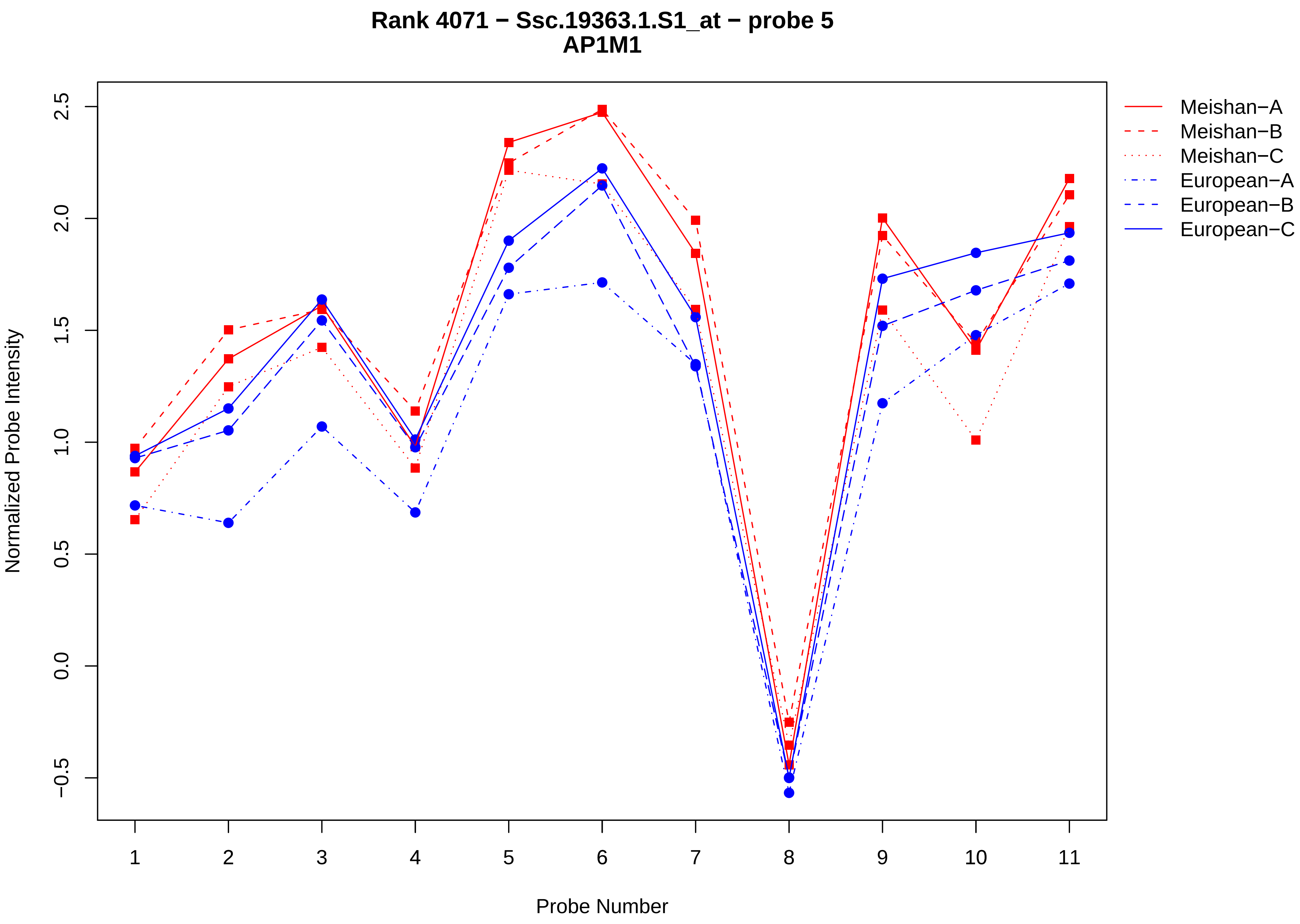

Rank 4071 − Ssc.19363.1.S1_at − probe 5
AP1M1
Normalized Probe Intensity
Probe Number
Meishan−A
Meishan−B
Meishan−C
European−A
European−B
European−C
−0.5
0.0
0.5
1.0
1.5
2.0
2.5
1
2
3
4
5
6
7
8
9
10
11

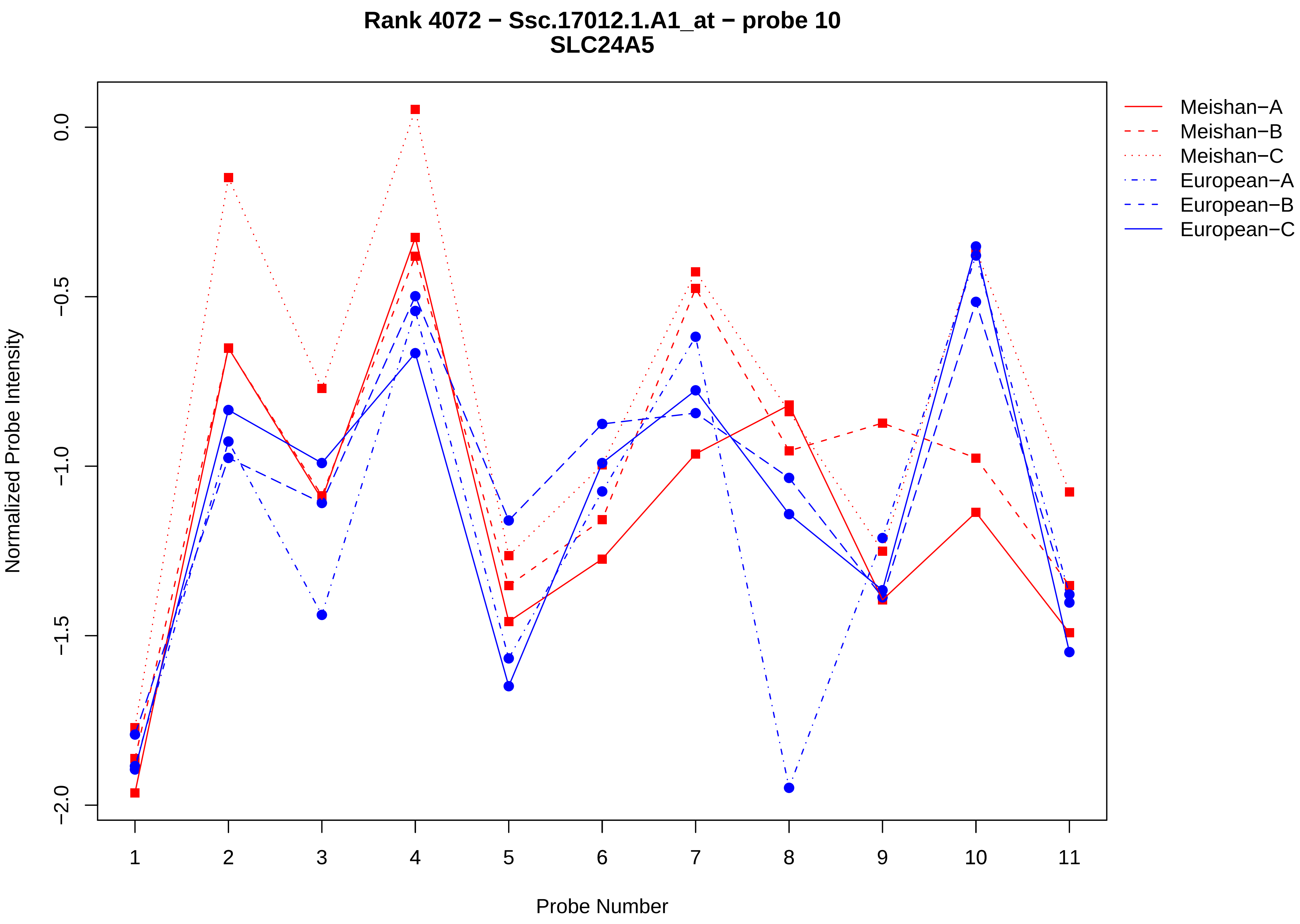

Rank 4072 − Ssc.17012.1.A1_at − probe 10
SLC24A5
Normalized Probe Intensity
Probe Number
0.0
−0.5
−1.0
−1.5
−2.0
1
2
3
4
5
6
7
8
9
10
11
Meishan−A
Meishan−B
Meishan−C
European−A
European−B
European−C

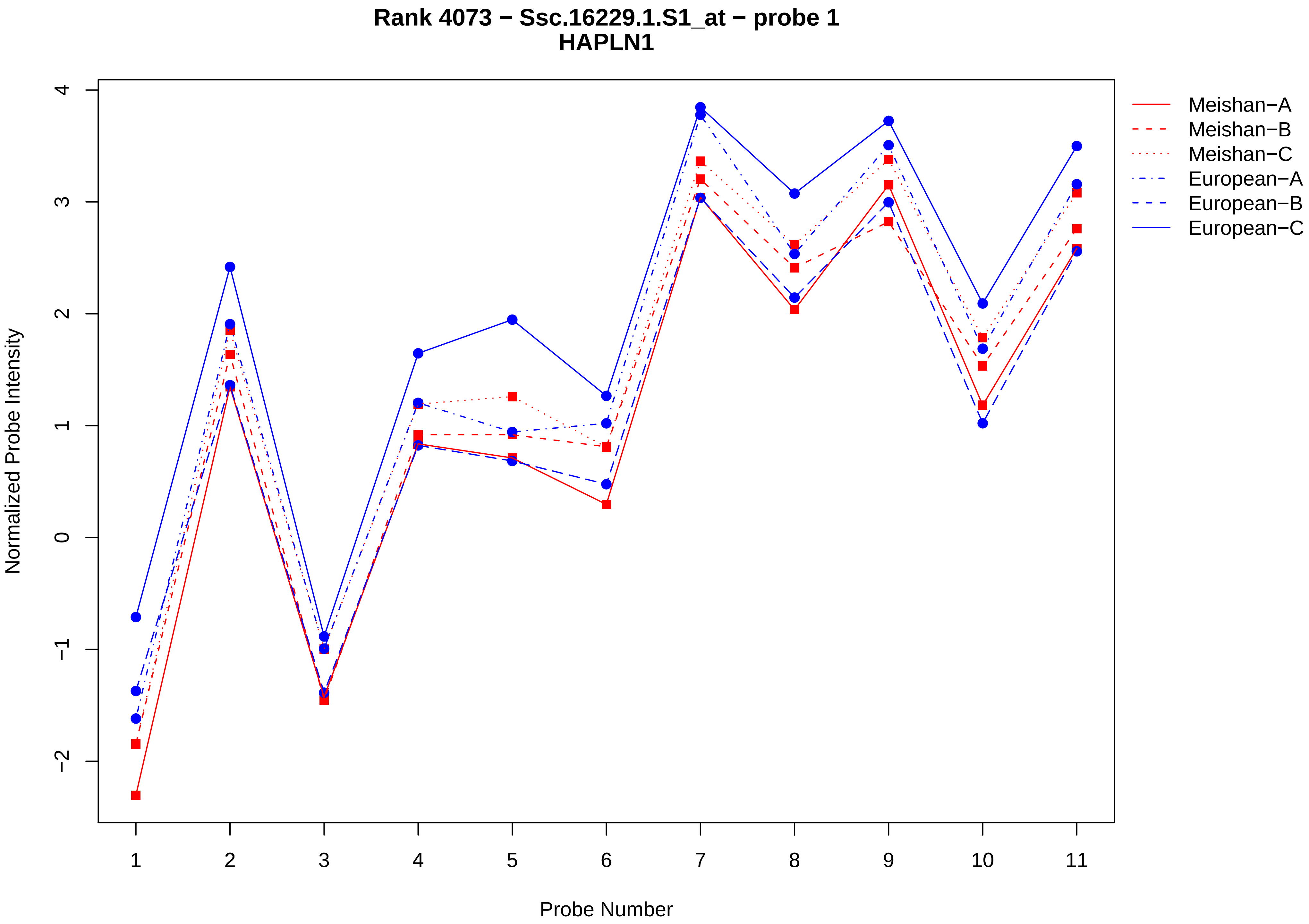
Rank 4073 − Ssc.16229.1.S1_at − probe 1
HAPLN1
Normalized Probe Intensity
Probe Number
Meishan−A
Meishan−B
Meishan−C
European−A
European−B
European−C

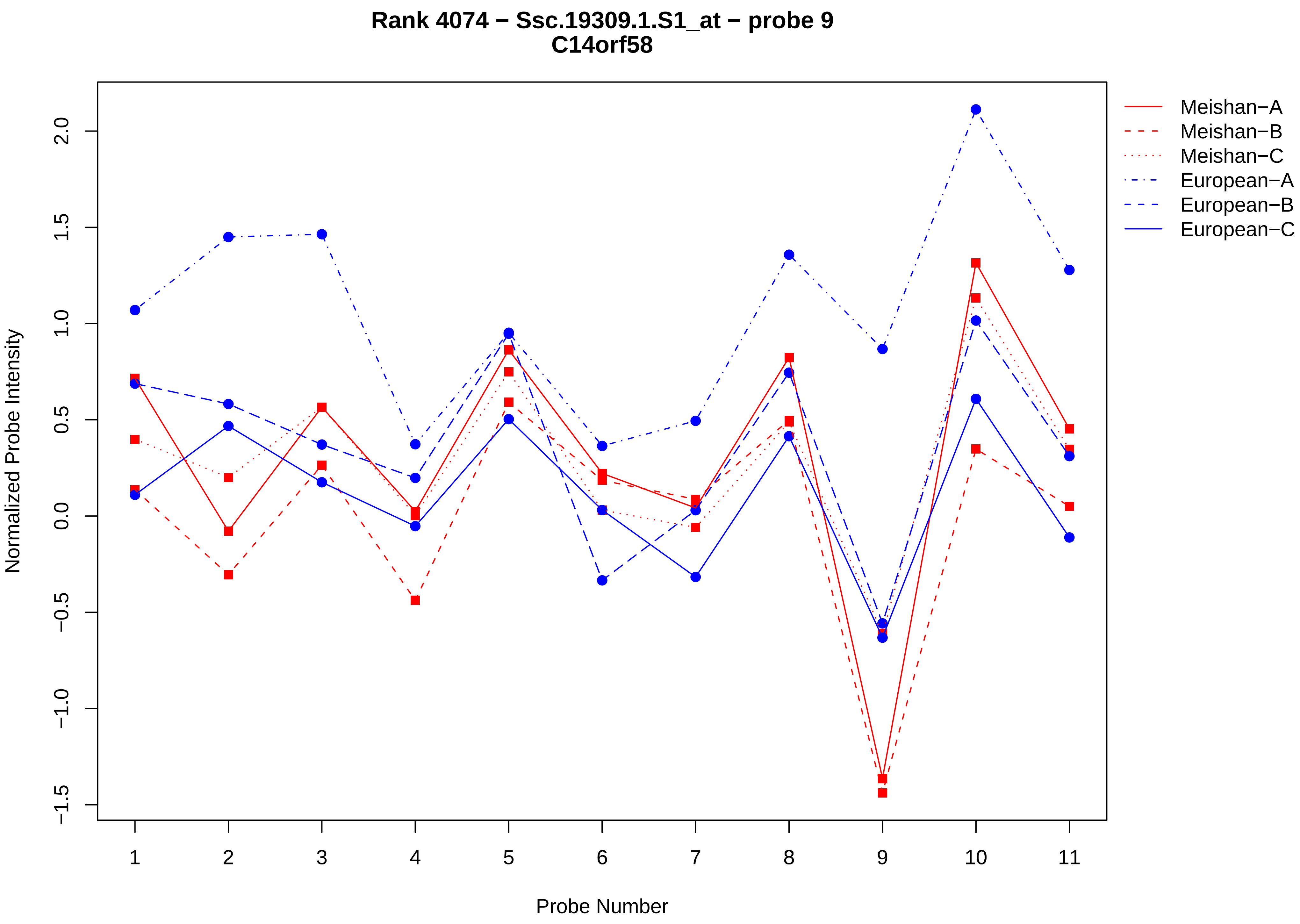

Rank 4074 − Ssc.19309.1.S1_at − probe 9
C14orf58
Normalized Probe Intensity
Probe Number
Meishan−A
Meishan−B
Meishan−C
European−A
European−B
European−C

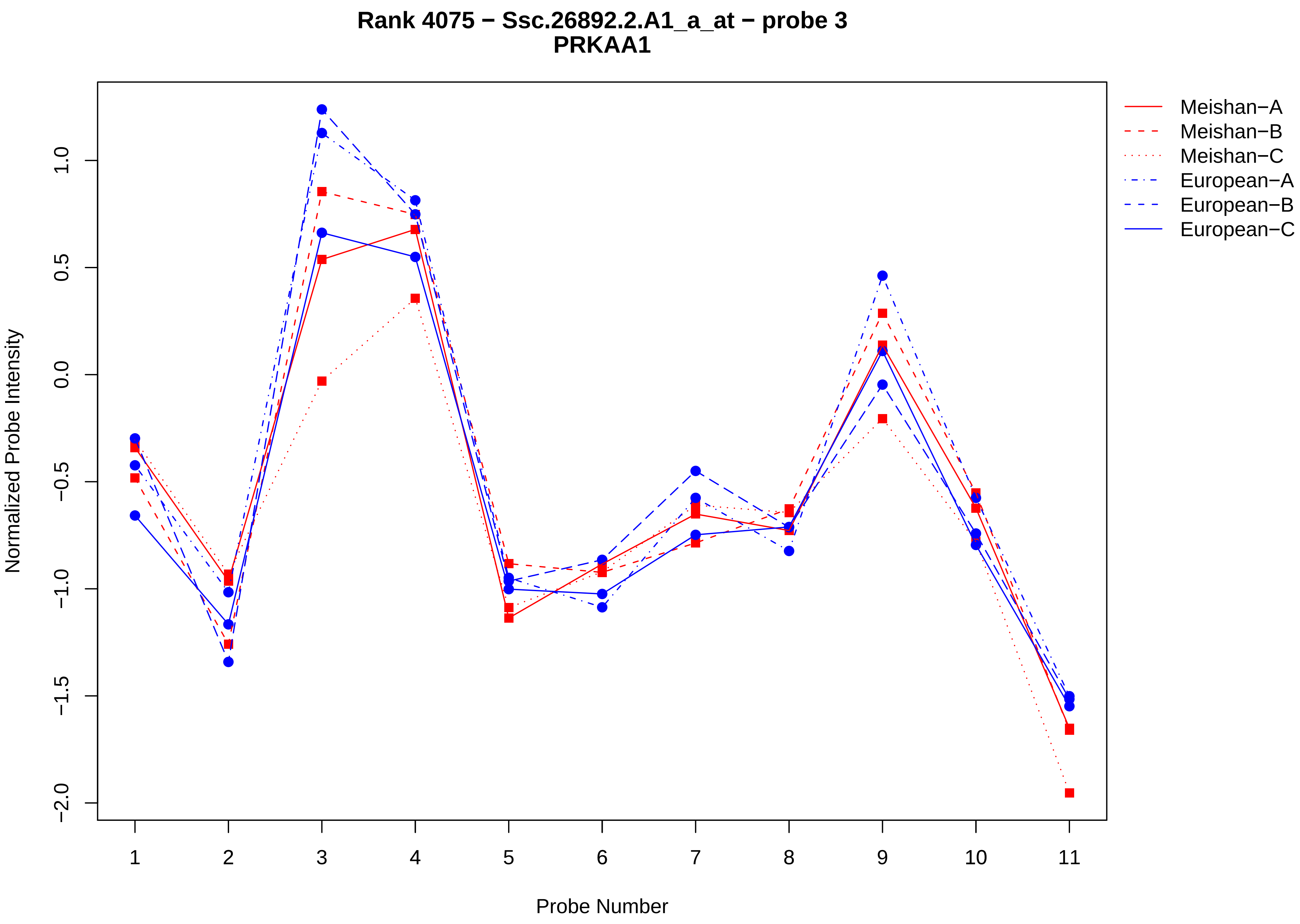
Rank 4075 − Ssc.26892.2.A1_a_at − probe 3
PRKAA1
Normalized Probe Intensity
Probe Number
1.0
0.5
0.0
−0.5
−1.0
−1.5
−2.0
1
2
3
4
5
6
7
8
9
10
11
Meishan−A
Meishan−B
Meishan−C
European−A
European−B
European−C

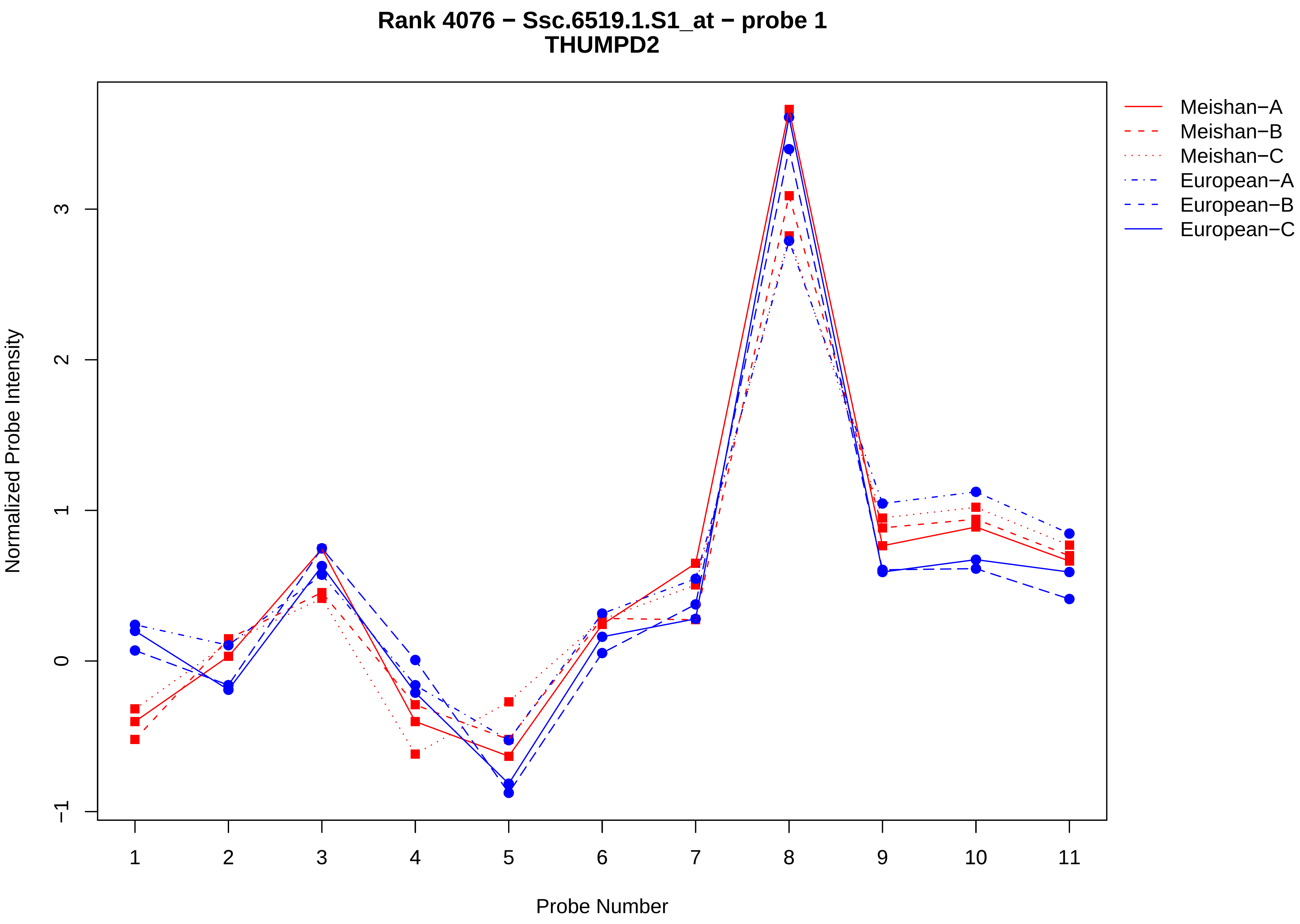
Rank 4076 − Ssc.6519.1.S1_at − probe 1
THUMPD2
Normalized Probe Intensity
Probe Number
−1
0
1
2
3
1
2
3
4
5
6
7
8
9
10
11
Meishan−A
Meishan−B
Meishan−C
European−A
European−B
European−C

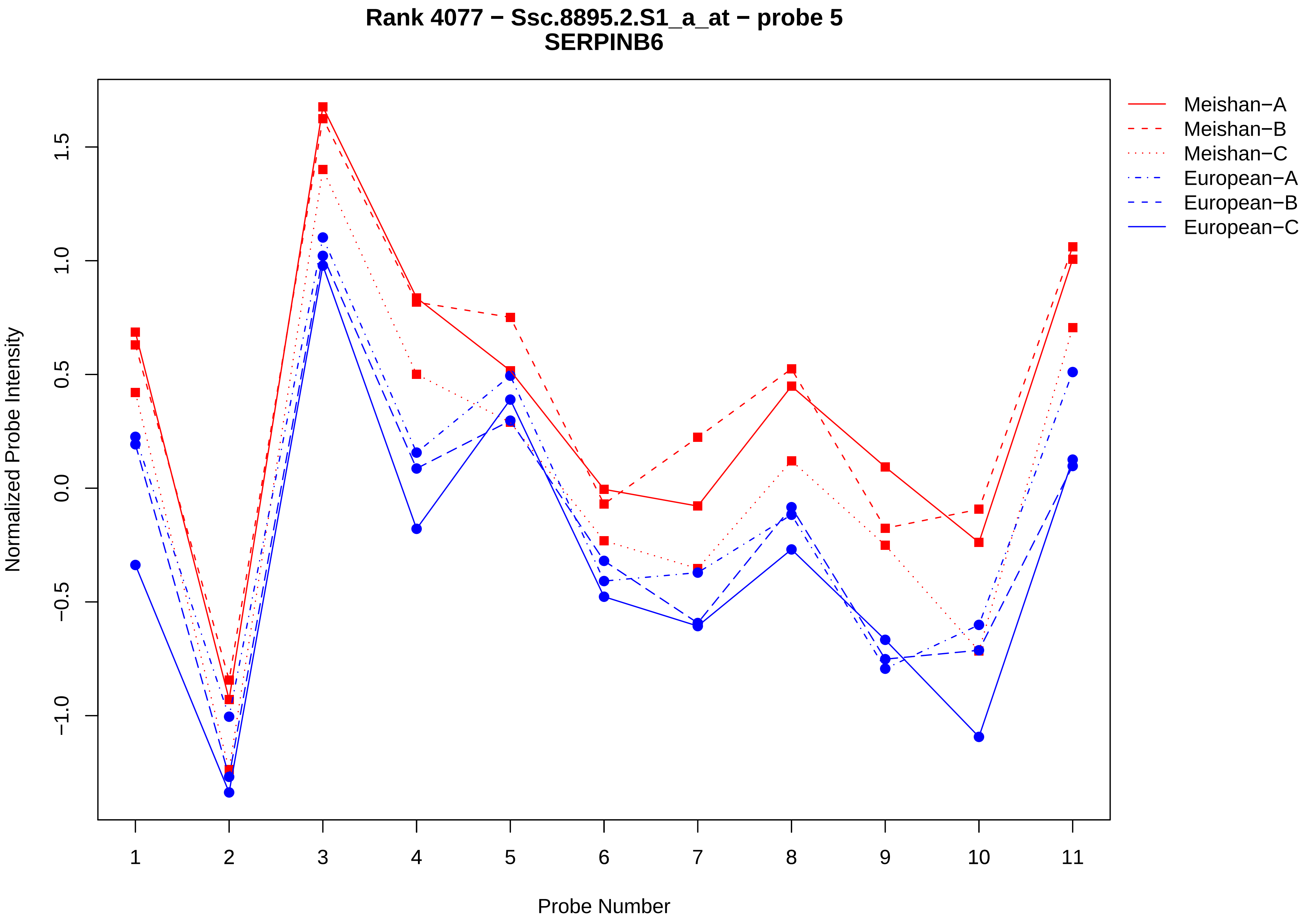

Rank 4077 − Ssc.8895.2.S1_a_at − probe 5
SERPINB6
Normalized Probe Intensity
Probe Number
−1.0
−0.5
0.0
0.5
1.0
1.5
1
2
3
4
5
6
7
8
9
10
11
Meishan−A
Meishan−B
Meishan−C
European−A
European−B
European−C

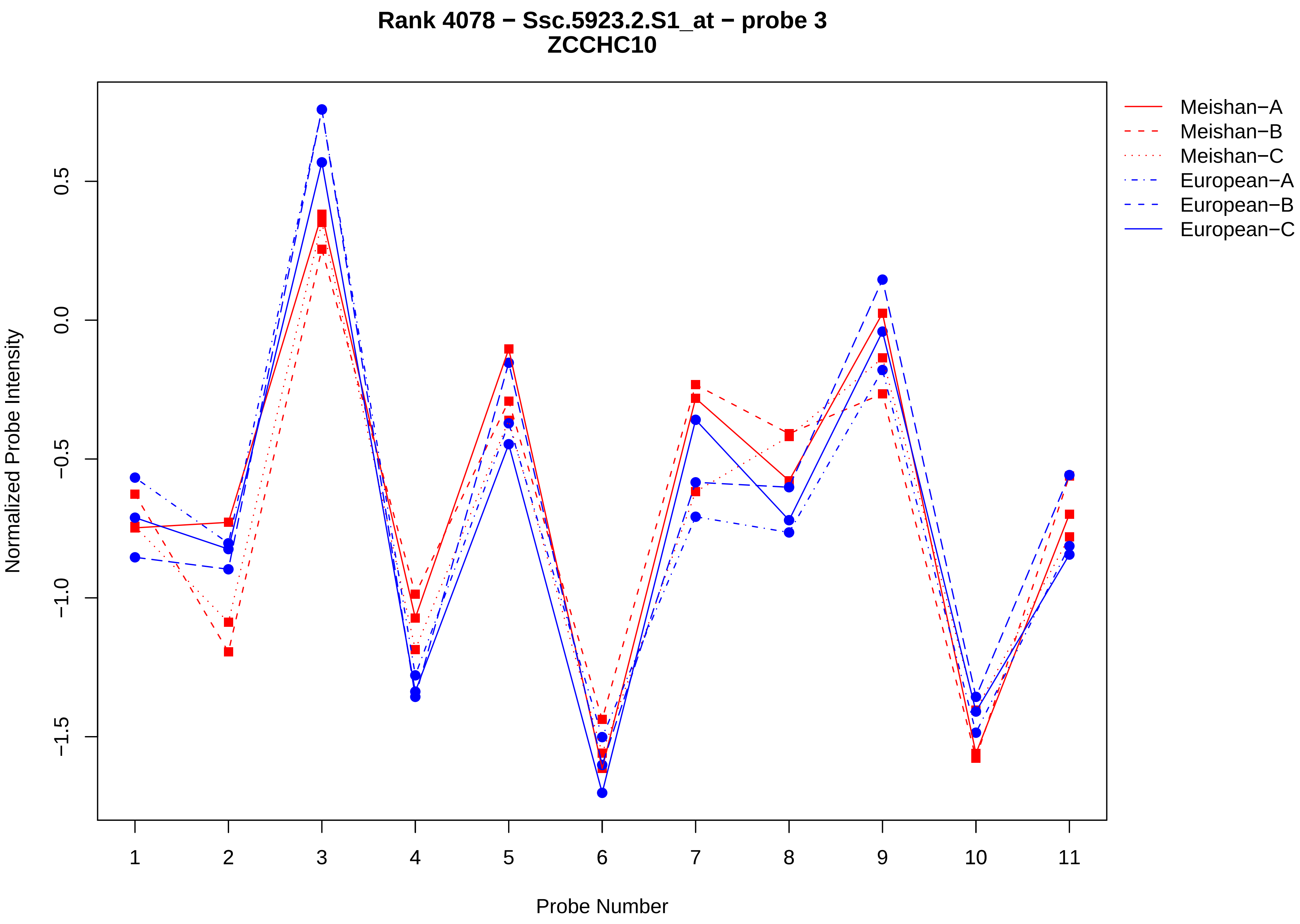

Rank 4078 − Ssc.5923.2.S1_at − probe 3
ZCCHC10
Normalized Probe Intensity
Probe Number
−1.5
−1.0
−0.5
0.0
0.5
1
2
3
4
5
6
7
8
9
10
11
Meishan−A
Meishan−B
Meishan−C
European−A
European−B
European−C

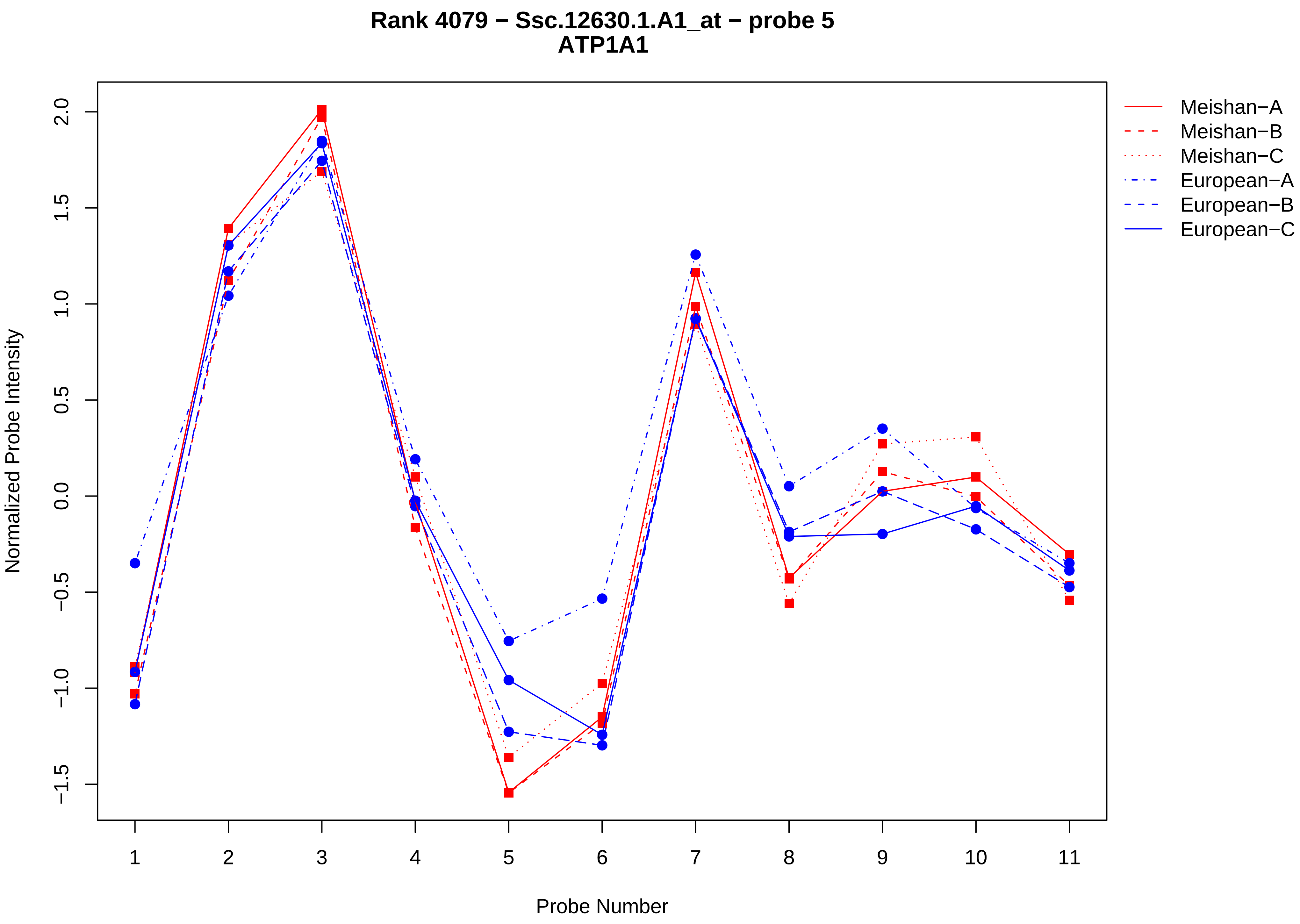
Rank 4079 − Ssc.12630.1.A1_at − probe 5
ATP1A1
Normalized Probe Intensity
Probe Number
−1.5
−1.0
−0.5
0.0
0.5
1.0
1.5
2.0
1
2
3
4
5
6
7
8
9
10
11
Meishan−A
Meishan−B
Meishan−C
European−A
European−B
European−C

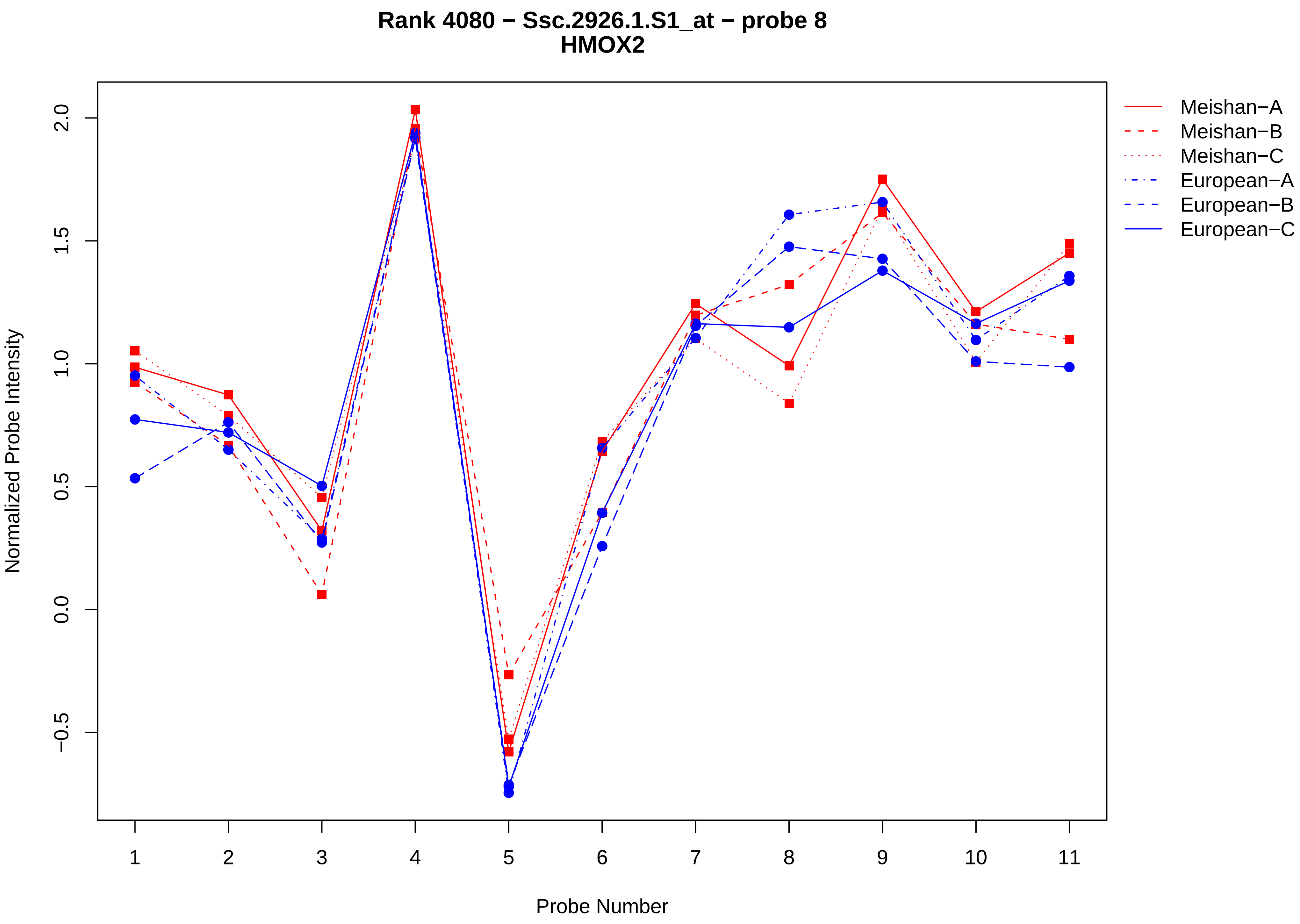

Rank 4080 − Ssc.2926.1.S1_at − probe 8
HMOX2
Normalized Probe Intensity
−0.5
0.0
0.5
1.0
1.5
2.0
Probe Number
1
2
3
4
5
6
7
8
9
10
11
Meishan−A
Meishan−B
Meishan−C
European−A
European−B
European−C

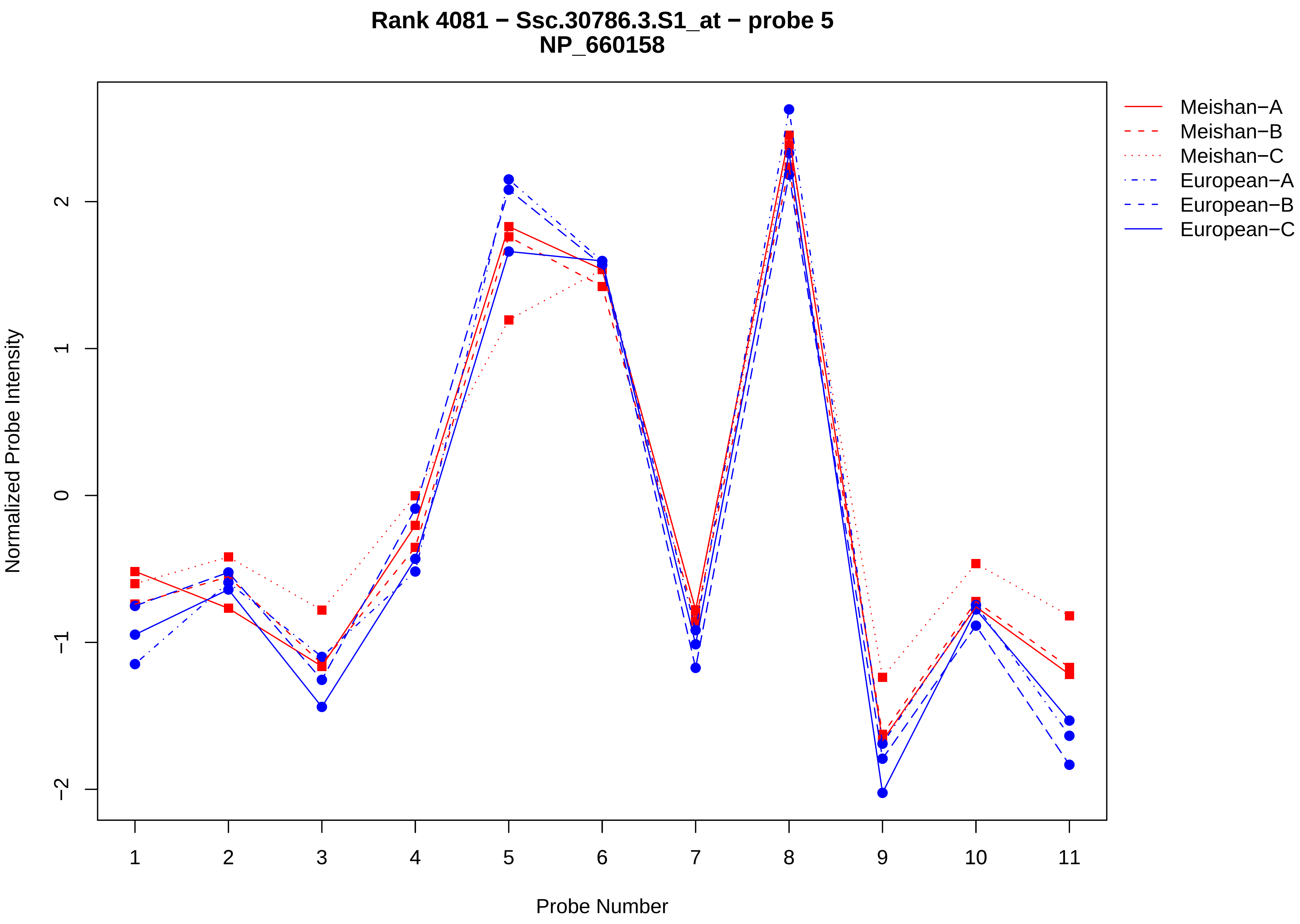

Rank 4081 − Ssc.30786.3.S1_at − probe 5
NP_660158
Normalized Probe Intensity
Probe Number
Meishan−A
Meishan−B
Meishan−C
European−A
European−B
European−C
−2
−1
0
1
2
1
2
3
4
5
6
7
8
9
10
11

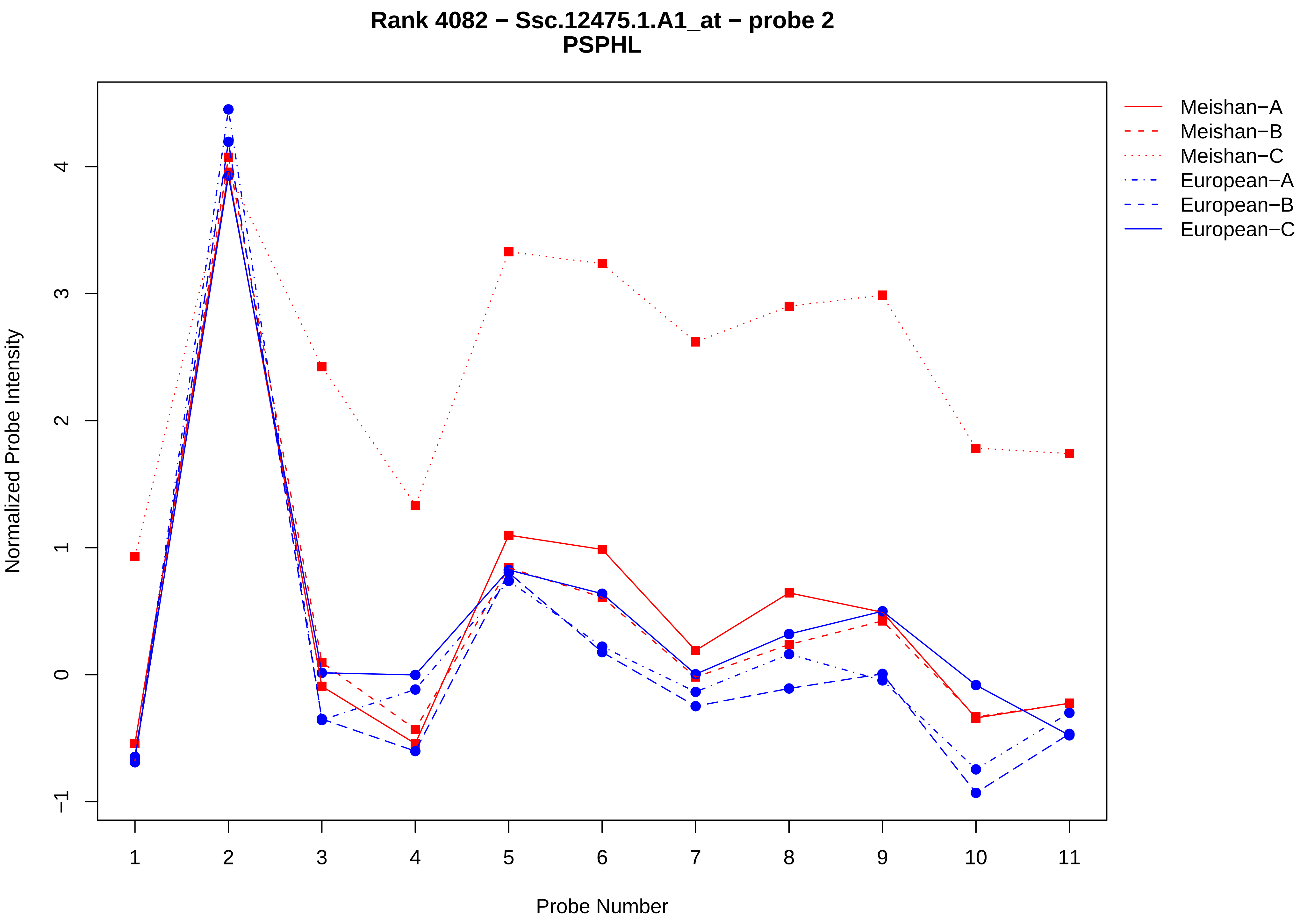

Rank 4082 − Ssc.12475.1.A1_at − probe 2
PSPHL
Normalized Probe Intensity
Probe Number
Meishan−A
Meishan−B
Meishan−C
European−A
European−B
European−C

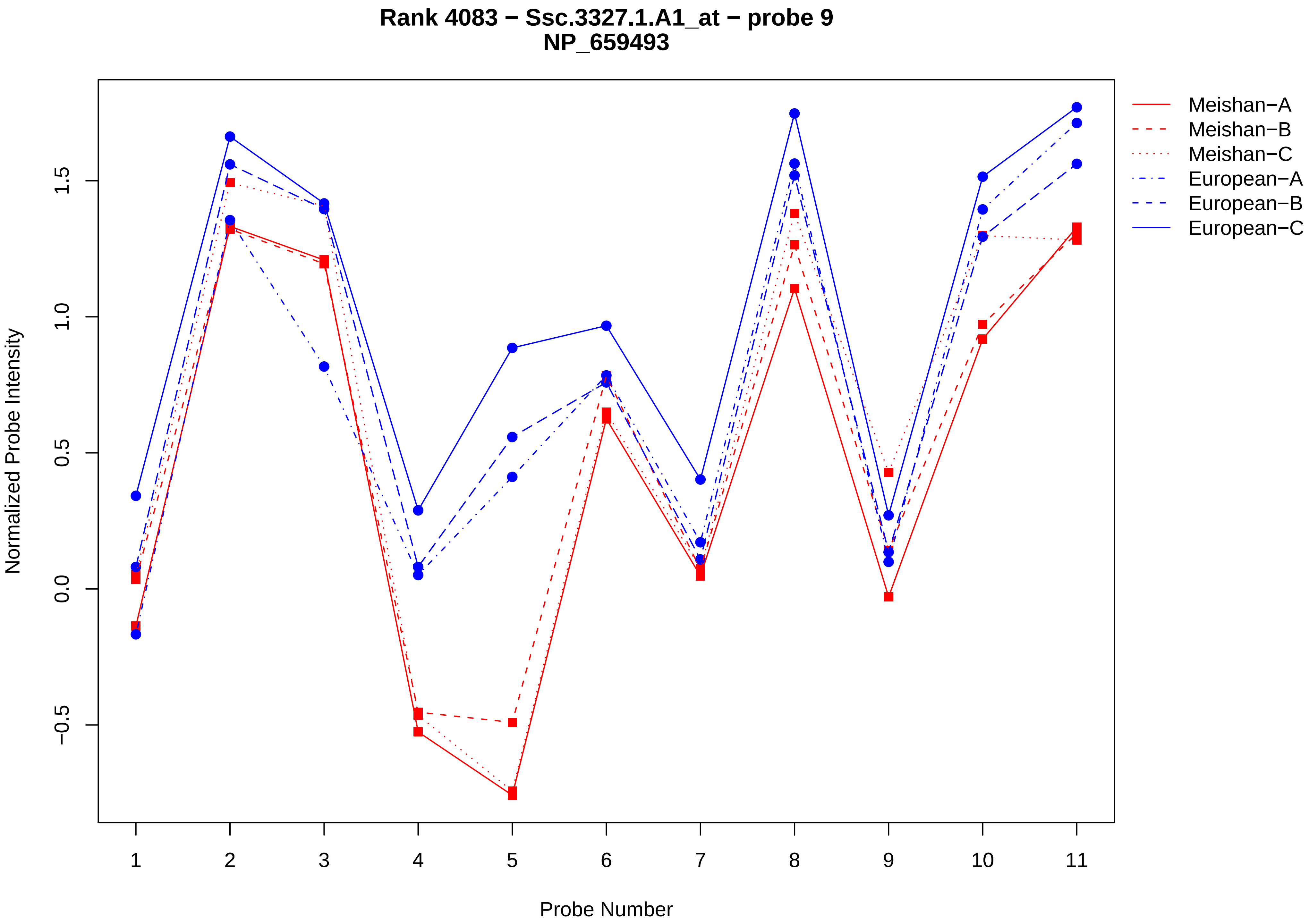

Rank 4083 − Ssc.3327.1.A1_at − probe 9
NP_659493
Normalized Probe Intensity
Probe Number
−0.5
0.0
0.5
1.0
1.5
1
2
3
4
5
6
7
8
9
10
11
Meishan−A
Meishan−B
Meishan−C
European−A
European−B
European−C

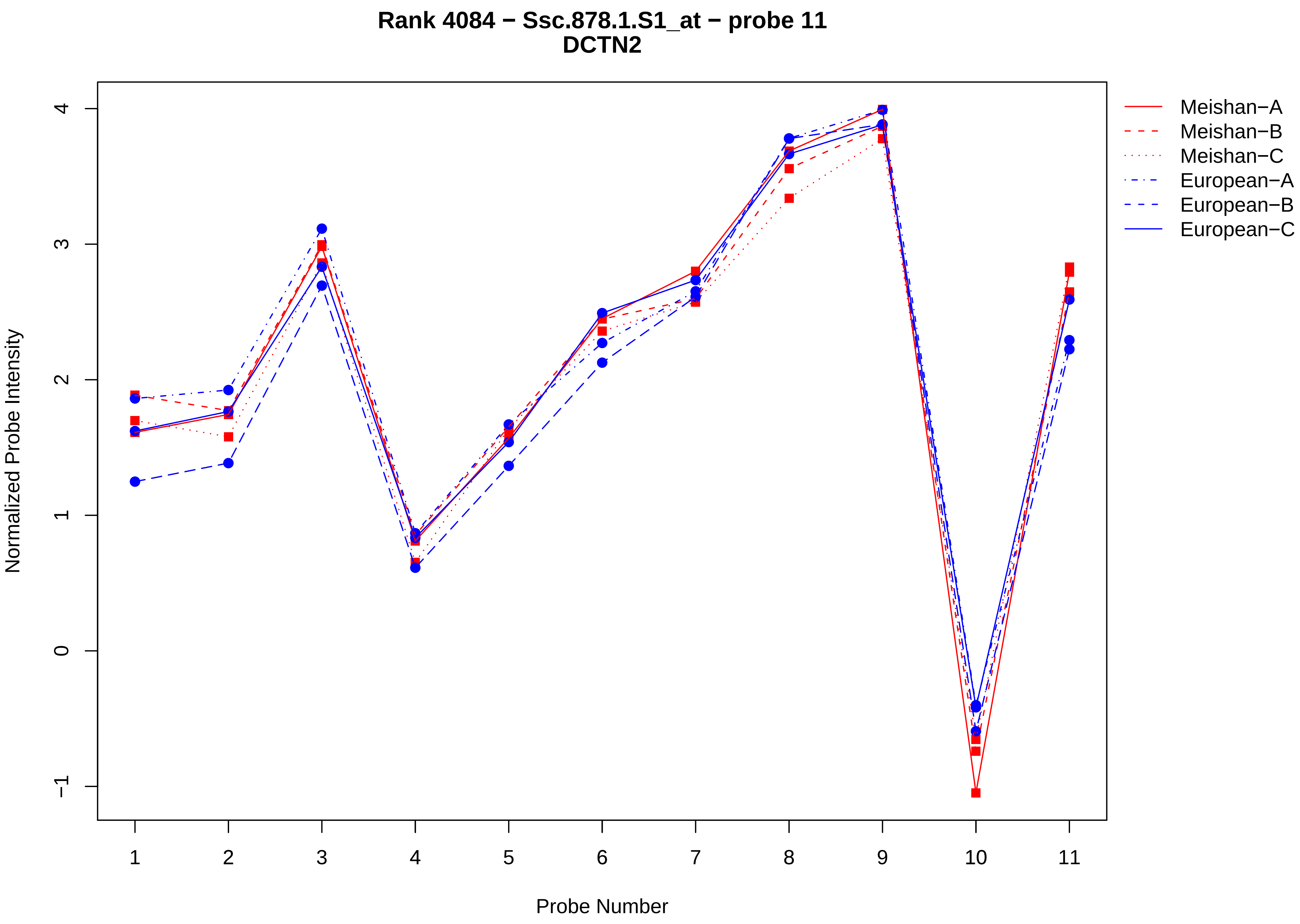
Rank 4084 − Ssc.878.1.S1_at − probe 11
DCTN2
Normalized Probe Intensity
Probe Number
−1
0
1
2
3
4
1
2
3
4
5
6
7
8
9
10
11
Meishan−A
Meishan−B
Meishan−C
European−A
European−B
European−C

Rank 4085 − Ssc.21637.1.S1_at − probe 6
DPYD

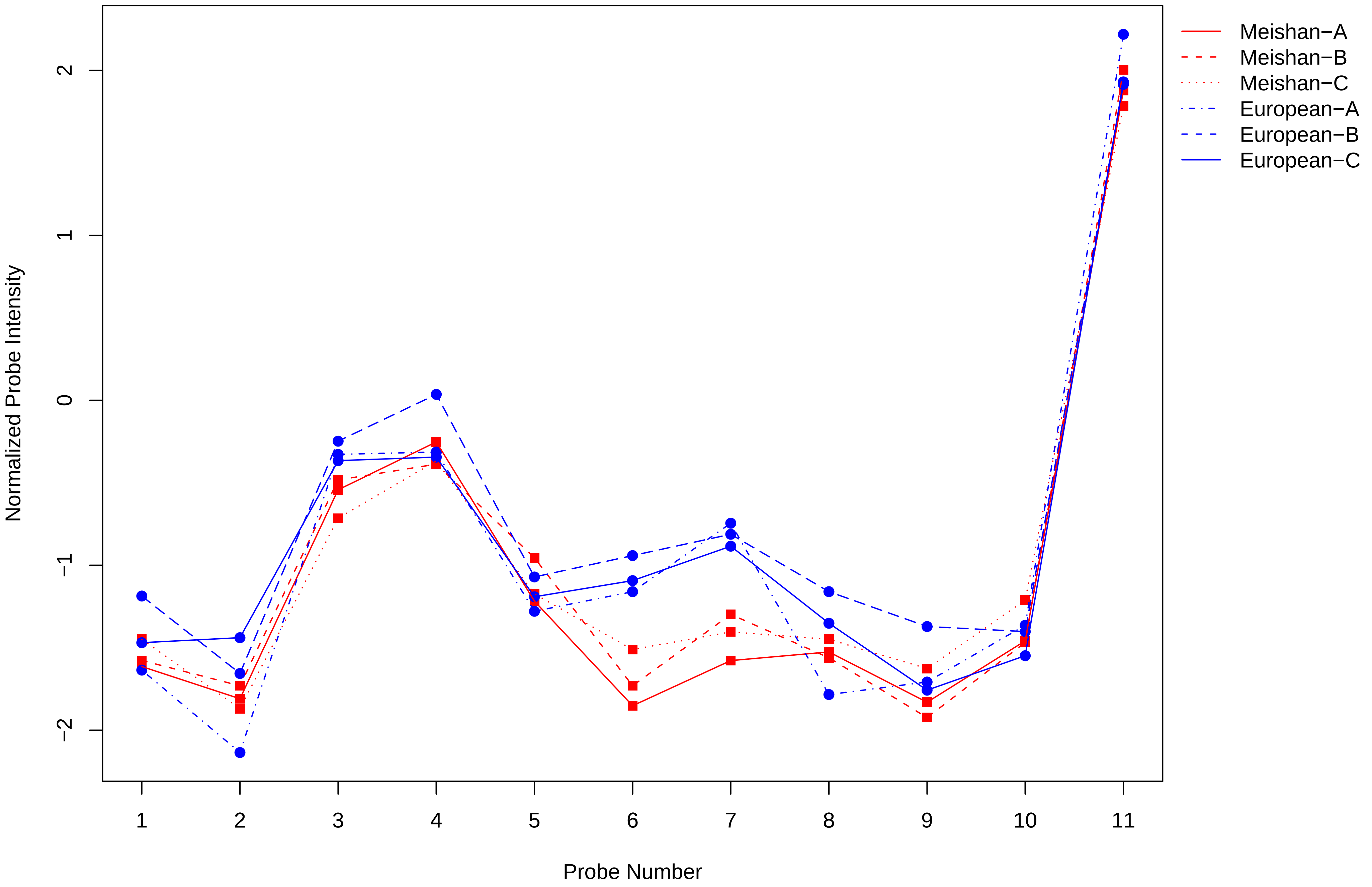

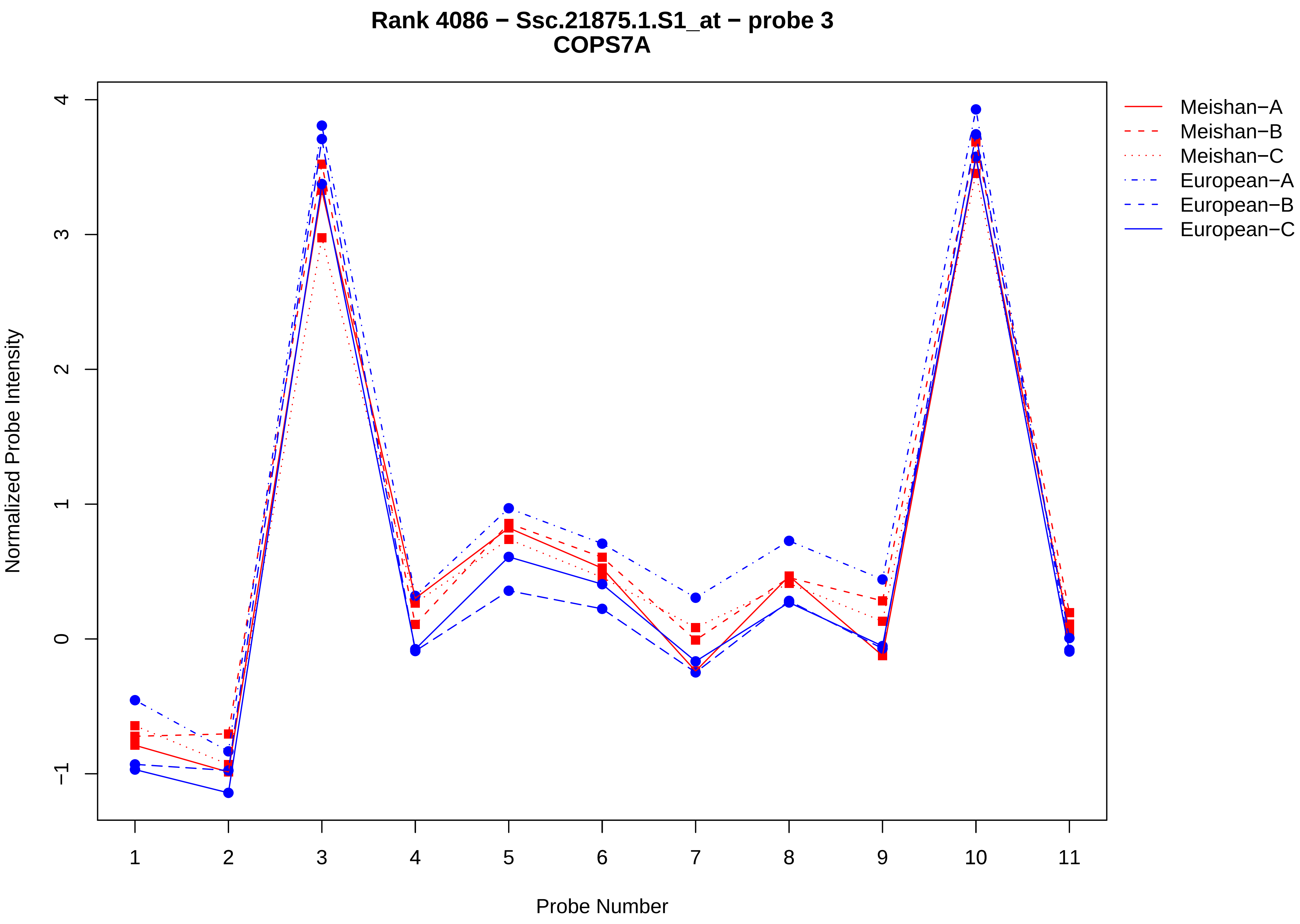
Rank 4086 − Ssc.21875.1.S1_at − probe 3
COPS7A
Normalized Probe Intensity
Probe Number
Meishan−A
Meishan−B
Meishan−C
European−A
European−B
European−C
−1
0
1
2
3
4
1
2
3
4
5
6
7
8
9
10
11

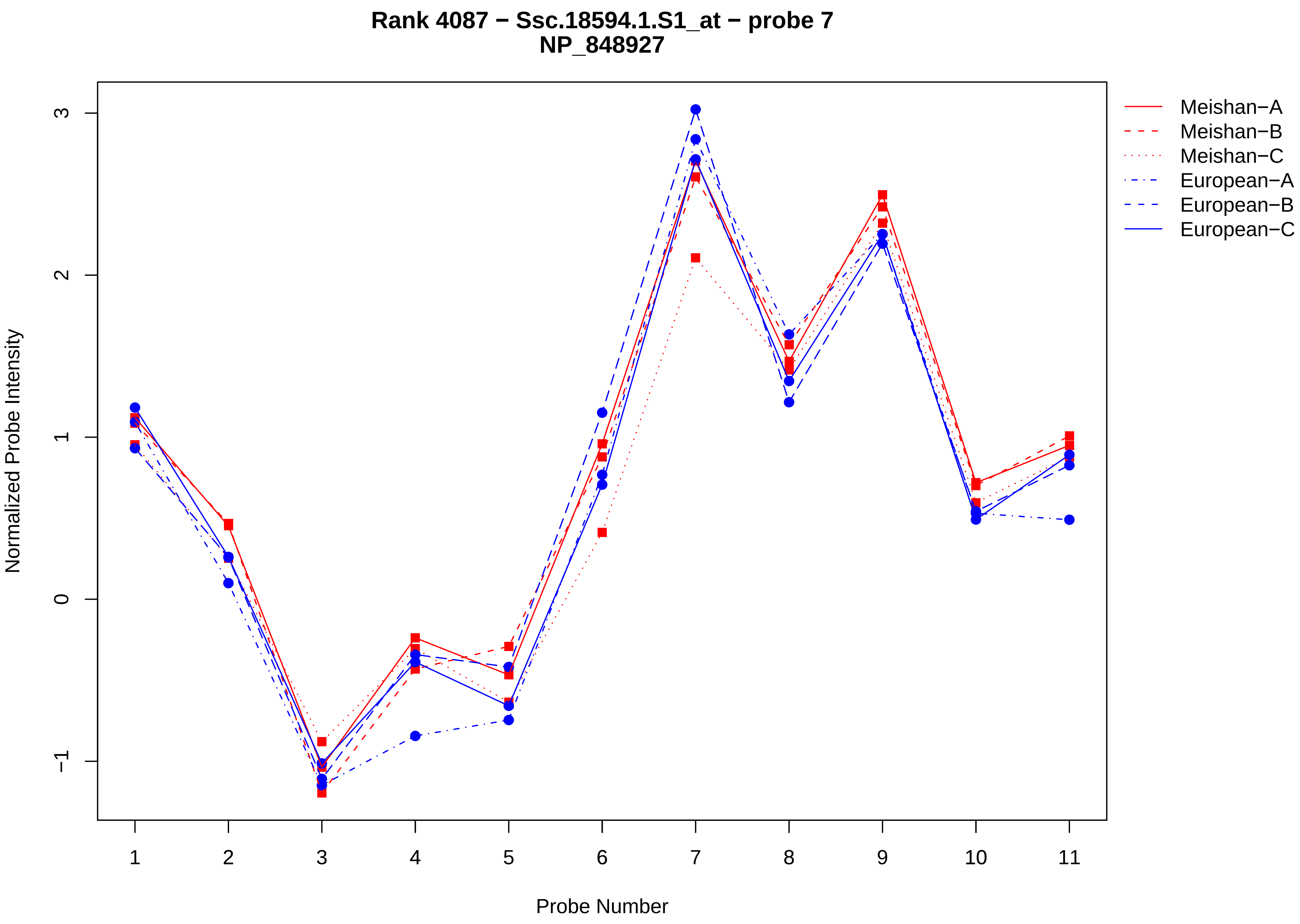

Rank 4087 − Ssc.18594.1.S1_at − probe 7
NP_848927
Normalized Probe Intensity
Probe Number
−1
0
1
2
3
1
2
3
4
5
6
7
8
9
10
11
Meishan−A
Meishan−B
Meishan−C
European−A
European−B
European−C

Rank 4088 − Ssc.4814.1.A1_at − probe 6
LRRC3B

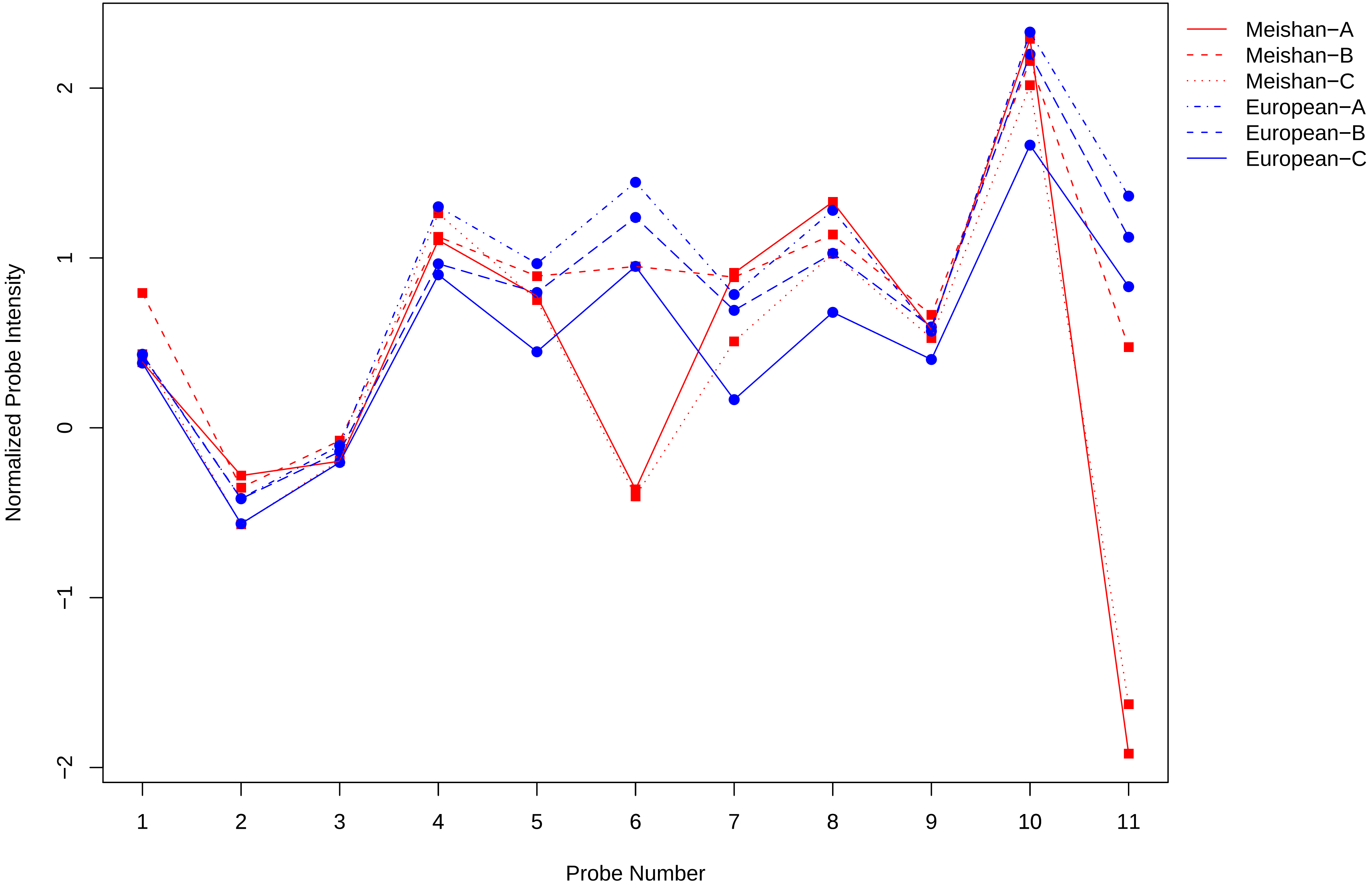

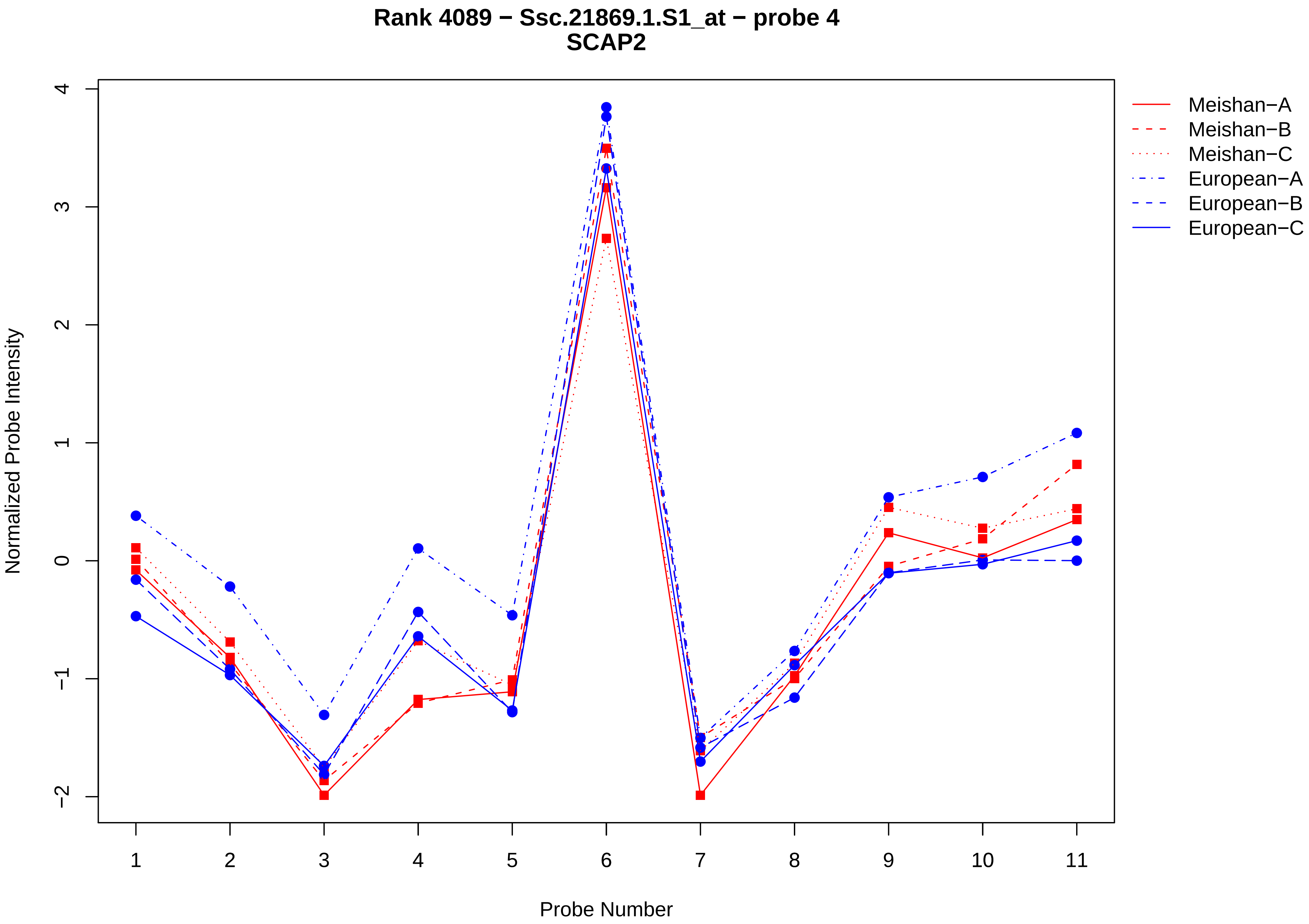

Rank 4089 − Ssc.21869.1.S1_at − probe 4
SCAP2
Normalized Probe Intensity
Probe Number
Meishan−A
Meishan−B
Meishan−C
European−A
European−B
European−C

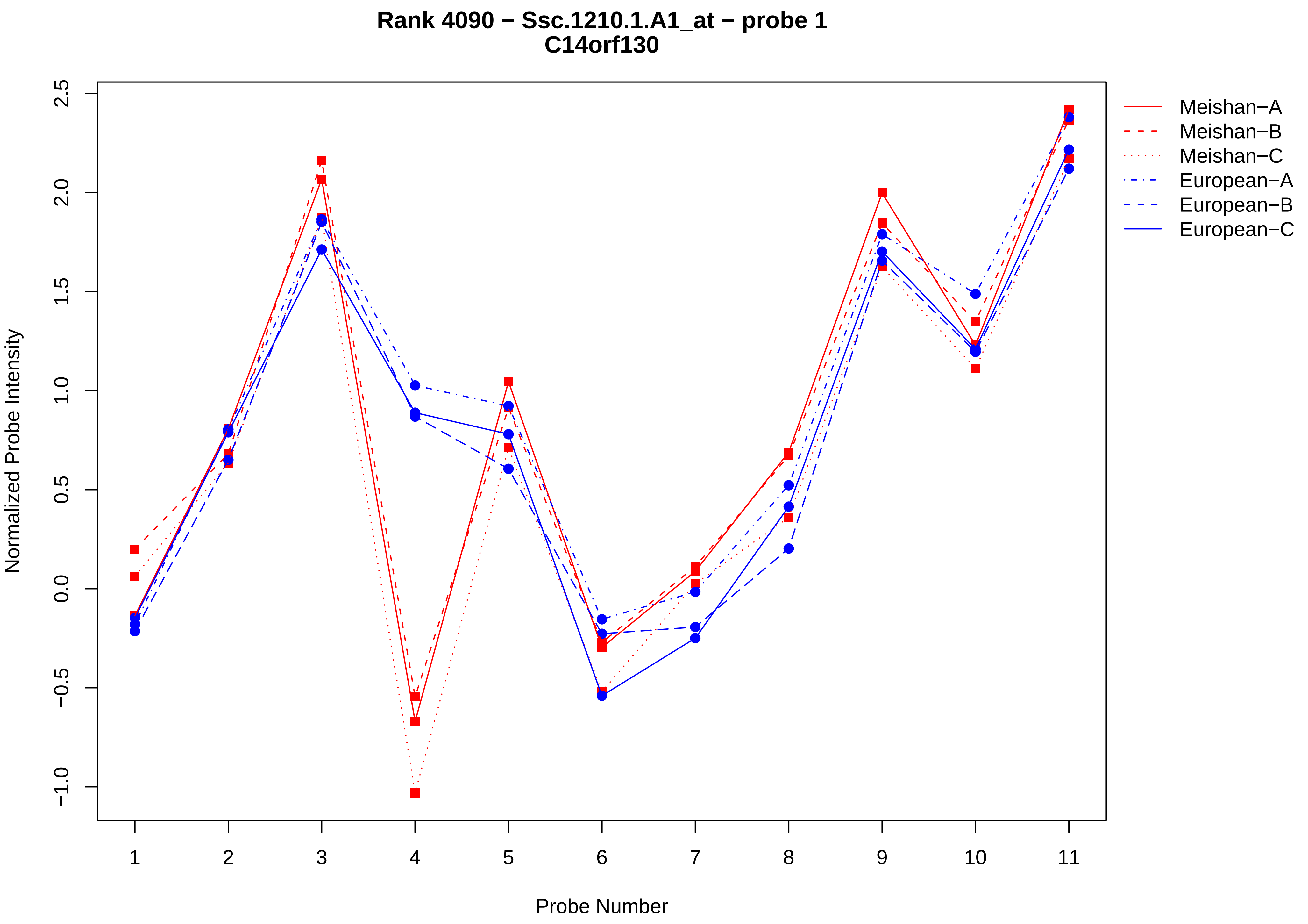

Rank 4090 − Ssc.1210.1.A1_at − probe 1
C14orf130
Normalized Probe Intensity
Probe Number
−1.0
−0.5
0.0
0.5
1.0
1.5
2.0
2.5
1
2
3
4
5
6
7
8
9
10
11
Meishan−A
Meishan−B
Meishan−C
European−A
European−B
European−C

Rank 4091 − Ssc.24549.1.S1_at − probe 2
LIG1

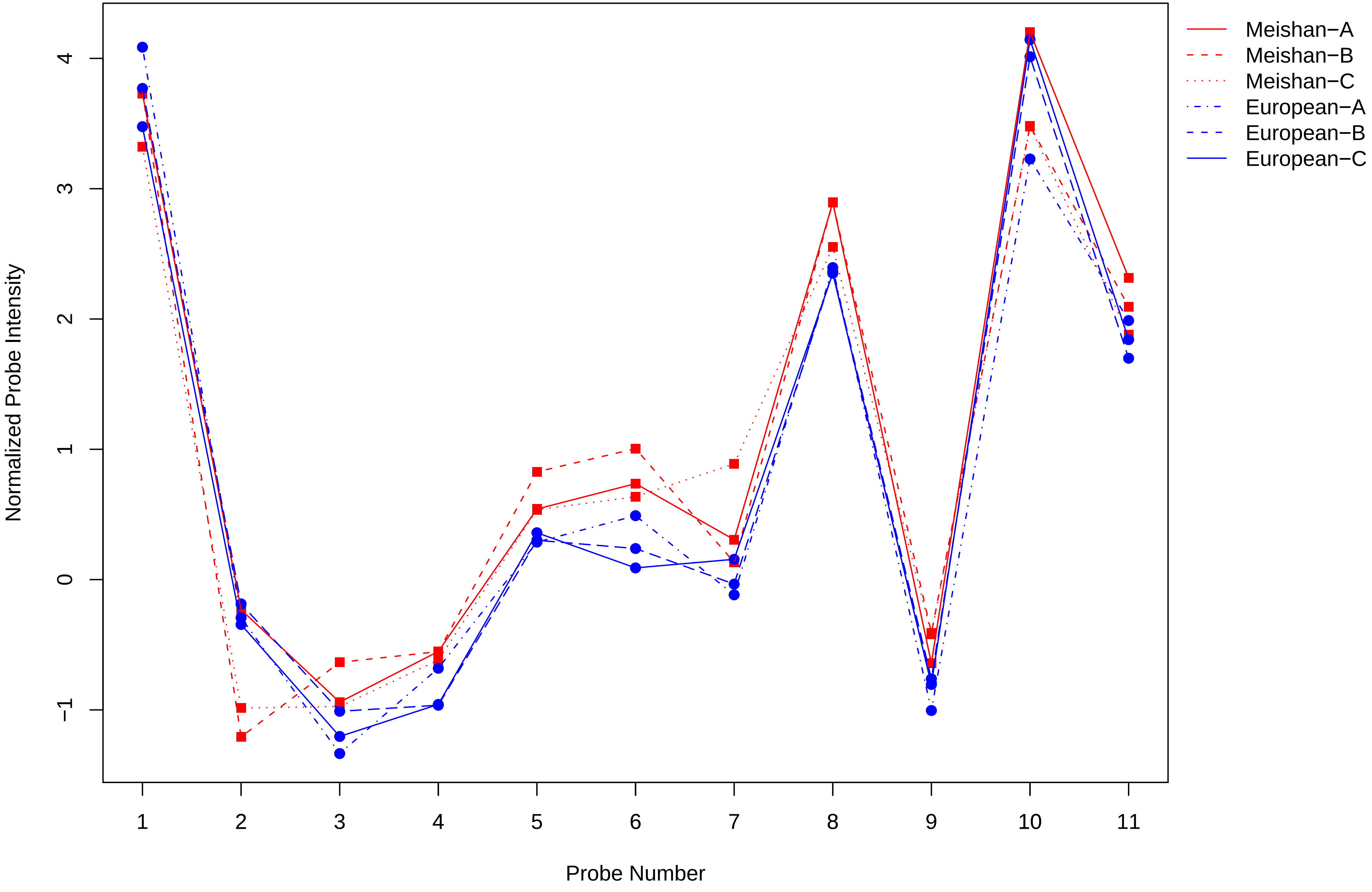

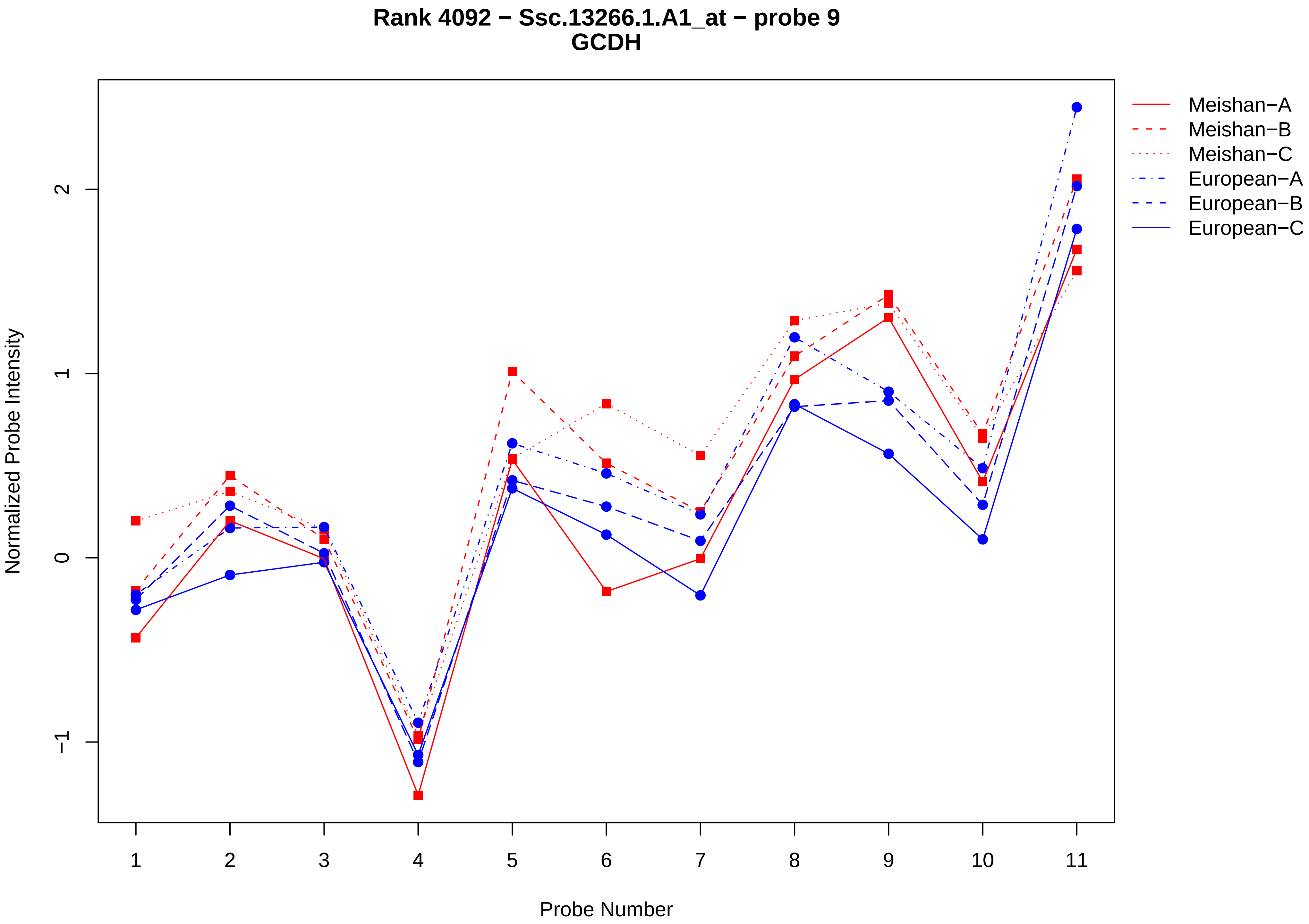

Rank 4092 − Ssc.13266.1.A1_at − probe 9
GCDH
Normalized Probe Intensity
−1
0
1
2
1
2
3
4
5
6
7
8
9
10
11
Probe Number
Meishan−A
Meishan−B
Meishan−C
European−A
European−B
European−C

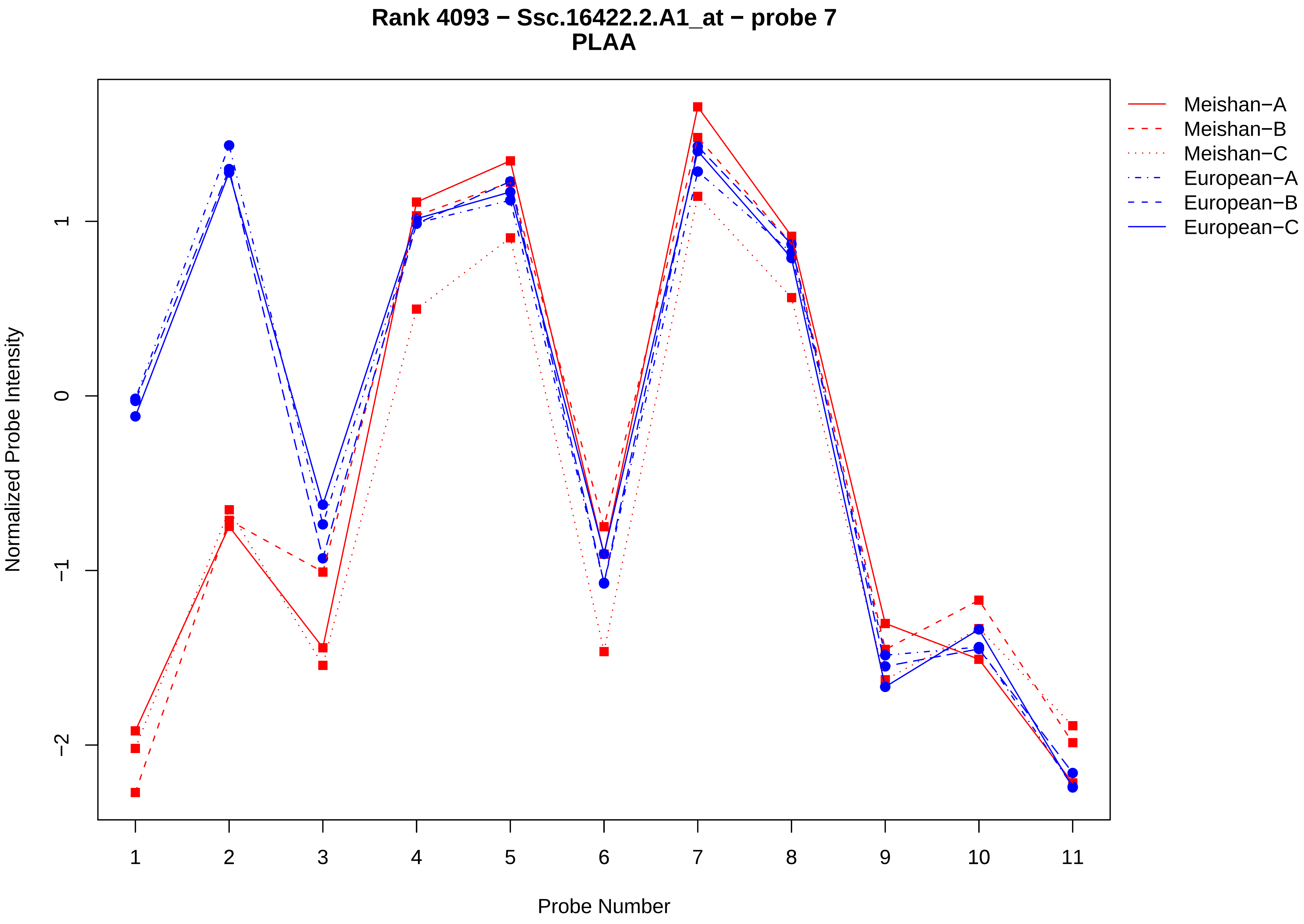

Rank 4093 − Ssc.16422.2.A1_at − probe 7
PLAA
Normalized Probe Intensity
Probe Number
−2
−1
0
1
1
2
3
4
5
6
7
8
9
10
11
Meishan−A
Meishan−B
Meishan−C
European−A
European−B
European−C

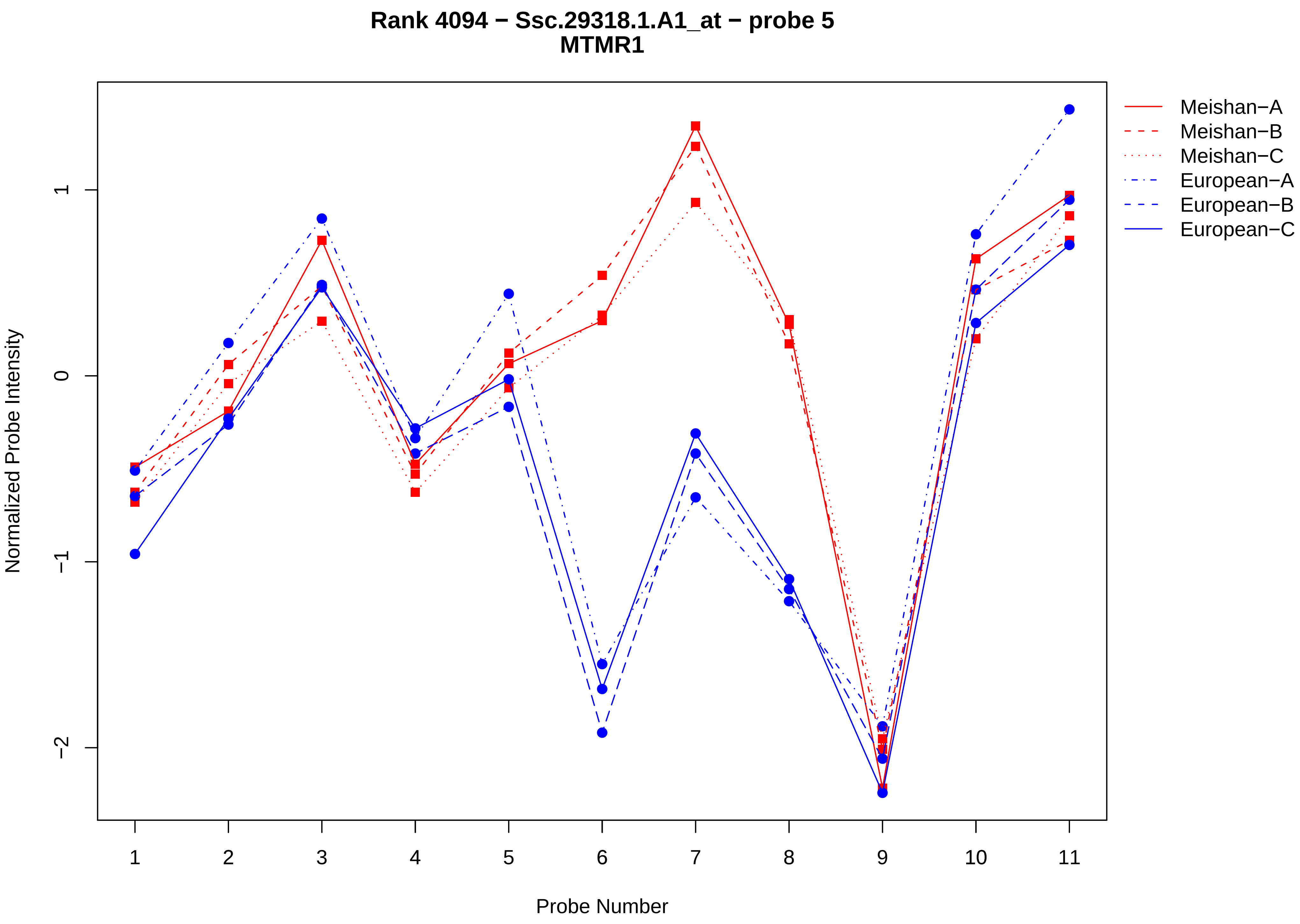

Rank 4094 − Ssc.29318.1.A1_at − probe 5
MTMR1
Normalized Probe Intensity
−2
−1
0
1
1
2
3
4
5
6
7
8
9
10
11
Probe Number
Meishan−A
Meishan−B
Meishan−C
European−A
European−B
European−C

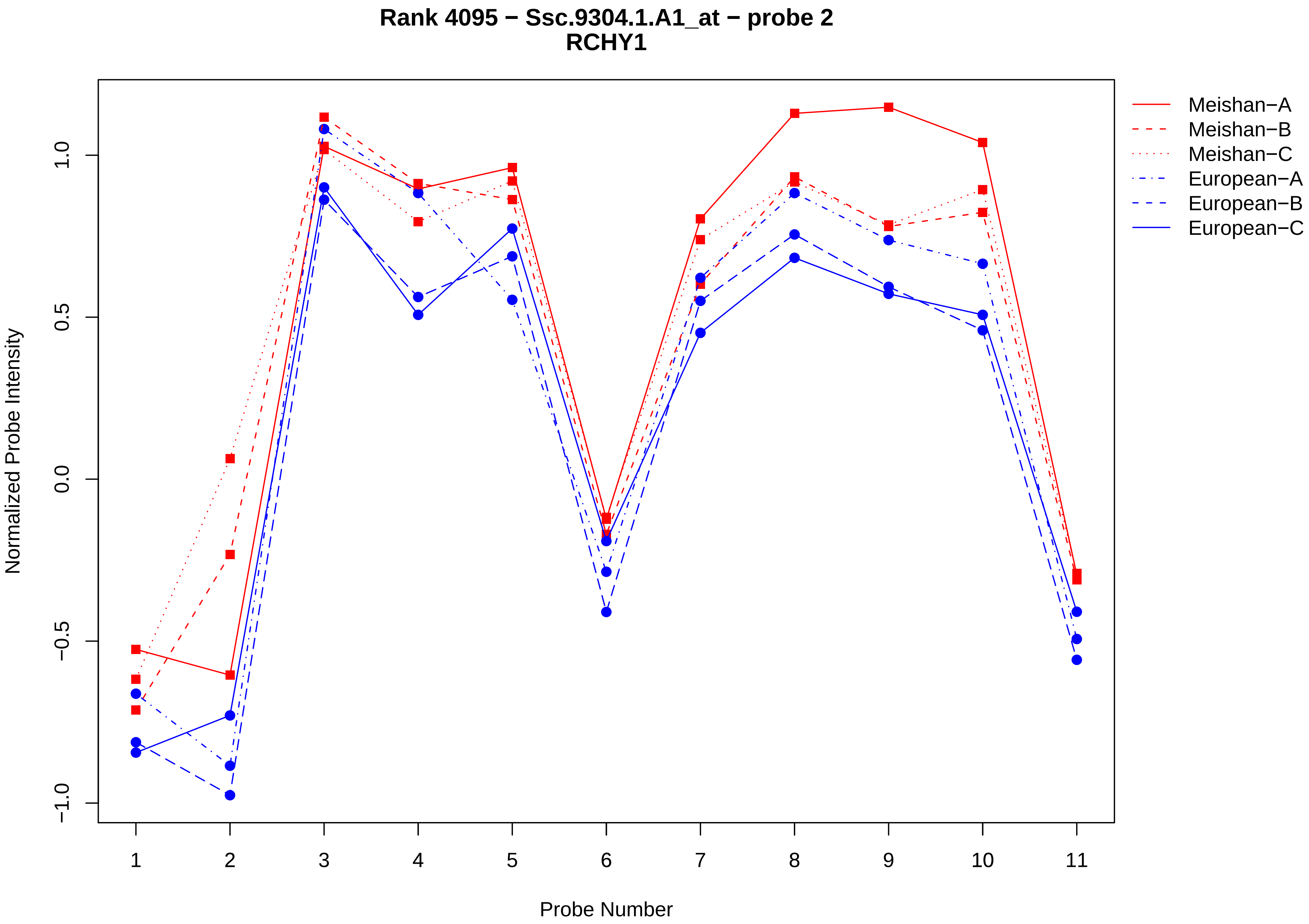
Rank 4095 − Ssc.9304.1.A1_at − probe 2
RCHY1
Normalized Probe Intensity
Probe Number
−1.0
−0.5
0.0
0.5
1.0
1
2
3
4
5
6
7
8
9
10
11
Meishan−A
Meishan−B
Meishan−C
European−A
European−B
European−C

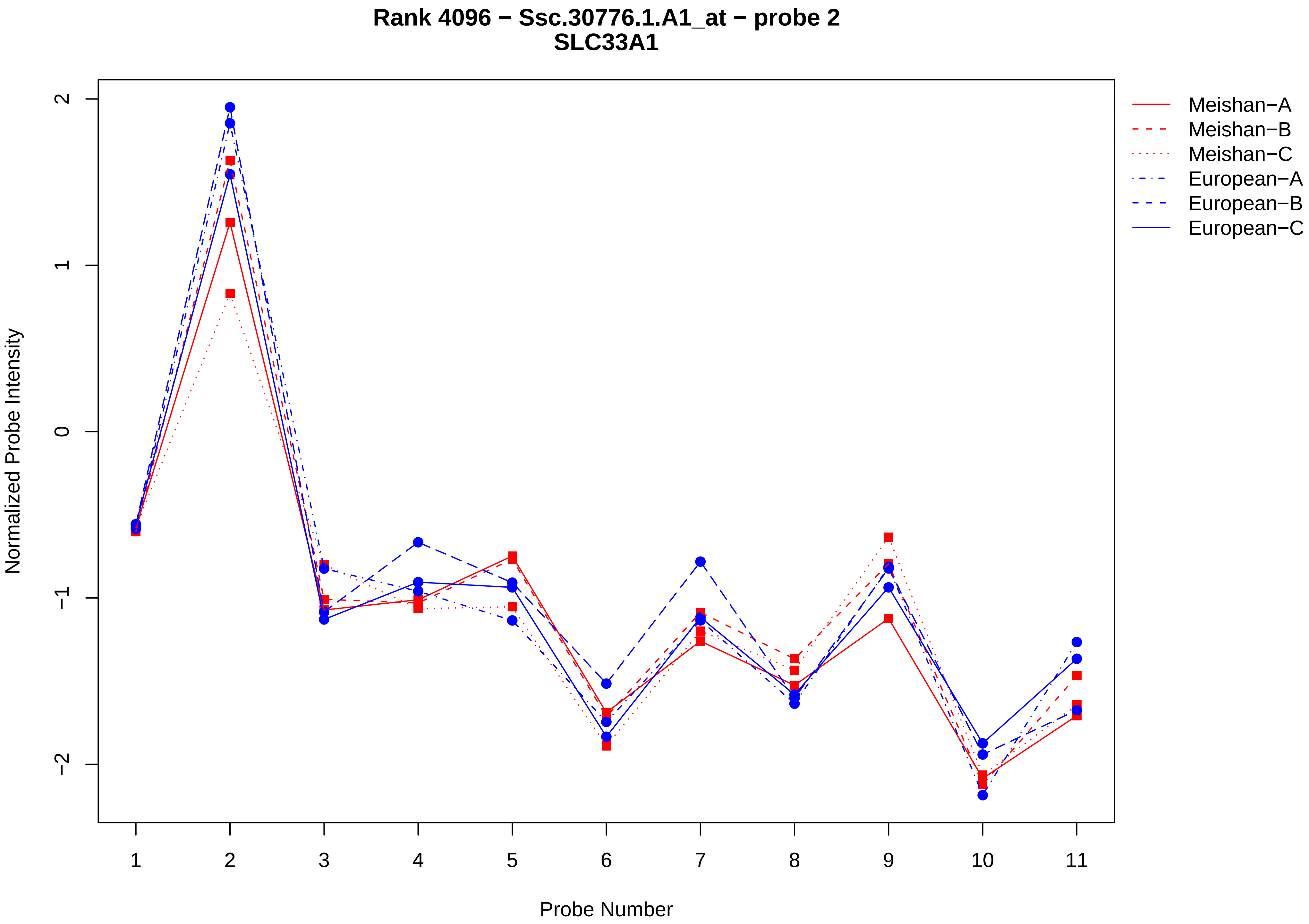

Rank 4096 − Ssc.30776.1.A1_at − probe 2
SLC33A1
Normalized Probe Intensity
Probe Number
−2
−1
0
1
2
1
2
3
4
5
6
7
8
9
10
11
Meishan−A
Meishan−B
Meishan−C
European−A
European−B
European−C

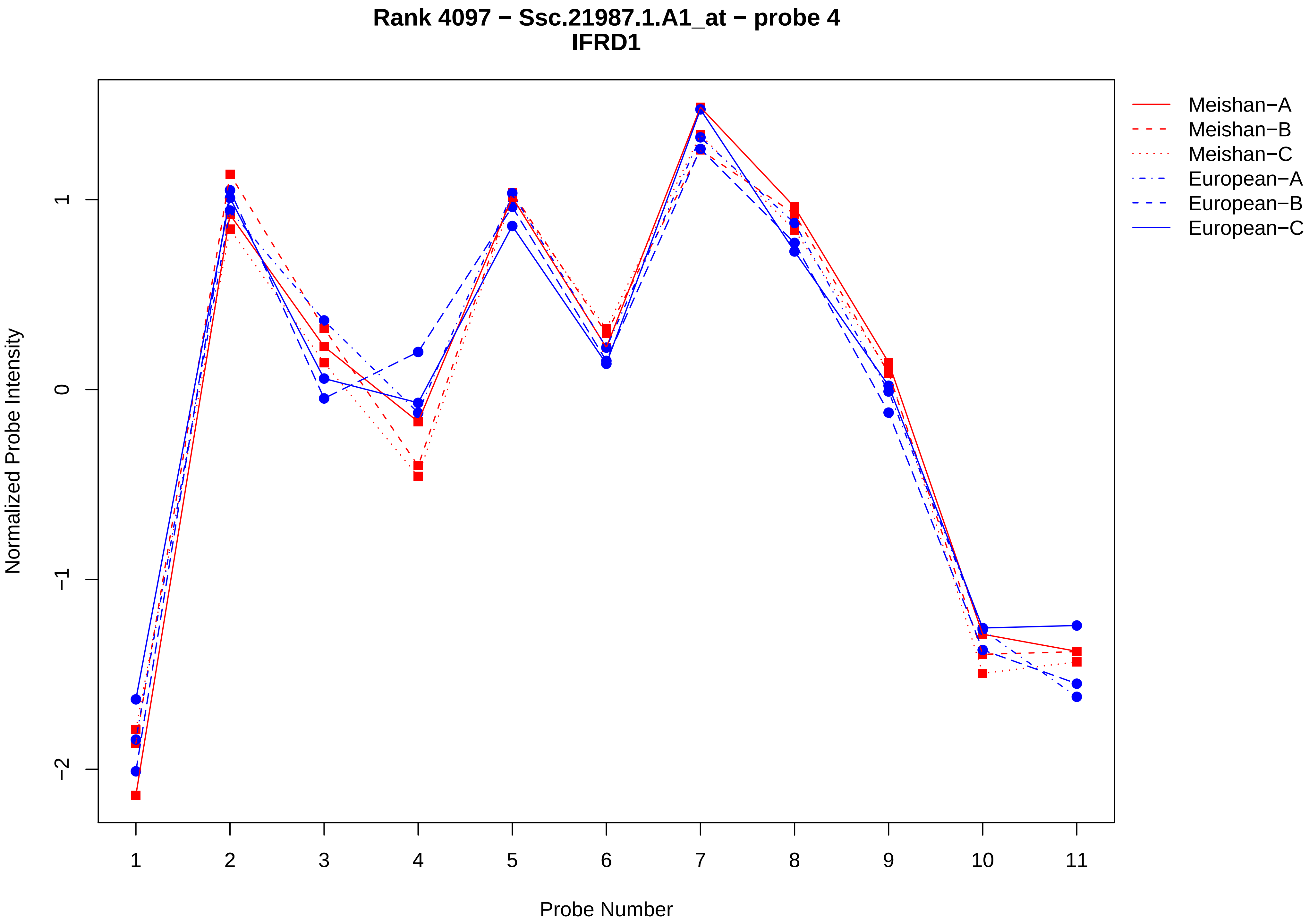

Rank 4097 − Ssc.21987.1.A1_at − probe 4
IFRD1
Normalized Probe Intensity
Probe Number
Meishan−A
Meishan−B
Meishan−C
European−A
European−B
European−C

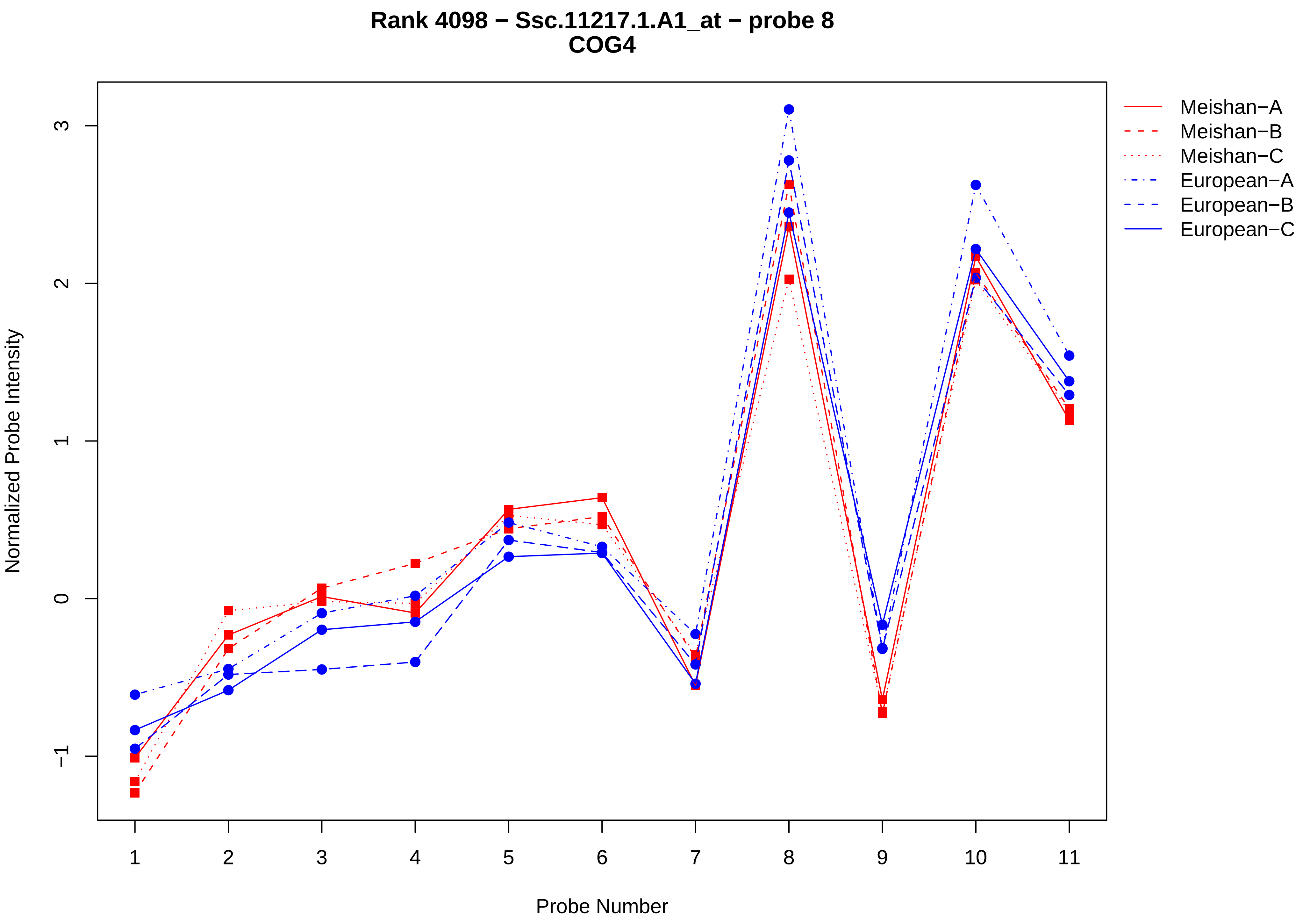

Rank 4098 − Ssc.11217.1.A1_at − probe 8
COG4
Normalized Probe Intensity
Probe Number
−1
0
1
2
3
1
2
3
4
5
6
7
8
9
10
11
Meishan−A
Meishan−B
Meishan−C
European−A
European−B
European−C

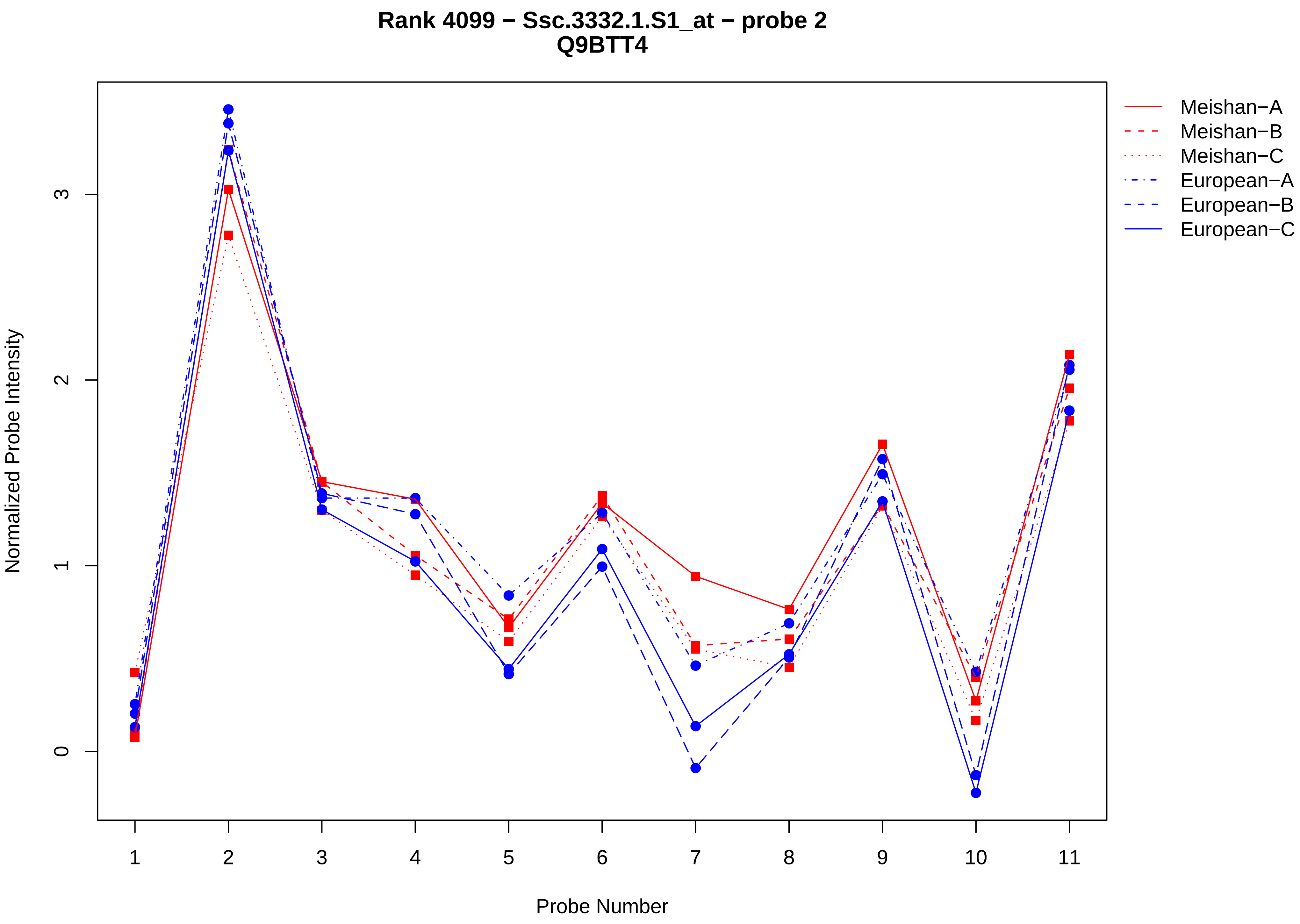
Rank 4099 − Ssc.3332.1.S1_at − probe 2
Q9BTT4
Normalized Probe Intensity
0
1
2
3
1
2
3
4
5
6
7
8
9
10
11
Probe Number
Meishan−A
Meishan−B
Meishan−C
European−A
European−B
European−C

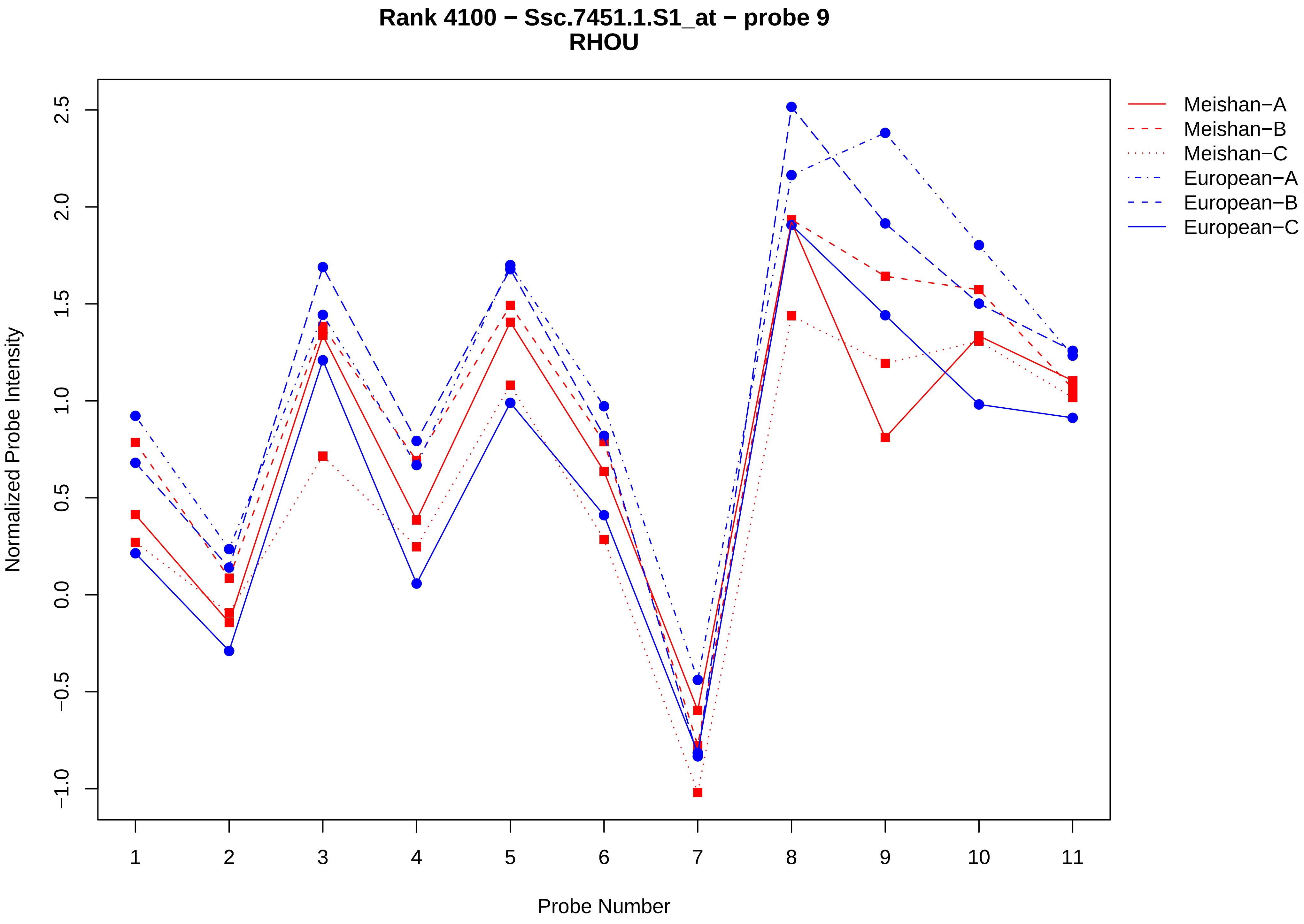

Rank 4100 − Ssc.7451.1.S1_at − probe 9
RHOU
Normalized Probe Intensity
Probe Number
−1.0
−0.5
0.0
0.5
1.0
1.5
2.0
2.5
1
2
3
4
5
6
7
8
9
10
11
Meishan−A
Meishan−B
Meishan−C
European−A
European−B
European−C

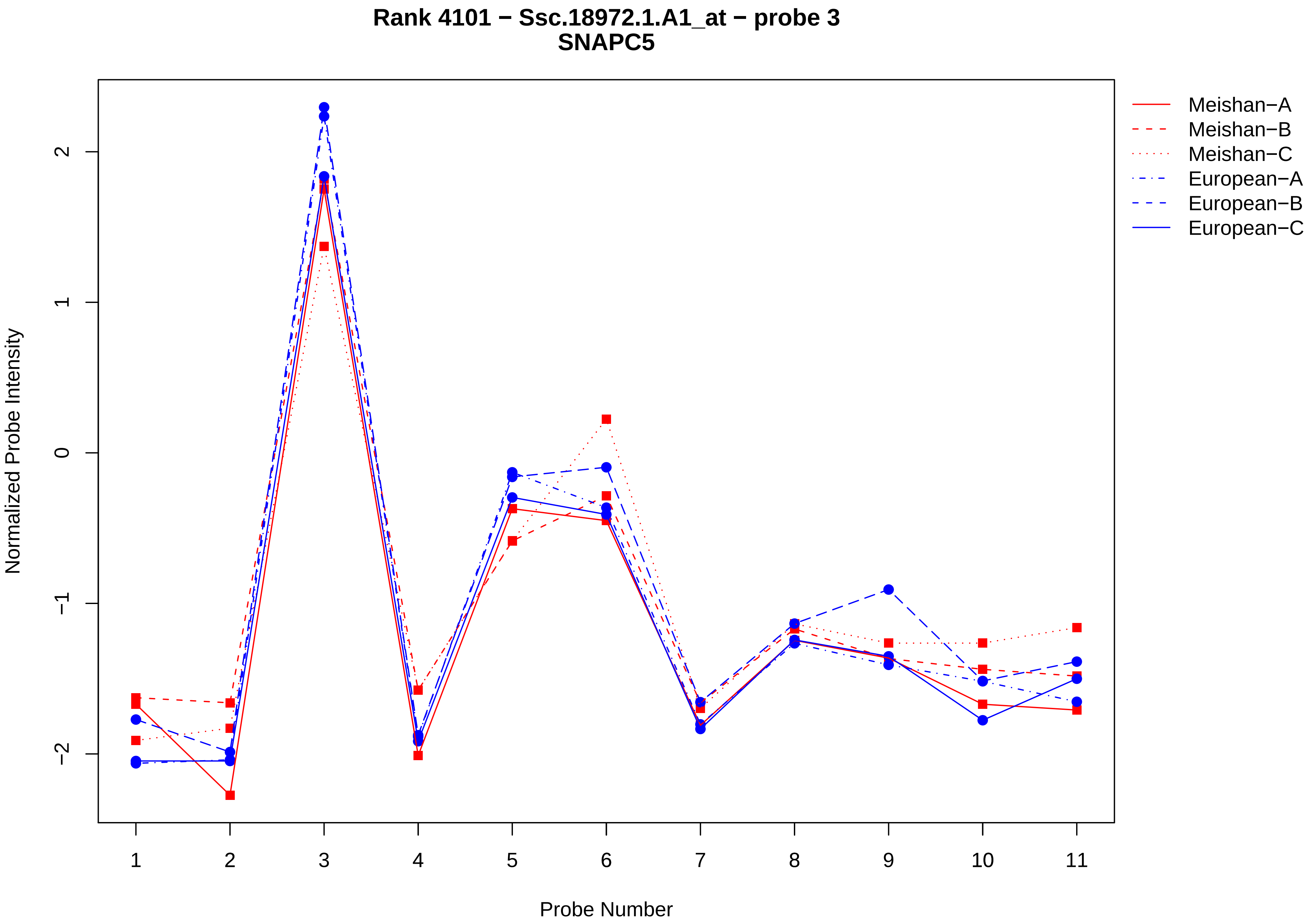

Rank 4101 − Ssc.18972.1.A1_at − probe 3
SNAPC5
Normalized Probe Intensity
Probe Number
−2
−1
0
1
2
1
2
3
4
5
6
7
8
9
10
11
Meishan−A
Meishan−B
Meishan−C
European−A
European−B
European−C

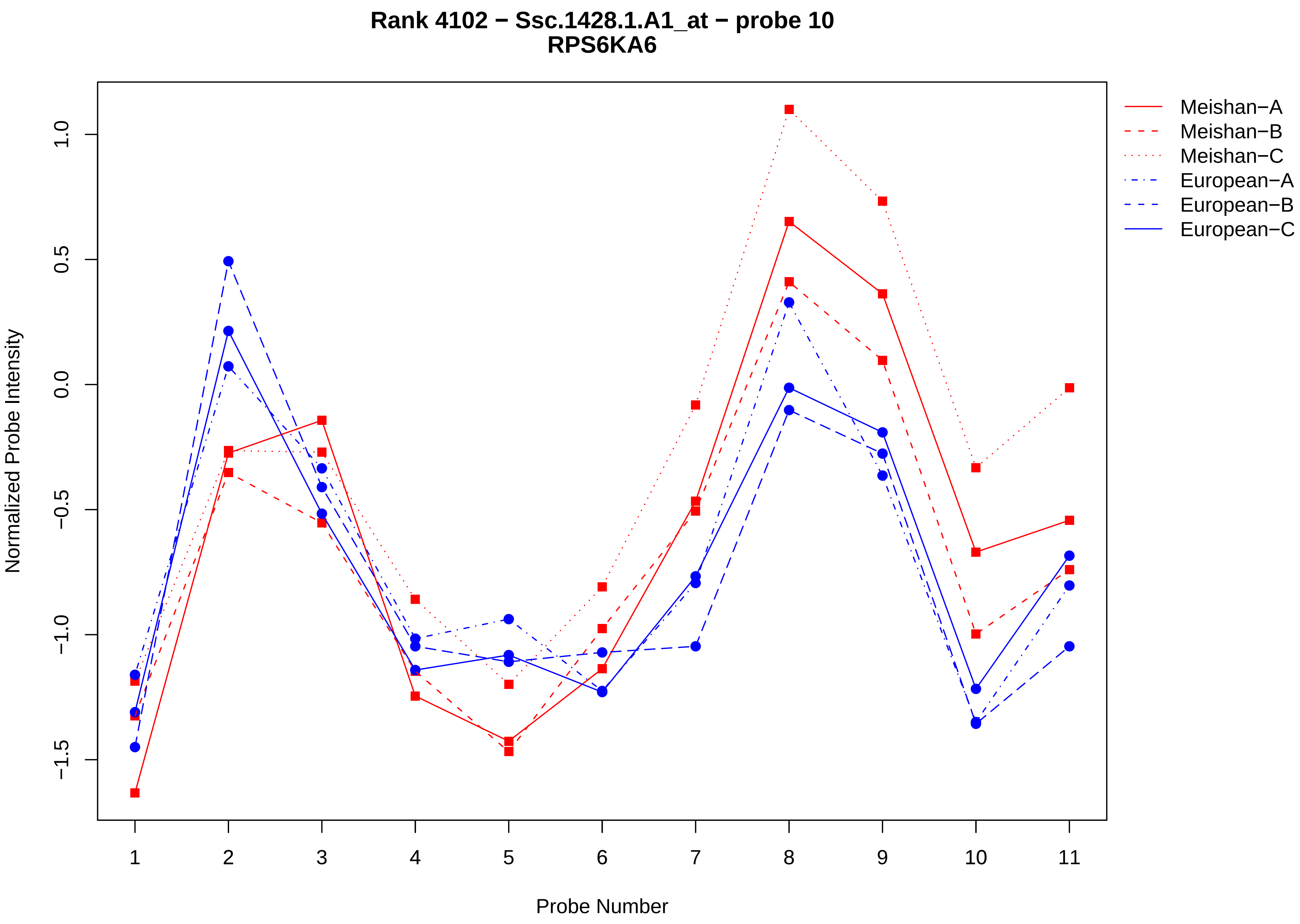

Rank 4102 − Ssc.1428.1.A1_at − probe 10
RPS6KA6
Normalized Probe Intensity
Probe Number
1.0
0.5
0.0
−0.5
−1.0
−1.5
1
2
3
4
5
6
7
8
9
10
11
Meishan−A
Meishan−B
Meishan−C
European−A
European−B
European−C

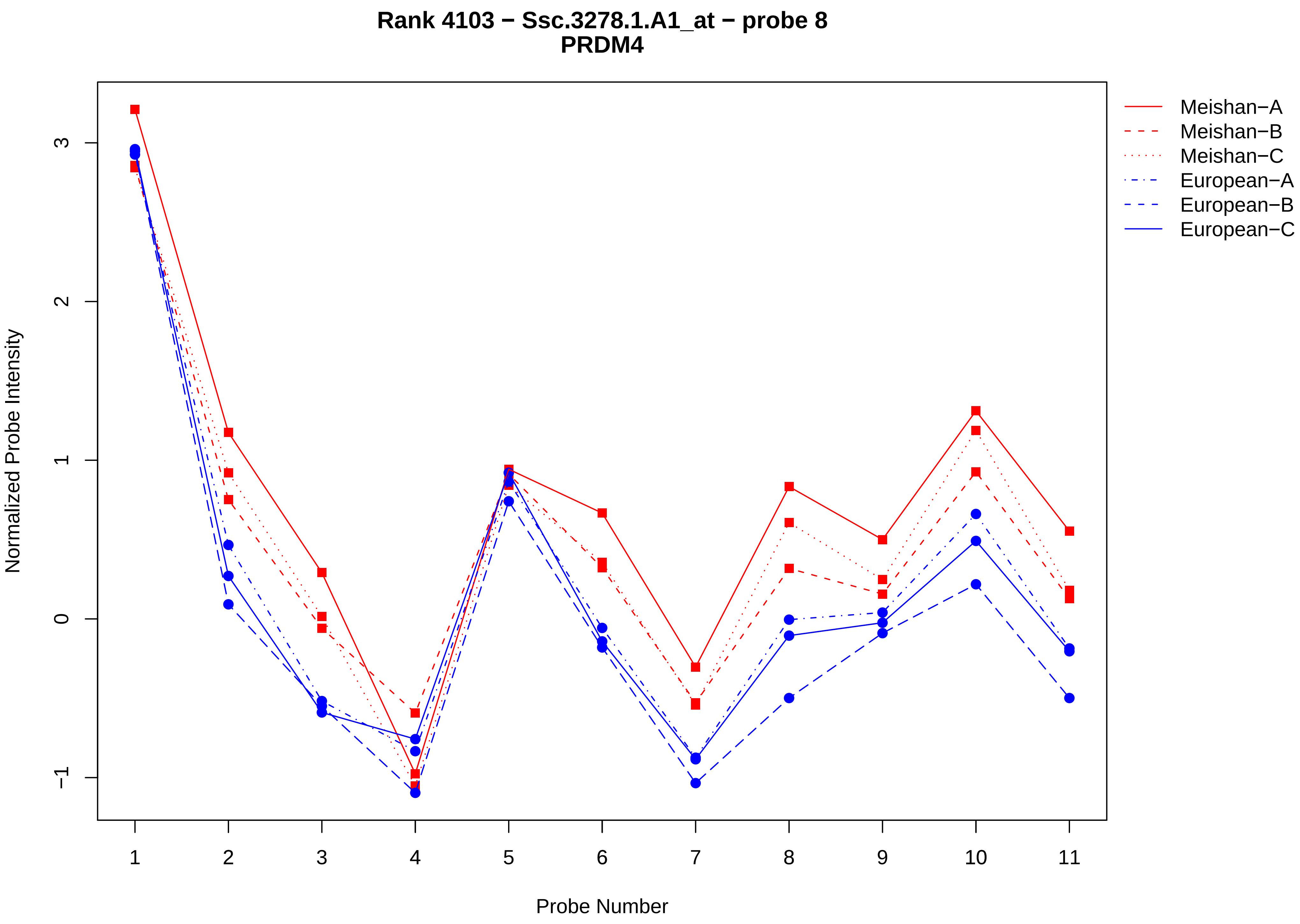
Rank 4103 − Ssc.3278.1.A1_at − probe 8
PRDM4
Normalized Probe Intensity
Probe Number
−1
0
1
2
3
1
2
3
4
5
6
7
8
9
10
11
Meishan−A
Meishan−B
Meishan−C
European−A
European−B
European−C

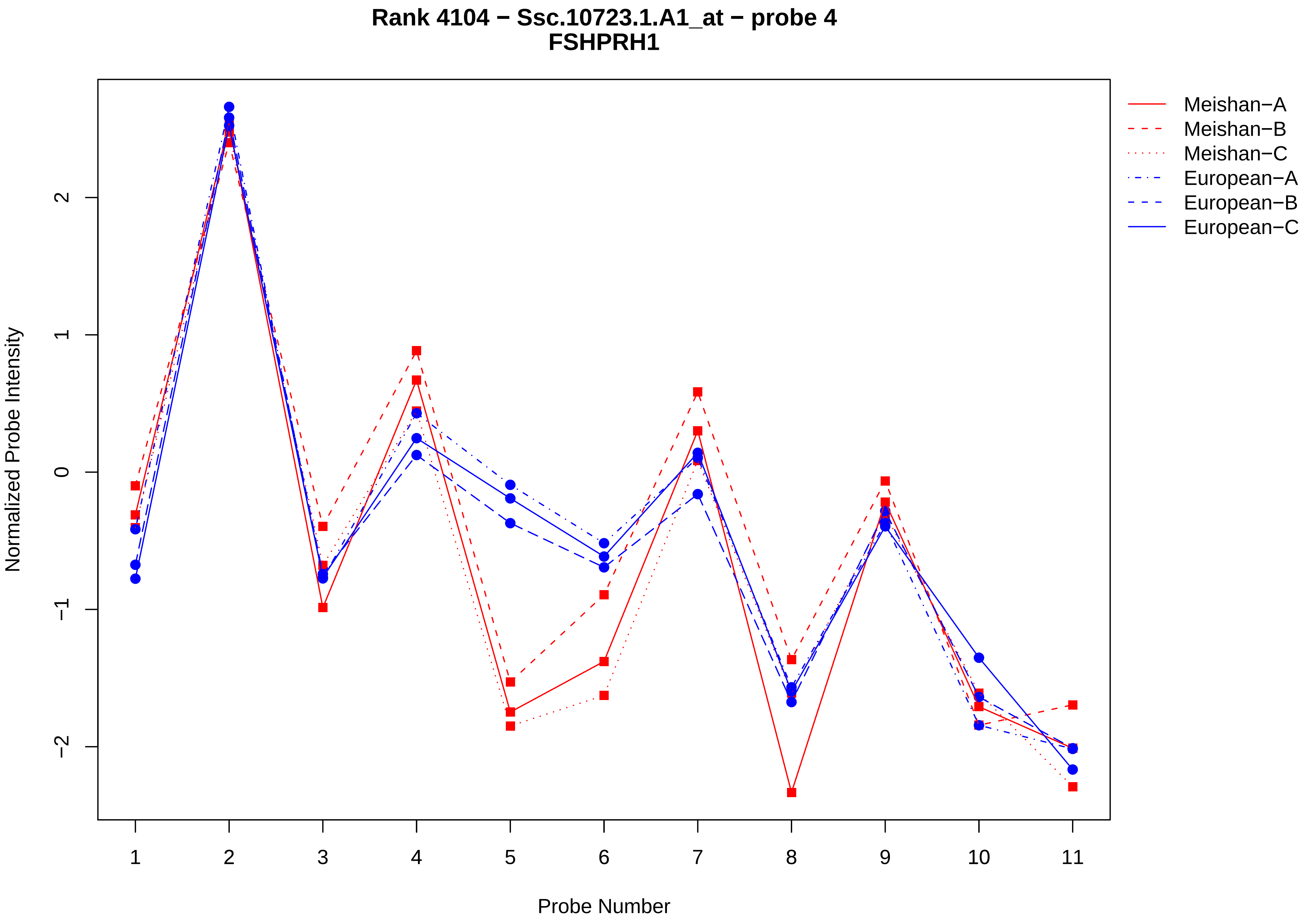

Rank 4104 − Ssc.10723.1.A1_at − probe 4
FSHPRH1
Normalized Probe Intensity
Probe Number
−2
−1
0
1
2
1
2
3
4
5
6
7
8
9
10
11
Meishan−A
Meishan−B
Meishan−C
European−A
European−B
European−C

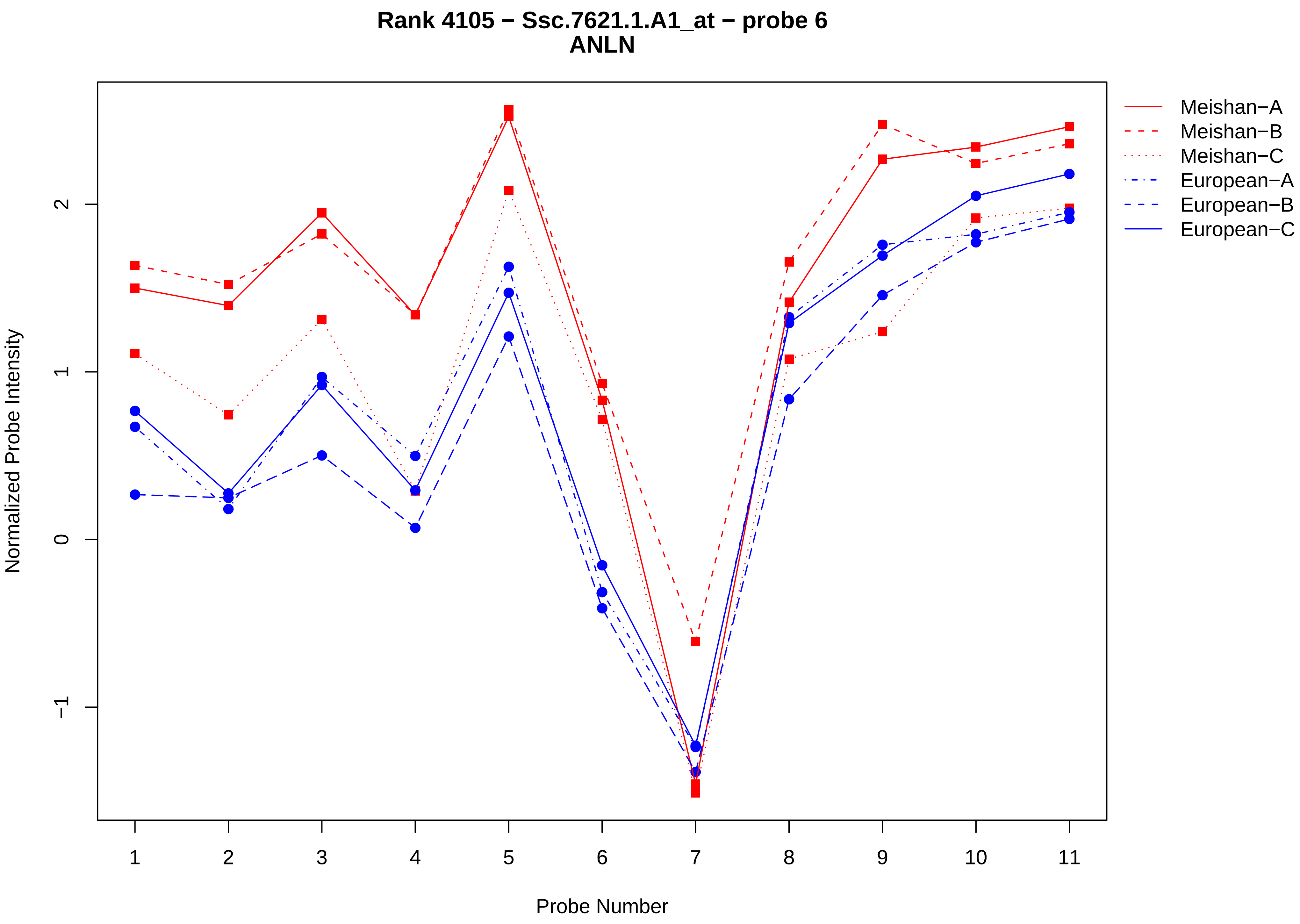

Rank 4105 − Ssc.7621.1.A1_at − probe 6
ANLN
Normalized Probe Intensity
Probe Number
−1
0
1
2
1
2
3
4
5
6
7
8
9
10
11
Meishan−A
Meishan−B
Meishan−C
European−A
European−B
European−C

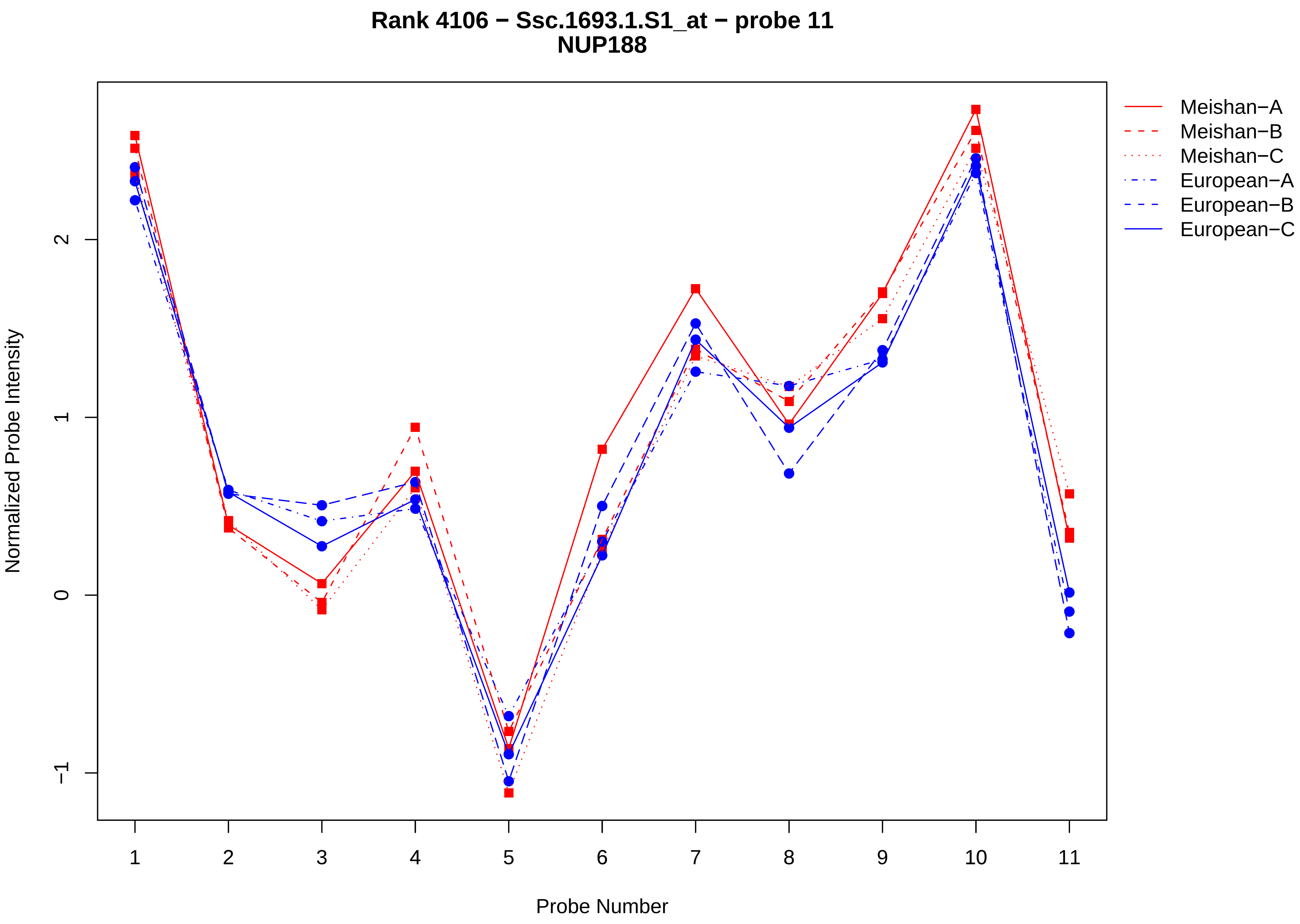

Rank 4106 − Ssc.1693.1.S1_at − probe 11
NUP188
Normalized Probe Intensity
Probe Number
−1
0
1
2
1
2
3
4
5
6
7
8
9
10
11
Meishan−A
Meishan−B
Meishan−C
European−A
European−B
European−C

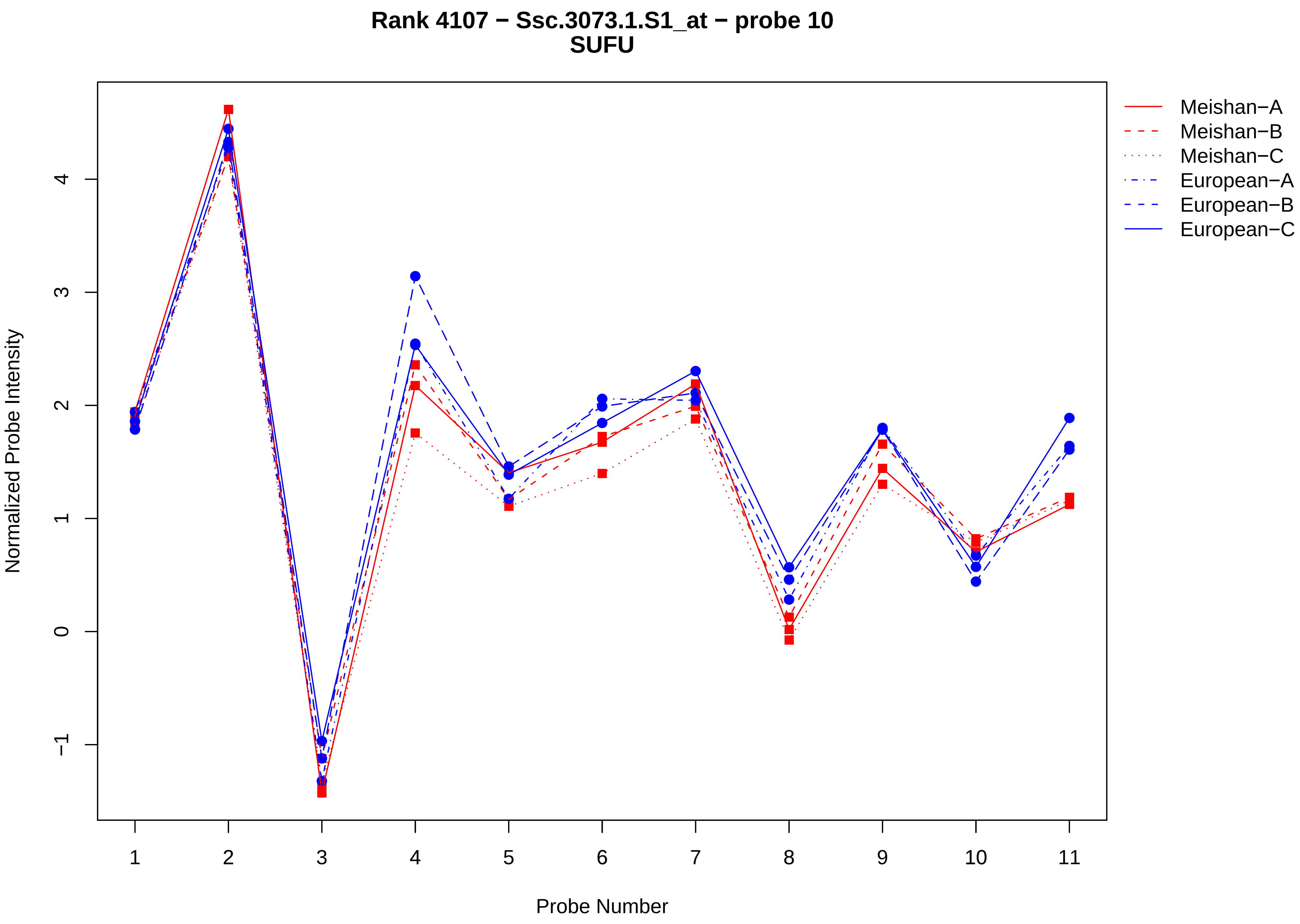

Rank 4107 − Ssc.3073.1.S1_at − probe 10
SUFU
Normalized Probe Intensity
Probe Number
−1
0
1
2
3
4
1
2
3
4
5
6
7
8
9
10
11
Meishan−A
Meishan−B
Meishan−C
European−A
European−B
European−C

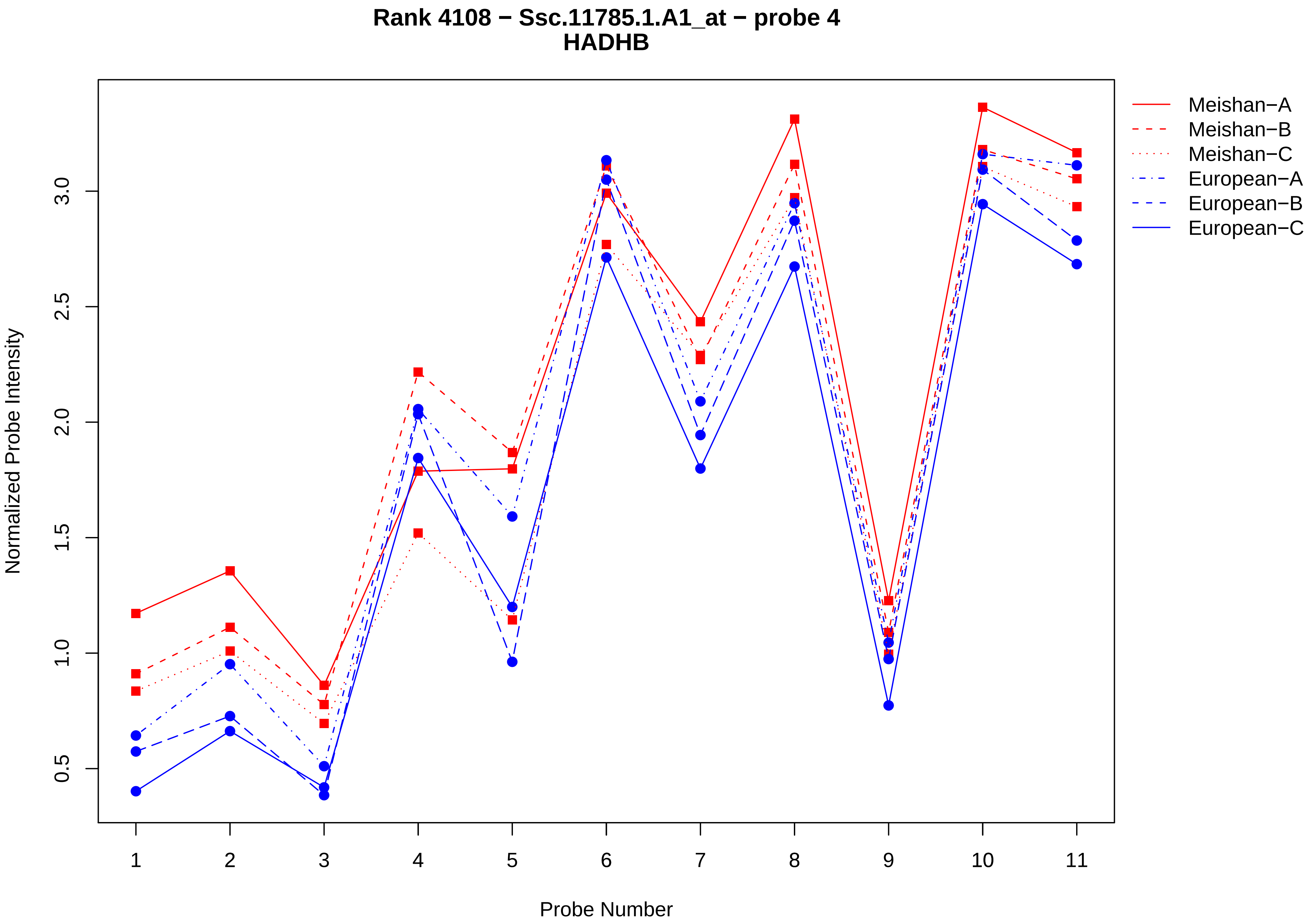

Rank 4108 − Ssc.11785.1.A1_at − probe 4
HADHB
Normalized Probe Intensity
Probe Number
0.5
1.0
1.5
2.0
2.5
3.0
1
2
3
4
5
6
7
8
9
10
11
Meishan−A
Meishan−B
Meishan−C
European−A
European−B
European−C

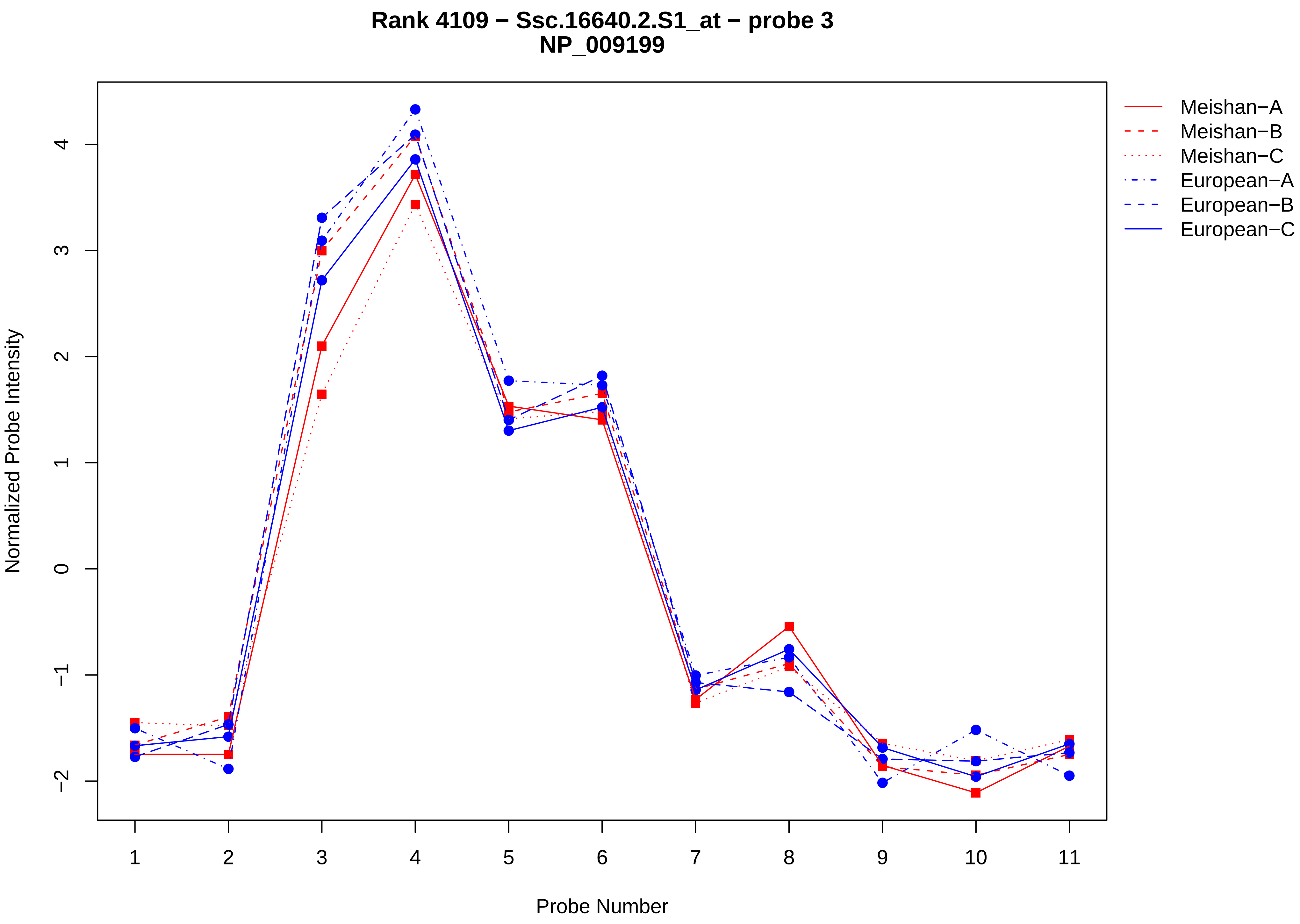

Rank 4109 − Ssc.16640.2.S1_at − probe 3
NP_009199
Normalized Probe Intensity
Probe Number
Meishan−A
Meishan−B
Meishan−C
European−A
European−B
European−C

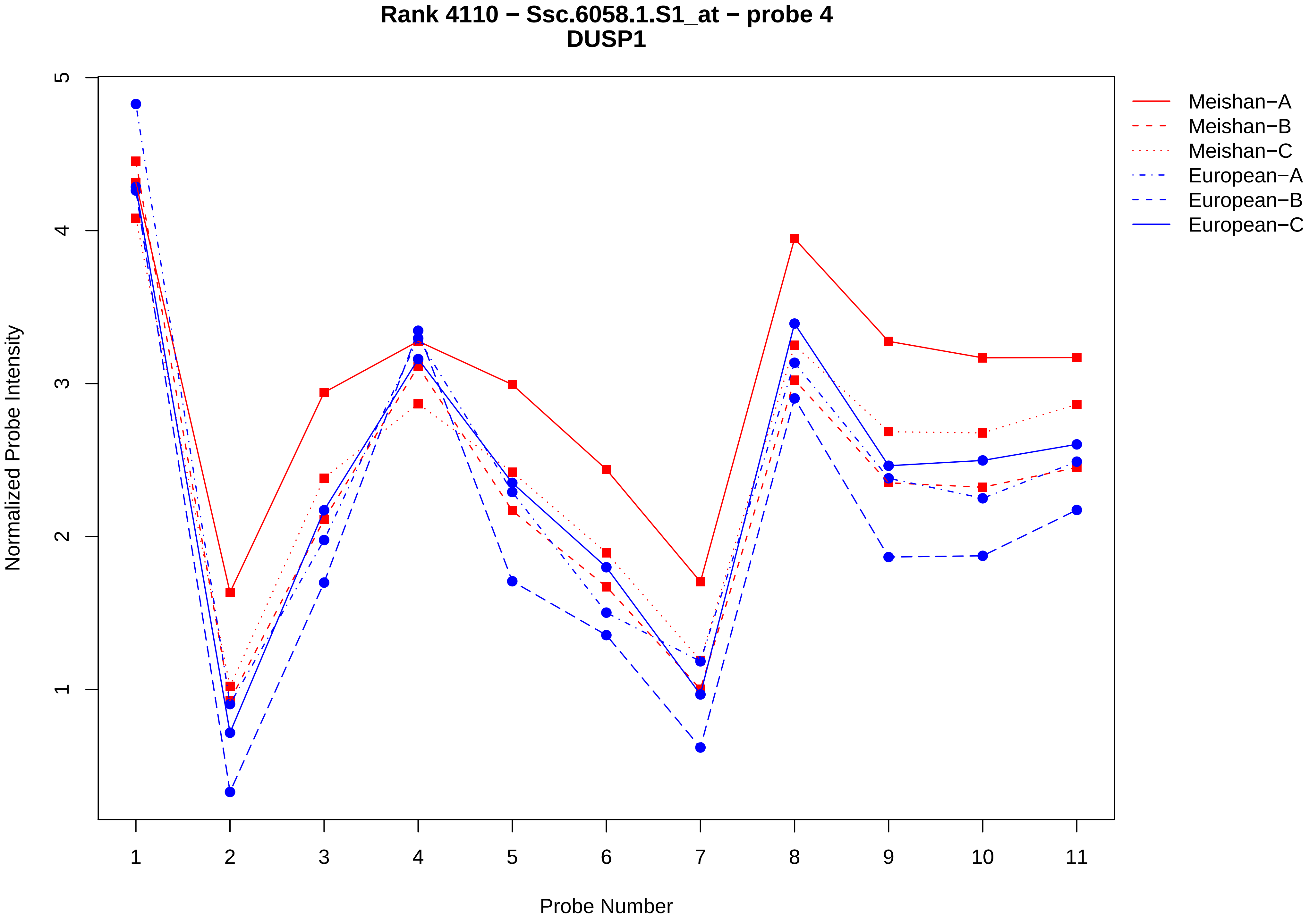
Rank 4110 − Ssc.6058.1.S1_at − probe 4
DUSP1
Normalized Probe Intensity
Probe Number
1
2
3
4
5
1
2
3
4
5
6
7
8
9
10
11
Meishan−A
Meishan−B
Meishan−C
European−A
European−B
European−C

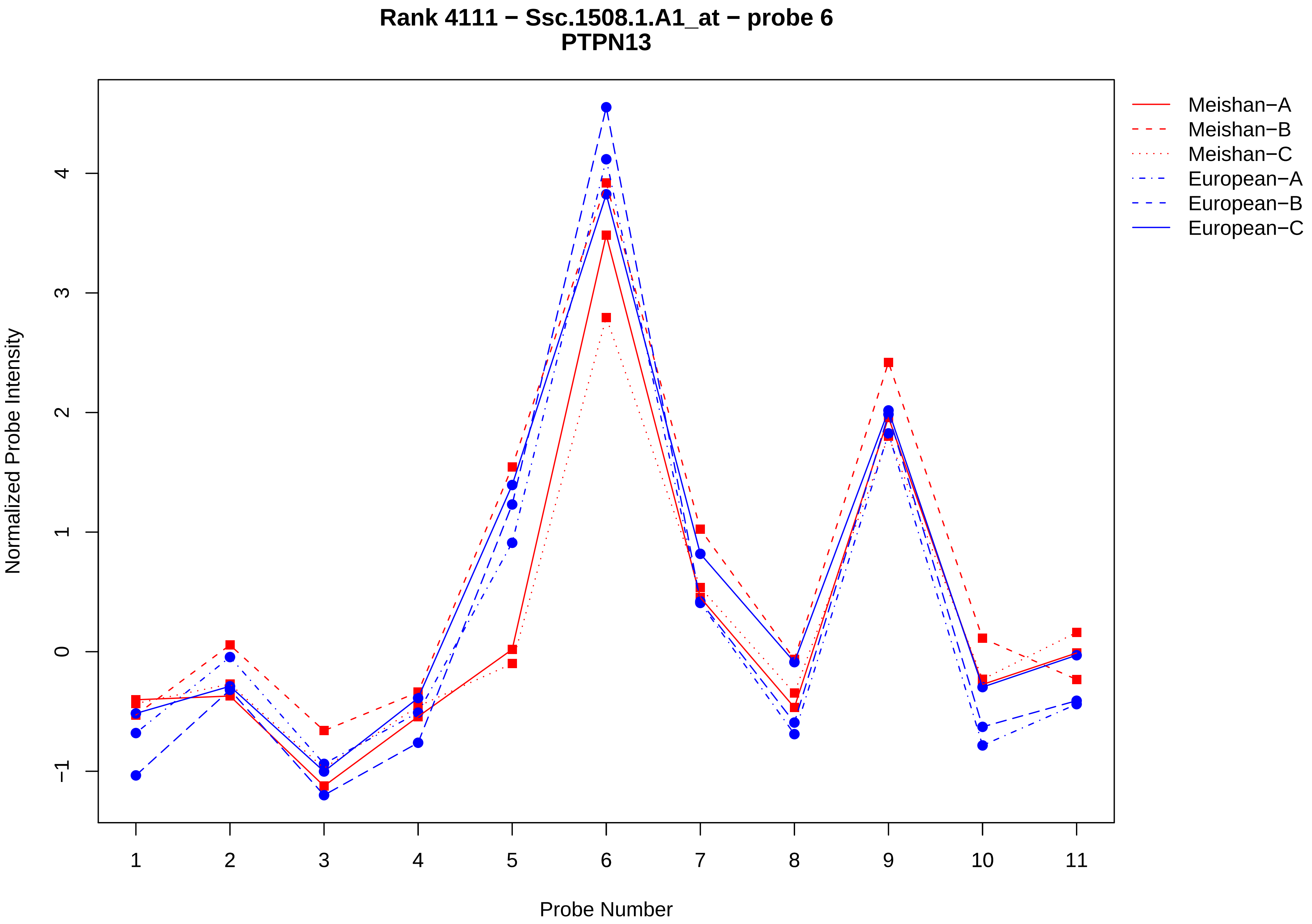
Rank 4111 − Ssc.1508.1.A1_at − probe 6
PTPN13
Normalized Probe Intensity
Probe Number
Meishan−A
Meishan−B
Meishan−C
European−A
European−B
European−C

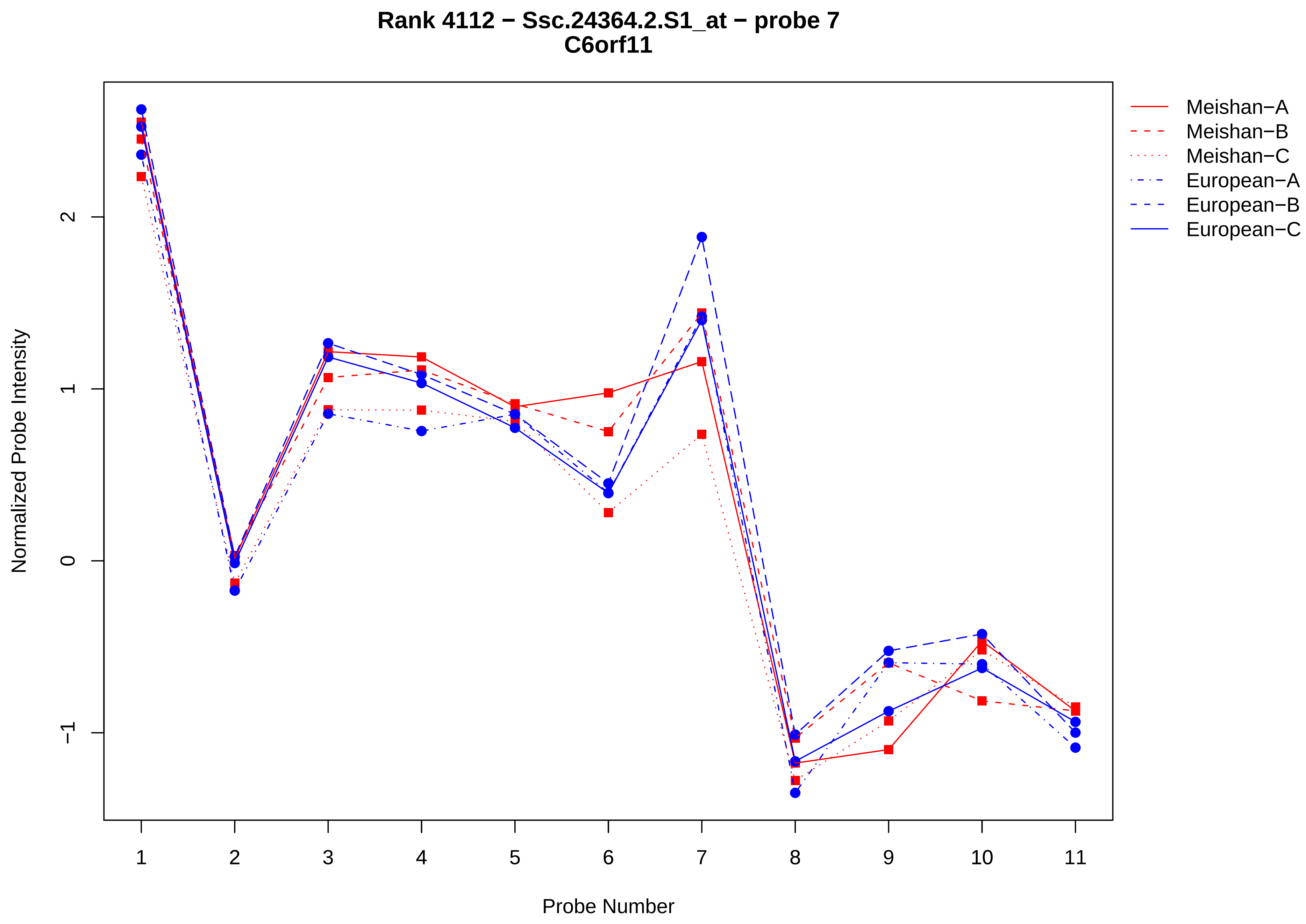

Rank 4112 − Ssc.24364.2.S1_at − probe 7
C6orf11
Normalized Probe Intensity
Probe Number
Meishan−A
Meishan−B
Meishan−C
European−A
European−B
European−C

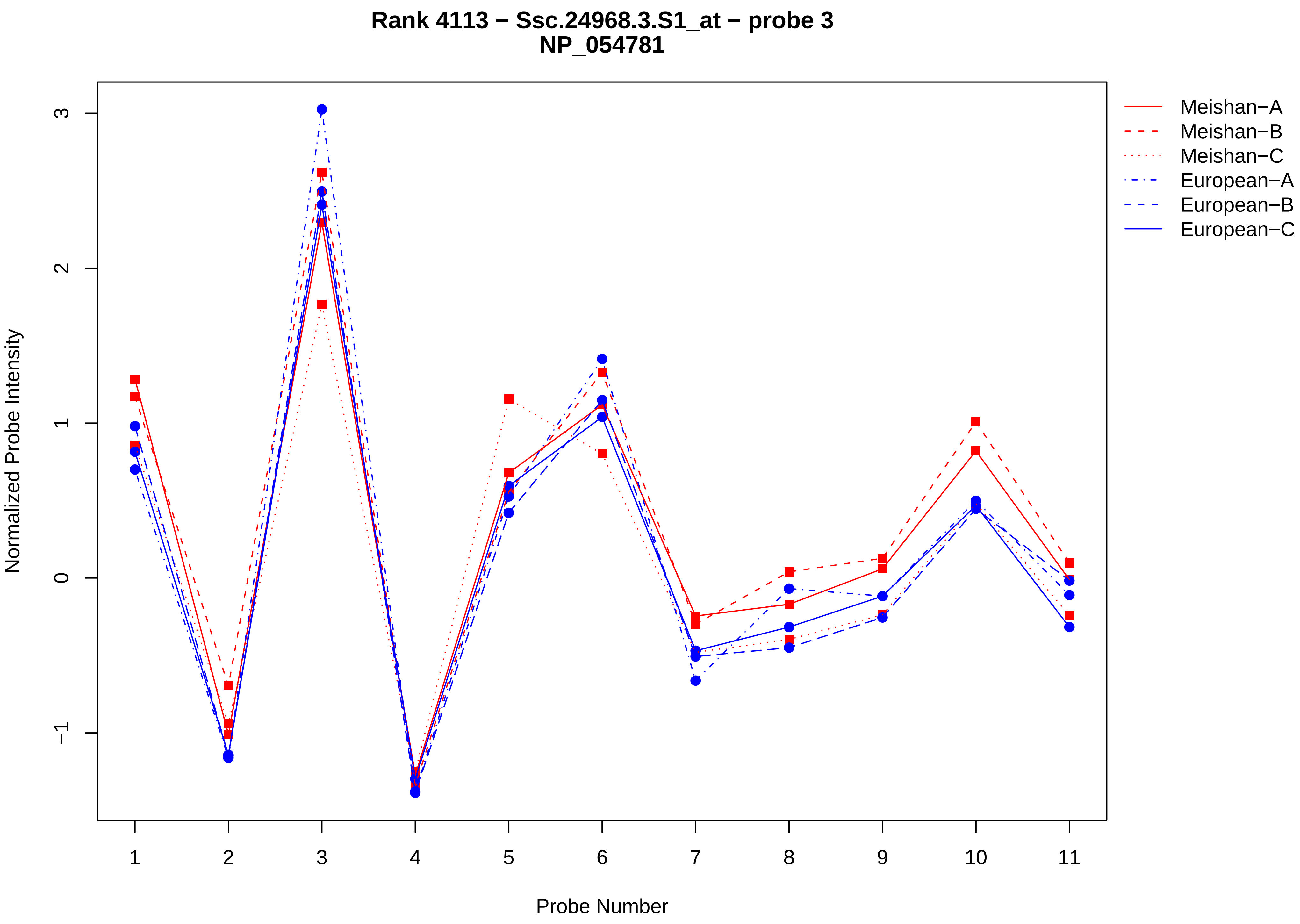
Rank 4113 − Ssc.24968.3.S1_at − probe 3
NP_054781
Normalized Probe Intensity
Probe Number
−1
0
1
2
3
1
2
3
4
5
6
7
8
9
10
11
Meishan−A
Meishan−B
Meishan−C
European−A
European−B
European−C

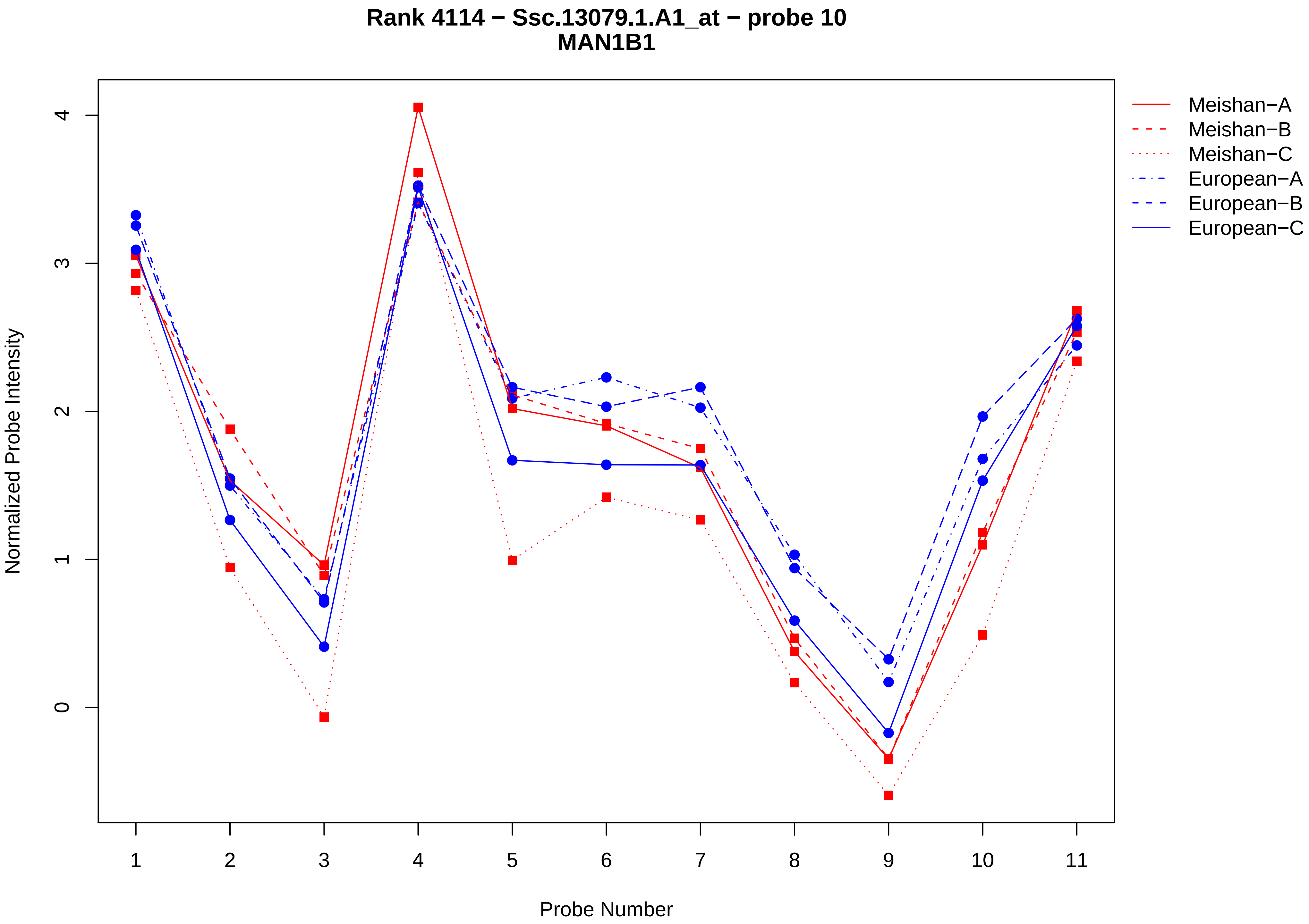

Rank 4114 − Ssc.13079.1.A1_at − probe 10
MAN1B1
Normalized Probe Intensity
Probe Number
0
1
2
3
4
1
2
3
4
5
6
7
8
9
10
11
Meishan−A
Meishan−B
Meishan−C
European−A
European−B
European−C

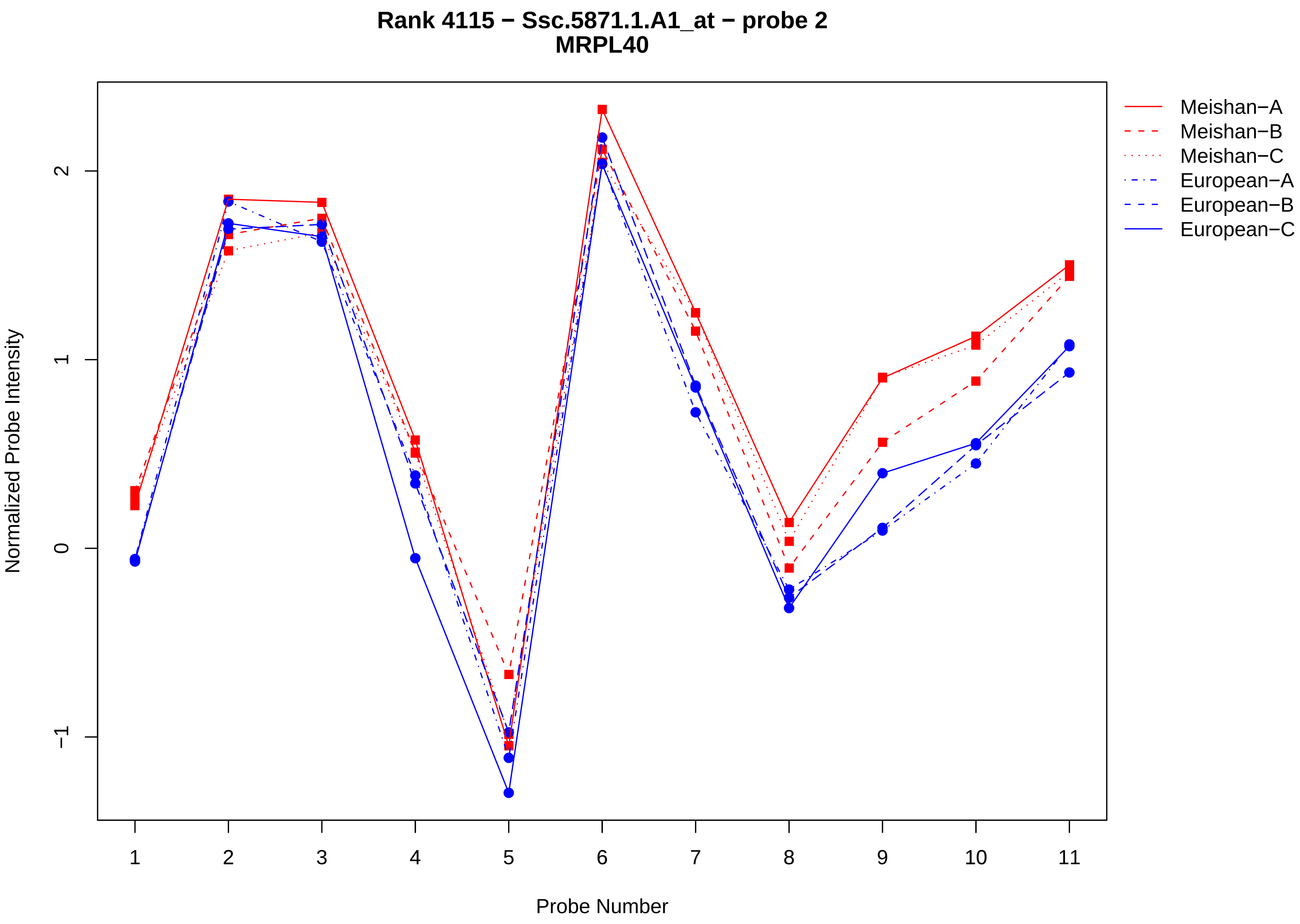

Rank 4115 − Ssc.5871.1.A1_at − probe 2
MRPL40
Normalized Probe Intensity
Probe Number
−1
0
1
2
1
2
3
4
5
6
7
8
9
10
11
Meishan−A
Meishan−B
Meishan−C
European−A
European−B
European−C

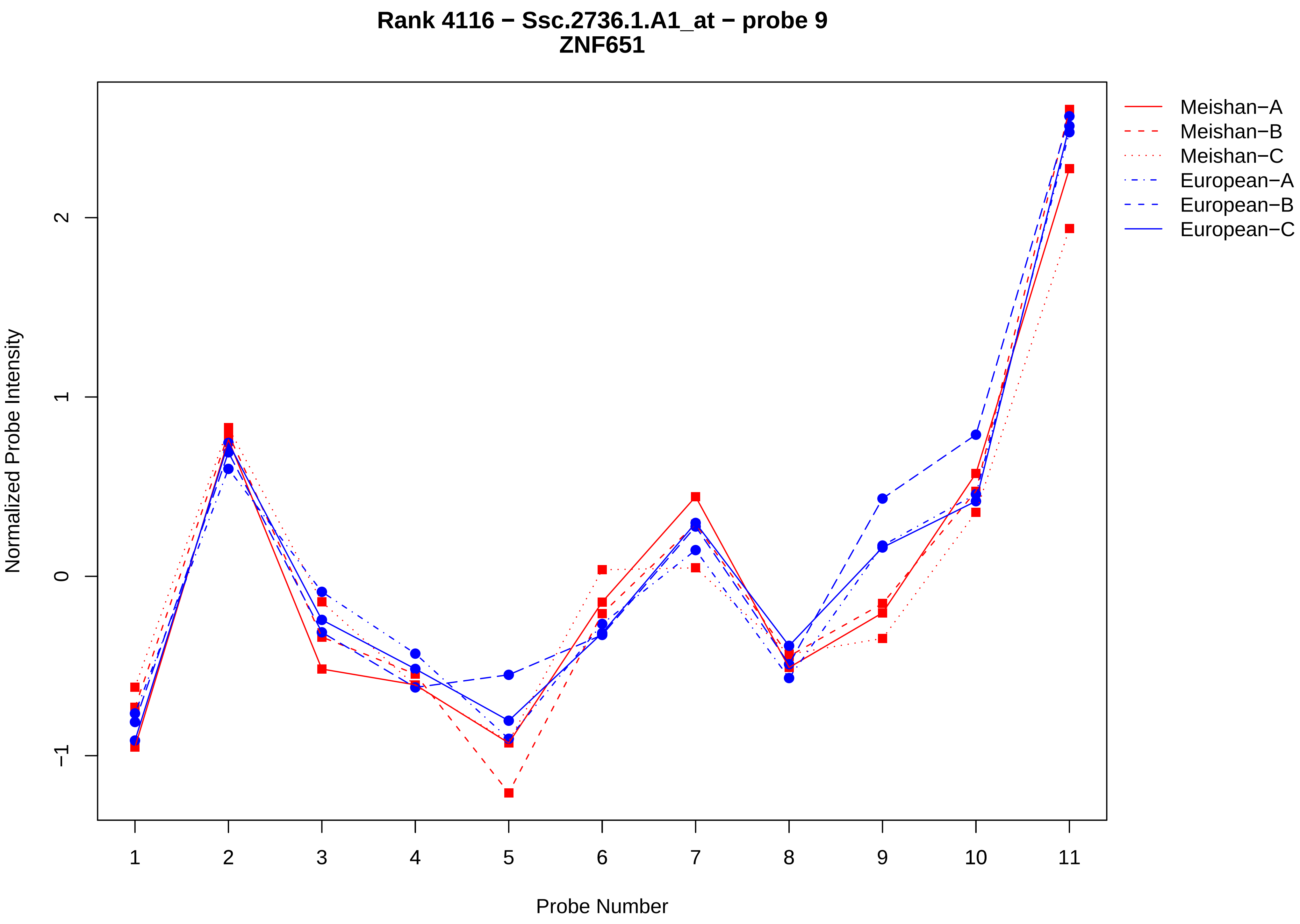

Rank 4116 − Ssc.2736.1.A1_at − probe 9
ZNF651
Normalized Probe Intensity
Probe Number
−1
0
1
2
1
2
3
4
5
6
7
8
9
10
11
Meishan−A
Meishan−B
Meishan−C
European−A
European−B
European−C

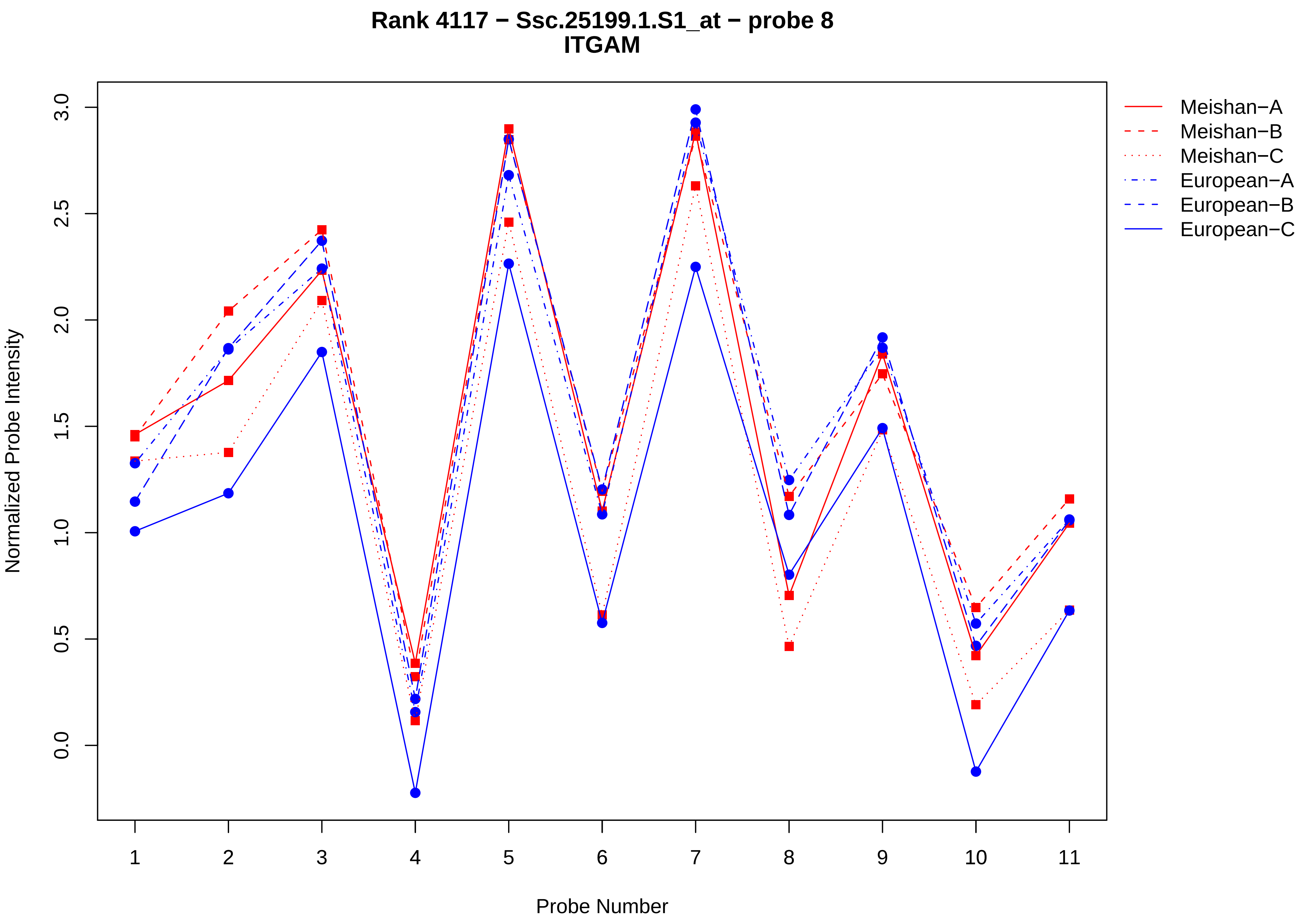

Rank 4117 − Ssc.25199.1.S1_at − probe 8
ITGAM
Normalized Probe Intensity
Probe Number
Meishan−A
Meishan−B
Meishan−C
European−A
European−B
European−C

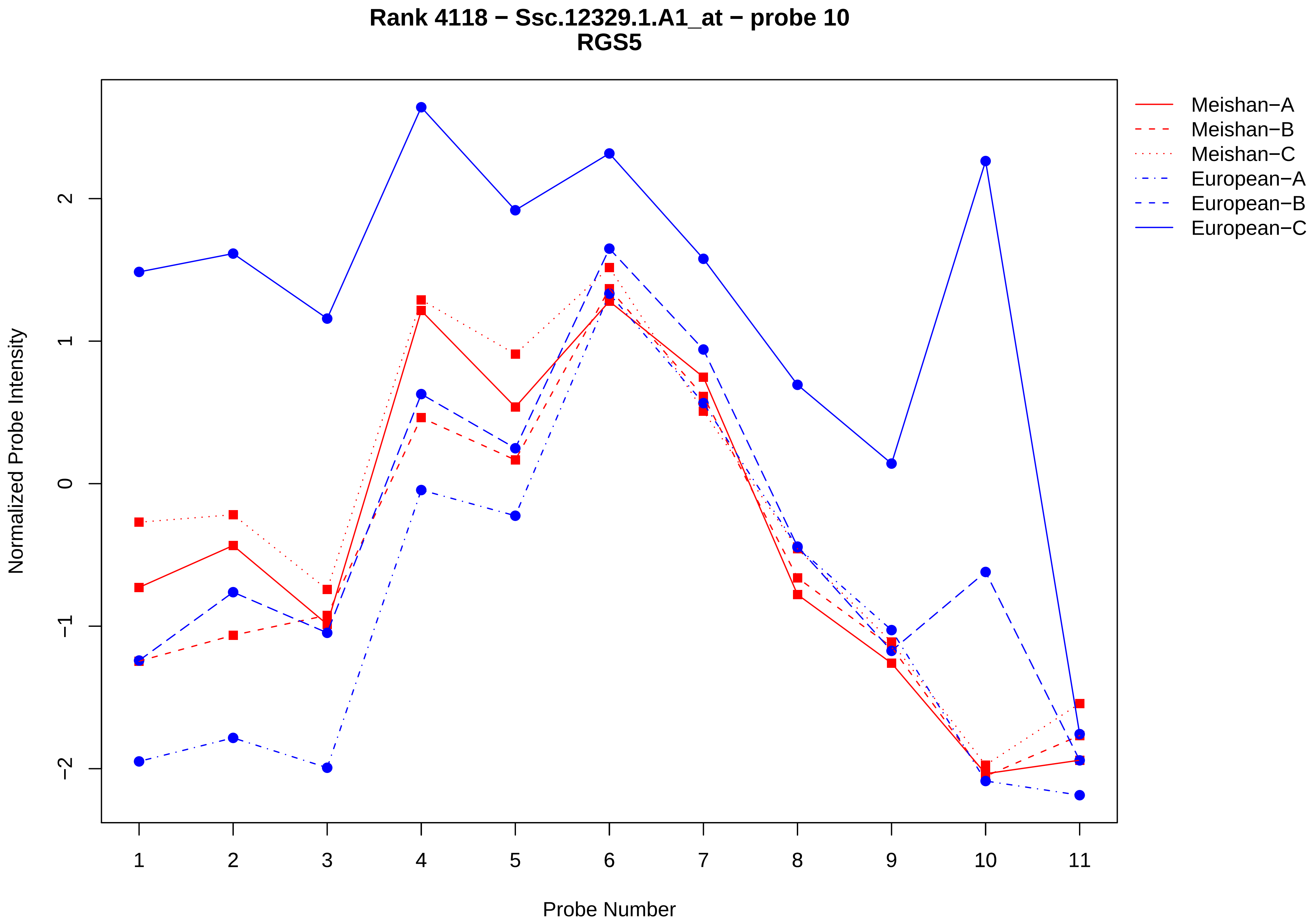

Rank 4118 − Ssc.12329.1.A1_at − probe 10
RGS5
Normalized Probe Intensity
Probe Number
−2
−1
0
1
2
1
2
3
4
5
6
7
8
9
10
11
Meishan−A
Meishan−B
Meishan−C
European−A
European−B
European−C

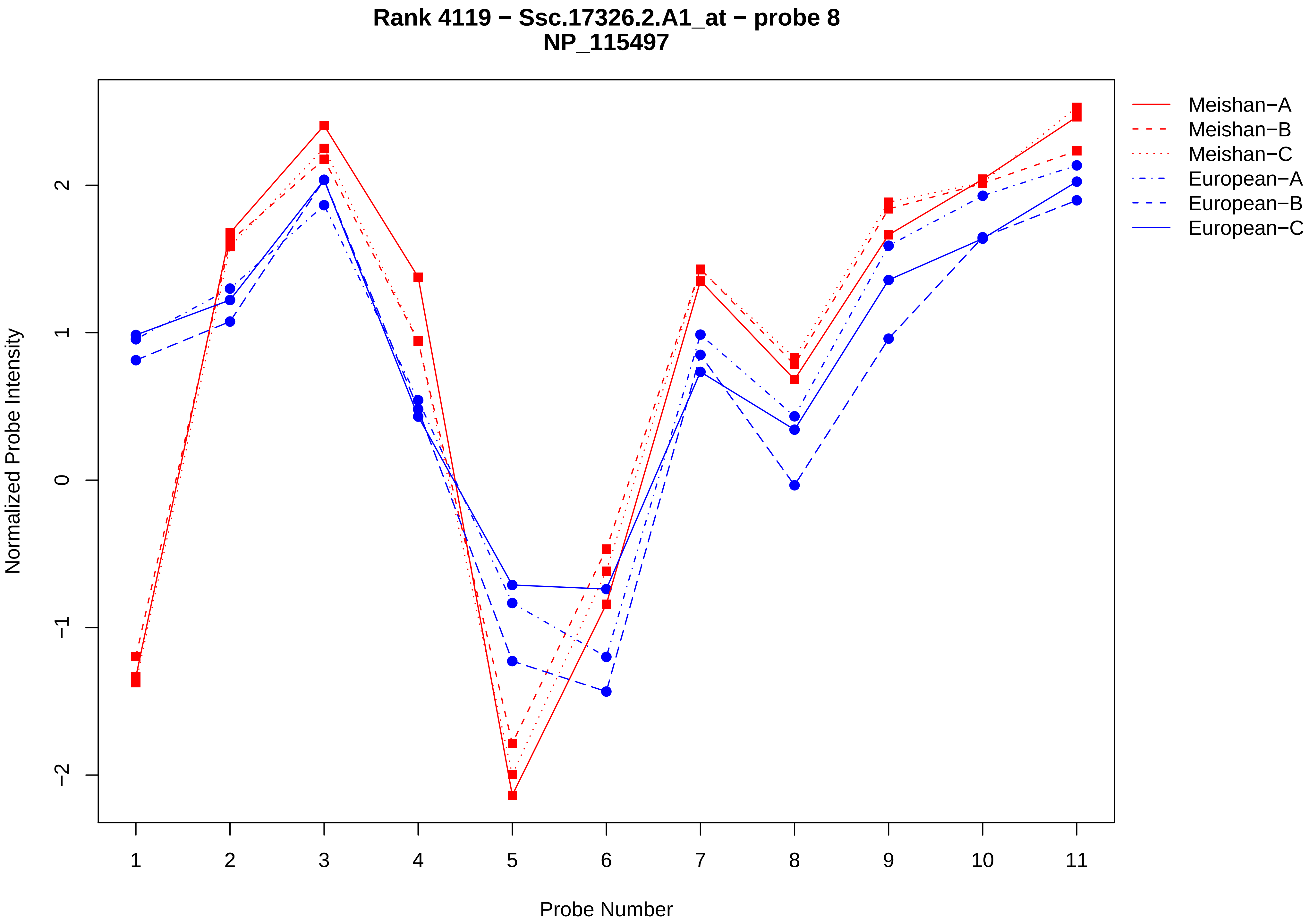

Rank 4119 − Ssc.17326.2.A1_at − probe 8
NP_115497
Normalized Probe Intensity
Probe Number
−2
−1
0
1
2
1
2
3
4
5
6
7
8
9
10
11
Meishan−A
Meishan−B
Meishan−C
European−A
European−B
European−C

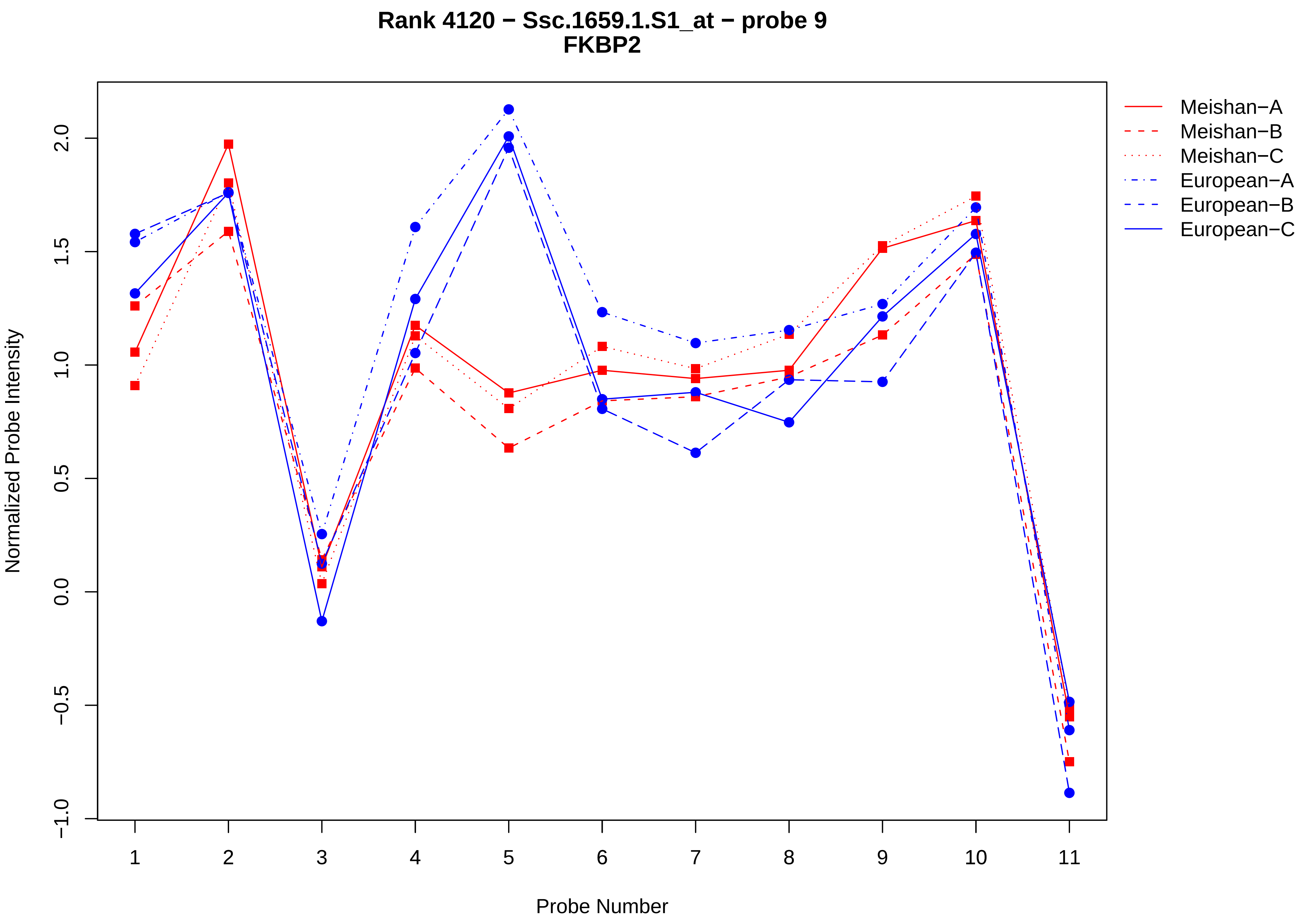

Rank 4120 − Ssc.1659.1.S1_at − probe 9
FKBP2
Normalized Probe Intensity
Probe Number
−1.0
−0.5
0.0
0.5
1.0
1.5
2.0
1
2
3
4
5
6
7
8
9
10
11
Meishan−A
Meishan−B
Meishan−C
European−A
European−B
European−C

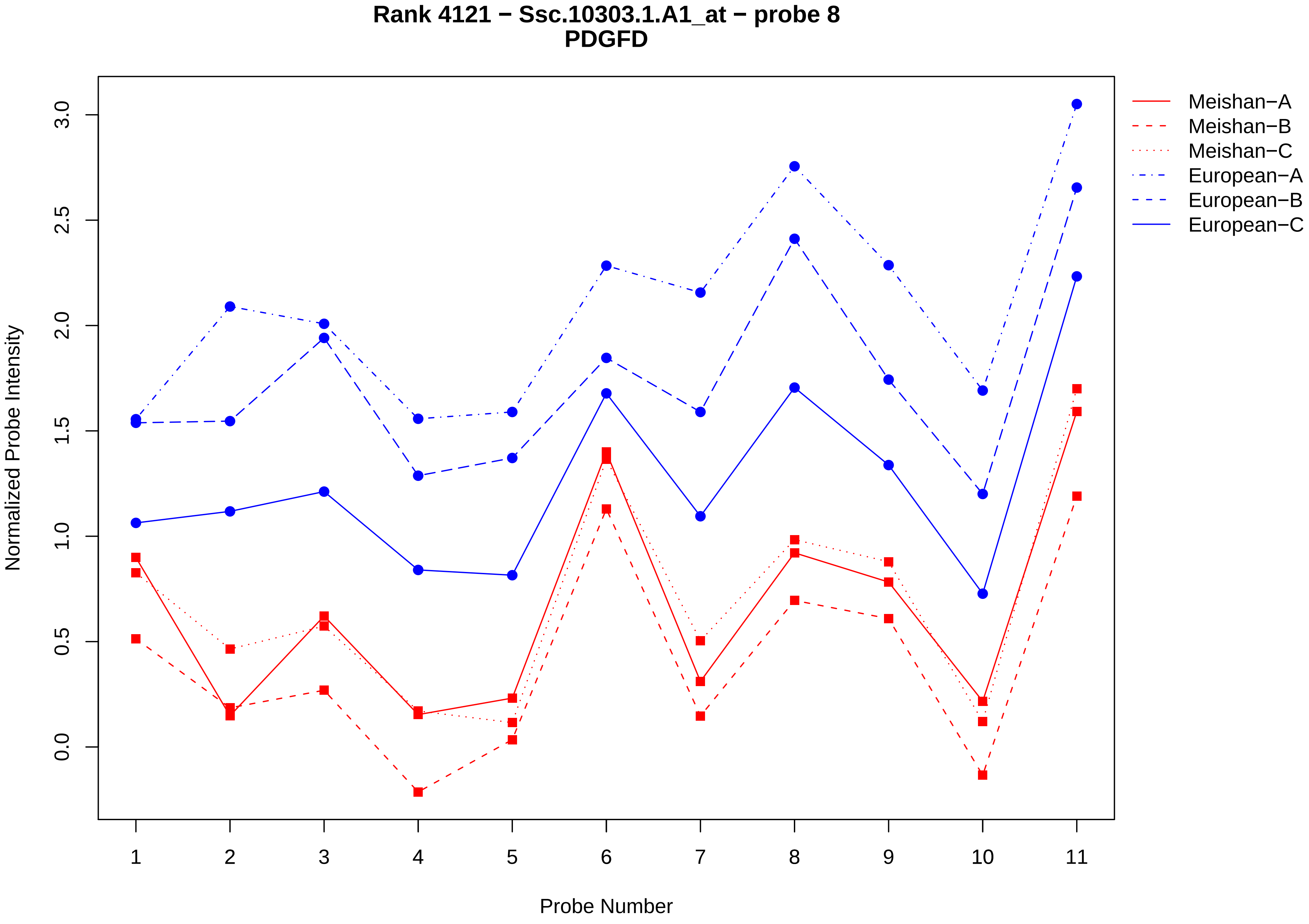

Rank 4121 − Ssc.10303.1.A1_at − probe 8
PDGFD
Normalized Probe Intensity
Probe Number
0.0
0.5
1.0
1.5
2.0
2.5
3.0
1 2 3 4 5 6 7 8 9 10 11
Meishan−A
Meishan−B
Meishan−C
European−A
European−B
European−C

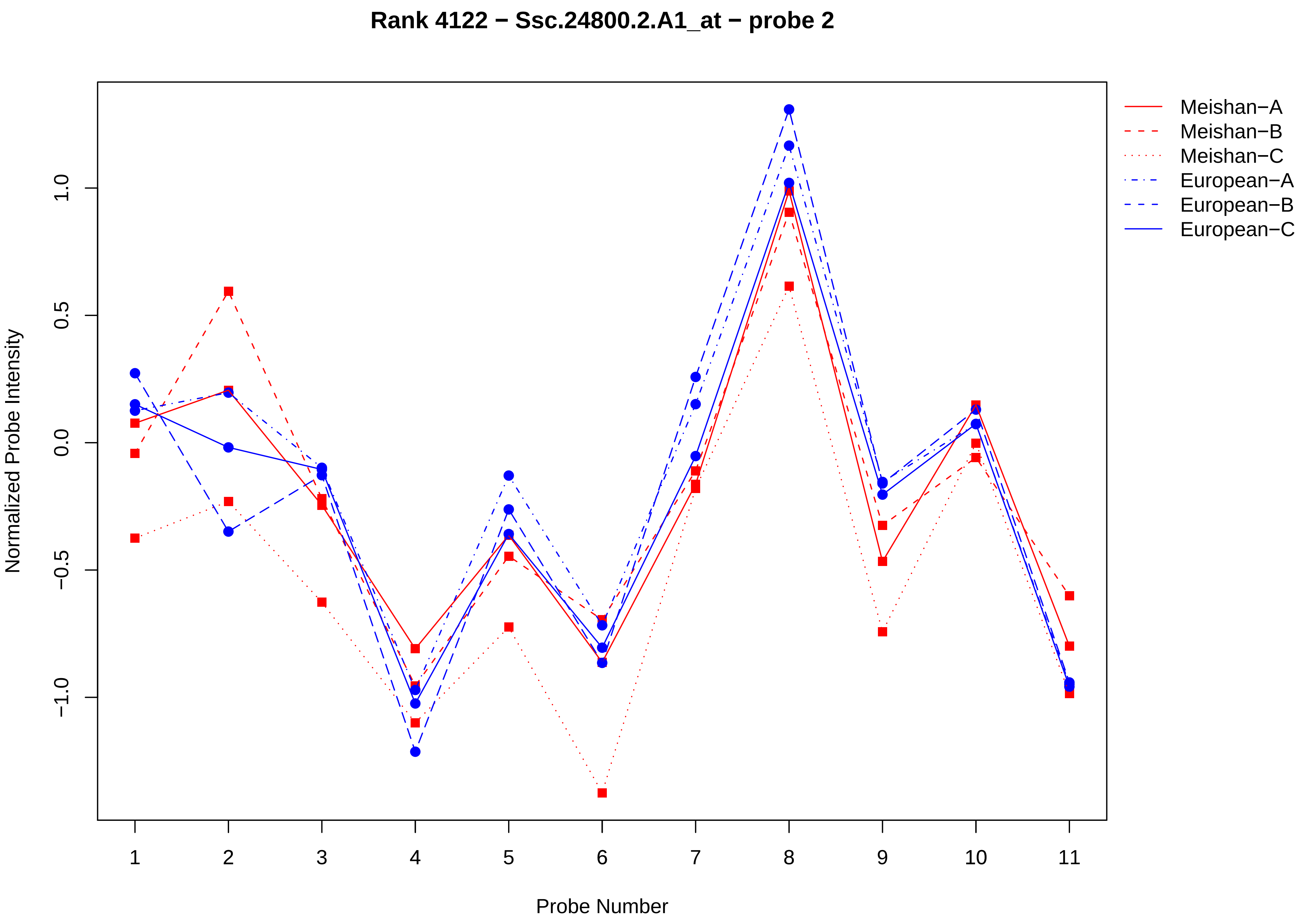
Rank 4122 − Ssc.24800.2.A1_at − probe 2
Normalized Probe Intensity
Probe Number
1.0
0.5
0.0
−0.5
−1.0
1
2
3
4
5
6
7
8
9
10
11
Meishan−A
Meishan−B
Meishan−C
European−A
European−B
European−C

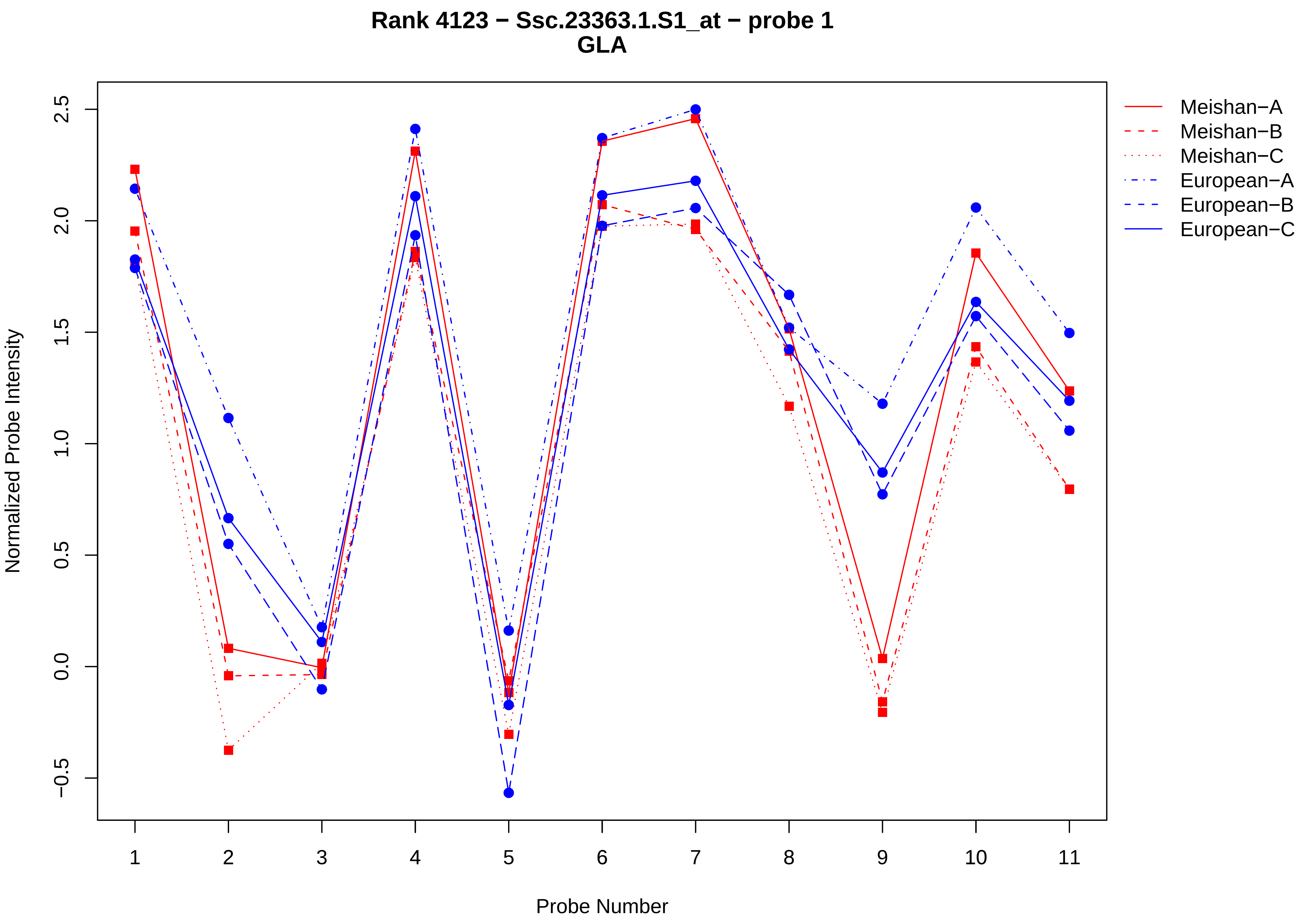

Rank 4123 − Ssc.23363.1.S1_at − probe 1
GLA
Normalized Probe Intensity
Probe Number
−0.5
0.0
0.5
1.0
1.5
2.0
2.5
1
2
3
4
5
6
7
8
9
10
11
Meishan−A
Meishan−B
Meishan−C
European−A
European−B
European−C

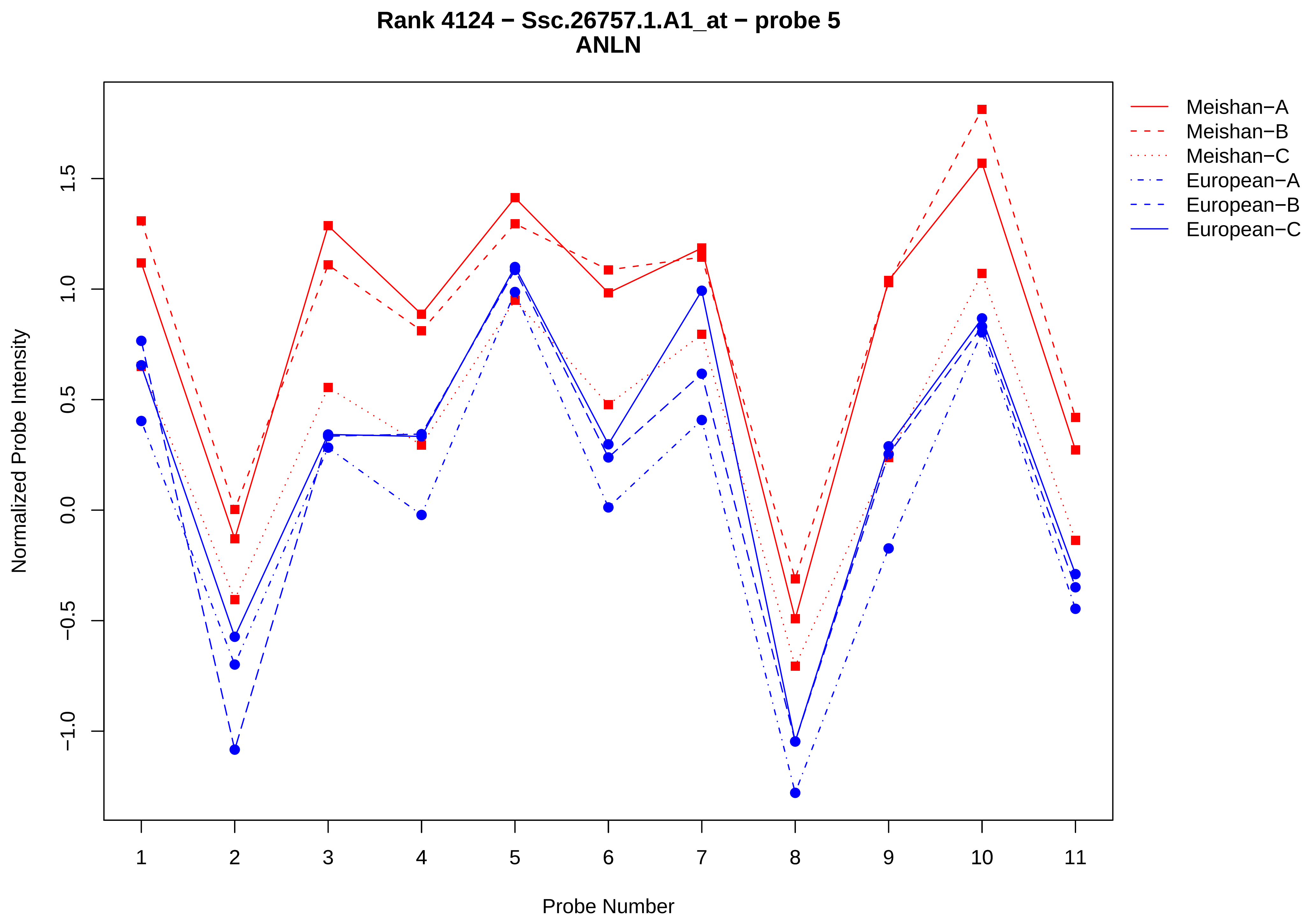

Rank 4124 − Ssc.26757.1.A1_at − probe 5
ANLN
Normalized Probe Intensity
Probe Number
−1.0
−0.5
0.0
0.5
1.0
1.5
1
2
3
4
5
6
7
8
9
10
11
Meishan−A
Meishan−B
Meishan−C
European−A
European−B
European−C

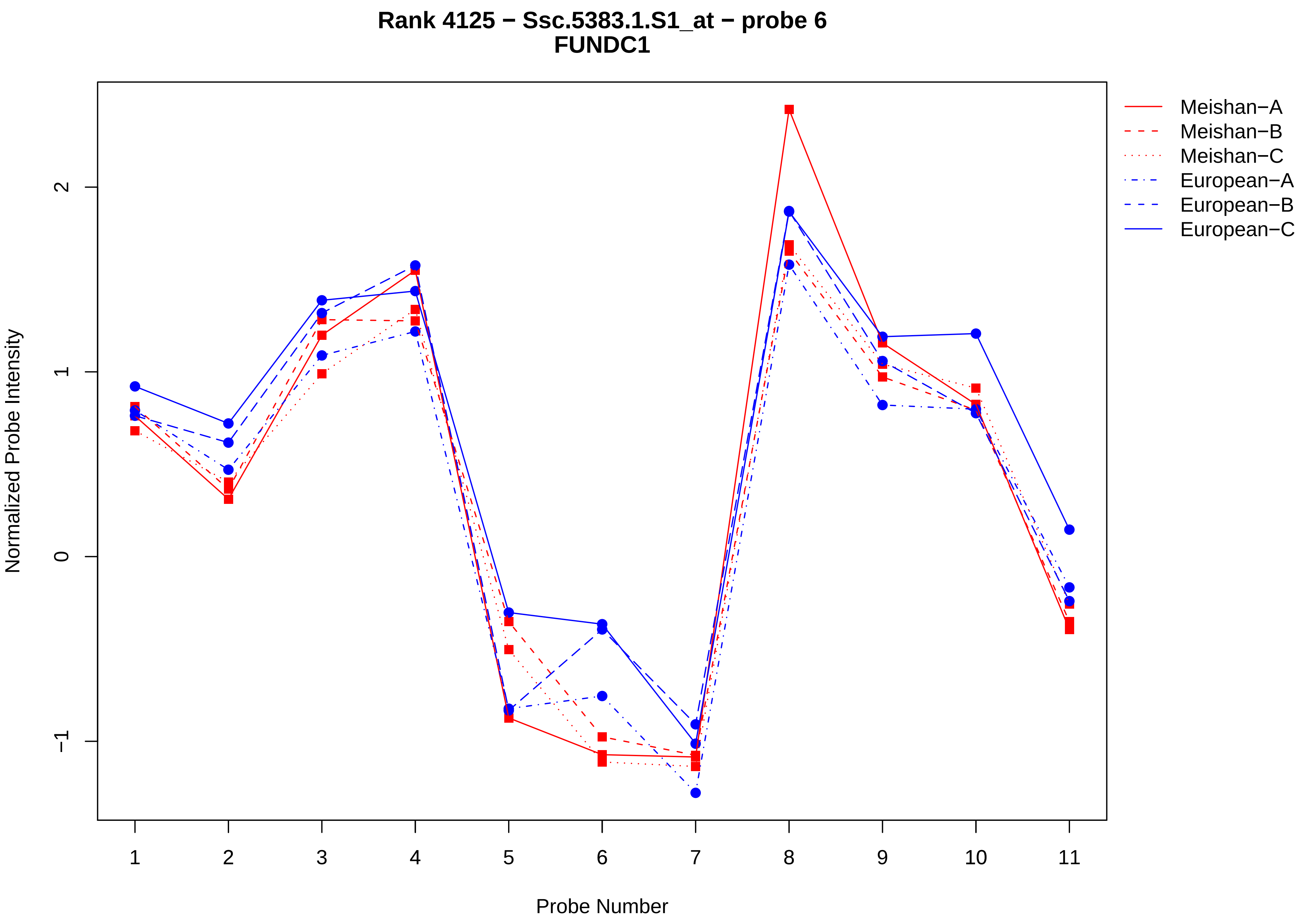
Rank 4125 − Ssc.5383.1.S1_at − probe 6
FUNDC1
Normalized Probe Intensity
Probe Number
−1
0
1
2
1
2
3
4
5
6
7
8
9
10
11
Meishan−A
Meishan−B
Meishan−C
European−A
European−B
European−C

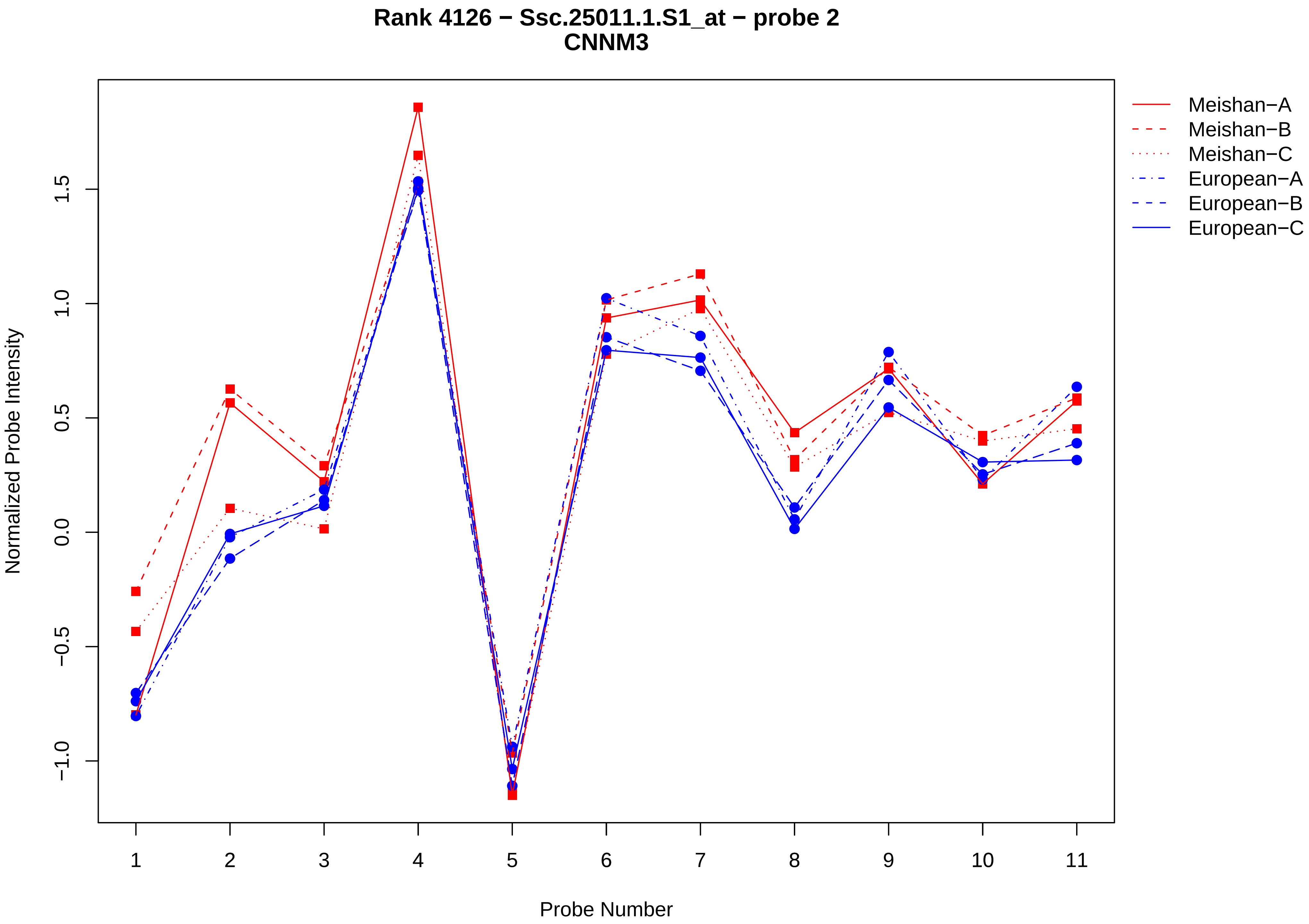

Rank 4126 − Ssc.25011.1.S1_at − probe 2
CNNM3
Normalized Probe Intensity
Probe Number
−1.0
−0.5
0.0
0.5
1.0
1.5
1
2
3
4
5
6
7
8
9
10
11
Meishan−A
Meishan−B
Meishan−C
European−A
European−B
European−C

Rank 4127 − Ssc.30956.1.A1_s_at − probe 9
IFITM3

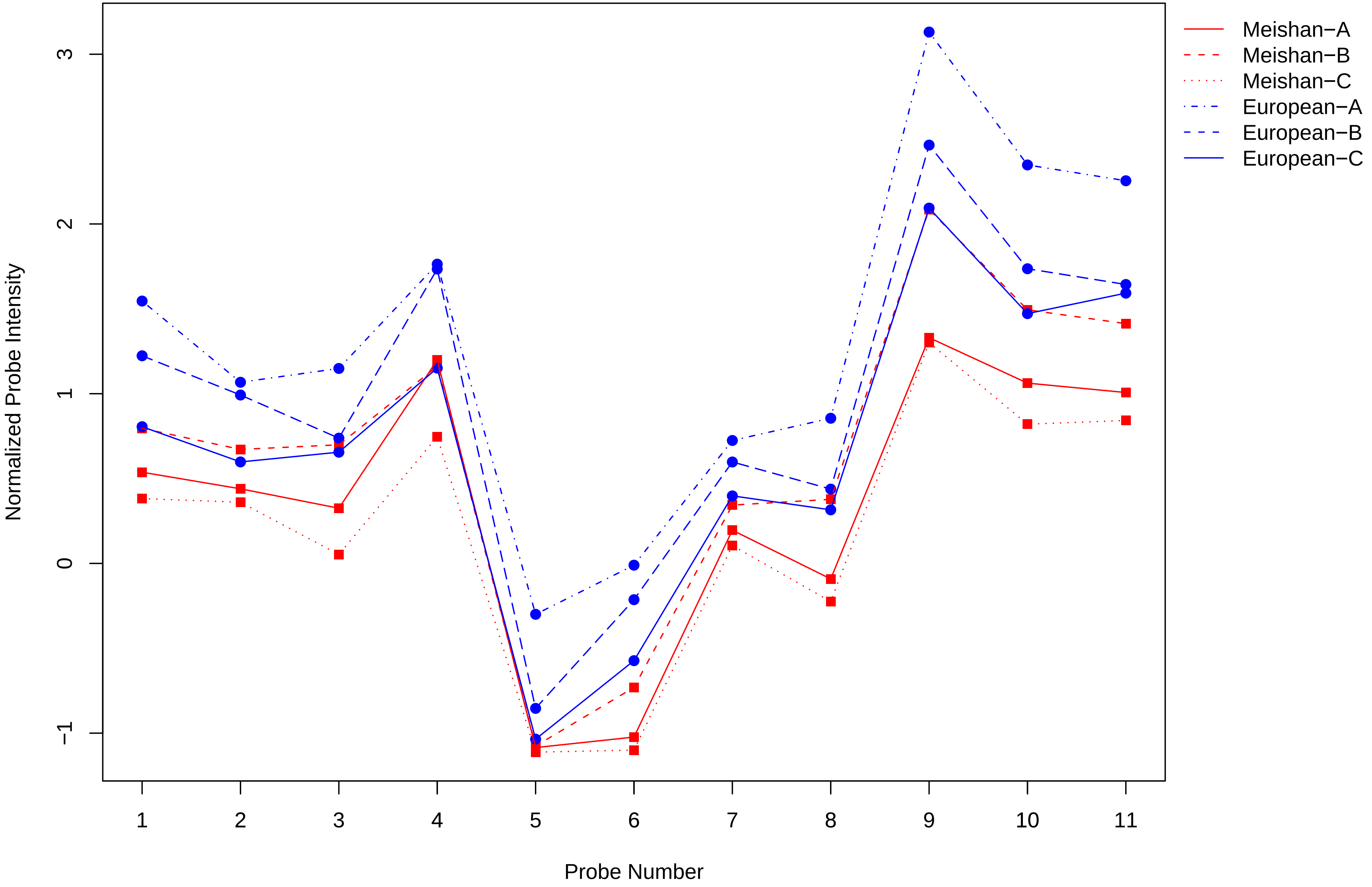

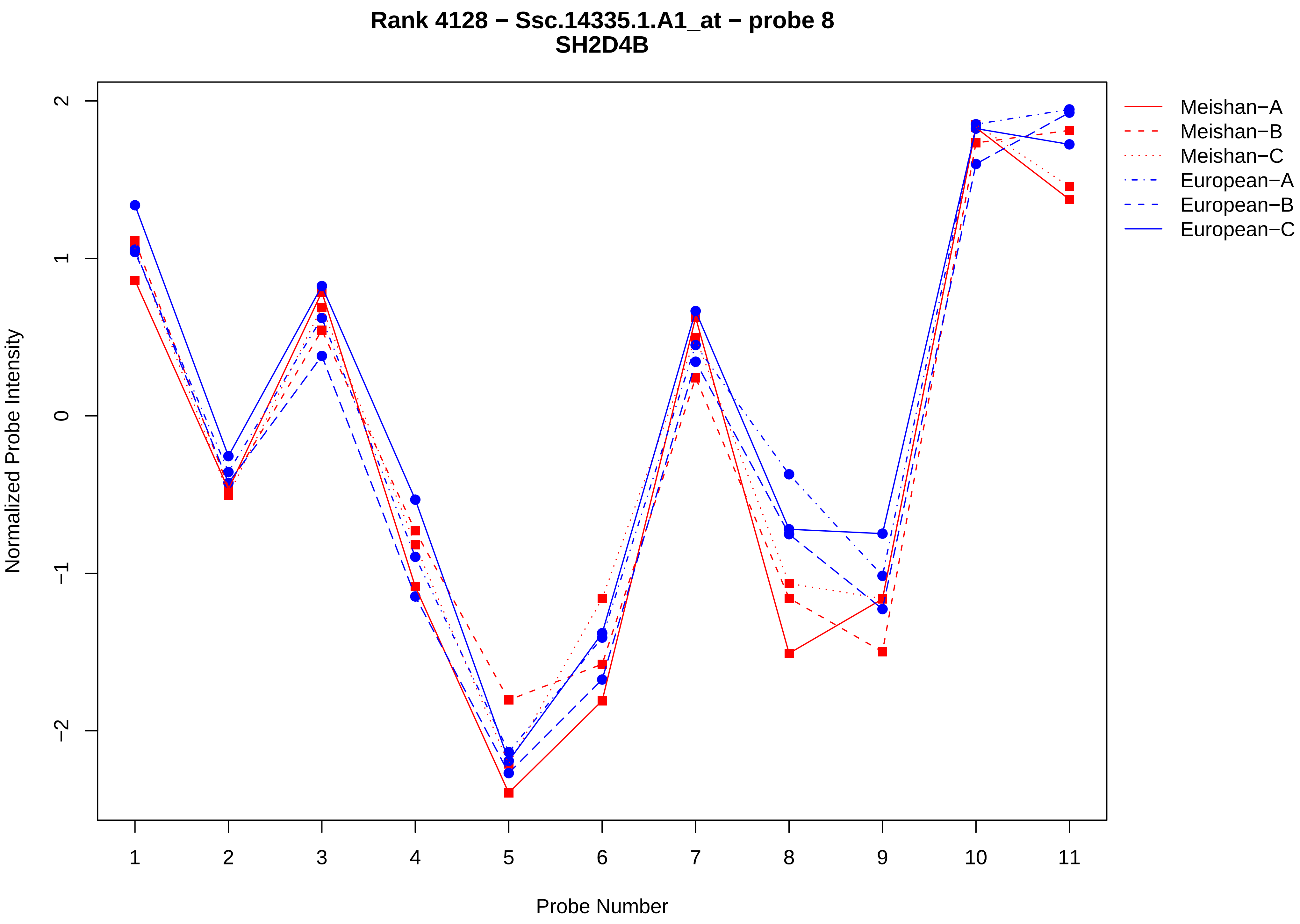
Rank 4128 − Ssc.14335.1.A1_at − probe 8
SH2D4B
Normalized Probe Intensity
Probe Number
2
1
0
−1
−2
1
2
3
4
5
6
7
8
9
10
11
Meishan−A
Meishan−B
Meishan−C
European−A
European−B
European−C

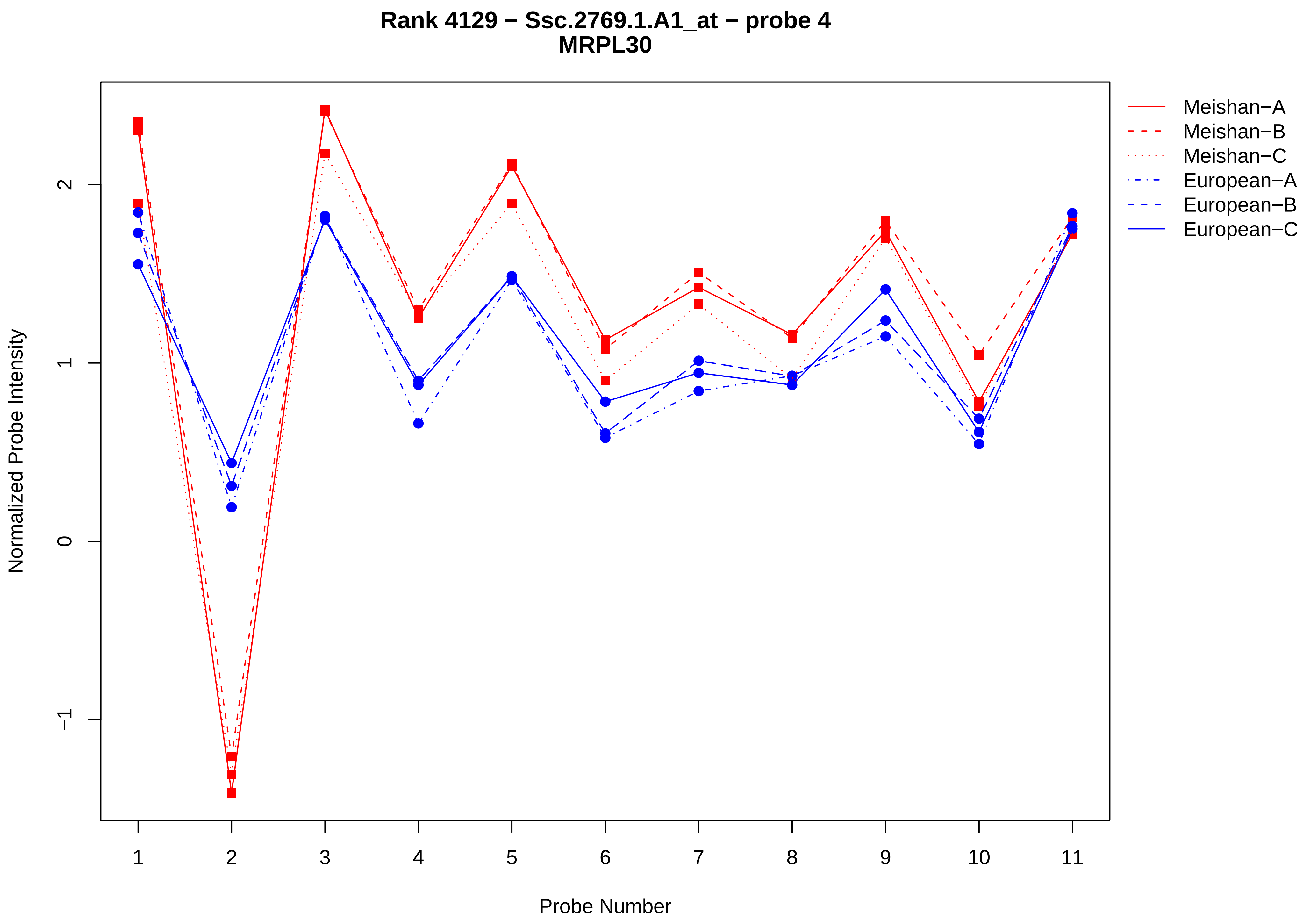

Rank 4129 − Ssc.2769.1.A1_at − probe 4
MRPL30
Normalized Probe Intensity
Probe Number
−1
0
1
2
1
2
3
4
5
6
7
8
9
10
11
Meishan−A
Meishan−B
Meishan−C
European−A
European−B
European−C

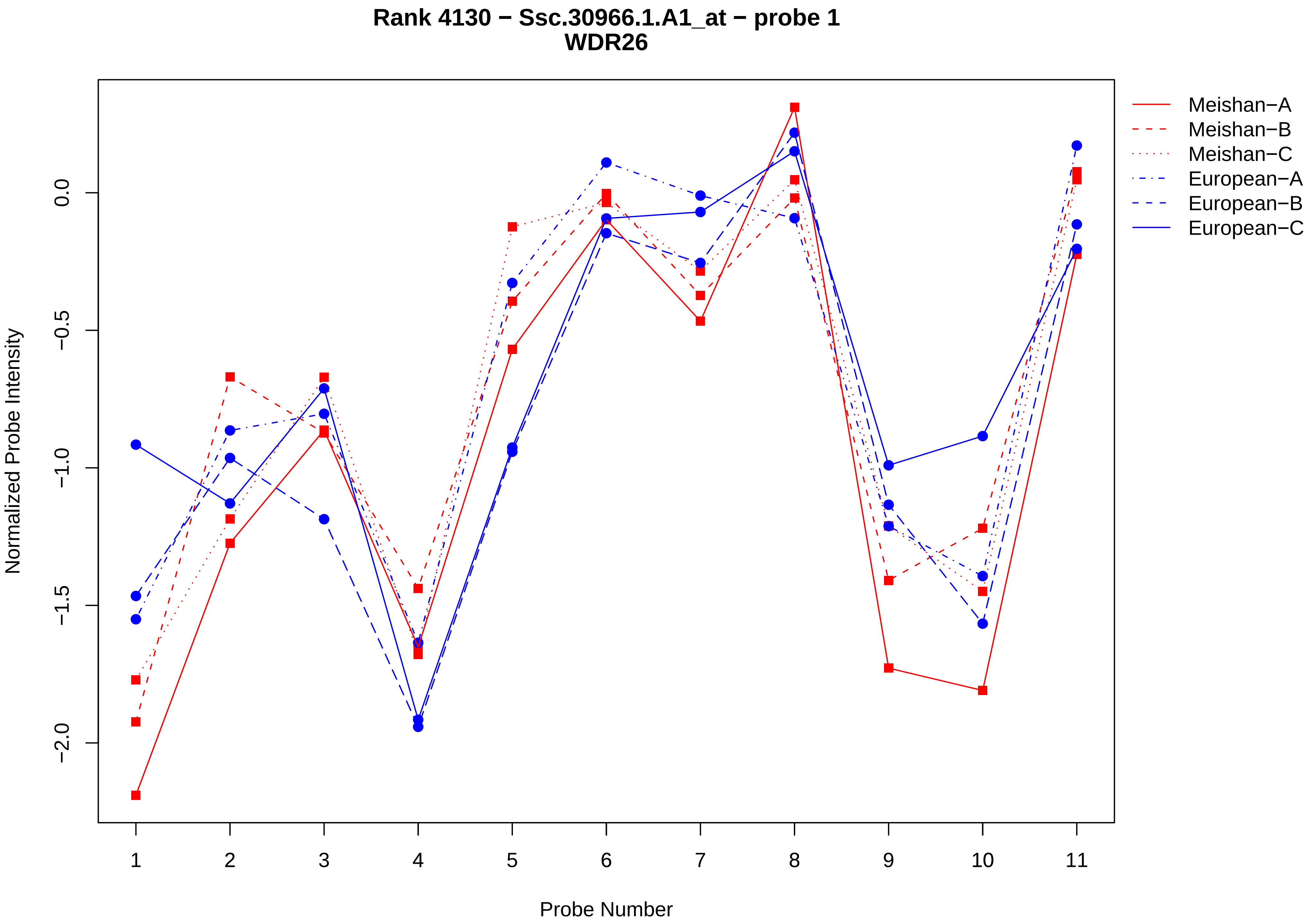

Rank 4130 − Ssc.30966.1.A1_at − probe 1
WDR26
Normalized Probe Intensity
0.0
−0.5
−1.0
−1.5
−2.0
1
2
3
4
5
6
7
8
9
10
11
Probe Number
Meishan−A
Meishan−B
Meishan−C
European−A
European−B
European−C

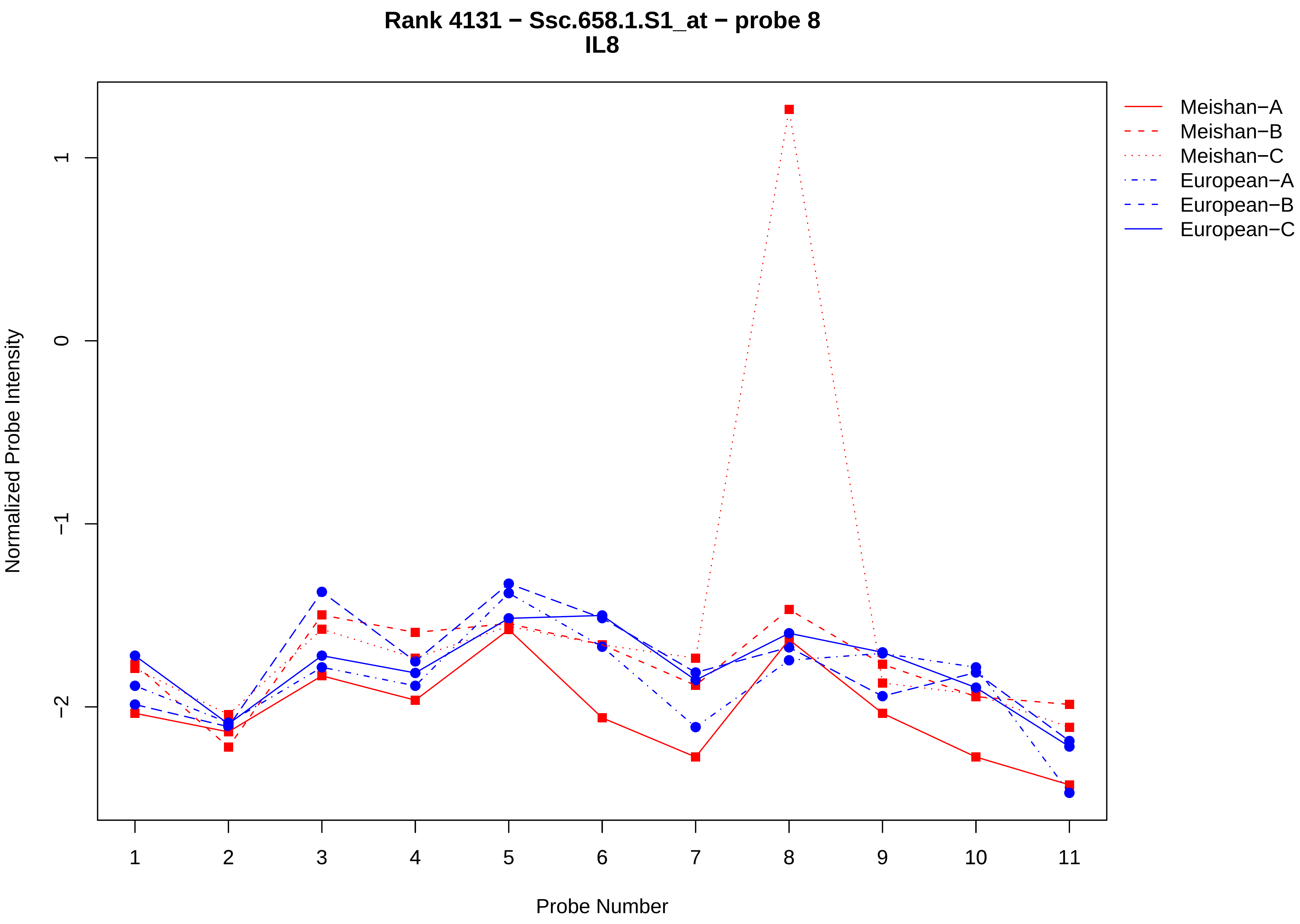

Rank 4131 − Ssc.658.1.S1_at − probe 8
IL8
Normalized Probe Intensity
Probe Number
1
0
−1
−2
1
2
3
4
5
6
7
8
9
10
11
Meishan−A
Meishan−B
Meishan−C
European−A
European−B
European−C

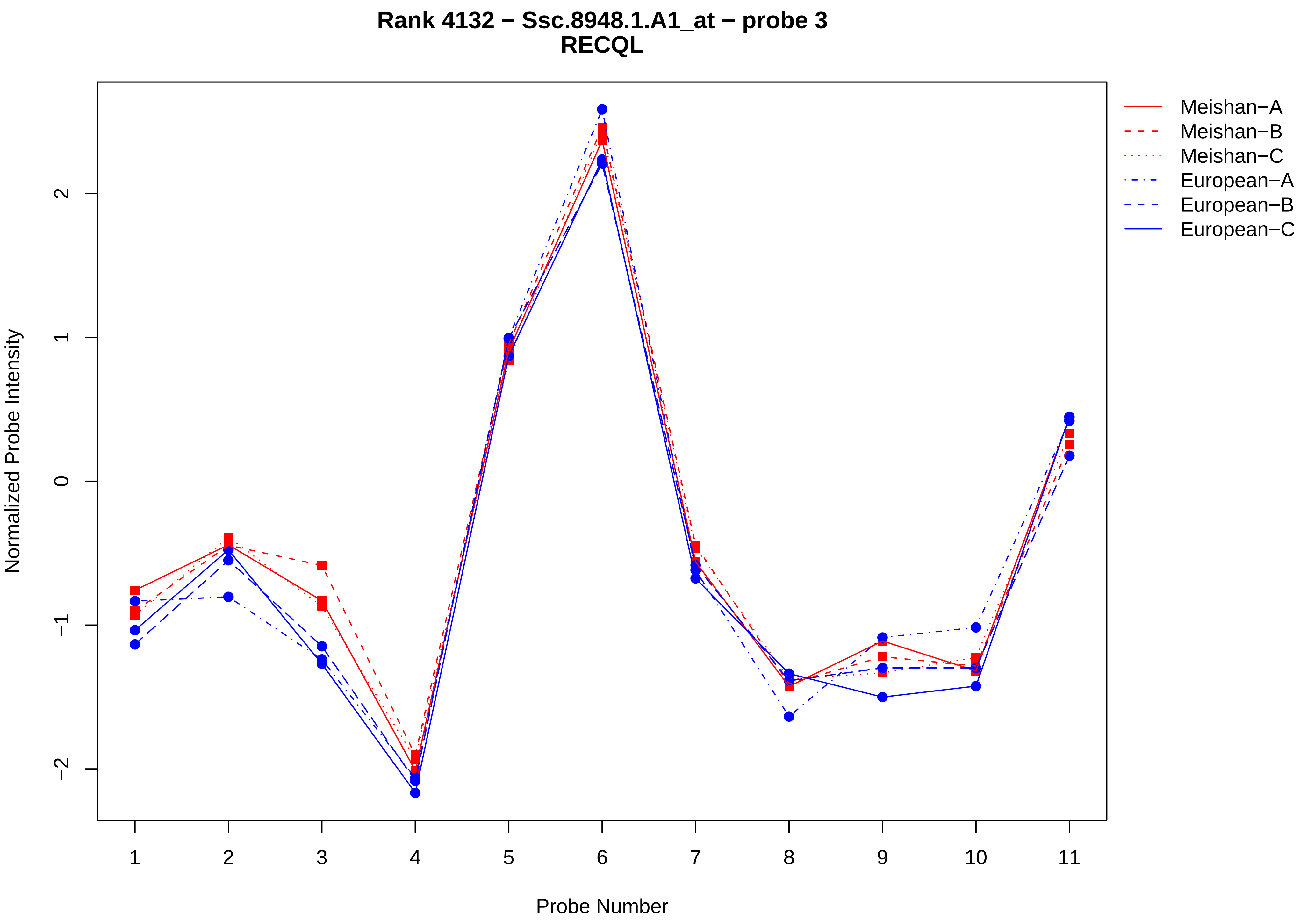

Rank 4132 − Ssc.8948.1.A1_at − probe 3
RECQL
Normalized Probe Intensity
Probe Number
Meishan−A
Meishan−B
Meishan−C
European−A
European−B
European−C
−2
−1
0
1
2
1
2
3
4
5
6
7
8
9
10
11

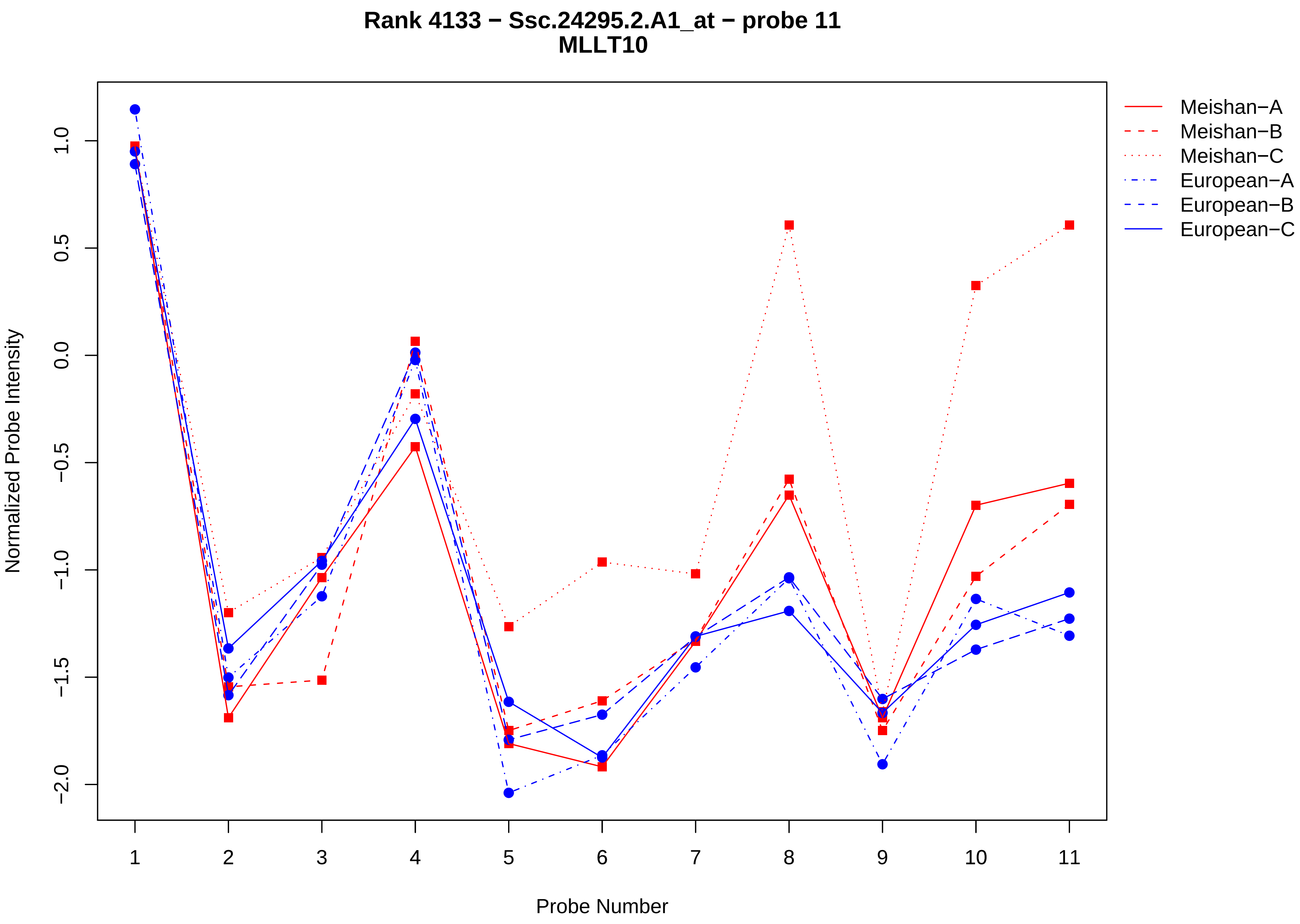
Rank 4133 − Ssc.24295.2.A1_at − probe 11
MLLT10
Normalized Probe Intensity
1.0
0.5
0.0
−0.5
−1.0
−1.5
−2.0
1
2
3
4
5
6
7
8
9
10
11
Probe Number
Meishan−A
Meishan−B
Meishan−C
European−A
European−B
European−C

Rank 4134 − Ssc.4035.1.A1_at − probe 3
C10orf68

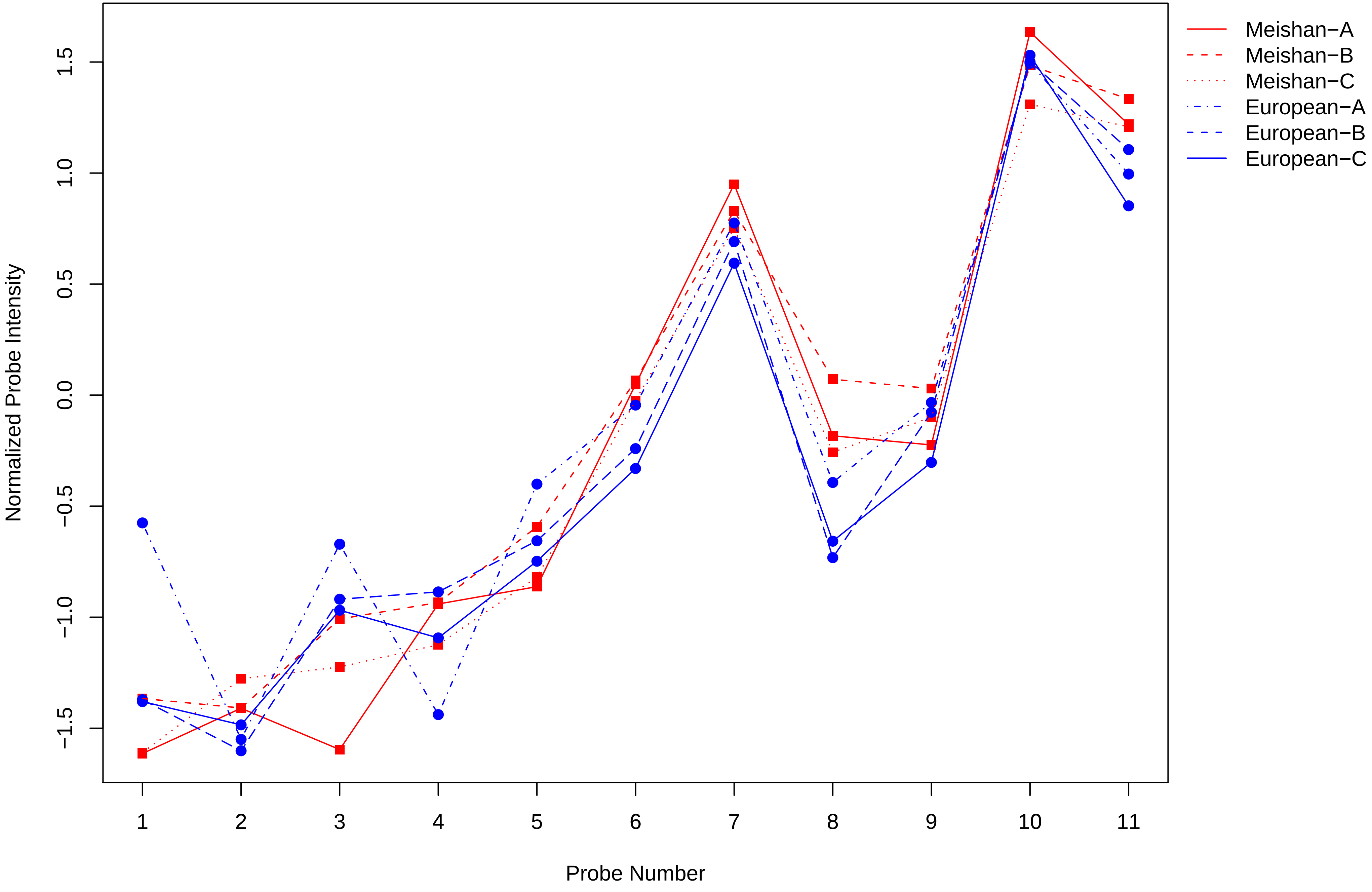

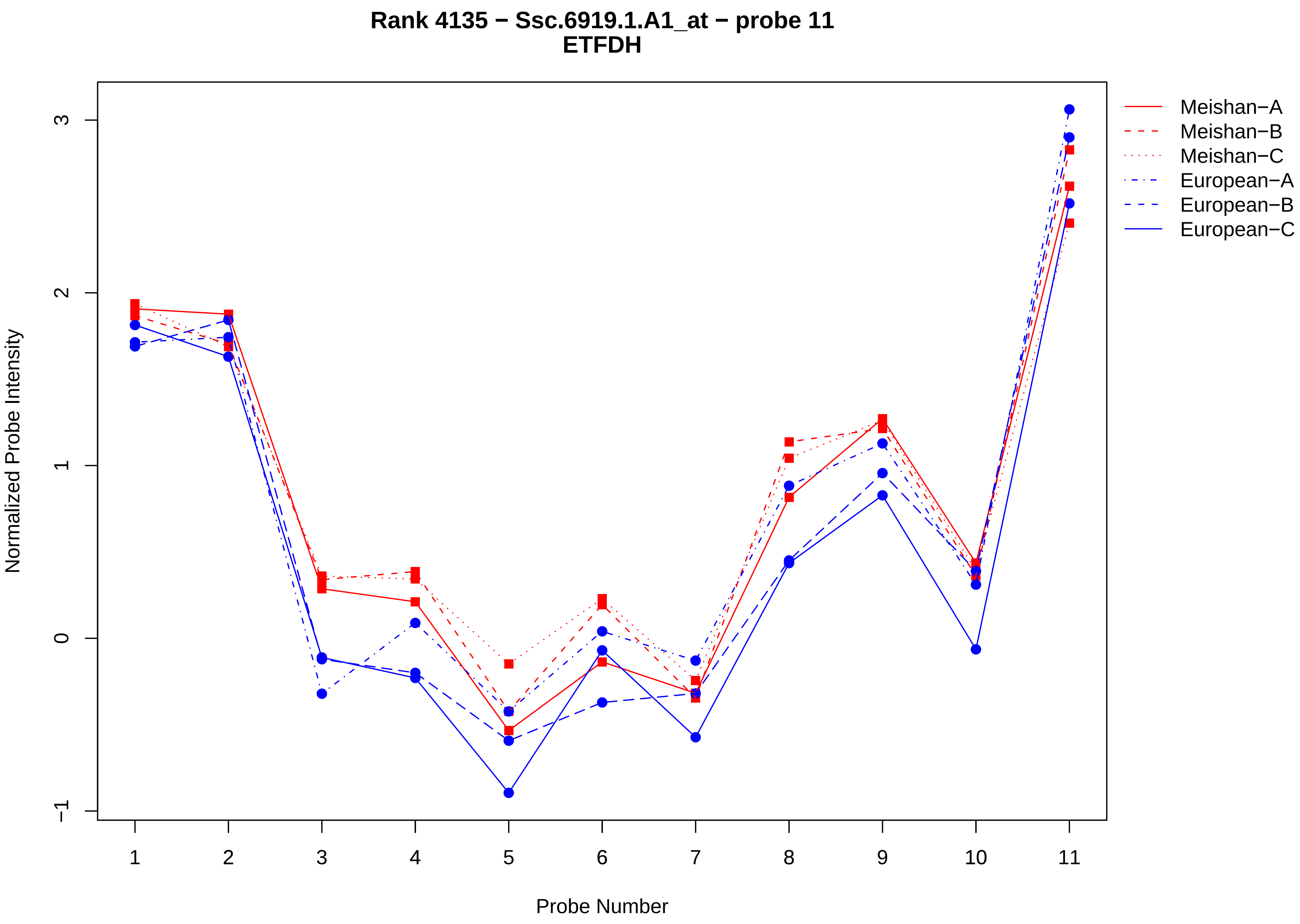

Rank 4135 − Ssc.6919.1.A1_at − probe 11
ETFDH
Normalized Probe Intensity
Probe Number
−1
0
1
2
3
1
2
3
4
5
6
7
8
9
10
11
Meishan−A
Meishan−B
Meishan−C
European−A
European−B
European−C

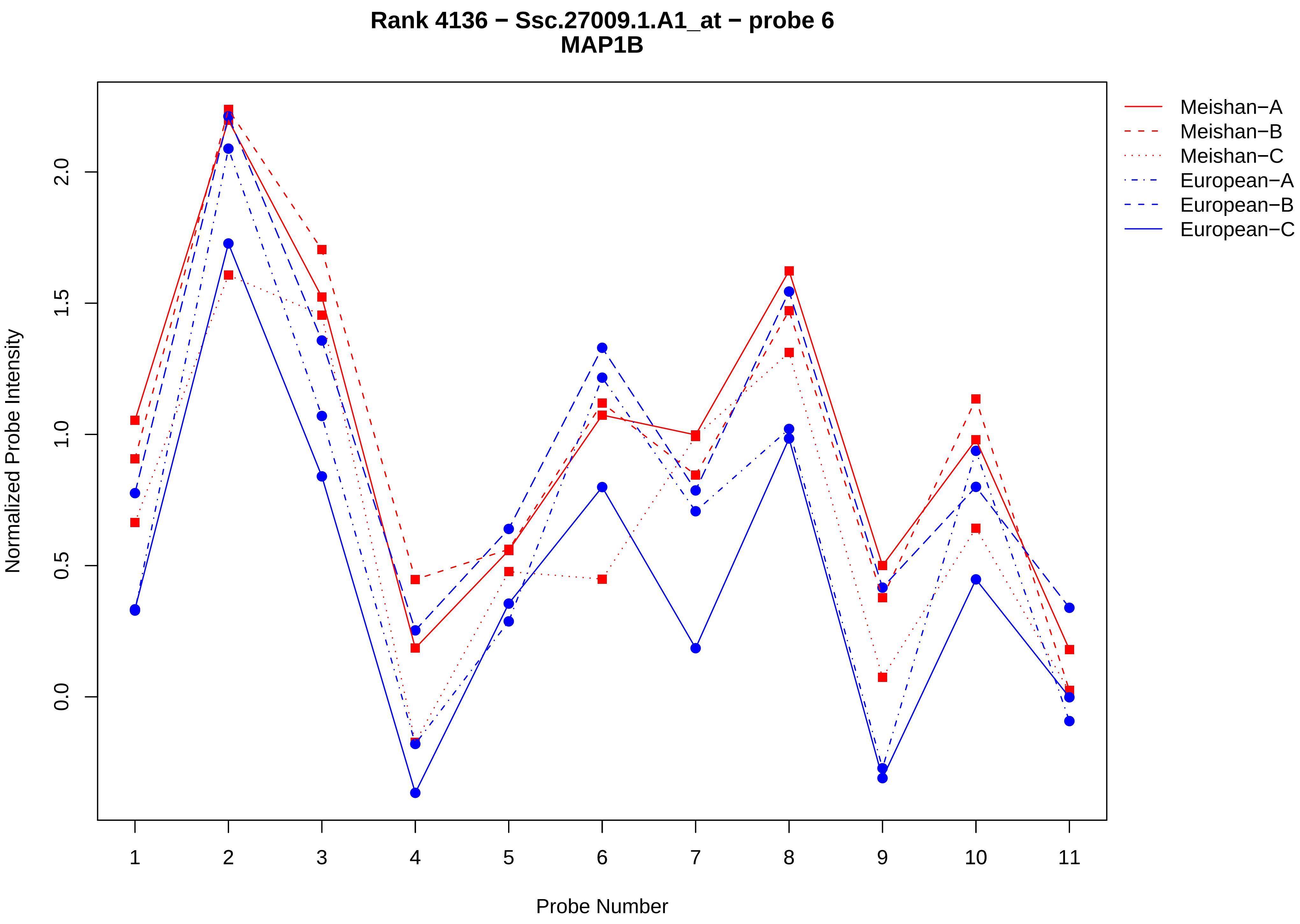

Rank 4136 − Ssc.27009.1.A1_at − probe 6
MAP1B
Normalized Probe Intensity
0.0
0.5
1.0
1.5
2.0
1
2
3
4
5
6
7
8
9
10
11
Probe Number
Meishan−A
Meishan−B
Meishan−C
European−A
European−B
European−C

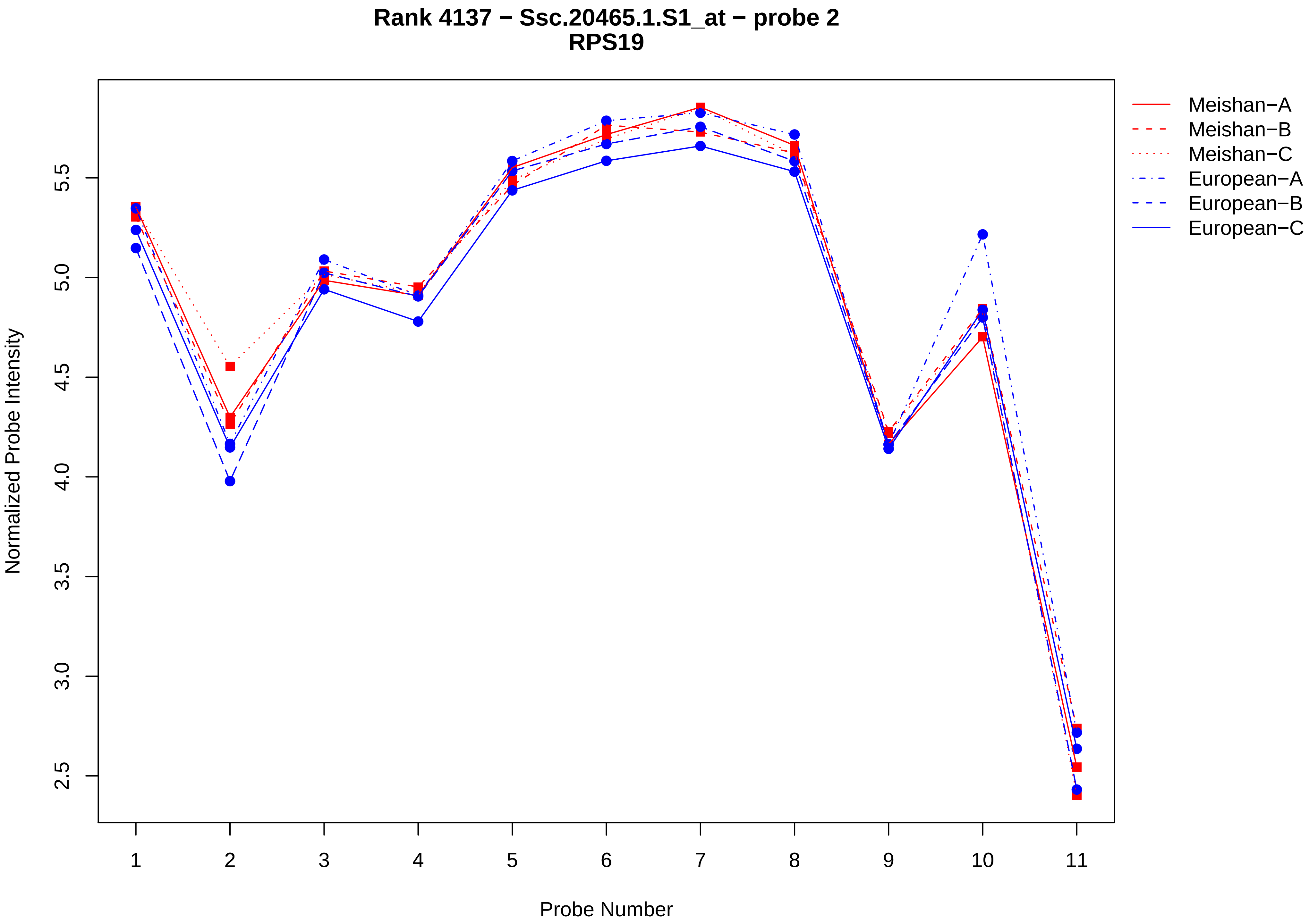

Rank 4137 − Ssc.20465.1.S1_at − probe 2
RPS19
Normalized Probe Intensity
Probe Number
2.5
3.0
3.5
4.0
4.5
5.0
5.5
1
2
3
4
5
6
7
8
9
10
11
Meishan−A
Meishan−B
Meishan−C
European−A
European−B
European−C

Rank 4138 − Ssc.20340.1.S1_a_at − probe 5
DTYMK

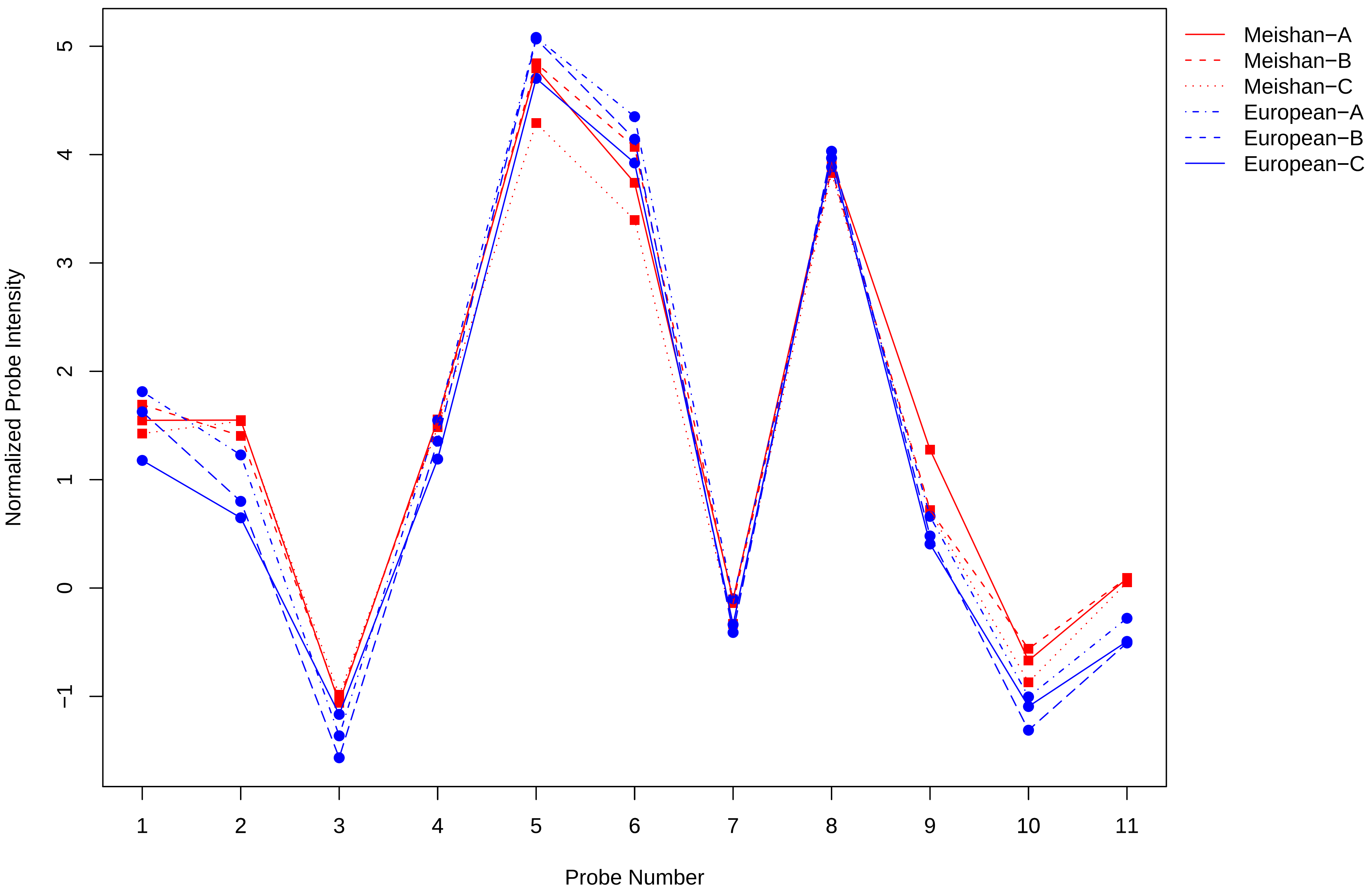

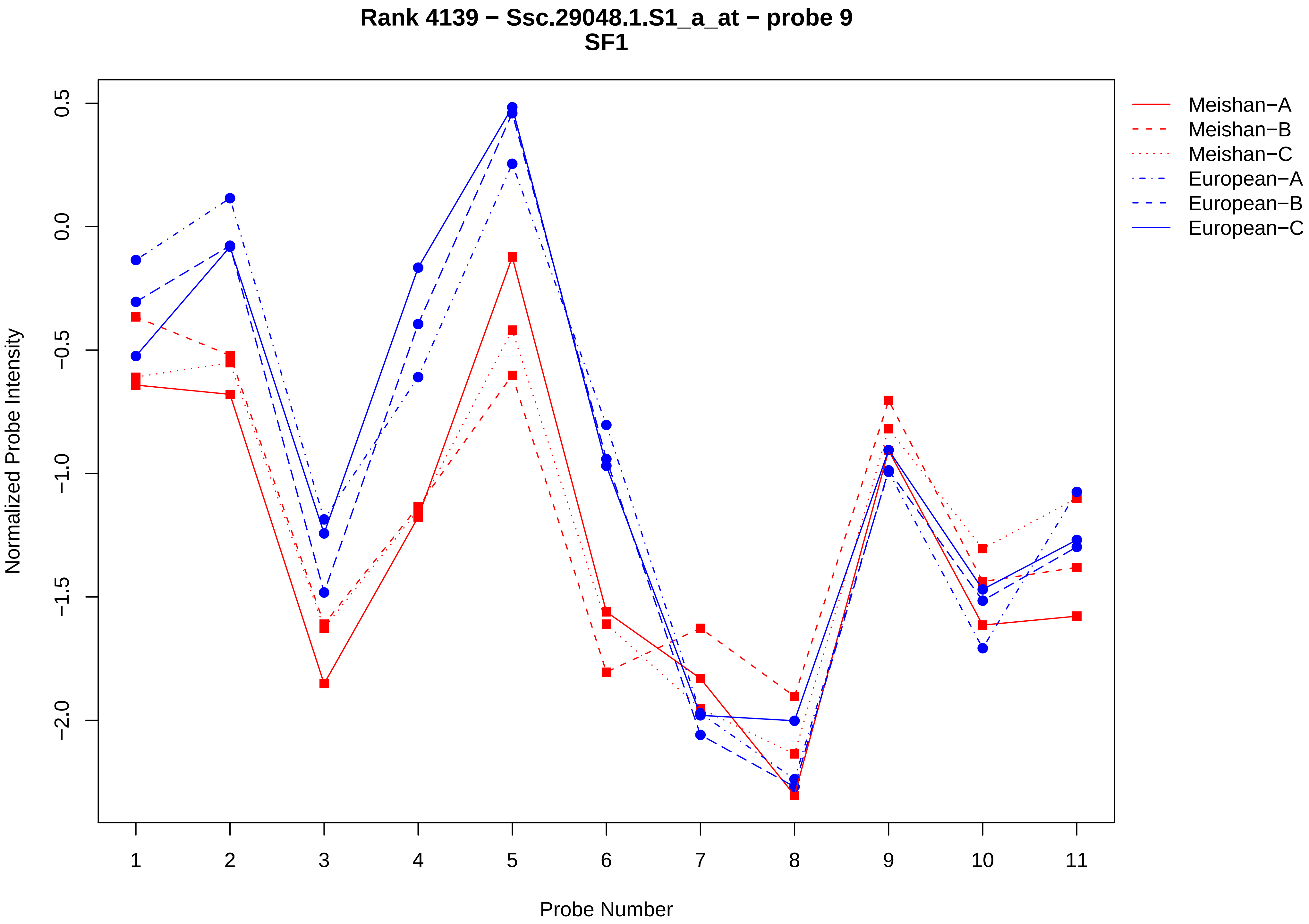

Rank 4139 − Ssc.29048.1.S1_a_at − probe 9
SF1
Normalized Probe Intensity
Probe Number
0.5
0.0
−0.5
−1.0
−1.5
−2.0
1
2
3
4
5
6
7
8
9
10
11
Meishan−A
Meishan−B
Meishan−C
European−A
European−B
European−C

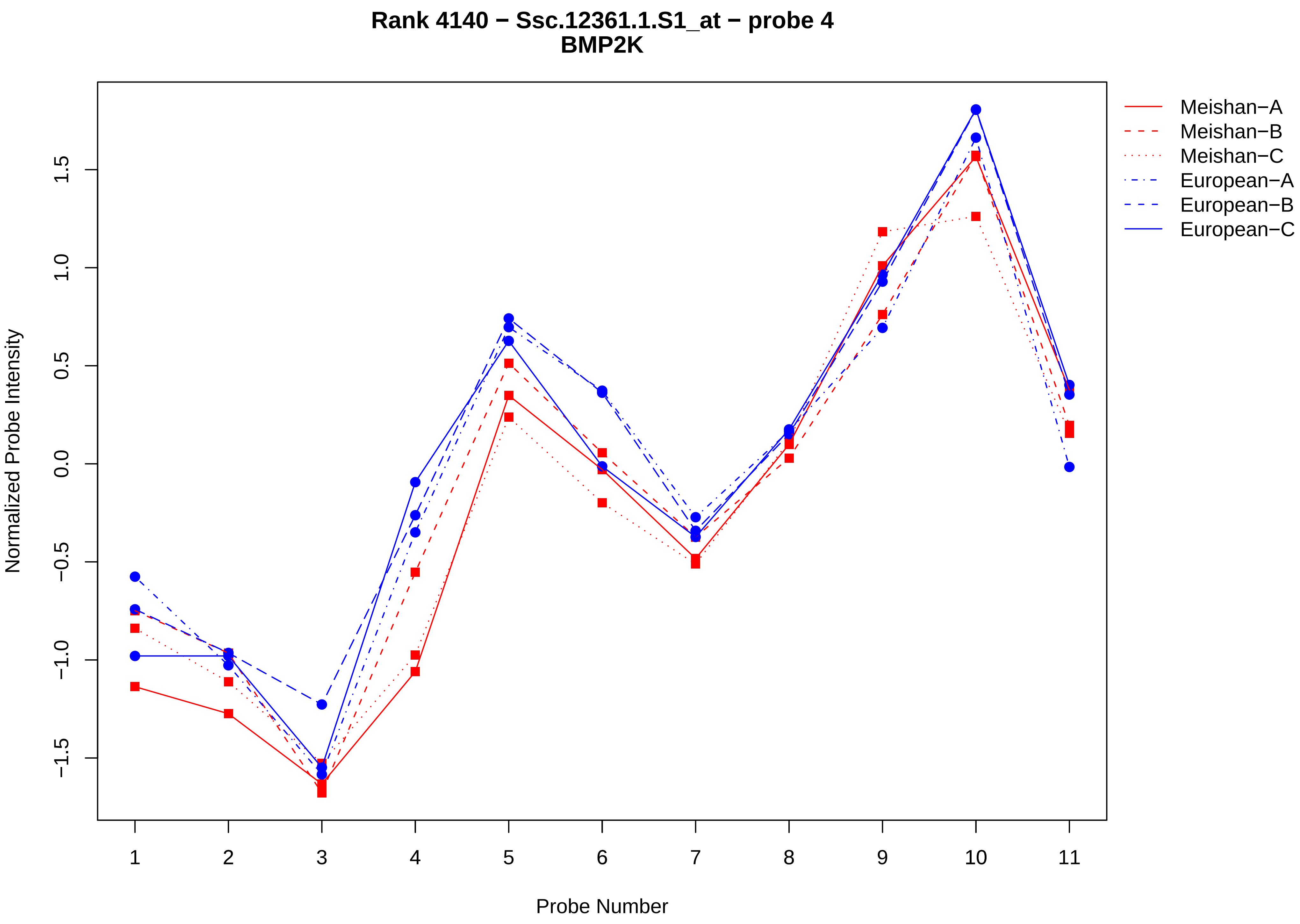

Rank 4140 − Ssc.12361.1.S1_at − probe 4
BMP2K
Normalized Probe Intensity
Probe Number
−1.5
−1.0
−0.5
0.0
0.5
1.0
1.5
1
2
3
4
5
6
7
8
9
10
11
Meishan−A
Meishan−B
Meishan−C
European−A
European−B
European−C

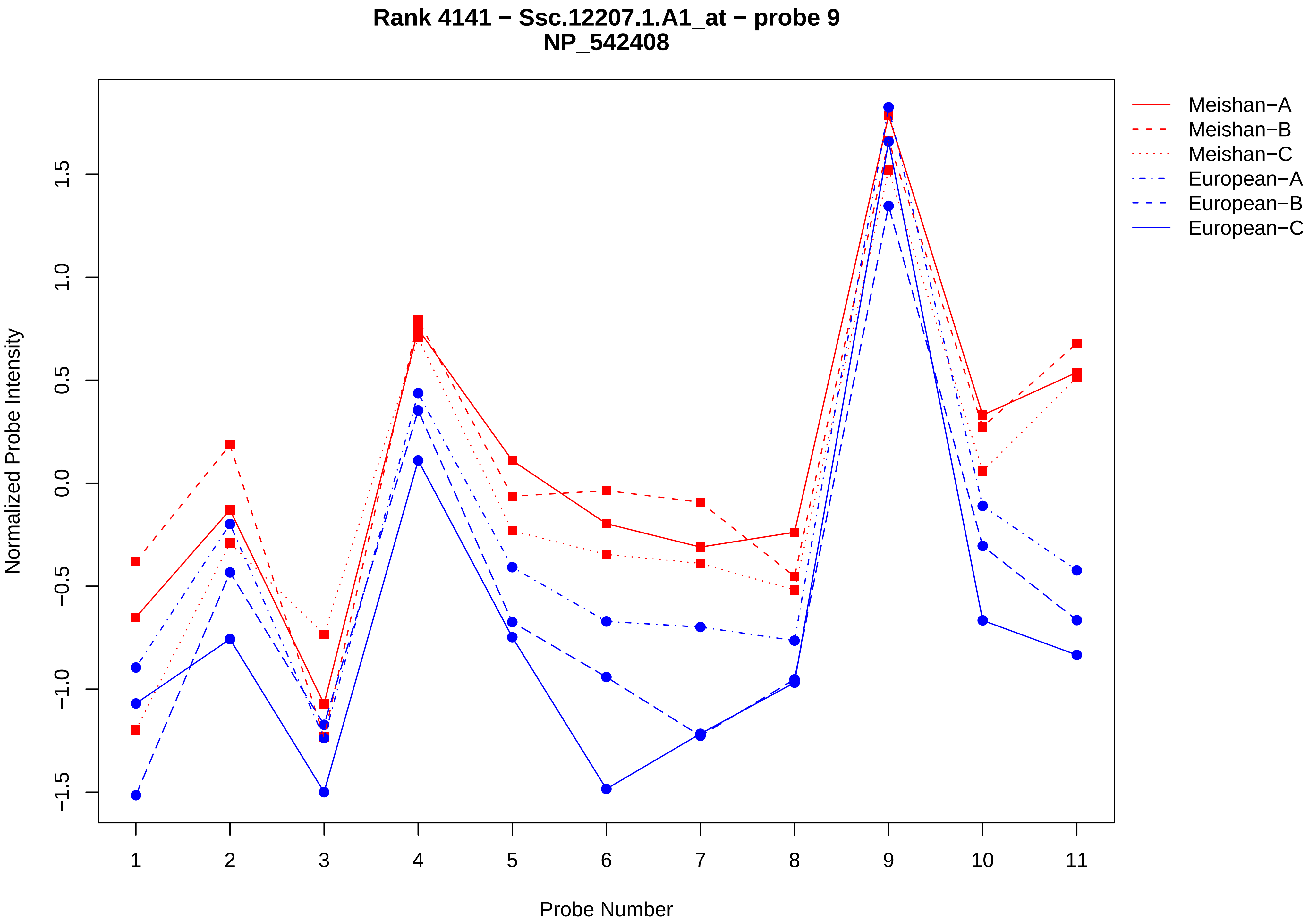

Rank 4141 − Ssc.12207.1.A1_at − probe 9
NP_542408
Normalized Probe Intensity
Probe Number
Meishan−A
Meishan−B
Meishan−C
European−A
European−B
European−C

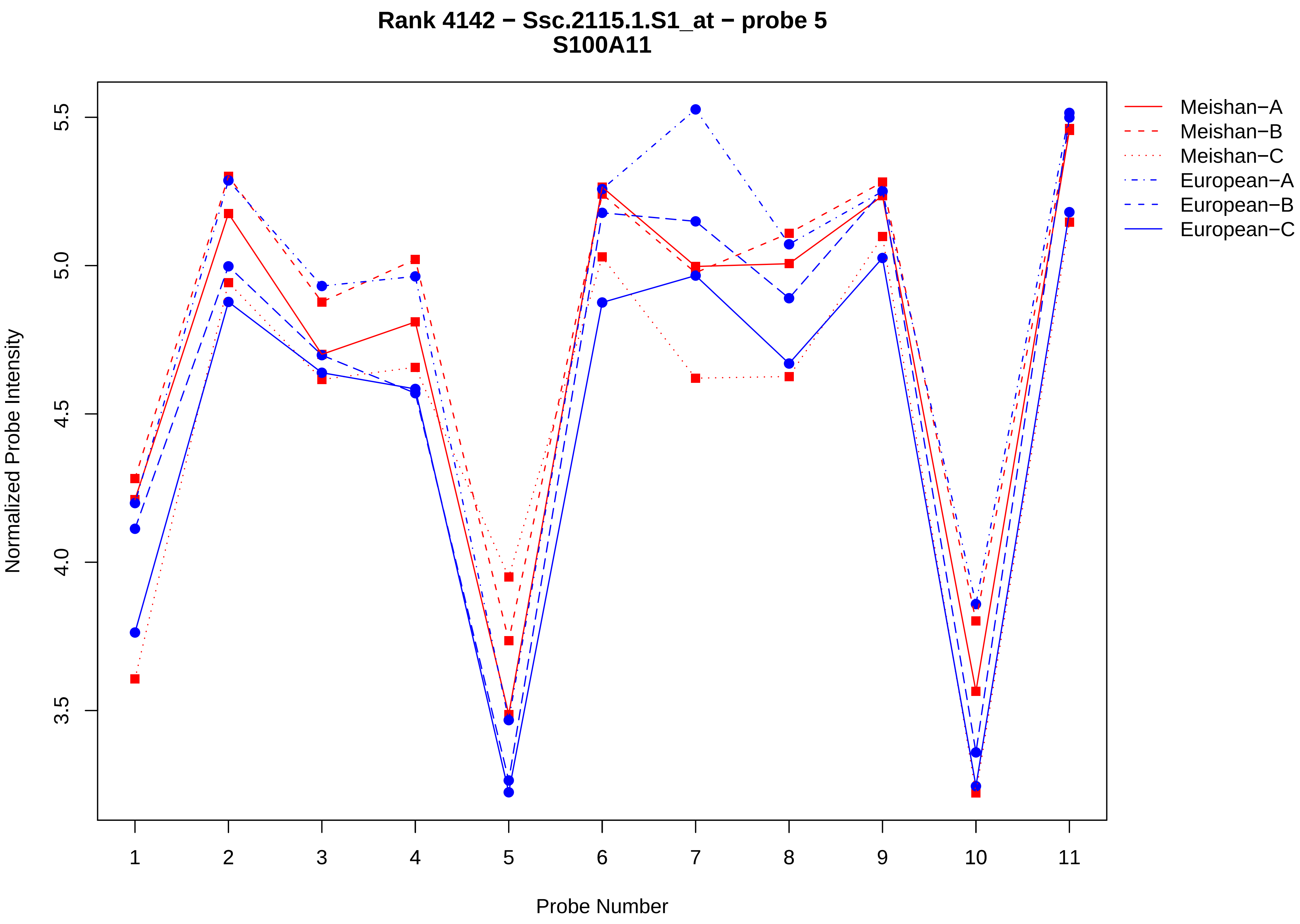
Rank 4142 − Ssc.2115.1.S1_at − probe 5
S100A11
Normalized Probe Intensity
3.5
4.0
4.5
5.0
5.5
1
2
3
4
5
6
7
8
9
10
11
Probe Number
Meishan−A
Meishan−B
Meishan−C
European−A
European−B
European−C

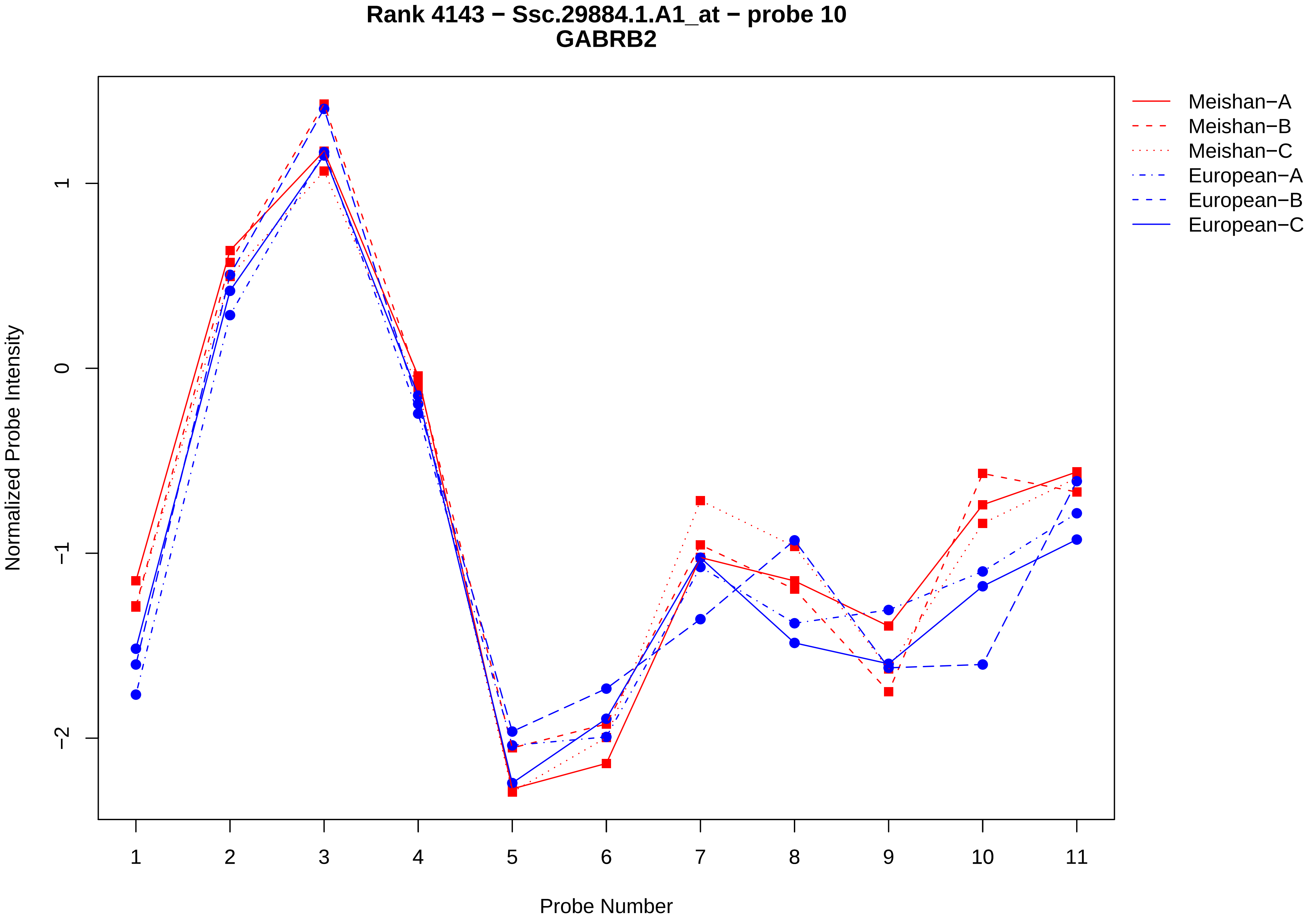

Rank 4143 − Ssc.29884.1.A1_at − probe 10
GABRB2
Normalized Probe Intensity
Probe Number
Meishan−A
Meishan−B
Meishan−C
European−A
European−B
European−C
1
0
−1
−2
1
2
3
4
5
6
7
8
9
10
11

Rank 4144 − Ssc.13095.1.A1_at − probe 7
RGS7

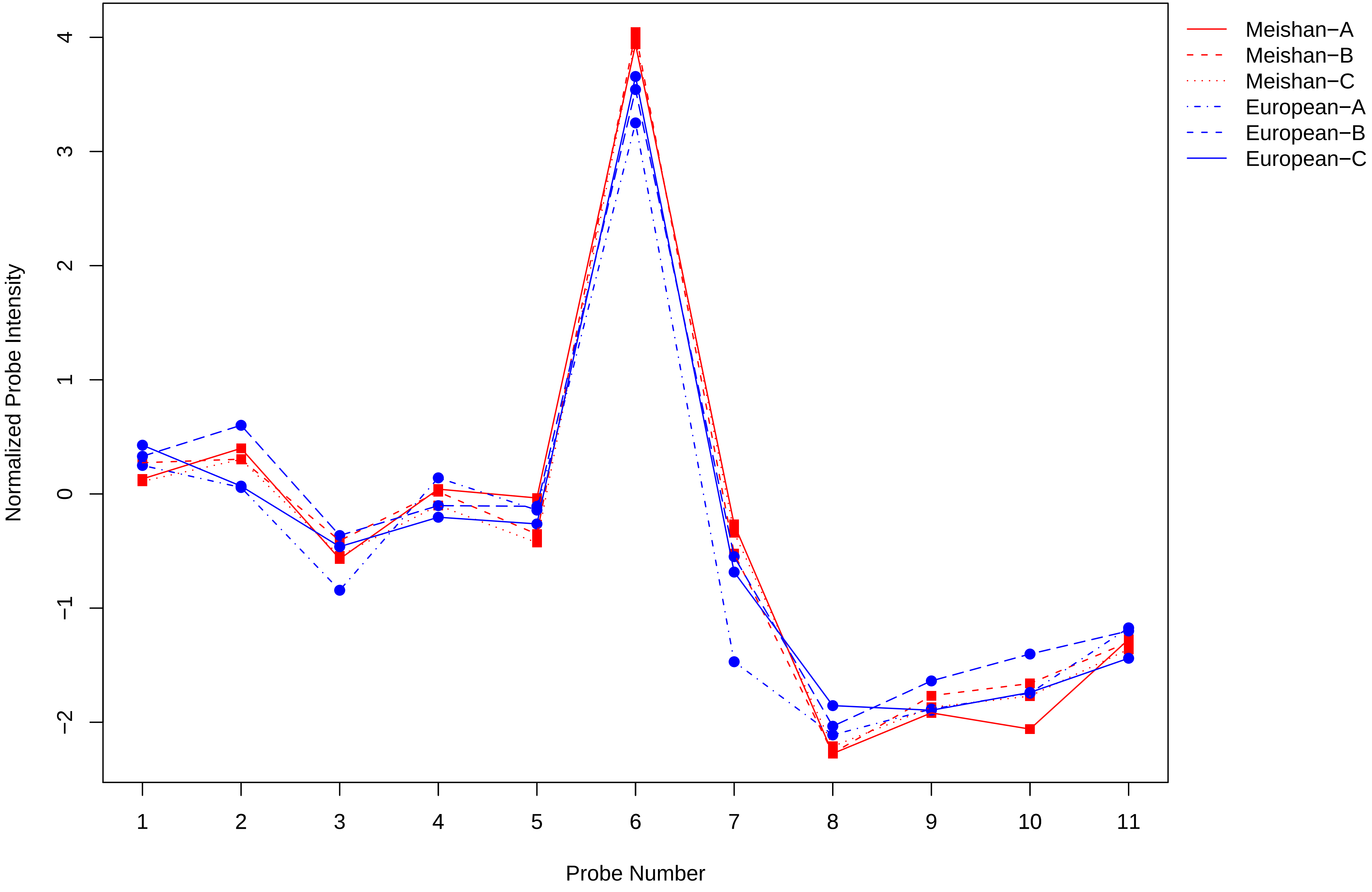

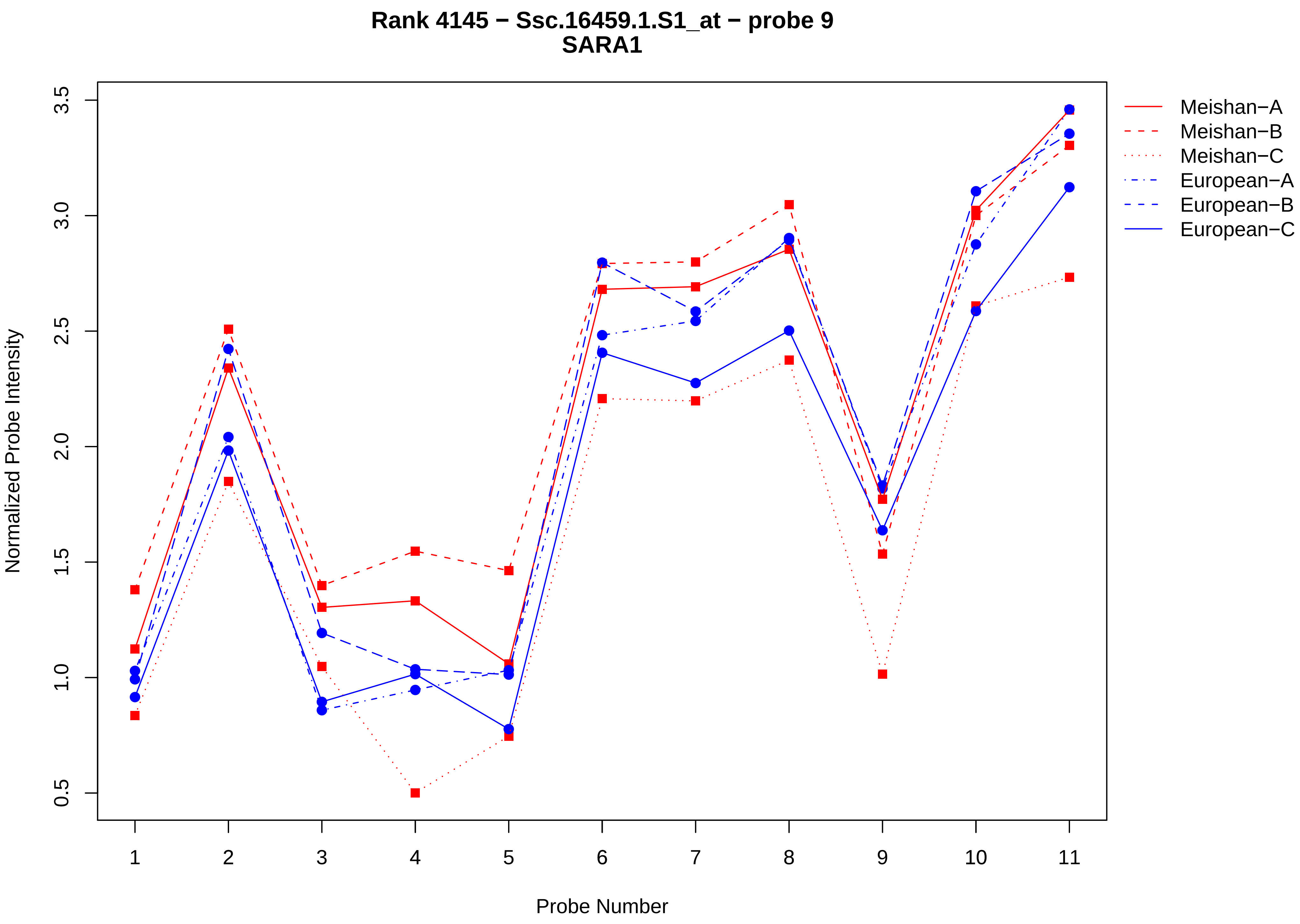
Rank 4145 − Ssc.16459.1.S1_at − probe 9
SARA1
Normalized Probe Intensity
Probe Number
Meishan−A
Meishan−B
Meishan−C
European−A
European−B
European−C
0.5
1.0
1.5
2.0
2.5
3.0
3.5
1
2
3
4
5
6
7
8
9
10
11

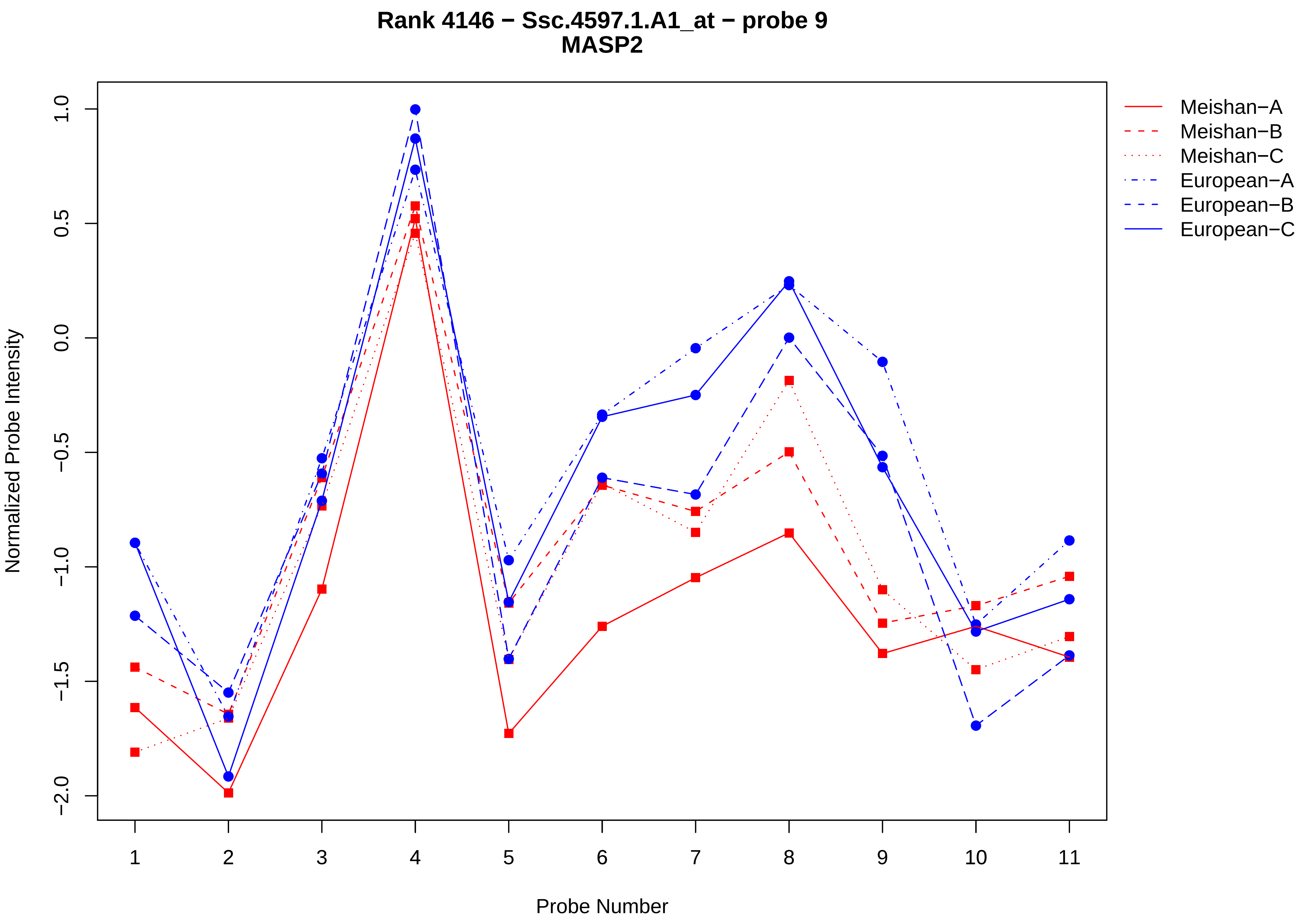

Rank 4146 − Ssc.4597.1.A1_at − probe 9
MASP2
Normalized Probe Intensity
Probe Number
1.0
0.5
0.0
−0.5
−1.0
−1.5
−2.0
1
2
3
4
5
6
7
8
9
10
11
Meishan−A
Meishan−B
Meishan−C
European−A
European−B
European−C

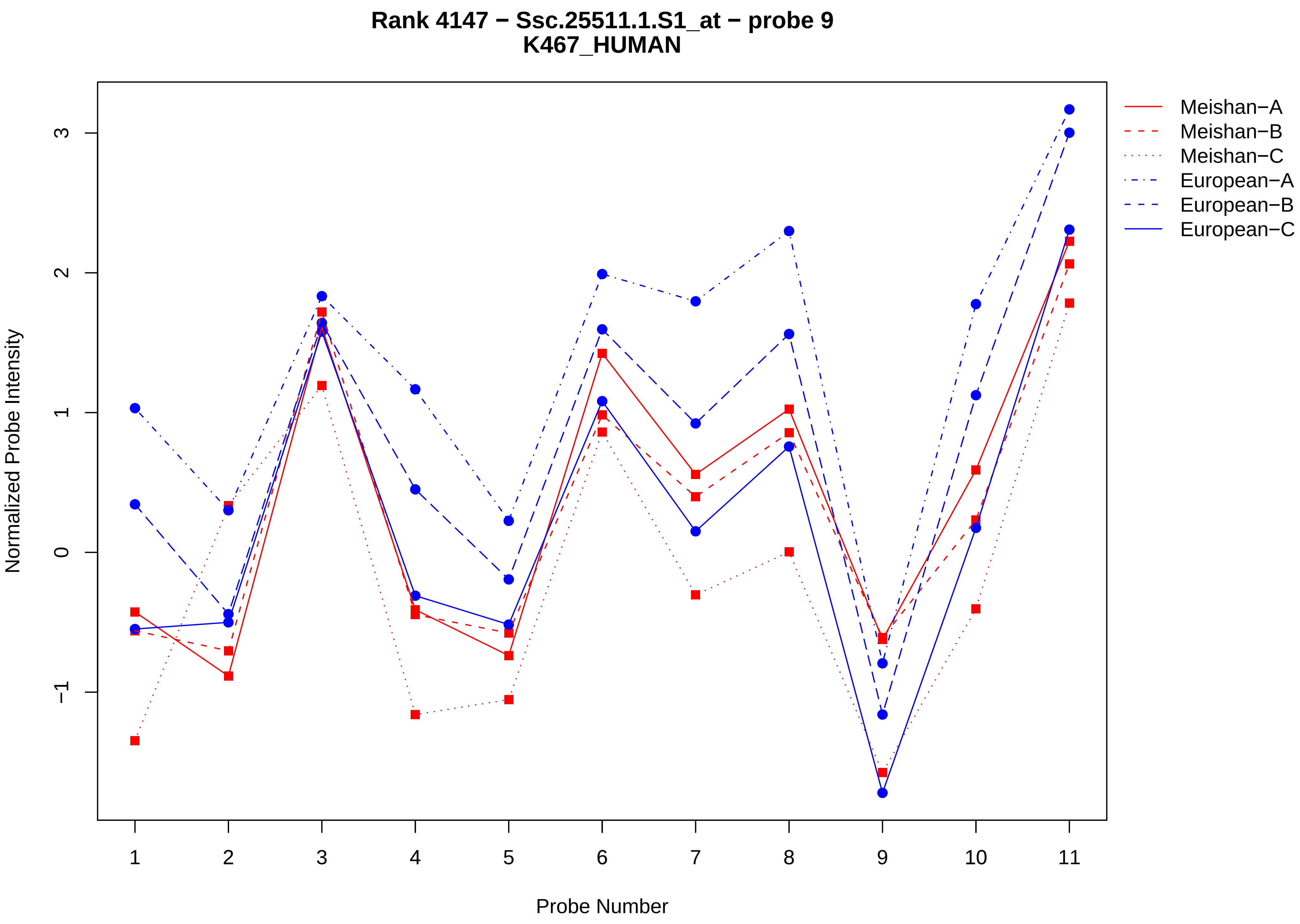

Rank 4147 − Ssc.25511.1.S1_at − probe 9
K467_HUMAN
Normalized Probe Intensity
Probe Number
−1
0
1
2
3
1
2
3
4
5
6
7
8
9
10
11
Meishan−A
Meishan−B
Meishan−C
European−A
European−B
European−C

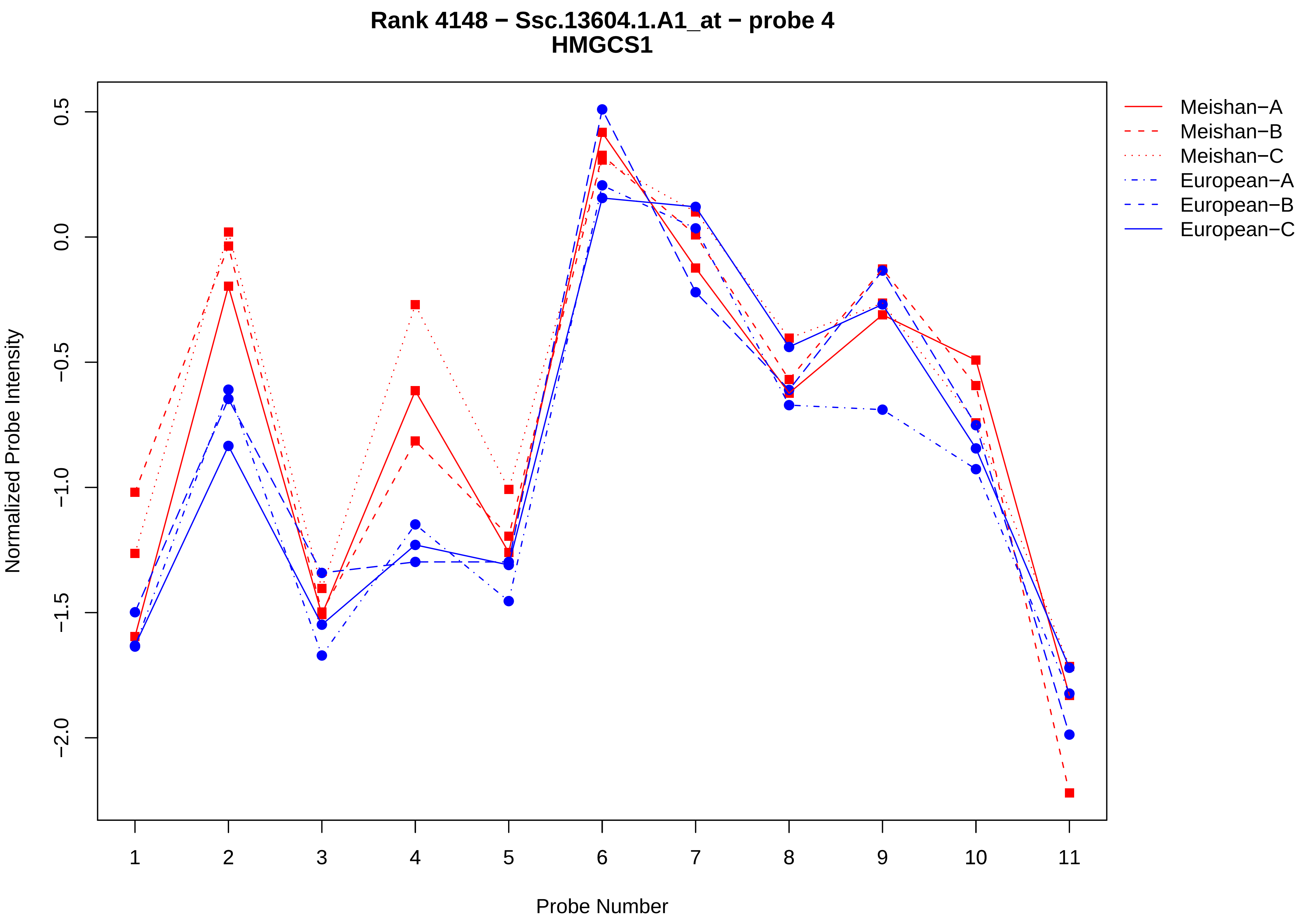
Rank 4148 − Ssc.13604.1.A1_at − probe 4
HMGCS1
Normalized Probe Intensity
Probe Number
0.5
0.0
−0.5
−1.0
−1.5
−2.0
1
2
3
4
5
6
7
8
9
10
11
Meishan−A
Meishan−B
Meishan−C
European−A
European−B
European−C

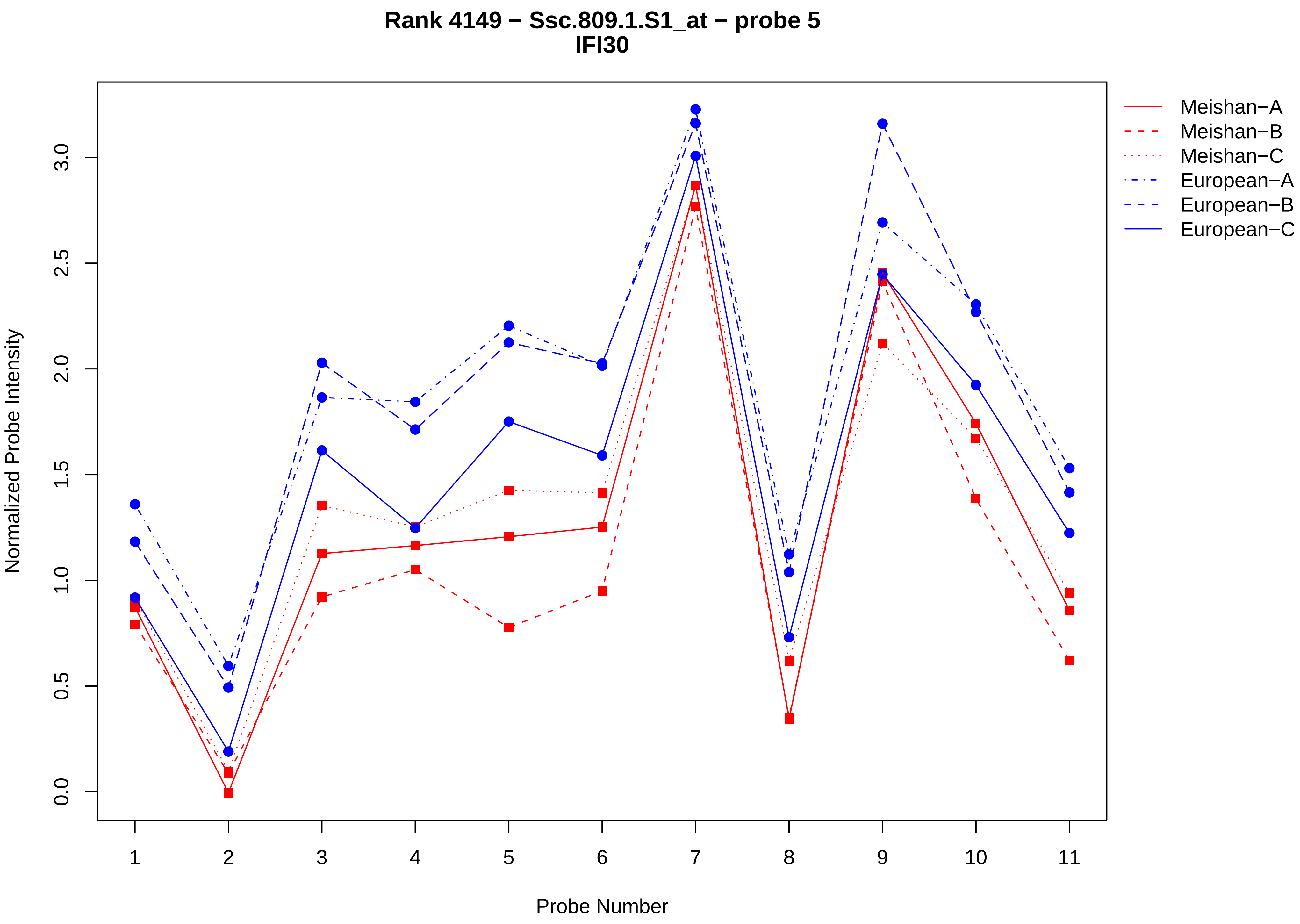

Rank 4149 − Ssc.809.1.S1_at − probe 5
IFI30
Normalized Probe Intensity
0.0
0.5
1.0
1.5
2.0
2.5
3.0
1
2
3
4
5
6
7
8
9
10
11
Probe Number
Meishan−A
Meishan−B
Meishan−C
European−A
European−B
European−C

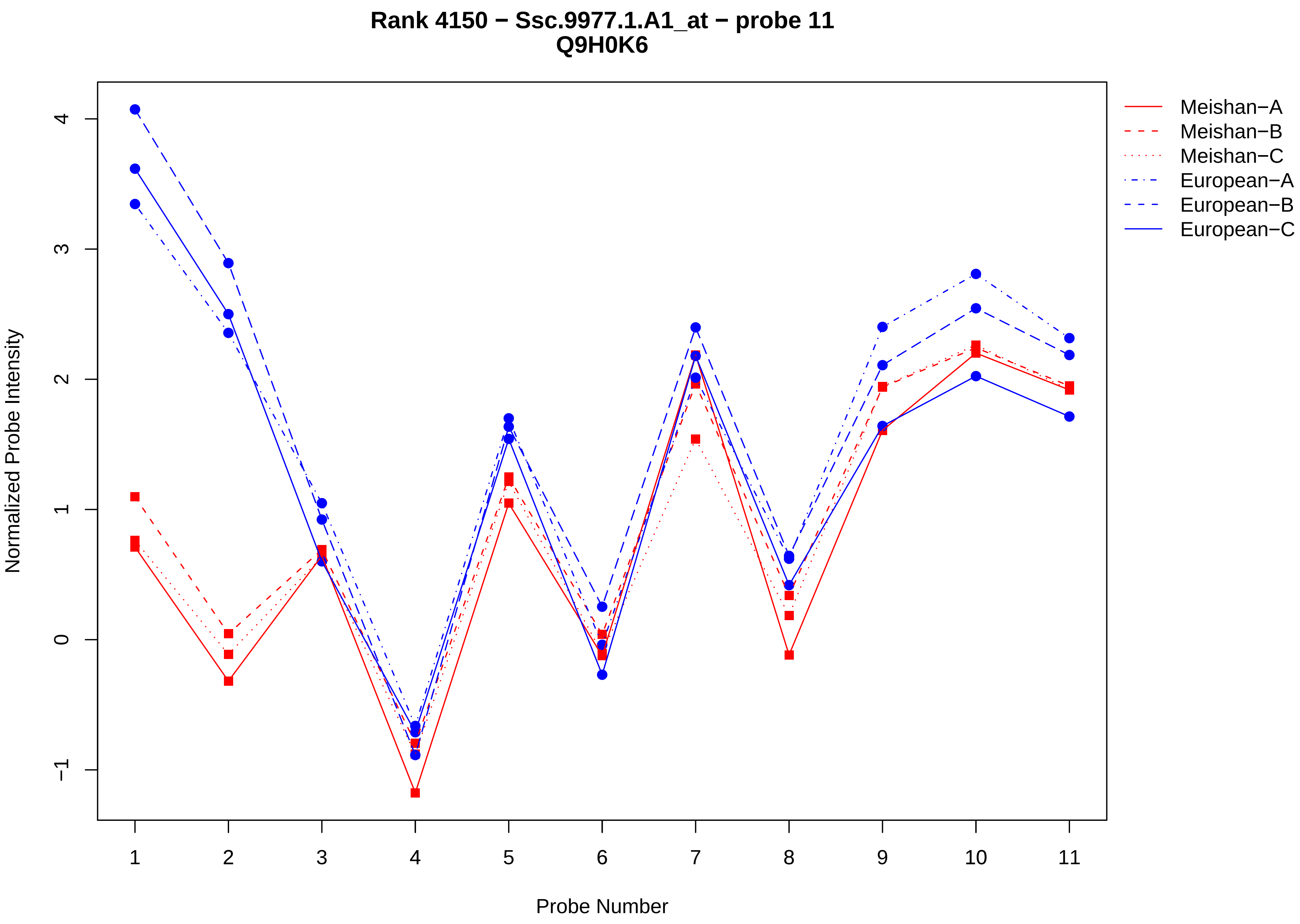

Rank 4150 − Ssc.9977.1.A1_at − probe 11
Q9H0K6
Normalized Probe Intensity
Probe Number
−1
0
1
2
3
4
1
2
3
4
5
6
7
8
9
10
11
Meishan−A
Meishan−B
Meishan−C
European−A
European−B
European−C

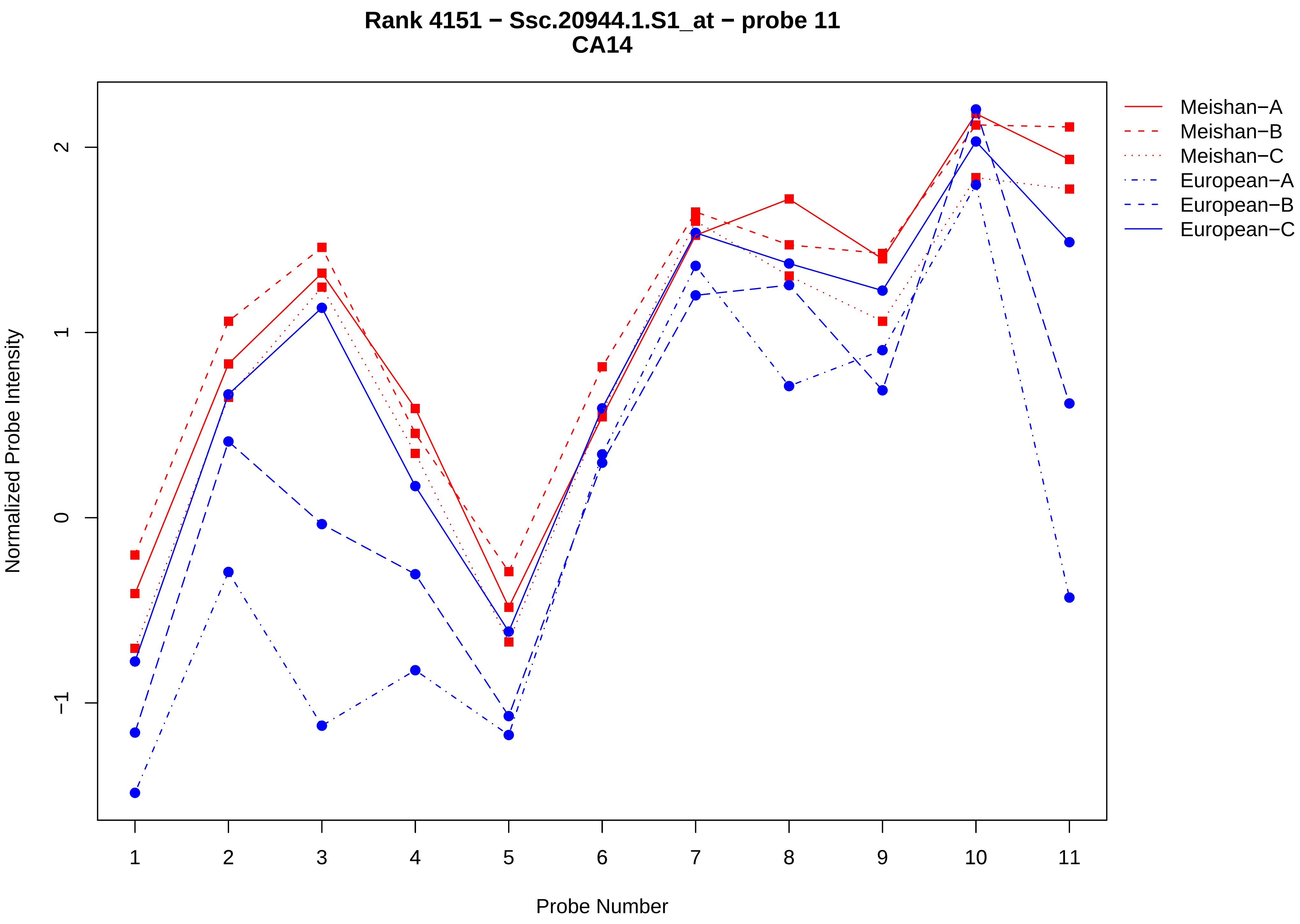

Rank 4151 − Ssc.20944.1.S1_at − probe 11
CA14
Normalized Probe Intensity
Probe Number
−1
0
1
2
1
2
3
4
5
6
7
8
9
10
11
Meishan−A
Meishan−B
Meishan−C
European−A
European−B
European−C

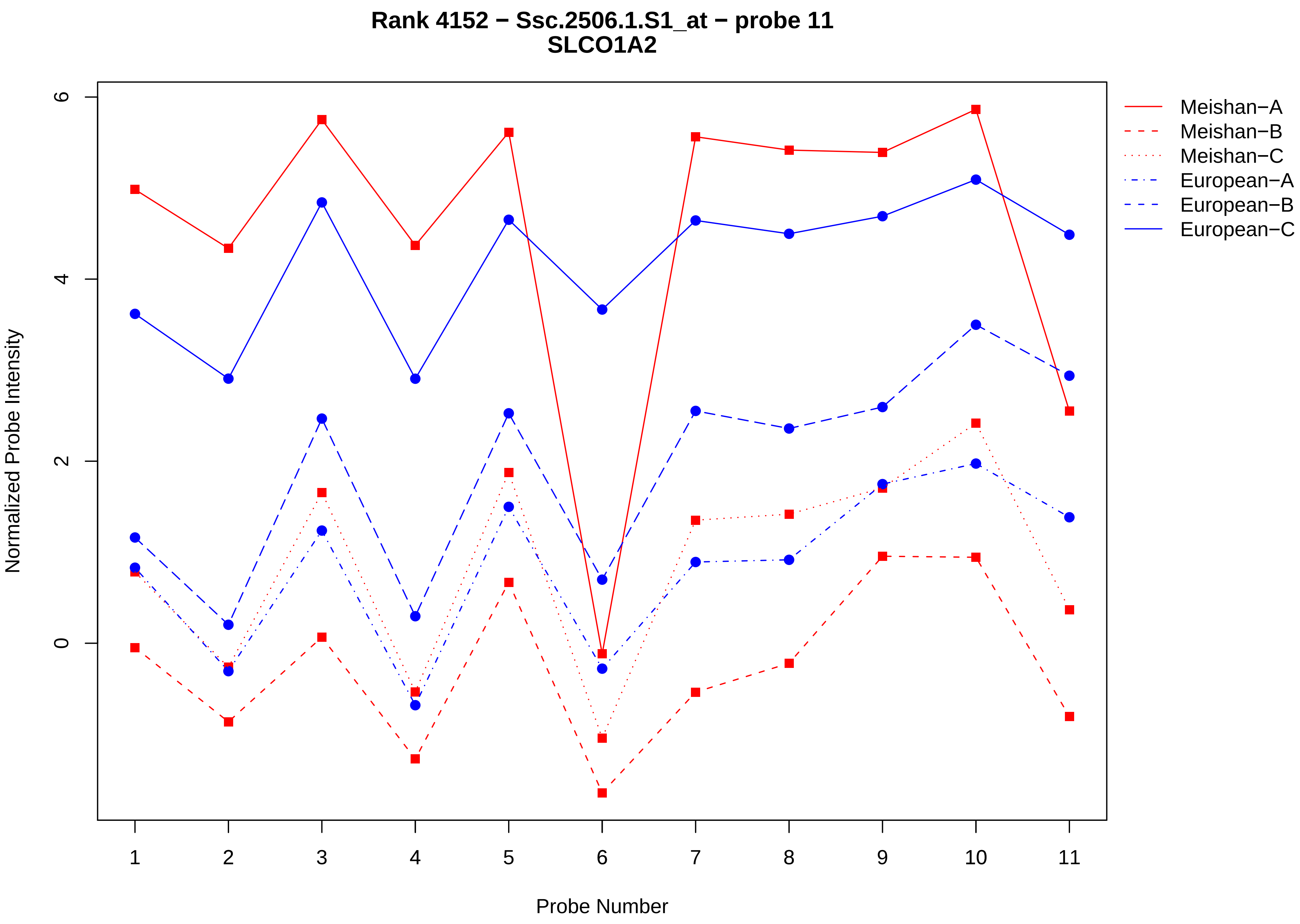

Rank 4152 − Ssc.2506.1.S1_at − probe 11
SLCO1A2
Normalized Probe Intensity
Probe Number
0
2
4
6
1
2
3
4
5
6
7
8
9
10
11
Meishan−A
Meishan−B
Meishan−C
European−A
European−B
European−C

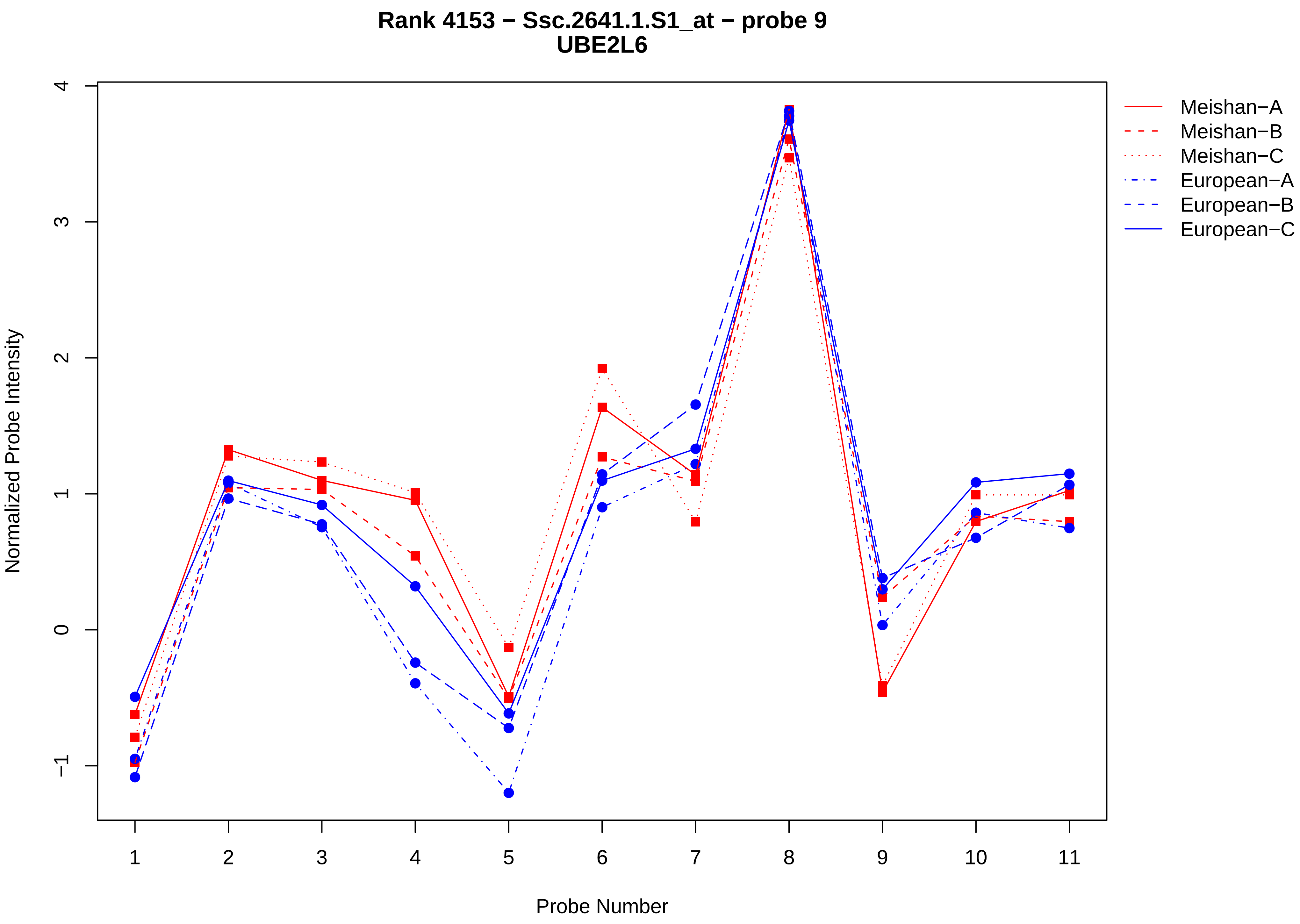

Rank 4153 − Ssc.2641.1.S1_at − probe 9
UBE2L6
Normalized Probe Intensity
Probe Number
Meishan−A
Meishan−B
Meishan−C
European−A
European−B
European−C

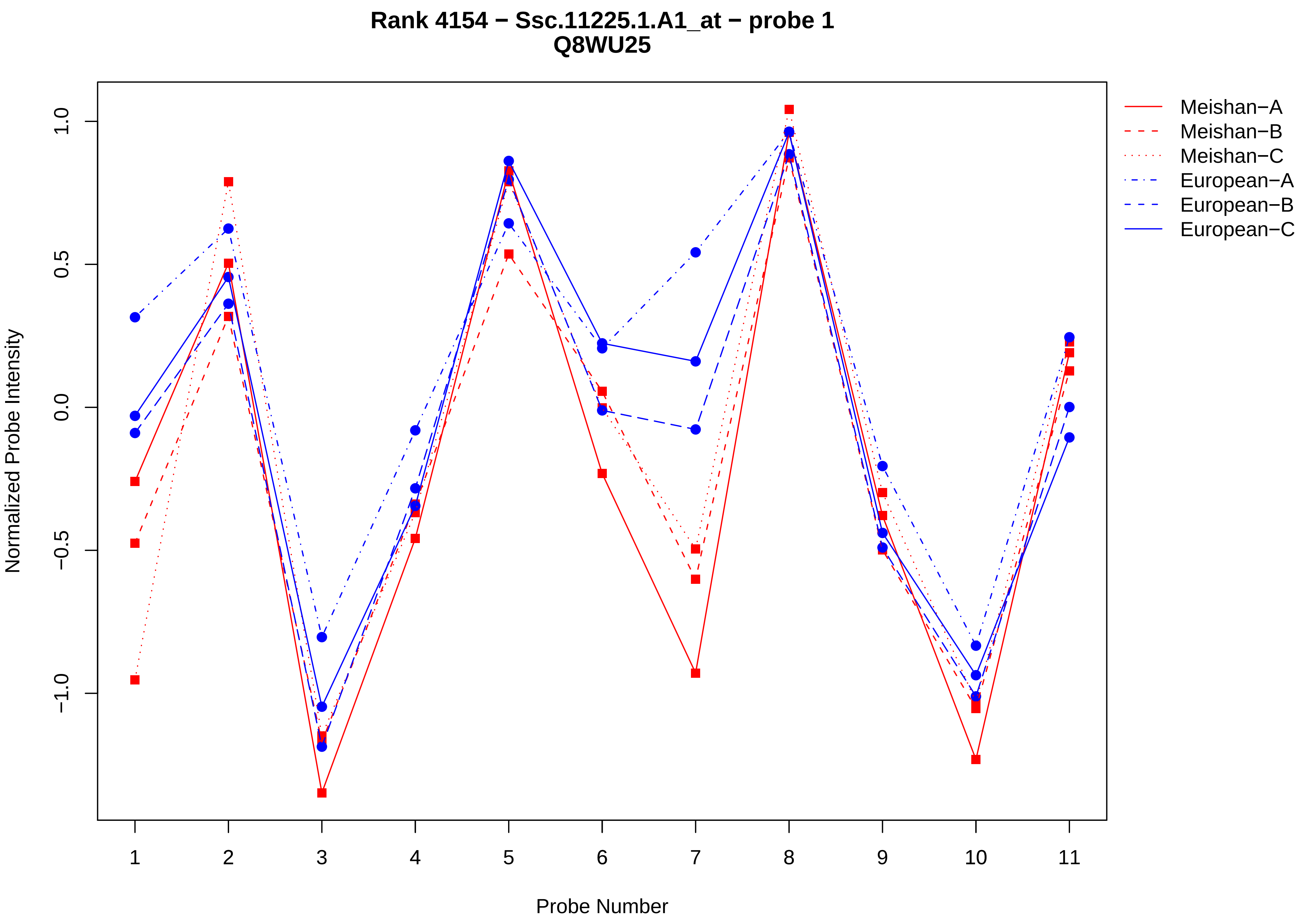

Rank 4154 − Ssc.11225.1.A1_at − probe 1
Q8WU25
Normalized Probe Intensity
Probe Number
1.0
0.5
0.0
−0.5
−1.0
1
2
3
4
5
6
7
8
9
10
11
Meishan−A
Meishan−B
Meishan−C
European−A
European−B
European−C

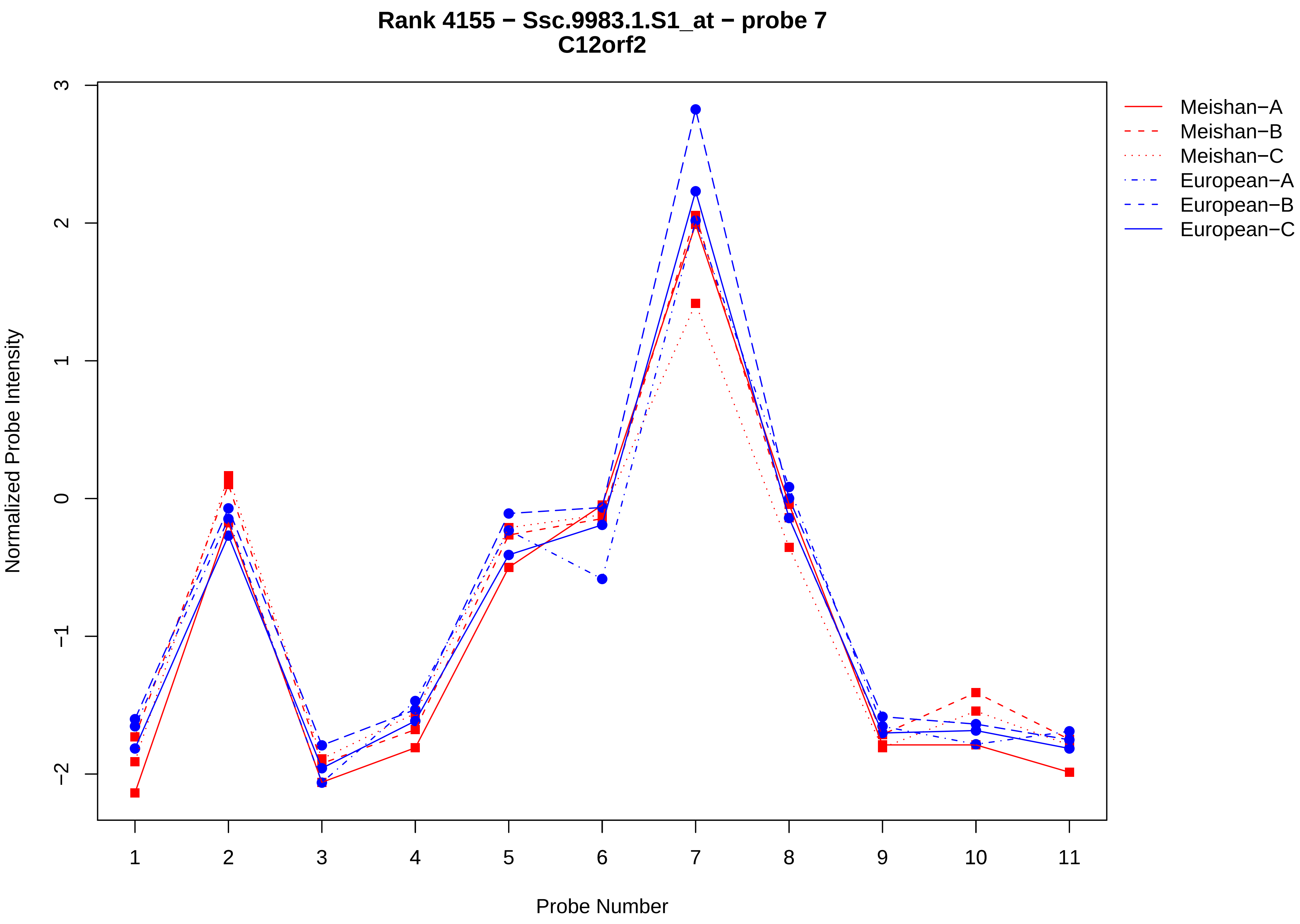
Rank 4155 − Ssc.9983.1.S1_at − probe 7
C12orf2
Normalized Probe Intensity
Probe Number
Meishan−A
Meishan−B
Meishan−C
European−A
European−B
European−C

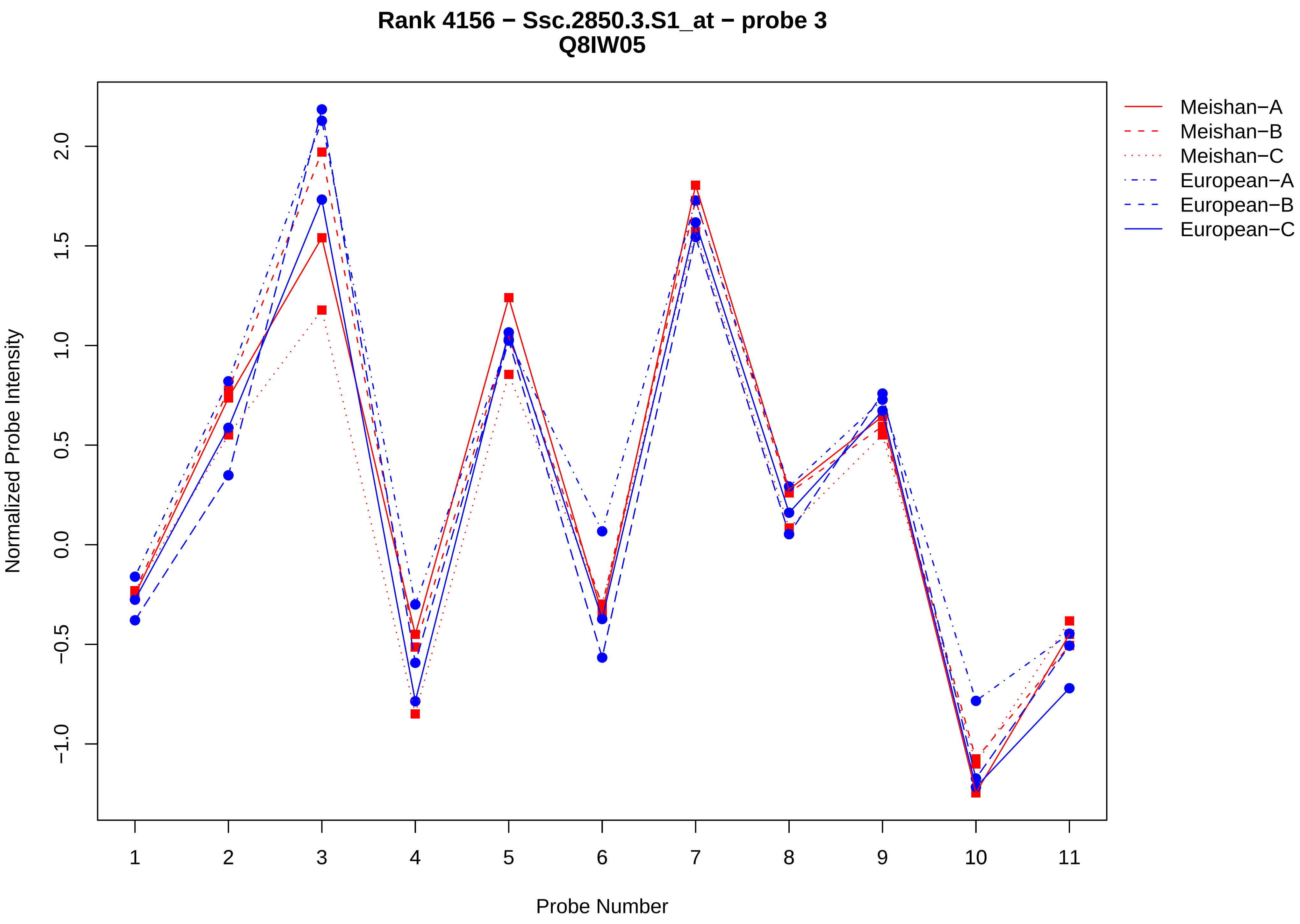
Rank 4156 − Ssc.2850.3.S1_at − probe 3
Q8IW05
Normalized Probe Intensity
−1.0
−0.5
0.0
0.5
1.0
1.5
2.0
1
2
3
4
5
6
7
8
9
10
11
Probe Number
Meishan−A
Meishan−B
Meishan−C
European−A
European−B
European−C

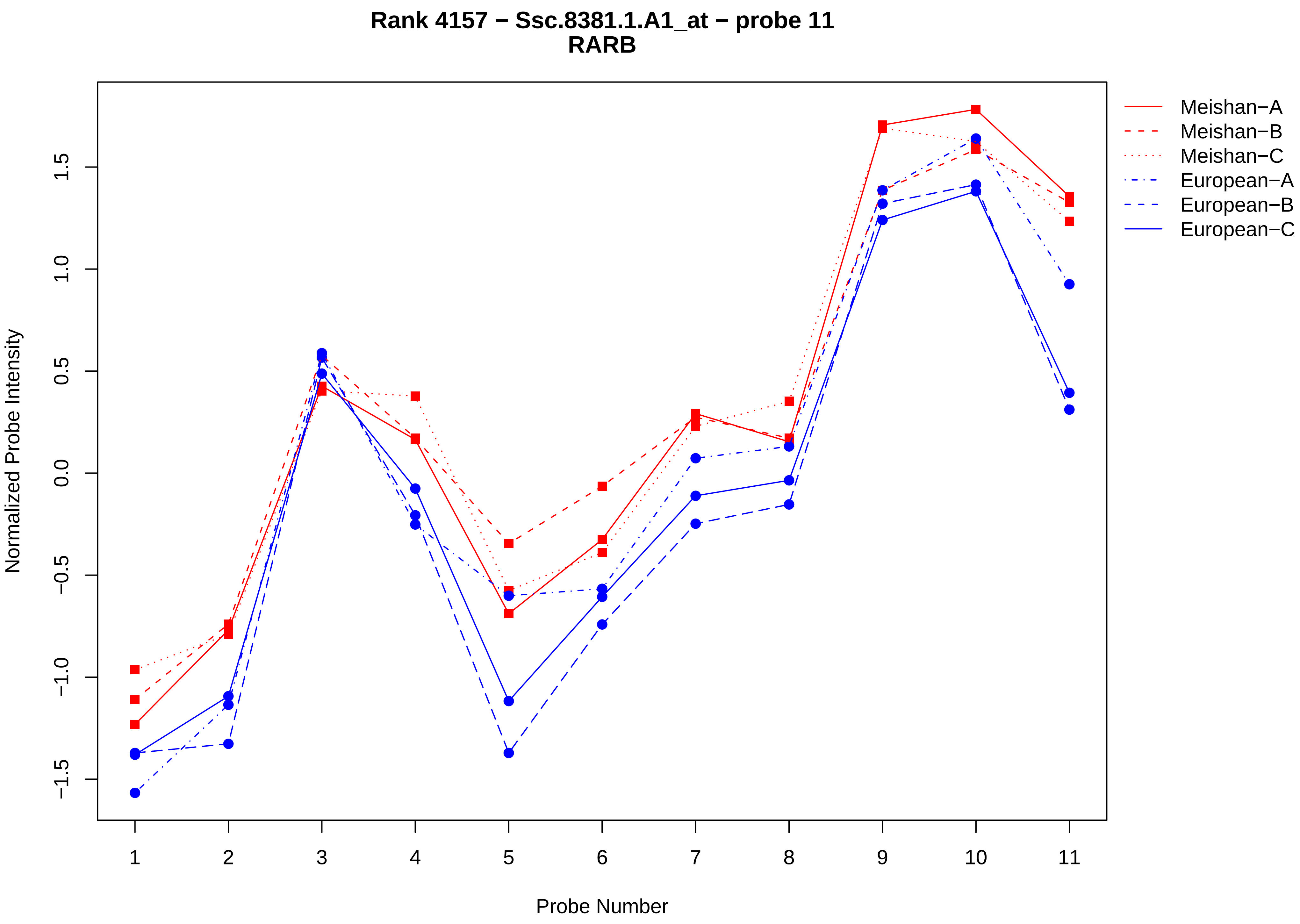

Rank 4157 − Ssc.8381.1.A1_at − probe 11
RARB
Normalized Probe Intensity
Probe Number
Meishan−A
Meishan−B
Meishan−C
European−A
European−B
European−C
−1.5
−1.0
−0.5
0.0
0.5
1.0
1.5
1
2
3
4
5
6
7
8
9
10
11

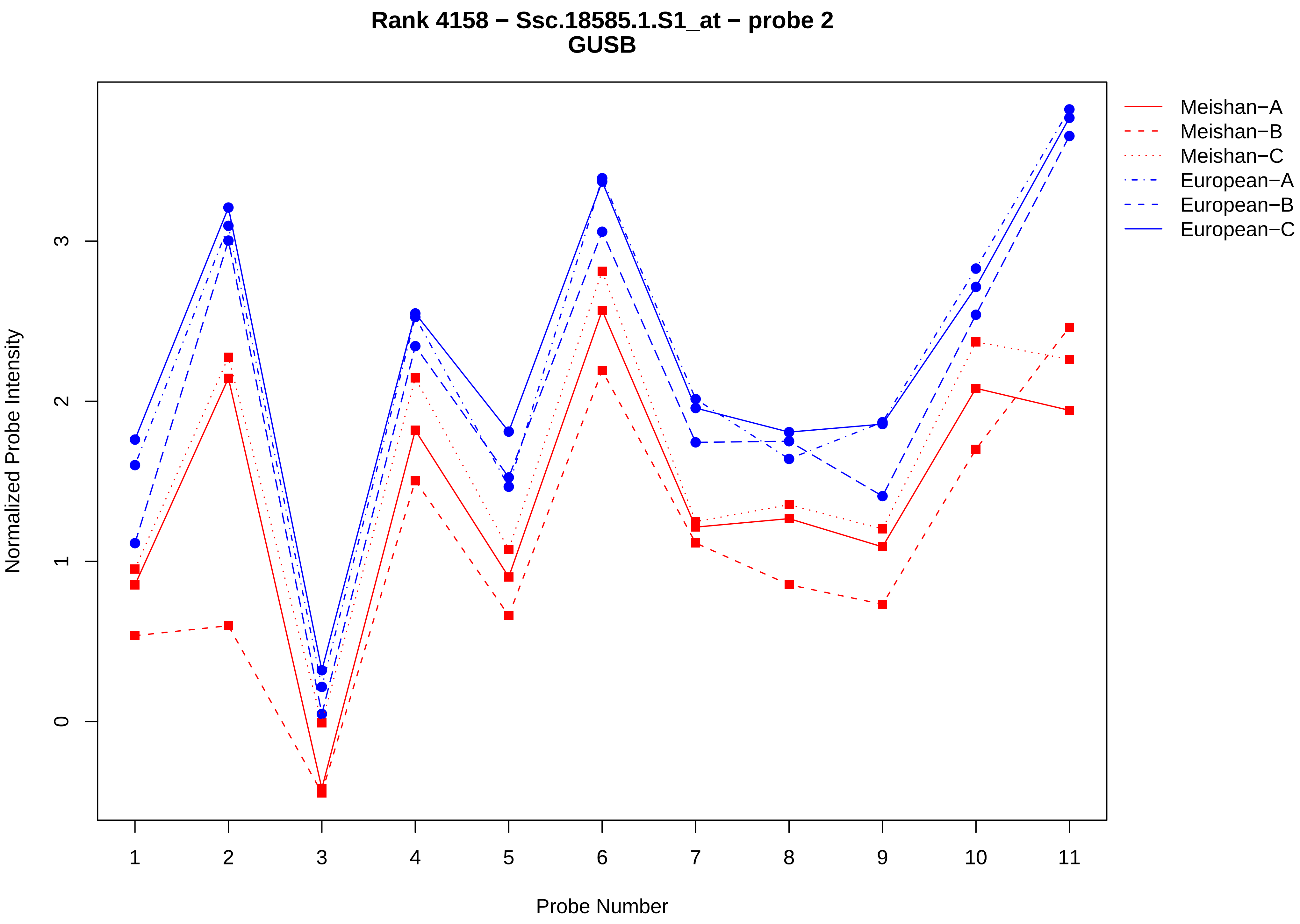

Rank 4158 − Ssc.18585.1.S1_at − probe 2
GUSB
Normalized Probe Intensity
Probe Number
0
1
2
3
1
2
3
4
5
6
7
8
9
10
11
Meishan−A
Meishan−B
Meishan−C
European−A
European−B
European−C

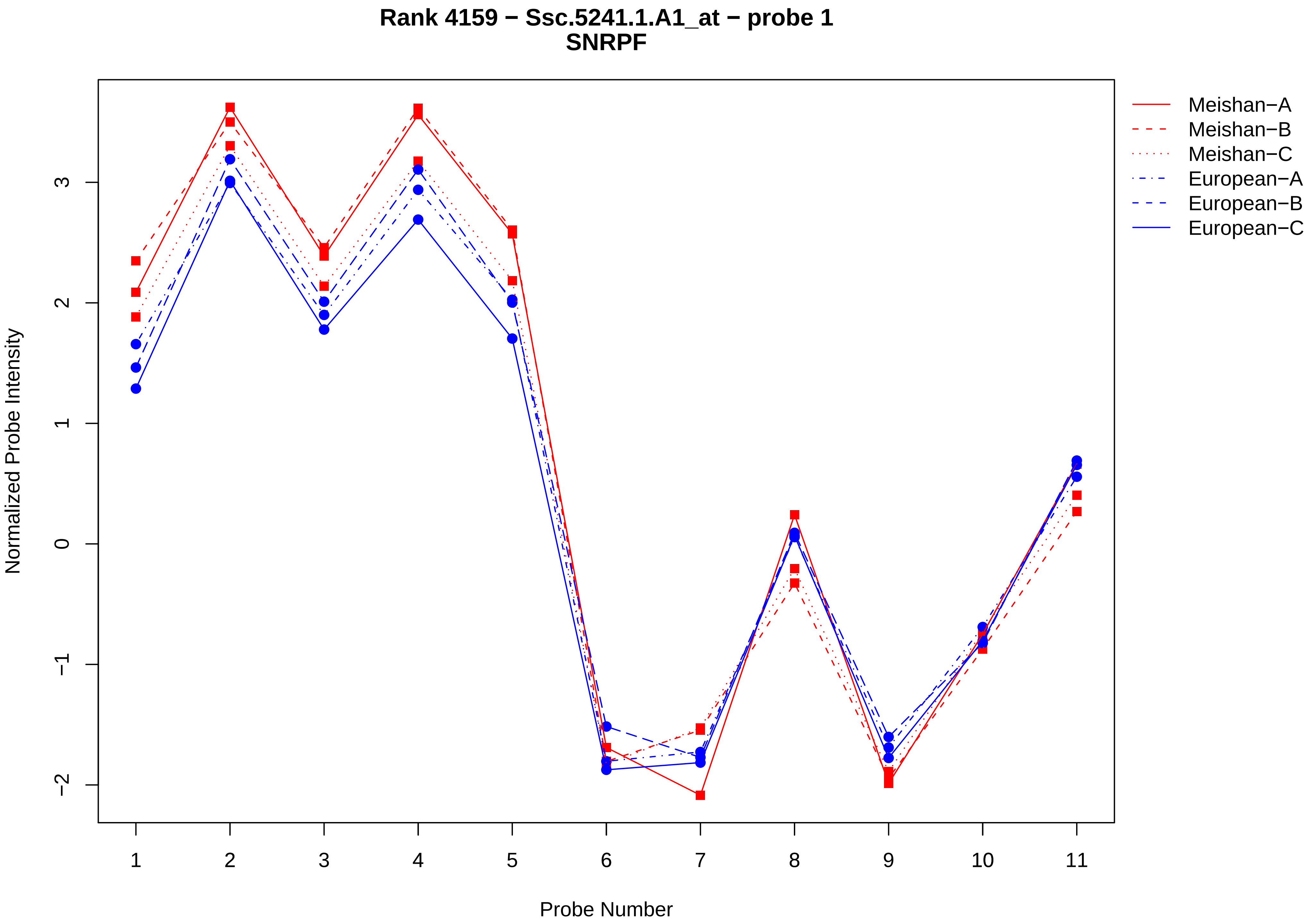

Rank 4159 − Ssc.5241.1.A1_at − probe 1
SNRPF
Normalized Probe Intensity
Probe Number
Meishan−A
Meishan−B
Meishan−C
European−A
European−B
European−C
−2
−1
0
1
2
3
1
2
3
4
5
6
7
8
9
10
11

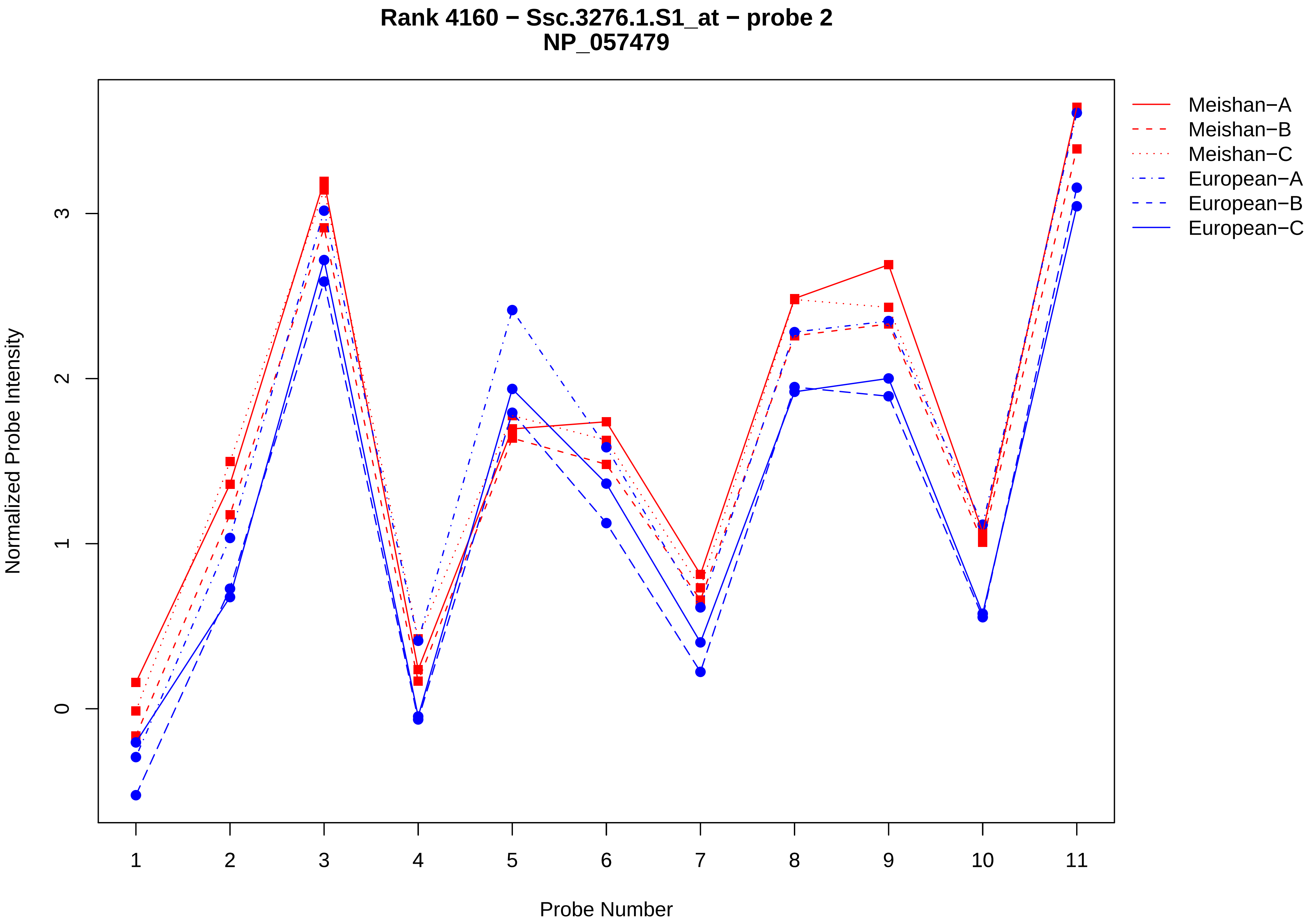
Rank 4160 − Ssc.3276.1.S1_at − probe 2
NP_057479
Normalized Probe Intensity
0
1
2
3
1
2
3
4
5
6
7
8
9
10
11
Probe Number
Meishan−A
Meishan−B
Meishan−C
European−A
European−B
European−C

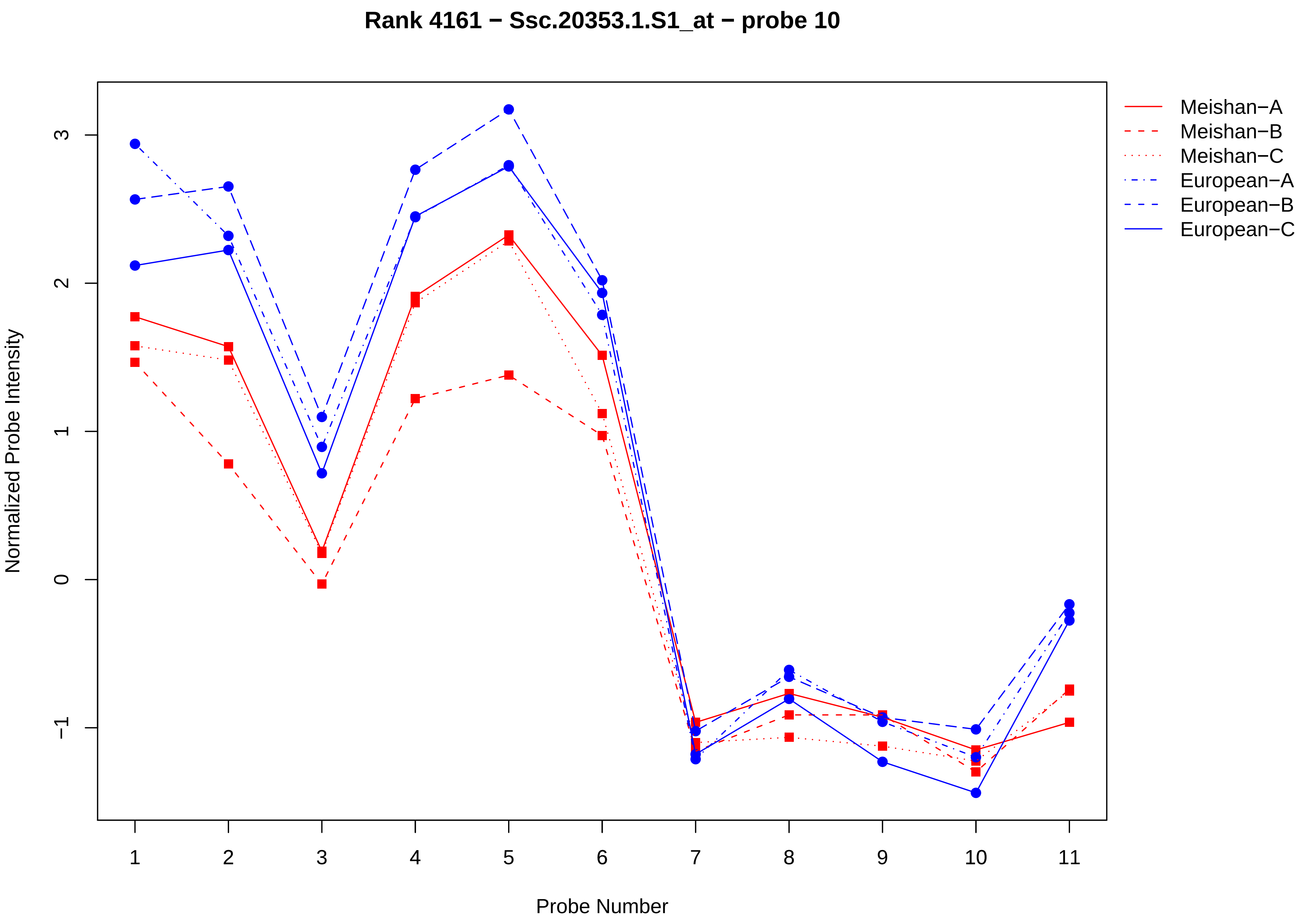

Rank 4161 − Ssc.20353.1.S1_at − probe 10
Normalized Probe Intensity
Probe Number
−1
0
1
2
3
1
2
3
4
5
6
7
8
9
10
11
Meishan−A
Meishan−B
Meishan−C
European−A
European−B
European−C

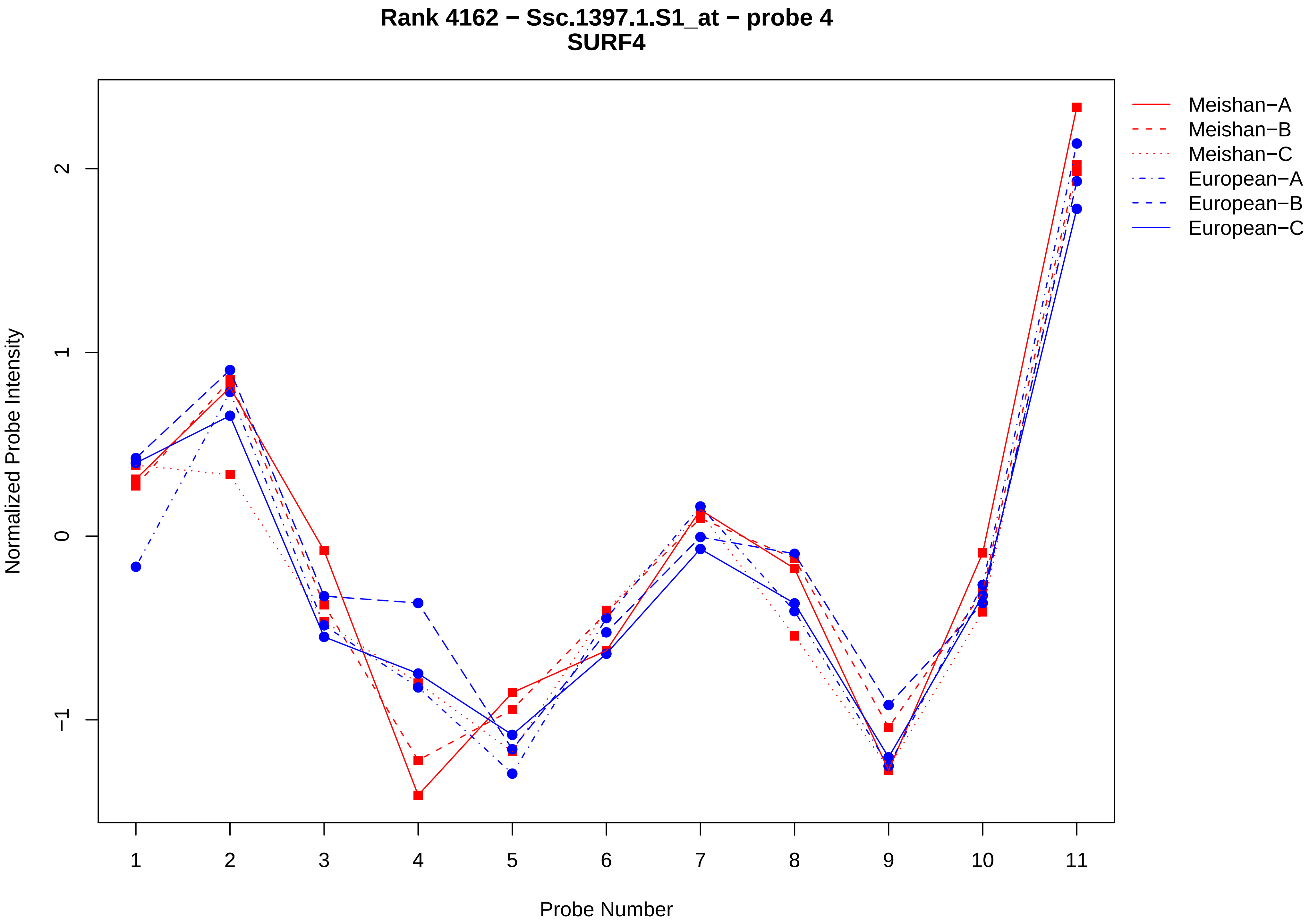

Rank 4162 − Ssc.1397.1.S1_at − probe 4
SURF4
Normalized Probe Intensity
Probe Number
Meishan−A
Meishan−B
Meishan−C
European−A
European−B
European−C
−1
0
1
2
1
2
3
4
5
6
7
8
9
10
11

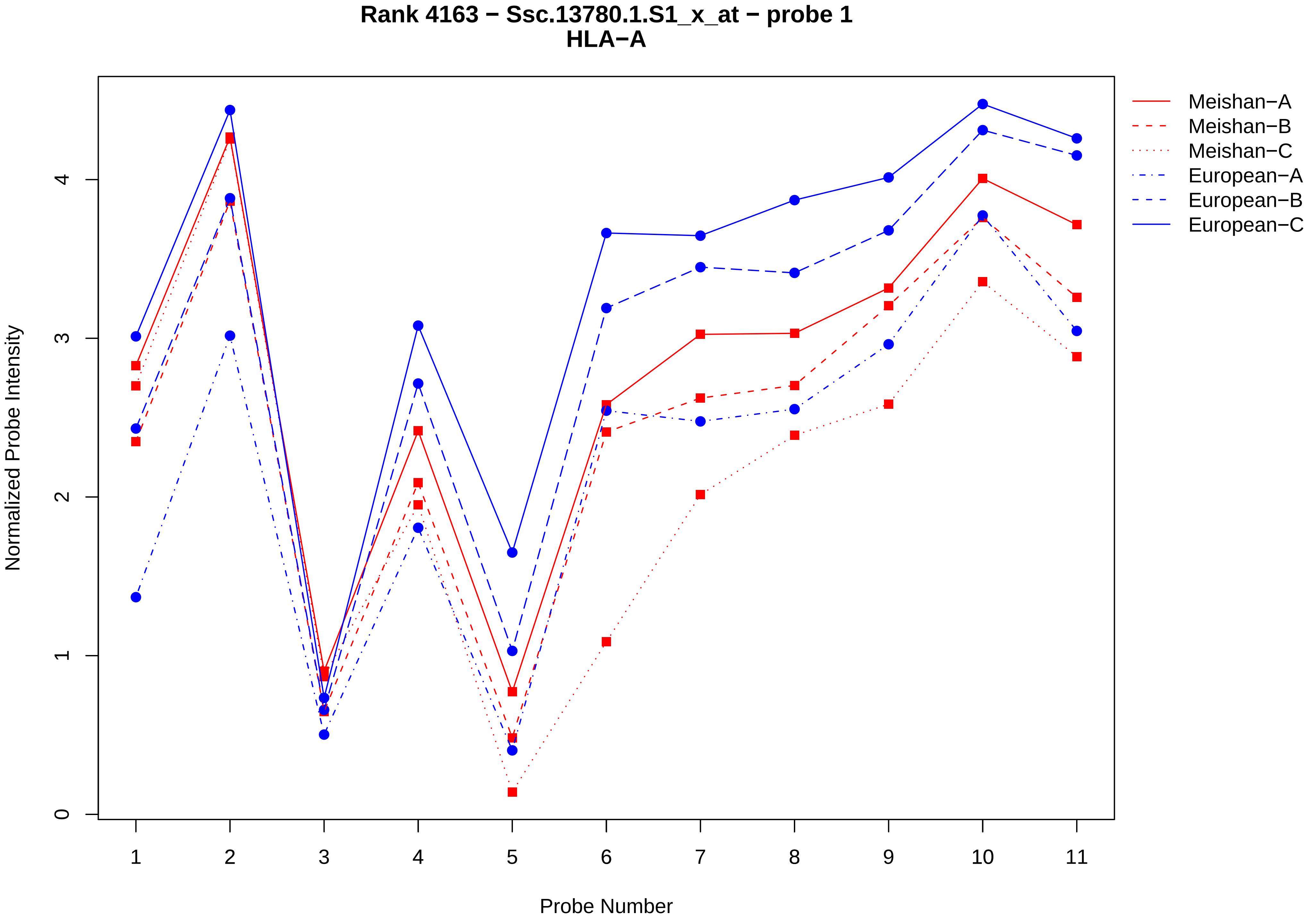

Rank 4163 − Ssc.13780.1.S1_x_at − probe 1
HLA−A
Normalized Probe Intensity
Probe Number
Meishan−A
Meishan−B
Meishan−C
European−A
European−B
European−C
0
1
2
3
4
1
2
3
4
5
6
7
8
9
10
11

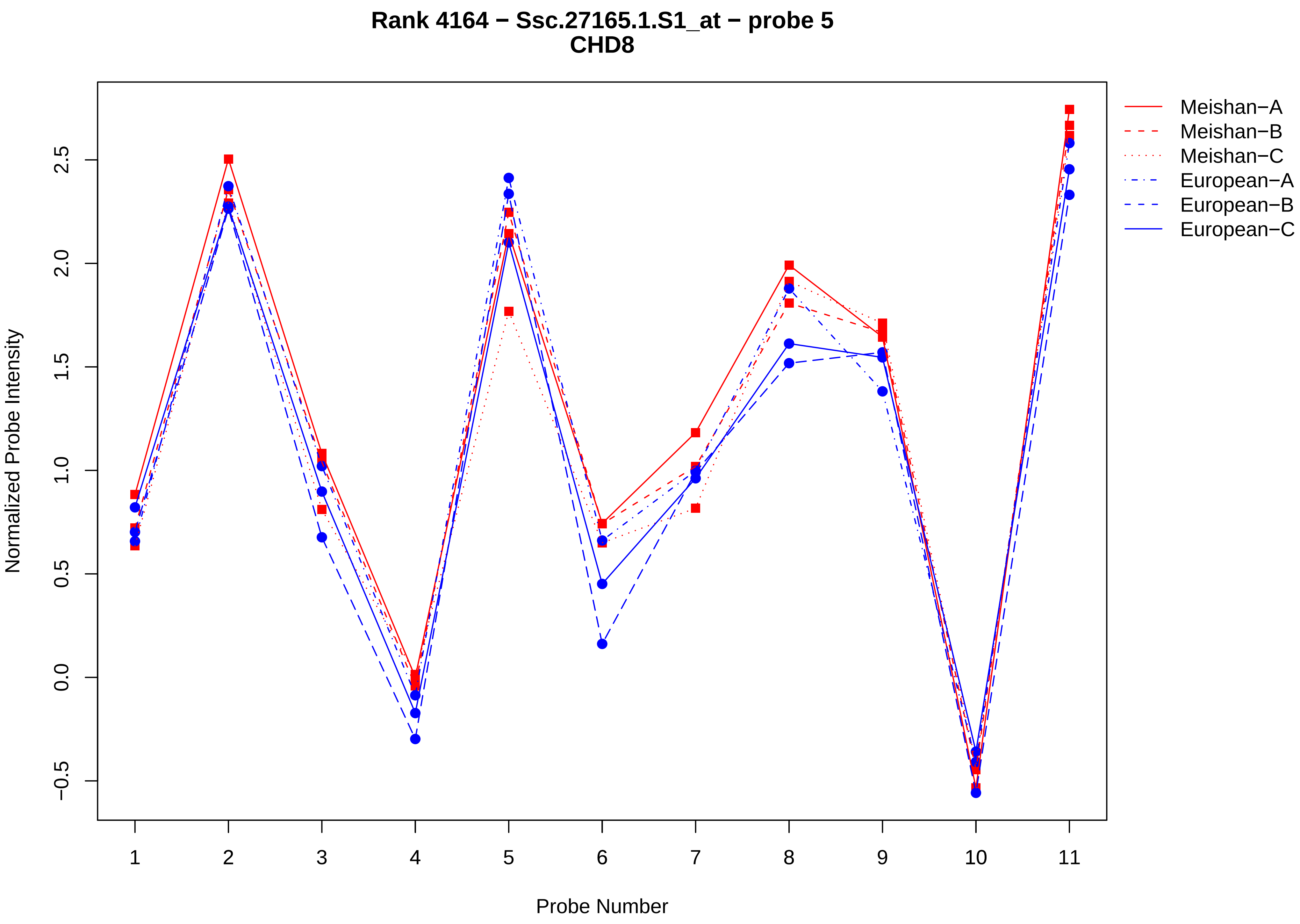

Rank 4164 − Ssc.27165.1.S1_at − probe 5
CHD8
Normalized Probe Intensity
Probe Number
Meishan−A
Meishan−B
Meishan−C
European−A
European−B
European−C
−0.5
0.0
0.5
1.0
1.5
2.0
2.5
1
2
3
4
5
6
7
8
9
10
11

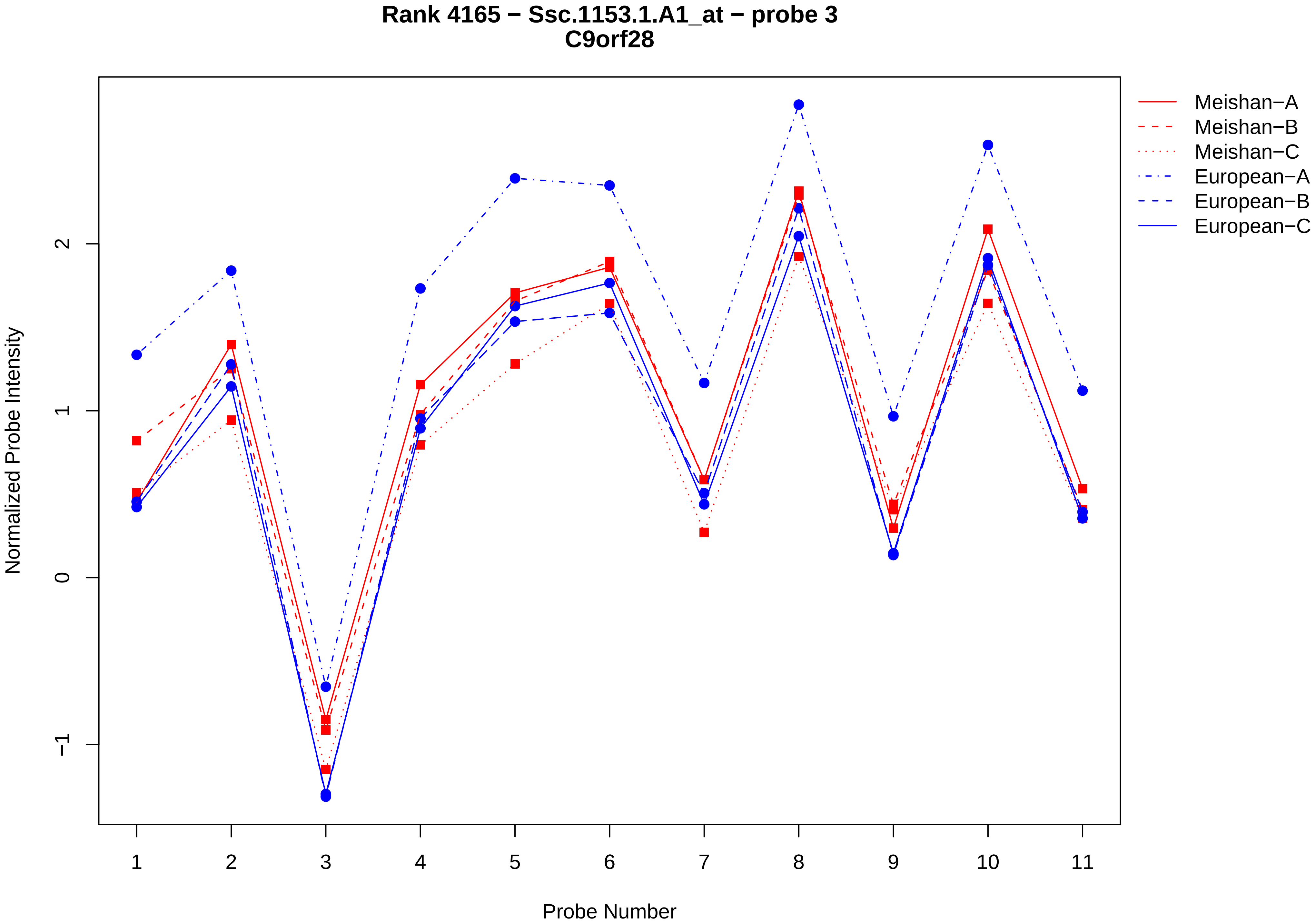

Rank 4165 − Ssc.1153.1.A1_at − probe 3
C9orf28
Normalized Probe Intensity
Probe Number
Meishan−A
Meishan−B
Meishan−C
European−A
European−B
European−C

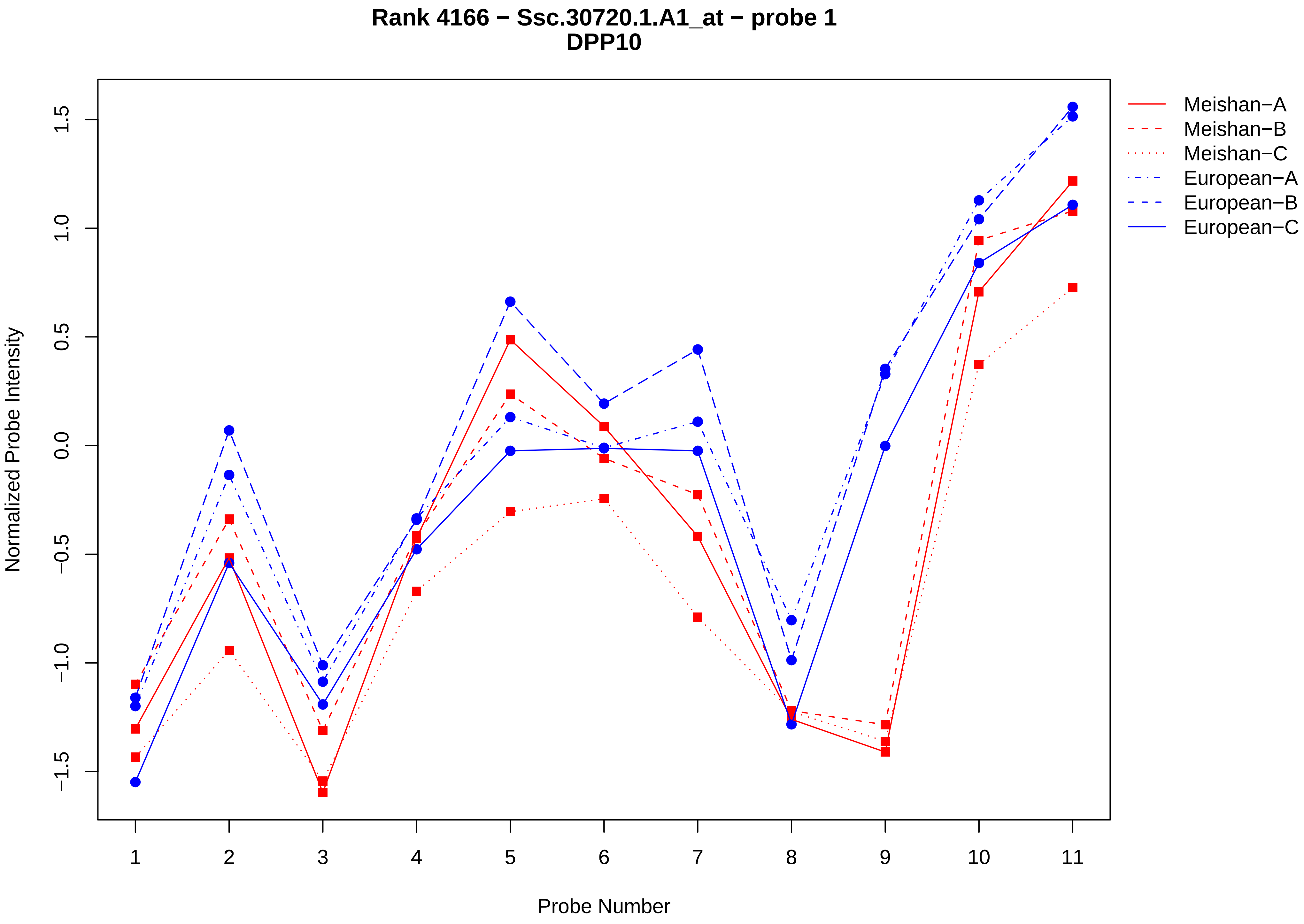

Rank 4166 − Ssc.30720.1.A1_at − probe 1
DPP10
Normalized Probe Intensity
Probe Number
Meishan−A
Meishan−B
Meishan−C
European−A
European−B
European−C
1.5
1.0
0.5
0.0
−0.5
−1.0
−1.5
1
2
3
4
5
6
7
8
9
10
11

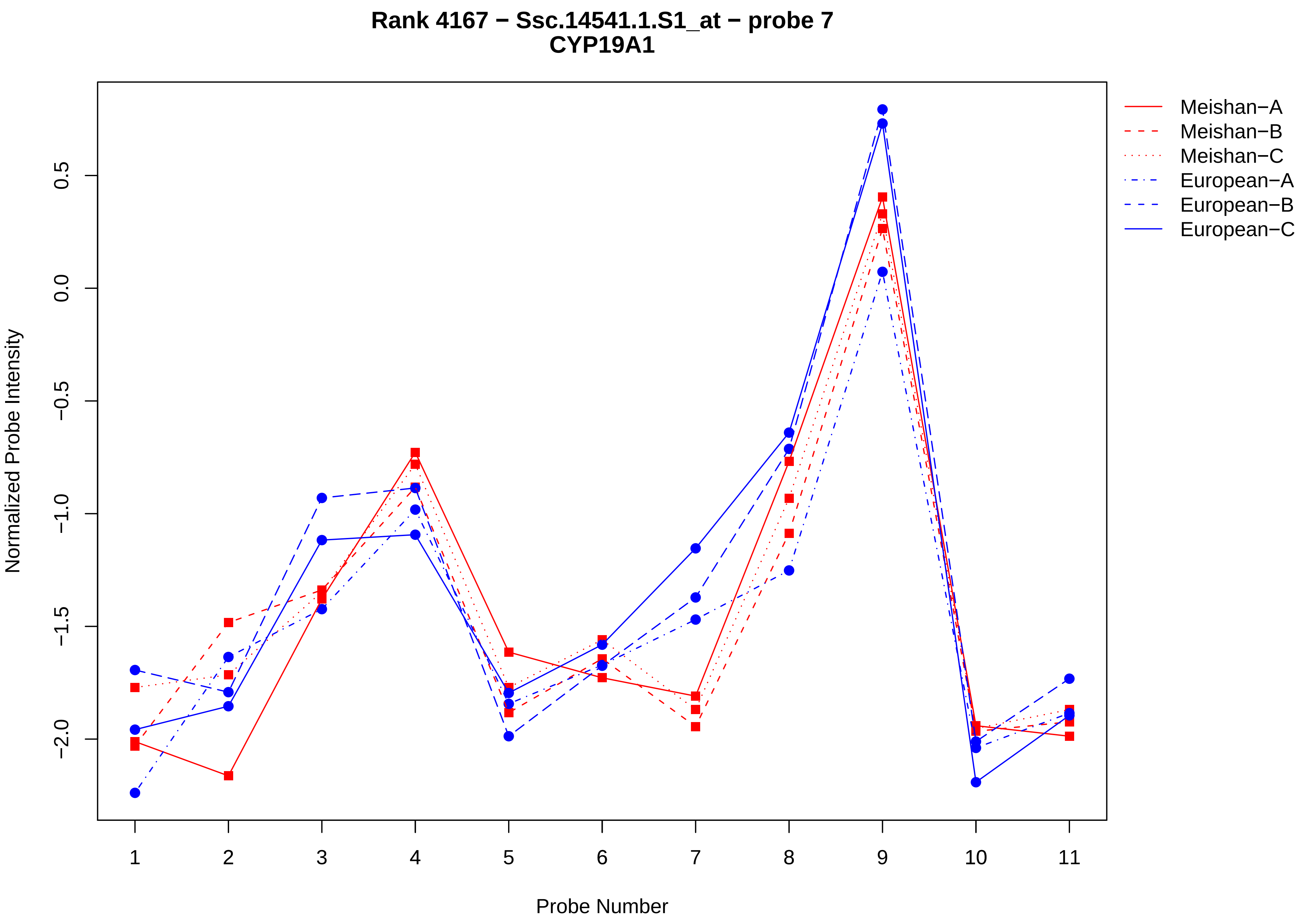

Rank 4167 − Ssc.14541.1.S1_at − probe 7
CYP19A1
Normalized Probe Intensity
Probe Number
−2.0
−1.5
−1.0
−0.5
0.0
0.5
1
2
3
4
5
6
7
8
9
10
11
Meishan−A
Meishan−B
Meishan−C
European−A
European−B
European−C

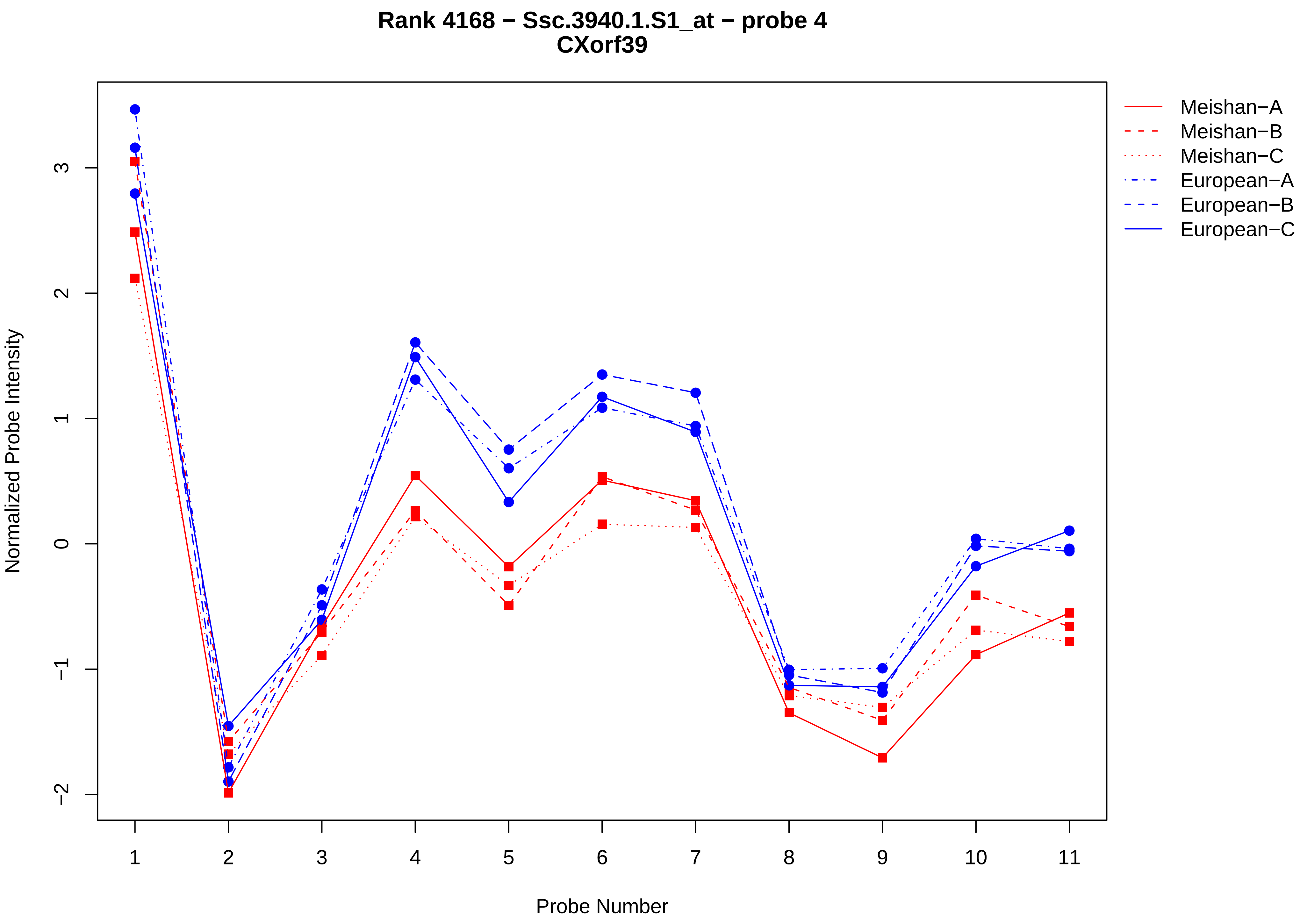

Rank 4168 − Ssc.3940.1.S1_at − probe 4
CXorf39
Normalized Probe Intensity
Probe Number
Meishan−A
Meishan−B
Meishan−C
European−A
European−B
European−C
−2
−1
0
1
2
3
1
2
3
4
5
6
7
8
9
10
11

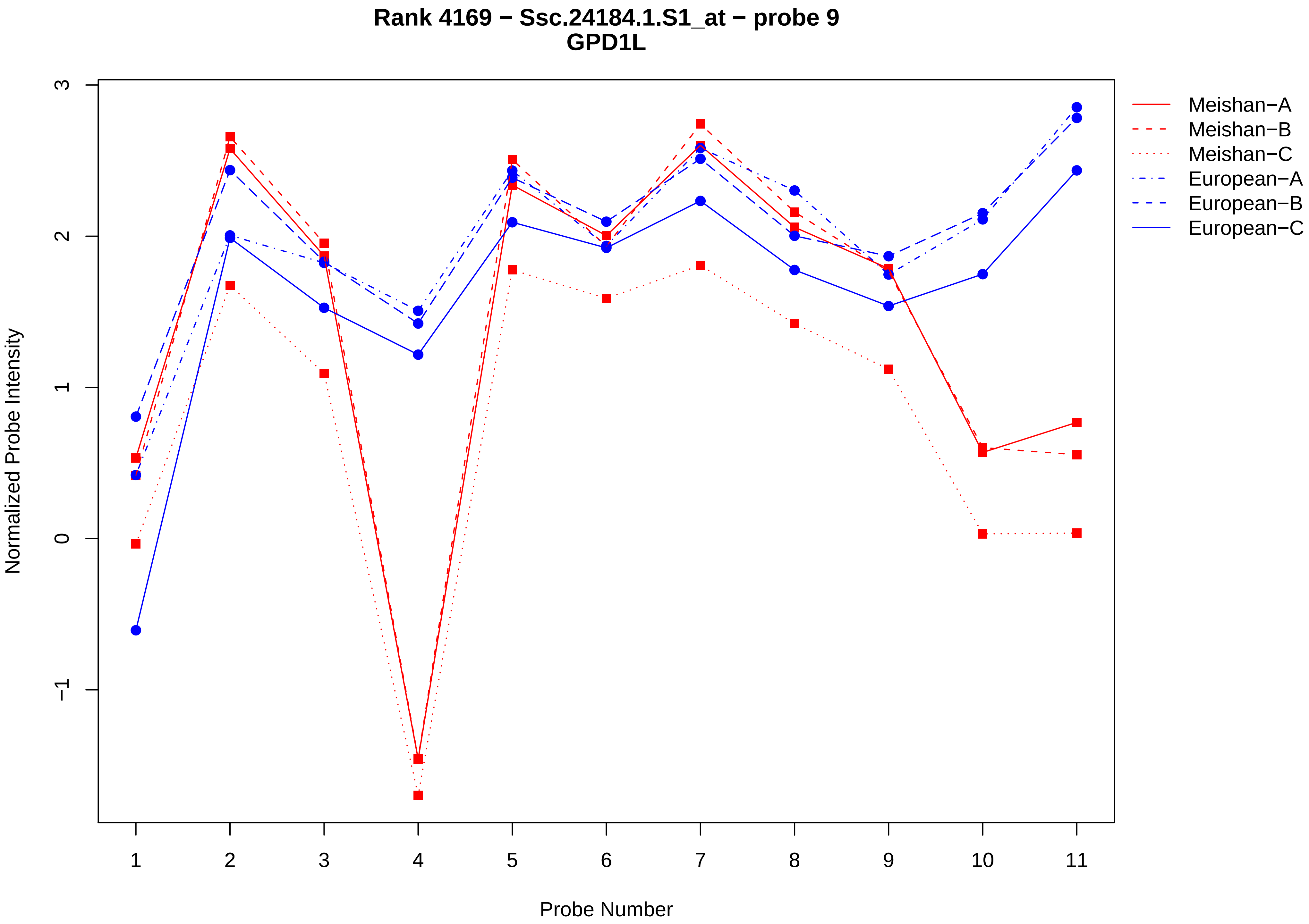
Rank 4169 − Ssc.24184.1.S1_at − probe 9
GPD1L
Normalized Probe Intensity
Probe Number
−1
0
1
2
3
1
2
3
4
5
6
7
8
9
10
11
Meishan−A
Meishan−B
Meishan−C
European−A
European−B
European−C

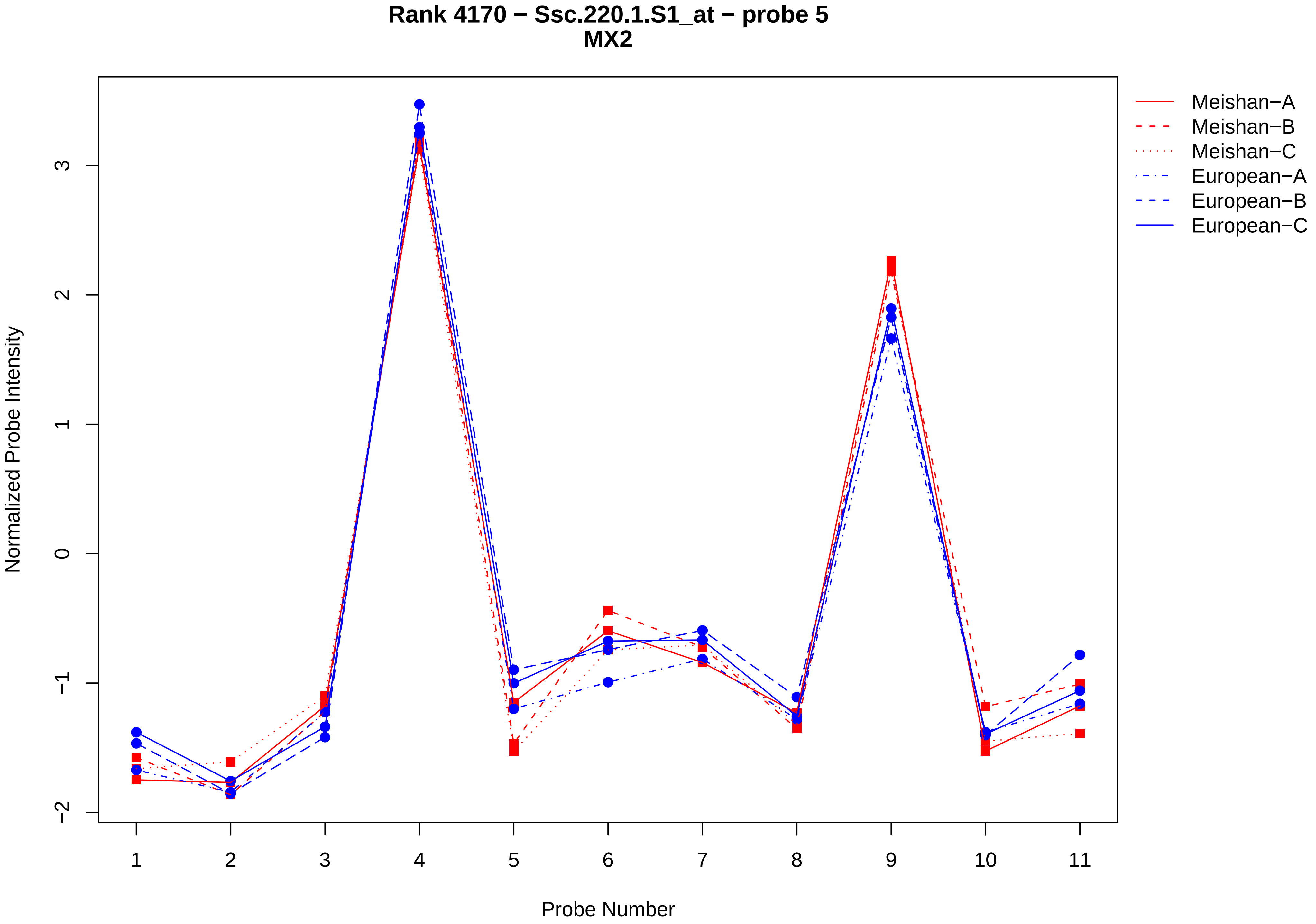

Rank 4170 − Ssc.220.1.S1_at − probe 5
MX2
Normalized Probe Intensity
Probe Number
−2
−1
0
1
2
3
1
2
3
4
5
6
7
8
9
10
11
Meishan−A
Meishan−B
Meishan−C
European−A
European−B
European−C

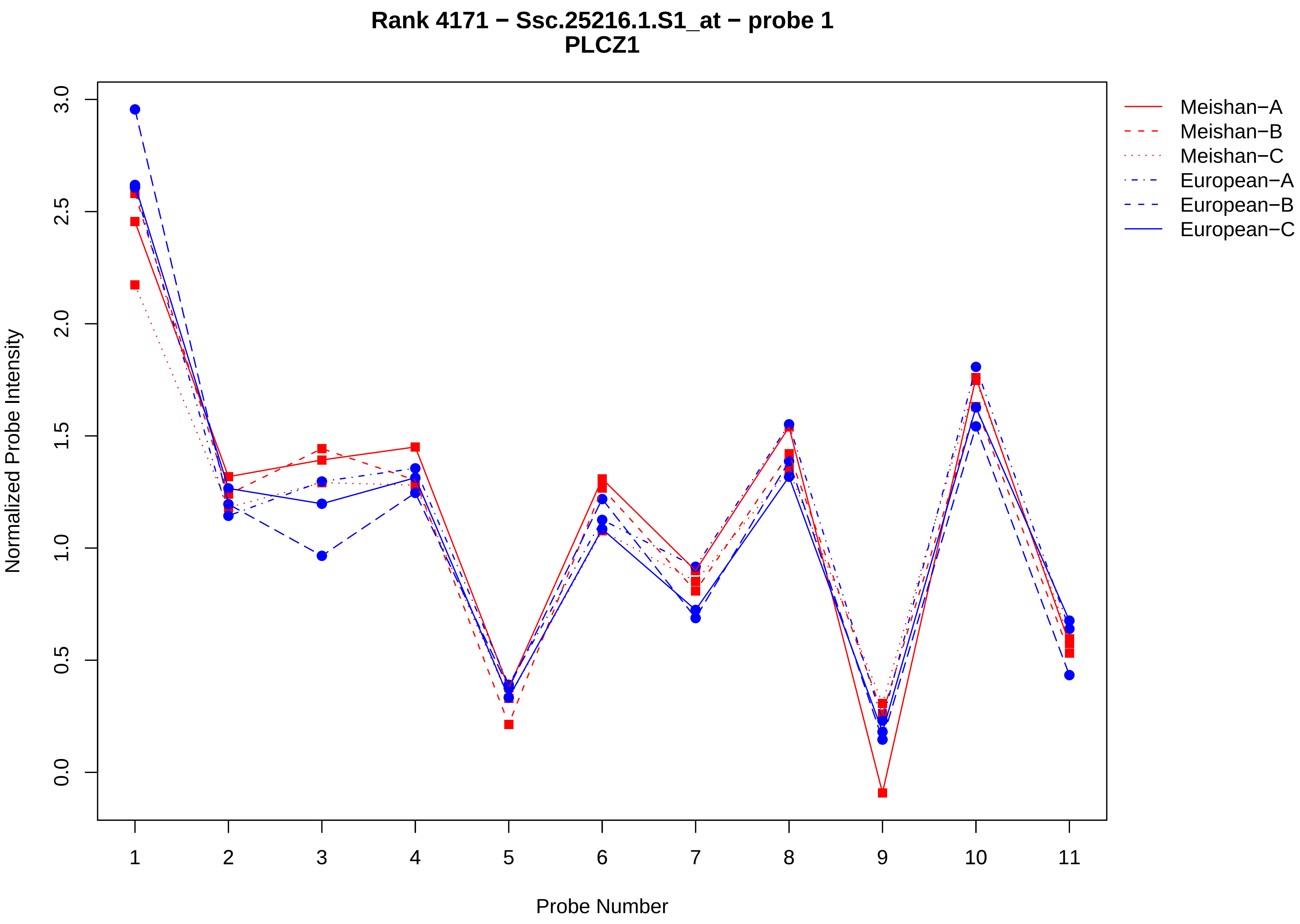
Rank 4171 − Ssc.25216.1.S1_at − probe 1
PLCZ1
Normalized Probe Intensity
0.0
0.5
1.0
1.5
2.0
2.5
3.0
Probe Number
1
2
3
4
5
6
7
8
9
10
11
Meishan−A
Meishan−B
Meishan−C
European−A
European−B
European−C

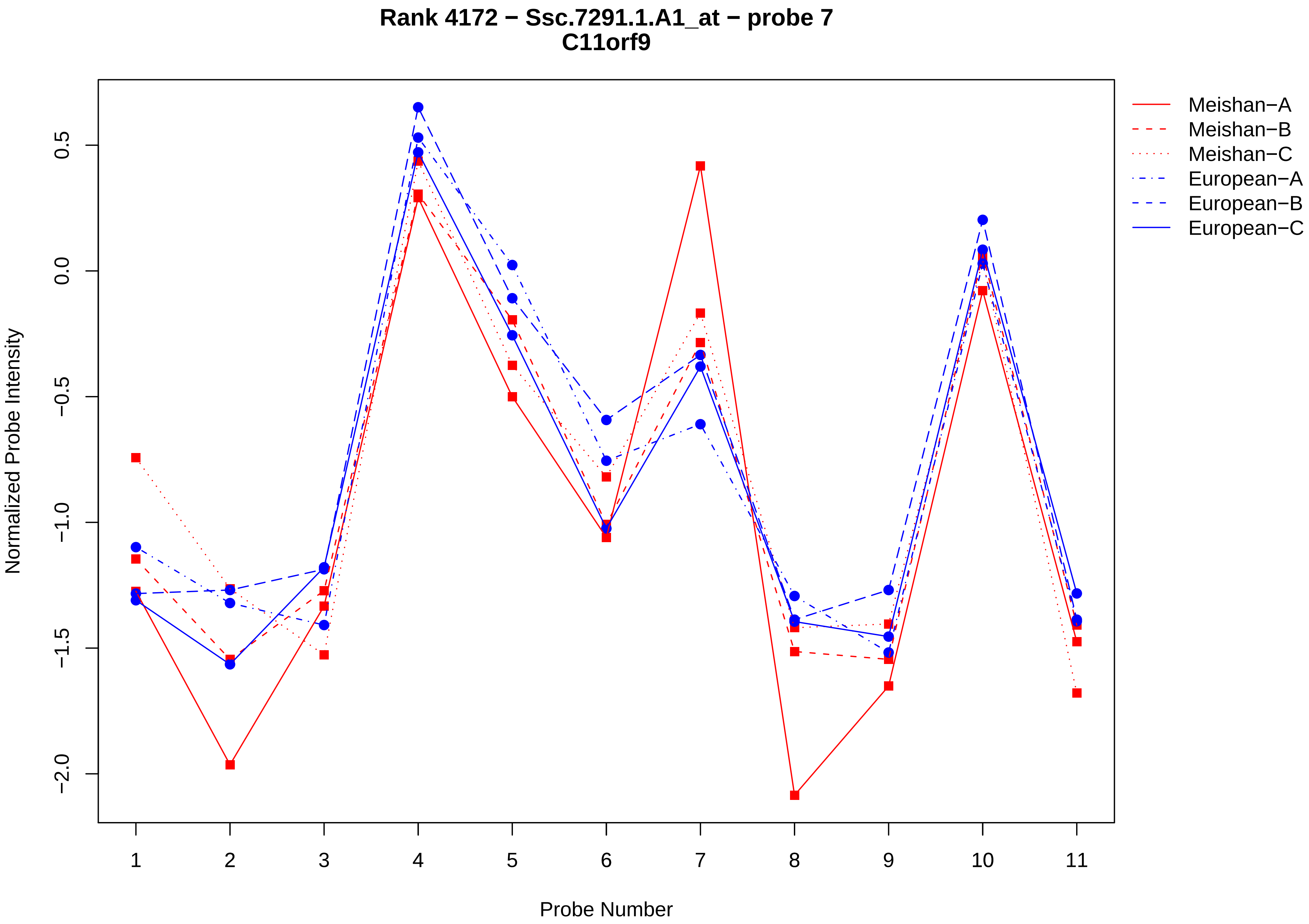

Rank 4172 − Ssc.7291.1.A1_at − probe 7
C11orf9
Normalized Probe Intensity
Probe Number
Meishan−A
Meishan−B
Meishan−C
European−A
European−B
European−C
0.5
0.0
−0.5
−1.0
−1.5
−2.0
1
2
3
4
5
6
7
8
9
10
11

Rank 4173 − Ssc.13451.1.A1_at − probe 9
EPIM

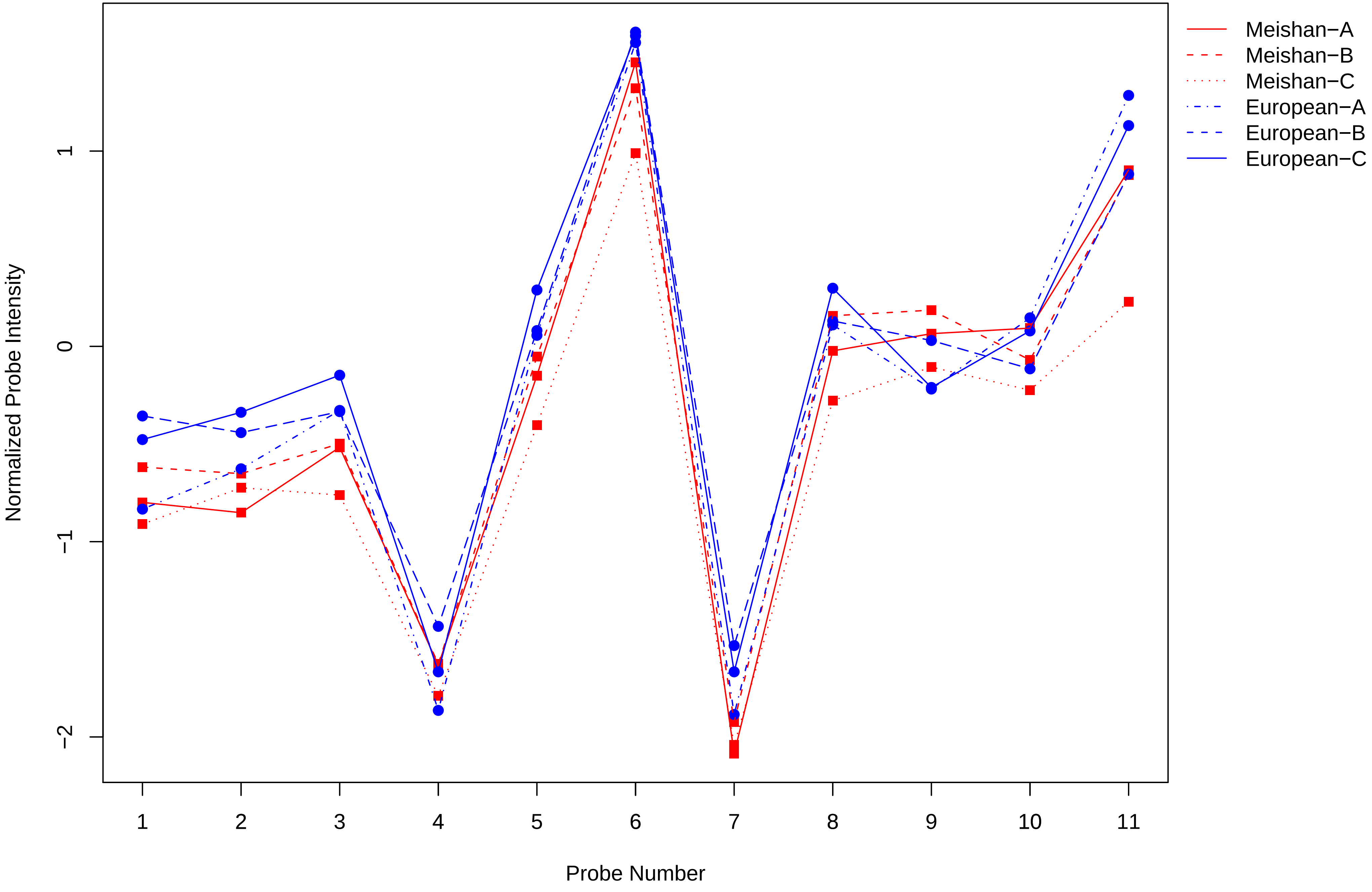

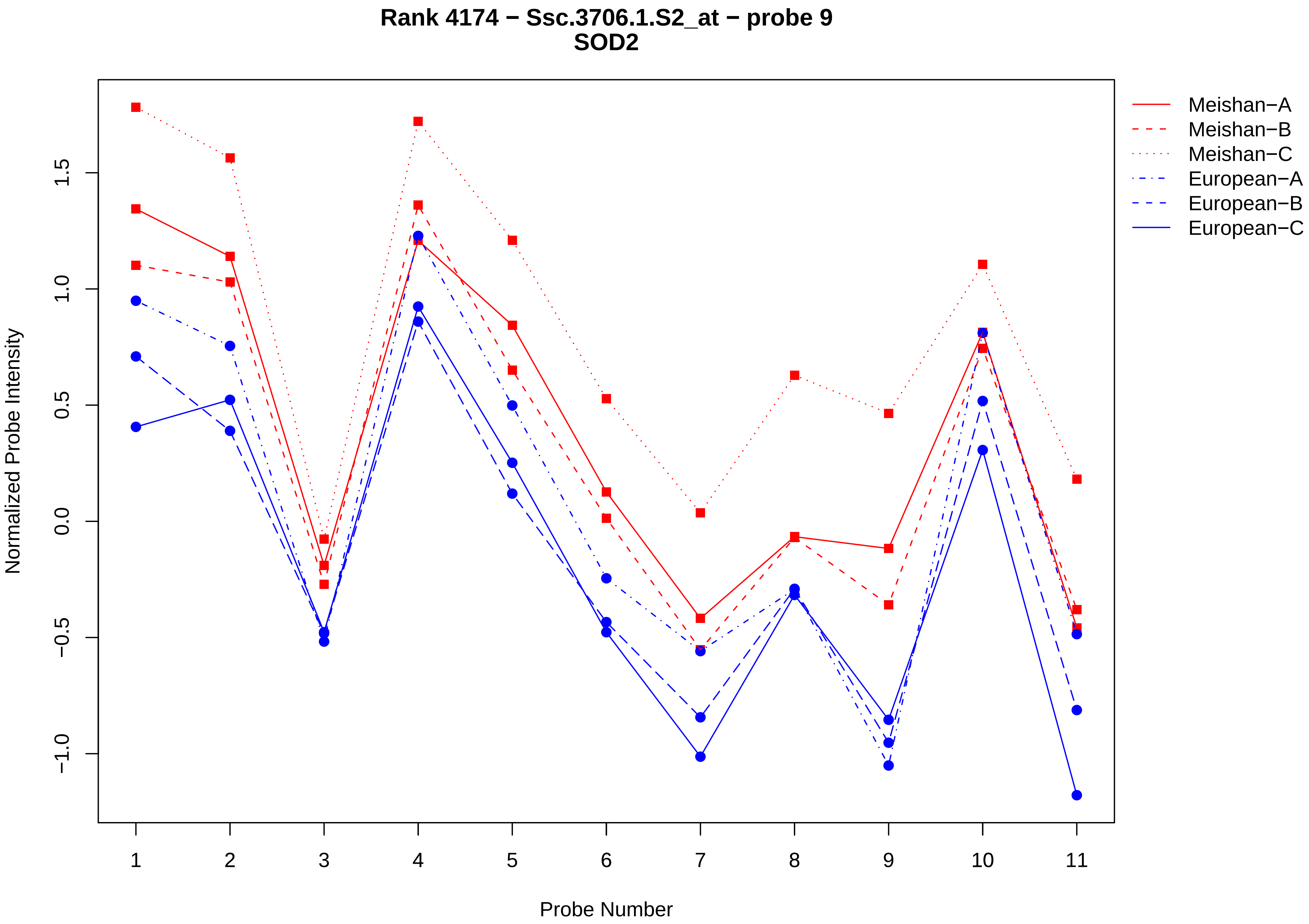
Rank 4174 − Ssc.3706.1.S2_at − probe 9
SOD2
Normalized Probe Intensity
Probe Number
−1.0
−0.5
0.0
0.5
1.0
1.5
1
2
3
4
5
6
7
8
9
10
11
Meishan−A
Meishan−B
Meishan−C
European−A
European−B
European−C

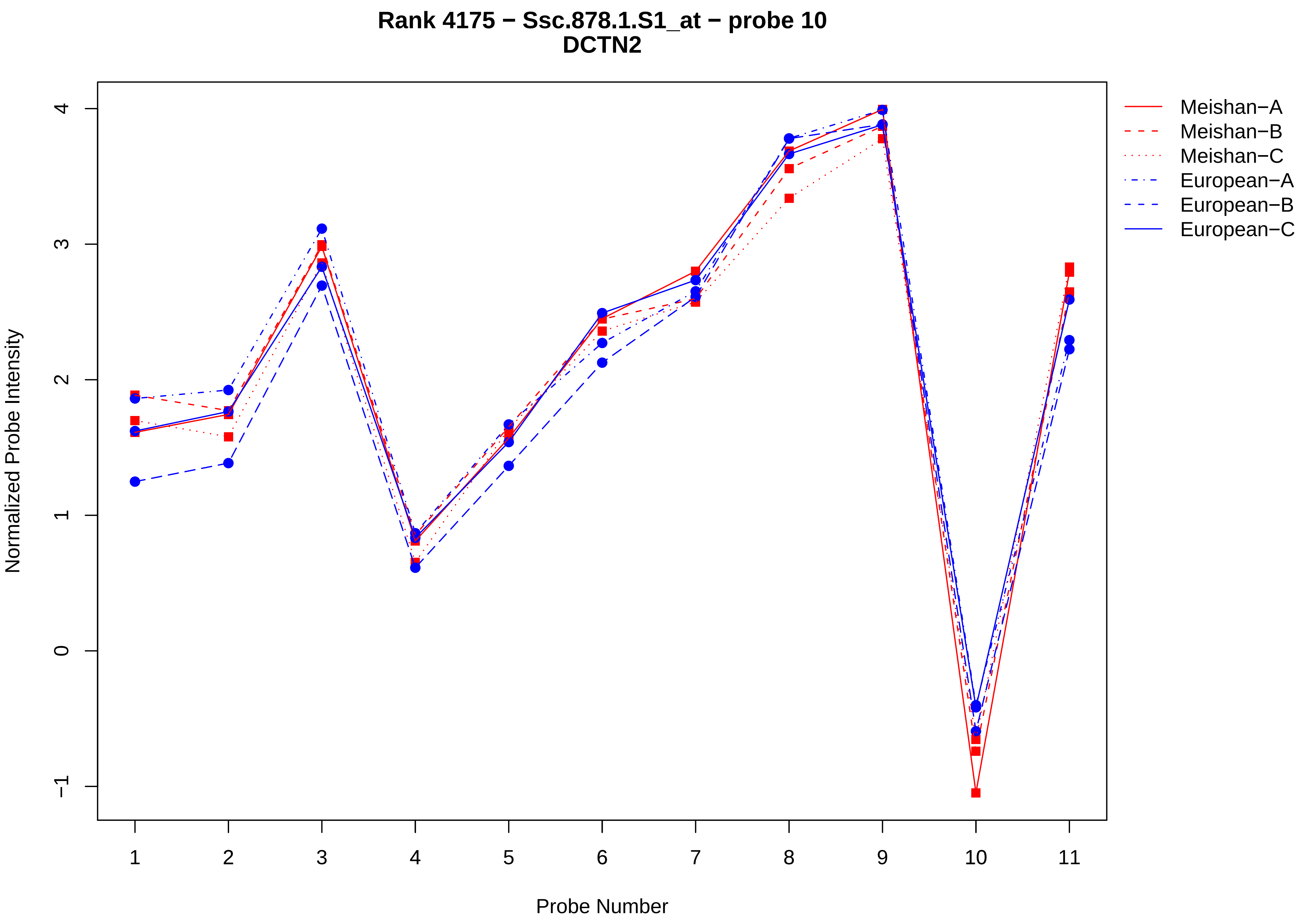
Rank 4175 − Ssc.878.1.S1_at − probe 10
DCTN2
Normalized Probe Intensity
Probe Number
−1
0
1
2
3
4
1
2
3
4
5
6
7
8
9
10
11
Meishan−A
Meishan−B
Meishan−C
European−A
European−B
European−C

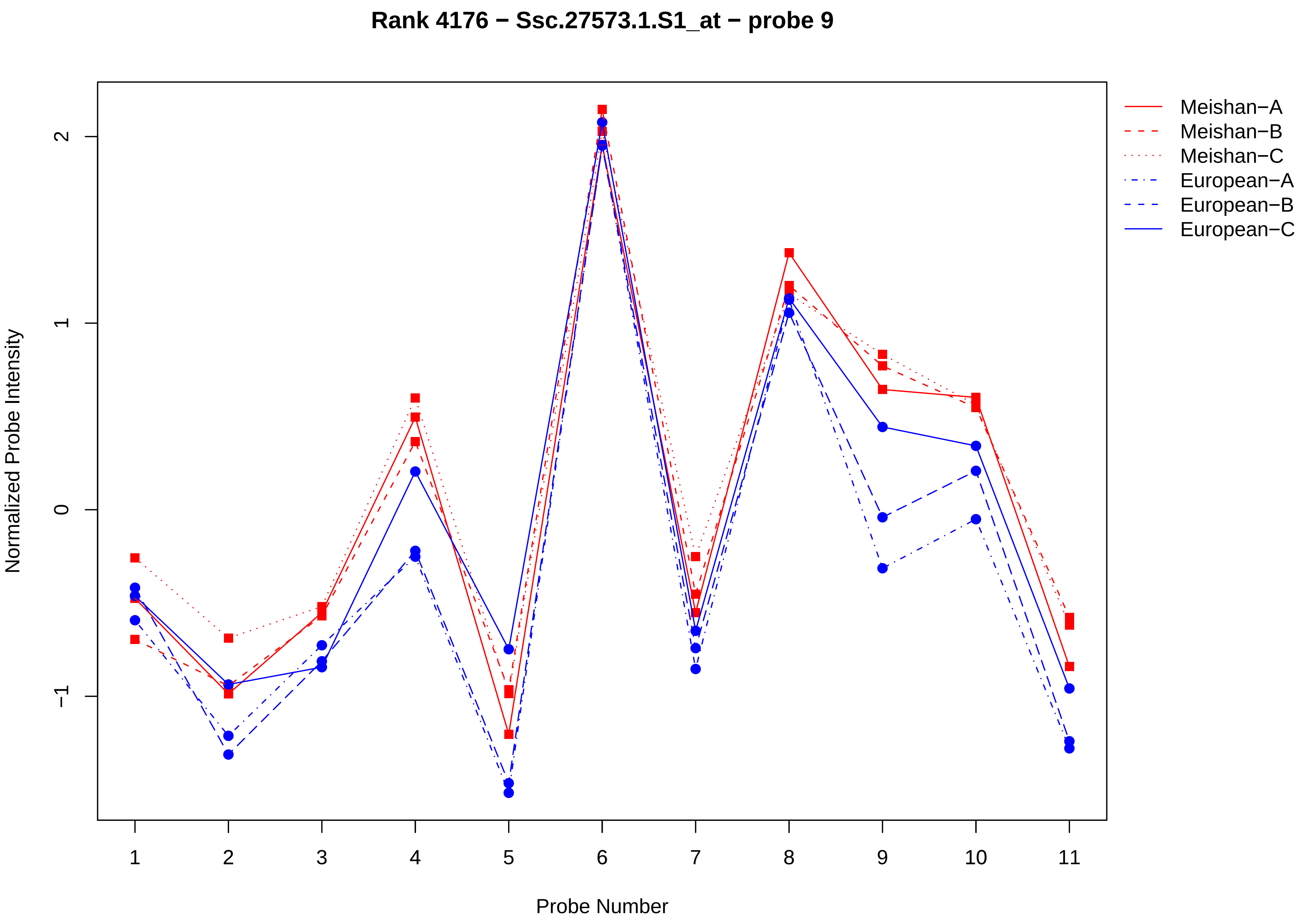

Rank 4176 − Ssc.27573.1.S1_at − probe 9
Normalized Probe Intensity
Probe Number
−1
0
1
2
1
2
3
4
5
6
7
8
9
10
11
Meishan−A
Meishan−B
Meishan−C
European−A
European−B
European−C

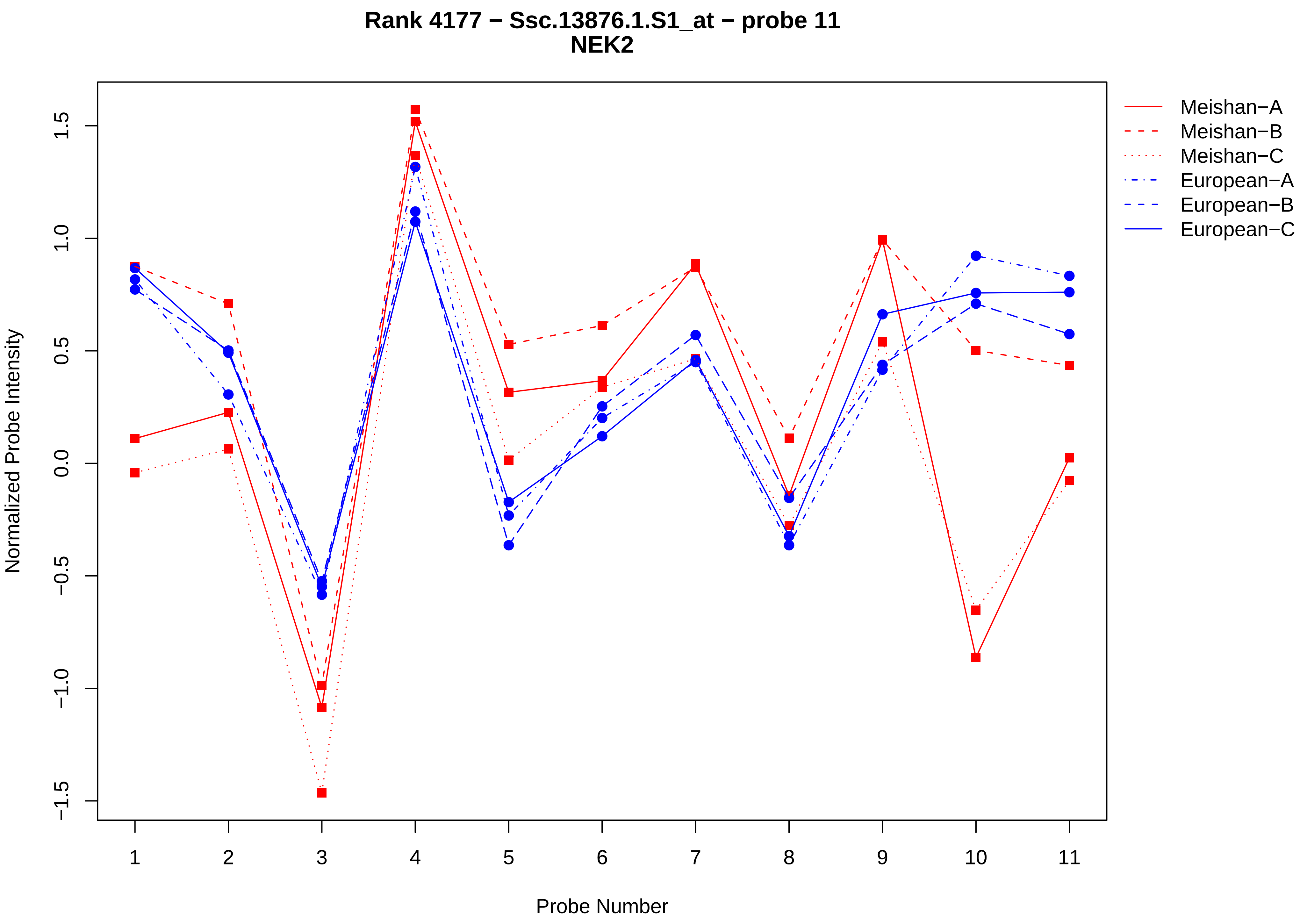

Rank 4177 − Ssc.13876.1.S1_at − probe 11
NEK2
Normalized Probe Intensity
Probe Number
−1.5
−1.0
−0.5
0.0
0.5
1.0
1.5
1
2
3
4
5
6
7
8
9
10
11
Meishan−A
Meishan−B
Meishan−C
European−A
European−B
European−C

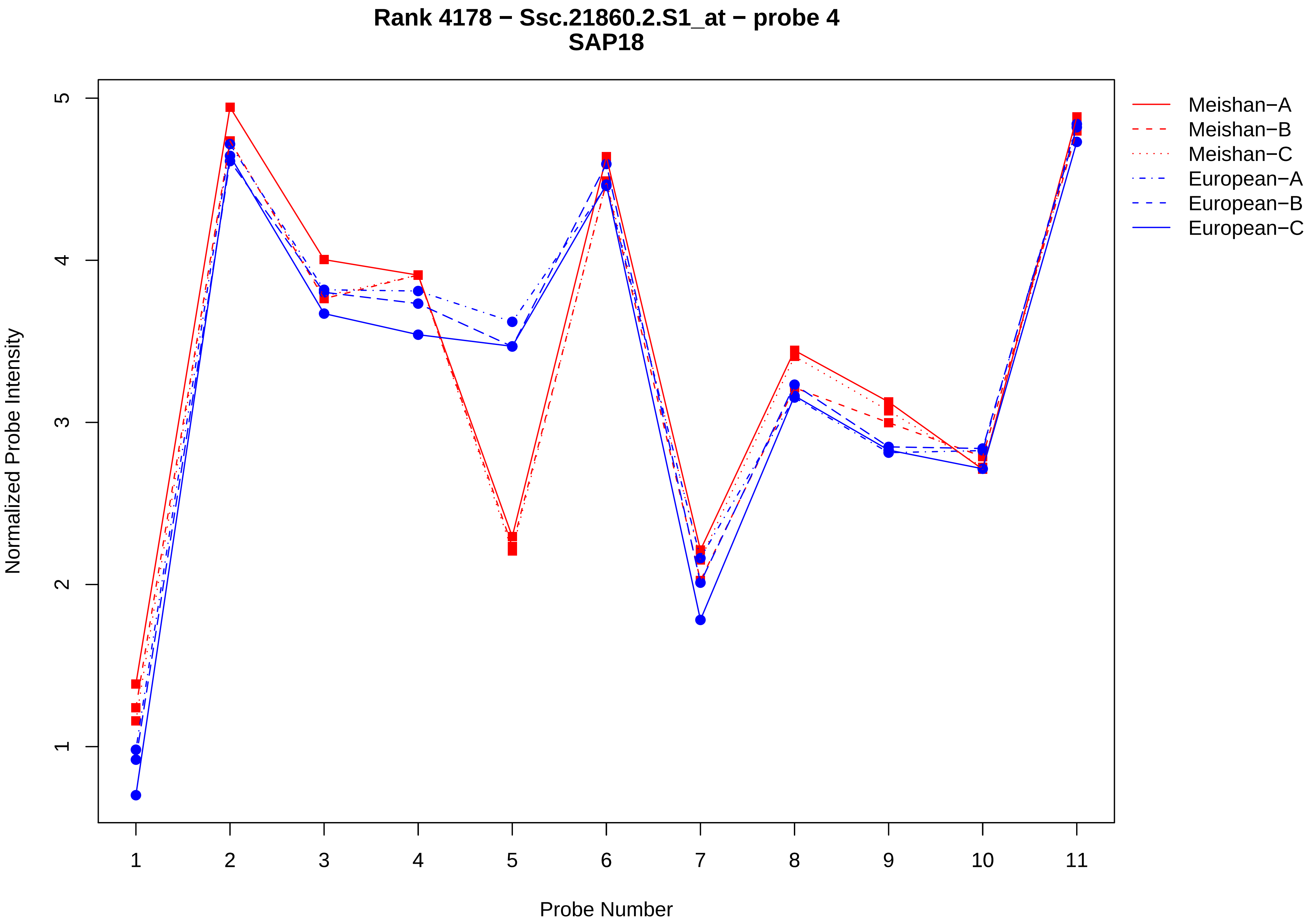
Rank 4178 − Ssc.21860.2.S1_at − probe 4
SAP18
Normalized Probe Intensity
Probe Number
1
2
3
4
5
1
2
3
4
5
6
7
8
9
10
11
Meishan−A
Meishan−B
Meishan−C
European−A
European−B
European−C

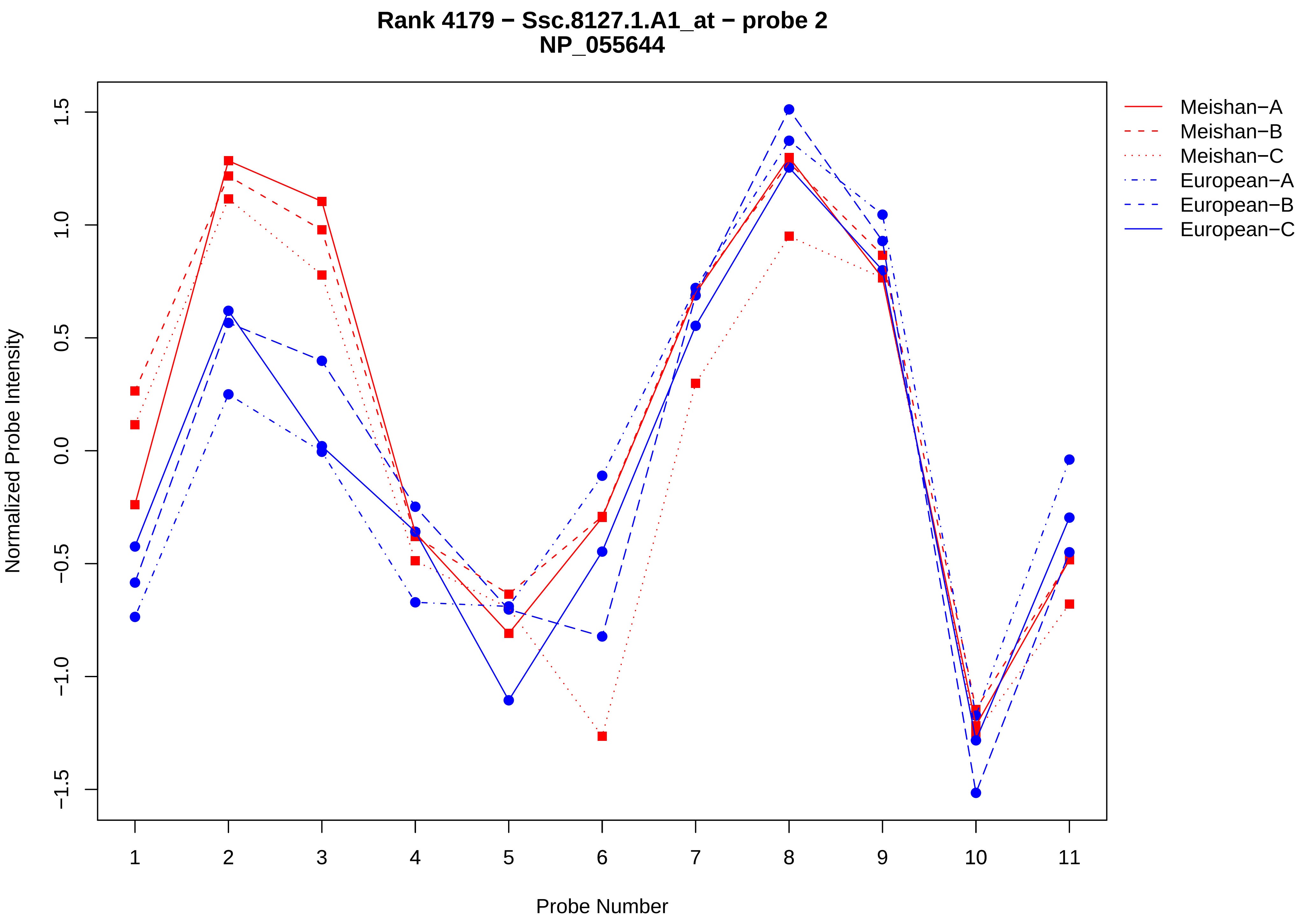

Rank 4179 − Ssc.8127.1.A1_at − probe 2
NP_055644
Normalized Probe Intensity
Probe Number
1.5
1.0
0.5
0.0
−0.5
−1.0
−1.5
1
2
3
4
5
6
7
8
9
10
11
Meishan−A
Meishan−B
Meishan−C
European−A
European−B
European−C

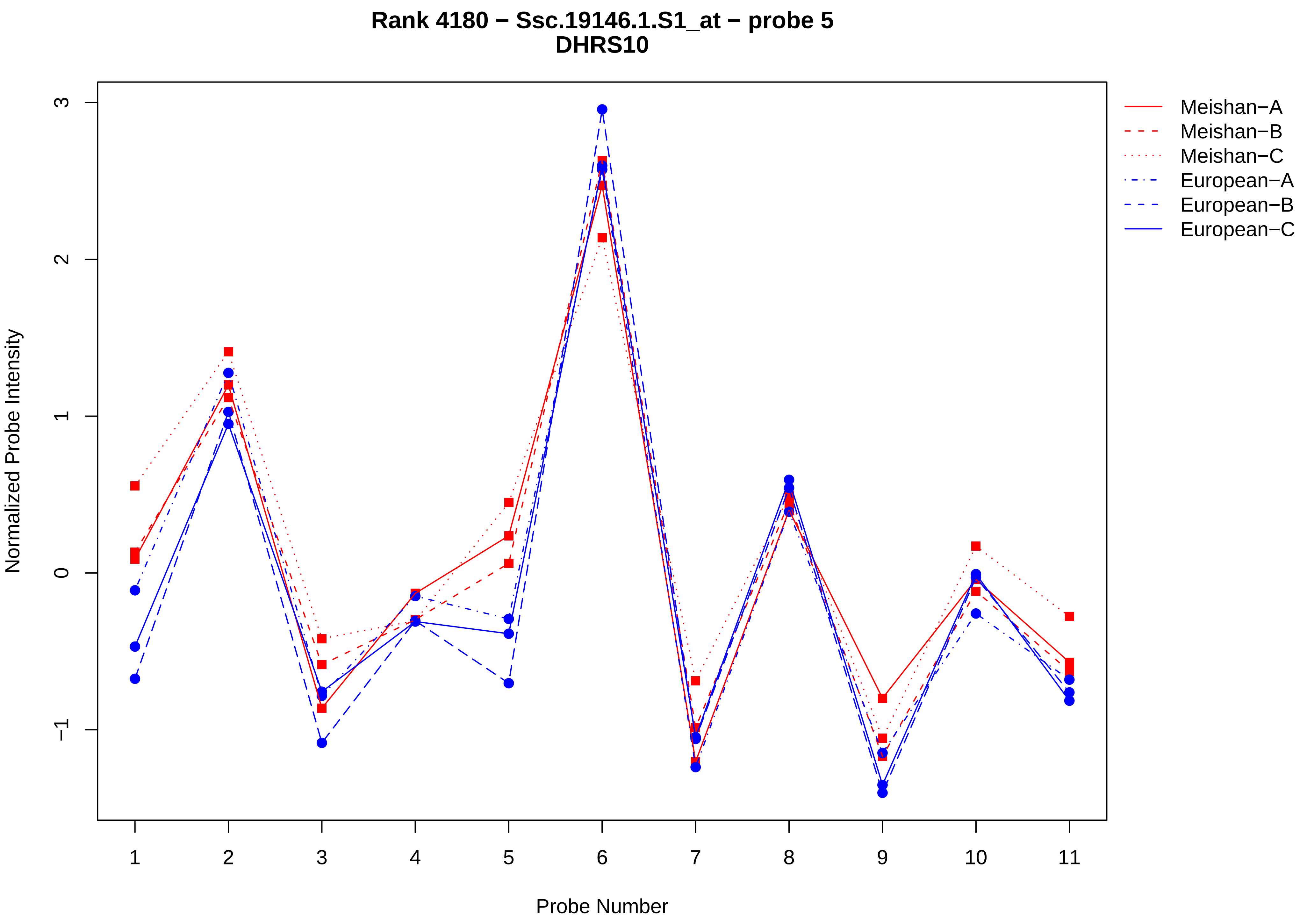

Rank 4180 − Ssc.19146.1.S1_at − probe 5
DHRS10
Normalized Probe Intensity
Probe Number
−1
0
1
2
3
1
2
3
4
5
6
7
8
9
10
11
Meishan−A
Meishan−B
Meishan−C
European−A
European−B
European−C

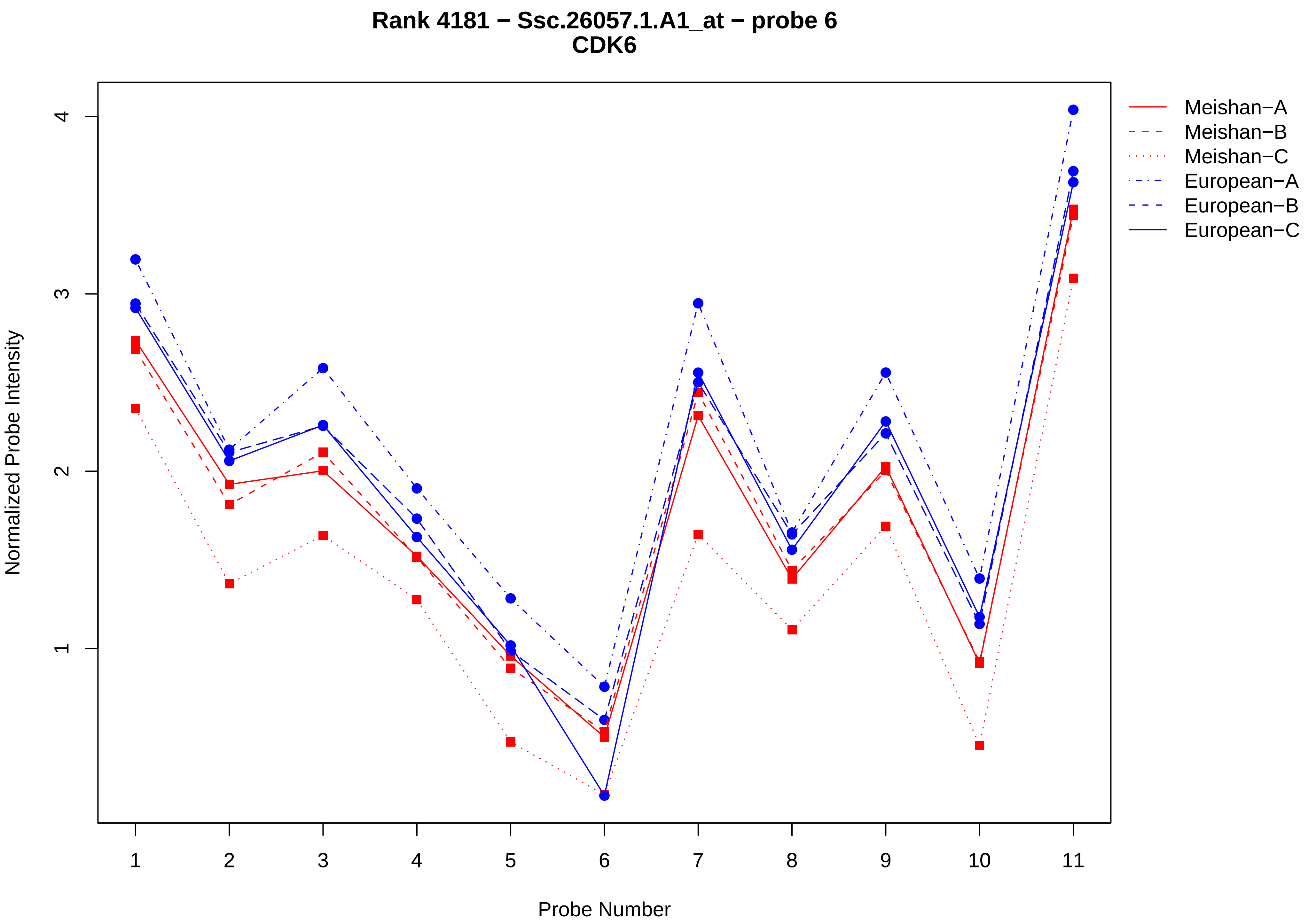
Rank 4181 − Ssc.26057.1.A1_at − probe 6
CDK6
Normalized Probe Intensity
Probe Number
1
2
3
4
1
2
3
4
5
6
7
8
9
10
11
Meishan−A
Meishan−B
Meishan−C
European−A
European−B
European−C

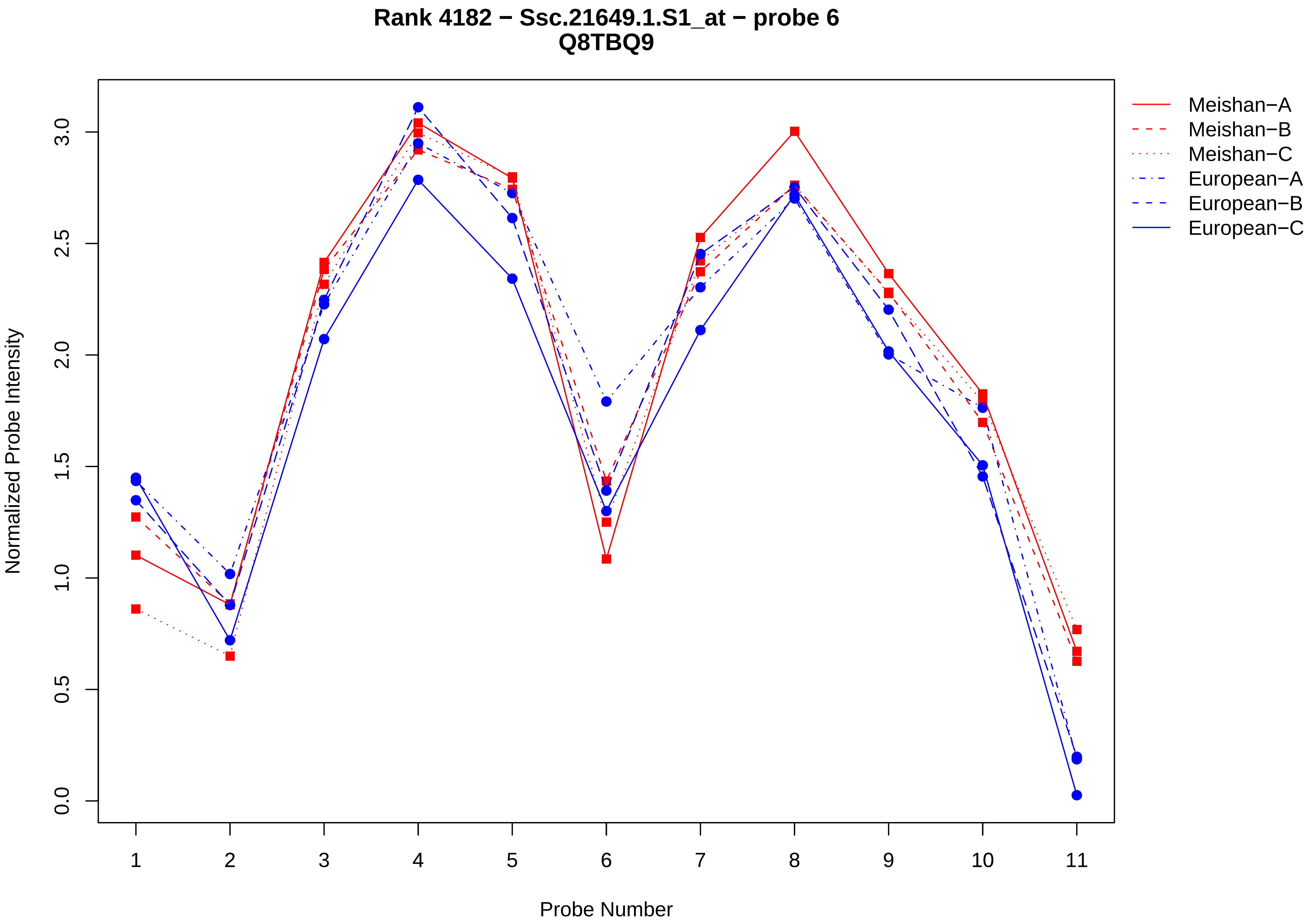
Rank 4182 − Ssc.21649.1.S1_at − probe 6
Q8TBQ9
Normalized Probe Intensity
0.0
0.5
1.0
1.5
2.0
2.5
3.0
1
2
3
4
5
6
7
8
9
10
11
Probe Number
Meishan−A
Meishan−B
Meishan−C
European−A
European−B
European−C

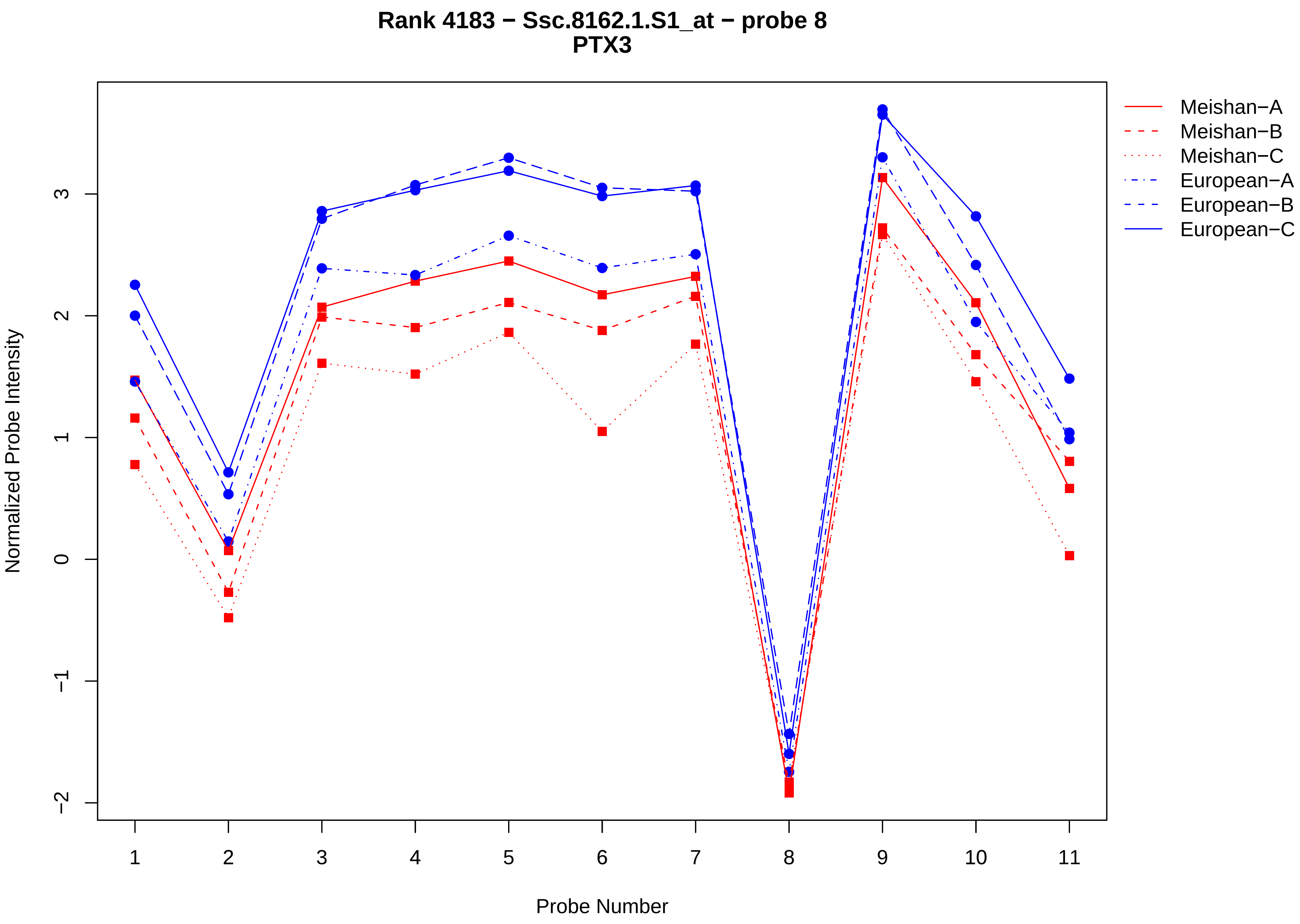

Rank 4183 − Ssc.8162.1.S1_at − probe 8
PTX3
Normalized Probe Intensity
Probe Number
Meishan−A
Meishan−B
Meishan−C
European−A
European−B
European−C
−2
−1
0
1
2
3
1
2
3
4
5
6
7
8
9
10
11

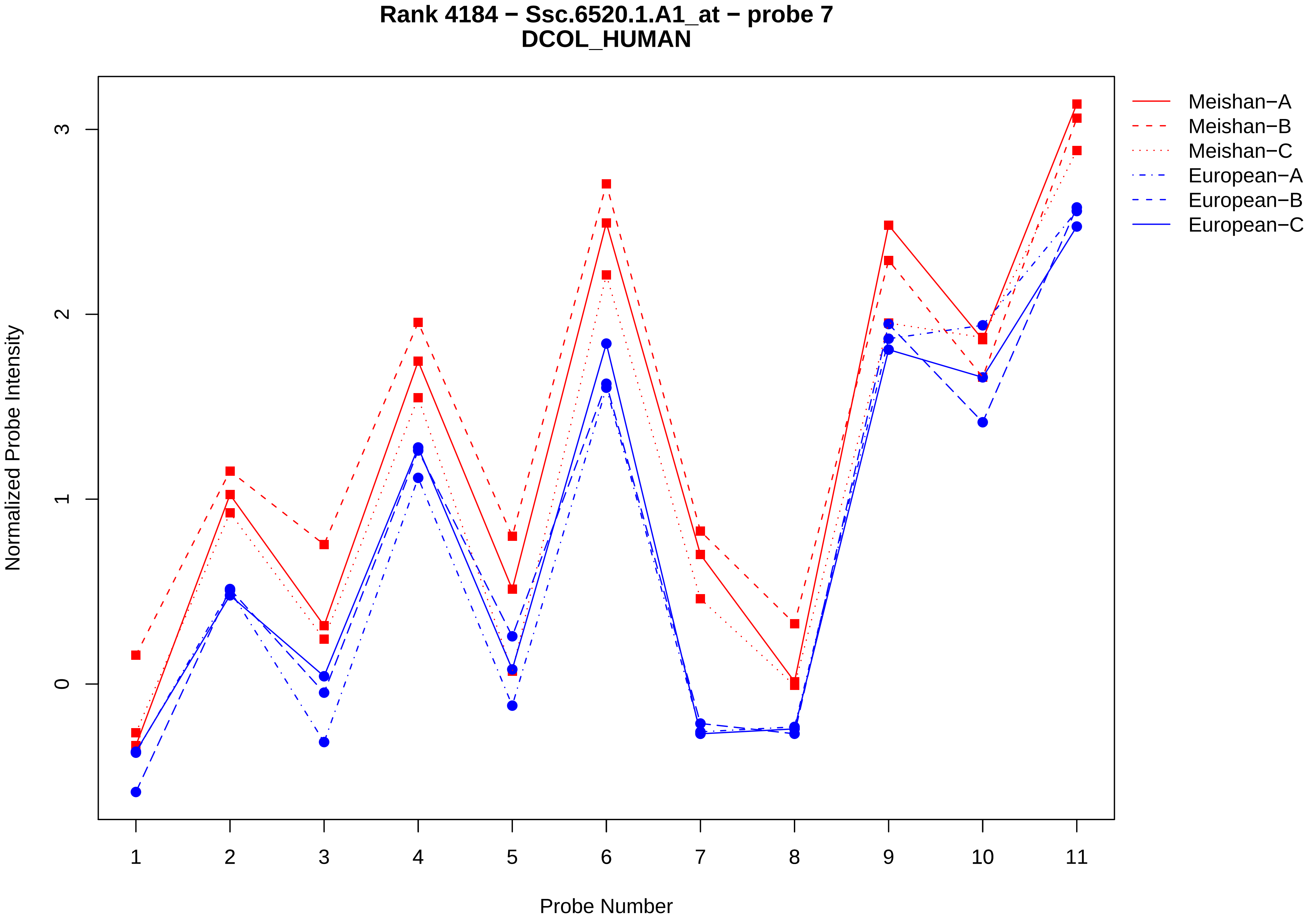

Rank 4184 − Ssc.6520.1.A1_at − probe 7
DCOL_HUMAN
Normalized Probe Intensity
Probe Number
0
1
2
3
1
2
3
4
5
6
7
8
9
10
11
Meishan−A
Meishan−B
Meishan−C
European−A
European−B
European−C

Rank 4185 − Ssc.1480.1.S1_at − probe 11
PSMD1

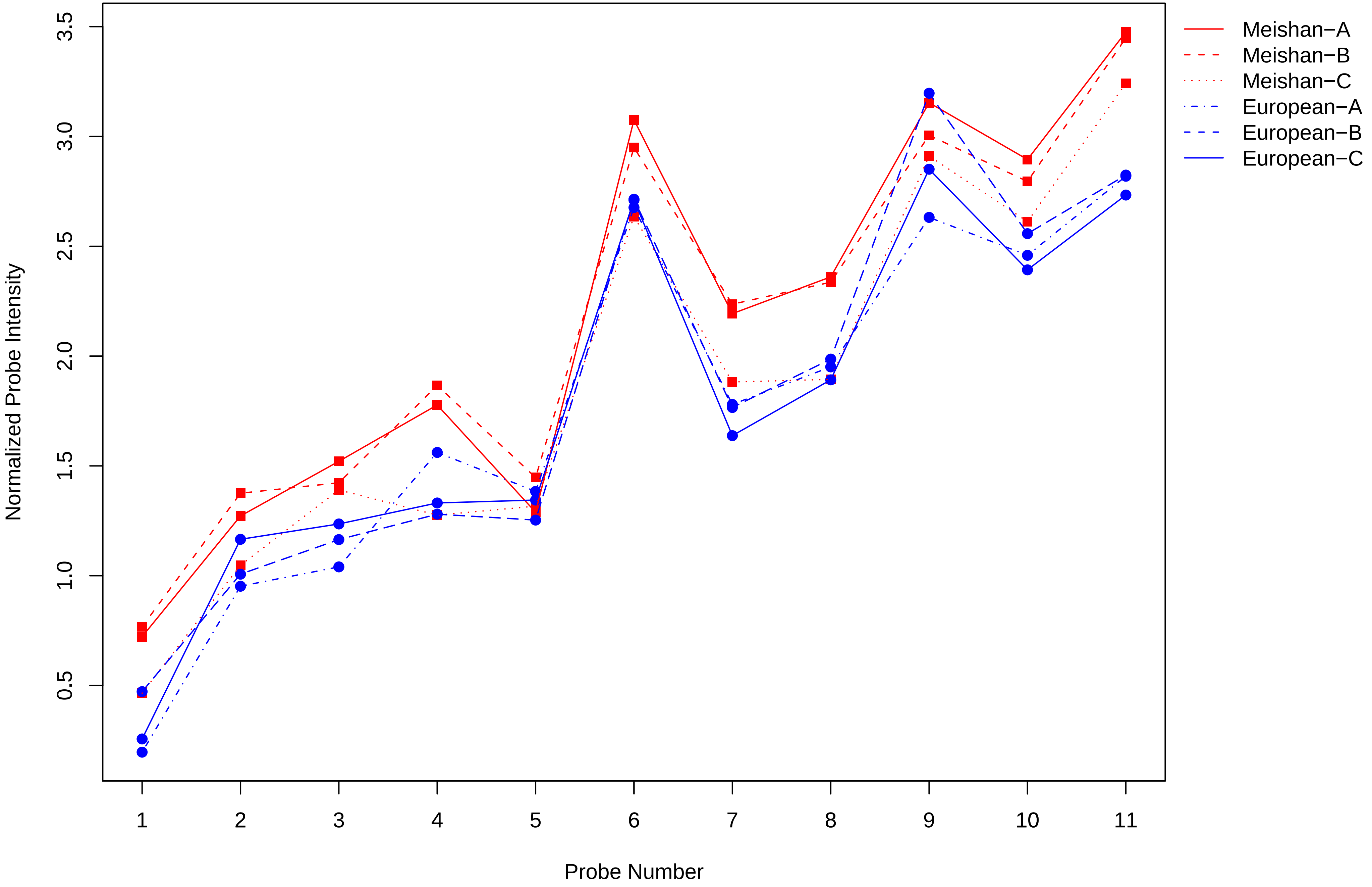

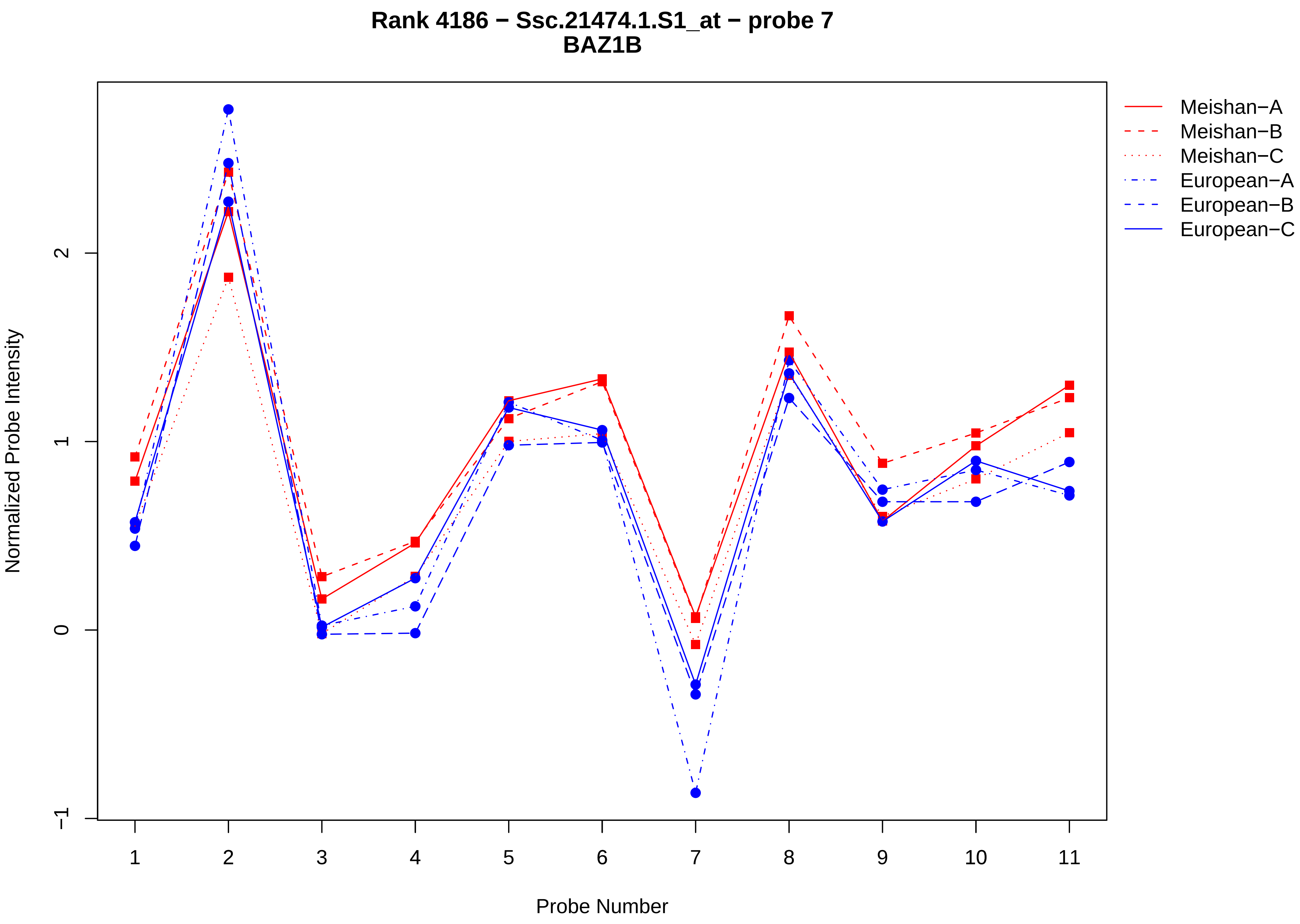

Rank 4186 − Ssc.21474.1.S1_at − probe 7
BAZ1B
Normalized Probe Intensity
Probe Number
−1
0
1
2
1
2
3
4
5
6
7
8
9
10
11
Meishan−A
Meishan−B
Meishan−C
European−A
European−B
European−C

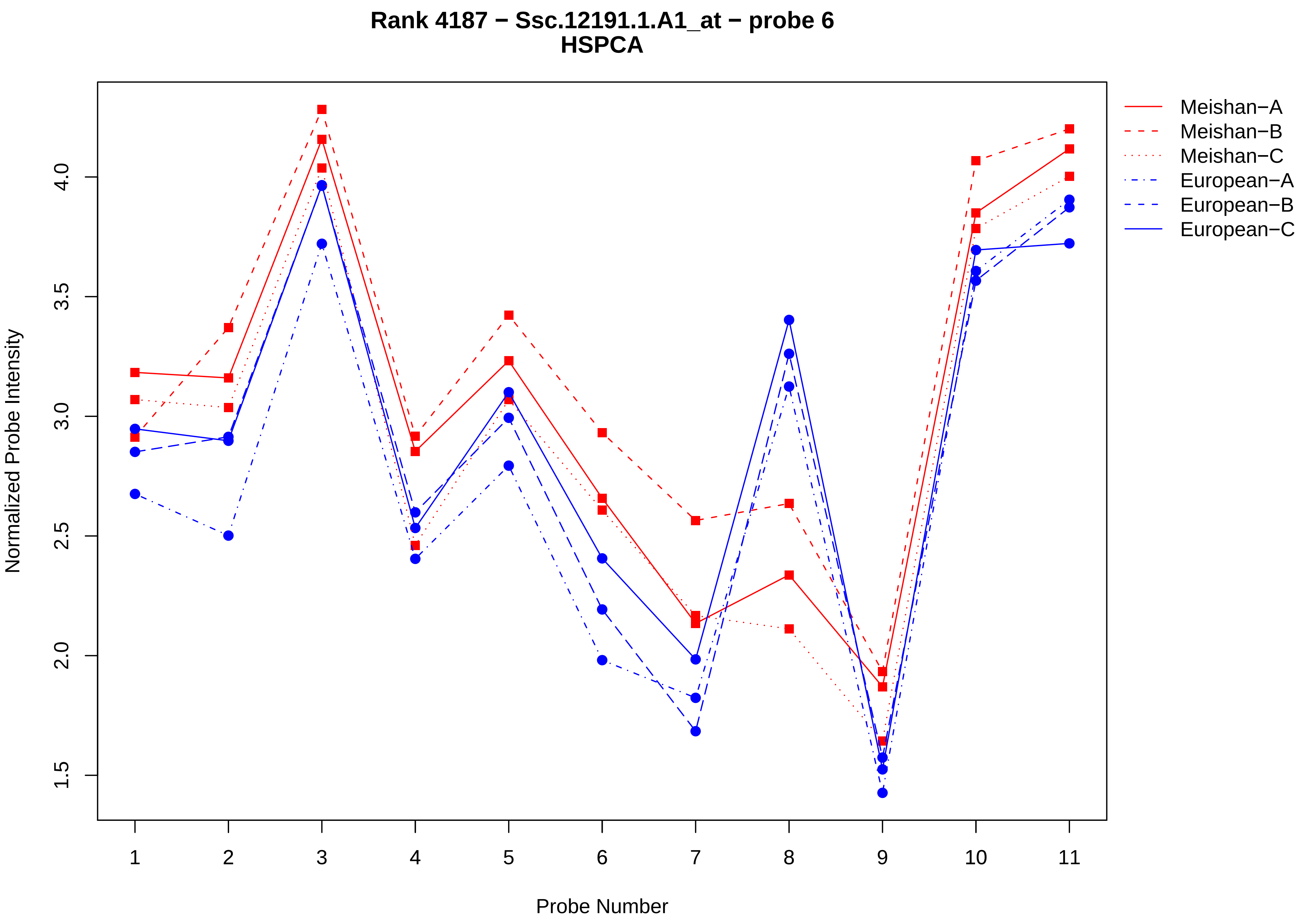

Rank 4187 − Ssc.12191.1.A1_at − probe 6
HSPCA
Normalized Probe Intensity
Probe Number
1.5
2.0
2.5
3.0
3.5
4.0
1
2
3
4
5
6
7
8
9
10
11
Meishan−A
Meishan−B
Meishan−C
European−A
European−B
European−C

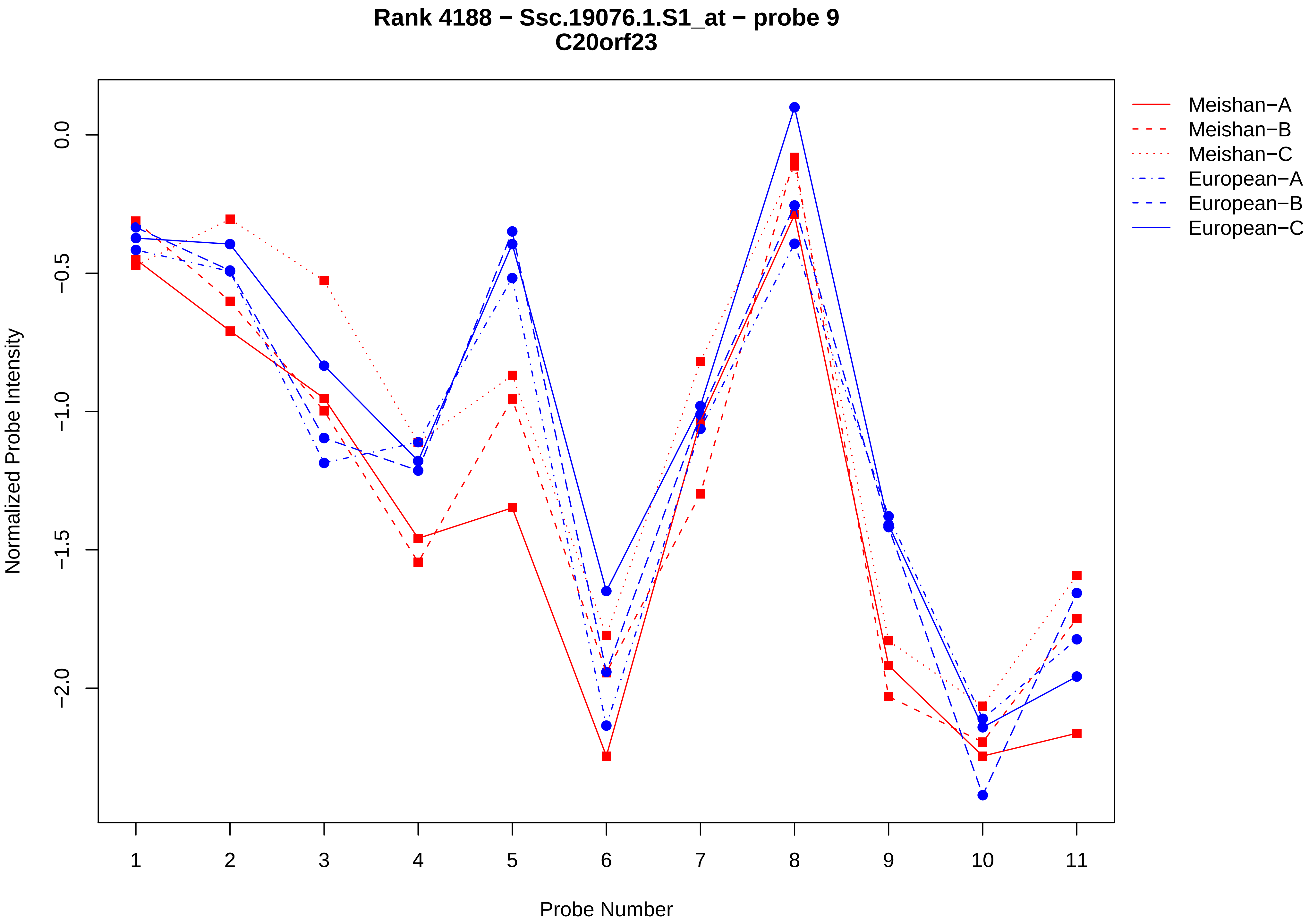

Rank 4188 − Ssc.19076.1.S1_at − probe 9
C20orf23
Normalized Probe Intensity
0.0
−0.5
−1.0
−1.5
−2.0
1
2
3
4
5
6
7
8
9
10
11
Probe Number
Meishan−A
Meishan−B
Meishan−C
European−A
European−B
European−C

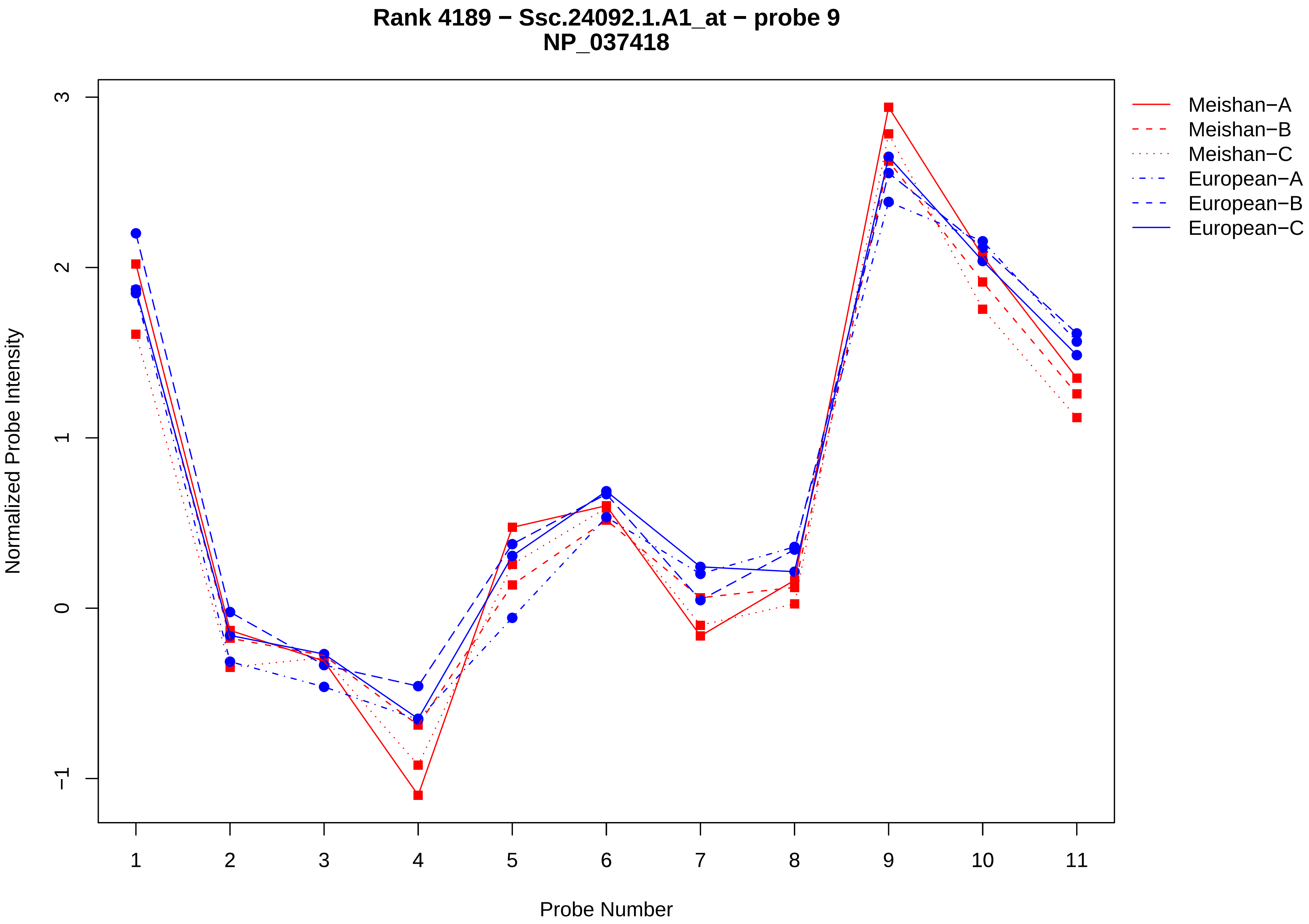

Rank 4189 − Ssc.24092.1.A1_at − probe 9
NP_037418
Normalized Probe Intensity
Probe Number
−1
0
1
2
3
1
2
3
4
5
6
7
8
9
10
11
Meishan−A
Meishan−B
Meishan−C
European−A
European−B
European−C

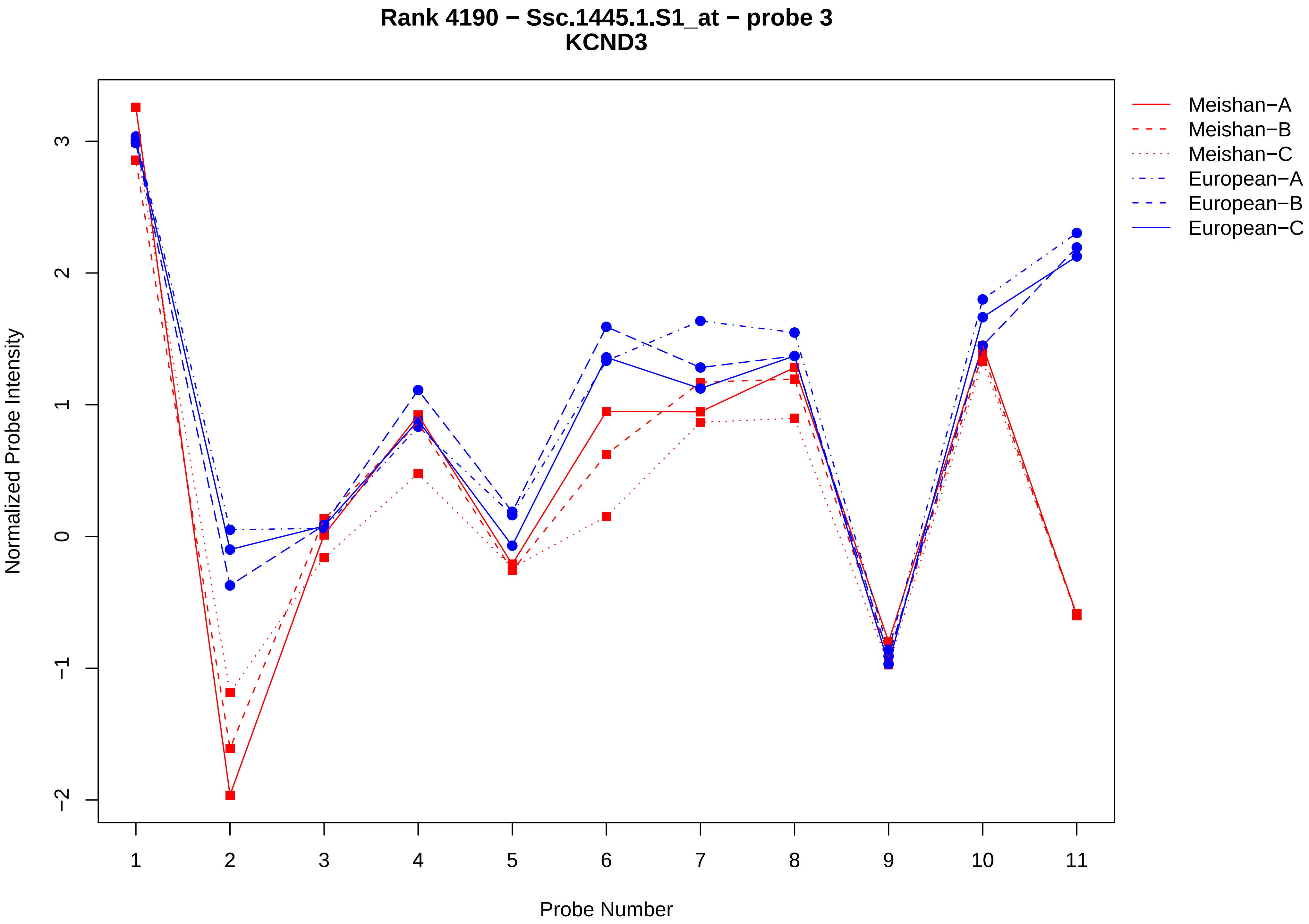

Rank 4190 − Ssc.1445.1.S1_at − probe 3
KCND3
Normalized Probe Intensity
Probe Number
−2
−1
0
1
2
3
1
2
3
4
5
6
7
8
9
10
11
Meishan−A
Meishan−B
Meishan−C
European−A
European−B
European−C

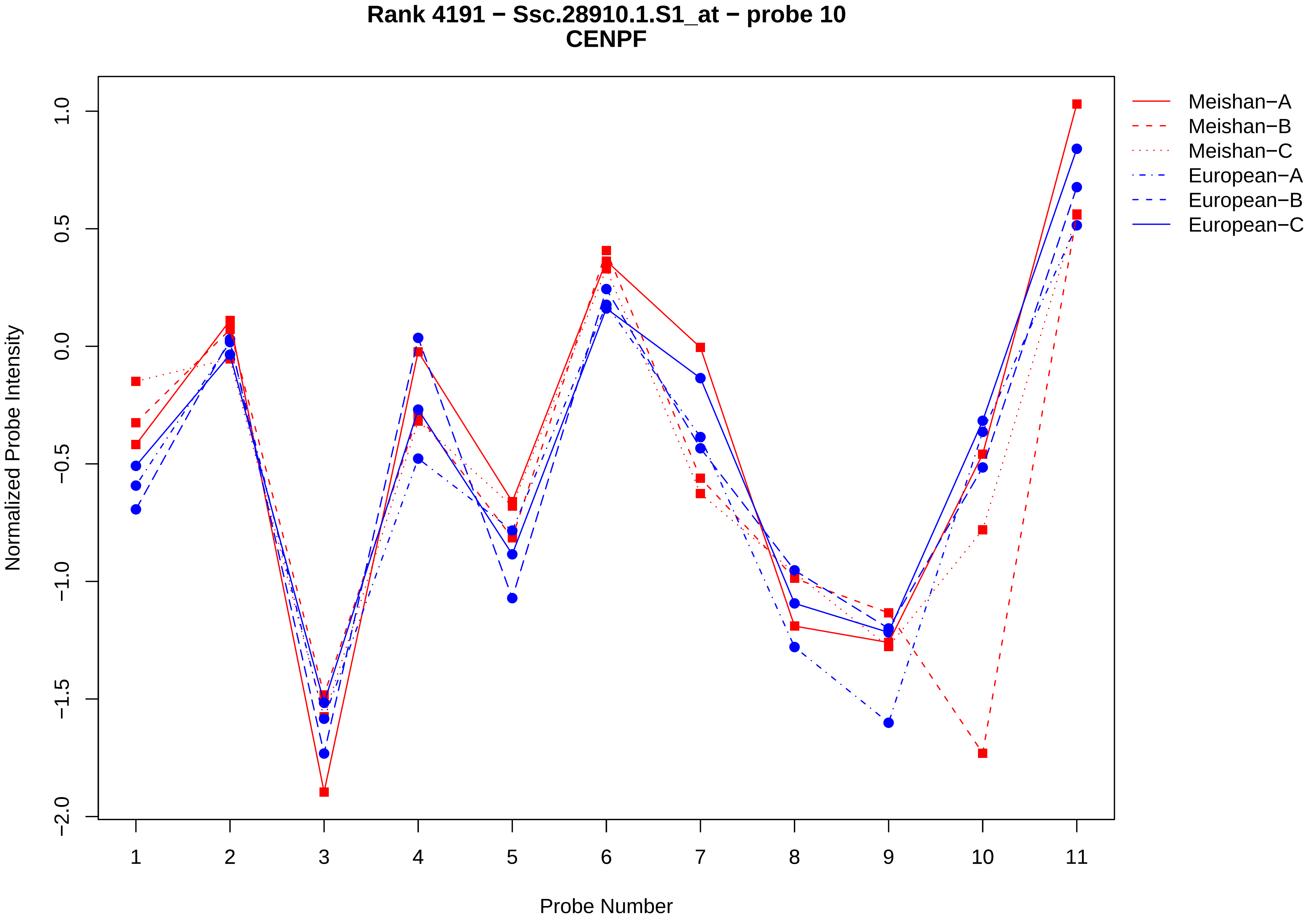

Rank 4191 − Ssc.28910.1.S1_at − probe 10
CENPF
Normalized Probe Intensity
Probe Number
1.0
0.5
0.0
−0.5
−1.0
−1.5
−2.0
1
2
3
4
5
6
7
8
9
10
11
Meishan−A
Meishan−B
Meishan−C
European−A
European−B
European−C

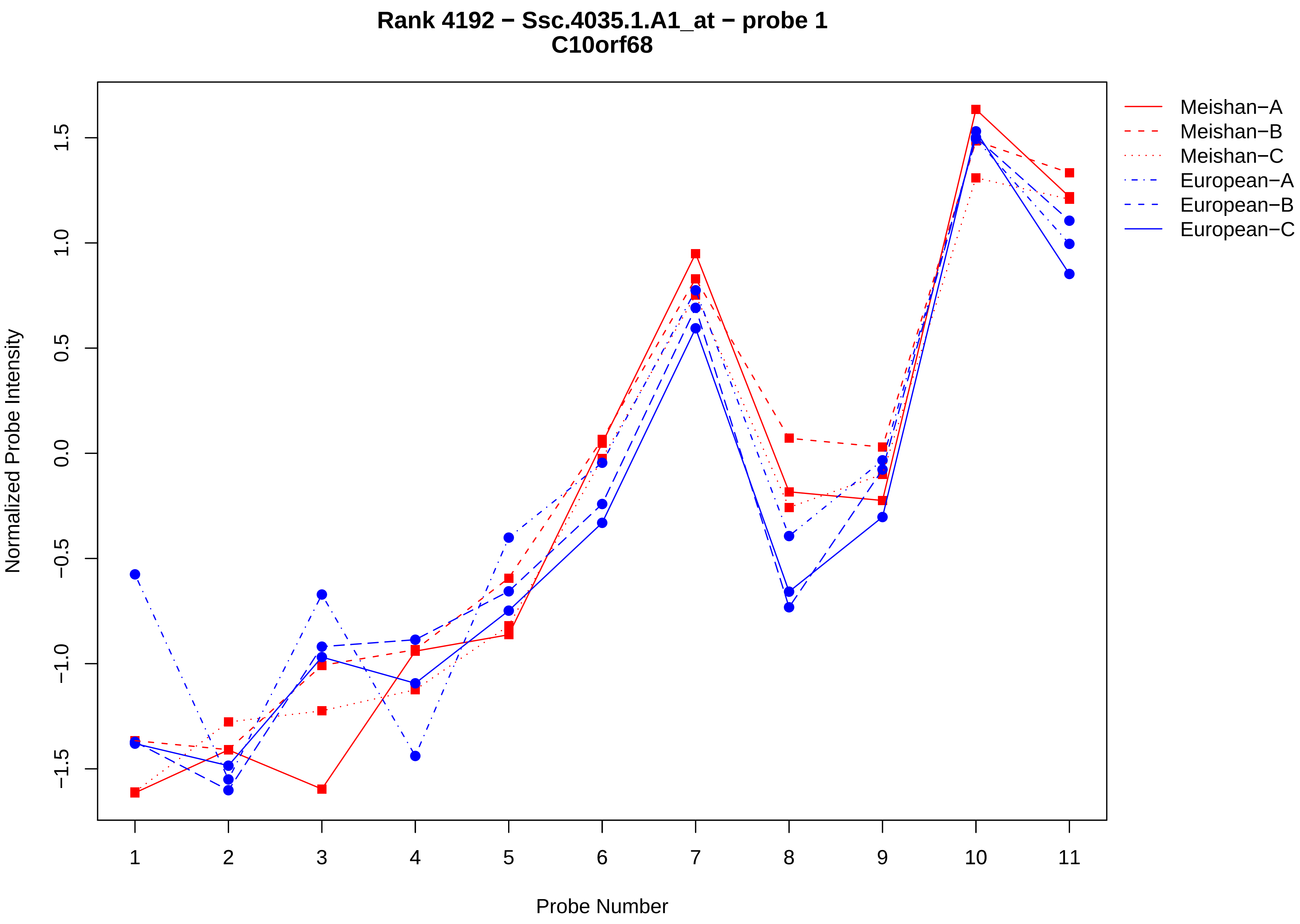

Rank 4192 − Ssc.4035.1.A1_at − probe 1
C10orf68
Normalized Probe Intensity
Probe Number
Meishan−A
Meishan−B
Meishan−C
European−A
European−B
European−C

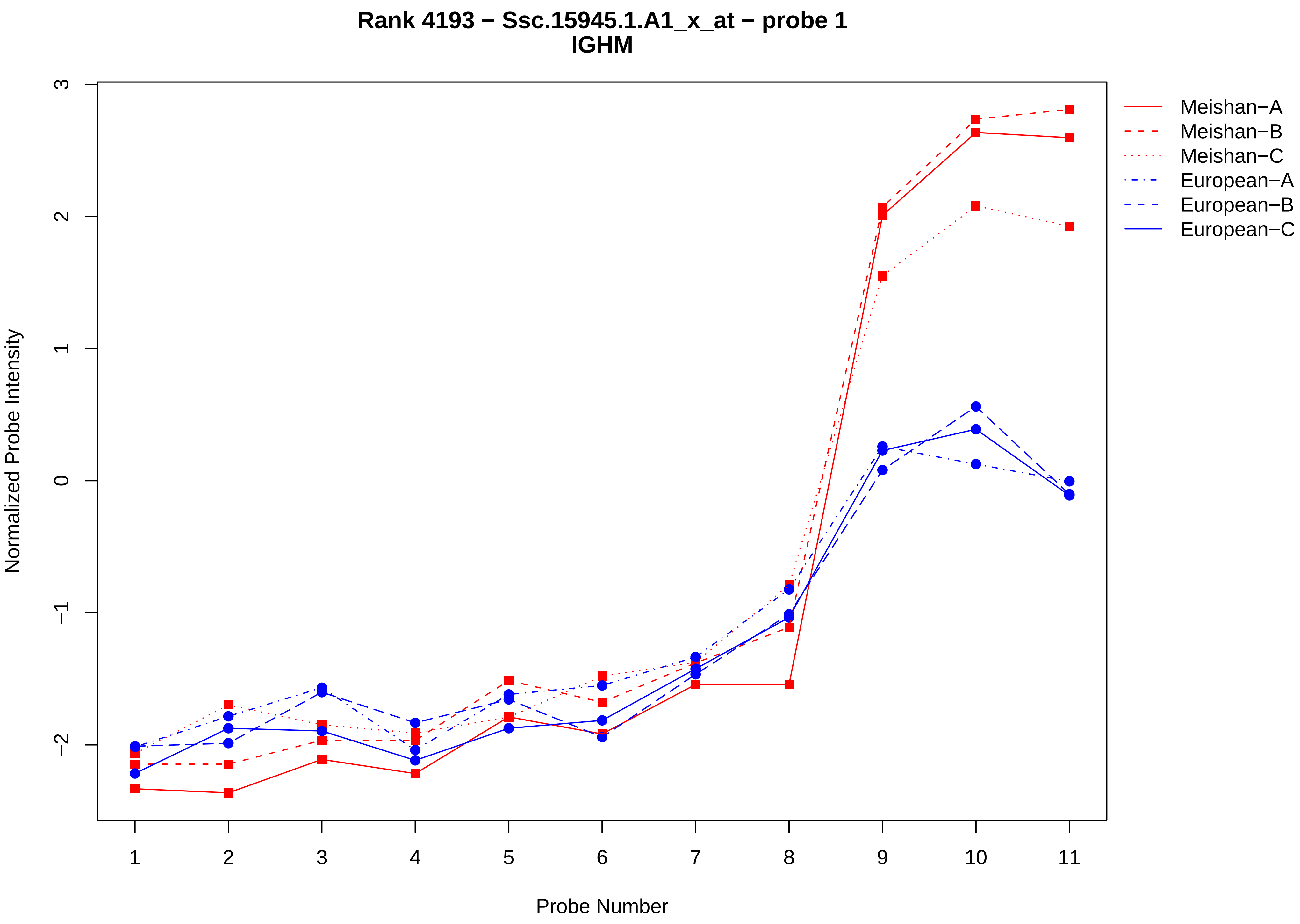

Rank 4193 − Ssc.15945.1.A1_x_at − probe 1
IGHM
Normalized Probe Intensity
Probe Number
Meishan−A
Meishan−B
Meishan−C
European−A
European−B
European−C
−2
−1
0
1
2
3
1
2
3
4
5
6
7
8
9
10
11

Rank 4194 − Ssc.30902.1.A1_at − probe 10
Q8IXR9

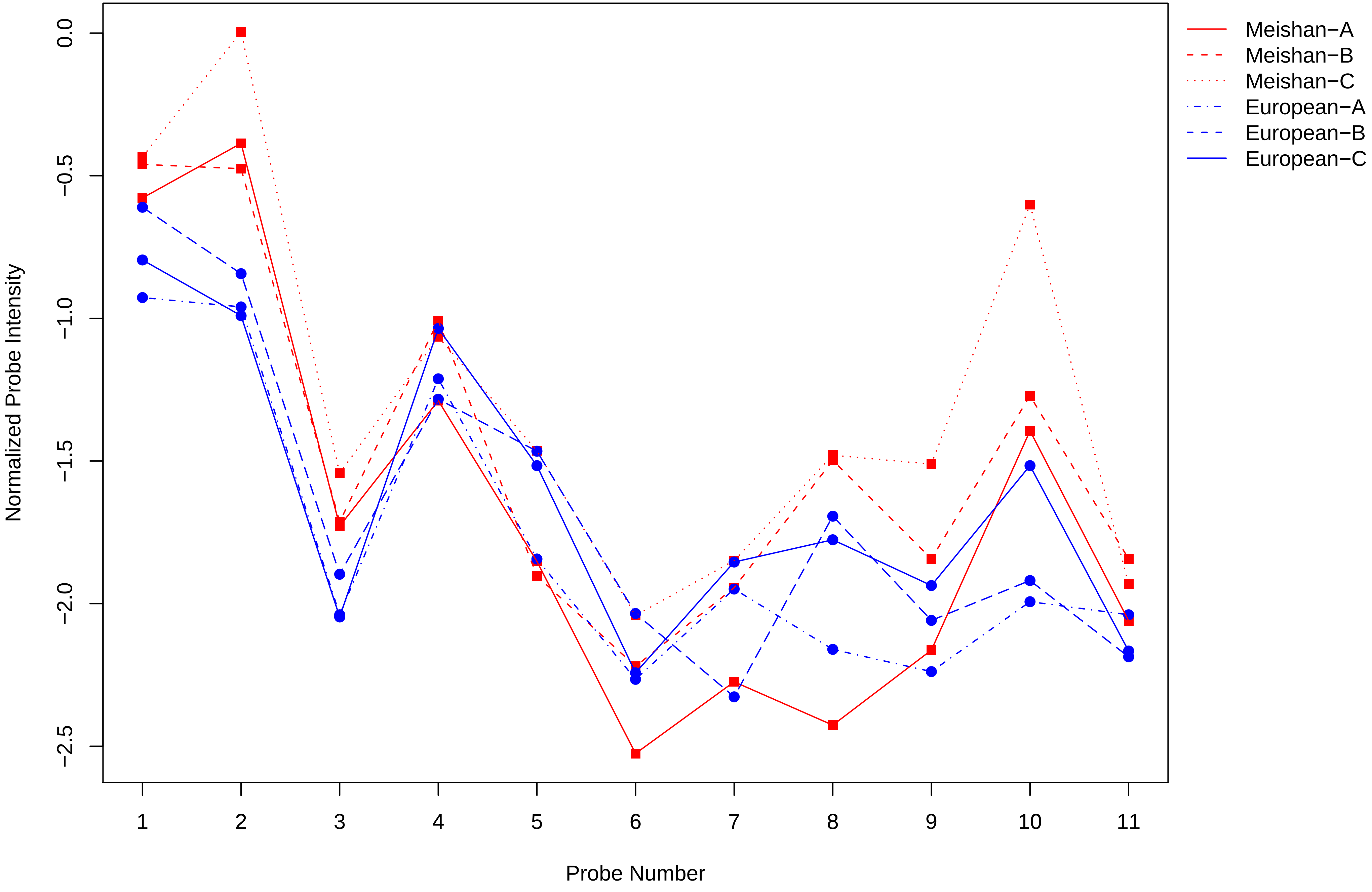

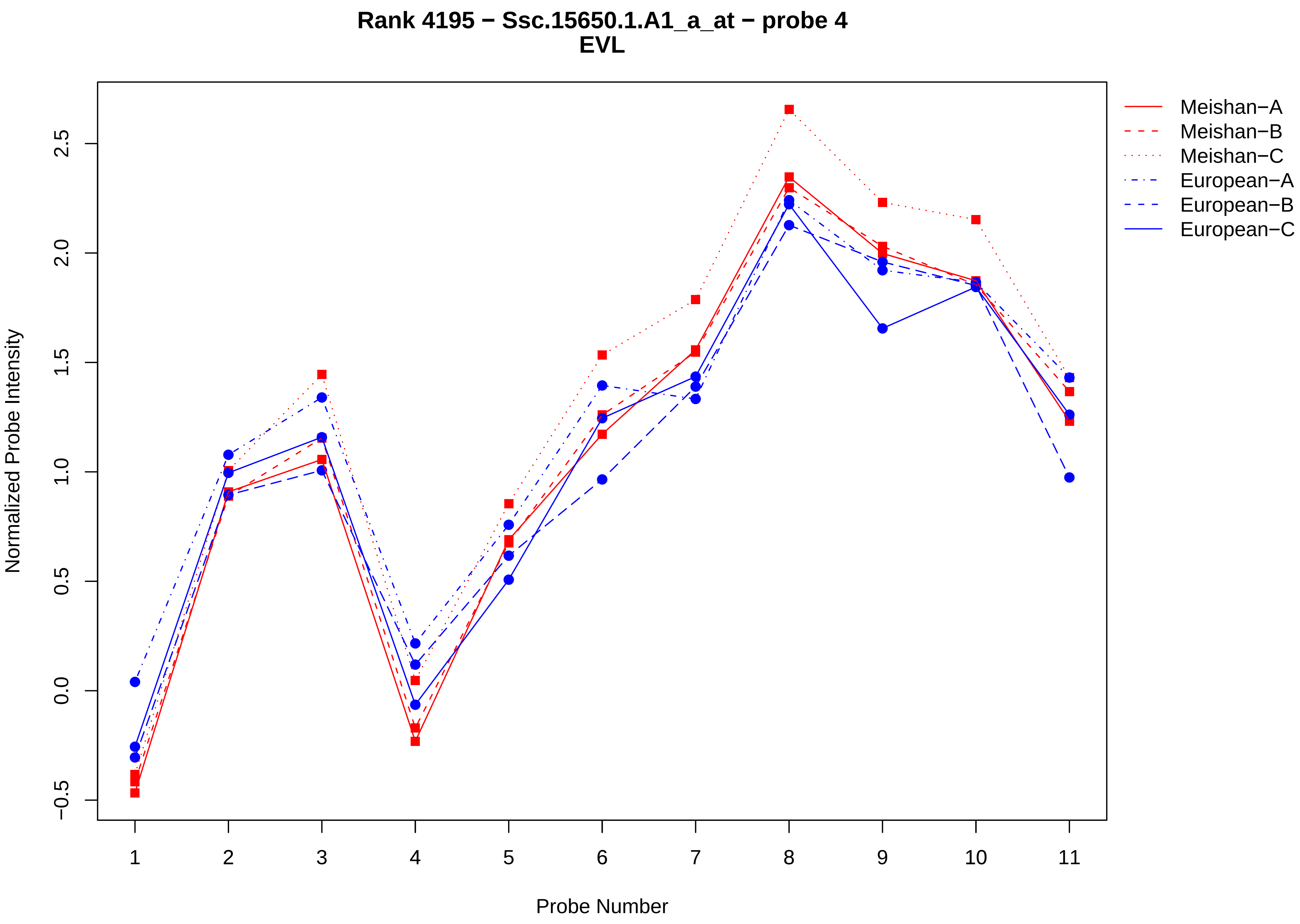
Rank 4195 − Ssc.15650.1.A1_a_at − probe 4
EVL
Normalized Probe Intensity
Probe Number
−0.5
0.0
0.5
1.0
1.5
2.0
2.5
1
2
3
4
5
6
7
8
9
10
11
Meishan−A
Meishan−B
Meishan−C
European−A
European−B
European−C

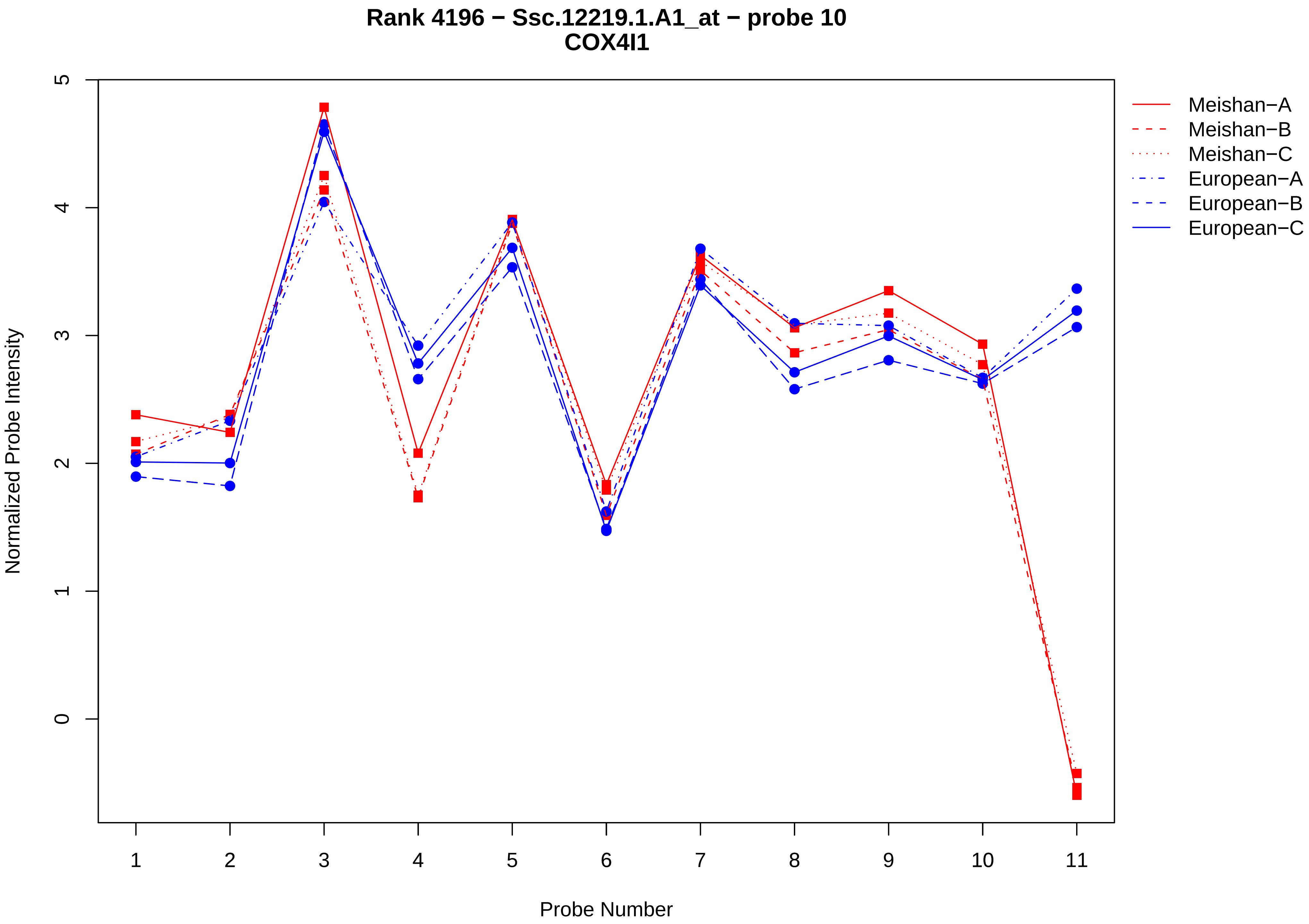

Rank 4196 − Ssc.12219.1.A1_at − probe 10
COX4I1
Normalized Probe Intensity
Probe Number
0
1
2
3
4
5
1
2
3
4
5
6
7
8
9
10
11
Meishan−A
Meishan−B
Meishan−C
European−A
European−B
European−C

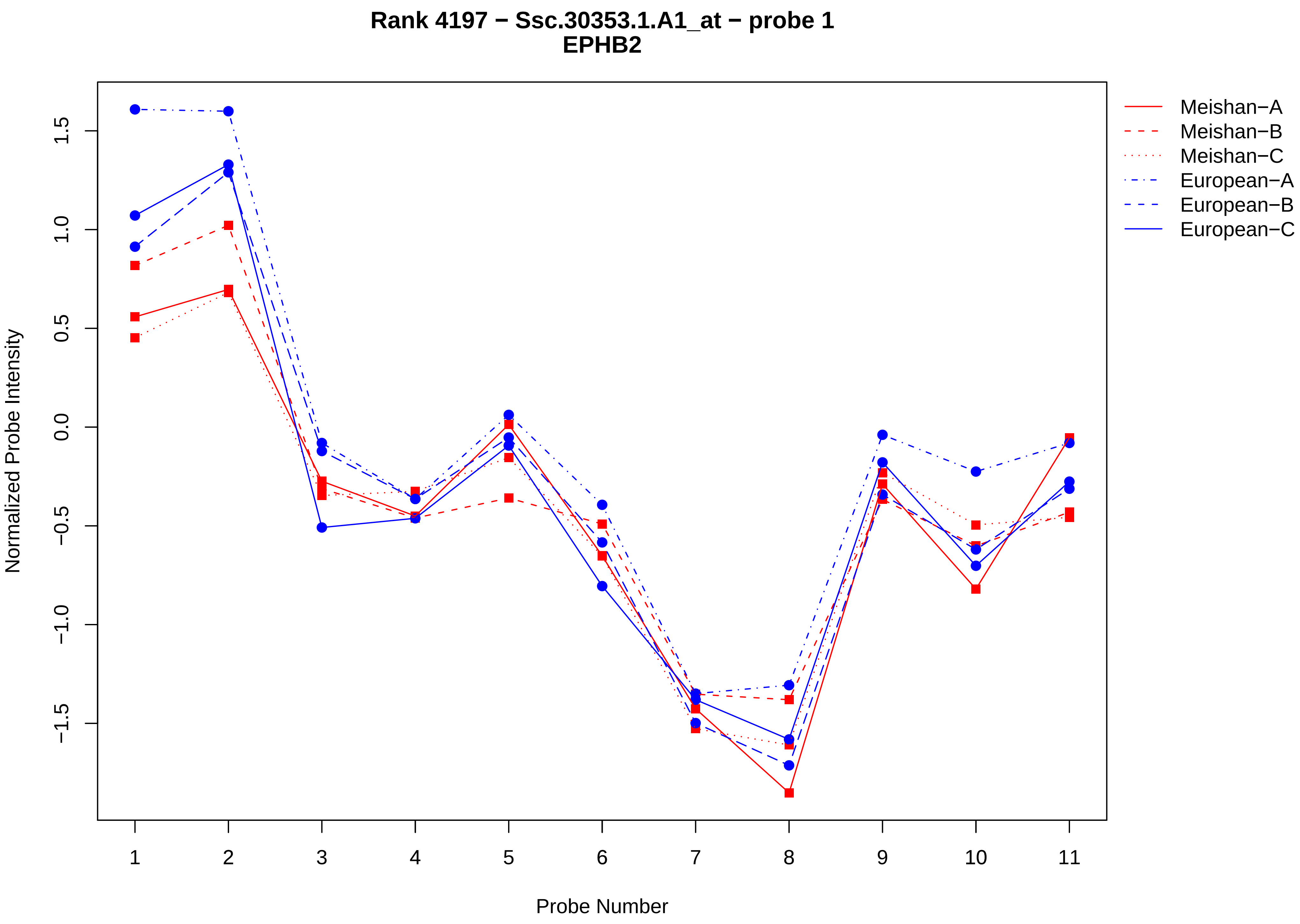

Rank 4197 − Ssc.30353.1.A1_at − probe 1
EPHB2
Normalized Probe Intensity
Probe Number
1.5
1.0
0.5
0.0
−0.5
−1.0
−1.5
1
2
3
4
5
6
7
8
9
10
11
Meishan−A
Meishan−B
Meishan−C
European−A
European−B
European−C

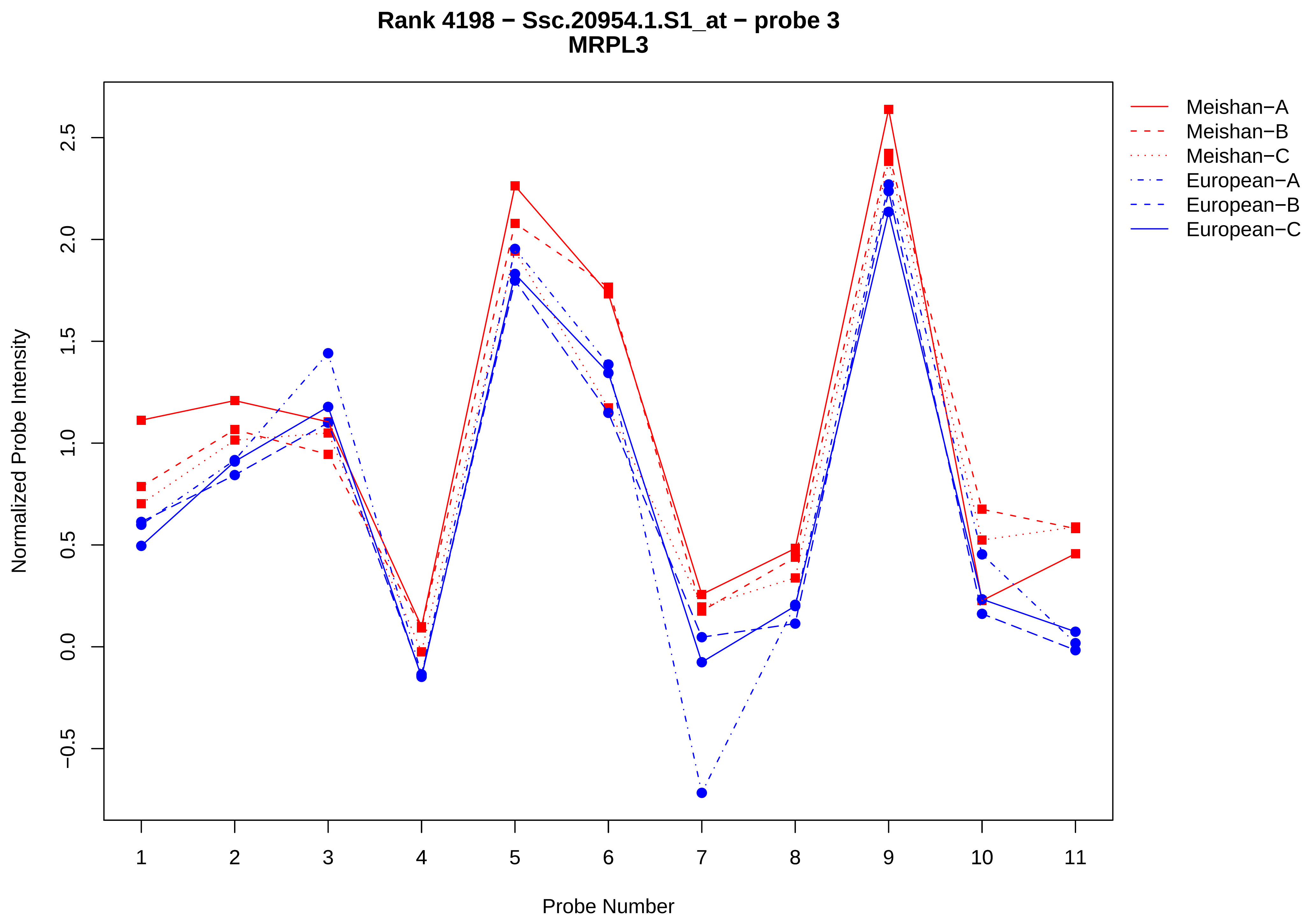

Rank 4198 − Ssc.20954.1.S1_at − probe 3
MRPL3
Normalized Probe Intensity
Probe Number
−0.5
0.0
0.5
1.0
1.5
2.0
2.5
1
2
3
4
5
6
7
8
9
10
11
Meishan−A
Meishan−B
Meishan−C
European−A
European−B
European−C

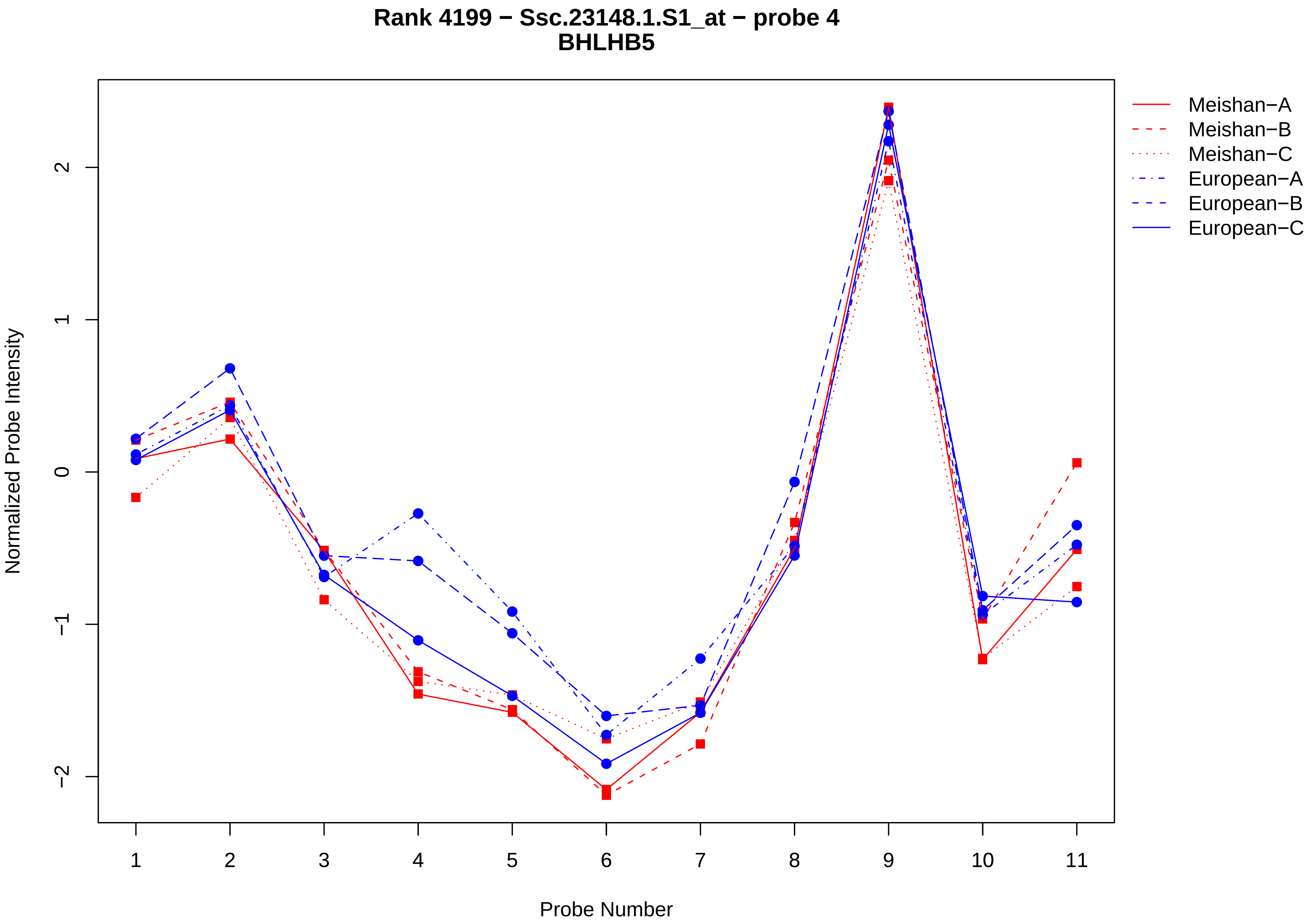
Rank 4199 − Ssc.23148.1.S1_at − probe 4
BHLHB5
Normalized Probe Intensity
Probe Number
−2
−1
0
1
2
1
2
3
4
5
6
7
8
9
10
11
Meishan−A
Meishan−B
Meishan−C
European−A
European−B
European−C

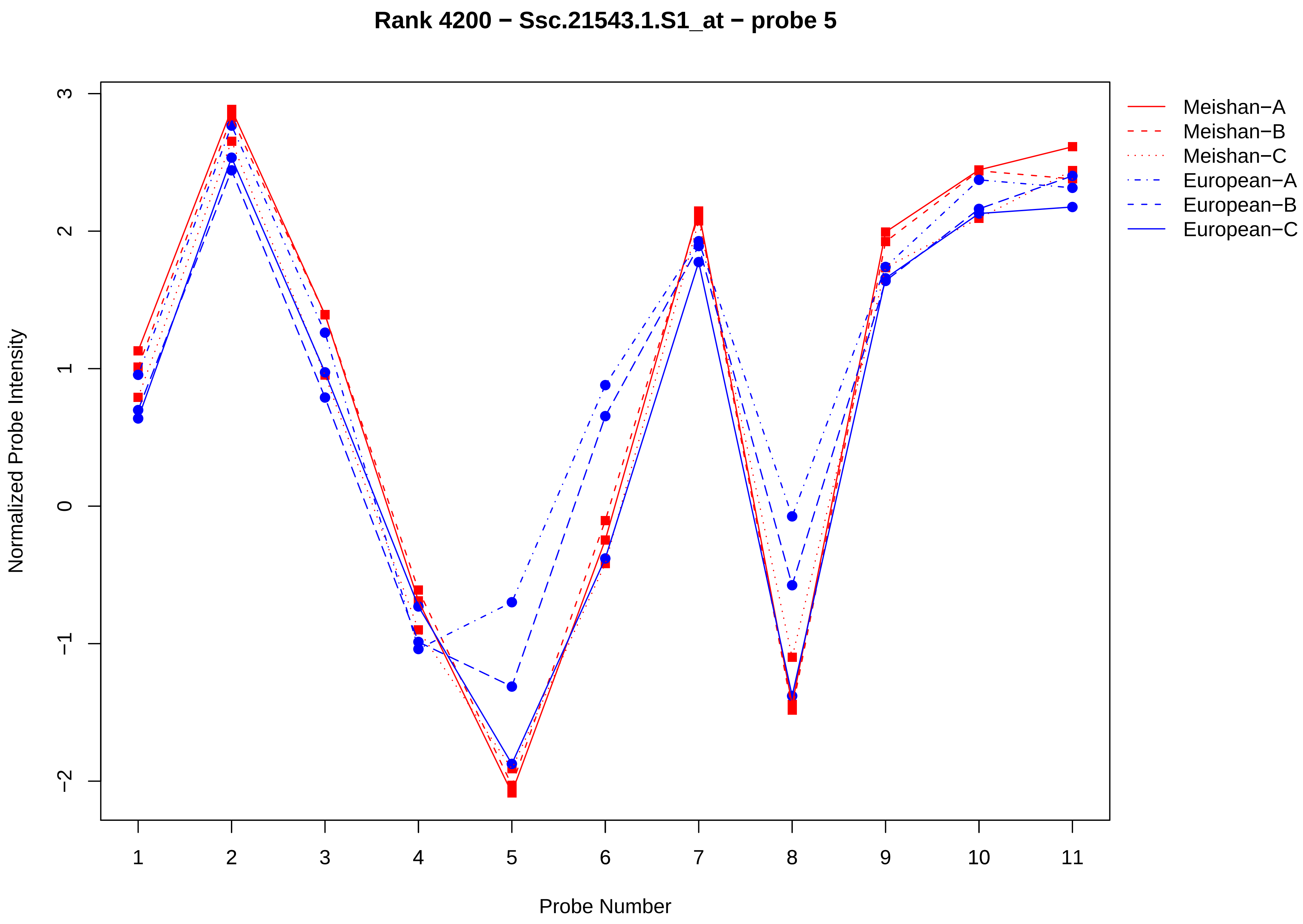

Rank 4200 − Ssc.21543.1.S1_at − probe 5
Normalized Probe Intensity
Probe Number
−2
−1
0
1
2
3
1
2
3
4
5
6
7
8
9
10
11
Meishan−A
Meishan−B
Meishan−C
European−A
European−B
European−C

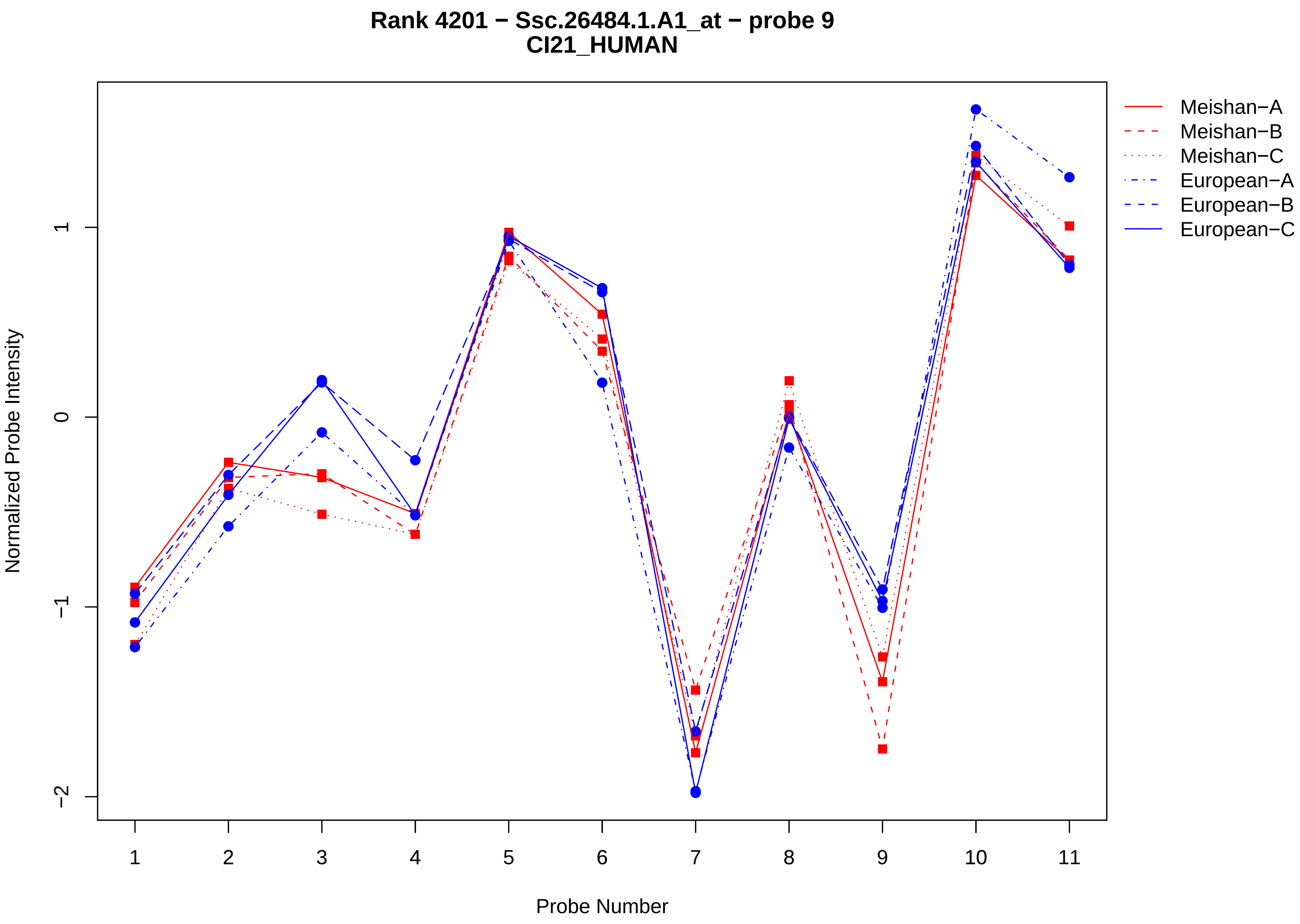

Rank 4201 − Ssc.26484.1.A1_at − probe 9
CI21_HUMAN
Normalized Probe Intensity
Probe Number
Meishan−A
Meishan−B
Meishan−C
European−A
European−B
European−C
1
0
−1
−2
1
2
3
4
5
6
7
8
9
10
11

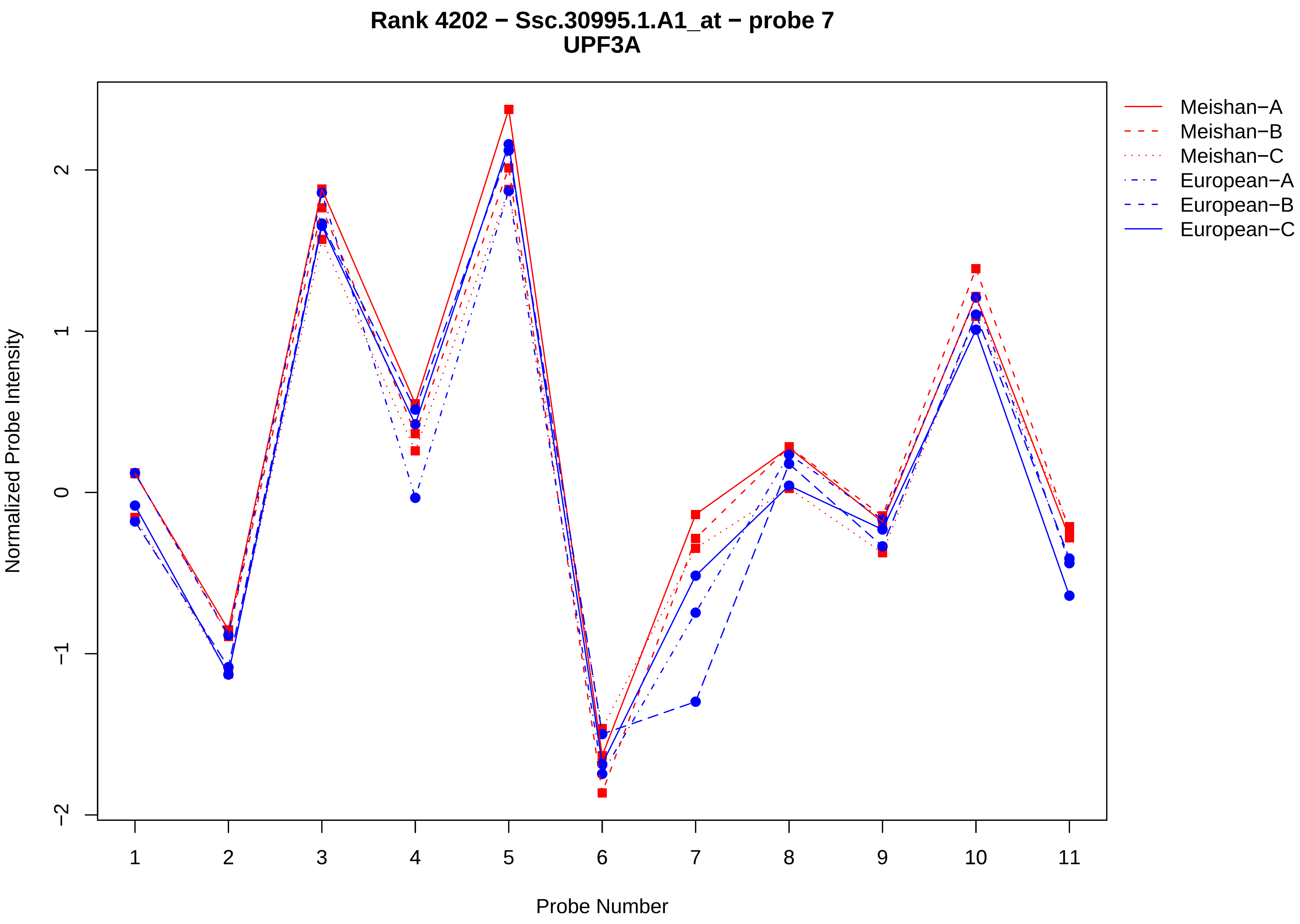

Rank 4202 − Ssc.30995.1.A1_at − probe 7
UPF3A
Normalized Probe Intensity
Probe Number
−2
−1
0
1
2
1
2
3
4
5
6
7
8
9
10
11
Meishan−A
Meishan−B
Meishan−C
European−A
European−B
European−C

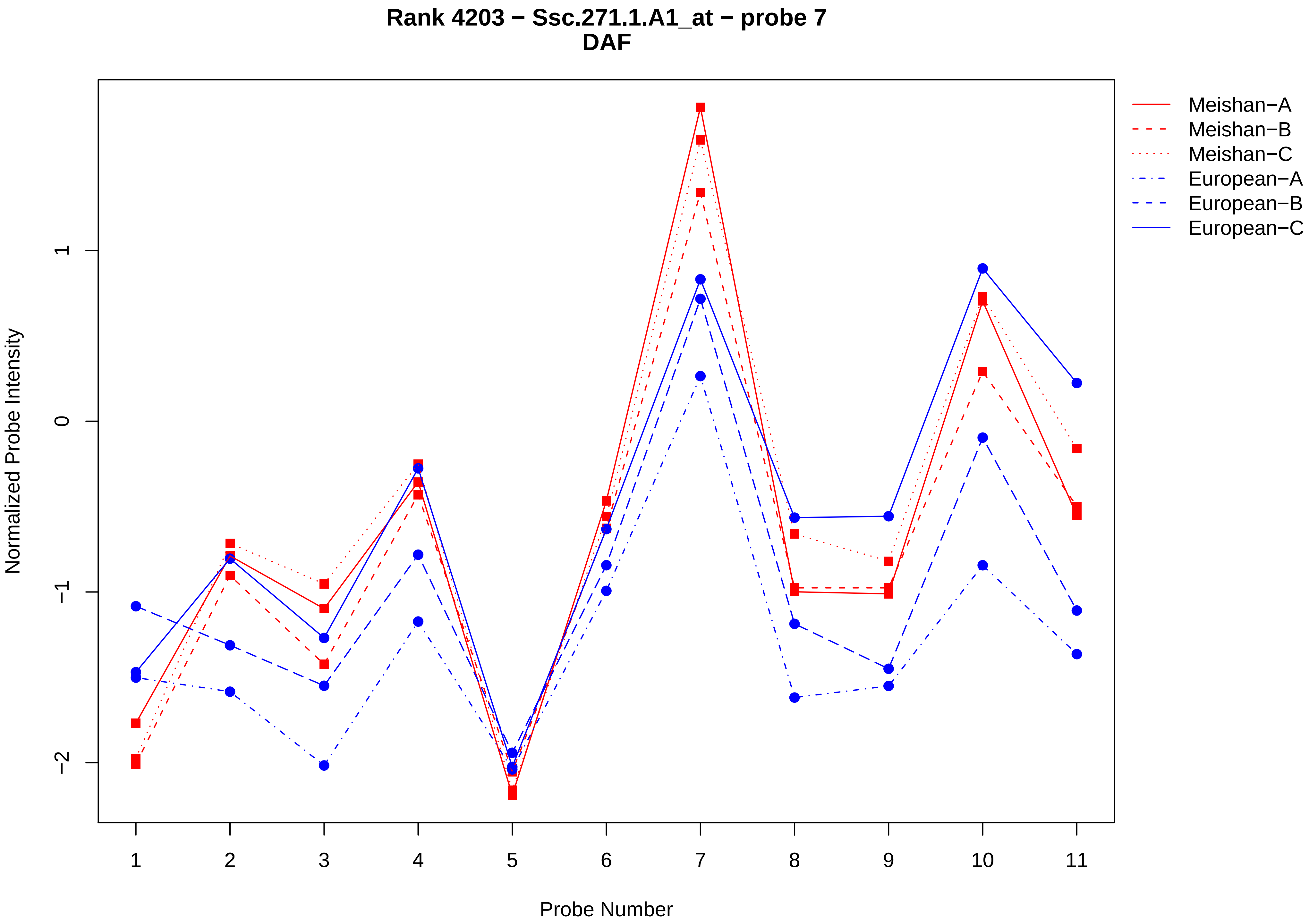

Rank 4203 − Ssc.271.1.A1_at − probe 7
DAF
Normalized Probe Intensity
Probe Number
1
0
−1
−2
1
2
3
4
5
6
7
8
9
10
11
Meishan−A
Meishan−B
Meishan−C
European−A
European−B
European−C

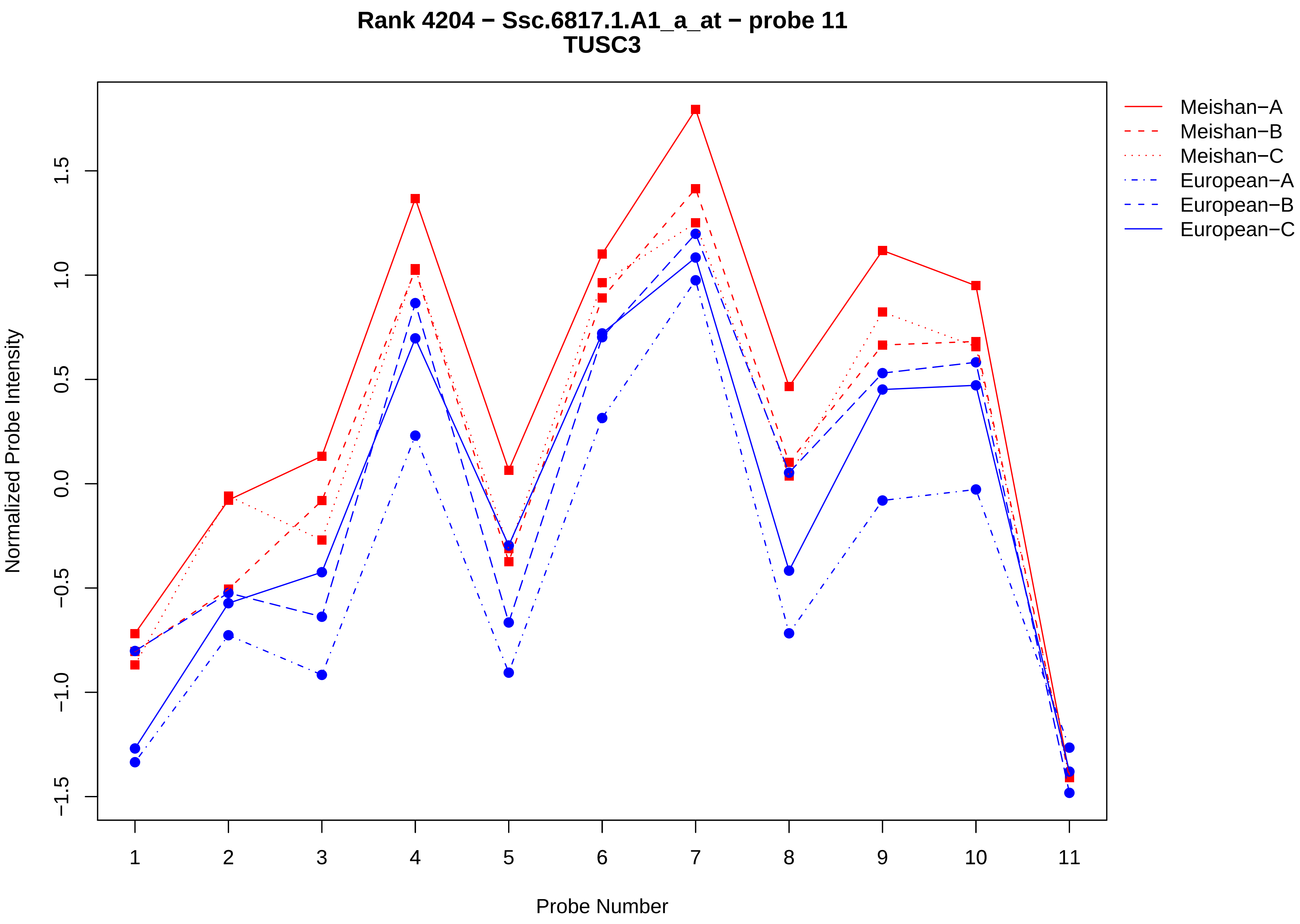
Rank 4204 − Ssc.6817.1.A1_a_at − probe 11
TUSC3
Normalized Probe Intensity
Probe Number
−1.5
−1.0
−0.5
0.0
0.5
1.0
1.5
1
2
3
4
5
6
7
8
9
10
11
Meishan−A
Meishan−B
Meishan−C
European−A
European−B
European−C

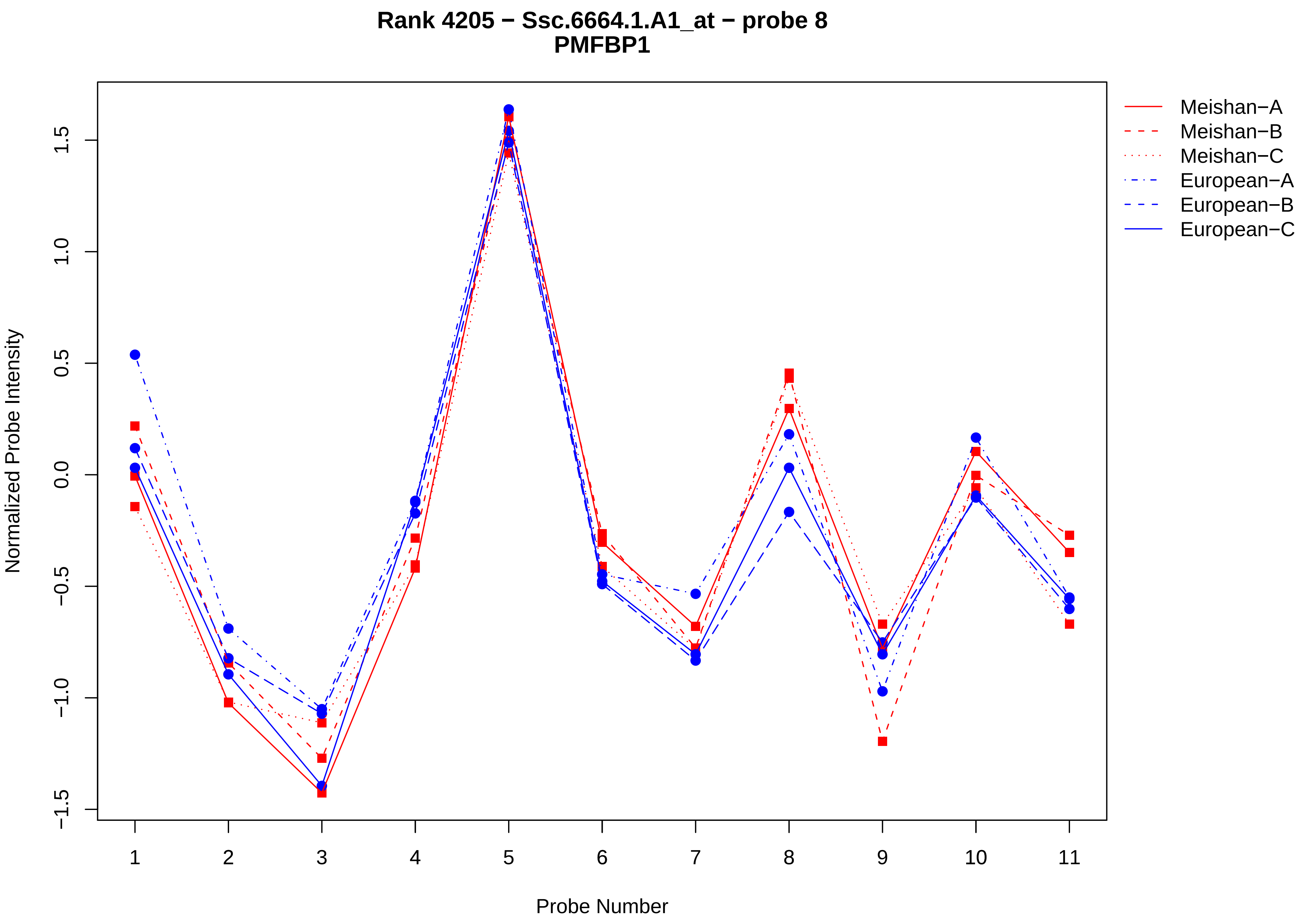

Rank 4205 − Ssc.6664.1.A1_at − probe 8
PMFBP1
Normalized Probe Intensity
Probe Number
−1.5
−1.0
−0.5
0.0
0.5
1.0
1.5
1
2
3
4
5
6
7
8
9
10
11
Meishan−A
Meishan−B
Meishan−C
European−A
European−B
European−C

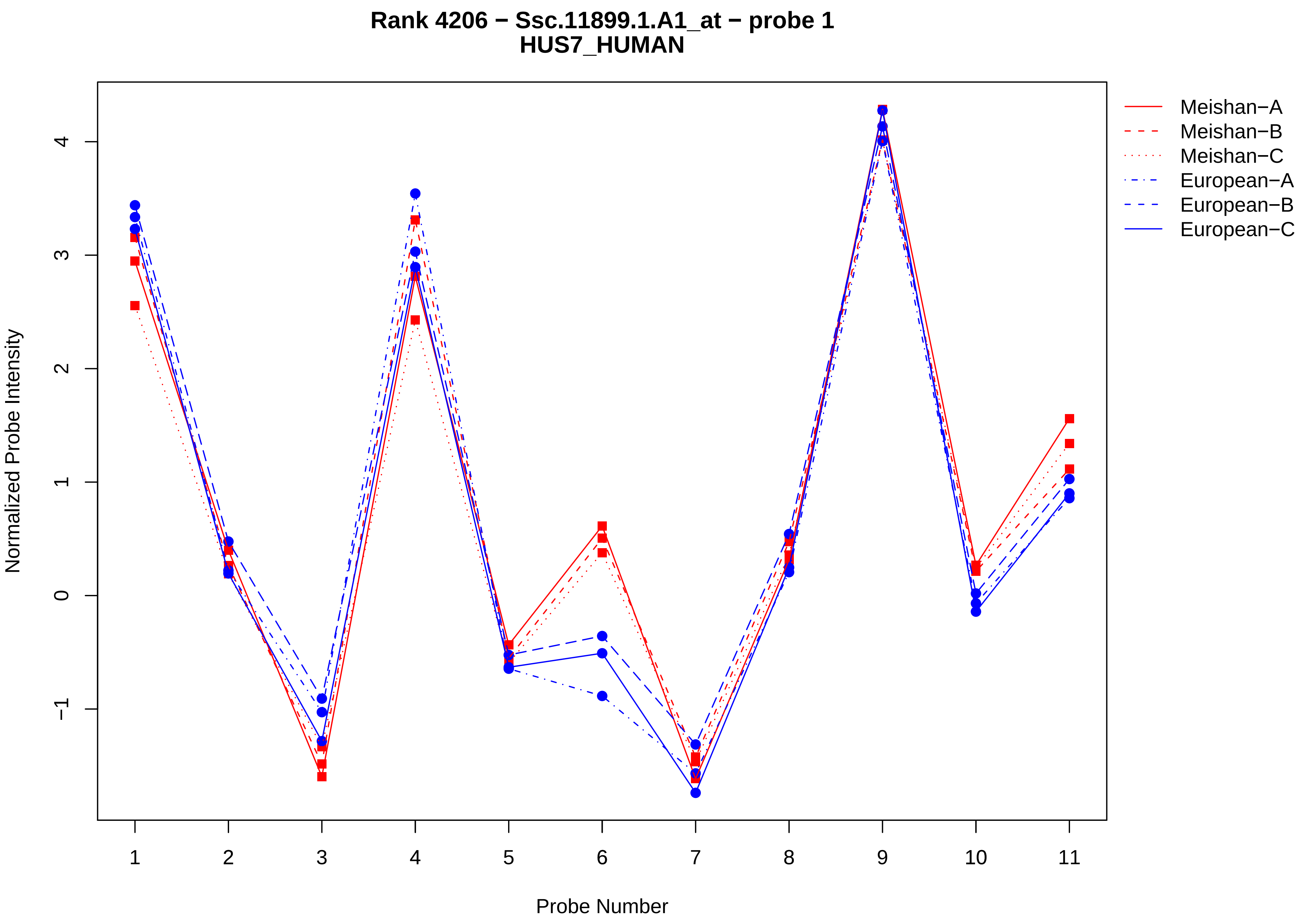

Rank 4206 − Ssc.11899.1.A1_at − probe 1
HUS7_HUMAN
Normalized Probe Intensity
Probe Number
Meishan−A
Meishan−B
Meishan−C
European−A
European−B
European−C
−1
0
1
2
3
4
1
2
3
4
5
6
7
8
9
10
11

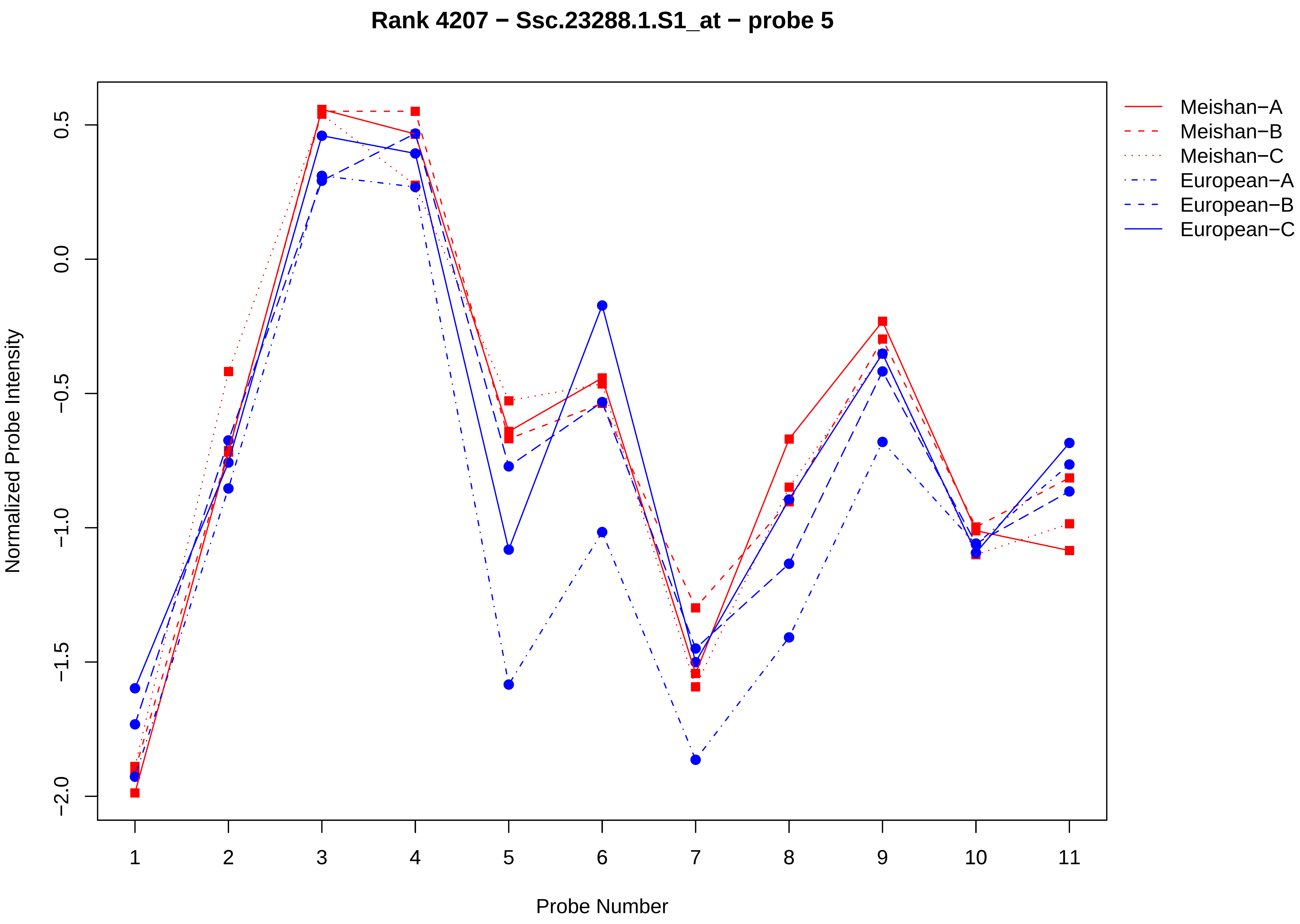

Rank 4207 − Ssc.23288.1.S1_at − probe 5
Normalized Probe Intensity
Probe Number
0.5
0.0
−0.5
−1.0
−1.5
−2.0
1
2
3
4
5
6
7
8
9
10
11
Meishan−A
Meishan−B
Meishan−C
European−A
European−B
European−C

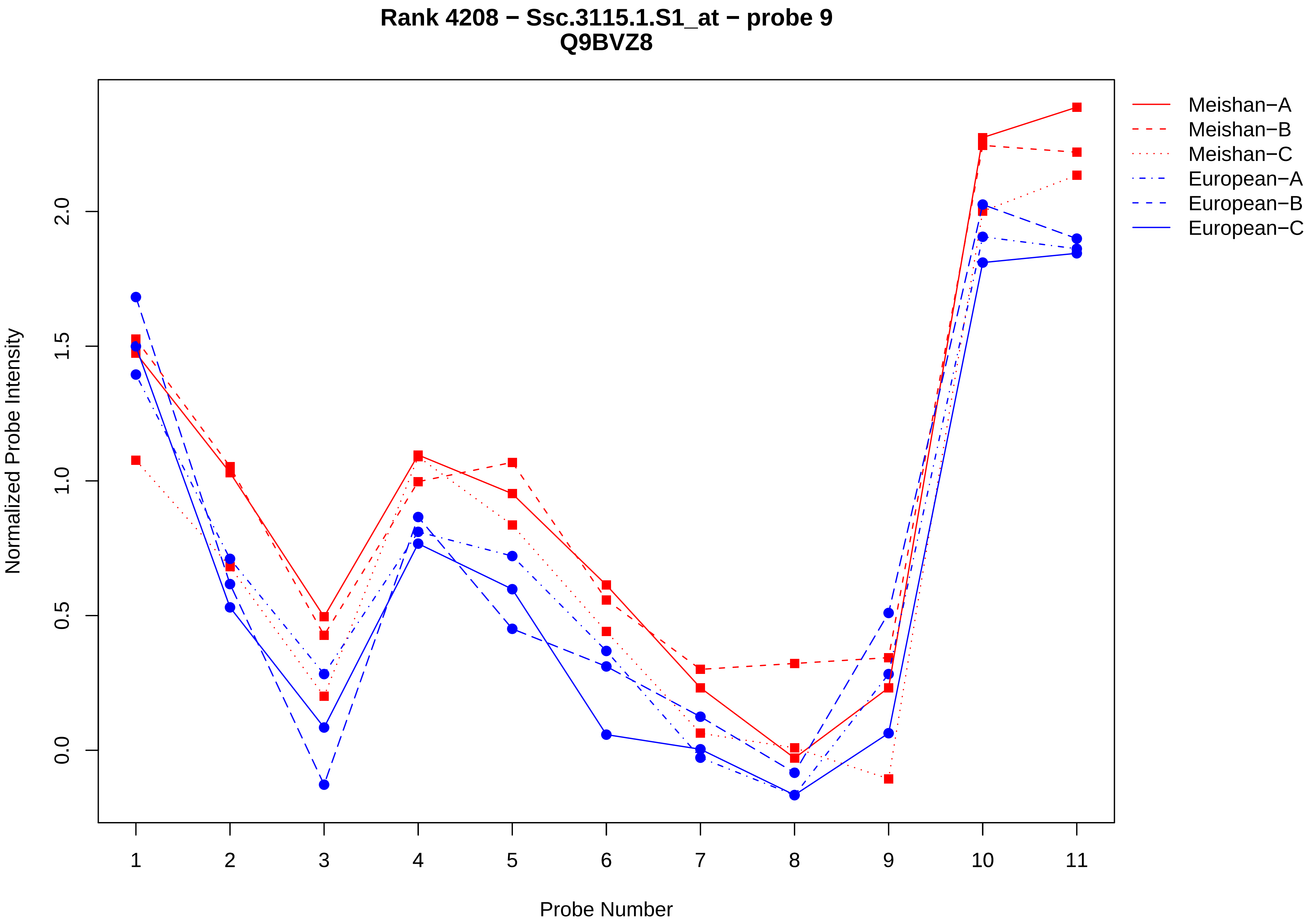

Rank 4208 − Ssc.3115.1.S1_at − probe 9
Q9BVZ8
Normalized Probe Intensity
Probe Number
0.0
0.5
1.0
1.5
2.0
1
2
3
4
5
6
7
8
9
10
11
Meishan−A
Meishan−B
Meishan−C
European−A
European−B
European−C

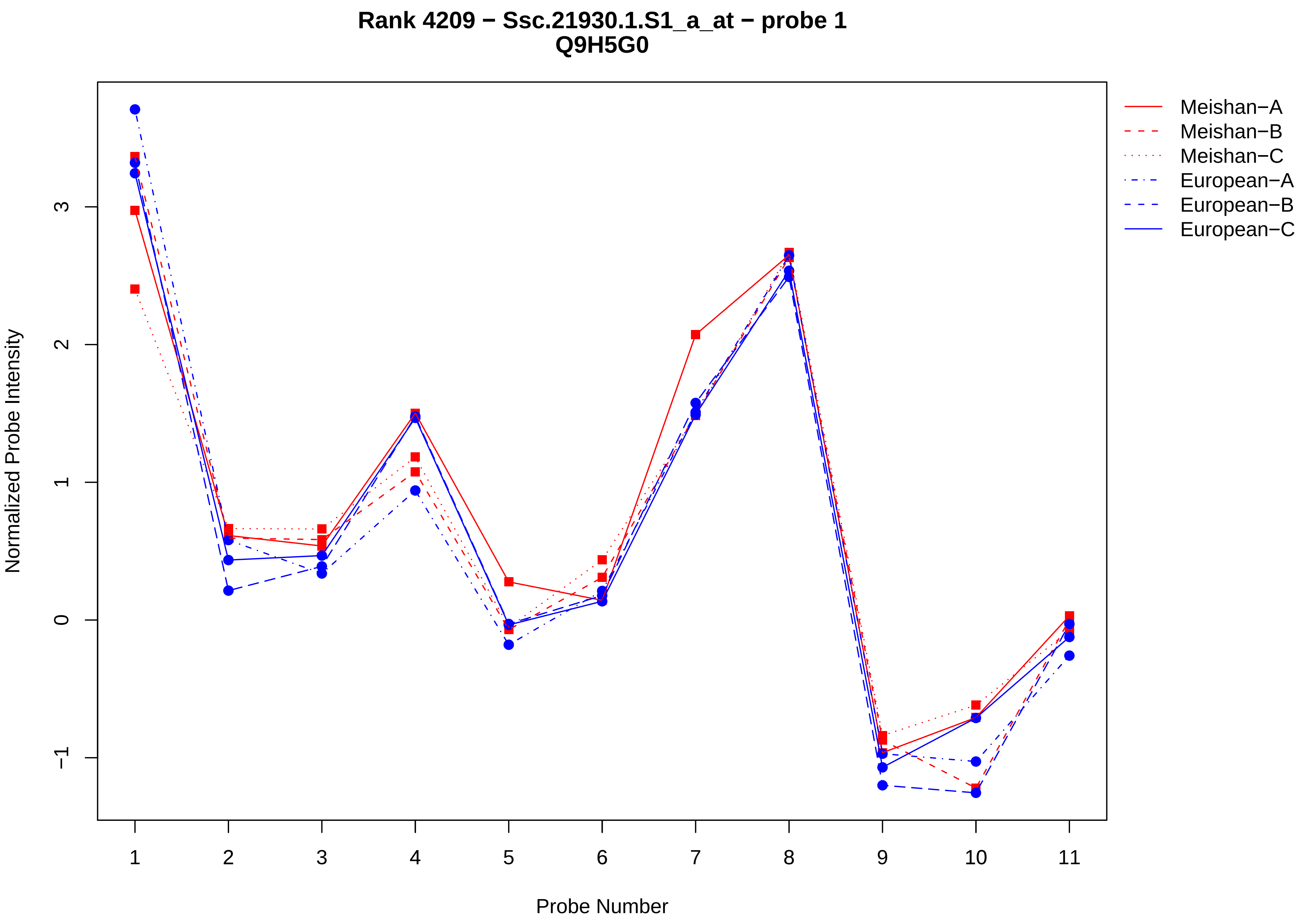

Rank 4209 − Ssc.21930.1.S1_a_at − probe 1
Q9H5G0
Normalized Probe Intensity
Probe Number
Meishan−A
Meishan−B
Meishan−C
European−A
European−B
European−C

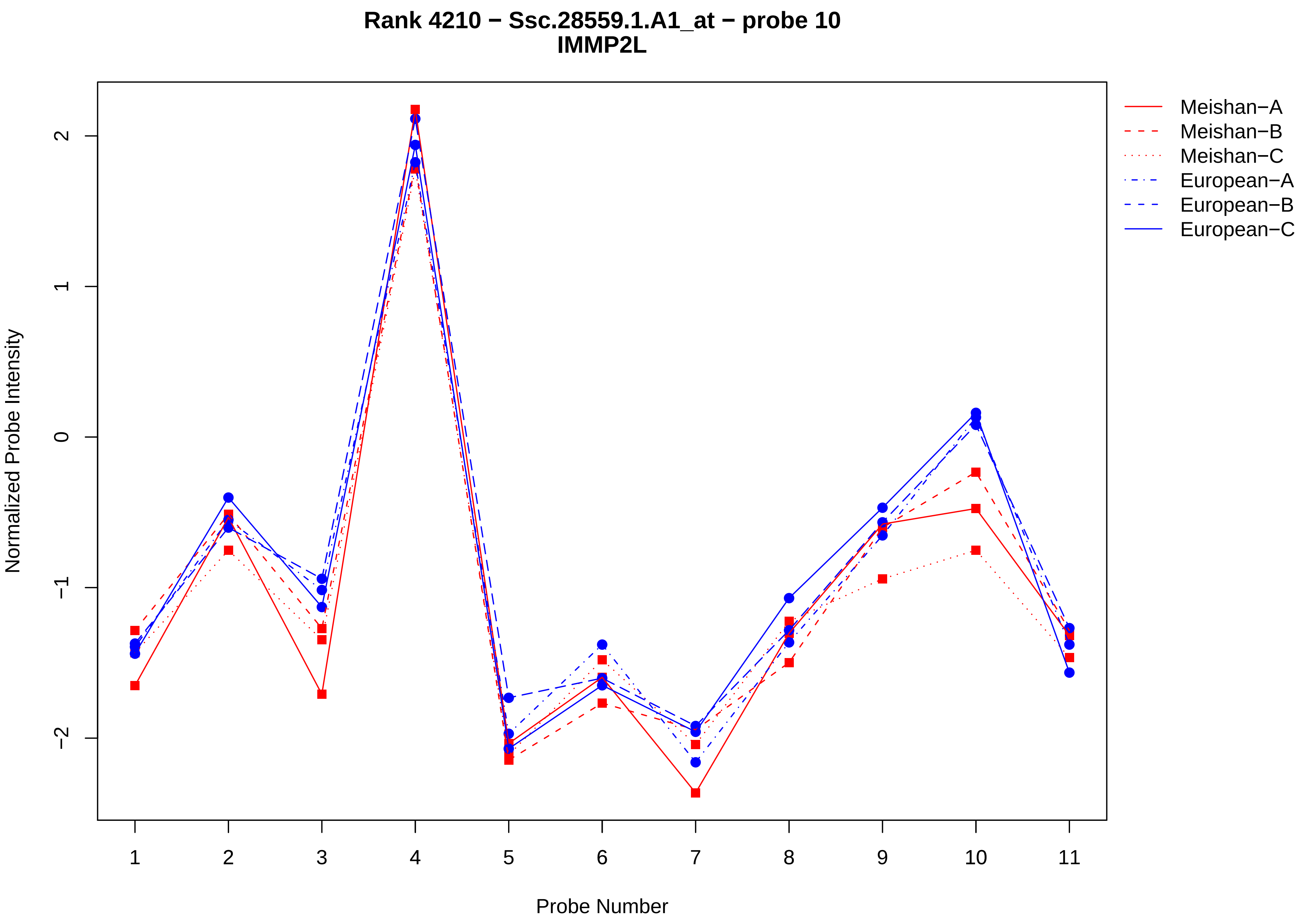

Rank 4210 − Ssc.28559.1.A1_at − probe 10
IMMP2L
Normalized Probe Intensity
Probe Number
2
1
0
−1
−2
1
2
3
4
5
6
7
8
9
10
11
Meishan−A
Meishan−B
Meishan−C
European−A
European−B
European−C

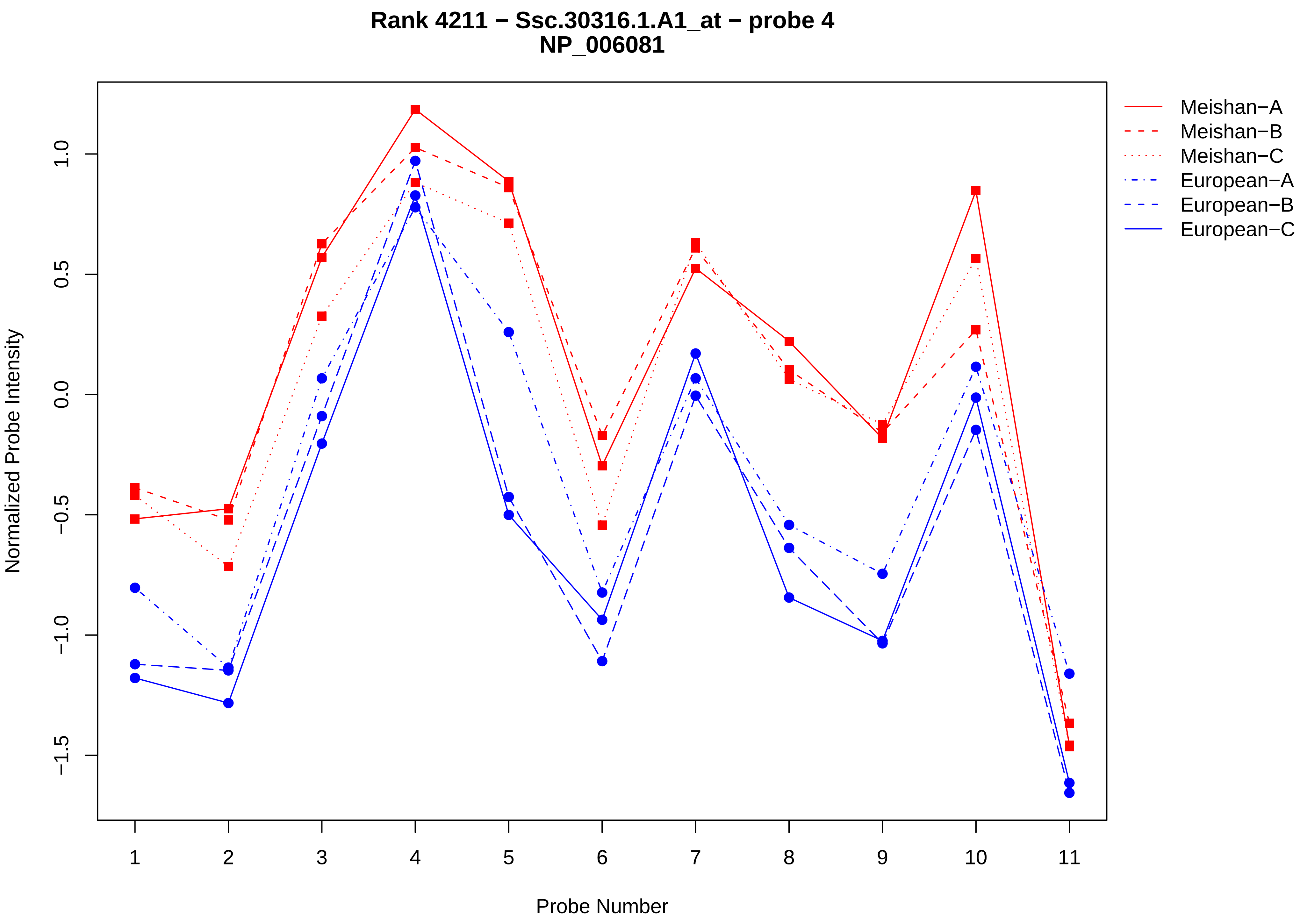
Rank 4211 − Ssc.30316.1.A1_at − probe 4
NP_006081
Normalized Probe Intensity
Probe Number
Meishan−A
Meishan−B
Meishan−C
European−A
European−B
European−C
1.0
0.5
0.0
−0.5
−1.0
−1.5
1
2
3
4
5
6
7
8
9
10
11

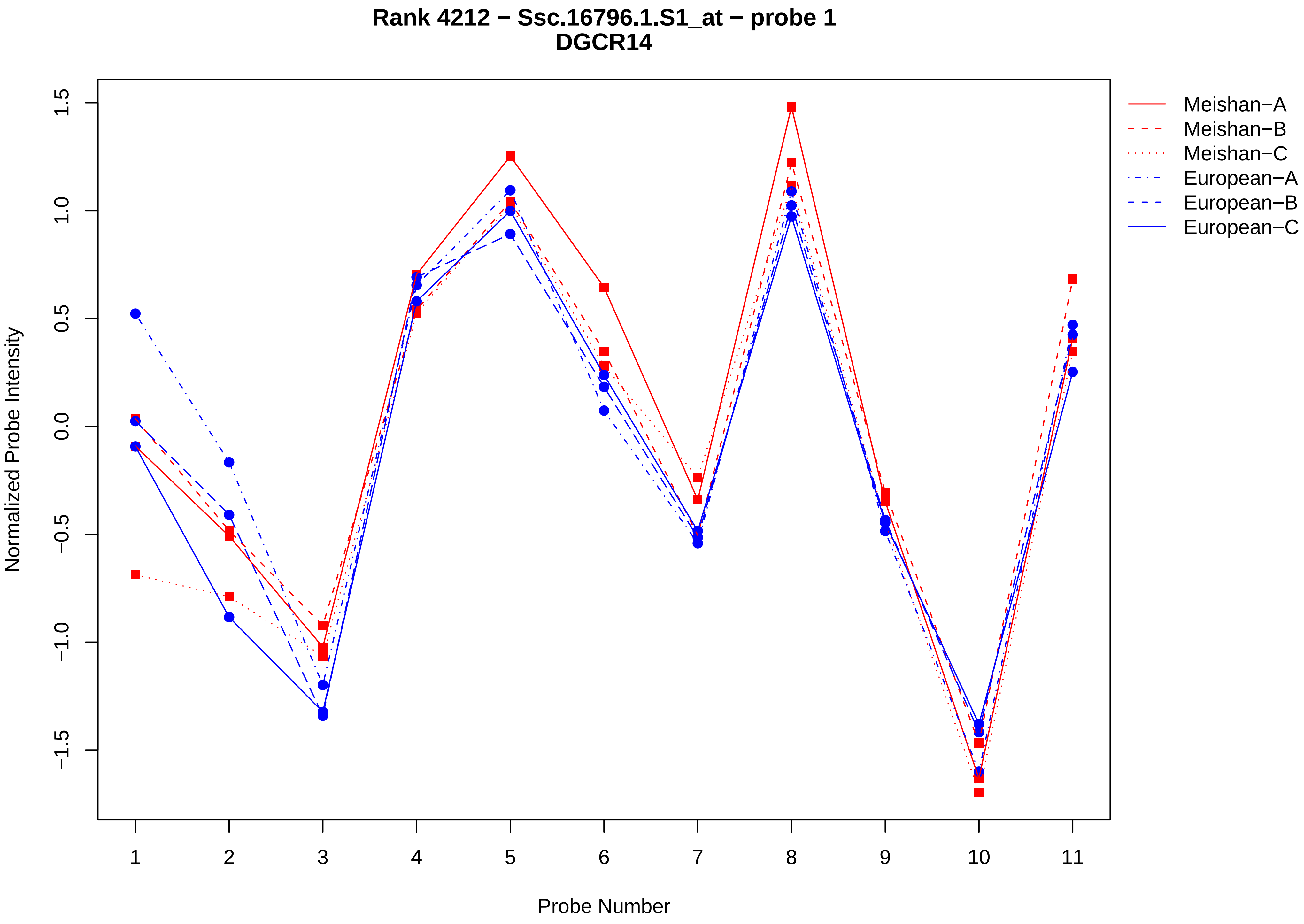

Rank 4212 − Ssc.16796.1.S1_at − probe 1
DGCR14
Normalized Probe Intensity
Probe Number
−1.5
−1.0
−0.5
0.0
0.5
1.0
1.5
1
2
3
4
5
6
7
8
9
10
11
Meishan−A
Meishan−B
Meishan−C
European−A
European−B
European−C

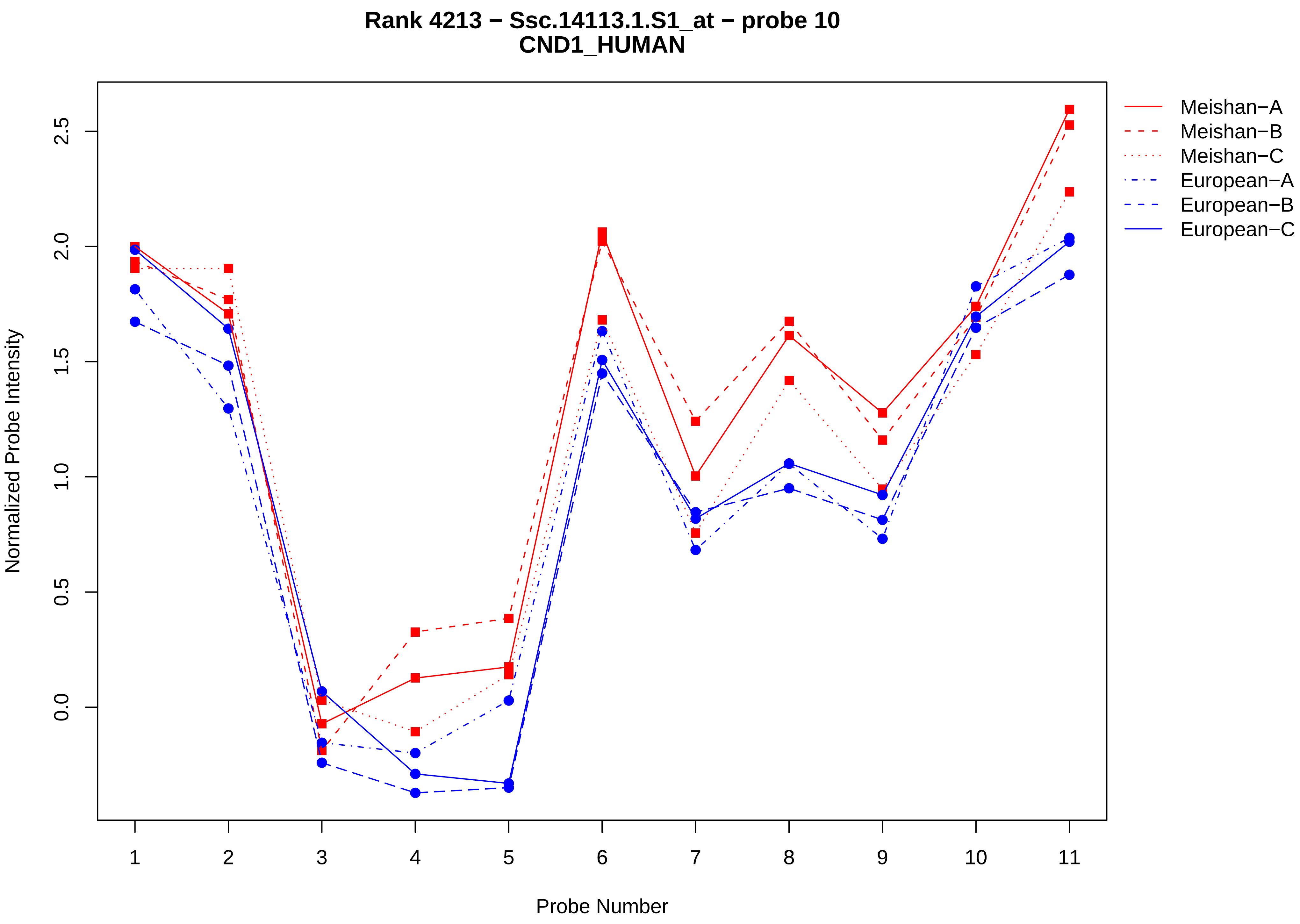

Rank 4213 − Ssc.14113.1.S1_at − probe 10
CND1_HUMAN
Normalized Probe Intensity
Probe Number
0.0
0.5
1.0
1.5
2.0
2.5
1
2
3
4
5
6
7
8
9
10
11
Meishan−A
Meishan−B
Meishan−C
European−A
European−B
European−C

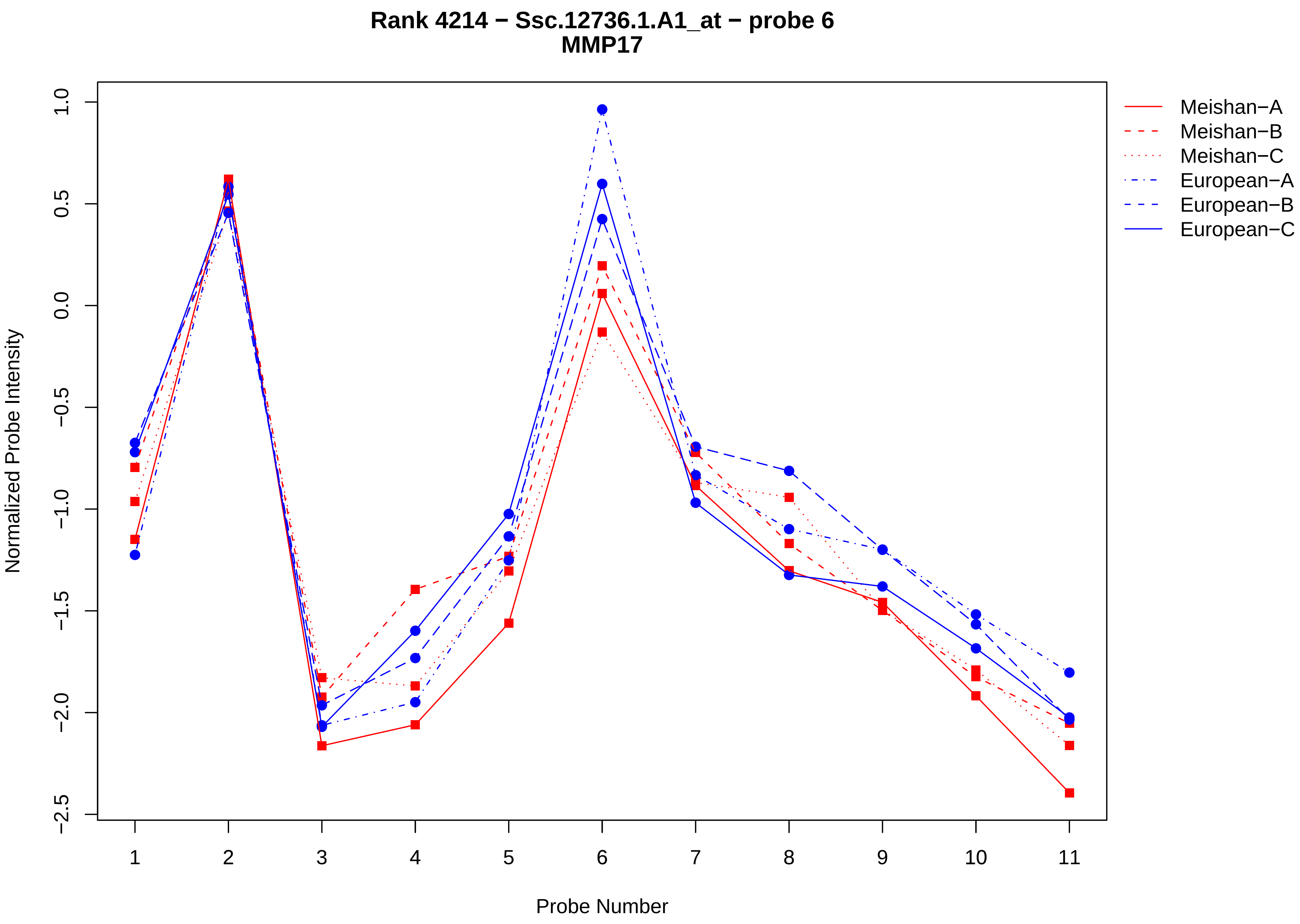

Rank 4214 − Ssc.12736.1.A1_at − probe 6
MMP17
Normalized Probe Intensity
Probe Number
1.0
0.5
0.0
−0.5
−1.0
−1.5
−2.0
−2.5
1
2
3
4
5
6
7
8
9
10
11
Meishan−A
Meishan−B
Meishan−C
European−A
European−B
European−C

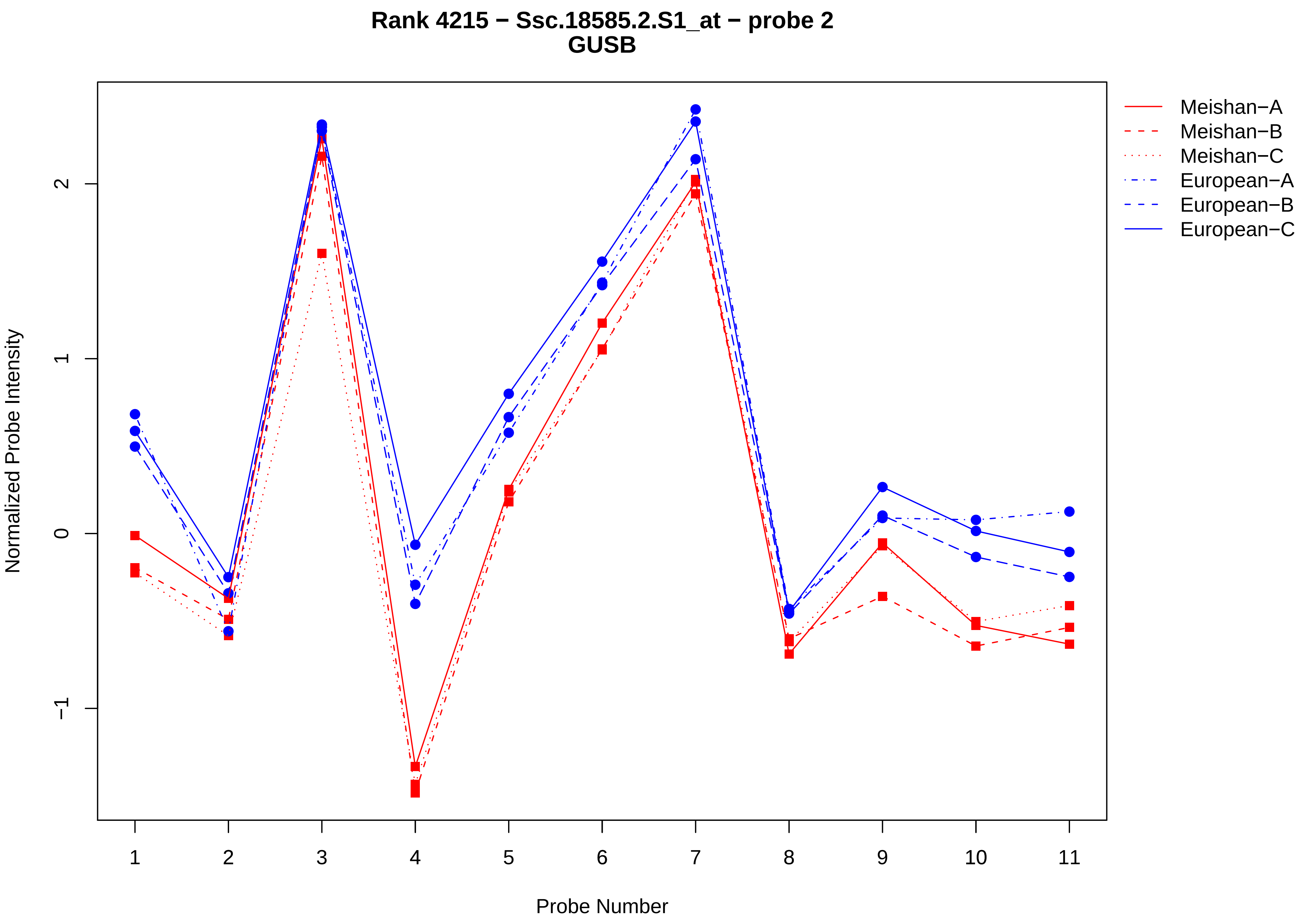

Rank 4215 − Ssc.18585.2.S1_at − probe 2
GUSB
Normalized Probe Intensity
Probe Number
−1
0
1
2
1
2
3
4
5
6
7
8
9
10
11
Meishan−A
Meishan−B
Meishan−C
European−A
European−B
European−C

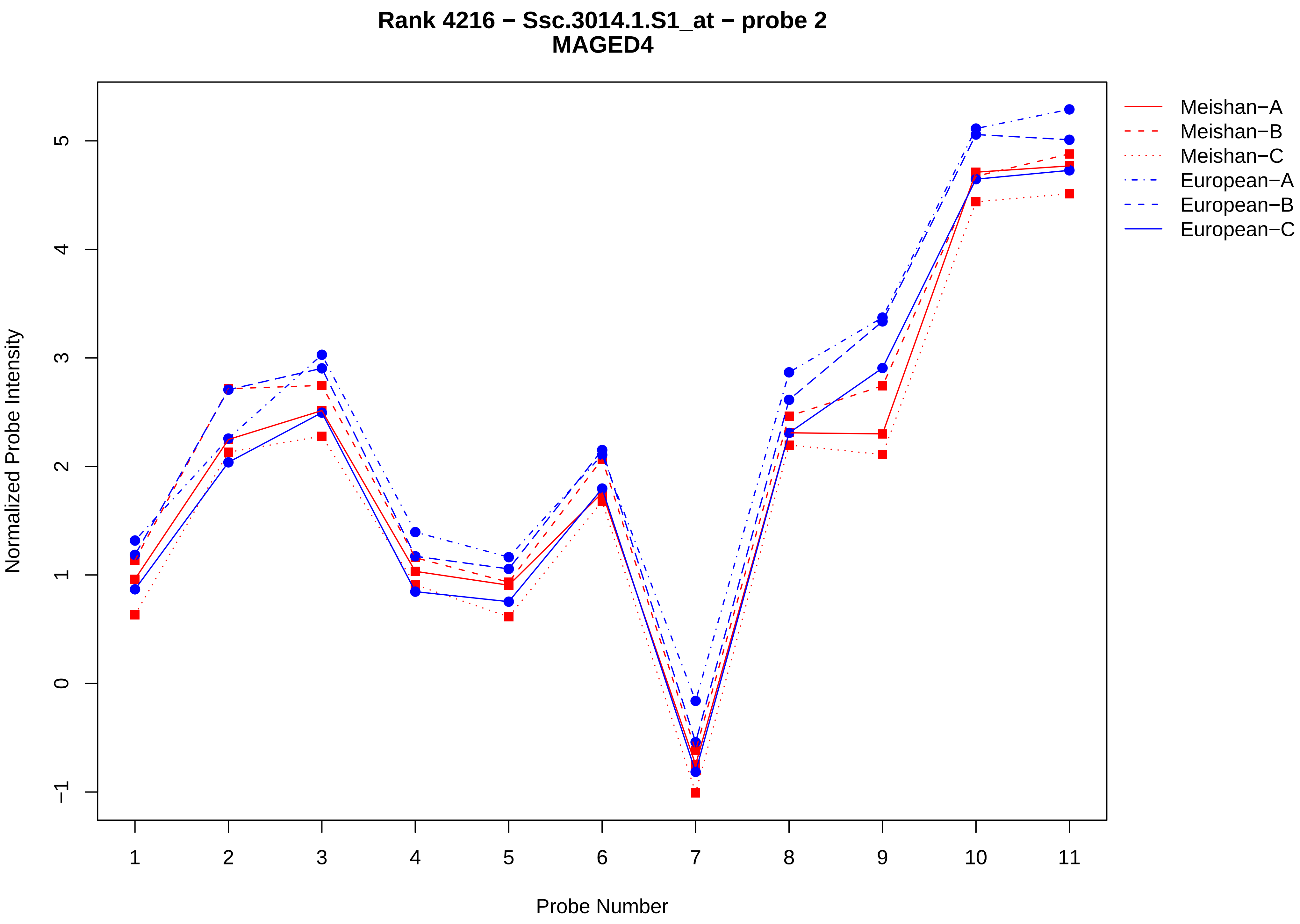
Rank 4216 − Ssc.3014.1.S1_at − probe 2
MAGED4
Normalized Probe Intensity
Probe Number
−1
0
1
2
3
4
5
1
2
3
4
5
6
7
8
9
10
11
Meishan−A
Meishan−B
Meishan−C
European−A
European−B
European−C

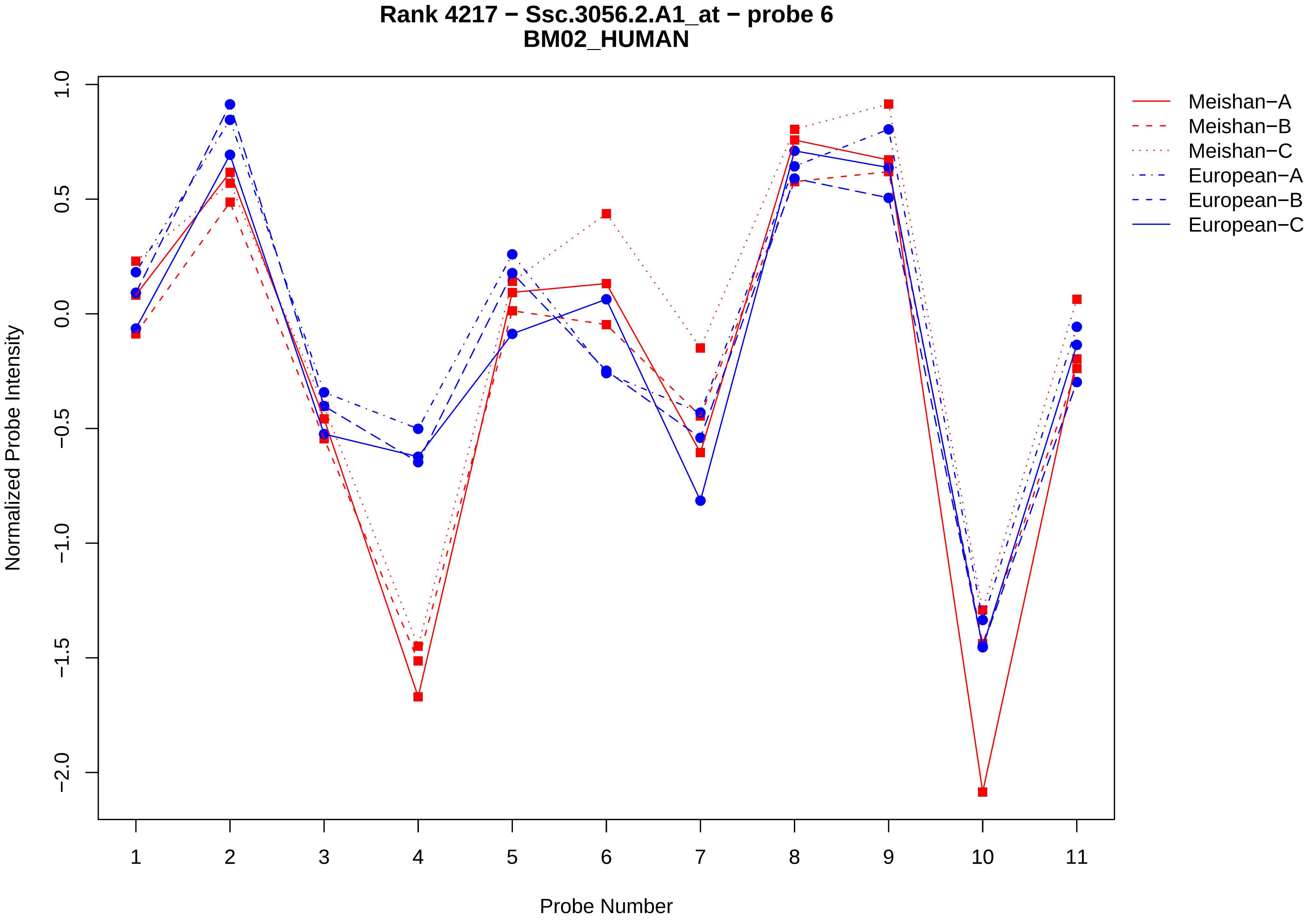
Rank 4217 − Ssc.3056.2.A1_at − probe 6
BM02_HUMAN
Normalized Probe Intensity
Probe Number
Meishan−A
Meishan−B
Meishan−C
European−A
European−B
European−C
1.0
0.5
0.0
−0.5
−1.0
−1.5
−2.0
1
2
3
4
5
6
7
8
9
10
11

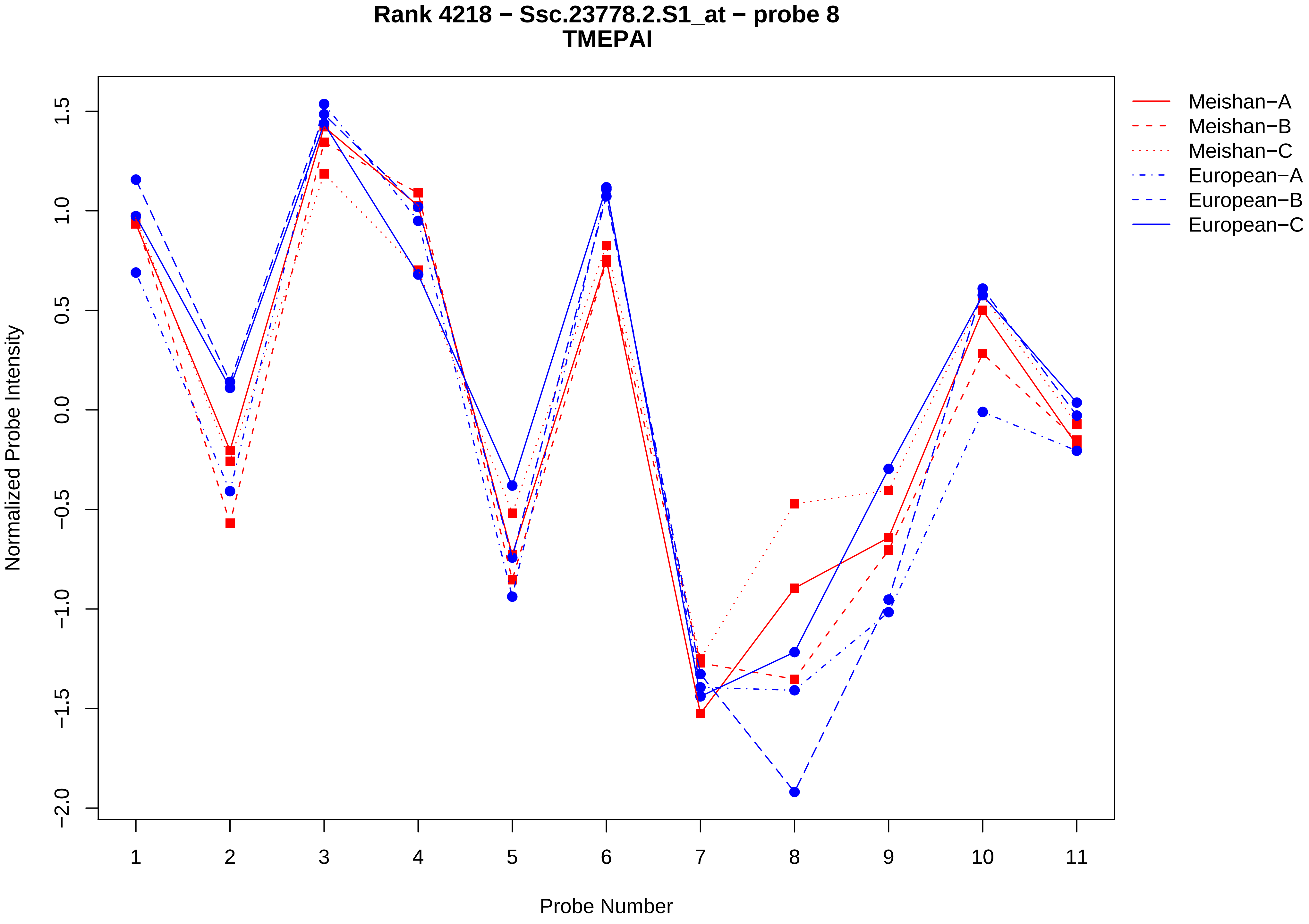
Rank 4218 − Ssc.23778.2.S1_at − probe 8
TMEPAI
Normalized Probe Intensity
Probe Number
1.5
1.0
0.5
0.0
−0.5
−1.0
−1.5
−2.0
1
2
3
4
5
6
7
8
9
10
11
Meishan−A
Meishan−B
Meishan−C
European−A
European−B
European−C

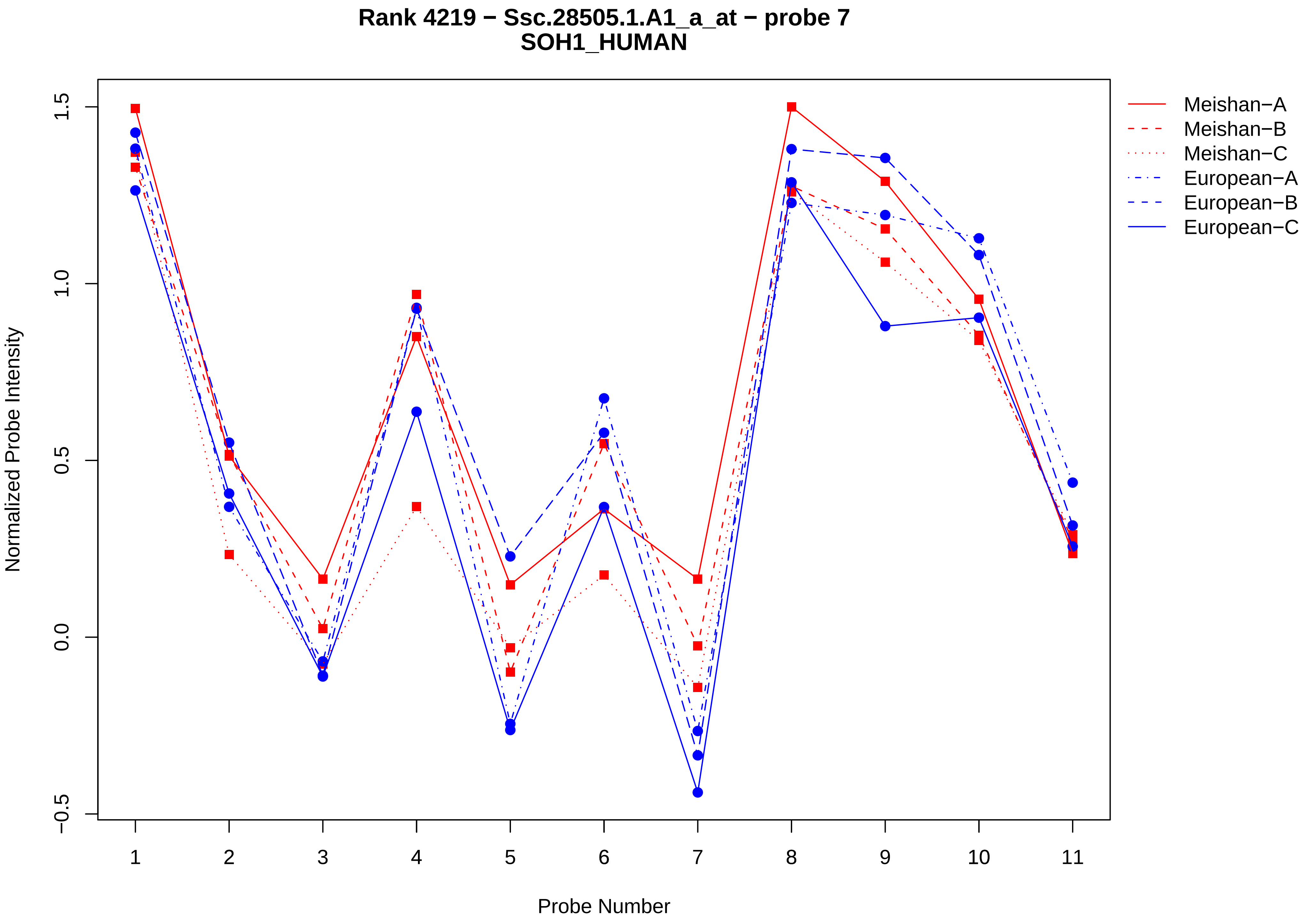
Rank 4219 − Ssc.28505.1.A1_a_at − probe 7
SOH1_HUMAN
Normalized Probe Intensity
Probe Number
−0.5
0.0
0.5
1.0
1.5
1
2
3
4
5
6
7
8
9
10
11
Meishan−A
Meishan−B
Meishan−C
European−A
European−B
European−C

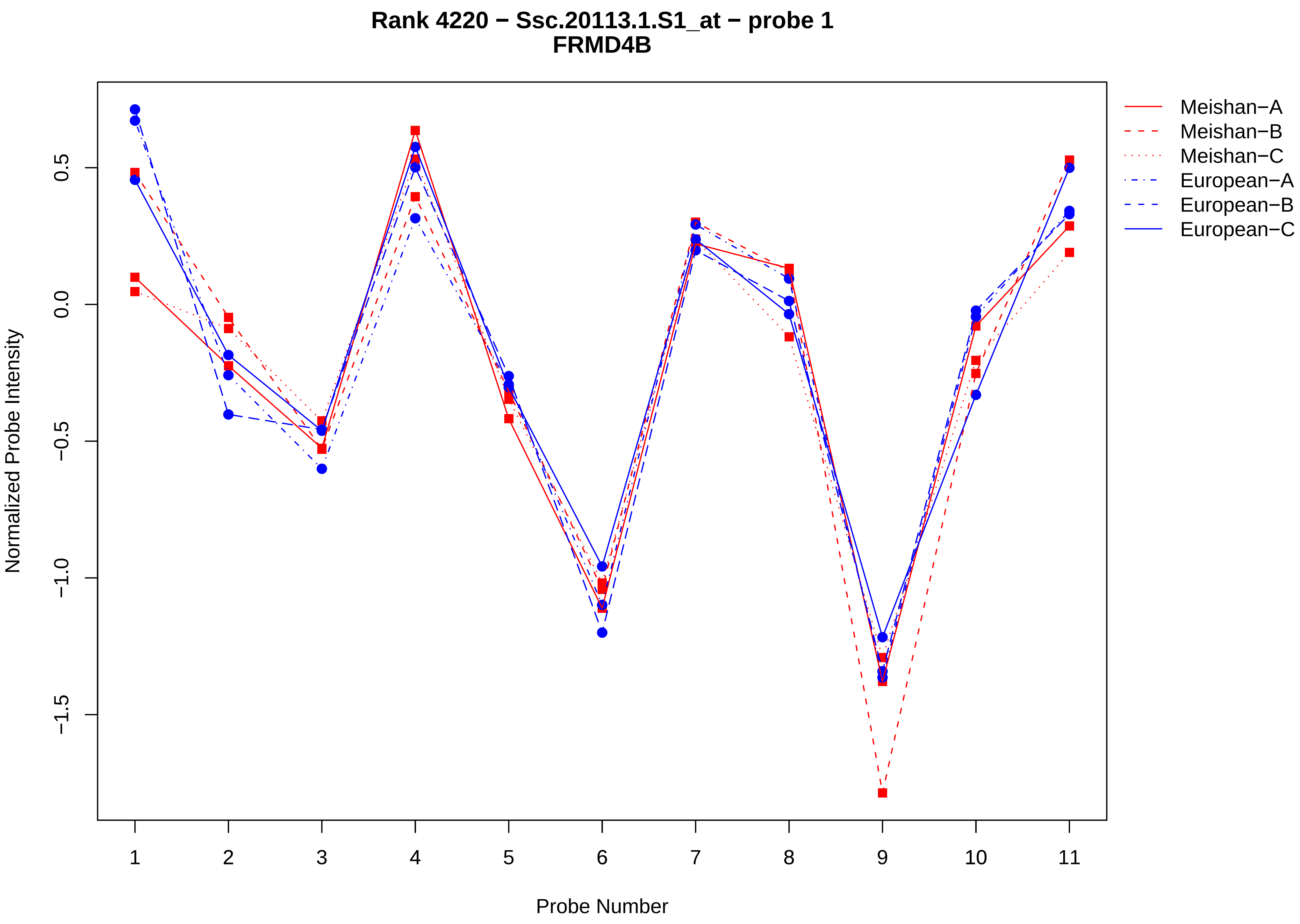

Rank 4220 − Ssc.20113.1.S1_at − probe 1
FRMD4B
Normalized Probe Intensity
Probe Number
0.5
0.0
−0.5
−1.0
−1.5
1
2
3
4
5
6
7
8
9
10
11
Meishan−A
Meishan−B
Meishan−C
European−A
European−B
European−C

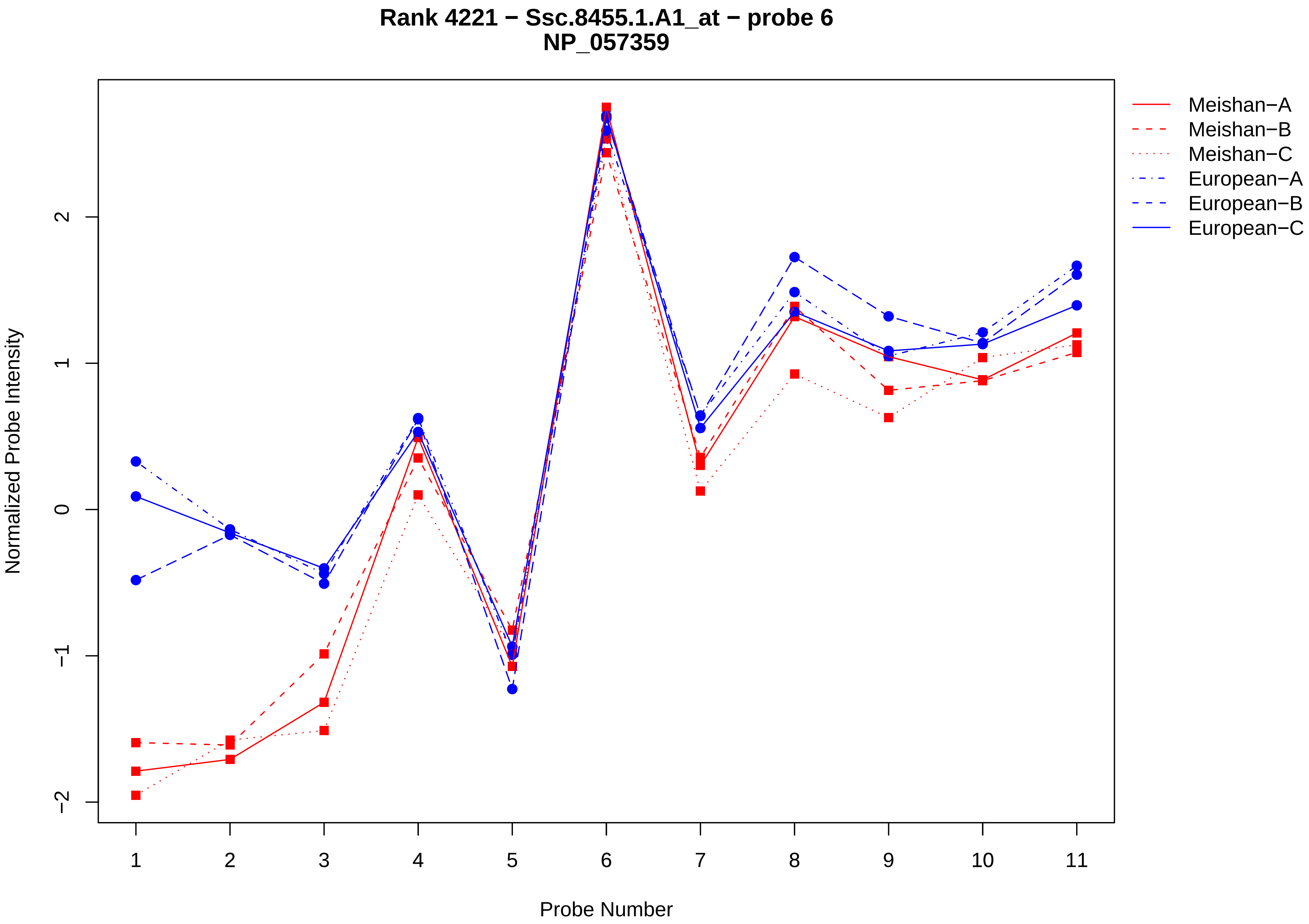

Rank 4221 − Ssc.8455.1.A1_at − probe 6
NP_057359
Normalized Probe Intensity
Probe Number
Meishan−A
Meishan−B
Meishan−C
European−A
European−B
European−C

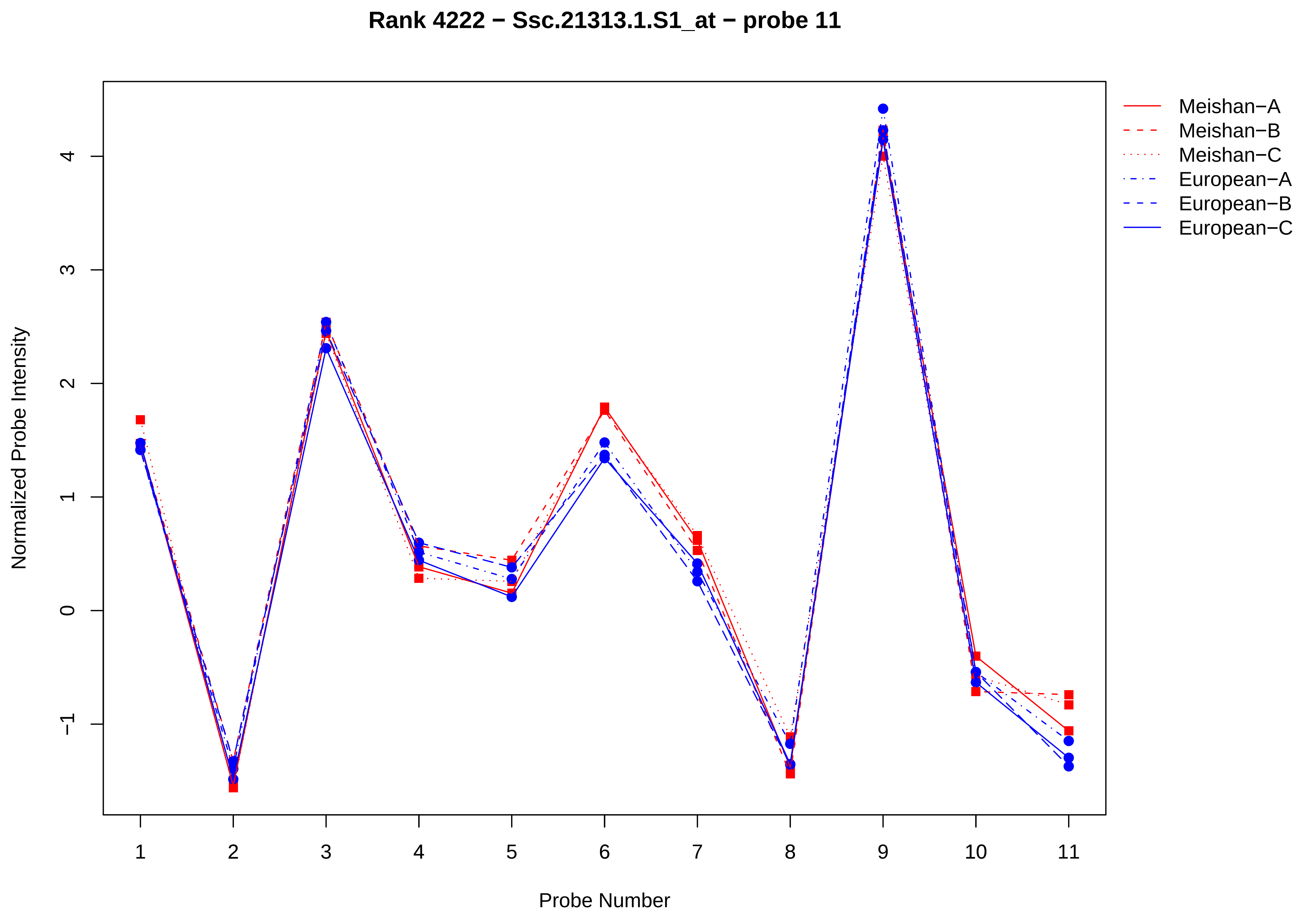

Rank 4222 − Ssc.21313.1.S1_at − probe 11
Normalized Probe Intensity
Probe Number
Meishan−A
Meishan−B
Meishan−C
European−A
European−B
European−C
−1
0
1
2
3
4
1
2
3
4
5
6
7
8
9
10
11

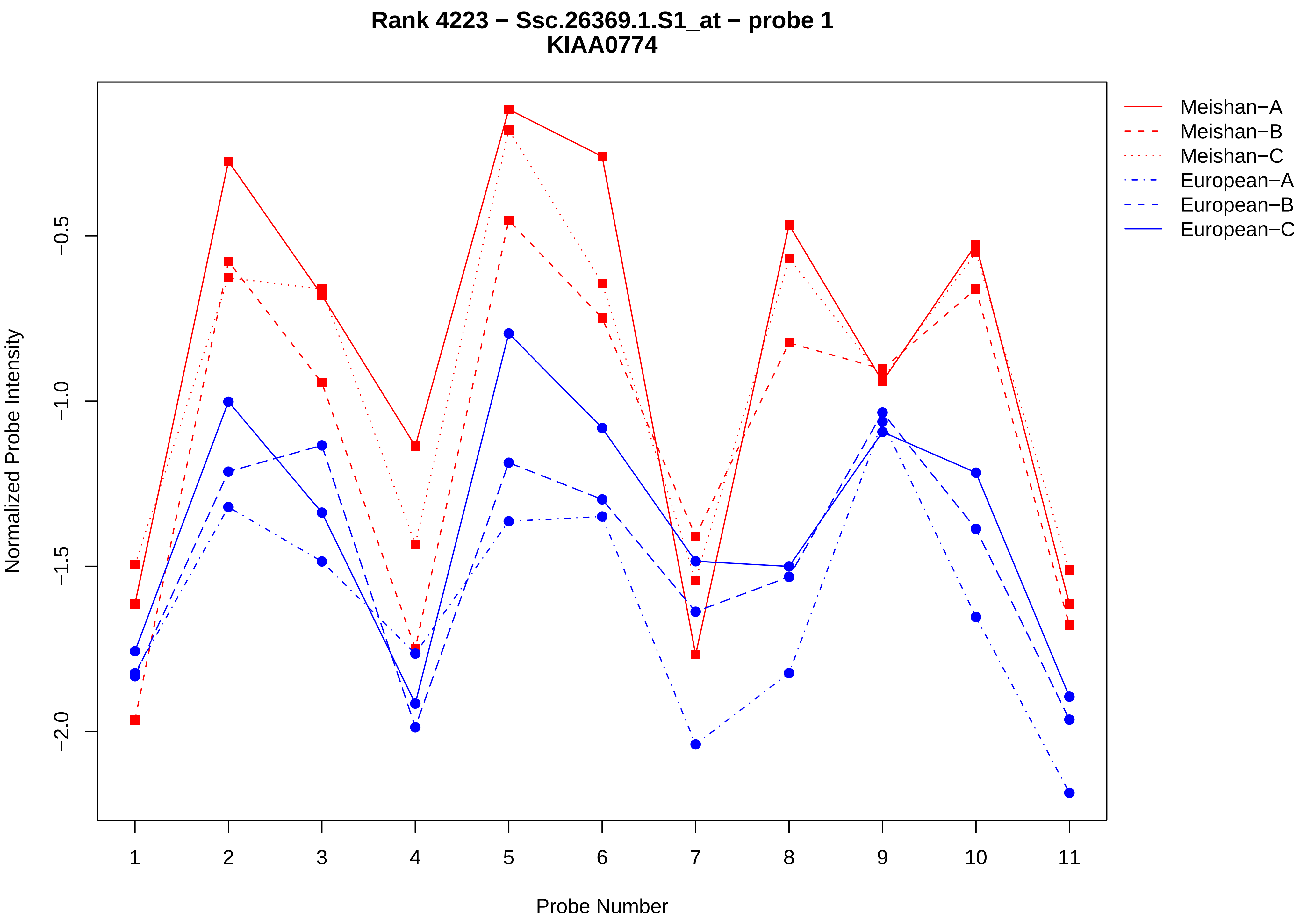

Rank 4223 − Ssc.26369.1.S1_at − probe 1
KIAA0774
Normalized Probe Intensity
−0.5
−1.0
−1.5
−2.0
1
2
3
4
5
6
7
8
9
10
11
Probe Number
Meishan−A
Meishan−B
Meishan−C
European−A
European−B
European−C

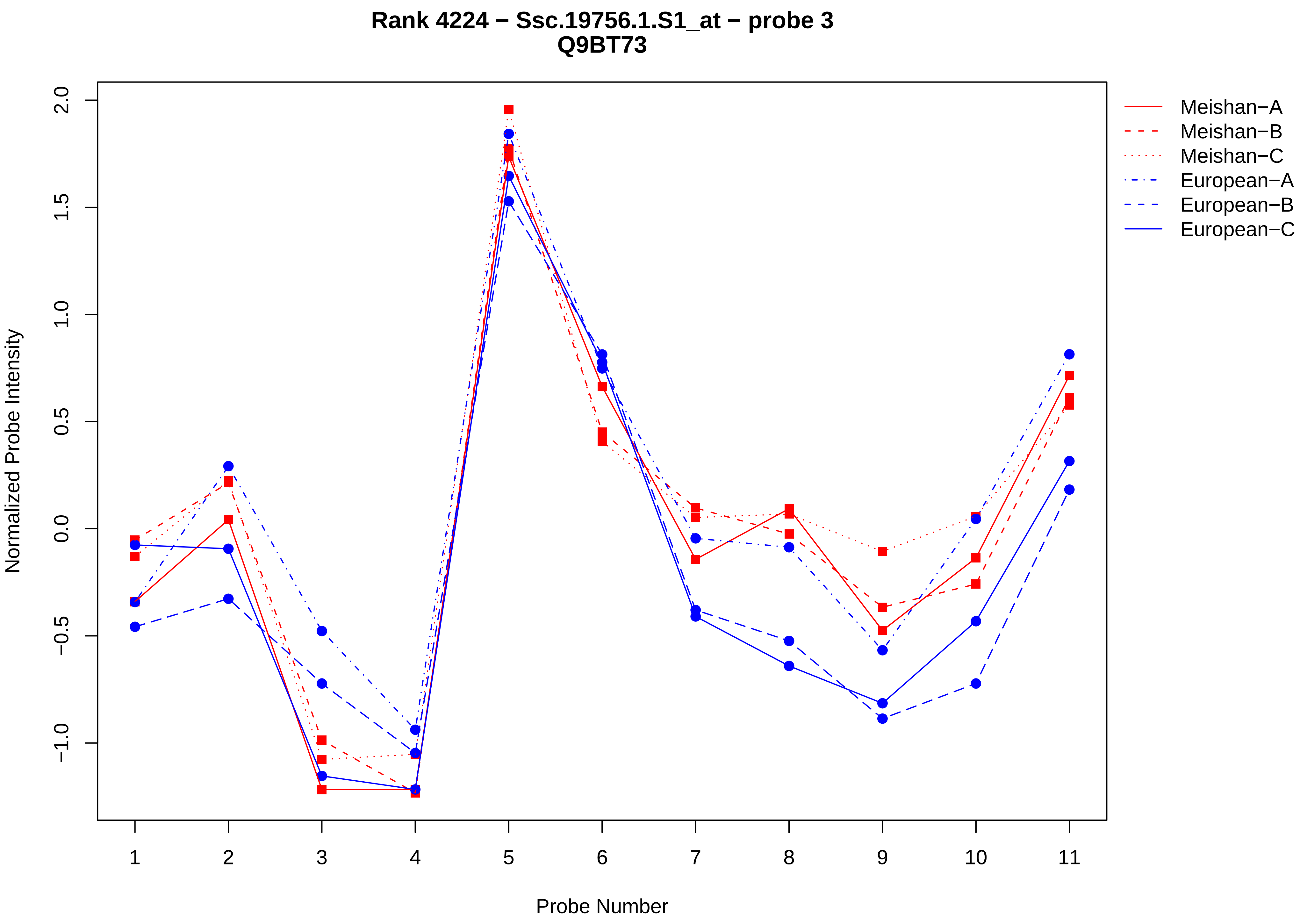
Rank 4224 − Ssc.19756.1.S1_at − probe 3
Q9BT73
Normalized Probe Intensity
Probe Number
Meishan−A
Meishan−B
Meishan−C
European−A
European−B
European−C
2.0
1.5
1.0
0.5
0.0
−0.5
−1.0
1
2
3
4
5
6
7
8
9
10
11

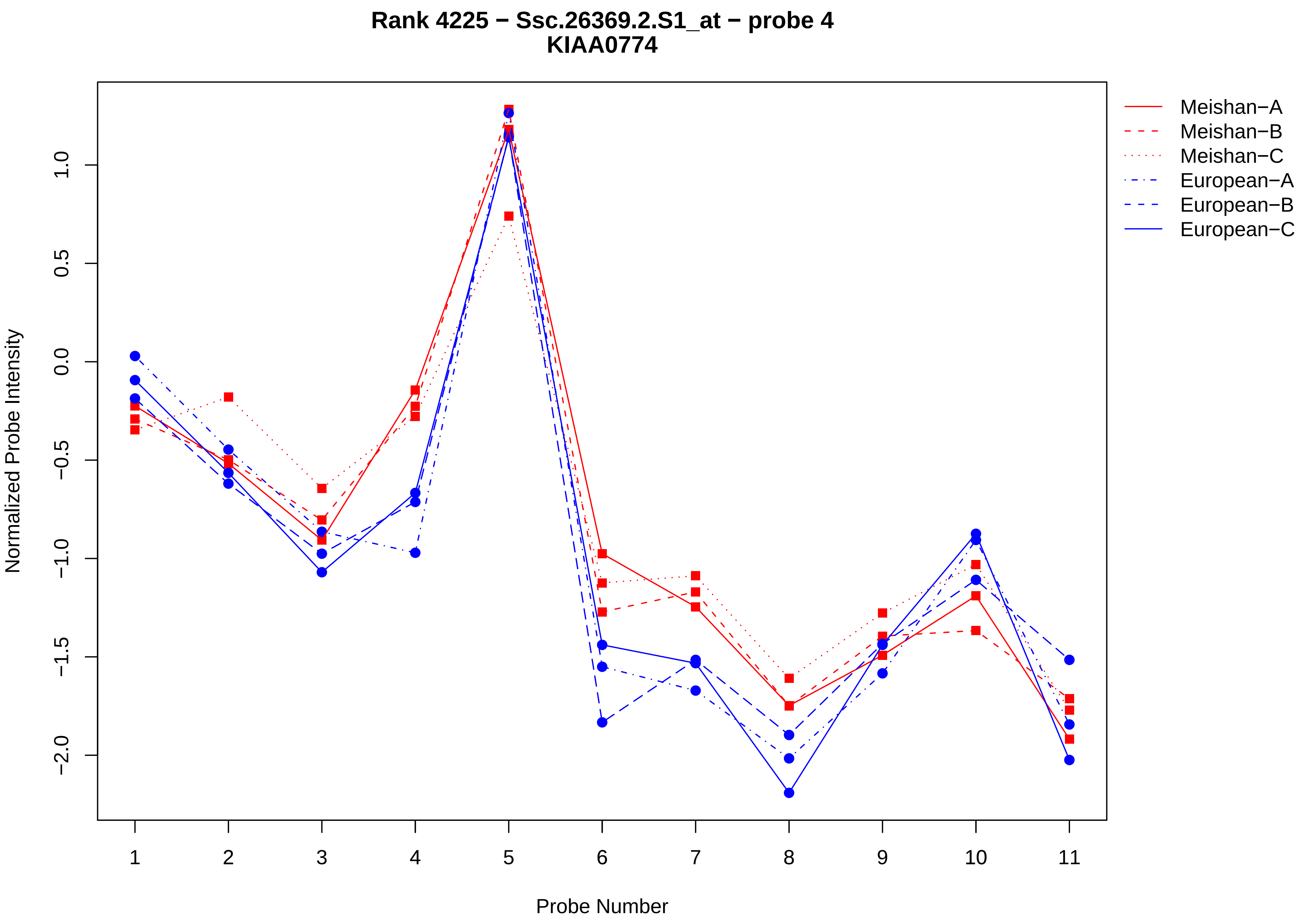

Rank 4225 − Ssc.26369.2.S1_at − probe 4
KIAA0774
Normalized Probe Intensity
Probe Number
1.0
0.5
0.0
−0.5
−1.0
−1.5
−2.0
1
2
3
4
5
6
7
8
9
10
11
Meishan−A
Meishan−B
Meishan−C
European−A
European−B
European−C

Rank 4226 − Ssc.6353.2.S1_at − probe 2
PSMF1

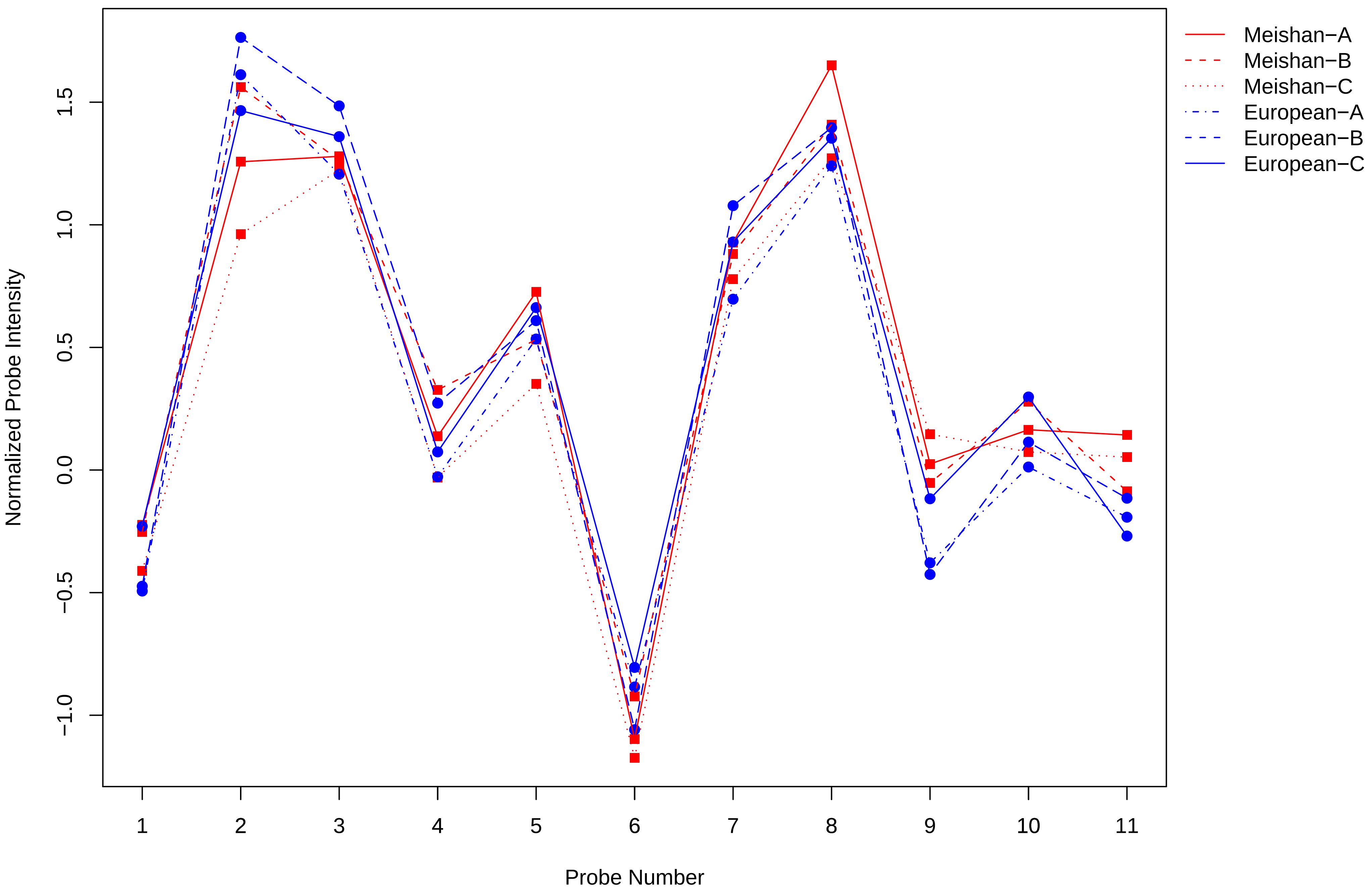

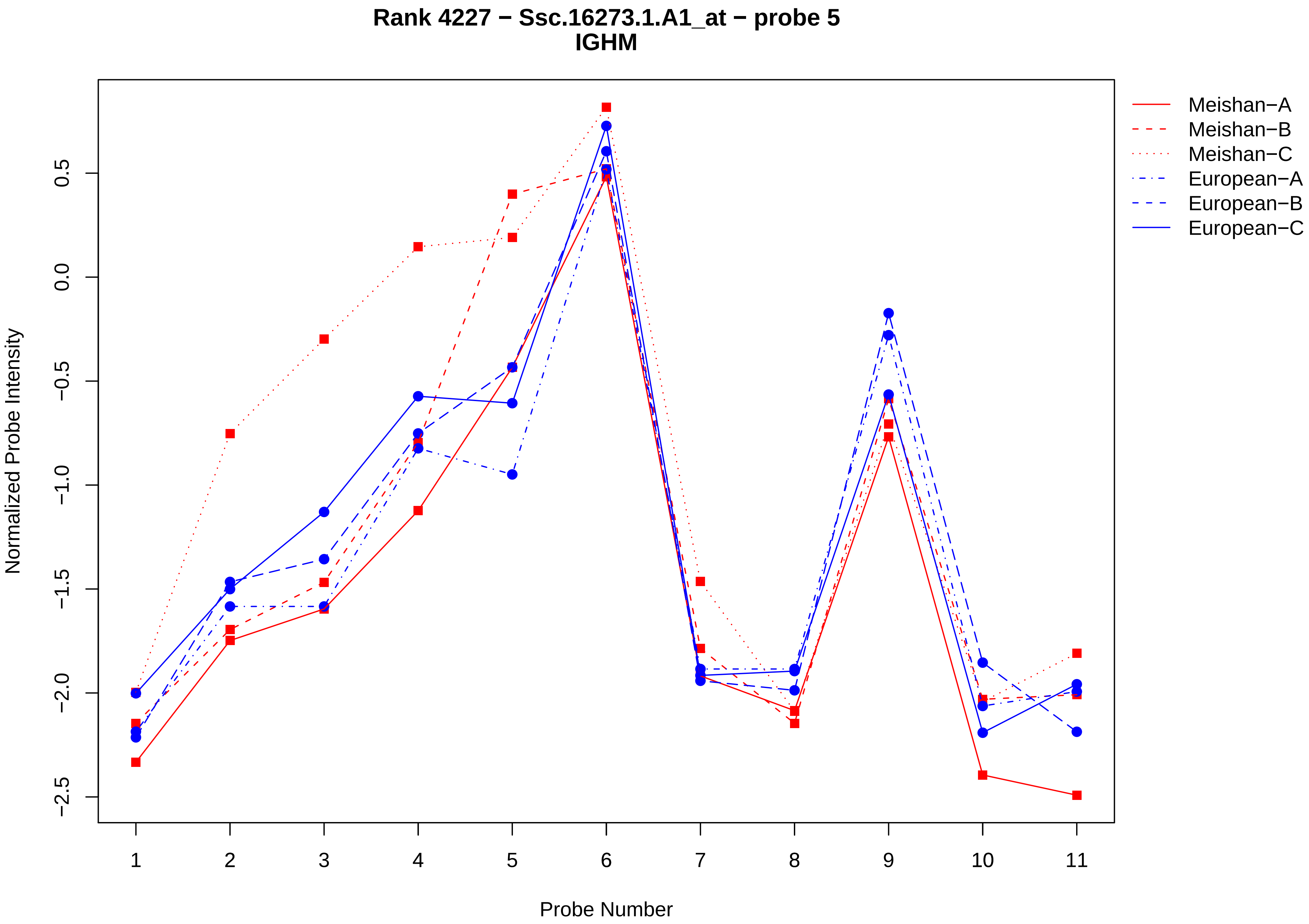

Rank 4227 − Ssc.16273.1.A1_at − probe 5
IGHM
Normalized Probe Intensity
Probe Number
Meishan−A
Meishan−B
Meishan−C
European−A
European−B
European−C
0.5
0.0
−0.5
−1.0
−1.5
−2.0
−2.5
1
2
3
4
5
6
7
8
9
10
11

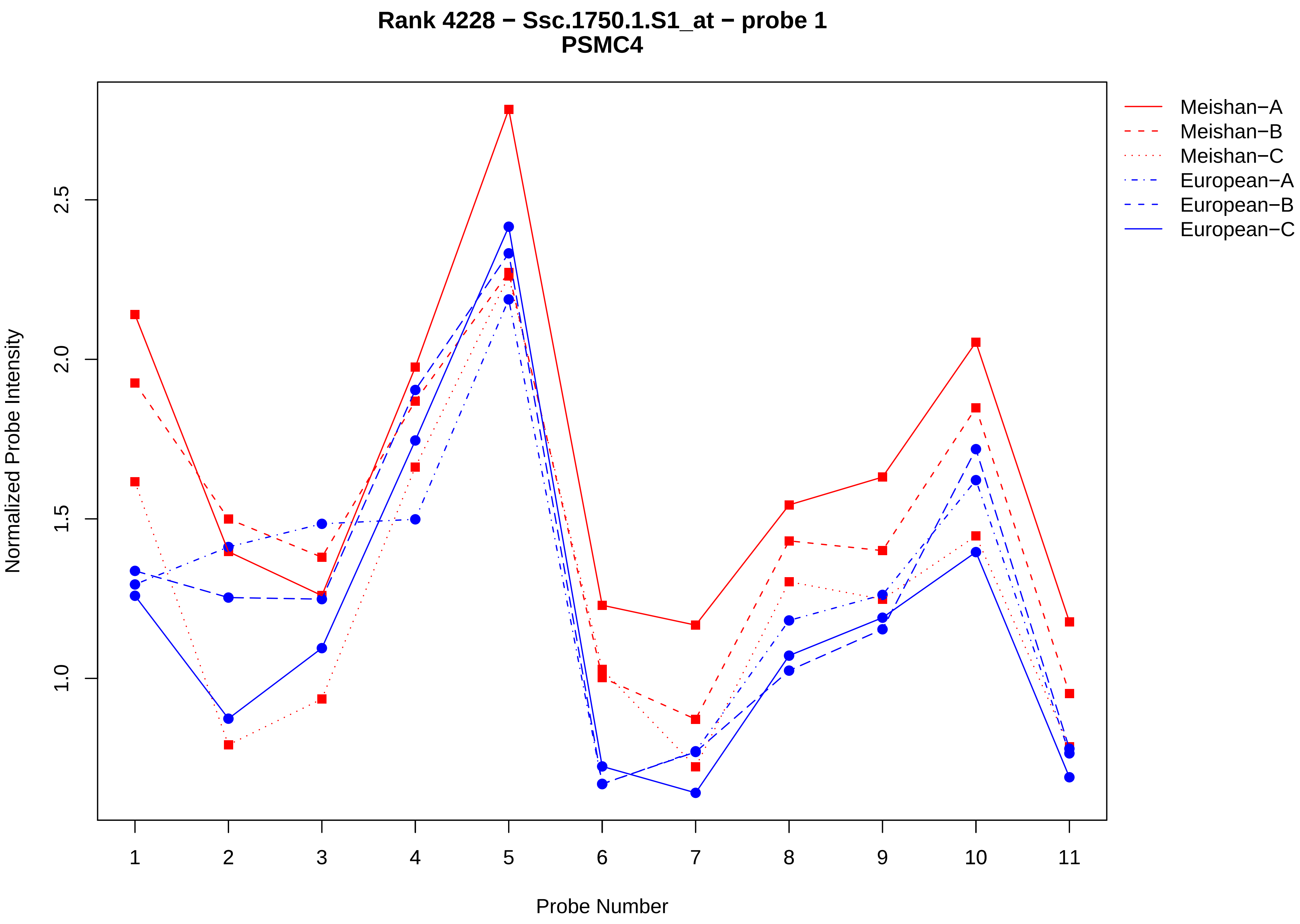

Rank 4228 − Ssc.1750.1.S1_at − probe 1
PSMC4
Normalized Probe Intensity
Probe Number
1.0
1.5
2.0
2.5
1
2
3
4
5
6
7
8
9
10
11
Meishan−A
Meishan−B
Meishan−C
European−A
European−B
European−C

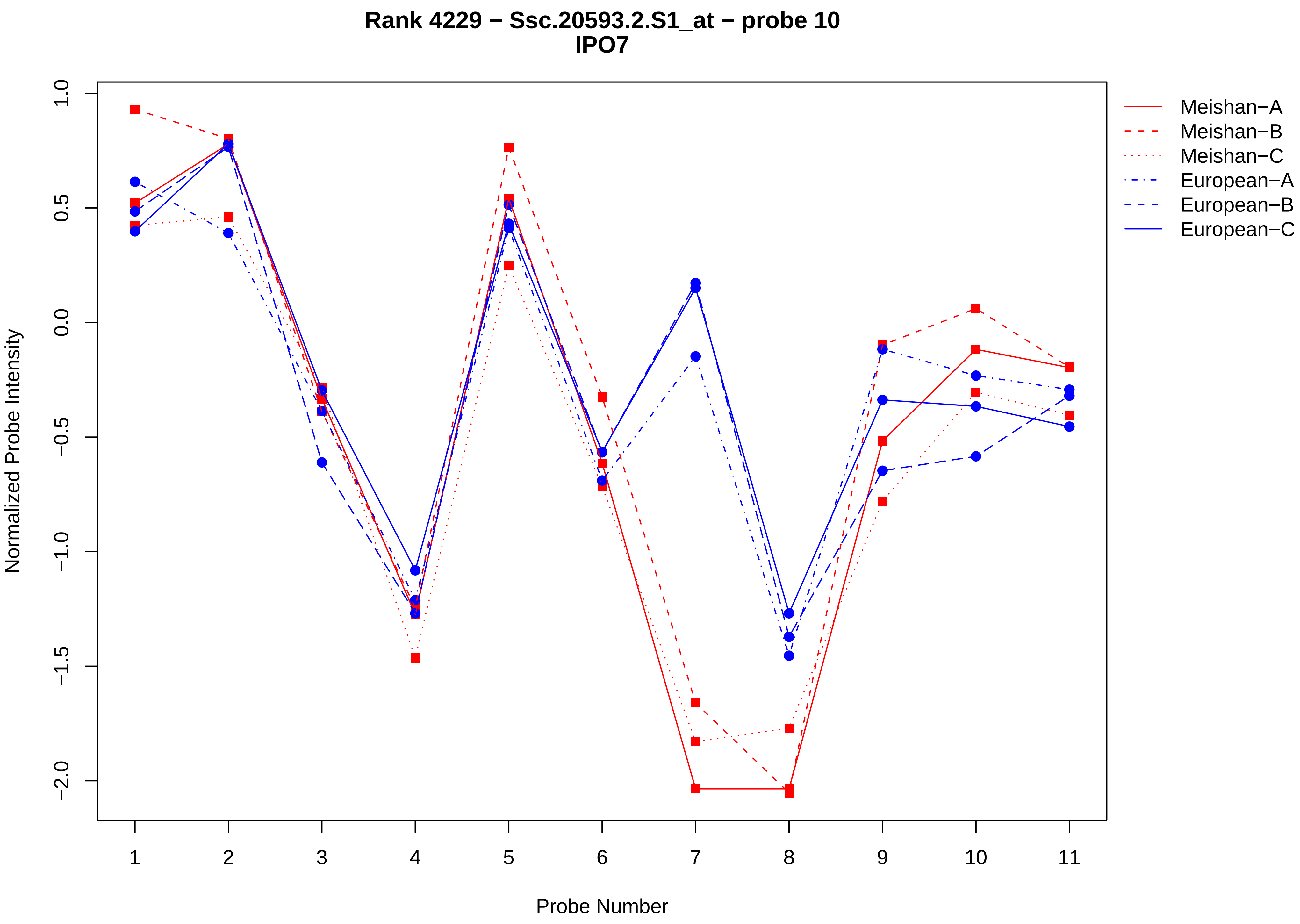

Rank 4229 − Ssc.20593.2.S1_at − probe 10
IPO7
Normalized Probe Intensity
Probe Number
1.0
0.5
0.0
−0.5
−1.0
−1.5
−2.0
1
2
3
4
5
6
7
8
9
10
11
Meishan−A
Meishan−B
Meishan−C
European−A
European−B
European−C

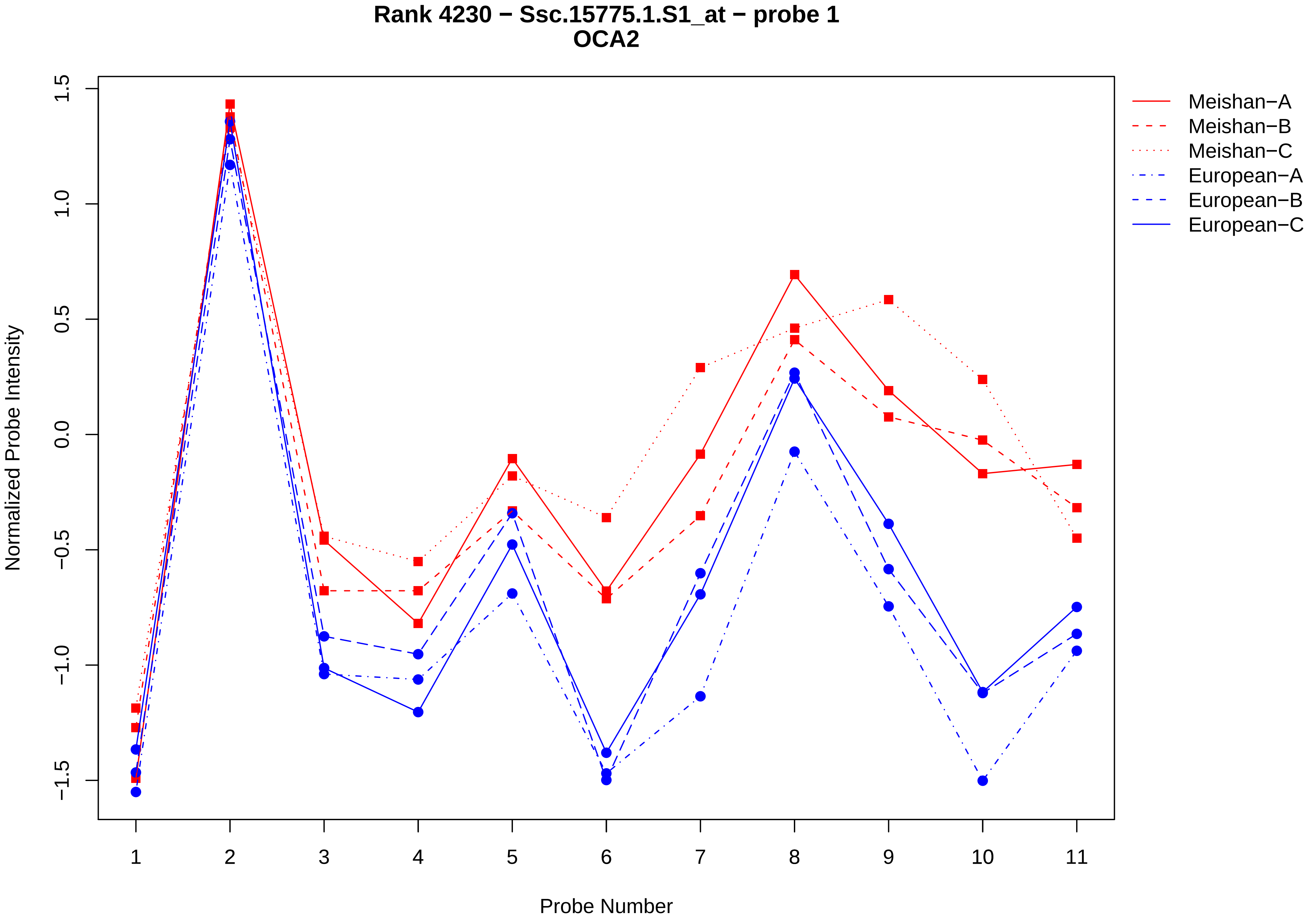

Rank 4230 − Ssc.15775.1.S1_at − probe 1
OCA2
Normalized Probe Intensity
Probe Number
−1.5
−1.0
−0.5
0.0
0.5
1.0
1.5
1
2
3
4
5
6
7
8
9
10
11
Meishan−A
Meishan−B
Meishan−C
European−A
European−B
European−C

Rank 4231 − Ssc.21216.2.A1_a_at − probe 11
PSMB3

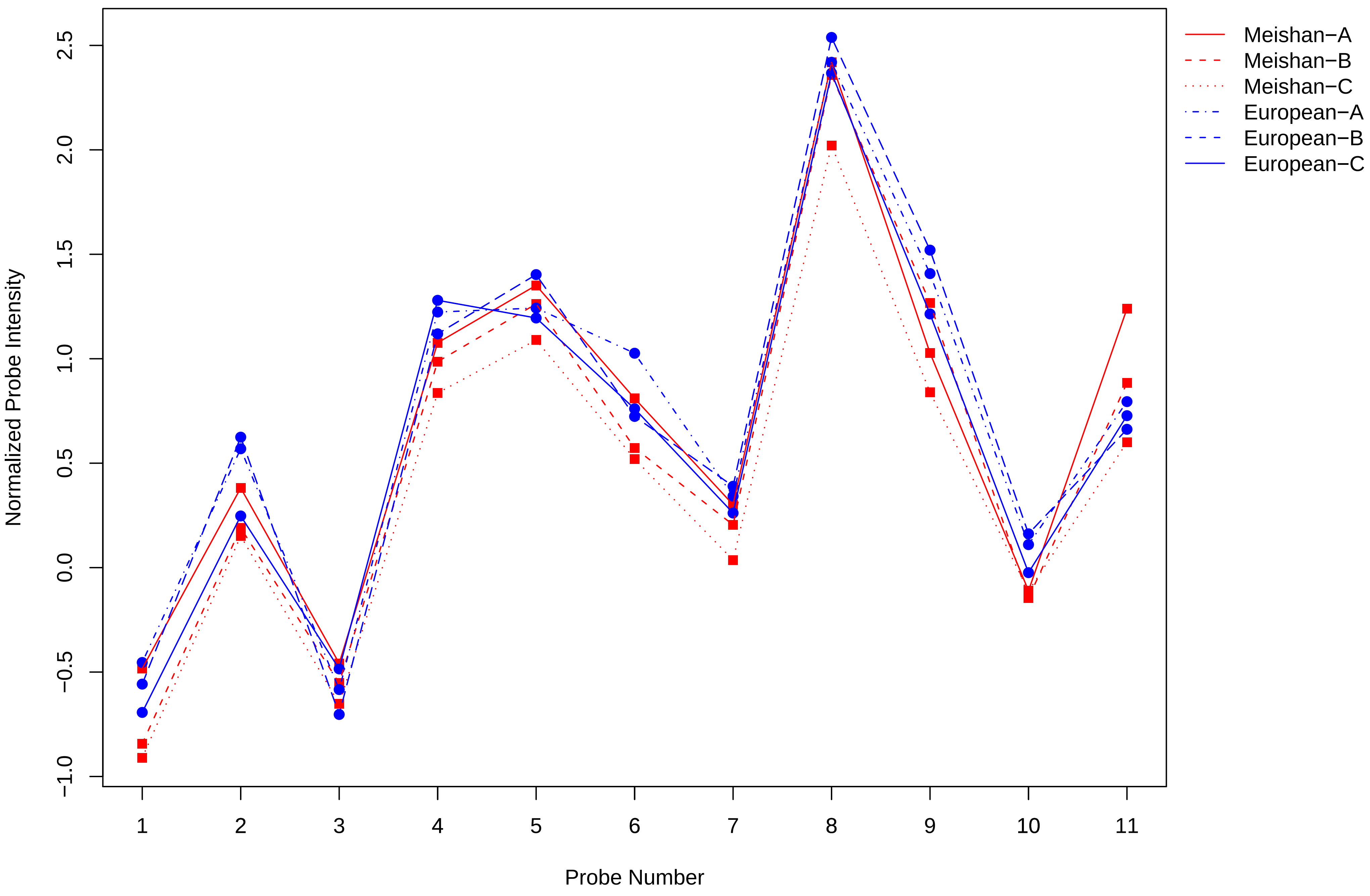

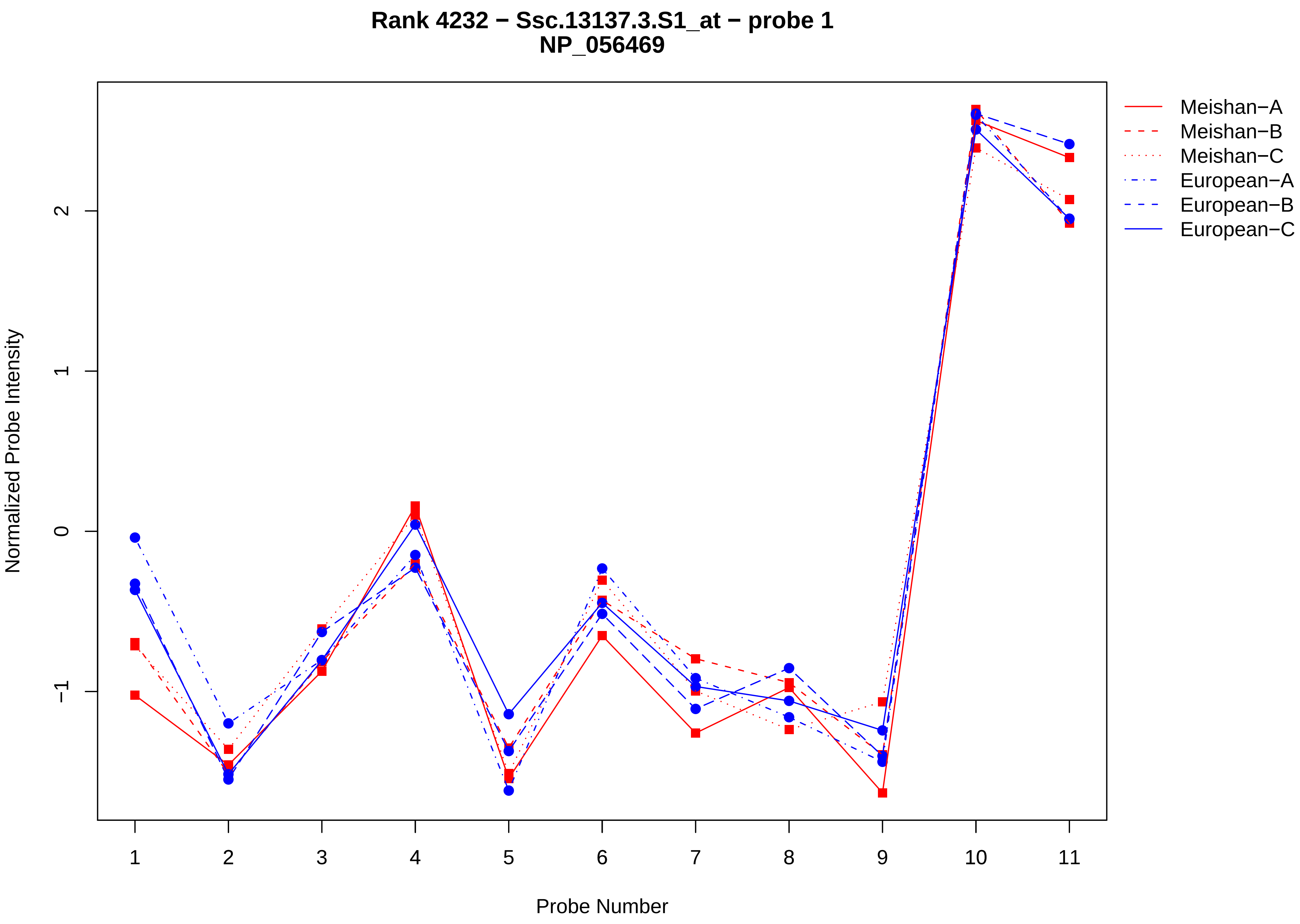
Rank 4232 − Ssc.13137.3.S1_at − probe 1
NP_056469
Normalized Probe Intensity
Probe Number
−1
0
1
2
1
2
3
4
5
6
7
8
9
10
11
Meishan−A
Meishan−B
Meishan−C
European−A
European−B
European−C

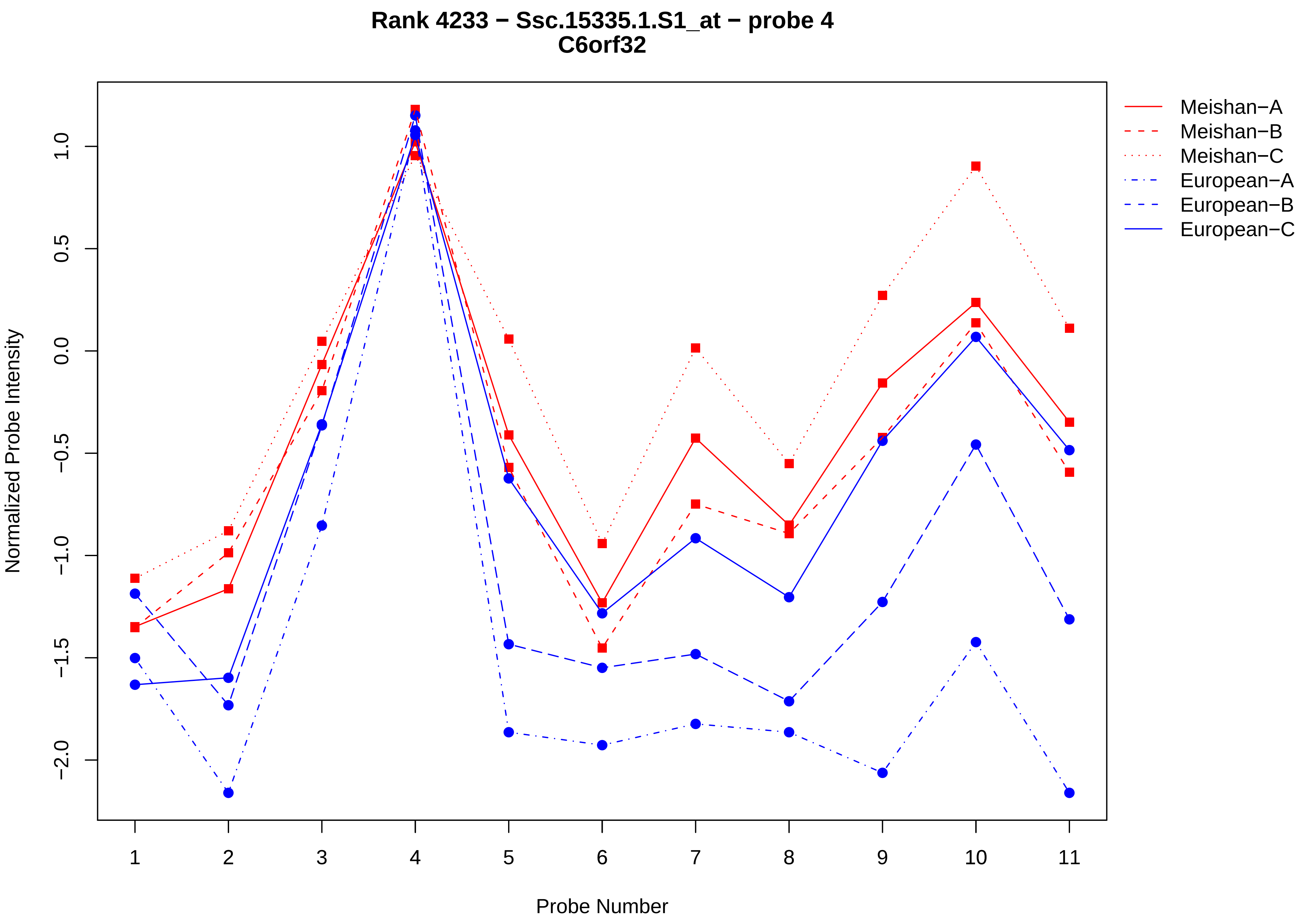

Rank 4233 − Ssc.15335.1.S1_at − probe 4
C6orf32
Normalized Probe Intensity
Probe Number
−2.0
−1.5
−1.0
−0.5
0.0
0.5
1.0
1
2
3
4
5
6
7
8
9
10
11
Meishan−A
Meishan−B
Meishan−C
European−A
European−B
European−C

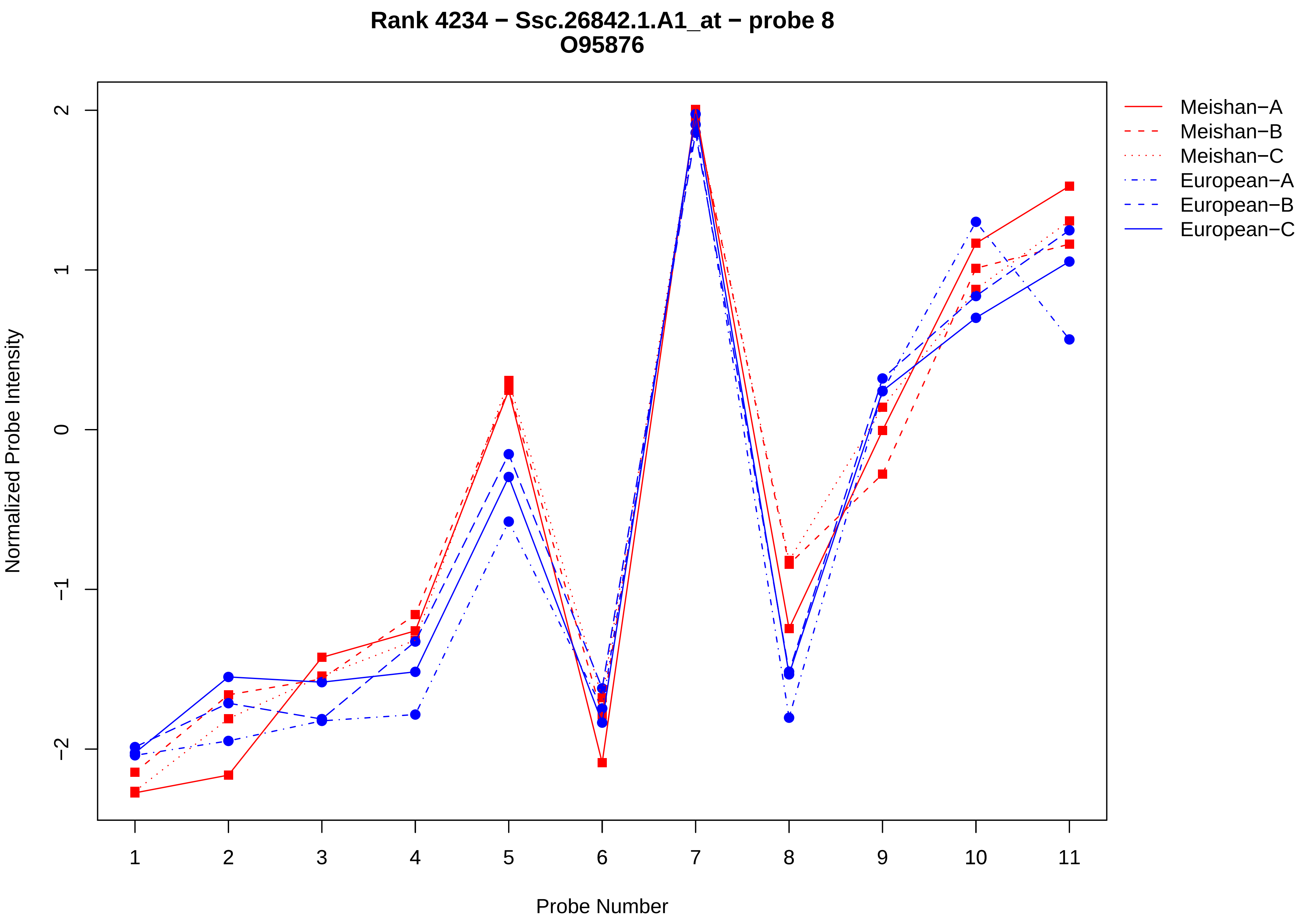
Rank 4234 − Ssc.26842.1.A1_at − probe 8
O95876
Normalized Probe Intensity
Probe Number
−2
−1
0
1
2
1
2
3
4
5
6
7
8
9
10
11
Meishan−A
Meishan−B
Meishan−C
European−A
European−B
European−C

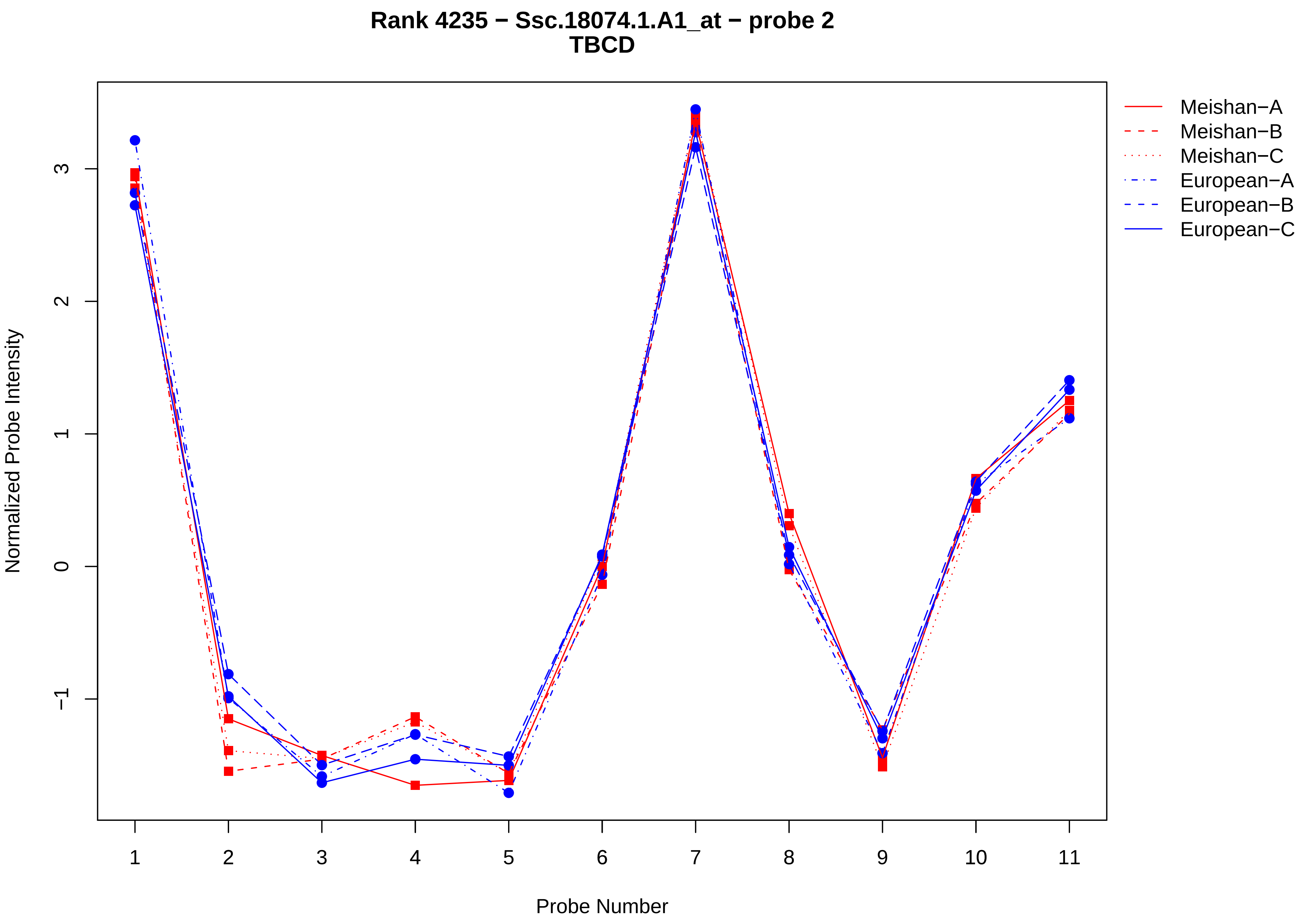

Rank 4235 − Ssc.18074.1.A1_at − probe 2
TBCD
Normalized Probe Intensity
Probe Number
Meishan−A
Meishan−B
Meishan−C
European−A
European−B
European−C

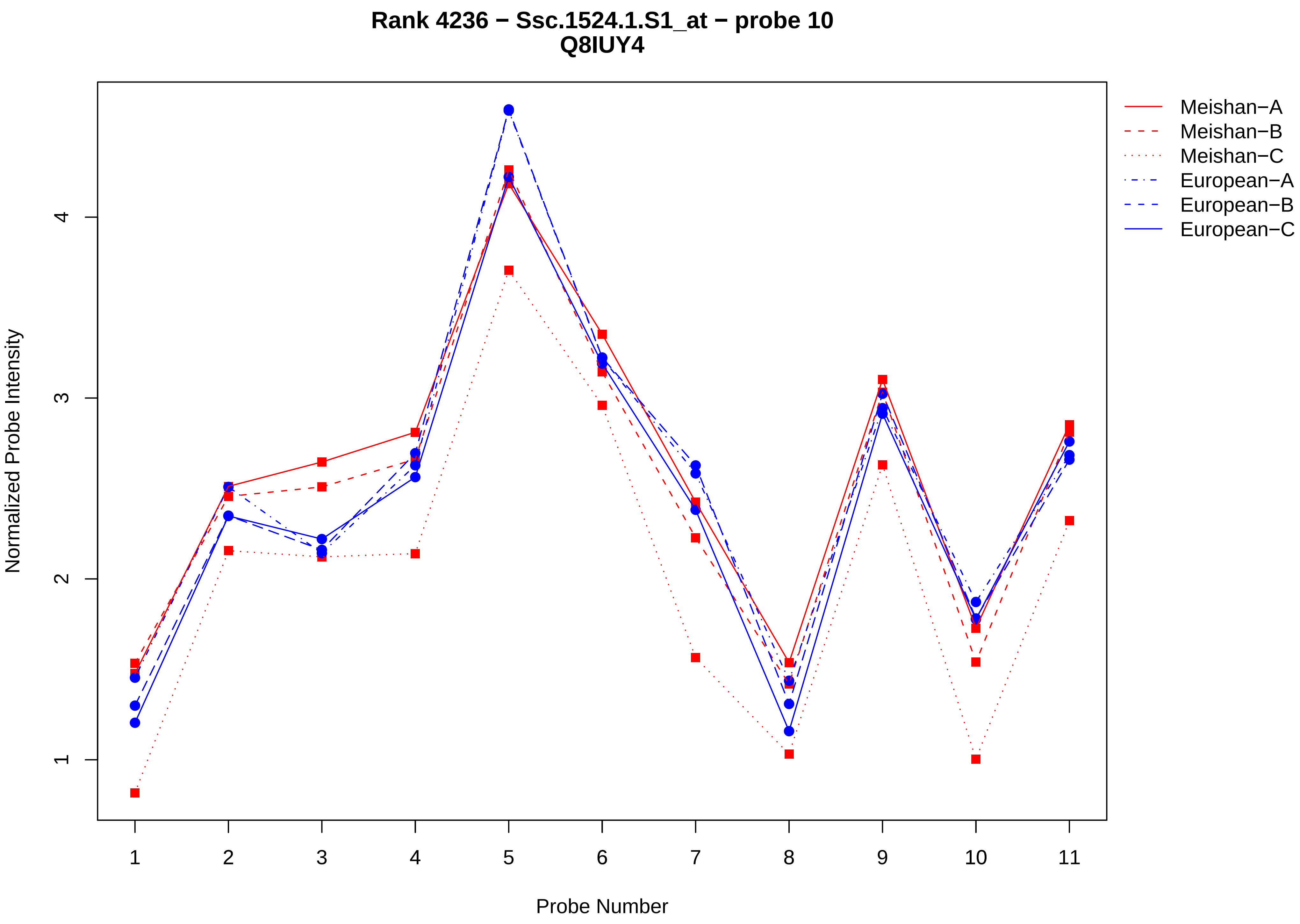

Rank 4236 − Ssc.1524.1.S1_at − probe 10
Q8IUY4
Normalized Probe Intensity
1
2
3
4
Probe Number
1
2
3
4
5
6
7
8
9
10
11
Meishan−A
Meishan−B
Meishan−C
European−A
European−B
European−C

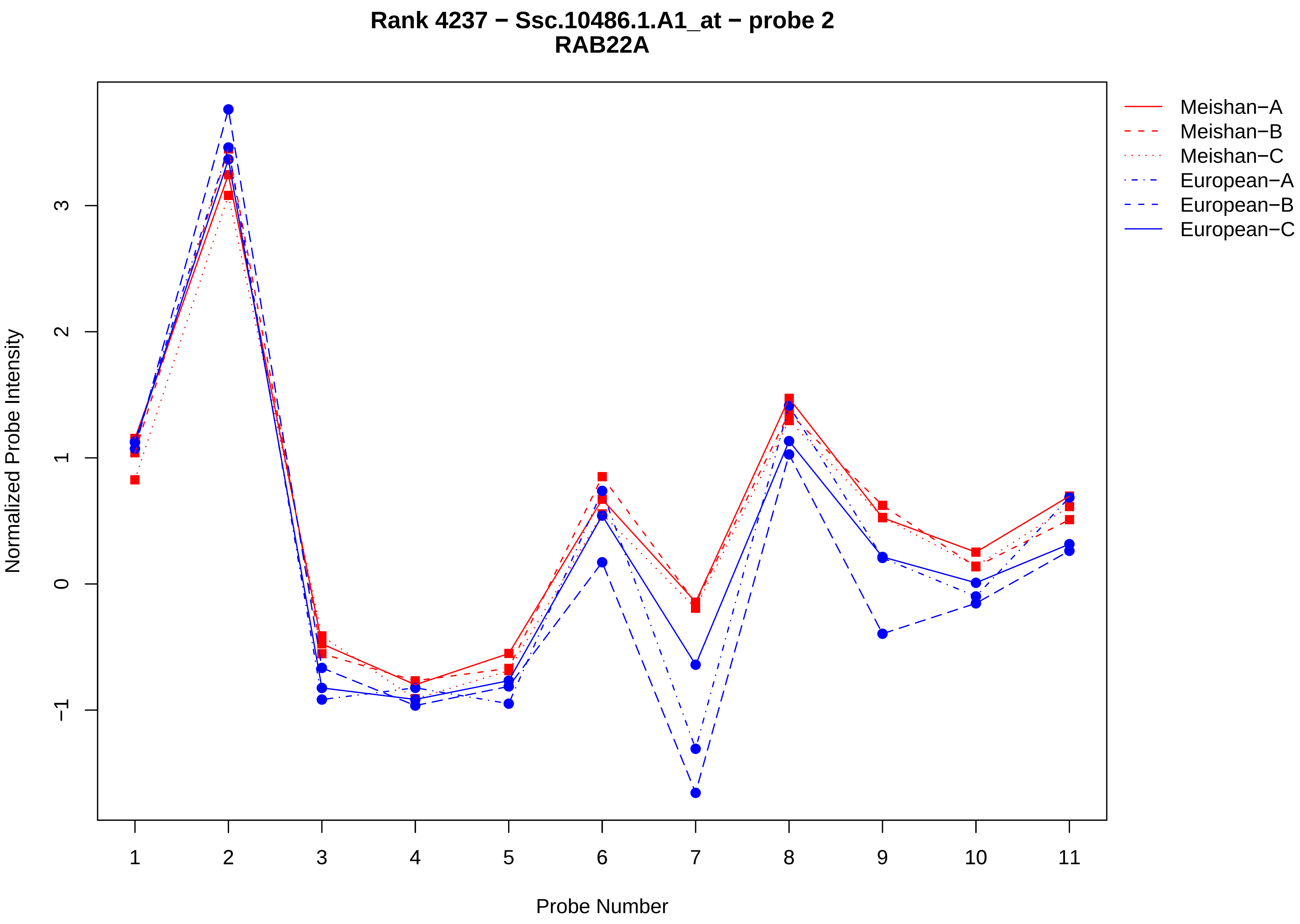

Rank 4237 − Ssc.10486.1.A1_at − probe 2
RAB22A
Normalized Probe Intensity
Probe Number
−1
0
1
2
3
1
2
3
4
5
6
7
8
9
10
11
Meishan−A
Meishan−B
Meishan−C
European−A
European−B
European−C

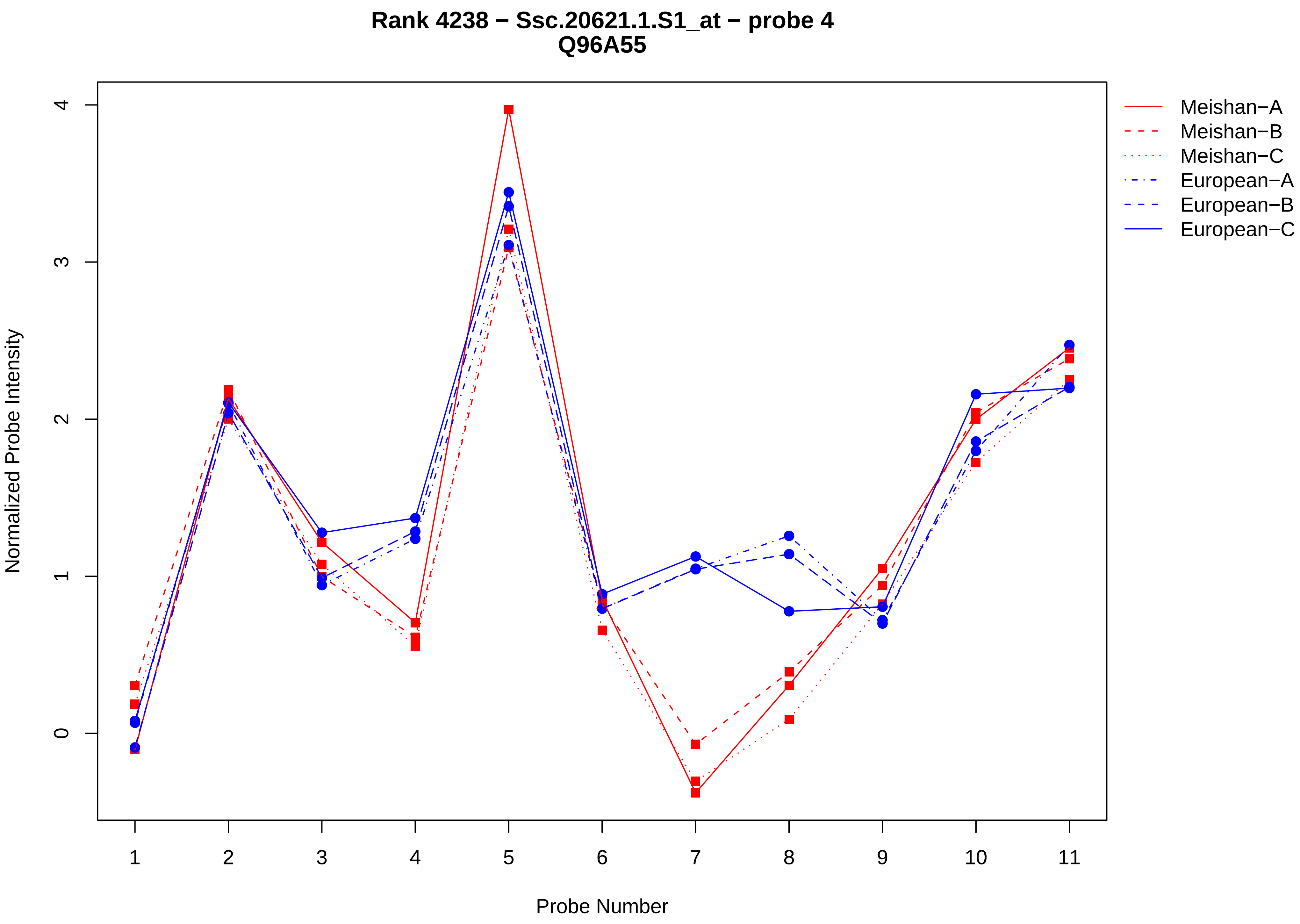

Rank 4238 − Ssc.20621.1.S1_at − probe 4
Q96A55
Normalized Probe Intensity
Probe Number
0
1
2
3
4
1
2
3
4
5
6
7
8
9
10
11
Meishan−A
Meishan−B
Meishan−C
European−A
European−B
European−C

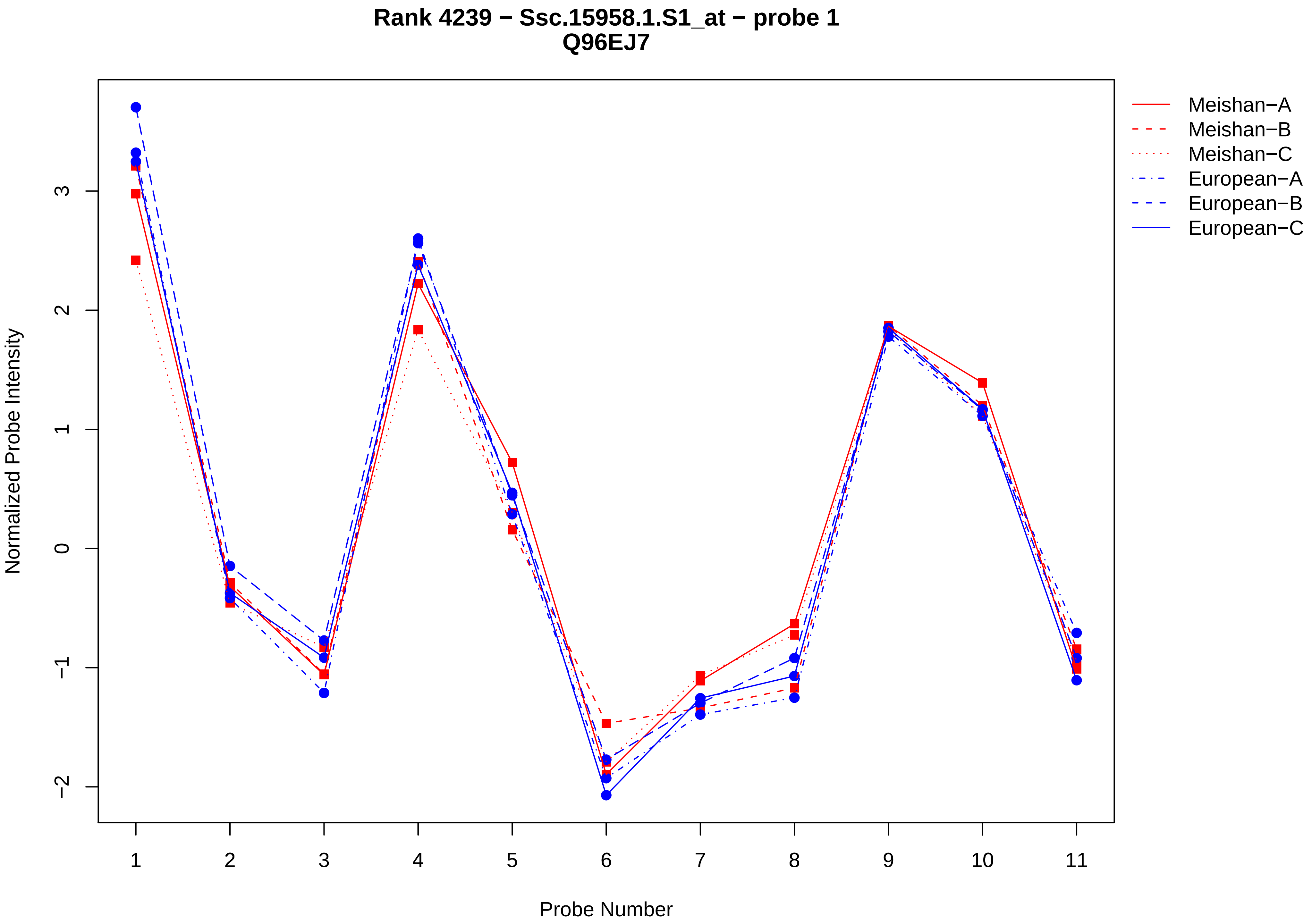

Rank 4239 − Ssc.15958.1.S1_at − probe 1
Q96EJ7
Normalized Probe Intensity
Probe Number
Meishan−A
Meishan−B
Meishan−C
European−A
European−B
European−C
−2
−1
0
1
2
3
1
2
3
4
5
6
7
8
9
10
11

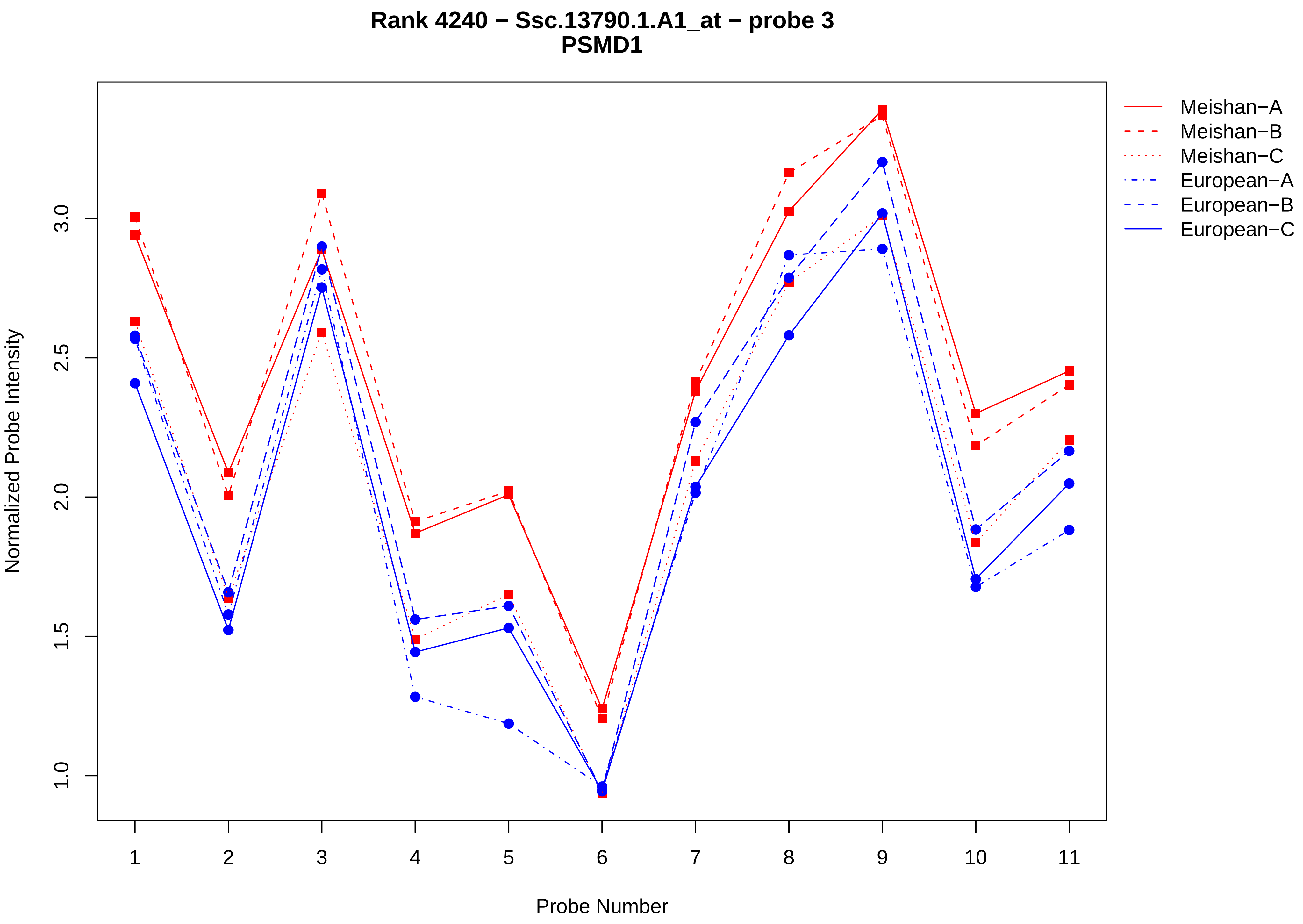
Rank 4240 − Ssc.13790.1.A1_at − probe 3
PSMD1
Normalized Probe Intensity
Probe Number
1.0
1.5
2.0
2.5
3.0
1
2
3
4
5
6
7
8
9
10
11
Meishan−A
Meishan−B
Meishan−C
European−A
European−B
European−C

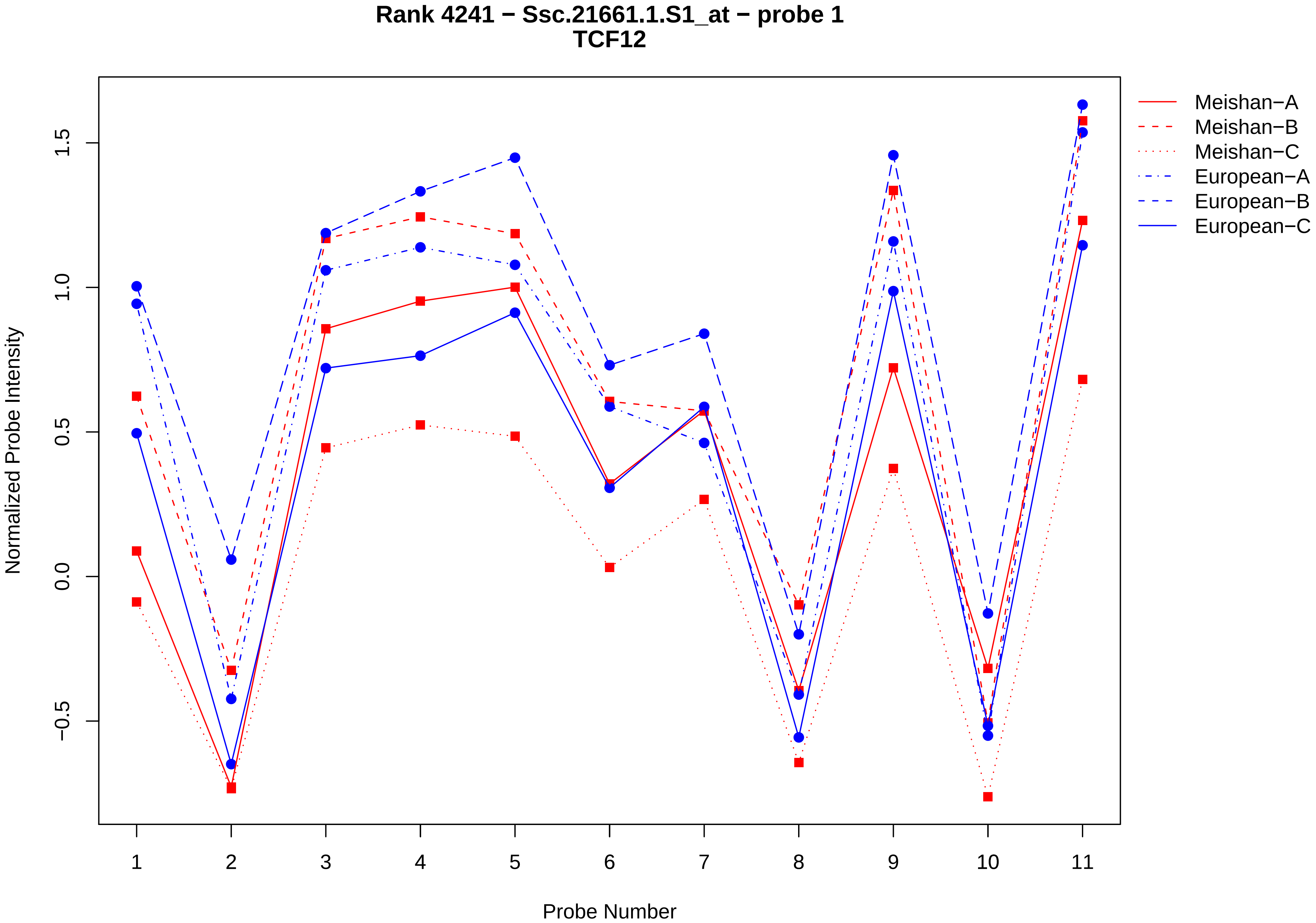

Rank 4241 − Ssc.21661.1.S1_at − probe 1
TCF12
Normalized Probe Intensity
Probe Number
−0.5
0.0
0.5
1.0
1.5
1
2
3
4
5
6
7
8
9
10
11
Meishan−A
Meishan−B
Meishan−C
European−A
European−B
European−C

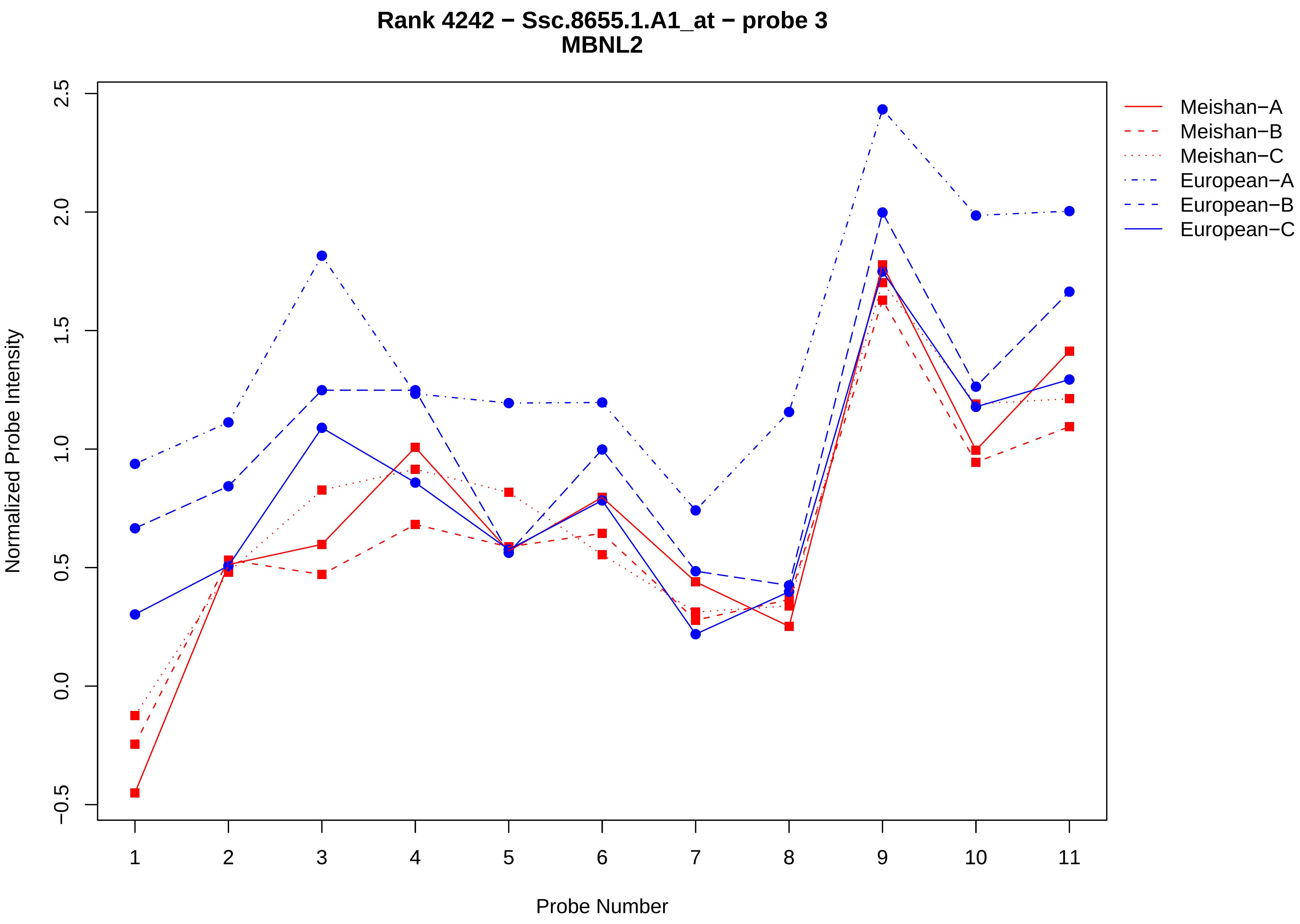

Rank 4242 − Ssc.8655.1.A1_at − probe 3
MBNL2
Normalized Probe Intensity
Probe Number
−0.5
0.0
0.5
1.0
1.5
2.0
2.5
1
2
3
4
5
6
7
8
9
10
11
Meishan−A
Meishan−B
Meishan−C
European−A
European−B
European−C

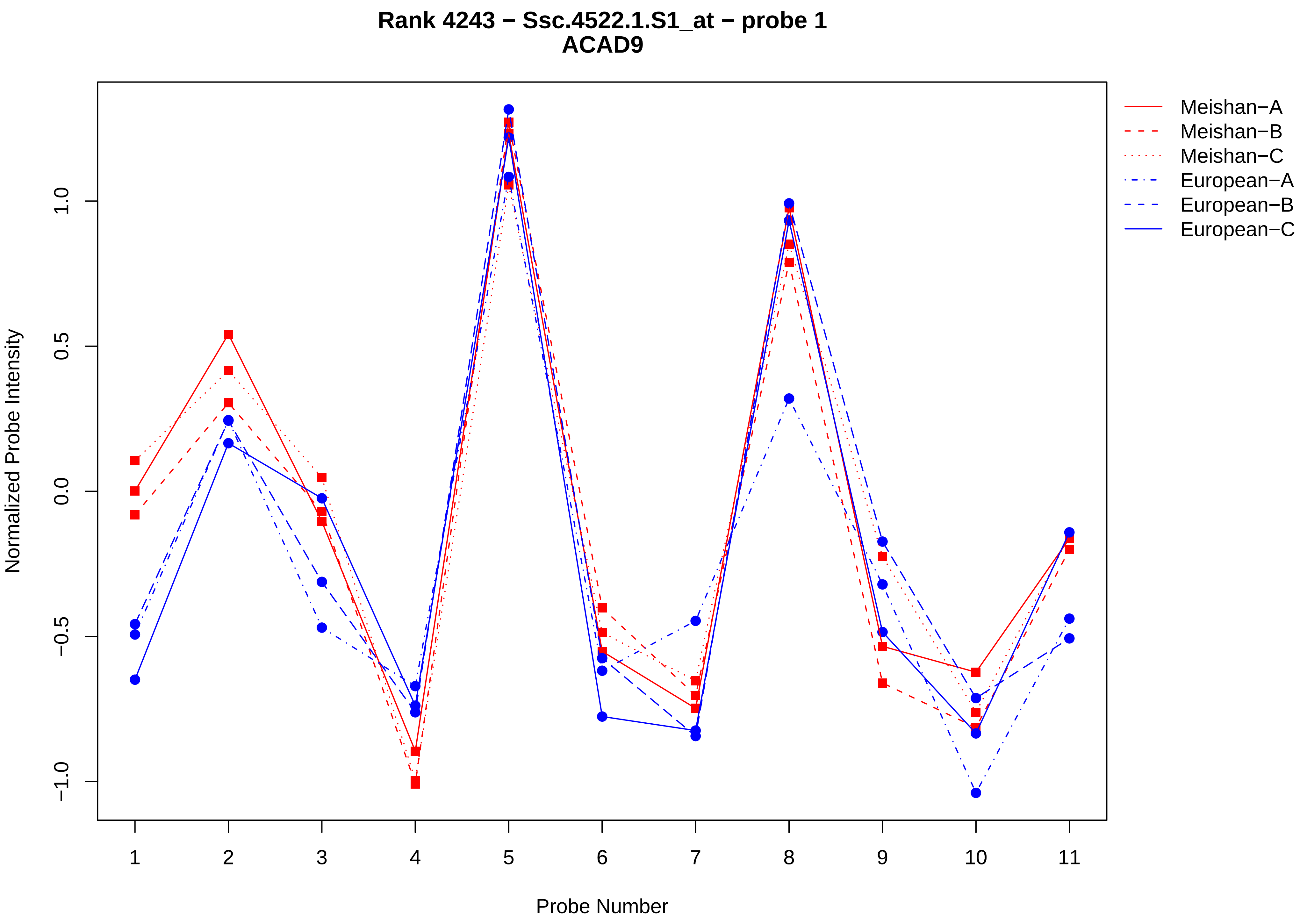

Rank 4243 − Ssc.4522.1.S1_at − probe 1
ACAD9
Normalized Probe Intensity
Probe Number
1.0
0.5
0.0
−0.5
−1.0
1
2
3
4
5
6
7
8
9
10
11
Meishan−A
Meishan−B
Meishan−C
European−A
European−B
European−C

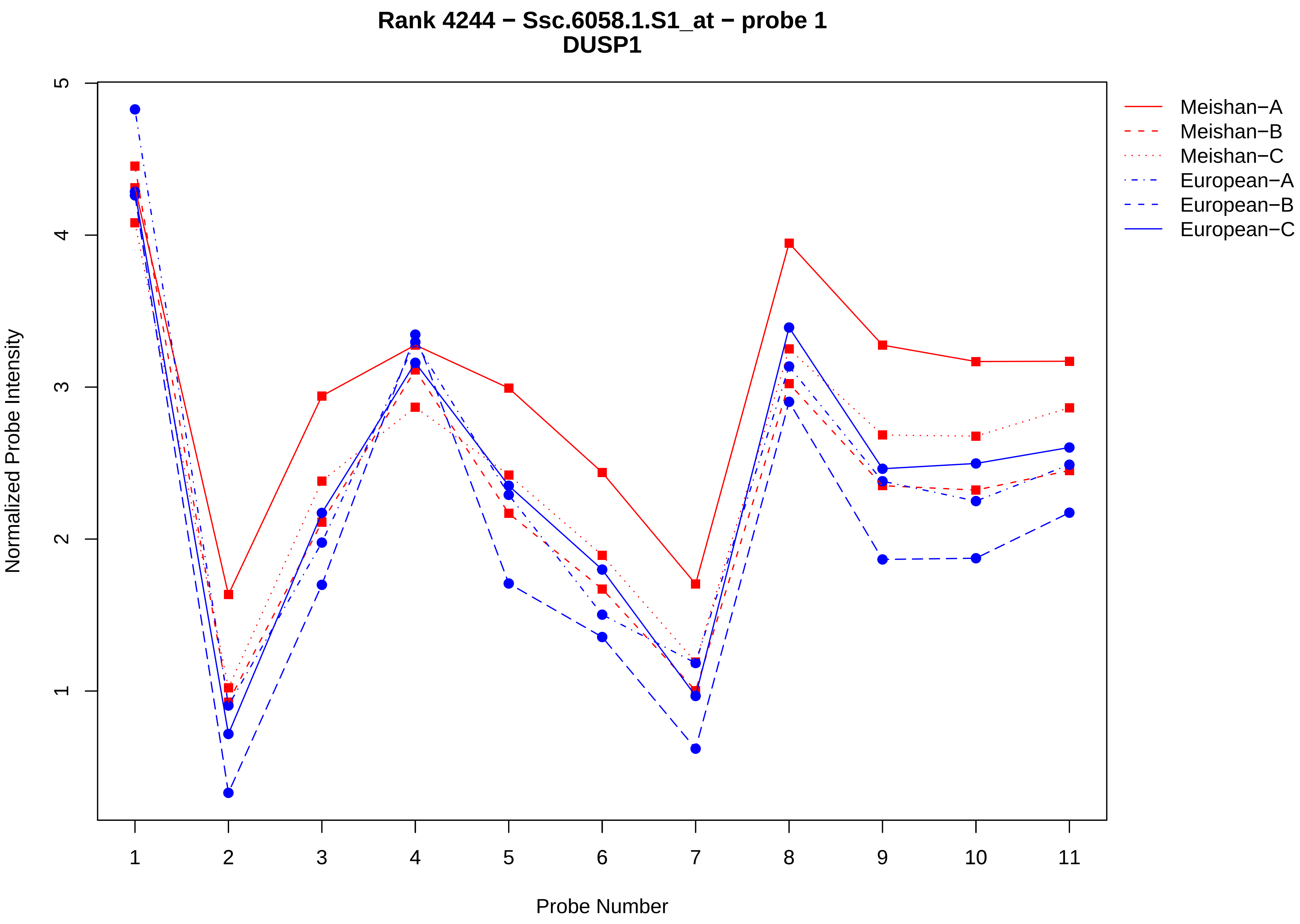
Rank 4244 − Ssc.6058.1.S1_at − probe 1
DUSP1
Normalized Probe Intensity
Probe Number
1
2
3
4
5
6
7
8
9
10
11
Meishan−A
Meishan−B
Meishan−C
European−A
European−B
European−C

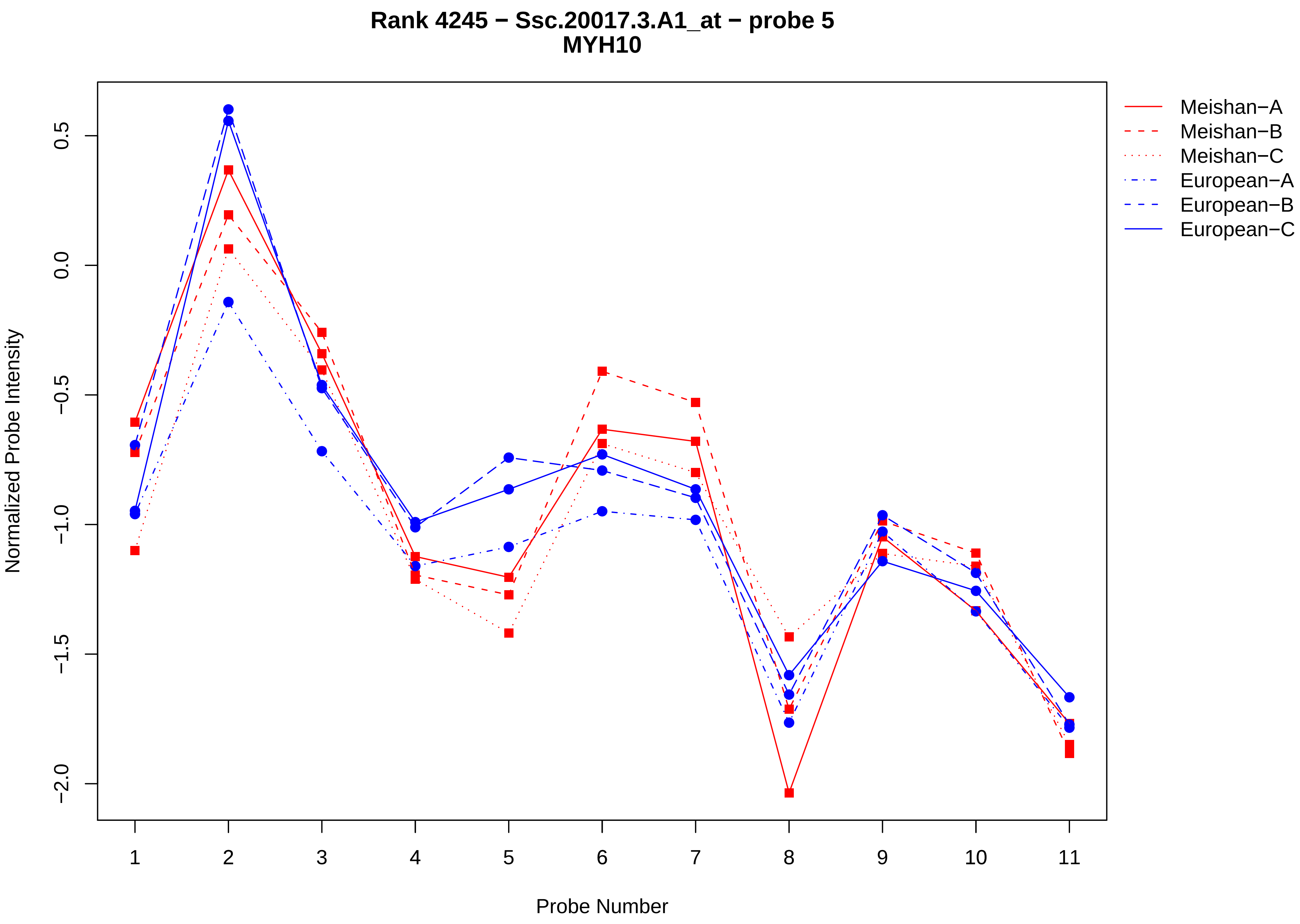

Rank 4245 − Ssc.20017.3.A1_at − probe 5
MYH10
Normalized Probe Intensity
Probe Number
0.5
0.0
−0.5
−1.0
−1.5
−2.0
1
2
3
4
5
6
7
8
9
10
11
Meishan−A
Meishan−B
Meishan−C
European−A
European−B
European−C

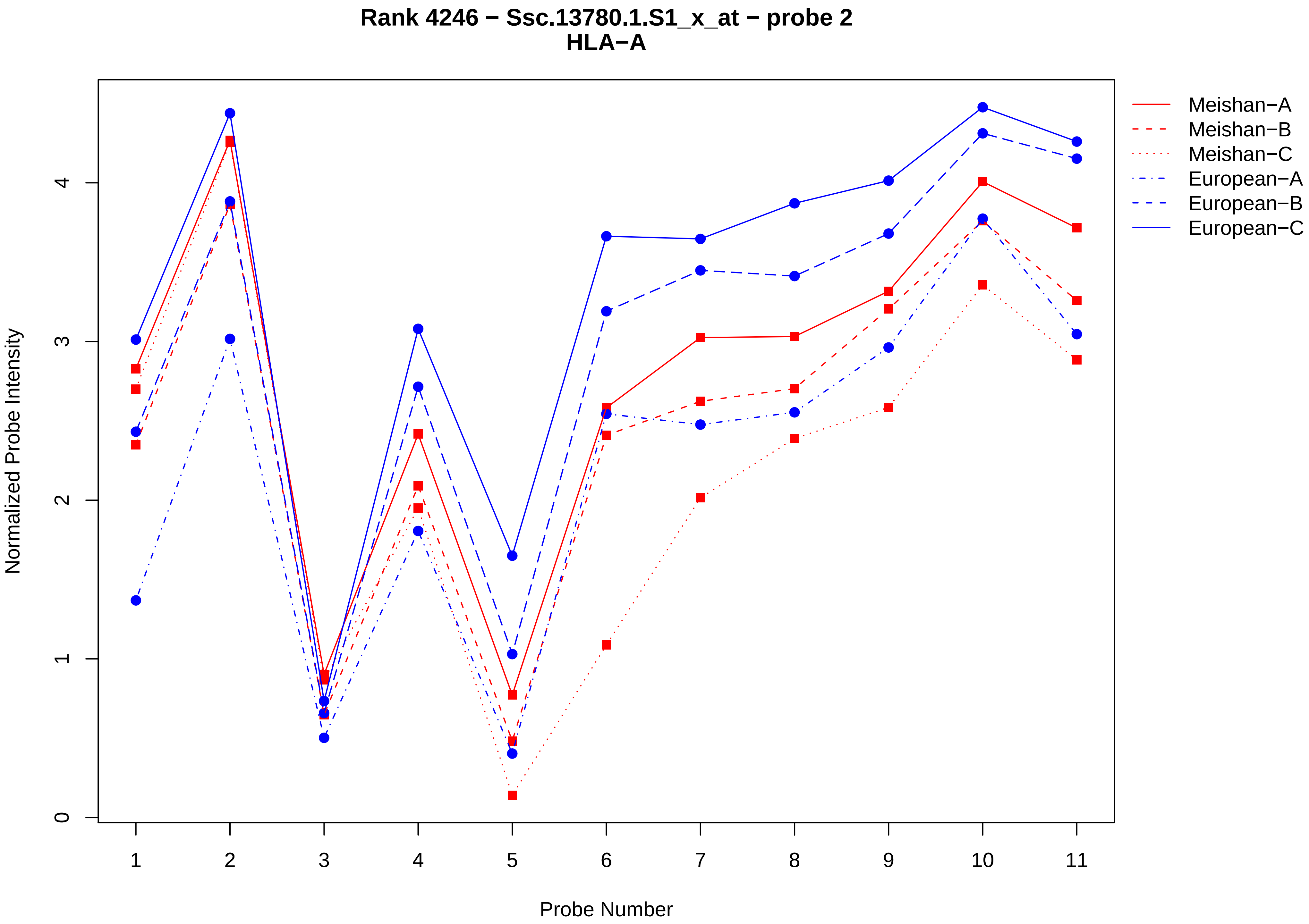
Rank 4246 − Ssc.13780.1.S1_x_at − probe 2
HLA−A
Normalized Probe Intensity
Probe Number
0
1
2
3
4
1
2
3
4
5
6
7
8
9
10
11
Meishan−A
Meishan−B
Meishan−C
European−A
European−B
European−C

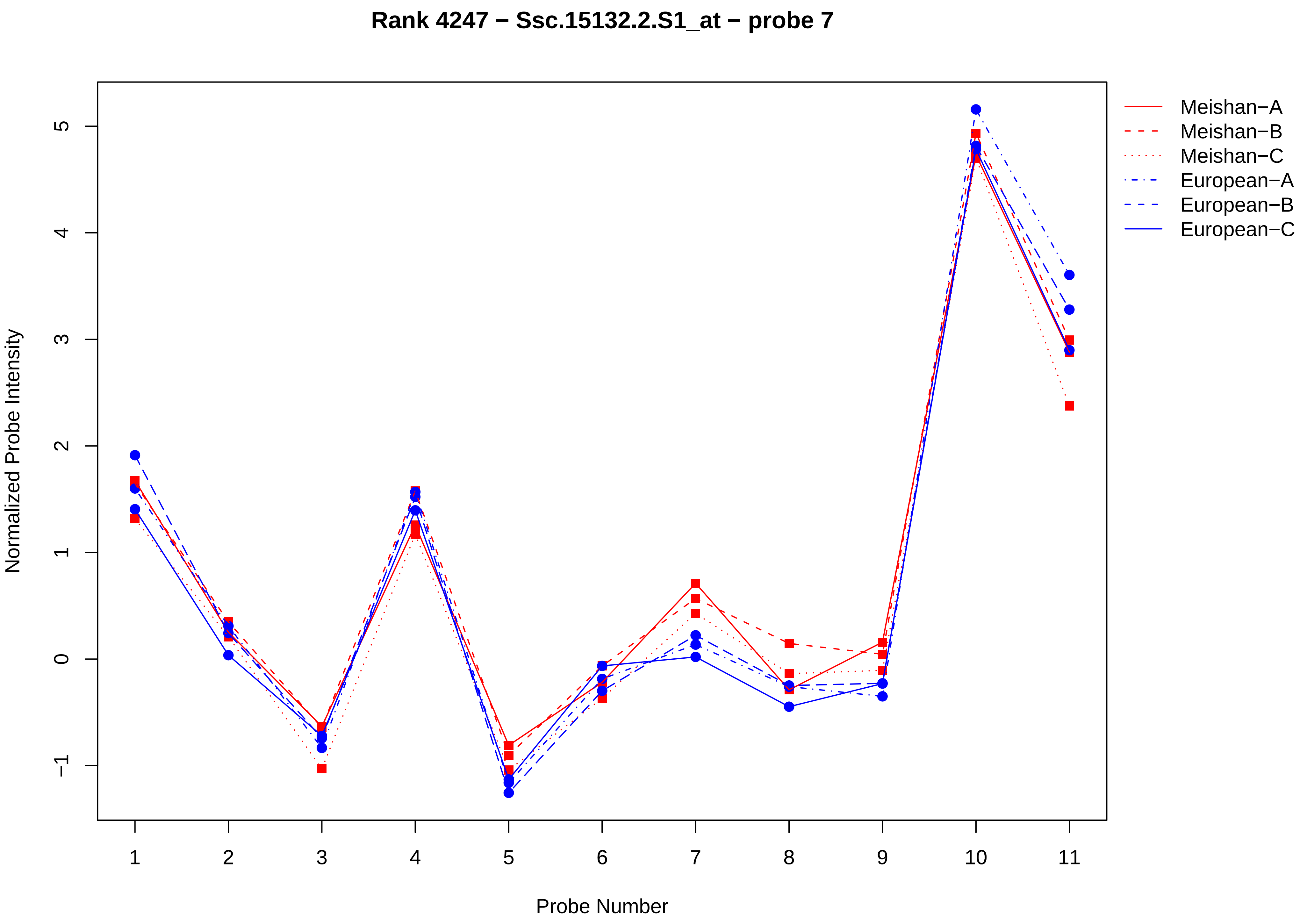

Rank 4247 − Ssc.15132.2.S1_at − probe 7
Normalized Probe Intensity
Probe Number
−1
0
1
2
3
4
5
1
2
3
4
5
6
7
8
9
10
11
Meishan−A
Meishan−B
Meishan−C
European−A
European−B
European−C

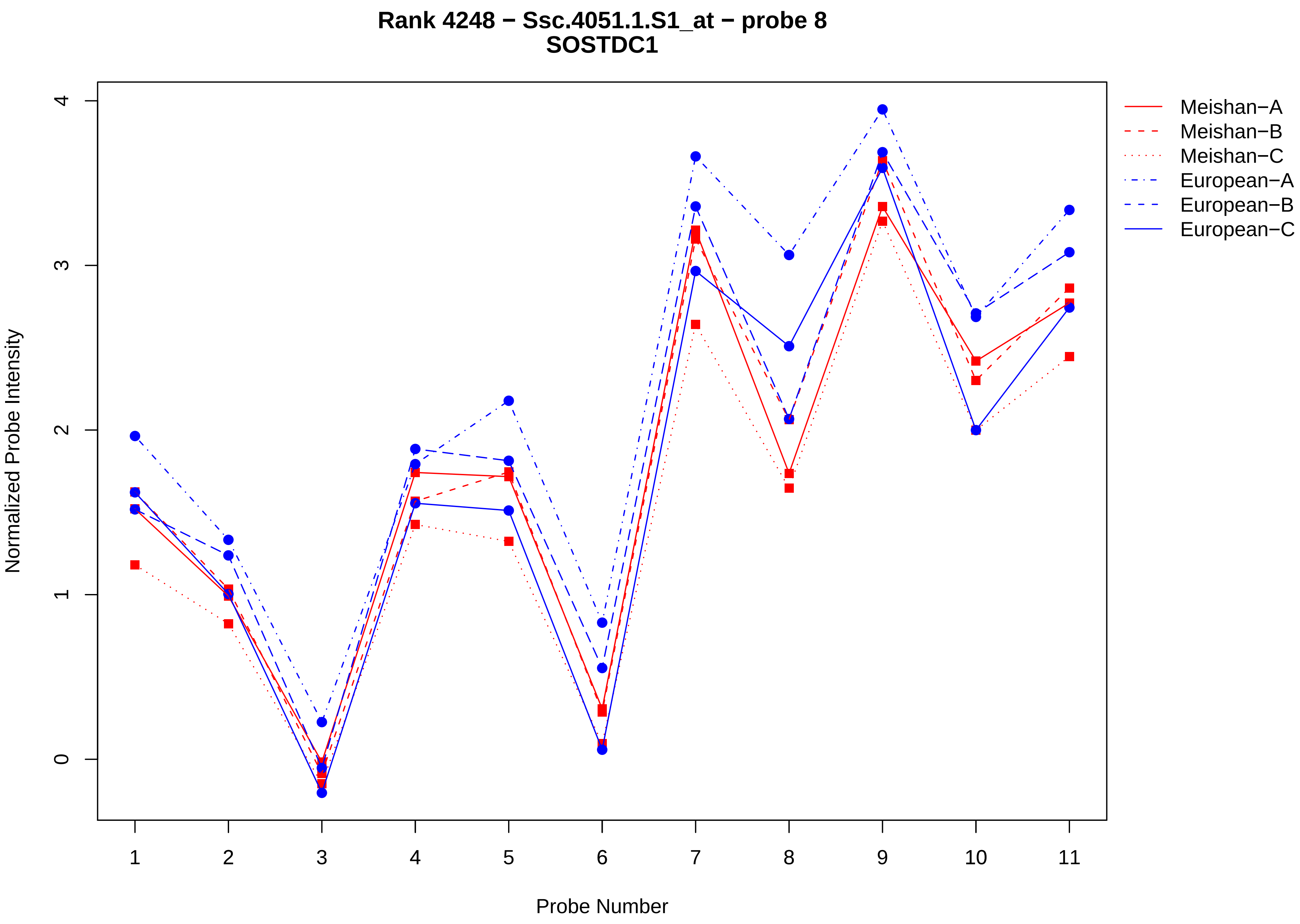
Rank 4248 − Ssc.4051.1.S1_at − probe 8
SOSTDC1
Normalized Probe Intensity
0
1
2
3
4
1
2
3
4
5
6
7
8
9
10
11
Probe Number
Meishan−A
Meishan−B
Meishan−C
European−A
European−B
European−C

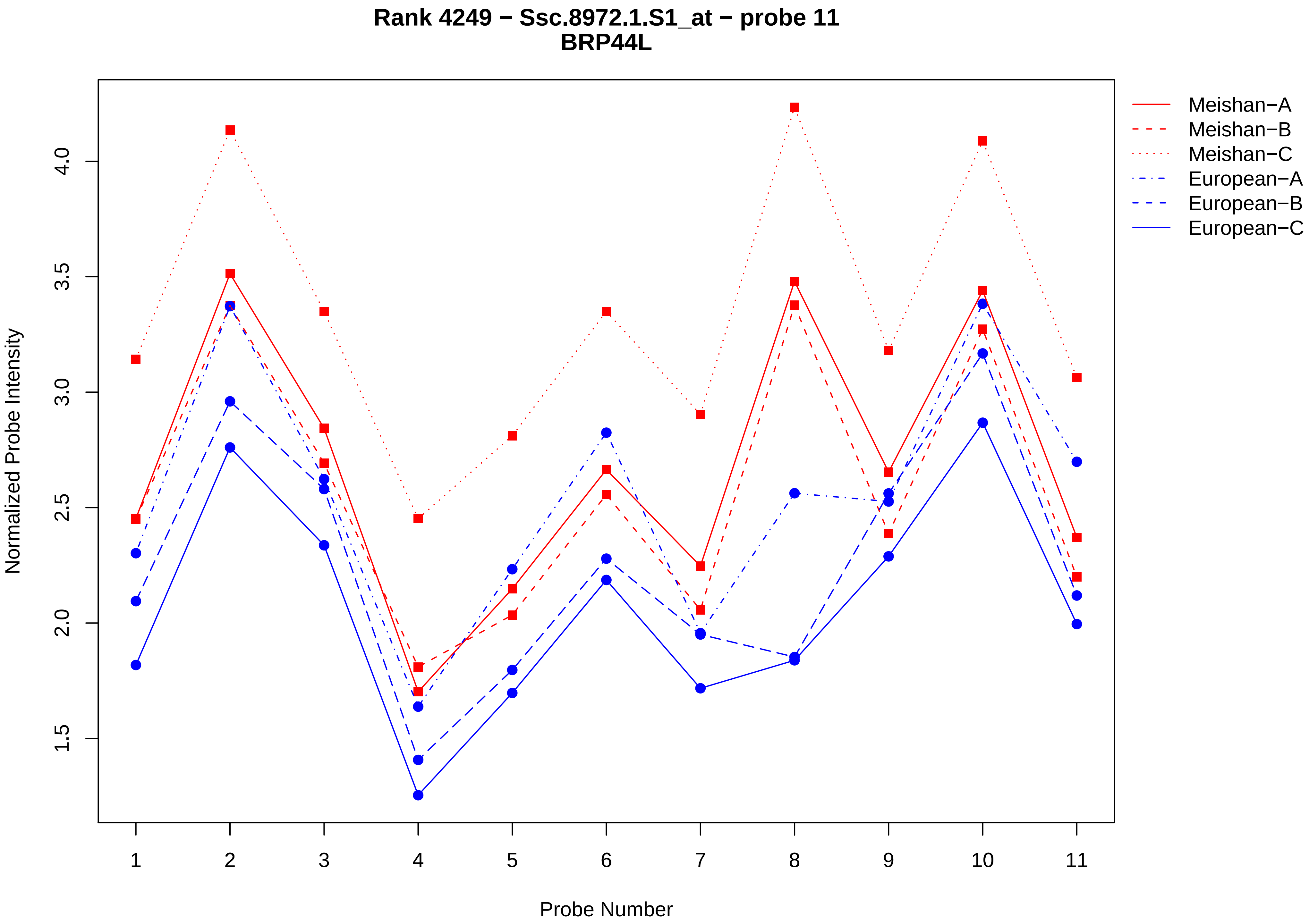

Rank 4249 − Ssc.8972.1.S1_at − probe 11
BRP44L
Normalized Probe Intensity
Probe Number
1.5
2.0
2.5
3.0
3.5
4.0
1
2
3
4
5
6
7
8
9
10
11
Meishan−A
Meishan−B
Meishan−C
European−A
European−B
European−C

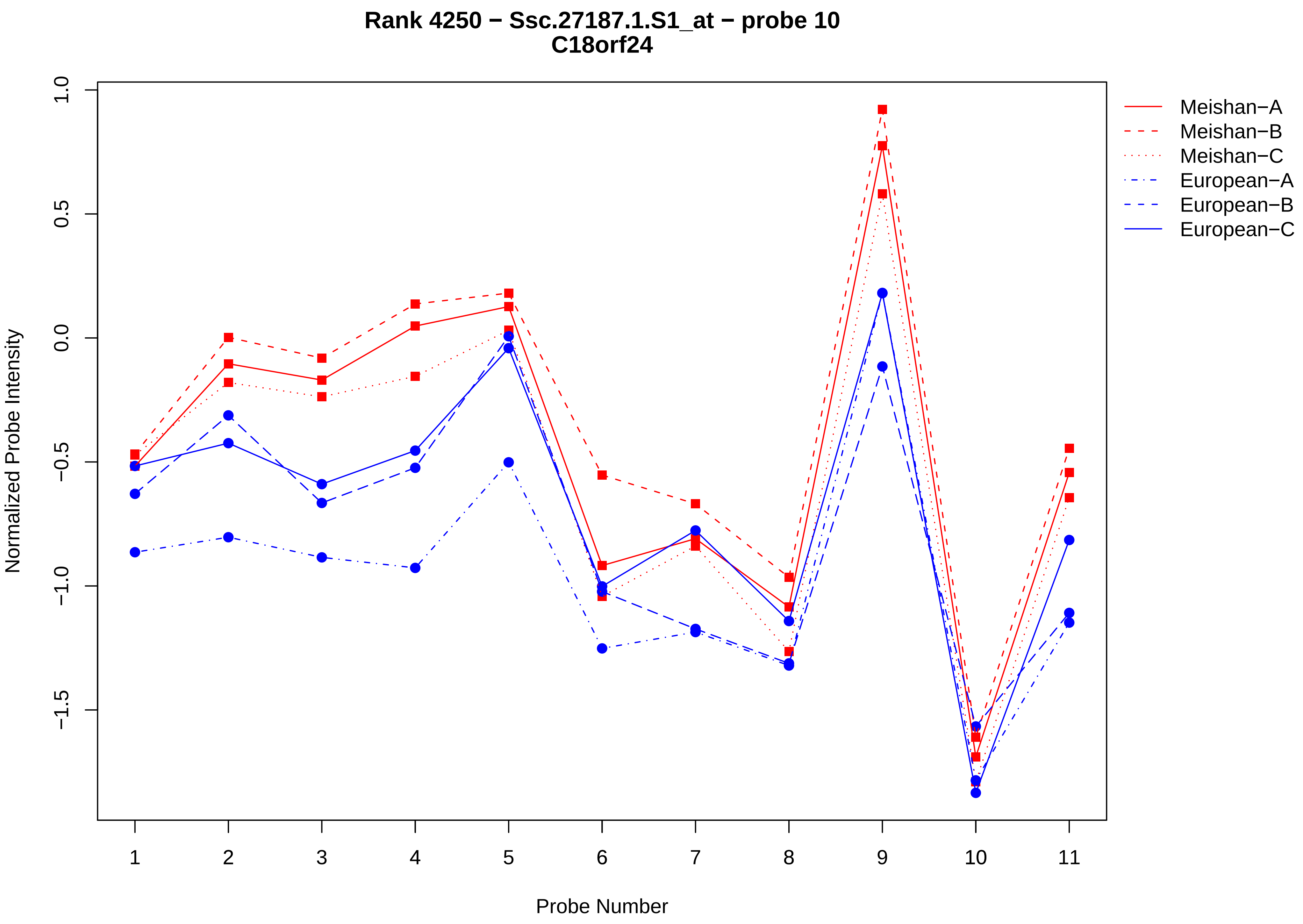

Rank 4250 − Ssc.27187.1.S1_at − probe 10
C18orf24
Normalized Probe Intensity
Probe Number
1.0
0.5
0.0
−0.5
−1.0
−1.5
1
2
3
4
5
6
7
8
9
10
11
Meishan−A
Meishan−B
Meishan−C
European−A
European−B
European−C

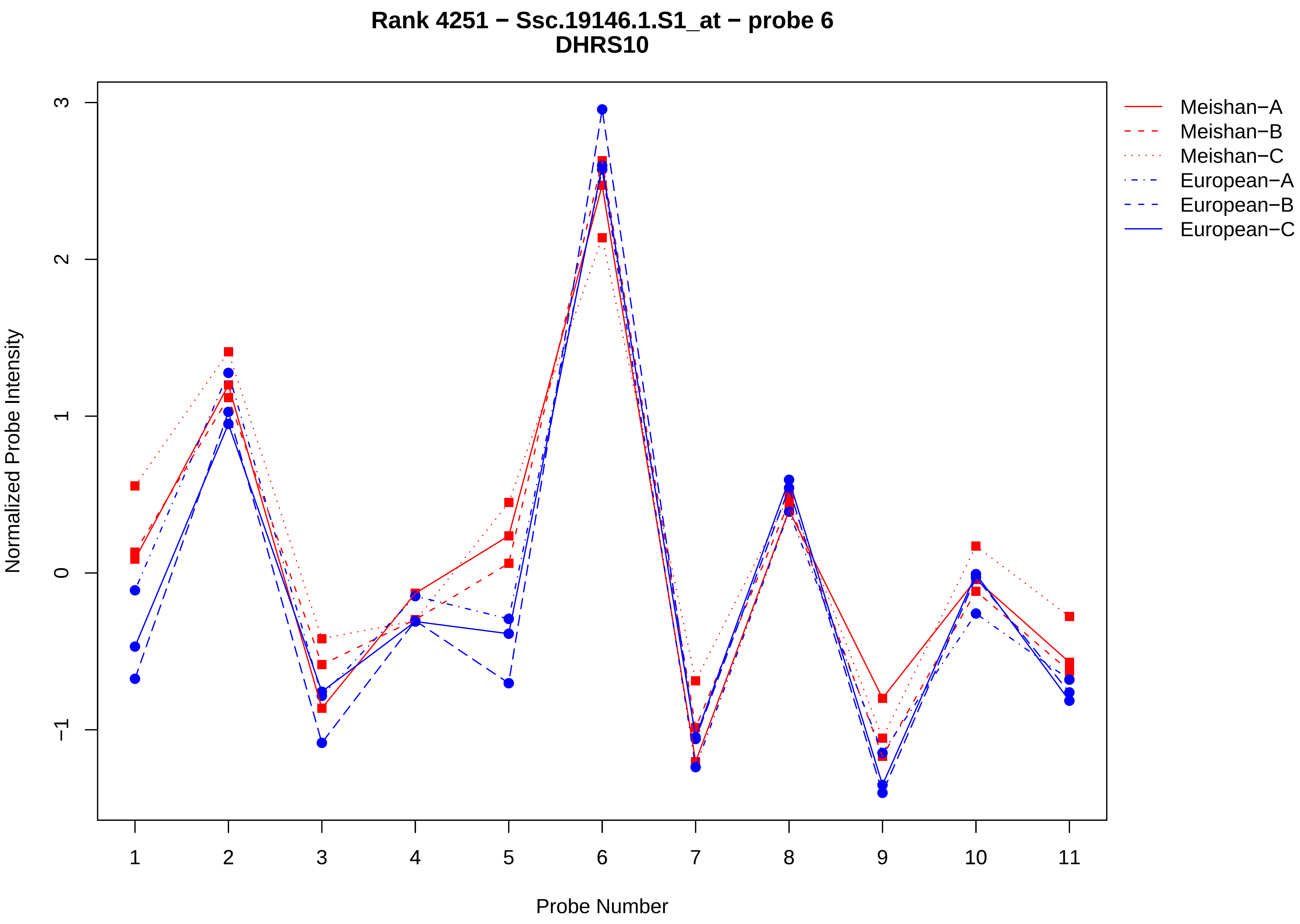
Rank 4251 − Ssc.19146.1.S1_at − probe 6
DHRS10
Normalized Probe Intensity
Probe Number
Meishan−A
Meishan−B
Meishan−C
European−A
European−B
European−C
−1
0
1
2
3
1
2
3
4
5
6
7
8
9
10
11

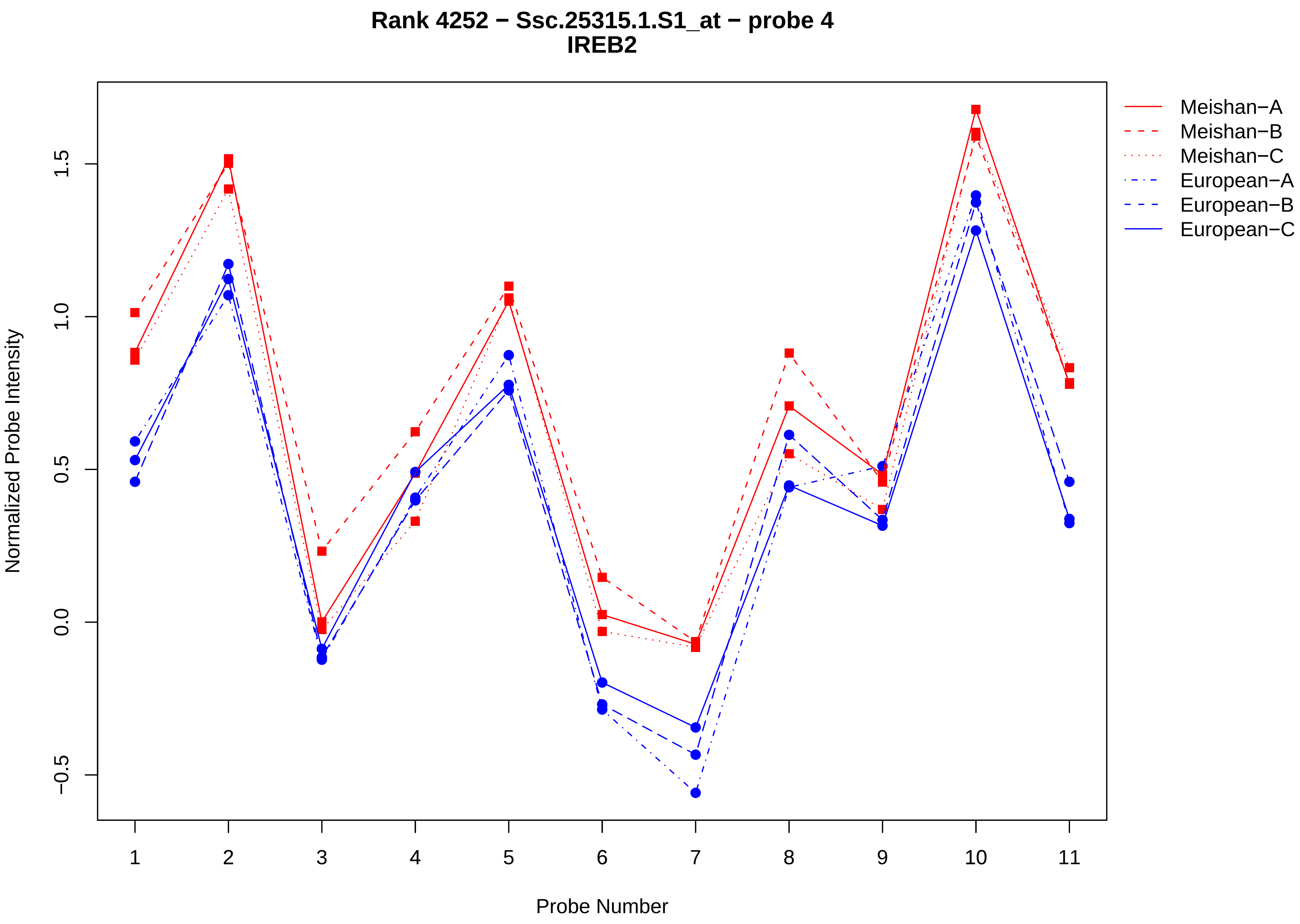

Rank 4252 − Ssc.25315.1.S1_at − probe 4
IREB2
Normalized Probe Intensity
Probe Number
−0.5
0.0
0.5
1.0
1.5
1
2
3
4
5
6
7
8
9
10
11
Meishan−A
Meishan−B
Meishan−C
European−A
European−B
European−C

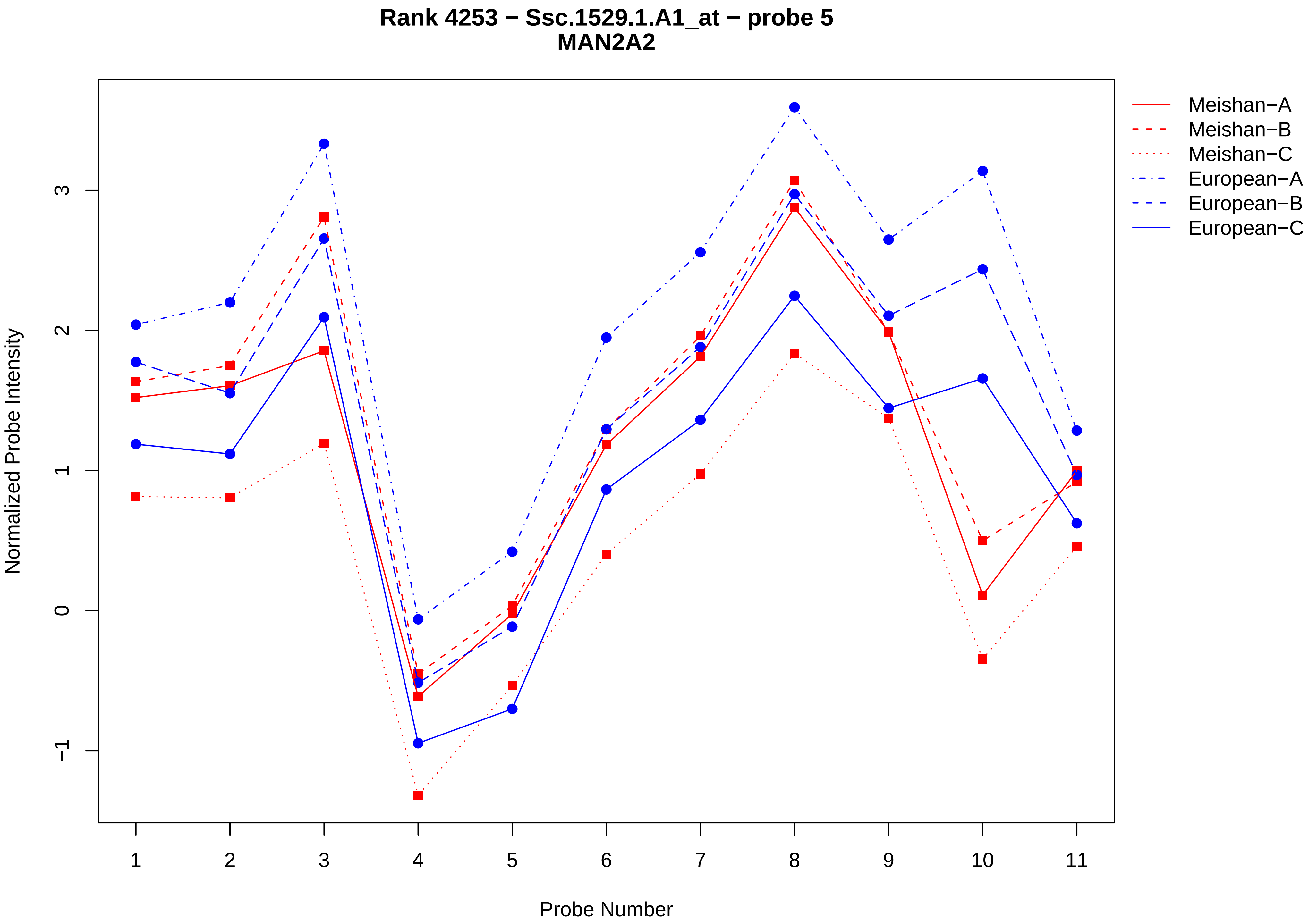

Rank 4253 − Ssc.1529.1.A1_at − probe 5
MAN2A2
Normalized Probe Intensity
Probe Number
−1
0
1
2
3
1
2
3
4
5
6
7
8
9
10
11
Meishan−A
Meishan−B
Meishan−C
European−A
European−B
European−C

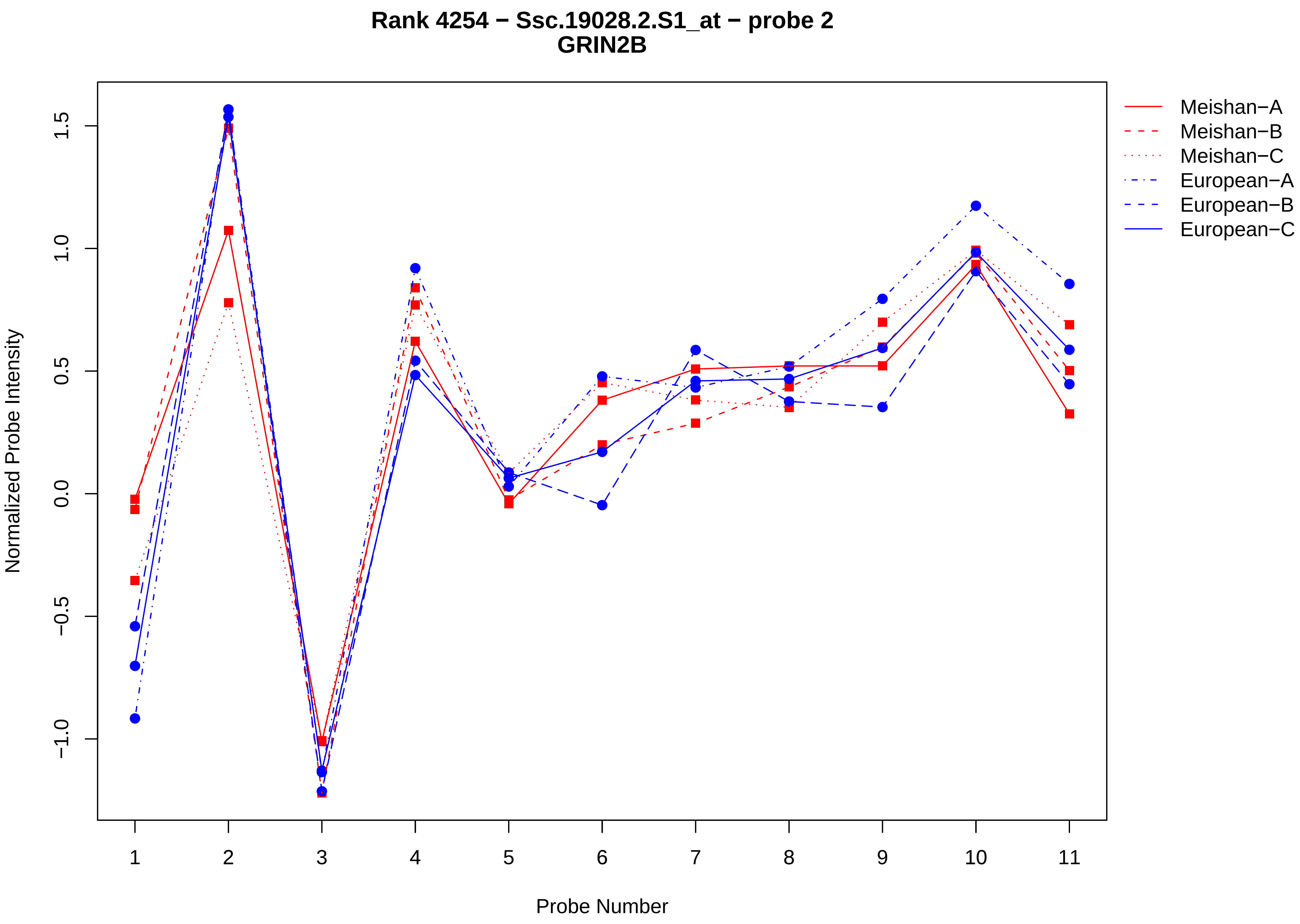
Rank 4254 − Ssc.19028.2.S1_at − probe 2
GRIN2B
Normalized Probe Intensity
Probe Number
Meishan−A
Meishan−B
Meishan−C
European−A
European−B
European−C

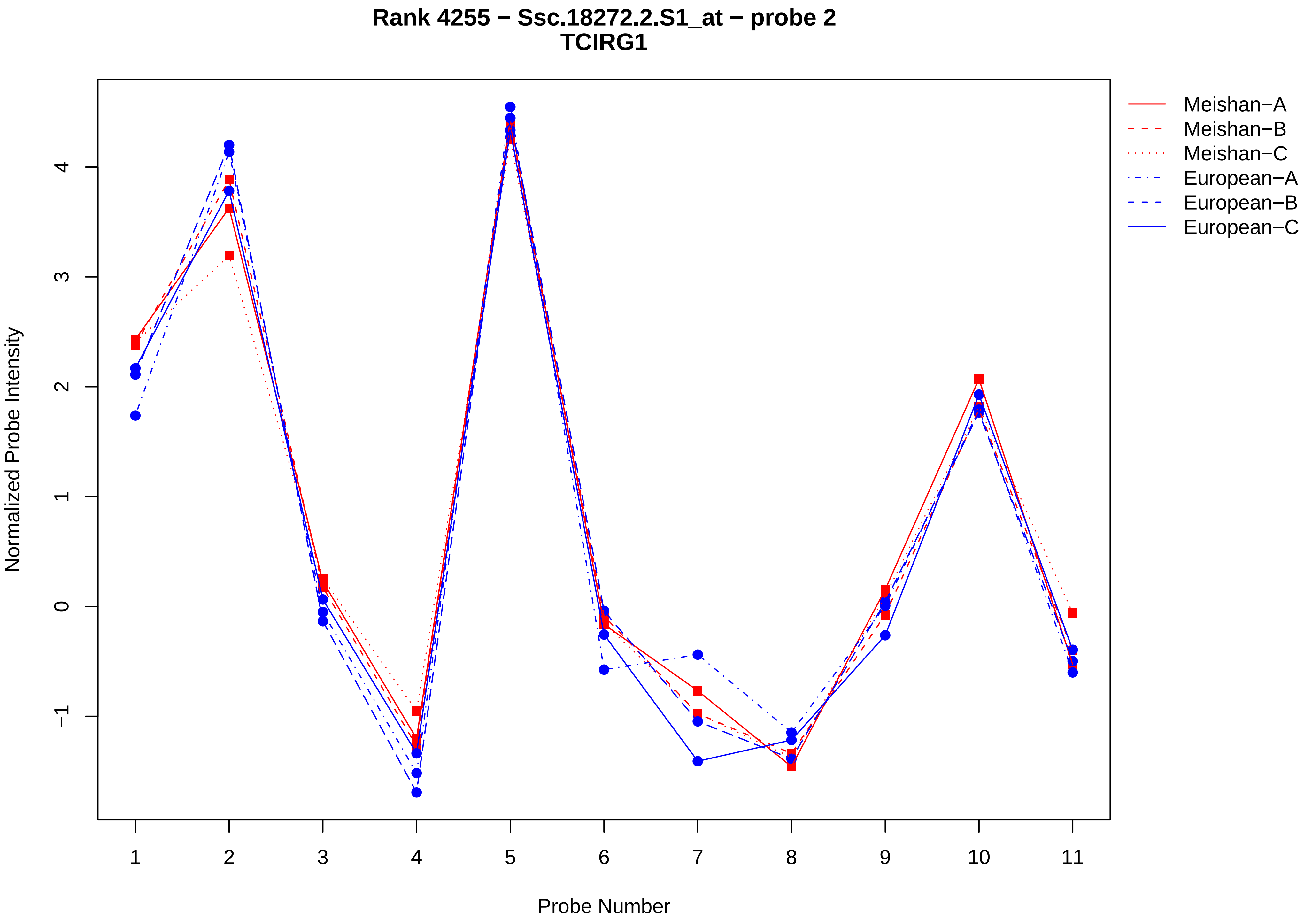

Rank 4255 − Ssc.18272.2.S1_at − probe 2
TCIRG1
Normalized Probe Intensity
Probe Number
−1
0
1
2
3
4
1
2
3
4
5
6
7
8
9
10
11
Meishan−A
Meishan−B
Meishan−C
European−A
European−B
European−C

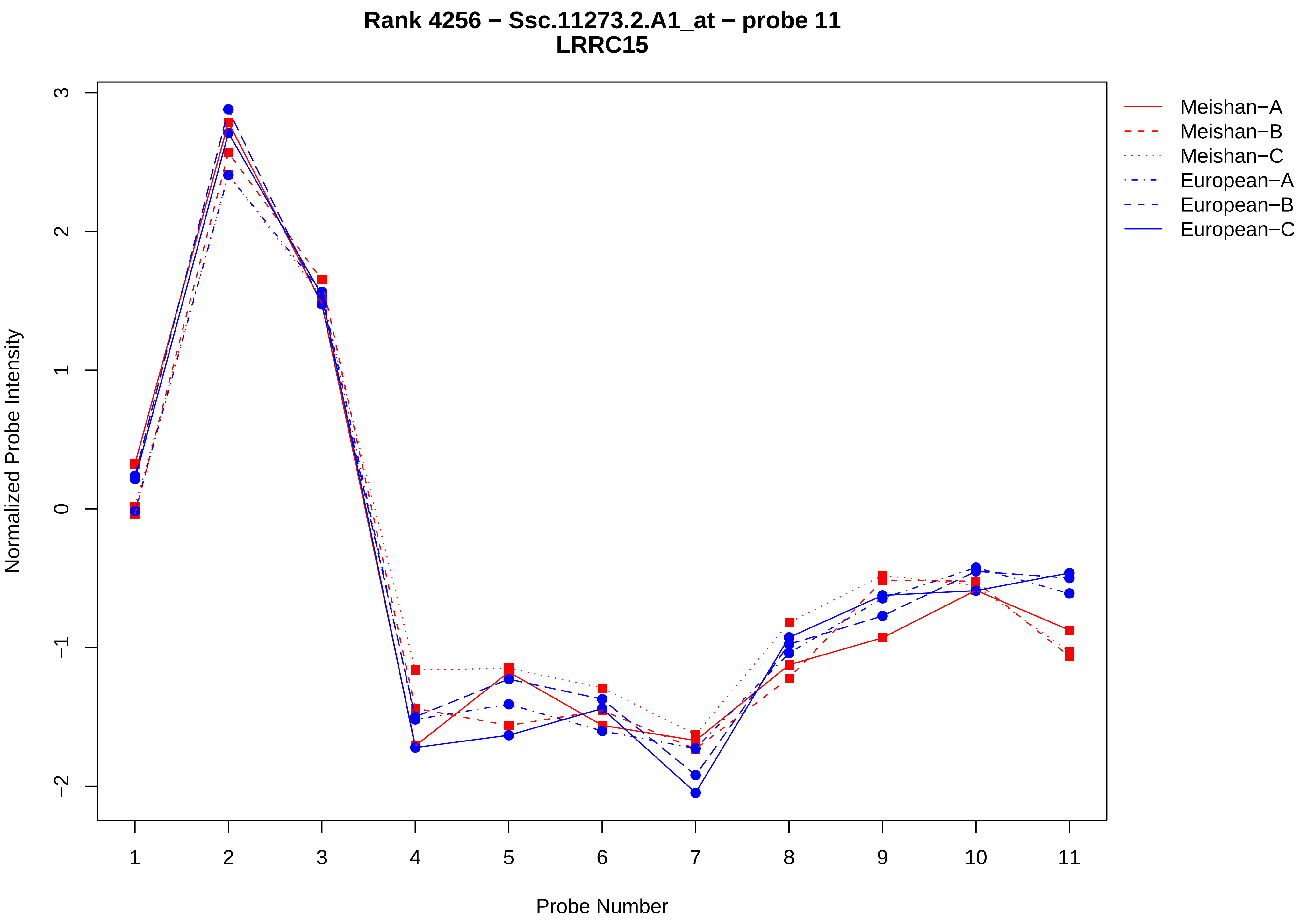

Rank 4256 − Ssc.11273.2.A1_at − probe 11
LRRC15
Normalized Probe Intensity
Probe Number
Meishan−A
Meishan−B
Meishan−C
European−A
European−B
European−C

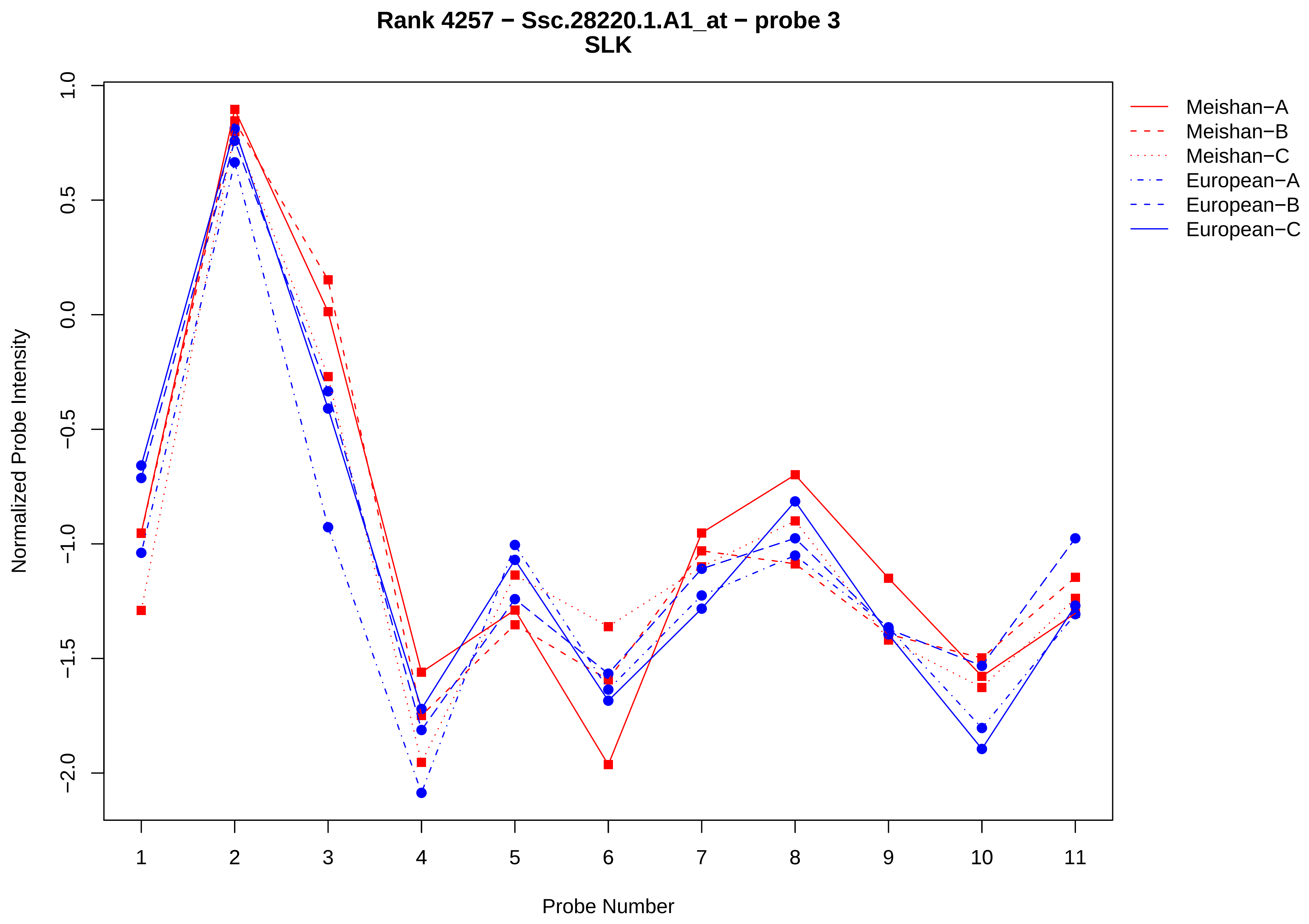

Rank 4257 − Ssc.28220.1.A1_at − probe 3
SLK
Normalized Probe Intensity
Probe Number
1.0
0.5
0.0
−0.5
−1.0
−1.5
−2.0
1
2
3
4
5
6
7
8
9
10
11
Meishan−A
Meishan−B
Meishan−C
European−A
European−B
European−C

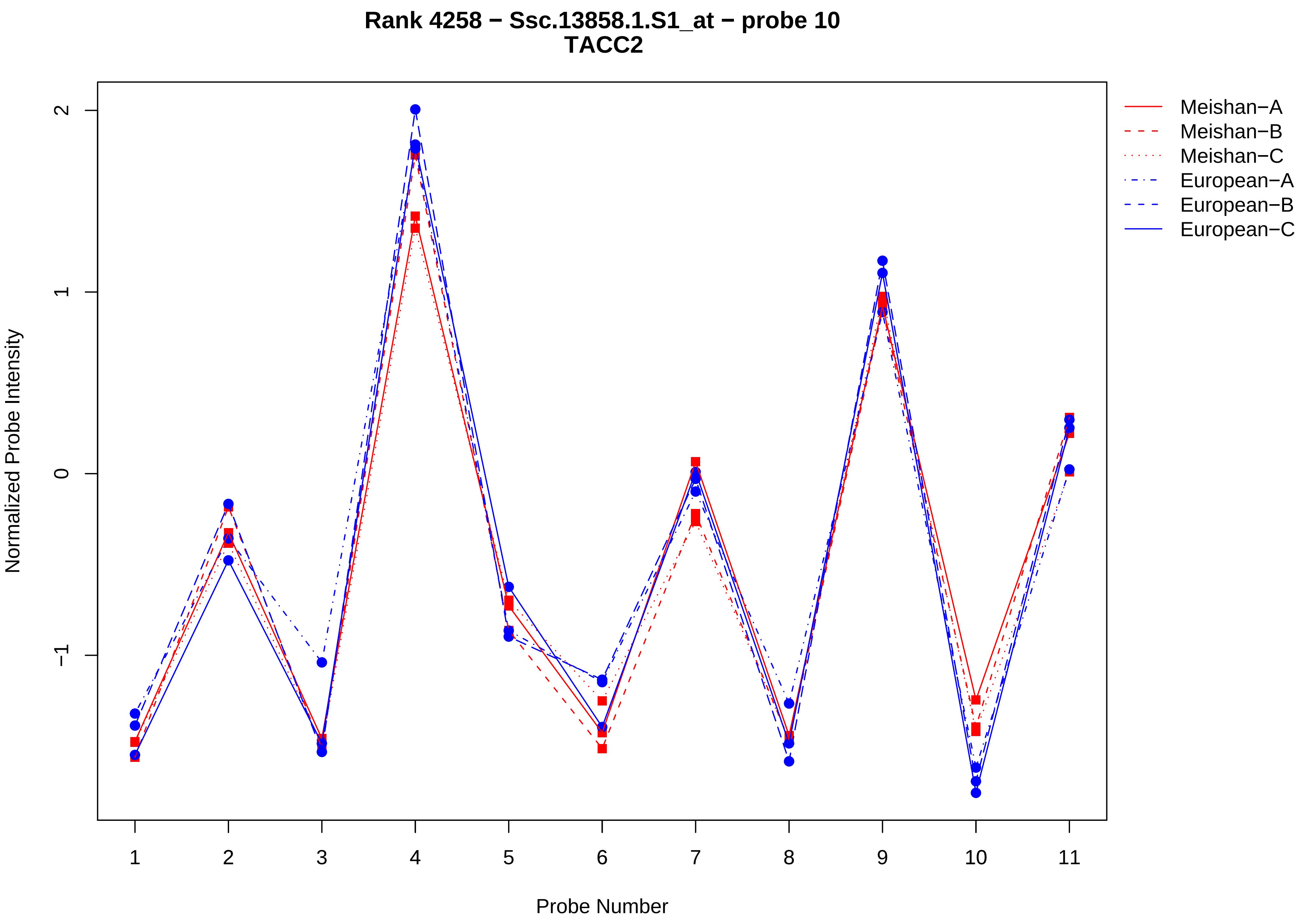

Rank 4258 − Ssc.13858.1.S1_at − probe 10
TACC2
Normalized Probe Intensity
Probe Number
Meishan−A
Meishan−B
Meishan−C
European−A
European−B
European−C

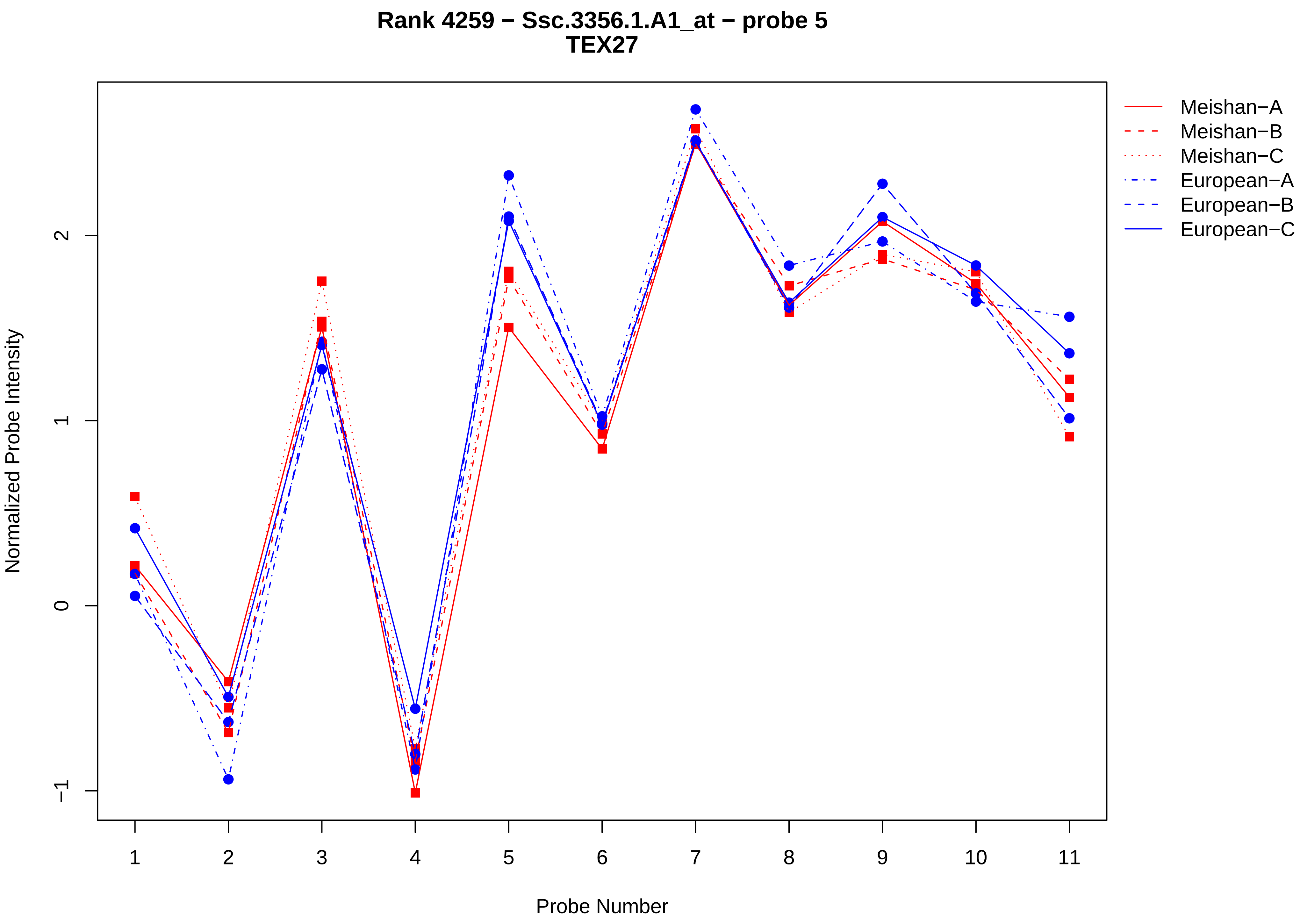

Rank 4259 − Ssc.3356.1.A1_at − probe 5
TEX27
Normalized Probe Intensity
Probe Number
−1
0
1
2
1
2
3
4
5
6
7
8
9
10
11
Meishan−A
Meishan−B
Meishan−C
European−A
European−B
European−C

Rank 4260 − Ssc.2087.1.S1_at − probe 6
HYOU1

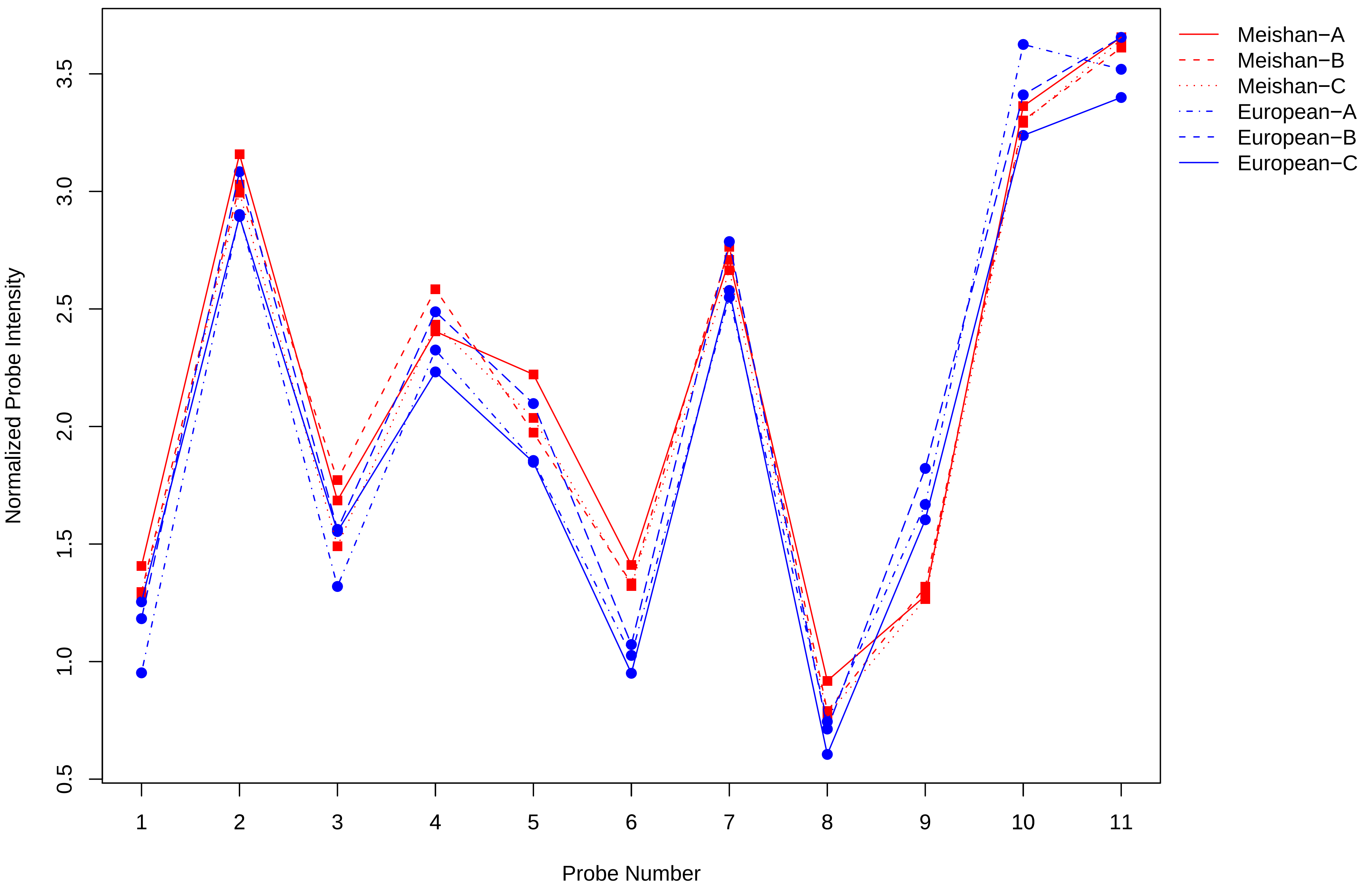

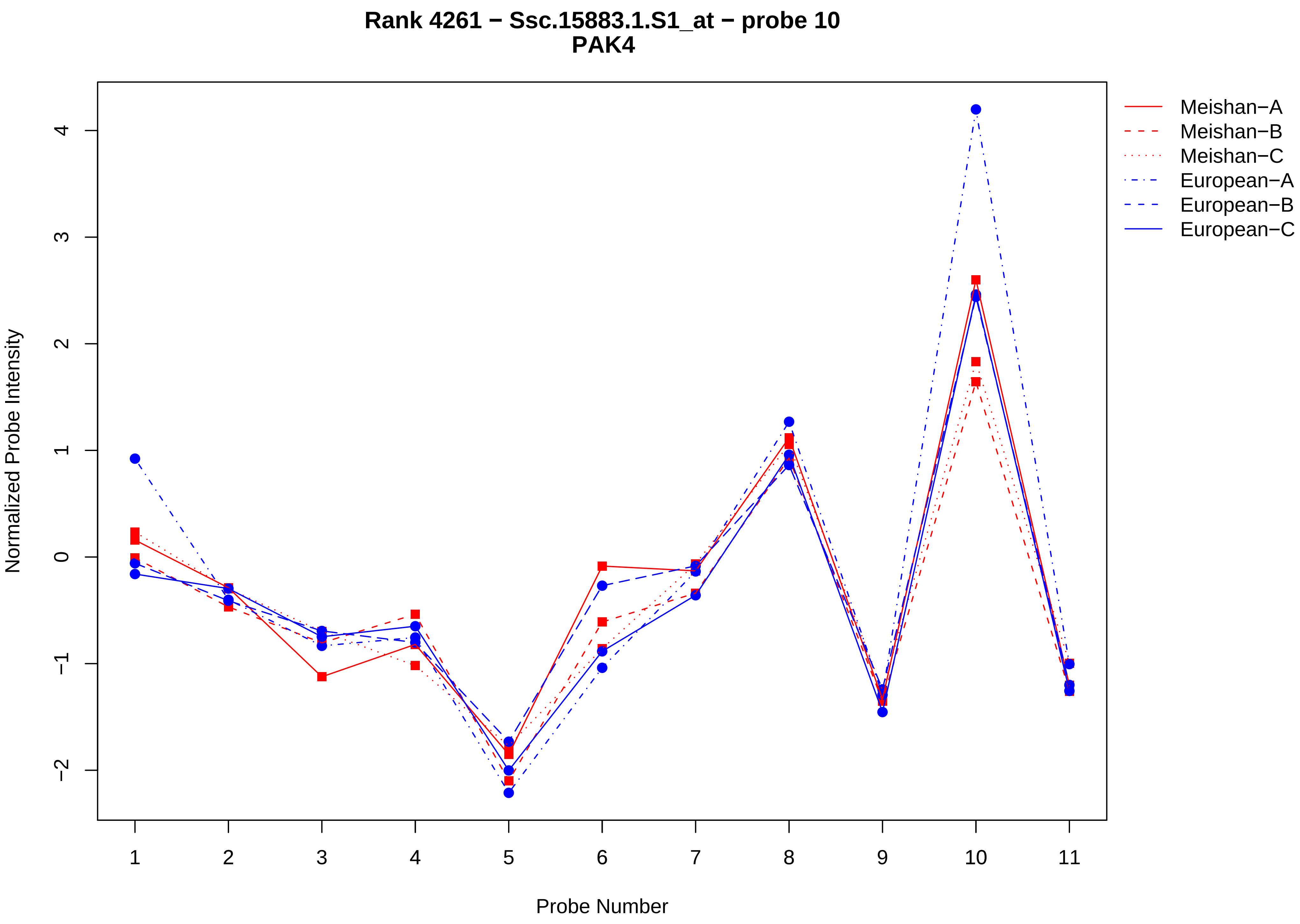

Rank 4261 − Ssc.15883.1.S1_at − probe 10
PAK4
Normalized Probe Intensity
Probe Number
−2
−1
0
1
2
3
4
1
2
3
4
5
6
7
8
9
10
11
Meishan−A
Meishan−B
Meishan−C
European−A
European−B
European−C

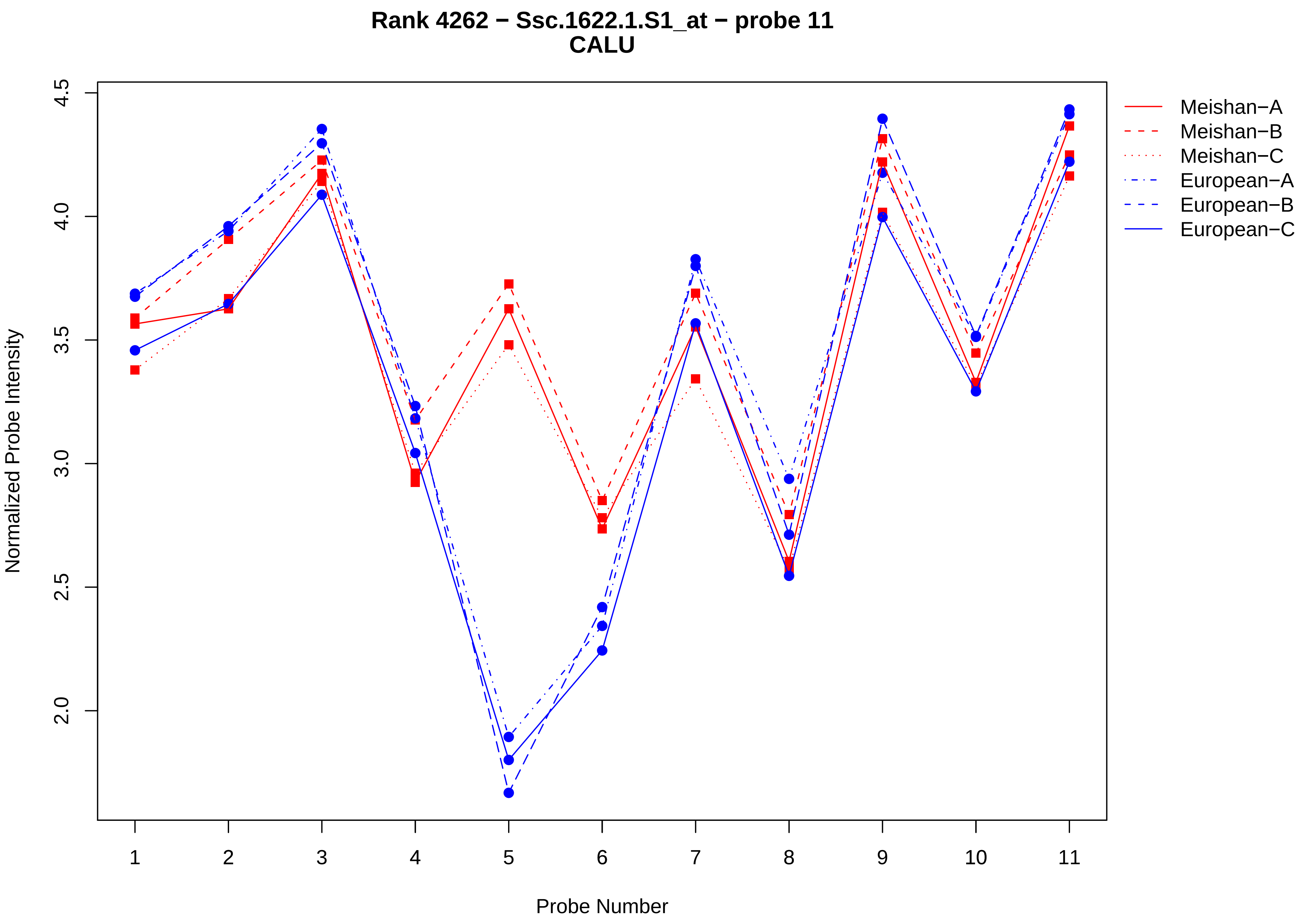

Rank 4262 − Ssc.1622.1.S1_at − probe 11
CALU
Normalized Probe Intensity
Probe Number
Meishan−A
Meishan−B
Meishan−C
European−A
European−B
European−C
4.5
4.0
3.5
3.0
2.5
2.0
1
2
3
4
5
6
7
8
9
10
11

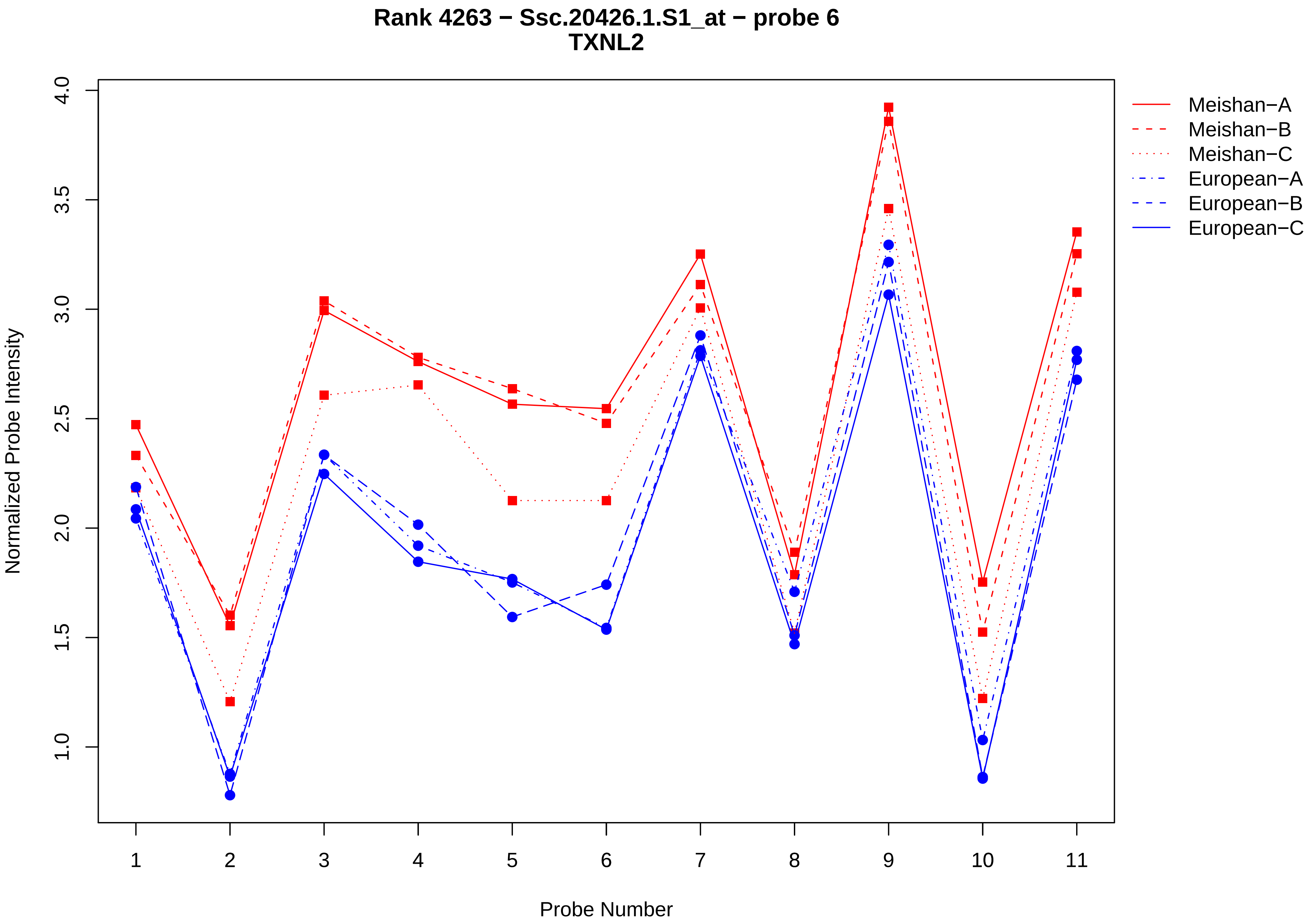

Rank 4263 − Ssc.20426.1.S1_at − probe 6
TXNL2
Normalized Probe Intensity
Probe Number
Meishan−A
Meishan−B
Meishan−C
European−A
European−B
European−C

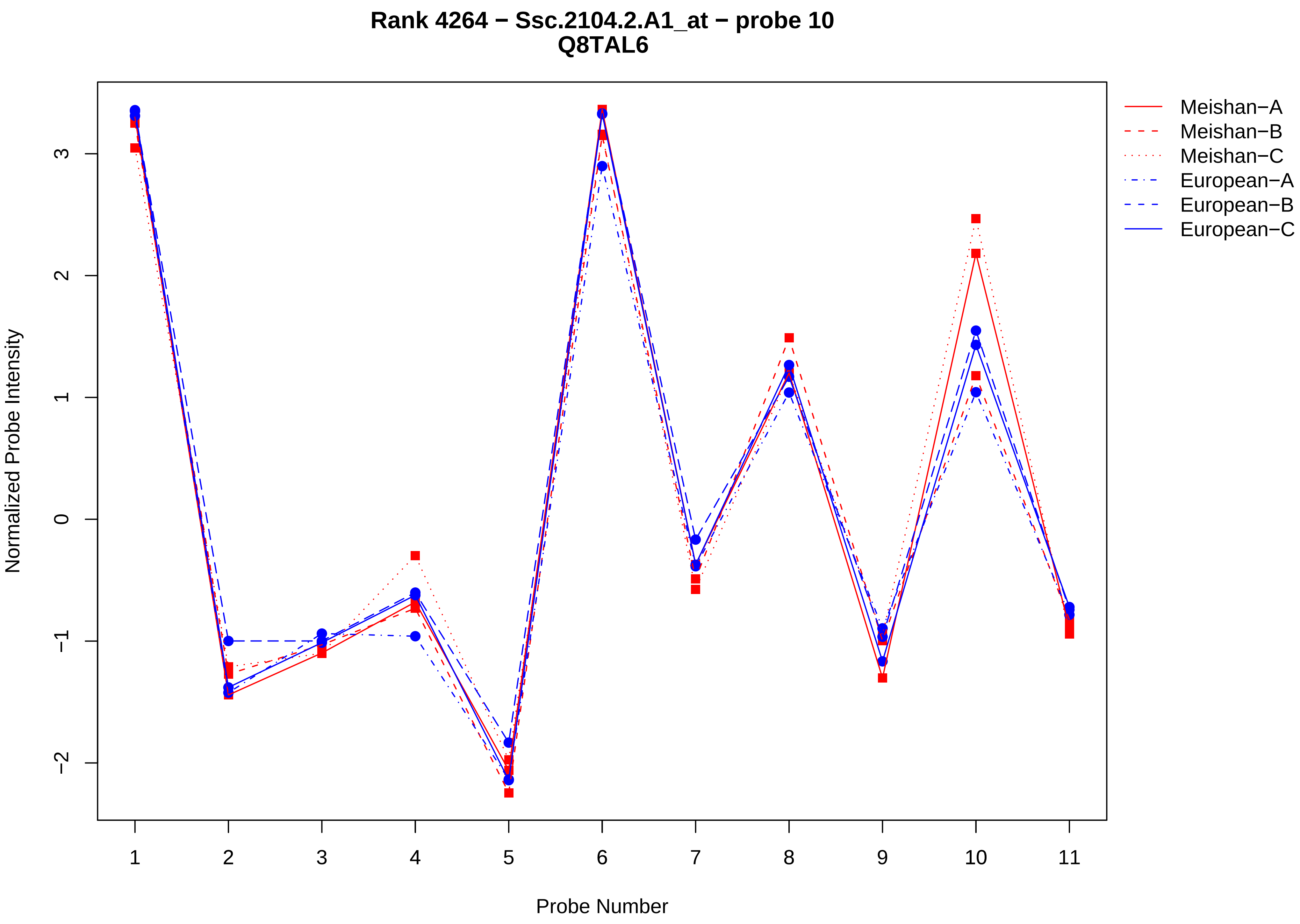

Rank 4264 − Ssc.2104.2.A1_at − probe 10
Q8TAL6
Normalized Probe Intensity
Probe Number
−2
−1
0
1
2
3
1
2
3
4
5
6
7
8
9
10
11
Meishan−A
Meishan−B
Meishan−C
European−A
European−B
European−C

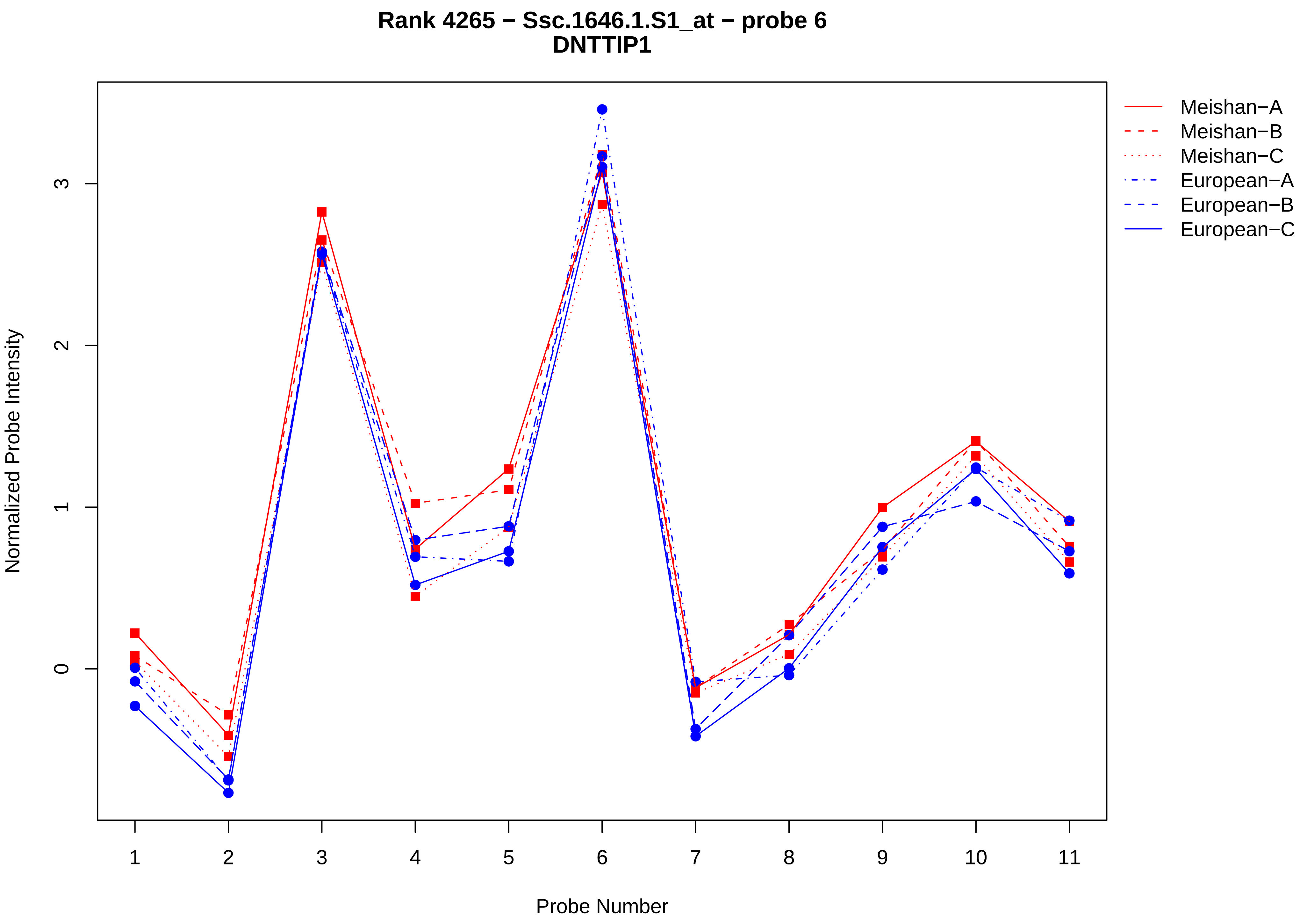

Rank 4265 − Ssc.1646.1.S1_at − probe 6
DNTTIP1
Normalized Probe Intensity
Probe Number
0
1
2
3
1
2
3
4
5
6
7
8
9
10
11
Meishan−A
Meishan−B
Meishan−C
European−A
European−B
European−C

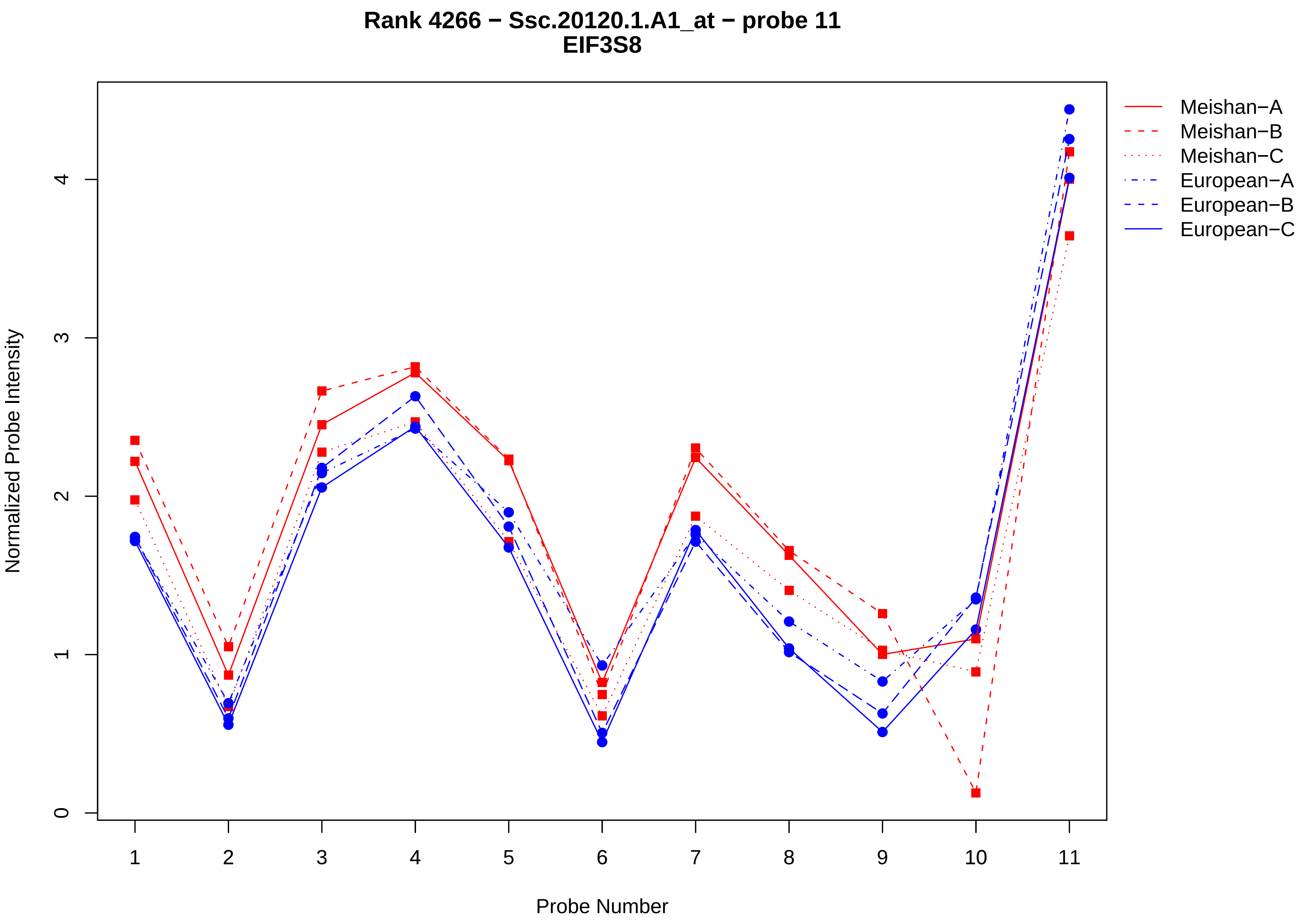
Rank 4266 − Ssc.20120.1.A1_at − probe 11
EIF3S8
Normalized Probe Intensity
0
1
2
3
4
1
2
3
4
5
6
7
8
9
10
11
Probe Number
Meishan−A
Meishan−B
Meishan−C
European−A
European−B
European−C

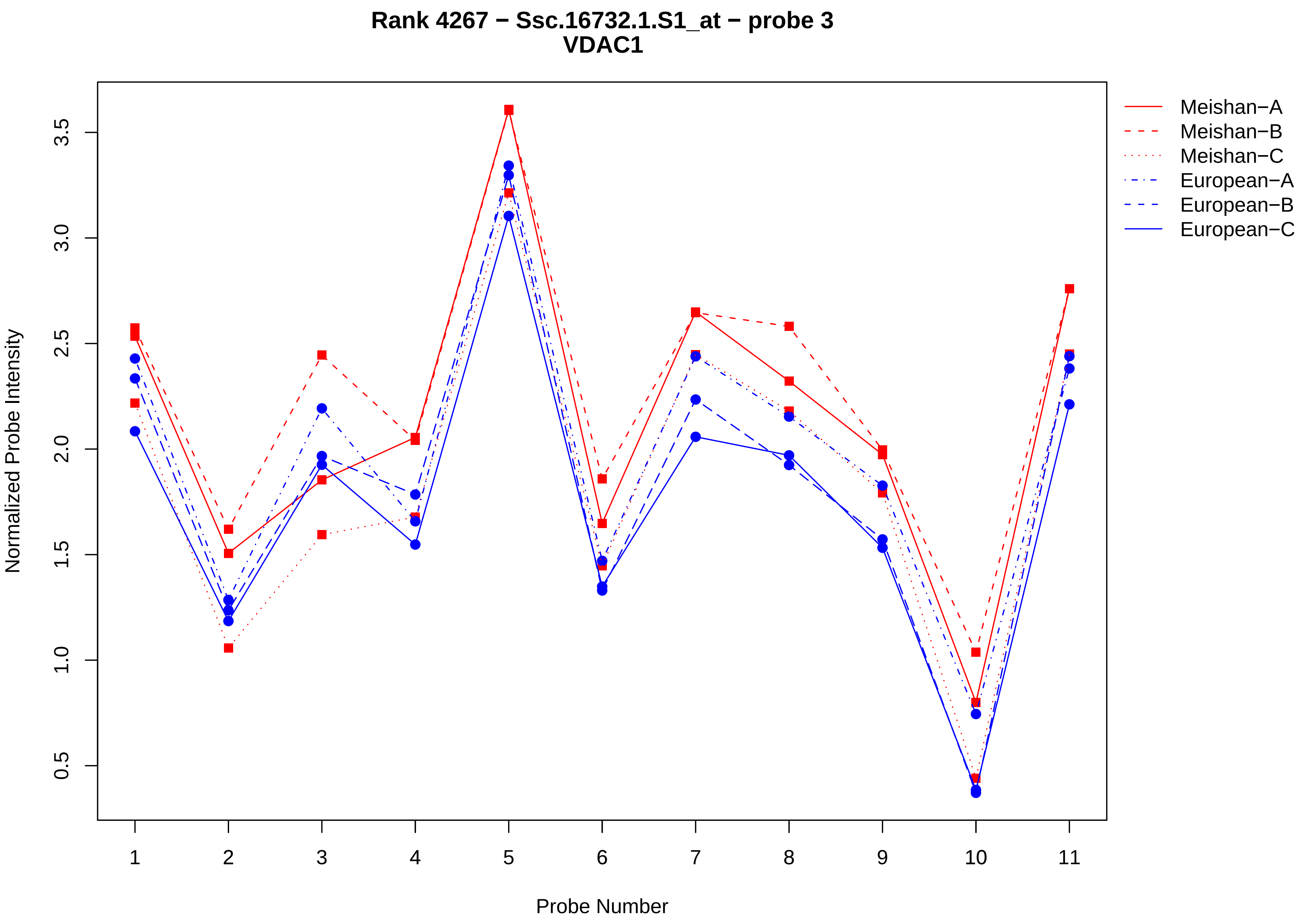

Rank 4267 − Ssc.16732.1.S1_at − probe 3
VDAC1
Meishan−A
Meishan−B
Meishan−C
European−A
European−B
European−C
Normalized Probe Intensity
0.5
1.0
1.5
2.0
2.5
3.0
3.5
1
2
3
4
5
6
7
8
9
10
11
Probe Number

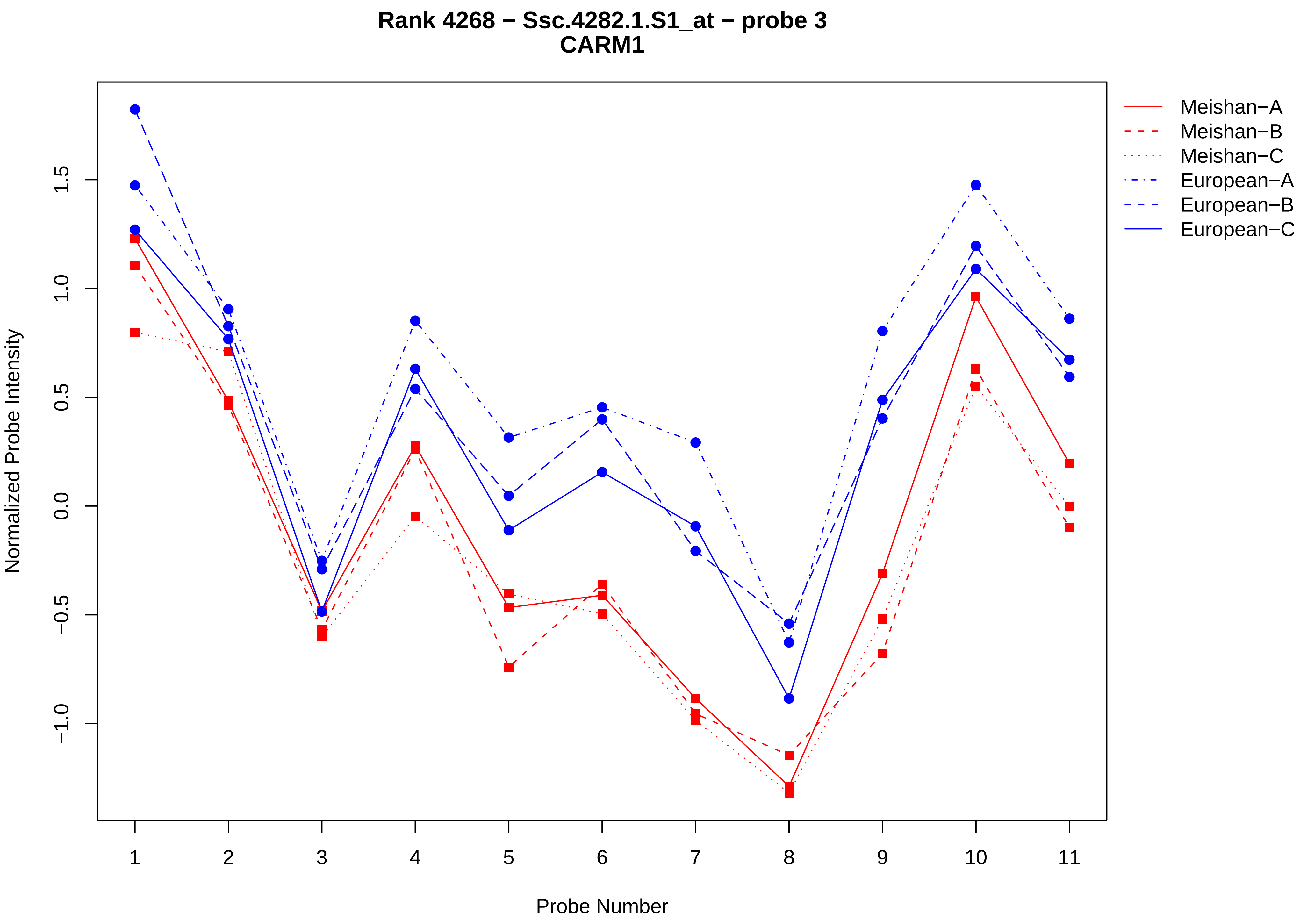

Rank 4268 − Ssc.4282.1.S1_at − probe 3
CARM1
Normalized Probe Intensity
Probe Number
−1.0
−0.5
0.0
0.5
1.0
1.5
1
2
3
4
5
6
7
8
9
10
11
Meishan−A
Meishan−B
Meishan−C
European−A
European−B
European−C

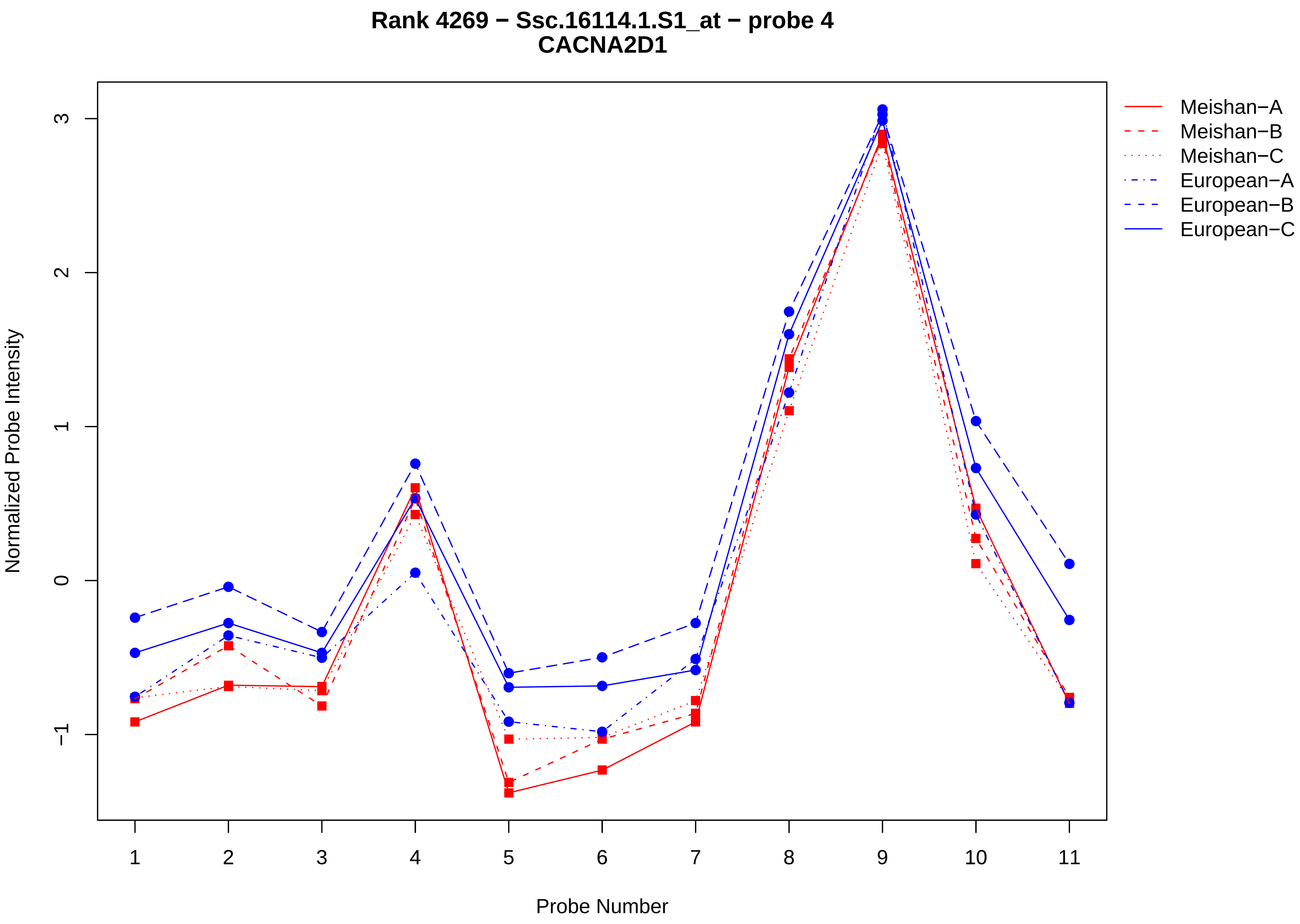

Rank 4269 − Ssc.16114.1.S1_at − probe 4
CACNA2D1
Normalized Probe Intensity
Probe Number
−1
0
1
2
3
1
2
3
4
5
6
7
8
9
10
11
Meishan−A
Meishan−B
Meishan−C
European−A
European−B
European−C

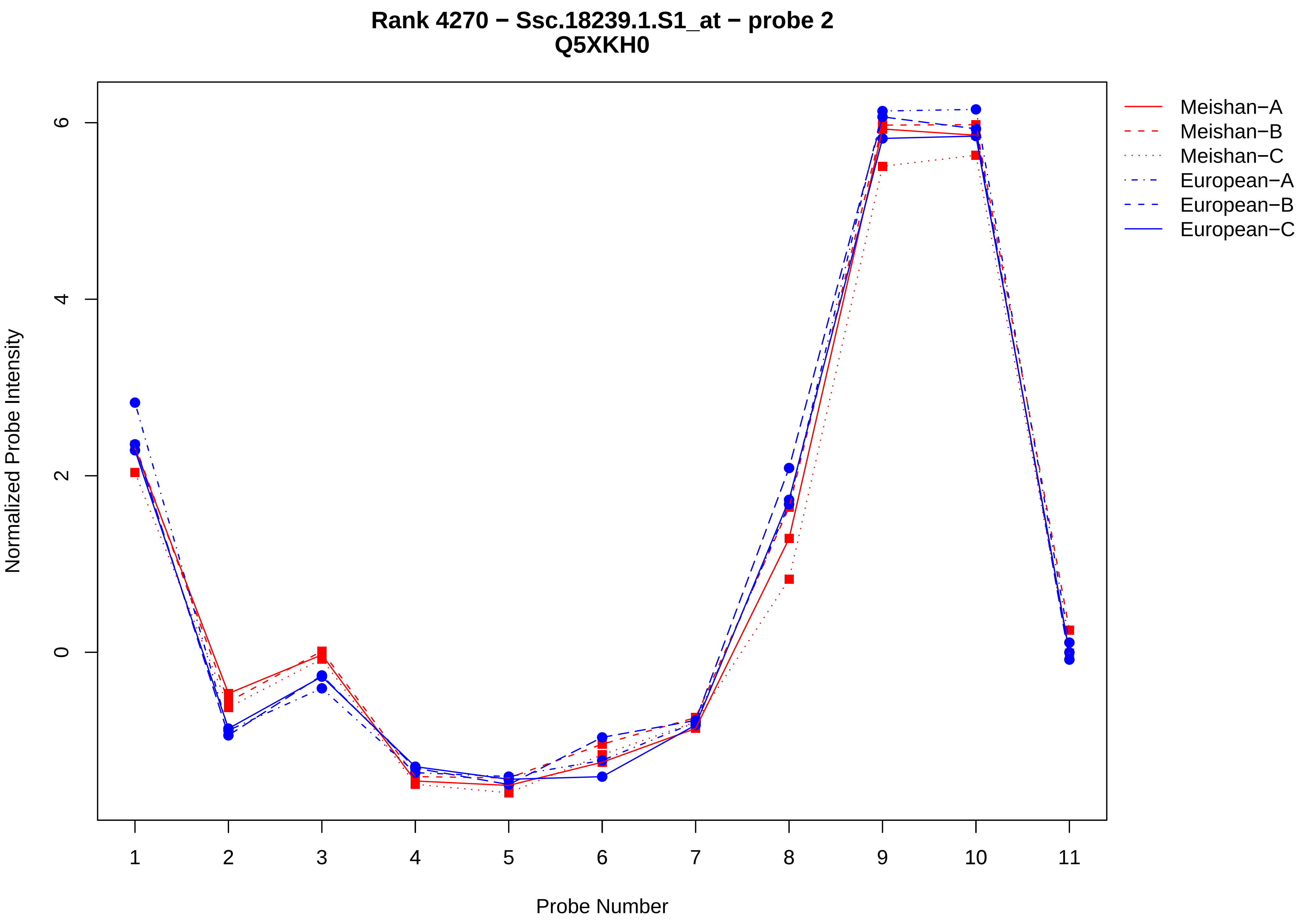

Rank 4270 − Ssc.18239.1.S1_at − probe 2
Q5XKH0
Normalized Probe Intensity
Probe Number
0
2
4
6
1
2
3
4
5
6
7
8
9
10
11
Meishan−A
Meishan−B
Meishan−C
European−A
European−B
European−C

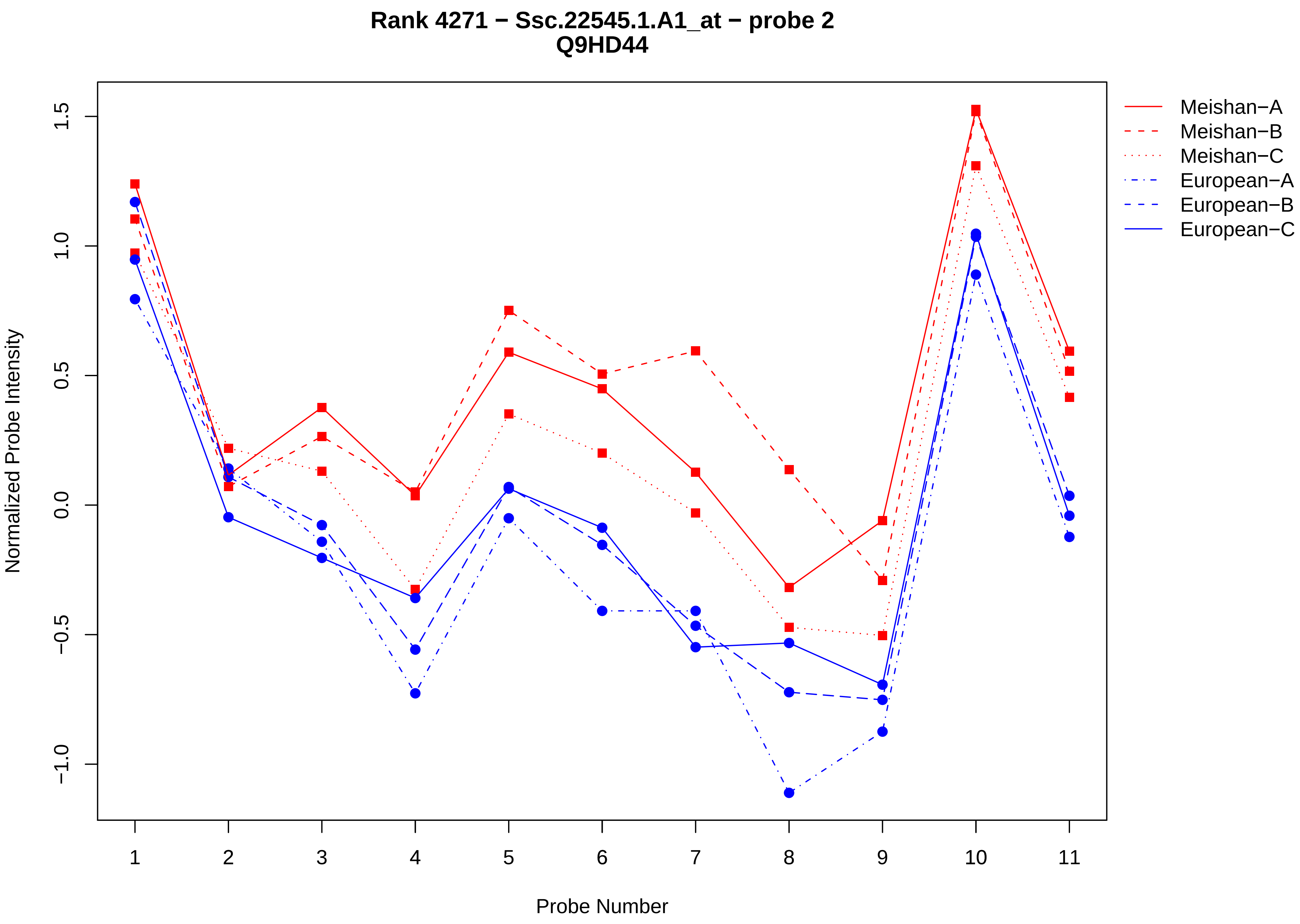

Rank 4271 − Ssc.22545.1.A1_at − probe 2
Q9HD44
Normalized Probe Intensity
Probe Number
Meishan−A
Meishan−B
Meishan−C
European−A
European−B
European−C
1.5
1.0
0.5
0.0
−0.5
−1.0
1
2
3
4
5
6
7
8
9
10
11

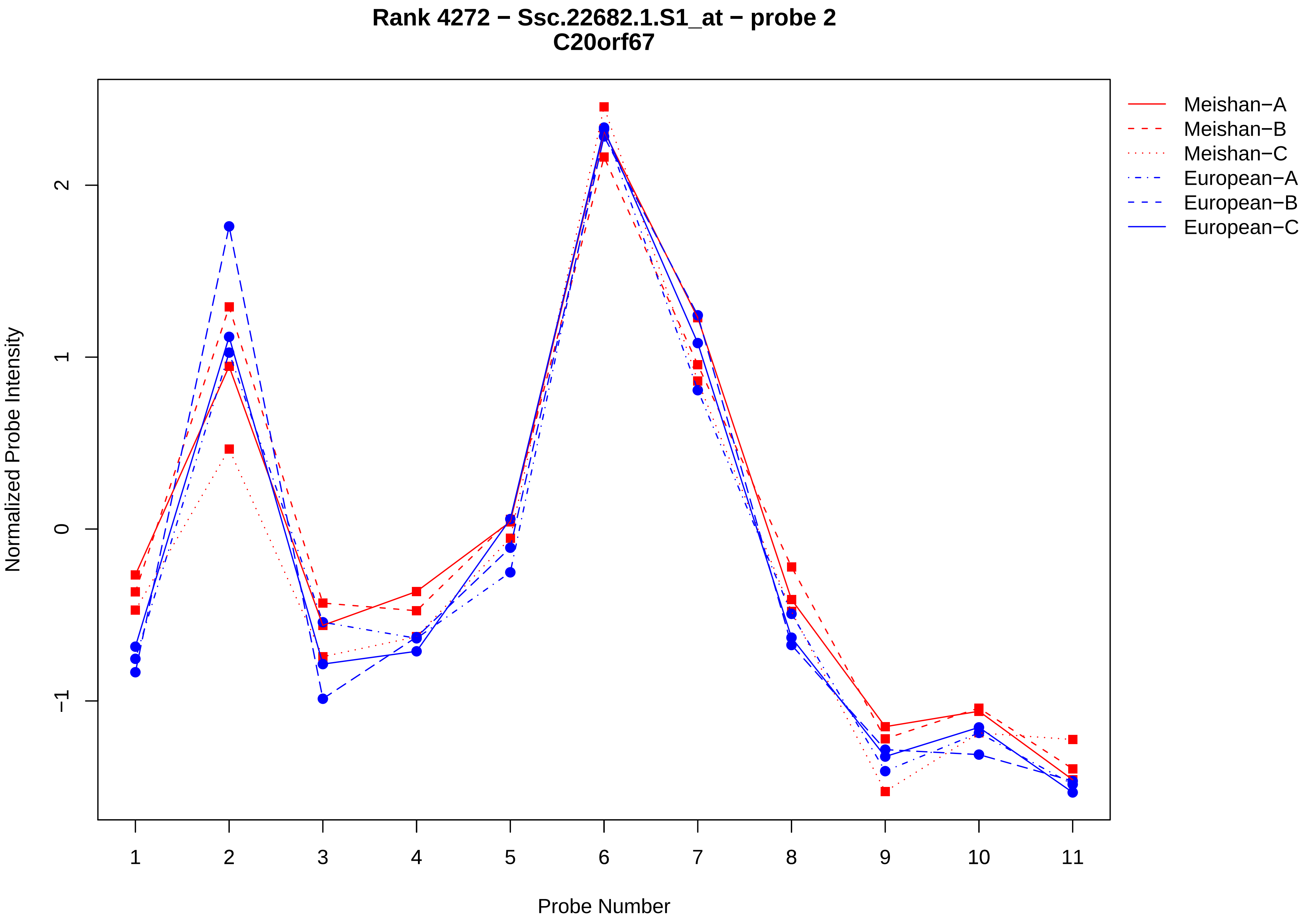

Rank 4272 − Ssc.22682.1.S1_at − probe 2
C20orf67
Normalized Probe Intensity
Probe Number
Meishan−A
Meishan−B
Meishan−C
European−A
European−B
European−C
−1
0
1
2
1
2
3
4
5
6
7
8
9
10
11

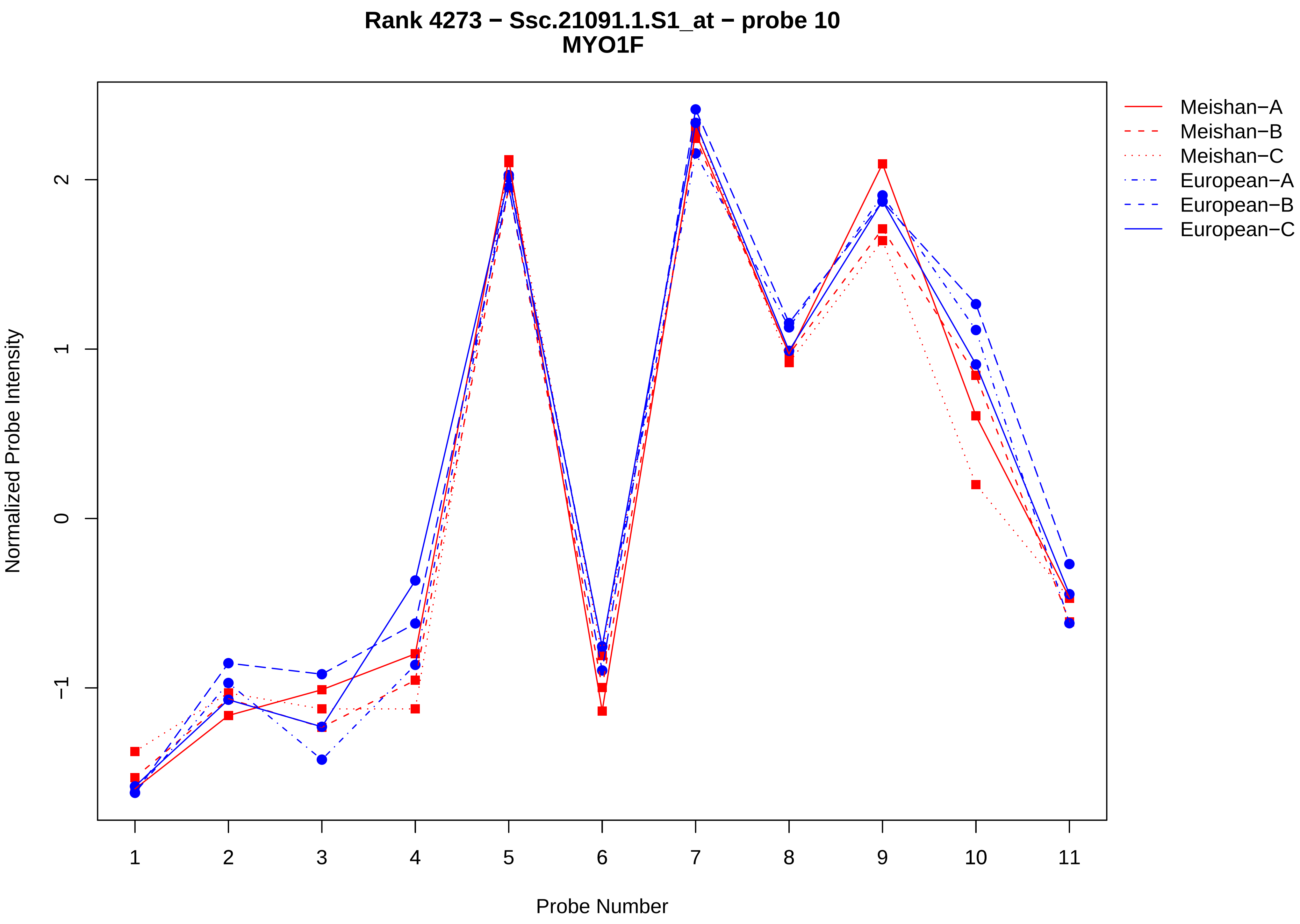

Rank 4273 − Ssc.21091.1.S1_at − probe 10
MYO1F
Normalized Probe Intensity
Probe Number
−1
0
1
2
1
2
3
4
5
6
7
8
9
10
11
Meishan−A
Meishan−B
Meishan−C
European−A
European−B
European−C

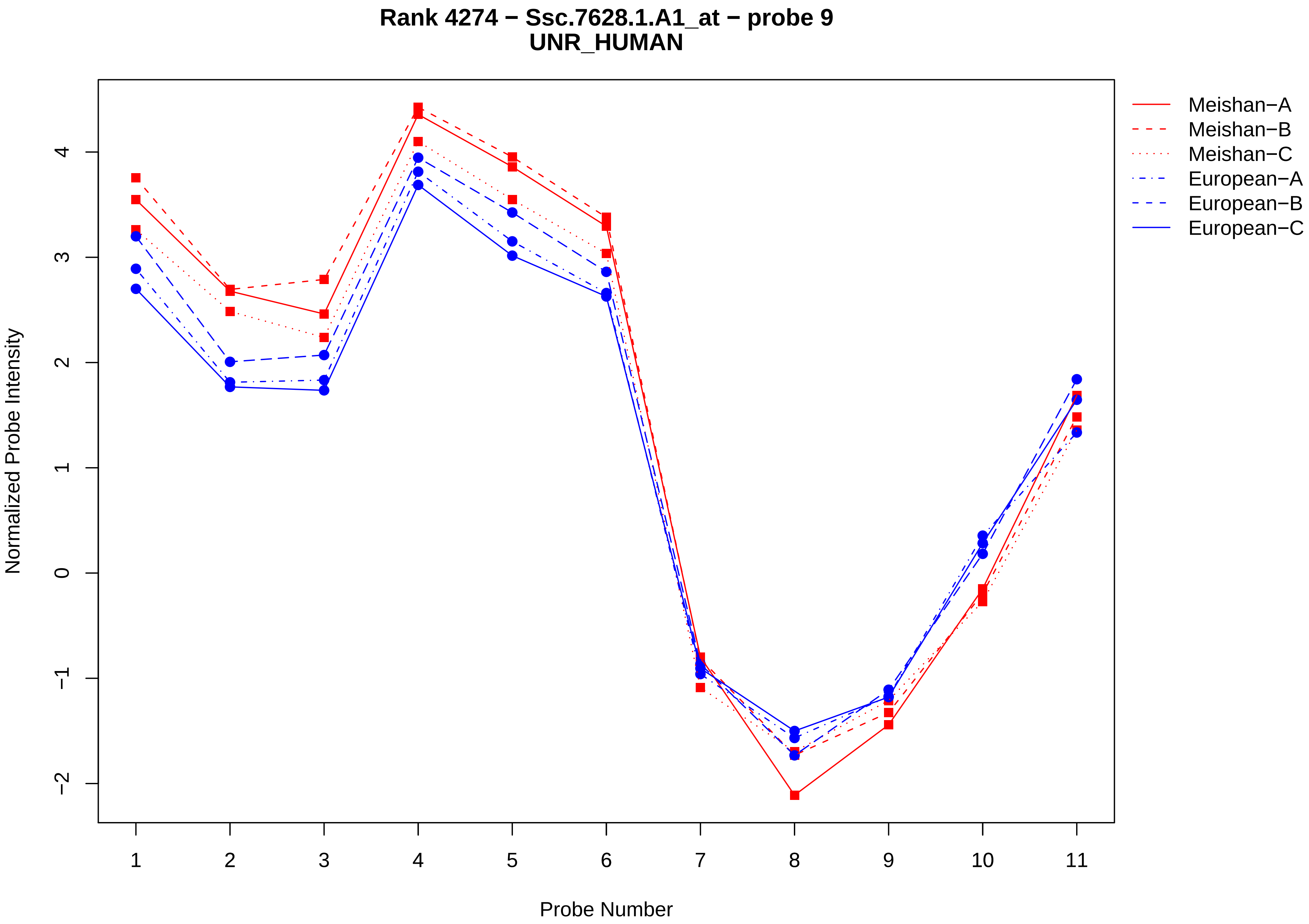
Rank 4274 − Ssc.7628.1.A1_at − probe 9
UNR_HUMAN
Normalized Probe Intensity
Probe Number
−2
−1
0
1
2
3
4
1
2
3
4
5
6
7
8
9
10
11
Meishan−A
Meishan−B
Meishan−C
European−A
European−B
European−C

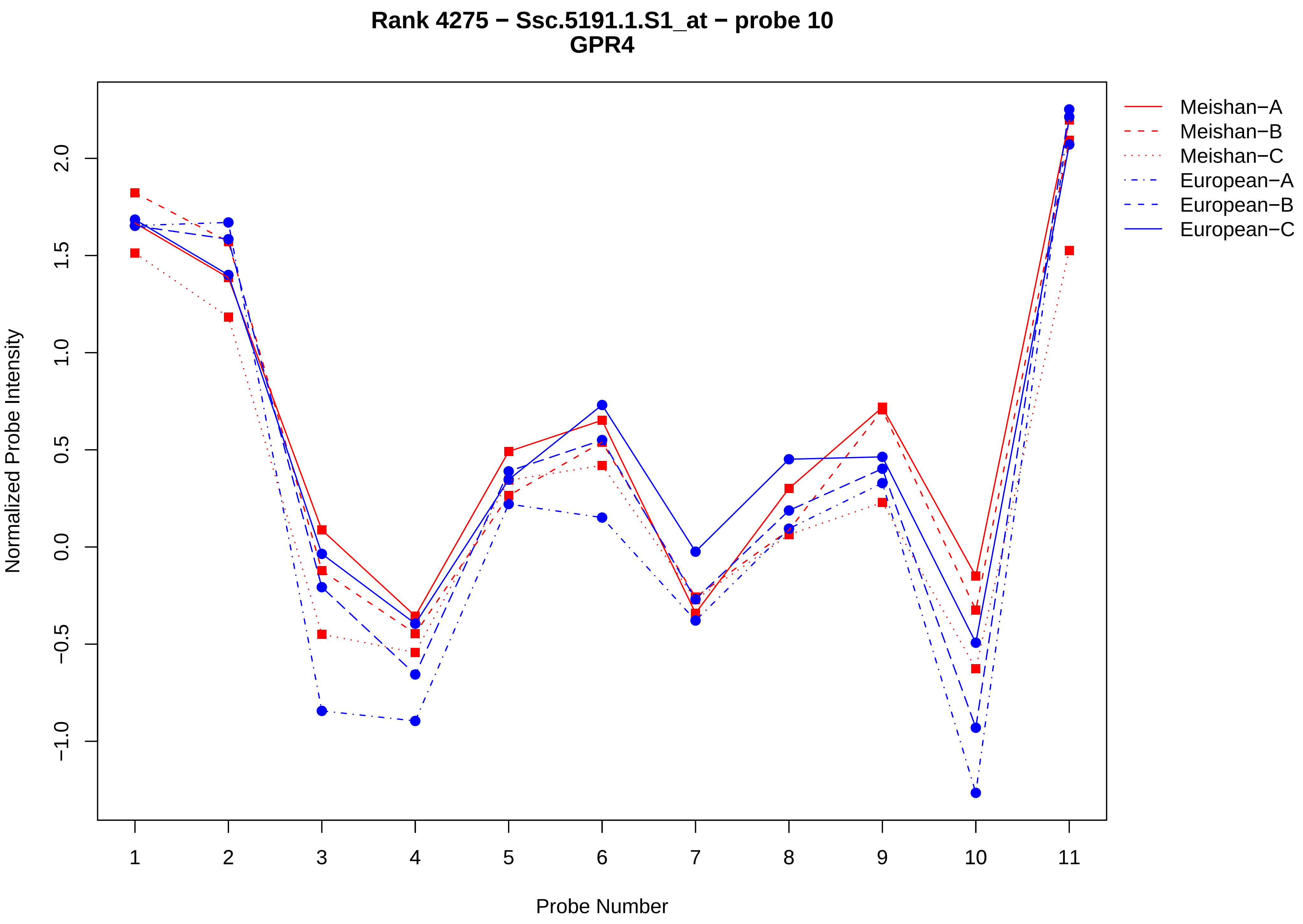

Rank 4275 − Ssc.5191.1.S1_at − probe 10
GPR4
Normalized Probe Intensity
Probe Number
Meishan−A
Meishan−B
Meishan−C
European−A
European−B
European−C

Rank 4276 − Ssc.21889.1.S1_at − probe 3
Q9NP48

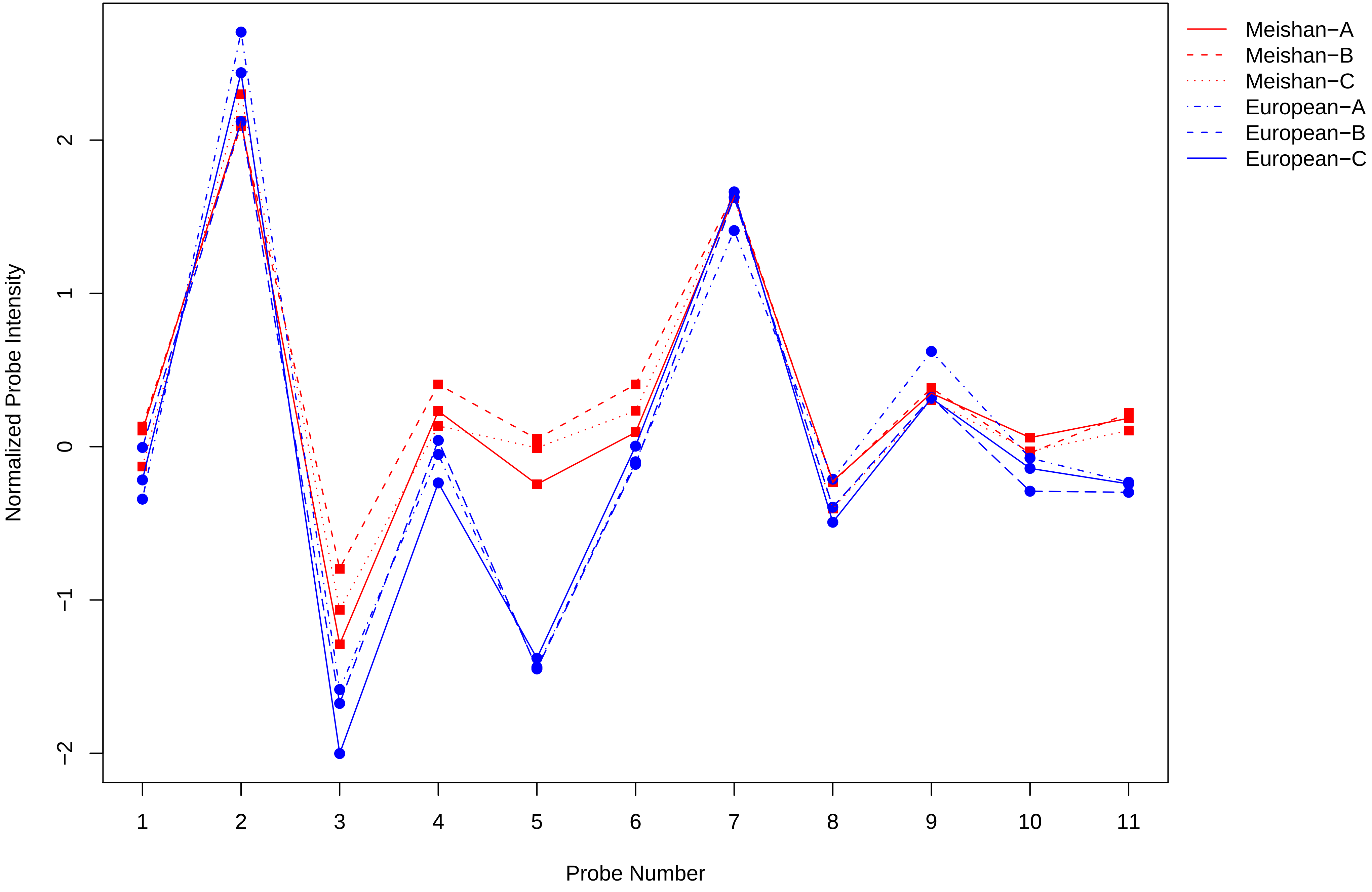

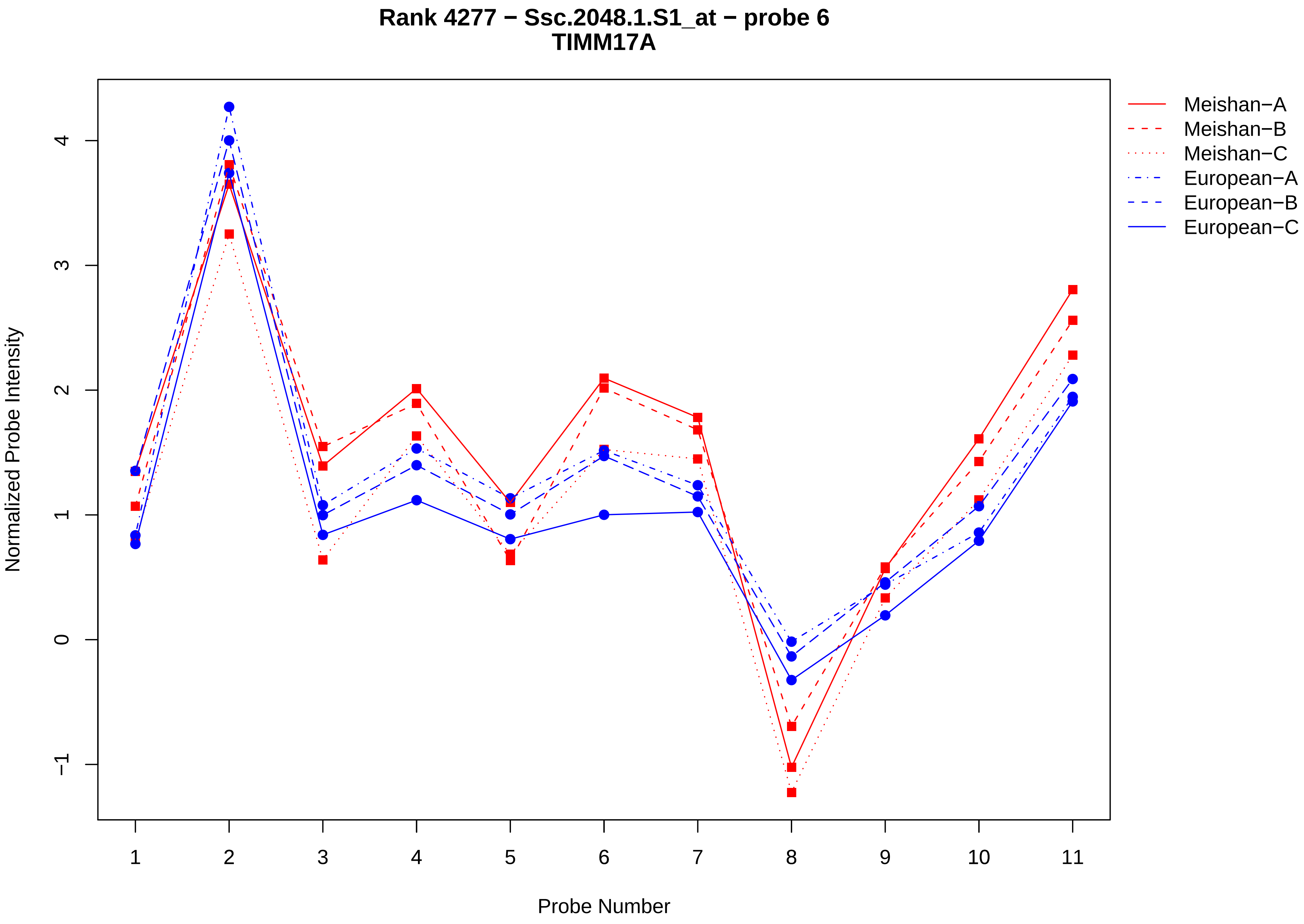

Rank 4277 − Ssc.2048.1.S1_at − probe 6
TIMM17A
Normalized Probe Intensity
Probe Number
Meishan−A
Meishan−B
Meishan−C
European−A
European−B
European−C
−1
0
1
2
3
4
1
2
3
4
5
6
7
8
9
10
11

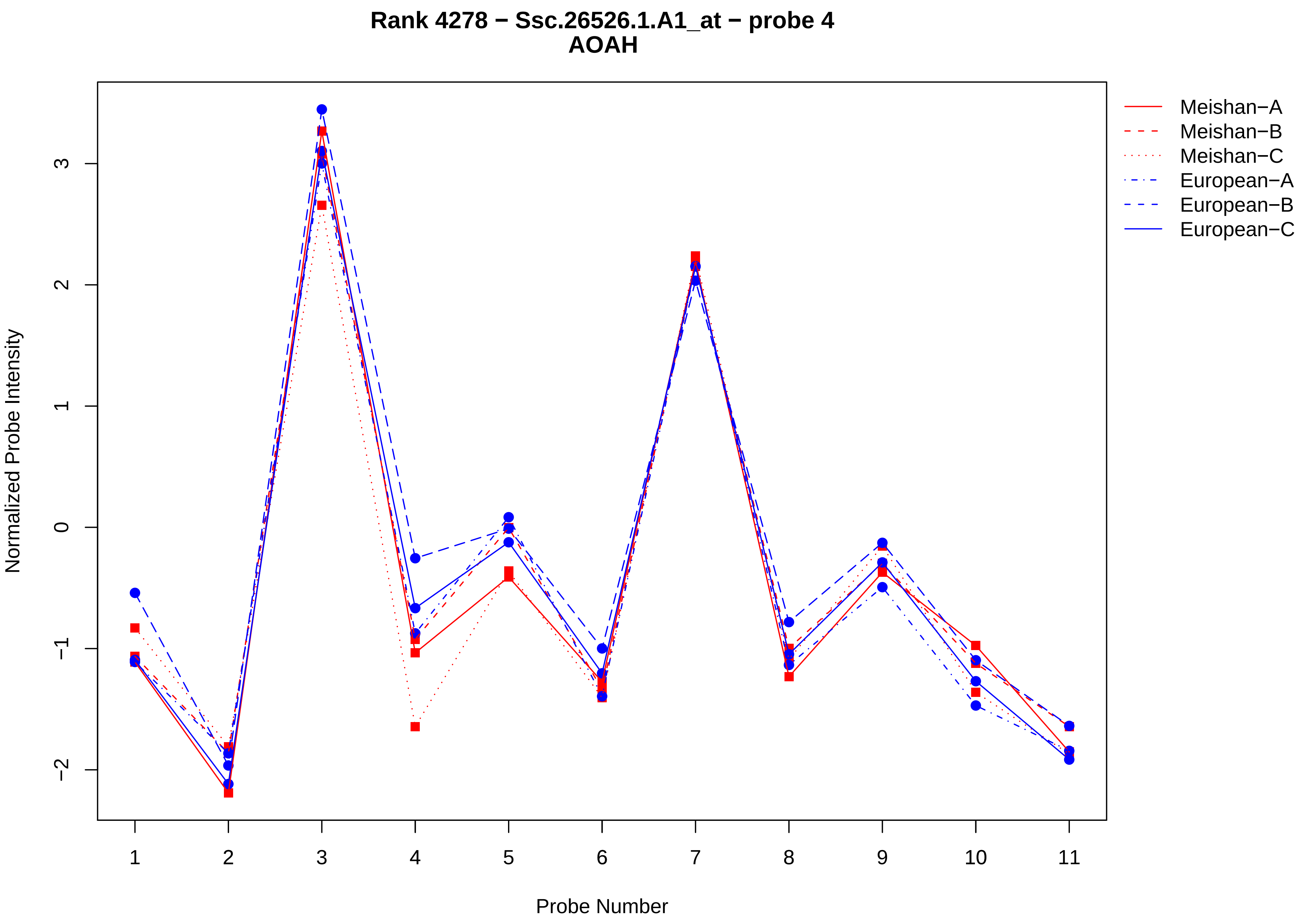

Rank 4278 − Ssc.26526.1.A1_at − probe 4
AOAH
Normalized Probe Intensity
Probe Number
−2
−1
0
1
2
3
1
2
3
4
5
6
7
8
9
10
11
Meishan−A
Meishan−B
Meishan−C
European−A
European−B
European−C

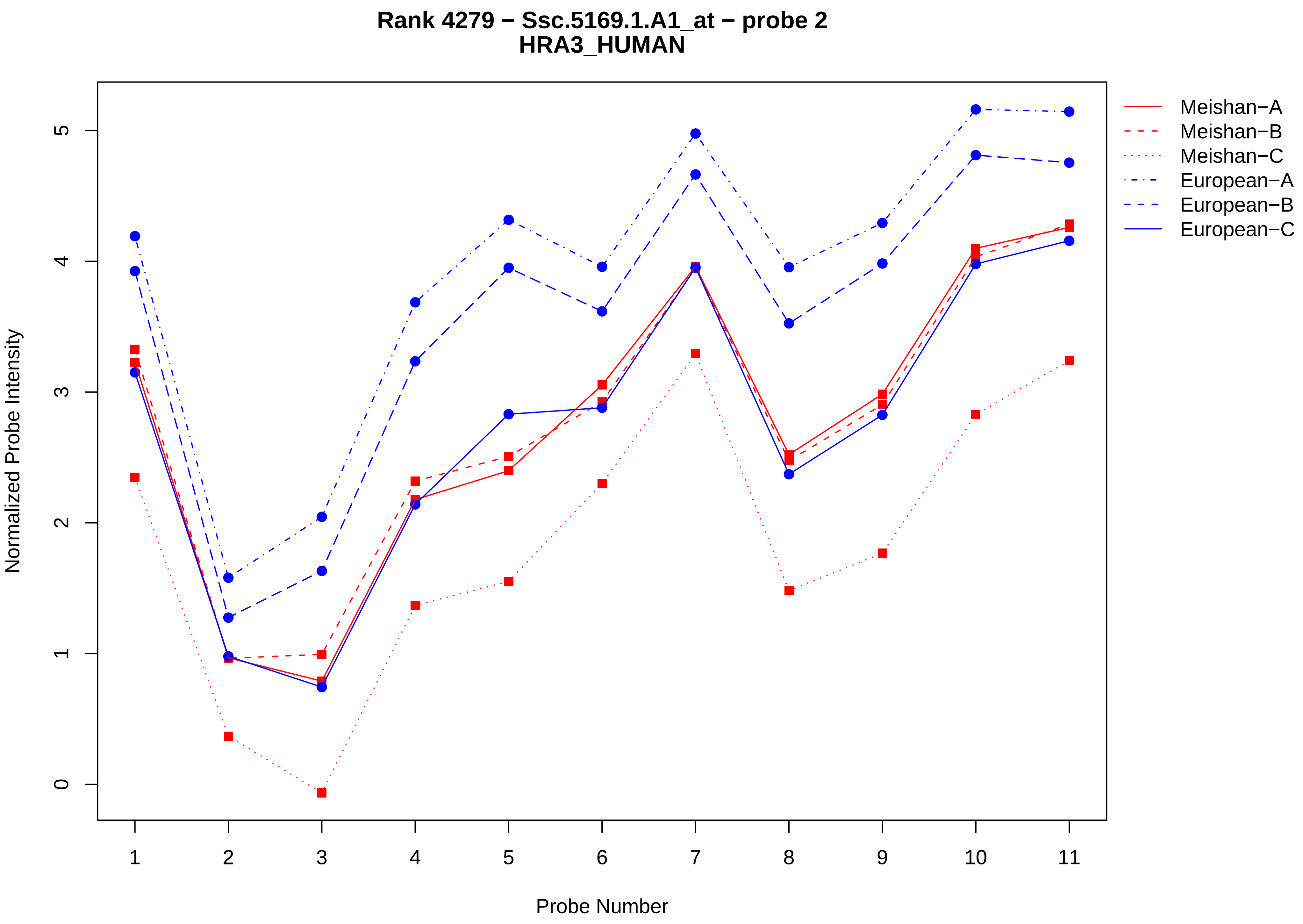
Rank 4279 − Ssc.5169.1.A1_at − probe 2
HRA3_HUMAN
Normalized Probe Intensity
Probe Number
0
1
2
3
4
5
1
2
3
4
5
6
7
8
9
10
11
Meishan−A
Meishan−B
Meishan−C
European−A
European−B
European−C

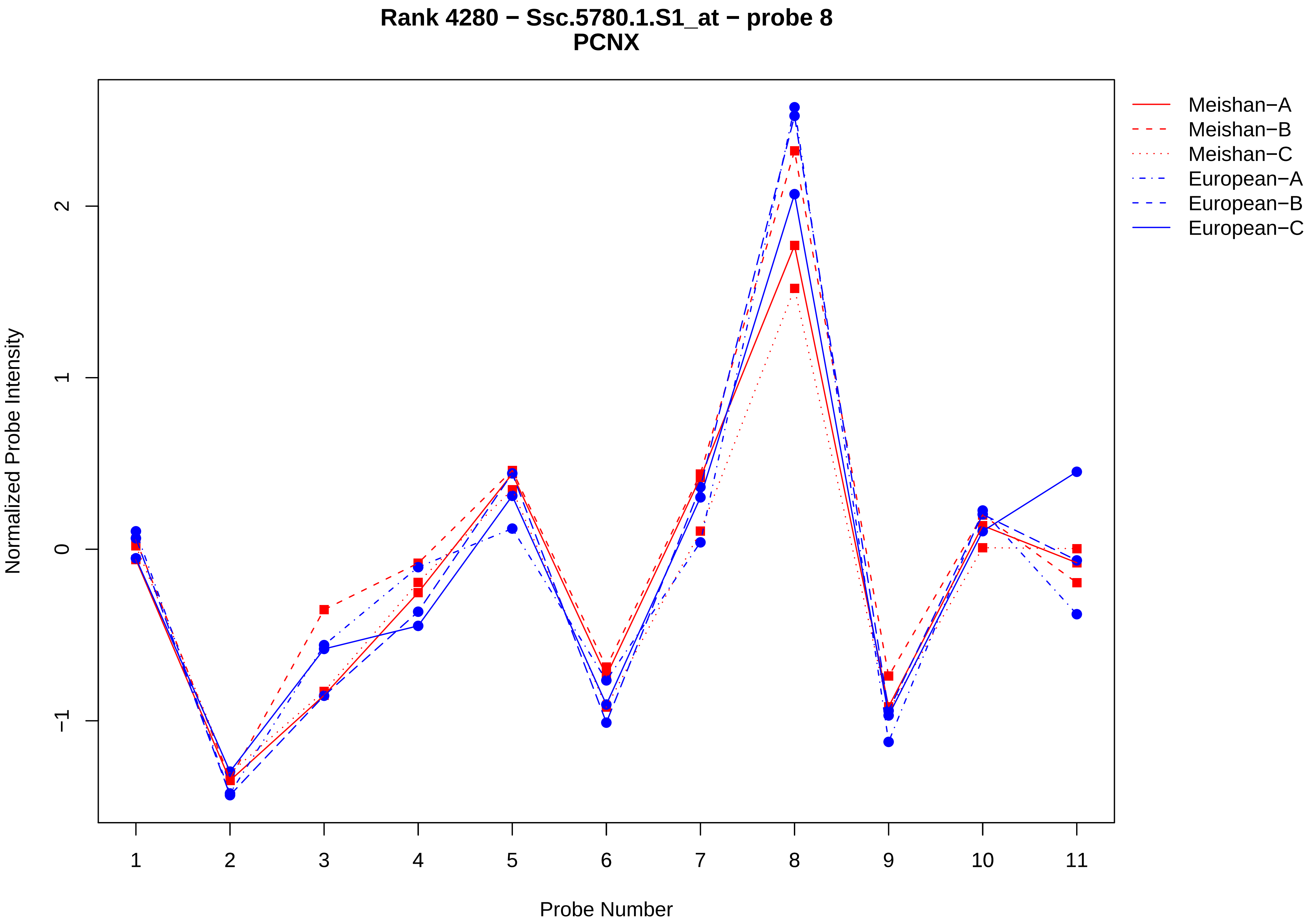

Rank 4280 − Ssc.5780.1.S1_at − probe 8
PCNX
Normalized Probe Intensity
Probe Number
−1
0
1
2
1
2
3
4
5
6
7
8
9
10
11
Meishan−A
Meishan−B
Meishan−C
European−A
European−B
European−C

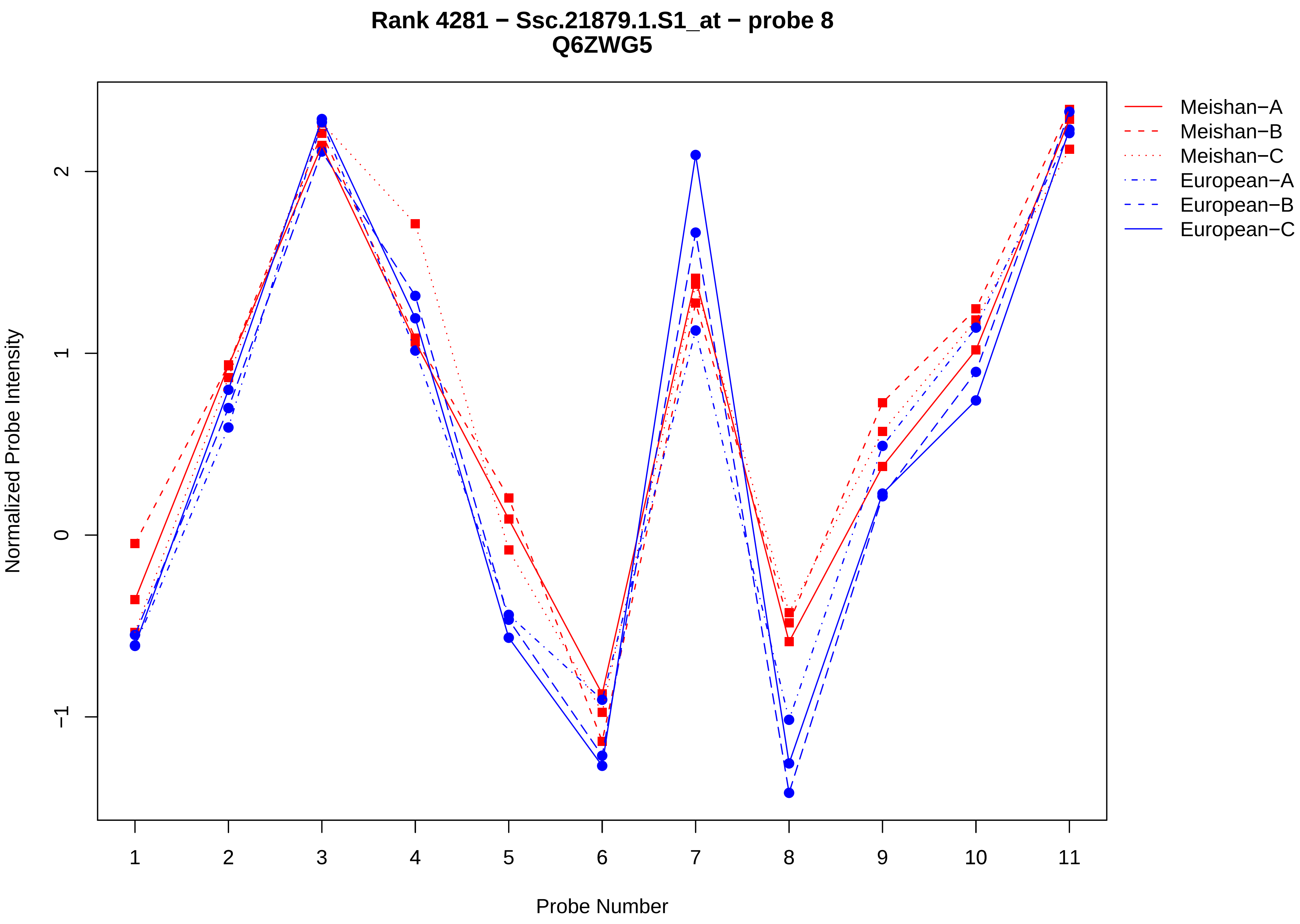

Rank 4281 − Ssc.21879.1.S1_at − probe 8
Q6ZWG5
Normalized Probe Intensity
−1
0
1
2
1
2
3
4
5
6
7
8
9
10
11
Probe Number
Meishan−A
Meishan−B
Meishan−C
European−A
European−B
European−C

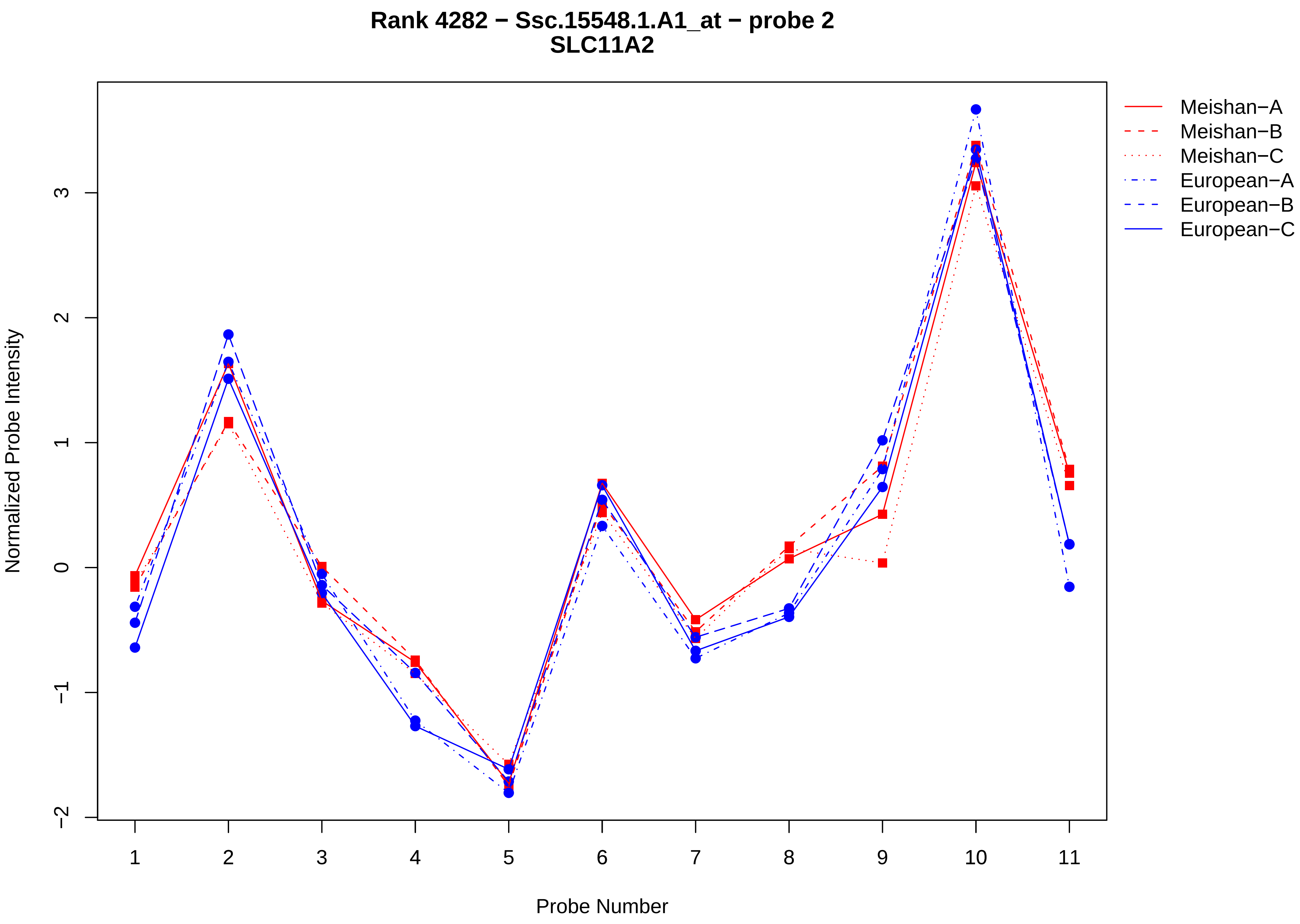

Rank 4282 − Ssc.15548.1.A1_at − probe 2
SLC11A2
Normalized Probe Intensity
Probe Number
−2
−1
0
1
2
3
1
2
3
4
5
6
7
8
9
10
11
Meishan−A
Meishan−B
Meishan−C
European−A
European−B
European−C

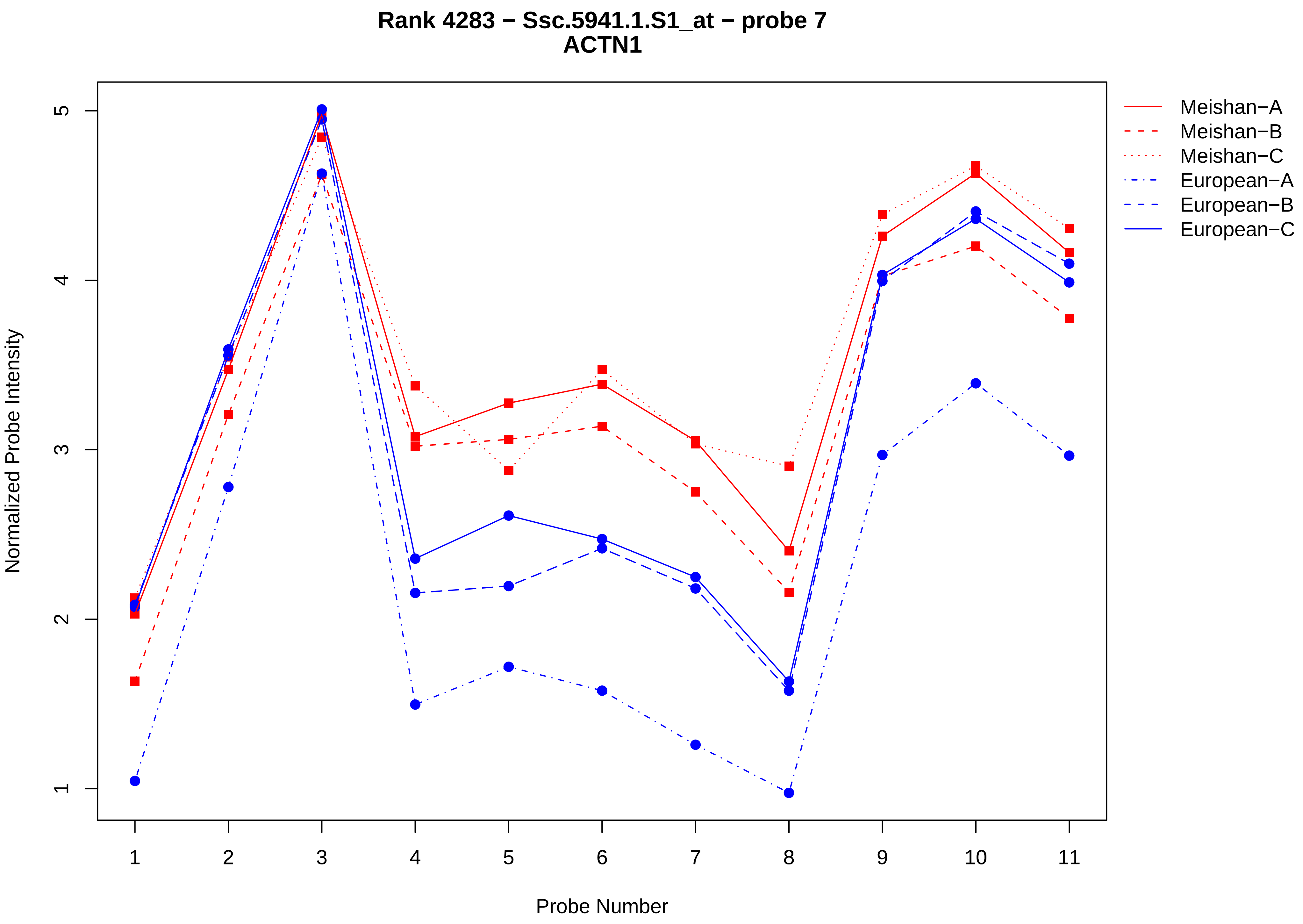
Rank 4283 − Ssc.5941.1.S1_at − probe 7
ACTN1
Normalized Probe Intensity
Probe Number
1
2
3
4
5
1
2
3
4
5
6
7
8
9
10
11
Meishan−A
Meishan−B
Meishan−C
European−A
European−B
European−C

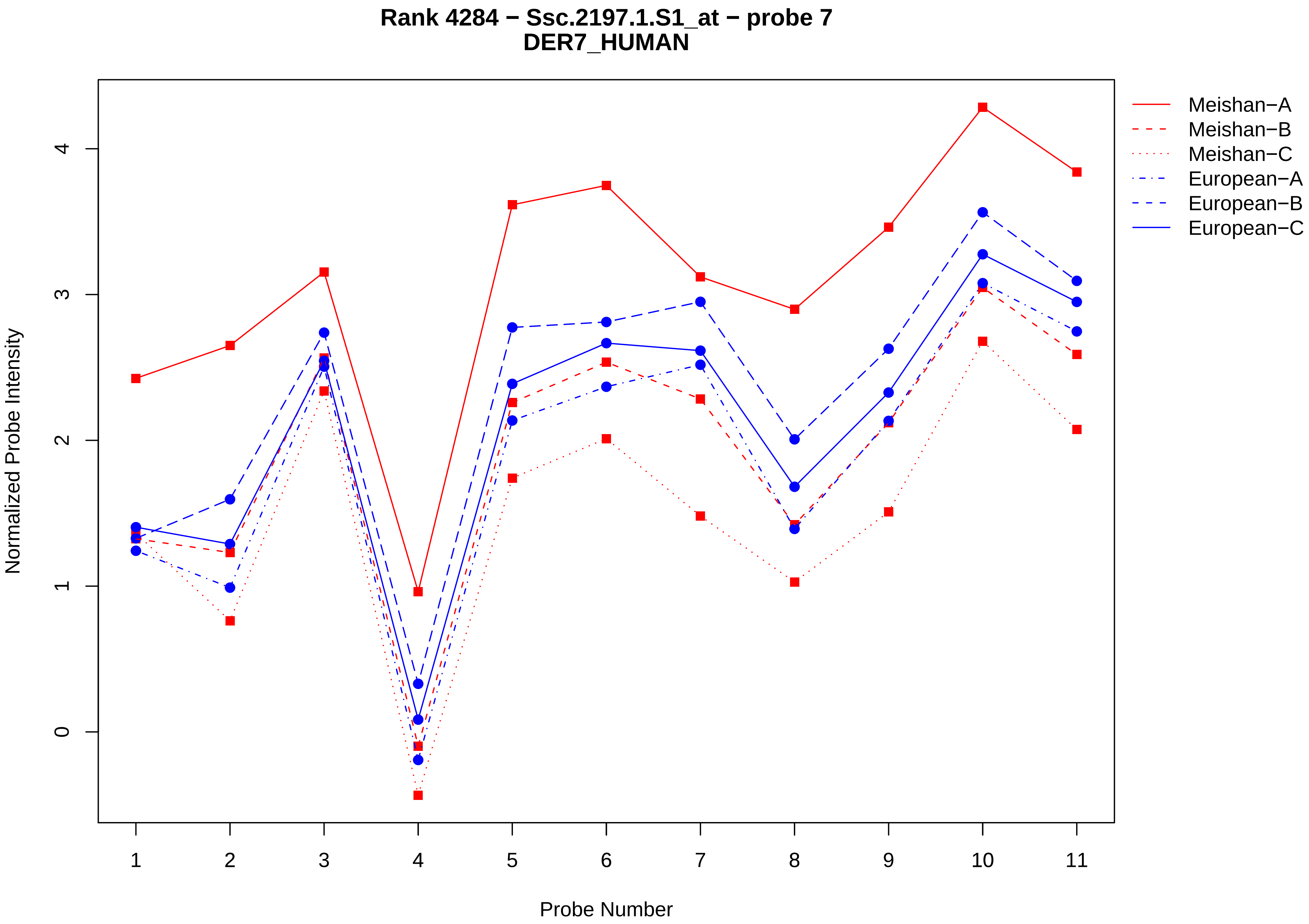

Rank 4284 − Ssc.2197.1.S1_at − probe 7
DER7_HUMAN
Normalized Probe Intensity
0
1
2
3
4
1
2
3
4
5
6
7
8
9
10
11
Probe Number
Meishan−A
Meishan−B
Meishan−C
European−A
European−B
European−C

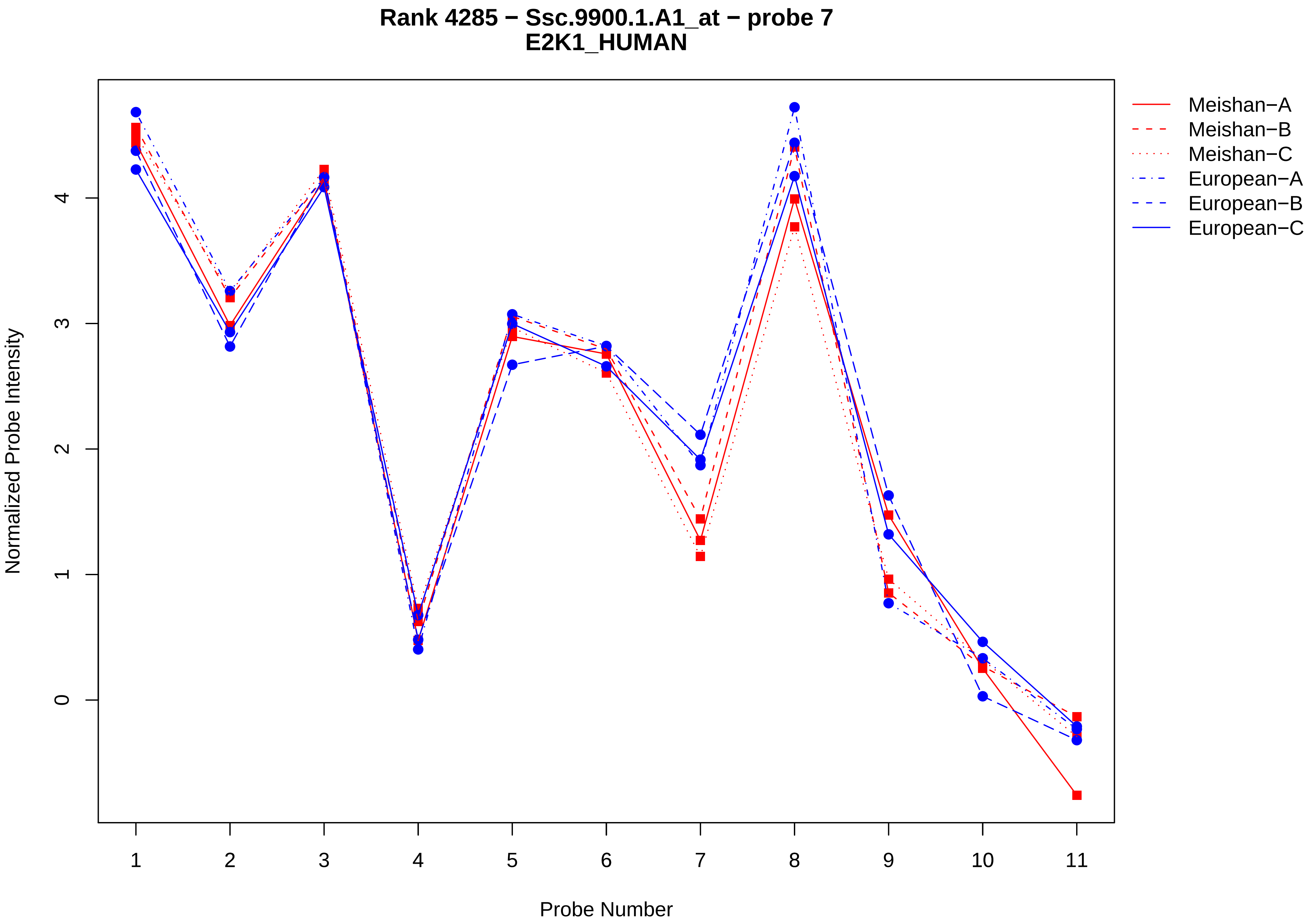

Rank 4285 − Ssc.9900.1.A1_at − probe 7
E2K1_HUMAN
Normalized Probe Intensity
Probe Number
0
1
2
3
4
1
2
3
4
5
6
7
8
9
10
11
Meishan−A
Meishan−B
Meishan−C
European−A
European−B
European−C

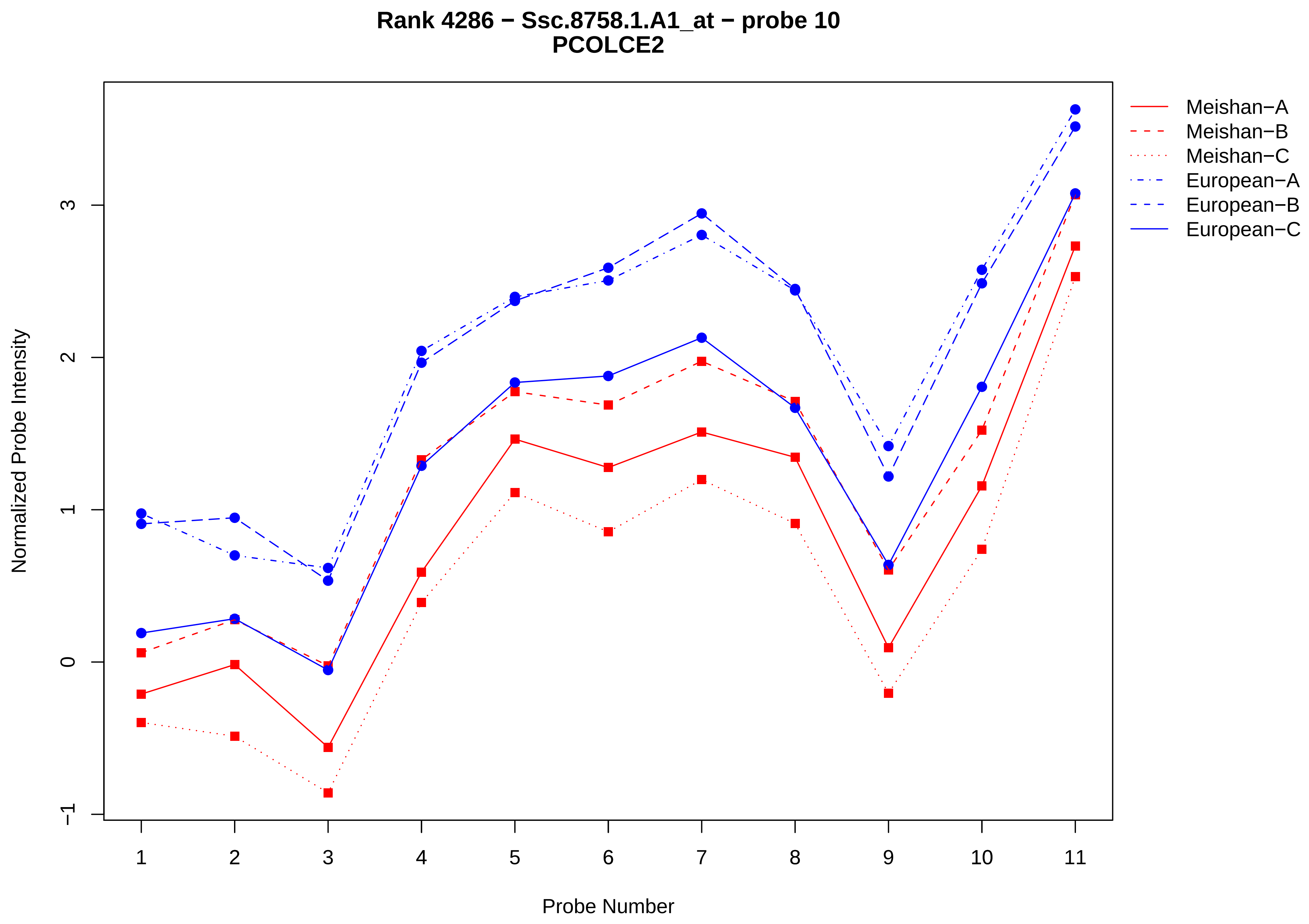

Rank 4286 − Ssc.8758.1.A1_at − probe 10
PCOLCE2
Normalized Probe Intensity
Probe Number
−1
0
1
2
3
1
2
3
4
5
6
7
8
9
10
11
Meishan−A
Meishan−B
Meishan−C
European−A
European−B
European−C

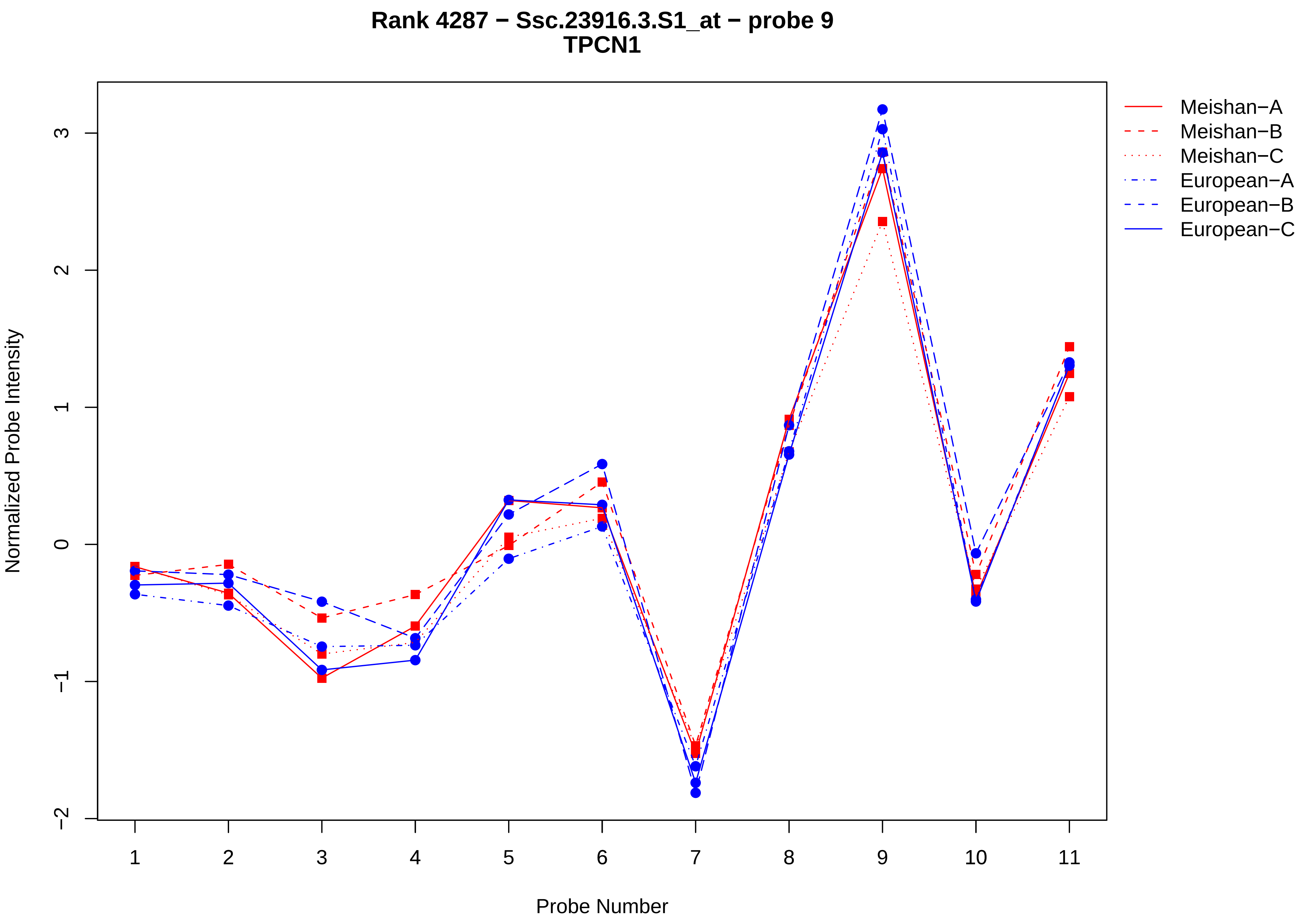

Rank 4287 − Ssc.23916.3.S1_at − probe 9
TPCN1
Normalized Probe Intensity
−2
−1
0
1
2
3
1
2
3
4
5
6
7
8
9
10
11
Probe Number
Meishan−A
Meishan−B
Meishan−C
European−A
European−B
European−C

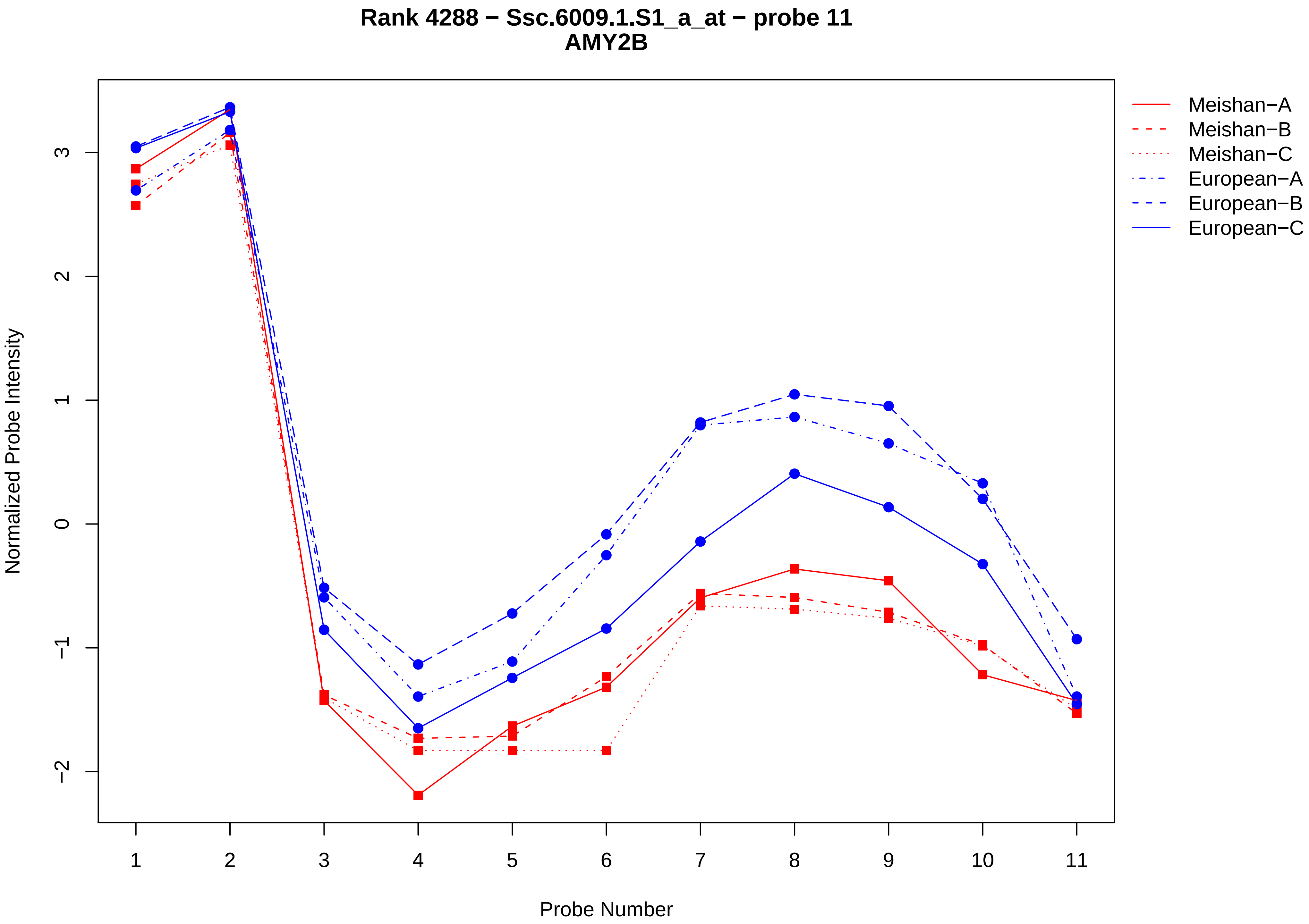

Rank 4288 − Ssc.6009.1.S1_a_at − probe 11
AMY2B
Normalized Probe Intensity
Probe Number
−2
−1
0
1
2
3
1
2
3
4
5
6
7
8
9
10
11
Meishan−A
Meishan−B
Meishan−C
European−A
European−B
European−C

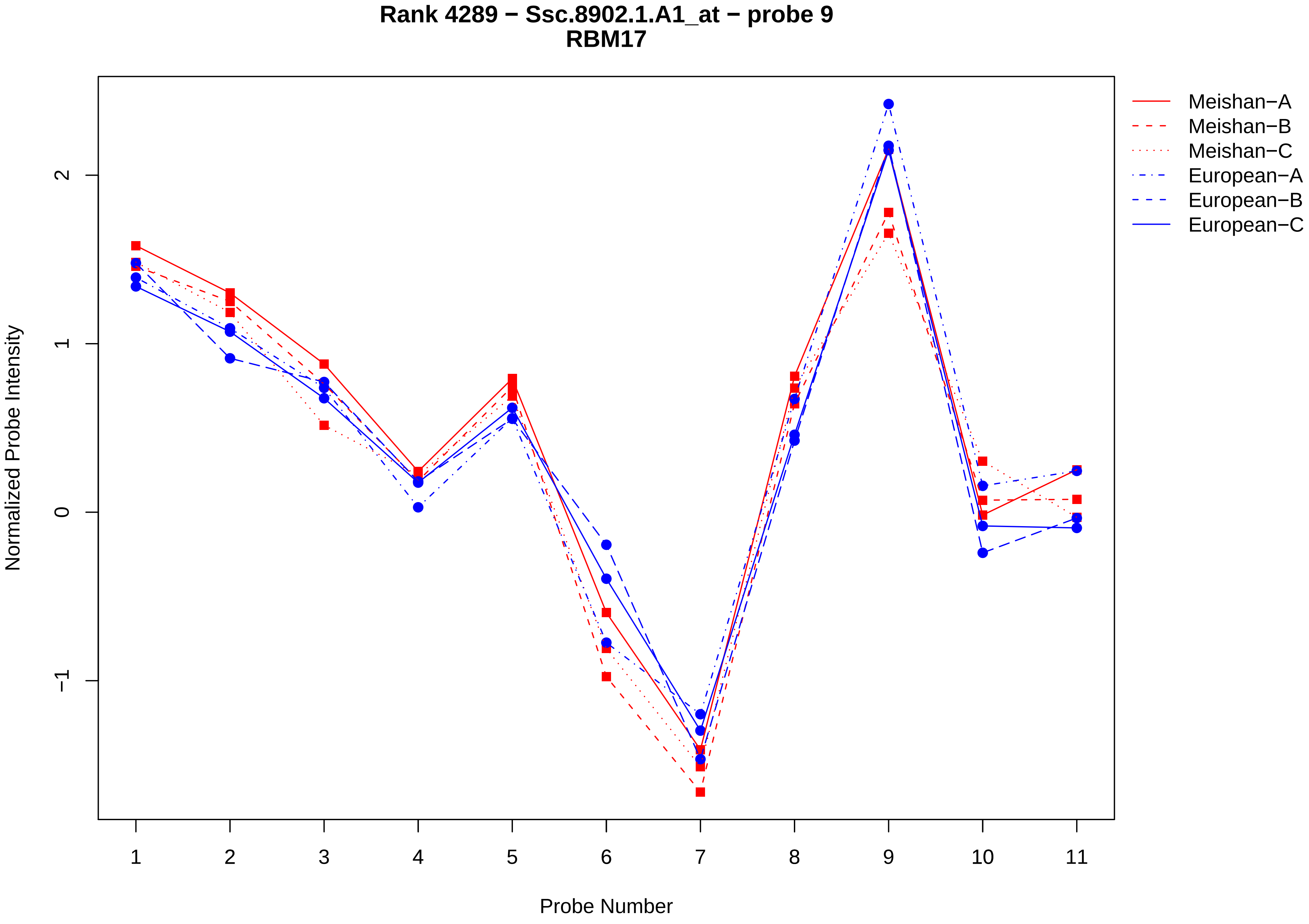

Rank 4289 − Ssc.8902.1.A1_at − probe 9
RBM17
Normalized Probe Intensity
Probe Number
Meishan−A
Meishan−B
Meishan−C
European−A
European−B
European−C
−1
0
1
2
1
2
3
4
5
6
7
8
9
10
11

Rank 4290 − Ssc.10297.3.S1_a_at − probe 11
CAPZB

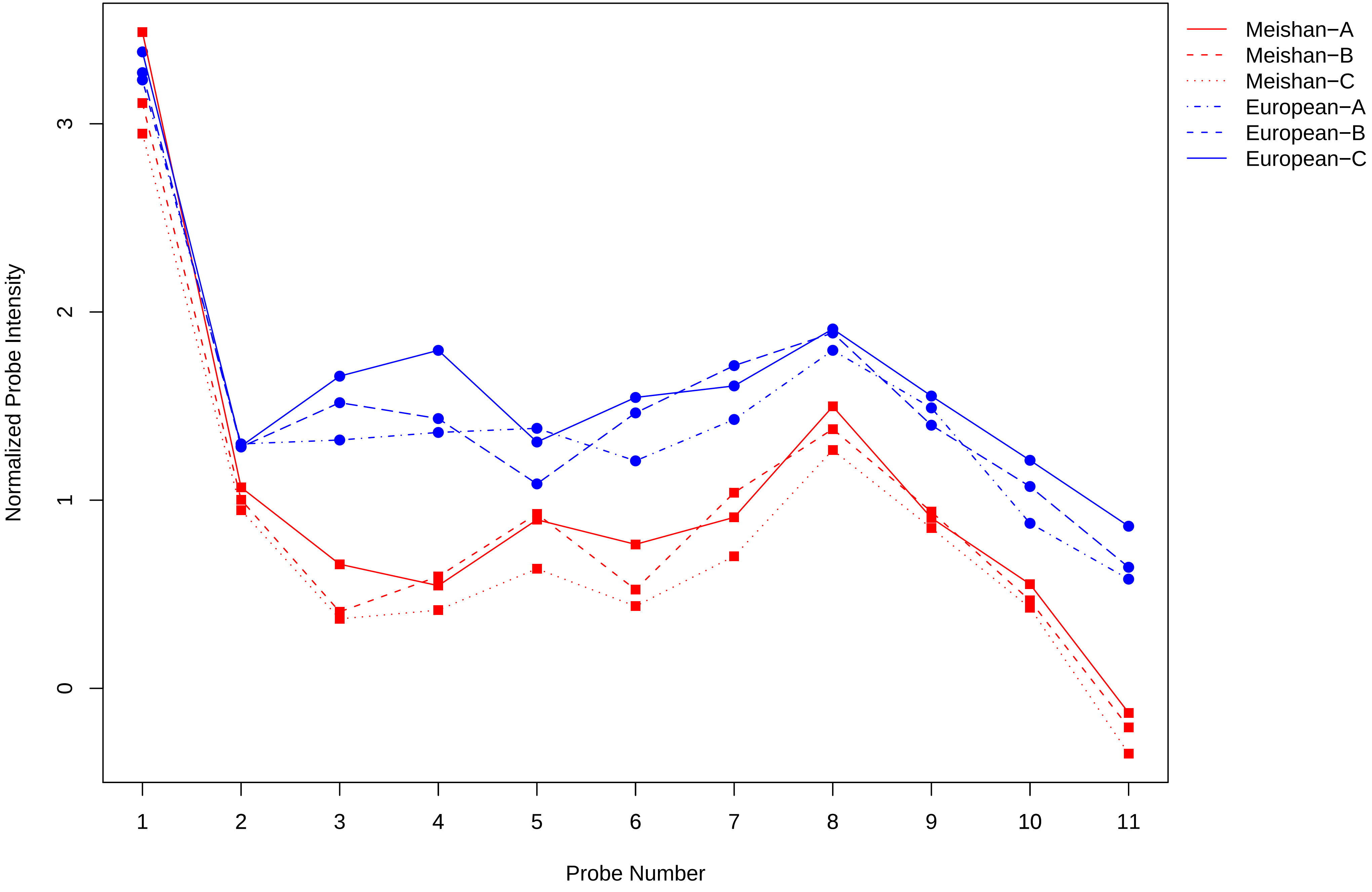

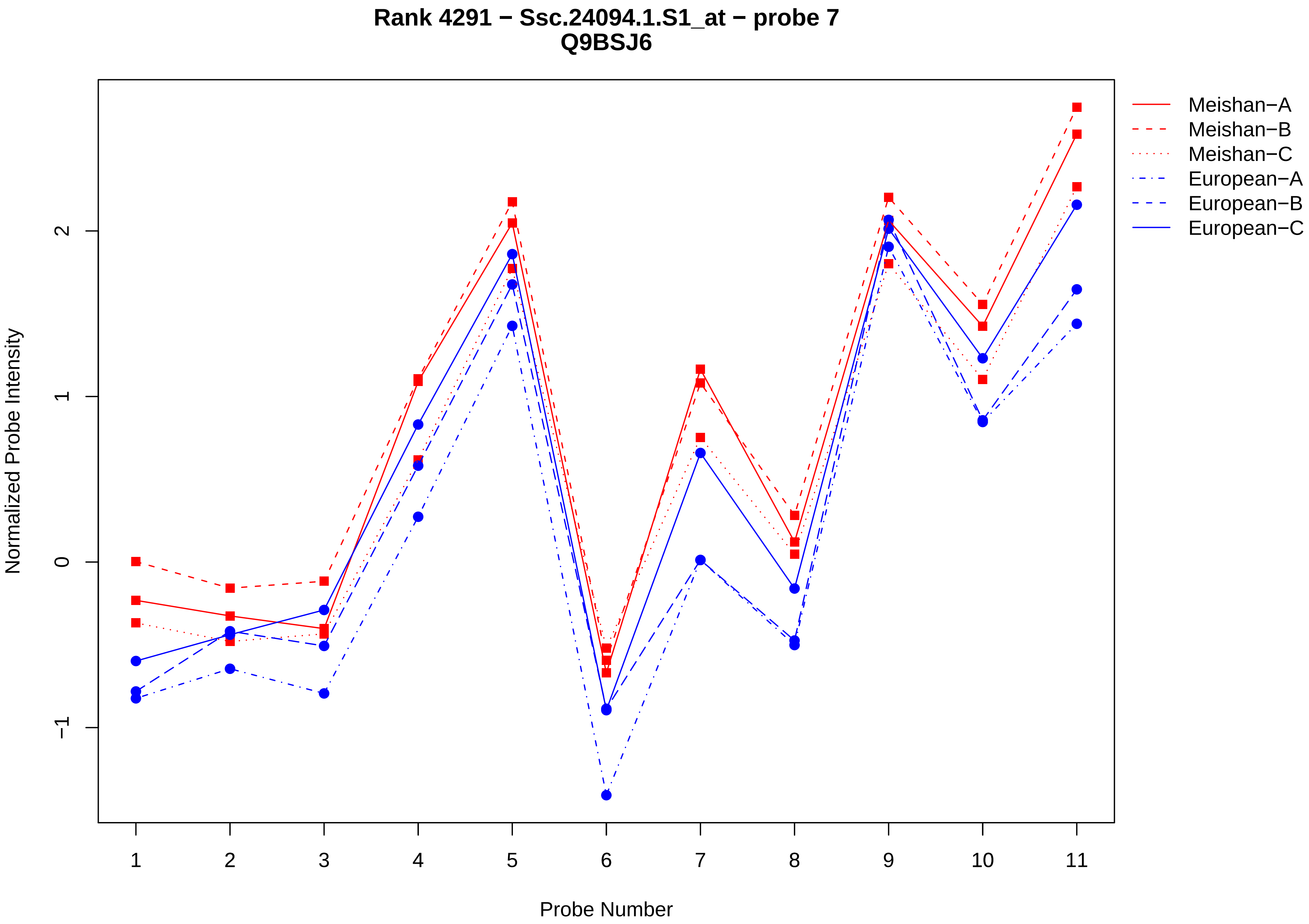

Rank 4291 − Ssc.24094.1.S1_at − probe 7
Q9BSJ6
Normalized Probe Intensity
Probe Number
−1
0
1
2
1
2
3
4
5
6
7
8
9
10
11
Meishan−A
Meishan−B
Meishan−C
European−A
European−B
European−C

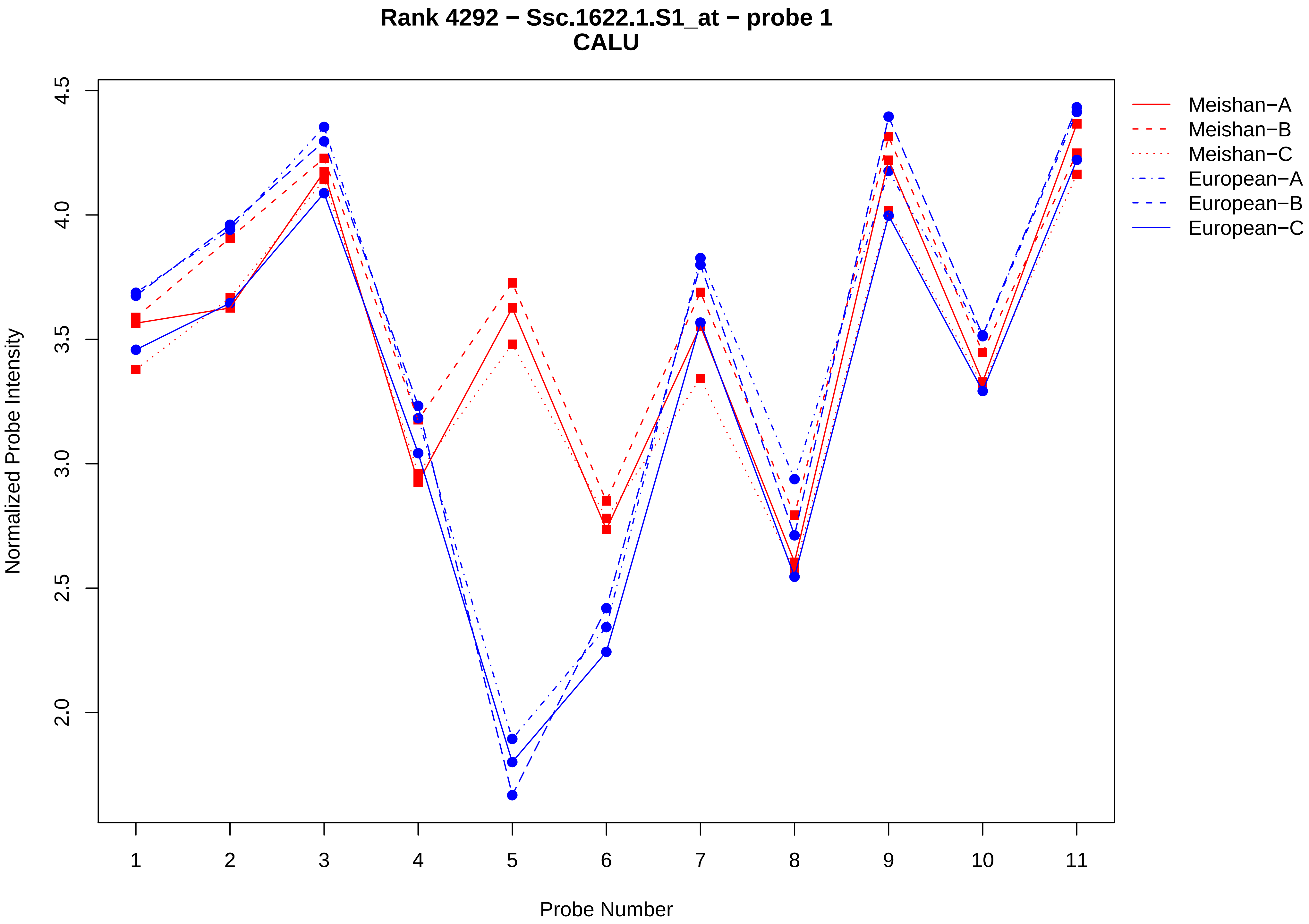
Rank 4292 − Ssc.1622.1.S1_at − probe 1
CALU
Normalized Probe Intensity
Probe Number
2.0
2.5
3.0
3.5
4.0
4.5
1
2
3
4
5
6
7
8
9
10
11
Meishan−A
Meishan−B
Meishan−C
European−A
European−B
European−C

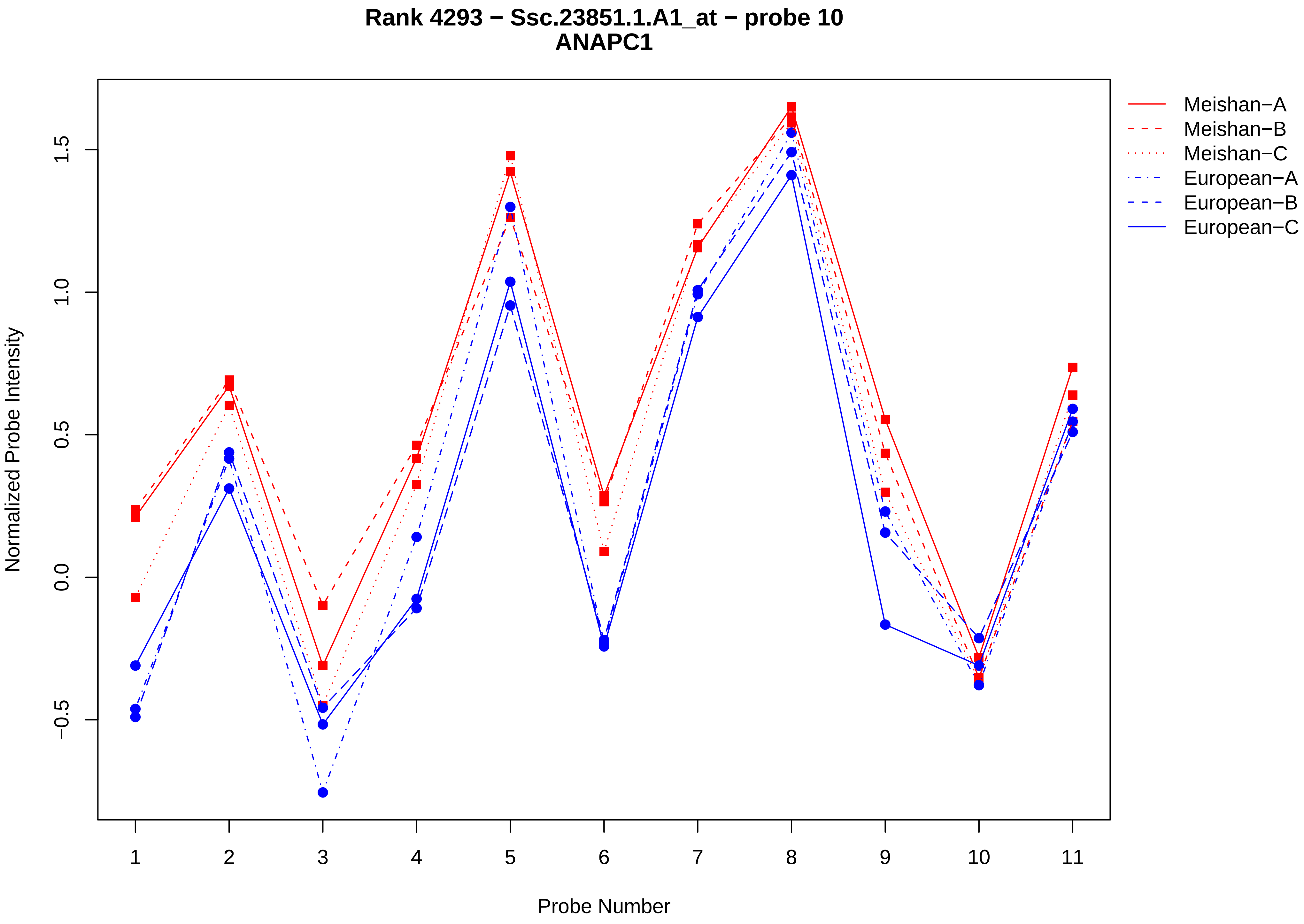

Rank 4293 − Ssc.23851.1.A1_at − probe 10
ANAPC1
Normalized Probe Intensity
Probe Number
−0.5
0.0
0.5
1.0
1.5
1
2
3
4
5
6
7
8
9
10
11
Meishan−A
Meishan−B
Meishan−C
European−A
European−B
European−C

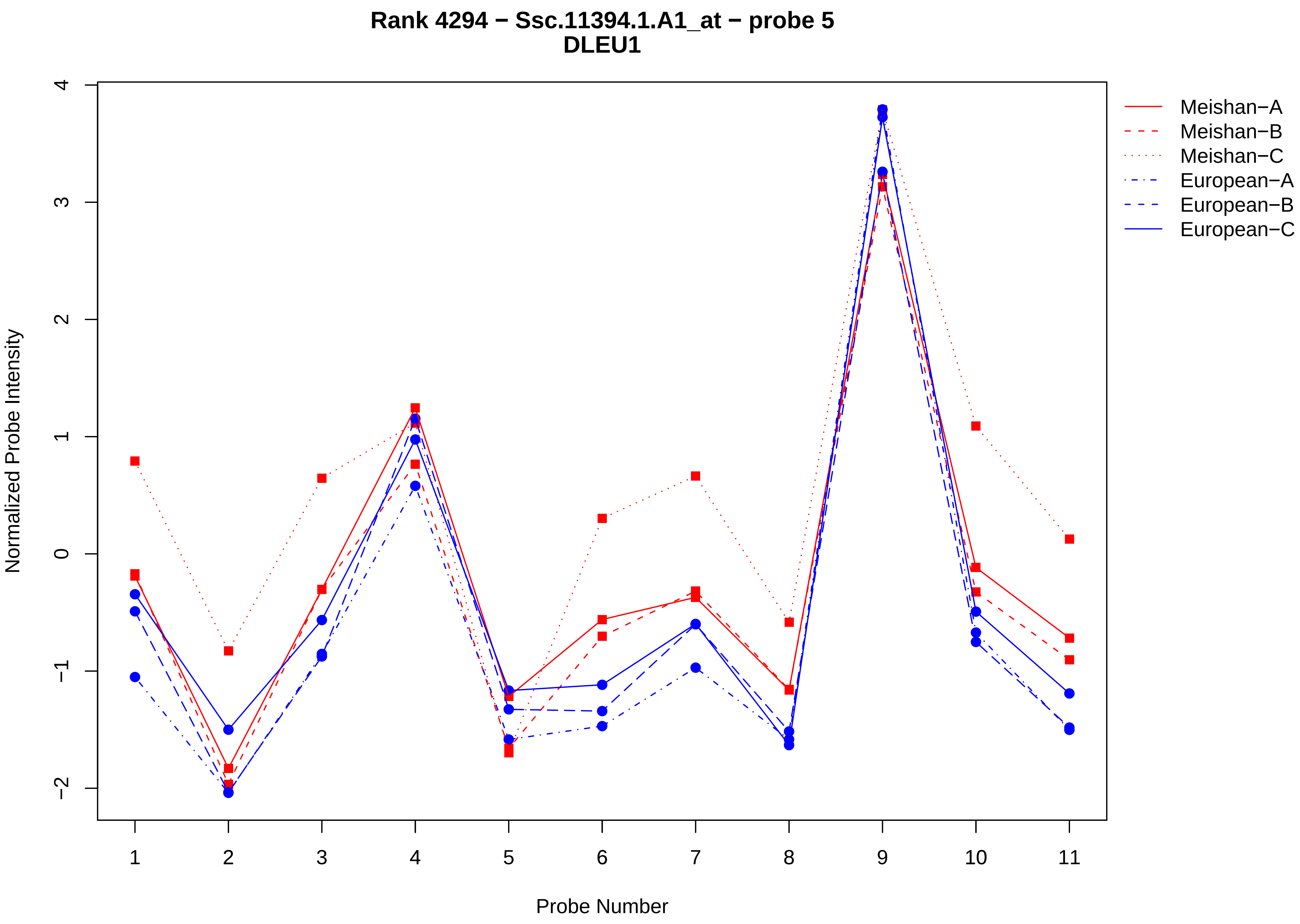

Rank 4294 − Ssc.11394.1.A1_at − probe 5
DLEU1
Normalized Probe Intensity
Probe Number
Meishan−A
Meishan−B
Meishan−C
European−A
European−B
European−C

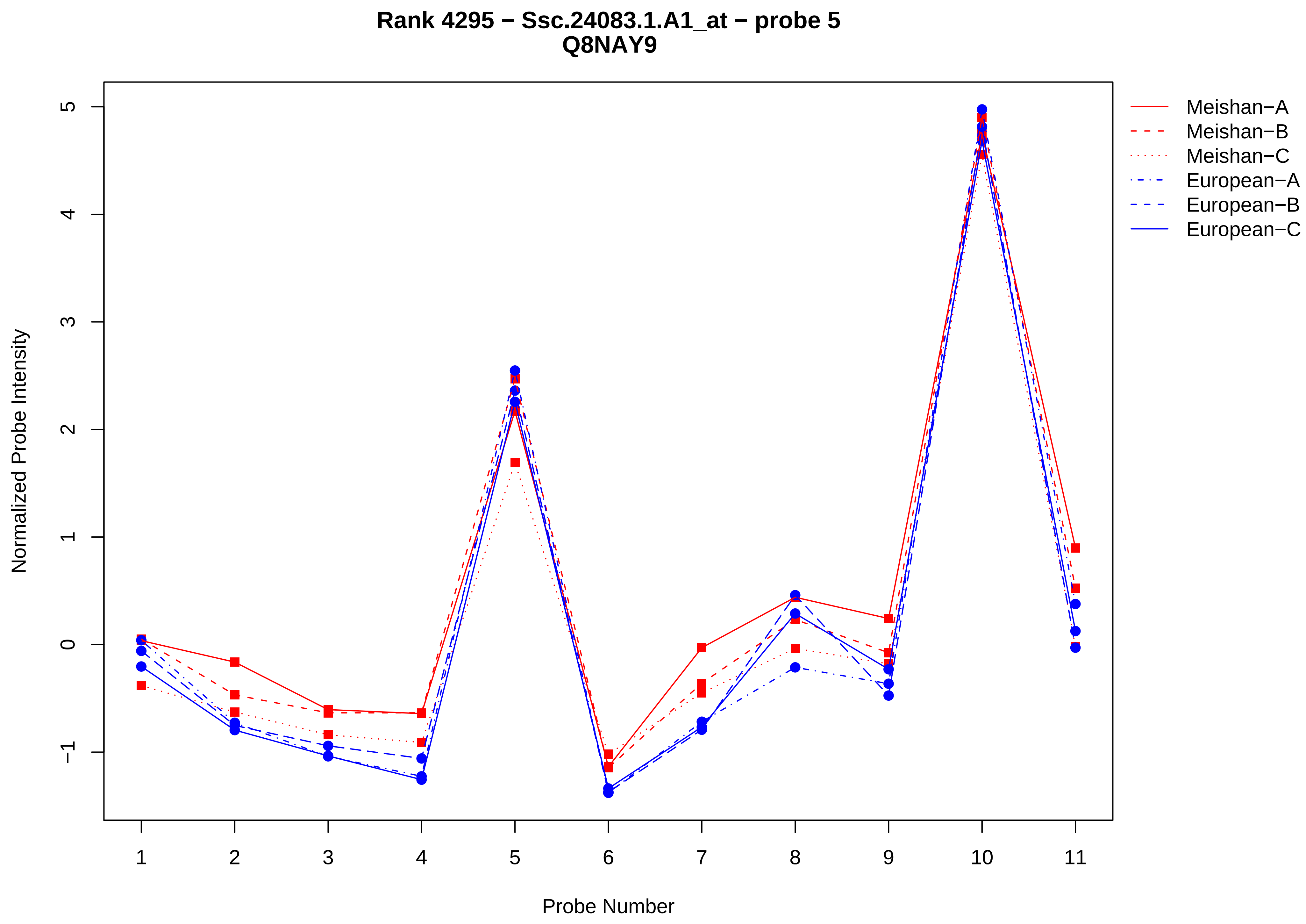

Rank 4295 − Ssc.24083.1.A1_at − probe 5
Q8NAY9
Normalized Probe Intensity
Probe Number
Meishan−A
Meishan−B
Meishan−C
European−A
European−B
European−C
−1
0
1
2
3
4
5
1
2
3
4
5
6
7
8
9
10
11

Rank 4296 − Ssc.2489.1.S1_at − probe 3
Q9Y3T6

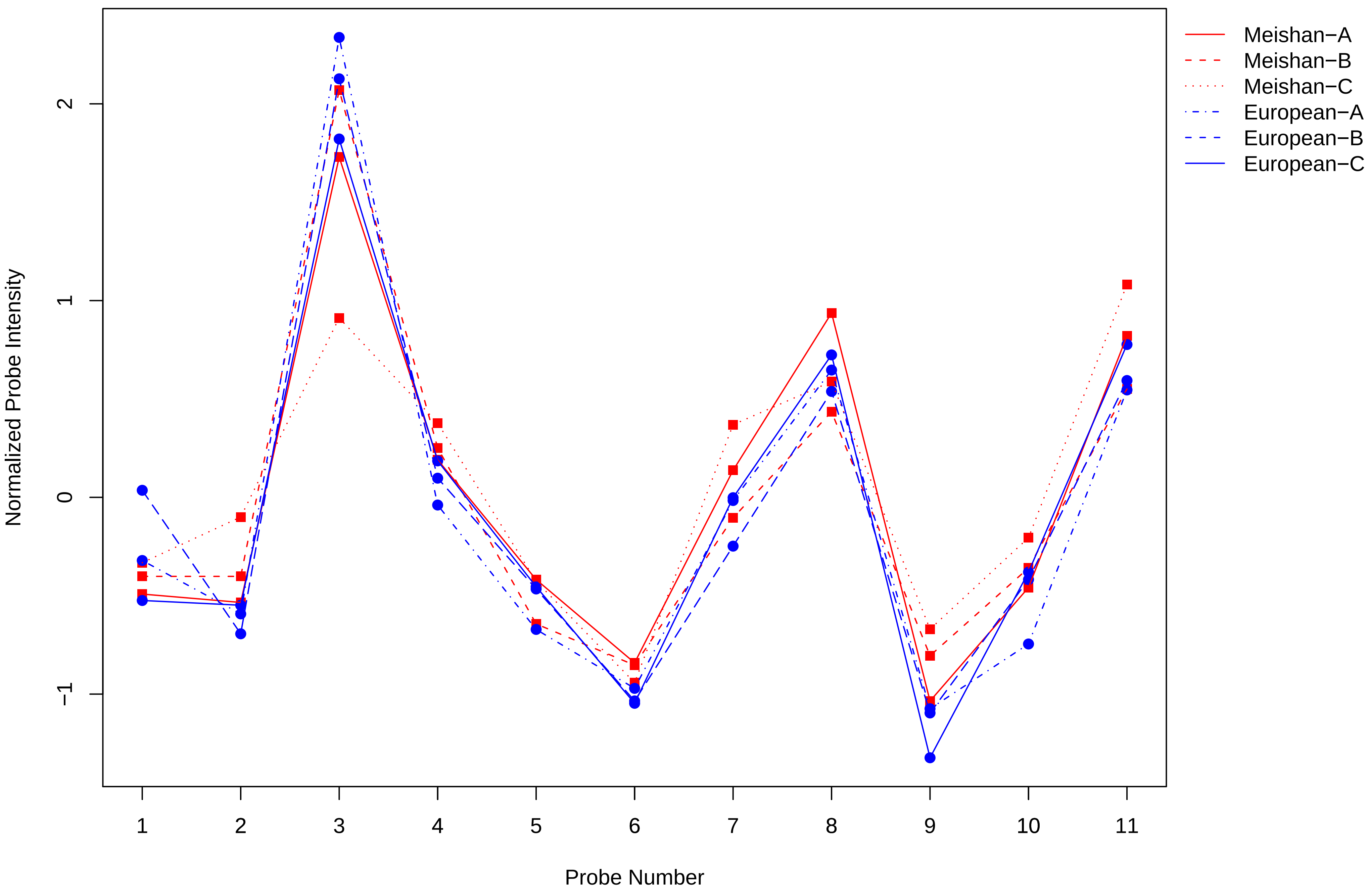

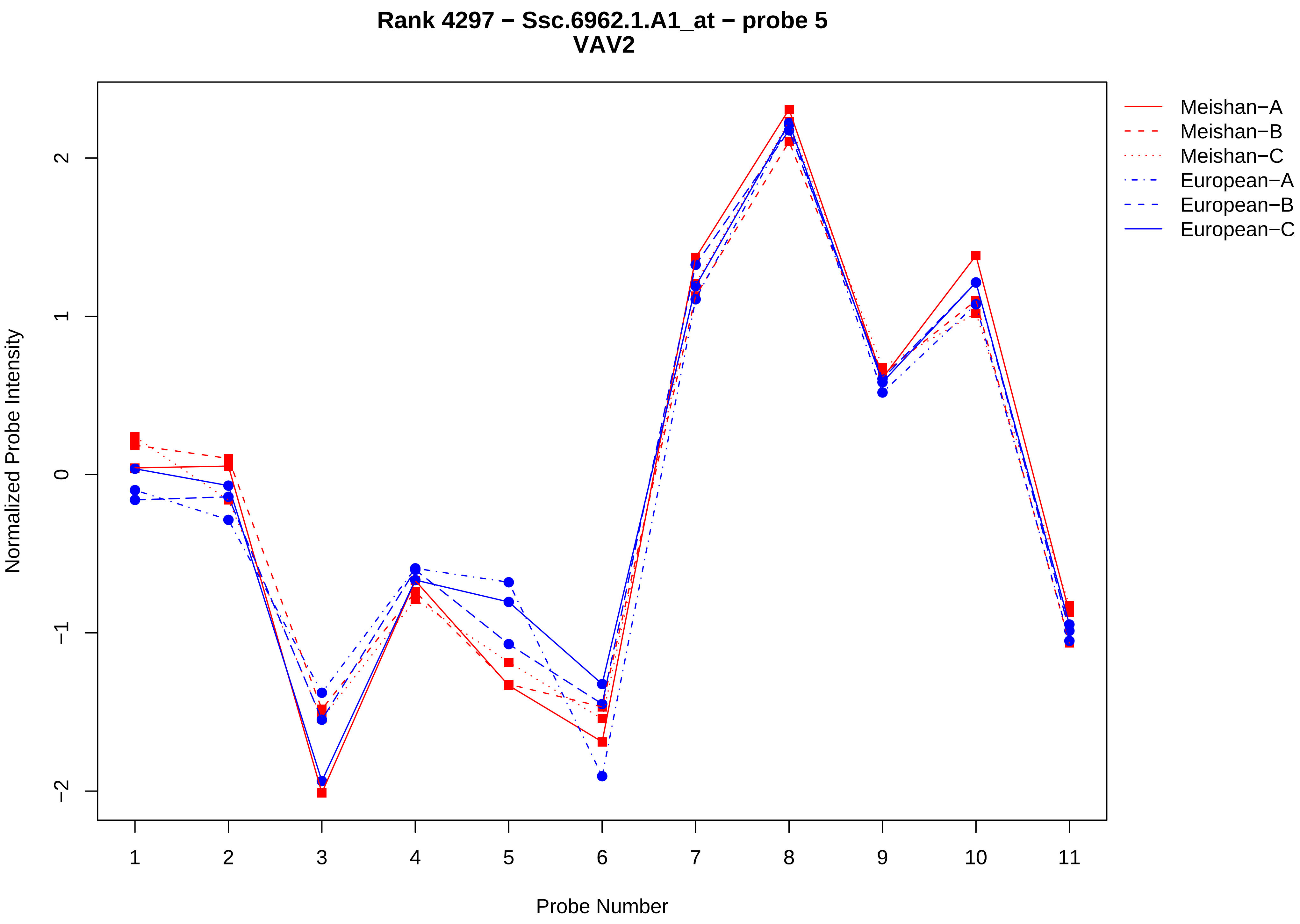

Rank 4297 − Ssc.6962.1.A1_at − probe 5
VAV2
Normalized Probe Intensity
Probe Number
−2
−1
0
1
2
1
2
3
4
5
6
7
8
9
10
11
Meishan−A
Meishan−B
Meishan−C
European−A
European−B
European−C

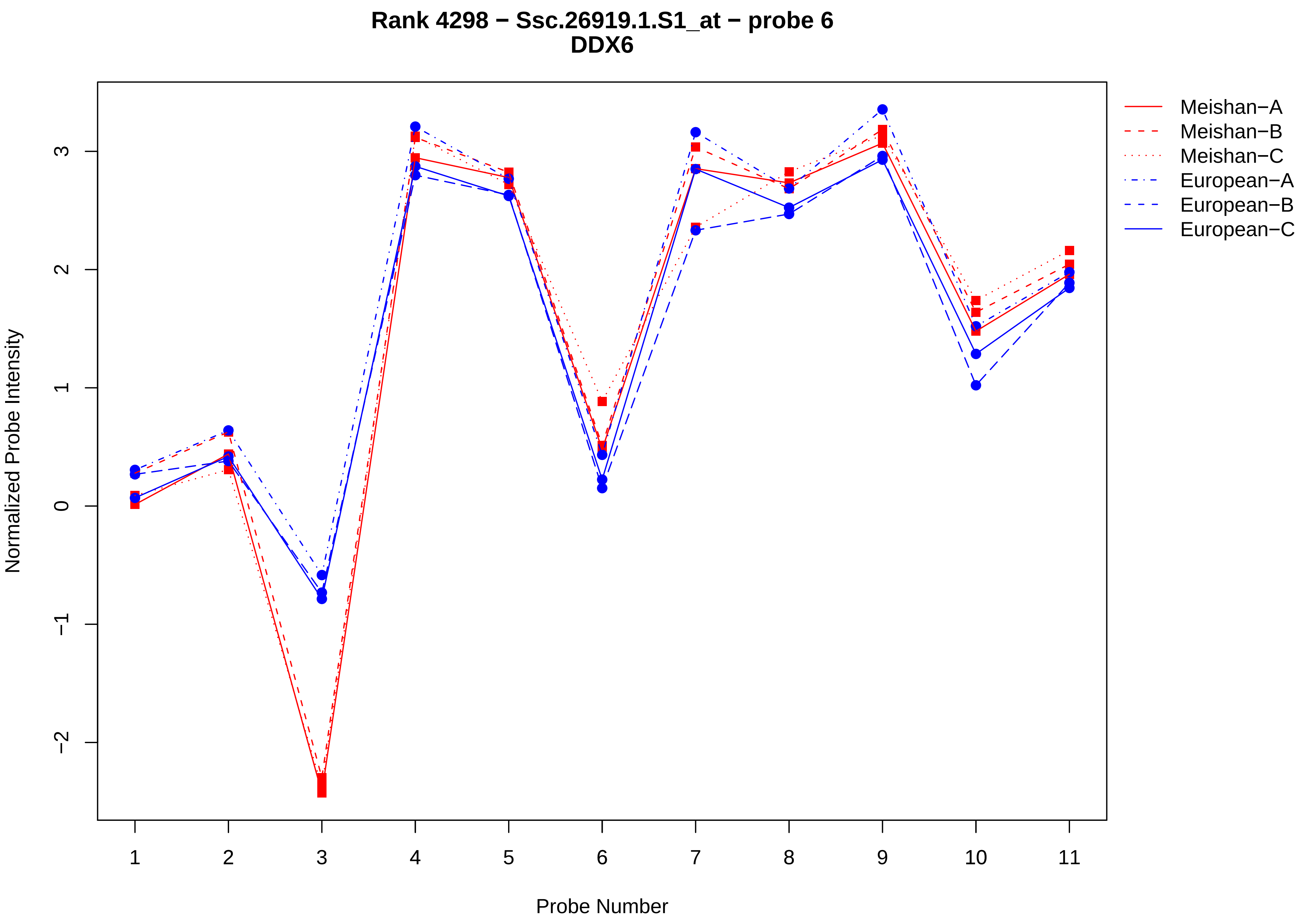

Rank 4298 − Ssc.26919.1.S1_at − probe 6
DDX6
Normalized Probe Intensity
Probe Number
−2
−1
0
1
2
3
1
2
3
4
5
6
7
8
9
10
11
Meishan−A
Meishan−B
Meishan−C
European−A
European−B
European−C

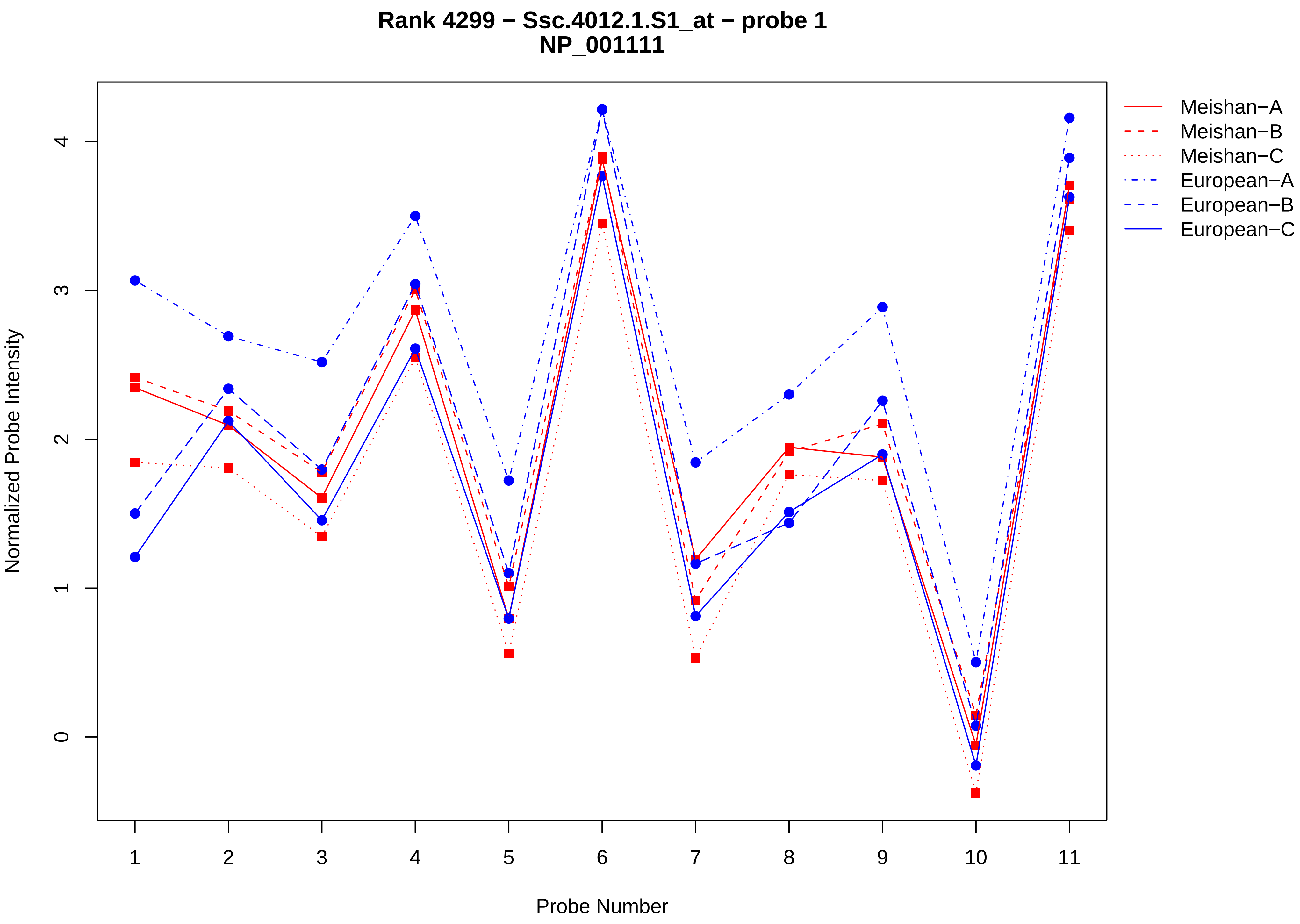
Rank 4299 − Ssc.4012.1.S1_at − probe 1
NP_001111
Normalized Probe Intensity
Probe Number
0
1
2
3
4
1
2
3
4
5
6
7
8
9
10
11
Meishan−A
Meishan−B
Meishan−C
European−A
European−B
European−C

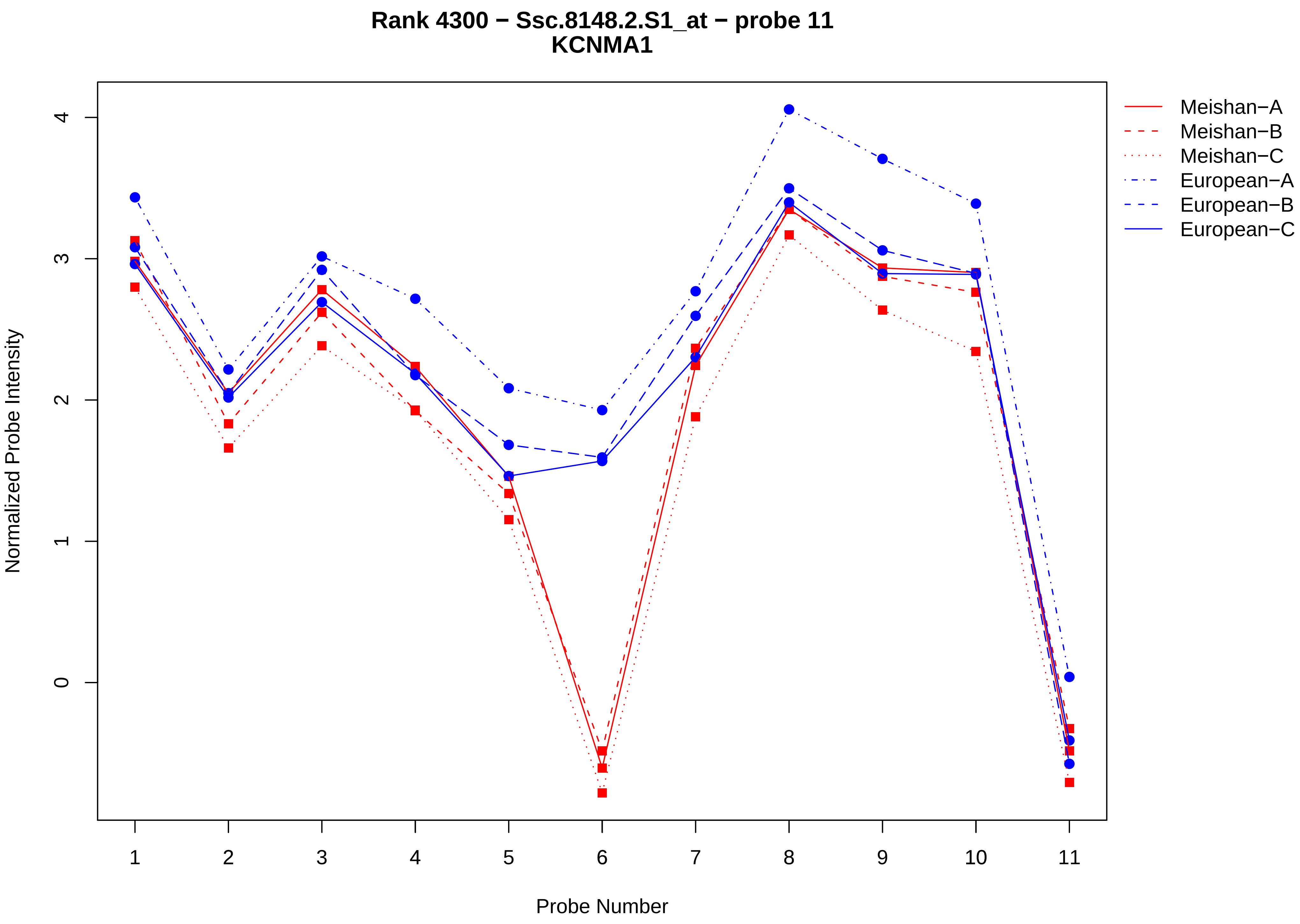

Rank 4300 − Ssc.8148.2.S1_at − probe 11
KCNMA1
Normalized Probe Intensity
Probe Number
0
1
2
3
4
1
2
3
4
5
6
7
8
9
10
11
Meishan−A
Meishan−B
Meishan−C
European−A
European−B
European−C

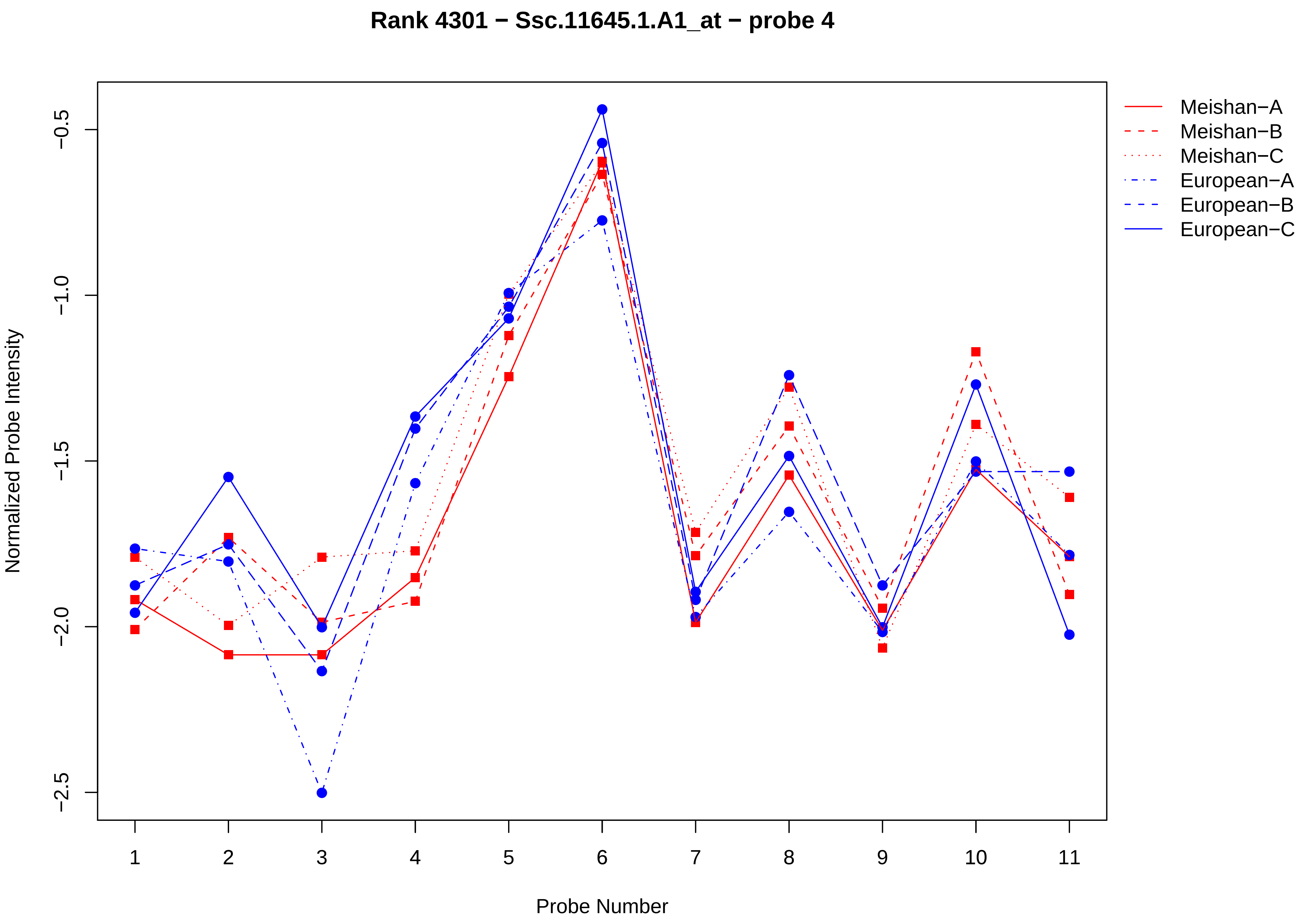

Rank 4301 − Ssc.11645.1.A1_at − probe 4
Normalized Probe Intensity
−0.5
−1.0
−1.5
−2.0
−2.5
1
2
3
4
5
6
7
8
9
10
11
Probe Number
Meishan−A
Meishan−B
Meishan−C
European−A
European−B
European−C

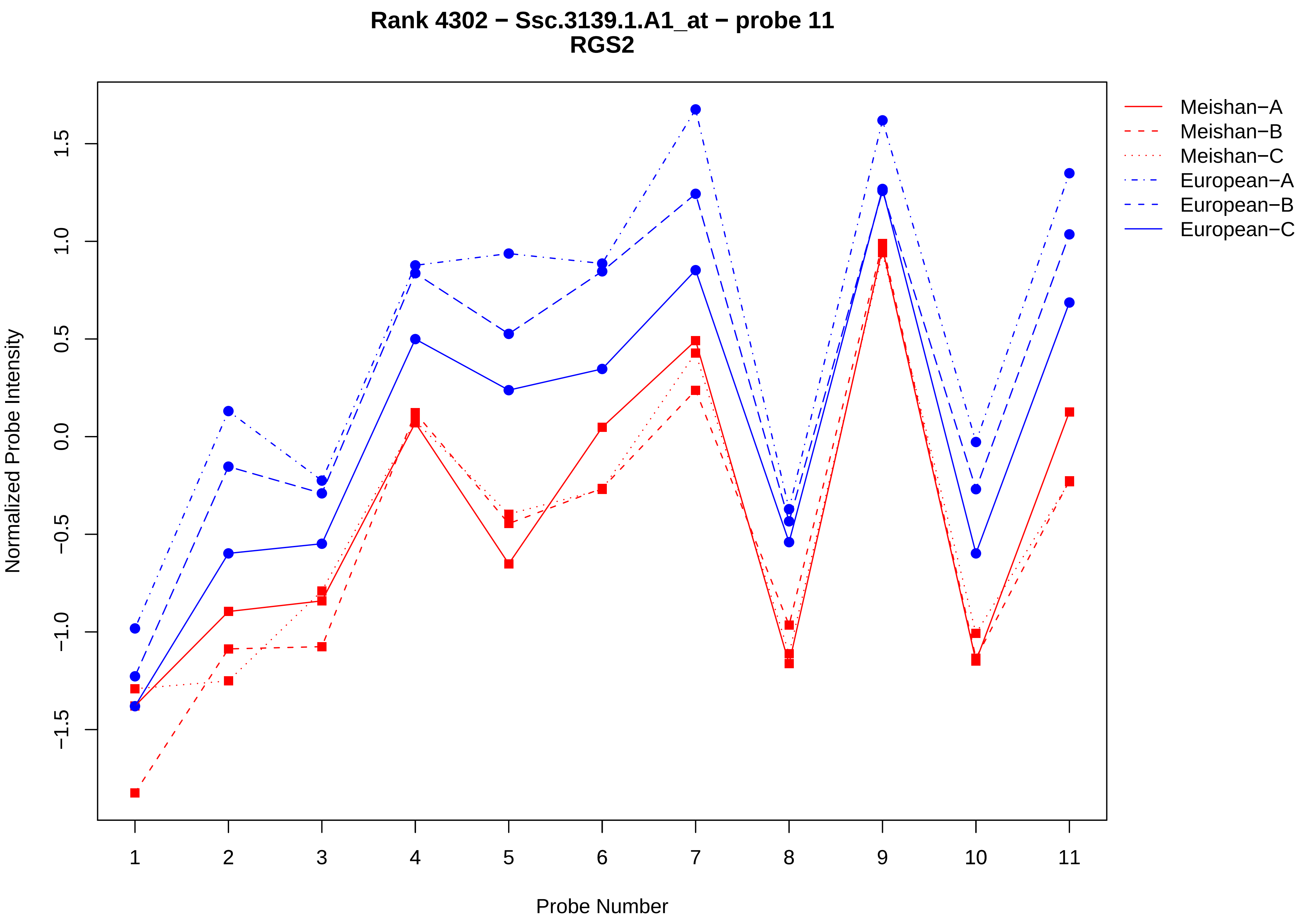

Rank 4302 − Ssc.3139.1.A1_at − probe 11
RGS2
Normalized Probe Intensity
Probe Number
−1.5
−1.0
−0.5
0.0
0.5
1.0
1.5
1
2
3
4
5
6
7
8
9
10
11
Meishan−A
Meishan−B
Meishan−C
European−A
European−B
European−C

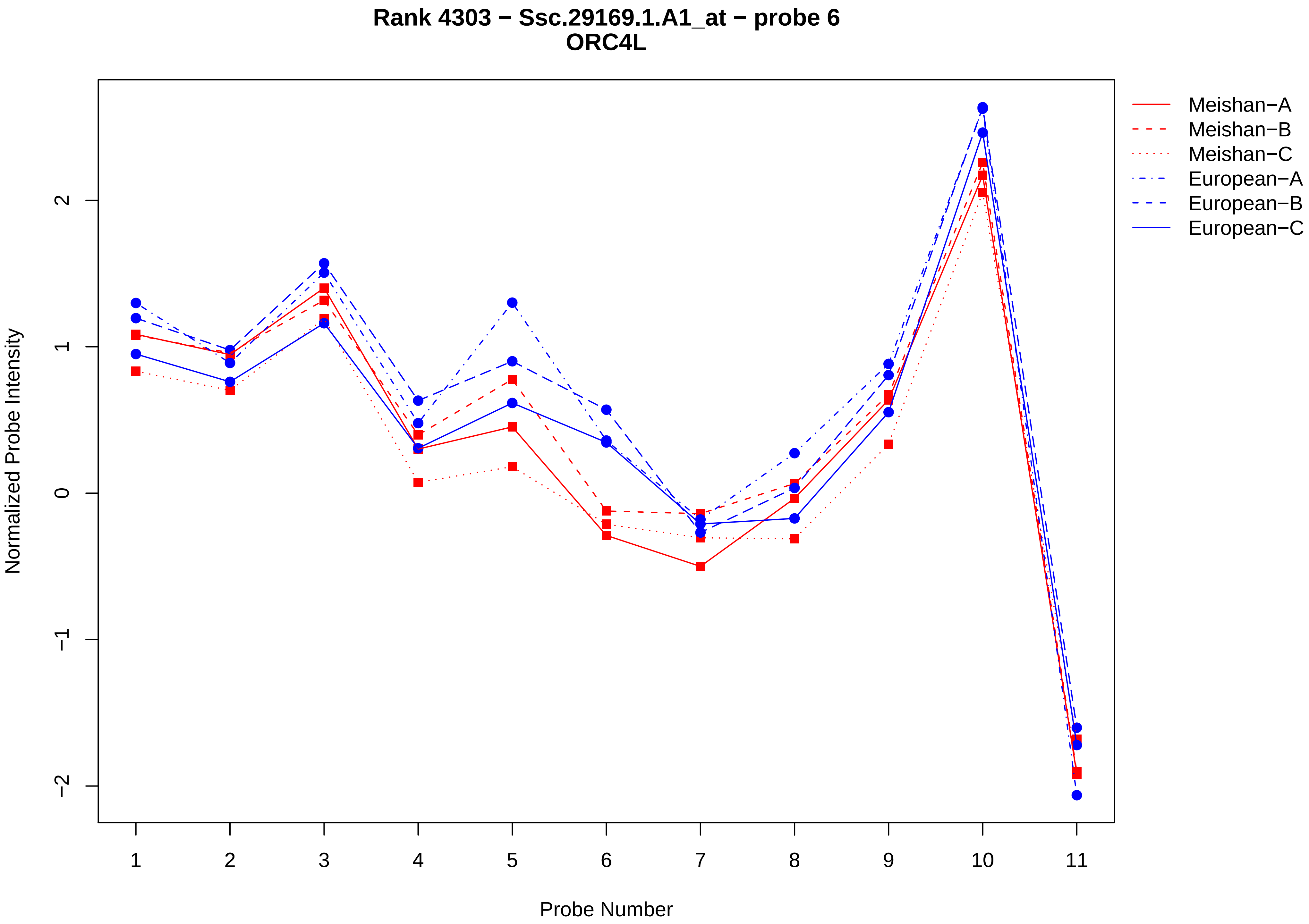

Rank 4303 − Ssc.29169.1.A1_at − probe 6
ORC4L
Normalized Probe Intensity
Probe Number
−2
−1
0
1
2
1
2
3
4
5
6
7
8
9
10
11
Meishan−A
Meishan−B
Meishan−C
European−A
European−B
European−C

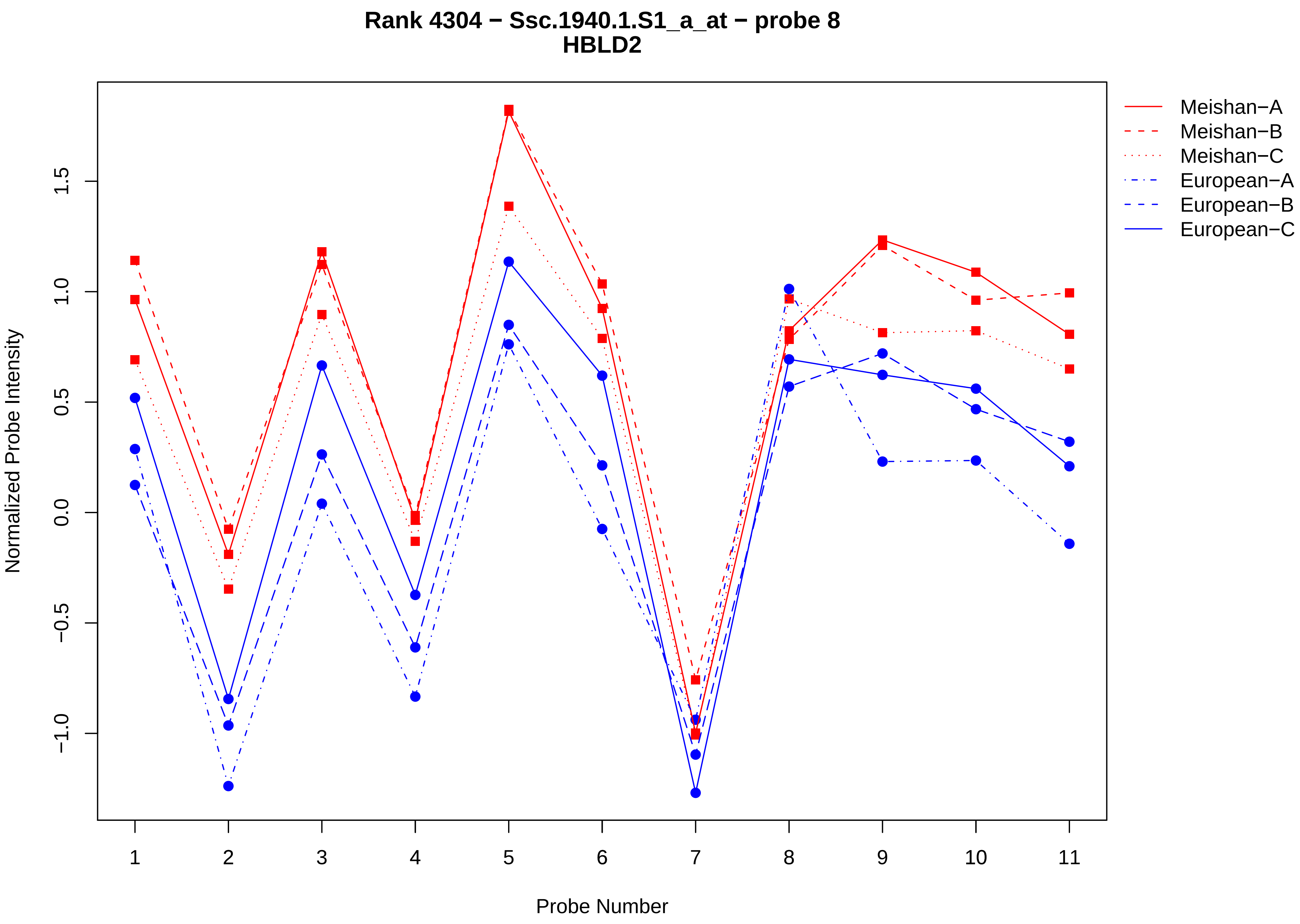

Rank 4304 − Ssc.1940.1.S1_a_at − probe 8
HBLD2
Normalized Probe Intensity
Probe Number
Meishan−A
Meishan−B
Meishan−C
European−A
European−B
European−C
−1.0
−0.5
0.0
0.5
1.0
1.5
1
2
3
4
5
6
7
8
9
10
11

Rank 4305 − Ssc.6493.2.S1_at − probe 3
RBMS1

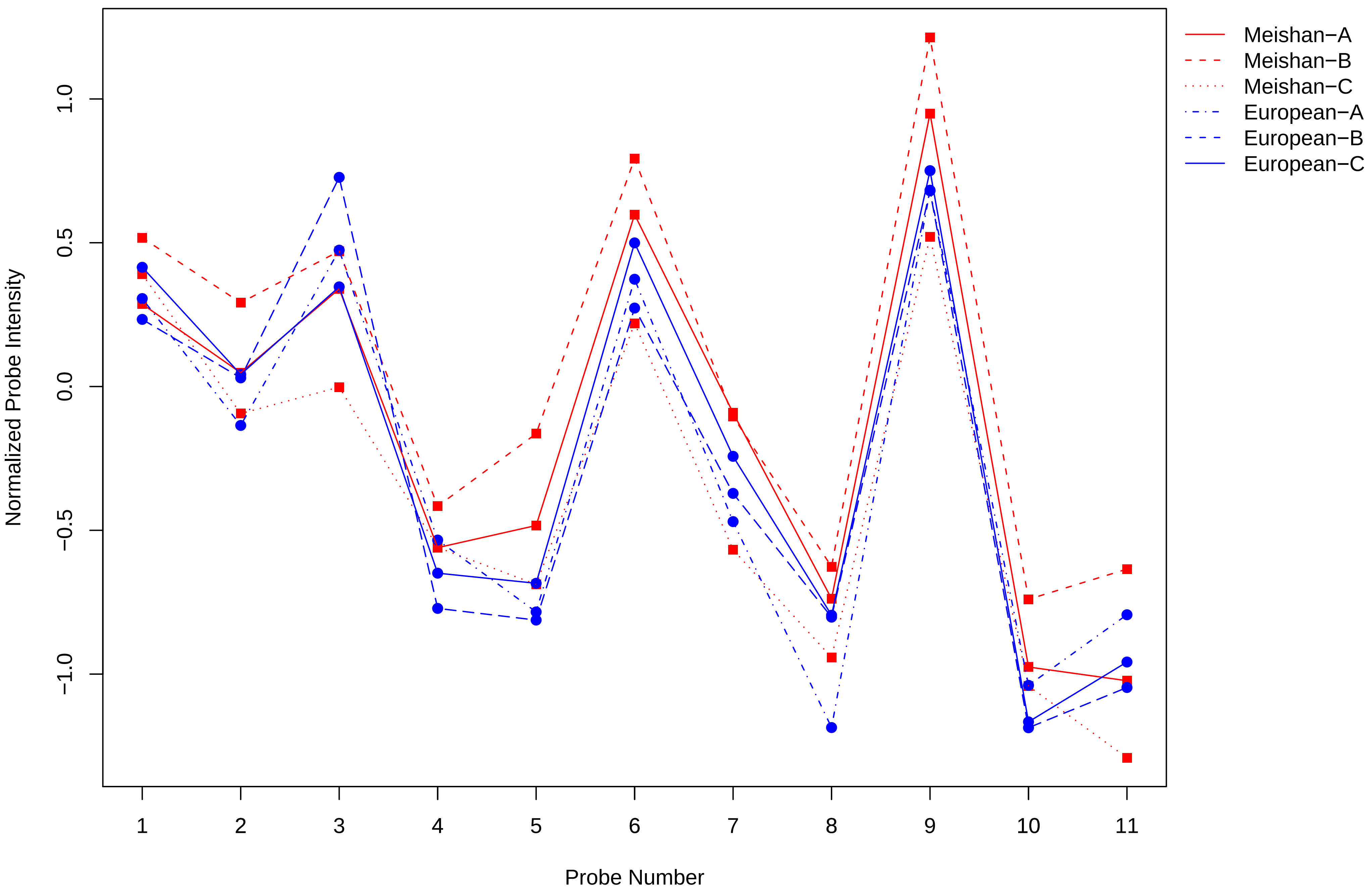

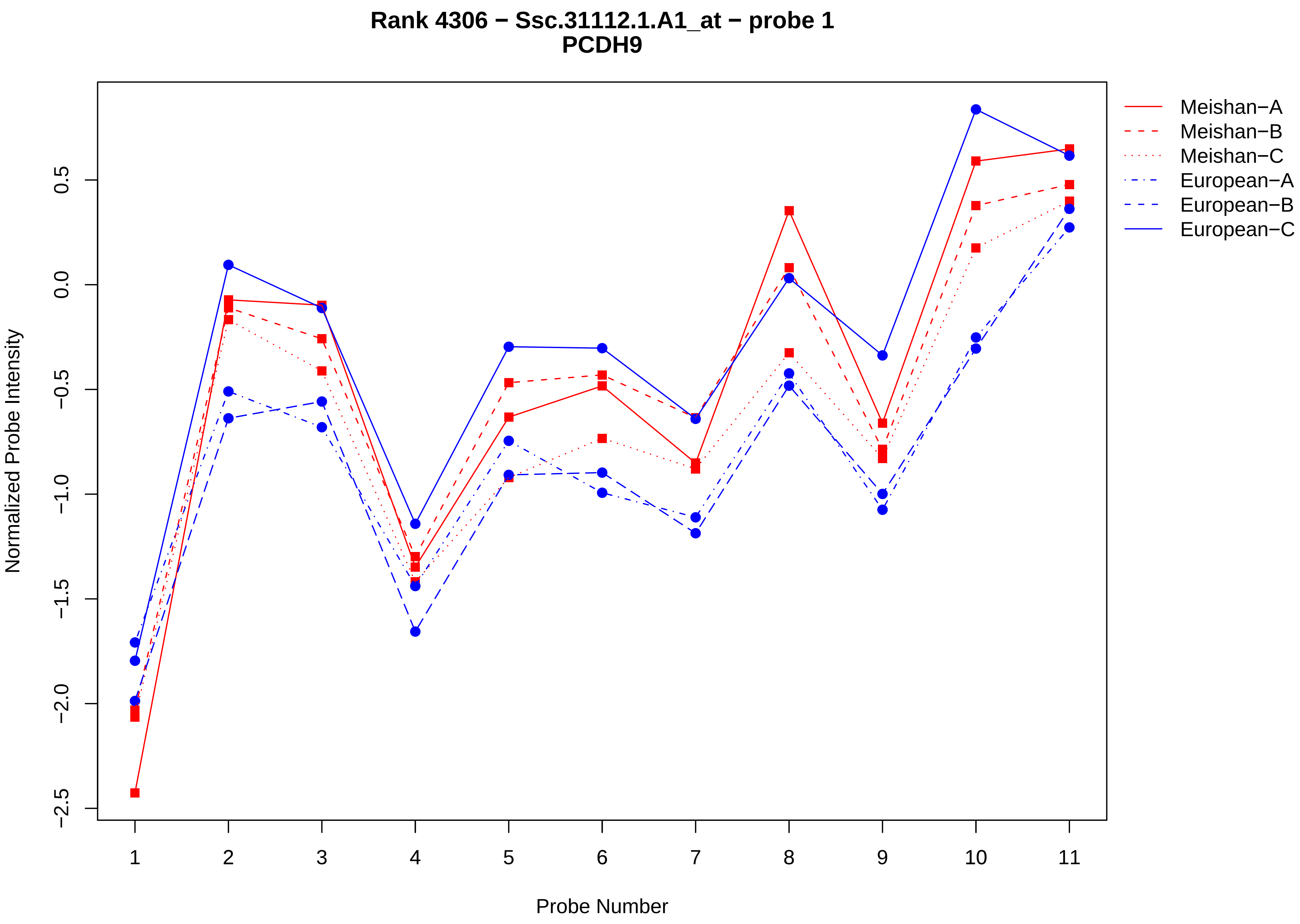

Rank 4306 − Ssc.31112.1.A1_at − probe 1
PCDH9
Normalized Probe Intensity
Probe Number
Meishan−A
Meishan−B
Meishan−C
European−A
European−B
European−C
0.5
0.0
−0.5
−1.0
−1.5
−2.0
−2.5
1
2
3
4
5
6
7
8
9
10
11

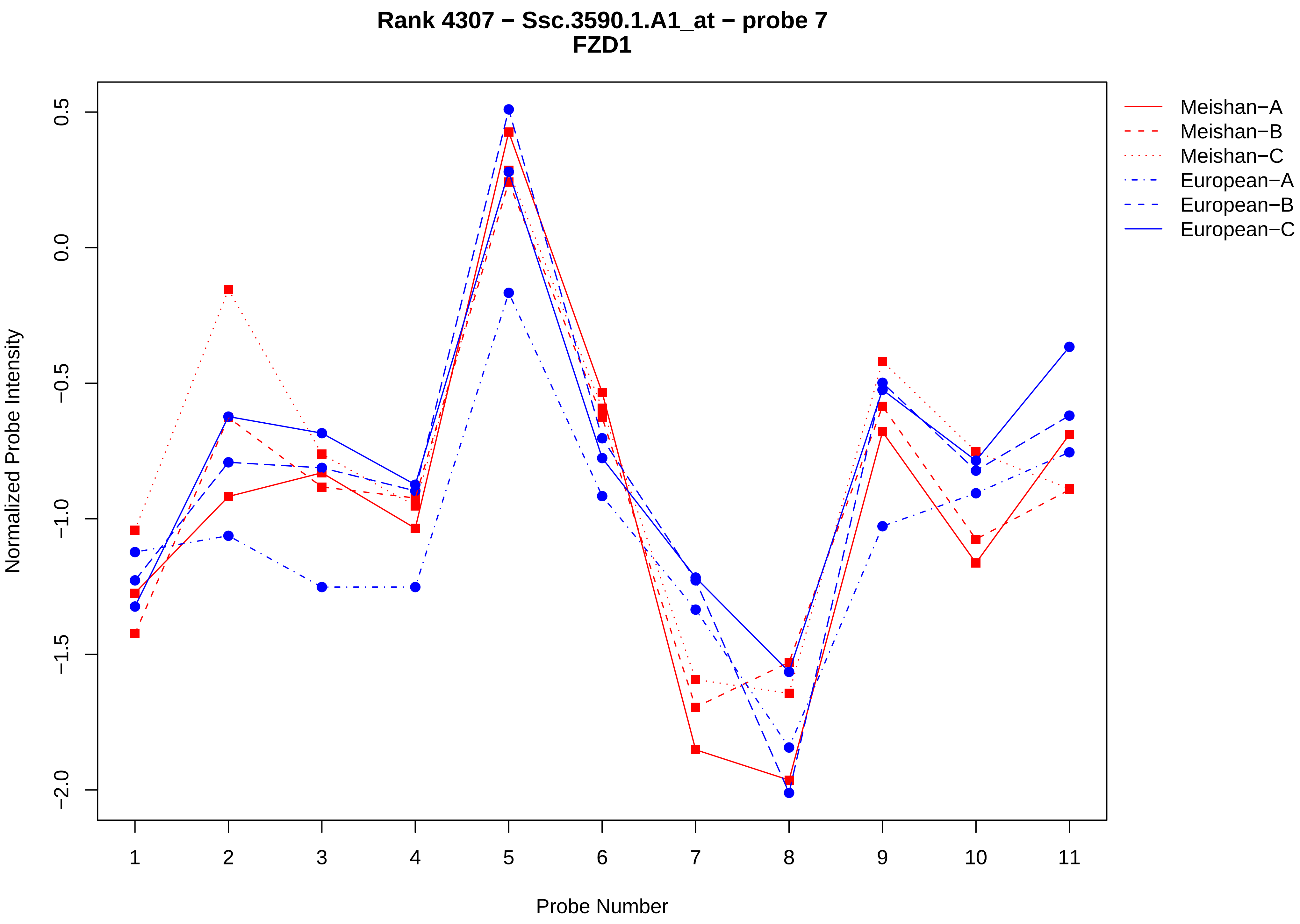

Rank 4307 − Ssc.3590.1.A1_at − probe 7
FZD1
Normalized Probe Intensity
Probe Number
0.5
0.0
−0.5
−1.0
−1.5
−2.0
1
2
3
4
5
6
7
8
9
10
11
Meishan−A
Meishan−B
Meishan−C
European−A
European−B
European−C

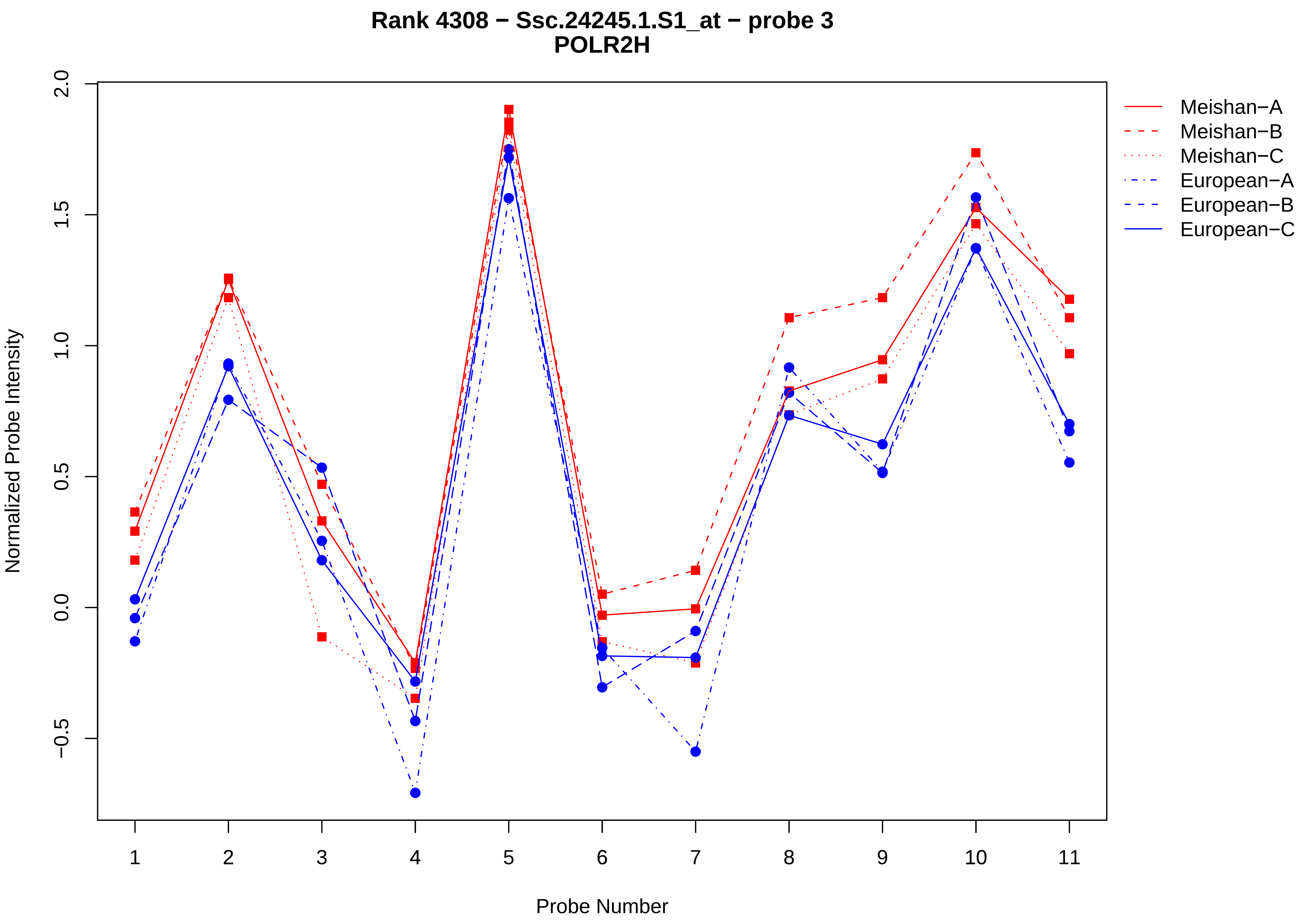
Rank 4308 − Ssc.24245.1.S1_at − probe 3
POLR2H
Normalized Probe Intensity
Probe Number
−0.5
0.0
0.5
1.0
1.5
2.0
1
2
3
4
5
6
7
8
9
10
11
Meishan−A
Meishan−B
Meishan−C
European−A
European−B
European−C

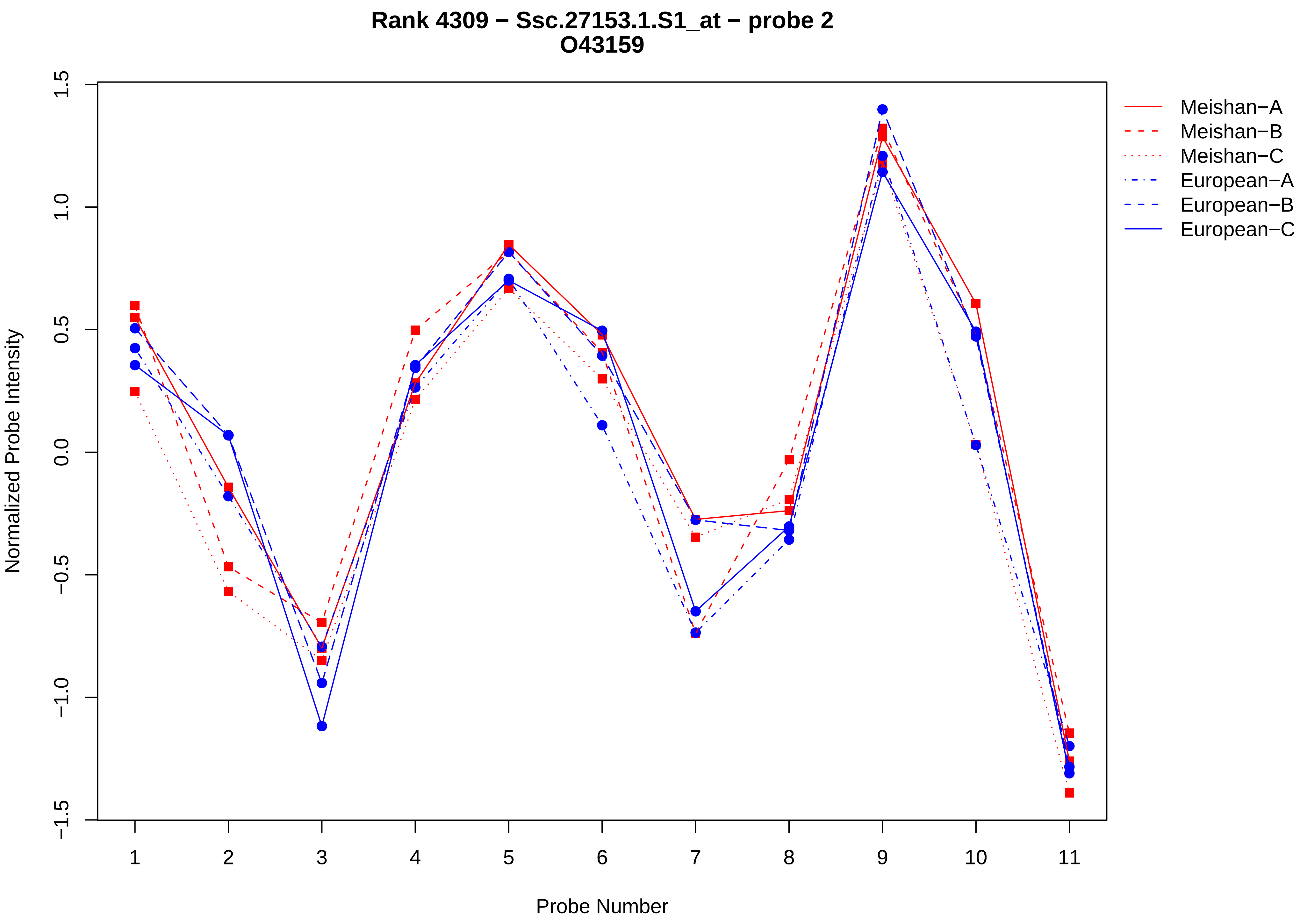

Rank 4309 − Ssc.27153.1.S1_at − probe 2
O43159
Normalized Probe Intensity
Probe Number
1.5
1.0
0.5
0.0
−0.5
−1.0
−1.5
1
2
3
4
5
6
7
8
9
10
11
Meishan−A
Meishan−B
Meishan−C
European−A
European−B
European−C

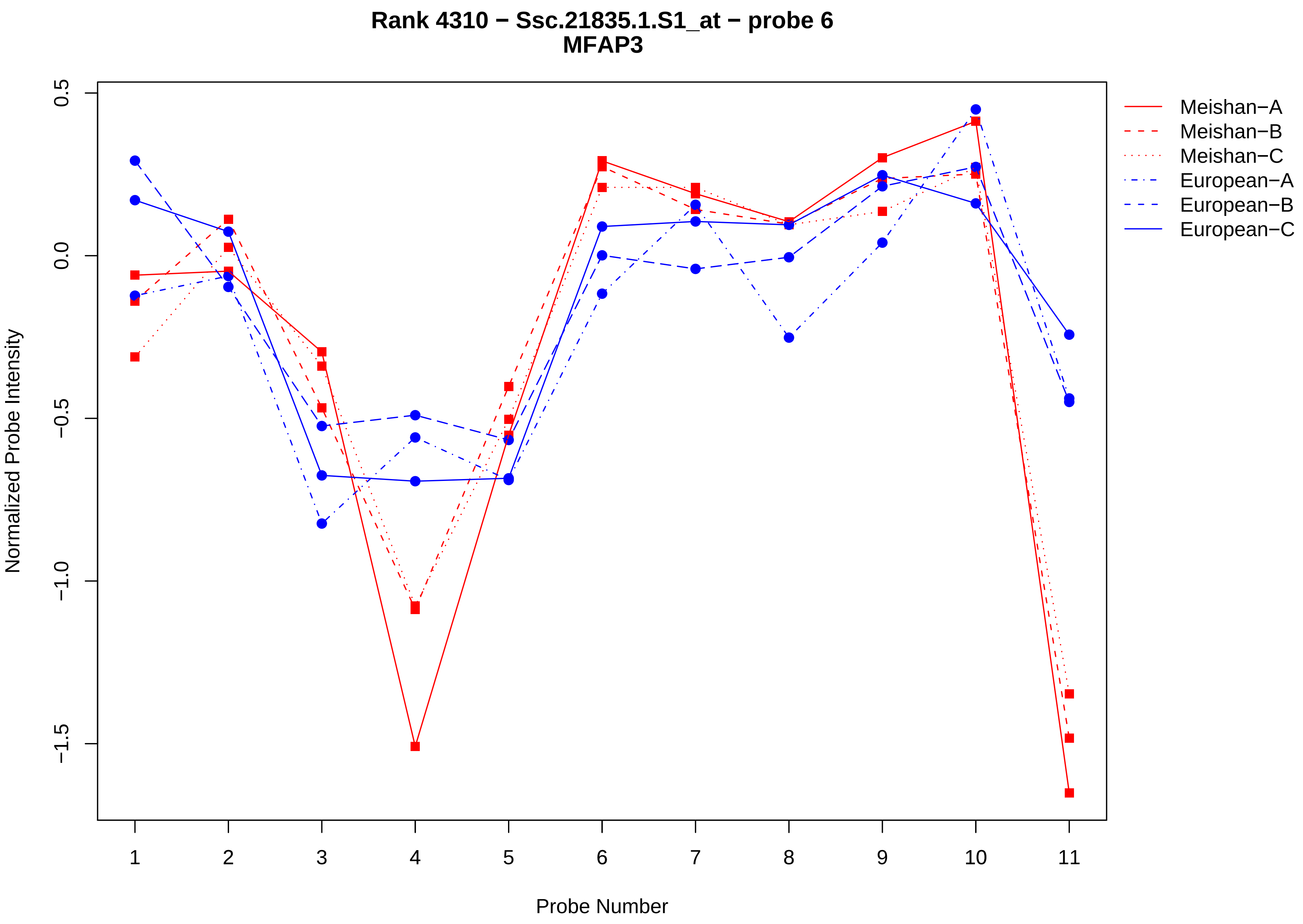

Rank 4310 − Ssc.21835.1.S1_at − probe 6
MFAP3
Normalized Probe Intensity
Probe Number
0.5
0.0
−0.5
−1.0
−1.5
1
2
3
4
5
6
7
8
9
10
11
Meishan−A
Meishan−B
Meishan−C
European−A
European−B
European−C

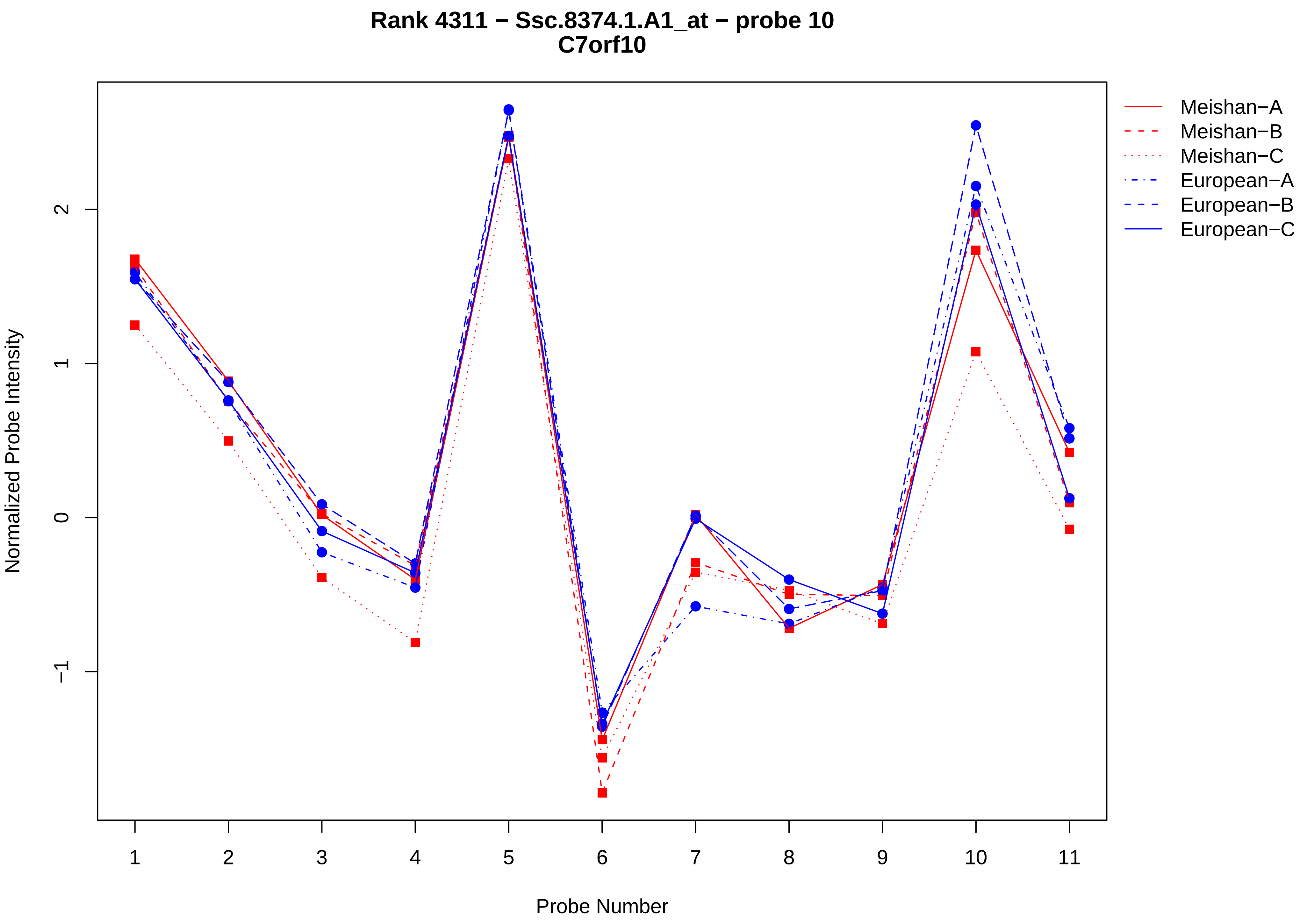
Rank 4311 − Ssc.8374.1.A1_at − probe 10
C7orf10
Normalized Probe Intensity
Probe Number
Meishan−A
Meishan−B
Meishan−C
European−A
European−B
European−C
−1
0
1
2
1
2
3
4
5
6
7
8
9
10
11

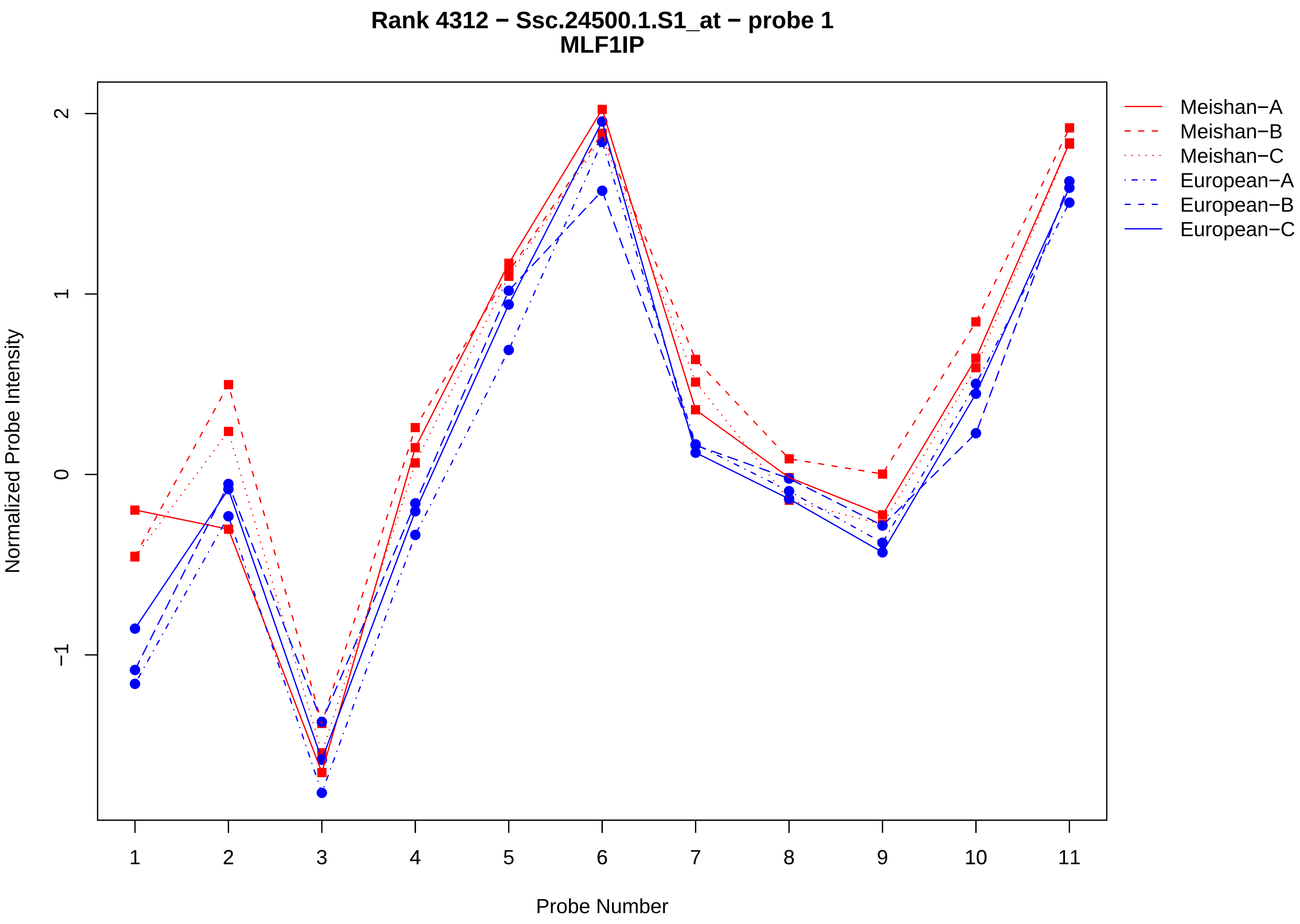
Rank 4312 − Ssc.24500.1.S1_at − probe 1
MLF1IP
Normalized Probe Intensity
Probe Number
−1
0
1
2
1
2
3
4
5
6
7
8
9
10
11
Meishan−A
Meishan−B
Meishan−C
European−A
European−B
European−C

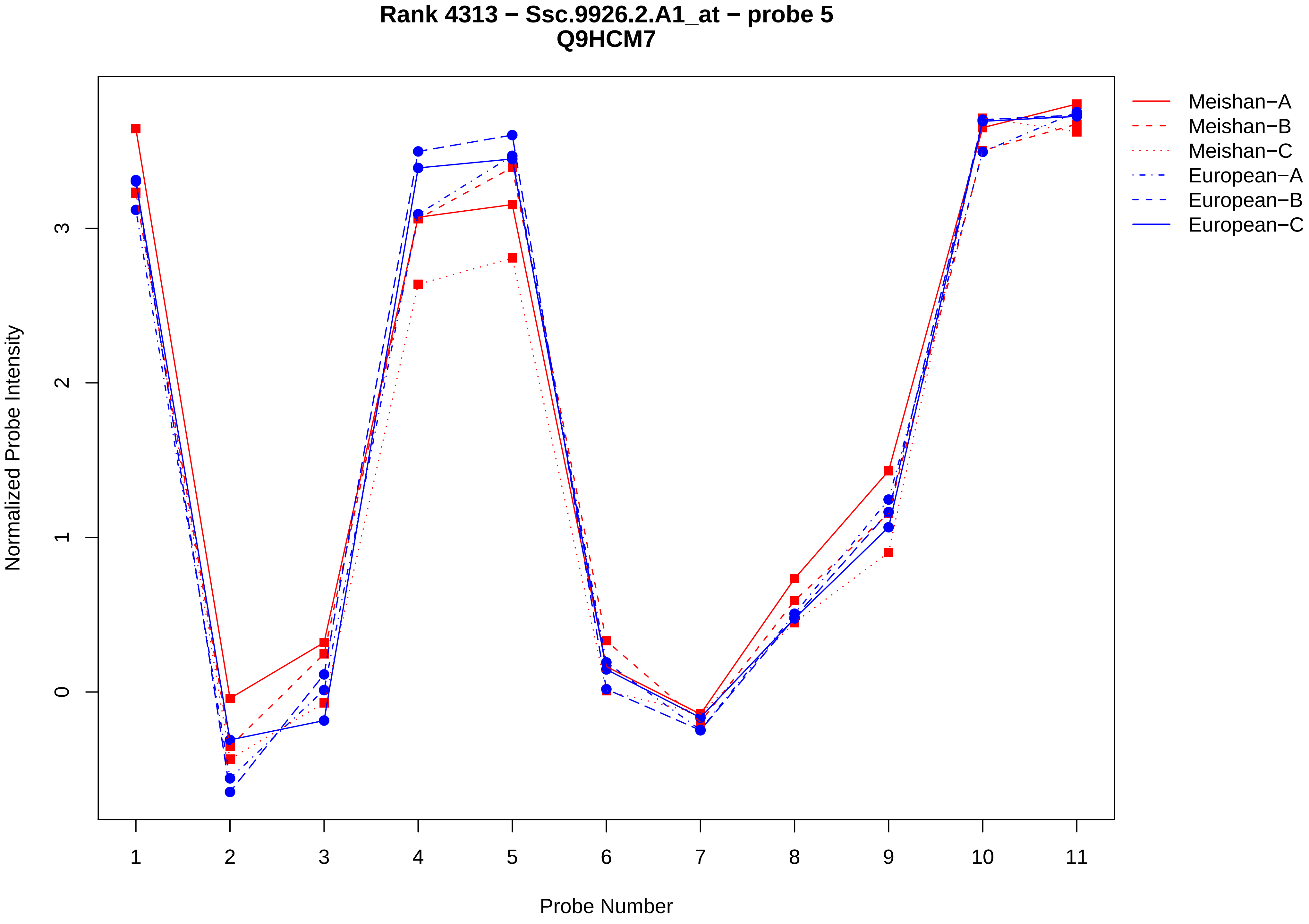
Rank 4313 − Ssc.9926.2.A1_at − probe 5
Q9HCM7
Normalized Probe Intensity
Probe Number
0
1
2
3
1
2
3
4
5
6
7
8
9
10
11
Meishan−A
Meishan−B
Meishan−C
European−A
European−B
European−C

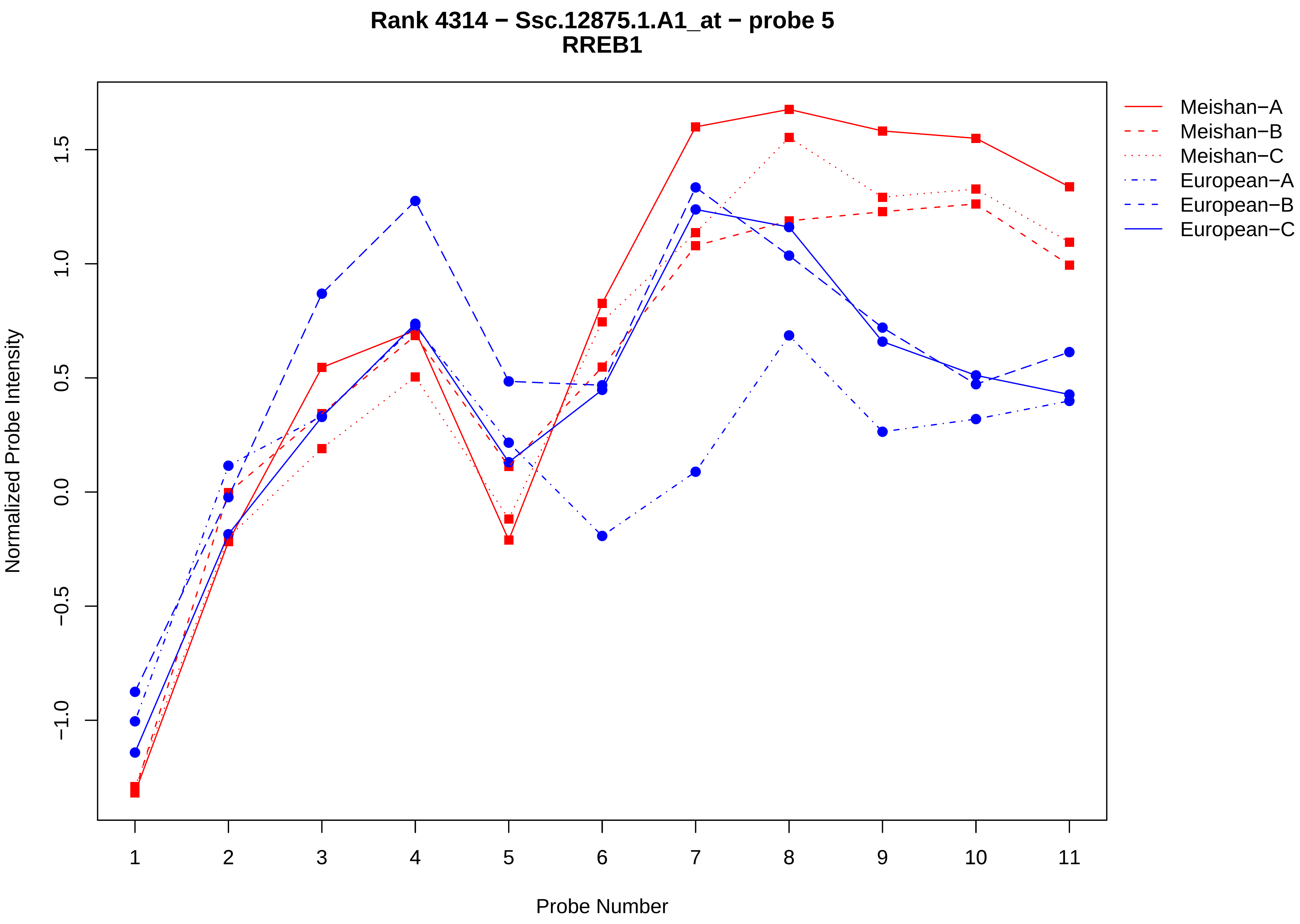
Rank 4314 − Ssc.12875.1.A1_at − probe 5
RREB1
Normalized Probe Intensity
Probe Number
−1.0
−0.5
0.0
0.5
1.0
1.5
1
2
3
4
5
6
7
8
9
10
11
Meishan−A
Meishan−B
Meishan−C
European−A
European−B
European−C

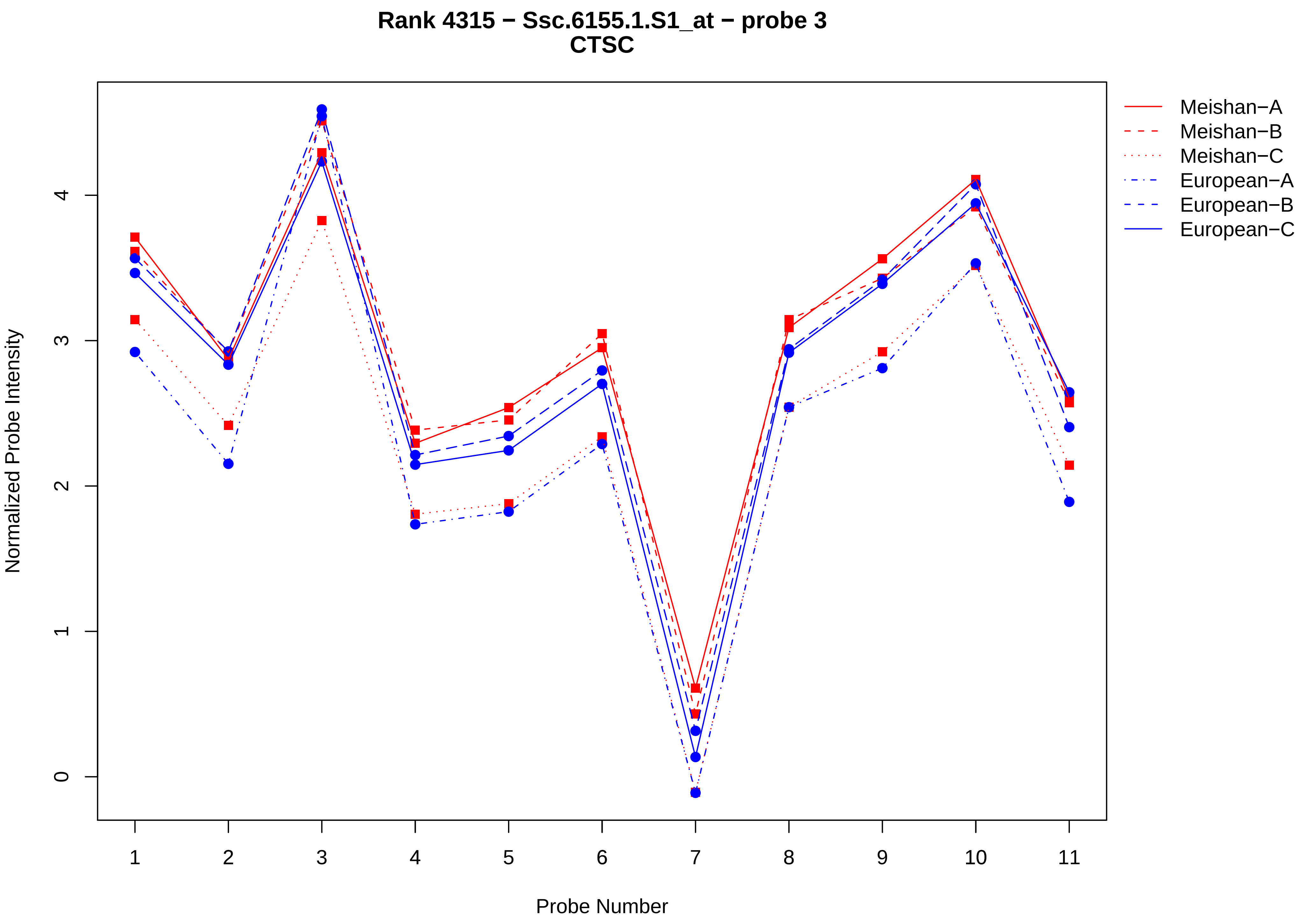

Rank 4315 − Ssc.6155.1.S1_at − probe 3
CTSC
Normalized Probe Intensity
0
1
2
3
4
Probe Number
1
2
3
4
5
6
7
8
9
10
11
Meishan−A
Meishan−B
Meishan−C
European−A
European−B
European−C

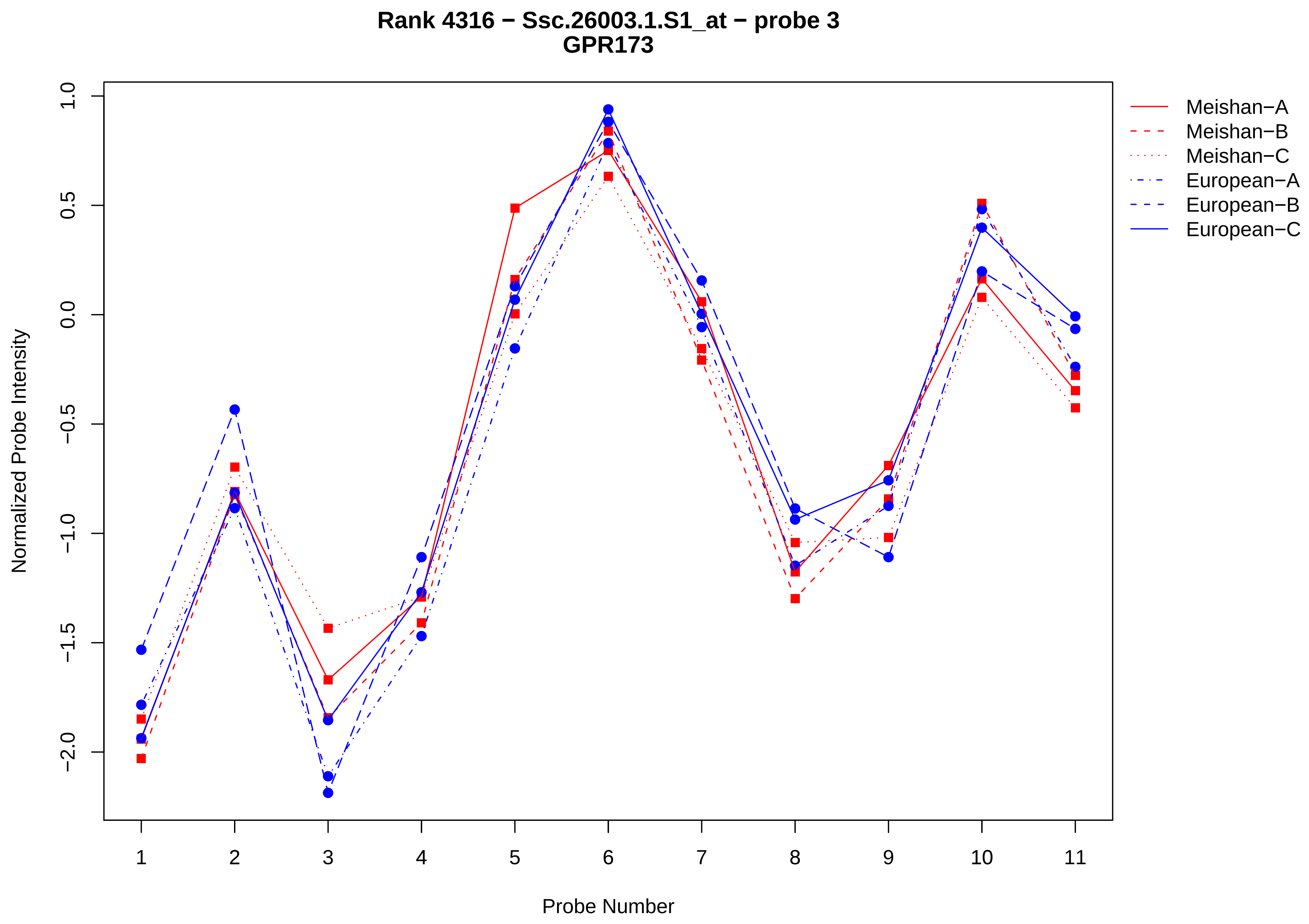

Rank 4316 − Ssc.26003.1.S1_at − probe 3
GPR173
Normalized Probe Intensity
Probe Number
1.0
0.5
0.0
−0.5
−1.0
−1.5
−2.0
1
2
3
4
5
6
7
8
9
10
11
Meishan−A
Meishan−B
Meishan−C
European−A
European−B
European−C

Rank 4317 − Ssc.6604.1.S1_at − probe 5
O75143

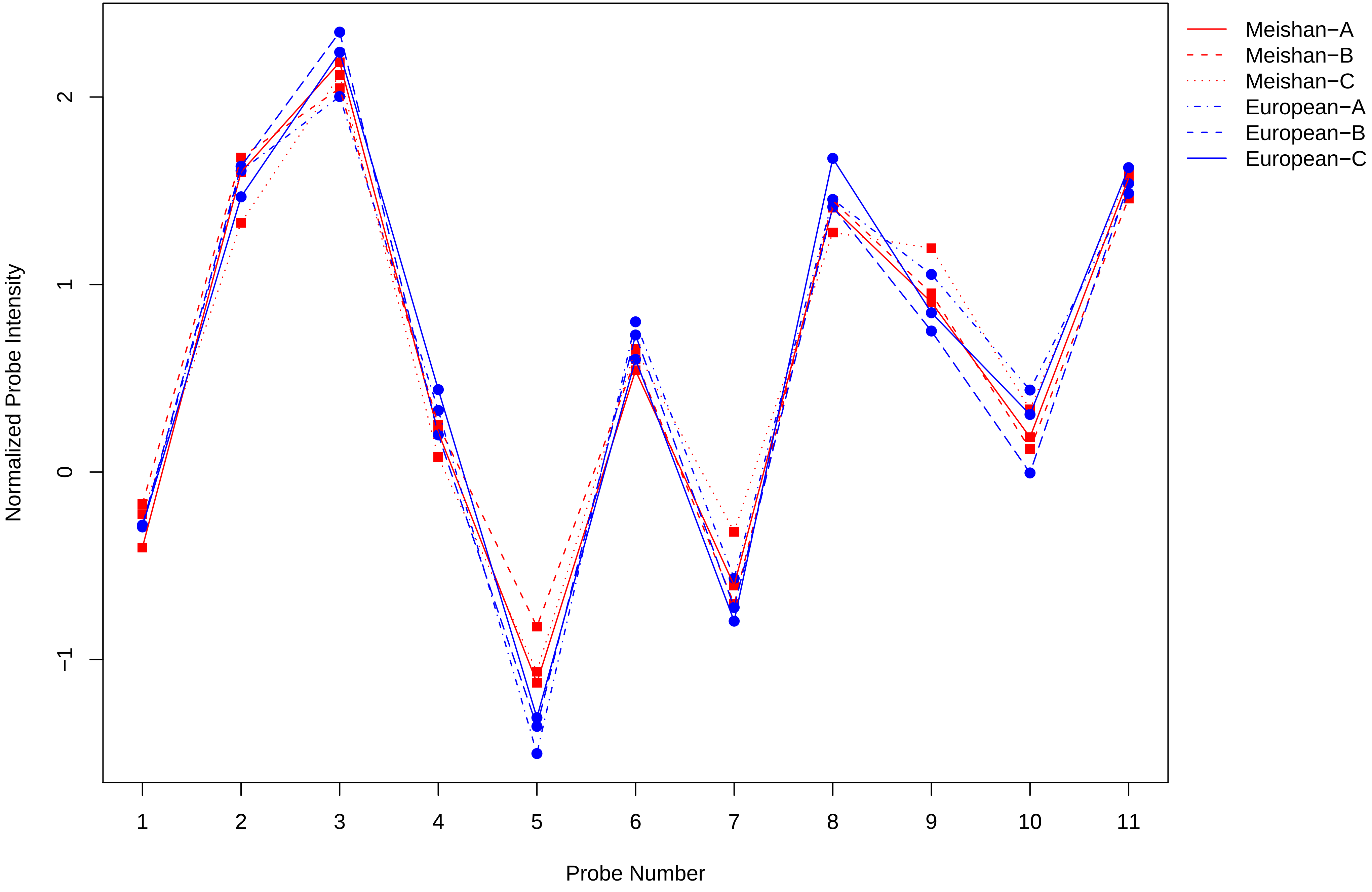

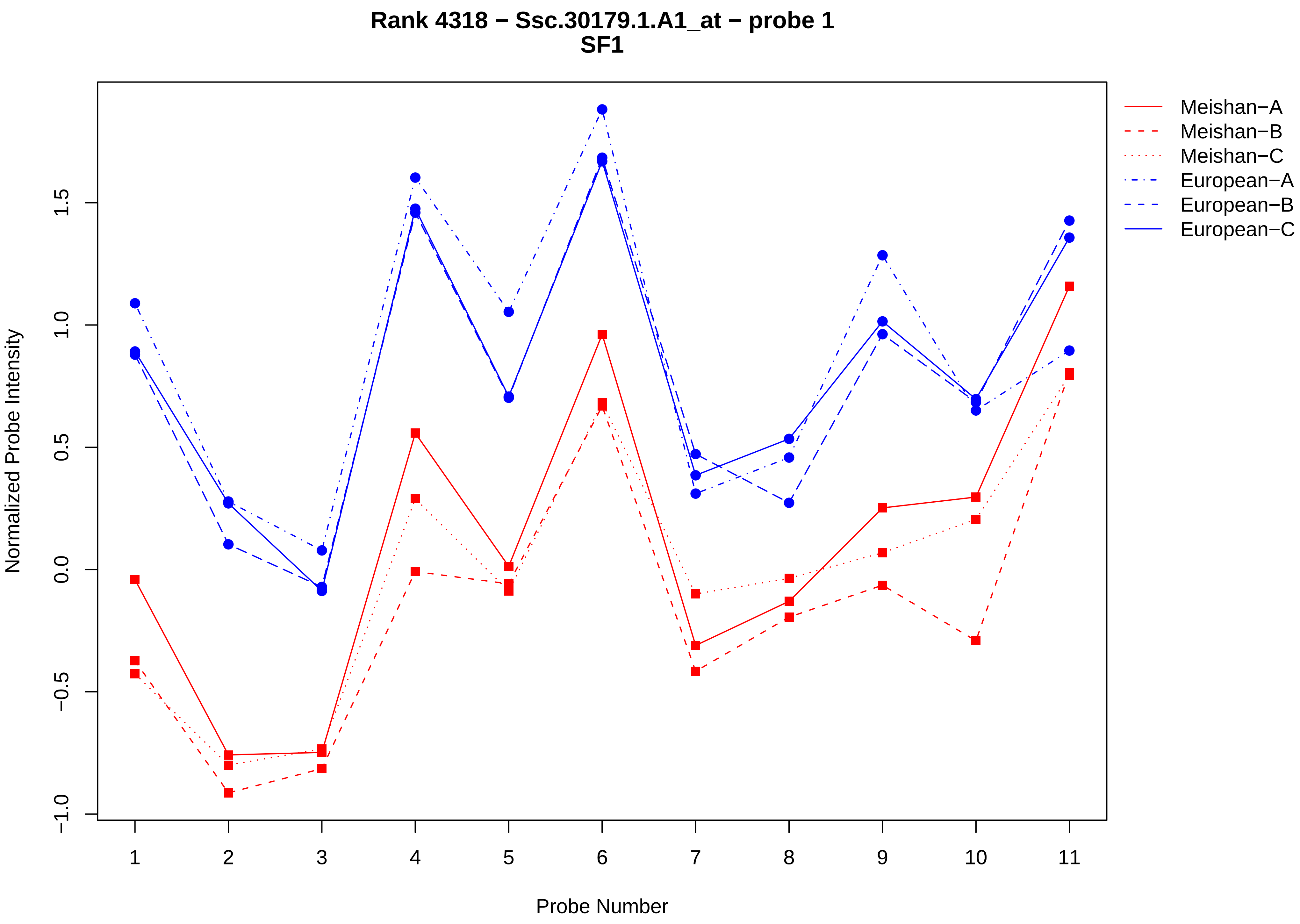

Rank 4318 − Ssc.30179.1.A1_at − probe 1
SF1
Normalized Probe Intensity
−1.0
−0.5
0.0
0.5
1.0
1.5
1
2
3
4
5
6
7
8
9
10
11
Probe Number
Meishan−A
Meishan−B
Meishan−C
European−A
European−B
European−C

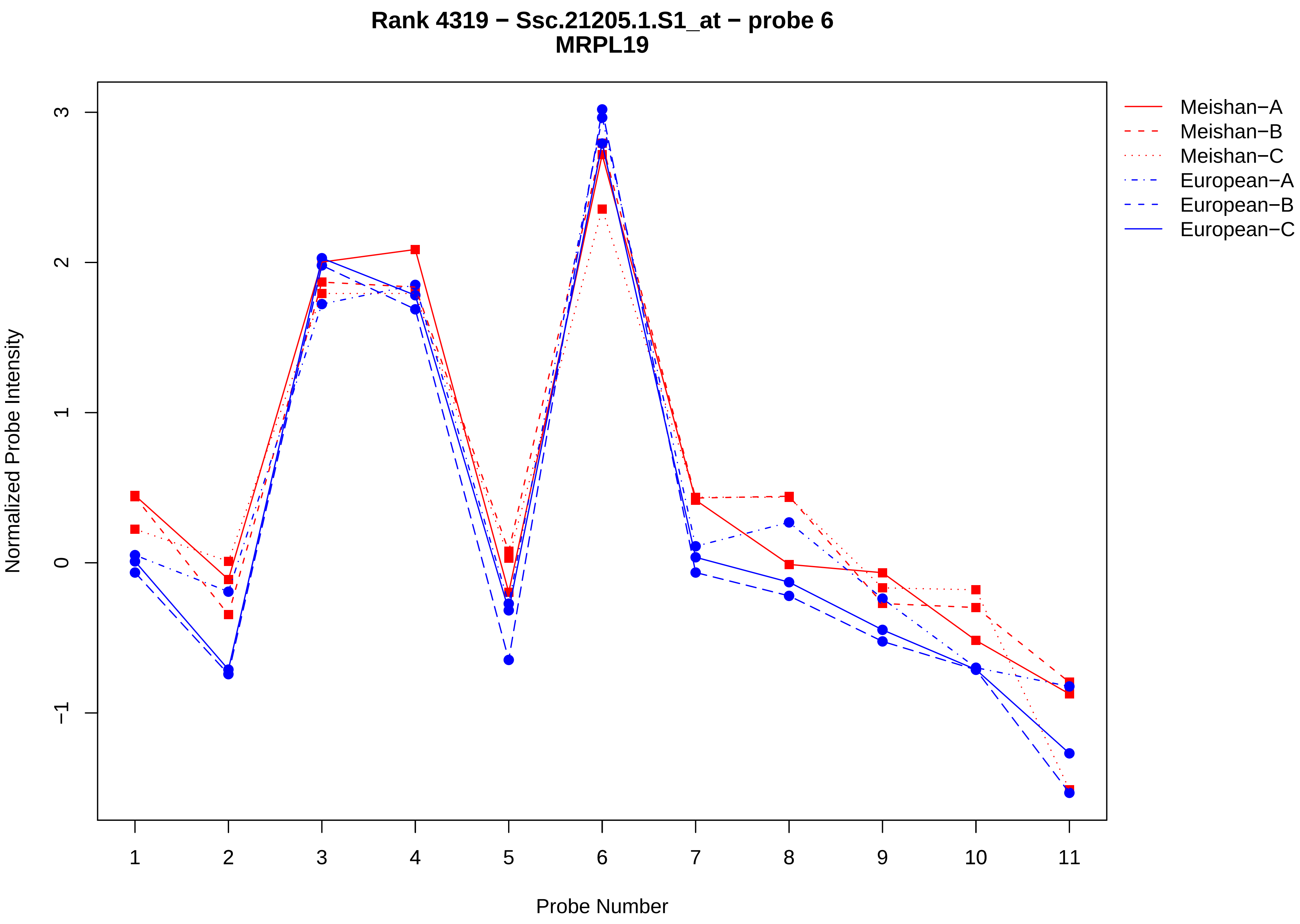

Rank 4319 − Ssc.21205.1.S1_at − probe 6
MRPL19
Normalized Probe Intensity
Probe Number
Meishan−A
Meishan−B
Meishan−C
European−A
European−B
European−C

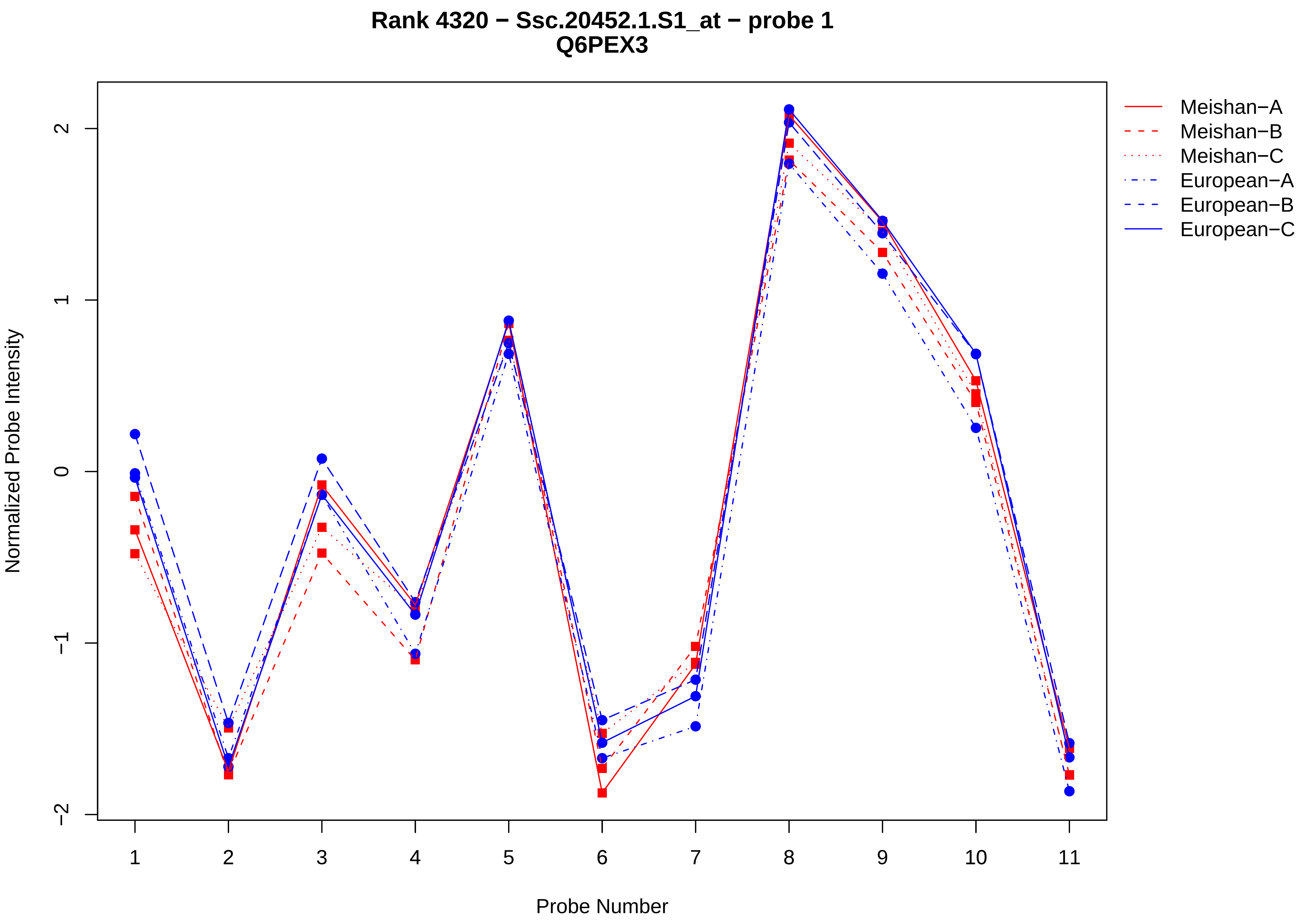

Rank 4320 − Ssc.20452.1.S1_at − probe 1
Q6PEX3
Normalized Probe Intensity
Probe Number
−2
−1
0
1
2
1
2
3
4
5
6
7
8
9
10
11
Meishan−A
Meishan−B
Meishan−C
European−A
European−B
European−C

Rank 4321 − Ssc.8148.2.S1_at − probe 1
KCNMA1

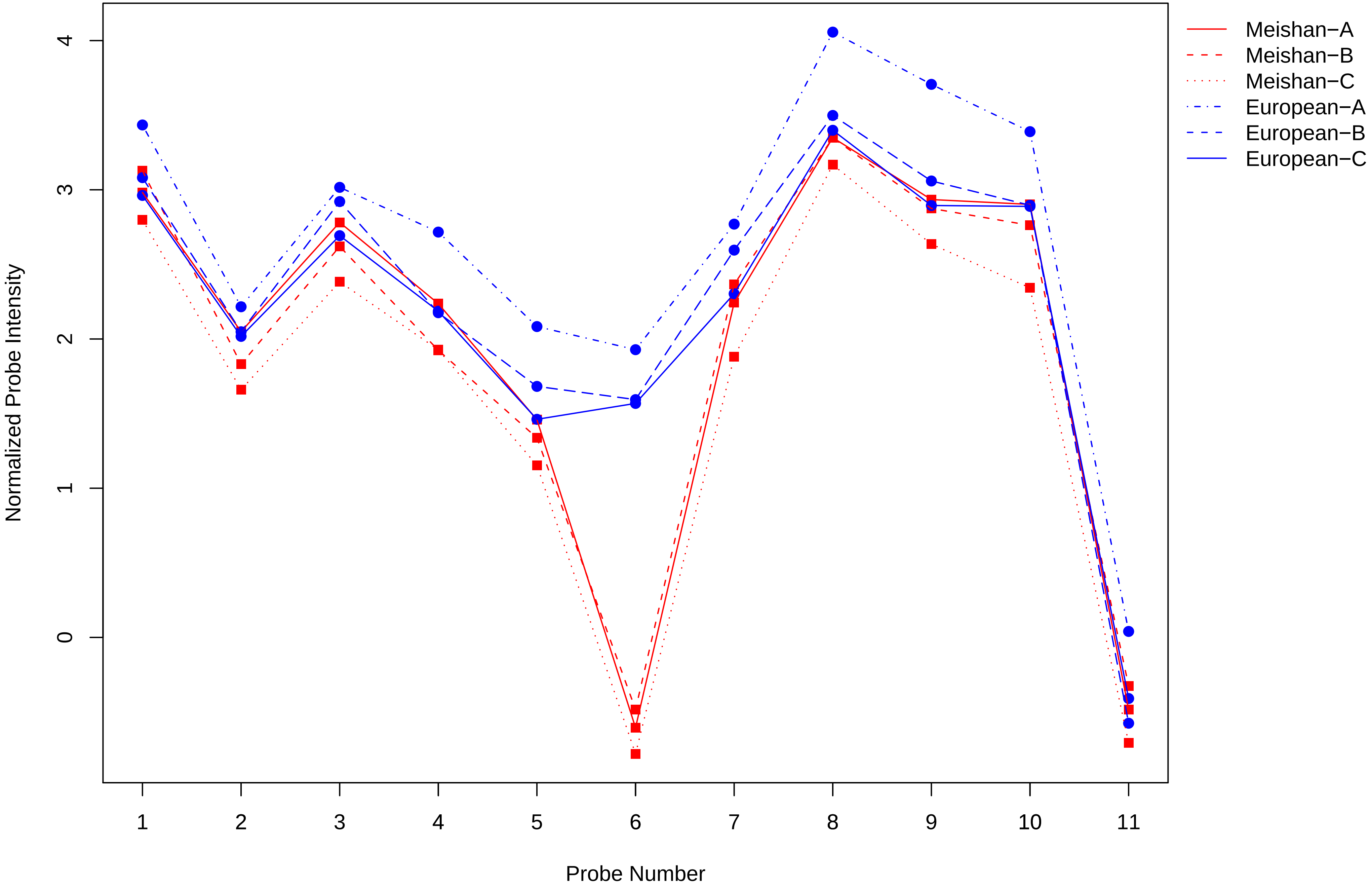

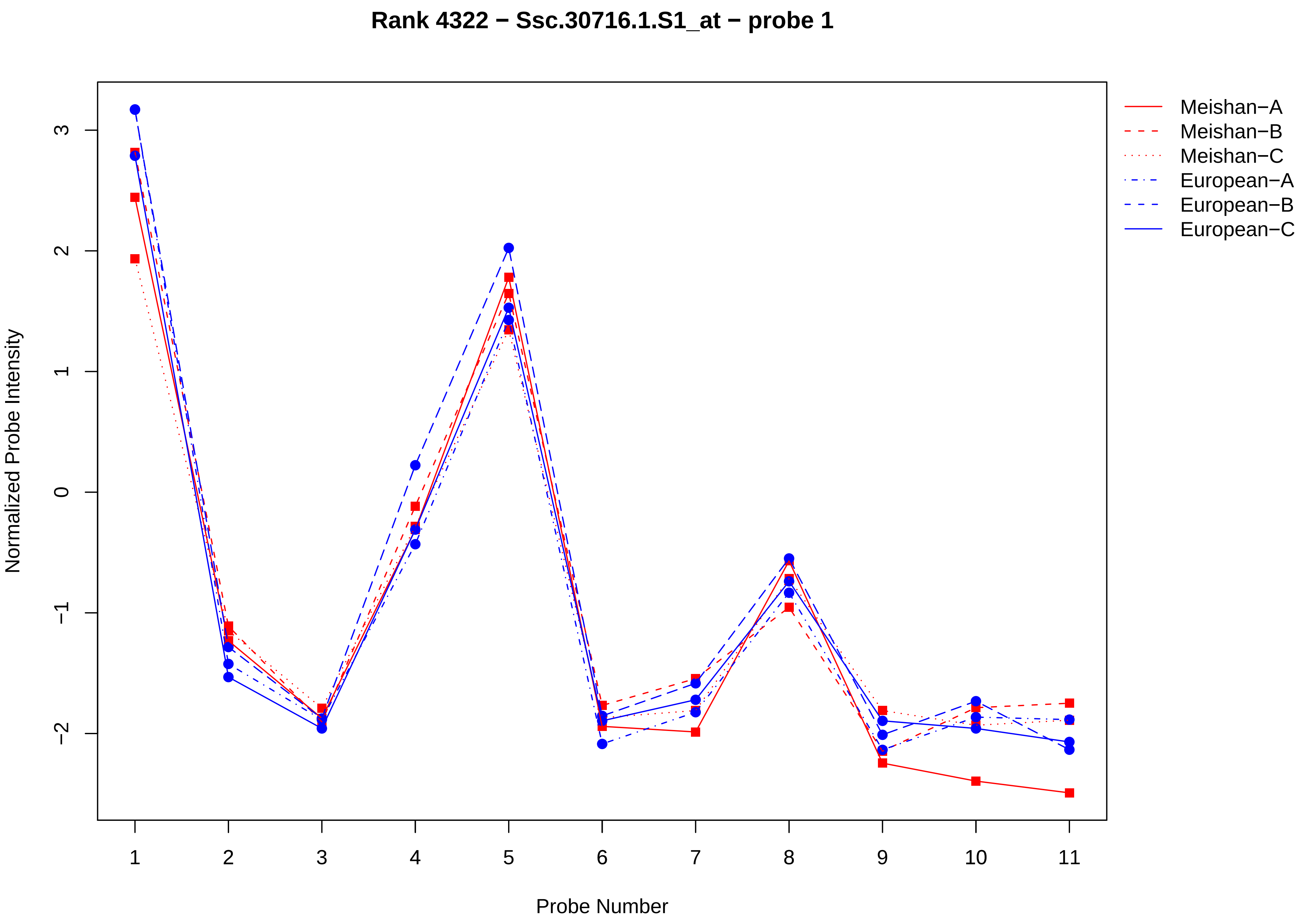

Rank 4322 − Ssc.30716.1.S1_at − probe 1
Normalized Probe Intensity
Probe Number
Meishan−A
Meishan−B
Meishan−C
European−A
European−B
European−C

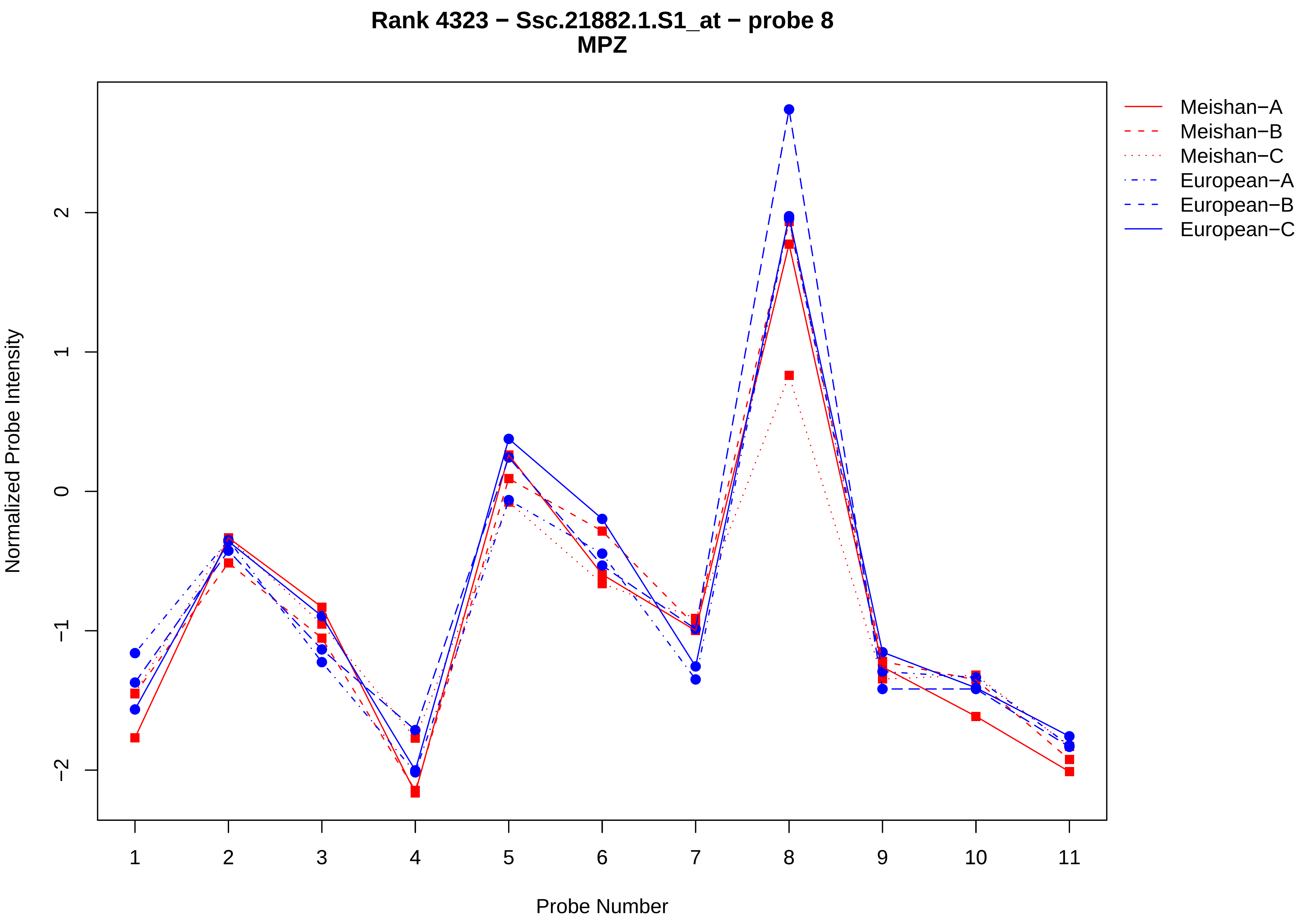

Rank 4323 − Ssc.21882.1.S1_at − probe 8
MPZ
Normalized Probe Intensity
Probe Number
−2
−1
0
1
2
1
2
3
4
5
6
7
8
9
10
11
Meishan−A
Meishan−B
Meishan−C
European−A
European−B
European−C

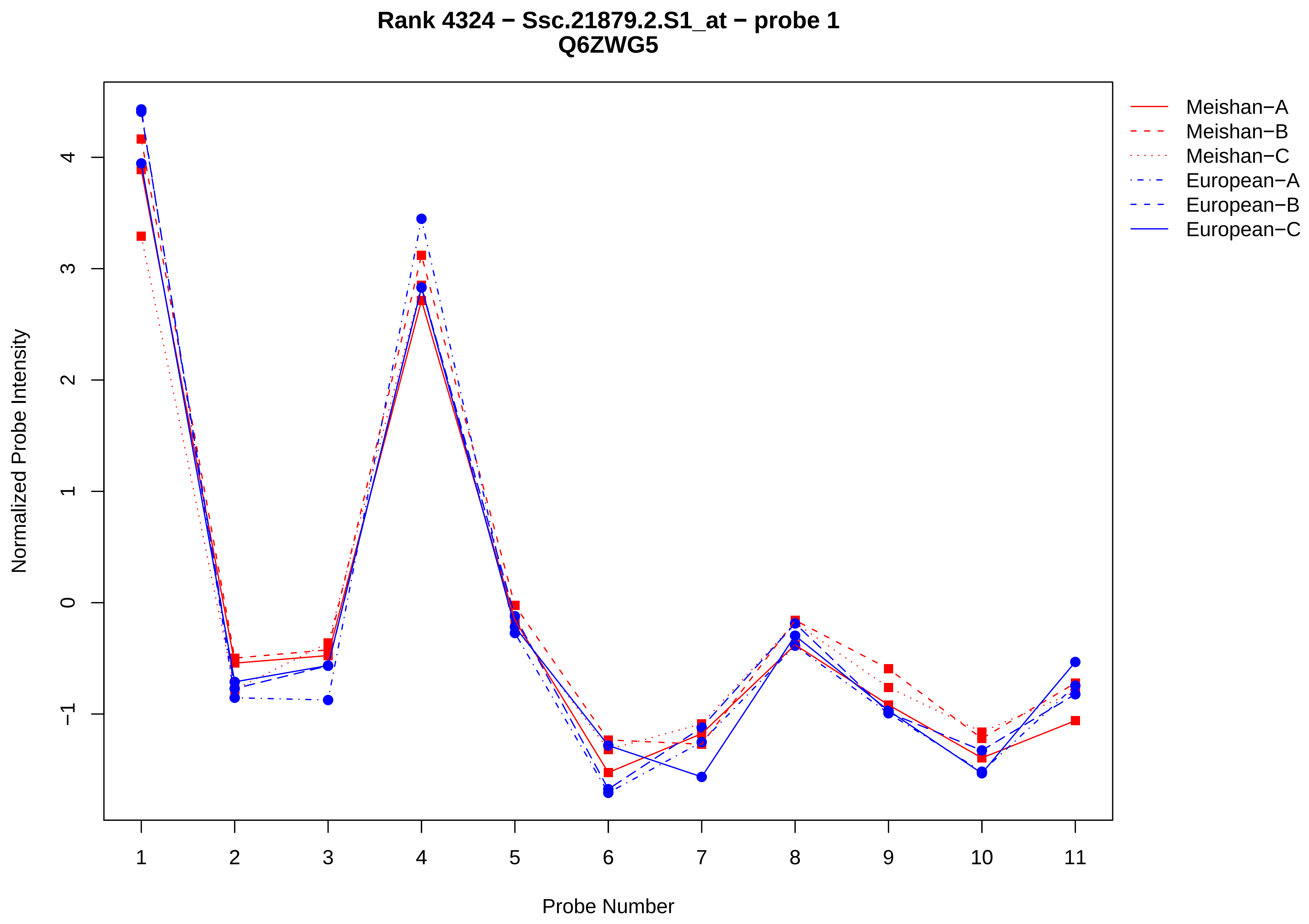

Rank 4324 − Ssc.21879.2.S1_at − probe 1
Q6ZWG5
Normalized Probe Intensity
Probe Number
Meishan−A
Meishan−B
Meishan−C
European−A
European−B
European−C

Rank 4325 − Ssc.3203.1.S1_at − probe 6
PCTK1

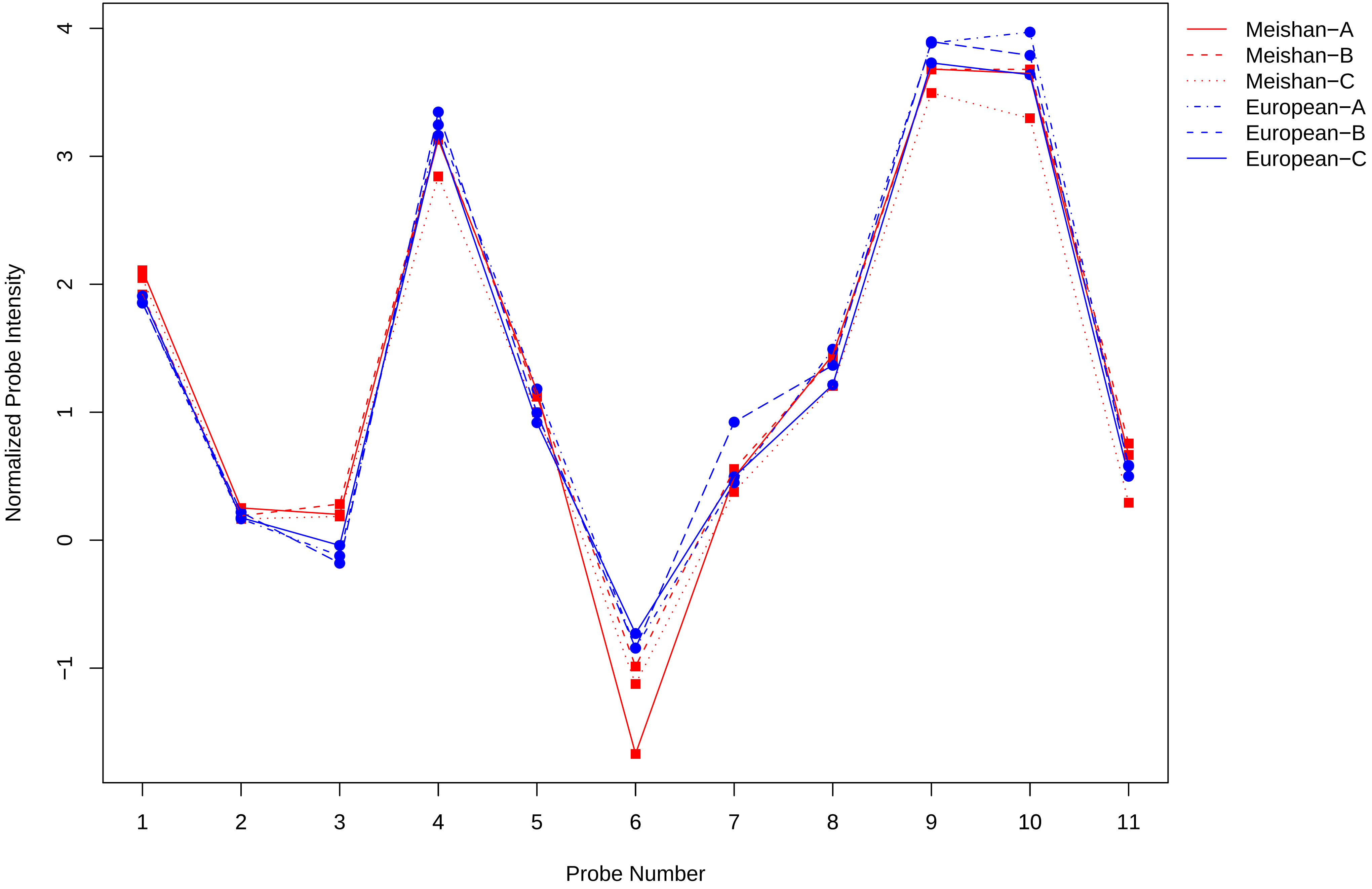

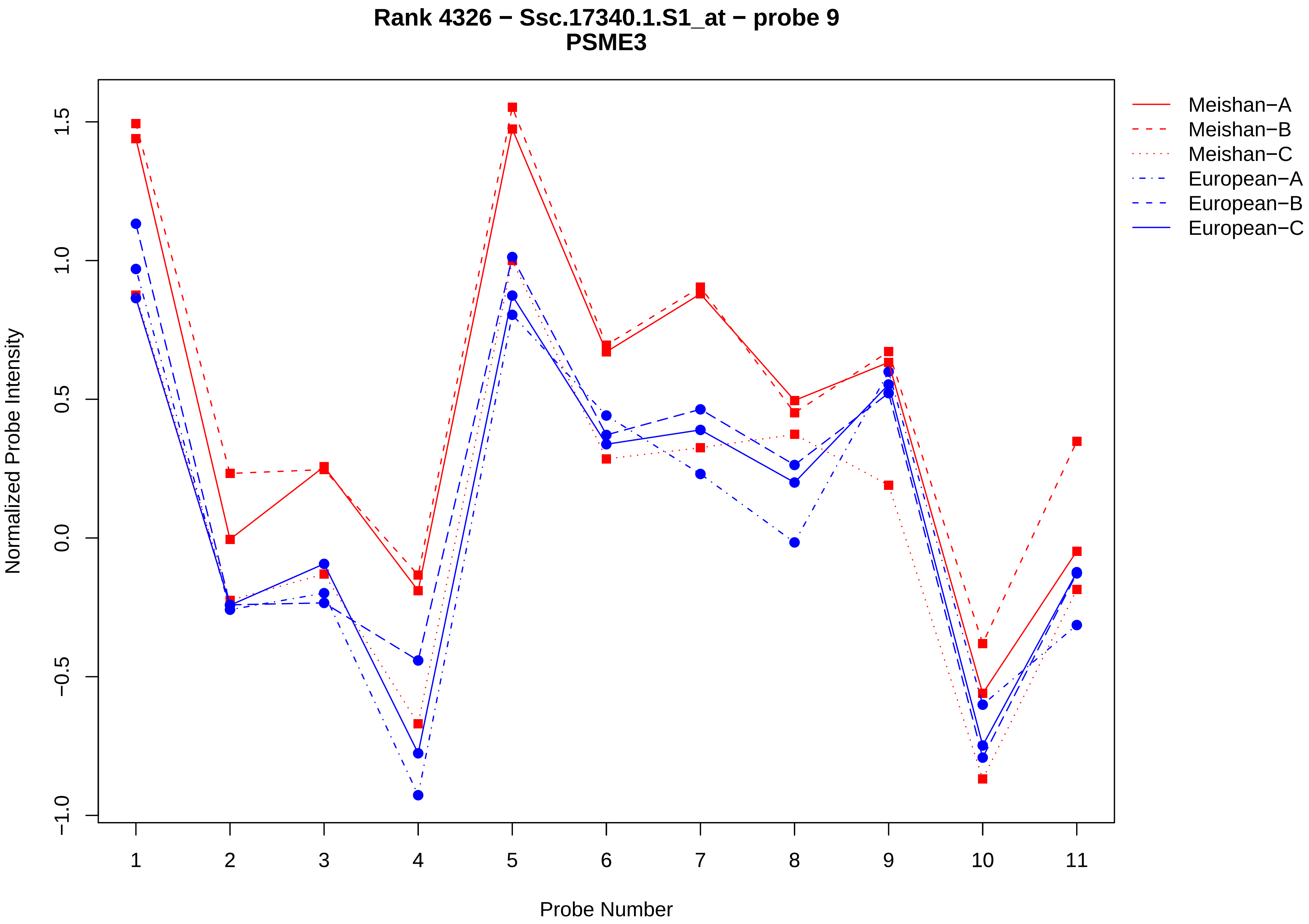

Rank 4326 − Ssc.17340.1.S1_at − probe 9
PSME3
Normalized Probe Intensity
−1.0
−0.5
0.0
0.5
1.0
1.5
1
2
3
4
5
6
7
8
9
10
11
Probe Number
Meishan−A
Meishan−B
Meishan−C
European−A
European−B
European−C

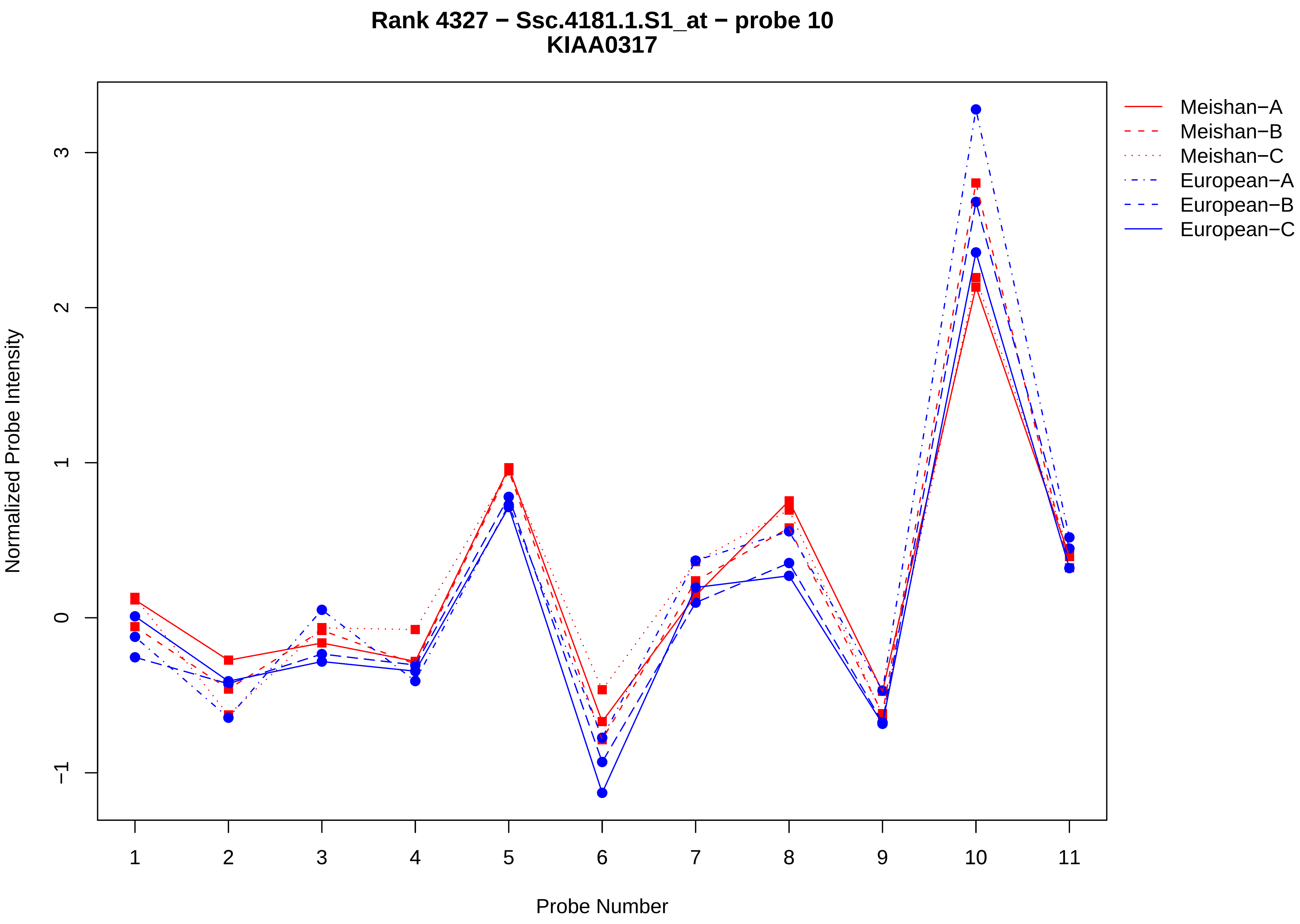

Rank 4327 − Ssc.4181.1.S1_at − probe 10
KIAA0317
Normalized Probe Intensity
Probe Number
−1
0
1
2
3
1
2
3
4
5
6
7
8
9
10
11
Meishan−A
Meishan−B
Meishan−C
European−A
European−B
European−C

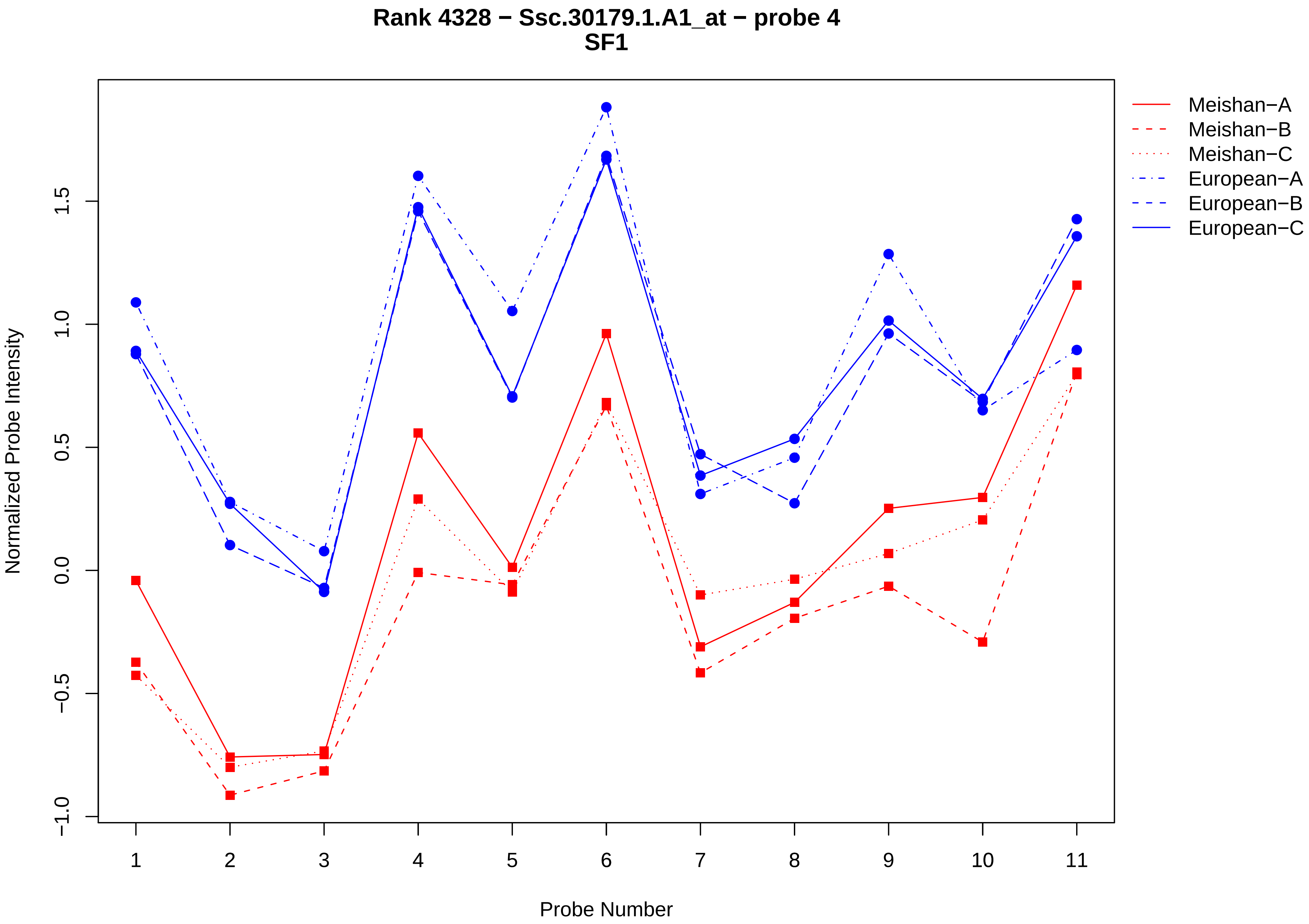

Rank 4328 − Ssc.30179.1.A1_at − probe 4
SF1
Normalized Probe Intensity
−1.0
−0.5
0.0
0.5
1.0
1.5
1
2
3
4
5
6
7
8
9
10
11
Probe Number
Meishan−A
Meishan−B
Meishan−C
European−A
European−B
European−C

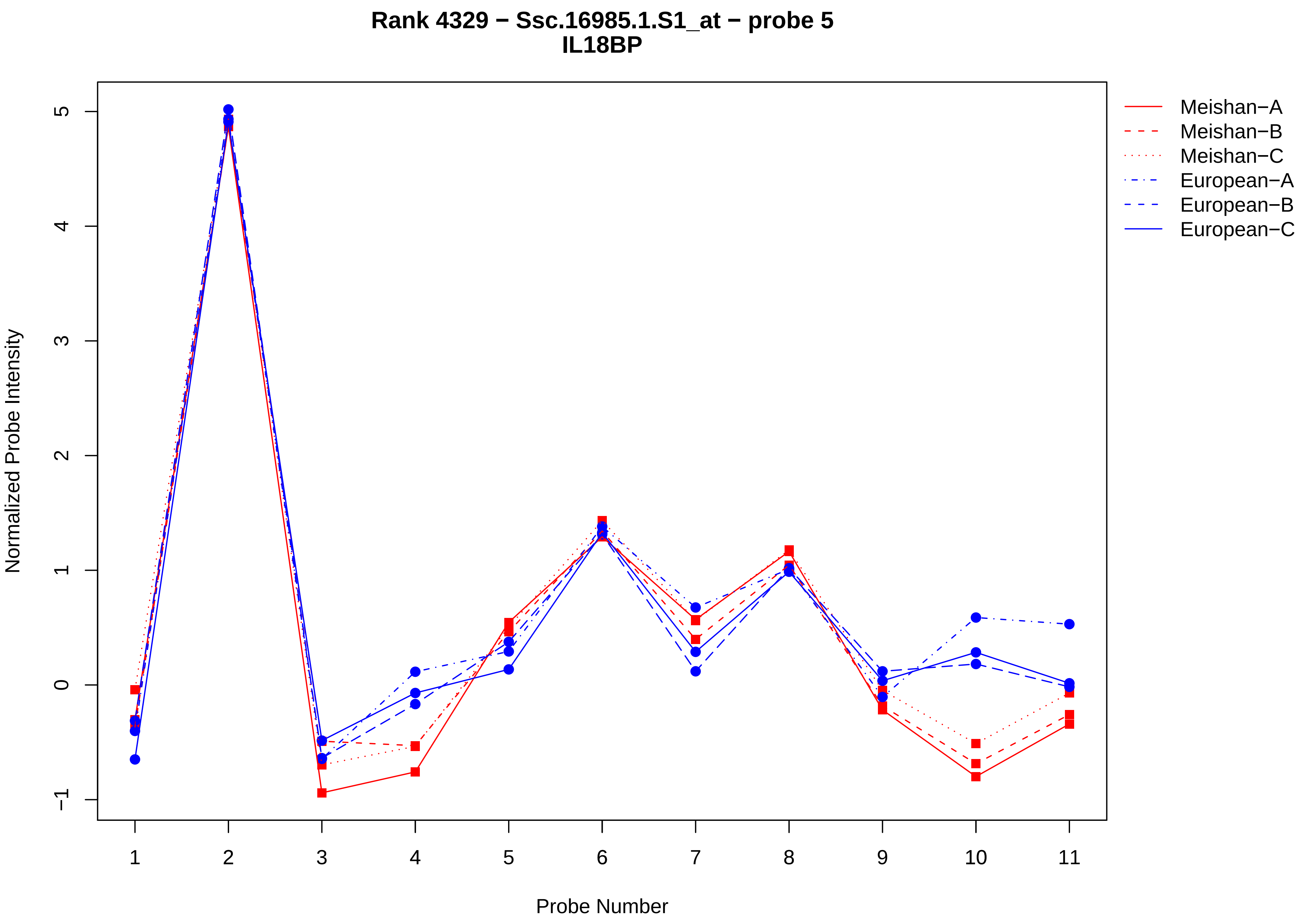

Rank 4329 − Ssc.16985.1.S1_at − probe 5
IL18BP
Normalized Probe Intensity
Probe Number
Meishan−A
Meishan−B
Meishan−C
European−A
European−B
European−C

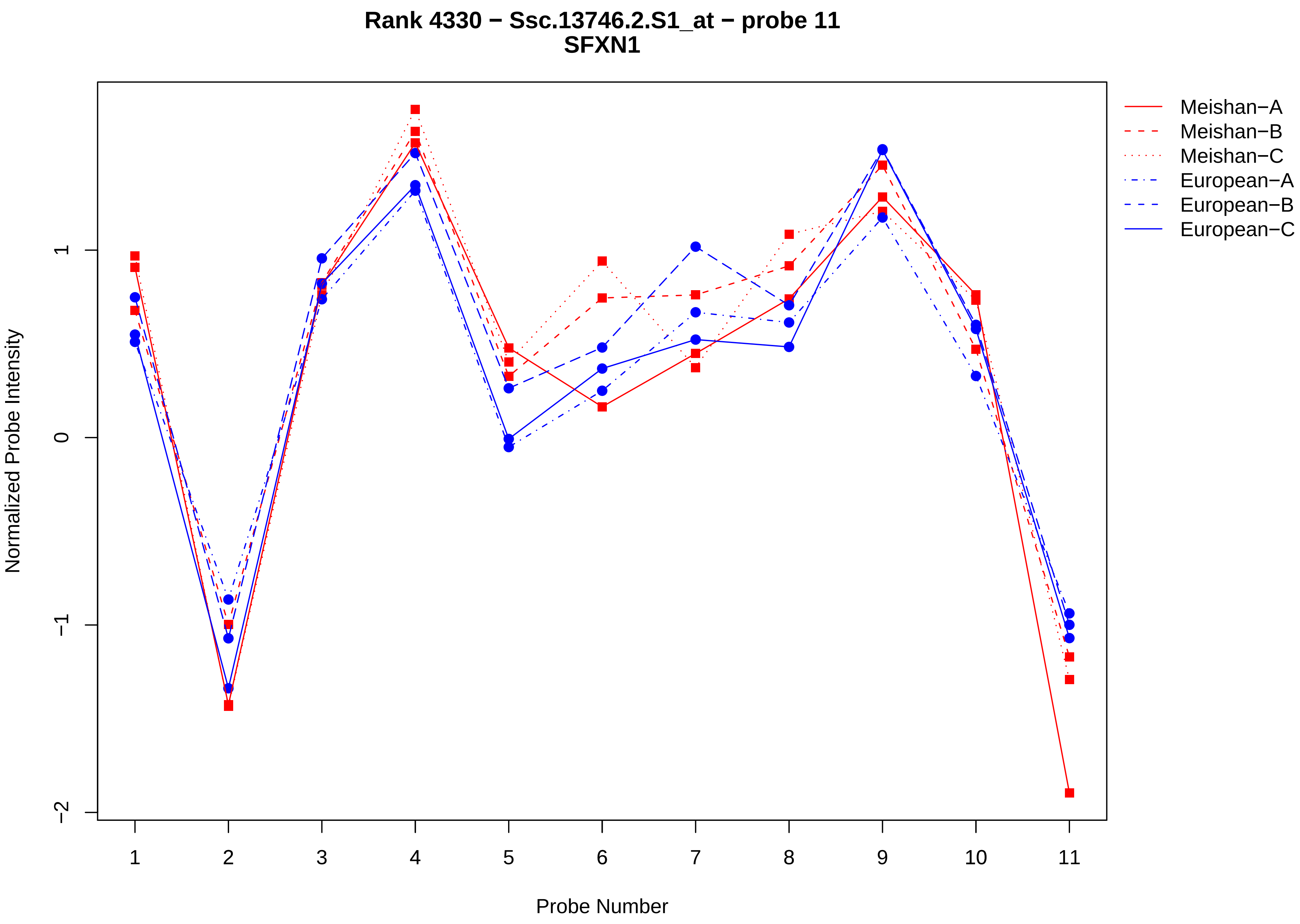

Rank 4330 − Ssc.13746.2.S1_at − probe 11
SFXN1
Normalized Probe Intensity
Probe Number
−2
−1
0
1
1
2
3
4
5
6
7
8
9
10
11
Meishan−A
Meishan−B
Meishan−C
European−A
European−B
European−C

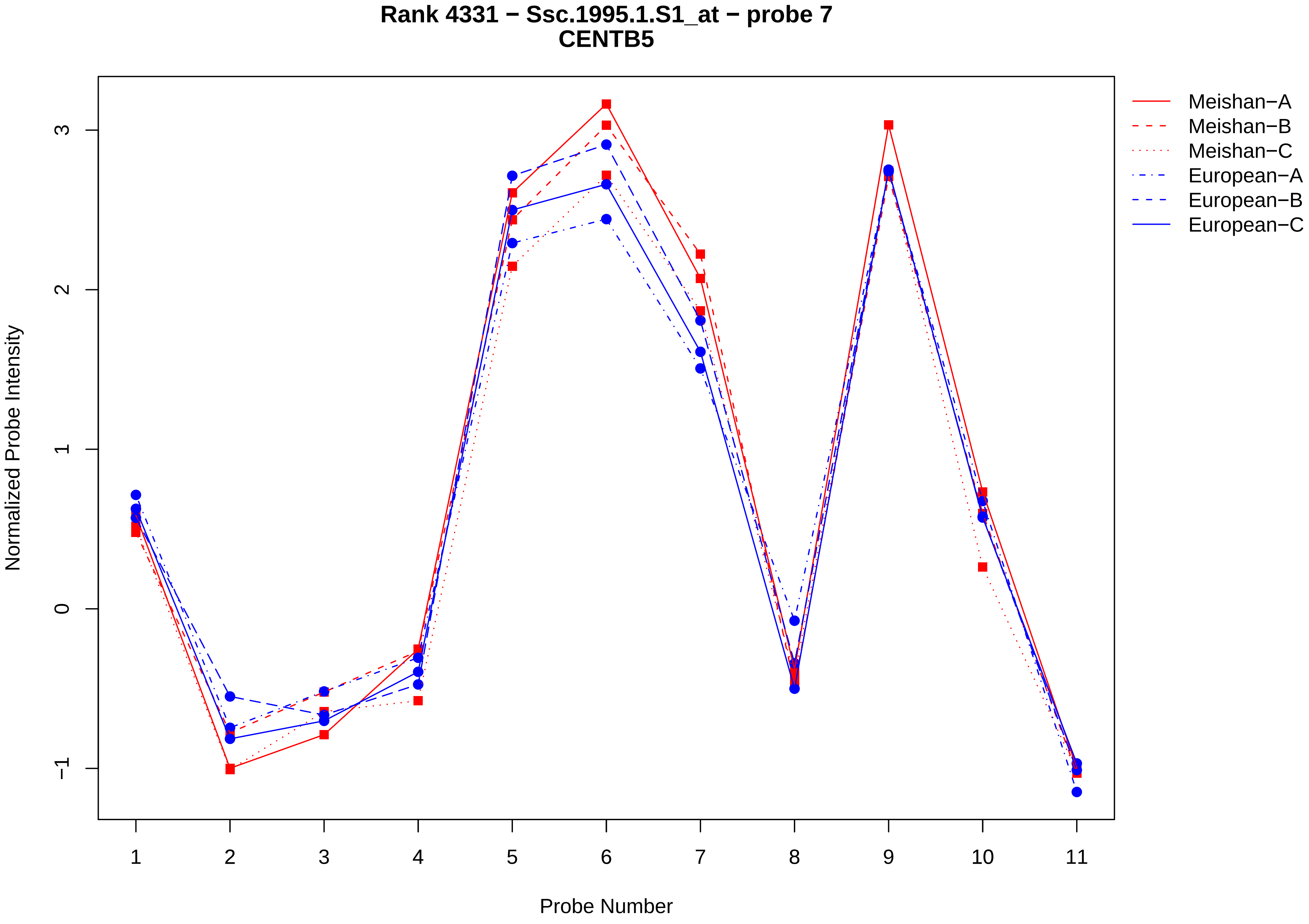

Rank 4331 − Ssc.1995.1.S1_at − probe 7
CENTB5
Normalized Probe Intensity
Probe Number
Meishan−A
Meishan−B
Meishan−C
European−A
European−B
European−C
−1
0
1
2
3
1
2
3
4
5
6
7
8
9
10
11

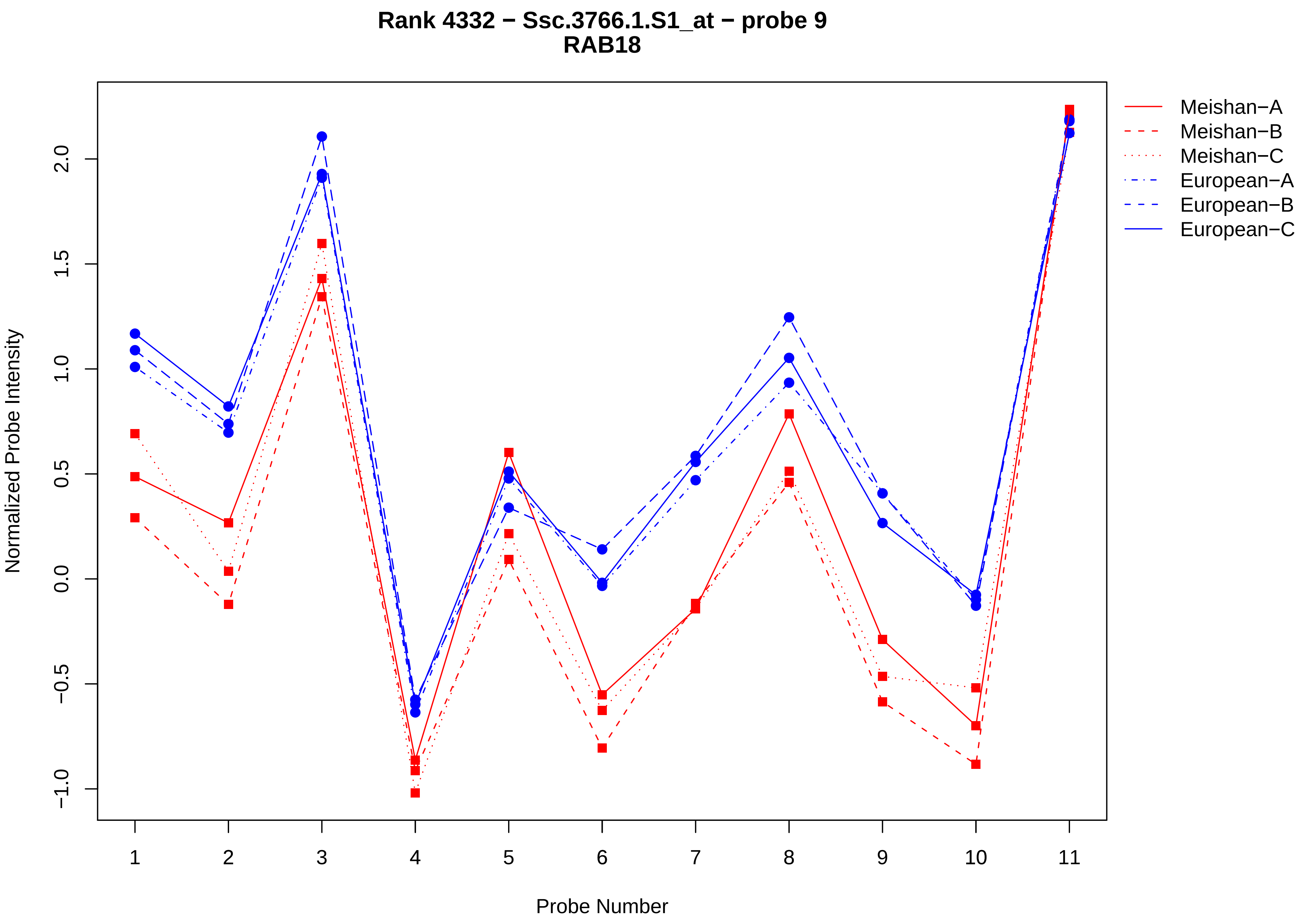

Rank 4332 − Ssc.3766.1.S1_at − probe 9
RAB18
Normalized Probe Intensity
Probe Number
−1.0
−0.5
0.0
0.5
1.0
1.5
2.0
1
2
3
4
5
6
7
8
9
10
11
Meishan−A
Meishan−B
Meishan−C
European−A
European−B
European−C

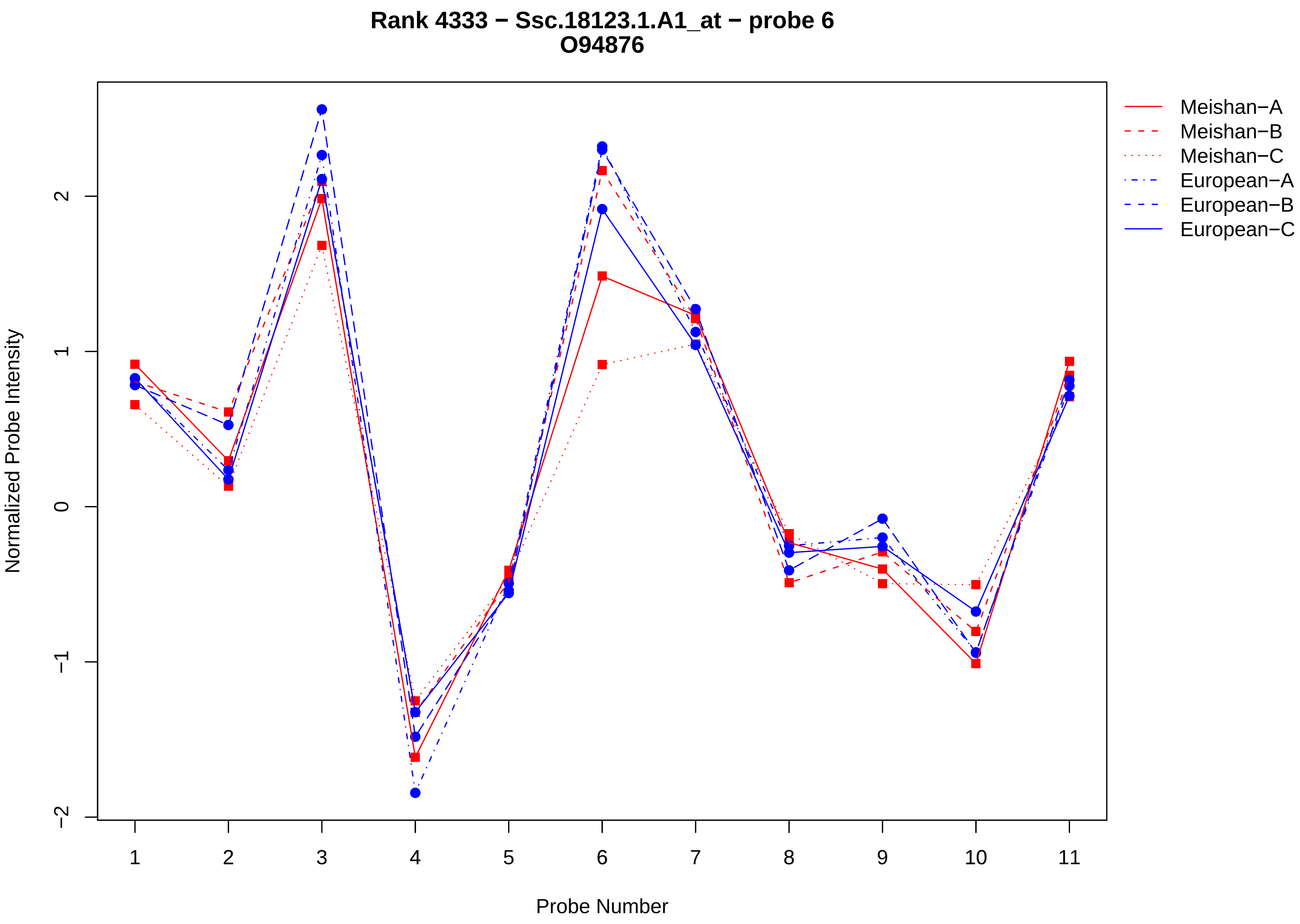

Rank 4333 − Ssc.18123.1.A1_at − probe 6
O94876
Normalized Probe Intensity
Probe Number
−2
−1
0
1
2
1
2
3
4
5
6
7
8
9
10
11
Meishan−A
Meishan−B
Meishan−C
European−A
European−B
European−C

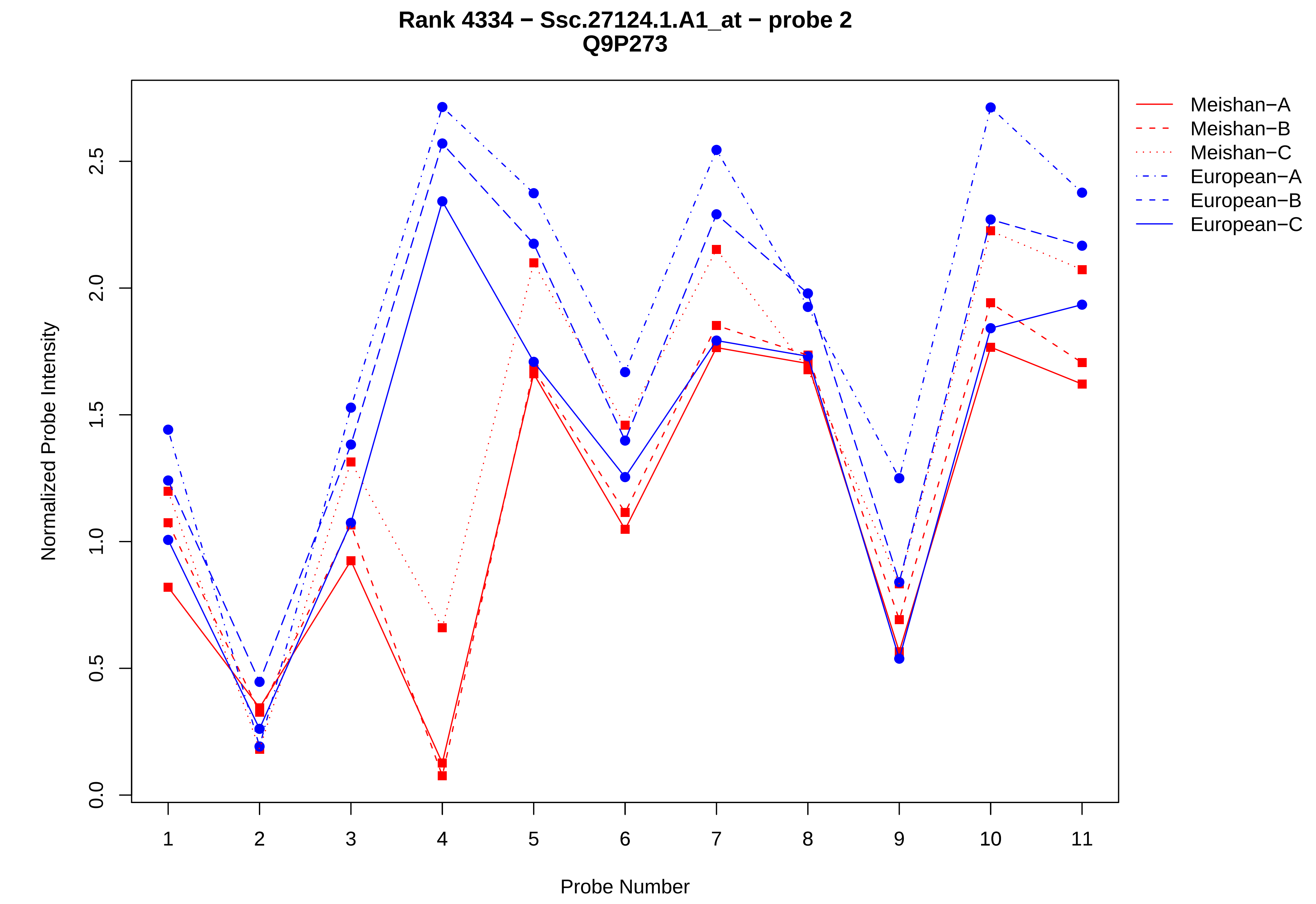

Rank 4334 − Ssc.27124.1.A1_at − probe 2
Q9P273
Normalized Probe Intensity
0.0
0.5
1.0
1.5
2.0
2.5
1
2
3
4
5
6
7
8
9
10
11
Probe Number
Meishan−A
Meishan−B
Meishan−C
European−A
European−B
European−C

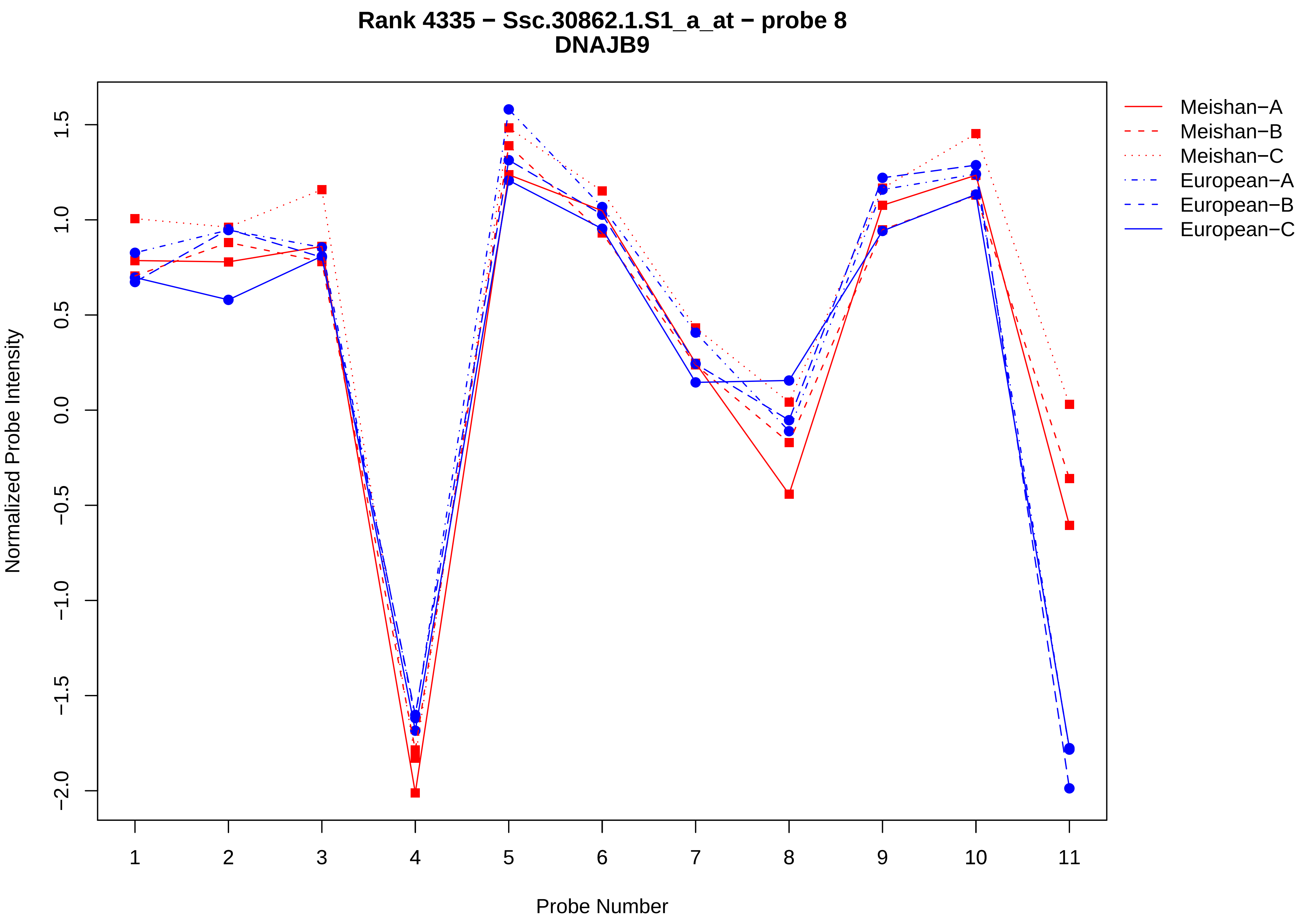

Rank 4335 − Ssc.30862.1.S1_a_at − probe 8
DNAJB9
Normalized Probe Intensity
Probe Number
−2.0
−1.5
−1.0
−0.5
0.0
0.5
1.0
1.5
1
2
3
4
5
6
7
8
9
10
11
Meishan−A
Meishan−B
Meishan−C
European−A
European−B
European−C

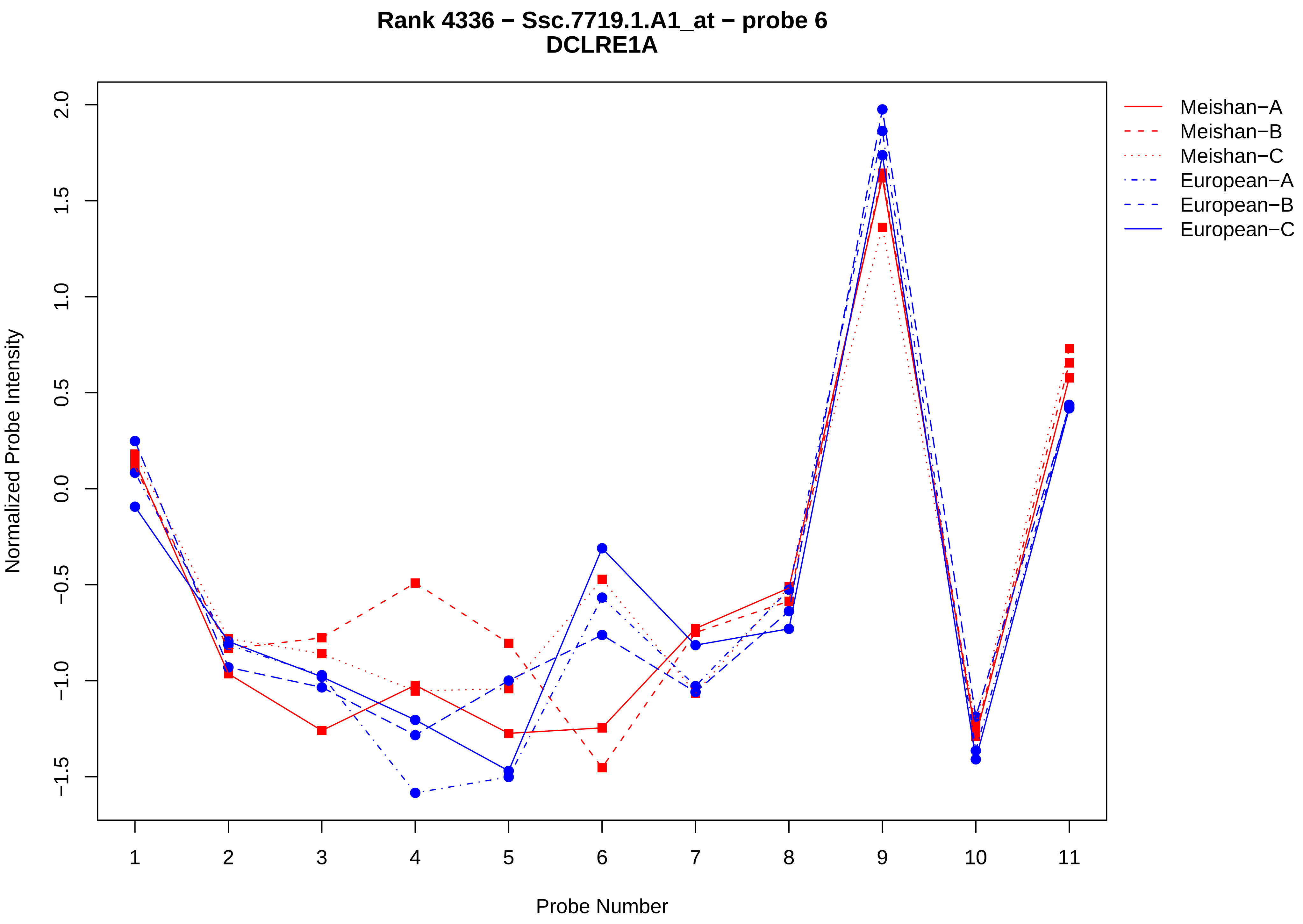

Rank 4336 − Ssc.7719.1.A1_at − probe 6
DCLRE1A
Normalized Probe Intensity
Probe Number
Meishan−A
Meishan−B
Meishan−C
European−A
European−B
European−C
−1.5
−1.0
−0.5
0.0
0.5
1.0
1.5
2.0
1
2
3
4
5
6
7
8
9
10
11

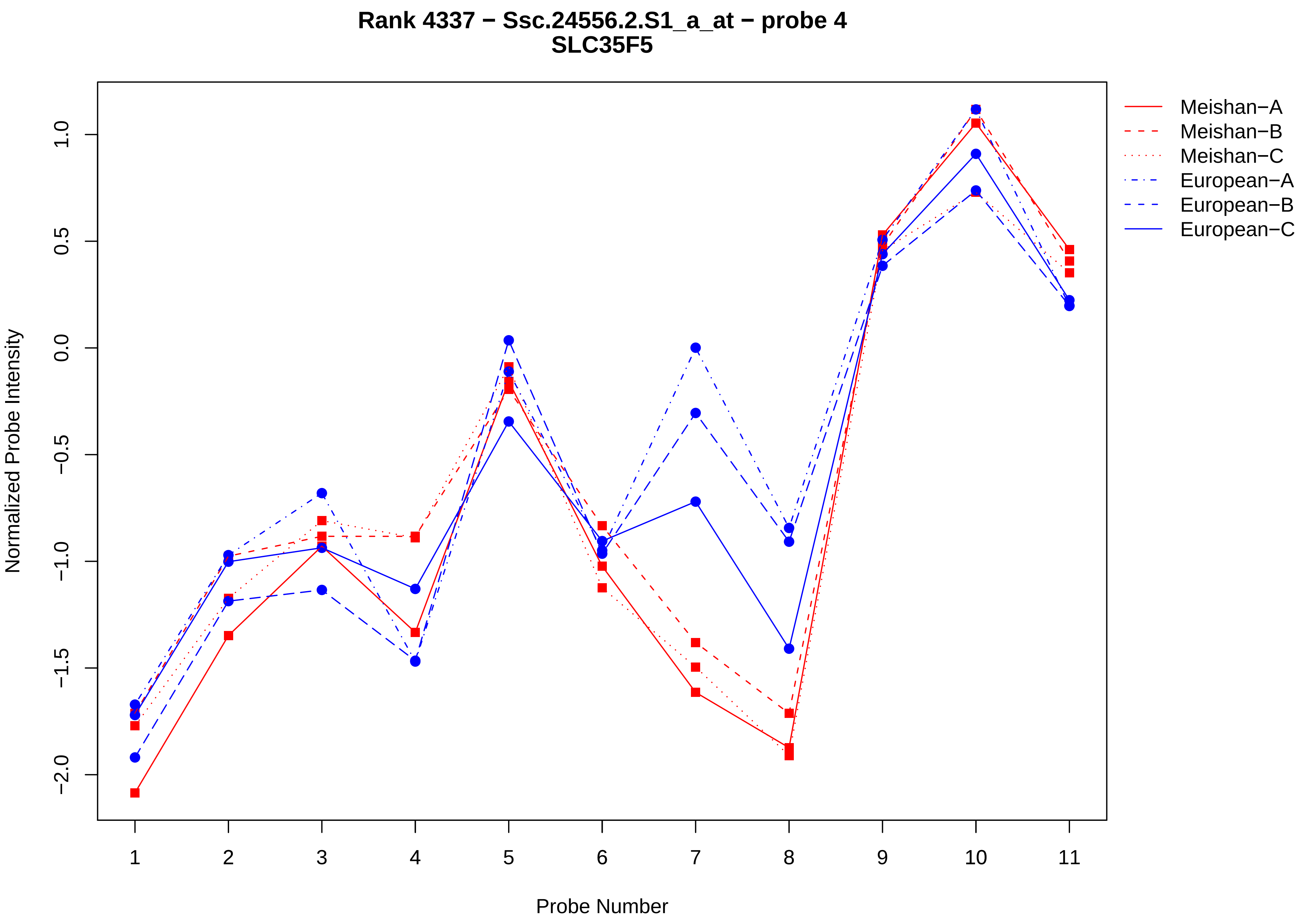

Rank 4337 − Ssc.24556.2.S1_a_at − probe 4
SLC35F5
Normalized Probe Intensity
Probe Number
−2.0
−1.5
−1.0
−0.5
0.0
0.5
1.0
1
2
3
4
5
6
7
8
9
10
11
Meishan−A
Meishan−B
Meishan−C
European−A
European−B
European−C

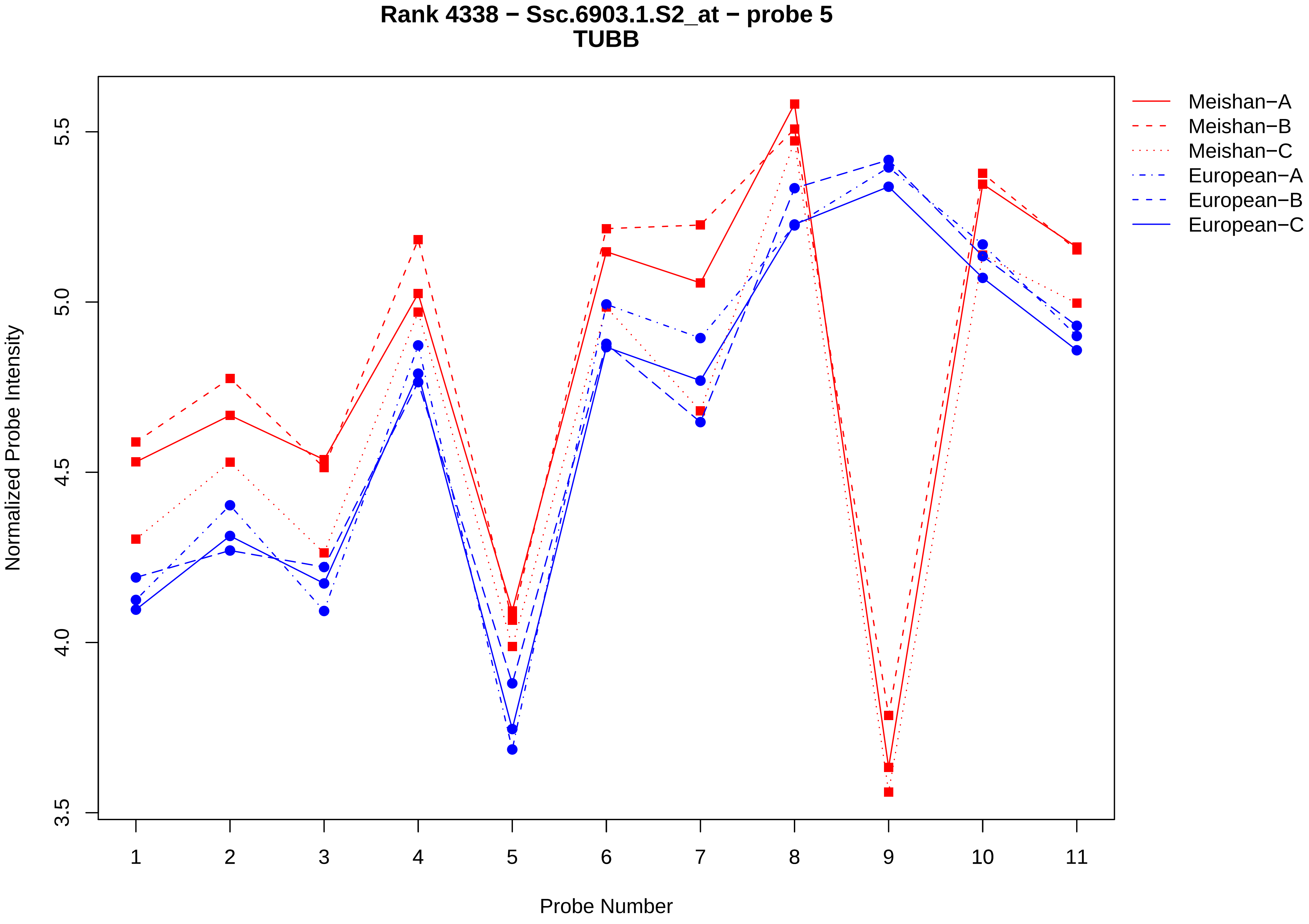

Rank 4338 − Ssc.6903.1.S2_at − probe 5
TUBB
Normalized Probe Intensity
Probe Number
3.5
4.0
4.5
5.0
5.5
1
2
3
4
5
6
7
8
9
10
11
Meishan−A
Meishan−B
Meishan−C
European−A
European−B
European−C

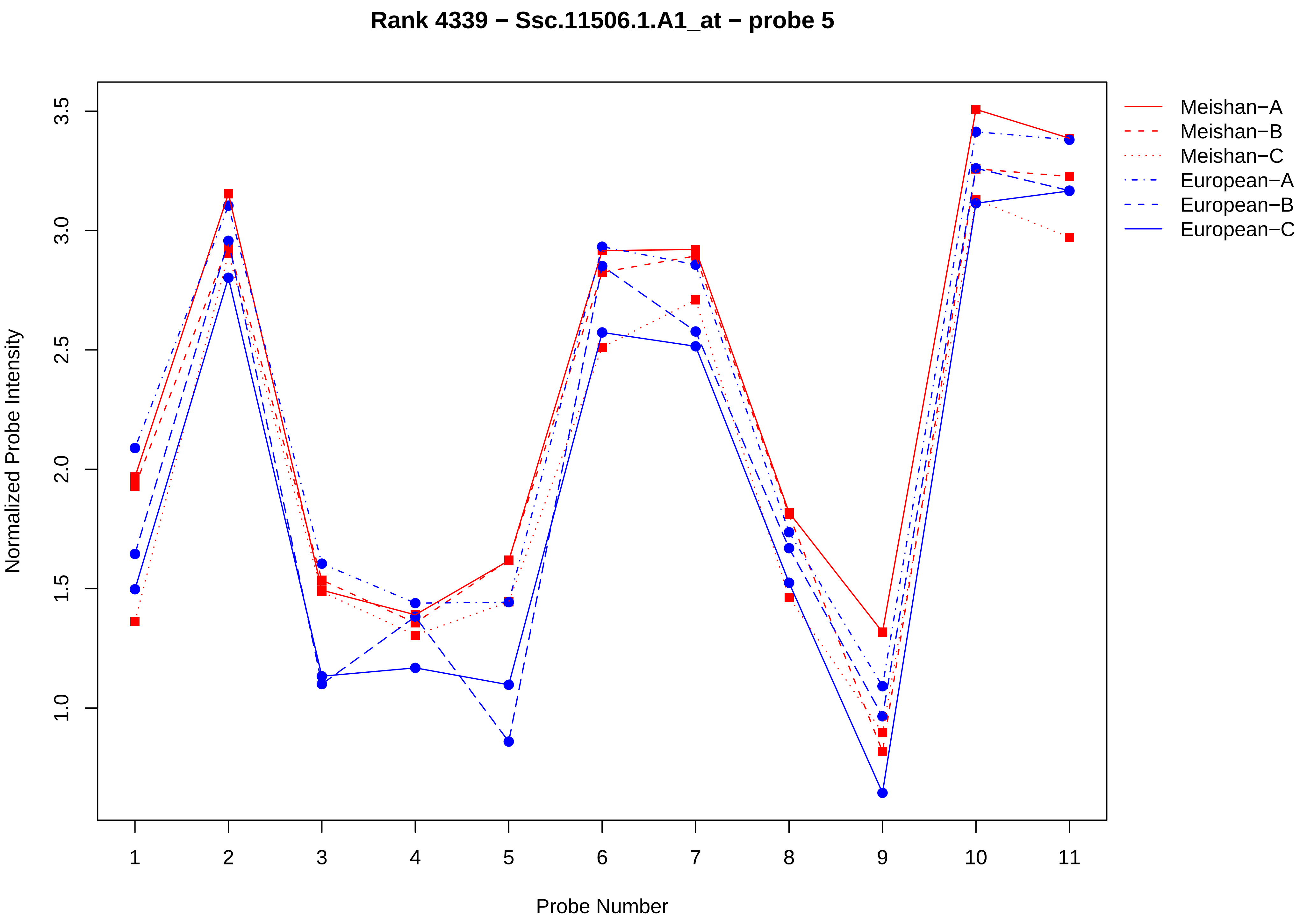

Rank 4339 − Ssc.11506.1.A1_at − probe 5
Normalized Probe Intensity
1.0
1.5
2.0
2.5
3.0
3.5
1
2
3
4
5
6
7
8
9
10
11
Probe Number
Meishan−A
Meishan−B
Meishan−C
European−A
European−B
European−C

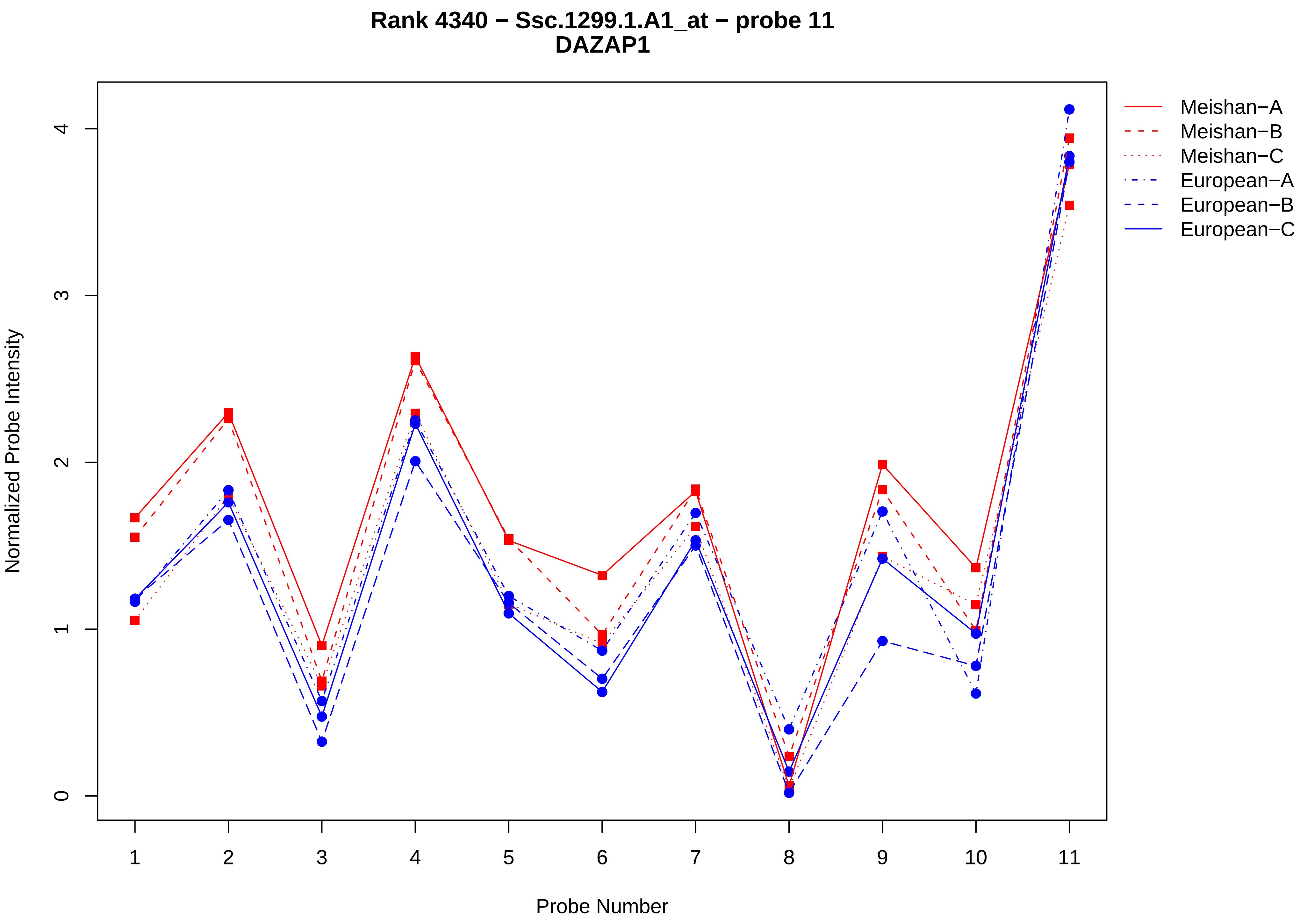

Rank 4340 − Ssc.1299.1.A1_at − probe 11
DAZAP1
Normalized Probe Intensity
Probe Number
0
1
2
3
4
1
2
3
4
5
6
7
8
9
10
11
Meishan−A
Meishan−B
Meishan−C
European−A
European−B
European−C

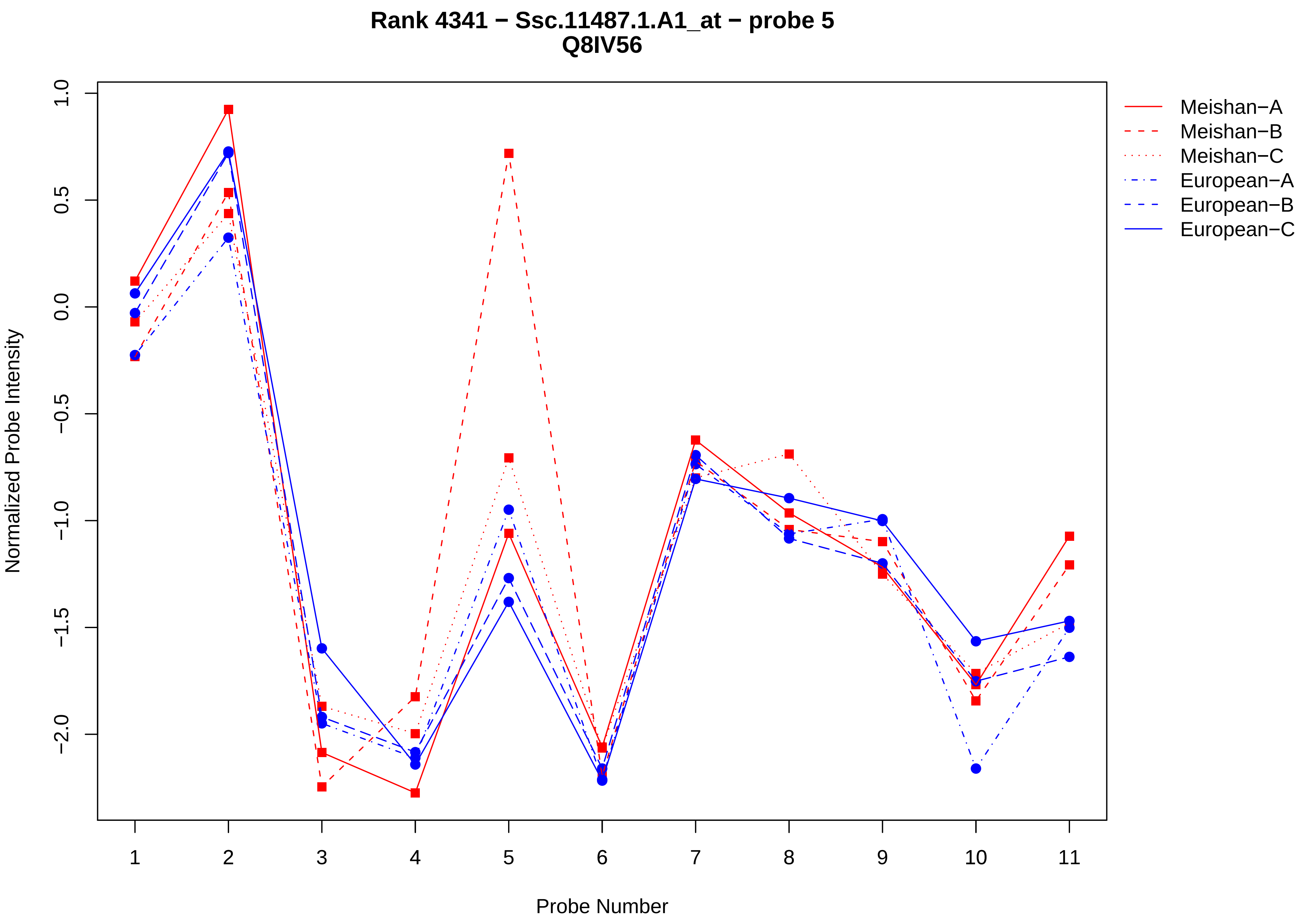

Rank 4341 − Ssc.11487.1.A1_at − probe 5
Q8IV56
Normalized Probe Intensity
Probe Number
1.0
0.5
0.0
−0.5
−1.0
−1.5
−2.0
1
2
3
4
5
6
7
8
9
10
11
Meishan−A
Meishan−B
Meishan−C
European−A
European−B
European−C

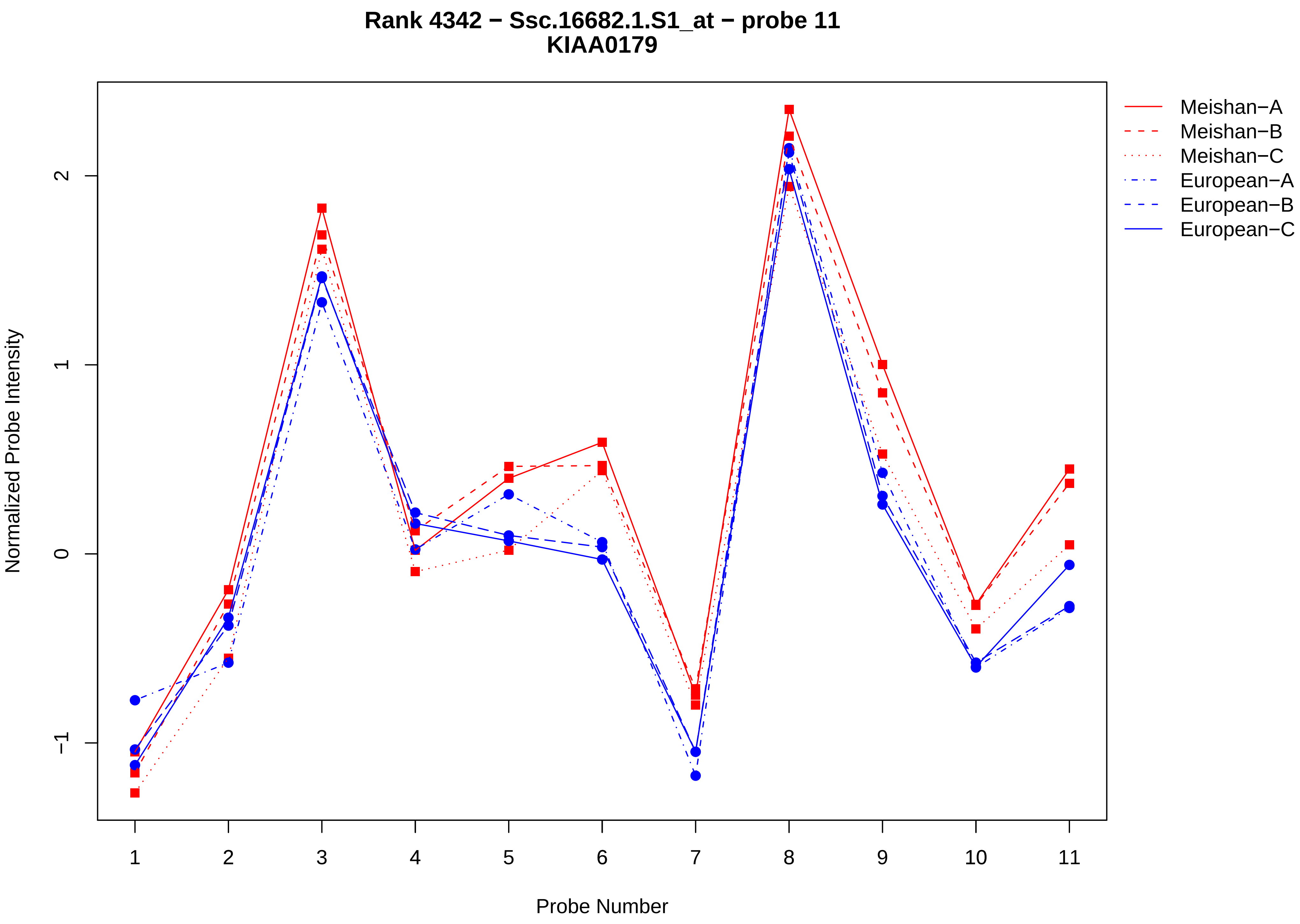

Rank 4342 − Ssc.16682.1.S1_at − probe 11
KIAA0179
Normalized Probe Intensity
Probe Number
Meishan−A
Meishan−B
Meishan−C
European−A
European−B
European−C
−1
0
1
2
1
2
3
4
5
6
7
8
9
10
11

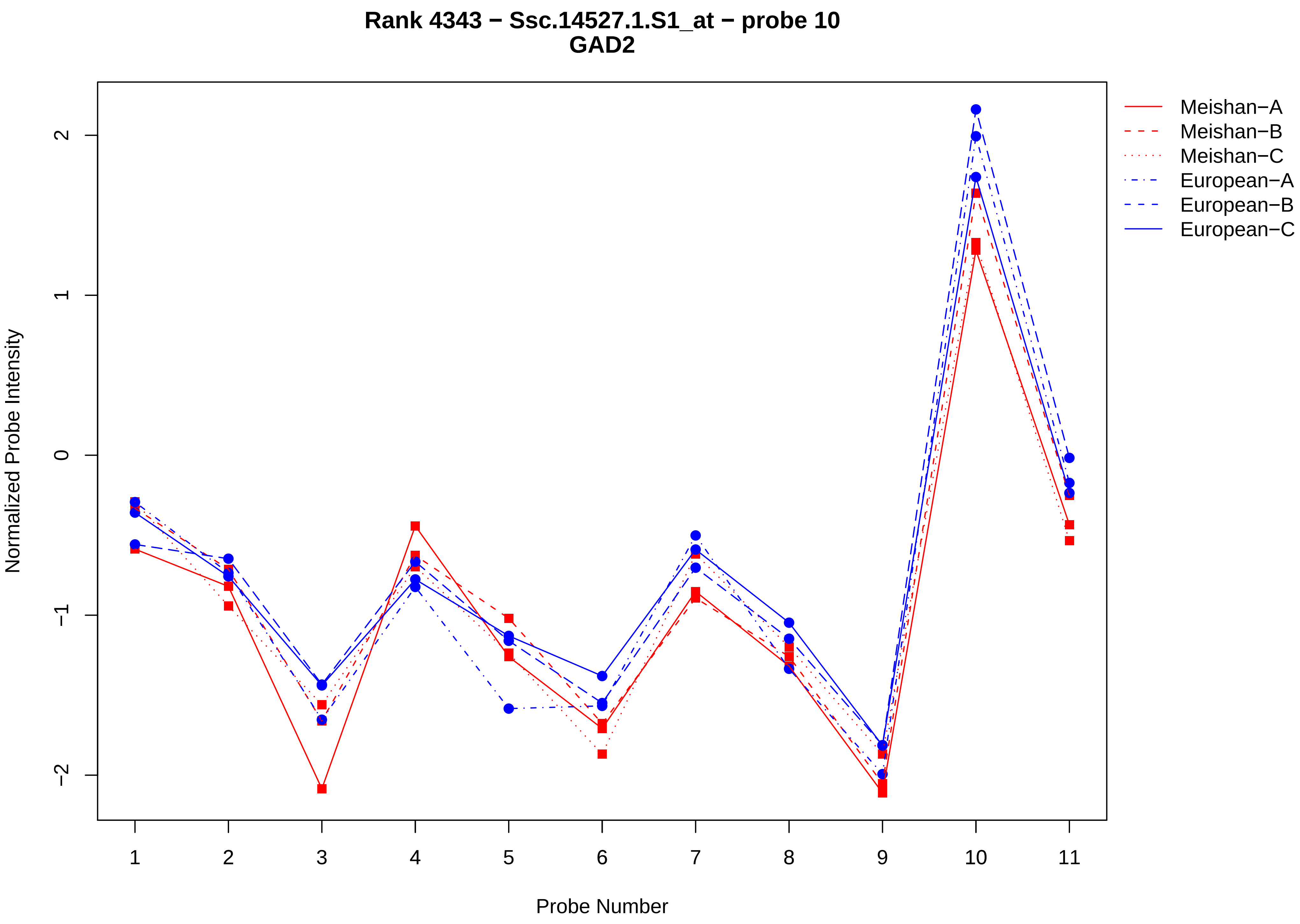

Rank 4343 − Ssc.14527.1.S1_at − probe 10
GAD2
Normalized Probe Intensity
Probe Number
Meishan−A
Meishan−B
Meishan−C
European−A
European−B
European−C

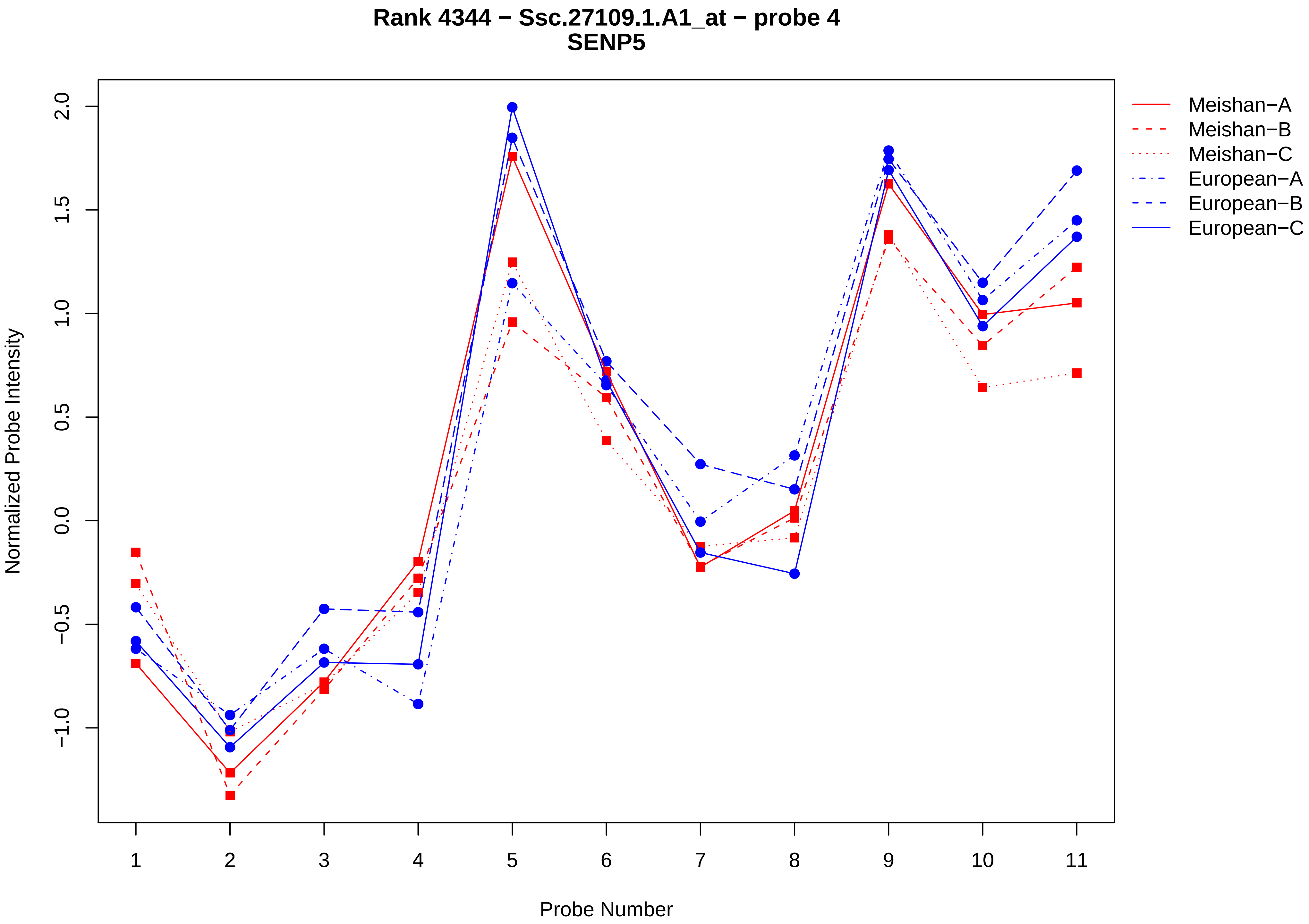

Rank 4344 − Ssc.27109.1.A1_at − probe 4
SENP5
Normalized Probe Intensity
Probe Number
−1.0
−0.5
0.0
0.5
1.0
1.5
2.0
1
2
3
4
5
6
7
8
9
10
11
Meishan−A
Meishan−B
Meishan−C
European−A
European−B
European−C

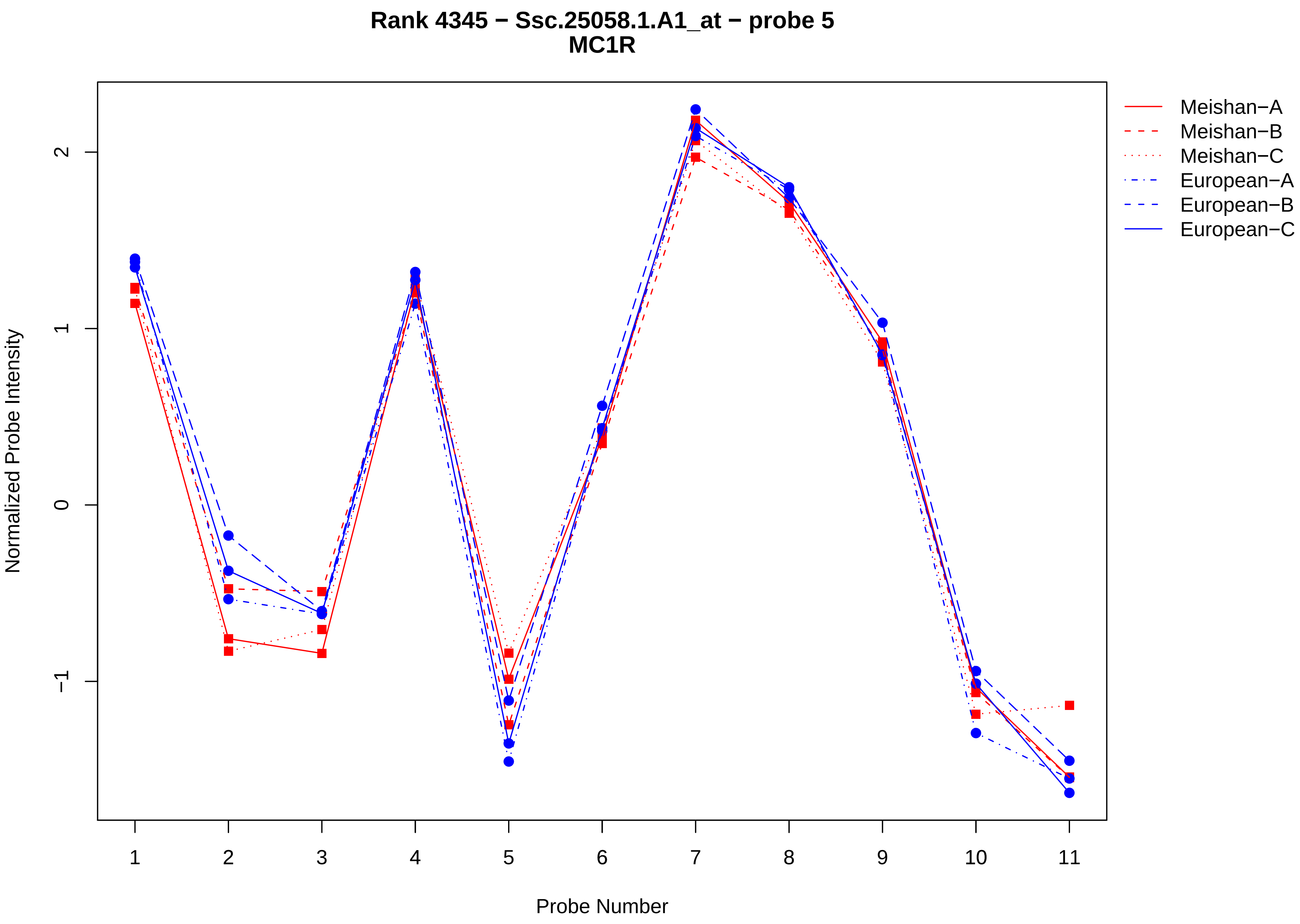
Rank 4345 − Ssc.25058.1.A1_at − probe 5
MC1R
Normalized Probe Intensity
Probe Number
−1
0
1
2
1
2
3
4
5
6
7
8
9
10
11
Meishan−A
Meishan−B
Meishan−C
European−A
European−B
European−C

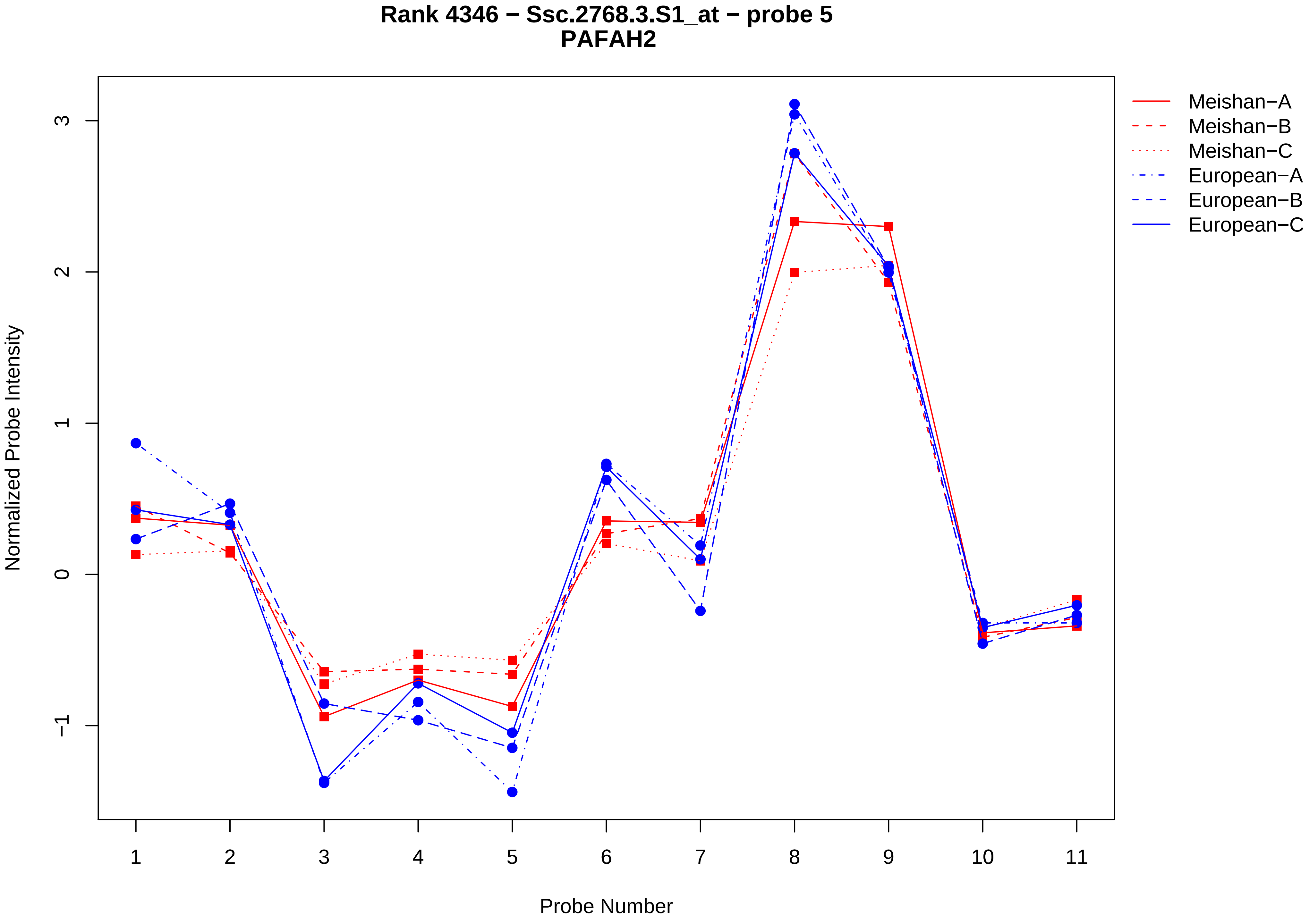

Rank 4346 − Ssc.2768.3.S1_at − probe 5
PAFAH2
Normalized Probe Intensity
Probe Number
Meishan−A
Meishan−B
Meishan−C
European−A
European−B
European−C

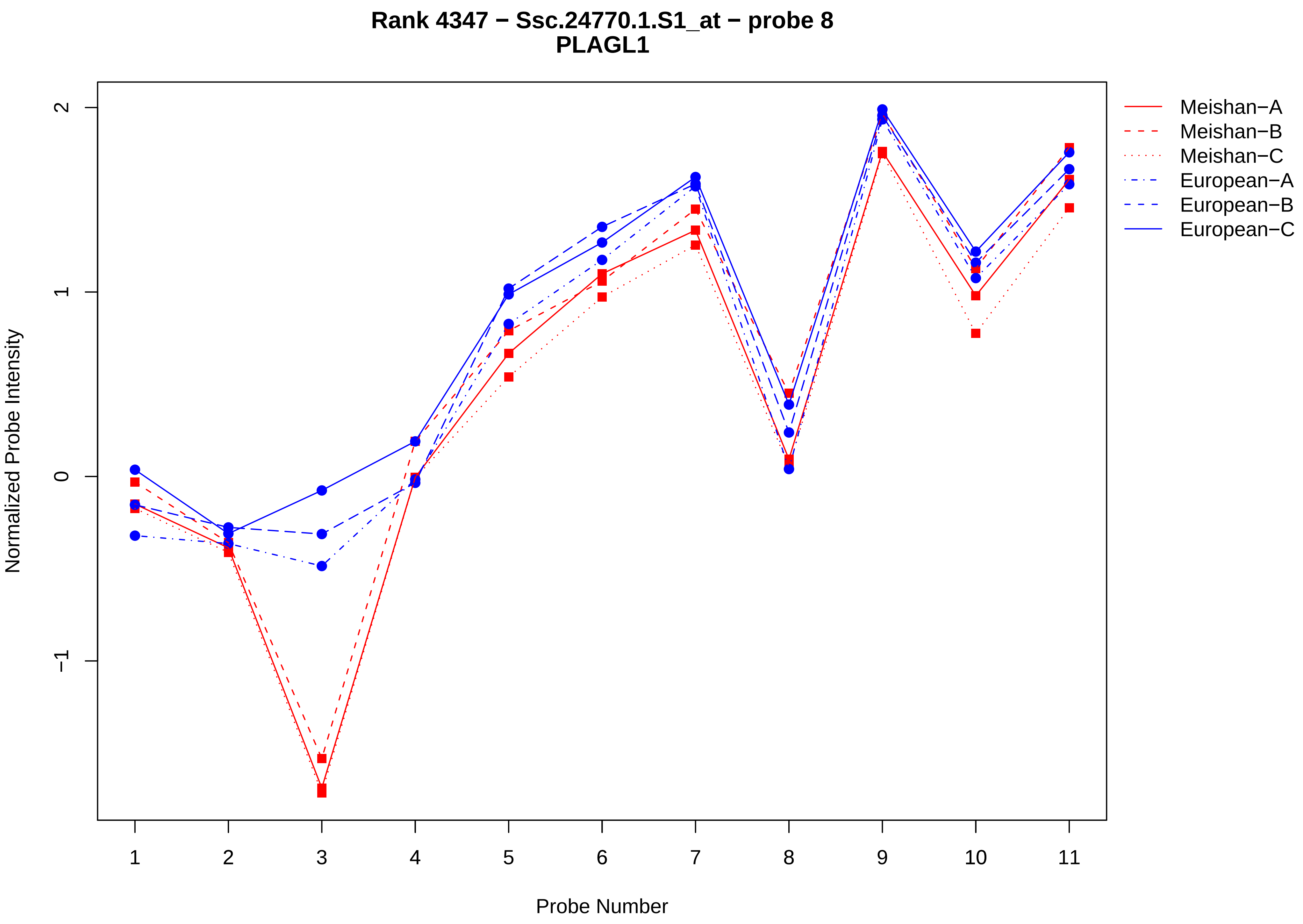

Rank 4347 − Ssc.24770.1.S1_at − probe 8
PLAGL1
Normalized Probe Intensity
Probe Number
−1
0
1
2
1
2
3
4
5
6
7
8
9
10
11
Meishan−A
Meishan−B
Meishan−C
European−A
European−B
European−C

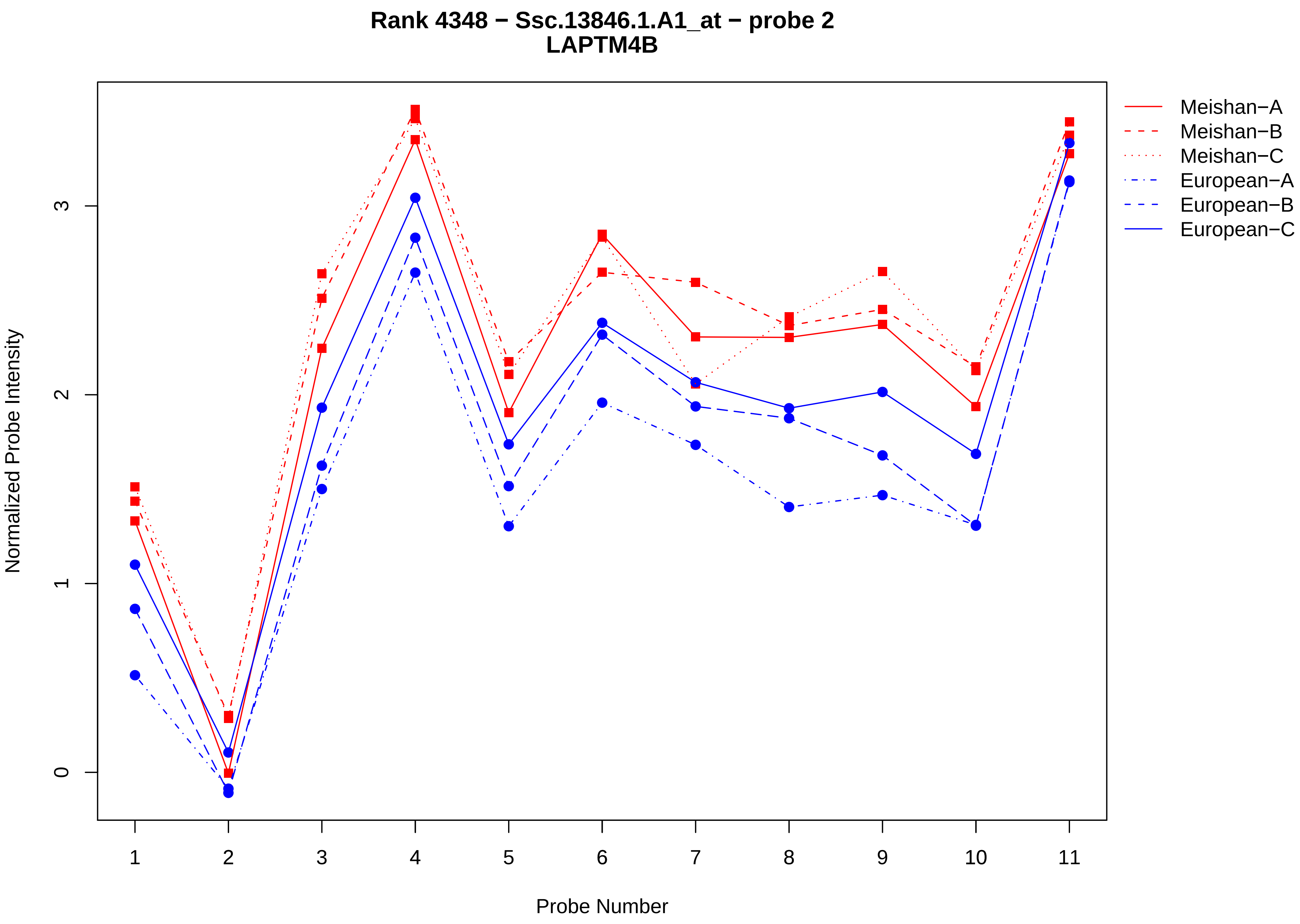

Rank 4348 − Ssc.13846.1.A1_at − probe 2
LAPTM4B
Normalized Probe Intensity
Probe Number
0
1
2
3
1
2
3
4
5
6
7
8
9
10
11
Meishan−A
Meishan−B
Meishan−C
European−A
European−B
European−C

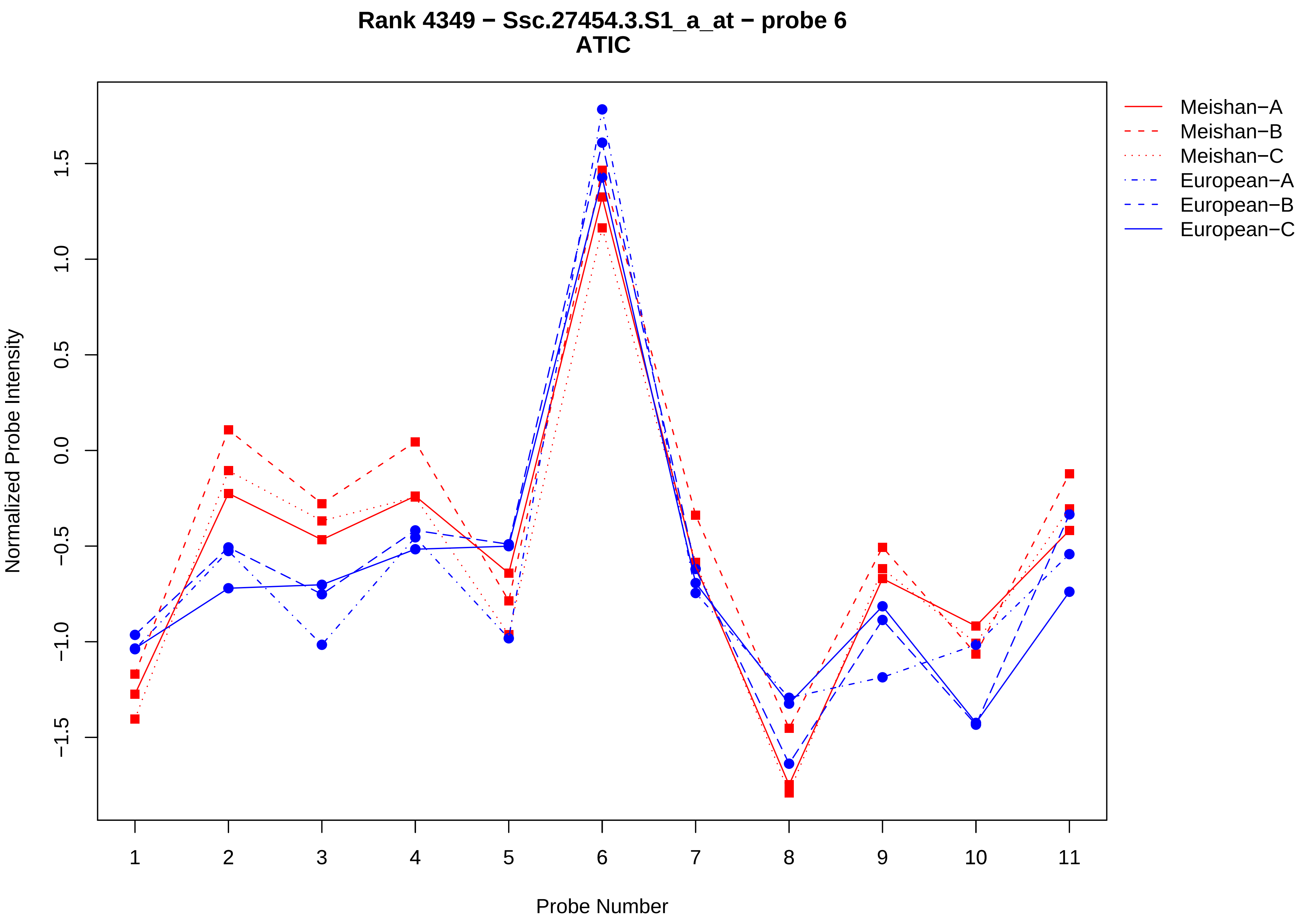

Rank 4349 − Ssc.27454.3.S1_a_at − probe 6
ATIC
Normalized Probe Intensity
Probe Number
−1.5
−1.0
−0.5
0.0
0.5
1.0
1.5
1
2
3
4
5
6
7
8
9
10
11
Meishan−A
Meishan−B
Meishan−C
European−A
European−B
European−C

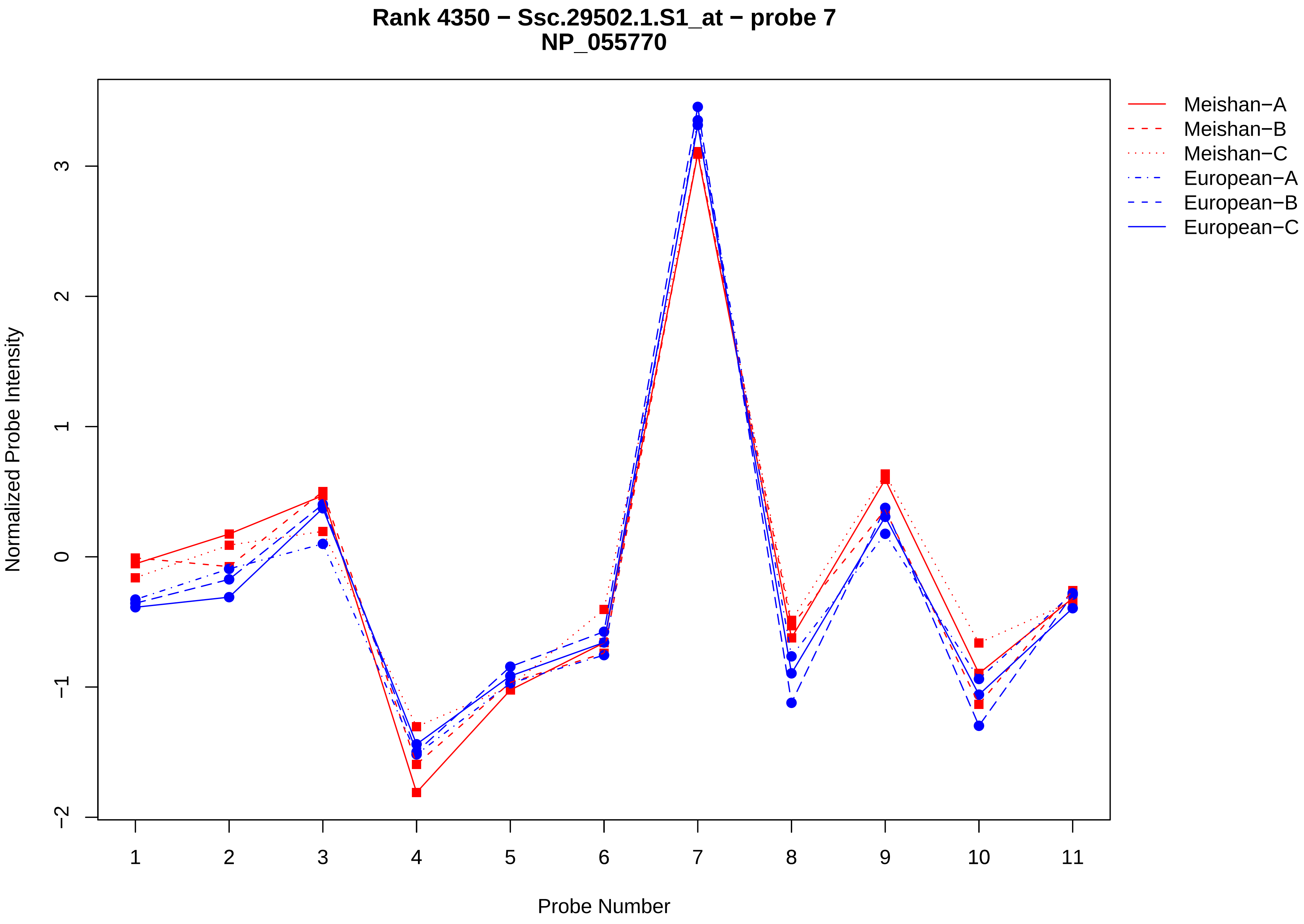

Rank 4350 − Ssc.29502.1.S1_at − probe 7
NP_055770
Normalized Probe Intensity
Probe Number
Meishan−A
Meishan−B
Meishan−C
European−A
European−B
European−C

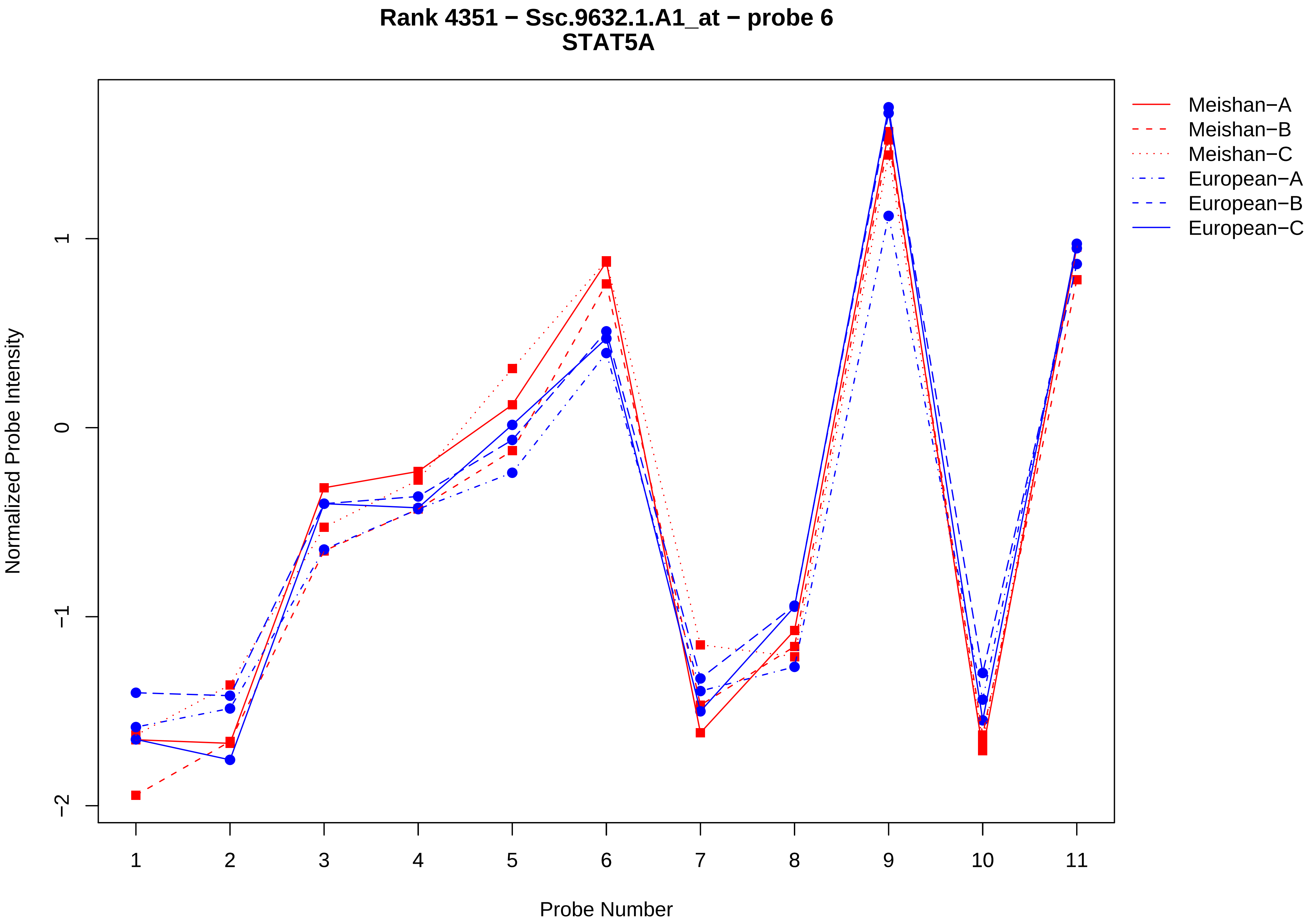

Rank 4351 − Ssc.9632.1.A1_at − probe 6
STAT5A
Normalized Probe Intensity
Probe Number
Meishan−A
Meishan−B
Meishan−C
European−A
European−B
European−C
−2
−1
0
1
1
2
3
4
5
6
7
8
9
10
11

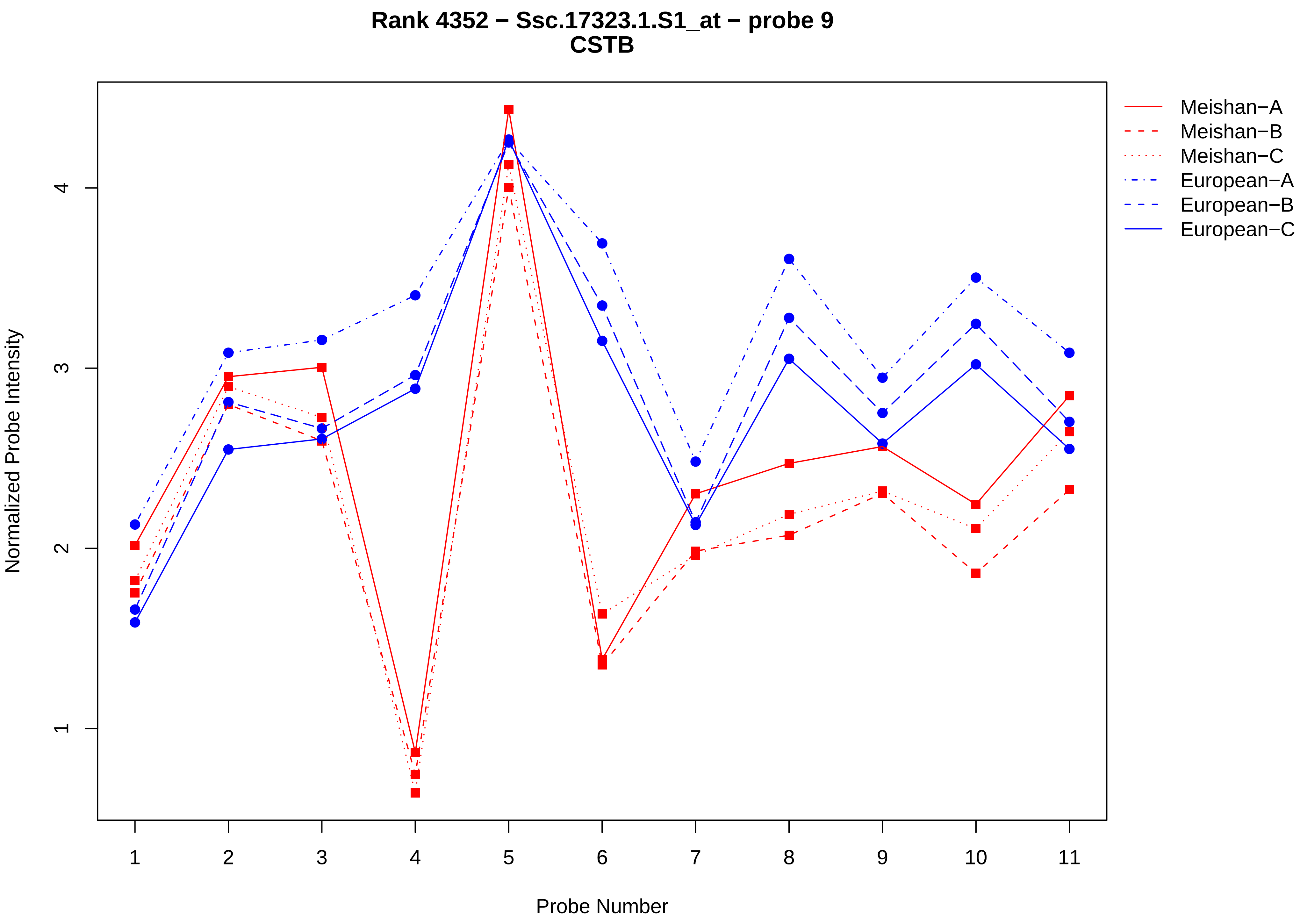

Rank 4352 − Ssc.17323.1.S1_at − probe 9
CSTB
Normalized Probe Intensity
Probe Number
1
2
3
4
1
2
3
4
5
6
7
8
9
10
11
Meishan−A
Meishan−B
Meishan−C
European−A
European−B
European−C

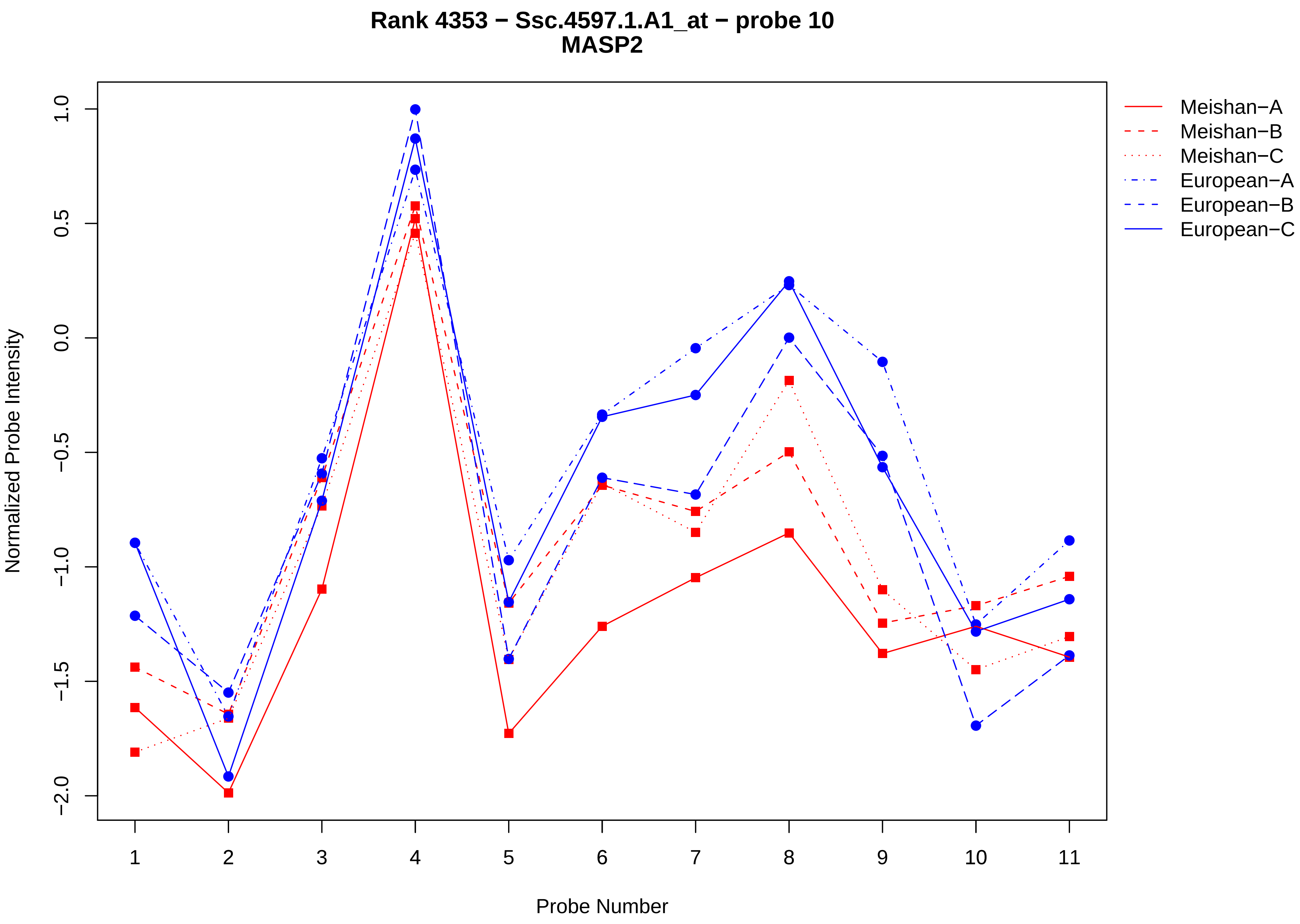

Rank 4353 − Ssc.4597.1.A1_at − probe 10
MASP2
Normalized Probe Intensity
Probe Number
1.0
0.5
0.0
−0.5
−1.0
−1.5
−2.0
1
2
3
4
5
6
7
8
9
10
11
Meishan−A
Meishan−B
Meishan−C
European−A
European−B
European−C

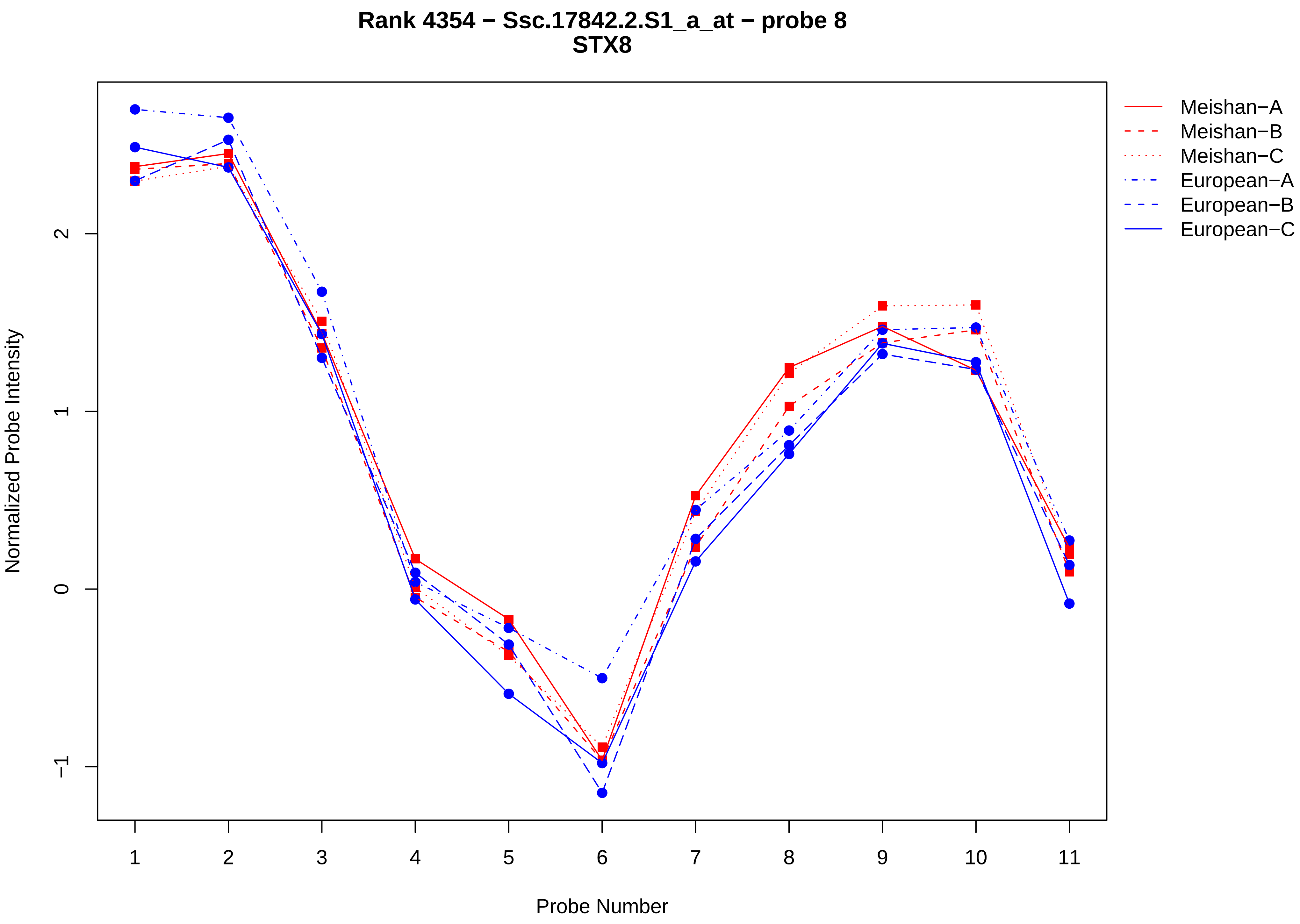

Rank 4354 − Ssc.17842.2.S1_a_at − probe 8
STX8
Normalized Probe Intensity
Probe Number
−1
0
1
2
1
2
3
4
5
6
7
8
9
10
11
Meishan−A
Meishan−B
Meishan−C
European−A
European−B
European−C

Rank 4355 − Ssc.19166.1.S1_s_at − probe 1
ASCL2

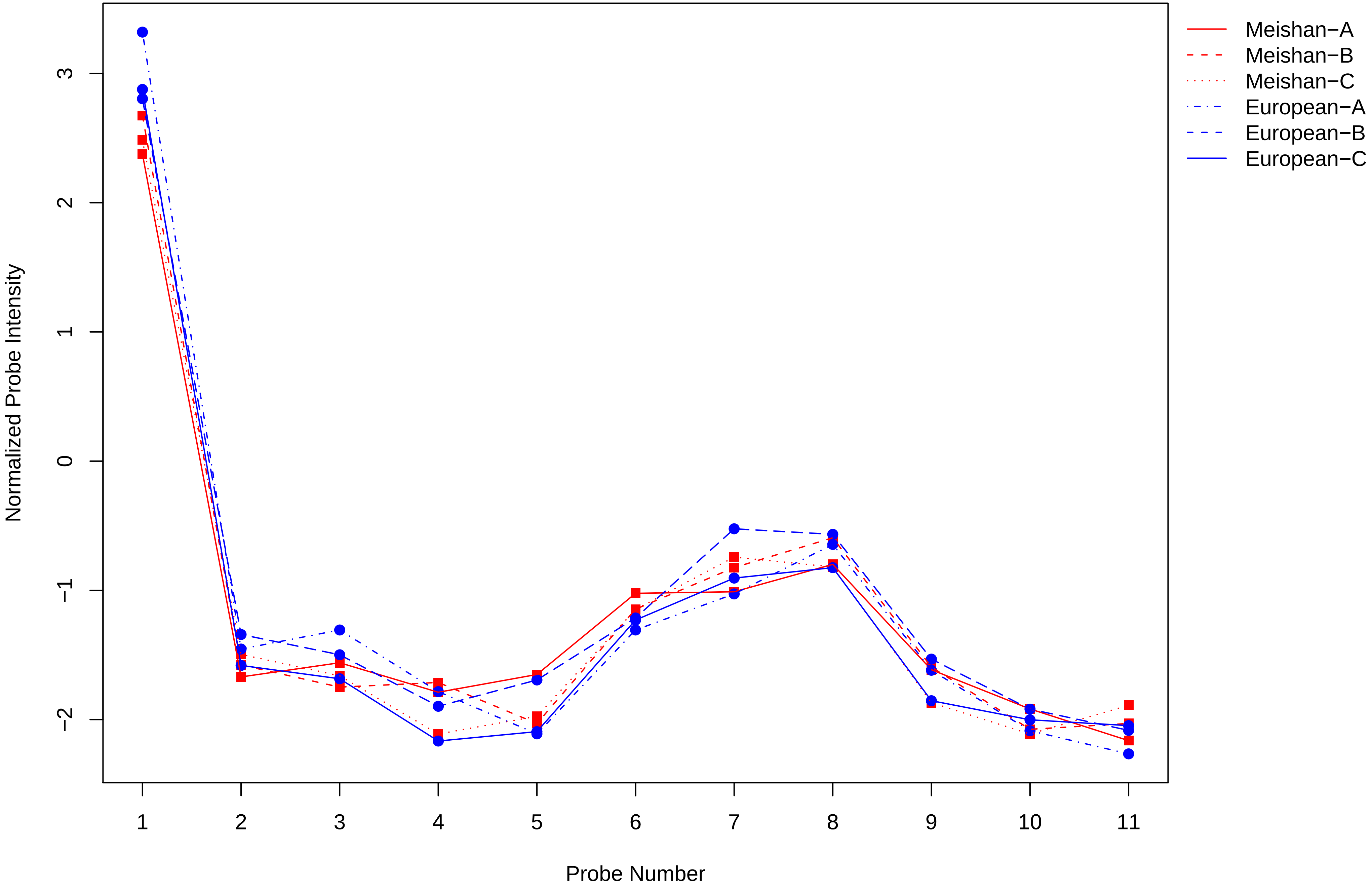

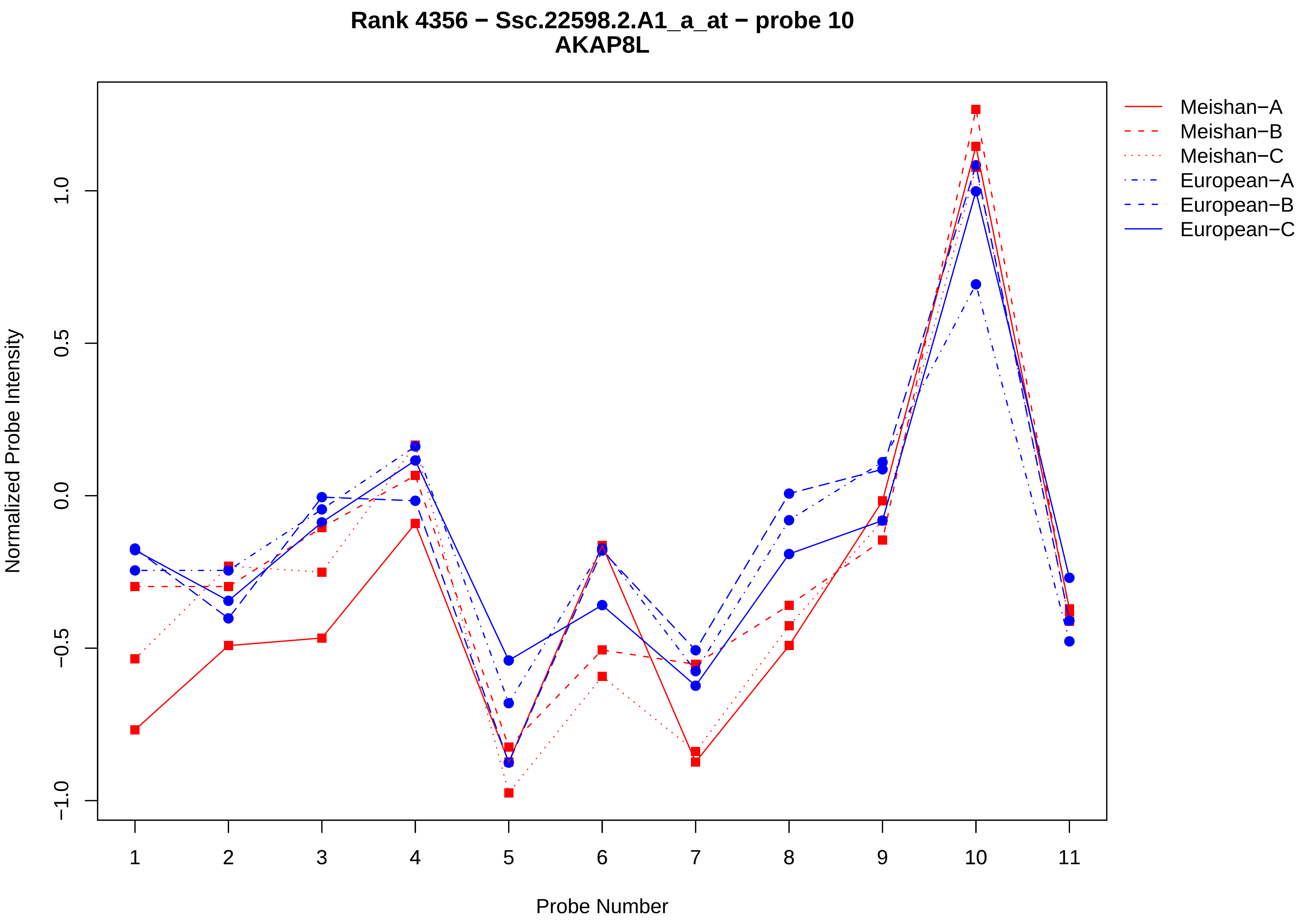
Rank 4356 − Ssc.22598.2.A1_a_at − probe 10
AKAP8L
Normalized Probe Intensity
Probe Number
Meishan−A
Meishan−B
Meishan−C
European−A
European−B
European−C
−1.0
−0.5
0.0
0.5
1.0
1
2
3
4
5
6
7
8
9
10
11

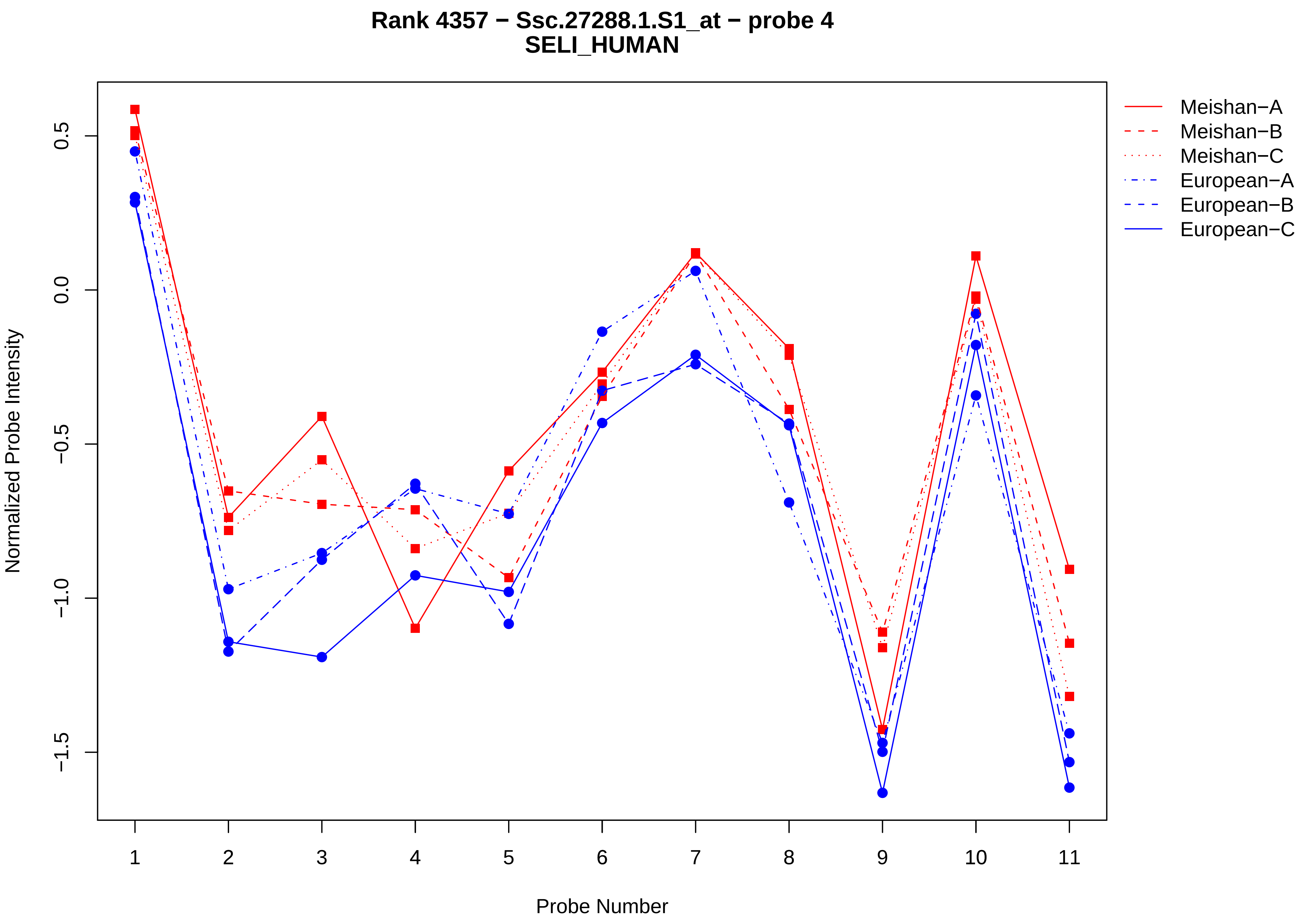

Rank 4357 − Ssc.27288.1.S1_at − probe 4
SELI_HUMAN
Normalized Probe Intensity
Probe Number
0.5
0.0
−0.5
−1.0
−1.5
1
2
3
4
5
6
7
8
9
10
11
Meishan−A
Meishan−B
Meishan−C
European−A
European−B
European−C

Rank 4358 − Ssc.17453.1.S1_at − probe 4
ATP2B4

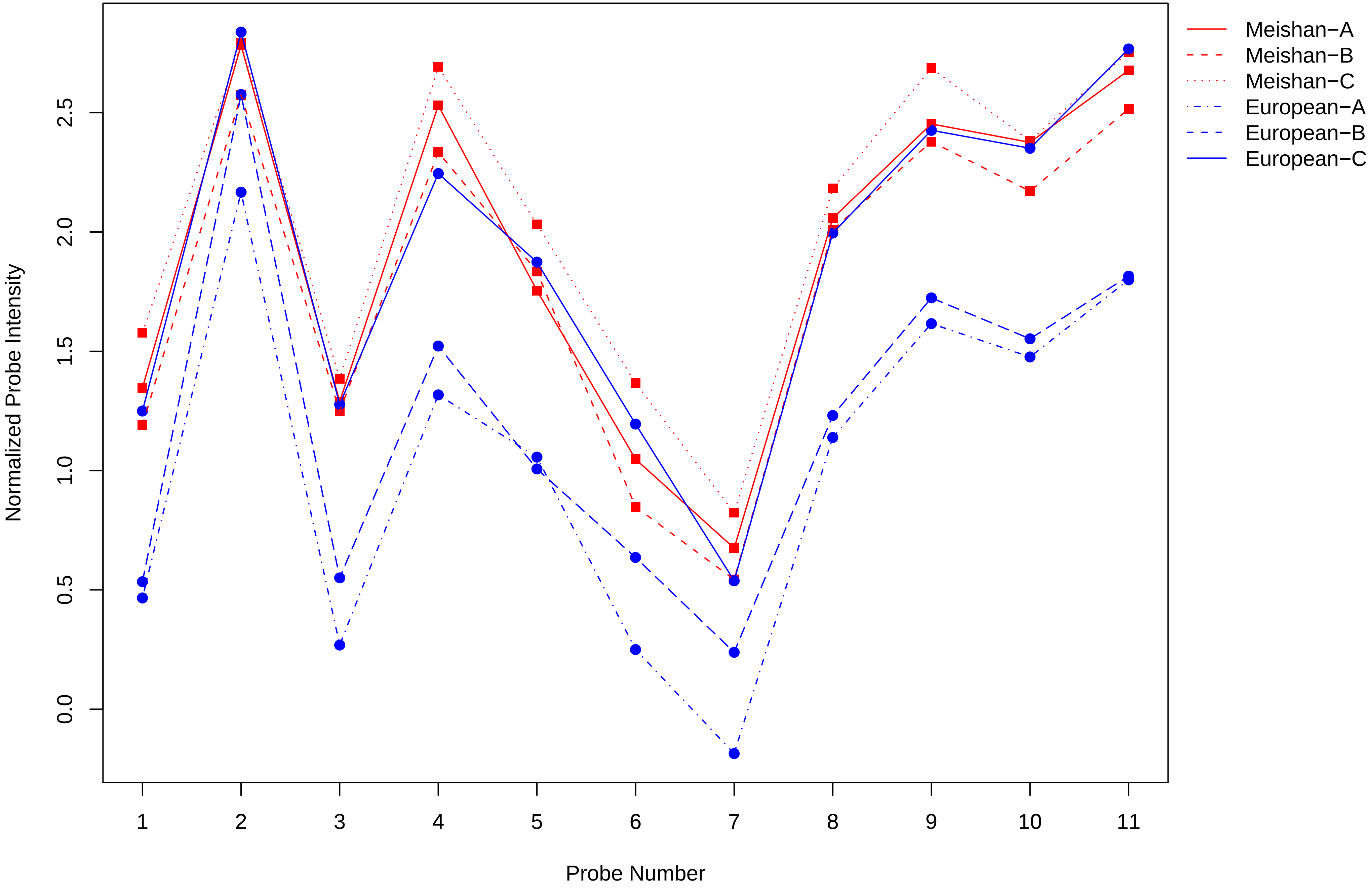

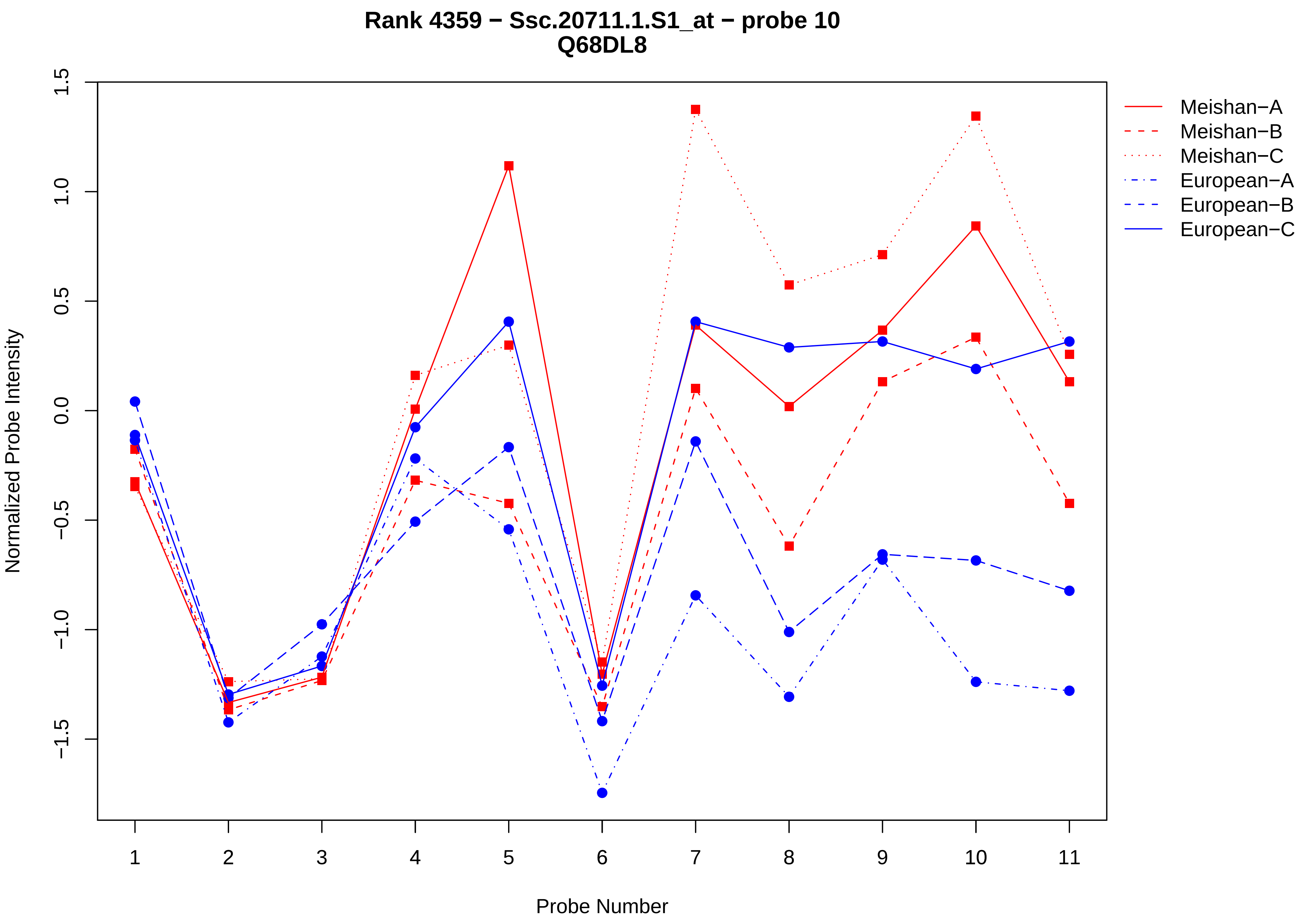

Rank 4359 − Ssc.20711.1.S1_at − probe 10
Q68DL8
Normalized Probe Intensity
Probe Number
1.5
1.0
0.5
0.0
−0.5
−1.0
−1.5
1
2
3
4
5
6
7
8
9
10
11
Meishan−A
Meishan−B
Meishan−C
European−A
European−B
European−C

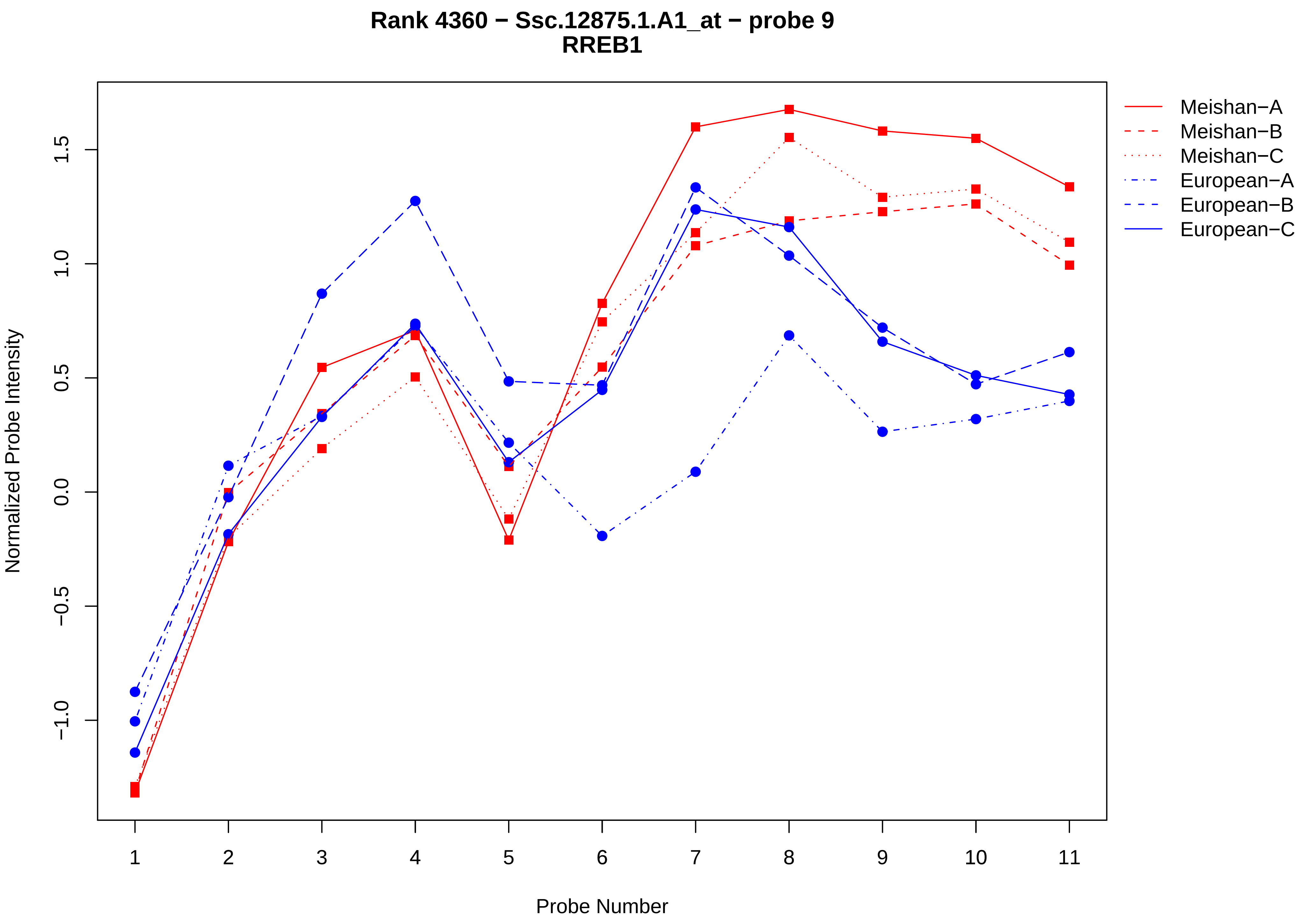
Rank 4360 − Ssc.12875.1.A1_at − probe 9
RREB1
Normalized Probe Intensity
Probe Number
Meishan−A
Meishan−B
Meishan−C
European−A
European−B
European−C
−1.0
−0.5
0.0
0.5
1.0
1.5
1
2
3
4
5
6
7
8
9
10
11

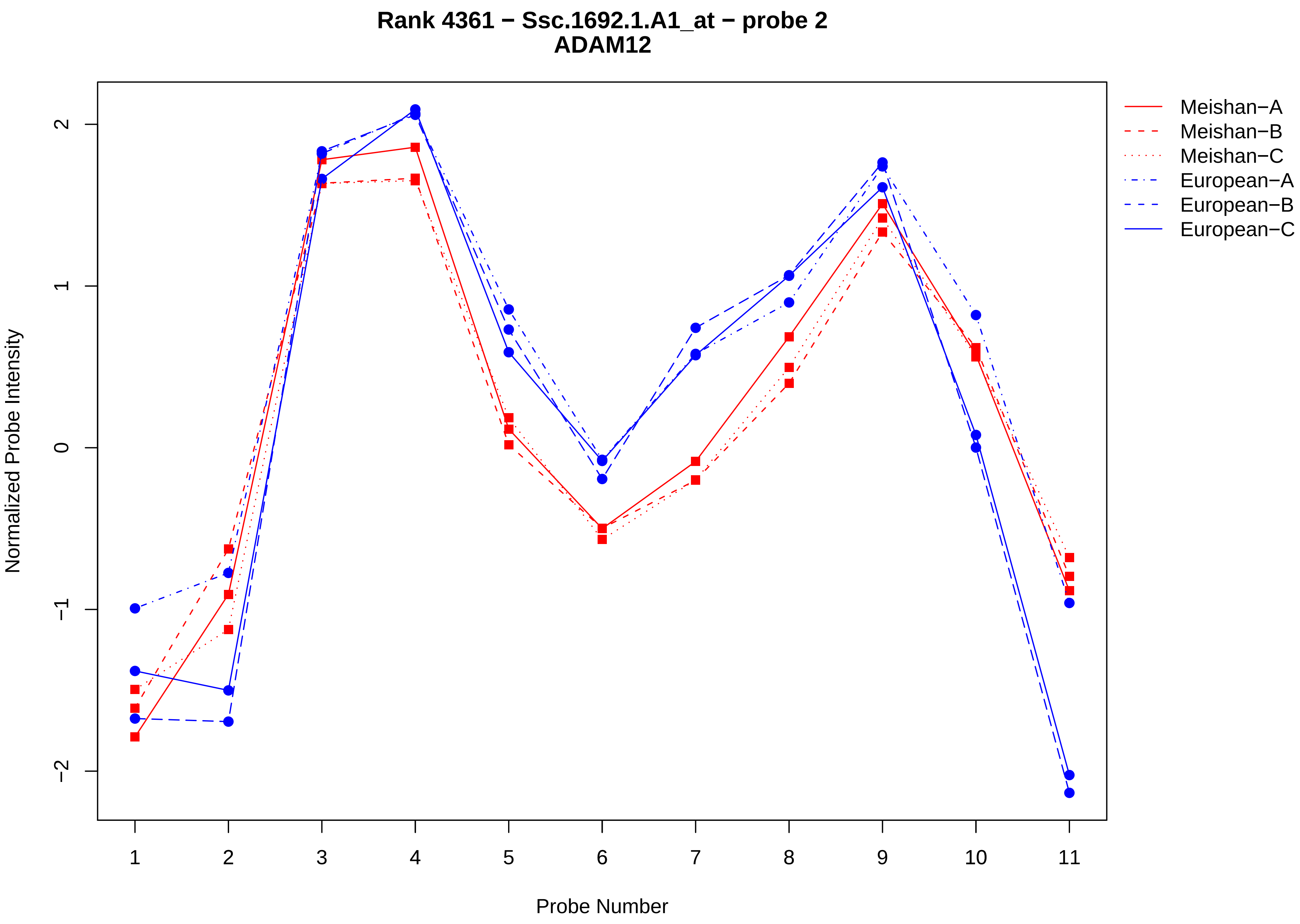

Rank 4361 − Ssc.1692.1.A1_at − probe 2
ADAM12
Normalized Probe Intensity
Probe Number
Meishan−A
Meishan−B
Meishan−C
European−A
European−B
European−C

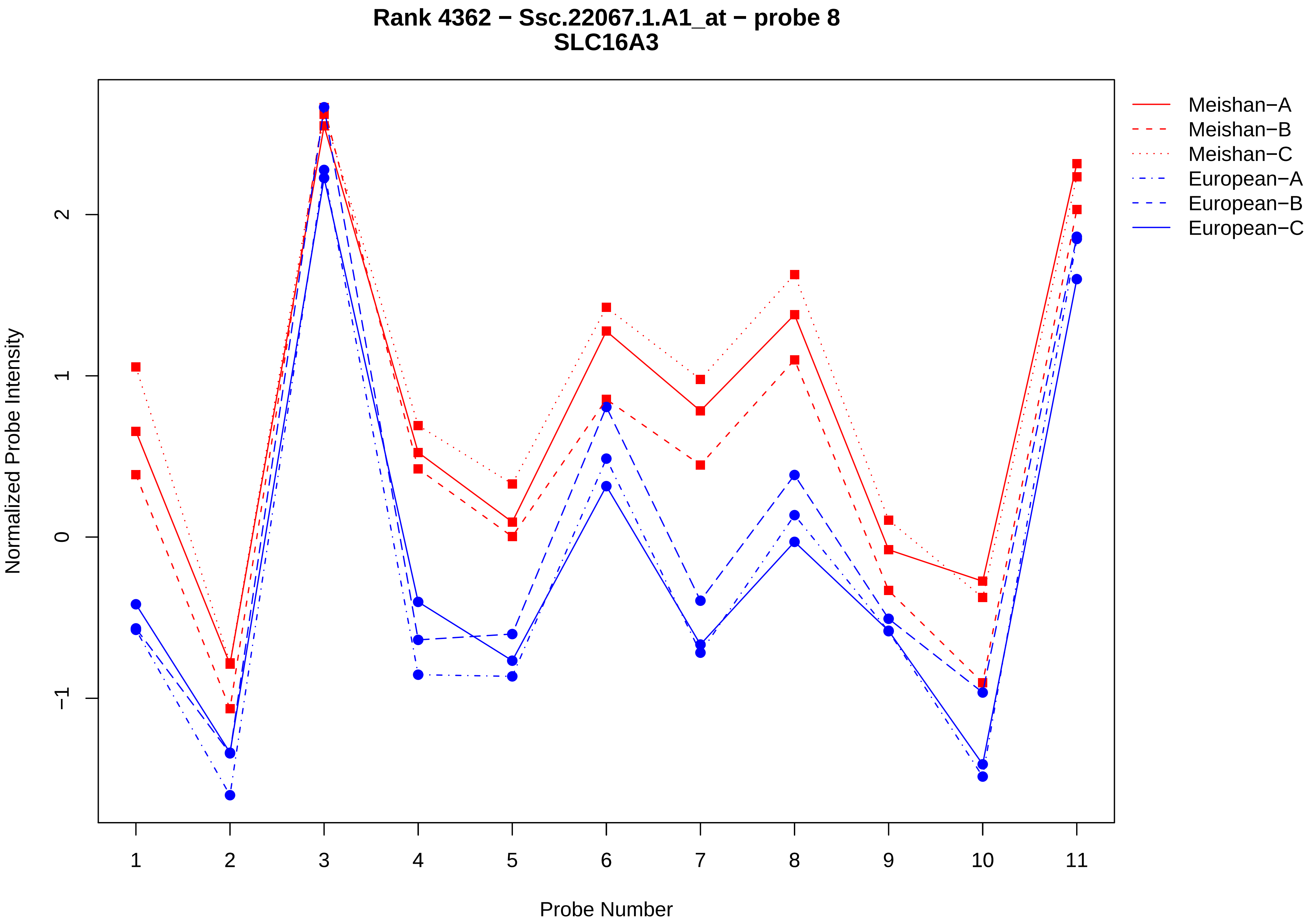

Rank 4362 − Ssc.22067.1.A1_at − probe 8
SLC16A3
Normalized Probe Intensity
Probe Number
Meishan−A
Meishan−B
Meishan−C
European−A
European−B
European−C

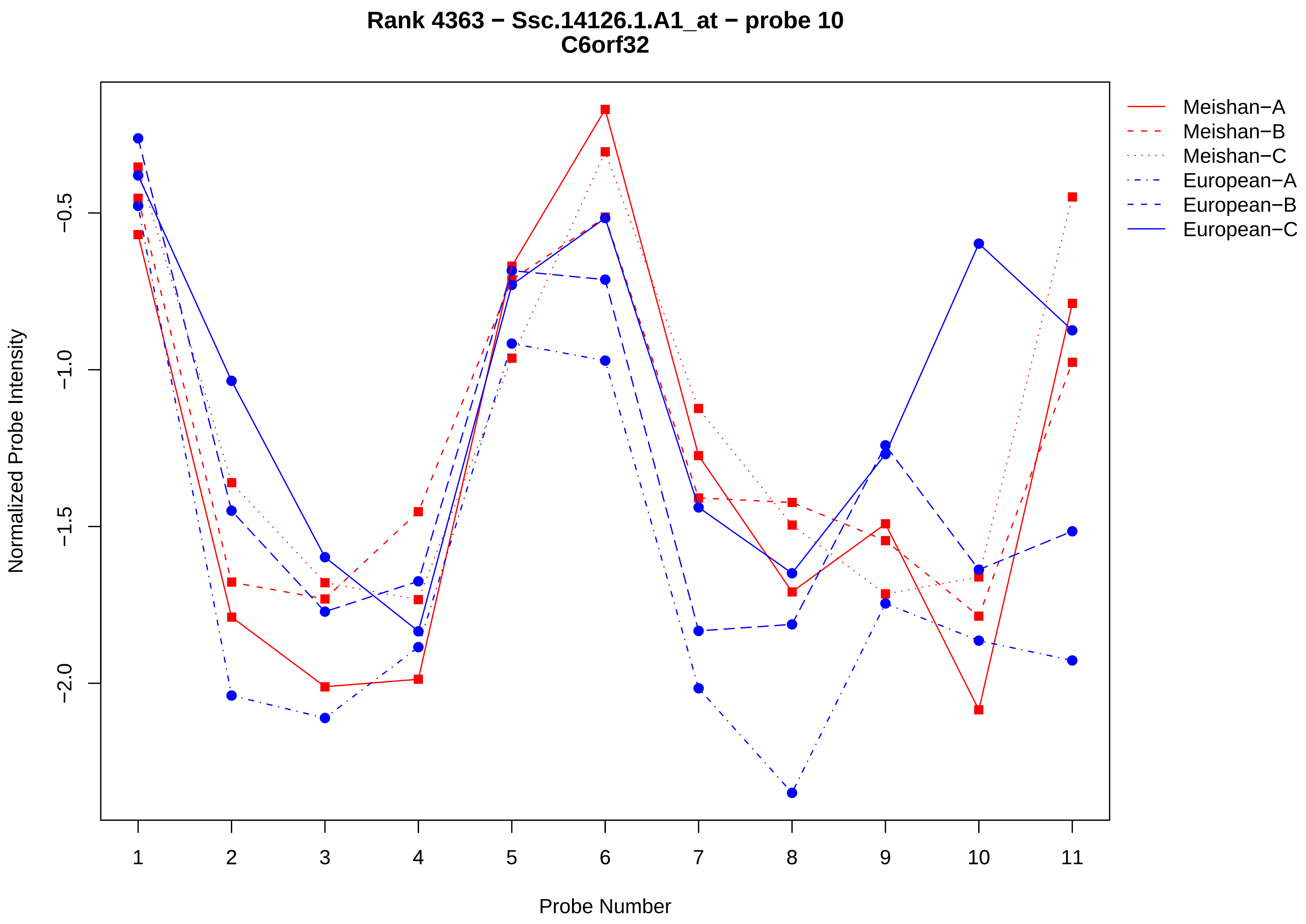
Rank 4363 − Ssc.14126.1.A1_at − probe 10
C6orf32
Normalized Probe Intensity
−0.5
−1.0
−1.5
−2.0
1
2
3
4
5
6
7
8
9
10
11
Probe Number
Meishan−A
Meishan−B
Meishan−C
European−A
European−B
European−C

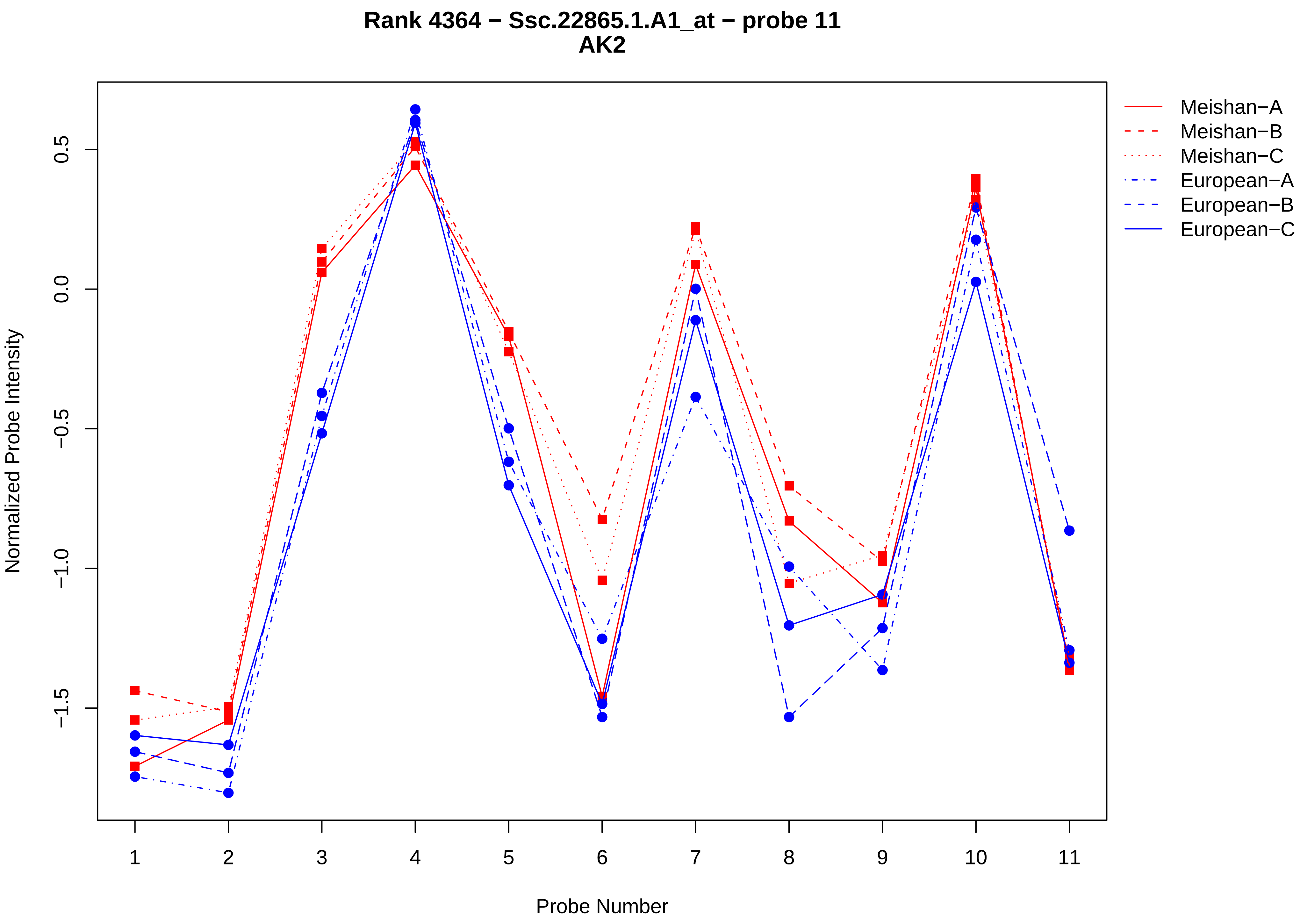

Rank 4364 − Ssc.22865.1.A1_at − probe 11
AK2
Normalized Probe Intensity
Probe Number
0.5
0.0
−0.5
−1.0
−1.5
1
2
3
4
5
6
7
8
9
10
11
Meishan−A
Meishan−B
Meishan−C
European−A
European−B
European−C

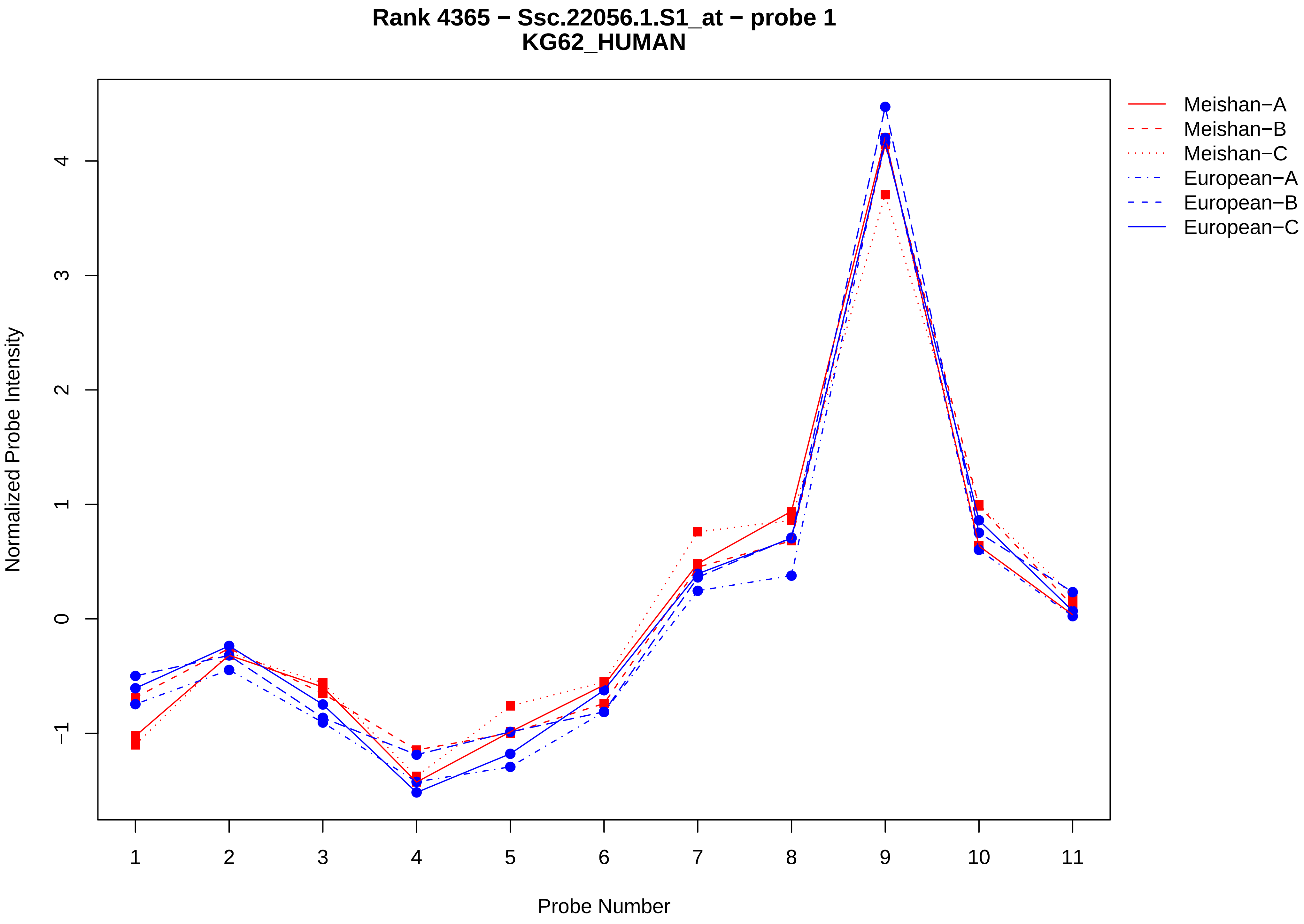

Rank 4365 − Ssc.22056.1.S1_at − probe 1
KG62_HUMAN
Normalized Probe Intensity
Probe Number
Meishan−A
Meishan−B
Meishan−C
European−A
European−B
European−C
−1
0
1
2
3
4
1
2
3
4
5
6
7
8
9
10
11

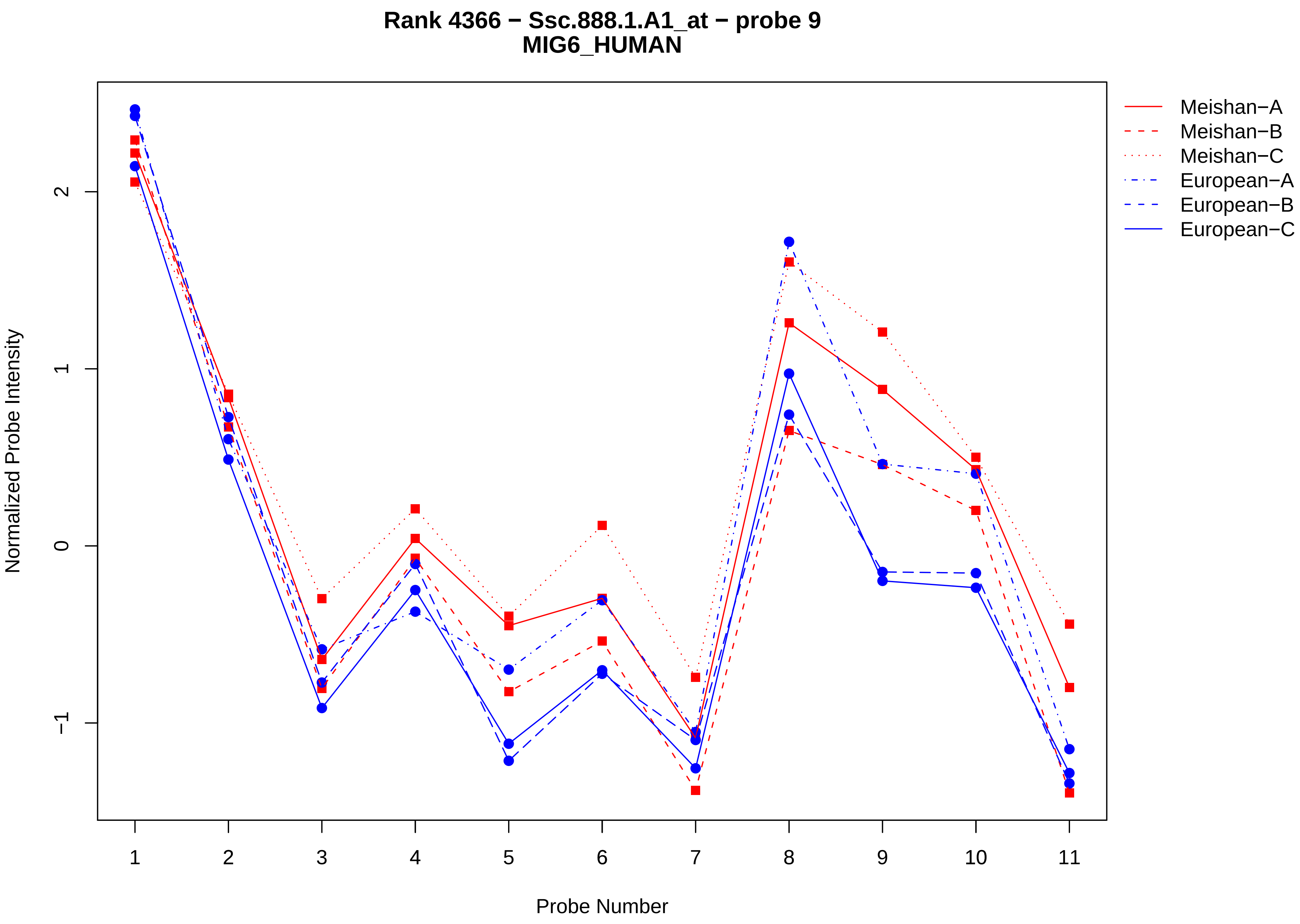

Rank 4366 − Ssc.888.1.A1_at − probe 9
MIG6_HUMAN
Normalized Probe Intensity
Probe Number
Meishan−A
Meishan−B
Meishan−C
European−A
European−B
European−C

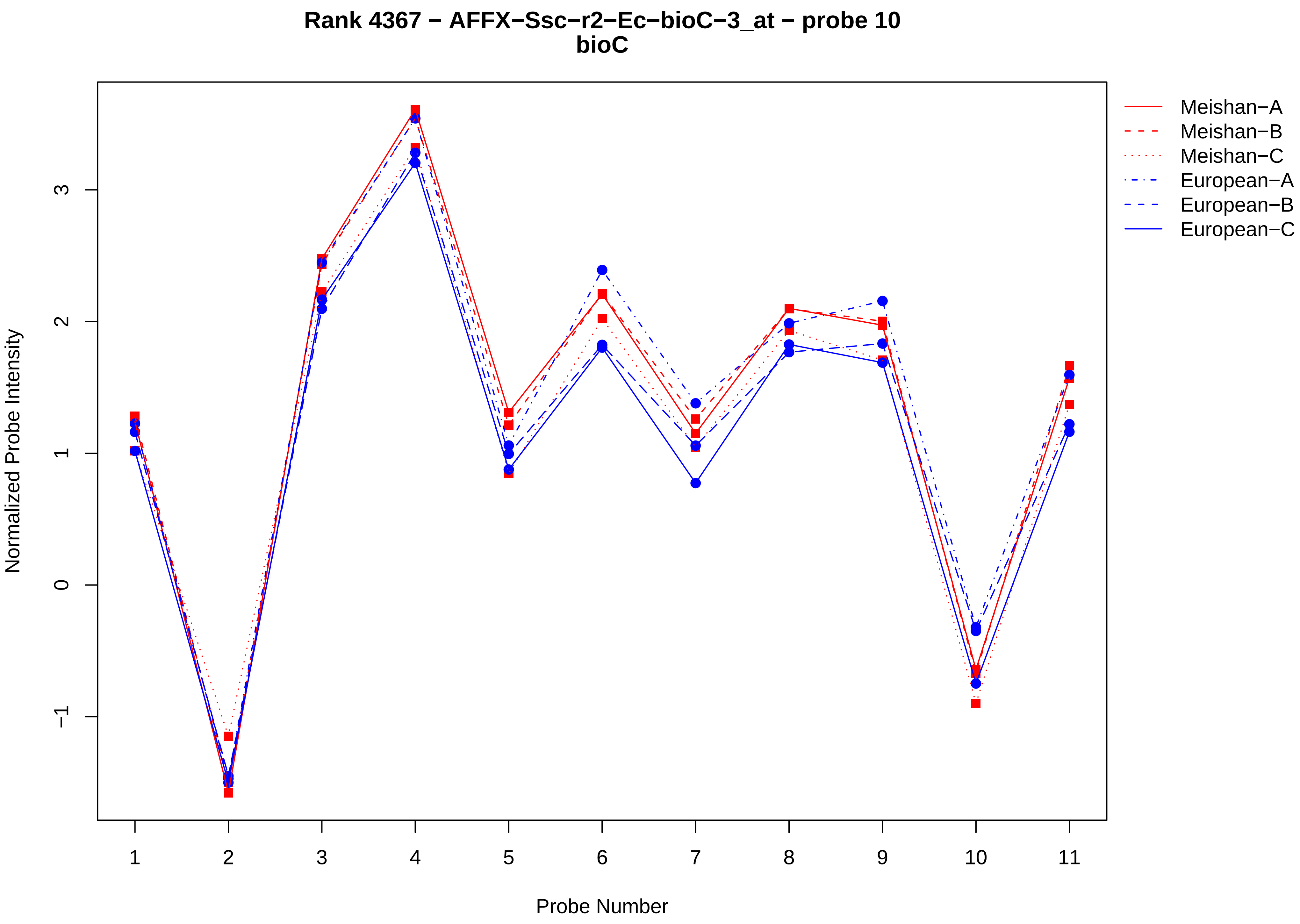

Rank 4367 − AFFX−Ssc−r2−Ec−bioC−3_at − probe 10
bioC
Normalized Probe Intensity
Probe Number
−1
0
1
2
3
1
2
3
4
5
6
7
8
9
10
11
Meishan−A
Meishan−B
Meishan−C
European−A
European−B
European−C

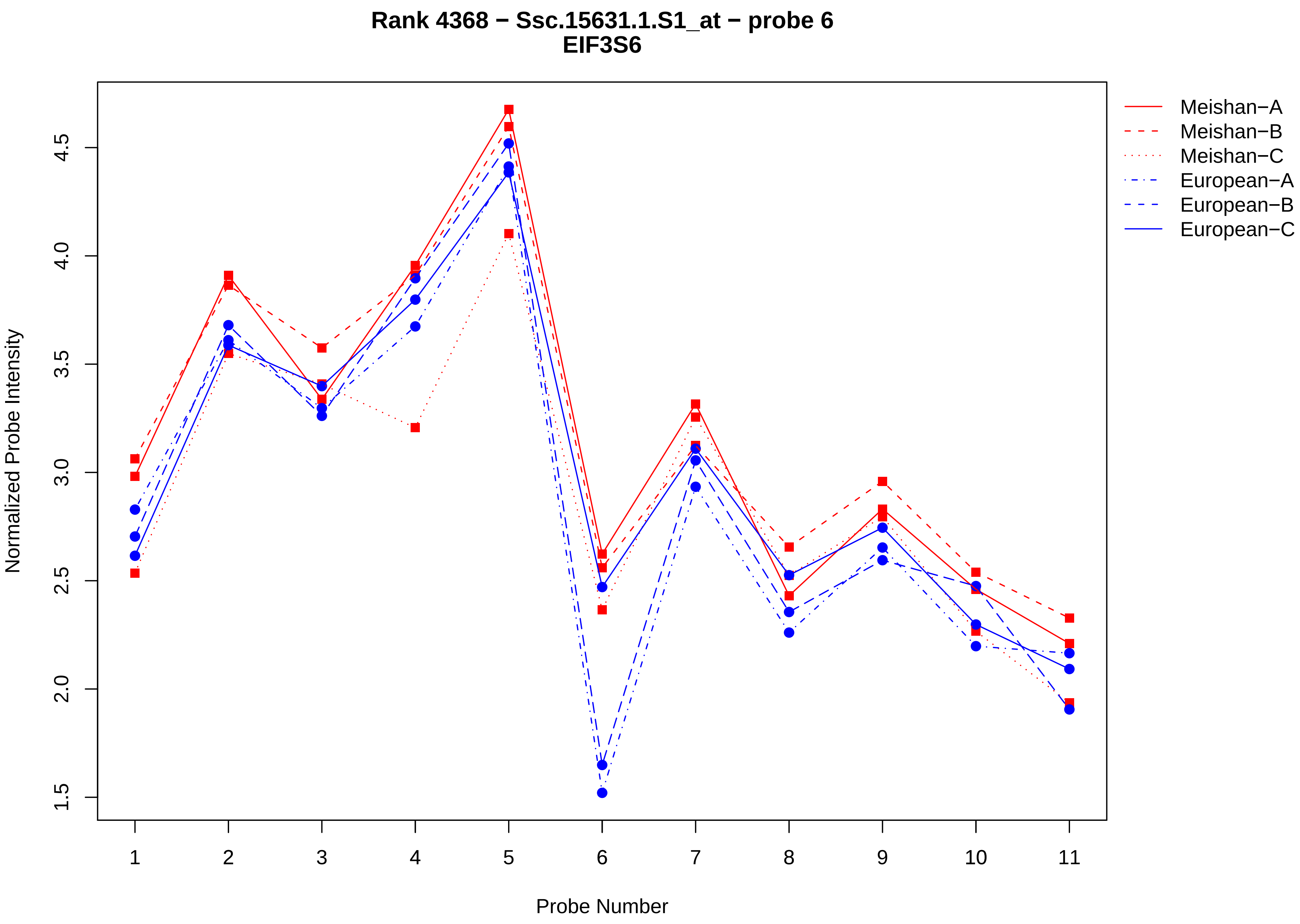

Rank 4368 − Ssc.15631.1.S1_at − probe 6
EIF3S6
Normalized Probe Intensity
Probe Number
1.5
2.0
2.5
3.0
3.5
4.0
4.5
1
2
3
4
5
6
7
8
9
10
11
Meishan−A
Meishan−B
Meishan−C
European−A
European−B
European−C

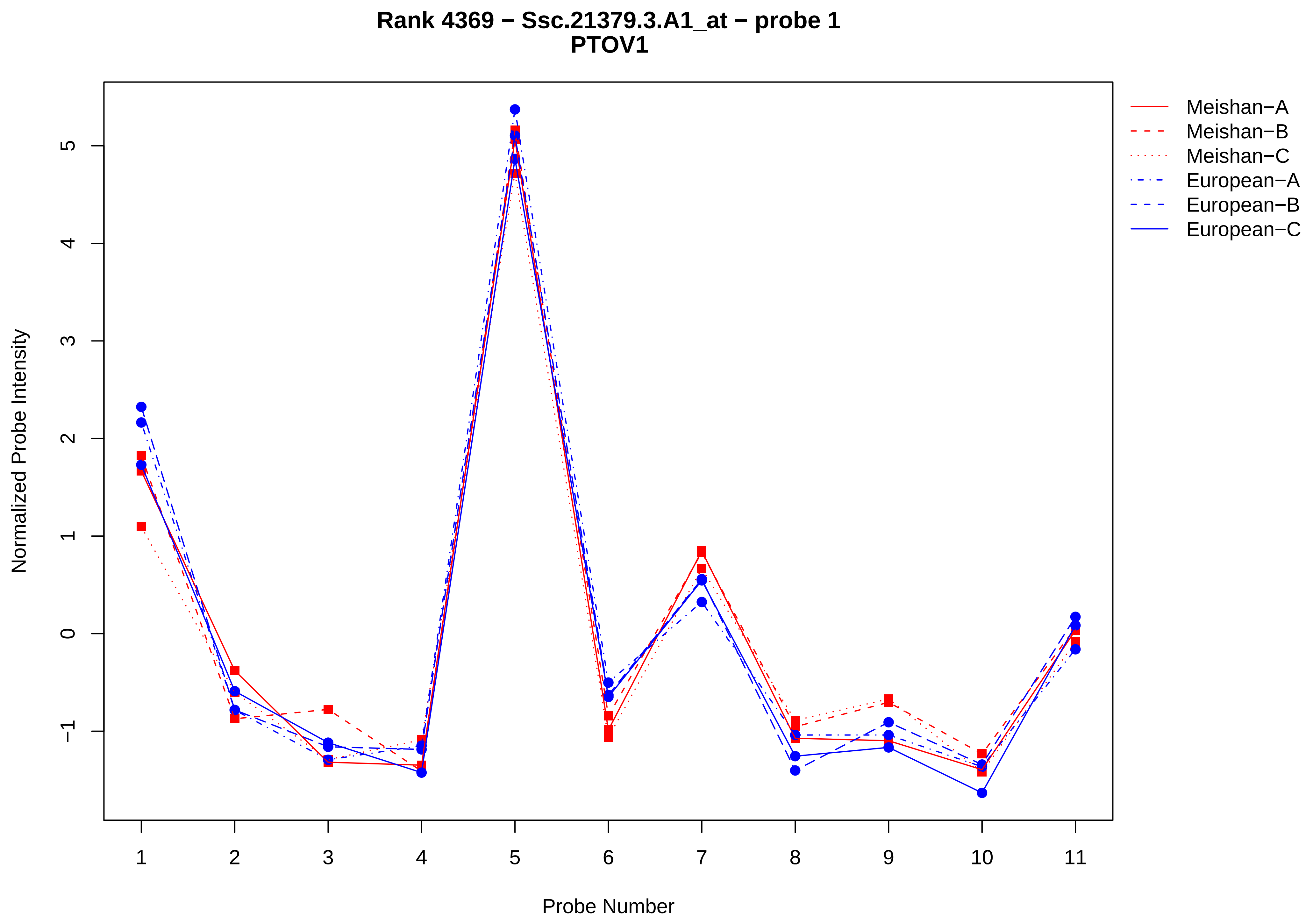

Rank 4369 − Ssc.21379.3.A1_at − probe 1
PTOV1
Normalized Probe Intensity
Probe Number
−1
0
1
2
3
4
5
1
2
3
4
5
6
7
8
9
10
11
Meishan−A
Meishan−B
Meishan−C
European−A
European−B
European−C

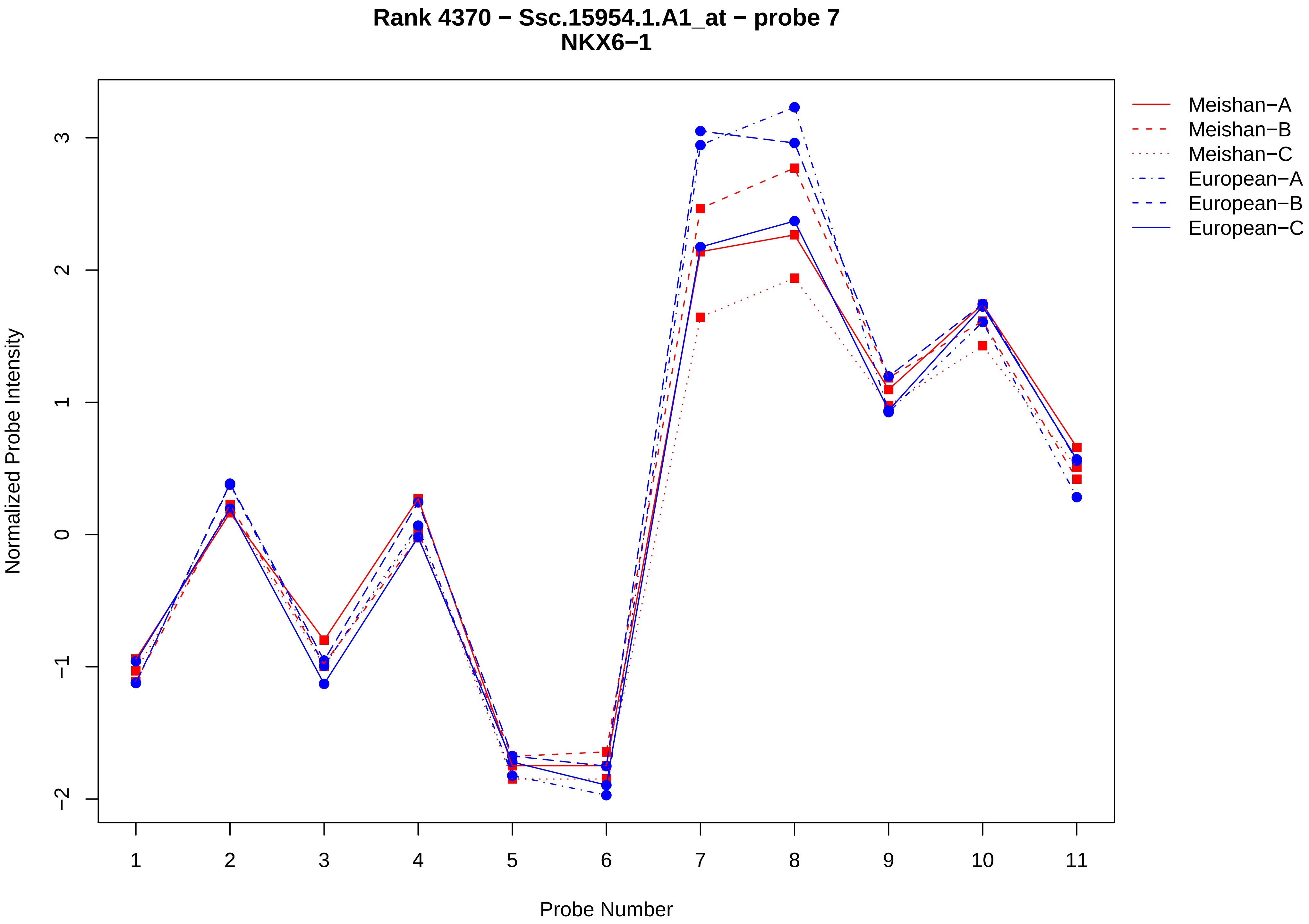

Rank 4370 − Ssc.15954.1.A1_at − probe 7
NKX6−1
Normalized Probe Intensity
Probe Number
Meishan−A
Meishan−B
Meishan−C
European−A
European−B
European−C

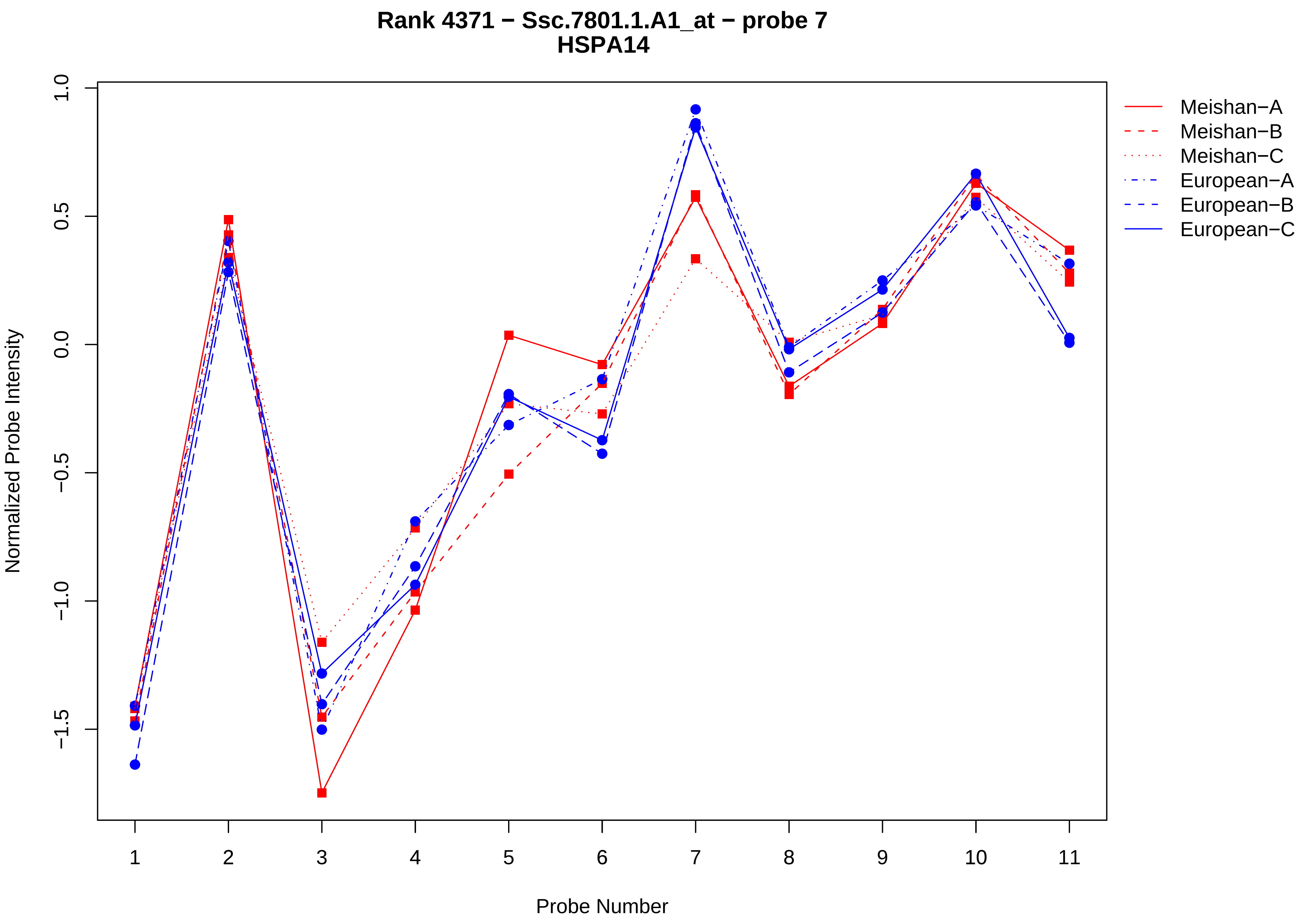
Rank 4371 − Ssc.7801.1.A1_at − probe 7
HSPA14
Normalized Probe Intensity
Probe Number
Meishan−A
Meishan−B
Meishan−C
European−A
European−B
European−C
1.0
0.5
0.0
−0.5
−1.0
−1.5
1
2
3
4
5
6
7
8
9
10
11

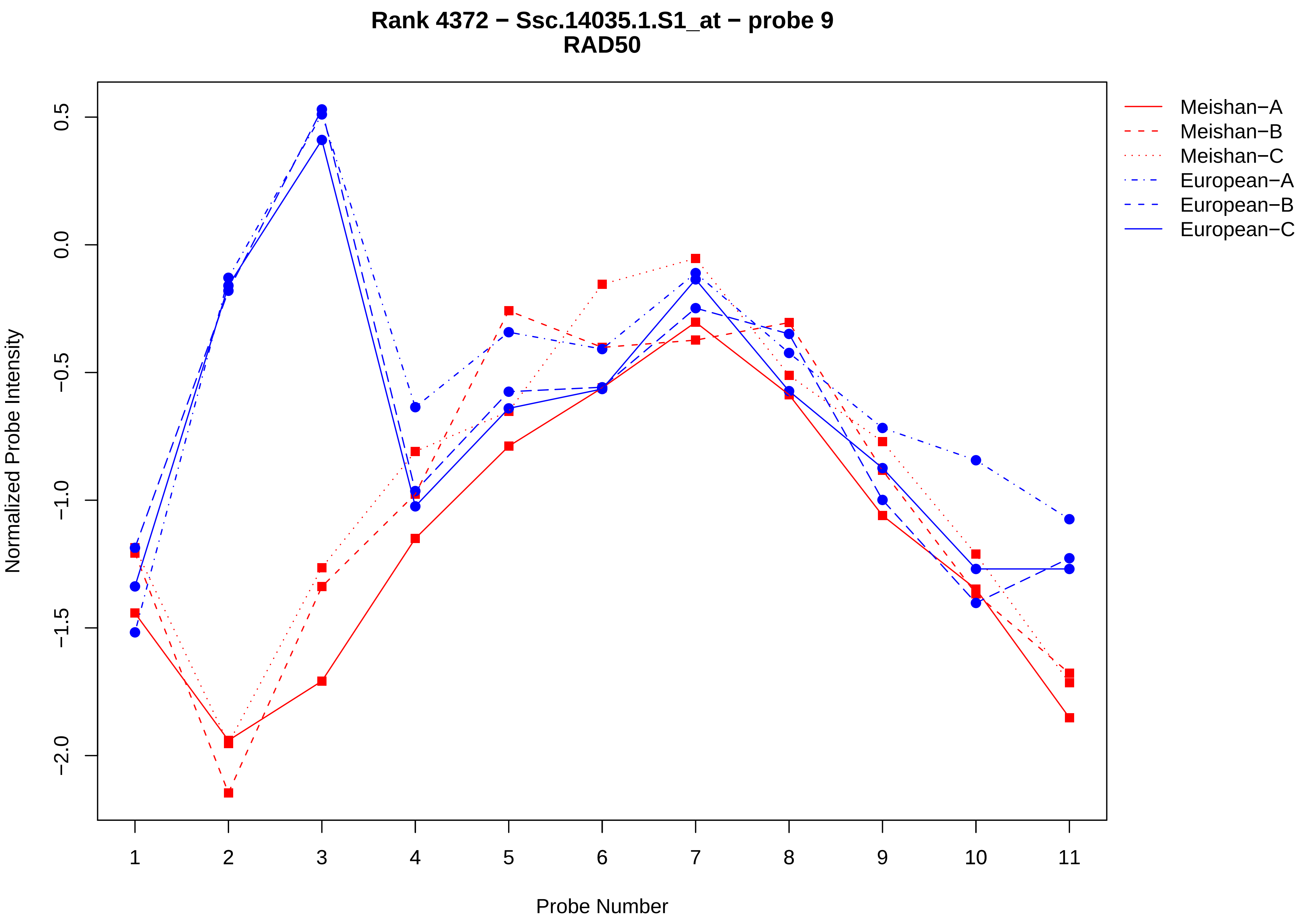
Rank 4372 − Ssc.14035.1.S1_at − probe 9
RAD50
Normalized Probe Intensity
Probe Number
0.5
0.0
−0.5
−1.0
−1.5
−2.0
1
2
3
4
5
6
7
8
9
10
11
Meishan−A
Meishan−B
Meishan−C
European−A
European−B
European−C

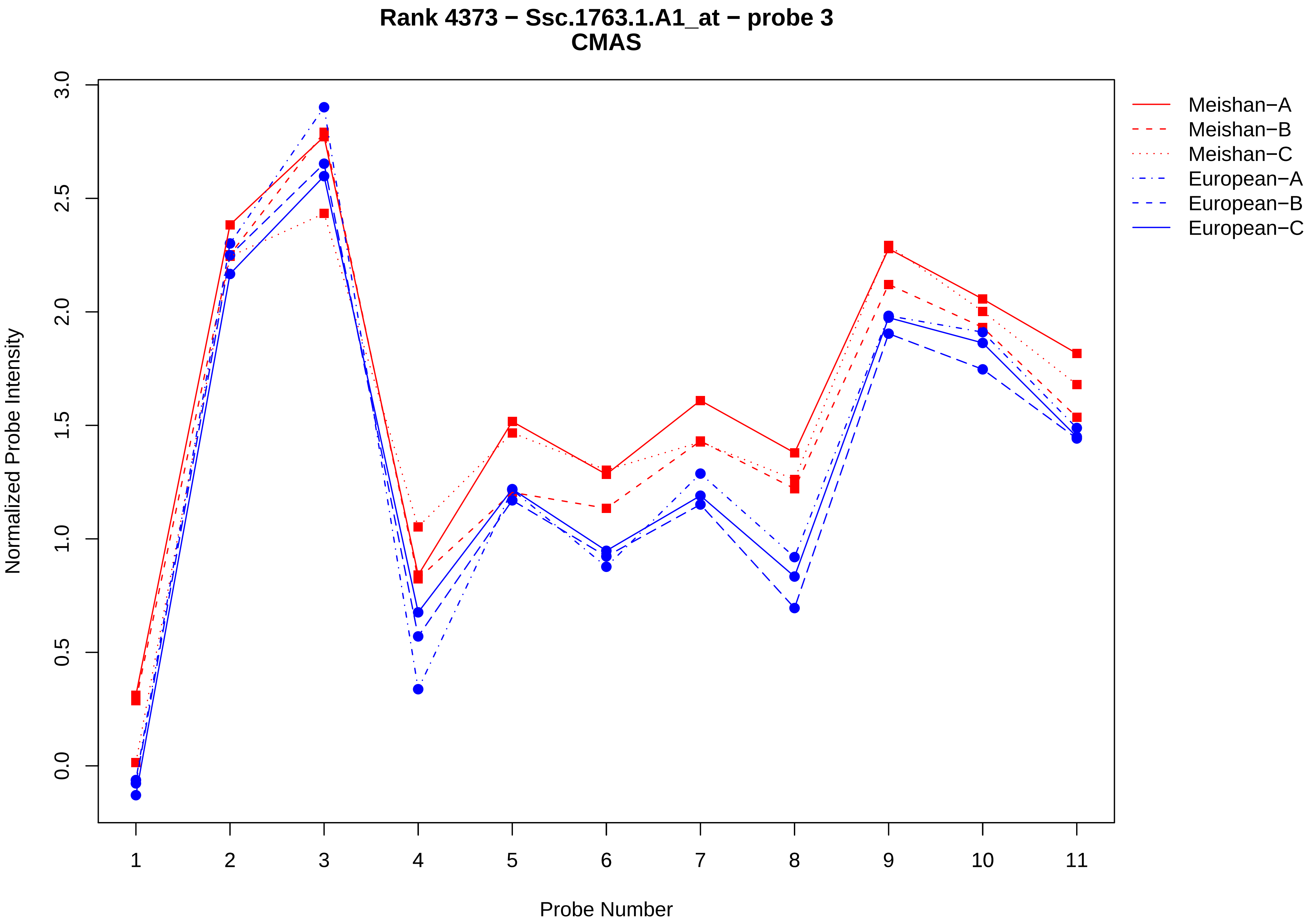
Rank 4373 − Ssc.1763.1.A1_at − probe 3
CMAS
Normalized Probe Intensity
Probe Number
Meishan−A
Meishan−B
Meishan−C
European−A
European−B
European−C
0.0
0.5
1.0
1.5
2.0
2.5
3.0
1
2
3
4
5
6
7
8
9
10
11

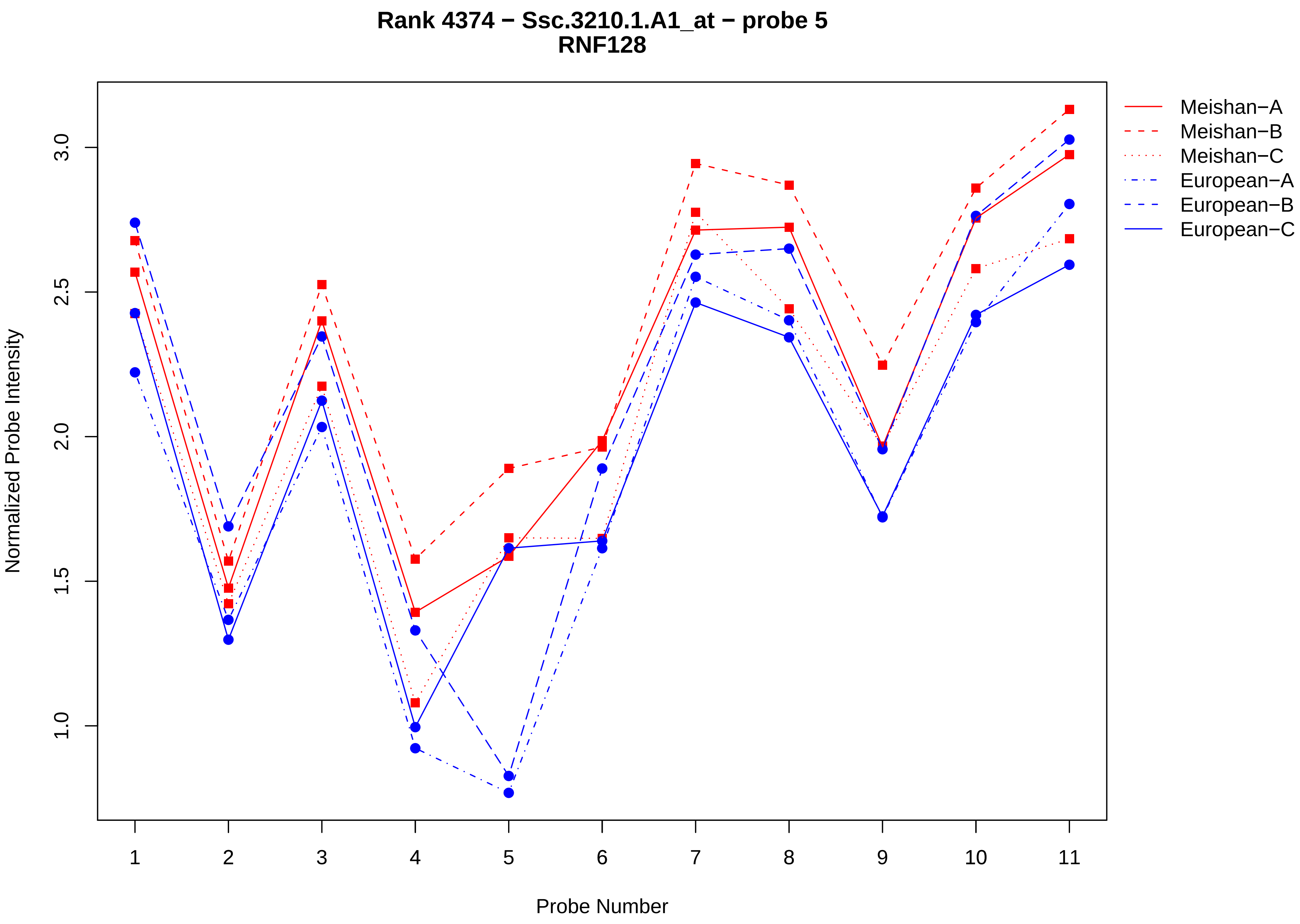

Rank 4374 − Ssc.3210.1.A1_at − probe 5
RNF128
Normalized Probe Intensity
1.0
1.5
2.0
2.5
3.0
1
2
3
4
5
6
7
8
9
10
11
Probe Number
Meishan−A
Meishan−B
Meishan−C
European−A
European−B
European−C

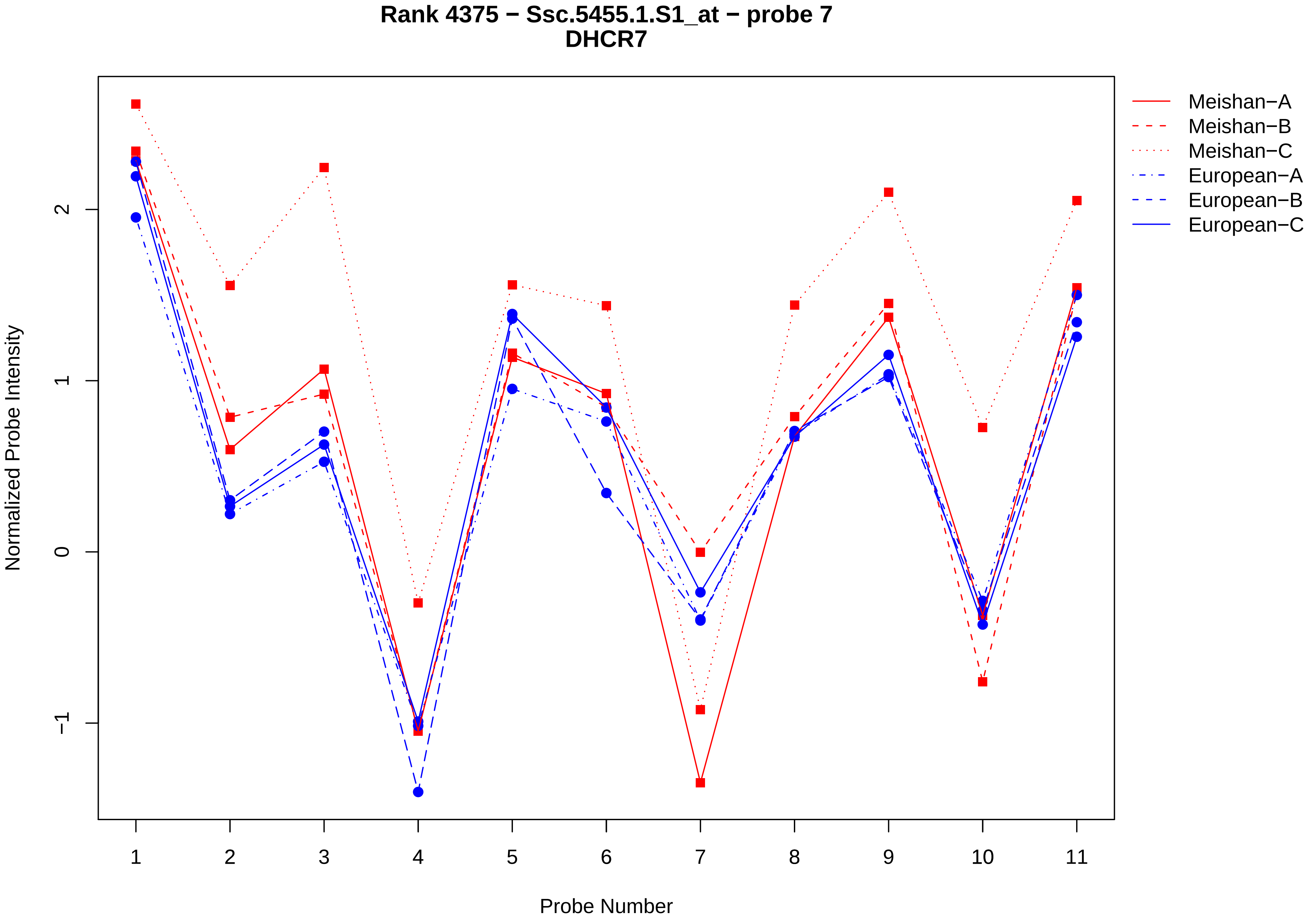

Rank 4375 − Ssc.5455.1.S1_at − probe 7
DHCR7
Normalized Probe Intensity
Probe Number
Meishan−A
Meishan−B
Meishan−C
European−A
European−B
European−C
−1
0
1
2
1
2
3
4
5
6
7
8
9
10
11

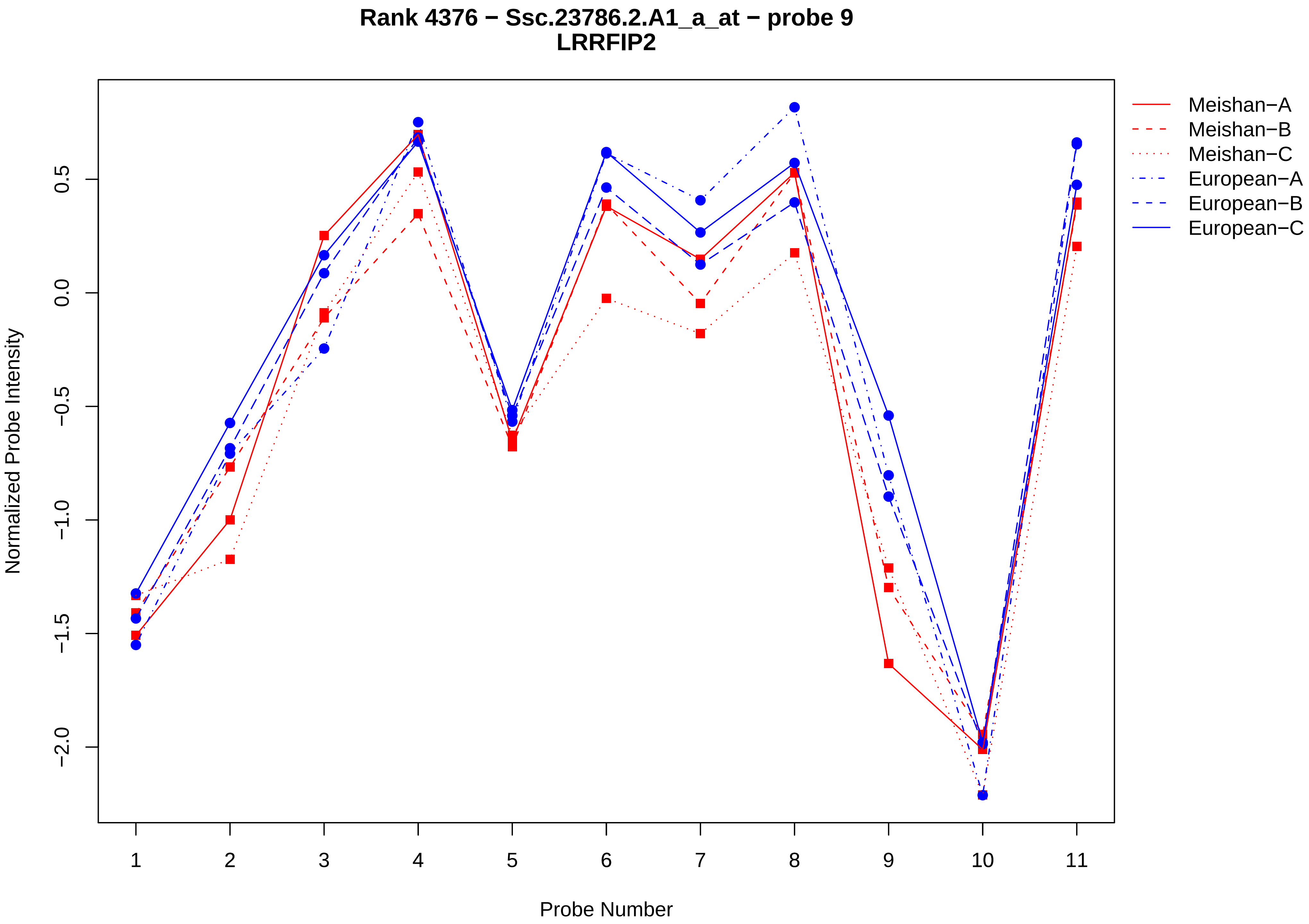

Rank 4376 − Ssc.23786.2.A1_a_at − probe 9
LRRFIP2
Normalized Probe Intensity
Probe Number
0.5
0.0
−0.5
−1.0
−1.5
−2.0
1
2
3
4
5
6
7
8
9
10
11
Meishan−A
Meishan−B
Meishan−C
European−A
European−B
European−C

Rank 4377 − Ssc.3629.1.S1_at − probe 5
OTUB1

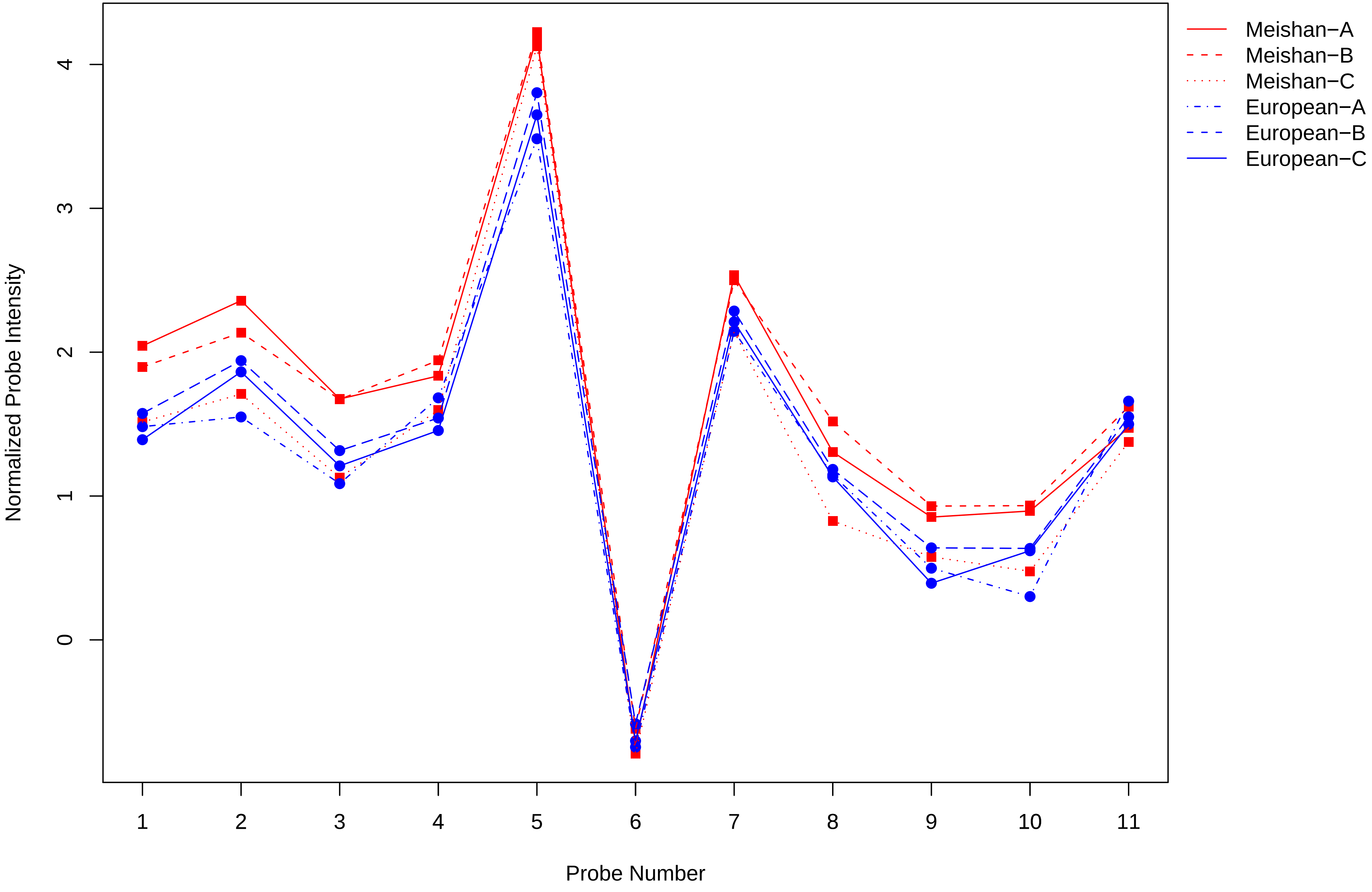

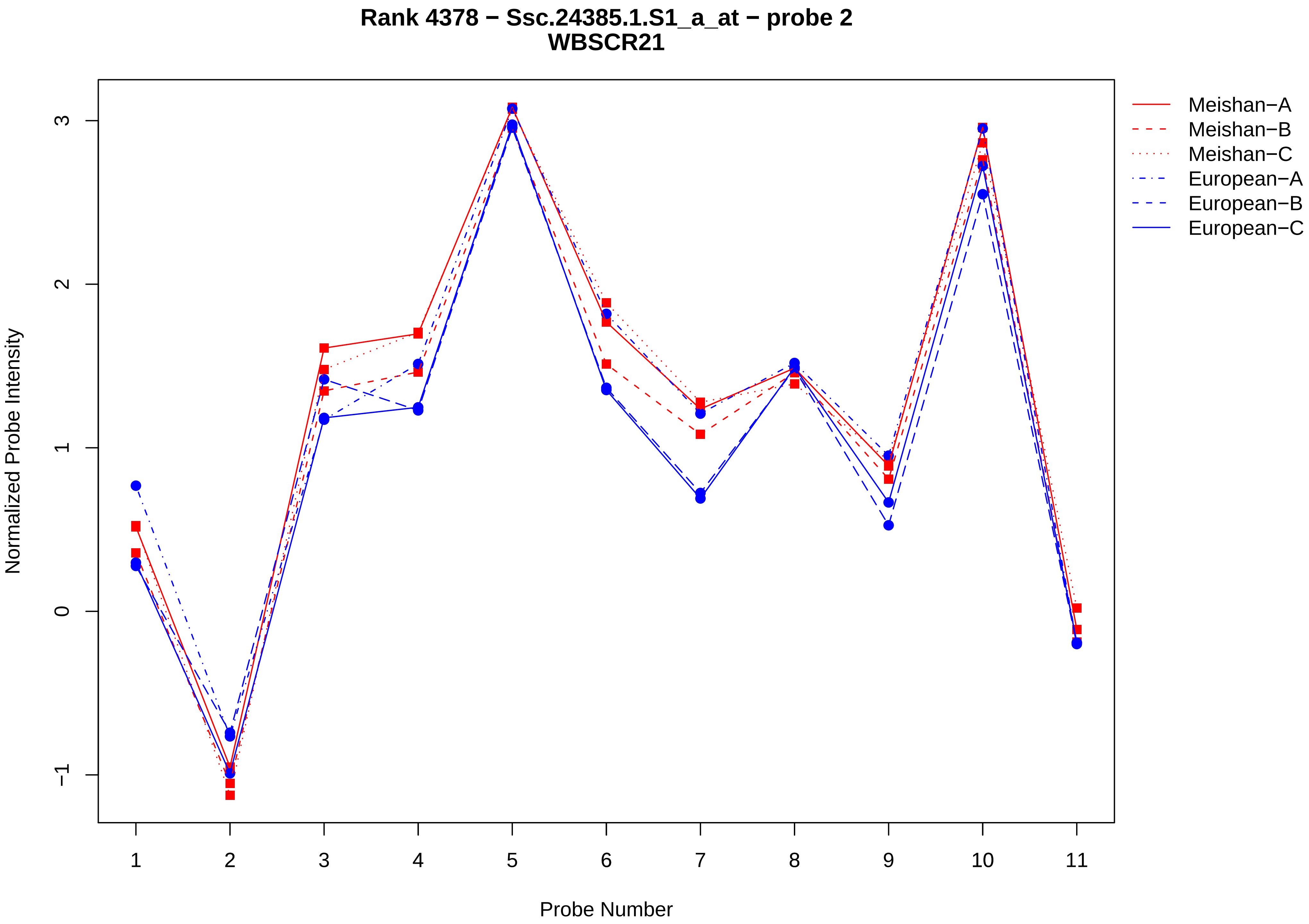

Rank 4378 − Ssc.24385.1.S1_a_at − probe 2
WBSCR21
Normalized Probe Intensity
Probe Number
Meishan−A
Meishan−B
Meishan−C
European−A
European−B
European−C
−1
0
1
2
3
1
2
3
4
5
6
7
8
9
10
11

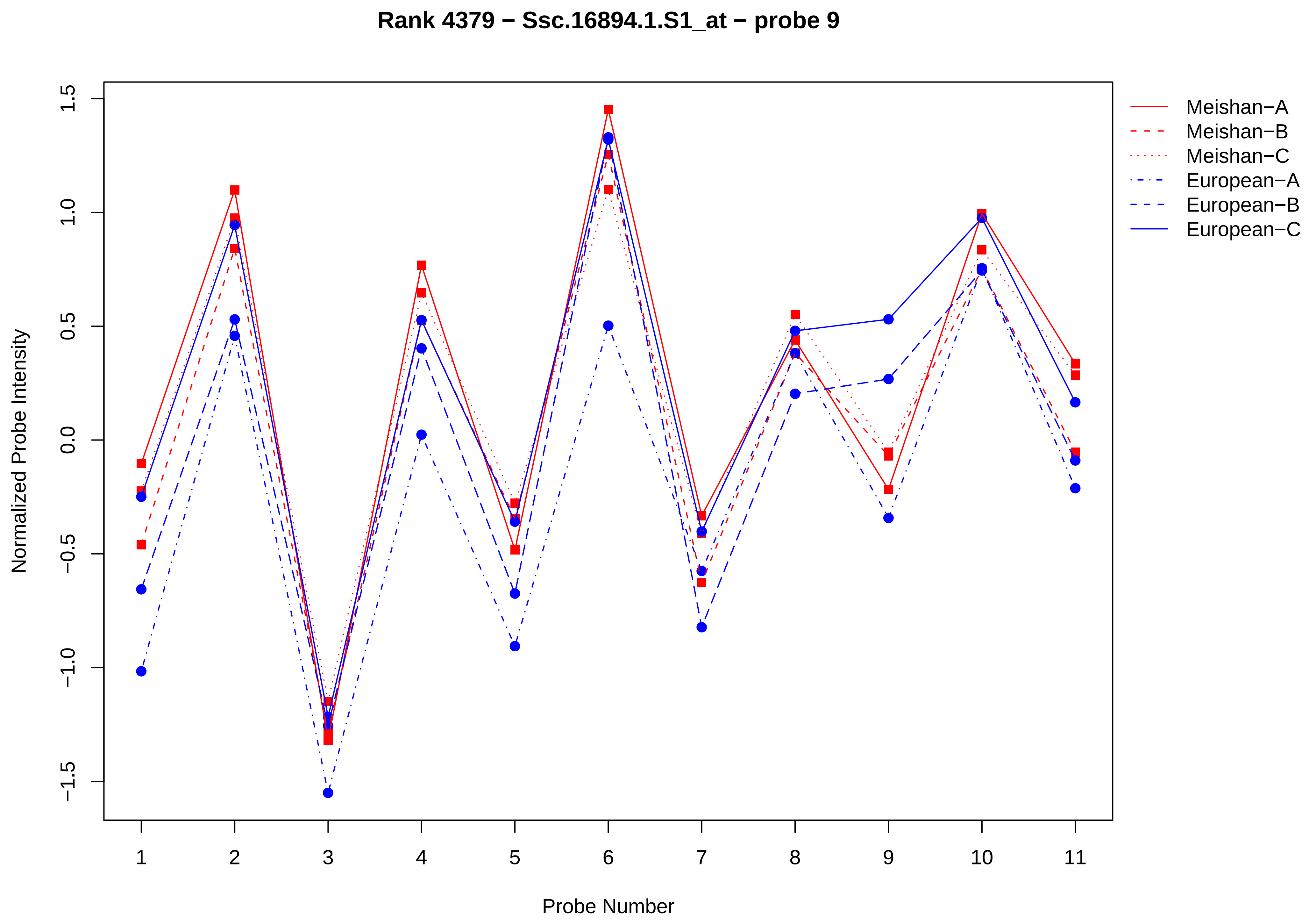

Rank 4379 − Ssc.16894.1.S1_at − probe 9
Normalized Probe Intensity
Probe Number
Meishan−A
Meishan−B
Meishan−C
European−A
European−B
European−C
1.5
1.0
0.5
0.0
−0.5
−1.0
−1.5
1
2
3
4
5
6
7
8
9
10
11

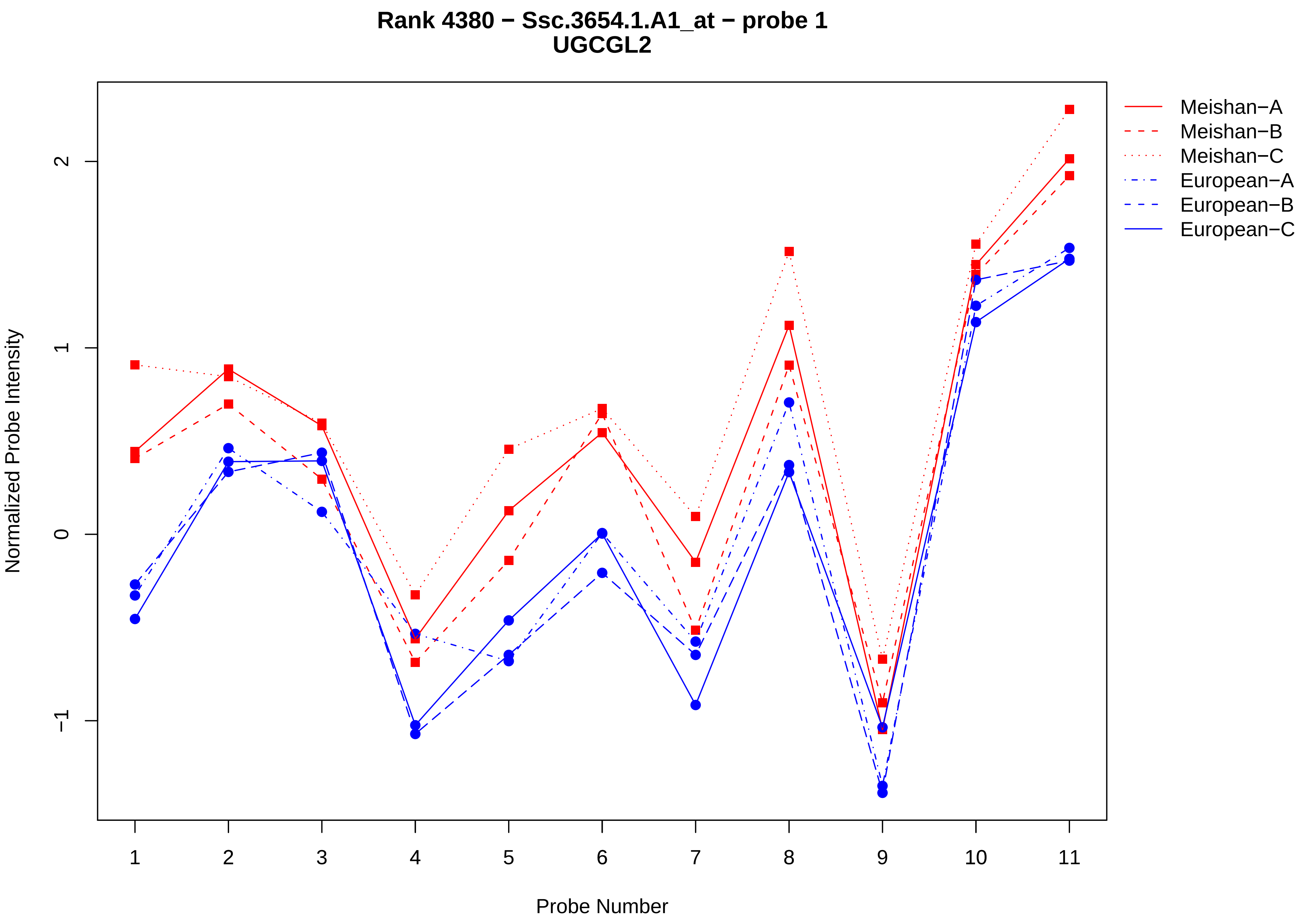
Rank 4380 − Ssc.3654.1.A1_at − probe 1
UGCGL2
Normalized Probe Intensity
Probe Number
Meishan−A
Meishan−B
Meishan−C
European−A
European−B
European−C
−1
0
1
2
1
2
3
4
5
6
7
8
9
10
11

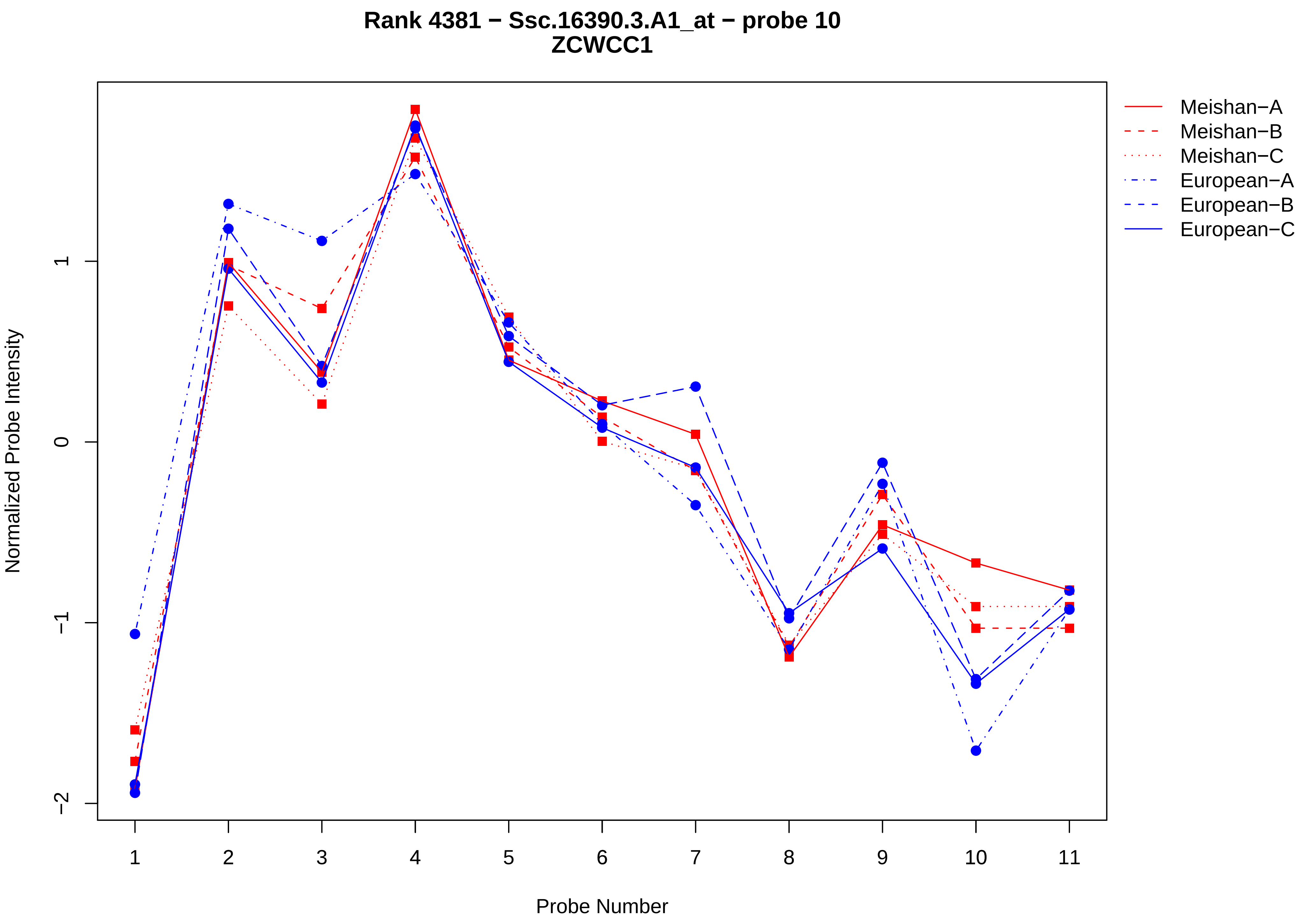
Rank 4381 − Ssc.16390.3.A1_at − probe 10
ZCWCC1
Normalized Probe Intensity
Probe Number
Meishan−A
Meishan−B
Meishan−C
European−A
European−B
European−C

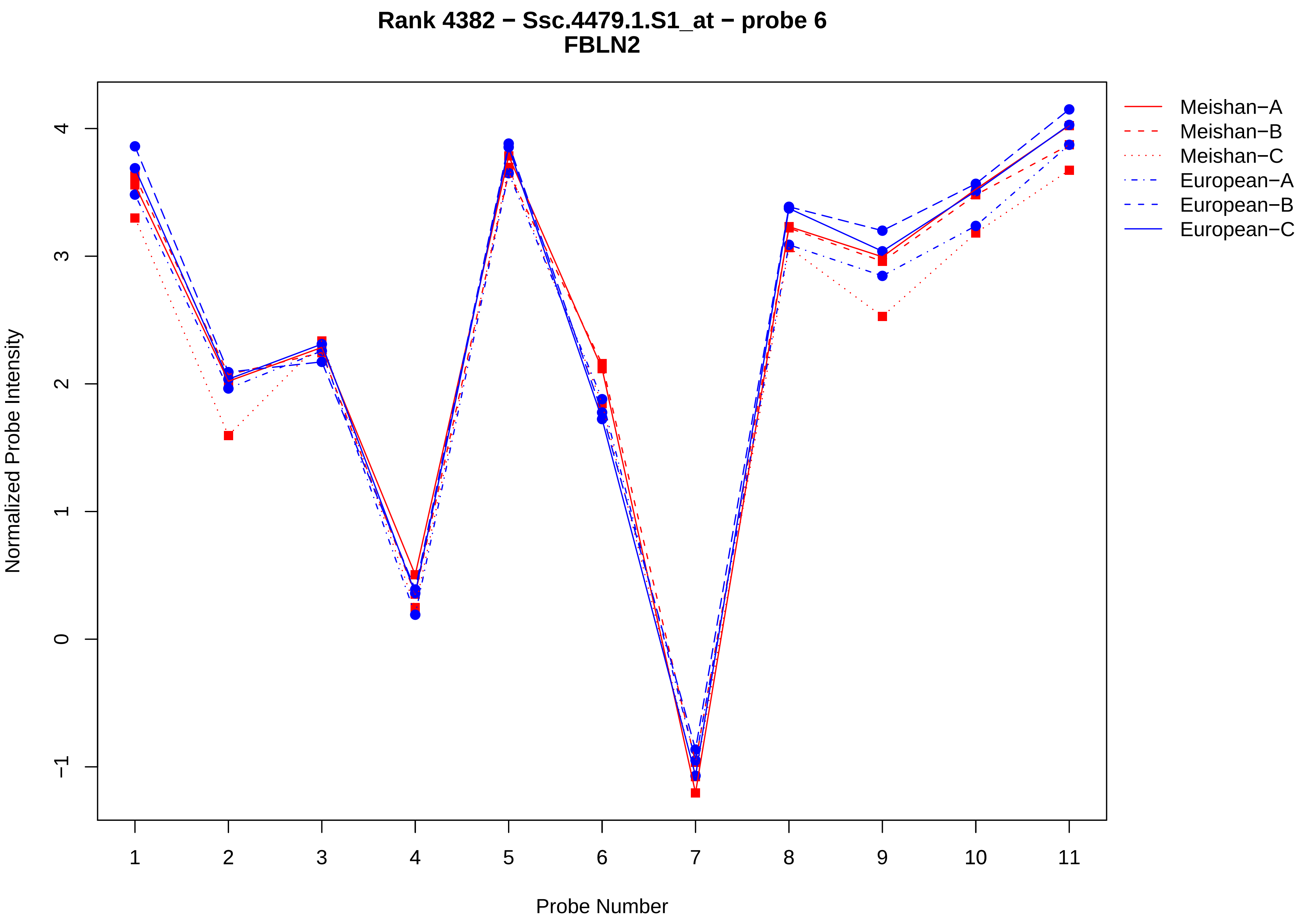

Rank 4382 − Ssc.4479.1.S1_at − probe 6
FBLN2
Normalized Probe Intensity
Probe Number
Meishan−A
Meishan−B
Meishan−C
European−A
European−B
European−C
−1
0
1
2
3
4
1
2
3
4
5
6
7
8
9
10
11

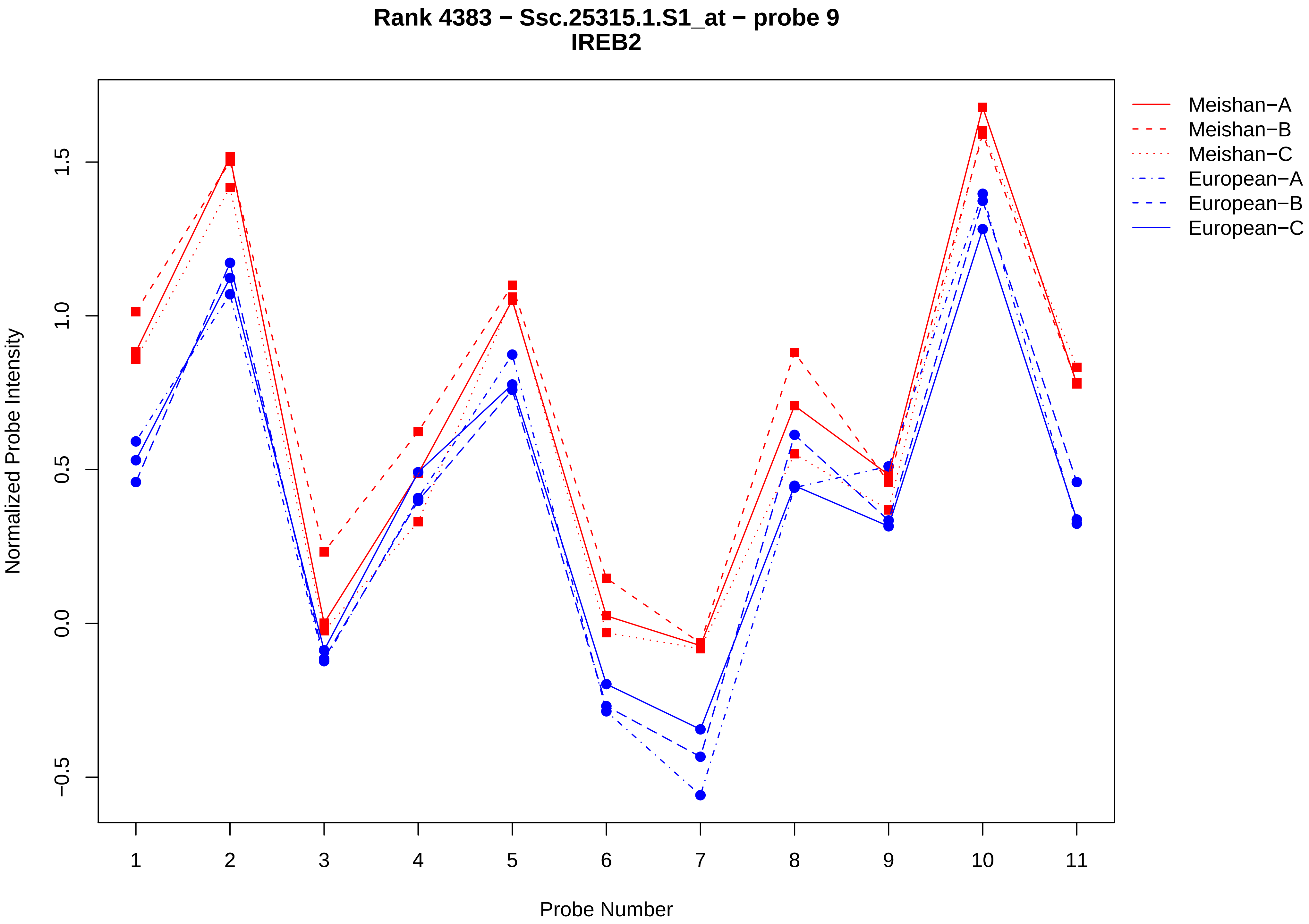
Rank 4383 − Ssc.25315.1.S1_at − probe 9
IREB2
Normalized Probe Intensity
Probe Number
−0.5
0.0
0.5
1.0
1.5
1
2
3
4
5
6
7
8
9
10
11
Meishan−A
Meishan−B
Meishan−C
European−A
European−B
European−C

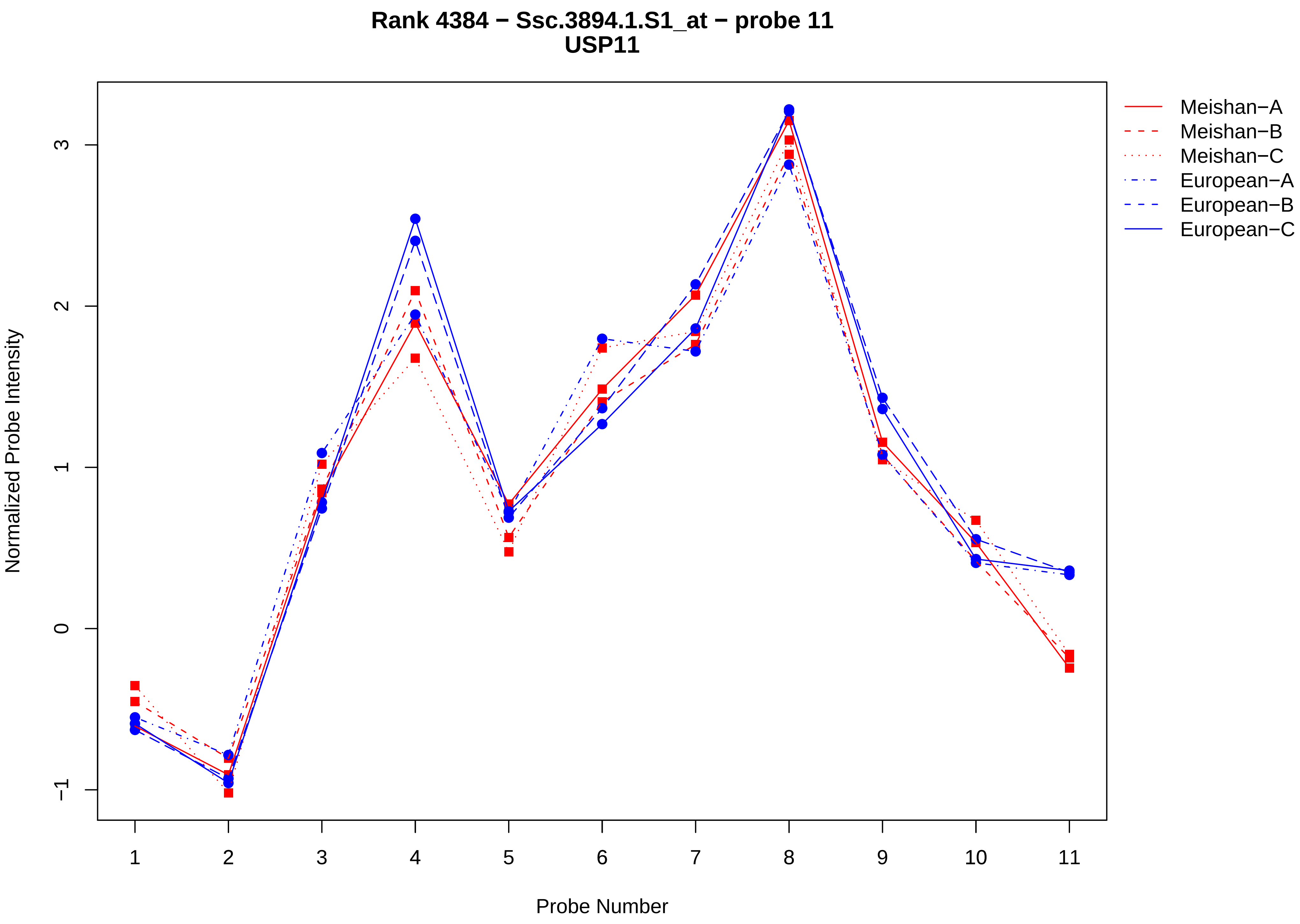

Rank 4384 − Ssc.3894.1.S1_at − probe 11
USP11
Normalized Probe Intensity
Probe Number
−1
0
1
2
3
1
2
3
4
5
6
7
8
9
10
11
Meishan−A
Meishan−B
Meishan−C
European−A
European−B
European−C

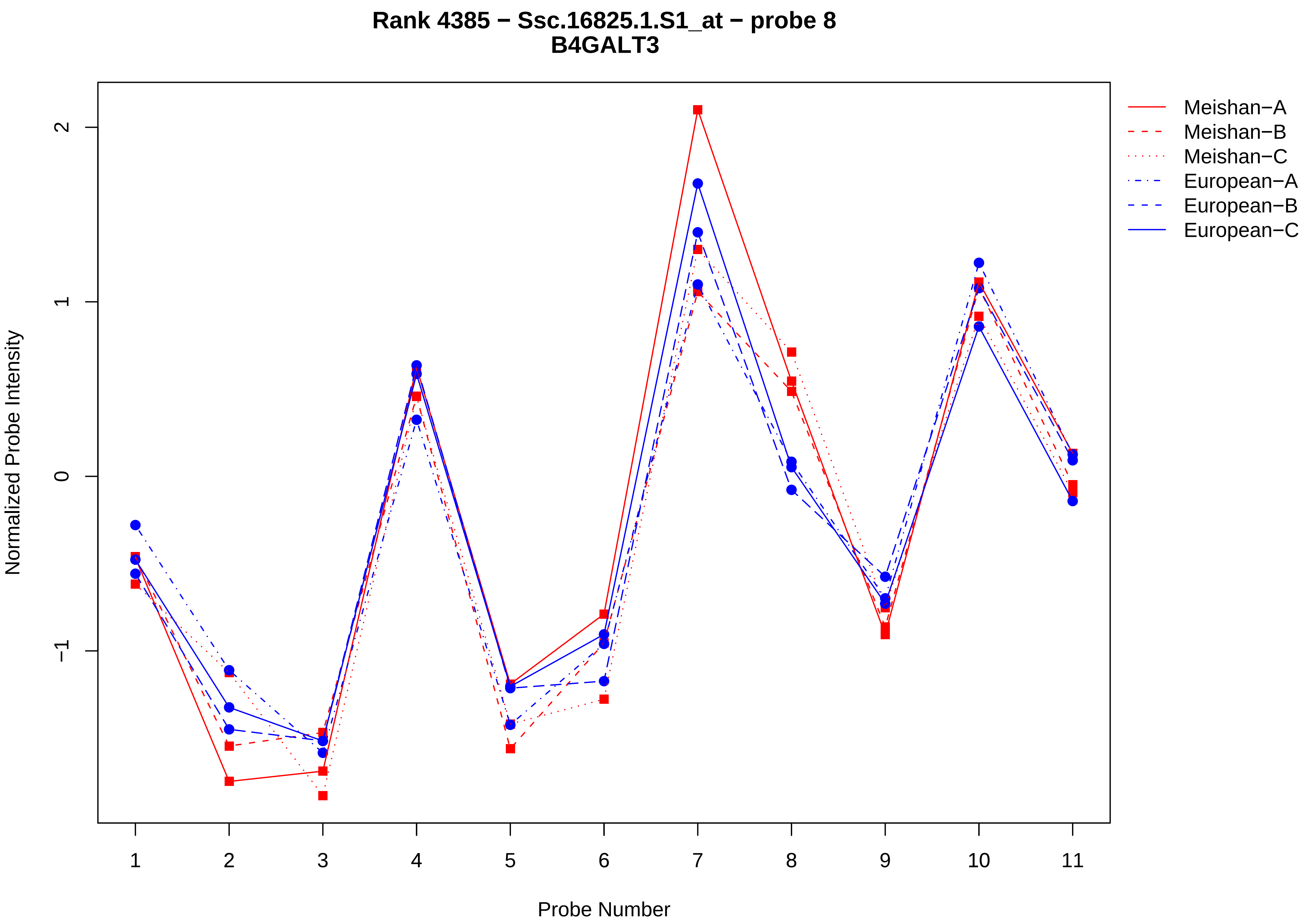

Rank 4385 − Ssc.16825.1.S1_at − probe 8
B4GALT3
Normalized Probe Intensity
Probe Number
Meishan−A
Meishan−B
Meishan−C
European−A
European−B
European−C
−1
0
1
2
1
2
3
4
5
6
7
8
9
10
11

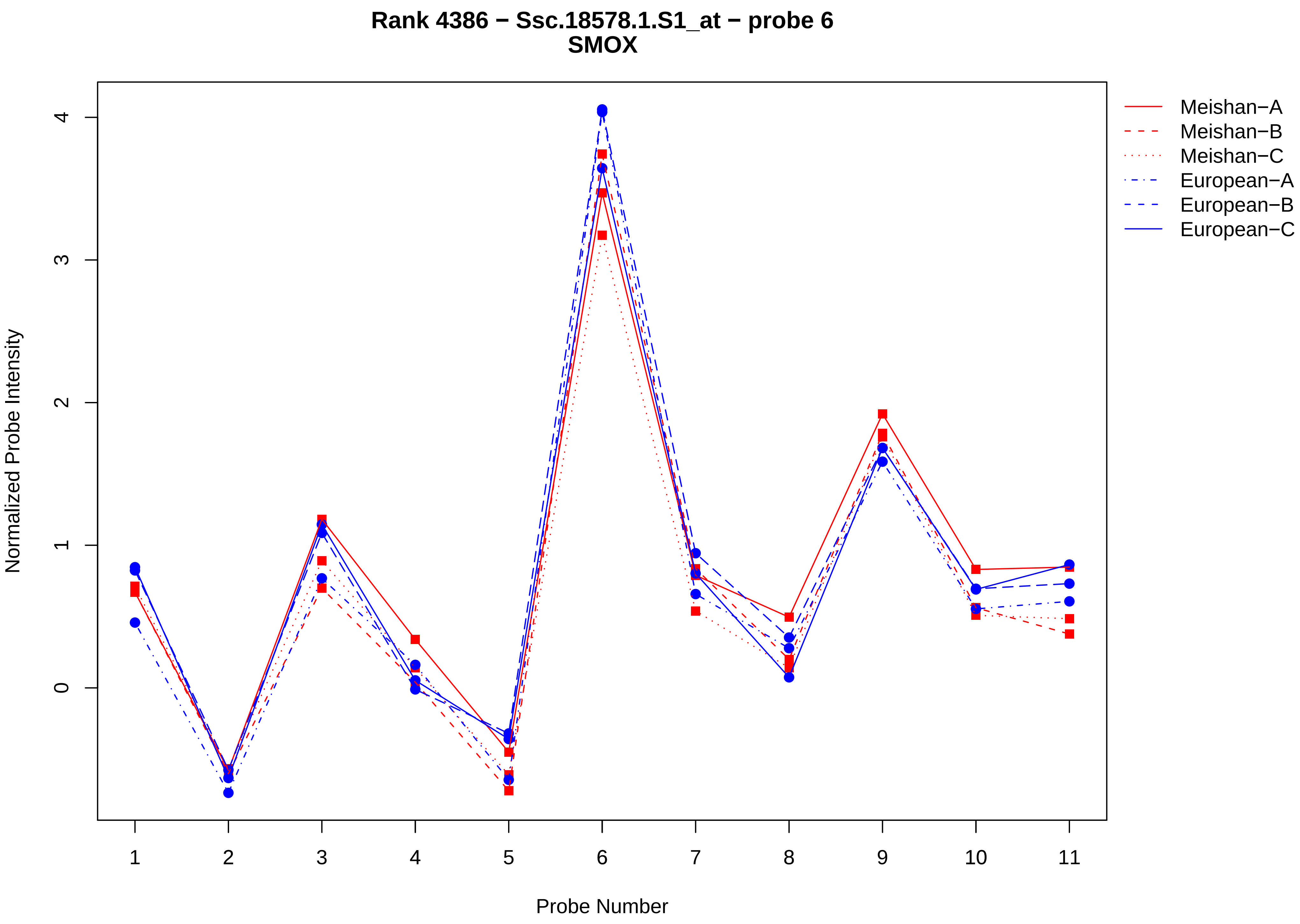
Rank 4386 − Ssc.18578.1.S1_at − probe 6
SMOX
Normalized Probe Intensity
Probe Number
0
1
2
3
4
1
2
3
4
5
6
7
8
9
10
11
Meishan−A
Meishan−B
Meishan−C
European−A
European−B
European−C

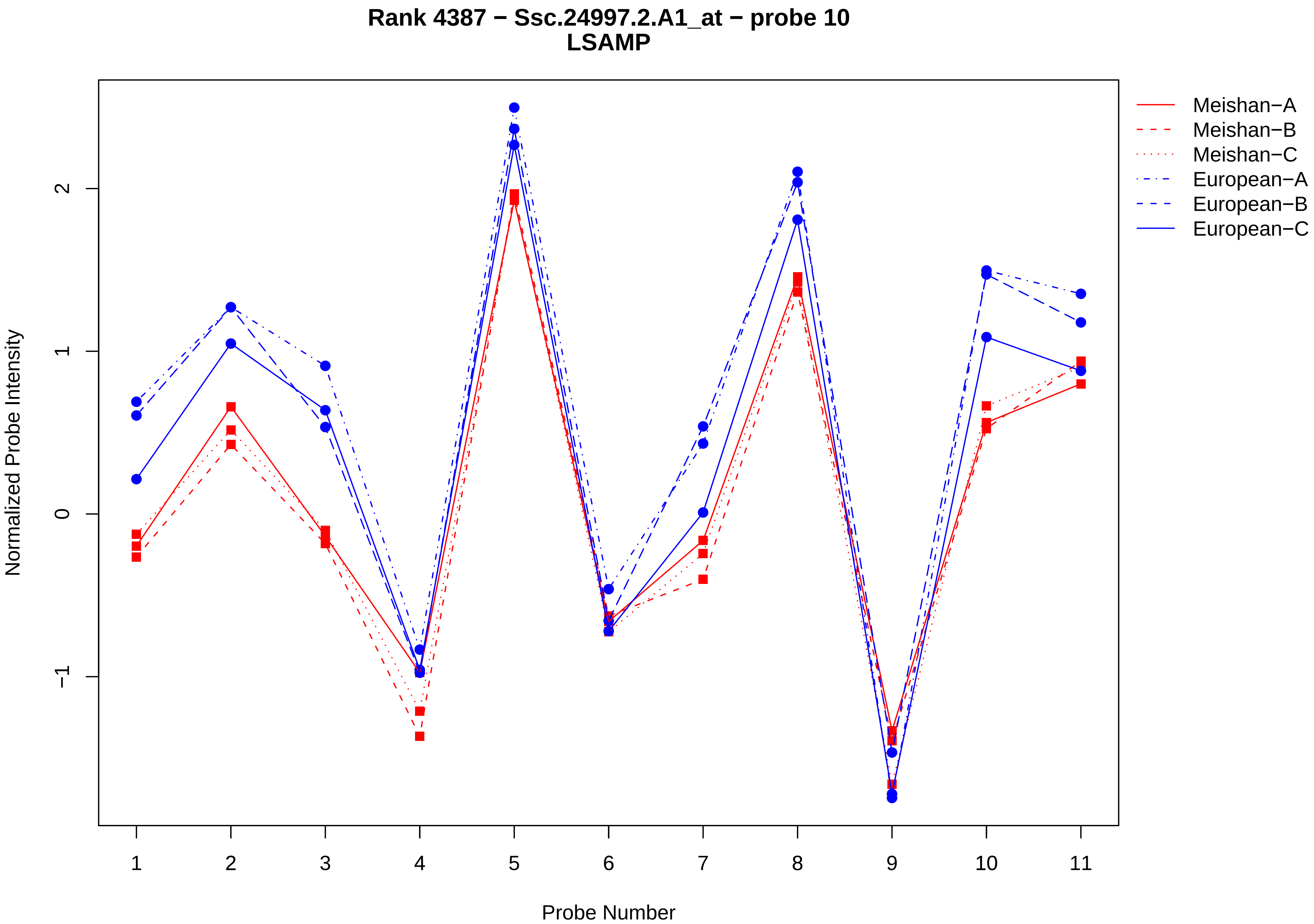
Rank 4387 − Ssc.24997.2.A1_at − probe 10
LSAMP
Normalized Probe Intensity
Probe Number
Meishan−A
Meishan−B
Meishan−C
European−A
European−B
European−C
−1
0
1
2
1
2
3
4
5
6
7
8
9
10
11

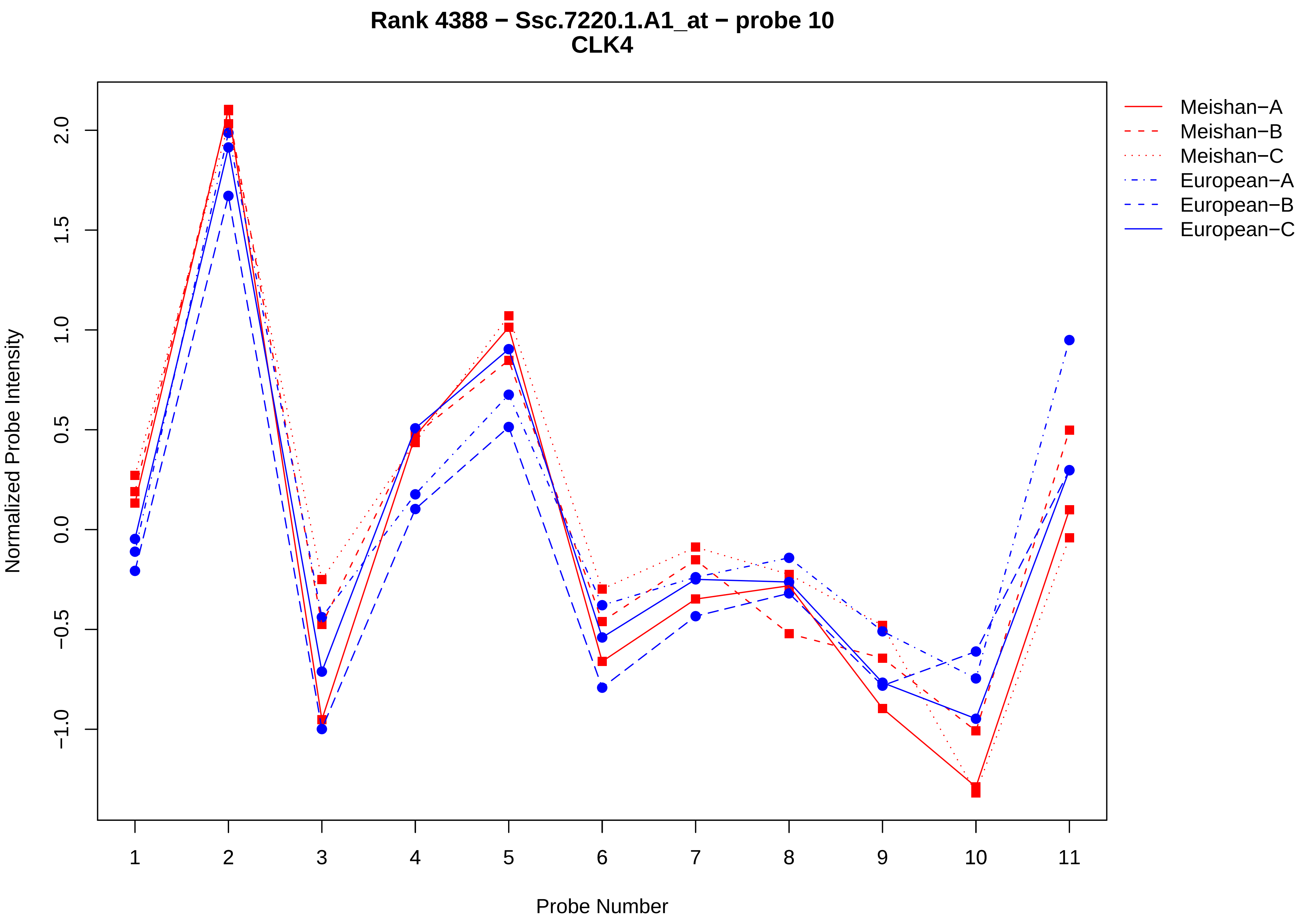

Rank 4388 − Ssc.7220.1.A1_at − probe 10
CLK4
Normalized Probe Intensity
Probe Number
2.0
1.5
1.0
0.5
0.0
−0.5
−1.0
1
2
3
4
5
6
7
8
9
10
11
Meishan−A
Meishan−B
Meishan−C
European−A
European−B
European−C

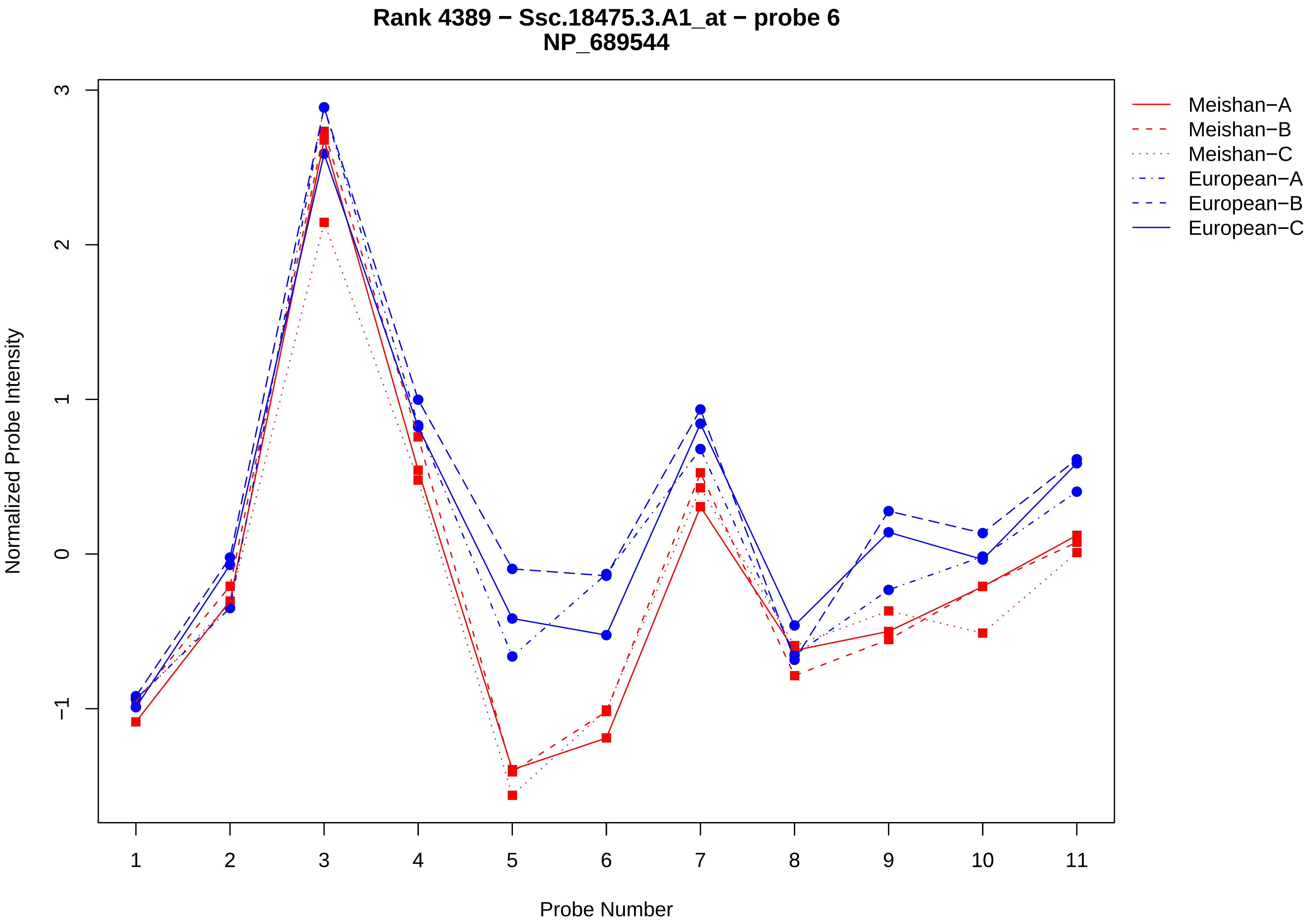
Rank 4389 − Ssc.18475.3.A1_at − probe 6
NP_689544
Normalized Probe Intensity
Probe Number
Meishan−A
Meishan−B
Meishan−C
European−A
European−B
European−C
−1
0
1
2
3
1
2
3
4
5
6
7
8
9
10
11

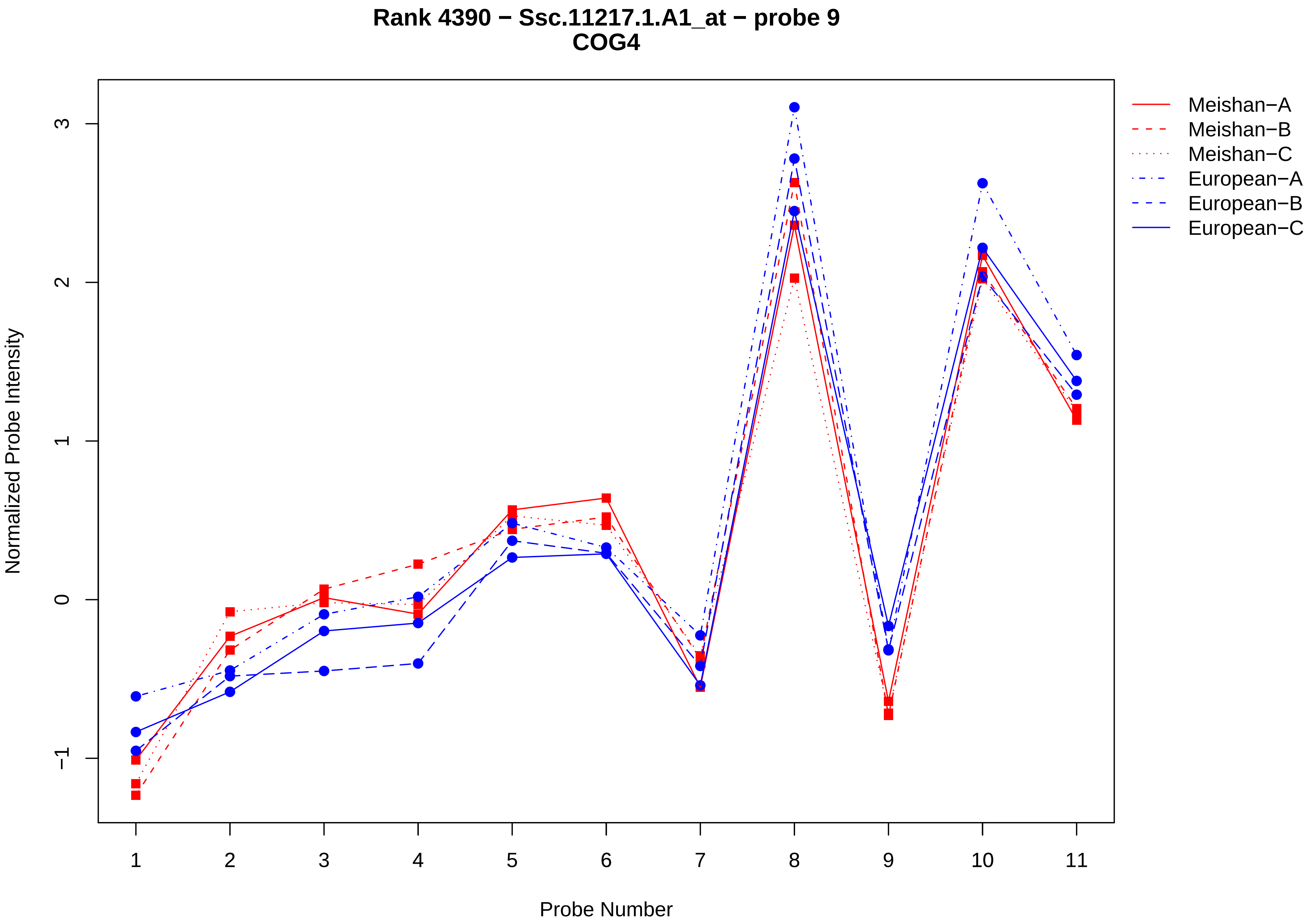

Rank 4390 − Ssc.11217.1.A1_at − probe 9
COG4
Normalized Probe Intensity
Probe Number
Meishan−A
Meishan−B
Meishan−C
European−A
European−B
European−C
−1
0
1
2
3
1
2
3
4
5
6
7
8
9
10
11

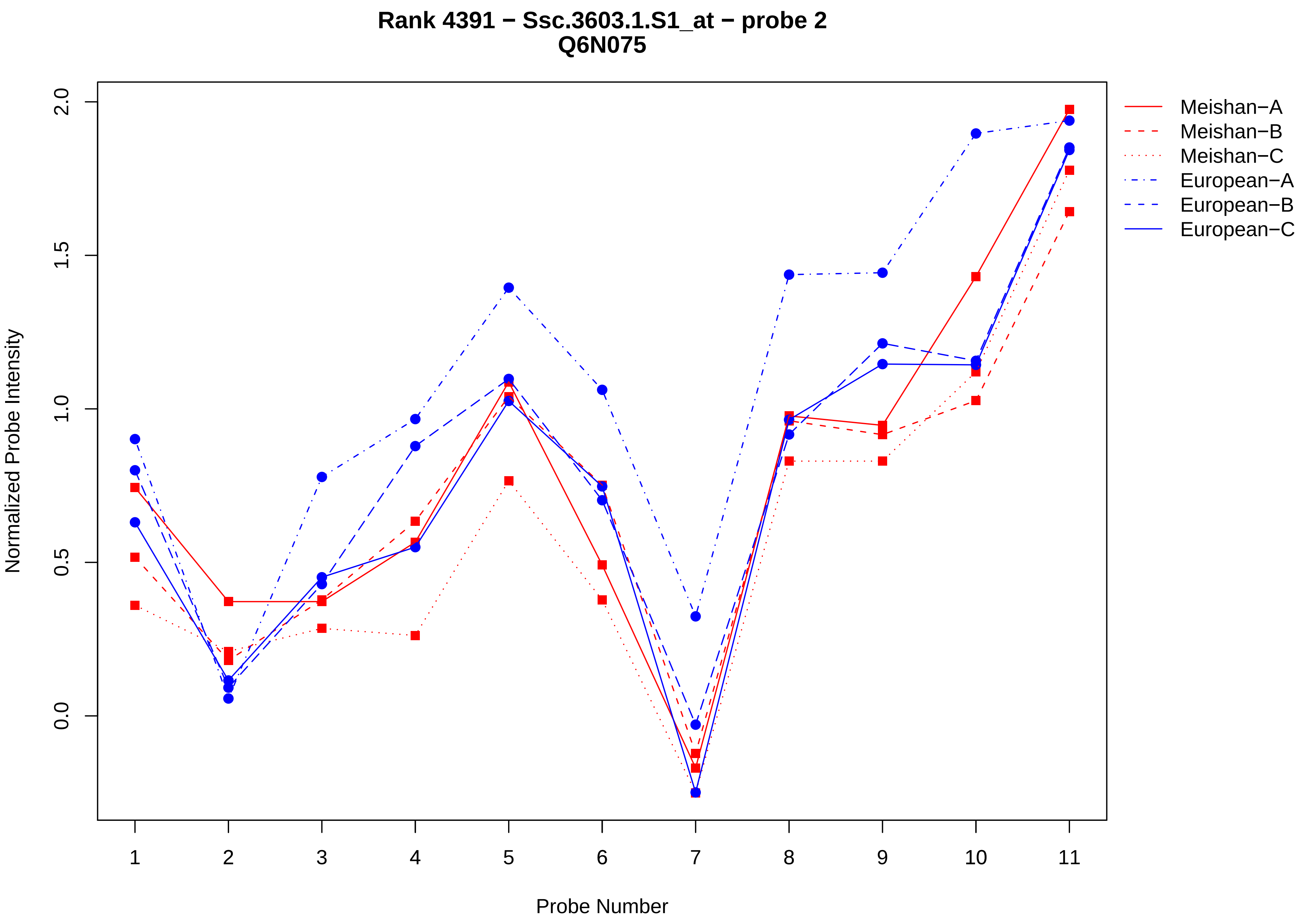

Rank 4391 − Ssc.3603.1.S1_at − probe 2
Q6N075
Normalized Probe Intensity
Probe Number
0.0
0.5
1.0
1.5
2.0
1
2
3
4
5
6
7
8
9
10
11
Meishan−A
Meishan−B
Meishan−C
European−A
European−B
European−C

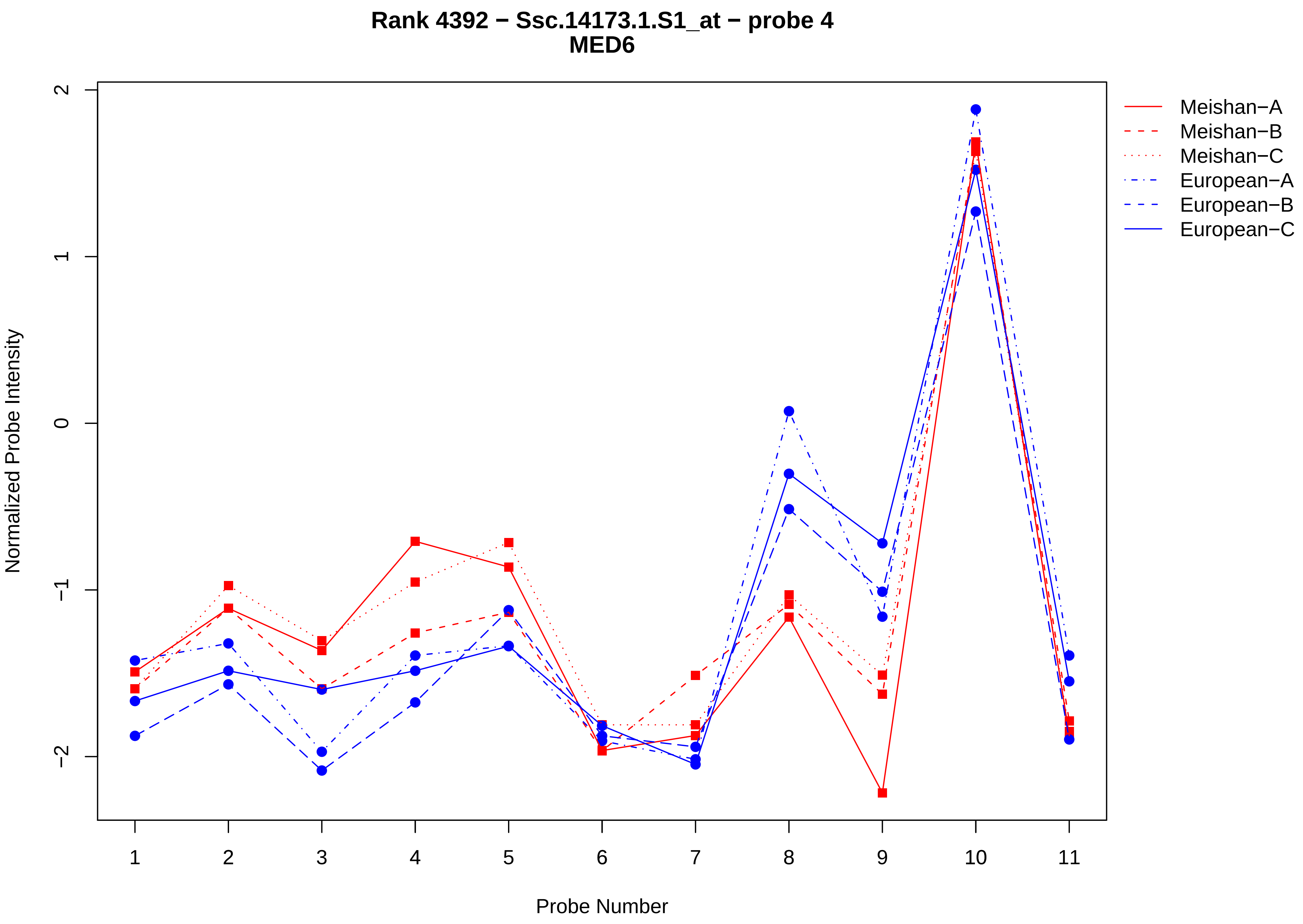
Rank 4392 − Ssc.14173.1.S1_at − probe 4
MED6
Normalized Probe Intensity
Probe Number
−2
−1
0
1
2
1
2
3
4
5
6
7
8
9
10
11
Meishan−A
Meishan−B
Meishan−C
European−A
European−B
European−C

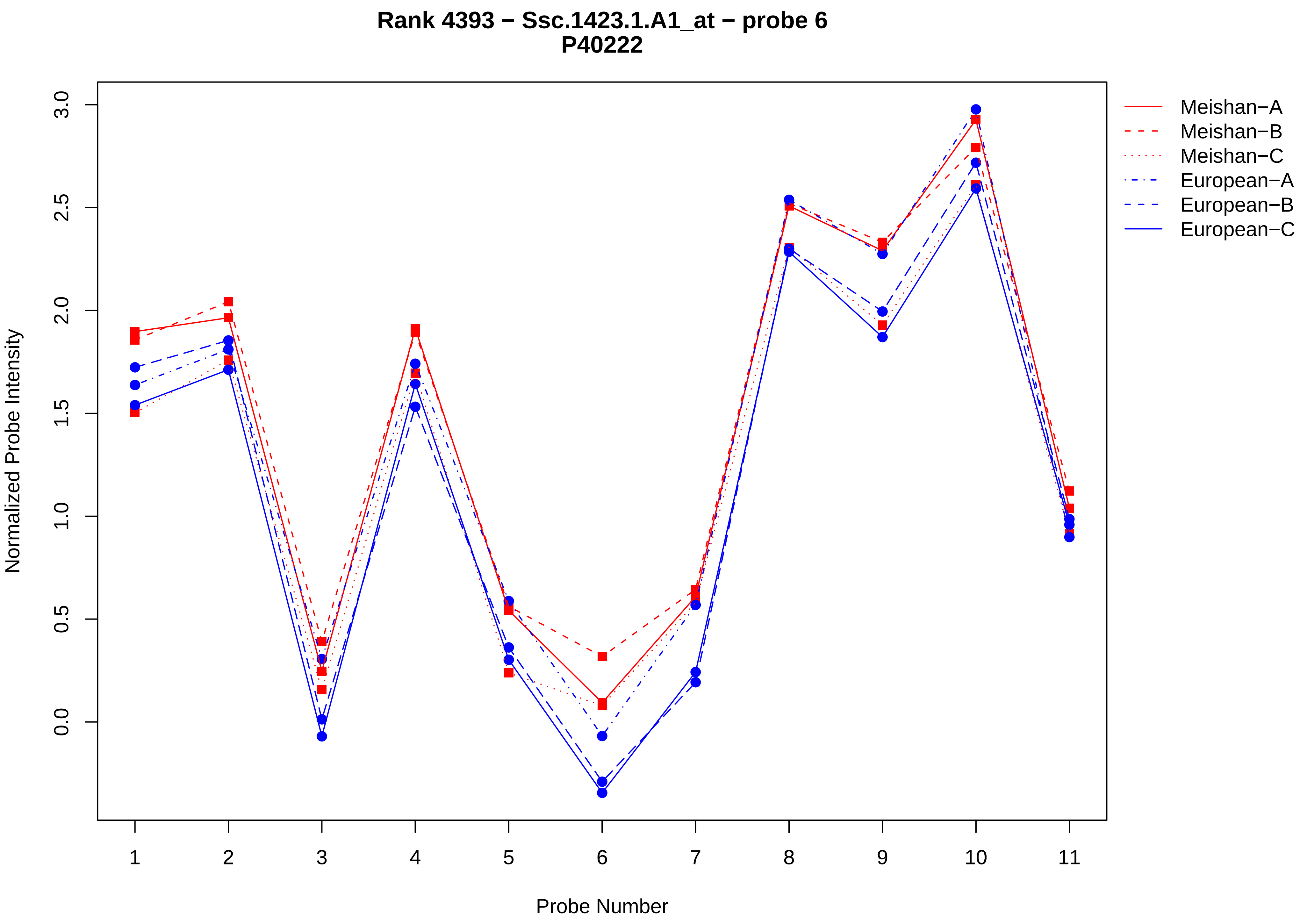
Rank 4393 − Ssc.1423.1.A1_at − probe 6
P40222
Normalized Probe Intensity
Probe Number
0.0
0.5
1.0
1.5
2.0
2.5
3.0
1
2
3
4
5
6
7
8
9
10
11
Meishan−A
Meishan−B
Meishan−C
European−A
European−B
European−C

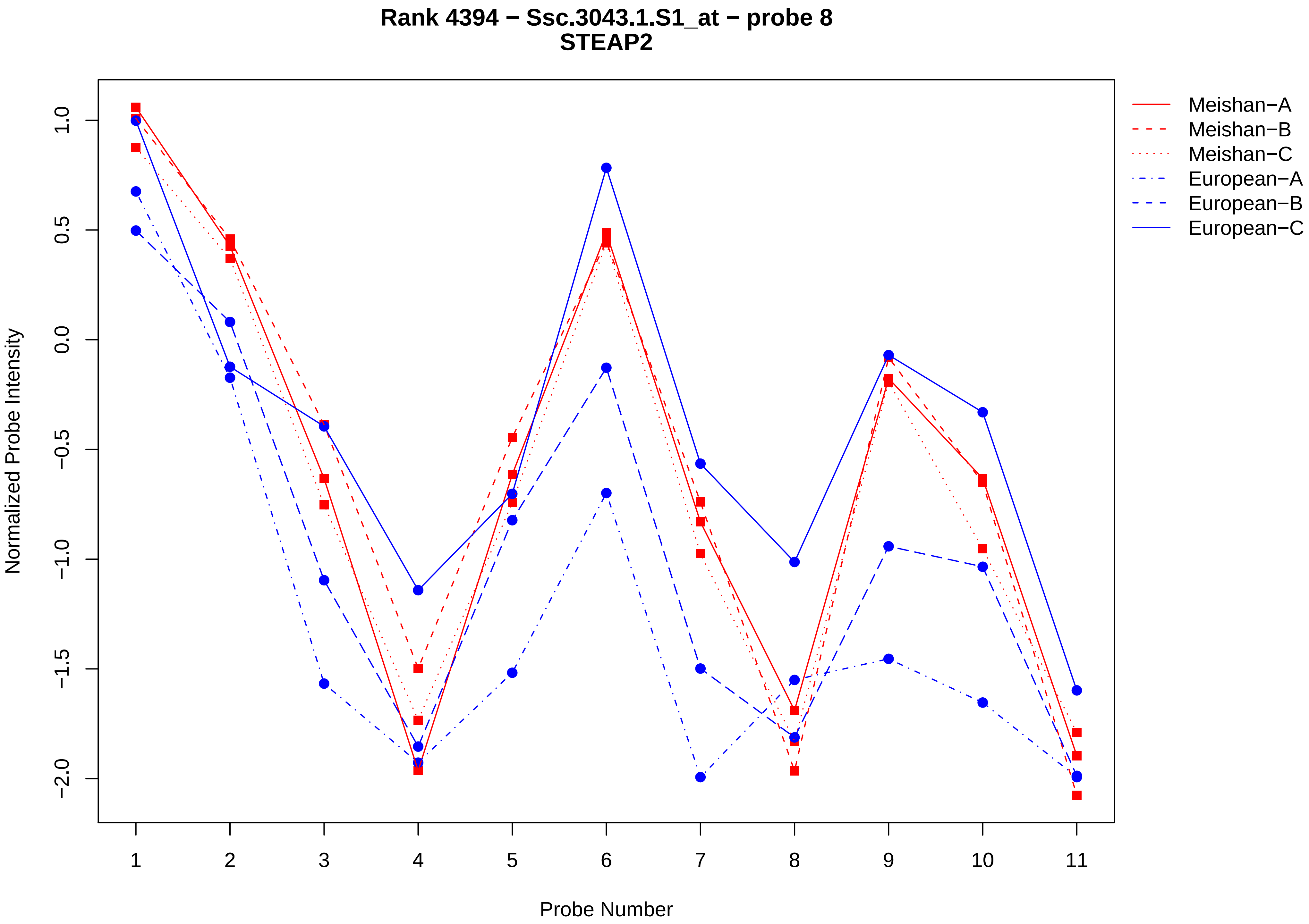

Rank 4394 − Ssc.3043.1.S1_at − probe 8
STEAP2
Normalized Probe Intensity
1.0
0.5
0.0
−0.5
−1.0
−1.5
−2.0
1
2
3
4
5
6
7
8
9
10
11
Probe Number
Meishan−A
Meishan−B
Meishan−C
European−A
European−B
European−C

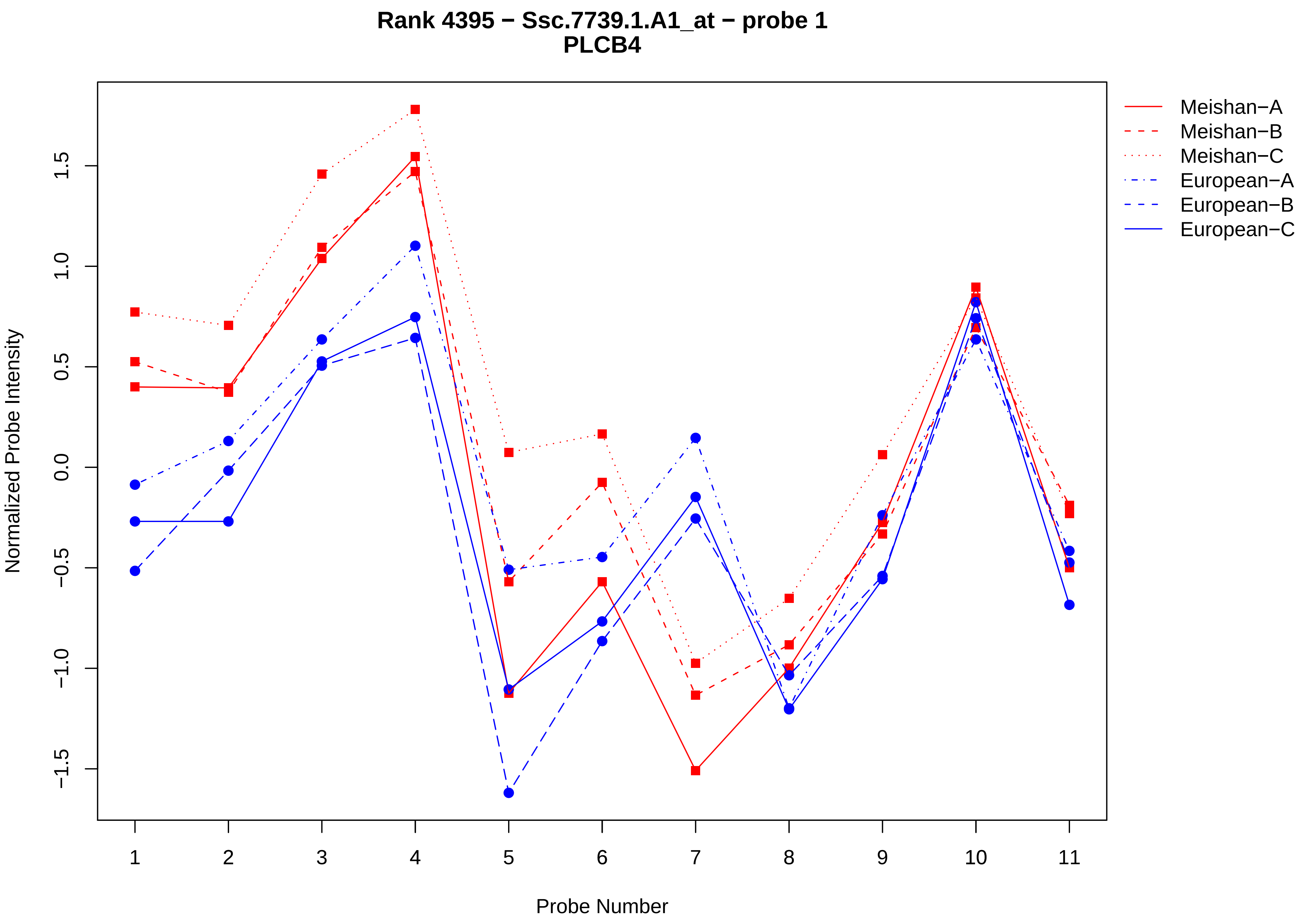

Rank 4395 − Ssc.7739.1.A1_at − probe 1
PLCB4
Normalized Probe Intensity
Probe Number
−1.5
−1.0
−0.5
0.0
0.5
1.0
1.5
1
2
3
4
5
6
7
8
9
10
11
Meishan−A
Meishan−B
Meishan−C
European−A
European−B
European−C

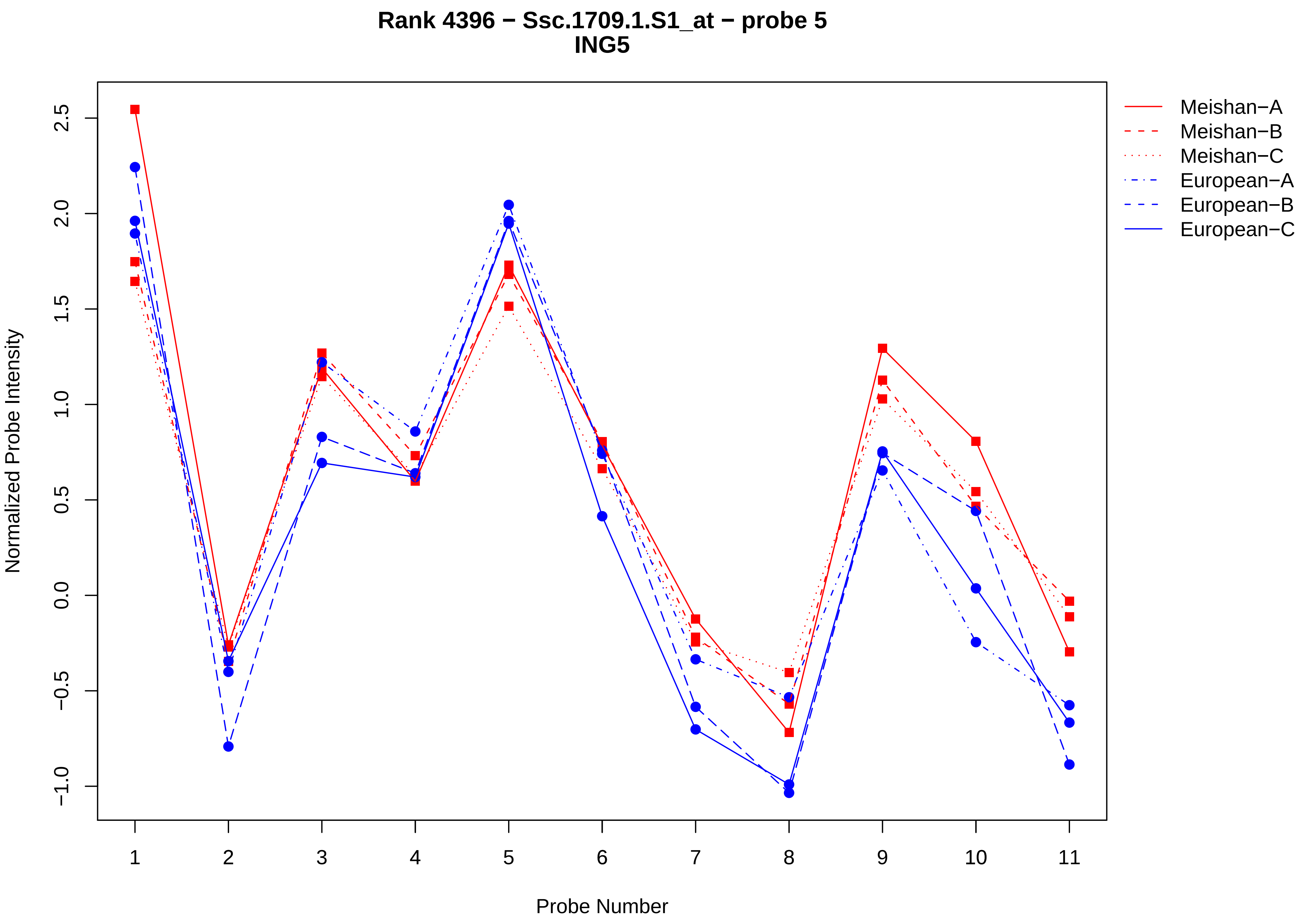

Rank 4396 − Ssc.1709.1.S1_at − probe 5
ING5
Normalized Probe Intensity
−1.0
−0.5
0.0
0.5
1.0
1.5
2.0
2.5
1
2
3
4
5
6
7
8
9
10
11
Probe Number
Meishan−A
Meishan−B
Meishan−C
European−A
European−B
European−C

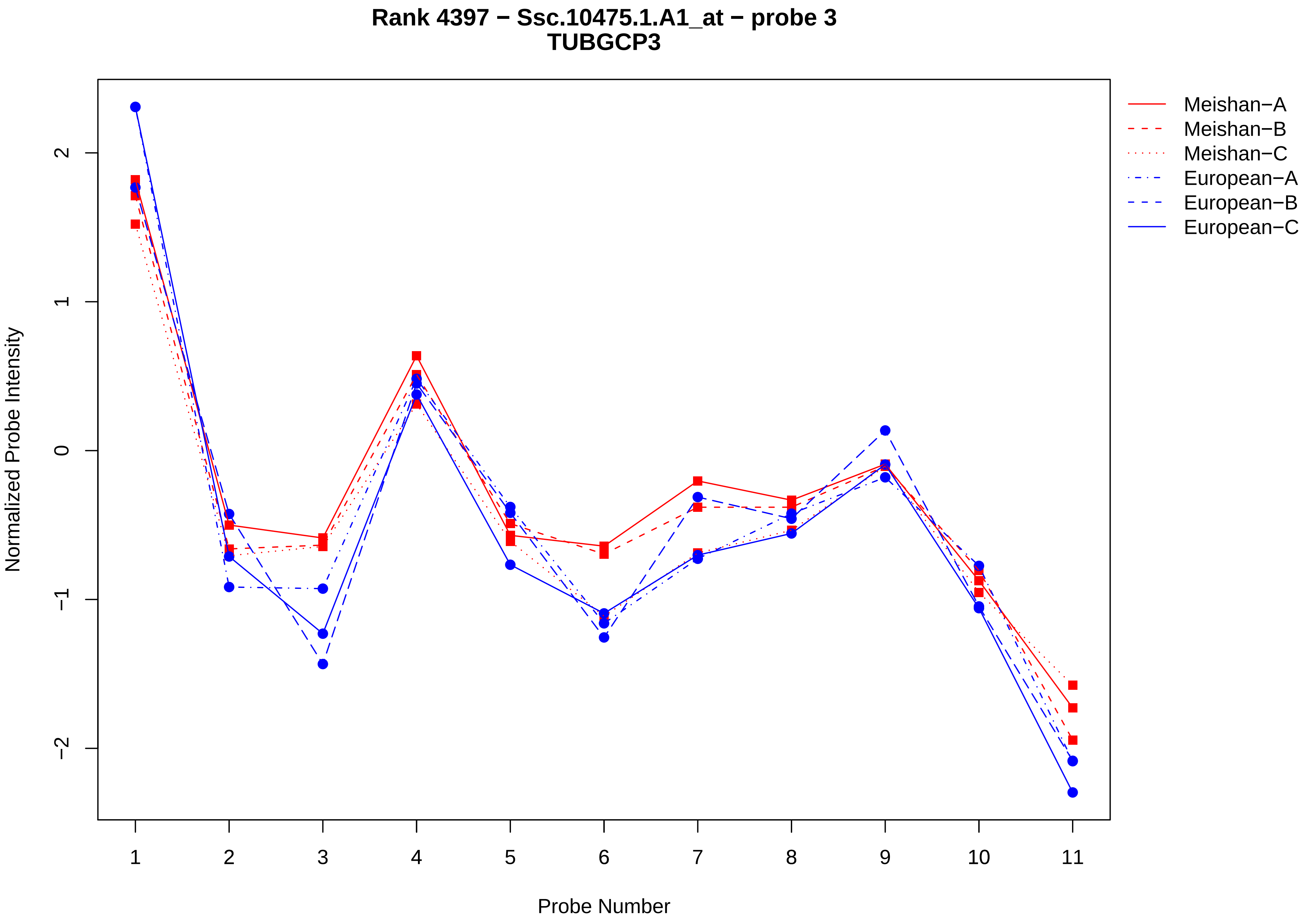

Rank 4397 − Ssc.10475.1.A1_at − probe 3
TUBGCP3
Normalized Probe Intensity
Probe Number
Meishan−A
Meishan−B
Meishan−C
European−A
European−B
European−C
2
1
0
−1
−2
1
2
3
4
5
6
7
8
9
10
11

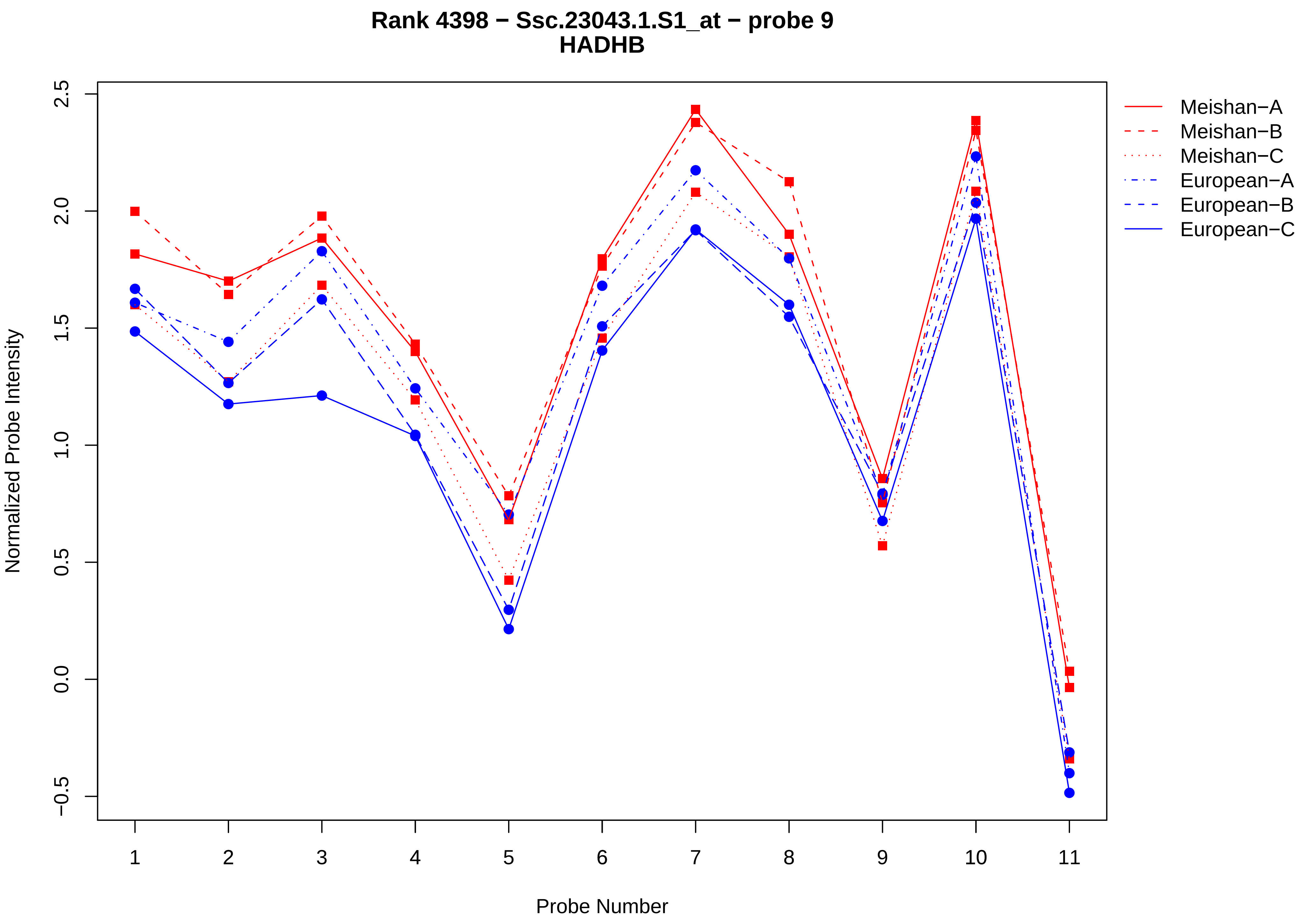

Rank 4398 − Ssc.23043.1.S1_at − probe 9
HADHB
Normalized Probe Intensity
Probe Number
−0.5
0.0
0.5
1.0
1.5
2.0
2.5
1
2
3
4
5
6
7
8
9
10
11
Meishan−A
Meishan−B
Meishan−C
European−A
European−B
European−C

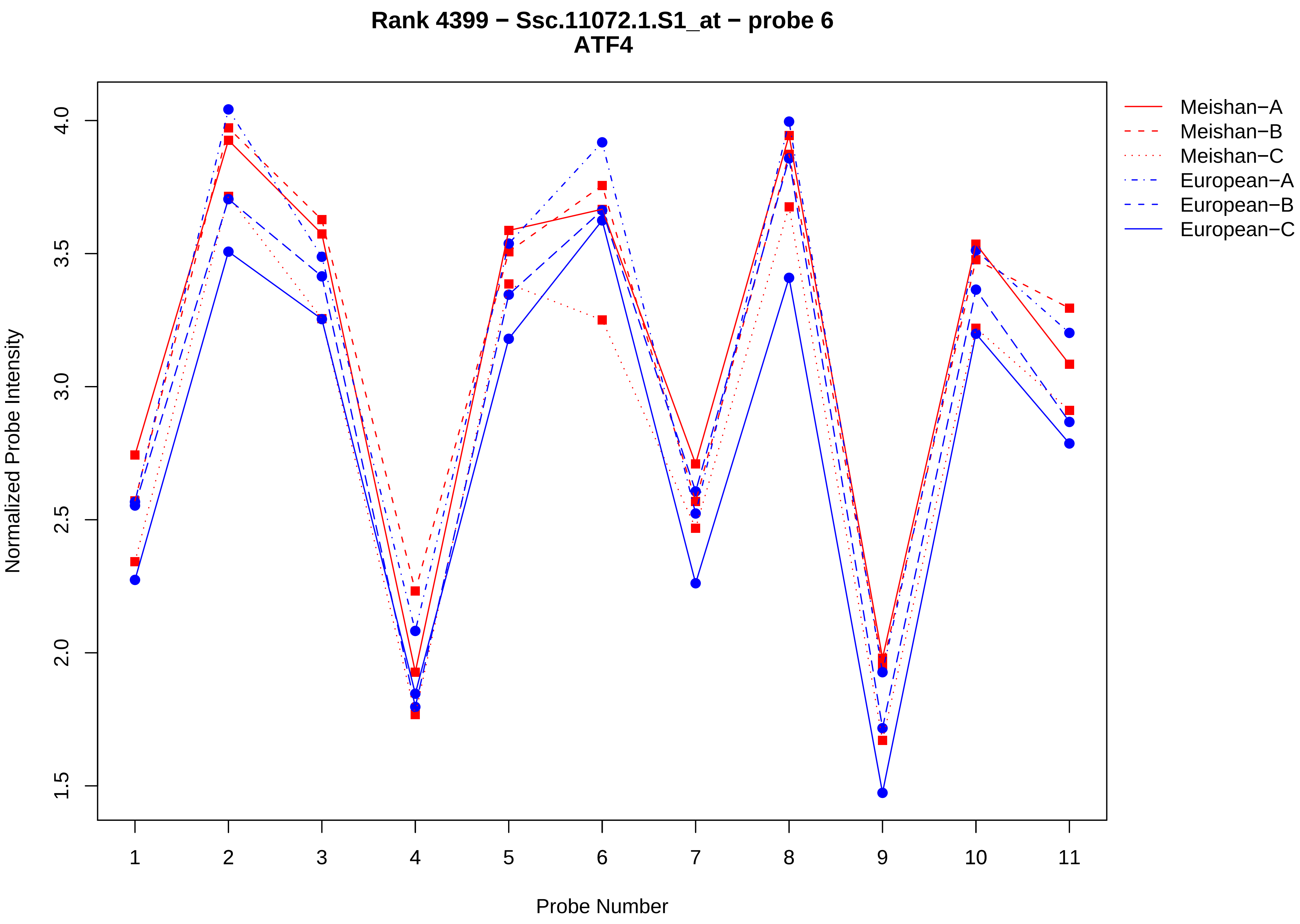

Rank 4399 − Ssc.11072.1.S1_at − probe 6
ATF4
Normalized Probe Intensity
Probe Number
1.5
2.0
2.5
3.0
3.5
4.0
1
2
3
4
5
6
7
8
9
10
11
Meishan−A
Meishan−B
Meishan−C
European−A
European−B
European−C

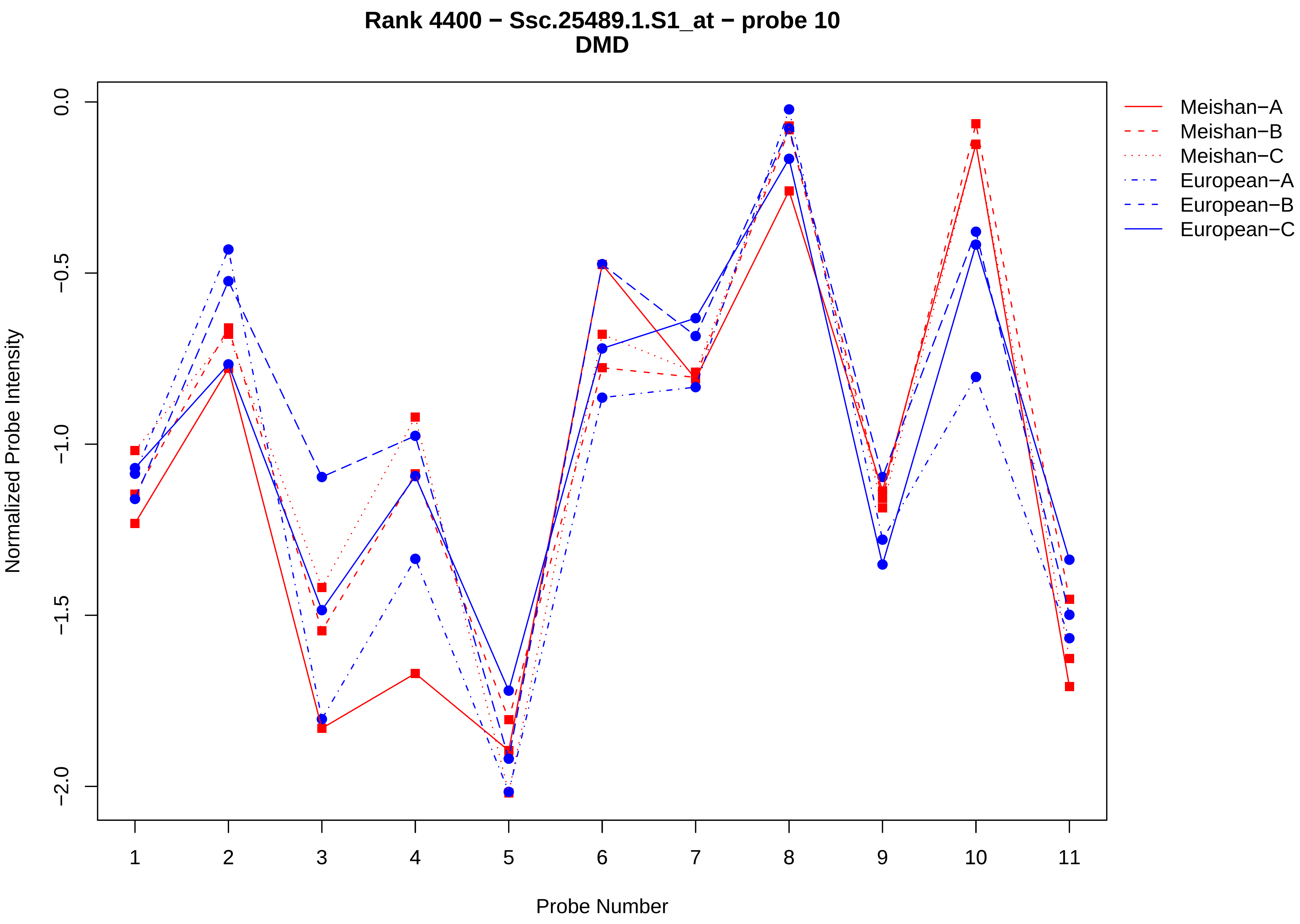

Rank 4400 − Ssc.25489.1.S1_at − probe 10
DMD
Normalized Probe Intensity
0.0
−0.5
−1.0
−1.5
−2.0
Probe Number
1
2
3
4
5
6
7
8
9
10
11
Meishan−A
Meishan−B
Meishan−C
European−A
European−B
European−C

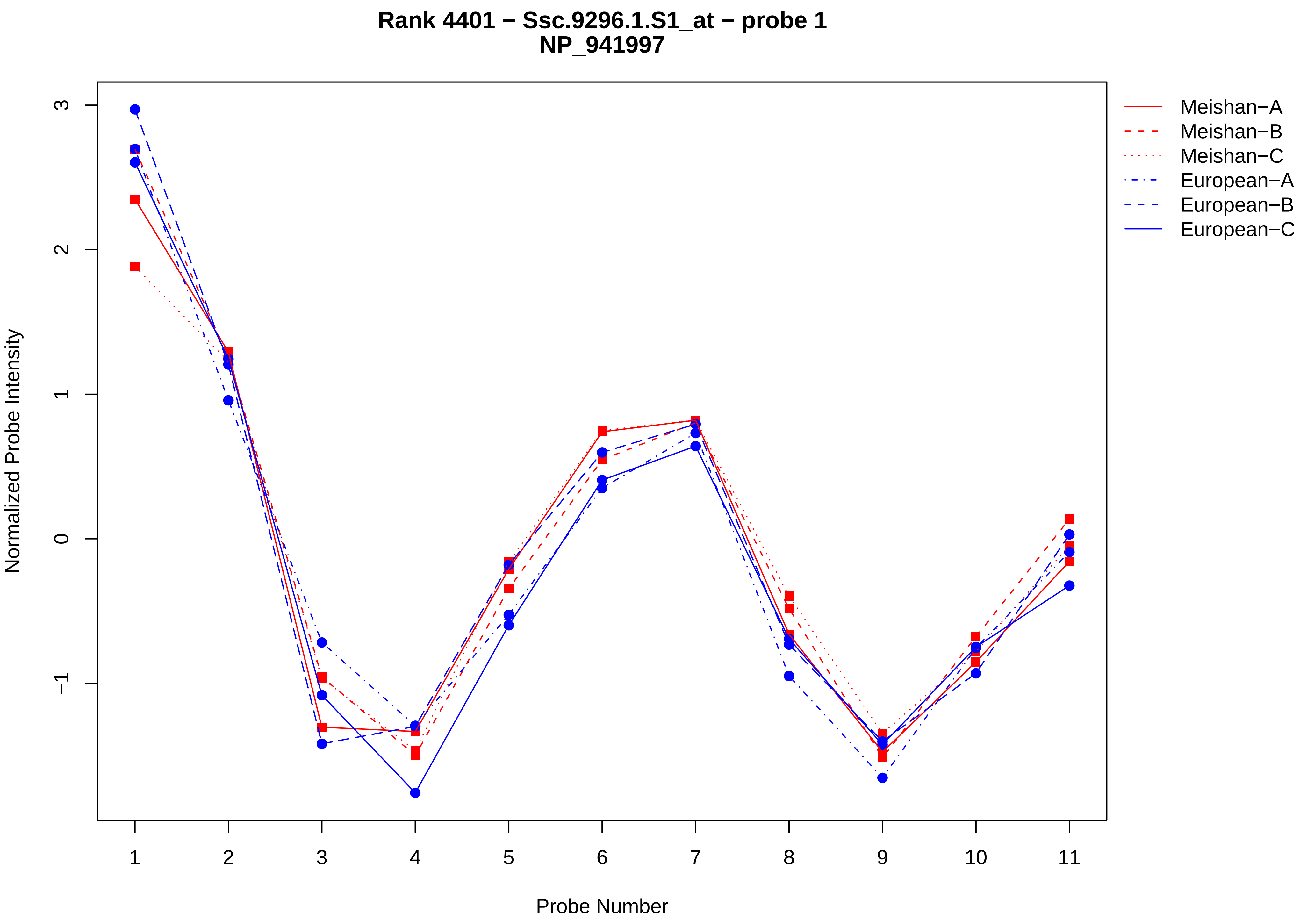
Rank 4401 − Ssc.9296.1.S1_at − probe 1
NP_941997
Normalized Probe Intensity
Probe Number
−1
0
1
2
3
1
2
3
4
5
6
7
8
9
10
11
Meishan−A
Meishan−B
Meishan−C
European−A
European−B
European−C

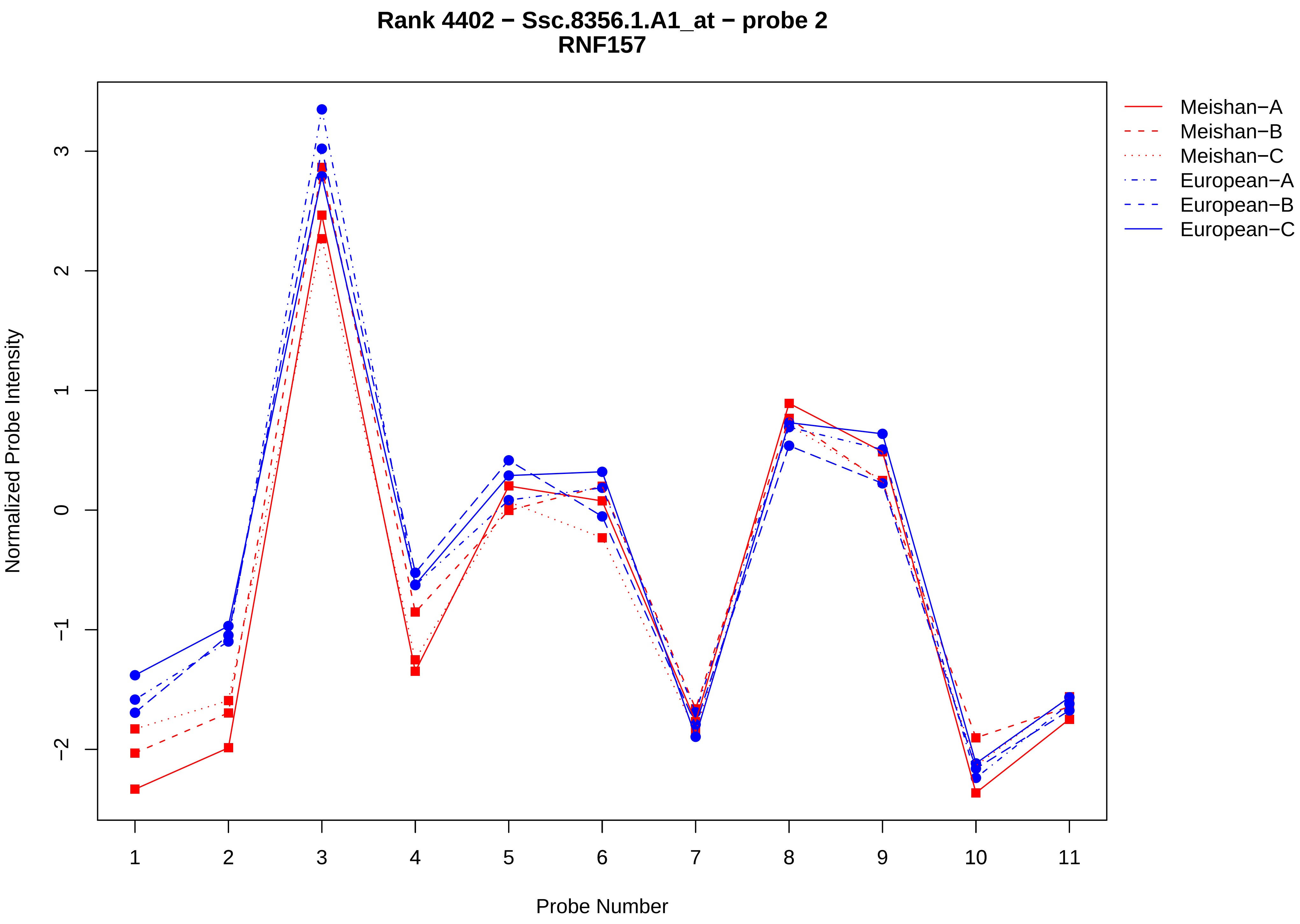

Rank 4402 − Ssc.8356.1.A1_at − probe 2
RNF157
Normalized Probe Intensity
Probe Number
Meishan−A
Meishan−B
Meishan−C
European−A
European−B
European−C
−2
−1
0
1
2
3
1
2
3
4
5
6
7
8
9
10
11

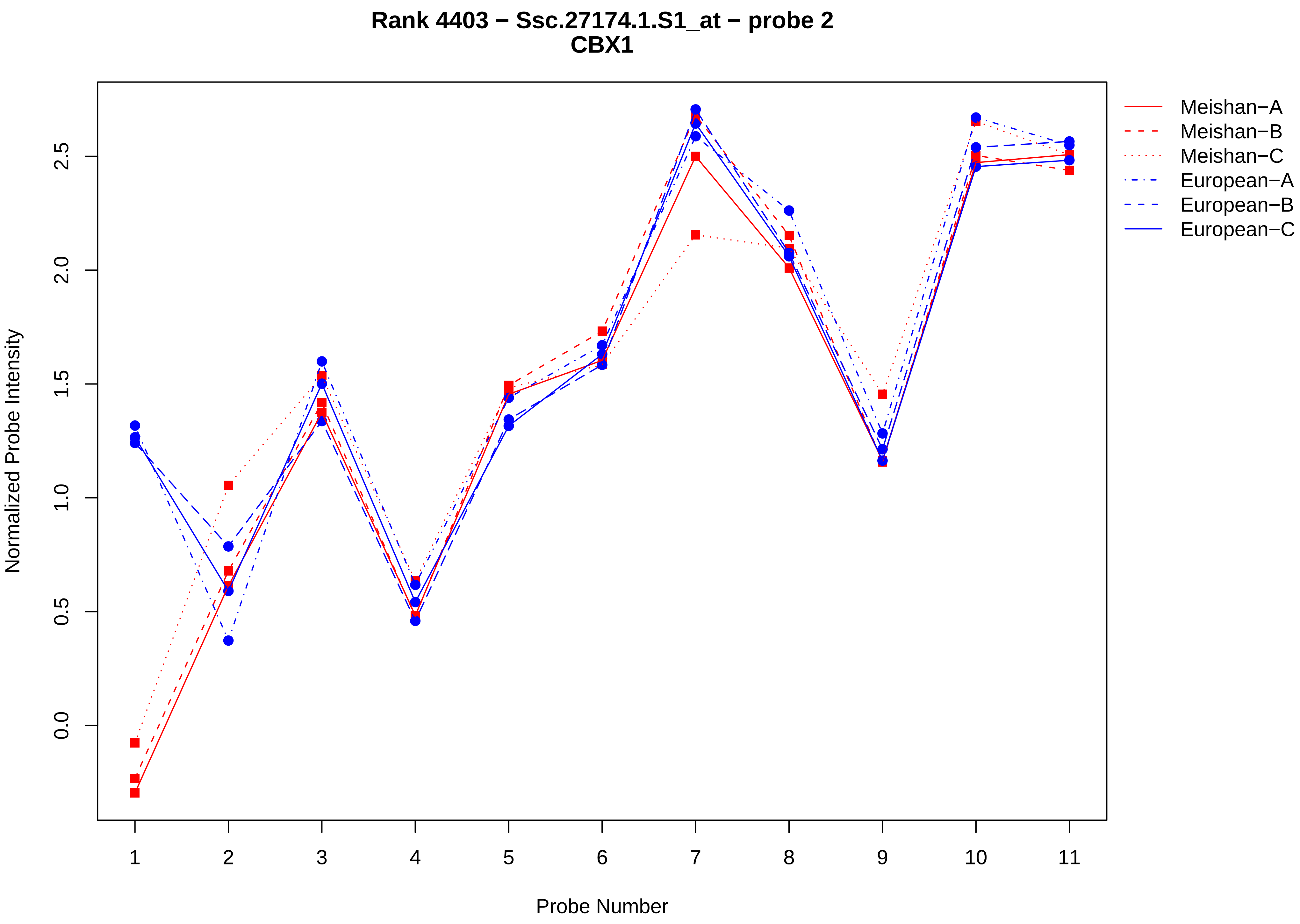

Rank 4403 − Ssc.27174.1.S1_at − probe 2
CBX1
Normalized Probe Intensity
Probe Number
0.0
0.5
1.0
1.5
2.0
2.5
1
2
3
4
5
6
7
8
9
10
11
Meishan−A
Meishan−B
Meishan−C
European−A
European−B
European−C

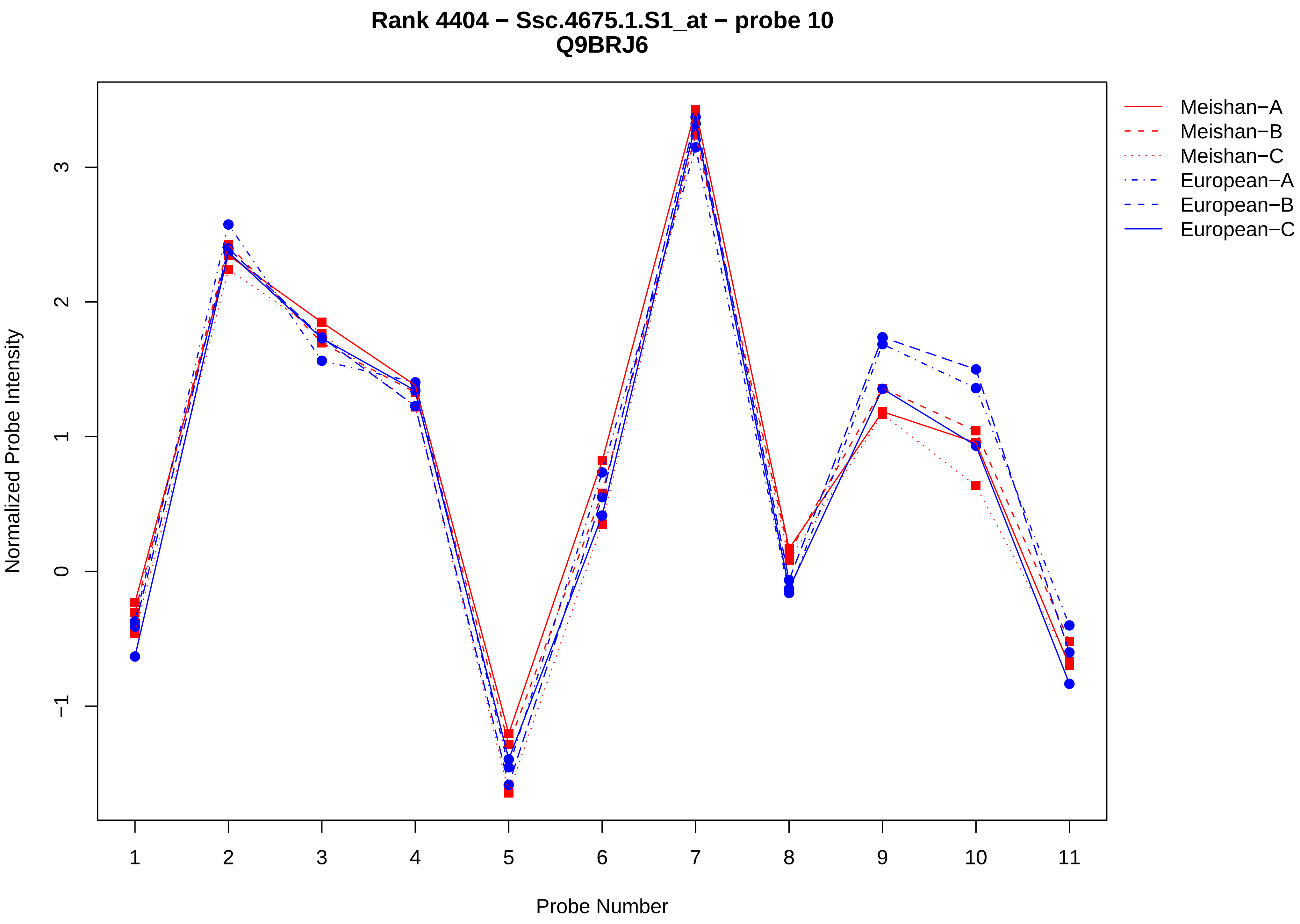

Rank 4404 − Ssc.4675.1.S1_at − probe 10
Q9BRJ6
Normalized Probe Intensity
Probe Number
−1
0
1
2
3
1
2
3
4
5
6
7
8
9
10
11
Meishan−A
Meishan−B
Meishan−C
European−A
European−B
European−C

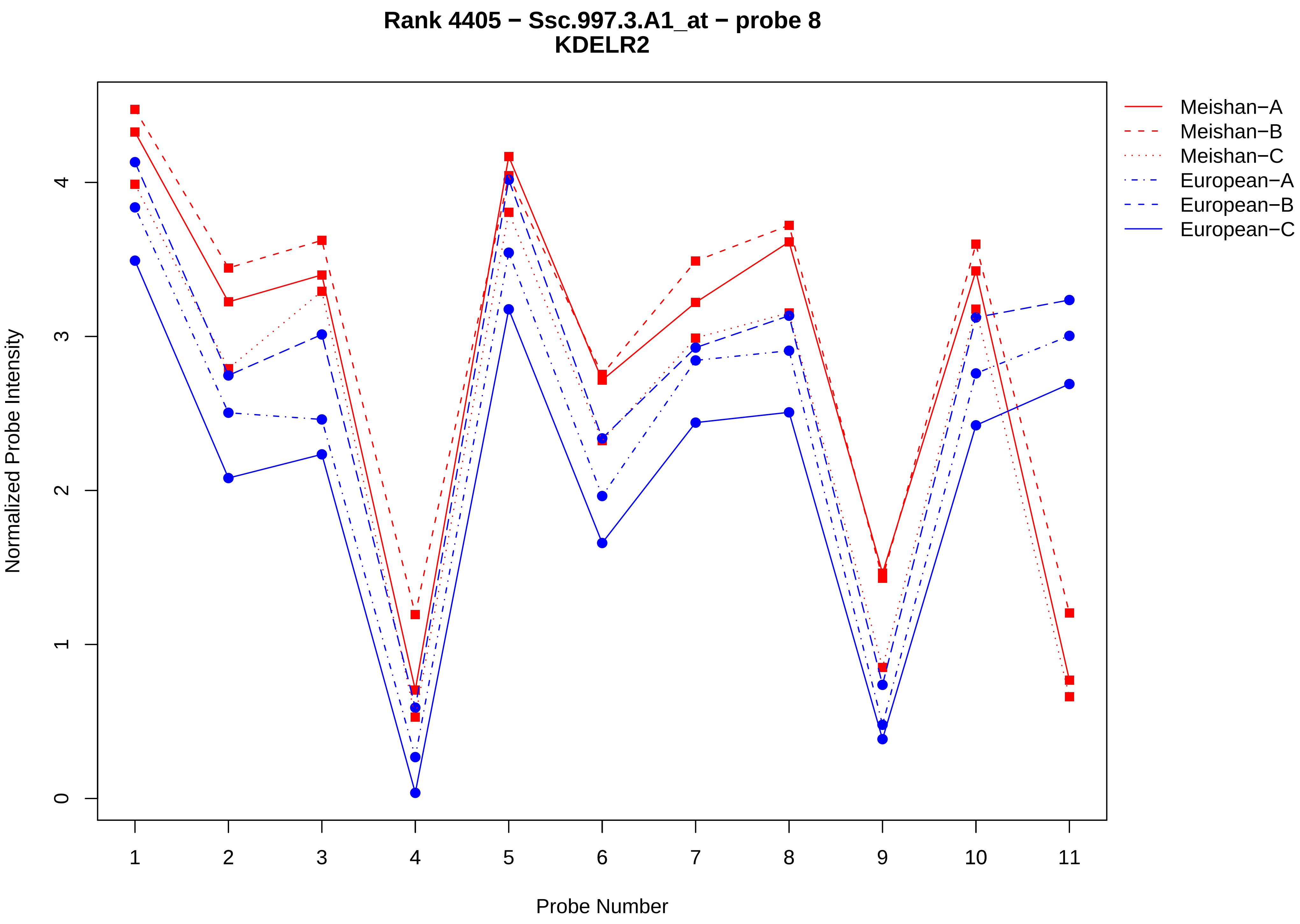

Rank 4405 − Ssc.997.3.A1_at − probe 8
KDELR2
Normalized Probe Intensity
Probe Number
0
1
2
3
4
1
2
3
4
5
6
7
8
9
10
11
Meishan−A
Meishan−B
Meishan−C
European−A
European−B
European−C

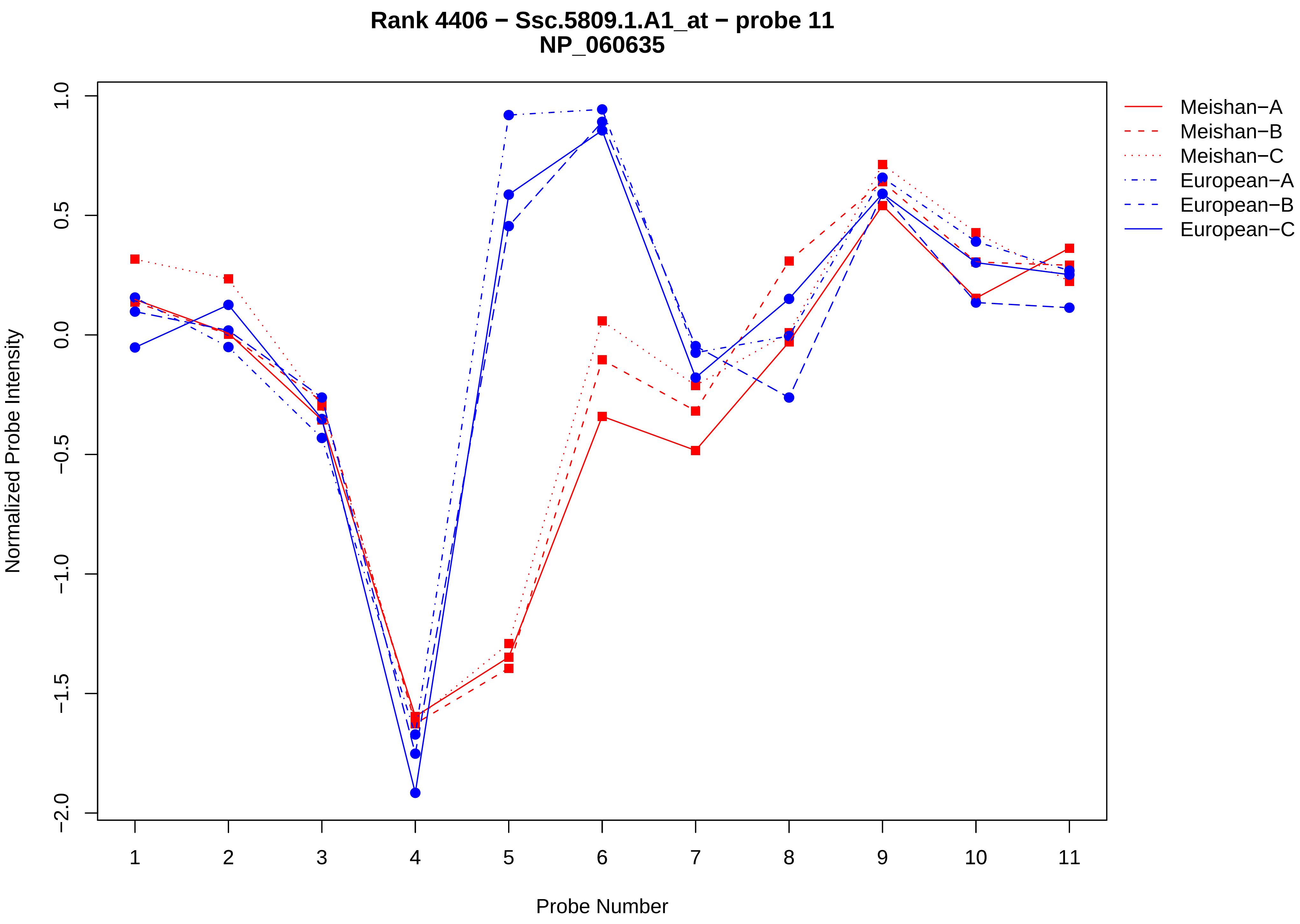

Rank 4406 − Ssc.5809.1.A1_at − probe 11
NP_060635
Normalized Probe Intensity
Probe Number
1.0
0.5
0.0
−0.5
−1.0
−1.5
−2.0
1
2
3
4
5
6
7
8
9
10
11
Meishan−A
Meishan−B
Meishan−C
European−A
European−B
European−C

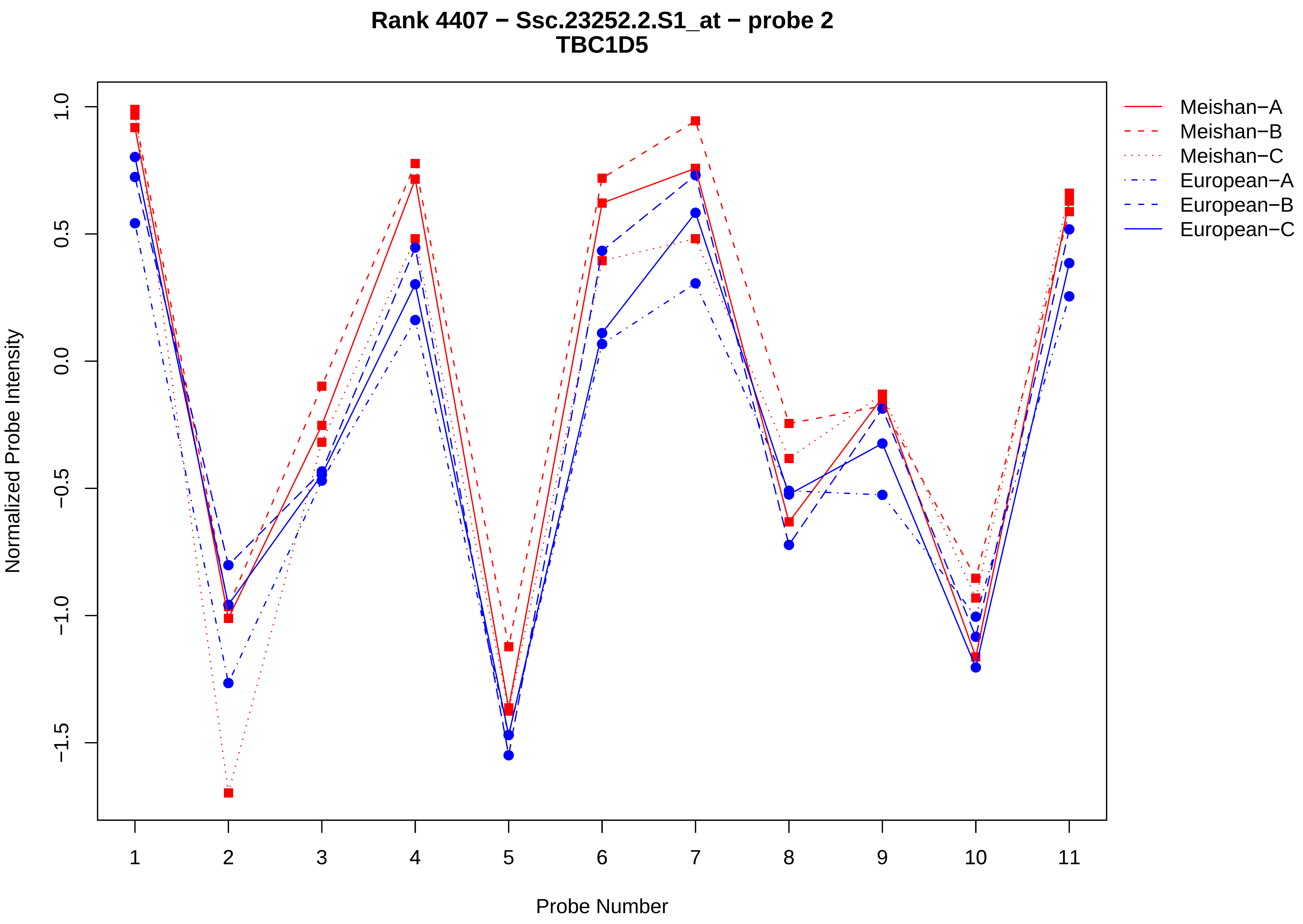
Rank 4407 − Ssc.23252.2.S1_at − probe 2
TBC1D5
Normalized Probe Intensity
Probe Number
Meishan−A
Meishan−B
Meishan−C
European−A
European−B
European−C
1.0
0.5
0.0
−0.5
−1.0
−1.5
1
2
3
4
5
6
7
8
9
10
11

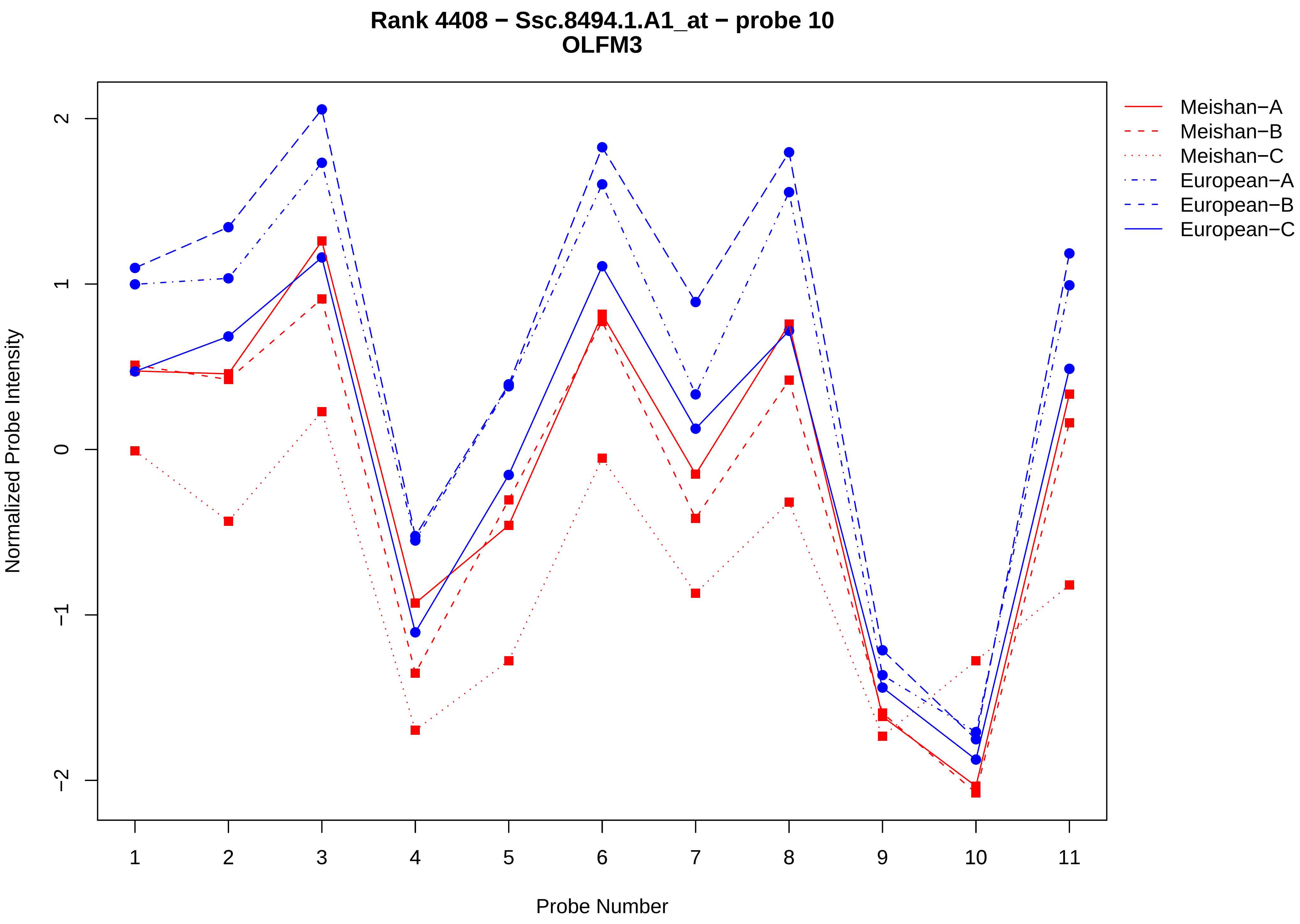
Rank 4408 − Ssc.8494.1.A1_at − probe 10
OLFM3
Normalized Probe Intensity
Probe Number
Meishan−A
Meishan−B
Meishan−C
European−A
European−B
European−C
−2
−1
0
1
2
1
2
3
4
5
6
7
8
9
10
11

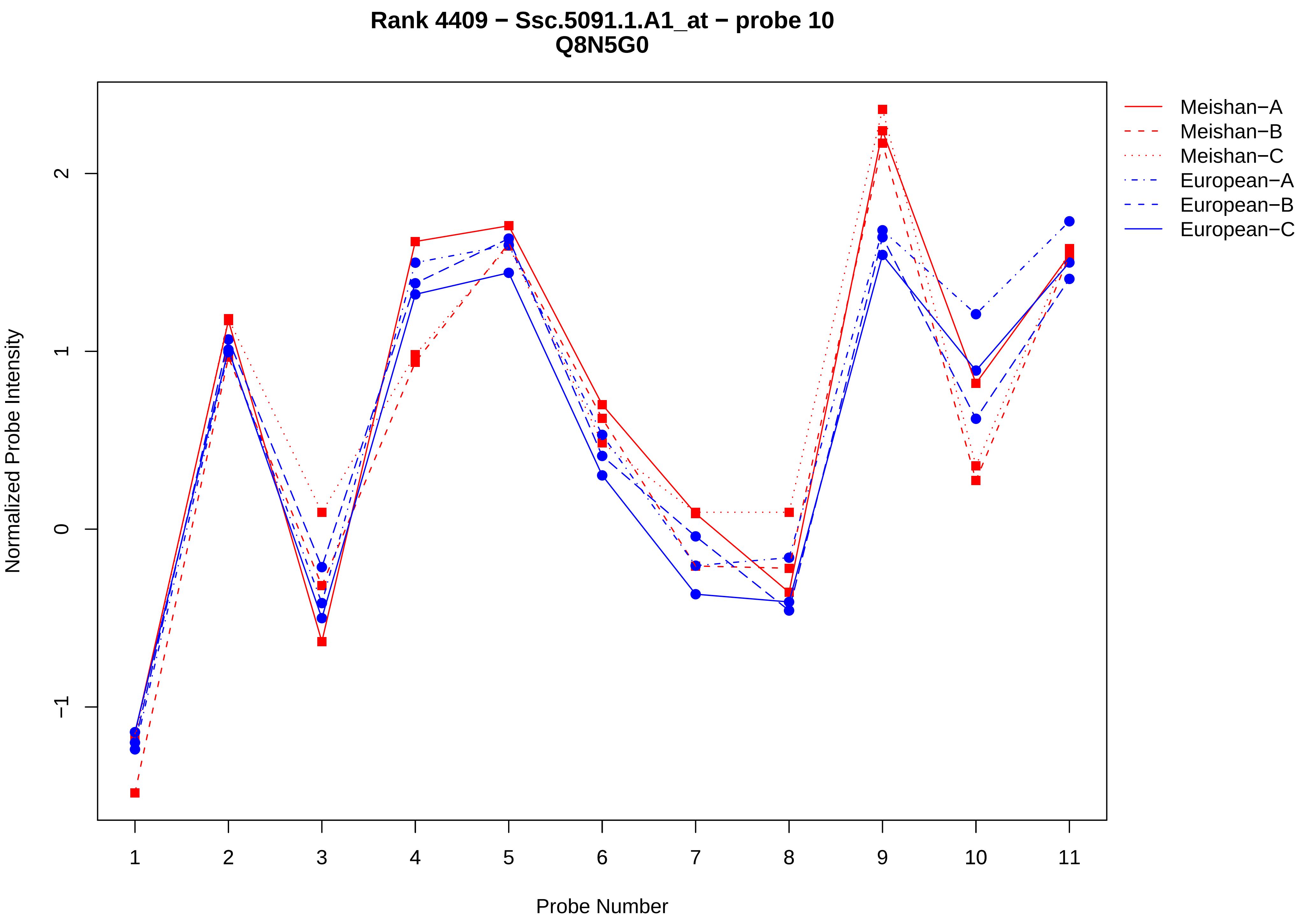
Rank 4409 − Ssc.5091.1.A1_at − probe 10
Q8N5G0
Normalized Probe Intensity
Probe Number
−1
0
1
2
1
2
3
4
5
6
7
8
9
10
11
Meishan−A
Meishan−B
Meishan−C
European−A
European−B
European−C

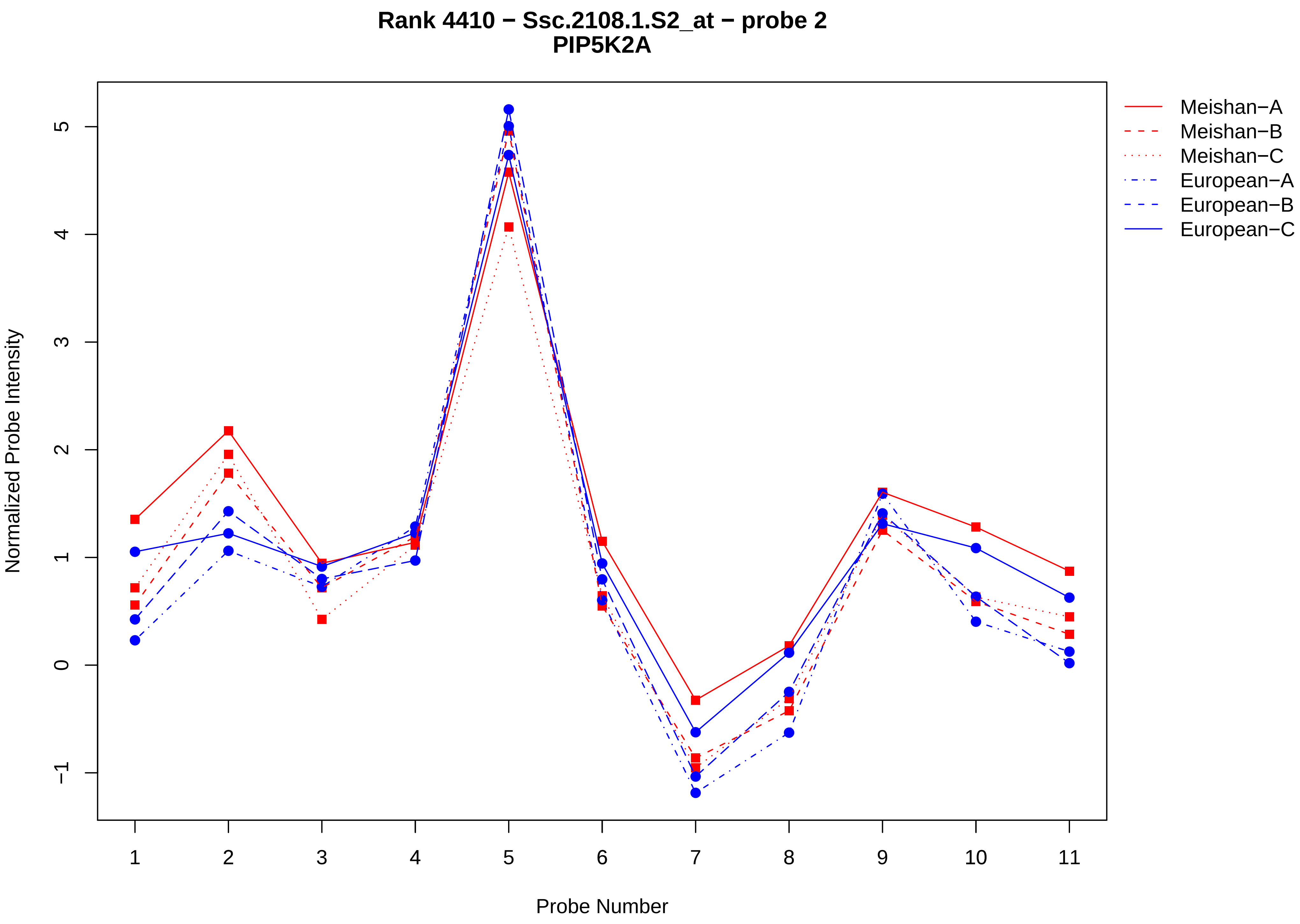

Rank 4410 − Ssc.2108.1.S2_at − probe 2
PIP5K2A
Normalized Probe Intensity
Probe Number
Meishan−A
Meishan−B
Meishan−C
European−A
European−B
European−C
−1
0
1
2
3
4
5
1
2
3
4
5
6
7
8
9
10
11

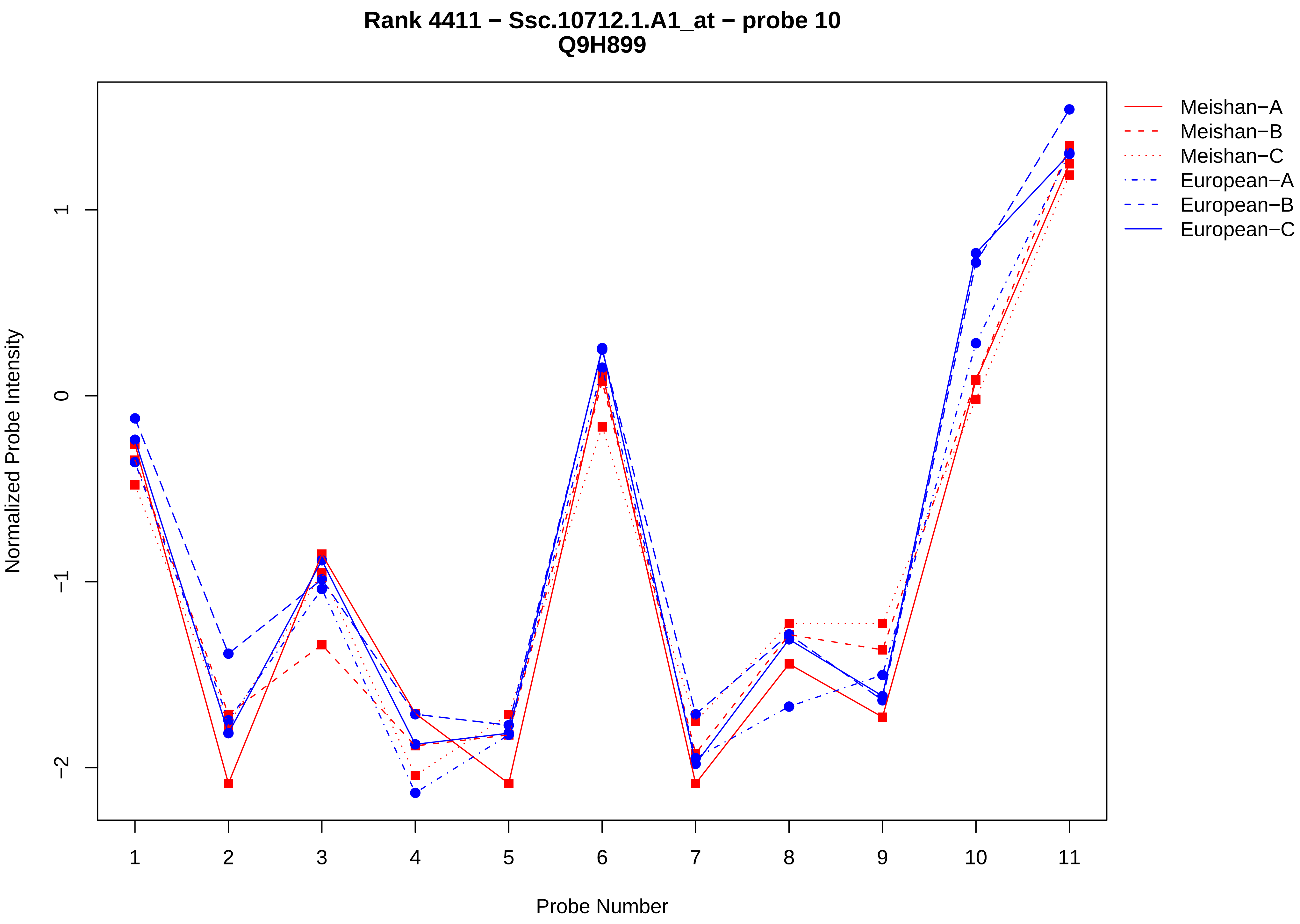

Rank 4411 − Ssc.10712.1.A1_at − probe 10
Q9H899
Normalized Probe Intensity
Probe Number
−2
−1
0
1
1
2
3
4
5
6
7
8
9
10
11
Meishan−A
Meishan−B
Meishan−C
European−A
European−B
European−C

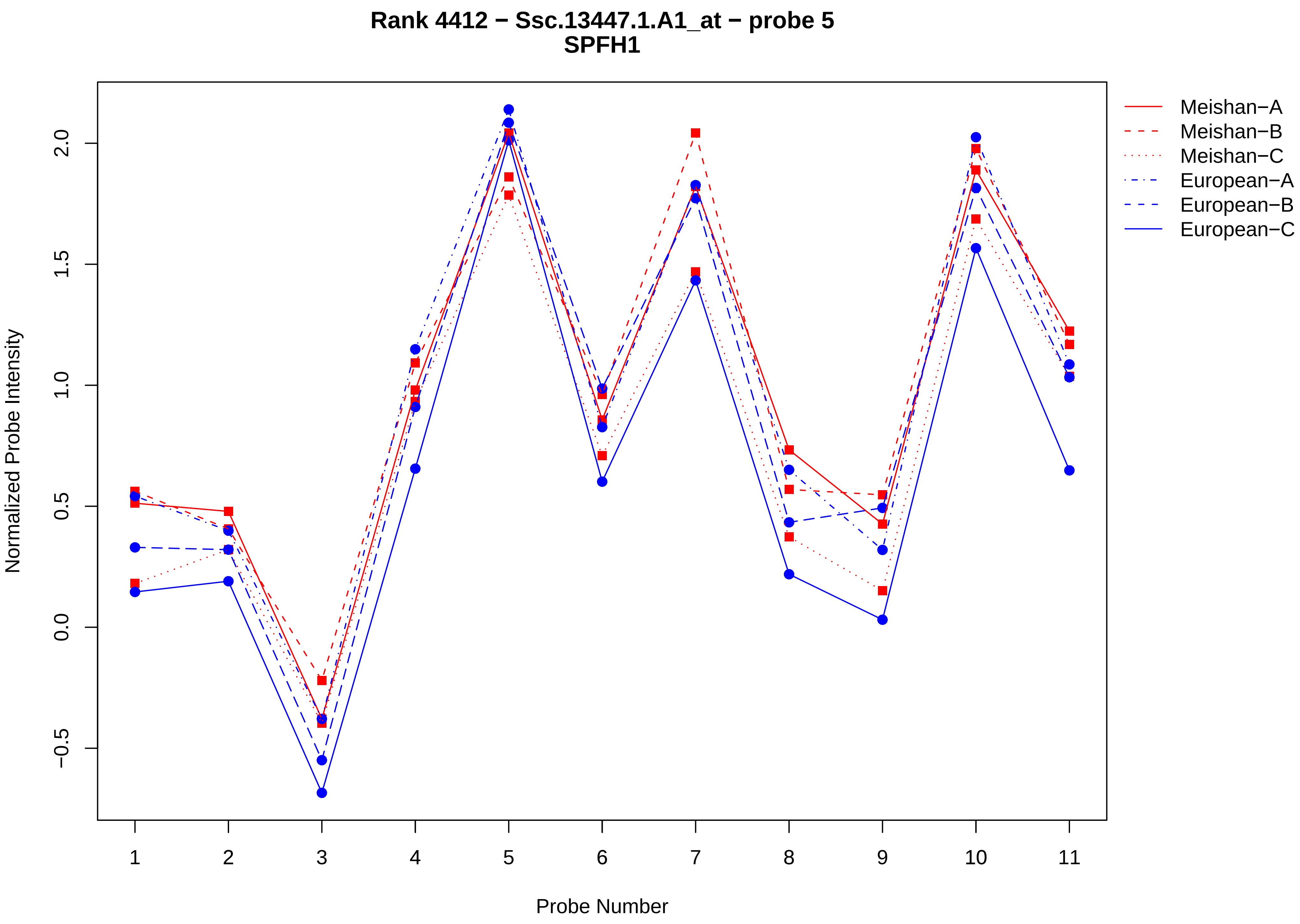

Rank 4412 − Ssc.13447.1.A1_at − probe 5
SPFH1
Normalized Probe Intensity
−0.5
0.0
0.5
1.0
1.5
2.0
1
2
3
4
5
6
7
8
9
10
11
Probe Number
Meishan−A
Meishan−B
Meishan−C
European−A
European−B
European−C

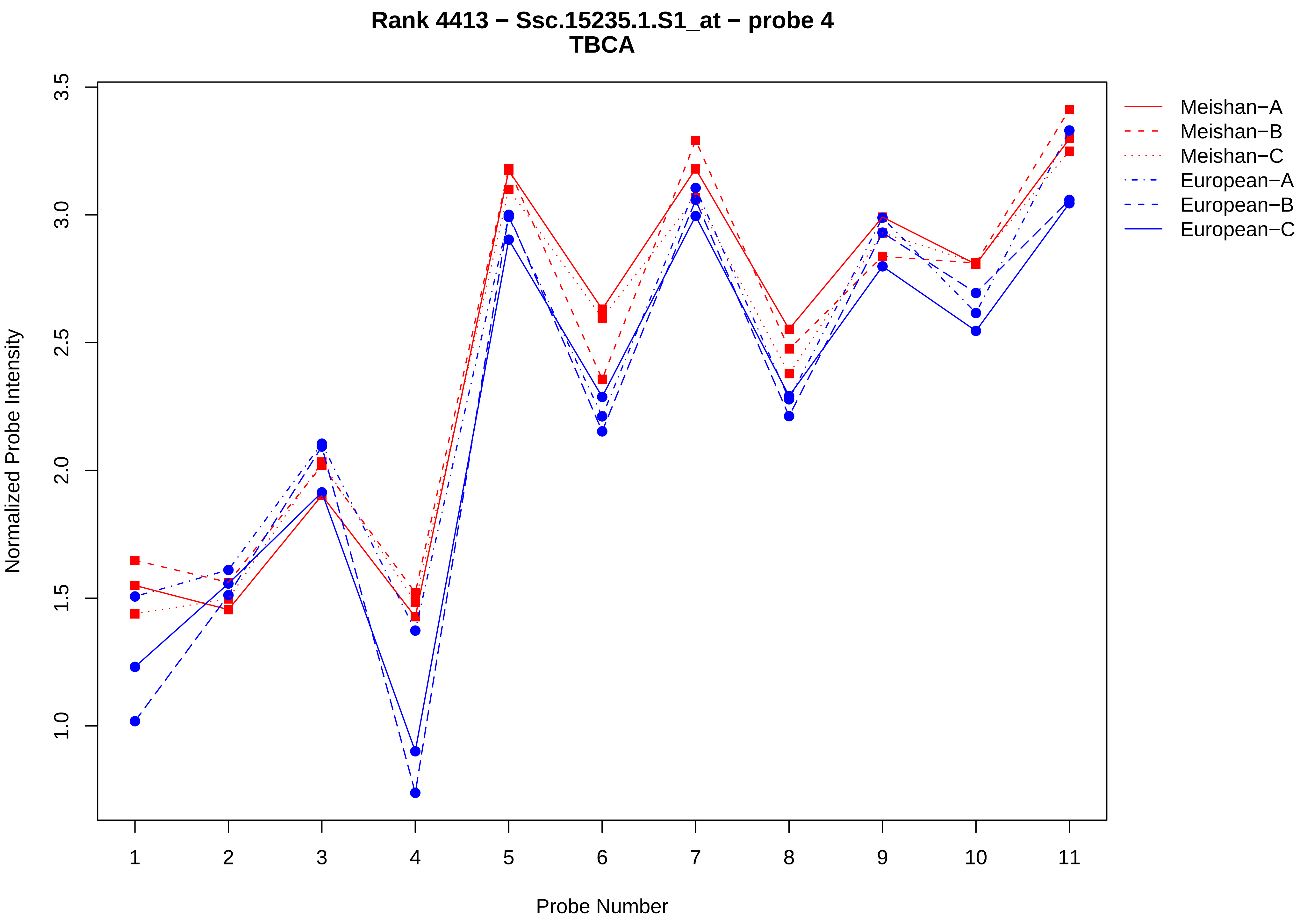
Rank 4413 − Ssc.15235.1.S1_at − probe 4
TBCA
Normalized Probe Intensity
Probe Number
1.0
1.5
2.0
2.5
3.0
3.5
1
2
3
4
5
6
7
8
9
10
11
Meishan−A
Meishan−B
Meishan−C
European−A
European−B
European−C

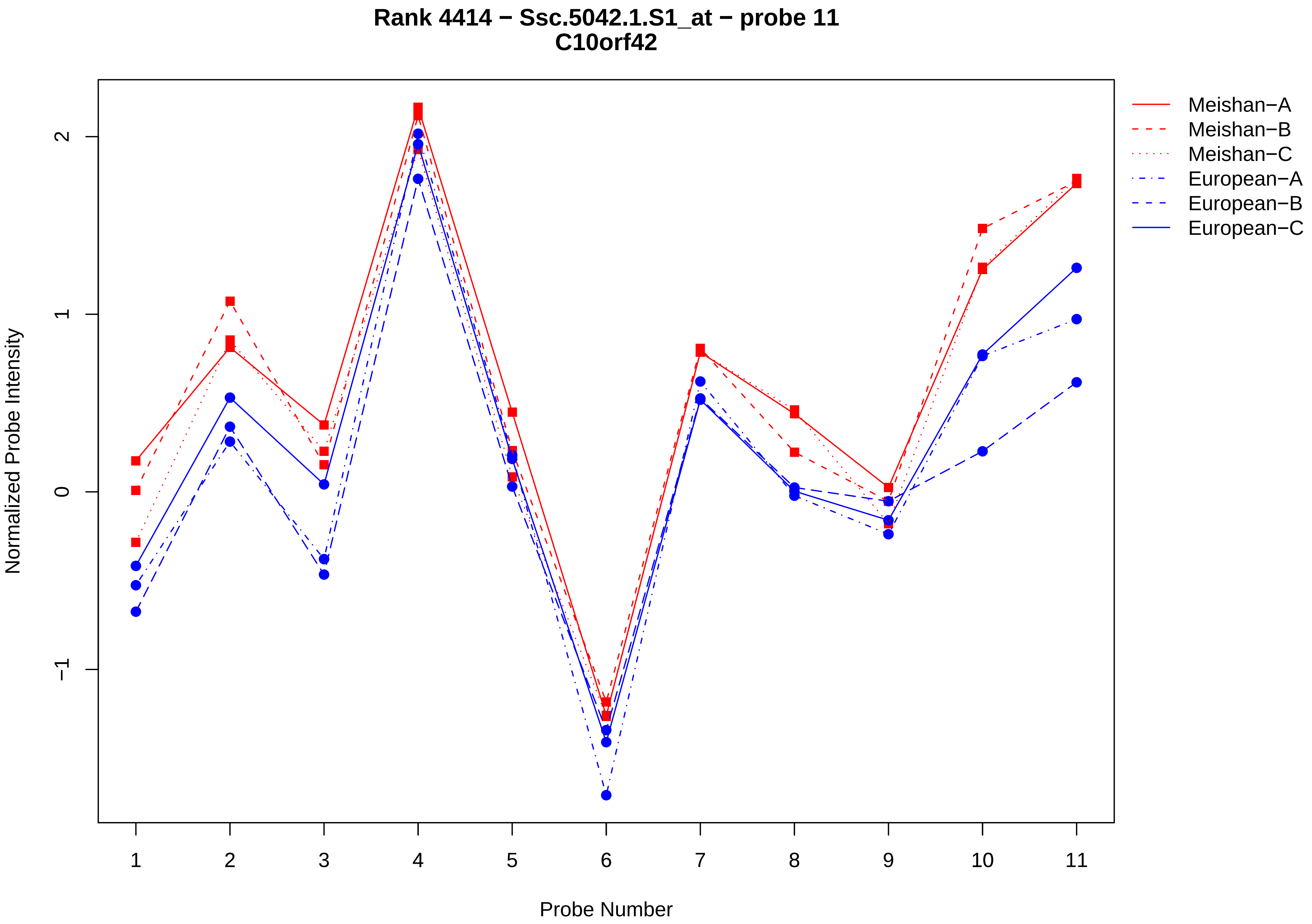
Rank 4414 − Ssc.5042.1.S1_at − probe 11
C10orf42
Normalized Probe Intensity
Probe Number
−1
0
1
2
1
2
3
4
5
6
7
8
9
10
11
Meishan−A
Meishan−B
Meishan−C
European−A
European−B
European−C

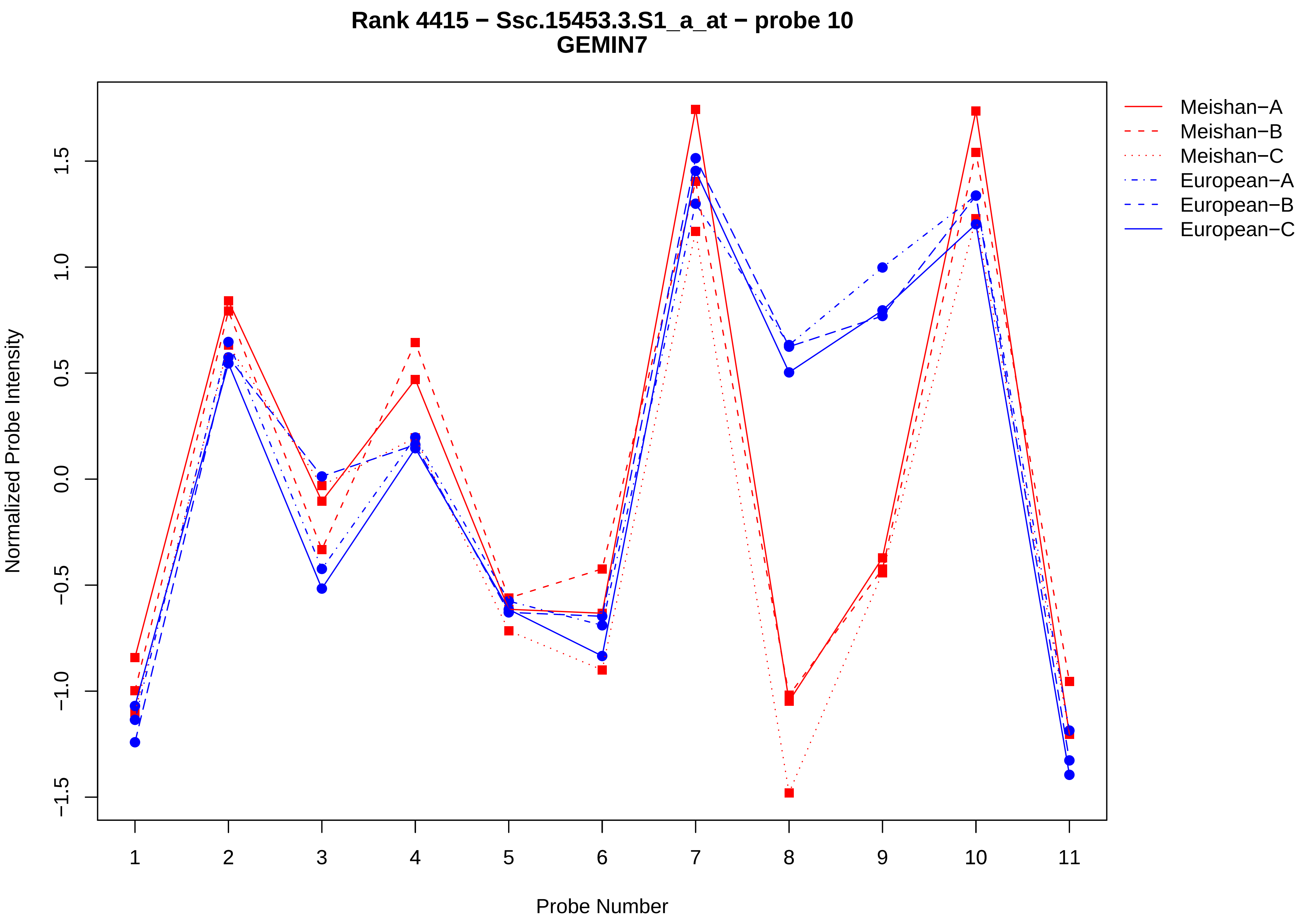

Rank 4415 − Ssc.15453.3.S1_a_at − probe 10
GEMIN7
Normalized Probe Intensity
Probe Number
−1.5
−1.0
−0.5
0.0
0.5
1.0
1.5
1
2
3
4
5
6
7
8
9
10
11
Meishan−A
Meishan−B
Meishan−C
European−A
European−B
European−C

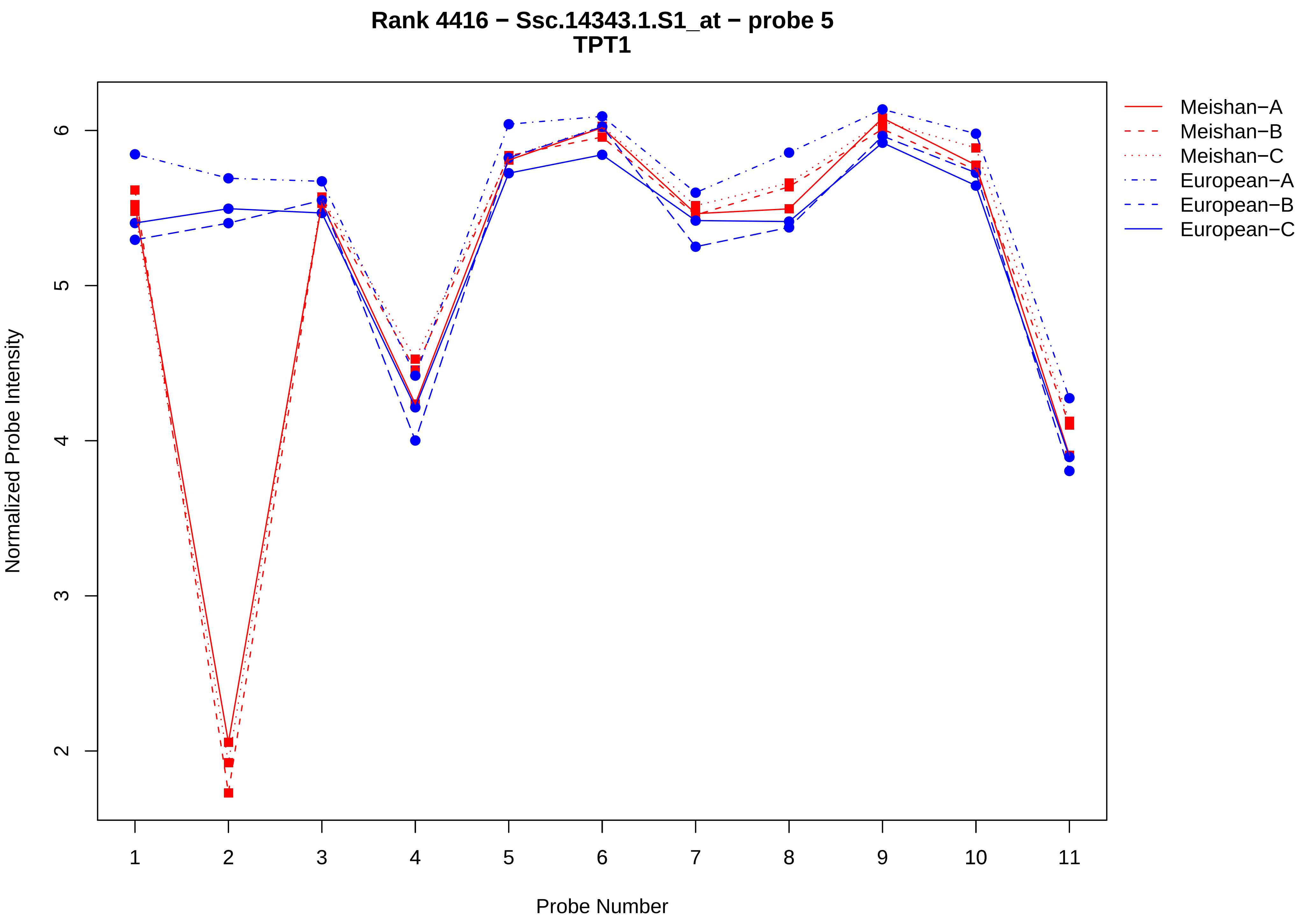

Rank 4416 − Ssc.14343.1.S1_at − probe 5
TPT1
Normalized Probe Intensity
Probe Number
Meishan−A
Meishan−B
Meishan−C
European−A
European−B
European−C

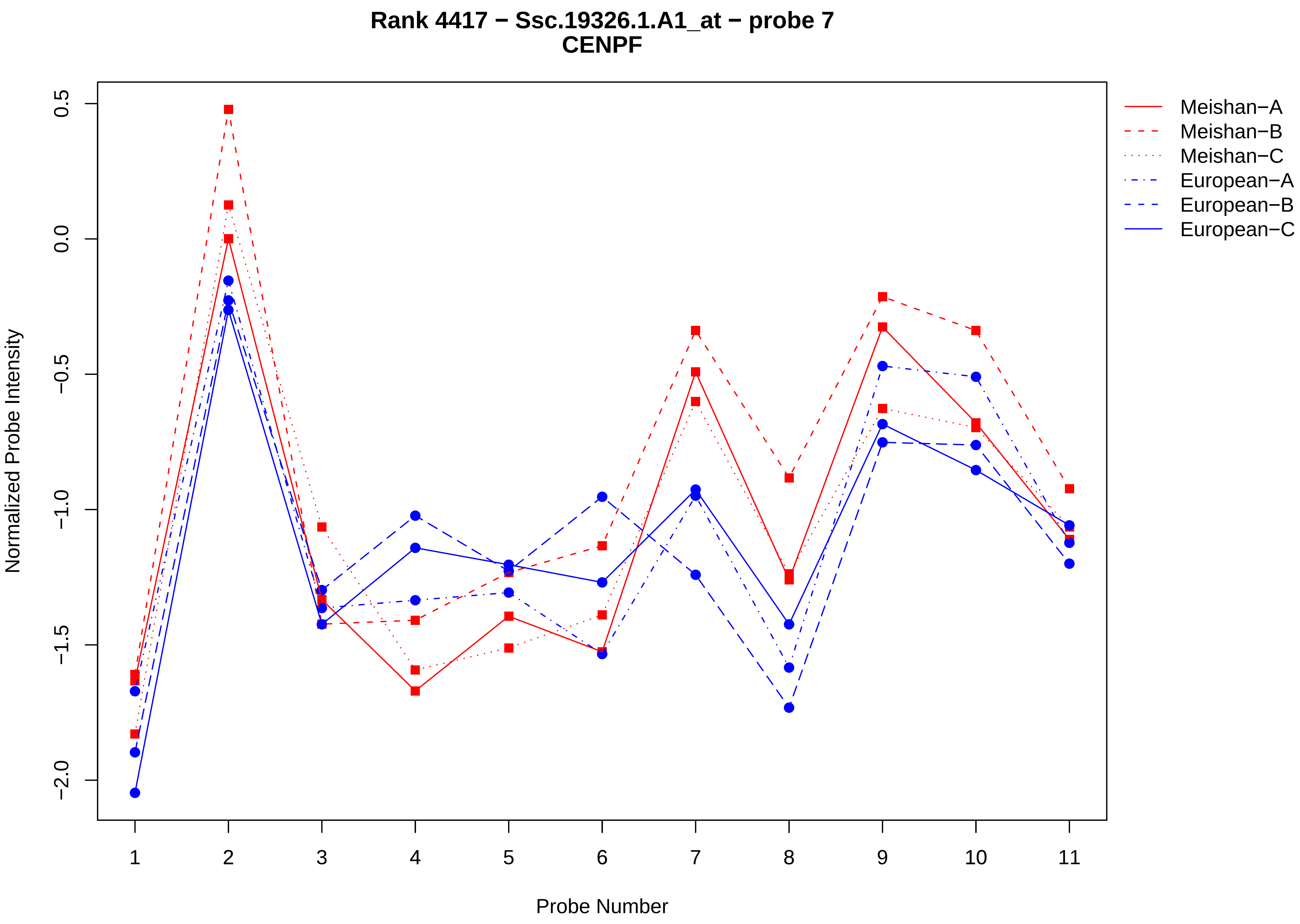

Rank 4417 − Ssc.19326.1.A1_at − probe 7
CENPF
Normalized Probe Intensity
0.5
0.0
−0.5
−1.0
−1.5
−2.0
1
2
3
4
5
6
7
8
9
10
11
Probe Number
Meishan−A
Meishan−B
Meishan−C
European−A
European−B
European−C

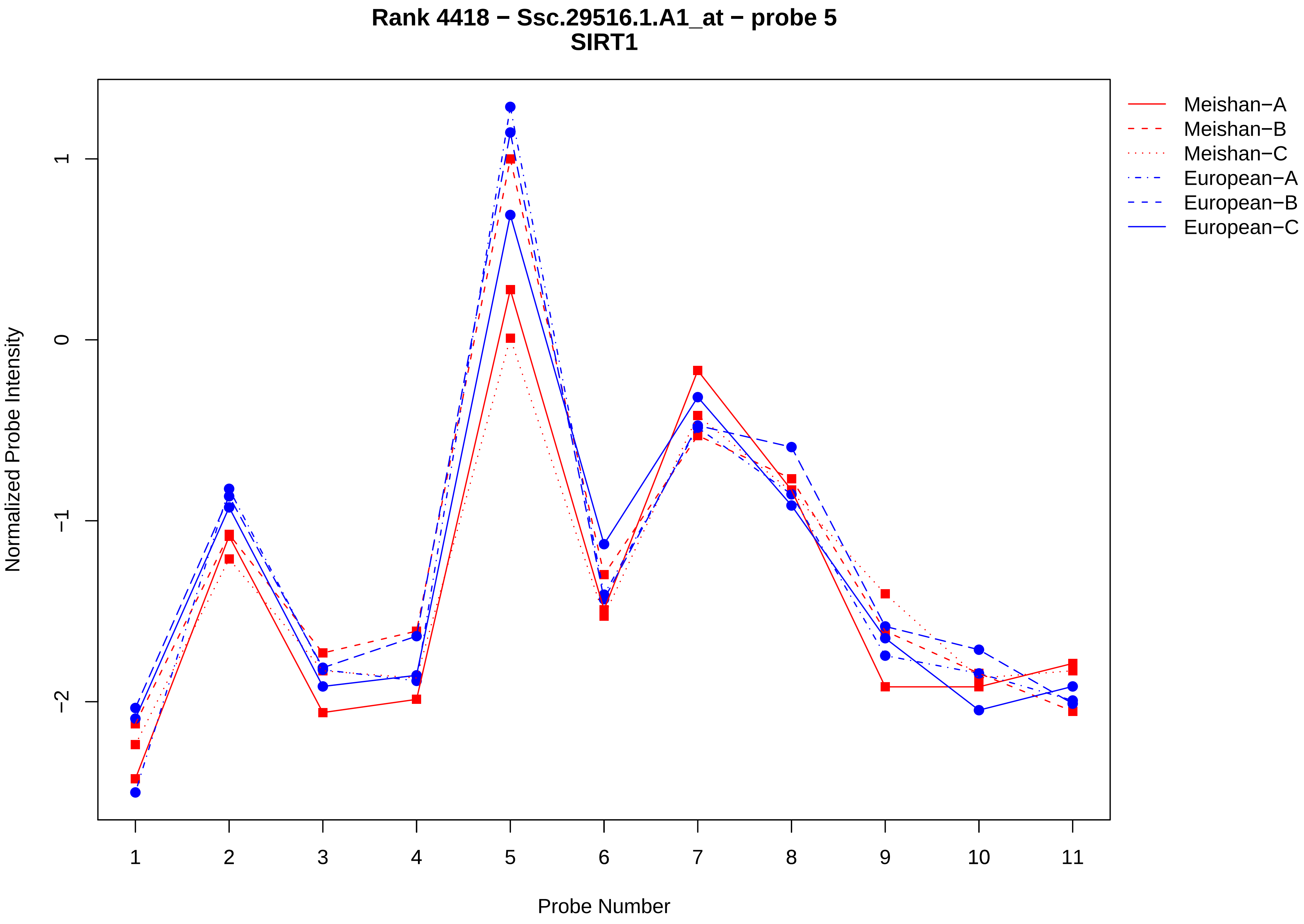

Rank 4418 − Ssc.29516.1.A1_at − probe 5
SIRT1
Normalized Probe Intensity
Probe Number
Meishan−A
Meishan−B
Meishan−C
European−A
European−B
European−C
1
0
−1
−2
1
2
3
4
5
6
7
8
9
10
11

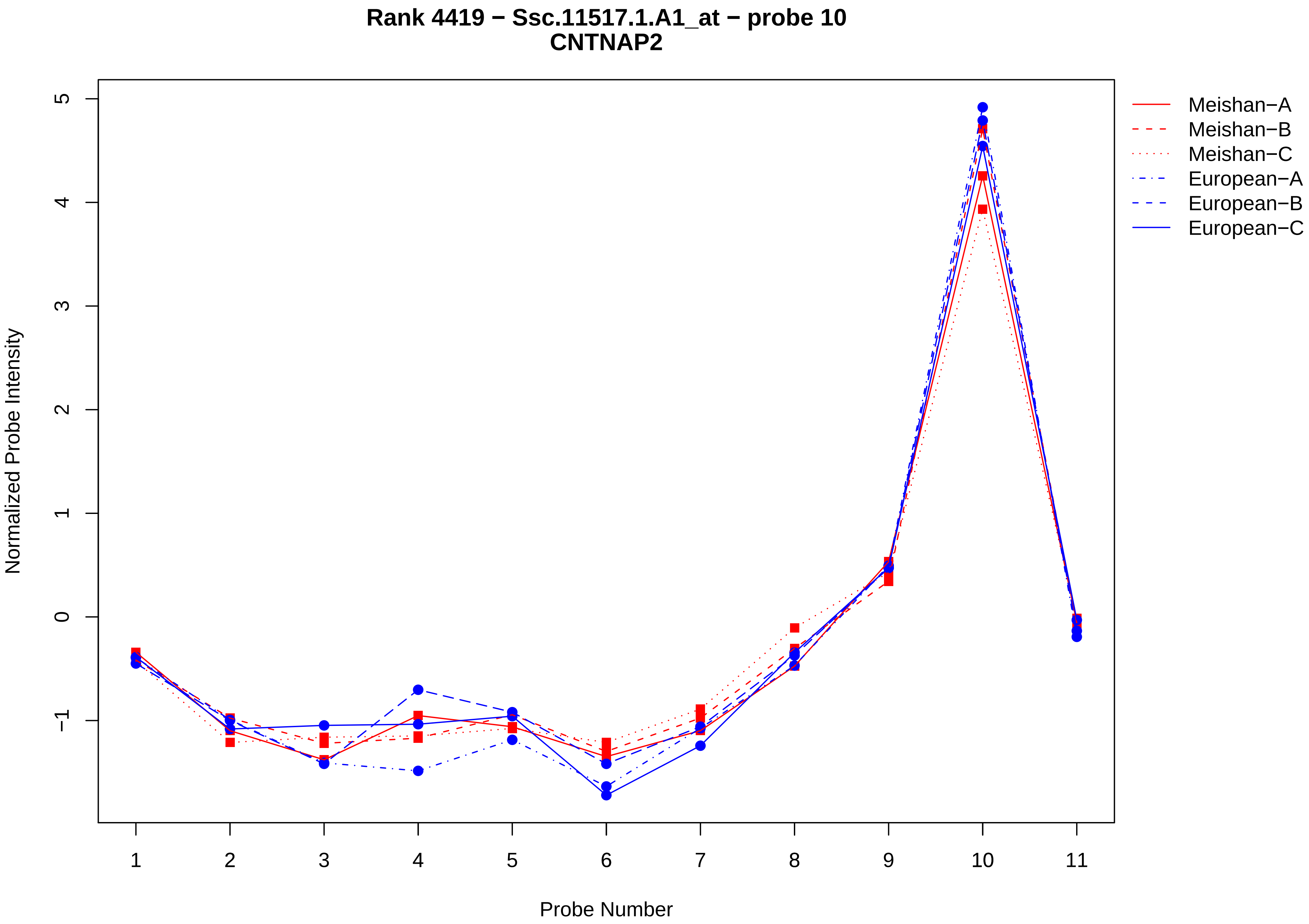

Rank 4419 − Ssc.11517.1.A1_at − probe 10
CNTNAP2
Normalized Probe Intensity
Probe Number
Meishan−A
Meishan−B
Meishan−C
European−A
European−B
European−C

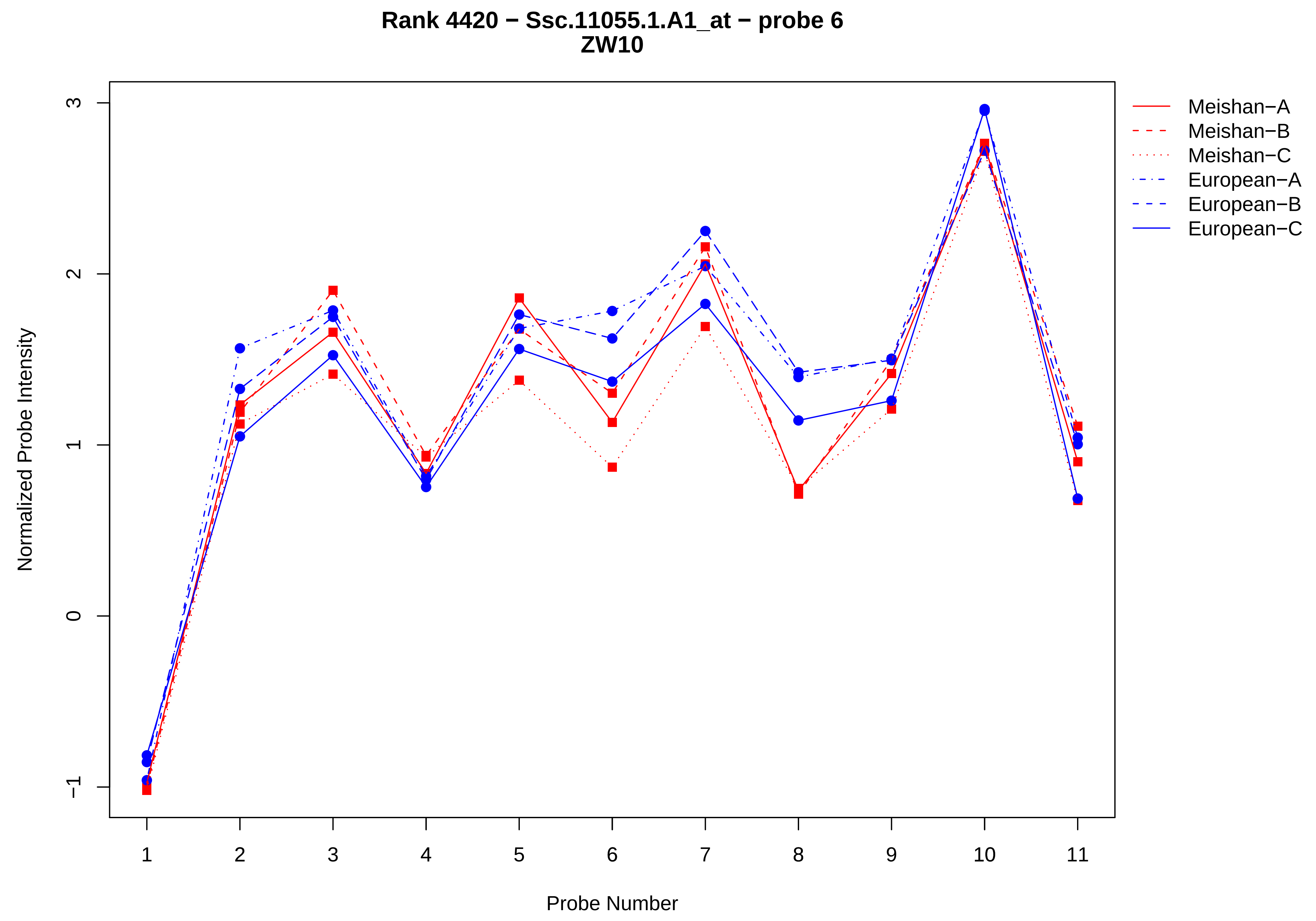

Rank 4420 − Ssc.11055.1.A1_at − probe 6
ZW10
Normalized Probe Intensity
Probe Number
−1
0
1
2
3
1
2
3
4
5
6
7
8
9
10
11
Meishan−A
Meishan−B
Meishan−C
European−A
European−B
European−C

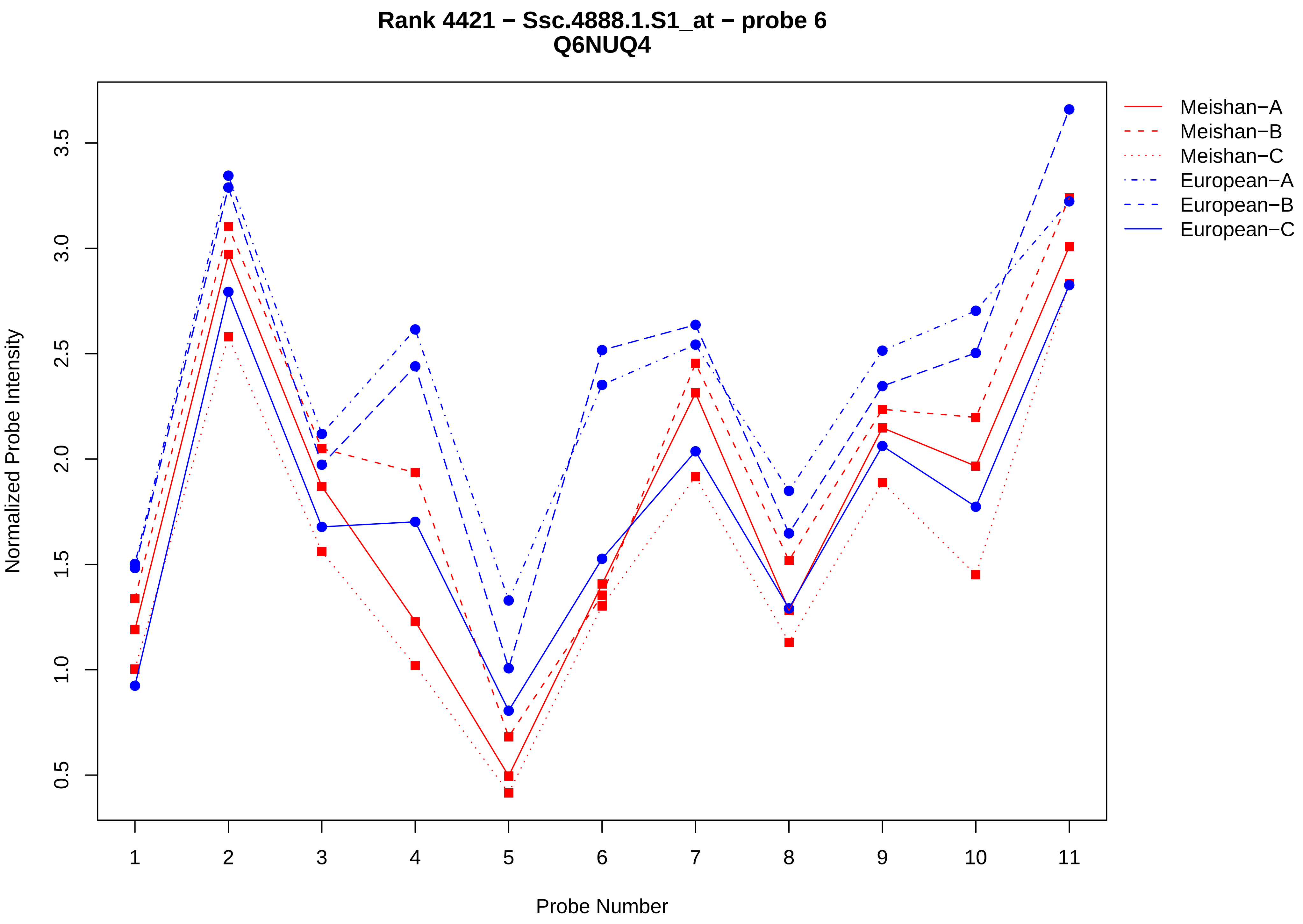

Rank 4421 − Ssc.4888.1.S1_at − probe 6
Q6NUQ4
Normalized Probe Intensity
Probe Number
0.5
1.0
1.5
2.0
2.5
3.0
3.5
1
2
3
4
5
6
7
8
9
10
11
Meishan−A
Meishan−B
Meishan−C
European−A
European−B
European−C

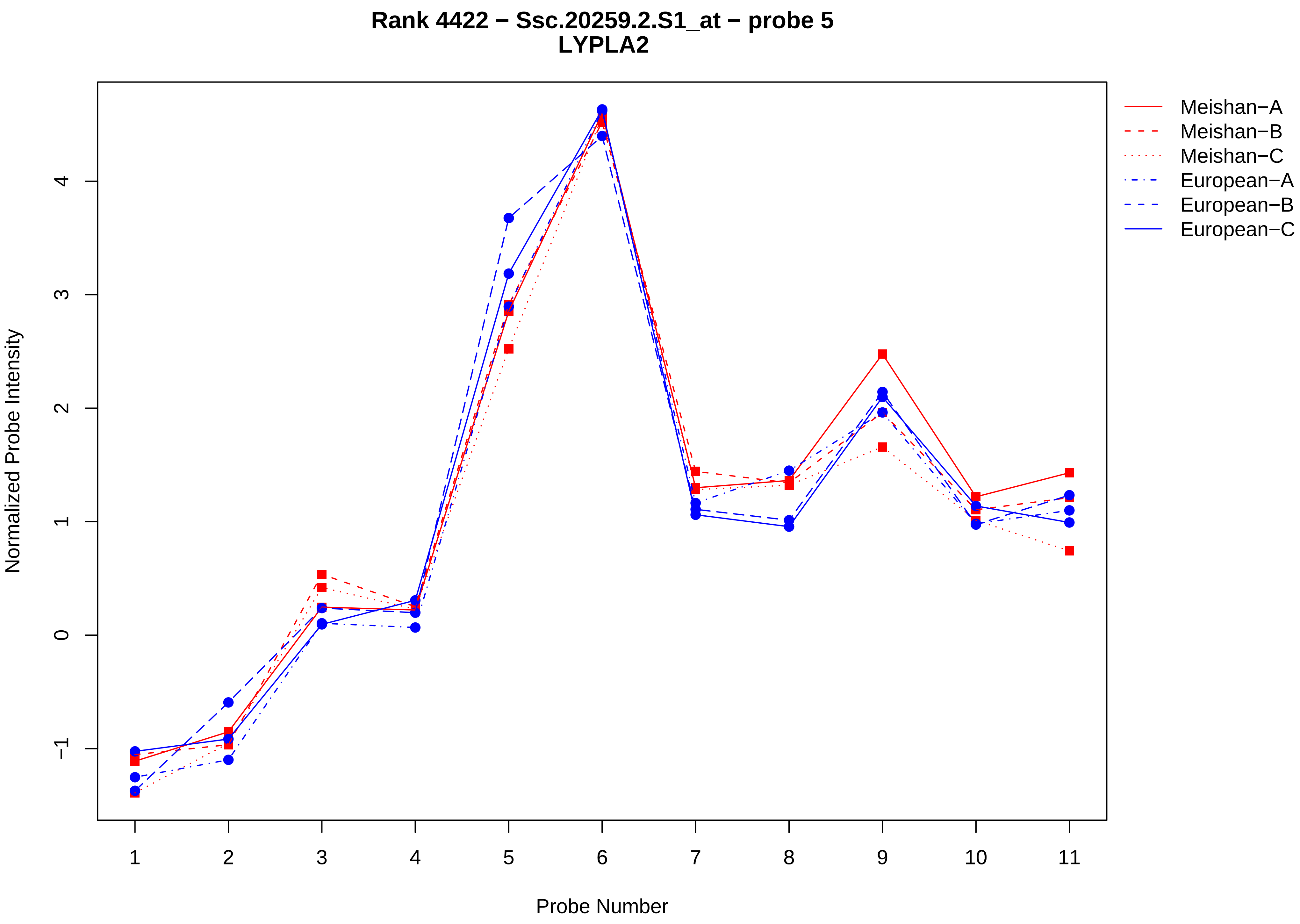

Rank 4422 − Ssc.20259.2.S1_at − probe 5
LYPLA2
Normalized Probe Intensity
Probe Number
−1
0
1
2
3
4
1
2
3
4
5
6
7
8
9
10
11
Meishan−A
Meishan−B
Meishan−C
European−A
European−B
European−C

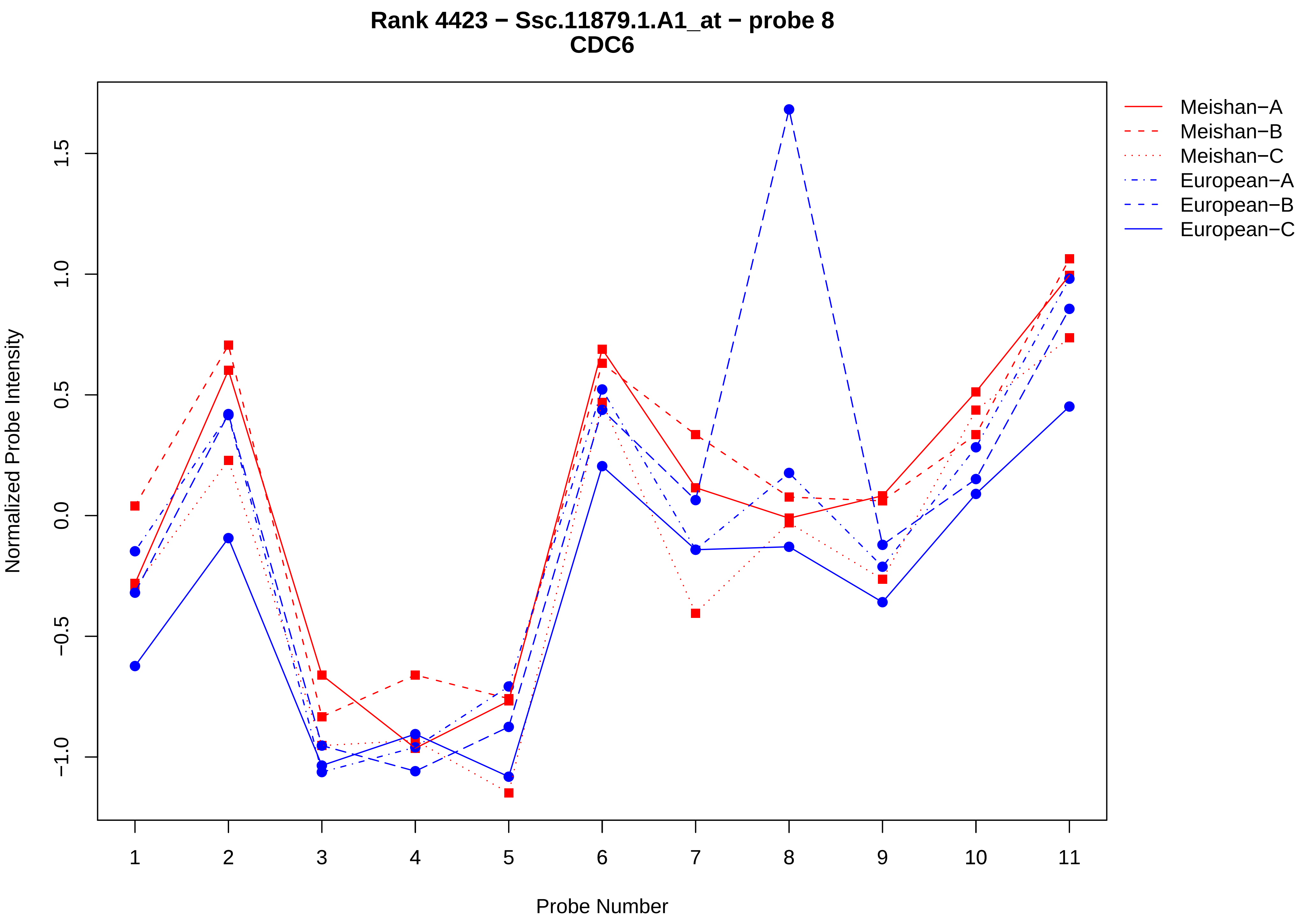

Rank 4423 − Ssc.11879.1.A1_at − probe 8
CDC6
Normalized Probe Intensity
Probe Number
−1.0
−0.5
0.0
0.5
1.0
1.5
1
2
3
4
5
6
7
8
9
10
11
Meishan−A
Meishan−B
Meishan−C
European−A
European−B
European−C

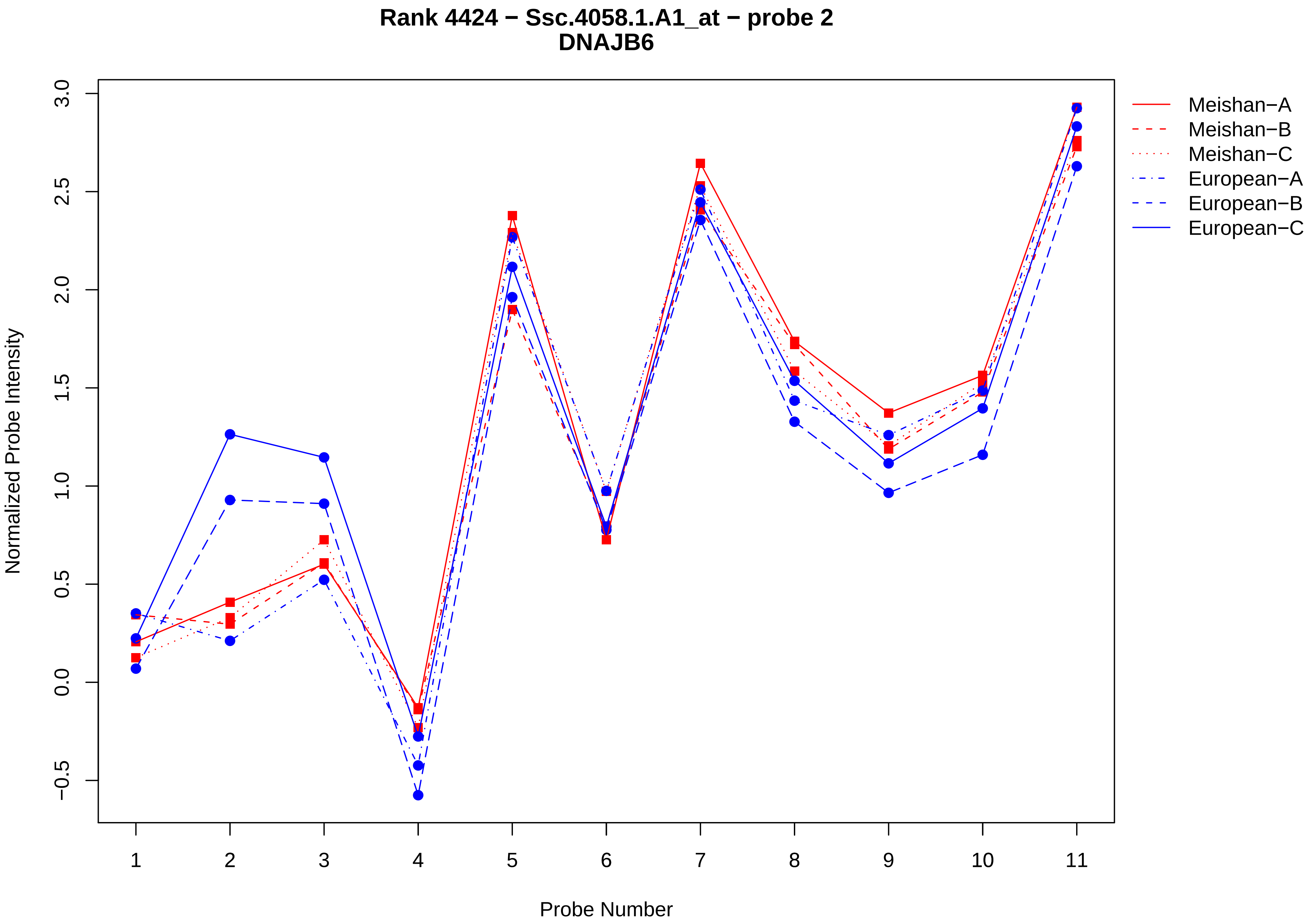

Rank 4424 − Ssc.4058.1.A1_at − probe 2
DNAJB6
Normalized Probe Intensity
Probe Number
−0.5
0.0
0.5
1.0
1.5
2.0
2.5
3.0
1
2
3
4
5
6
7
8
9
10
11
Meishan−A
Meishan−B
Meishan−C
European−A
European−B
European−C

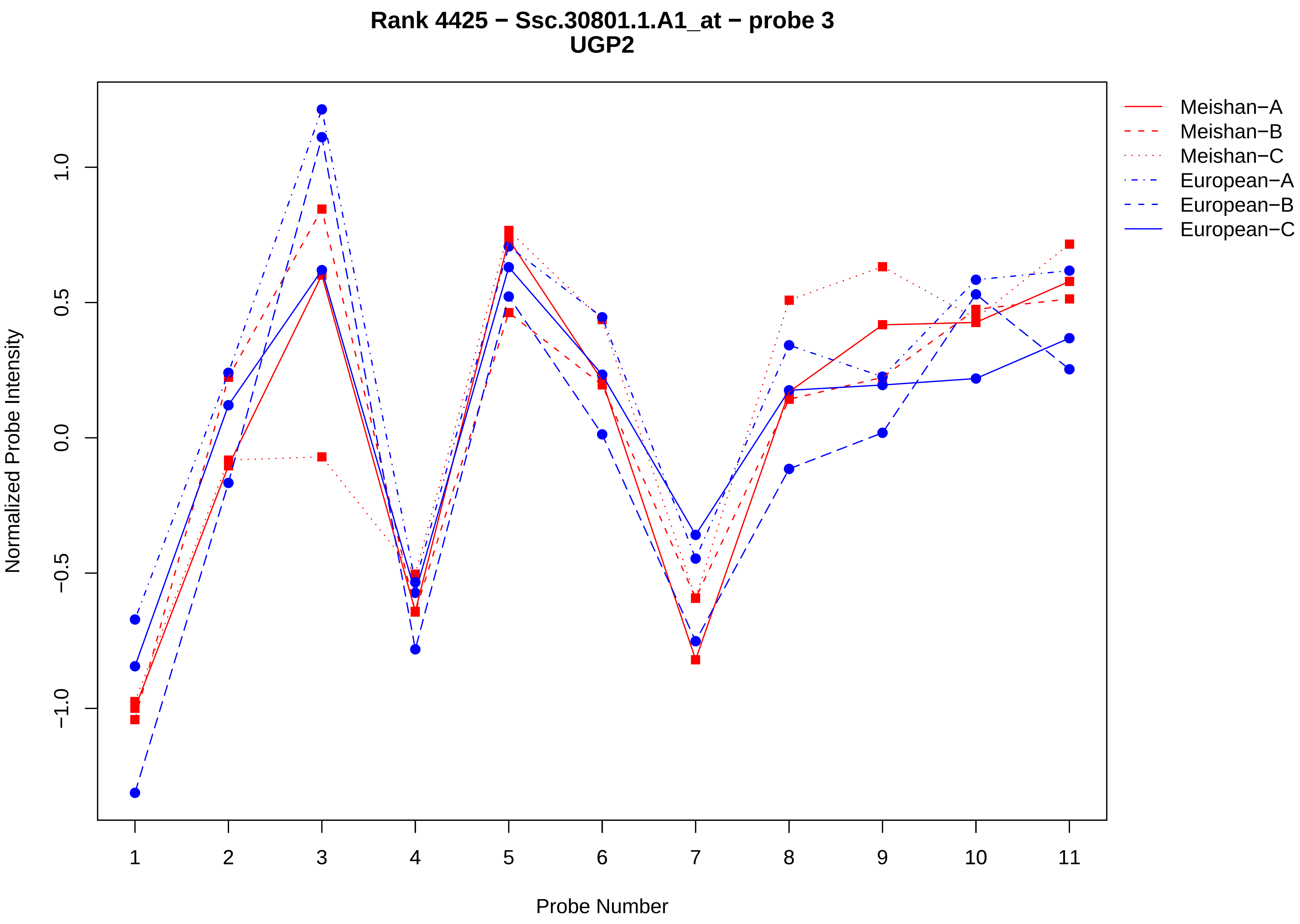
Rank 4425 − Ssc.30801.1.A1_at − probe 3
UGP2
Normalized Probe Intensity
Probe Number
Meishan−A
Meishan−B
Meishan−C
European−A
European−B
European−C
−1.0
−0.5
0.0
0.5
1.0
1
2
3
4
5
6
7
8
9
10
11

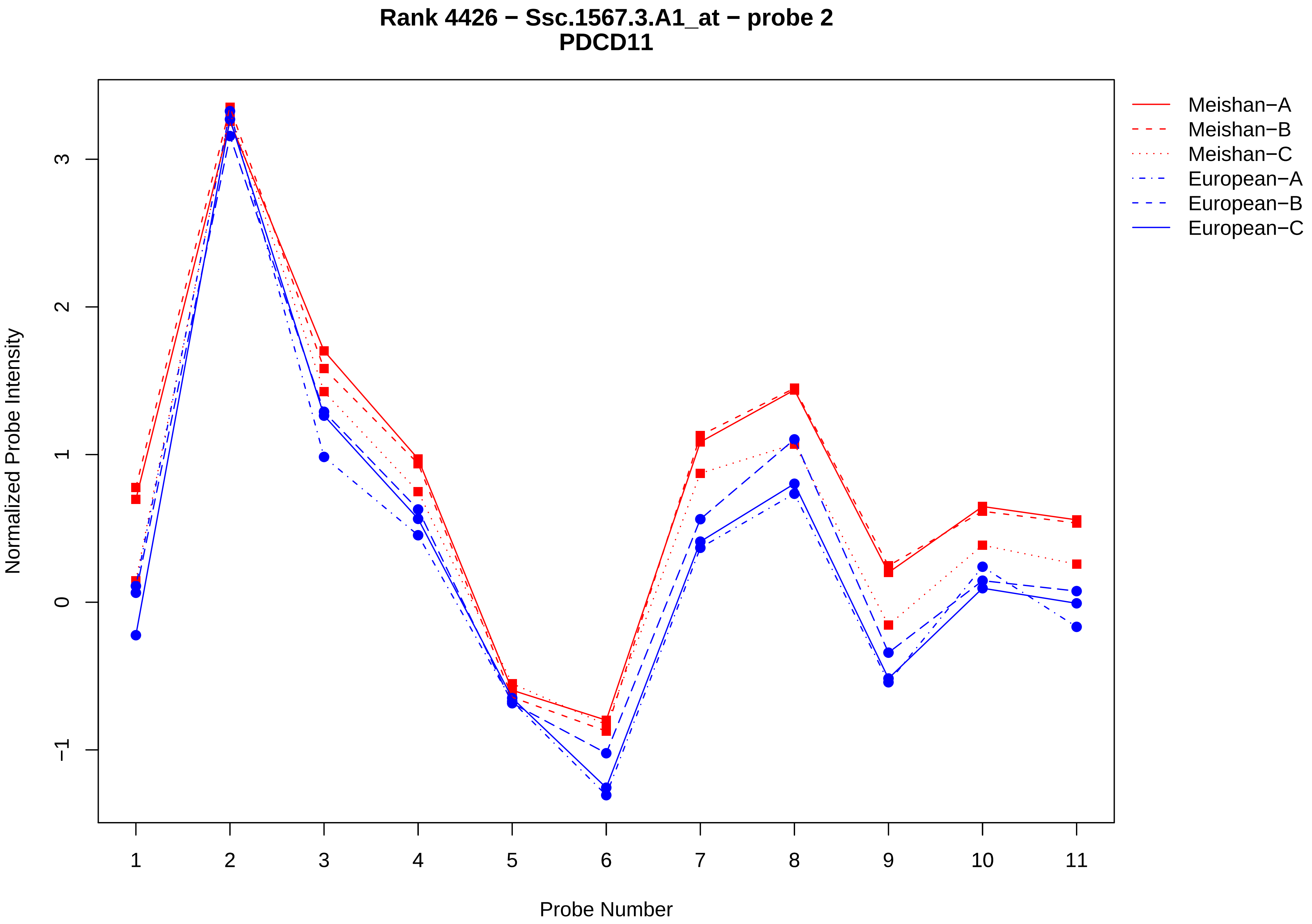
Rank 4426 − Ssc.1567.3.A1_at − probe 2
PDCD11
Normalized Probe Intensity
Probe Number
−1
0
1
2
3
1
2
3
4
5
6
7
8
9
10
11
Meishan−A
Meishan−B
Meishan−C
European−A
European−B
European−C

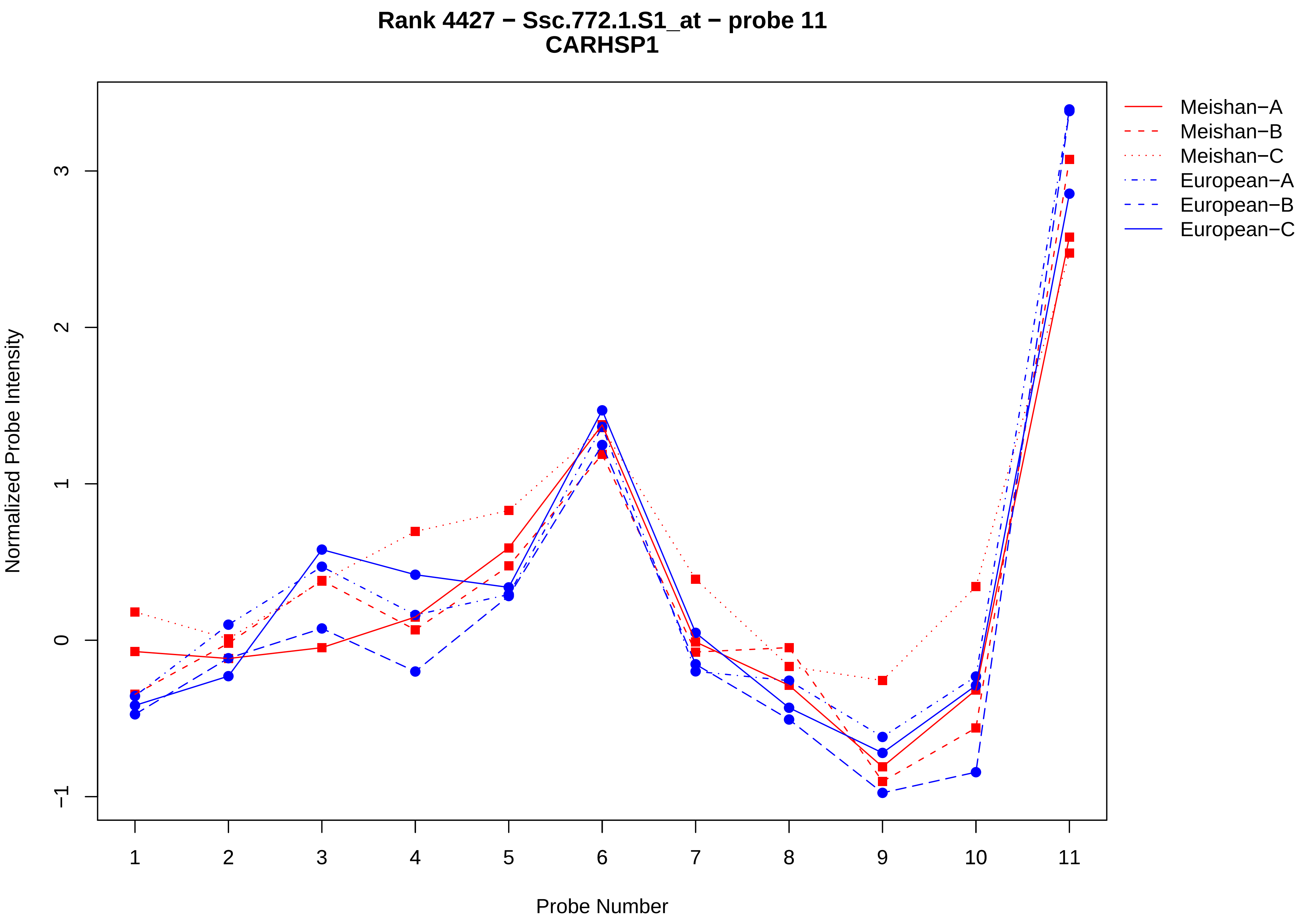

Rank 4427 − Ssc.772.1.S1_at − probe 11
CARHSP1
Normalized Probe Intensity
−1
0
1
2
3
1
2
3
4
5
6
7
8
9
10
11
Probe Number
Meishan−A
Meishan−B
Meishan−C
European−A
European−B
European−C

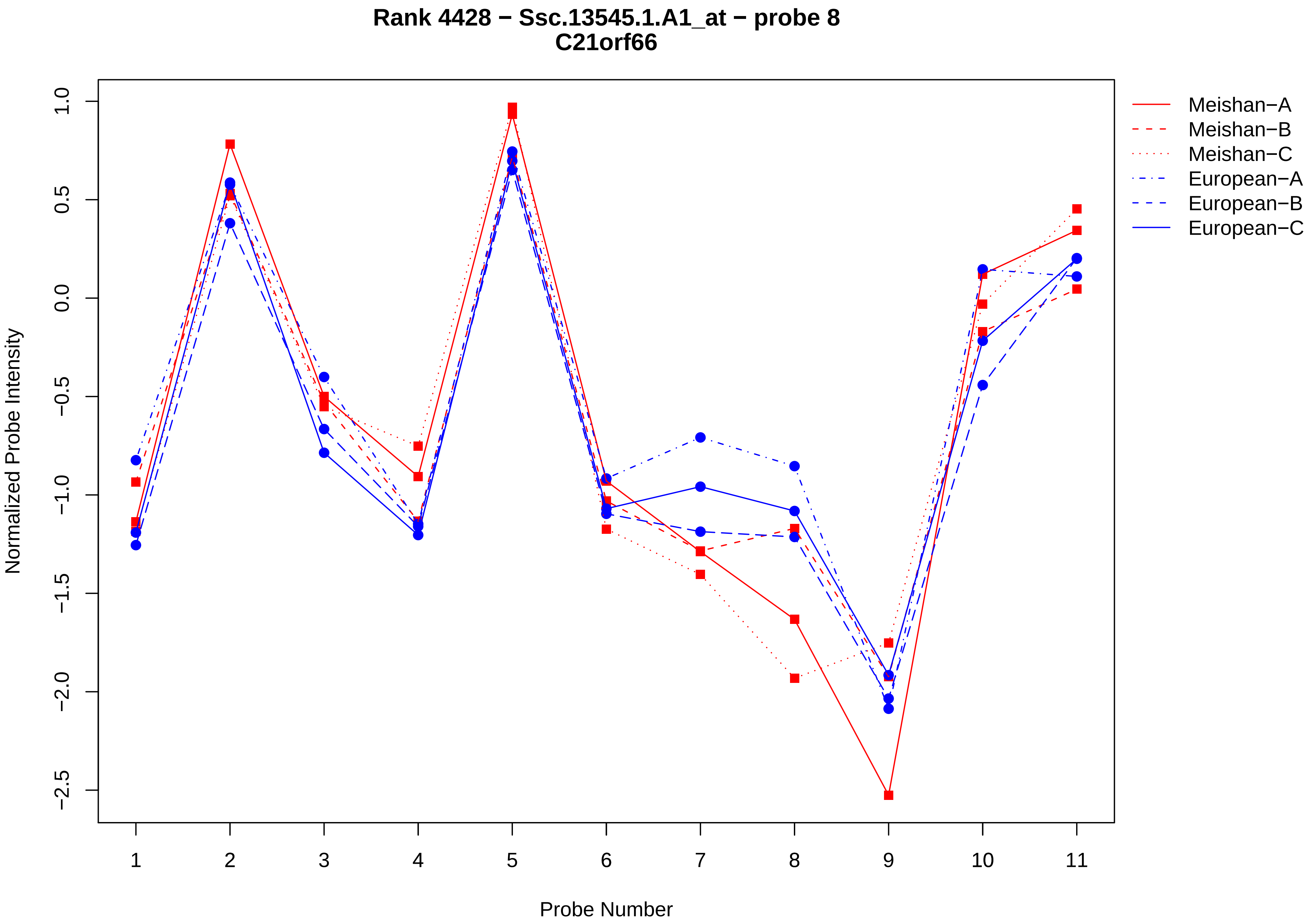
Rank 4428 − Ssc.13545.1.A1_at − probe 8
C21orf66
Normalized Probe Intensity
Probe Number
1.0
0.5
0.0
−0.5
−1.0
−1.5
−2.0
−2.5
1
2
3
4
5
6
7
8
9
10
11
Meishan−A
Meishan−B
Meishan−C
European−A
European−B
European−C

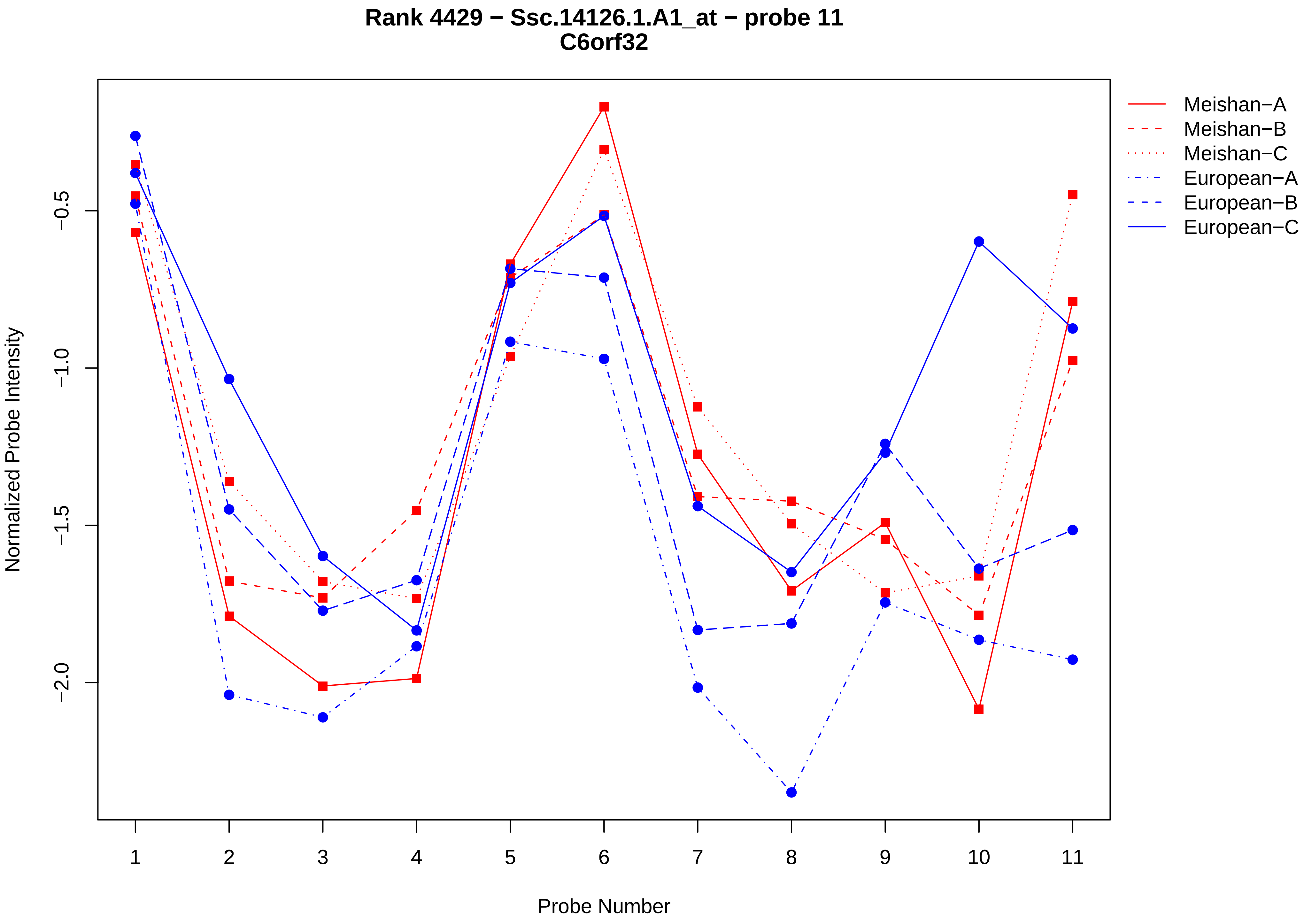
Rank 4429 − Ssc.14126.1.A1_at − probe 11
C6orf32
Normalized Probe Intensity
−0.5
−1.0
−1.5
−2.0
1
2
3
4
5
6
7
8
9
10
11
Probe Number
Meishan−A
Meishan−B
Meishan−C
European−A
European−B
European−C

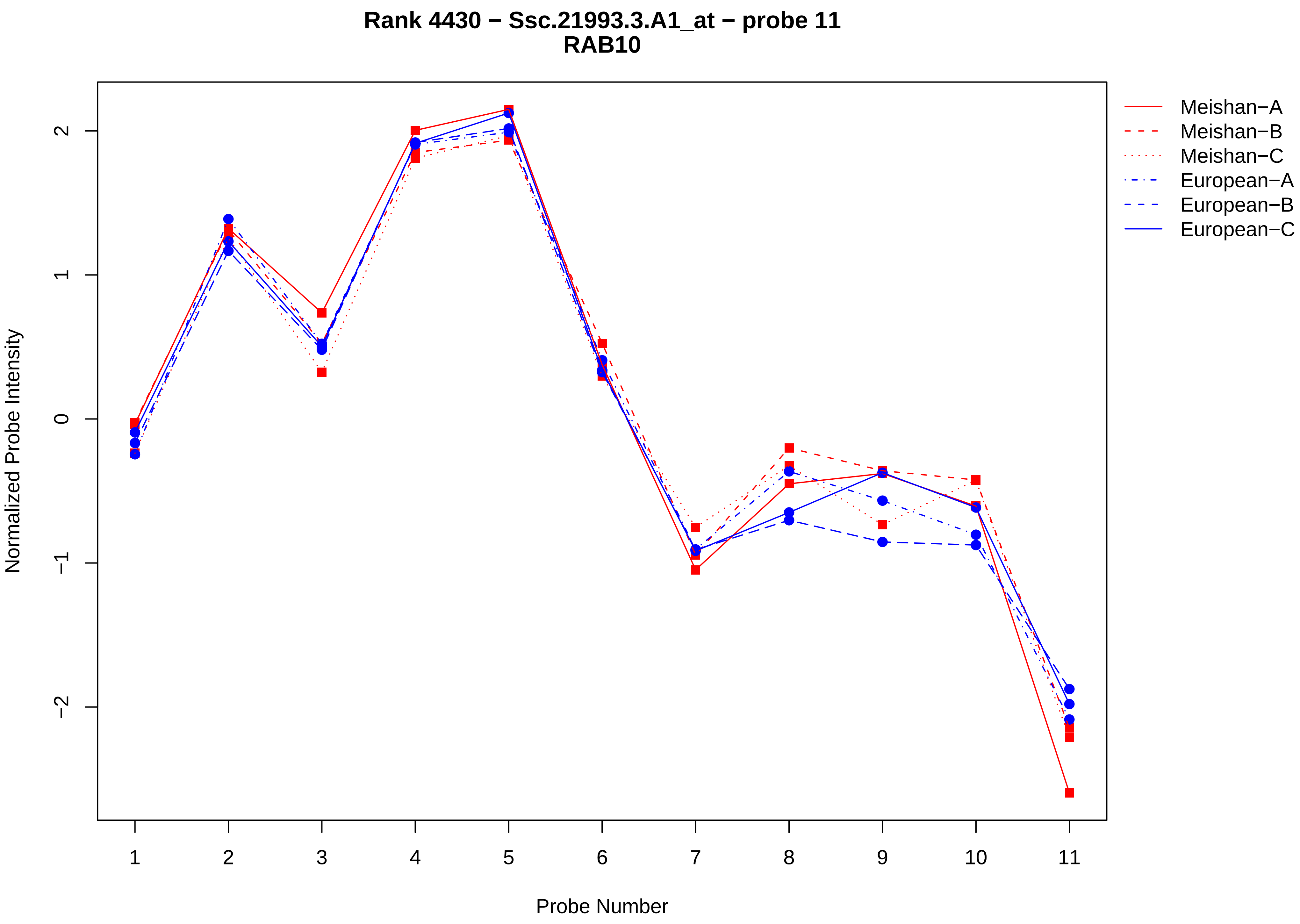

Rank 4430 − Ssc.21993.3.A1_at − probe 11
RAB10
Normalized Probe Intensity
Probe Number
−2
−1
0
1
2
1
2
3
4
5
6
7
8
9
10
11
Meishan−A
Meishan−B
Meishan−C
European−A
European−B
European−C

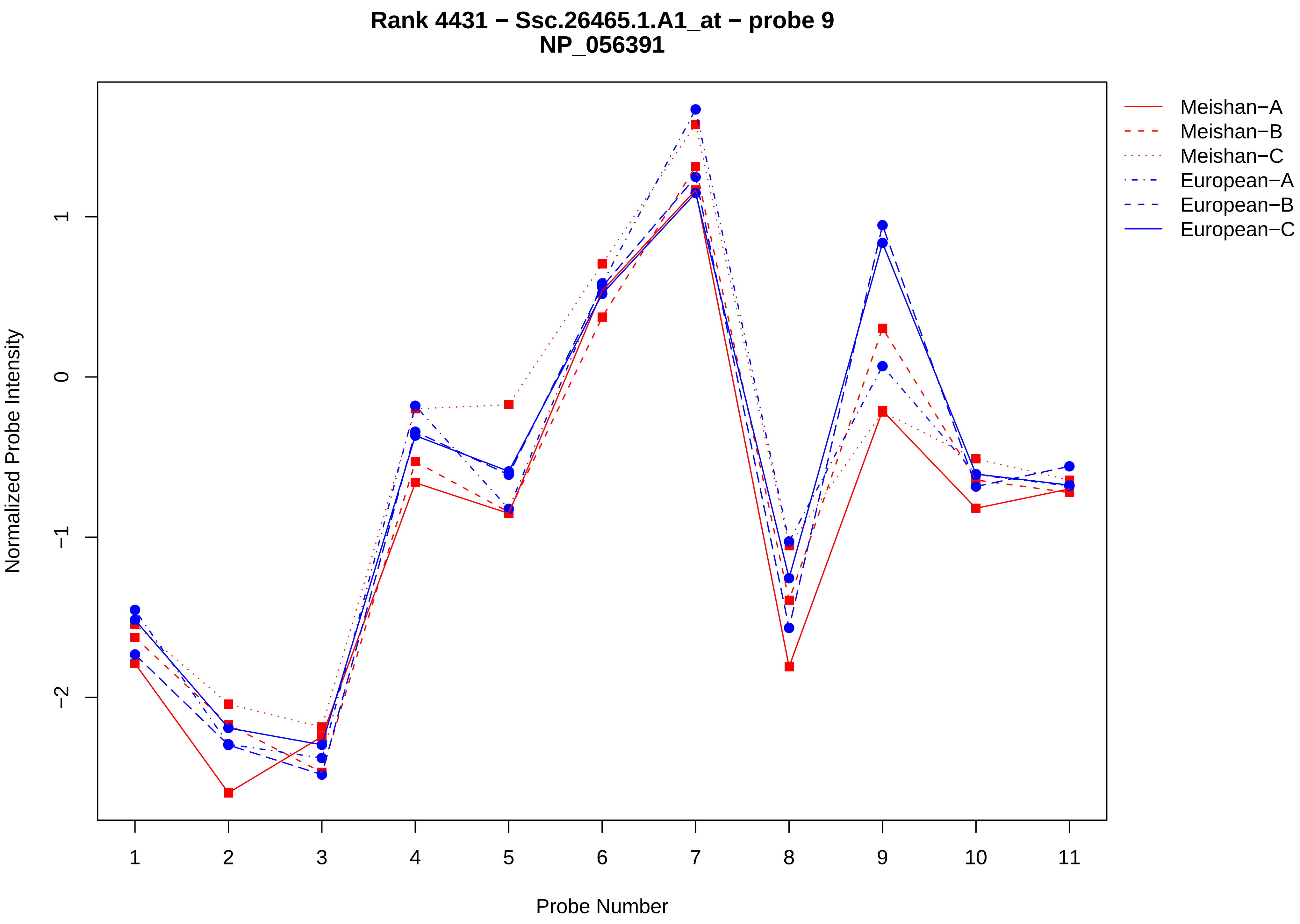

Rank 4431 − Ssc.26465.1.A1_at − probe 9
NP_056391
Meishan−A
Meishan−B
Meishan−C
European−A
European−B
European−C
Normalized Probe Intensity
Probe Number
1
0
−1
−2
1
2
3
4
5
6
7
8
9
10
11

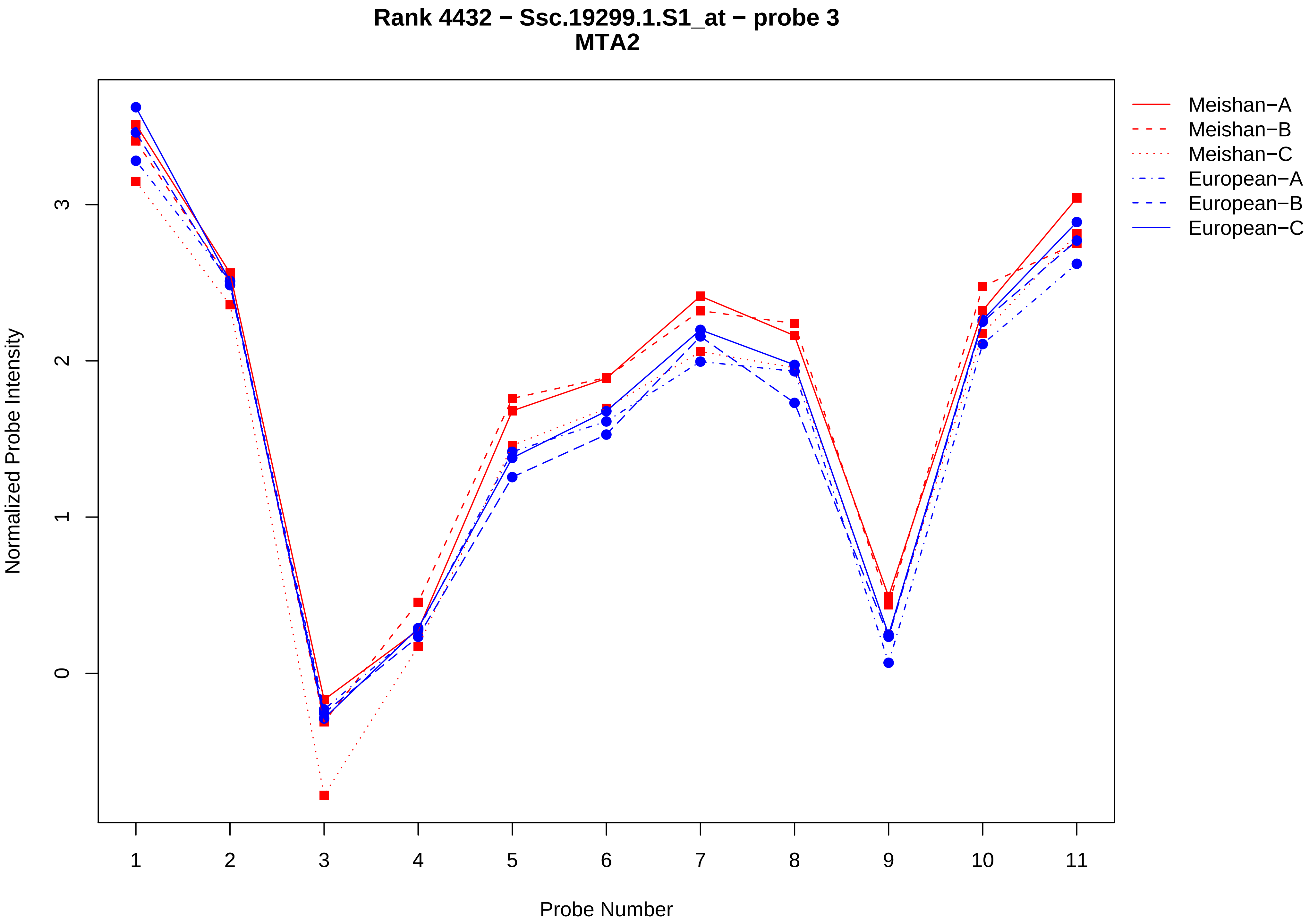
Rank 4432 − Ssc.19299.1.S1_at − probe 3
MTA2
Normalized Probe Intensity
Probe Number
0
1
2
3
1
2
3
4
5
6
7
8
9
10
11
Meishan−A
Meishan−B
Meishan−C
European−A
European−B
European−C

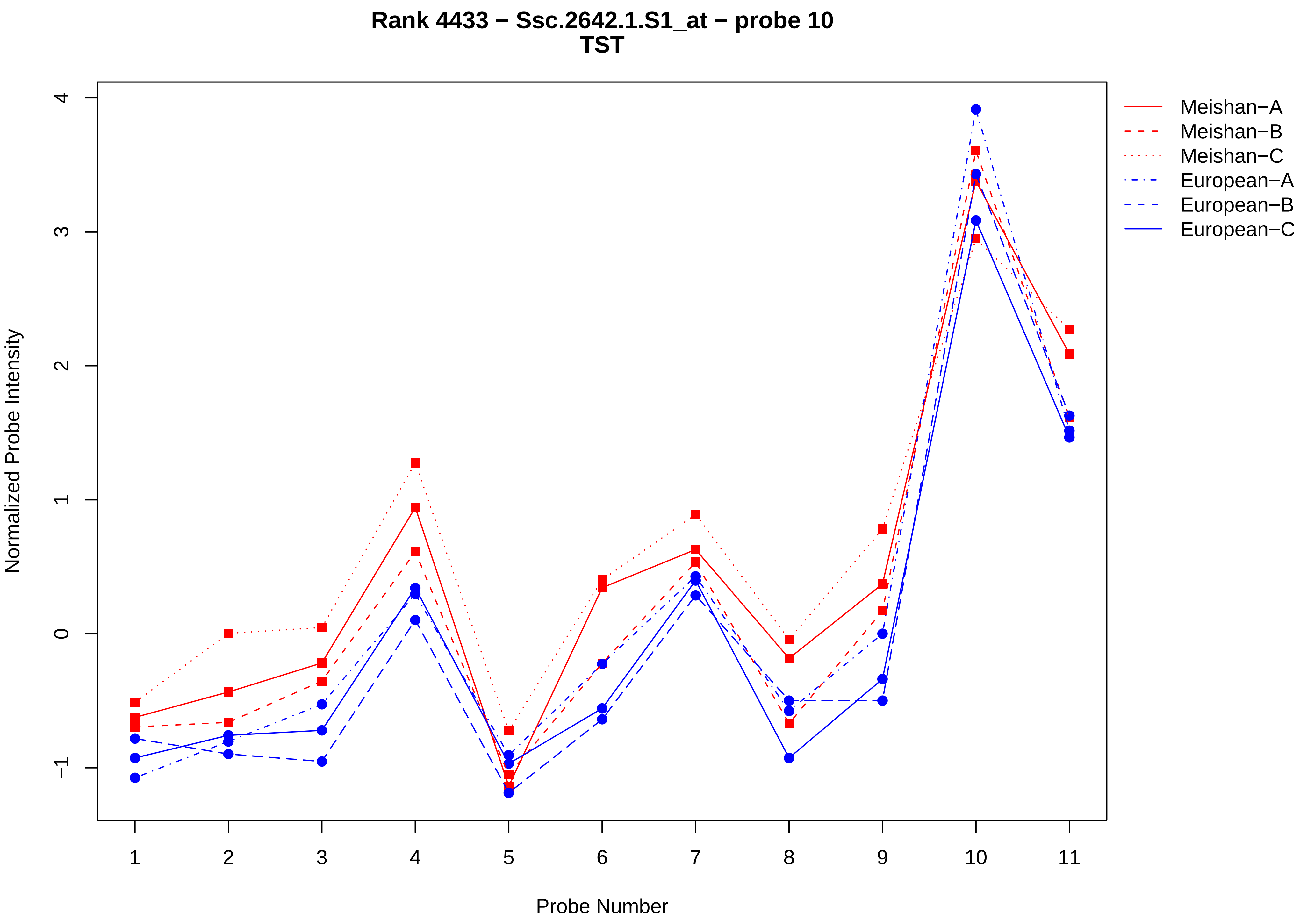

Rank 4433 − Ssc.2642.1.S1_at − probe 10
TST
Normalized Probe Intensity
Probe Number
Meishan−A
Meishan−B
Meishan−C
European−A
European−B
European−C
−1
0
1
2
3
4
1
2
3
4
5
6
7
8
9
10
11

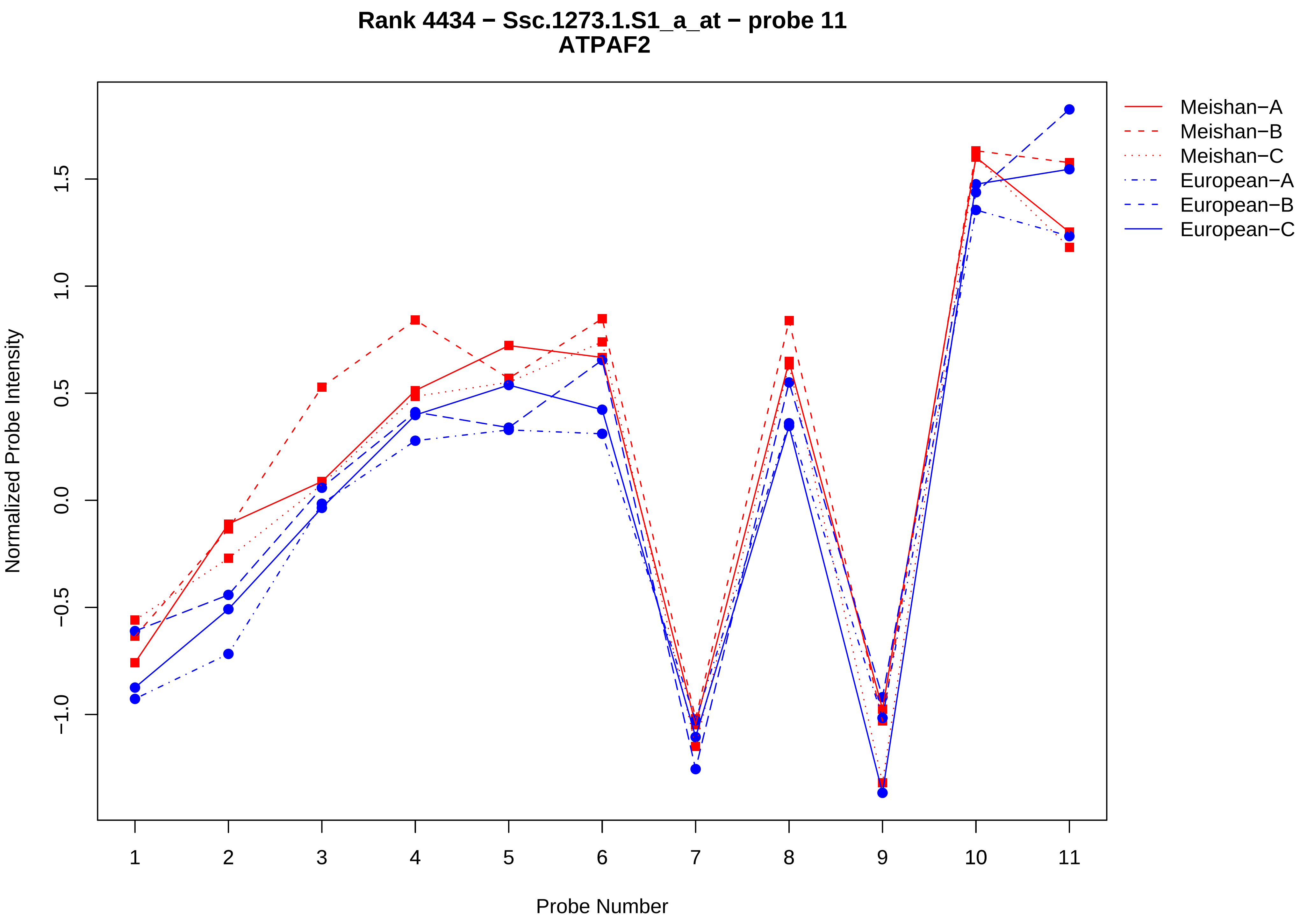

Rank 4434 − Ssc.1273.1.S1_a_at − probe 11
ATPAF2
Normalized Probe Intensity
Probe Number
−1.0
−0.5
0.0
0.5
1.0
1.5
1
2
3
4
5
6
7
8
9
10
11
Meishan−A
Meishan−B
Meishan−C
European−A
European−B
European−C

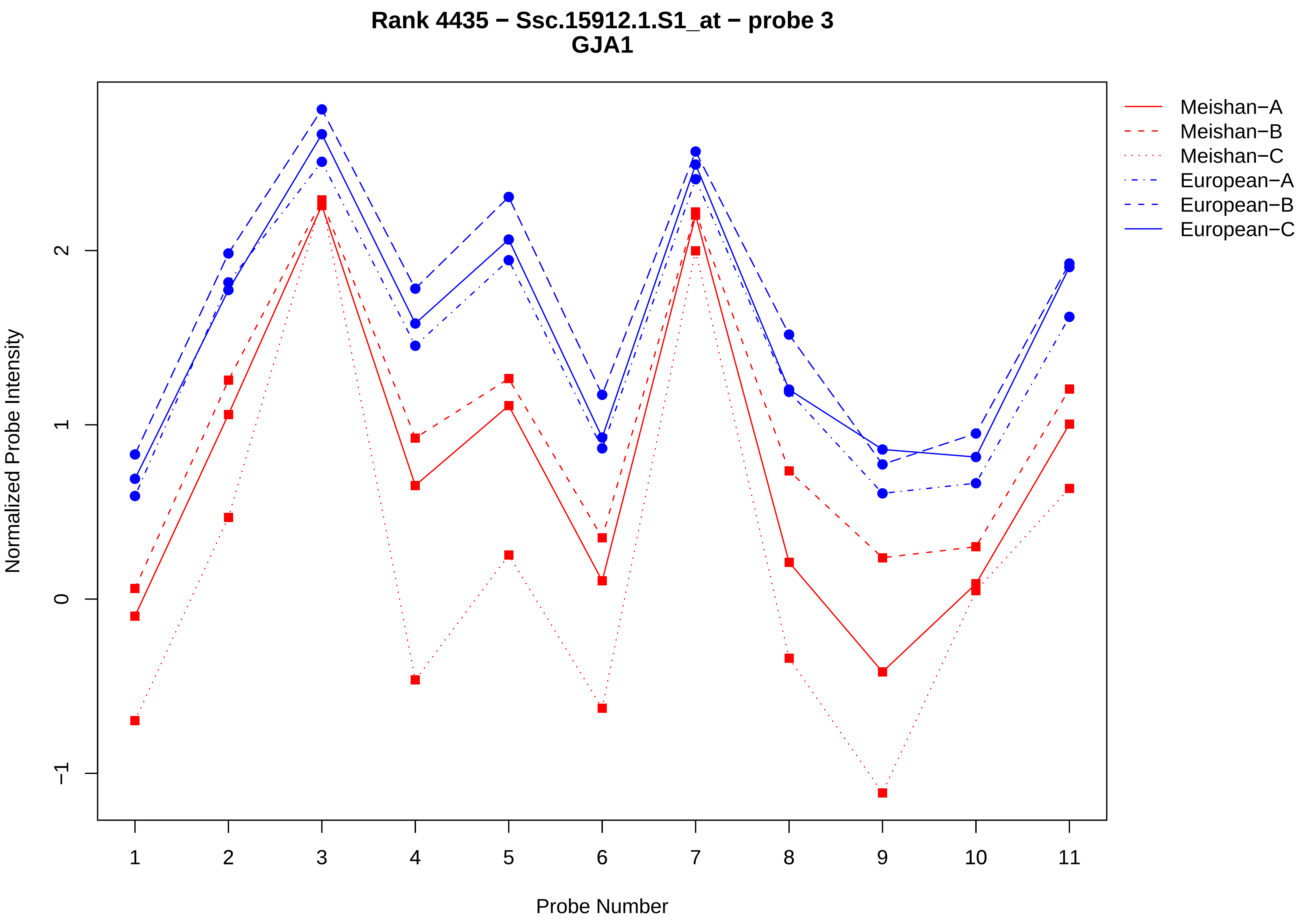

Rank 4435 − Ssc.15912.1.S1_at − probe 3
GJA1
Normalized Probe Intensity
−1
0
1
2
1
2
3
4
5
6
7
8
9
10
11
Probe Number
Meishan−A
Meishan−B
Meishan−C
European−A
European−B
European−C

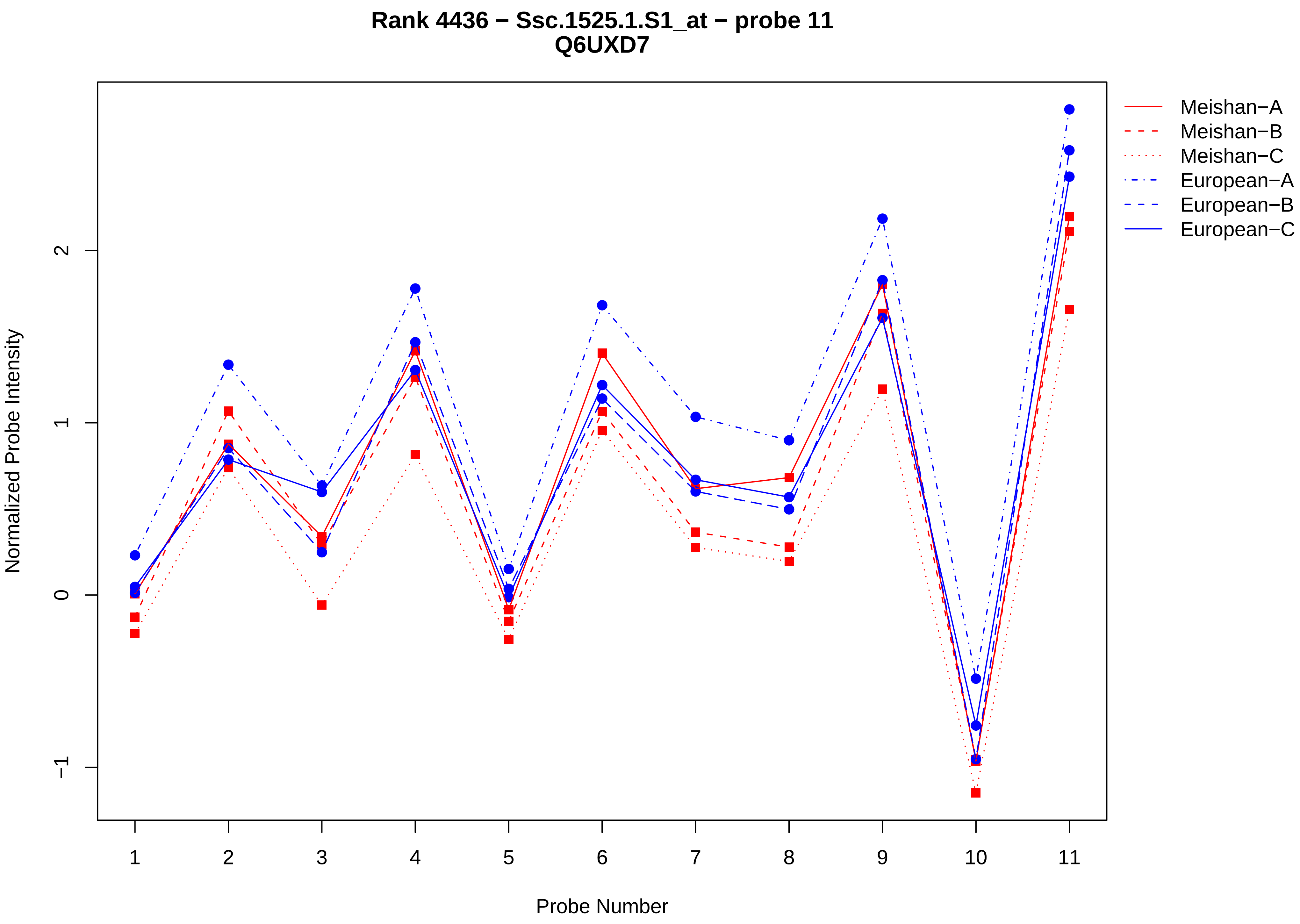
Rank 4436 − Ssc.1525.1.S1_at − probe 11
Q6UXD7
Normalized Probe Intensity
Probe Number
Meishan−A
Meishan−B
Meishan−C
European−A
European−B
European−C
−1
0
1
2
1
2
3
4
5
6
7
8
9
10
11

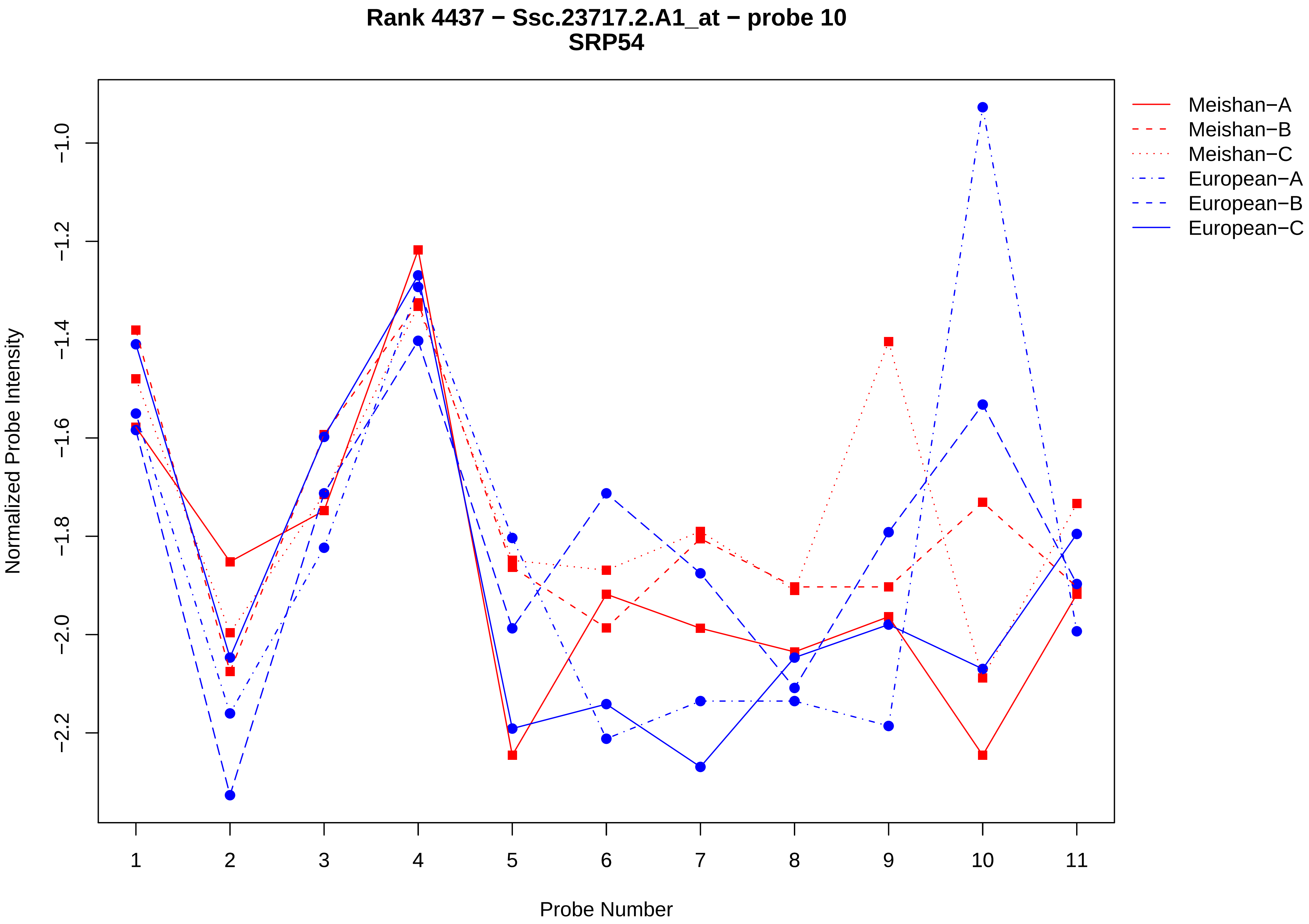

Rank 4437 − Ssc.23717.2.A1_at − probe 10
SRP54
Normalized Probe Intensity
Probe Number
−1.0
−1.2
−1.4
−1.6
−1.8
−2.0
−2.2
1
2
3
4
5
6
7
8
9
10
11
Meishan−A
Meishan−B
Meishan−C
European−A
European−B
European−C

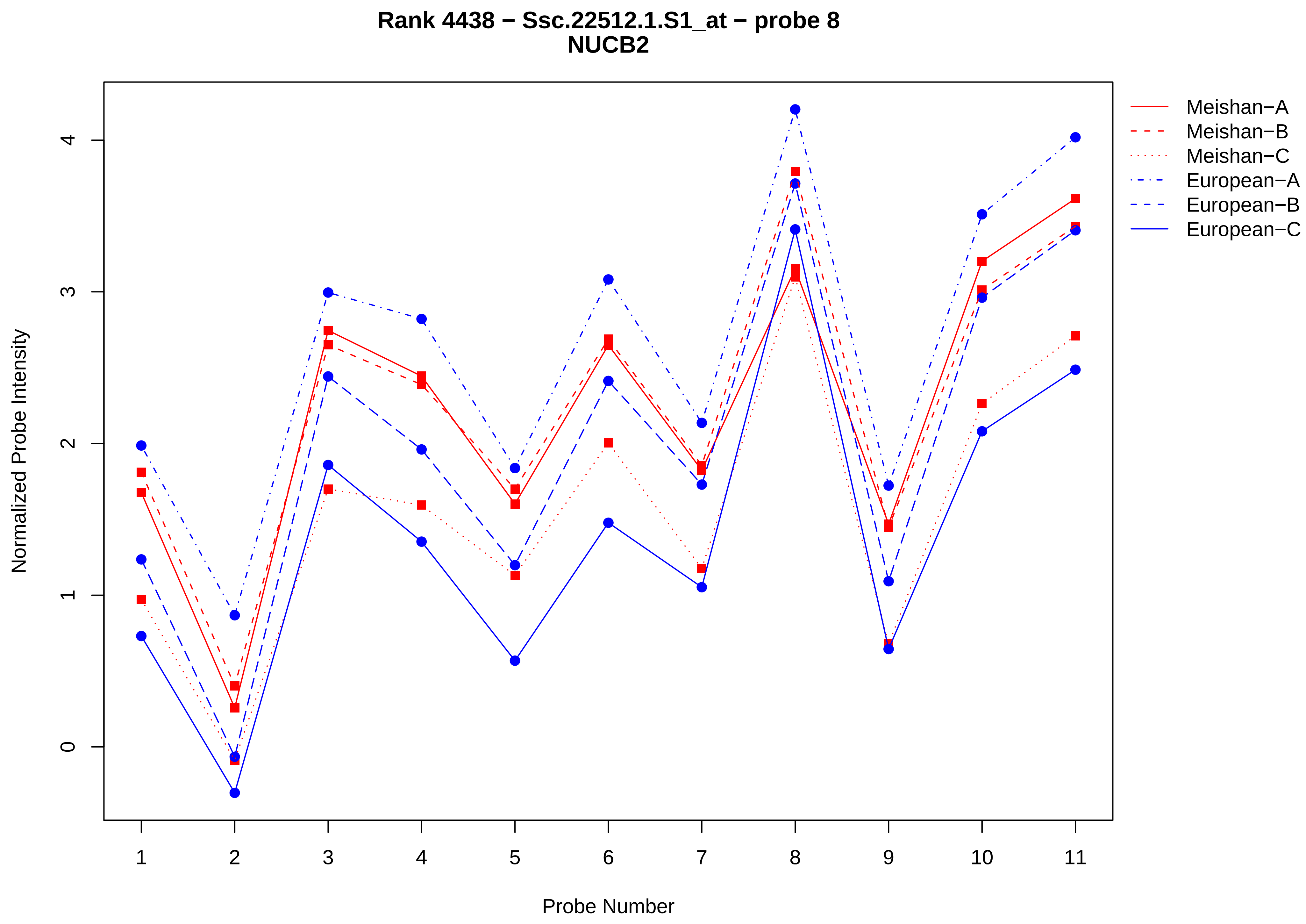
Rank 4438 − Ssc.22512.1.S1_at − probe 8
NUCB2
Normalized Probe Intensity
Probe Number
0
1
2
3
4
1
2
3
4
5
6
7
8
9
10
11
Meishan−A
Meishan−B
Meishan−C
European−A
European−B
European−C

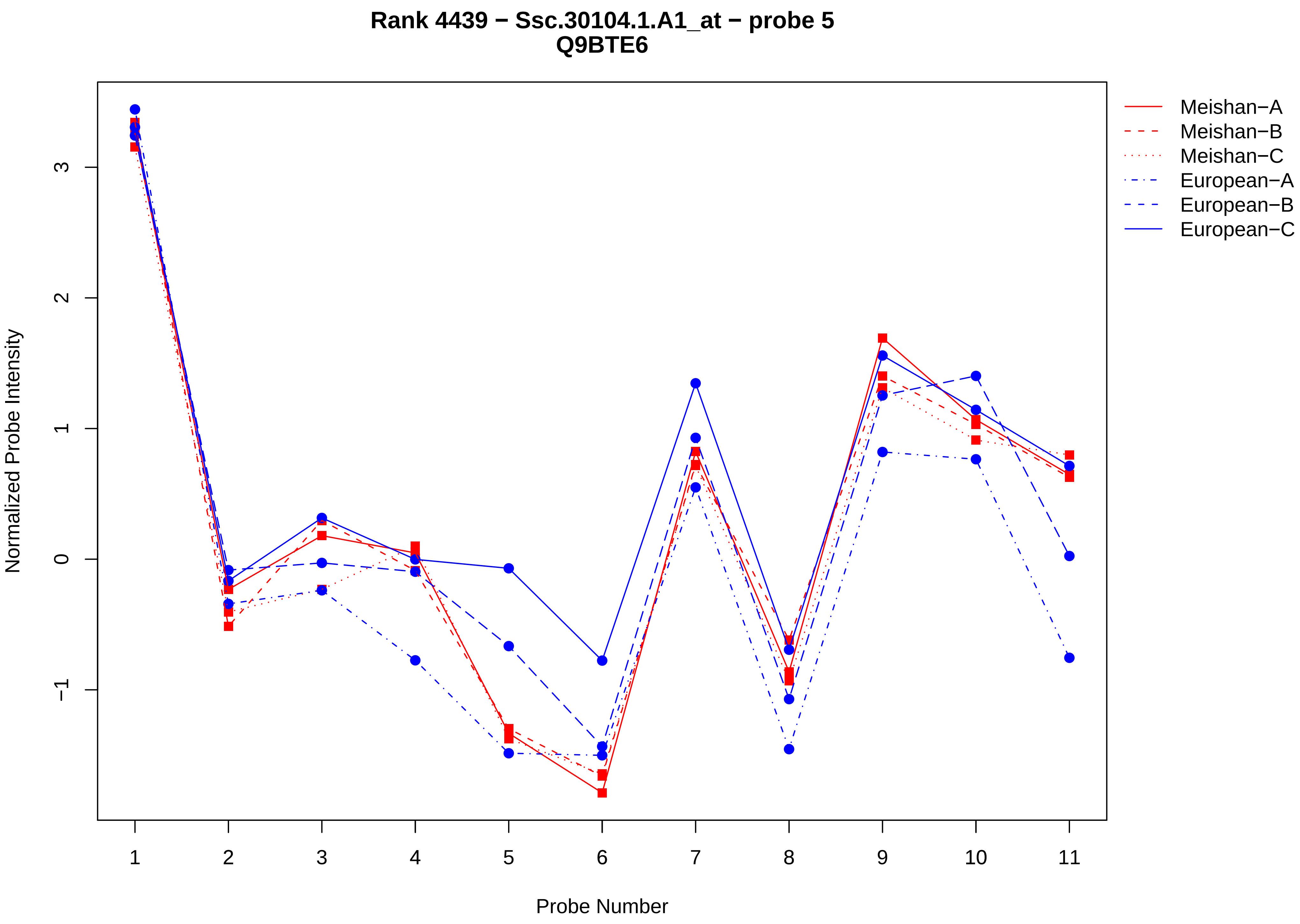

Rank 4439 − Ssc.30104.1.A1_at − probe 5
Q9BTE6
Normalized Probe Intensity
Probe Number
−1
0
1
2
3
1
2
3
4
5
6
7
8
9
10
11
Meishan−A
Meishan−B
Meishan−C
European−A
European−B
European−C

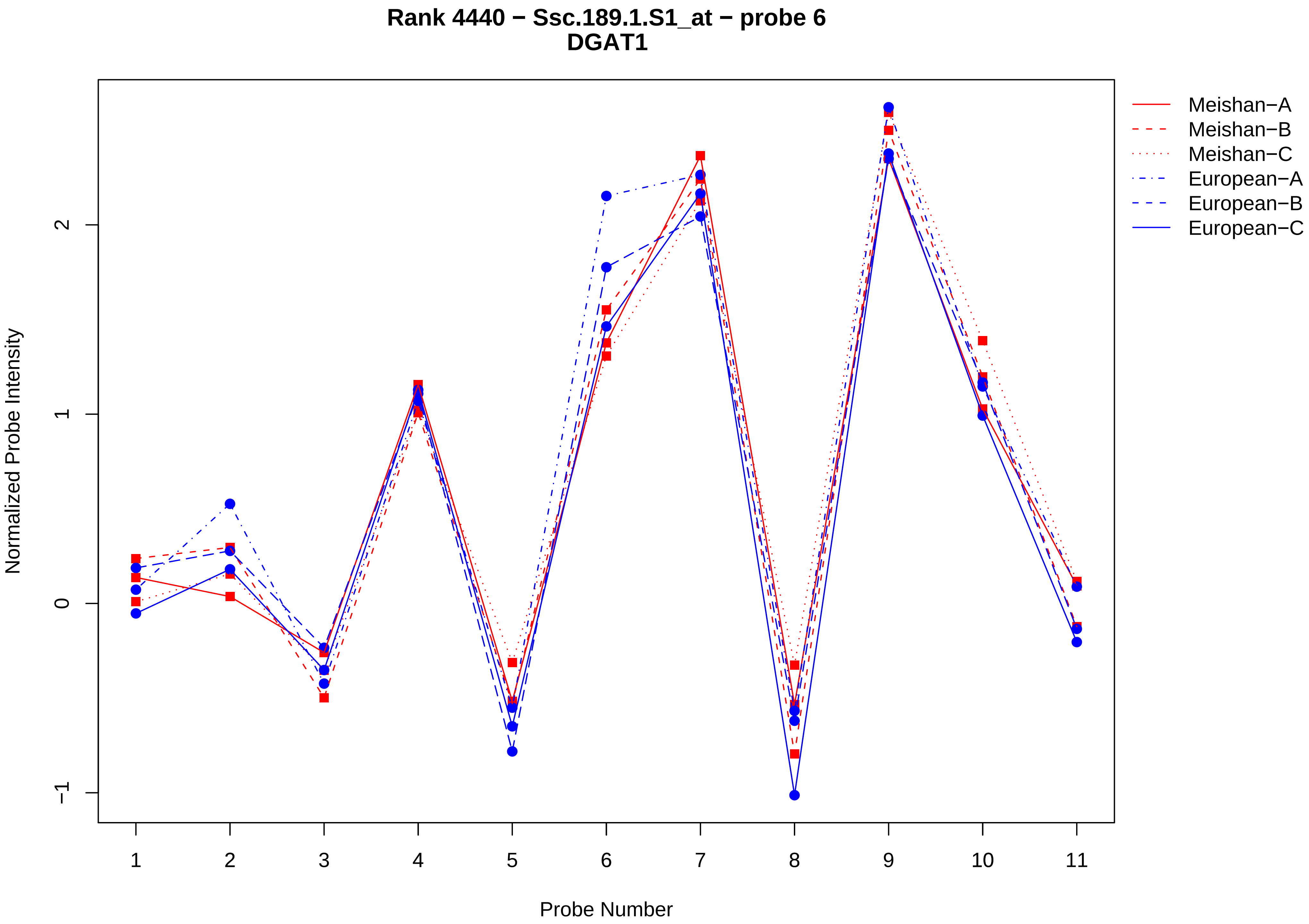

Rank 4440 − Ssc.189.1.S1_at − probe 6
DGAT1
Normalized Probe Intensity
−1
0
1
2
1
2
3
4
5
6
7
8
9
10
11
Probe Number
Meishan−A
Meishan−B
Meishan−C
European−A
European−B
European−C

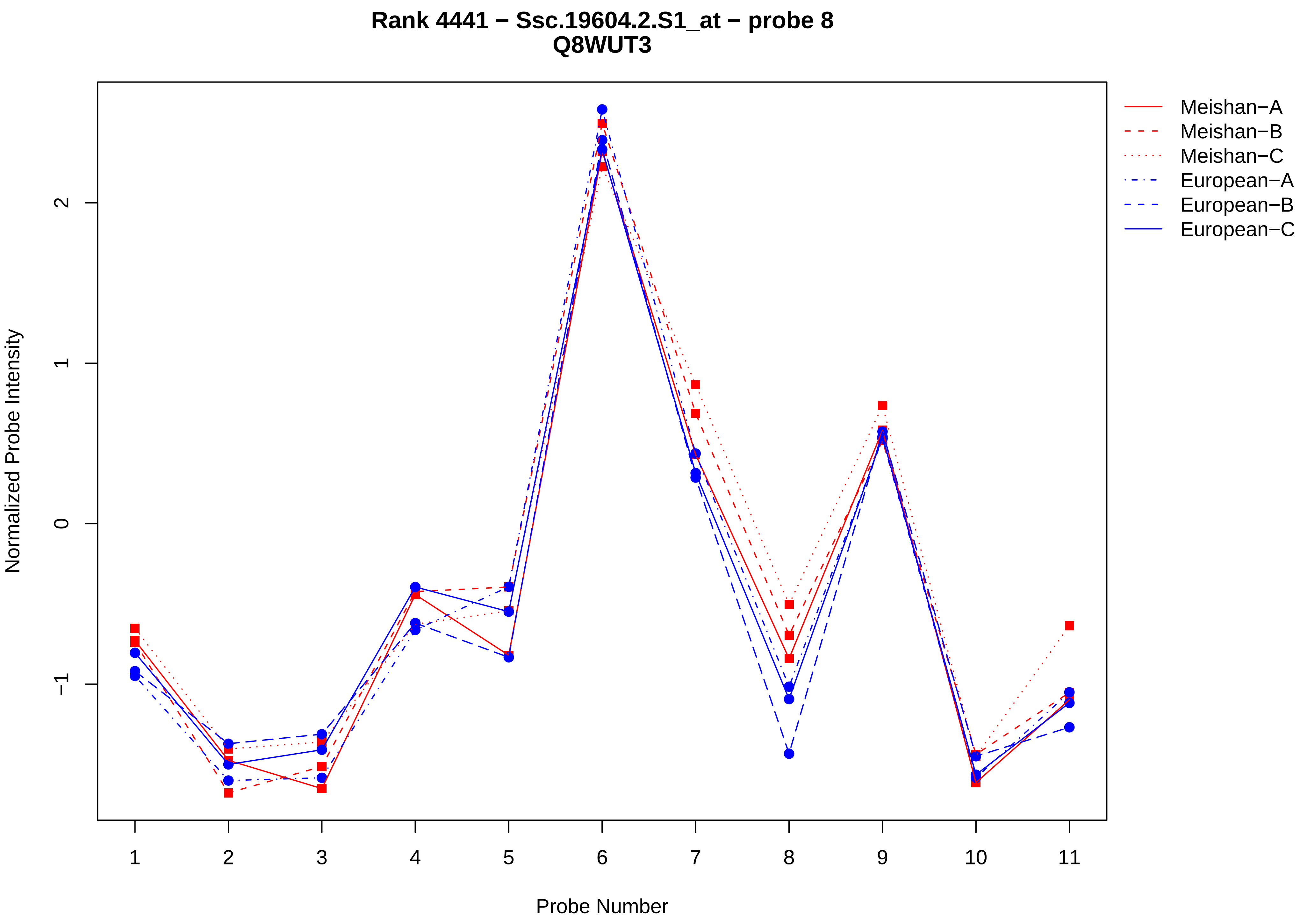
Rank 4441 − Ssc.19604.2.S1_at − probe 8
Q8WUT3
Normalized Probe Intensity
Probe Number
Meishan−A
Meishan−B
Meishan−C
European−A
European−B
European−C
−1
0
1
2
1
2
3
4
5
6
7
8
9
10
11

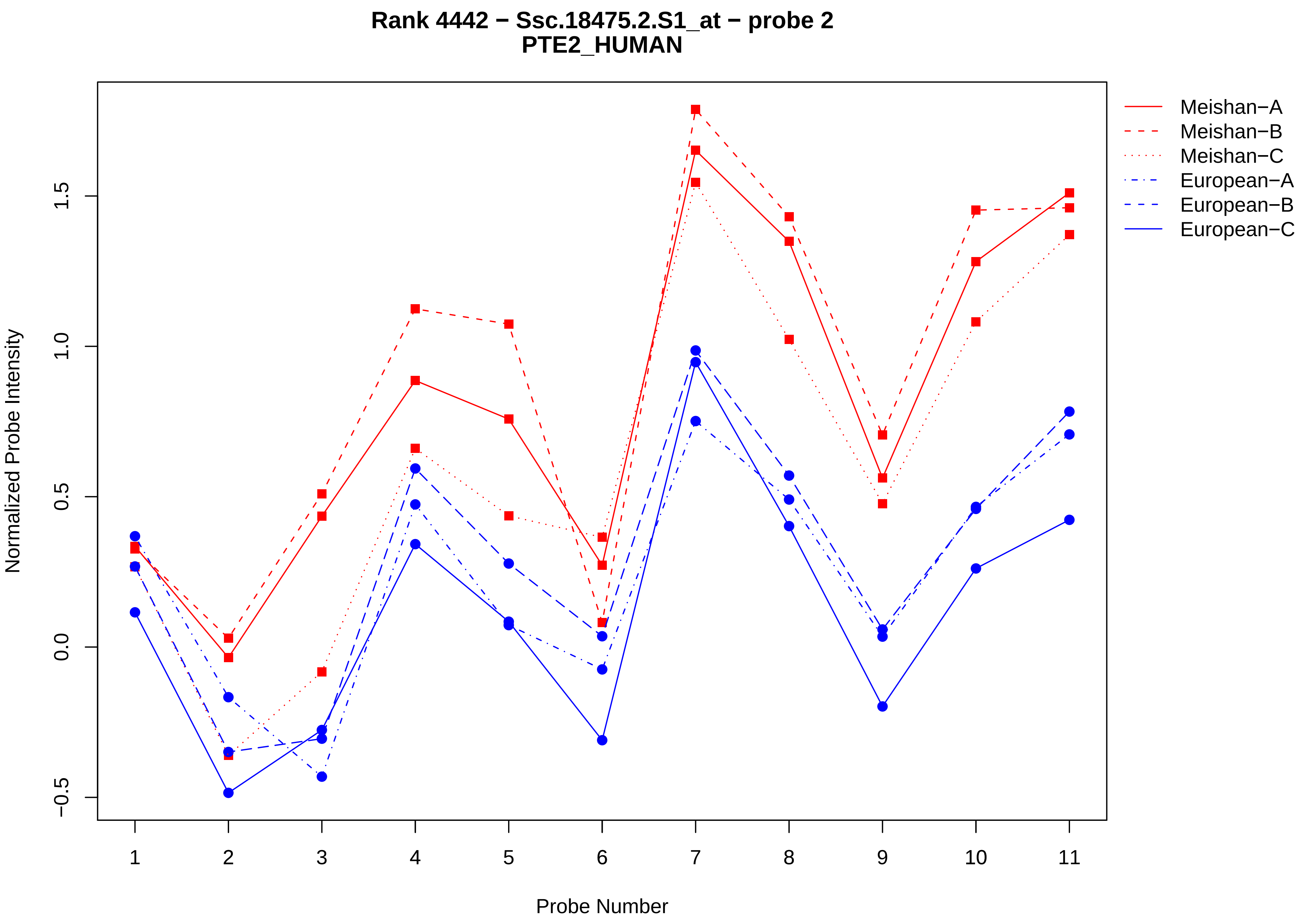

Rank 4442 − Ssc.18475.2.S1_at − probe 2
PTE2_HUMAN
Normalized Probe Intensity
Probe Number
−0.5
0.0
0.5
1.0
1.5
1
2
3
4
5
6
7
8
9
10
11
Meishan−A
Meishan−B
Meishan−C
European−A
European−B
European−C

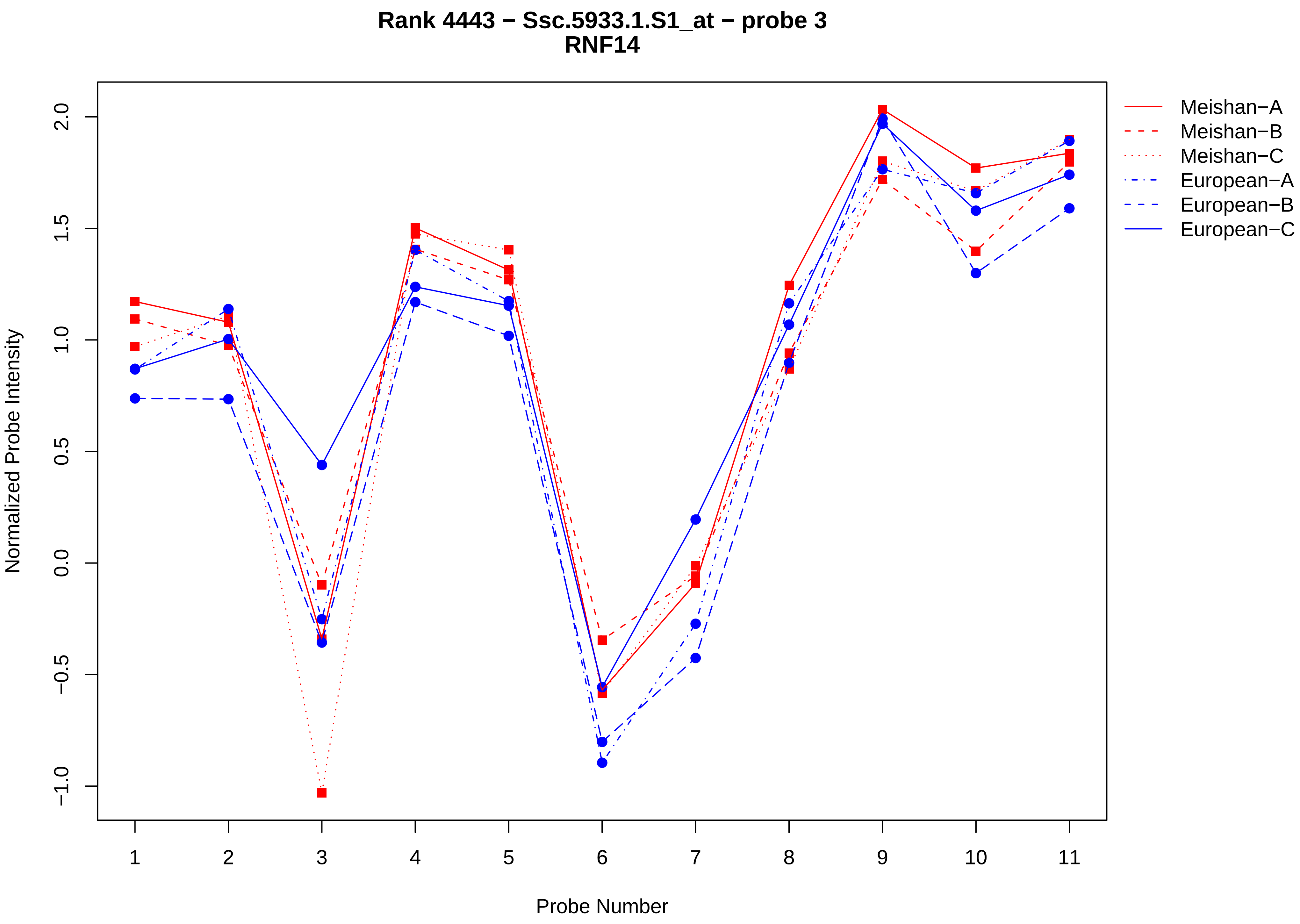

Rank 4443 − Ssc.5933.1.S1_at − probe 3
RNF14
Normalized Probe Intensity
Probe Number
−1.0
−0.5
0.0
0.5
1.0
1.5
2.0
1
2
3
4
5
6
7
8
9
10
11
Meishan−A
Meishan−B
Meishan−C
European−A
European−B
European−C

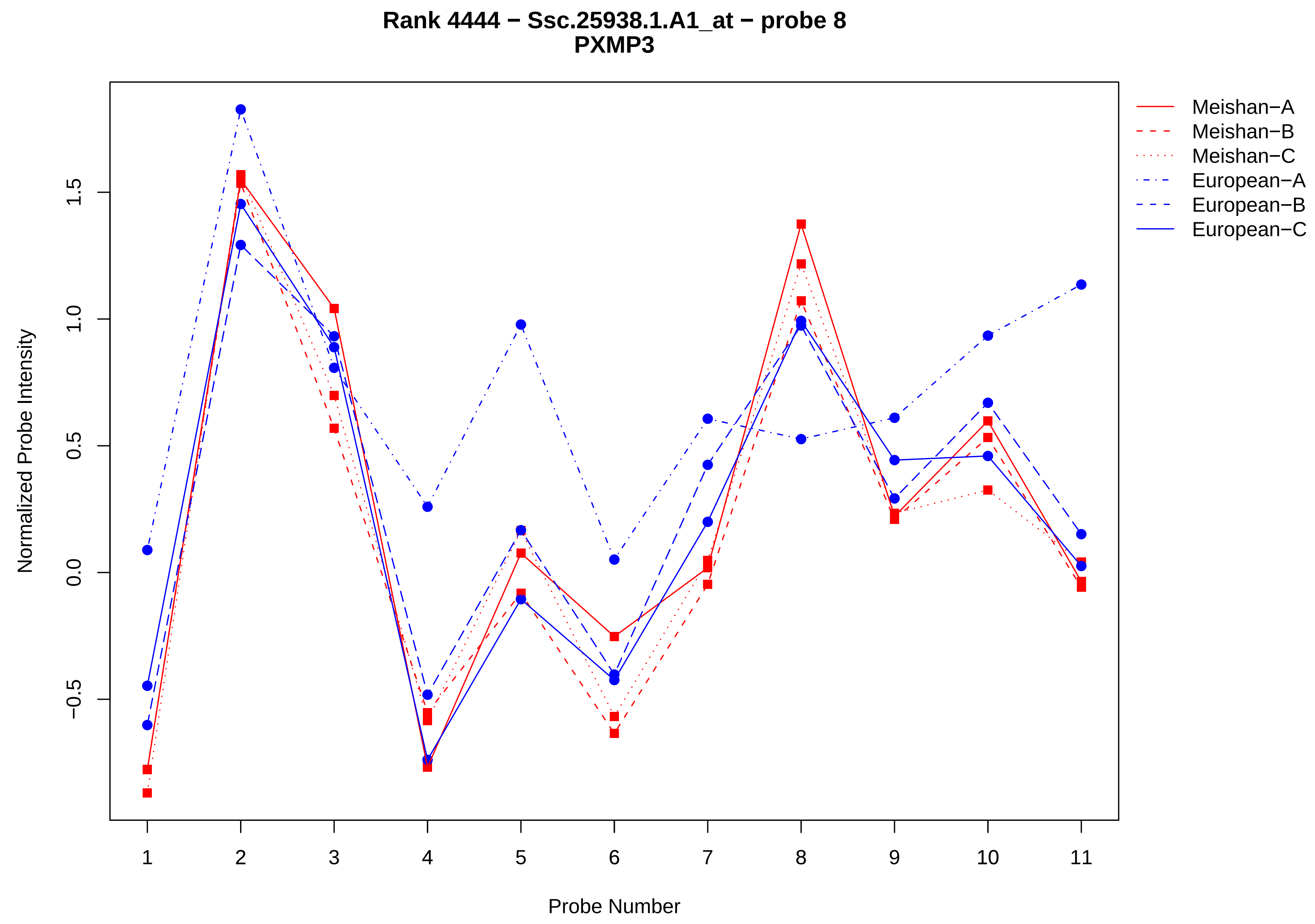

Rank 4444 − Ssc.25938.1.A1_at − probe 8
PXMP3
Normalized Probe Intensity
Probe Number
−0.5
0.0
0.5
1.0
1.5
1
2
3
4
5
6
7
8
9
10
11
Meishan−A
Meishan−B
Meishan−C
European−A
European−B
European−C

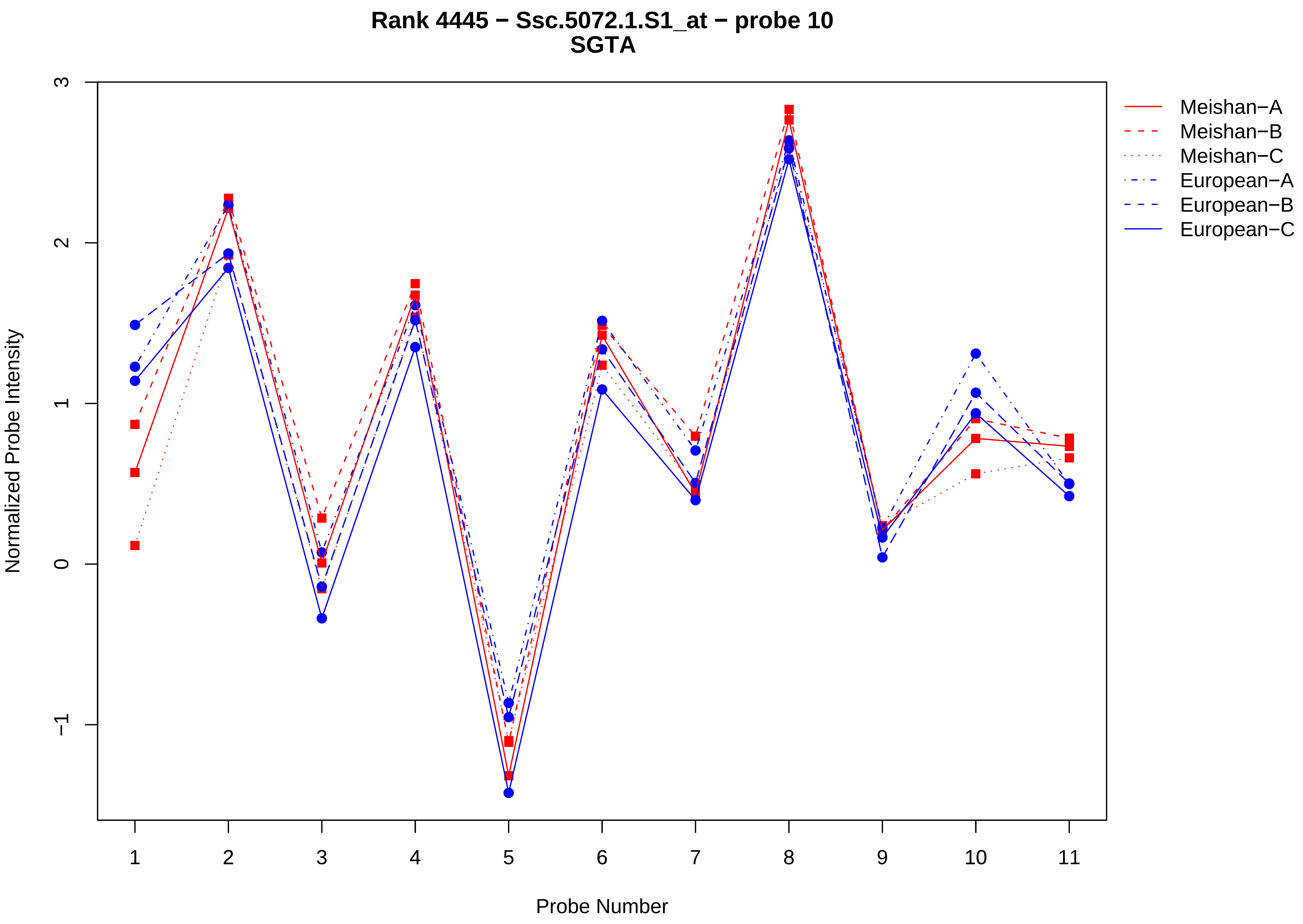

Rank 4445 − Ssc.5072.1.S1_at − probe 10
SGTA
Normalized Probe Intensity
Probe Number
−1
0
1
2
3
1
2
3
4
5
6
7
8
9
10
11
Meishan−A
Meishan−B
Meishan−C
European−A
European−B
European−C

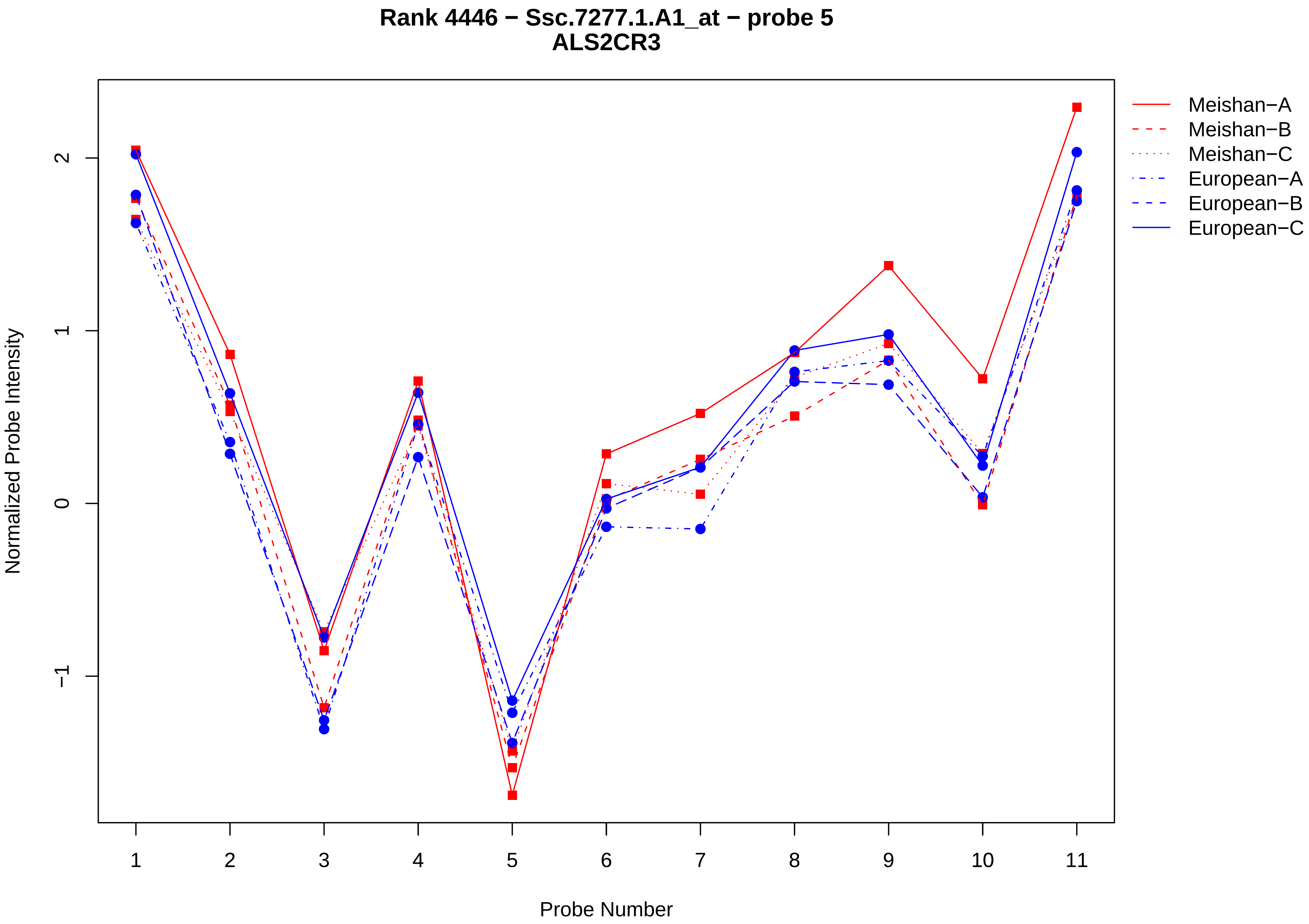

Rank 4446 − Ssc.7277.1.A1_at − probe 5
ALS2CR3
Normalized Probe Intensity
Probe Number
Meishan−A
Meishan−B
Meishan−C
European−A
European−B
European−C

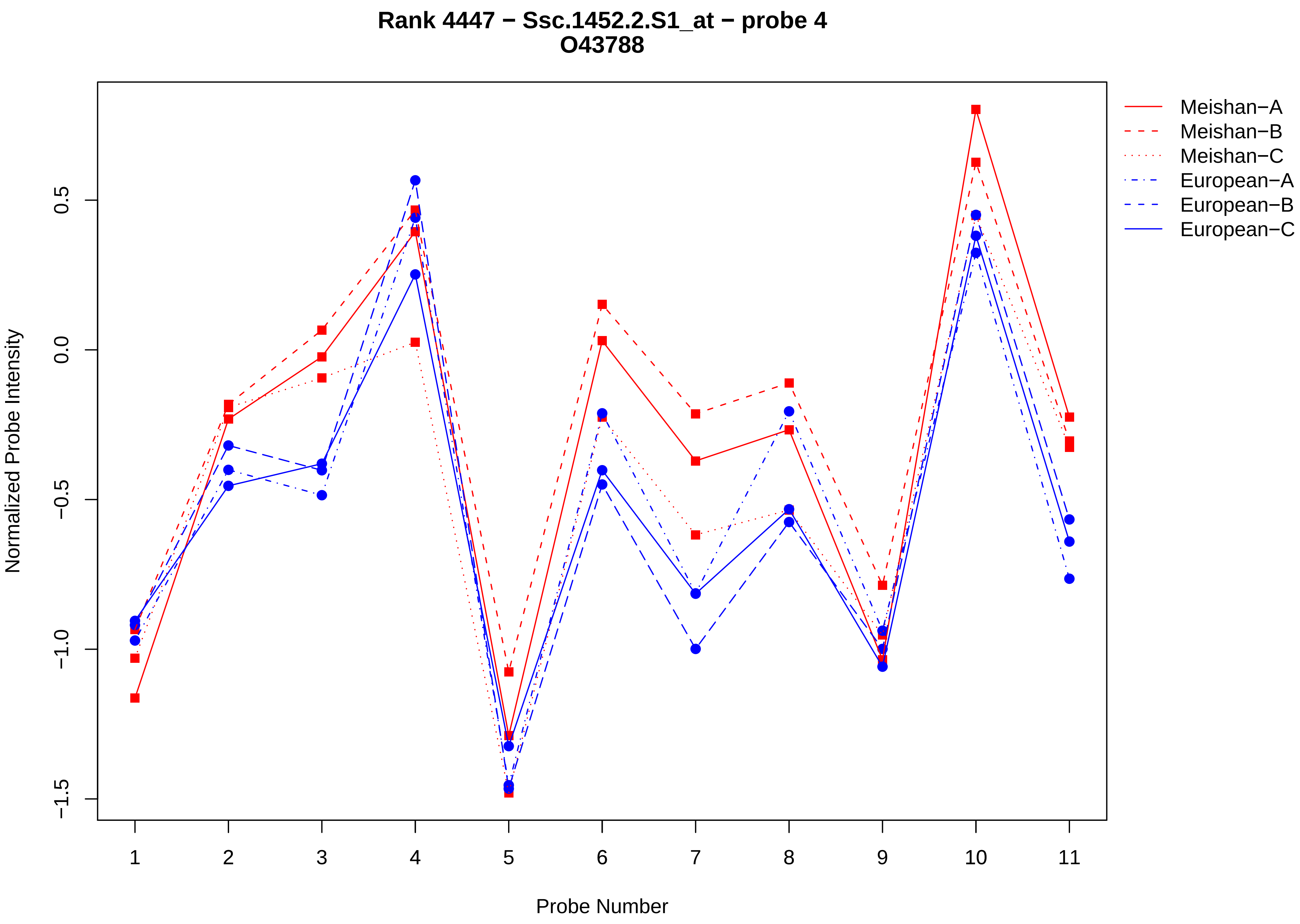

Rank 4447 − Ssc.1452.2.S1_at − probe 4
O43788
Normalized Probe Intensity
0.5
0.0
−0.5
−1.0
−1.5
1
2
3
4
5
6
7
8
9
10
11
Probe Number
Meishan−A
Meishan−B
Meishan−C
European−A
European−B
European−C

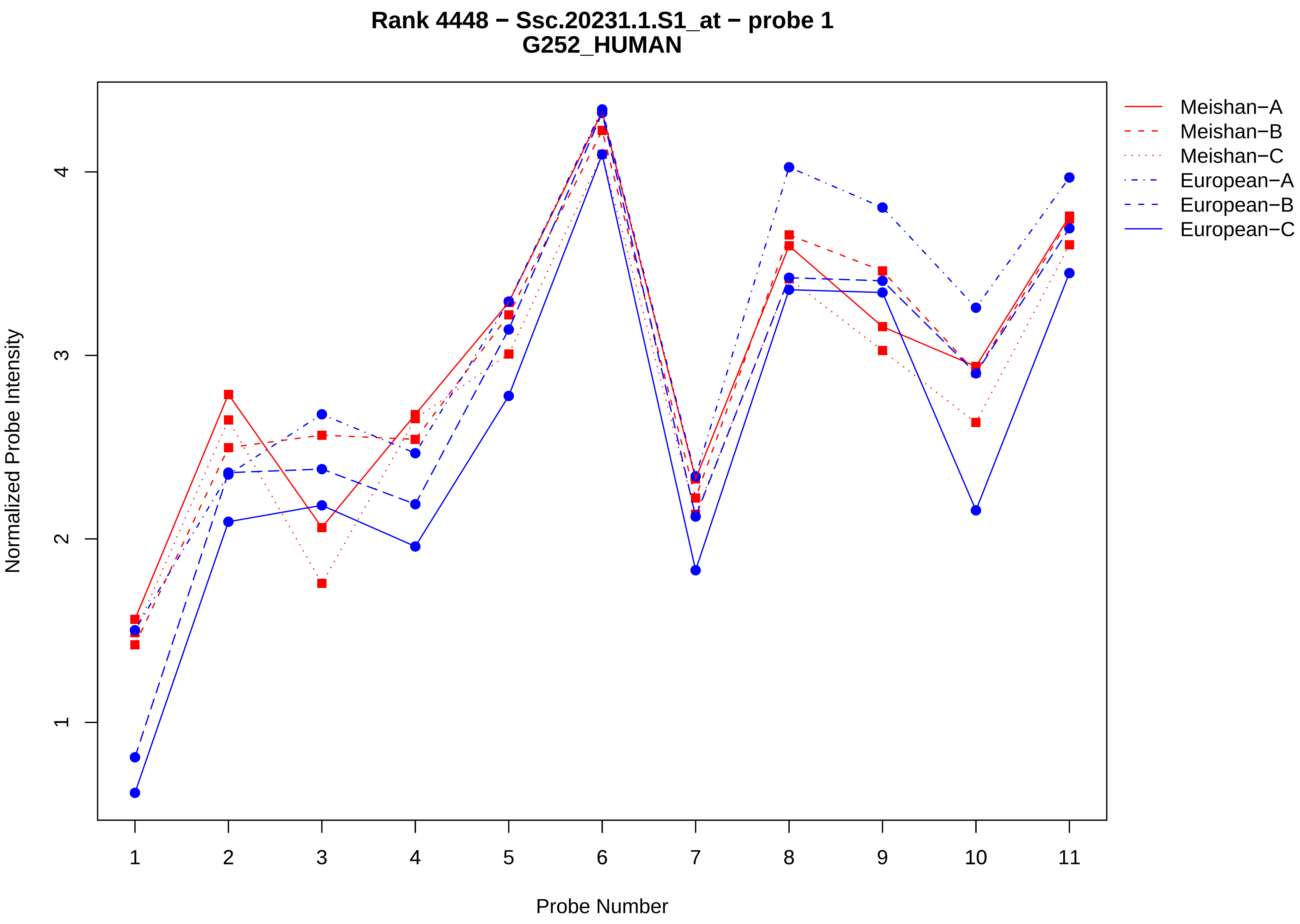

Rank 4448 − Ssc.20231.1.S1_at − probe 1
G252_HUMAN
Normalized Probe Intensity
Probe Number
1
2
3
4
1
2
3
4
5
6
7
8
9
10
11
Meishan−A
Meishan−B
Meishan−C
European−A
European−B
European−C

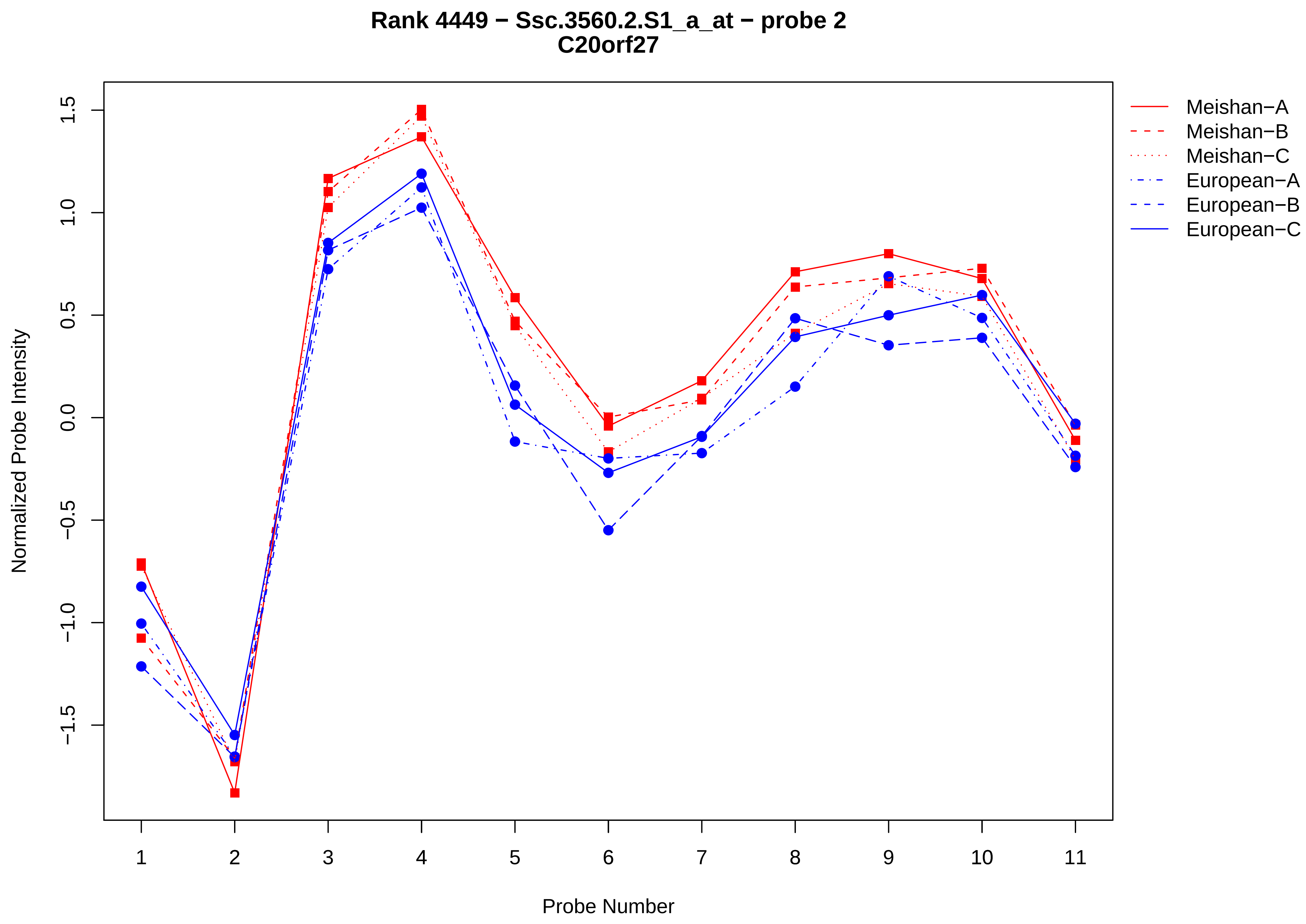

Rank 4449 − Ssc.3560.2.S1_a_at − probe 2
C20orf27
Normalized Probe Intensity
Probe Number
1.5
1.0
0.5
0.0
−0.5
−1.0
−1.5
1
2
3
4
5
6
7
8
9
10
11
Meishan−A
Meishan−B
Meishan−C
European−A
European−B
European−C

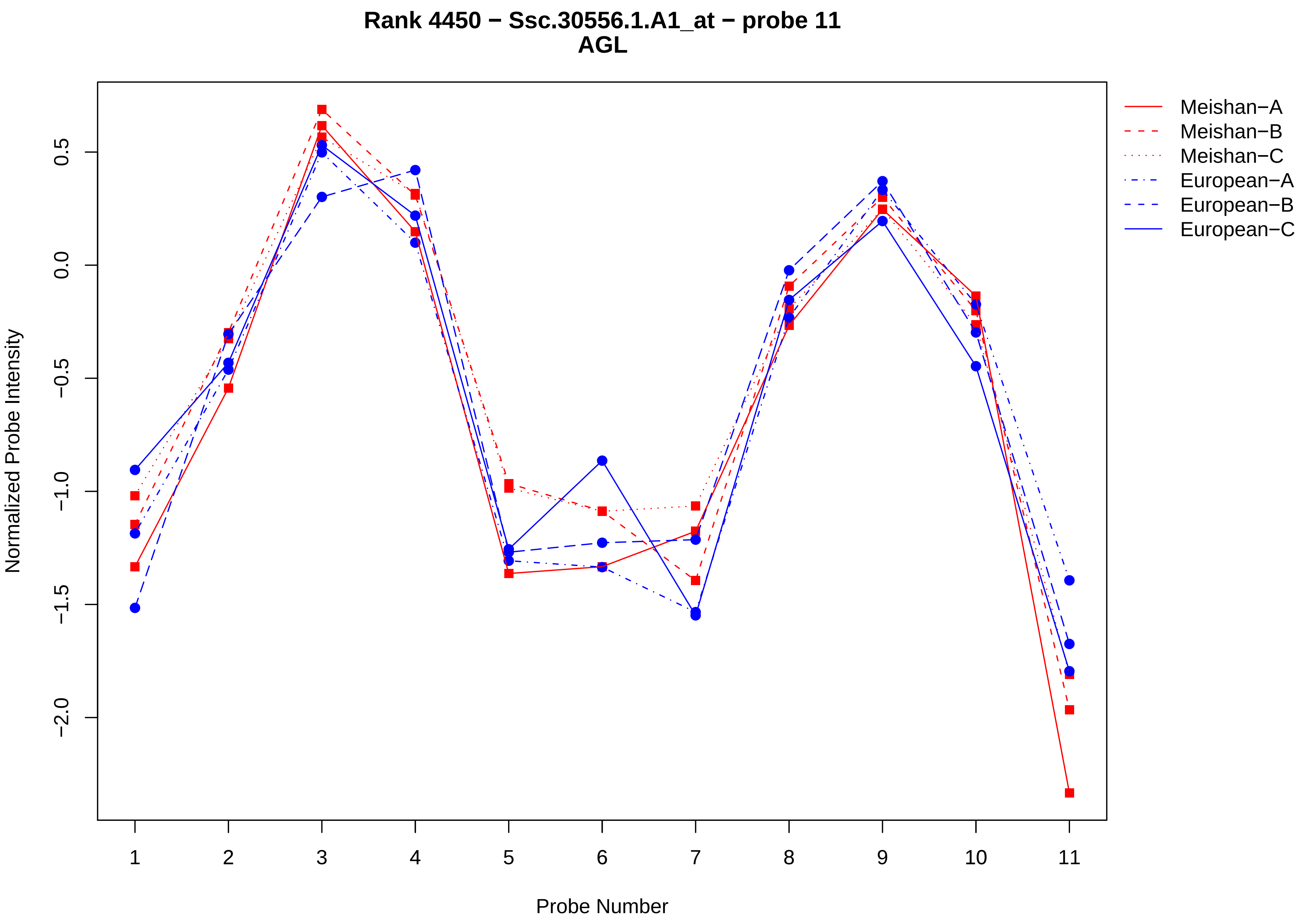
Rank 4450 − Ssc.30556.1.A1_at − probe 11
AGL
Normalized Probe Intensity
Probe Number
0.5
0.0
−0.5
−1.0
−1.5
−2.0
1
2
3
4
5
6
7
8
9
10
11
Meishan−A
Meishan−B
Meishan−C
European−A
European−B
European−C

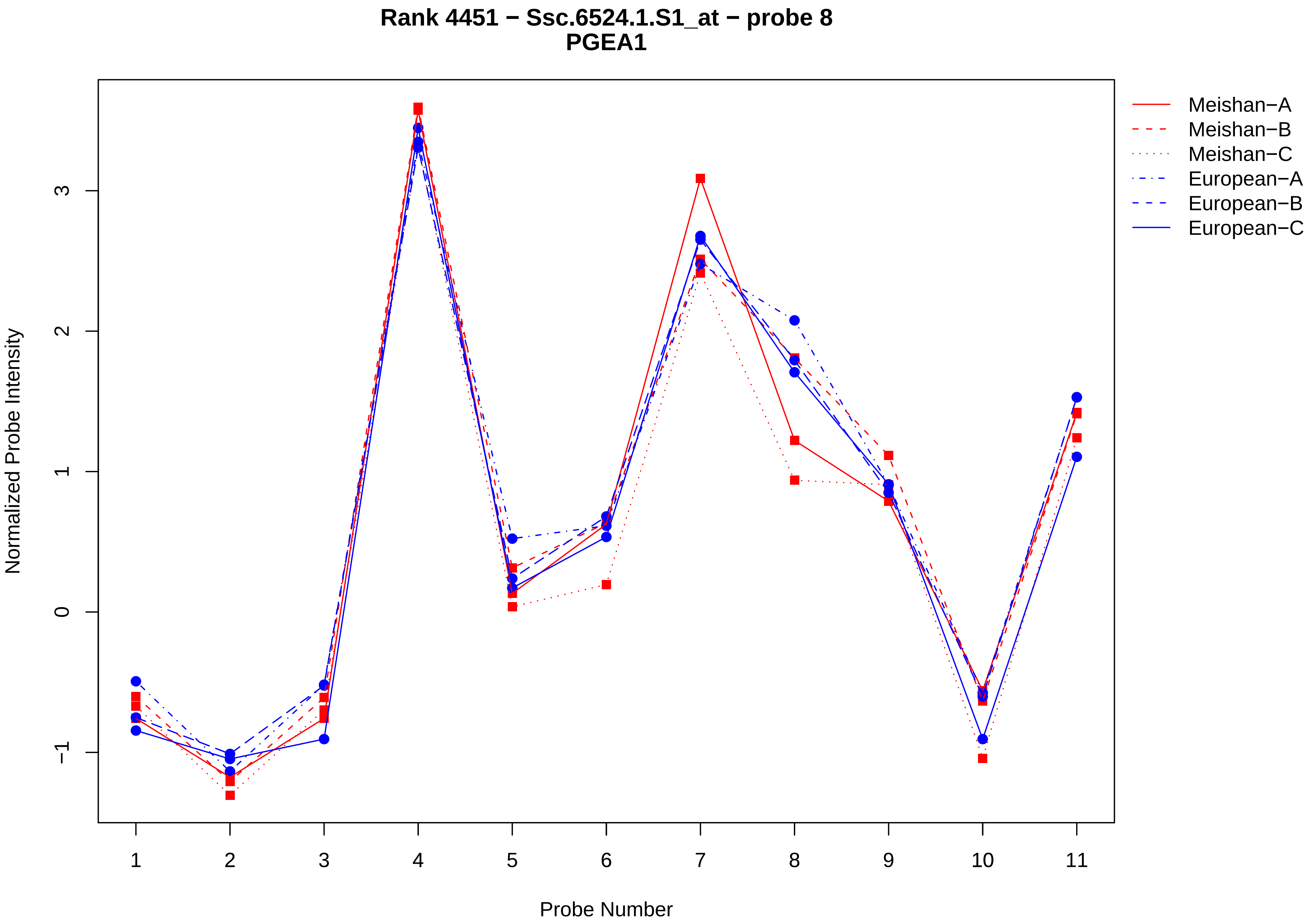

Rank 4451 − Ssc.6524.1.S1_at − probe 8
PGEA1
Normalized Probe Intensity
Probe Number
−1
0
1
2
3
1
2
3
4
5
6
7
8
9
10
11
Meishan−A
Meishan−B
Meishan−C
European−A
European−B
European−C

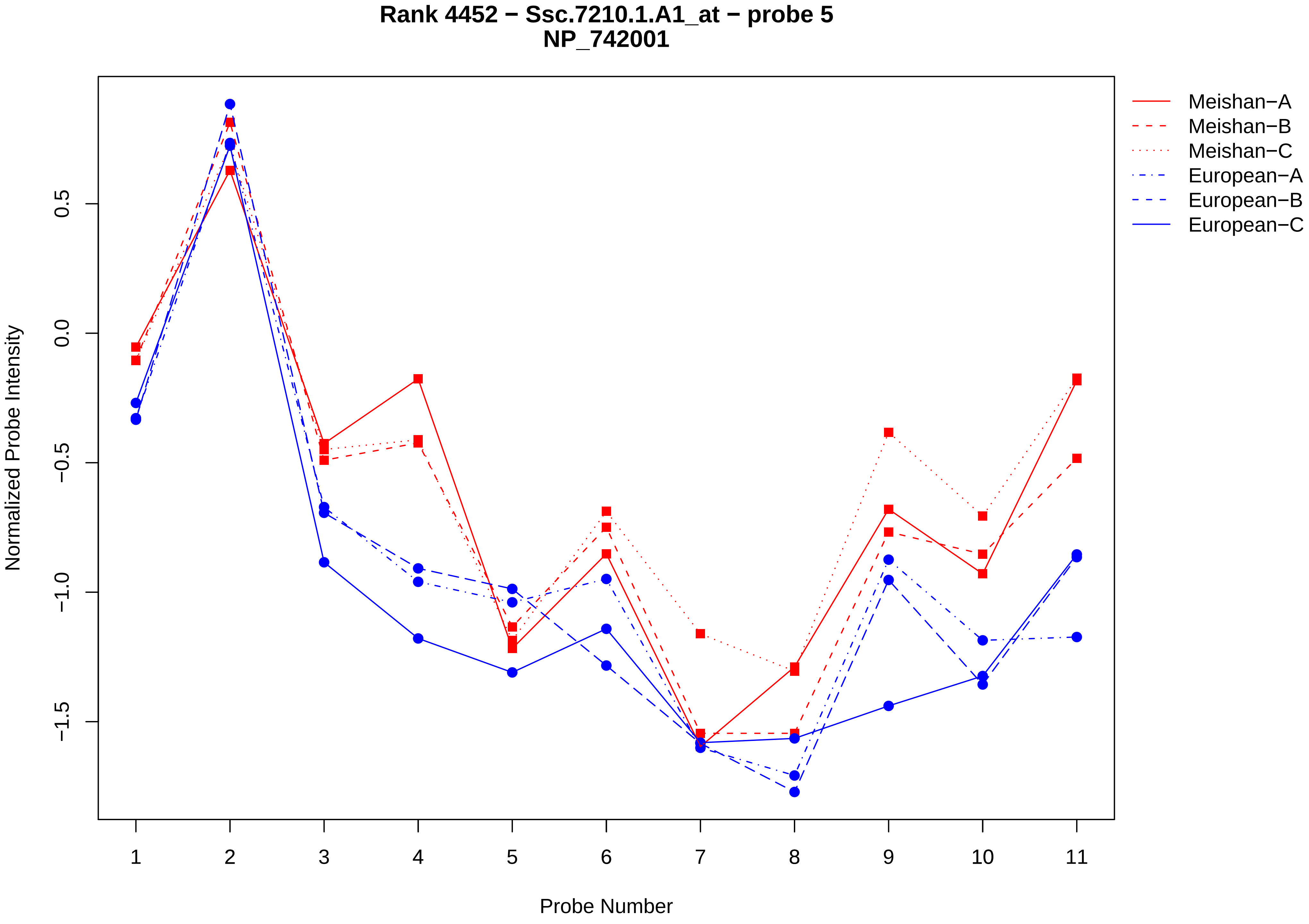

Rank 4452 − Ssc.7210.1.A1_at − probe 5
NP_742001
Normalized Probe Intensity
0.5
0.0
−0.5
−1.0
−1.5
1
2
3
4
5
6
7
8
9
10
11
Probe Number
Meishan−A
Meishan−B
Meishan−C
European−A
European−B
European−C

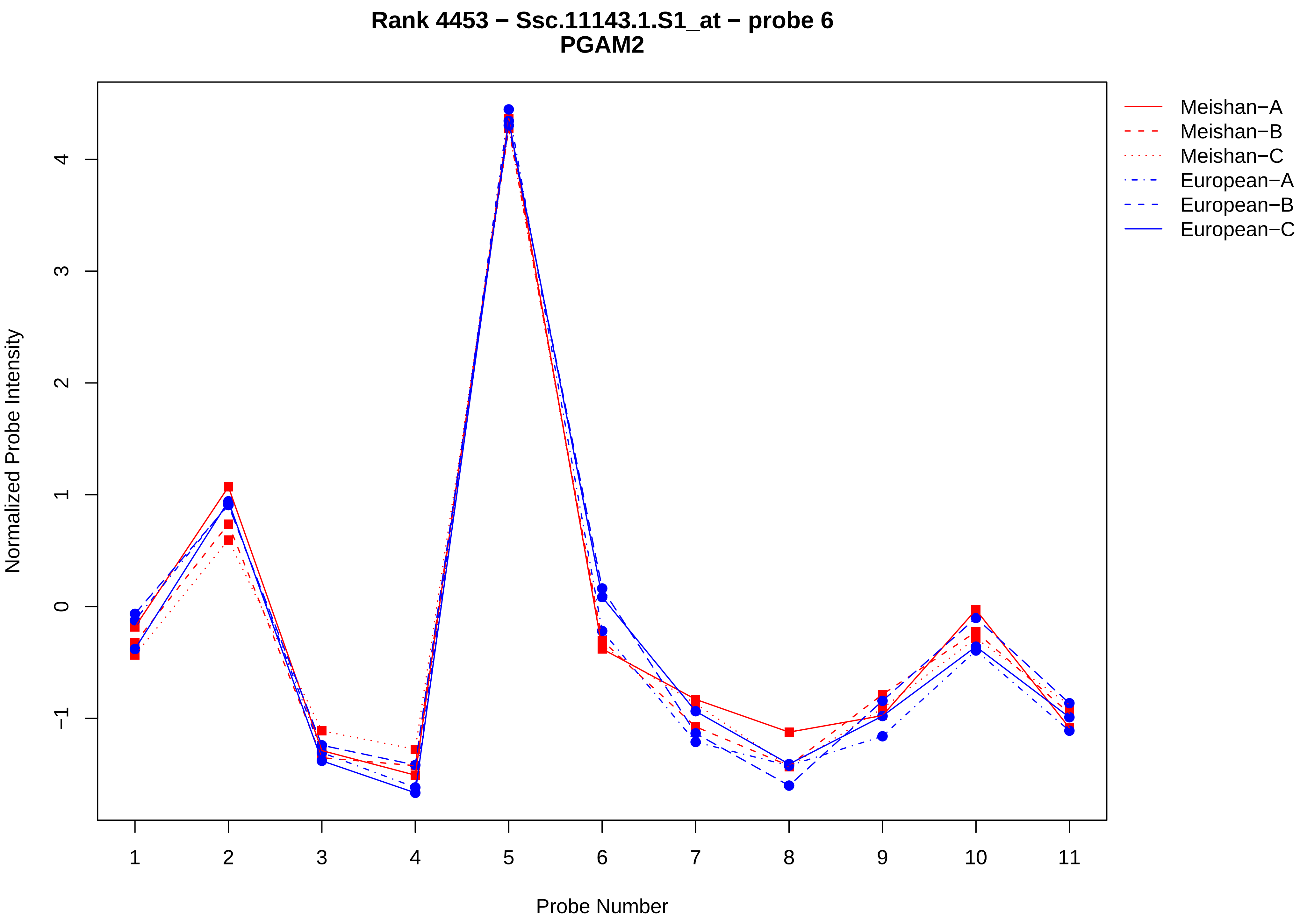

Rank 4453 − Ssc.11143.1.S1_at − probe 6
PGAM2
Normalized Probe Intensity
Probe Number
Meishan−A
Meishan−B
Meishan−C
European−A
European−B
European−C

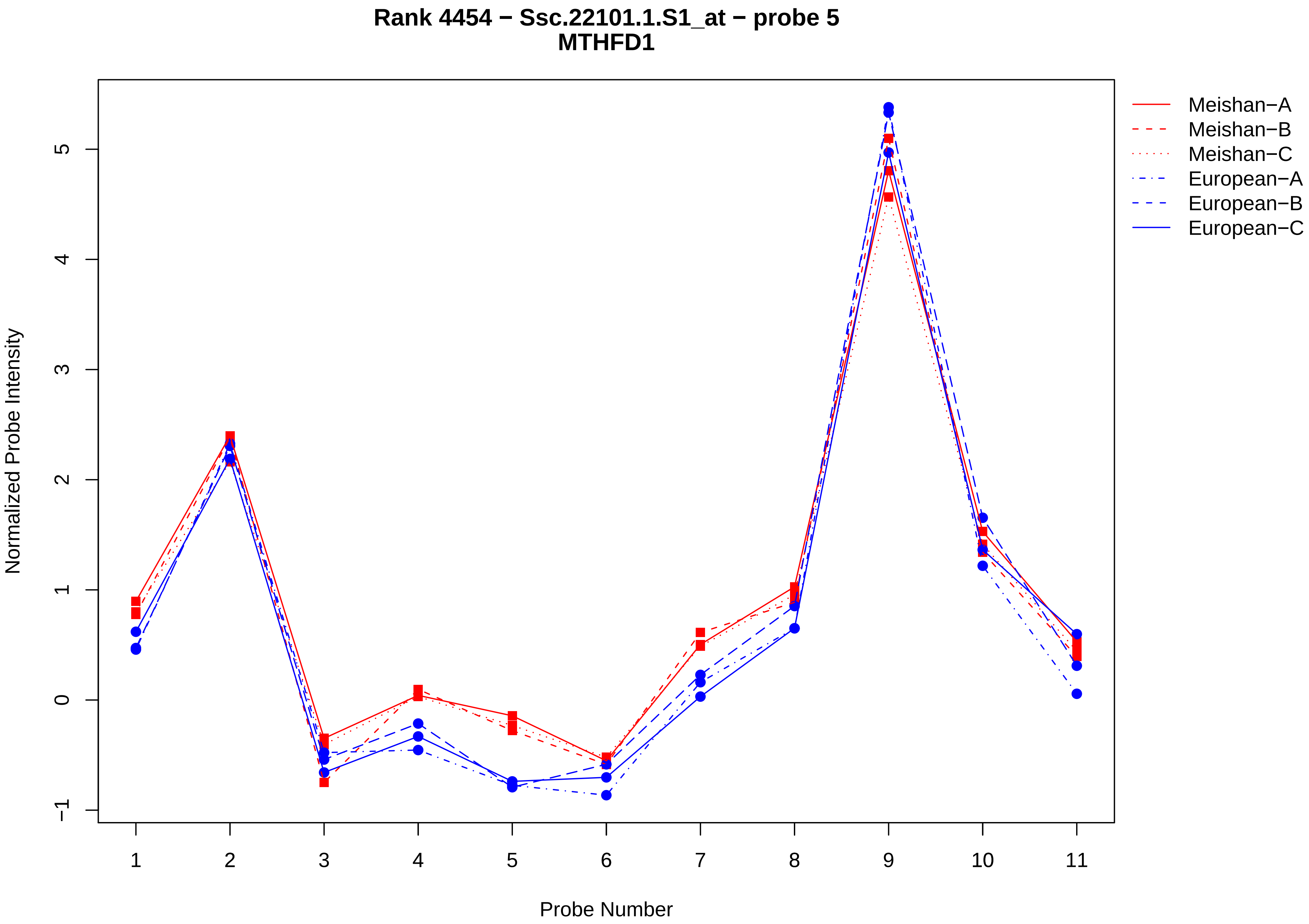

Rank 4454 − Ssc.22101.1.S1_at − probe 5
MTHFD1
Normalized Probe Intensity
Probe Number
−1
0
1
2
3
4
5
1
2
3
4
5
6
7
8
9
10
11
Meishan−A
Meishan−B
Meishan−C
European−A
European−B
European−C

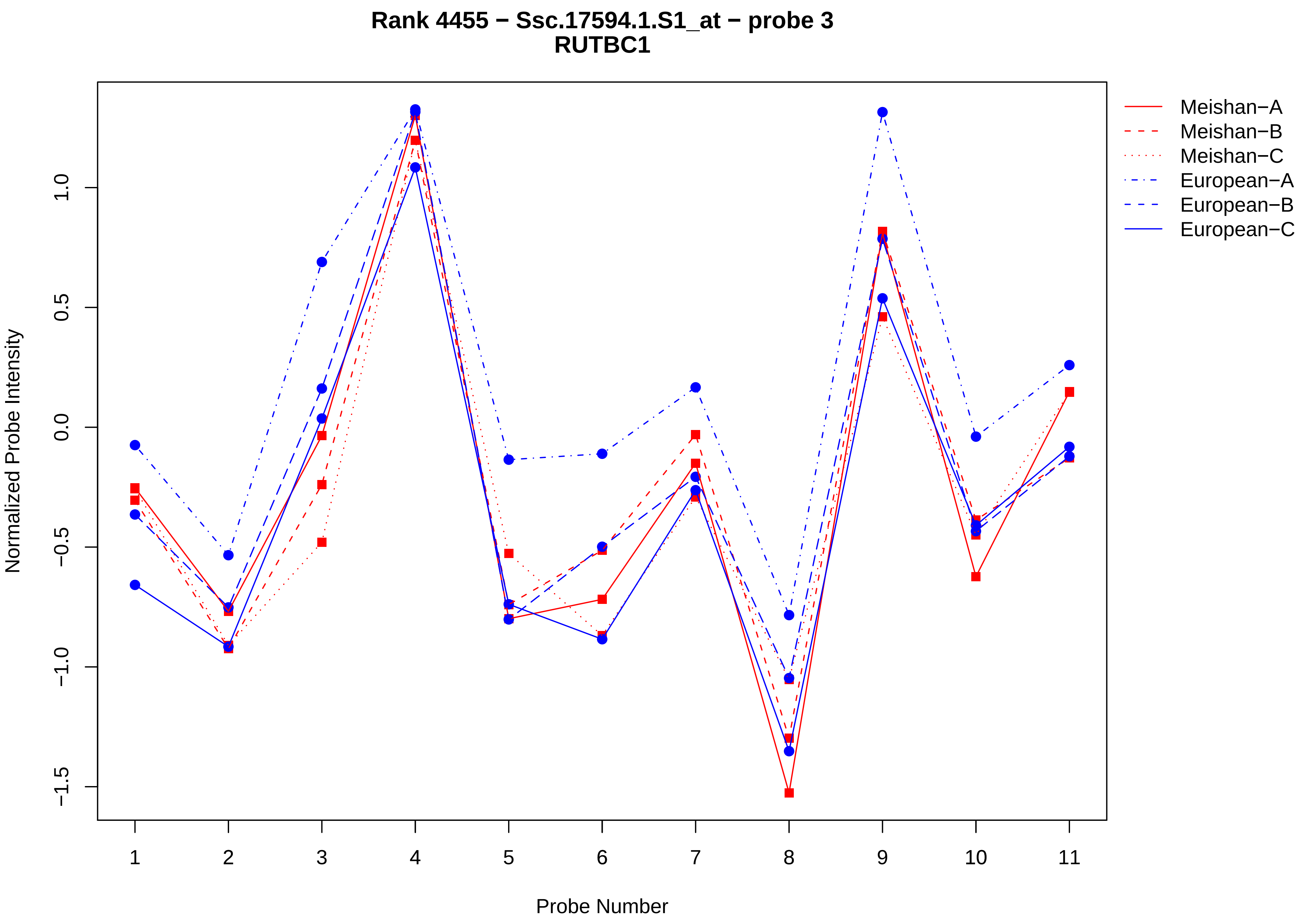

Rank 4455 − Ssc.17594.1.S1_at − probe 3
RUTBC1
Normalized Probe Intensity
−1.5
−1.0
−0.5
0.0
0.5
1.0
1
2
3
4
5
6
7
8
9
10
11
Probe Number
Meishan−A
Meishan−B
Meishan−C
European−A
European−B
European−C

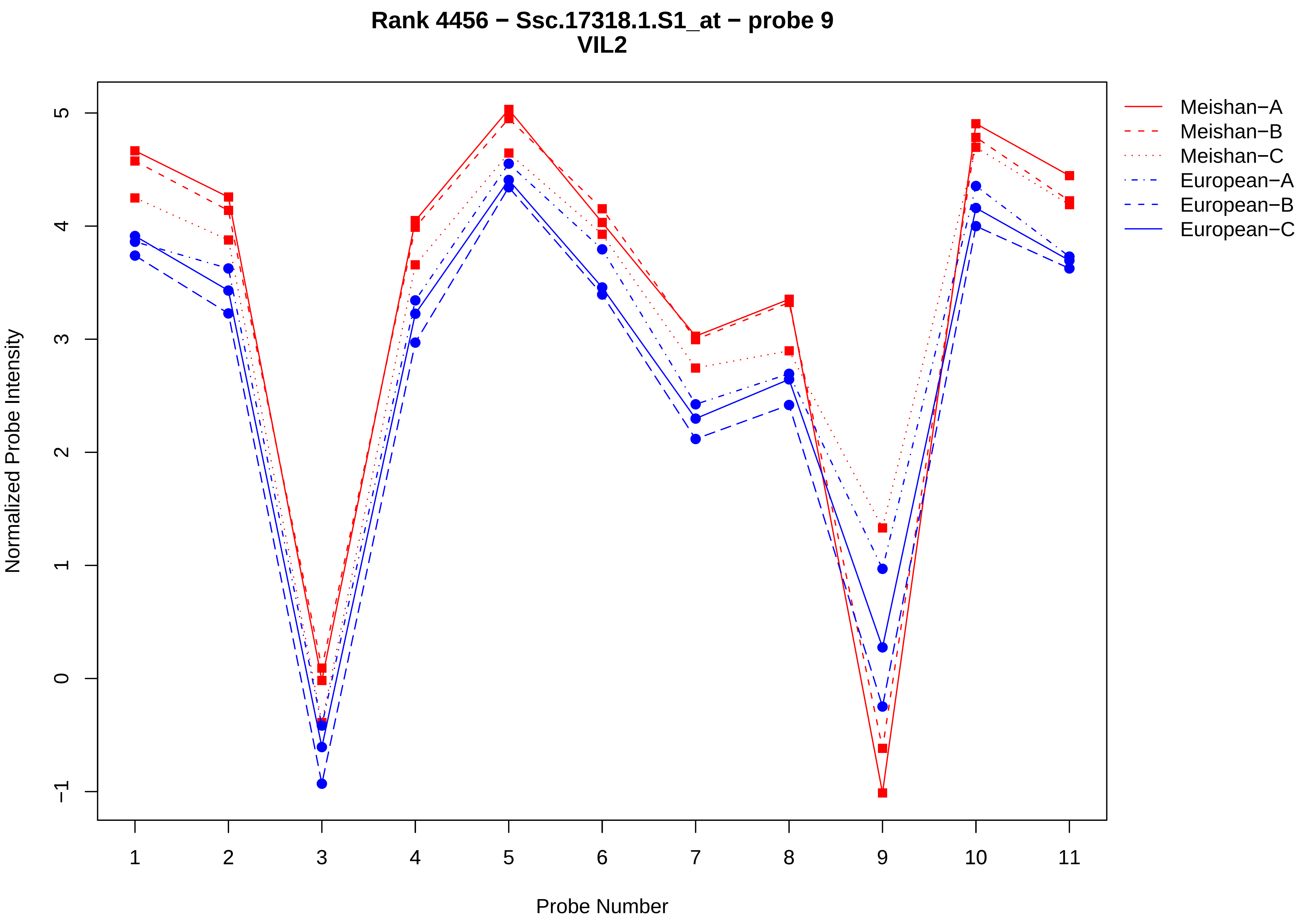

Rank 4456 − Ssc.17318.1.S1_at − probe 9
VIL2
Normalized Probe Intensity
Probe Number
−1
0
1
2
3
4
5
1
2
3
4
5
6
7
8
9
10
11
Meishan−A
Meishan−B
Meishan−C
European−A
European−B
European−C

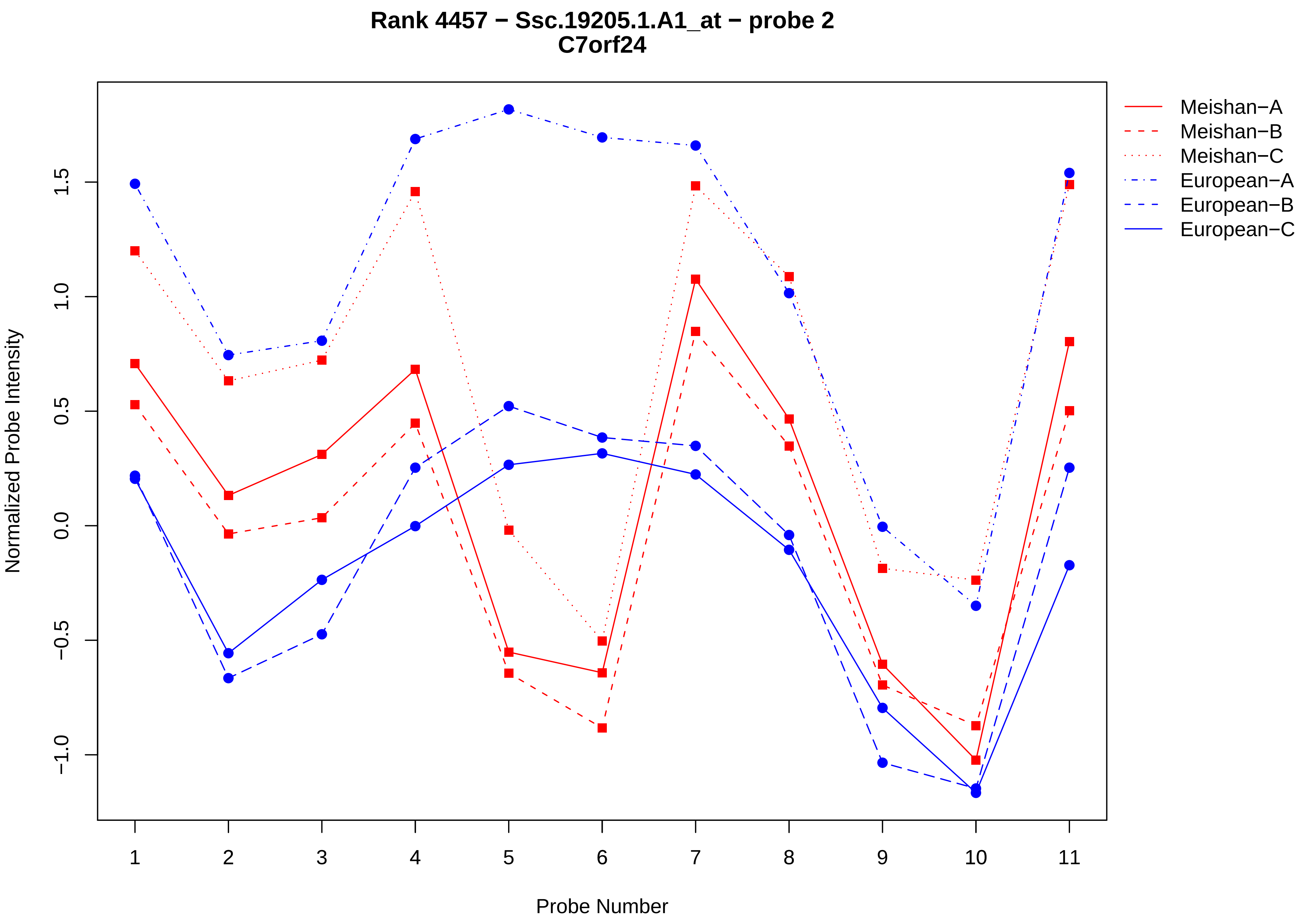

Rank 4457 − Ssc.19205.1.A1_at − probe 2
C7orf24
Normalized Probe Intensity
Probe Number
−1.0
−0.5
0.0
0.5
1.0
1.5
1
2
3
4
5
6
7
8
9
10
11
Meishan−A
Meishan−B
Meishan−C
European−A
European−B
European−C

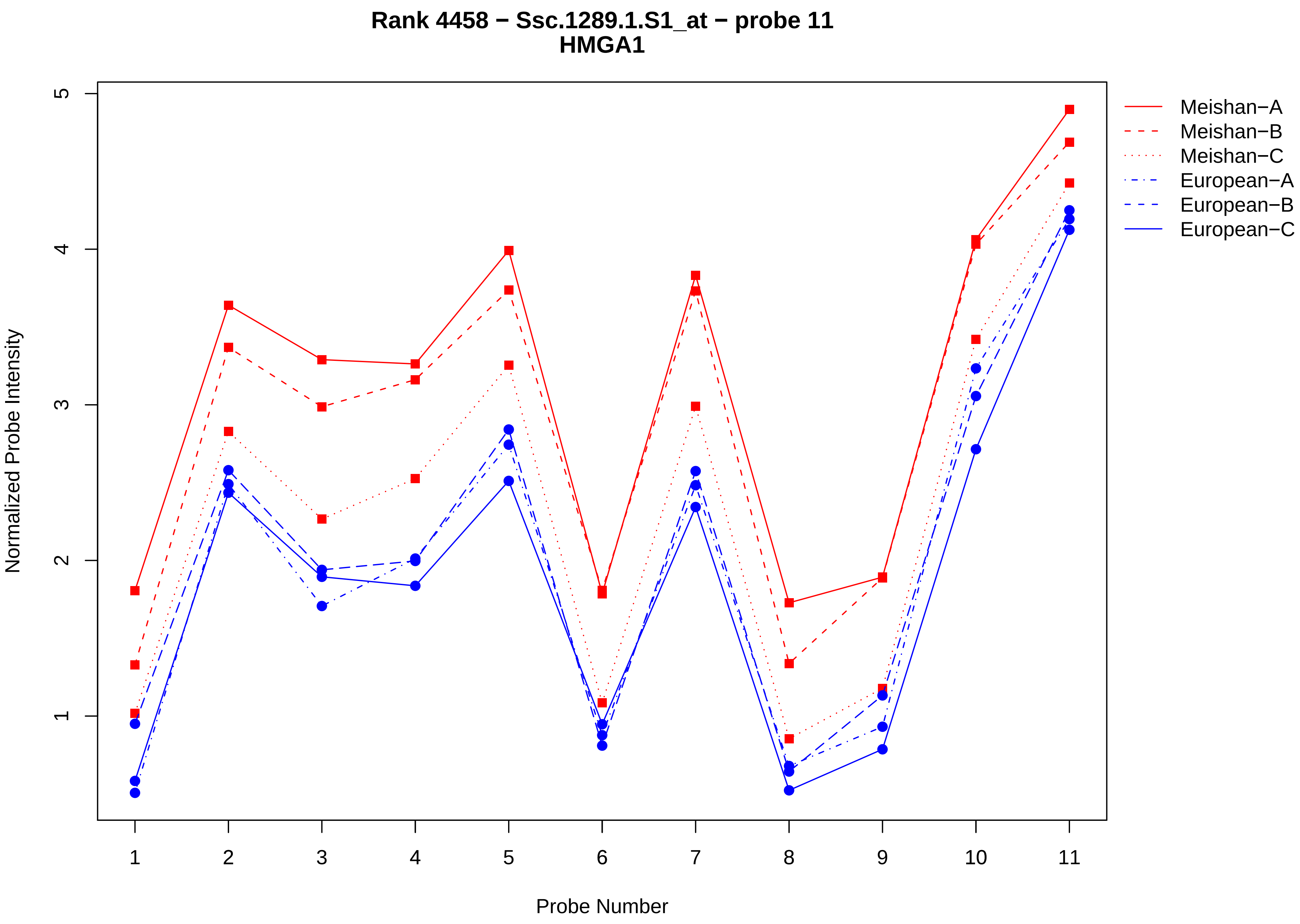

Rank 4458 − Ssc.1289.1.S1_at − probe 11
HMGA1
Normalized Probe Intensity
Probe Number
1
2
3
4
5
1
2
3
4
5
6
7
8
9
10
11
Meishan−A
Meishan−B
Meishan−C
European−A
European−B
European−C

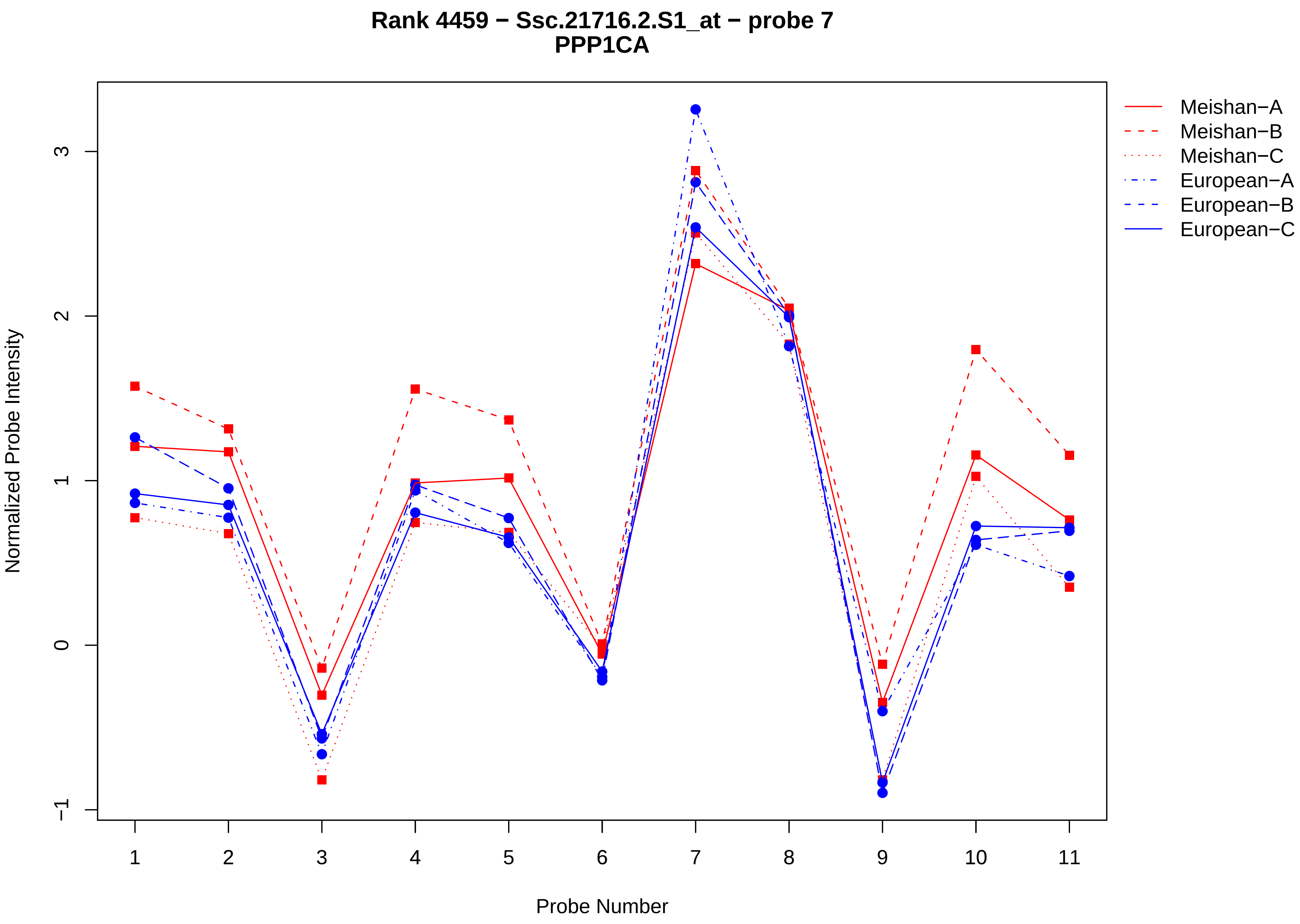

Rank 4459 − Ssc.21716.2.S1_at − probe 7
PPP1CA
Normalized Probe Intensity
−1
0
1
2
3
1
2
3
4
5
6
7
8
9
10
11
Probe Number
Meishan−A
Meishan−B
Meishan−C
European−A
European−B
European−C

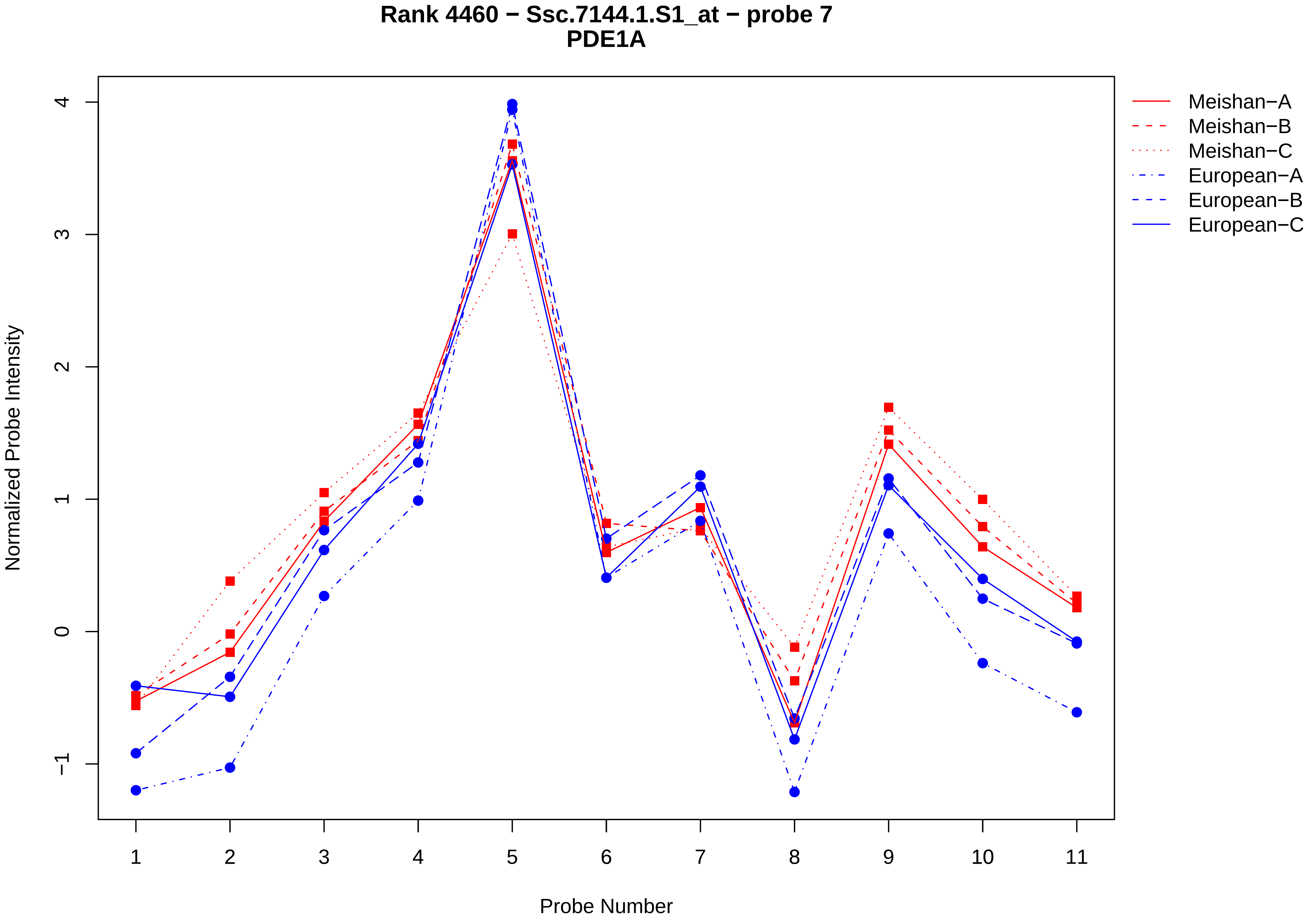

Rank 4460 − Ssc.7144.1.S1_at − probe 7
PDE1A
Normalized Probe Intensity
Probe Number
Meishan−A
Meishan−B
Meishan−C
European−A
European−B
European−C

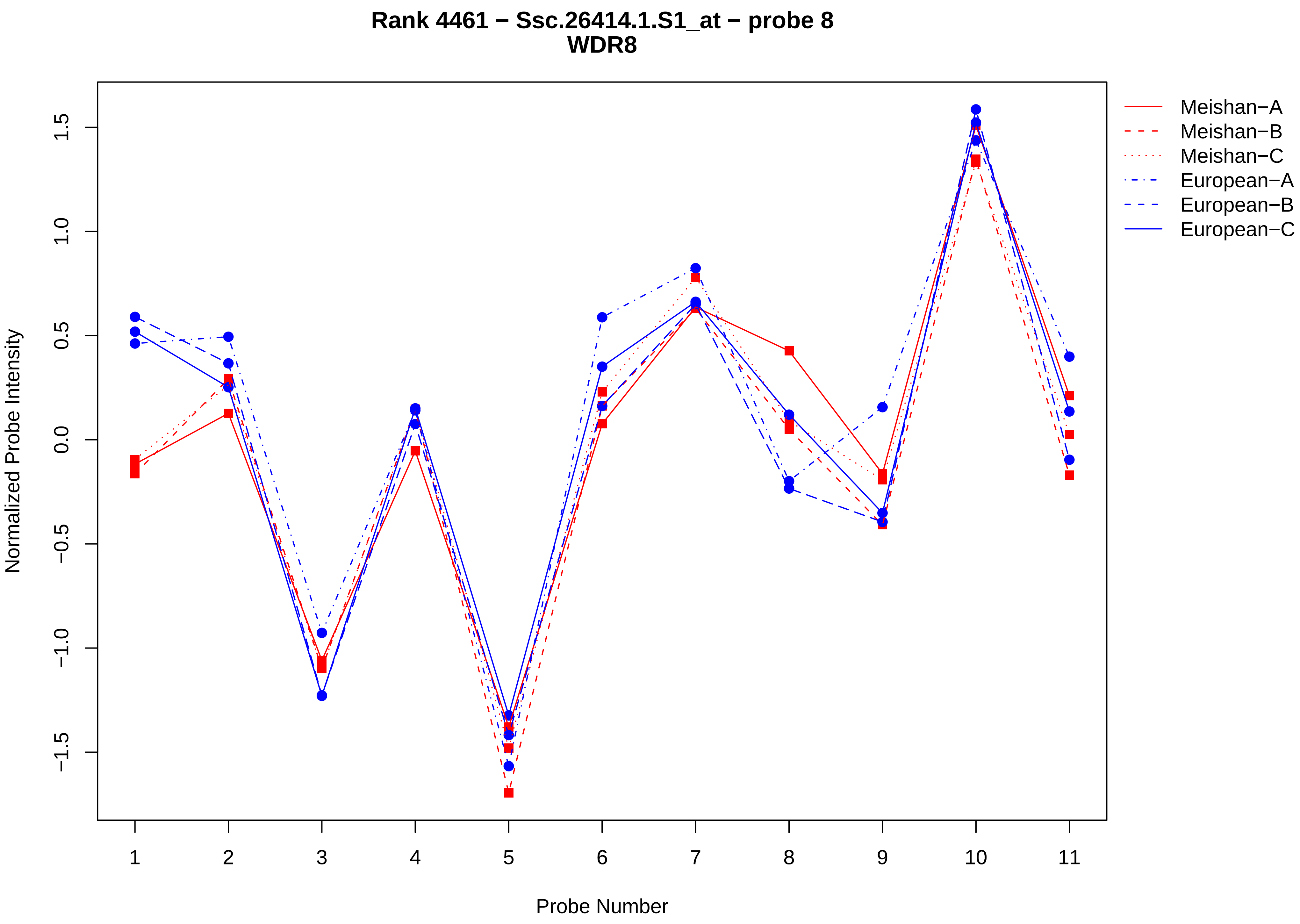

Rank 4461 − Ssc.26414.1.S1_at − probe 8
WDR8
Normalized Probe Intensity
Probe Number
1.5
1.0
0.5
0.0
−0.5
−1.0
−1.5
1
2
3
4
5
6
7
8
9
10
11
Meishan−A
Meishan−B
Meishan−C
European−A
European−B
European−C

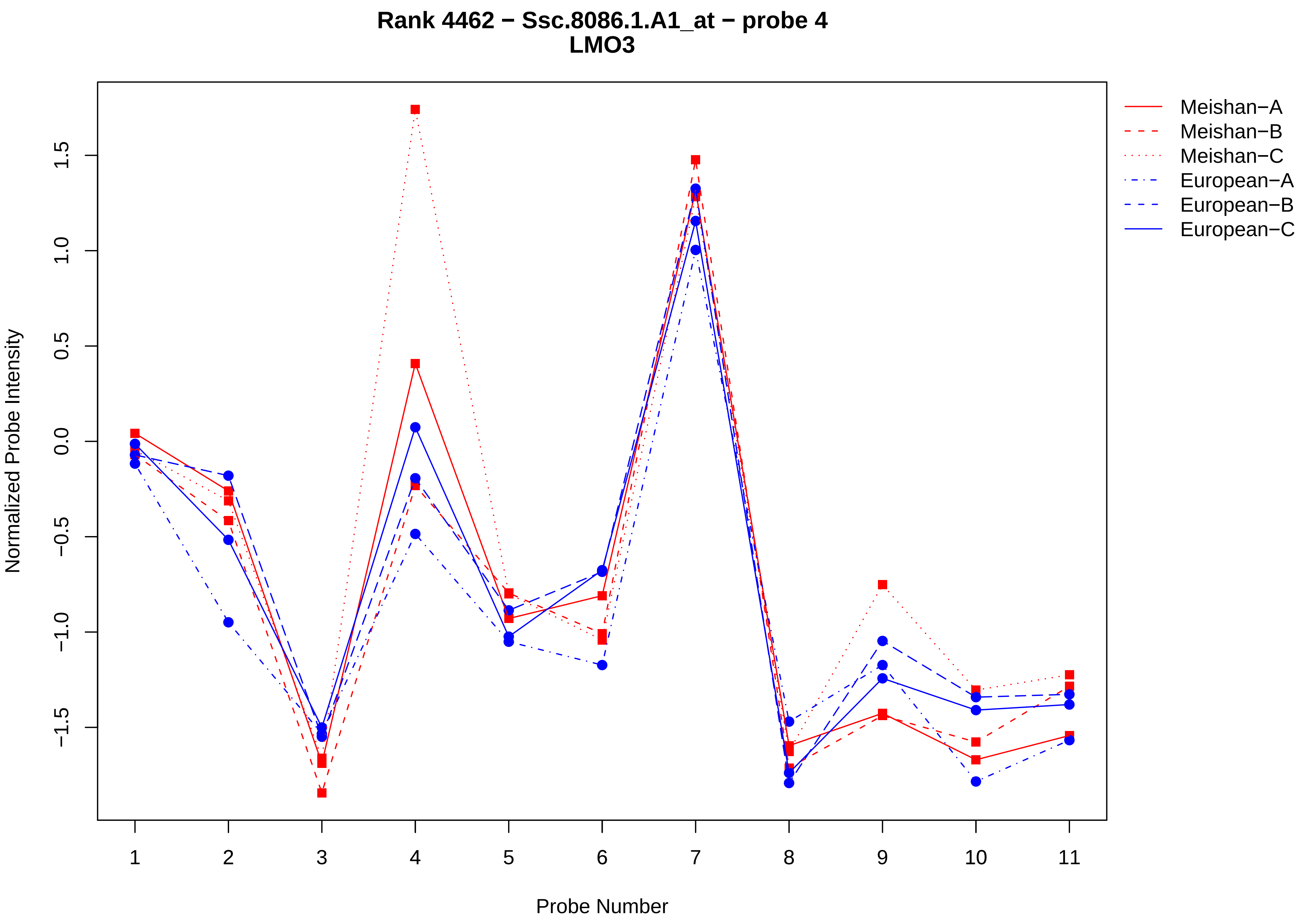

Rank 4462 − Ssc.8086.1.A1_at − probe 4
LMO3
Normalized Probe Intensity
Probe Number
1.5
1.0
0.5
0.0
−0.5
−1.0
−1.5
1
2
3
4
5
6
7
8
9
10
11
Meishan−A
Meishan−B
Meishan−C
European−A
European−B
European−C

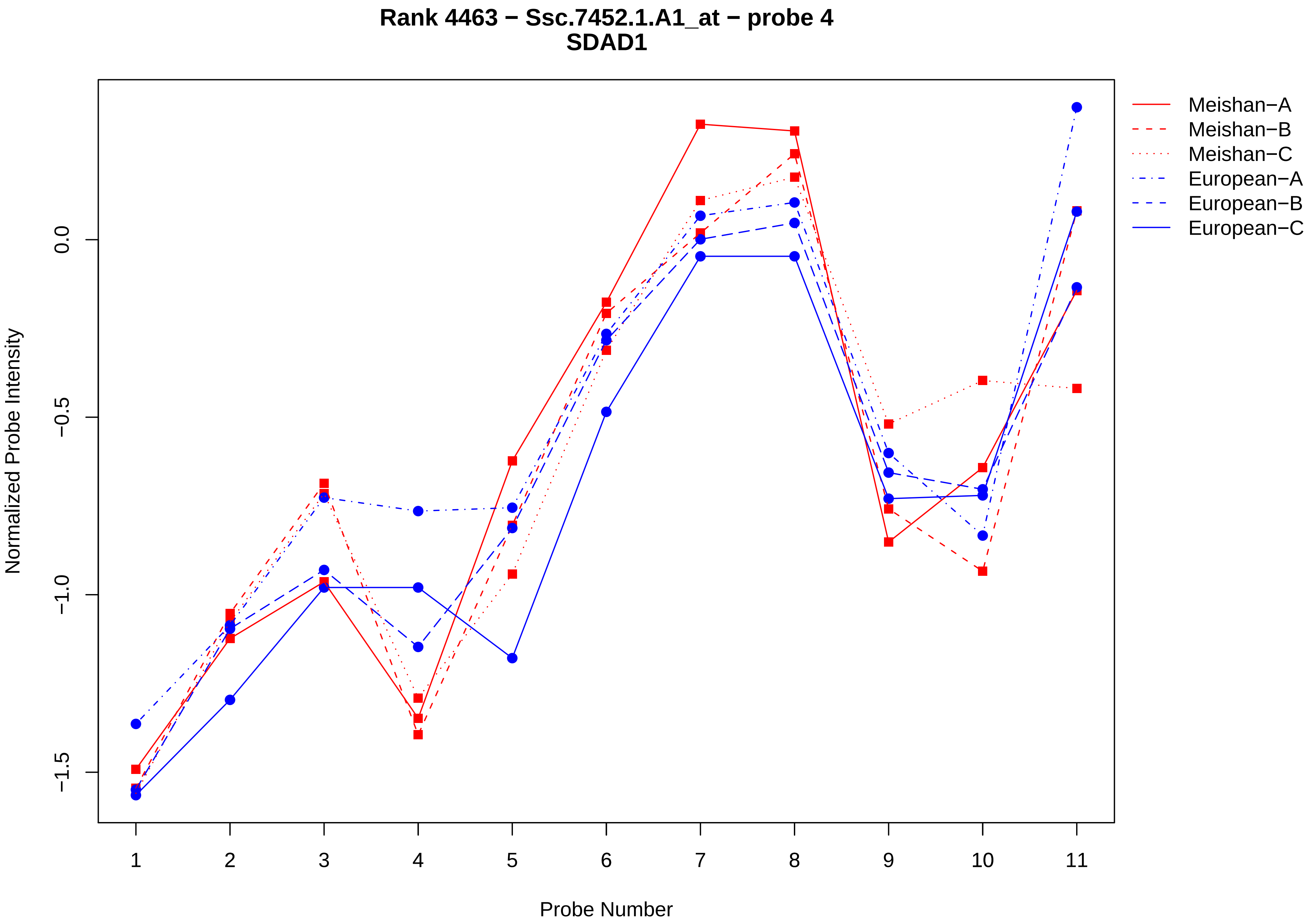

Rank 4463 − Ssc.7452.1.A1_at − probe 4
SDAD1
Normalized Probe Intensity
Probe Number
0.0
−0.5
−1.0
−1.5
1
2
3
4
5
6
7
8
9
10
11
Meishan−A
Meishan−B
Meishan−C
European−A
European−B
European−C

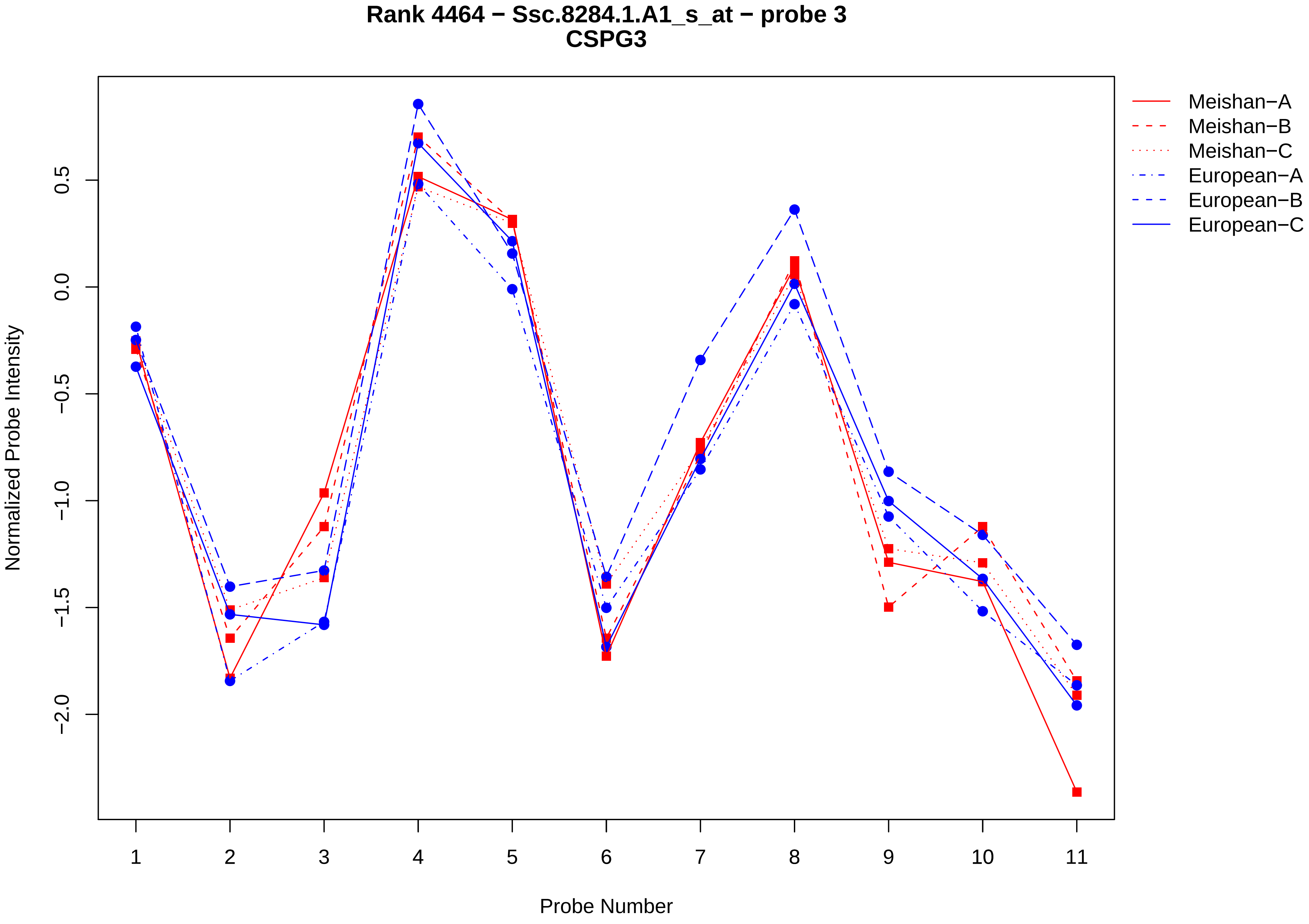

Rank 4464 − Ssc.8284.1.A1_s_at − probe 3
CSPG3
Normalized Probe Intensity
Probe Number
Meishan−A
Meishan−B
Meishan−C
European−A
European−B
European−C
0.5
0.0
−0.5
−1.0
−1.5
−2.0
1
2
3
4
5
6
7
8
9
10
11

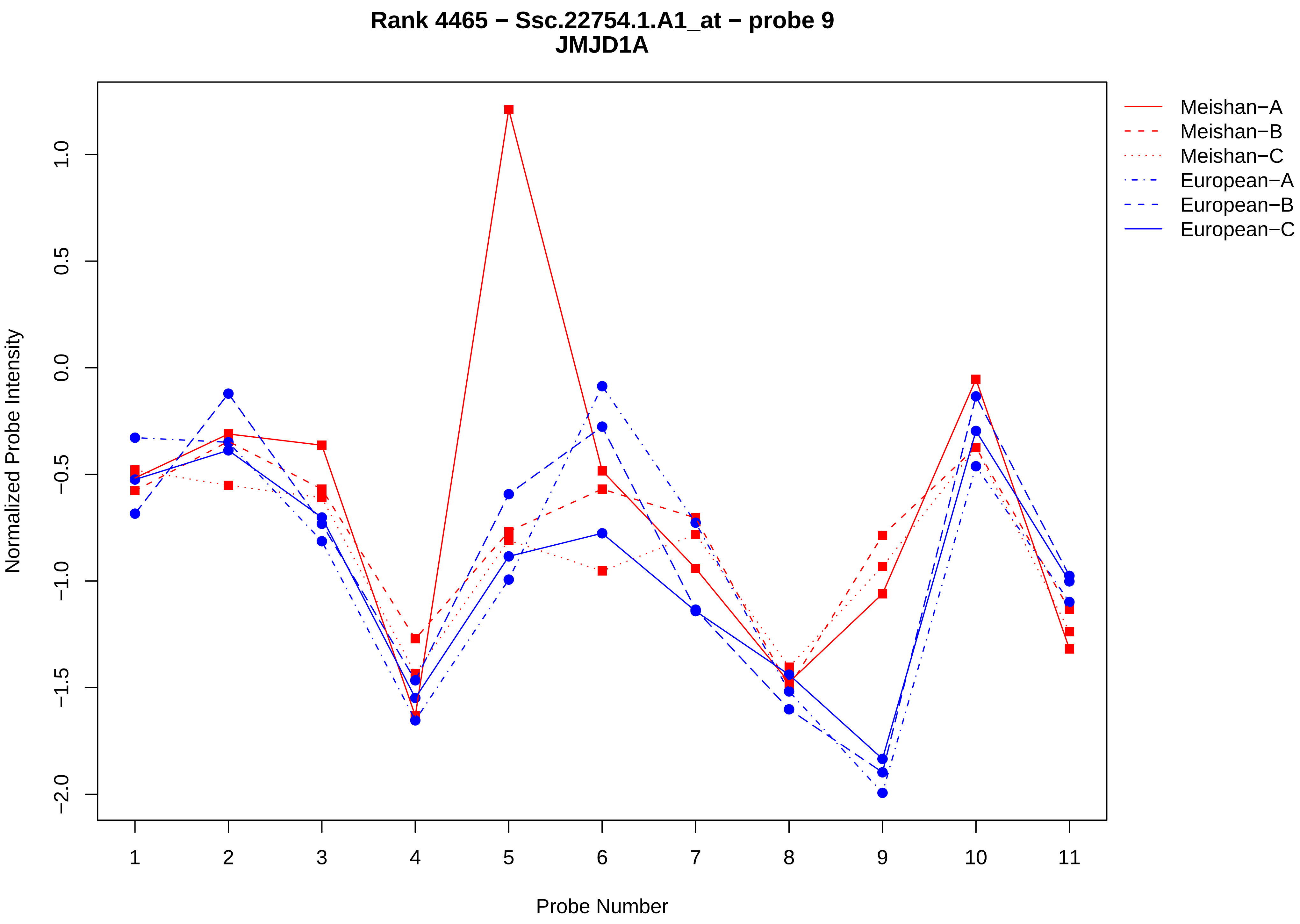

Rank 4465 − Ssc.22754.1.A1_at − probe 9
JMJD1A
Normalized Probe Intensity
Probe Number
1.0
0.5
0.0
−0.5
−1.0
−1.5
−2.0
1
2
3
4
5
6
7
8
9
10
11
Meishan−A
Meishan−B
Meishan−C
European−A
European−B
European−C

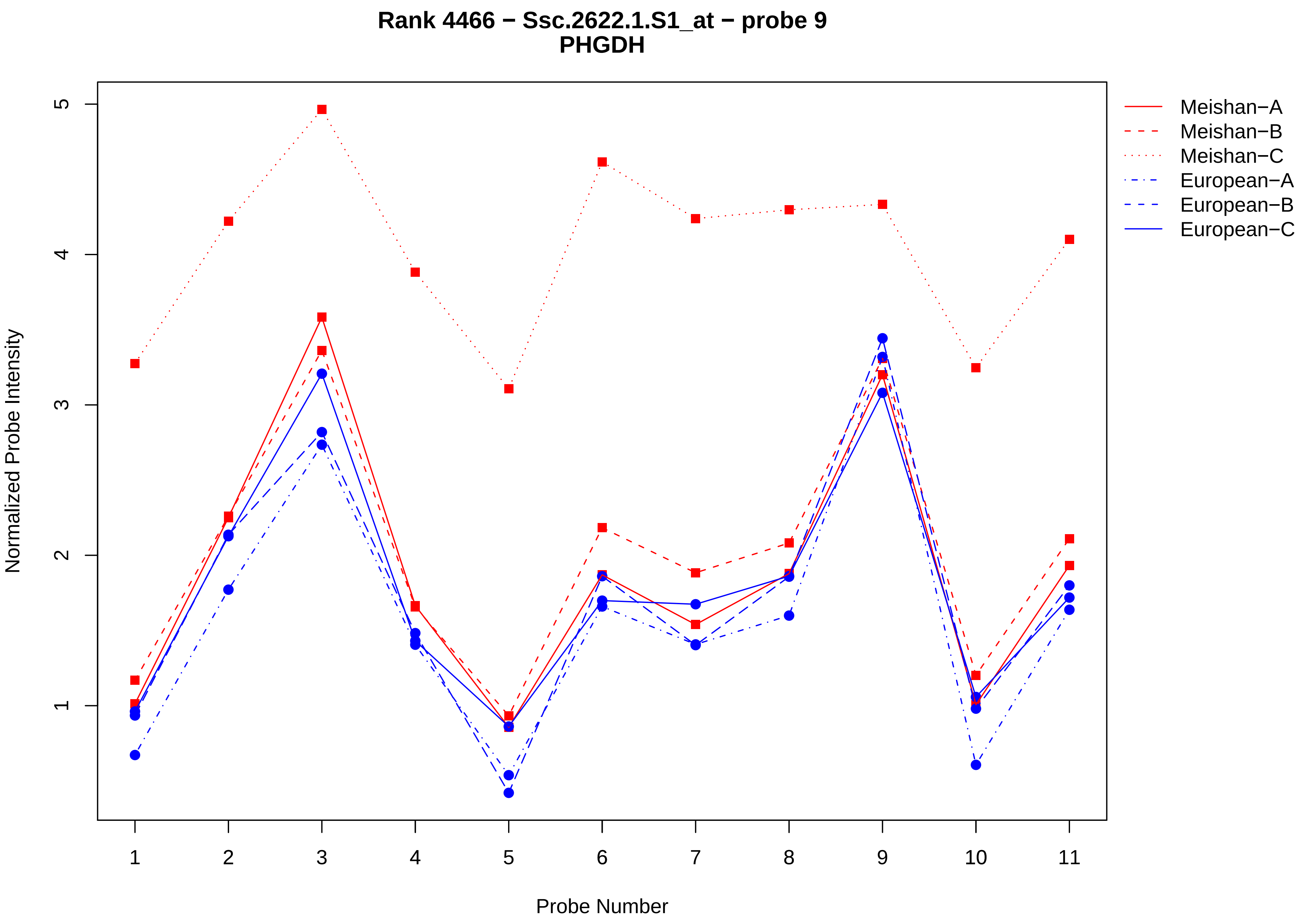

Rank 4466 − Ssc.2622.1.S1_at − probe 9
PHGDH
Normalized Probe Intensity
Probe Number
1
2
3
4
5
1
2
3
4
5
6
7
8
9
10
11
Meishan−A
Meishan−B
Meishan−C
European−A
European−B
European−C

Rank 4467 − AFFX−Ssc−r2−Ec−bioB−3_at − probe 2
bioB

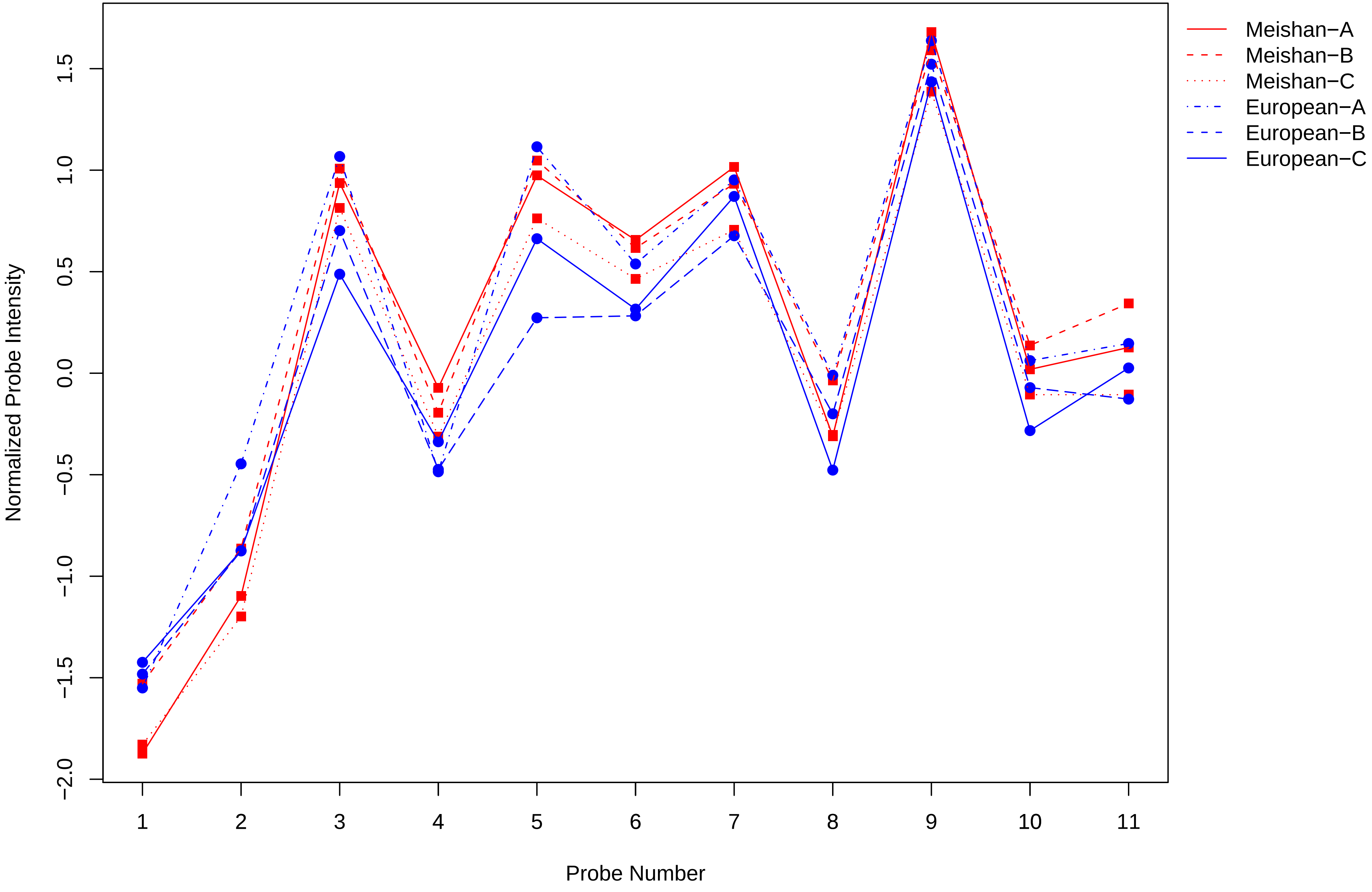

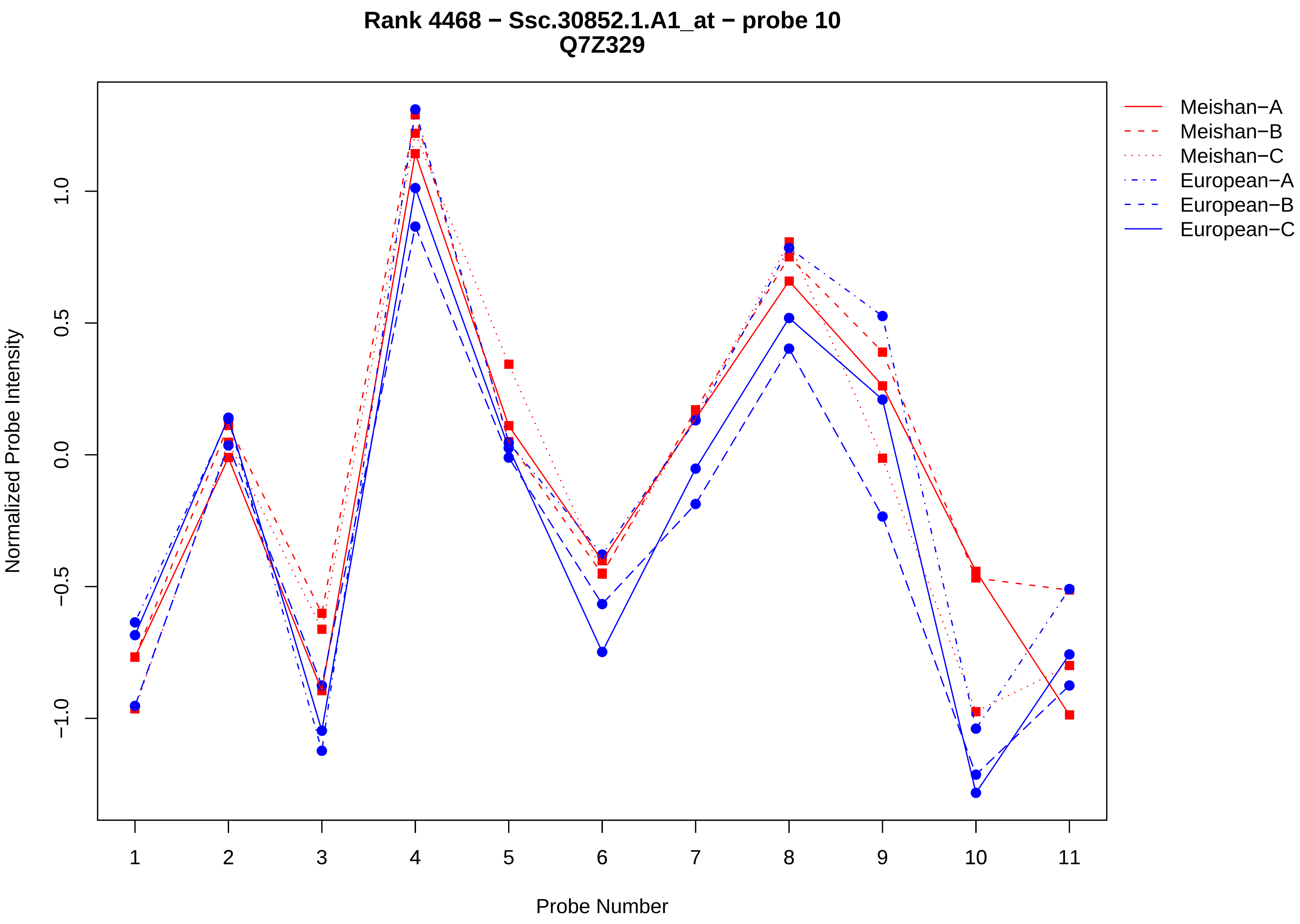

Rank 4468 − Ssc.30852.1.A1_at − probe 10
Q7Z329
Normalized Probe Intensity
Probe Number
−1.0
−0.5
0.0
0.5
1.0
1
2
3
4
5
6
7
8
9
10
11
Meishan−A
Meishan−B
Meishan−C
European−A
European−B
European−C

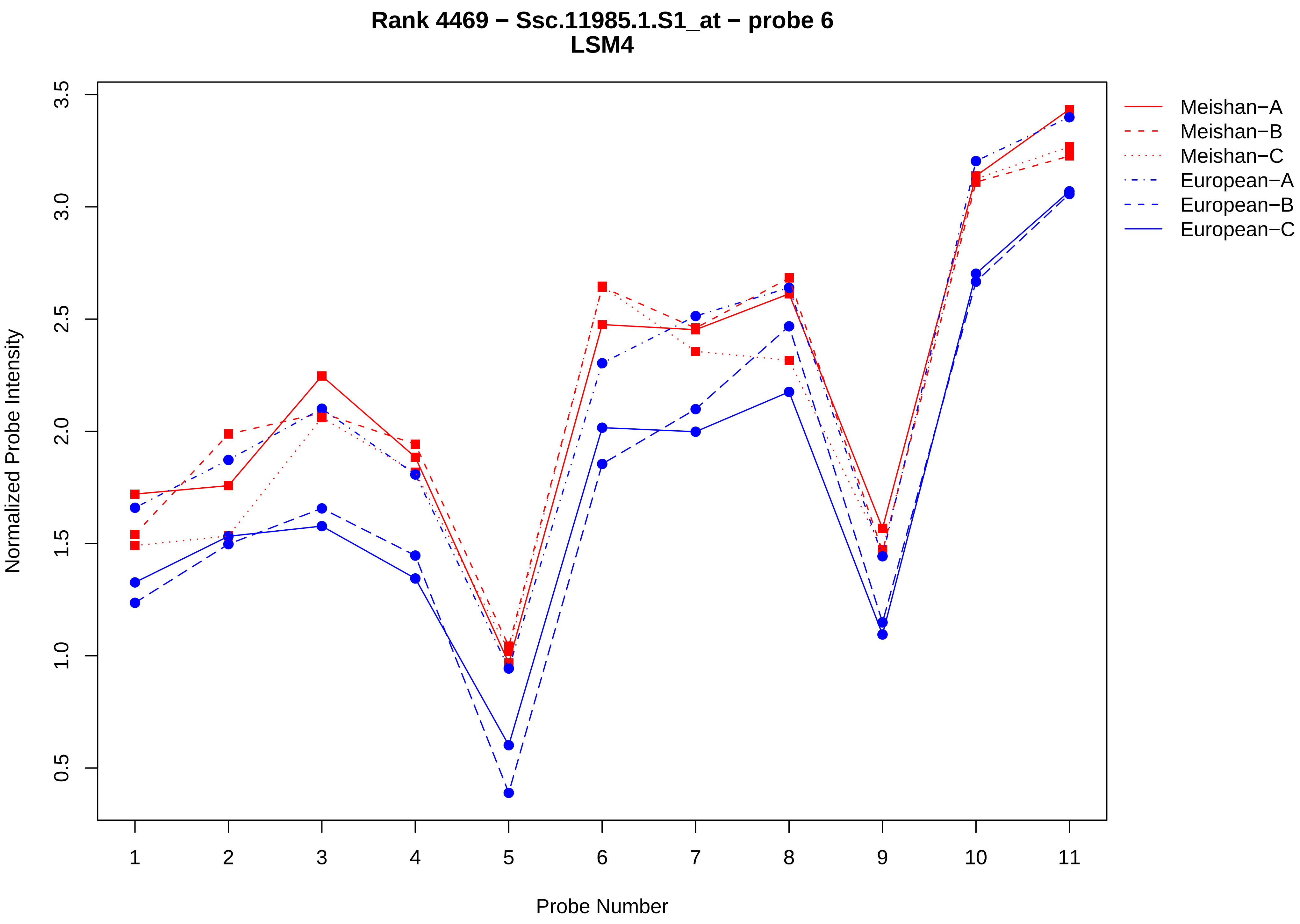

Rank 4469 − Ssc.11985.1.S1_at − probe 6
LSM4
Normalized Probe Intensity
0.5
1.0
1.5
2.0
2.5
3.0
3.5
1
2
3
4
5
6
7
8
9
10
11
Probe Number
Meishan−A
Meishan−B
Meishan−C
European−A
European−B
European−C

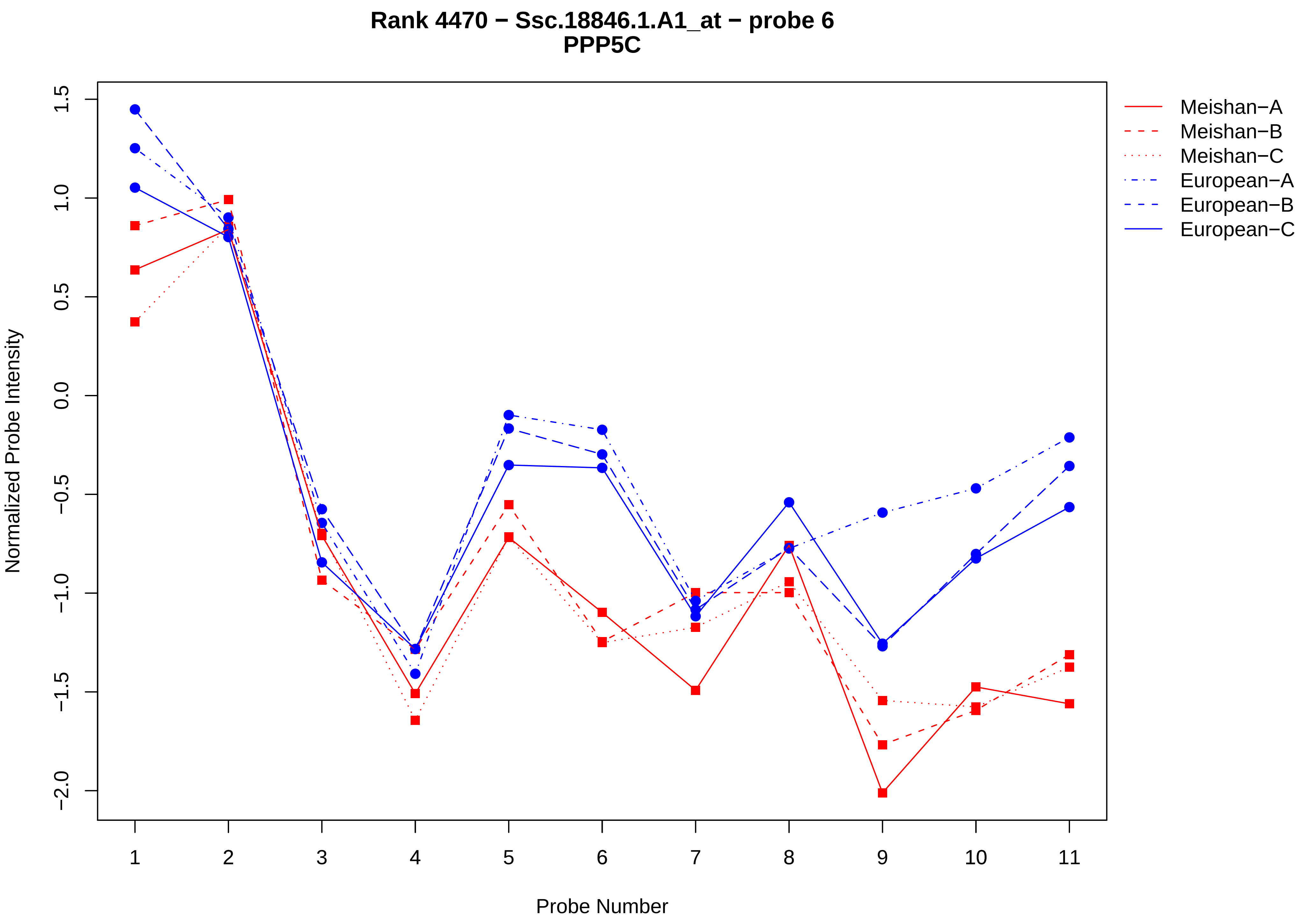

Rank 4470 − Ssc.18846.1.A1_at − probe 6
PPP5C
Normalized Probe Intensity
Probe Number
1.5
1.0
0.5
0.0
−0.5
−1.0
−1.5
−2.0
1
2
3
4
5
6
7
8
9
10
11
Meishan−A
Meishan−B
Meishan−C
European−A
European−B
European−C

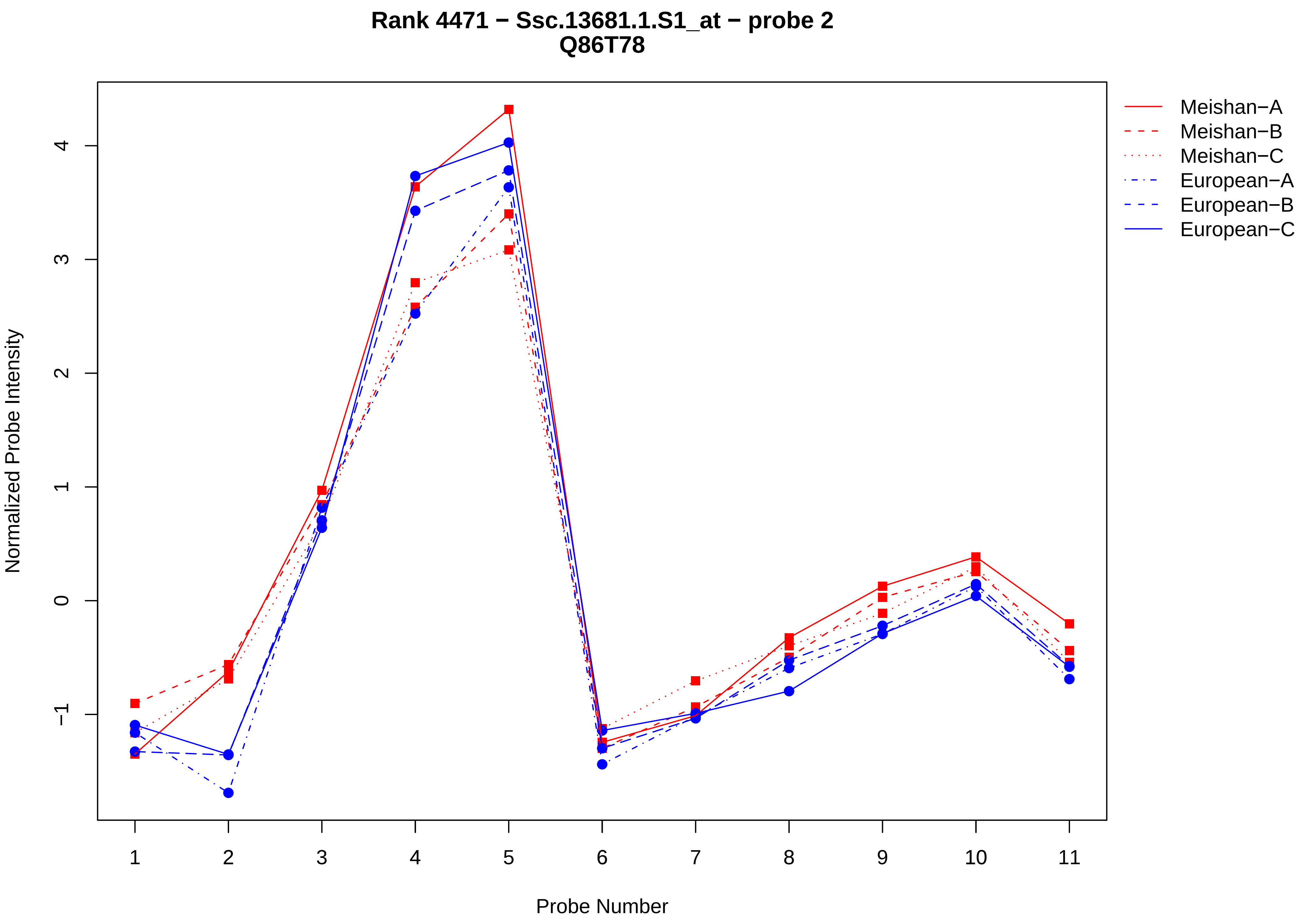

Rank 4471 − Ssc.13681.1.S1_at − probe 2
Q86T78
Normalized Probe Intensity
Probe Number
Meishan−A
Meishan−B
Meishan−C
European−A
European−B
European−C
−1
0
1
2
3
4
1
2
3
4
5
6
7
8
9
10
11

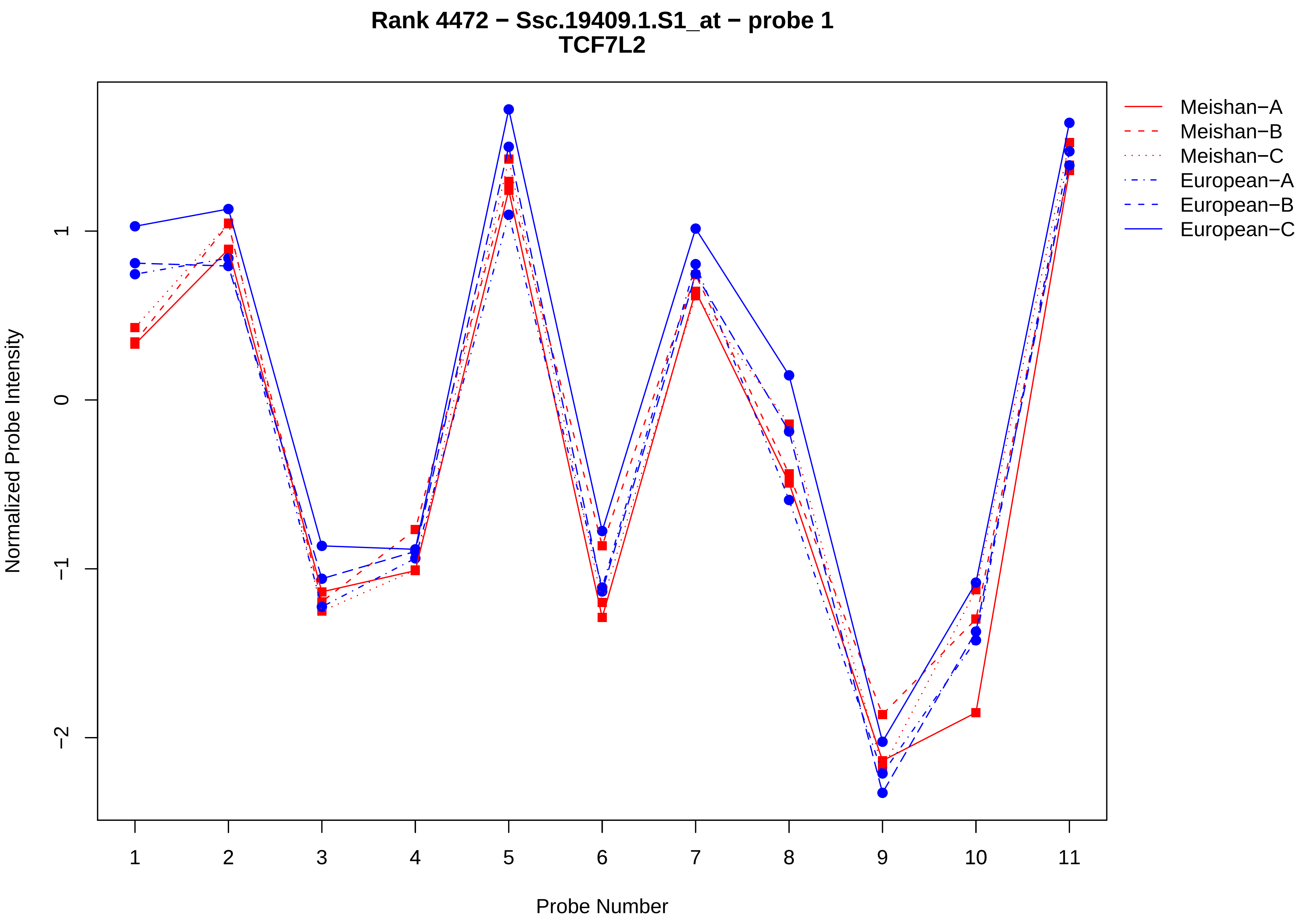

Rank 4472 − Ssc.19409.1.S1_at − probe 1
TCF7L2
Normalized Probe Intensity
Probe Number
Meishan−A
Meishan−B
Meishan−C
European−A
European−B
European−C
−2
−1
0
1
1
2
3
4
5
6
7
8
9
10
11

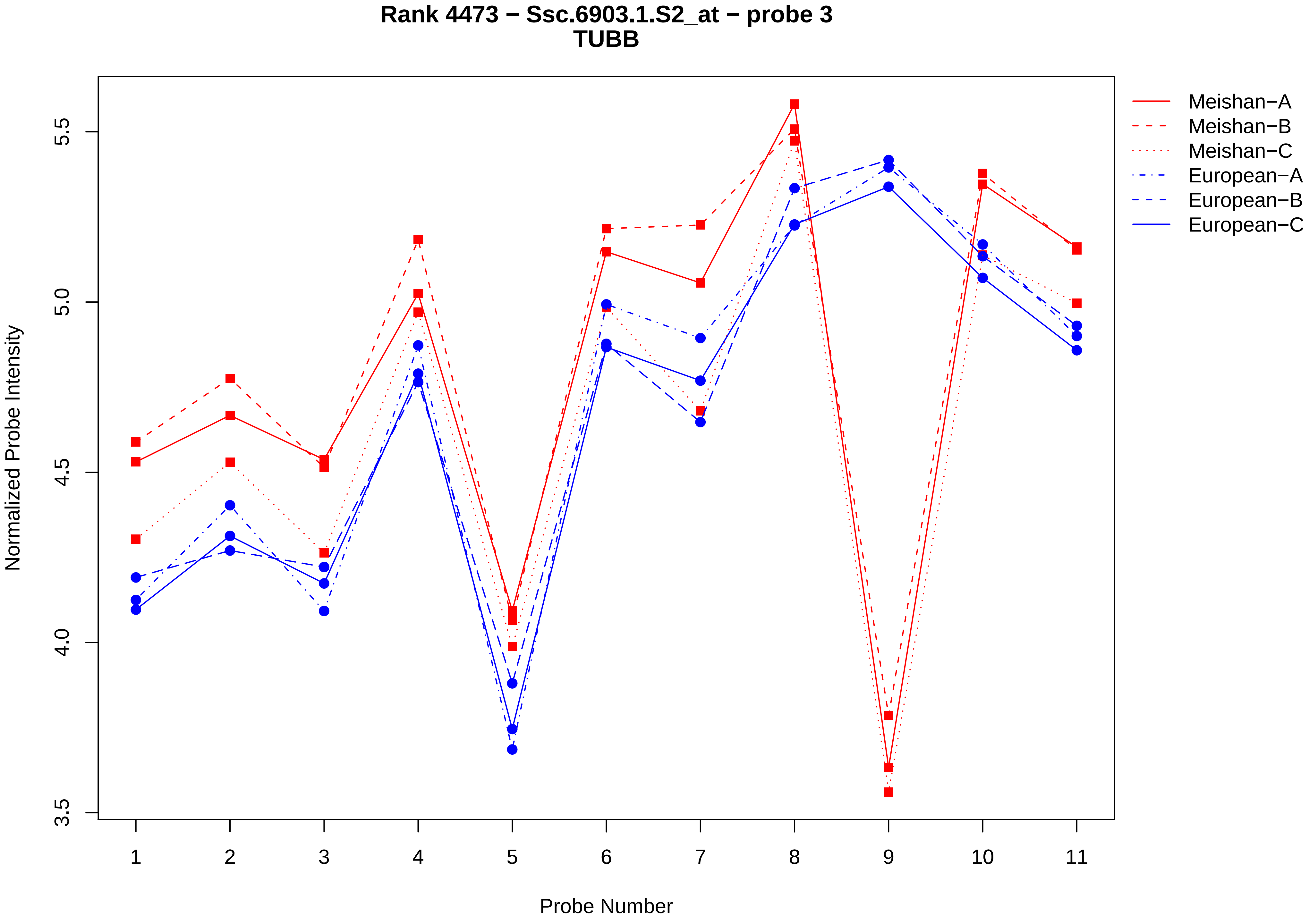
Rank 4473 − Ssc.6903.1.S2_at − probe 3
TUBB
Normalized Probe Intensity
Probe Number
3.5
4.0
4.5
5.0
5.5
1
2
3
4
5
6
7
8
9
10
11
Meishan−A
Meishan−B
Meishan−C
European−A
European−B
European−C

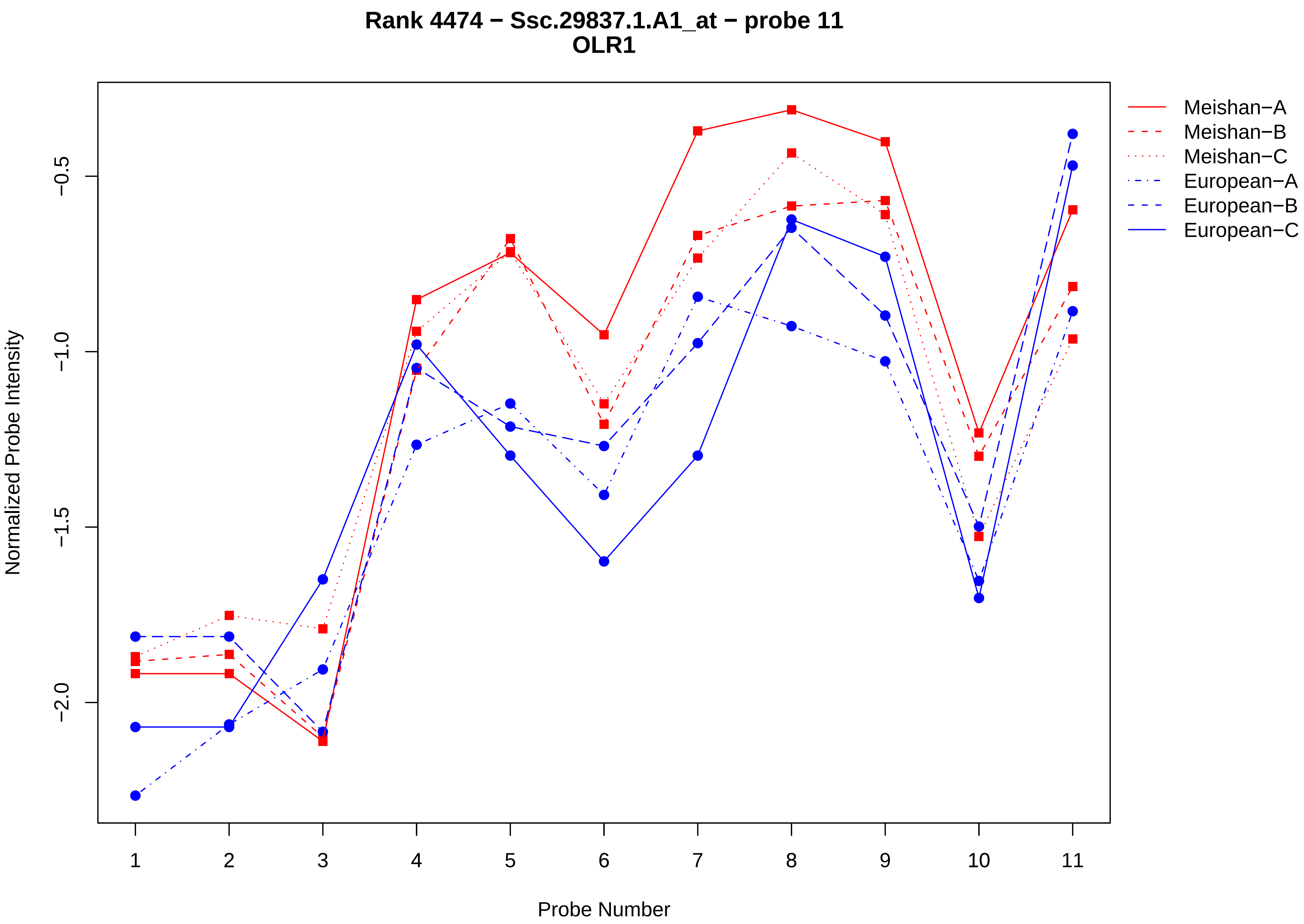

Rank 4474 − Ssc.29837.1.A1_at − probe 11
OLR1
Normalized Probe Intensity
−0.5
−1.0
−1.5
−2.0
1
2
3
4
5
6
7
8
9
10
11
Probe Number
Meishan−A
Meishan−B
Meishan−C
European−A
European−B
European−C

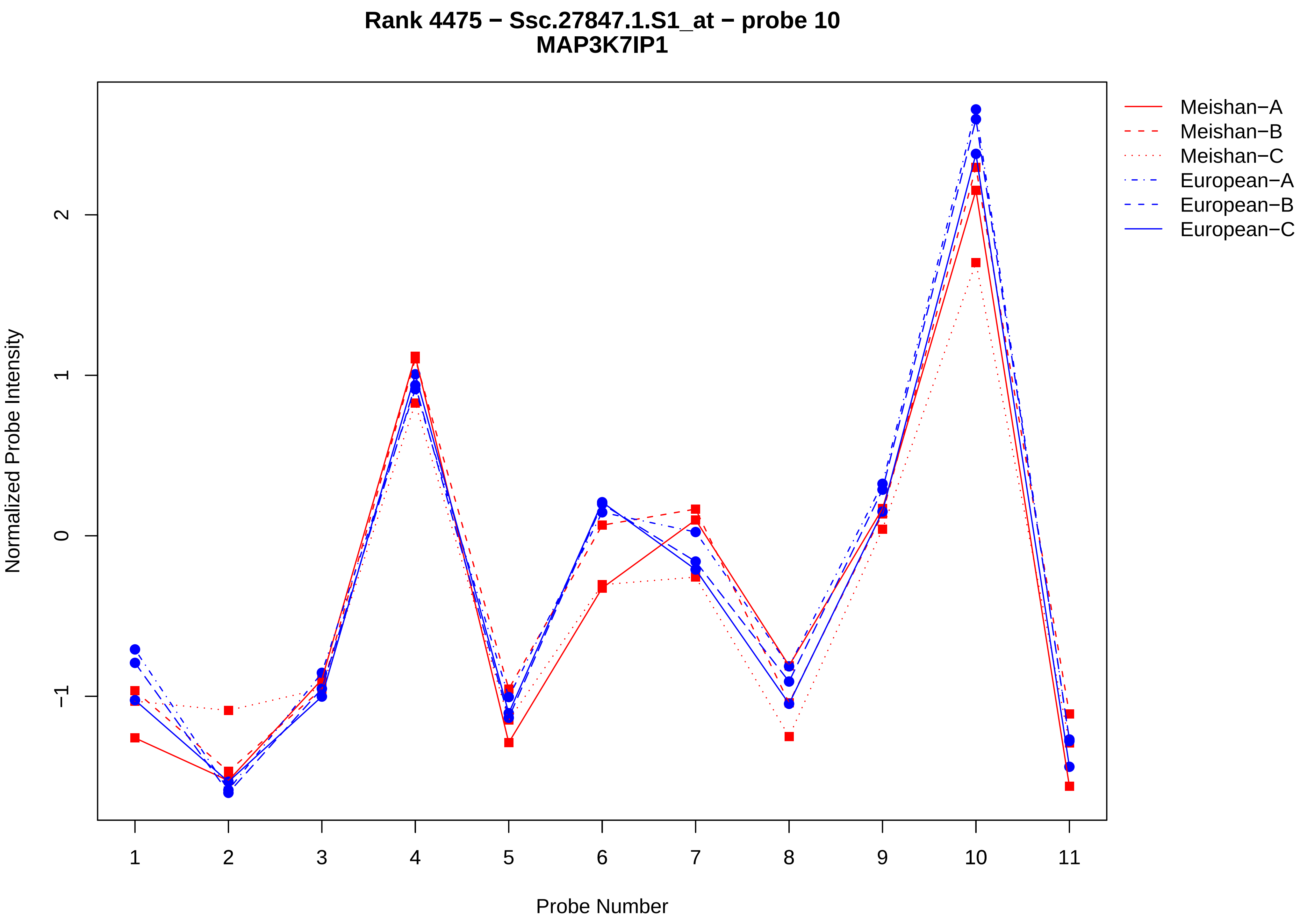
Rank 4475 − Ssc.27847.1.S1_at − probe 10
MAP3K7IP1
Normalized Probe Intensity
Probe Number
Meishan−A
Meishan−B
Meishan−C
European−A
European−B
European−C
−1
0
1
2
1
2
3
4
5
6
7
8
9
10
11

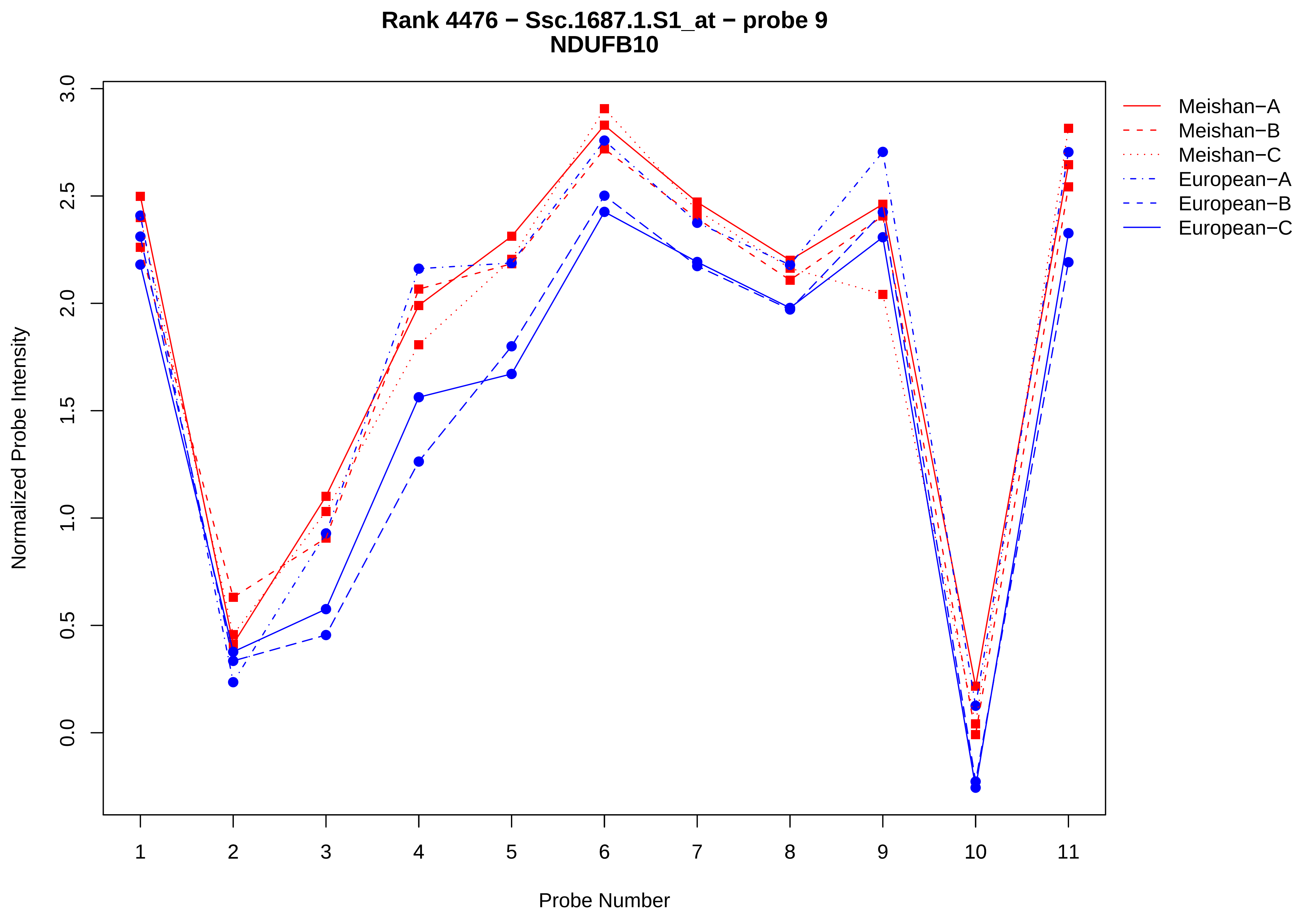

Rank 4476 − Ssc.1687.1.S1_at − probe 9
NDUFB10
Normalized Probe Intensity
Probe Number
Meishan−A
Meishan−B
Meishan−C
European−A
European−B
European−C

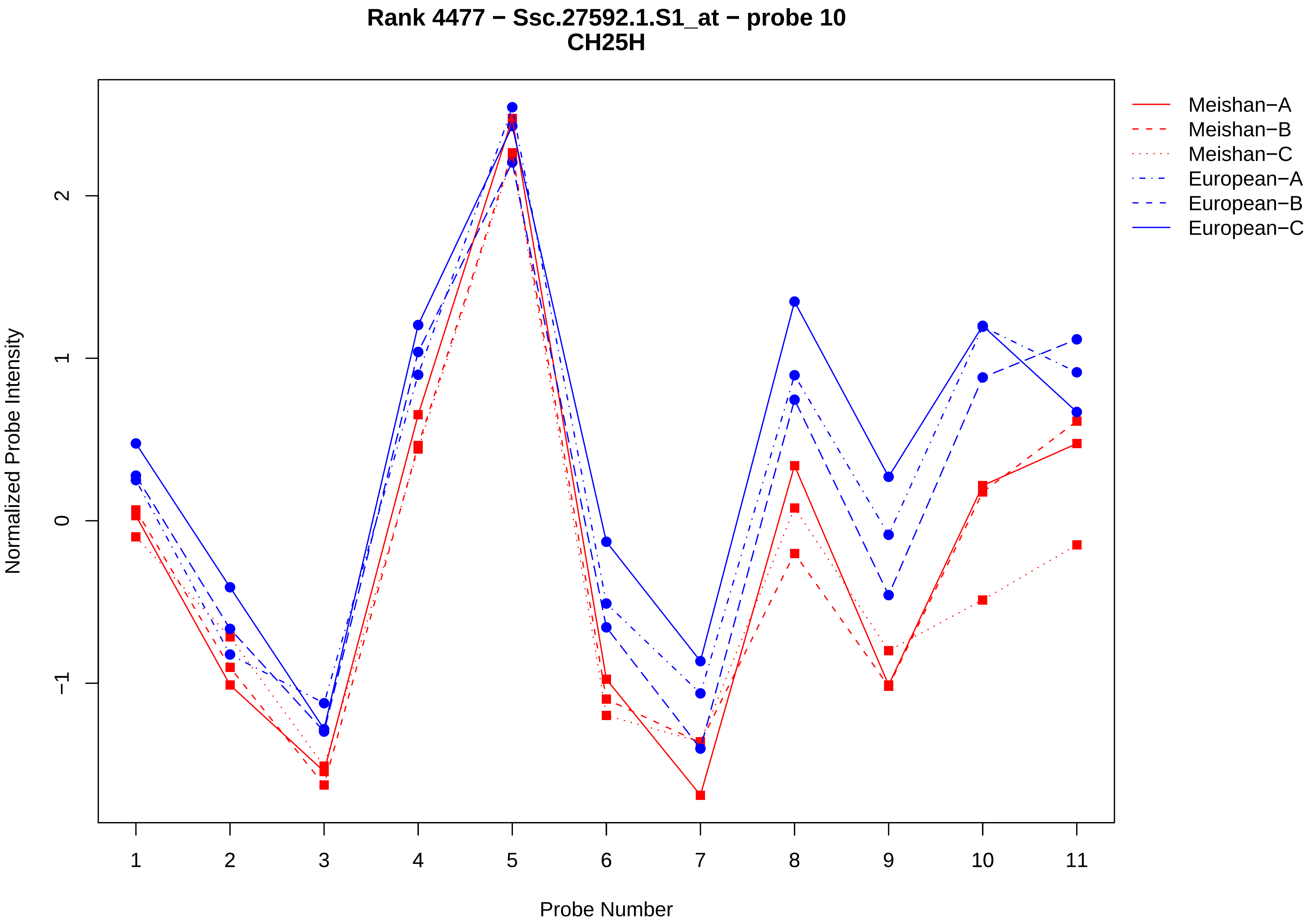
Rank 4477 − Ssc.27592.1.S1_at − probe 10
CH25H
Normalized Probe Intensity
Probe Number
Meishan−A
Meishan−B
Meishan−C
European−A
European−B
European−C
−1
0
1
2
1
2
3
4
5
6
7
8
9
10
11

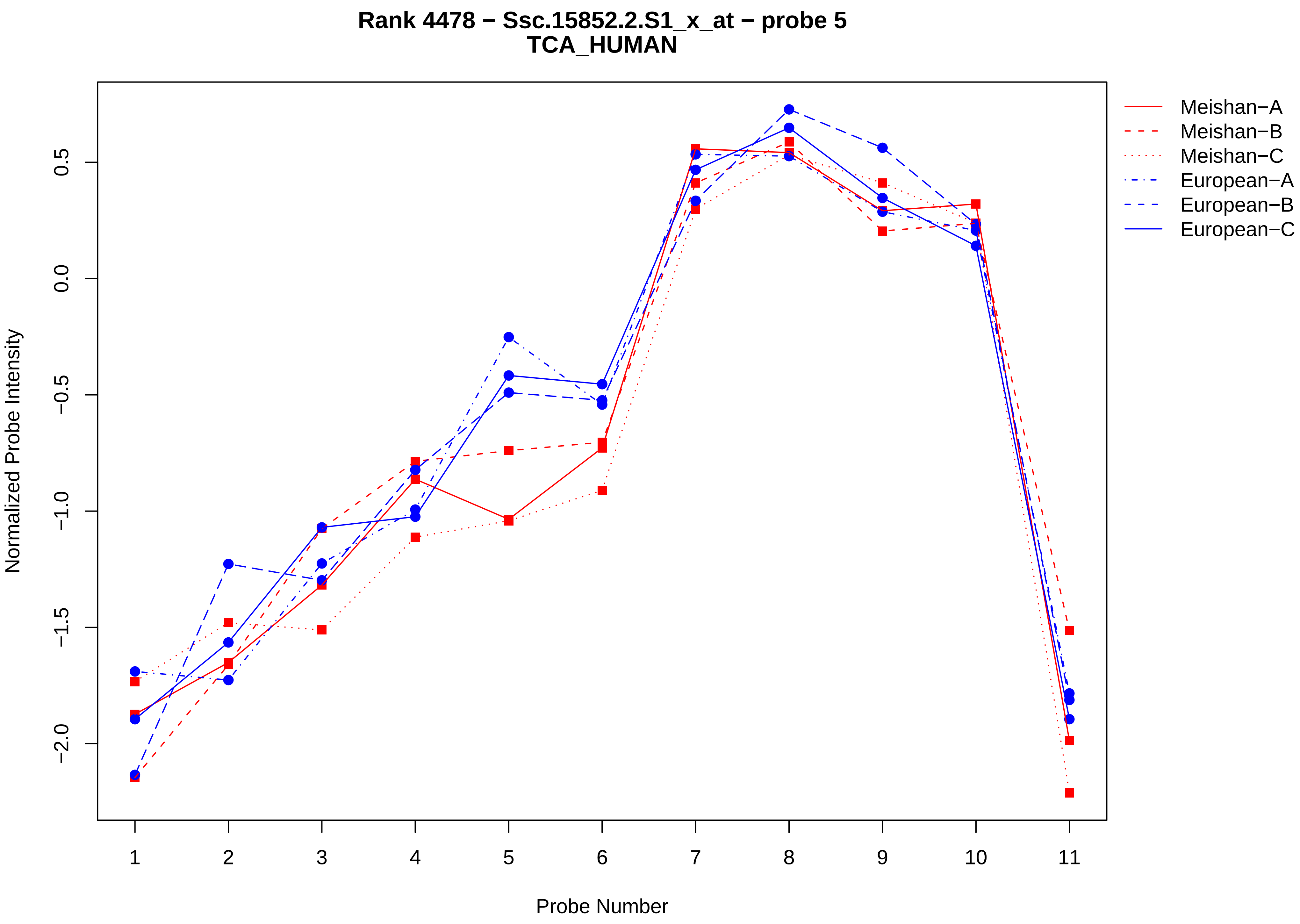

Rank 4478 − Ssc.15852.2.S1_x_at − probe 5
TCA_HUMAN
Normalized Probe Intensity
Probe Number
0.5
0.0
−0.5
−1.0
−1.5
−2.0
1
2
3
4
5
6
7
8
9
10
11
Meishan−A
Meishan−B
Meishan−C
European−A
European−B
European−C

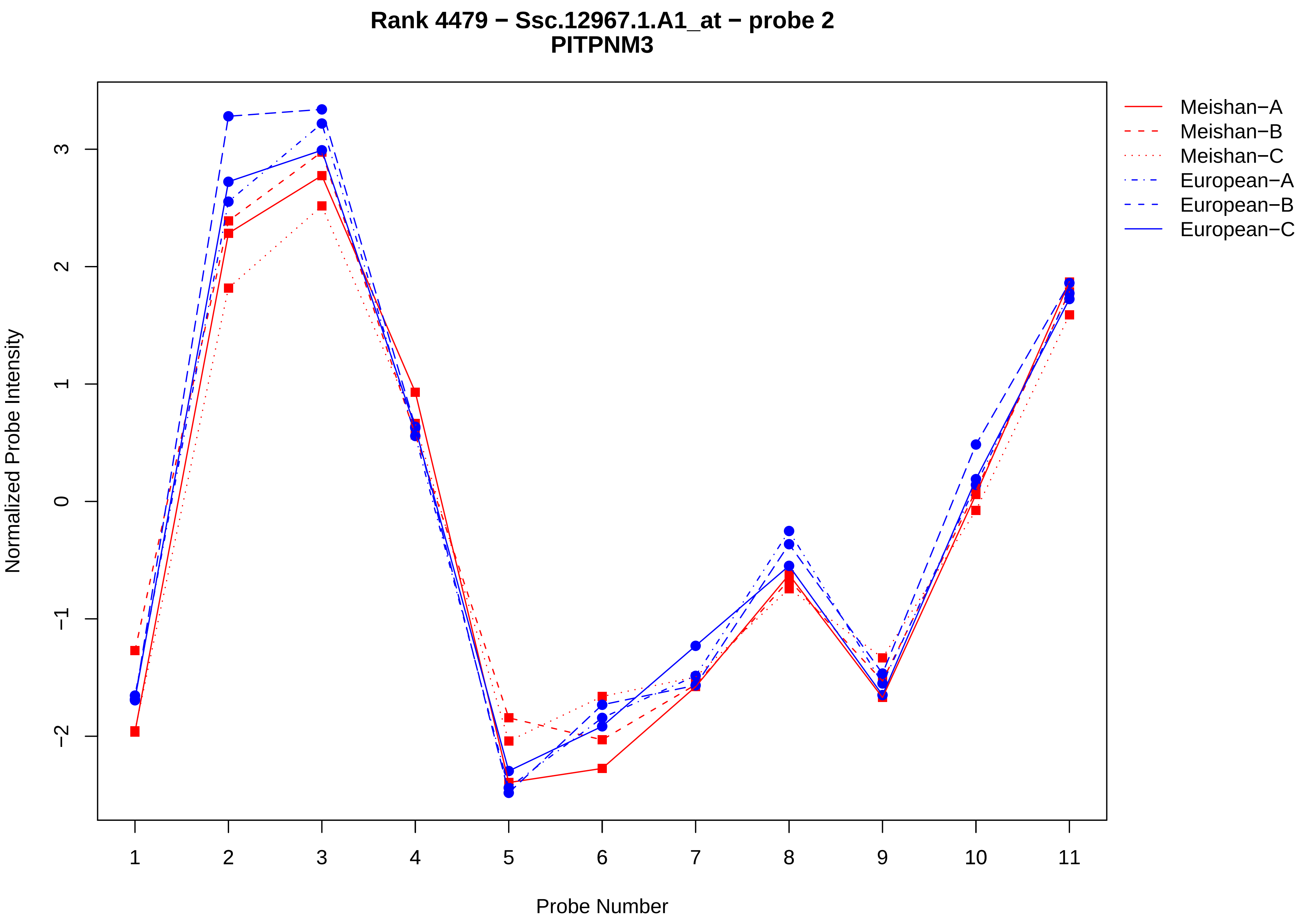

Rank 4479 − Ssc.12967.1.A1_at − probe 2
PITPNM3
Normalized Probe Intensity
Probe Number
Meishan−A
Meishan−B
Meishan−C
European−A
European−B
European−C

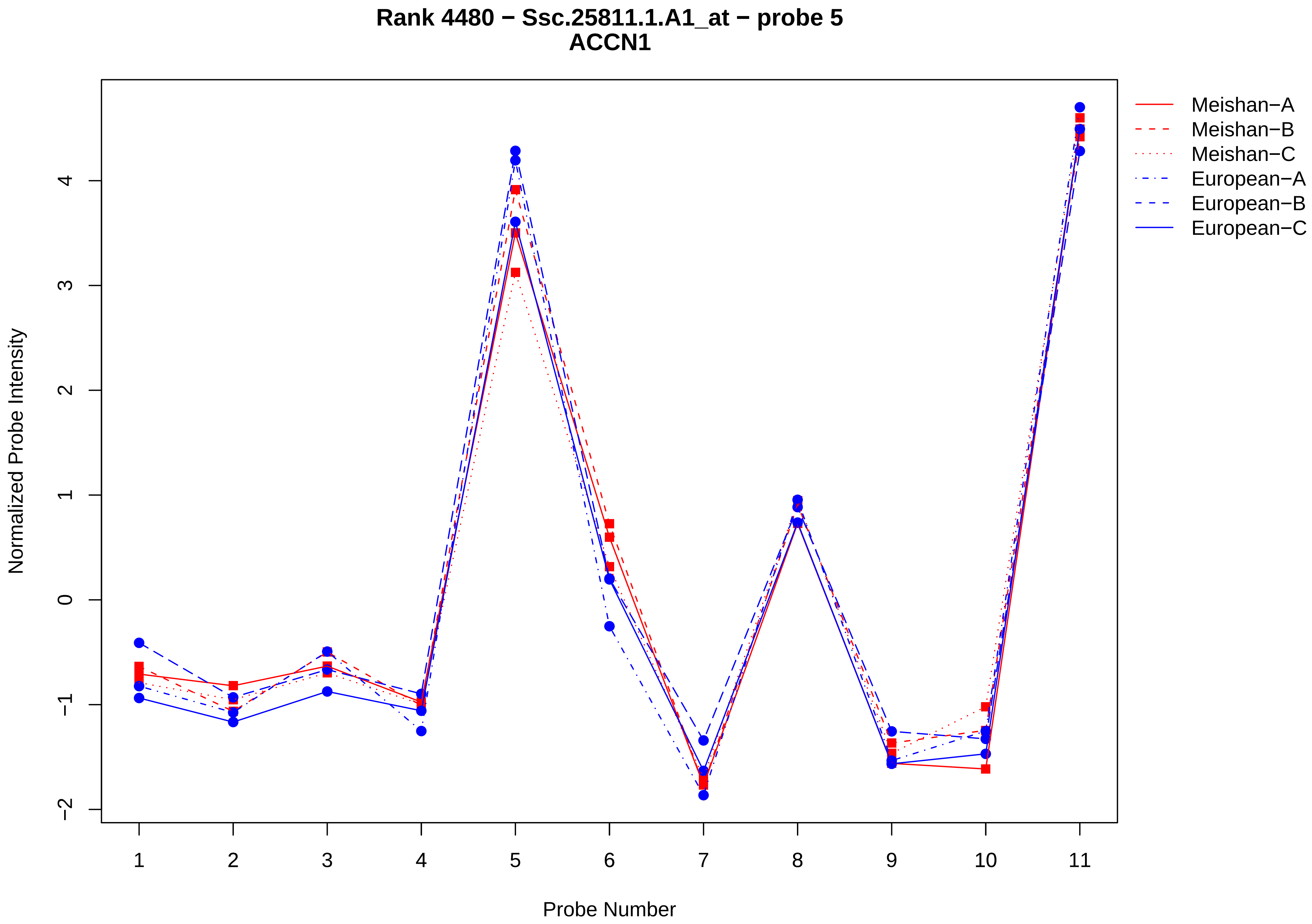

Rank 4480 − Ssc.25811.1.A1_at − probe 5
ACCN1
Normalized Probe Intensity
Probe Number
−2
−1
0
1
2
3
4
1
2
3
4
5
6
7
8
9
10
11
Meishan−A
Meishan−B
Meishan−C
European−A
European−B
European−C

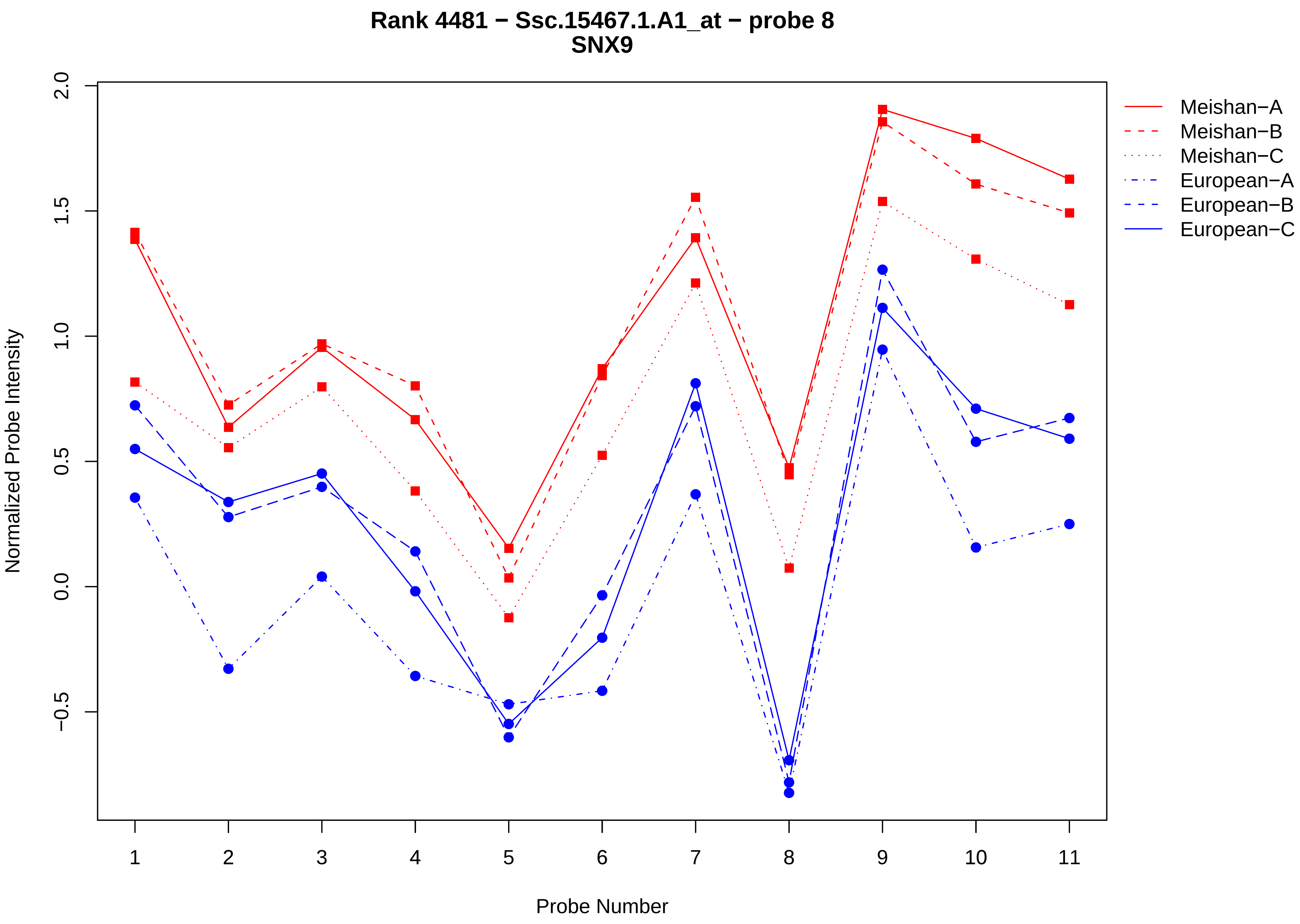

Rank 4481 − Ssc.15467.1.A1_at − probe 8
SNX9
Normalized Probe Intensity
Probe Number
−0.5
0.0
0.5
1.0
1.5
2.0
1
2
3
4
5
6
7
8
9
10
11
Meishan−A
Meishan−B
Meishan−C
European−A
European−B
European−C

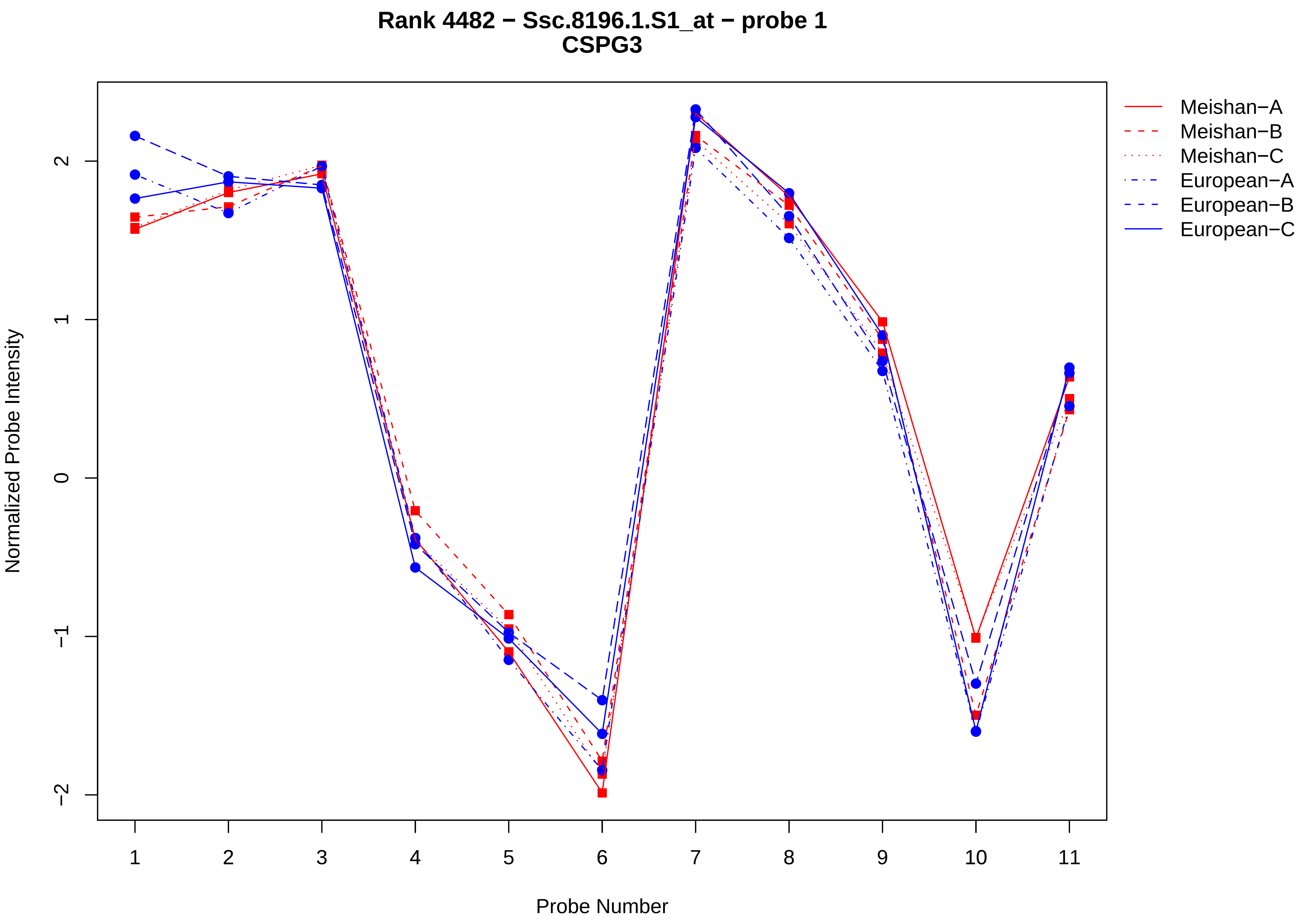

Rank 4482 − Ssc.8196.1.S1_at − probe 1
CSPG3
Normalized Probe Intensity
Probe Number
−2
−1
0
1
2
1
2
3
4
5
6
7
8
9
10
11
Meishan−A
Meishan−B
Meishan−C
European−A
European−B
European−C

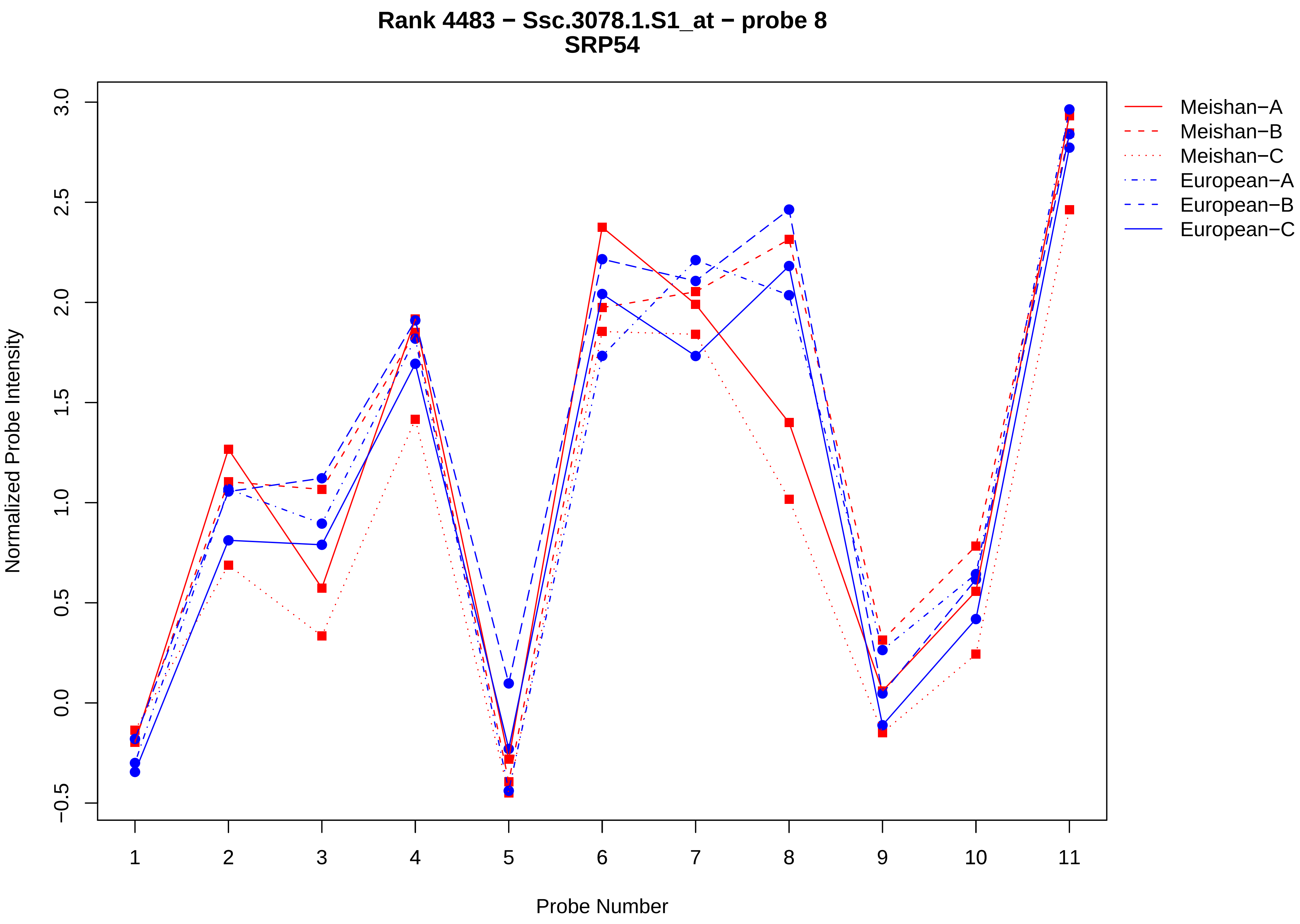

Rank 4483 − Ssc.3078.1.S1_at − probe 8
SRP54
Normalized Probe Intensity
Probe Number
−0.5
0.0
0.5
1.0
1.5
2.0
2.5
3.0
1
2
3
4
5
6
7
8
9
10
11
Meishan−A
Meishan−B
Meishan−C
European−A
European−B
European−C

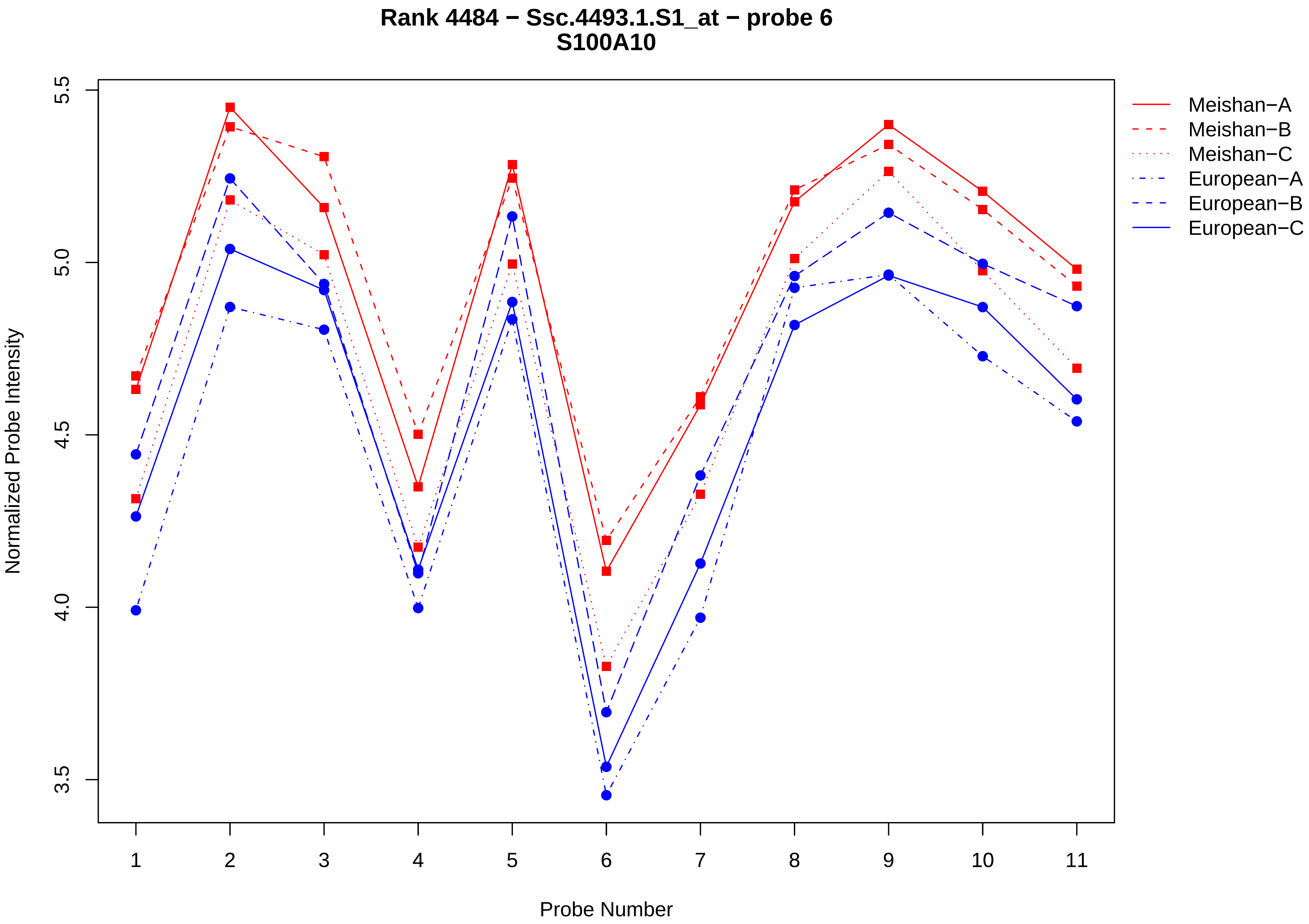
Rank 4484 − Ssc.4493.1.S1_at − probe 6
S100A10
Normalized Probe Intensity
3.5
4.0
4.5
5.0
5.5
1
2
3
4
5
6
7
8
9
10
11
Probe Number
Meishan−A
Meishan−B
Meishan−C
European−A
European−B
European−C

Rank 4485 − Ssc.21552.2.S1_at − probe 5
PRPSAP1

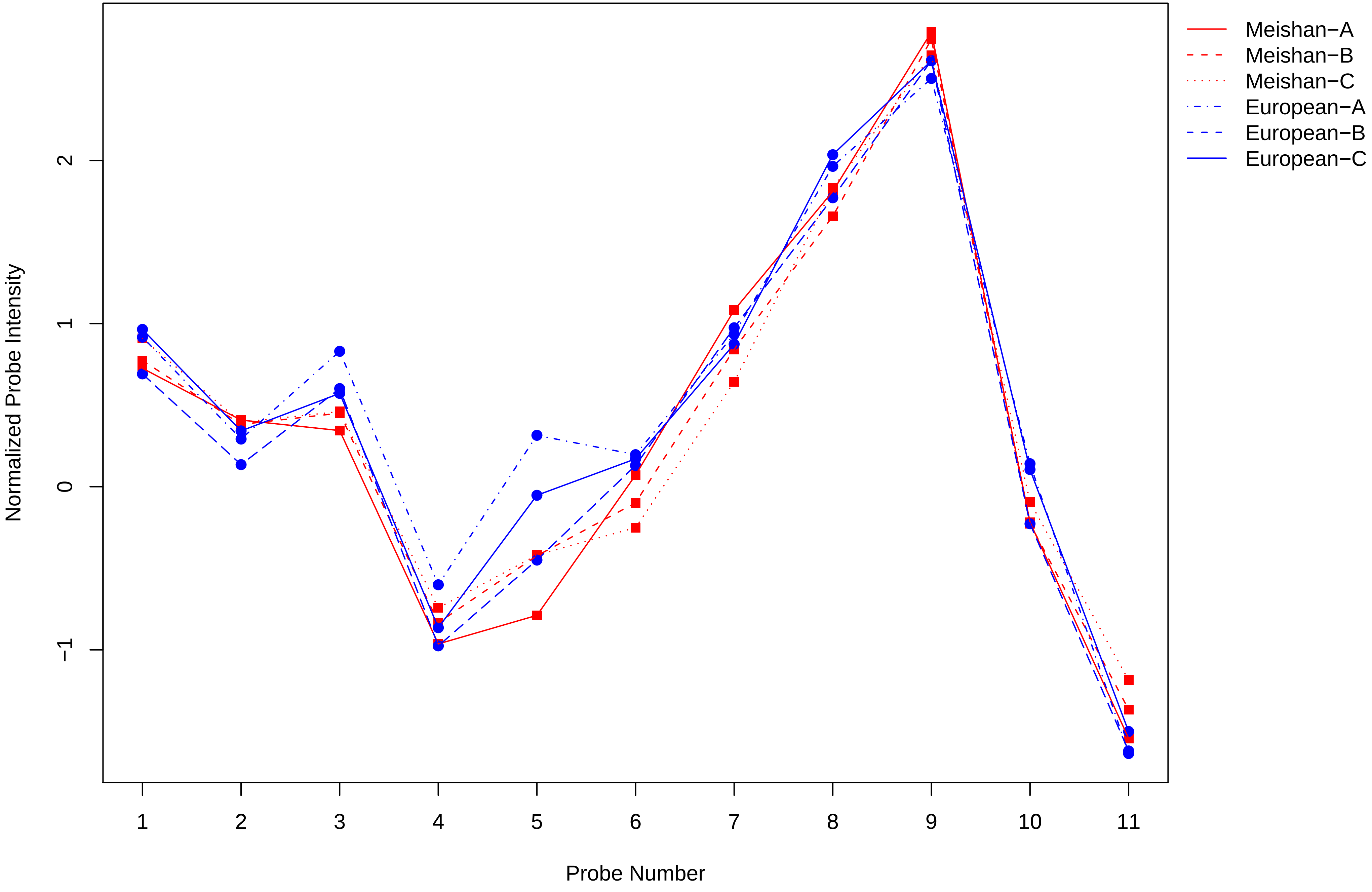

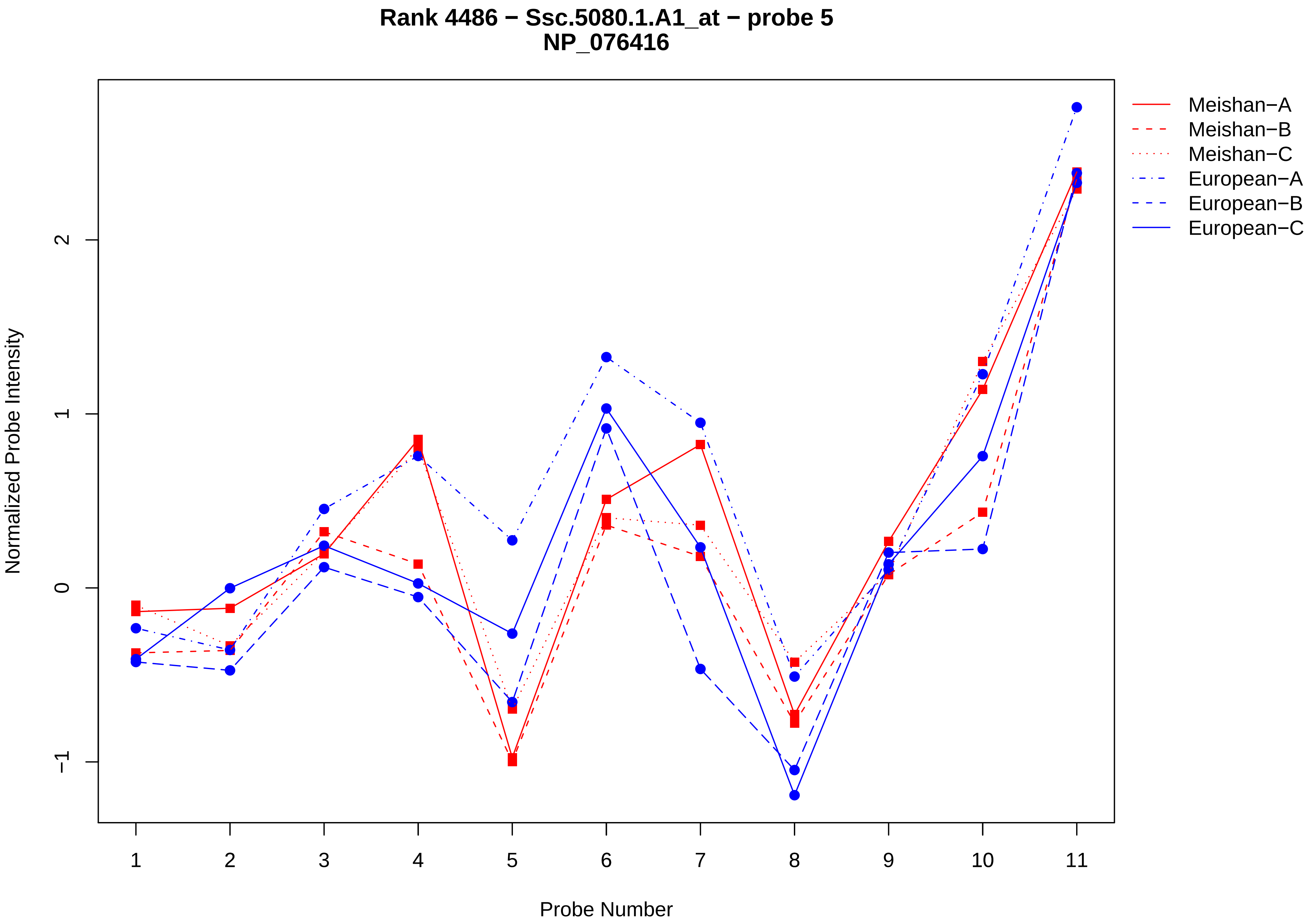

Rank 4486 − Ssc.5080.1.A1_at − probe 5
NP_076416
Normalized Probe Intensity
Probe Number
−1
0
1
2
1
2
3
4
5
6
7
8
9
10
11
Meishan−A
Meishan−B
Meishan−C
European−A
European−B
European−C

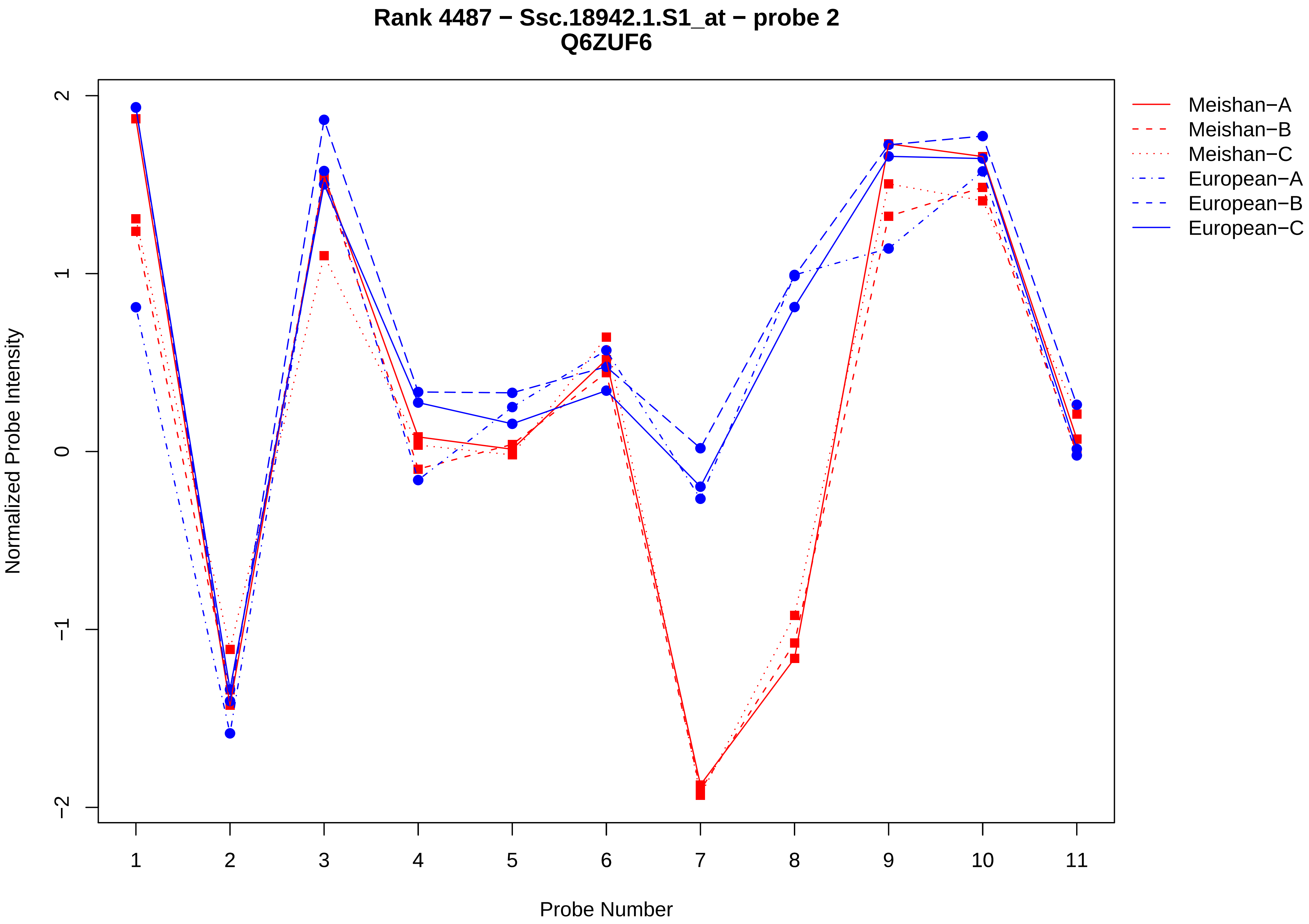

Rank 4487 − Ssc.18942.1.S1_at − probe 2
Q6ZUF6
Normalized Probe Intensity
Probe Number
Meishan−A
Meishan−B
Meishan−C
European−A
European−B
European−C
−2
−1
0
1
2
1
2
3
4
5
6
7
8
9
10
11

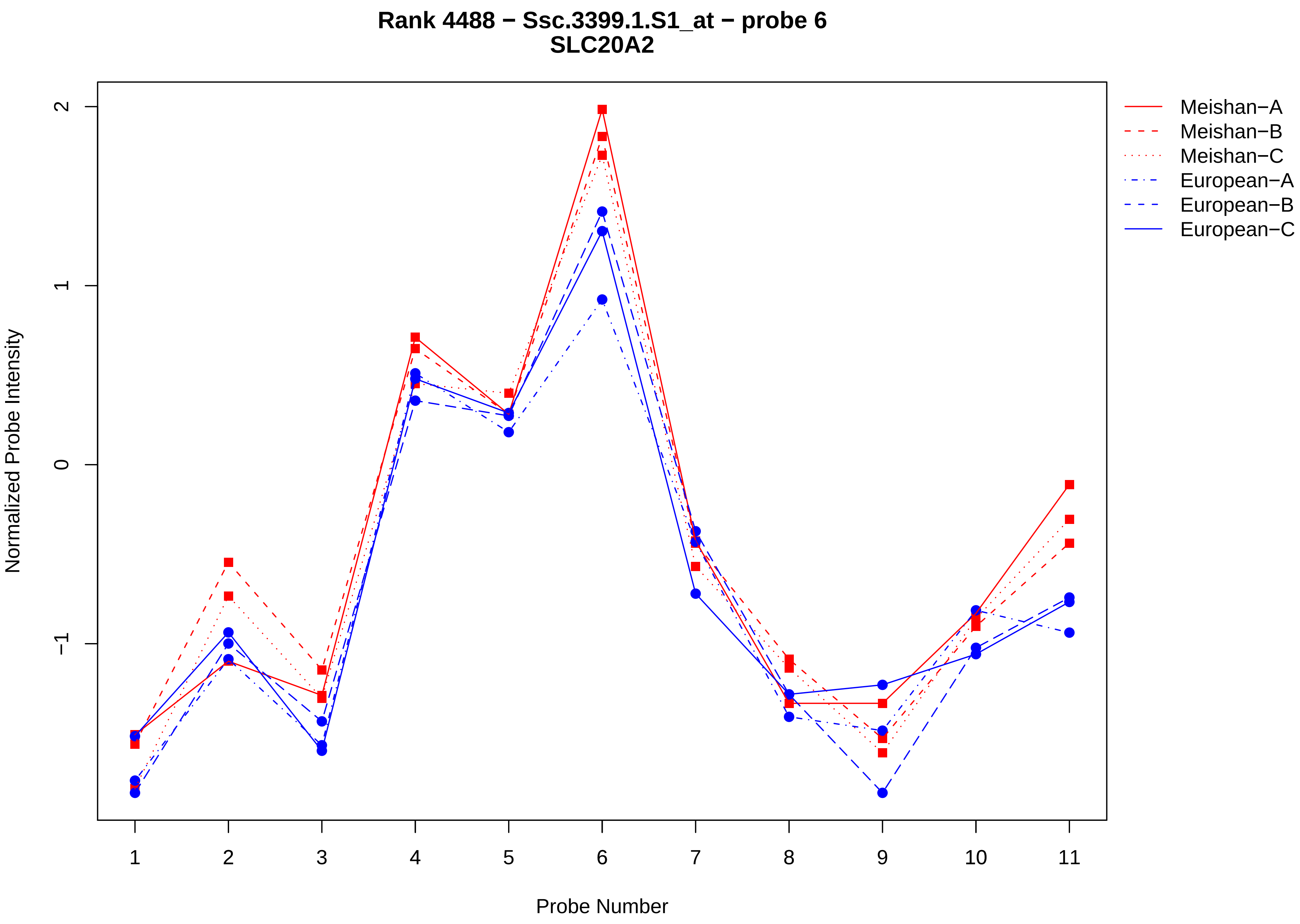

Rank 4488 − Ssc.3399.1.S1_at − probe 6
SLC20A2
Normalized Probe Intensity
Probe Number
−1
0
1
2
1
2
3
4
5
6
7
8
9
10
11
Meishan−A
Meishan−B
Meishan−C
European−A
European−B
European−C

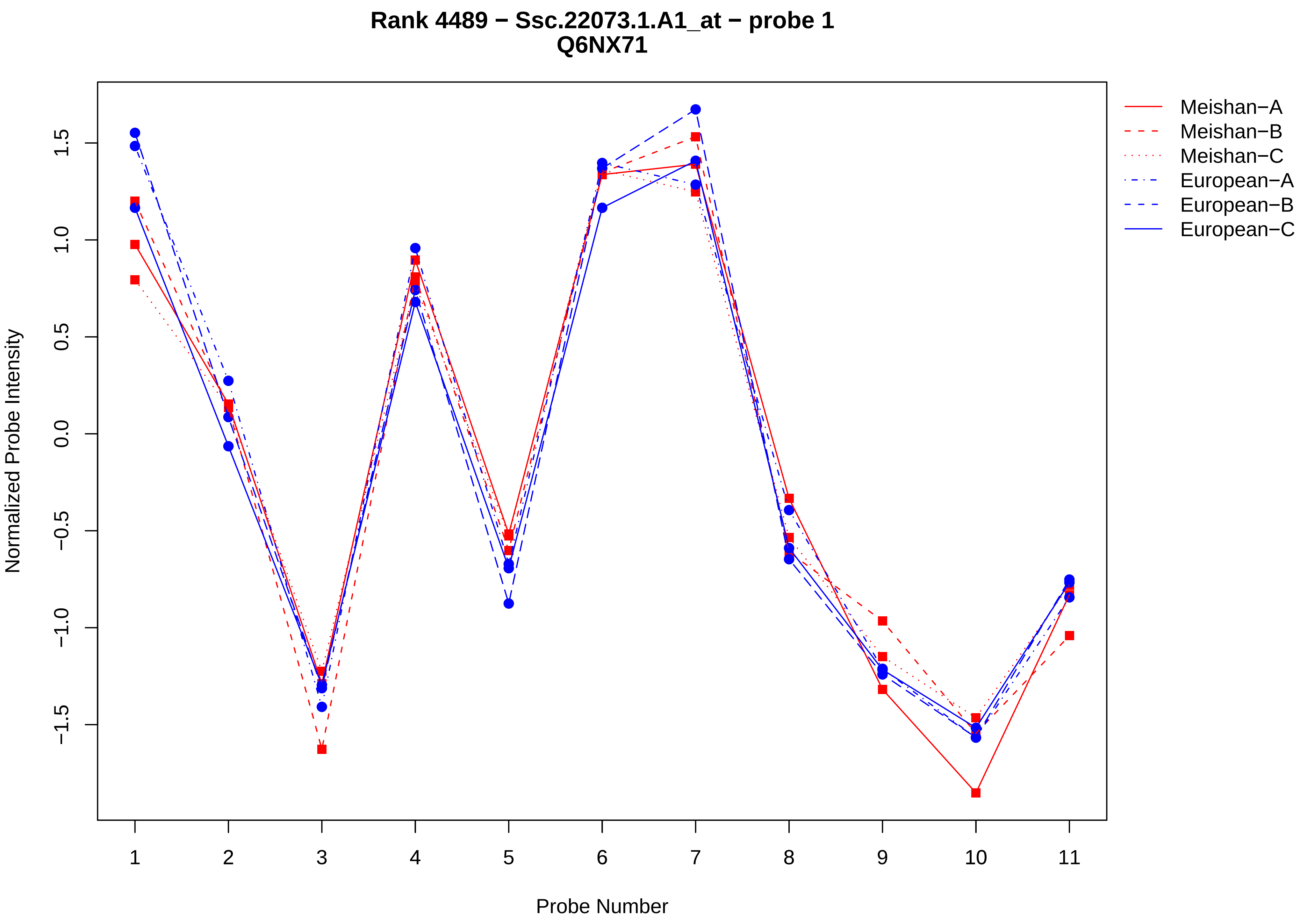

Rank 4489 − Ssc.22073.1.A1_at − probe 1
Q6NX71
Normalized Probe Intensity
Probe Number
Meishan−A
Meishan−B
Meishan−C
European−A
European−B
European−C

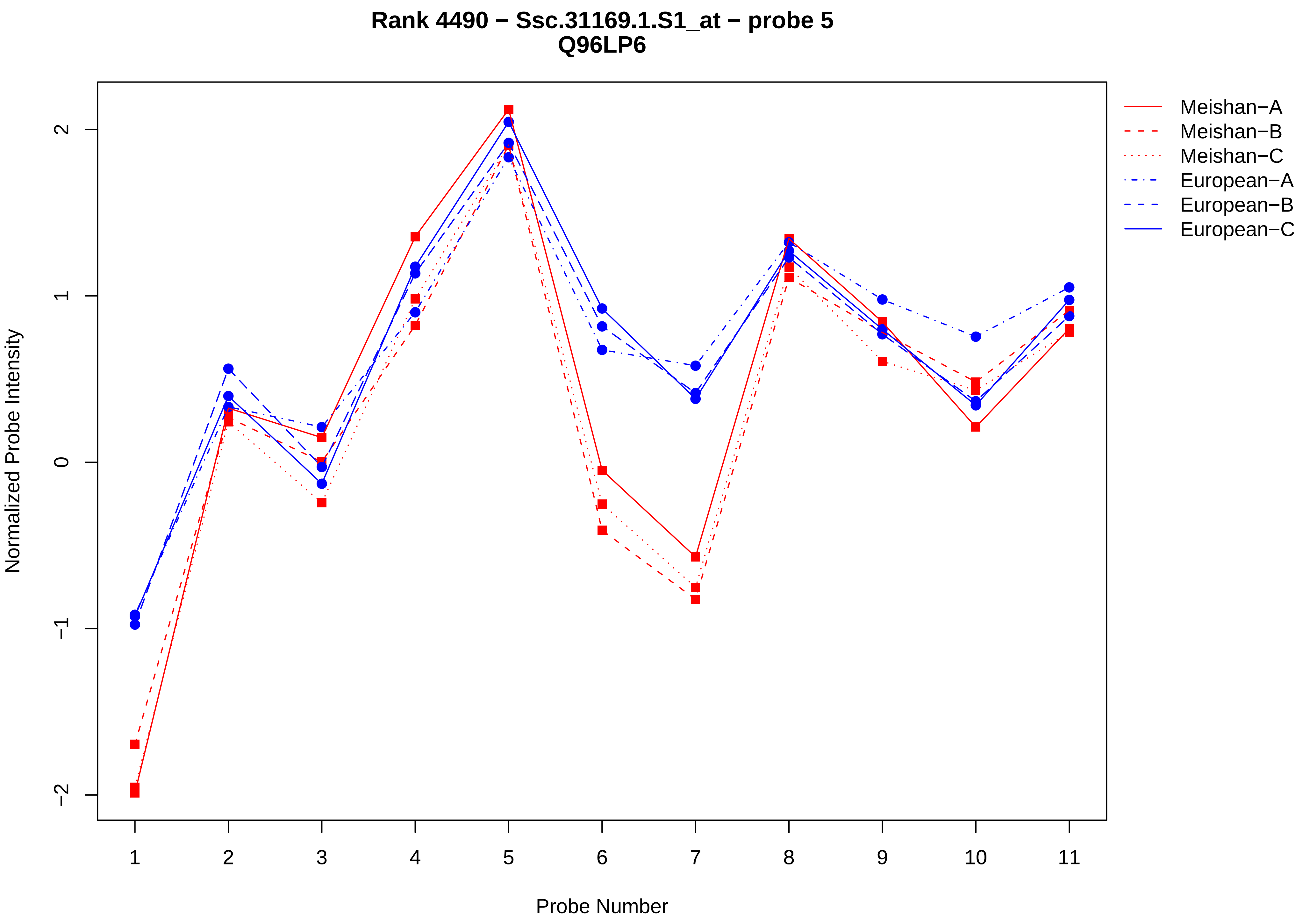

Rank 4490 − Ssc.31169.1.S1_at − probe 5
Q96LP6
Normalized Probe Intensity
Probe Number
−2
−1
0
1
2
1
2
3
4
5
6
7
8
9
10
11
Meishan−A
Meishan−B
Meishan−C
European−A
European−B
European−C

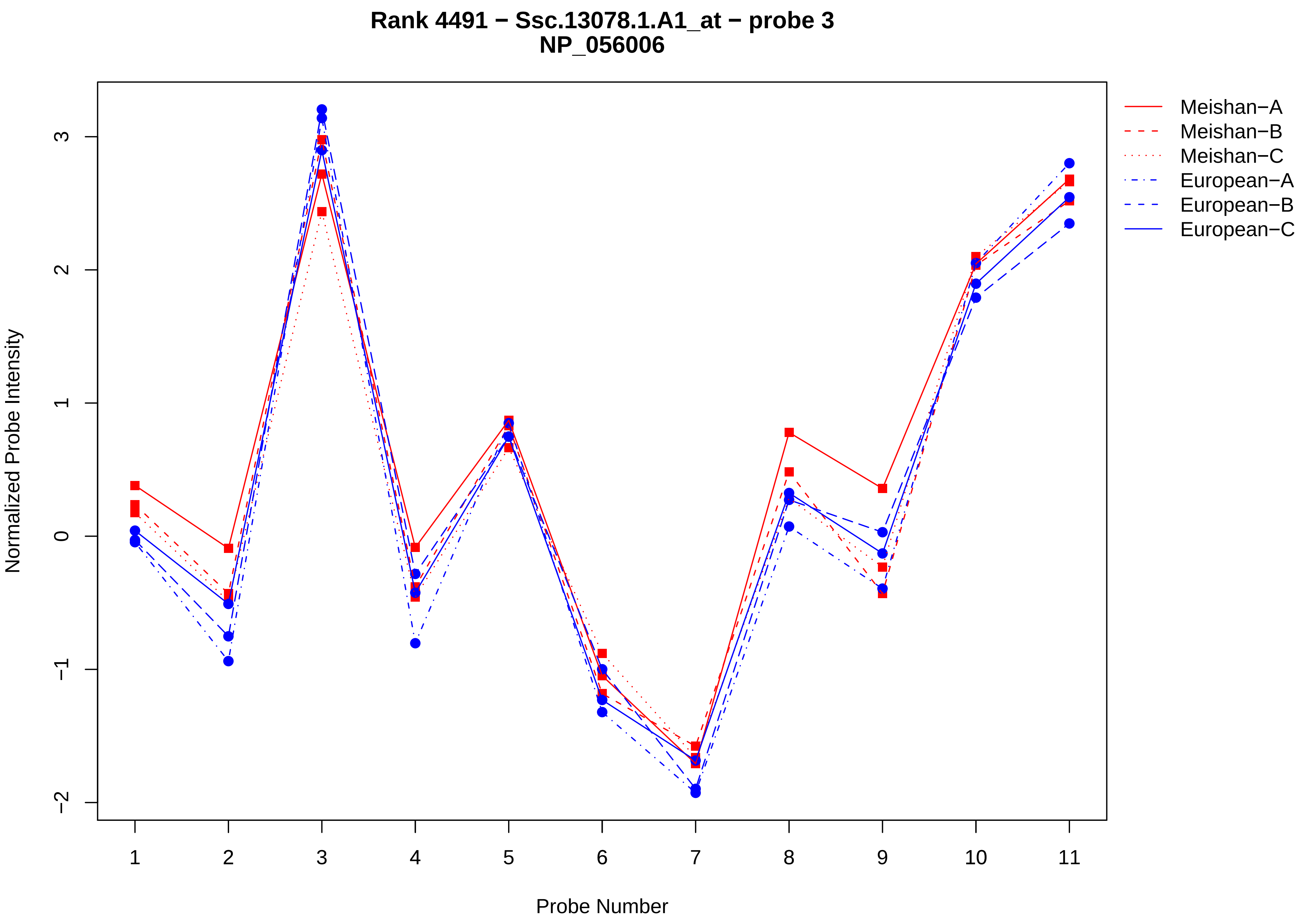

Rank 4491 − Ssc.13078.1.A1_at − probe 3
NP_056006
Normalized Probe Intensity
Probe Number
Meishan−A
Meishan−B
Meishan−C
European−A
European−B
European−C
−2
−1
0
1
2
3
1
2
3
4
5
6
7
8
9
10
11

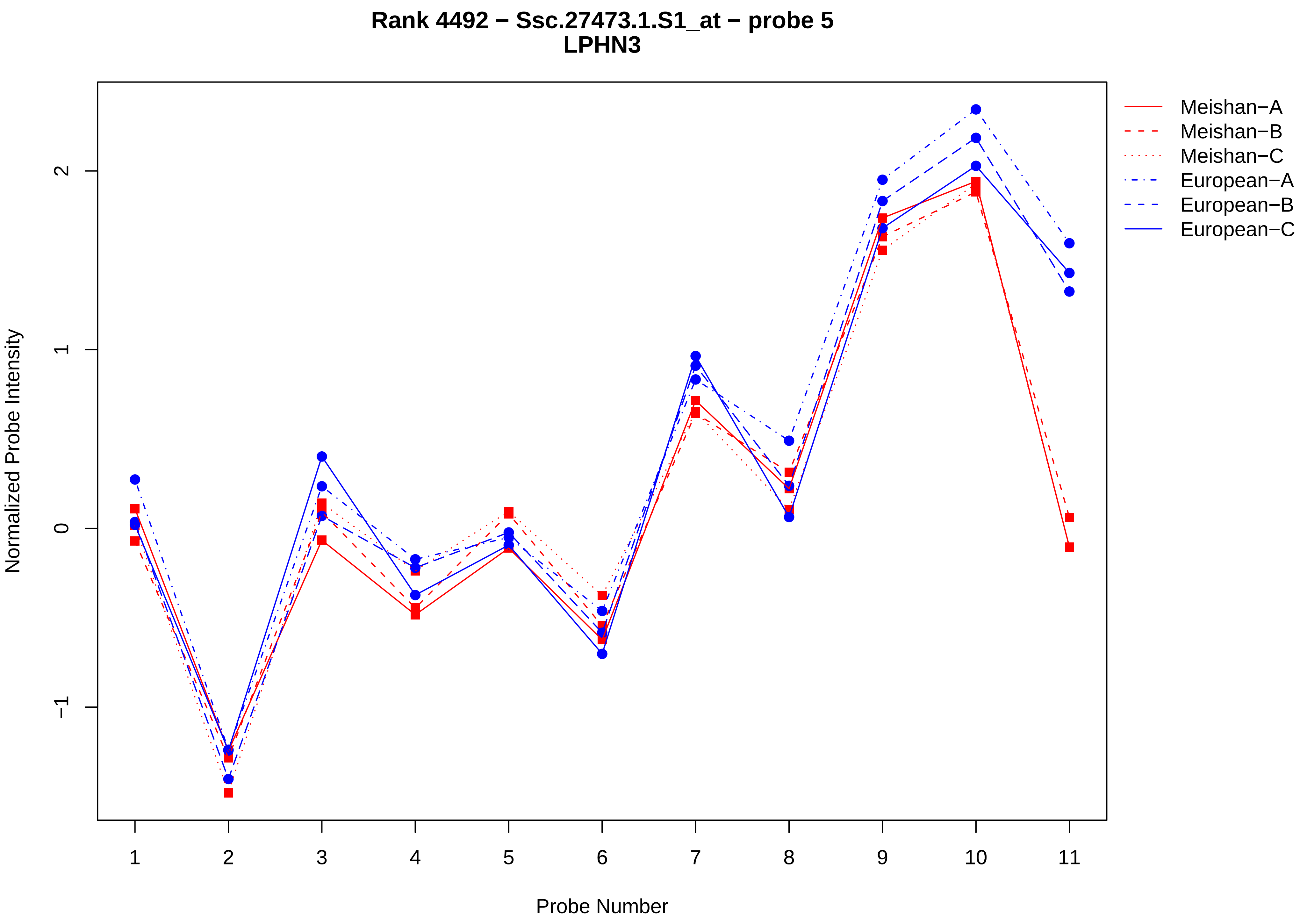
Rank 4492 − Ssc.27473.1.S1_at − probe 5
LPHN3
Normalized Probe Intensity
Probe Number
Meishan−A
Meishan−B
Meishan−C
European−A
European−B
European−C
−1
0
1
2
1
2
3
4
5
6
7
8
9
10
11

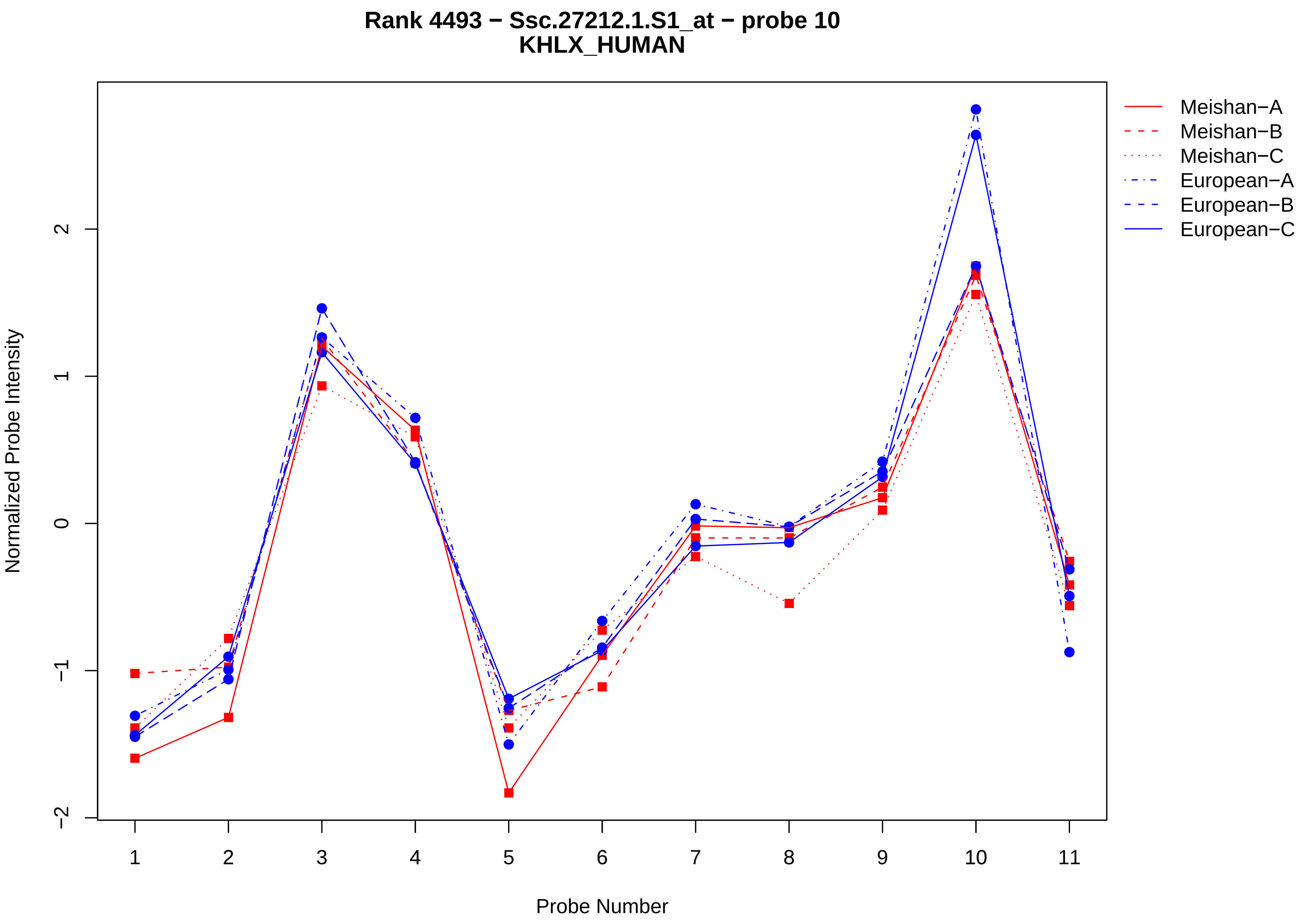

Rank 4493 − Ssc.27212.1.S1_at − probe 10
KHLX_HUMAN
Normalized Probe Intensity
Probe Number
Meishan−A
Meishan−B
Meishan−C
European−A
European−B
European−C
−2
−1
0
1
2
1
2
3
4
5
6
7
8
9
10
11

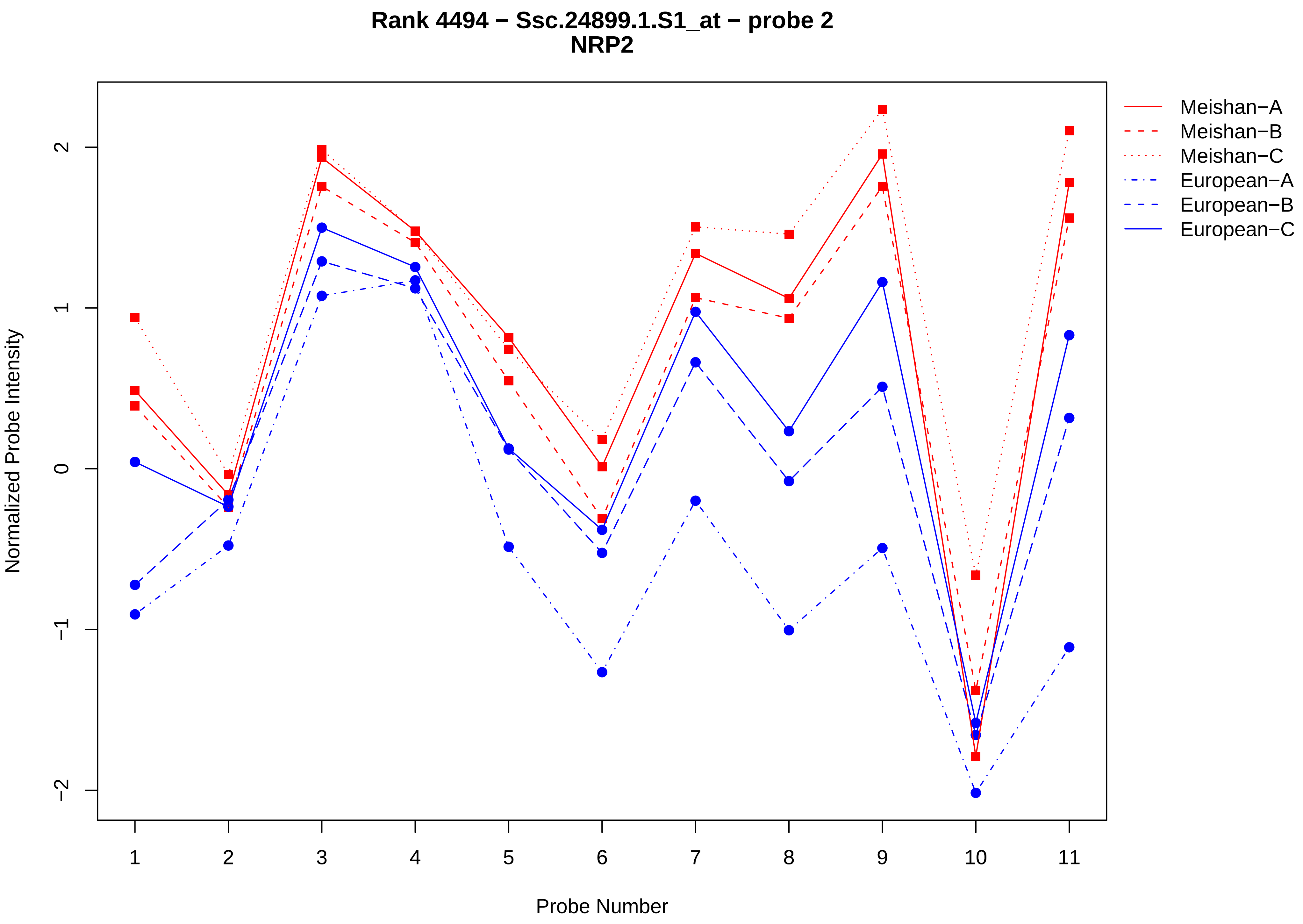

Rank 4494 − Ssc.24899.1.S1_at − probe 2
NRP2
Normalized Probe Intensity
Probe Number
−2
−1
0
1
2
1
2
3
4
5
6
7
8
9
10
11
Meishan−A
Meishan−B
Meishan−C
European−A
European−B
European−C

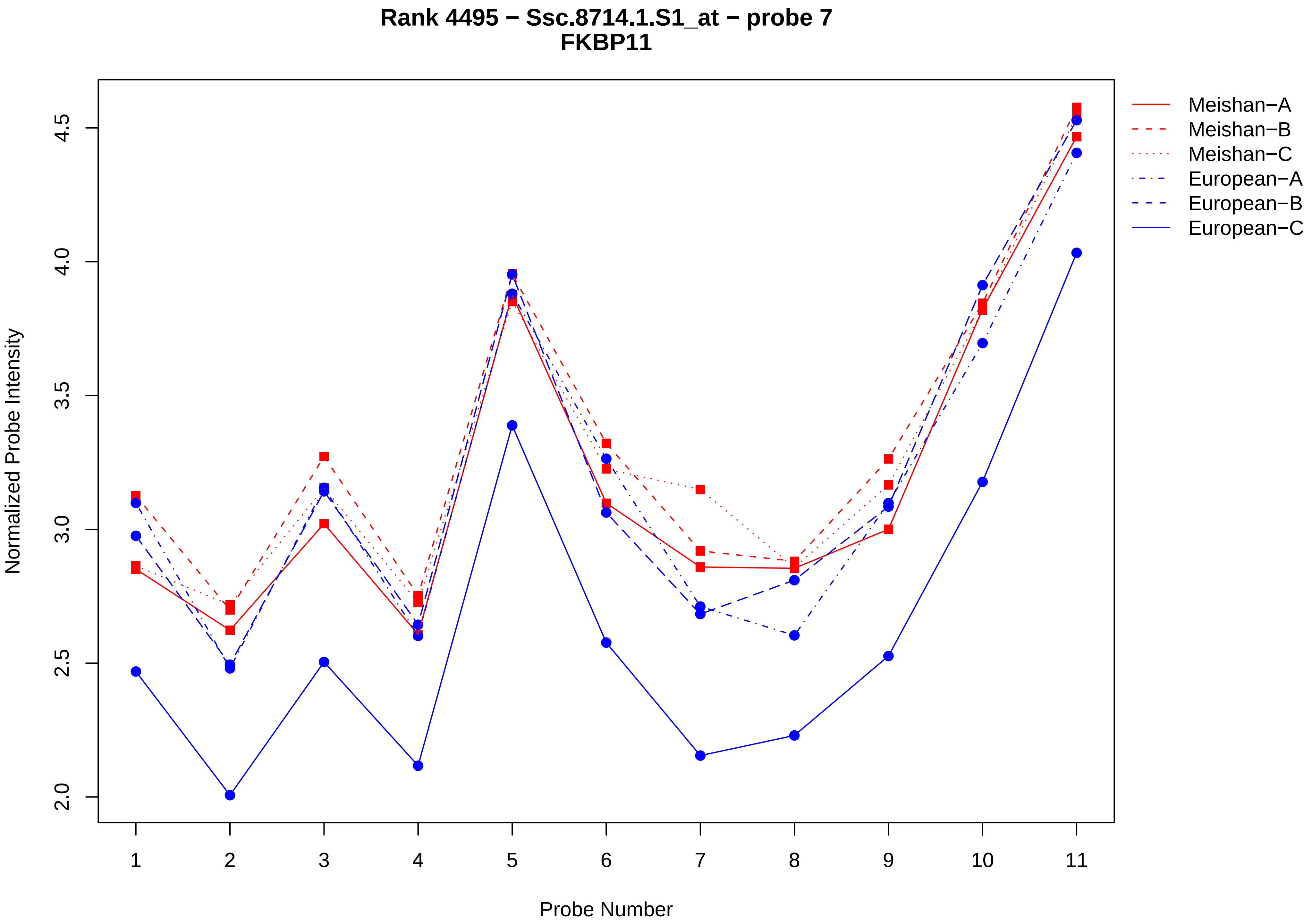
Rank 4495 − Ssc.8714.1.S1_at − probe 7
FKBP11
Normalized Probe Intensity
2.0
2.5
3.0
3.5
4.0
4.5
1
2
3
4
5
6
7
8
9
10
11
Probe Number
Meishan−A
Meishan−B
Meishan−C
European−A
European−B
European−C

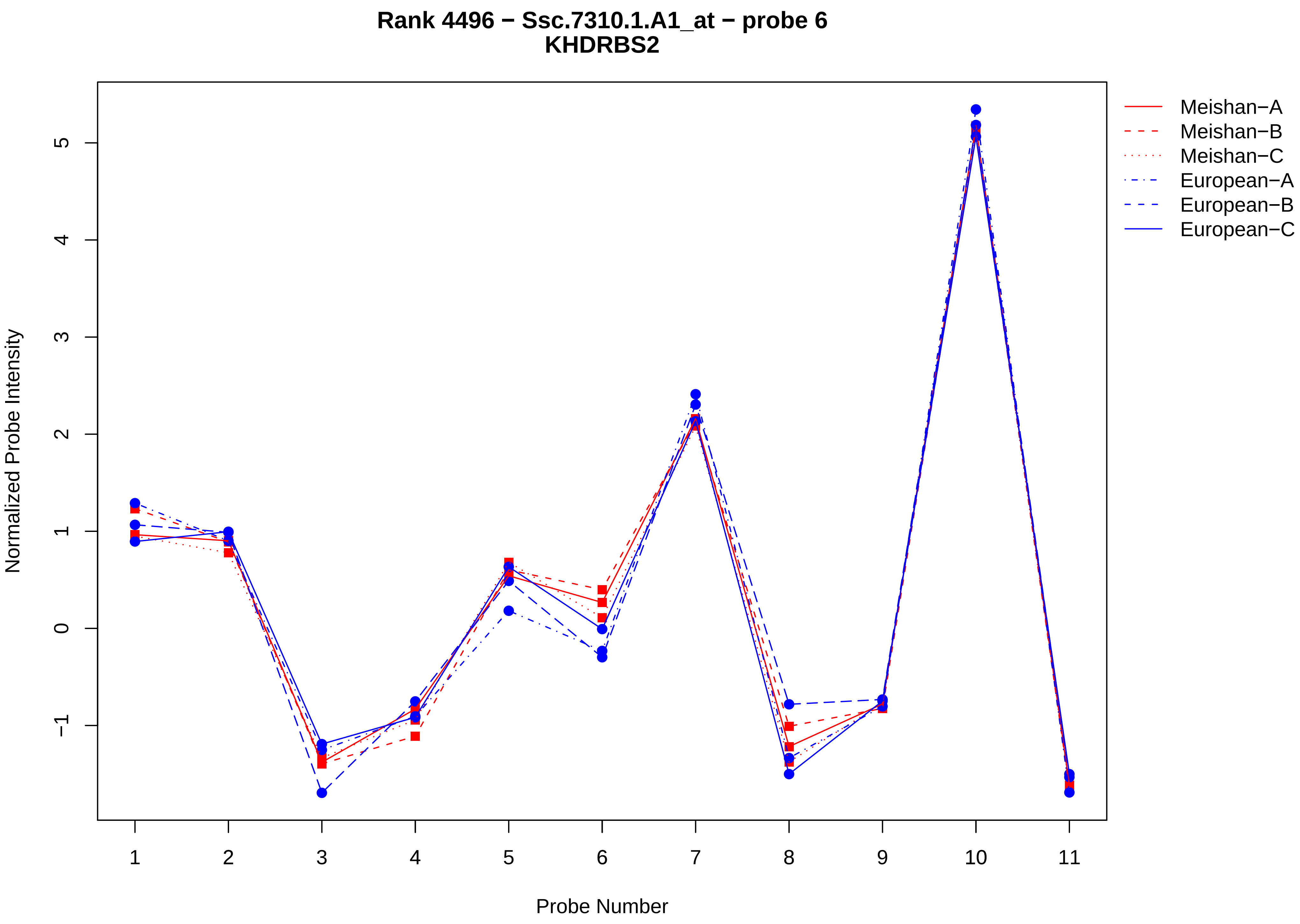

Rank 4496 − Ssc.7310.1.A1_at − probe 6
KHDRBS2
Normalized Probe Intensity
Probe Number
Meishan−A
Meishan−B
Meishan−C
European−A
European−B
European−C

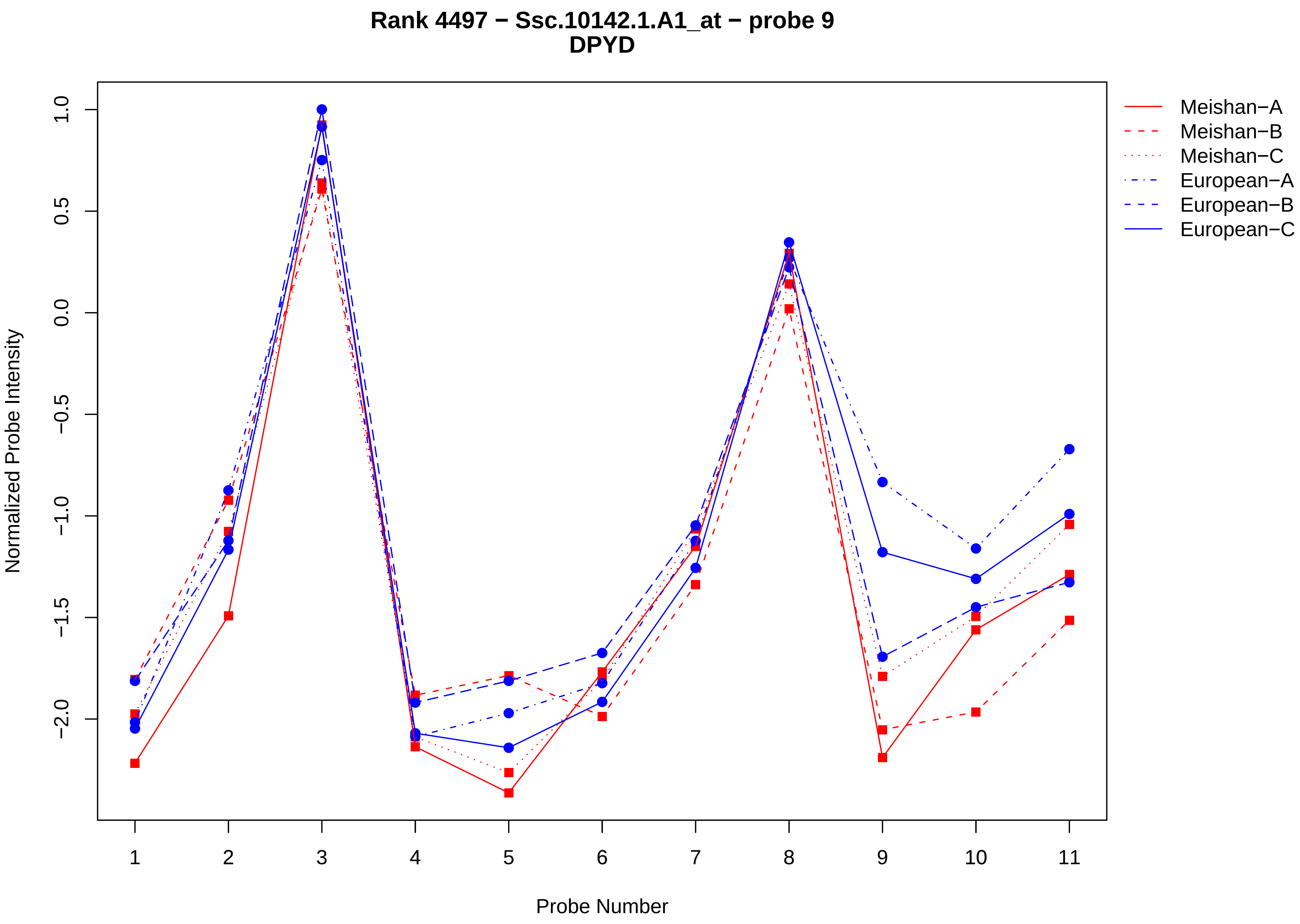

Rank 4497 − Ssc.10142.1.A1_at − probe 9
DPYD
Normalized Probe Intensity
Probe Number
1.0
0.5
0.0
−0.5
−1.0
−1.5
−2.0
1
2
3
4
5
6
7
8
9
10
11
Meishan−A
Meishan−B
Meishan−C
European−A
European−B
European−C

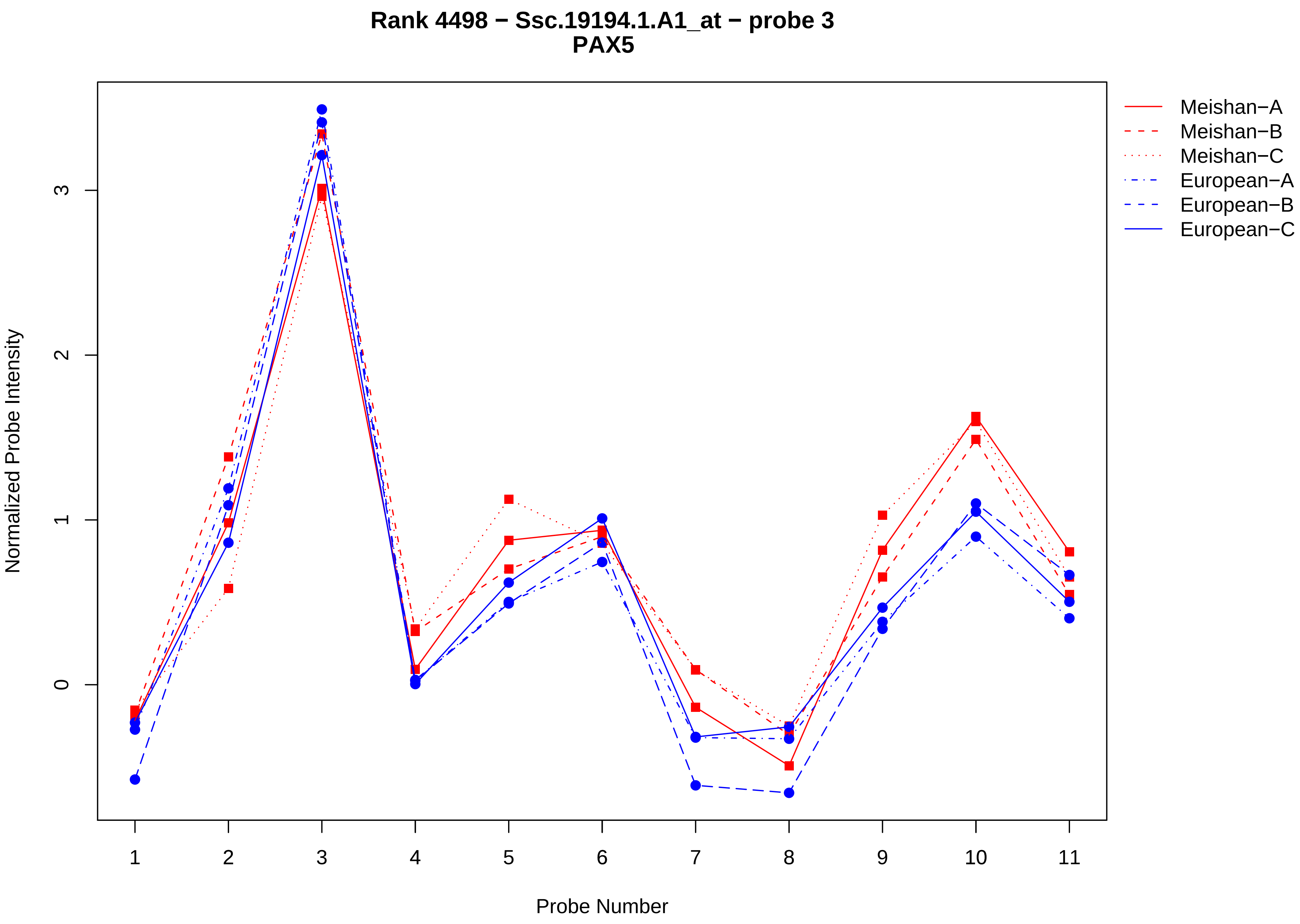

Rank 4498 − Ssc.19194.1.A1_at − probe 3
PAX5
Normalized Probe Intensity
Probe Number
0
1
2
3
1
2
3
4
5
6
7
8
9
10
11
Meishan−A
Meishan−B
Meishan−C
European−A
European−B
European−C

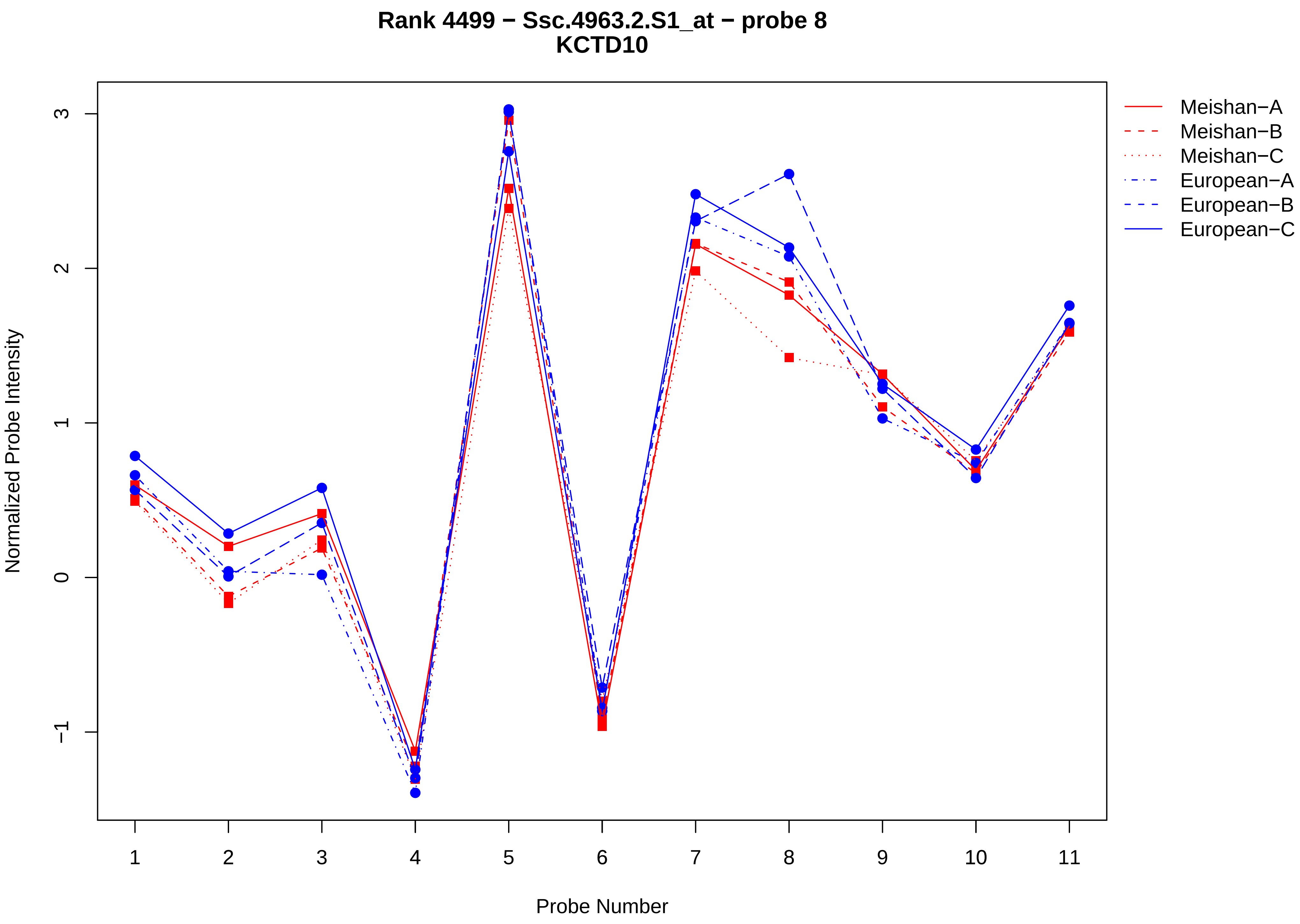

Rank 4499 − Ssc.4963.2.S1_at − probe 8
KCTD10
Normalized Probe Intensity
Probe Number
−1
0
1
2
3
1
2
3
4
5
6
7
8
9
10
11
Meishan−A
Meishan−B
Meishan−C
European−A
European−B
European−C

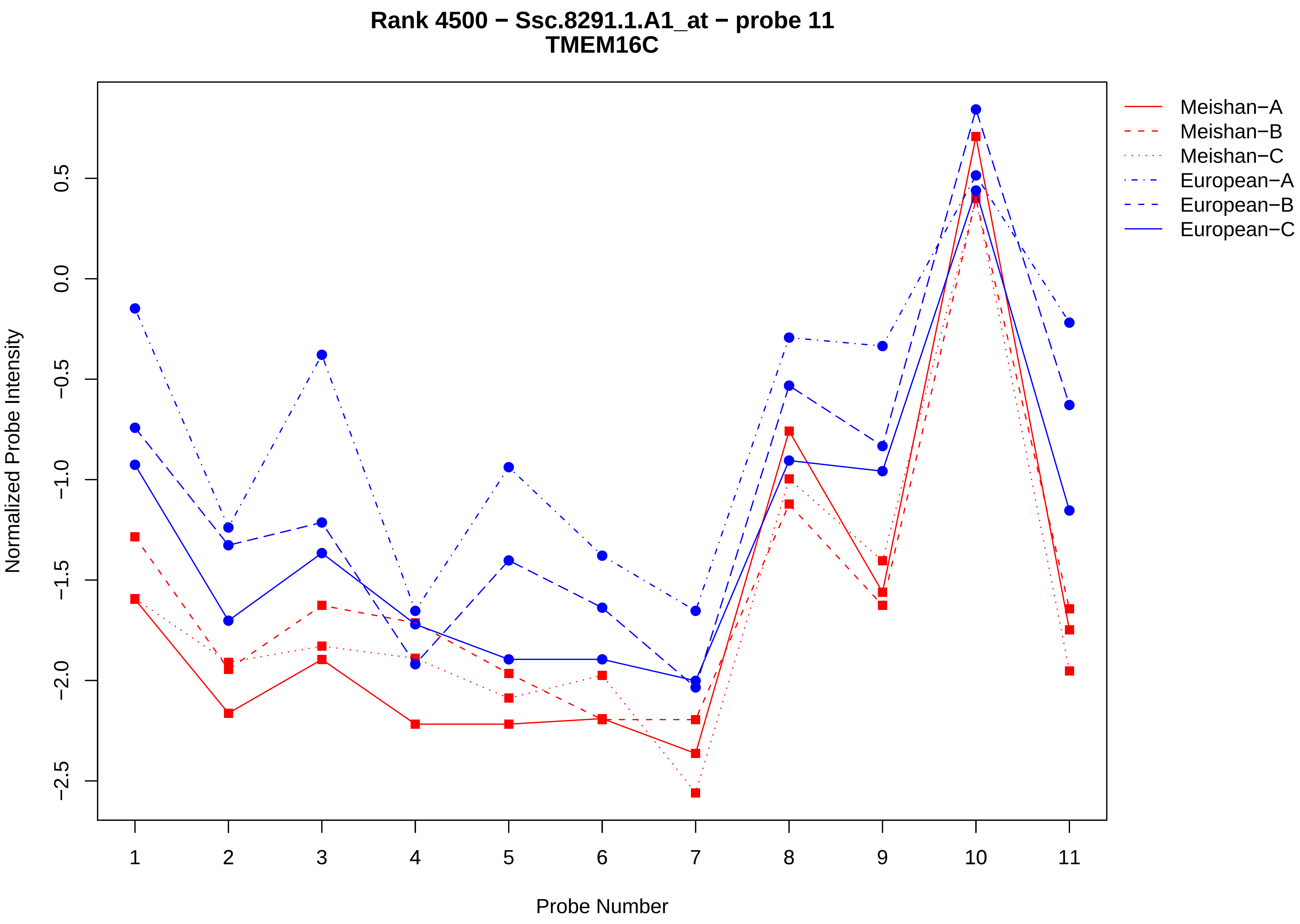

Rank 4500 − Ssc.8291.1.A1_at − probe 11
TMEM16C
Normalized Probe Intensity
Probe Number
0.5
0.0
−0.5
−1.0
−1.5
−2.0
−2.5
1
2
3
4
5
6
7
8
9
10
11
Meishan−A
Meishan−B
Meishan−C
European−A
European−B
European−C

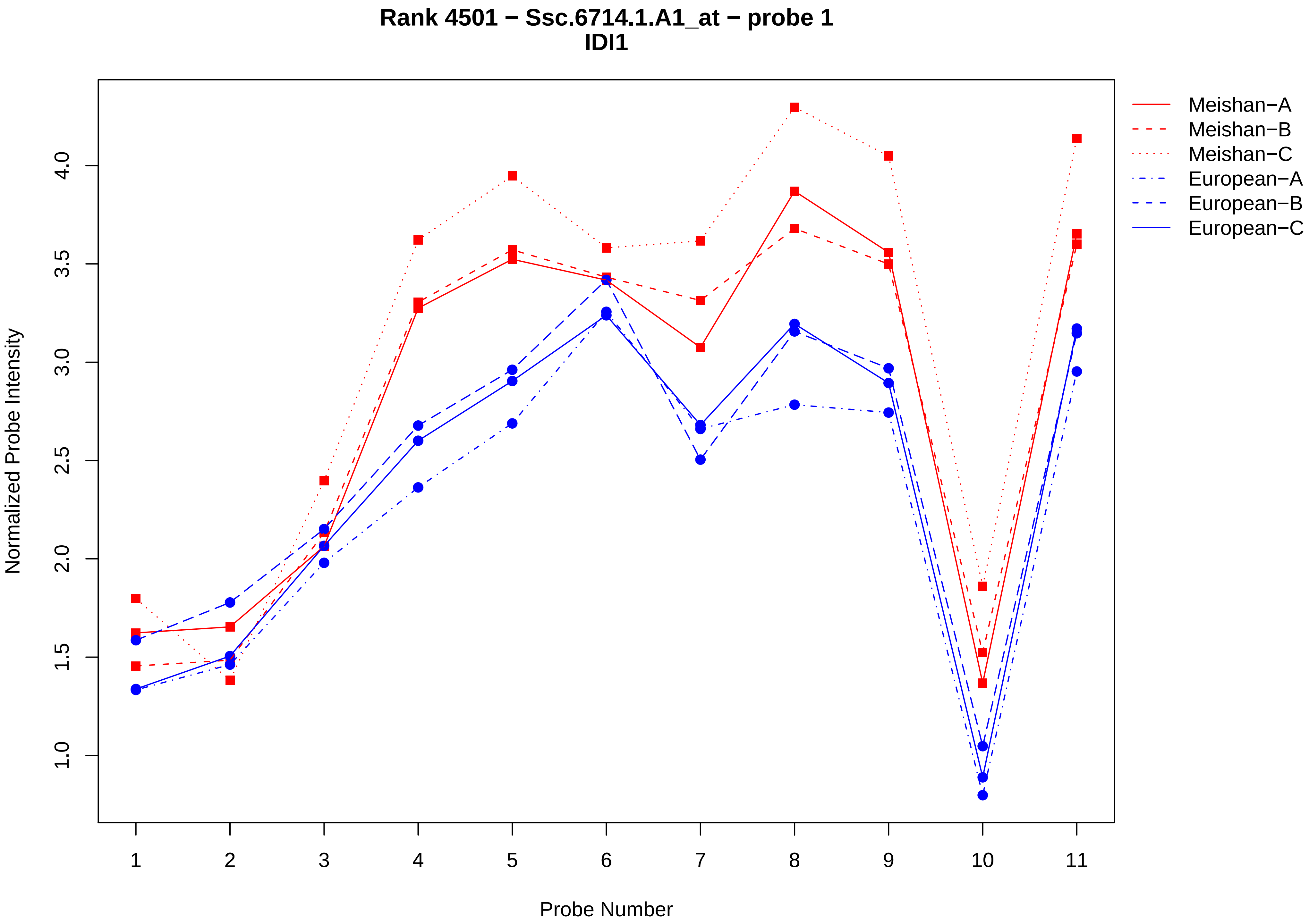

Rank 4501 − Ssc.6714.1.A1_at − probe 1
IDI1
Normalized Probe Intensity
1.0
1.5
2.0
2.5
3.0
3.5
4.0
1
2
3
4
5
6
7
8
9
10
11
Probe Number
Meishan−A
Meishan−B
Meishan−C
European−A
European−B
European−C

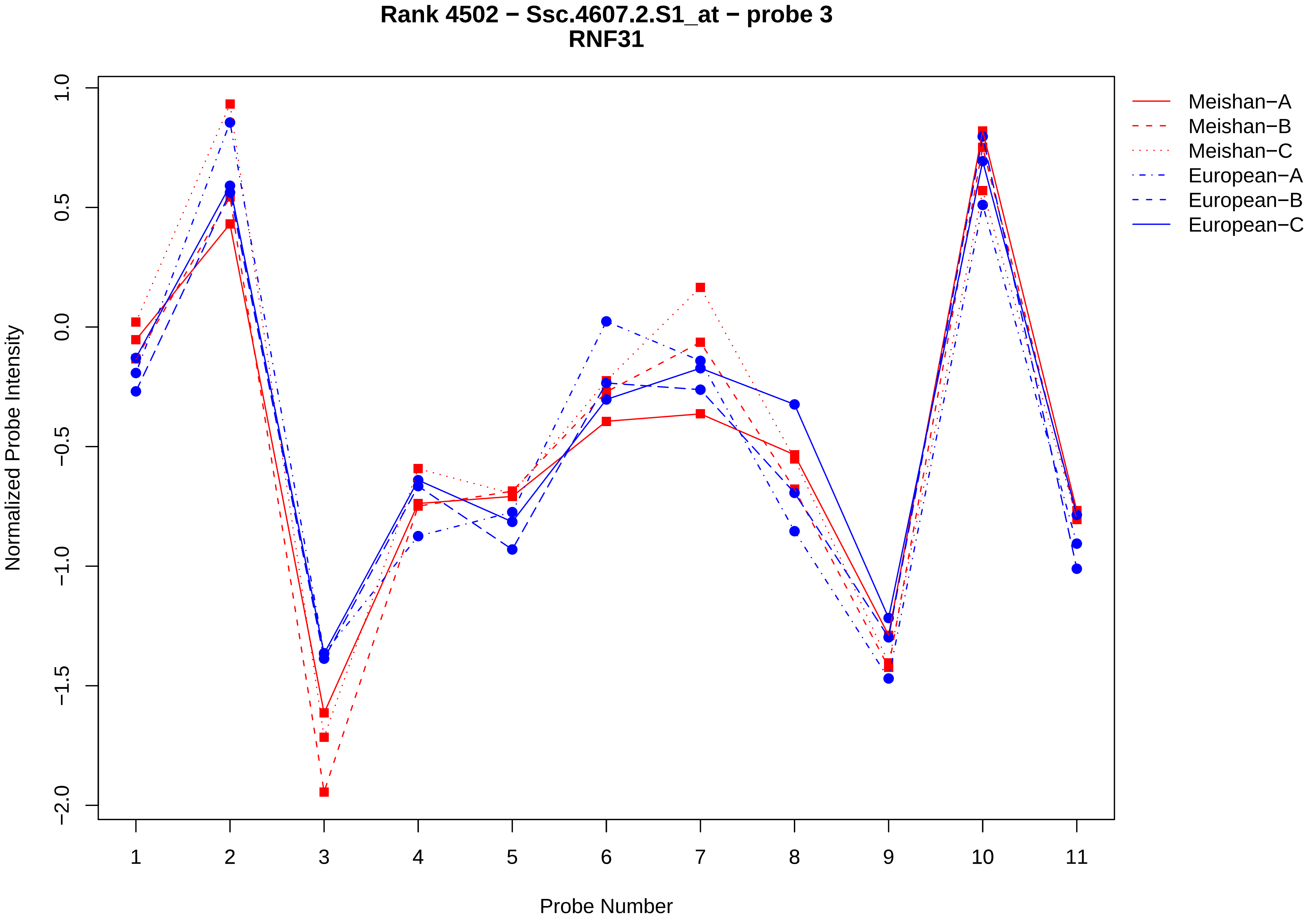
Rank 4502 − Ssc.4607.2.S1_at − probe 3
RNF31
Normalized Probe Intensity
Probe Number
Meishan−A
Meishan−B
Meishan−C
European−A
European−B
European−C
1.0
0.5
0.0
−0.5
−1.0
−1.5
−2.0
1
2
3
4
5
6
7
8
9
10
11

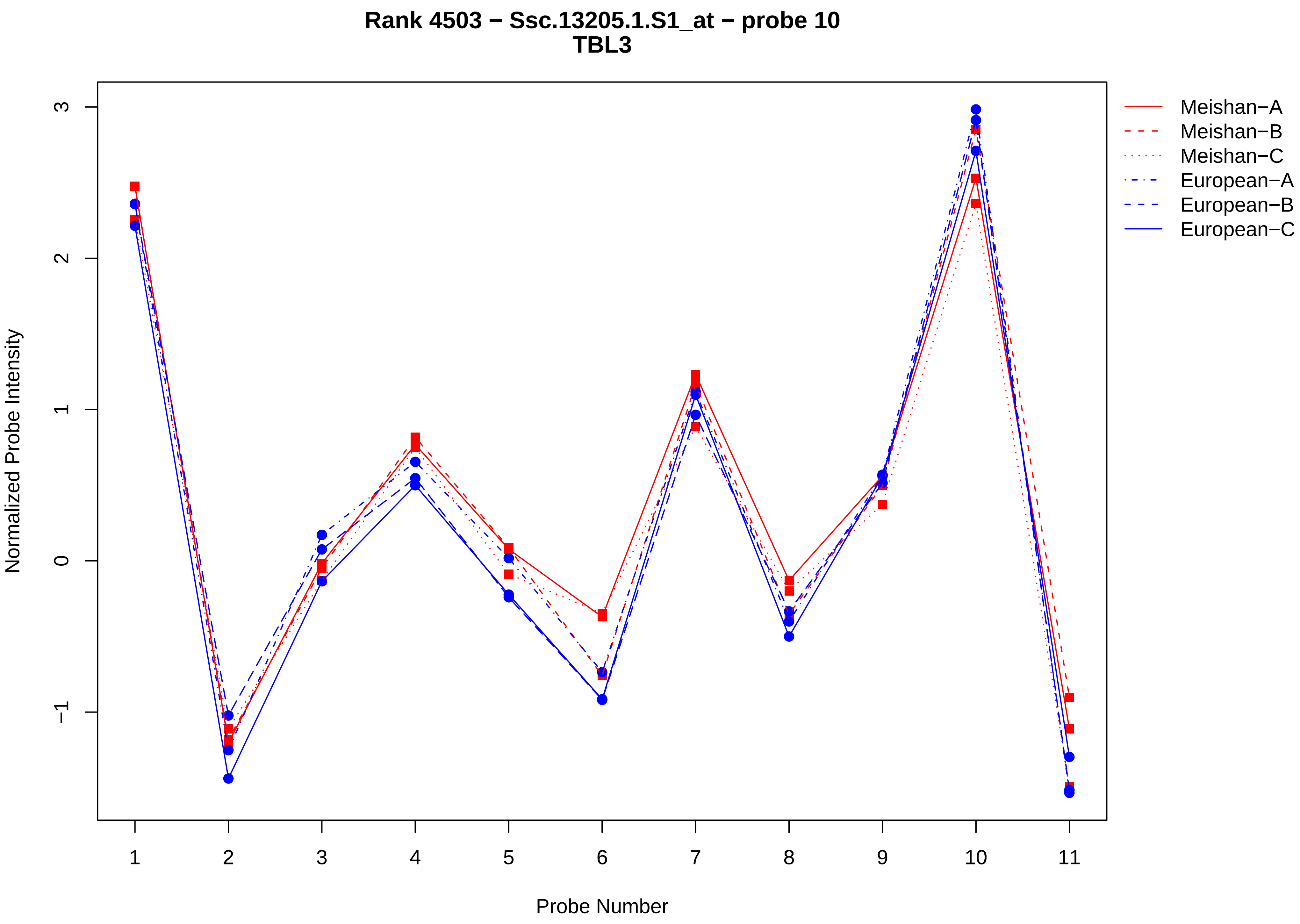

Rank 4503 − Ssc.13205.1.S1_at − probe 10
TBL3
Normalized Probe Intensity
Probe Number
−1
0
1
2
3
1
2
3
4
5
6
7
8
9
10
11
Meishan−A
Meishan−B
Meishan−C
European−A
European−B
European−C

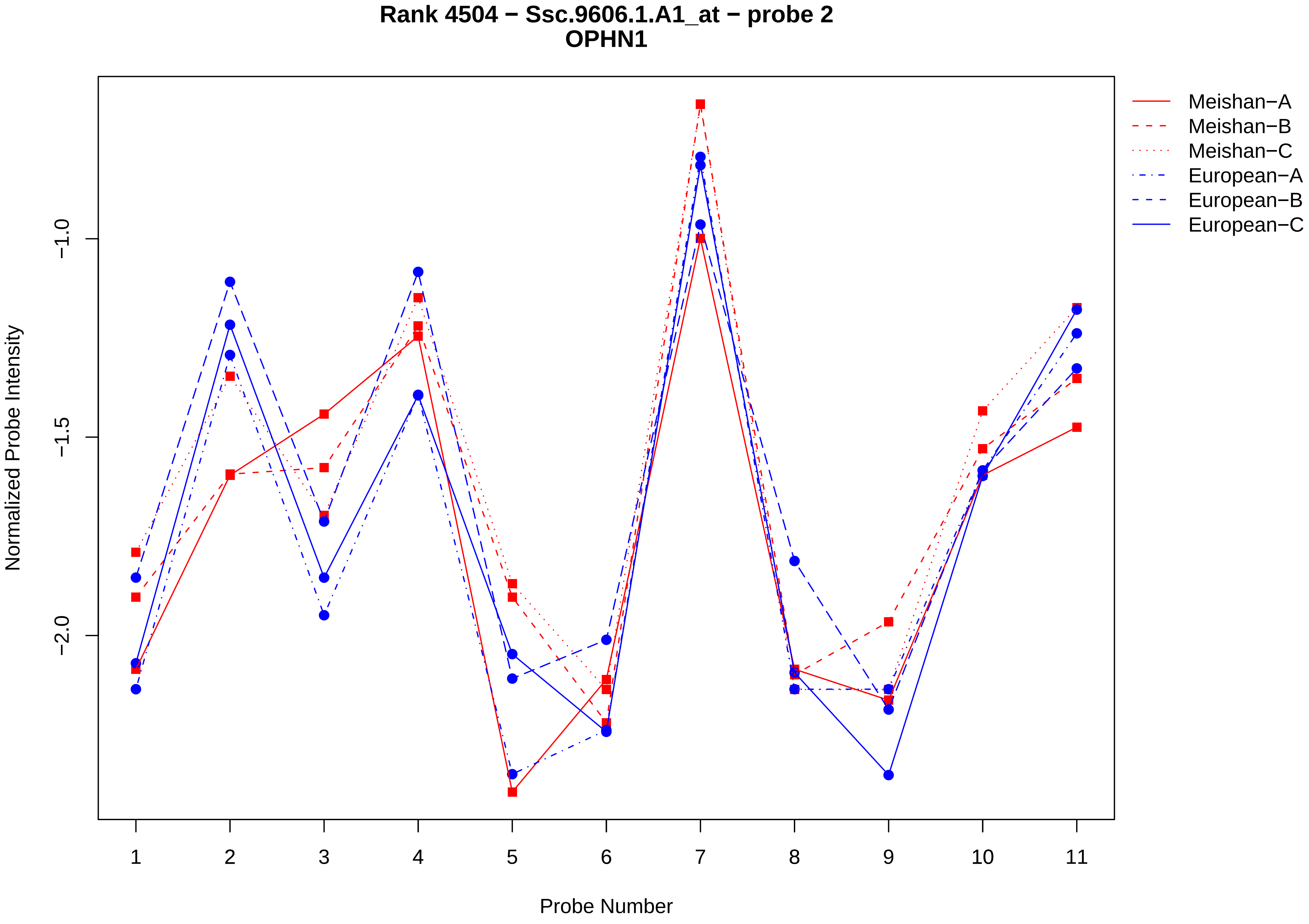

Rank 4504 − Ssc.9606.1.A1_at − probe 2
OPHN1
Normalized Probe Intensity
−1.0
−1.5
−2.0
Probe Number
1
2
3
4
5
6
7
8
9
10
11
Meishan−A
Meishan−B
Meishan−C
European−A
European−B
European−C

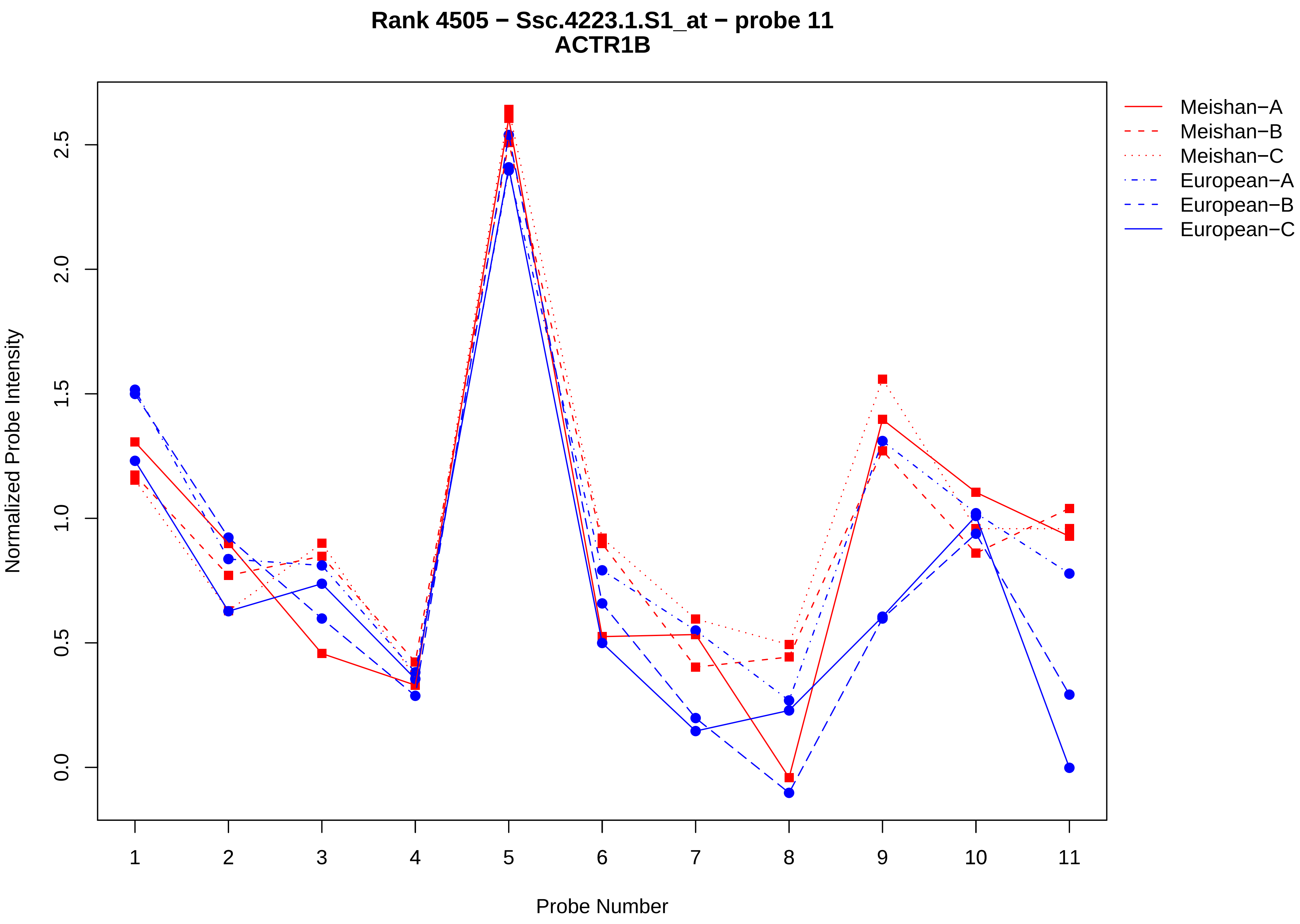

Rank 4505 − Ssc.4223.1.S1_at − probe 11
ACTR1B
Normalized Probe Intensity
Probe Number
0.0
0.5
1.0
1.5
2.0
2.5
1
2
3
4
5
6
7
8
9
10
11
Meishan−A
Meishan−B
Meishan−C
European−A
European−B
European−C

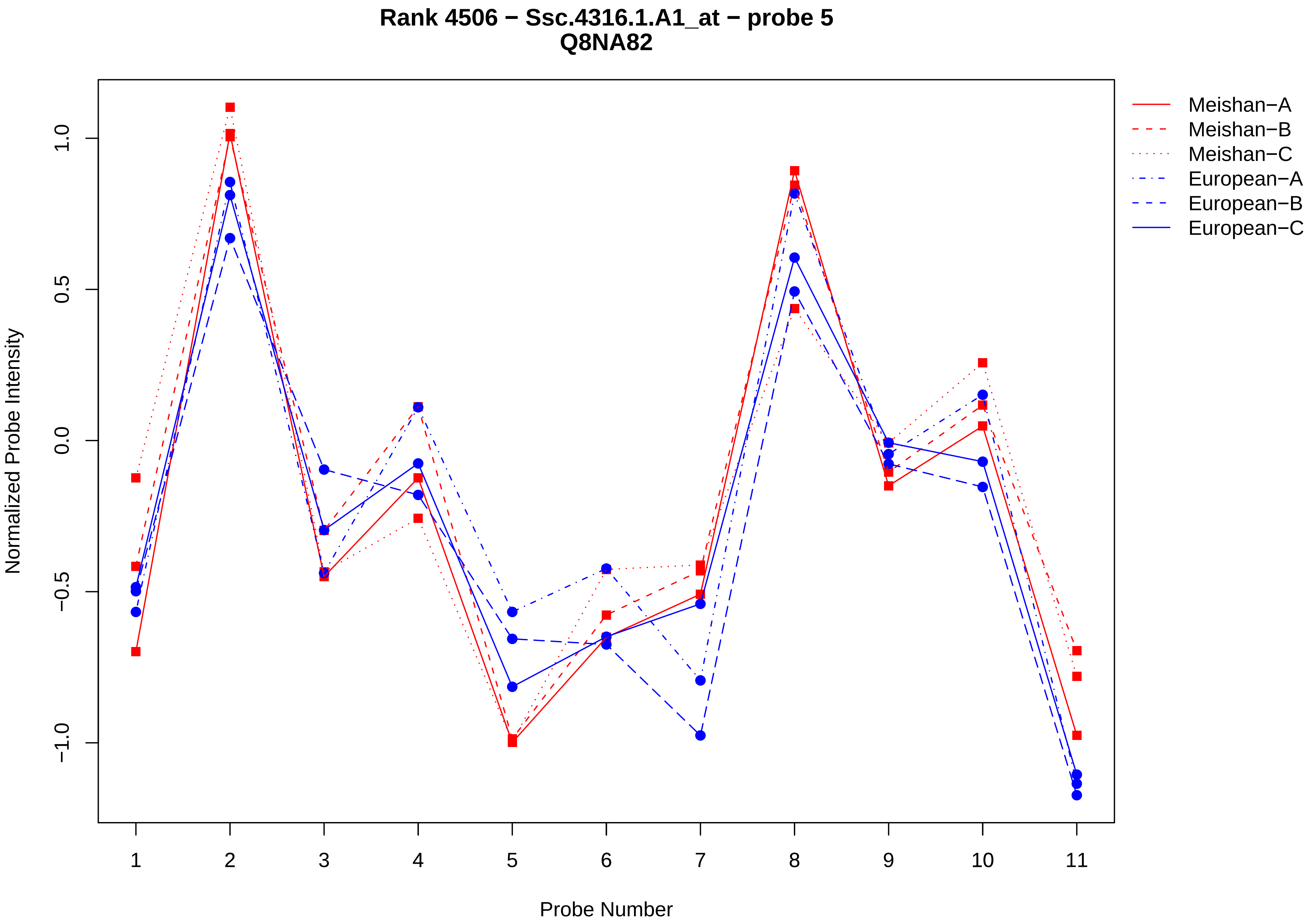

Rank 4506 − Ssc.4316.1.A1_at − probe 5
Q8NA82
Normalized Probe Intensity
Probe Number
−1.0
−0.5
0.0
0.5
1.0
1
2
3
4
5
6
7
8
9
10
11
Meishan−A
Meishan−B
Meishan−C
European−A
European−B
European−C

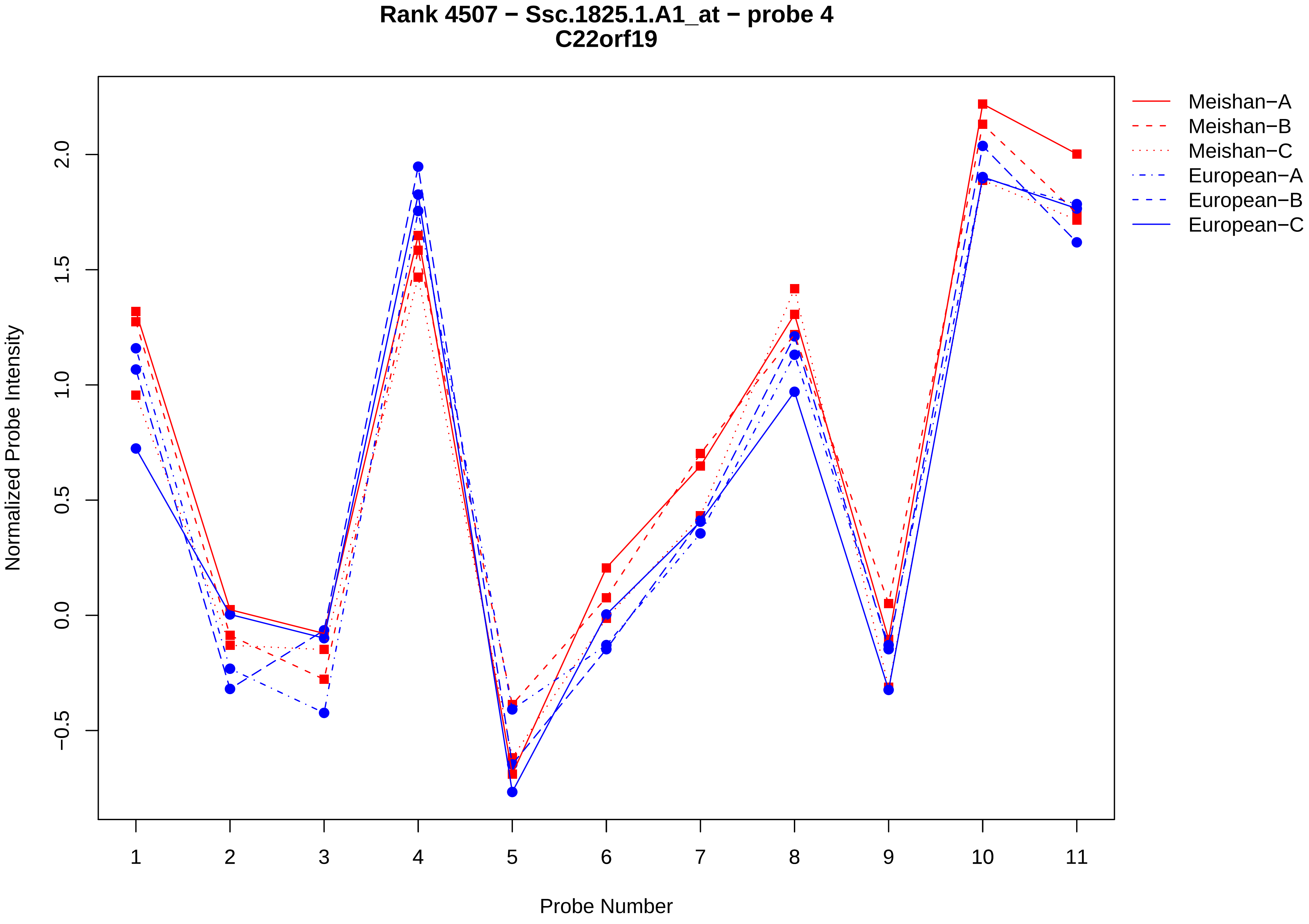

Rank 4507 − Ssc.1825.1.A1_at − probe 4
C22orf19
Normalized Probe Intensity
Probe Number
−0.5
0.0
0.5
1.0
1.5
2.0
1
2
3
4
5
6
7
8
9
10
11
Meishan−A
Meishan−B
Meishan−C
European−A
European−B
European−C

Rank 4508 − Ssc.17160.1.S1_at − probe 5
POFUT2

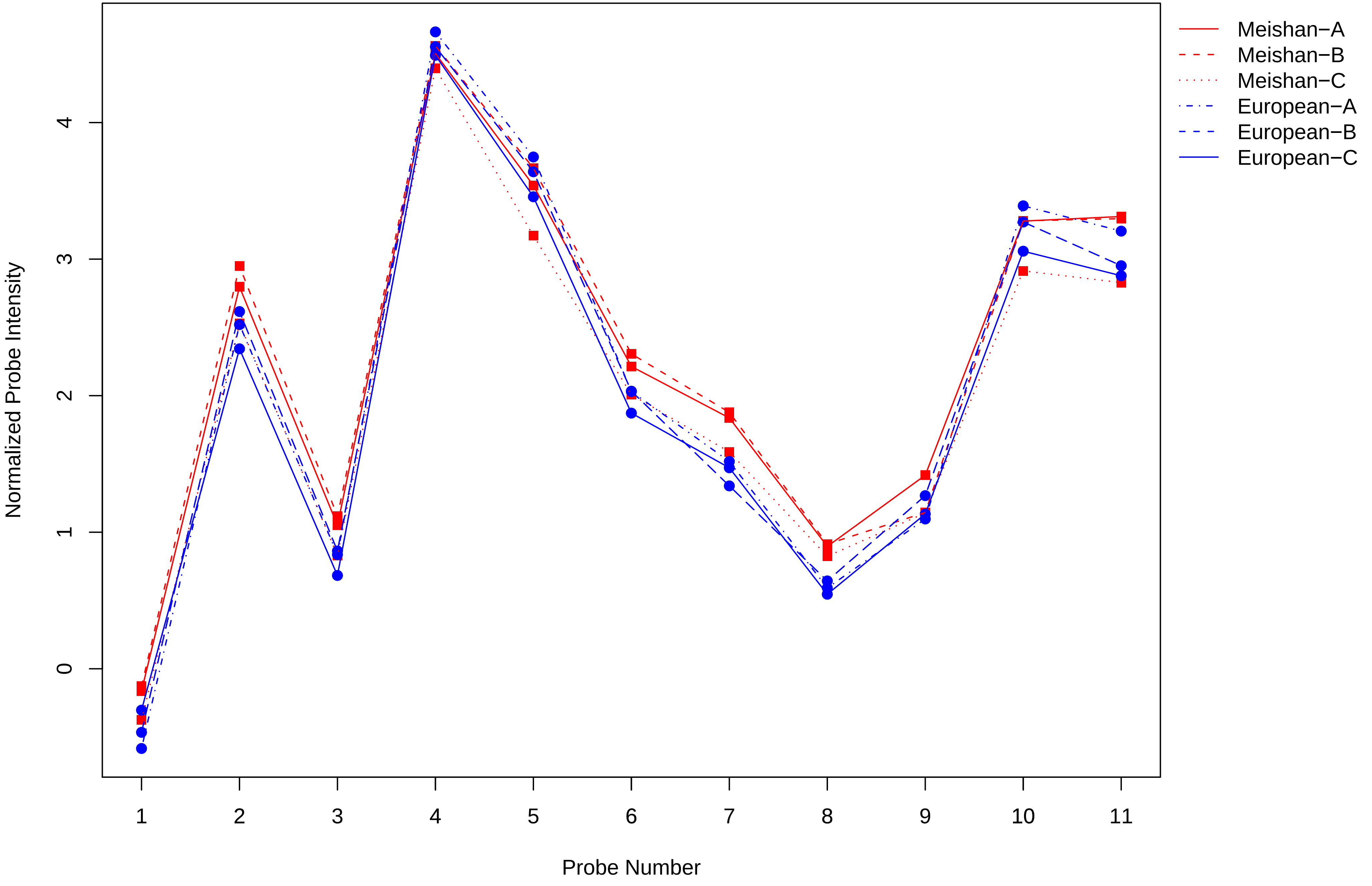

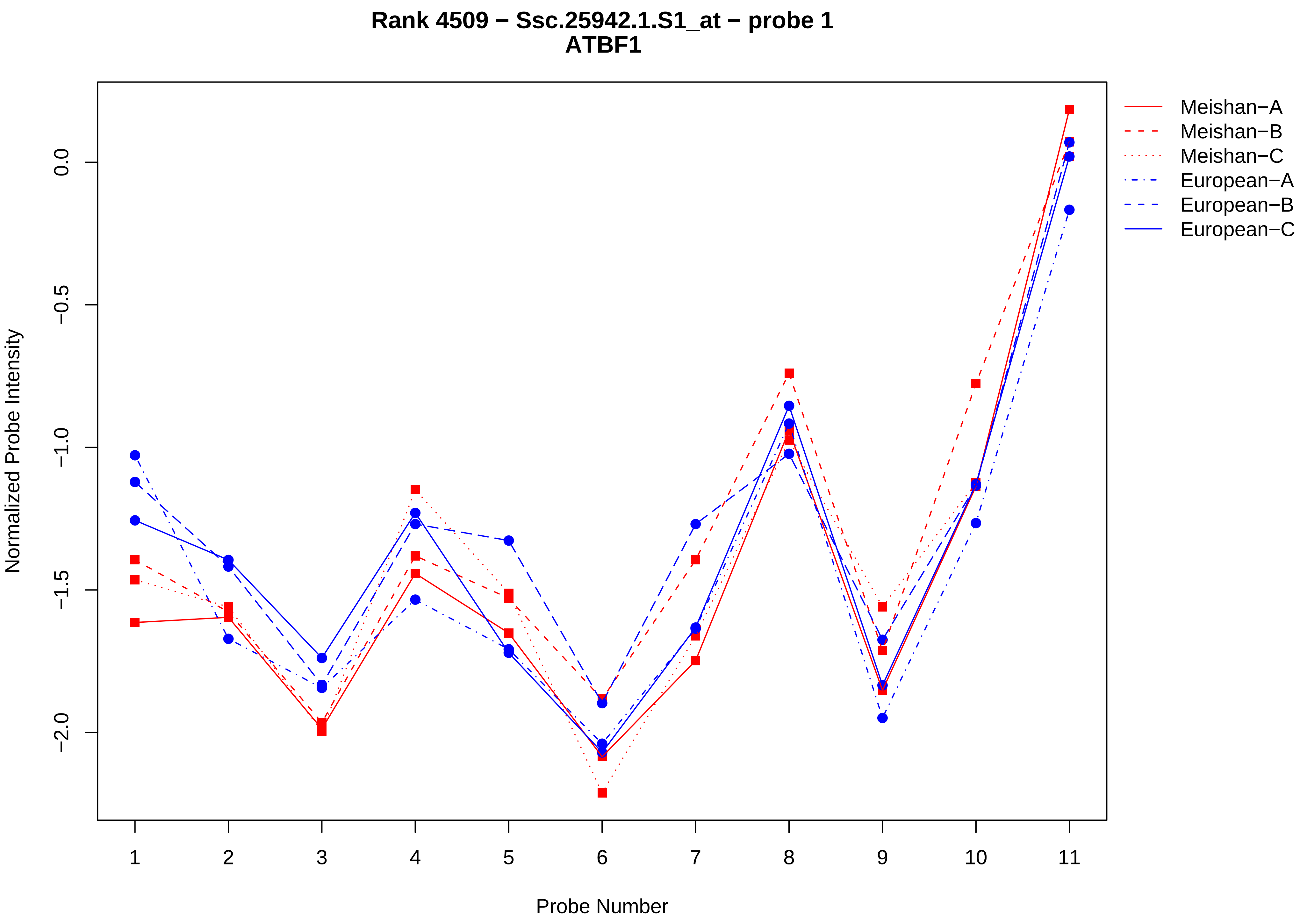

Rank 4509 − Ssc.25942.1.S1_at − probe 1
ATBF1
Normalized Probe Intensity
0.0
−0.5
−1.0
−1.5
−2.0
1
2
3
4
5
6
7
8
9
10
11
Probe Number
Meishan−A
Meishan−B
Meishan−C
European−A
European−B
European−C

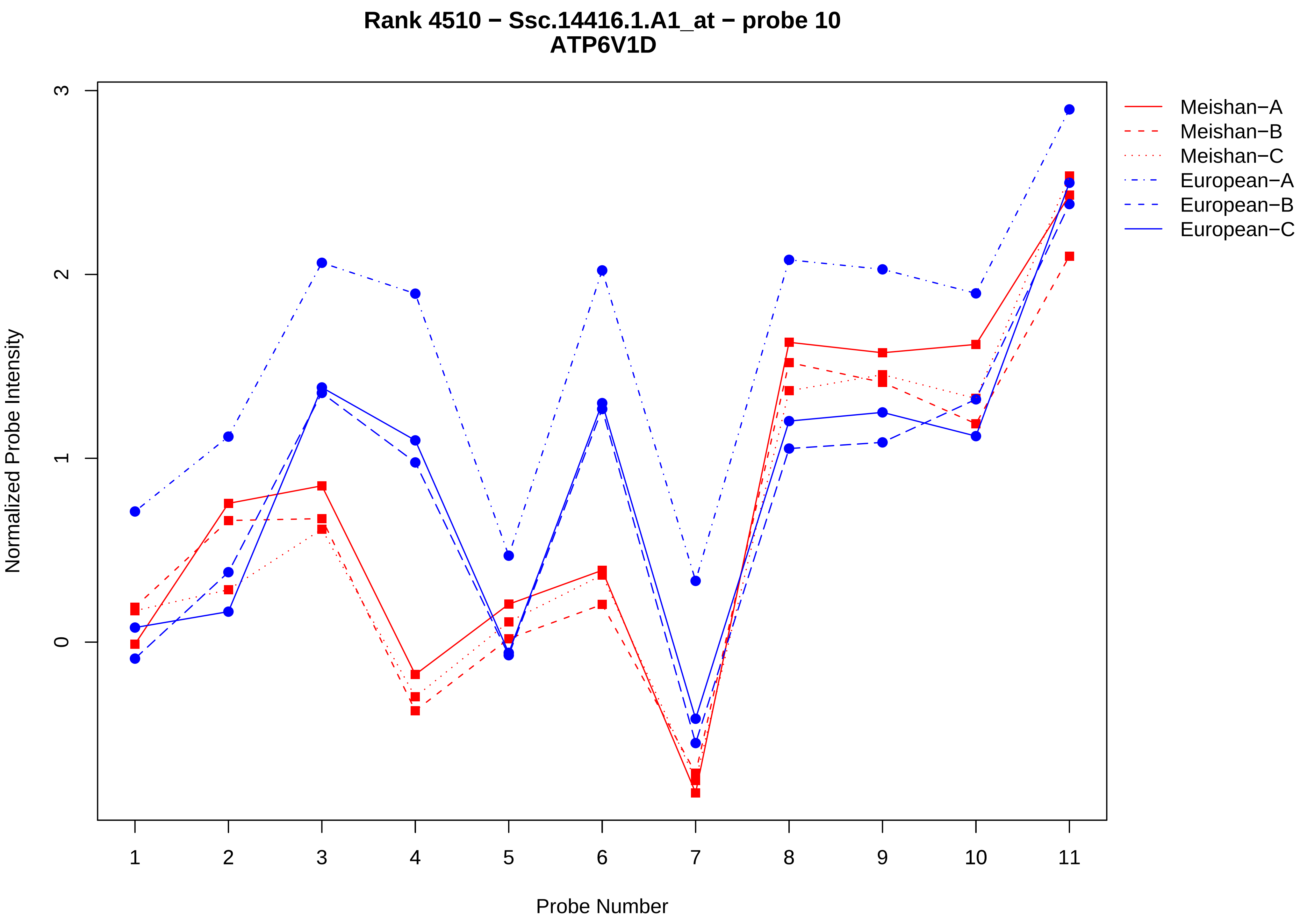

Rank 4510 − Ssc.14416.1.A1_at − probe 10
ATP6V1D
Normalized Probe Intensity
Probe Number
0
1
2
3
1
2
3
4
5
6
7
8
9
10
11
Meishan−A
Meishan−B
Meishan−C
European−A
European−B
European−C

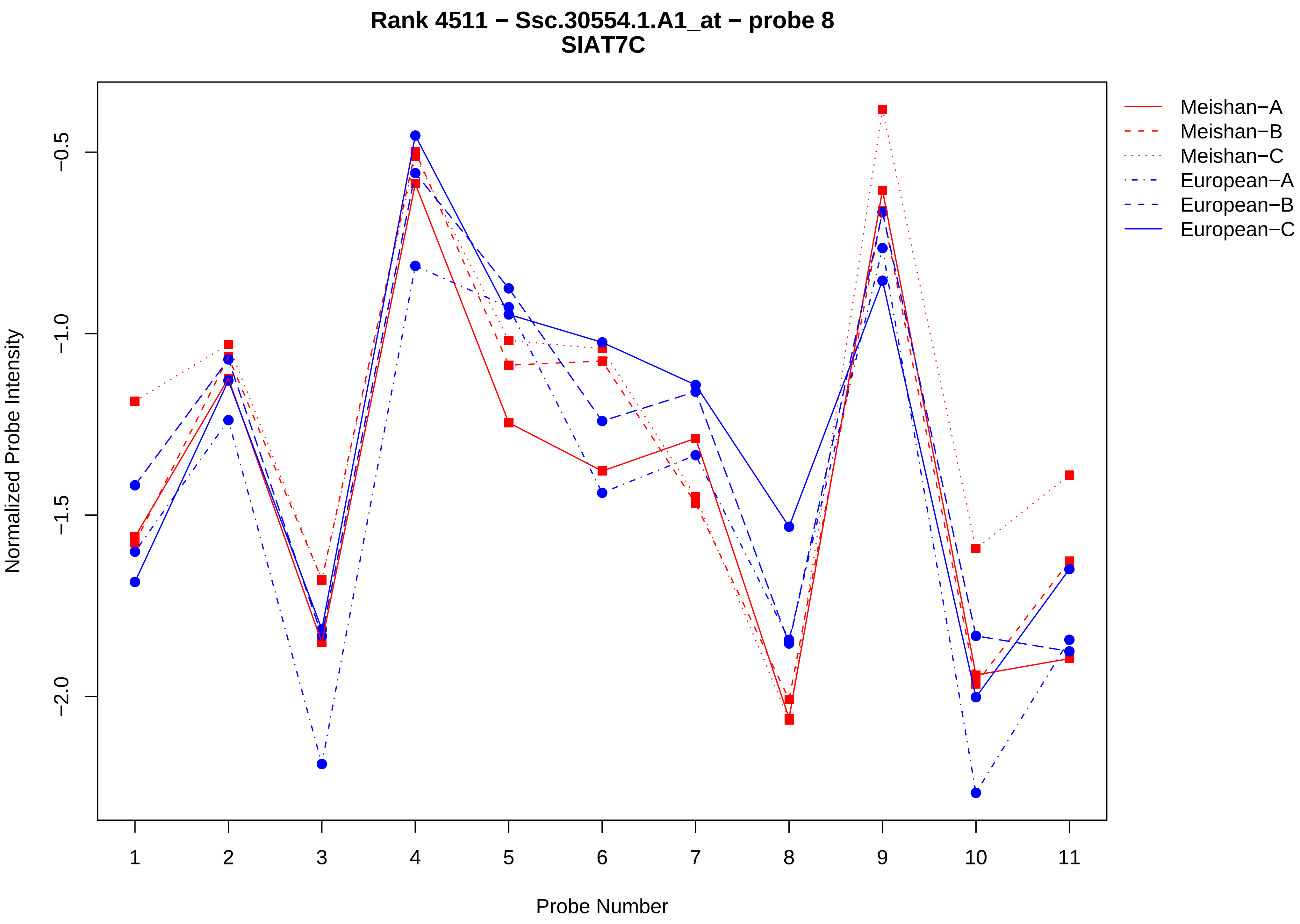

Rank 4511 − Ssc.30554.1.A1_at − probe 8
SIAT7C
Normalized Probe Intensity
−0.5
−1.0
−1.5
−2.0
1
2
3
4
5
6
7
8
9
10
11
Probe Number
Meishan−A
Meishan−B
Meishan−C
European−A
European−B
European−C

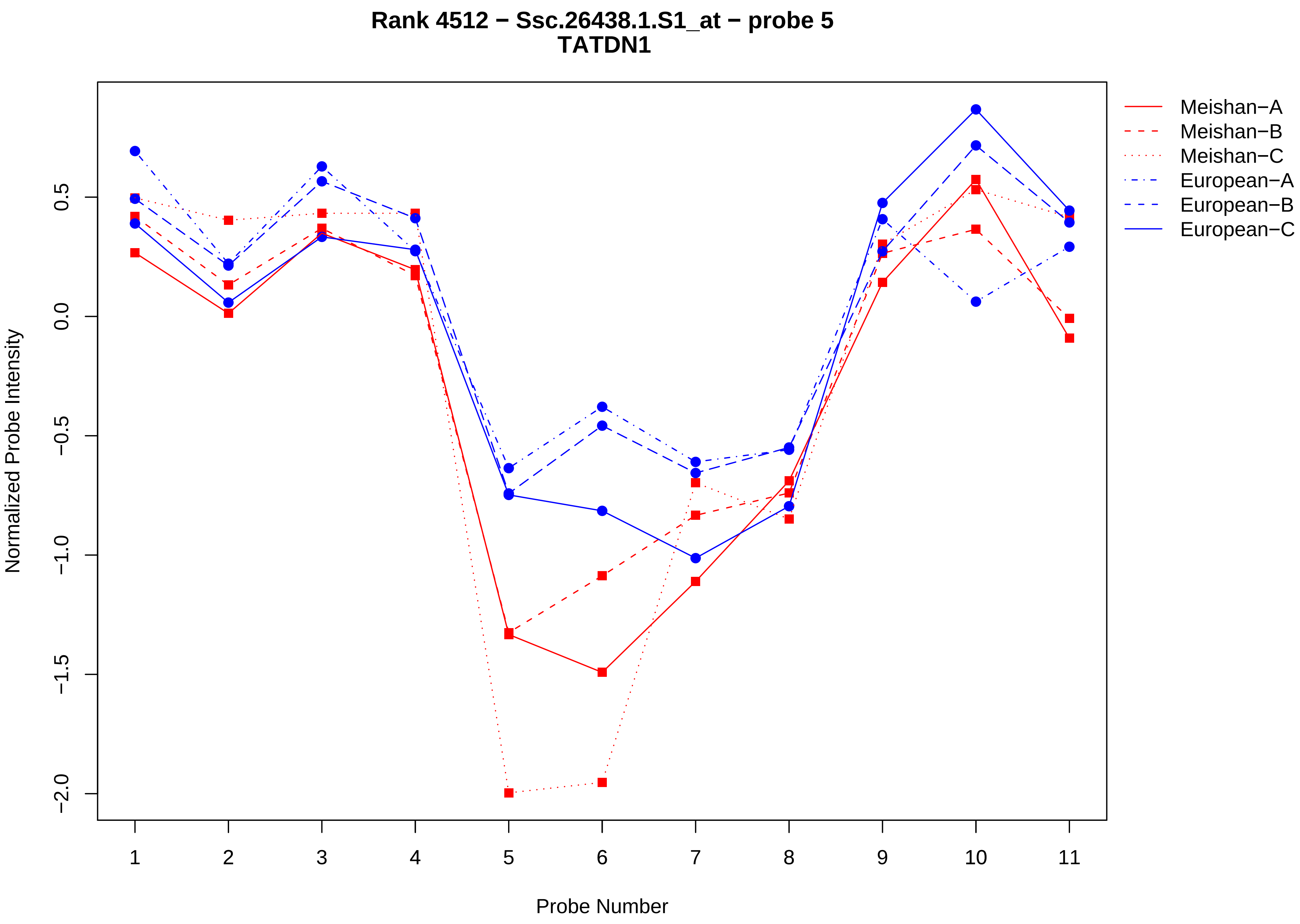
Rank 4512 − Ssc.26438.1.S1_at − probe 5
TATDN1
Normalized Probe Intensity
Probe Number
0.5
0.0
−0.5
−1.0
−1.5
−2.0
1
2
3
4
5
6
7
8
9
10
11
Meishan−A
Meishan−B
Meishan−C
European−A
European−B
European−C

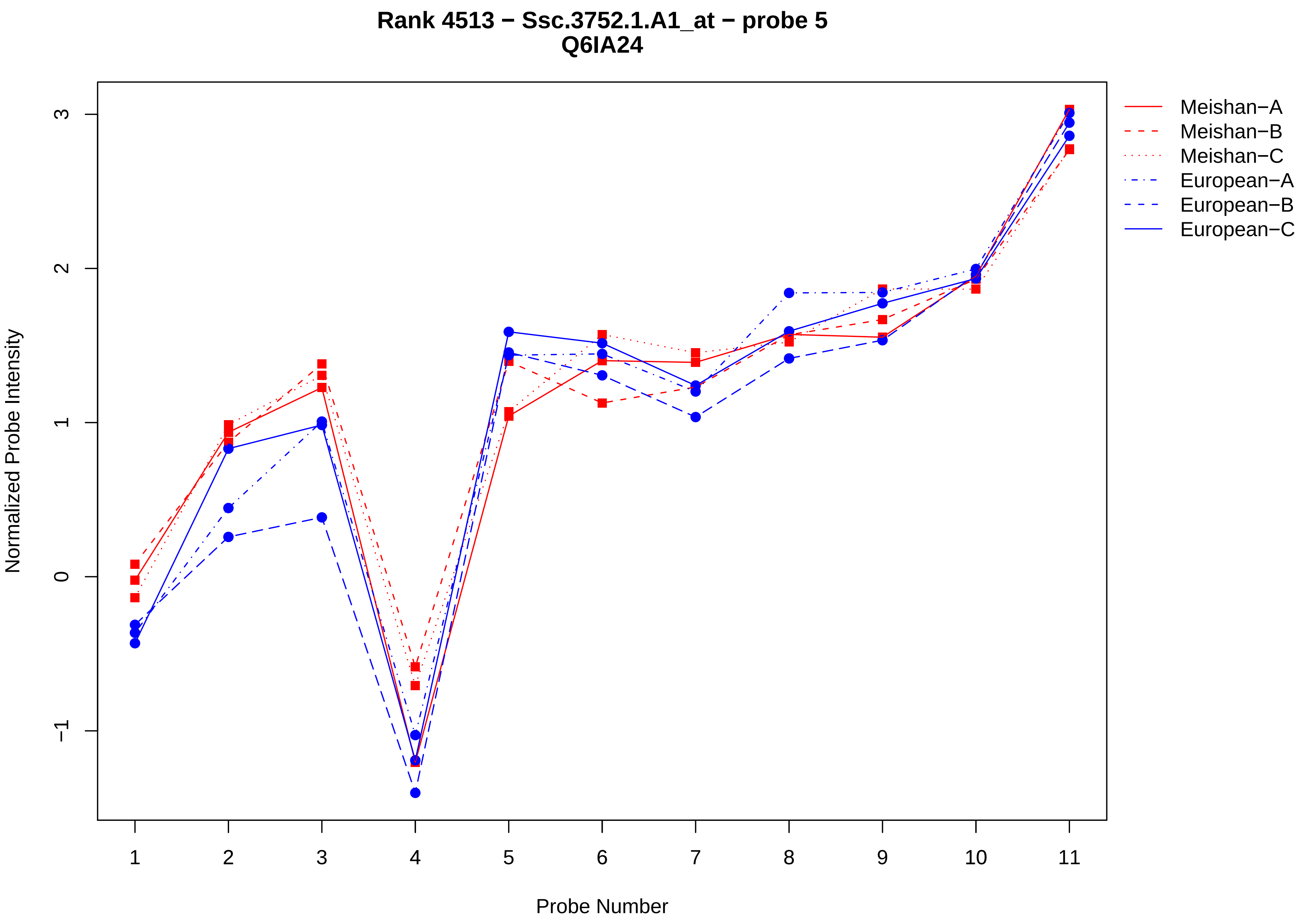

Rank 4513 − Ssc.3752.1.A1_at − probe 5
Q6IA24
Normalized Probe Intensity
Probe Number
−1
0
1
2
3
1
2
3
4
5
6
7
8
9
10
11
Meishan−A
Meishan−B
Meishan−C
European−A
European−B
European−C

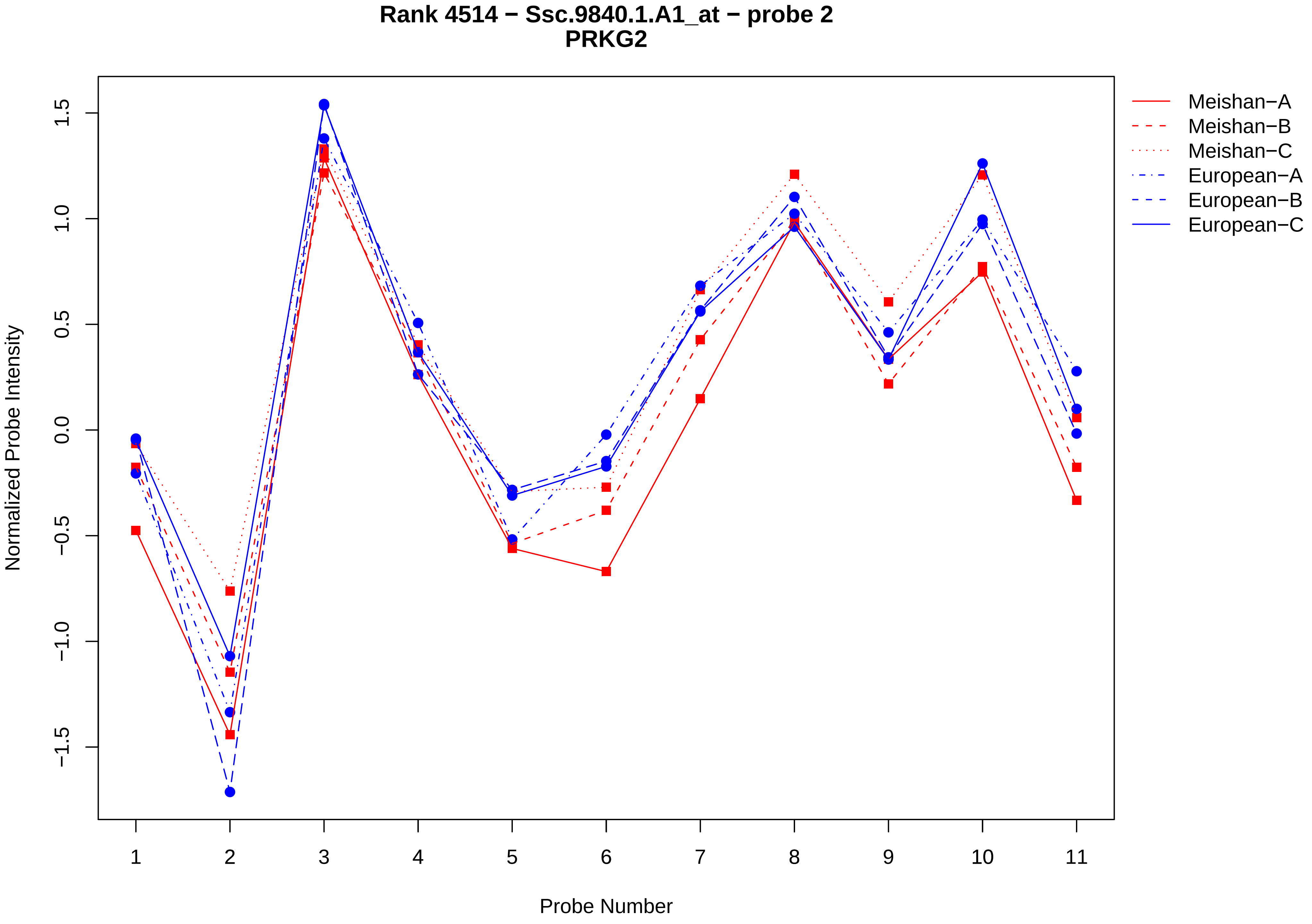

Rank 4514 − Ssc.9840.1.A1_at − probe 2
PRKG2
Normalized Probe Intensity
Probe Number
1.5
1.0
0.5
0.0
−0.5
−1.0
−1.5
1
2
3
4
5
6
7
8
9
10
11
Meishan−A
Meishan−B
Meishan−C
European−A
European−B
European−C

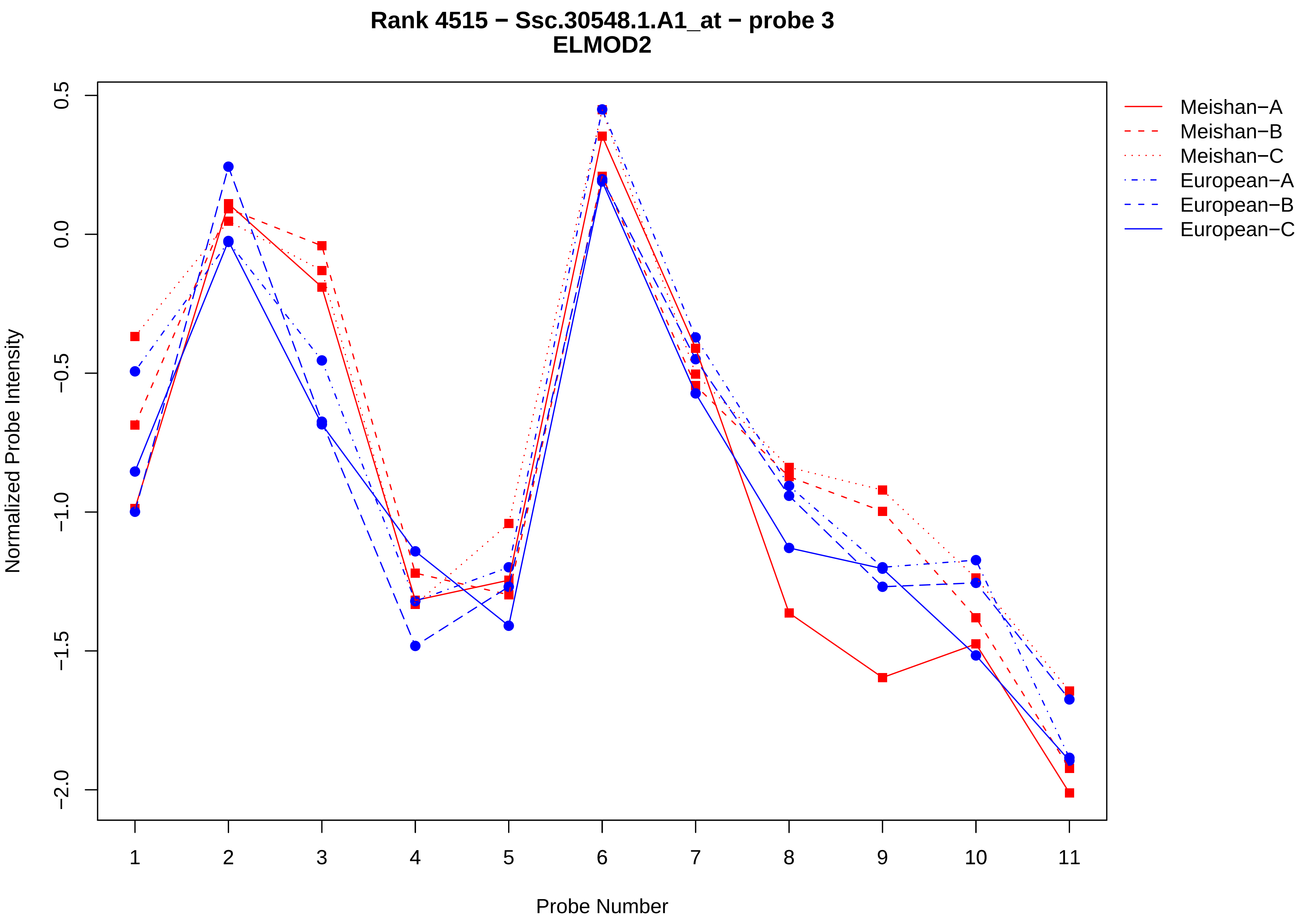
Rank 4515 − Ssc.30548.1.A1_at − probe 3
ELMOD2
Normalized Probe Intensity
0.5
0.0
−0.5
−1.0
−1.5
−2.0
1
2
3
4
5
6
7
8
9
10
11
Probe Number
Meishan−A
Meishan−B
Meishan−C
European−A
European−B
European−C

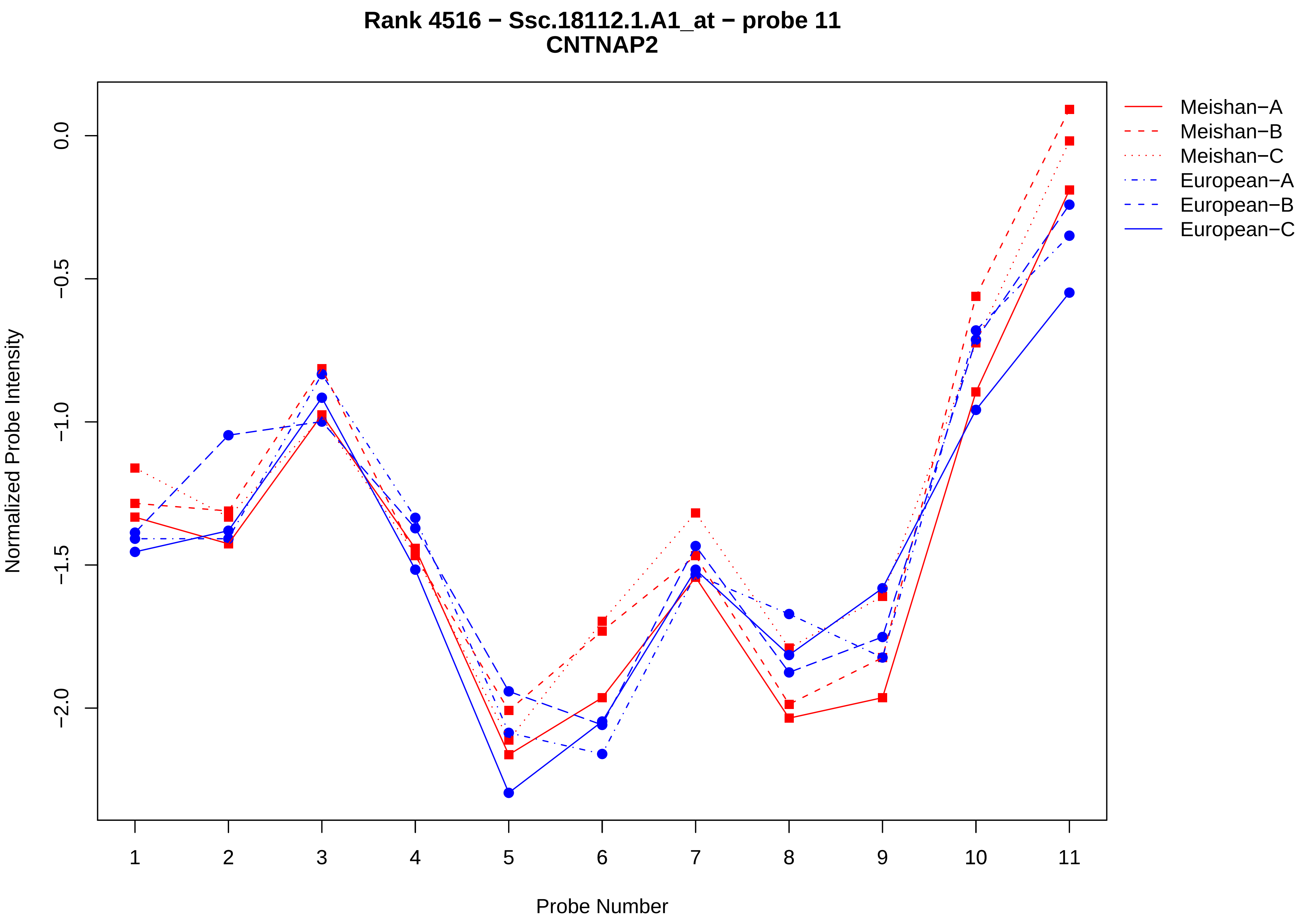

Rank 4516 − Ssc.18112.1.A1_at − probe 11
CNTNAP2
Normalized Probe Intensity
0.0
−0.5
−1.0
−1.5
−2.0
1
2
3
4
5
6
7
8
9
10
11
Probe Number
Meishan−A
Meishan−B
Meishan−C
European−A
European−B
European−C

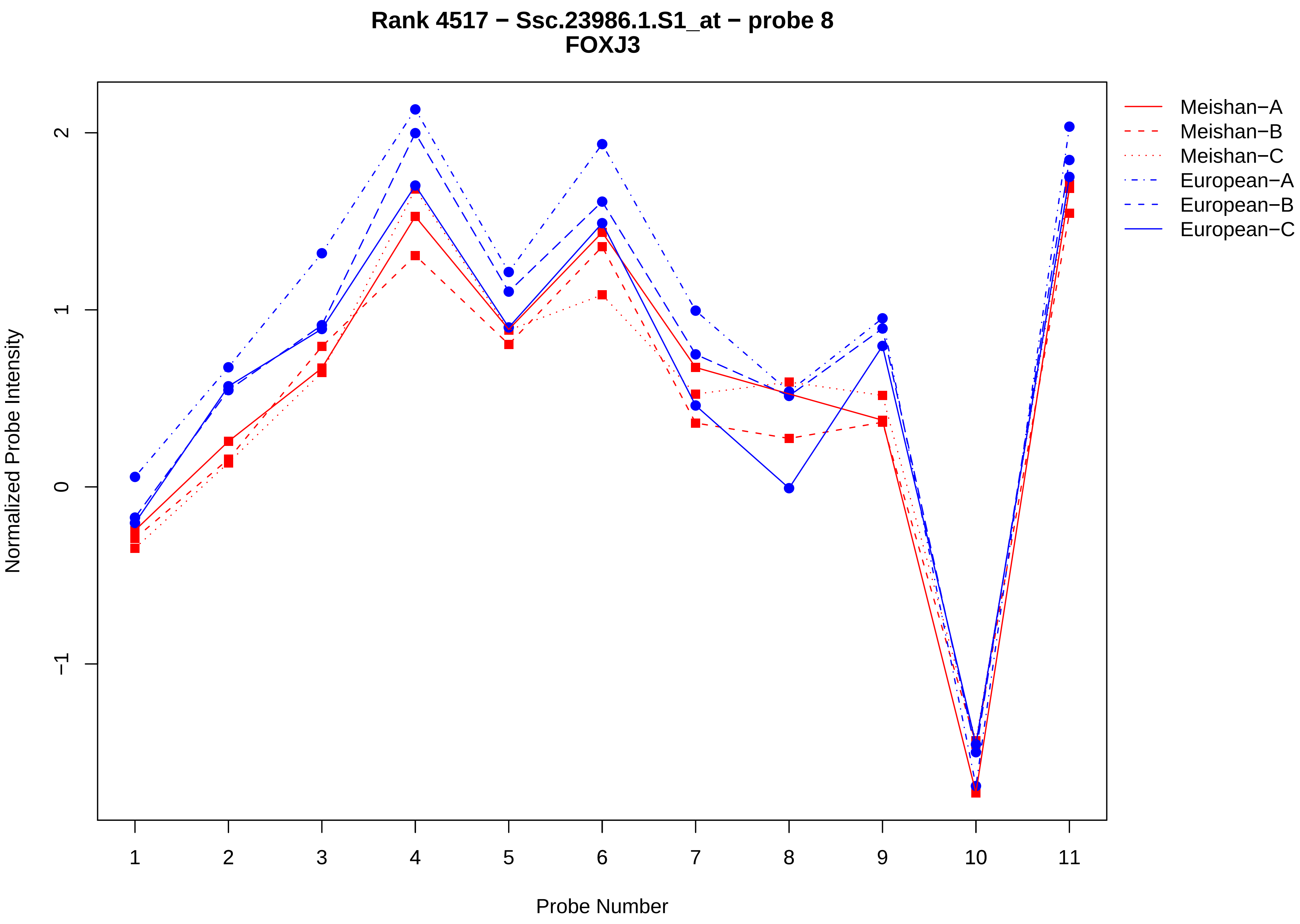

Rank 4517 − Ssc.23986.1.S1_at − probe 8
FOXJ3
Normalized Probe Intensity
Probe Number
Meishan−A
Meishan−B
Meishan−C
European−A
European−B
European−C
−1
0
1
2
1
2
3
4
5
6
7
8
9
10
11

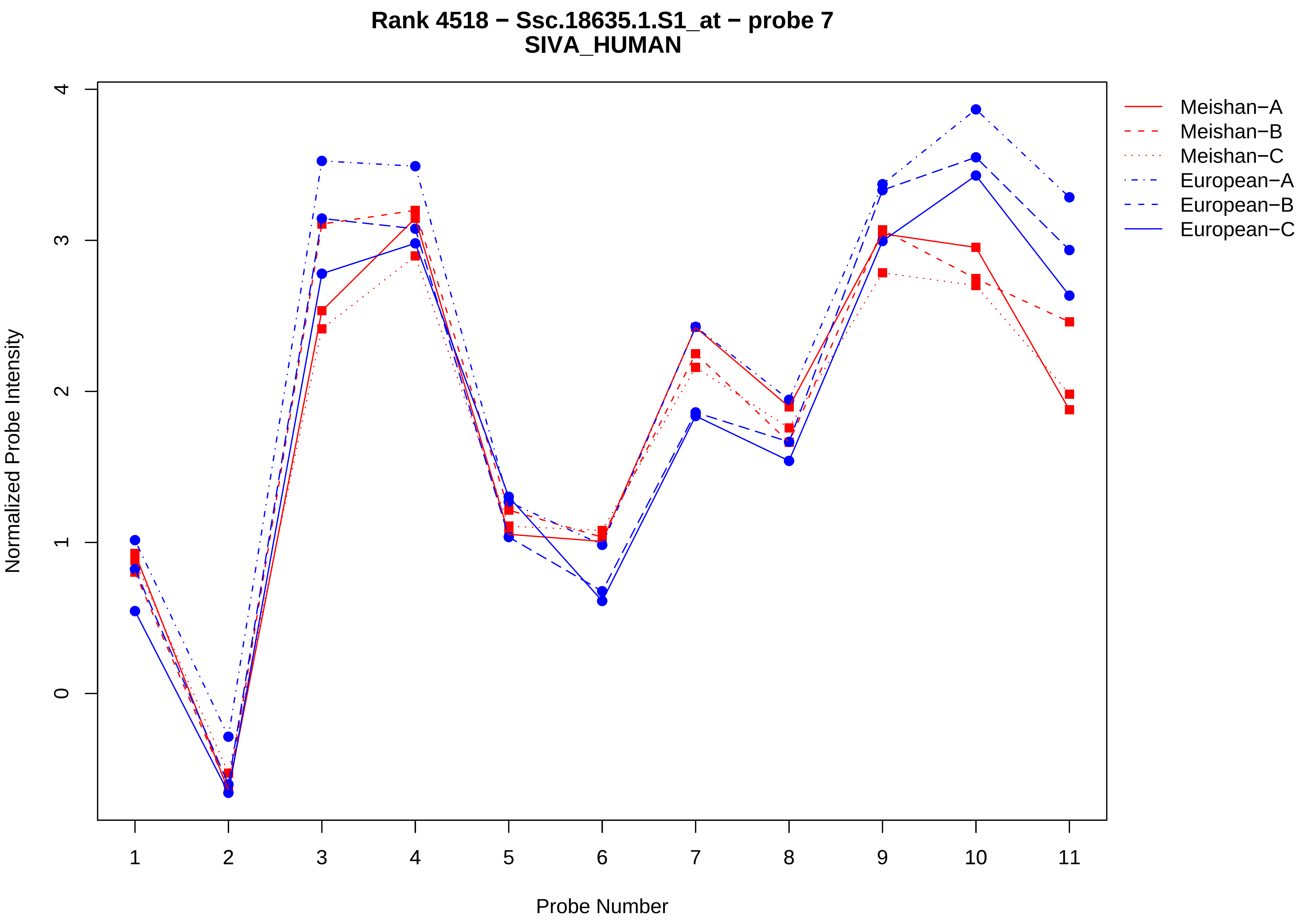

Rank 4518 − Ssc.18635.1.S1_at − probe 7
SIVA_HUMAN
Normalized Probe Intensity
Probe Number
0
1
2
3
4
1
2
3
4
5
6
7
8
9
10
11
Meishan−A
Meishan−B
Meishan−C
European−A
European−B
European−C

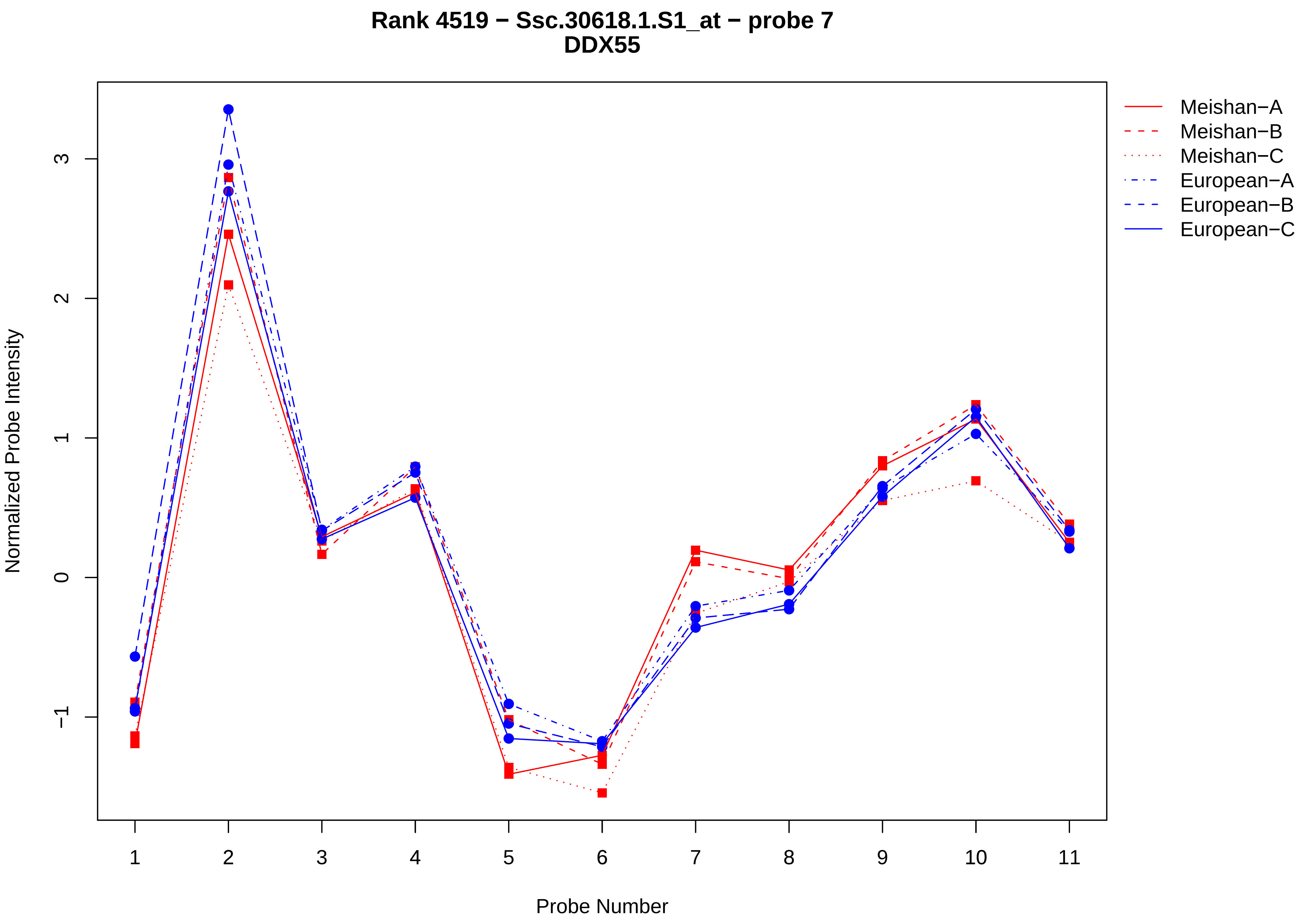

Rank 4519 − Ssc.30618.1.S1_at − probe 7
DDX55
Normalized Probe Intensity
Probe Number
−1
0
1
2
3
1
2
3
4
5
6
7
8
9
10
11
Meishan−A
Meishan−B
Meishan−C
European−A
European−B
European−C

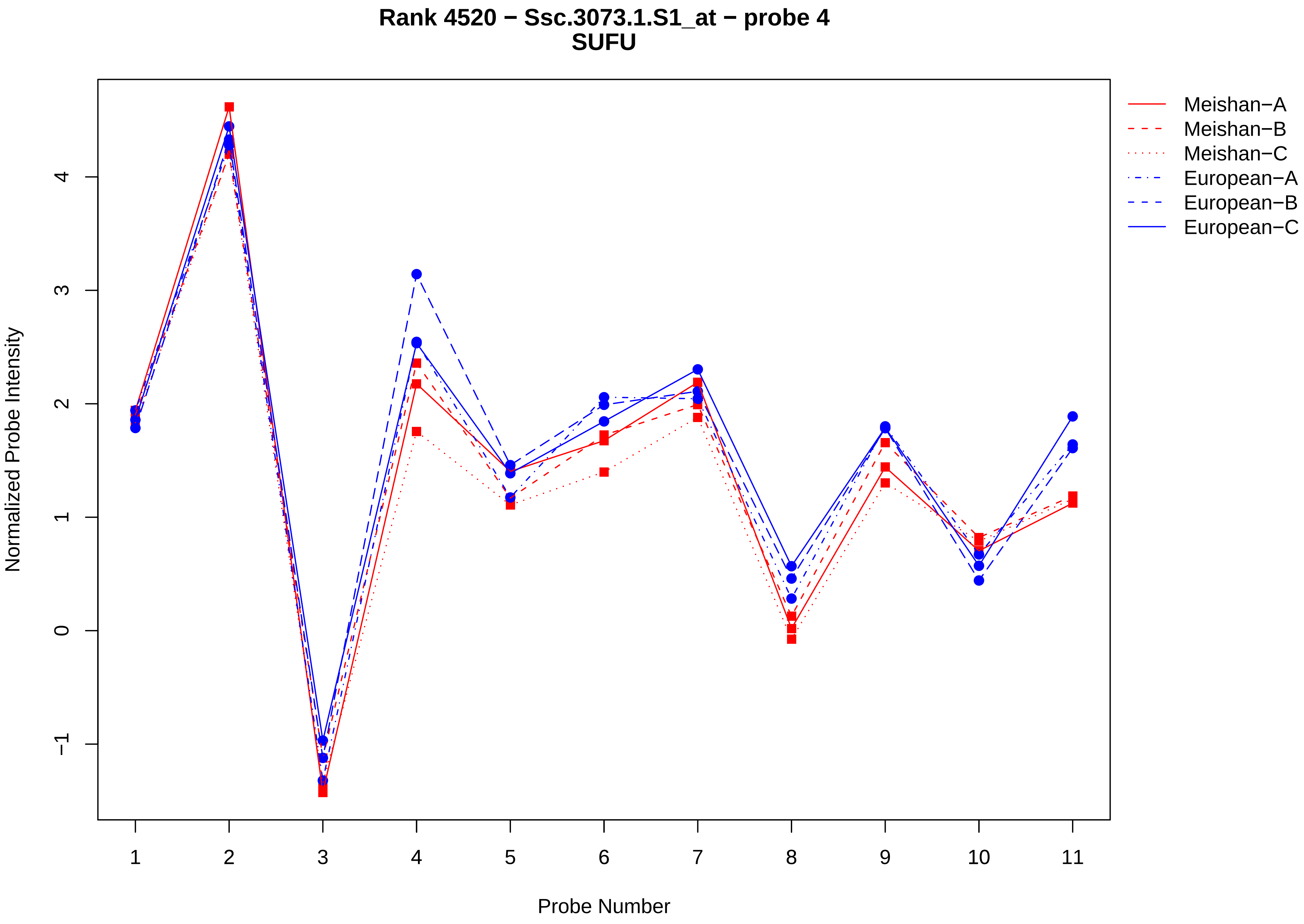

Rank 4520 − Ssc.3073.1.S1_at − probe 4
SUFU
Normalized Probe Intensity
Probe Number
Meishan−A
Meishan−B
Meishan−C
European−A
European−B
European−C
−1
0
1
2
3
4
1
2
3
4
5
6
7
8
9
10
11

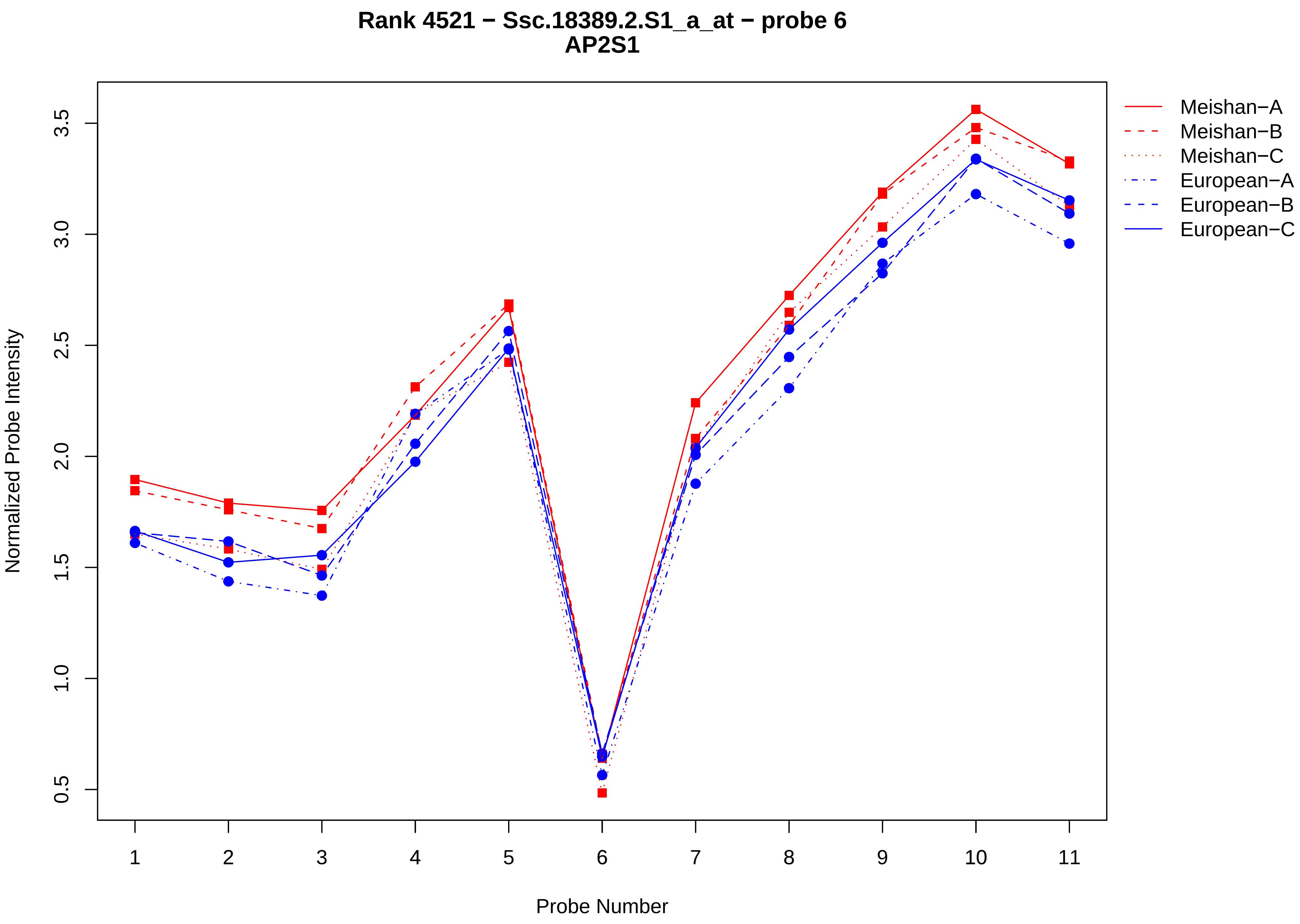
Rank 4521 − Ssc.18389.2.S1_a_at − probe 6
AP2S1
Normalized Probe Intensity
Probe Number
Meishan−A
Meishan−B
Meishan−C
European−A
European−B
European−C
0.5
1.0
1.5
2.0
2.5
3.0
3.5
1
2
3
4
5
6
7
8
9
10
11

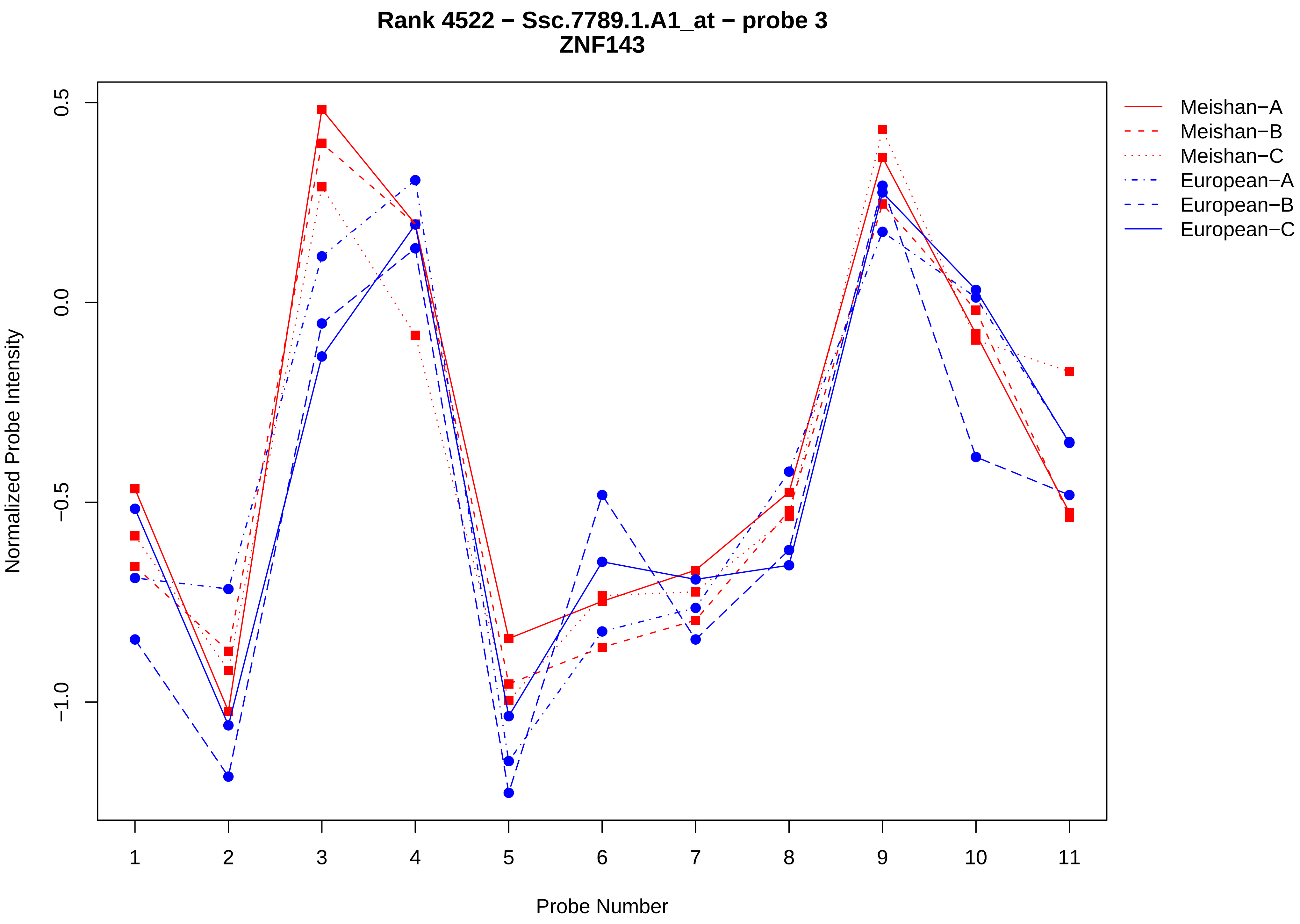

Rank 4522 − Ssc.7789.1.A1_at − probe 3
ZNF143
Normalized Probe Intensity
Probe Number
0.5
0.0
−0.5
−1.0
1
2
3
4
5
6
7
8
9
10
11
Meishan−A
Meishan−B
Meishan−C
European−A
European−B
European−C

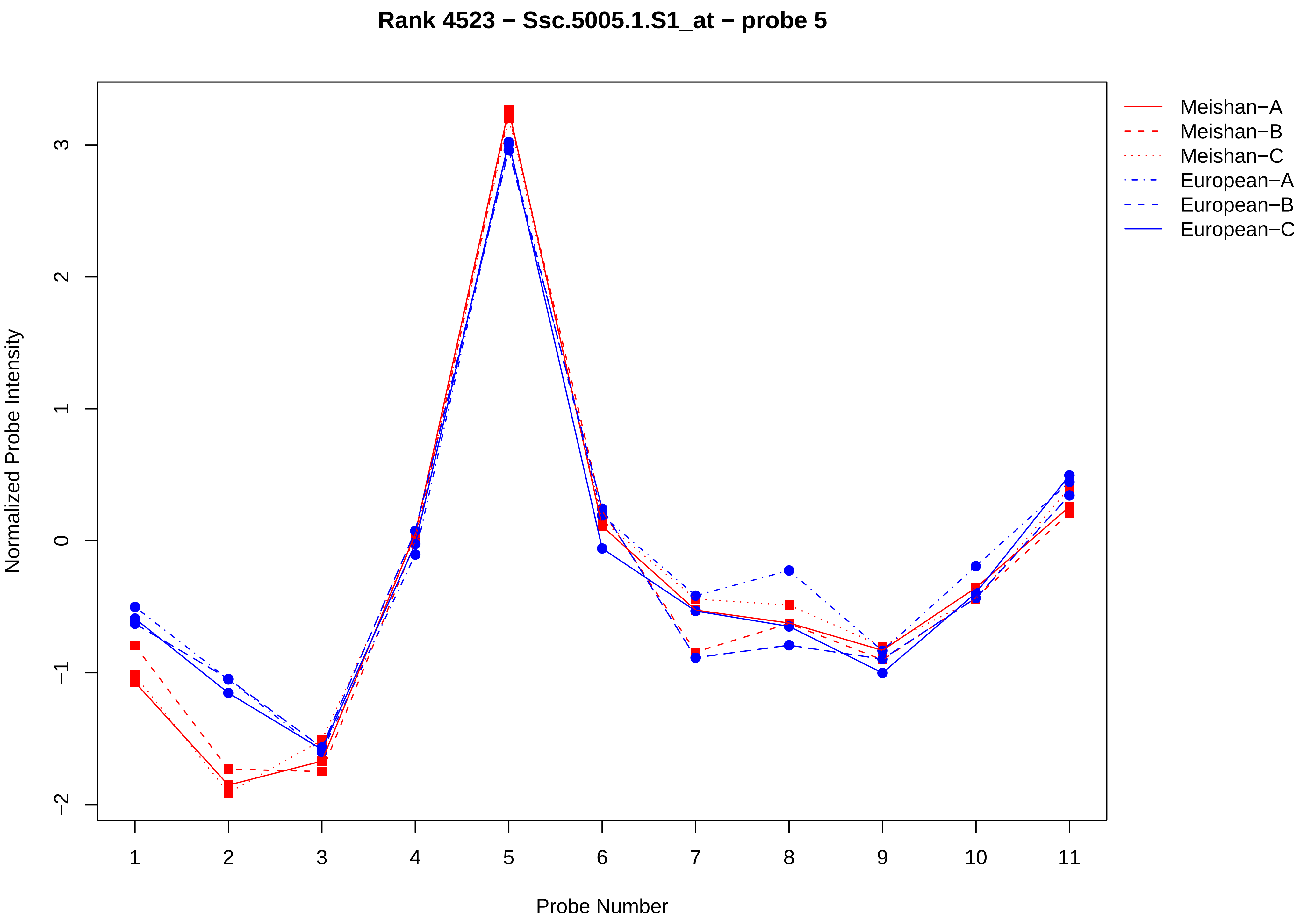

Rank 4523 − Ssc.5005.1.S1_at − probe 5
Normalized Probe Intensity
Probe Number
−2
−1
0
1
2
3
1
2
3
4
5
6
7
8
9
10
11
Meishan−A
Meishan−B
Meishan−C
European−A
European−B
European−C

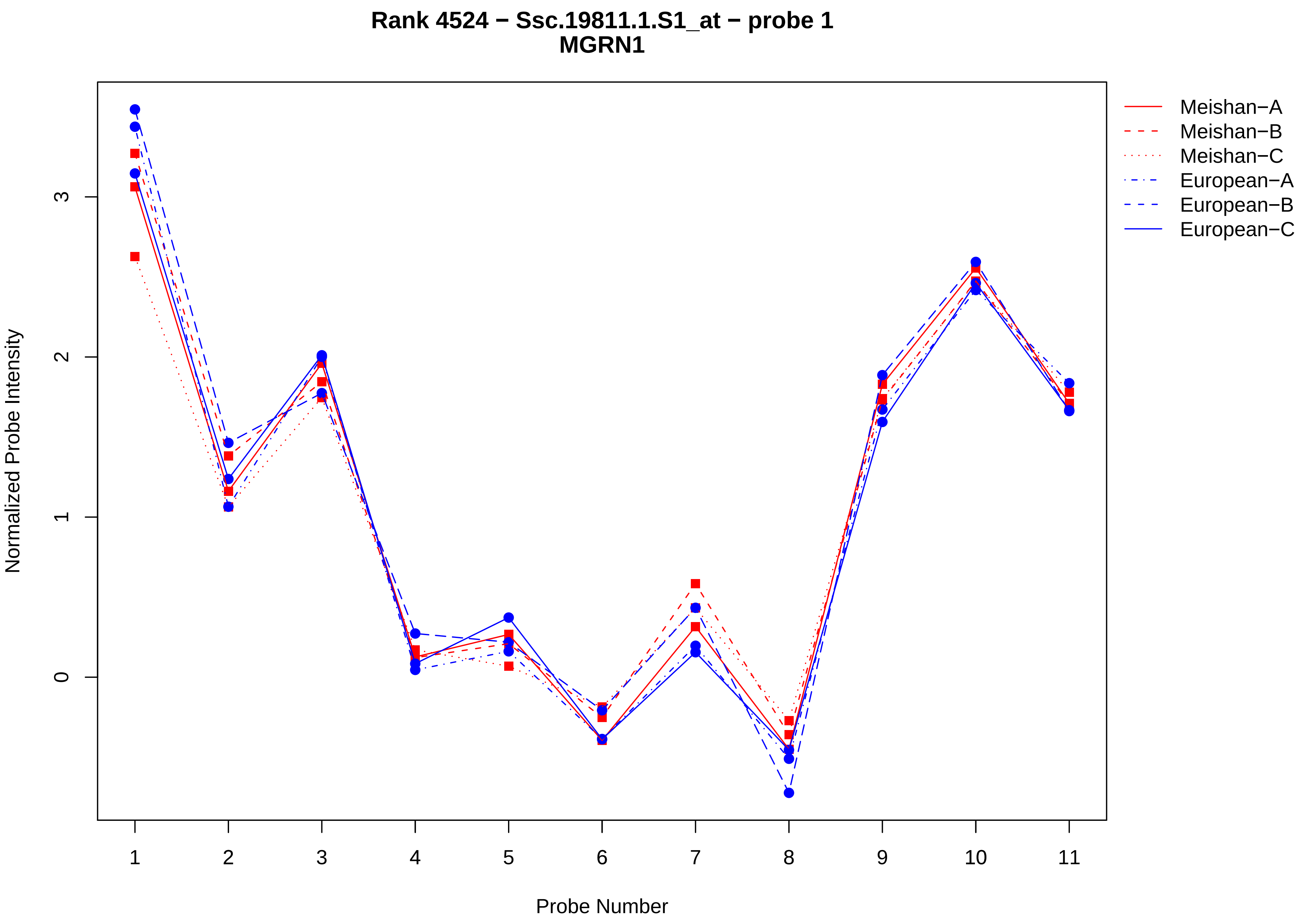

Rank 4524 − Ssc.19811.1.S1_at − probe 1
MGRN1
Normalized Probe Intensity
Probe Number
0
1
2
3
1
2
3
4
5
6
7
8
9
10
11
Meishan−A
Meishan−B
Meishan−C
European−A
European−B
European−C

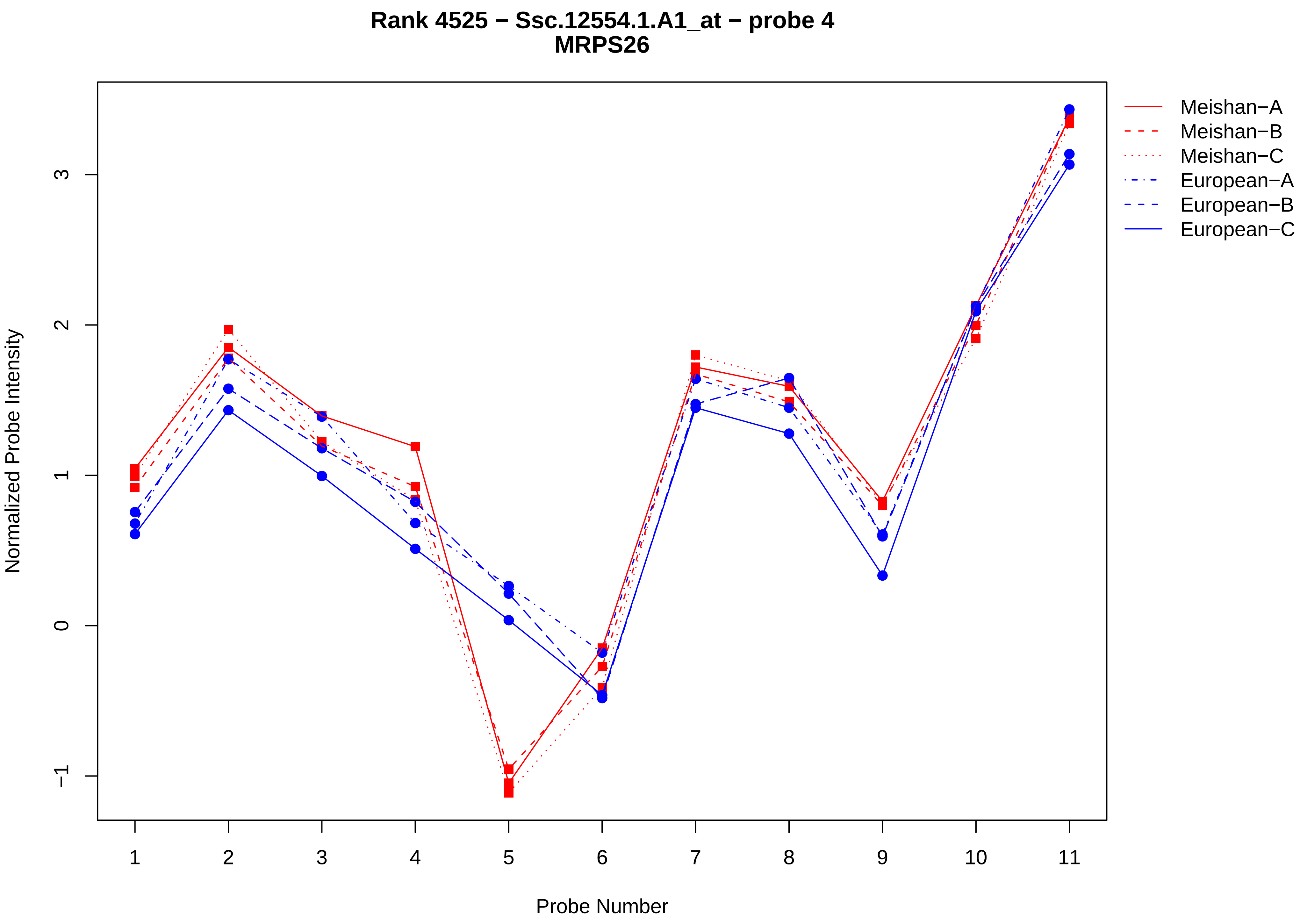

Rank 4525 − Ssc.12554.1.A1_at − probe 4
MRPS26
Normalized Probe Intensity
Probe Number
−1
0
1
2
3
1
2
3
4
5
6
7
8
9
10
11
Meishan−A
Meishan−B
Meishan−C
European−A
European−B
European−C

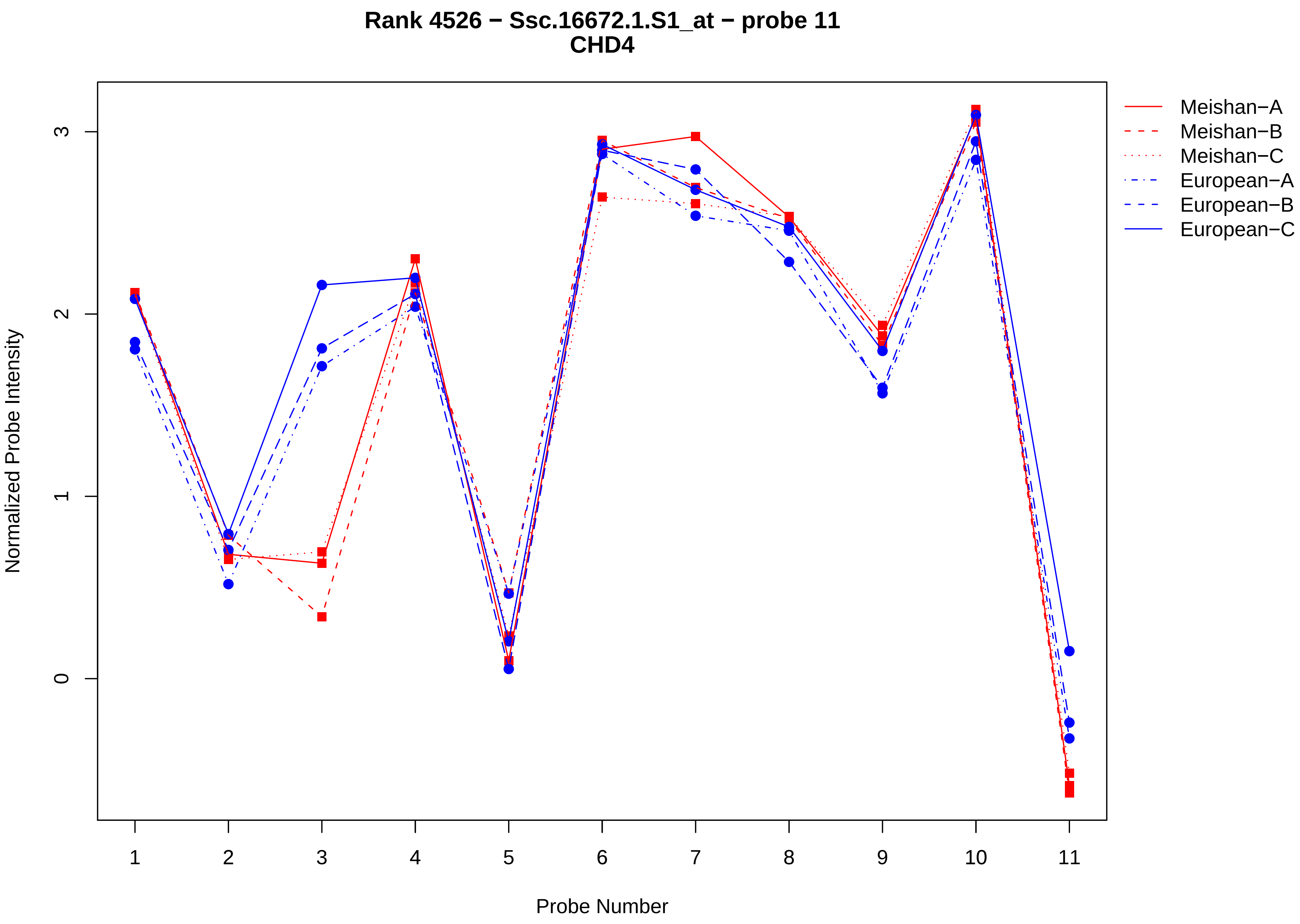
Rank 4526 − Ssc.16672.1.S1_at − probe 11
CHD4
Normalized Probe Intensity
Probe Number
0
1
2
3
1
2
3
4
5
6
7
8
9
10
11
Meishan−A
Meishan−B
Meishan−C
European−A
European−B
European−C

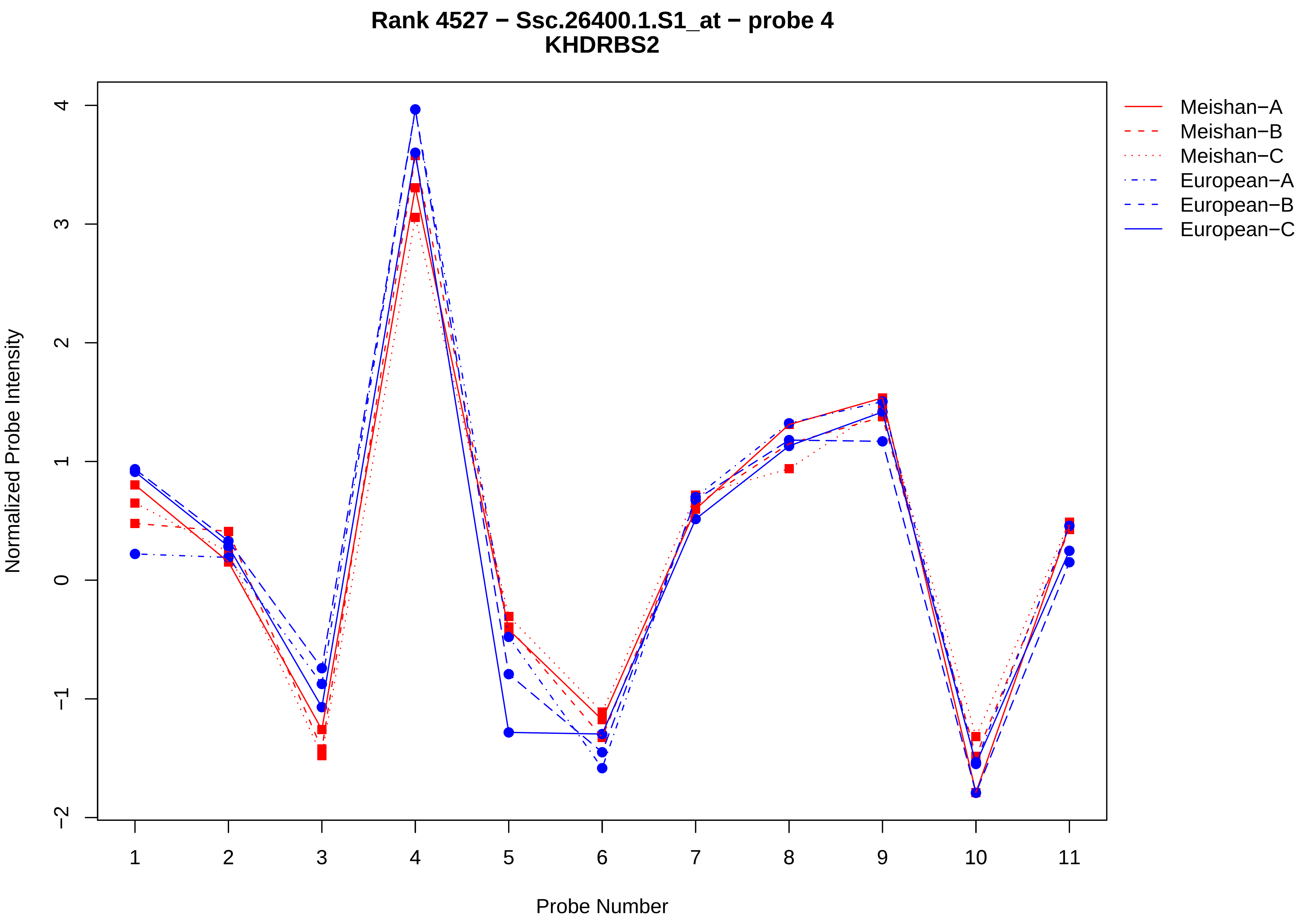
Rank 4527 − Ssc.26400.1.S1_at − probe 4
KHDRBS2
Normalized Probe Intensity
Probe Number
Meishan−A
Meishan−B
Meishan−C
European−A
European−B
European−C
−2
−1
0
1
2
3
4
1
2
3
4
5
6
7
8
9
10
11

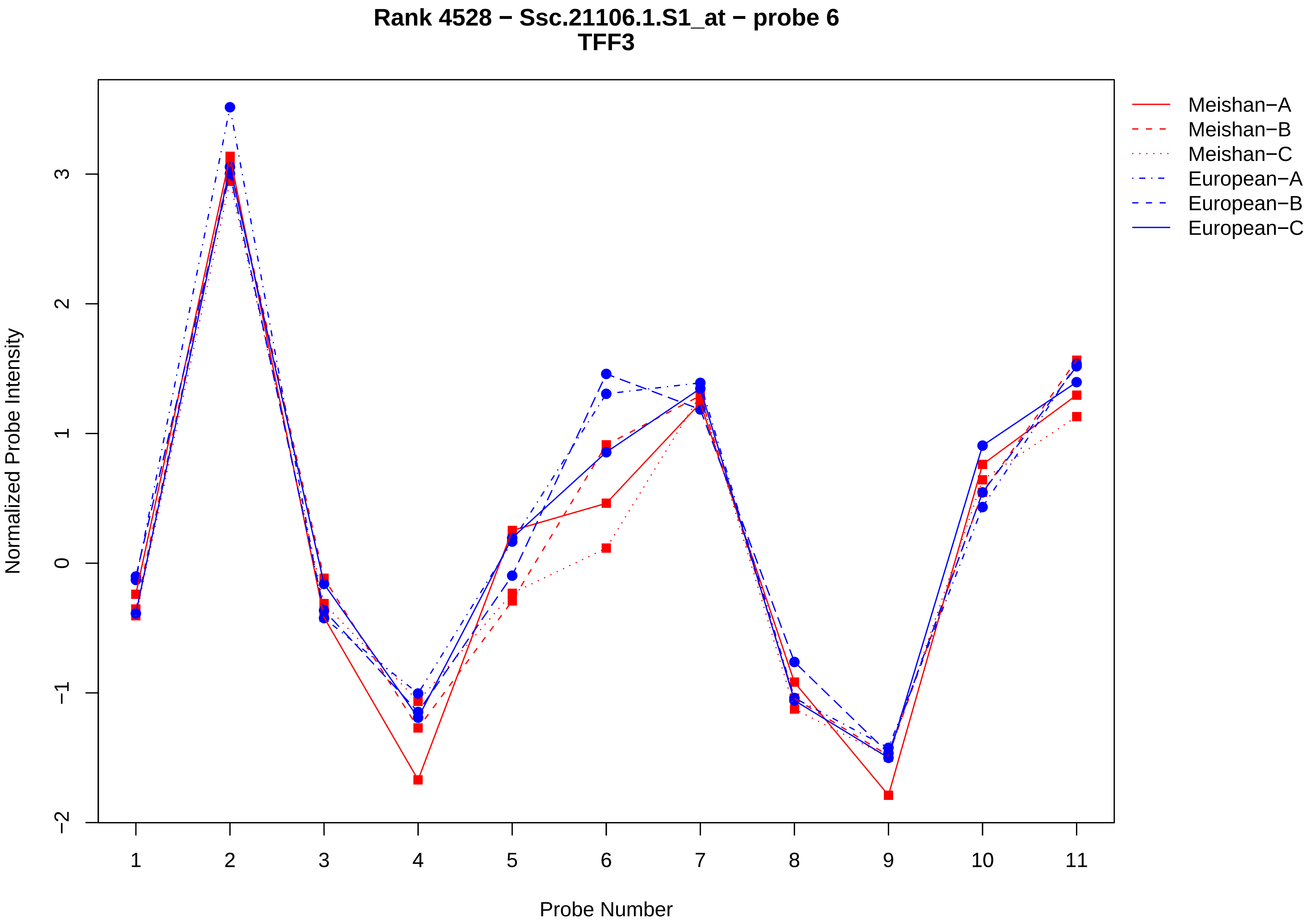
Rank 4528 − Ssc.21106.1.S1_at − probe 6
TFF3
Normalized Probe Intensity
Probe Number
Meishan−A
Meishan−B
Meishan−C
European−A
European−B
European−C
−2
−1
0
1
2
3
1
2
3
4
5
6
7
8
9
10
11

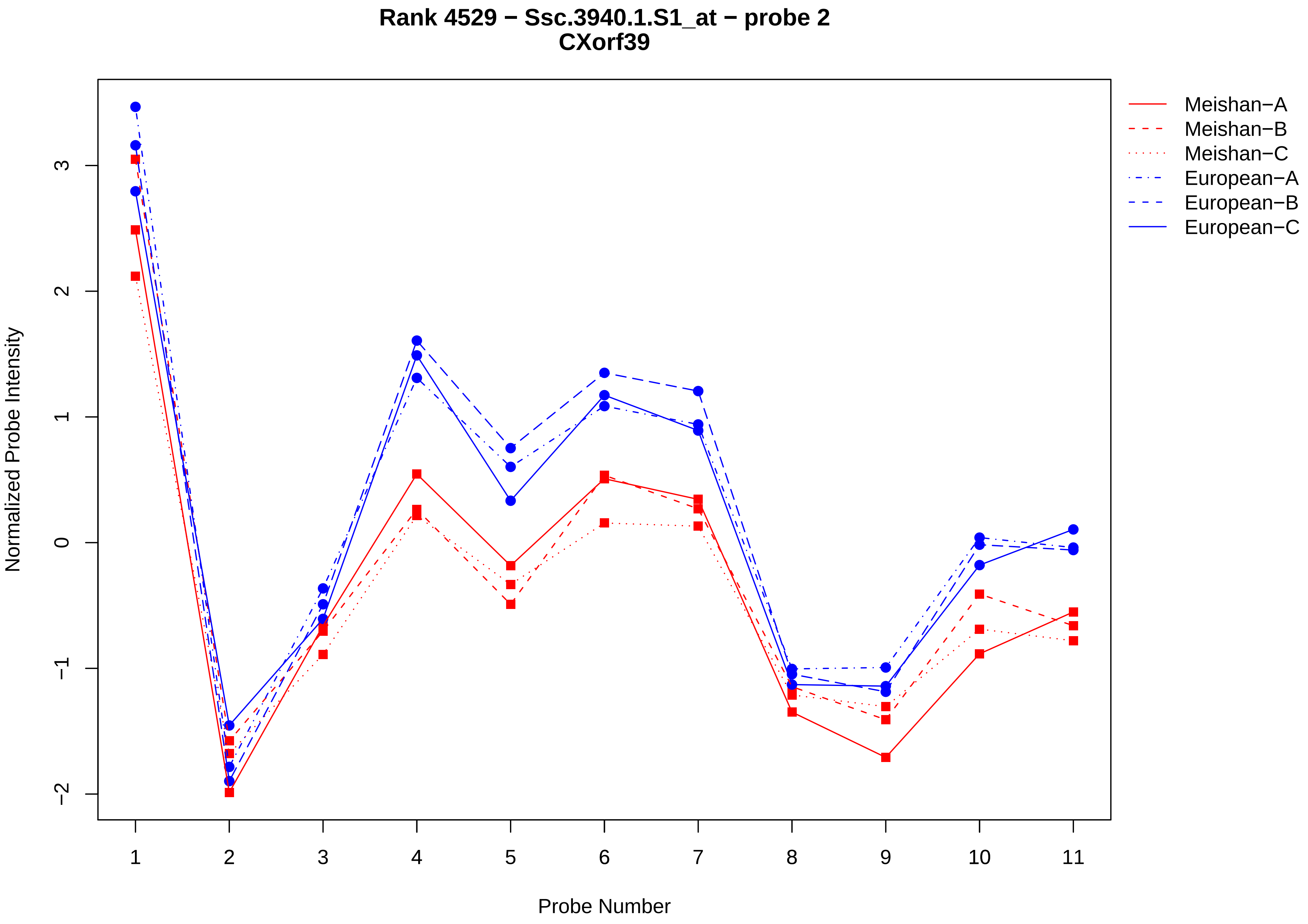

Rank 4529 − Ssc.3940.1.S1_at − probe 2
CXorf39
Normalized Probe Intensity
Probe Number
−2
−1
0
1
2
3
1
2
3
4
5
6
7
8
9
10
11
Meishan−A
Meishan−B
Meishan−C
European−A
European−B
European−C

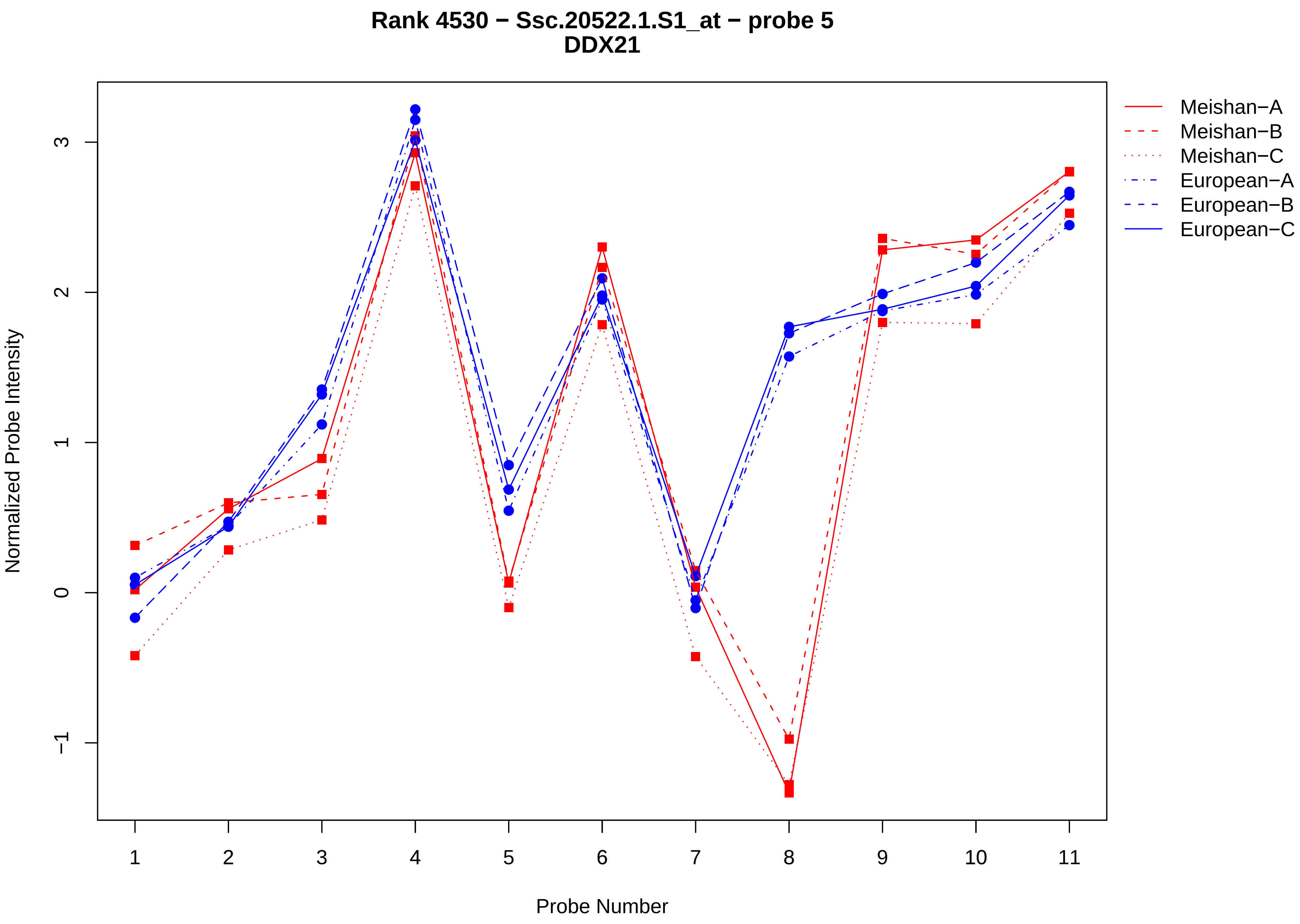
Rank 4530 − Ssc.20522.1.S1_at − probe 5
DDX21
Normalized Probe Intensity
Probe Number
Meishan−A
Meishan−B
Meishan−C
European−A
European−B
European−C

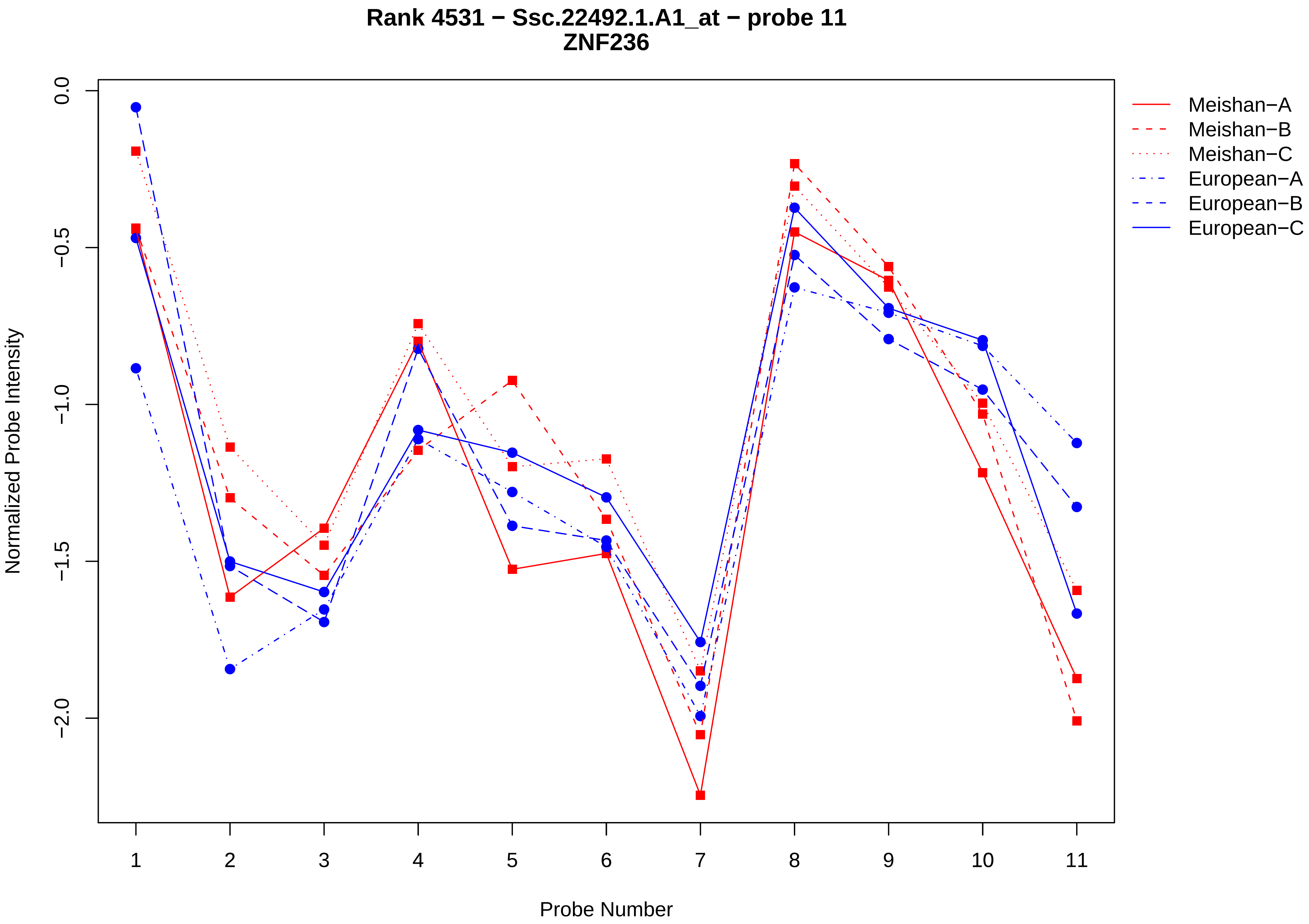

Rank 4531 − Ssc.22492.1.A1_at − probe 11
ZNF236
Normalized Probe Intensity
Probe Number
0.0
−0.5
−1.0
−1.5
−2.0
1
2
3
4
5
6
7
8
9
10
11
Meishan−A
Meishan−B
Meishan−C
European−A
European−B
European−C

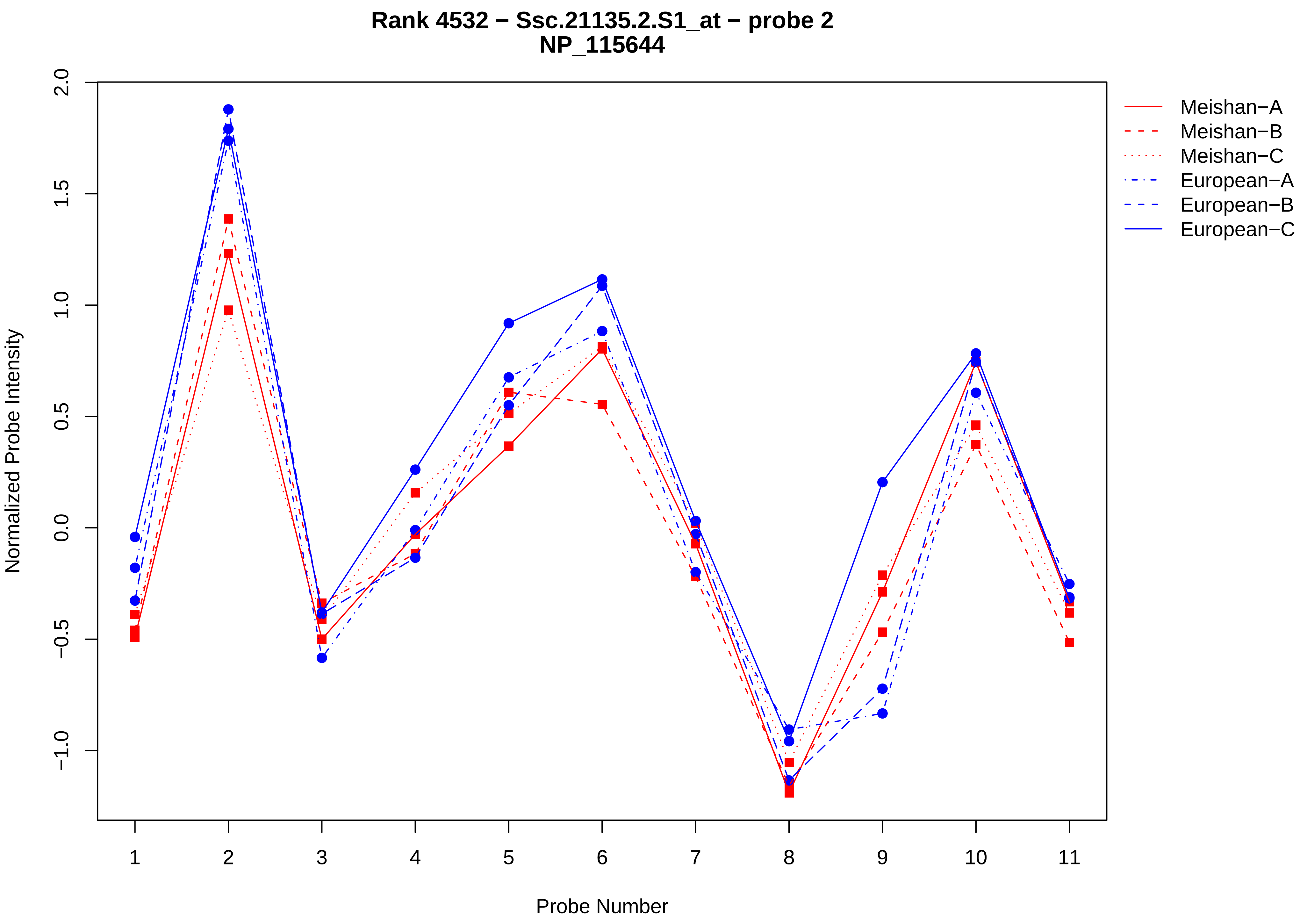
Rank 4532 − Ssc.21135.2.S1_at − probe 2
NP_115644
Normalized Probe Intensity
Probe Number
2.0
1.5
1.0
0.5
0.0
−0.5
−1.0
1
2
3
4
5
6
7
8
9
10
11
Meishan−A
Meishan−B
Meishan−C
European−A
European−B
European−C

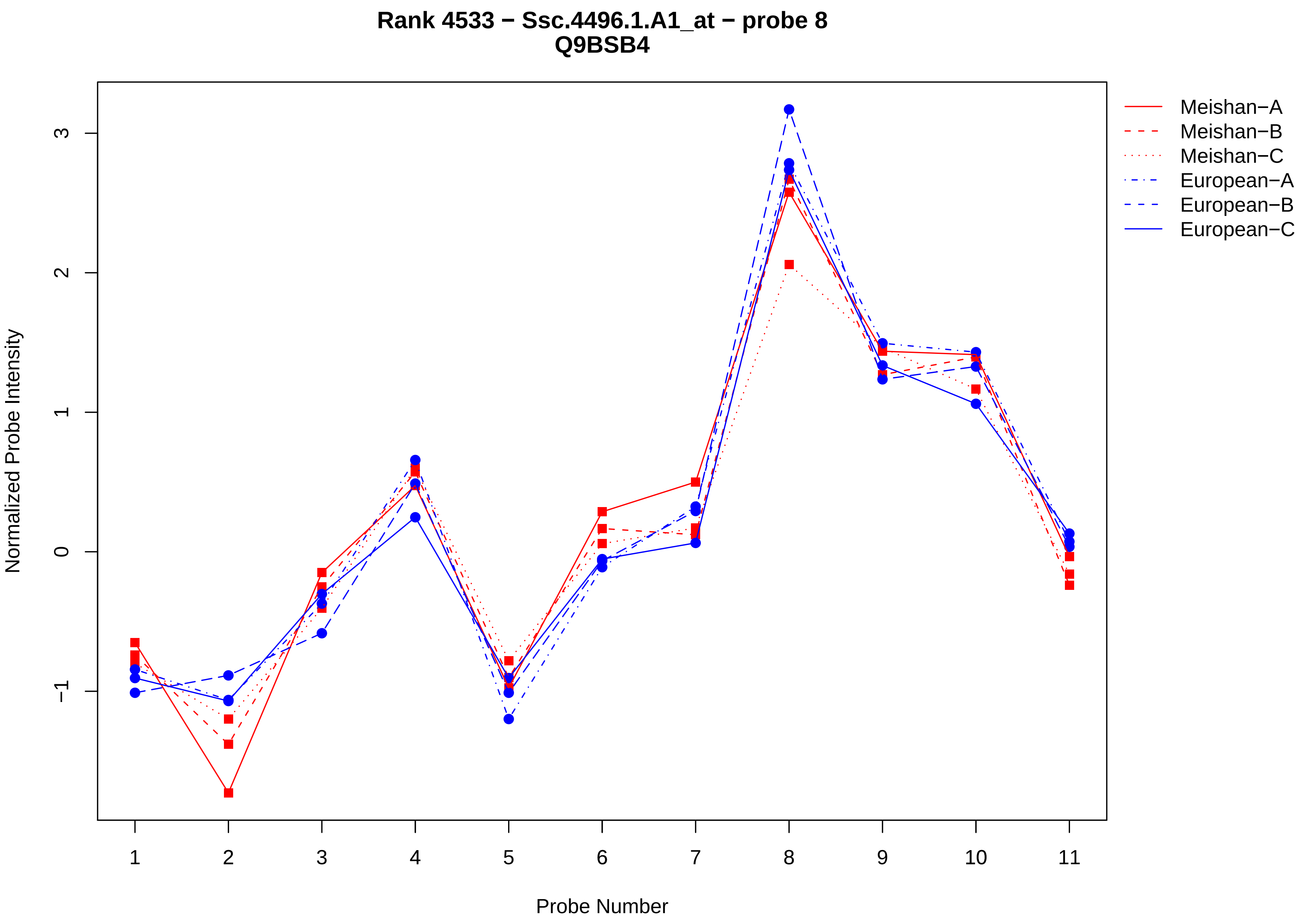
Rank 4533 − Ssc.4496.1.A1_at − probe 8
Q9BSB4
Normalized Probe Intensity
Probe Number
−1
0
1
2
3
1
2
3
4
5
6
7
8
9
10
11
Meishan−A
Meishan−B
Meishan−C
European−A
European−B
European−C

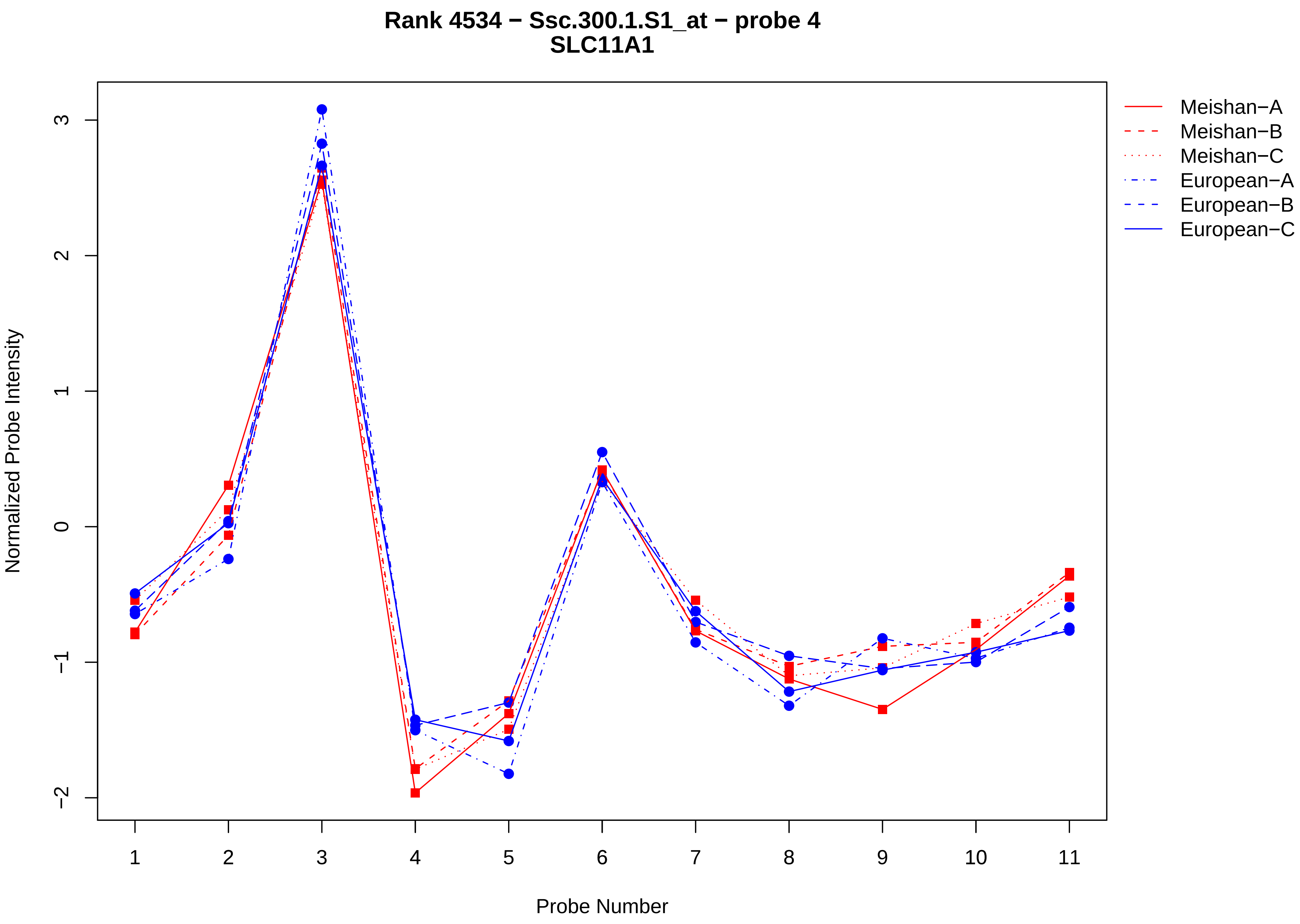

Rank 4534 − Ssc.300.1.S1_at − probe 4
SLC11A1
Normalized Probe Intensity
Probe Number
−2
−1
0
1
2
3
1
2
3
4
5
6
7
8
9
10
11
Meishan−A
Meishan−B
Meishan−C
European−A
European−B
European−C

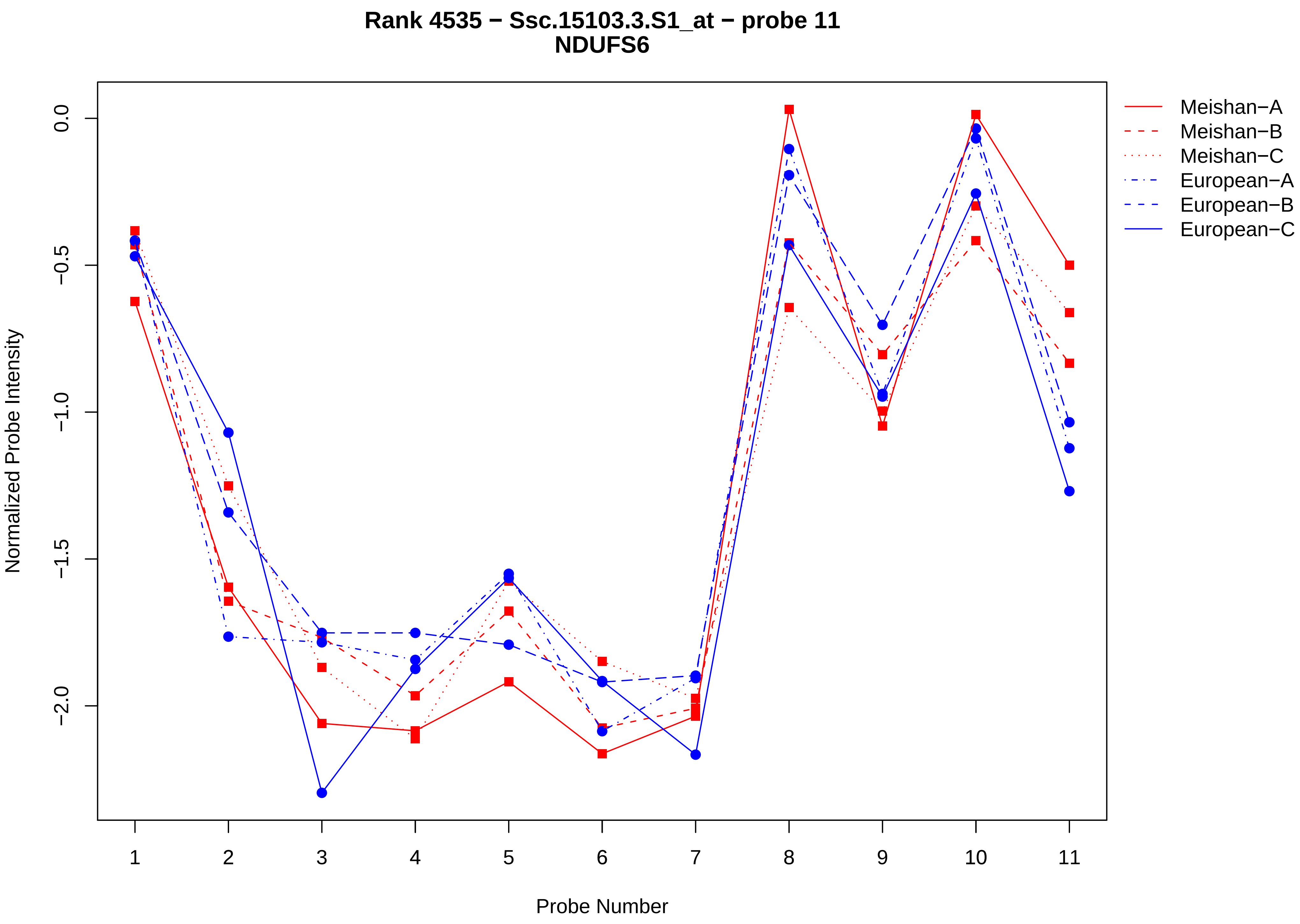

Rank 4535 − Ssc.15103.3.S1_at − probe 11
NDUFS6
Normalized Probe Intensity
Probe Number
0.0
−0.5
−1.0
−1.5
−2.0
1
2
3
4
5
6
7
8
9
10
11
Meishan−A
Meishan−B
Meishan−C
European−A
European−B
European−C

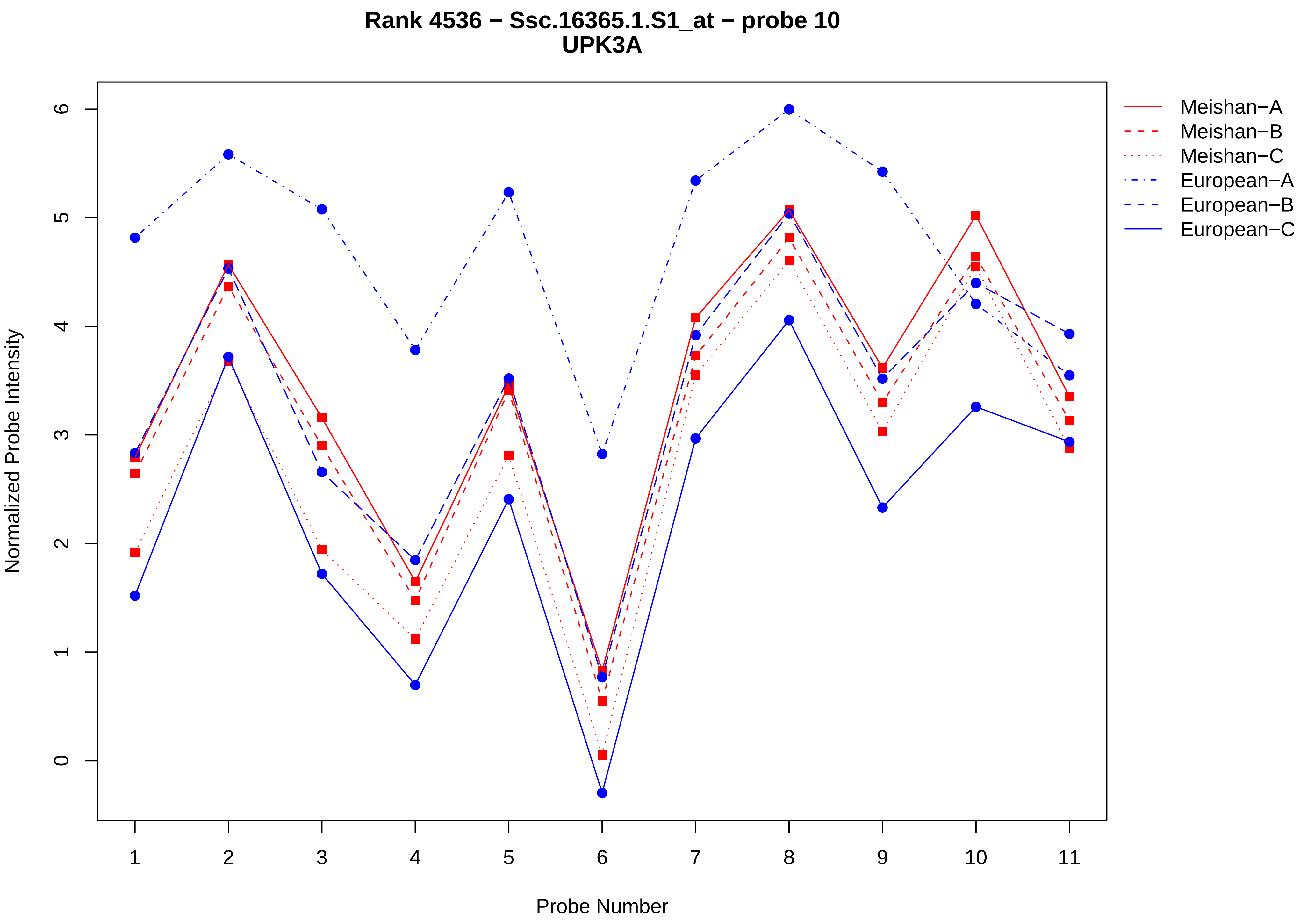

Rank 4536 − Ssc.16365.1.S1_at − probe 10
UPK3A
Normalized Probe Intensity
Probe Number
0
1
2
3
4
5
6
1
2
3
4
5
6
7
8
9
10
11
Meishan−A
Meishan−B
Meishan−C
European−A
European−B
European−C

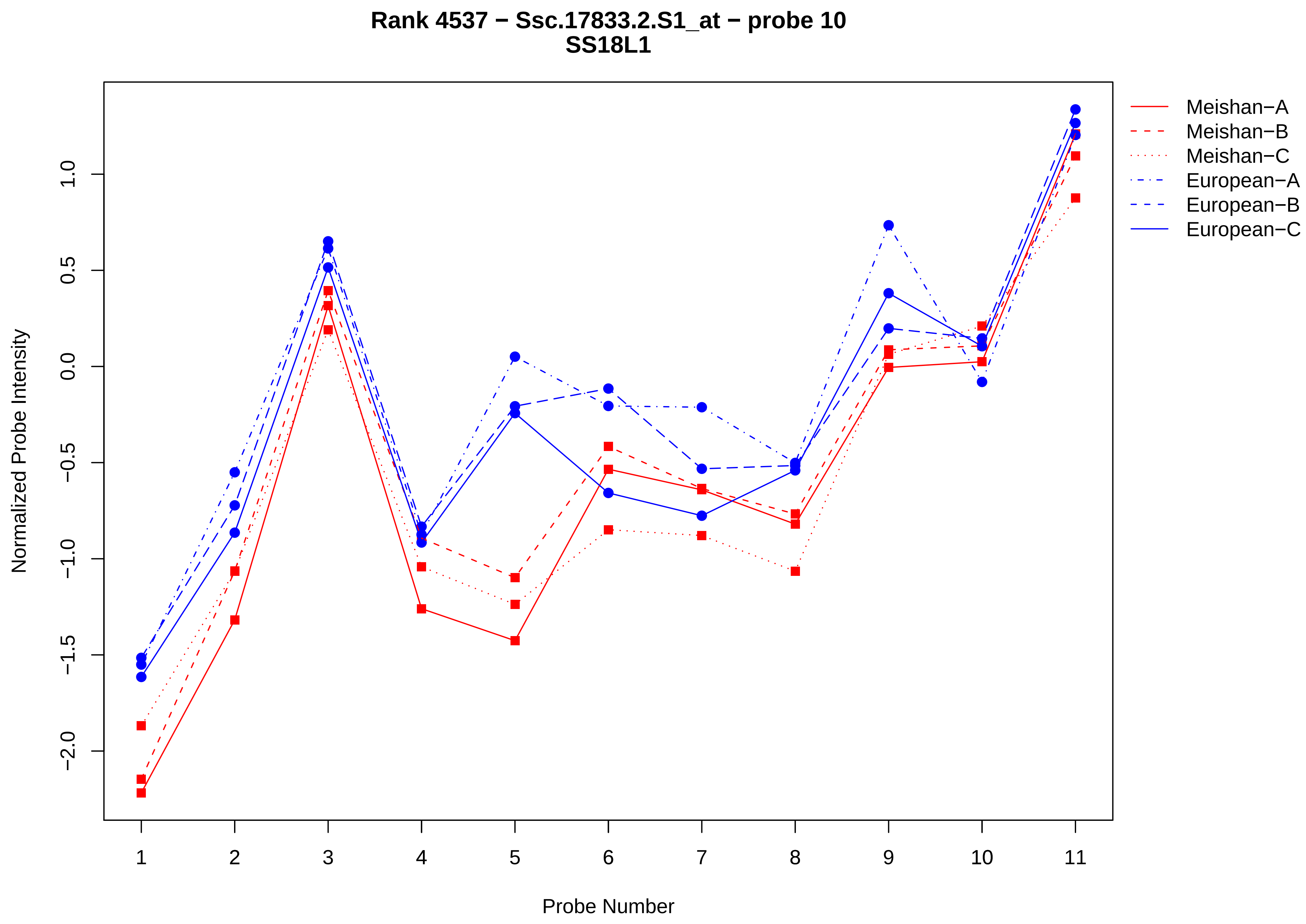

Rank 4537 − Ssc.17833.2.S1_at − probe 10
SS18L1
Normalized Probe Intensity
Probe Number
−2.0
−1.5
−1.0
−0.5
0.0
0.5
1.0
1
2
3
4
5
6
7
8
9
10
11
Meishan−A
Meishan−B
Meishan−C
European−A
European−B
European−C

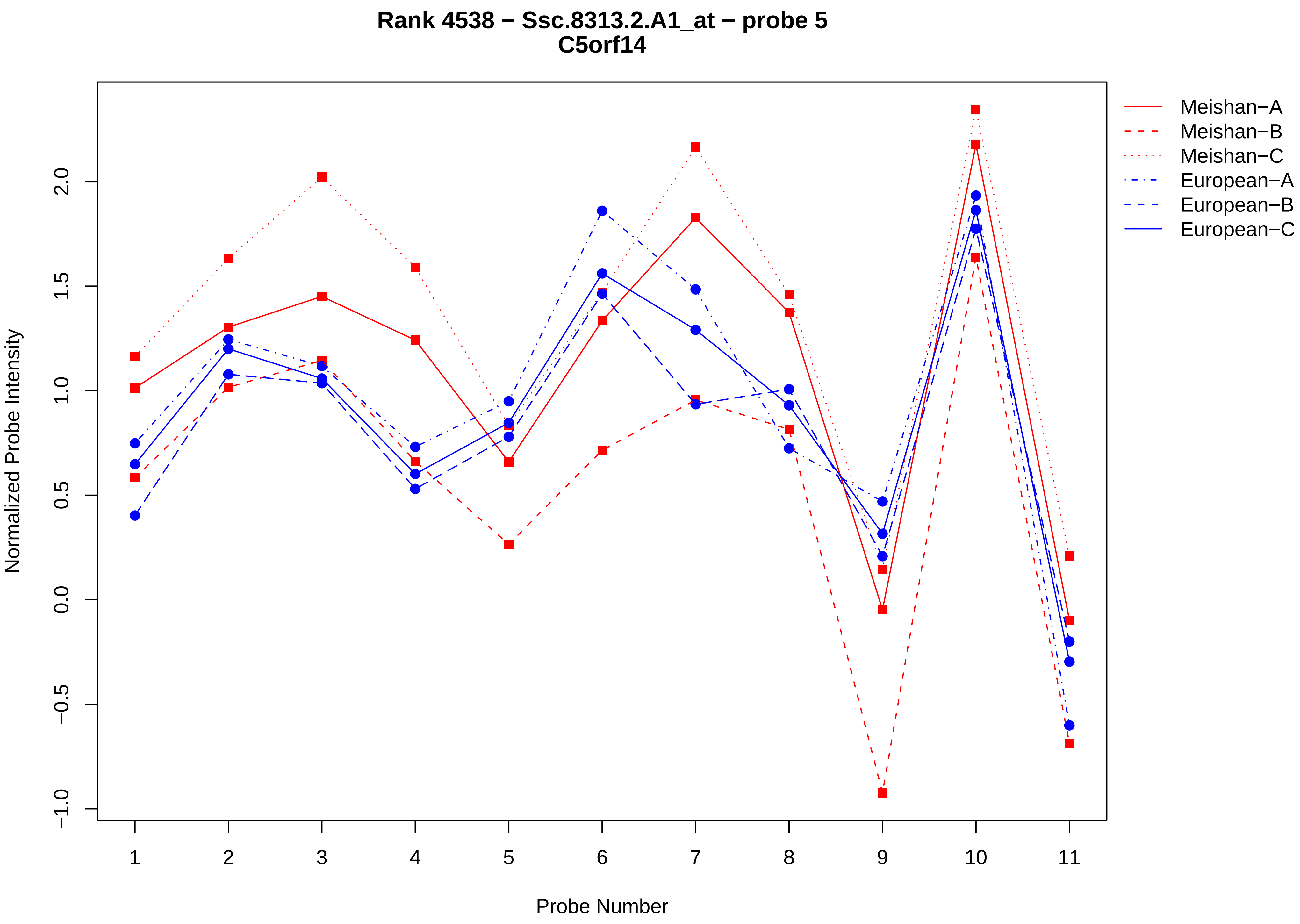

Rank 4538 − Ssc.8313.2.A1_at − probe 5
C5orf14
Normalized Probe Intensity
−1.0
−0.5
0.0
0.5
1.0
1.5
2.0
Probe Number
1
2
3
4
5
6
7
8
9
10
11
Meishan−A
Meishan−B
Meishan−C
European−A
European−B
European−C

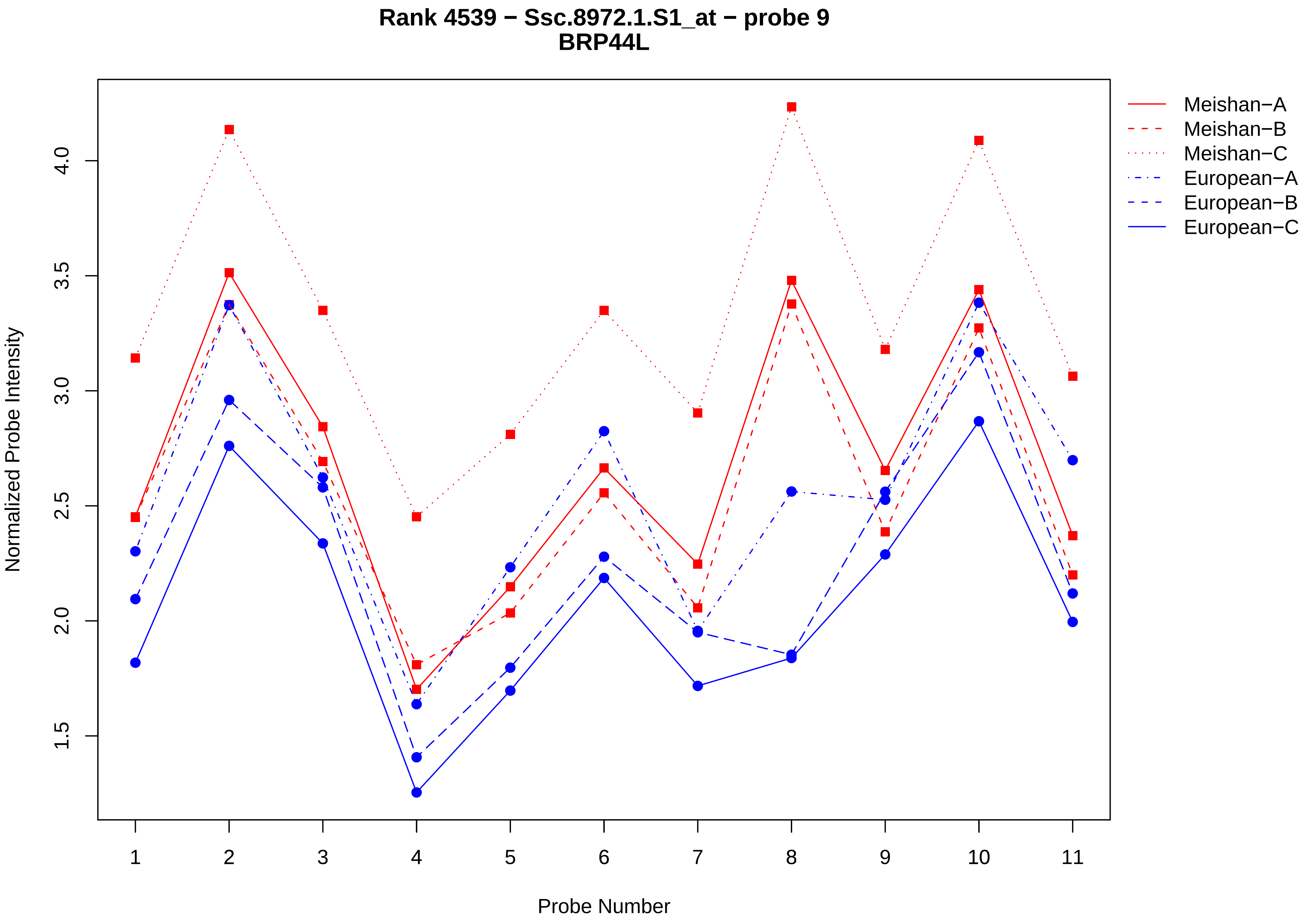

Rank 4539 − Ssc.8972.1.S1_at − probe 9
BRP44L
Normalized Probe Intensity
1.5
2.0
2.5
3.0
3.5
4.0
1
2
3
4
5
6
7
8
9
10
11
Probe Number
Meishan−A
Meishan−B
Meishan−C
European−A
European−B
European−C

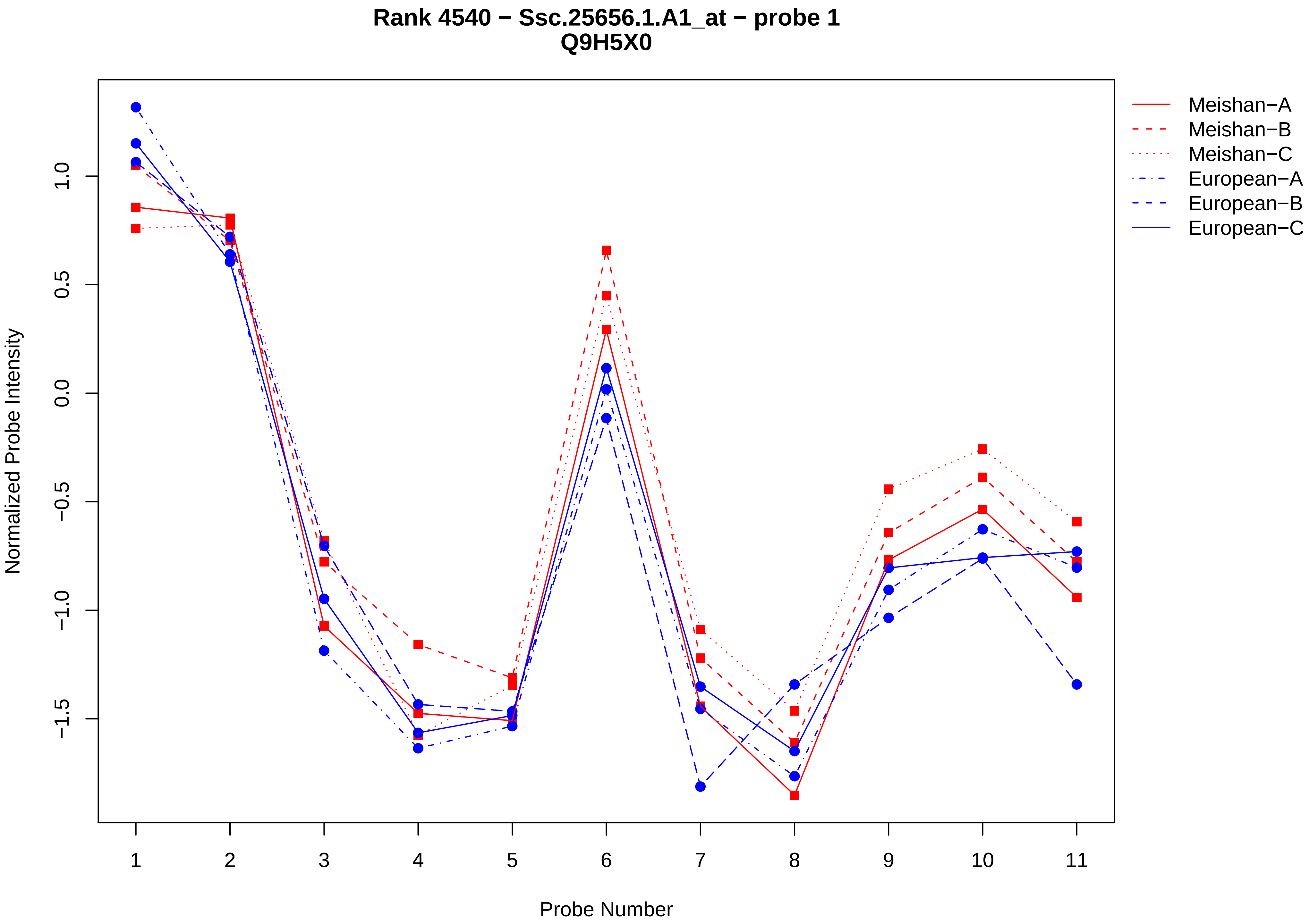

Rank 4540 − Ssc.25656.1.A1_at − probe 1
Q9H5X0
Normalized Probe Intensity
Probe Number
1.0
0.5
0.0
−0.5
−1.0
−1.5
1
2
3
4
5
6
7
8
9
10
11
Meishan−A
Meishan−B
Meishan−C
European−A
European−B
European−C

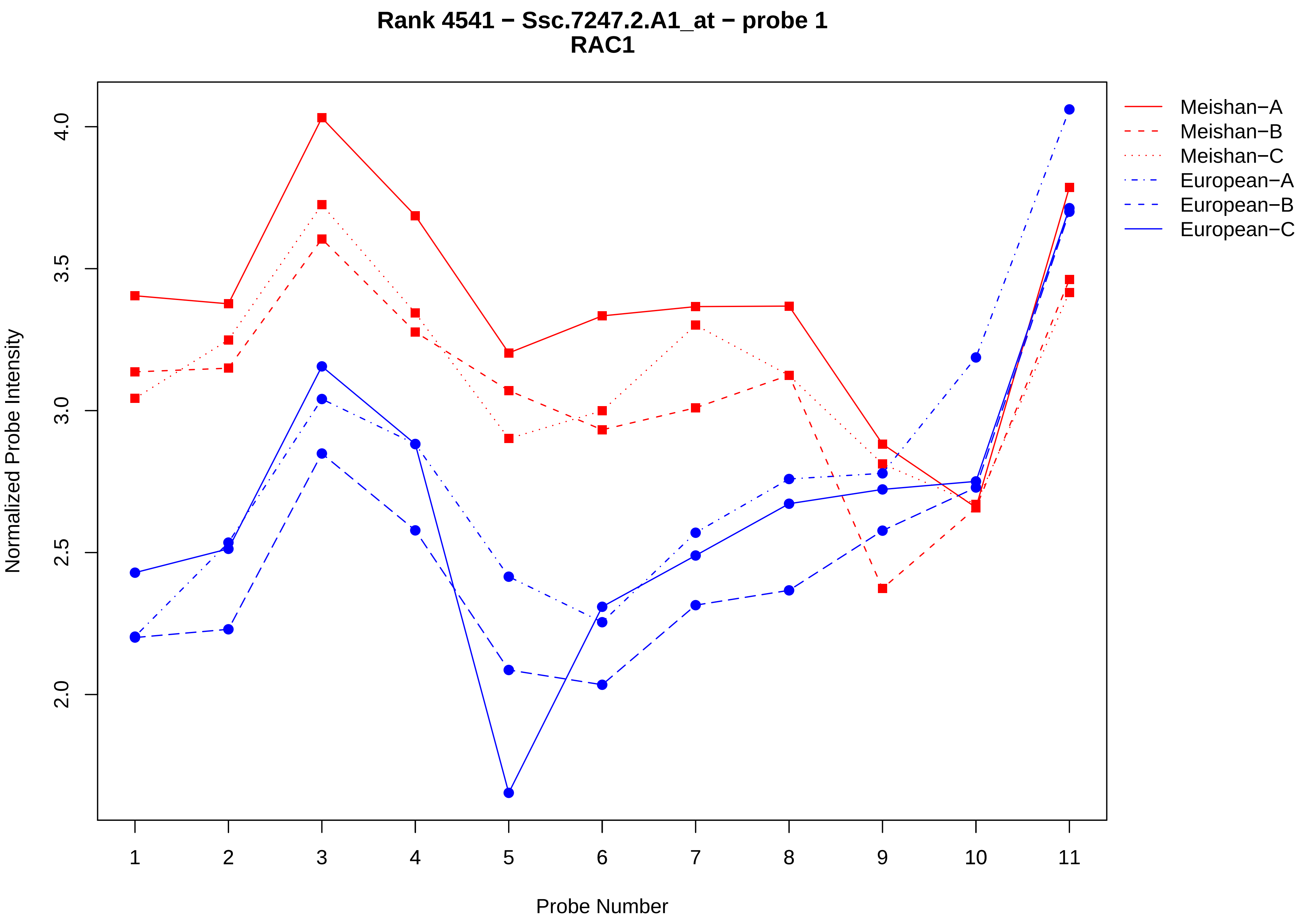

Rank 4541 − Ssc.7247.2.A1_at − probe 1
RAC1
Normalized Probe Intensity
Probe Number
2.0
2.5
3.0
3.5
4.0
1
2
3
4
5
6
7
8
9
10
11
Meishan−A
Meishan−B
Meishan−C
European−A
European−B
European−C

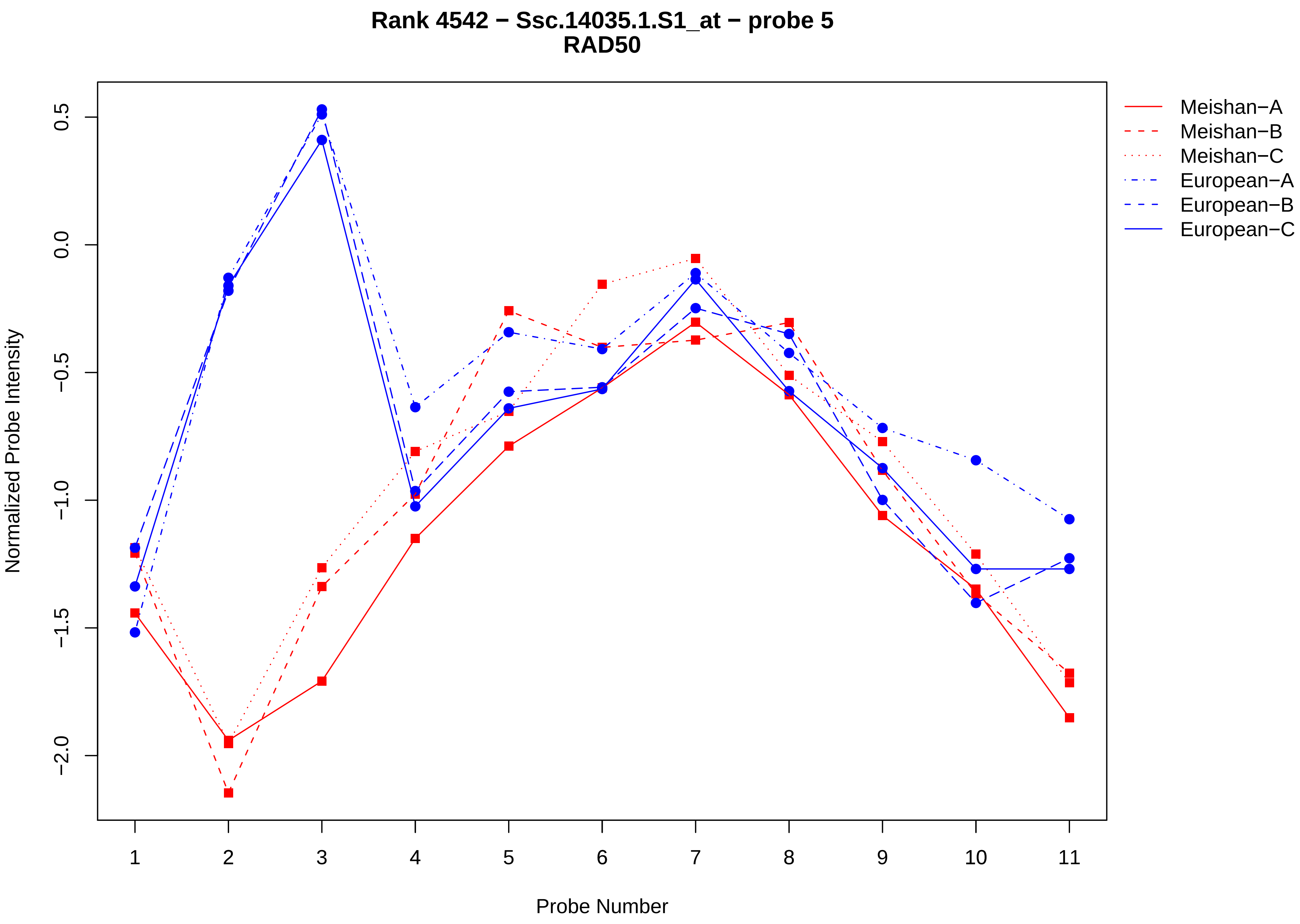

Rank 4542 − Ssc.14035.1.S1_at − probe 5
RAD50
Normalized Probe Intensity
Probe Number
Meishan−A
Meishan−B
Meishan−C
European−A
European−B
European−C

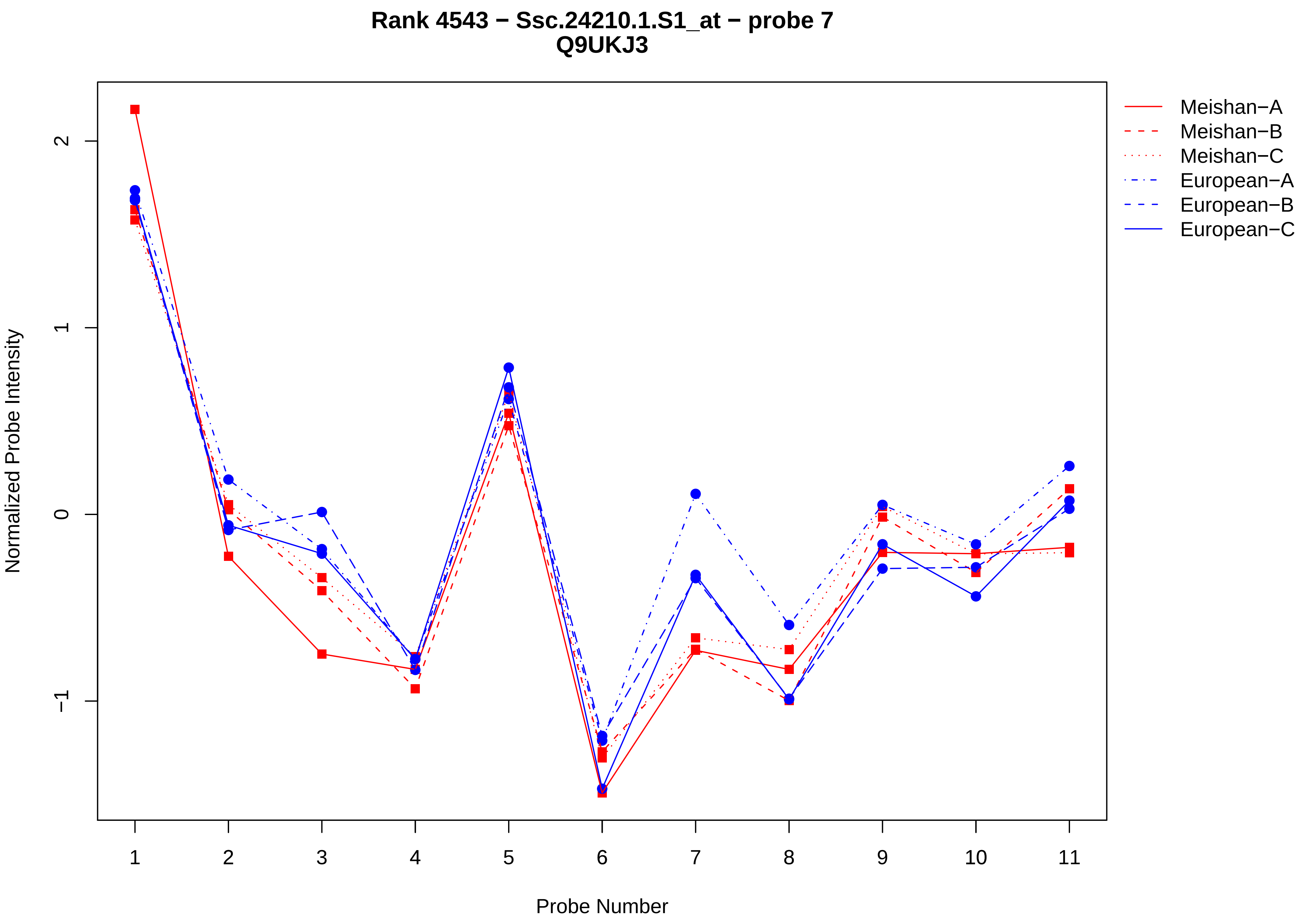
Rank 4543 − Ssc.24210.1.S1_at − probe 7
Q9UKJ3
Normalized Probe Intensity
Probe Number
−1
0
1
2
1
2
3
4
5
6
7
8
9
10
11
Meishan−A
Meishan−B
Meishan−C
European−A
European−B
European−C

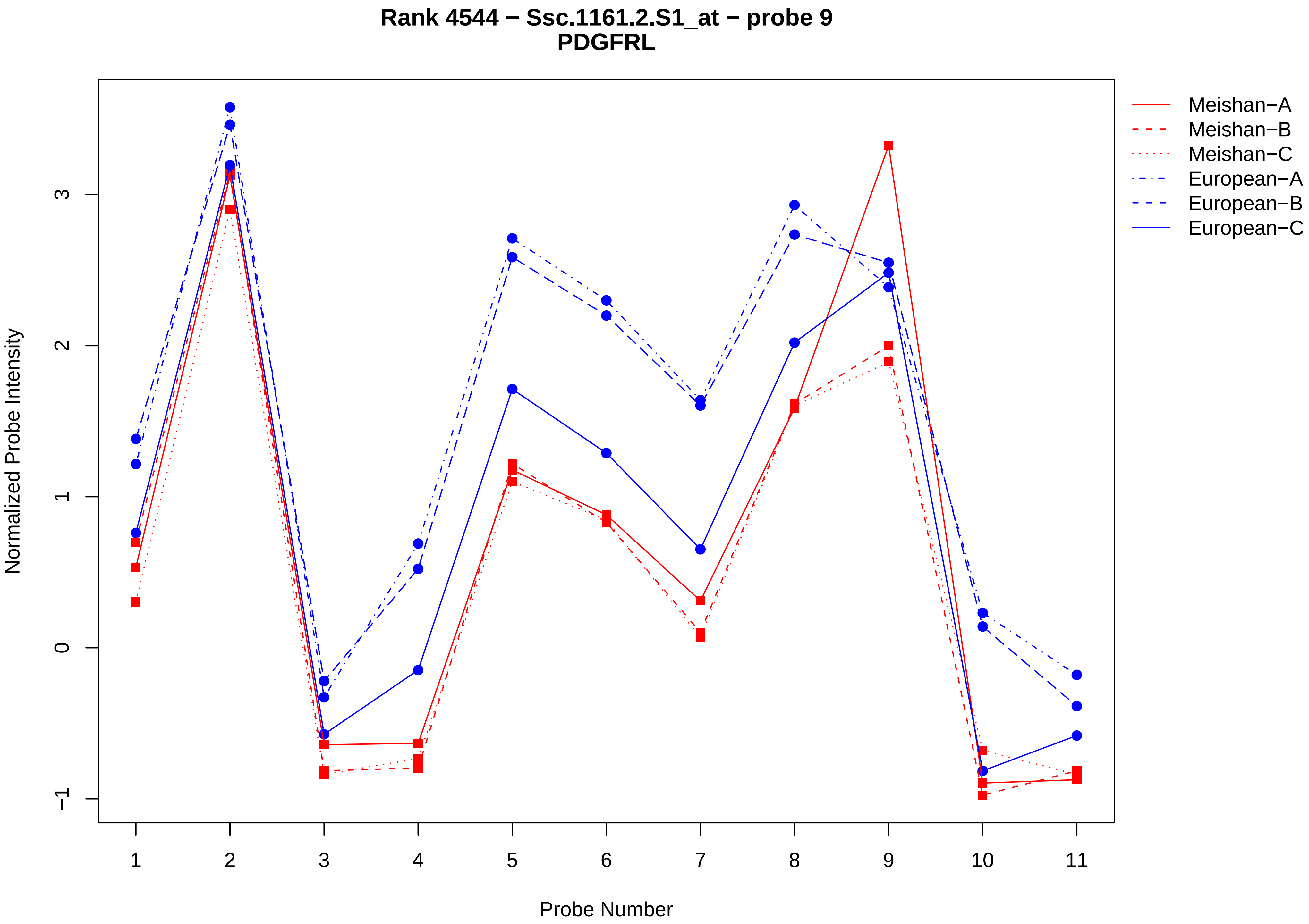

Rank 4544 − Ssc.1161.2.S1_at − probe 9
PDGFRL
Normalized Probe Intensity
Probe Number
−1
0
1
2
3
1
2
3
4
5
6
7
8
9
10
11
Meishan−A
Meishan−B
Meishan−C
European−A
European−B
European−C

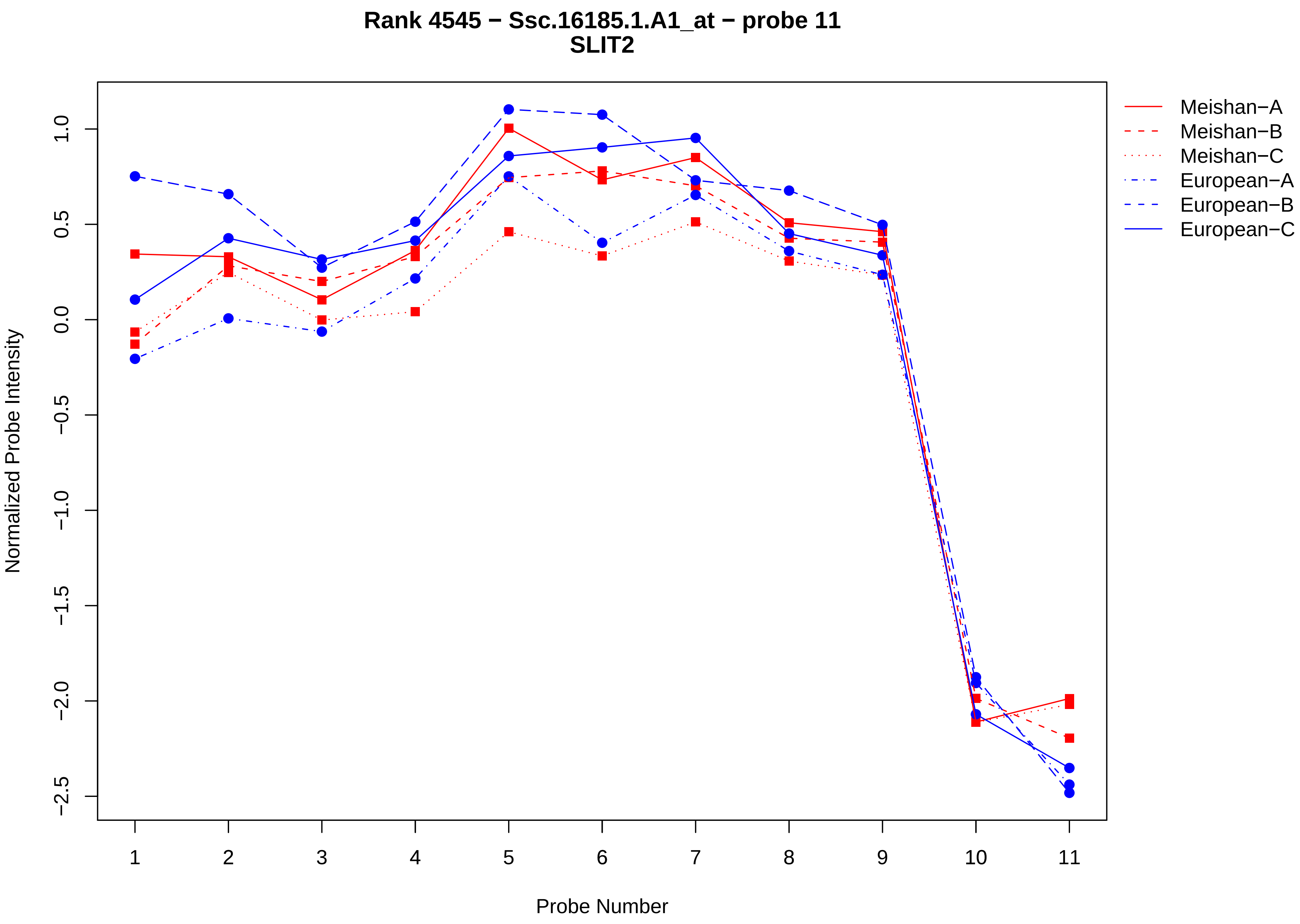

Rank 4545 − Ssc.16185.1.A1_at − probe 11
SLIT2
Normalized Probe Intensity
Probe Number
1.0
0.5
0.0
−0.5
−1.0
−1.5
−2.0
−2.5
1
2
3
4
5
6
7
8
9
10
11
Meishan−A
Meishan−B
Meishan−C
European−A
European−B
European−C

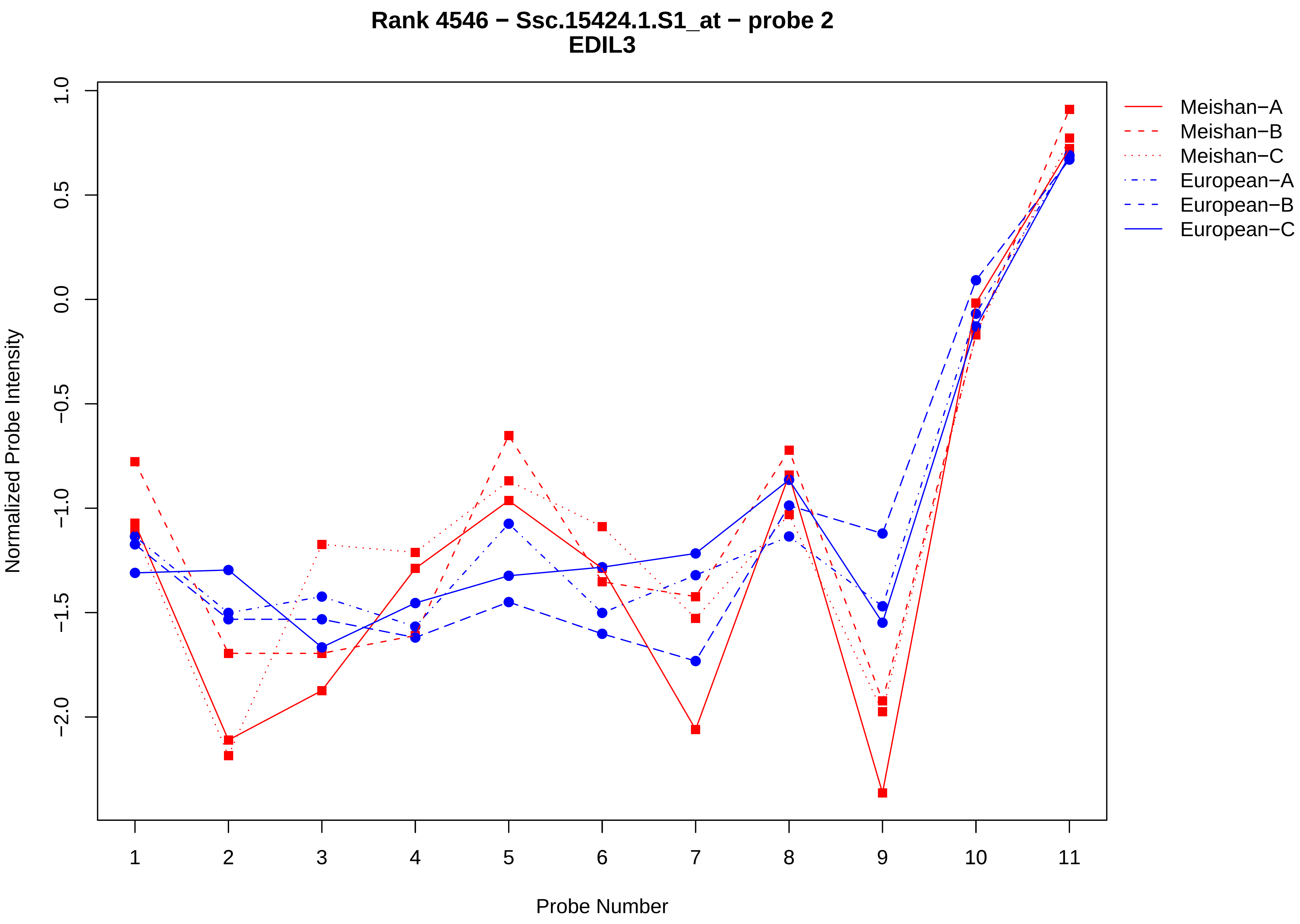

Rank 4546 − Ssc.15424.1.S1_at − probe 2
EDIL3
Normalized Probe Intensity
Probe Number
1.0
0.5
0.0
−0.5
−1.0
−1.5
−2.0
1
2
3
4
5
6
7
8
9
10
11
Meishan−A
Meishan−B
Meishan−C
European−A
European−B
European−C

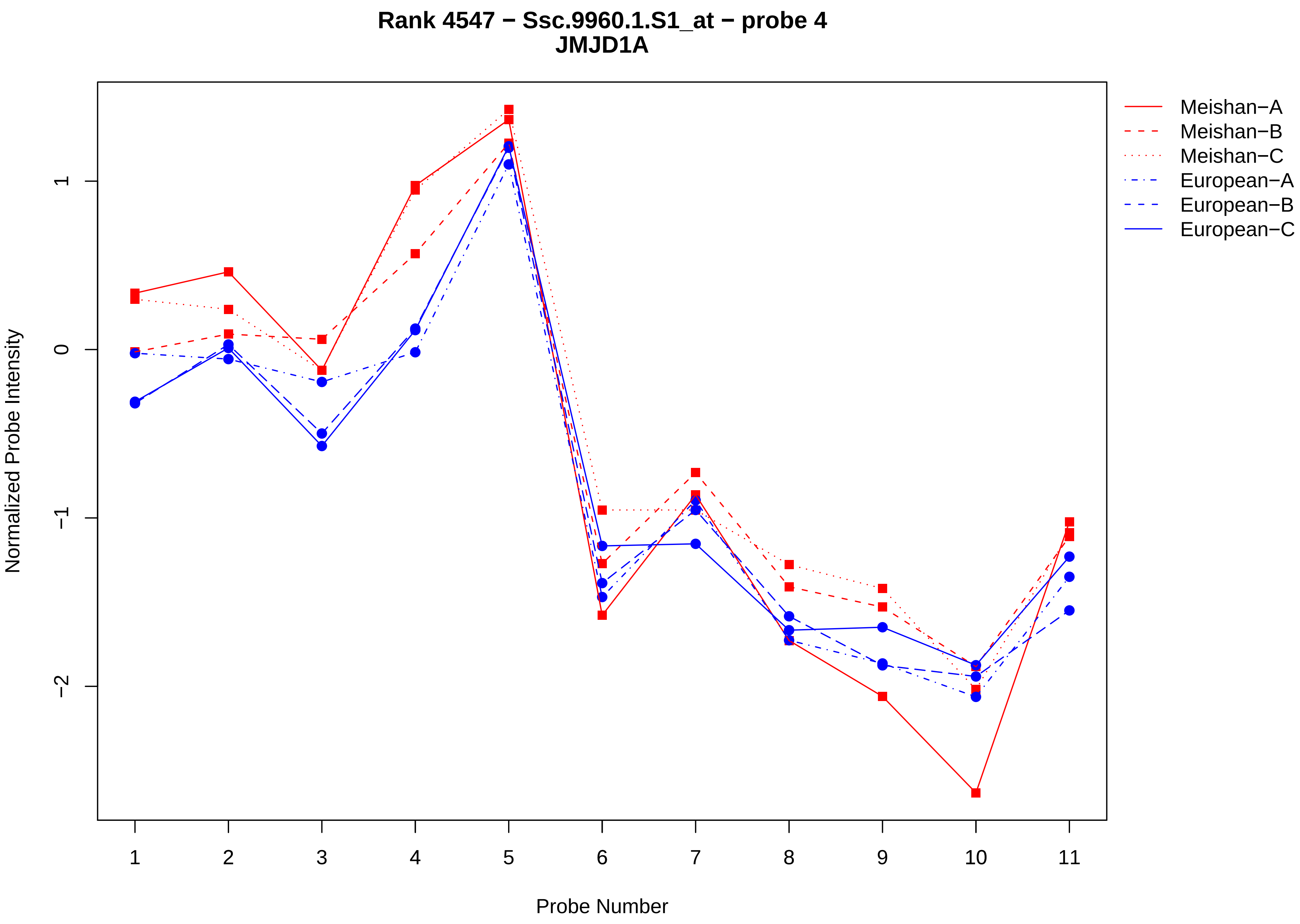

Rank 4547 − Ssc.9960.1.S1_at − probe 4
JMJD1A
Normalized Probe Intensity
Probe Number
1
0
−1
−2
1
2
3
4
5
6
7
8
9
10
11
Meishan−A
Meishan−B
Meishan−C
European−A
European−B
European−C

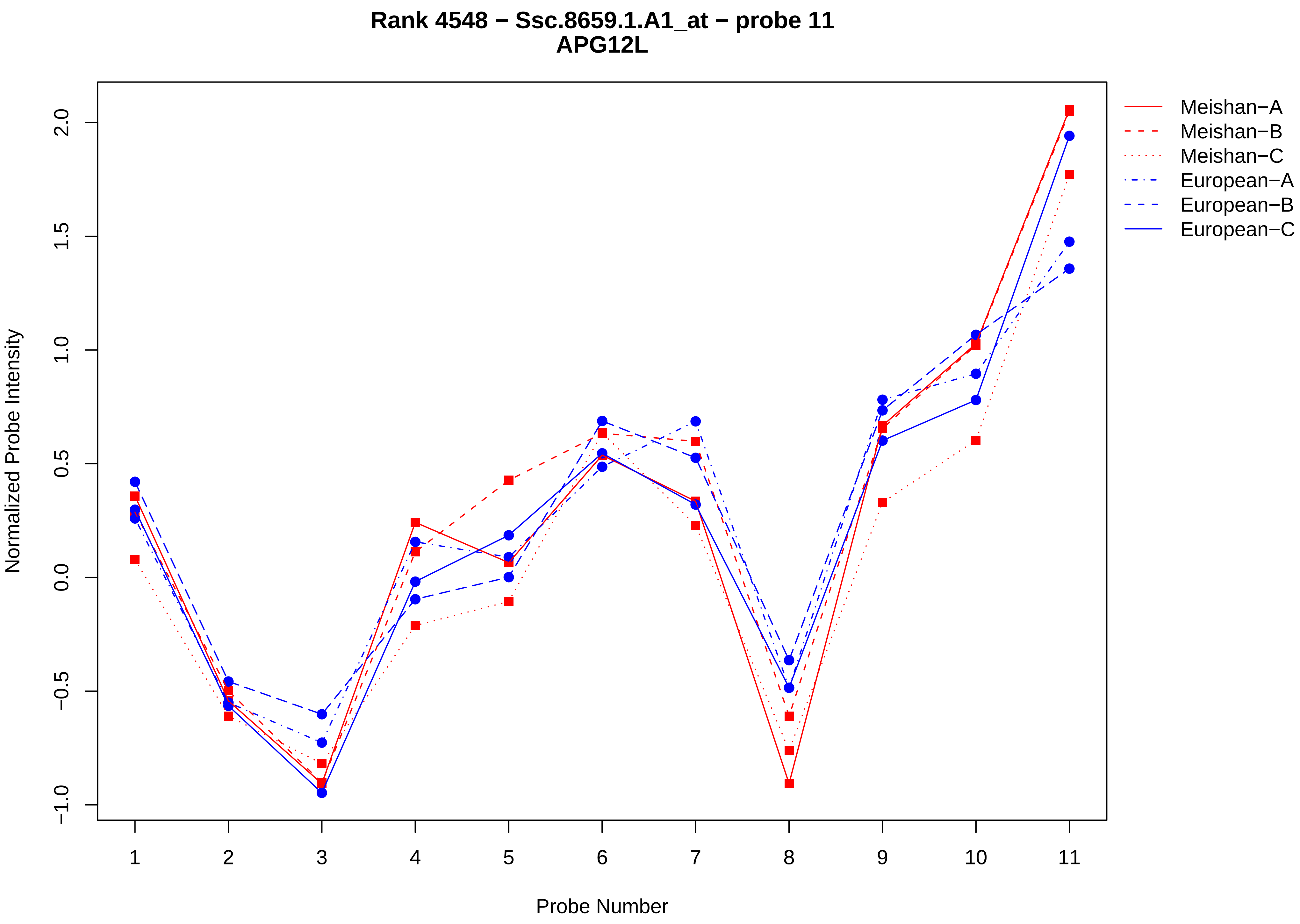
Rank 4548 − Ssc.8659.1.A1_at − probe 11
APG12L
Normalized Probe Intensity
Probe Number
−1.0
−0.5
0.0
0.5
1.0
1.5
2.0
1
2
3
4
5
6
7
8
9
10
11
Meishan−A
Meishan−B
Meishan−C
European−A
European−B
European−C

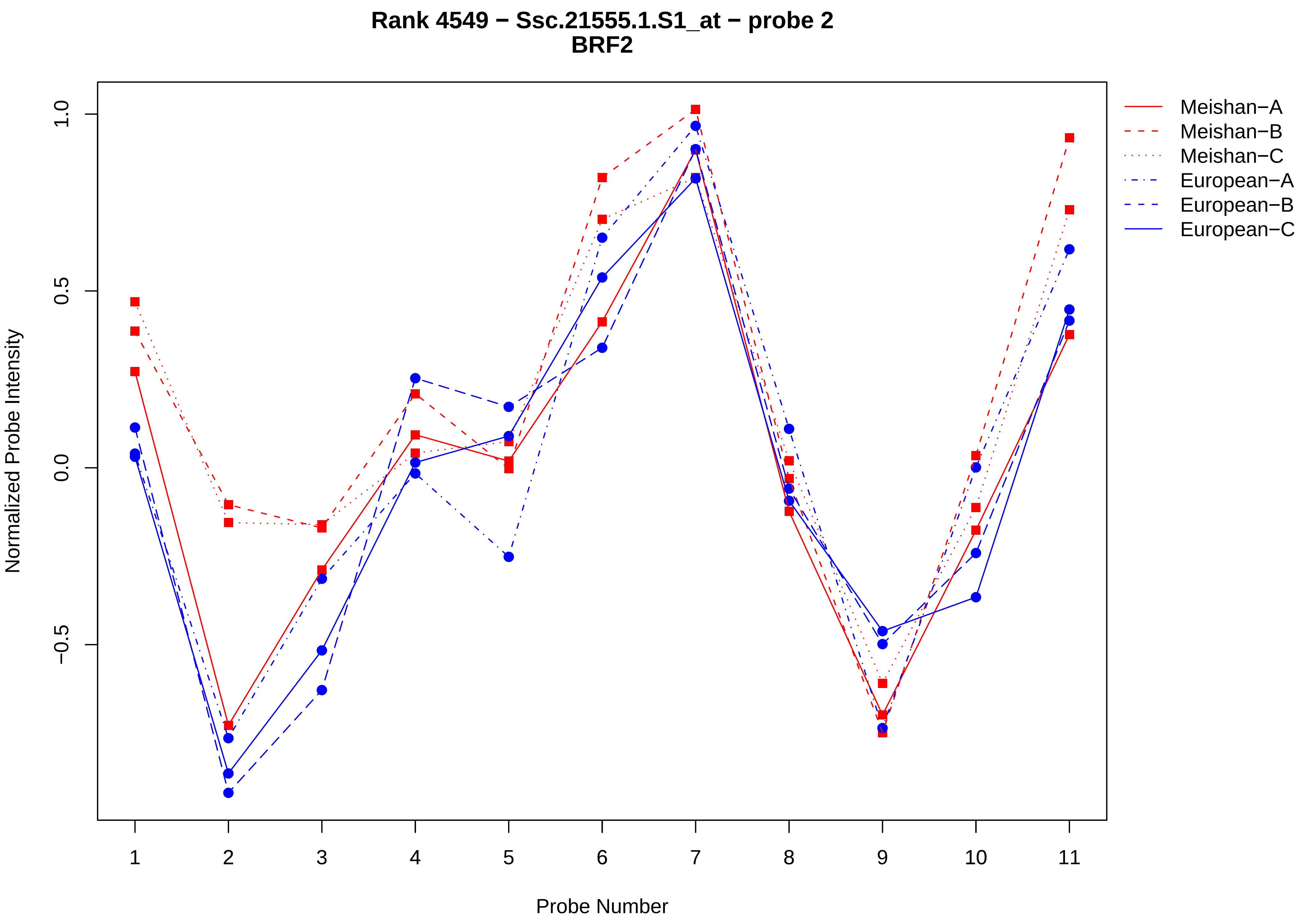
Rank 4549 − Ssc.21555.1.S1_at − probe 2
BRF2
Normalized Probe Intensity
Probe Number
1.0
0.5
0.0
−0.5
1
2
3
4
5
6
7
8
9
10
11
Meishan−A
Meishan−B
Meishan−C
European−A
European−B
European−C

Rank 4550 − Ssc.10698.1.S1_at − probe 10
SDCCAG10

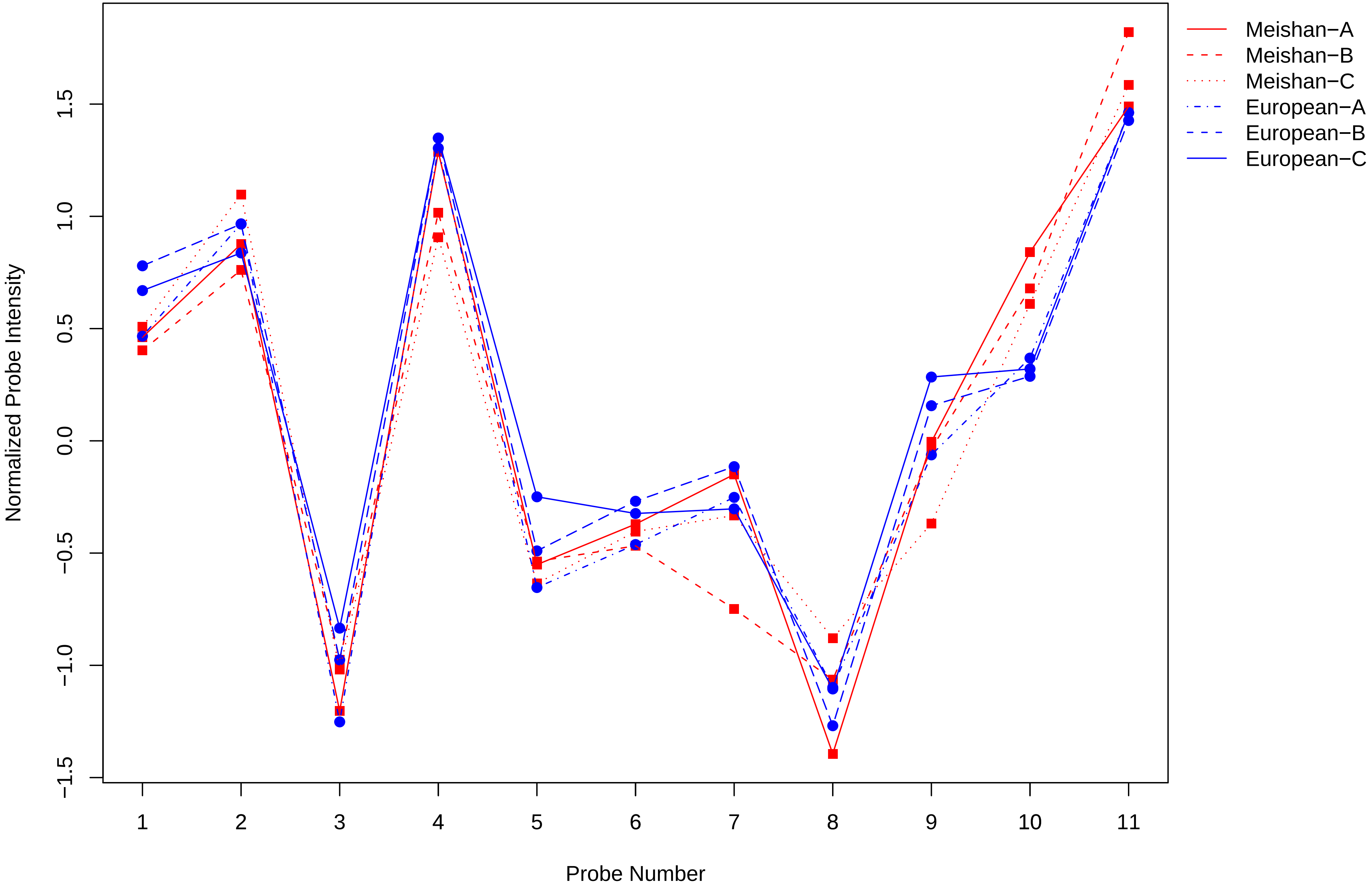

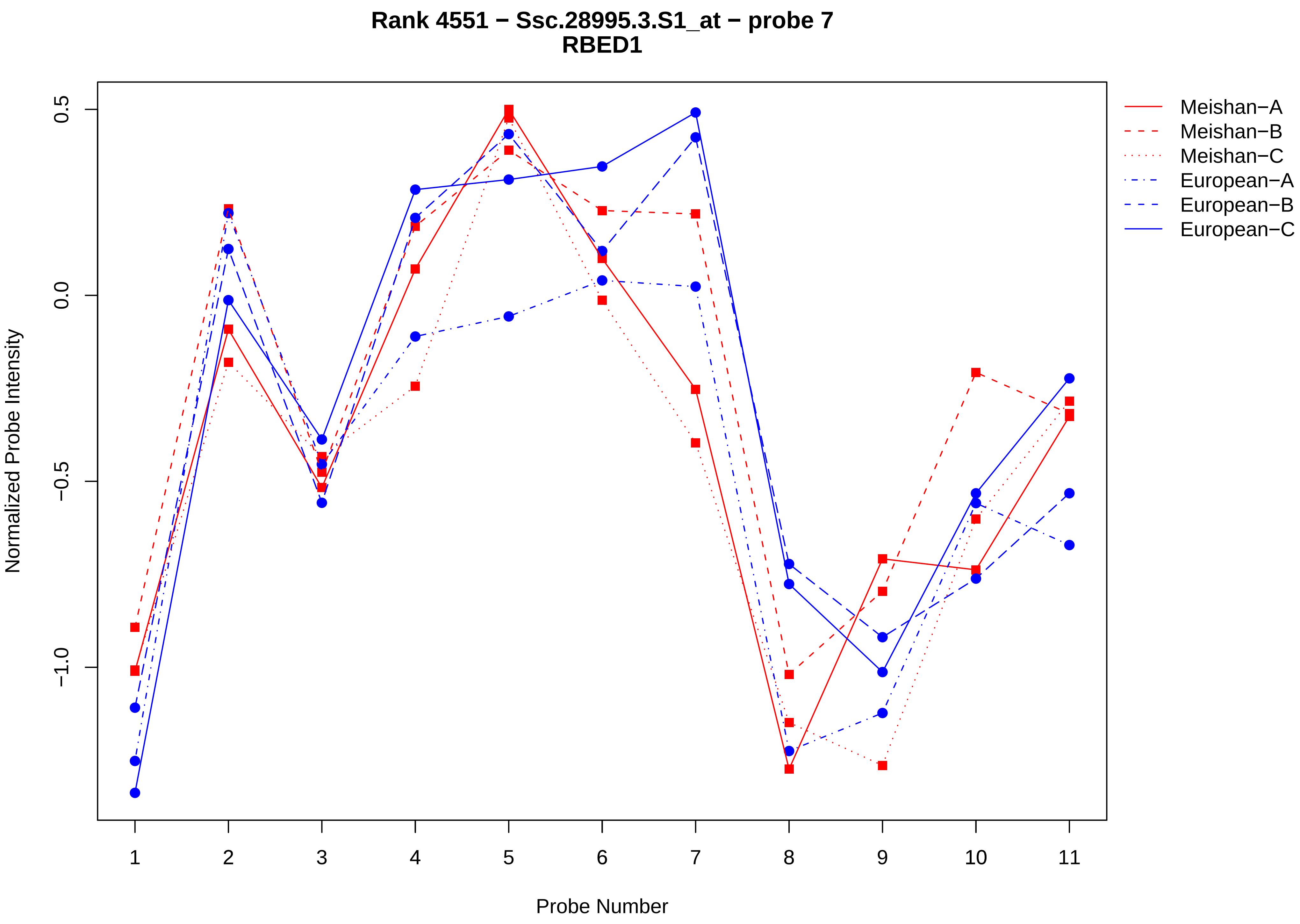
Rank 4551 − Ssc.28995.3.S1_at − probe 7
RBED1
Normalized Probe Intensity
0.5
0.0
−0.5
−1.0
Probe Number
1
2
3
4
5
6
7
8
9
10
11
Meishan−A
Meishan−B
Meishan−C
European−A
European−B
European−C

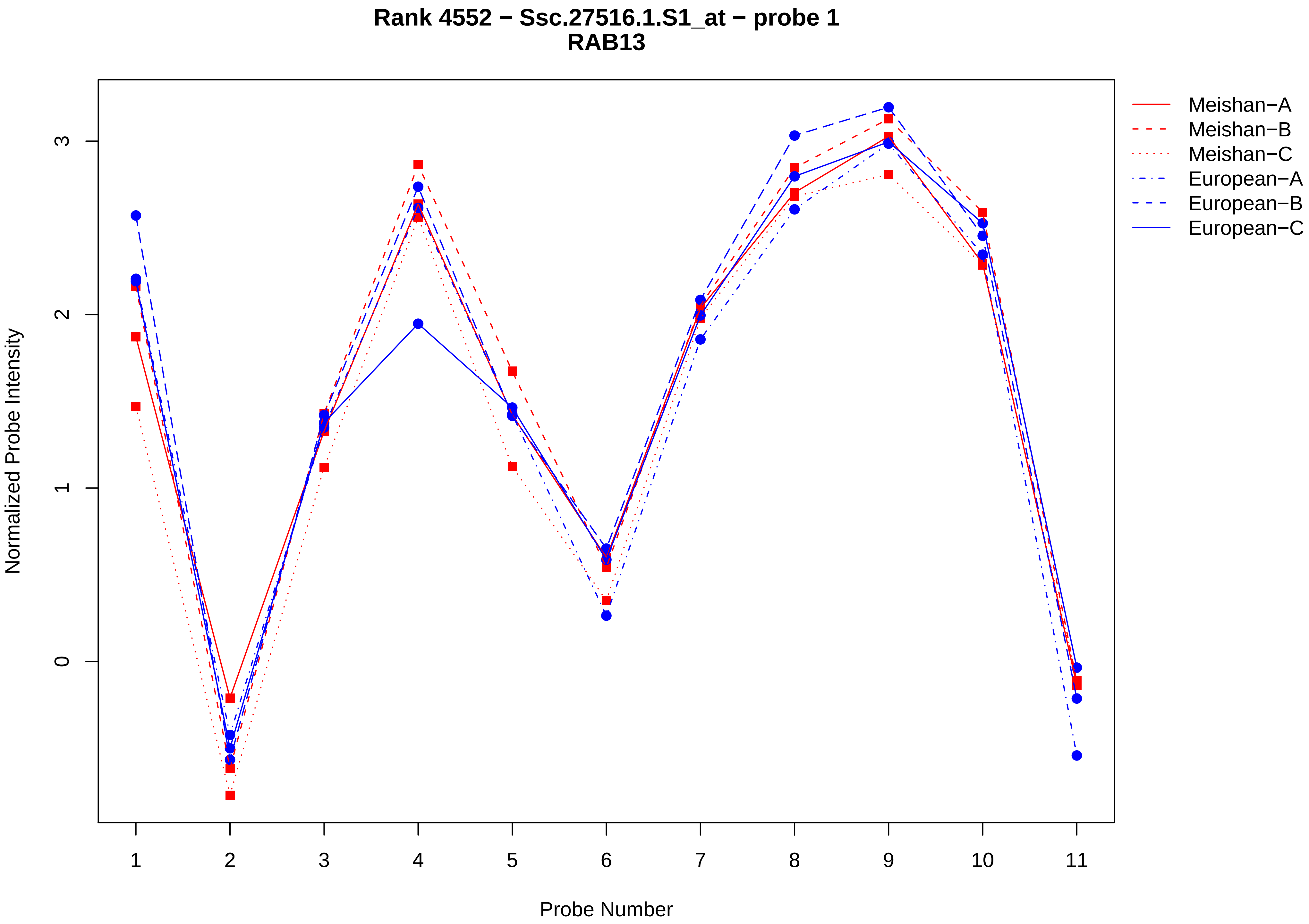

Rank 4552 − Ssc.27516.1.S1_at − probe 1
RAB13
Normalized Probe Intensity
Probe Number
0
1
2
3
1
2
3
4
5
6
7
8
9
10
11
Meishan−A
Meishan−B
Meishan−C
European−A
European−B
European−C

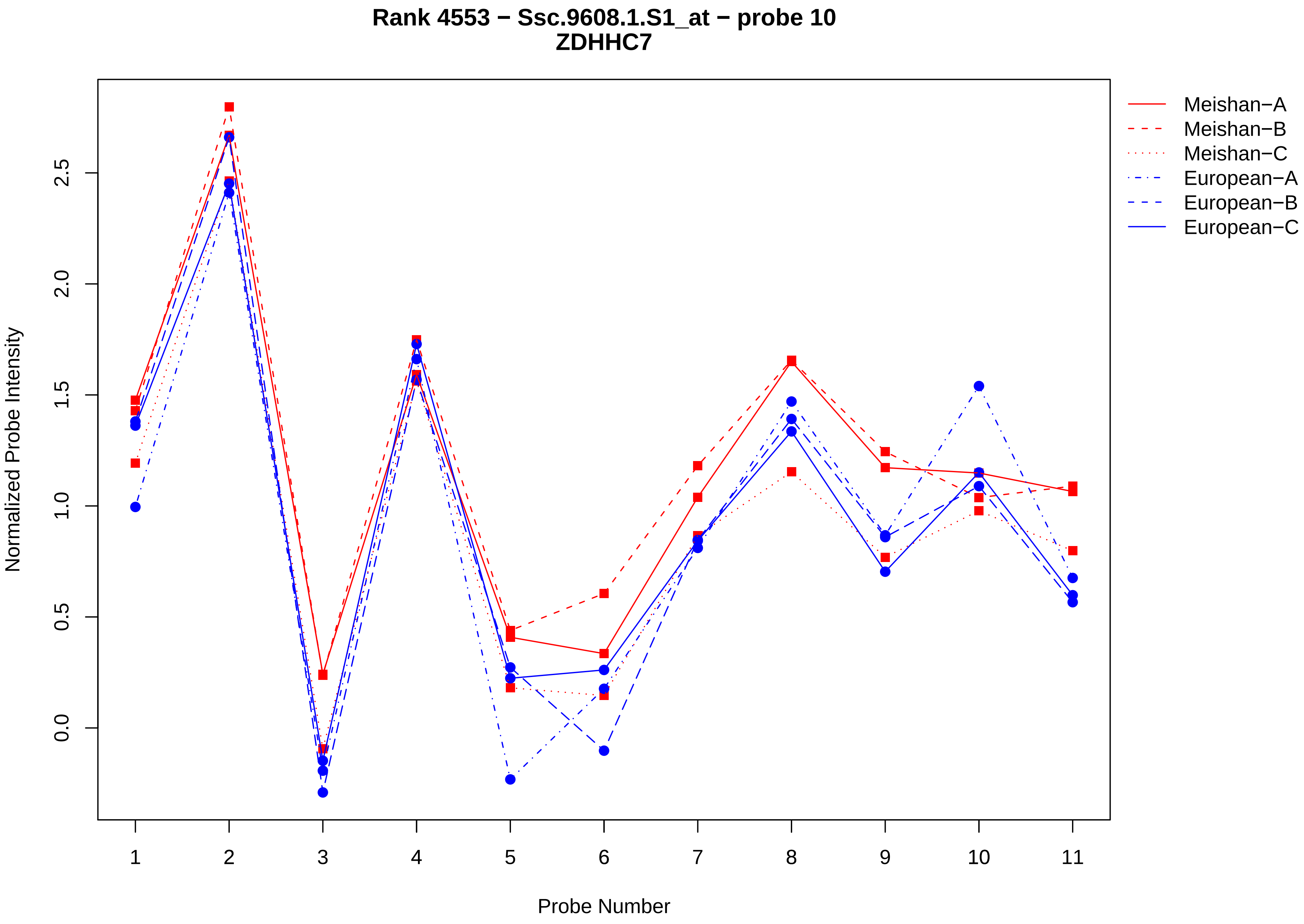

Rank 4553 − Ssc.9608.1.S1_at − probe 10
ZDHHC7
Normalized Probe Intensity
Probe Number
0.0
0.5
1.0
1.5
2.0
2.5
1
2
3
4
5
6
7
8
9
10
11
Meishan−A
Meishan−B
Meishan−C
European−A
European−B
European−C

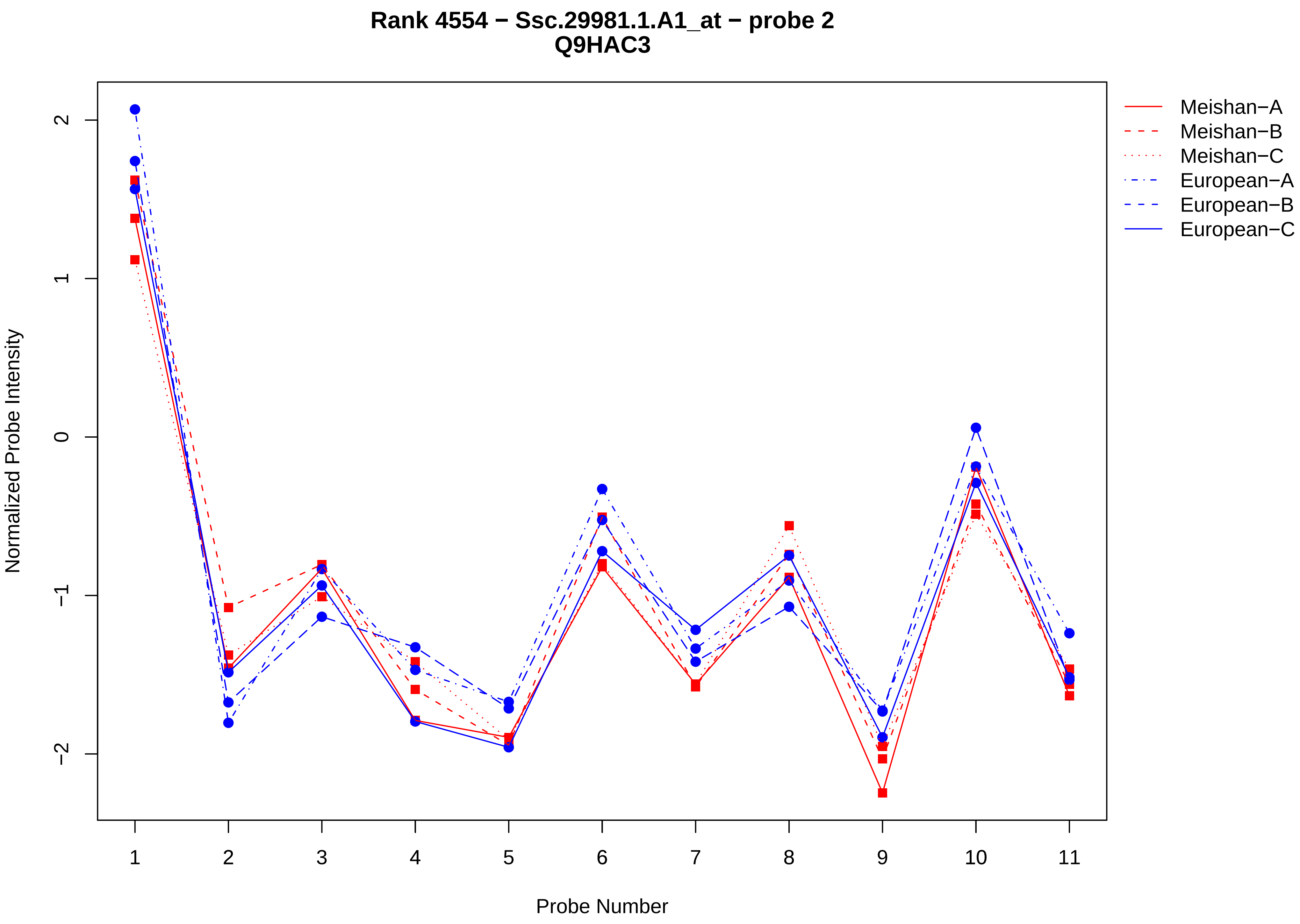
Rank 4554 − Ssc.29981.1.A1_at − probe 2
Q9HAC3
Normalized Probe Intensity
Probe Number
2
1
0
−1
−2
1
2
3
4
5
6
7
8
9
10
11
Meishan−A
Meishan−B
Meishan−C
European−A
European−B
European−C

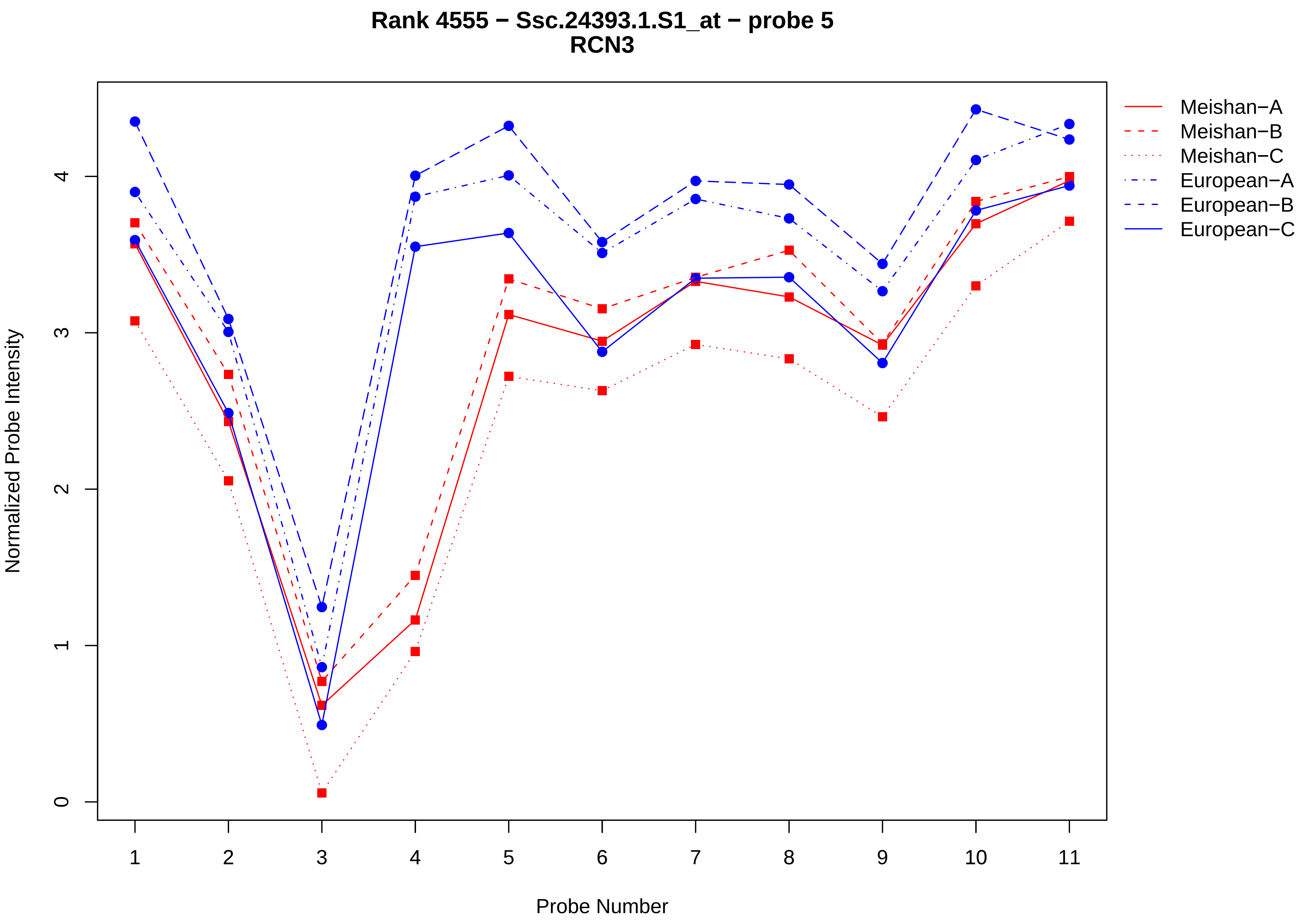

Rank 4555 − Ssc.24393.1.S1_at − probe 5
RCN3
Normalized Probe Intensity
Probe Number
0
1
2
3
4
1
2
3
4
5
6
7
8
9
10
11
Meishan−A
Meishan−B
Meishan−C
European−A
European−B
European−C

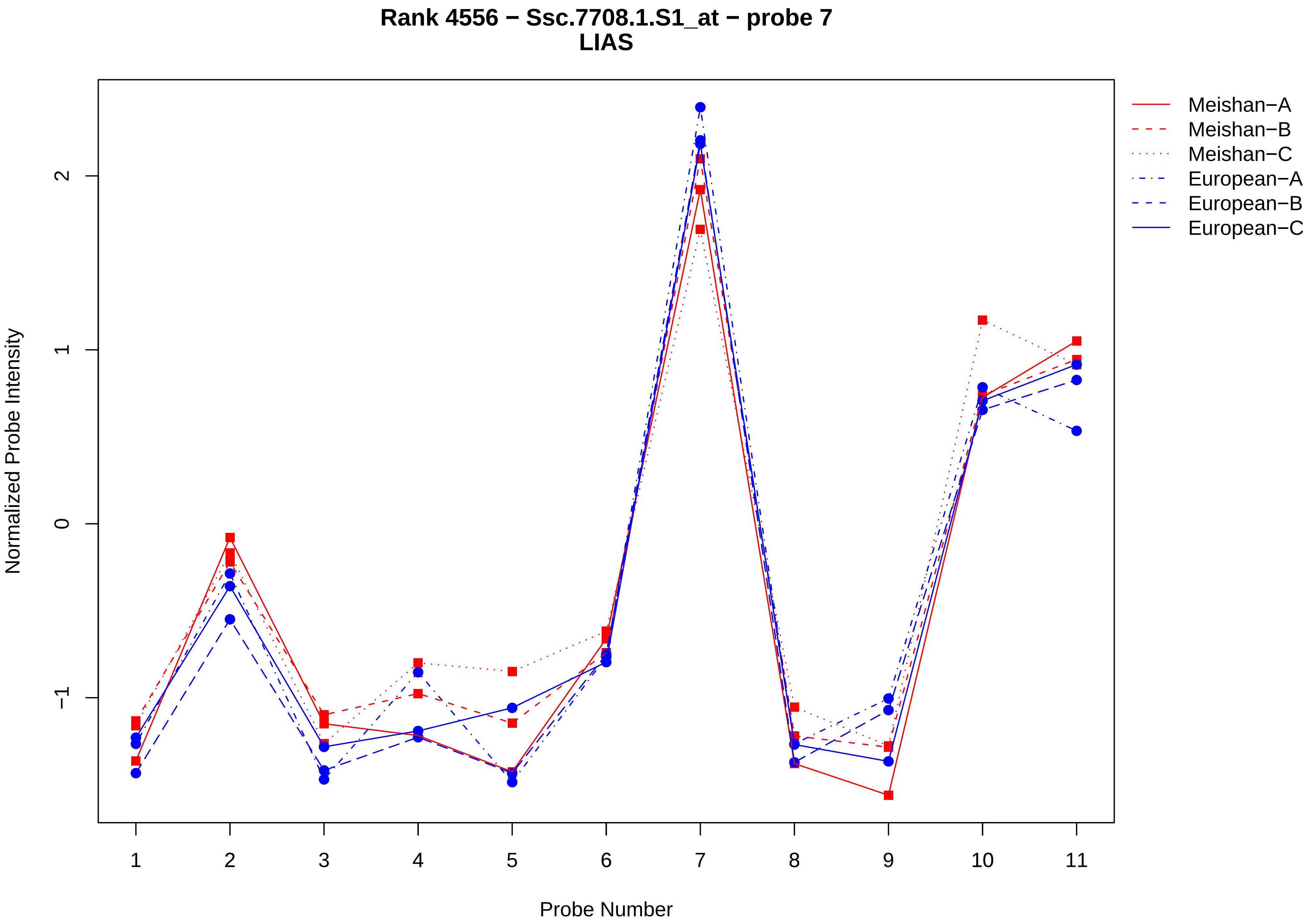

Rank 4556 − Ssc.7708.1.S1_at − probe 7
LIAS
Normalized Probe Intensity
Probe Number
−1
0
1
2
1
2
3
4
5
6
7
8
9
10
11
Meishan−A
Meishan−B
Meishan−C
European−A
European−B
European−C

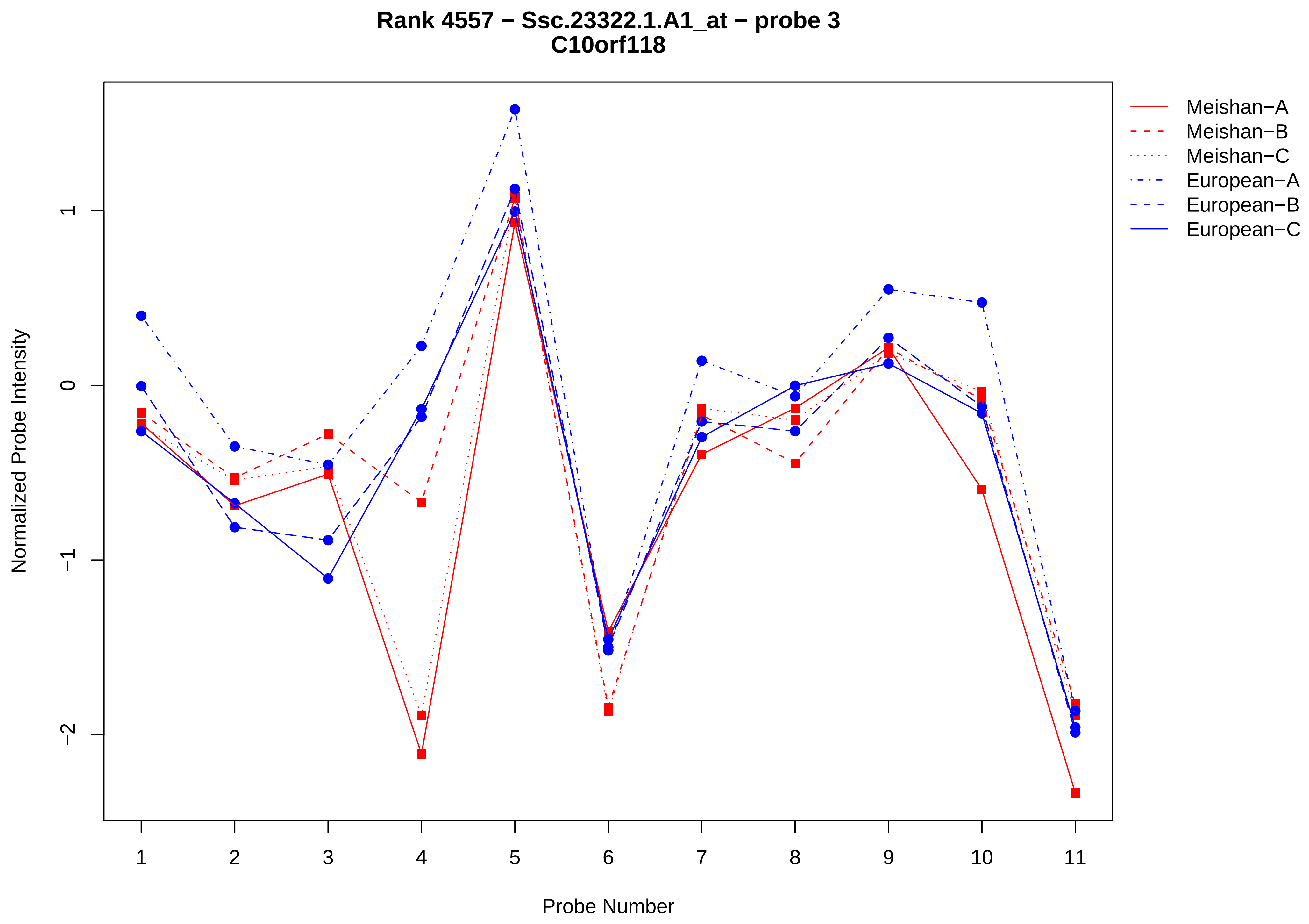
Rank 4557 − Ssc.23322.1.A1_at − probe 3
C10orf118
Normalized Probe Intensity
Probe Number
1
0
−1
−2
1
2
3
4
5
6
7
8
9
10
11
Meishan−A
Meishan−B
Meishan−C
European−A
European−B
European−C

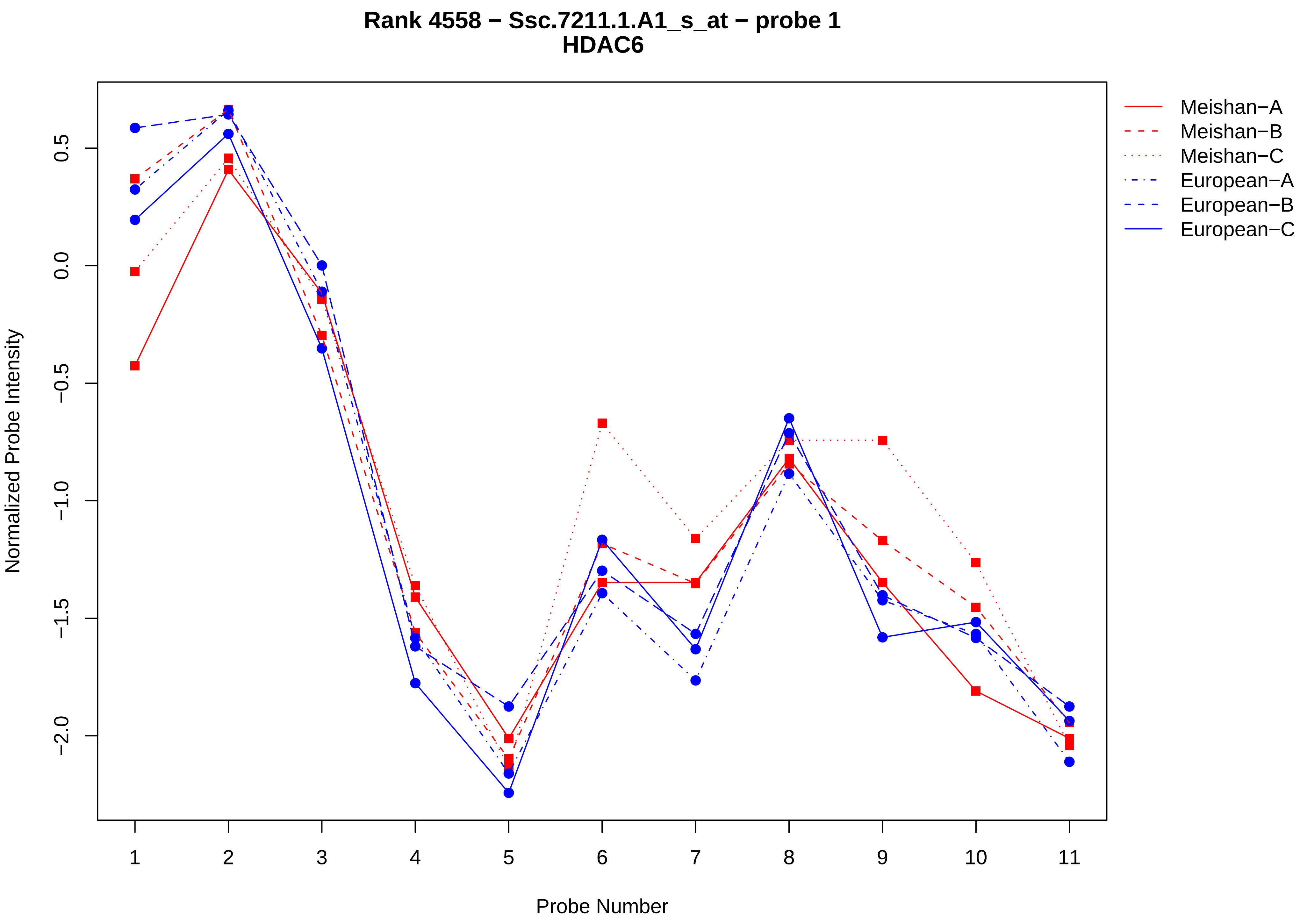

Rank 4558 − Ssc.7211.1.A1_s_at − probe 1
HDAC6
Normalized Probe Intensity
Probe Number
0.5
0.0
−0.5
−1.0
−1.5
−2.0
1
2
3
4
5
6
7
8
9
10
11
Meishan−A
Meishan−B
Meishan−C
European−A
European−B
European−C

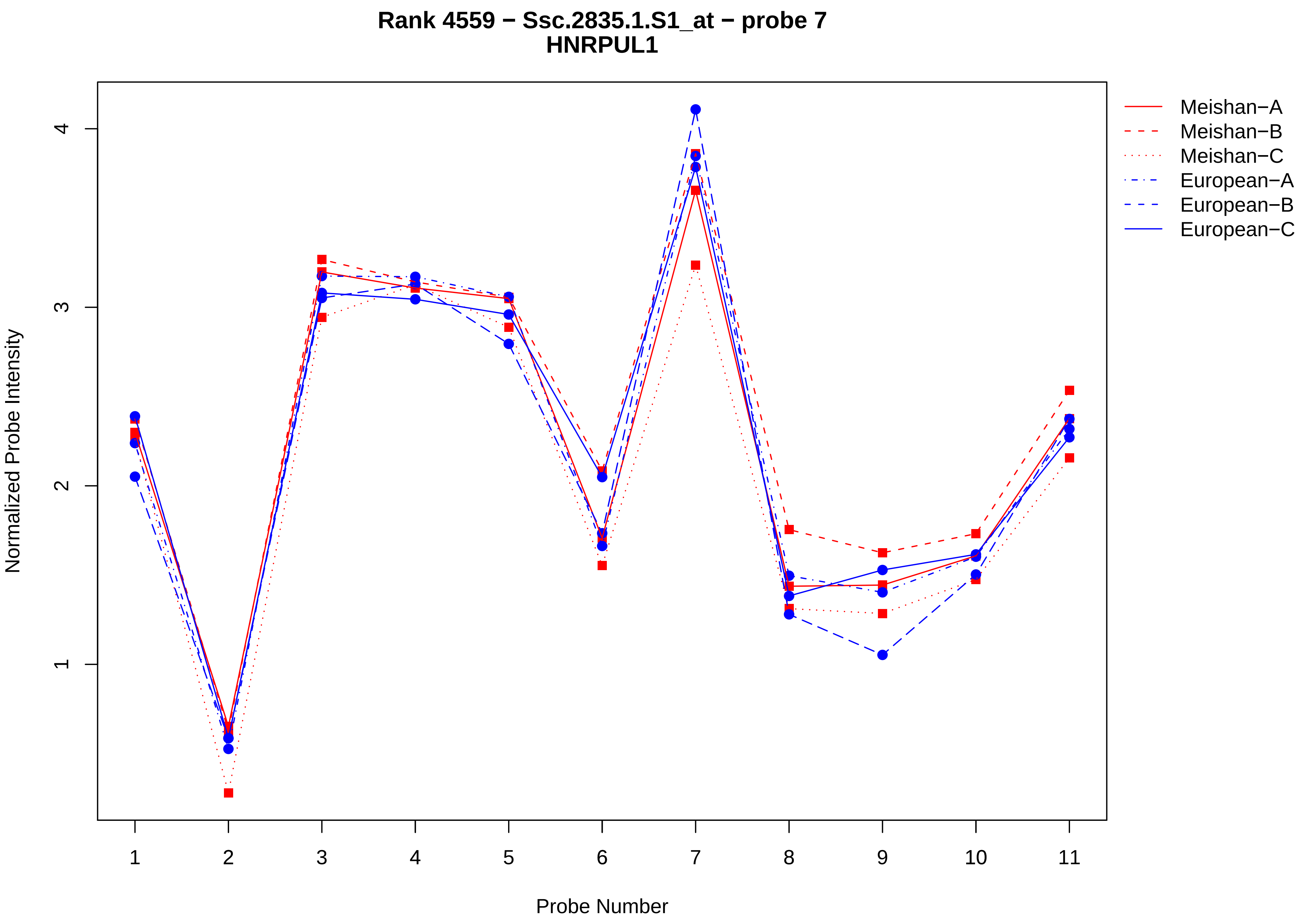

Rank 4559 − Ssc.2835.1.S1_at − probe 7
HNRPUL1
Normalized Probe Intensity
Probe Number
1
2
3
4
5
6
7
8
9
10
11
Meishan−A
Meishan−B
Meishan−C
European−A
European−B
European−C

Rank 4560 − Ssc.22623.3.A1_at − probe 5
PEG3

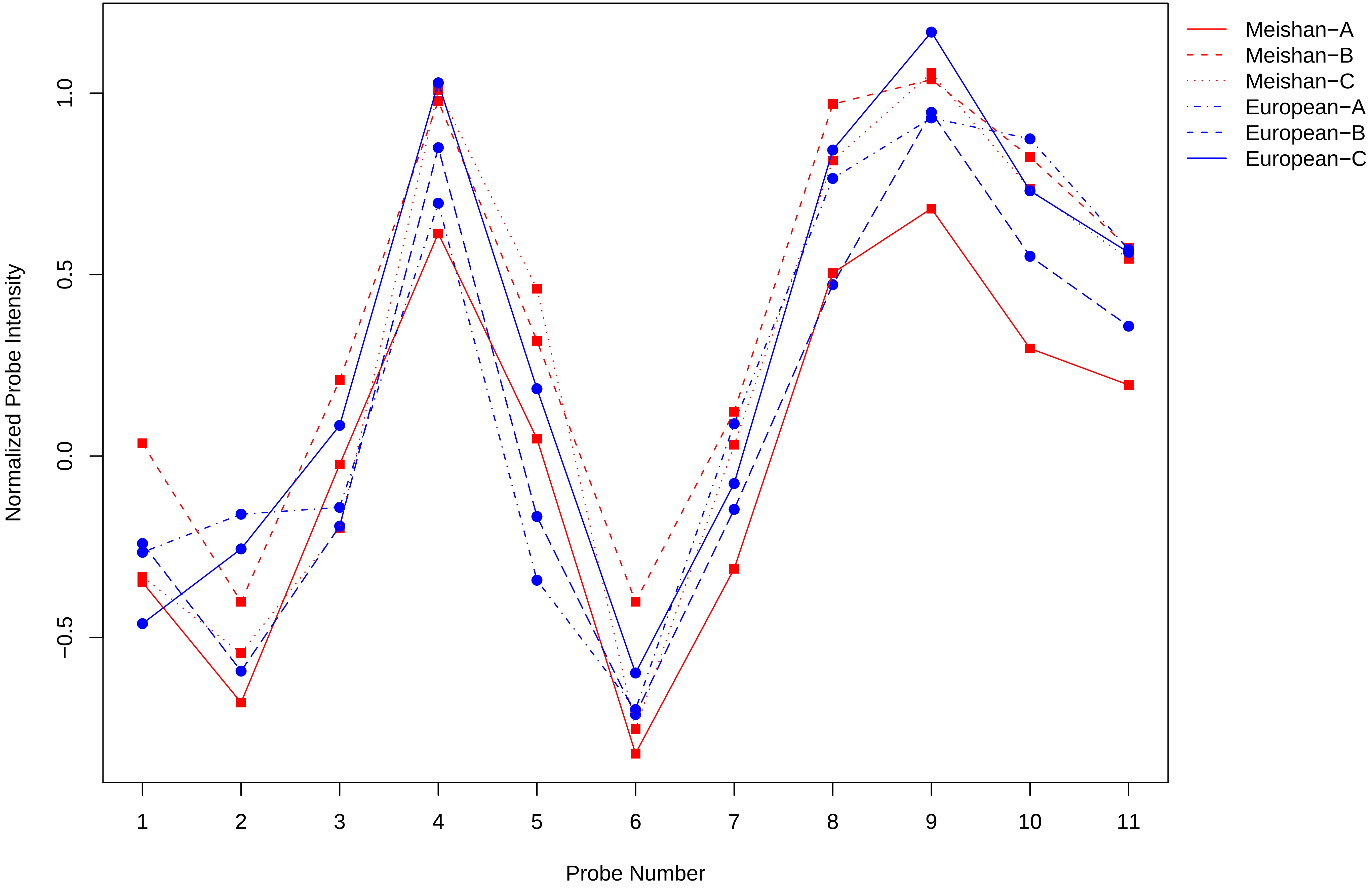

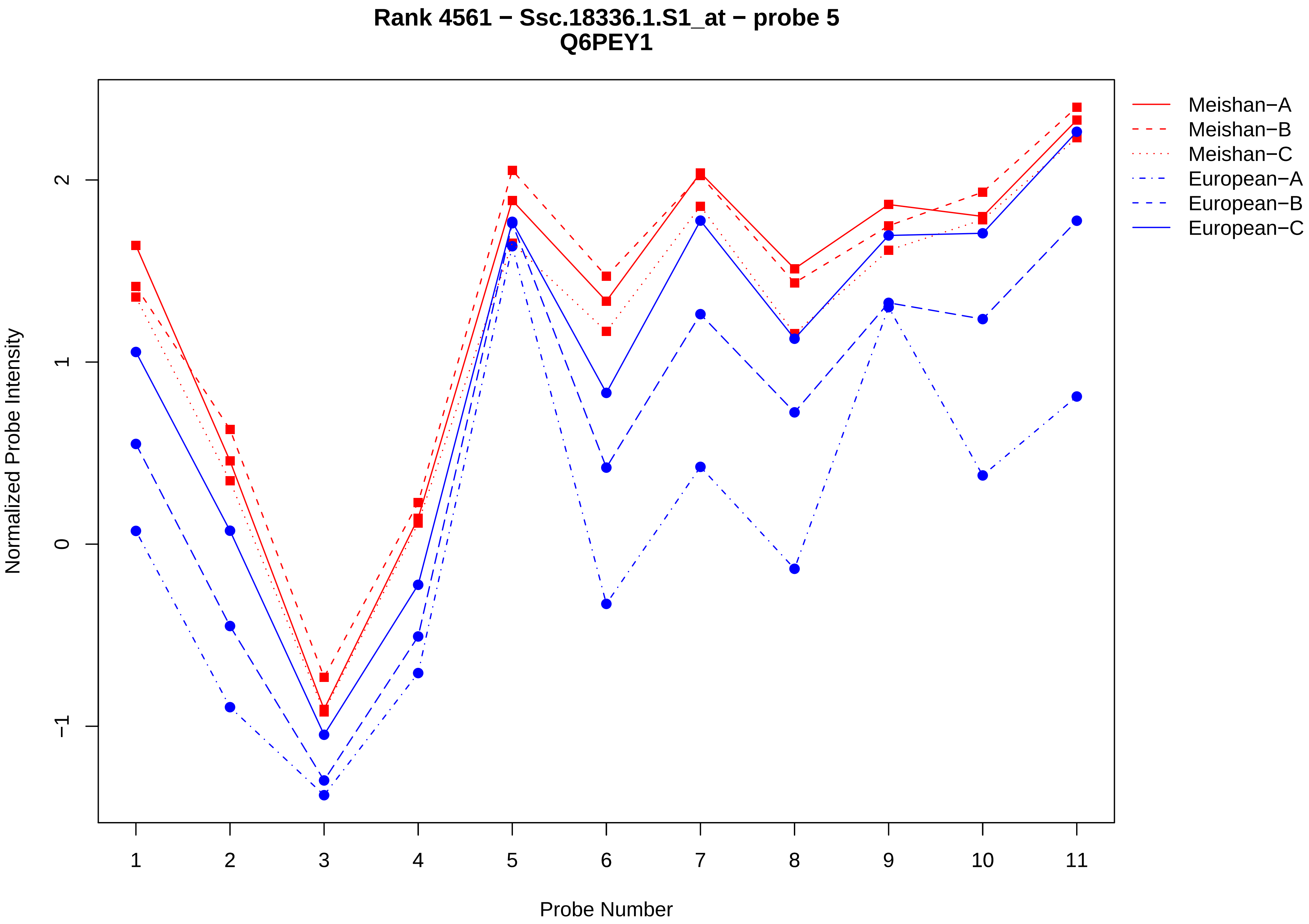

Rank 4561 − Ssc.18336.1.S1_at − probe 5
Q6PEY1
Normalized Probe Intensity
Probe Number
−1
0
1
2
1
2
3
4
5
6
7
8
9
10
11
Meishan−A
Meishan−B
Meishan−C
European−A
European−B
European−C

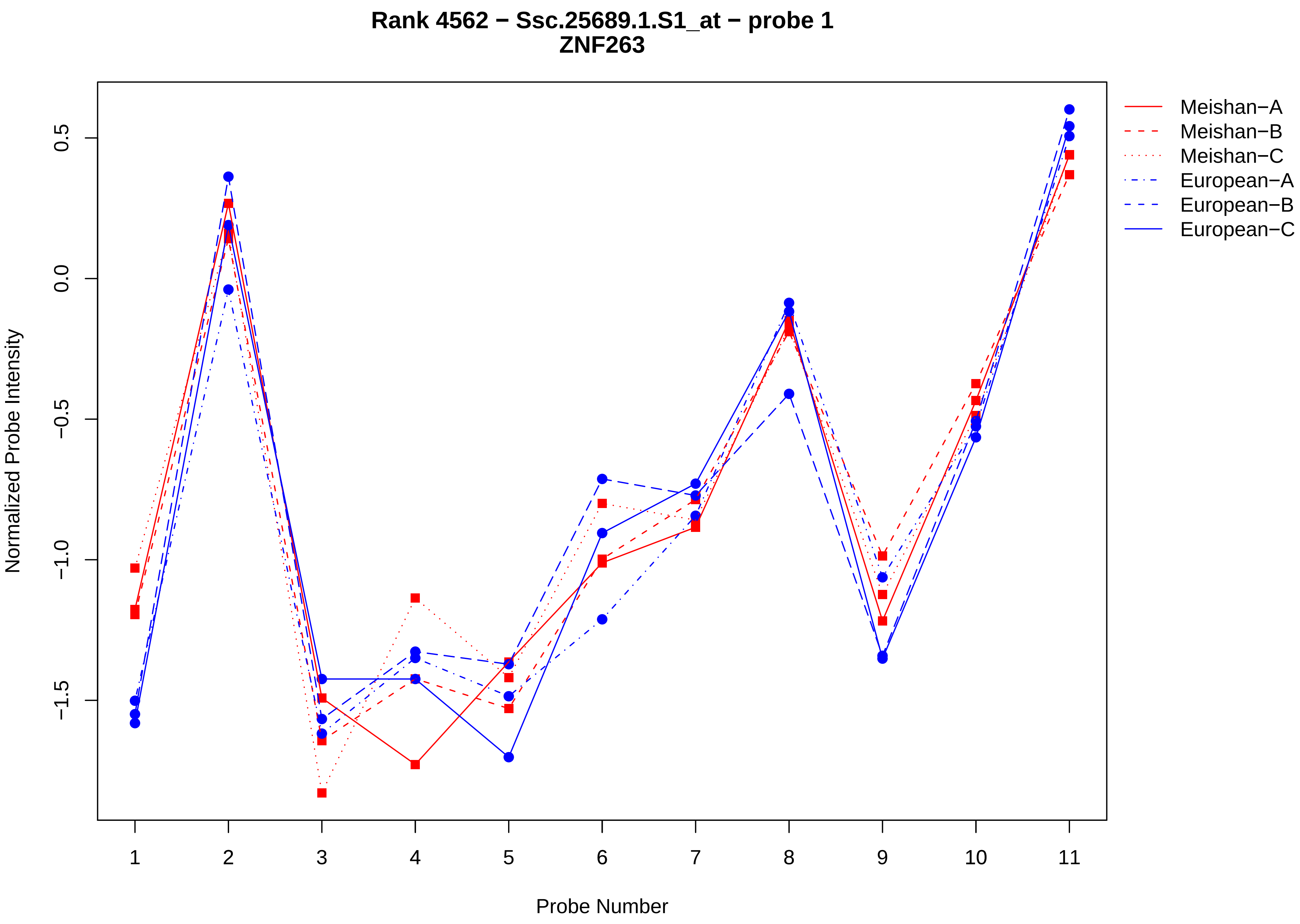

Rank 4562 − Ssc.25689.1.S1_at − probe 1
ZNF263
Normalized Probe Intensity
0.5
0.0
−0.5
−1.0
−1.5
1
2
3
4
5
6
7
8
9
10
11
Probe Number
Meishan−A
Meishan−B
Meishan−C
European−A
European−B
European−C

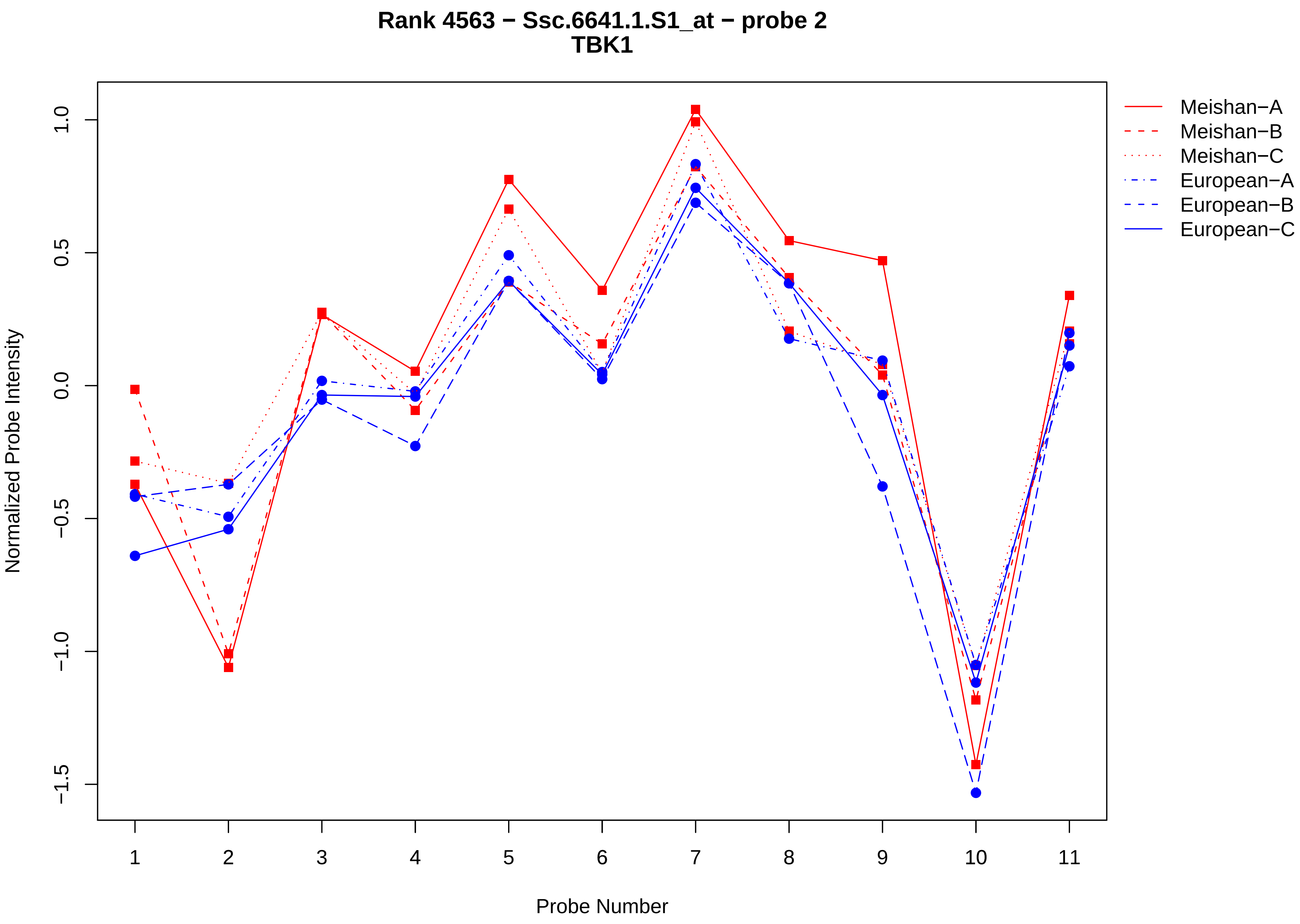
Rank 4563 − Ssc.6641.1.S1_at − probe 2
TBK1
Normalized Probe Intensity
1.0
0.5
0.0
−0.5
−1.0
−1.5
1
2
3
4
5
6
7
8
9
10
11
Probe Number
Meishan−A
Meishan−B
Meishan−C
European−A
European−B
European−C

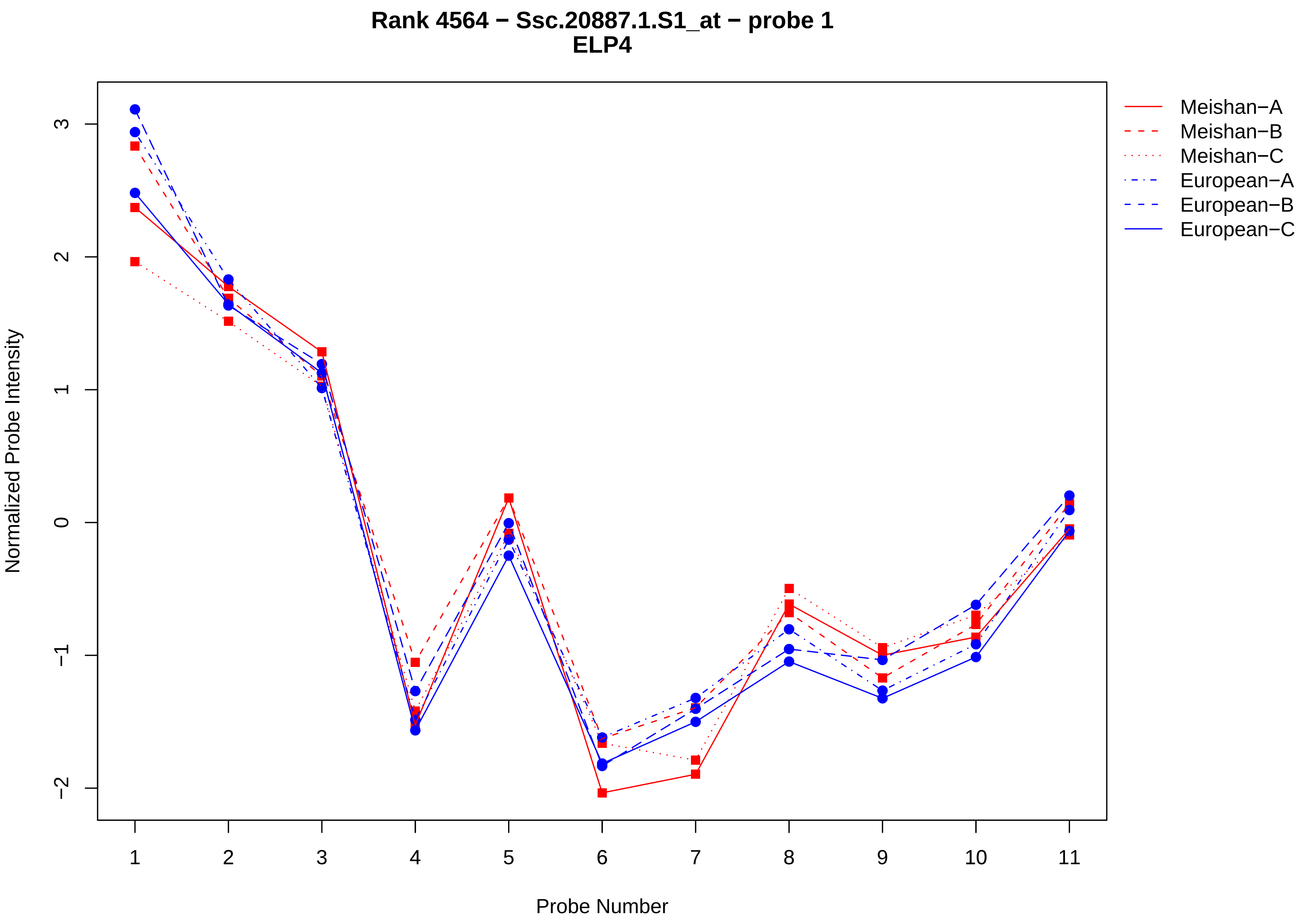
Rank 4564 − Ssc.20887.1.S1_at − probe 1
ELP4
Normalized Probe Intensity
Probe Number
−2
−1
0
1
2
3
1
2
3
4
5
6
7
8
9
10
11
Meishan−A
Meishan−B
Meishan−C
European−A
European−B
European−C

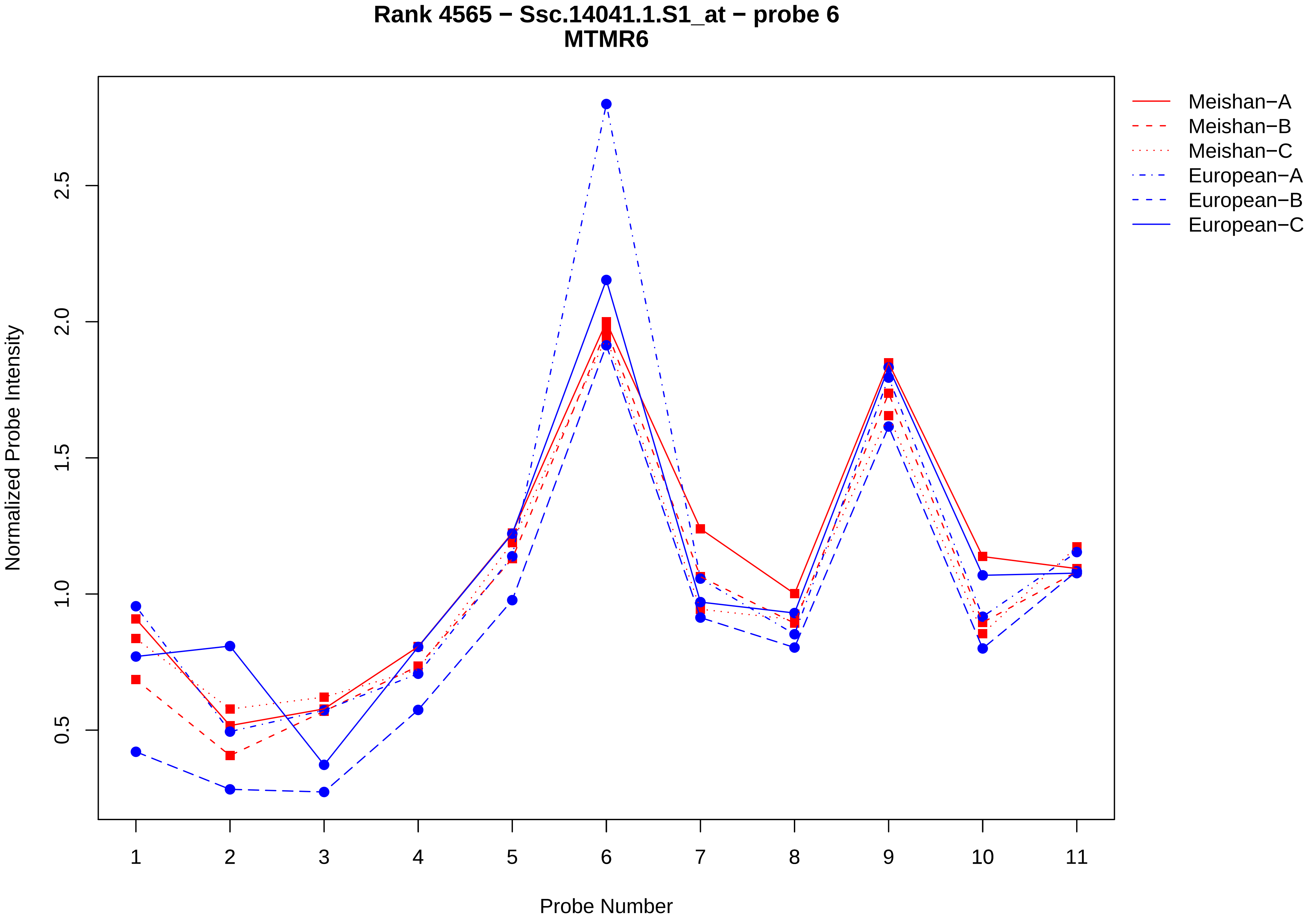

Rank 4565 − Ssc.14041.1.S1_at − probe 6
MTMR6
Normalized Probe Intensity
0.5
1.0
1.5
2.0
2.5
Probe Number
1
2
3
4
5
6
7
8
9
10
11
Meishan−A
Meishan−B
Meishan−C
European−A
European−B
European−C

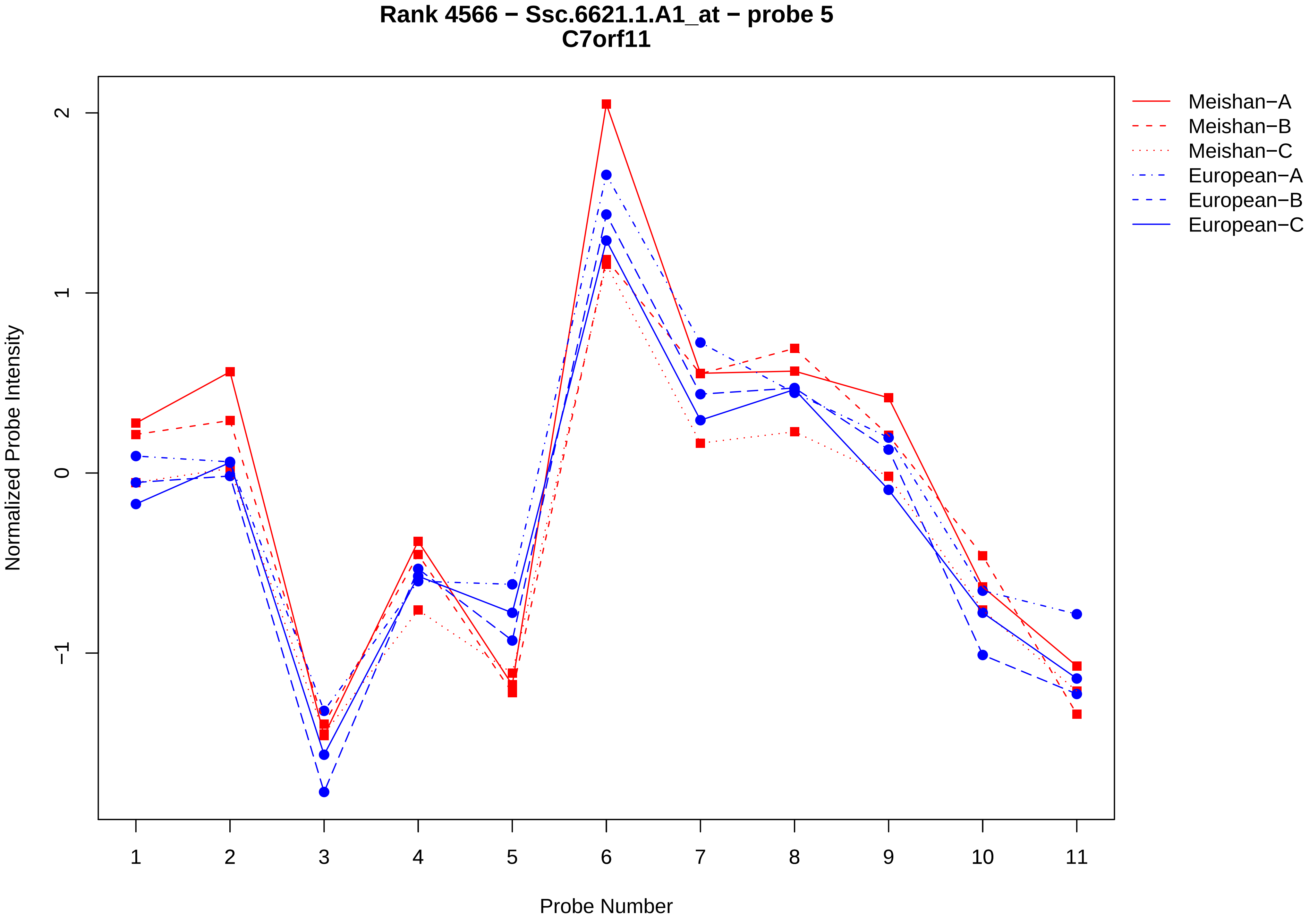

Rank 4566 − Ssc.6621.1.A1_at − probe 5
C7orf11
Normalized Probe Intensity
Probe Number
−1
0
1
2
1
2
3
4
5
6
7
8
9
10
11
Meishan−A
Meishan−B
Meishan−C
European−A
European−B
European−C

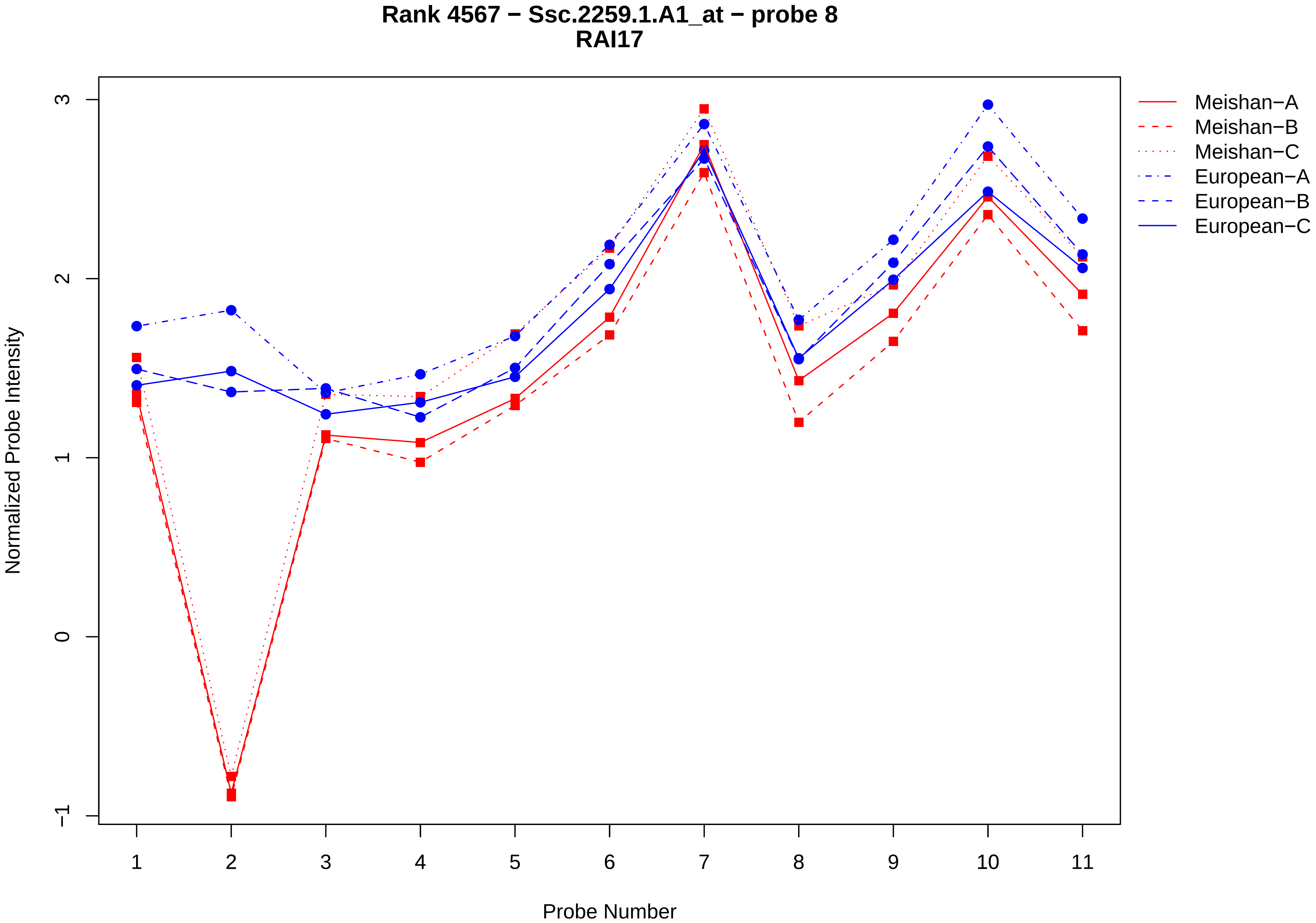

Rank 4567 − Ssc.2259.1.A1_at − probe 8
RAI17
Normalized Probe Intensity
Probe Number
−1
0
1
2
3
1
2
3
4
5
6
7
8
9
10
11
Meishan−A
Meishan−B
Meishan−C
European−A
European−B
European−C

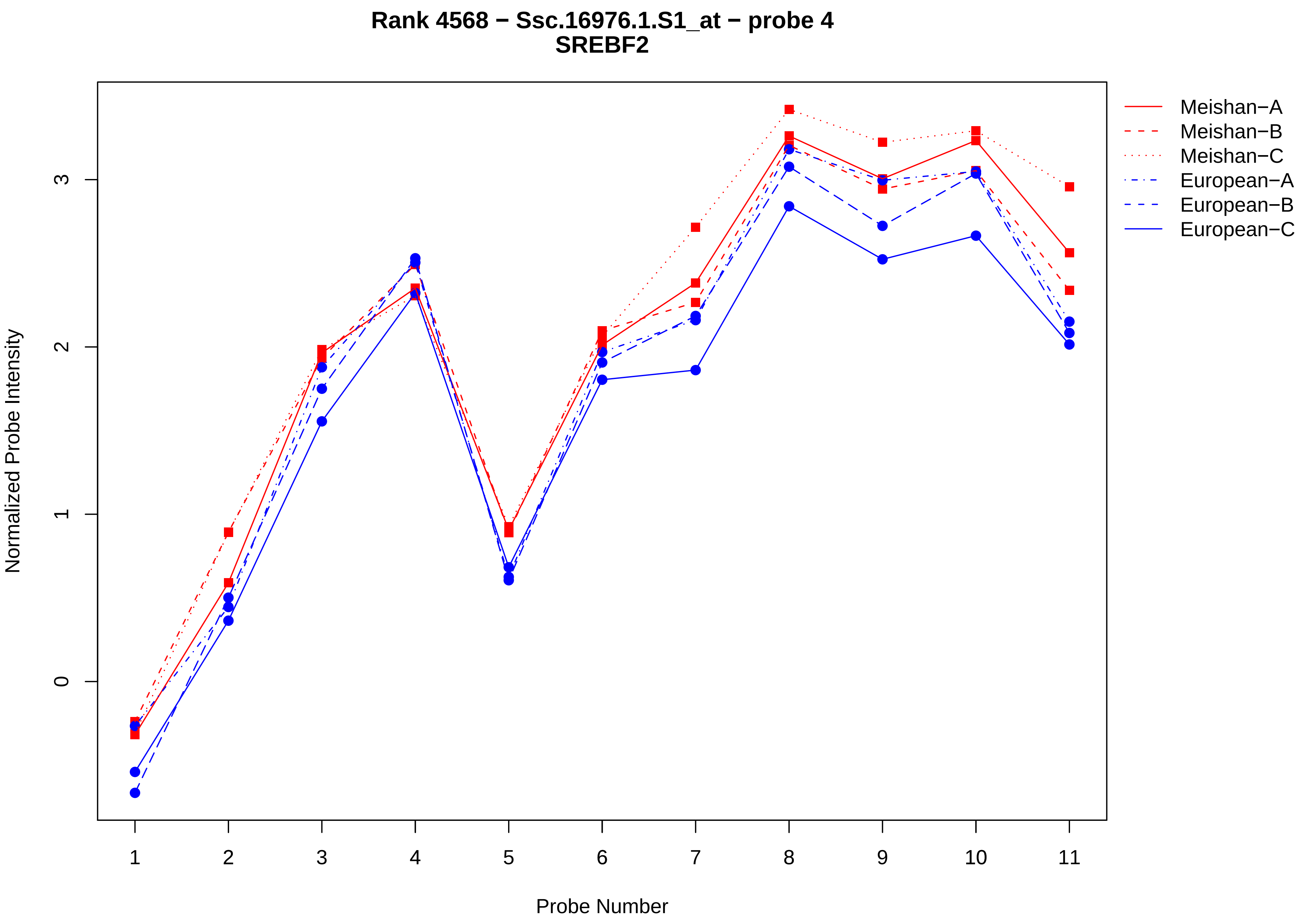
Rank 4568 − Ssc.16976.1.S1_at − probe 4
SREBF2
Normalized Probe Intensity
Probe Number
0
1
2
3
1
2
3
4
5
6
7
8
9
10
11
Meishan−A
Meishan−B
Meishan−C
European−A
European−B
European−C

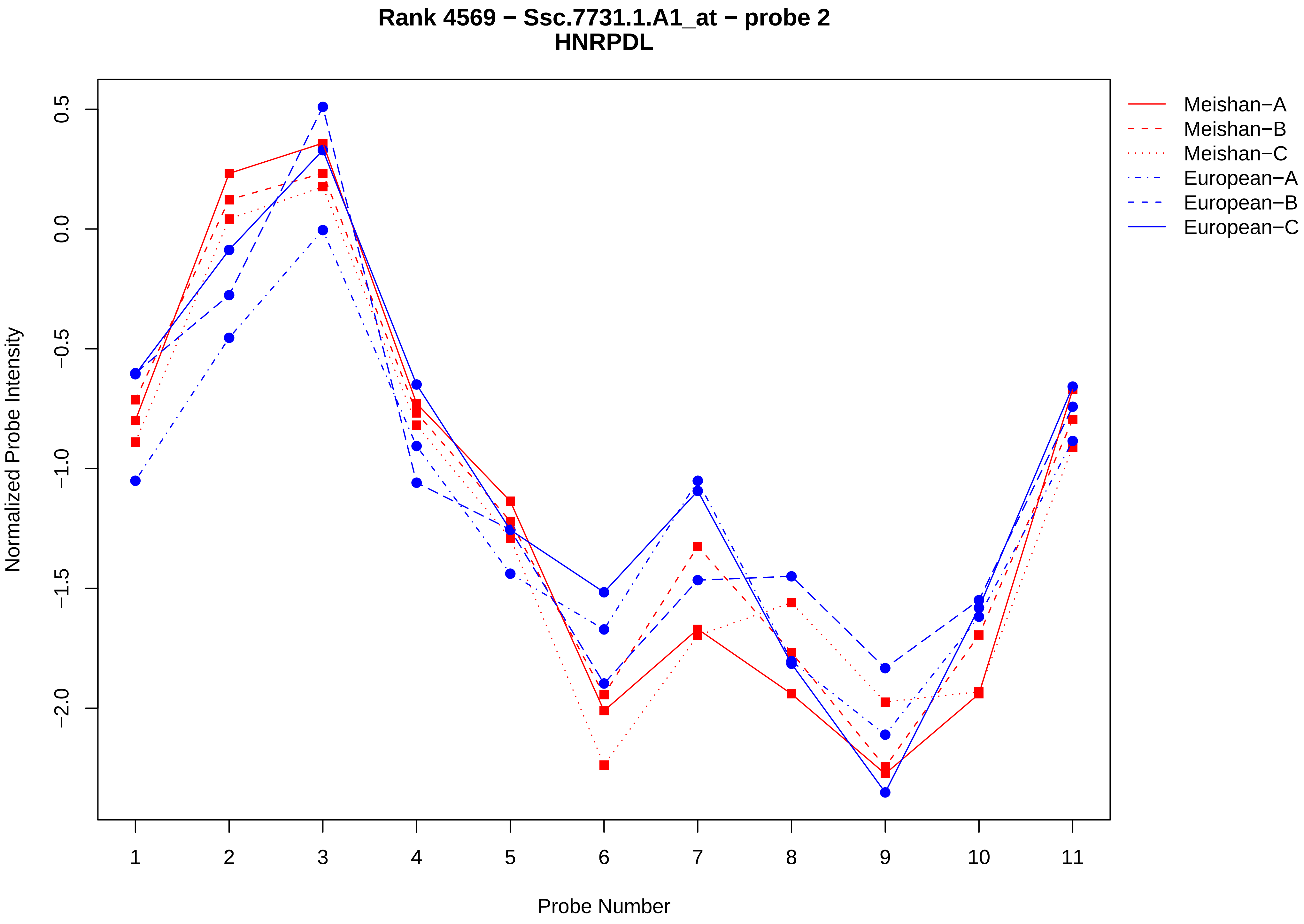
Rank 4569 − Ssc.7731.1.A1_at − probe 2
HNRPDL
Normalized Probe Intensity
Probe Number
0.5
0.0
−0.5
−1.0
−1.5
−2.0
1
2
3
4
5
6
7
8
9
10
11
Meishan−A
Meishan−B
Meishan−C
European−A
European−B
European−C

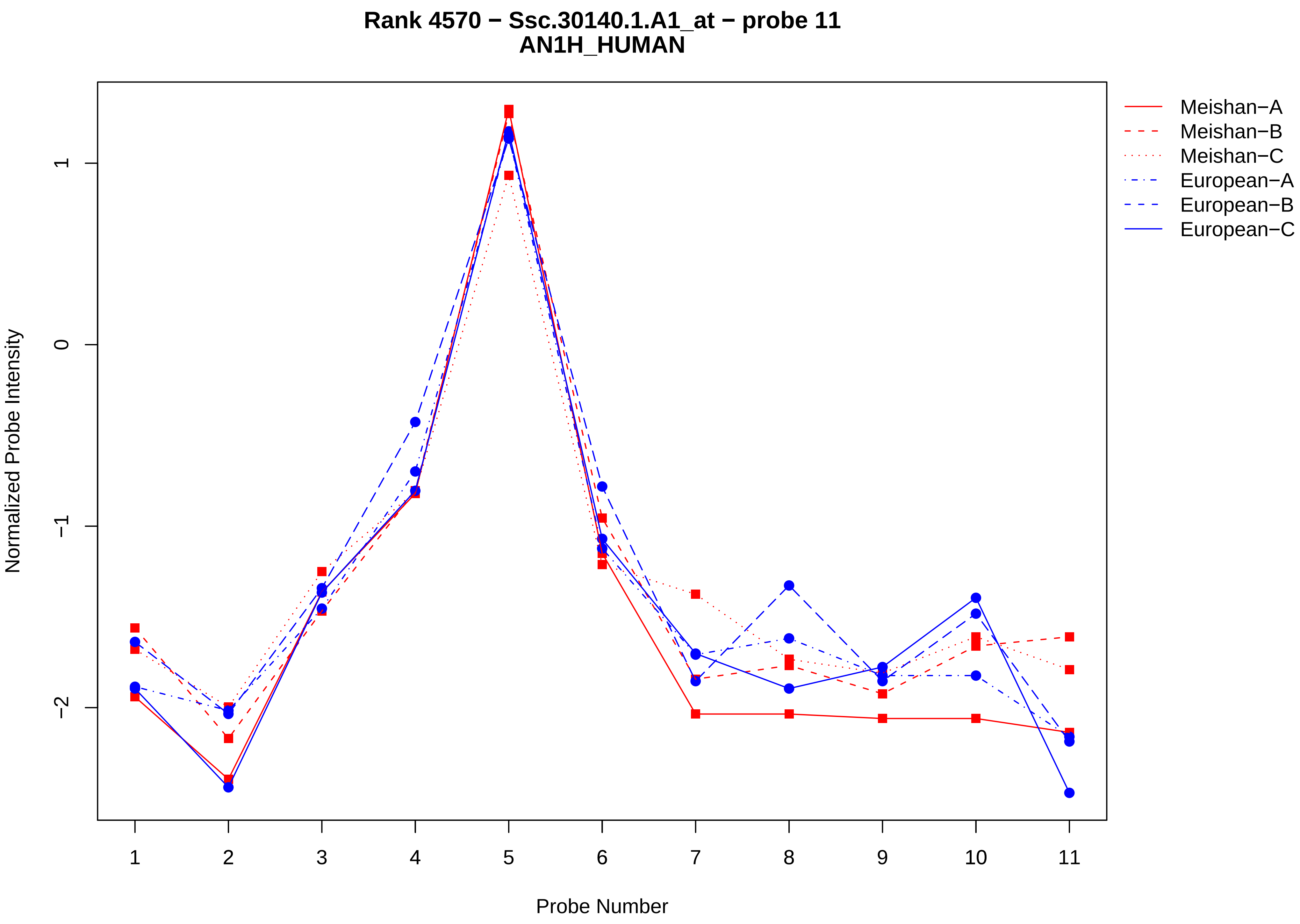
Rank 4570 − Ssc.30140.1.A1_at − probe 11
AN1H_HUMAN
Normalized Probe Intensity
Probe Number
1
0
−1
−2
1
2
3
4
5
6
7
8
9
10
11
Meishan−A
Meishan−B
Meishan−C
European−A
European−B
European−C

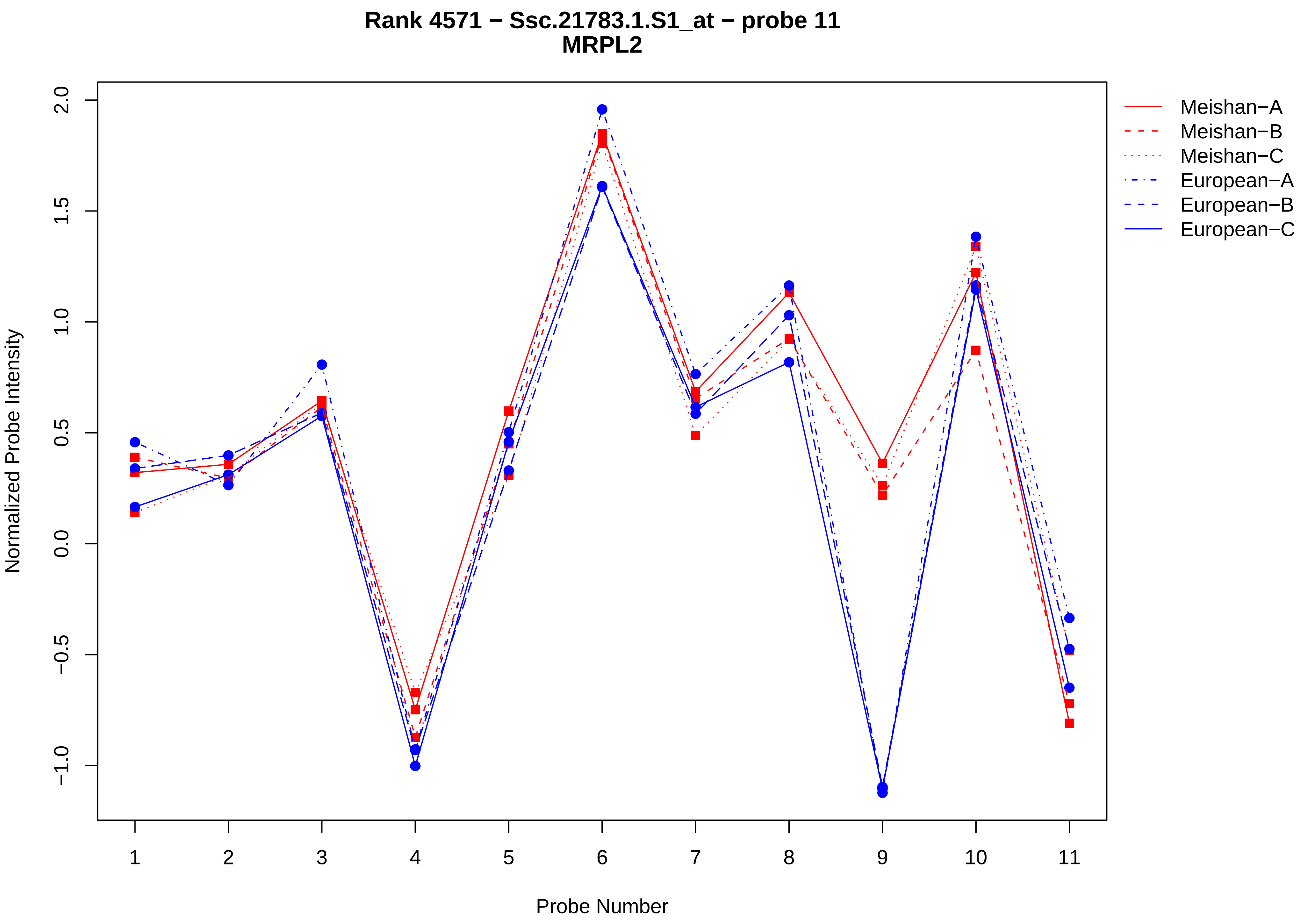

Rank 4571 − Ssc.21783.1.S1_at − probe 11
MRPL2
Normalized Probe Intensity
Probe Number
−1.0
−0.5
0.0
0.5
1.0
1.5
2.0
1
2
3
4
5
6
7
8
9
10
11
Meishan−A
Meishan−B
Meishan−C
European−A
European−B
European−C

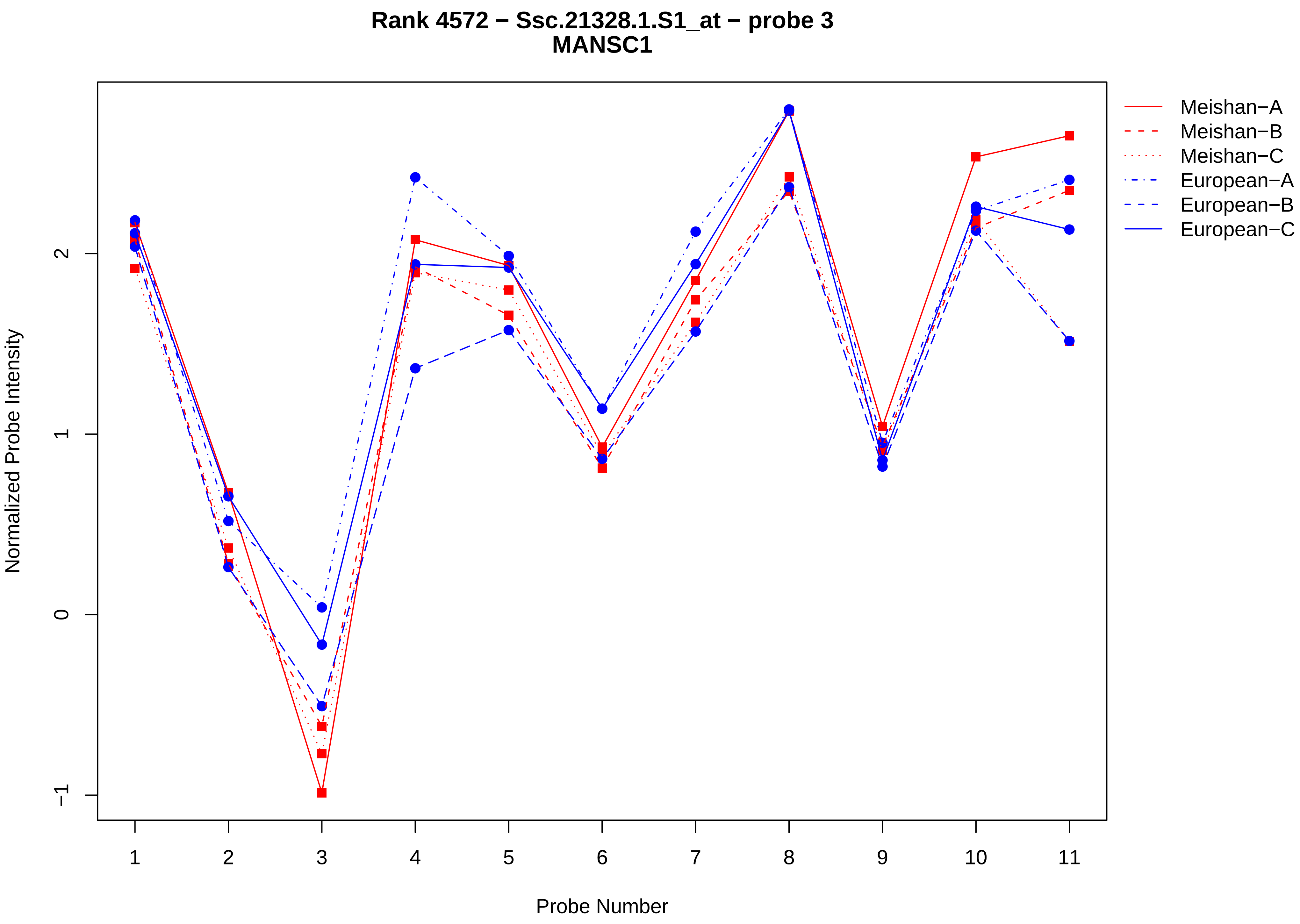

Rank 4572 − Ssc.21328.1.S1_at − probe 3
MANSC1
Normalized Probe Intensity
Probe Number
−1
0
1
2
1
2
3
4
5
6
7
8
9
10
11
Meishan−A
Meishan−B
Meishan−C
European−A
European−B
European−C

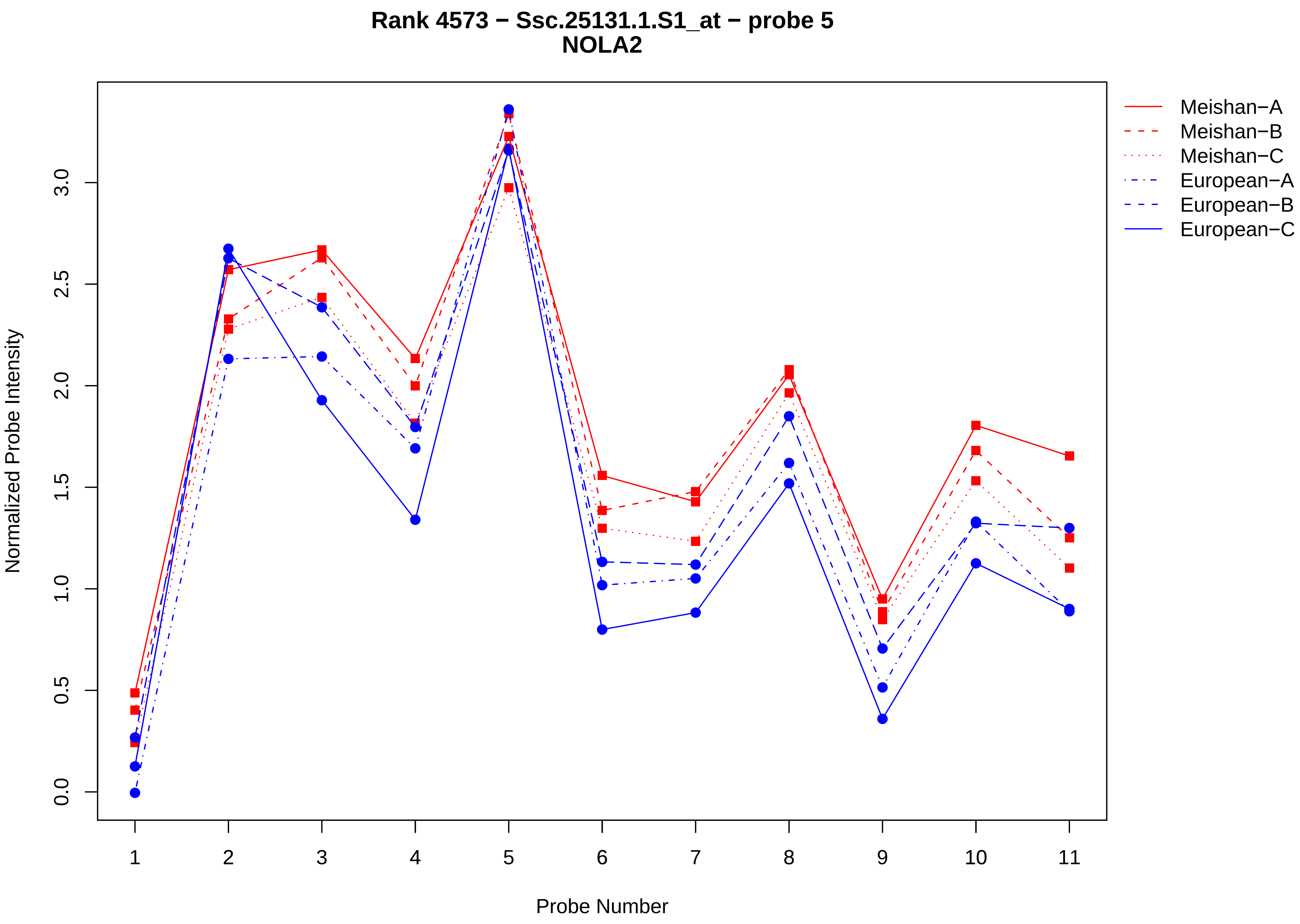
Rank 4573 − Ssc.25131.1.S1_at − probe 5
NOLA2
Normalized Probe Intensity
0.0
0.5
1.0
1.5
2.0
2.5
3.0
Probe Number
1
2
3
4
5
6
7
8
9
10
11
Meishan−A
Meishan−B
Meishan−C
European−A
European−B
European−C

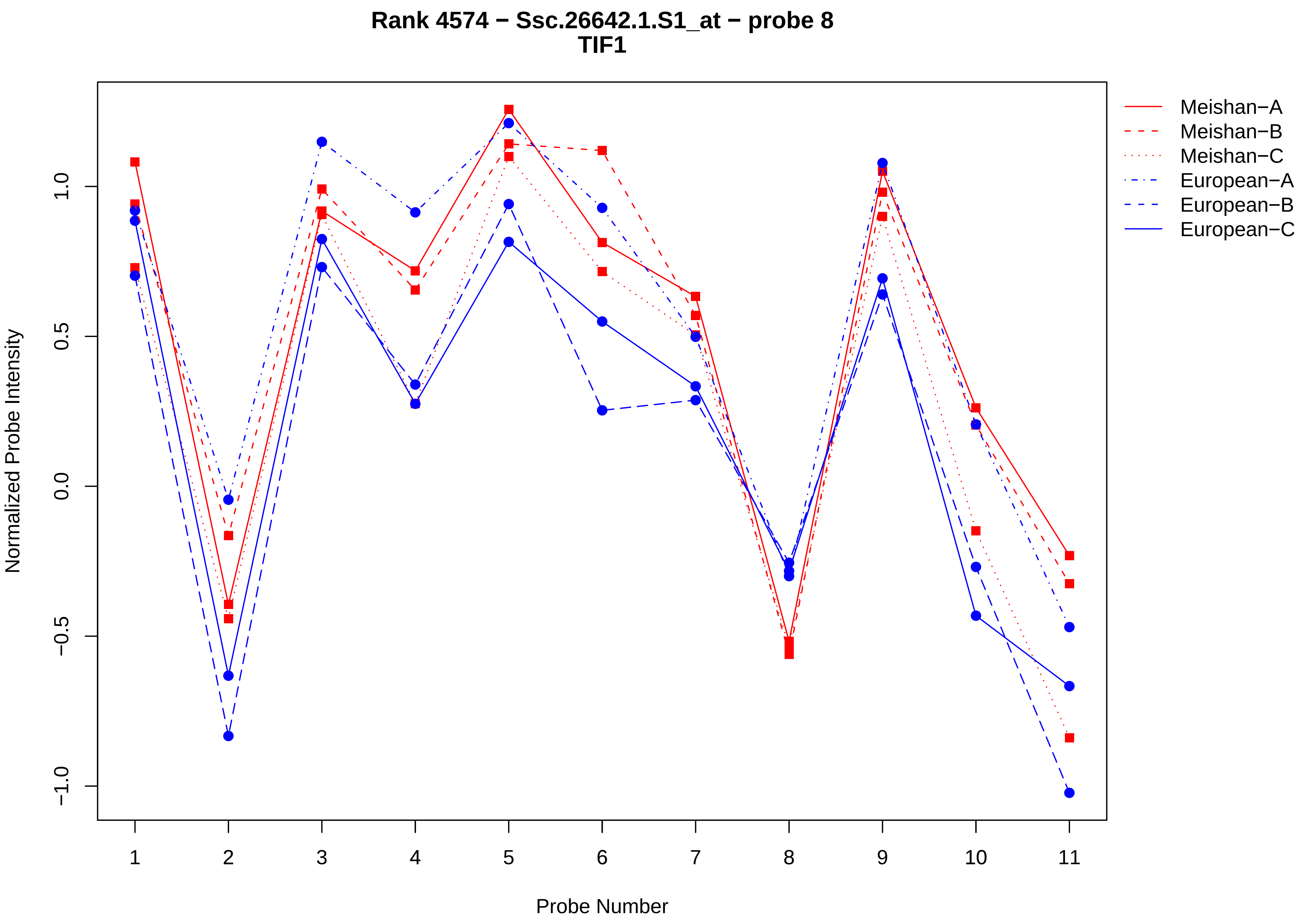

Rank 4574 − Ssc.26642.1.S1_at − probe 8
TIF1
Normalized Probe Intensity
Probe Number
−1.0
−0.5
0.0
0.5
1.0
1
2
3
4
5
6
7
8
9
10
11
Meishan−A
Meishan−B
Meishan−C
European−A
European−B
European−C

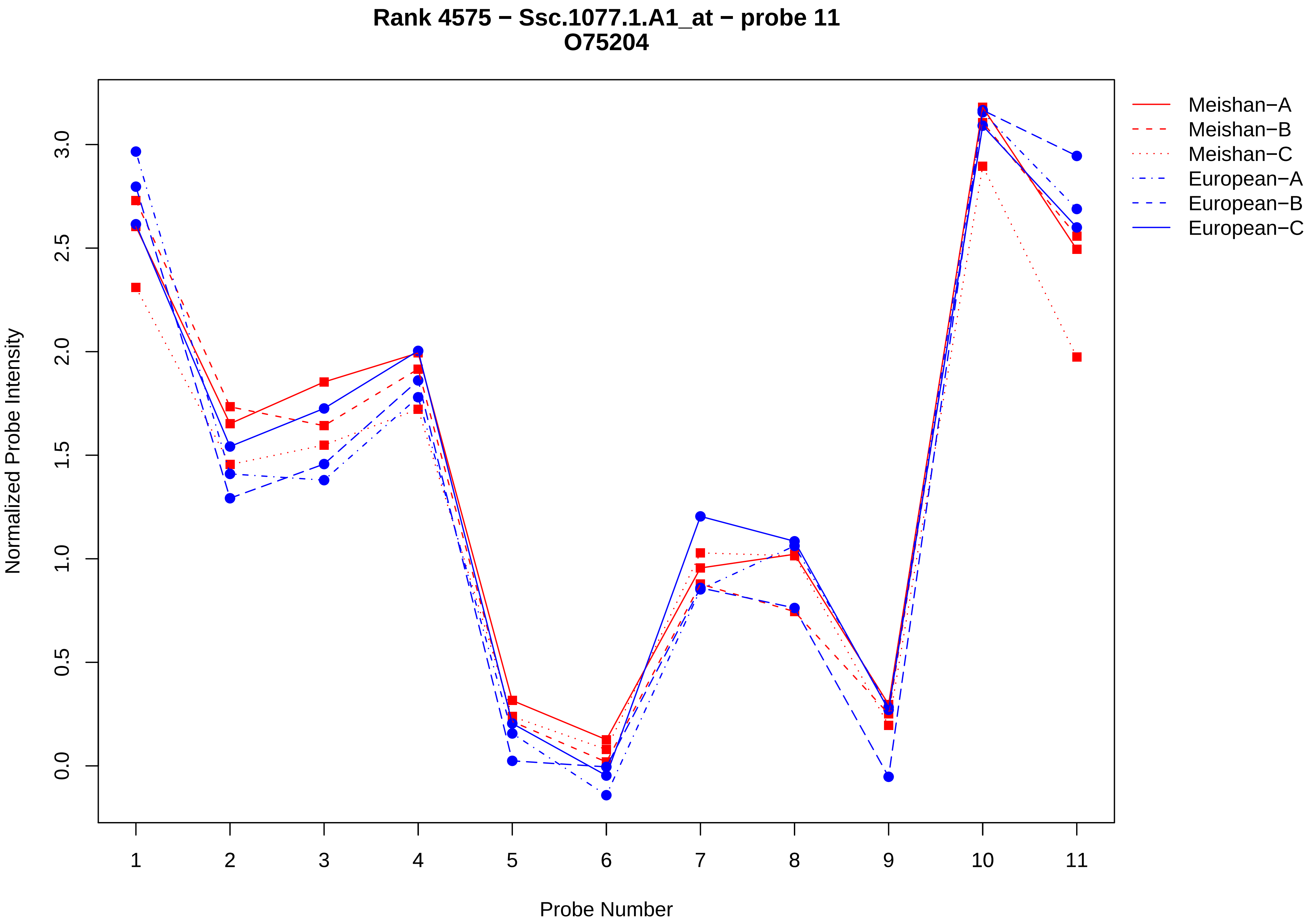

Rank 4575 − Ssc.1077.1.A1_at − probe 11
O75204
Normalized Probe Intensity
Probe Number
Meishan−A
Meishan−B
Meishan−C
European−A
European−B
European−C
0.0
0.5
1.0
1.5
2.0
2.5
3.0
1
2
3
4
5
6
7
8
9
10
11

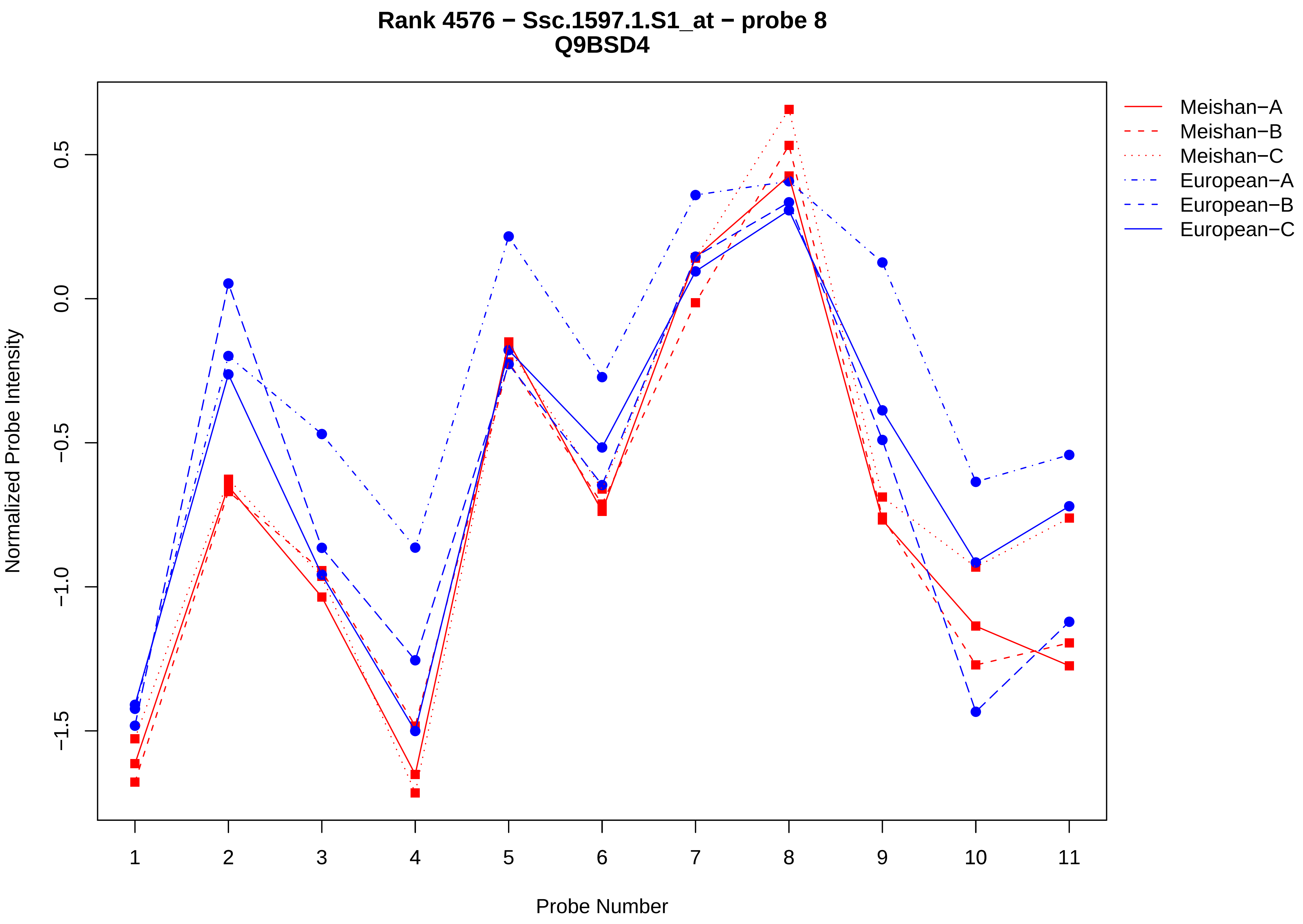
Rank 4576 − Ssc.1597.1.S1_at − probe 8
Q9BSD4
Normalized Probe Intensity
Probe Number
−1.5
−1.0
−0.5
0.0
0.5
1
2
3
4
5
6
7
8
9
10
11
Meishan−A
Meishan−B
Meishan−C
European−A
European−B
European−C

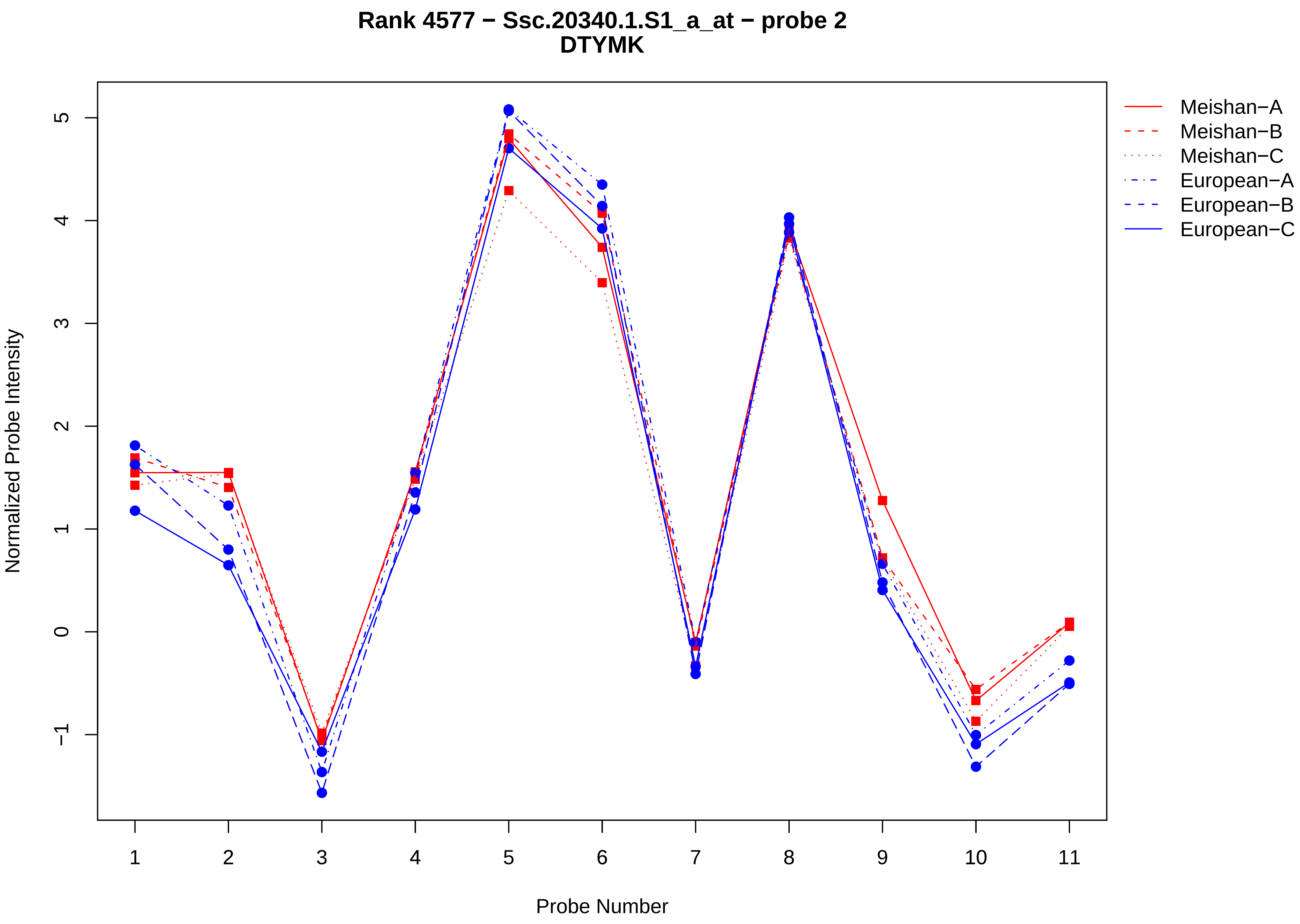

Rank 4577 − Ssc.20340.1.S1_a_at − probe 2
DTYMK
Normalized Probe Intensity
Probe Number
−1
0
1
2
3
4
5
1
2
3
4
5
6
7
8
9
10
11
Meishan−A
Meishan−B
Meishan−C
European−A
European−B
European−C

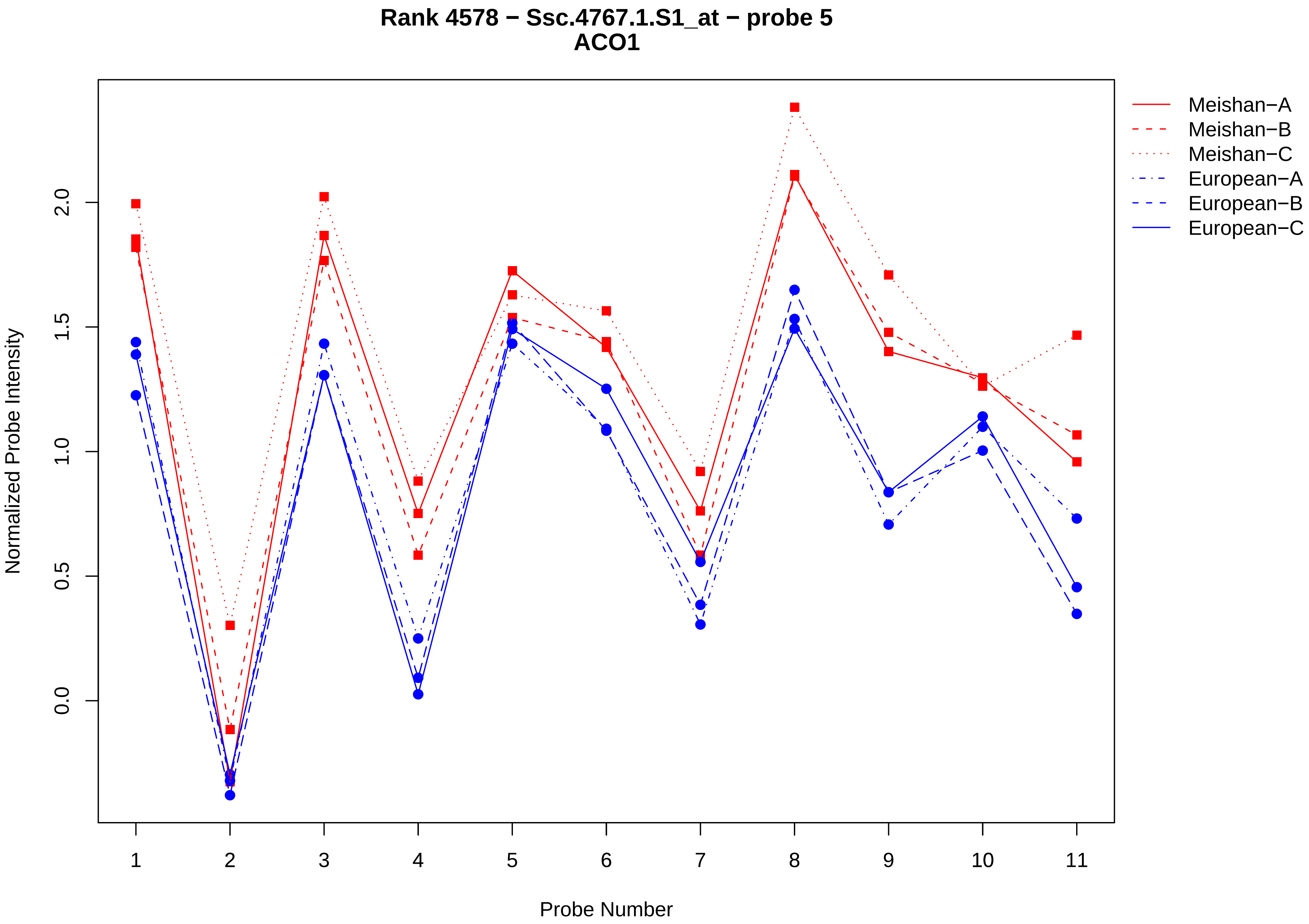

Rank 4578 − Ssc.4767.1.S1_at − probe 5
ACO1
Normalized Probe Intensity
Probe Number
0.0
0.5
1.0
1.5
2.0
1
2
3
4
5
6
7
8
9
10
11
Meishan−A
Meishan−B
Meishan−C
European−A
European−B
European−C

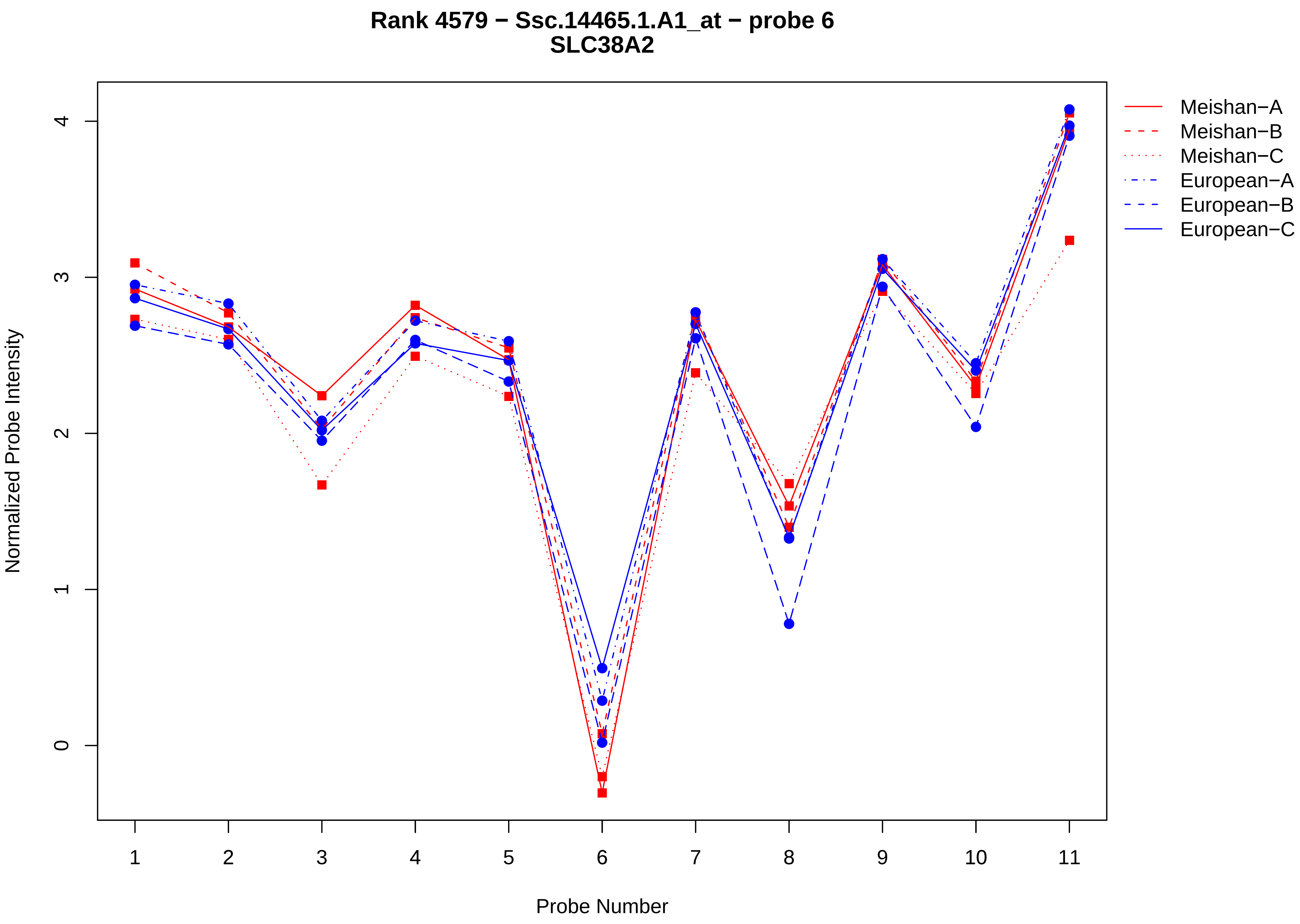

Rank 4579 − Ssc.14465.1.A1_at − probe 6
SLC38A2
Normalized Probe Intensity
Probe Number
0
1
2
3
4
1
2
3
4
5
6
7
8
9
10
11
Meishan−A
Meishan−B
Meishan−C
European−A
European−B
European−C

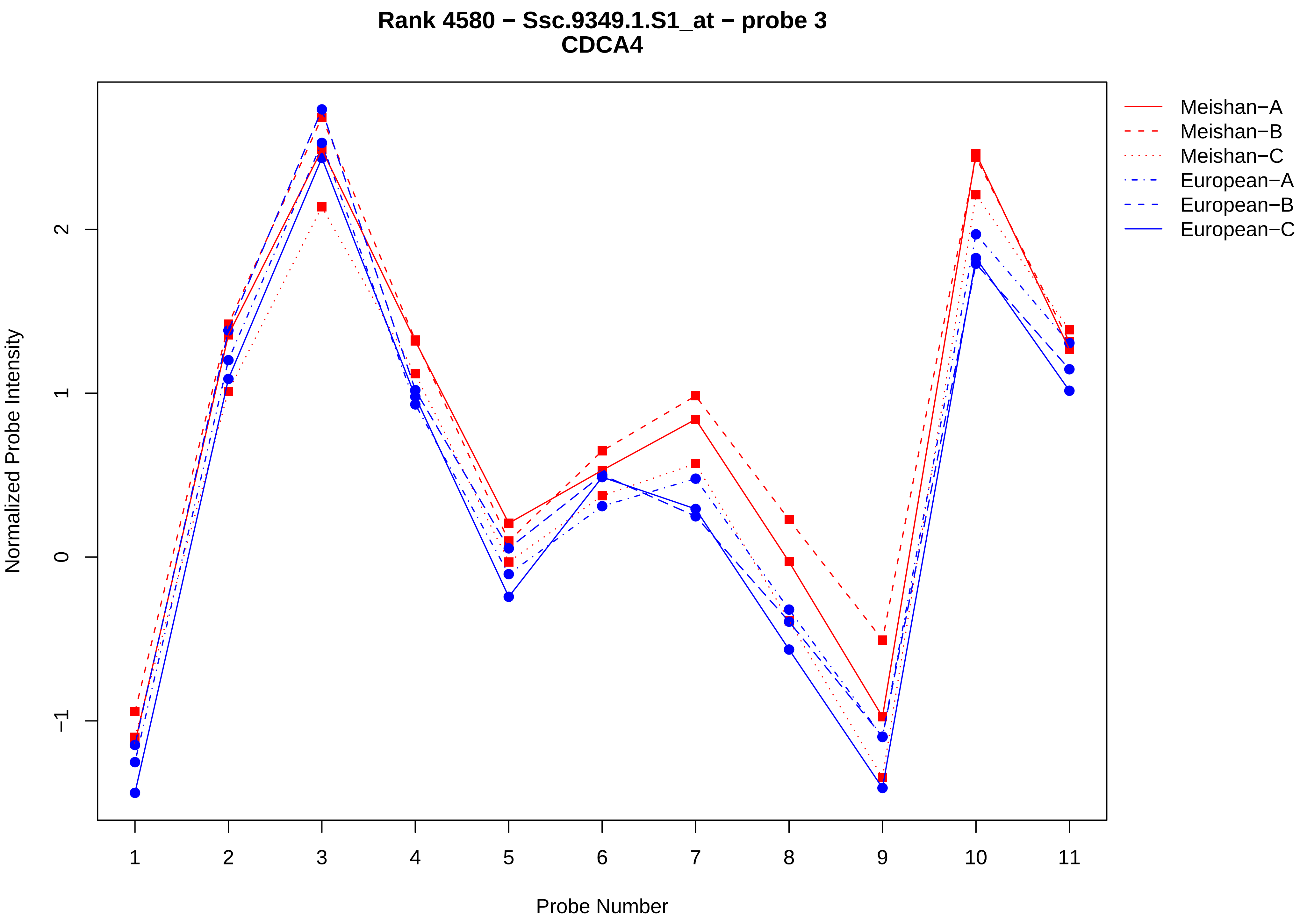

Rank 4580 − Ssc.9349.1.S1_at − probe 3
CDCA4
Normalized Probe Intensity
Probe Number
−1
0
1
2
1
2
3
4
5
6
7
8
9
10
11
Meishan−A
Meishan−B
Meishan−C
European−A
European−B
European−C

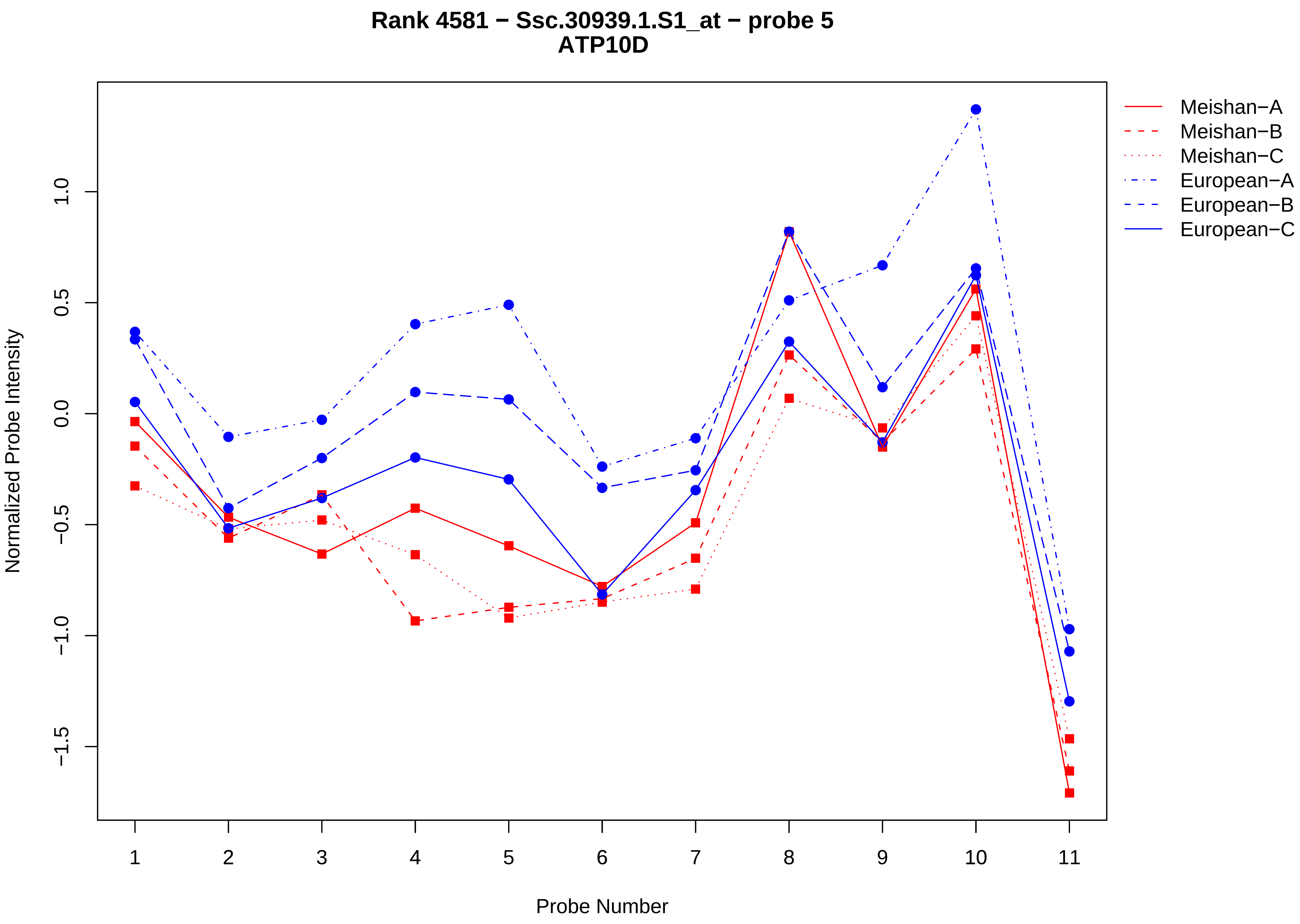

Rank 4581 − Ssc.30939.1.S1_at − probe 5
ATP10D
Normalized Probe Intensity
Probe Number
−1.5
−1.0
−0.5
0.0
0.5
1.0
1
2
3
4
5
6
7
8
9
10
11
Meishan−A
Meishan−B
Meishan−C
European−A
European−B
European−C

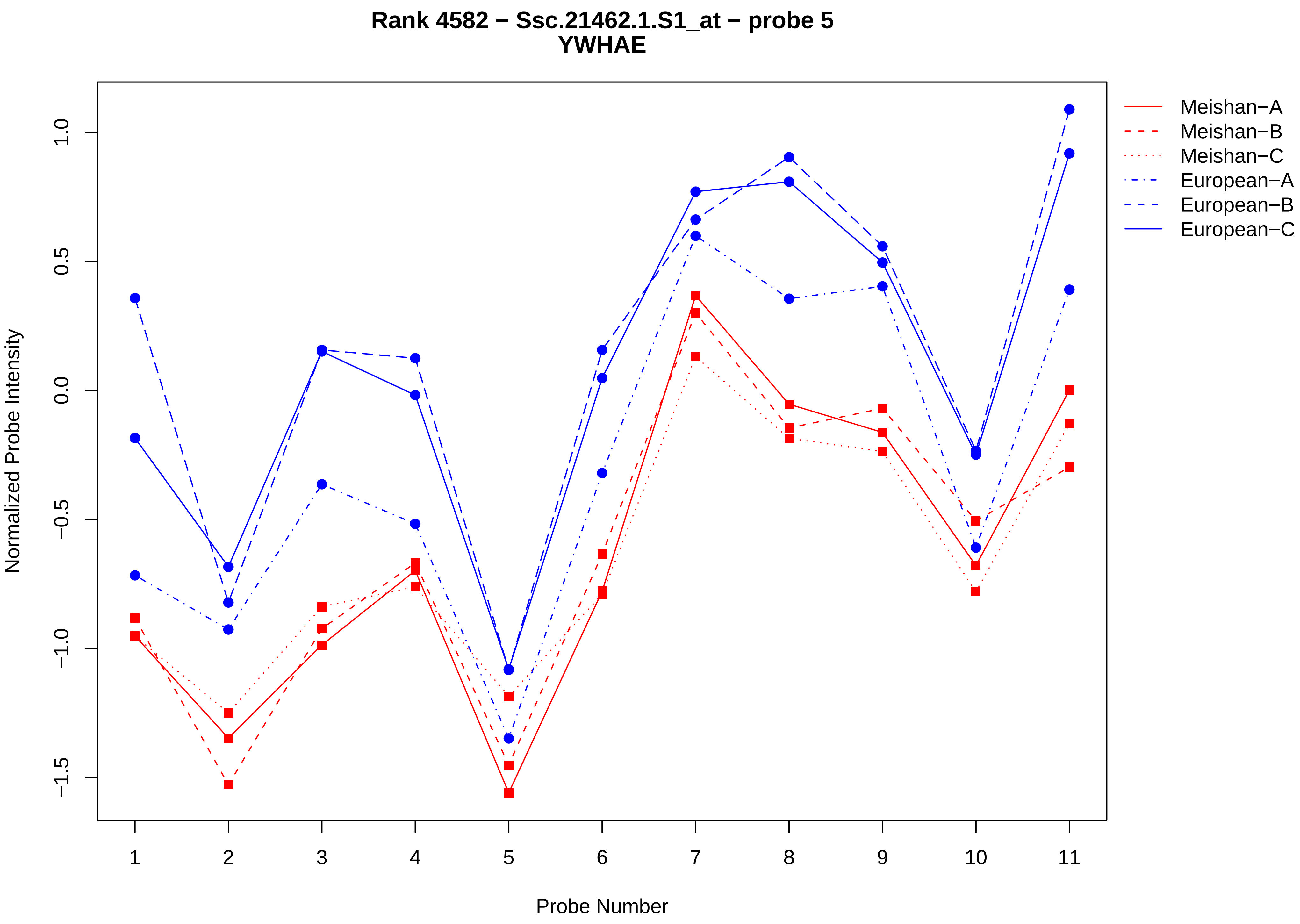

Rank 4582 − Ssc.21462.1.S1_at − probe 5
YWHAE
Normalized Probe Intensity
Probe Number
Meishan−A
Meishan−B
Meishan−C
European−A
European−B
European−C
1.0
0.5
0.0
−0.5
−1.0
−1.5
1
2
3
4
5
6
7
8
9
10
11

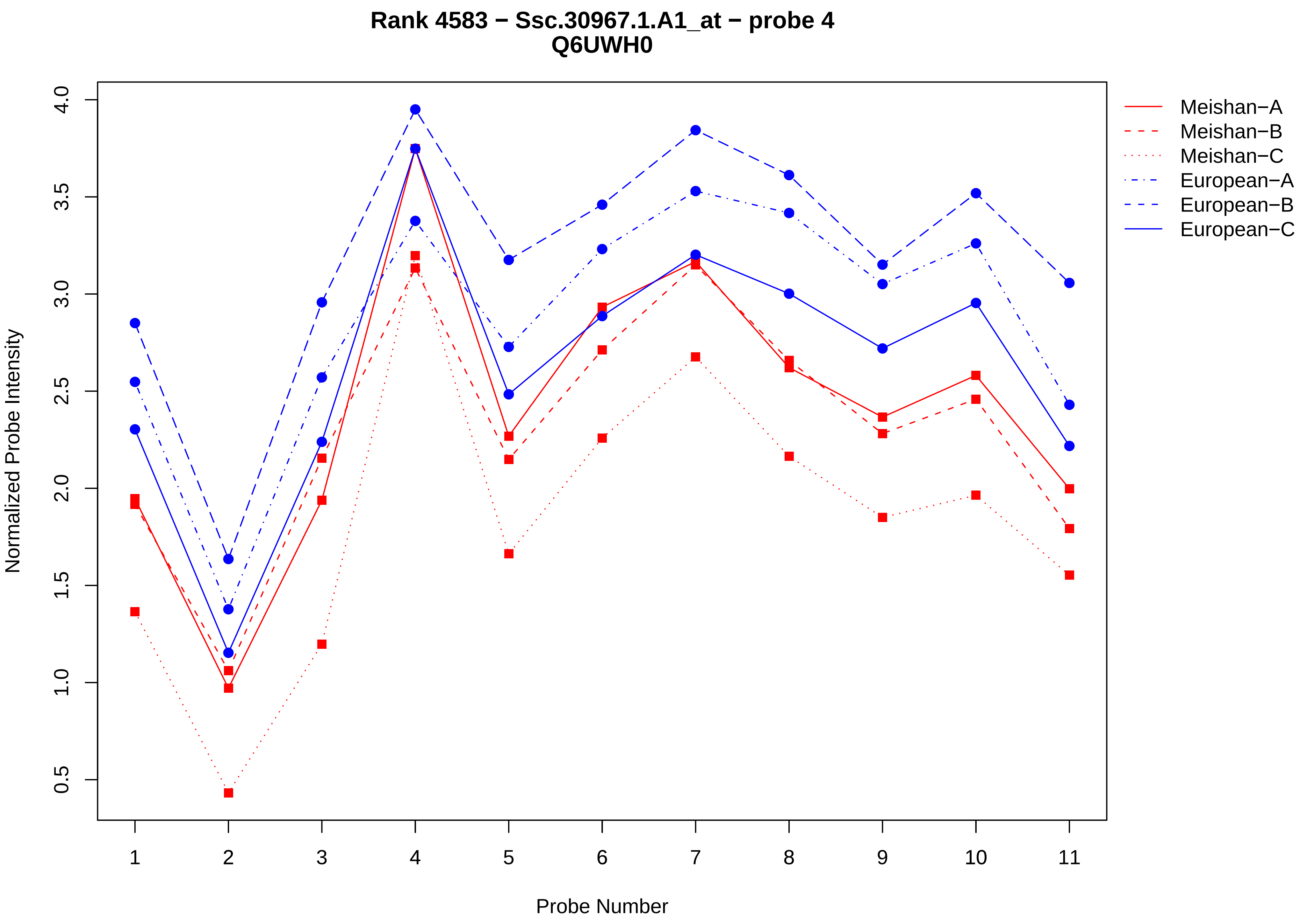

Rank 4583 − Ssc.30967.1.A1_at − probe 4
Q6UWH0
Normalized Probe Intensity
Probe Number
0.5
1.0
1.5
2.0
2.5
3.0
3.5
4.0
1
2
3
4
5
6
7
8
9
10
11
Meishan−A
Meishan−B
Meishan−C
European−A
European−B
European−C

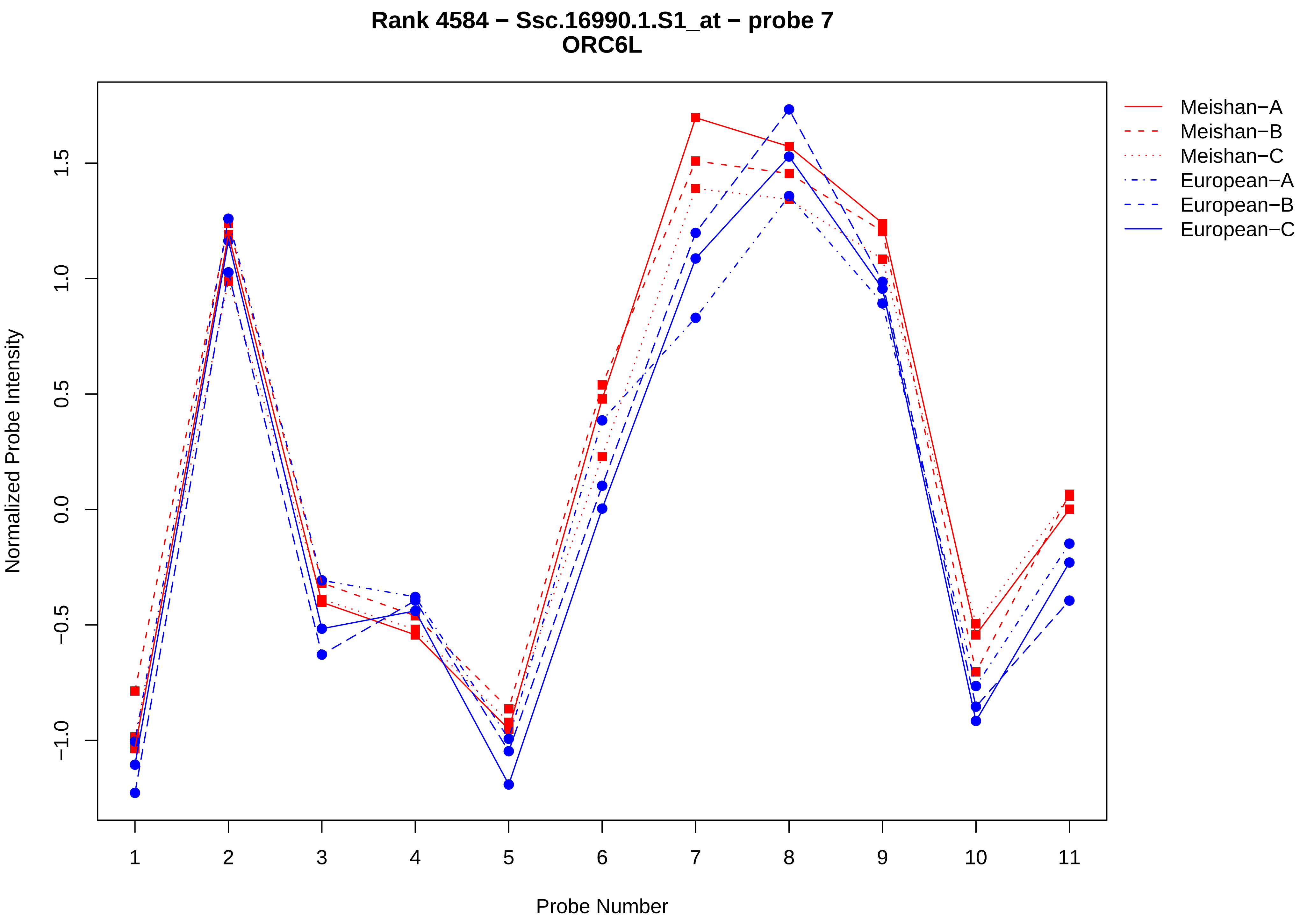

Rank 4584 − Ssc.16990.1.S1_at − probe 7
ORC6L
Normalized Probe Intensity
Probe Number
Meishan−A
Meishan−B
Meishan−C
European−A
European−B
European−C

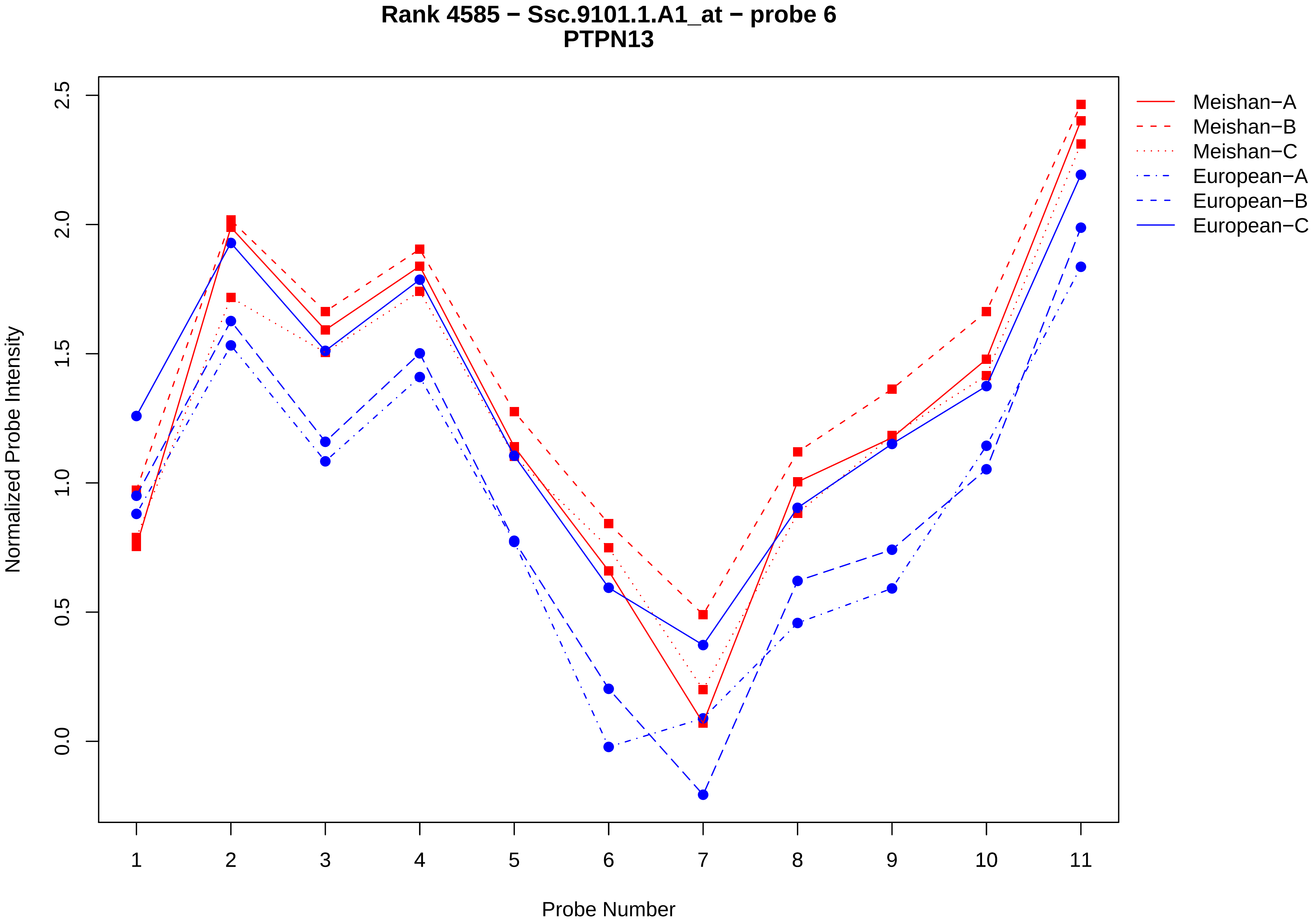

Rank 4585 − Ssc.9101.1.A1_at − probe 6
PTPN13
Normalized Probe Intensity
Probe Number
Meishan−A
Meishan−B
Meishan−C
European−A
European−B
European−C
0.0
0.5
1.0
1.5
2.0
2.5
1
2
3
4
5
6
7
8
9
10
11

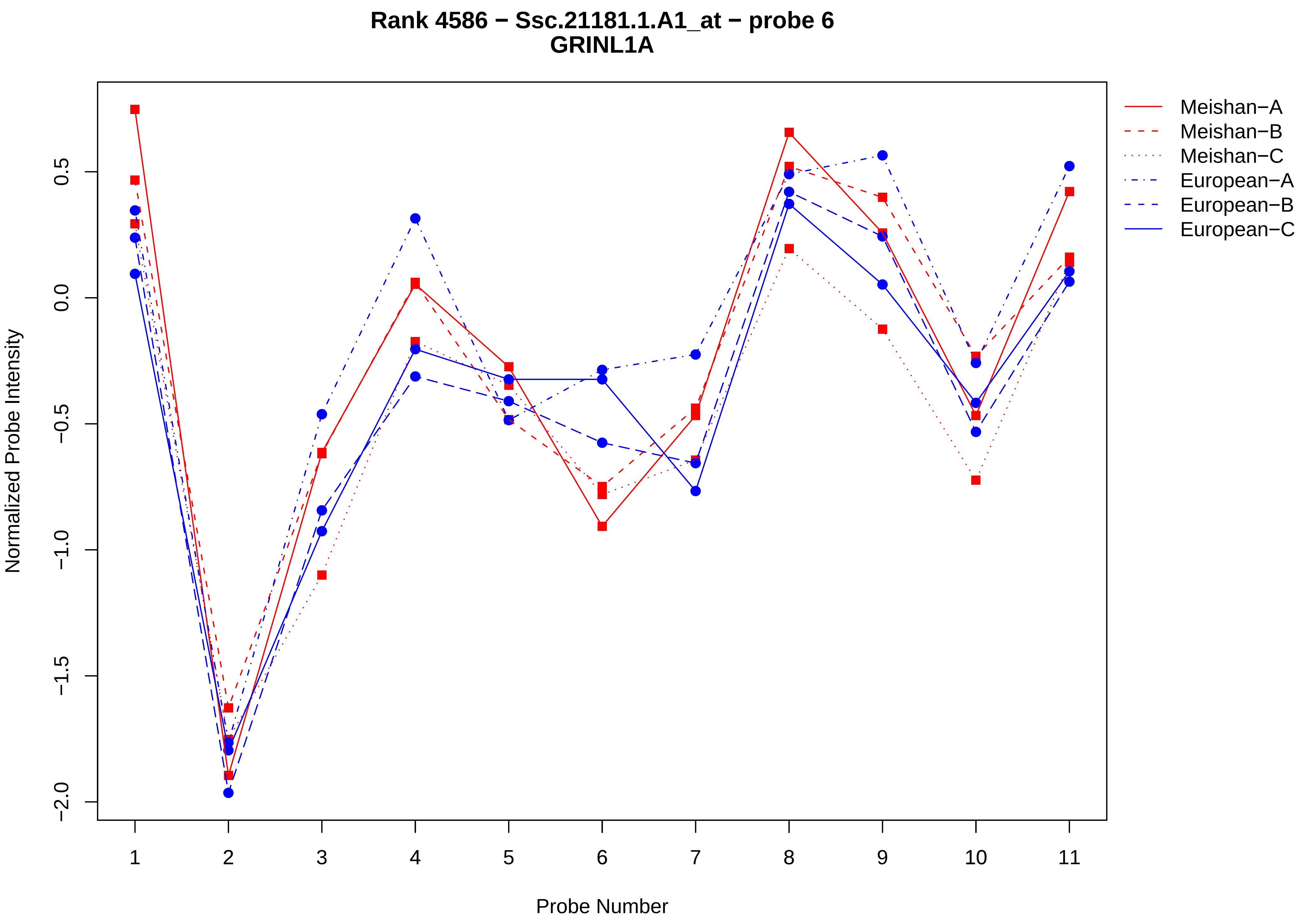

Rank 4586 − Ssc.21181.1.A1_at − probe 6
GRINL1A
Normalized Probe Intensity
Probe Number
0.5
0.0
−0.5
−1.0
−1.5
−2.0
1
2
3
4
5
6
7
8
9
10
11
Meishan−A
Meishan−B
Meishan−C
European−A
European−B
European−C

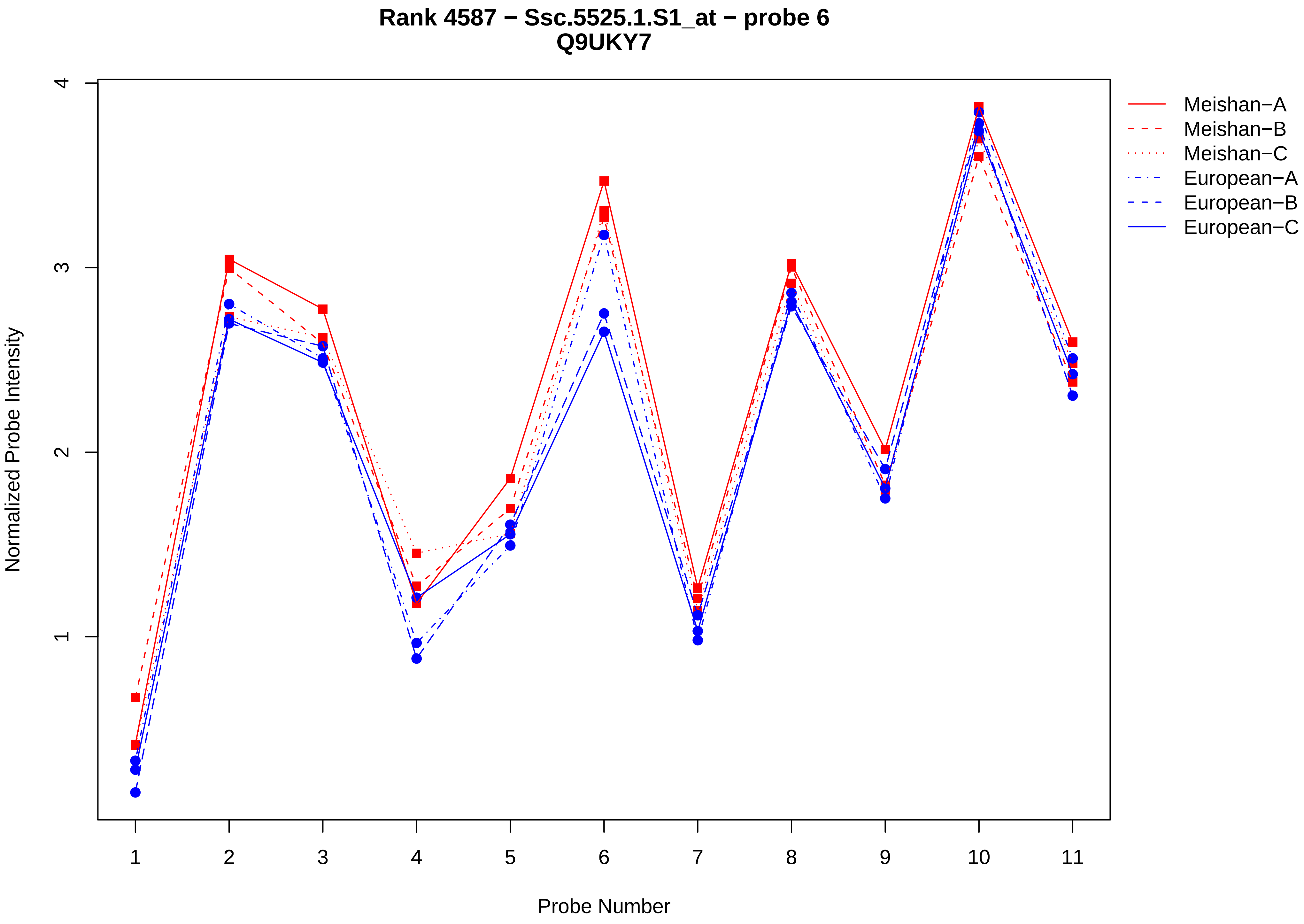
Rank 4587 − Ssc.5525.1.S1_at − probe 6
Q9UKY7
Normalized Probe Intensity
Probe Number
1
2
3
4
1
2
3
4
5
6
7
8
9
10
11
Meishan−A
Meishan−B
Meishan−C
European−A
European−B
European−C

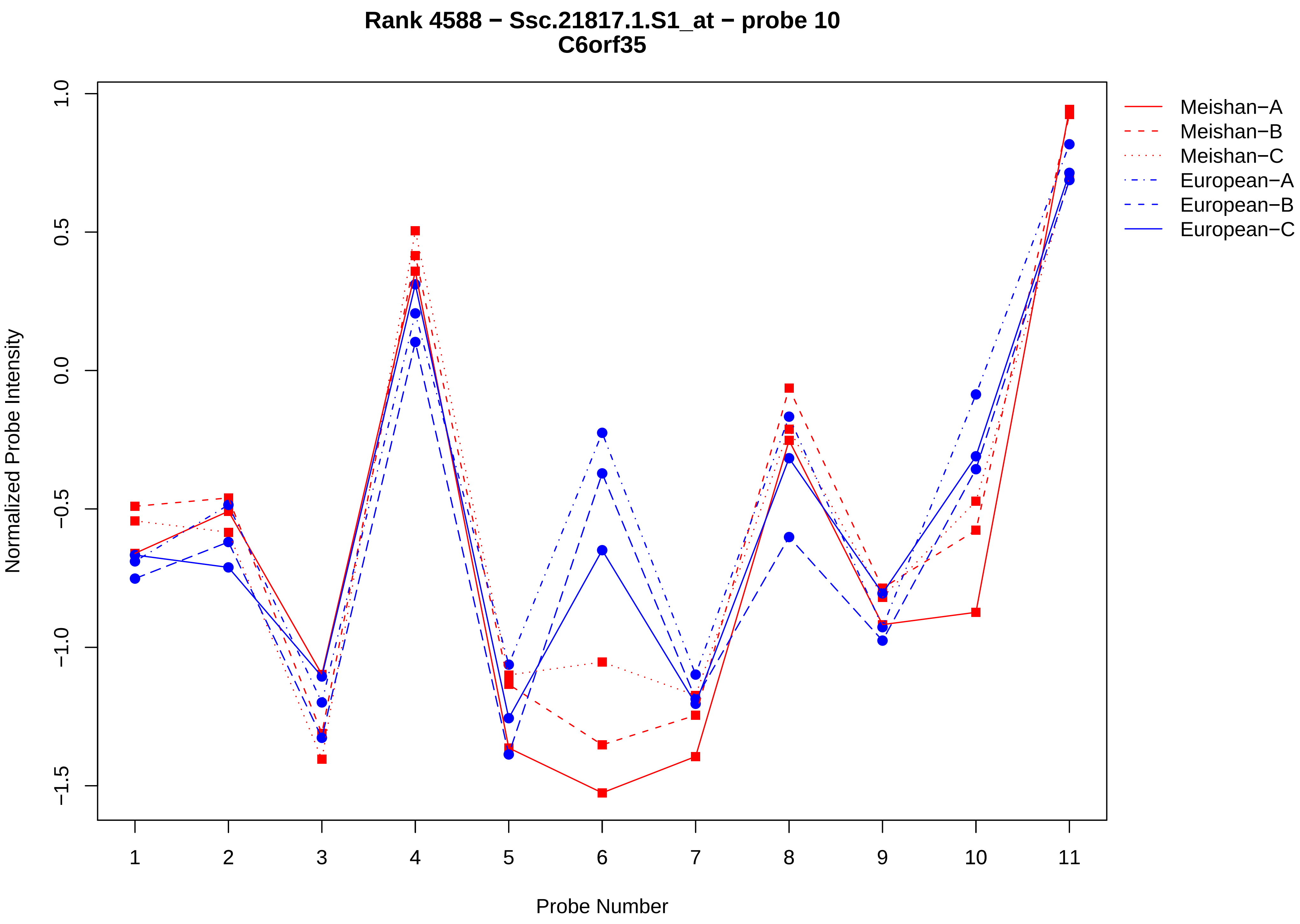
Rank 4588 − Ssc.21817.1.S1_at − probe 10
C6orf35
Normalized Probe Intensity
Probe Number
1.0
0.5
0.0
−0.5
−1.0
−1.5
1
2
3
4
5
6
7
8
9
10
11
Meishan−A
Meishan−B
Meishan−C
European−A
European−B
European−C

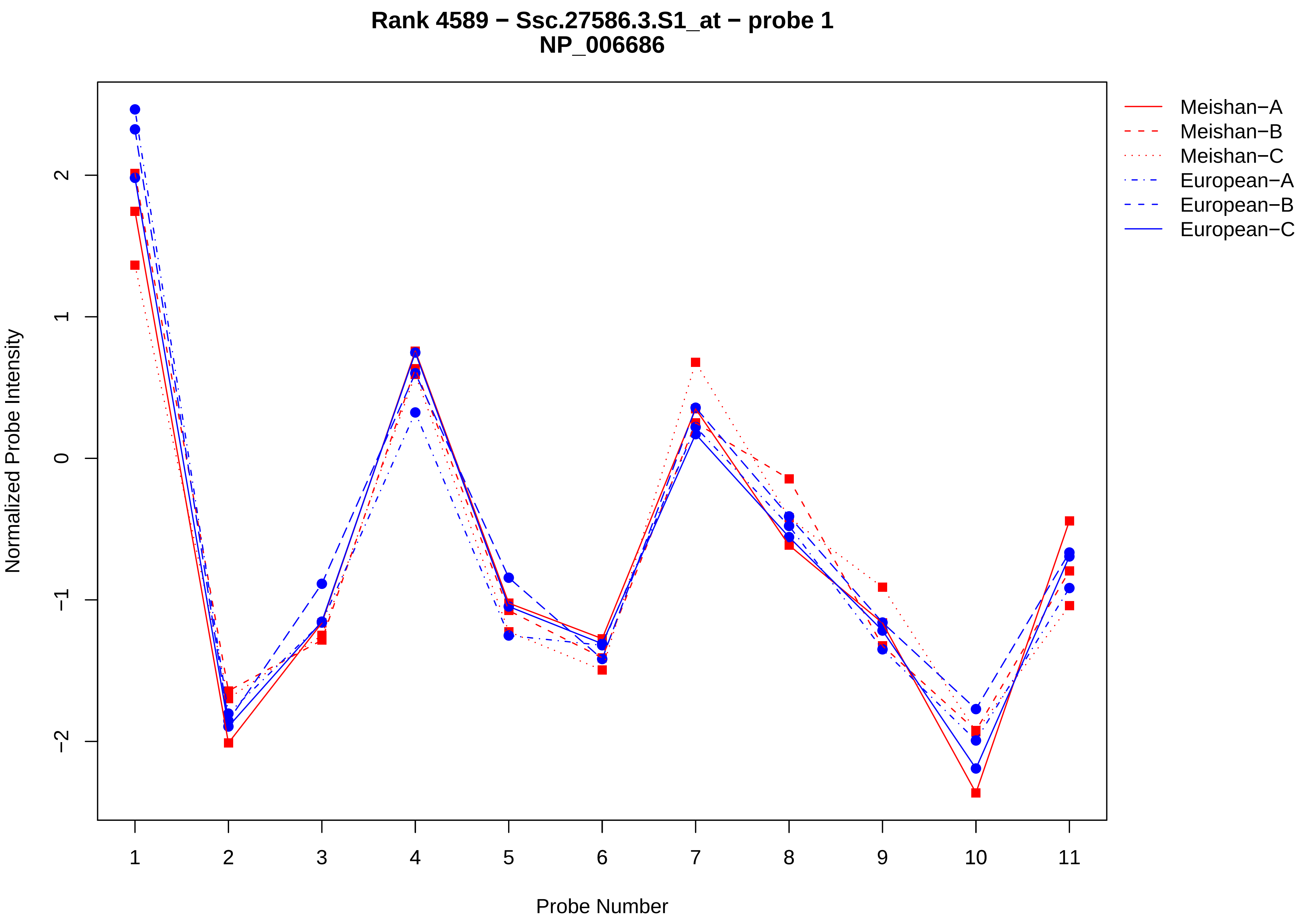

Rank 4589 − Ssc.27586.3.S1_at − probe 1
NP_006686
Normalized Probe Intensity
Probe Number
−2
−1
0
1
2
1
2
3
4
5
6
7
8
9
10
11
Meishan−A
Meishan−B
Meishan−C
European−A
European−B
European−C

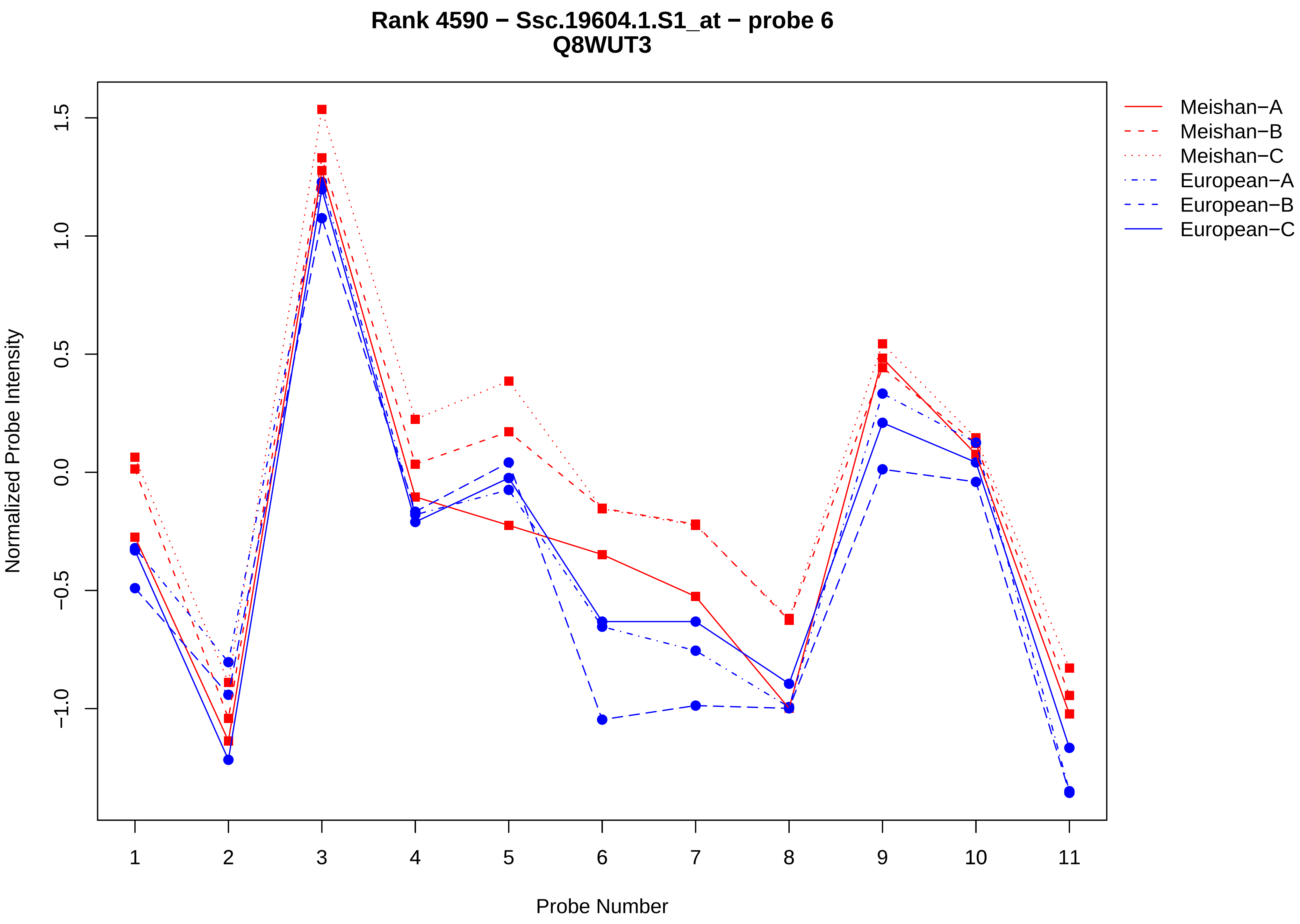

Rank 4590 − Ssc.19604.1.S1_at − probe 6
Q8WUT3
Normalized Probe Intensity
Probe Number
−1.0
−0.5
0.0
0.5
1.0
1.5
1
2
3
4
5
6
7
8
9
10
11
Meishan−A
Meishan−B
Meishan−C
European−A
European−B
European−C

Rank 4591 − Ssc.29811.1.A1_at − probe 5
AKAP2

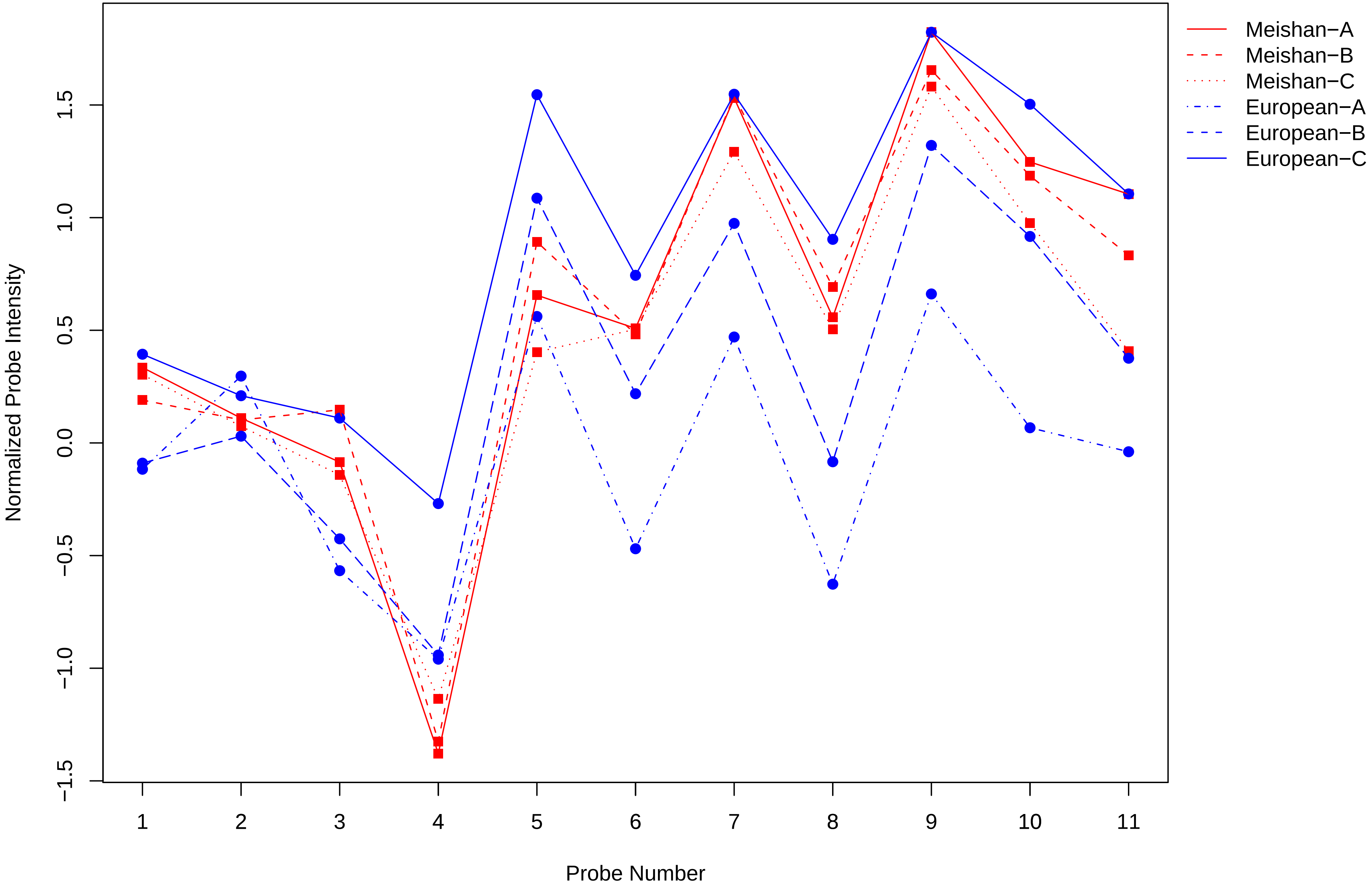

Rank 4592 − Ssc.2554.3.S1_at − probe 2
NP_149118

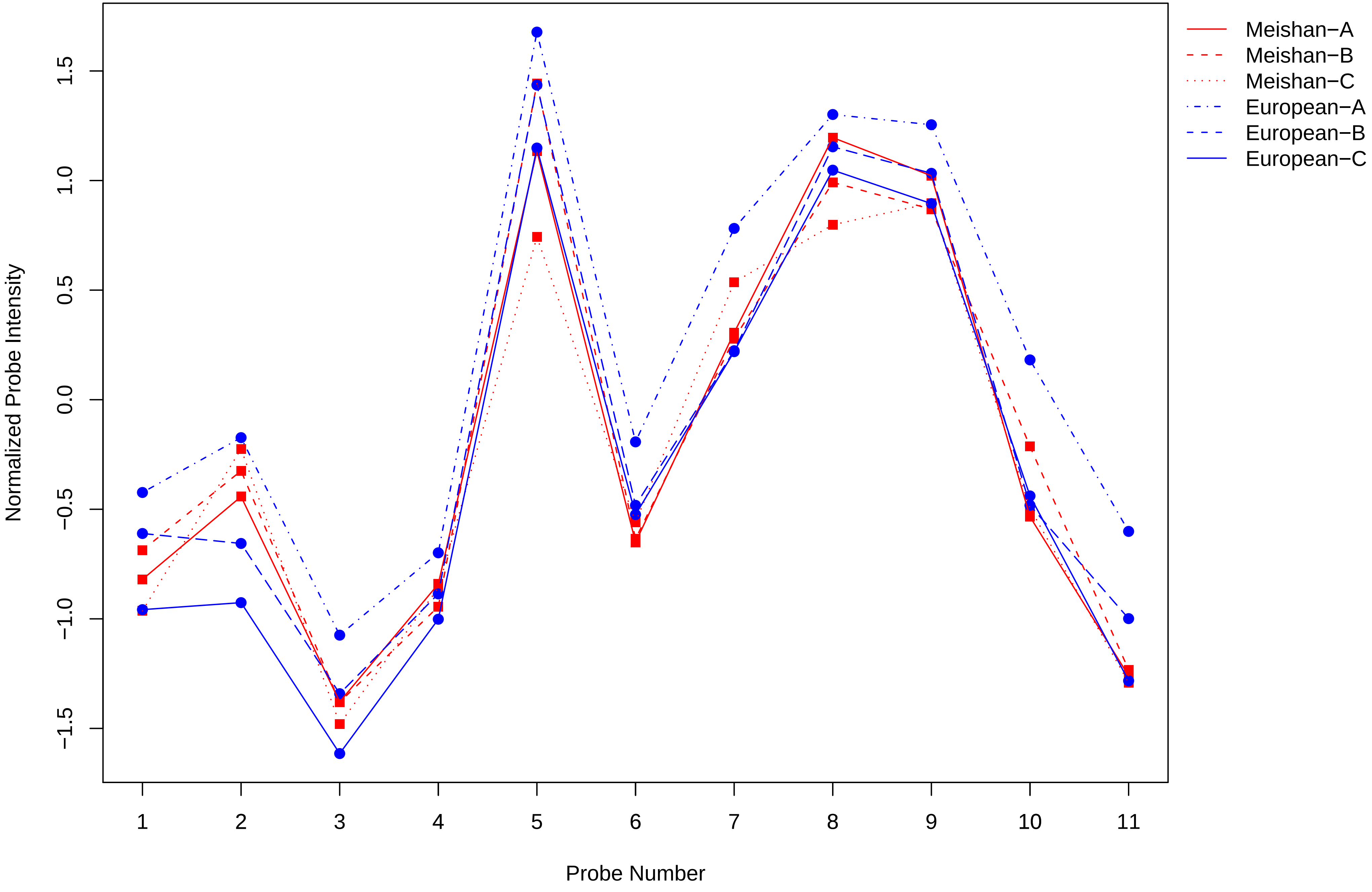

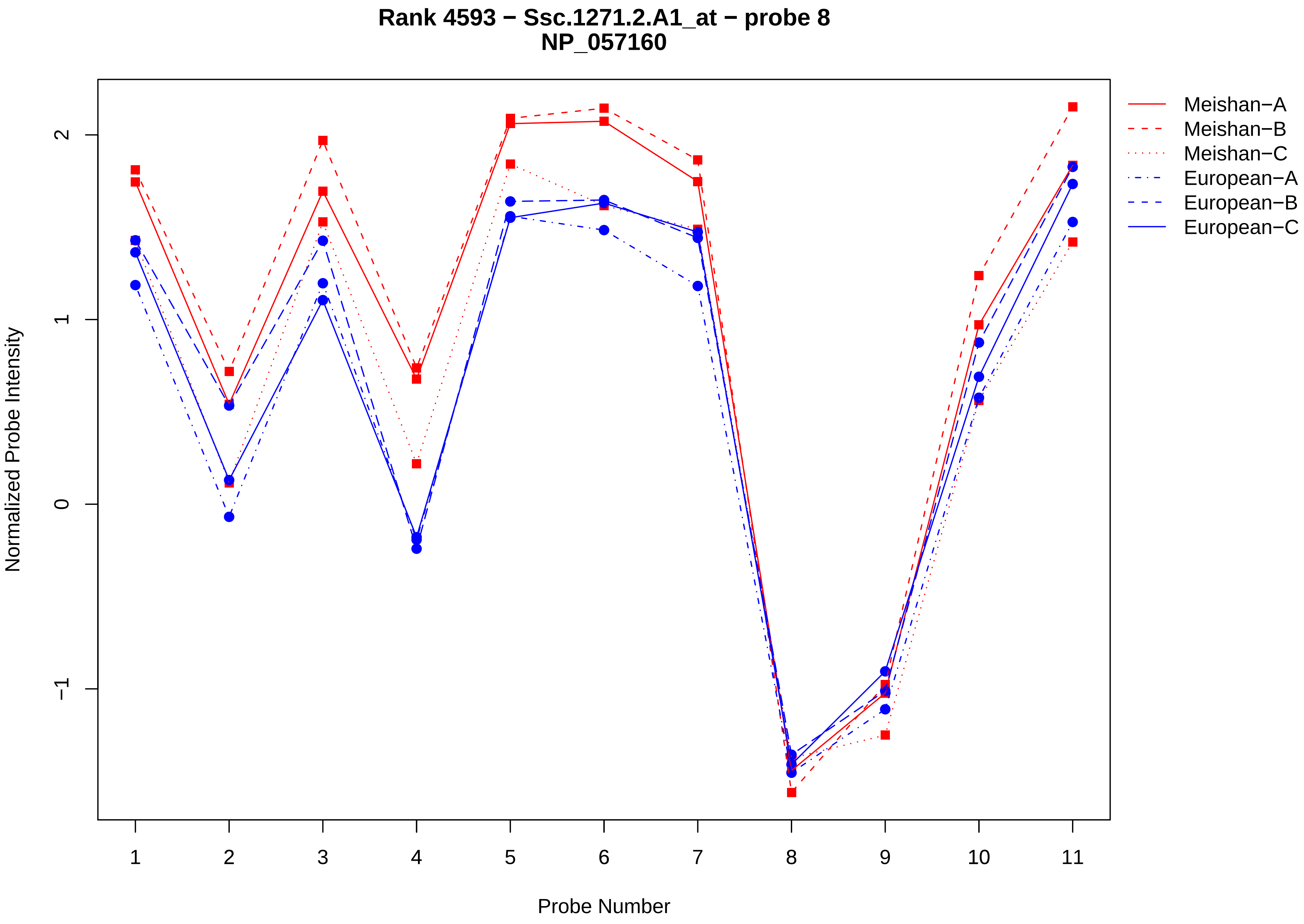

Rank 4593 − Ssc.1271.2.A1_at − probe 8
NP_057160
Normalized Probe Intensity
Probe Number
−1
0
1
2
1
2
3
4
5
6
7
8
9
10
11
Meishan−A
Meishan−B
Meishan−C
European−A
European−B
European−C

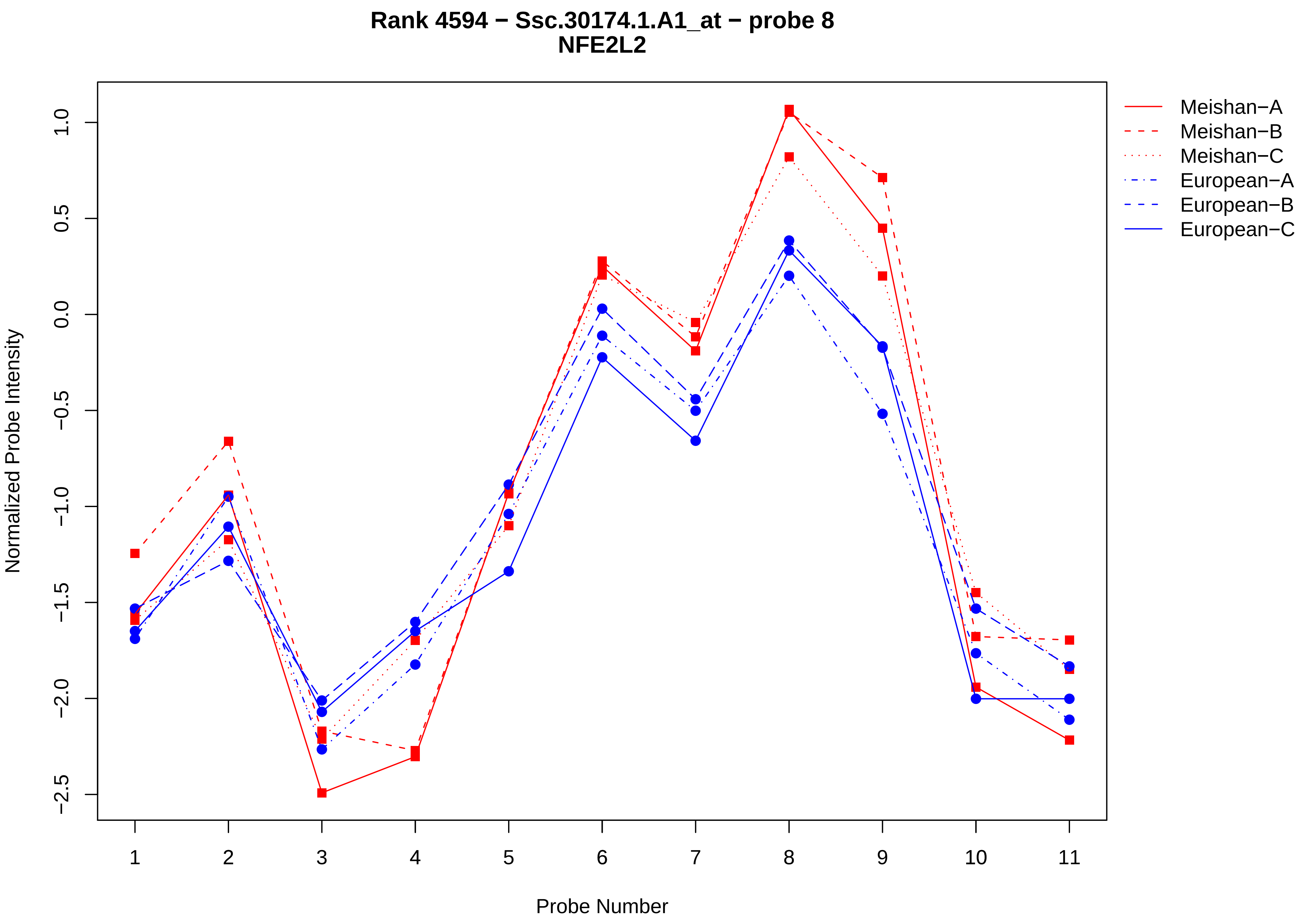

Rank 4594 − Ssc.30174.1.A1_at − probe 8
NFE2L2
Normalized Probe Intensity
Probe Number
1.0
0.5
0.0
−0.5
−1.0
−1.5
−2.0
−2.5
1
2
3
4
5
6
7
8
9
10
11
Meishan−A
Meishan−B
Meishan−C
European−A
European−B
European−C

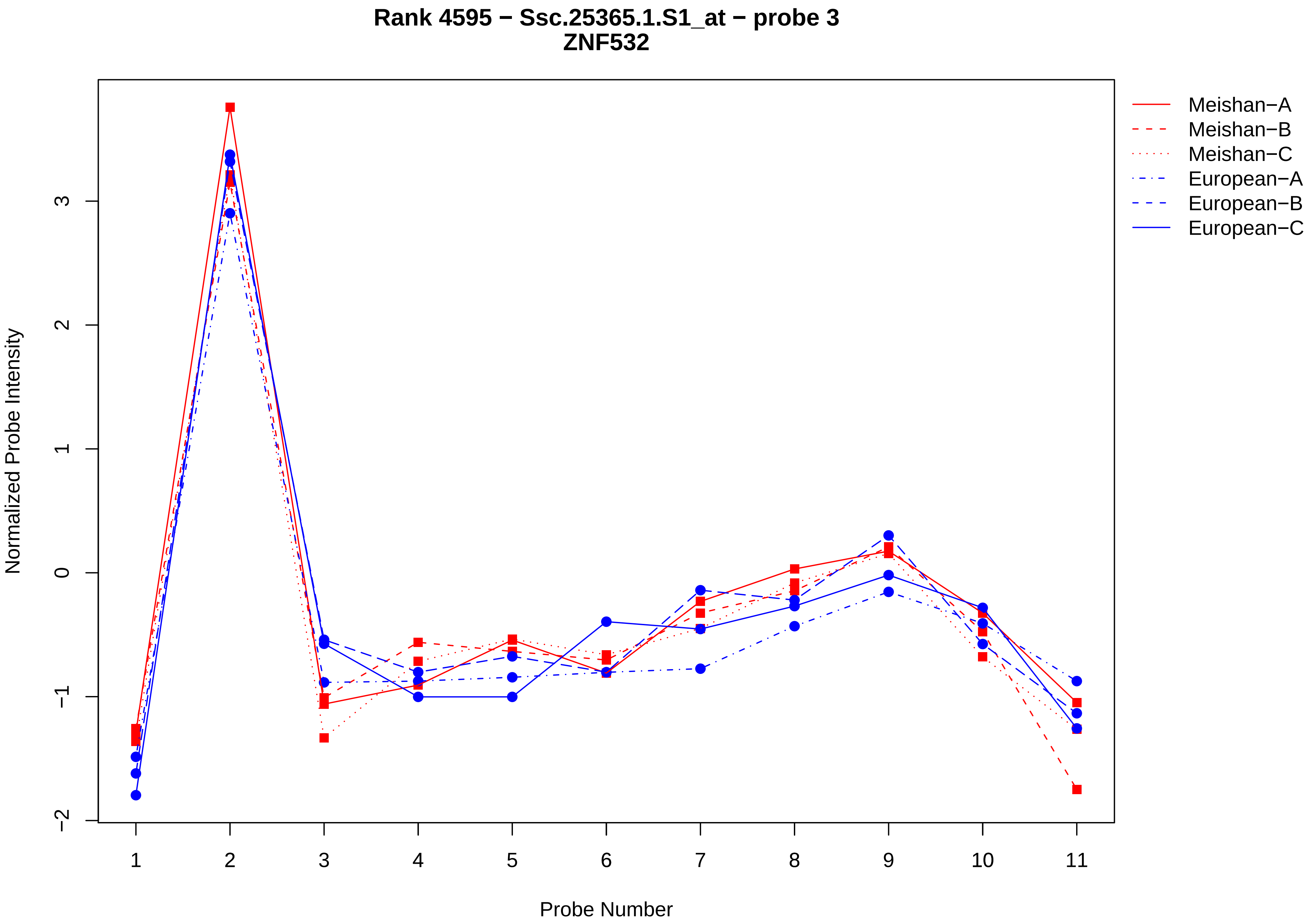

Rank 4595 − Ssc.25365.1.S1_at − probe 3
ZNF532
Normalized Probe Intensity
Probe Number
−2
−1
0
1
2
3
1
2
3
4
5
6
7
8
9
10
11
Meishan−A
Meishan−B
Meishan−C
European−A
European−B
European−C

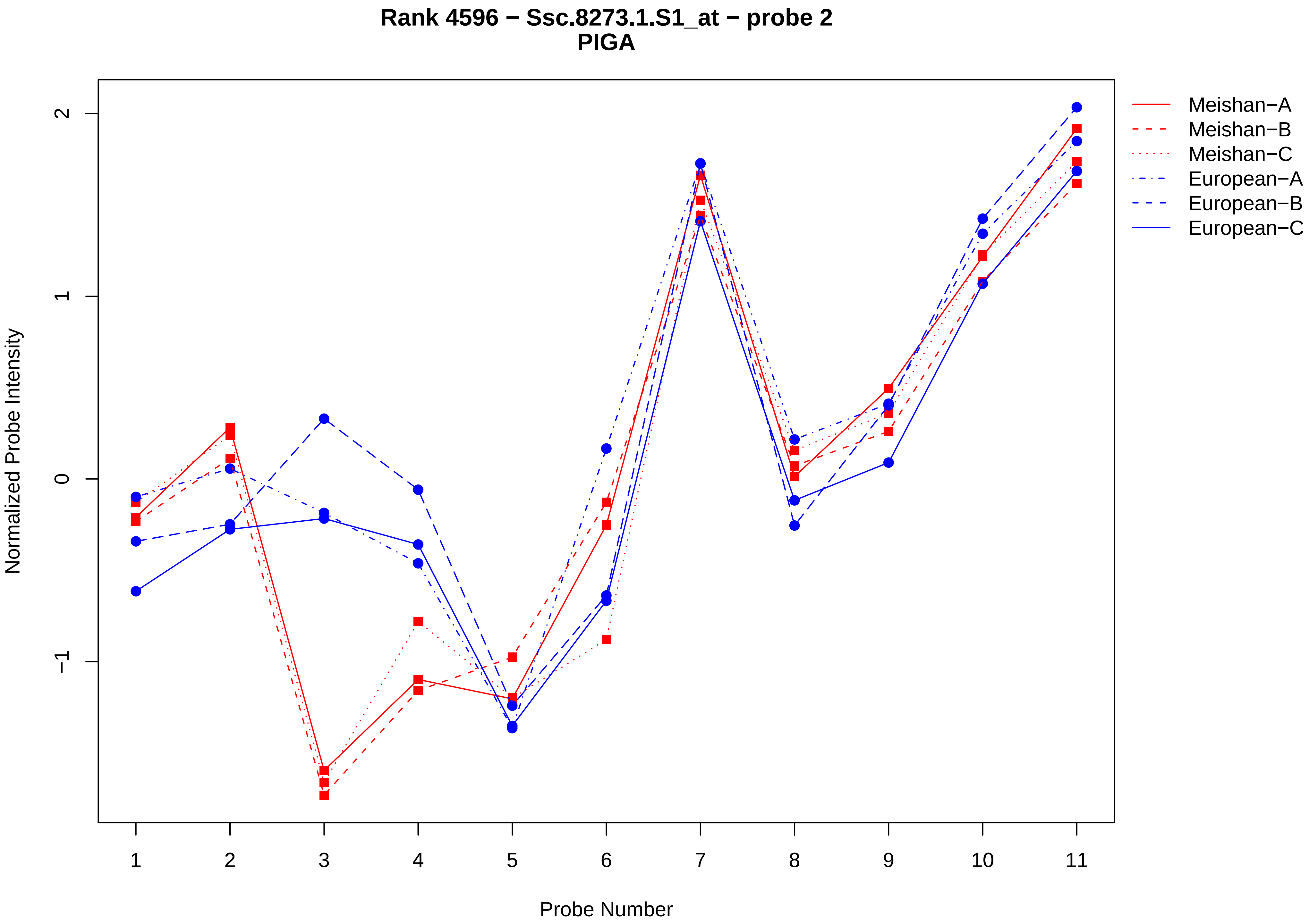

Rank 4596 − Ssc.8273.1.S1_at − probe 2
PIGA
Normalized Probe Intensity
Probe Number
−1
0
1
2
1
2
3
4
5
6
7
8
9
10
11
Meishan−A
Meishan−B
Meishan−C
European−A
European−B
European−C

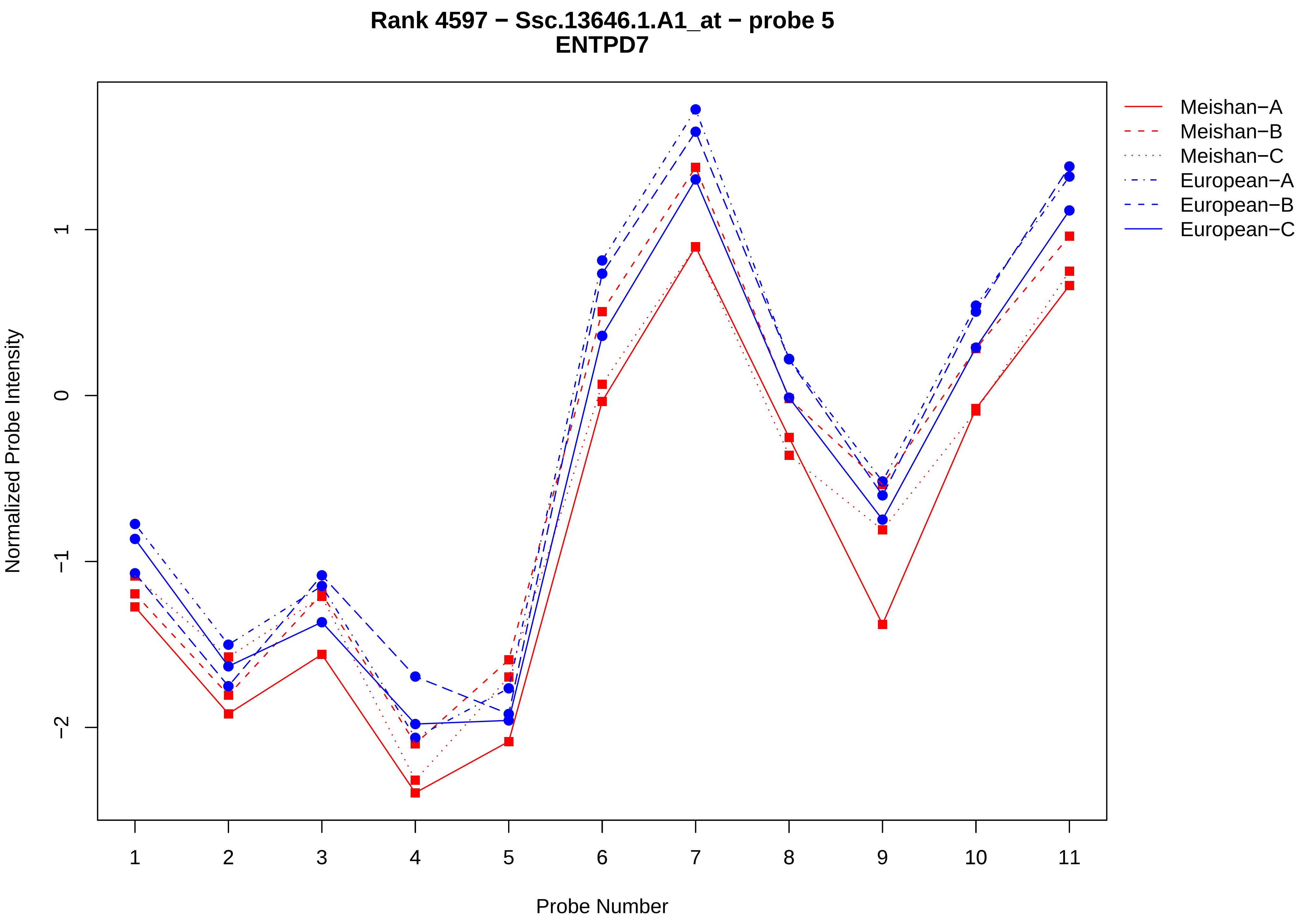

Rank 4597 − Ssc.13646.1.A1_at − probe 5
ENTPD7
Normalized Probe Intensity
Probe Number
−2
−1
0
1
1
2
3
4
5
6
7
8
9
10
11
Meishan−A
Meishan−B
Meishan−C
European−A
European−B
European−C

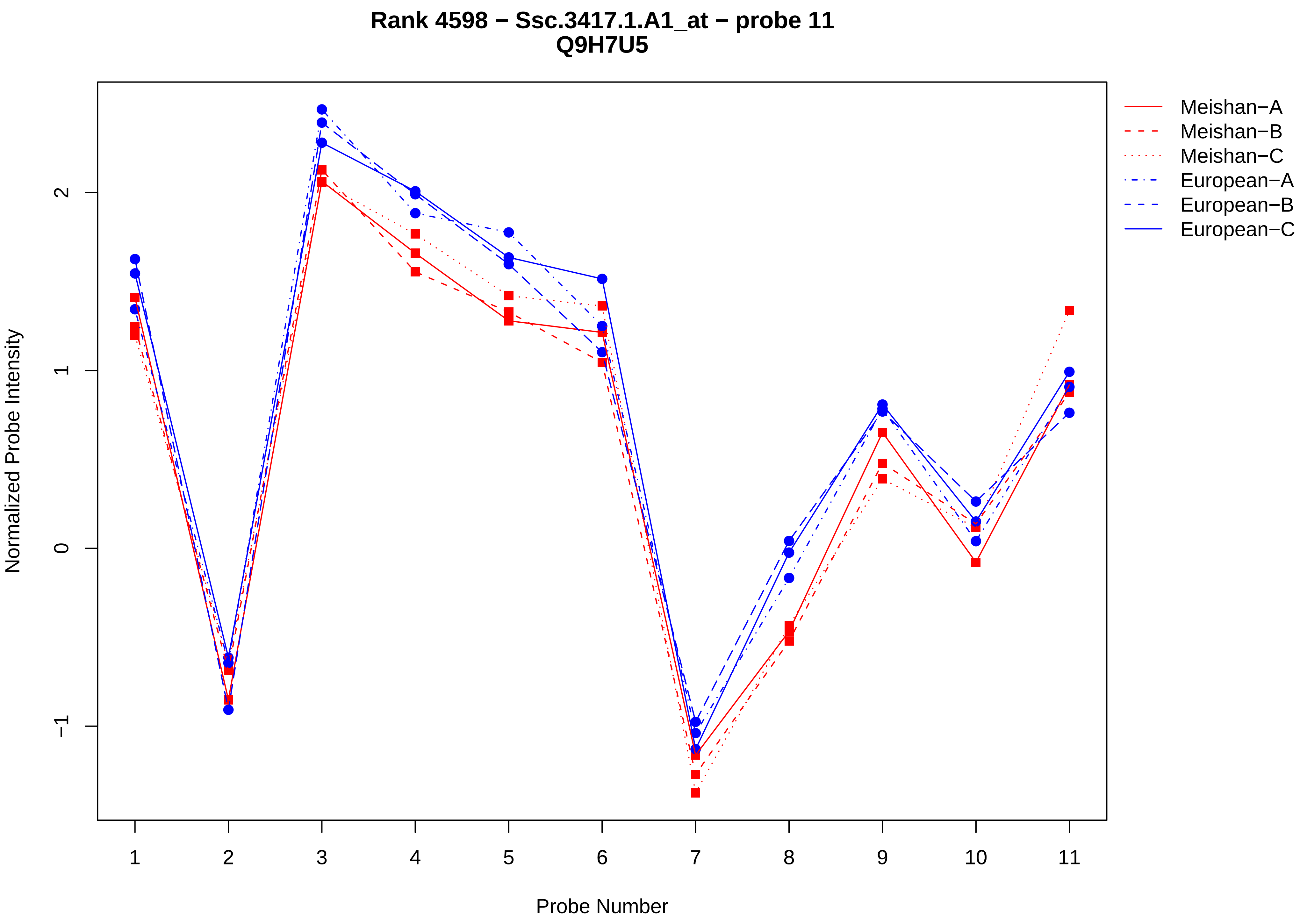
Rank 4598 − Ssc.3417.1.A1_at − probe 11
Q9H7U5
Normalized Probe Intensity
Probe Number
−1
0
1
2
1
2
3
4
5
6
7
8
9
10
11
Meishan−A
Meishan−B
Meishan−C
European−A
European−B
European−C

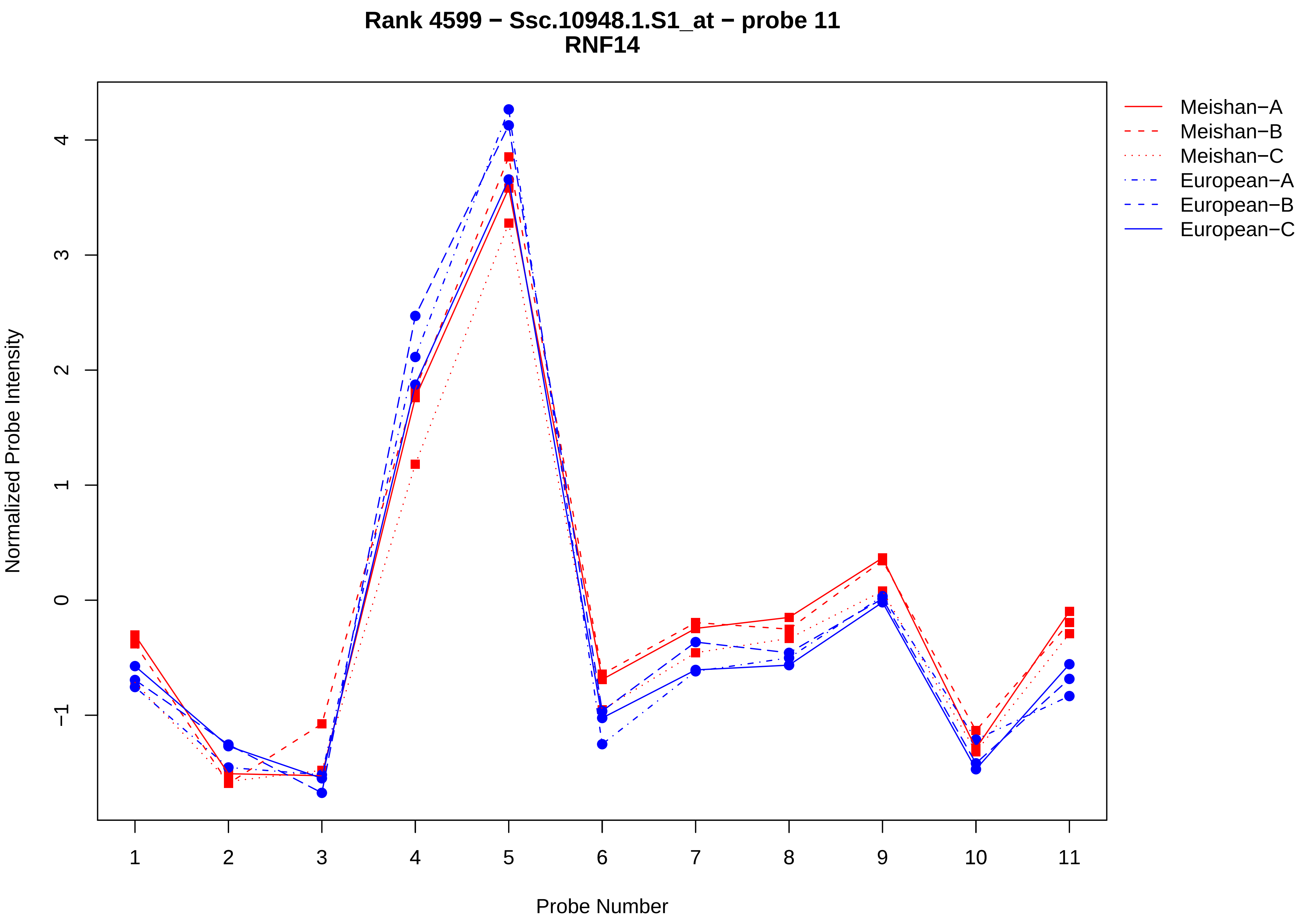

Rank 4599 − Ssc.10948.1.S1_at − probe 11
RNF14
Normalized Probe Intensity
Probe Number
Meishan−A
Meishan−B
Meishan−C
European−A
European−B
European−C
−1
0
1
2
3
4
1
2
3
4
5
6
7
8
9
10
11

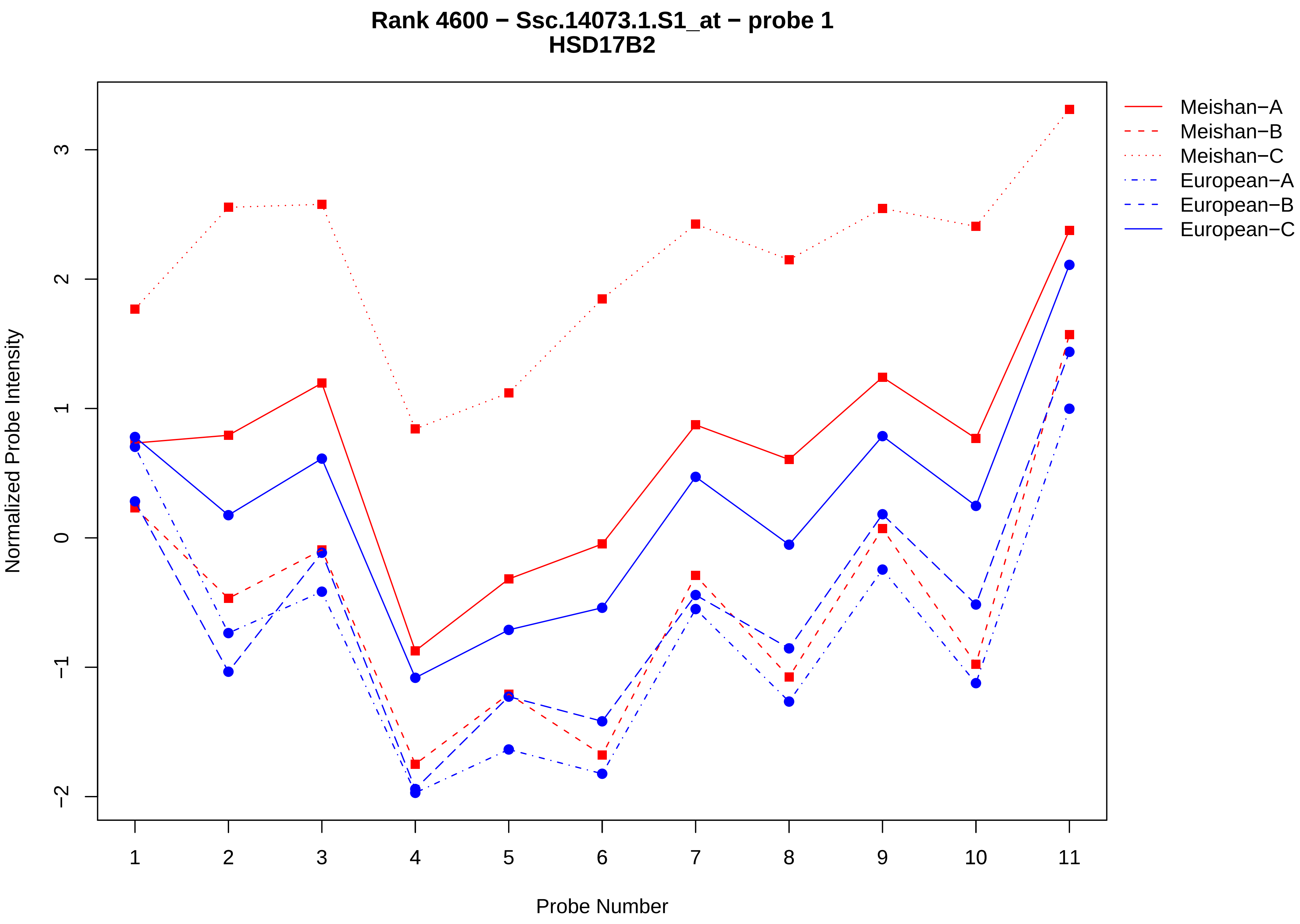
Rank 4600 − Ssc.14073.1.S1_at − probe 1
HSD17B2
Normalized Probe Intensity
Probe Number
Meishan−A
Meishan−B
Meishan−C
European−A
European−B
European−C
−2
−1
0
1
2
3
1
2
3
4
5
6
7
8
9
10
11

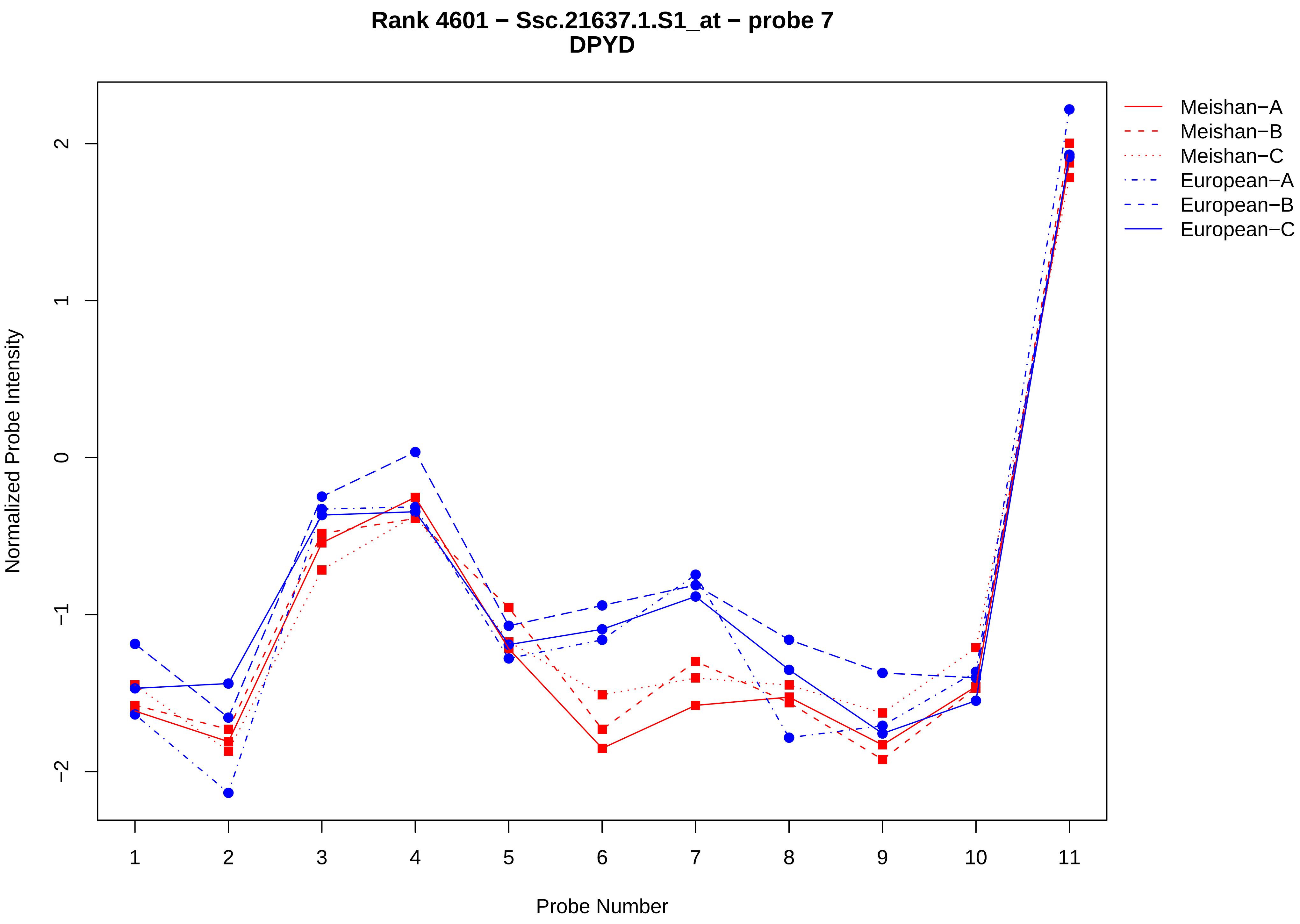

Rank 4601 − Ssc.21637.1.S1_at − probe 7
DPYD
Normalized Probe Intensity
Probe Number
−2
−1
0
1
2
1
2
3
4
5
6
7
8
9
10
11
Meishan−A
Meishan−B
Meishan−C
European−A
European−B
European−C

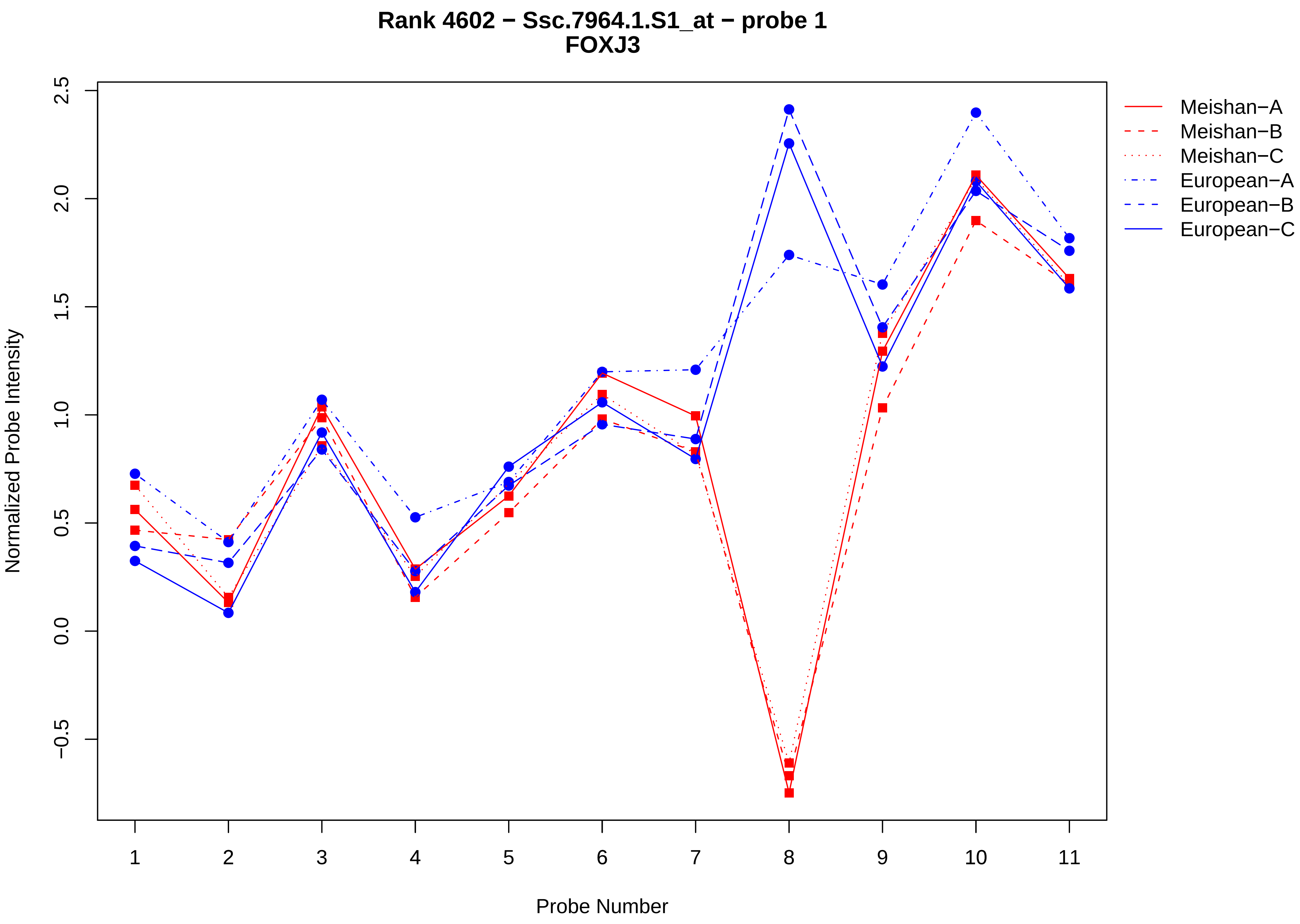

Rank 4602 − Ssc.7964.1.S1_at − probe 1
FOXJ3
Normalized Probe Intensity
Probe Number
−0.5
0.0
0.5
1.0
1.5
2.0
2.5
1
2
3
4
5
6
7
8
9
10
11
Meishan−A
Meishan−B
Meishan−C
European−A
European−B
European−C

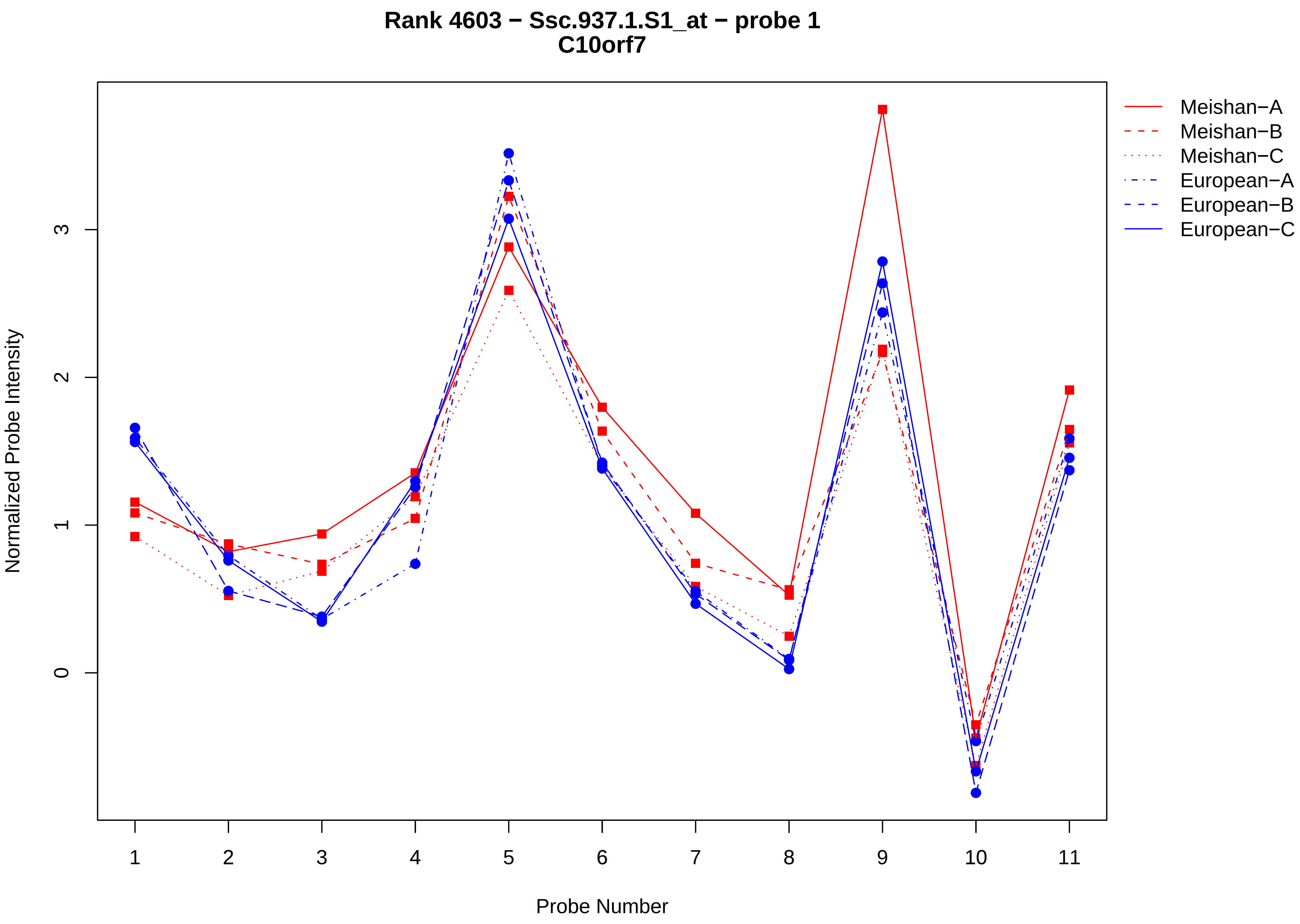
Rank 4603 − Ssc.937.1.S1_at − probe 1
C10orf7
Normalized Probe Intensity
Probe Number
0
1
2
3
1
2
3
4
5
6
7
8
9
10
11
Meishan−A
Meishan−B
Meishan−C
European−A
European−B
European−C

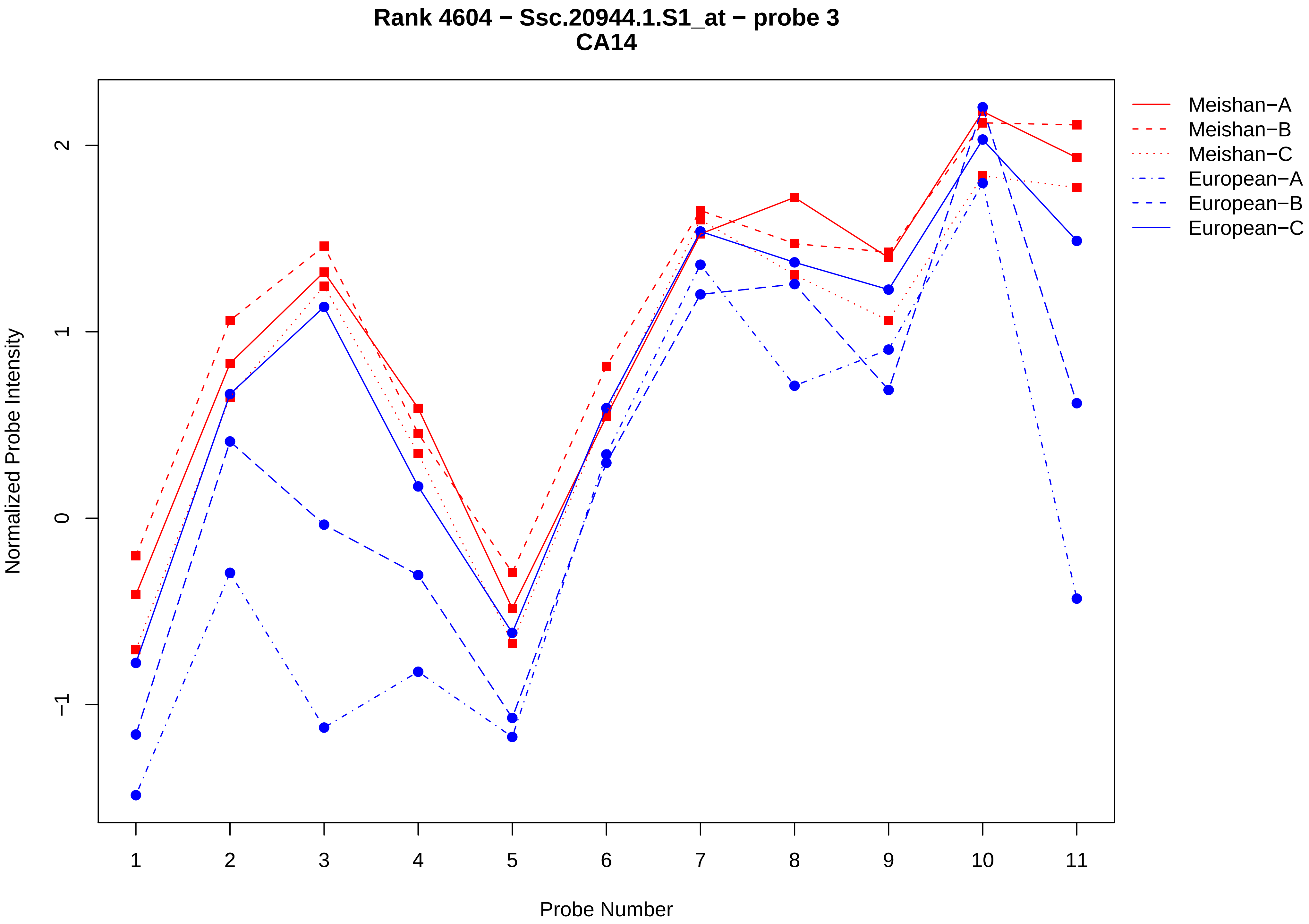

Rank 4604 − Ssc.20944.1.S1_at − probe 3
CA14
Normalized Probe Intensity
Probe Number
−1
0
1
2
1
2
3
4
5
6
7
8
9
10
11
Meishan−A
Meishan−B
Meishan−C
European−A
European−B
European−C

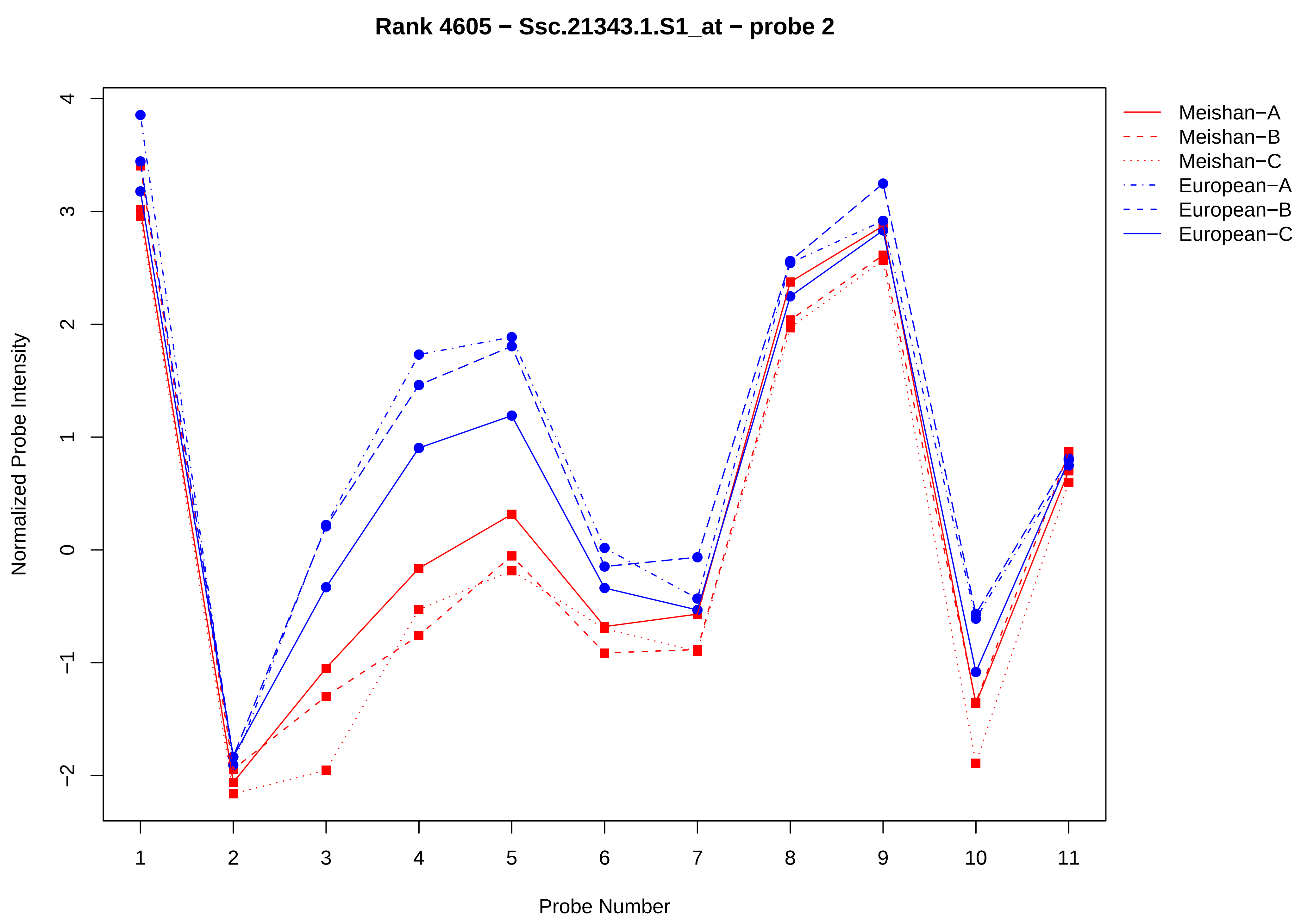

Rank 4605 − Ssc.21343.1.S1_at − probe 2
Normalized Probe Intensity
Probe Number
Meishan−A
Meishan−B
Meishan−C
European−A
European−B
European−C
−2
−1
0
1
2
3
4
1
2
3
4
5
6
7
8
9
10
11

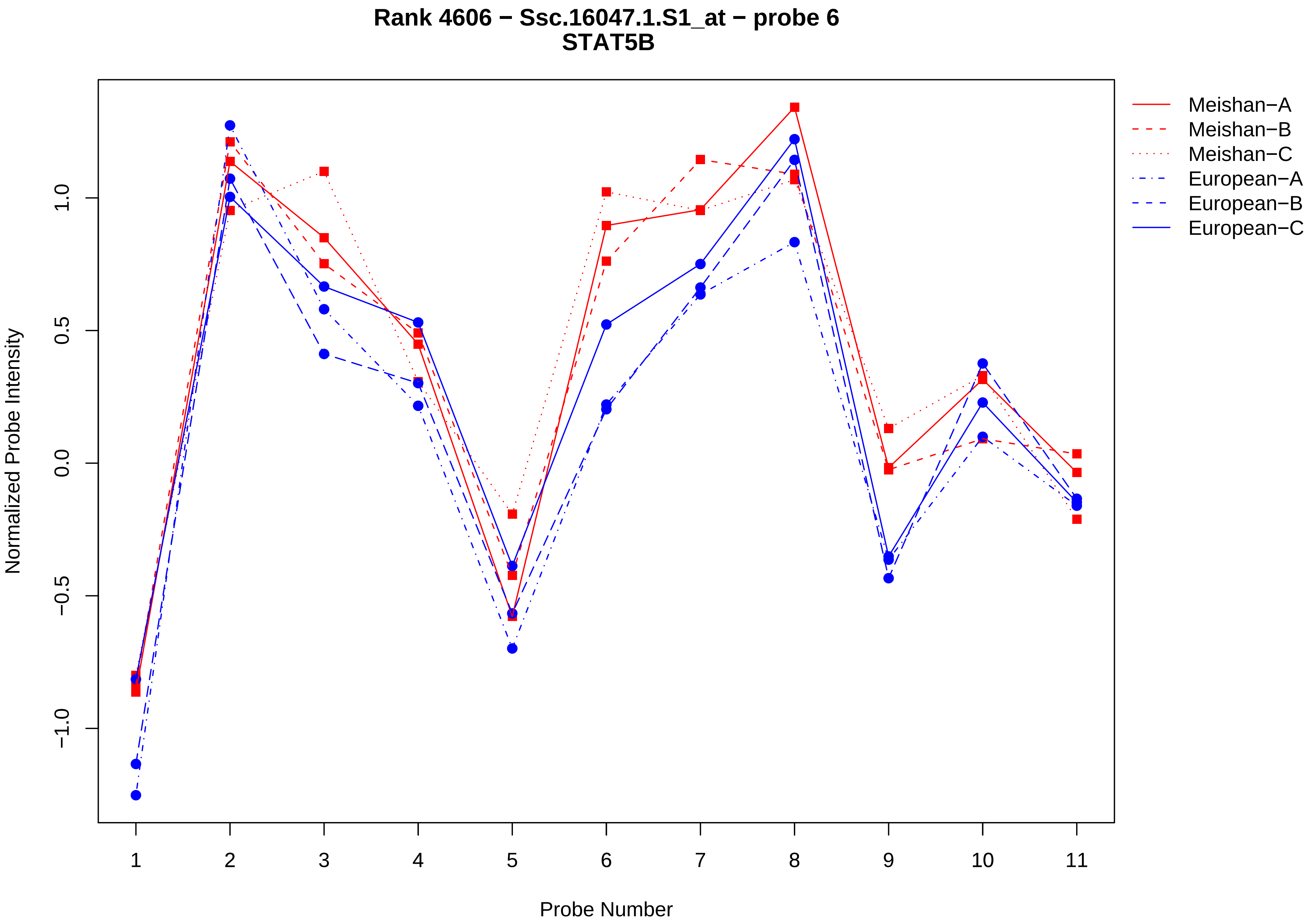
Rank 4606 − Ssc.16047.1.S1_at − probe 6
STAT5B
Normalized Probe Intensity
−1.0
−0.5
0.0
0.5
1.0
Probe Number
1
2
3
4
5
6
7
8
9
10
11
Meishan−A
Meishan−B
Meishan−C
European−A
European−B
European−C

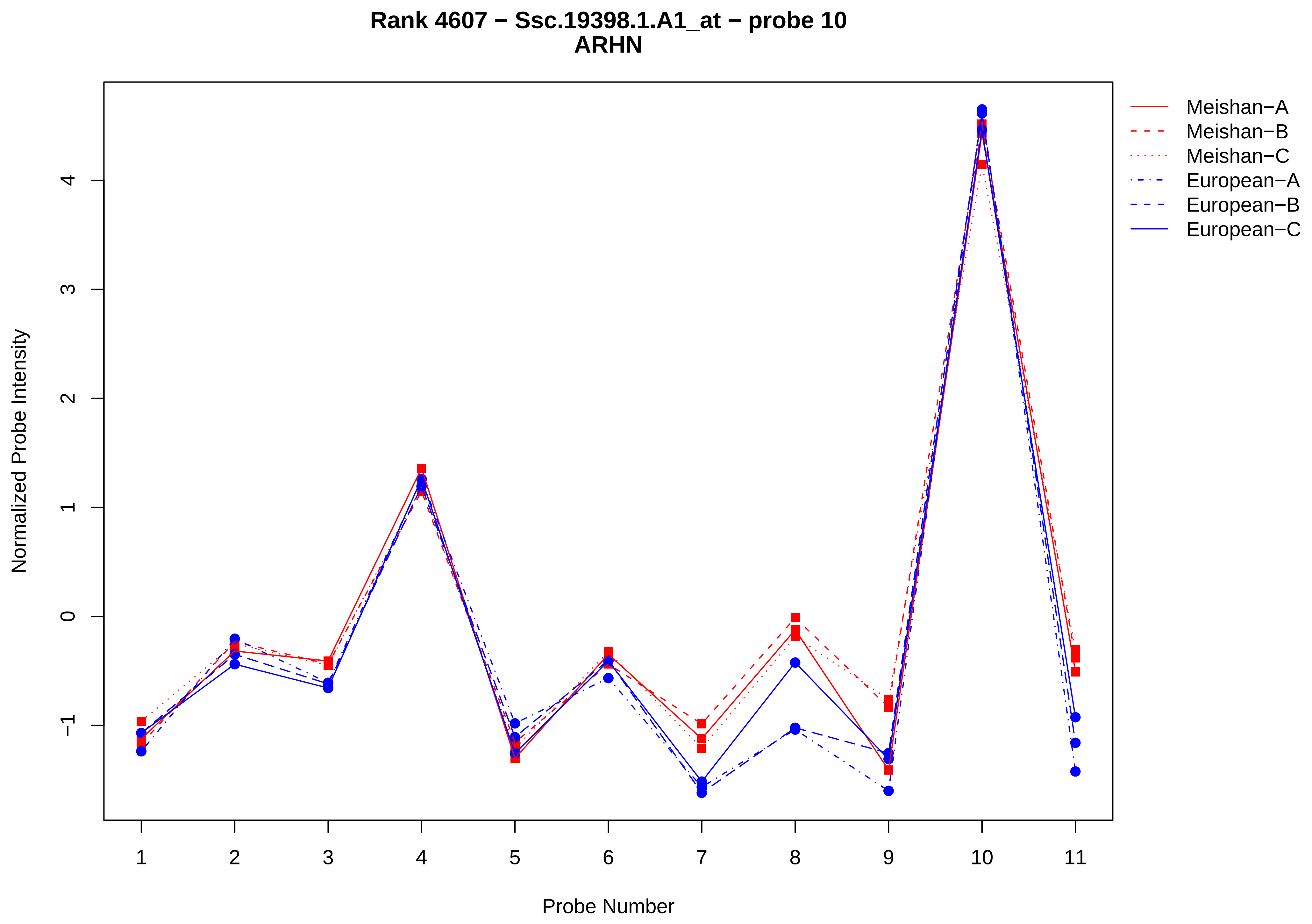

Rank 4607 − Ssc.19398.1.A1_at − probe 10
ARHN
Normalized Probe Intensity
Probe Number
Meishan−A
Meishan−B
Meishan−C
European−A
European−B
European−C

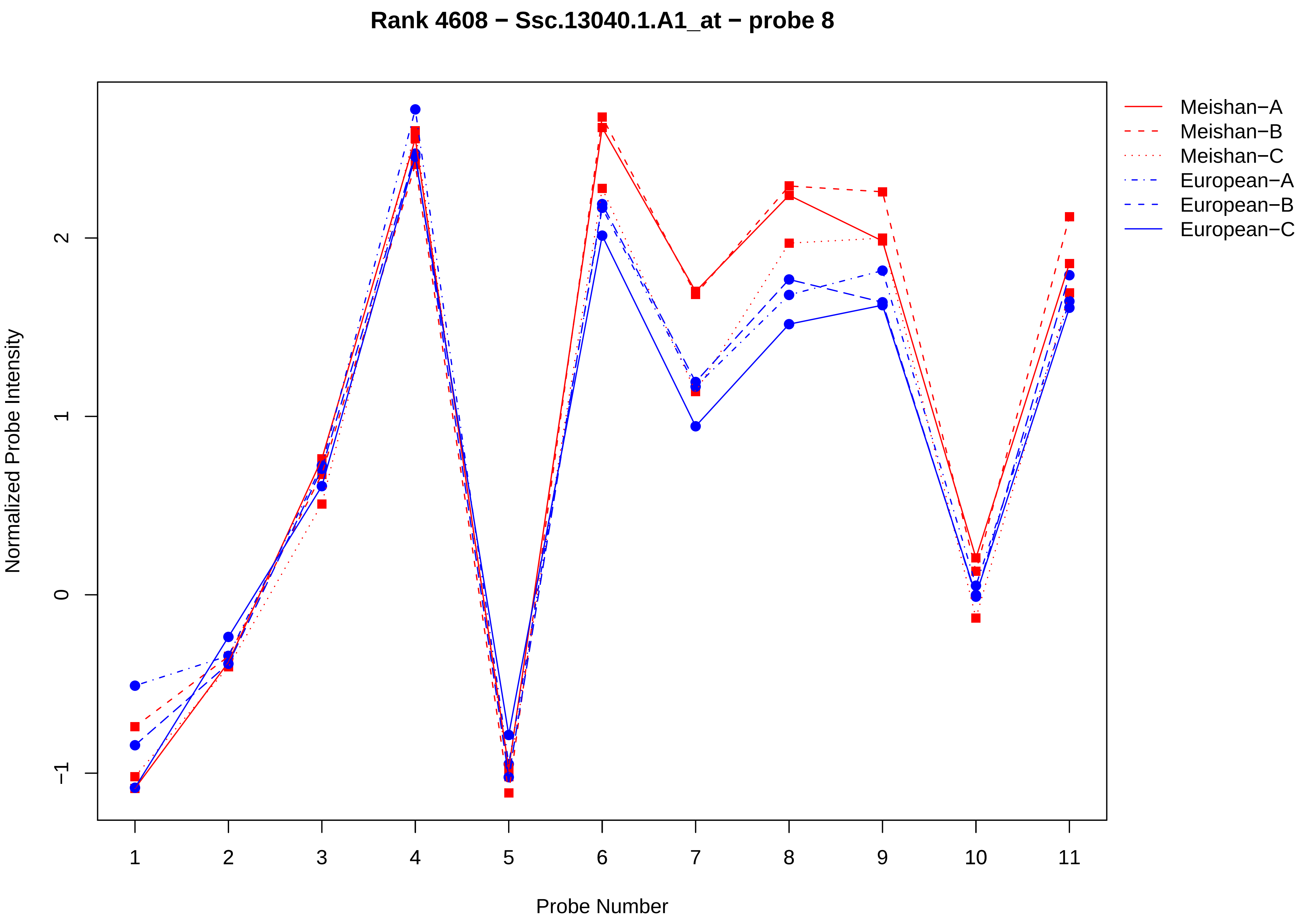
Rank 4608 − Ssc.13040.1.A1_at − probe 8
Normalized Probe Intensity
Probe Number
−1
0
1
2
1
2
3
4
5
6
7
8
9
10
11
Meishan−A
Meishan−B
Meishan−C
European−A
European−B
European−C

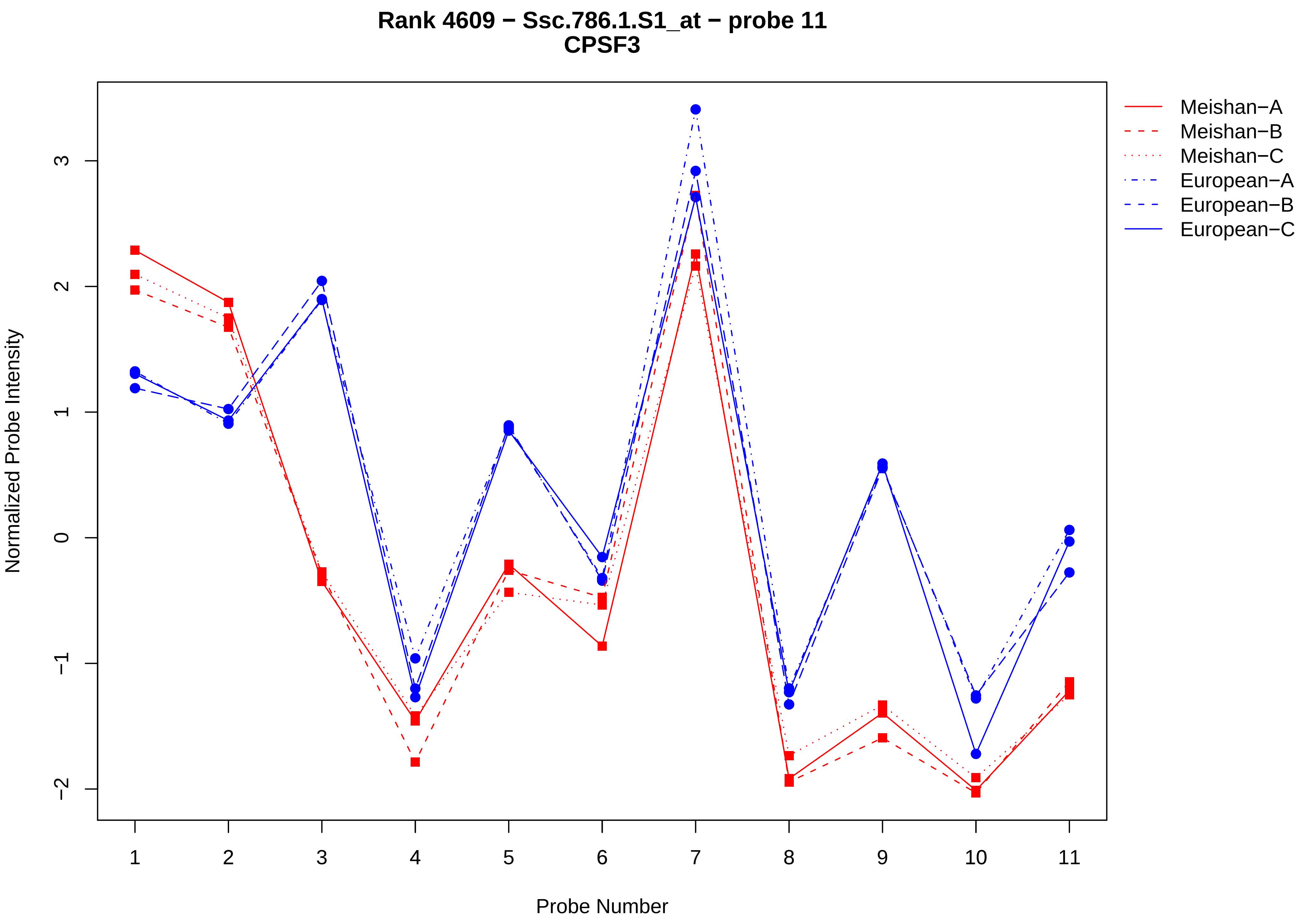

Rank 4609 − Ssc.786.1.S1_at − probe 11
CPSF3
Normalized Probe Intensity
Probe Number
Meishan−A
Meishan−B
Meishan−C
European−A
European−B
European−C
−2
−1
0
1
2
3
1
2
3
4
5
6
7
8
9
10
11

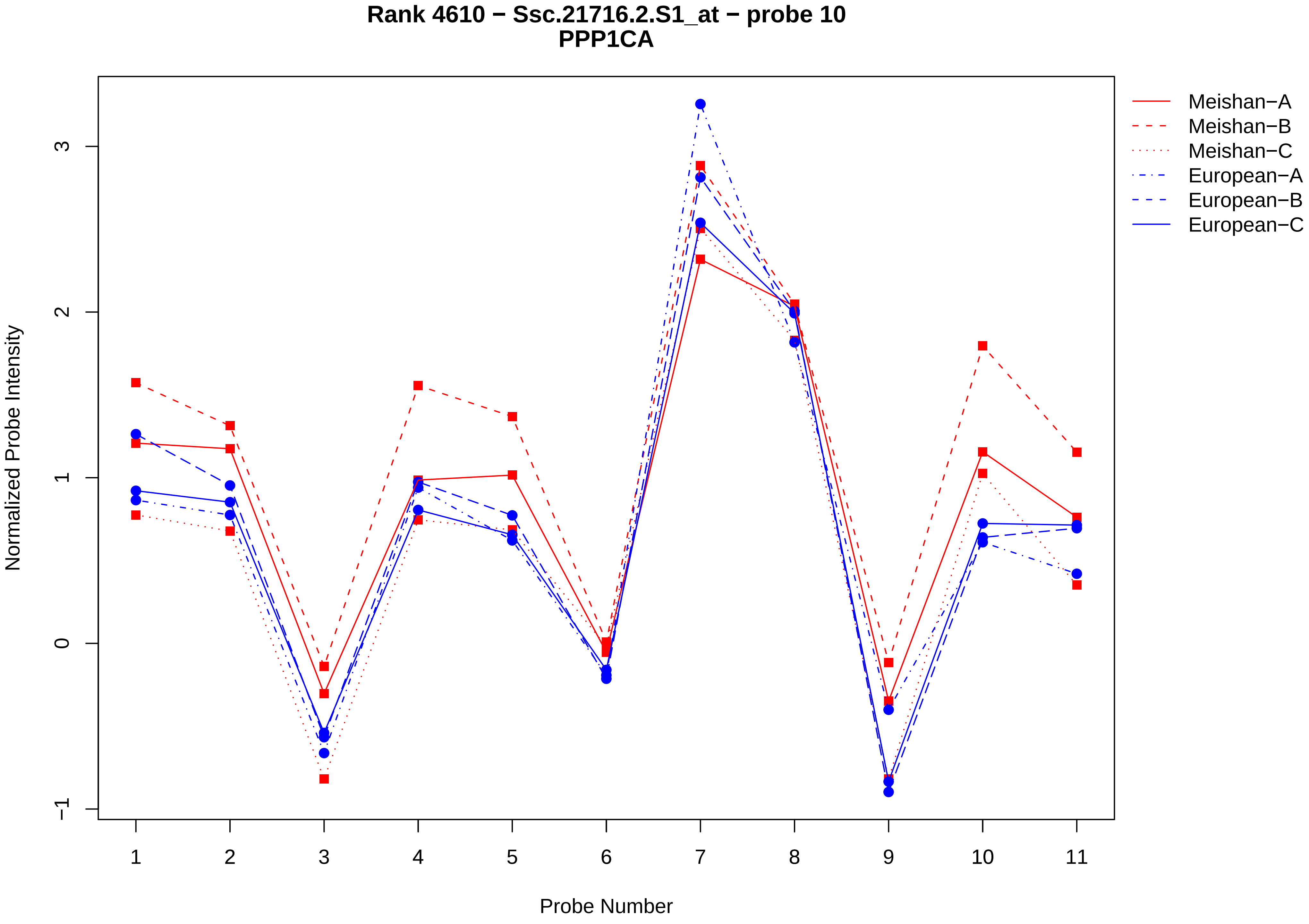
Rank 4610 − Ssc.21716.2.S1_at − probe 10
PPP1CA
Normalized Probe Intensity
Probe Number
Meishan−A
Meishan−B
Meishan−C
European−A
European−B
European−C
−1
0
1
2
3
1
2
3
4
5
6
7
8
9
10
11

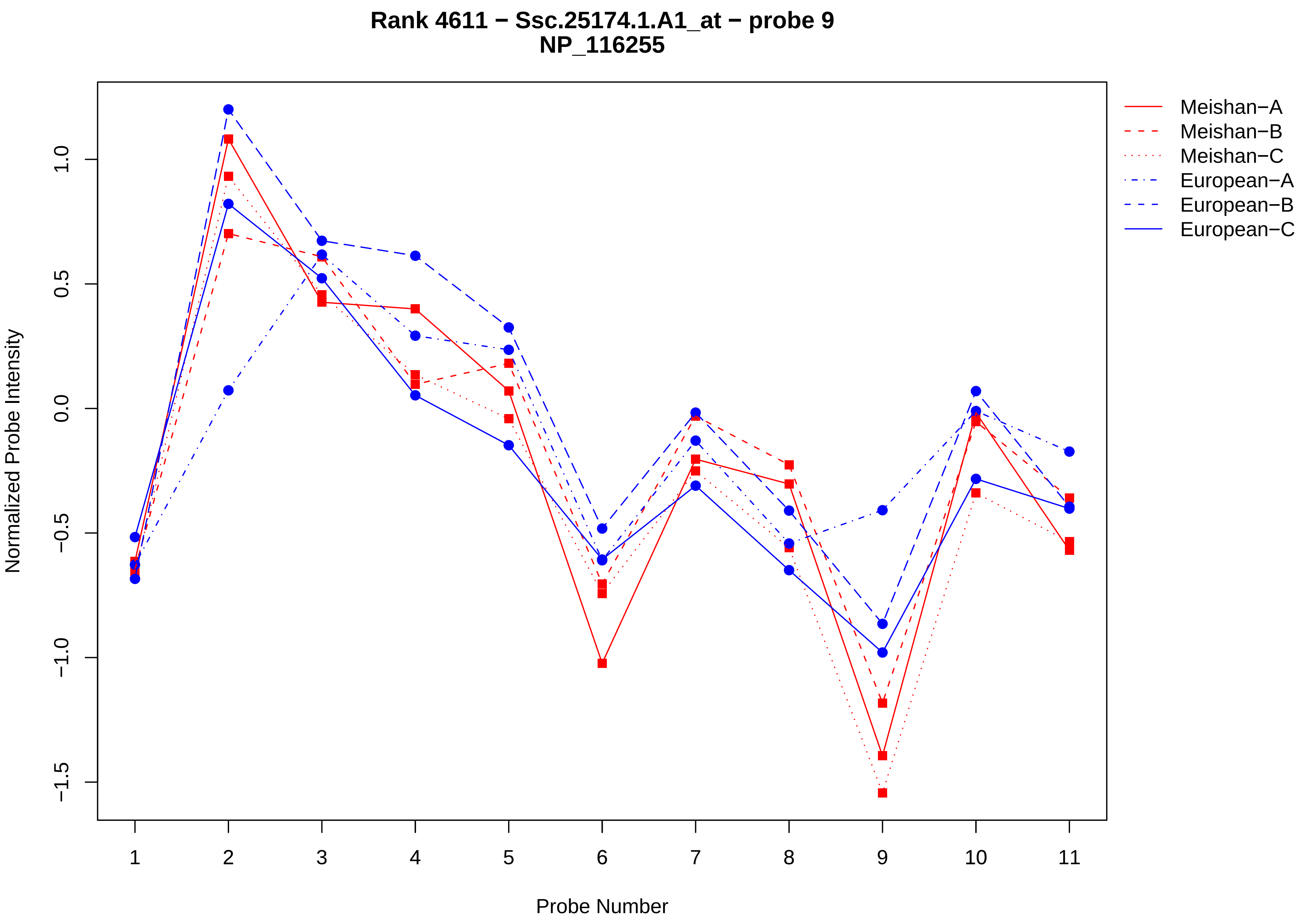

Rank 4611 − Ssc.25174.1.A1_at − probe 9
NP_116255
Normalized Probe Intensity
Probe Number
1.0
0.5
0.0
−0.5
−1.0
−1.5
1
2
3
4
5
6
7
8
9
10
11
Meishan−A
Meishan−B
Meishan−C
European−A
European−B
European−C

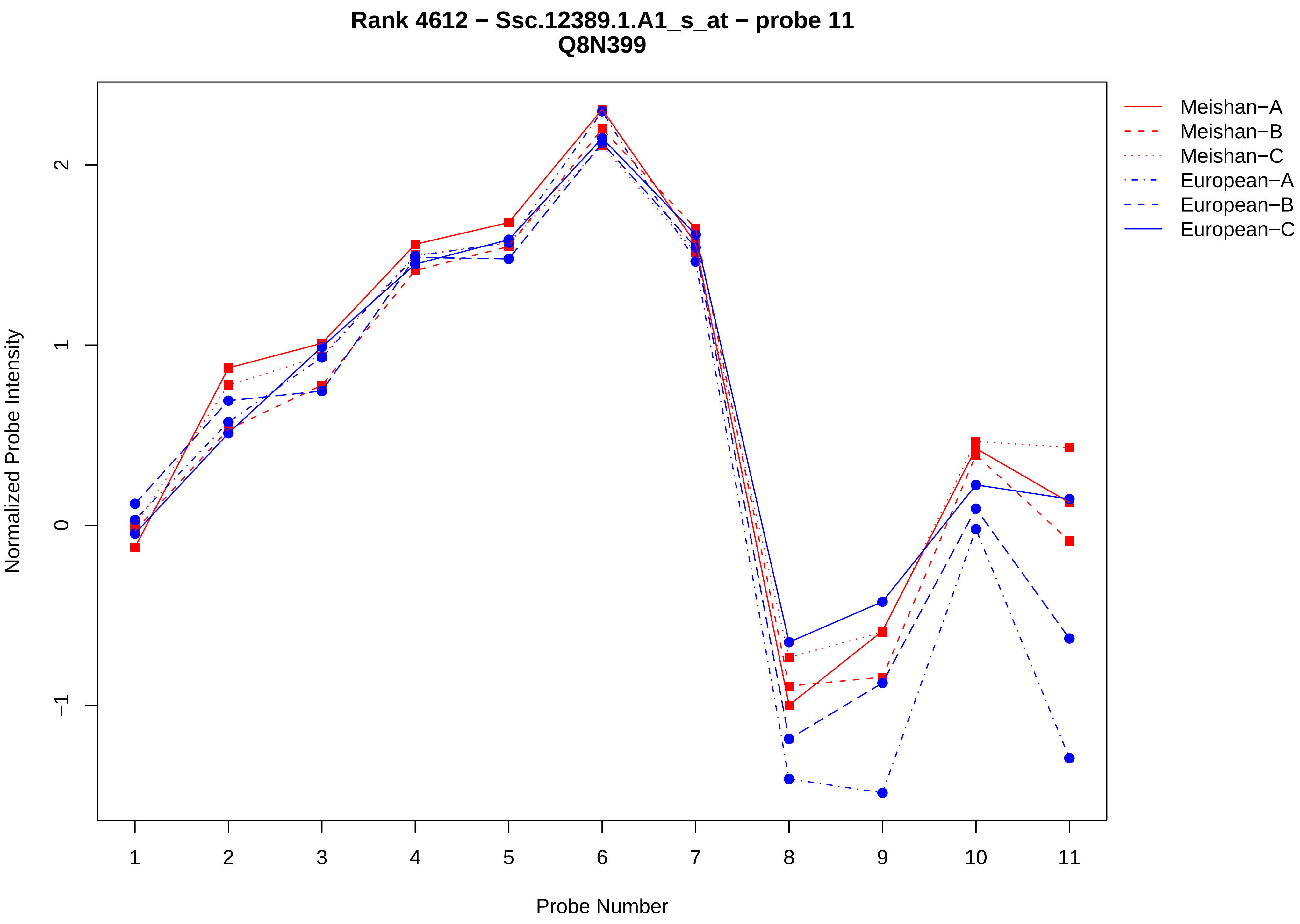

Rank 4612 − Ssc.12389.1.A1_s_at − probe 11
Q8N399
Normalized Probe Intensity
Probe Number
−1
0
1
2
1
2
3
4
5
6
7
8
9
10
11
Meishan−A
Meishan−B
Meishan−C
European−A
European−B
European−C

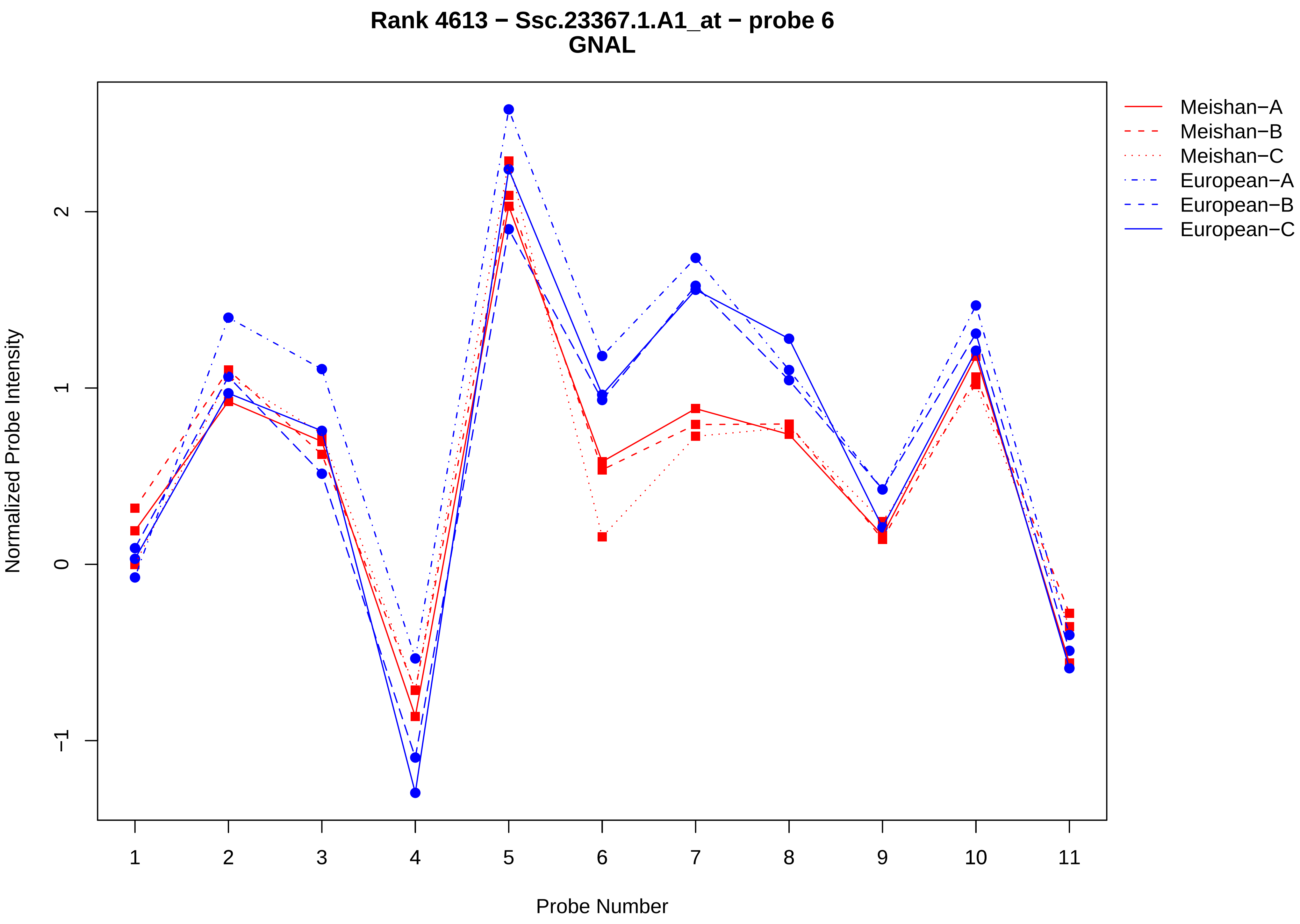

Rank 4613 − Ssc.23367.1.A1_at − probe 6
GNAL
Normalized Probe Intensity
Probe Number
Meishan−A
Meishan−B
Meishan−C
European−A
European−B
European−C

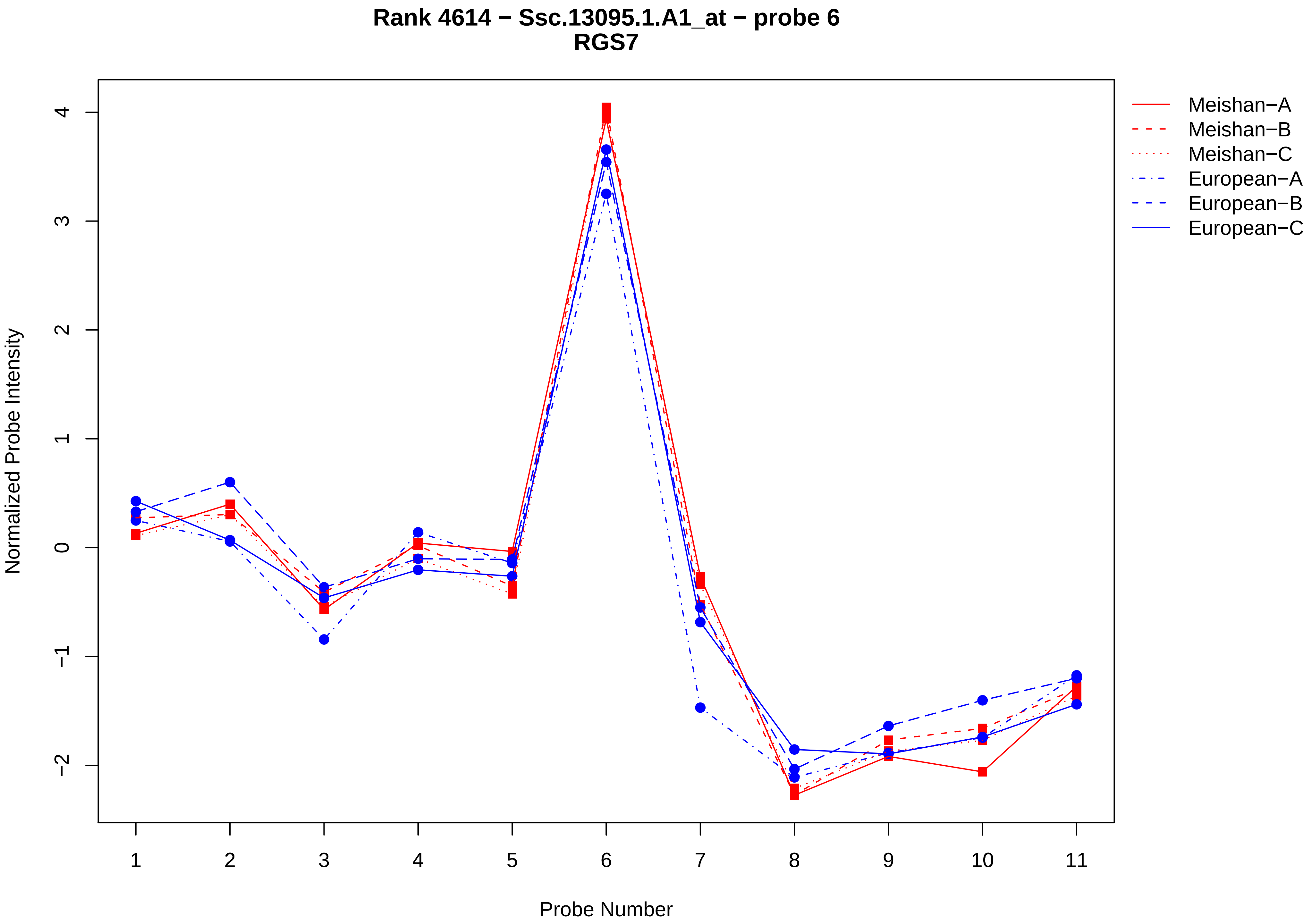
Rank 4614 − Ssc.13095.1.A1_at − probe 6
RGS7
Normalized Probe Intensity
Probe Number
Meishan−A
Meishan−B
Meishan−C
European−A
European−B
European−C

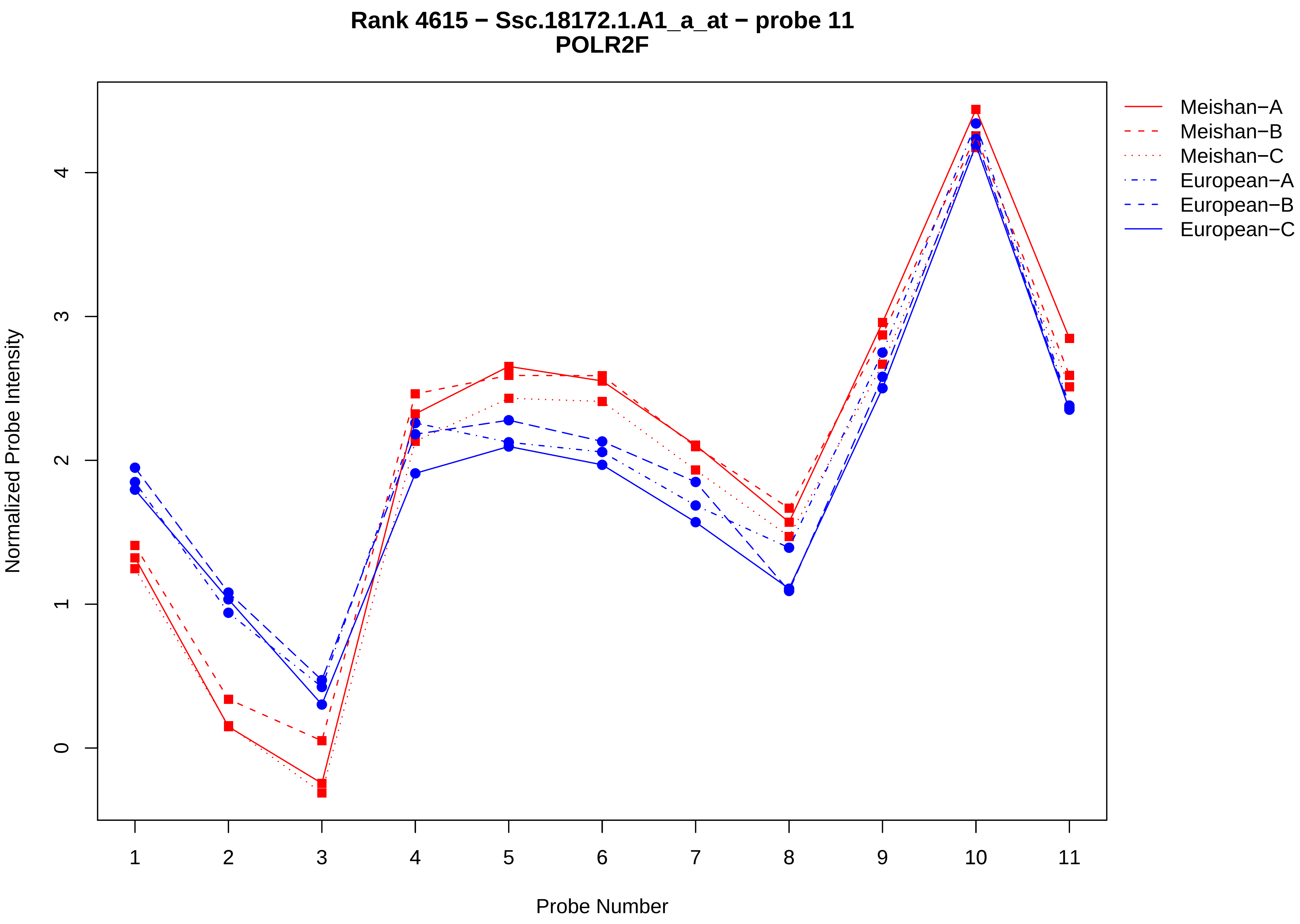
Rank 4615 − Ssc.18172.1.A1_a_at − probe 11
POLR2F
Normalized Probe Intensity
0
1
2
3
4
Probe Number
1
2
3
4
5
6
7
8
9
10
11
Meishan−A
Meishan−B
Meishan−C
European−A
European−B
European−C

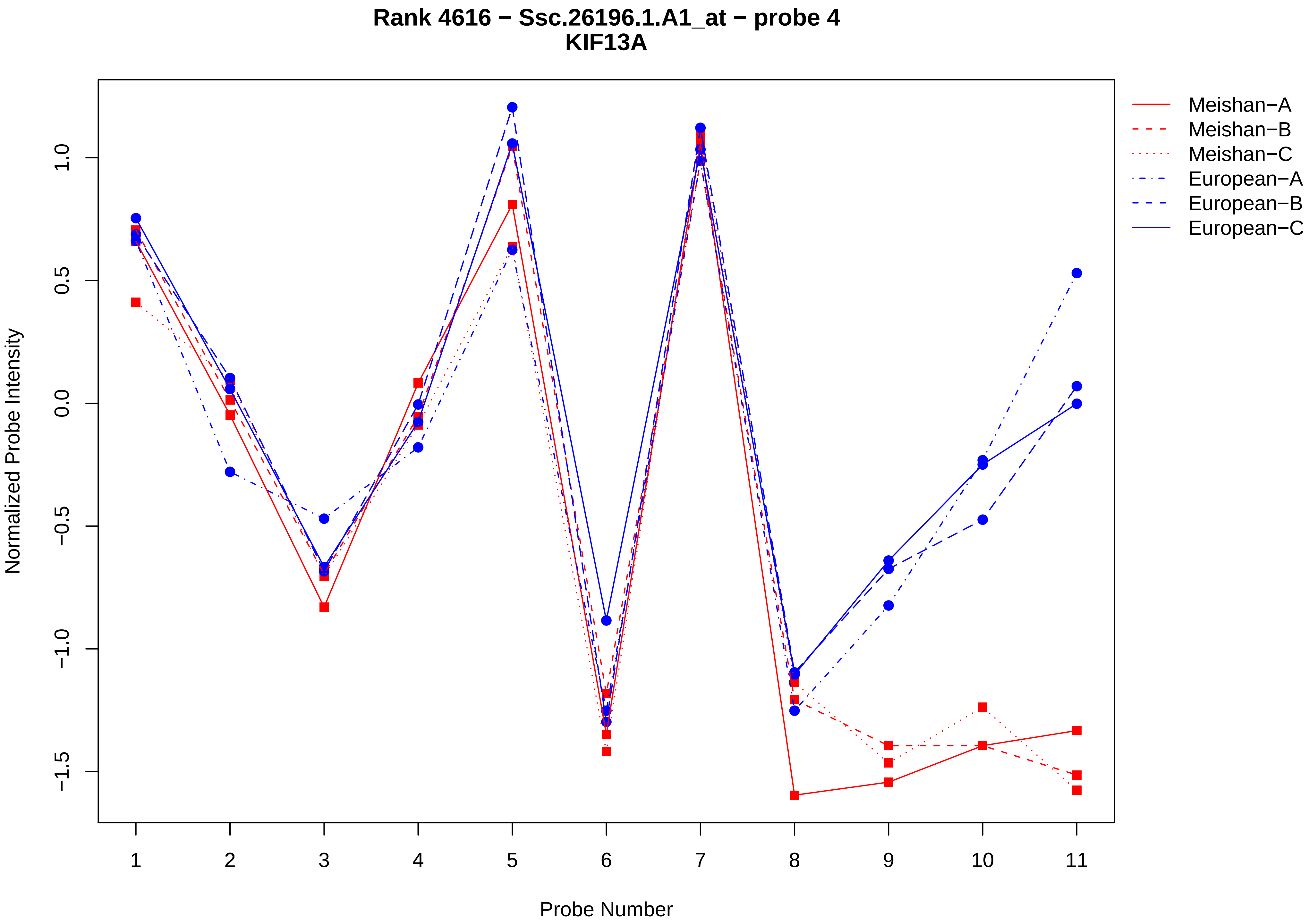

Rank 4616 − Ssc.26196.1.A1_at − probe 4
KIF13A
Normalized Probe Intensity
Probe Number
1.0
0.5
0.0
−0.5
−1.0
−1.5
1
2
3
4
5
6
7
8
9
10
11
Meishan−A
Meishan−B
Meishan−C
European−A
European−B
European−C

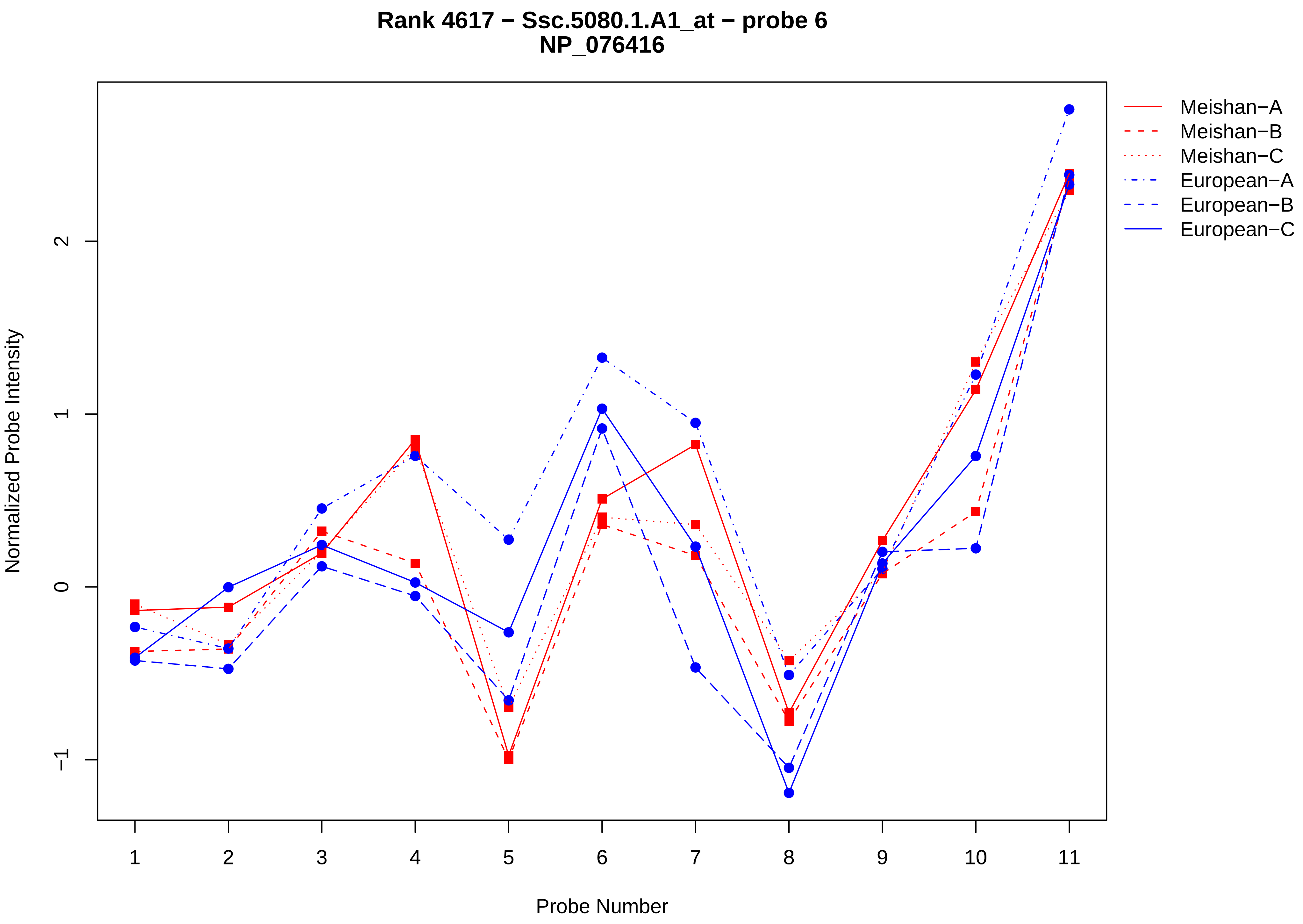

Rank 4617 − Ssc.5080.1.A1_at − probe 6
NP_076416
Normalized Probe Intensity
Probe Number
−1
0
1
2
1
2
3
4
5
6
7
8
9
10
11
Meishan−A
Meishan−B
Meishan−C
European−A
European−B
European−C

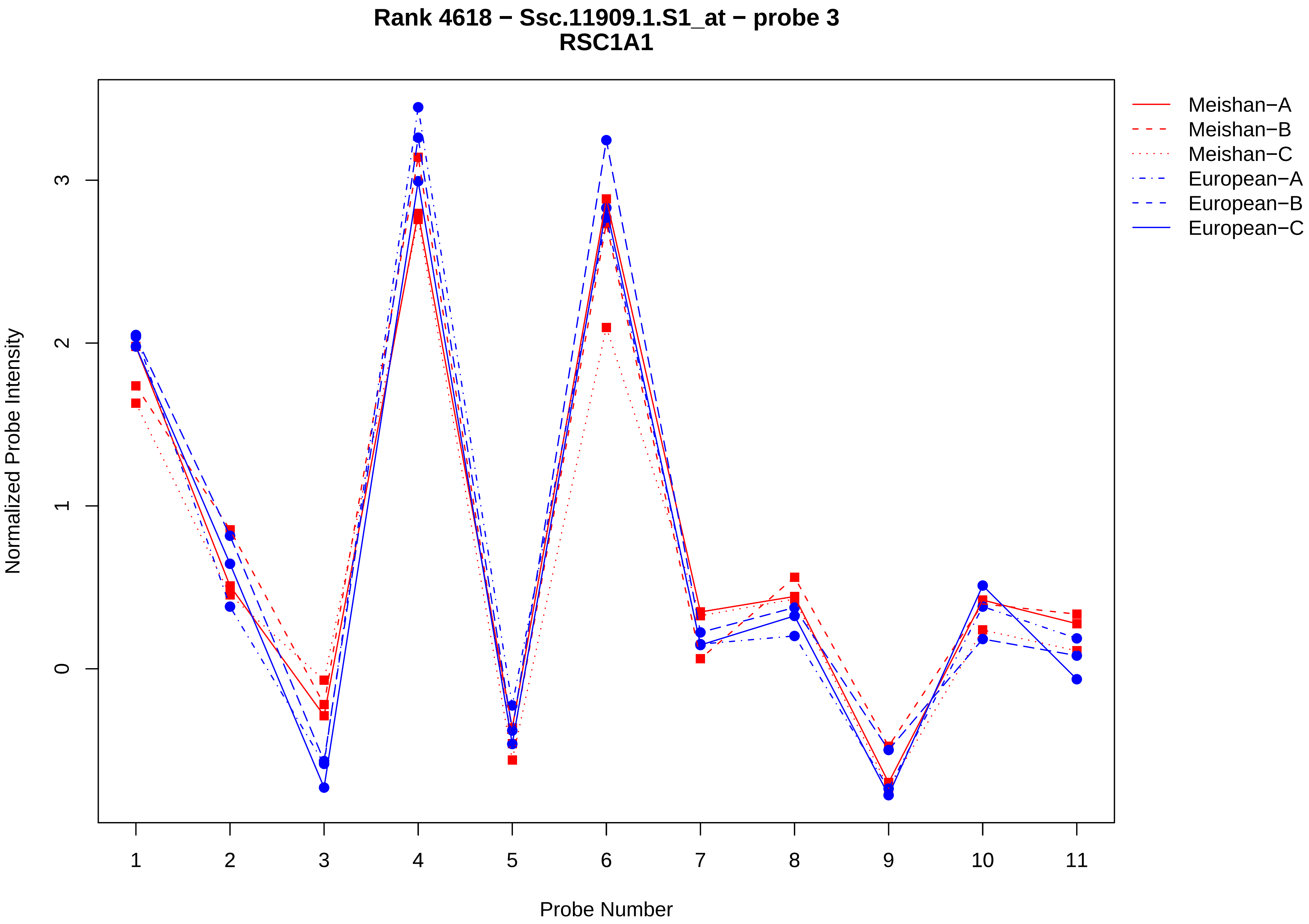

Rank 4618 − Ssc.11909.1.S1_at − probe 3
RSC1A1
Normalized Probe Intensity
Probe Number
0
1
2
3
1
2
3
4
5
6
7
8
9
10
11
Meishan−A
Meishan−B
Meishan−C
European−A
European−B
European−C

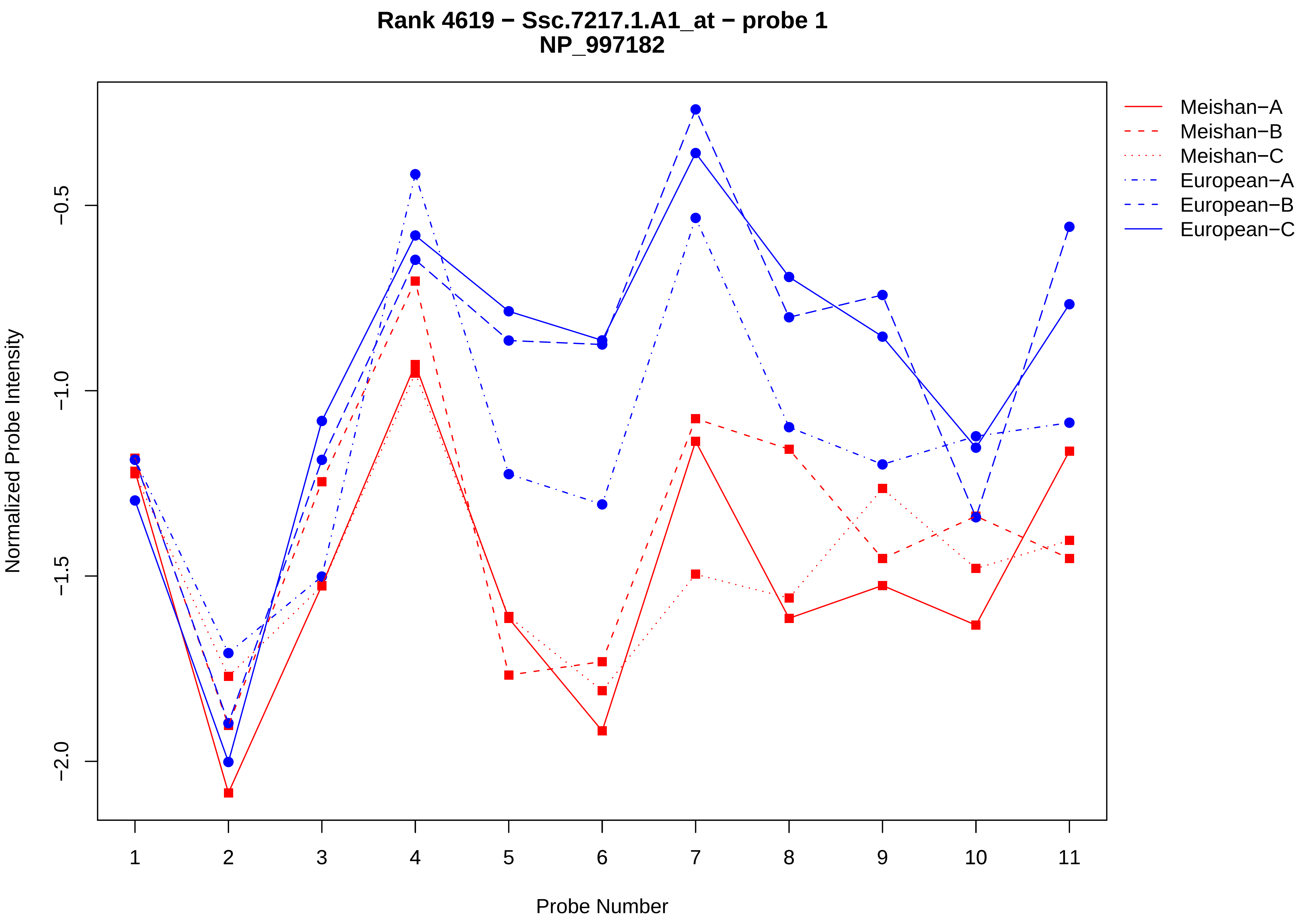

Rank 4619 − Ssc.7217.1.A1_at − probe 1
NP_997182
Normalized Probe Intensity
−0.5
−1.0
−1.5
−2.0
1
2
3
4
5
6
7
8
9
10
11
Probe Number
Meishan−A
Meishan−B
Meishan−C
European−A
European−B
European−C

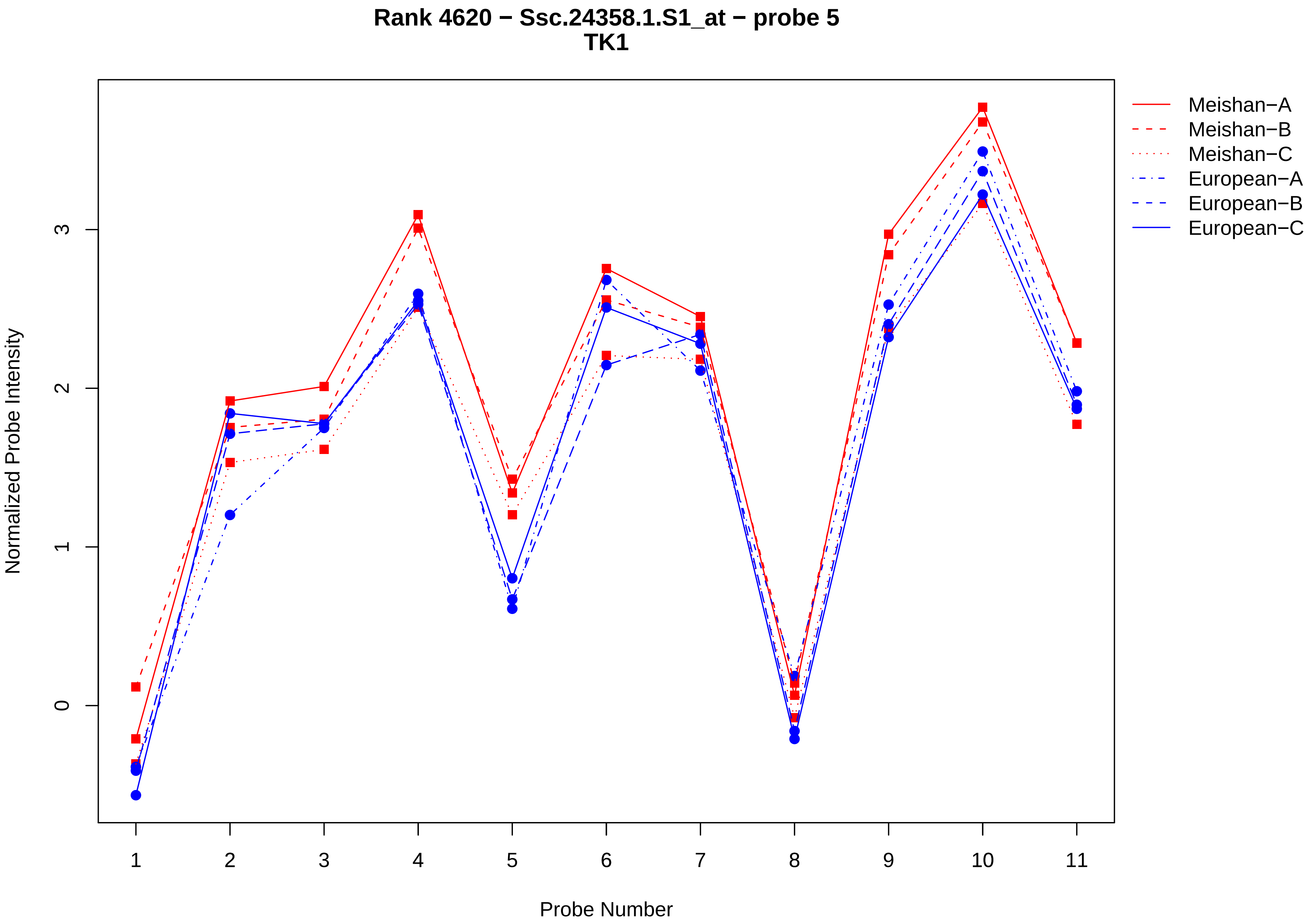
Rank 4620 − Ssc.24358.1.S1_at − probe 5
TK1
Normalized Probe Intensity
Probe Number
0
1
2
3
1
2
3
4
5
6
7
8
9
10
11
Meishan−A
Meishan−B
Meishan−C
European−A
European−B
European−C

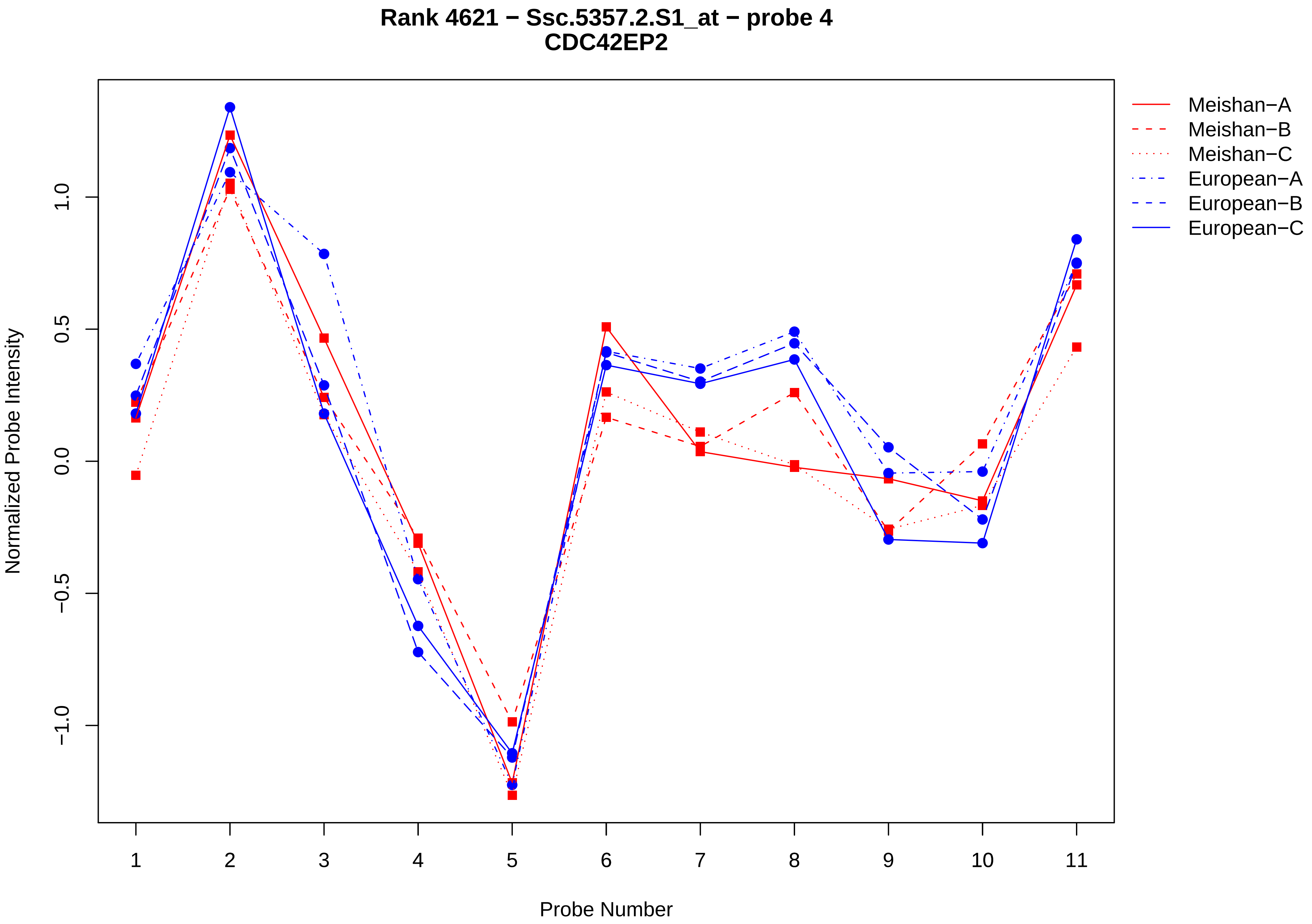

Rank 4621 − Ssc.5357.2.S1_at − probe 4
CDC42EP2
Normalized Probe Intensity
Probe Number
−1.0
−0.5
0.0
0.5
1.0
1
2
3
4
5
6
7
8
9
10
11
Meishan−A
Meishan−B
Meishan−C
European−A
European−B
European−C

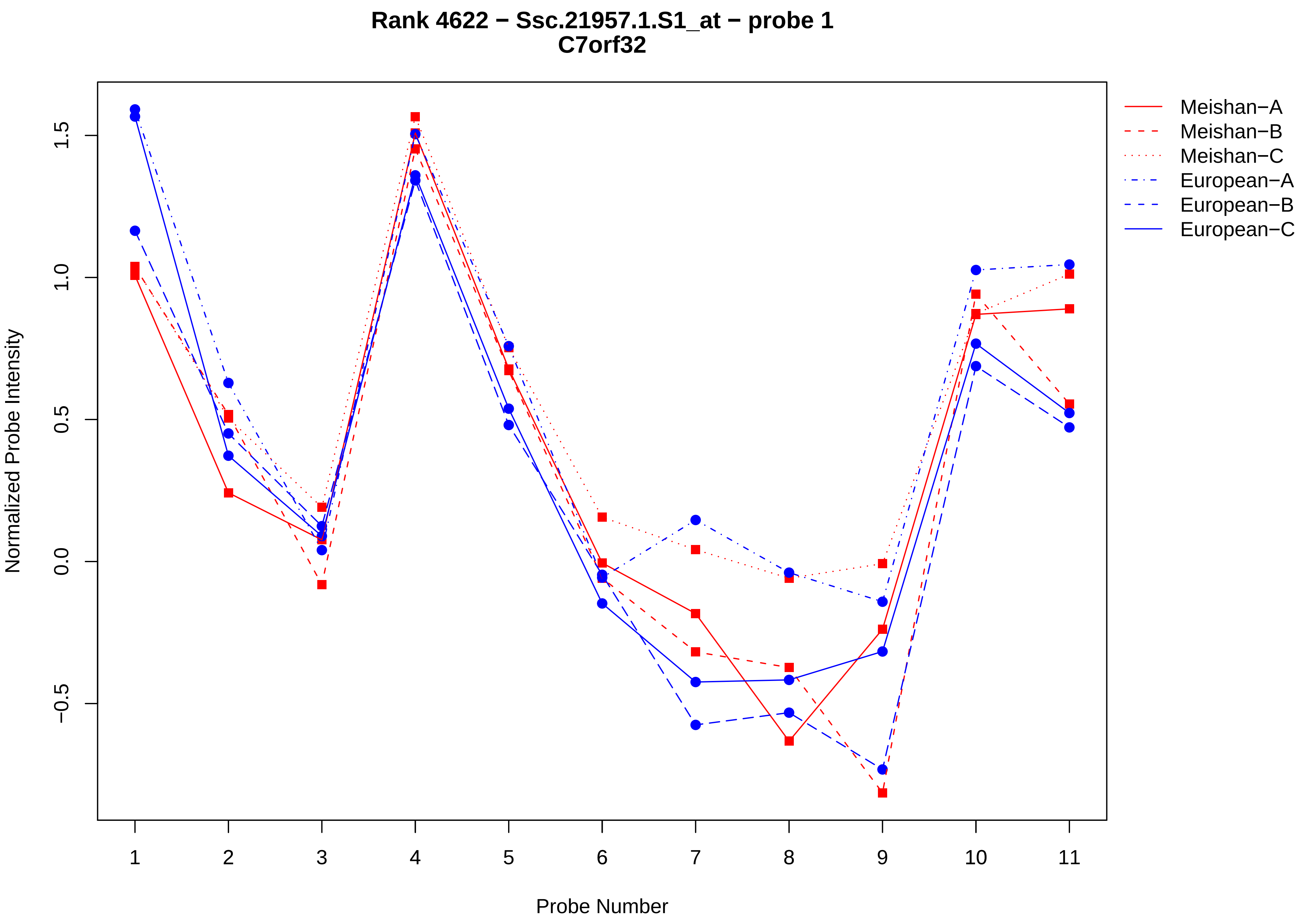

Rank 4622 − Ssc.21957.1.S1_at − probe 1
C7orf32
Normalized Probe Intensity
Probe Number
Meishan−A
Meishan−B
Meishan−C
European−A
European−B
European−C

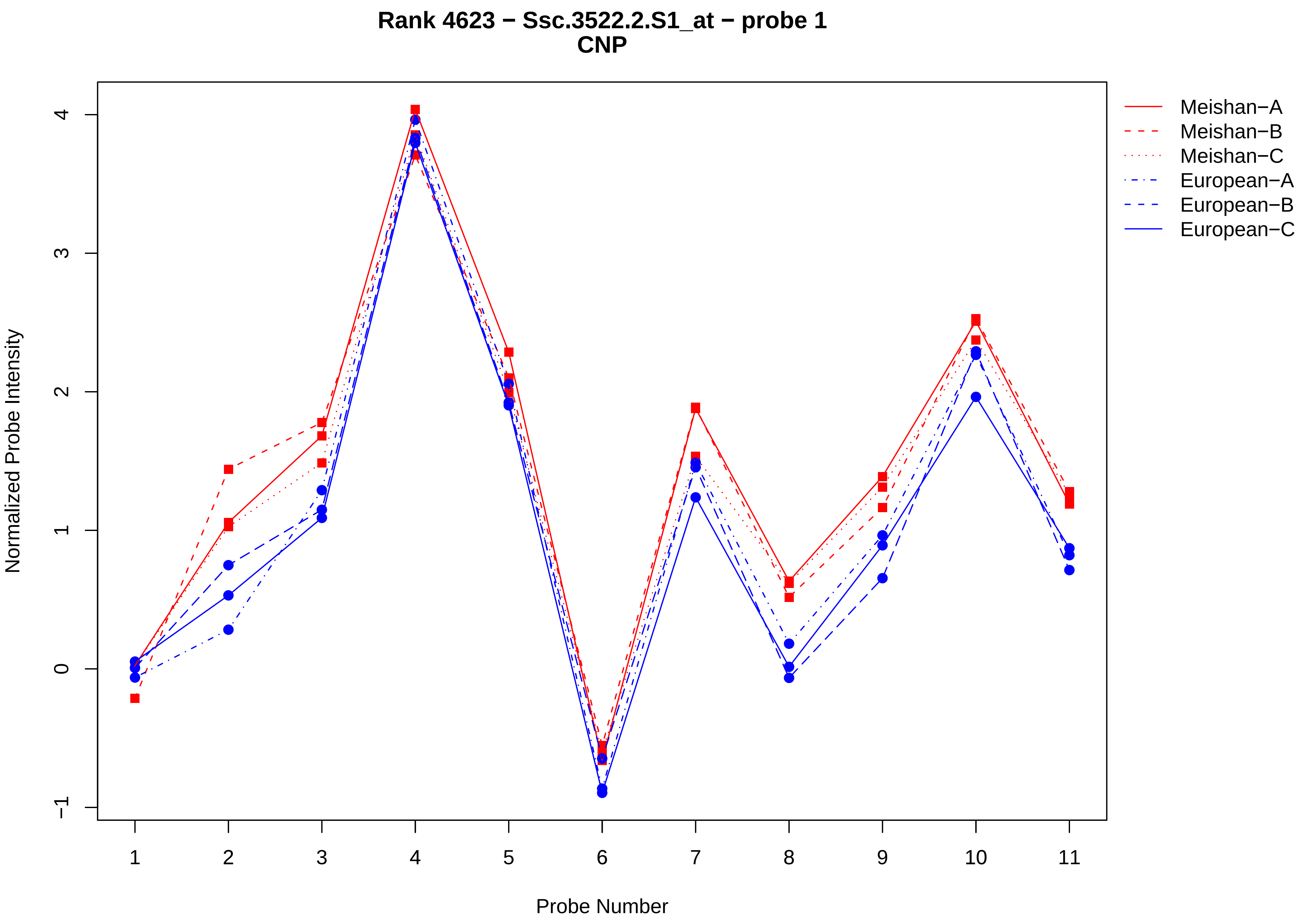

Rank 4623 − Ssc.3522.2.S1_at − probe 1
CNP
Normalized Probe Intensity
Probe Number
Meishan−A
Meishan−B
Meishan−C
European−A
European−B
European−C

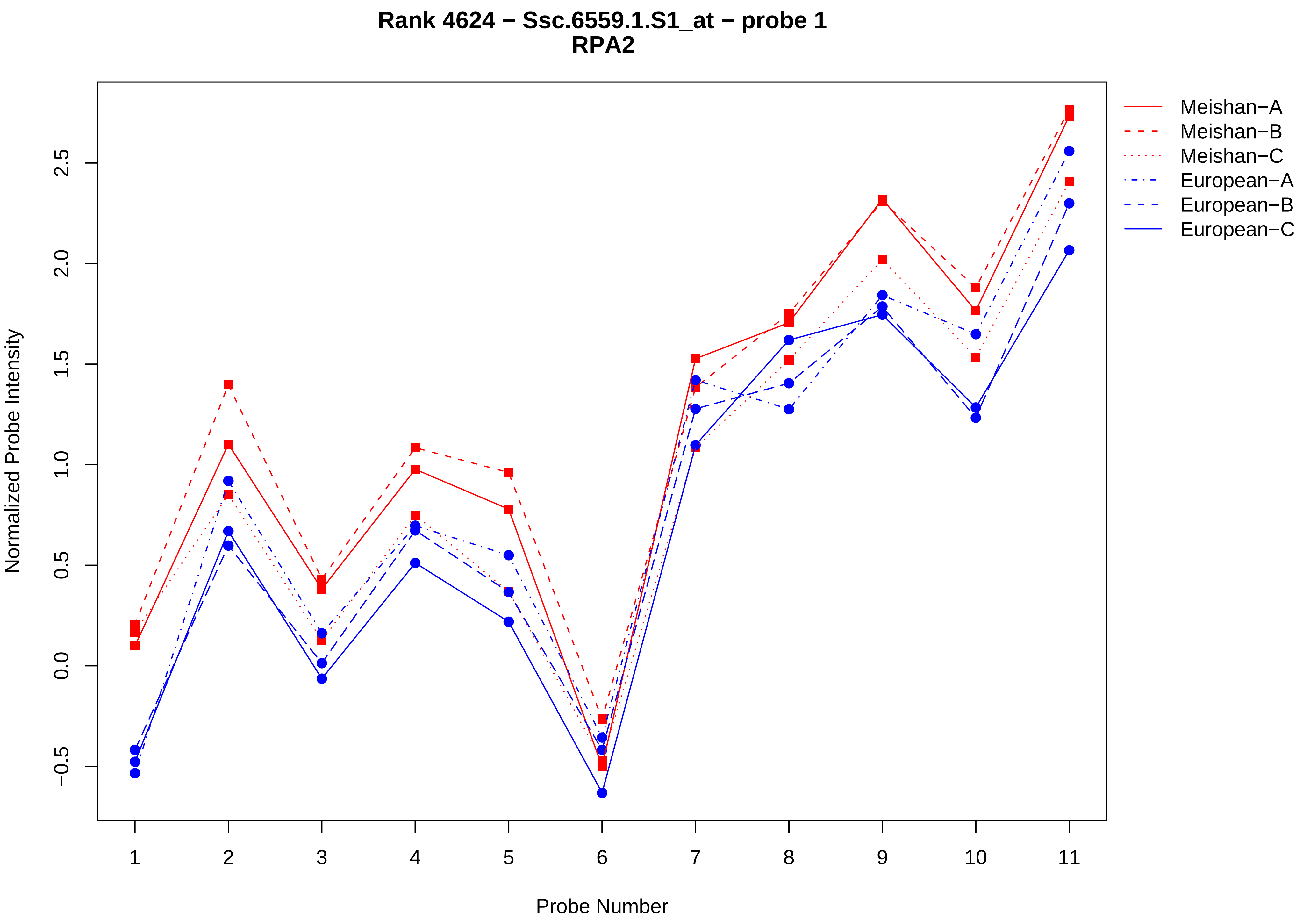

Rank 4624 − Ssc.6559.1.S1_at − probe 1
RPA2
Normalized Probe Intensity
Probe Number
−0.5
0.0
0.5
1.0
1.5
2.0
2.5
1
2
3
4
5
6
7
8
9
10
11
Meishan−A
Meishan−B
Meishan−C
European−A
European−B
European−C

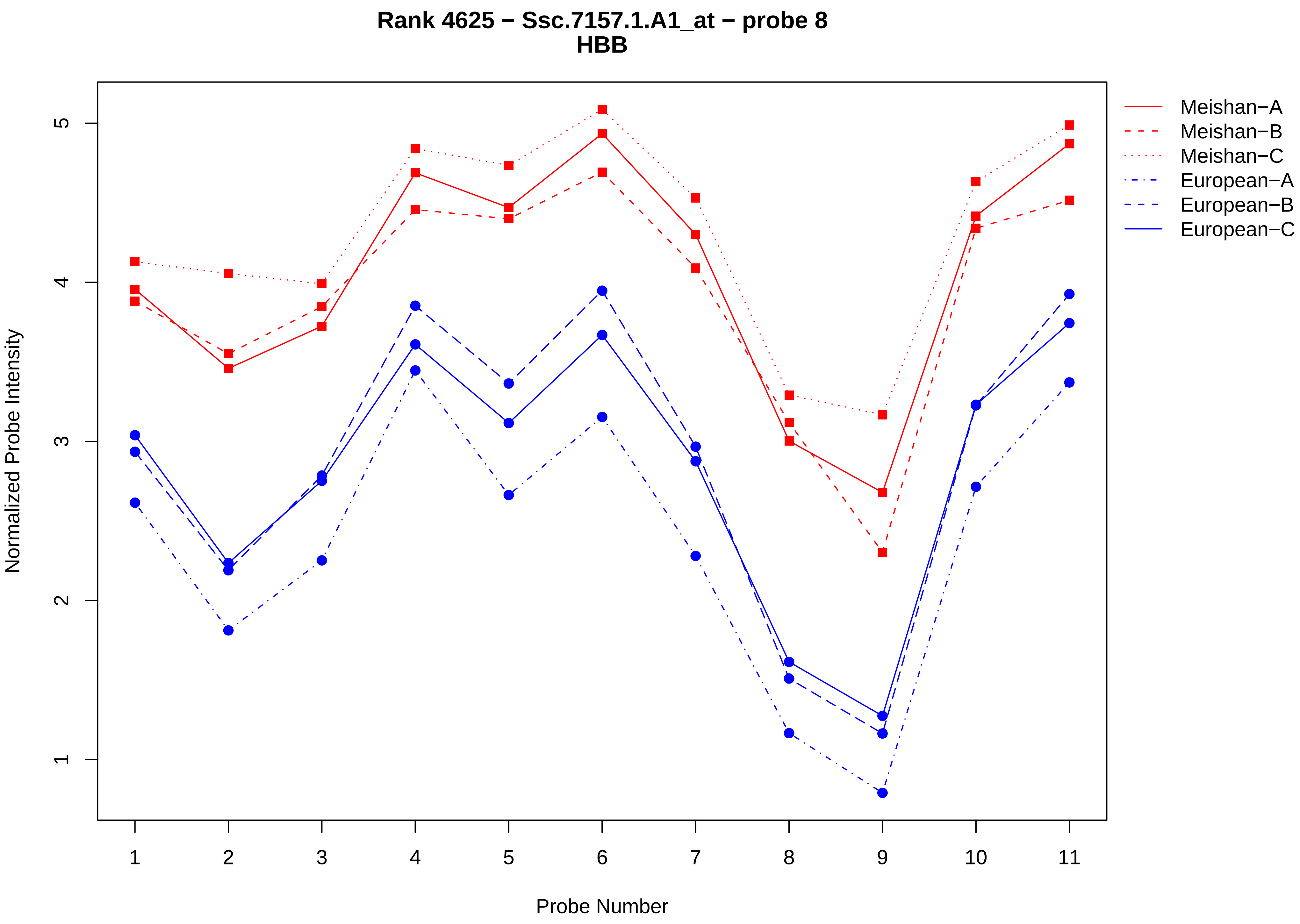

Rank 4625 − Ssc.7157.1.A1_at − probe 8
HBB
Normalized Probe Intensity
Probe Number
1
2
3
4
5
1
2
3
4
5
6
7
8
9
10
11
Meishan−A
Meishan−B
Meishan−C
European−A
European−B
European−C

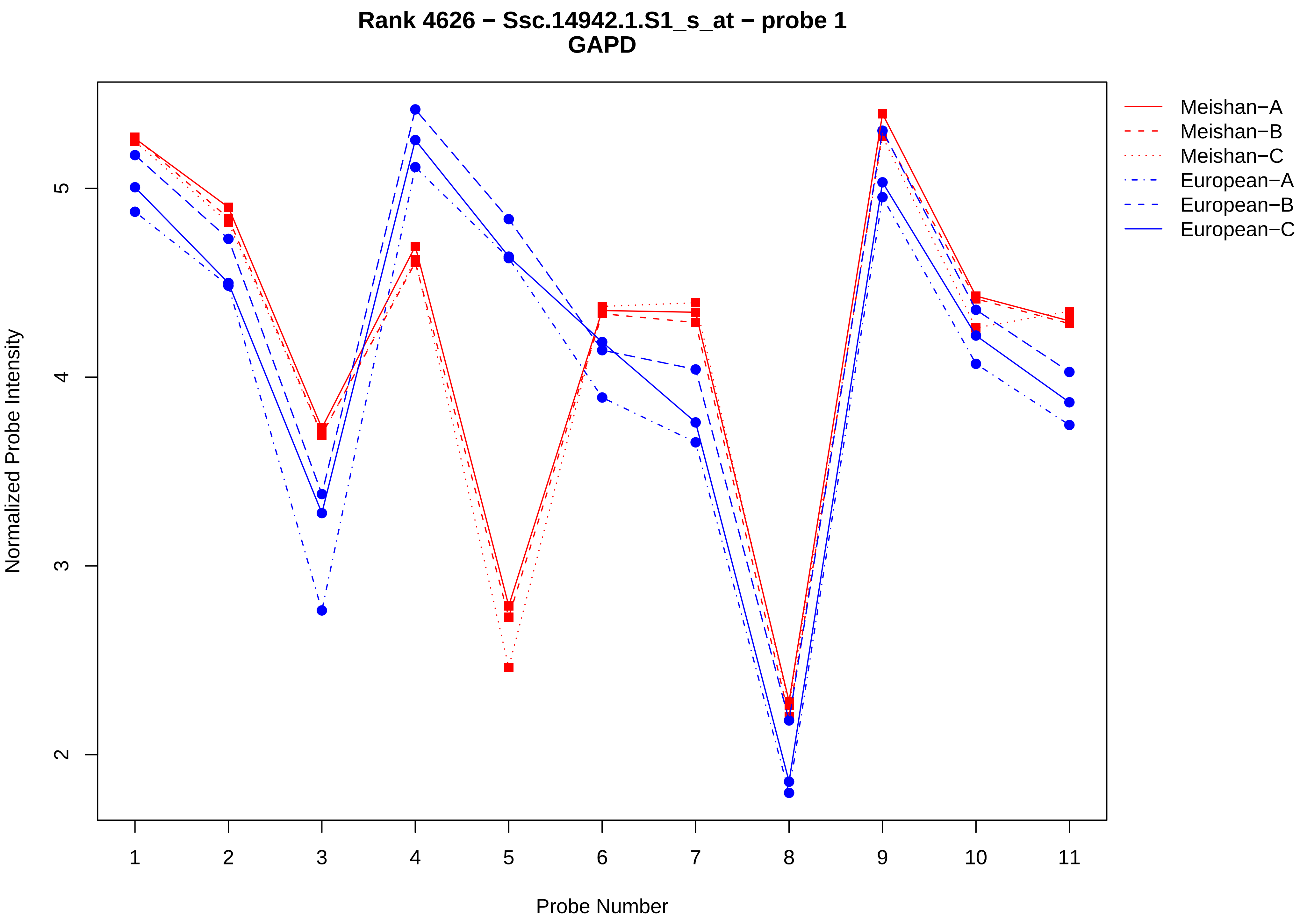

Rank 4626 − Ssc.14942.1.S1_s_at − probe 1
GAPD
Normalized Probe Intensity
Probe Number
2
3
4
5
1
2
3
4
5
6
7
8
9
10
11
Meishan−A
Meishan−B
Meishan−C
European−A
European−B
European−C

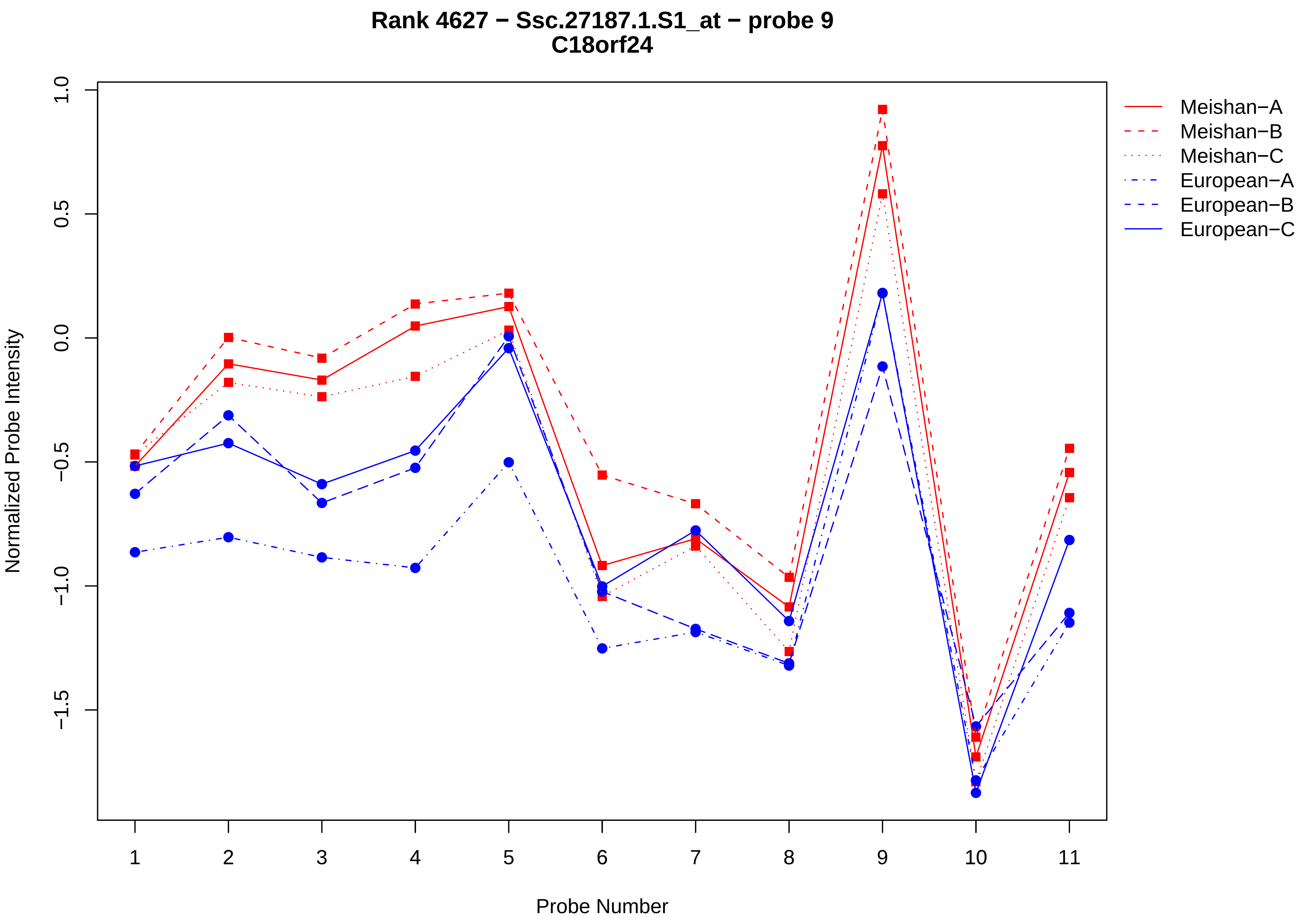

Rank 4627 − Ssc.27187.1.S1_at − probe 9
C18orf24
Normalized Probe Intensity
Probe Number
1.0
0.5
0.0
−0.5
−1.0
−1.5
1
2
3
4
5
6
7
8
9
10
11
Meishan−A
Meishan−B
Meishan−C
European−A
European−B
European−C

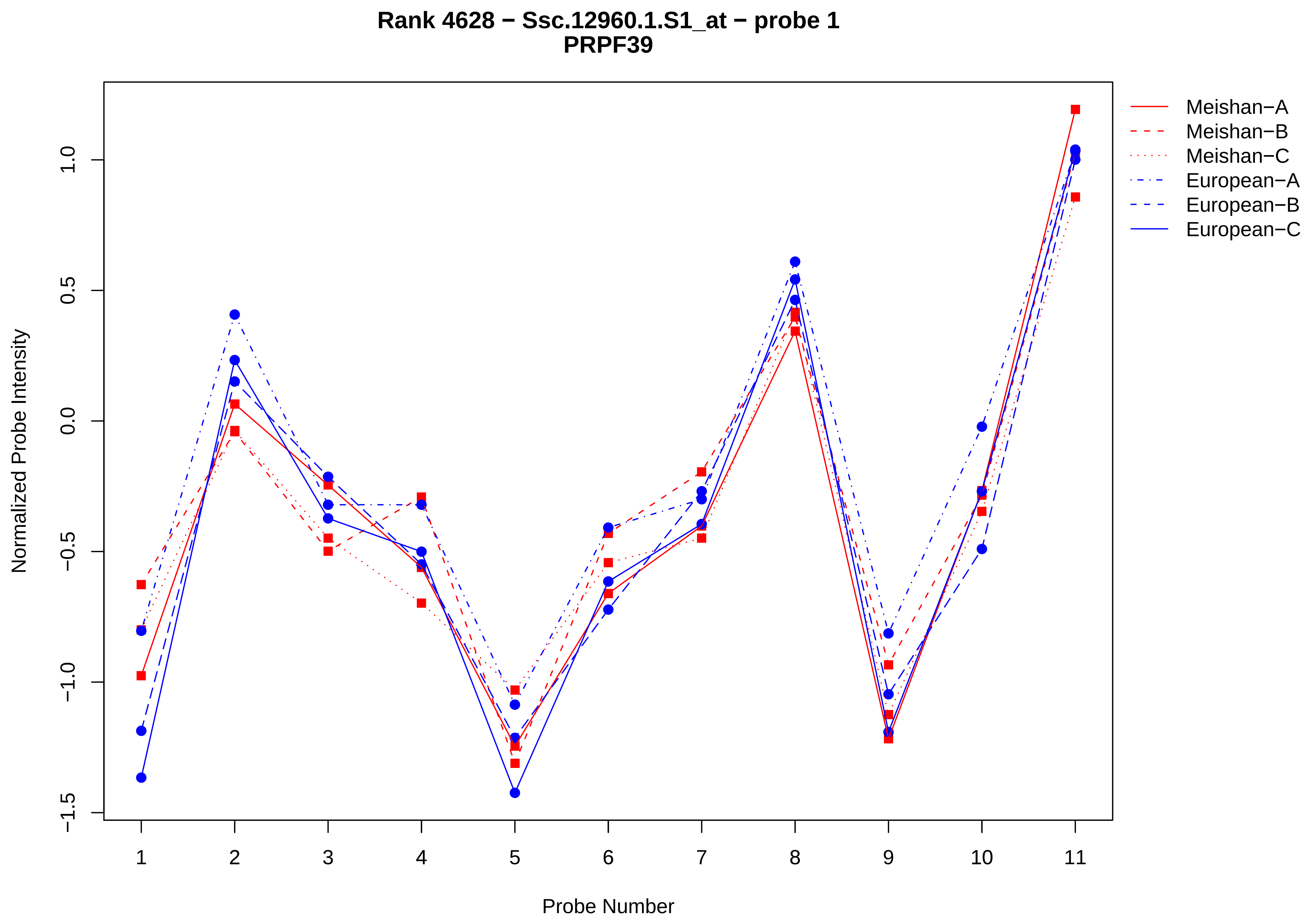

Rank 4628 − Ssc.12960.1.S1_at − probe 1
PRPF39
Normalized Probe Intensity
Probe Number
−1.5
−1.0
−0.5
0.0
0.5
1.0
1
2
3
4
5
6
7
8
9
10
11
Meishan−A
Meishan−B
Meishan−C
European−A
European−B
European−C

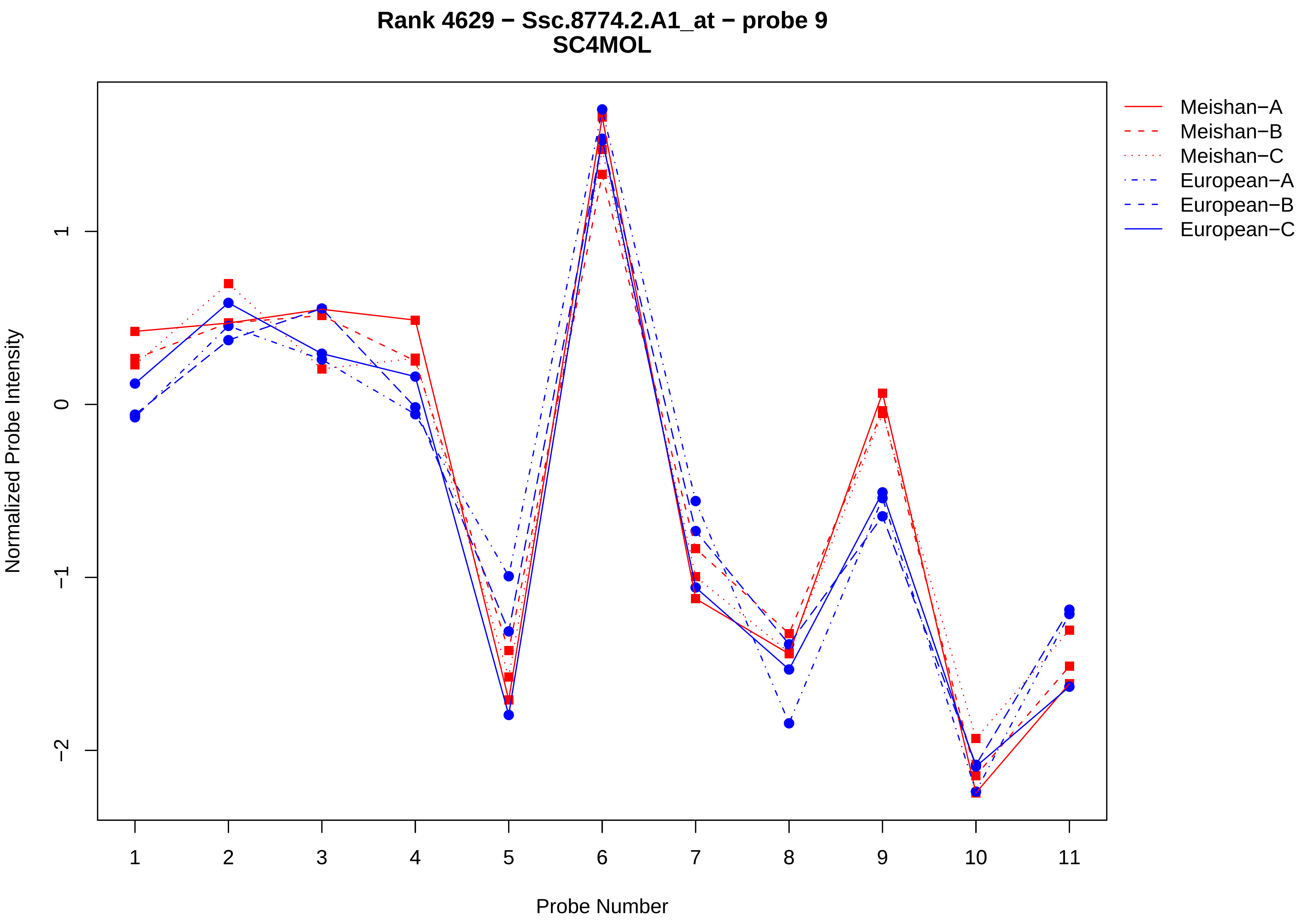
Rank 4629 − Ssc.8774.2.A1_at − probe 9
SC4MOL
Normalized Probe Intensity
Probe Number
Meishan−A
Meishan−B
Meishan−C
European−A
European−B
European−C
−2
−1
0
1
1
2
3
4
5
6
7
8
9
10
11

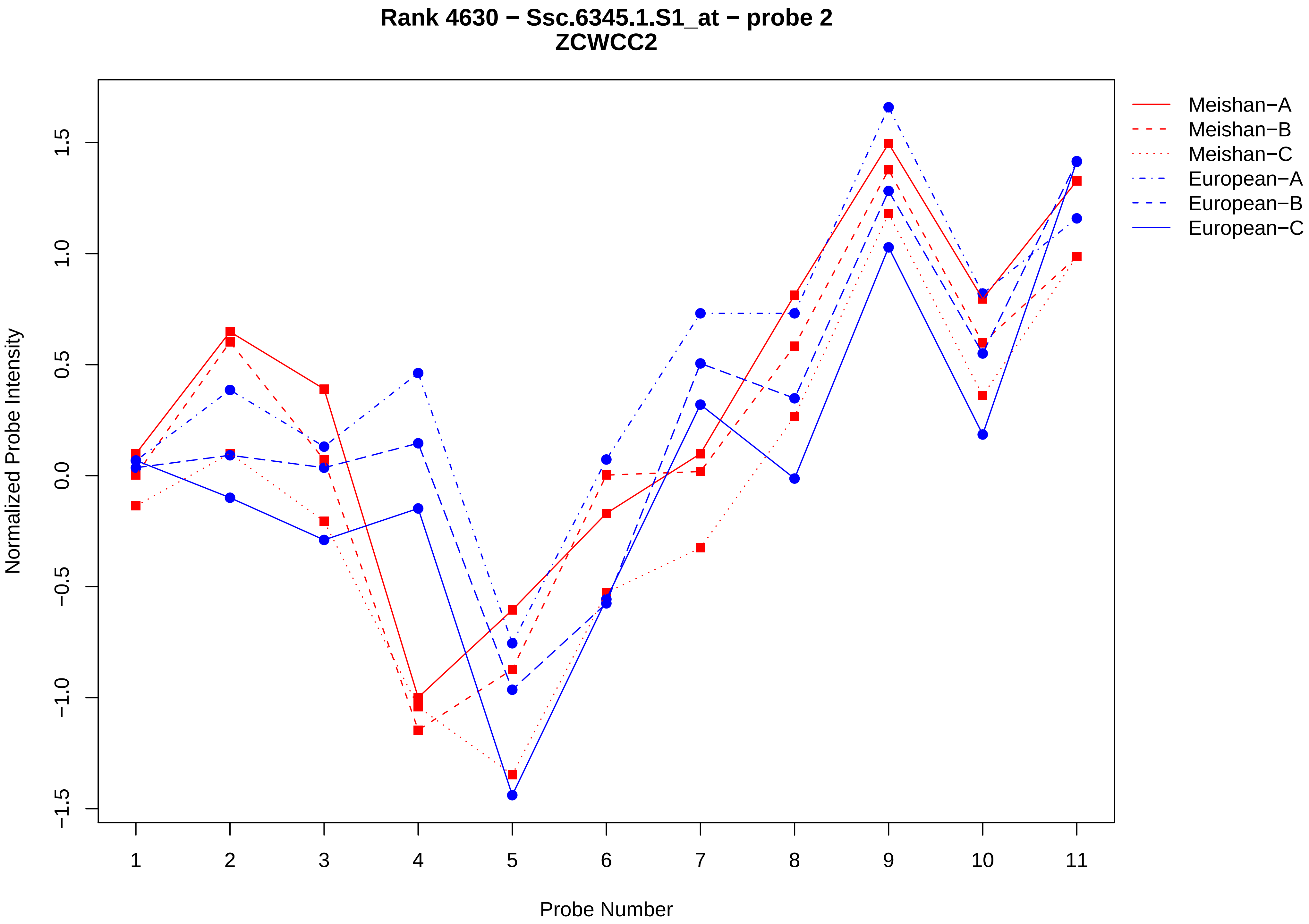

Rank 4630 − Ssc.6345.1.S1_at − probe 2
ZCWCC2
Normalized Probe Intensity
Probe Number
−1.5
−1.0
−0.5
0.0
0.5
1.0
1.5
1
2
3
4
5
6
7
8
9
10
11
Meishan−A
Meishan−B
Meishan−C
European−A
European−B
European−C

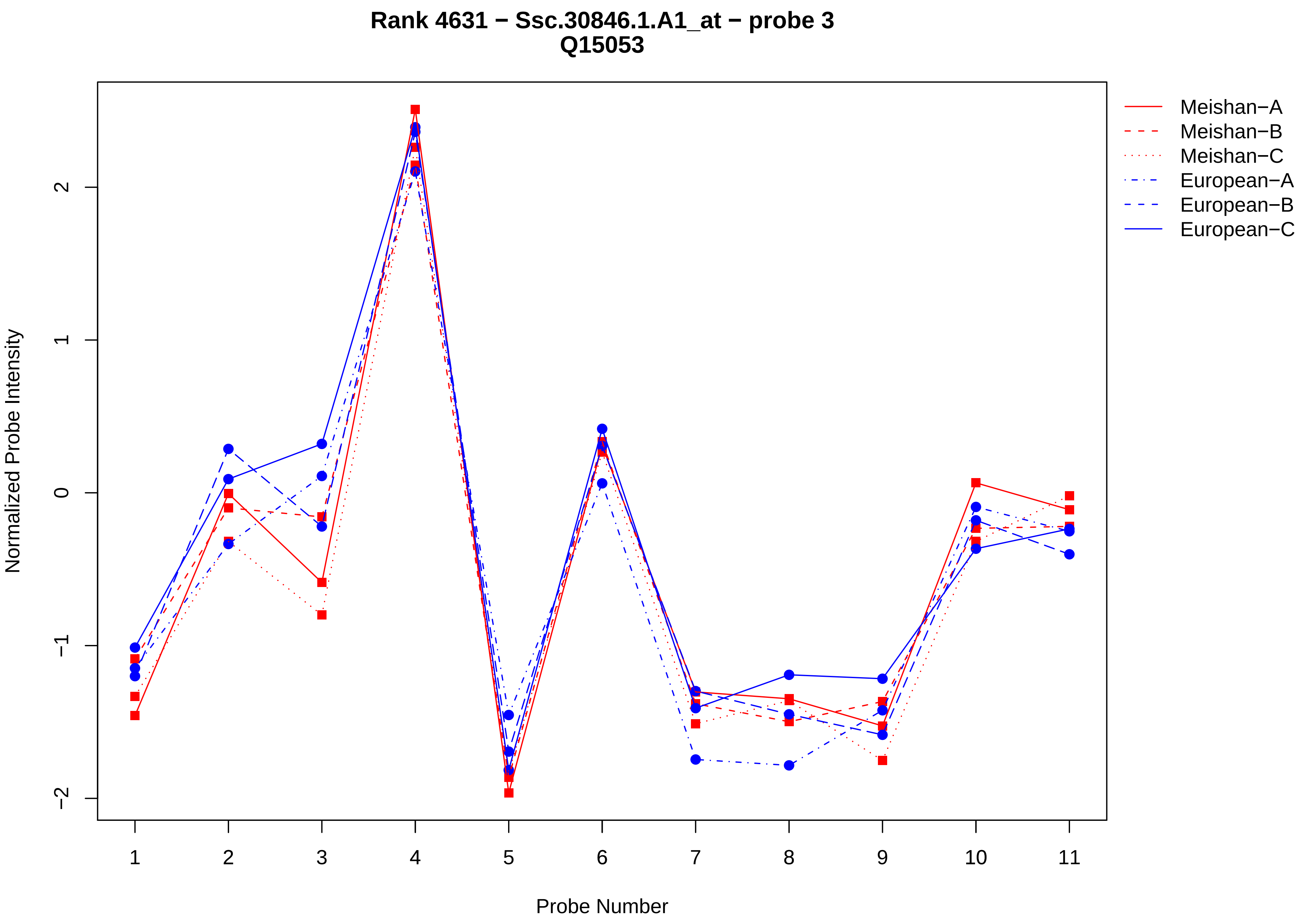

Rank 4631 − Ssc.30846.1.A1_at − probe 3
Q15053
Normalized Probe Intensity
Probe Number
−2
−1
0
1
2
1
2
3
4
5
6
7
8
9
10
11
Meishan−A
Meishan−B
Meishan−C
European−A
European−B
European−C

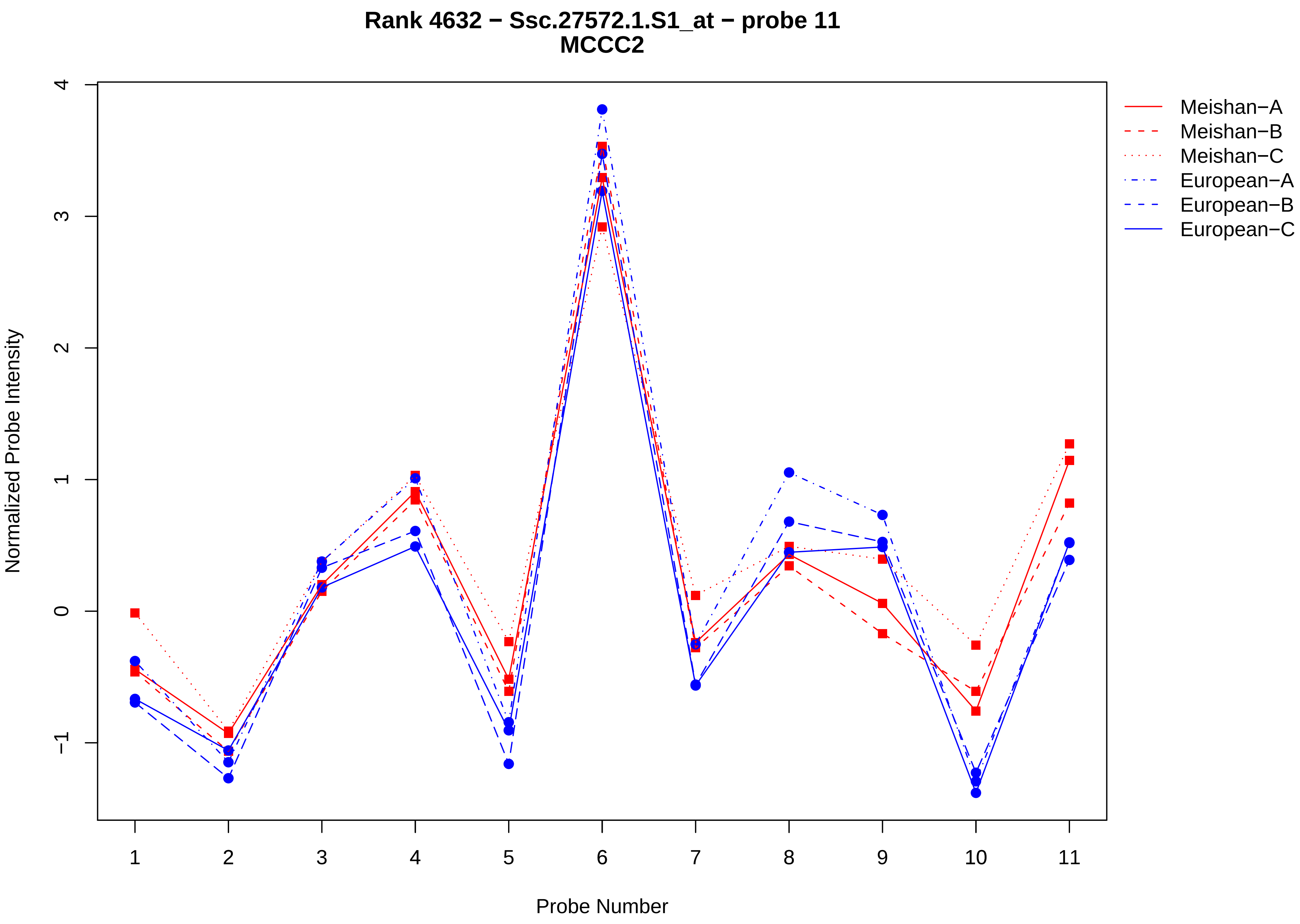

Rank 4632 − Ssc.27572.1.S1_at − probe 11
MCCC2
Normalized Probe Intensity
Probe Number
−1
0
1
2
3
4
1
2
3
4
5
6
7
8
9
10
11
Meishan−A
Meishan−B
Meishan−C
European−A
European−B
European−C

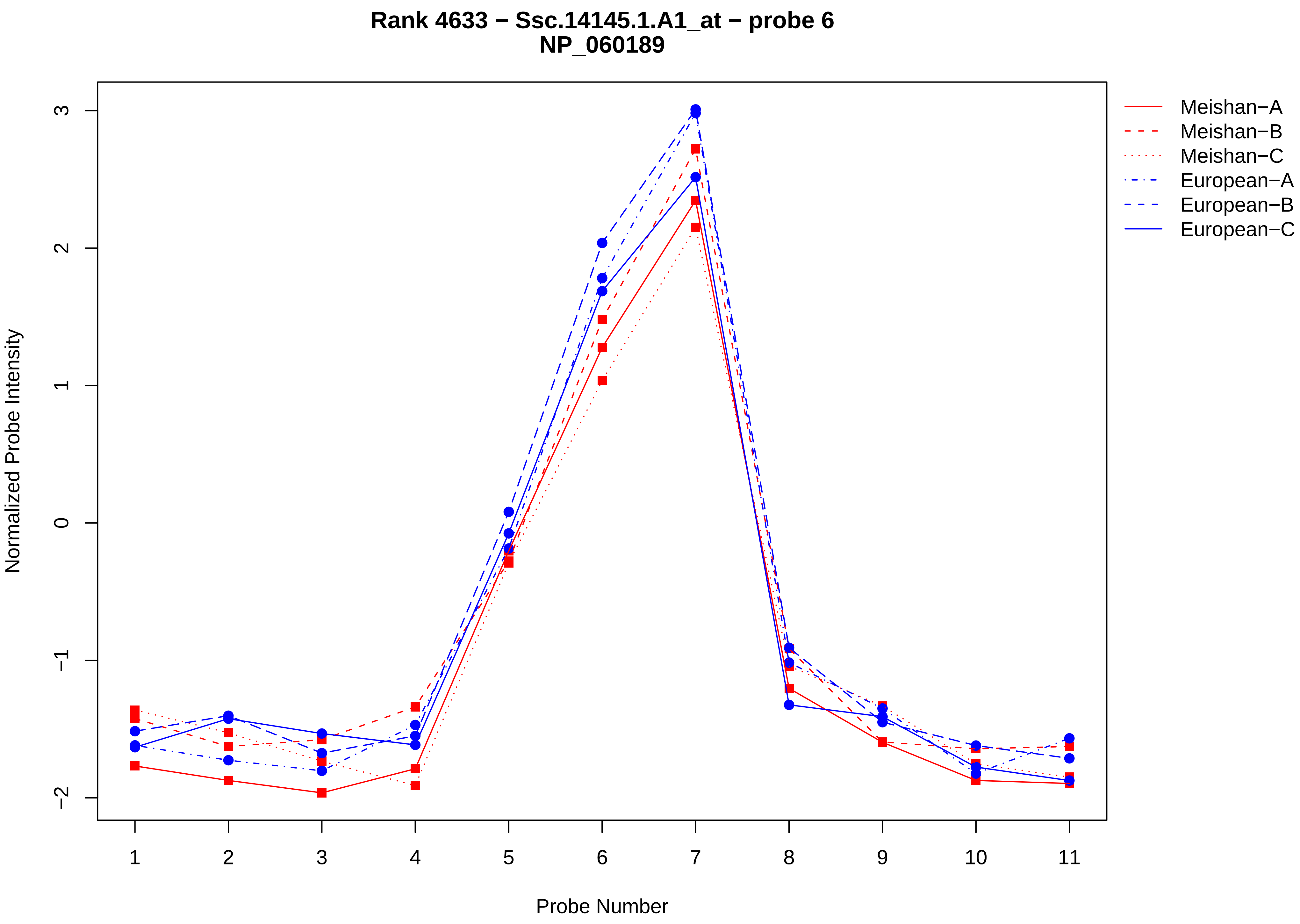

Rank 4633 − Ssc.14145.1.A1_at − probe 6
NP_060189
Normalized Probe Intensity
Probe Number
Meishan−A
Meishan−B
Meishan−C
European−A
European−B
European−C

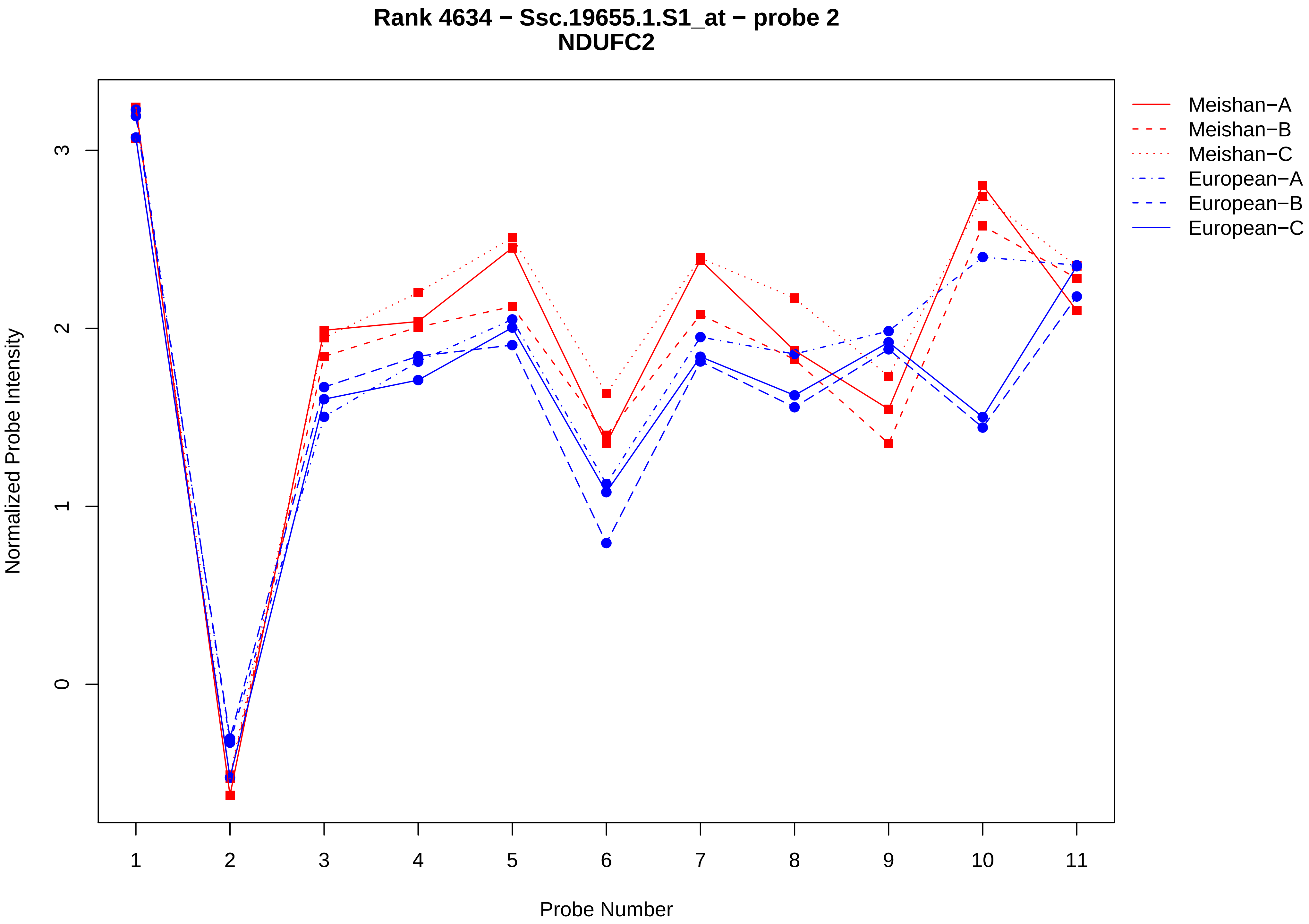

Rank 4634 − Ssc.19655.1.S1_at − probe 2
NDUFC2
Normalized Probe Intensity
Probe Number
0
1
2
3
1
2
3
4
5
6
7
8
9
10
11
Meishan−A
Meishan−B
Meishan−C
European−A
European−B
European−C

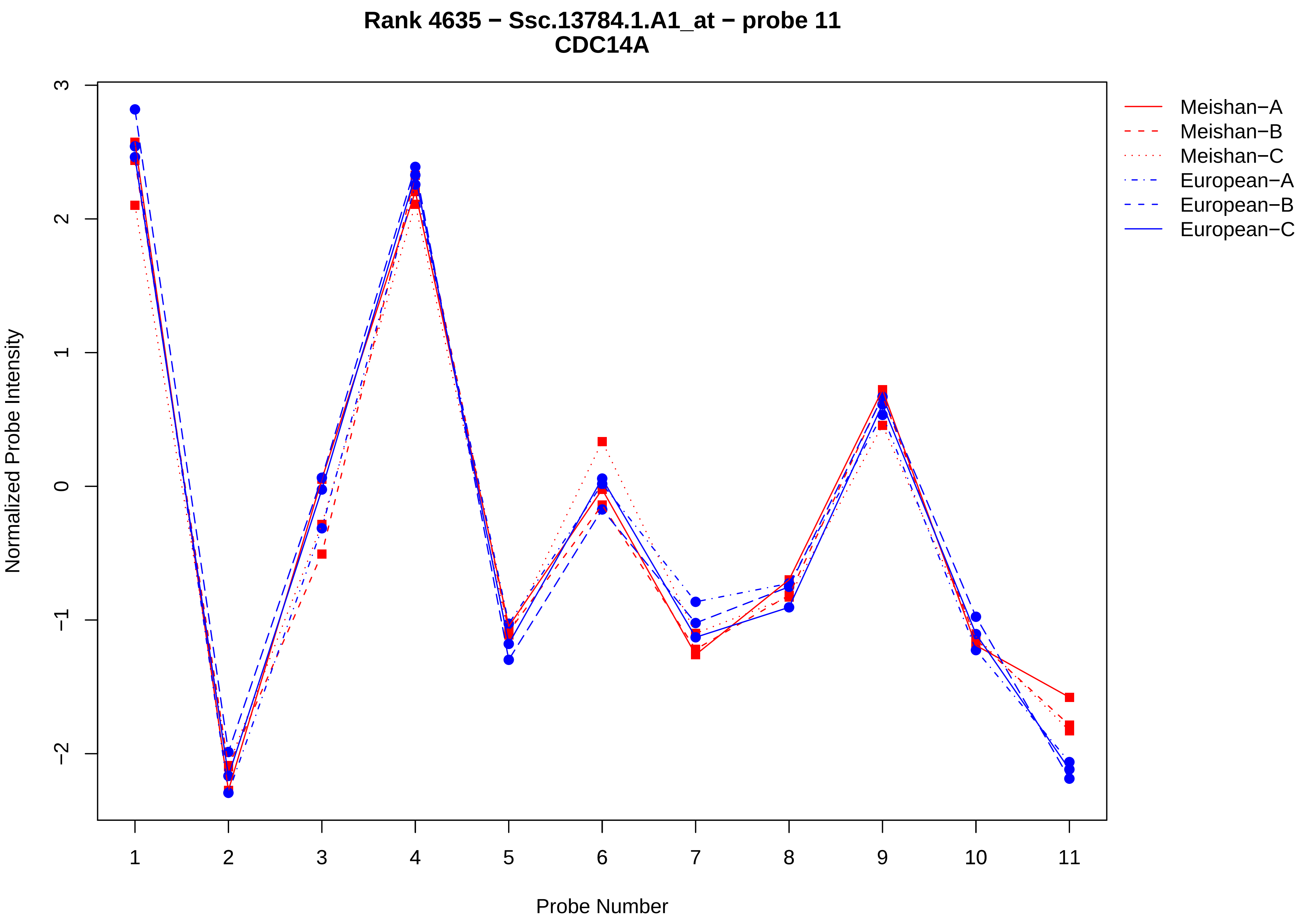

Rank 4635 − Ssc.13784.1.A1_at − probe 11
CDC14A
Normalized Probe Intensity
Probe Number
−2
−1
0
1
2
3
1
2
3
4
5
6
7
8
9
10
11
Meishan−A
Meishan−B
Meishan−C
European−A
European−B
European−C
