# Supplementary material for: Identification of SNPs and INDELS in swine transcribed sequences using short oligonucleotide microarrays
Source: BMC Genomics. 2008 May 29;9:252. doi: 10.1186/1471-2164-9-252 (PMC2442091; doi:10.1186/1471-2164-9-252)
Supplement: Additional File 3 — Porcine_Candidate_SFPs_2FC. Intensity plots of SFPs with q < 0.5 with |fold change| > 2. [file 1471-2164-9-252-S3.pdf]

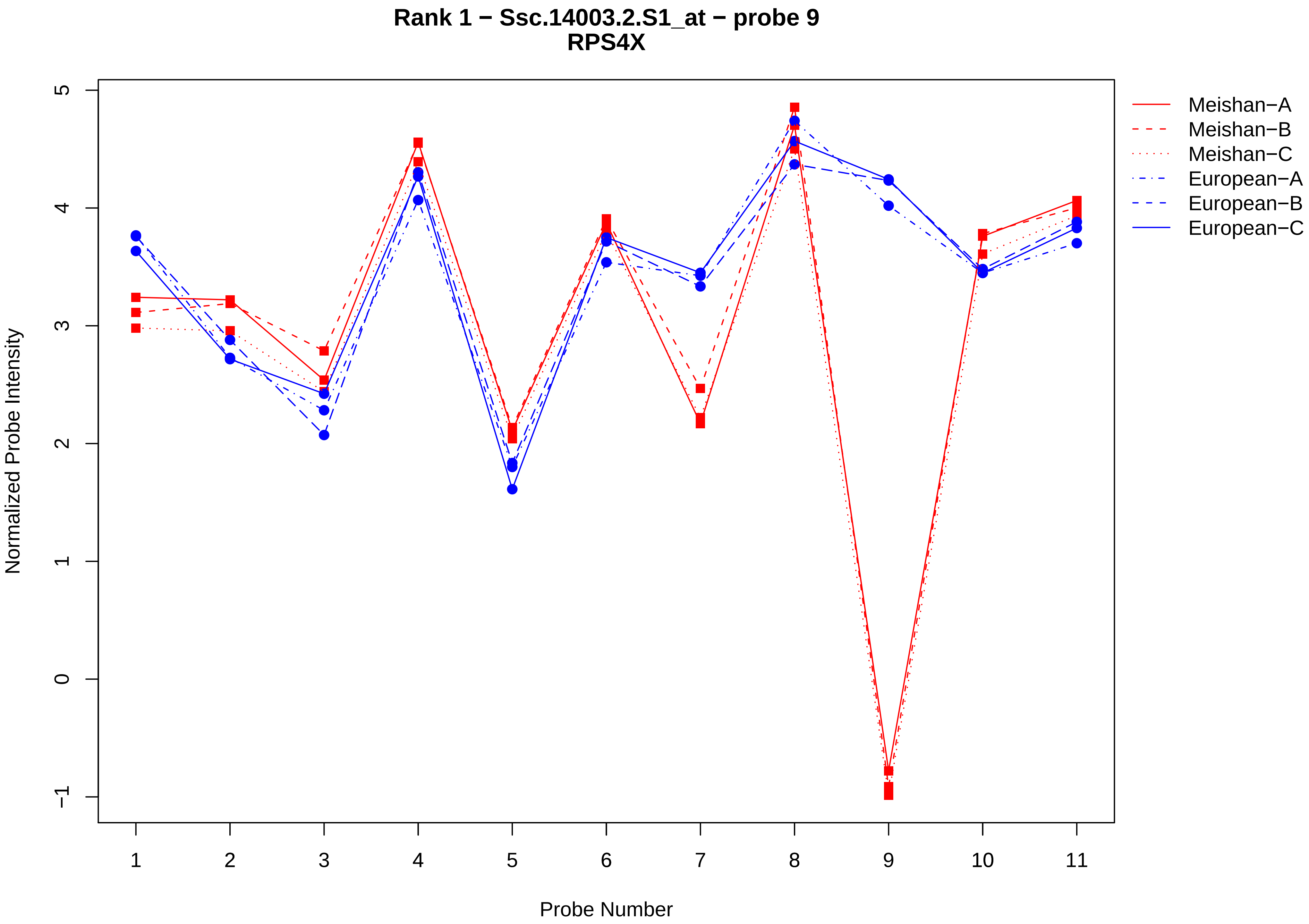

Rank 1 − Ssc.14003.2.S1_at − probe 9
RPS4X
Normalized Probe Intensity
Probe Number
−1
0
1
2
3
4
5
1
2
3
4
5
6
7
8
9
10
11
Meishan−A
Meishan−B
Meishan−C
European−A
European−B
European−C

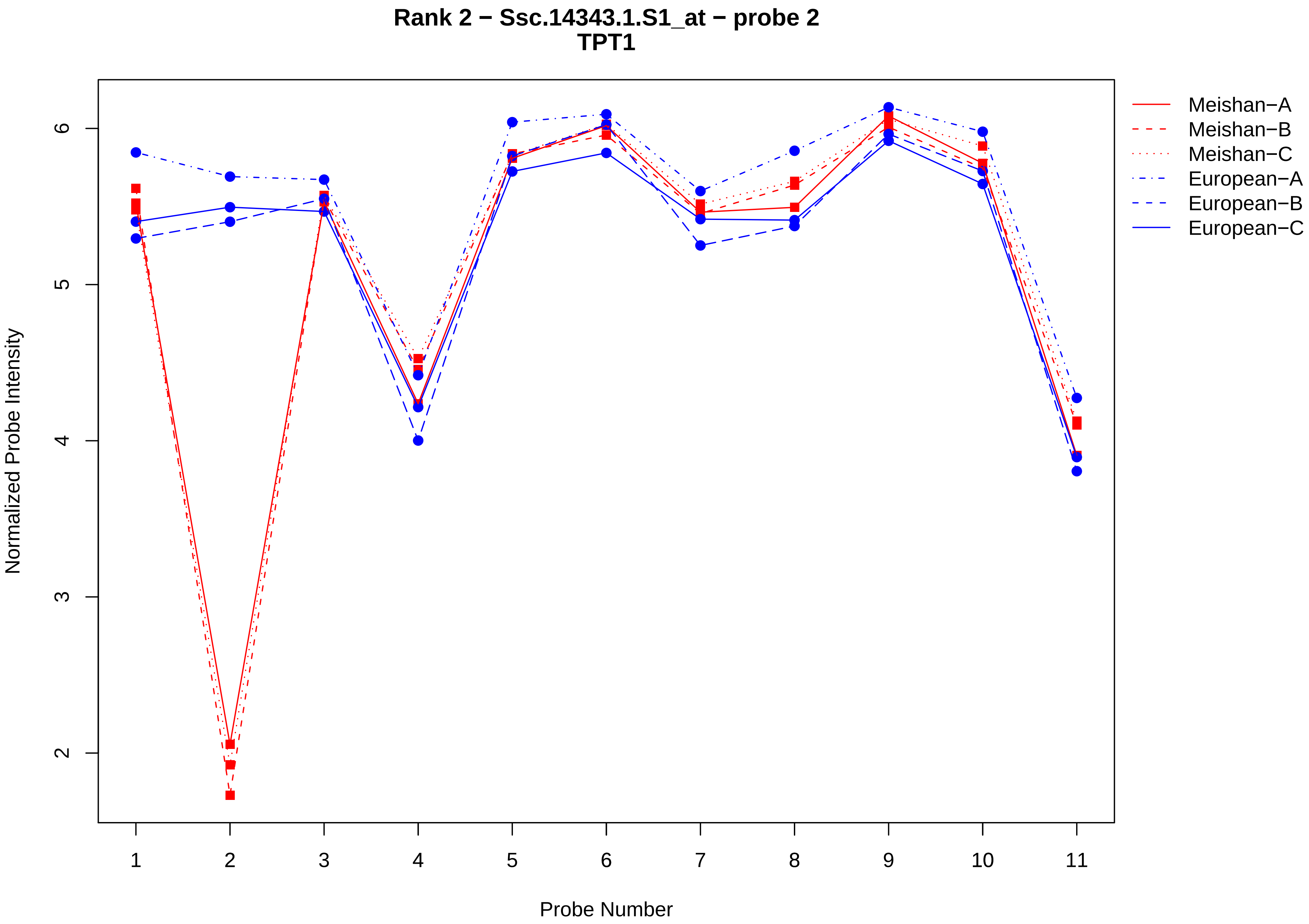

Rank 2 − Ssc.14343.1.S1_at − probe 2
TPT1
Normalized Probe Intensity
Probe Number
Meishan−A
Meishan−B
Meishan−C
European−A
European−B
European−C

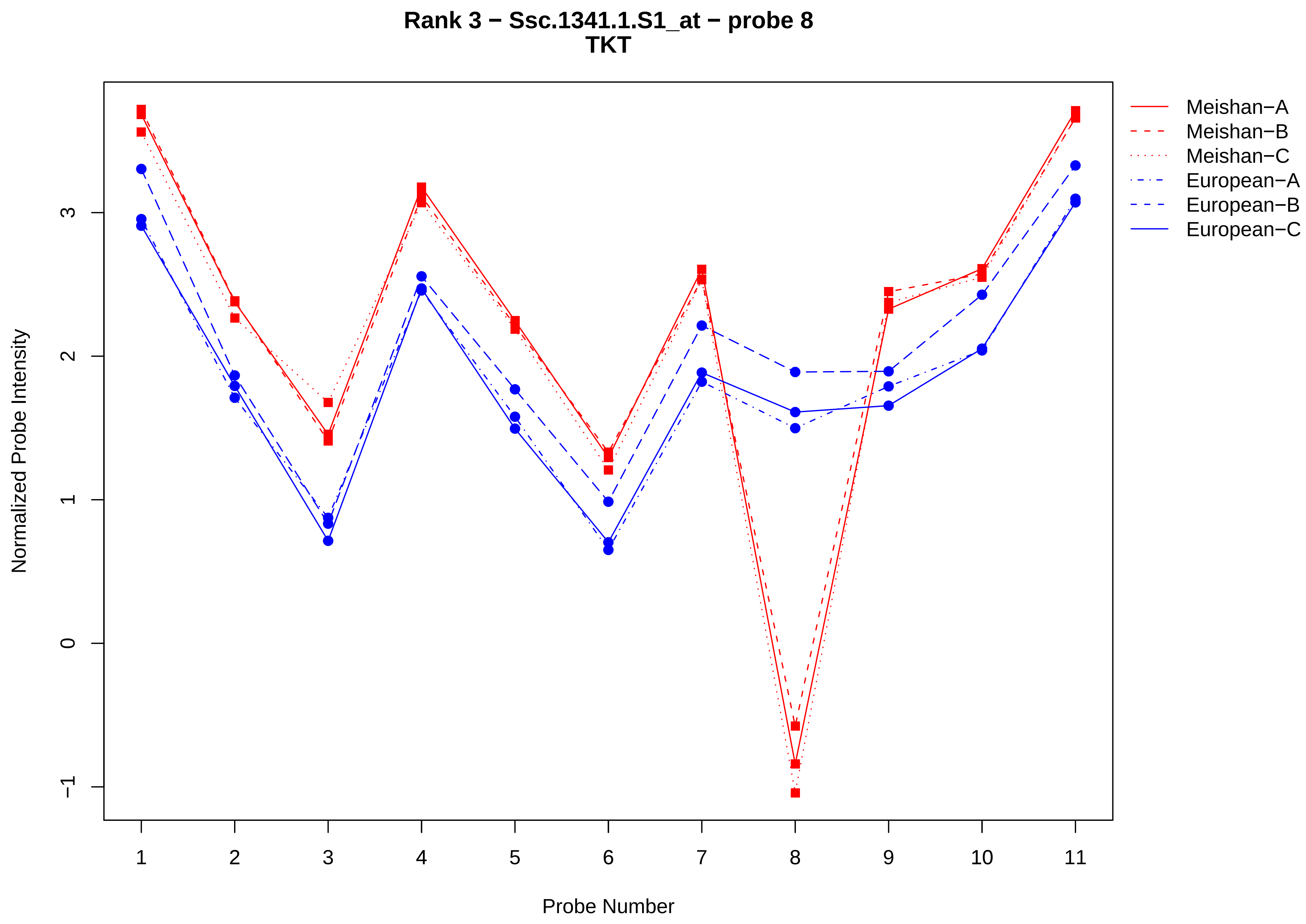
Rank 3 − Ssc.1341.1.S1_at − probe 8
TKT
Normalized Probe Intensity
Probe Number
−1
0
1
2
3
1
2
3
4
5
6
7
8
9
10
11
Meishan−A
Meishan−B
Meishan−C
European−A
European−B
European−C

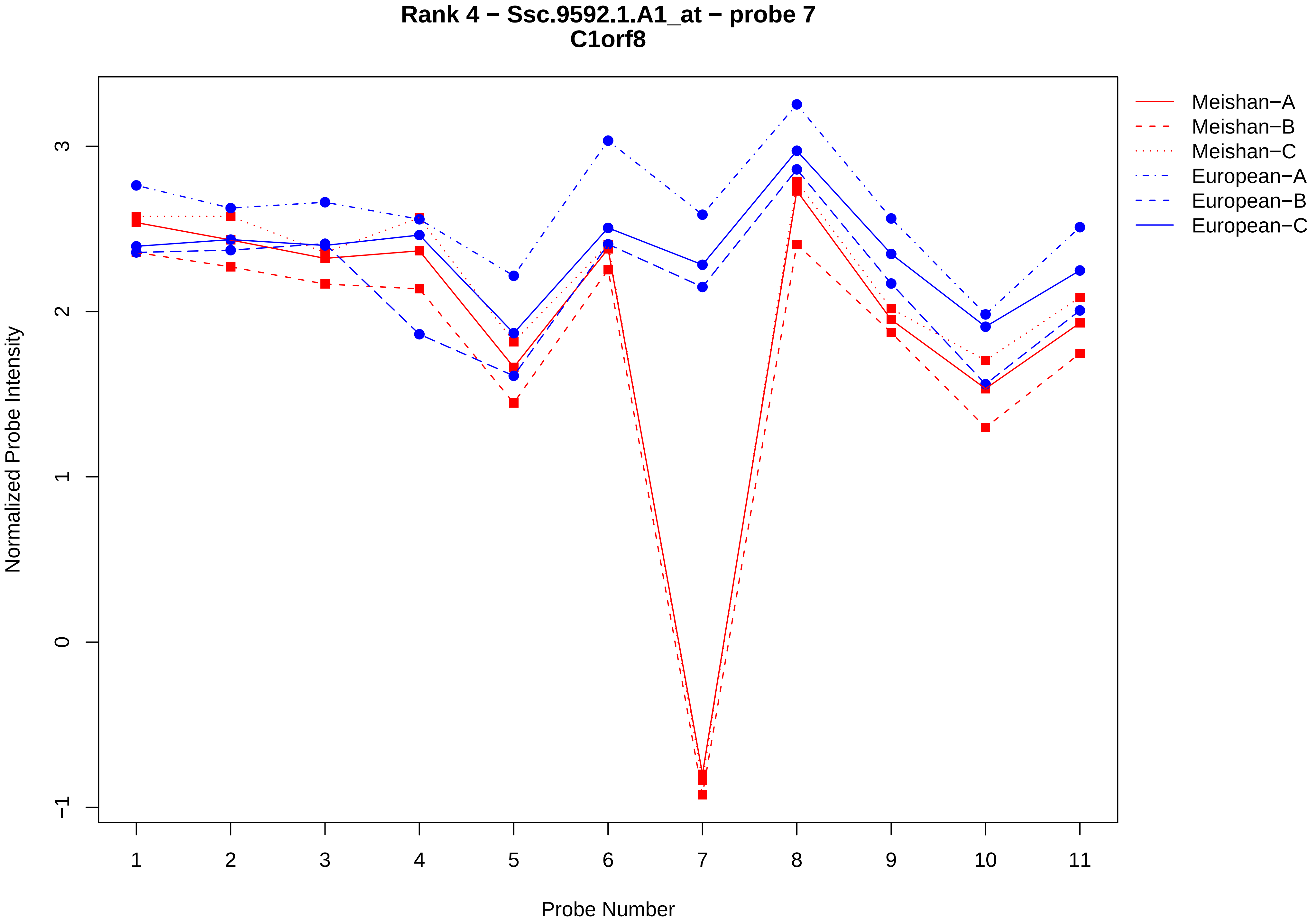
Rank 4 − Ssc.9592.1.A1_at − probe 7
C1orf8
Normalized Probe Intensity
Probe Number
Meishan−A
Meishan−B
Meishan−C
European−A
European−B
European−C

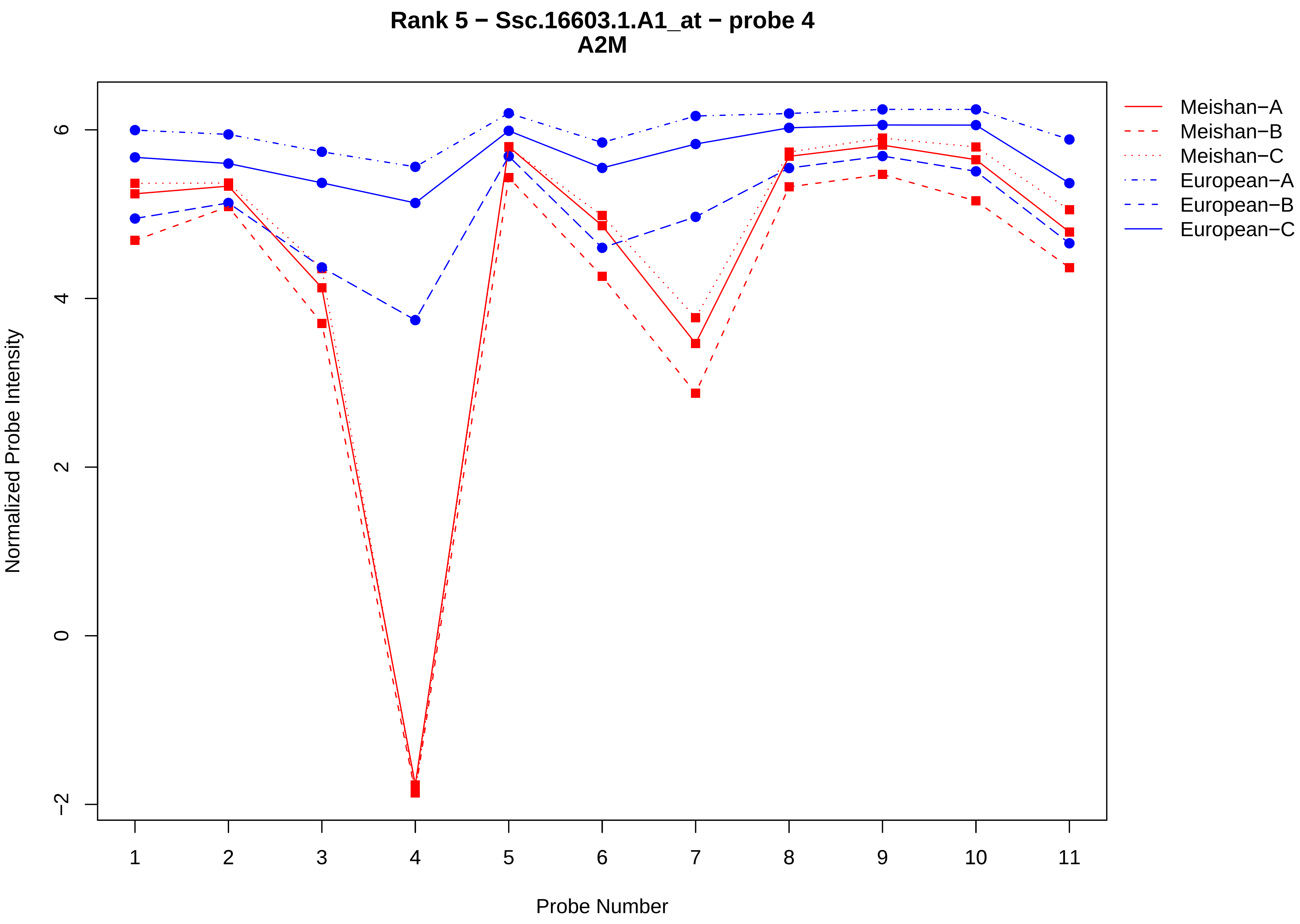

Rank 5 − Ssc.16603.1.A1_at − probe 4
A2M
Normalized Probe Intensity
Probe Number
−2
0
2
4
6
1
2
3
4
5
6
7
8
9
10
11
Meishan−A
Meishan−B
Meishan−C
European−A
European−B
European−C

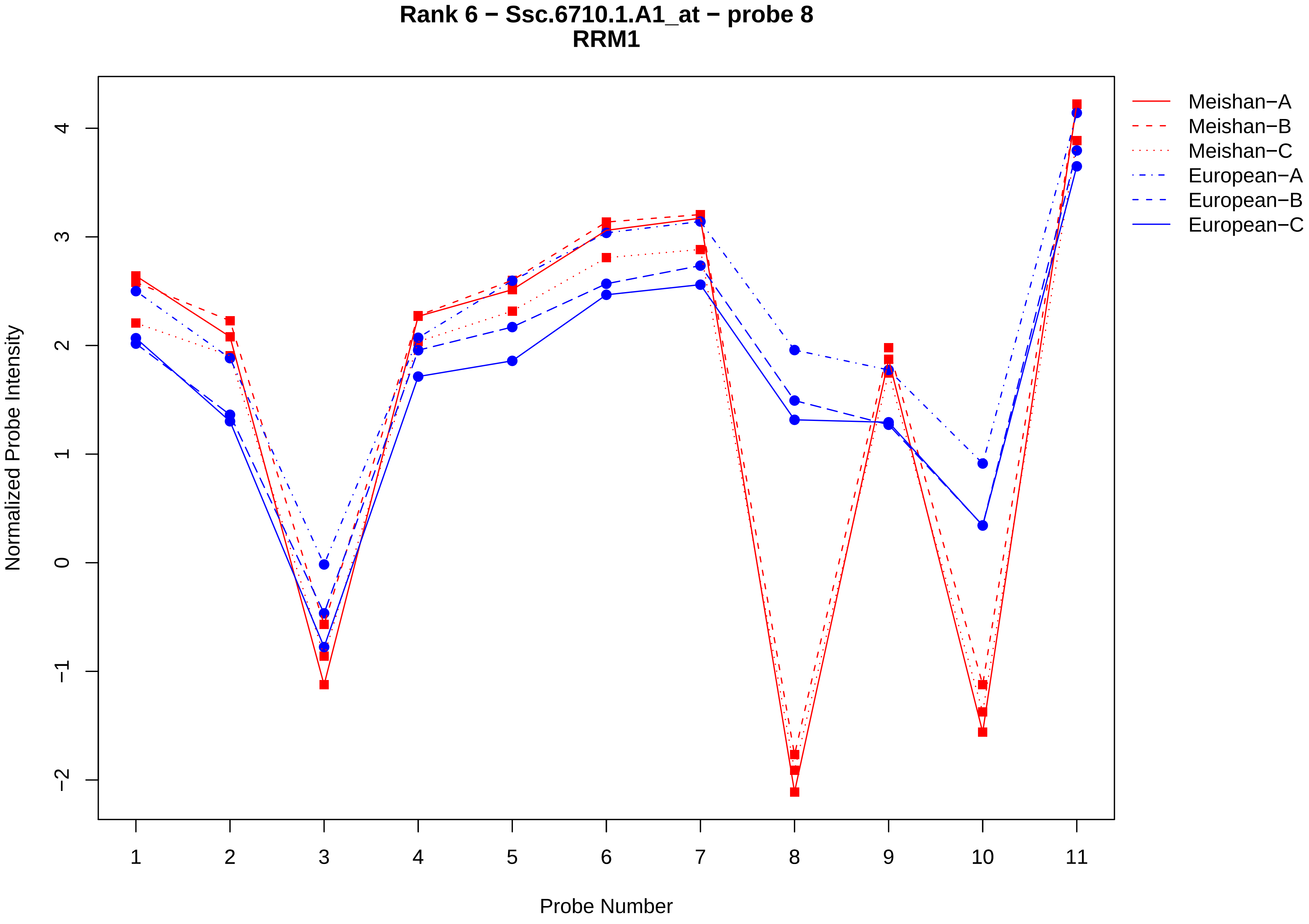

Rank 6 − Ssc.6710.1.A1_at − probe 8
RRM1
Normalized Probe Intensity
Probe Number
−2
−1
0
1
2
3
4
1
2
3
4
5
6
7
8
9
10
11
Meishan−A
Meishan−B
Meishan−C
European−A
European−B
European−C

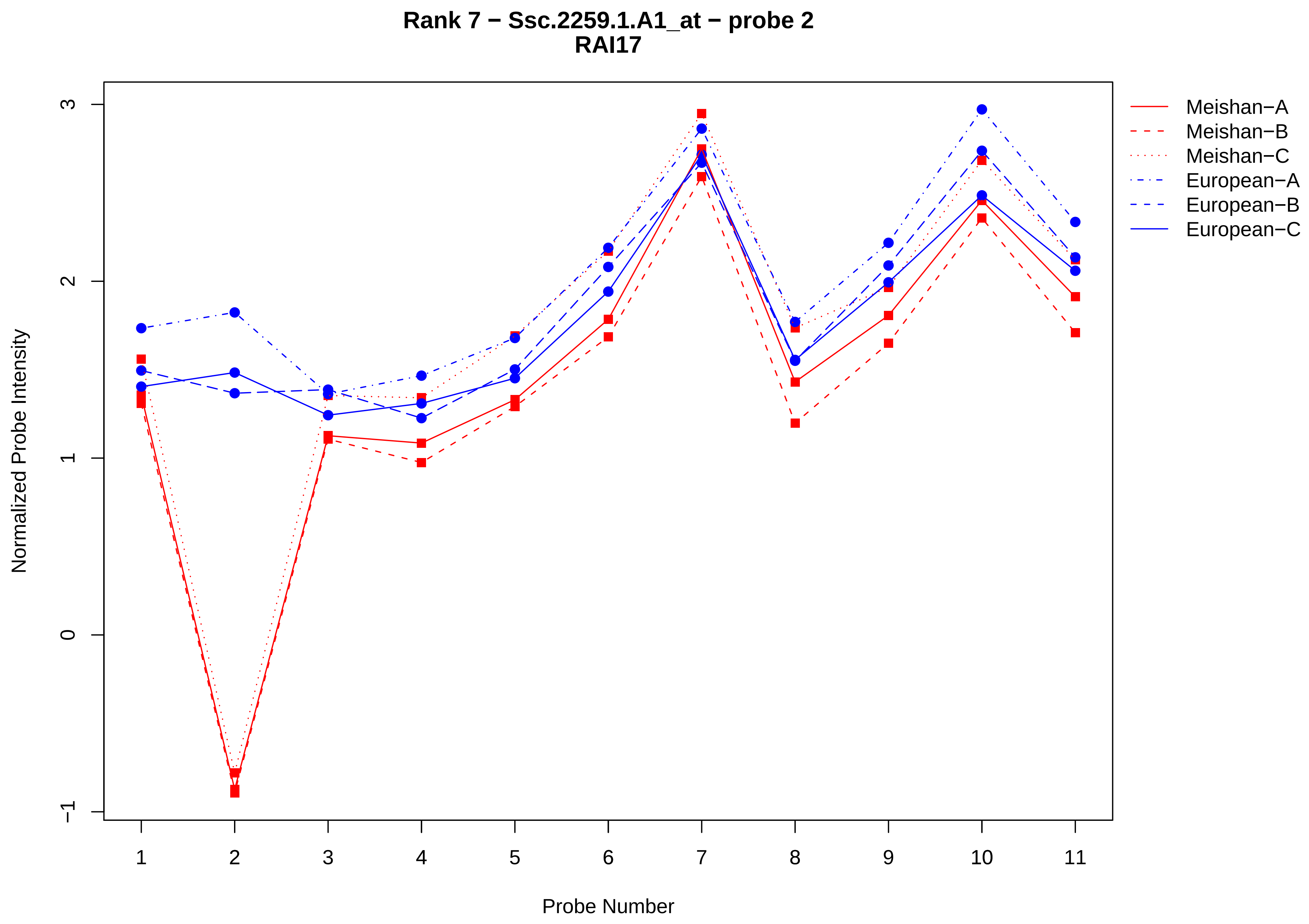

Rank 7 − Ssc.2259.1.A1_at − probe 2
RAI17
Normalized Probe Intensity
Probe Number
Meishan−A
Meishan−B
Meishan−C
European−A
European−B
European−C

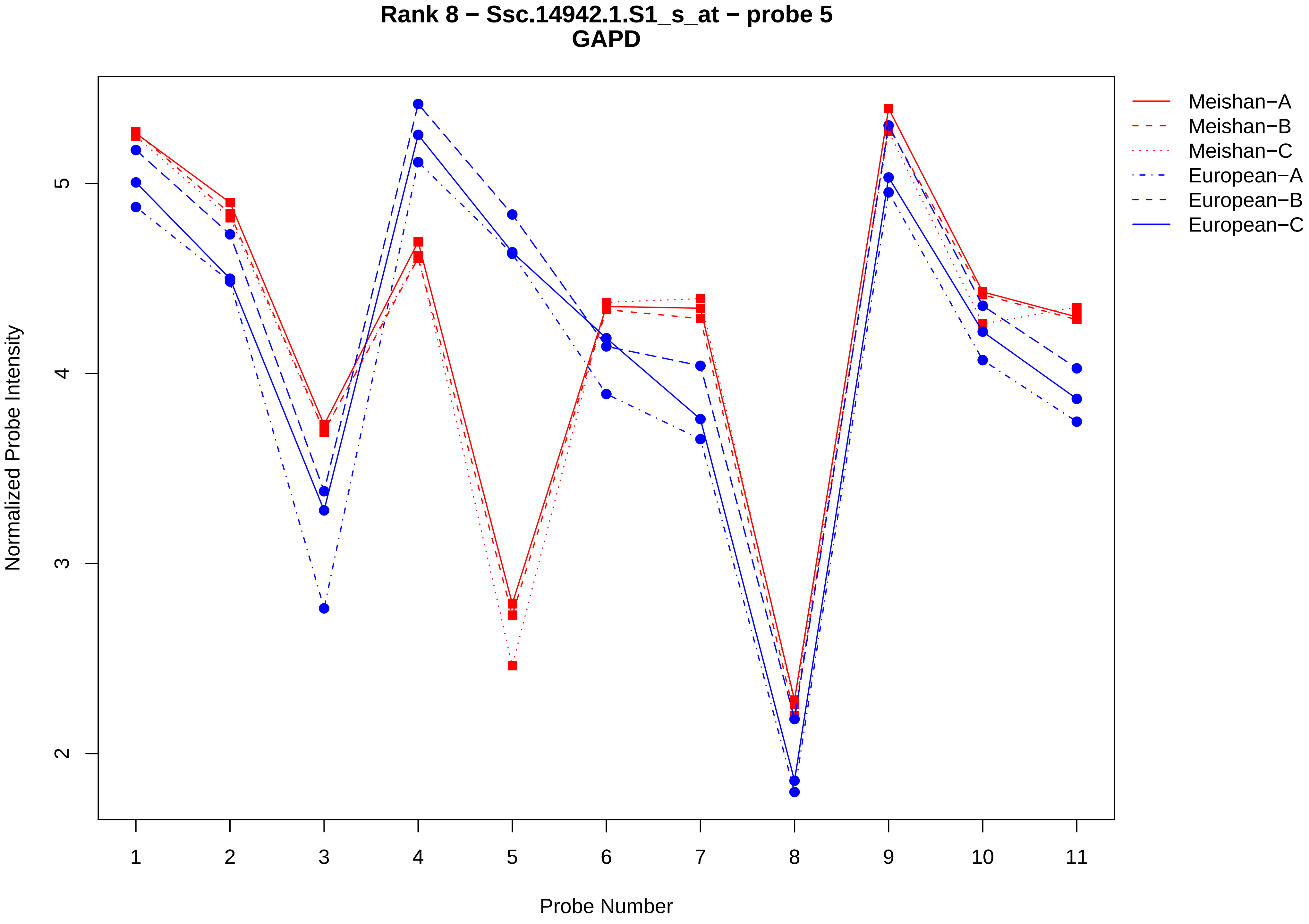

Rank 8 − Ssc.14942.1.S1_s_at − probe 5
GAPD
Normalized Probe Intensity
Probe Number
2
3
4
5
1
2
3
4
5
6
7
8
9
10
11
Meishan−A
Meishan−B
Meishan−C
European−A
European−B
European−C

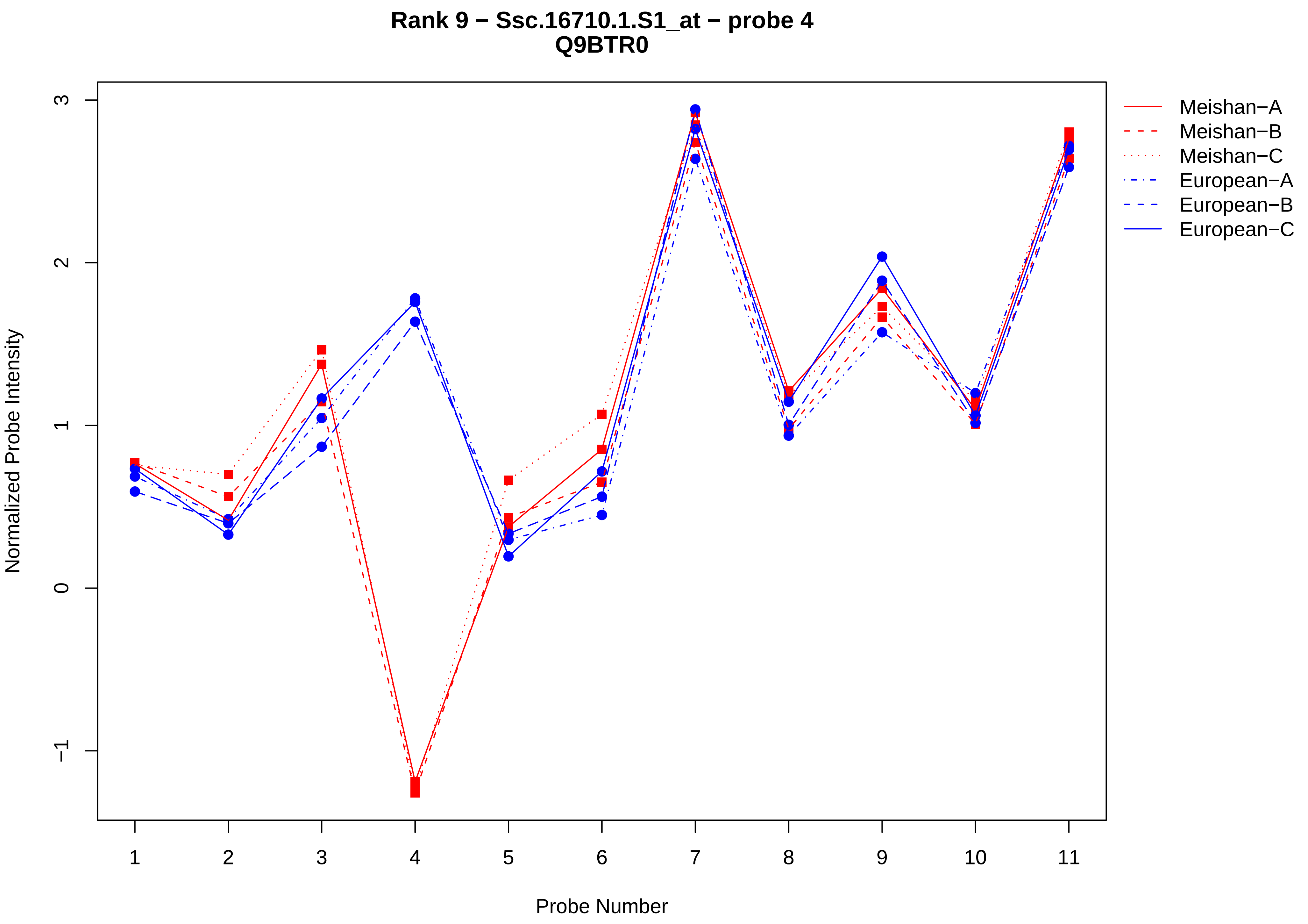

Rank 9 − Ssc.16710.1.S1_at − probe 4
Q9BTR0
Normalized Probe Intensity
Probe Number
Meishan−A
Meishan−B
Meishan−C
European−A
European−B
European−C
−1
0
1
2
3
1
2
3
4
5
6
7
8
9
10
11

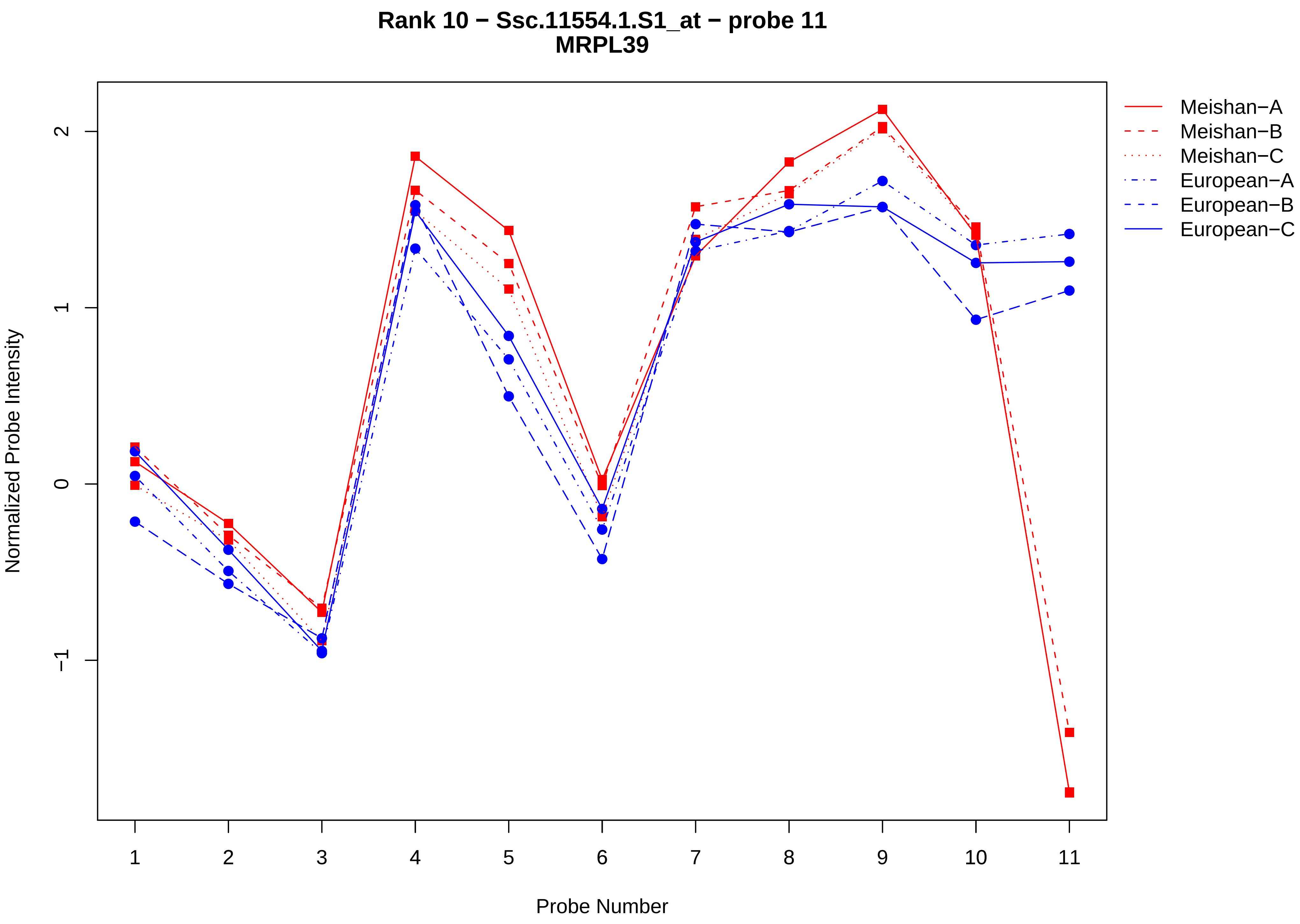

Rank 10 − Ssc.11554.1.S1_at − probe 11
MRPL39
Normalized Probe Intensity
Probe Number
−1
0
1
2
1
2
3
4
5
6
7
8
9
10
11
Meishan−A
Meishan−B
Meishan−C
European−A
European−B
European−C

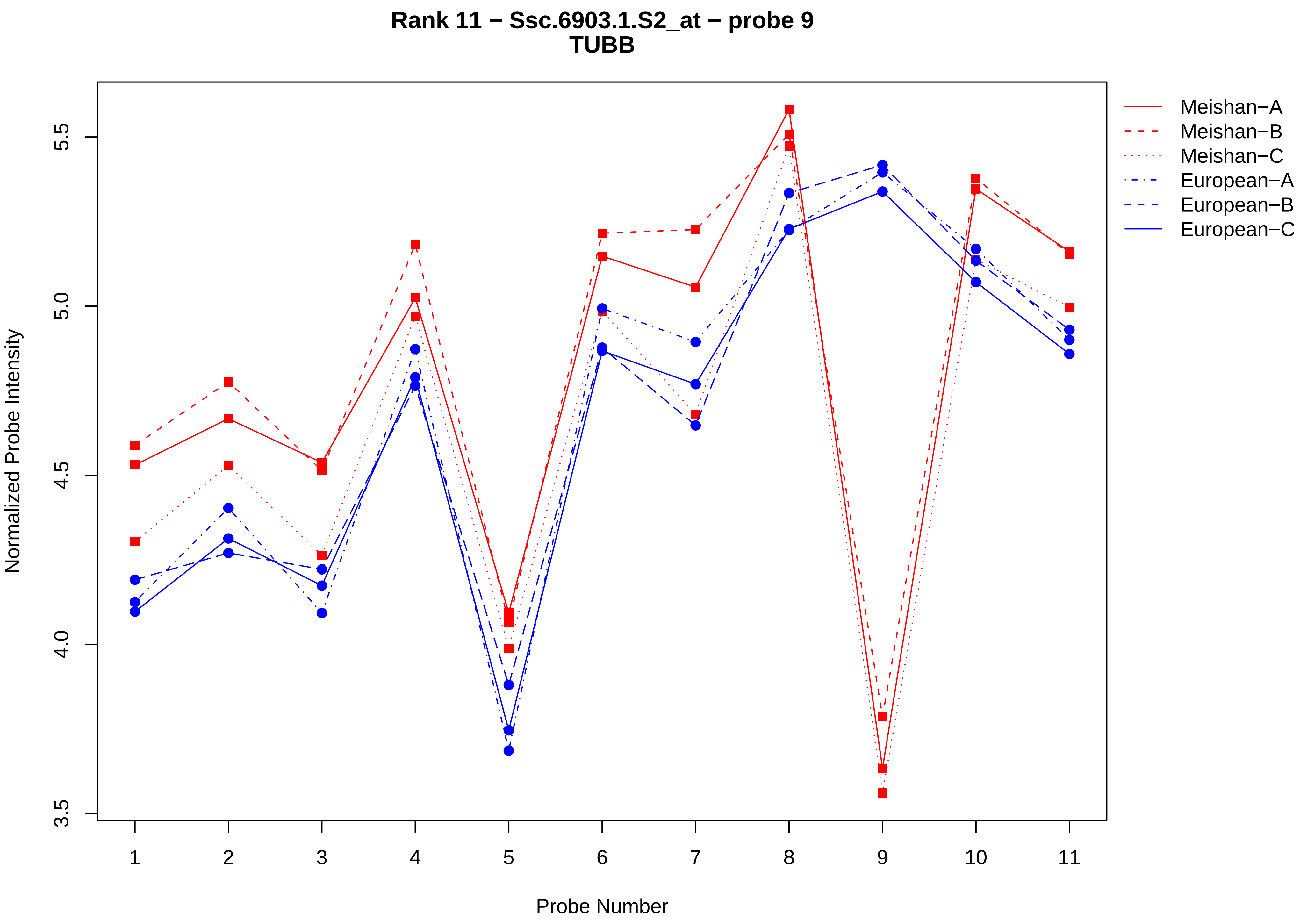

Rank 11 − Ssc.6903.1.S2_at − probe 9
TUBB
Normalized Probe Intensity
Probe Number
3.5
4.0
4.5
5.0
5.5
1
2
3
4
5
6
7
8
9
10
11
Meishan−A
Meishan−B
Meishan−C
European−A
European−B
European−C

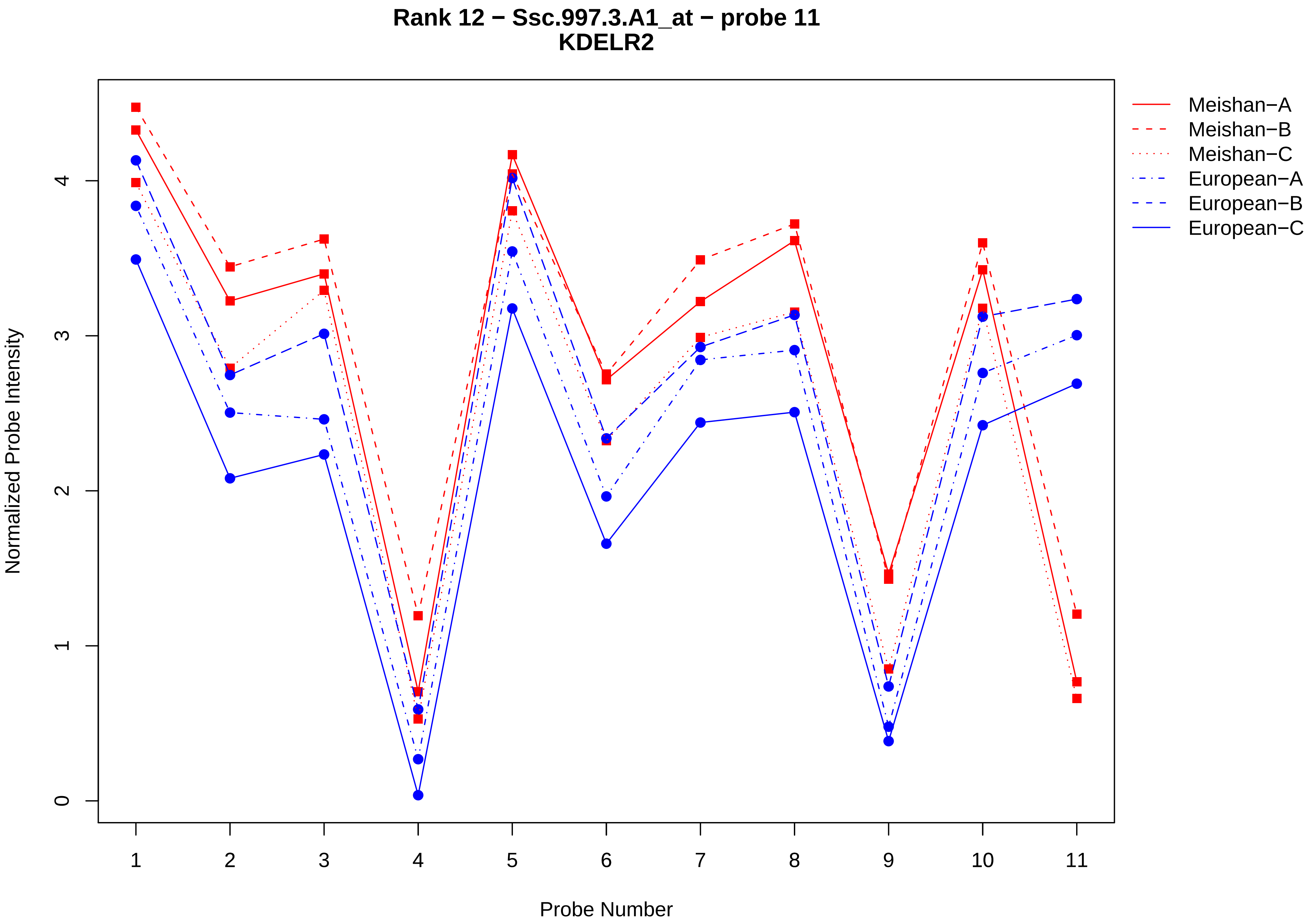
Rank 12 − Ssc.997.3.A1_at − probe 11
KDELR2
Normalized Probe Intensity
0
1
2
3
4
1
2
3
4
5
6
7
8
9
10
11
Probe Number
Meishan−A
Meishan−B
Meishan−C
European−A
European−B
European−C

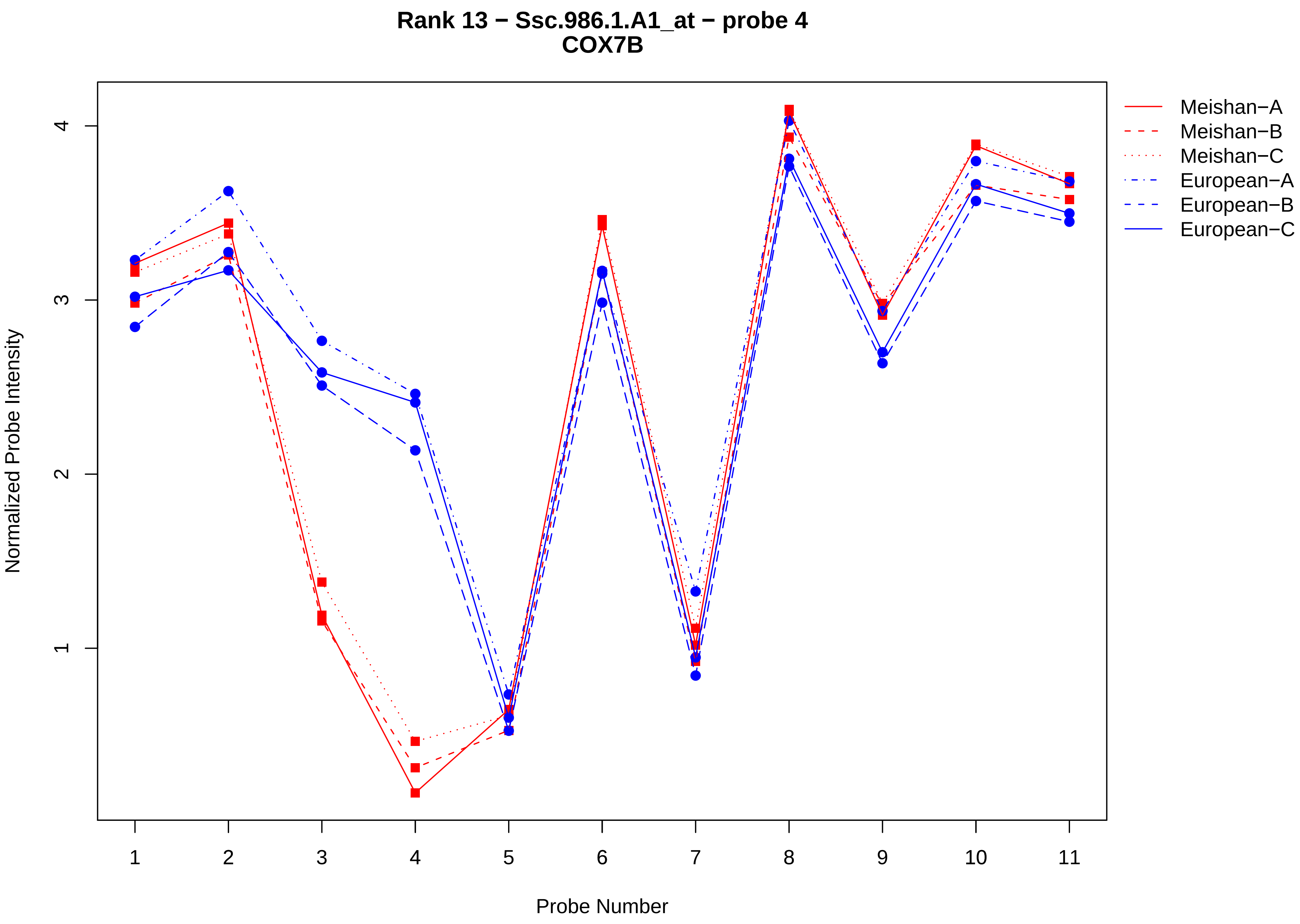
Rank 13 − Ssc.986.1.A1_at − probe 4
COX7B
Normalized Probe Intensity
1
2
3
4
1
2
3
4
5
6
7
8
9
10
11
Probe Number
Meishan−A
Meishan−B
Meishan−C
European−A
European−B
European−C

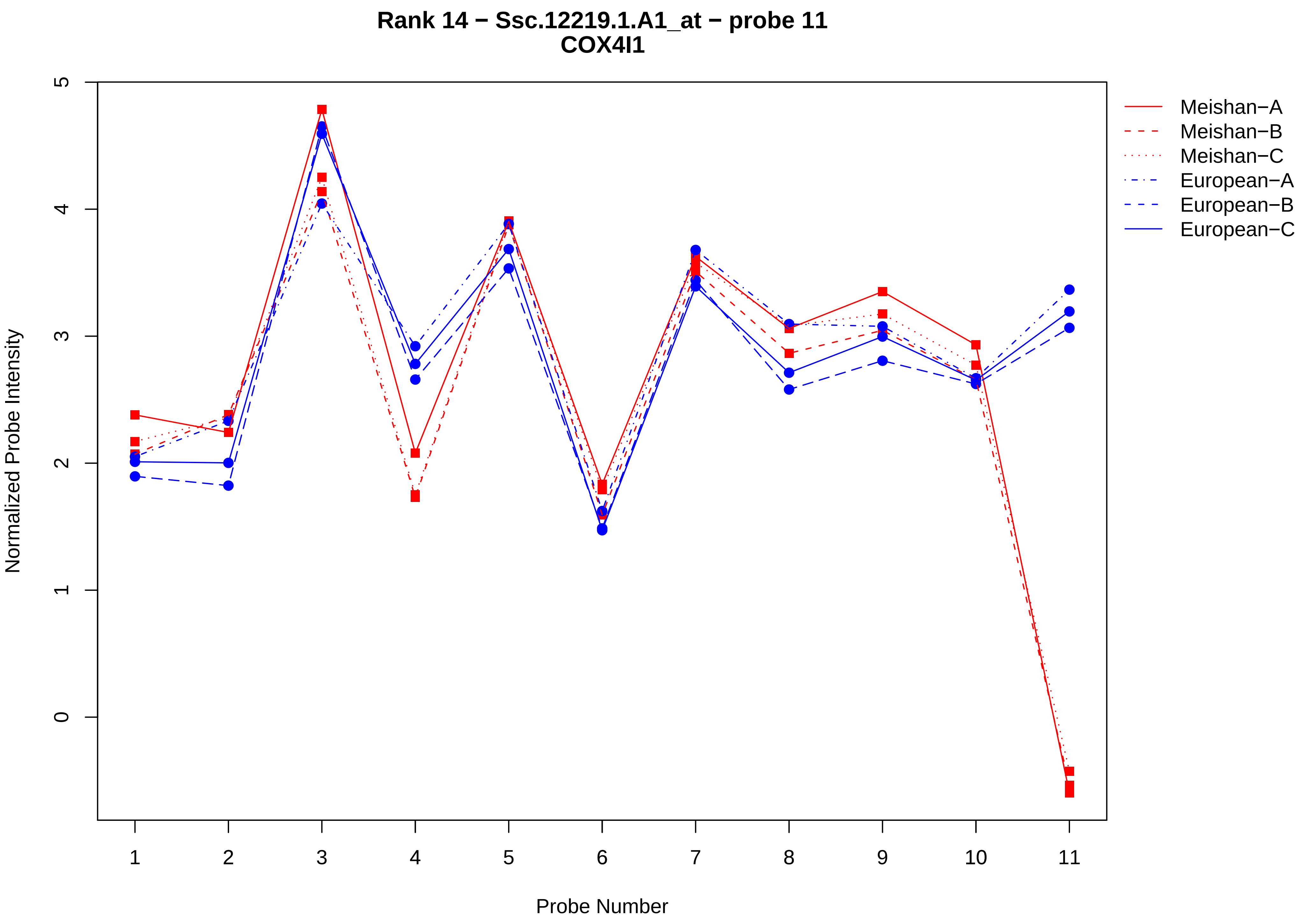

Rank 14 − Ssc.12219.1.A1_at − probe 11
COX4I1
Normalized Probe Intensity
Probe Number
0
1
2
3
4
5
1
2
3
4
5
6
7
8
9
10
11
Meishan−A
Meishan−B
Meishan−C
European−A
European−B
European−C

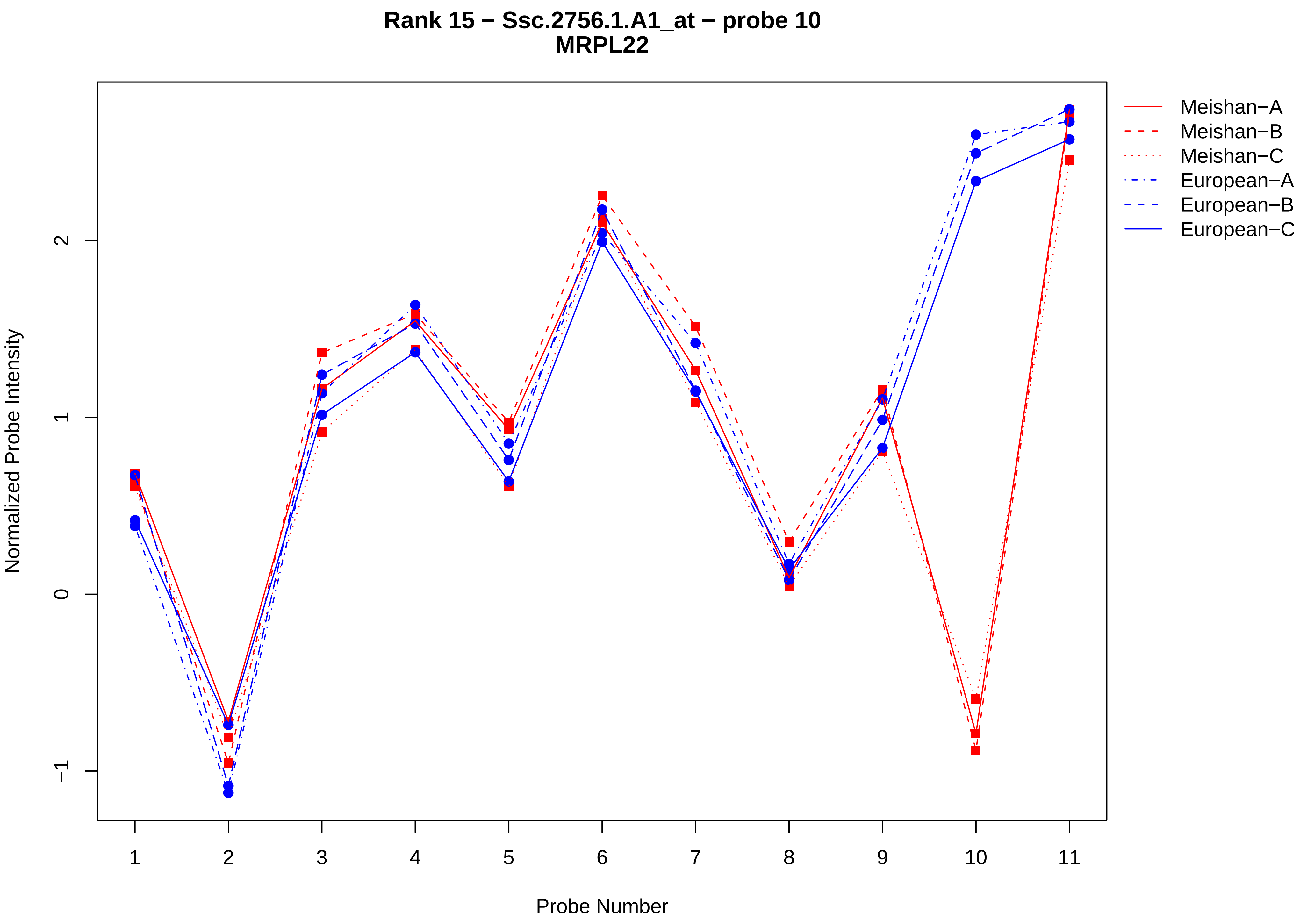

Rank 15 − Ssc.2756.1.A1_at − probe 10
MRPL22
Normalized Probe Intensity
Probe Number
−1
0
1
2
1
2
3
4
5
6
7
8
9
10
11
Meishan−A
Meishan−B
Meishan−C
European−A
European−B
European−C

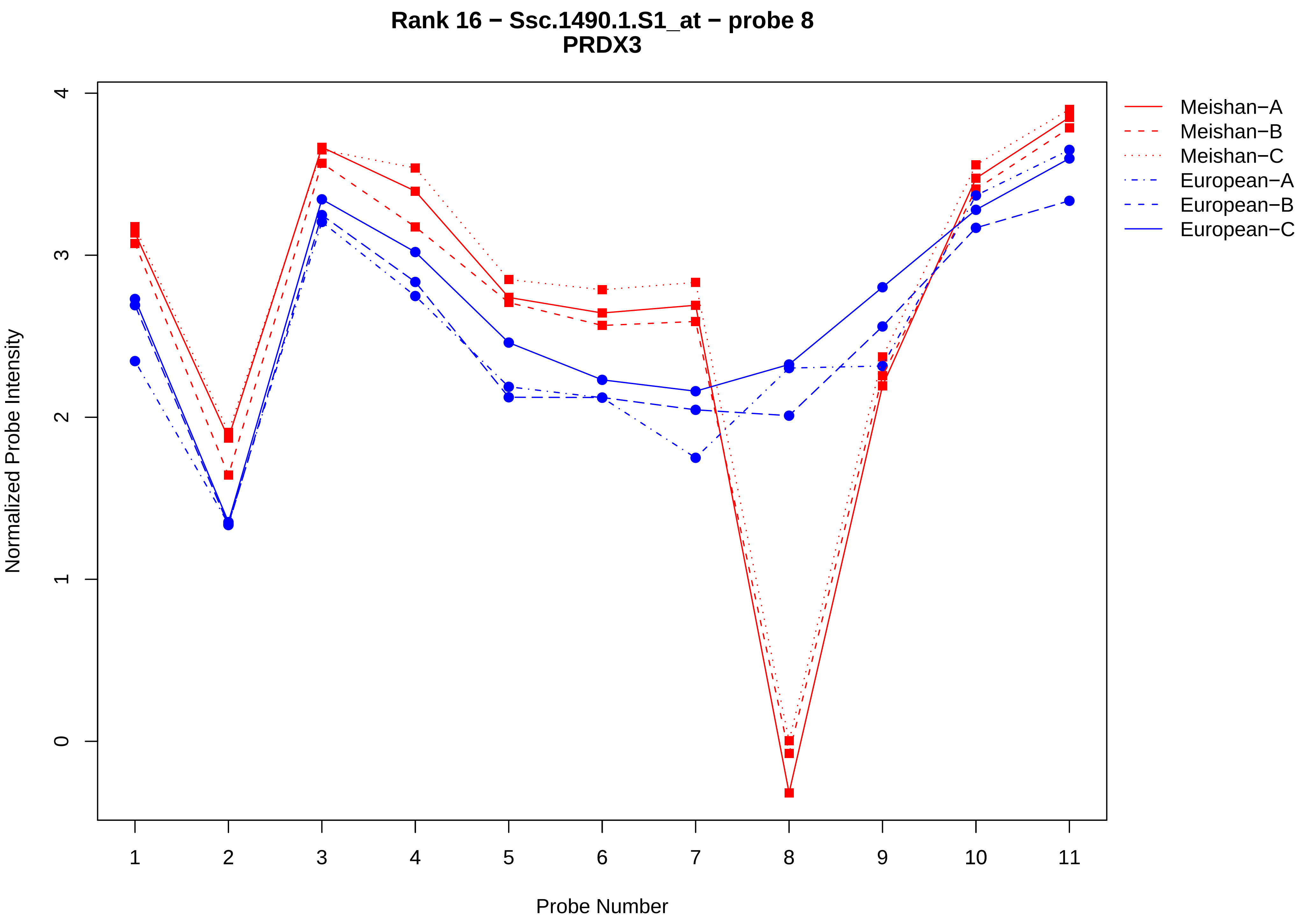
Rank 16 − Ssc.1490.1.S1_at − probe 8
PRDX3
Normalized Probe Intensity
Probe Number
0
1
2
3
4
1
2
3
4
5
6
7
8
9
10
11
Meishan−A
Meishan−B
Meishan−C
European−A
European−B
European−C

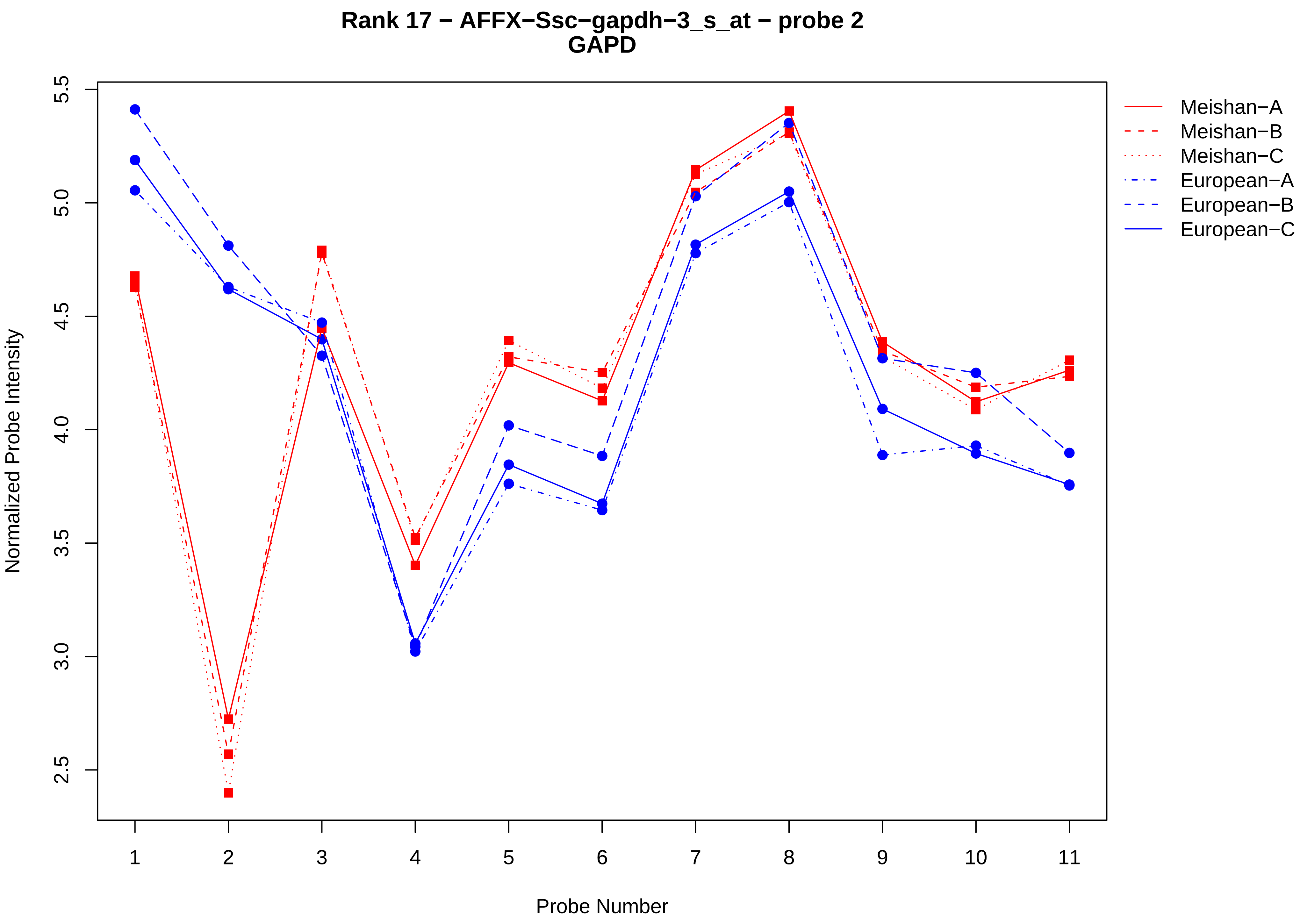
Rank 17 − AFFX−Ssc−gapdh−3_s_at − probe 2
GAPD
Normalized Probe Intensity
Probe Number
2.5
3.0
3.5
4.0
4.5
5.0
5.5
1
2
3
4
5
6
7
8
9
10
11
Meishan−A
Meishan−B
Meishan−C
European−A
European−B
European−C

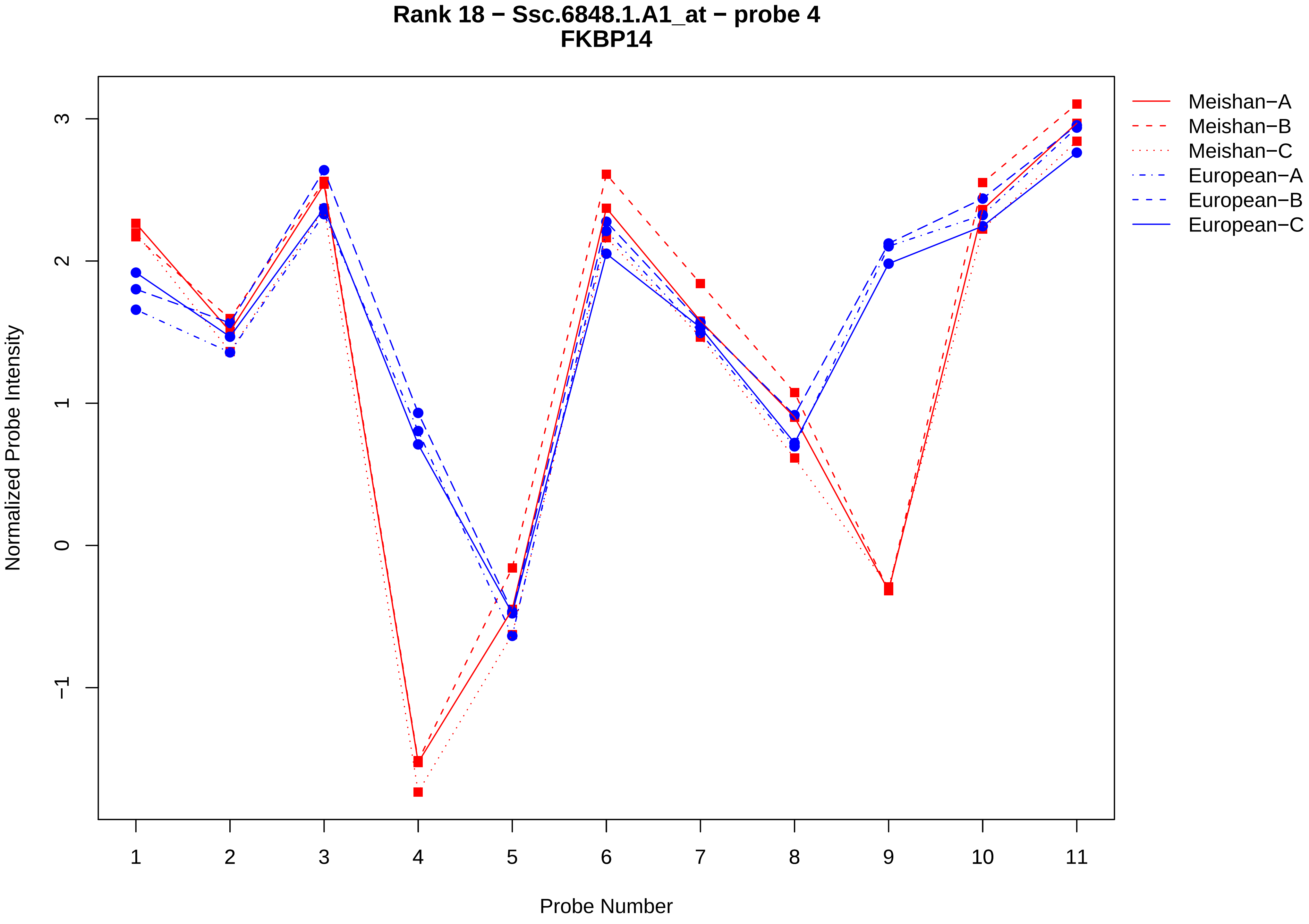
Rank 18 − Ssc.6848.1.A1_at − probe 4
FKBP14
Normalized Probe Intensity
Probe Number
Meishan−A
Meishan−B
Meishan−C
European−A
European−B
European−C

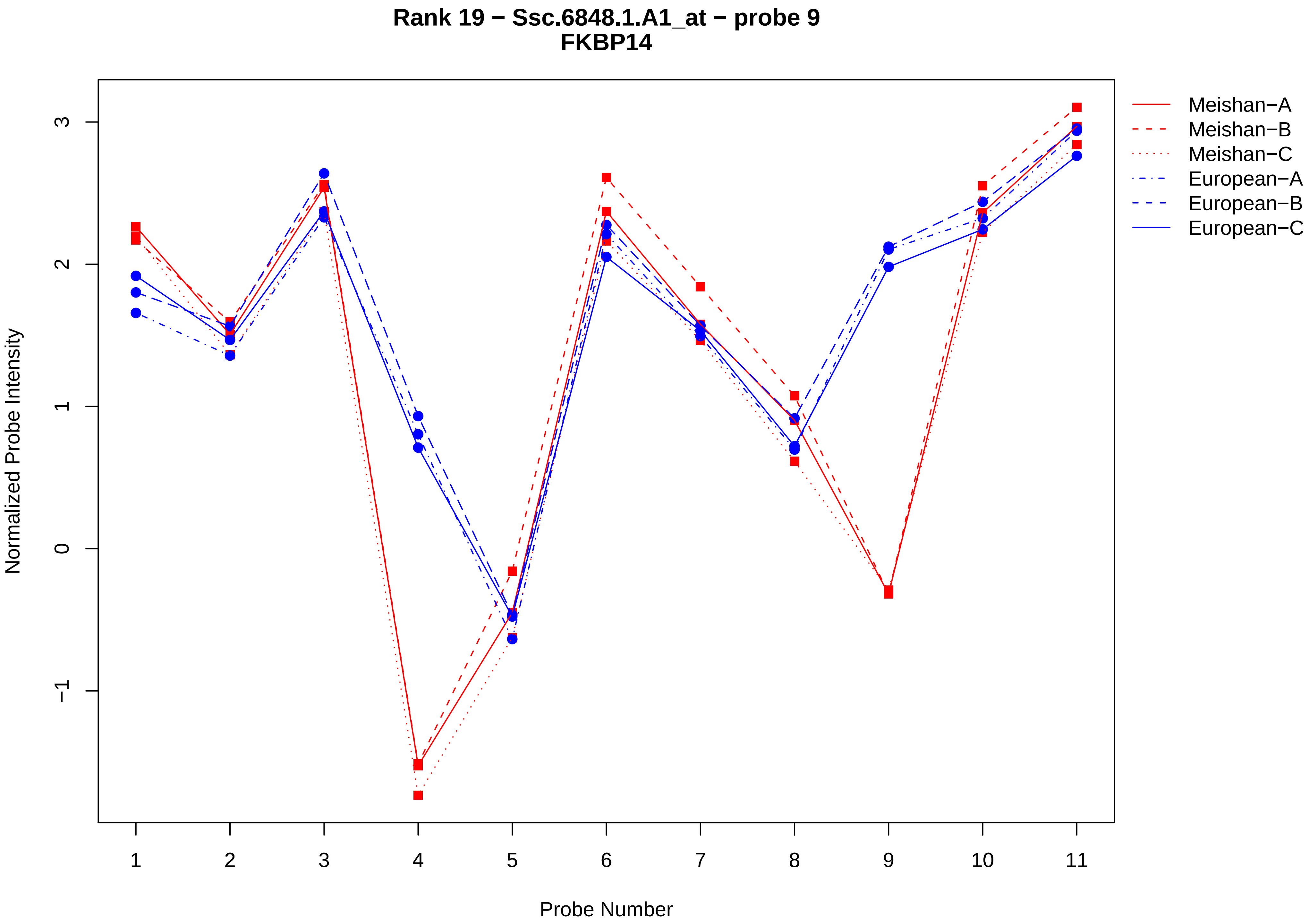

Rank 19 − Ssc.6848.1.A1_at − probe 9
FKBP14
Normalized Probe Intensity
Probe Number
Meishan−A
Meishan−B
Meishan−C
European−A
European−B
European−C

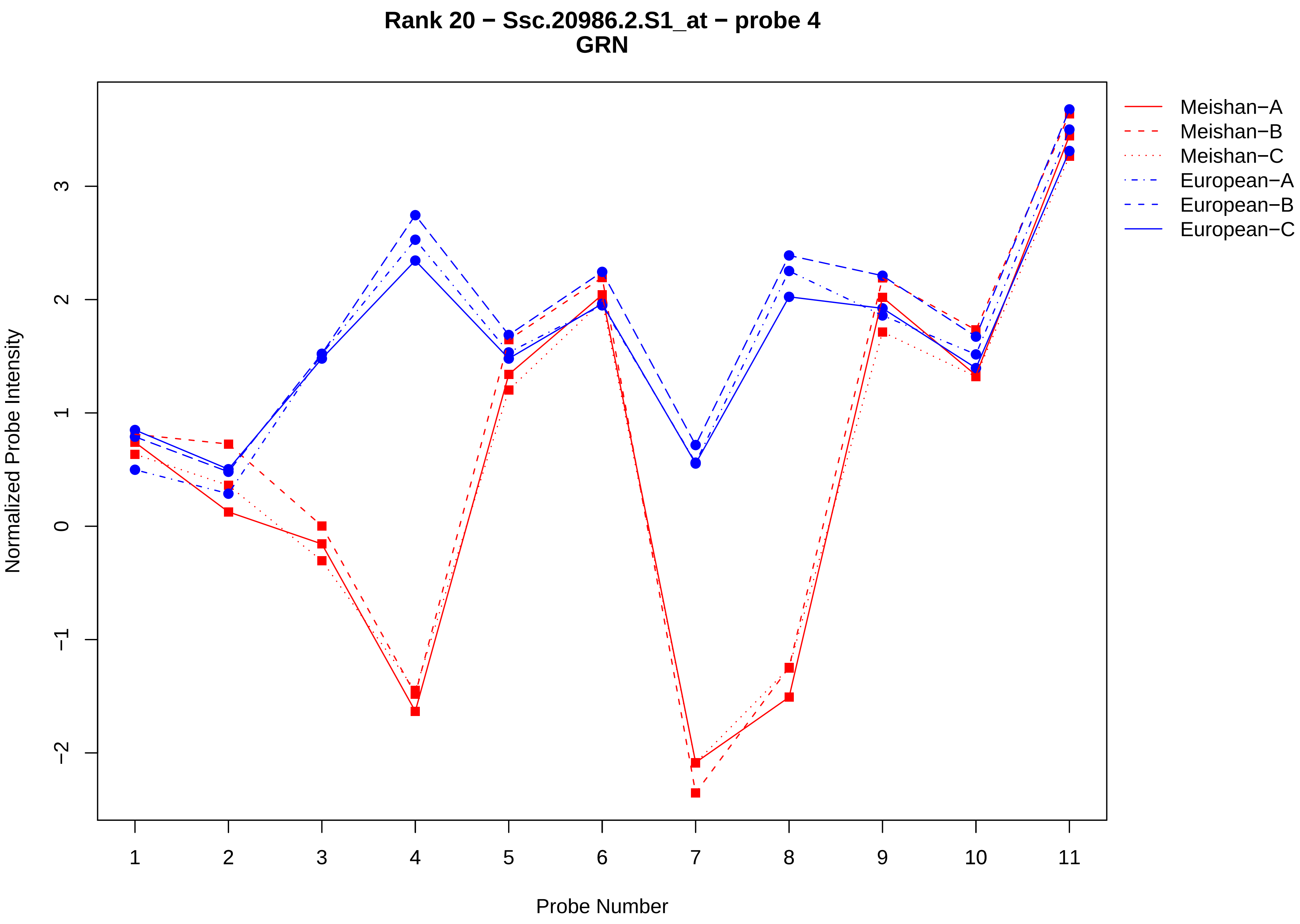

Rank 20 − Ssc.20986.2.S1_at − probe 4
GRN
Normalized Probe Intensity
Probe Number
−2
−1
0
1
2
3
1
2
3
4
5
6
7
8
9
10
11
Meishan−A
Meishan−B
Meishan−C
European−A
European−B
European−C

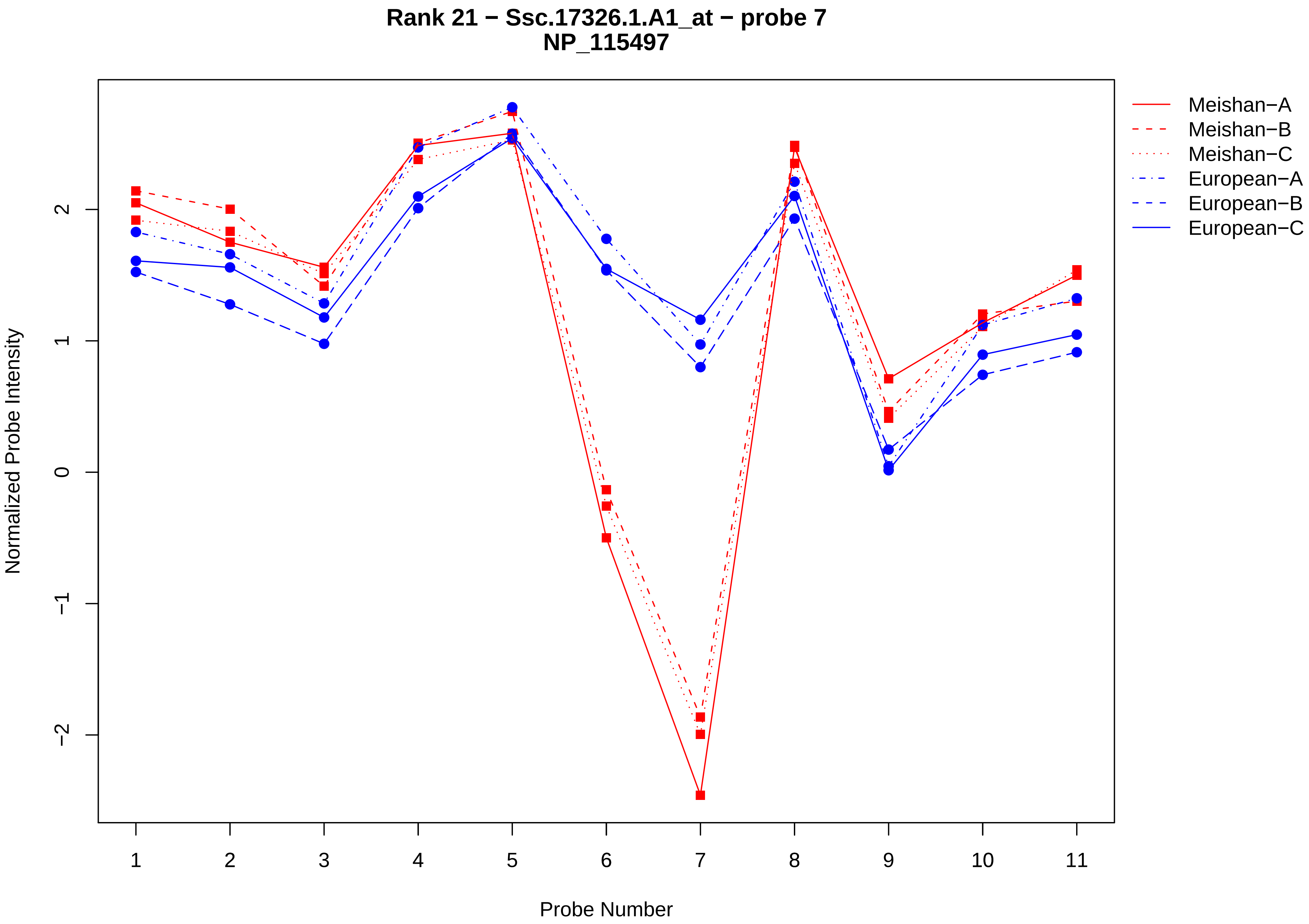

Rank 21 − Ssc.17326.1.A1_at − probe 7
NP_115497
Normalized Probe Intensity
Probe Number
−2
−1
0
1
2
1
2
3
4
5
6
7
8
9
10
11
Meishan−A
Meishan−B
Meishan−C
European−A
European−B
European−C

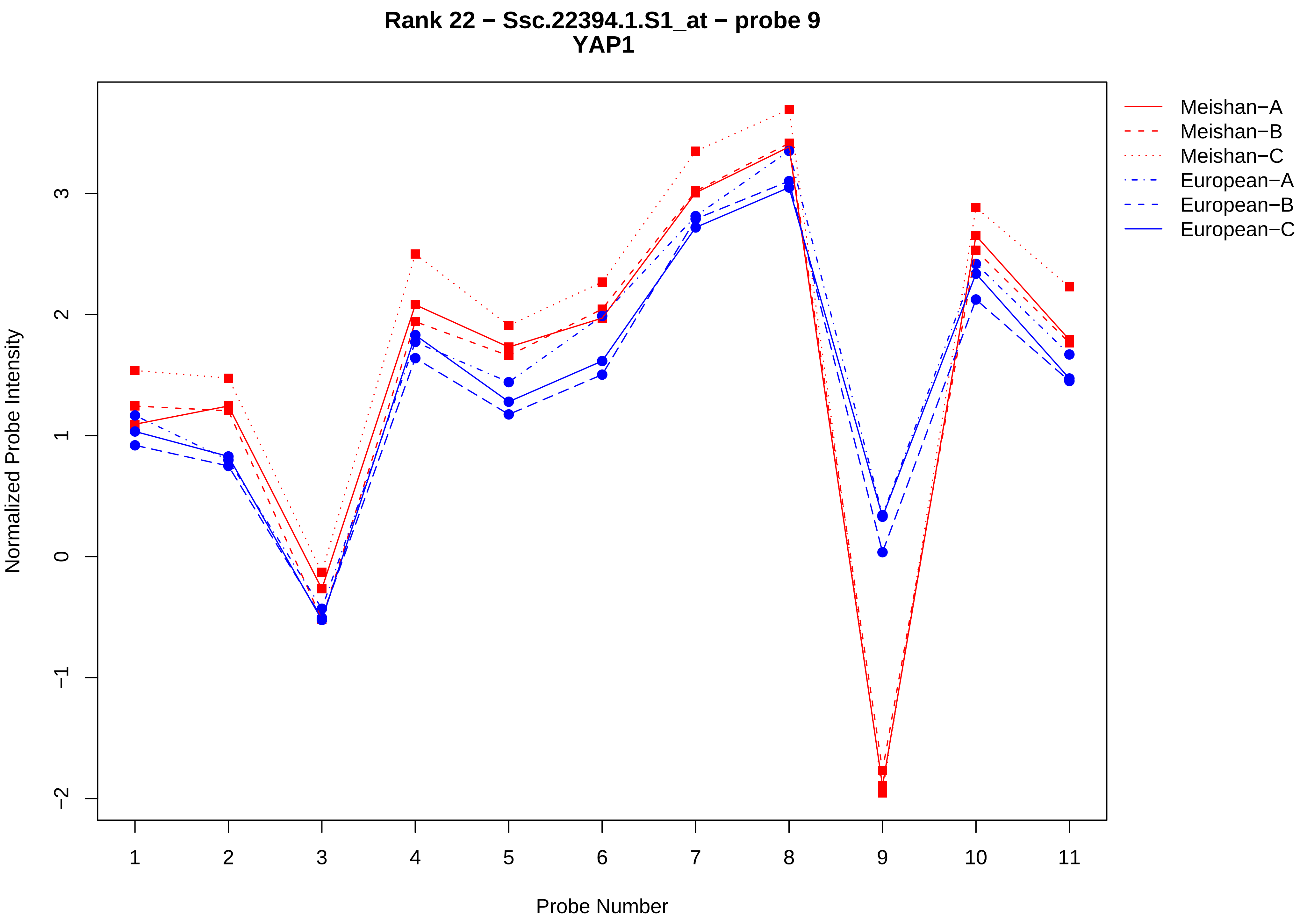

Rank 22 − Ssc.22394.1.S1_at − probe 9
YAP1
Normalized Probe Intensity
Probe Number
−2
−1
0
1
2
3
1
2
3
4
5
6
7
8
9
10
11
Meishan−A
Meishan−B
Meishan−C
European−A
European−B
European−C

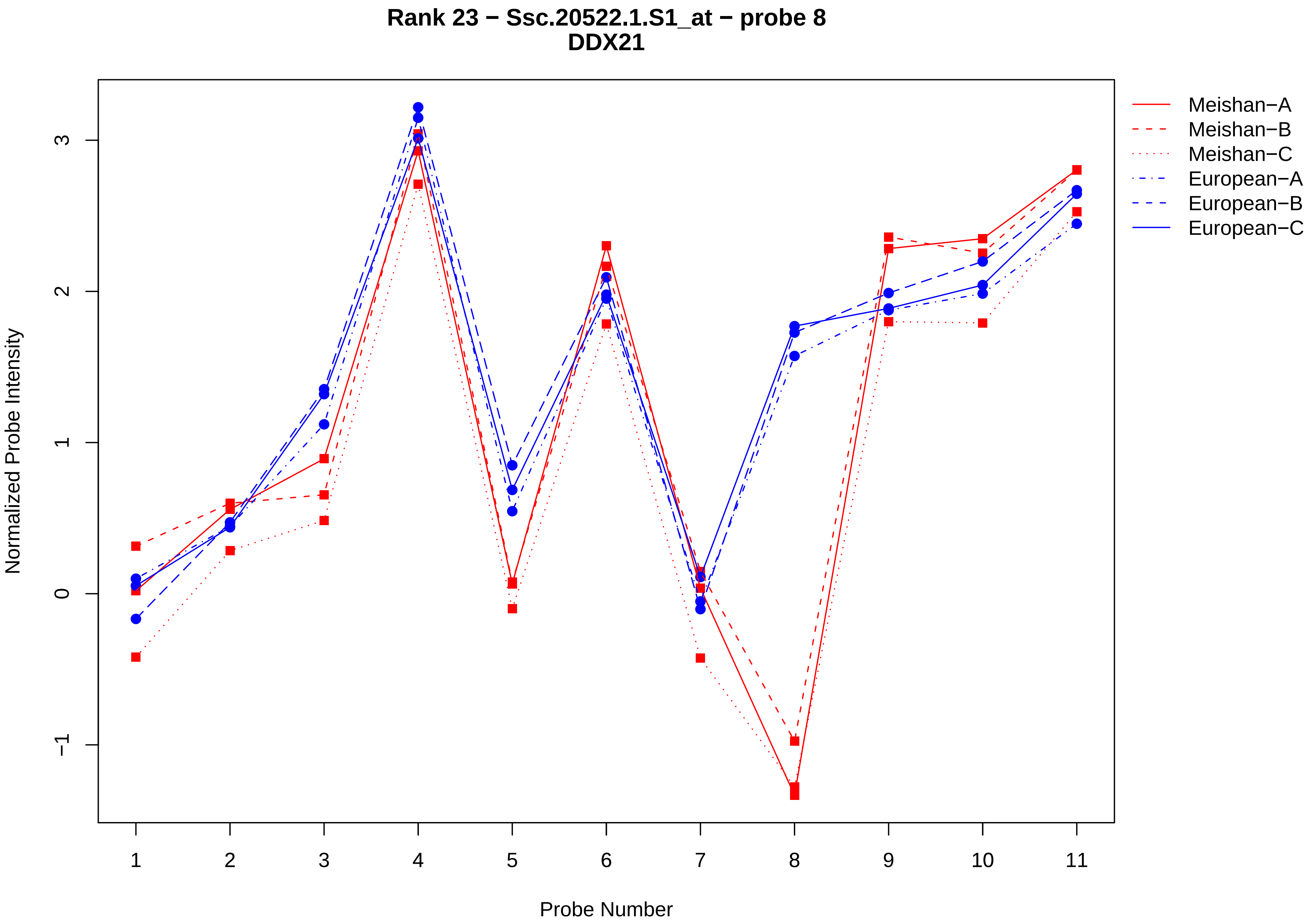

Rank 23 − Ssc.20522.1.S1_at − probe 8
DDX21
Normalized Probe Intensity
Probe Number
Meishan−A
Meishan−B
Meishan−C
European−A
European−B
European−C
−1
0
1
2
3
1
2
3
4
5
6
7
8
9
10
11

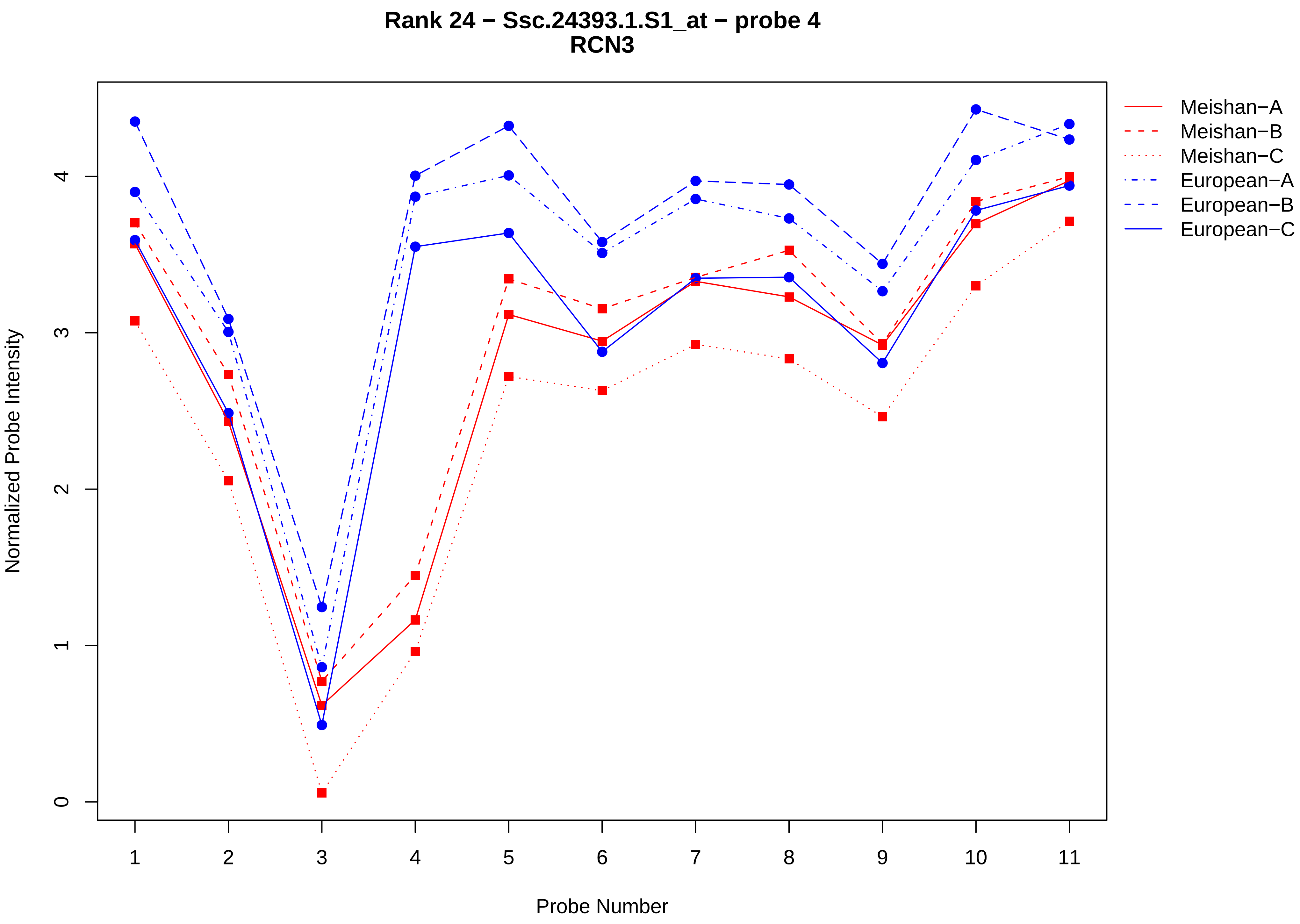
Rank 24 − Ssc.24393.1.S1_at − probe 4
RCN3
Normalized Probe Intensity
Probe Number
0
1
2
3
4
1
2
3
4
5
6
7
8
9
10
11
Meishan−A
Meishan−B
Meishan−C
European−A
European−B
European−C

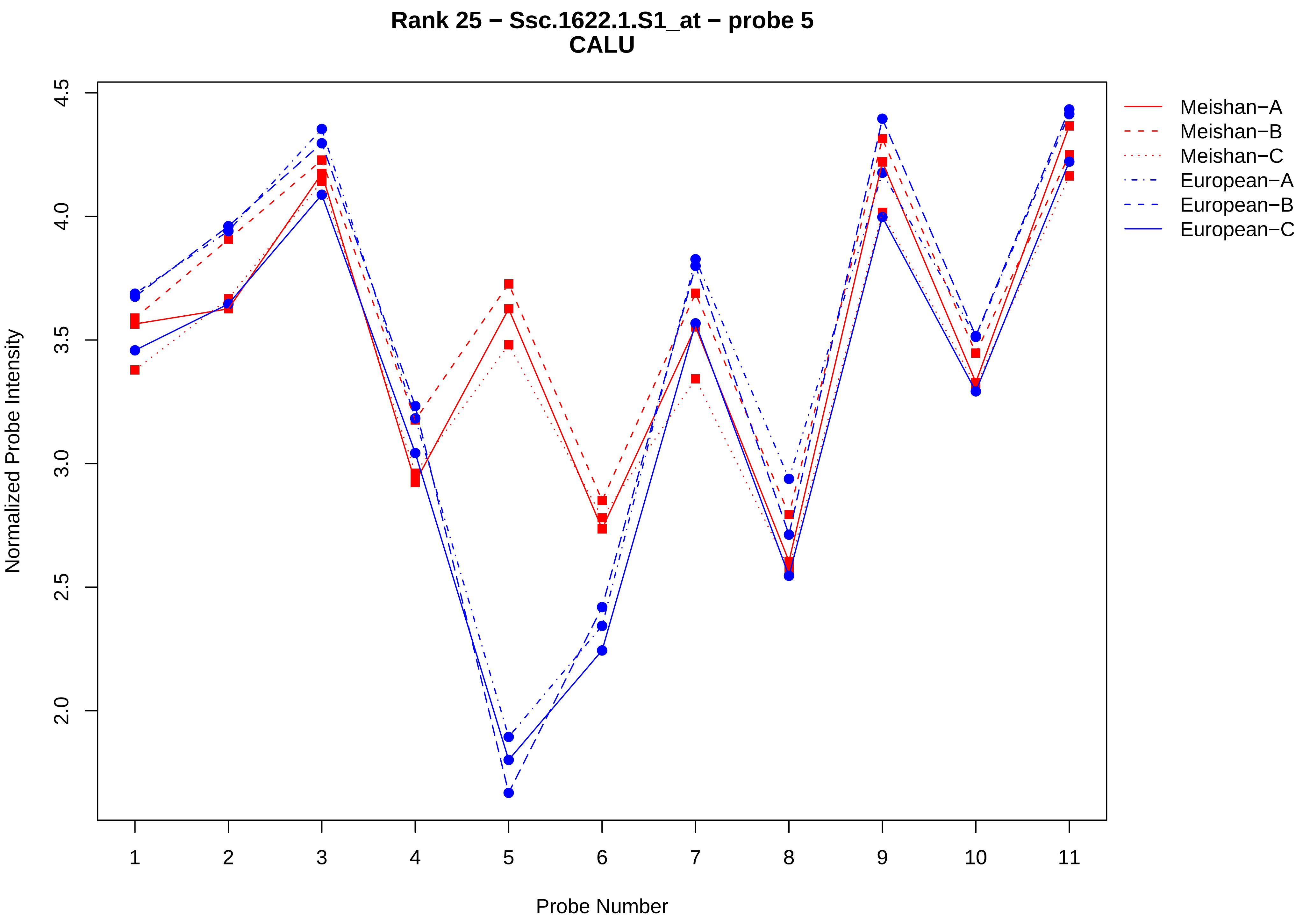

Rank 25 − Ssc.1622.1.S1_at − probe 5
CALU
Normalized Probe Intensity
Probe Number
4.5
4.0
3.5
3.0
2.5
2.0
1
2
3
4
5
6
7
8
9
10
11
Meishan−A
Meishan−B
Meishan−C
European−A
European−B
European−C

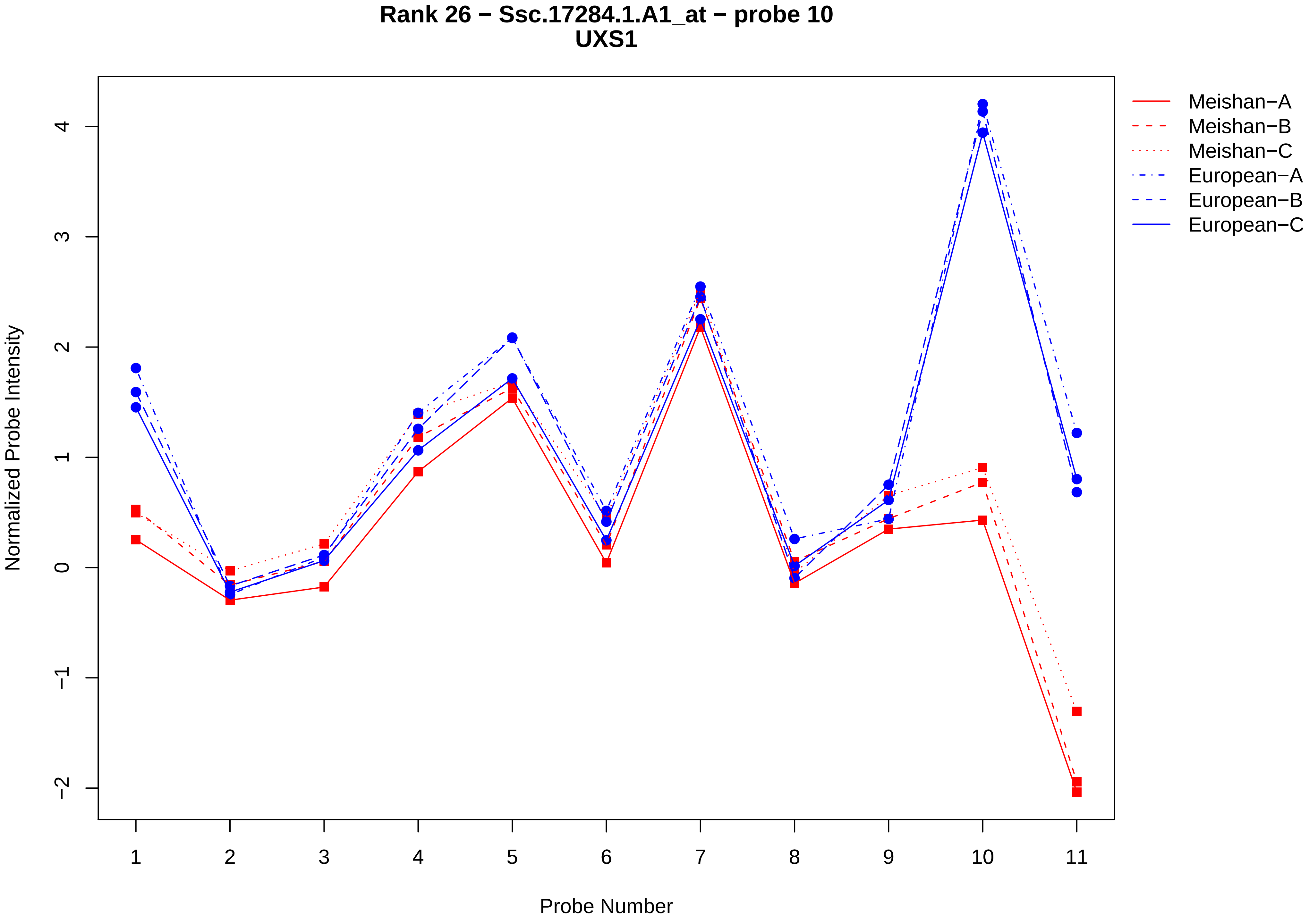

Rank 26 − Ssc.17284.1.A1_at − probe 10
UXS1
Normalized Probe Intensity
Probe Number
Meishan−A
Meishan−B
Meishan−C
European−A
European−B
European−C

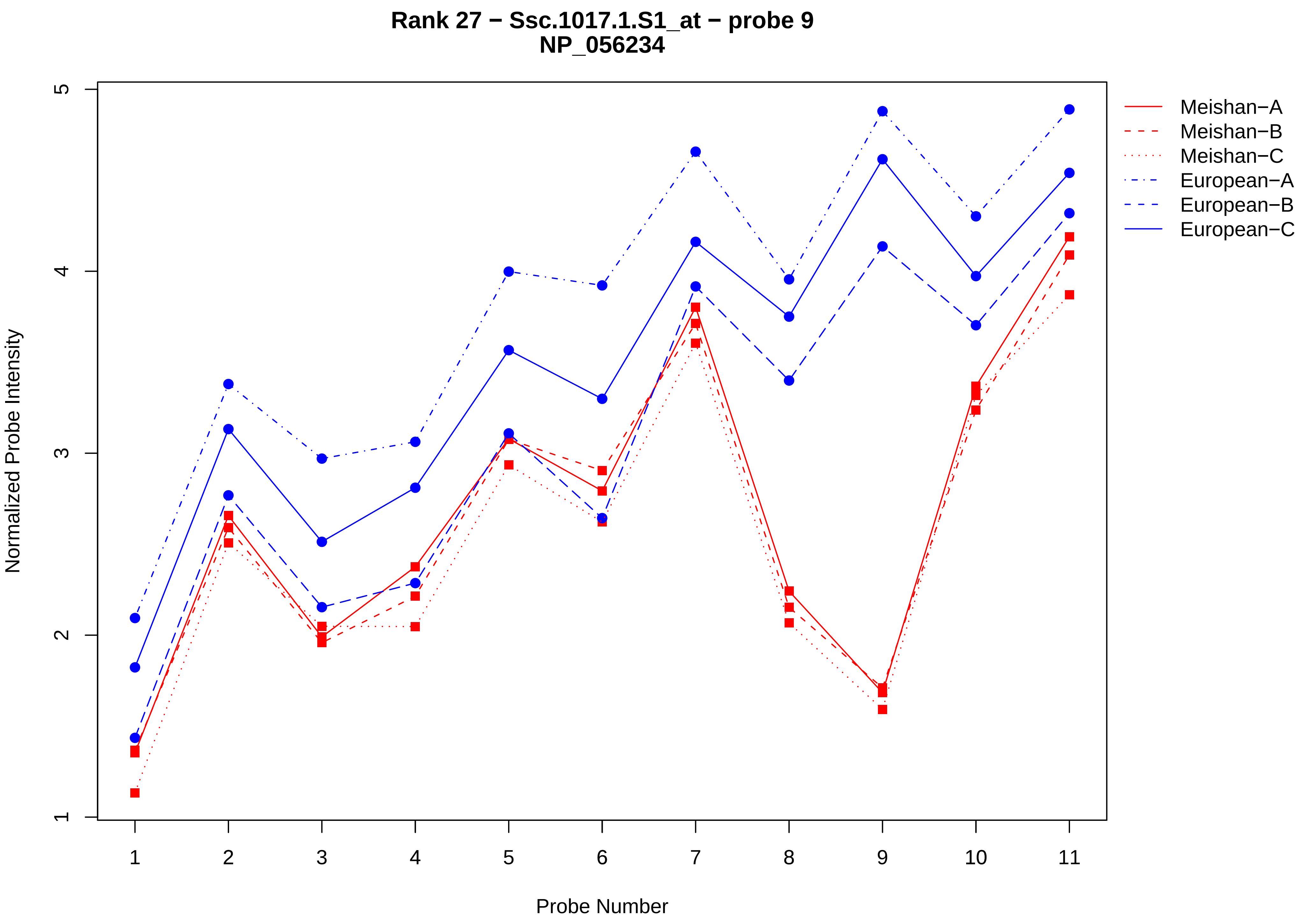

Rank 27 − Ssc.1017.1.S1_at − probe 9
NP_056234
Normalized Probe Intensity
Probe Number
Meishan−A
Meishan−B
Meishan−C
European−A
European−B
European−C
1
2
3
4
5
6
7
8
9
10
11

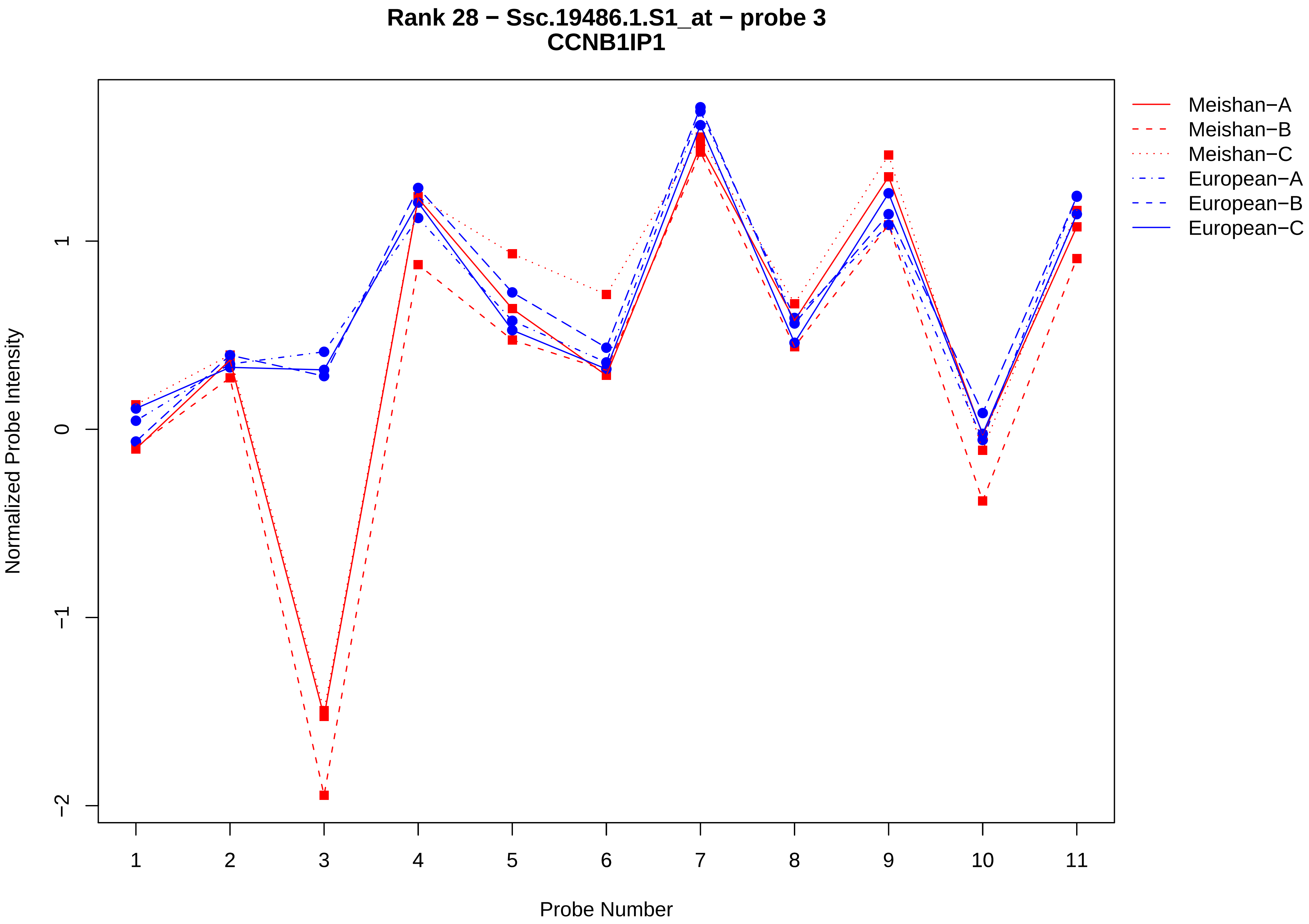
Rank 28 − Ssc.19486.1.S1_at − probe 3
CCNB1IP1
Normalized Probe Intensity
Probe Number
−2
−1
0
1
1
2
3
4
5
6
7
8
9
10
11
Meishan−A
Meishan−B
Meishan−C
European−A
European−B
European−C

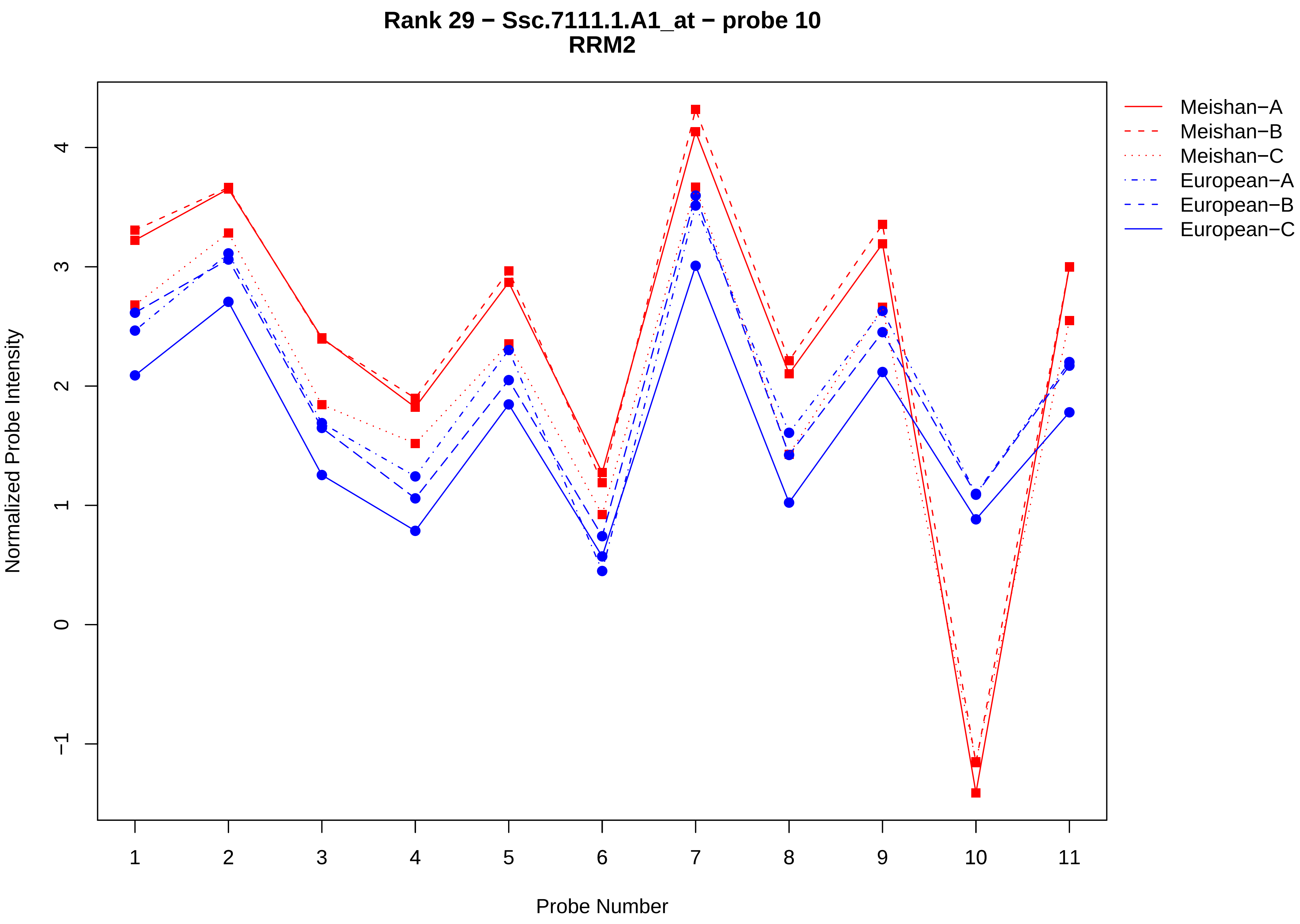

Rank 29 − Ssc.7111.1.A1_at − probe 10
RRM2
Normalized Probe Intensity
Probe Number
−1
0
1
2
3
4
1
2
3
4
5
6
7
8
9
10
11
Meishan−A
Meishan−B
Meishan−C
European−A
European−B
European−C

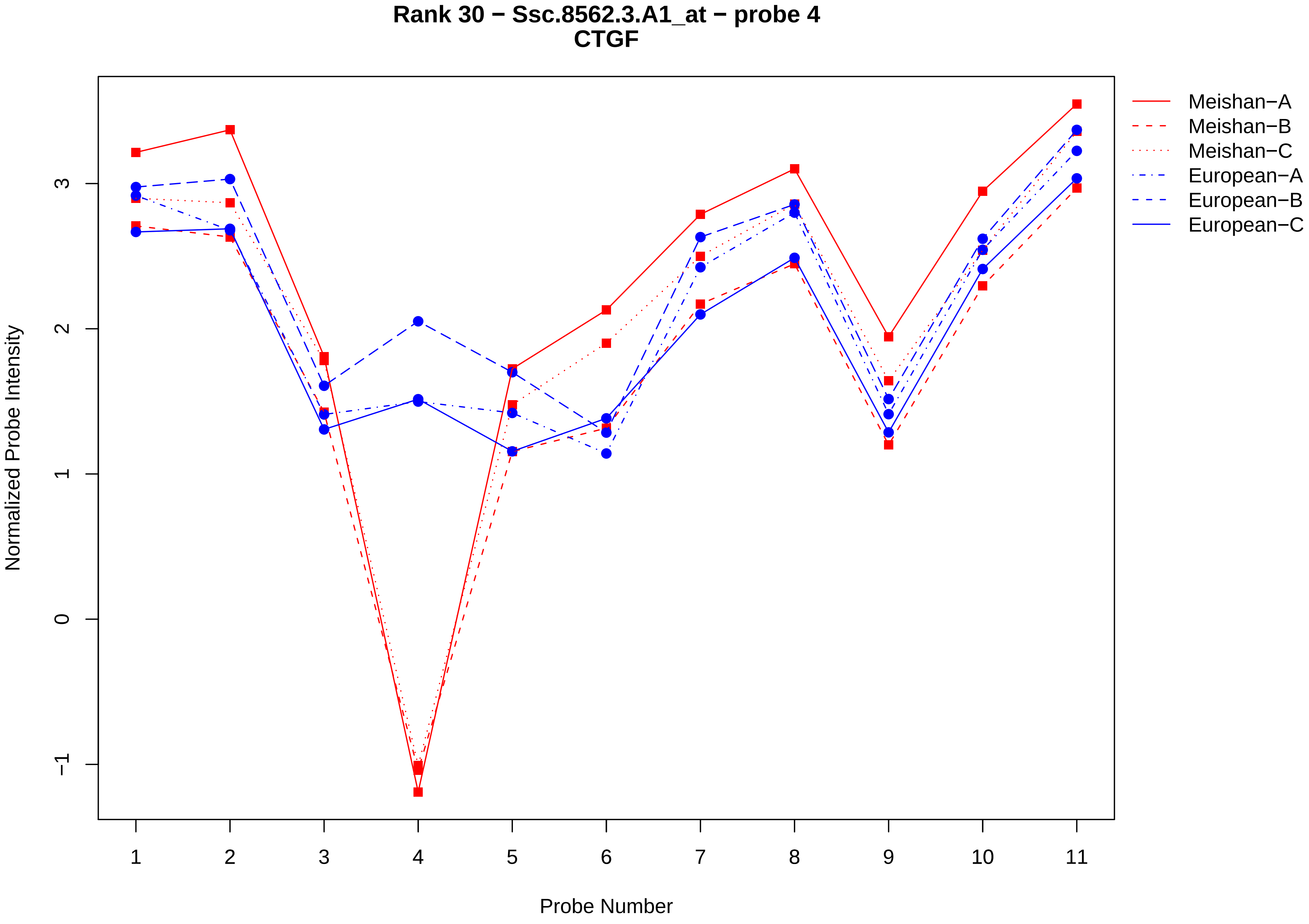
Rank 30 − Ssc.8562.3.A1_at − probe 4
CTGF
Normalized Probe Intensity
−1
0
1
2
3
Probe Number
1
2
3
4
5
6
7
8
9
10
11
Meishan−A
Meishan−B
Meishan−C
European−A
European−B
European−C

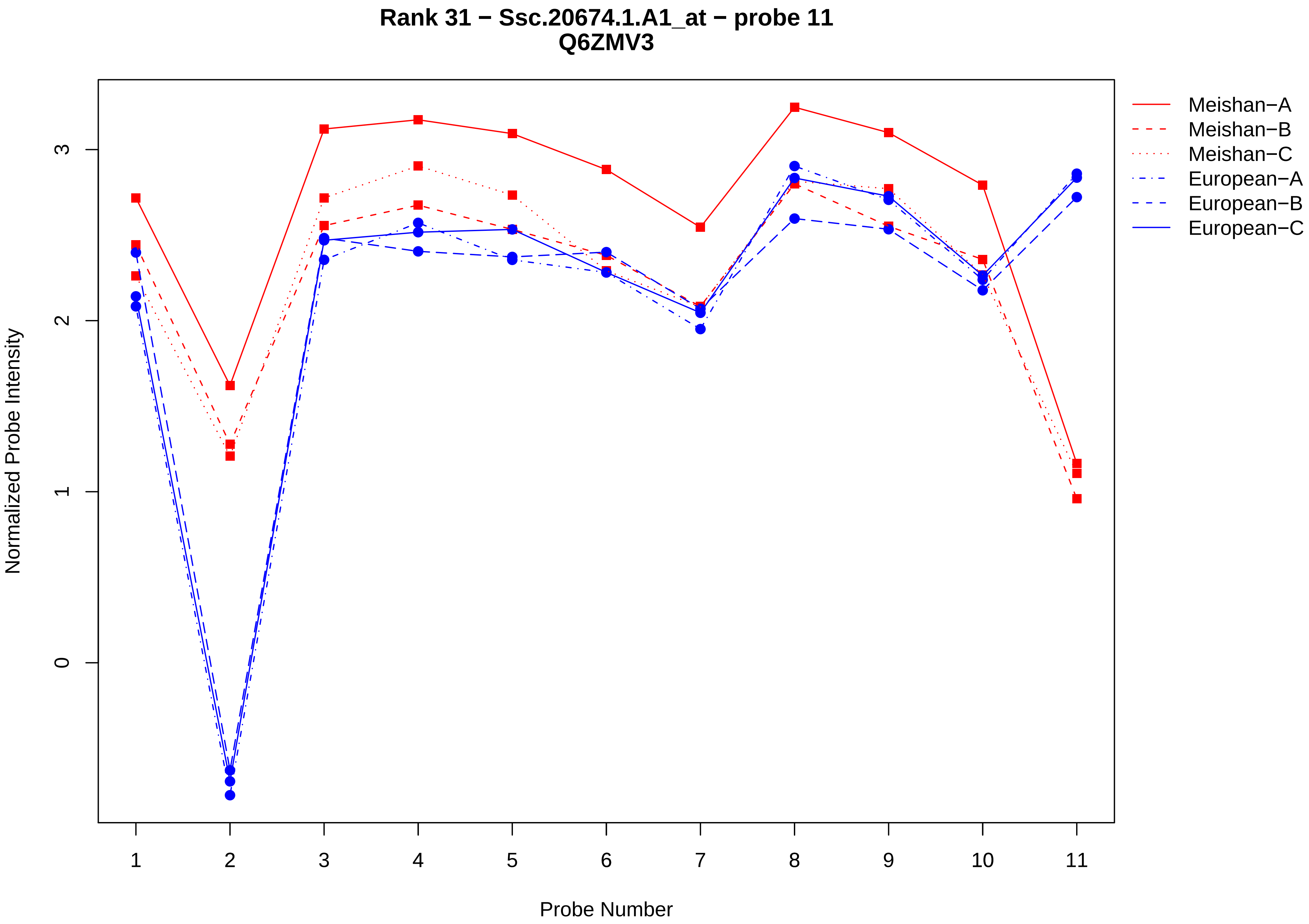
Rank 31 − Ssc.20674.1.A1_at − probe 11
Q6ZMV3
Normalized Probe Intensity
Probe Number
0
1
2
3
1
2
3
4
5
6
7
8
9
10
11
Meishan−A
Meishan−B
Meishan−C
European−A
European−B
European−C

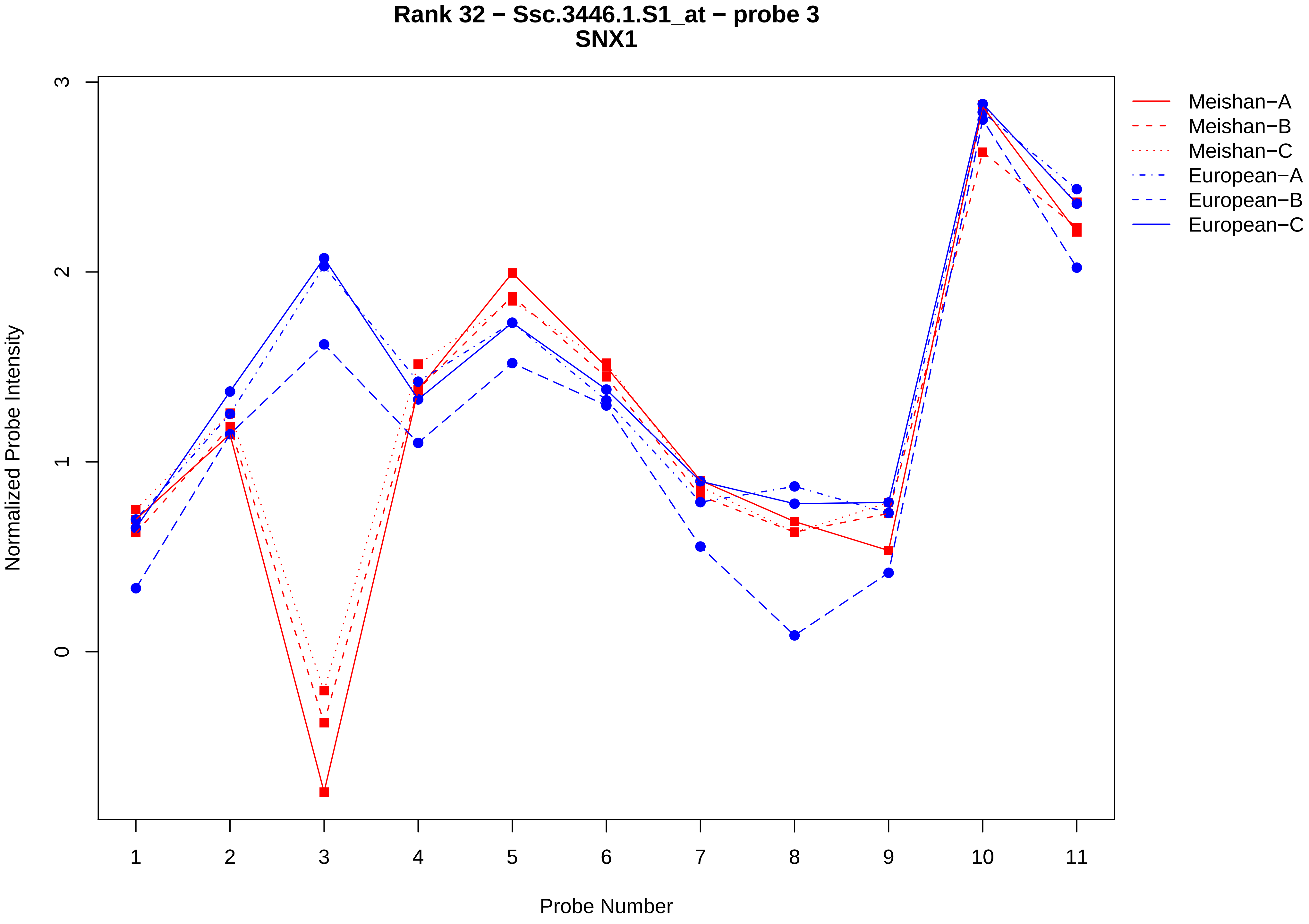

Rank 32 − Ssc.3446.1.S1_at − probe 3
SNX1
Normalized Probe Intensity
Probe Number
Meishan−A
Meishan−B
Meishan−C
European−A
European−B
European−C

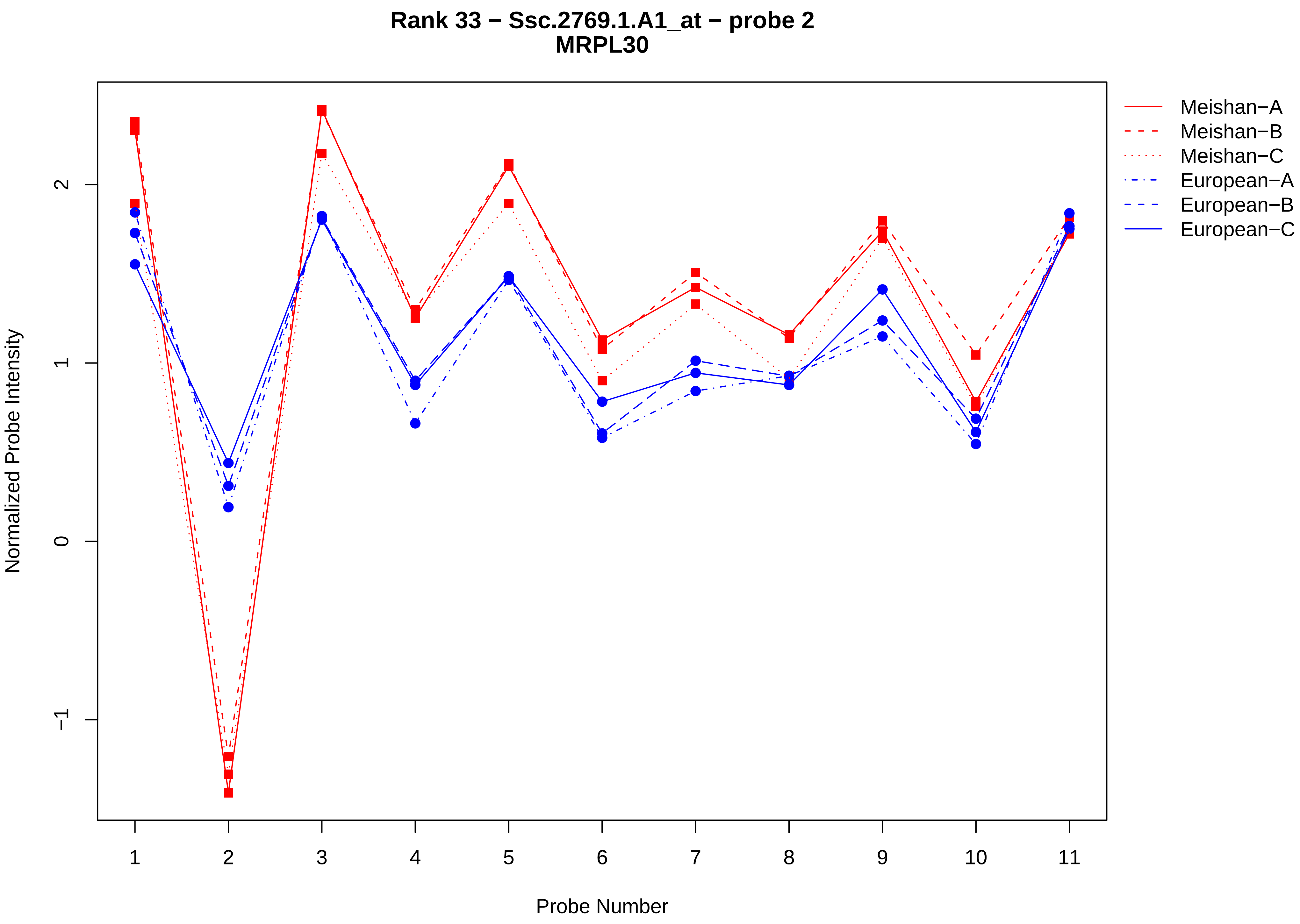
Rank 33 − Ssc.2769.1.A1_at − probe 2
MRPL30
Normalized Probe Intensity
Probe Number
−1
0
1
2
1
2
3
4
5
6
7
8
9
10
11
Meishan−A
Meishan−B
Meishan−C
European−A
European−B
European−C

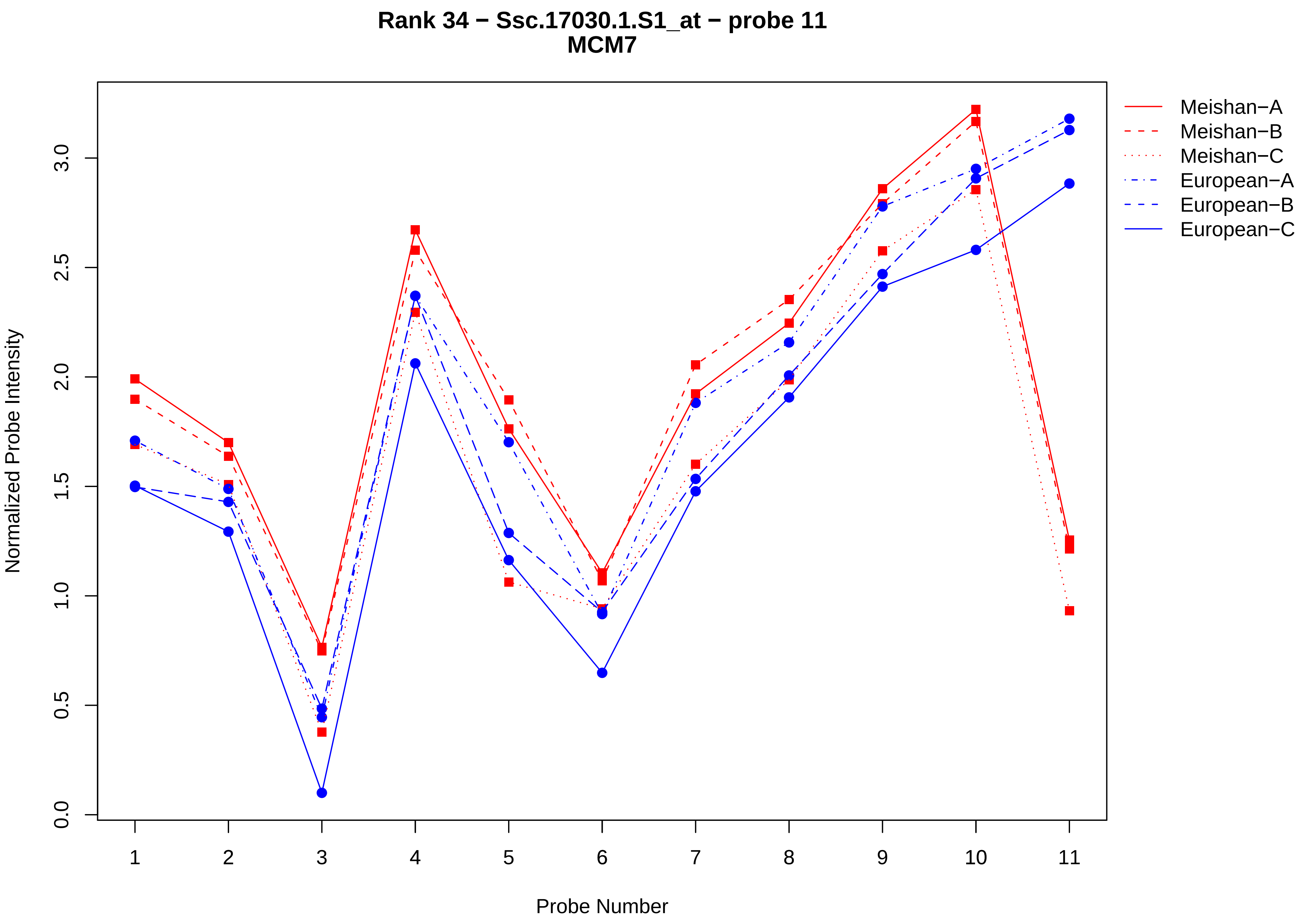

Rank 34 − Ssc.17030.1.S1_at − probe 11
MCM7
Normalized Probe Intensity
Probe Number
0.0
0.5
1.0
1.5
2.0
2.5
3.0
1
2
3
4
5
6
7
8
9
10
11
Meishan−A
Meishan−B
Meishan−C
European−A
European−B
European−C

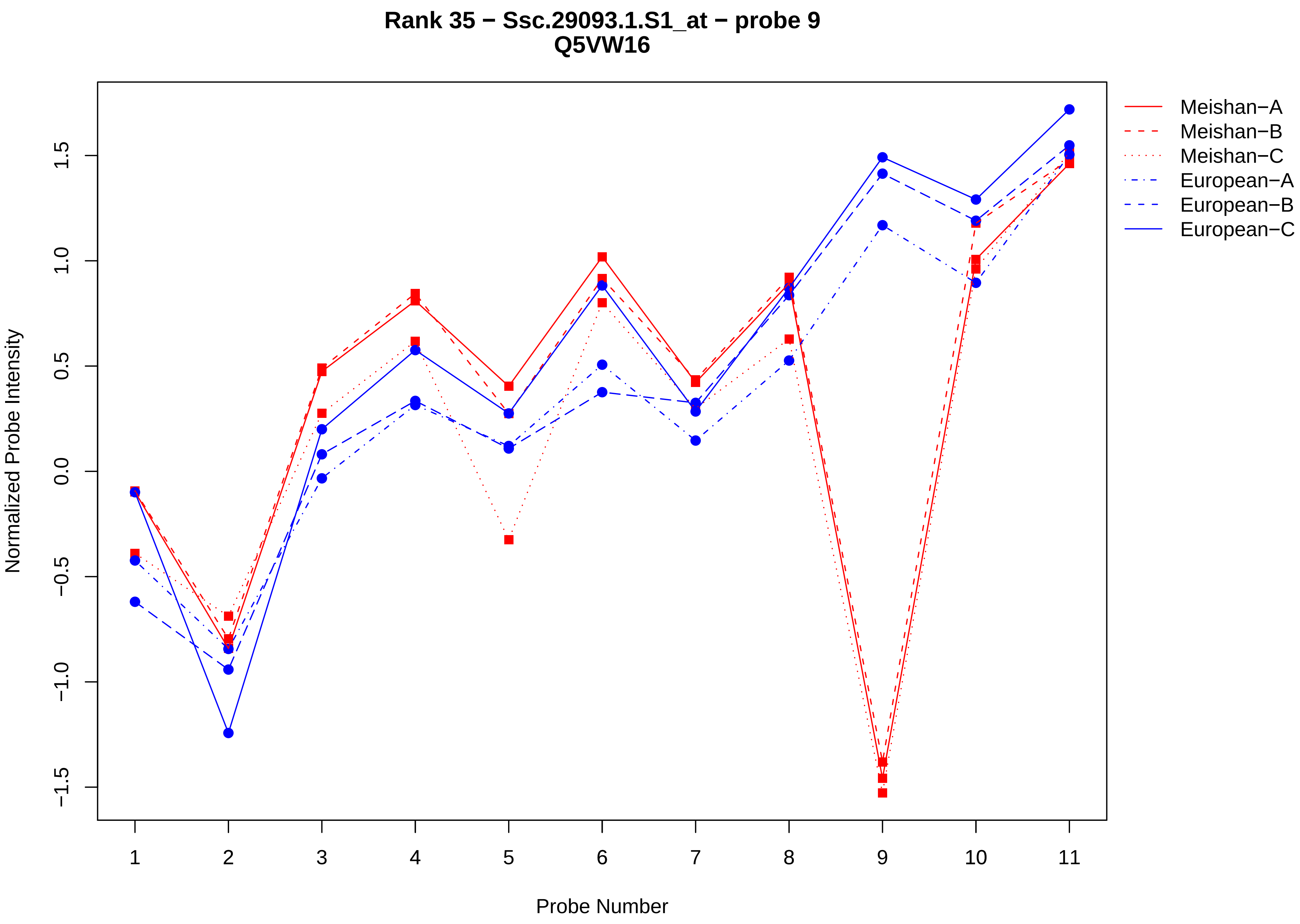

Rank 35 − Ssc.29093.1.S1_at − probe 9
Q5VW16
Normalized Probe Intensity
−1.5
−1.0
−0.5
0.0
0.5
1.0
1.5
Probe Number
1
2
3
4
5
6
7
8
9
10
11
Meishan−A
Meishan−B
Meishan−C
European−A
European−B
European−C

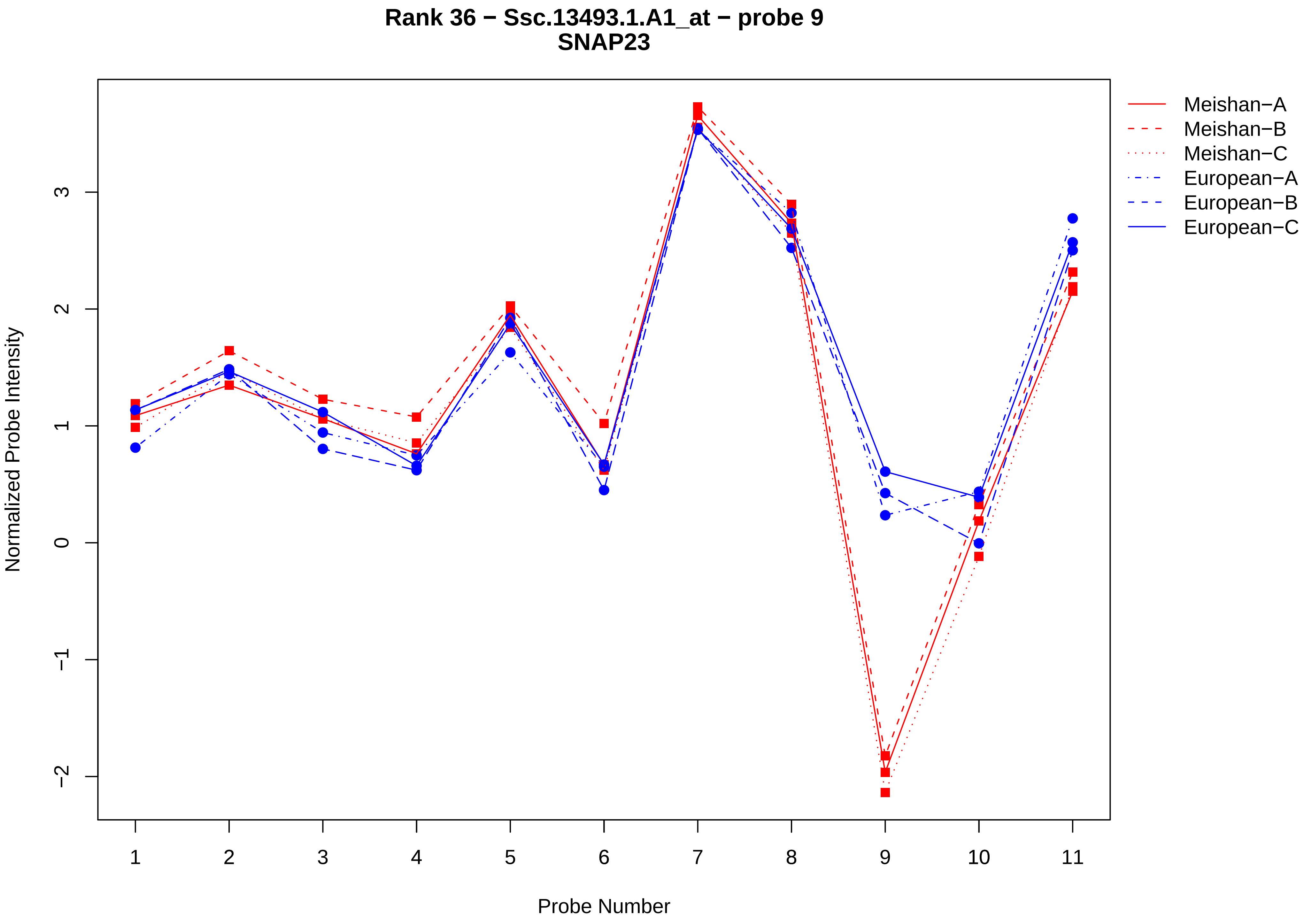

Rank 36 − Ssc.13493.1.A1_at − probe 9
SNAP23
Normalized Probe Intensity
−2
−1
0
1
2
3
1
2
3
4
5
6
7
8
9
10
11
Probe Number
Meishan−A
Meishan−B
Meishan−C
European−A
European−B
European−C

Rank 37 − Ssc.3128.1.A1_at − probe 9
C6orf82

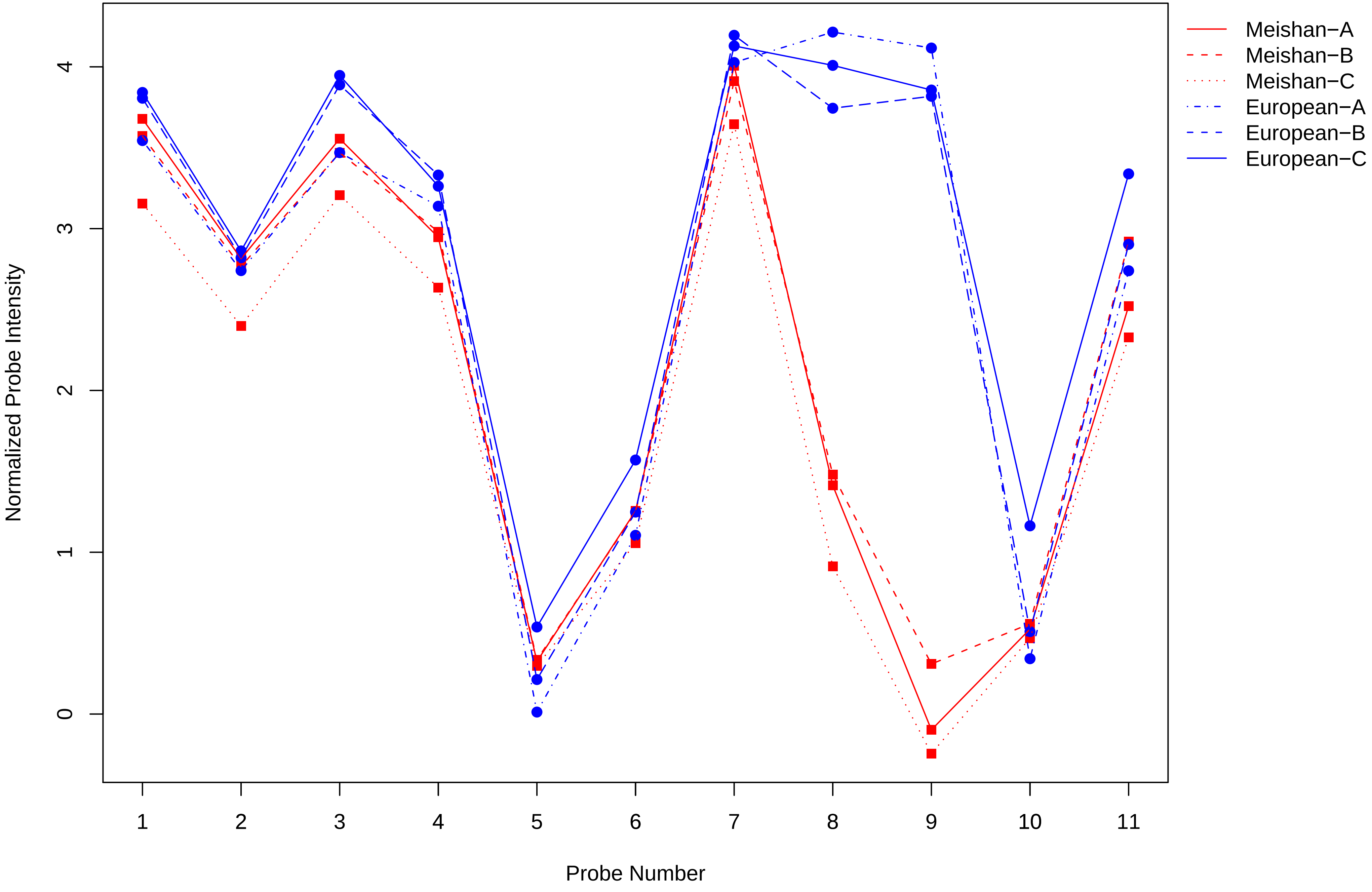

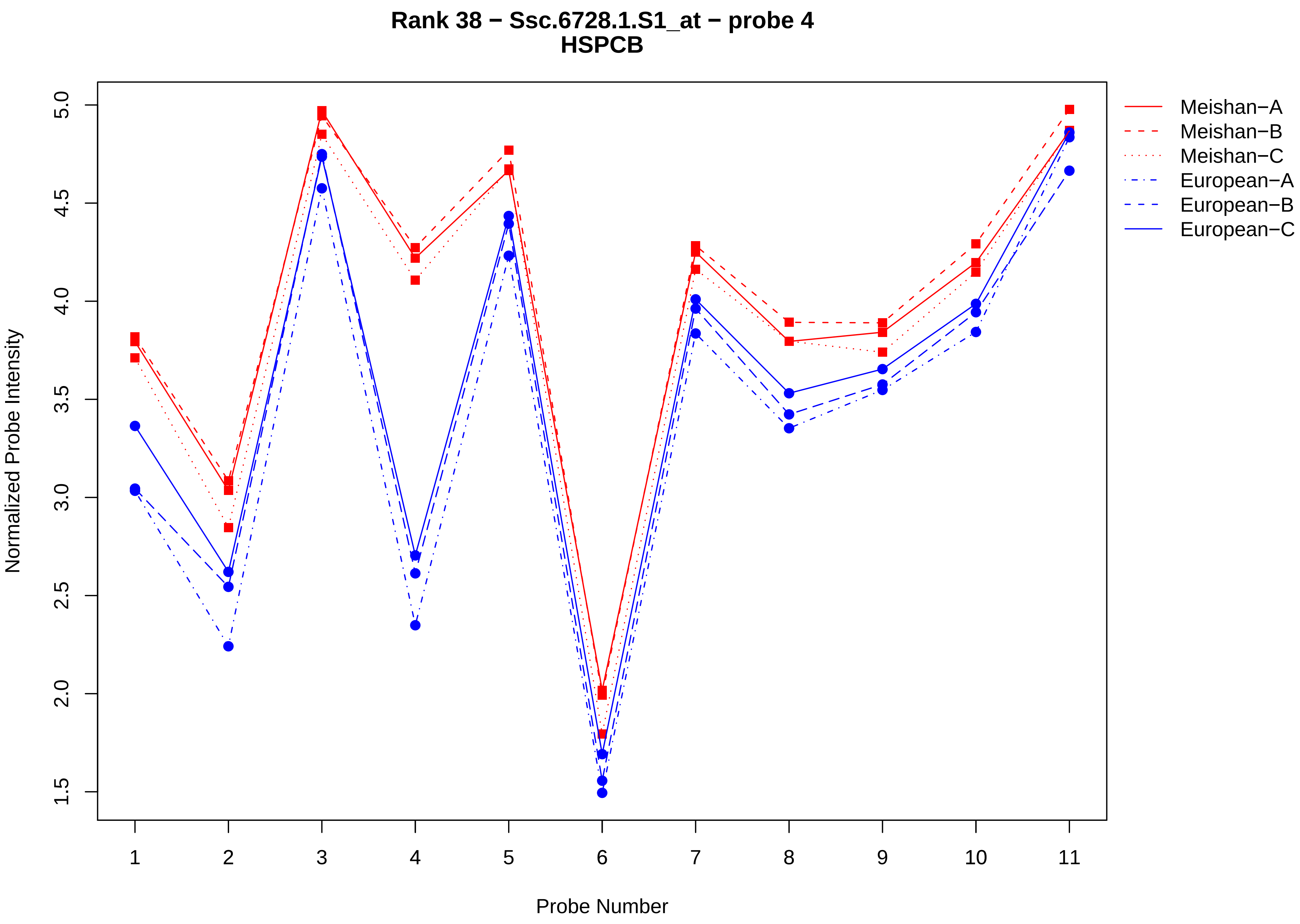

Rank 38 − Ssc.6728.1.S1_at − probe 4
HSPCB
Normalized Probe Intensity
Probe Number
1.5
2.0
2.5
3.0
3.5
4.0
4.5
5.0
1
2
3
4
5
6
7
8
9
10
11
Meishan−A
Meishan−B
Meishan−C
European−A
European−B
European−C

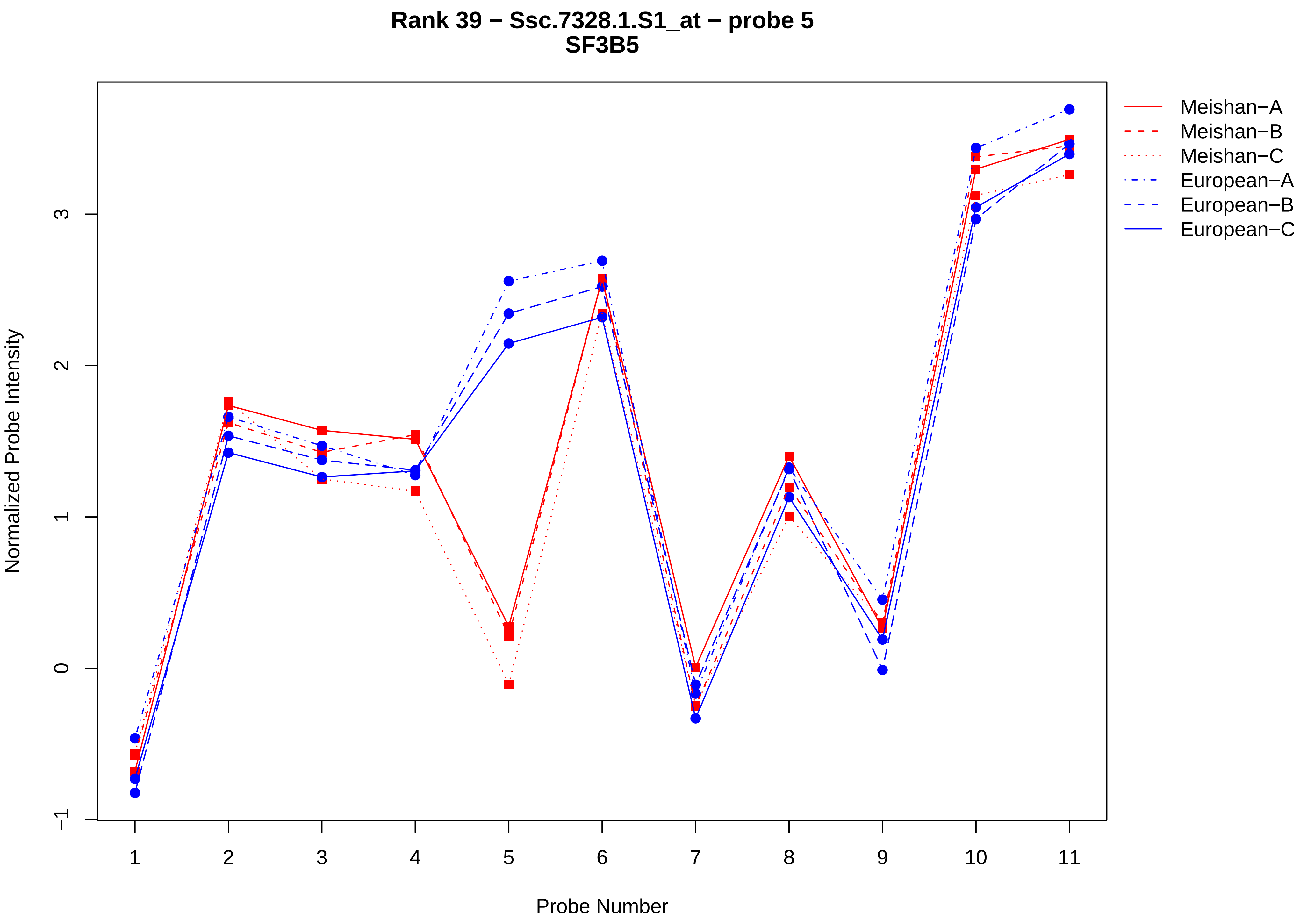

Rank 39 − Ssc.7328.1.S1_at − probe 5
SF3B5
Normalized Probe Intensity
−1
0
1
2
3
1
2
3
4
5
6
7
8
9
10
11
Probe Number
Meishan−A
Meishan−B
Meishan−C
European−A
European−B
European−C

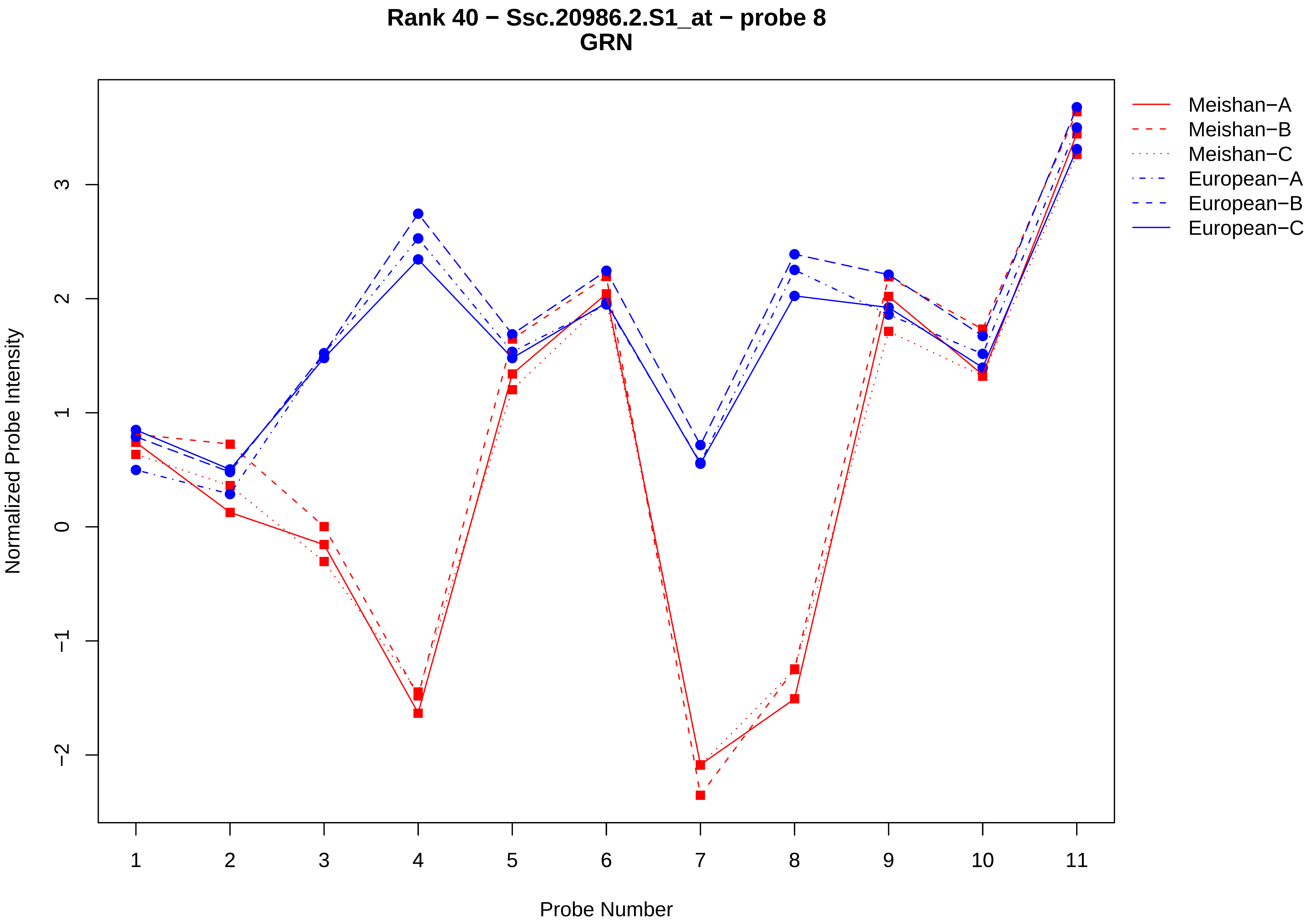

Rank 40 − Ssc.20986.2.S1_at − probe 8
GRN
Normalized Probe Intensity
Probe Number
−2
−1
0
1
2
3
1
2
3
4
5
6
7
8
9
10
11
Meishan−A
Meishan−B
Meishan−C
European−A
European−B
European−C

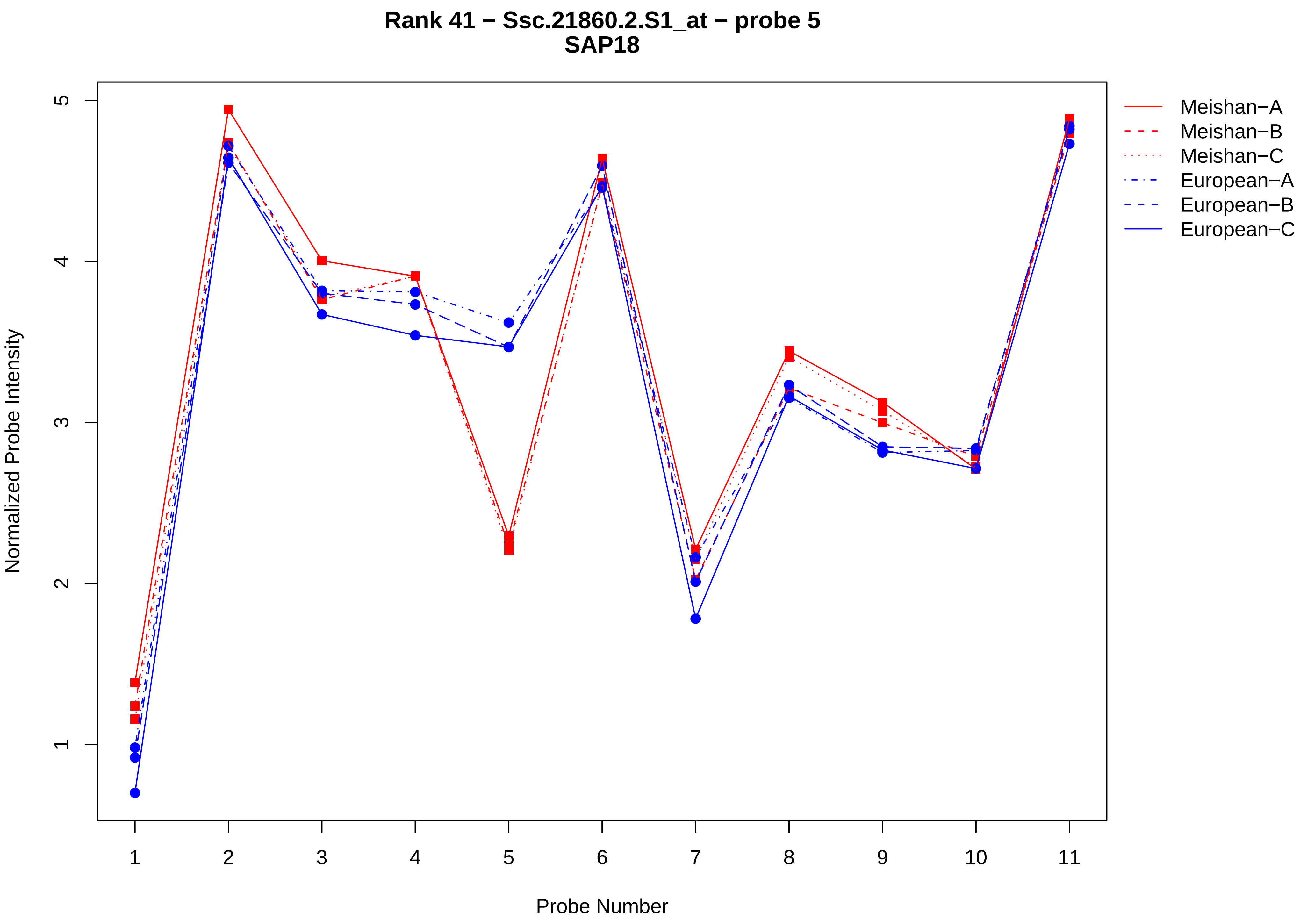
Rank 41 − Ssc.21860.2.S1_at − probe 5
SAP18
Normalized Probe Intensity
Probe Number
1
2
3
4
5
6
7
8
9
10
11
Meishan−A
Meishan−B
Meishan−C
European−A
European−B
European−C

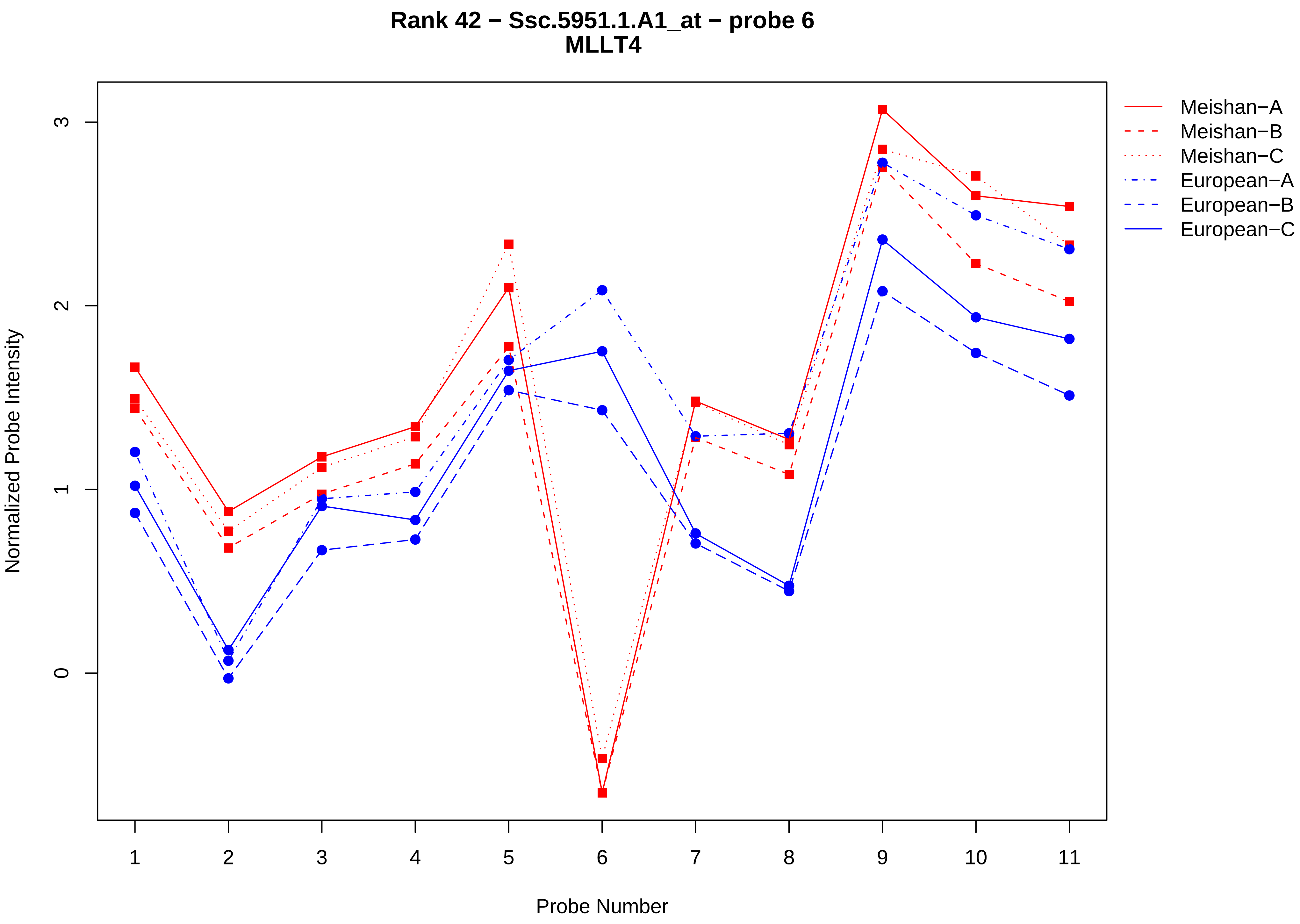

Rank 42 − Ssc.5951.1.A1_at − probe 6
MLLT4
Normalized Probe Intensity
Probe Number
0
1
2
3
1
2
3
4
5
6
7
8
9
10
11
Meishan−A
Meishan−B
Meishan−C
European−A
European−B
European−C

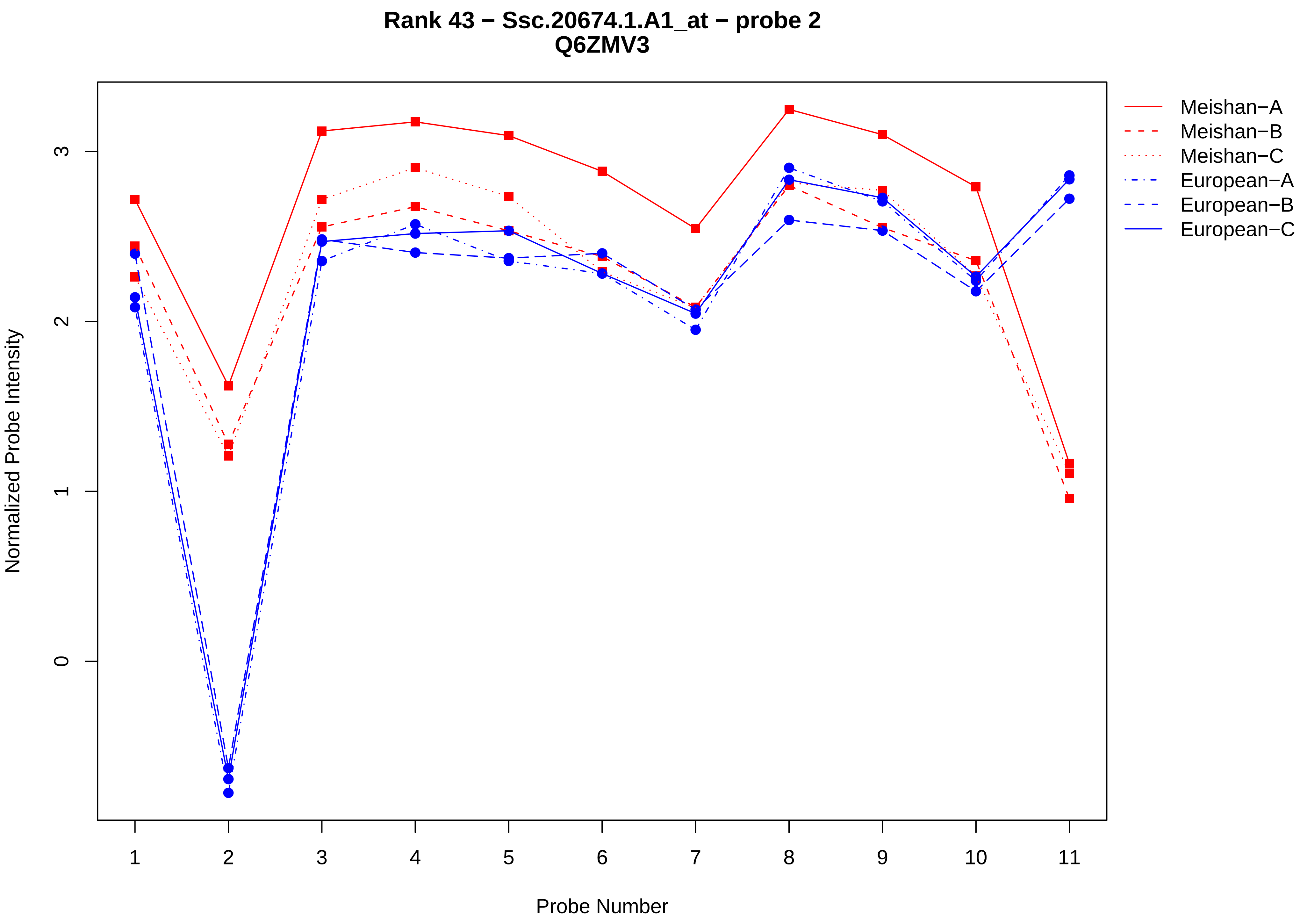

Rank 43 − Ssc.20674.1.A1_at − probe 2
Q6ZMV3
Normalized Probe Intensity
Probe Number
0
1
2
3
1
2
3
4
5
6
7
8
9
10
11
Meishan−A
Meishan−B
Meishan−C
European−A
European−B
European−C

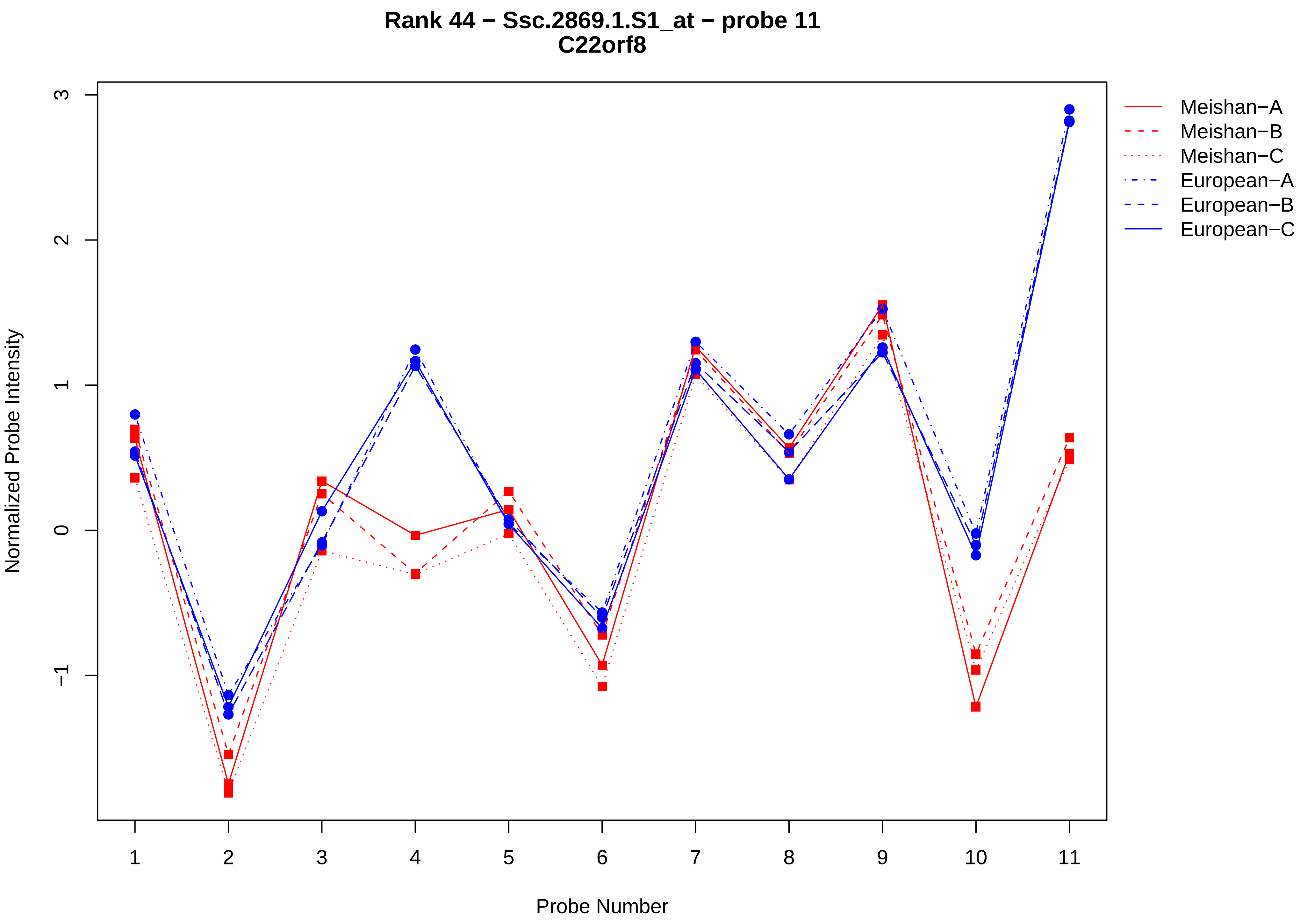
Rank 44 − Ssc.2869.1.S1_at − probe 11
C22orf8
Normalized Probe Intensity
Probe Number
Meishan−A
Meishan−B
Meishan−C
European−A
European−B
European−C
−1
0
1
2
3
1
2
3
4
5
6
7
8
9
10
11

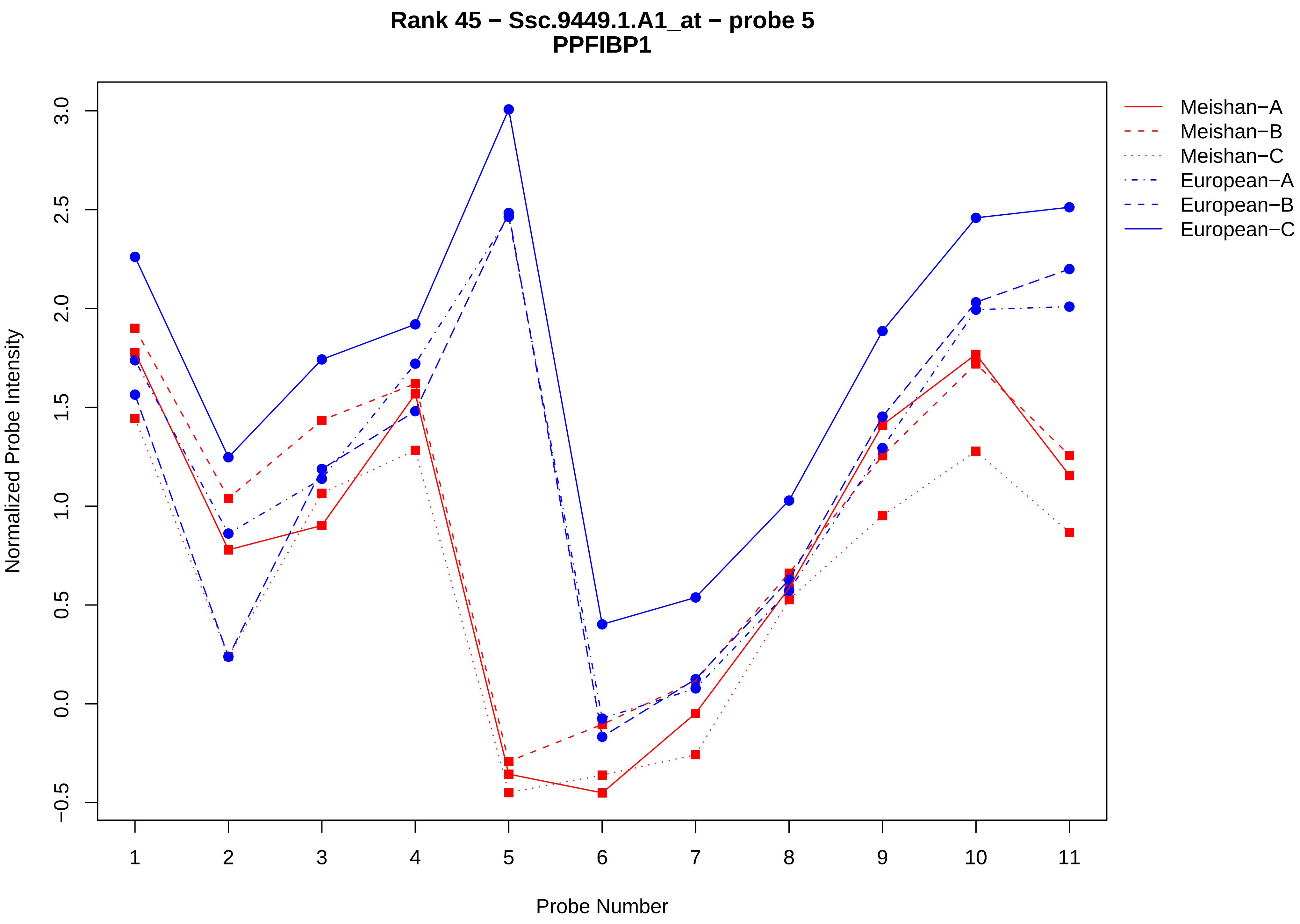
Rank 45 − Ssc.9449.1.A1_at − probe 5
PPFIBP1
Normalized Probe Intensity
Probe Number
−0.5
0.0
0.5
1.0
1.5
2.0
2.5
3.0
1
2
3
4
5
6
7
8
9
10
11
Meishan−A
Meishan−B
Meishan−C
European−A
European−B
European−C

Rank 46 − Ssc.7040.2.A1_at − probe 10
SLC30A9

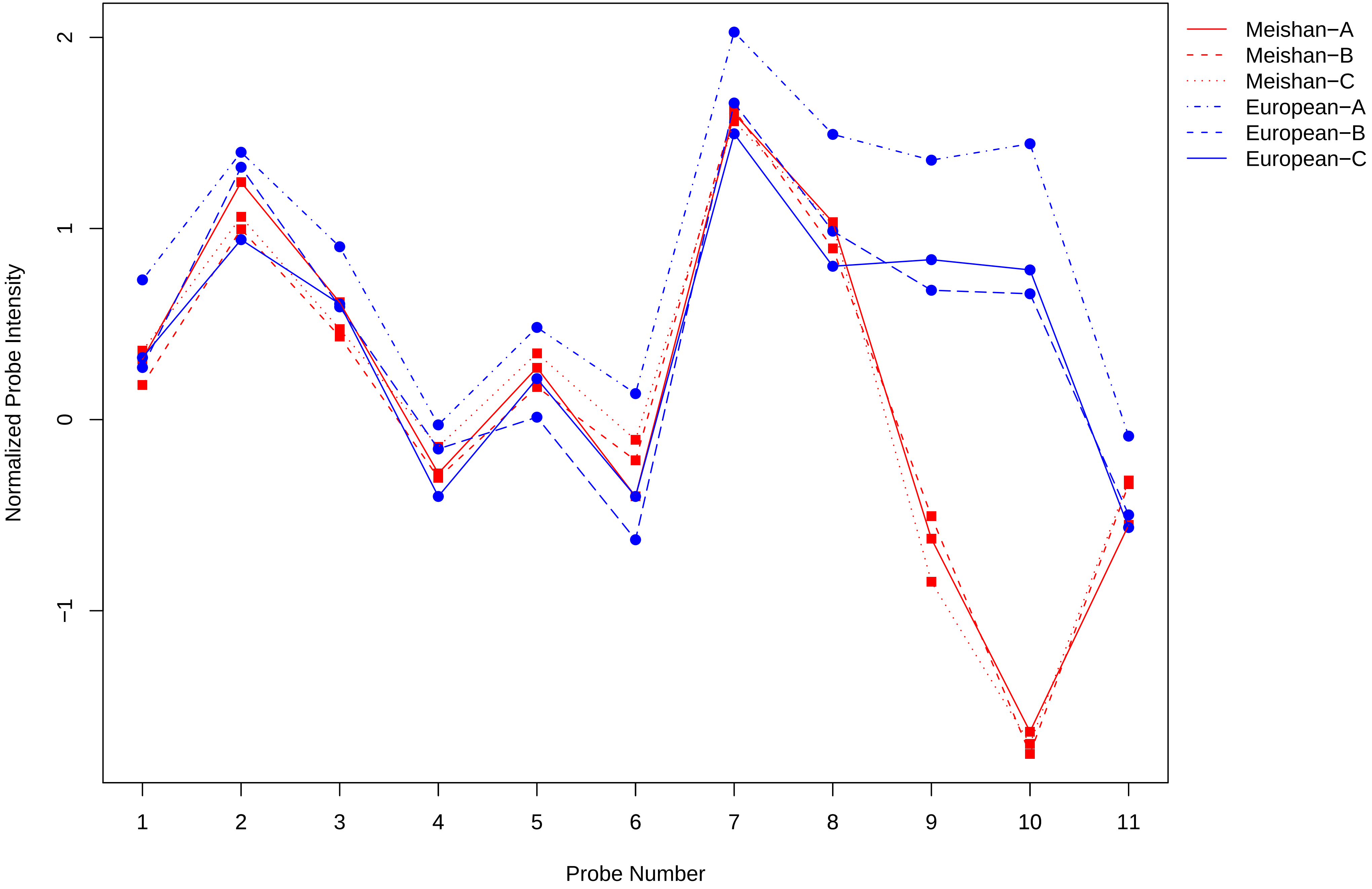

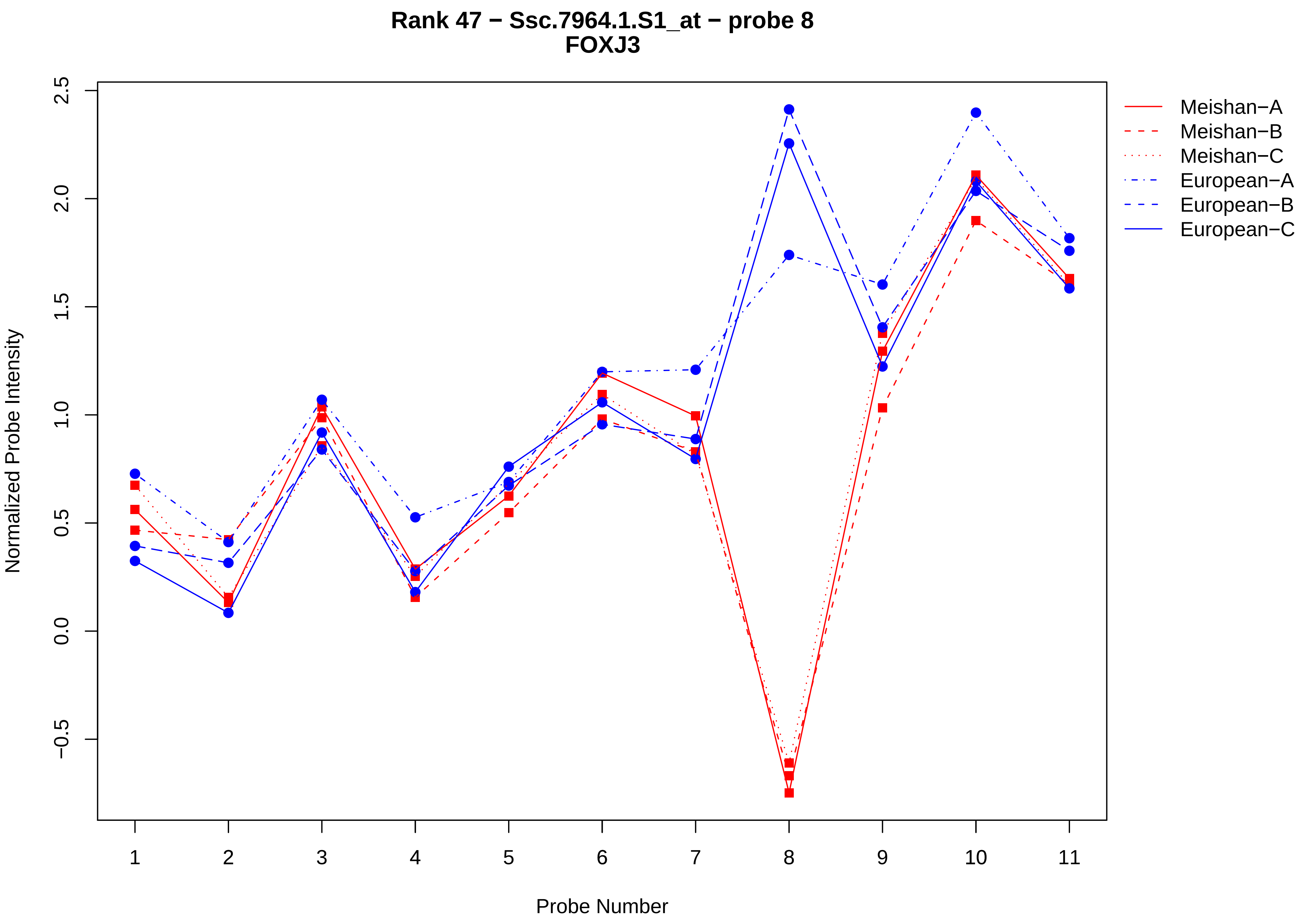

Rank 47 − Ssc.7964.1.S1_at − probe 8
FOXJ3
Normalized Probe Intensity
−0.5
0.0
0.5
1.0
1.5
2.0
2.5
Probe Number
1
2
3
4
5
6
7
8
9
10
11
Meishan−A
Meishan−B
Meishan−C
European−A
European−B
European−C

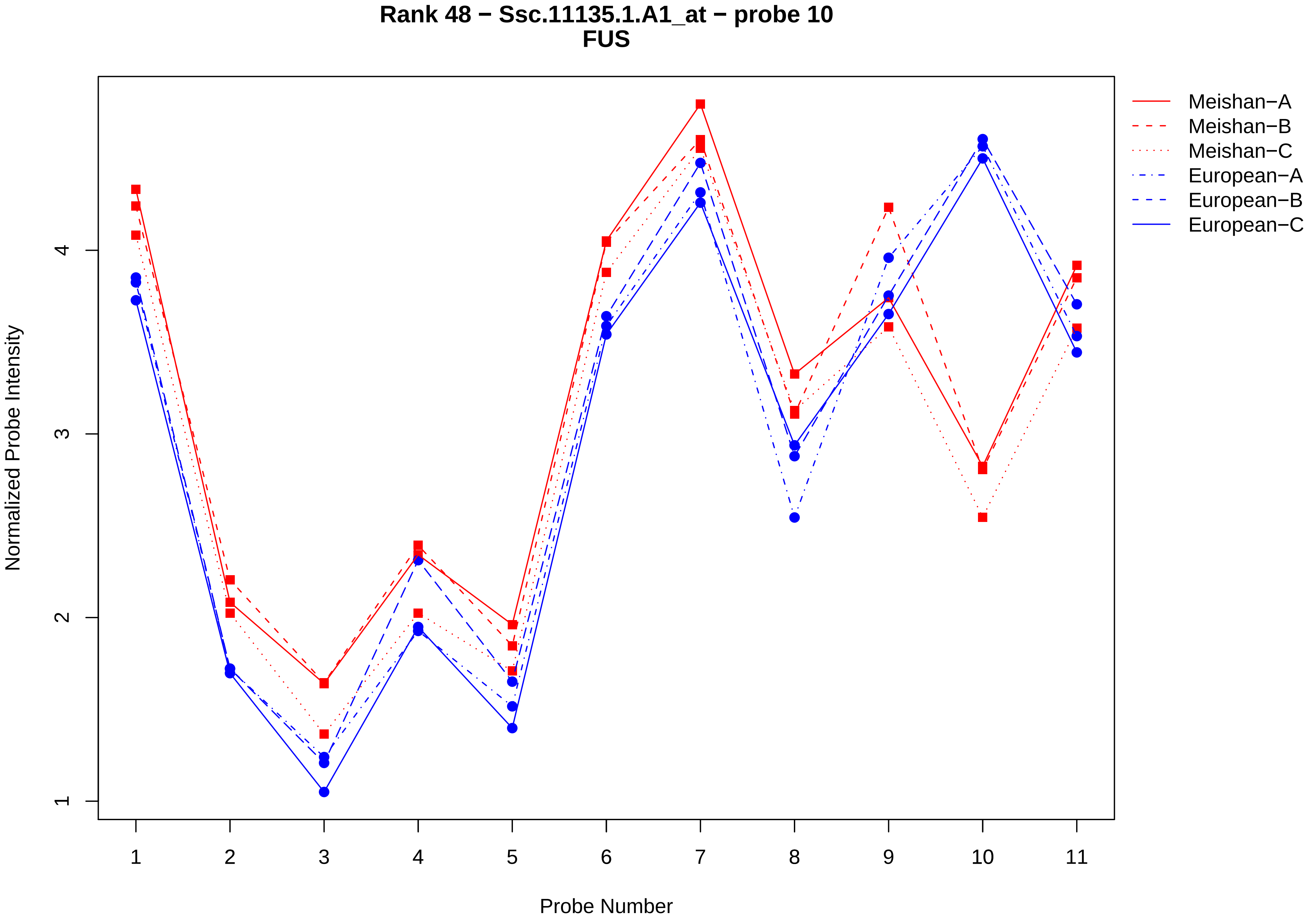
Rank 48 − Ssc.11135.1.A1_at − probe 10
FUS
Normalized Probe Intensity
Probe Number
1
2
3
4
5
6
7
8
9
10
11
Meishan−A
Meishan−B
Meishan−C
European−A
European−B
European−C

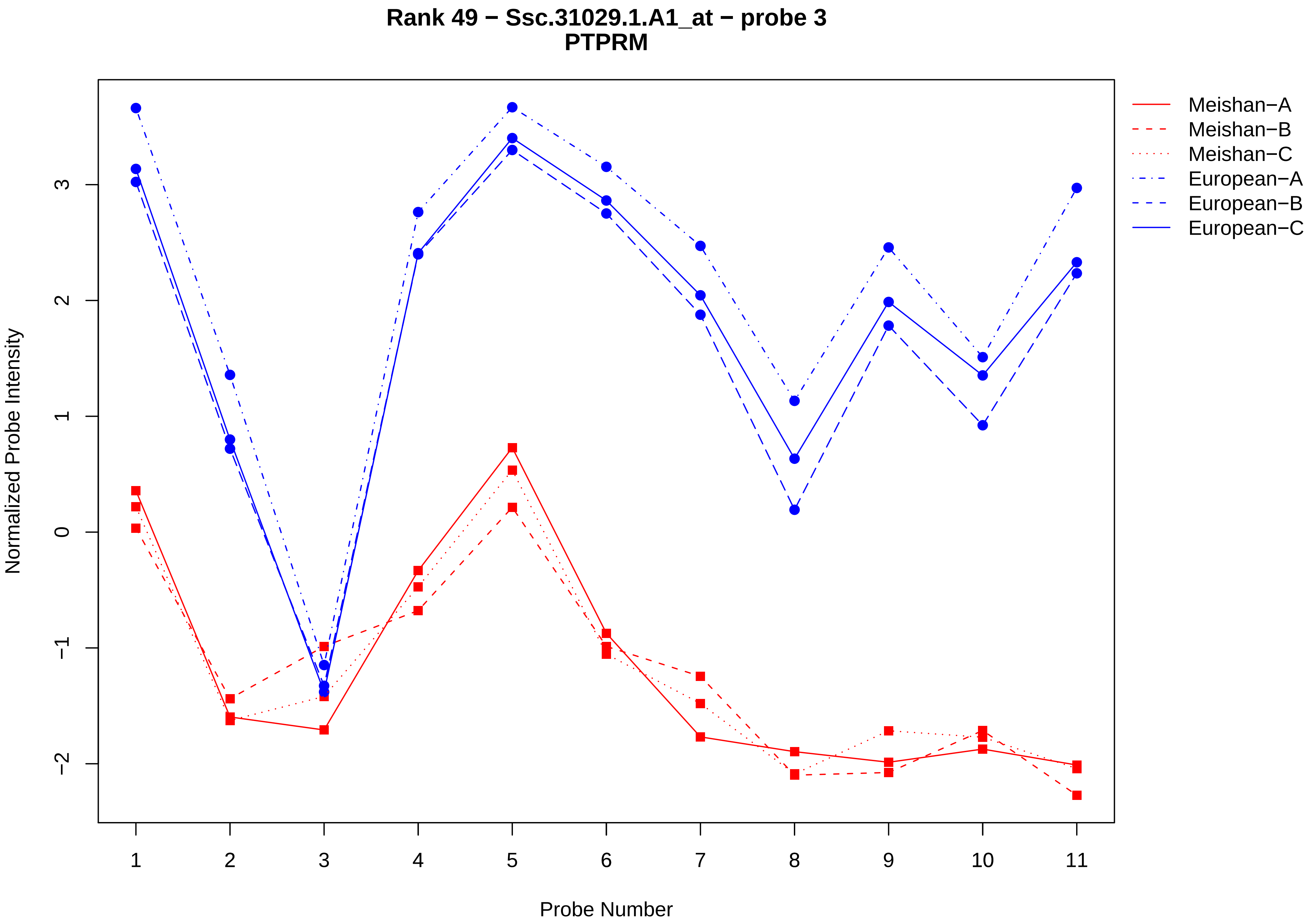

Rank 49 − Ssc.31029.1.A1_at − probe 3
PTPRM
Normalized Probe Intensity
Probe Number
Meishan−A
Meishan−B
Meishan−C
European−A
European−B
European−C

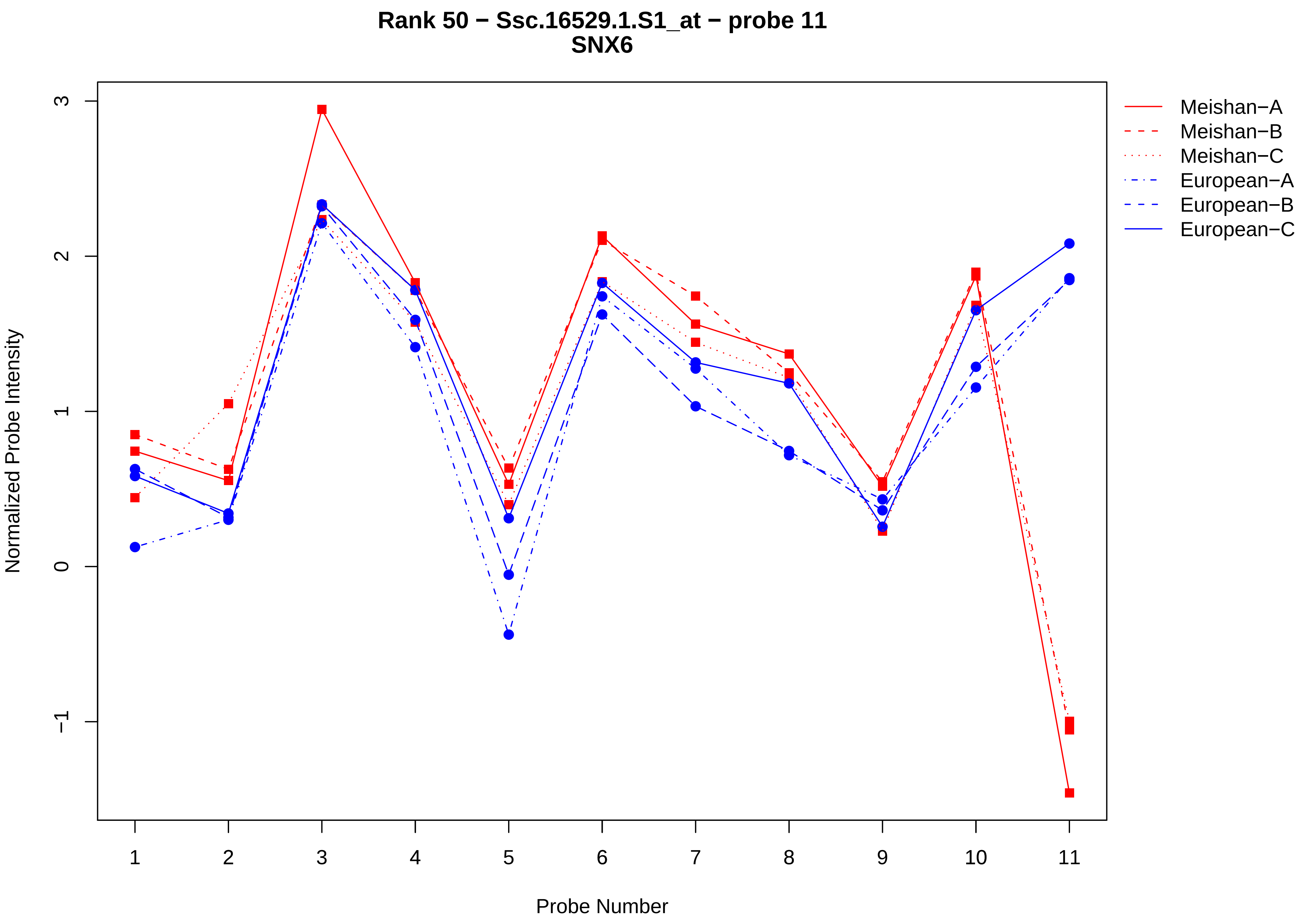

Rank 50 − Ssc.16529.1.S1_at − probe 11
SNX6
Normalized Probe Intensity
Probe Number
Meishan−A
Meishan−B
Meishan−C
European−A
European−B
European−C

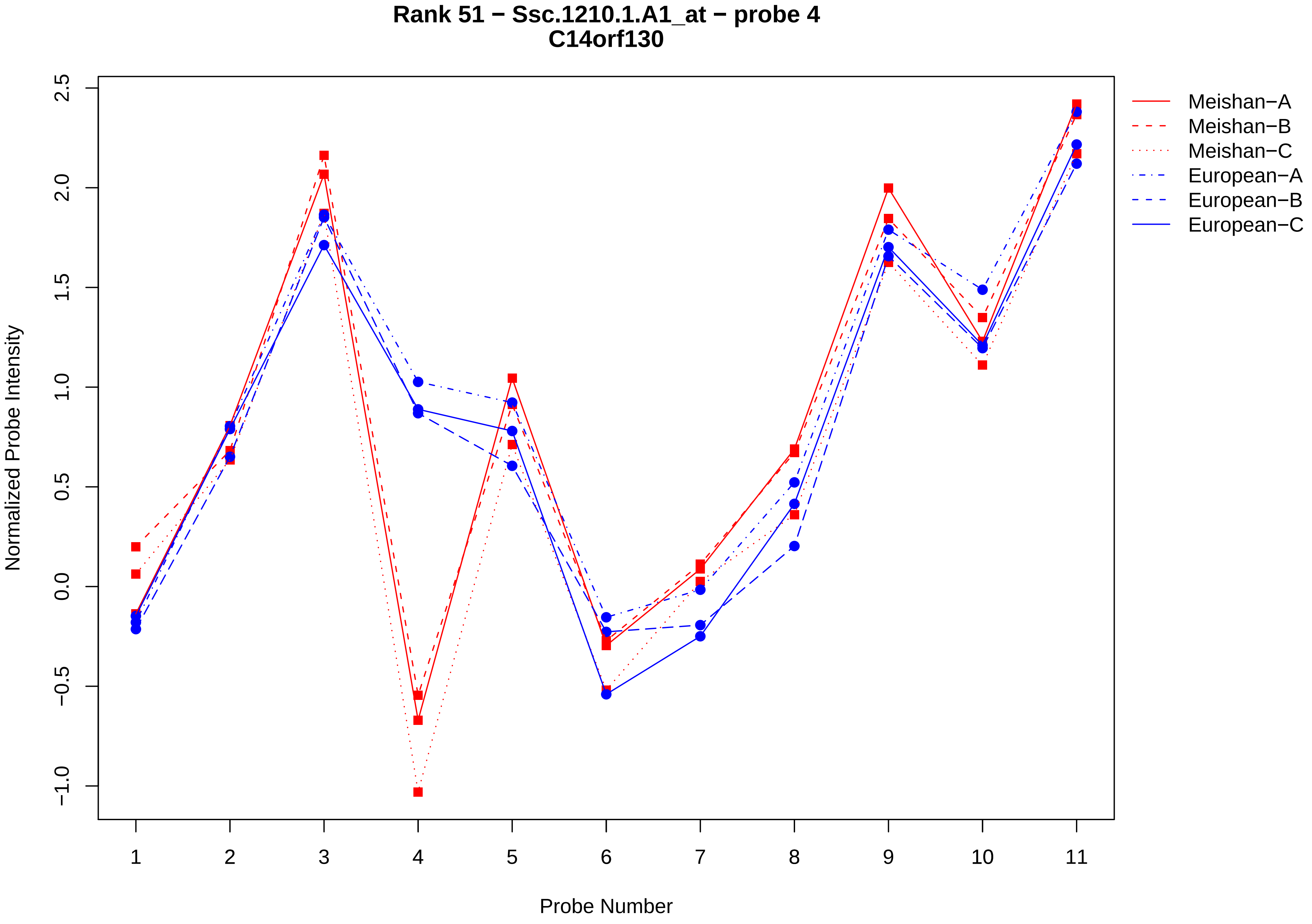

Rank 51 − Ssc.1210.1.A1_at − probe 4
C14orf130
Normalized Probe Intensity
Probe Number
Meishan−A
Meishan−B
Meishan−C
European−A
European−B
European−C
2.5
2.0
1.5
1.0
0.5
0.0
−0.5
−1.0
1
2
3
4
5
6
7
8
9
10
11

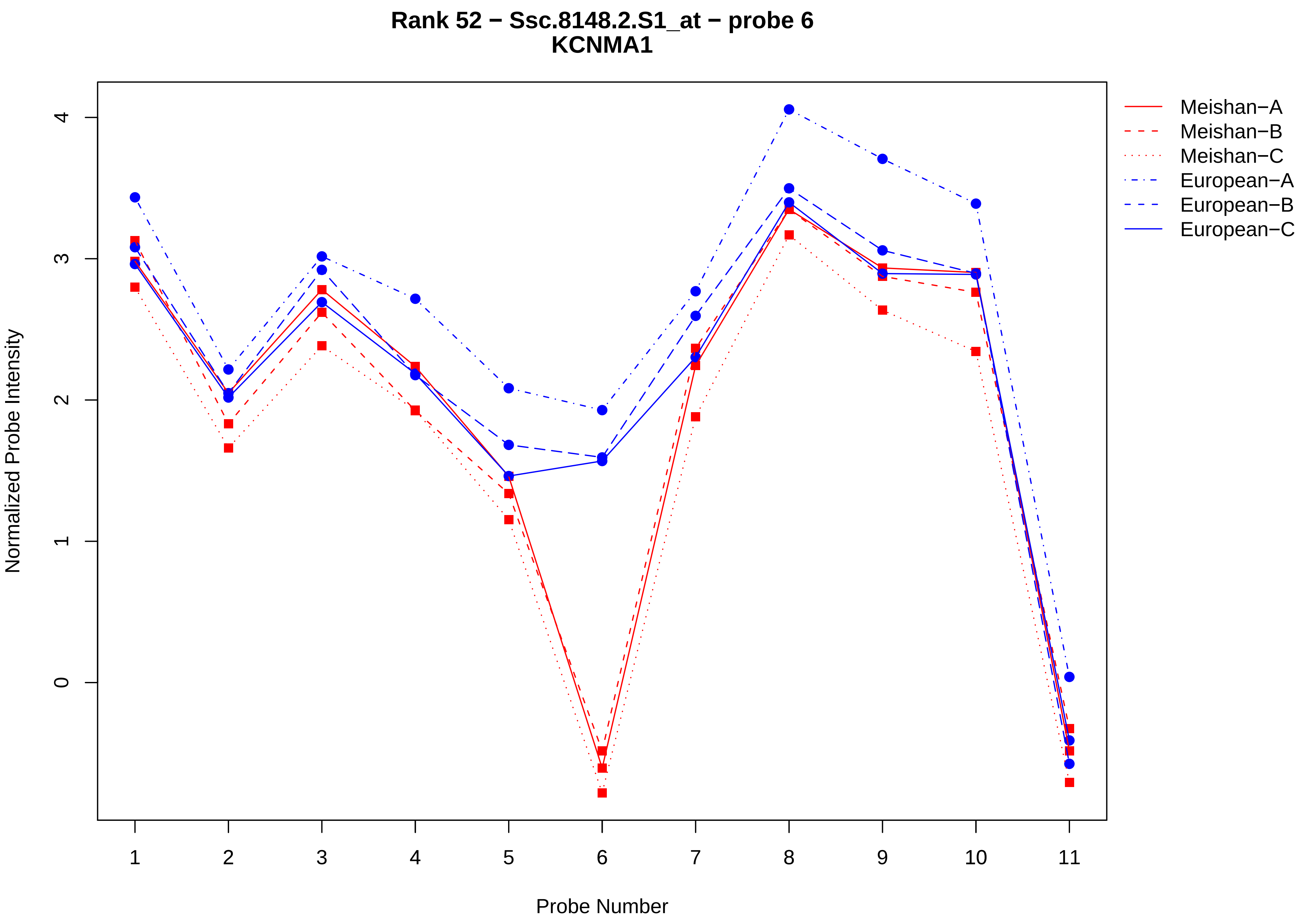

Rank 52 − Ssc.8148.2.S1_at − probe 6
KCNMA1
Normalized Probe Intensity
Probe Number
0
1
2
3
4
1
2
3
4
5
6
7
8
9
10
11
Meishan−A
Meishan−B
Meishan−C
European−A
European−B
European−C

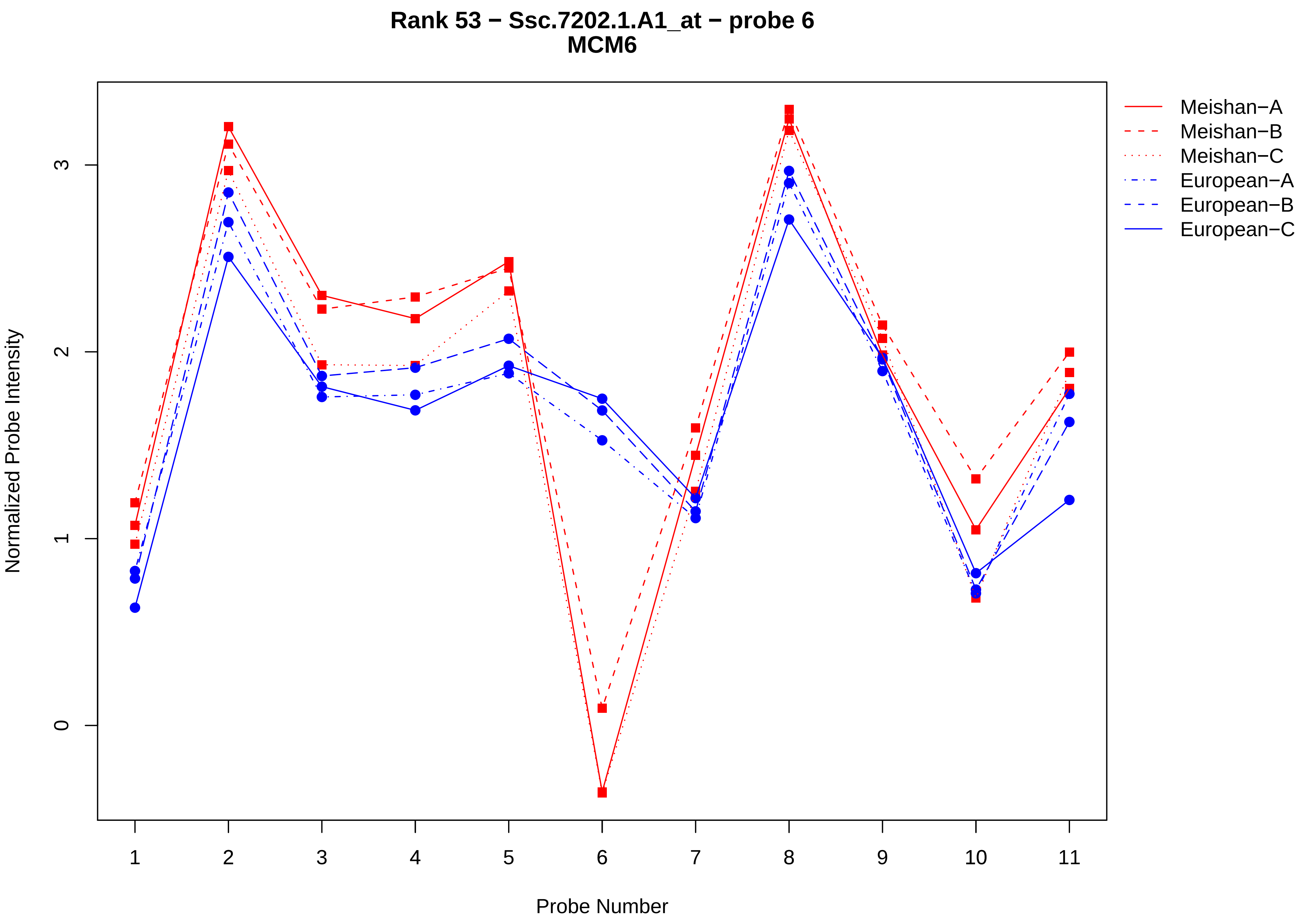

Rank 53 − Ssc.7202.1.A1_at − probe 6
MCM6
Normalized Probe Intensity
Probe Number
0
1
2
3
1
2
3
4
5
6
7
8
9
10
11
Meishan−A
Meishan−B
Meishan−C
European−A
European−B
European−C

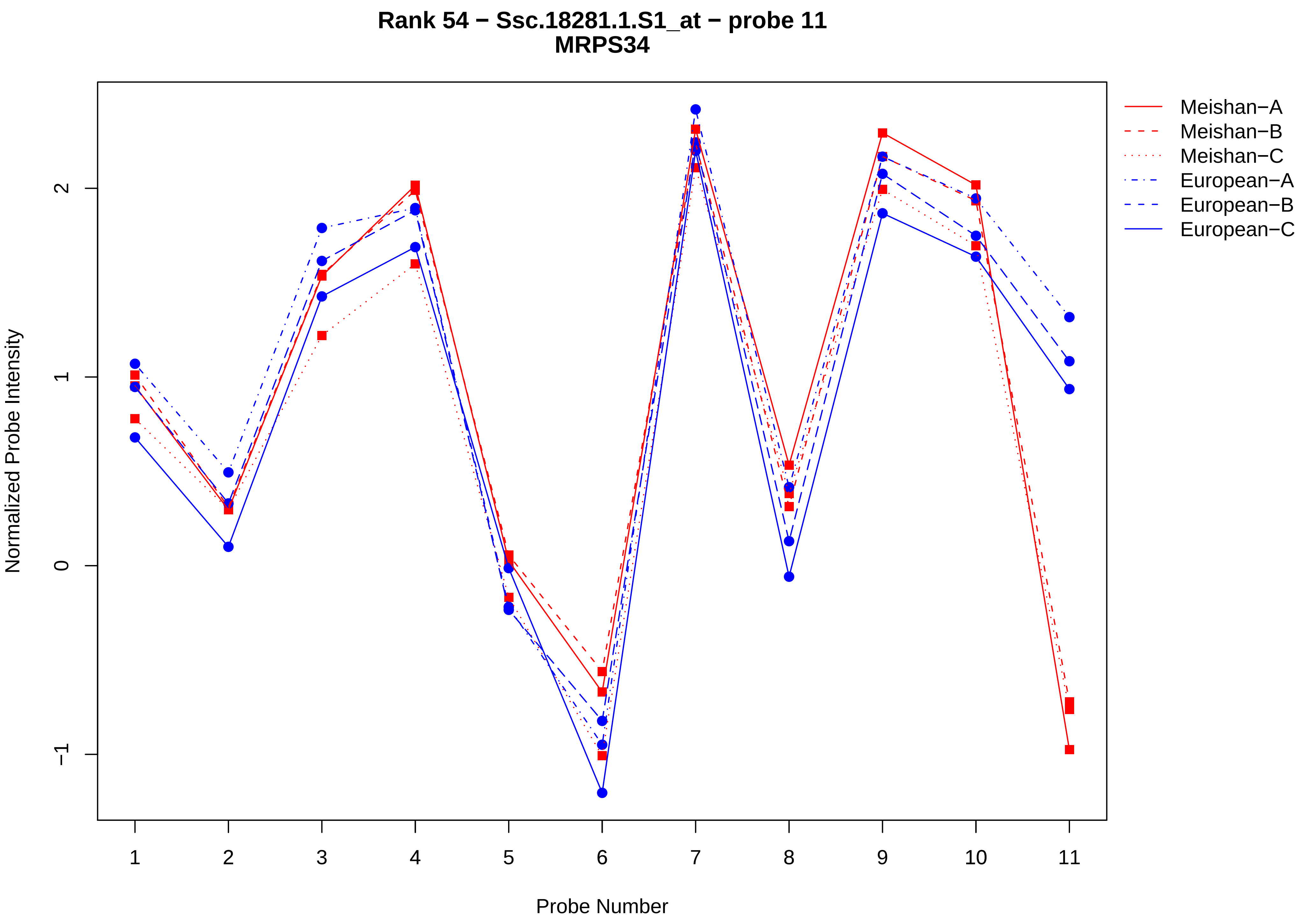

Rank 54 − Ssc.18281.1.S1_at − probe 11
MRPS34
Normalized Probe Intensity
Probe Number
−1
0
1
2
1
2
3
4
5
6
7
8
9
10
11
Meishan−A
Meishan−B
Meishan−C
European−A
European−B
European−C

Rank 55 − Ssc.17323.1.S1_at − probe 4
CSTB

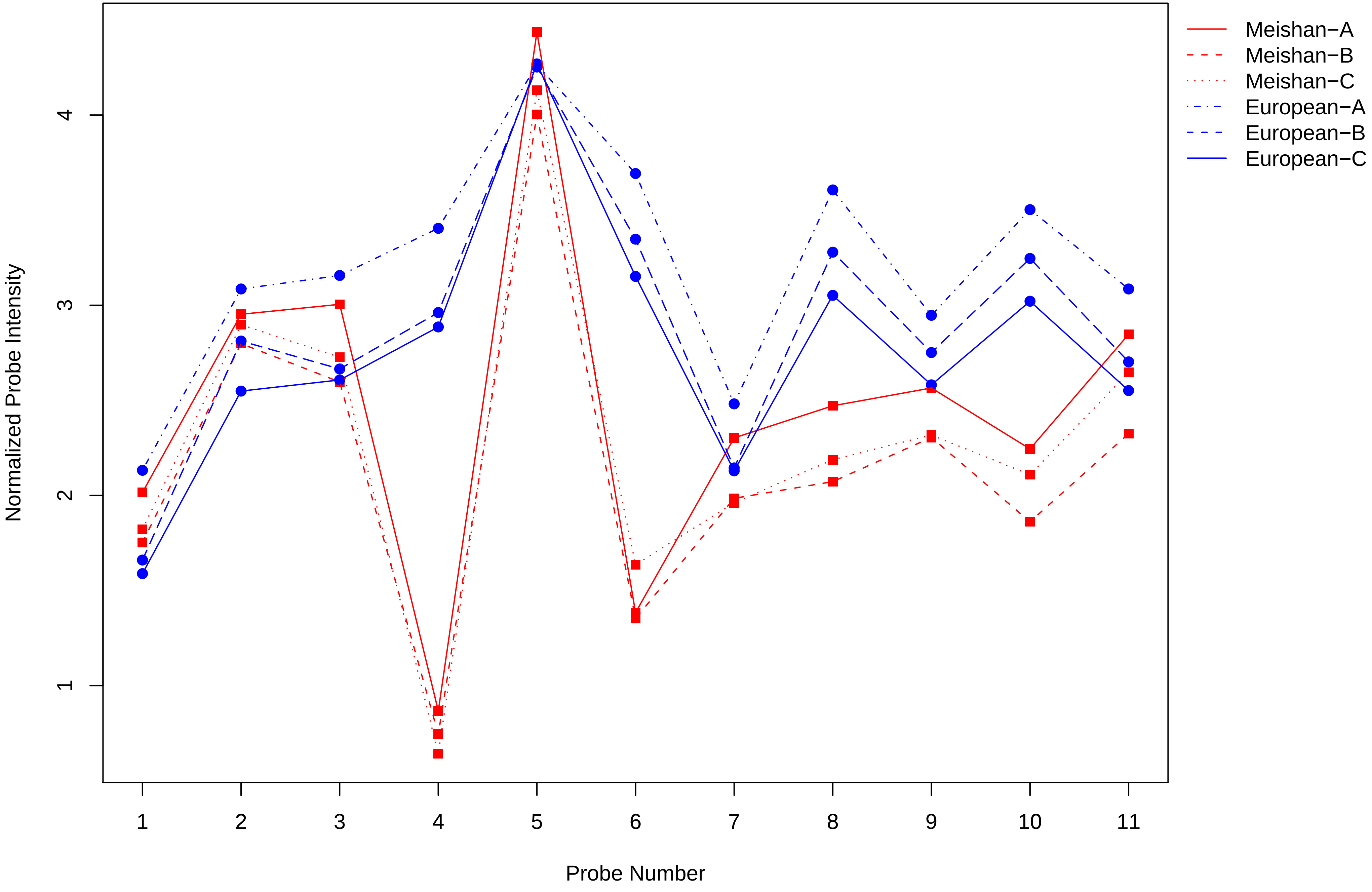

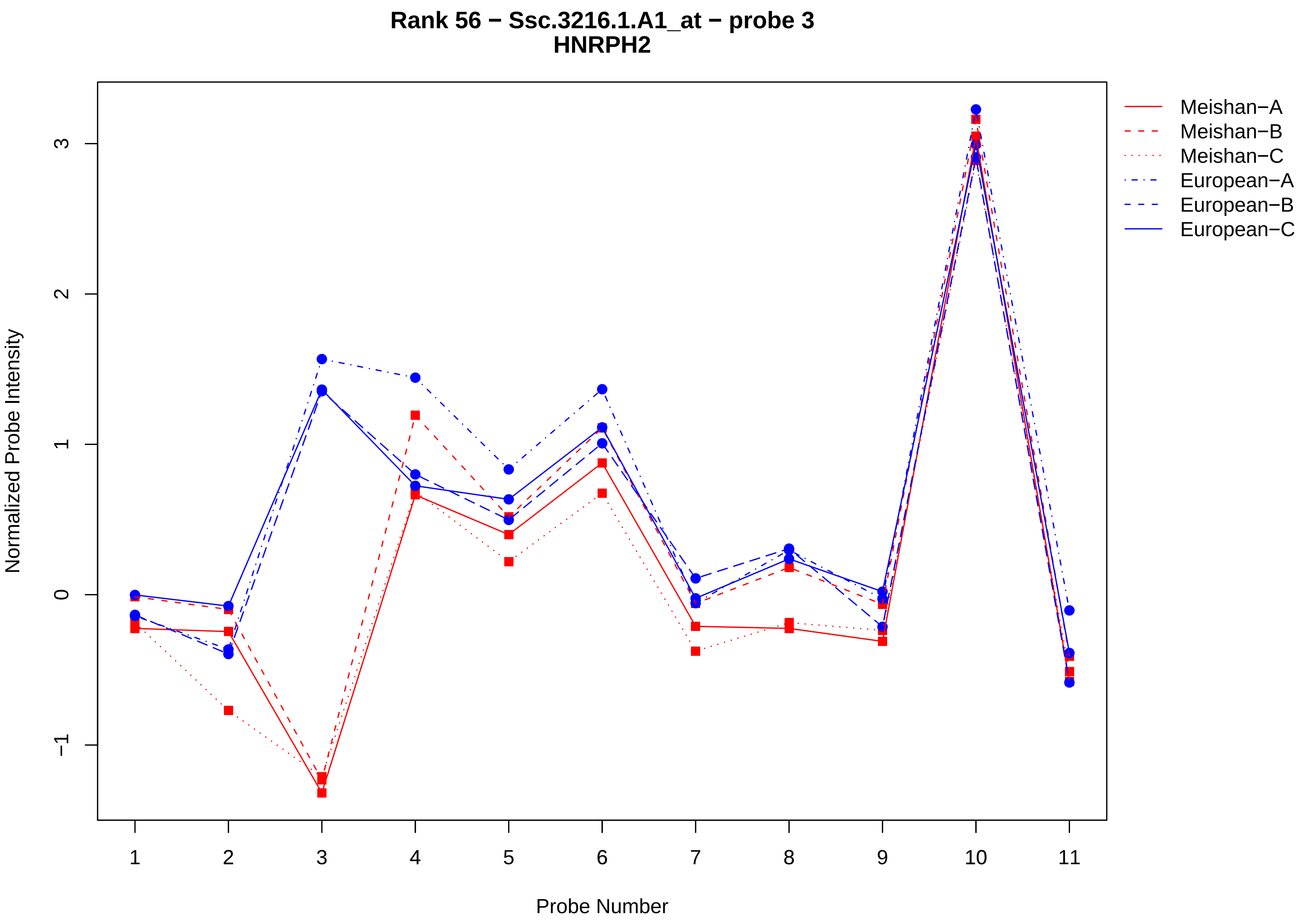

Rank 56 − Ssc.3216.1.A1_at − probe 3
HNRPH2
Normalized Probe Intensity
Probe Number
−1
0
1
2
3
1
2
3
4
5
6
7
8
9
10
11
Meishan−A
Meishan−B
Meishan−C
European−A
European−B
European−C

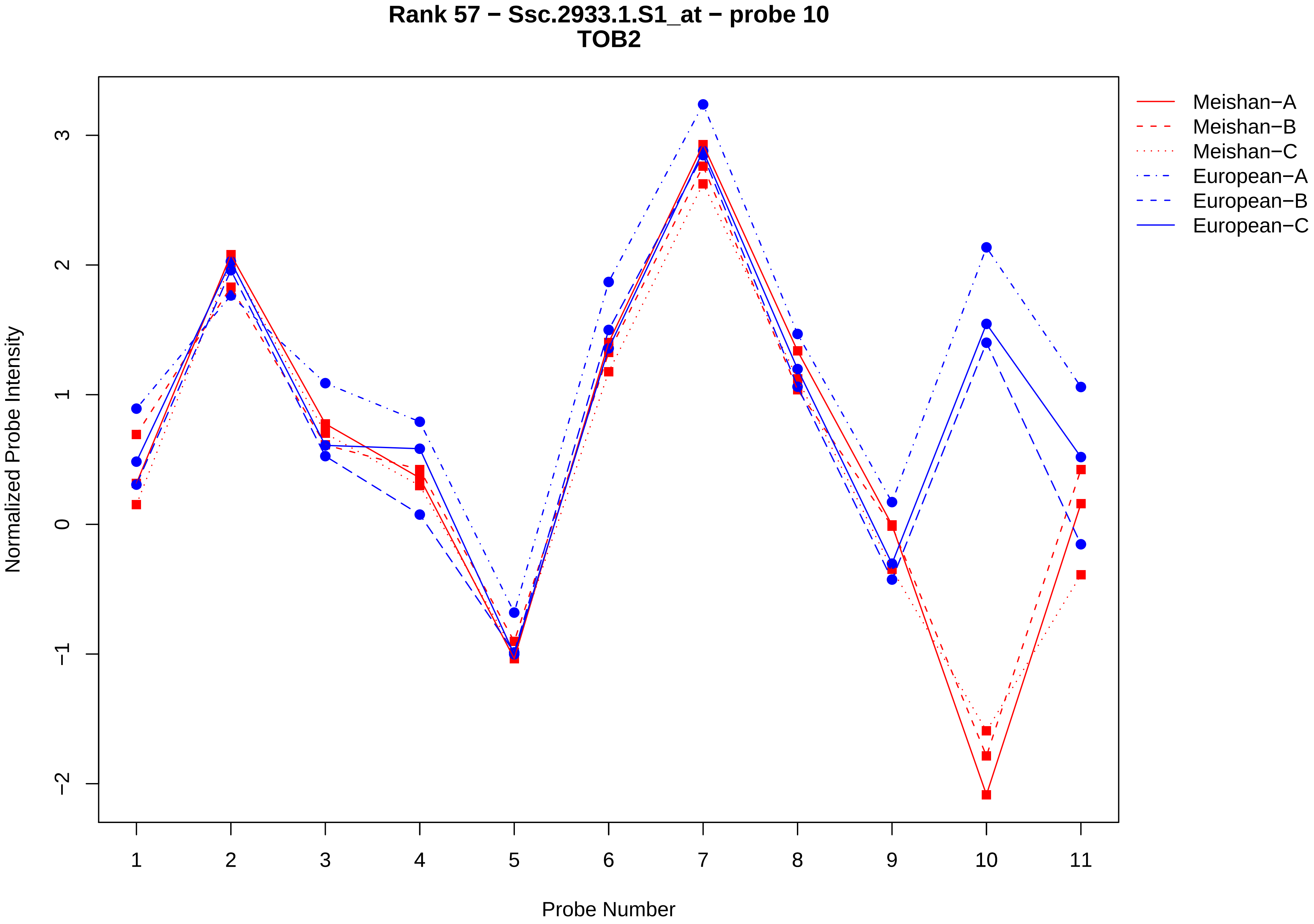
Rank 57 − Ssc.2933.1.S1_at − probe 10
TOB2
Normalized Probe Intensity
Probe Number
−2
−1
0
1
2
3
1
2
3
4
5
6
7
8
9
10
11
Meishan−A
Meishan−B
Meishan−C
European−A
European−B
European−C

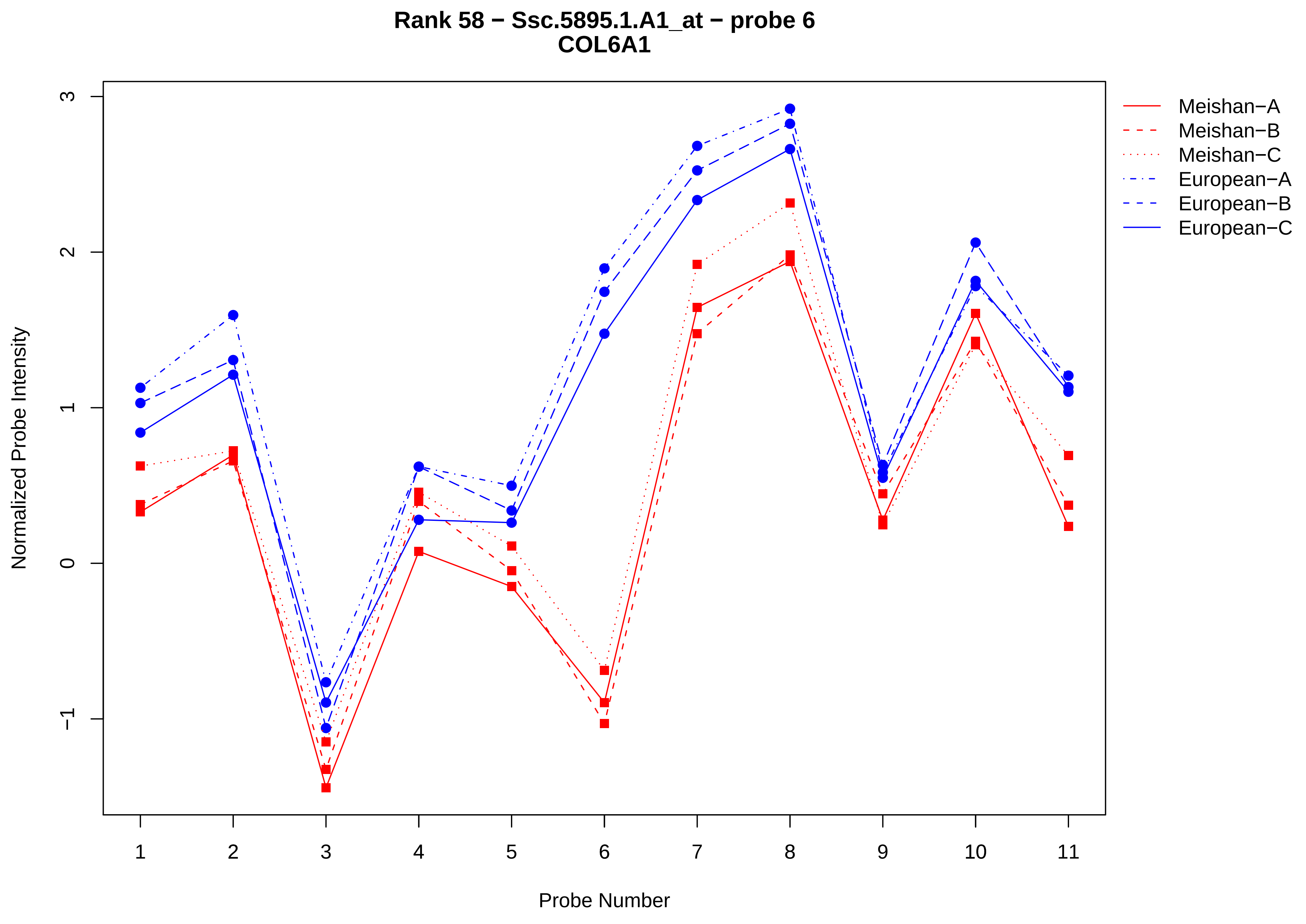

Rank 58 − Ssc.5895.1.A1_at − probe 6
COL6A1
Normalized Probe Intensity
Probe Number
Meishan−A
Meishan−B
Meishan−C
European−A
European−B
European−C

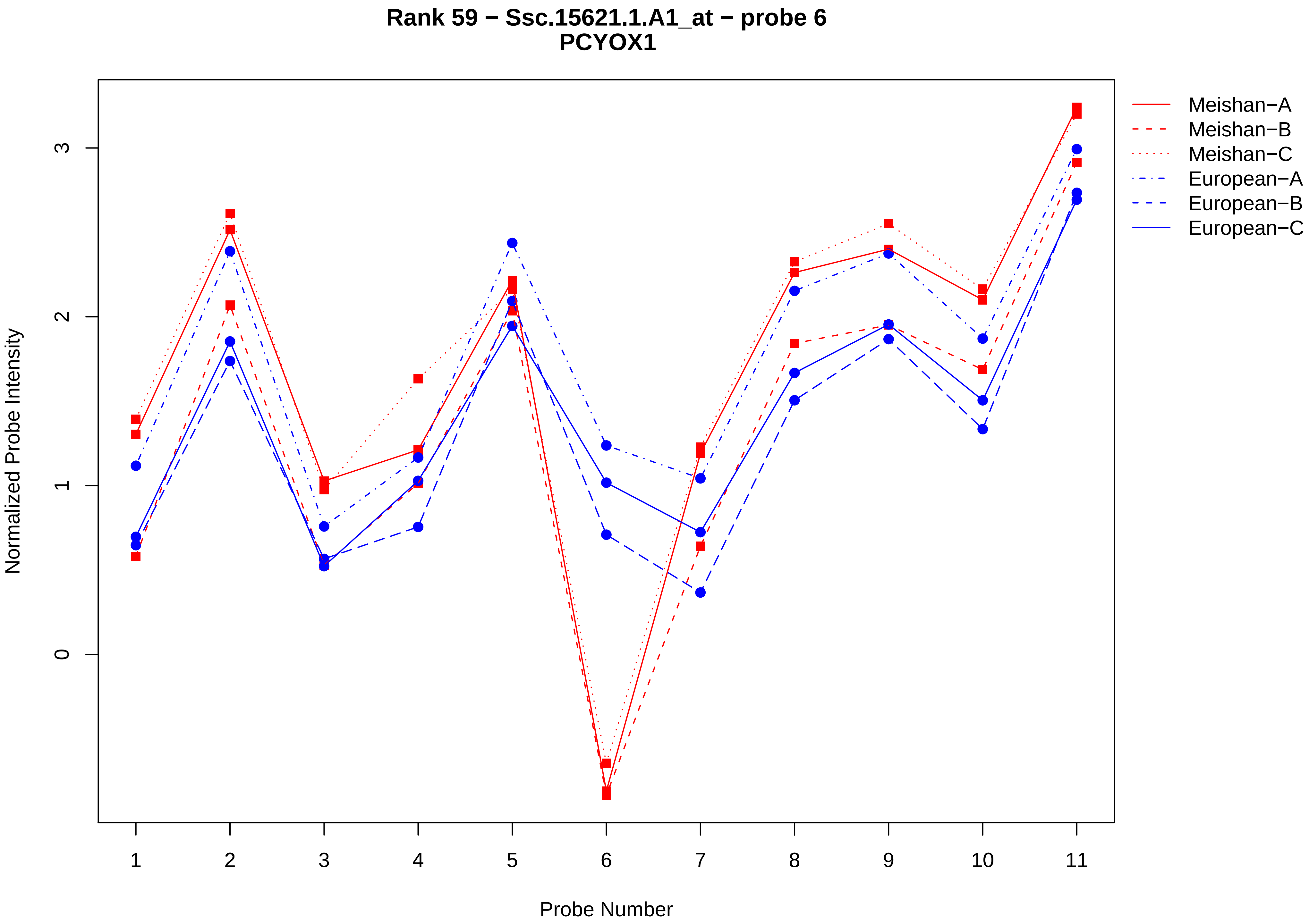

Rank 59 − Ssc.15621.1.A1_at − probe 6
PCYOX1
Normalized Probe Intensity
0
1
2
3
1
2
3
4
5
6
7
8
9
10
11
Probe Number
Meishan−A
Meishan−B
Meishan−C
European−A
European−B
European−C

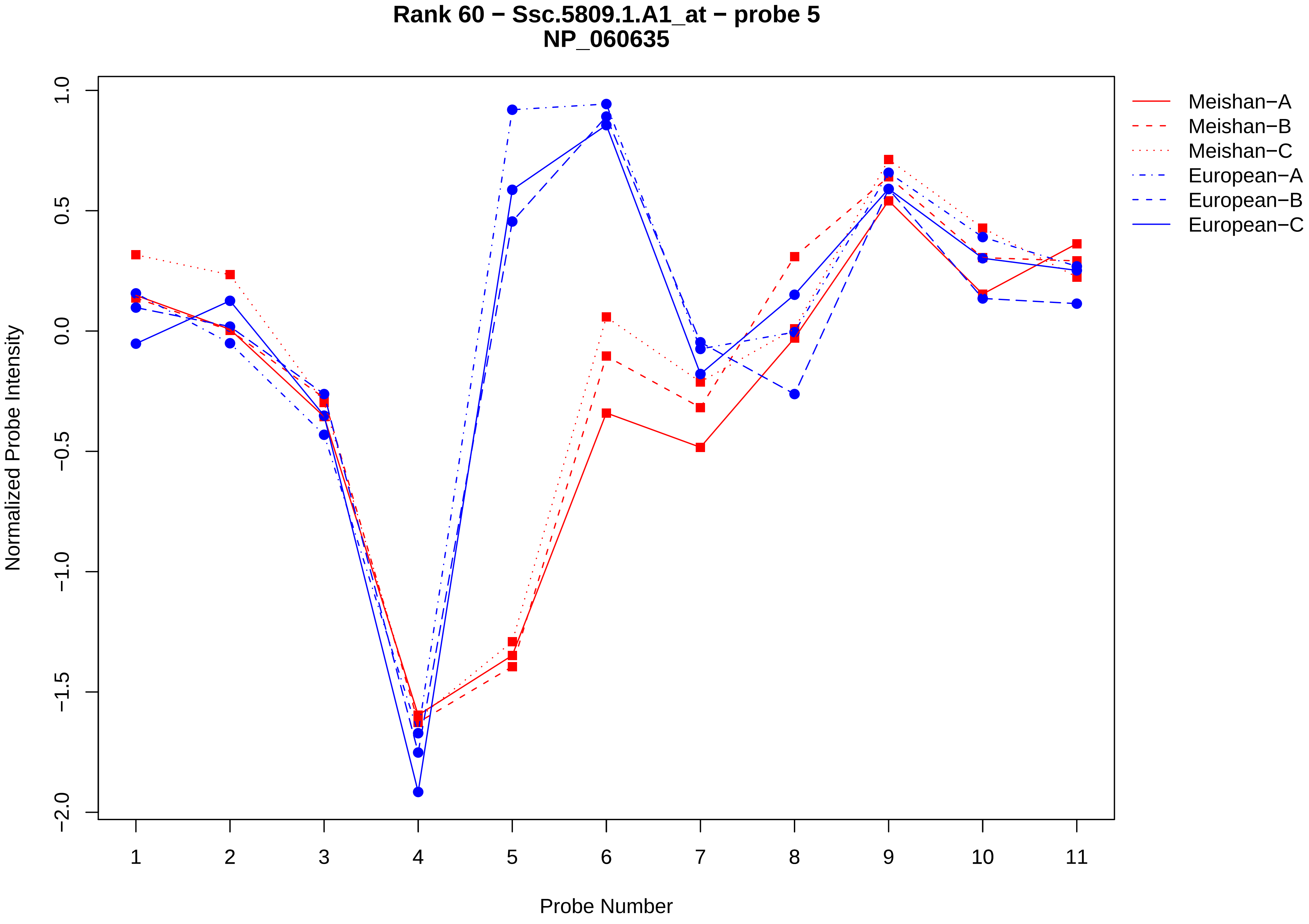
Rank 60 − Ssc.5809.1.A1_at − probe 5
NP_060635
Normalized Probe Intensity
Probe Number
Meishan−A
Meishan−B
Meishan−C
European−A
European−B
European−C
1.0
0.5
0.0
−0.5
−1.0
−1.5
−2.0
1
2
3
4
5
6
7
8
9
10
11

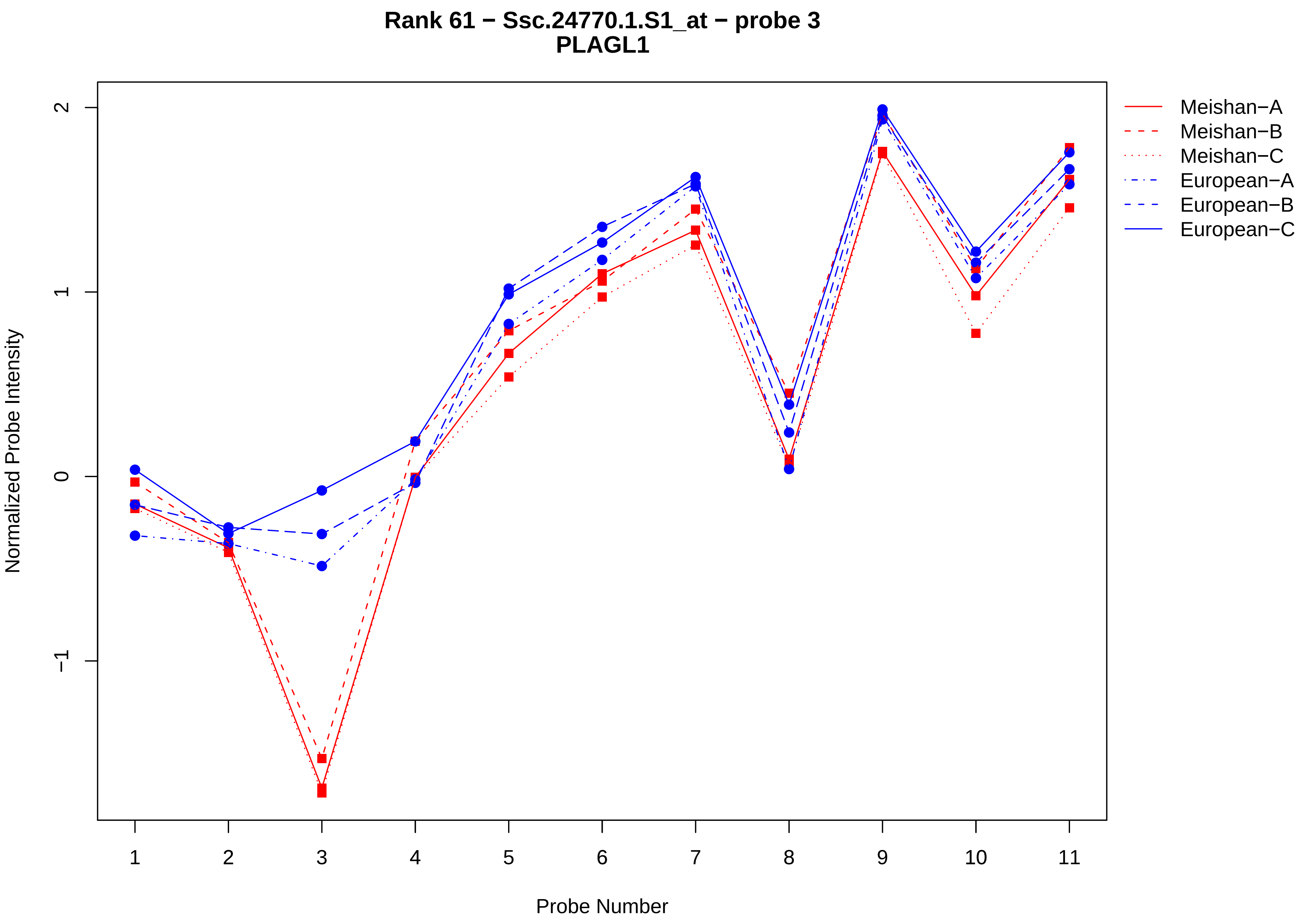
Rank 61 − Ssc.24770.1.S1_at − probe 3
PLAGL1
Normalized Probe Intensity
Probe Number
−1
0
1
2
1
2
3
4
5
6
7
8
9
10
11
Meishan−A
Meishan−B
Meishan−C
European−A
European−B
European−C

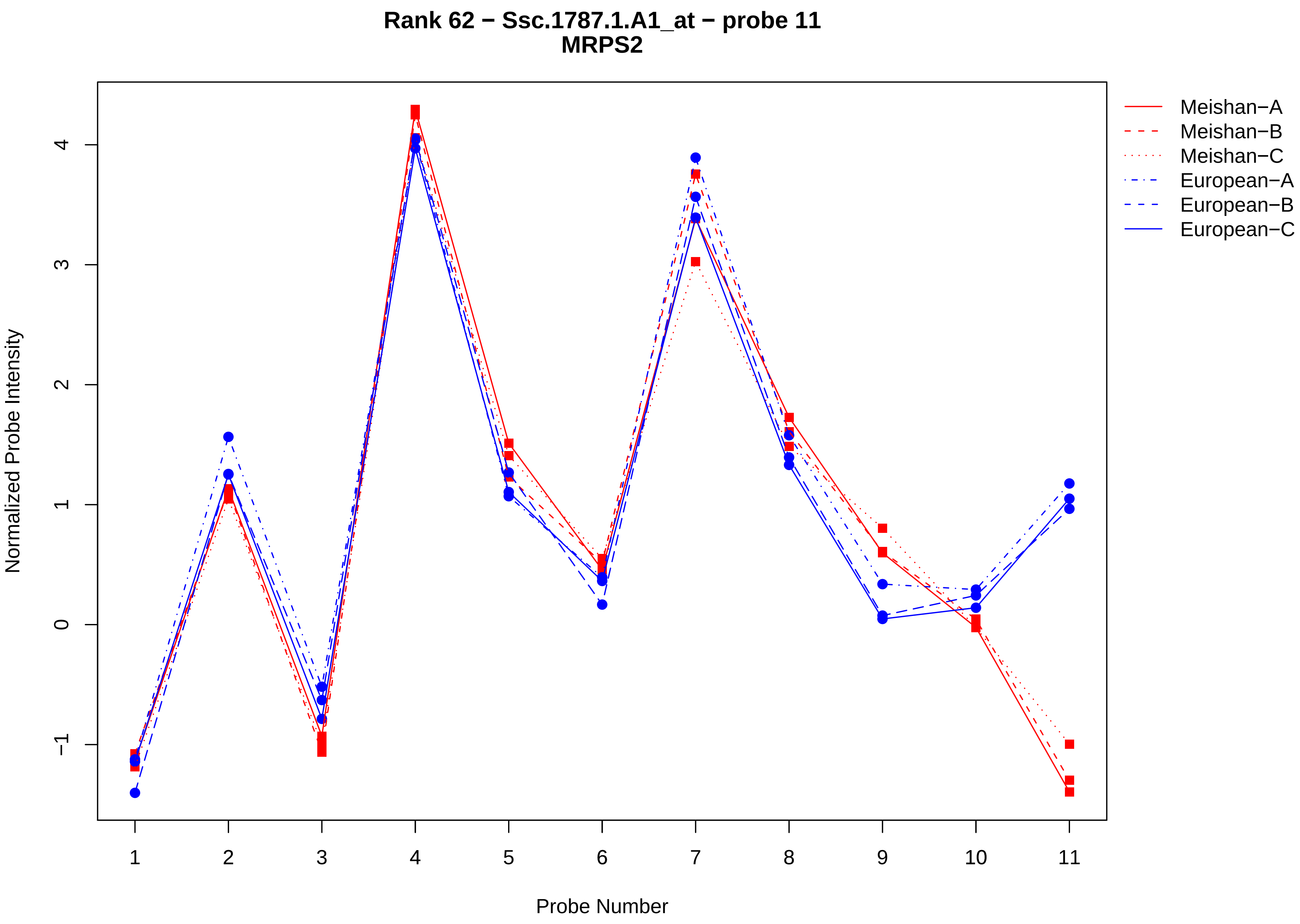

Rank 62 − Ssc.1787.1.A1_at − probe 11
MRPS2
Normalized Probe Intensity
Probe Number
Meishan−A
Meishan−B
Meishan−C
European−A
European−B
European−C
−1
0
1
2
3
4
1
2
3
4
5
6
7
8
9
10
11

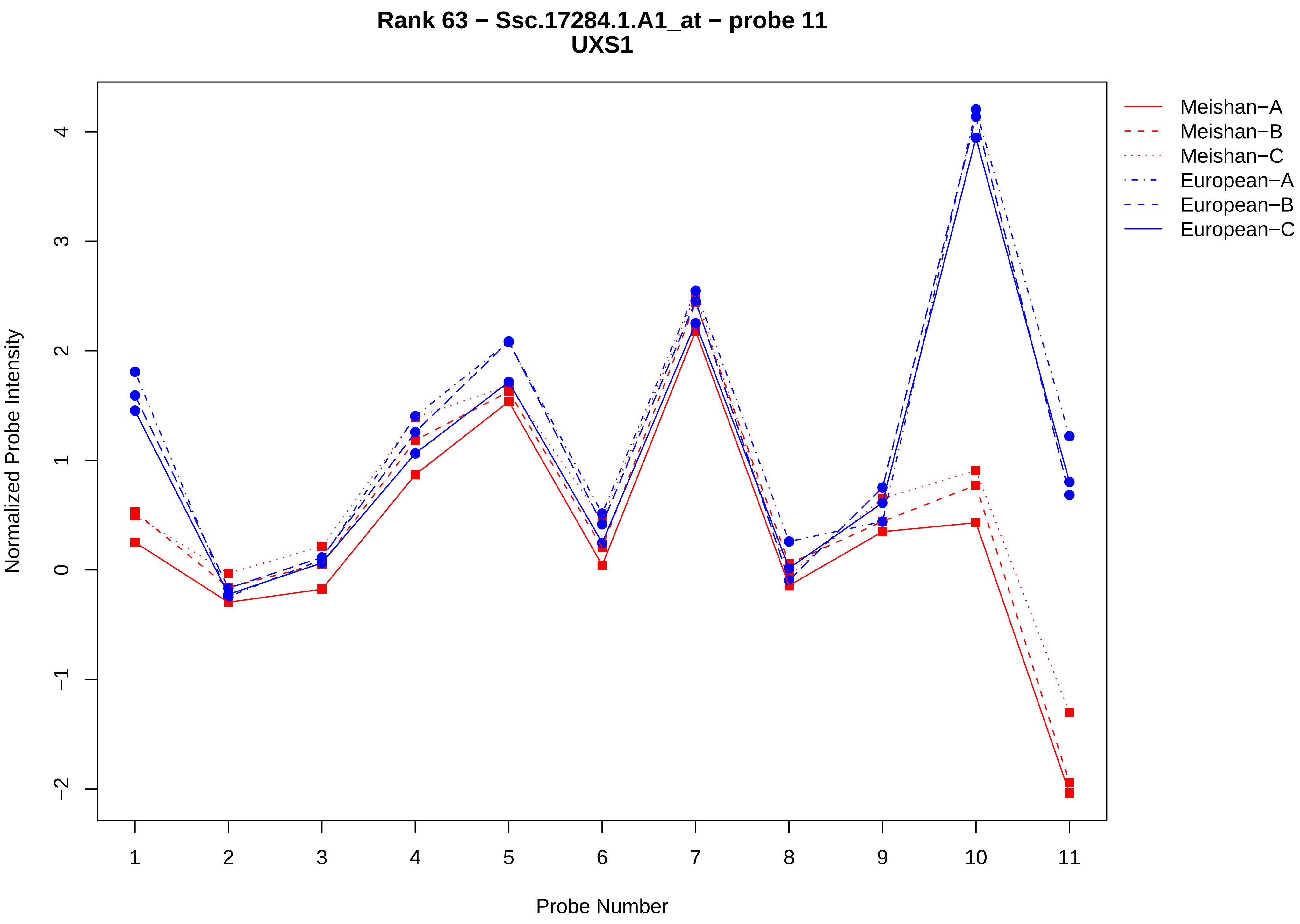

Rank 63 − Ssc.17284.1.A1_at − probe 11
UXS1
Normalized Probe Intensity
Probe Number
−2
−1
0
1
2
3
4
1
2
3
4
5
6
7
8
9
10
11
Meishan−A
Meishan−B
Meishan−C
European−A
European−B
European−C

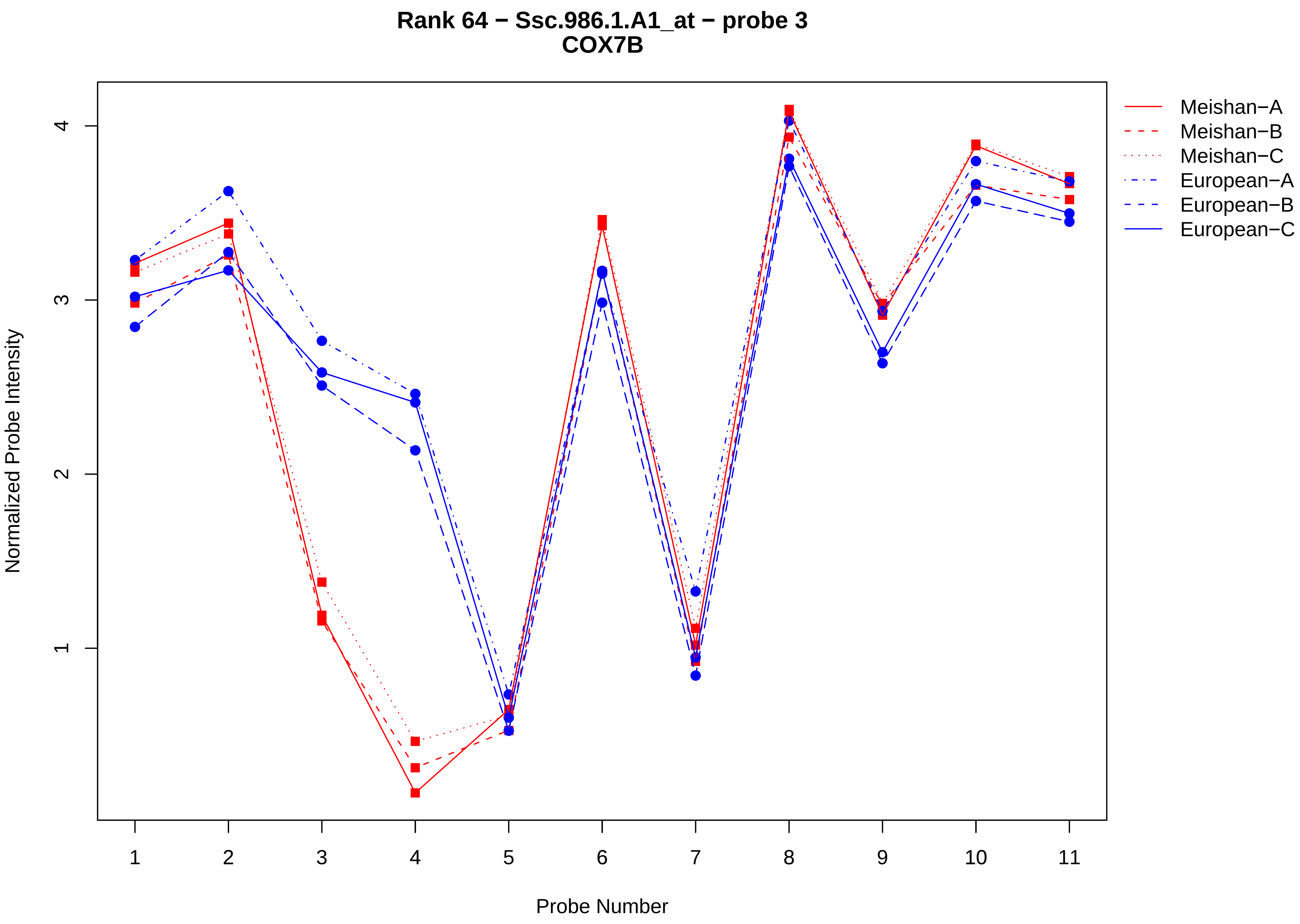

Rank 64 − Ssc.986.1.A1_at − probe 3
COX7B
Normalized Probe Intensity
1
2
3
4
Probe Number
1
2
3
4
5
6
7
8
9
10
11
Meishan−A
Meishan−B
Meishan−C
European−A
European−B
European−C

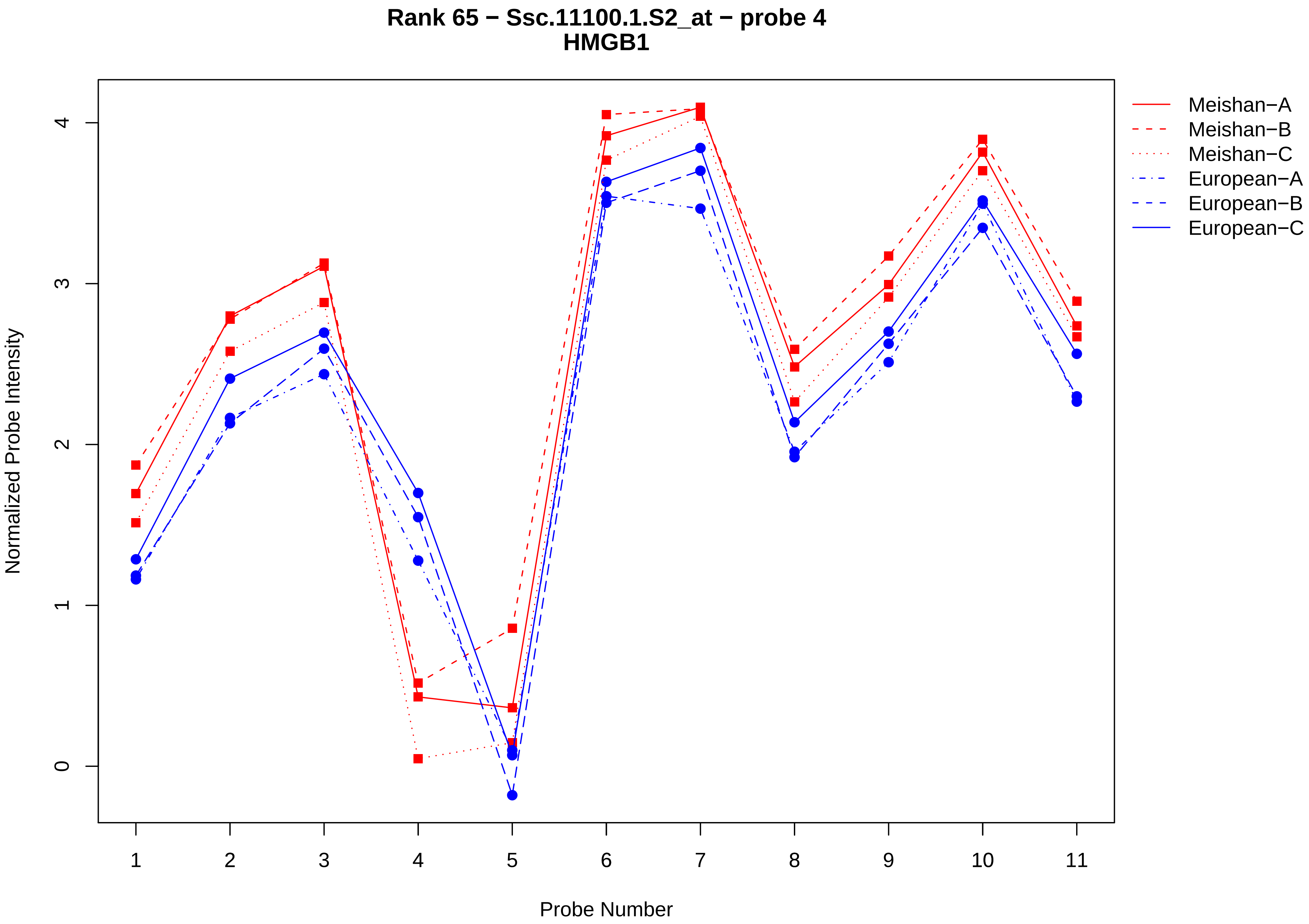

Rank 65 − Ssc.11100.1.S2_at − probe 4
HMGB1
Normalized Probe Intensity
Probe Number
0
1
2
3
4
1
2
3
4
5
6
7
8
9
10
11
Meishan−A
Meishan−B
Meishan−C
European−A
European−B
European−C

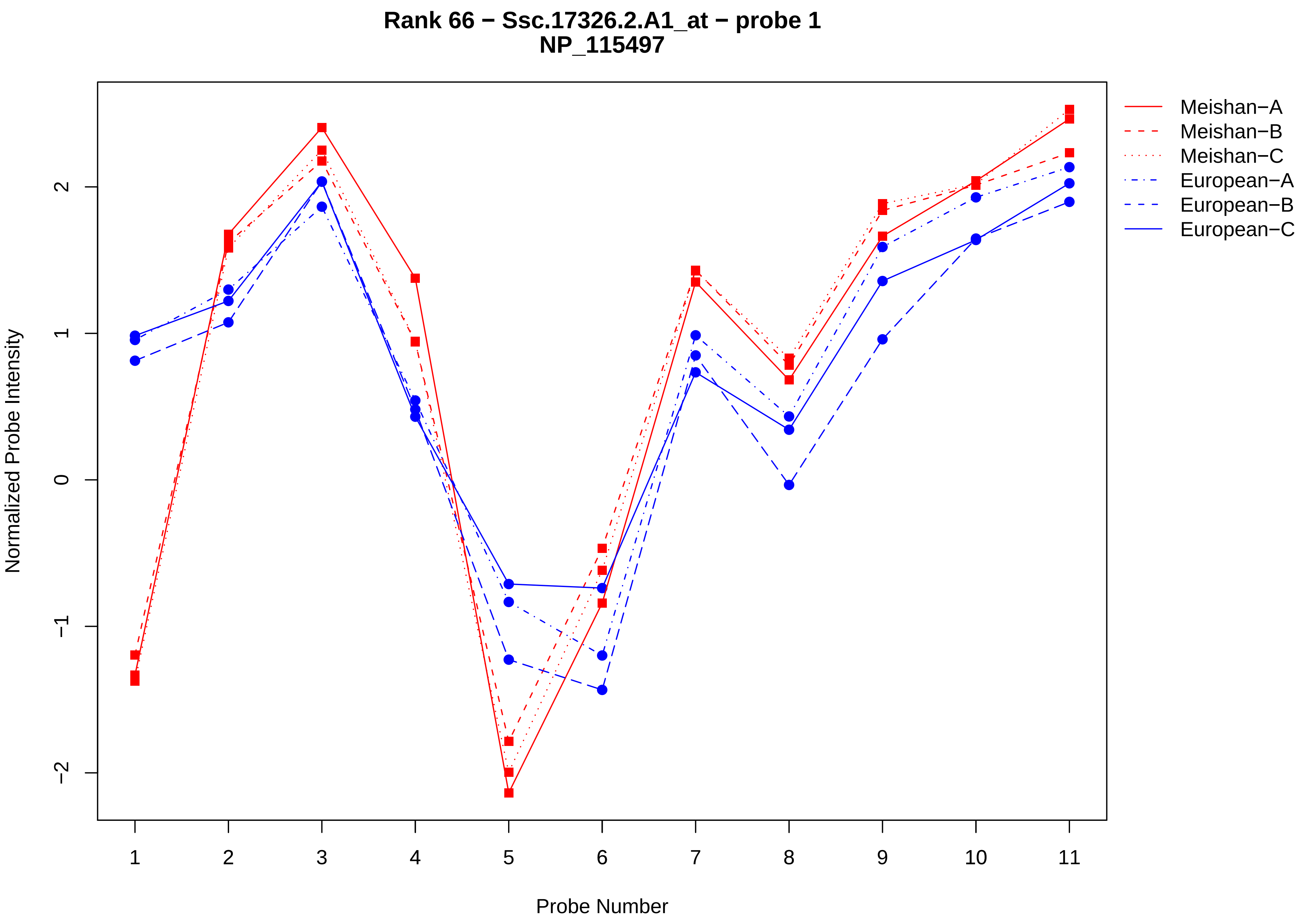

Rank 66 − Ssc.17326.2.A1_at − probe 1
NP_115497
Normalized Probe Intensity
Probe Number
−2
−1
0
1
2
1
2
3
4
5
6
7
8
9
10
11
Meishan−A
Meishan−B
Meishan−C
European−A
European−B
European−C

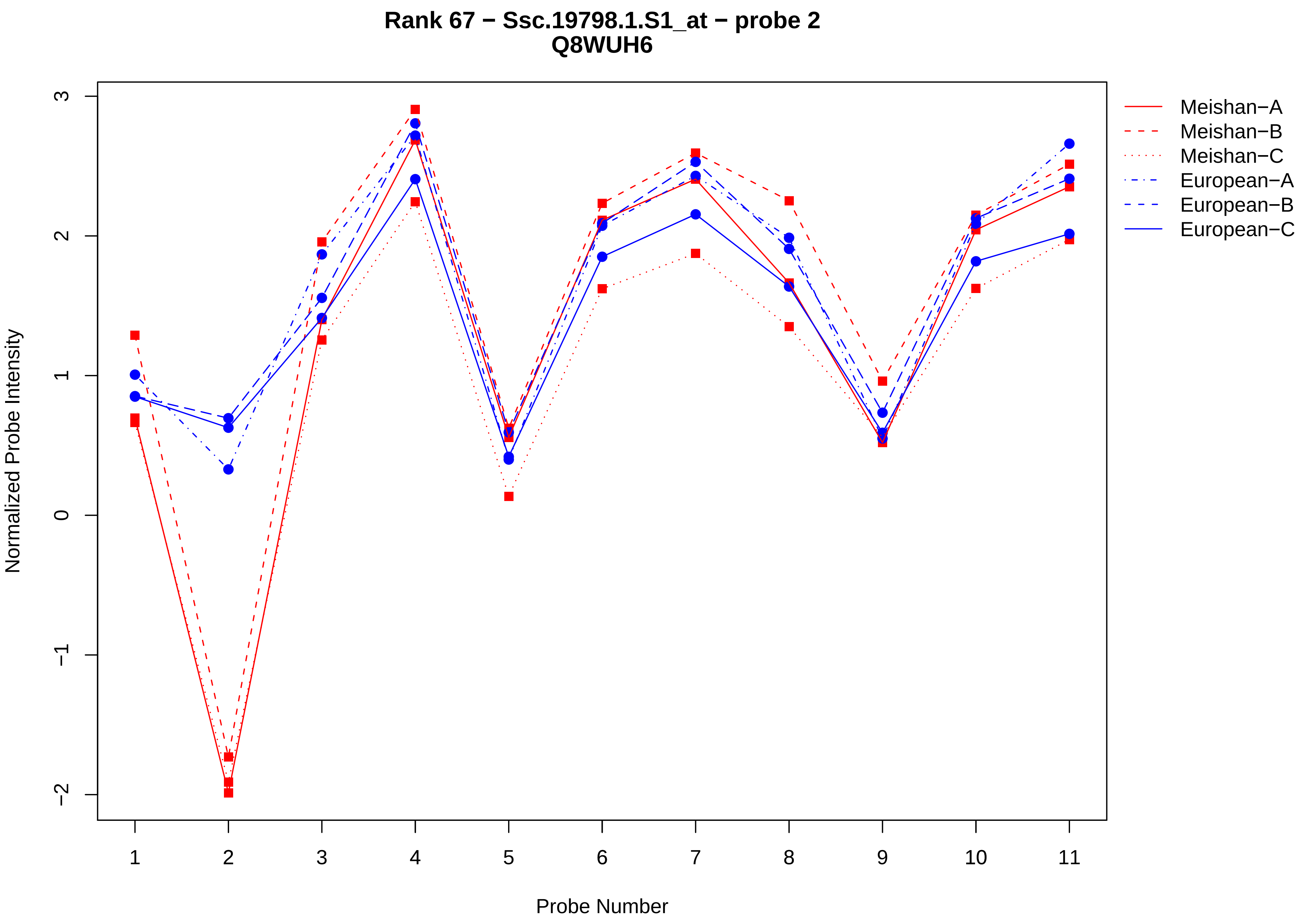

Rank 67 − Ssc.19798.1.S1_at − probe 2
Q8WUH6
Normalized Probe Intensity
Probe Number
−2
−1
0
1
2
3
1
2
3
4
5
6
7
8
9
10
11
Meishan−A
Meishan−B
Meishan−C
European−A
European−B
European−C

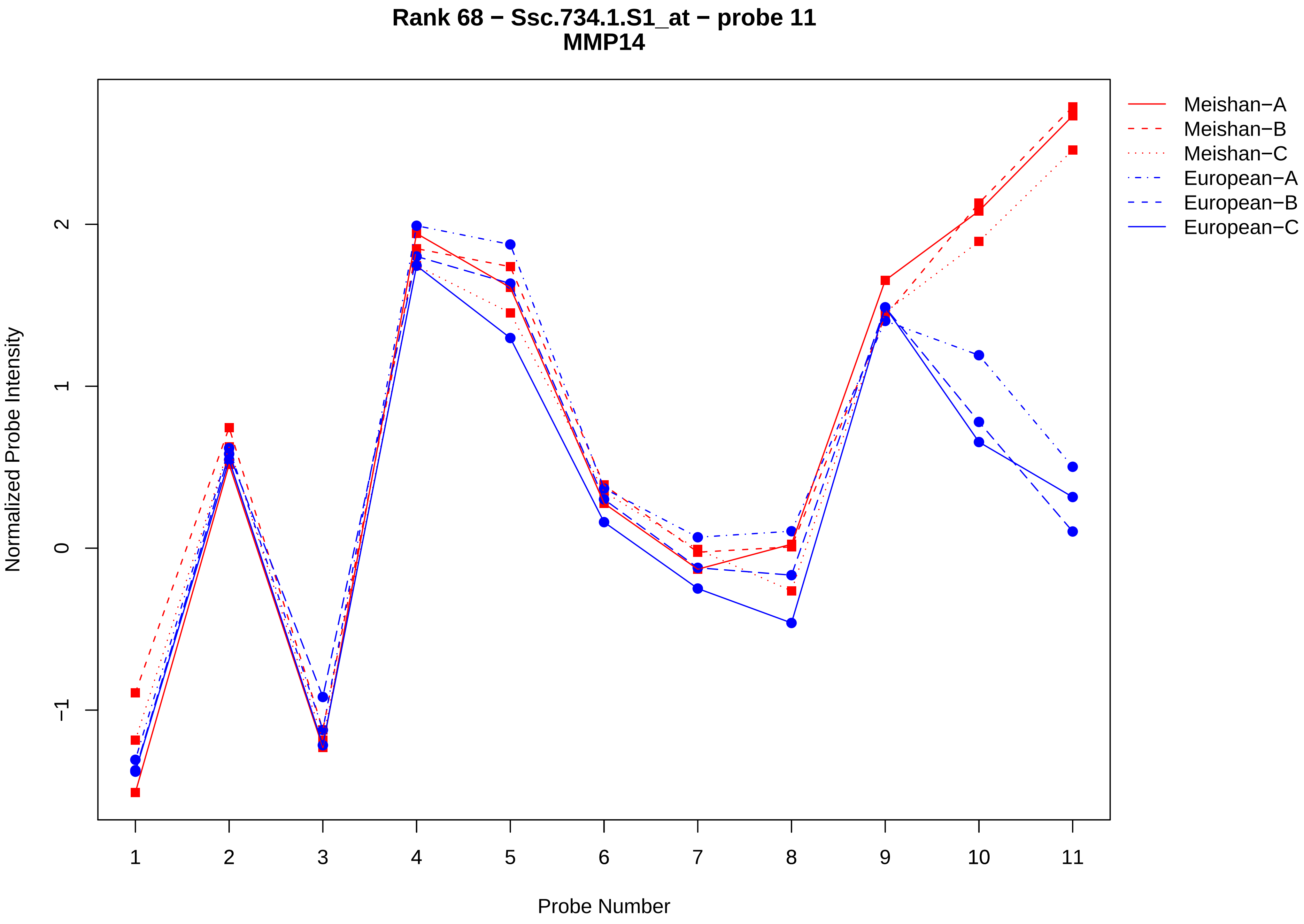

Rank 68 − Ssc.734.1.S1_at − probe 11
MMP14
Normalized Probe Intensity
Probe Number
−1
0
1
2
1
2
3
4
5
6
7
8
9
10
11
Meishan−A
Meishan−B
Meishan−C
European−A
European−B
European−C

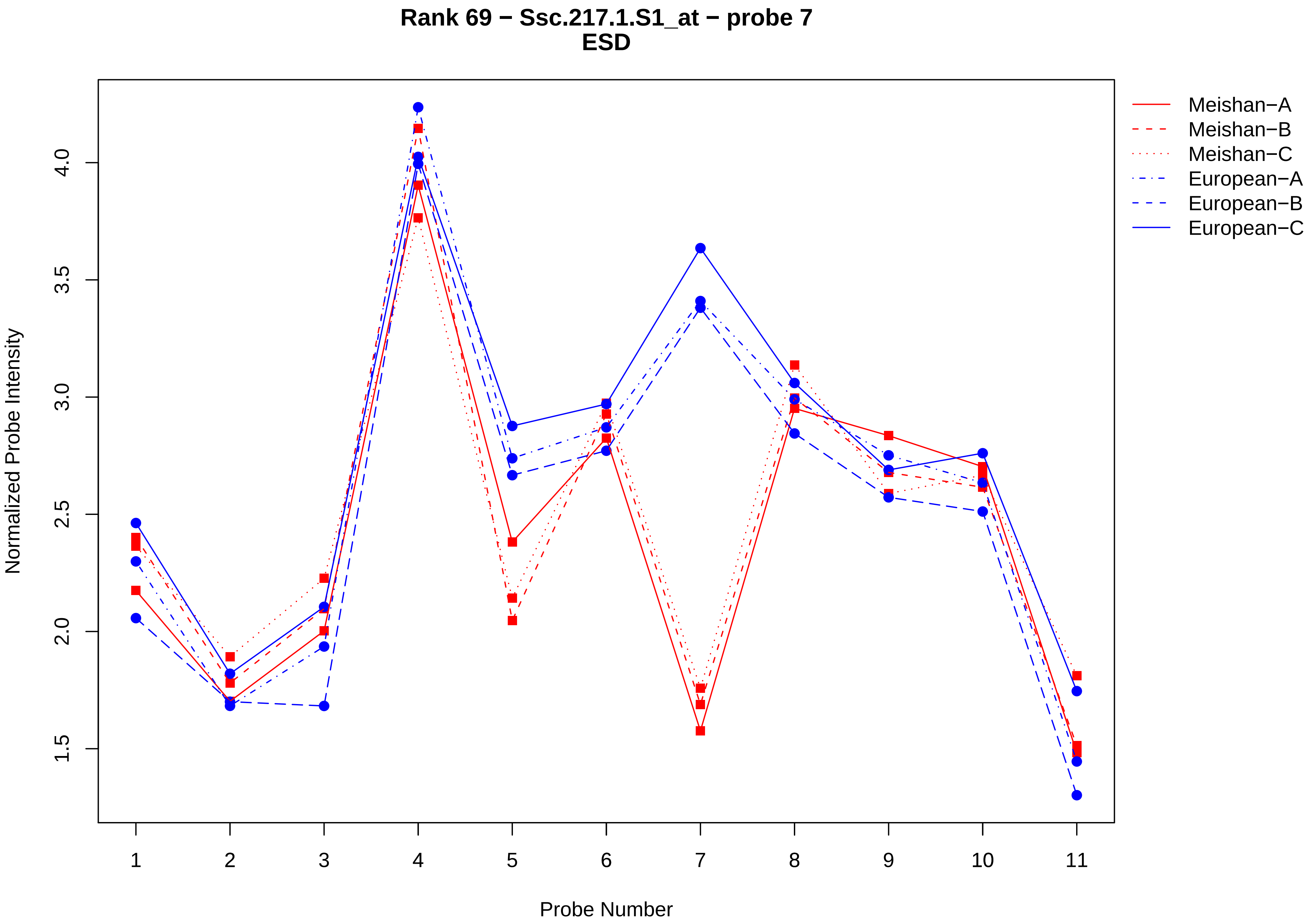

Rank 69 − Ssc.217.1.S1_at − probe 7
ESD
Normalized Probe Intensity
1.5
2.0
2.5
3.0
3.5
4.0
1
2
3
4
5
6
7
8
9
10
11
Probe Number
Meishan−A
Meishan−B
Meishan−C
European−A
European−B
European−C

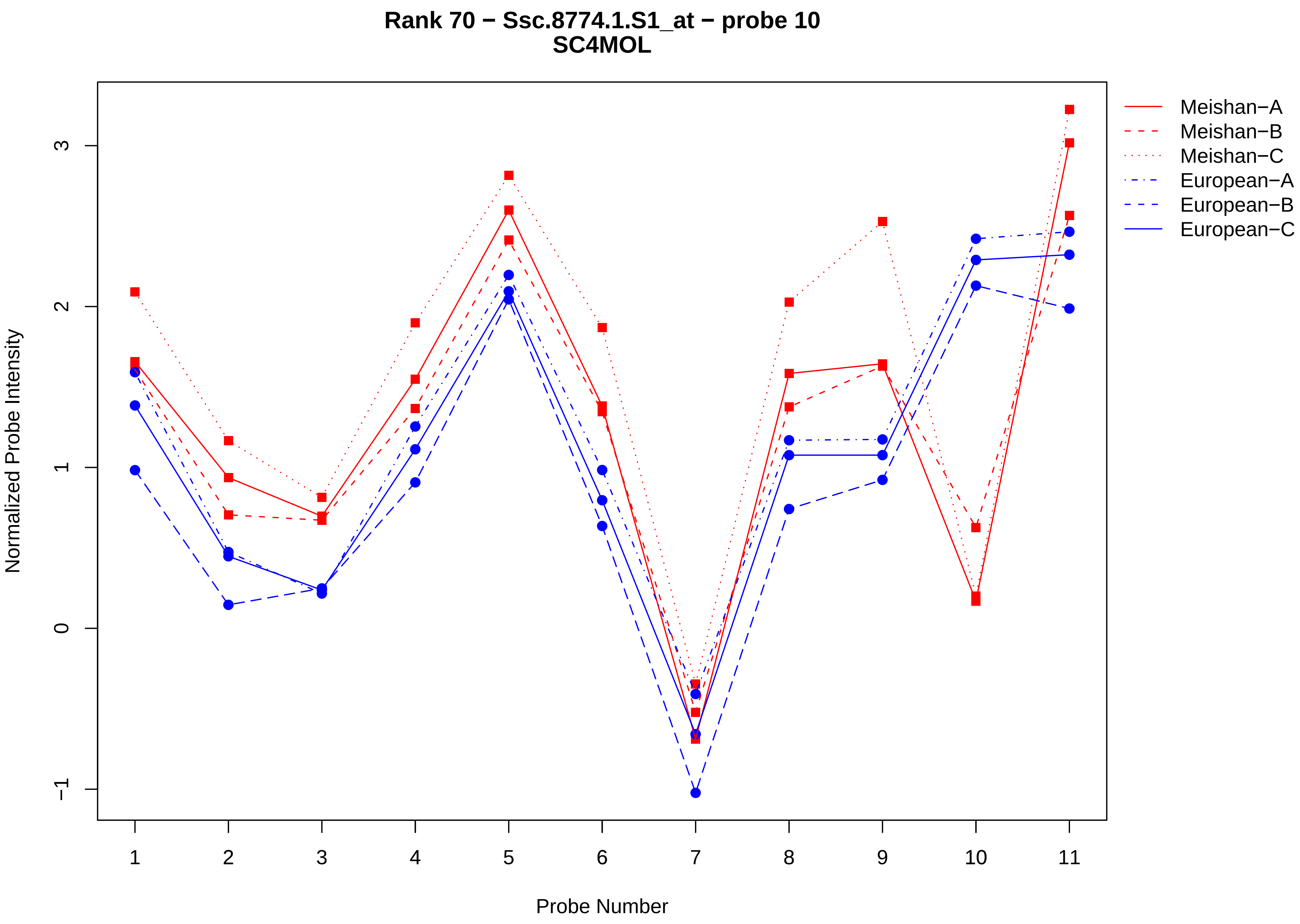

Rank 70 − Ssc.8774.1.S1_at − probe 10
SC4MOL
Normalized Probe Intensity
Probe Number
−1
0
1
2
3
1
2
3
4
5
6
7
8
9
10
11
Meishan−A
Meishan−B
Meishan−C
European−A
European−B
European−C

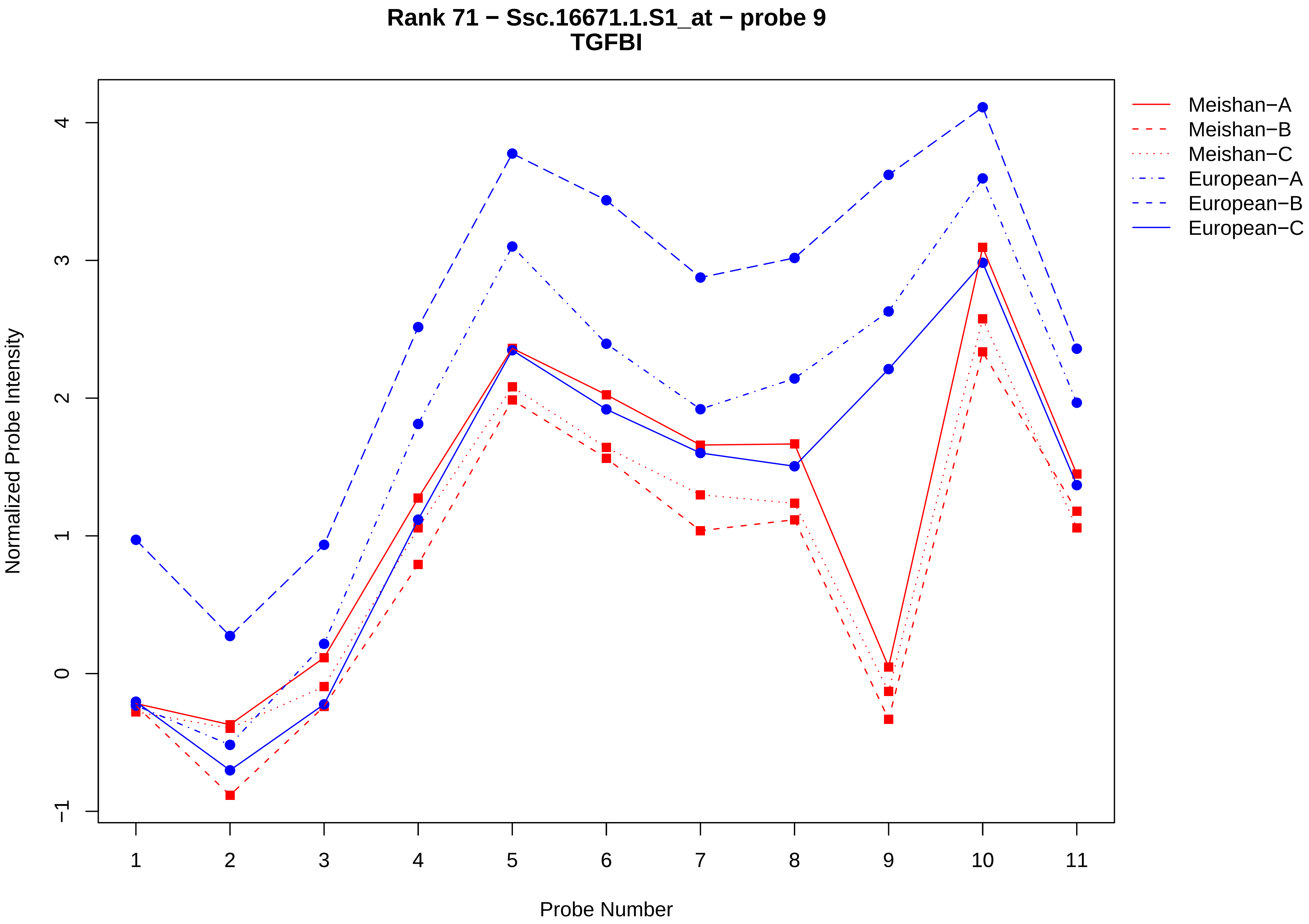
Rank 71 − Ssc.16671.1.S1_at − probe 9
TGFBI
Normalized Probe Intensity
Probe Number
Meishan−A
Meishan−B
Meishan−C
European−A
European−B
European−C

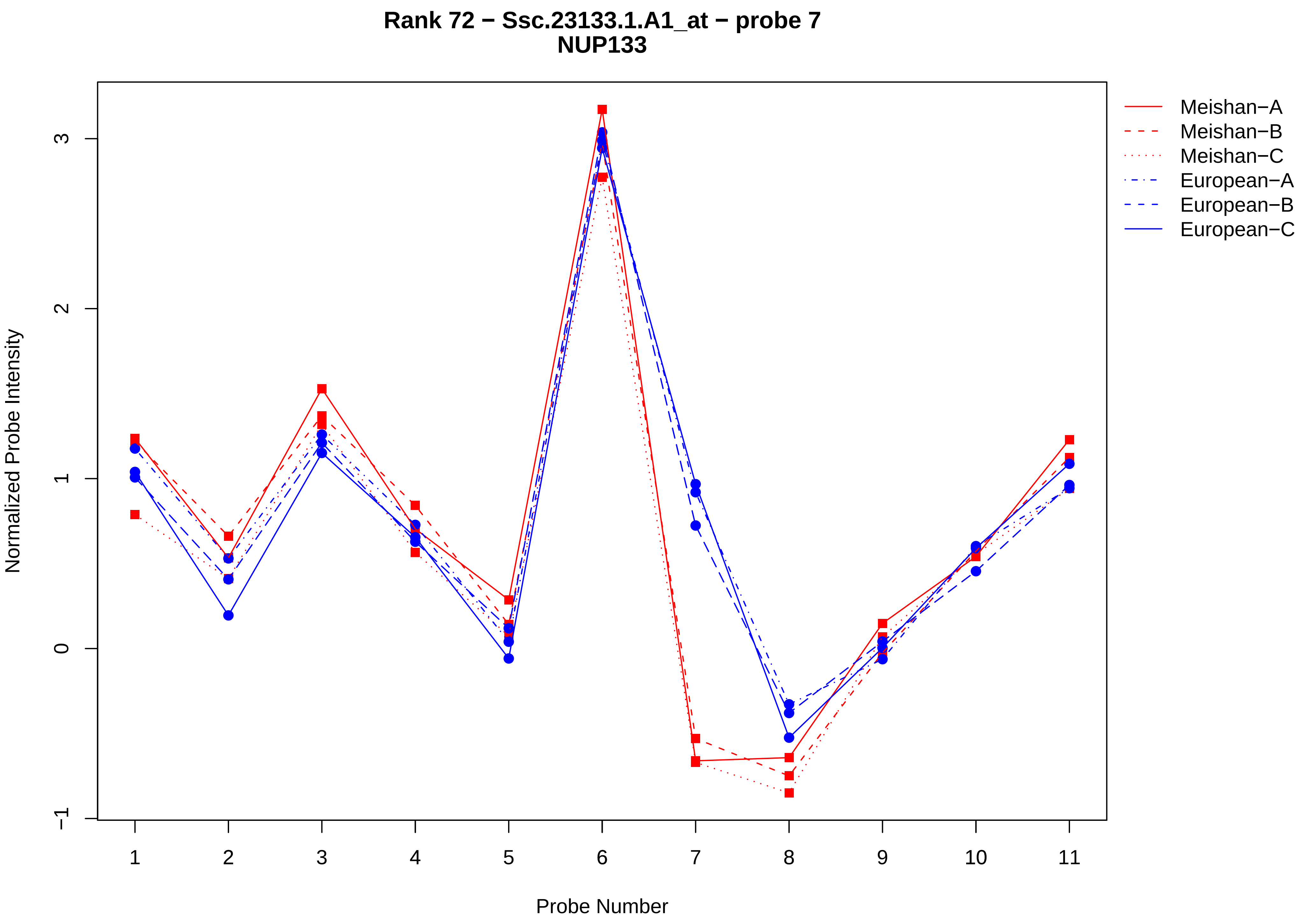

Rank 72 − Ssc.23133.1.A1_at − probe 7
NUP133
Normalized Probe Intensity
Probe Number
Meishan−A
Meishan−B
Meishan−C
European−A
European−B
European−C
−1
0
1
2
3
1
2
3
4
5
6
7
8
9
10
11

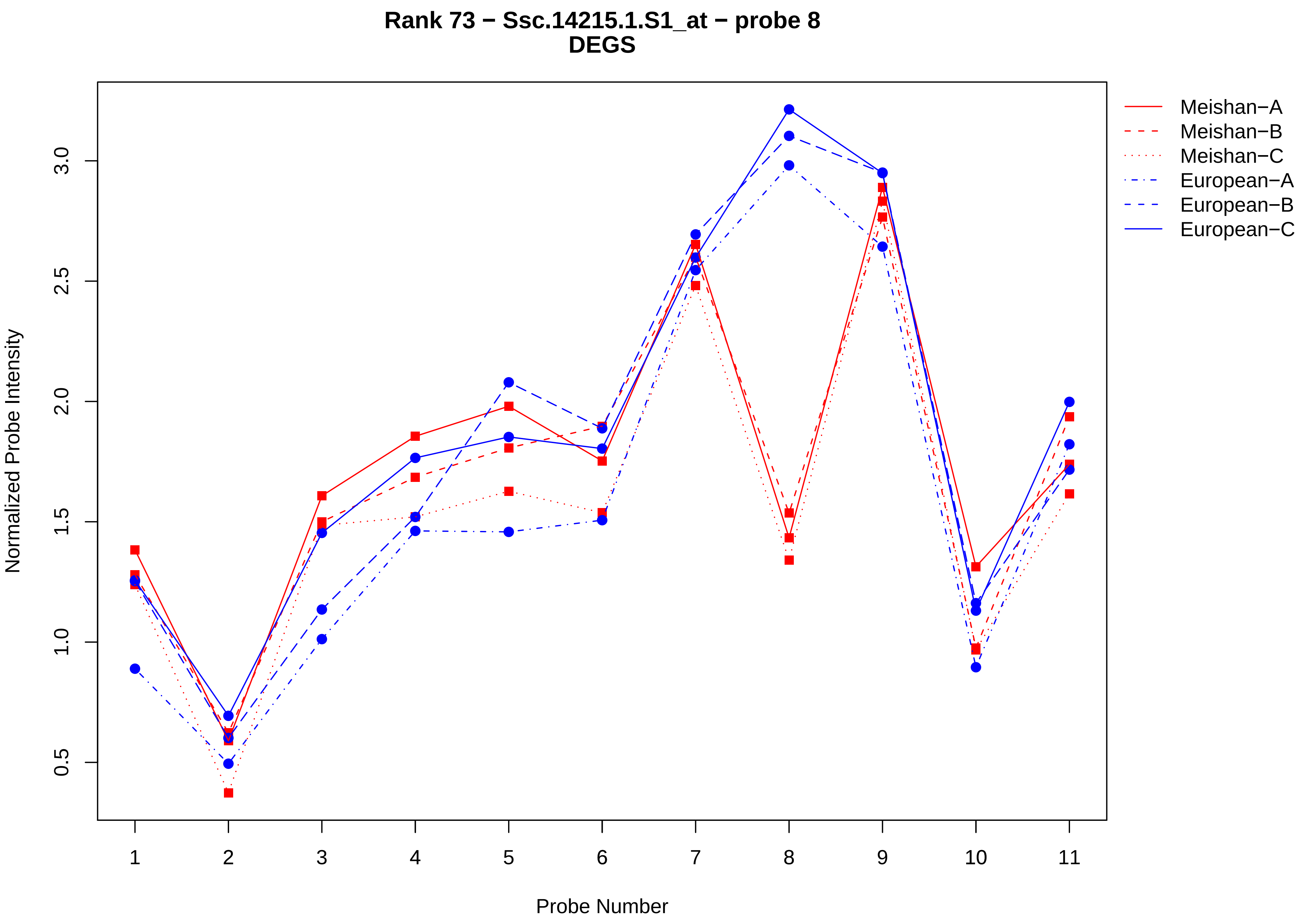
Rank 73 − Ssc.14215.1.S1_at − probe 8
DEGS
Normalized Probe Intensity
0.5
1.0
1.5
2.0
2.5
3.0
Probe Number
1
2
3
4
5
6
7
8
9
10
11
Meishan−A
Meishan−B
Meishan−C
European−A
European−B
European−C

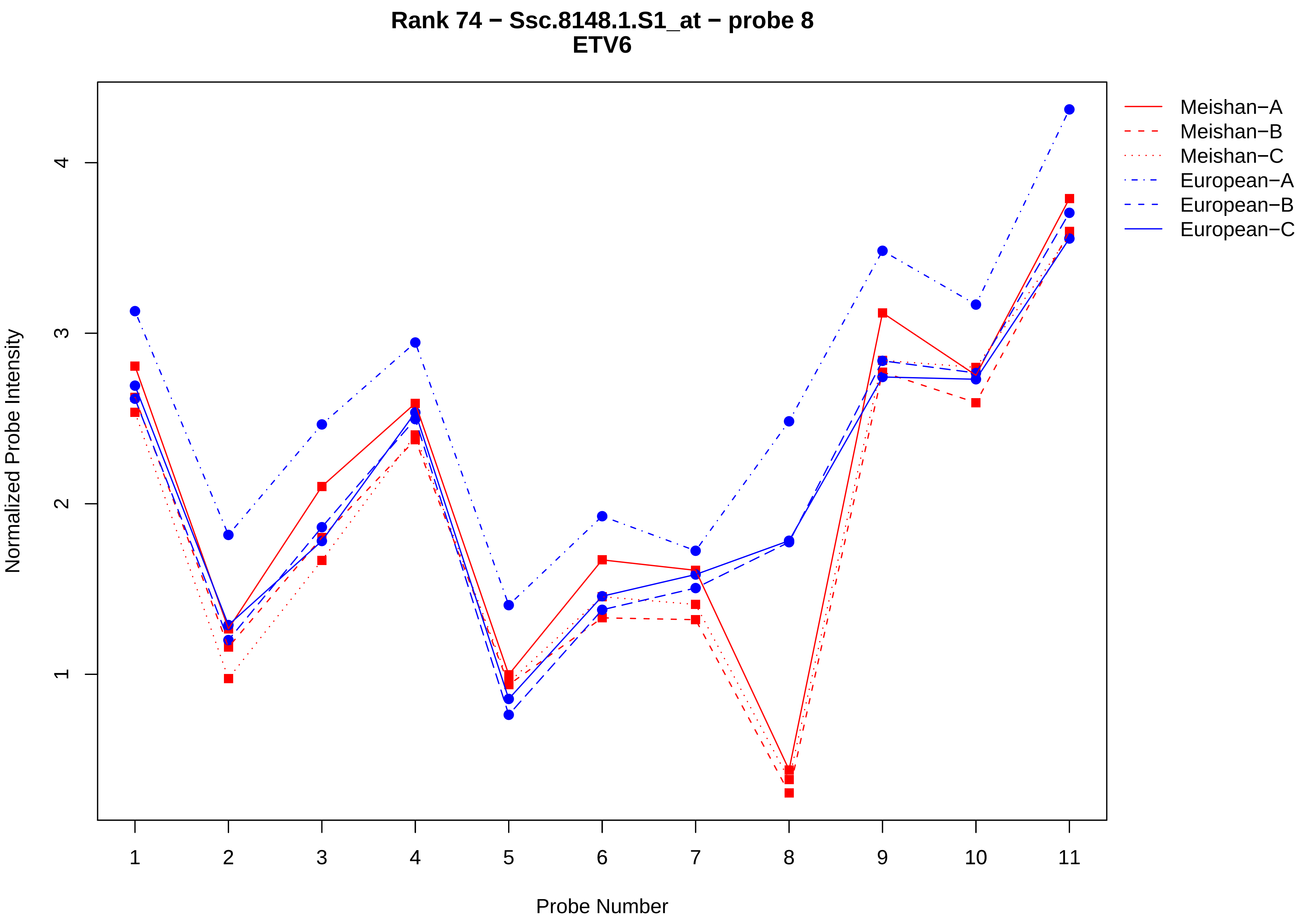

Rank 74 − Ssc.8148.1.S1_at − probe 8
ETV6
Normalized Probe Intensity
Probe Number
1
2
3
4
1
2
3
4
5
6
7
8
9
10
11
Meishan−A
Meishan−B
Meishan−C
European−A
European−B
European−C

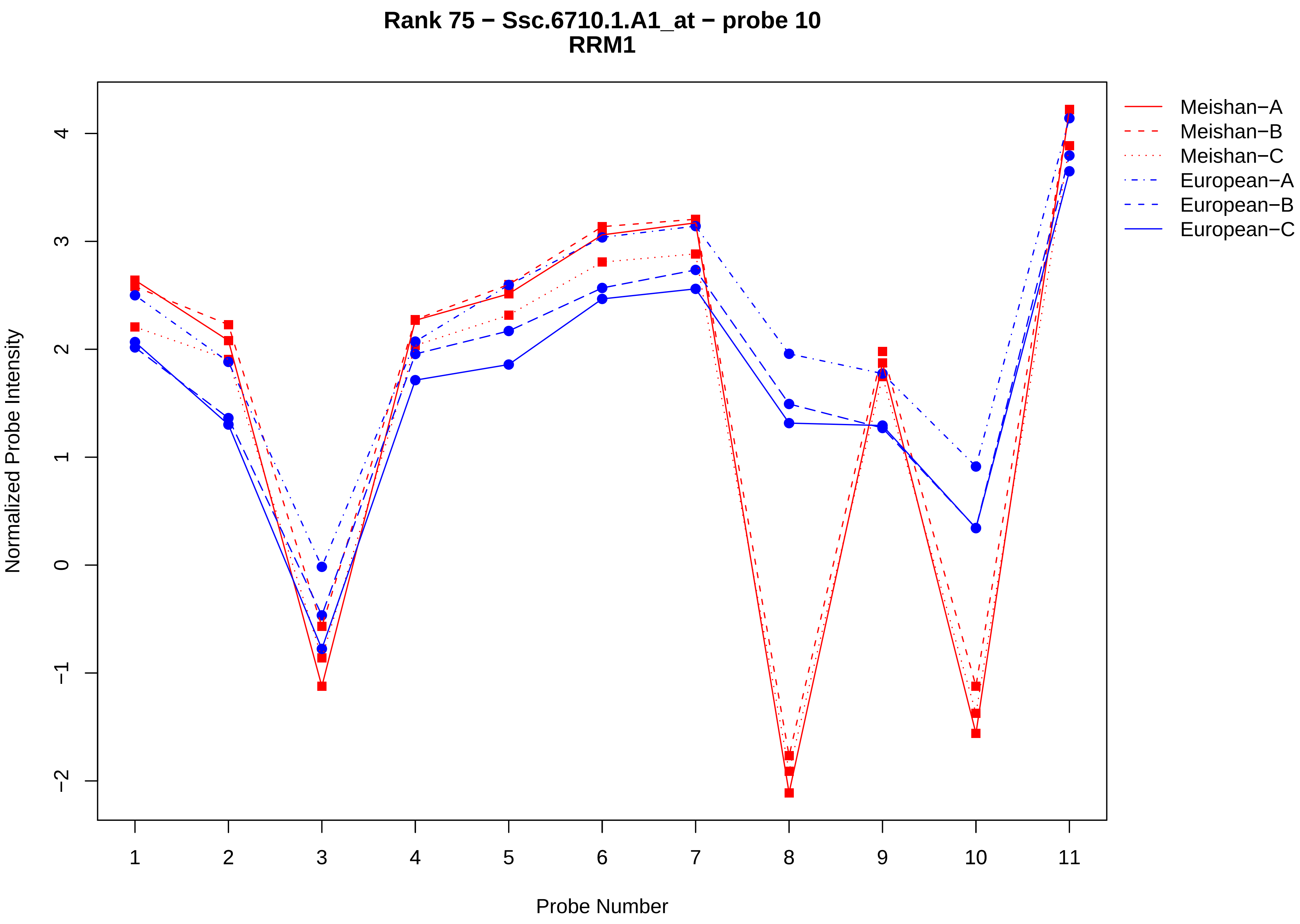

Rank 75 − Ssc.6710.1.A1_at − probe 10
RRM1
Normalized Probe Intensity
Probe Number
−2
−1
0
1
2
3
4
1
2
3
4
5
6
7
8
9
10
11
Meishan−A
Meishan−B
Meishan−C
European−A
European−B
European−C

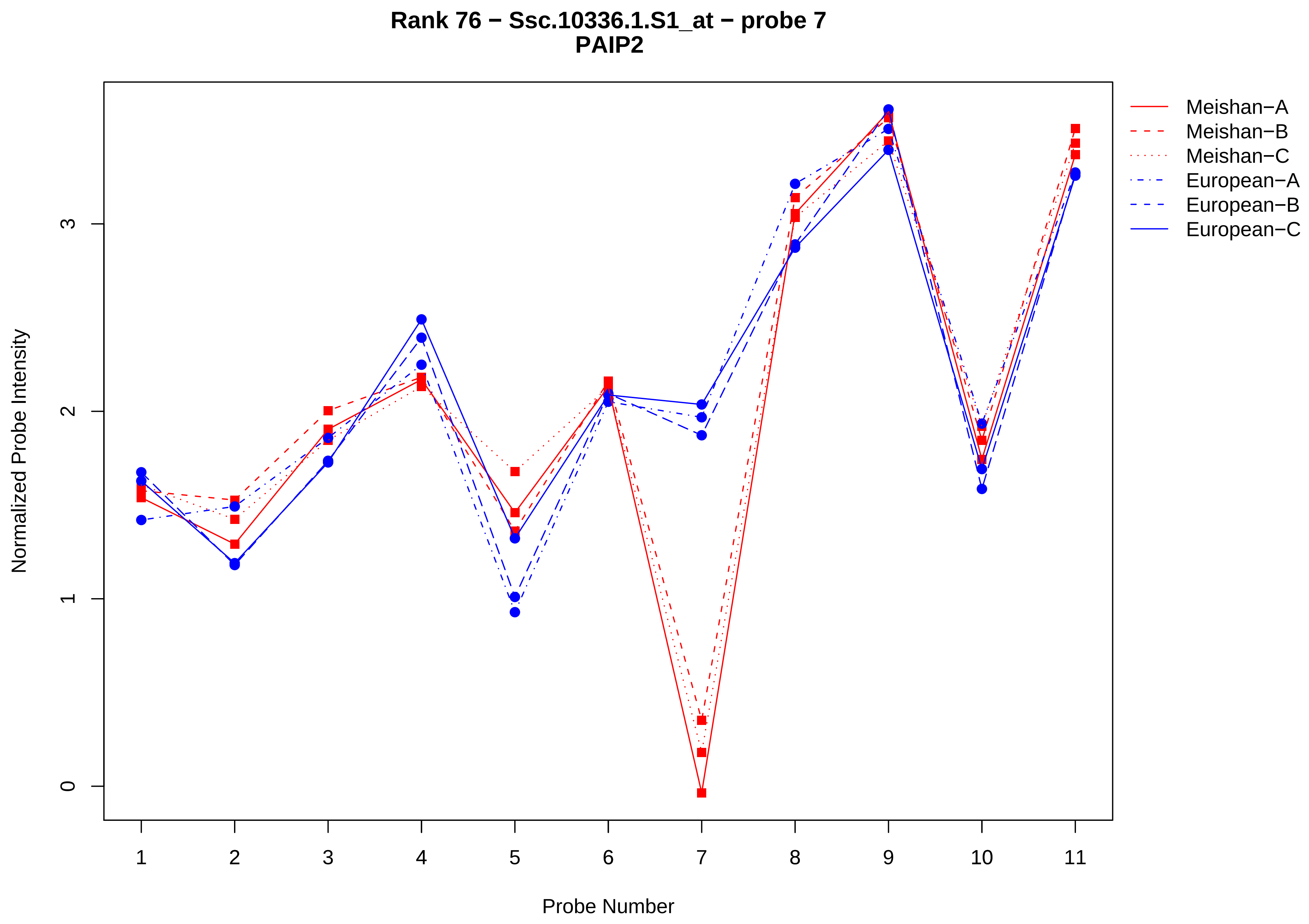

Rank 76 − Ssc.10336.1.S1_at − probe 7
PAIP2
Normalized Probe Intensity
Probe Number
0
1
2
3
1
2
3
4
5
6
7
8
9
10
11
Meishan−A
Meishan−B
Meishan−C
European−A
European−B
European−C

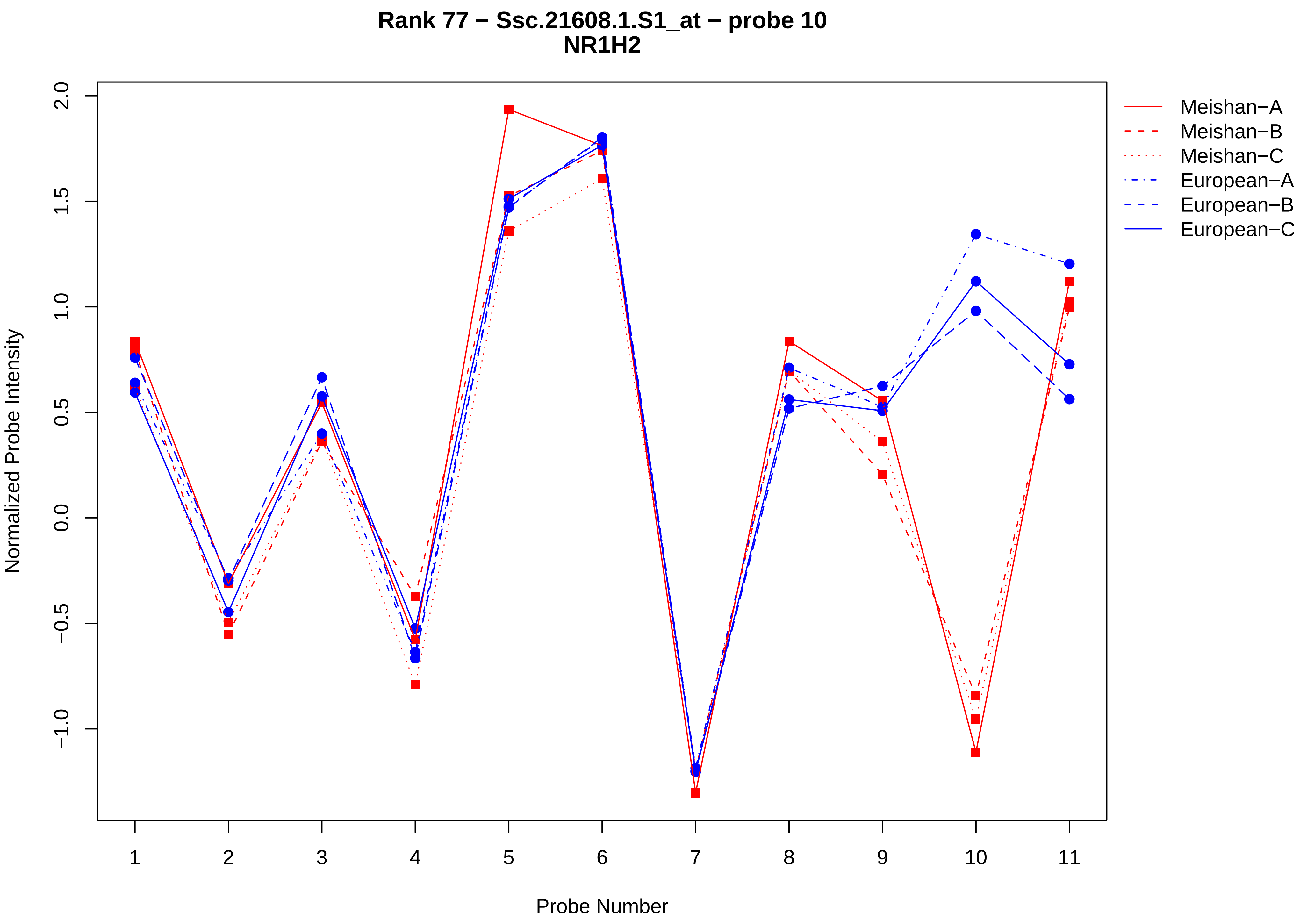

Rank 77 − Ssc.21608.1.S1_at − probe 10
NR1H2
Normalized Probe Intensity
Probe Number
Meishan−A
Meishan−B
Meishan−C
European−A
European−B
European−C

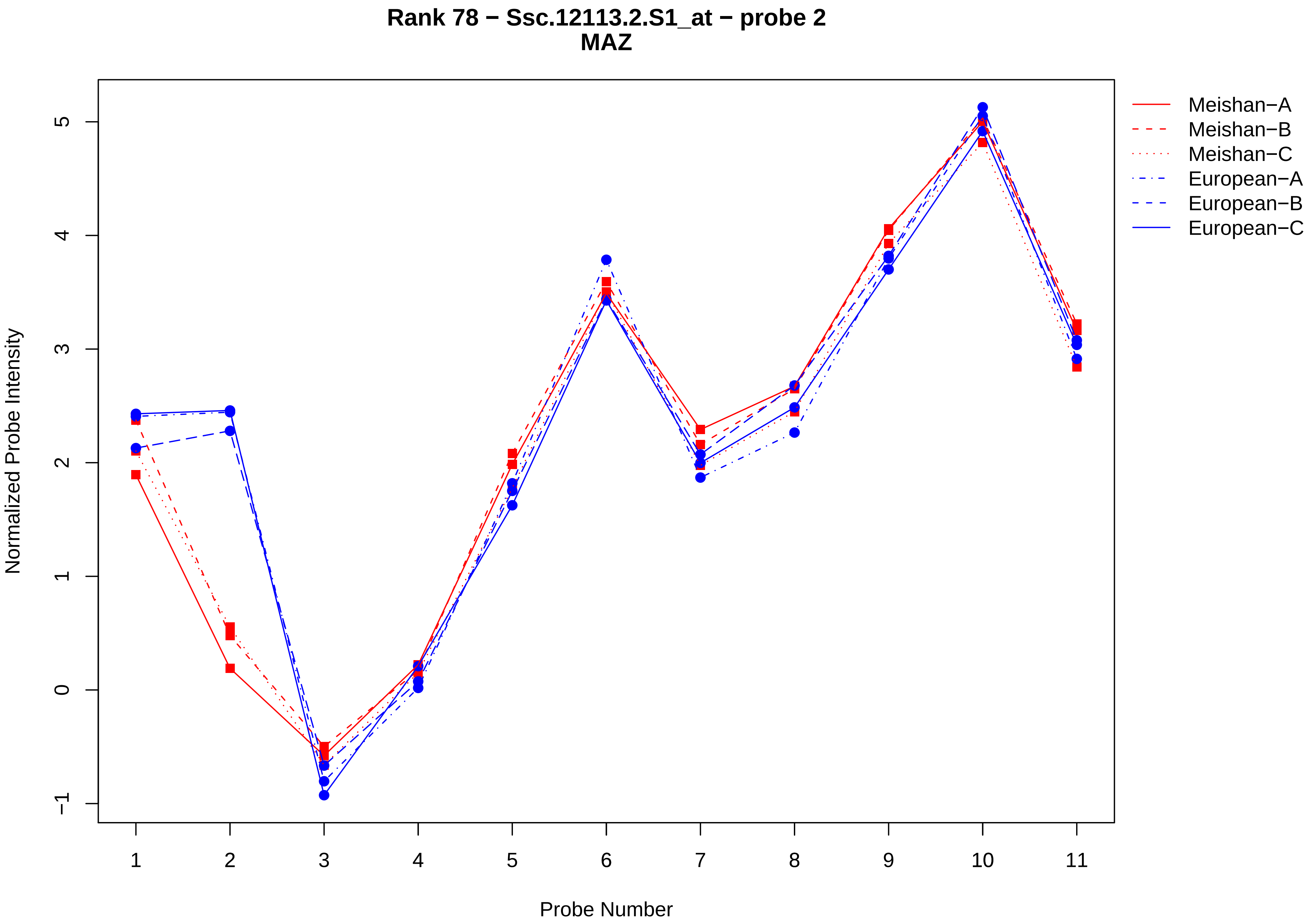
Rank 78 − Ssc.12113.2.S1_at − probe 2
MAZ
Normalized Probe Intensity
Probe Number
−1
0
1
2
3
4
5
1
2
3
4
5
6
7
8
9
10
11
Meishan−A
Meishan−B
Meishan−C
European−A
European−B
European−C

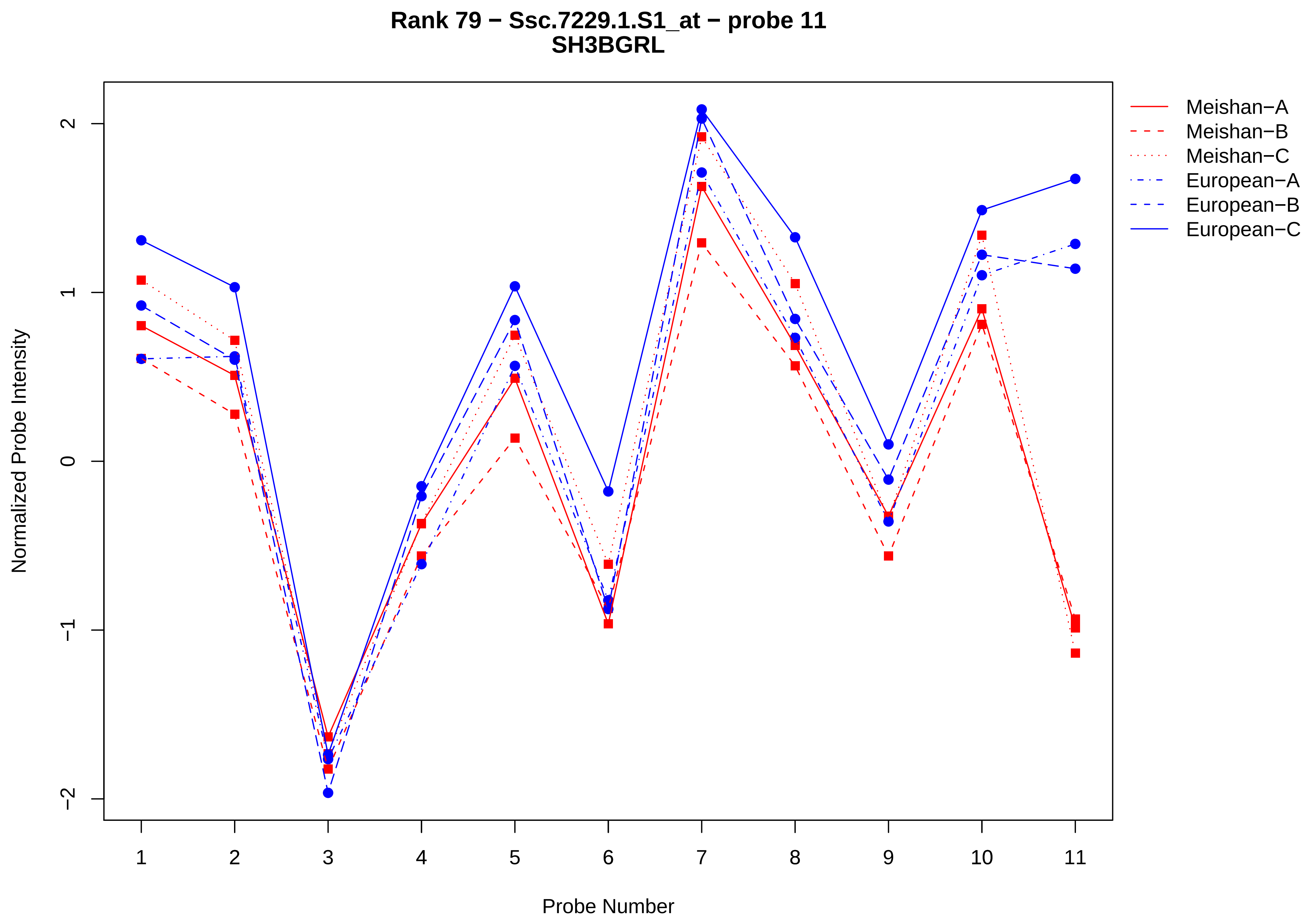

Rank 79 − Ssc.7229.1.S1_at − probe 11
SH3BGRL
Normalized Probe Intensity
Probe Number
−2
−1
0
1
2
1
2
3
4
5
6
7
8
9
10
11
Meishan−A
Meishan−B
Meishan−C
European−A
European−B
European−C

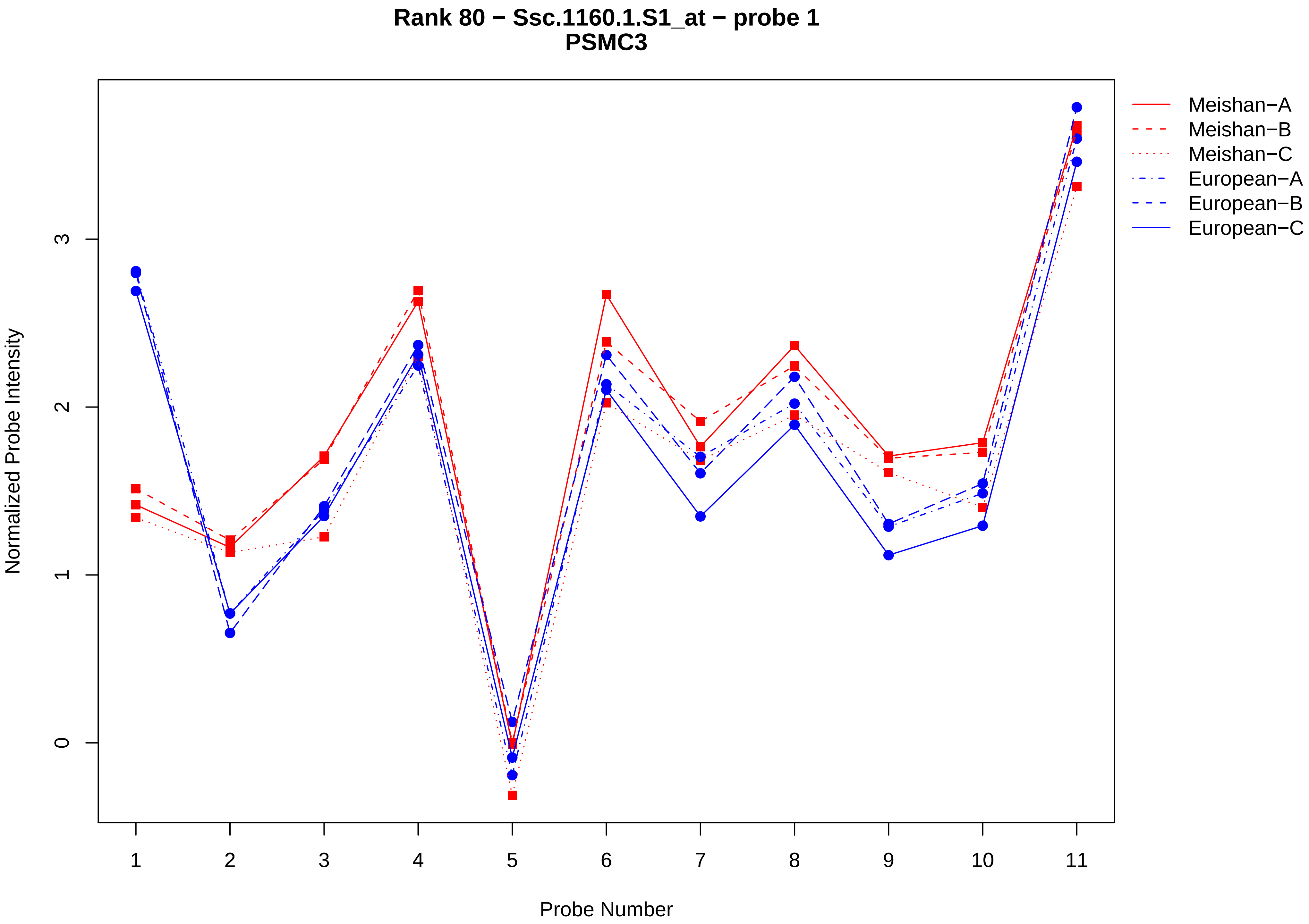

Rank 80 − Ssc.1160.1.S1_at − probe 1
PSMC3
Normalized Probe Intensity
Probe Number
0
1
2
3
1
2
3
4
5
6
7
8
9
10
11
Meishan−A
Meishan−B
Meishan−C
European−A
European−B
European−C

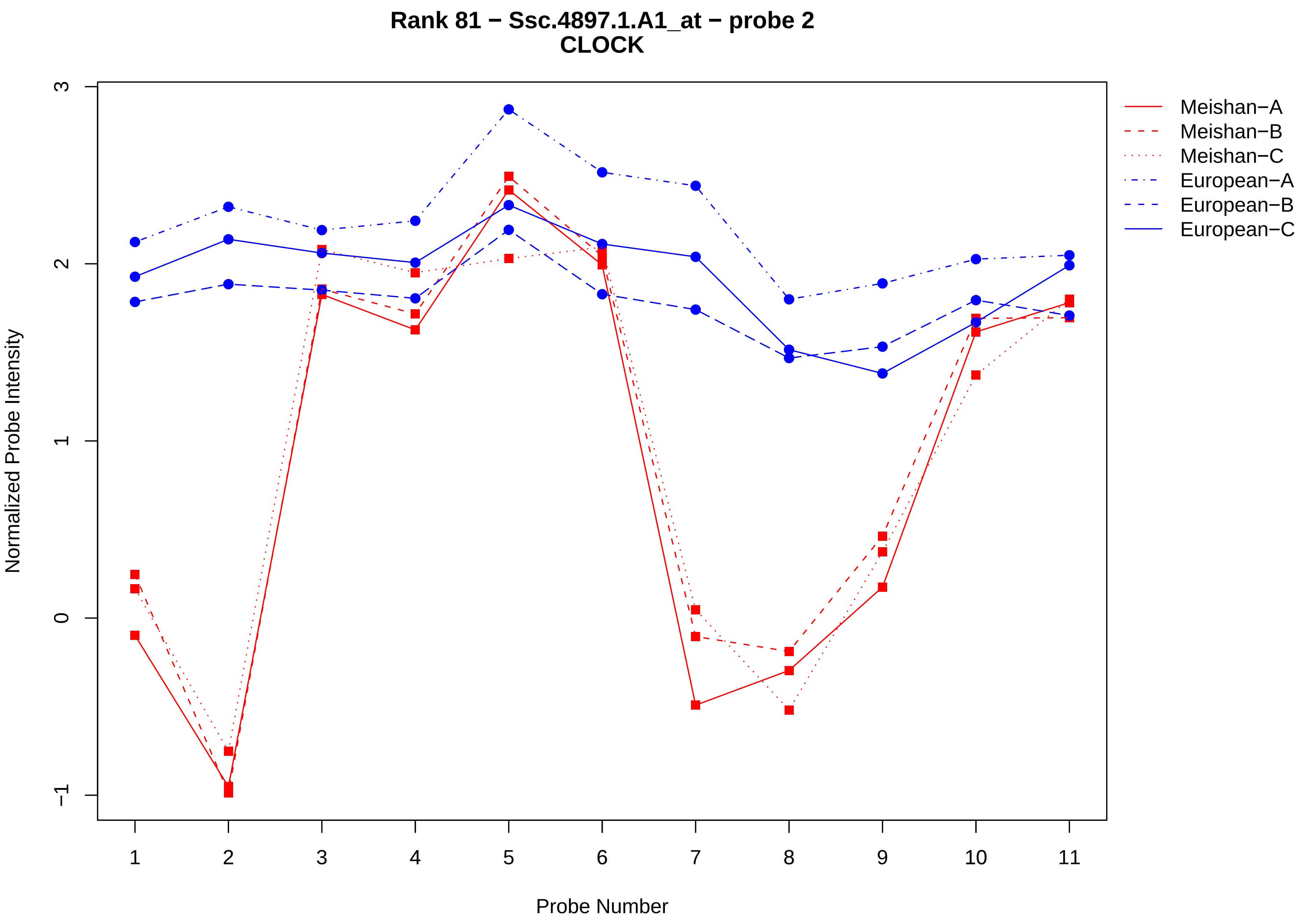
Rank 81 − Ssc.4897.1.A1_at − probe 2
CLOCK
Normalized Probe Intensity
Probe Number
−1
0
1
2
3
1
2
3
4
5
6
7
8
9
10
11
Meishan−A
Meishan−B
Meishan−C
European−A
European−B
European−C

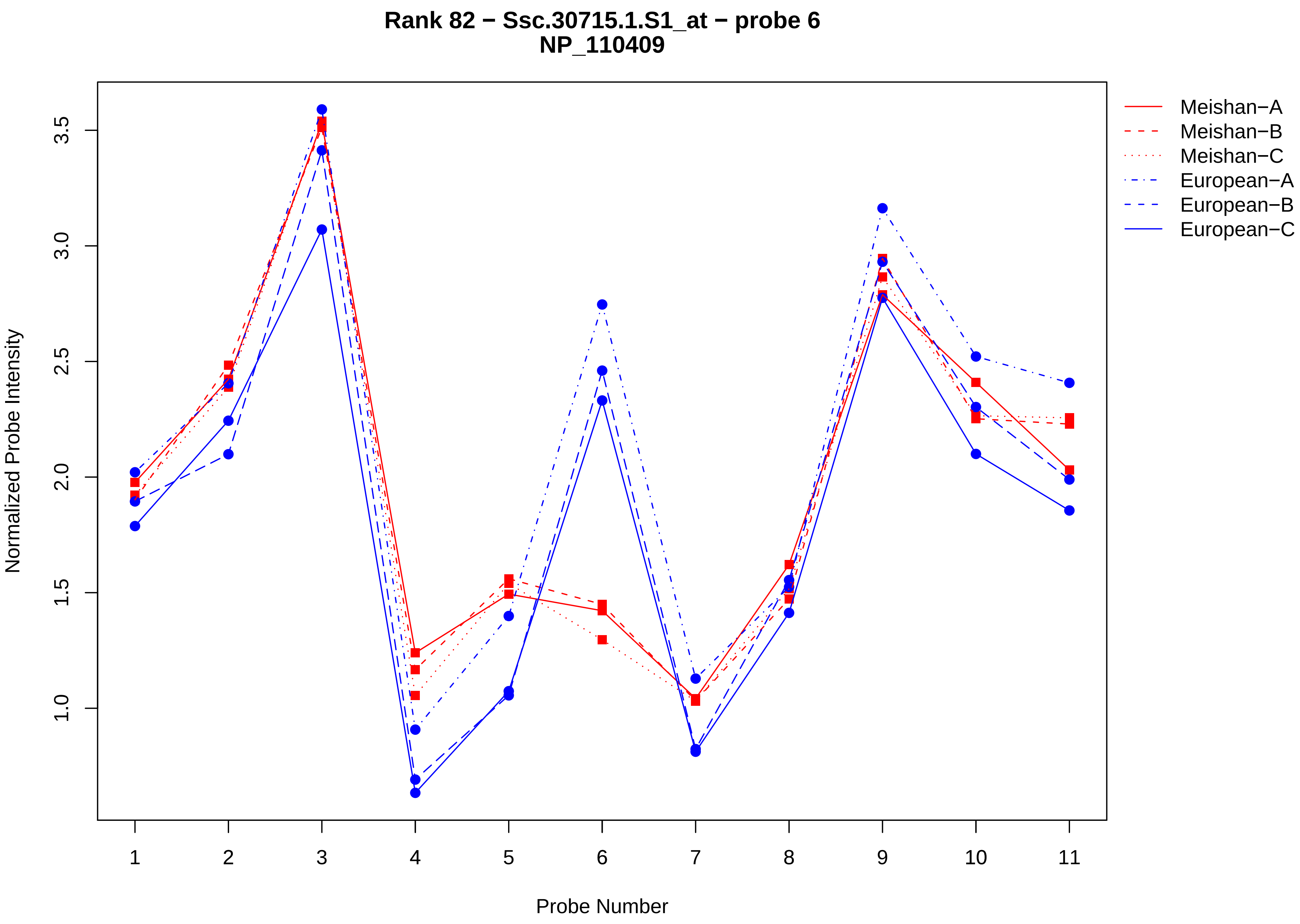

Rank 82 − Ssc.30715.1.S1_at − probe 6
NP_110409
Normalized Probe Intensity
1.0
1.5
2.0
2.5
3.0
3.5
1
2
3
4
5
6
7
8
9
10
11
Probe Number
Meishan−A
Meishan−B
Meishan−C
European−A
European−B
European−C

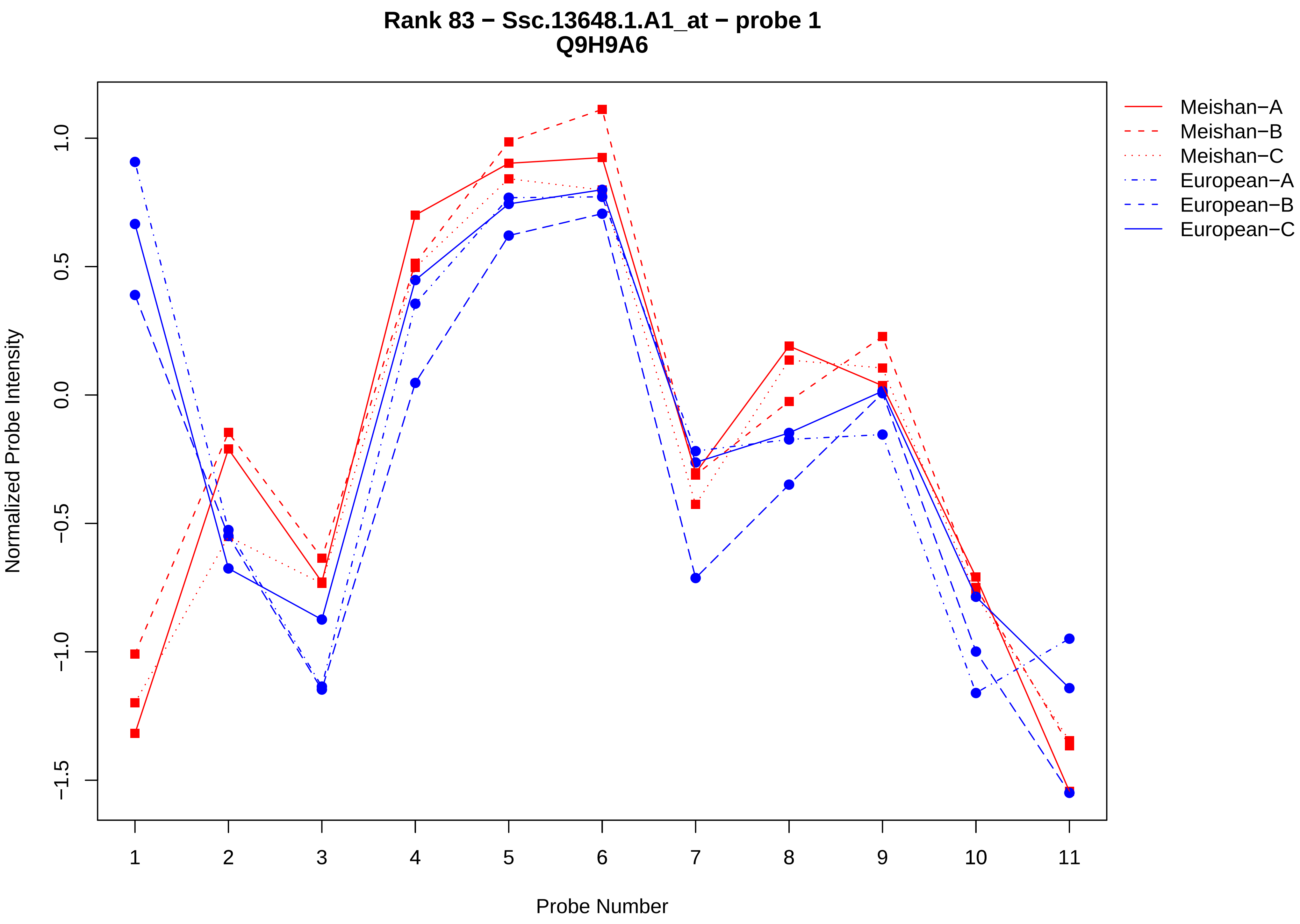

Rank 83 − Ssc.13648.1.A1_at − probe 1
Q9H9A6
Normalized Probe Intensity
Probe Number
1.0
0.5
0.0
−0.5
−1.0
−1.5
1
2
3
4
5
6
7
8
9
10
11
Meishan−A
Meishan−B
Meishan−C
European−A
European−B
European−C

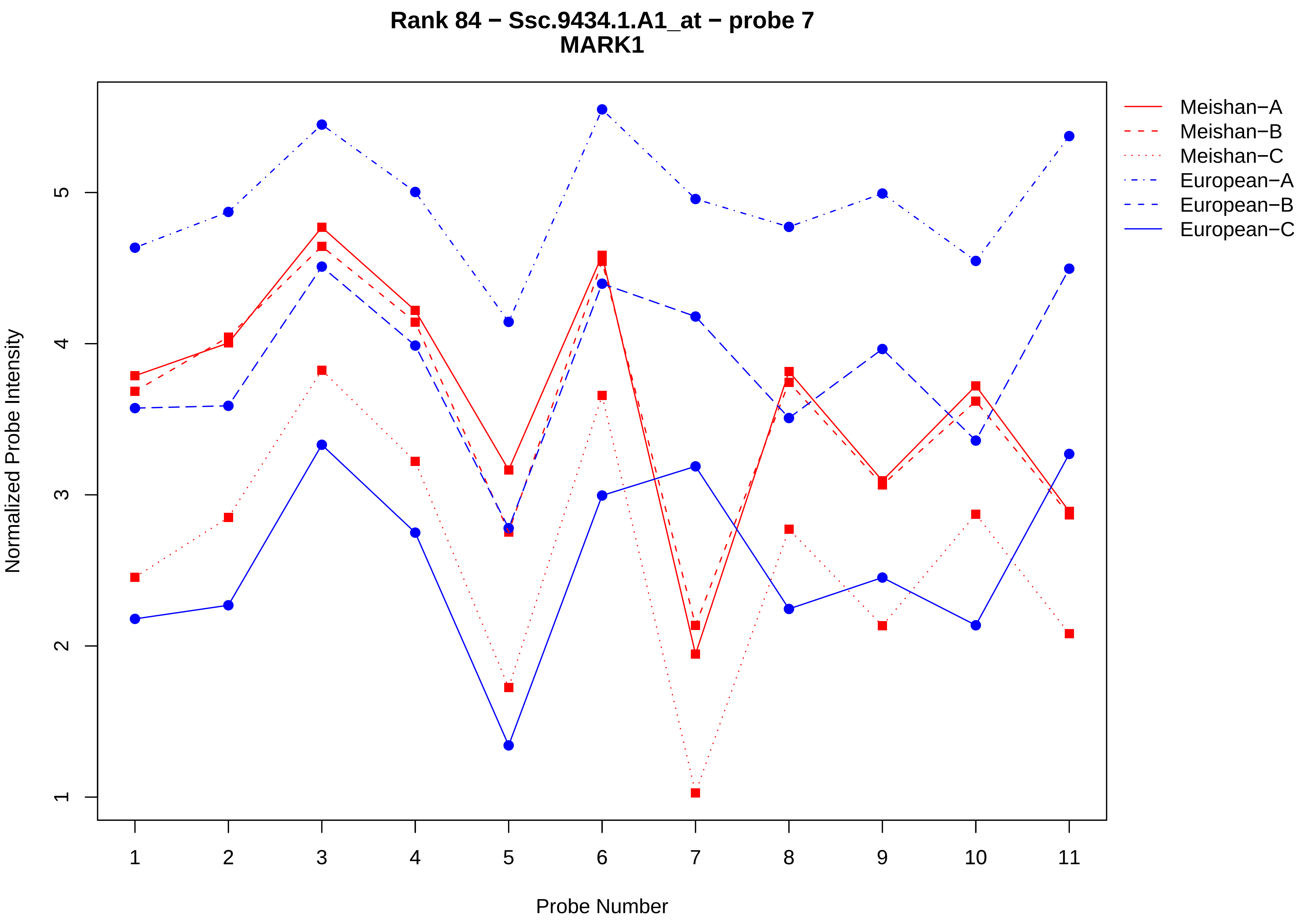

Rank 84 − Ssc.9434.1.A1_at − probe 7
MARK1
Normalized Probe Intensity
Probe Number
1
2
3
4
5
1
2
3
4
5
6
7
8
9
10
11
Meishan−A
Meishan−B
Meishan−C
European−A
European−B
European−C

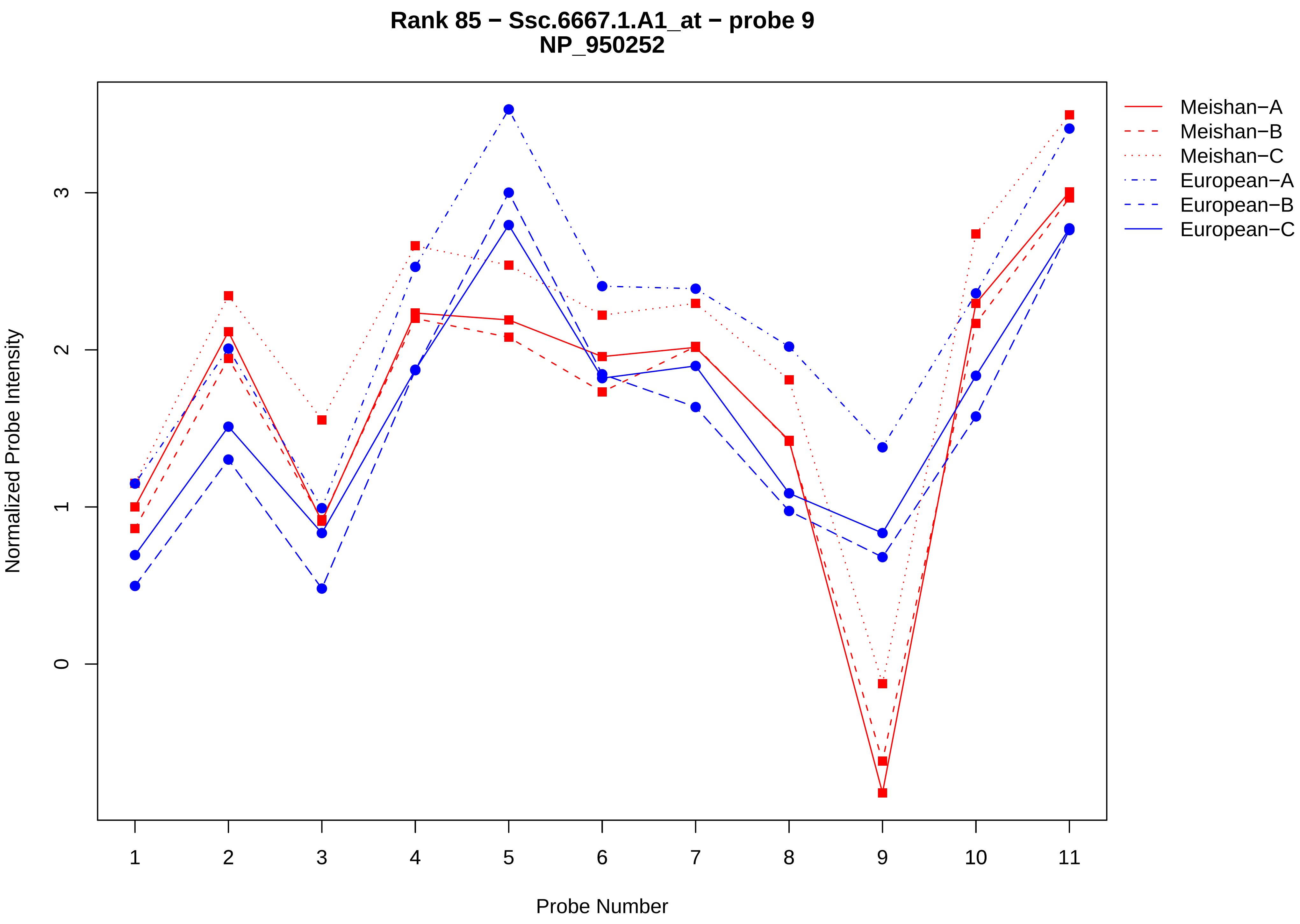

Rank 85 − Ssc.6667.1.A1_at − probe 9
NP_950252
Normalized Probe Intensity
0
1
2
3
1
2
3
4
5
6
7
8
9
10
11
Probe Number
Meishan−A
Meishan−B
Meishan−C
European−A
European−B
European−C

Rank 86 − Ssc.7556.1.S1_at − probe 10
SRP9

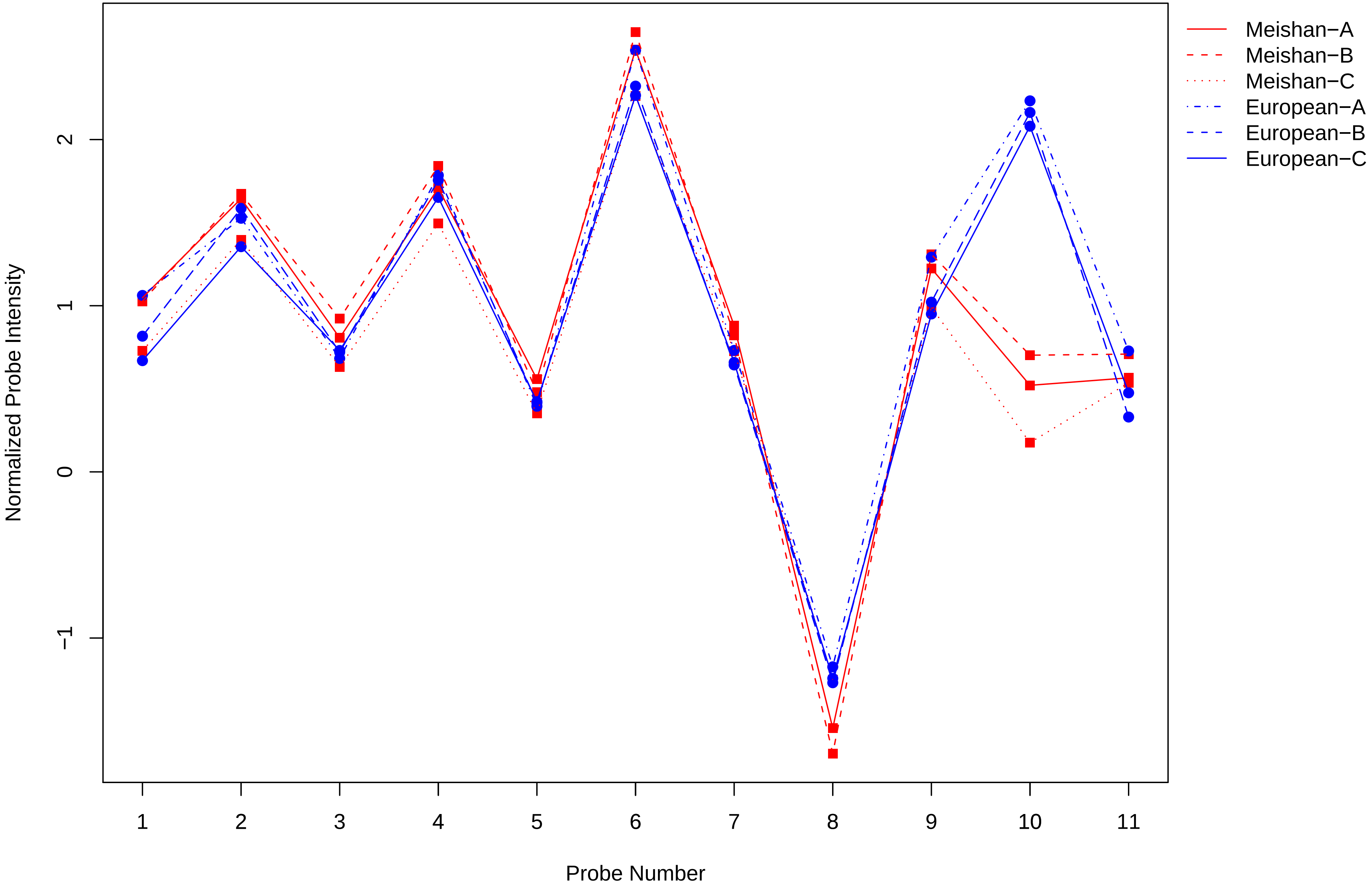

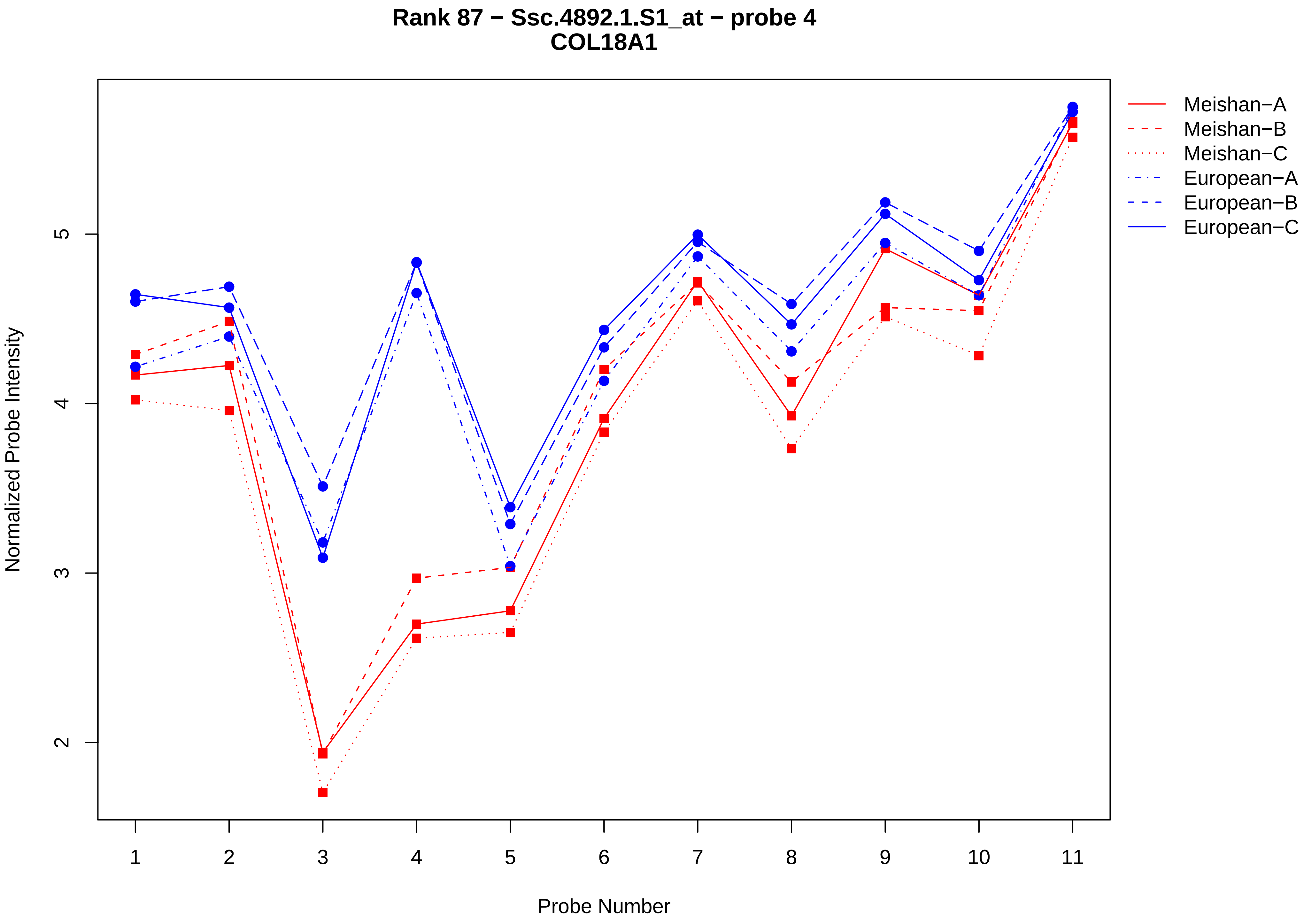

Rank 87 − Ssc.4892.1.S1_at − probe 4
COL18A1
Normalized Probe Intensity
Probe Number
2
3
4
5
1
2
3
4
5
6
7
8
9
10
11
Meishan−A
Meishan−B
Meishan−C
European−A
European−B
European−C

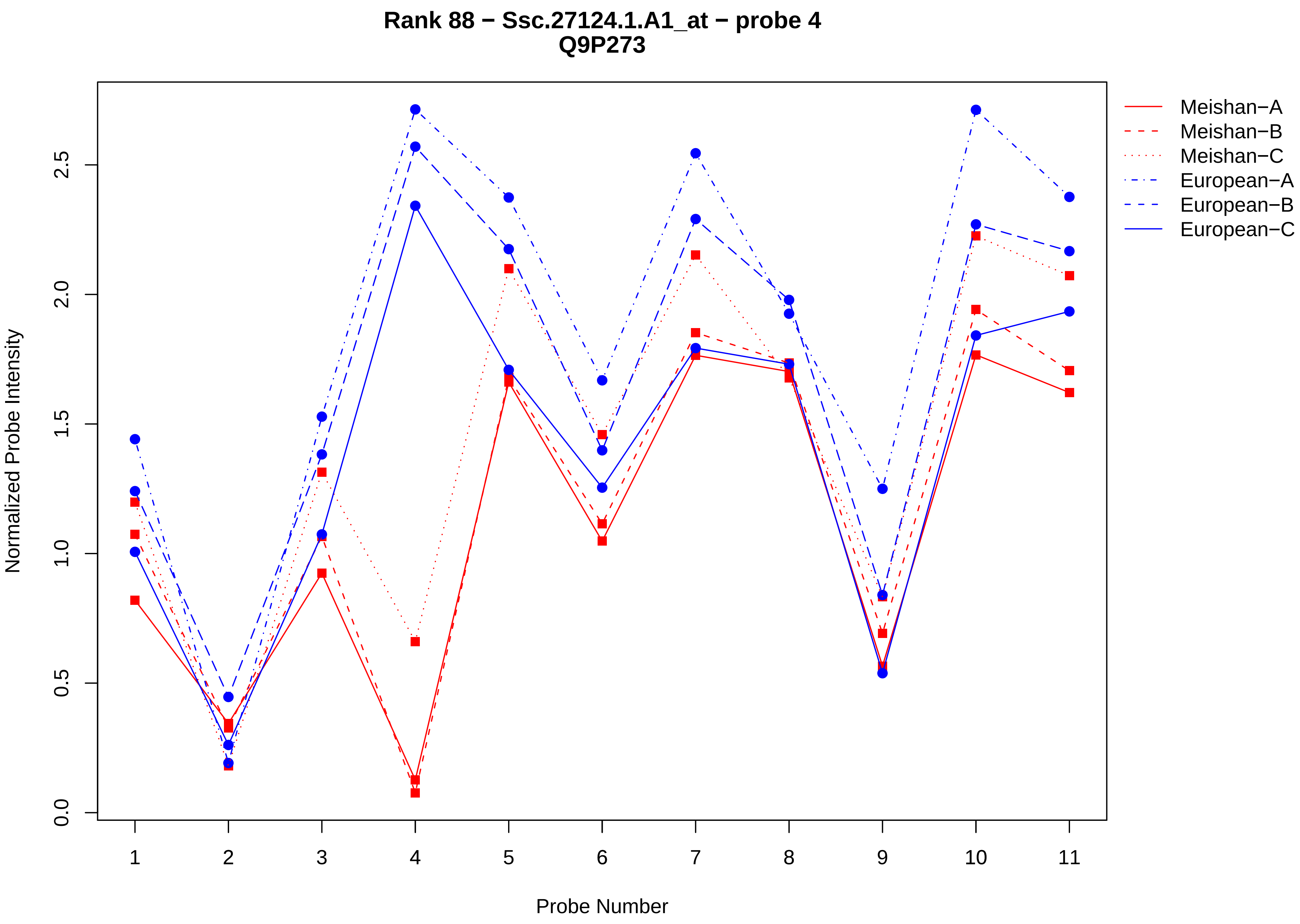

Rank 88 − Ssc.27124.1.A1_at − probe 4
Q9P273
Normalized Probe Intensity
0.0
0.5
1.0
1.5
2.0
2.5
Probe Number
1
2
3
4
5
6
7
8
9
10
11
Meishan−A
Meishan−B
Meishan−C
European−A
European−B
European−C

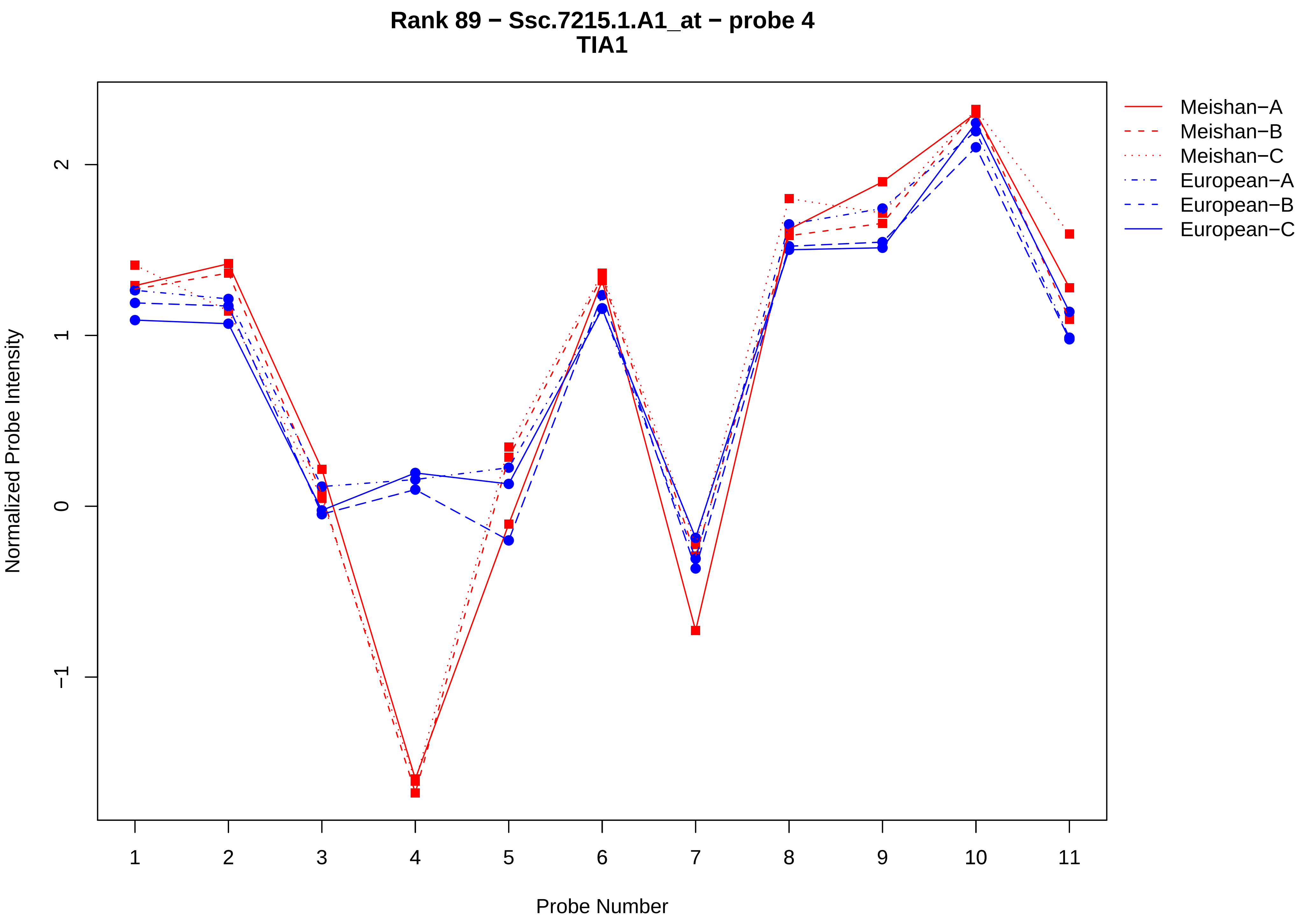

Rank 89 − Ssc.7215.1.A1_at − probe 4
TIA1
Normalized Probe Intensity
Probe Number
−1
0
1
2
1
2
3
4
5
6
7
8
9
10
11
Meishan−A
Meishan−B
Meishan−C
European−A
European−B
European−C

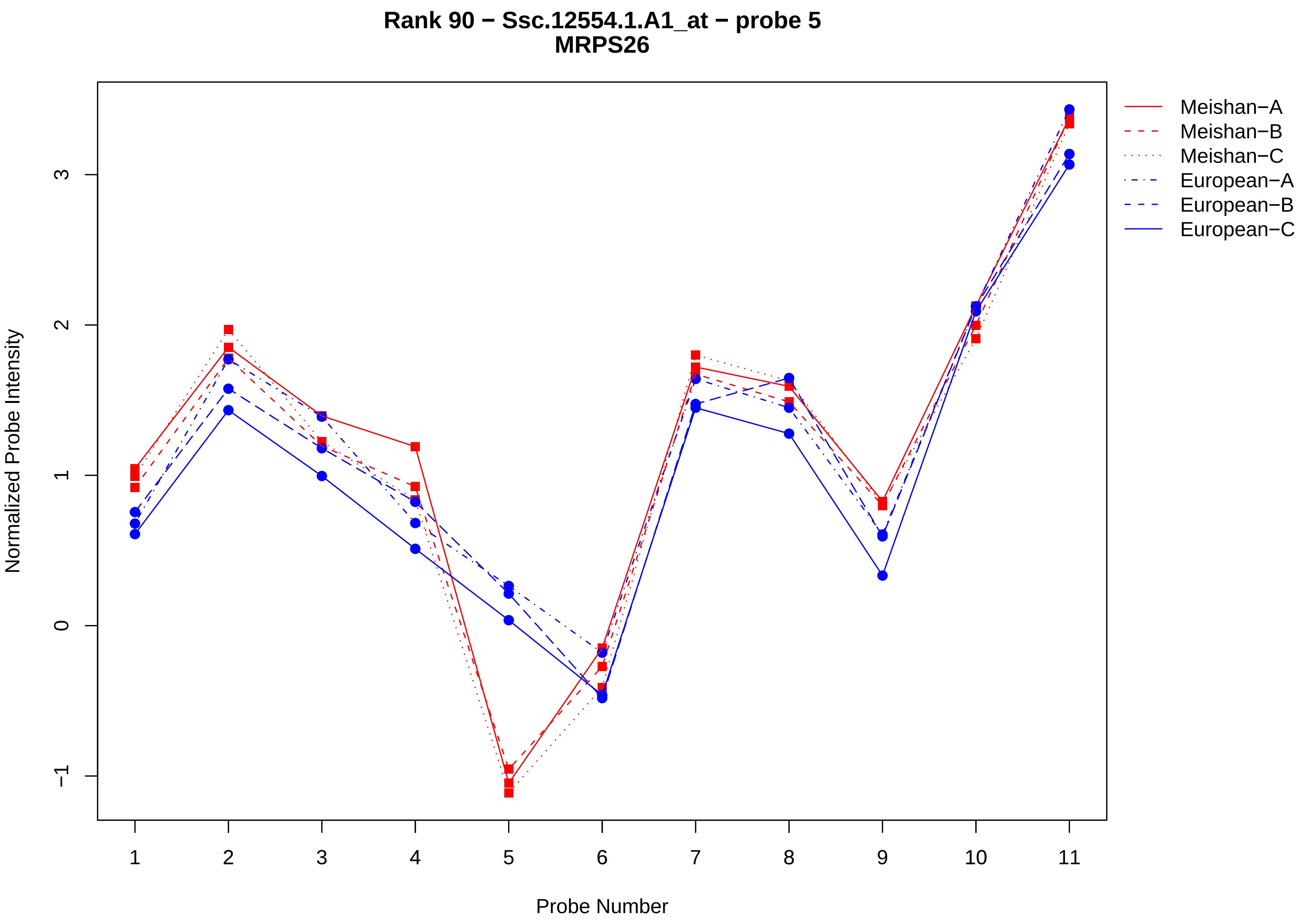
Rank 90 − Ssc.12554.1.A1_at − probe 5
MRPS26
Normalized Probe Intensity
Probe Number
Meishan−A
Meishan−B
Meishan−C
European−A
European−B
European−C

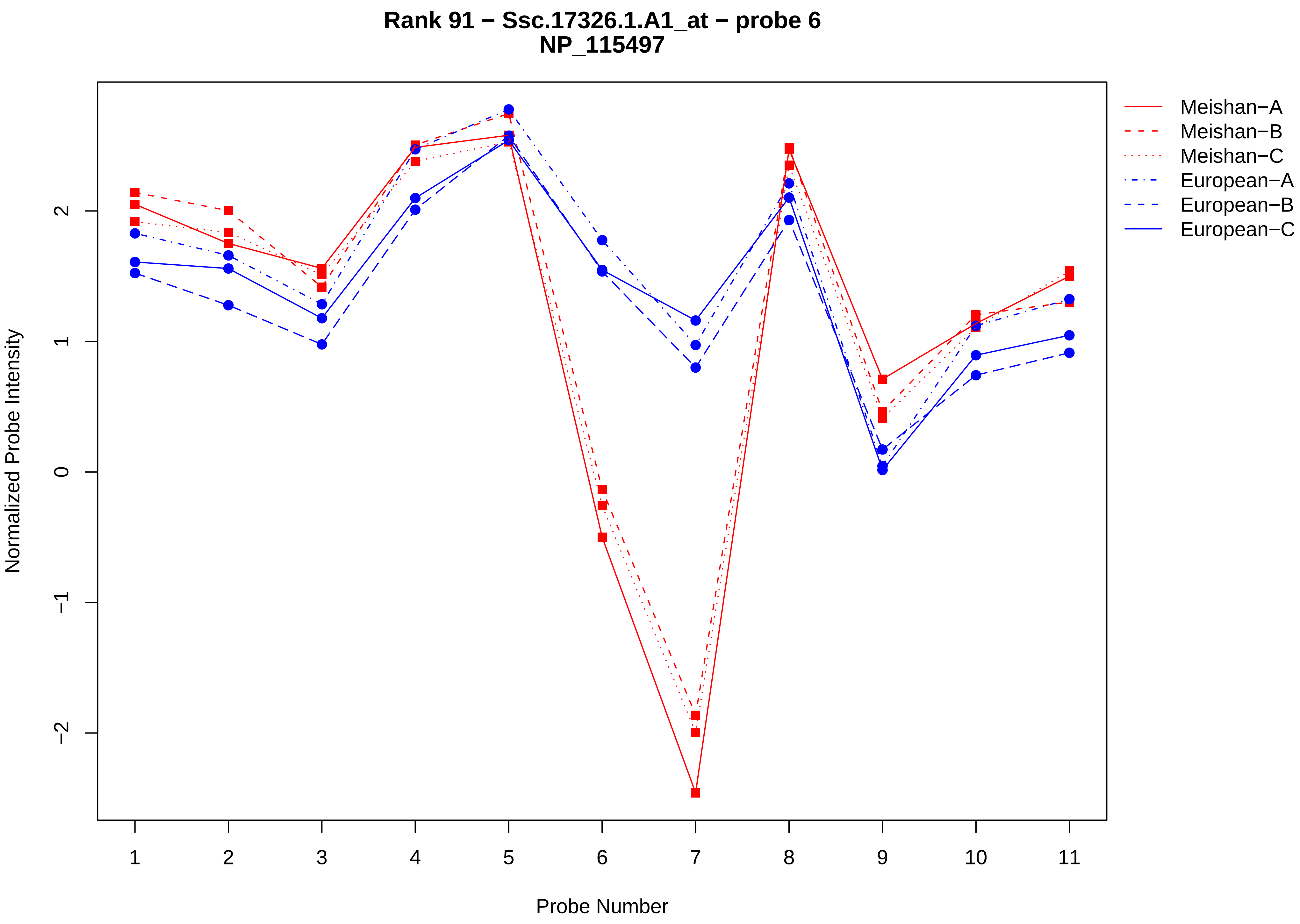

Rank 91 − Ssc.17326.1.A1_at − probe 6
NP_115497
Normalized Probe Intensity
Probe Number
−2
−1
0
1
2
1
2
3
4
5
6
7
8
9
10
11
Meishan−A
Meishan−B
Meishan−C
European−A
European−B
European−C

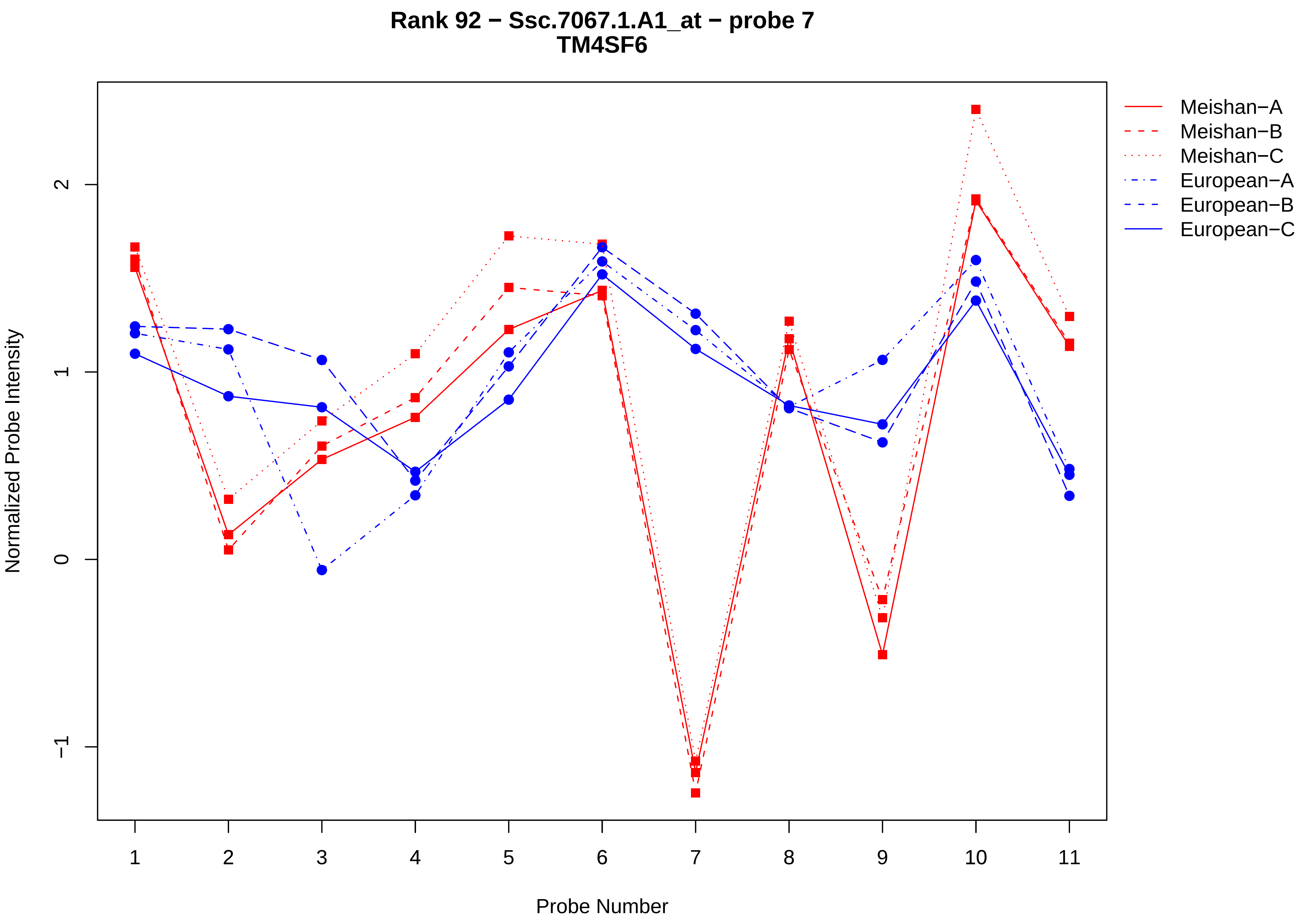
Rank 92 − Ssc.7067.1.A1_at − probe 7
TM4SF6
Normalized Probe Intensity
Probe Number
Meishan−A
Meishan−B
Meishan−C
European−A
European−B
European−C
−1
0
1
2
1
2
3
4
5
6
7
8
9
10
11

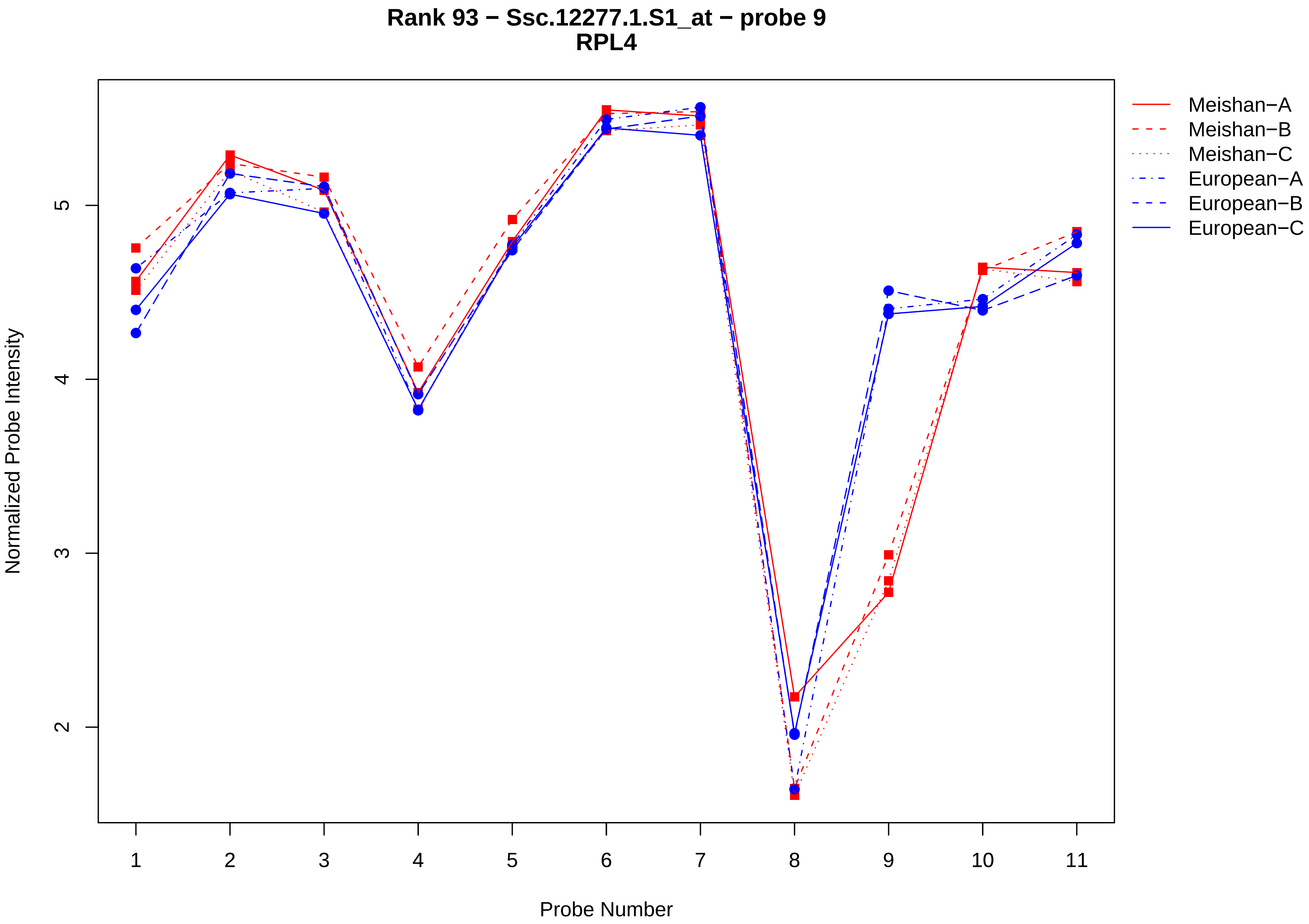

Rank 93 − Ssc.12277.1.S1_at − probe 9
RPL4
Normalized Probe Intensity
2
3
4
5
1
2
3
4
5
6
7
8
9
10
11
Probe Number
Meishan−A
Meishan−B
Meishan−C
European−A
European−B
European−C

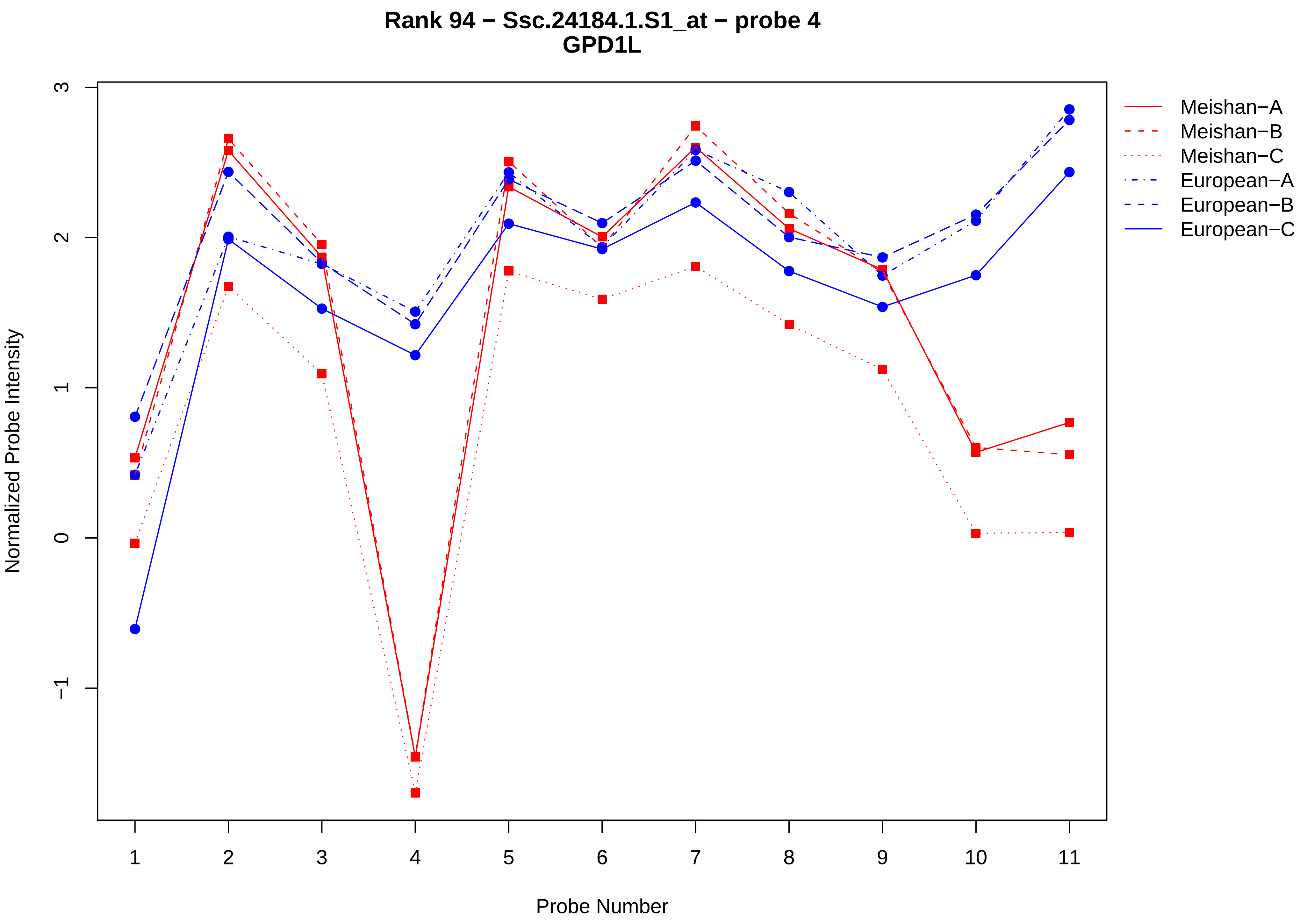

Rank 94 − Ssc.24184.1.S1_at − probe 4
GPD1L
Normalized Probe Intensity
Probe Number
3
2
1
0
−1
1
2
3
4
5
6
7
8
9
10
11
Meishan−A
Meishan−B
Meishan−C
European−A
European−B
European−C

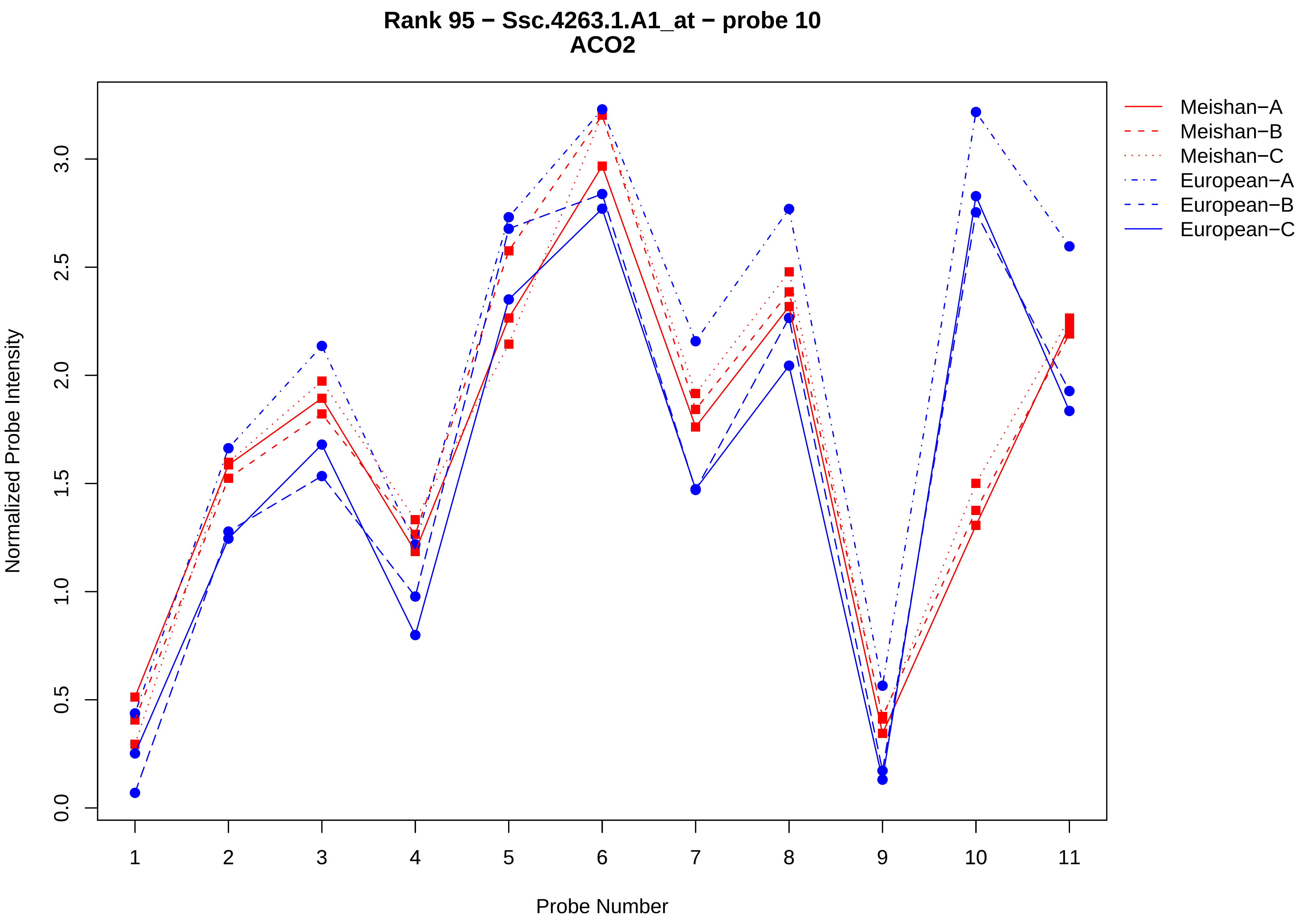

Rank 95 − Ssc.4263.1.A1_at − probe 10
ACO2
Normalized Probe Intensity
Probe Number
Meishan−A
Meishan−B
Meishan−C
European−A
European−B
European−C
0.0
0.5
1.0
1.5
2.0
2.5
3.0
1
2
3
4
5
6
7
8
9
10
11

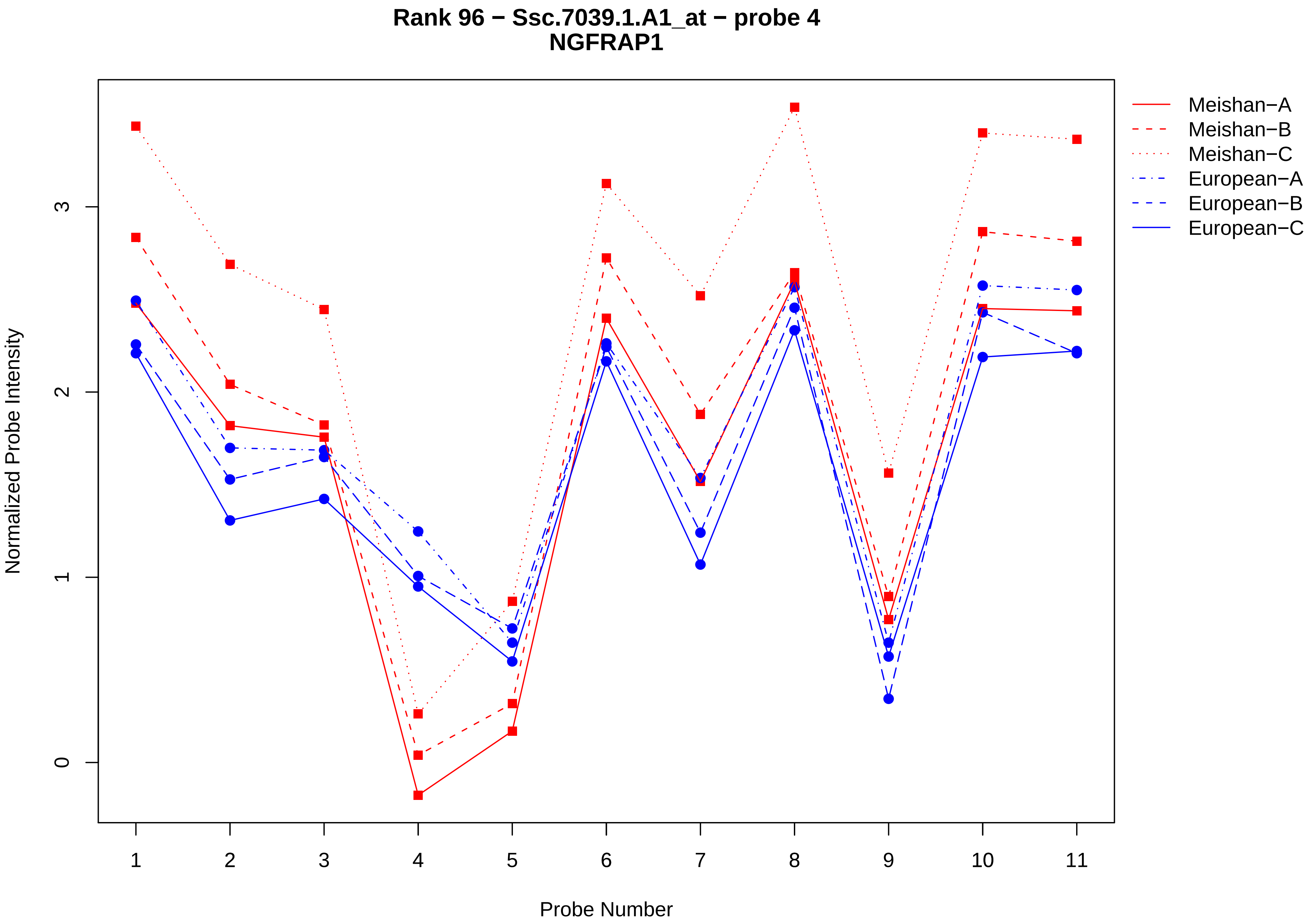

Rank 96 − Ssc.7039.1.A1_at − probe 4
NGFRAP1
Normalized Probe Intensity
Probe Number
0
1
2
3
1
2
3
4
5
6
7
8
9
10
11
Meishan−A
Meishan−B
Meishan−C
European−A
European−B
European−C

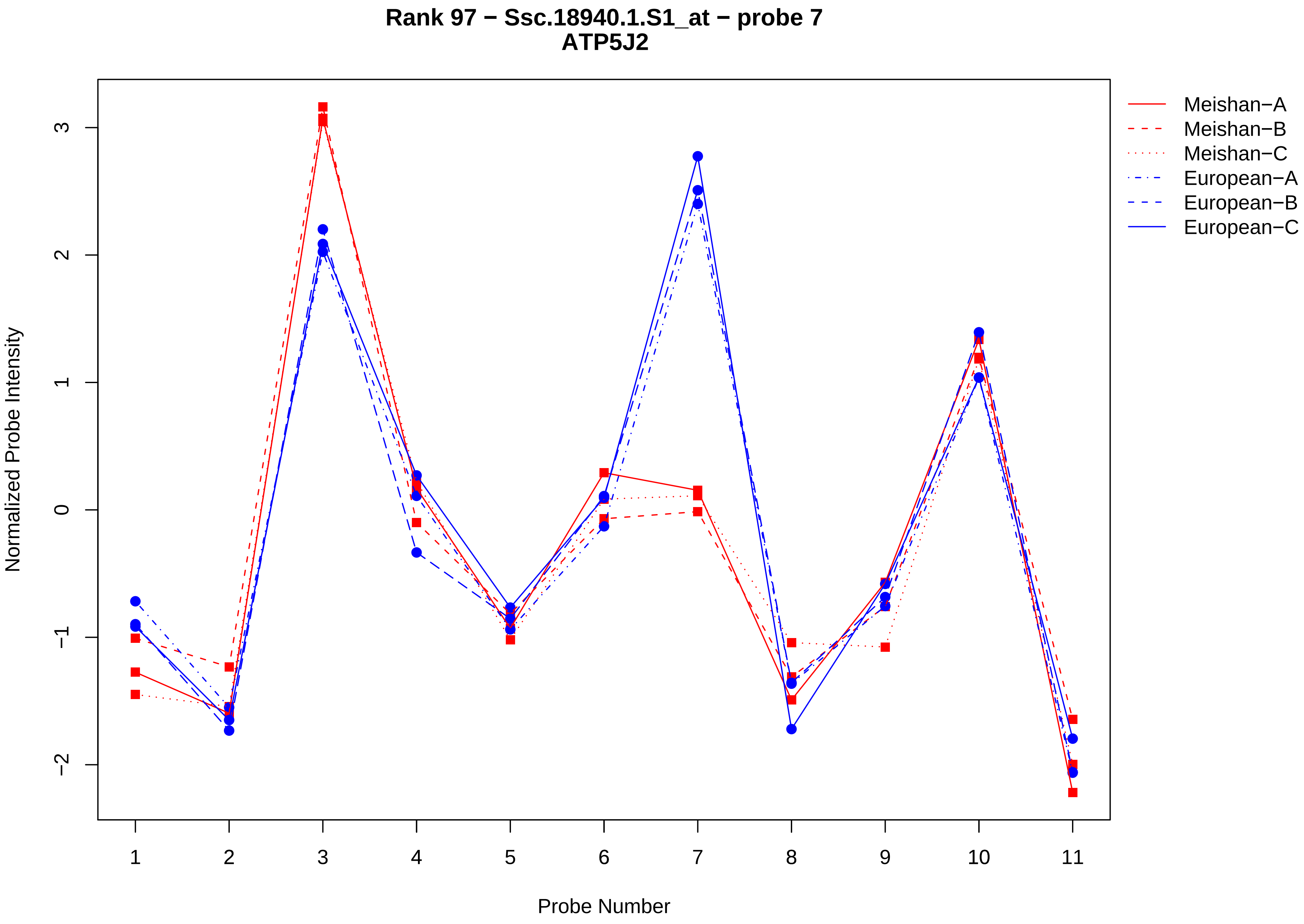

Rank 97 − Ssc.18940.1.S1_at − probe 7
ATP5J2
Normalized Probe Intensity
Probe Number
−2
−1
0
1
2
3
1
2
3
4
5
6
7
8
9
10
11
Meishan−A
Meishan−B
Meishan−C
European−A
European−B
European−C

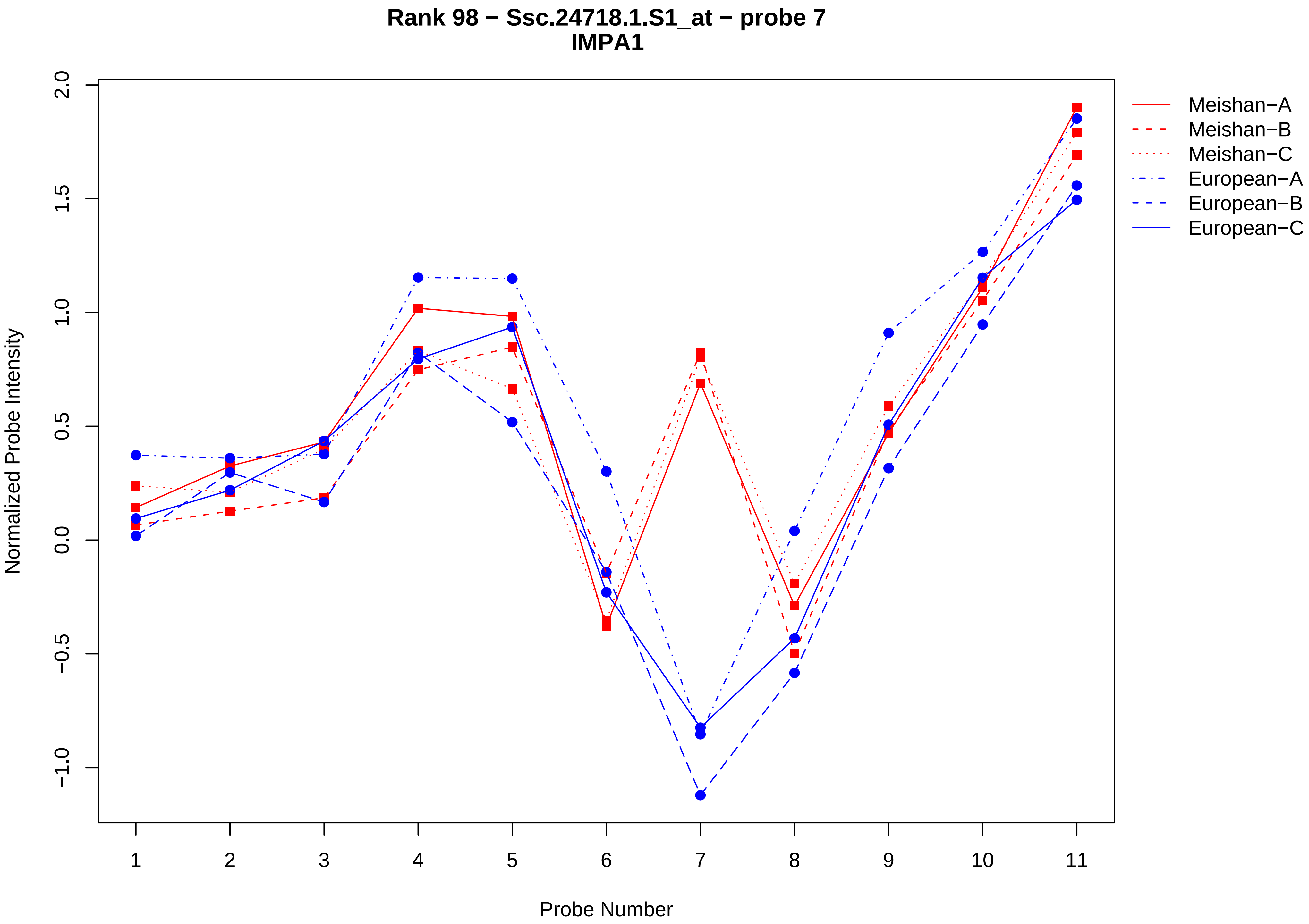

Rank 98 − Ssc.24718.1.S1_at − probe 7
IMPA1
Normalized Probe Intensity
Probe Number
2.0
1.5
1.0
0.5
0.0
−0.5
−1.0
1
2
3
4
5
6
7
8
9
10
11
Meishan−A
Meishan−B
Meishan−C
European−A
European−B
European−C

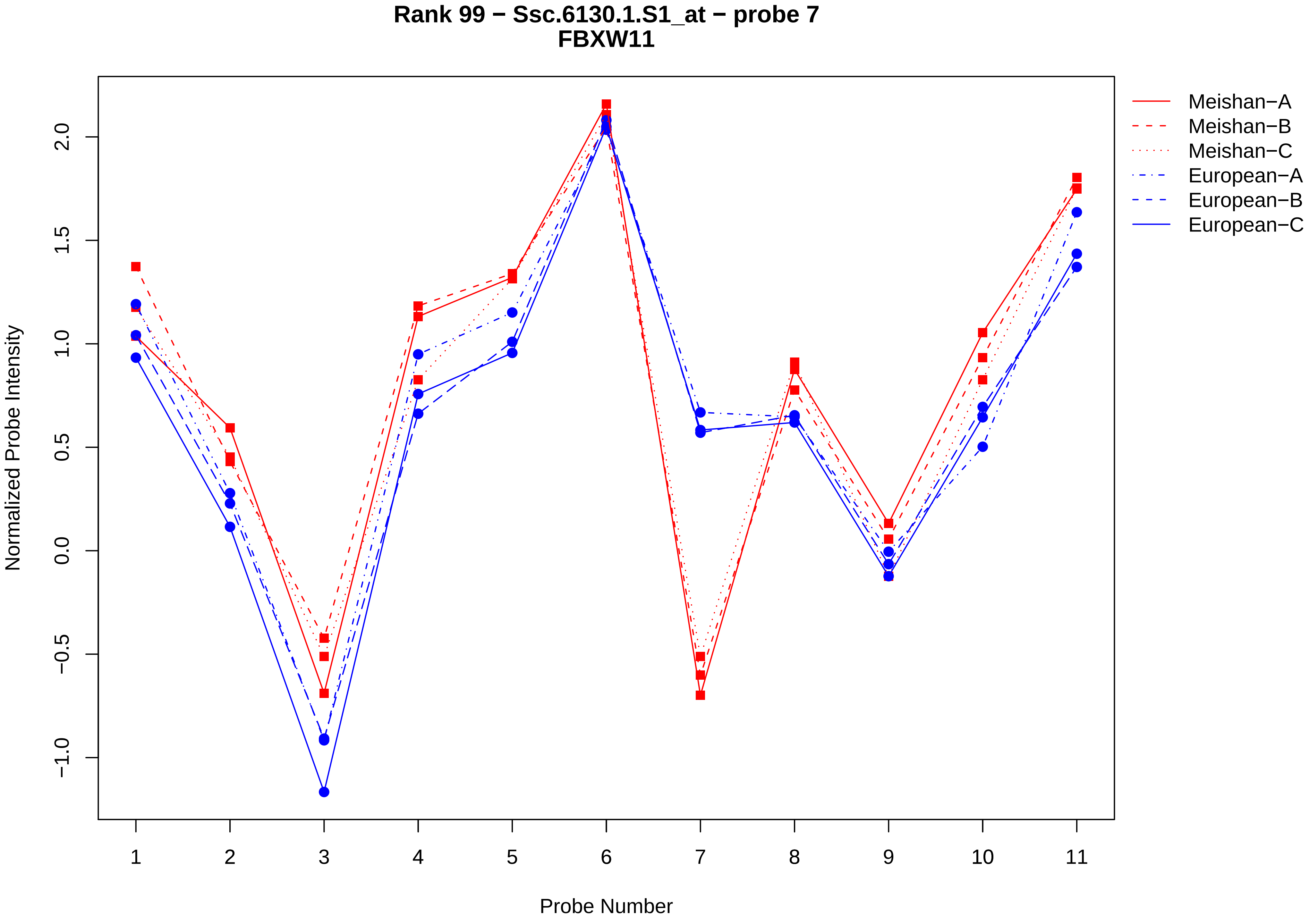

Rank 99 − Ssc.6130.1.S1_at − probe 7
FBXW11
Normalized Probe Intensity
Probe Number
−1.0
−0.5
0.0
0.5
1.0
1.5
2.0
1
2
3
4
5
6
7
8
9
10
11
Meishan−A
Meishan−B
Meishan−C
European−A
European−B
European−C

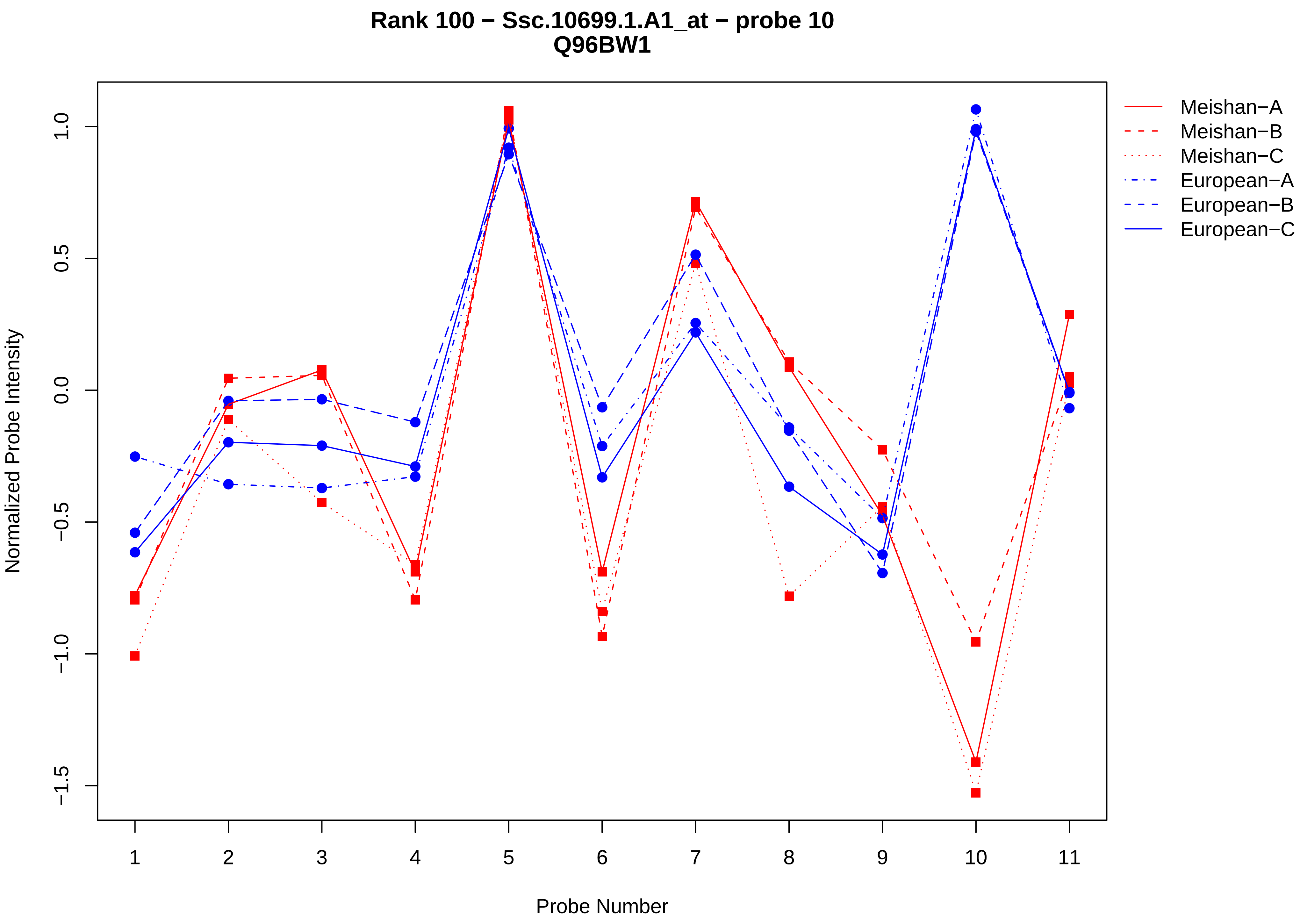

Rank 100 − Ssc.10699.1.A1_at − probe 10
Q96BW1
Normalized Probe Intensity
Probe Number
1.0
0.5
0.0
−0.5
−1.0
−1.5
1
2
3
4
5
6
7
8
9
10
11
Meishan−A
Meishan−B
Meishan−C
European−A
European−B
European−C

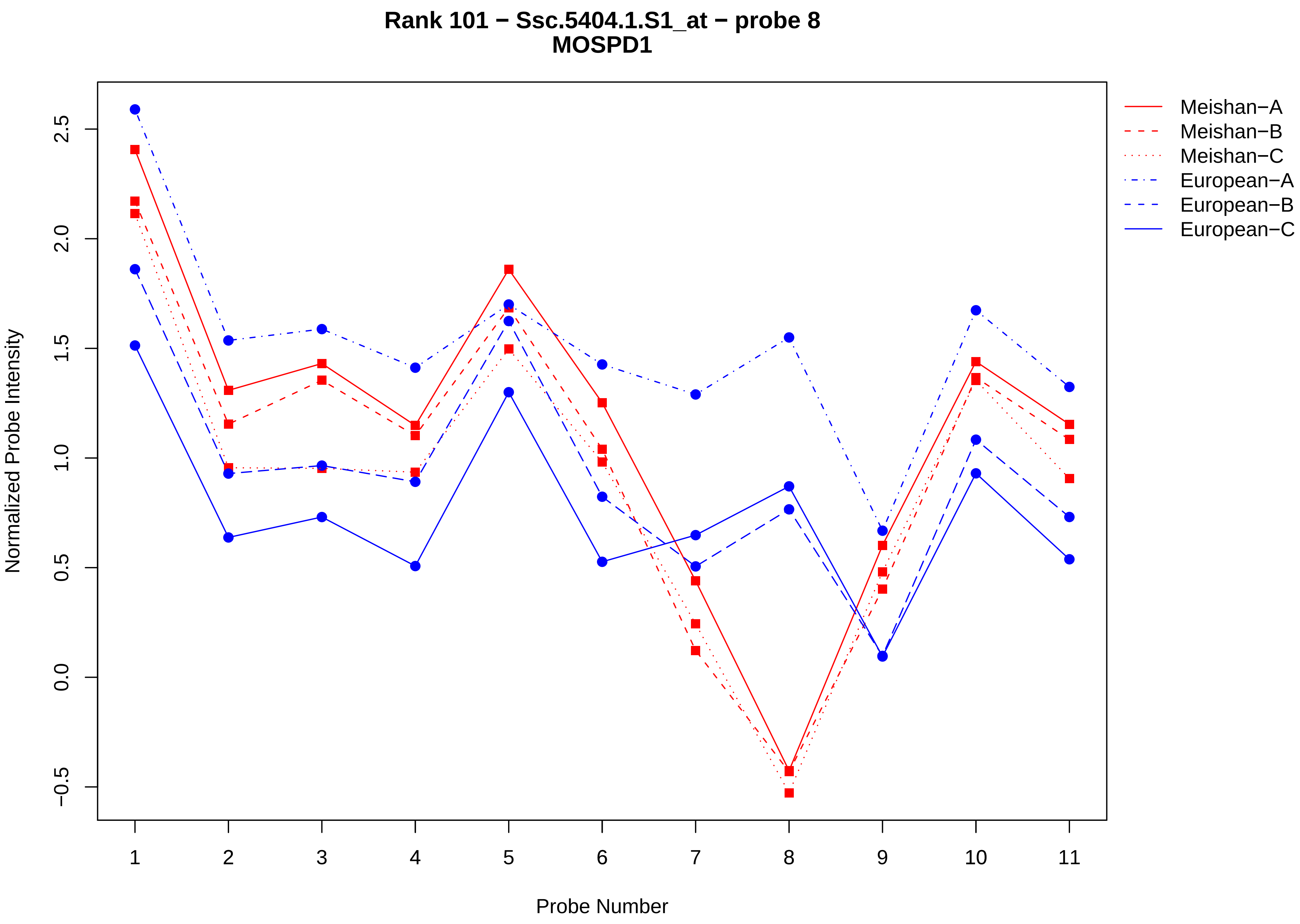

Rank 101 − Ssc.5404.1.S1_at − probe 8
MOSPD1
Normalized Probe Intensity
Probe Number
Meishan−A
Meishan−B
Meishan−C
European−A
European−B
European−C

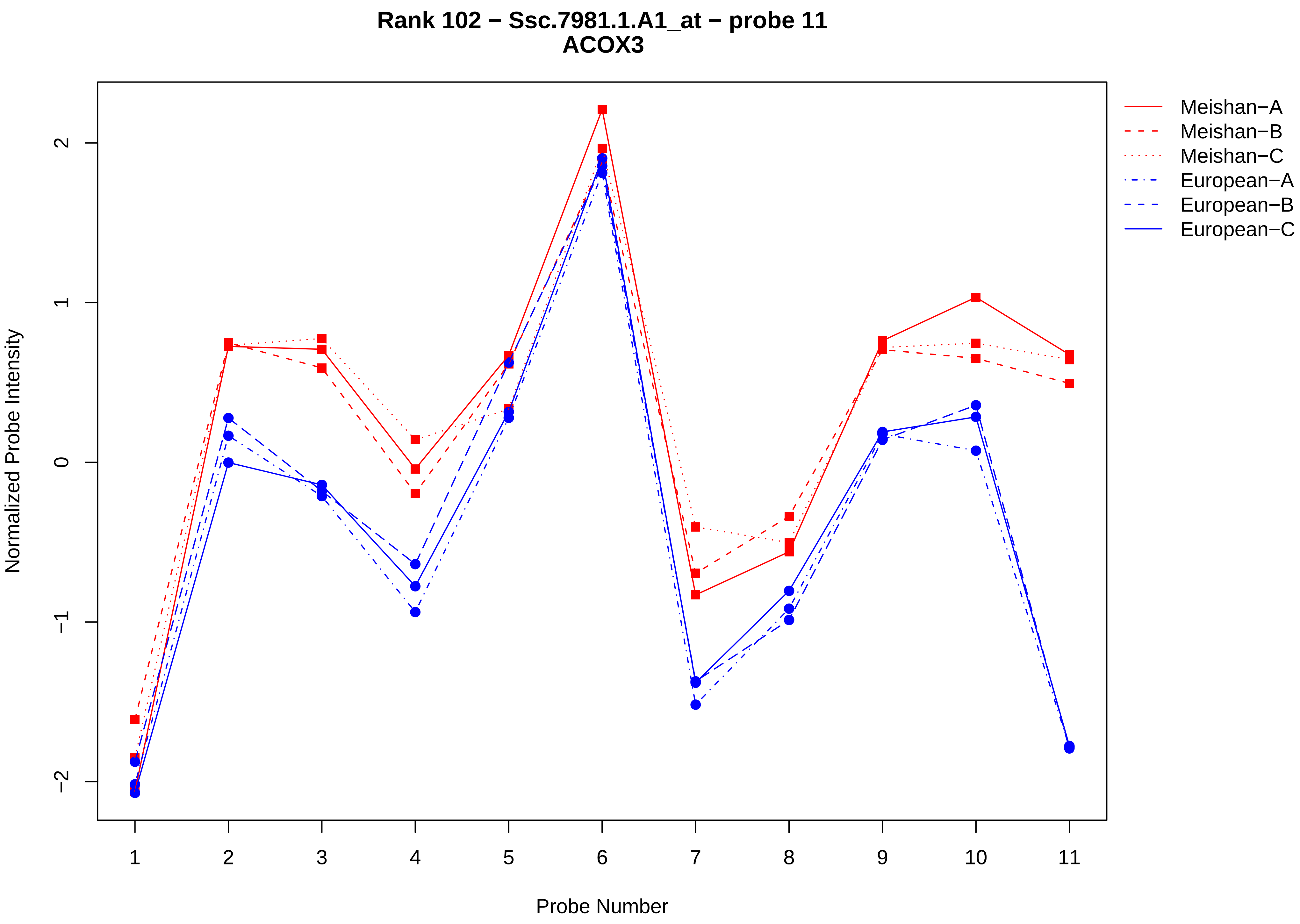

Rank 102 − Ssc.7981.1.A1_at − probe 11
ACOX3
Meishan−A
Meishan−B
Meishan−C
European−A
European−B
European−C
Normalized Probe Intensity
−2
−1
0
1
2
1
2
3
4
5
6
7
8
9
10
11
Probe Number

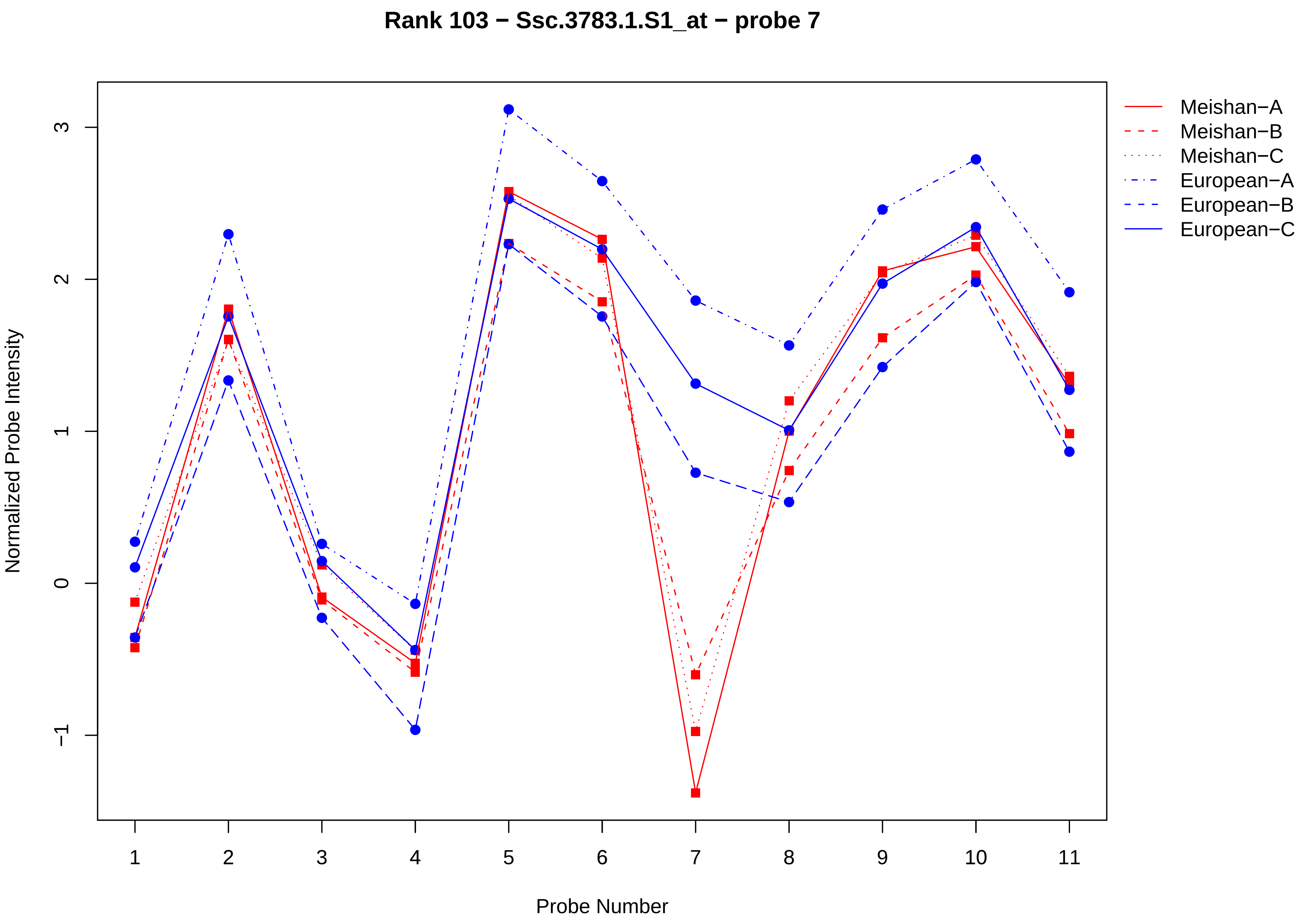
Rank 103 − Ssc.3783.1.S1_at − probe 7
Normalized Probe Intensity
−1
0
1
2
3
Probe Number
1
2
3
4
5
6
7
8
9
10
11
Meishan−A
Meishan−B
Meishan−C
European−A
European−B
European−C

Rank 104 − Ssc.20329.1.S1_at − probe 5
CAB39

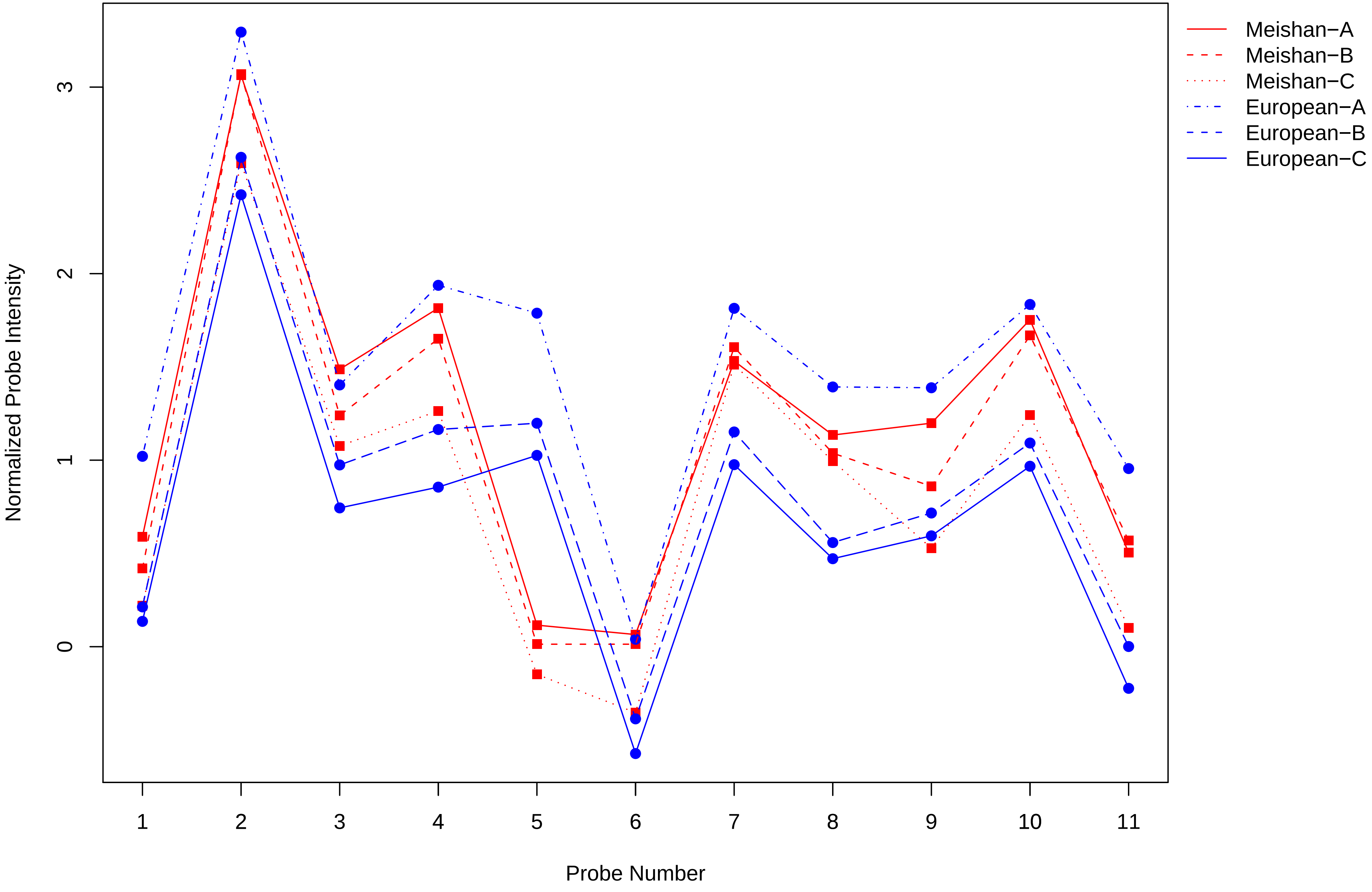

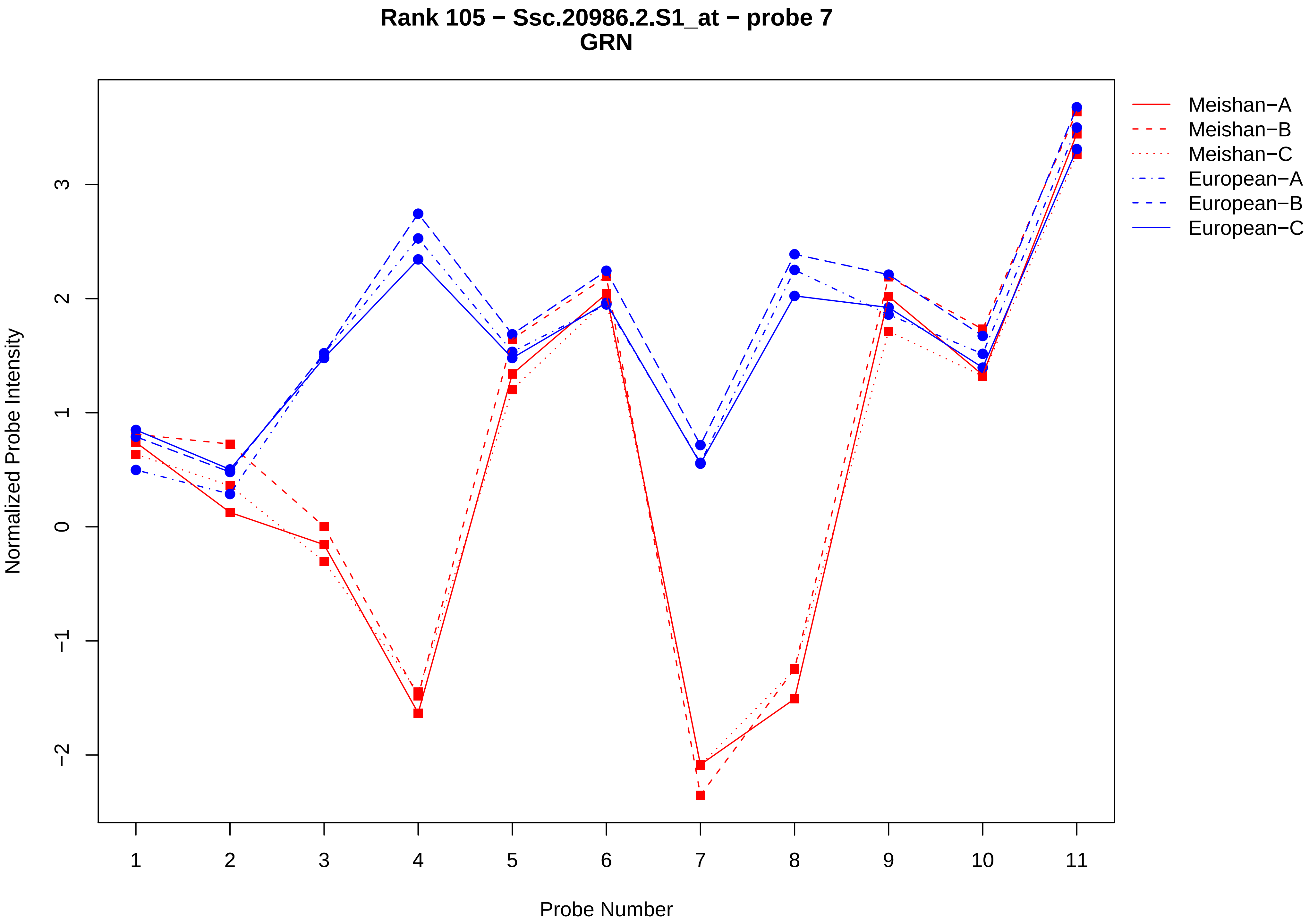

Rank 105 − Ssc.20986.2.S1_at − probe 7
GRN
Normalized Probe Intensity
Probe Number
−2
−1
0
1
2
3
1
2
3
4
5
6
7
8
9
10
11
Meishan−A
Meishan−B
Meishan−C
European−A
European−B
European−C

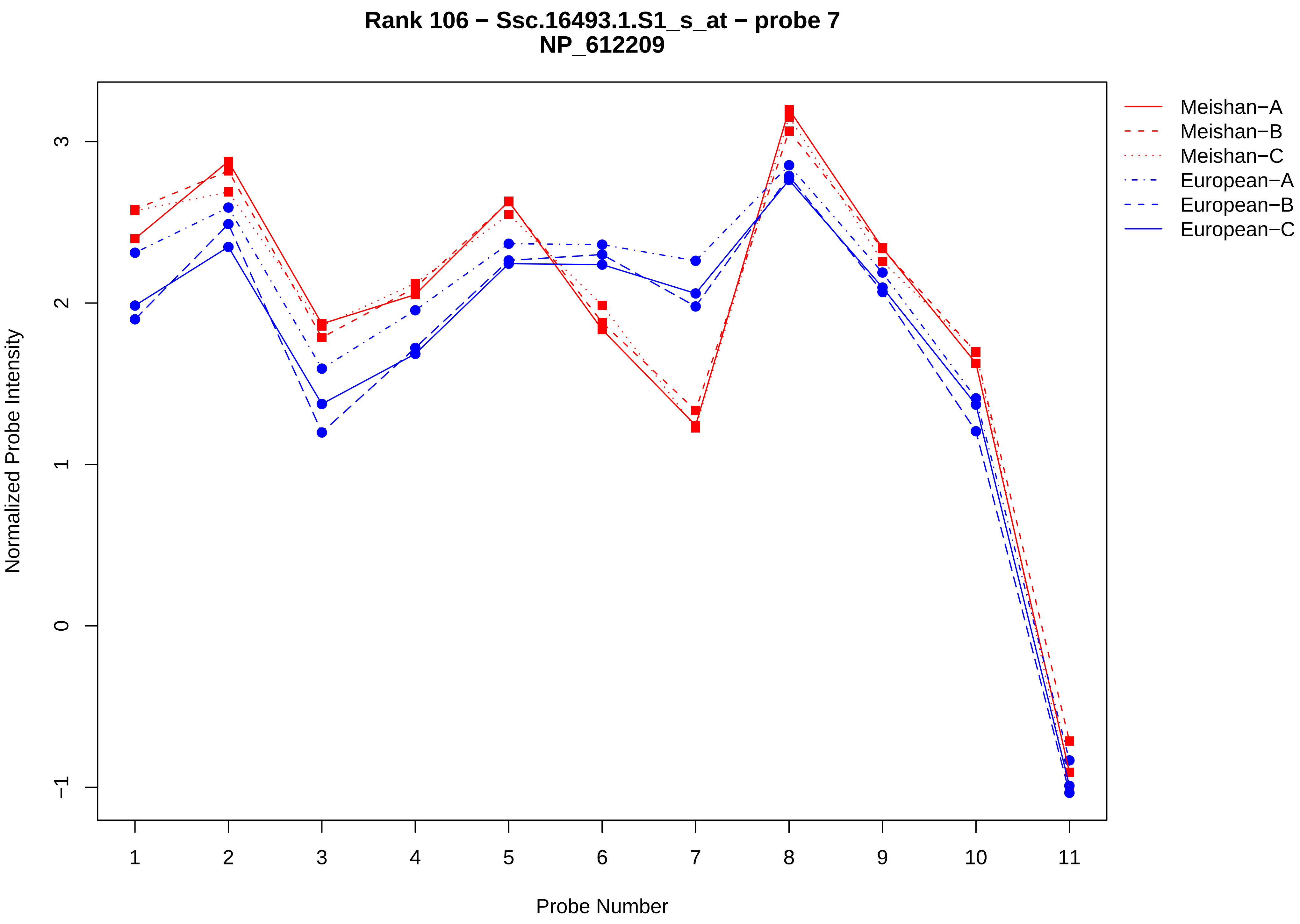

Rank 106 − Ssc.16493.1.S1_s_at − probe 7
NP_612209
Normalized Probe Intensity
Probe Number
−1
0
1
2
3
1
2
3
4
5
6
7
8
9
10
11
Meishan−A
Meishan−B
Meishan−C
European−A
European−B
European−C

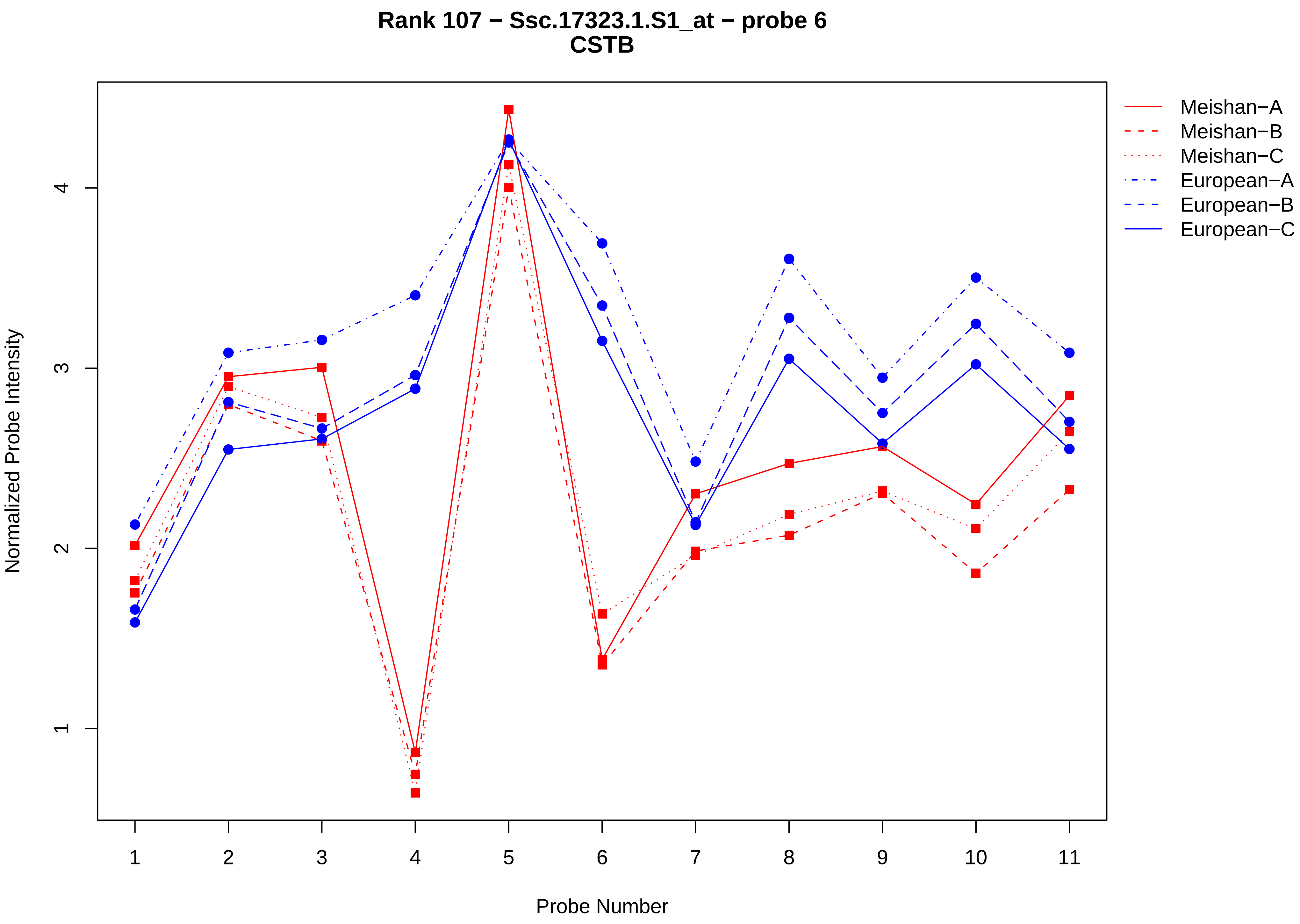

Rank 107 − Ssc.17323.1.S1_at − probe 6
CSTB
Normalized Probe Intensity
1
2
3
4
1
2
3
4
5
6
7
8
9
10
11
Probe Number
Meishan−A
Meishan−B
Meishan−C
European−A
European−B
European−C

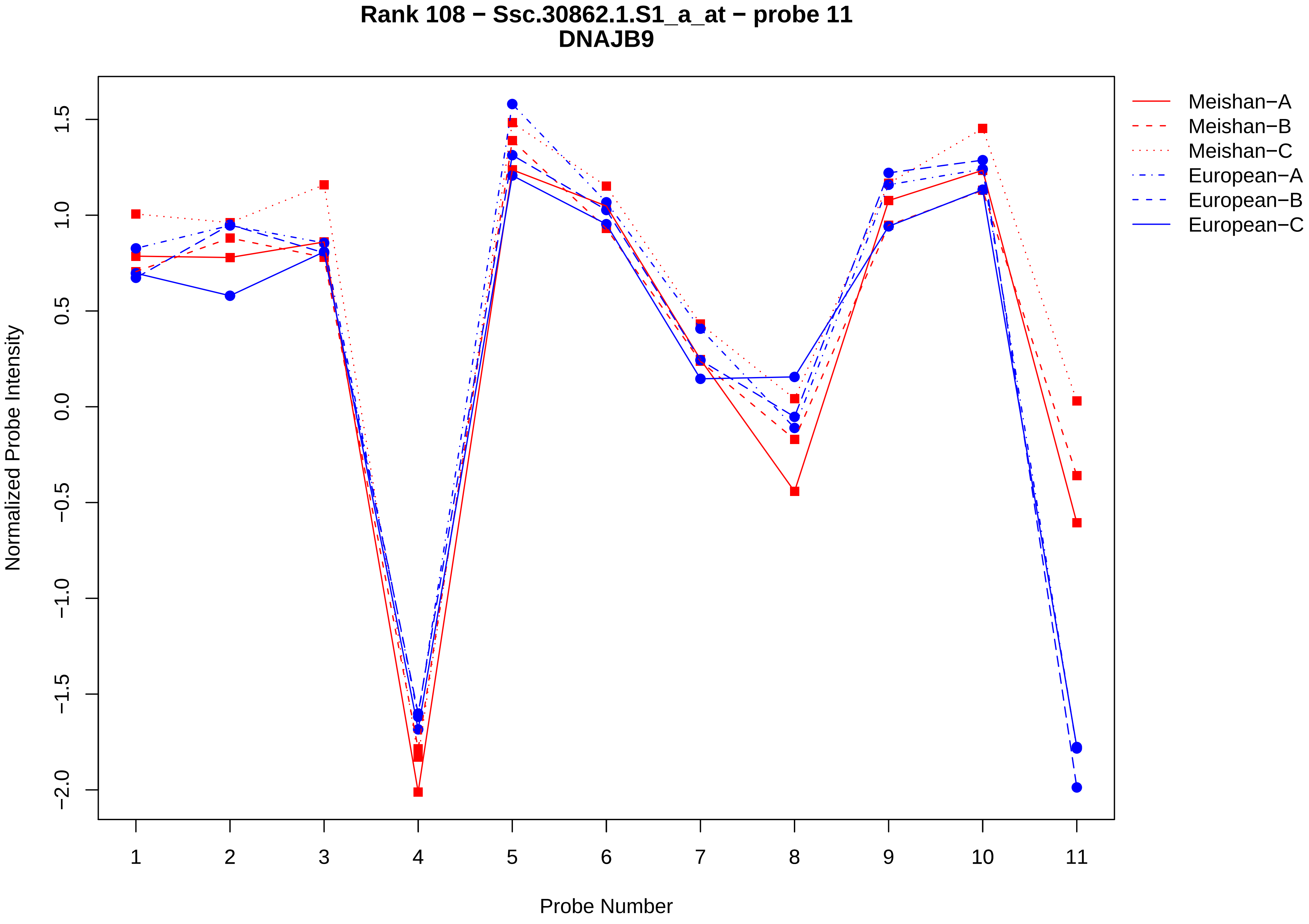
Rank 108 − Ssc.30862.1.S1_a_at − probe 11
DNAJB9
Normalized Probe Intensity
Probe Number
−2.0
−1.5
−1.0
−0.5
0.0
0.5
1.0
1.5
1
2
3
4
5
6
7
8
9
10
11
Meishan−A
Meishan−B
Meishan−C
European−A
European−B
European−C

Rank 109 − Ssc.9272.1.S1_at − probe 11
TACSTD1

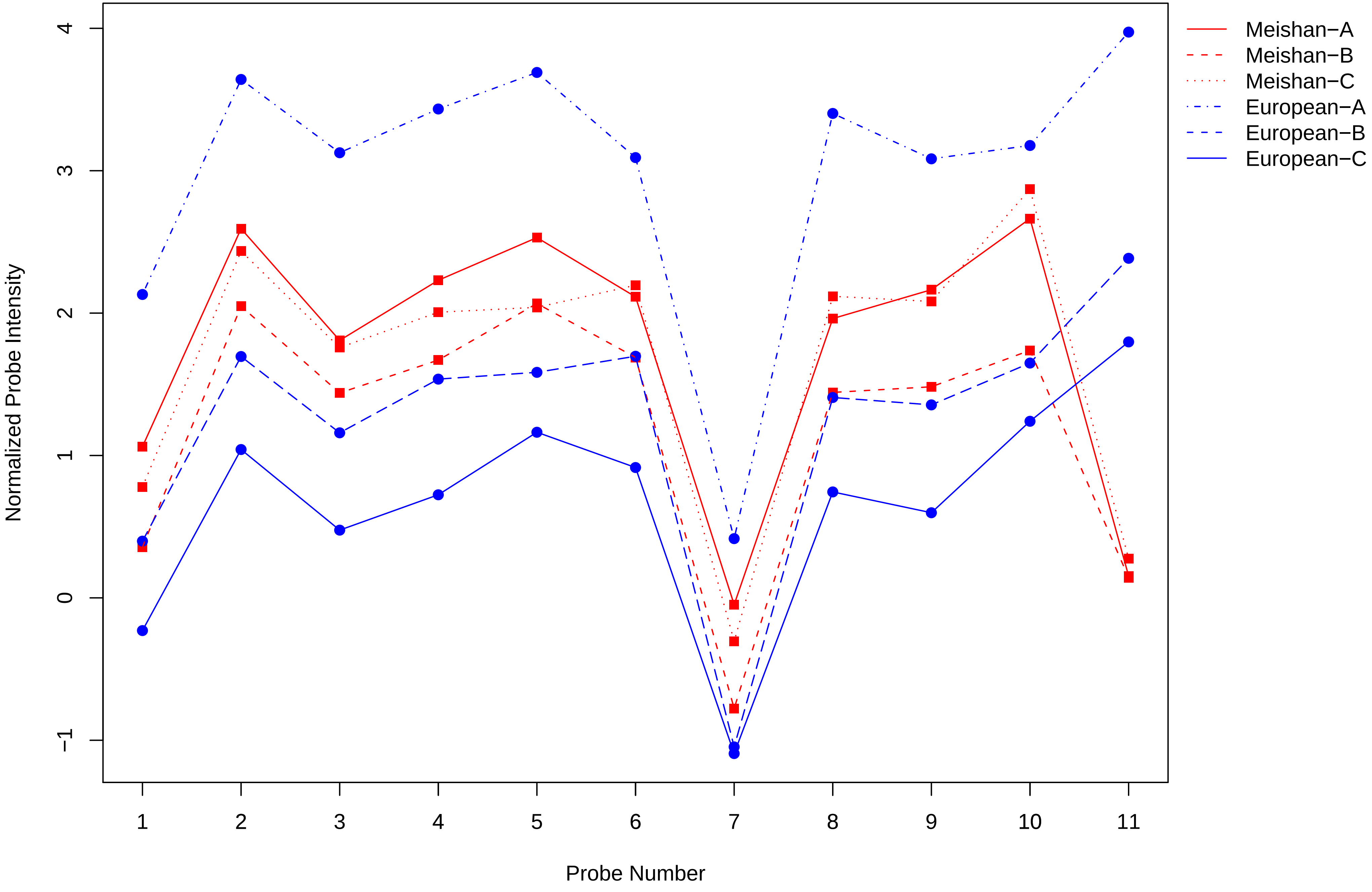

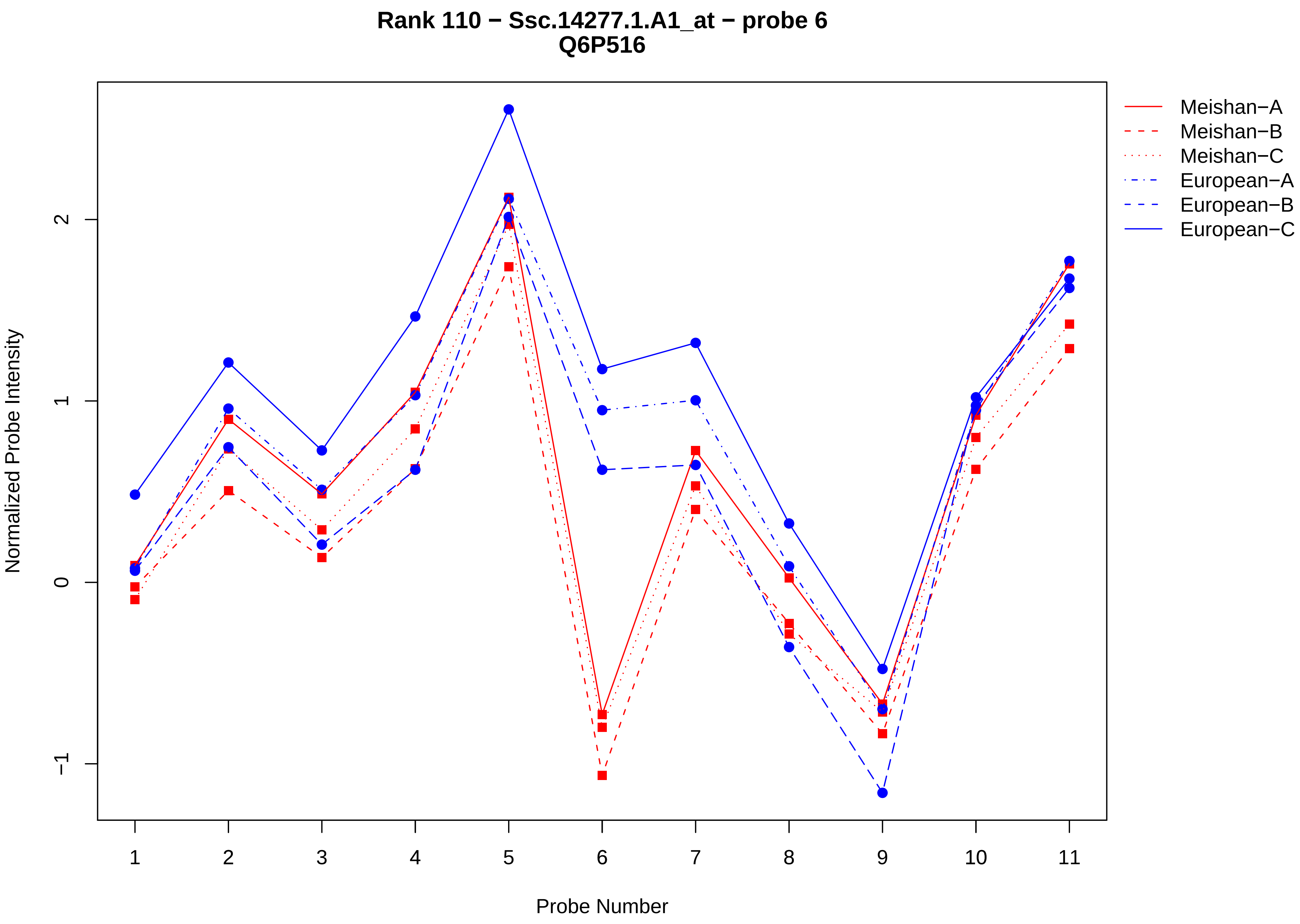

Rank 110 − Ssc.14277.1.A1_at − probe 6
Q6P516
Normalized Probe Intensity
Probe Number
−1
0
1
2
1
2
3
4
5
6
7
8
9
10
11
Meishan−A
Meishan−B
Meishan−C
European−A
European−B
European−C

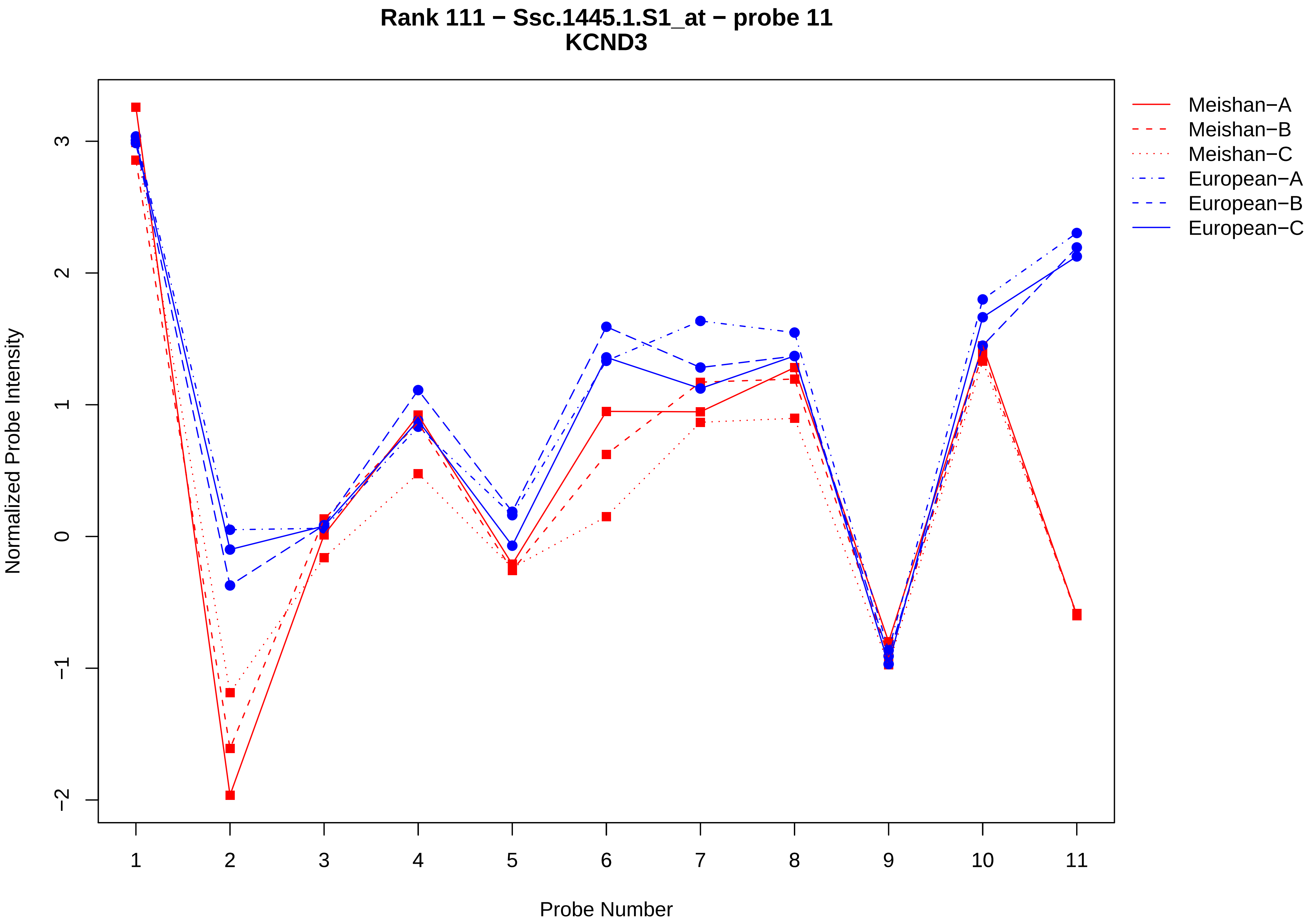

Rank 111 − Ssc.1445.1.S1_at − probe 11
KCND3
Normalized Probe Intensity
Probe Number
−2
−1
0
1
2
3
1
2
3
4
5
6
7
8
9
10
11
Meishan−A
Meishan−B
Meishan−C
European−A
European−B
European−C

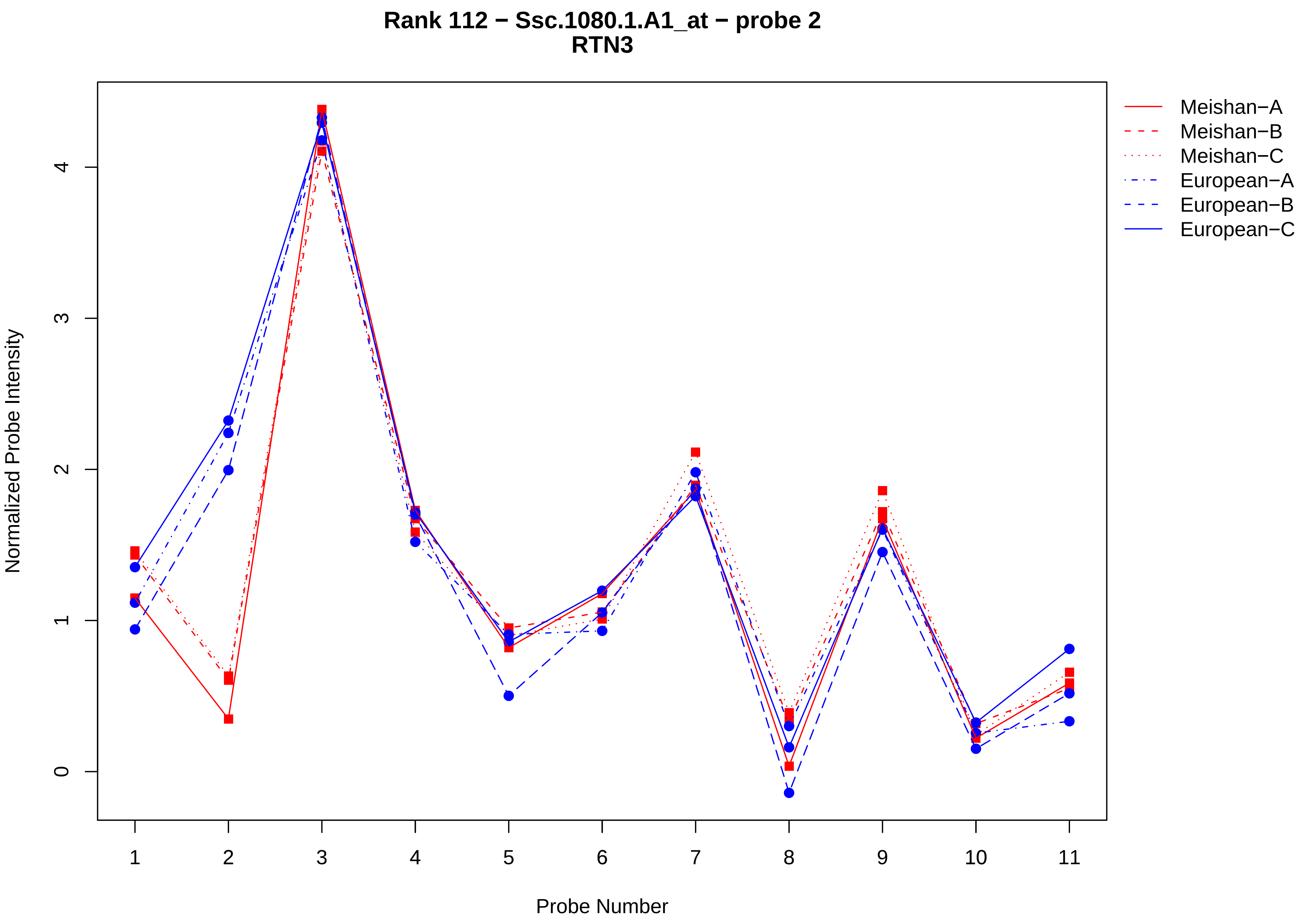
Rank 112 − Ssc.1080.1.A1_at − probe 2
RTN3
Normalized Probe Intensity
0
1
2
3
4
1
2
3
4
5
6
7
8
9
10
11
Probe Number
Meishan−A
Meishan−B
Meishan−C
European−A
European−B
European−C

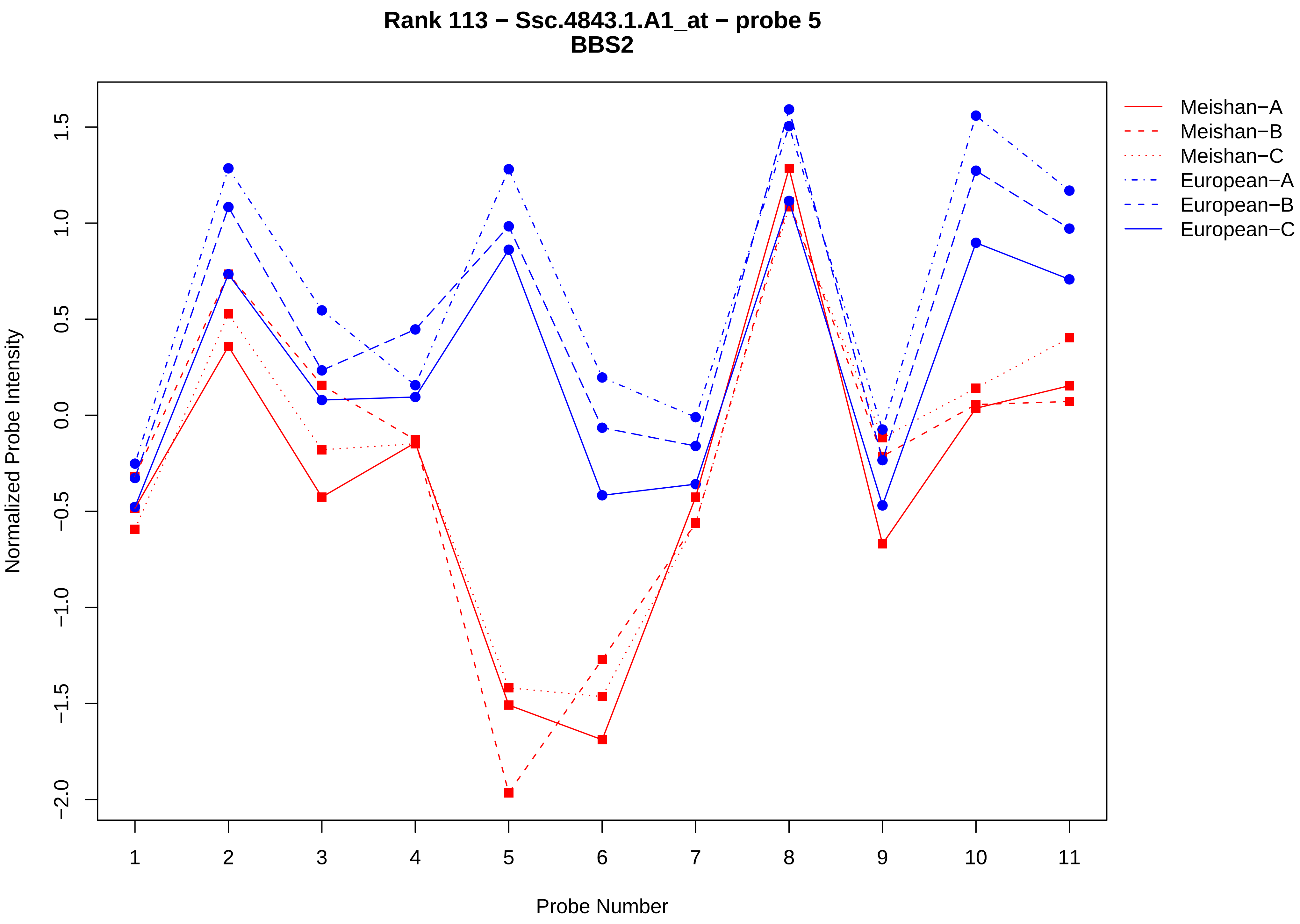
Rank 113 − Ssc.4843.1.A1_at − probe 5
BBS2
Normalized Probe Intensity
Probe Number
−2.0
−1.5
−1.0
−0.5
0.0
0.5
1.0
1.5
1
2
3
4
5
6
7
8
9
10
11
Meishan−A
Meishan−B
Meishan−C
European−A
European−B
European−C

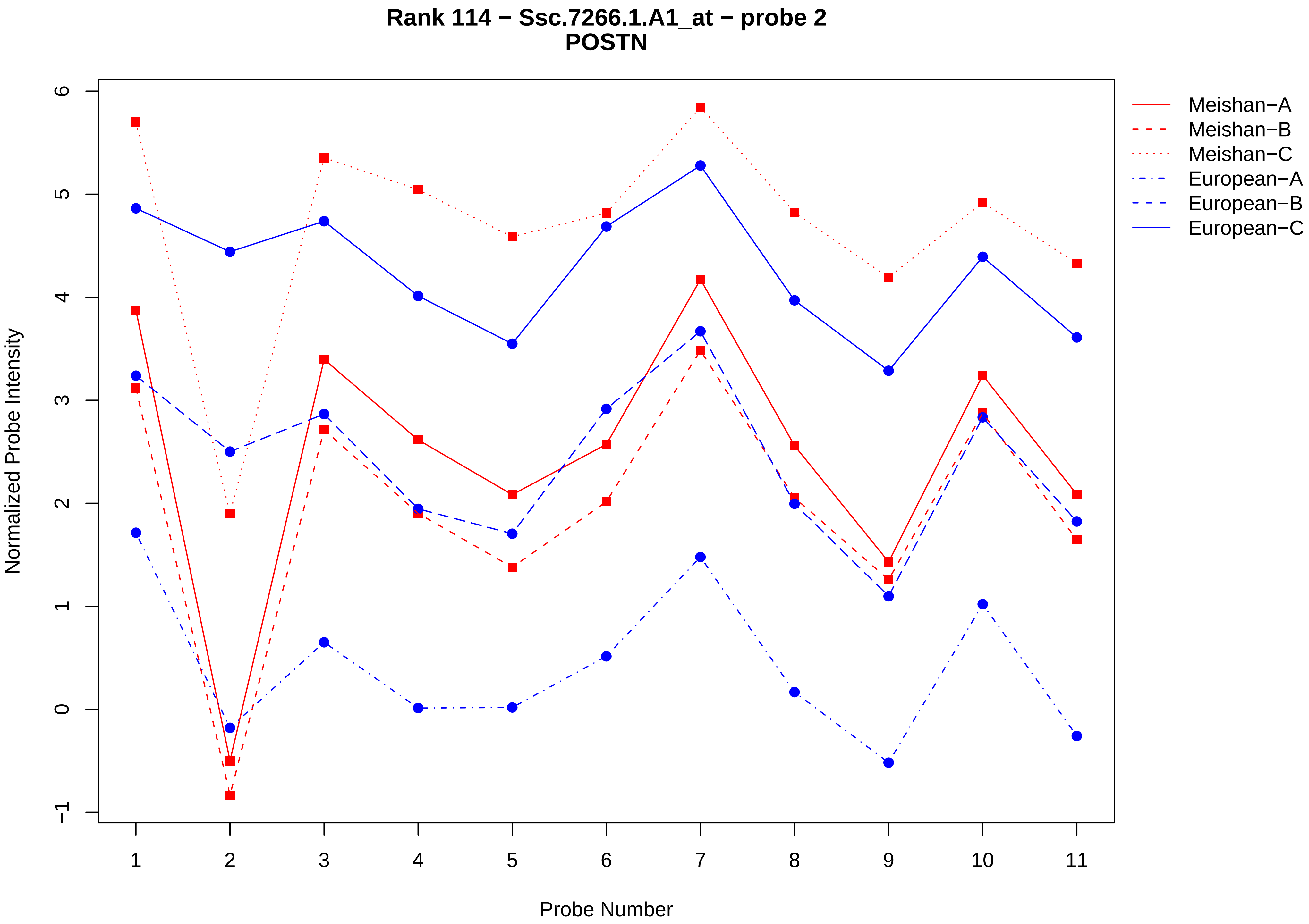

Rank 114 − Ssc.7266.1.A1_at − probe 2
POSTN
Normalized Probe Intensity
Probe Number
Meishan−A
Meishan−B
Meishan−C
European−A
European−B
European−C

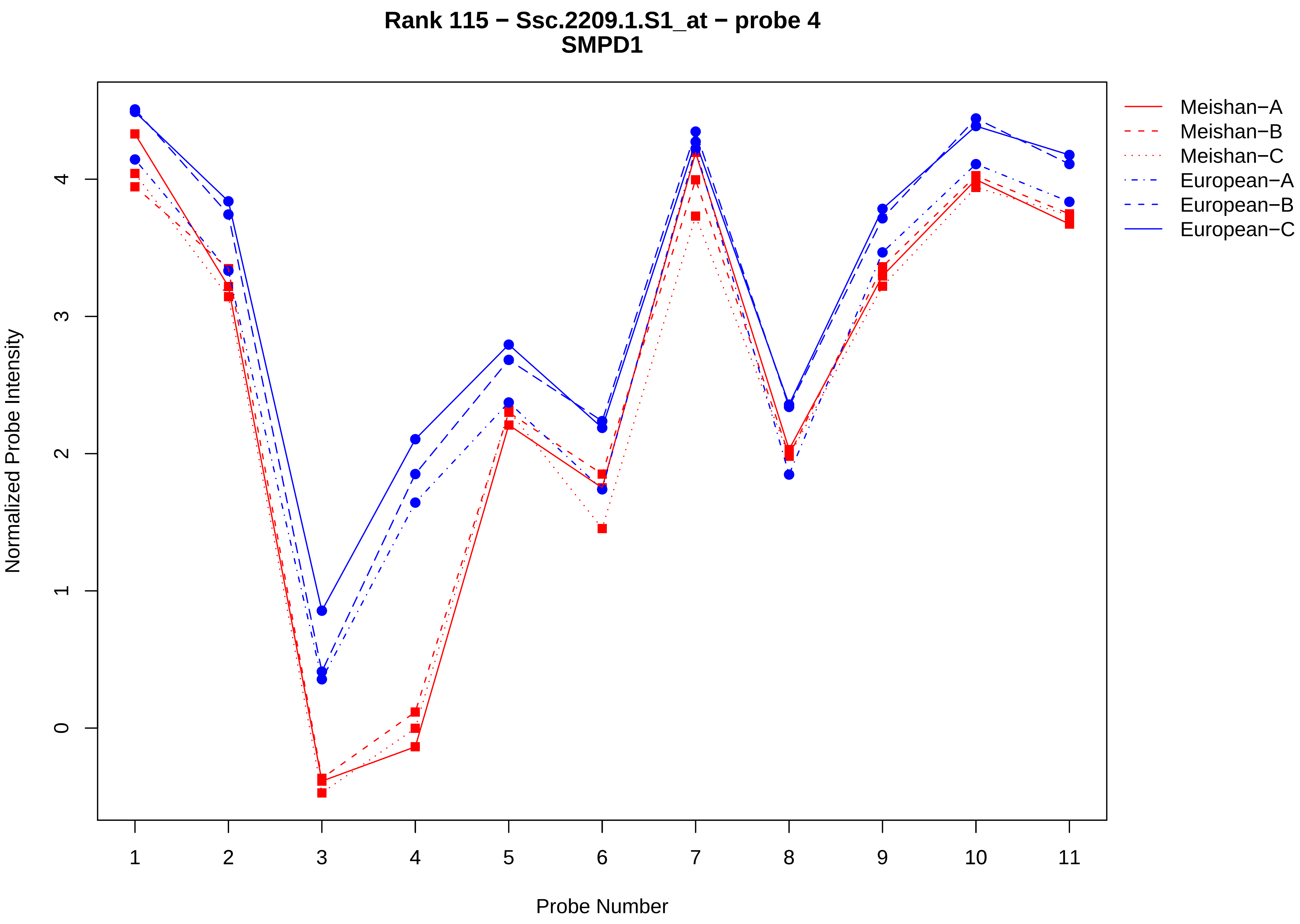
Rank 115 − Ssc.2209.1.S1_at − probe 4
SMPD1
Normalized Probe Intensity
Probe Number
0
1
2
3
4
1
2
3
4
5
6
7
8
9
10
11
Meishan−A
Meishan−B
Meishan−C
European−A
European−B
European−C

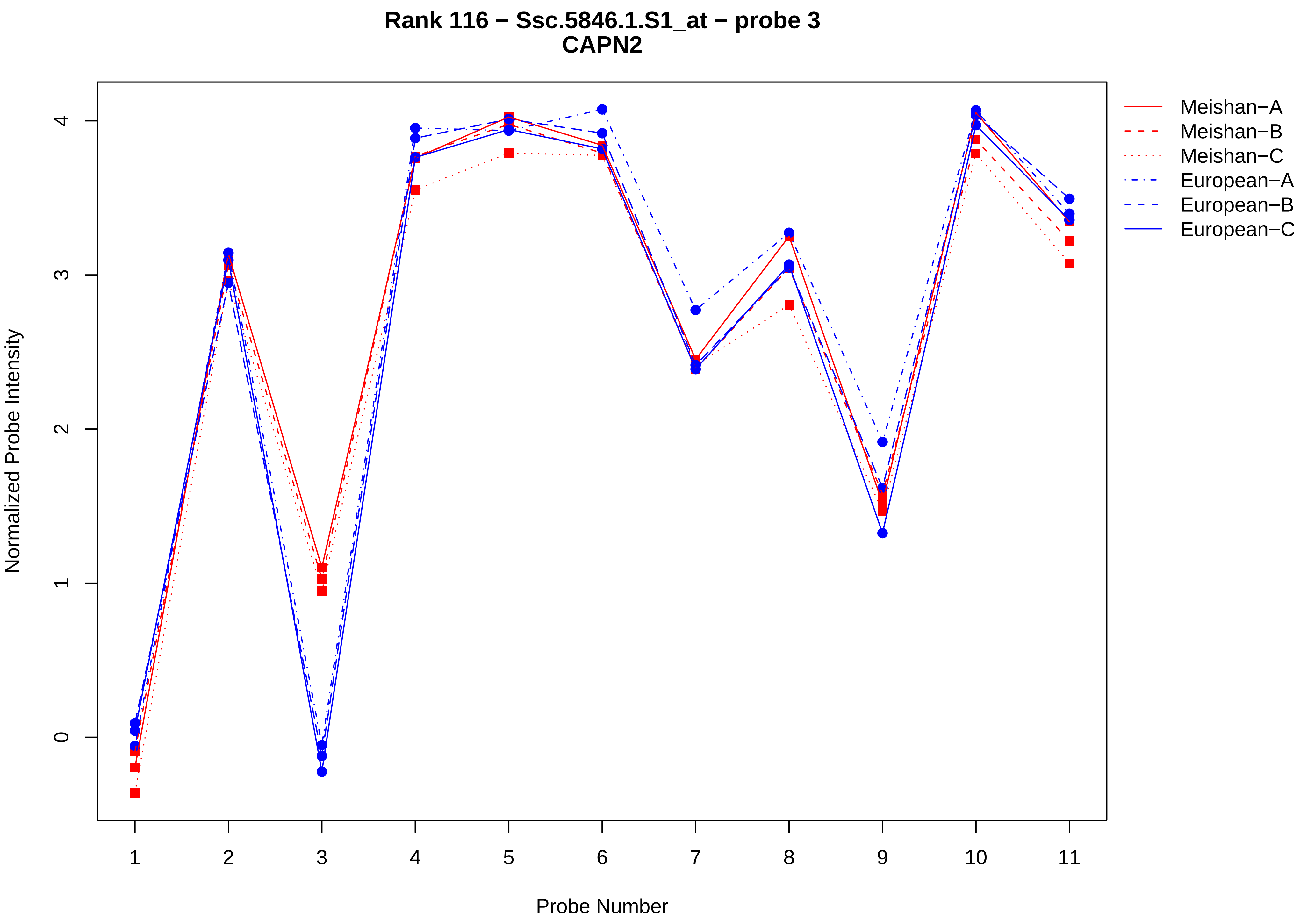

Rank 116 − Ssc.5846.1.S1_at − probe 3
CAPN2
Normalized Probe Intensity
Probe Number
0
1
2
3
4
1
2
3
4
5
6
7
8
9
10
11
Meishan−A
Meishan−B
Meishan−C
European−A
European−B
European−C

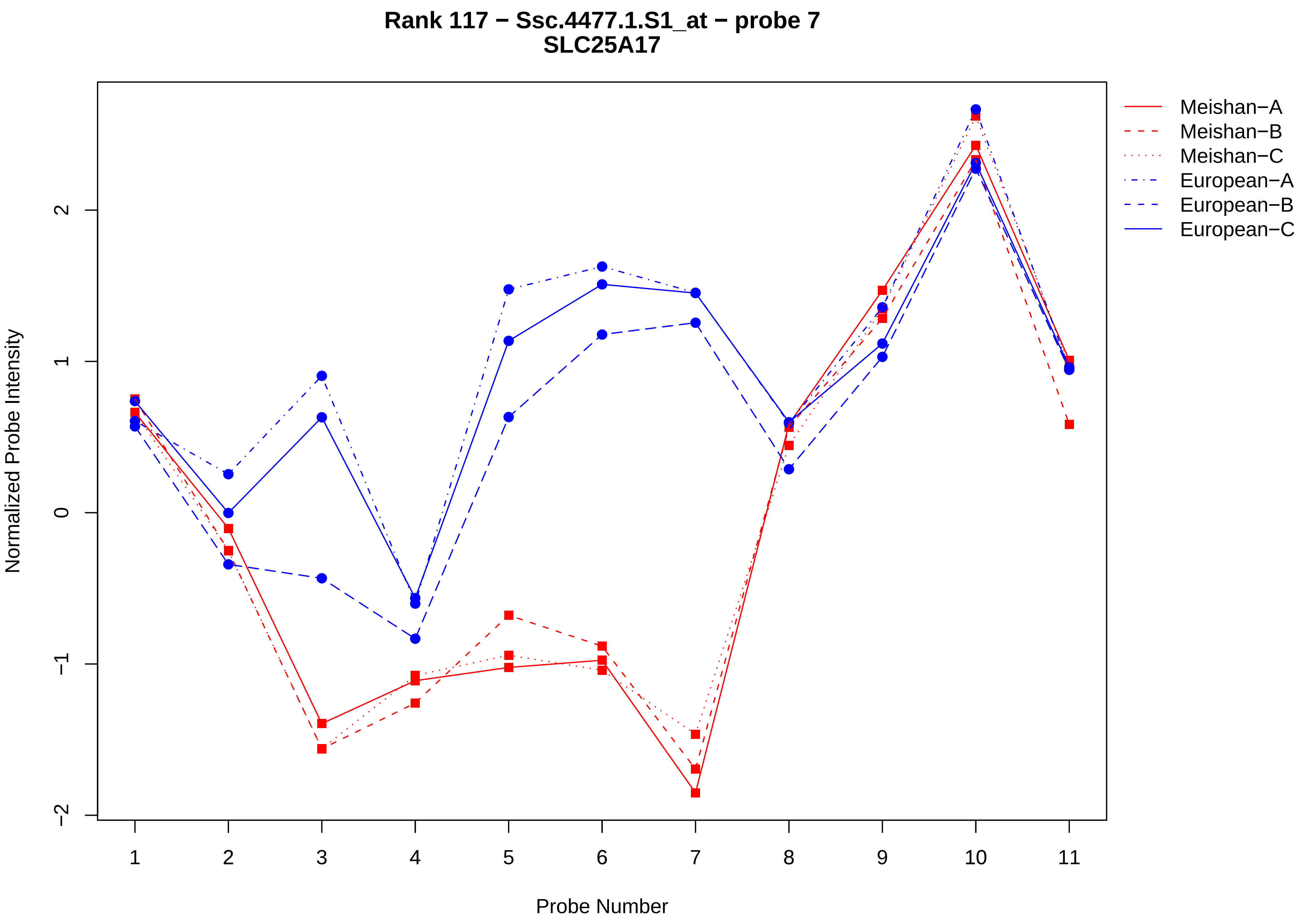
Rank 117 − Ssc.4477.1.S1_at − probe 7
SLC25A17
Normalized Probe Intensity
−2
−1
0
1
2
1
2
3
4
5
6
7
8
9
10
11
Probe Number
Meishan−A
Meishan−B
Meishan−C
European−A
European−B
European−C

Rank 118 − Ssc.26377.2.A1_at − probe 6
DGKA

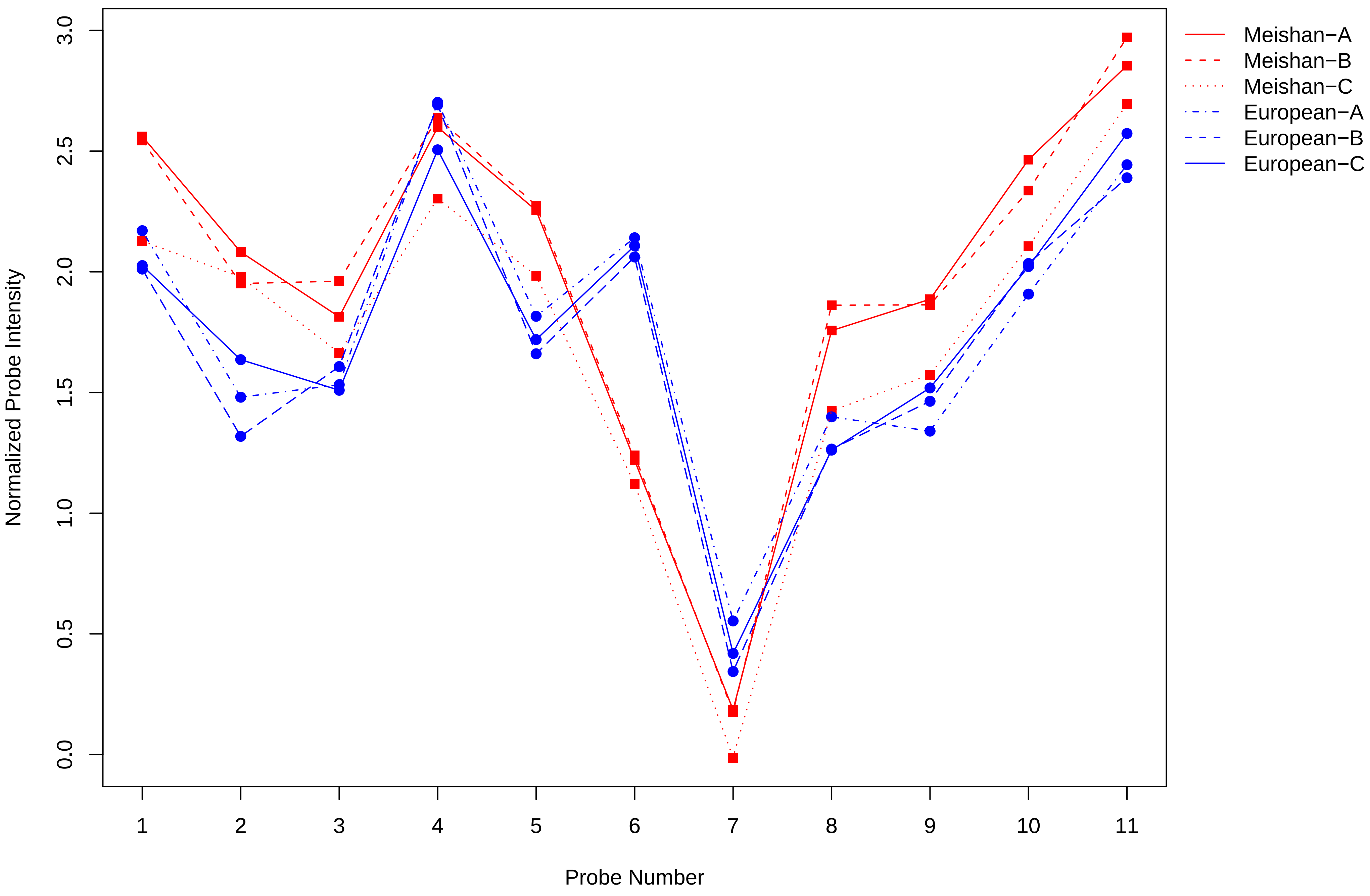

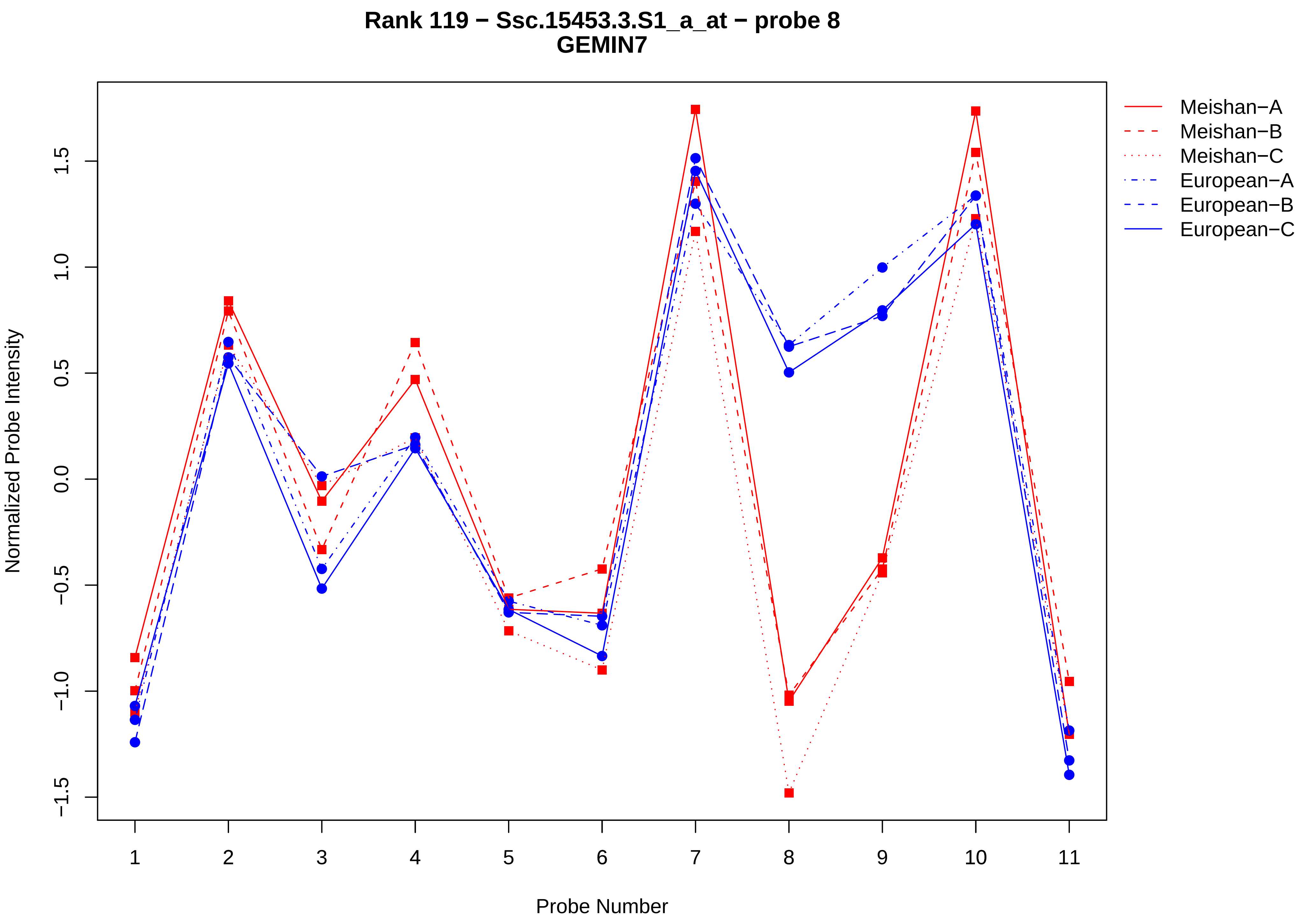

Rank 119 − Ssc.15453.3.S1_a_at − probe 8
GEMIN7
Normalized Probe Intensity
Probe Number
Meishan−A
Meishan−B
Meishan−C
European−A
European−B
European−C
−1.5
−1.0
−0.5
0.0
0.5
1.0
1.5
1
2
3
4
5
6
7
8
9
10
11

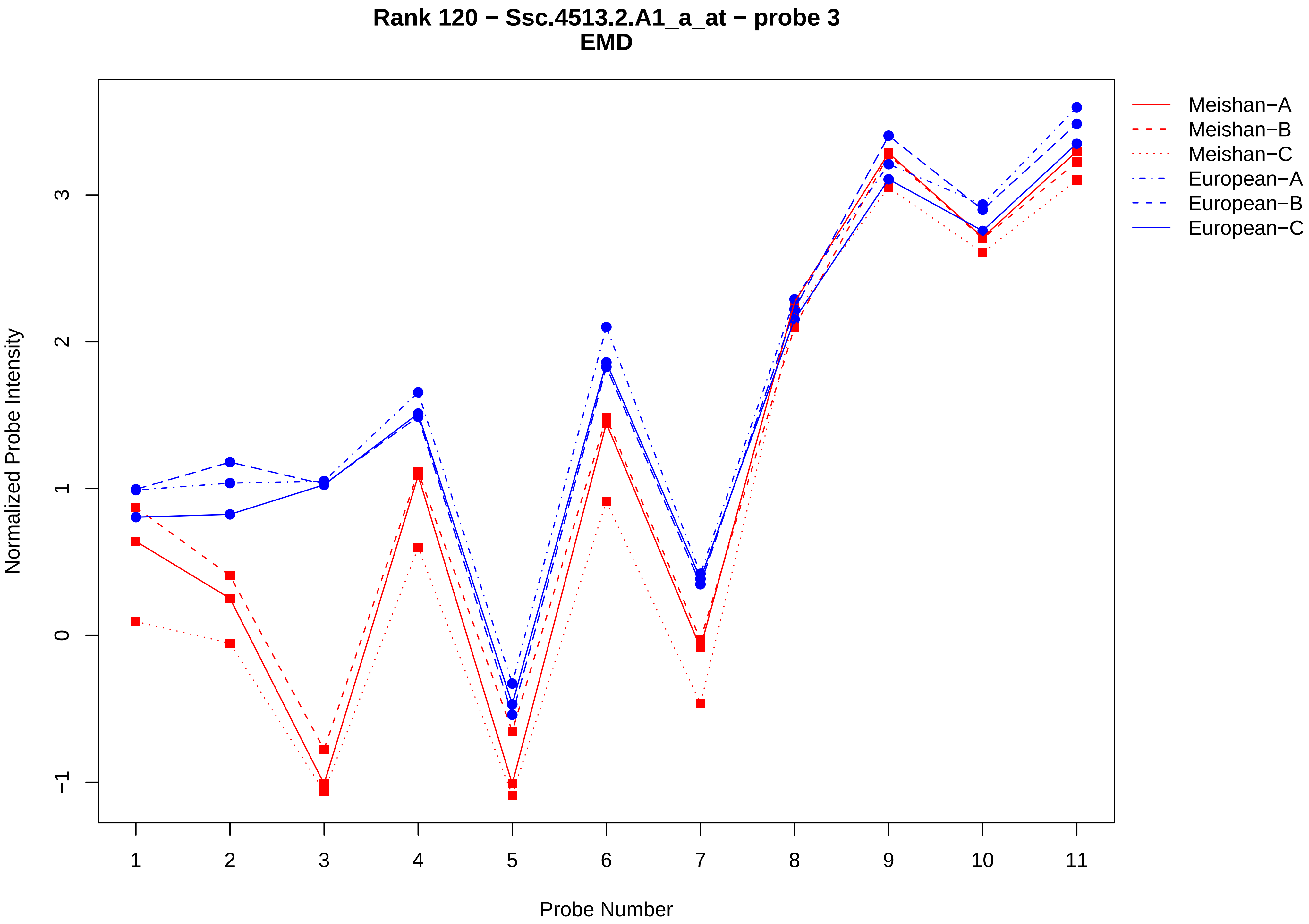
Rank 120 − Ssc.4513.2.A1_a_at − probe 3
EMD
Normalized Probe Intensity
Probe Number
−1
0
1
2
3
1
2
3
4
5
6
7
8
9
10
11
Meishan−A
Meishan−B
Meishan−C
European−A
European−B
European−C

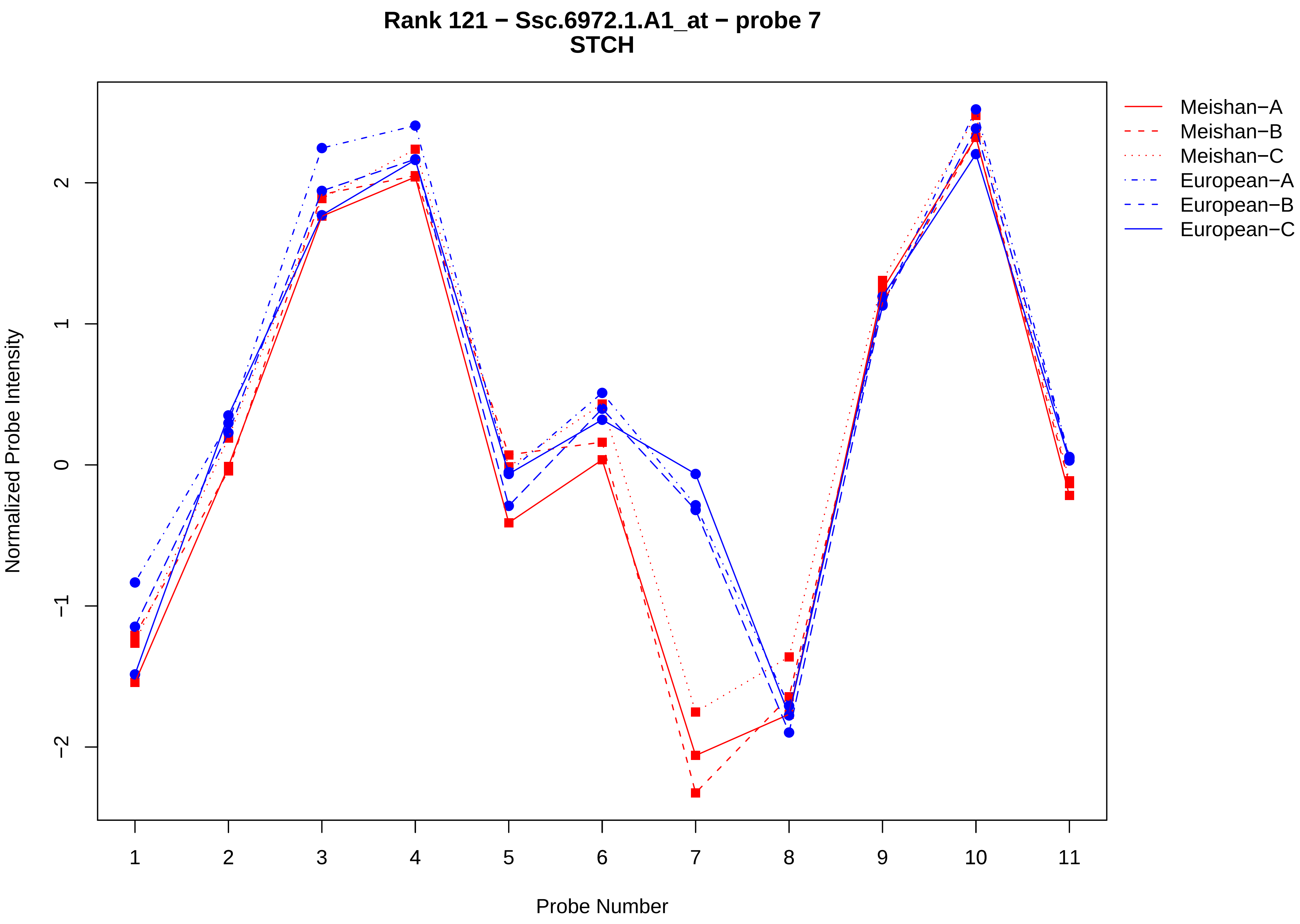

Rank 121 − Ssc.6972.1.A1_at − probe 7
STCH
Normalized Probe Intensity
Probe Number
Meishan−A
Meishan−B
Meishan−C
European−A
European−B
European−C

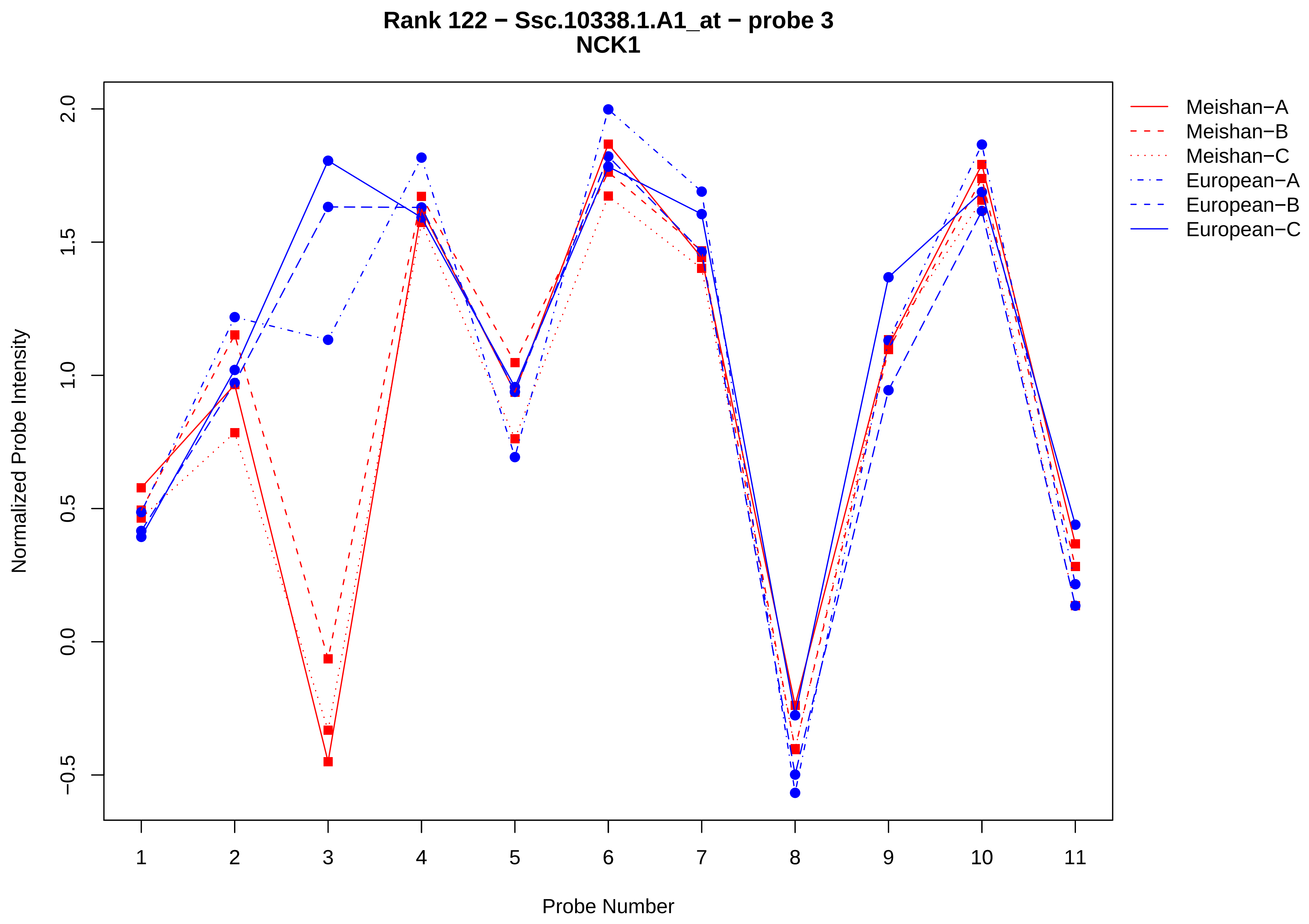

Rank 122 − Ssc.10338.1.A1_at − probe 3
NCK1
Normalized Probe Intensity
Probe Number
2.0
1.5
1.0
0.5
0.0
−0.5
1
2
3
4
5
6
7
8
9
10
11
Meishan−A
Meishan−B
Meishan−C
European−A
European−B
European−C

Rank 123 − Ssc.3581.1.S1_at − probe 2
DNPEP

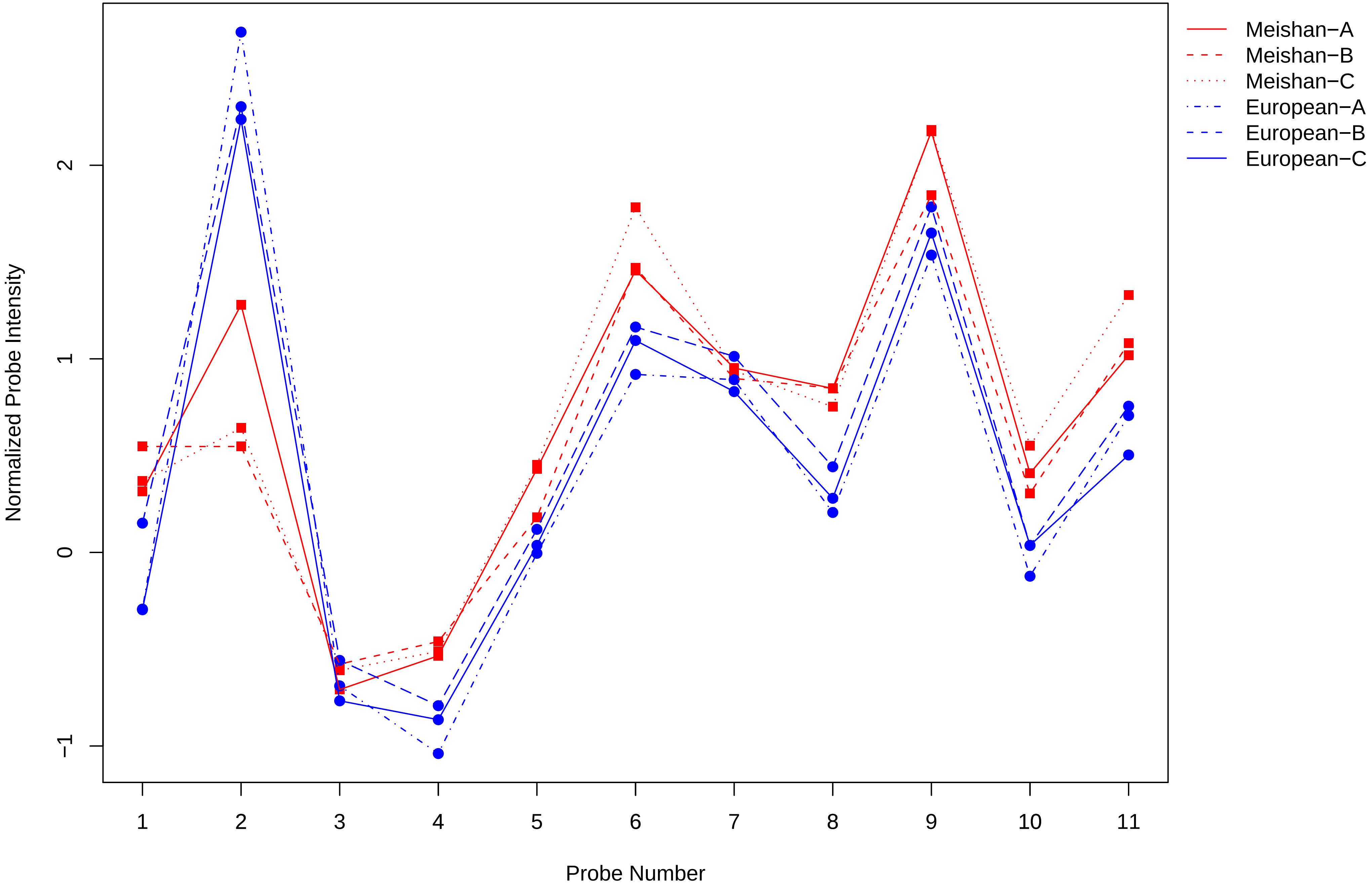

Rank 124 − Ssc.4324.2.A1_at − probe 4
CAMLG

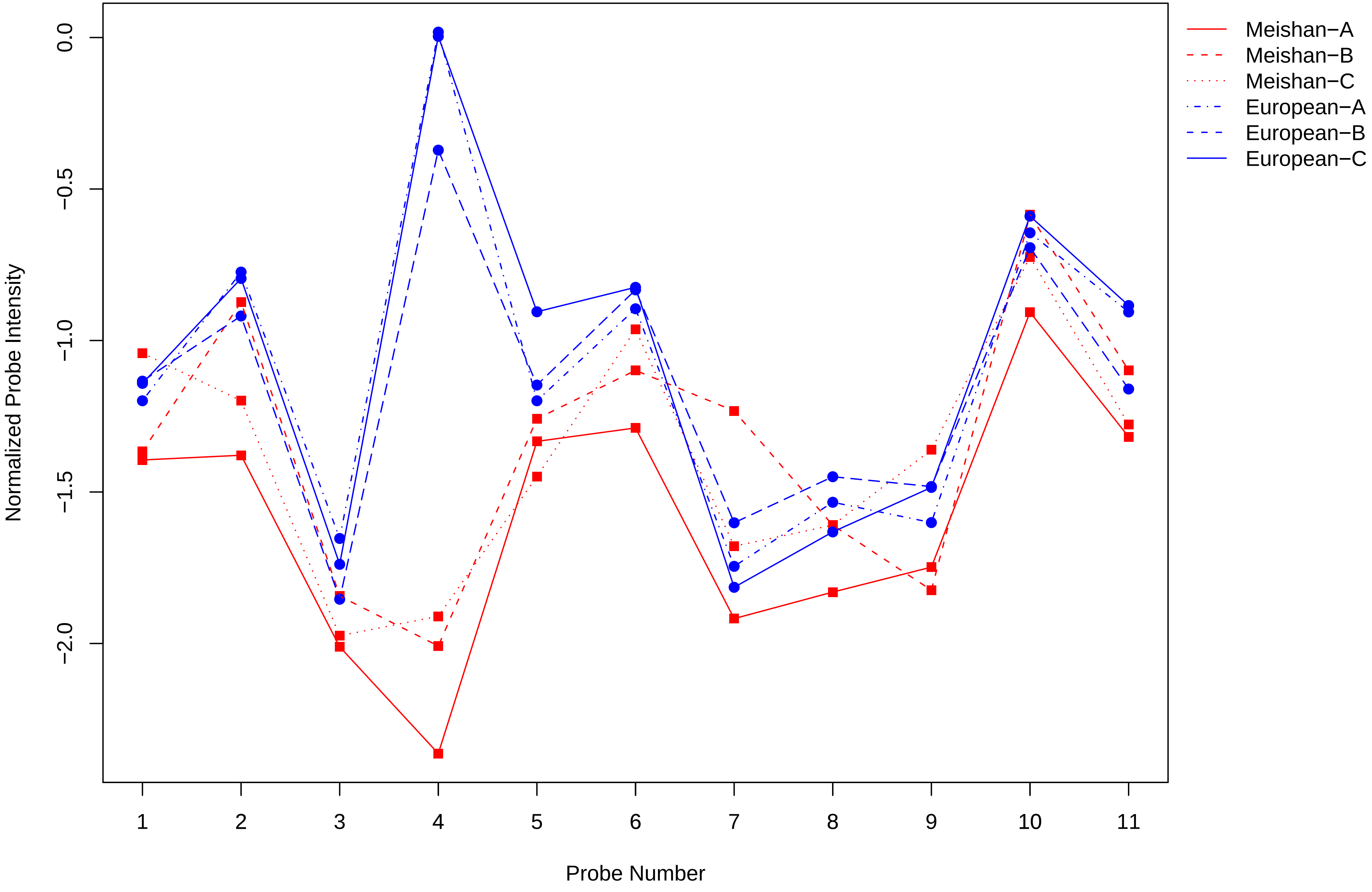

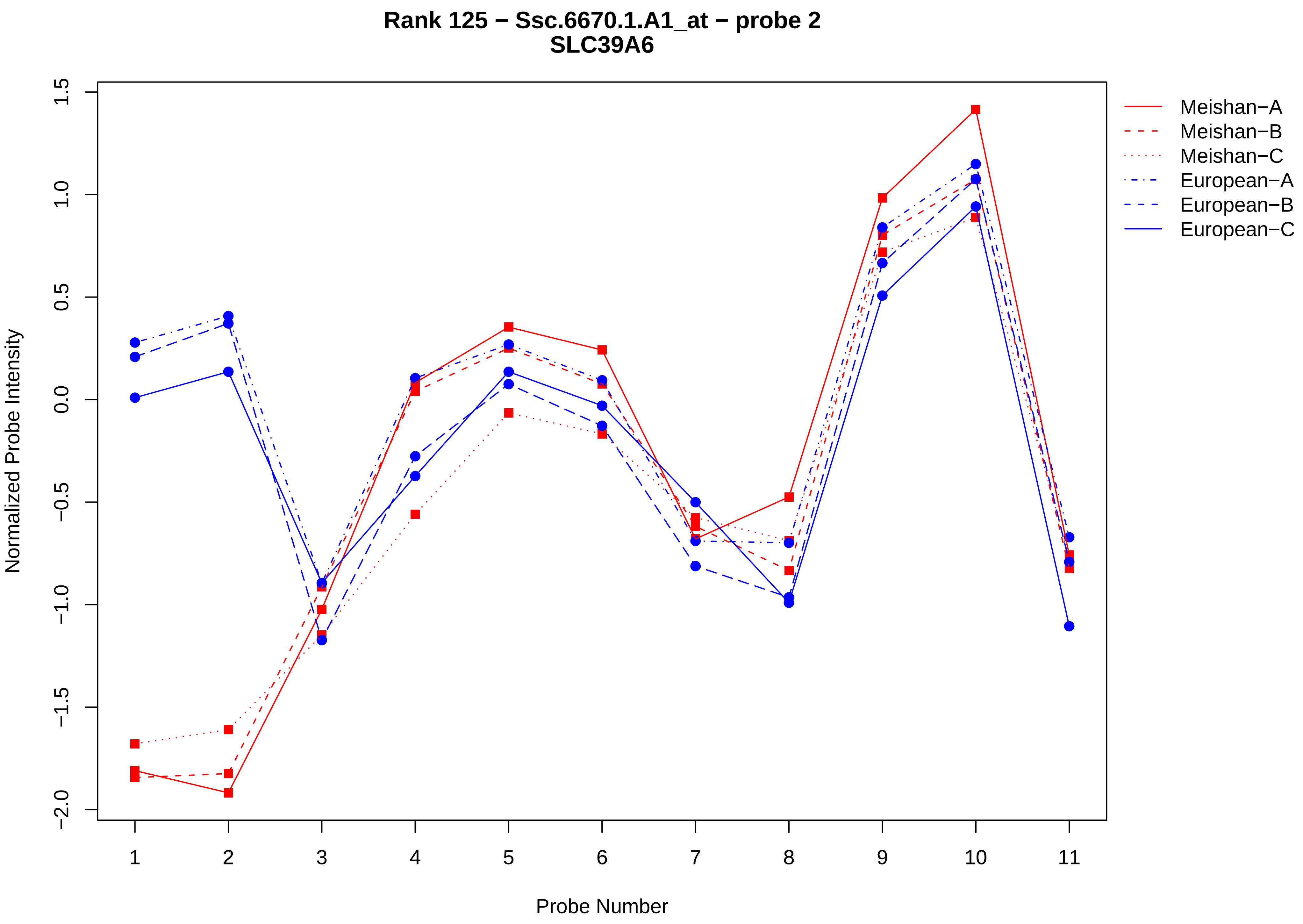

Rank 125 − Ssc.6670.1.A1_at − probe 2
SLC39A6
Normalized Probe Intensity
Probe Number
1.5
1.0
0.5
0.0
−0.5
−1.0
−1.5
−2.0
1
2
3
4
5
6
7
8
9
10
11
Meishan−A
Meishan−B
Meishan−C
European−A
European−B
European−C

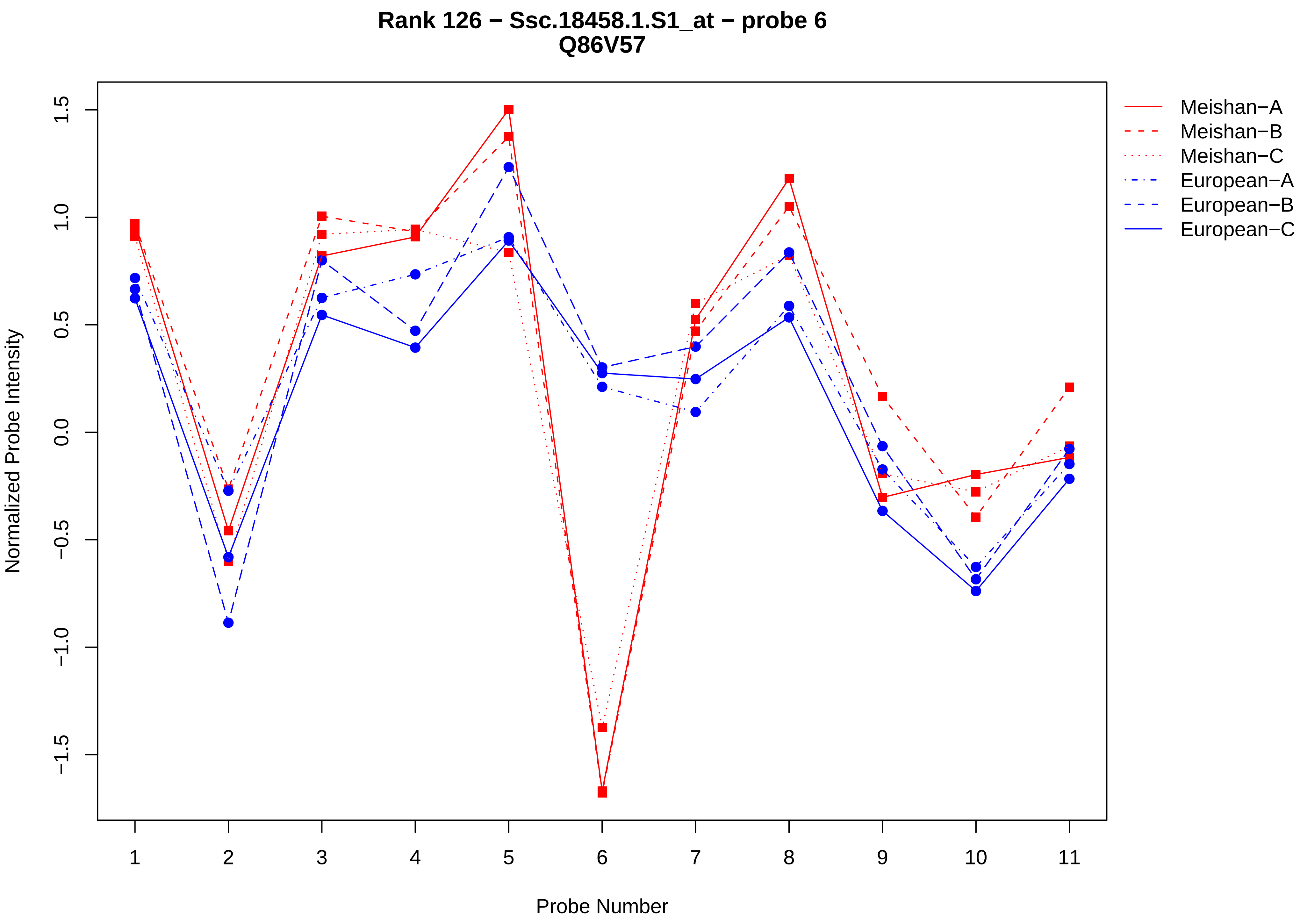

Rank 126 − Ssc.18458.1.S1_at − probe 6
Q86V57
Normalized Probe Intensity
Probe Number
1.5
1.0
0.5
0.0
−0.5
−1.0
−1.5
1
2
3
4
5
6
7
8
9
10
11
Meishan−A
Meishan−B
Meishan−C
European−A
European−B
European−C

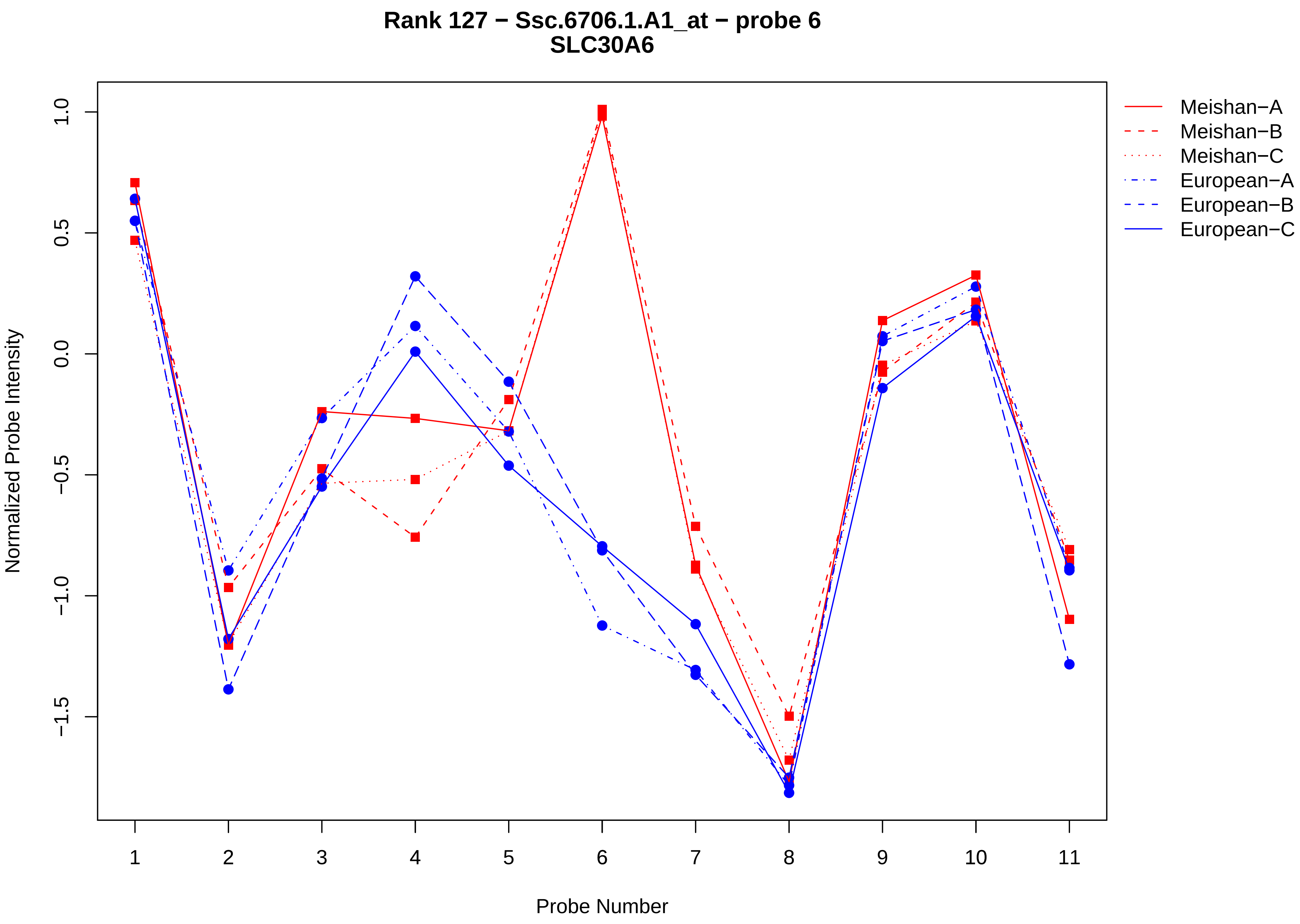

Rank 127 − Ssc.6706.1.A1_at − probe 6
SLC30A6
Normalized Probe Intensity
Probe Number
1.0
0.5
0.0
−0.5
−1.0
−1.5
1
2
3
4
5
6
7
8
9
10
11
Meishan−A
Meishan−B
Meishan−C
European−A
European−B
European−C

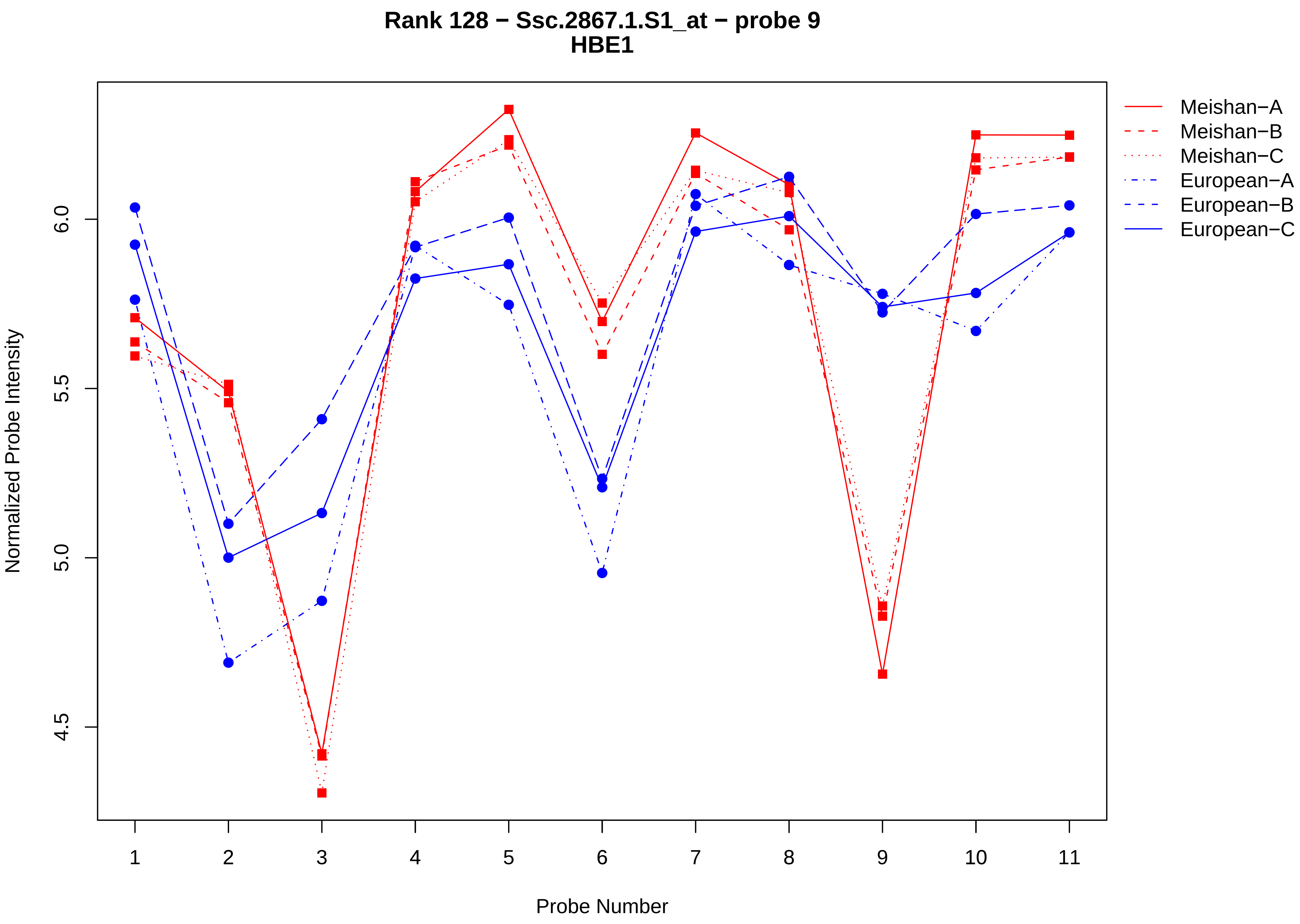

Rank 128 − Ssc.2867.1.S1_at − probe 9
HBE1
Normalized Probe Intensity
Probe Number
4.5
5.0
5.5
6.0
1
2
3
4
5
6
7
8
9
10
11
Meishan−A
Meishan−B
Meishan−C
European−A
European−B
European−C

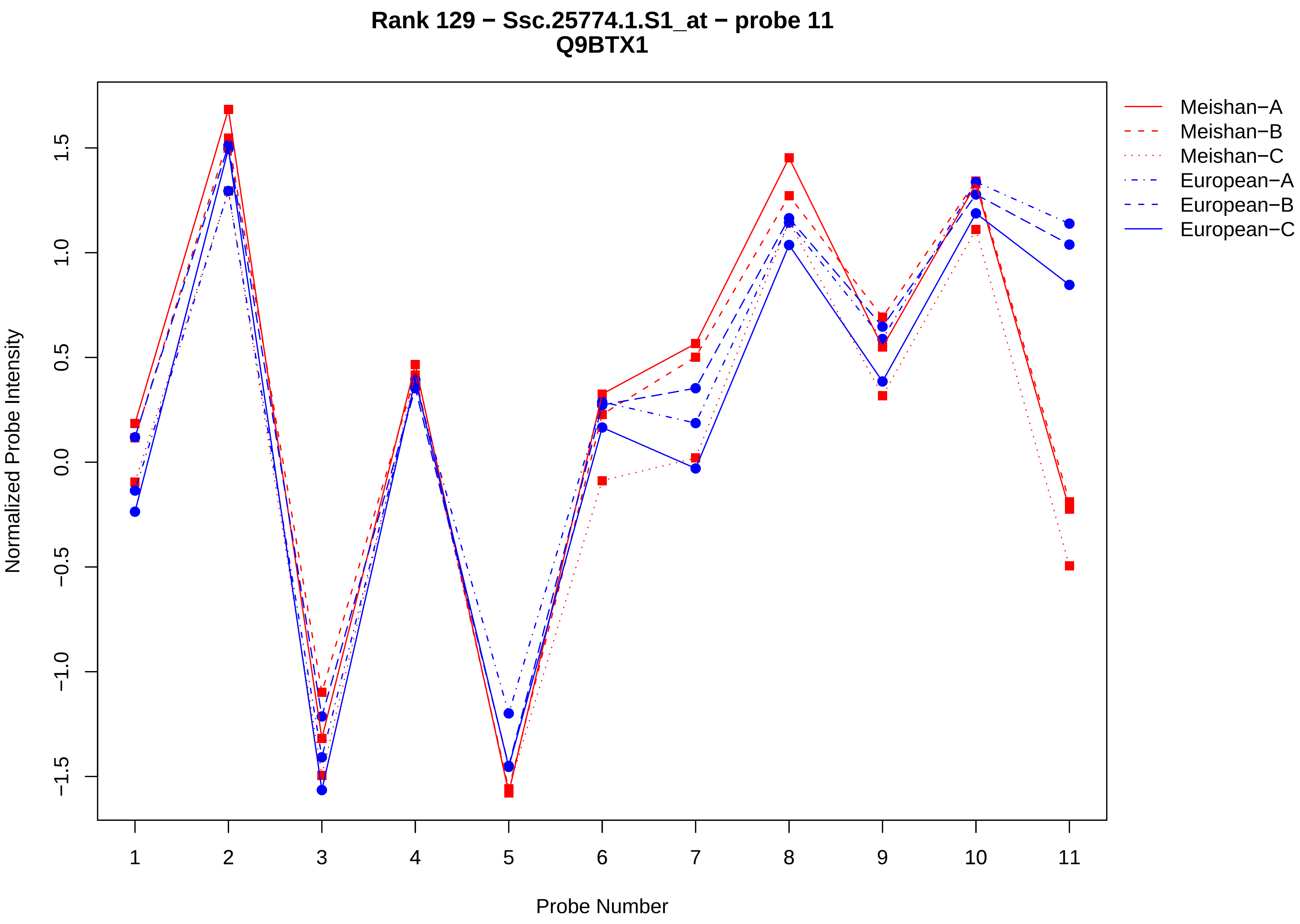

Rank 129 − Ssc.25774.1.S1_at − probe 11
Q9BTX1
Normalized Probe Intensity
Probe Number
1.5
1.0
0.5
0.0
−0.5
−1.0
−1.5
1
2
3
4
5
6
7
8
9
10
11
Meishan−A
Meishan−B
Meishan−C
European−A
European−B
European−C

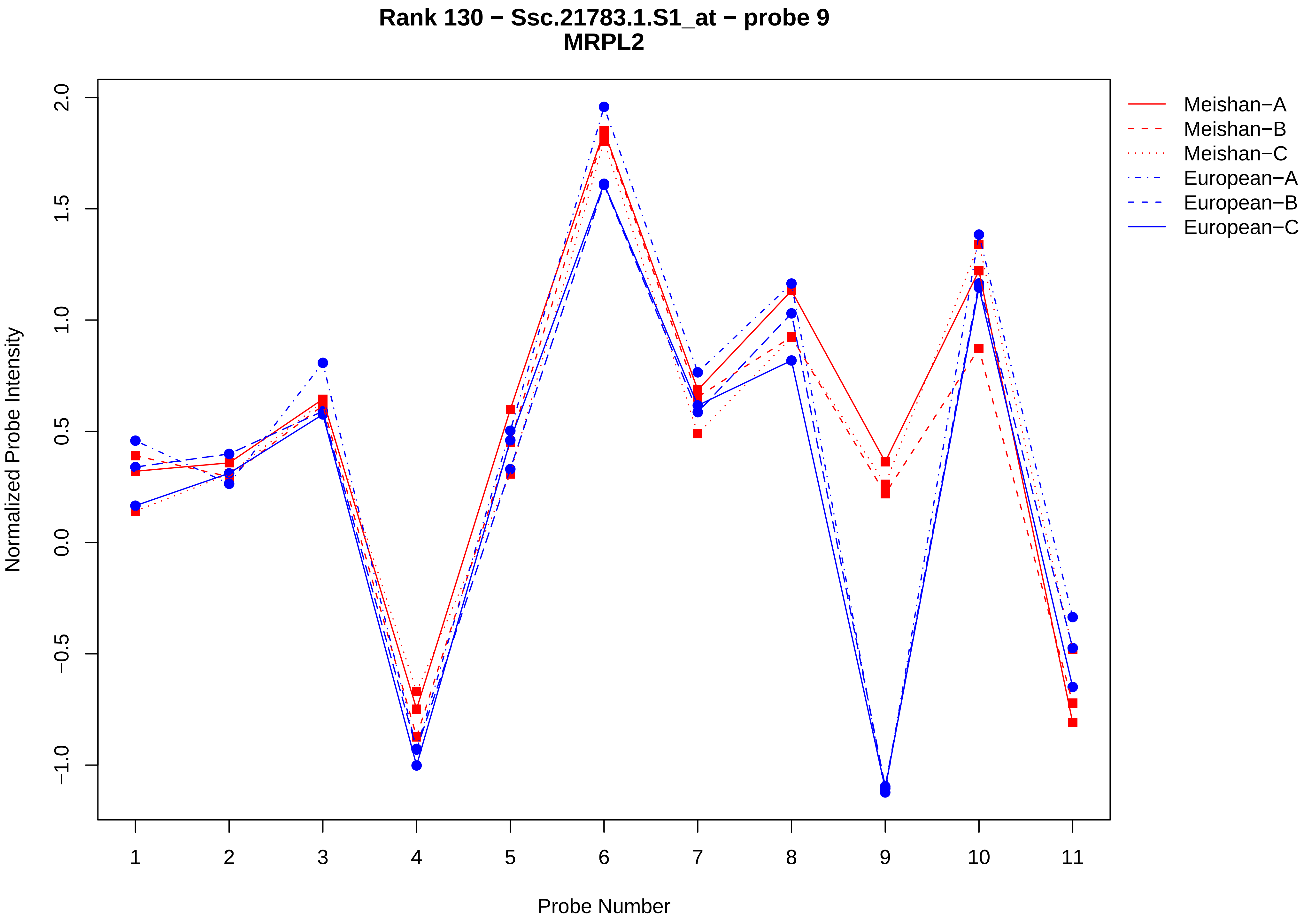

Rank 130 − Ssc.21783.1.S1_at − probe 9
MRPL2
Normalized Probe Intensity
Probe Number
2.0
1.5
1.0
0.5
0.0
−0.5
−1.0
1
2
3
4
5
6
7
8
9
10
11
Meishan−A
Meishan−B
Meishan−C
European−A
European−B
European−C

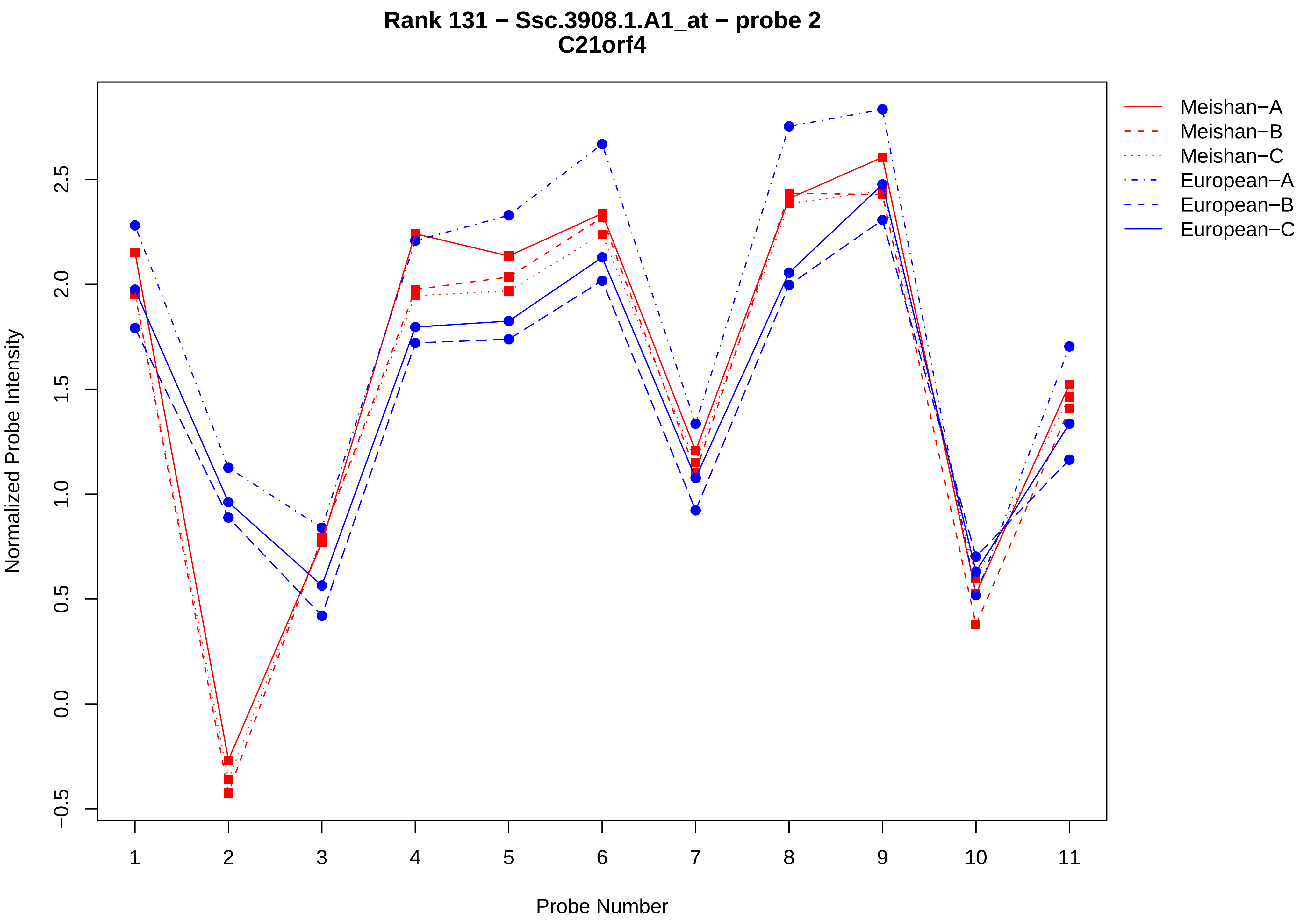

Rank 131 − Ssc.3908.1.A1_at − probe 2
C21orf4
Normalized Probe Intensity
−0.5
0.0
0.5
1.0
1.5
2.0
2.5
1
2
3
4
5
6
7
8
9
10
11
Probe Number
Meishan−A
Meishan−B
Meishan−C
European−A
European−B
European−C

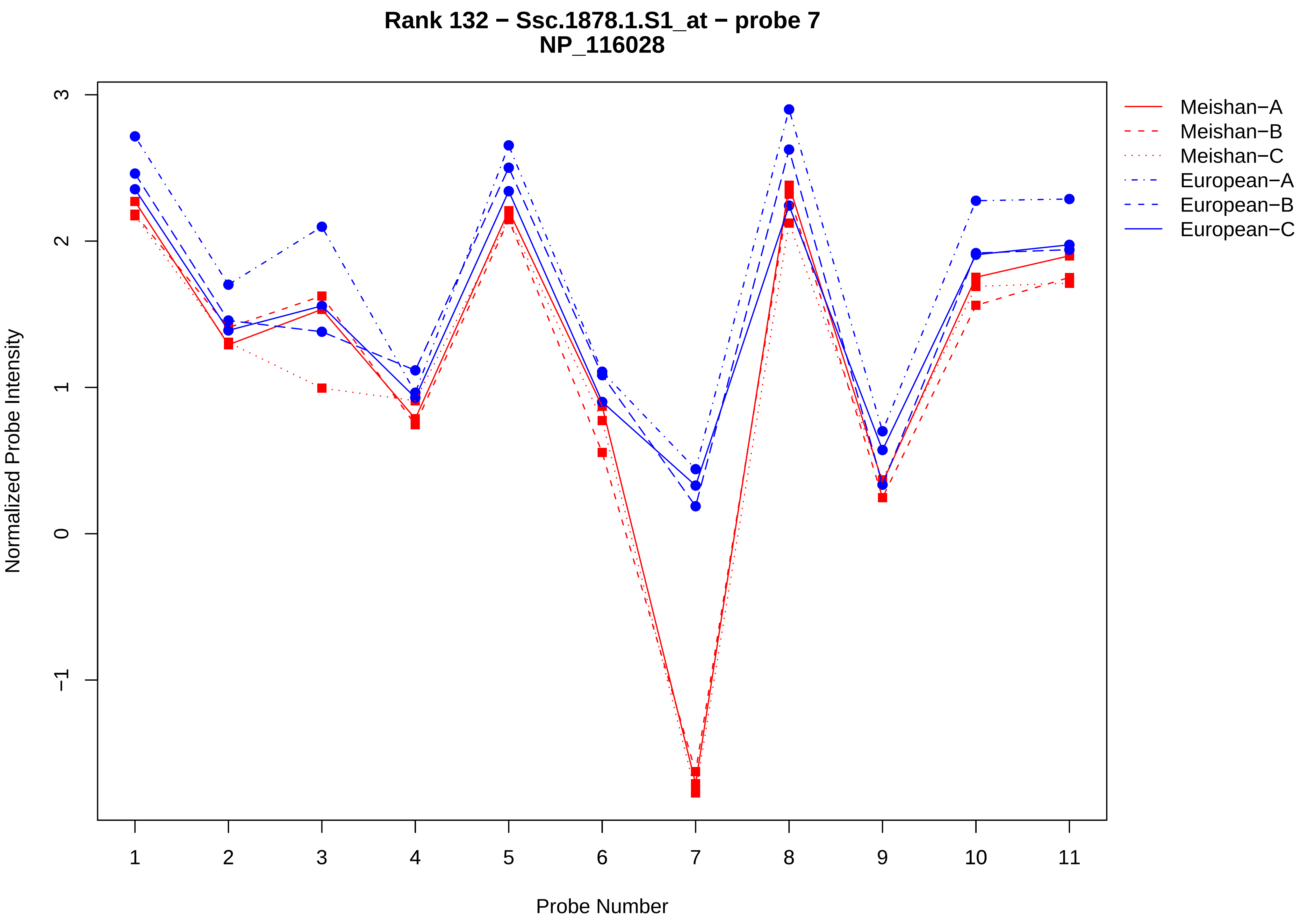

Rank 132 − Ssc.1878.1.S1_at − probe 7
NP_116028
Normalized Probe Intensity
Probe Number
3
2
1
0
−1
1
2
3
4
5
6
7
8
9
10
11
Meishan−A
Meishan−B
Meishan−C
European−A
European−B
European−C

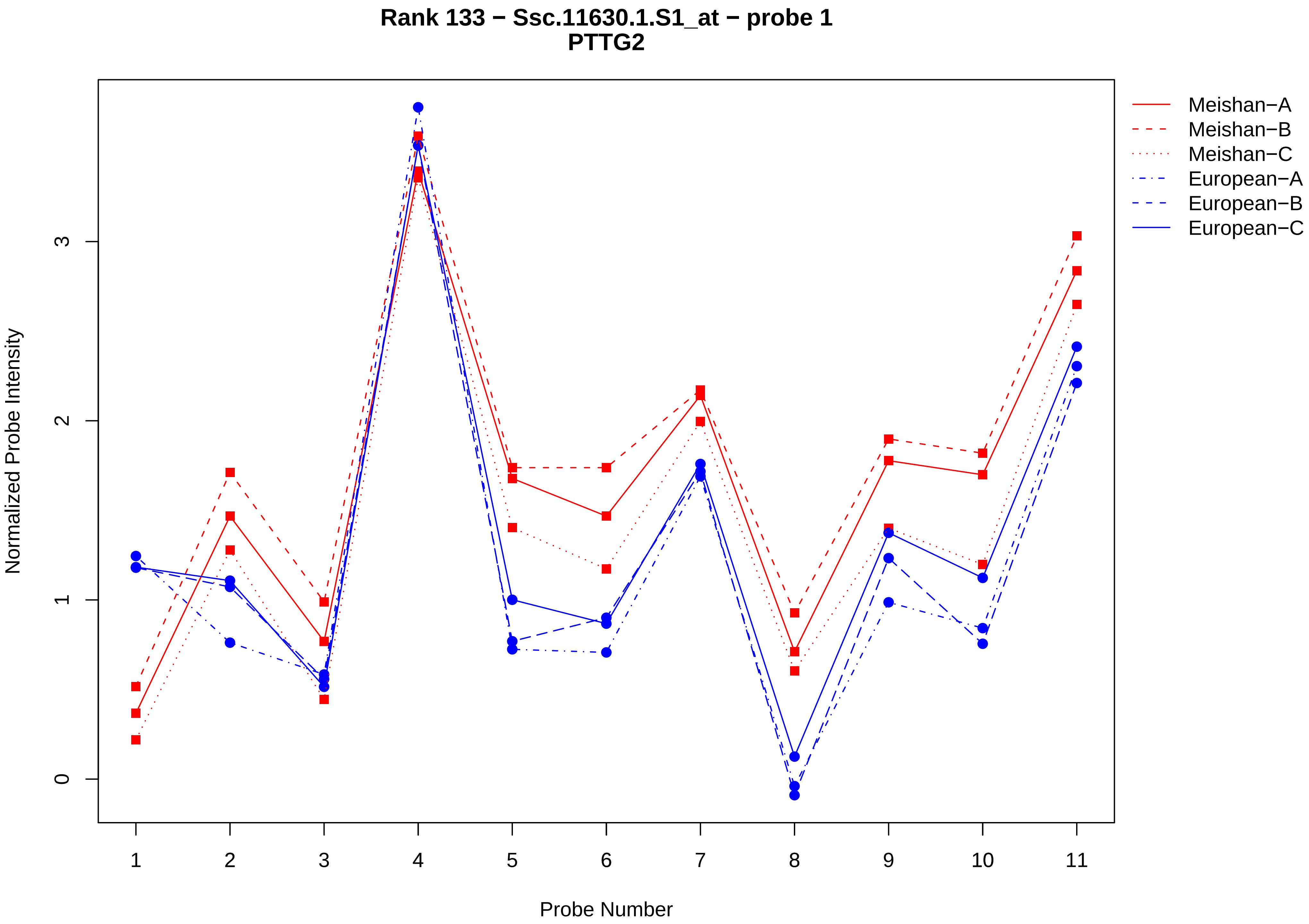

Rank 133 − Ssc.11630.1.S1_at − probe 1
PTTG2
Normalized Probe Intensity
Probe Number
0
1
2
3
1
2
3
4
5
6
7
8
9
10
11
Meishan−A
Meishan−B
Meishan−C
European−A
European−B
European−C

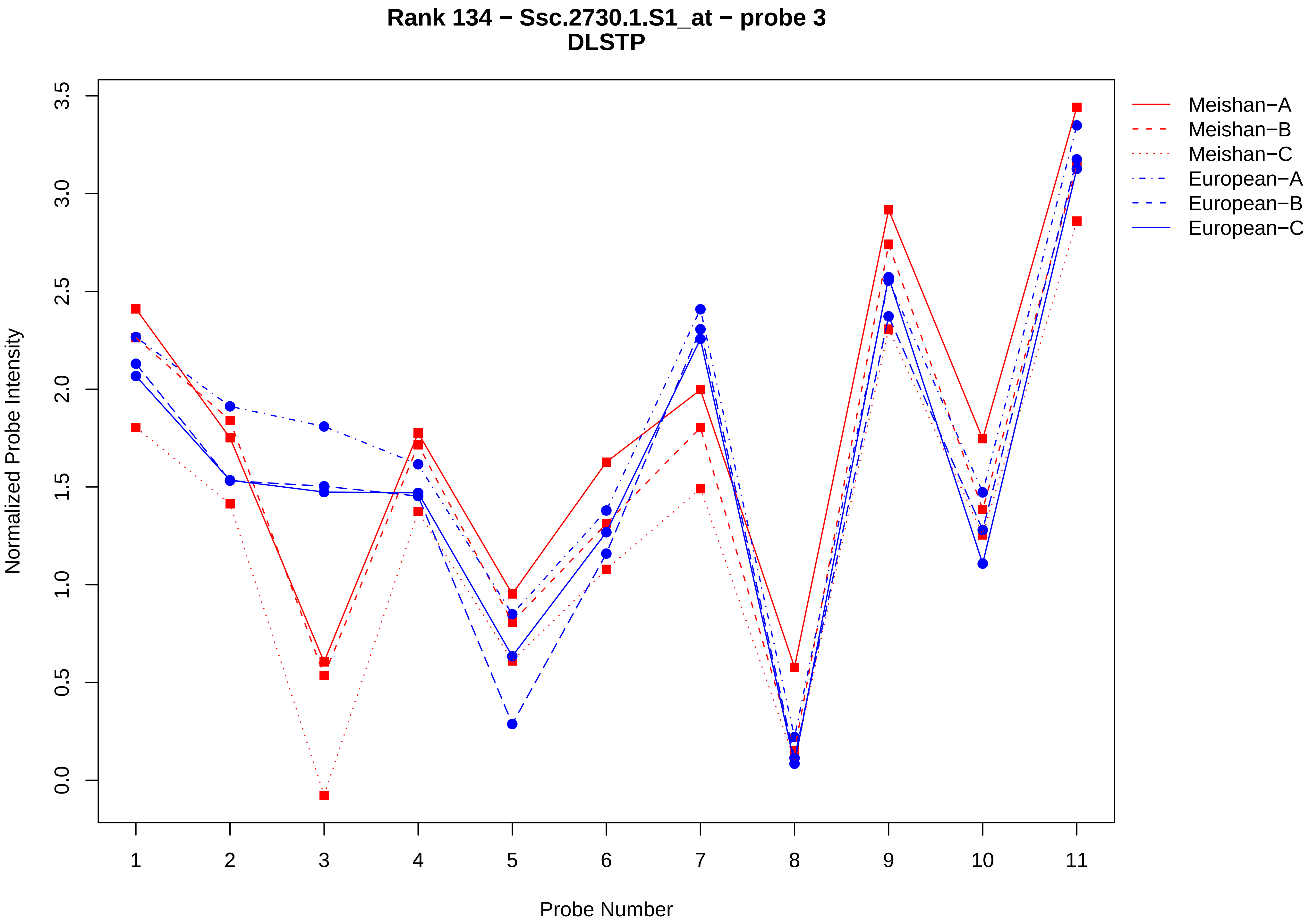

Rank 134 − Ssc.2730.1.S1_at − probe 3
DLSTP
Normalized Probe Intensity
Probe Number
Meishan−A
Meishan−B
Meishan−C
European−A
European−B
European−C
0.0
0.5
1.0
1.5
2.0
2.5
3.0
3.5
1
2
3
4
5
6
7
8
9
10
11

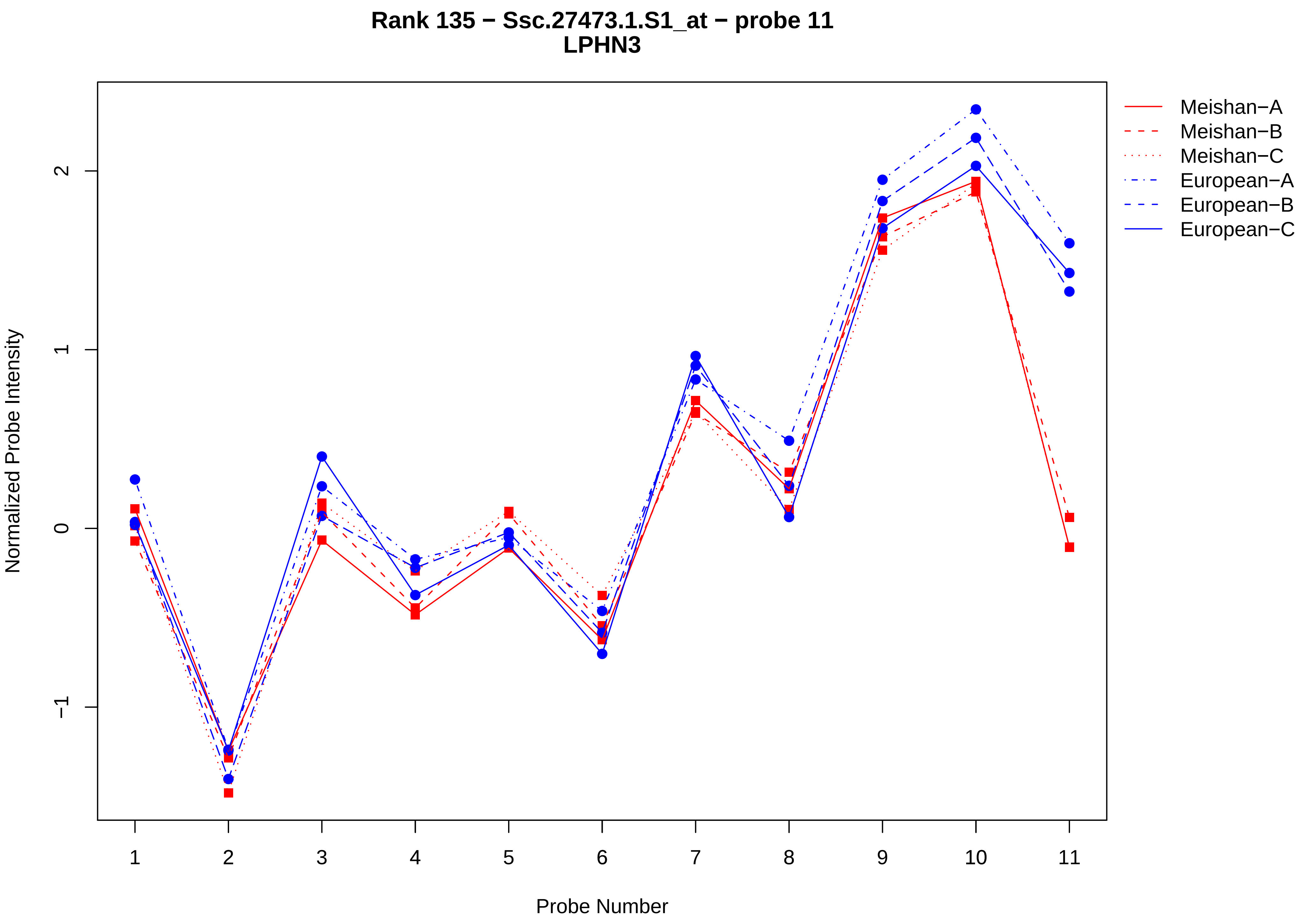

Rank 135 − Ssc.27473.1.S1_at − probe 11
LPHN3
Normalized Probe Intensity
Probe Number
Meishan−A
Meishan−B
Meishan−C
European−A
European−B
European−C
−1
0
1
2
1
2
3
4
5
6
7
8
9
10
11

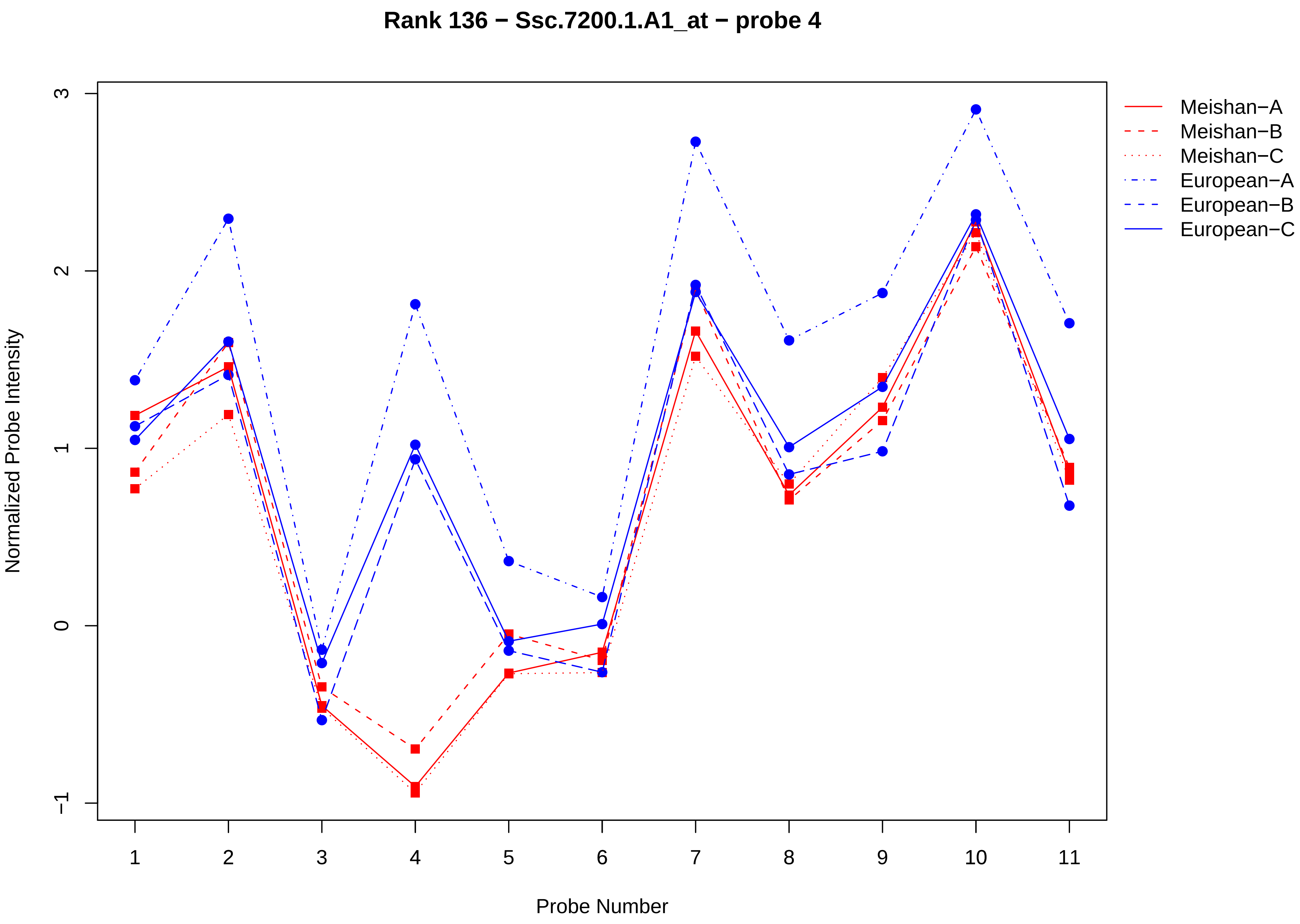

Rank 136 − Ssc.7200.1.A1_at − probe 4
Normalized Probe Intensity
Probe Number
Meishan−A
Meishan−B
Meishan−C
European−A
European−B
European−C
−1
0
1
2
3
1
2
3
4
5
6
7
8
9
10
11

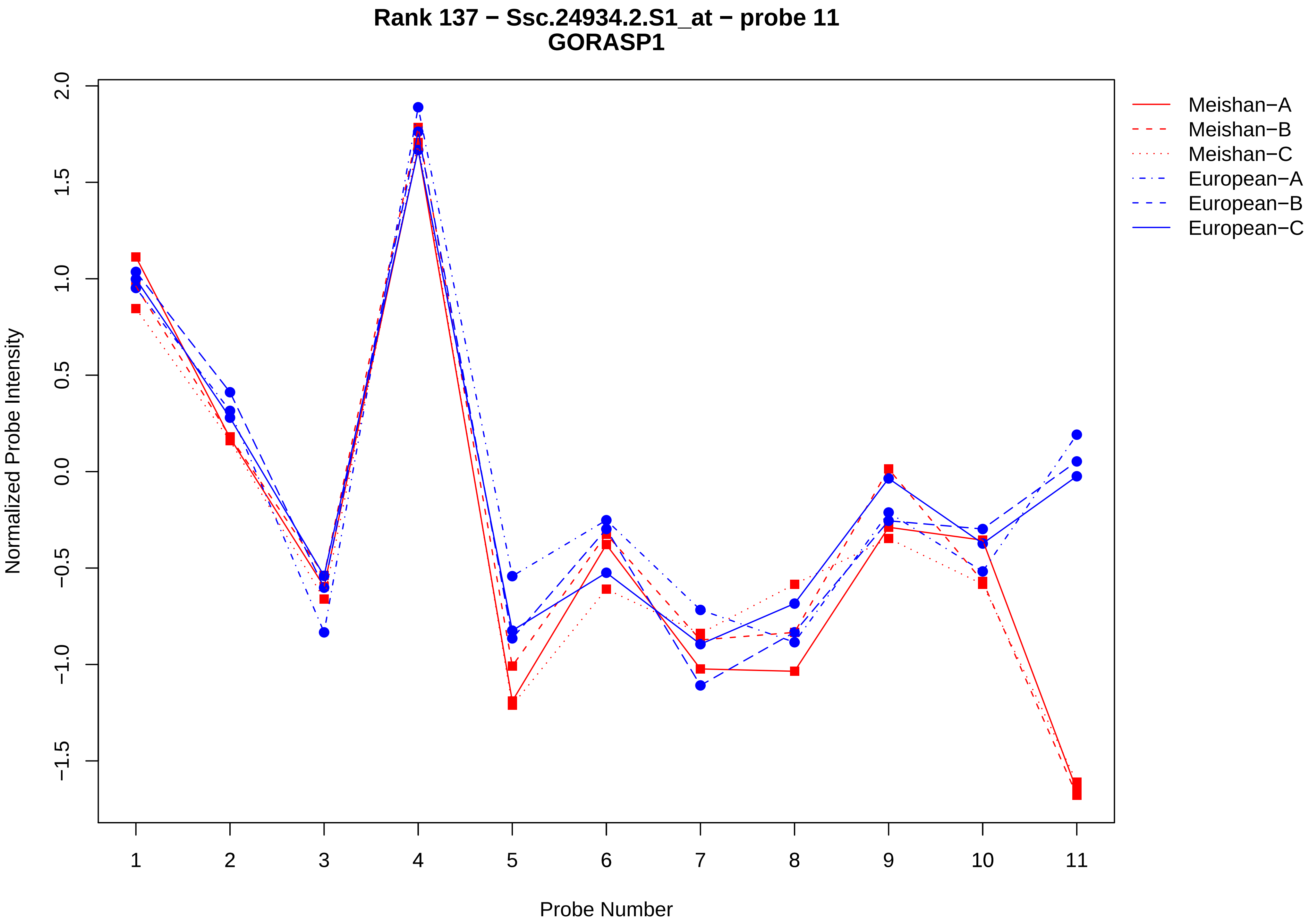

Rank 137 − Ssc.24934.2.S1_at − probe 11
GORASP1
Normalized Probe Intensity
Probe Number
Meishan−A
Meishan−B
Meishan−C
European−A
European−B
European−C

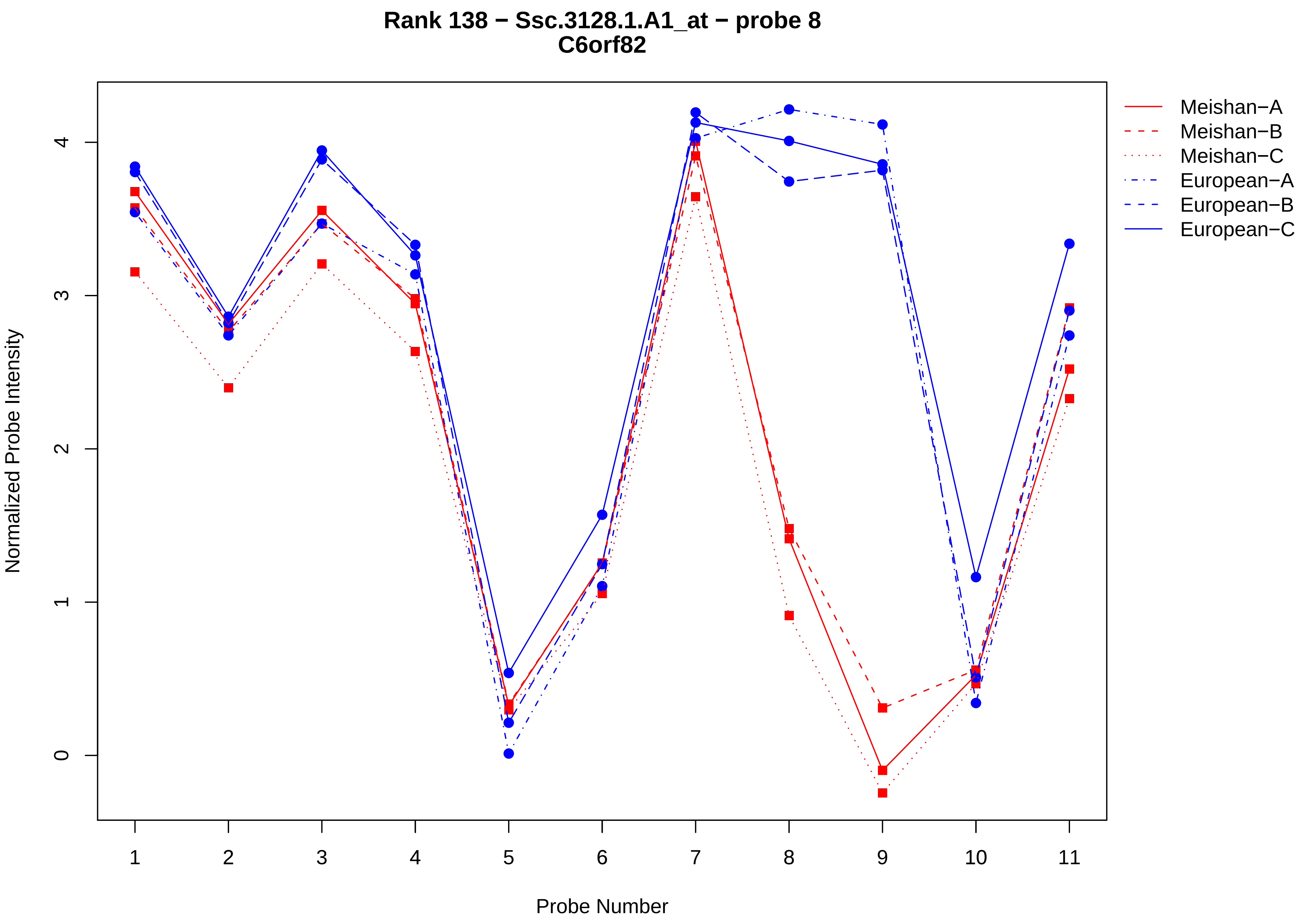

Rank 138 − Ssc.3128.1.A1_at − probe 8
C6orf82
Normalized Probe Intensity
Probe Number
0
1
2
3
4
1
2
3
4
5
6
7
8
9
10
11
Meishan−A
Meishan−B
Meishan−C
European−A
European−B
European−C

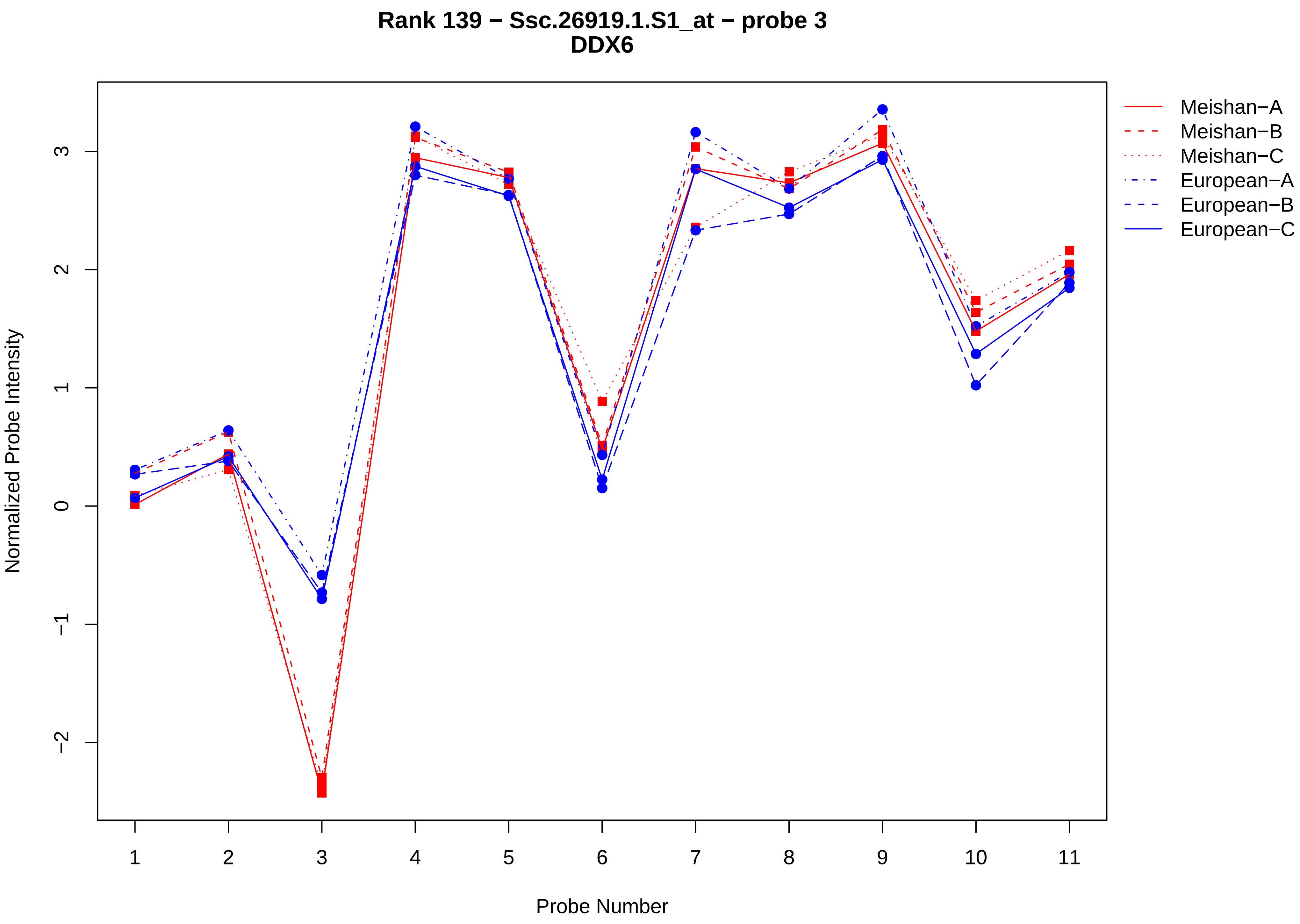
Rank 139 − Ssc.26919.1.S1_at − probe 3
DDX6
Normalized Probe Intensity
Probe Number
−2
−1
0
1
2
3
1
2
3
4
5
6
7
8
9
10
11
Meishan−A
Meishan−B
Meishan−C
European−A
European−B
European−C

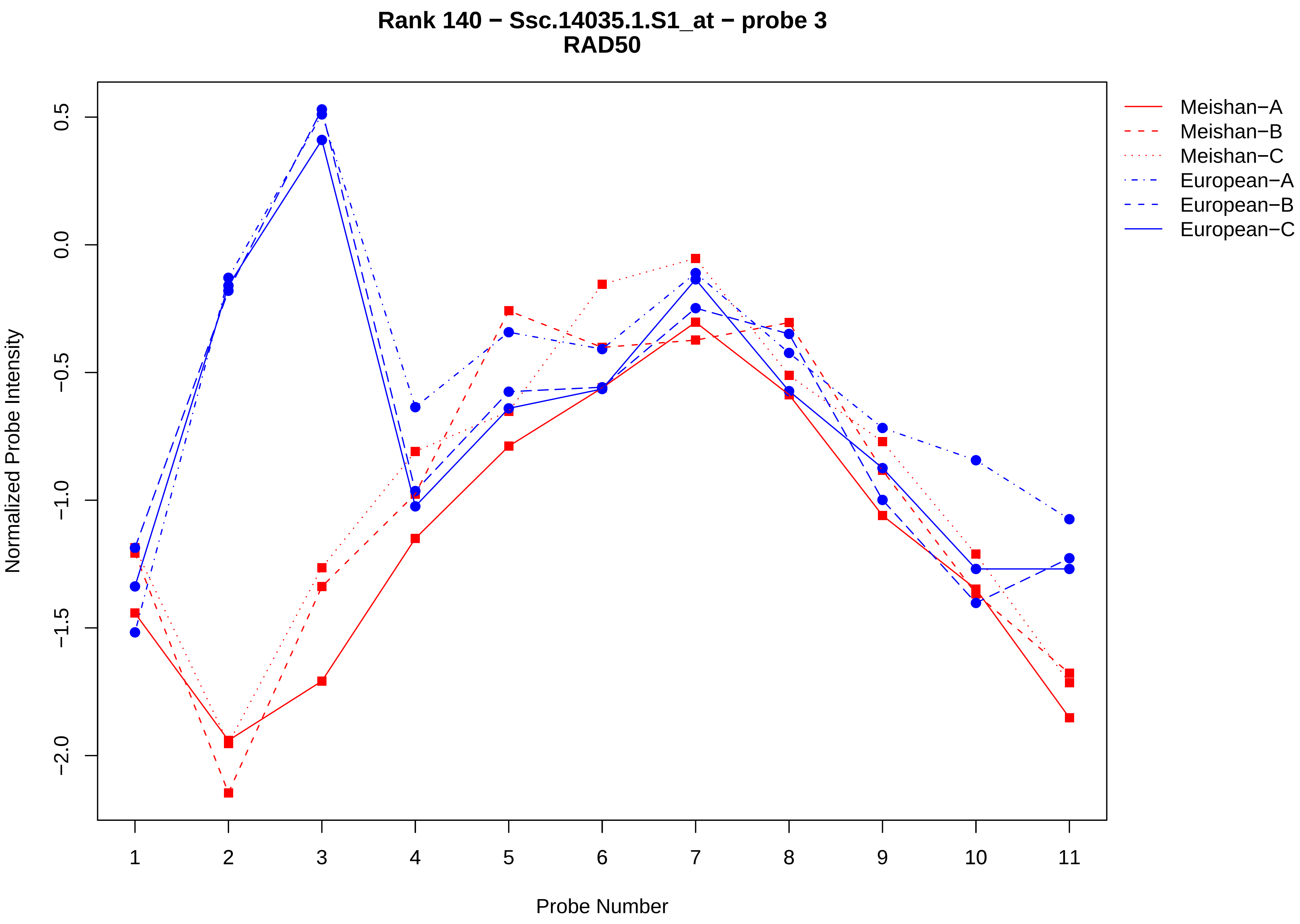

Rank 140 − Ssc.14035.1.S1_at − probe 3
RAD50
Normalized Probe Intensity
0.5
0.0
−0.5
−1.0
−1.5
−2.0
1
2
3
4
5
6
7
8
9
10
11
Probe Number
Meishan−A
Meishan−B
Meishan−C
European−A
European−B
European−C

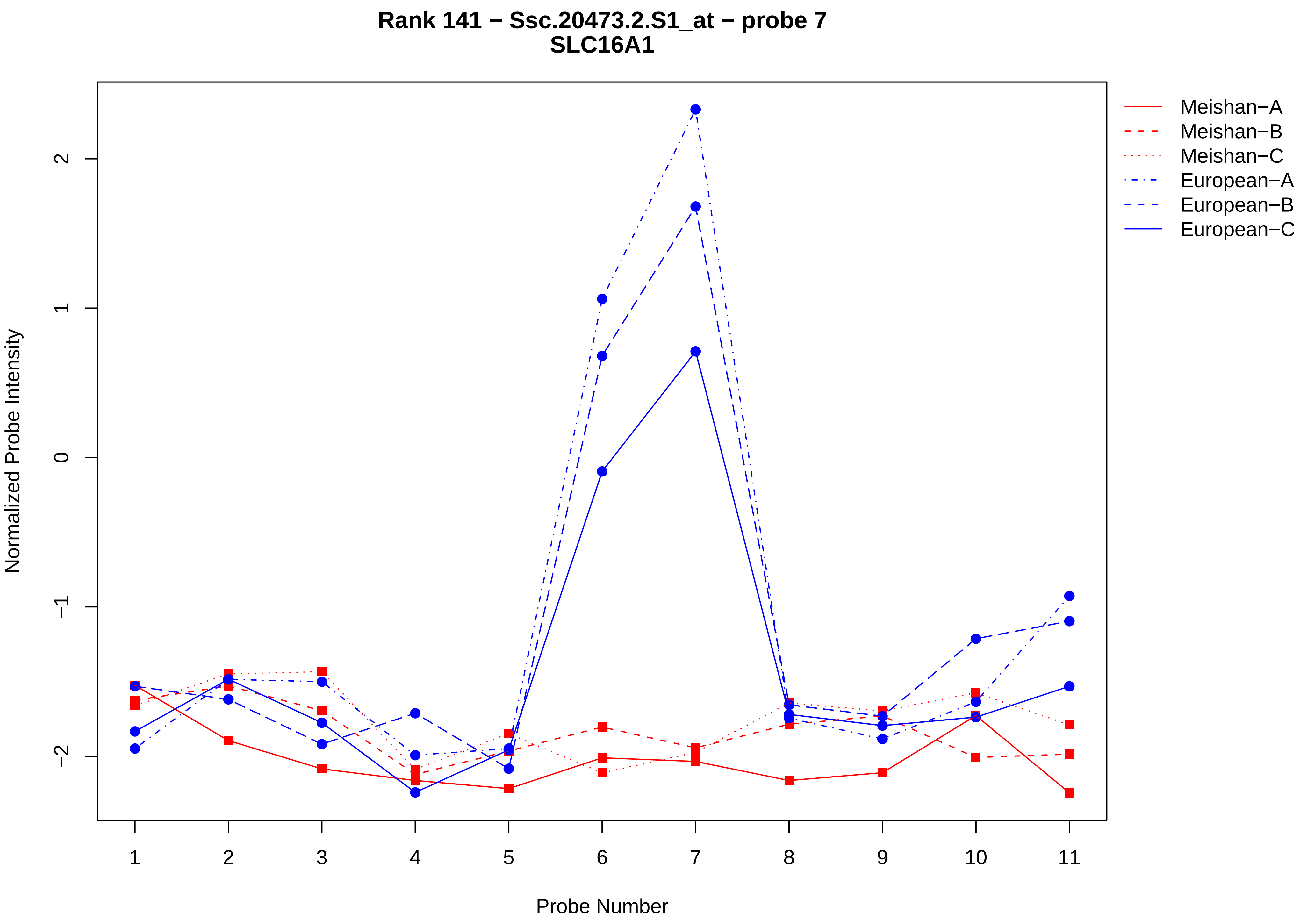

Rank 141 − Ssc.20473.2.S1_at − probe 7
SLC16A1
Normalized Probe Intensity
Probe Number
−2
−1
0
1
2
1
2
3
4
5
6
7
8
9
10
11
Meishan−A
Meishan−B
Meishan−C
European−A
European−B
European−C

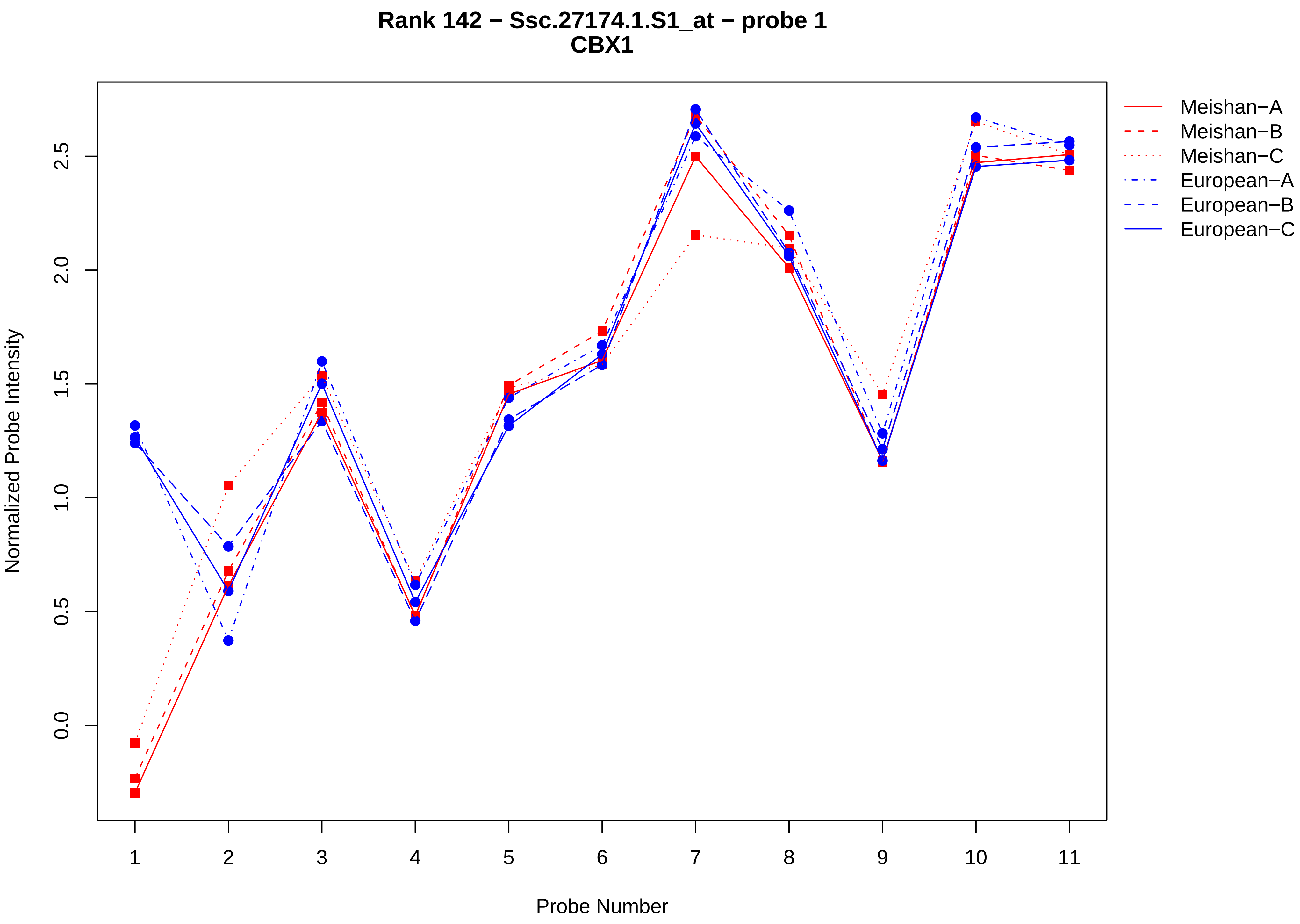

Rank 142 − Ssc.27174.1.S1_at − probe 1
CBX1
Normalized Probe Intensity
Probe Number
0.0
0.5
1.0
1.5
2.0
2.5
1
2
3
4
5
6
7
8
9
10
11
Meishan−A
Meishan−B
Meishan−C
European−A
European−B
European−C

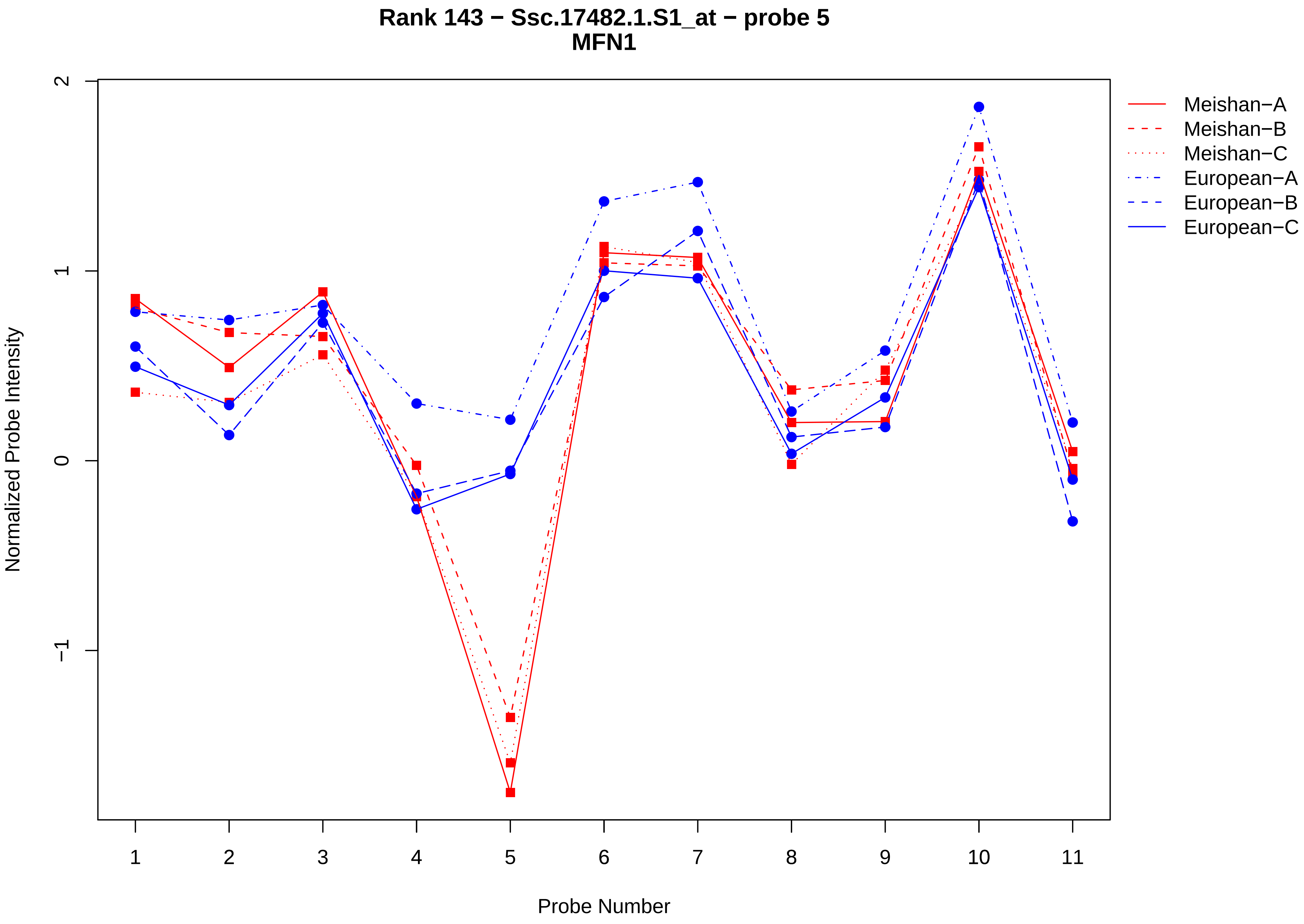

Rank 143 − Ssc.17482.1.S1_at − probe 5
MFN1
Normalized Probe Intensity
2
1
0
−1
1
2
3
4
5
6
7
8
9
10
11
Probe Number
Meishan−A
Meishan−B
Meishan−C
European−A
European−B
European−C

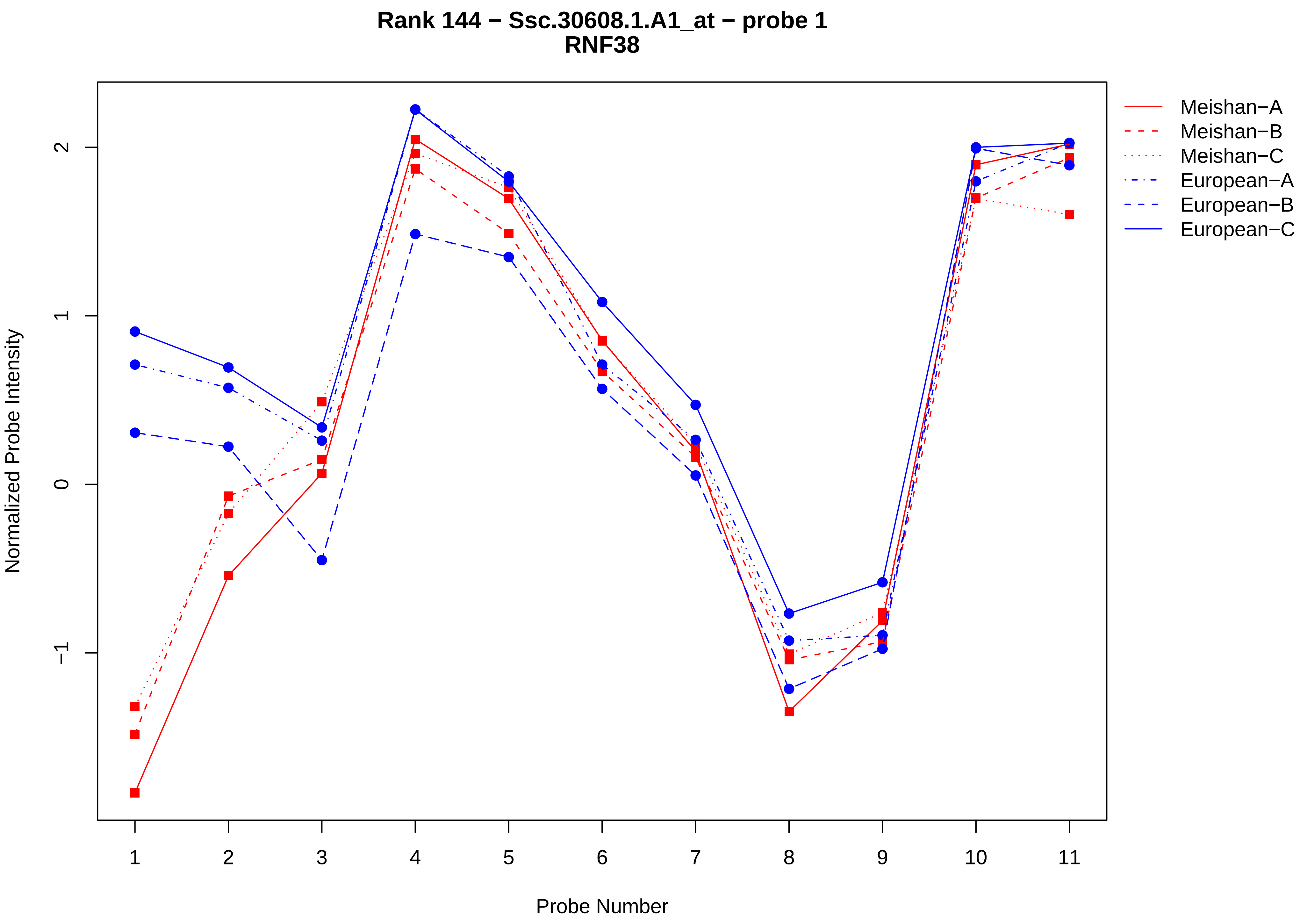
Rank 144 − Ssc.30608.1.A1_at − probe 1
RNF38
Normalized Probe Intensity
Probe Number
Meishan−A
Meishan−B
Meishan−C
European−A
European−B
European−C
−1
0
1
2
1
2
3
4
5
6
7
8
9
10
11

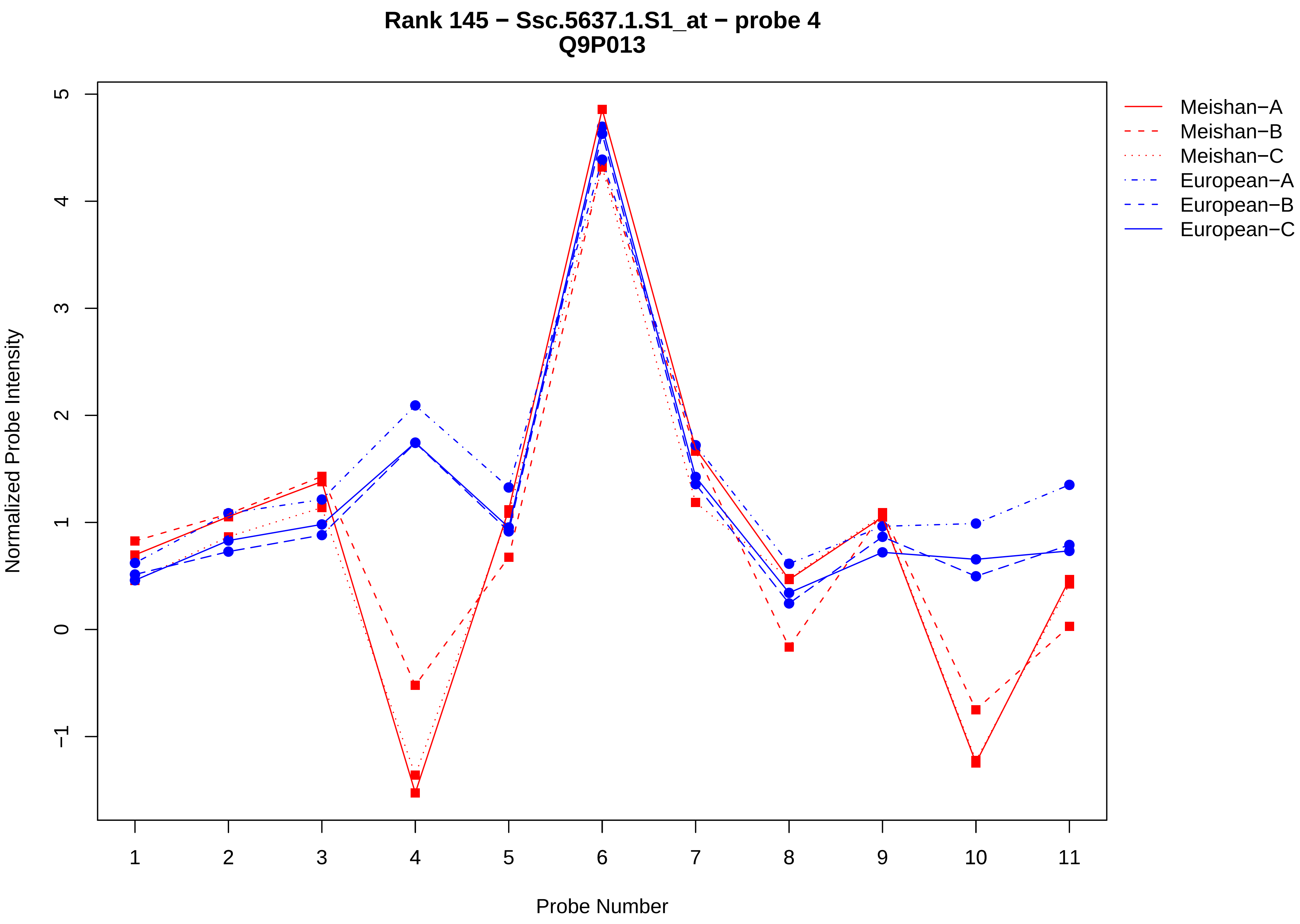
Rank 145 − Ssc.5637.1.S1_at − probe 4
Q9P013
Normalized Probe Intensity
Probe Number
−1
0
1
2
3
4
5
1
2
3
4
5
6
7
8
9
10
11
Meishan−A
Meishan−B
Meishan−C
European−A
European−B
European−C

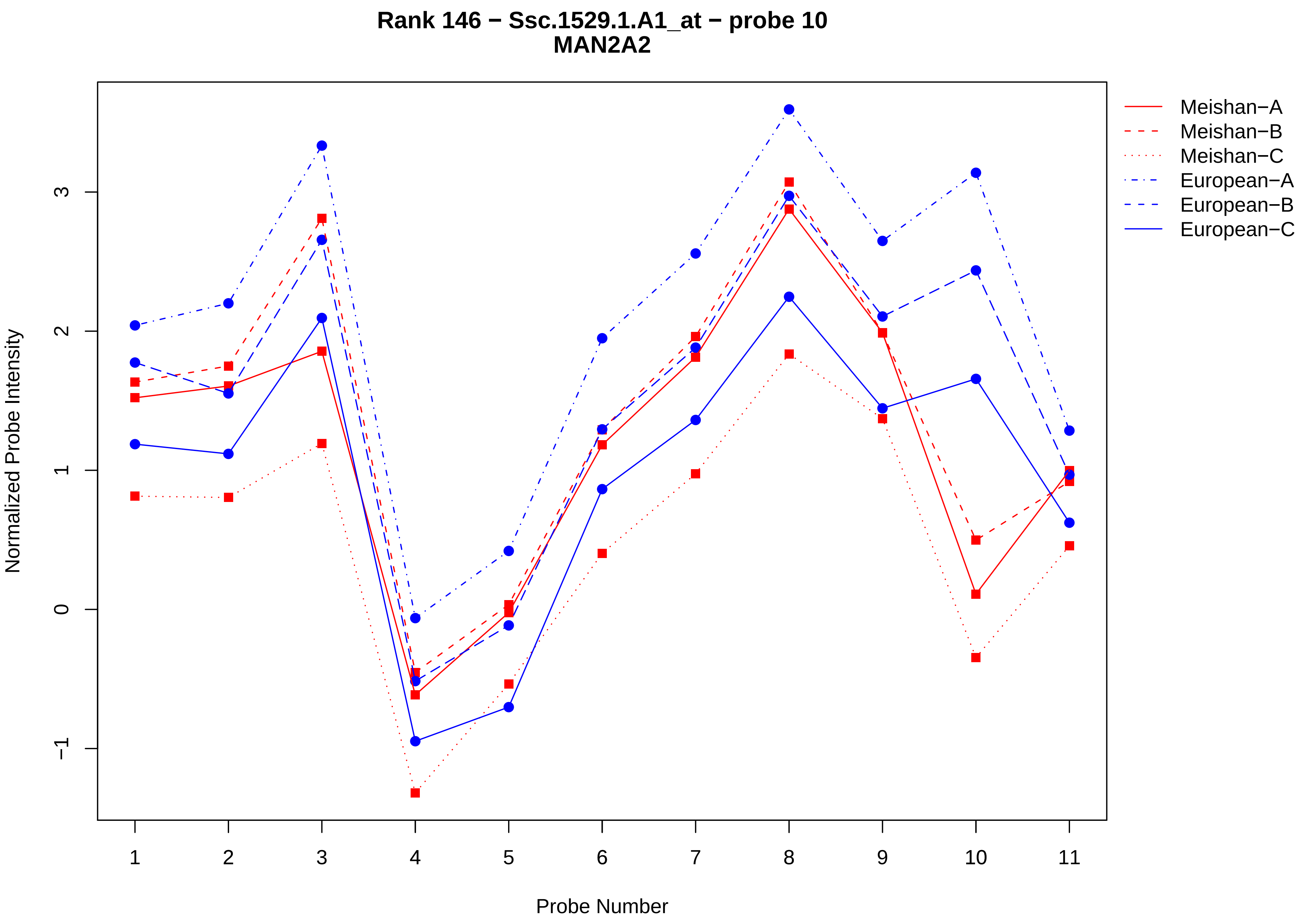

Rank 146 − Ssc.1529.1.A1_at − probe 10
MAN2A2
Normalized Probe Intensity
Probe Number
−1
0
1
2
3
1
2
3
4
5
6
7
8
9
10
11
Meishan−A
Meishan−B
Meishan−C
European−A
European−B
European−C

Rank 147 − Ssc.4221.1.S1_at − probe 10
COPZ2

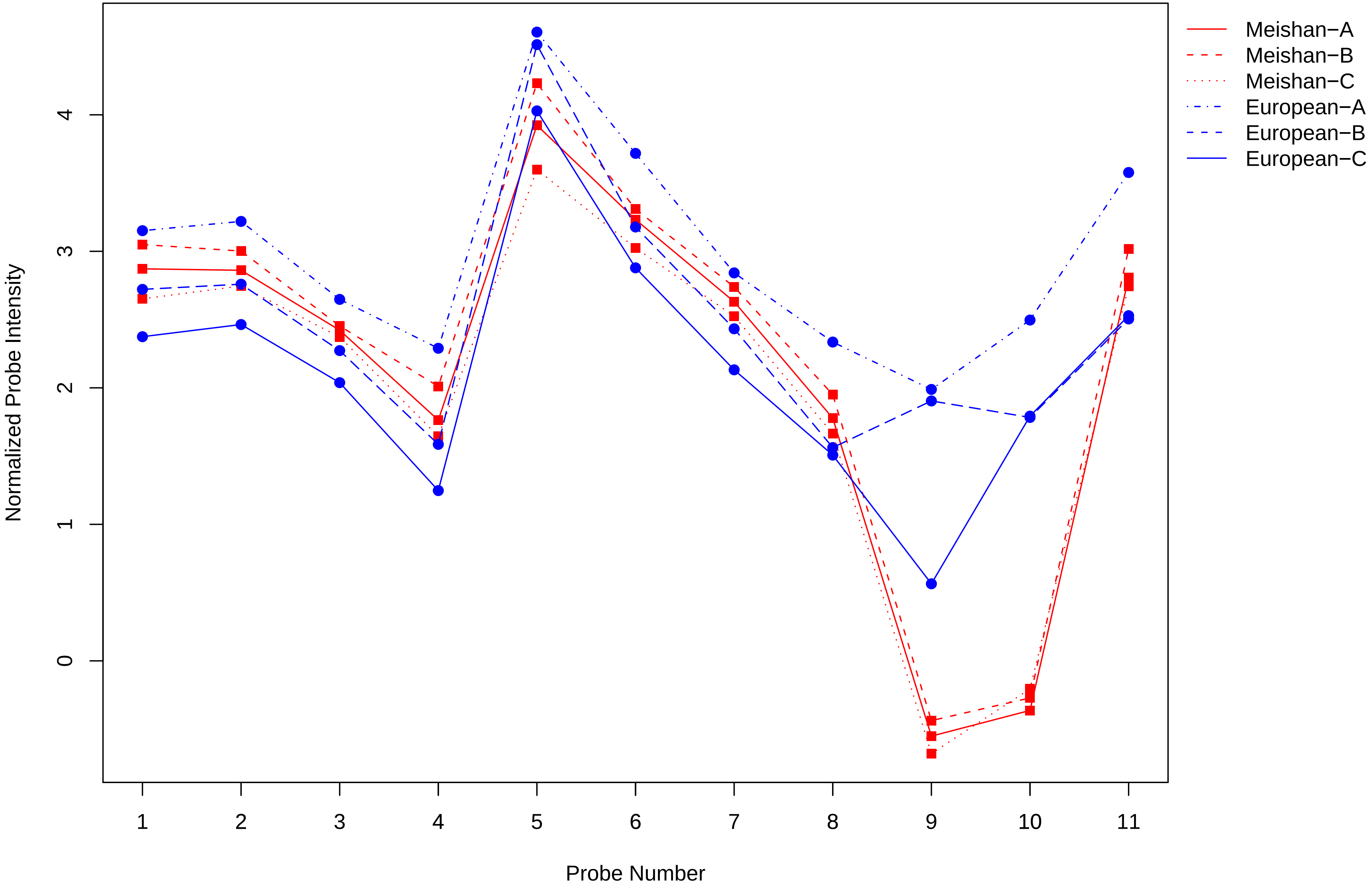

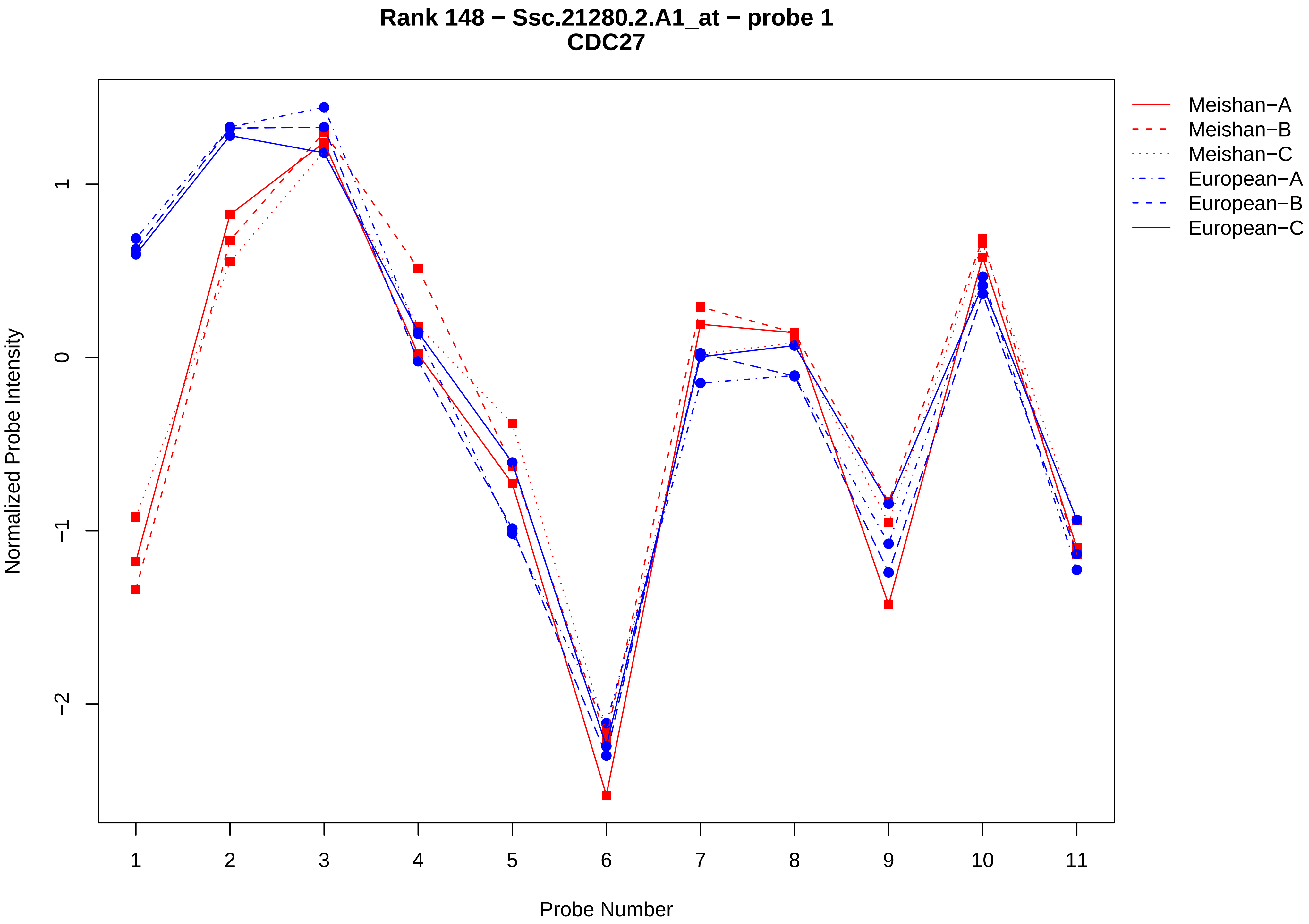

Rank 148 − Ssc.21280.2.A1_at − probe 1
CDC27
Normalized Probe Intensity
Probe Number
1
0
−1
−2
1
2
3
4
5
6
7
8
9
10
11
Meishan−A
Meishan−B
Meishan−C
European−A
European−B
European−C

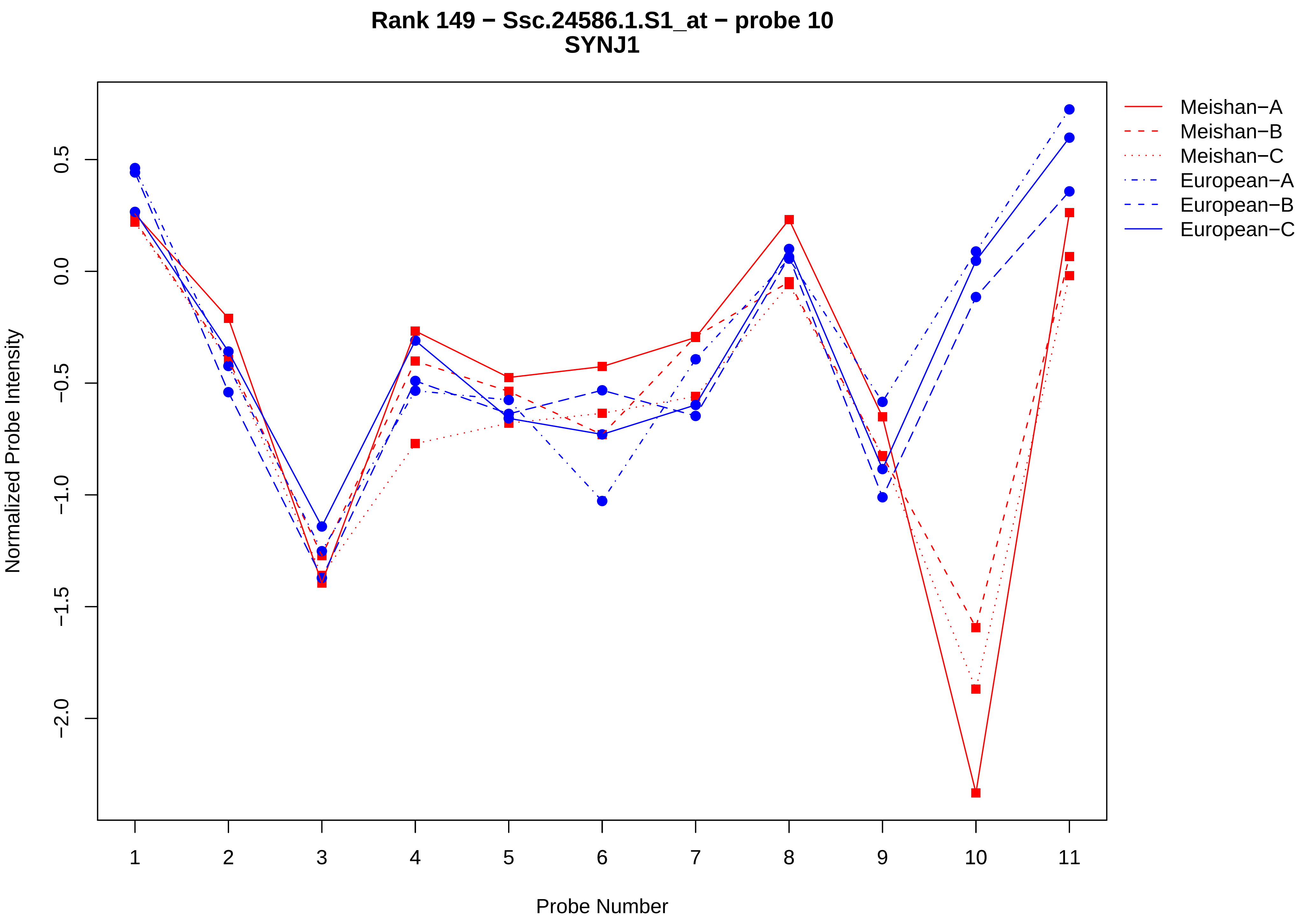
Rank 149 − Ssc.24586.1.S1_at − probe 10
SYNJ1
Normalized Probe Intensity
Probe Number
0.5
0.0
−0.5
−1.0
−1.5
−2.0
1
2
3
4
5
6
7
8
9
10
11
Meishan−A
Meishan−B
Meishan−C
European−A
European−B
European−C

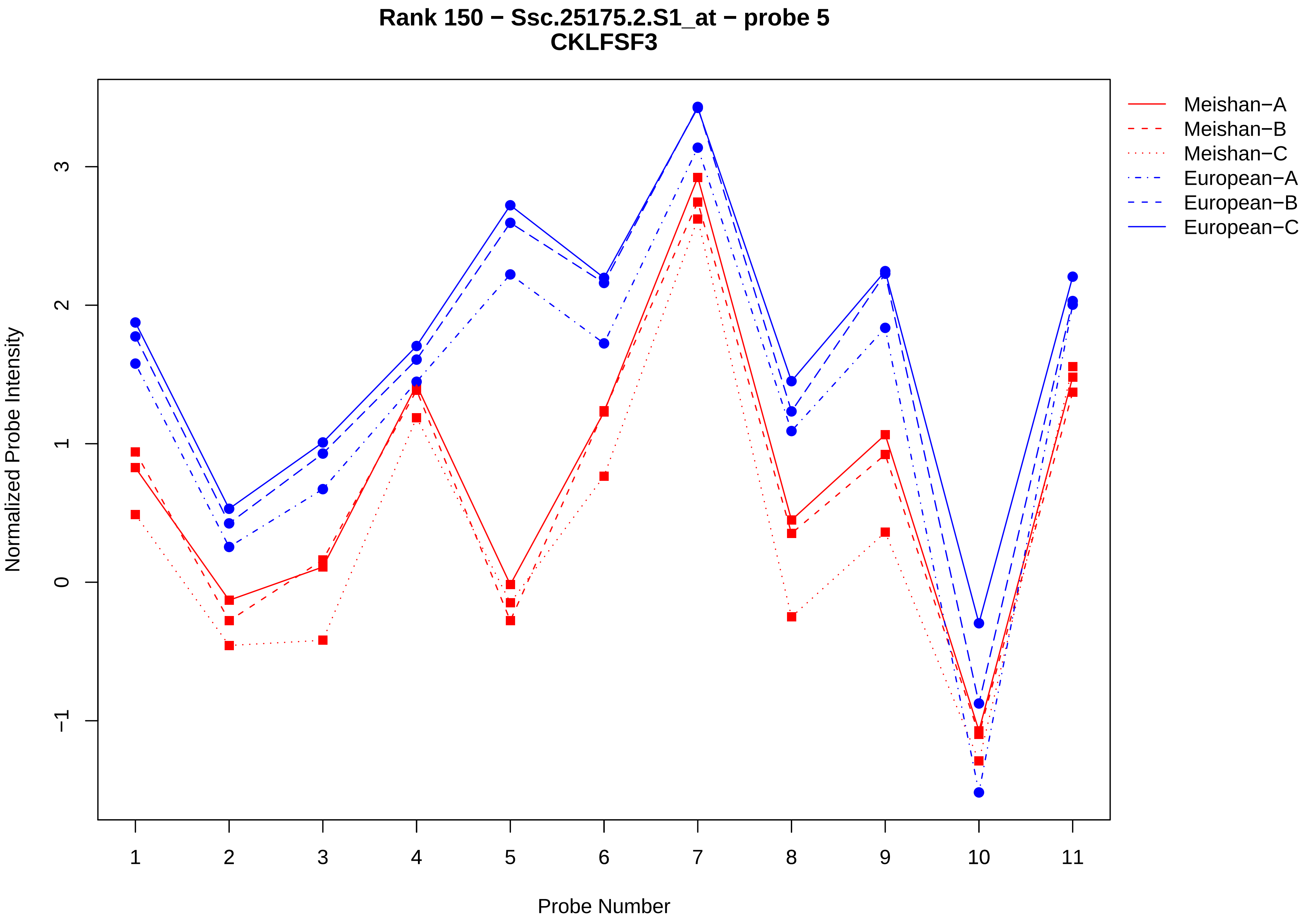

Rank 150 − Ssc.25175.2.S1_at − probe 5
CKLFSF3
Normalized Probe Intensity
Probe Number
Meishan−A
Meishan−B
Meishan−C
European−A
European−B
European−C
−1
0
1
2
3
1
2
3
4
5
6
7
8
9
10
11

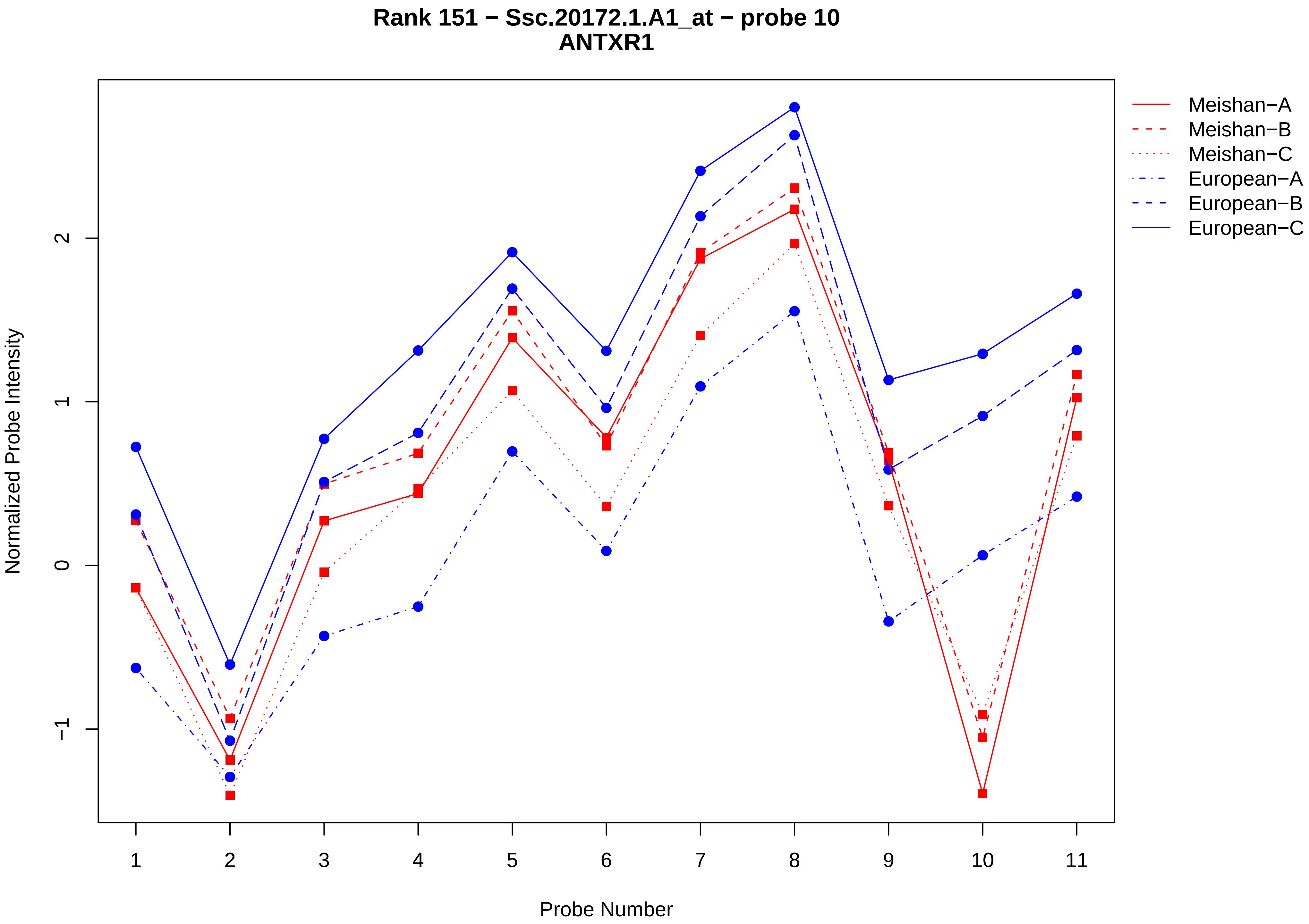

Rank 151 − Ssc.20172.1.A1_at − probe 10
ANTXR1
Normalized Probe Intensity
Probe Number
−1
0
1
2
1
2
3
4
5
6
7
8
9
10
11
Meishan−A
Meishan−B
Meishan−C
European−A
European−B
European−C

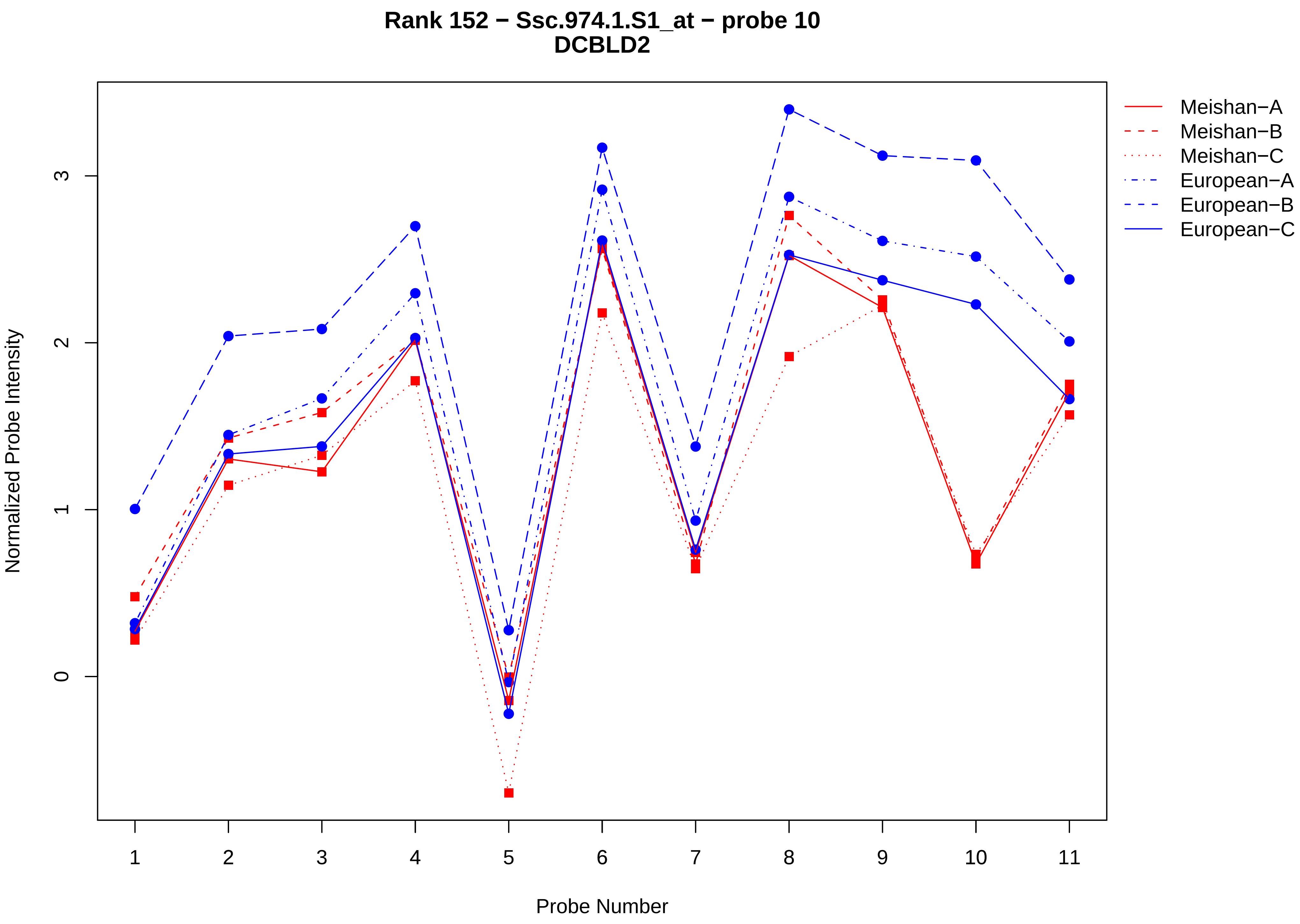
Rank 152 − Ssc.974.1.S1_at − probe 10
DCBLD2
Normalized Probe Intensity
Probe Number
0
1
2
3
1
2
3
4
5
6
7
8
9
10
11
Meishan−A
Meishan−B
Meishan−C
European−A
European−B
European−C

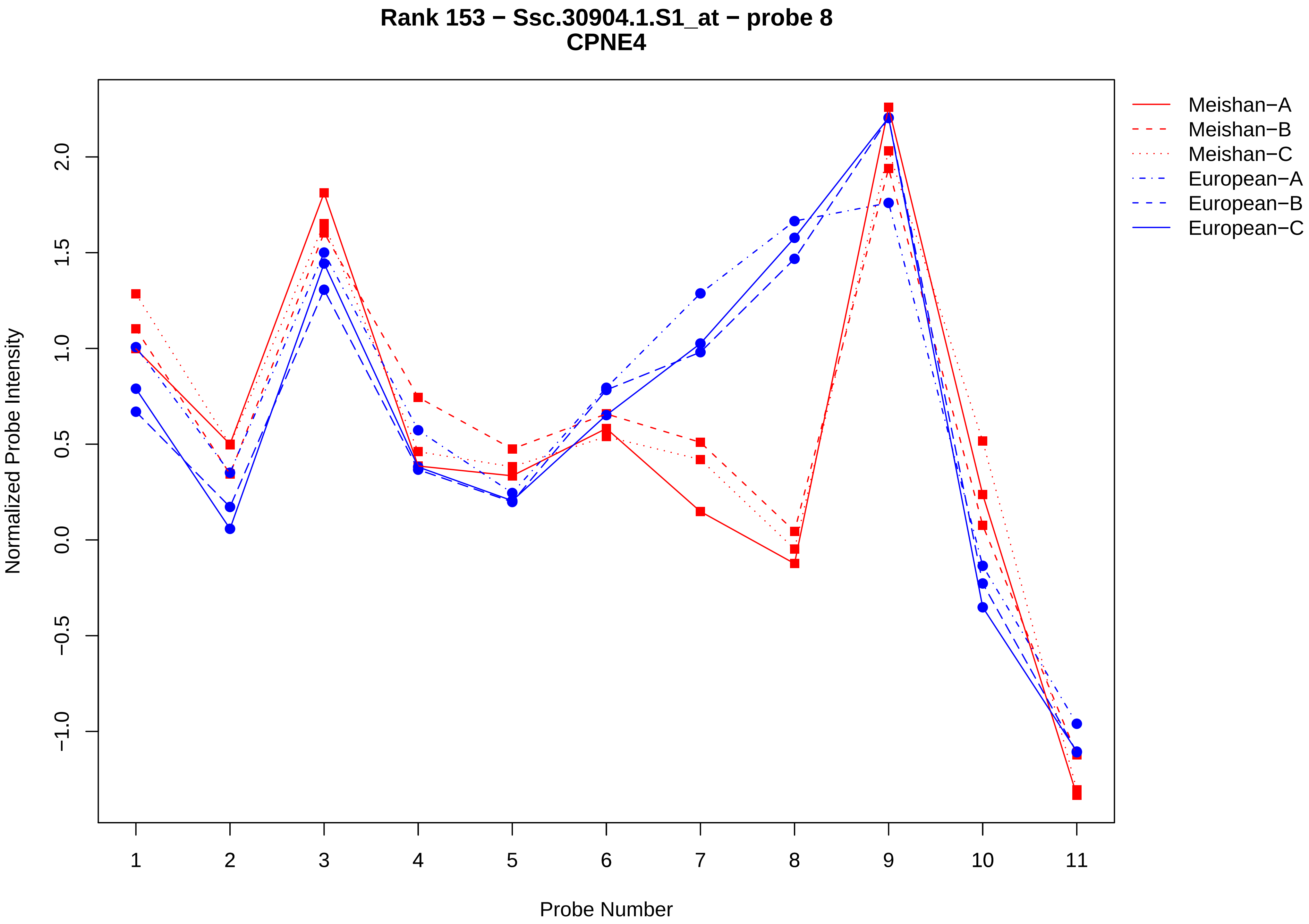
Rank 153 − Ssc.30904.1.S1_at − probe 8
CPNE4
Normalized Probe Intensity
Probe Number
Meishan−A
Meishan−B
Meishan−C
European−A
European−B
European−C
−1.0
−0.5
0.0
0.5
1.0
1.5
2.0
1
2
3
4
5
6
7
8
9
10
11

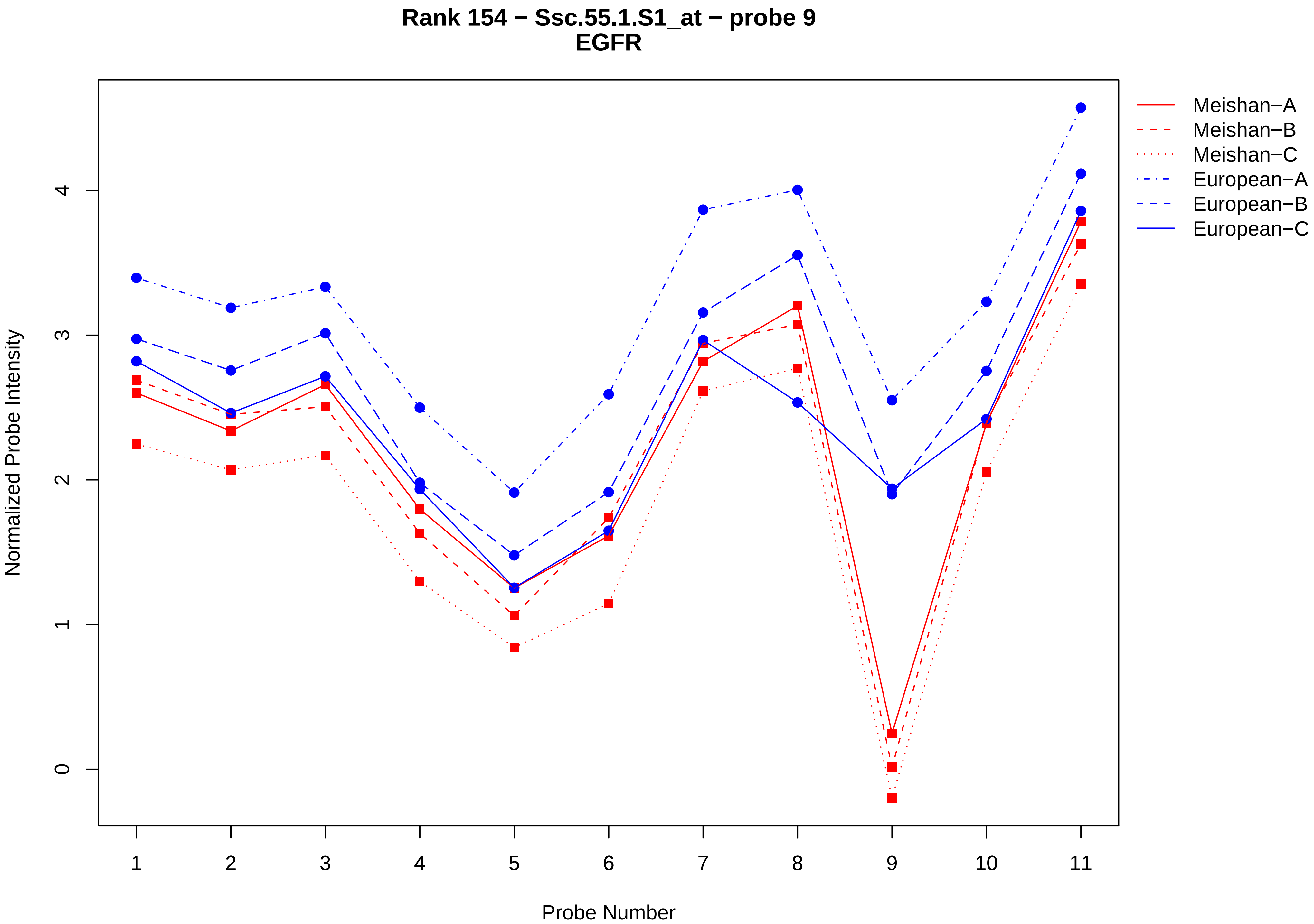

Rank 154 − Ssc.55.1.S1_at − probe 9
EGFR
Normalized Probe Intensity
Probe Number
0
1
2
3
4
1
2
3
4
5
6
7
8
9
10
11
Meishan−A
Meishan−B
Meishan−C
European−A
European−B
European−C

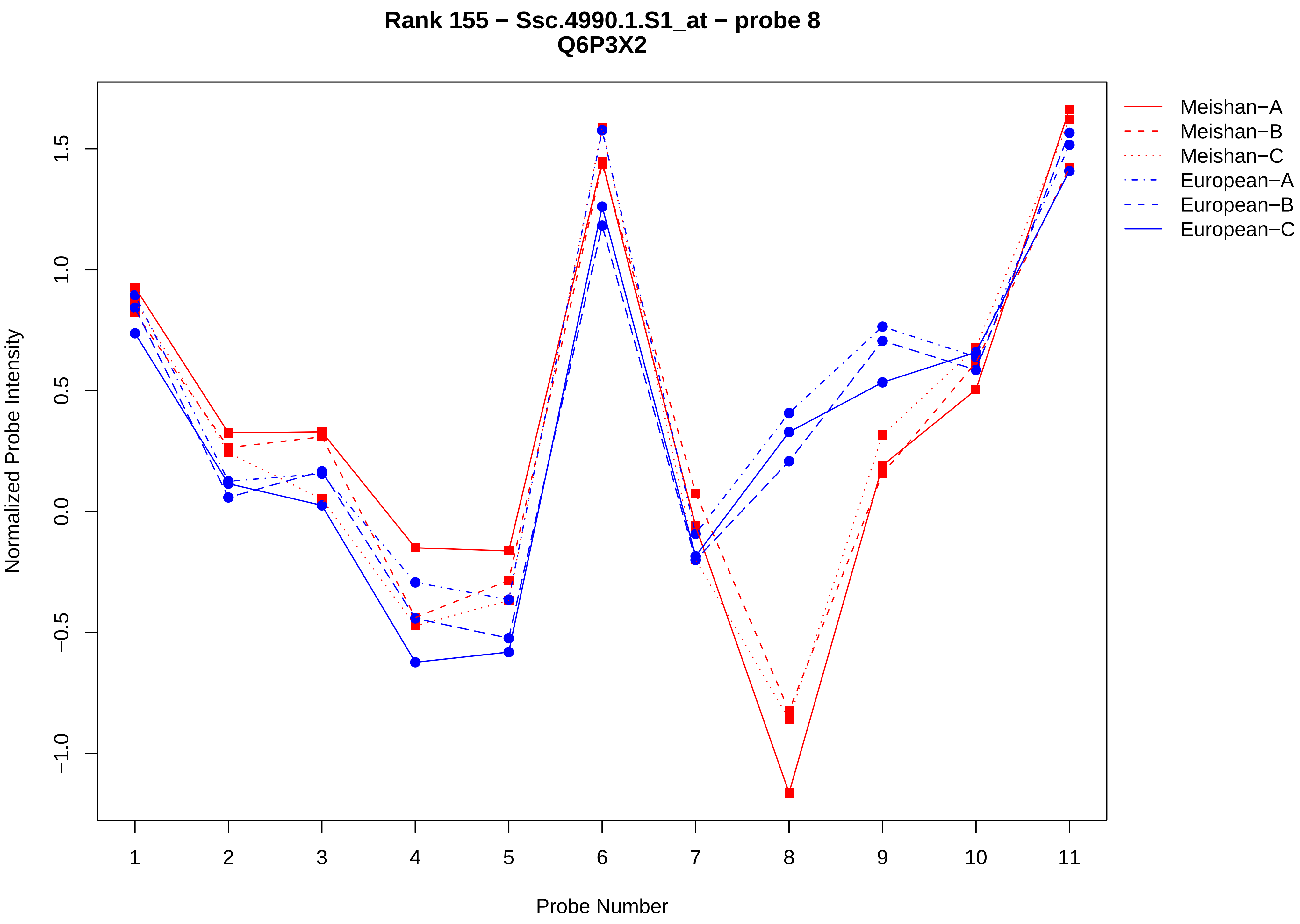

Rank 155 − Ssc.4990.1.S1_at − probe 8
Q6P3X2
Normalized Probe Intensity
Probe Number
−1.0
−0.5
0.0
0.5
1.0
1.5
1
2
3
4
5
6
7
8
9
10
11
Meishan−A
Meishan−B
Meishan−C
European−A
European−B
European−C

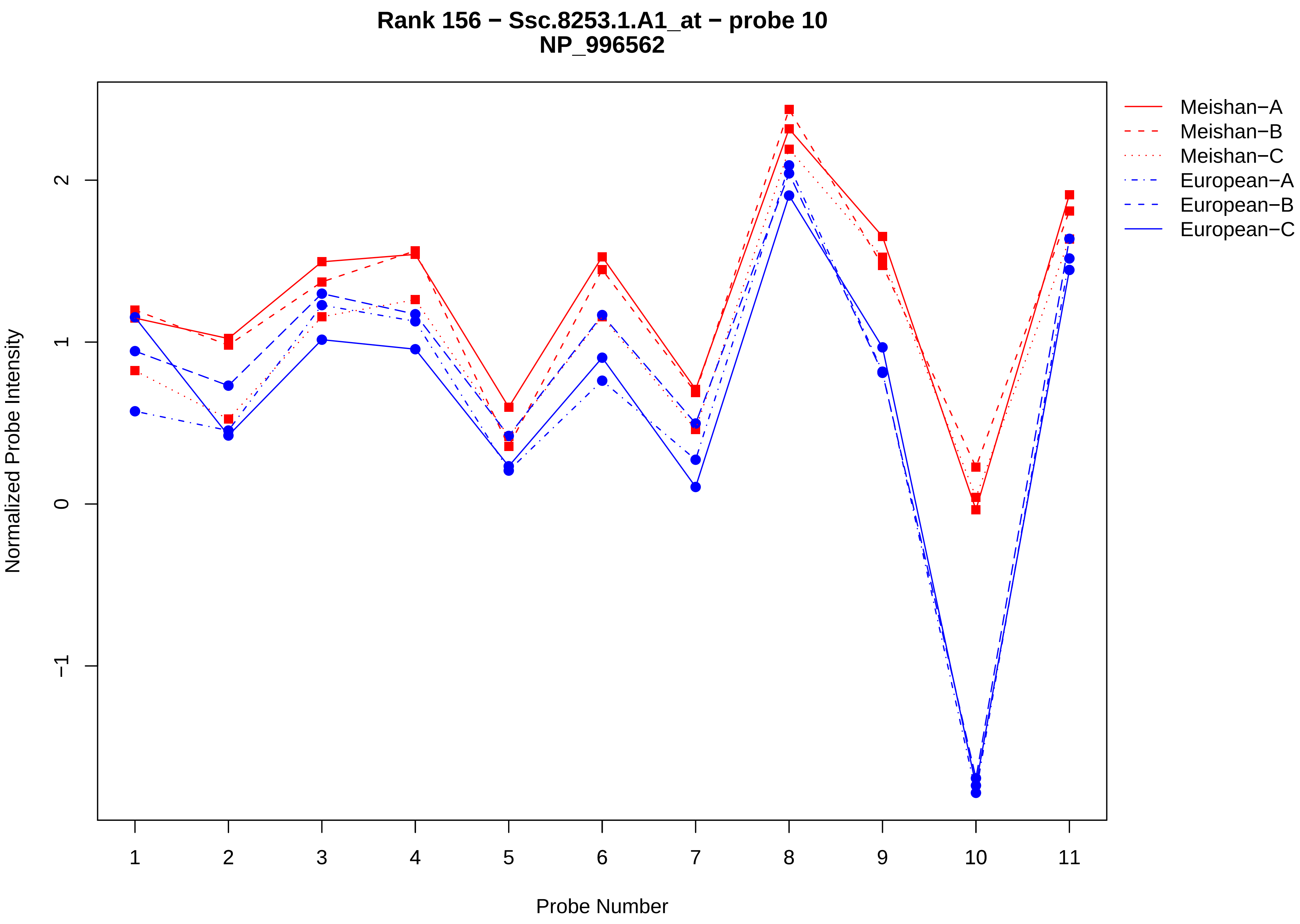
Rank 156 − Ssc.8253.1.A1_at − probe 10
NP_996562
Normalized Probe Intensity
Probe Number
−1
0
1
2
1
2
3
4
5
6
7
8
9
10
11
Meishan−A
Meishan−B
Meishan−C
European−A
European−B
European−C

Rank 157 − Ssc.14035.1.S1_at − probe 2
RAD50

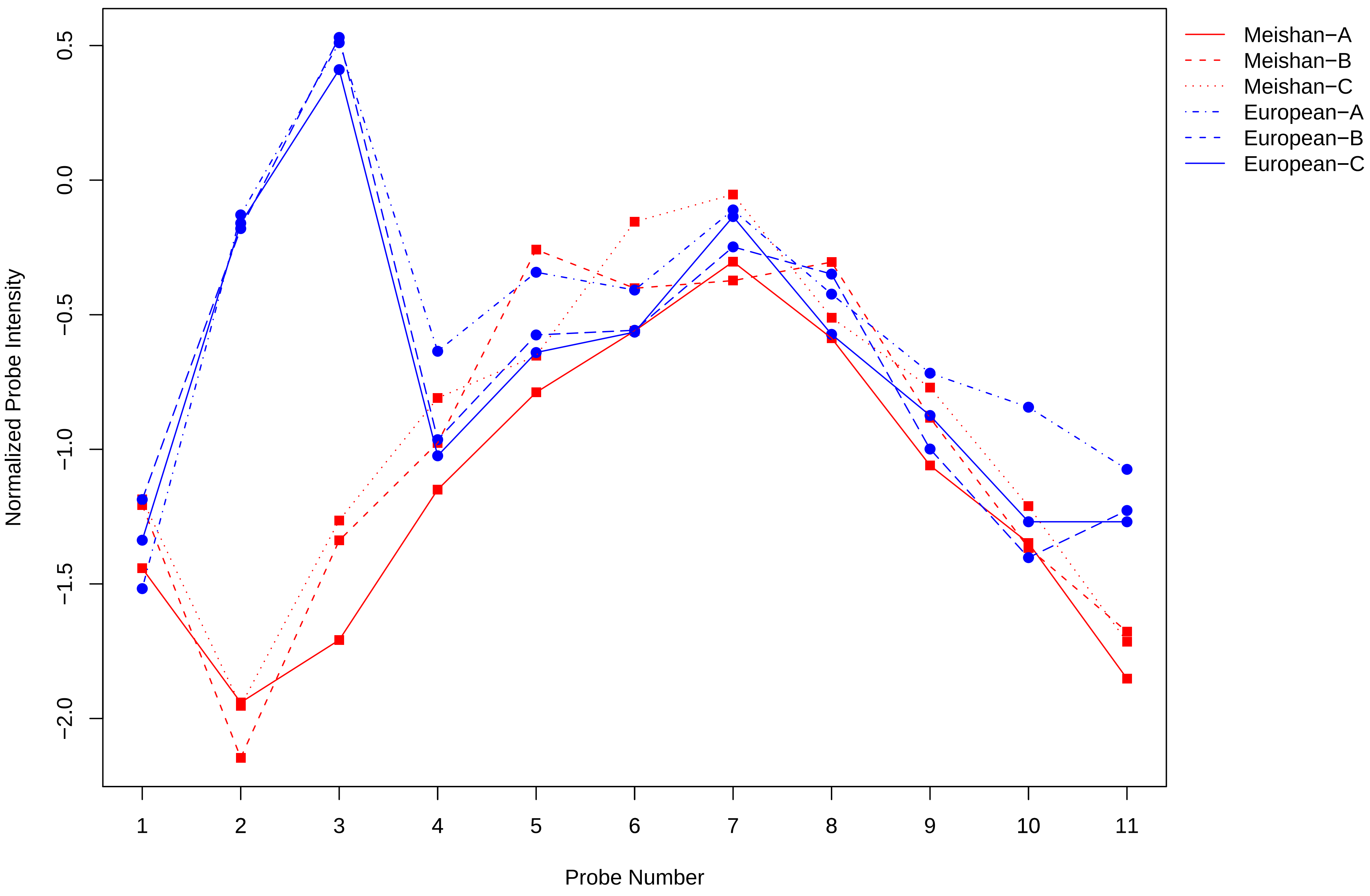

Rank 158 − Ssc.8577.1.A1_at − probe 11
Q6PJ77

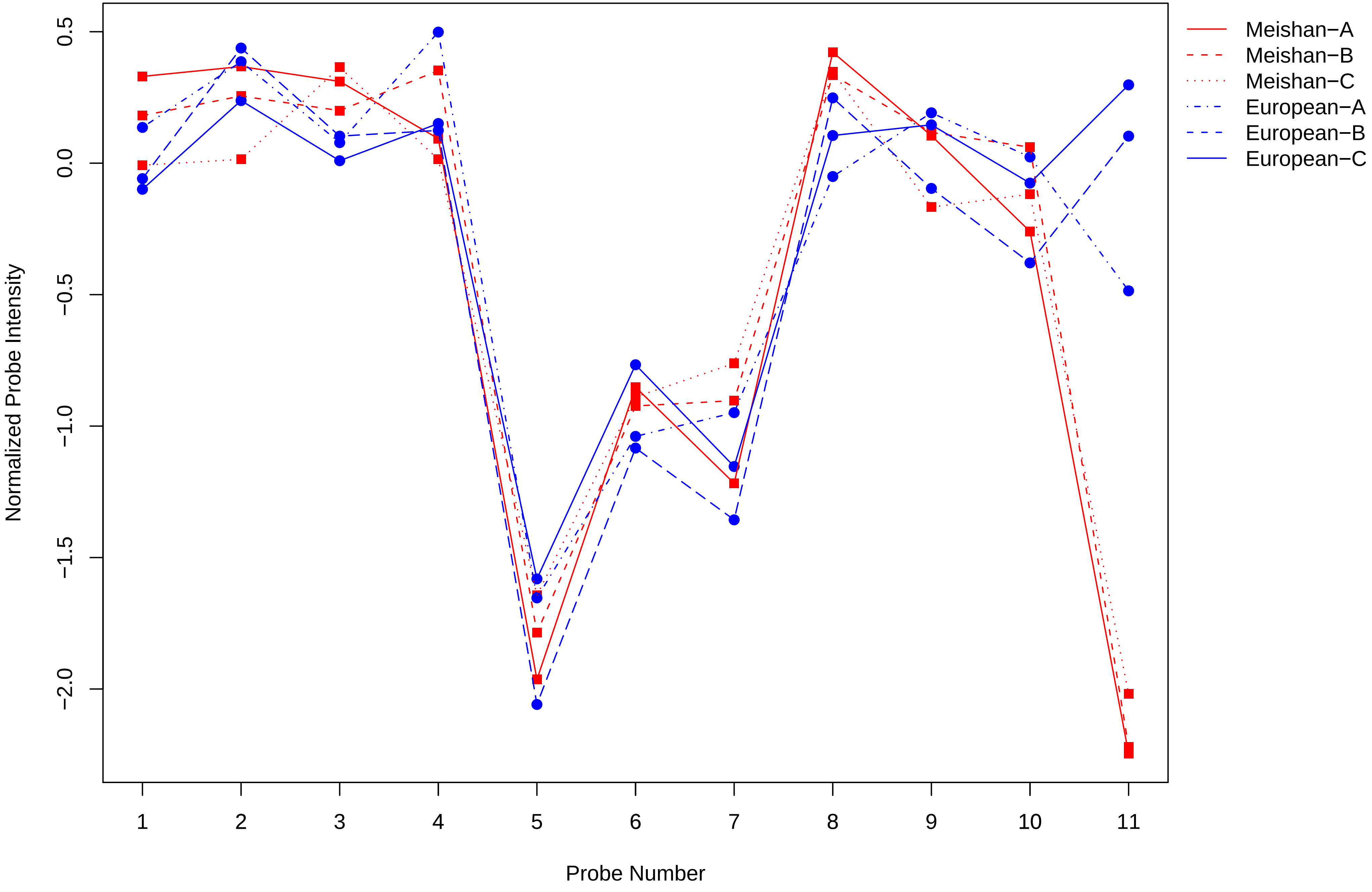

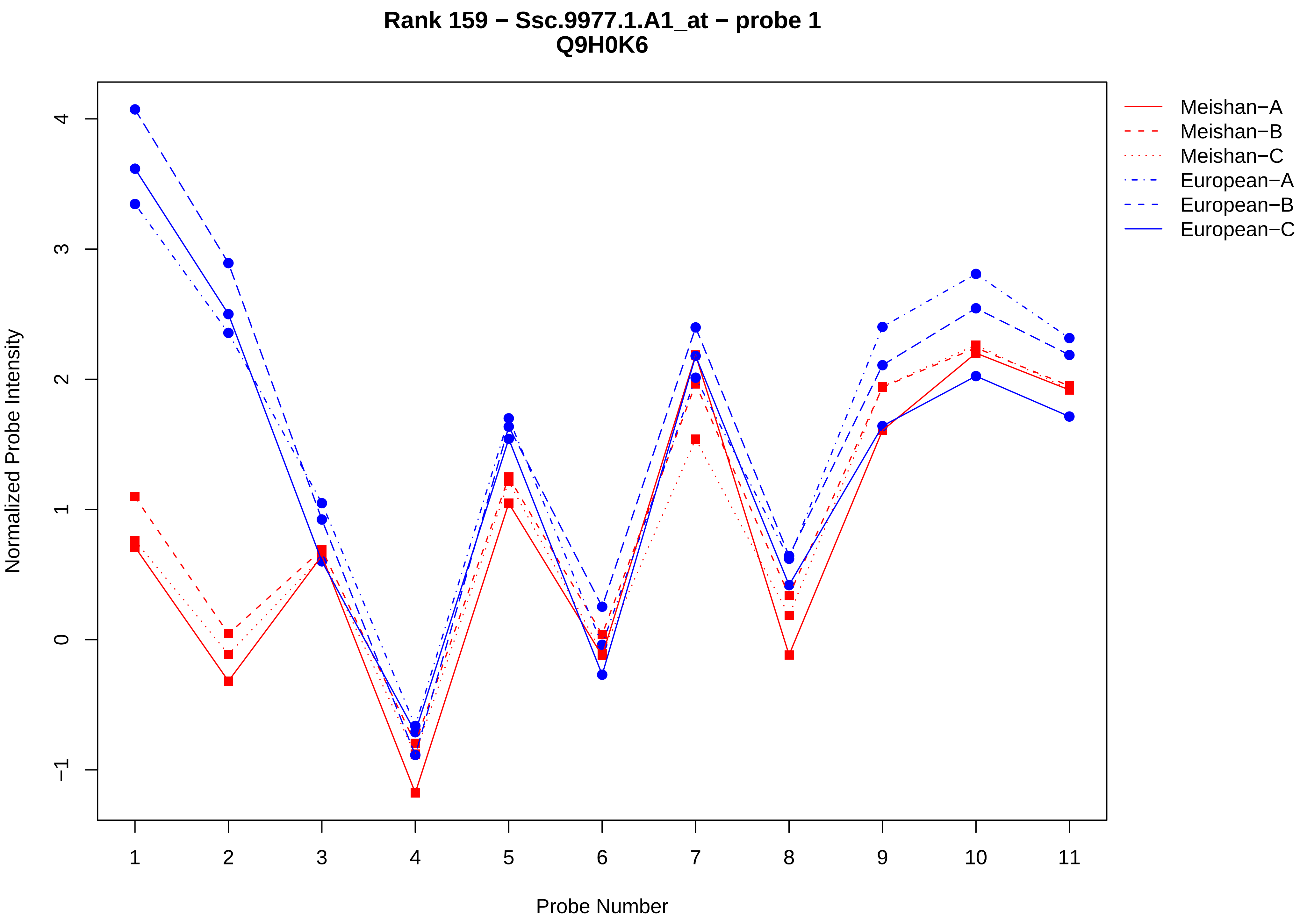

Rank 159 − Ssc.9977.1.A1_at − probe 1
Q9H0K6
Normalized Probe Intensity
Probe Number
−1
0
1
2
3
4
1
2
3
4
5
6
7
8
9
10
11
Meishan−A
Meishan−B
Meishan−C
European−A
European−B
European−C

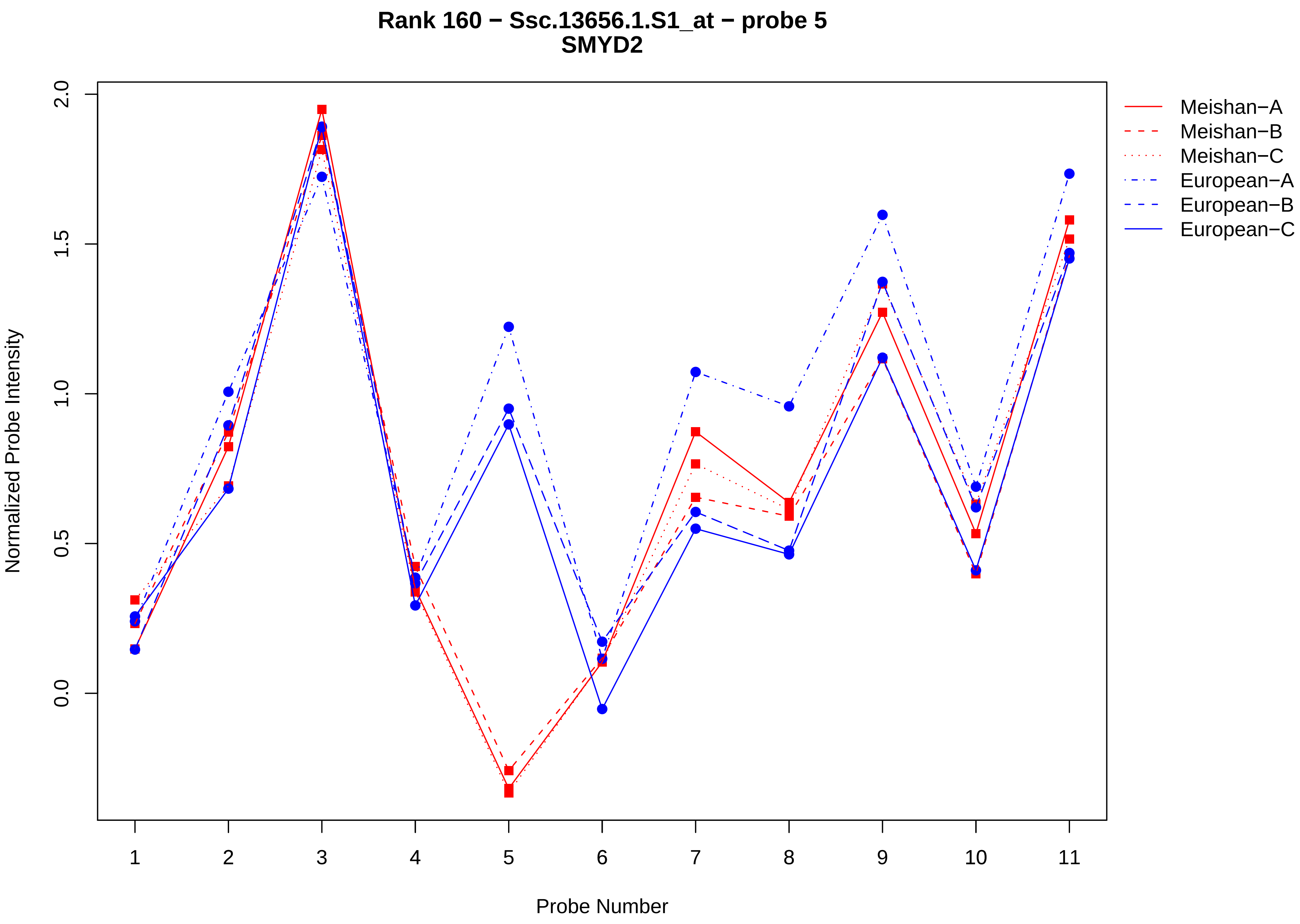

Rank 160 − Ssc.13656.1.S1_at − probe 5
SMYD2
Normalized Probe Intensity
Probe Number
0.0
0.5
1.0
1.5
2.0
1
2
3
4
5
6
7
8
9
10
11
Meishan−A
Meishan−B
Meishan−C
European−A
European−B
European−C

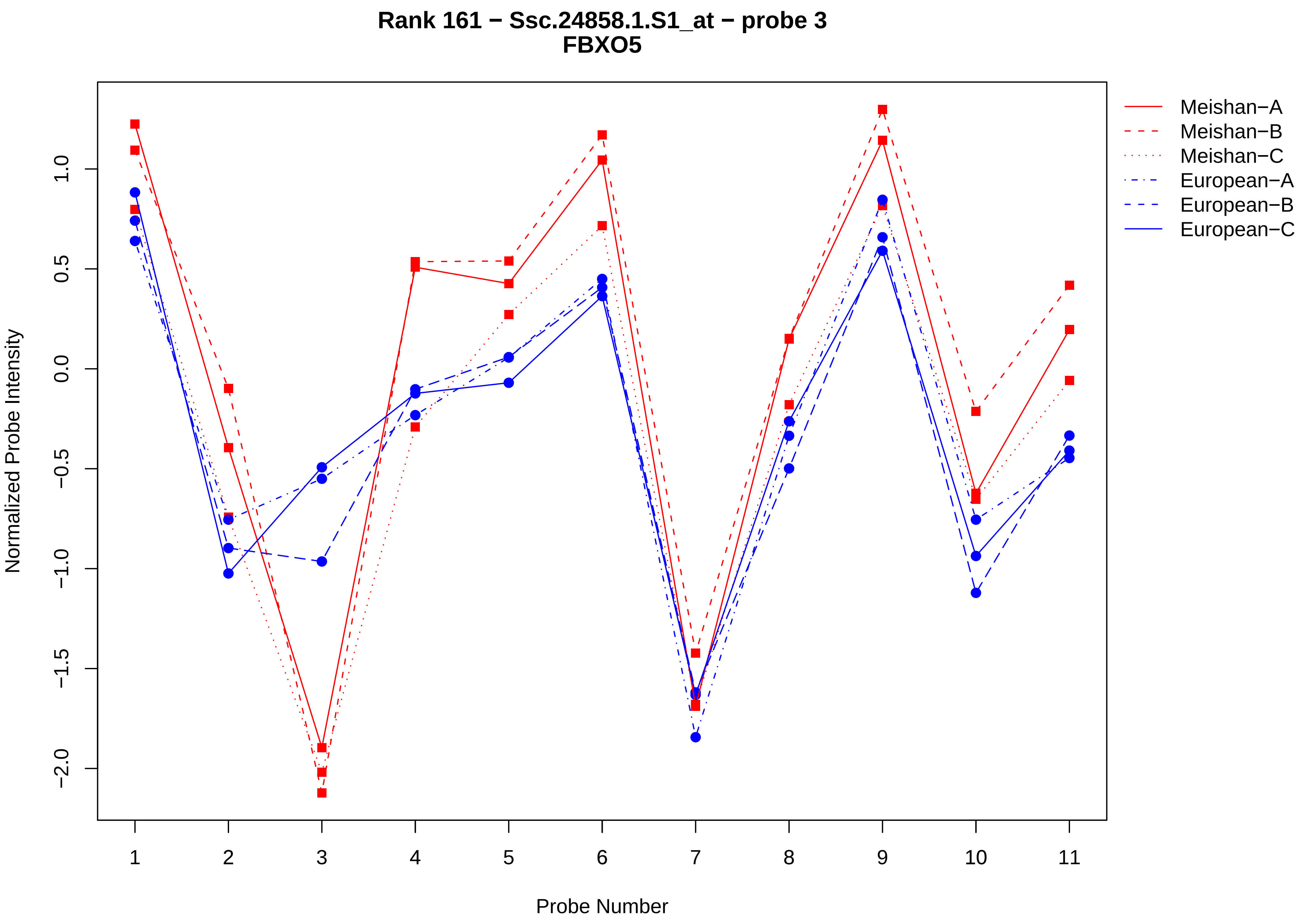
Rank 161 − Ssc.24858.1.S1_at − probe 3
FBXO5
Normalized Probe Intensity
Probe Number
−2.0
−1.5
−1.0
−0.5
0.0
0.5
1.0
1
2
3
4
5
6
7
8
9
10
11
Meishan−A
Meishan−B
Meishan−C
European−A
European−B
European−C

Rank 162 − Ssc.30797.1.S1_at − probe 10
NP_116028

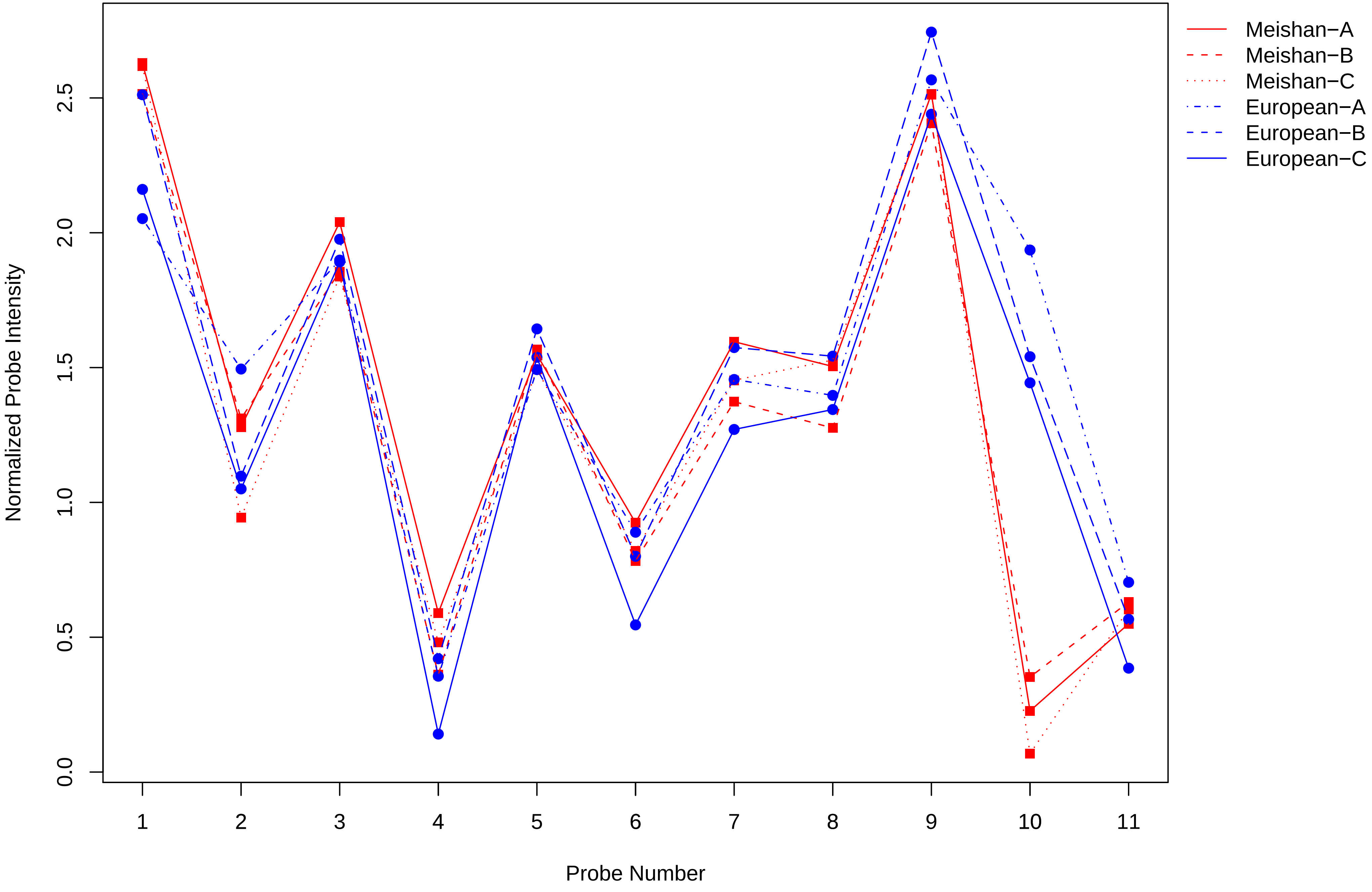

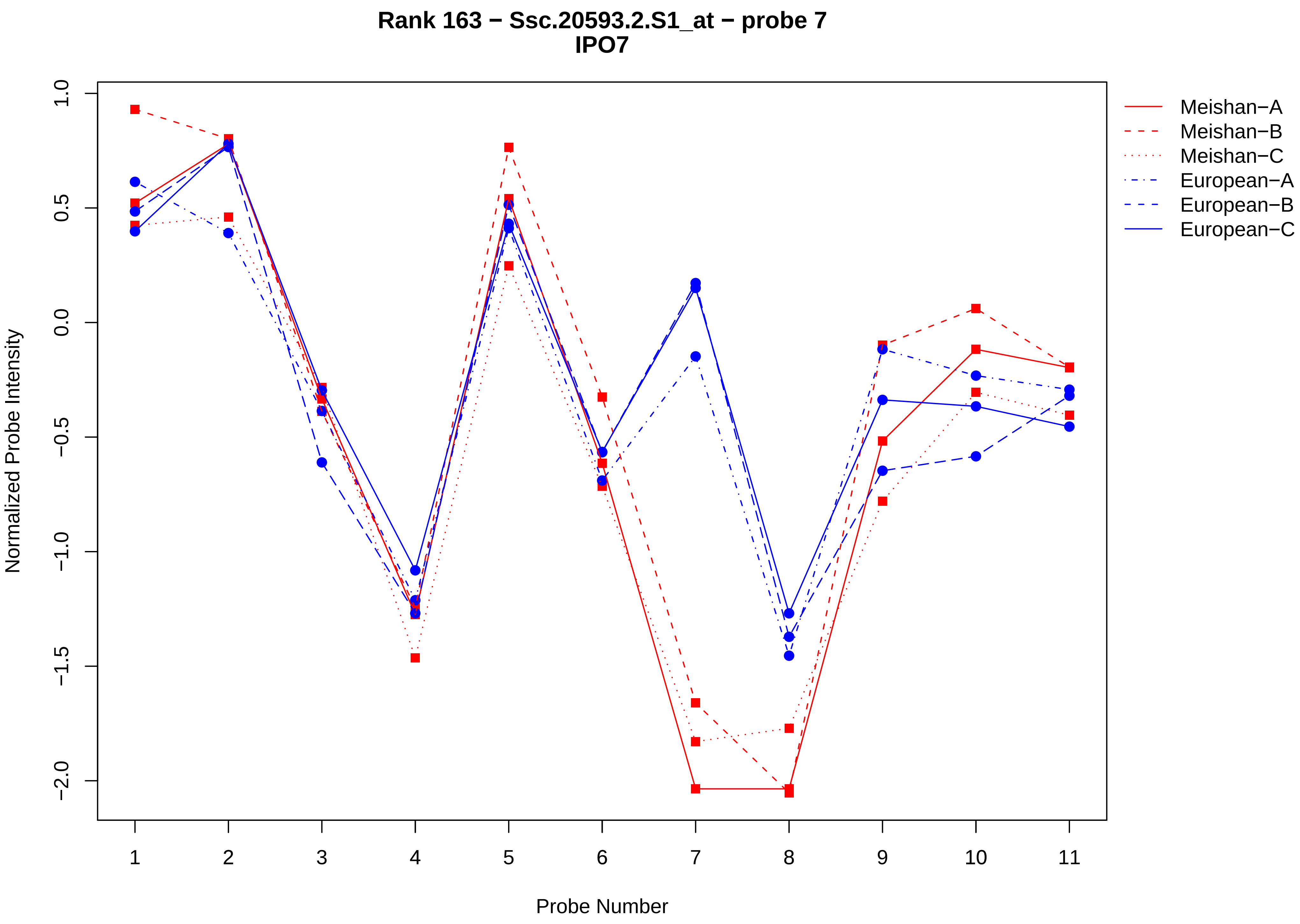

Rank 163 − Ssc.20593.2.S1_at − probe 7
IPO7
Normalized Probe Intensity
Probe Number
1.0
0.5
0.0
−0.5
−1.0
−1.5
−2.0
1
2
3
4
5
6
7
8
9
10
11
Meishan−A
Meishan−B
Meishan−C
European−A
European−B
European−C

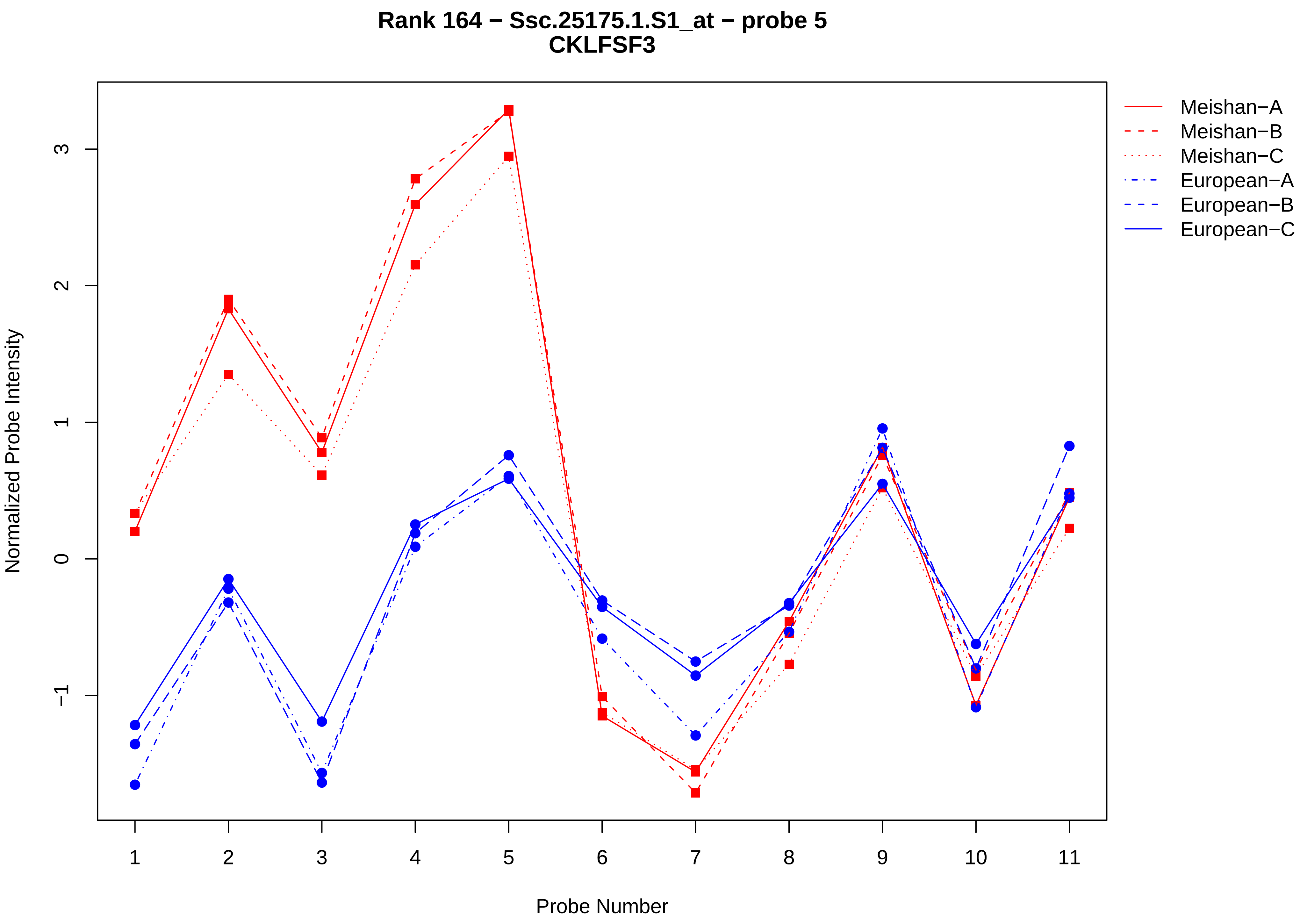

Rank 164 − Ssc.25175.1.S1_at − probe 5
CKLFSF3
Normalized Probe Intensity
Probe Number
Meishan−A
Meishan−B
Meishan−C
European−A
European−B
European−C

Rank 165 − Ssc.18436.3.A1_a_at − probe 11
RNF7

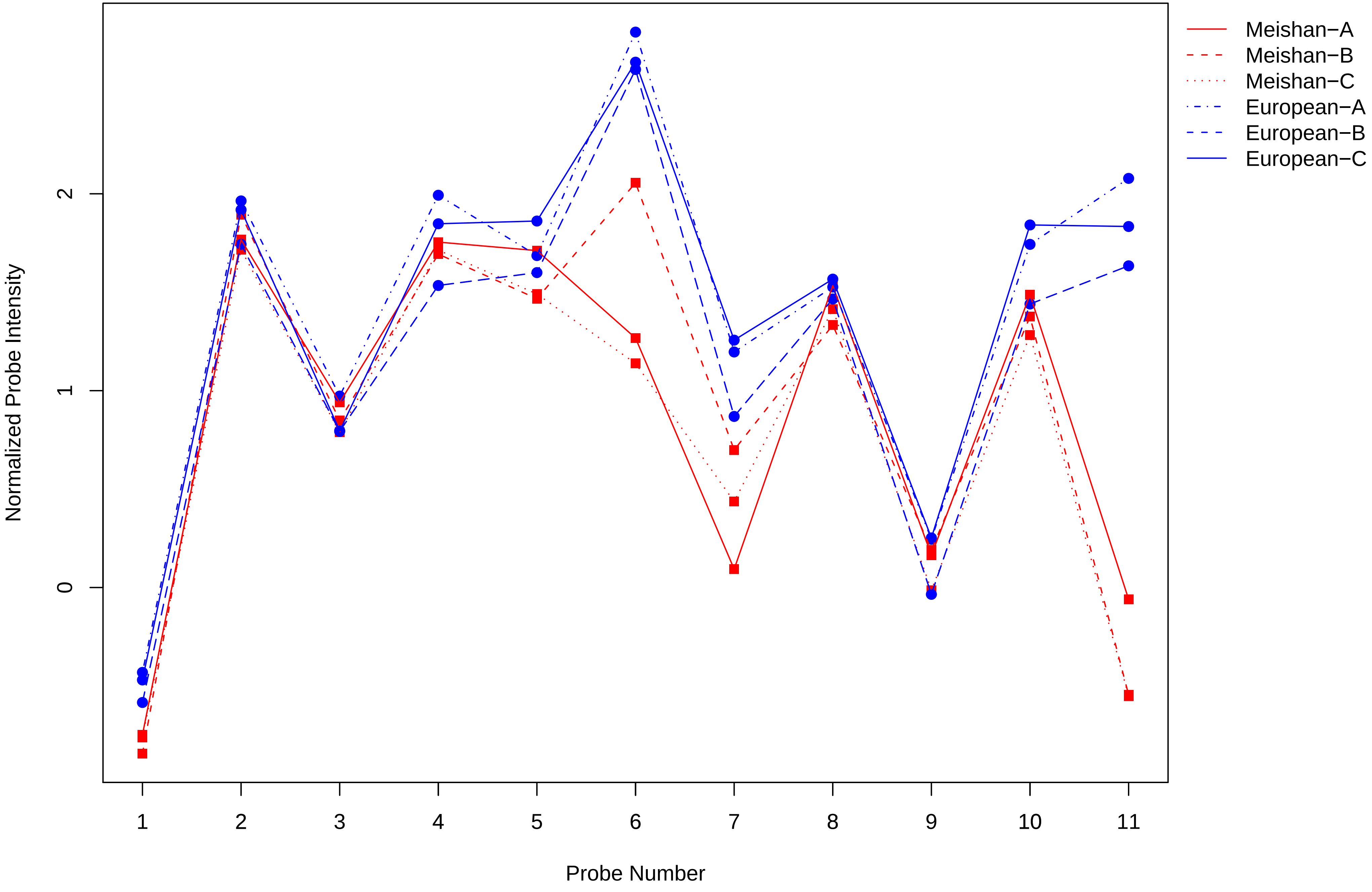

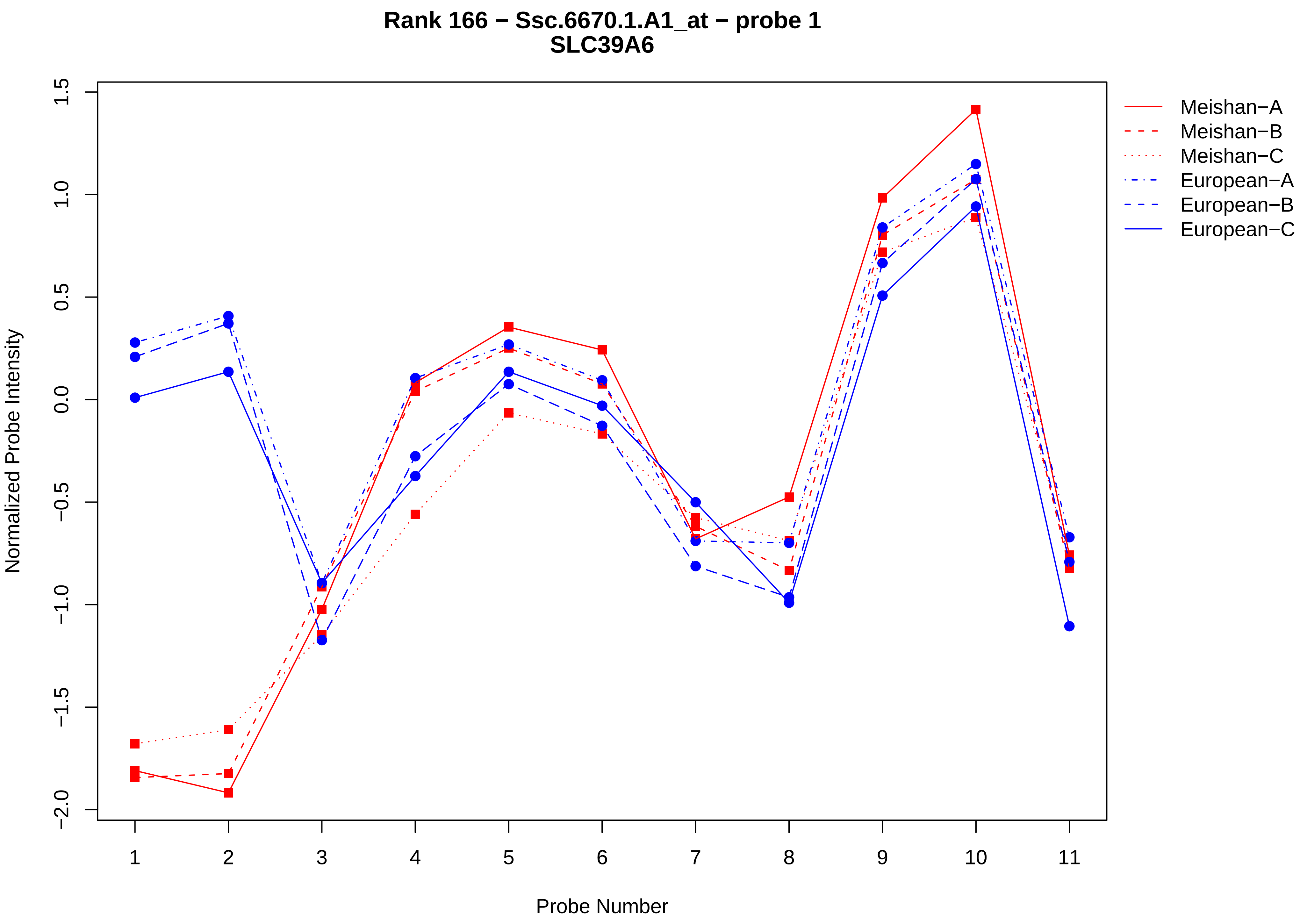

Rank 166 − Ssc.6670.1.A1_at − probe 1
SLC39A6
Normalized Probe Intensity
Probe Number
Meishan−A
Meishan−B
Meishan−C
European−A
European−B
European−C
1.5
1.0
0.5
0.0
−0.5
−1.0
−1.5
−2.0
1
2
3
4
5
6
7
8
9
10
11

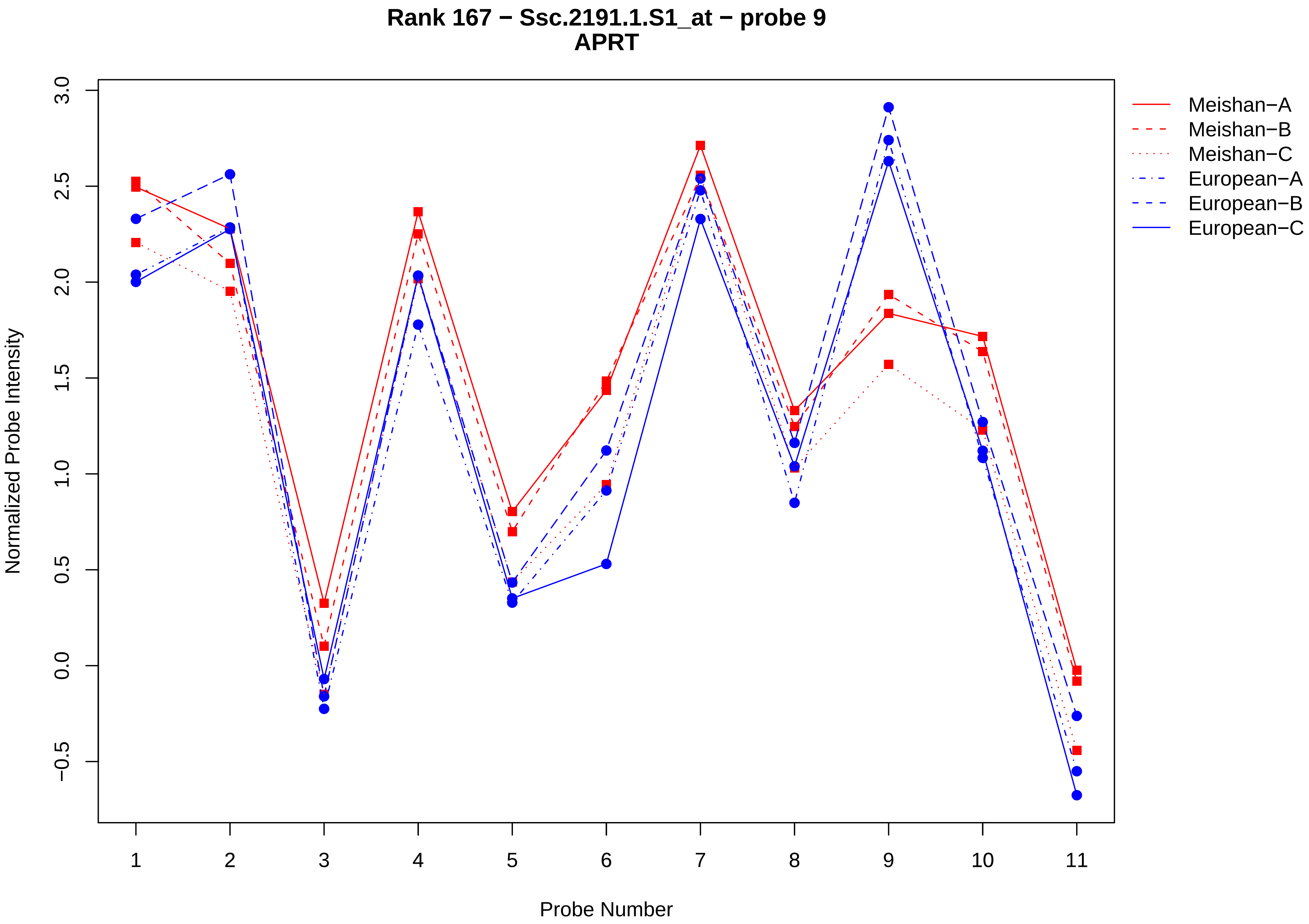
Rank 167 − Ssc.2191.1.S1_at − probe 9
APRT
Normalized Probe Intensity
Probe Number
−0.5
0.0
0.5
1.0
1.5
2.0
2.5
3.0
1
2
3
4
5
6
7
8
9
10
11
Meishan−A
Meishan−B
Meishan−C
European−A
European−B
European−C

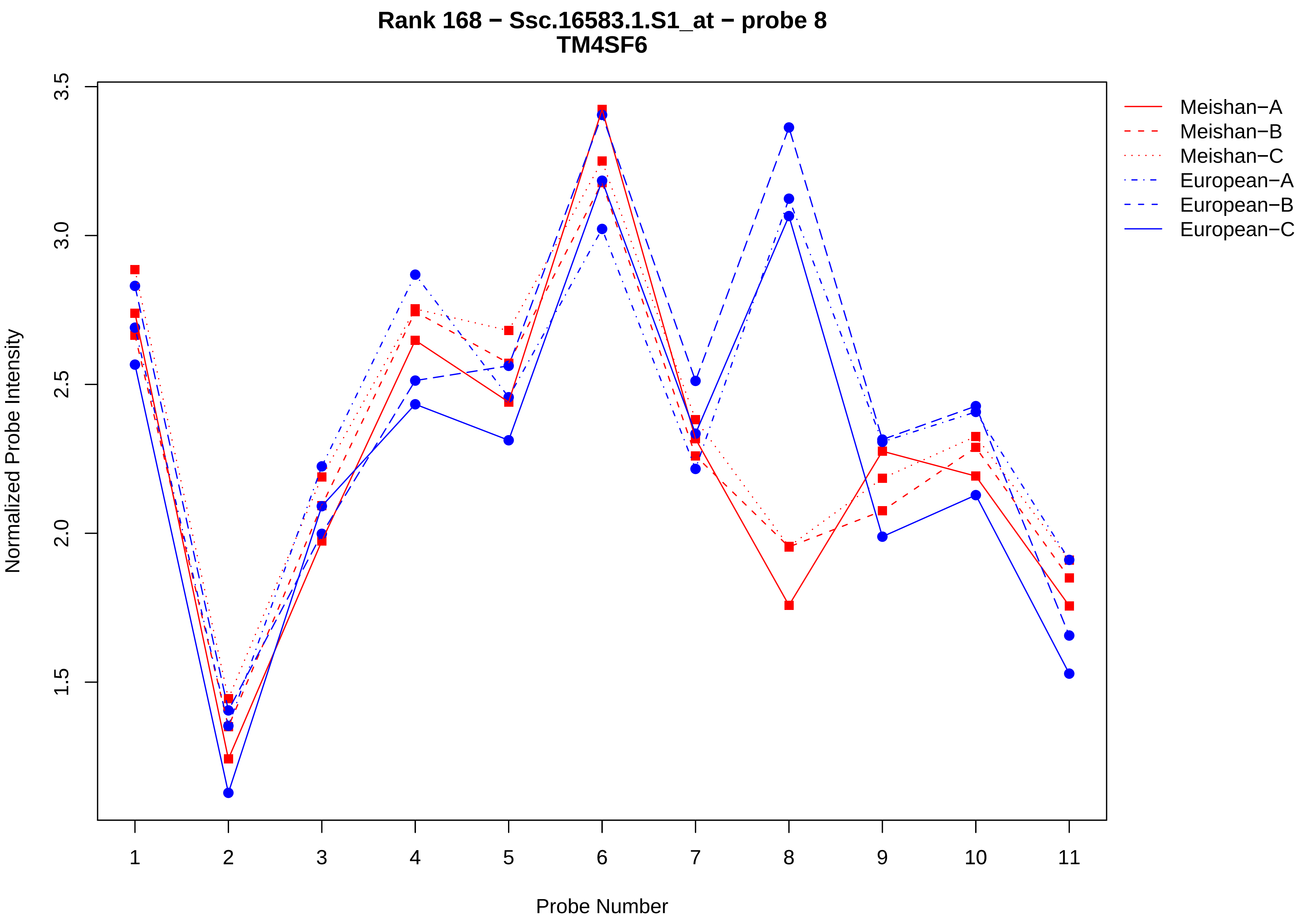

Rank 168 − Ssc.16583.1.S1_at − probe 8
TM4SF6
Normalized Probe Intensity
Probe Number
1.5
2.0
2.5
3.0
3.5
1
2
3
4
5
6
7
8
9
10
11
Meishan−A
Meishan−B
Meishan−C
European−A
European−B
European−C

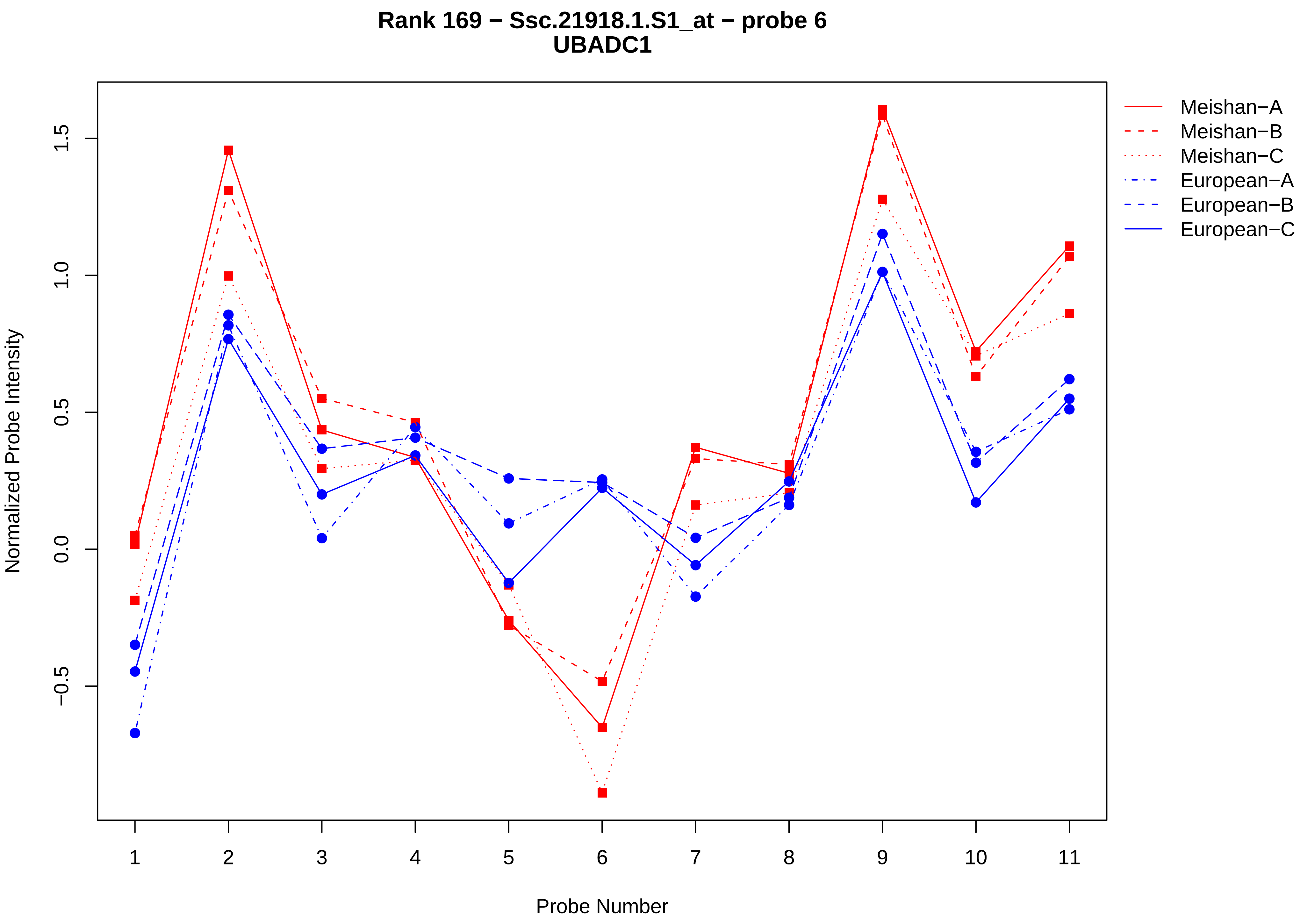

Rank 169 − Ssc.21918.1.S1_at − probe 6
UBADC1
Normalized Probe Intensity
−0.5
0.0
0.5
1.0
1.5
1
2
3
4
5
6
7
8
9
10
11
Probe Number
Meishan−A
Meishan−B
Meishan−C
European−A
European−B
European−C

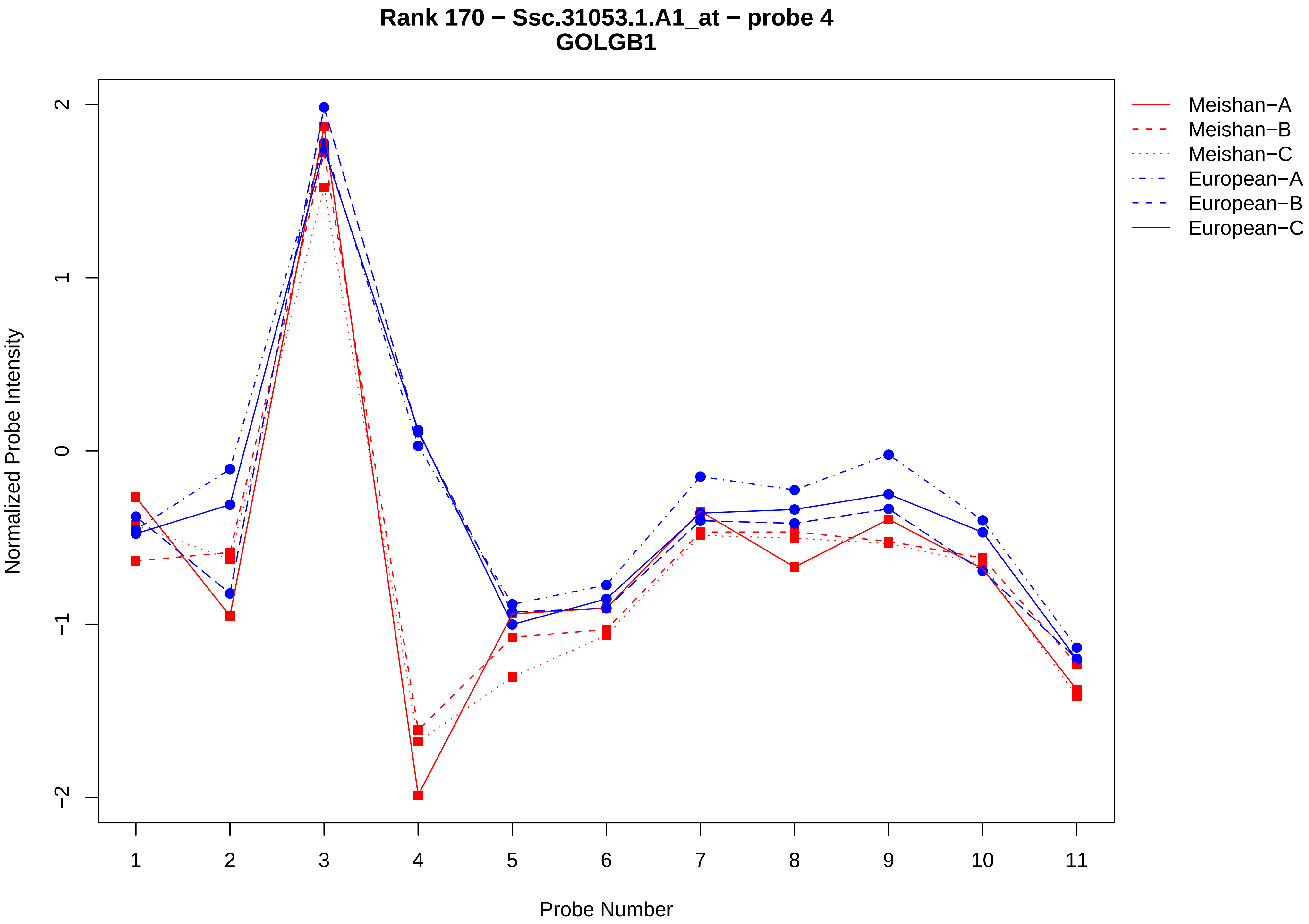

Rank 170 − Ssc.31053.1.A1_at − probe 4
GOLGB1
Normalized Probe Intensity
Probe Number
Meishan−A
Meishan−B
Meishan−C
European−A
European−B
European−C

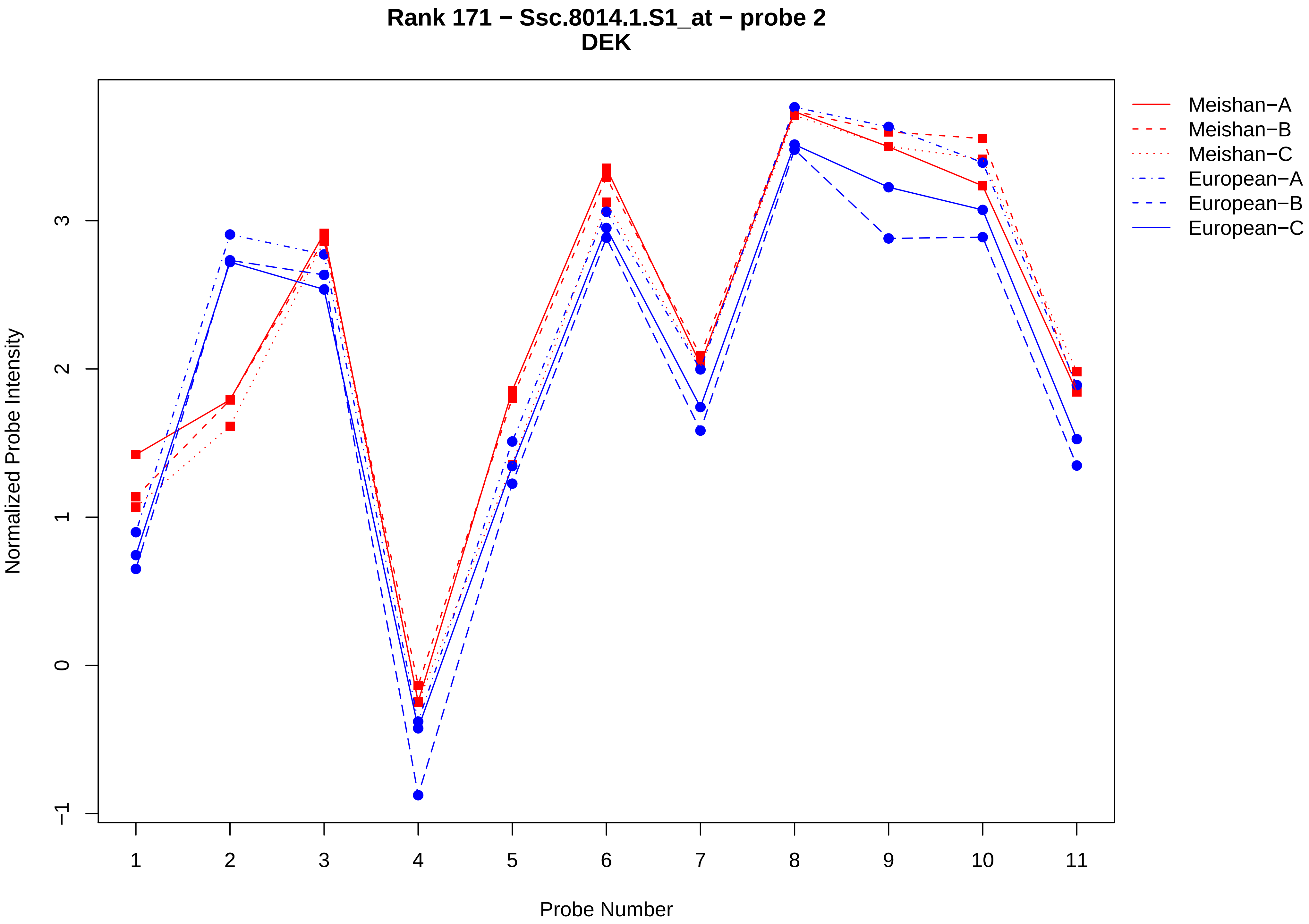
Rank 171 − Ssc.8014.1.S1_at − probe 2
DEK
Normalized Probe Intensity
Probe Number
−1
0
1
2
3
1
2
3
4
5
6
7
8
9
10
11
Meishan−A
Meishan−B
Meishan−C
European−A
European−B
European−C

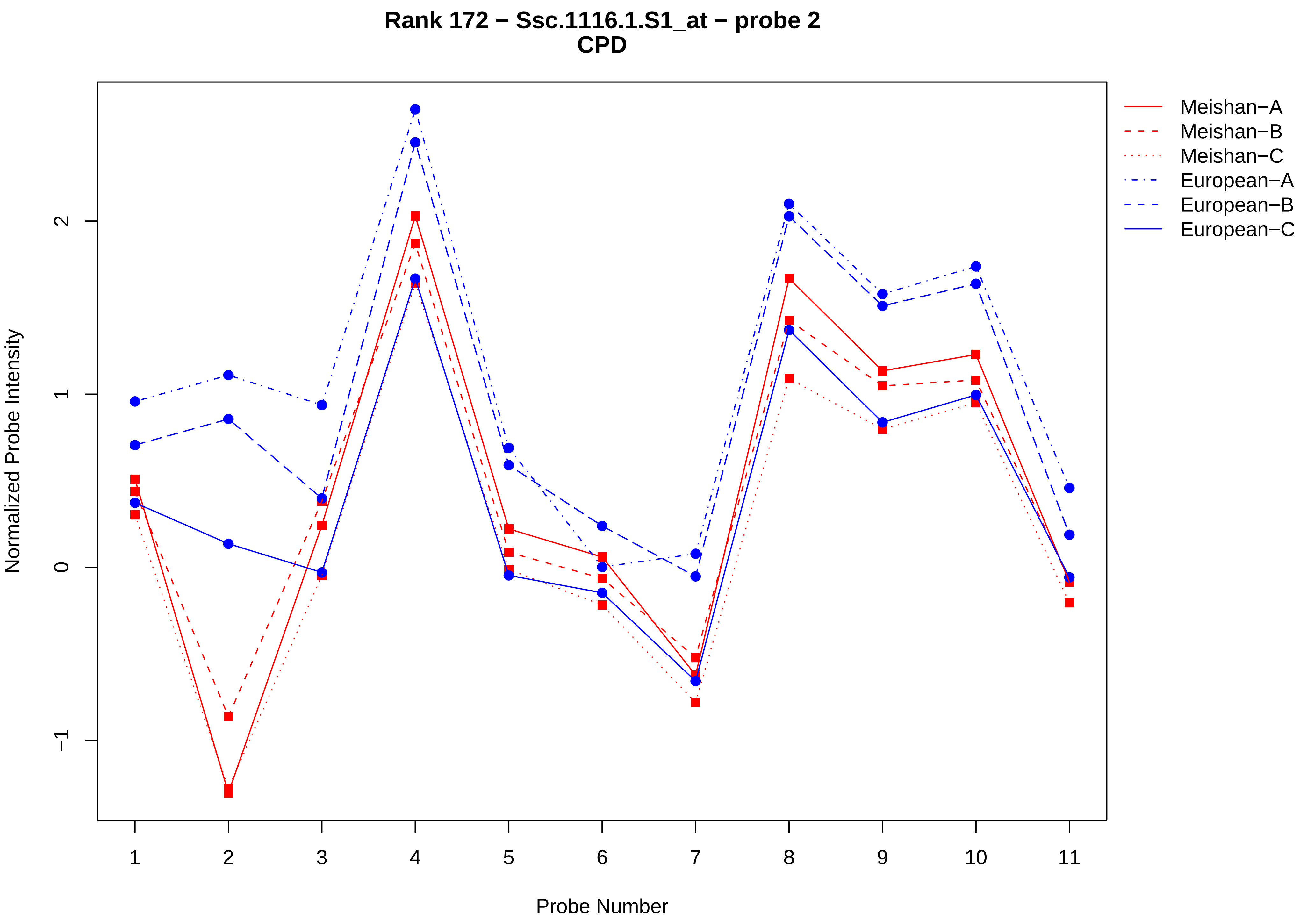

Rank 172 − Ssc.1116.1.S1_at − probe 2
CPD
Normalized Probe Intensity
Probe Number
−1
0
1
2
1
2
3
4
5
6
7
8
9
10
11
Meishan−A
Meishan−B
Meishan−C
European−A
European−B
European−C

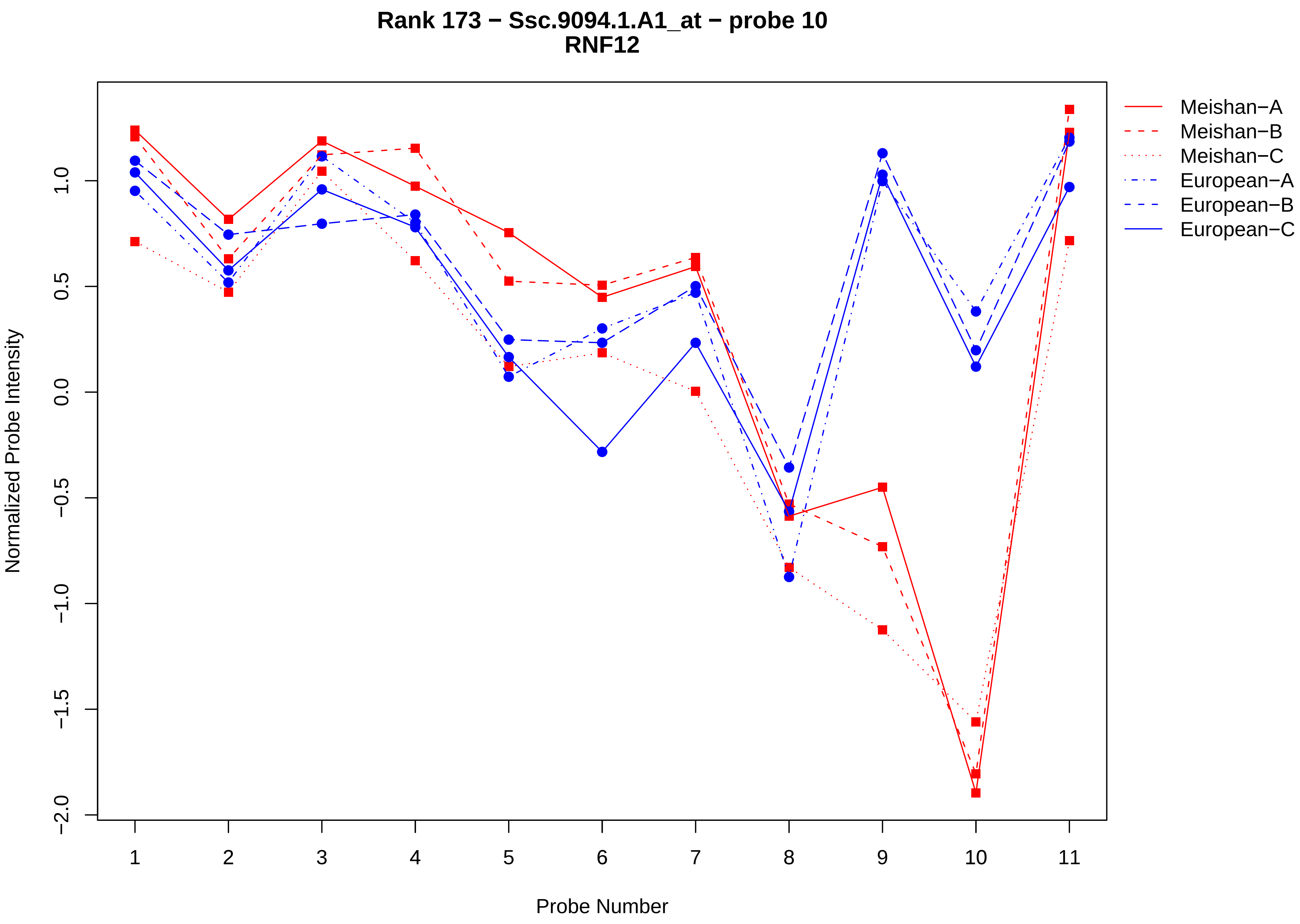

Rank 173 − Ssc.9094.1.A1_at − probe 10
RNF12
Normalized Probe Intensity
Probe Number
Meishan−A
Meishan−B
Meishan−C
European−A
European−B
European−C
1.0
0.5
0.0
−0.5
−1.0
−1.5
−2.0
1
2
3
4
5
6
7
8
9
10
11

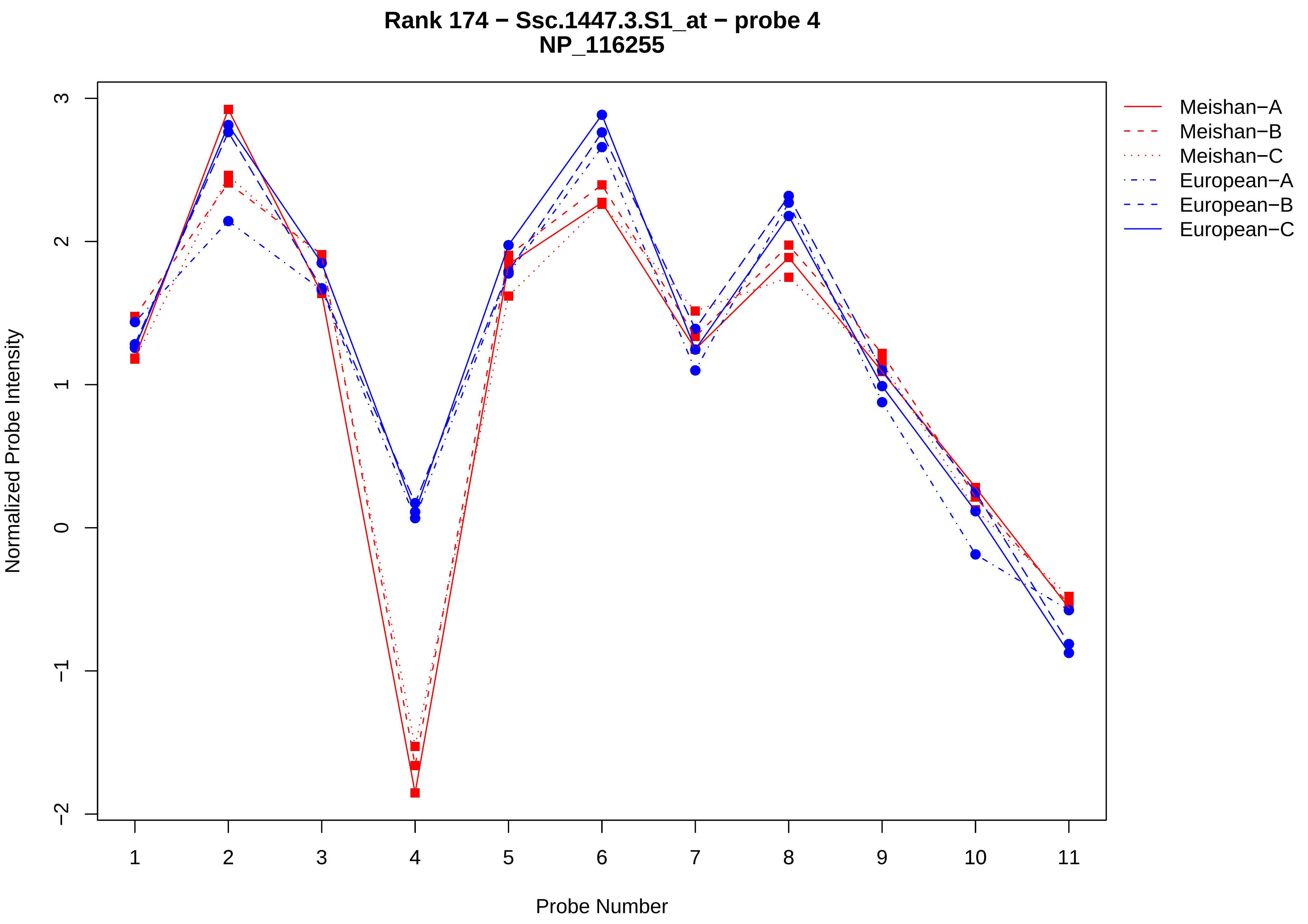

Rank 174 − Ssc.1447.3.S1_at − probe 4
NP_116255
Normalized Probe Intensity
Probe Number
−2
−1
0
1
2
3
1
2
3
4
5
6
7
8
9
10
11
Meishan−A
Meishan−B
Meishan−C
European−A
European−B
European−C

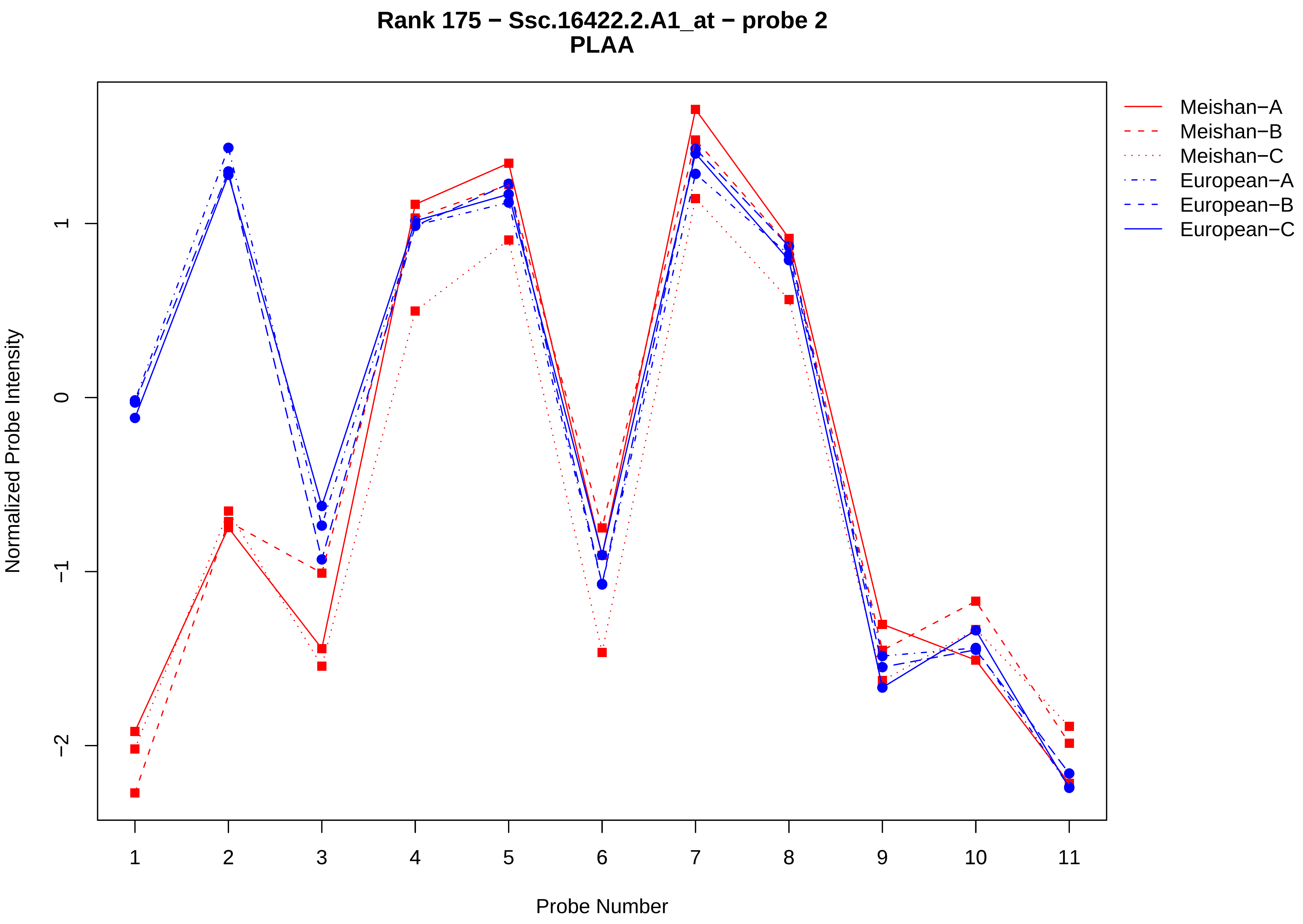

Rank 175 − Ssc.16422.2.A1_at − probe 2
PLAA
Normalized Probe Intensity
Probe Number
−2
−1
0
1
1
2
3
4
5
6
7
8
9
10
11
Meishan−A
Meishan−B
Meishan−C
European−A
European−B
European−C

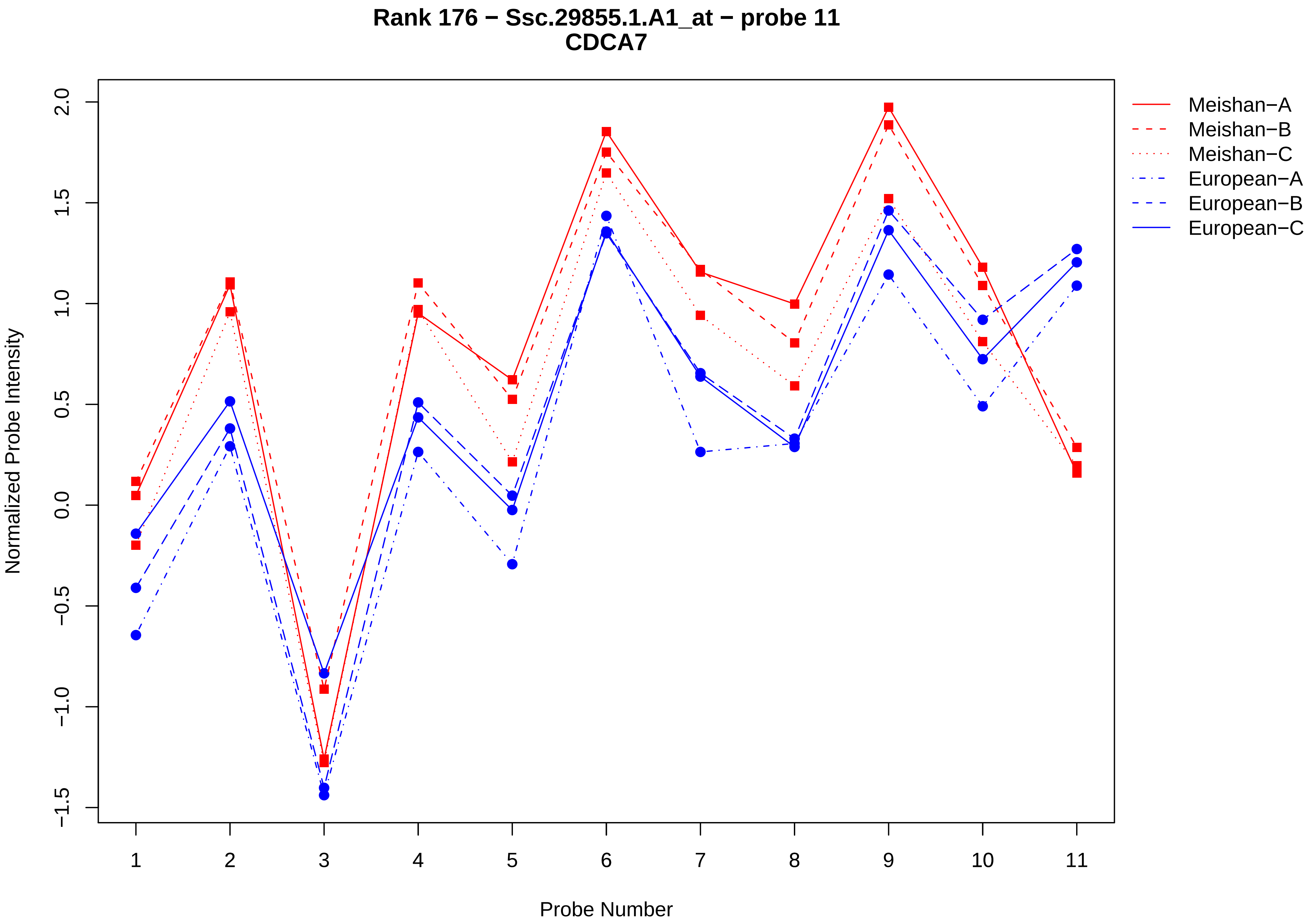
Rank 176 − Ssc.29855.1.A1_at − probe 11
CDCA7
Normalized Probe Intensity
Probe Number
Meishan−A
Meishan−B
Meishan−C
European−A
European−B
European−C
−1.5
−1.0
−0.5
0.0
0.5
1.0
1.5
2.0
1
2
3
4
5
6
7
8
9
10
11

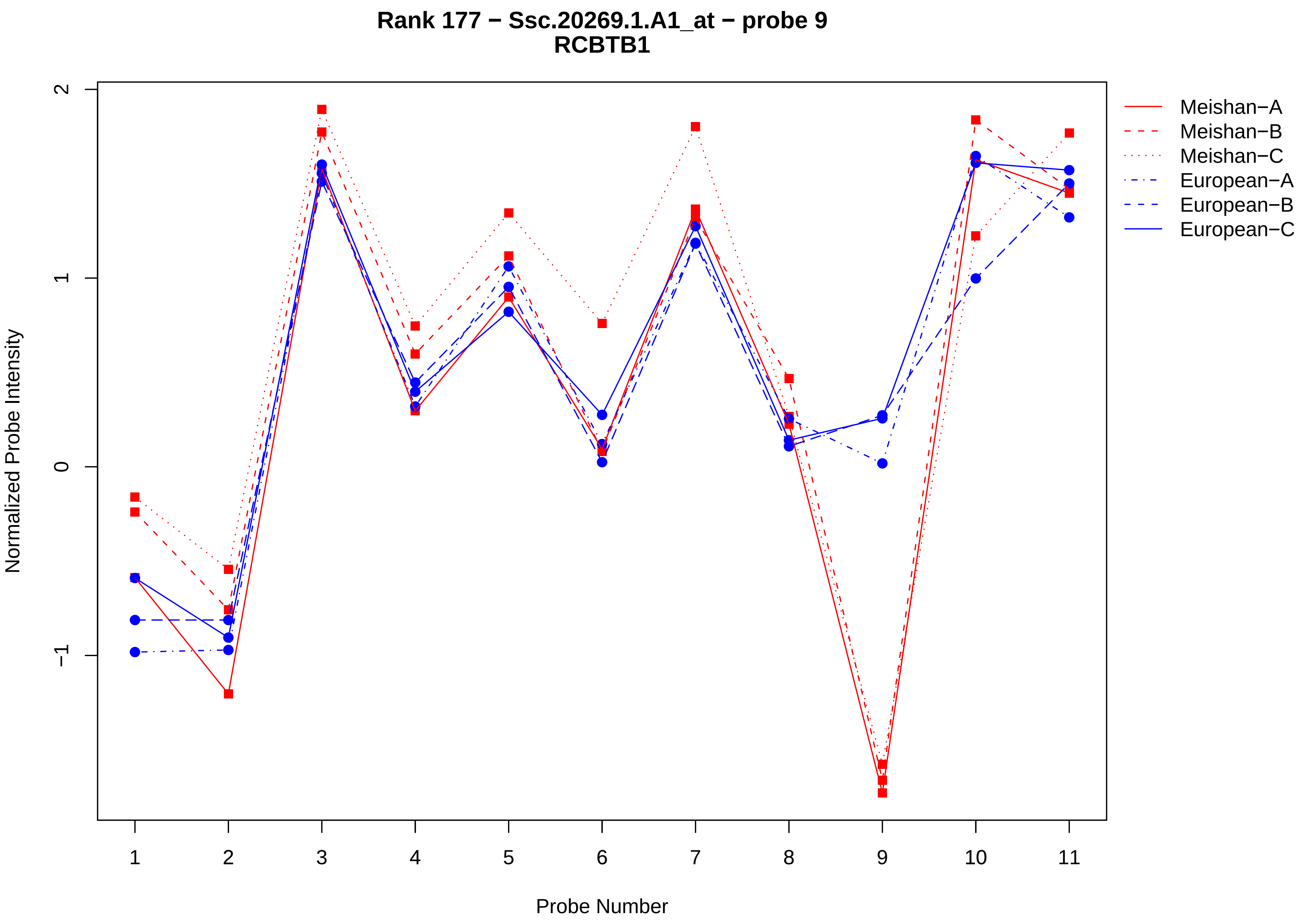

Rank 177 − Ssc.20269.1.A1_at − probe 9
RCBTB1
Normalized Probe Intensity
Probe Number
2
1
0
−1
1
2
3
4
5
6
7
8
9
10
11
Meishan−A
Meishan−B
Meishan−C
European−A
European−B
European−C

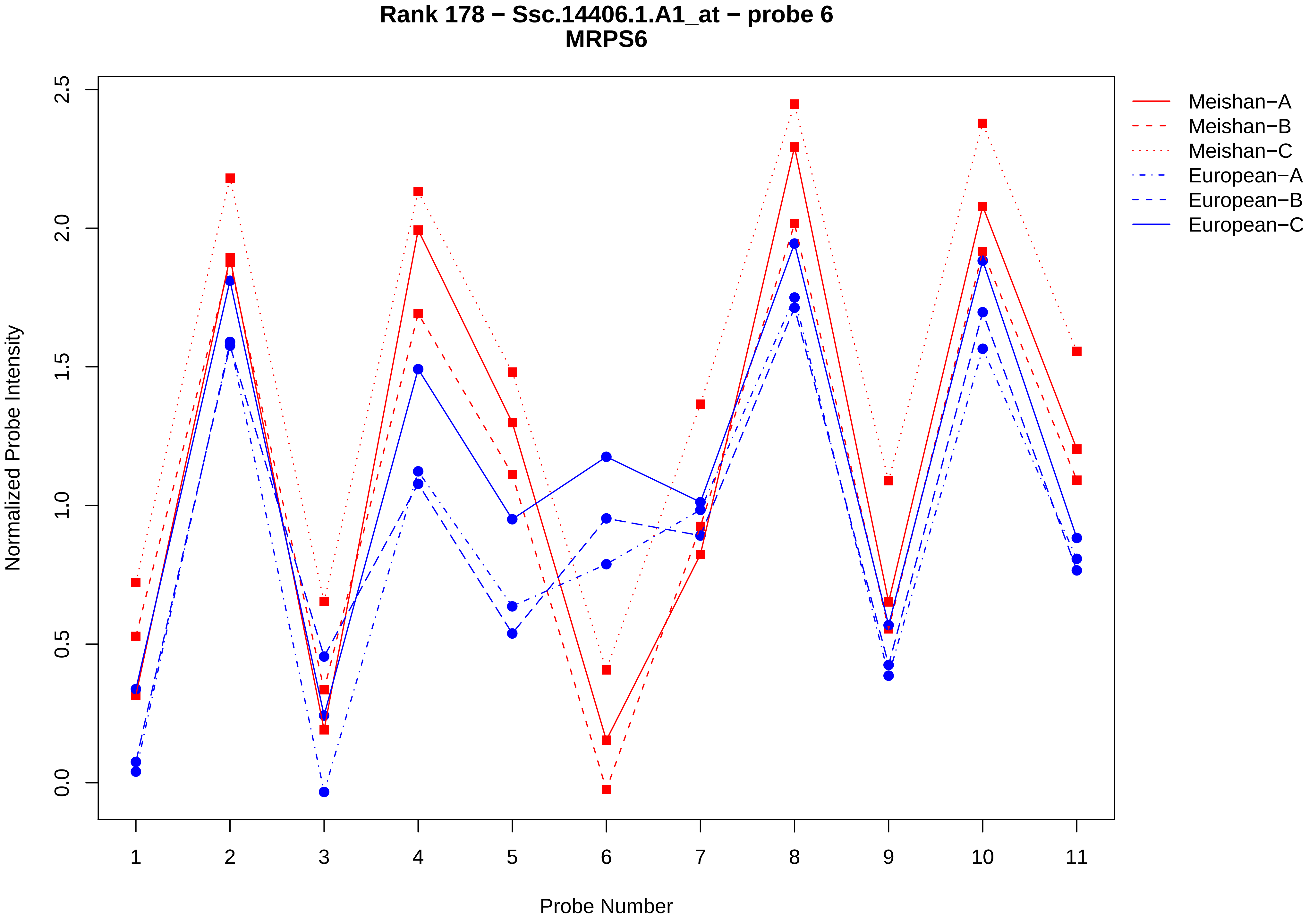

Rank 178 − Ssc.14406.1.A1_at − probe 6
MRPS6
Normalized Probe Intensity
Probe Number
0.0
0.5
1.0
1.5
2.0
2.5
1
2
3
4
5
6
7
8
9
10
11
Meishan−A
Meishan−B
Meishan−C
European−A
European−B
European−C

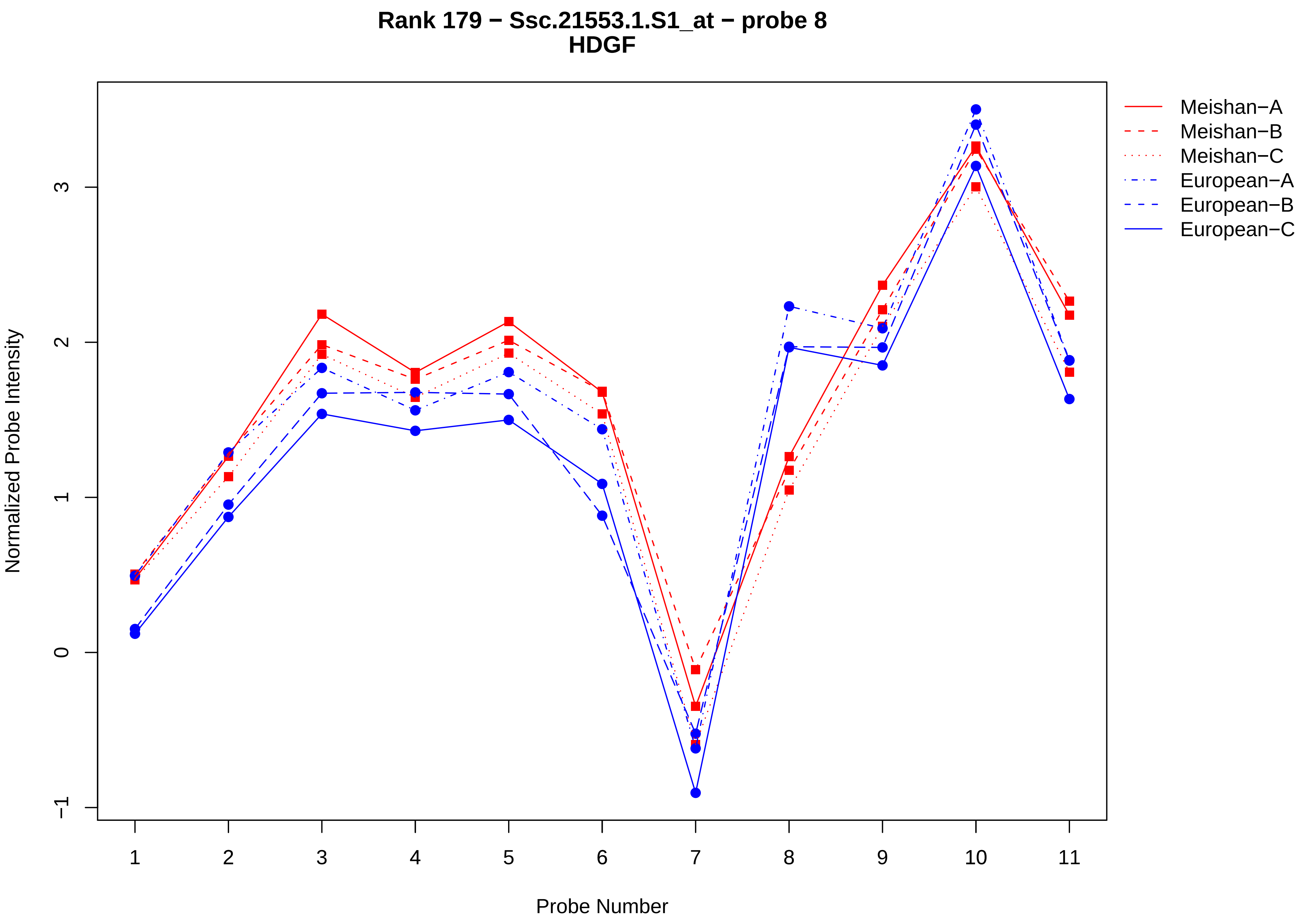

Rank 179 − Ssc.21553.1.S1_at − probe 8
HDGF
Normalized Probe Intensity
Probe Number
−1
0
1
2
3
1
2
3
4
5
6
7
8
9
10
11
Meishan−A
Meishan−B
Meishan−C
European−A
European−B
European−C

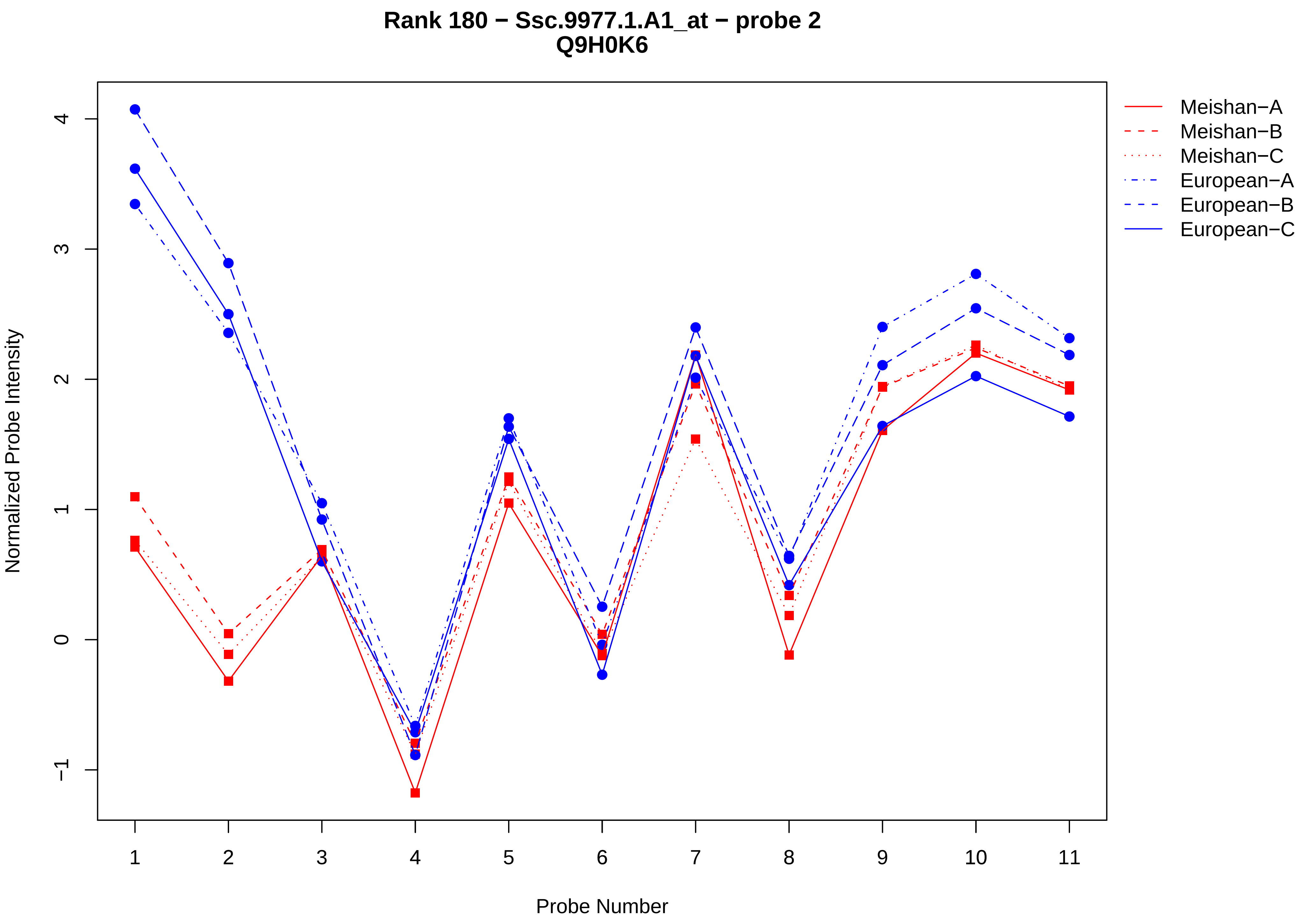

Rank 180 − Ssc.9977.1.A1_at − probe 2
Q9H0K6
Normalized Probe Intensity
Probe Number
Meishan−A
Meishan−B
Meishan−C
European−A
European−B
European−C

Rank 181 − Ssc.29318.1.A1_at − probe 6
MTMR1

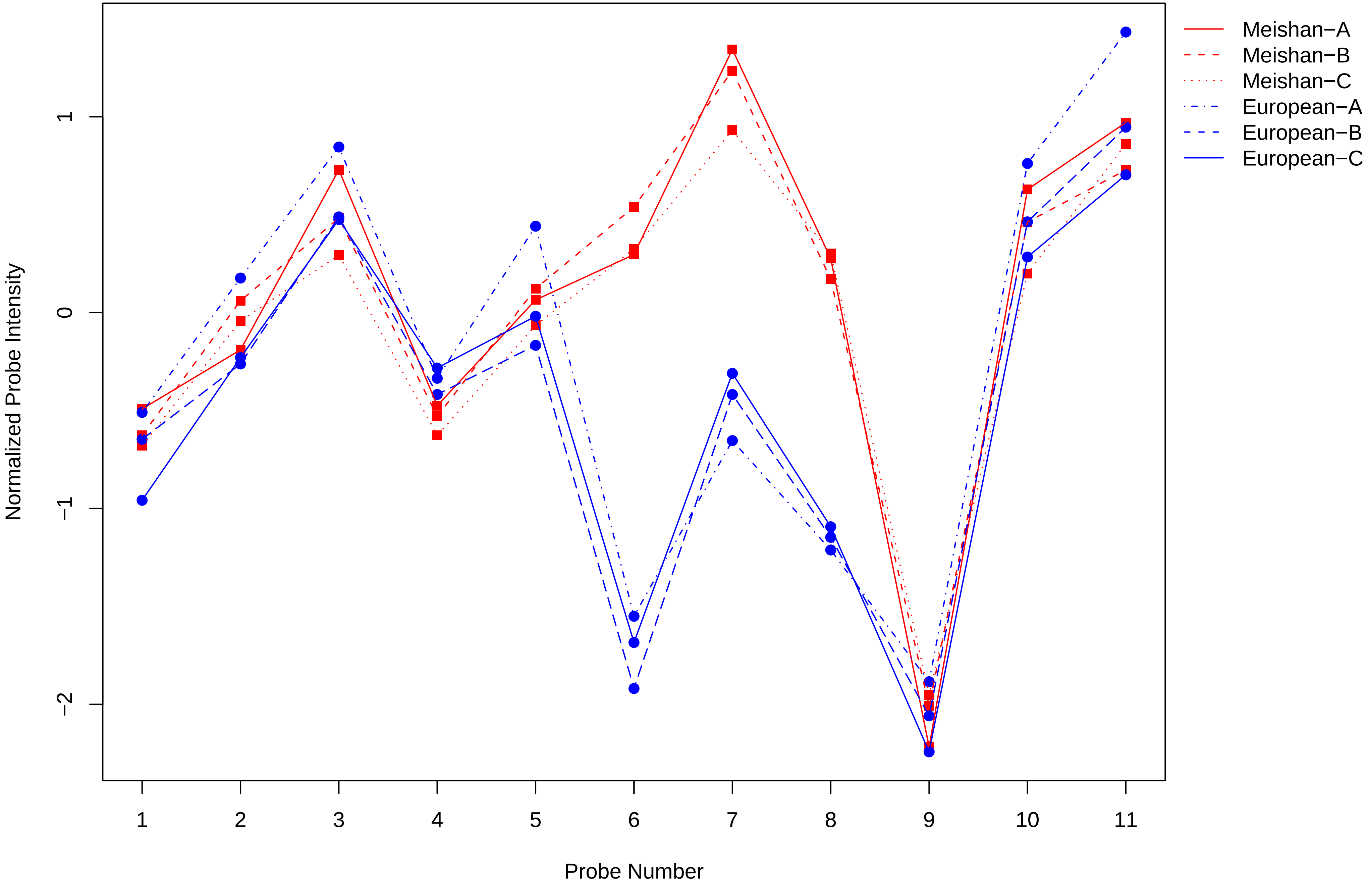

Rank 182 − Ssc.8000.1.A1_at − probe 8
OGT

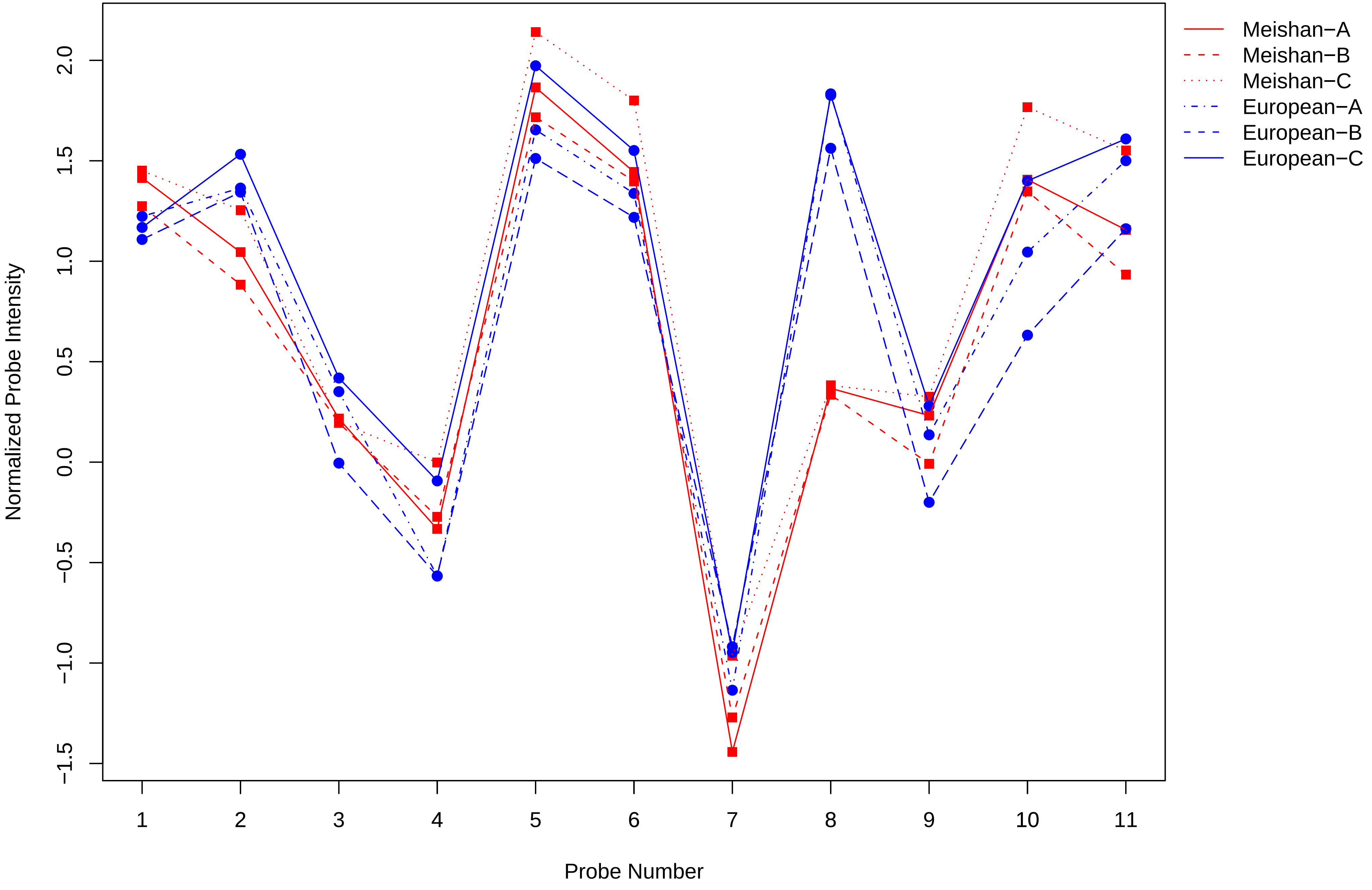

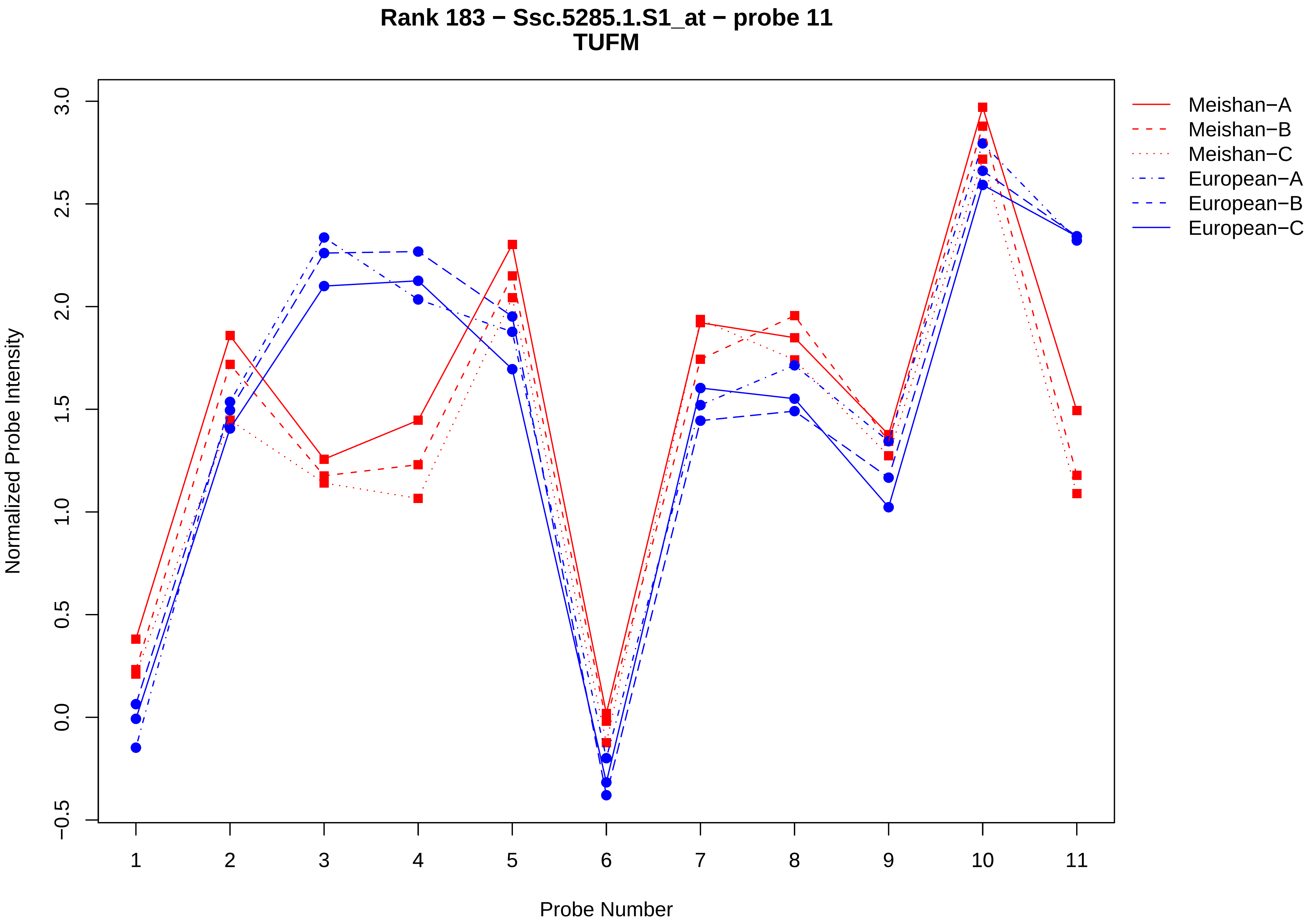

Rank 183 − Ssc.5285.1.S1_at − probe 11
TUFM
Normalized Probe Intensity
Probe Number
−0.5
0.0
0.5
1.0
1.5
2.0
2.5
3.0
1
2
3
4
5
6
7
8
9
10
11
Meishan−A
Meishan−B
Meishan−C
European−A
European−B
European−C

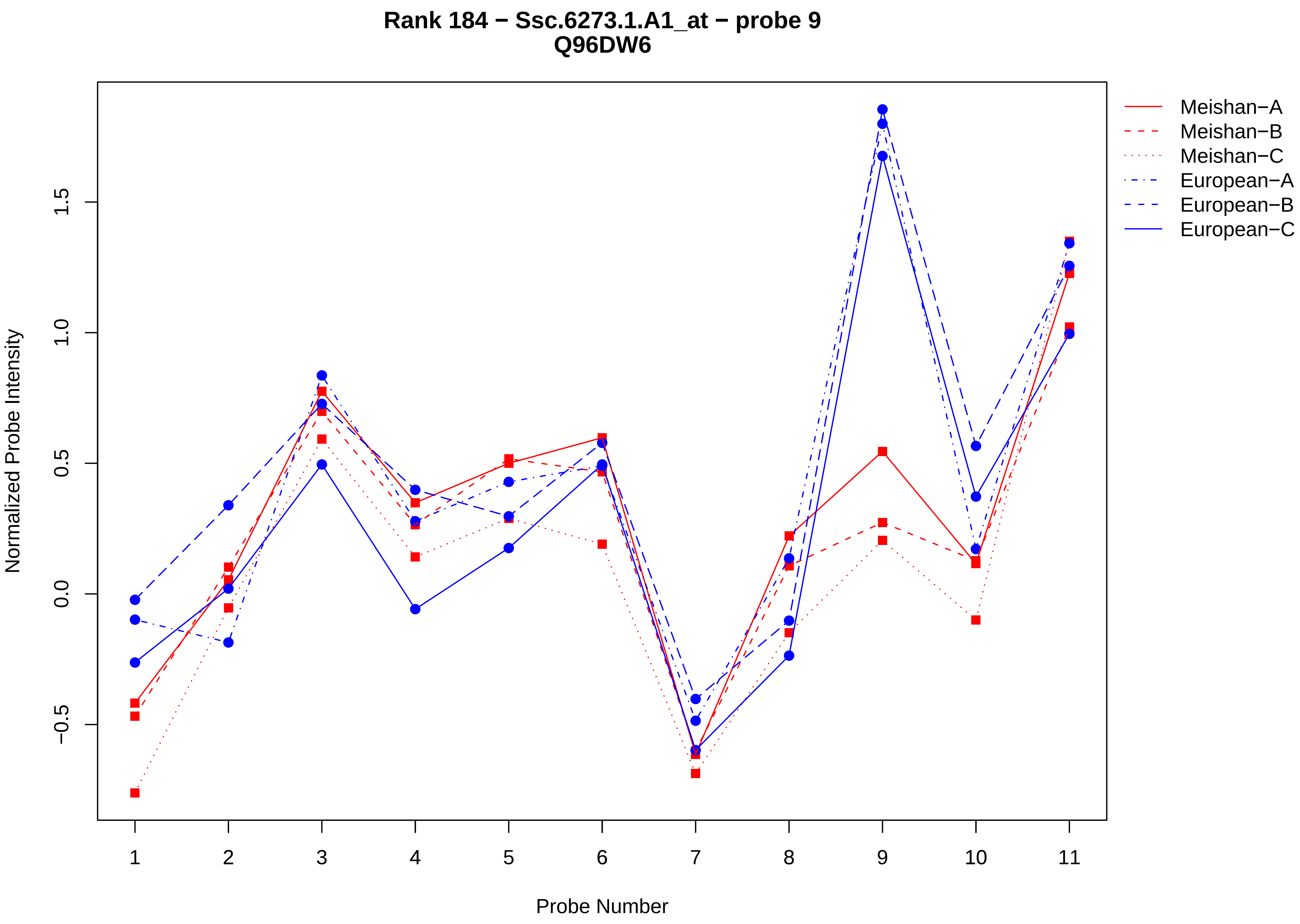

Rank 184 − Ssc.6273.1.A1_at − probe 9
Q96DW6
Normalized Probe Intensity
−0.5
0.0
0.5
1.0
1.5
1
2
3
4
5
6
7
8
9
10
11
Probe Number
Meishan−A
Meishan−B
Meishan−C
European−A
European−B
European−C

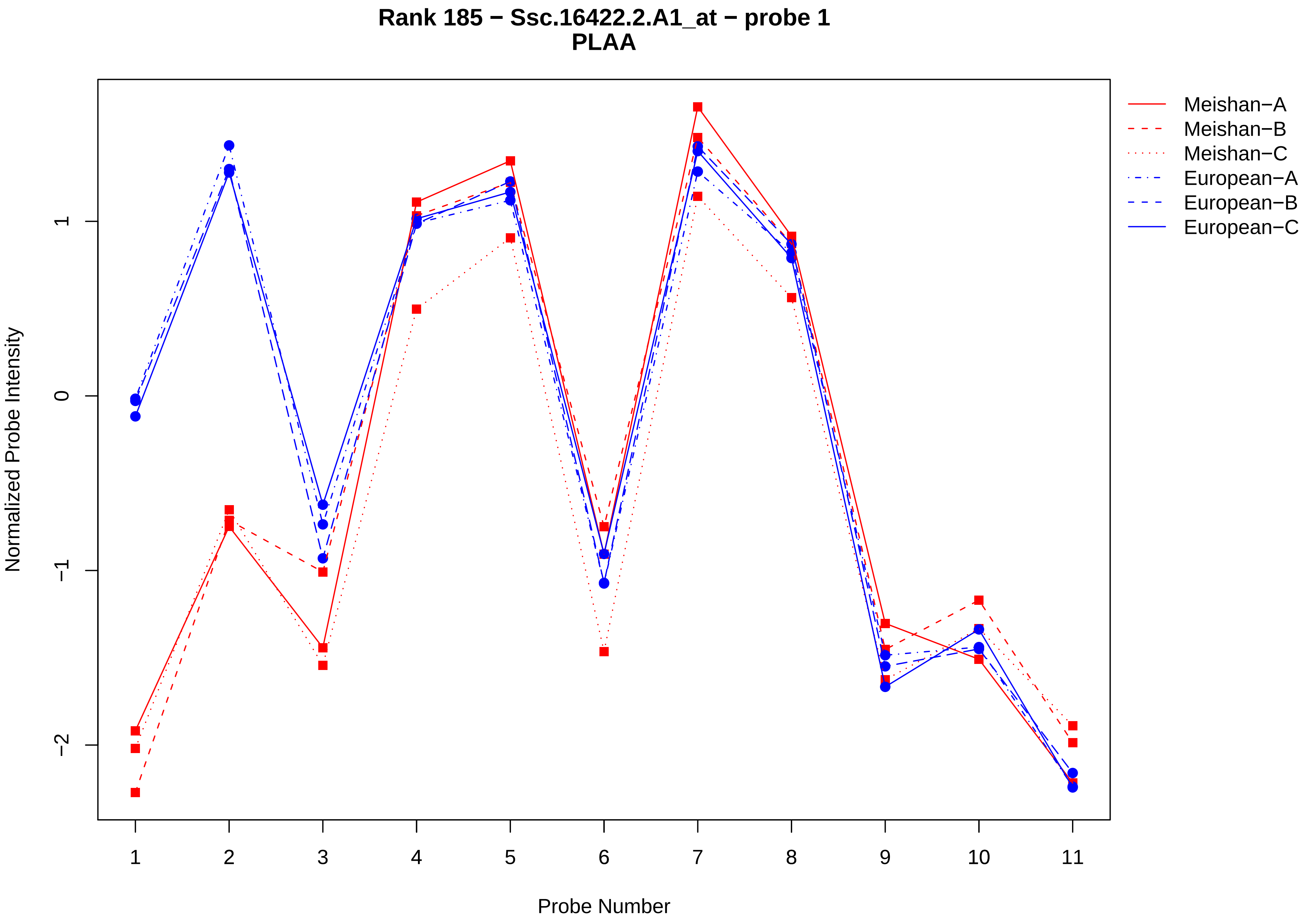

Rank 185 − Ssc.16422.2.A1_at − probe 1
PLAA
Normalized Probe Intensity
Probe Number
Meishan−A
Meishan−B
Meishan−C
European−A
European−B
European−C
−2
−1
0
1
1
2
3
4
5
6
7
8
9
10
11

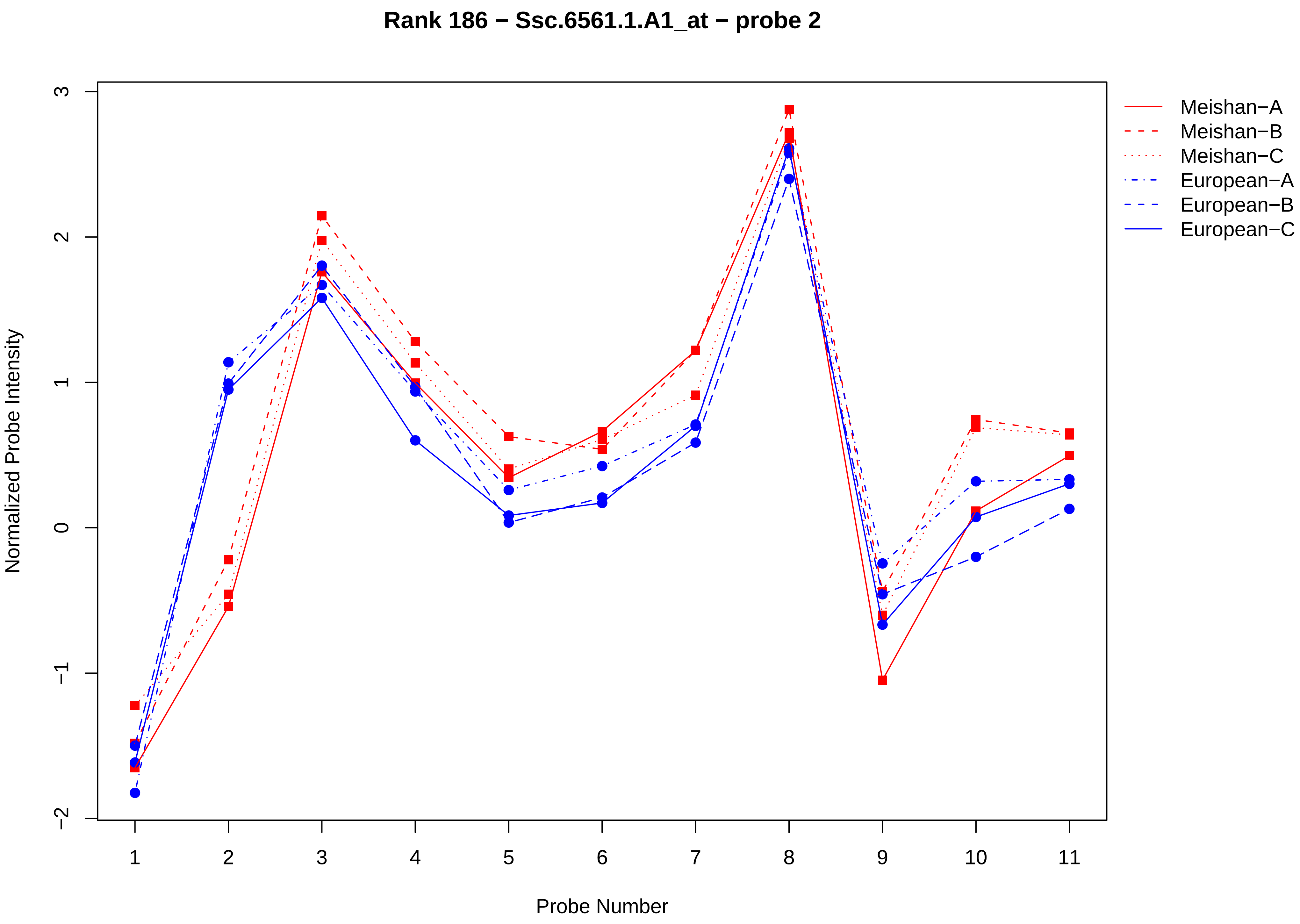

Rank 186 − Ssc.6561.1.A1_at − probe 2
Normalized Probe Intensity
Probe Number
3
2
1
0
−1
−2
1
2
3
4
5
6
7
8
9
10
11
Meishan−A
Meishan−B
Meishan−C
European−A
European−B
European−C

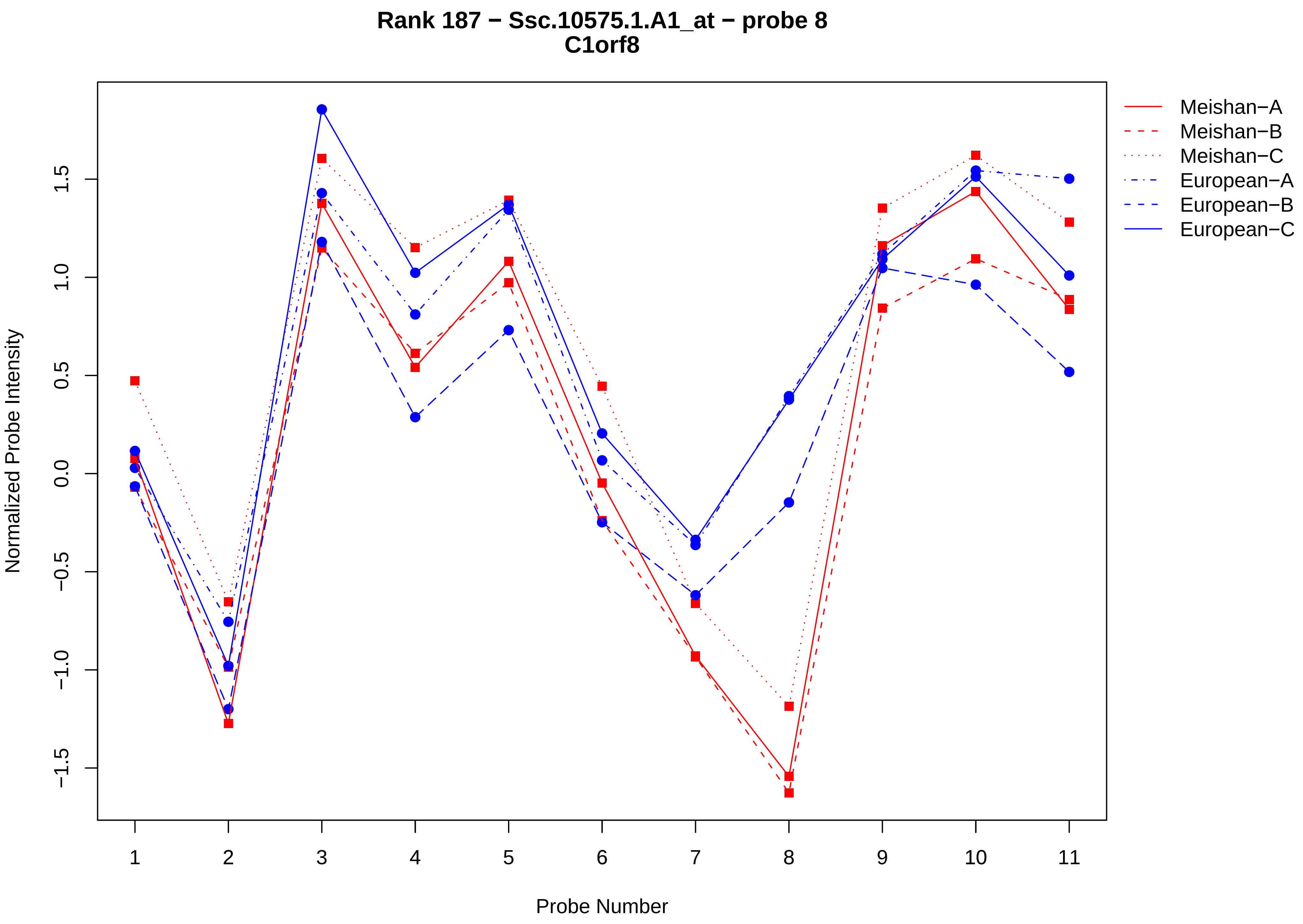
Rank 187 − Ssc.10575.1.A1_at − probe 8
C1orf8
Normalized Probe Intensity
Probe Number
−1.5
−1.0
−0.5
0.0
0.5
1.0
1.5
1
2
3
4
5
6
7
8
9
10
11
Meishan−A
Meishan−B
Meishan−C
European−A
European−B
European−C

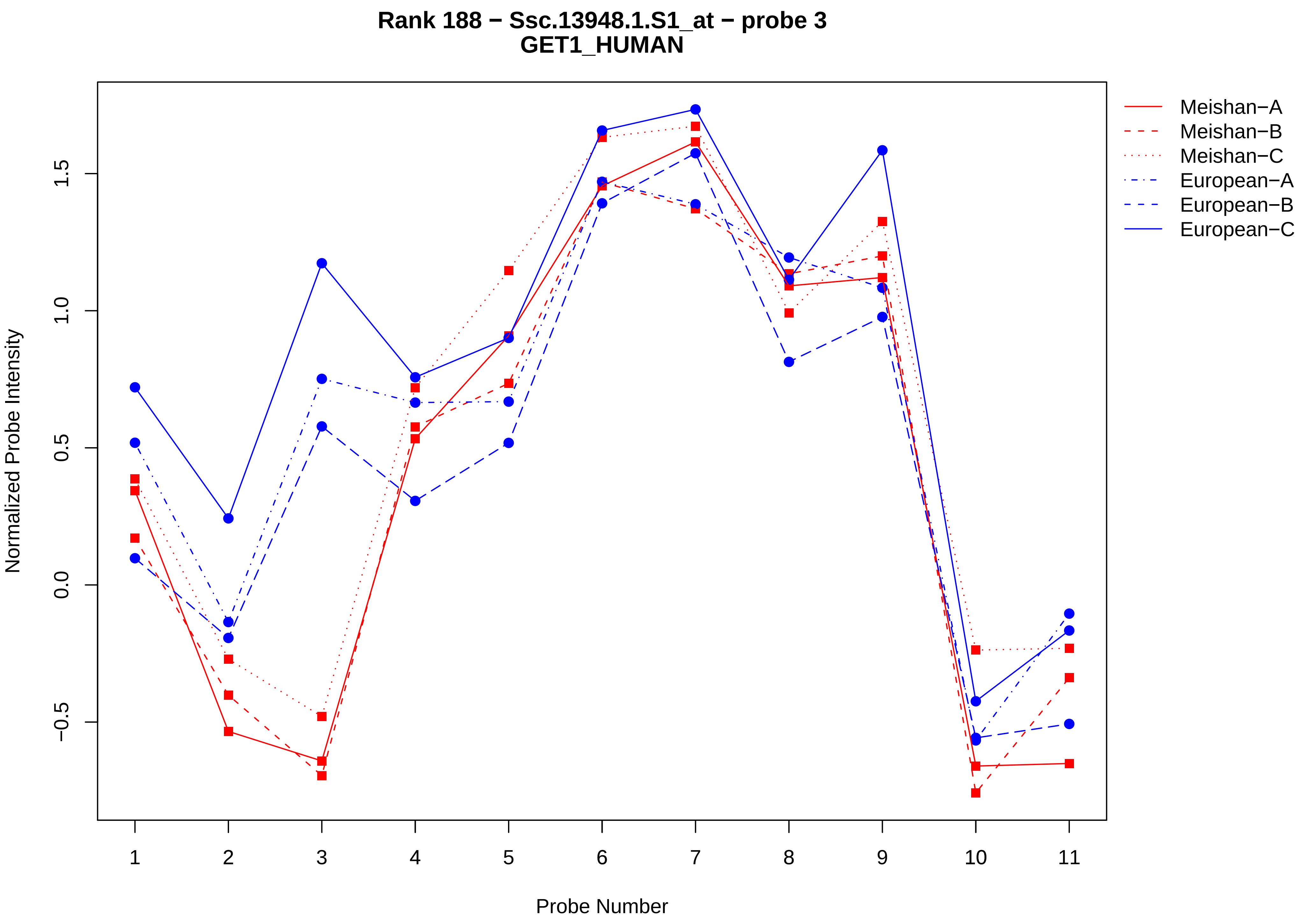
Rank 188 − Ssc.13948.1.S1_at − probe 3
GET1_HUMAN
Normalized Probe Intensity
Probe Number
−0.5
0.0
0.5
1.0
1.5
1
2
3
4
5
6
7
8
9
10
11
Meishan−A
Meishan−B
Meishan−C
European−A
European−B
European−C

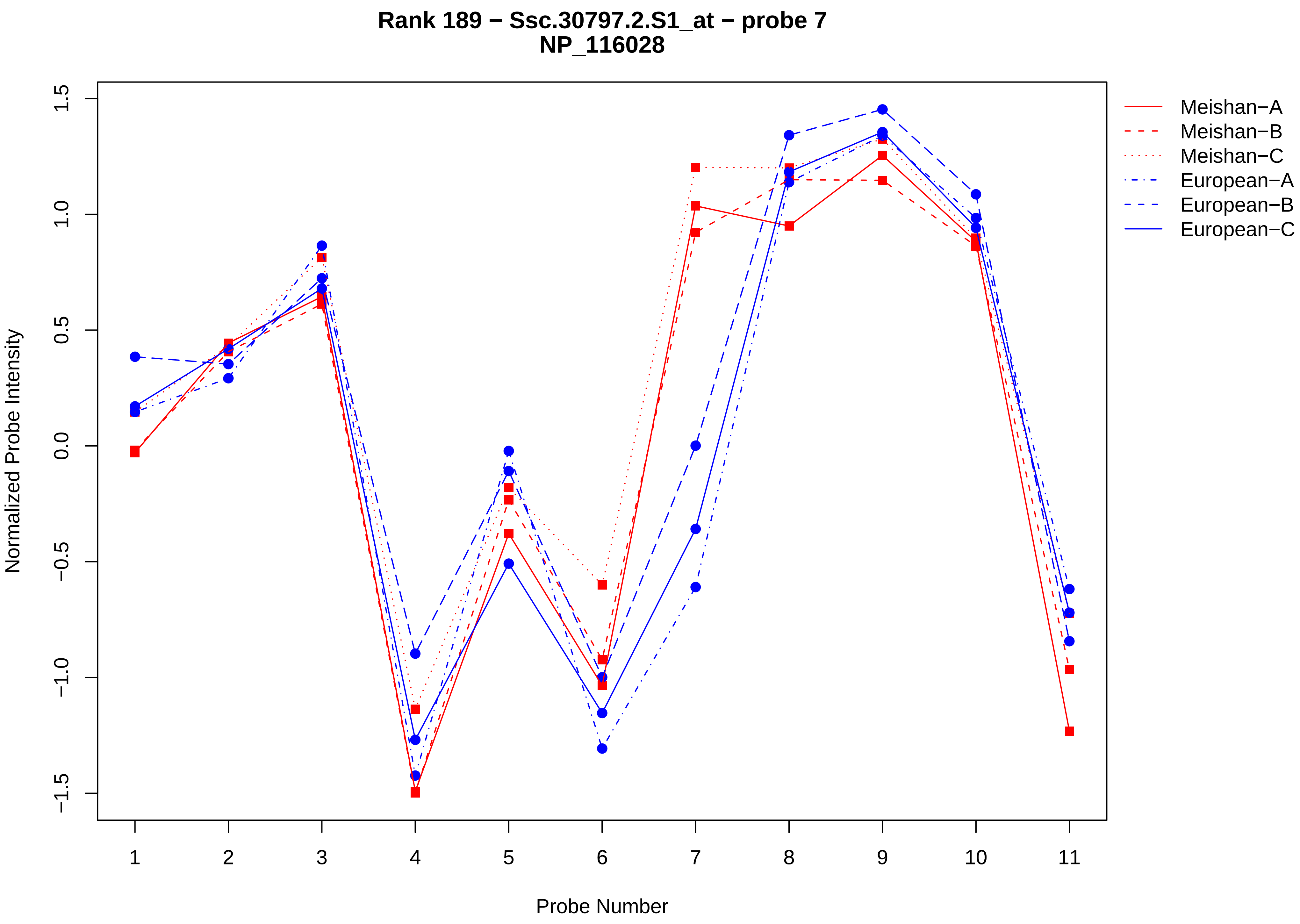
Rank 189 − Ssc.30797.2.S1_at − probe 7
NP_116028
Normalized Probe Intensity
Probe Number
1.5
1.0
0.5
0.0
−0.5
−1.0
−1.5
1
2
3
4
5
6
7
8
9
10
11
Meishan−A
Meishan−B
Meishan−C
European−A
European−B
European−C

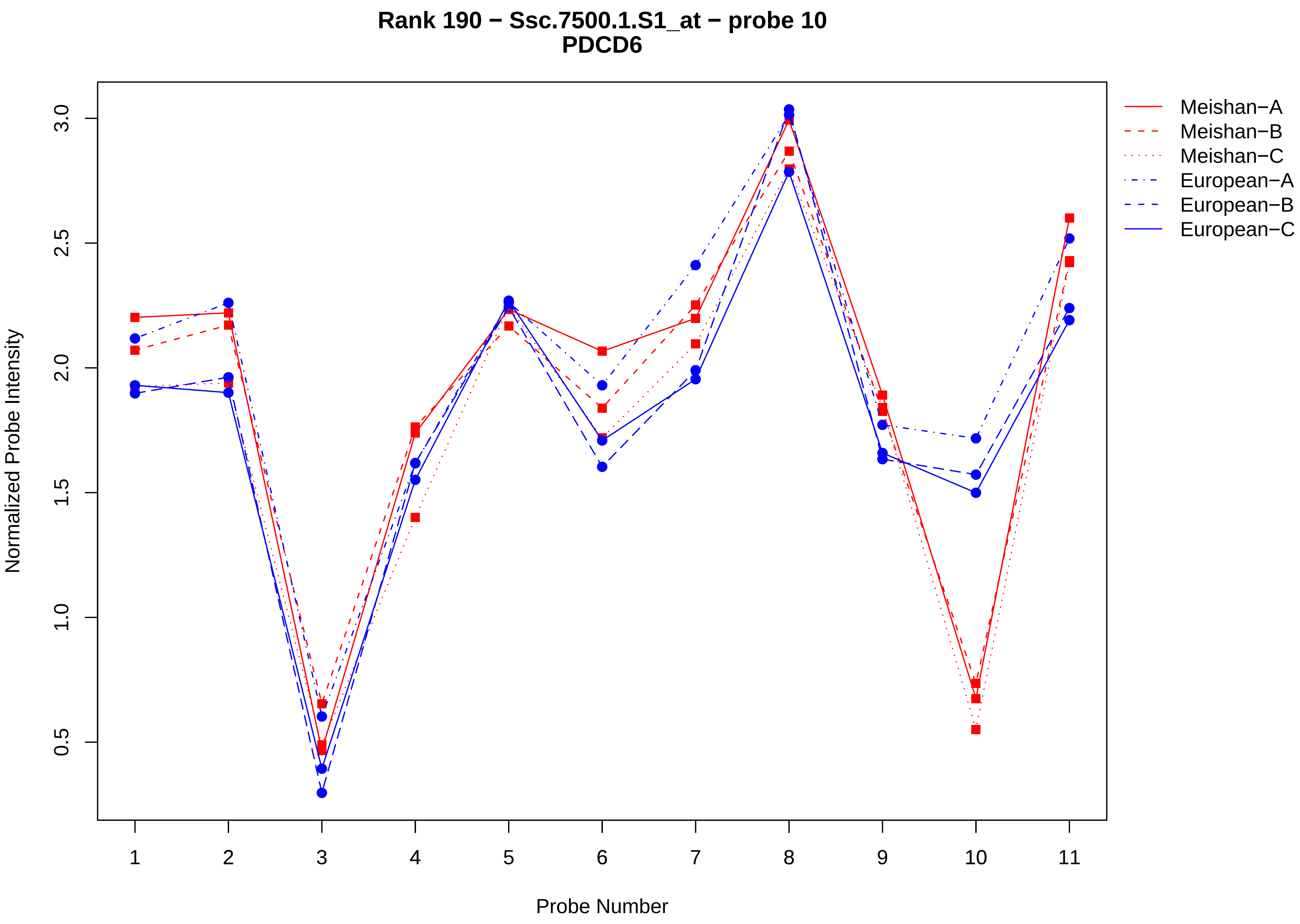

Rank 190 − Ssc.7500.1.S1_at − probe 10
PDCD6
Normalized Probe Intensity
0.5
1.0
1.5
2.0
2.5
3.0
Probe Number
1
2
3
4
5
6
7
8
9
10
11
Meishan−A
Meishan−B
Meishan−C
European−A
European−B
European−C

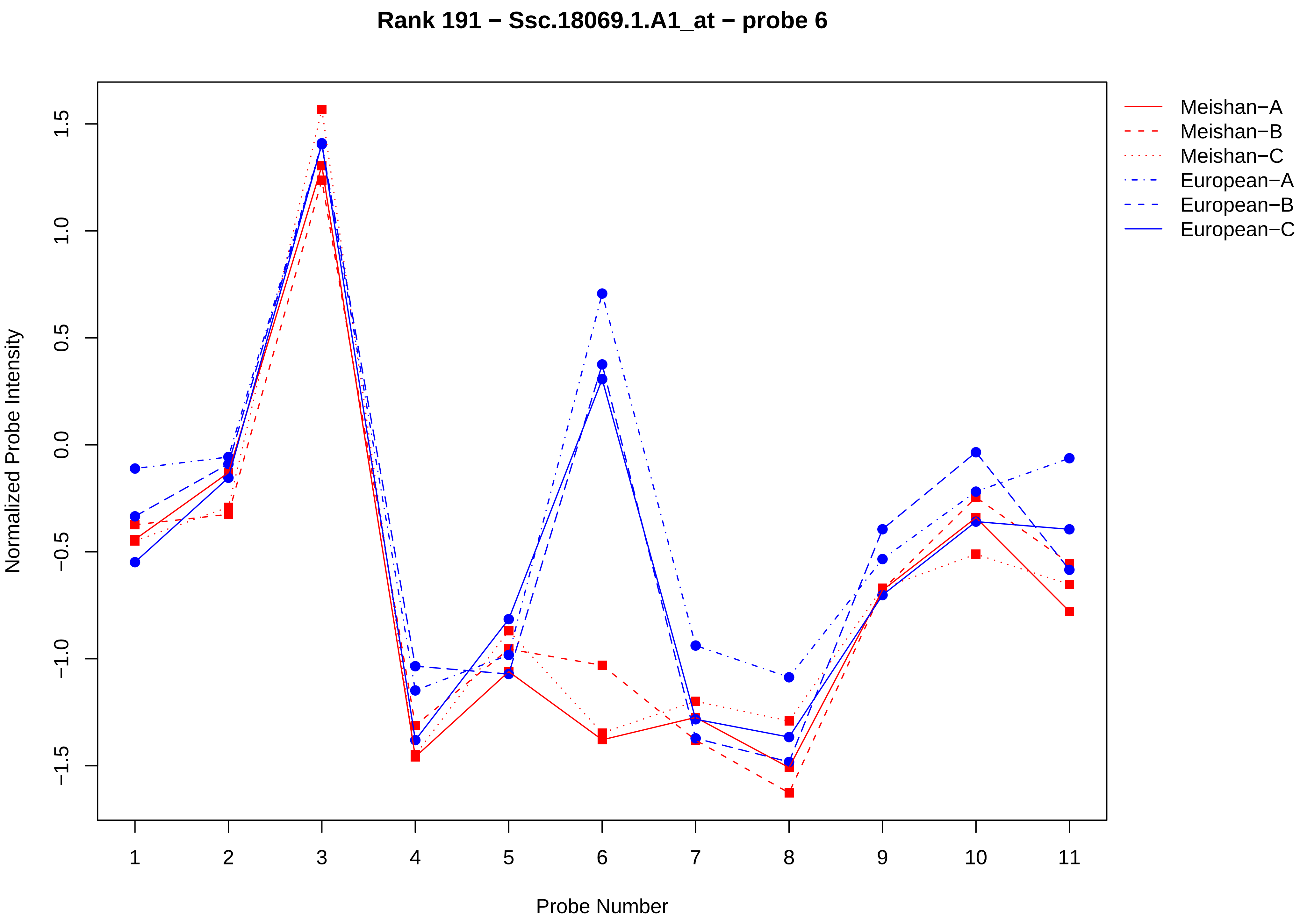

Rank 191 − Ssc.18069.1.A1_at − probe 6
Normalized Probe Intensity
−1.5
−1.0
−0.5
0.0
0.5
1.0
1.5
1
2
3
4
5
6
7
8
9
10
11
Probe Number
Meishan−A
Meishan−B
Meishan−C
European−A
European−B
European−C

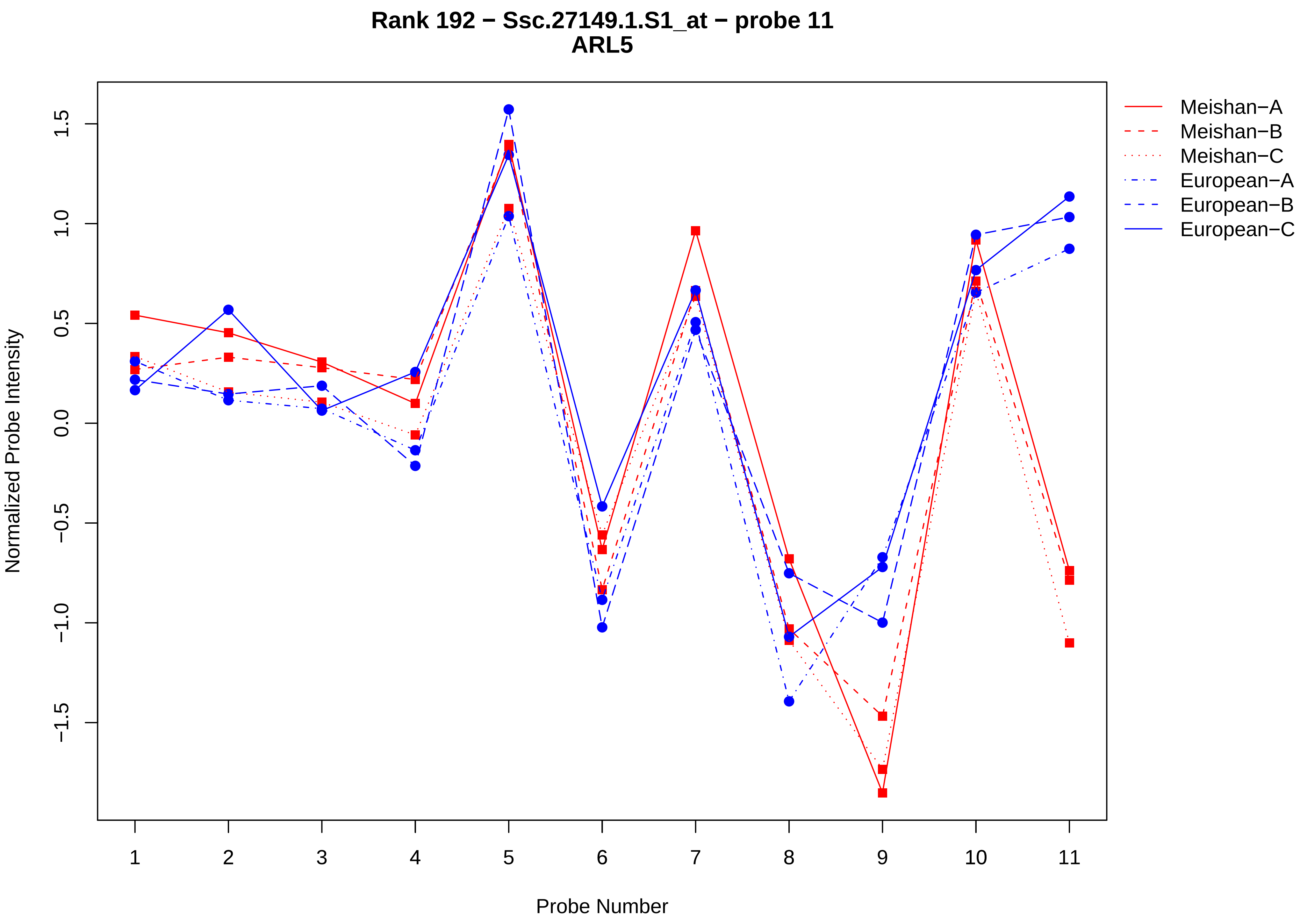

Rank 192 − Ssc.27149.1.S1_at − probe 11
ARL5
Normalized Probe Intensity
Probe Number
Meishan−A
Meishan−B
Meishan−C
European−A
European−B
European−C

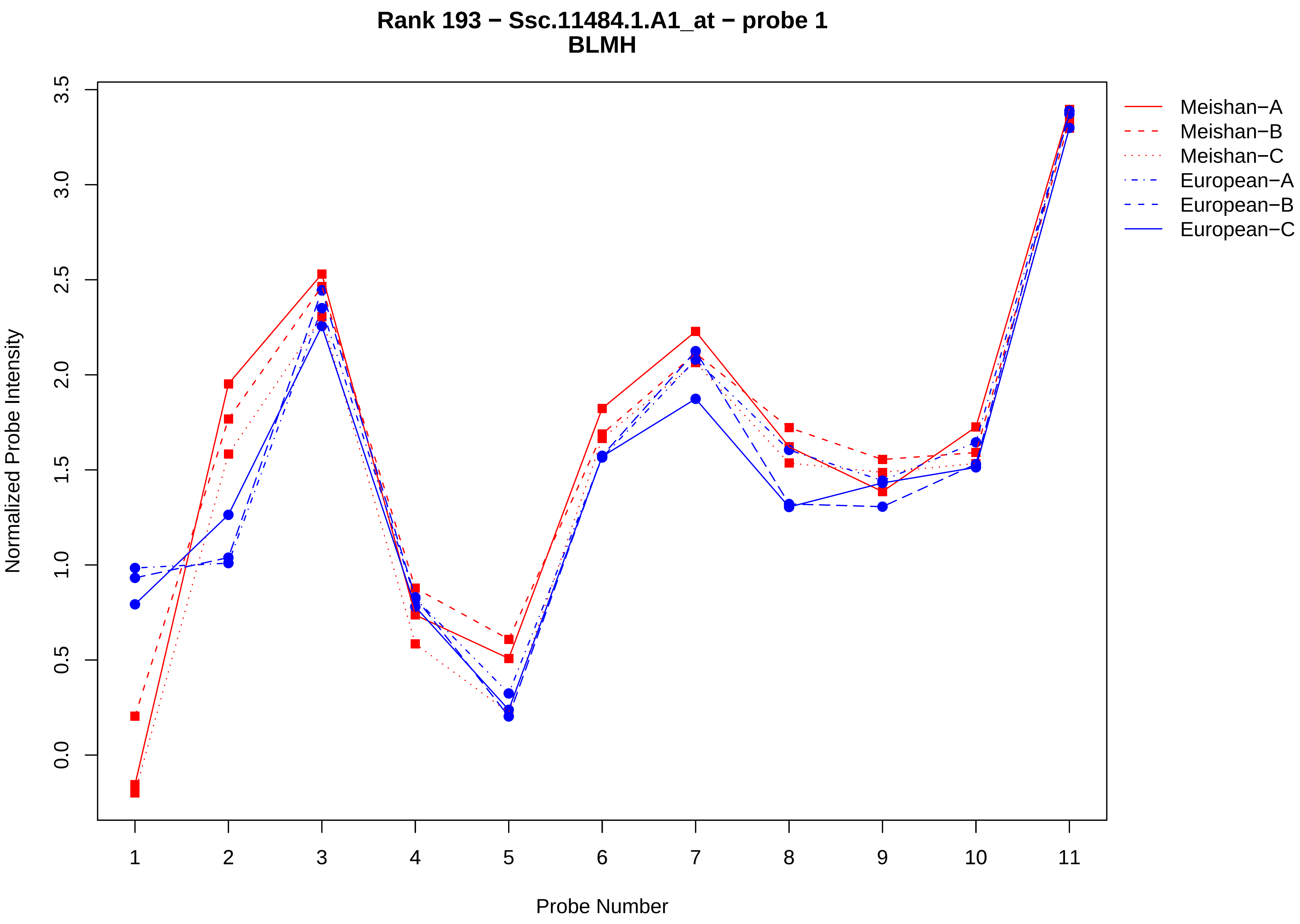
Rank 193 − Ssc.11484.1.A1_at − probe 1
BLMH
Normalized Probe Intensity
0.0
0.5
1.0
1.5
2.0
2.5
3.0
3.5
Probe Number
1
2
3
4
5
6
7
8
9
10
11
Meishan−A
Meishan−B
Meishan−C
European−A
European−B
European−C

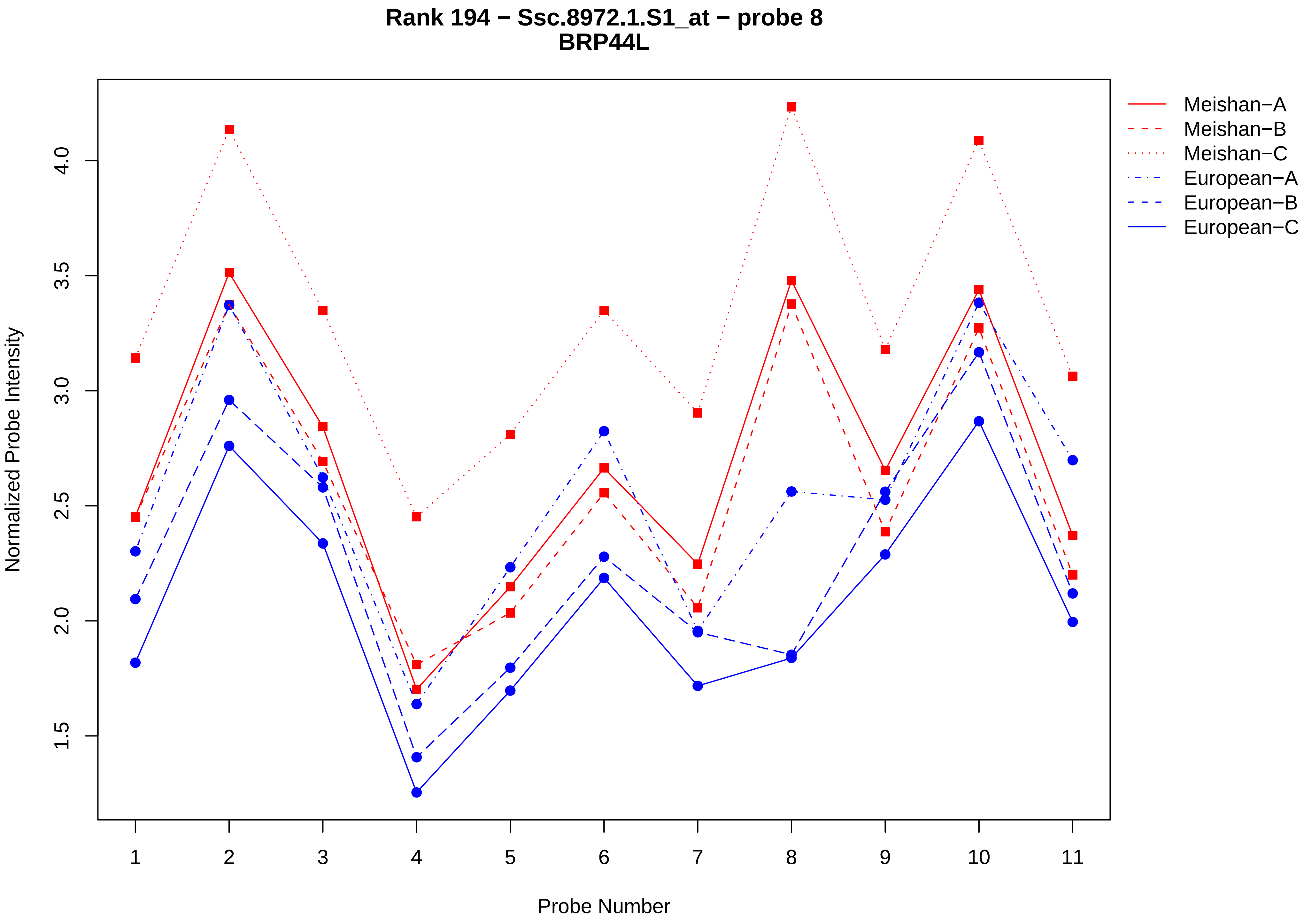

Rank 194 − Ssc.8972.1.S1_at − probe 8
BRP44L
Normalized Probe Intensity
Probe Number
1.5
2.0
2.5
3.0
3.5
4.0
1
2
3
4
5
6
7
8
9
10
11
Meishan−A
Meishan−B
Meishan−C
European−A
European−B
European−C

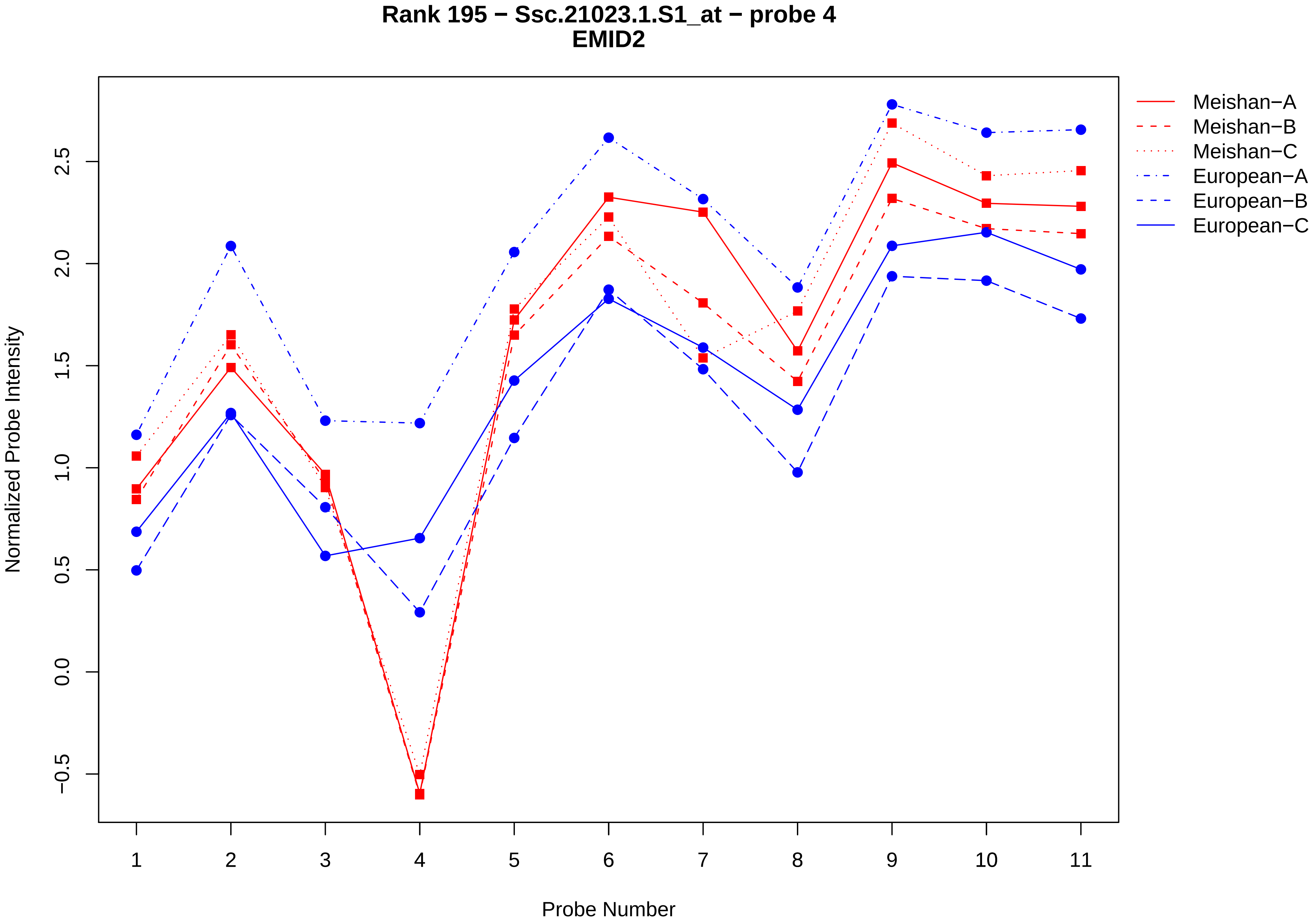

Rank 195 − Ssc.21023.1.S1_at − probe 4
EMID2
Normalized Probe Intensity
Probe Number
−0.5
0.0
0.5
1.0
1.5
2.0
2.5
1
2
3
4
5
6
7
8
9
10
11
Meishan−A
Meishan−B
Meishan−C
European−A
European−B
European−C

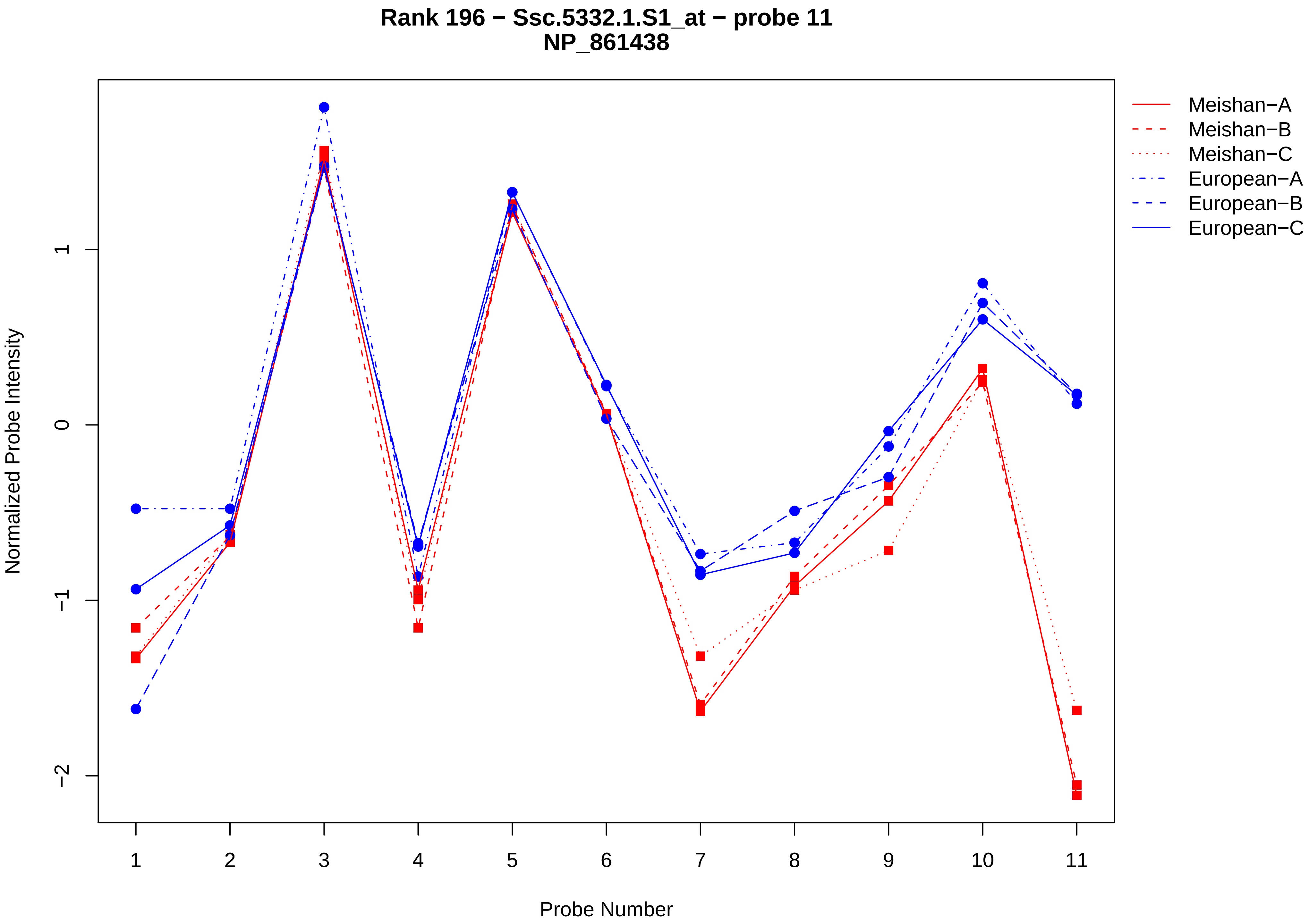

Rank 196 − Ssc.5332.1.S1_at − probe 11
NP_861438
Normalized Probe Intensity
Probe Number
−2
−1
0
1
1
2
3
4
5
6
7
8
9
10
11
Meishan−A
Meishan−B
Meishan−C
European−A
European−B
European−C

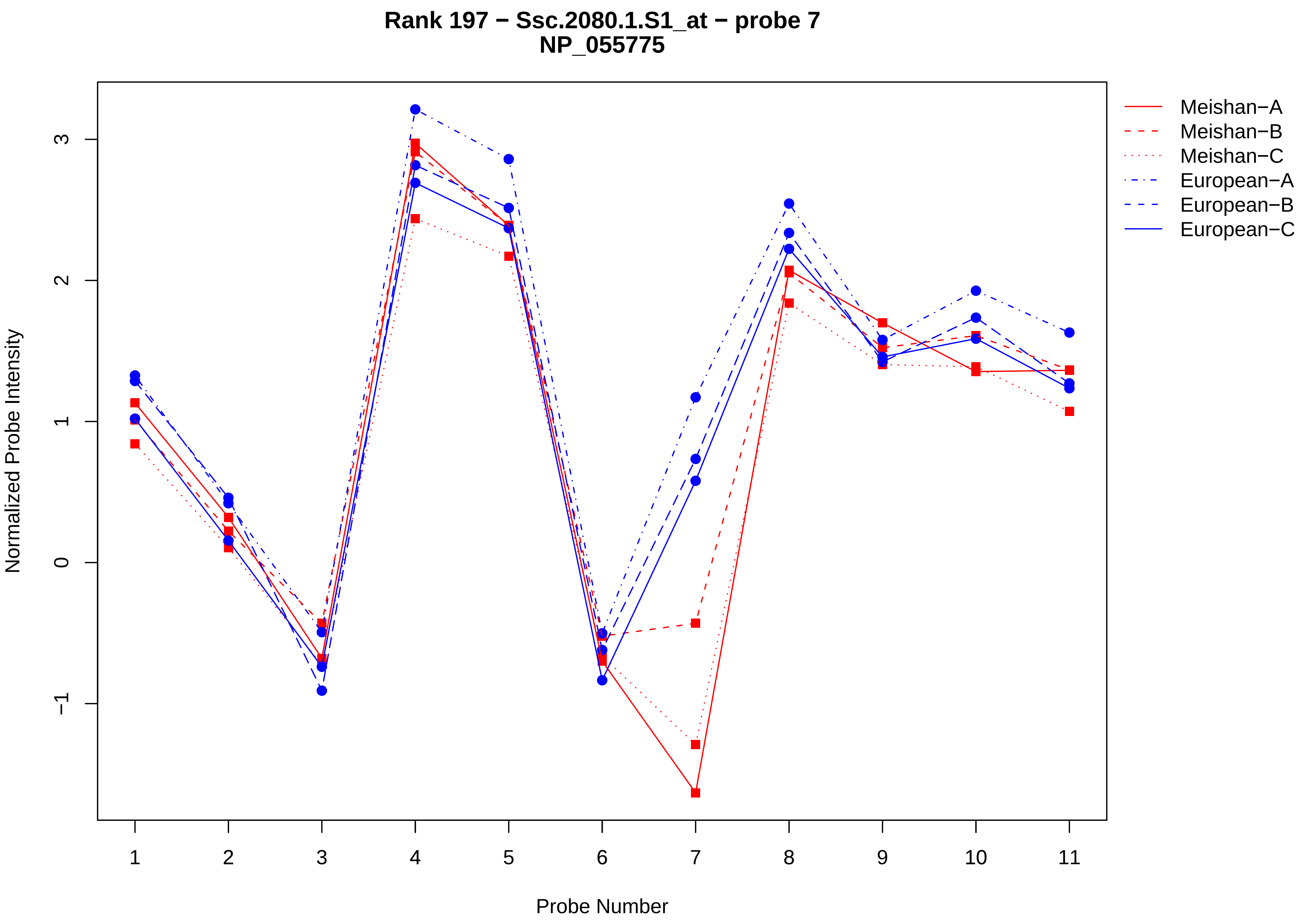
Rank 197 − Ssc.2080.1.S1_at − probe 7
NP_055775
Normalized Probe Intensity
Probe Number
−1
0
1
2
3
1
2
3
4
5
6
7
8
9
10
11
Meishan−A
Meishan−B
Meishan−C
European−A
European−B
European−C

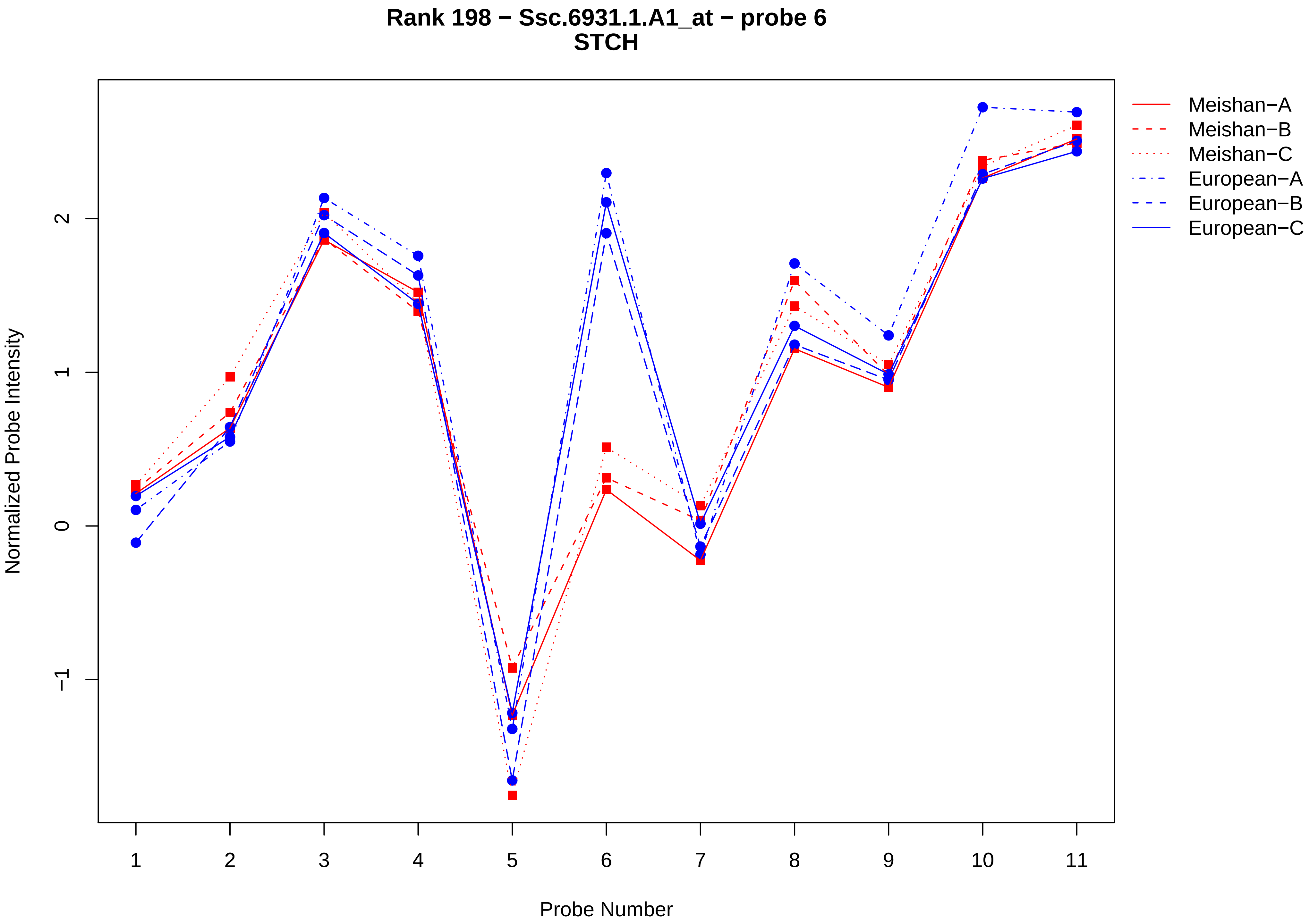

Rank 198 − Ssc.6931.1.A1_at − probe 6
STCH
Normalized Probe Intensity
Probe Number
−1
0
1
2
1
2
3
4
5
6
7
8
9
10
11
Meishan−A
Meishan−B
Meishan−C
European−A
European−B
European−C

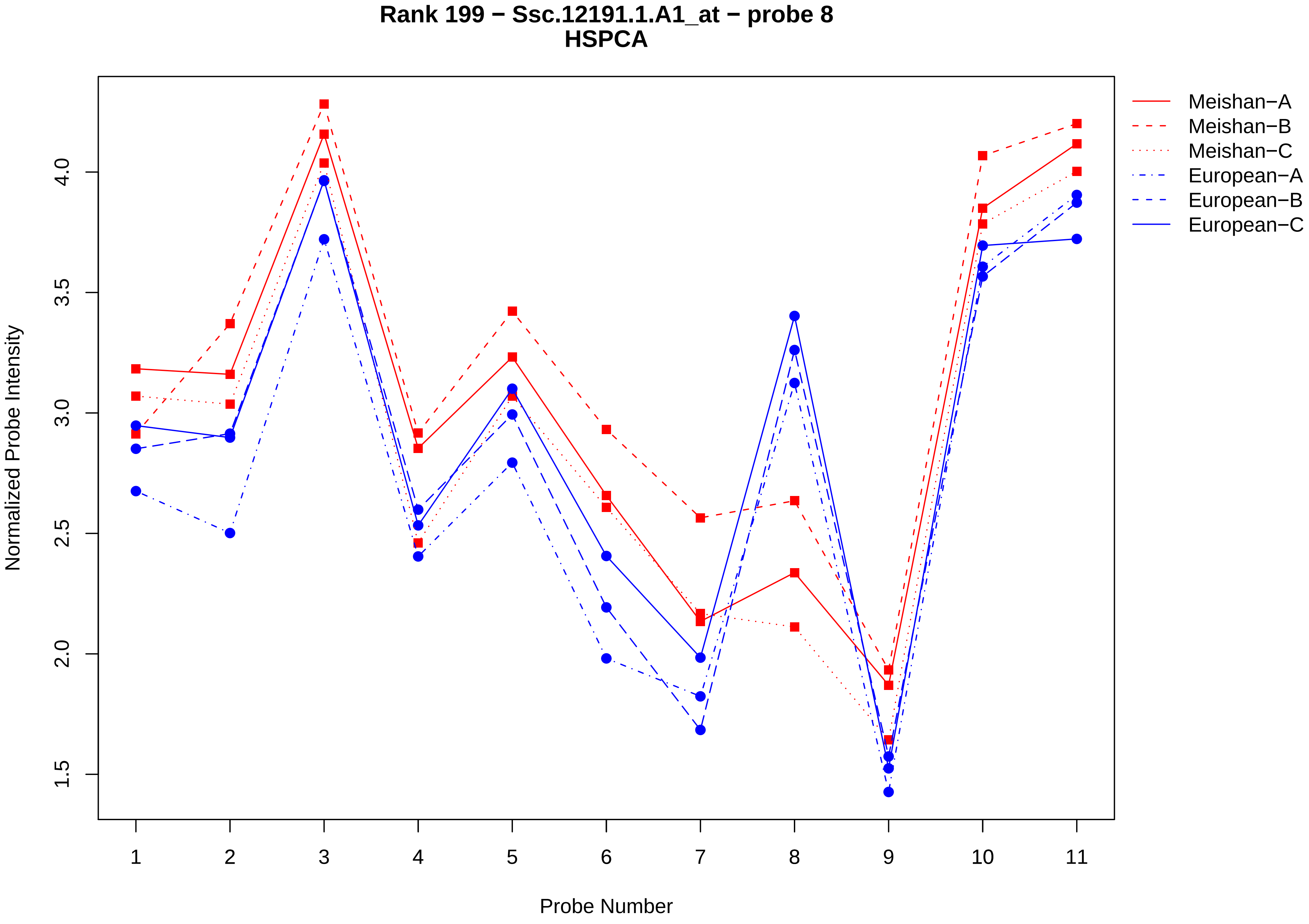

Rank 199 − Ssc.12191.1.A1_at − probe 8
HSPCA
Normalized Probe Intensity
1.5
2.0
2.5
3.0
3.5
4.0
Probe Number
1
2
3
4
5
6
7
8
9
10
11
Meishan−A
Meishan−B
Meishan−C
European−A
European−B
European−C

Rank 200 − Ssc.31190.1.S1_at − probe 11
PDE10A

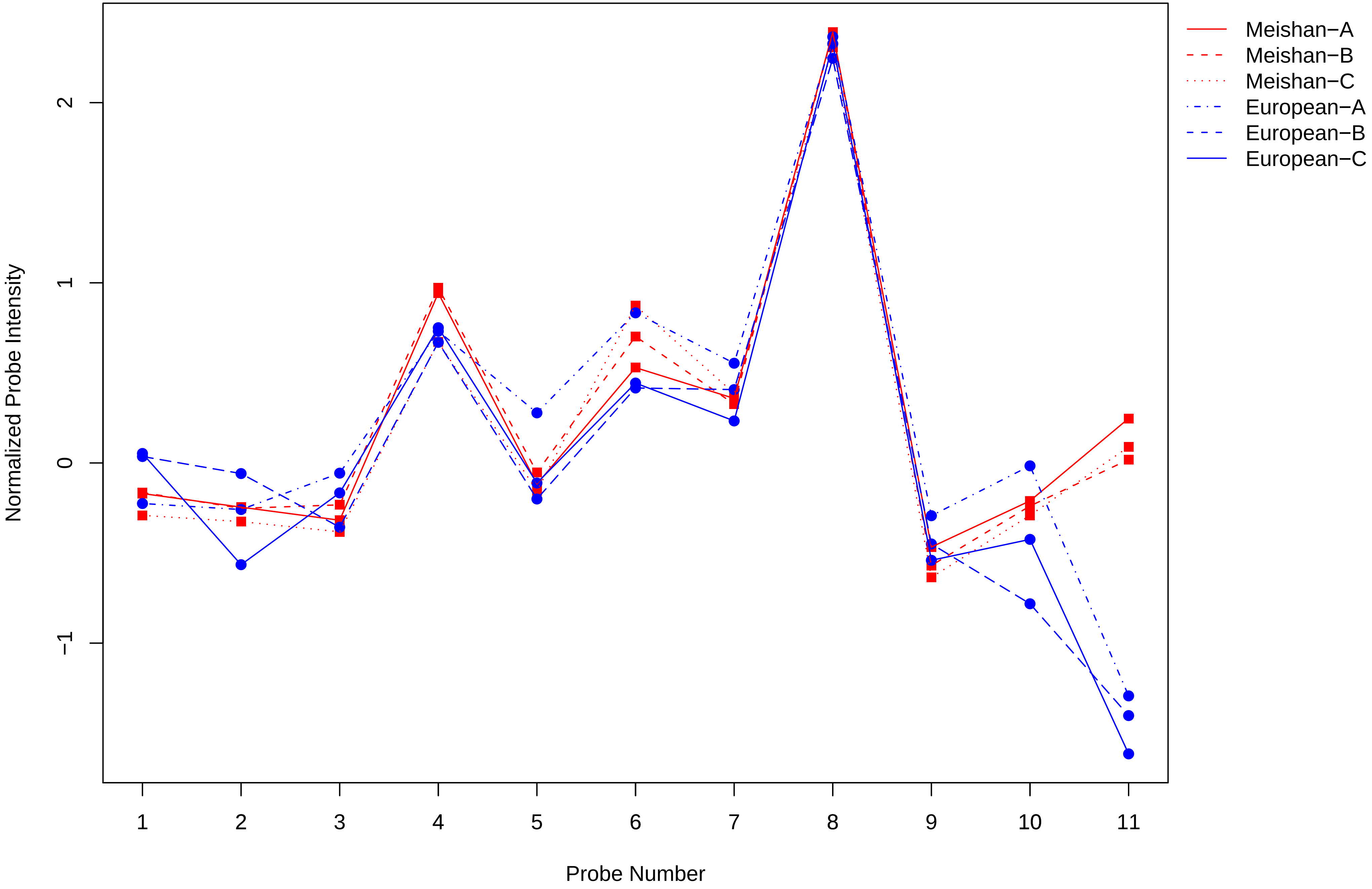

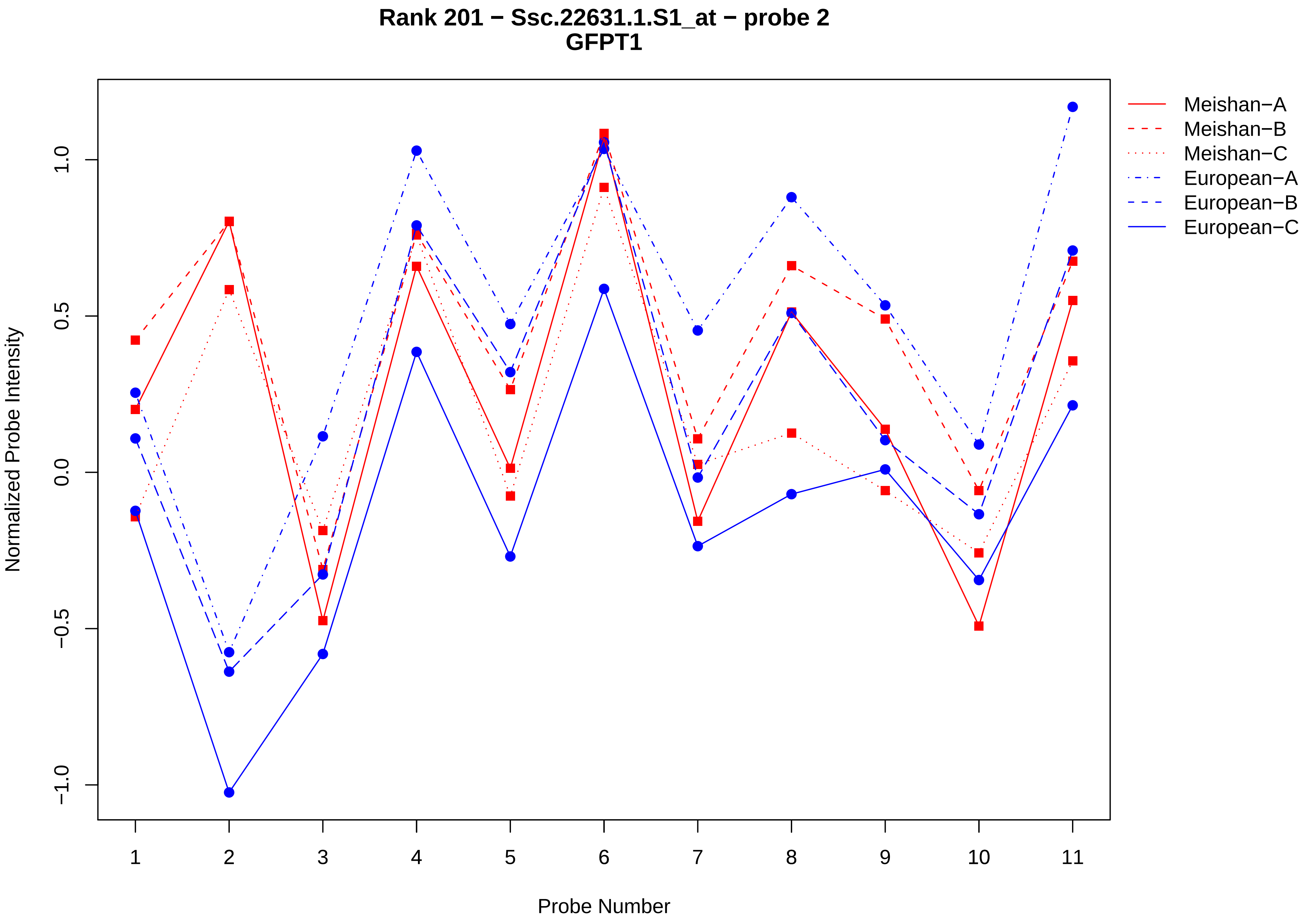

Rank 201 − Ssc.22631.1.S1_at − probe 2
GFPT1
Normalized Probe Intensity
Probe Number
Meishan−A
Meishan−B
Meishan−C
European−A
European−B
European−C
−1.0
−0.5
0.0
0.5
1.0
1
2
3
4
5
6
7
8
9
10
11

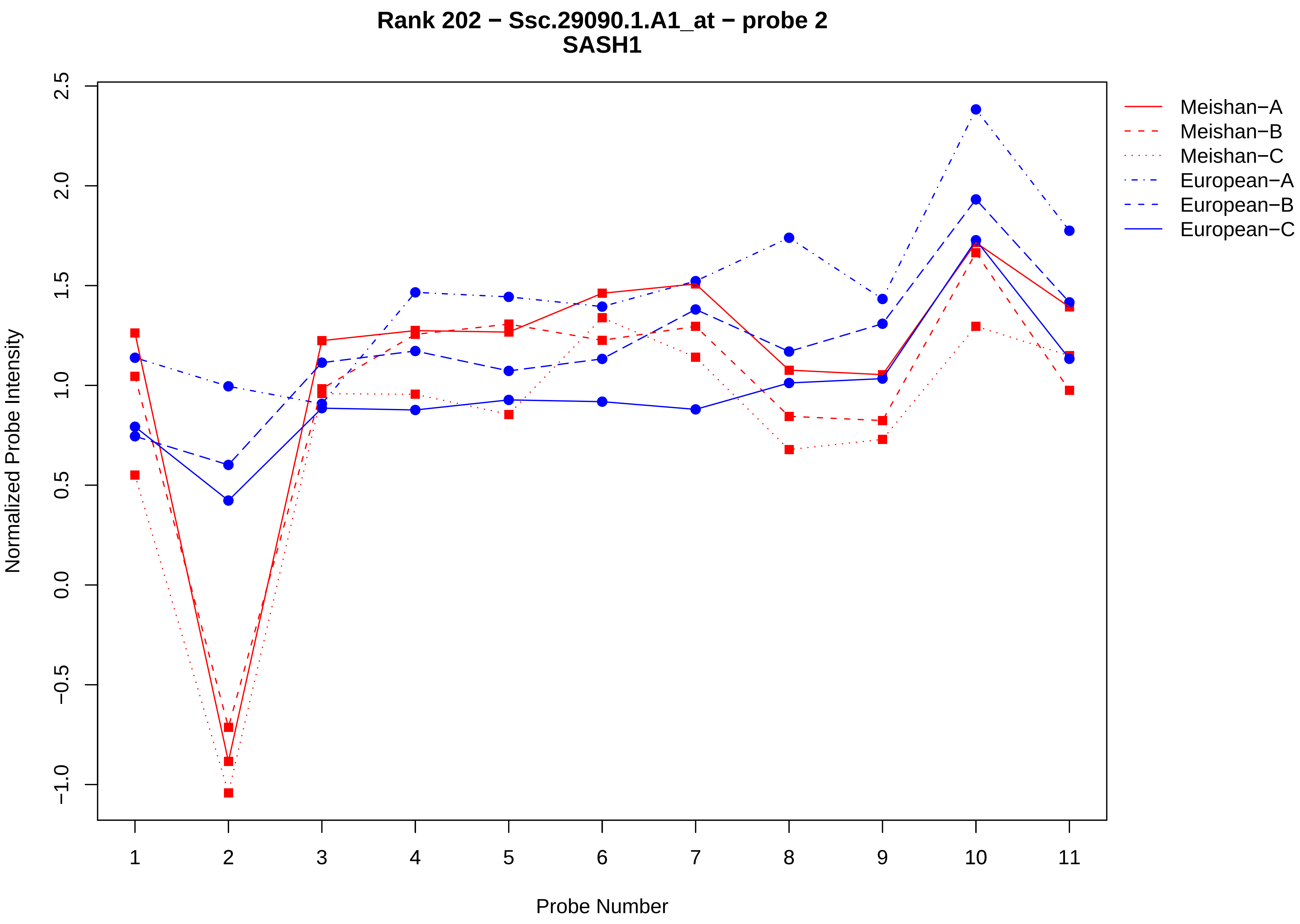

Rank 202 − Ssc.29090.1.A1_at − probe 2
SASH1
Normalized Probe Intensity
Probe Number
−1.0
−0.5
0.0
0.5
1.0
1.5
2.0
2.5
1
2
3
4
5
6
7
8
9
10
11
Meishan−A
Meishan−B
Meishan−C
European−A
European−B
European−C

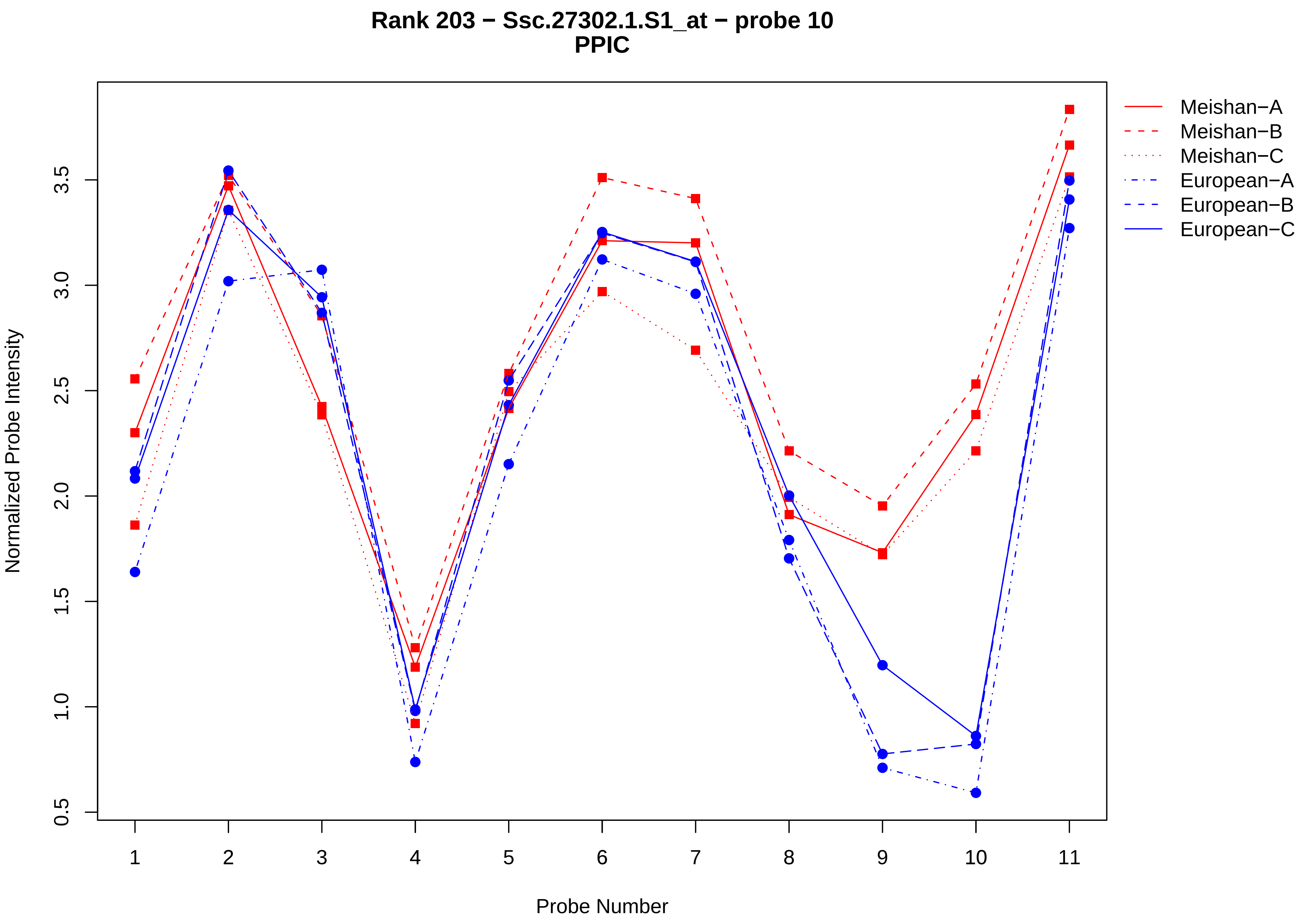
Rank 203 − Ssc.27302.1.S1_at − probe 10
PPIC
Normalized Probe Intensity
Probe Number
0.5
1.0
1.5
2.0
2.5
3.0
3.5
1
2
3
4
5
6
7
8
9
10
11
Meishan−A
Meishan−B
Meishan−C
European−A
European−B
European−C

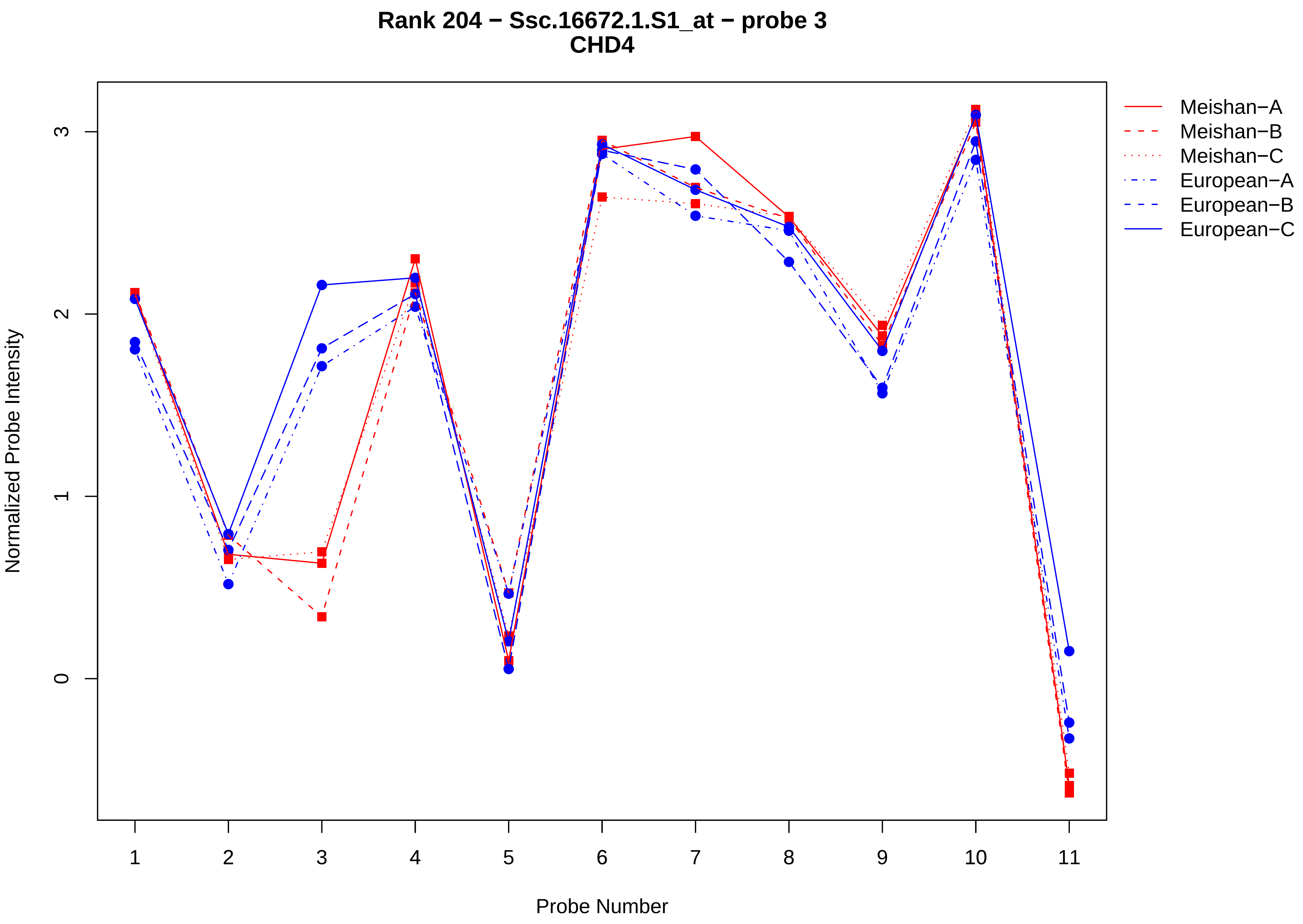

Rank 204 − Ssc.16672.1.S1_at − probe 3
CHD4
Normalized Probe Intensity
Probe Number
0
1
2
3
1
2
3
4
5
6
7
8
9
10
11
Meishan−A
Meishan−B
Meishan−C
European−A
European−B
European−C

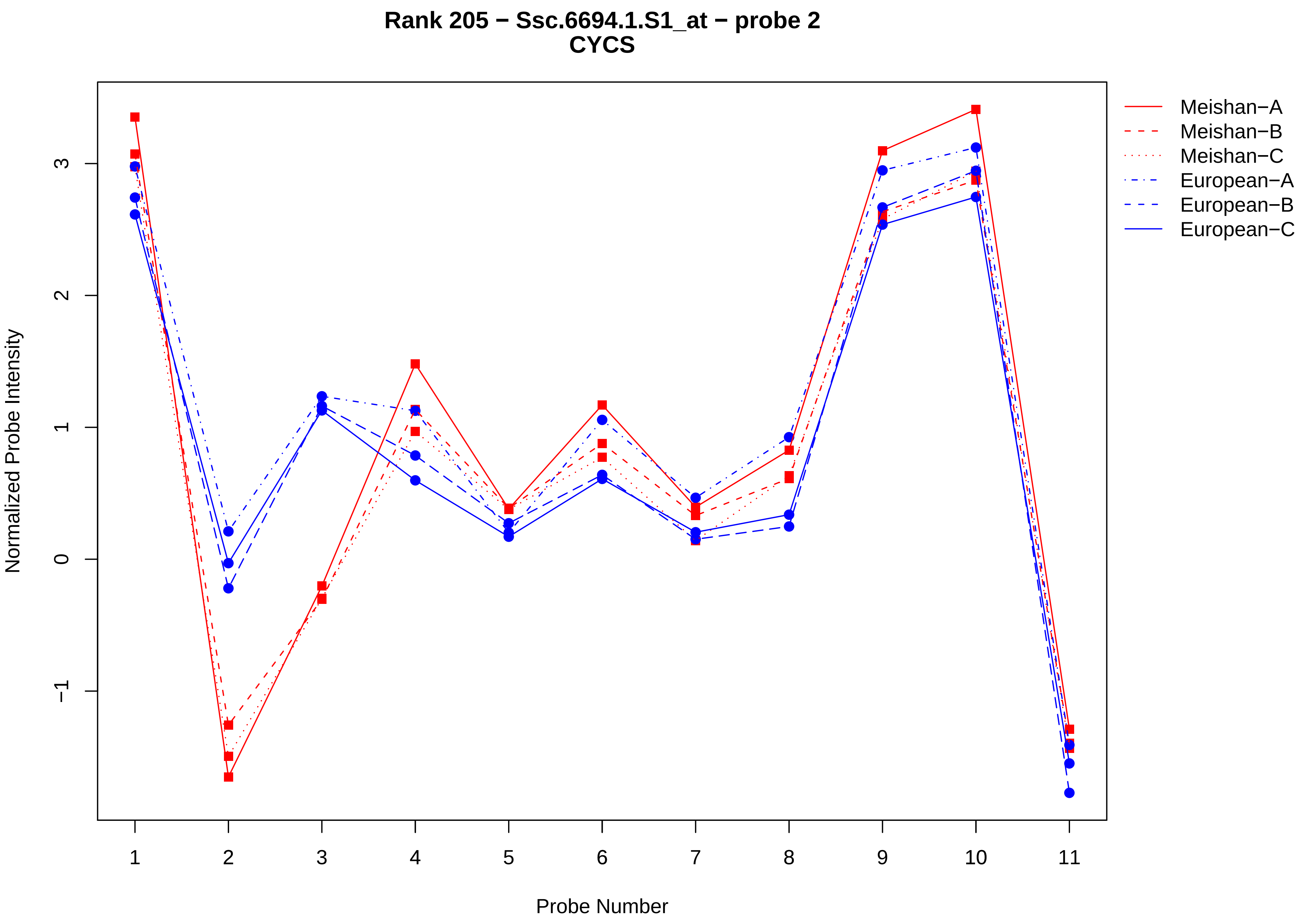

Rank 205 − Ssc.6694.1.S1_at − probe 2
CYCS
Normalized Probe Intensity
Probe Number
−1
0
1
2
3
1
2
3
4
5
6
7
8
9
10
11
Meishan−A
Meishan−B
Meishan−C
European−A
European−B
European−C

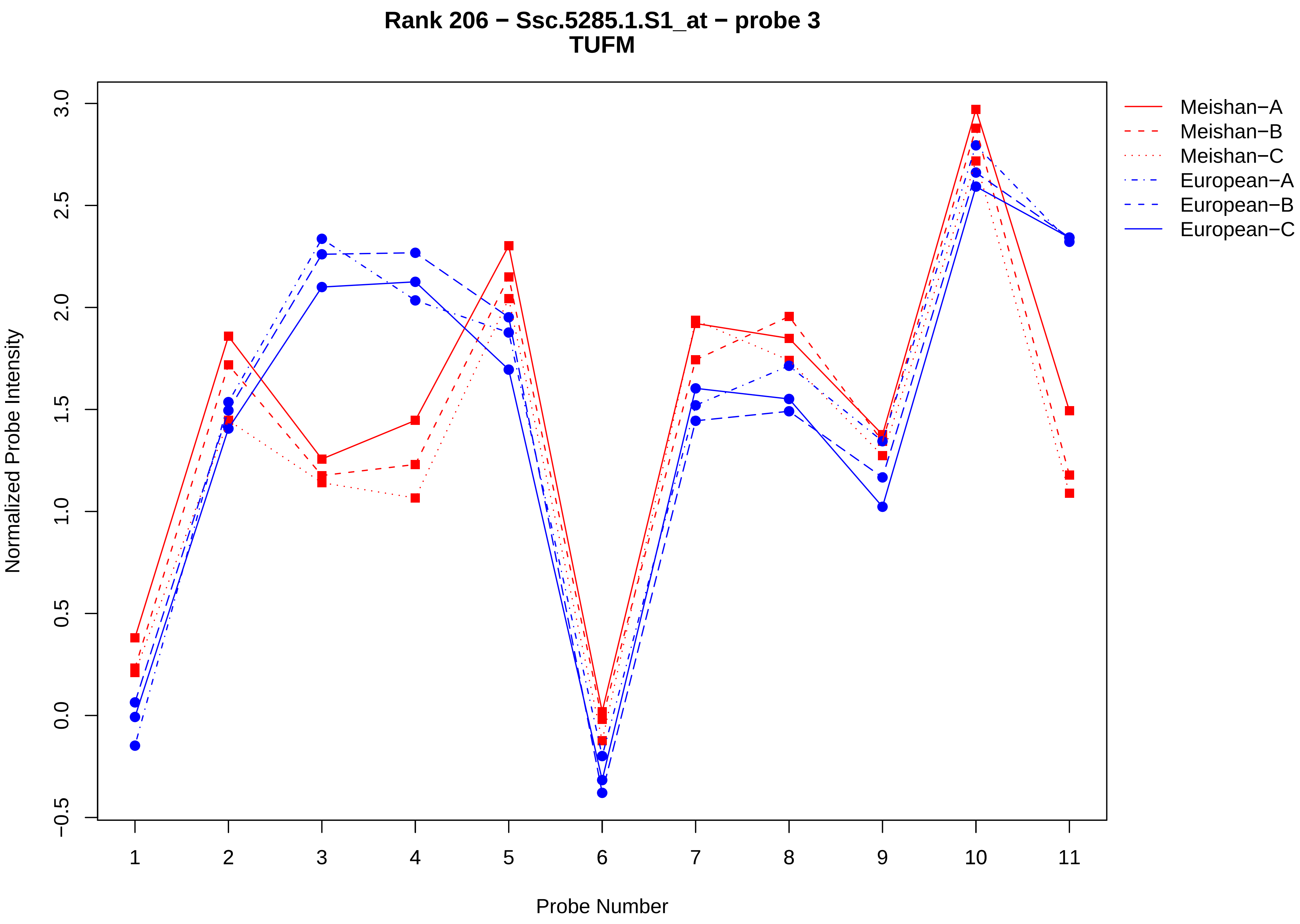

Rank 206 − Ssc.5285.1.S1_at − probe 3
TUFM
Normalized Probe Intensity
Probe Number
−0.5
0.0
0.5
1.0
1.5
2.0
2.5
3.0
1
2
3
4
5
6
7
8
9
10
11
Meishan−A
Meishan−B
Meishan−C
European−A
European−B
European−C

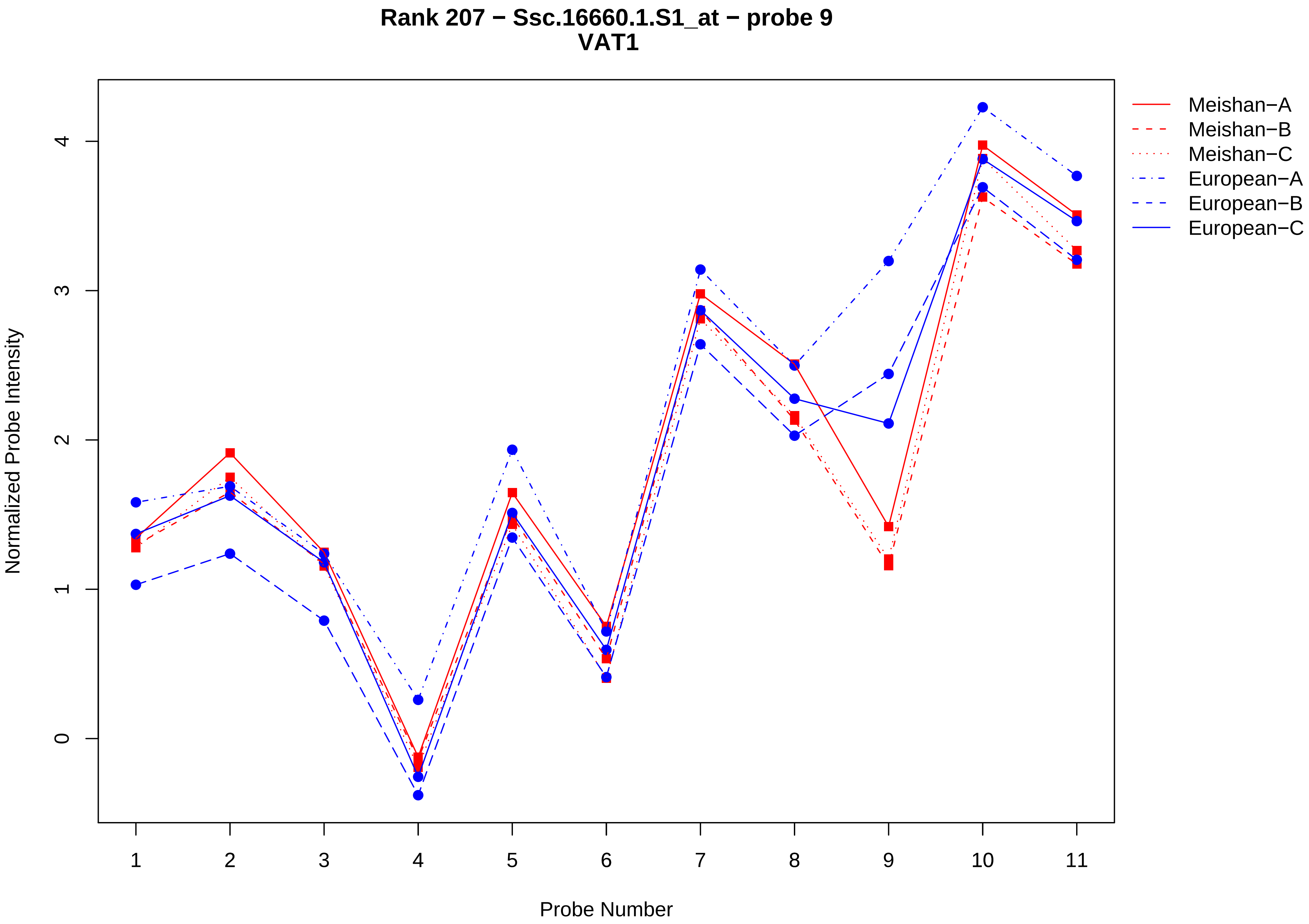

Rank 207 − Ssc.16660.1.S1_at − probe 9
VAT1
Normalized Probe Intensity
Probe Number
0
1
2
3
4
1
2
3
4
5
6
7
8
9
10
11
Meishan−A
Meishan−B
Meishan−C
European−A
European−B
European−C

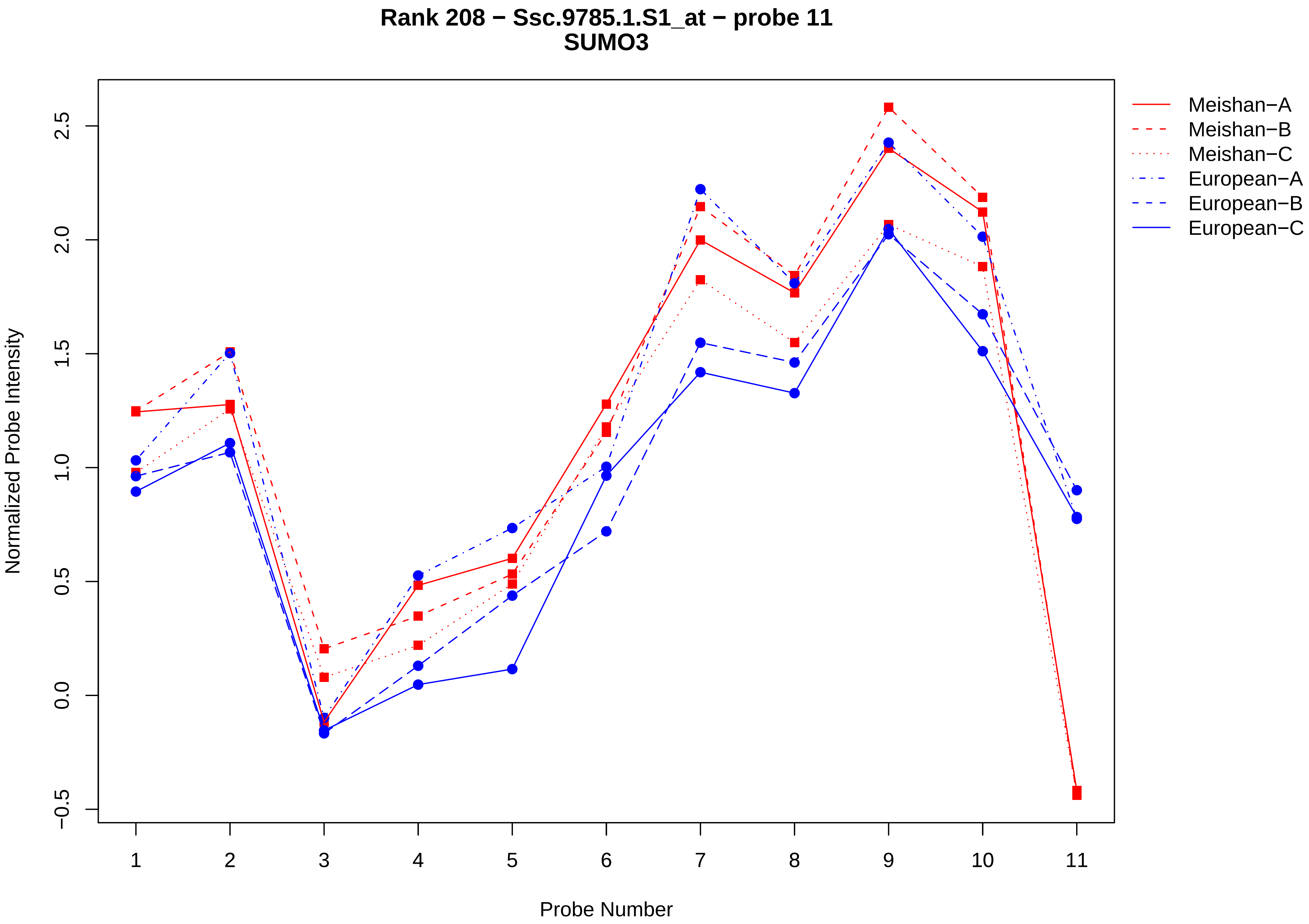

Rank 208 − Ssc.9785.1.S1_at − probe 11
SUMO3
Normalized Probe Intensity
Probe Number
−0.5
0.0
0.5
1.0
1.5
2.0
2.5
1
2
3
4
5
6
7
8
9
10
11
Meishan−A
Meishan−B
Meishan−C
European−A
European−B
European−C

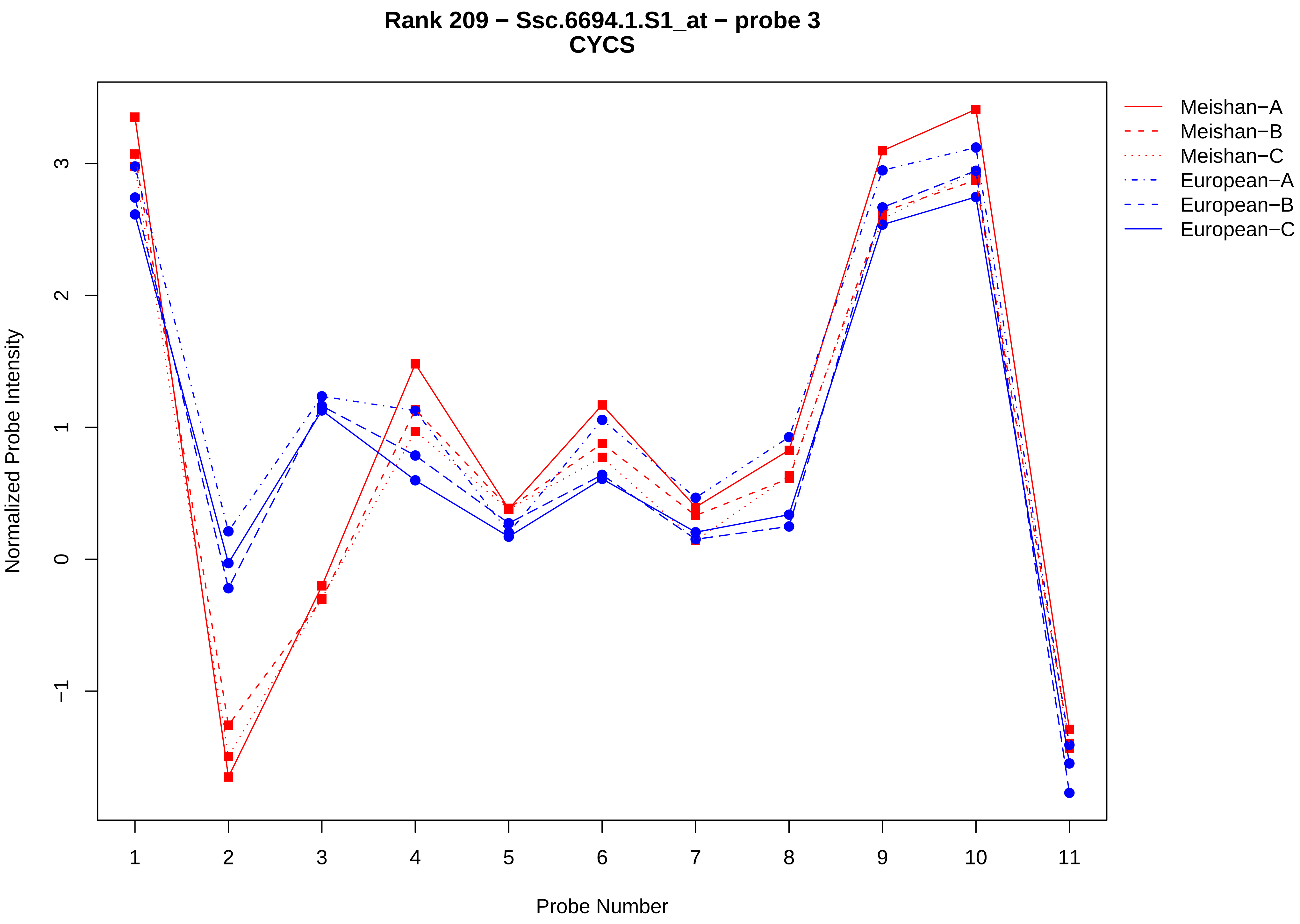

Rank 209 − Ssc.6694.1.S1_at − probe 3
CYCS
Normalized Probe Intensity
Probe Number
Meishan−A
Meishan−B
Meishan−C
European−A
European−B
European−C

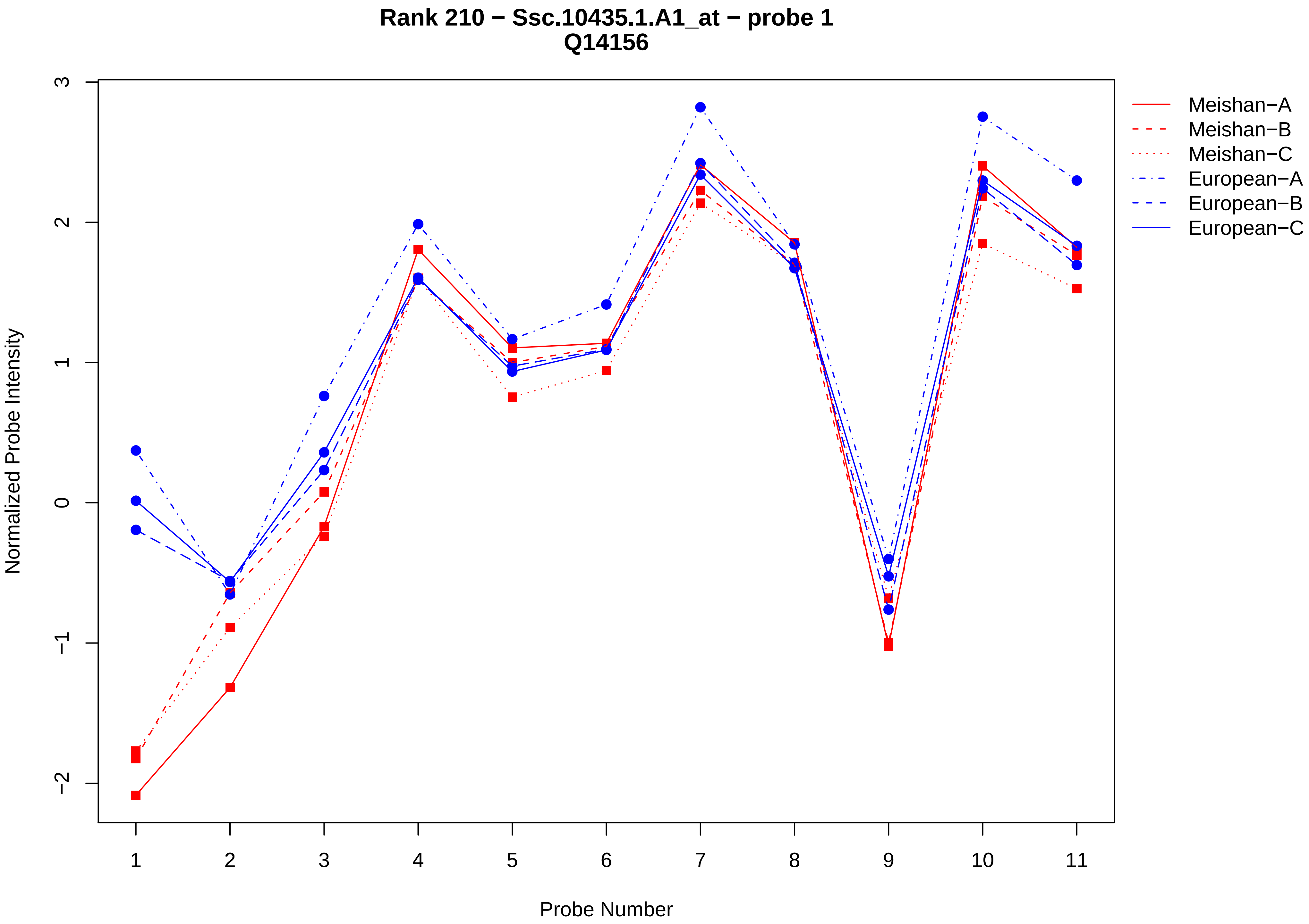

Rank 210 − Ssc.10435.1.A1_at − probe 1
Q14156
Normalized Probe Intensity
Probe Number
Meishan−A
Meishan−B
Meishan−C
European−A
European−B
European−C

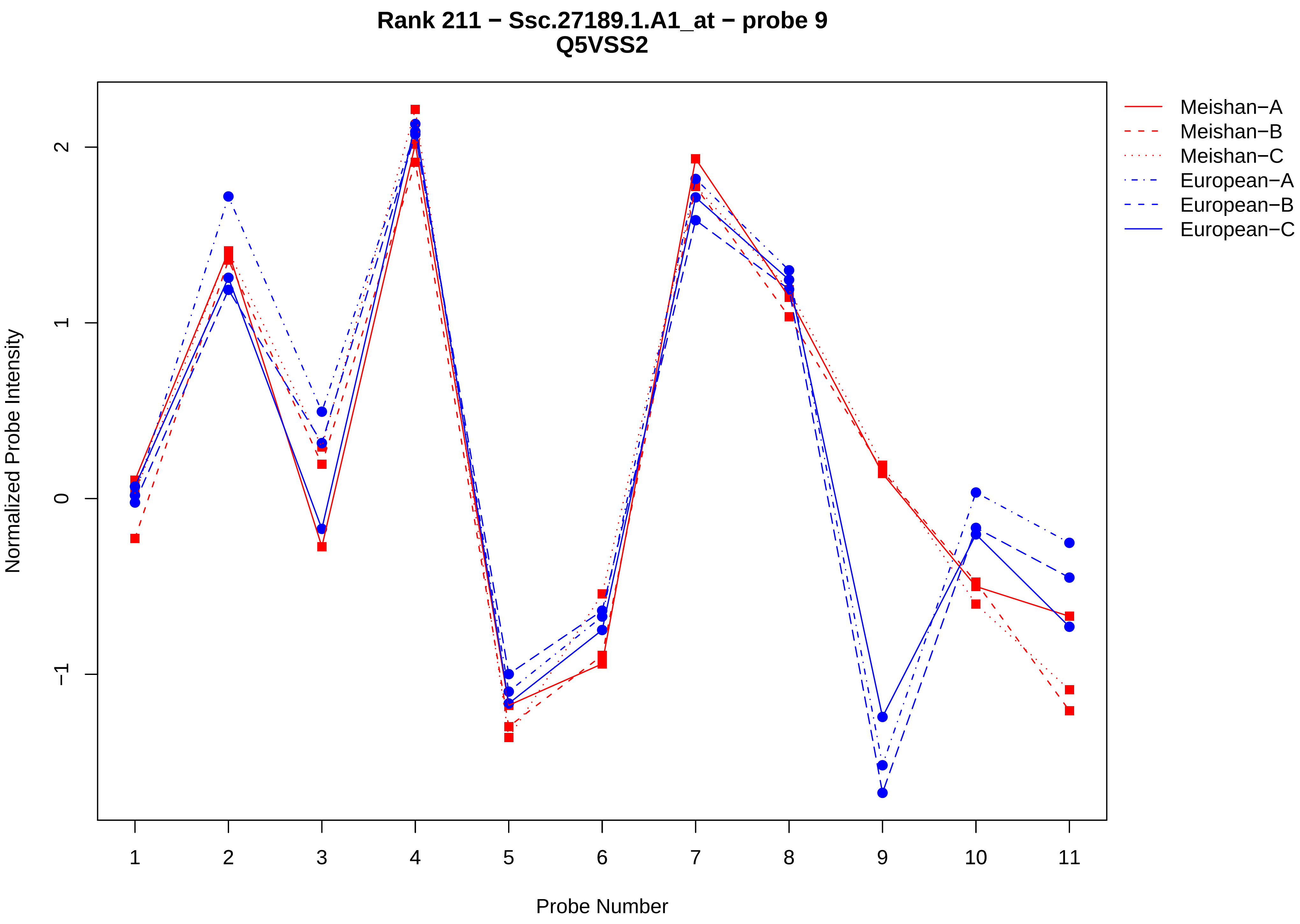

Rank 211 − Ssc.27189.1.A1_at − probe 9
Q5VSS2
Normalized Probe Intensity
Probe Number
−1
0
1
2
1
2
3
4
5
6
7
8
9
10
11
Meishan−A
Meishan−B
Meishan−C
European−A
European−B
European−C

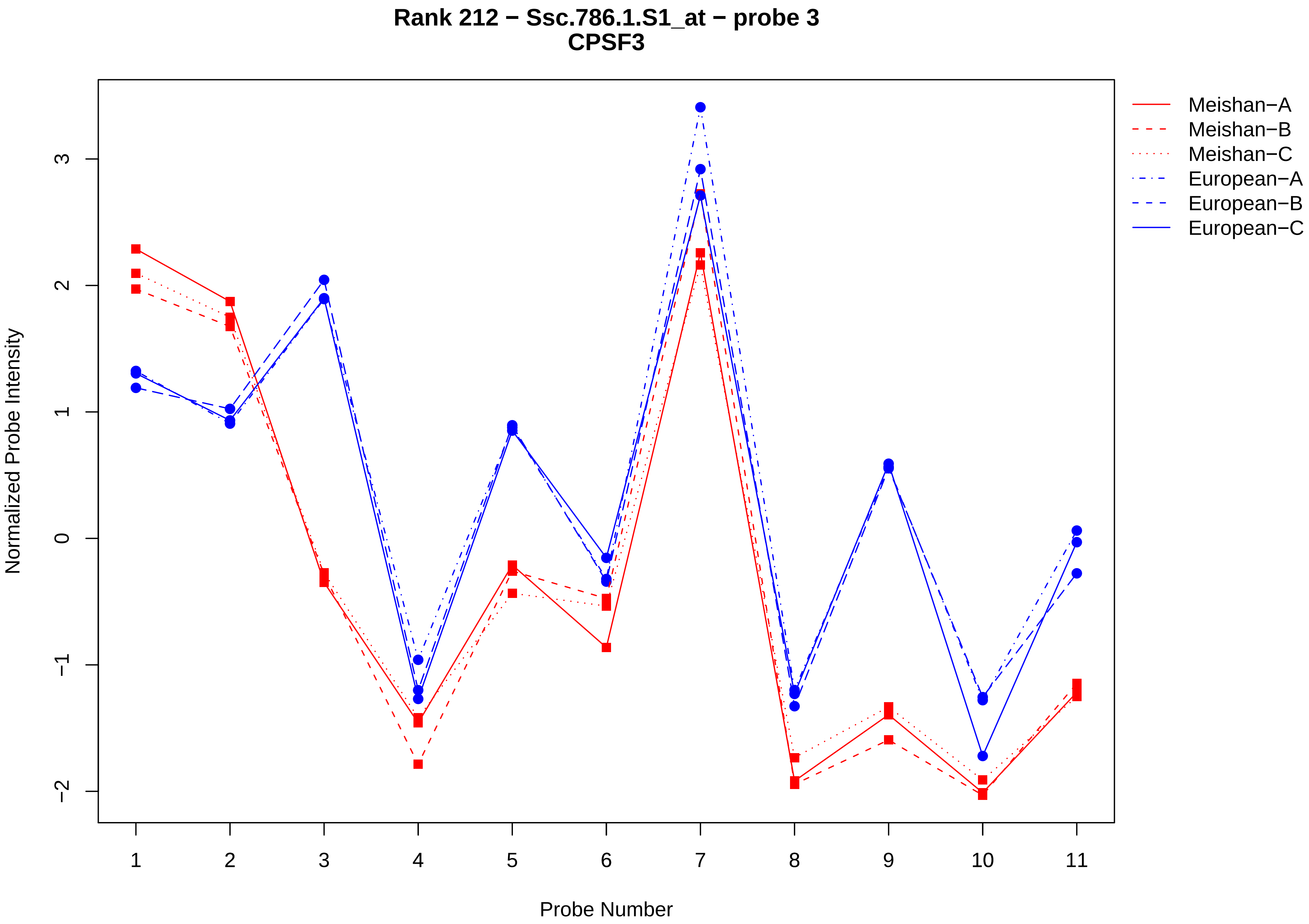

Rank 212 − Ssc.786.1.S1_at − probe 3
CPSF3
Normalized Probe Intensity
Probe Number
−2
−1
0
1
2
3
1
2
3
4
5
6
7
8
9
10
11
Meishan−A
Meishan−B
Meishan−C
European−A
European−B
European−C

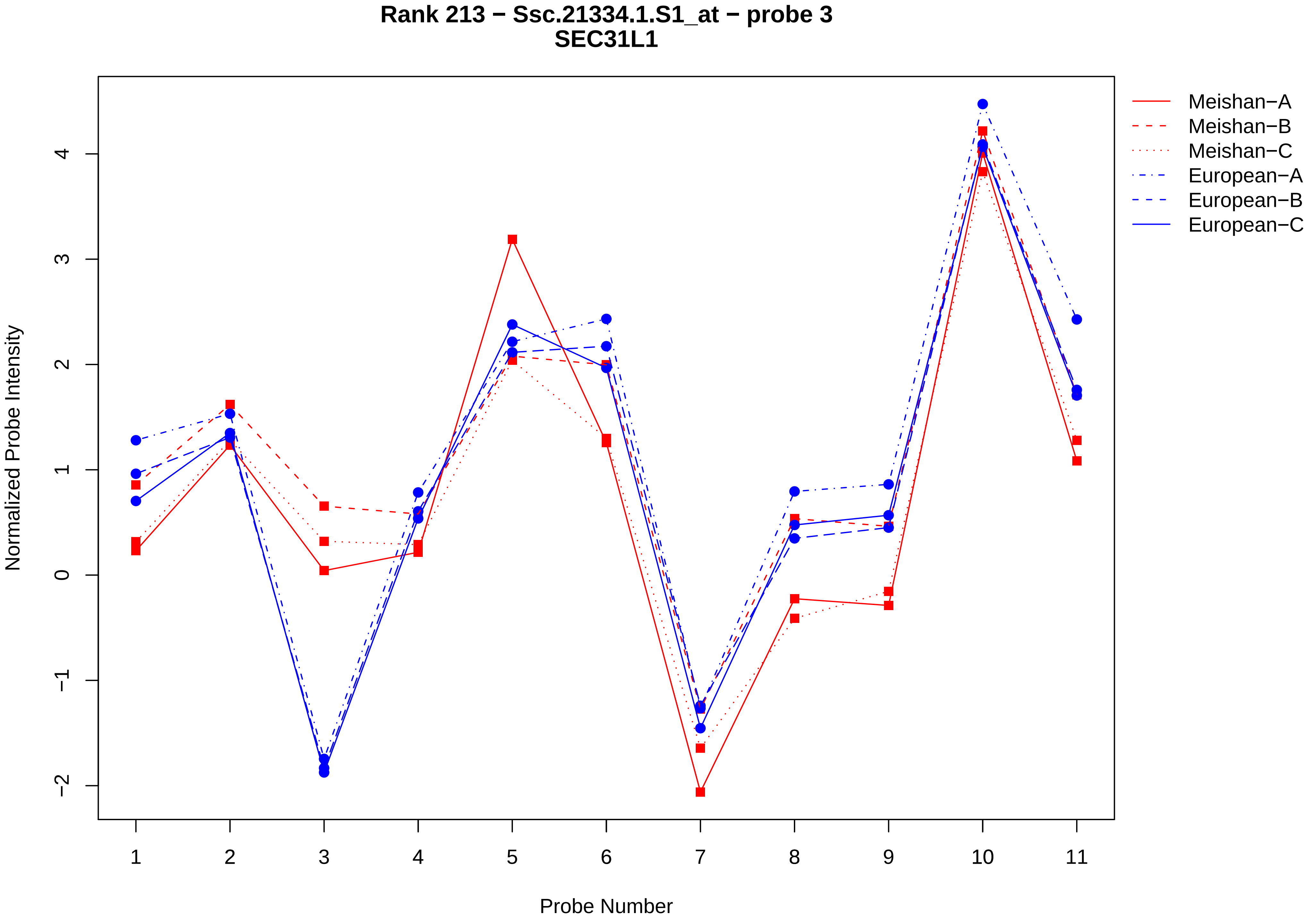

Rank 213 − Ssc.21334.1.S1_at − probe 3
SEC31L1
Normalized Probe Intensity
Probe Number
−2
−1
0
1
2
3
4
1
2
3
4
5
6
7
8
9
10
11
Meishan−A
Meishan−B
Meishan−C
European−A
European−B
European−C

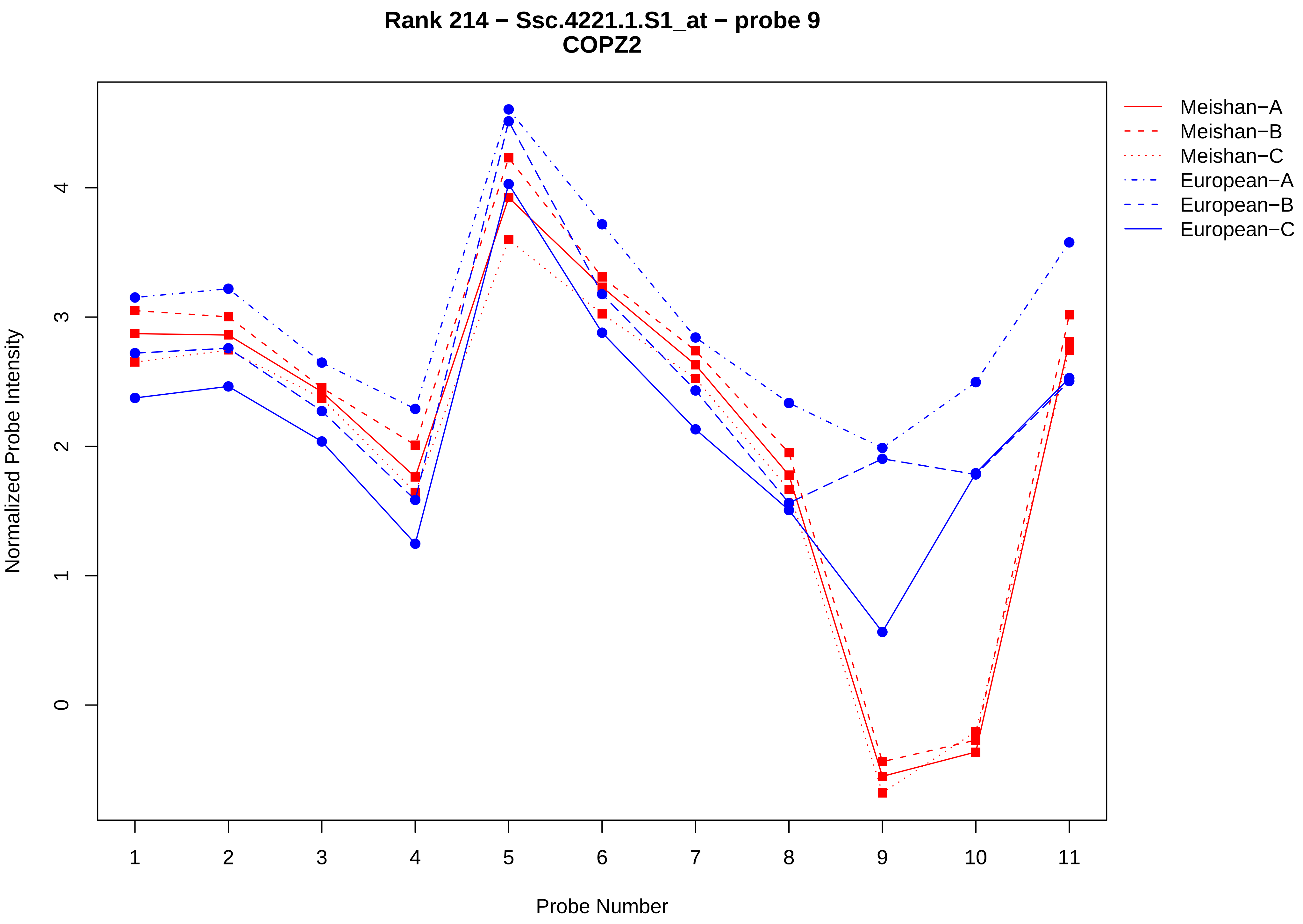
Rank 214 − Ssc.4221.1.S1_at − probe 9
COPZ2
Normalized Probe Intensity
0
1
2
3
4
Probe Number
1
2
3
4
5
6
7
8
9
10
11
Meishan−A
Meishan−B
Meishan−C
European−A
European−B
European−C

Rank 215 − Ssc.8464.1.A1_at − probe 6
TM4SF10

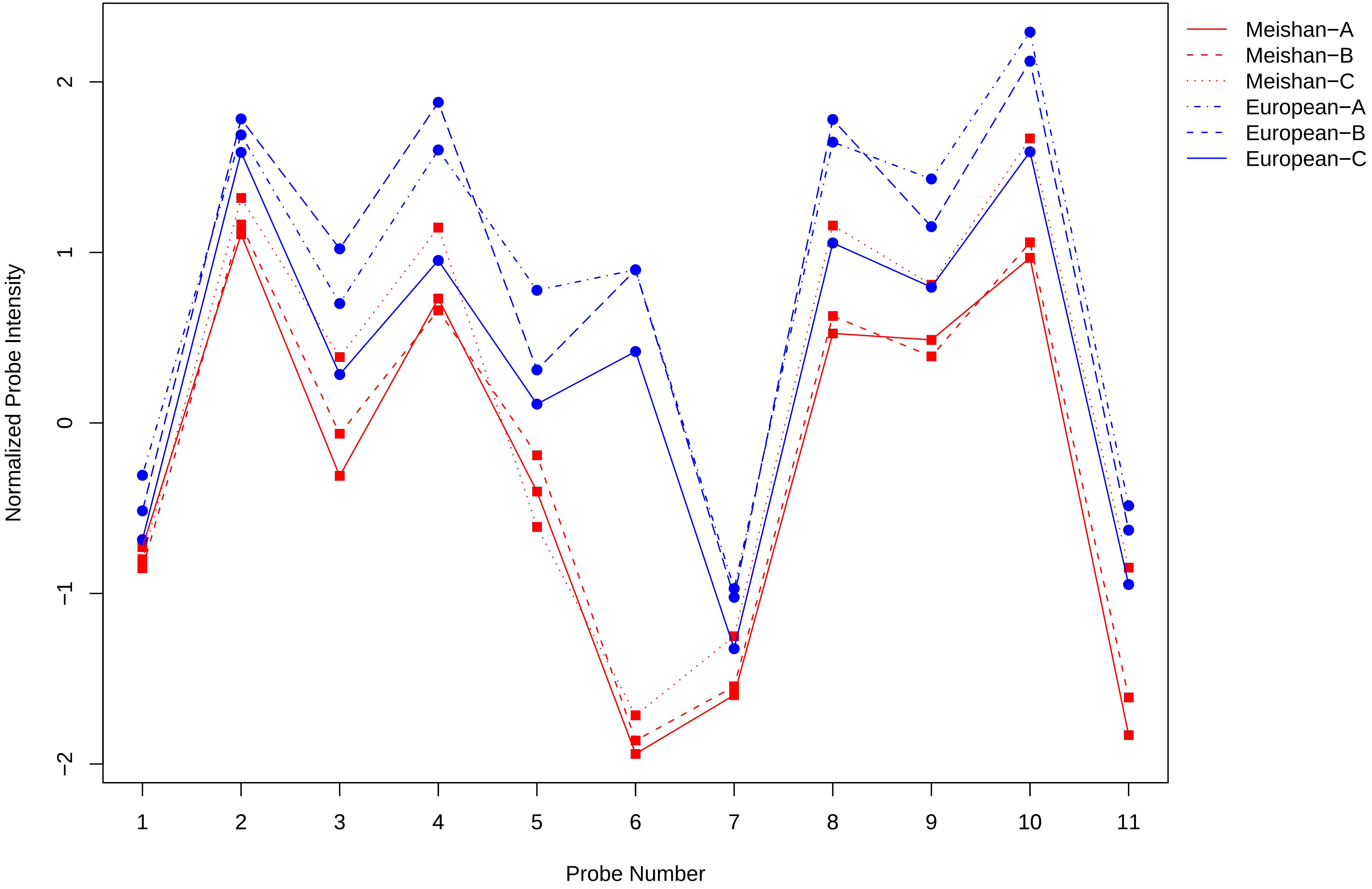

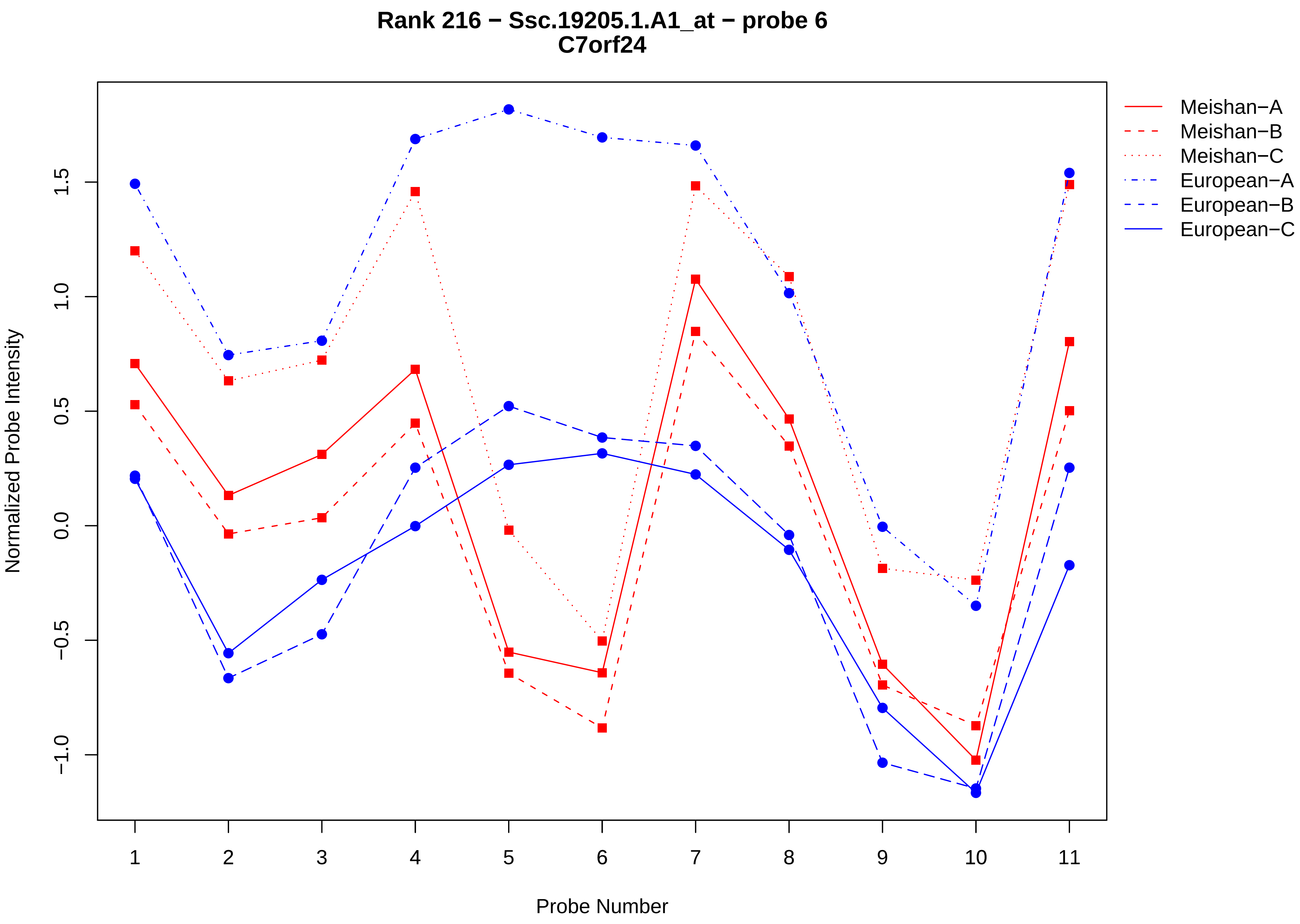
Rank 216 − Ssc.19205.1.A1_at − probe 6
C7orf24
Normalized Probe Intensity
Probe Number
Meishan−A
Meishan−B
Meishan−C
European−A
European−B
European−C
−1.0
−0.5
0.0
0.5
1.0
1.5
1
2
3
4
5
6
7
8
9
10
11

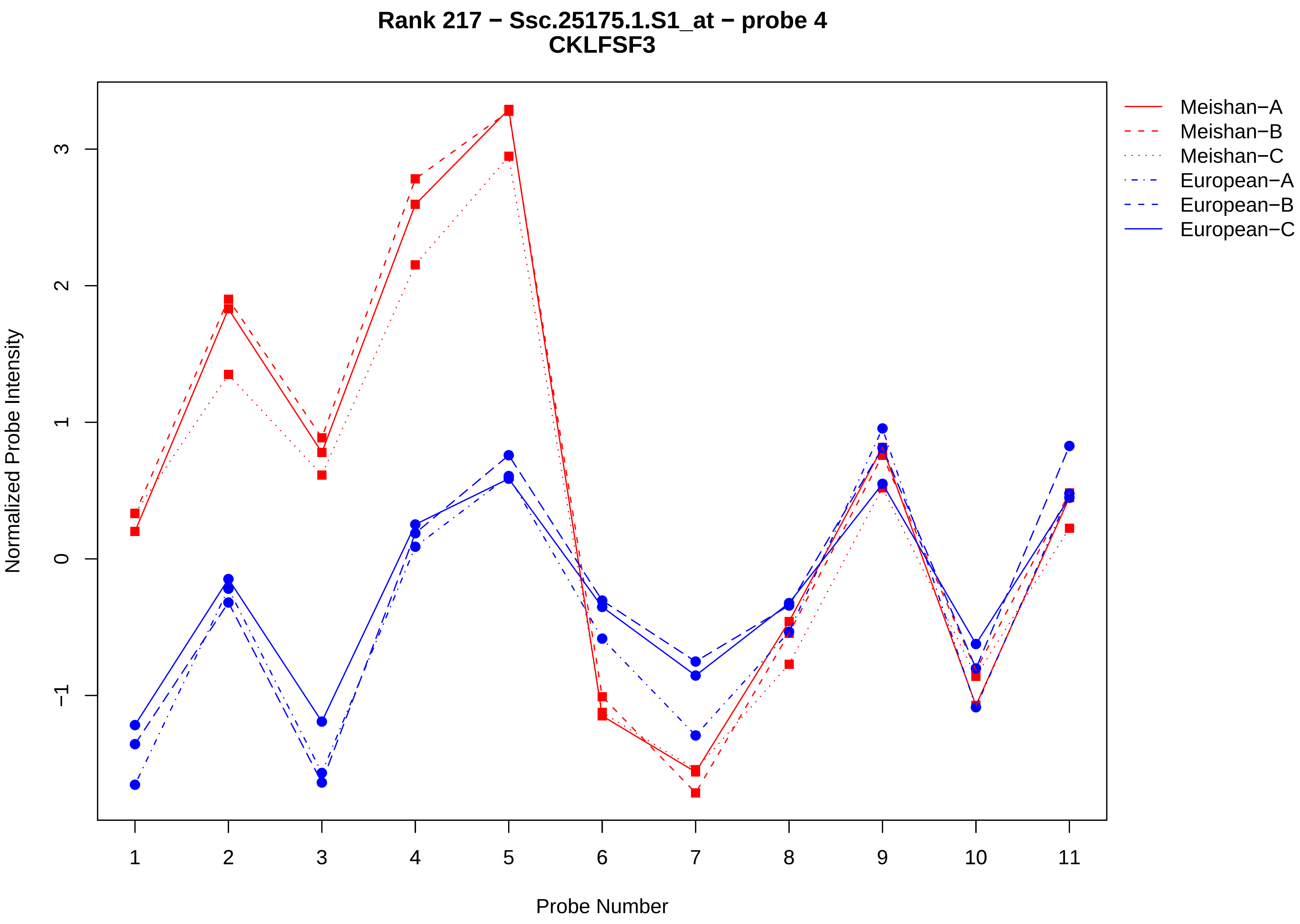

Rank 217 − Ssc.25175.1.S1_at − probe 4
CKLFSF3
Normalized Probe Intensity
Probe Number
Meishan−A
Meishan−B
Meishan−C
European−A
European−B
European−C
−1
0
1
2
3
1
2
3
4
5
6
7
8
9
10
11

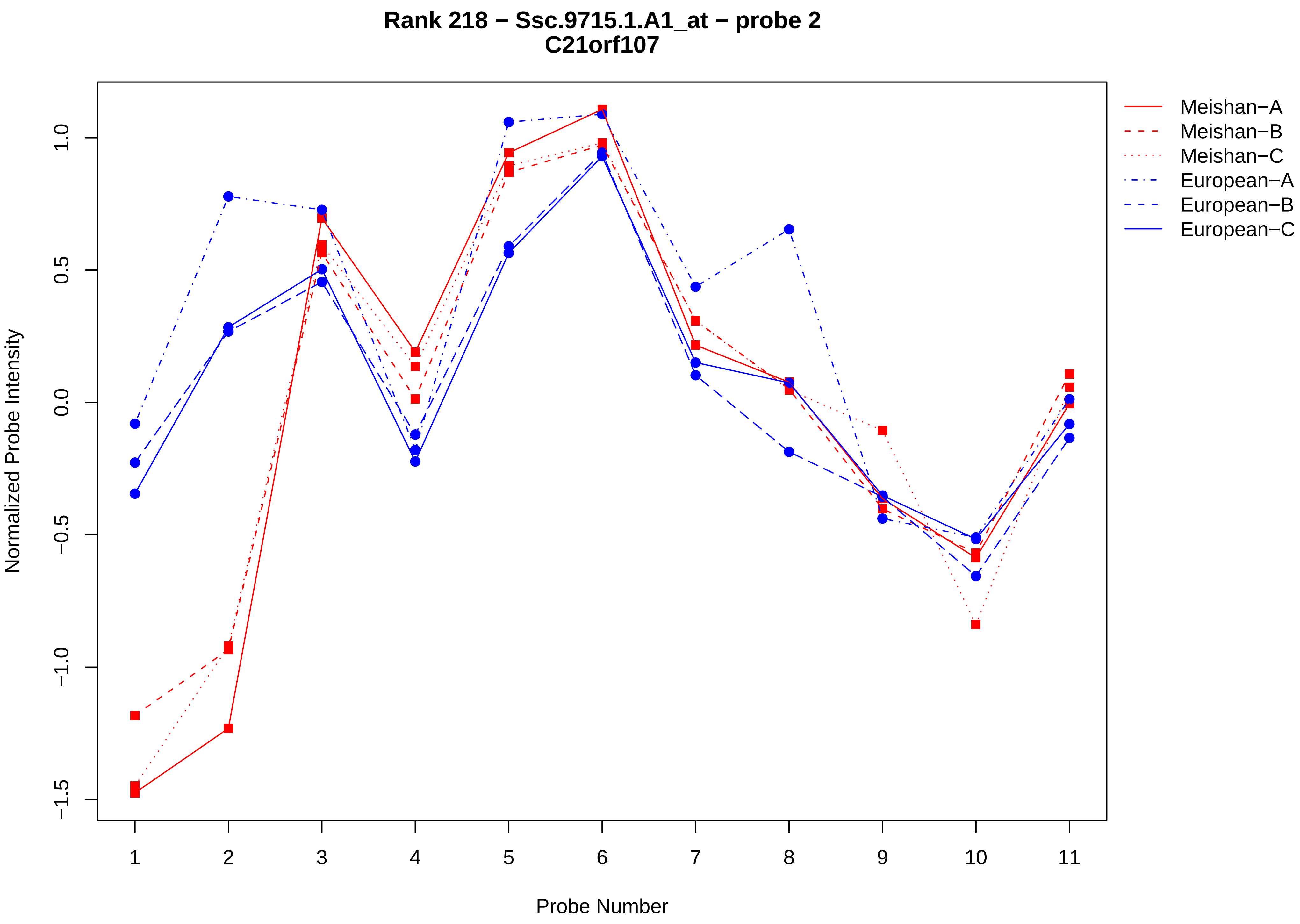

Rank 218 − Ssc.9715.1.A1_at − probe 2
C21orf107
Normalized Probe Intensity
Probe Number
1.0
0.5
0.0
−0.5
−1.0
−1.5
1
2
3
4
5
6
7
8
9
10
11
Meishan−A
Meishan−B
Meishan−C
European−A
European−B
European−C

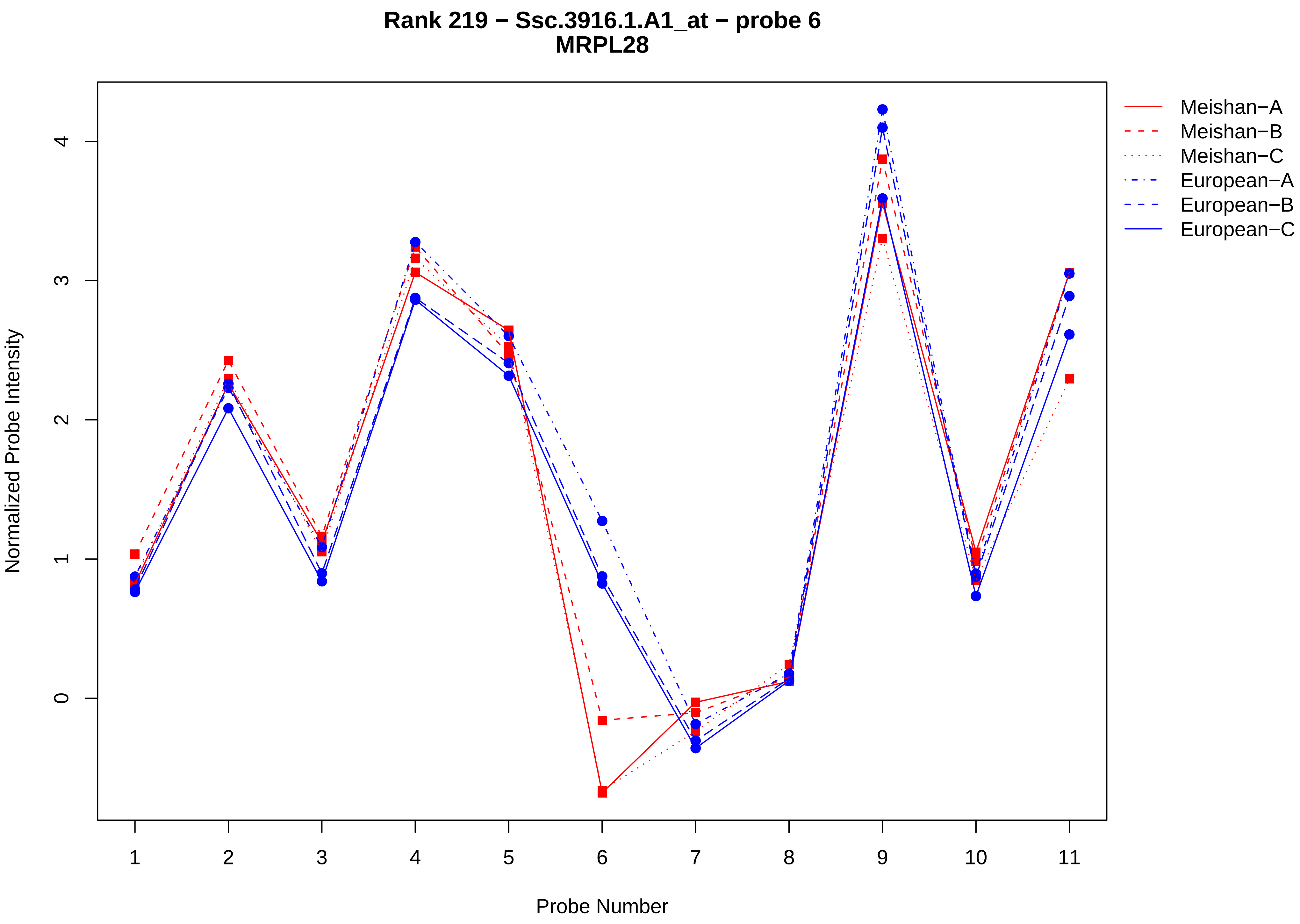

Rank 219 − Ssc.3916.1.A1_at − probe 6
MRPL28
Normalized Probe Intensity
Probe Number
0
1
2
3
4
1
2
3
4
5
6
7
8
9
10
11
Meishan−A
Meishan−B
Meishan−C
European−A
European−B
European−C

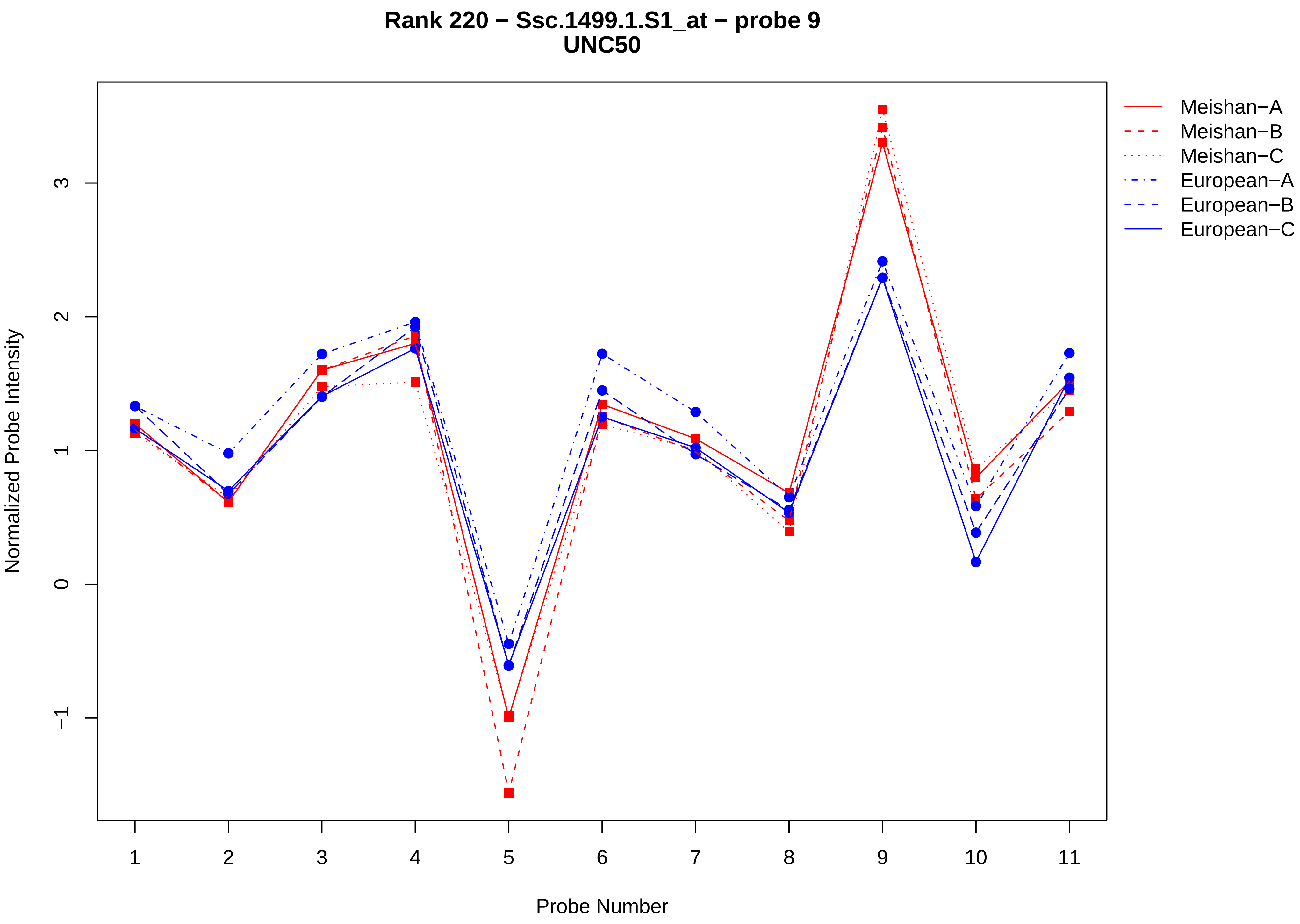

Rank 220 − Ssc.1499.1.S1_at − probe 9
UNC50
Normalized Probe Intensity
Probe Number
Meishan−A
Meishan−B
Meishan−C
European−A
European−B
European−C
−1
0
1
2
3
1
2
3
4
5
6
7
8
9
10
11

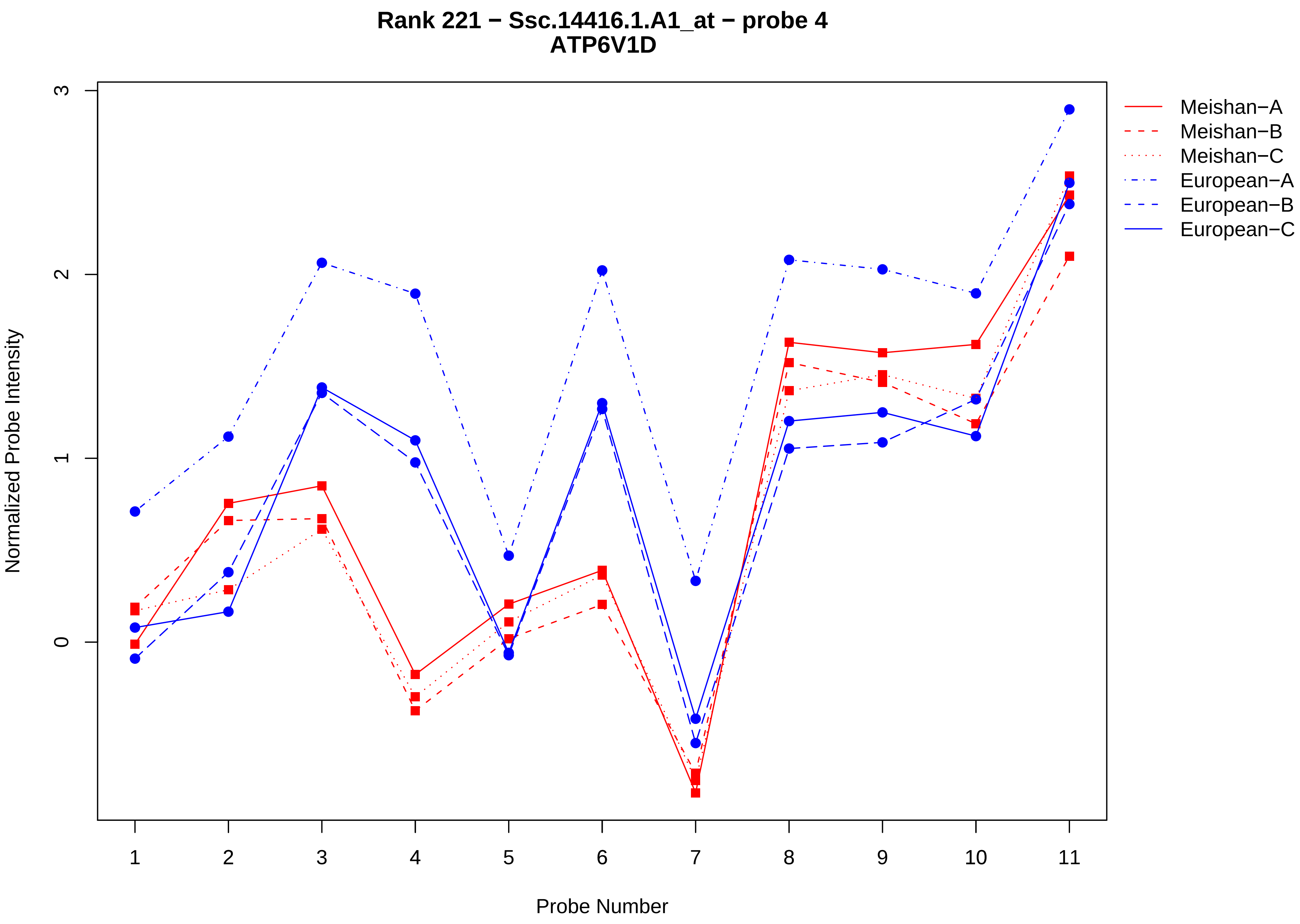

Rank 221 − Ssc.14416.1.A1_at − probe 4
ATP6V1D
Normalized Probe Intensity
Probe Number
Meishan−A
Meishan−B
Meishan−C
European−A
European−B
European−C
0
1
2
3
1
2
3
4
5
6
7
8
9
10
11

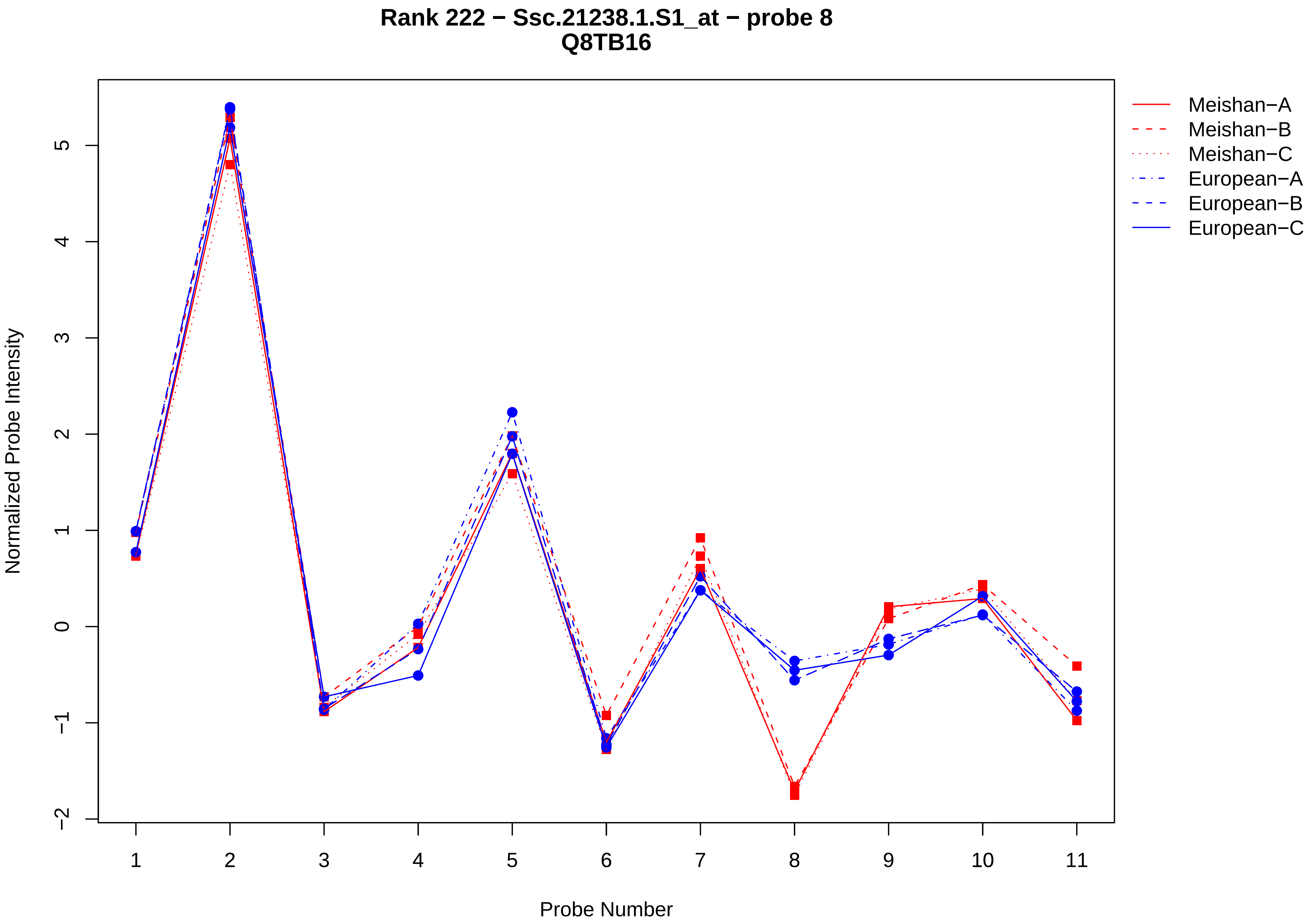

Rank 222 − Ssc.21238.1.S1_at − probe 8
Q8TB16
Normalized Probe Intensity
Probe Number
−2
−1
0
1
2
3
4
5
1
2
3
4
5
6
7
8
9
10
11
Meishan−A
Meishan−B
Meishan−C
European−A
European−B
European−C

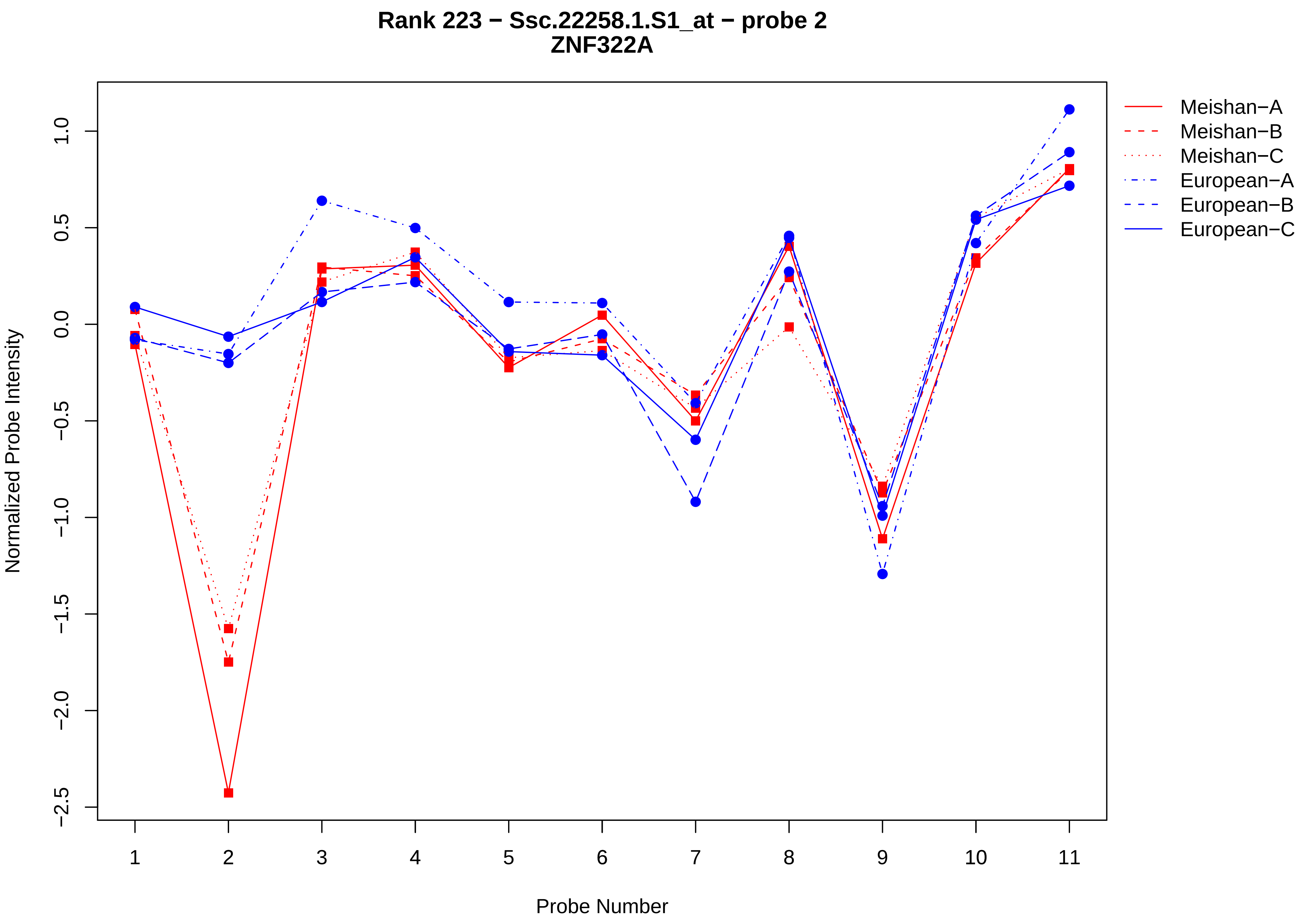
Rank 223 − Ssc.22258.1.S1_at − probe 2
ZNF322A
Normalized Probe Intensity
Probe Number
Meishan−A
Meishan−B
Meishan−C
European−A
European−B
European−C

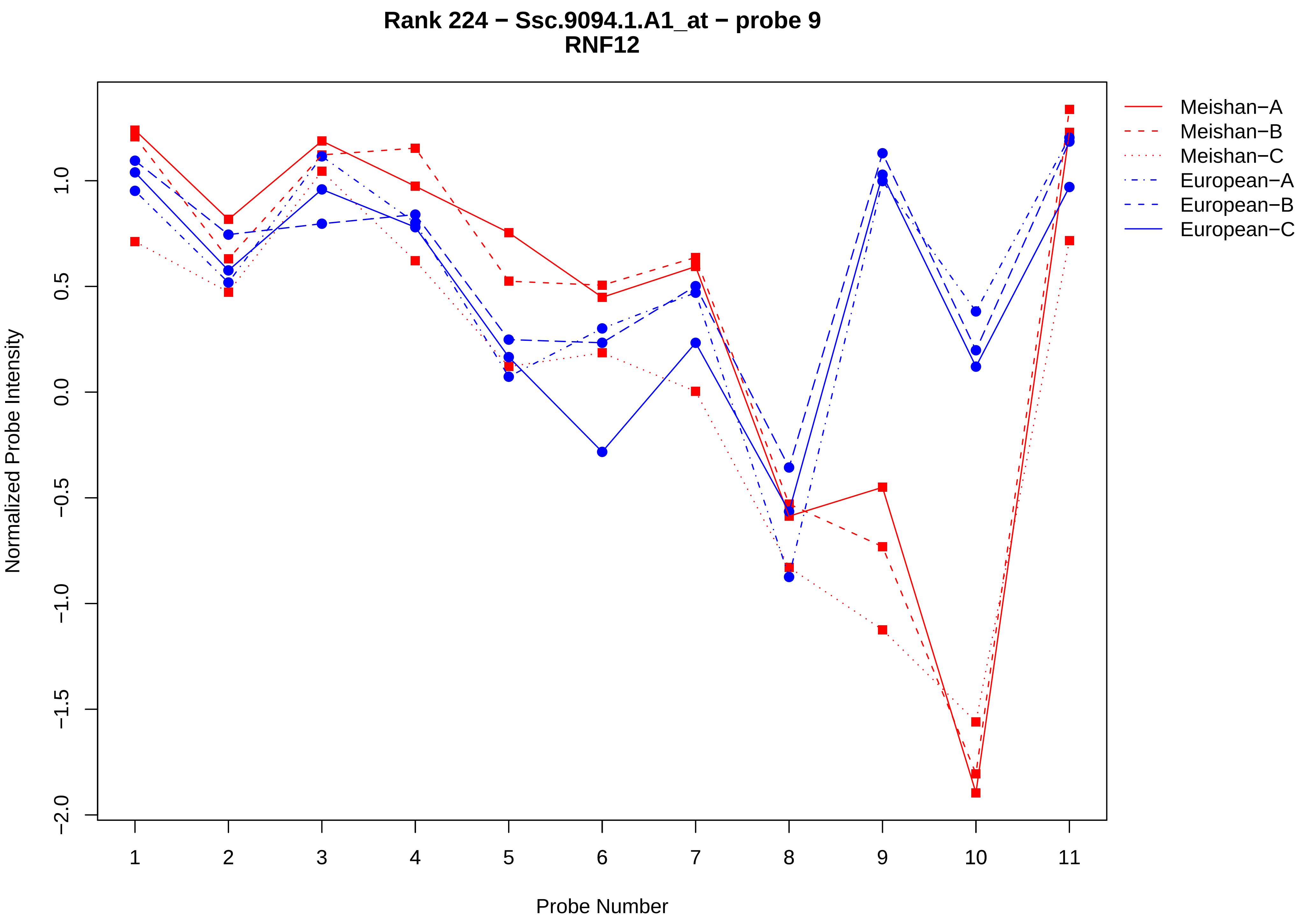

Rank 224 − Ssc.9094.1.A1_at − probe 9
RNF12
Normalized Probe Intensity
Probe Number
1.0
0.5
0.0
−0.5
−1.0
−1.5
−2.0
1
2
3
4
5
6
7
8
9
10
11
Meishan−A
Meishan−B
Meishan−C
European−A
European−B
European−C

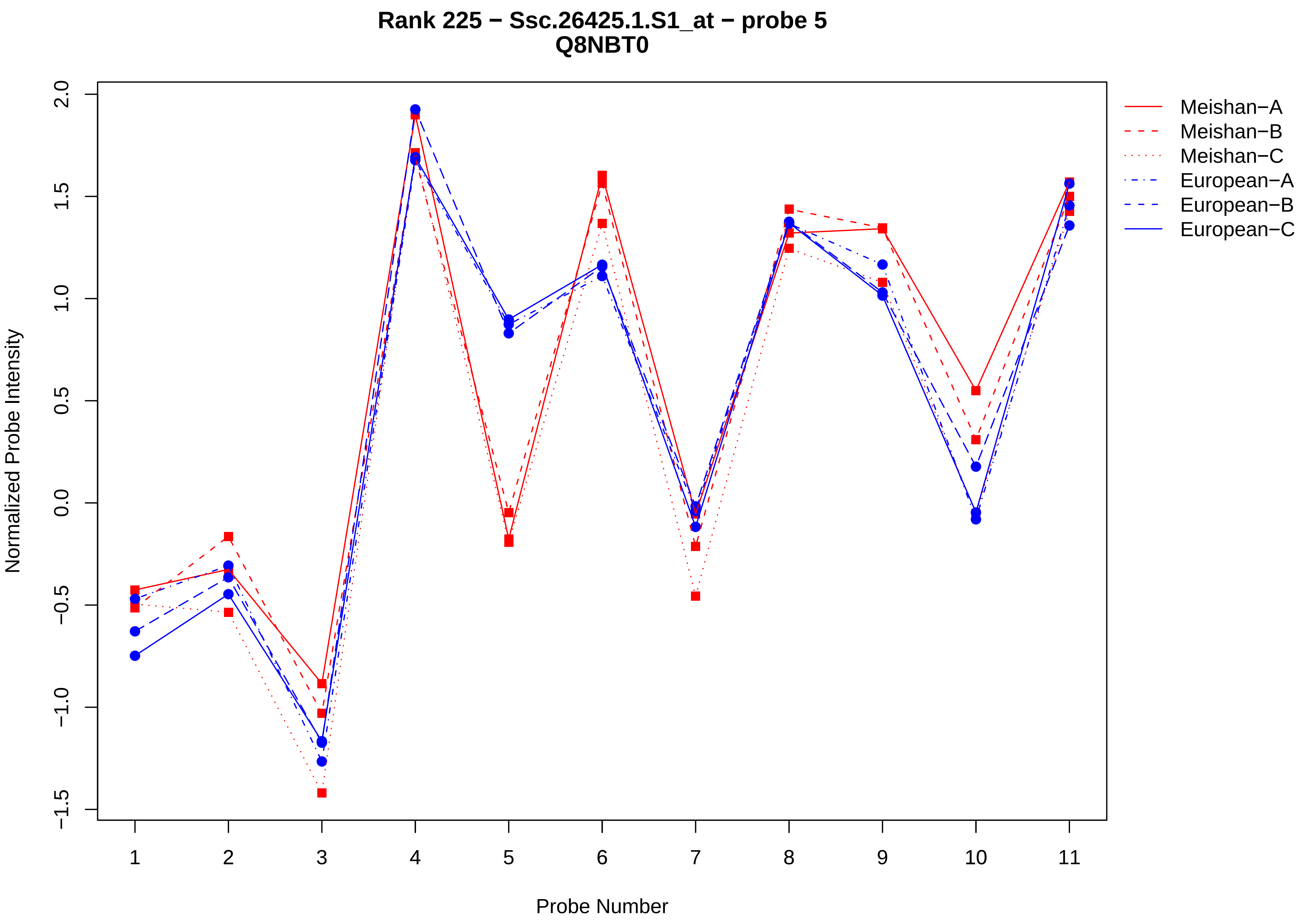
Rank 225 − Ssc.26425.1.S1_at − probe 5
Q8NBT0
Normalized Probe Intensity
Probe Number
−1.5
−1.0
−0.5
0.0
0.5
1.0
1.5
2.0
1
2
3
4
5
6
7
8
9
10
11
Meishan−A
Meishan−B
Meishan−C
European−A
European−B
European−C

Rank 226 − Ssc.786.1.S1_at − probe 1
CPSF3

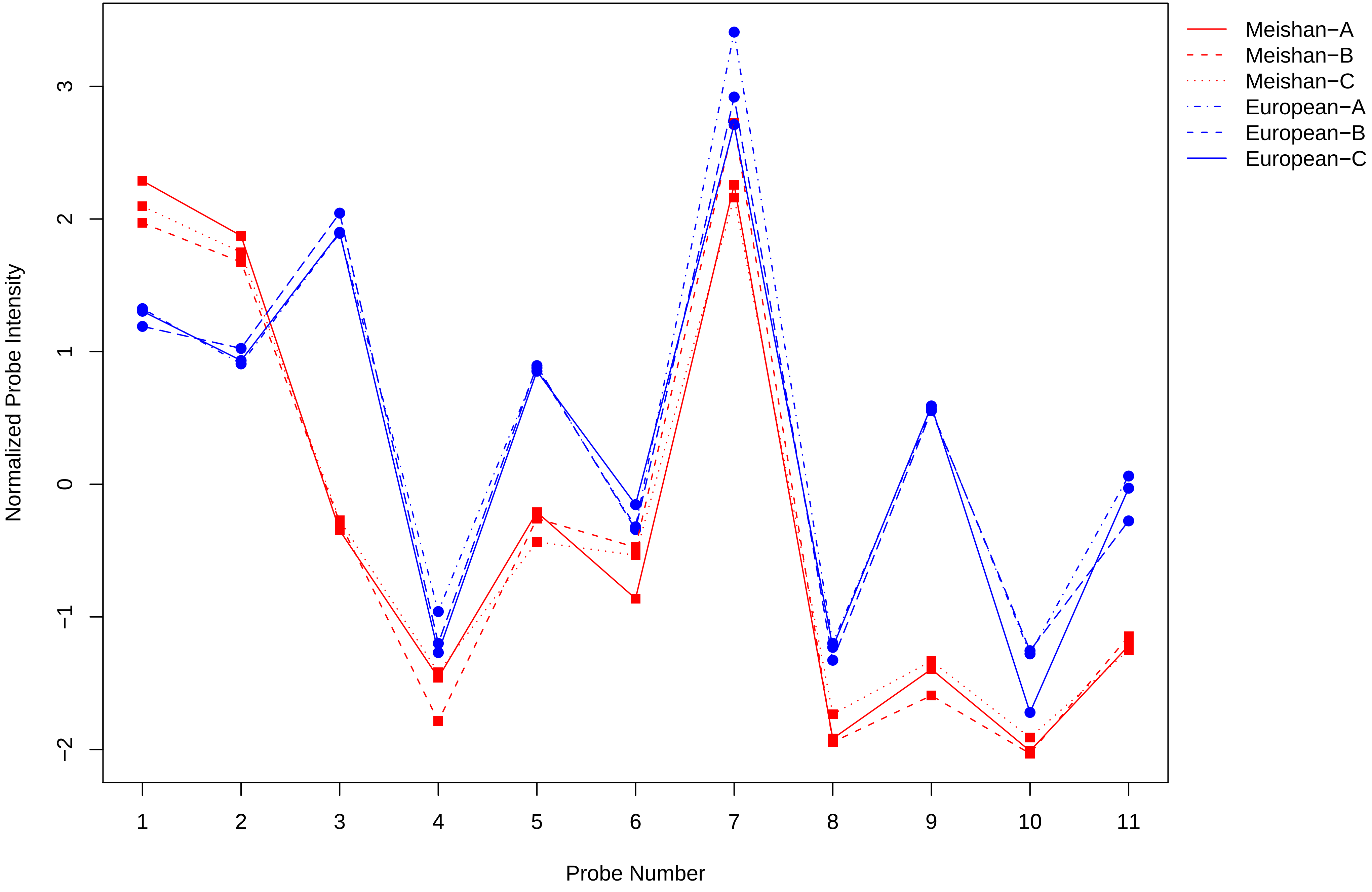

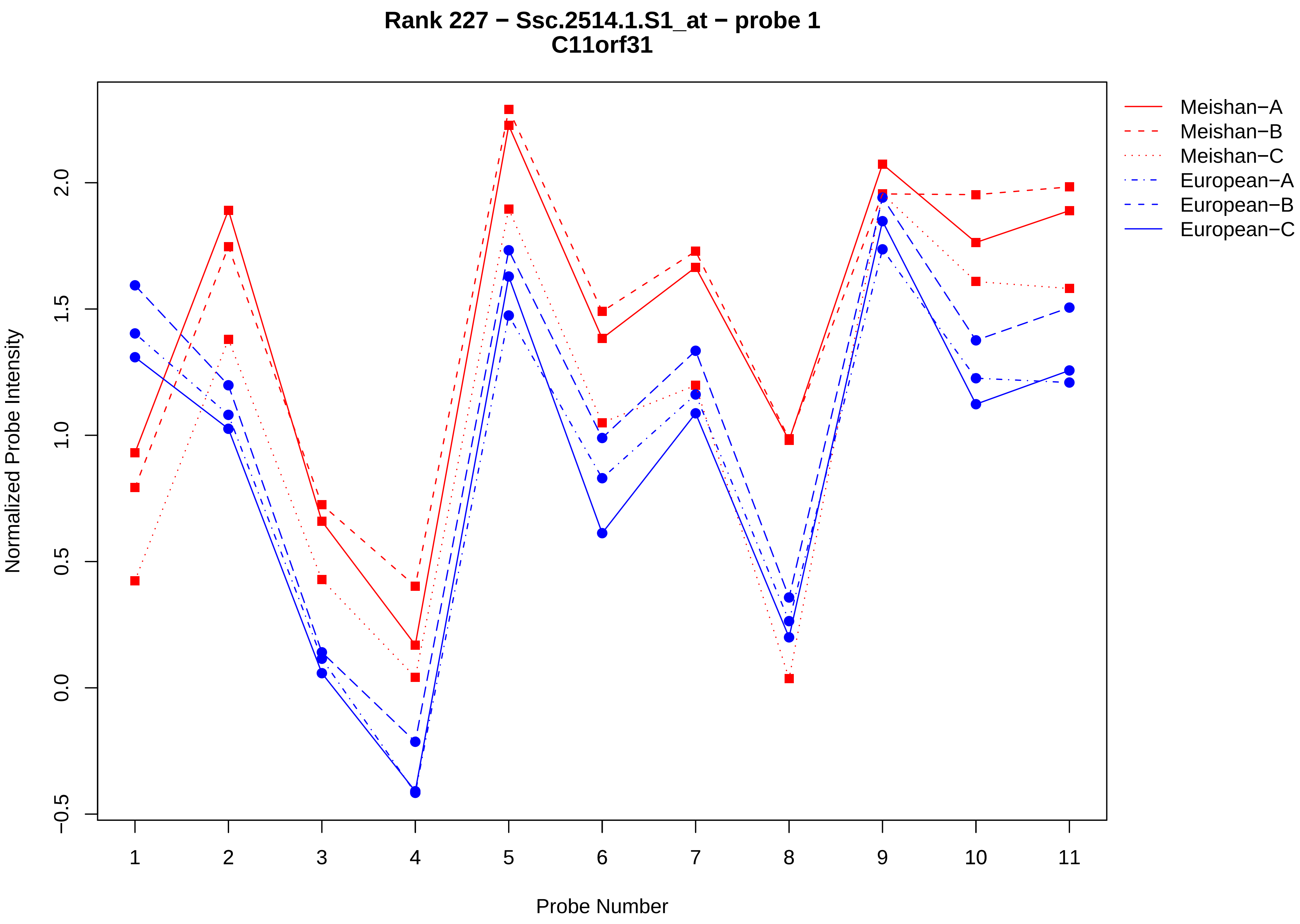

Rank 227 − Ssc.2514.1.S1_at − probe 1
C11orf31
Normalized Probe Intensity
−0.5
0.0
0.5
1.0
1.5
2.0
1
2
3
4
5
6
7
8
9
10
11
Probe Number
Meishan−A
Meishan−B
Meishan−C
European−A
European−B
European−C

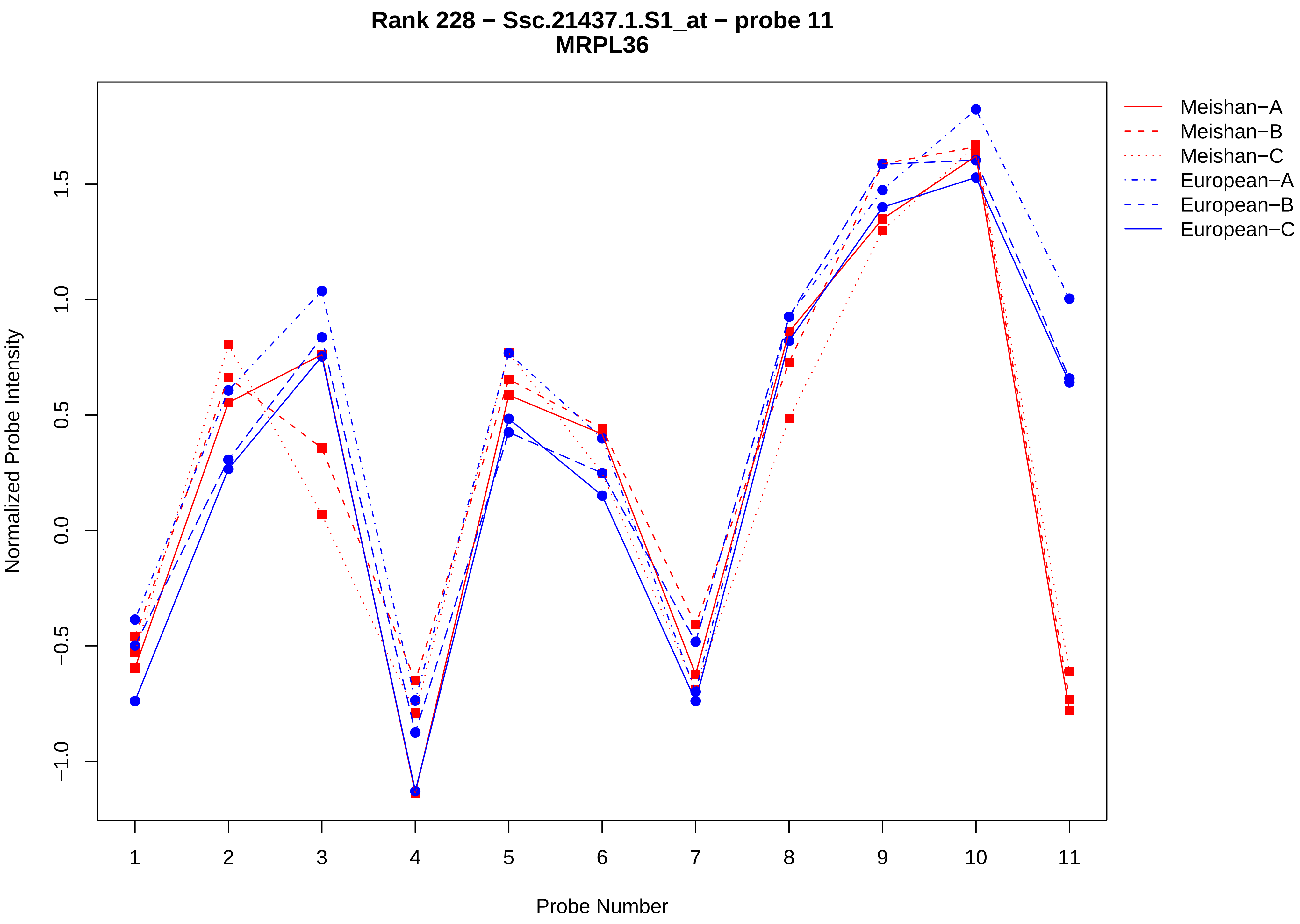

Rank 228 − Ssc.21437.1.S1_at − probe 11
MRPL36
Normalized Probe Intensity
Probe Number
−1.0
−0.5
0.0
0.5
1.0
1.5
1
2
3
4
5
6
7
8
9
10
11
Meishan−A
Meishan−B
Meishan−C
European−A
European−B
European−C

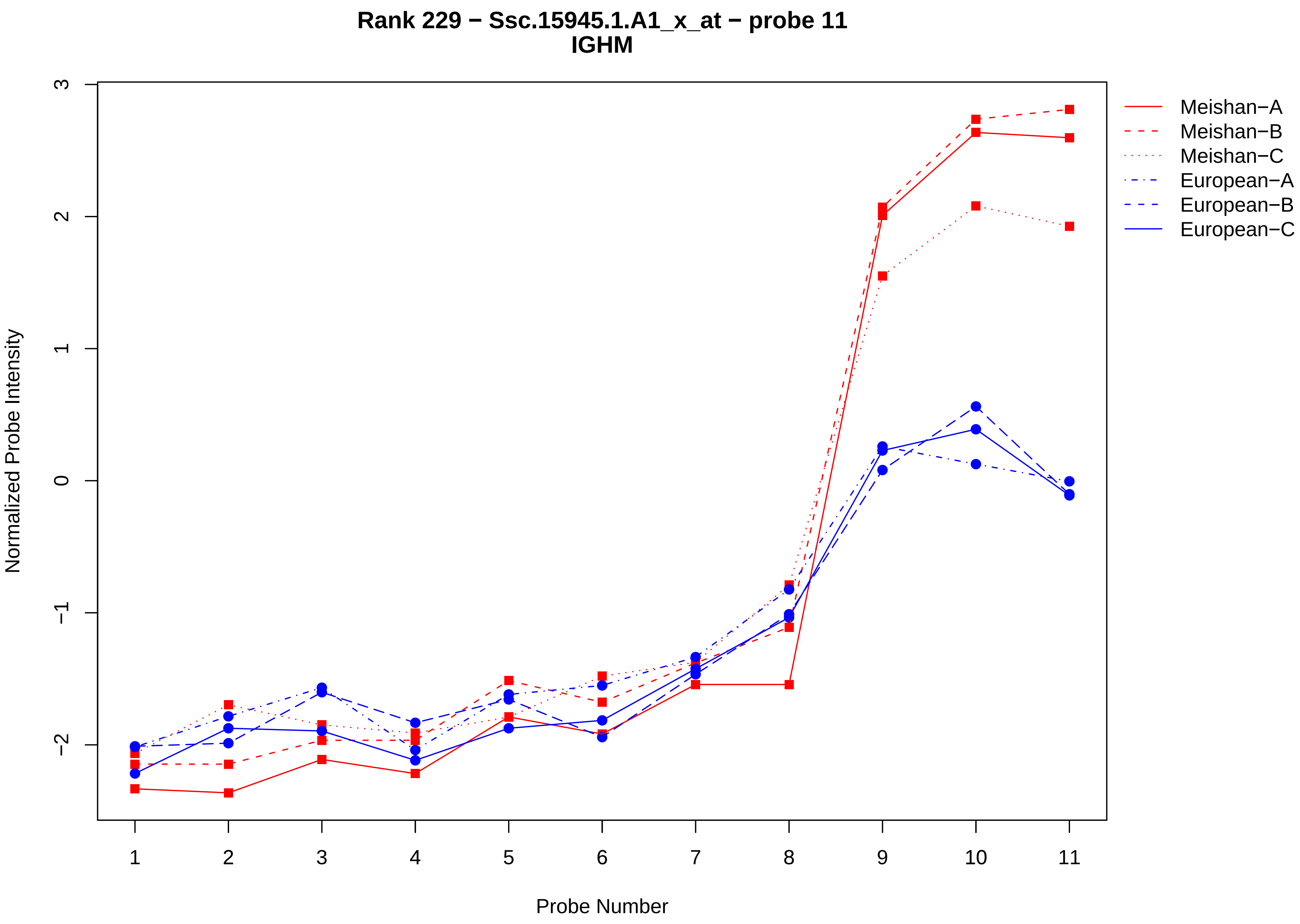

Rank 229 − Ssc.15945.1.A1_x_at − probe 11
IGHM
Normalized Probe Intensity
Probe Number
−2
−1
0
1
2
3
1
2
3
4
5
6
7
8
9
10
11
Meishan−A
Meishan−B
Meishan−C
European−A
European−B
European−C

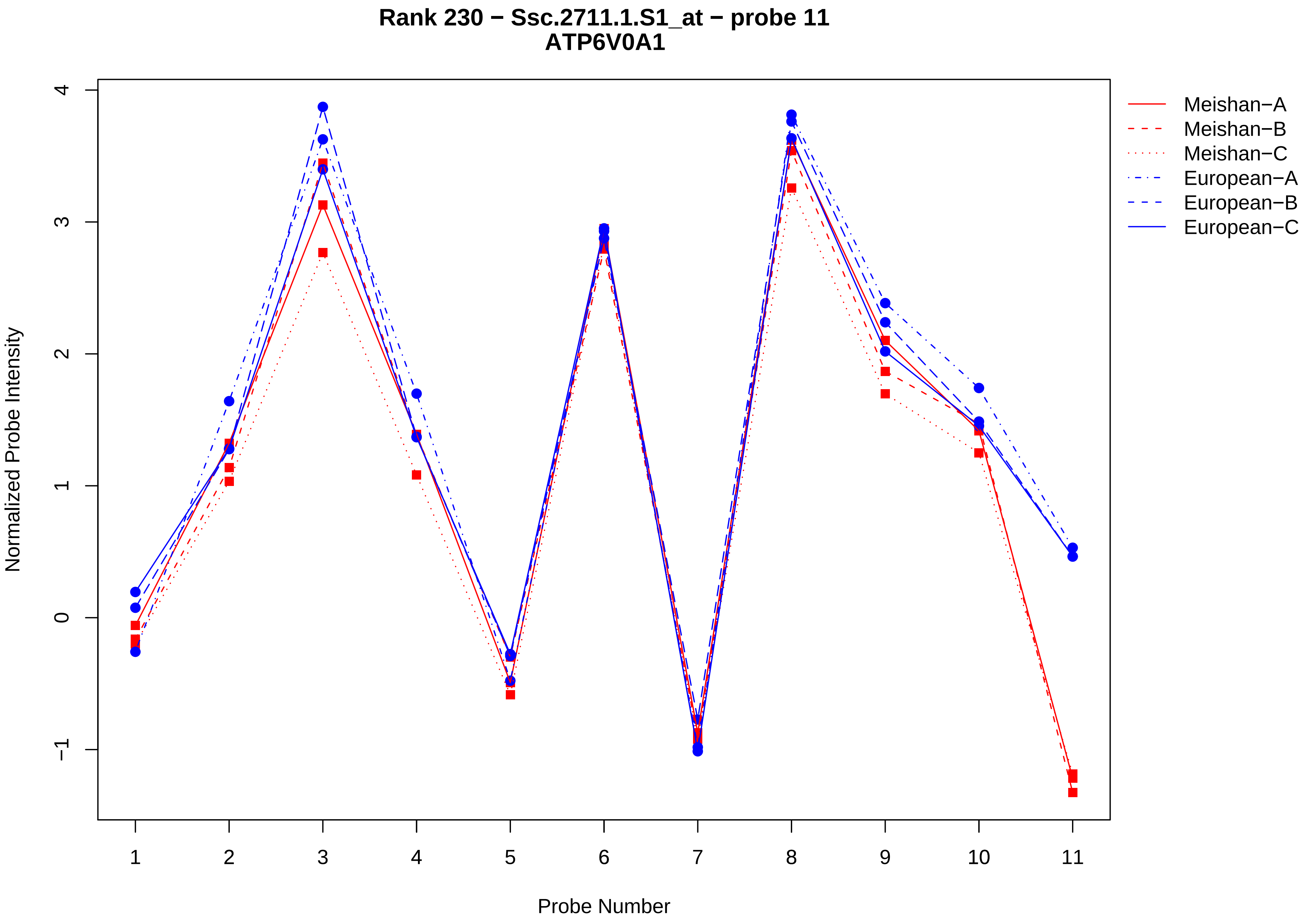

Rank 230 − Ssc.2711.1.S1_at − probe 11
ATP6V0A1
Normalized Probe Intensity
Probe Number
Meishan−A
Meishan−B
Meishan−C
European−A
European−B
European−C

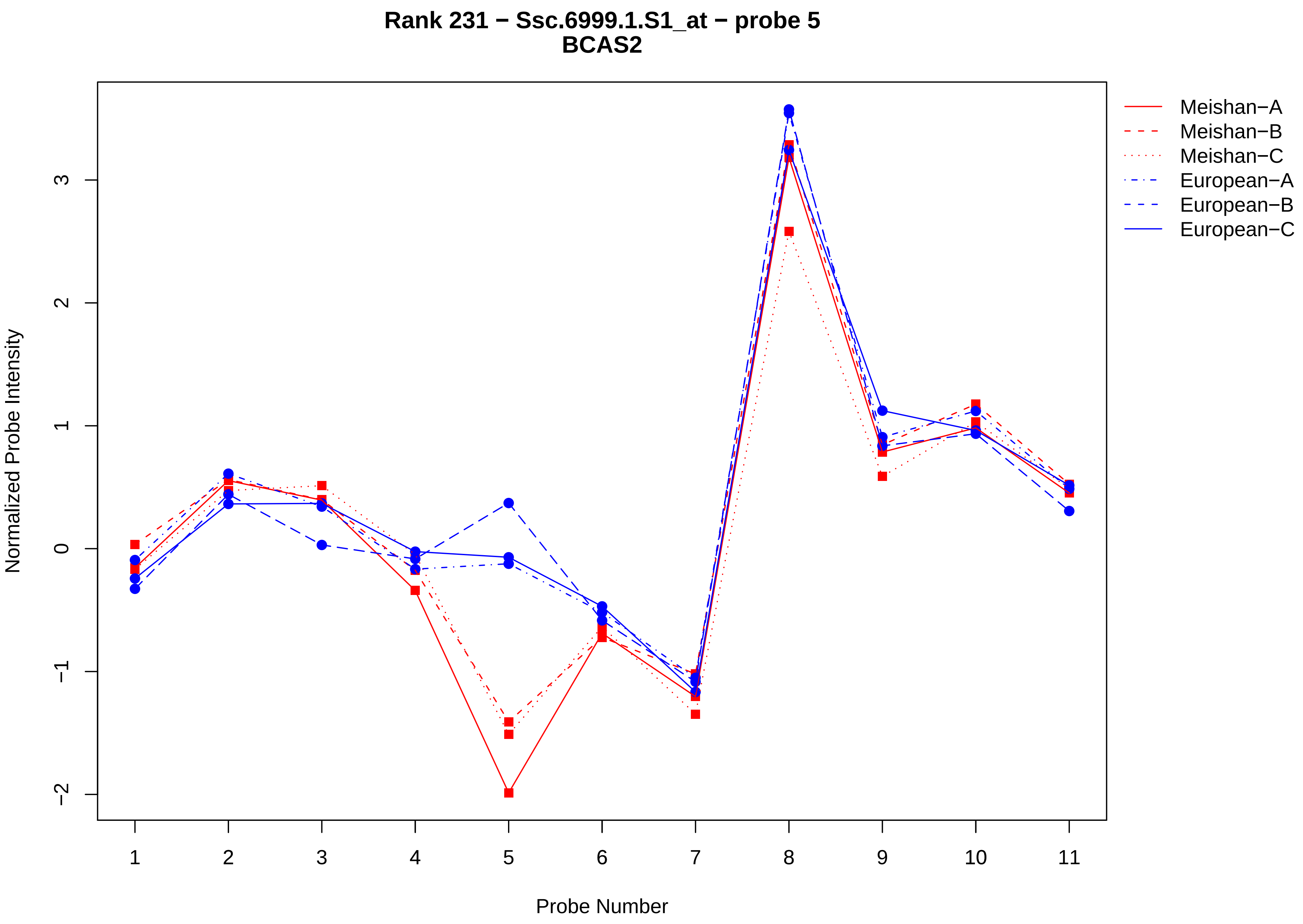
Rank 231 − Ssc.6999.1.S1_at − probe 5
BCAS2
Normalized Probe Intensity
Probe Number
Meishan−A
Meishan−B
Meishan−C
European−A
European−B
European−C
−2
−1
0
1
2
3
1
2
3
4
5
6
7
8
9
10
11

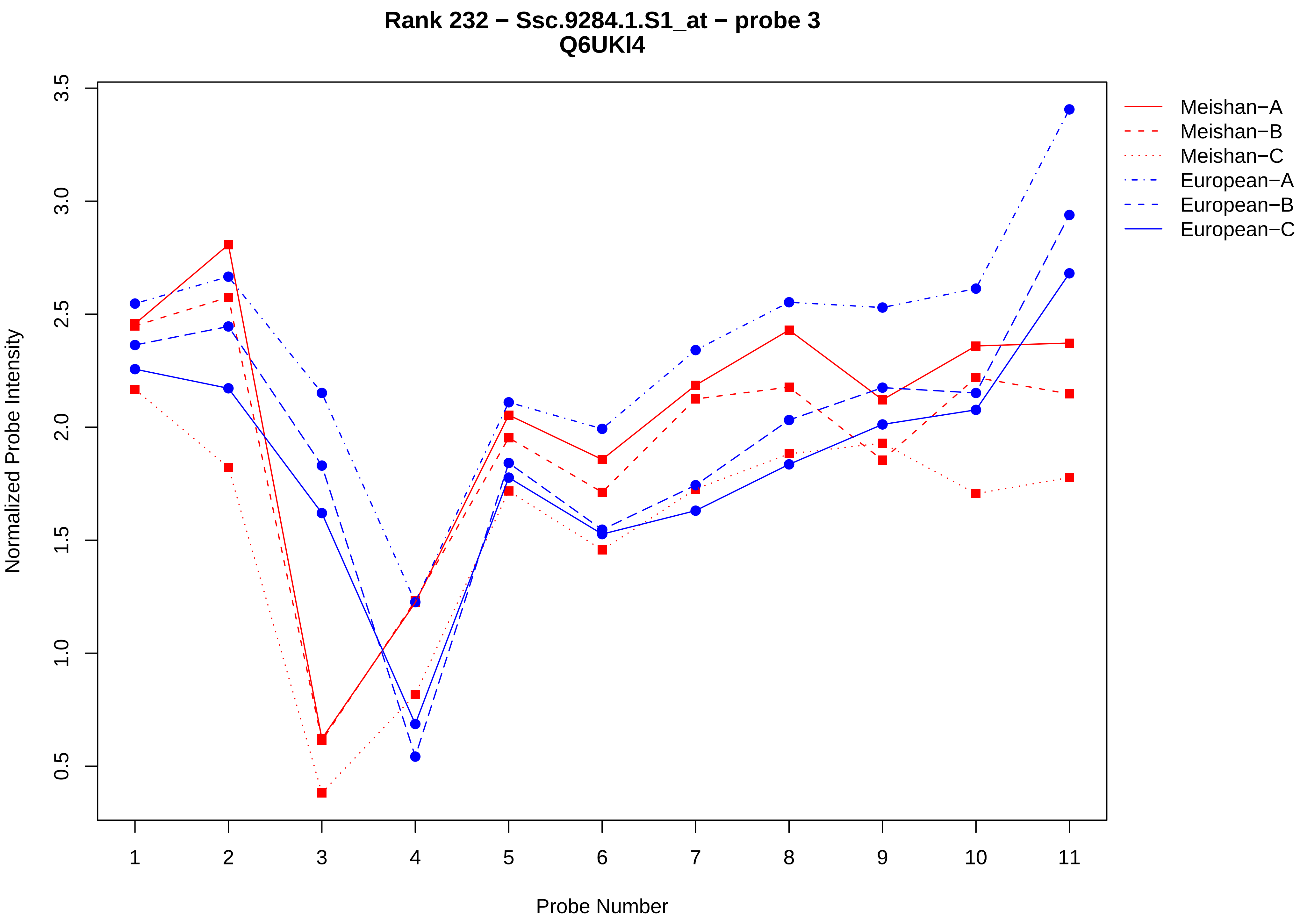

Rank 232 − Ssc.9284.1.S1_at − probe 3
Q6UKI4
Normalized Probe Intensity
Probe Number
0.5
1.0
1.5
2.0
2.5
3.0
3.5
1
2
3
4
5
6
7
8
9
10
11
Meishan−A
Meishan−B
Meishan−C
European−A
European−B
European−C

Rank 233 − Ssc.17315.1.S1_at − probe 3
EIF3S2

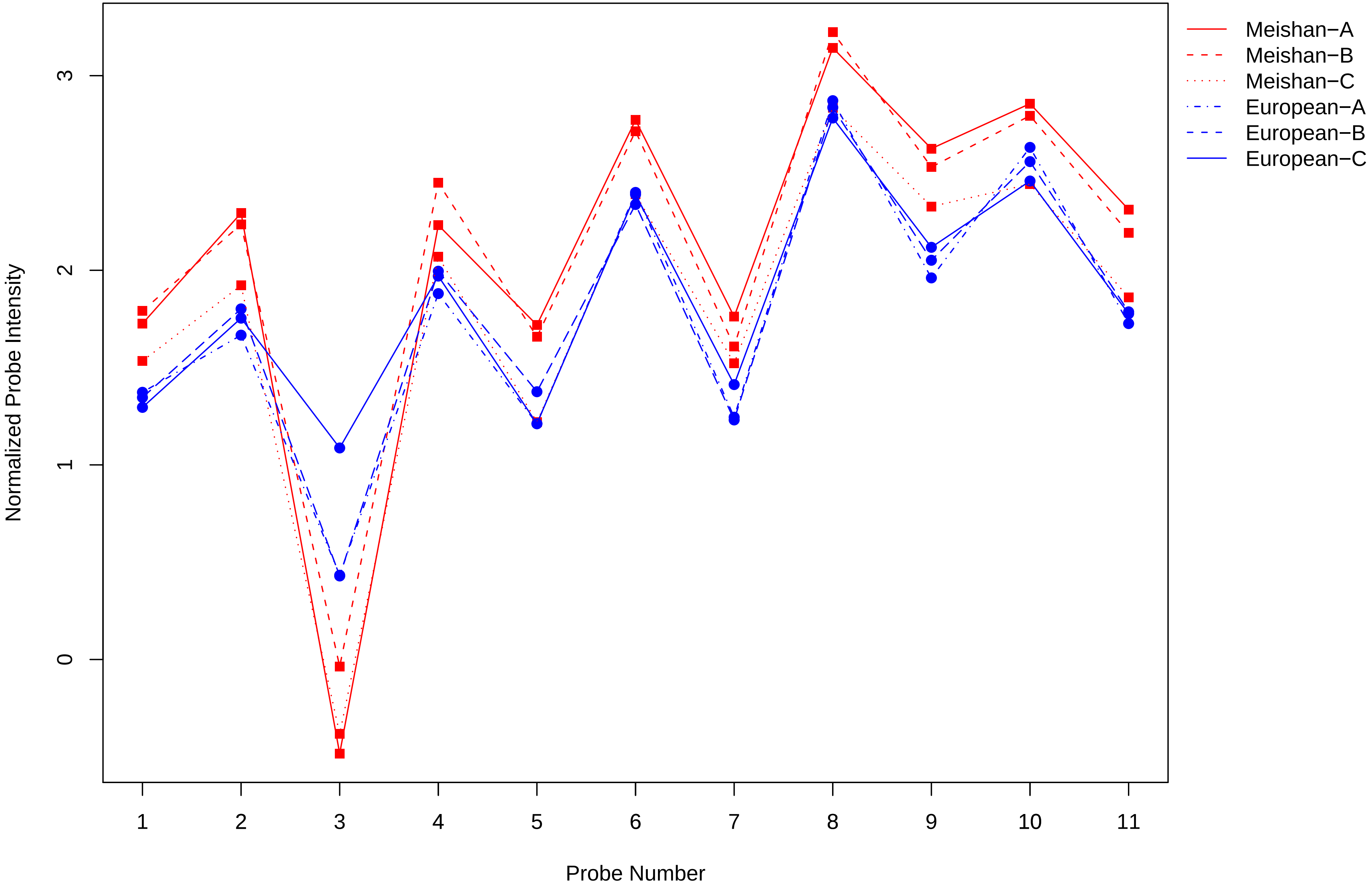

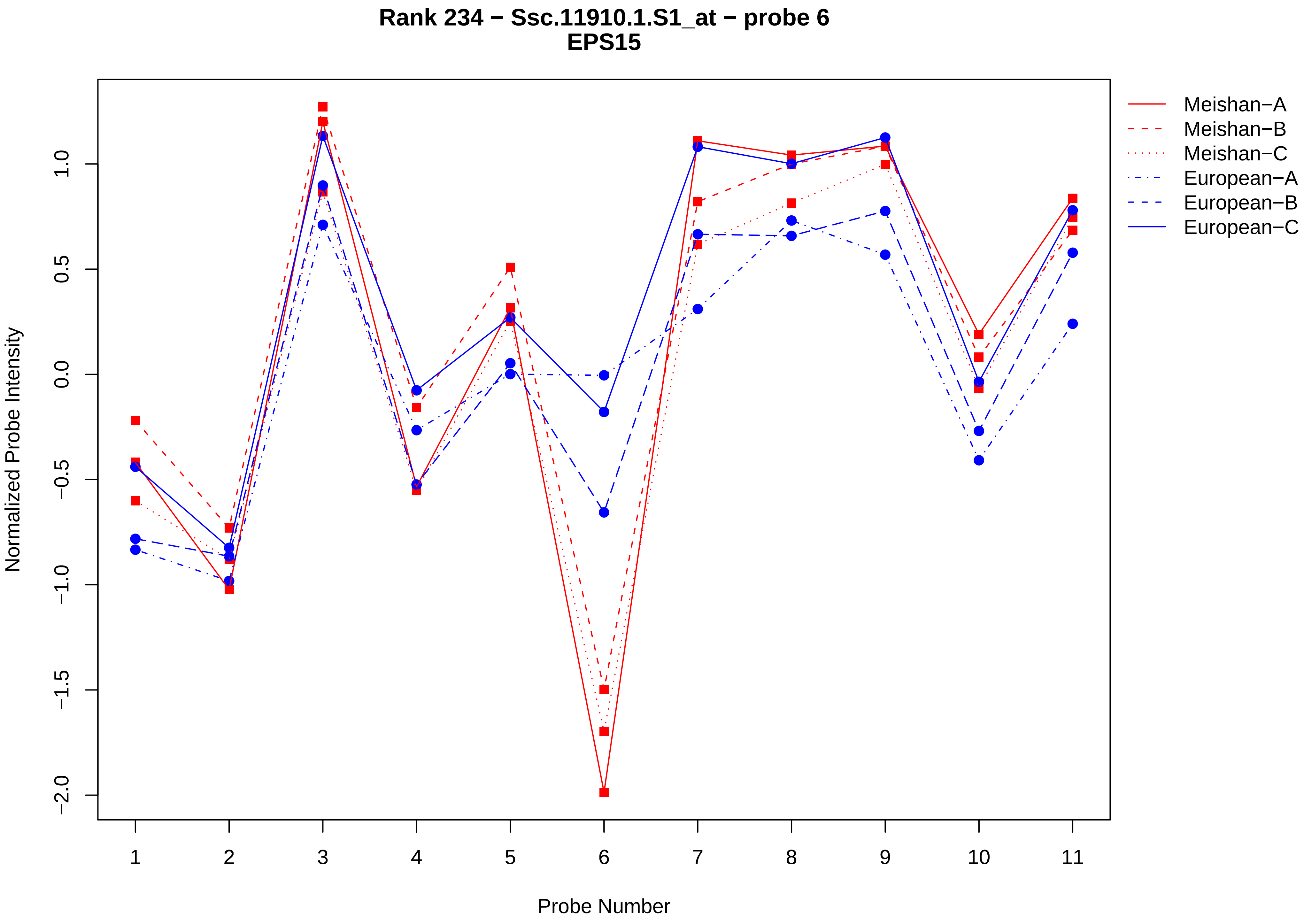

Rank 234 − Ssc.11910.1.S1_at − probe 6
EPS15
Normalized Probe Intensity
Probe Number
1.0
0.5
0.0
−0.5
−1.0
−1.5
−2.0
1
2
3
4
5
6
7
8
9
10
11
Meishan−A
Meishan−B
Meishan−C
European−A
European−B
European−C

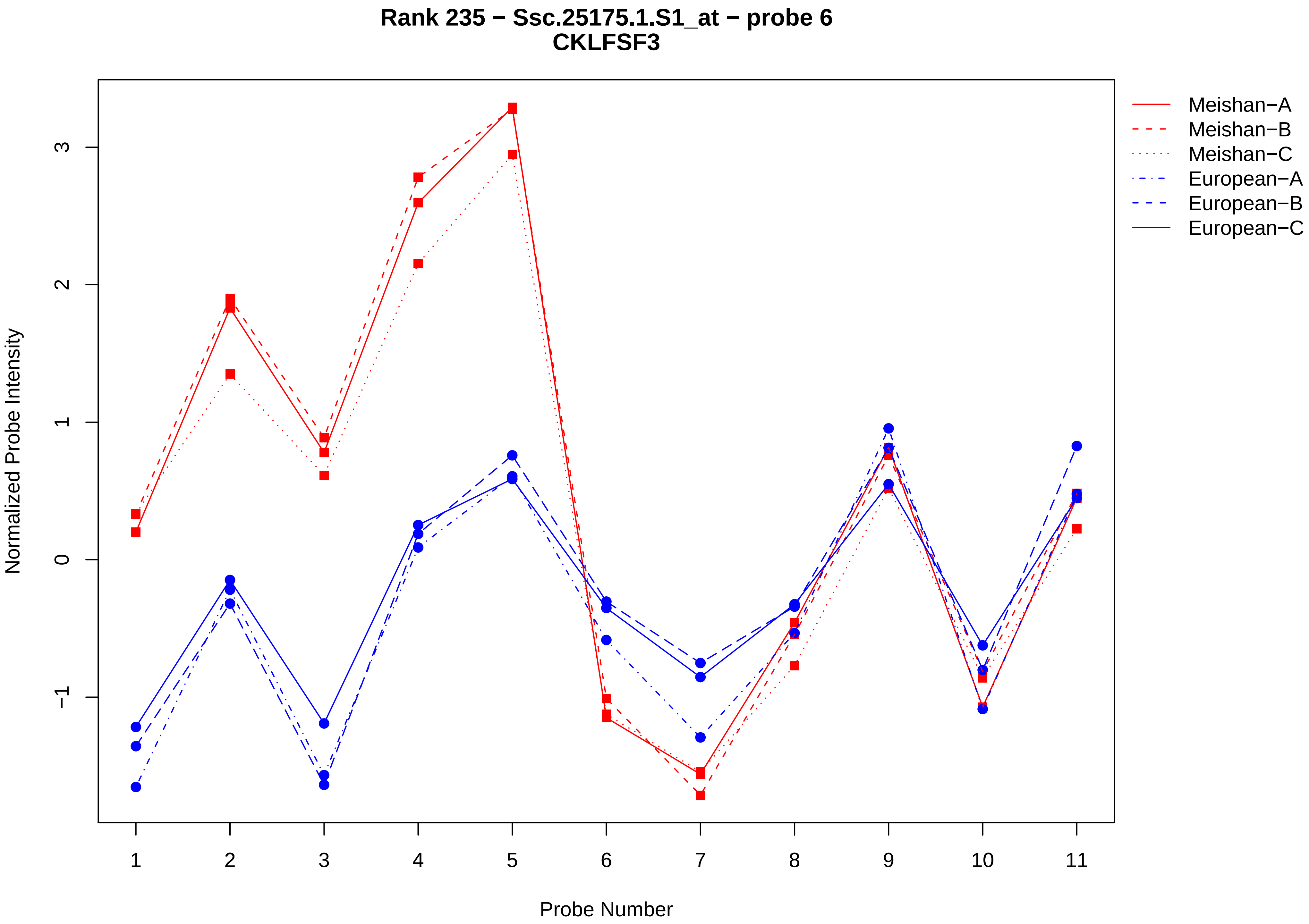

Rank 235 − Ssc.25175.1.S1_at − probe 6
CKLFSF3
Normalized Probe Intensity
Probe Number
−1
0
1
2
3
1
2
3
4
5
6
7
8
9
10
11
Meishan−A
Meishan−B
Meishan−C
European−A
European−B
European−C

Rank 236 − Ssc.786.1.S1_at − probe 2
CPSF3

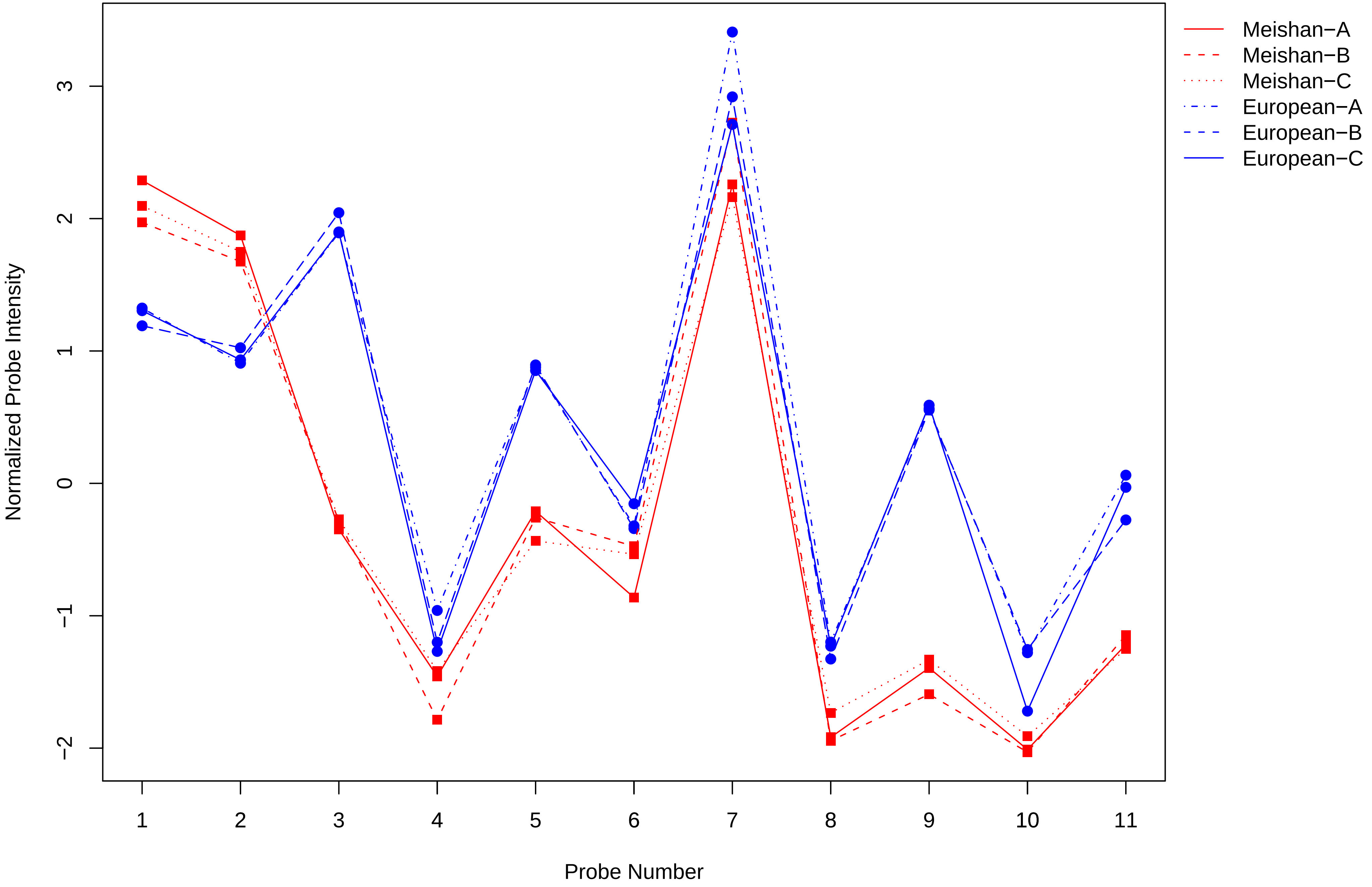

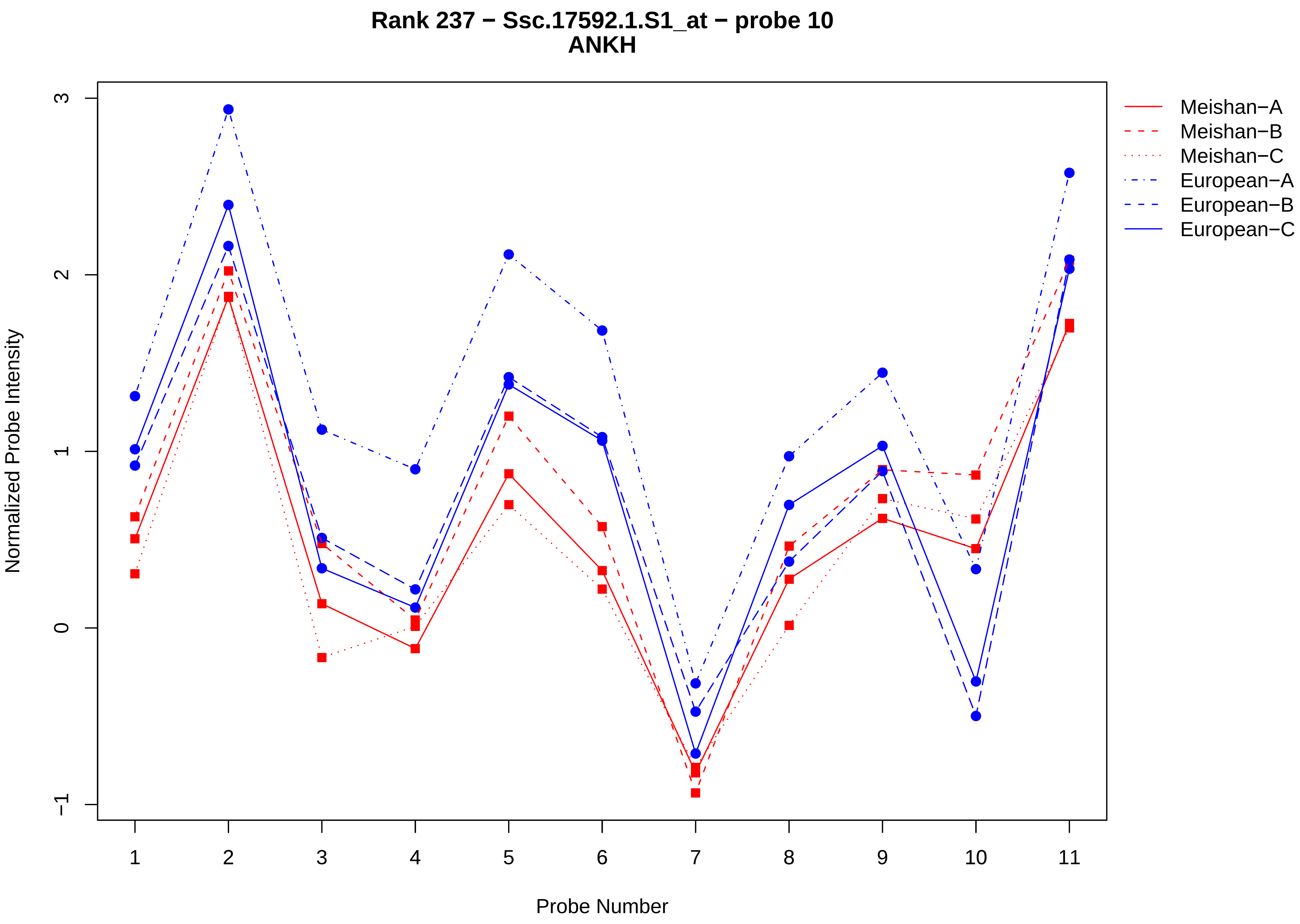

Rank 237 − Ssc.17592.1.S1_at − probe 10
ANKH
Normalized Probe Intensity
Probe Number
Meishan−A
Meishan−B
Meishan−C
European−A
European−B
European−C
−1
0
1
2
3
1
2
3
4
5
6
7
8
9
10
11

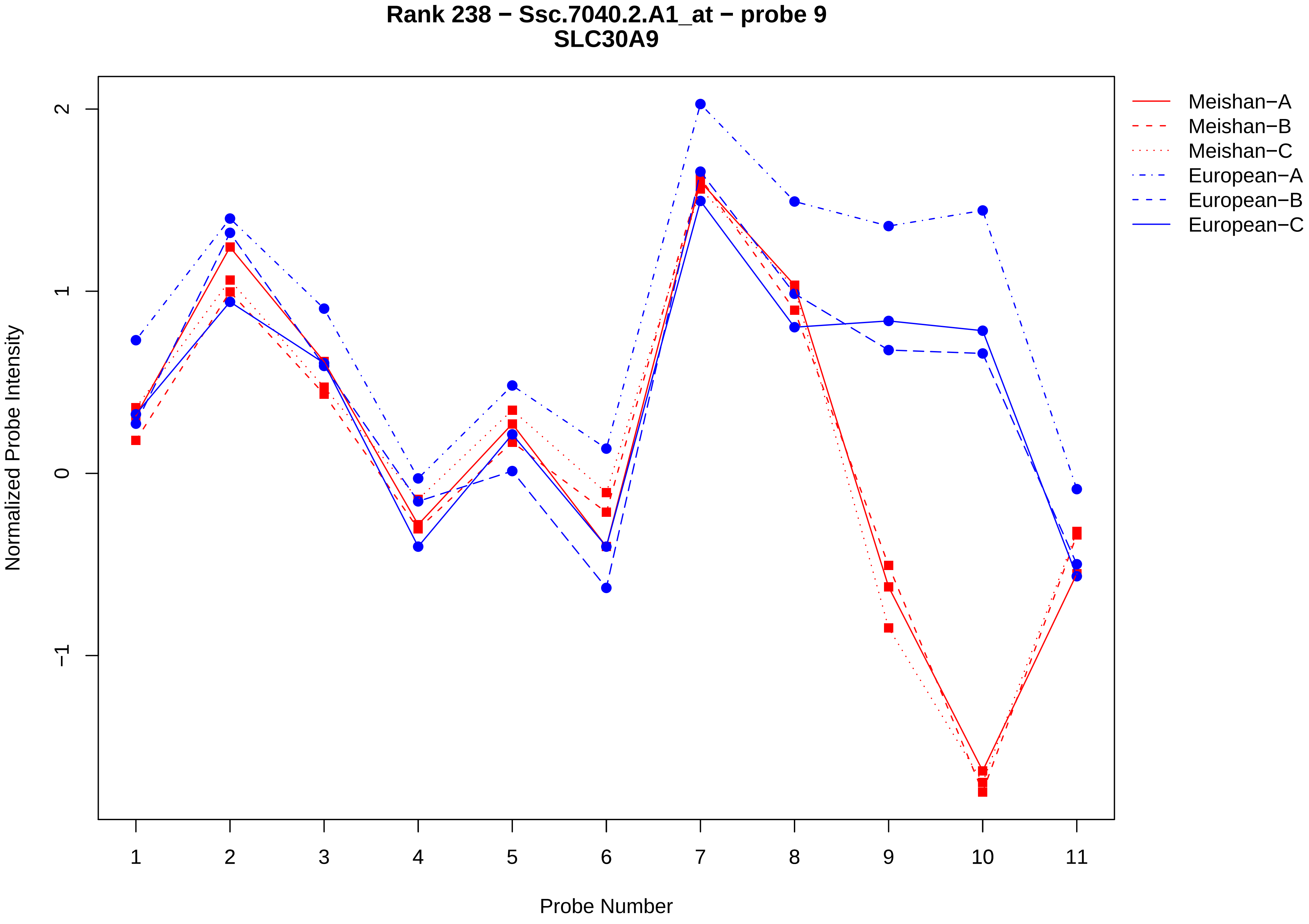

Rank 238 − Ssc.7040.2.A1_at − probe 9
SLC30A9
Normalized Probe Intensity
Probe Number
Meishan−A
Meishan−B
Meishan−C
European−A
European−B
European−C
−1
0
1
2
1
2
3
4
5
6
7
8
9
10
11

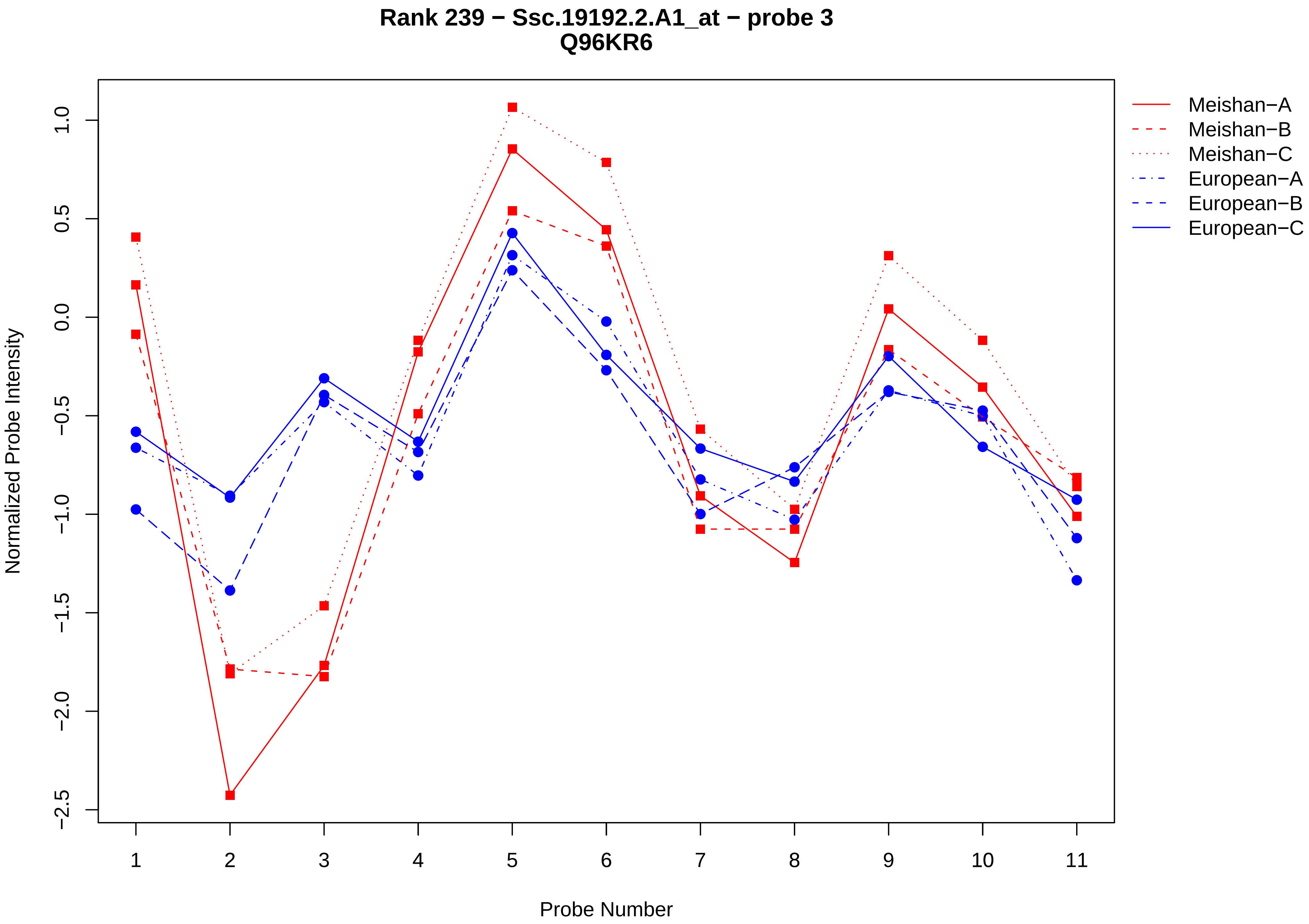

Rank 239 − Ssc.19192.2.A1_at − probe 3
Q96KR6
Normalized Probe Intensity
Probe Number
1.0
0.5
0.0
−0.5
−1.0
−1.5
−2.0
−2.5
1
2
3
4
5
6
7
8
9
10
11
Meishan−A
Meishan−B
Meishan−C
European−A
European−B
European−C

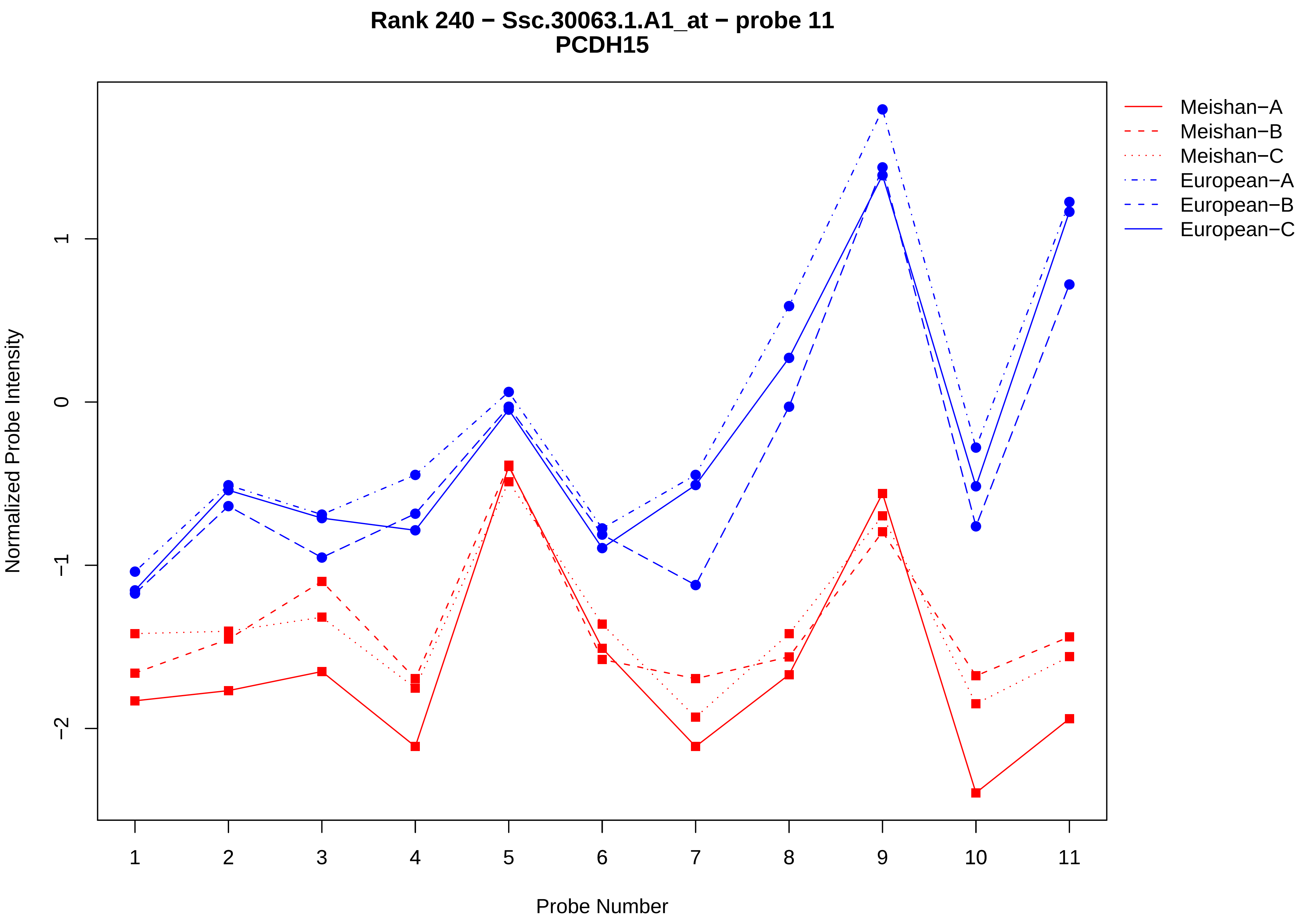

Rank 240 − Ssc.30063.1.A1_at − probe 11
PCDH15
Normalized Probe Intensity
−2
−1
0
1
1
2
3
4
5
6
7
8
9
10
11
Probe Number
Meishan−A
Meishan−B
Meishan−C
European−A
European−B
European−C

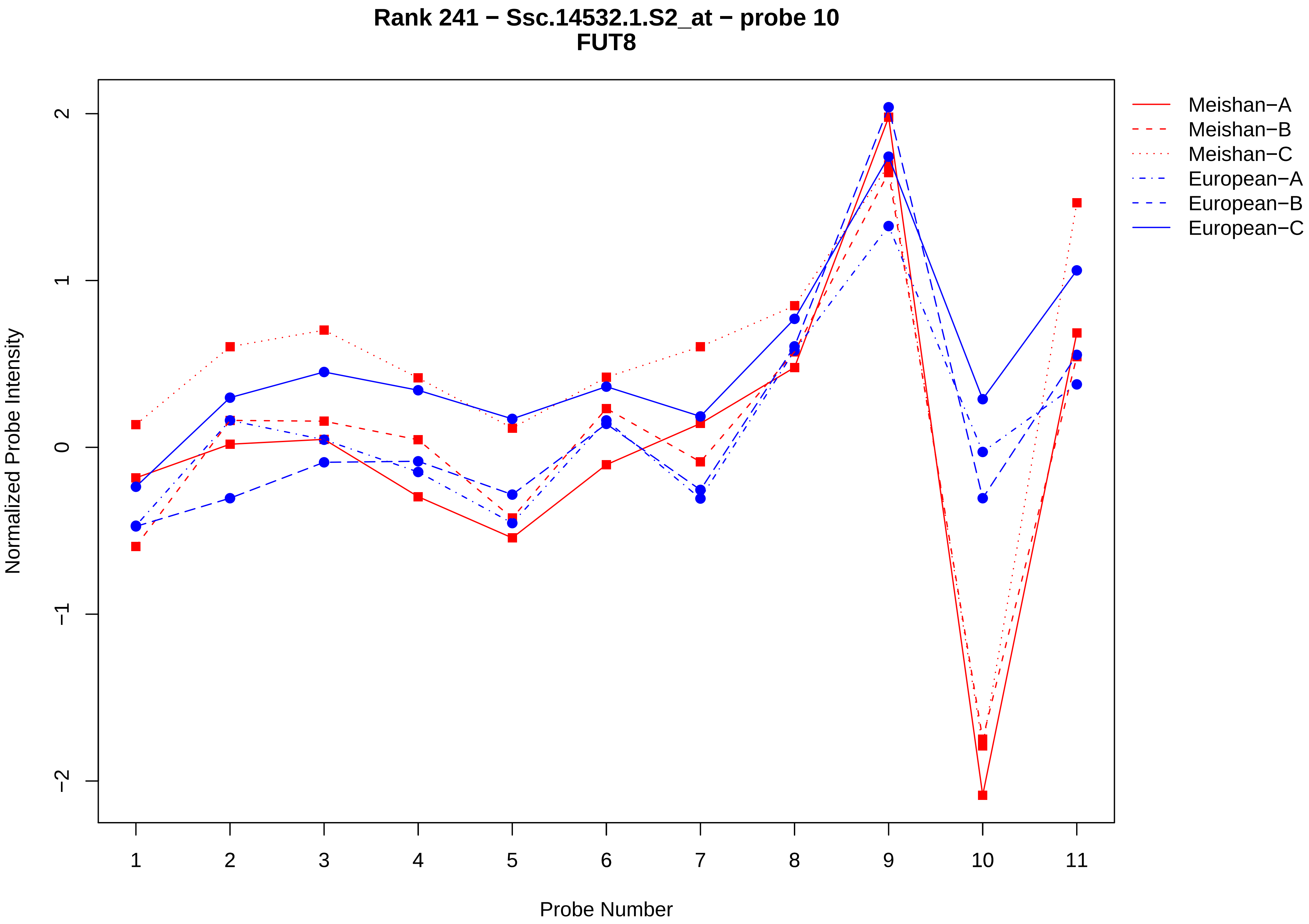
Rank 241 − Ssc.14532.1.S2_at − probe 10
FUT8
Normalized Probe Intensity
Probe Number
Meishan−A
Meishan−B
Meishan−C
European−A
European−B
European−C
2
1
0
−1
−2
1
2
3
4
5
6
7
8
9
10
11

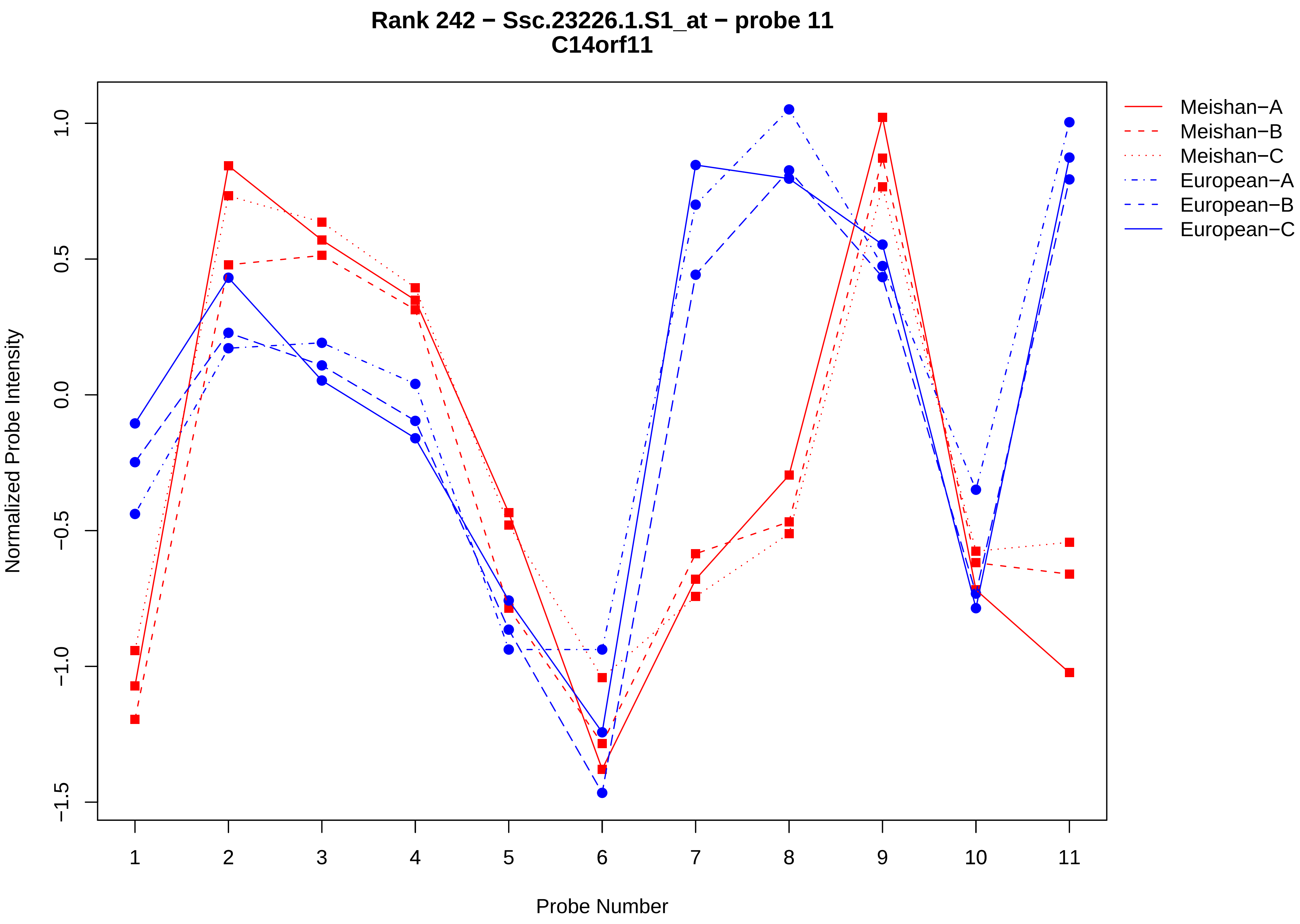
Rank 242 − Ssc.23226.1.S1_at − probe 11
C14orf11
Normalized Probe Intensity
1.0
0.5
0.0
−0.5
−1.0
−1.5
1
2
3
4
5
6
7
8
9
10
11
Probe Number
Meishan−A
Meishan−B
Meishan−C
European−A
European−B
European−C

Rank 243 − Ssc.24184.1.S1_at − probe 11
GPD1L

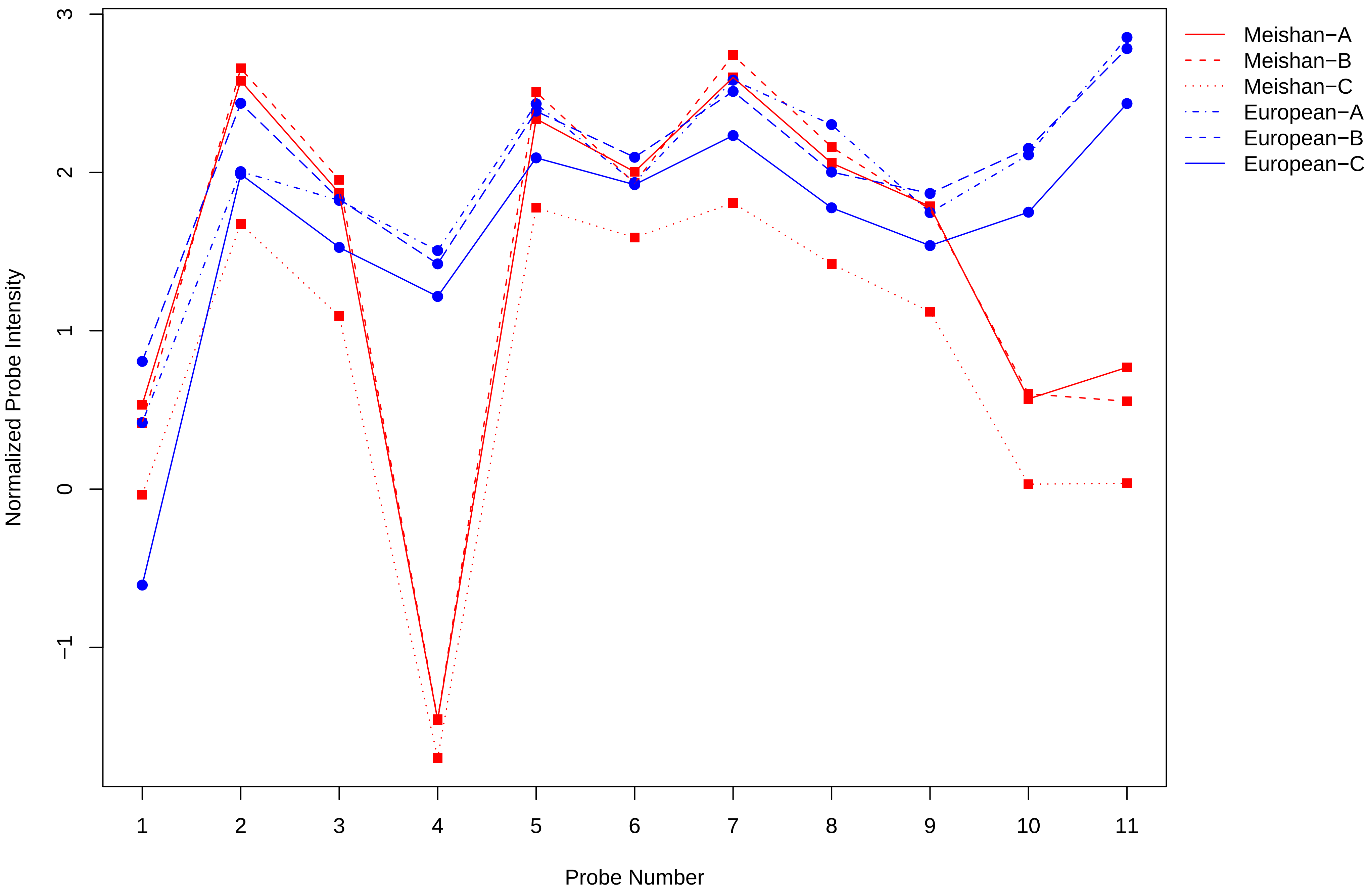

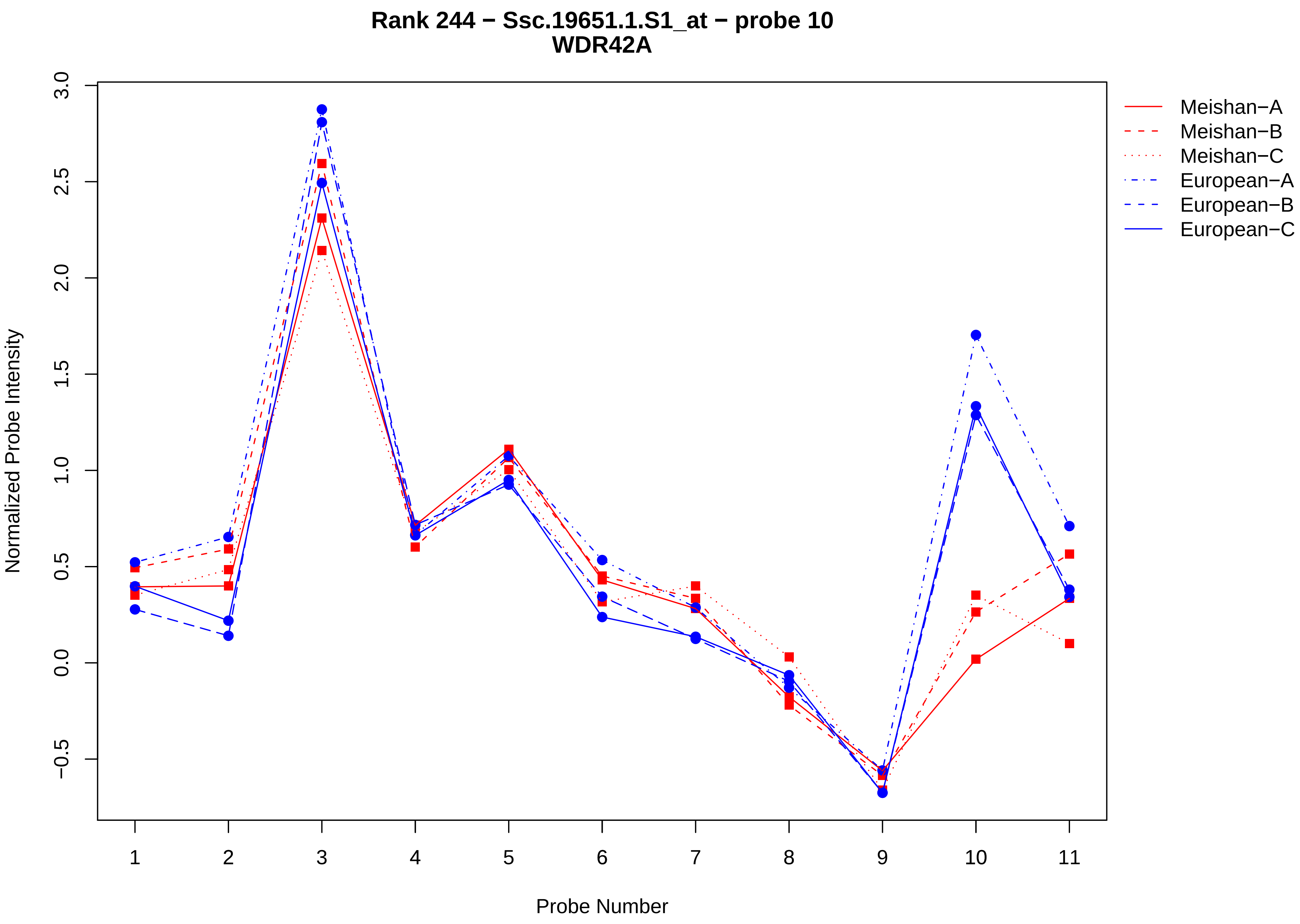

Rank 244 − Ssc.19651.1.S1_at − probe 10
WDR42A
Normalized Probe Intensity
Probe Number
−0.5
0.0
0.5
1.0
1.5
2.0
2.5
3.0
1
2
3
4
5
6
7
8
9
10
11
Meishan−A
Meishan−B
Meishan−C
European−A
European−B
European−C

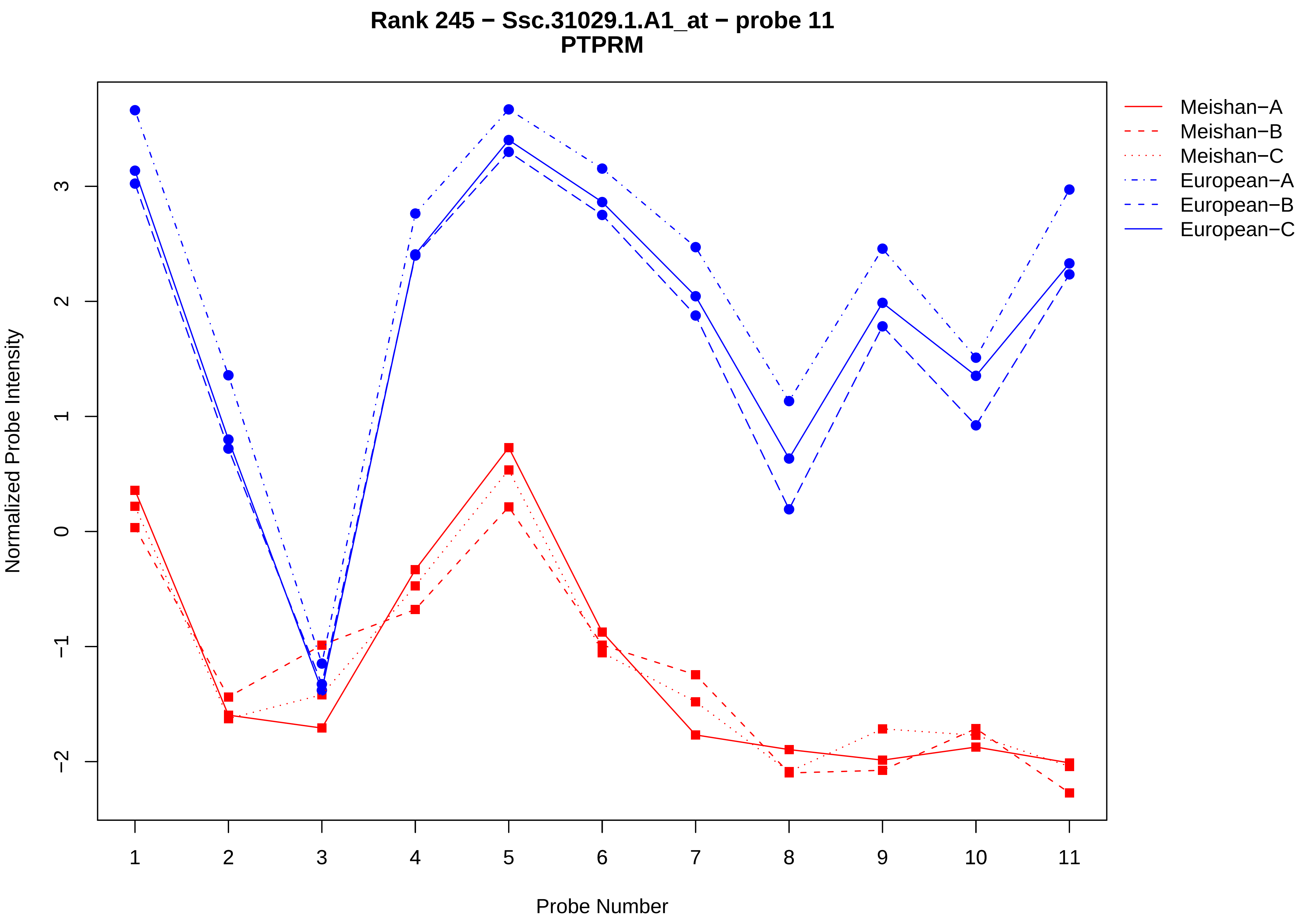

Rank 245 − Ssc.31029.1.A1_at − probe 11
PTPRM
Normalized Probe Intensity
Probe Number
Meishan−A
Meishan−B
Meishan−C
European−A
European−B
European−C

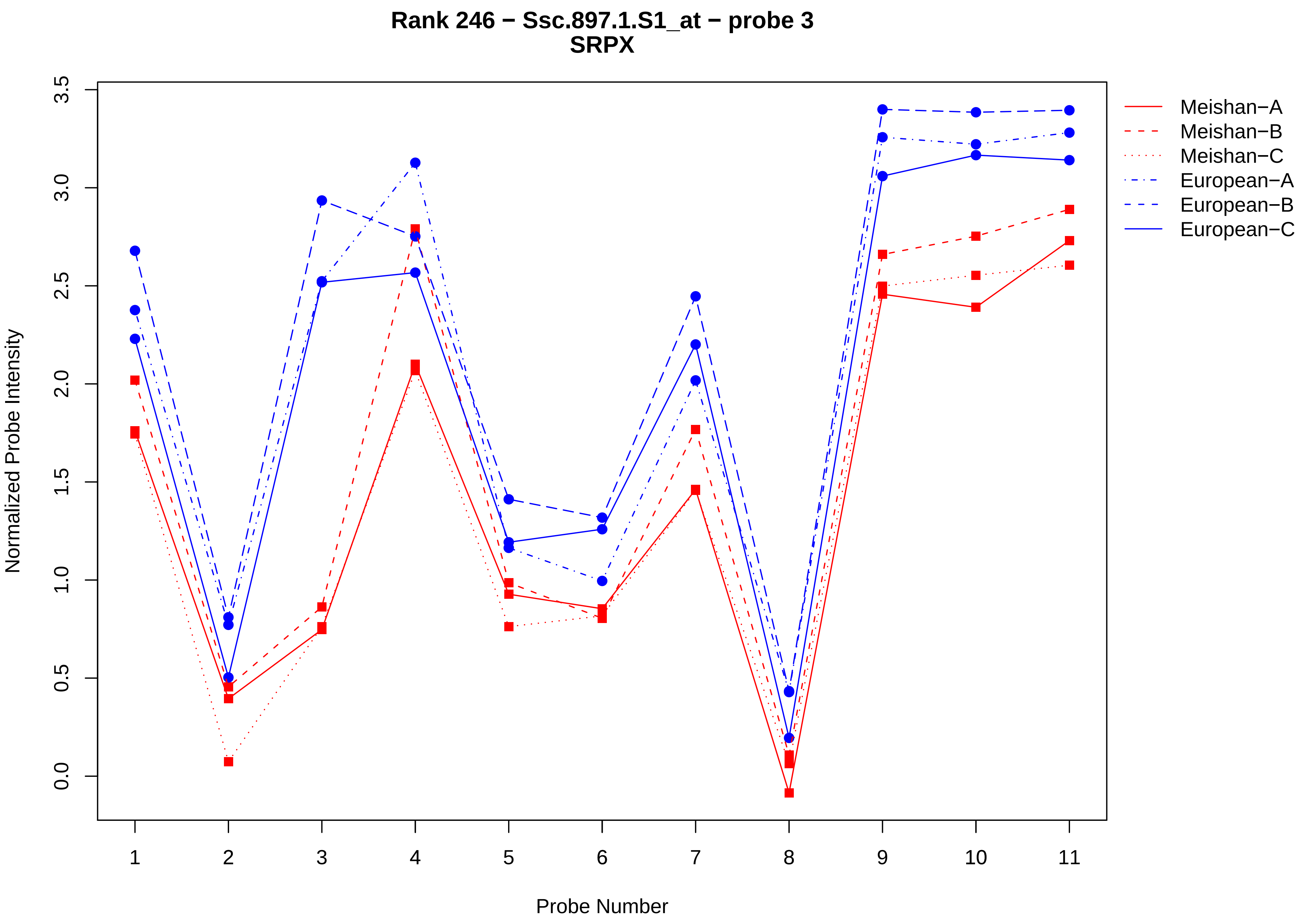

Rank 246 − Ssc.897.1.S1_at − probe 3
SRPX
Normalized Probe Intensity
0.0
0.5
1.0
1.5
2.0
2.5
3.0
3.5
1
2
3
4
5
6
7
8
9
10
11
Probe Number
Meishan−A
Meishan−B
Meishan−C
European−A
European−B
European−C

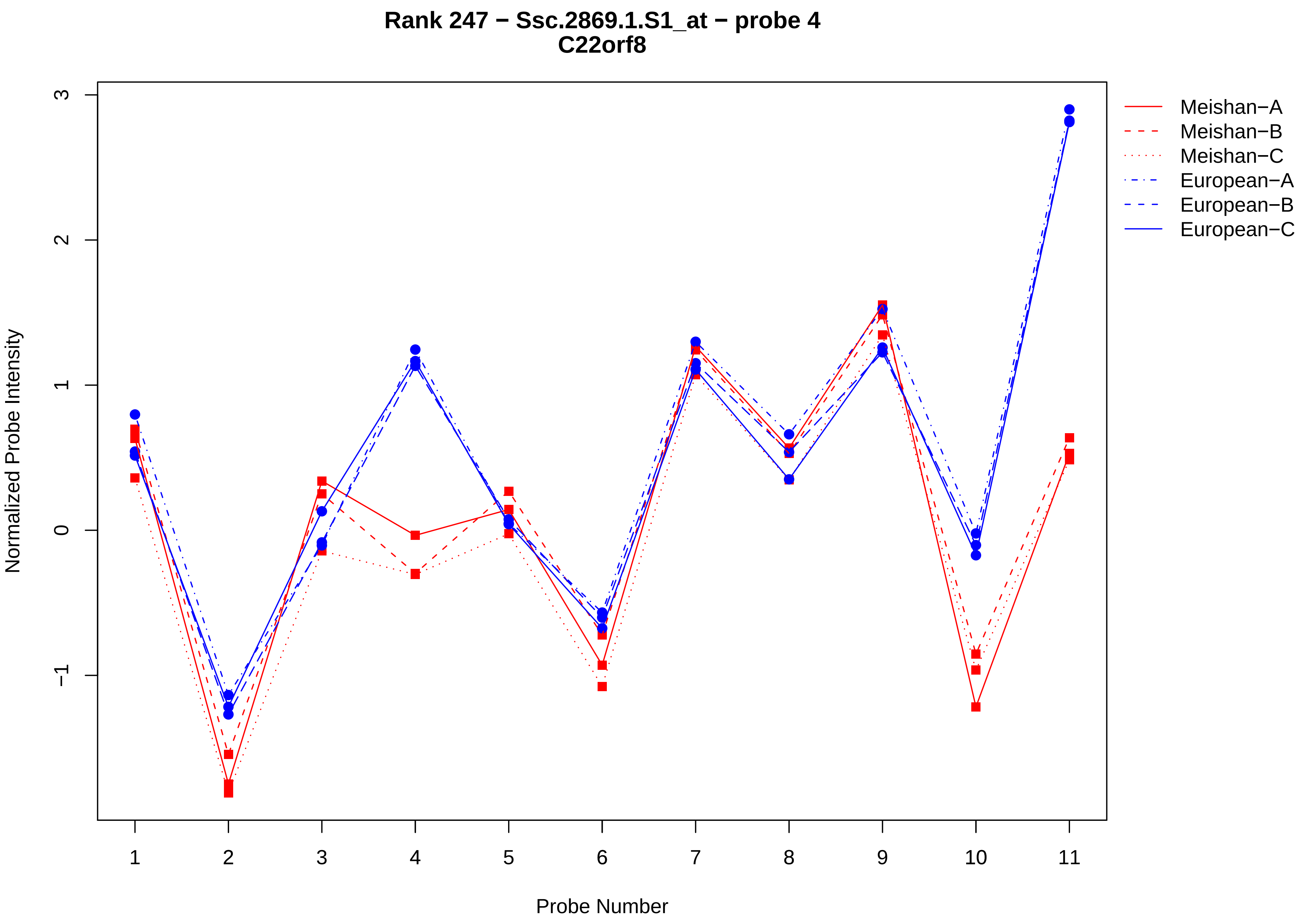

Rank 247 − Ssc.2869.1.S1_at − probe 4
C22orf8
Normalized Probe Intensity
Probe Number
Meishan−A
Meishan−B
Meishan−C
European−A
European−B
European−C

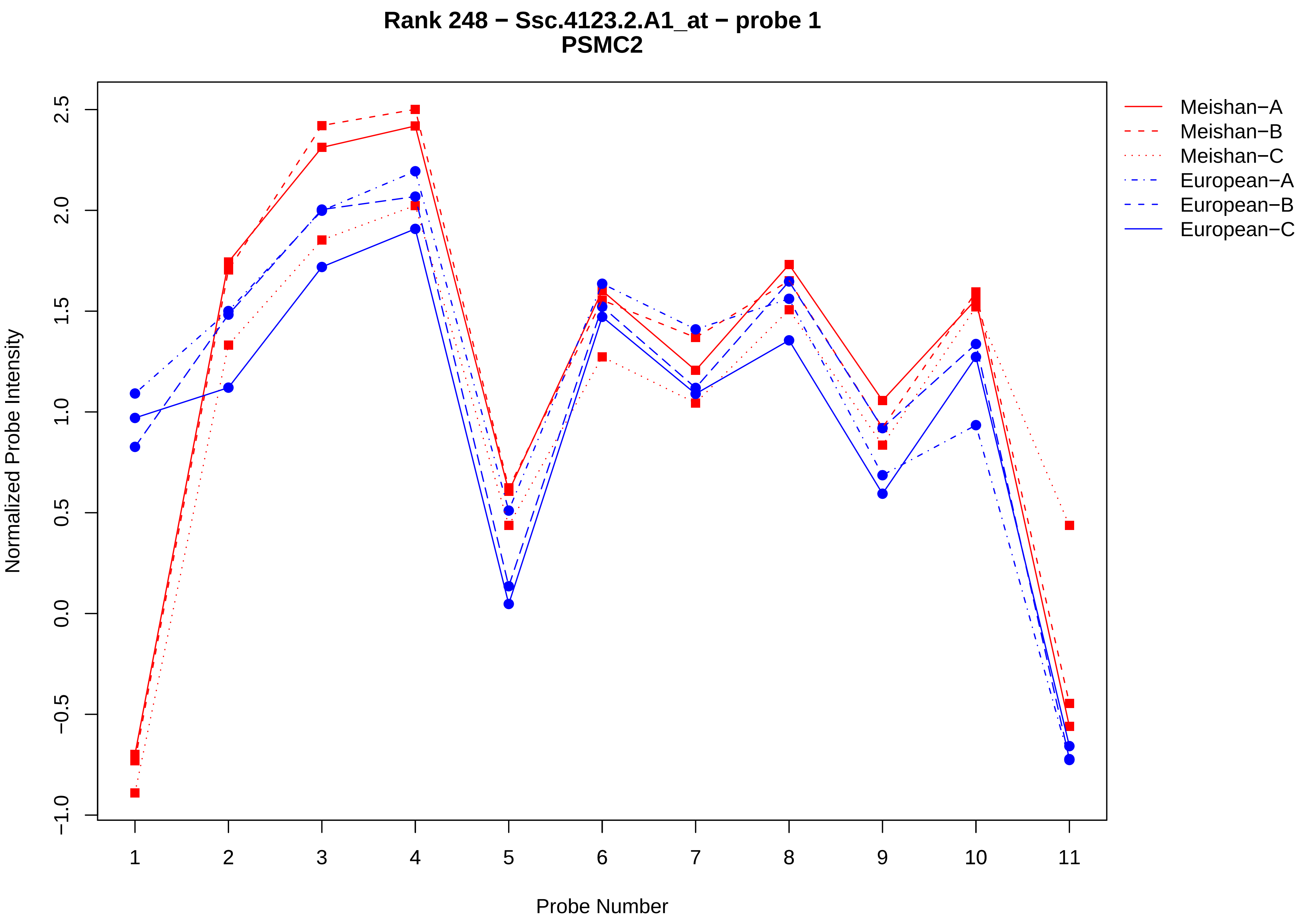

Rank 248 − Ssc.4123.2.A1_at − probe 1
PSMC2
Normalized Probe Intensity
Probe Number
Meishan−A
Meishan−B
Meishan−C
European−A
European−B
European−C
−1.0
−0.5
0.0
0.5
1.0
1.5
2.0
2.5
1
2
3
4
5
6
7
8
9
10
11

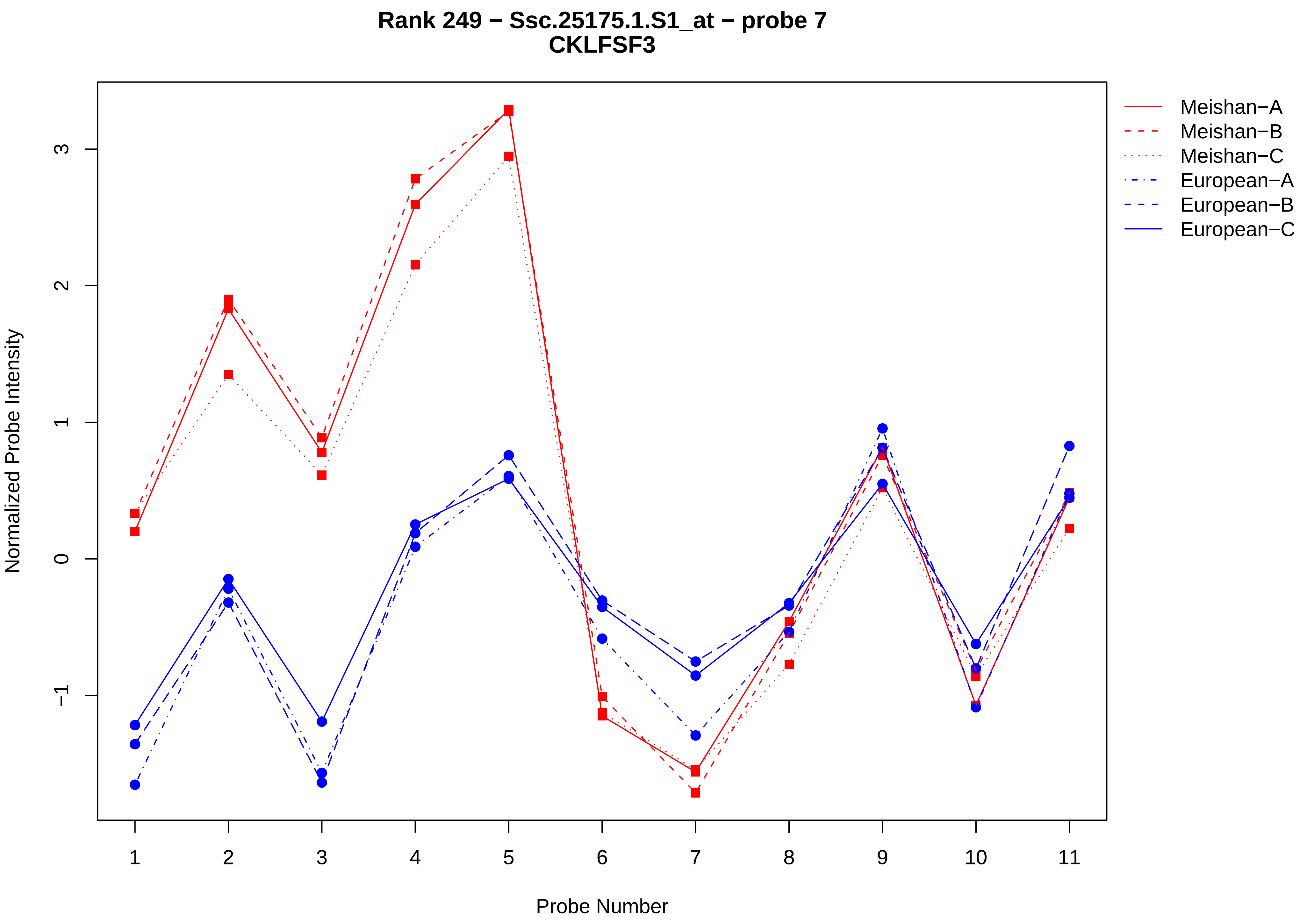

Rank 249 − Ssc.25175.1.S1_at − probe 7
CKLFSF3
Normalized Probe Intensity
Probe Number
−1
0
1
2
3
1
2
3
4
5
6
7
8
9
10
11
Meishan−A
Meishan−B
Meishan−C
European−A
European−B
European−C

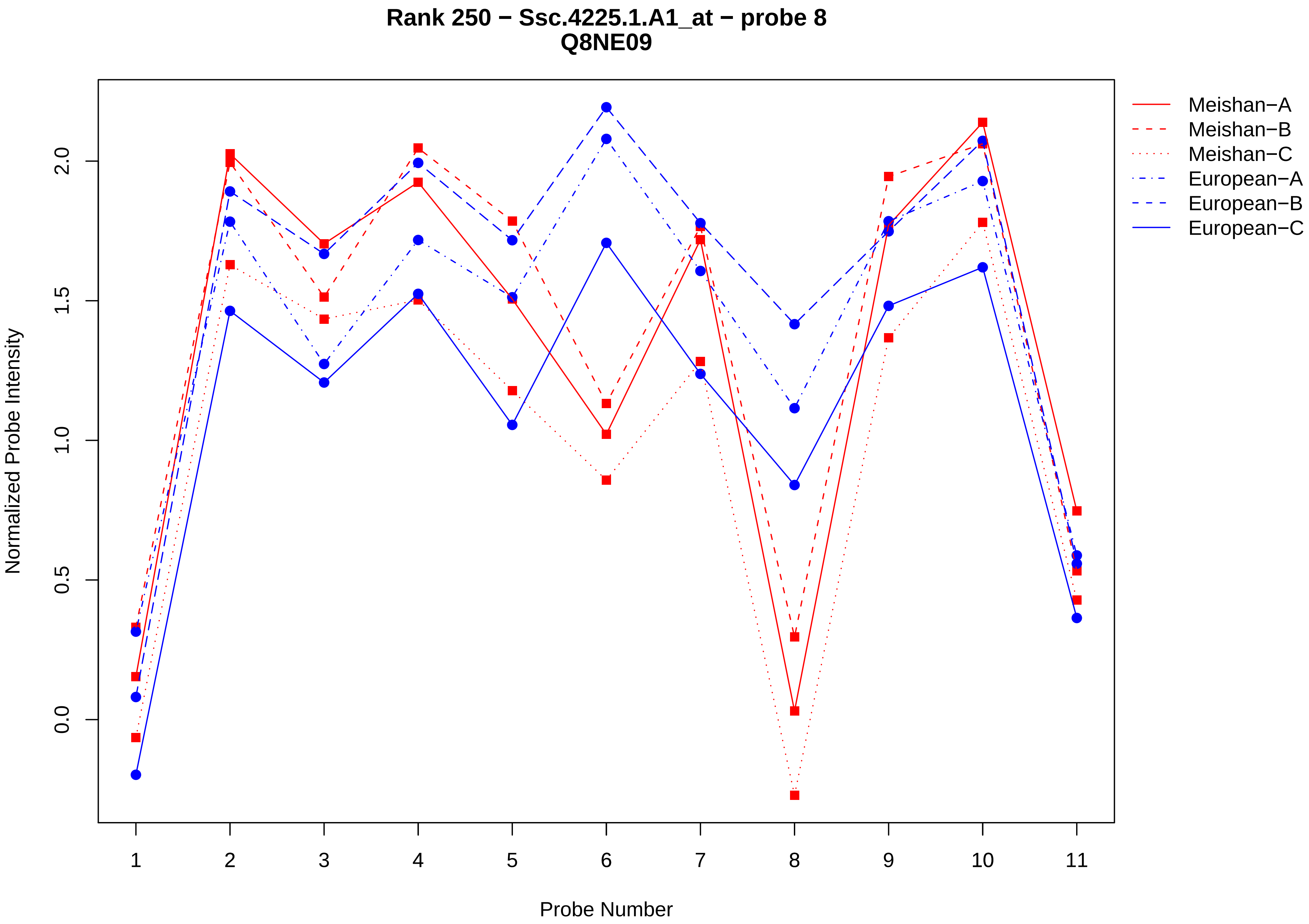

Rank 250 − Ssc.4225.1.A1_at − probe 8
Q8NE09
Normalized Probe Intensity
0.0
0.5
1.0
1.5
2.0
1
2
3
4
5
6
7
8
9
10
11
Probe Number
Meishan−A
Meishan−B
Meishan−C
European−A
European−B
European−C

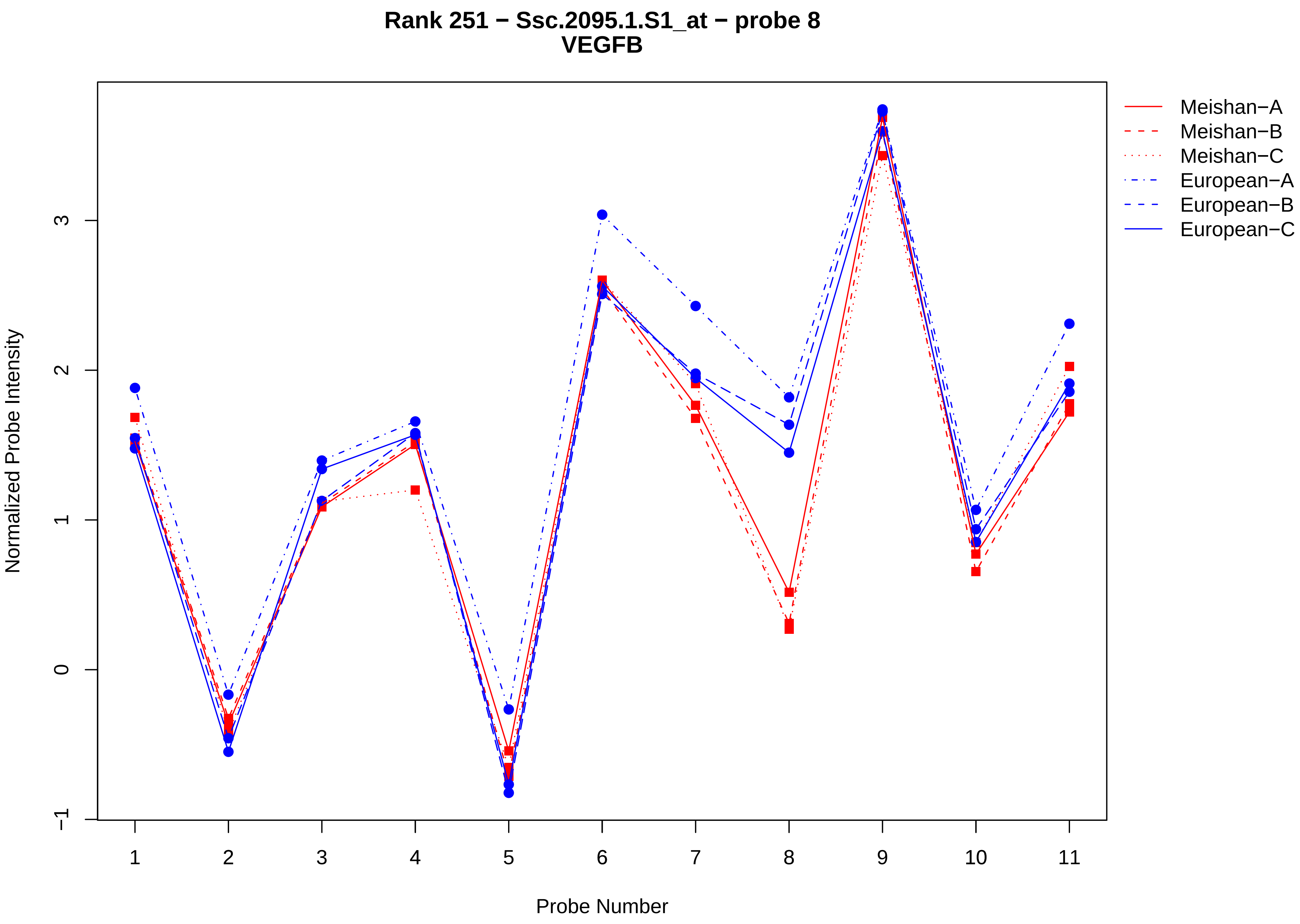

Rank 251 − Ssc.2095.1.S1_at − probe 8
VEGFB
Normalized Probe Intensity
Probe Number
Meishan−A
Meishan−B
Meishan−C
European−A
European−B
European−C
−1
0
1
2
3
1
2
3
4
5
6
7
8
9
10
11

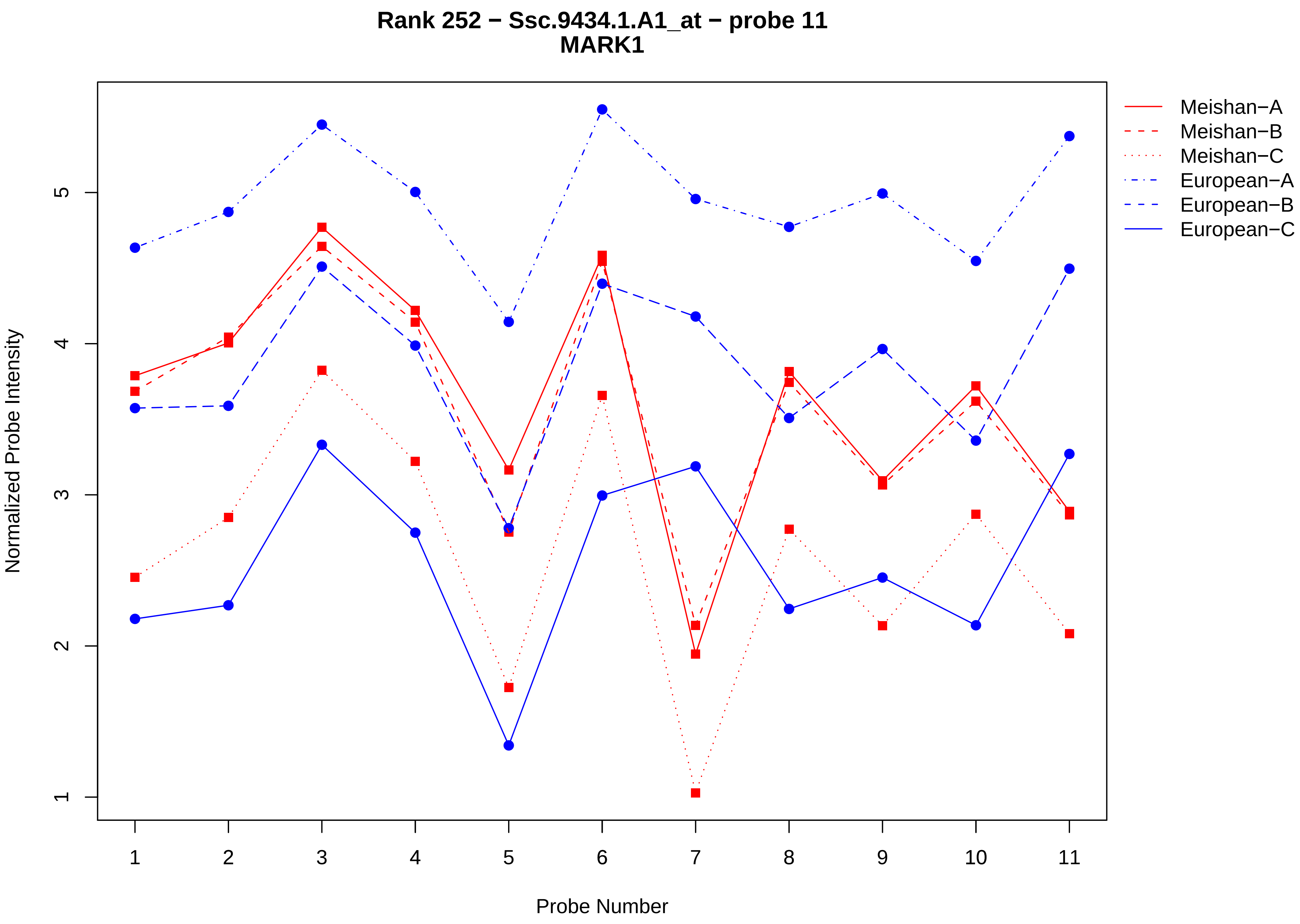

Rank 252 − Ssc.9434.1.A1_at − probe 11
MARK1
Normalized Probe Intensity
Probe Number
1
2
3
4
5
1
2
3
4
5
6
7
8
9
10
11
Meishan−A
Meishan−B
Meishan−C
European−A
European−B
European−C

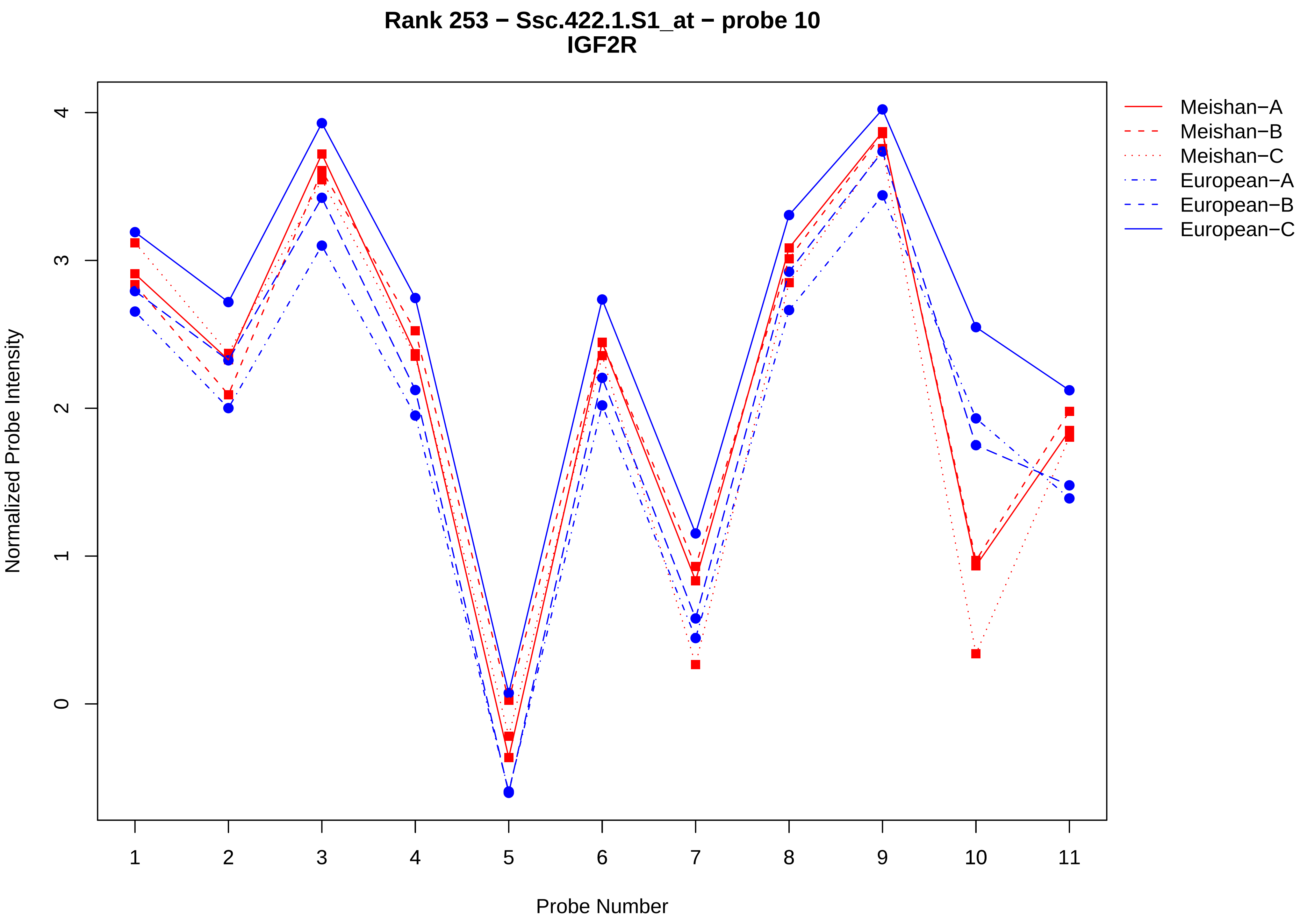

Rank 253 − Ssc.422.1.S1_at − probe 10
IGF2R
Normalized Probe Intensity
Probe Number
0
1
2
3
4
1
2
3
4
5
6
7
8
9
10
11
Meishan−A
Meishan−B
Meishan−C
European−A
European−B
European−C

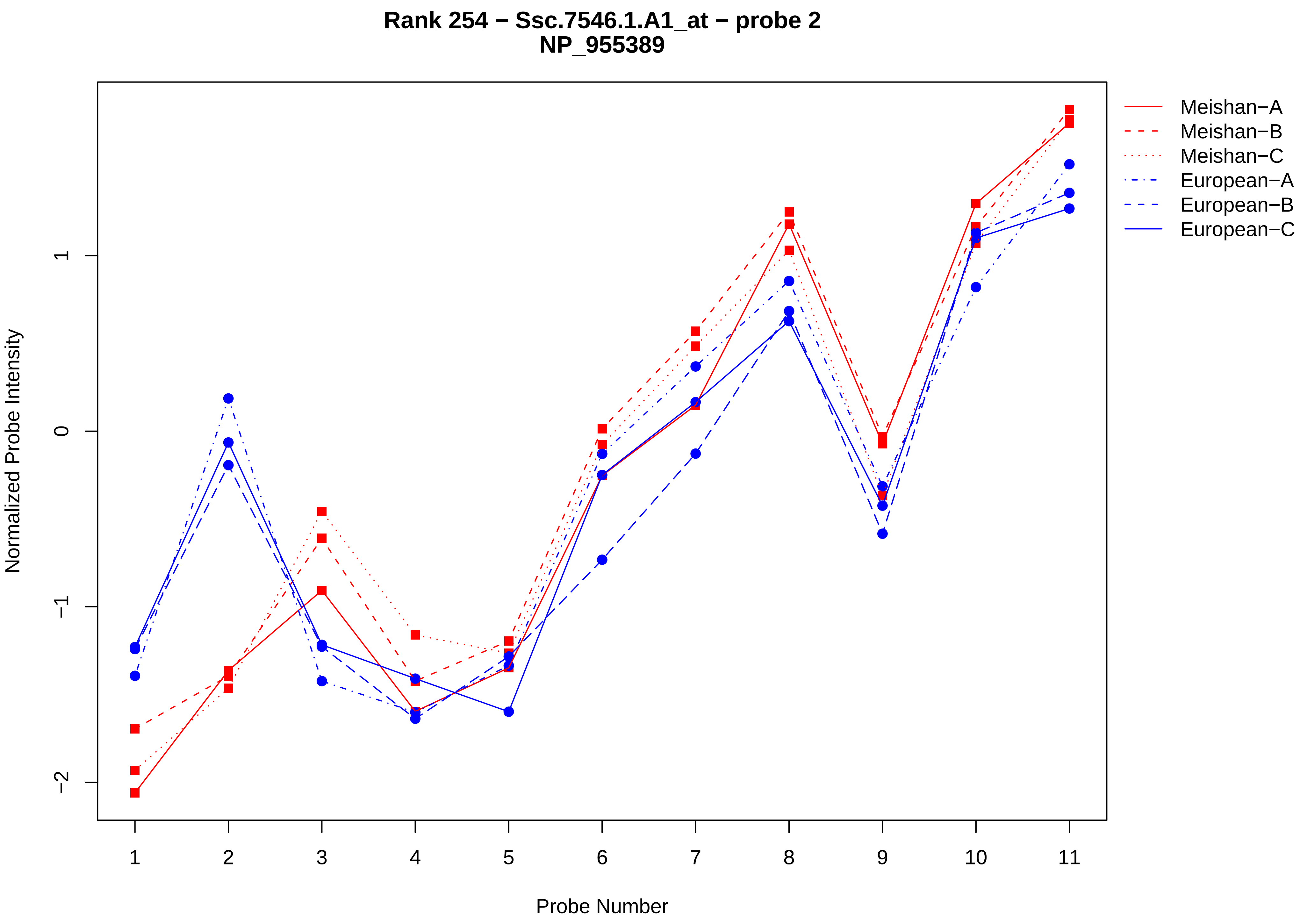
Rank 254 − Ssc.7546.1.A1_at − probe 2
NP_955389
Normalized Probe Intensity
Probe Number
−2
−1
0
1
1
2
3
4
5
6
7
8
9
10
11
Meishan−A
Meishan−B
Meishan−C
European−A
European−B
European−C

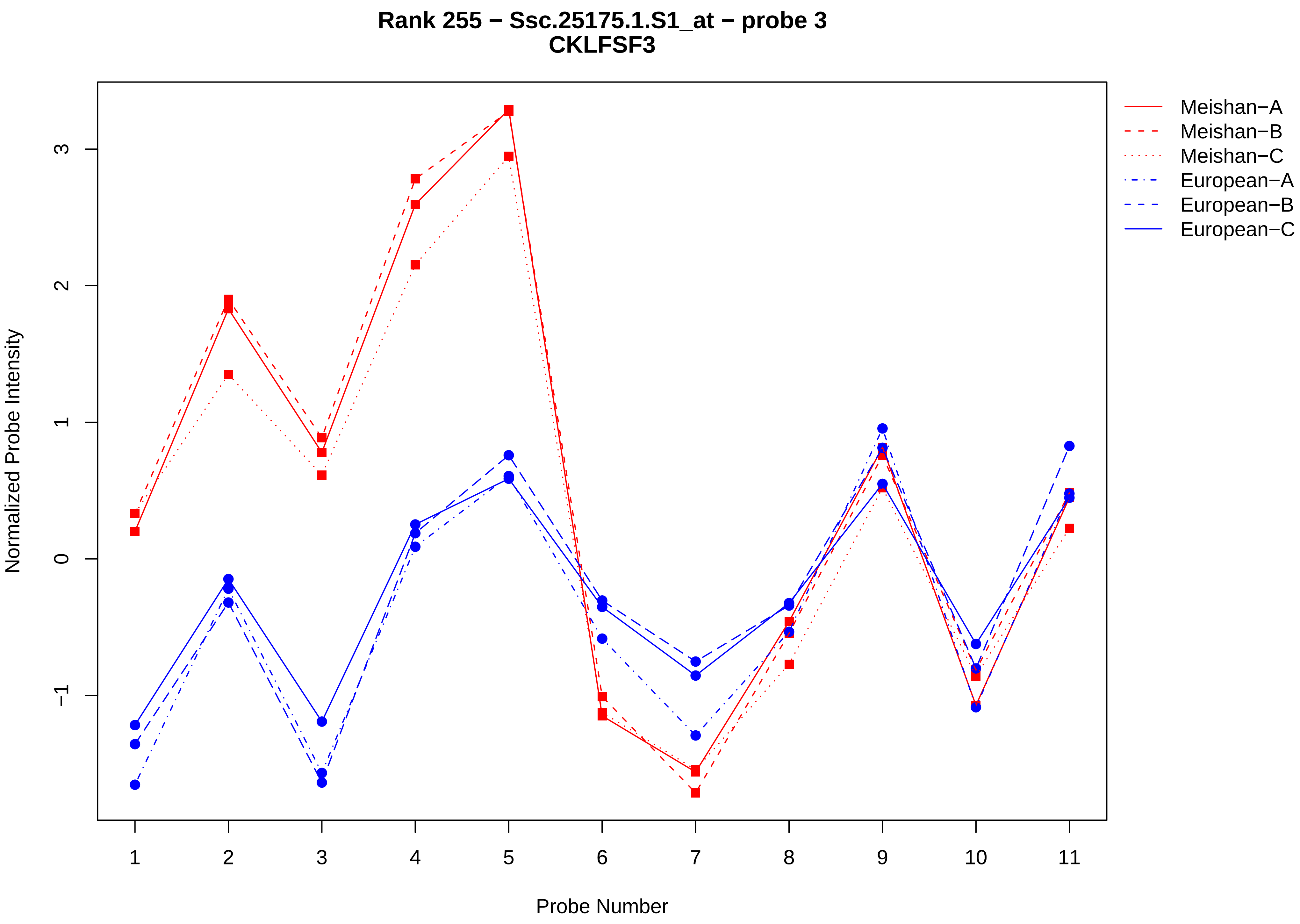

Rank 255 − Ssc.25175.1.S1_at − probe 3
CKLFSF3
Normalized Probe Intensity
Probe Number
Meishan−A
Meishan−B
Meishan−C
European−A
European−B
European−C

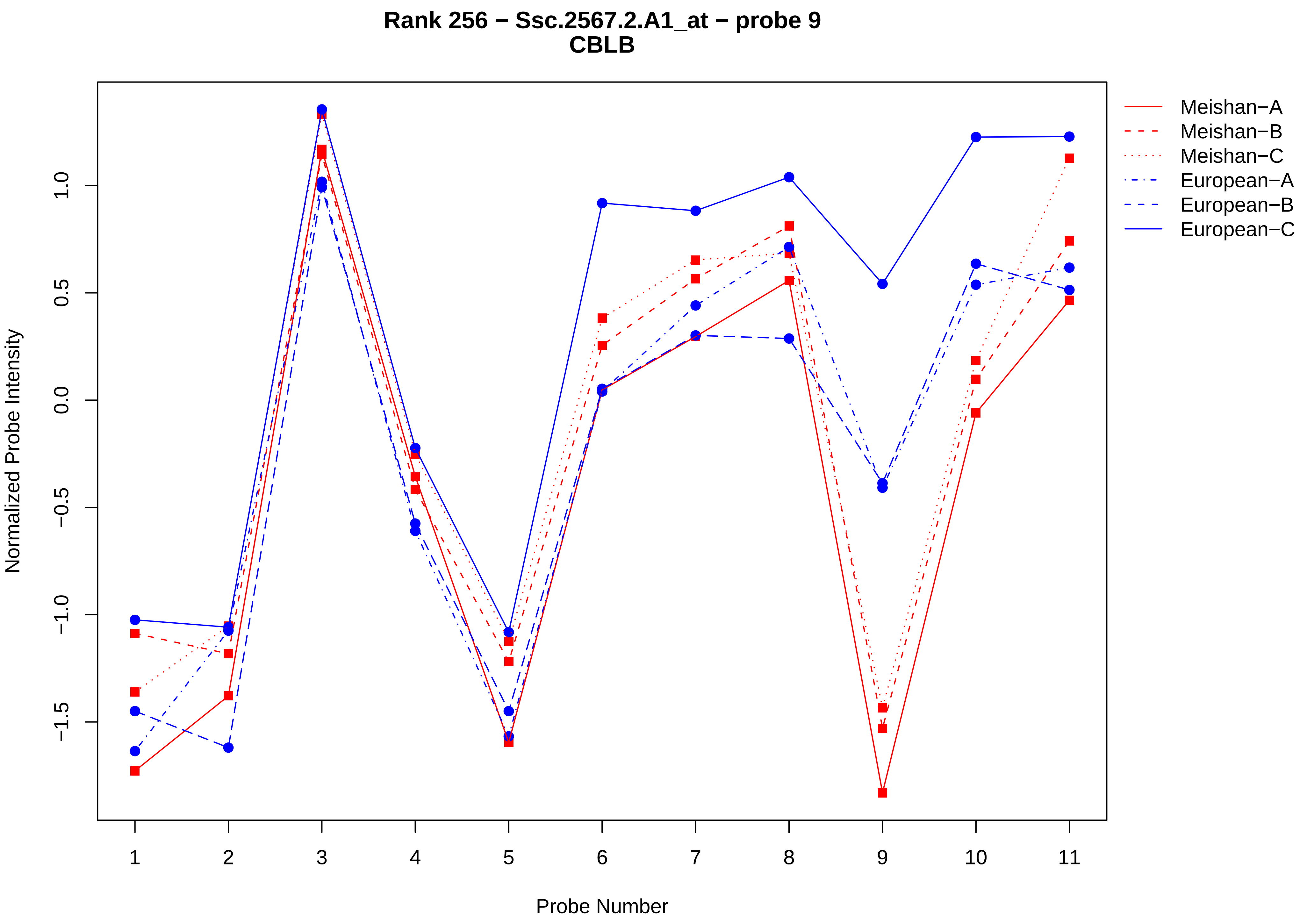

Rank 256 − Ssc.2567.2.A1_at − probe 9
CBLB
Normalized Probe Intensity
−1.5
−1.0
−0.5
0.0
0.5
1.0
Probe Number
1
2
3
4
5
6
7
8
9
10
11
Meishan−A
Meishan−B
Meishan−C
European−A
European−B
European−C

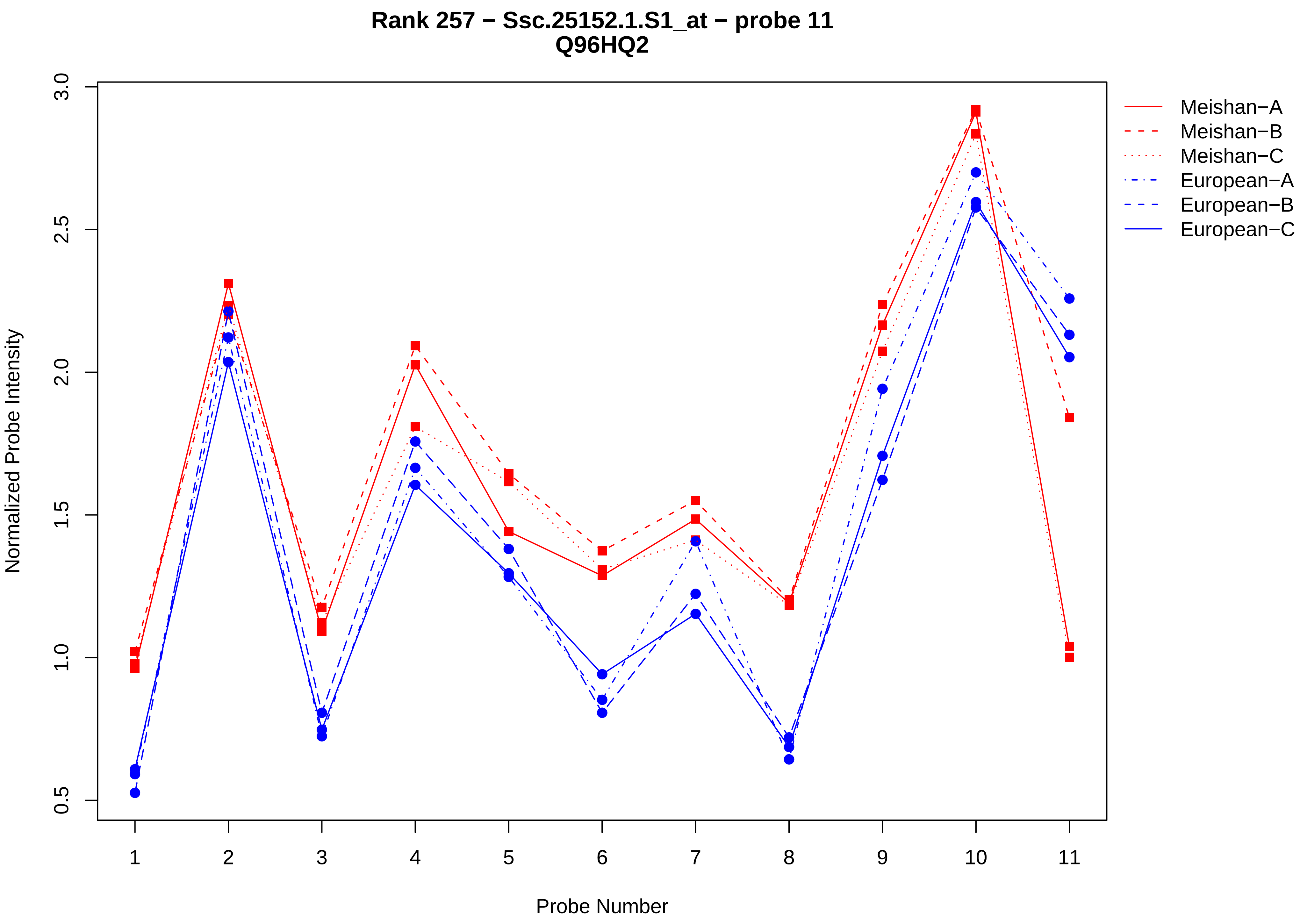

Rank 257 − Ssc.25152.1.S1_at − probe 11
Q96HQ2
Normalized Probe Intensity
Probe Number
0.5
1.0
1.5
2.0
2.5
3.0
1
2
3
4
5
6
7
8
9
10
11
Meishan−A
Meishan−B
Meishan−C
European−A
European−B
European−C

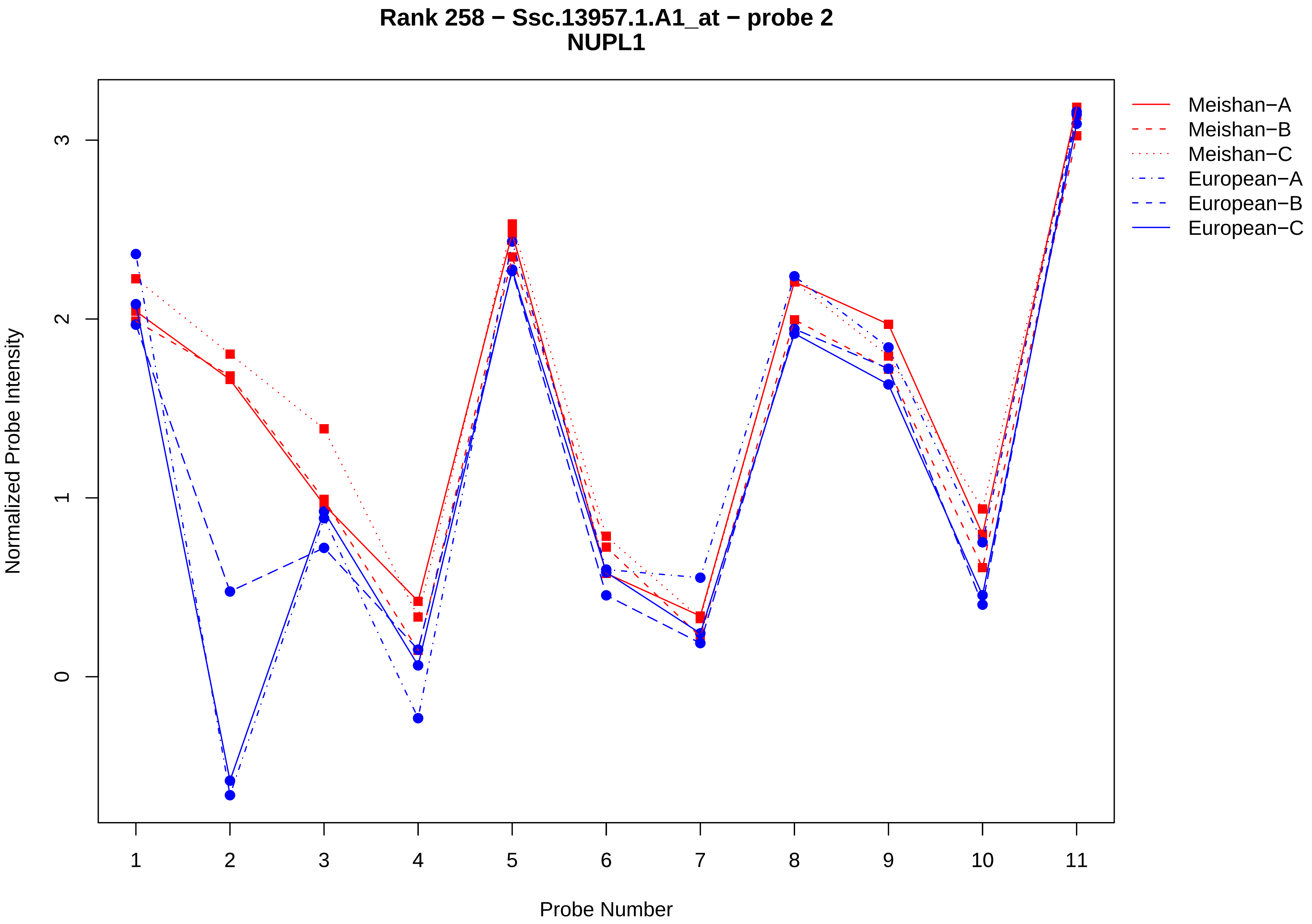

Rank 258 − Ssc.13957.1.A1_at − probe 2
NUPL1
Normalized Probe Intensity
Probe Number
0
1
2
3
1
2
3
4
5
6
7
8
9
10
11
Meishan−A
Meishan−B
Meishan−C
European−A
European−B
European−C

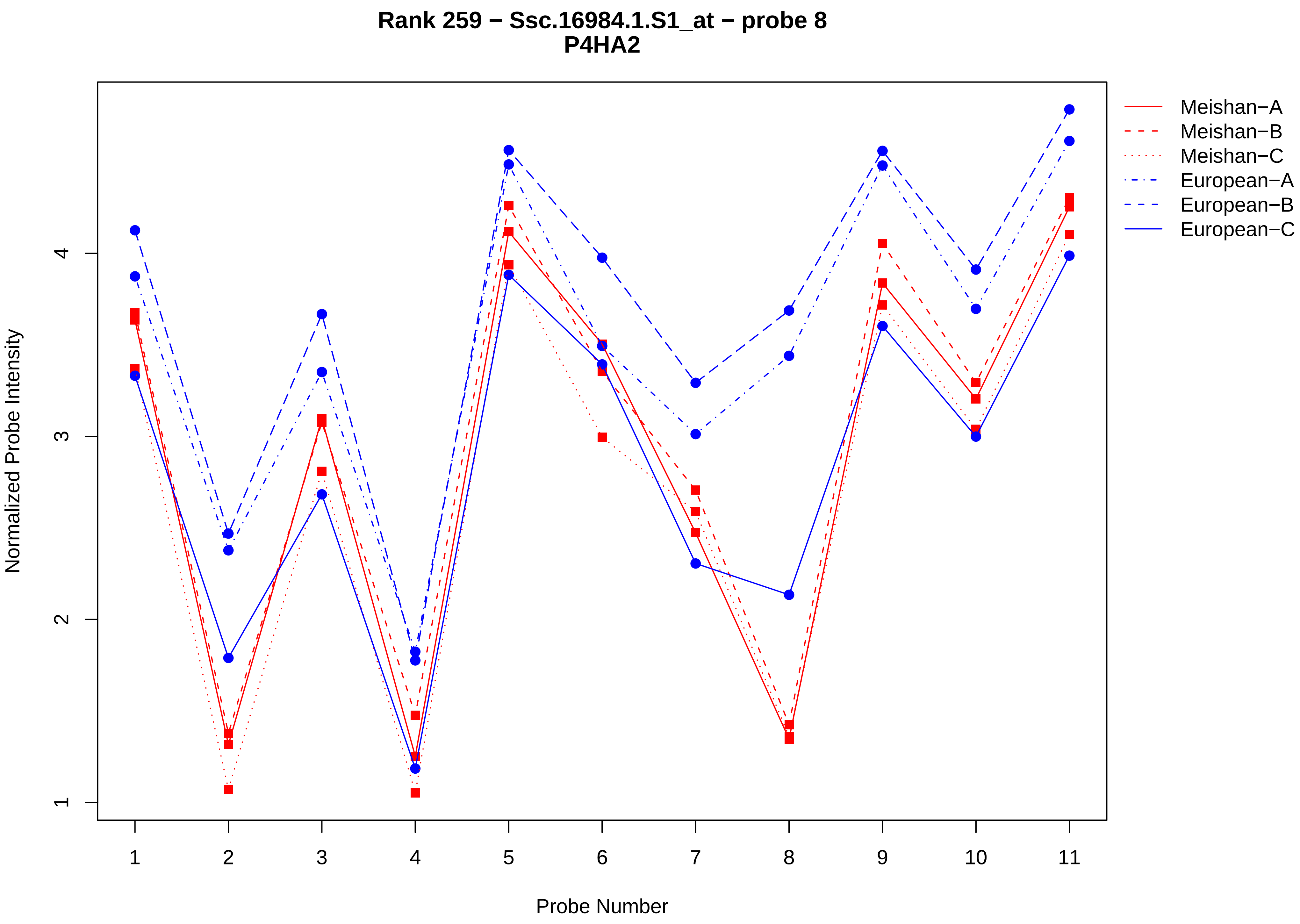

Rank 259 − Ssc.16984.1.S1_at − probe 8
P4HA2
Normalized Probe Intensity
Probe Number
1
2
3
4
1
2
3
4
5
6
7
8
9
10
11
Meishan−A
Meishan−B
Meishan−C
European−A
European−B
European−C

Rank 260 − Ssc.6512.1.S1_at − probe 9
Q6ZMJ2

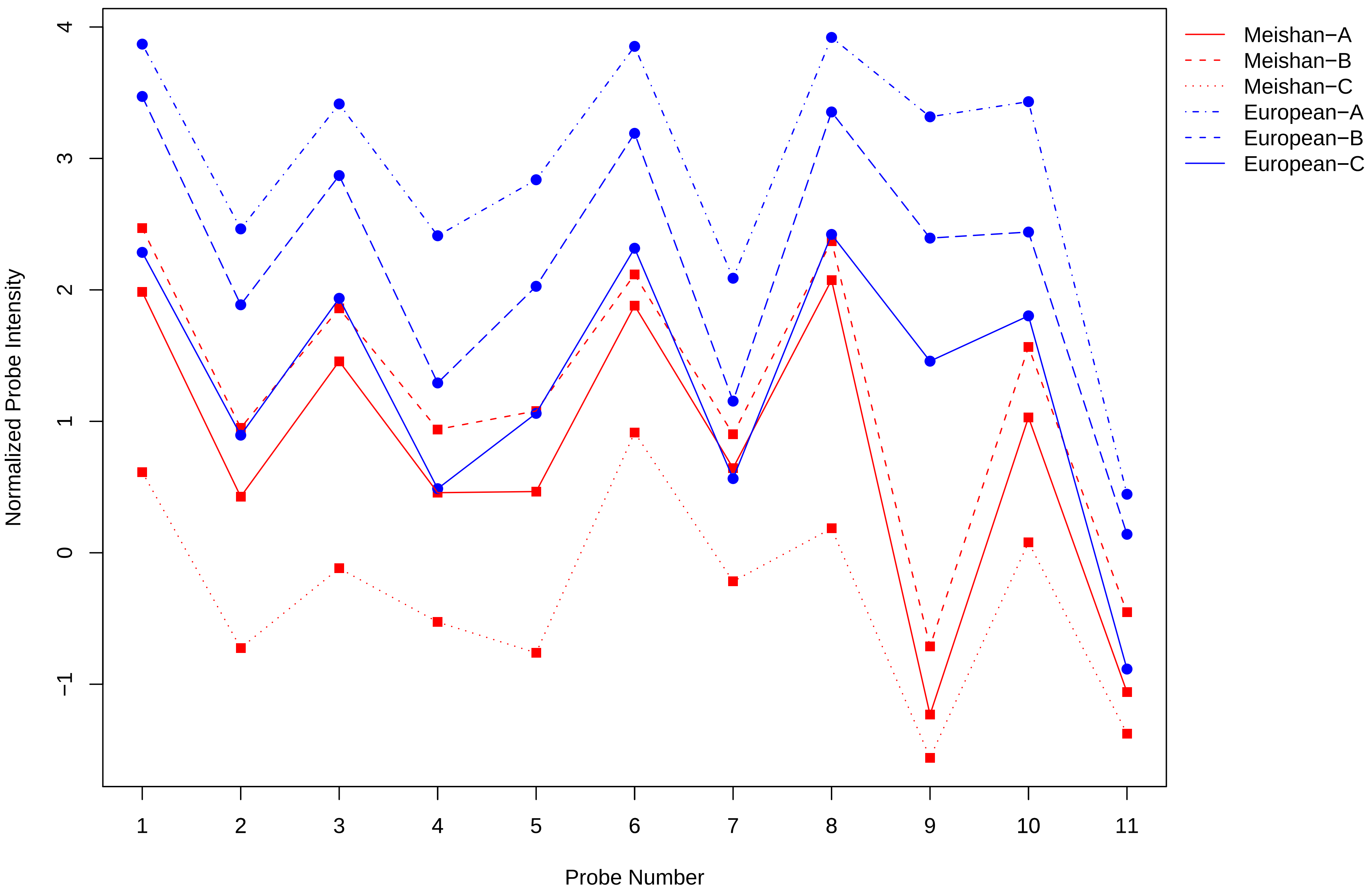

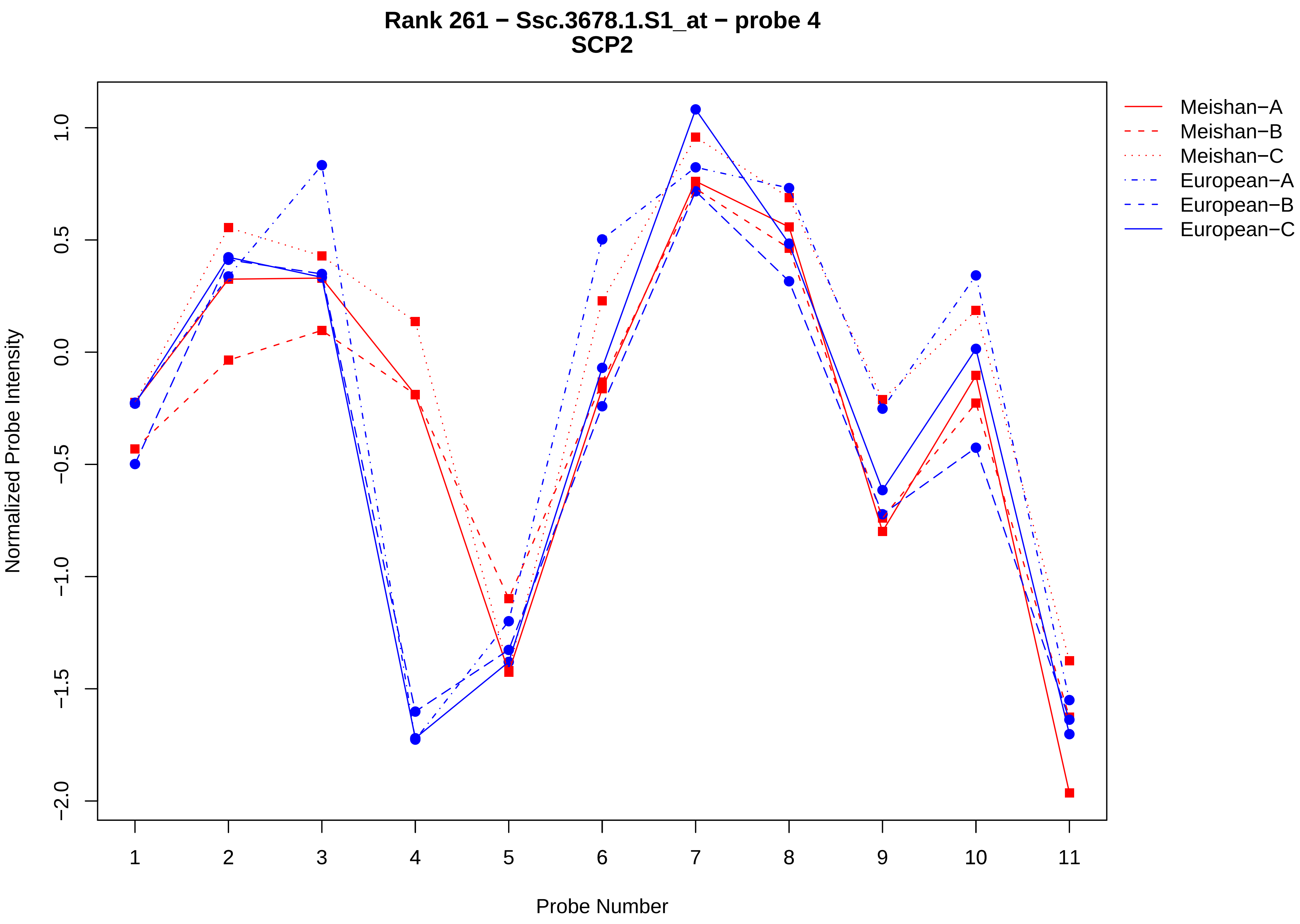
Rank 261 − Ssc.3678.1.S1_at − probe 4
SCP2
Normalized Probe Intensity
1.0
0.5
0.0
−0.5
−1.0
−1.5
−2.0
1
2
3
4
5
6
7
8
9
10
11
Probe Number
Meishan−A
Meishan−B
Meishan−C
European−A
European−B
European−C

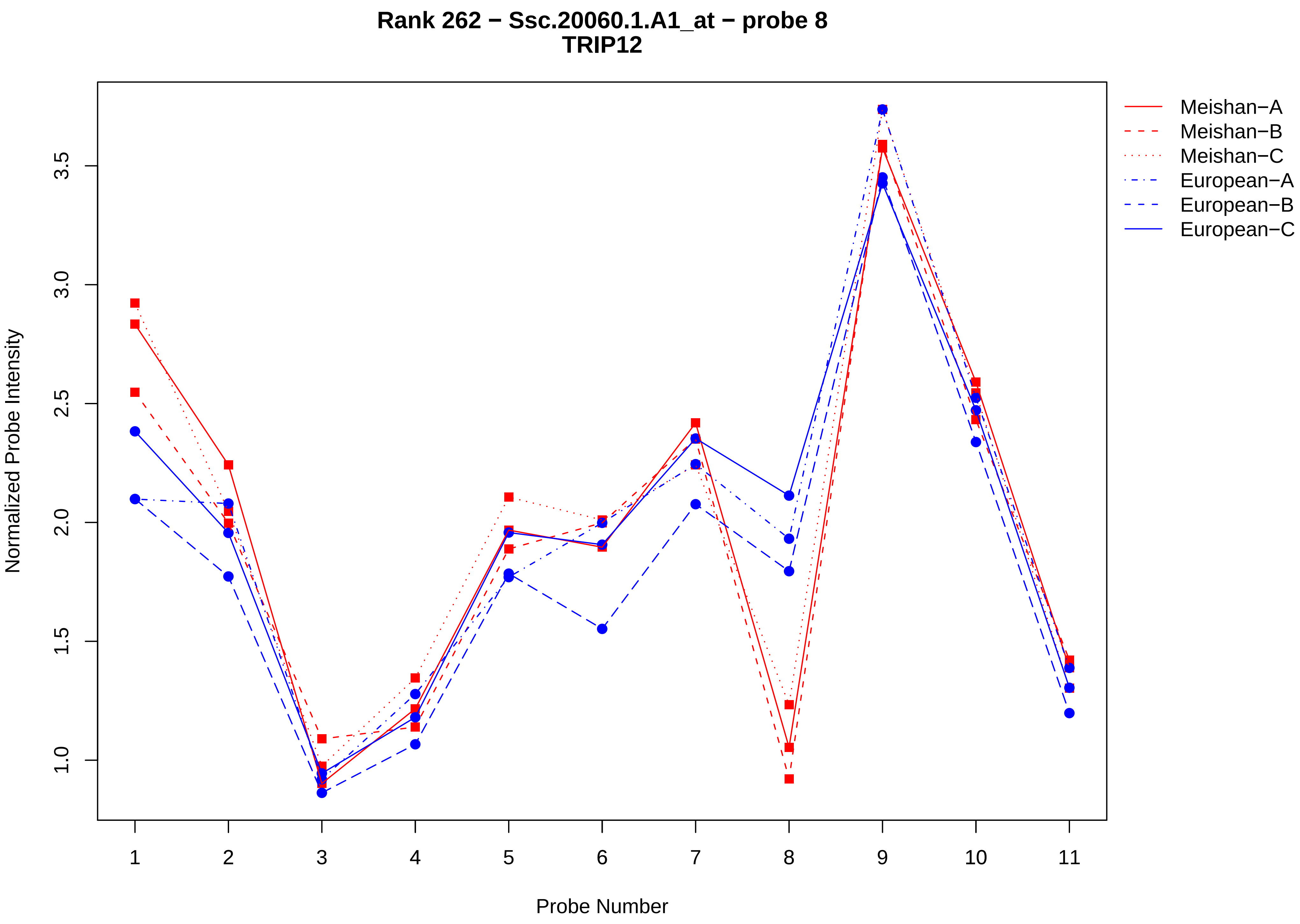

Rank 262 − Ssc.20060.1.A1_at − probe 8
TRIP12
Normalized Probe Intensity
Probe Number
Meishan−A
Meishan−B
Meishan−C
European−A
European−B
European−C
1.0
1.5
2.0
2.5
3.0
3.5
1
2
3
4
5
6
7
8
9
10
11

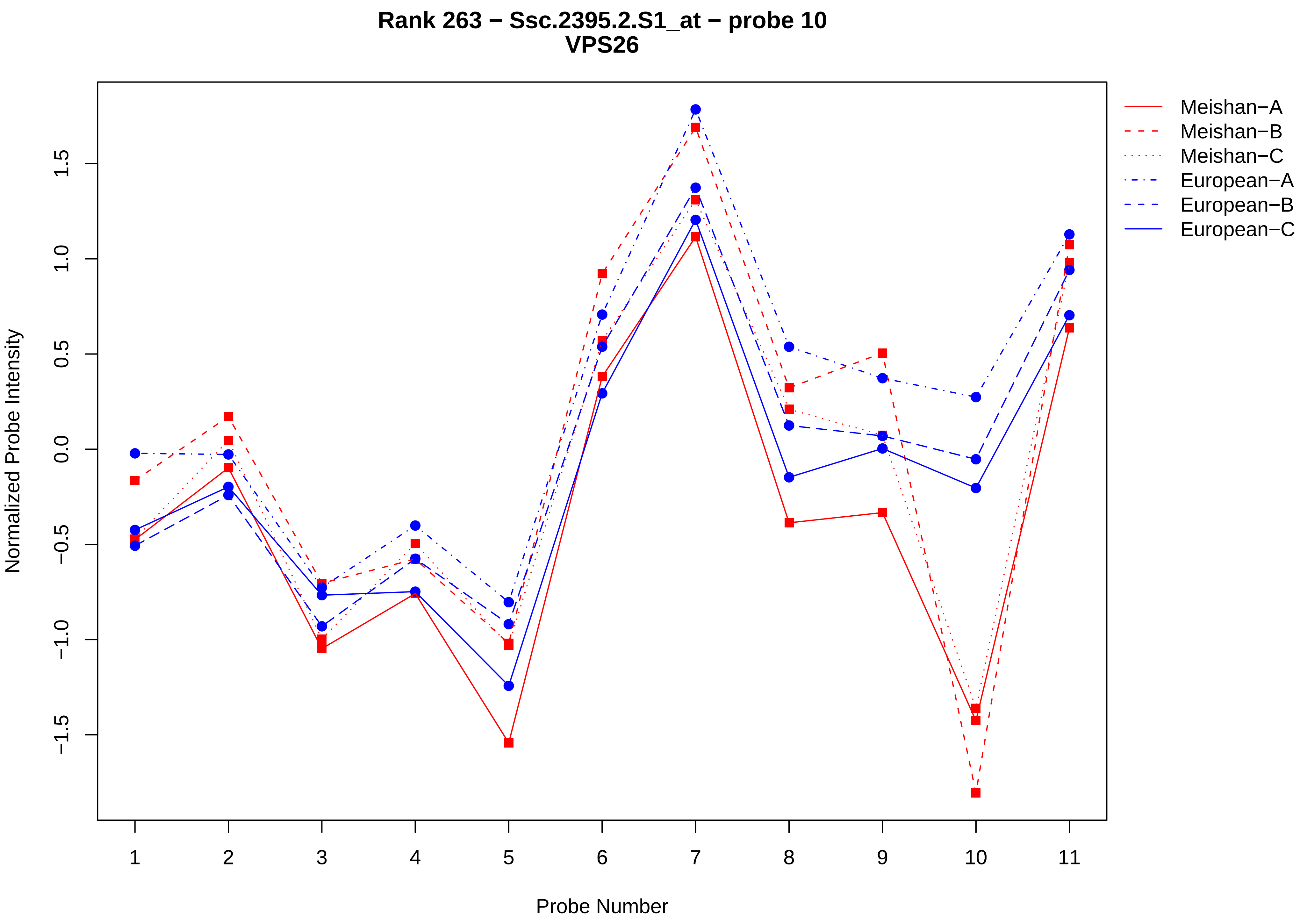

Rank 263 − Ssc.2395.2.S1_at − probe 10
VPS26
Normalized Probe Intensity
Probe Number
1.5
1.0
0.5
0.0
−0.5
−1.0
−1.5
1
2
3
4
5
6
7
8
9
10
11
Meishan−A
Meishan−B
Meishan−C
European−A
European−B
European−C

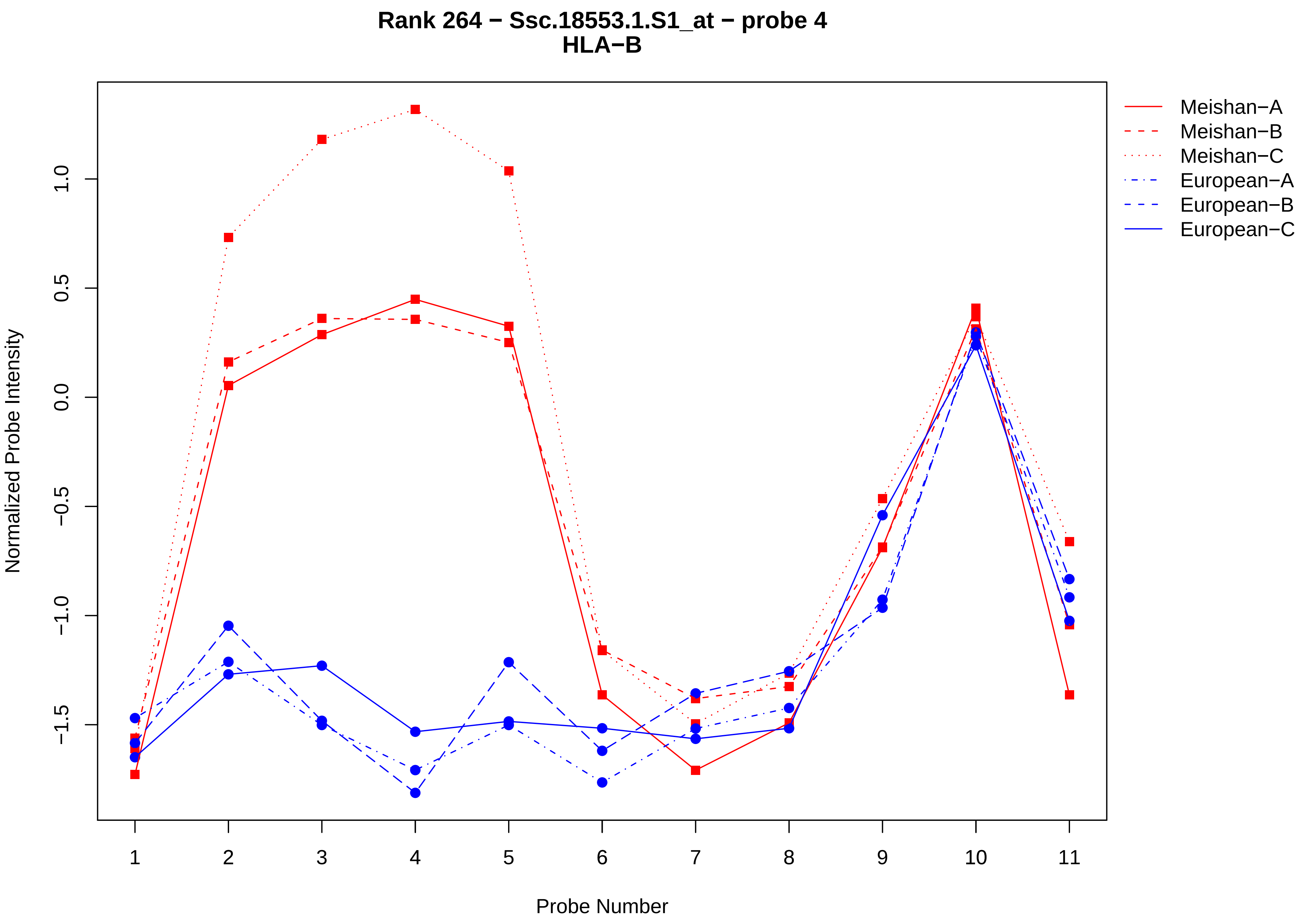
Rank 264 − Ssc.18553.1.S1_at − probe 4
HLA−B
Normalized Probe Intensity
Probe Number
Meishan−A
Meishan−B
Meishan−C
European−A
European−B
European−C
−1.5
−1.0
−0.5
0.0
0.5
1.0
1
2
3
4
5
6
7
8
9
10
11

Rank 265 − Ssc.6277.1.A1_at − probe 8
PCNT2

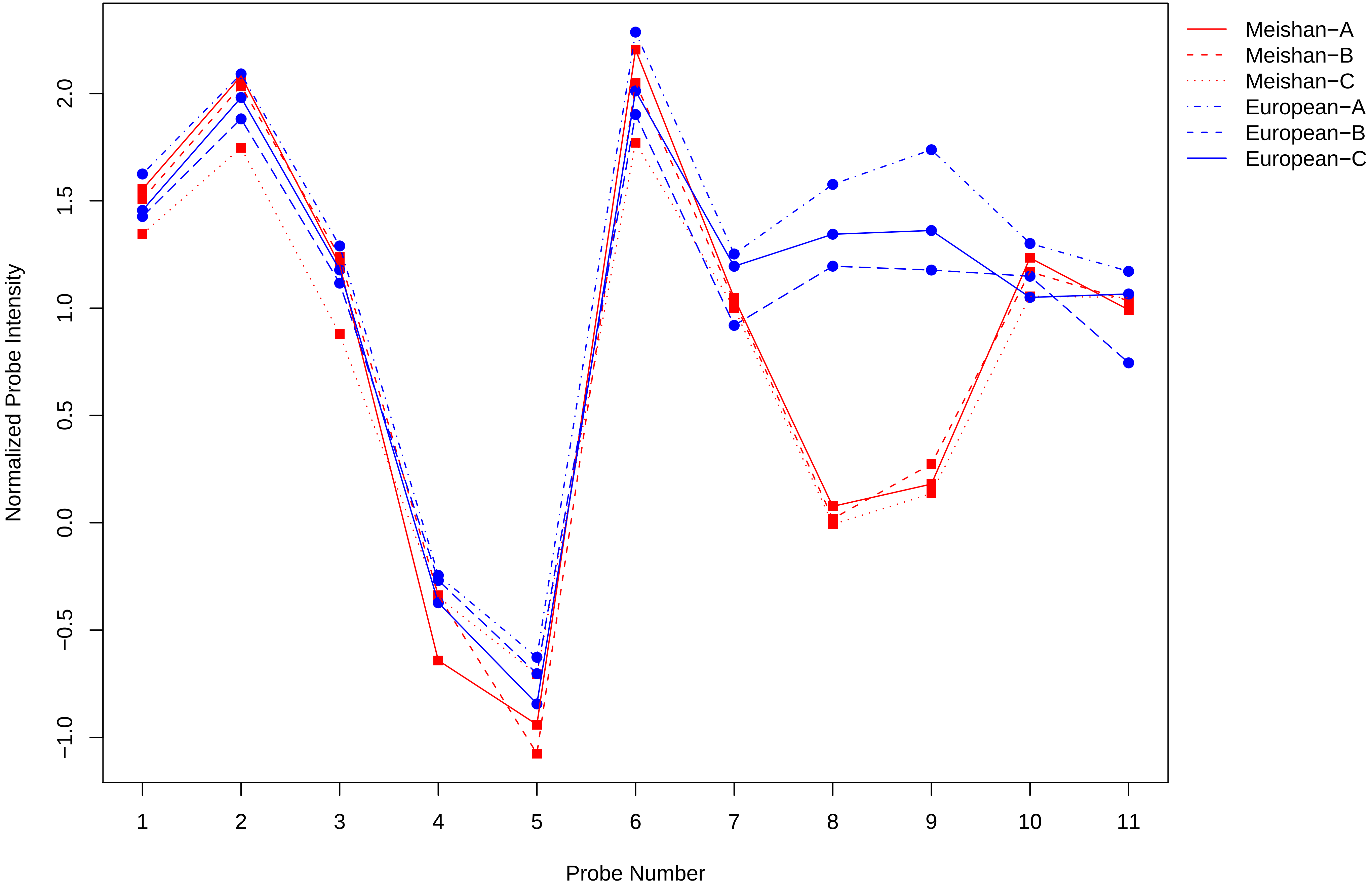

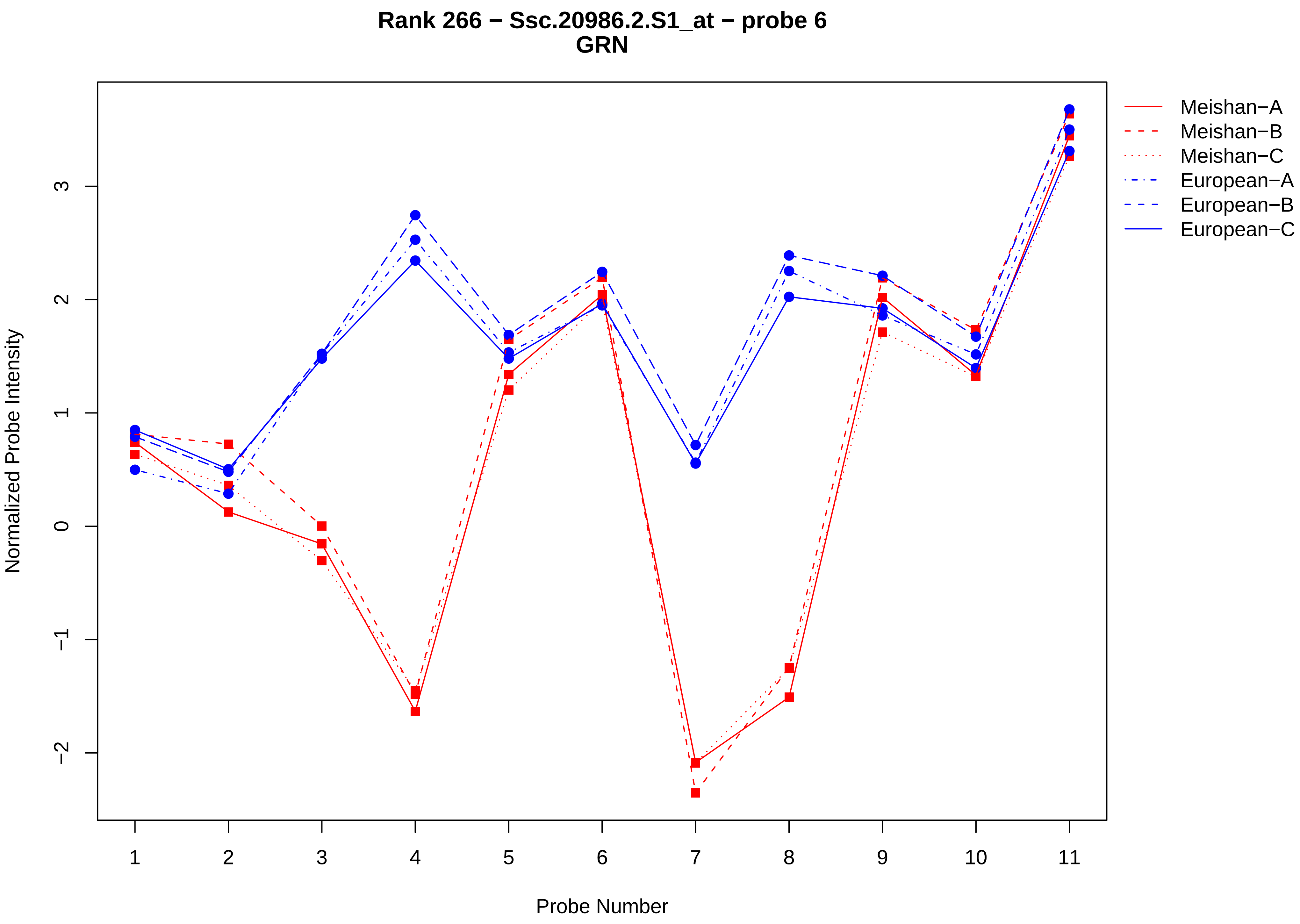

Rank 266 − Ssc.20986.2.S1_at − probe 6
GRN
Normalized Probe Intensity
Probe Number
−2
−1
0
1
2
3
1
2
3
4
5
6
7
8
9
10
11
Meishan−A
Meishan−B
Meishan−C
European−A
European−B
European−C

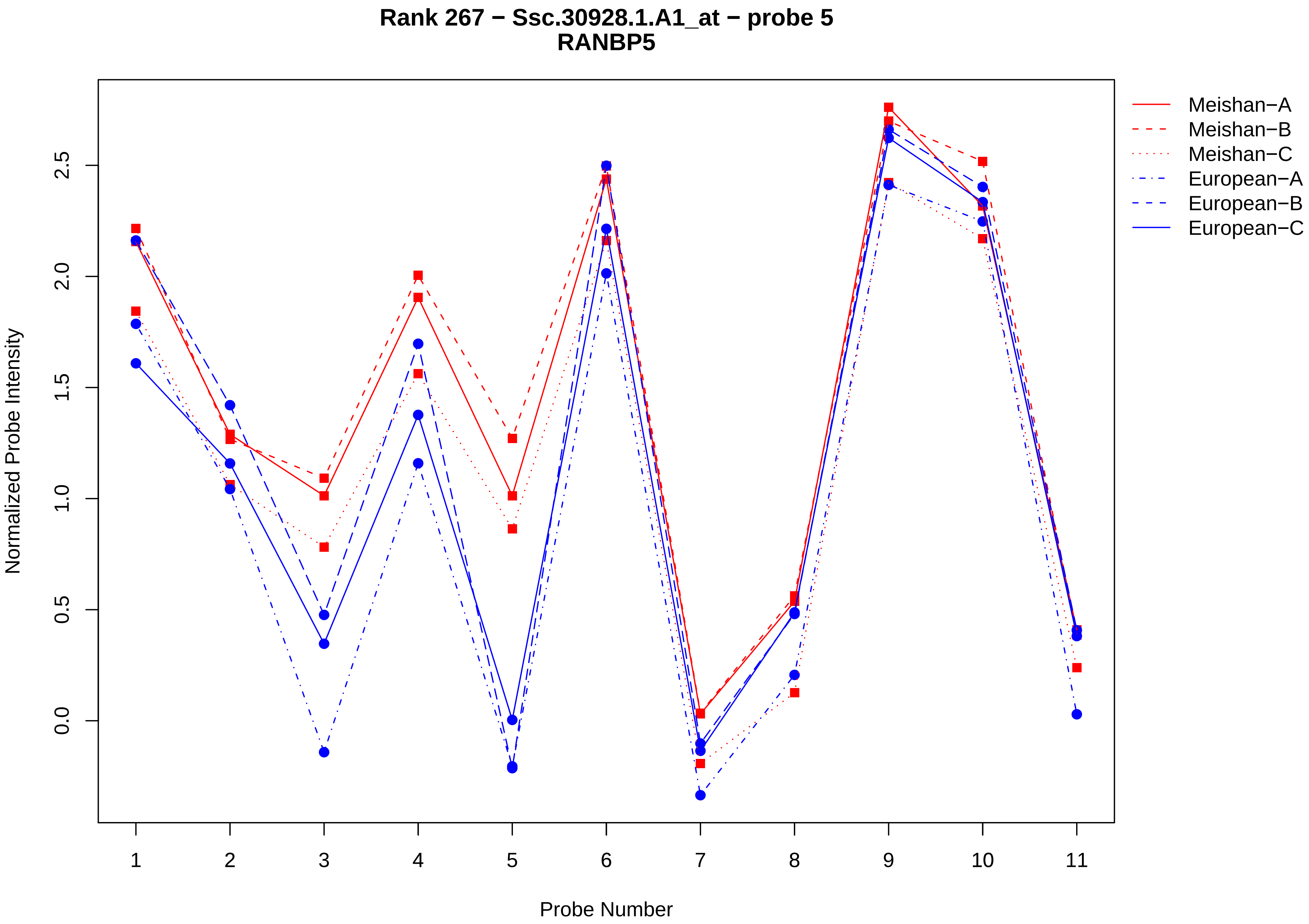

Rank 267 − Ssc.30928.1.A1_at − probe 5
RANBP5
Normalized Probe Intensity
0.0
0.5
1.0
1.5
2.0
2.5
1
2
3
4
5
6
7
8
9
10
11
Probe Number
Meishan−A
Meishan−B
Meishan−C
European−A
European−B
European−C

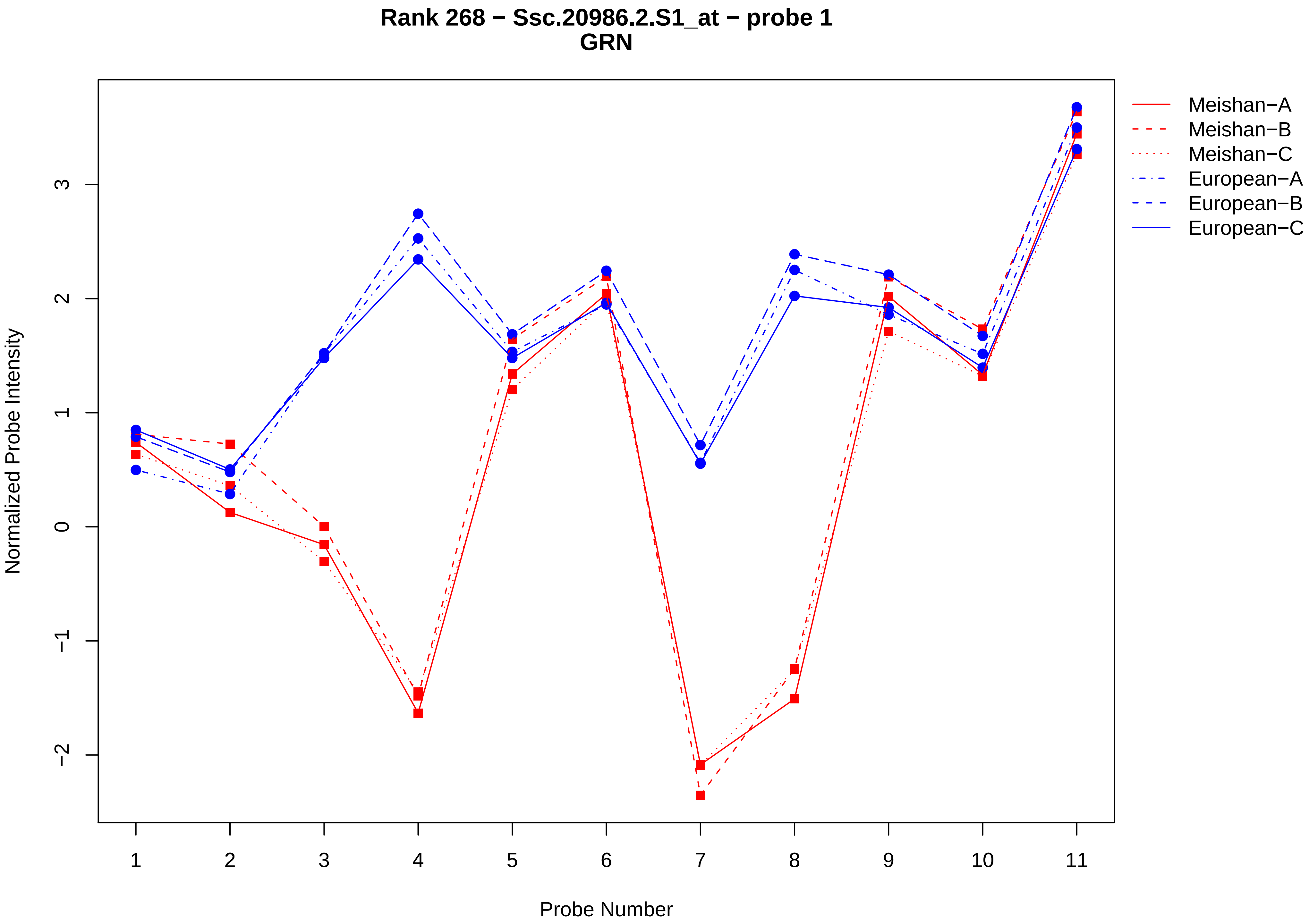

Rank 268 − Ssc.20986.2.S1_at − probe 1
GRN
Normalized Probe Intensity
Probe Number
−2
−1
0
1
2
3
1
2
3
4
5
6
7
8
9
10
11
Meishan−A
Meishan−B
Meishan−C
European−A
European−B
European−C

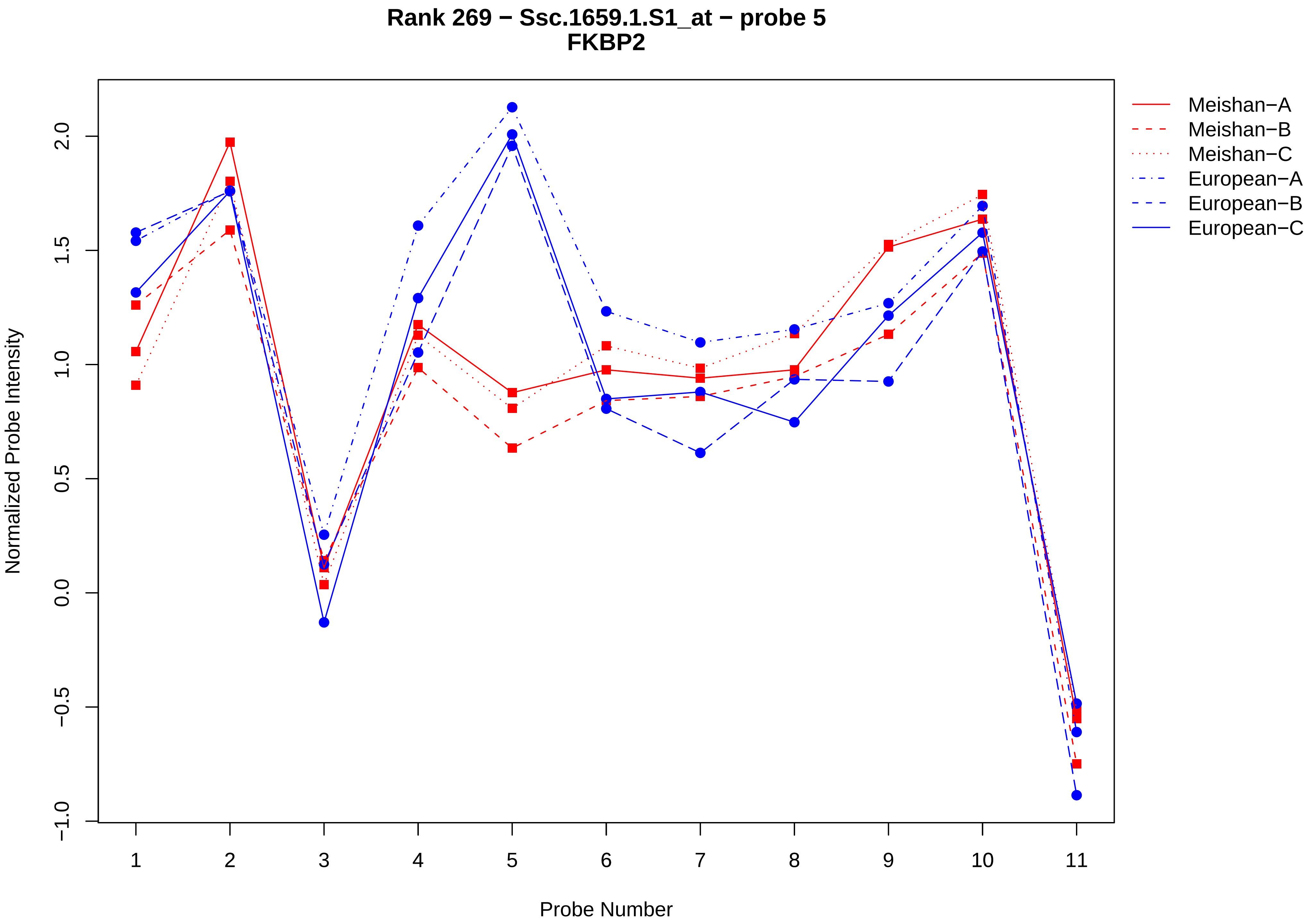

Rank 269 − Ssc.1659.1.S1_at − probe 5
FKBP2
Normalized Probe Intensity
Probe Number
−1.0
−0.5
0.0
0.5
1.0
1.5
2.0
1
2
3
4
5
6
7
8
9
10
11
Meishan−A
Meishan−B
Meishan−C
European−A
European−B
European−C

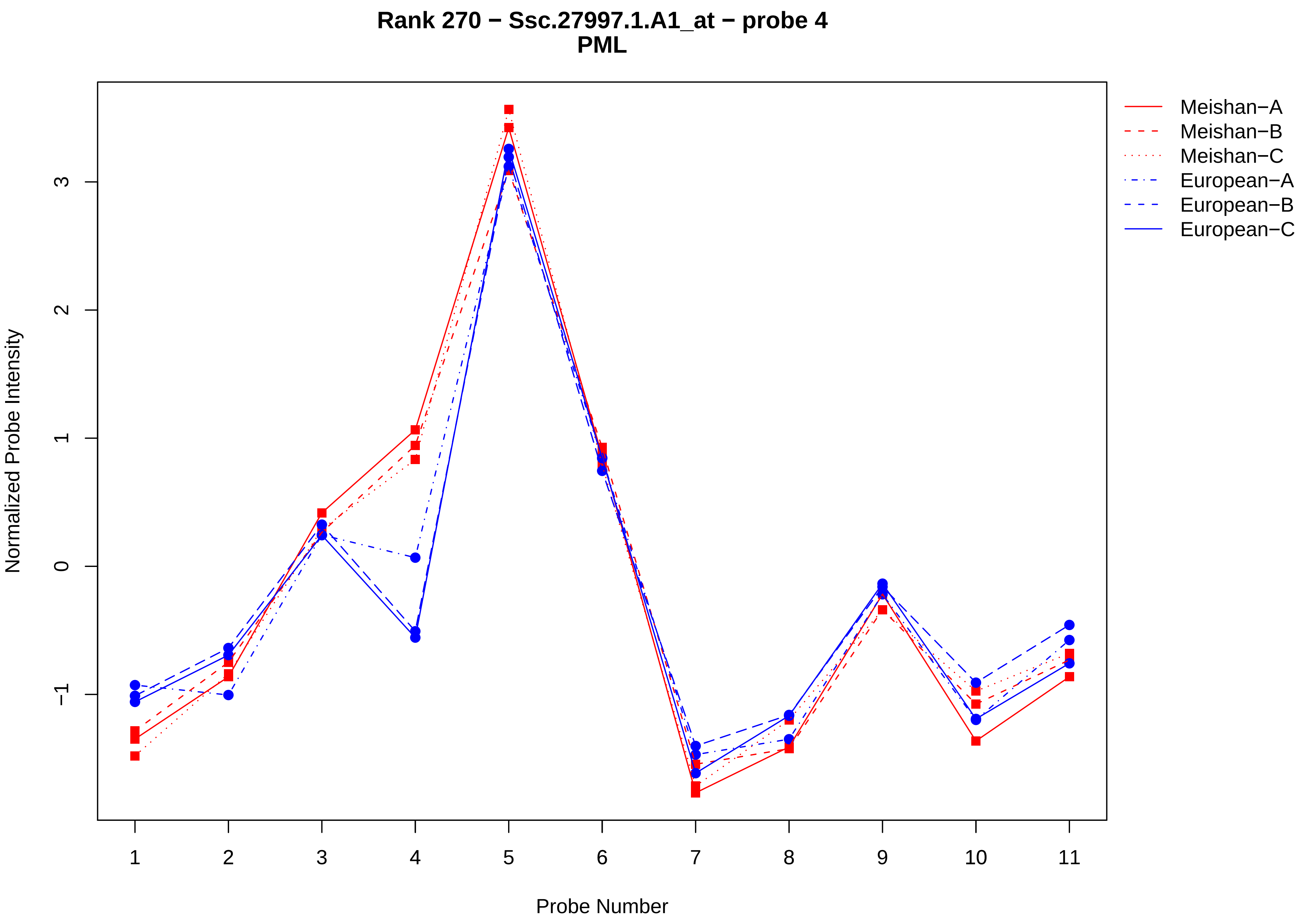

Rank 270 − Ssc.27997.1.A1_at − probe 4
PML
Normalized Probe Intensity
Probe Number
−1
0
1
2
3
1
2
3
4
5
6
7
8
9
10
11
Meishan−A
Meishan−B
Meishan−C
European−A
European−B
European−C

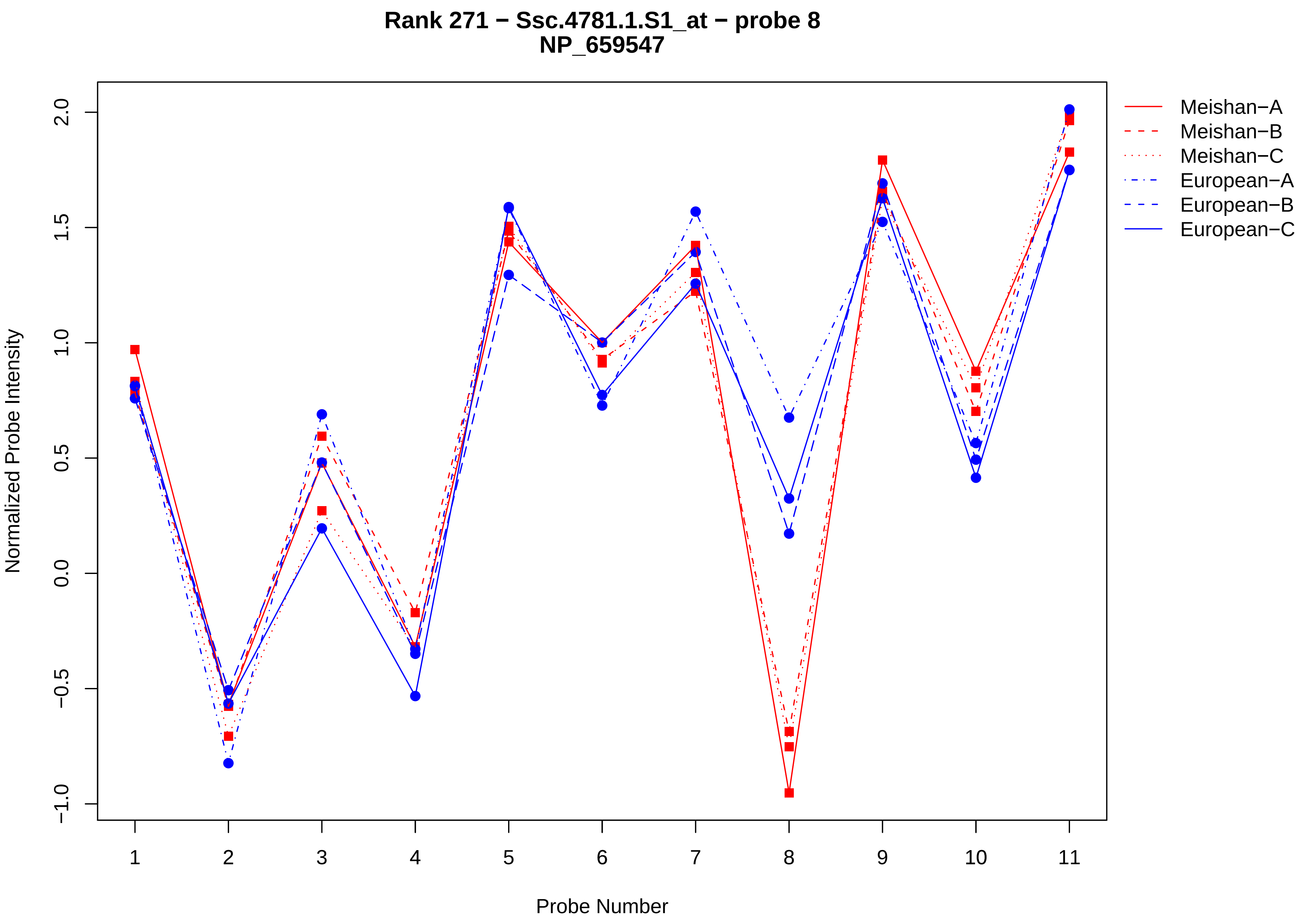

Rank 271 − Ssc.4781.1.S1_at − probe 8
NP_659547
Normalized Probe Intensity
Probe Number
−1.0
−0.5
0.0
0.5
1.0
1.5
2.0
1
2
3
4
5
6
7
8
9
10
11
Meishan−A
Meishan−B
Meishan−C
European−A
European−B
European−C

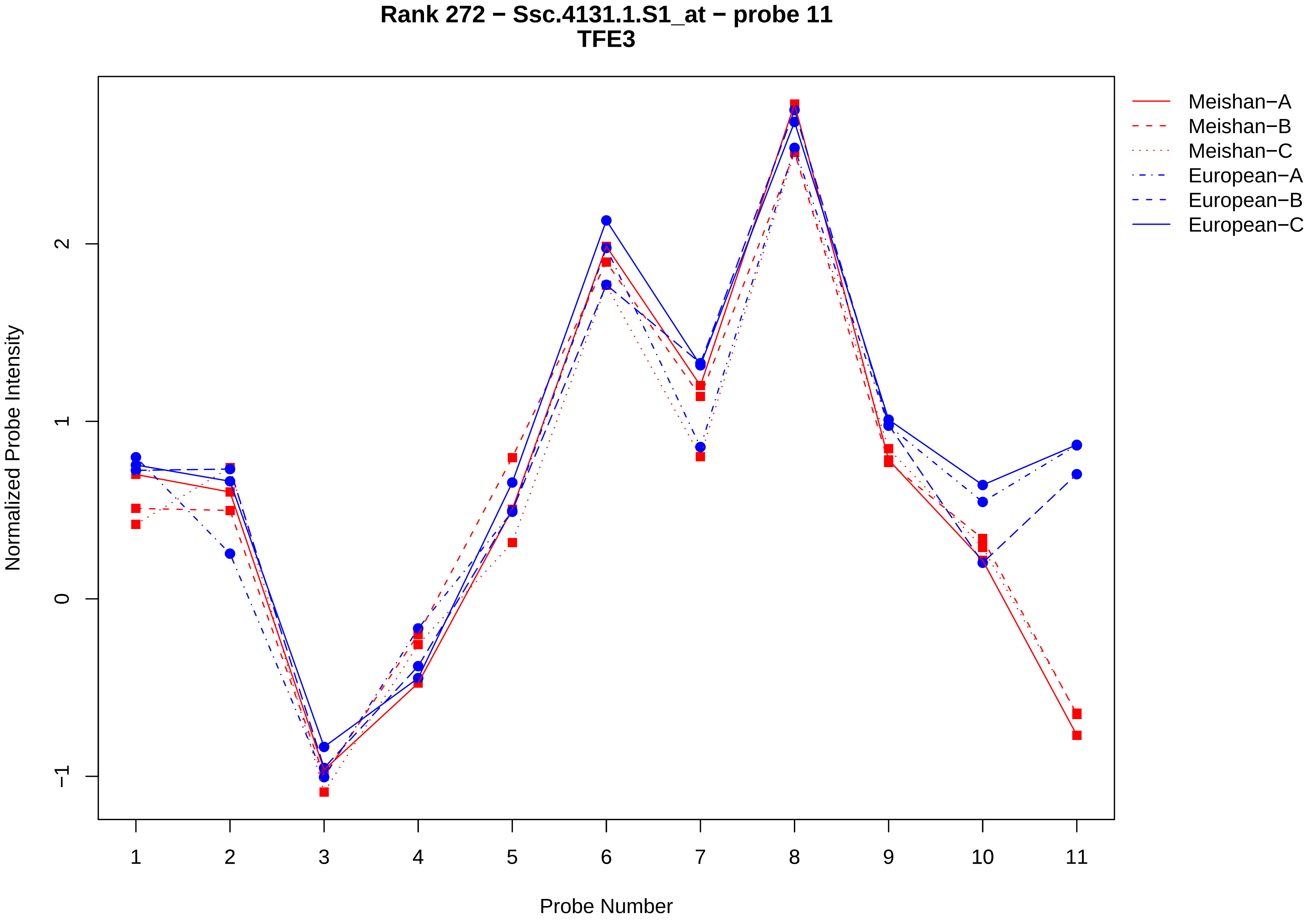

Rank 272 − Ssc.4131.1.S1_at − probe 11
TFE3
Normalized Probe Intensity
Probe Number
−1
0
1
2
1
2
3
4
5
6
7
8
9
10
11
Meishan−A
Meishan−B
Meishan−C
European−A
European−B
European−C

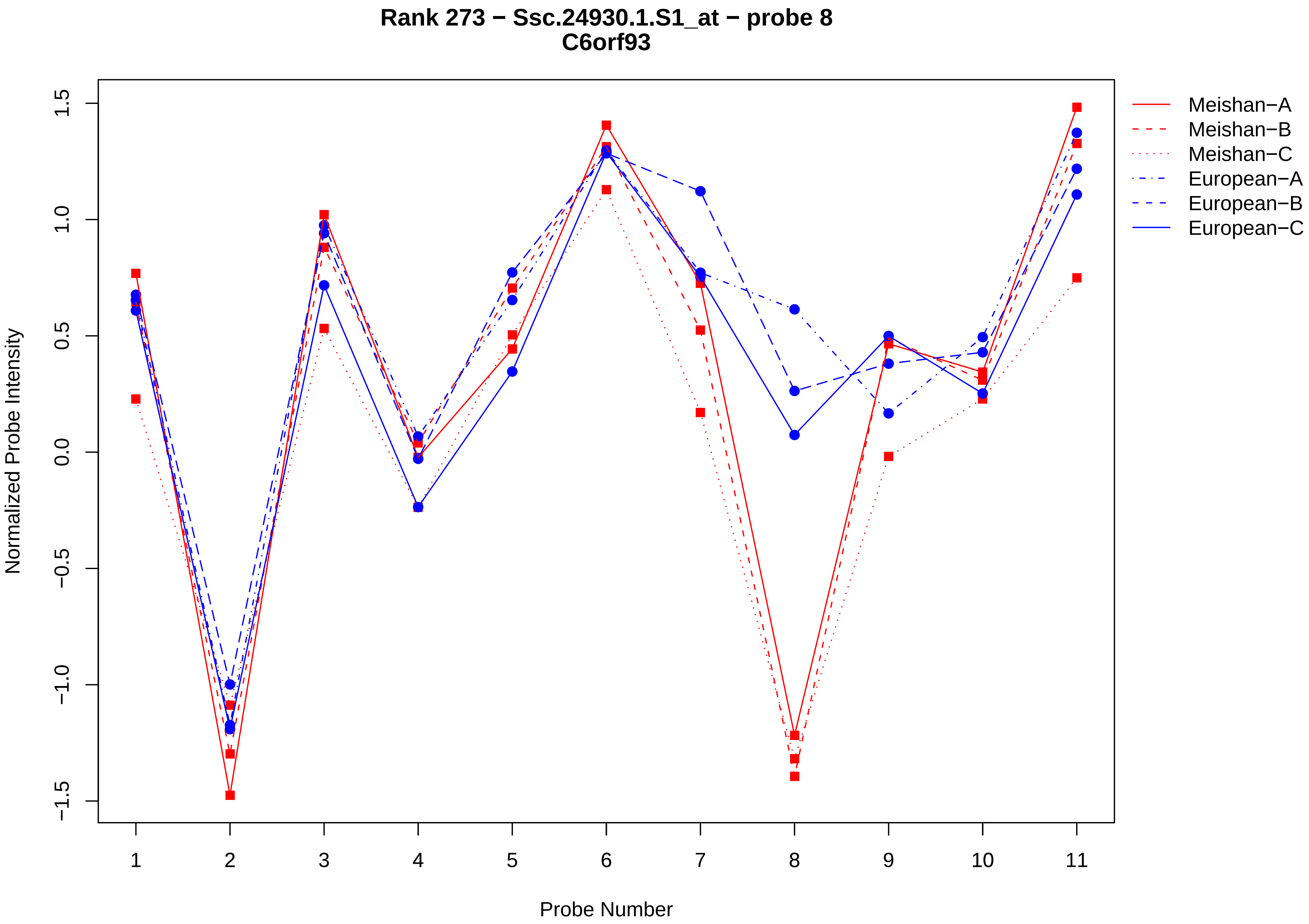

Rank 273 − Ssc.24930.1.S1_at − probe 8
C6orf93
Normalized Probe Intensity
−1.5
−1.0
−0.5
0.0
0.5
1.0
1.5
1
2
3
4
5
6
7
8
9
10
11
Probe Number
Meishan−A
Meishan−B
Meishan−C
European−A
European−B
European−C

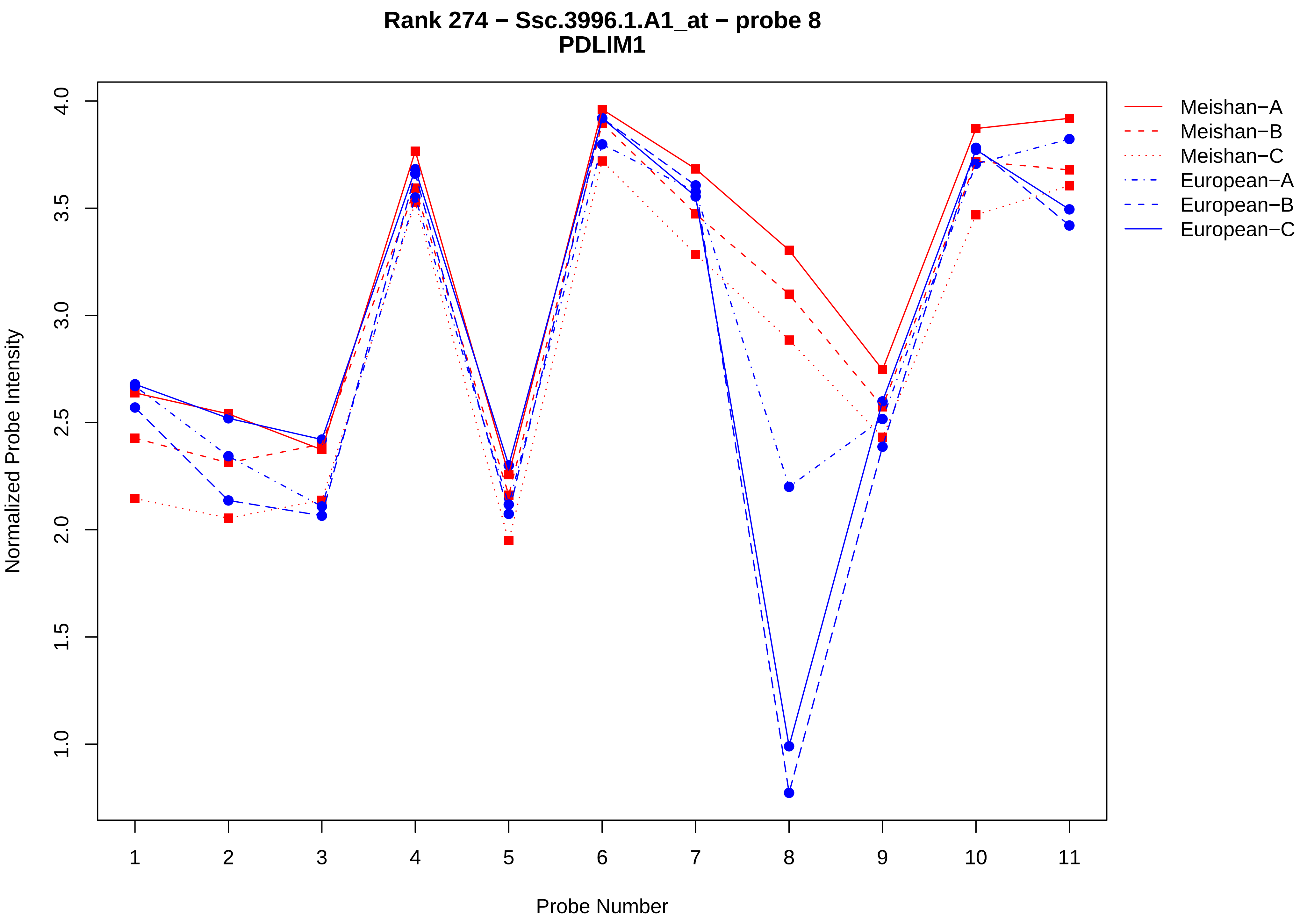

Rank 274 − Ssc.3996.1.A1_at − probe 8
PDLIM1
Normalized Probe Intensity
Probe Number
1.0
1.5
2.0
2.5
3.0
3.5
4.0
1
2
3
4
5
6
7
8
9
10
11
Meishan−A
Meishan−B
Meishan−C
European−A
European−B
European−C

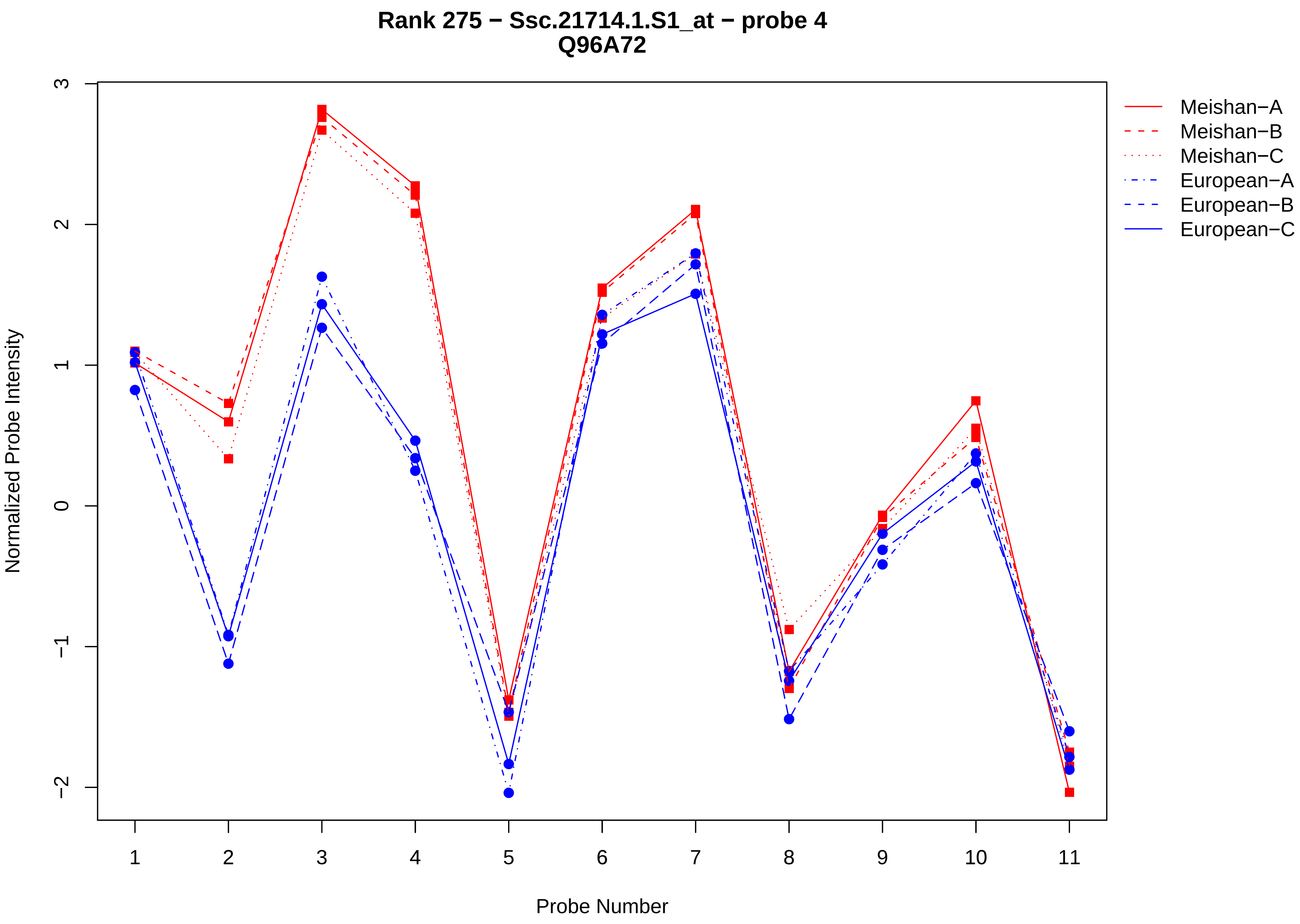
Rank 275 − Ssc.21714.1.S1_at − probe 4
Q96A72
Normalized Probe Intensity
3
2
1
0
−1
−2
1
2
3
4
5
6
7
8
9
10
11
Probe Number
Meishan−A
Meishan−B
Meishan−C
European−A
European−B
European−C

Rank 276 − Ssc.13817.1.A1_at − probe 6
VDAC3

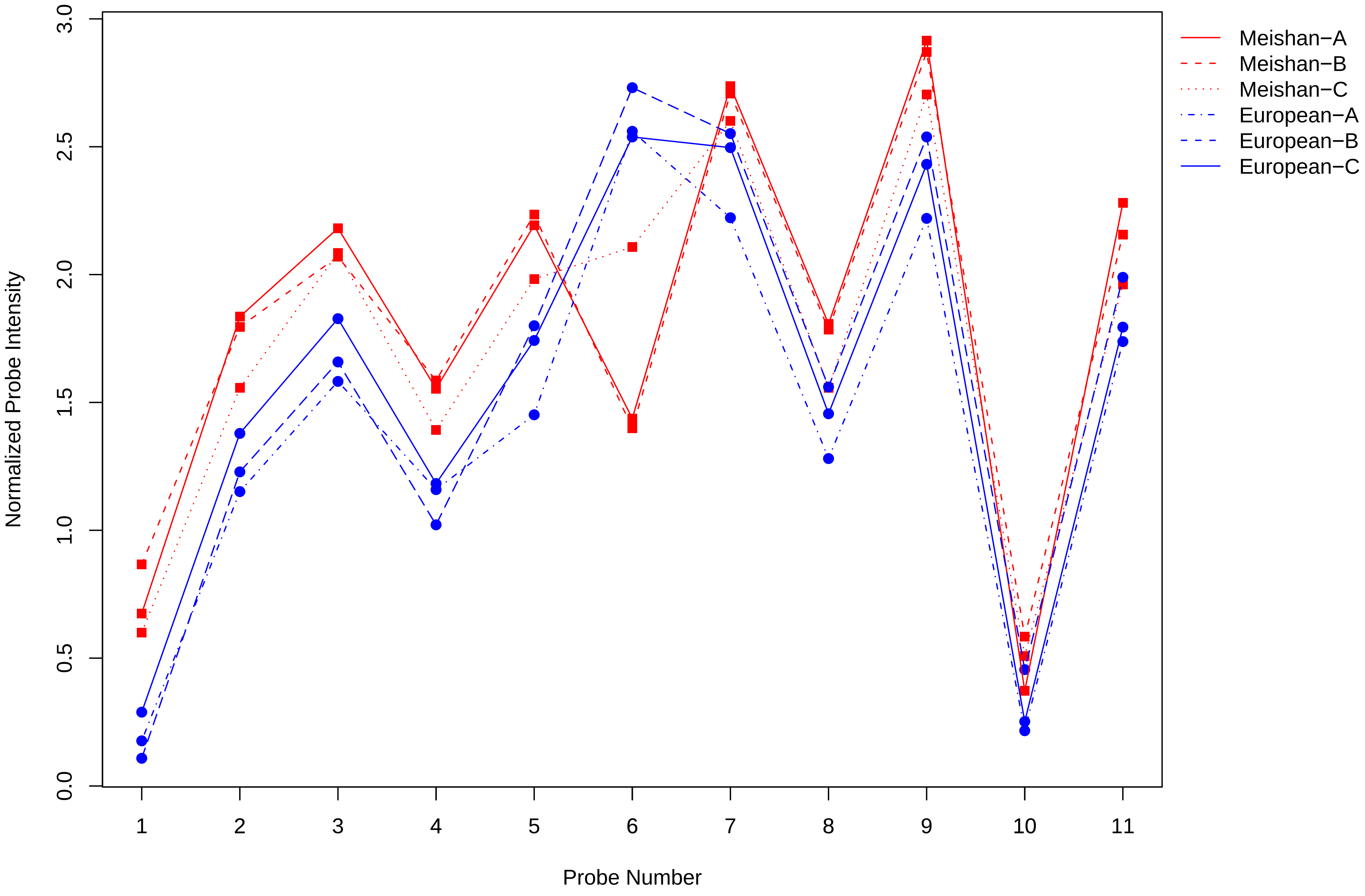

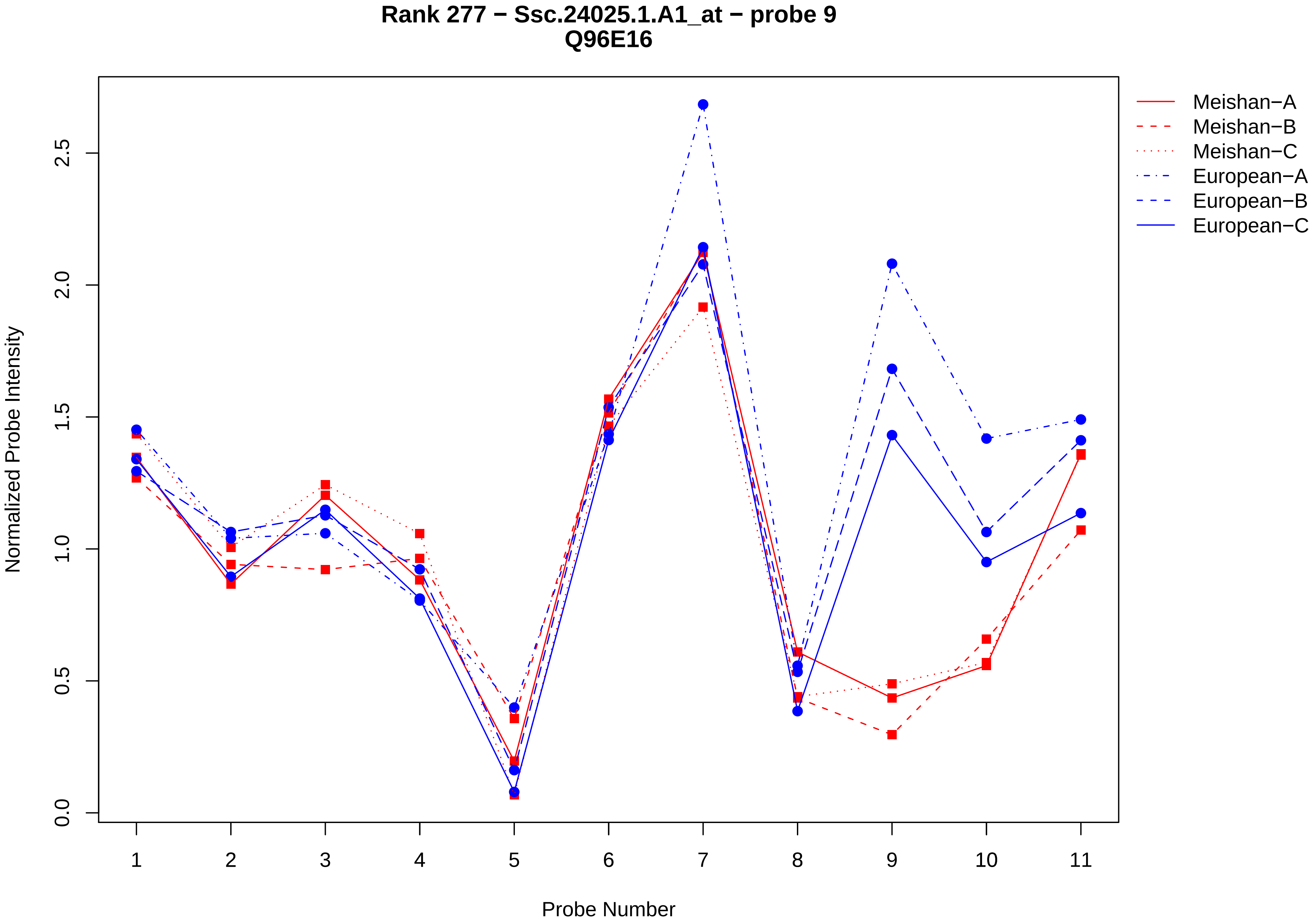

Rank 277 − Ssc.24025.1.A1_at − probe 9
Q96E16
Normalized Probe Intensity
0.0
0.5
1.0
1.5
2.0
2.5
1
2
3
4
5
6
7
8
9
10
11
Probe Number
Meishan−A
Meishan−B
Meishan−C
European−A
European−B
European−C

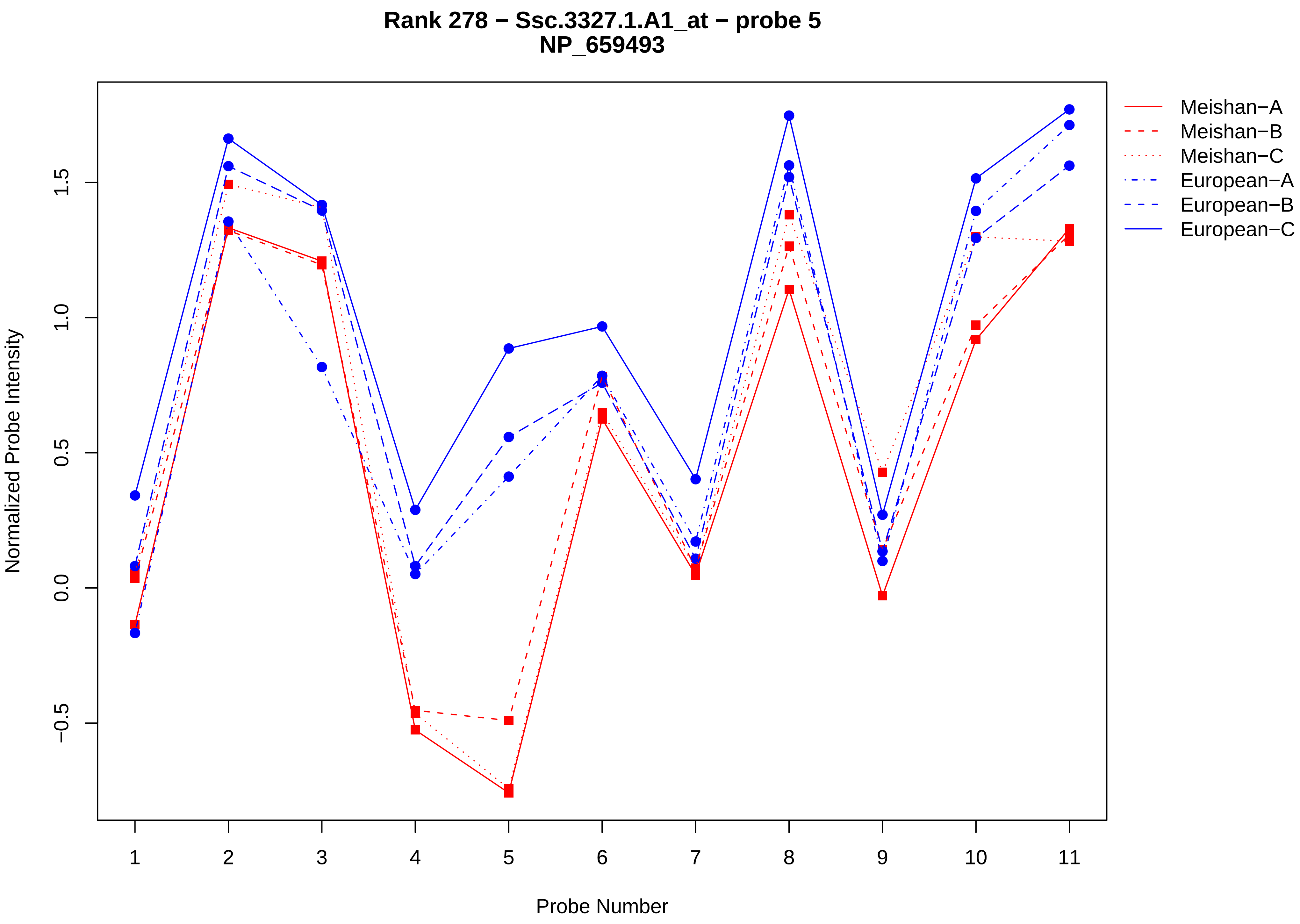

Rank 278 − Ssc.3327.1.A1_at − probe 5
NP_659493
Normalized Probe Intensity
Probe Number
−0.5
0.0
0.5
1.0
1.5
1
2
3
4
5
6
7
8
9
10
11
Meishan−A
Meishan−B
Meishan−C
European−A
European−B
European−C

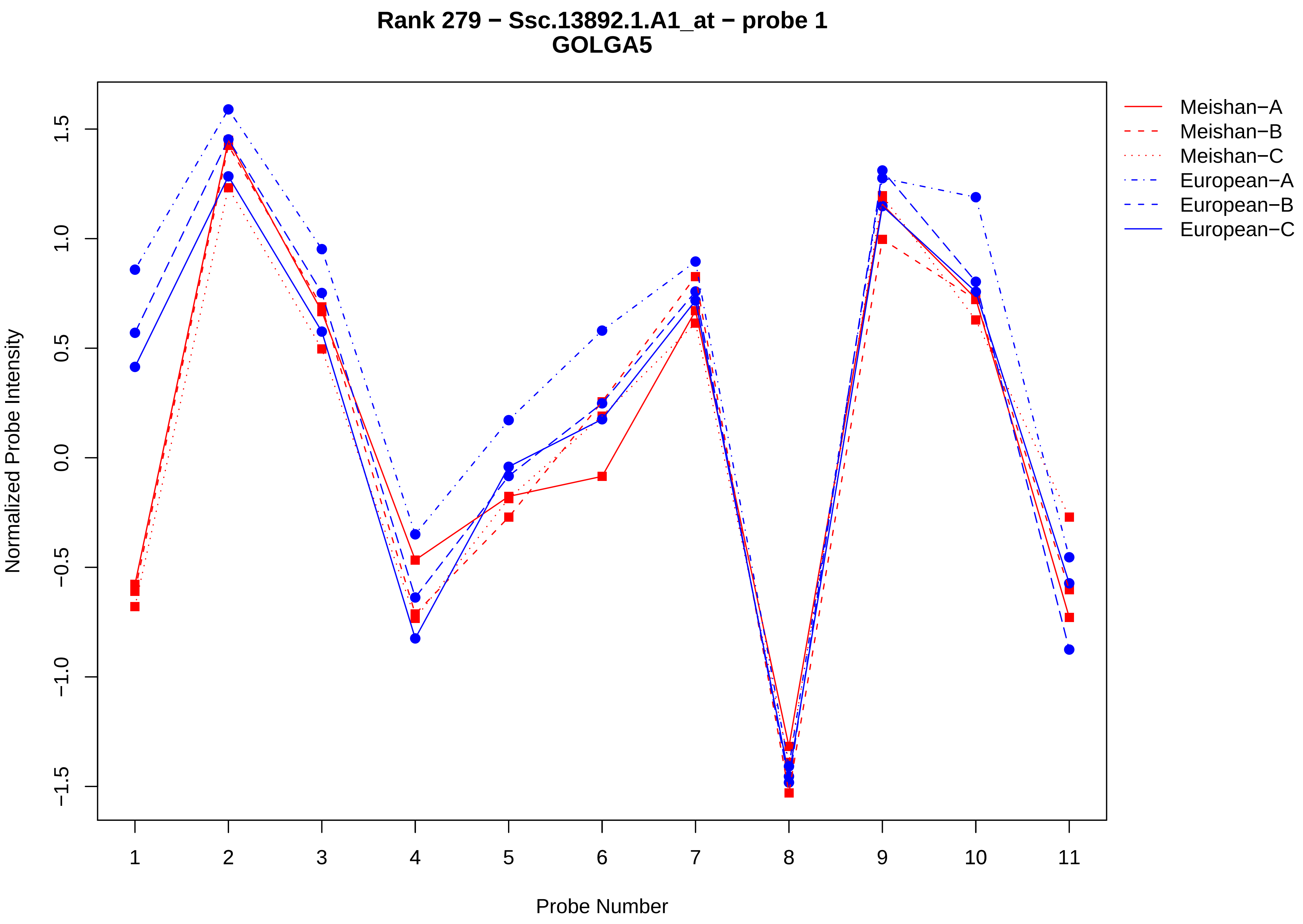

Rank 279 − Ssc.13892.1.A1_at − probe 1
GOLGA5
Normalized Probe Intensity
Probe Number
Meishan−A
Meishan−B
Meishan−C
European−A
European−B
European−C

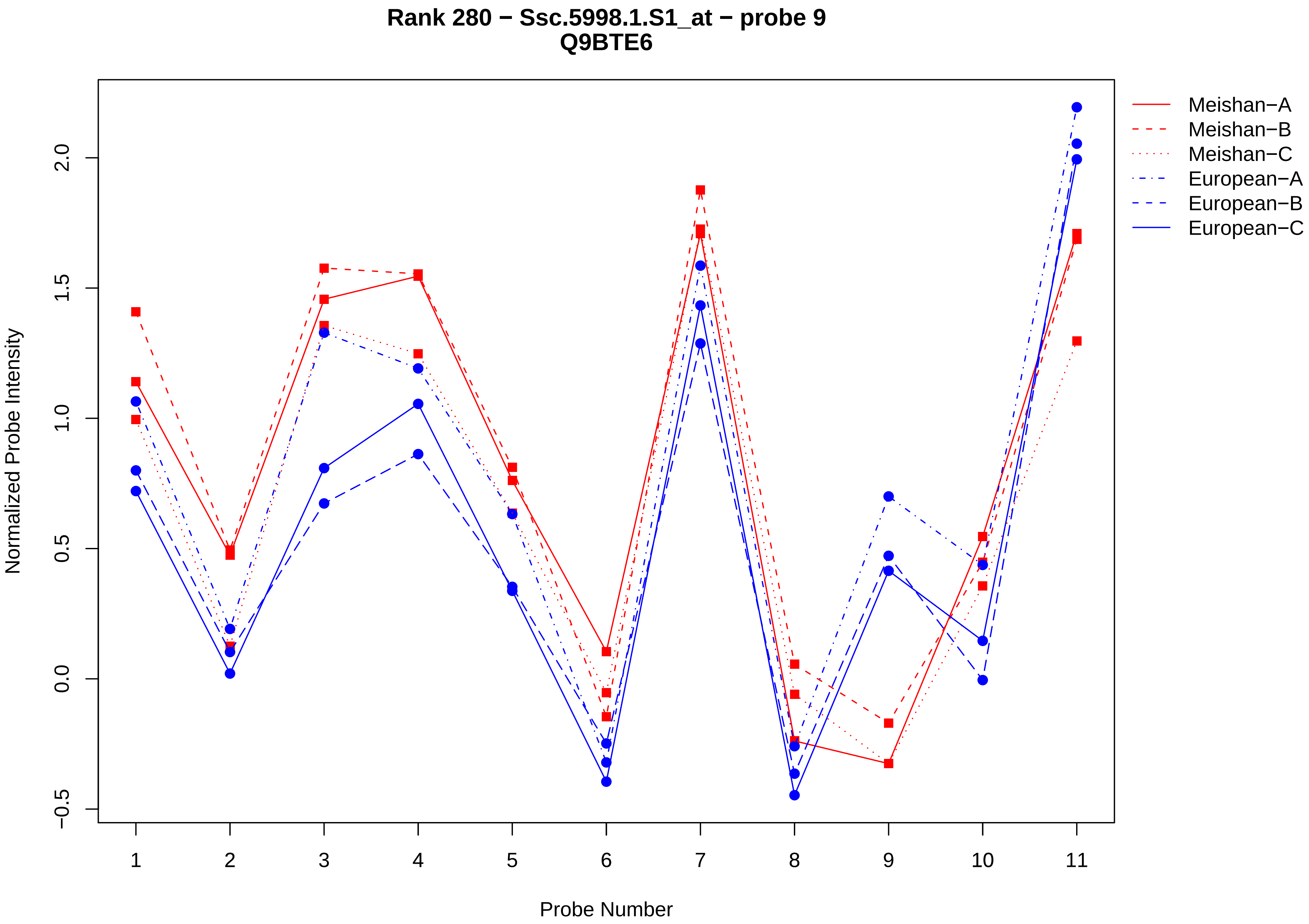

Rank 280 − Ssc.5998.1.S1_at − probe 9
Q9BTE6
Normalized Probe Intensity
Probe Number
−0.5
0.0
0.5
1.0
1.5
2.0
1
2
3
4
5
6
7
8
9
10
11
Meishan−A
Meishan−B
Meishan−C
European−A
European−B
European−C

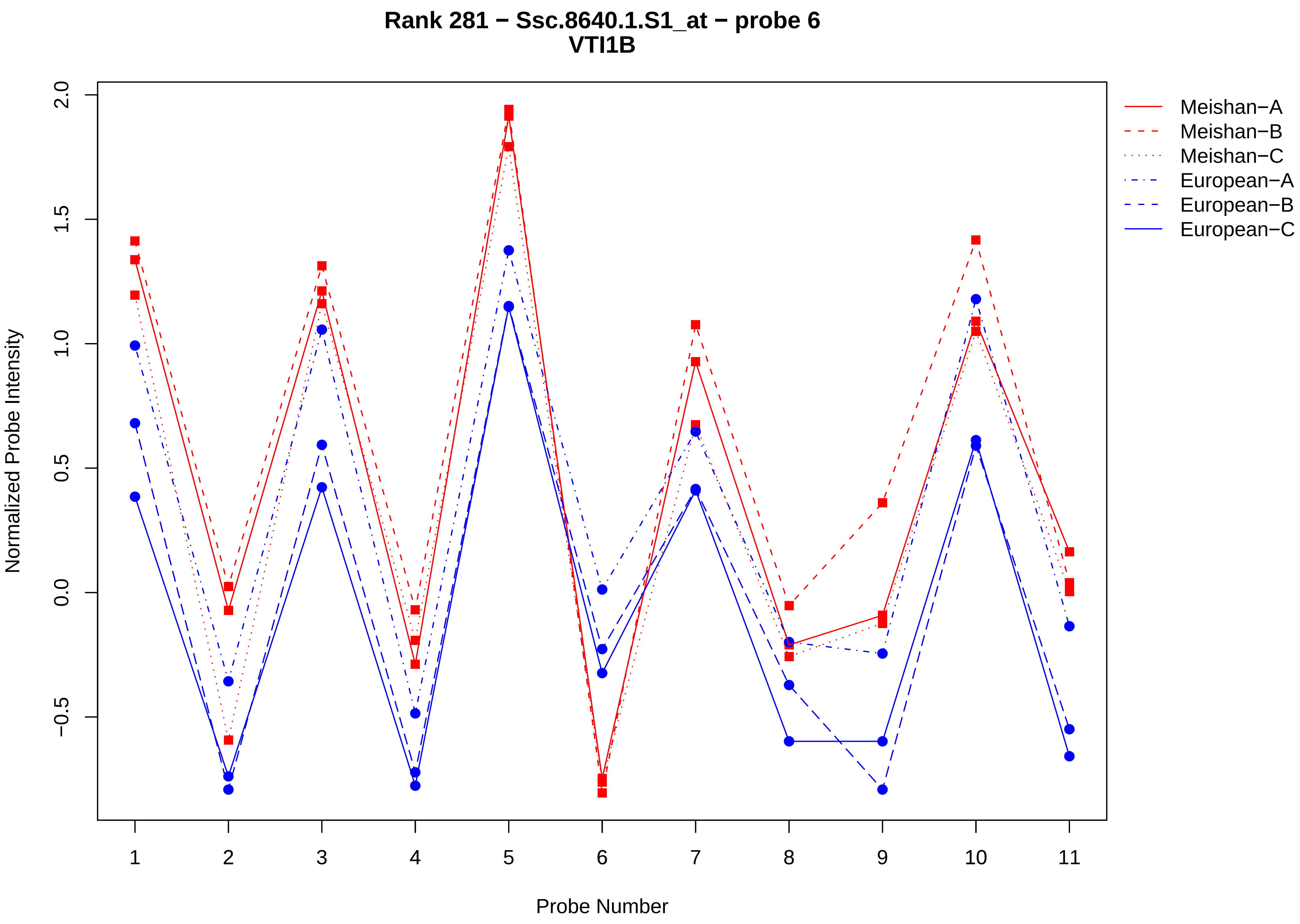
Rank 281 − Ssc.8640.1.S1_at − probe 6
VTI1B
Normalized Probe Intensity
Probe Number
−0.5
0.0
0.5
1.0
1.5
2.0
1
2
3
4
5
6
7
8
9
10
11
Meishan−A
Meishan−B
Meishan−C
European−A
European−B
European−C

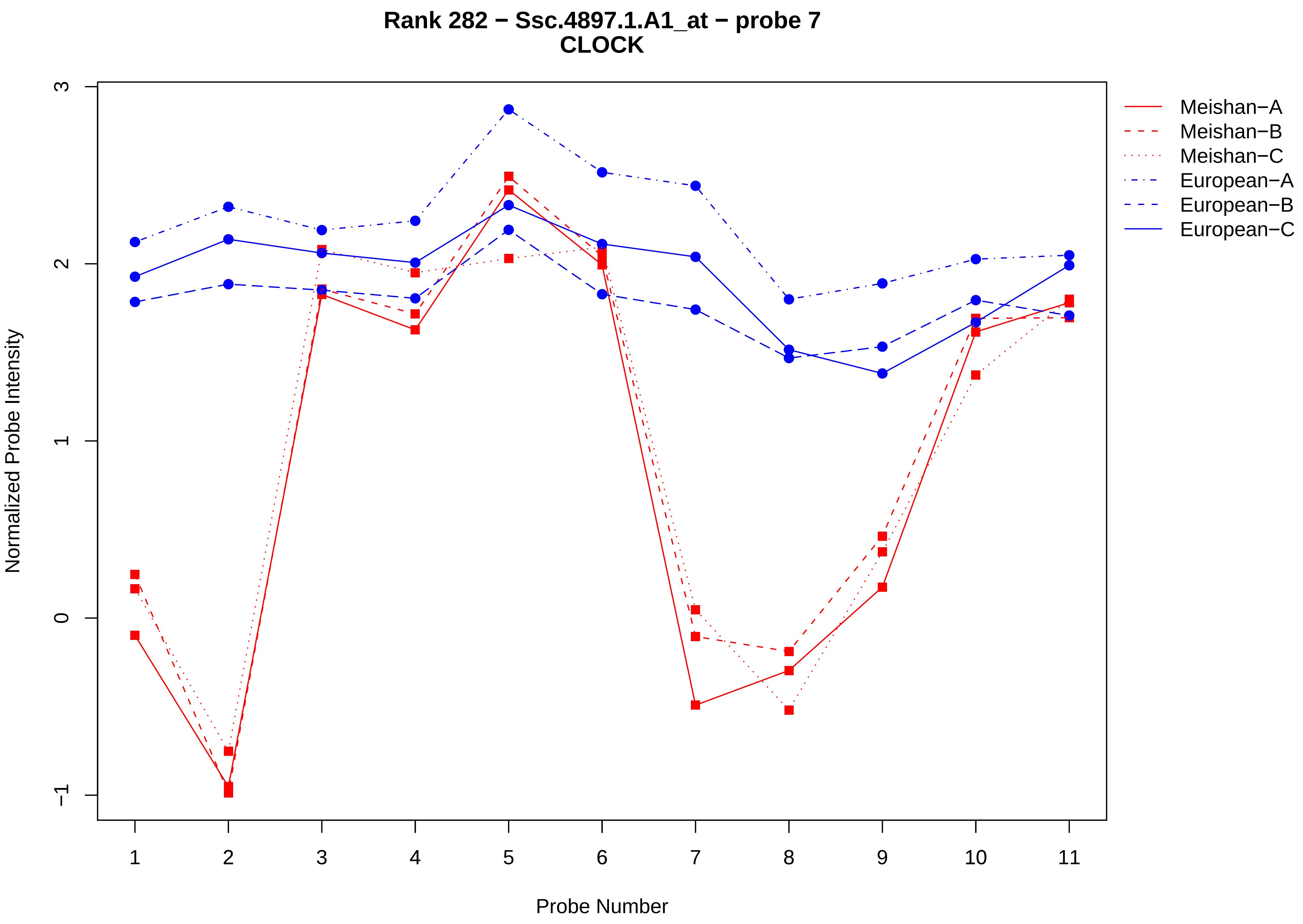
Rank 282 − Ssc.4897.1.A1_at − probe 7
CLOCK
Normalized Probe Intensity
Probe Number
−1
0
1
2
3
1
2
3
4
5
6
7
8
9
10
11
Meishan−A
Meishan−B
Meishan−C
European−A
European−B
European−C

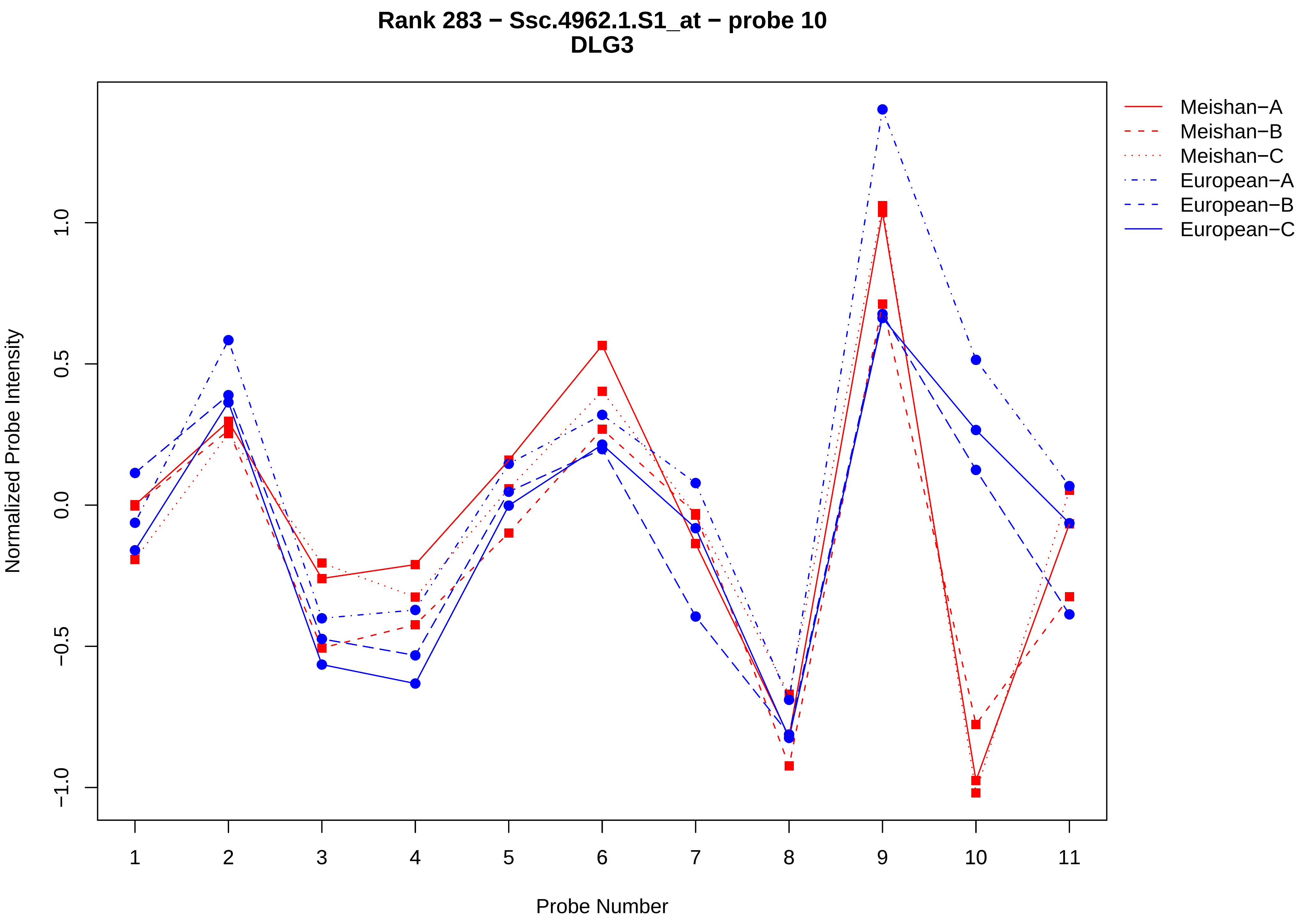

Rank 283 − Ssc.4962.1.S1_at − probe 10
DLG3
Normalized Probe Intensity
Probe Number
Meishan−A
Meishan−B
Meishan−C
European−A
European−B
European−C
−1.0
−0.5
0.0
0.5
1.0
1
2
3
4
5
6
7
8
9
10
11

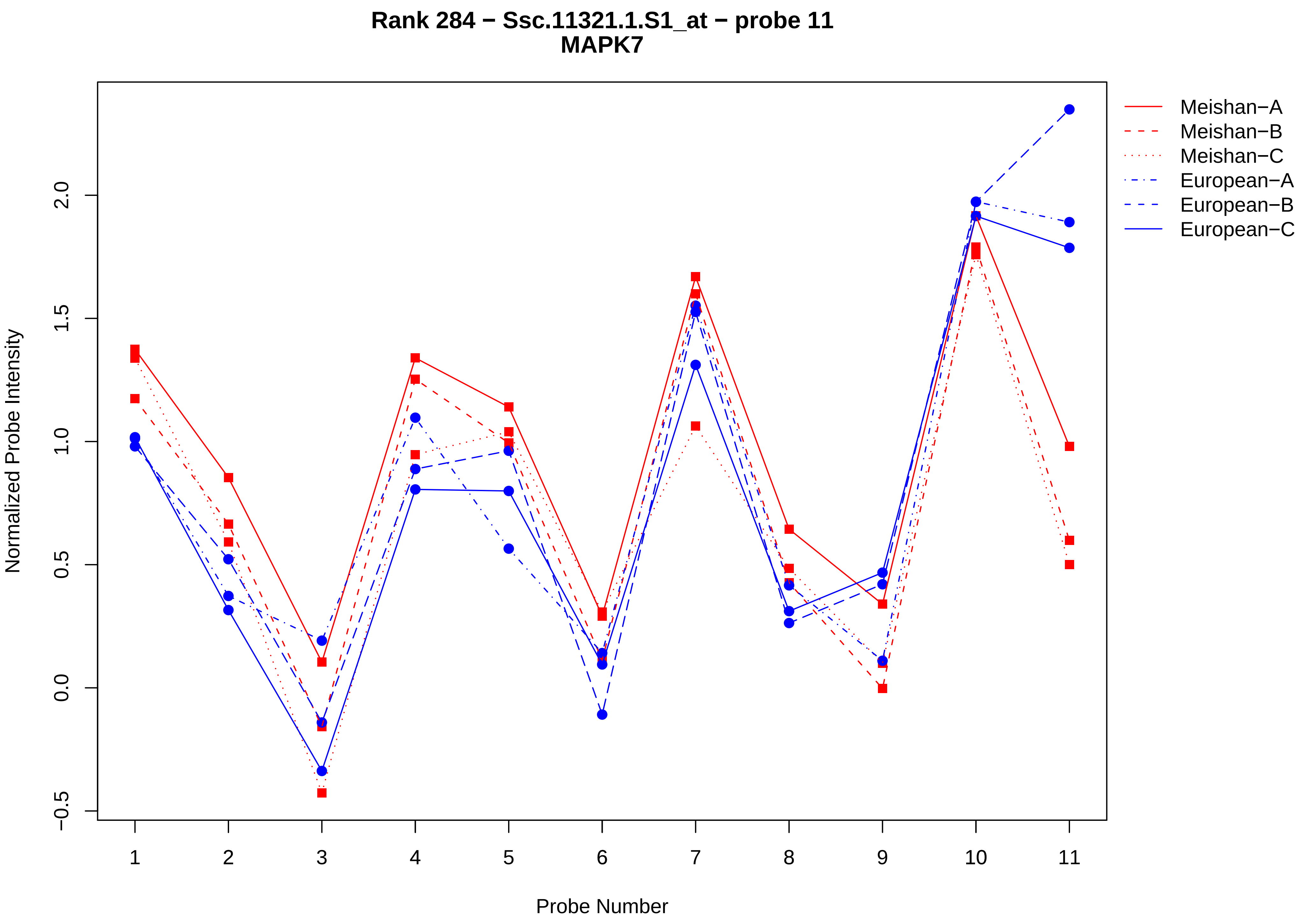

Rank 284 − Ssc.11321.1.S1_at − probe 11
MAPK7
Normalized Probe Intensity
Probe Number
−0.5
0.0
0.5
1.0
1.5
2.0
1
2
3
4
5
6
7
8
9
10
11
Meishan−A
Meishan−B
Meishan−C
European−A
European−B
European−C

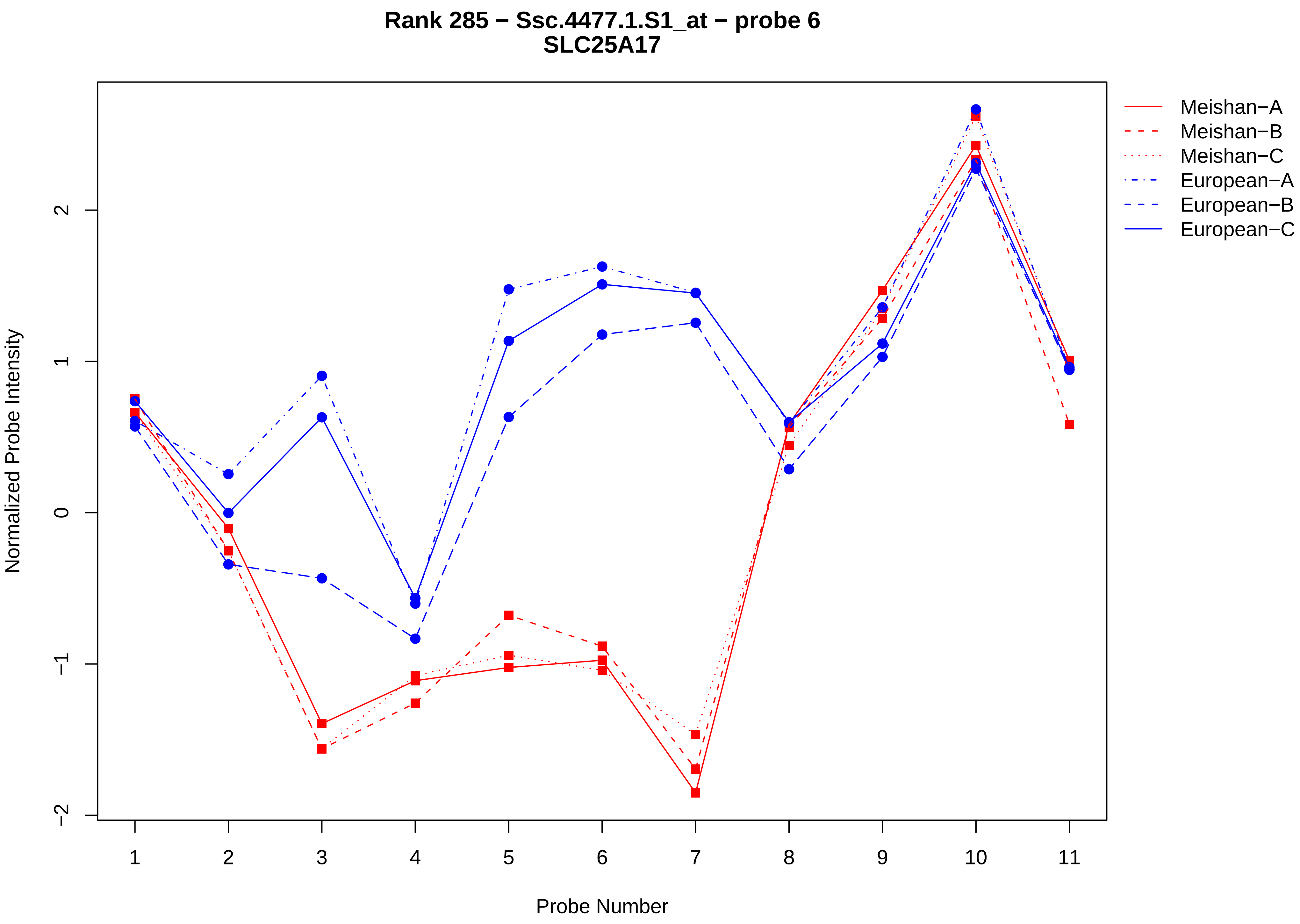
Rank 285 − Ssc.4477.1.S1_at − probe 6
SLC25A17
Normalized Probe Intensity
Probe Number
−2
−1
0
1
2
1
2
3
4
5
6
7
8
9
10
11
Meishan−A
Meishan−B
Meishan−C
European−A
European−B
European−C

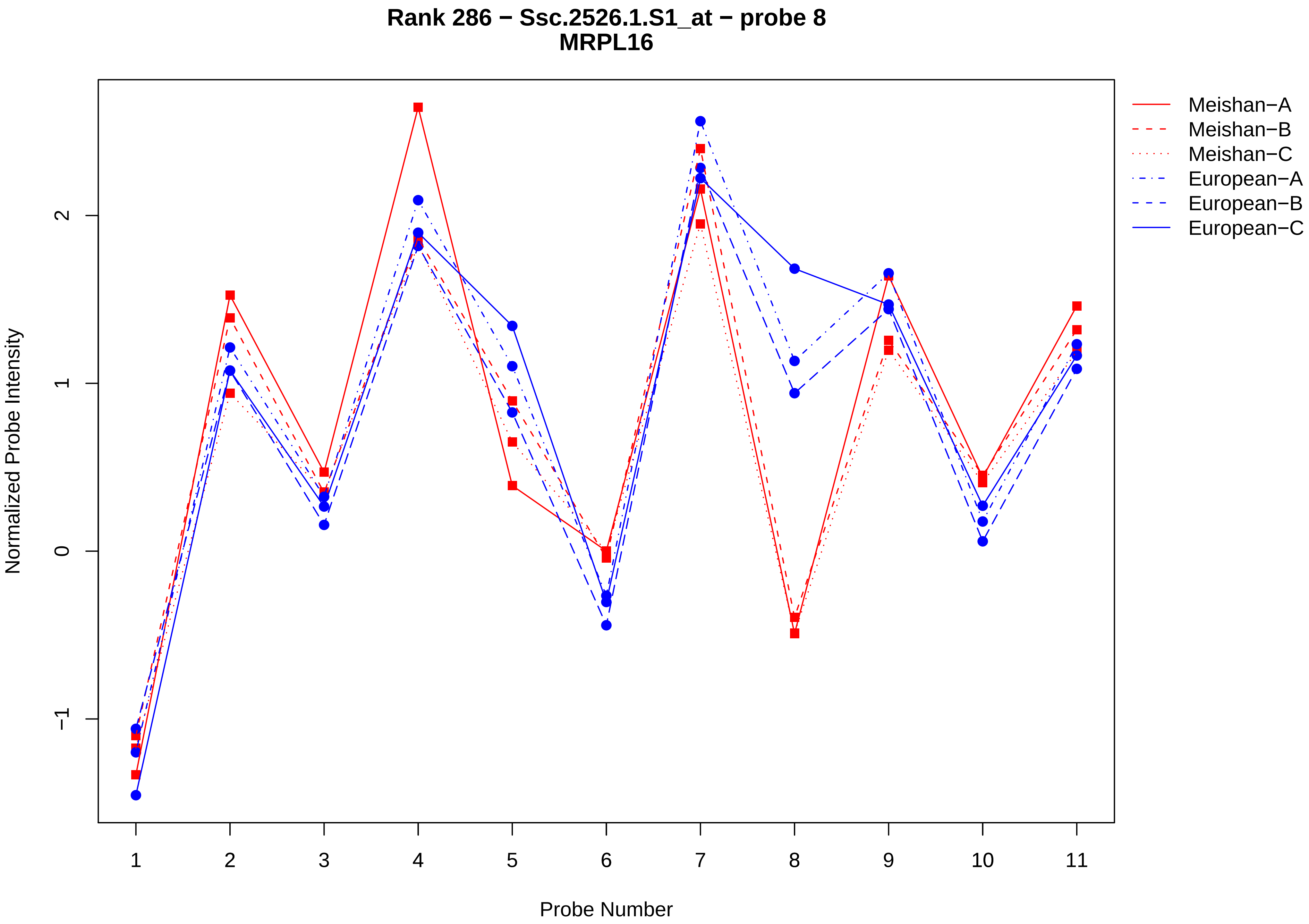
Rank 286 − Ssc.2526.1.S1_at − probe 8
MRPL16
Normalized Probe Intensity
Probe Number
−1
0
1
2
1
2
3
4
5
6
7
8
9
10
11
Meishan−A
Meishan−B
Meishan−C
European−A
European−B
European−C

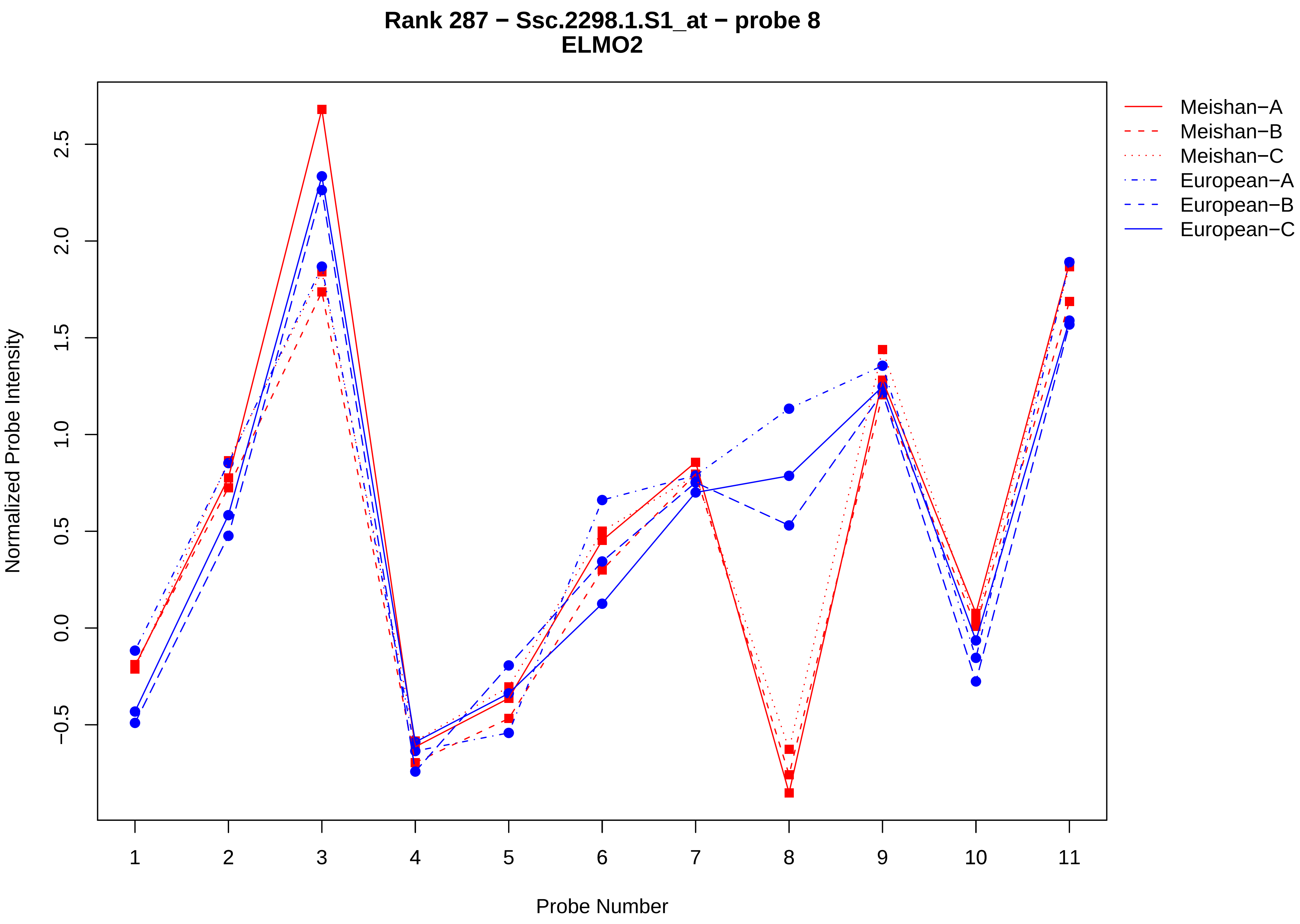

Rank 287 − Ssc.2298.1.S1_at − probe 8
ELMO2
Normalized Probe Intensity
Probe Number
−0.5
0.0
0.5
1.0
1.5
2.0
2.5
1
2
3
4
5
6
7
8
9
10
11
Meishan−A
Meishan−B
Meishan−C
European−A
European−B
European−C

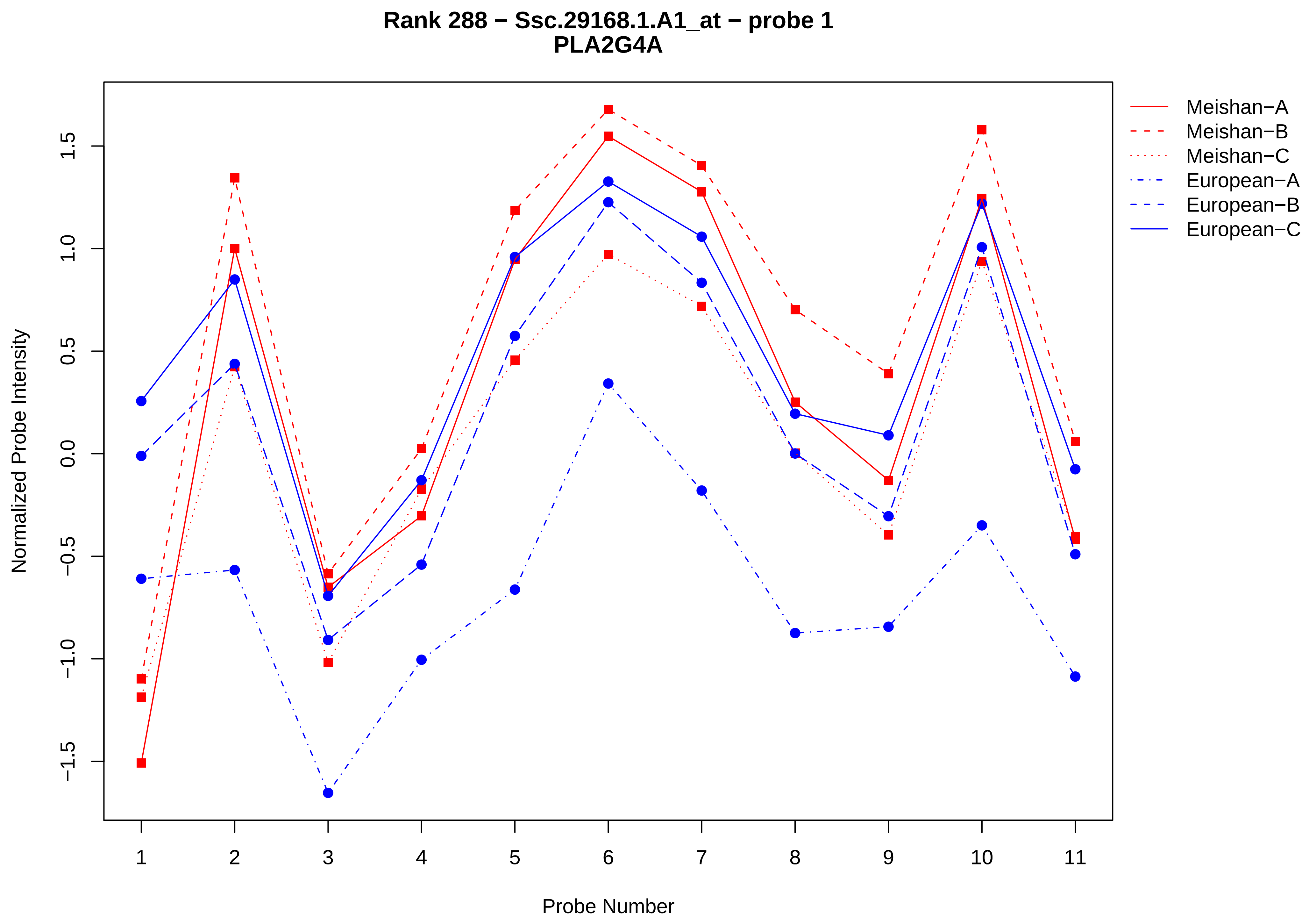

Rank 288 − Ssc.29168.1.A1_at − probe 1
PLA2G4A
Normalized Probe Intensity
Probe Number
−1.5
−1.0
−0.5
0.0
0.5
1.0
1.5
1
2
3
4
5
6
7
8
9
10
11
Meishan−A
Meishan−B
Meishan−C
European−A
European−B
European−C

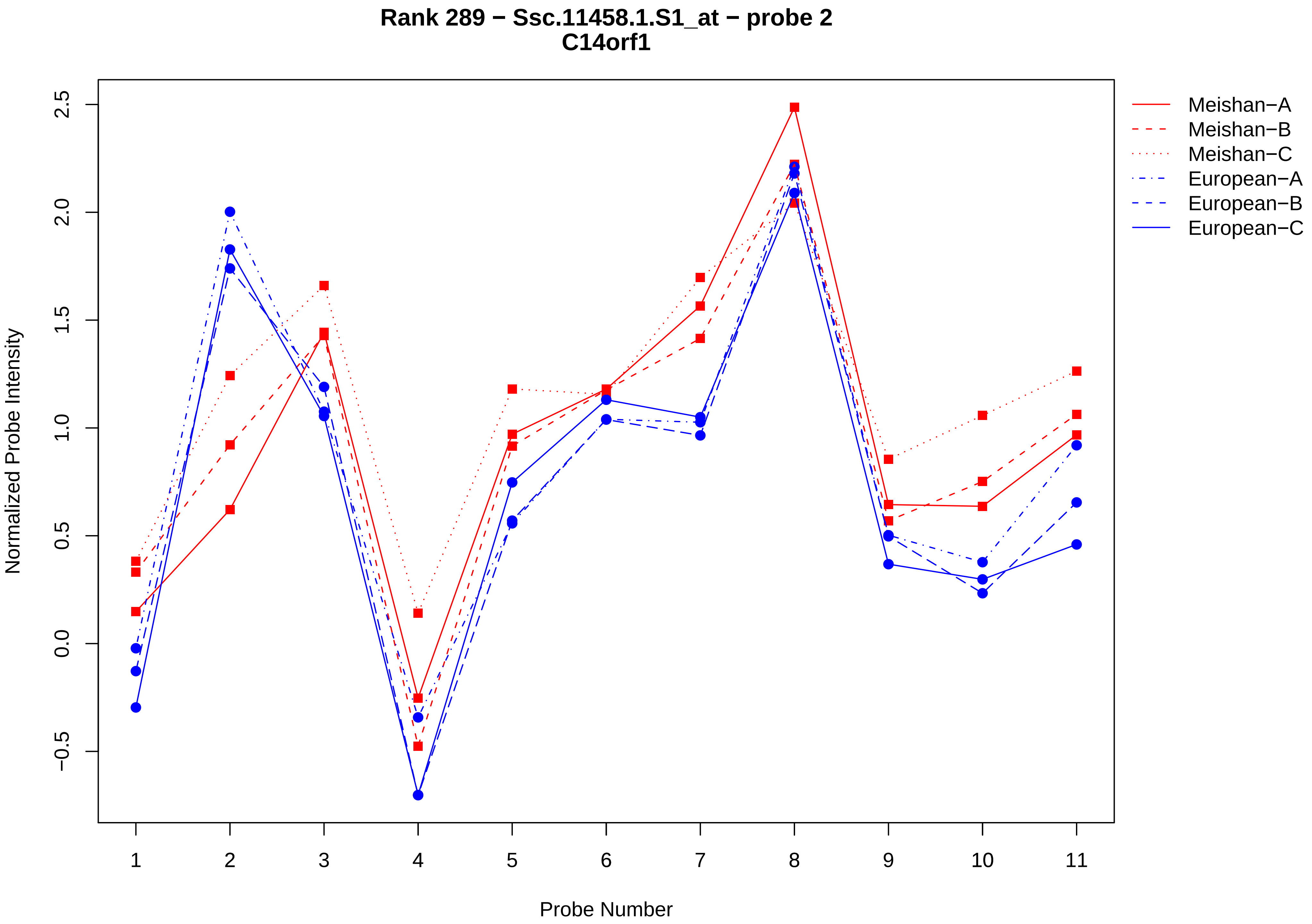

Rank 289 − Ssc.11458.1.S1_at − probe 2
C14orf1
Normalized Probe Intensity
Probe Number
−0.5
0.0
0.5
1.0
1.5
2.0
2.5
1
2
3
4
5
6
7
8
9
10
11
Meishan−A
Meishan−B
Meishan−C
European−A
European−B
European−C

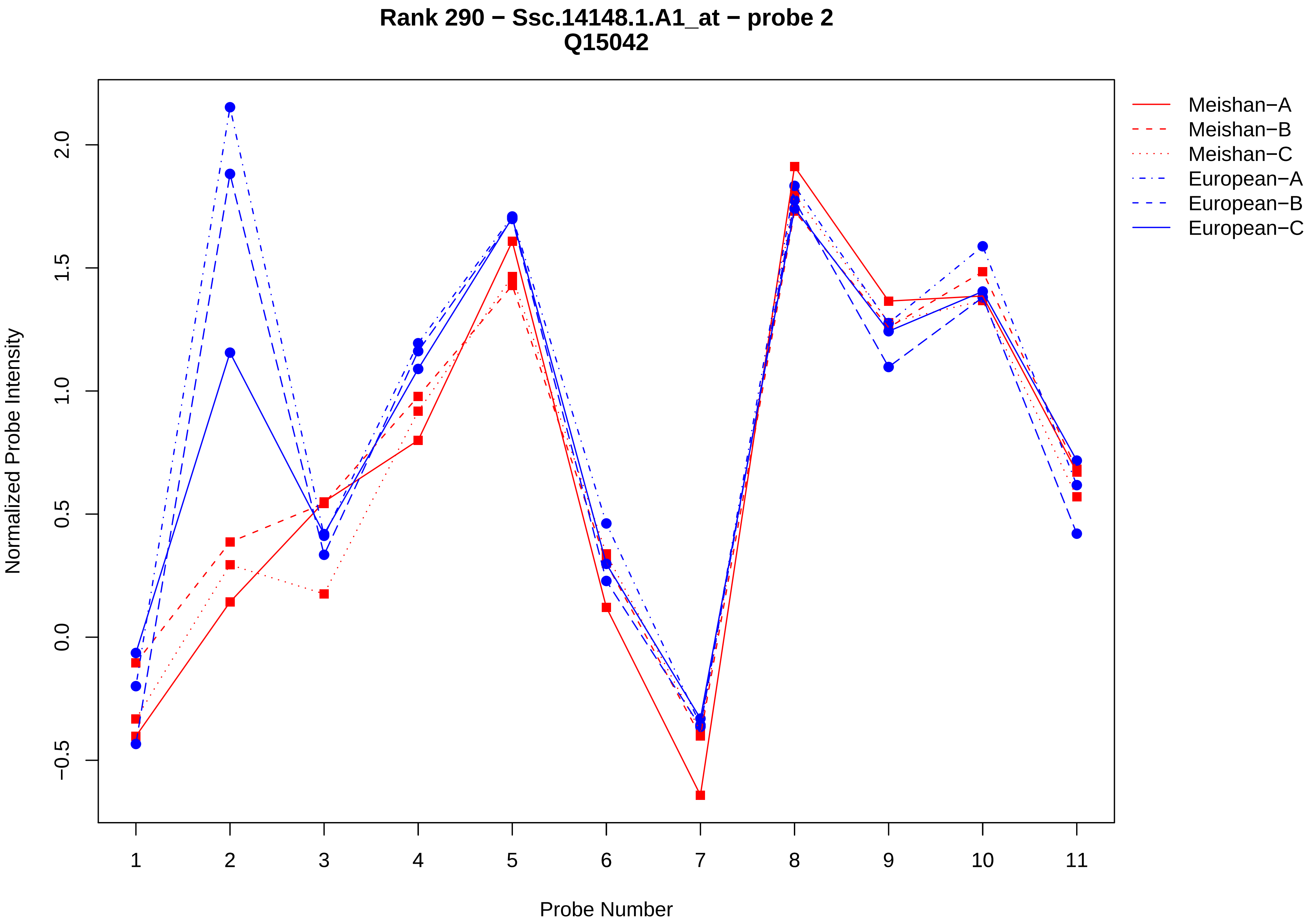

Rank 290 − Ssc.14148.1.A1_at − probe 2
Q15042
Normalized Probe Intensity
Probe Number
Meishan−A
Meishan−B
Meishan−C
European−A
European−B
European−C

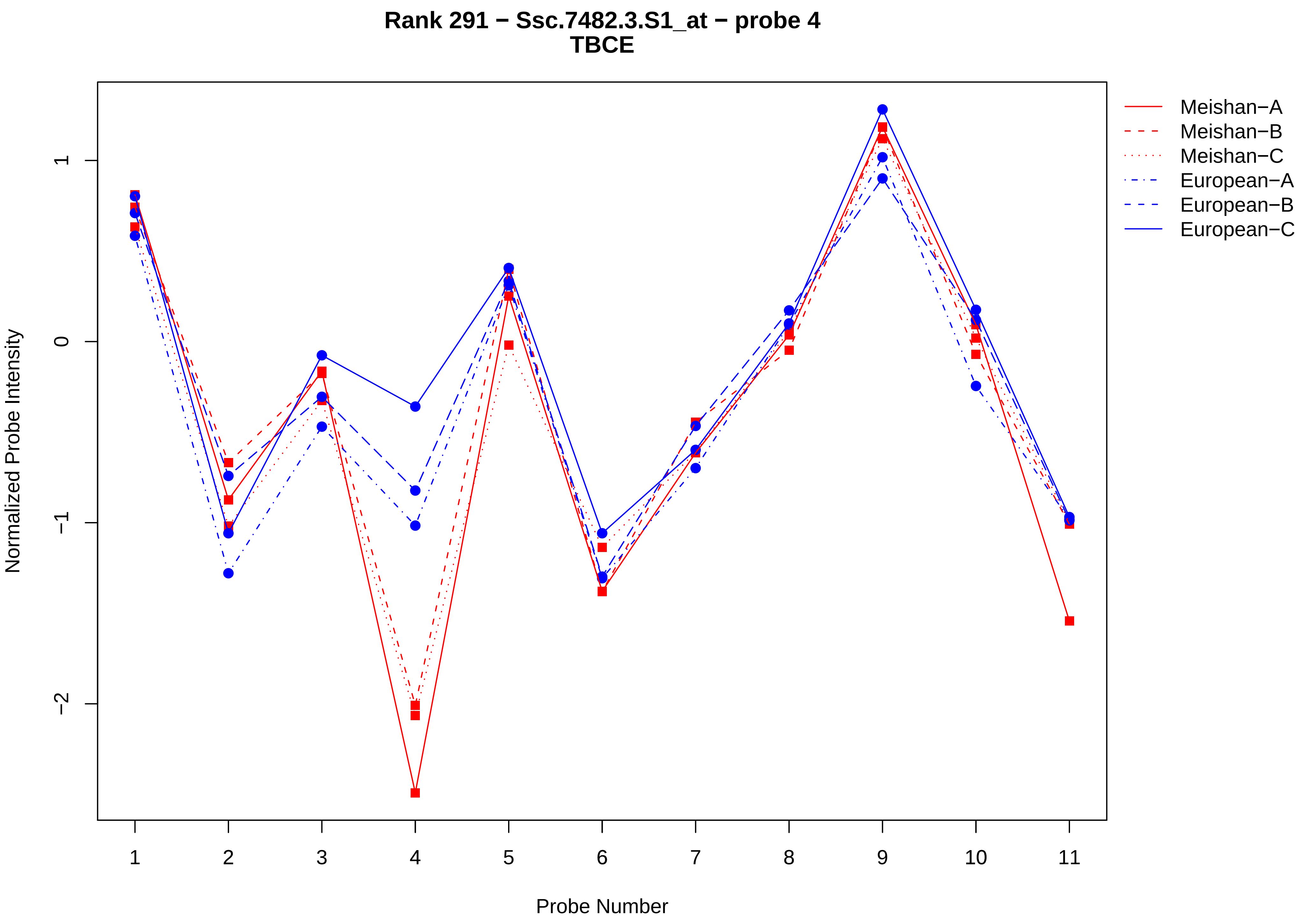
Rank 291 − Ssc.7482.3.S1_at − probe 4
TBCE
Normalized Probe Intensity
Probe Number
1
0
−1
−2
1
2
3
4
5
6
7
8
9
10
11
Meishan−A
Meishan−B
Meishan−C
European−A
European−B
European−C

Rank 292 − Ssc.20986.2.S1_at − probe 2
GRN

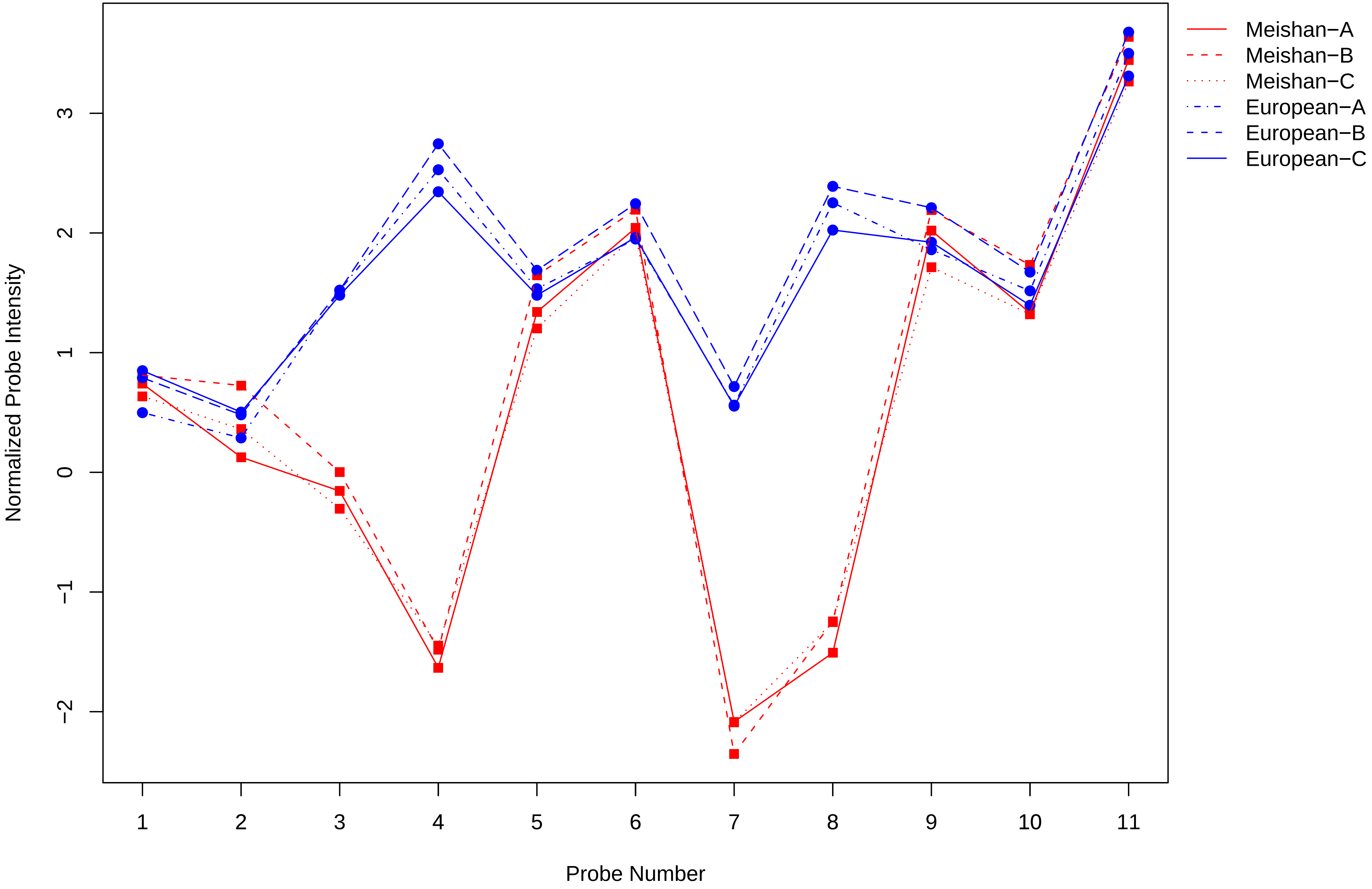

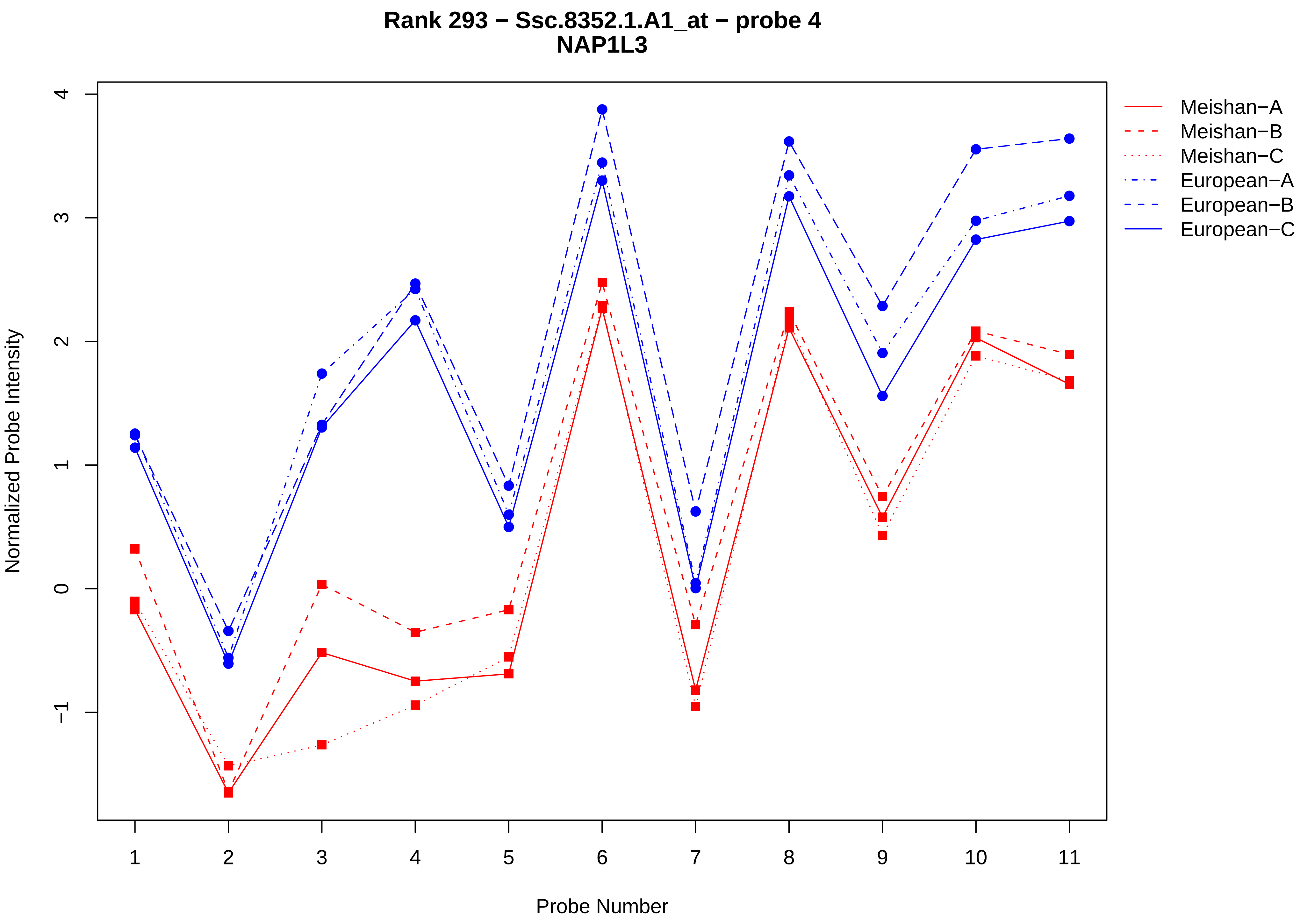

Rank 293 − Ssc.8352.1.A1_at − probe 4
NAP1L3
Normalized Probe Intensity
Probe Number
−1
0
1
2
3
4
1
2
3
4
5
6
7
8
9
10
11
Meishan−A
Meishan−B
Meishan−C
European−A
European−B
European−C

Rank 294 − Ssc.20986.2.S1_at − probe 9
GRN

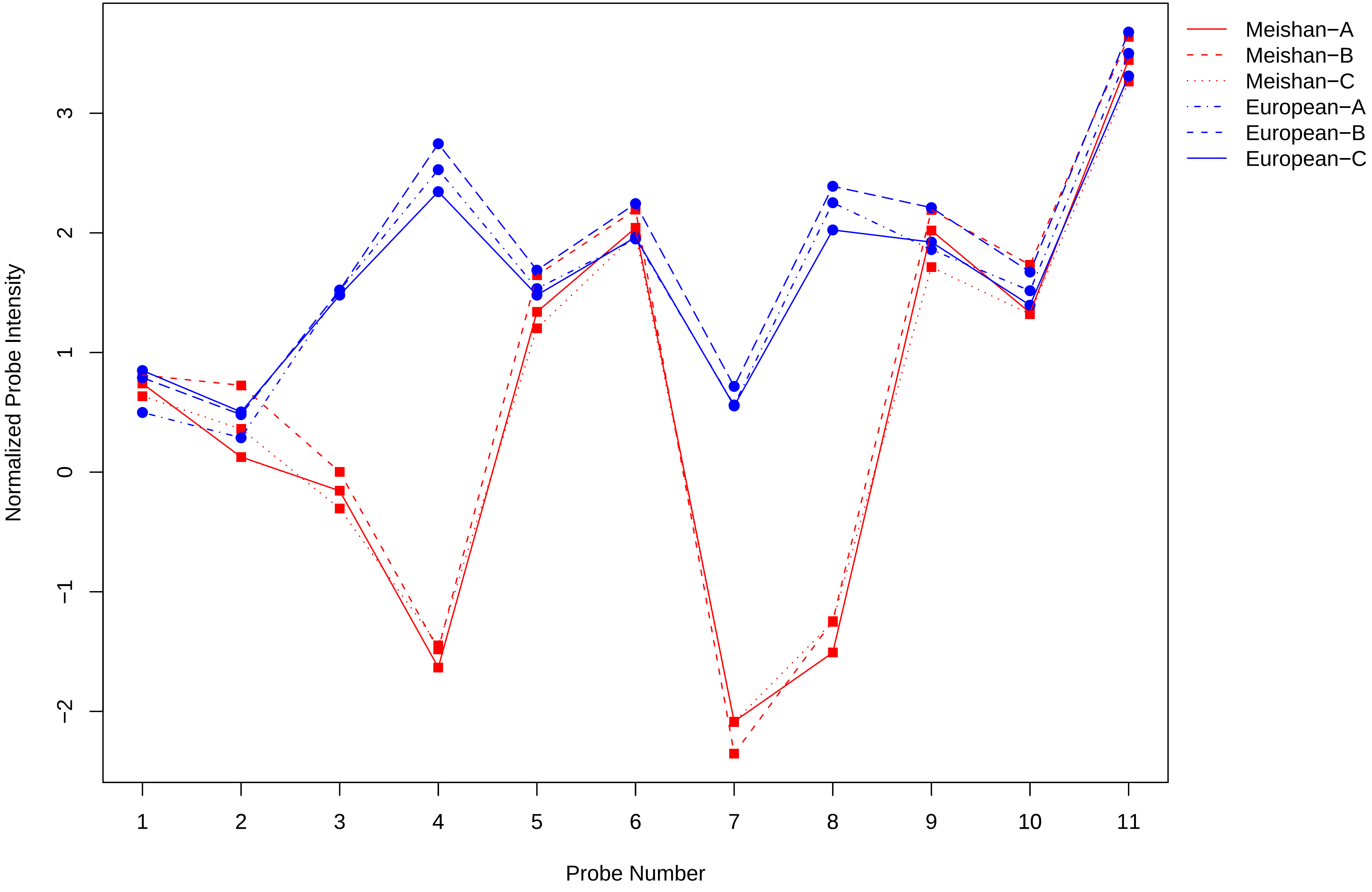

Rank 295 − Ssc.18454.1.A1_at − probe 2
TLE1

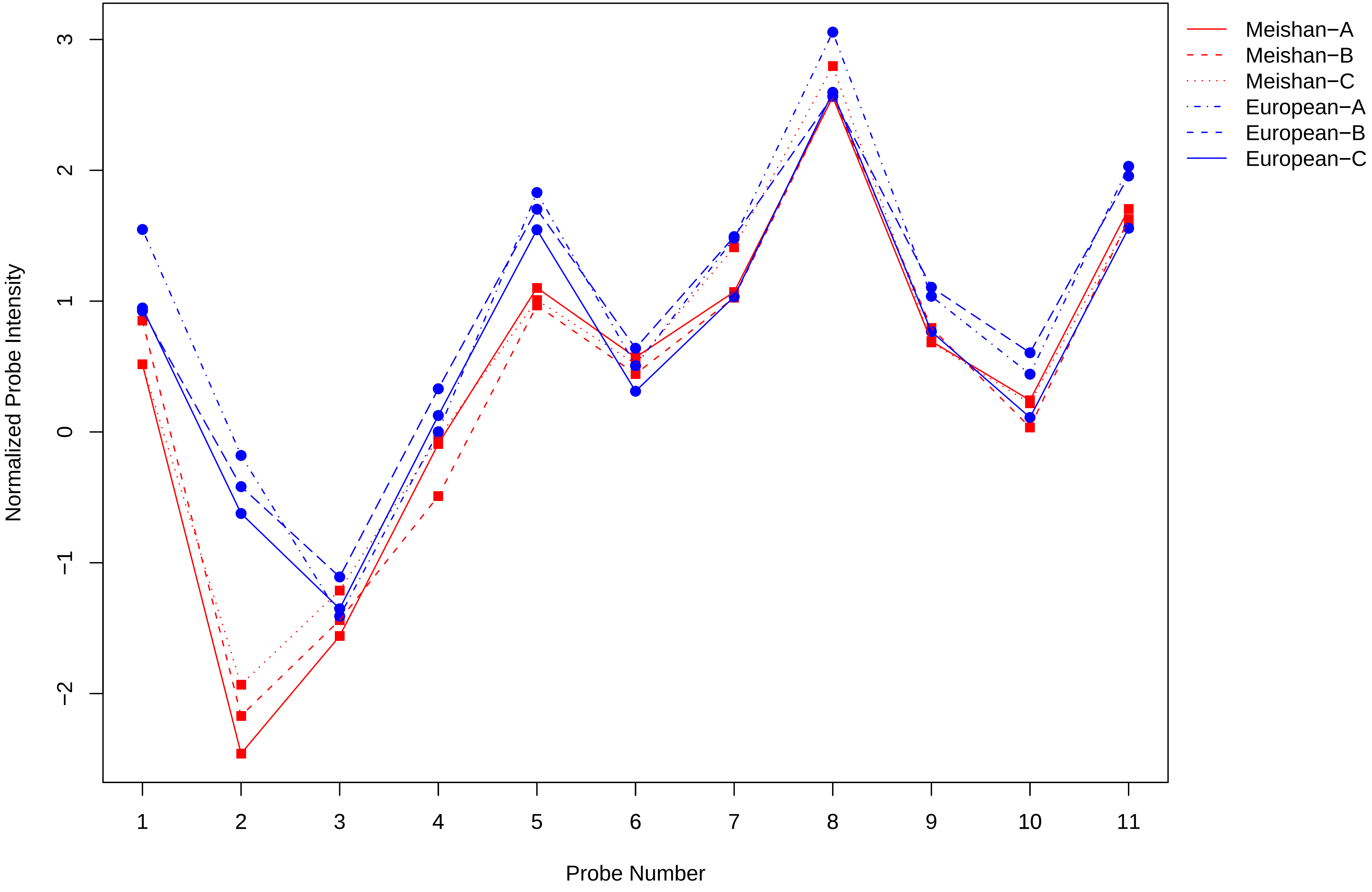

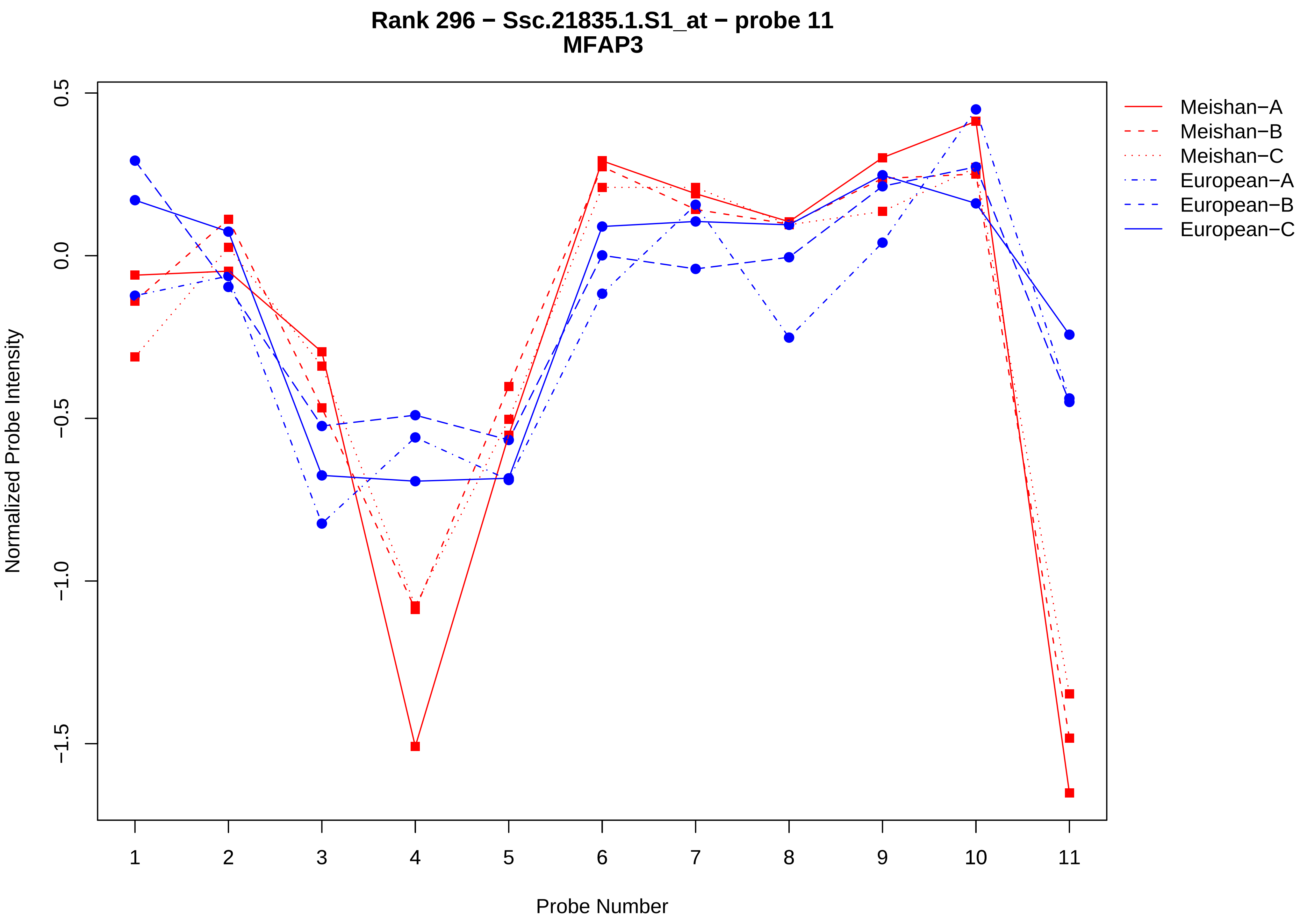

Rank 296 − Ssc.21835.1.S1_at − probe 11
MFAP3
Normalized Probe Intensity
Probe Number
0.5
0.0
−0.5
−1.0
−1.5
1
2
3
4
5
6
7
8
9
10
11
Meishan−A
Meishan−B
Meishan−C
European−A
European−B
European−C

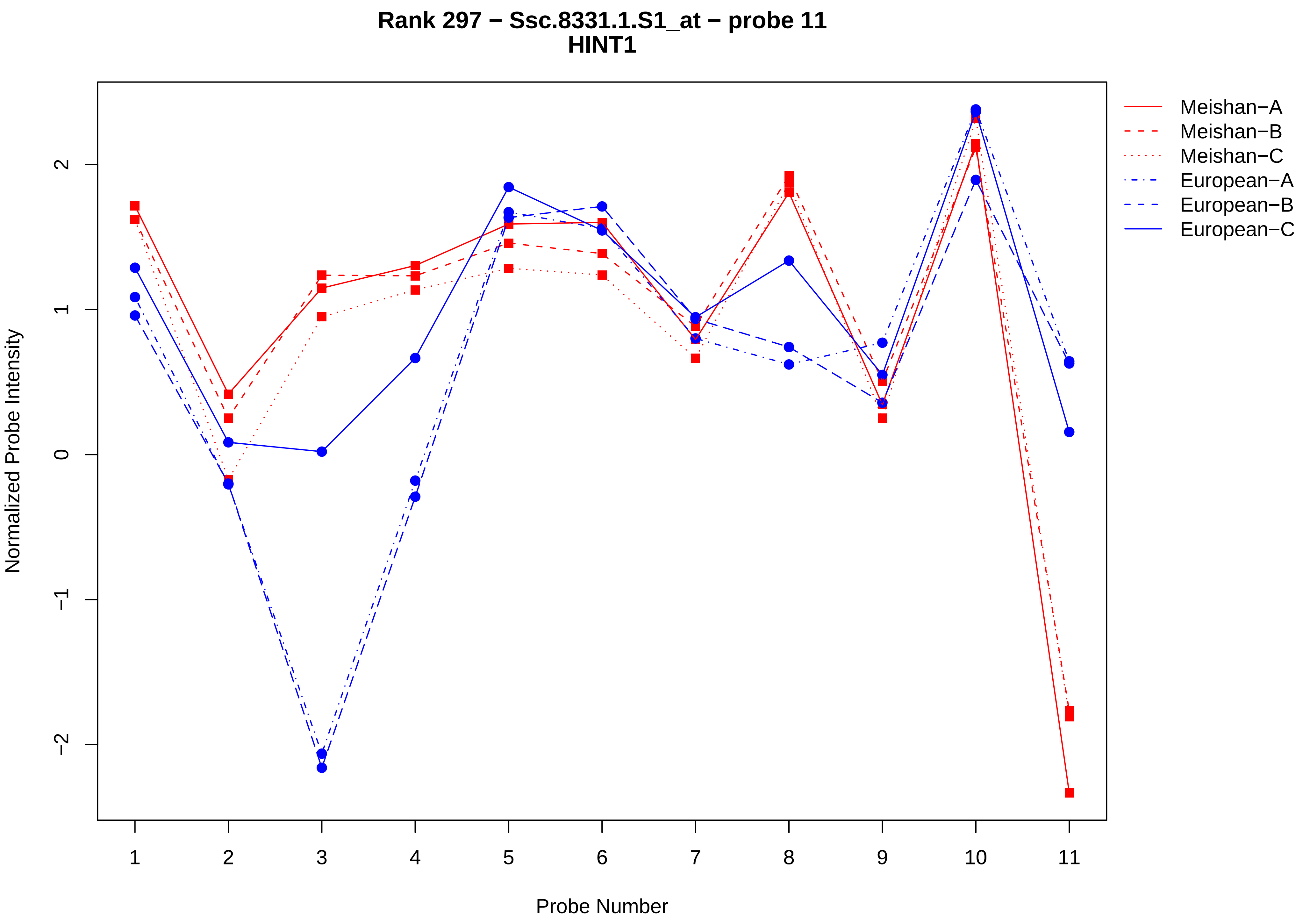
Rank 297 − Ssc.8331.1.S1_at − probe 11
HINT1
Normalized Probe Intensity
Probe Number
Meishan−A
Meishan−B
Meishan−C
European−A
European−B
European−C
−2
−1
0
1
2
1
2
3
4
5
6
7
8
9
10
11

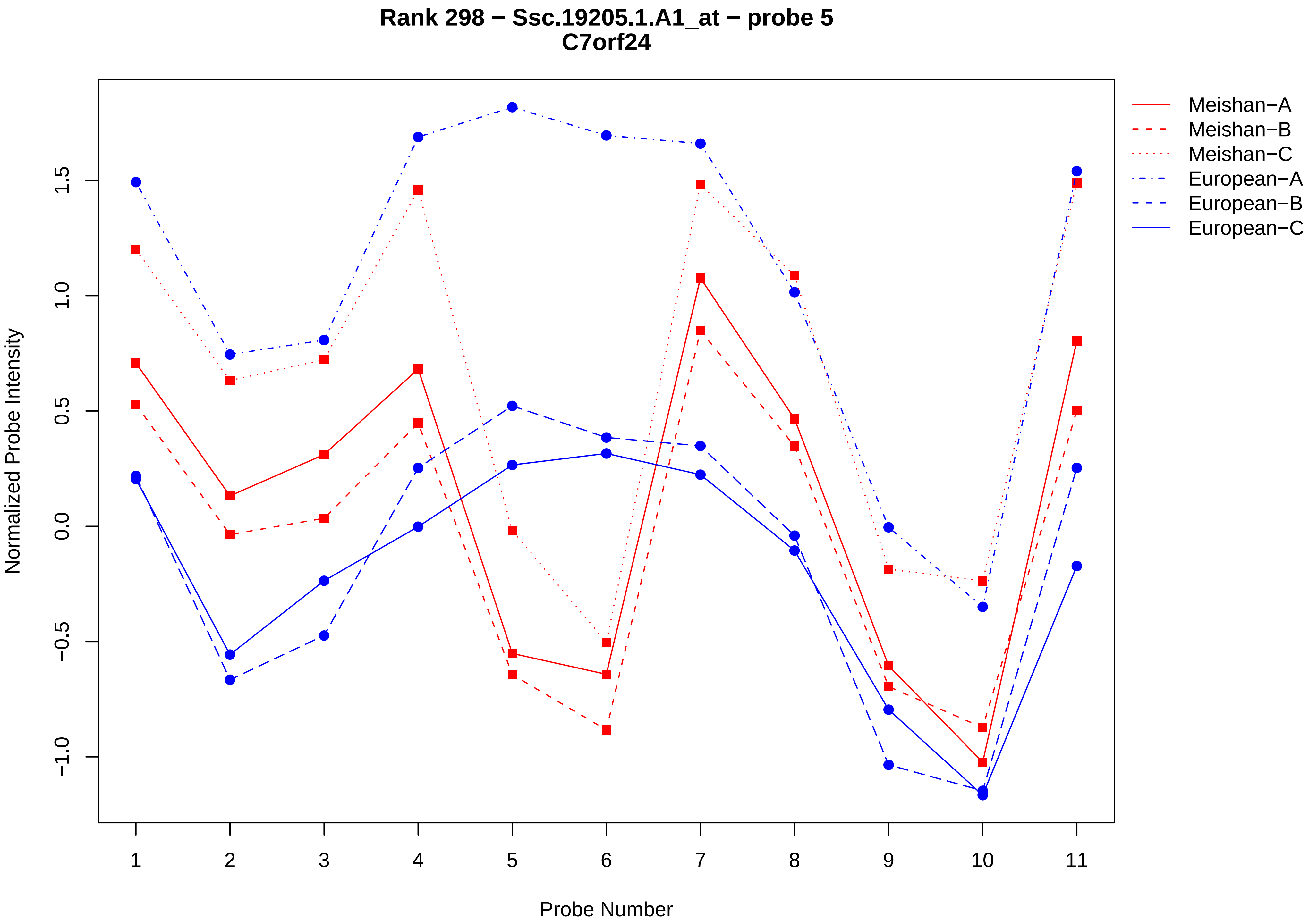

Rank 298 − Ssc.19205.1.A1_at − probe 5
C7orf24
Normalized Probe Intensity
Probe Number
Meishan−A
Meishan−B
Meishan−C
European−A
European−B
European−C
−1.0
−0.5
0.0
0.5
1.0
1.5
1
2
3
4
5
6
7
8
9
10
11

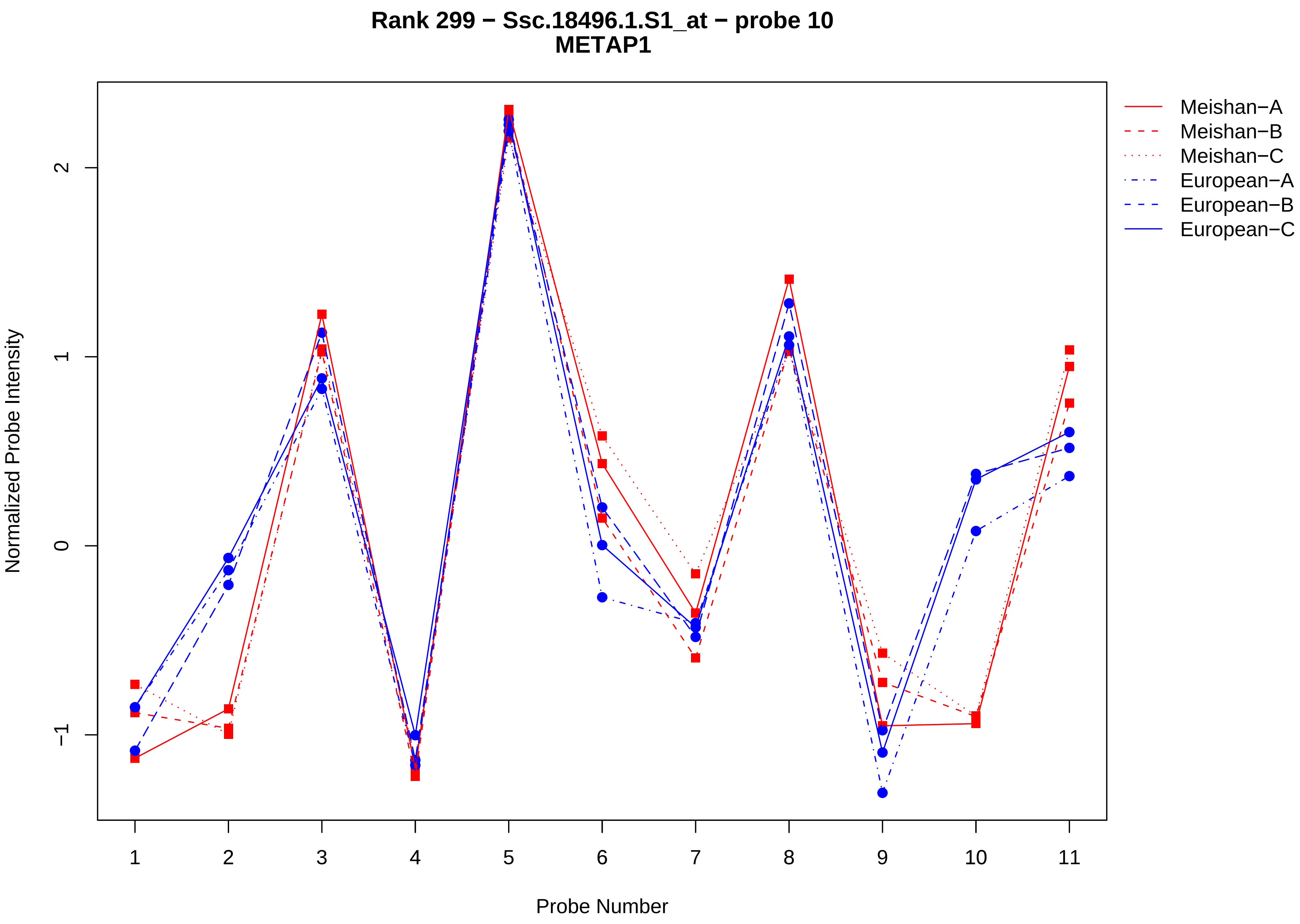

Rank 299 − Ssc.18496.1.S1_at − probe 10
METAP1
Normalized Probe Intensity
Probe Number
−1
0
1
2
1
2
3
4
5
6
7
8
9
10
11
Meishan−A
Meishan−B
Meishan−C
European−A
European−B
European−C

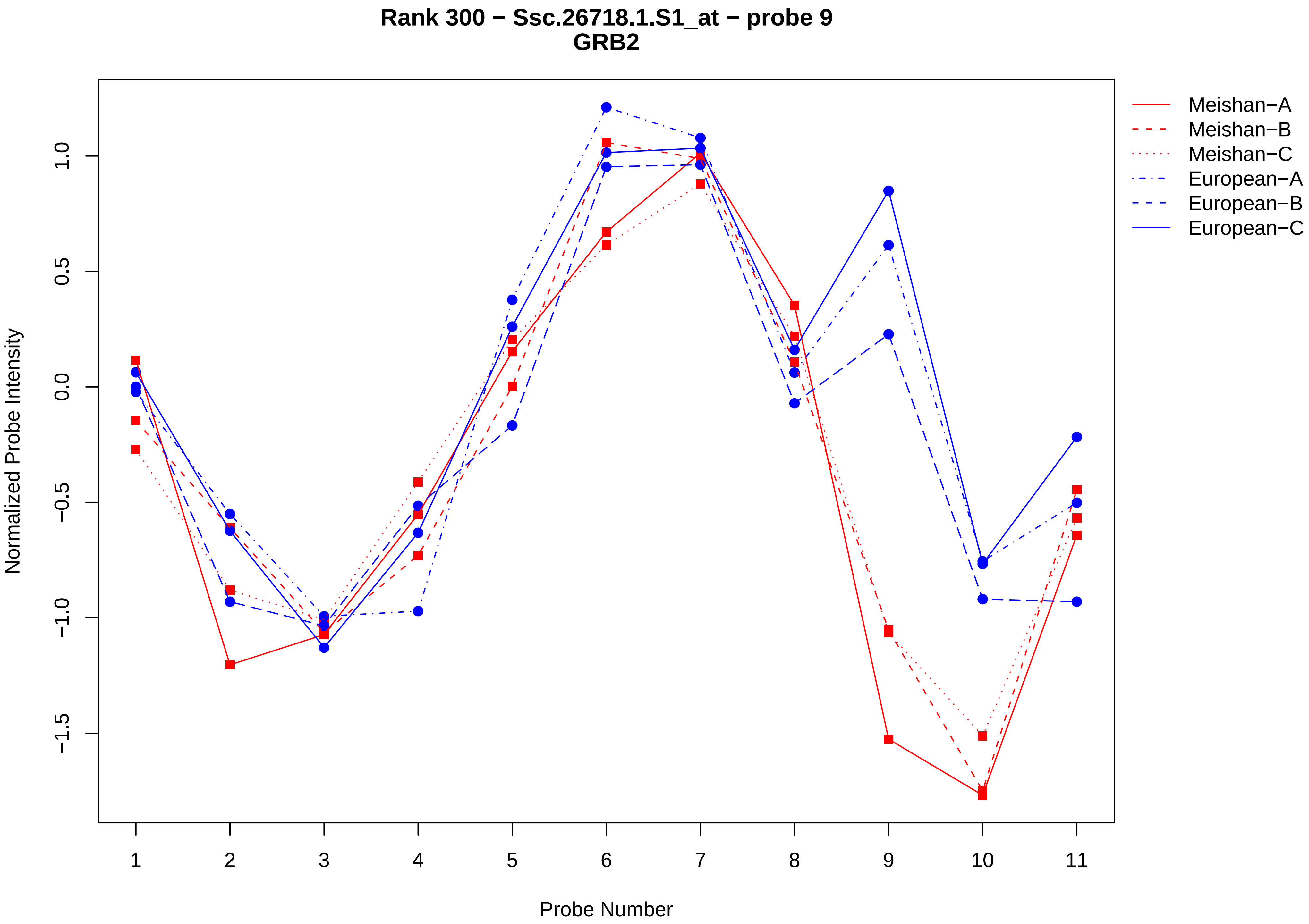

Rank 300 − Ssc.26718.1.S1_at − probe 9
GRB2
Normalized Probe Intensity
Probe Number
1.0
0.5
0.0
−0.5
−1.0
−1.5
1
2
3
4
5
6
7
8
9
10
11
Meishan−A
Meishan−B
Meishan−C
European−A
European−B
European−C

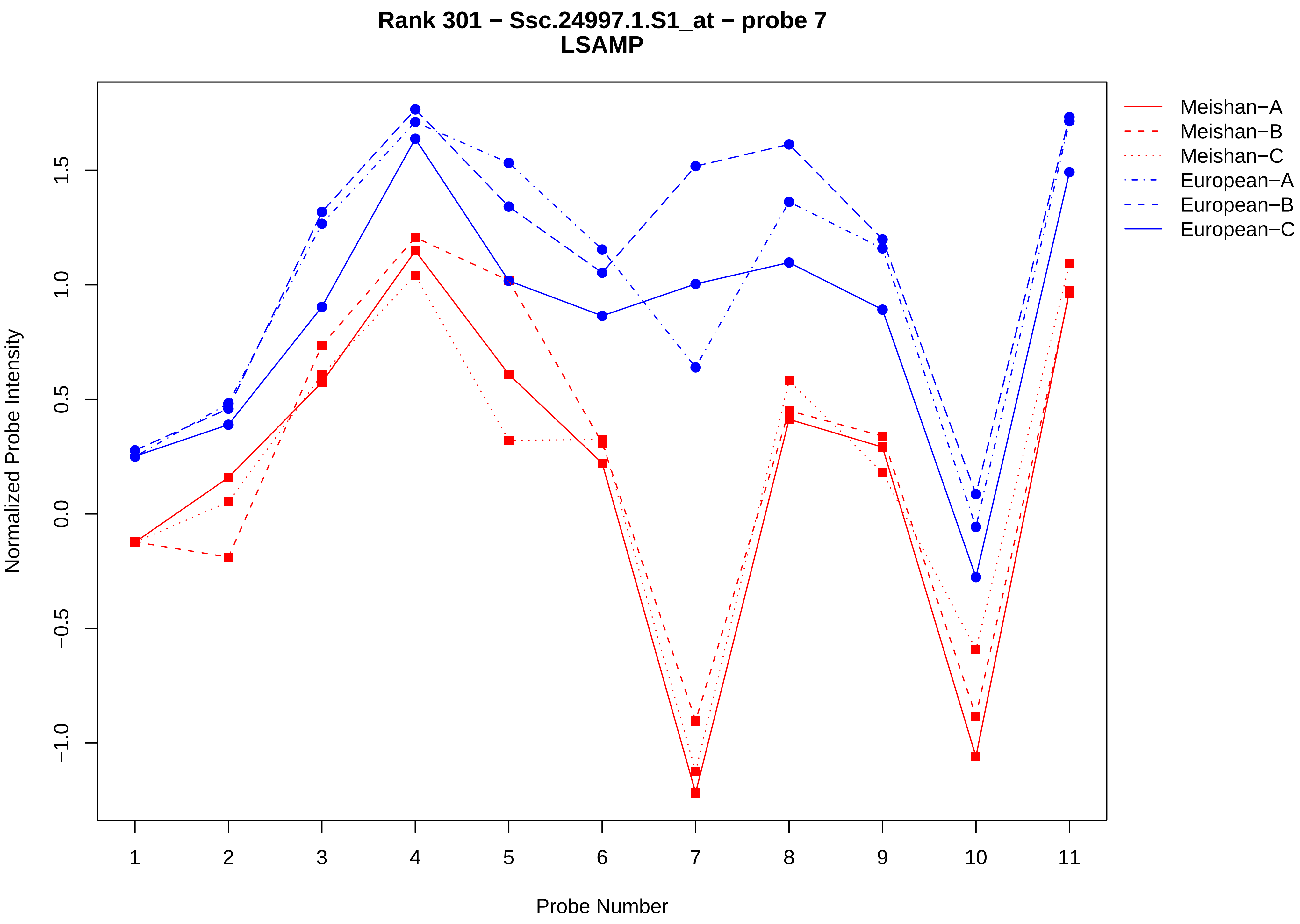

Rank 301 − Ssc.24997.1.S1_at − probe 7
LSAMP
Normalized Probe Intensity
Probe Number
−1.0
−0.5
0.0
0.5
1.0
1.5
1
2
3
4
5
6
7
8
9
10
11
Meishan−A
Meishan−B
Meishan−C
European−A
European−B
European−C

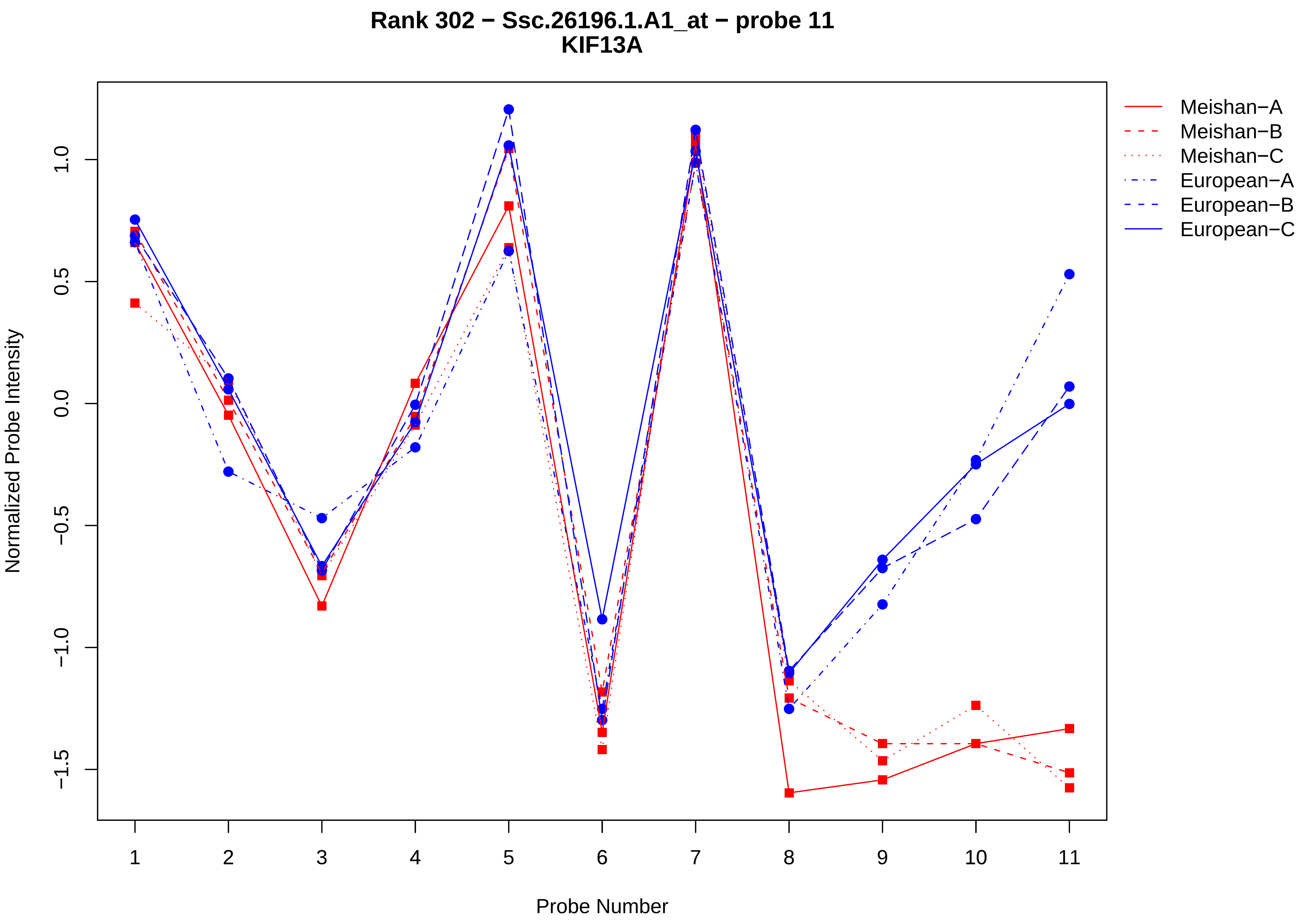
Rank 302 − Ssc.26196.1.A1_at − probe 11
KIF13A
Normalized Probe Intensity
Probe Number
Meishan−A
Meishan−B
Meishan−C
European−A
European−B
European−C
−1.5
−1.0
−0.5
0.0
0.5
1.0
1
2
3
4
5
6
7
8
9
10
11

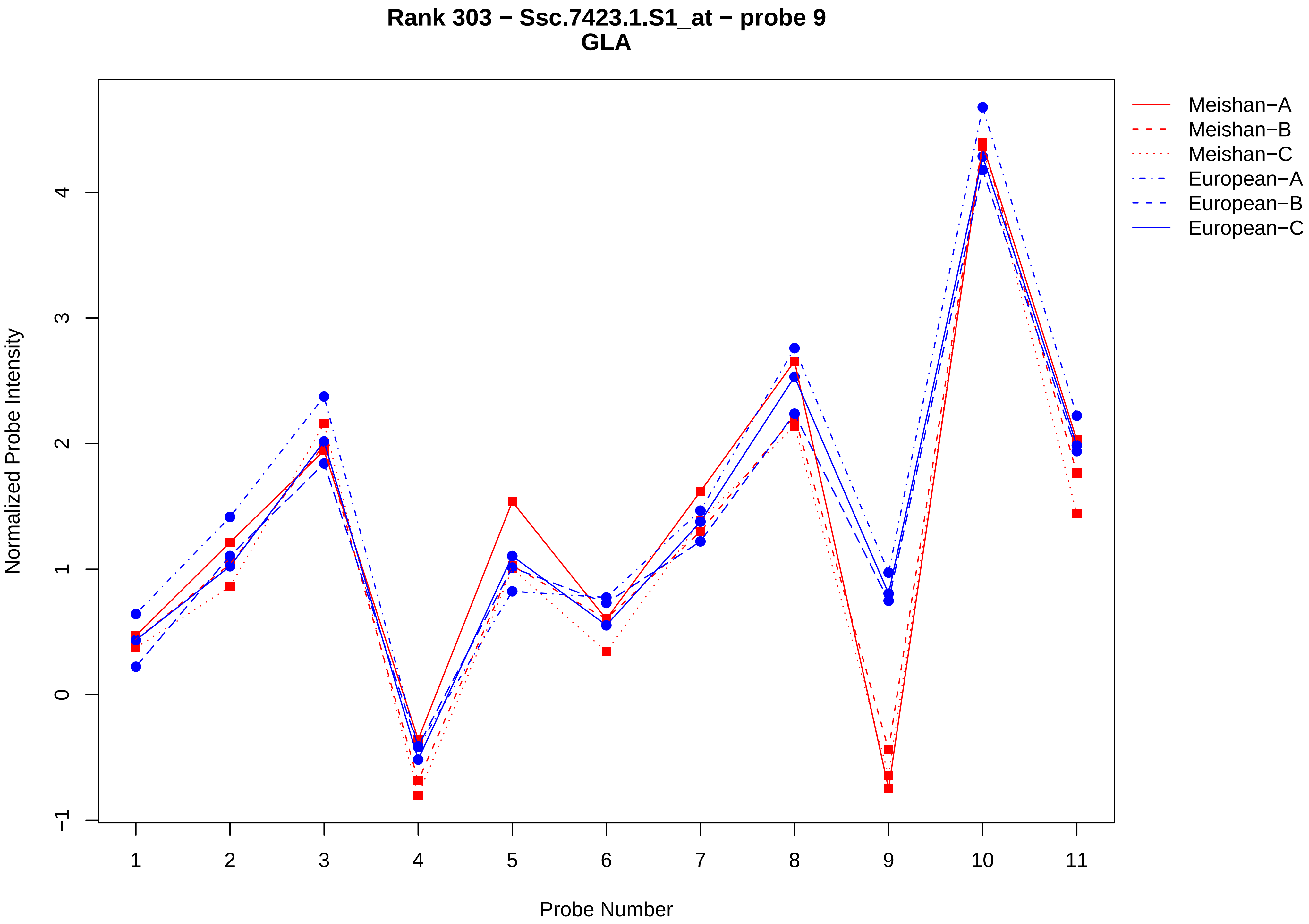
Rank 303 − Ssc.7423.1.S1_at − probe 9
GLA
Normalized Probe Intensity
Probe Number
Meishan−A
Meishan−B
Meishan−C
European−A
European−B
European−C
−1
0
1
2
3
4
1
2
3
4
5
6
7
8
9
10
11

Rank 304 − Ssc.786.1.S1_at − probe 9
CPSF3

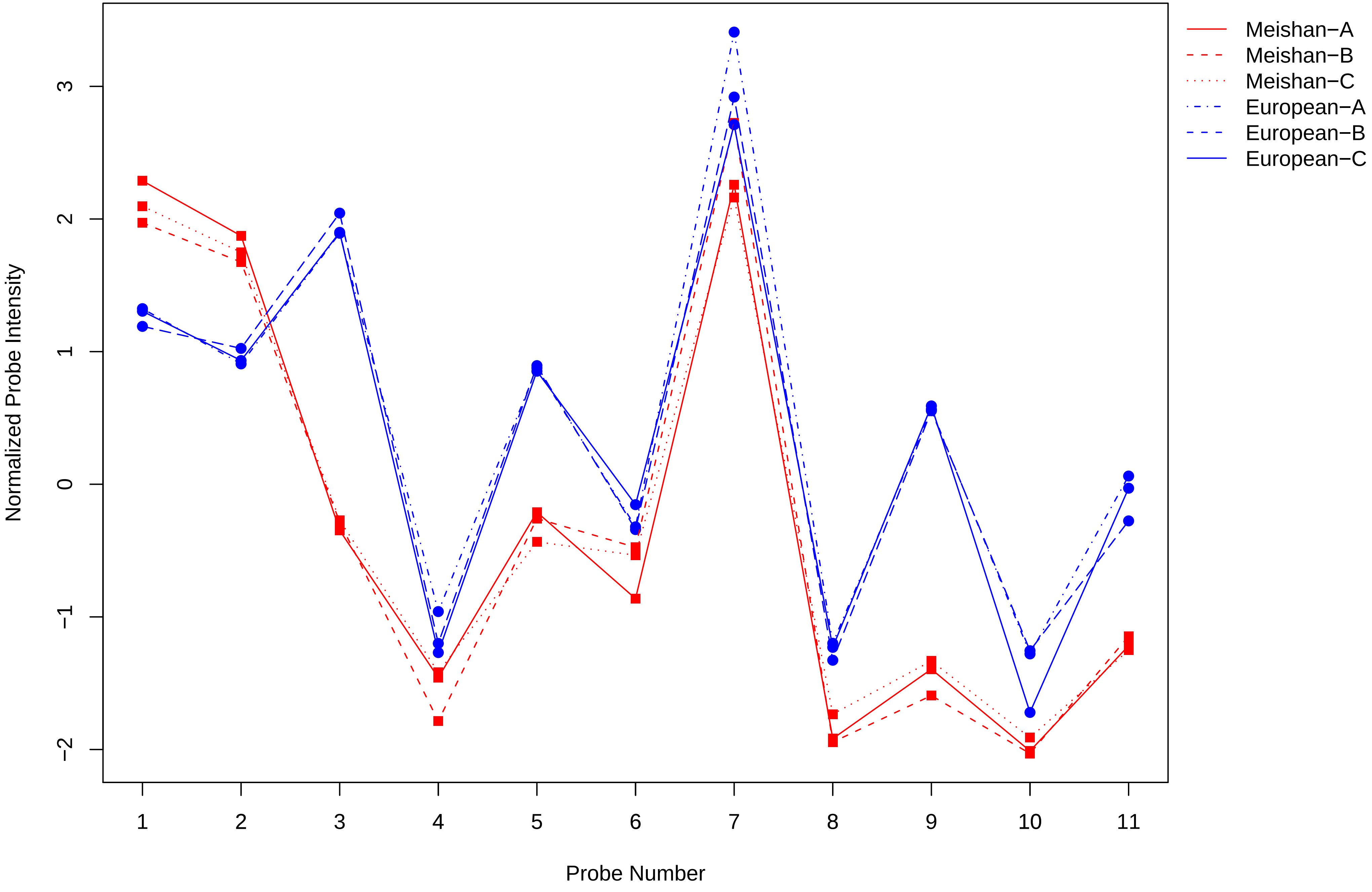

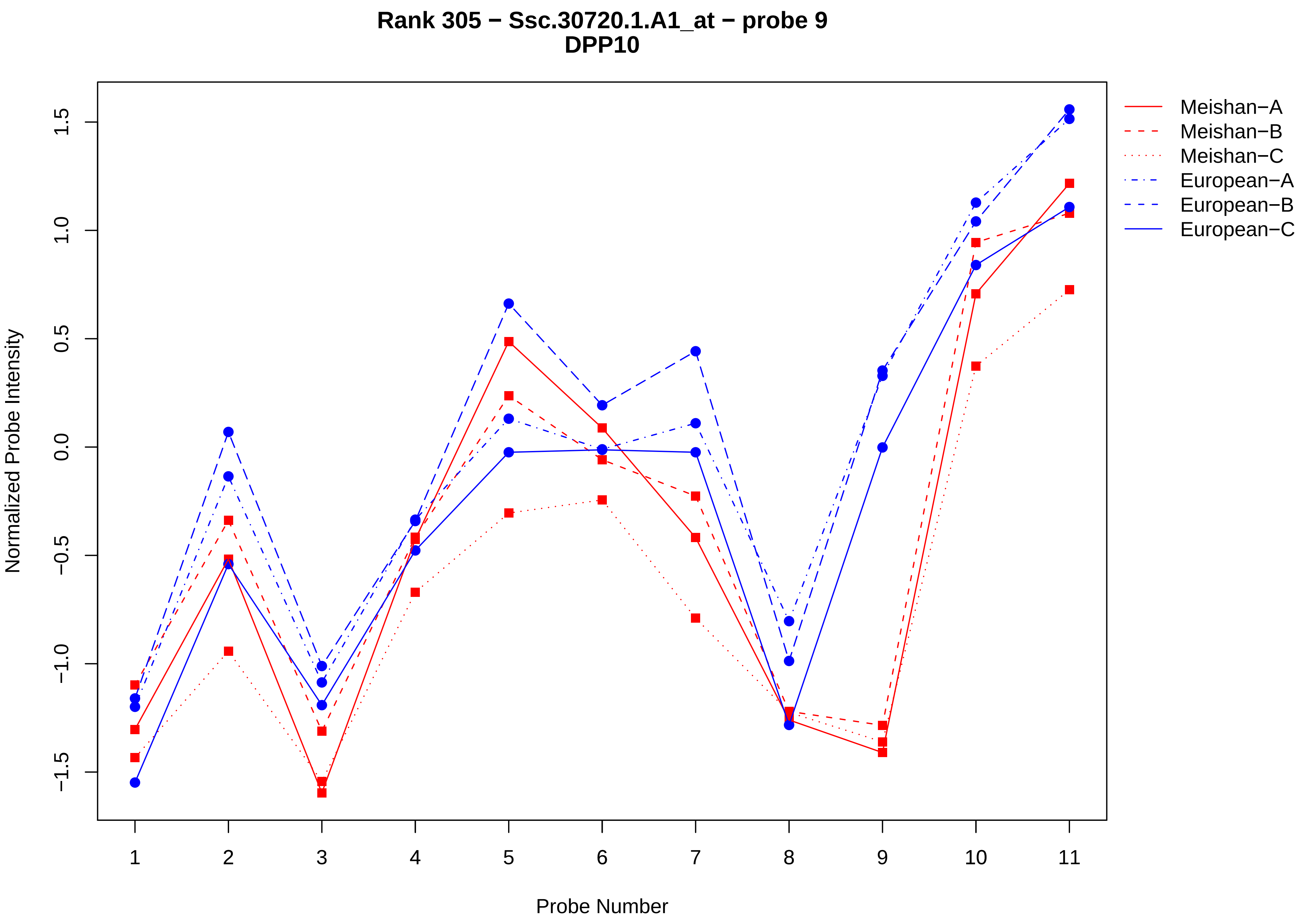

Rank 305 − Ssc.30720.1.A1_at − probe 9
DPP10
Normalized Probe Intensity
Probe Number
1.5
1.0
0.5
0.0
−0.5
−1.0
−1.5
1
2
3
4
5
6
7
8
9
10
11
Meishan−A
Meishan−B
Meishan−C
European−A
European−B
European−C

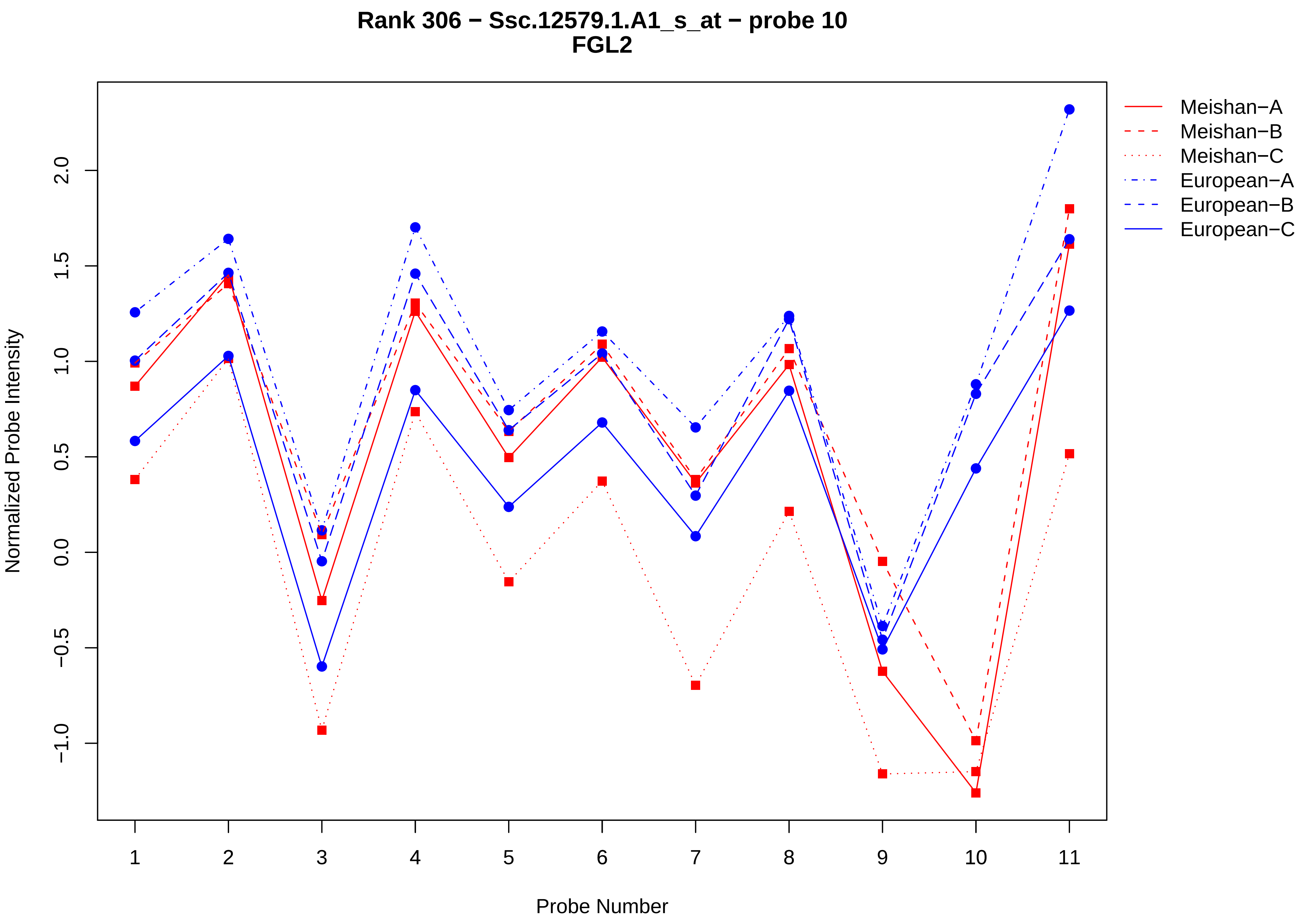

Rank 306 − Ssc.12579.1.A1_s_at − probe 10
FGL2
Normalized Probe Intensity
Probe Number
−1.0
−0.5
0.0
0.5
1.0
1.5
2.0
1
2
3
4
5
6
7
8
9
10
11
Meishan−A
Meishan−B
Meishan−C
European−A
European−B
European−C

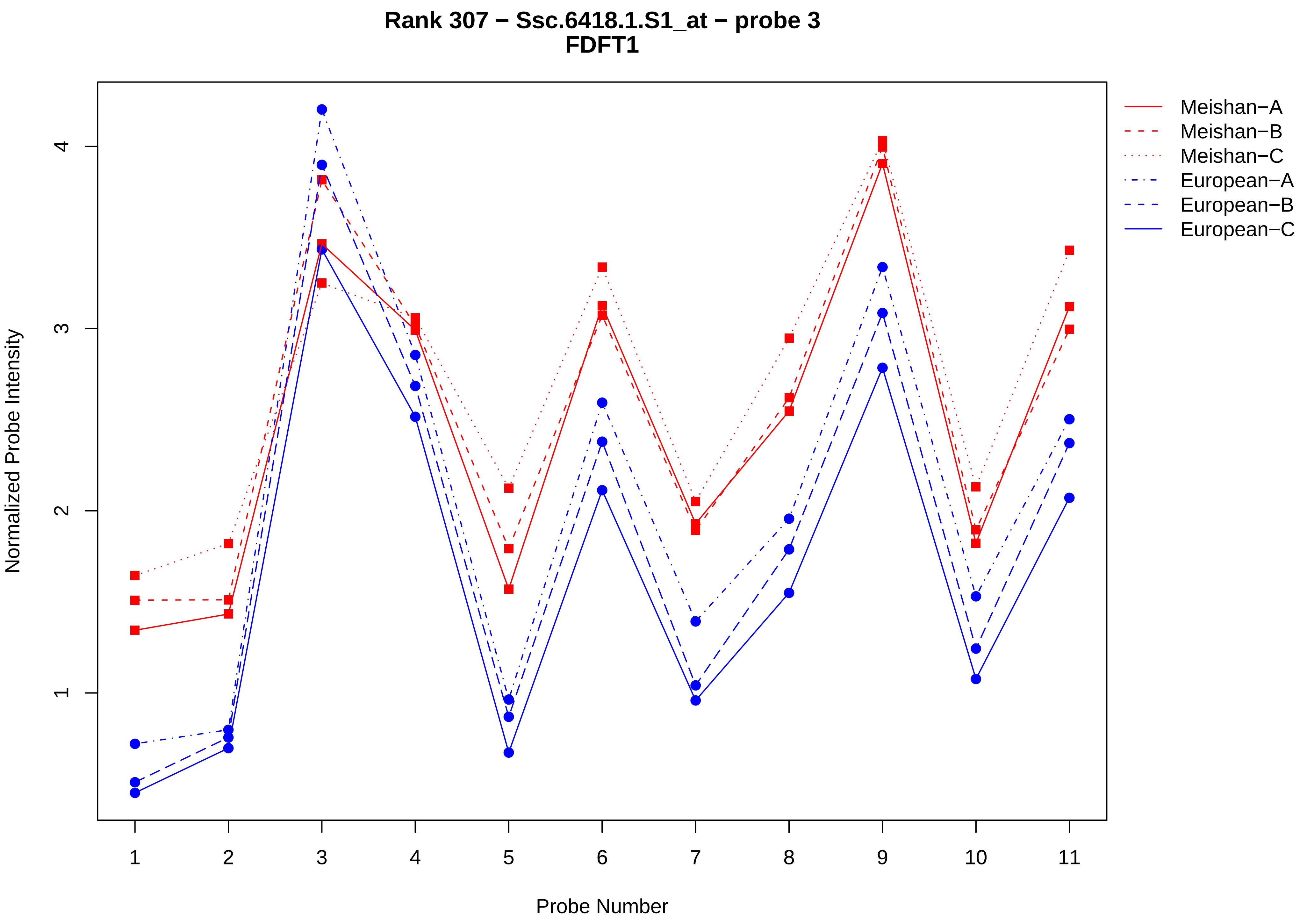
Rank 307 − Ssc.6418.1.S1_at − probe 3
FDFT1
Normalized Probe Intensity
Probe Number
1
2
3
4
1
2
3
4
5
6
7
8
9
10
11
Meishan−A
Meishan−B
Meishan−C
European−A
European−B
European−C

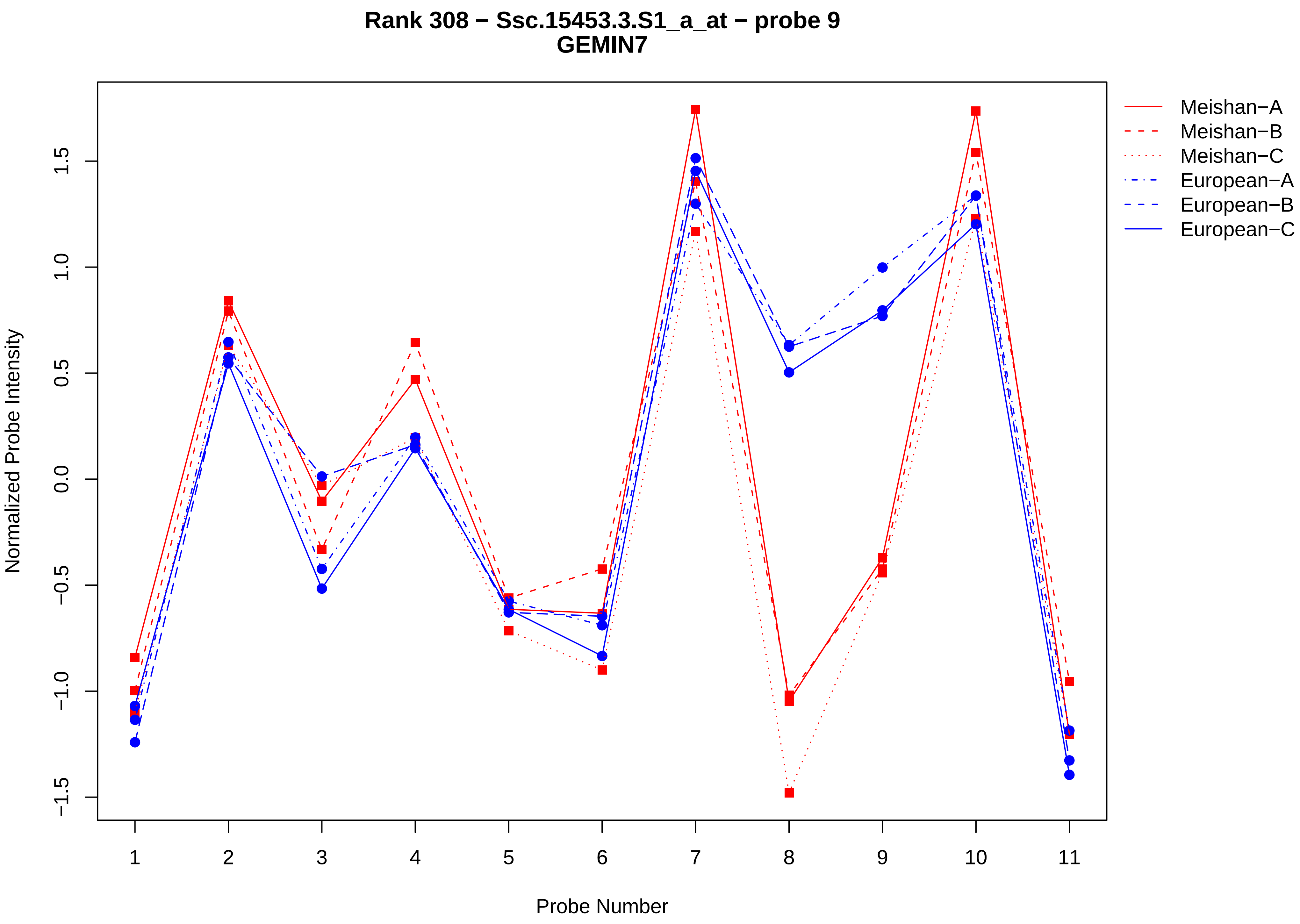

Rank 308 − Ssc.15453.3.S1_a_at − probe 9
GEMIN7
Normalized Probe Intensity
Probe Number
Meishan−A
Meishan−B
Meishan−C
European−A
European−B
European−C
−1.5
−1.0
−0.5
0.0
0.5
1.0
1.5
1
2
3
4
5
6
7
8
9
10
11

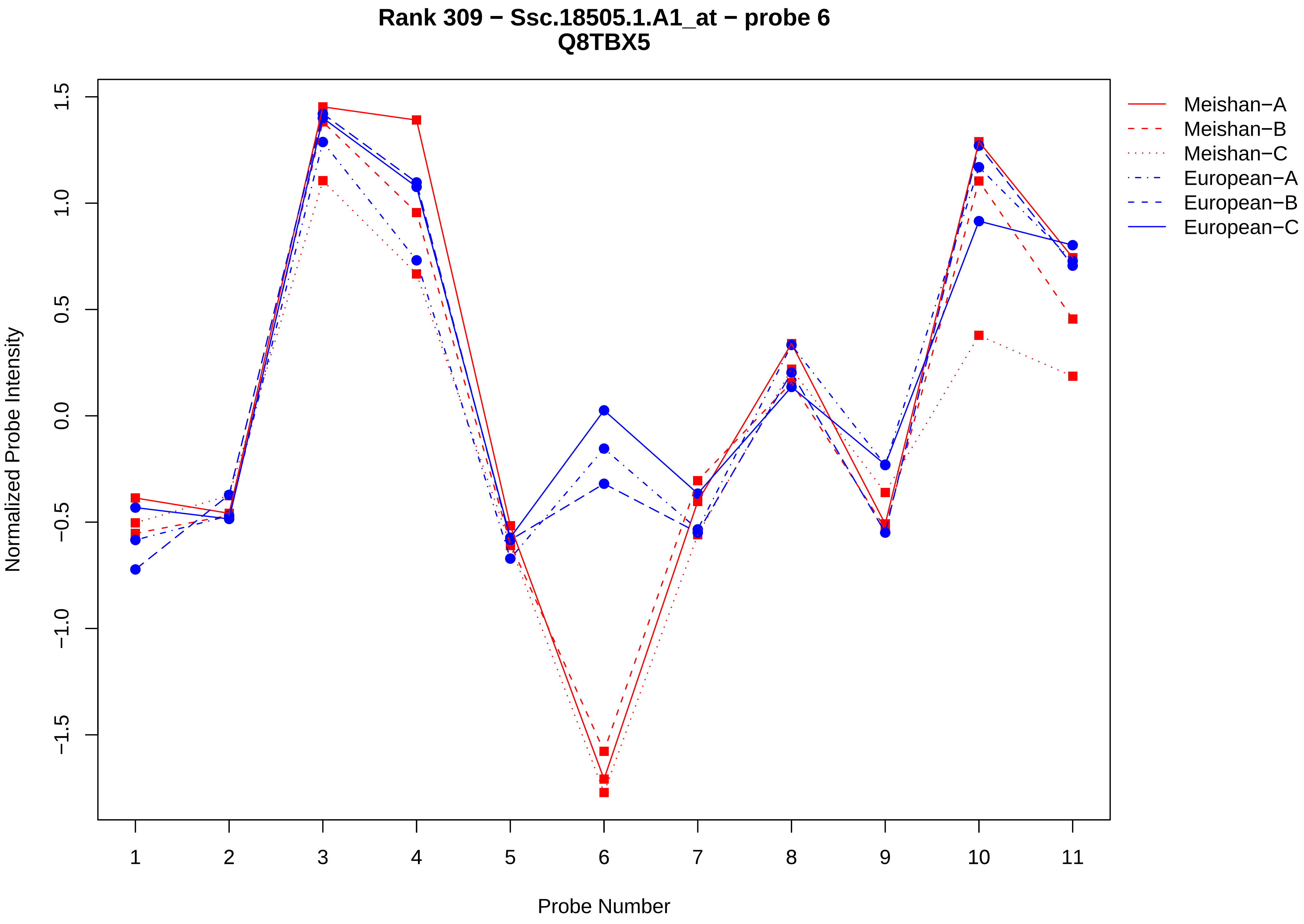

Rank 309 − Ssc.18505.1.A1_at − probe 6
Q8TBX5
Normalized Probe Intensity
Probe Number
1.5
1.0
0.5
0.0
−0.5
−1.0
−1.5
1
2
3
4
5
6
7
8
9
10
11
Meishan−A
Meishan−B
Meishan−C
European−A
European−B
European−C

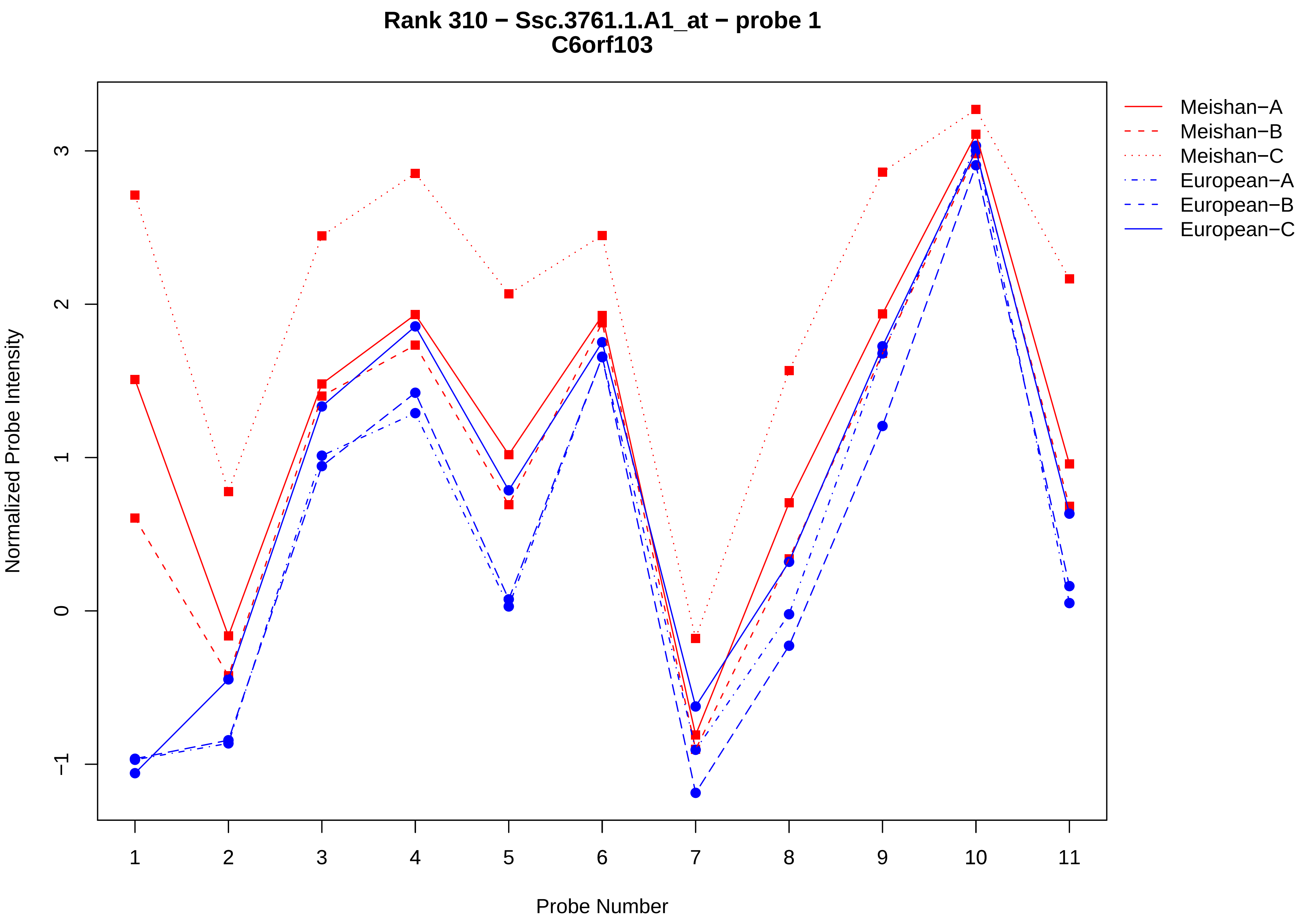

Rank 310 − Ssc.3761.1.A1_at − probe 1
C6orf103
Normalized Probe Intensity
Probe Number
−1
0
1
2
3
1
2
3
4
5
6
7
8
9
10
11
Meishan−A
Meishan−B
Meishan−C
European−A
European−B
European−C

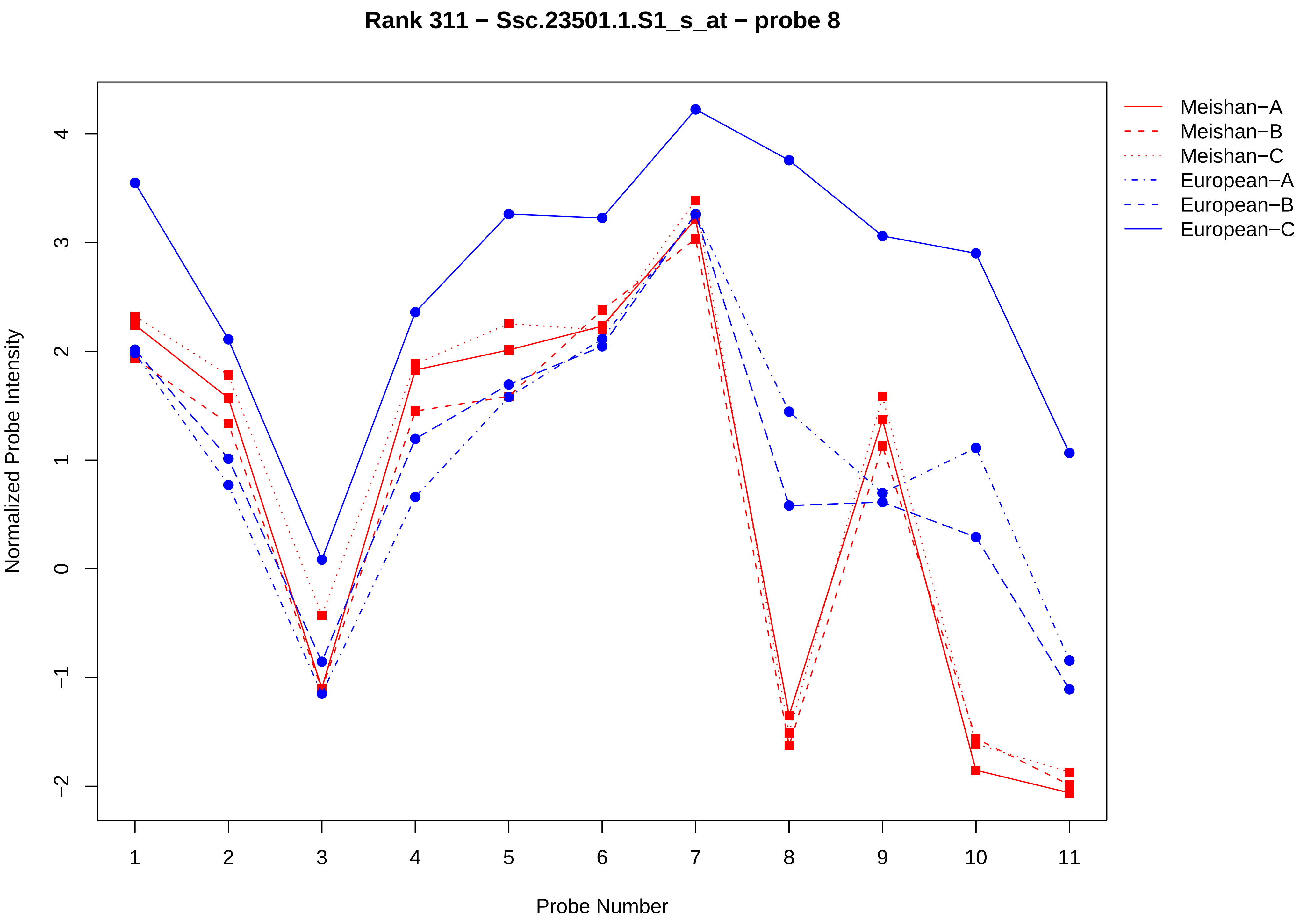
Rank 311 − Ssc.23501.1.S1_s_at − probe 8
Normalized Probe Intensity
Probe Number
−2
−1
0
1
2
3
4
1
2
3
4
5
6
7
8
9
10
11
Meishan−A
Meishan−B
Meishan−C
European−A
European−B
European−C

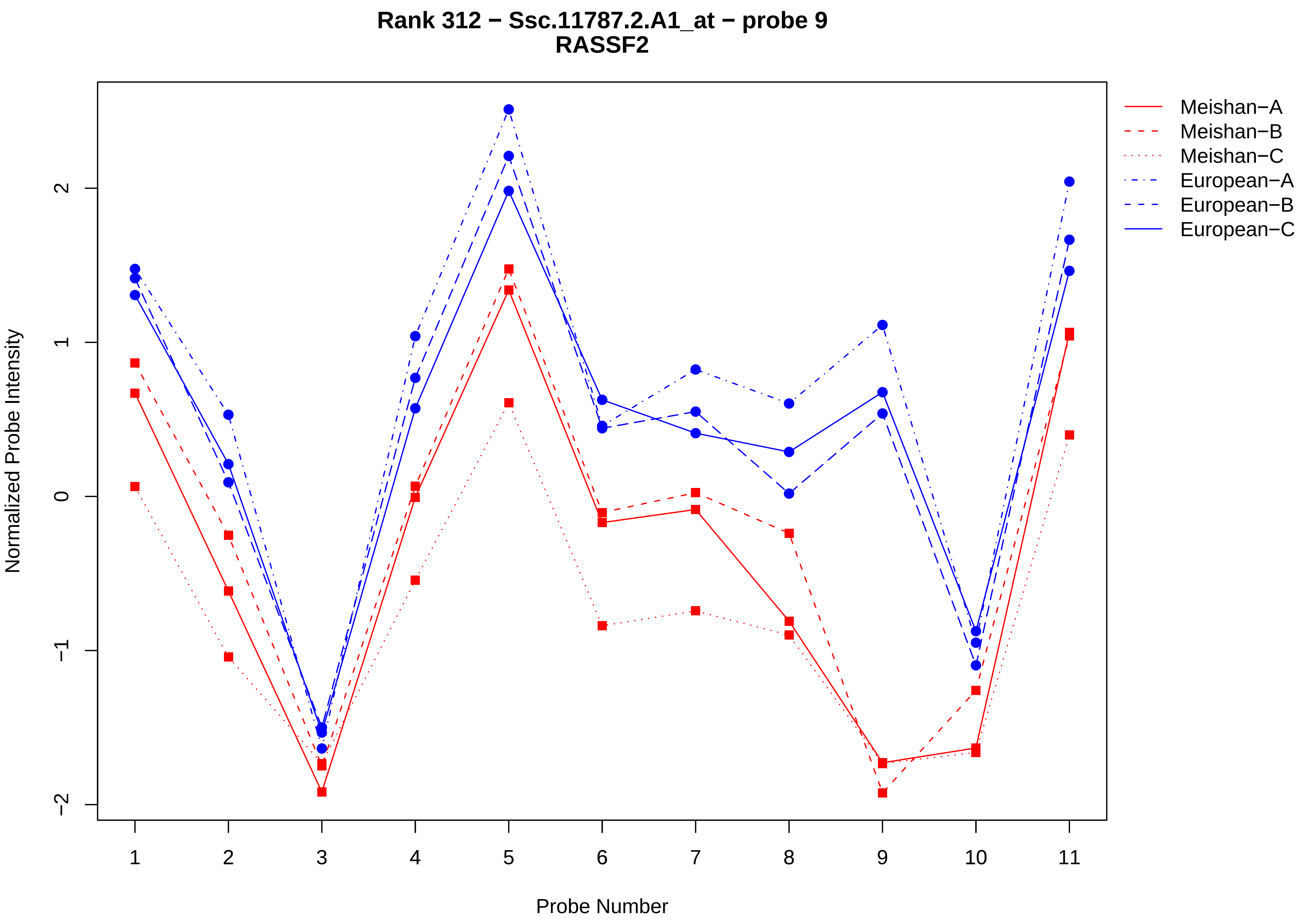

Rank 312 − Ssc.11787.2.A1_at − probe 9
RASSF2
Normalized Probe Intensity
Probe Number
−2
−1
0
1
2
1
2
3
4
5
6
7
8
9
10
11
Meishan−A
Meishan−B
Meishan−C
European−A
European−B
European−C

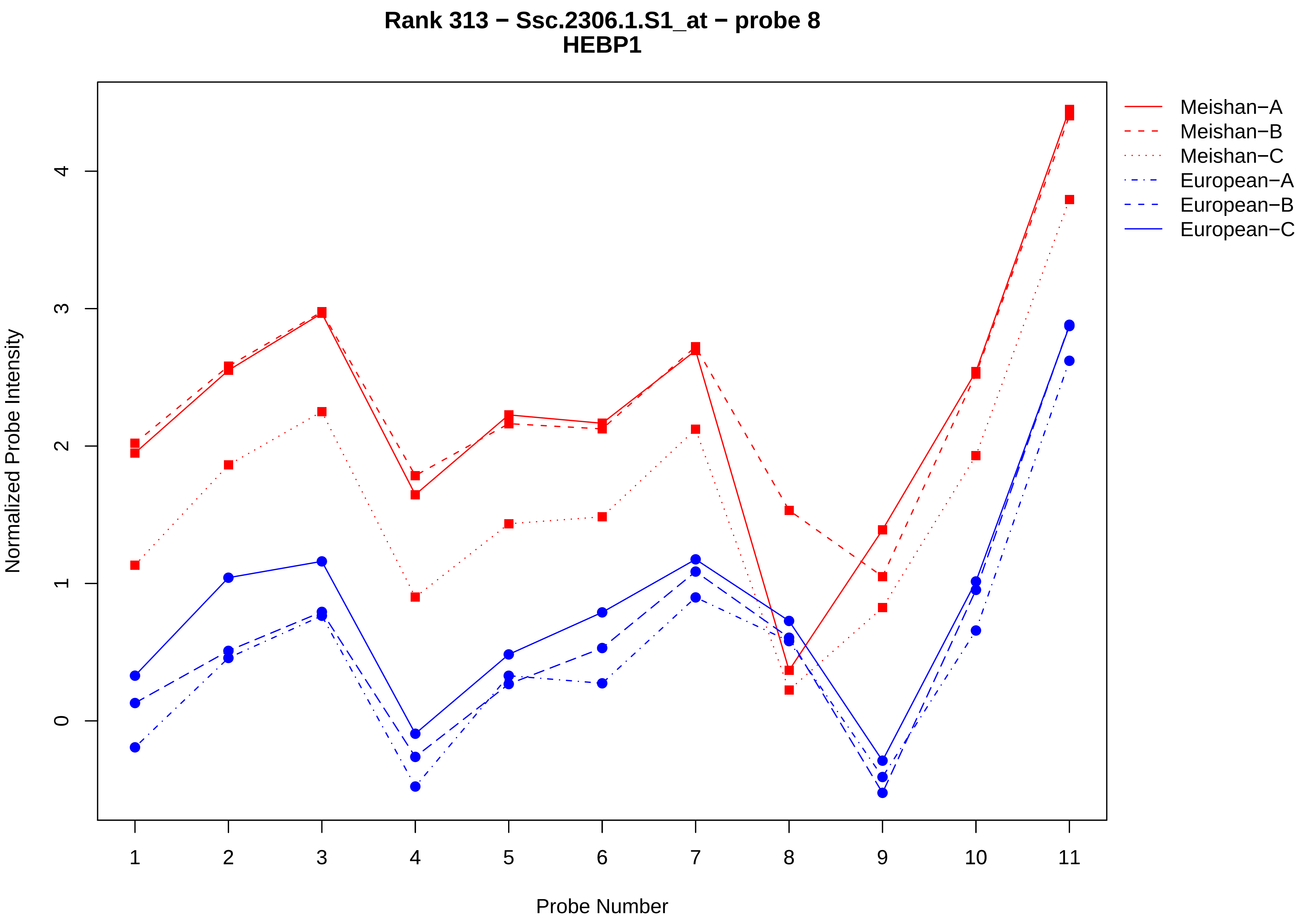

Rank 313 − Ssc.2306.1.S1_at − probe 8
HEBP1
Normalized Probe Intensity
Probe Number
0
1
2
3
4
1
2
3
4
5
6
7
8
9
10
11
Meishan−A
Meishan−B
Meishan−C
European−A
European−B
European−C

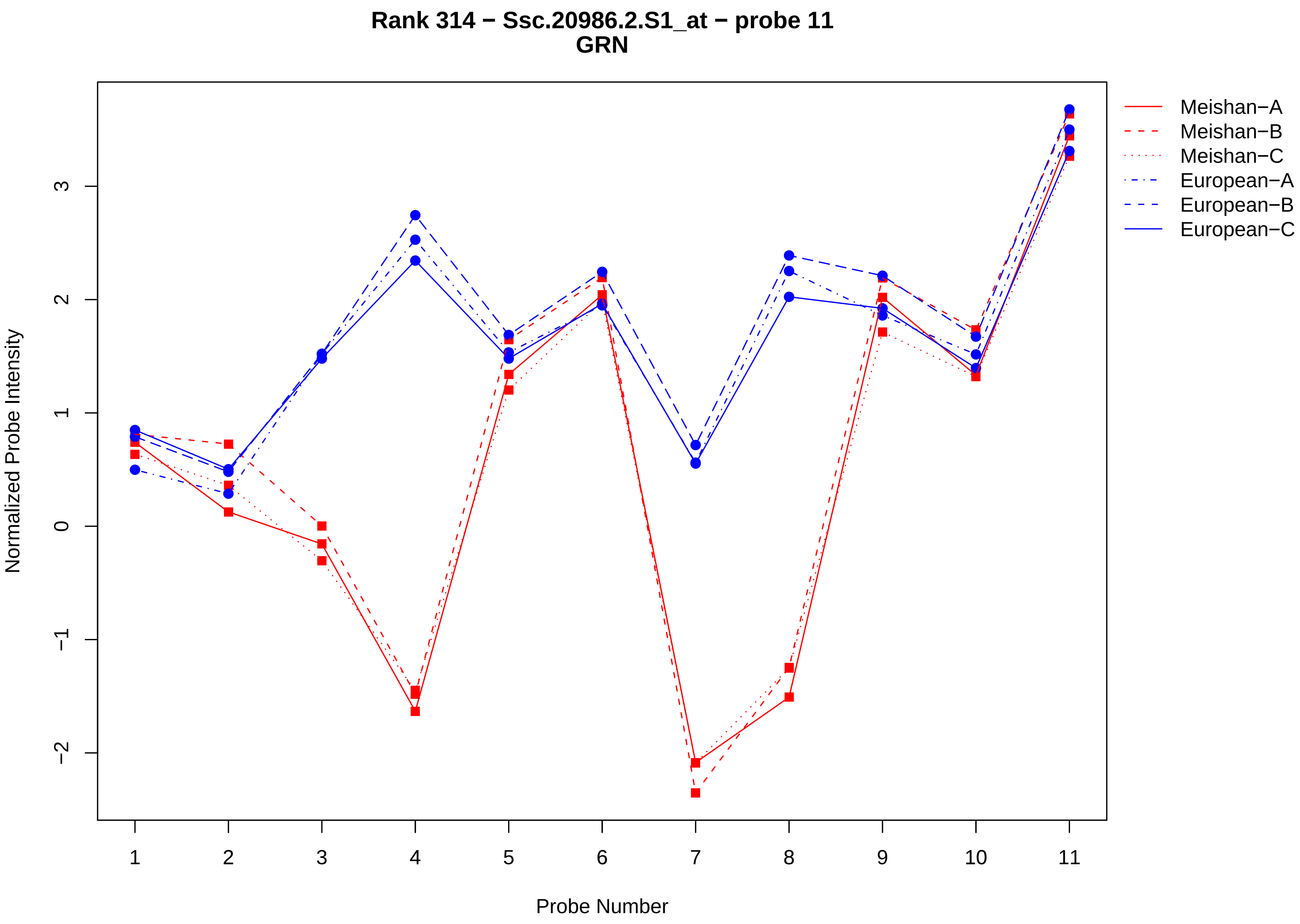

Rank 314 − Ssc.20986.2.S1_at − probe 11
GRN
Normalized Probe Intensity
Probe Number
−2
−1
0
1
2
3
1
2
3
4
5
6
7
8
9
10
11
Meishan−A
Meishan−B
Meishan−C
European−A
European−B
European−C

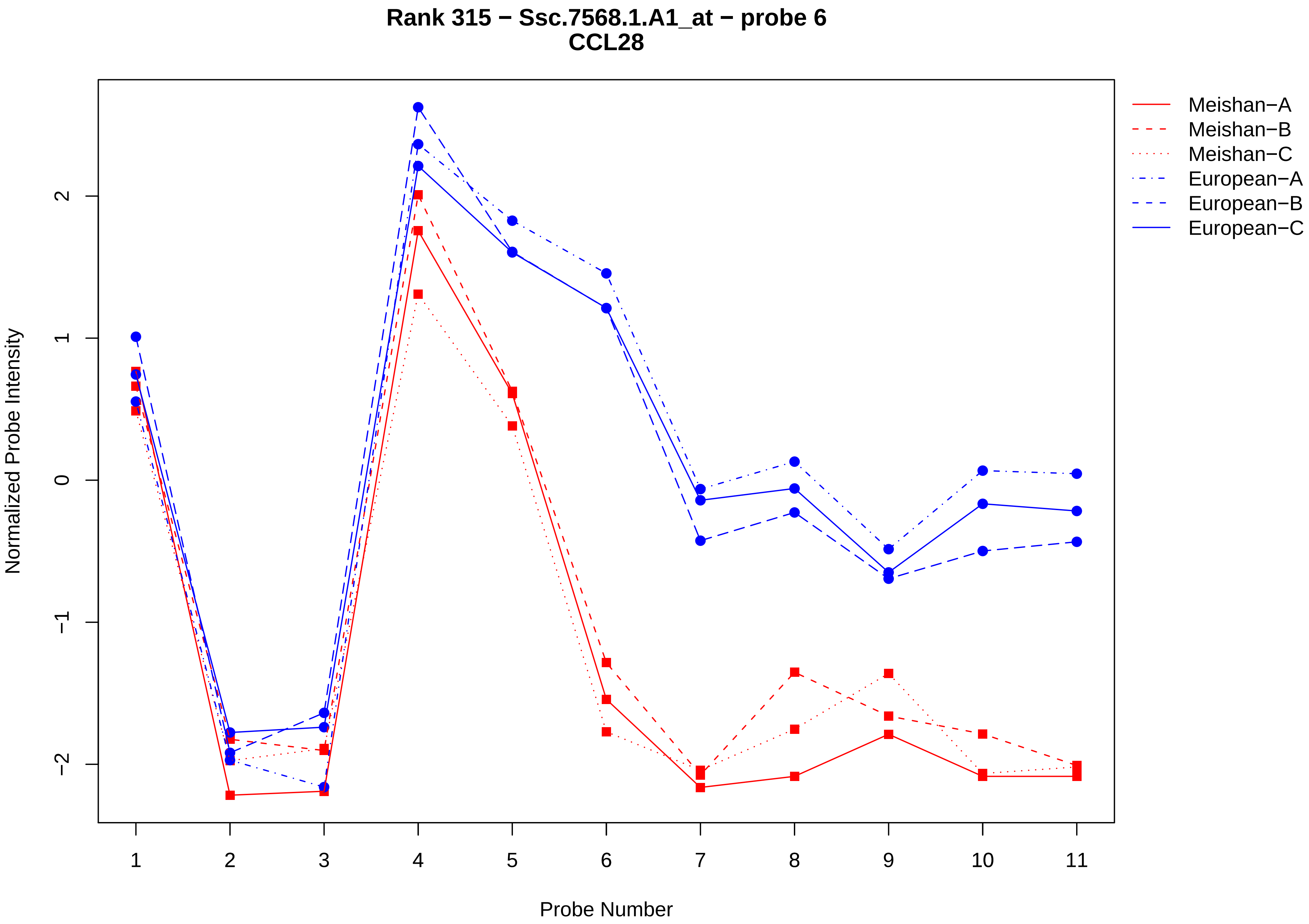

Rank 315 − Ssc.7568.1.A1_at − probe 6
CCL28
Normalized Probe Intensity
Probe Number
−2
−1
0
1
2
1
2
3
4
5
6
7
8
9
10
11
Meishan−A
Meishan−B
Meishan−C
European−A
European−B
European−C

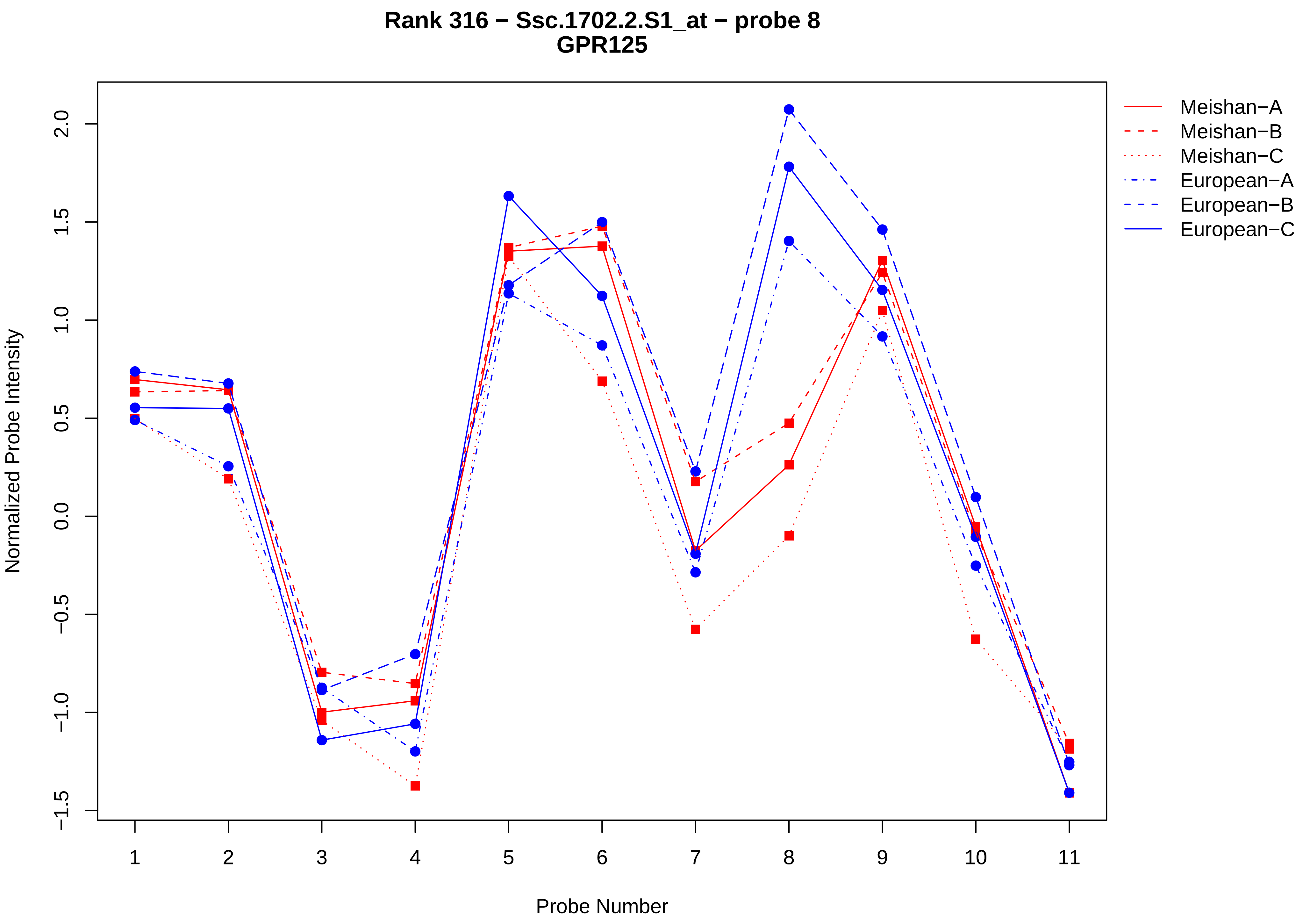

Rank 316 − Ssc.1702.2.S1_at − probe 8
GPR125
Normalized Probe Intensity
Probe Number
Meishan−A
Meishan−B
Meishan−C
European−A
European−B
European−C
−1.5
−1.0
−0.5
0.0
0.5
1.0
1.5
2.0
1
2
3
4
5
6
7
8
9
10
11

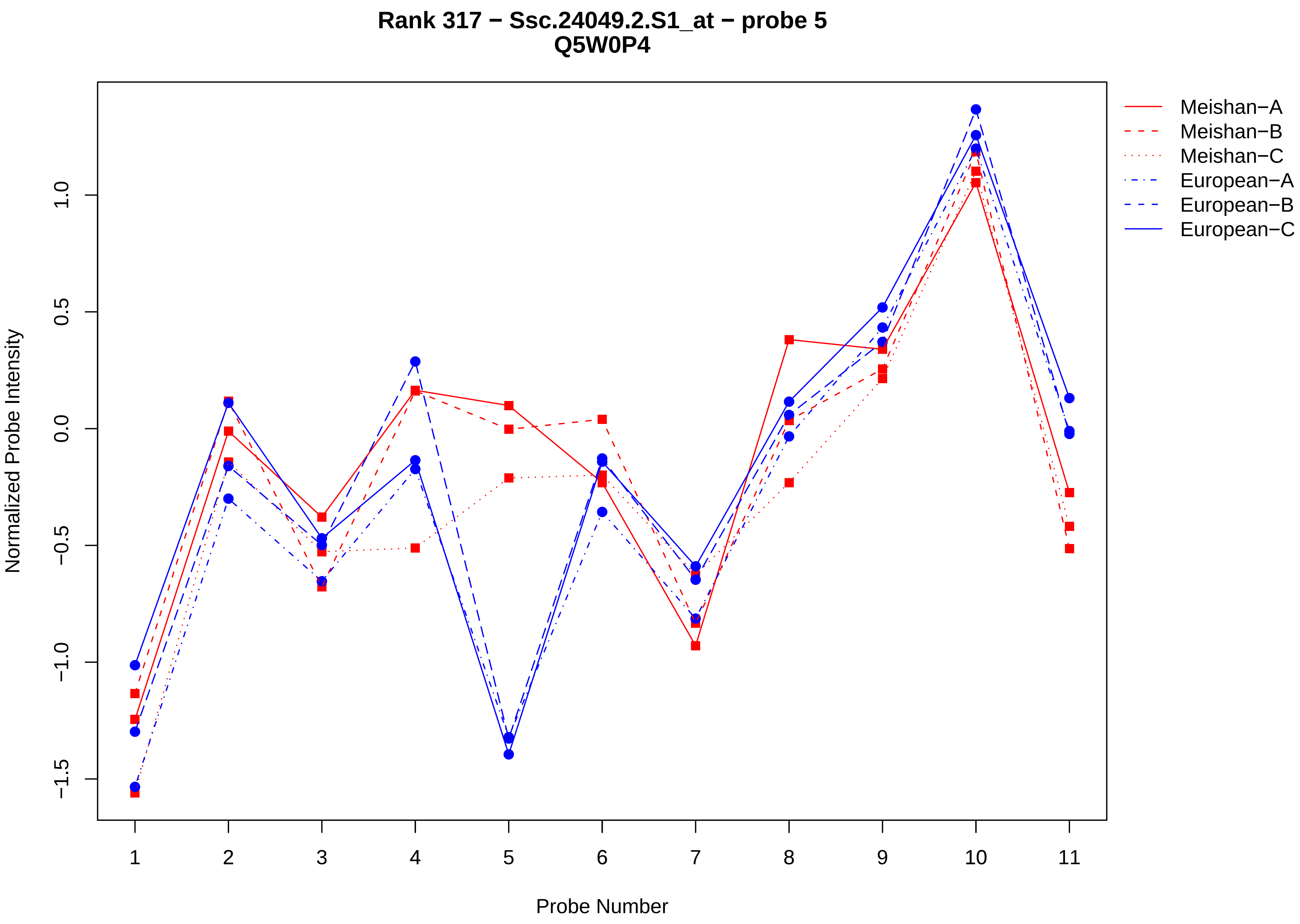

Rank 317 − Ssc.24049.2.S1_at − probe 5
Q5W0P4
Normalized Probe Intensity
Probe Number
Meishan−A
Meishan−B
Meishan−C
European−A
European−B
European−C

Rank 318 − Ssc.2147.1.A1_at − probe 6
CKAP4

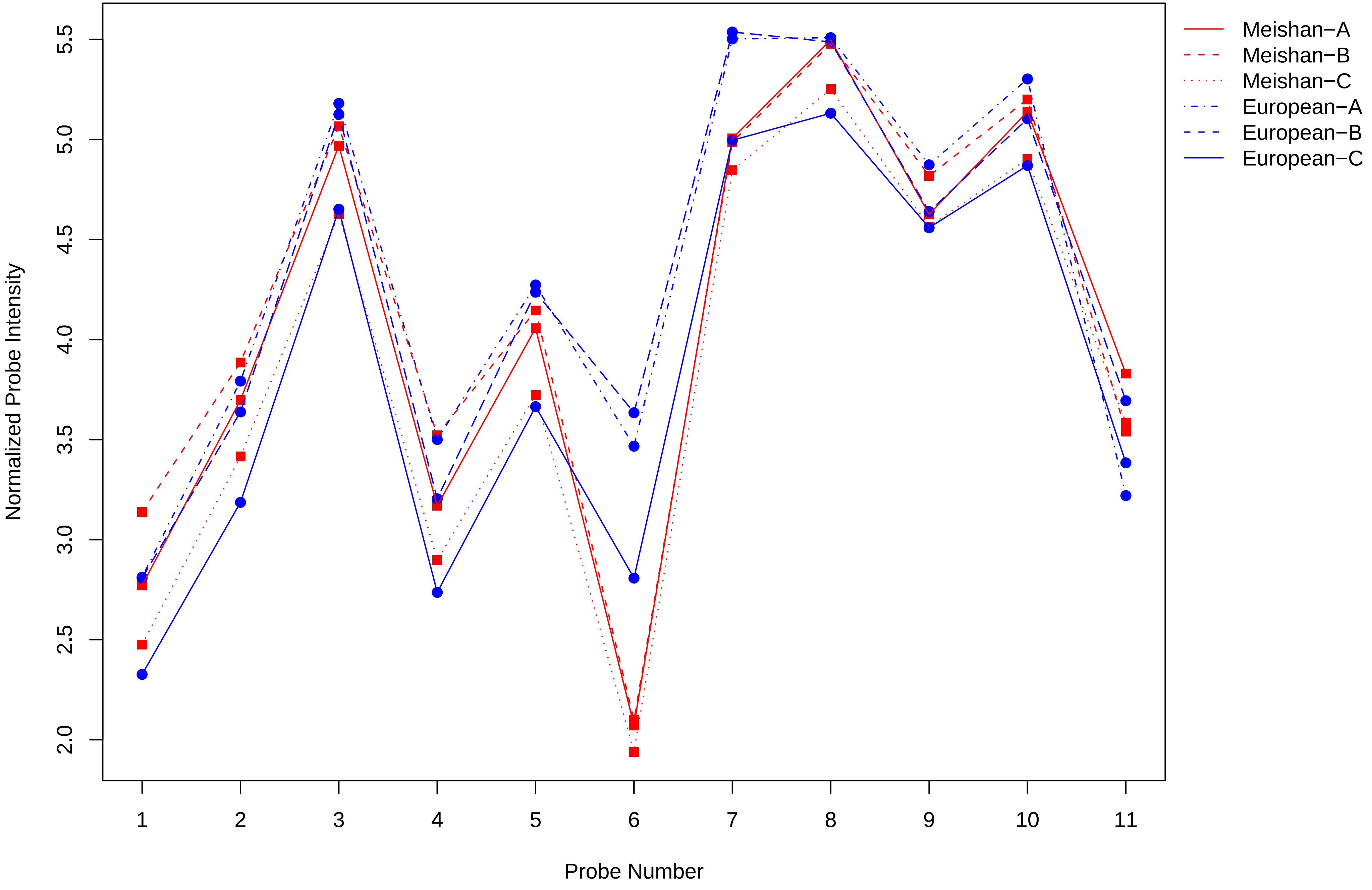

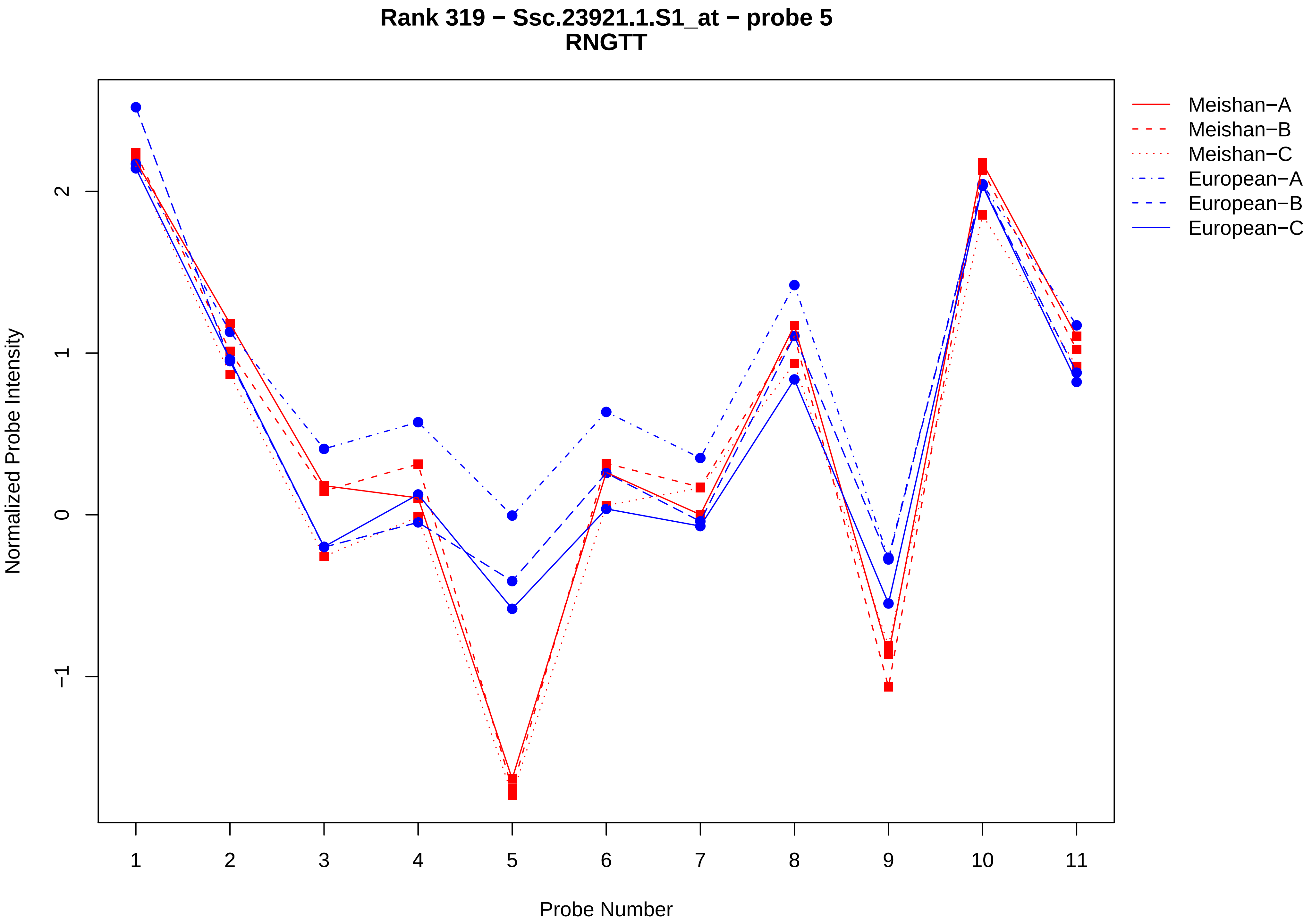

Rank 319 − Ssc.23921.1.S1_at − probe 5
RNGTT
Normalized Probe Intensity
Probe Number
Meishan−A
Meishan−B
Meishan−C
European−A
European−B
European−C
−1
0
1
2
1
2
3
4
5
6
7
8
9
10
11

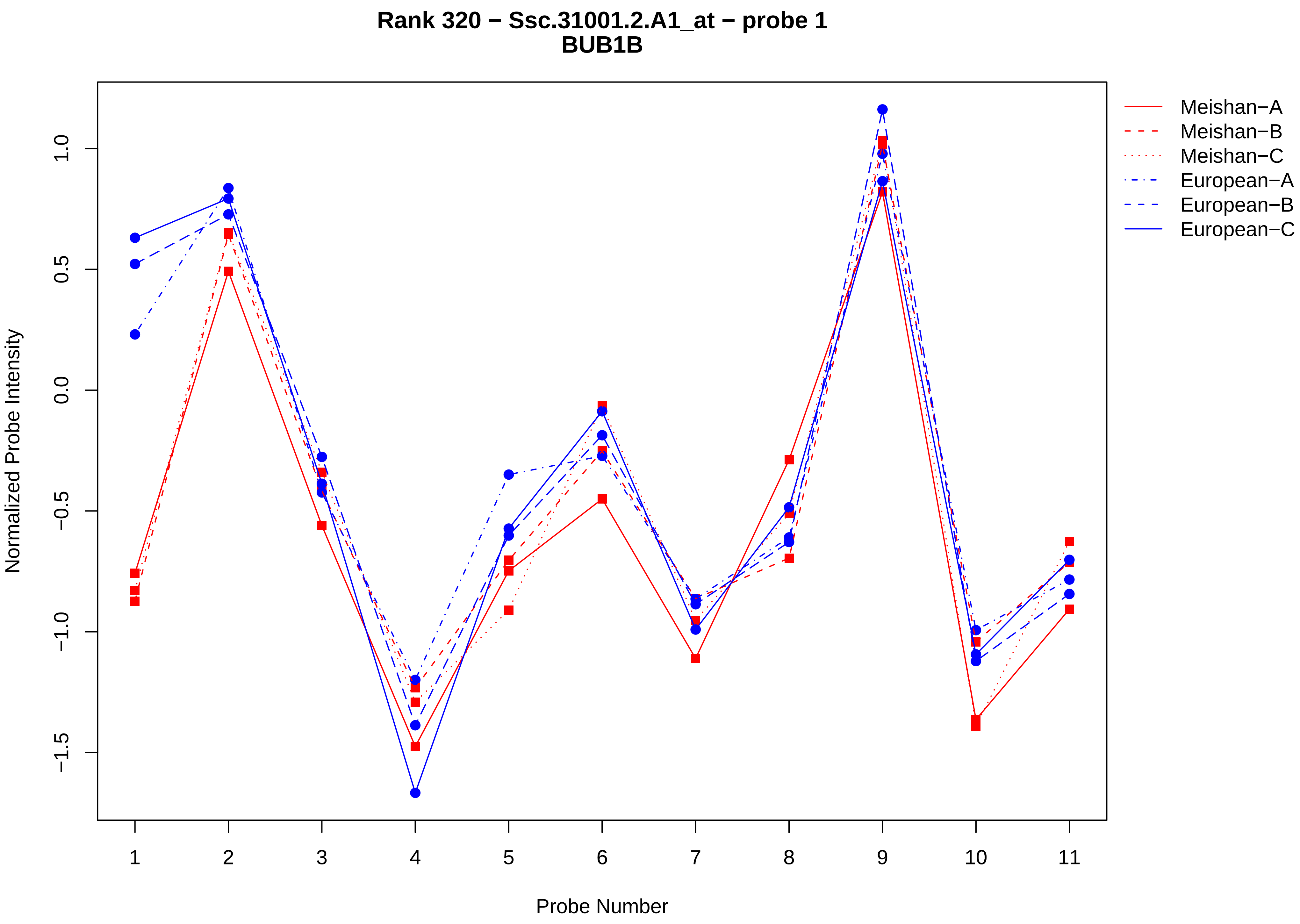

Rank 320 − Ssc.31001.2.A1_at − probe 1
BUB1B
Normalized Probe Intensity
Probe Number
1.0
0.5
0.0
−0.5
−1.0
−1.5
1
2
3
4
5
6
7
8
9
10
11
Meishan−A
Meishan−B
Meishan−C
European−A
European−B
European−C

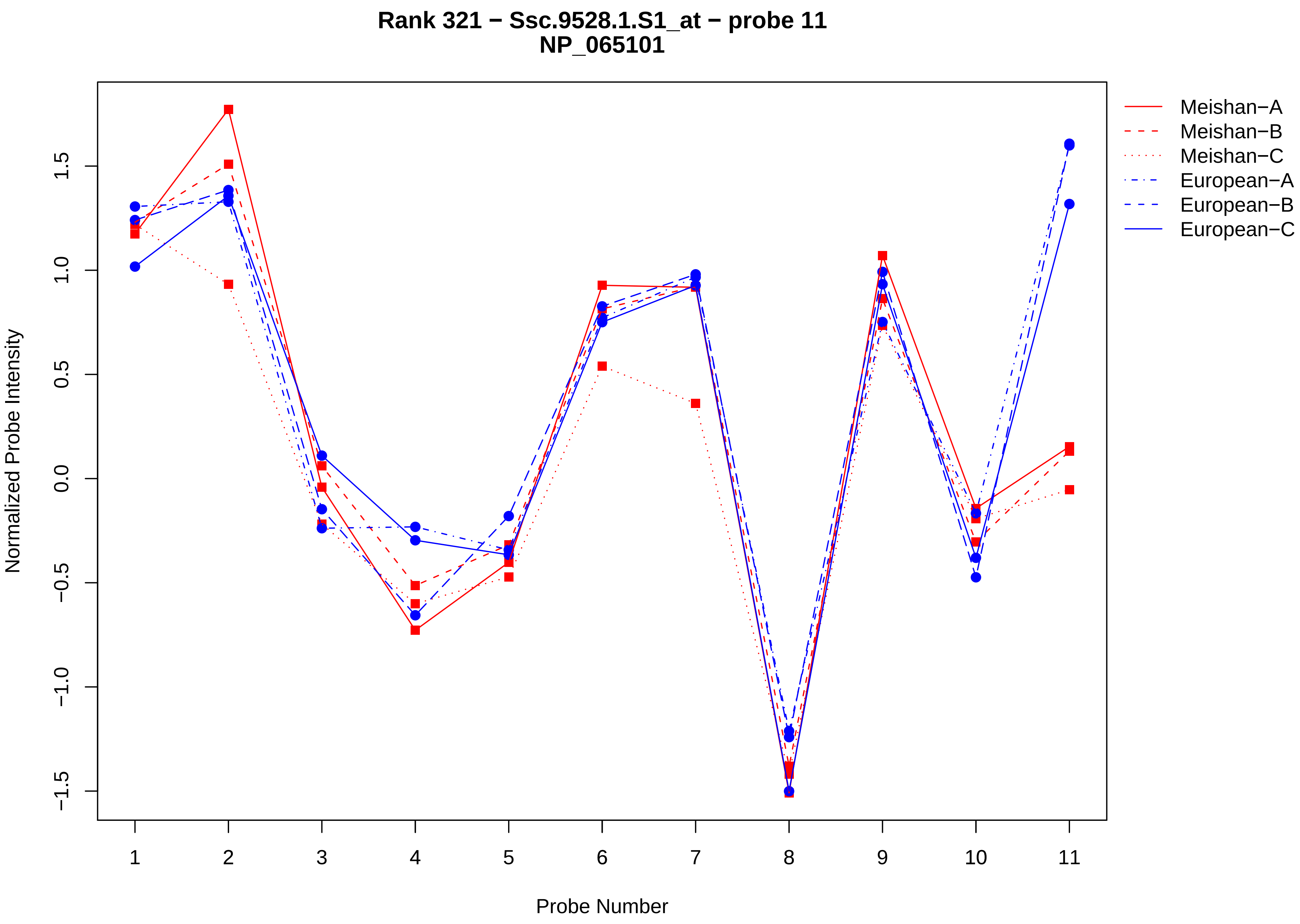
Rank 321 − Ssc.9528.1.S1_at − probe 11
NP_065101
Normalized Probe Intensity
Probe Number
Meishan−A
Meishan−B
Meishan−C
European−A
European−B
European−C
−1.5
−1.0
−0.5
0.0
0.5
1.0
1.5
1
2
3
4
5
6
7
8
9
10
11

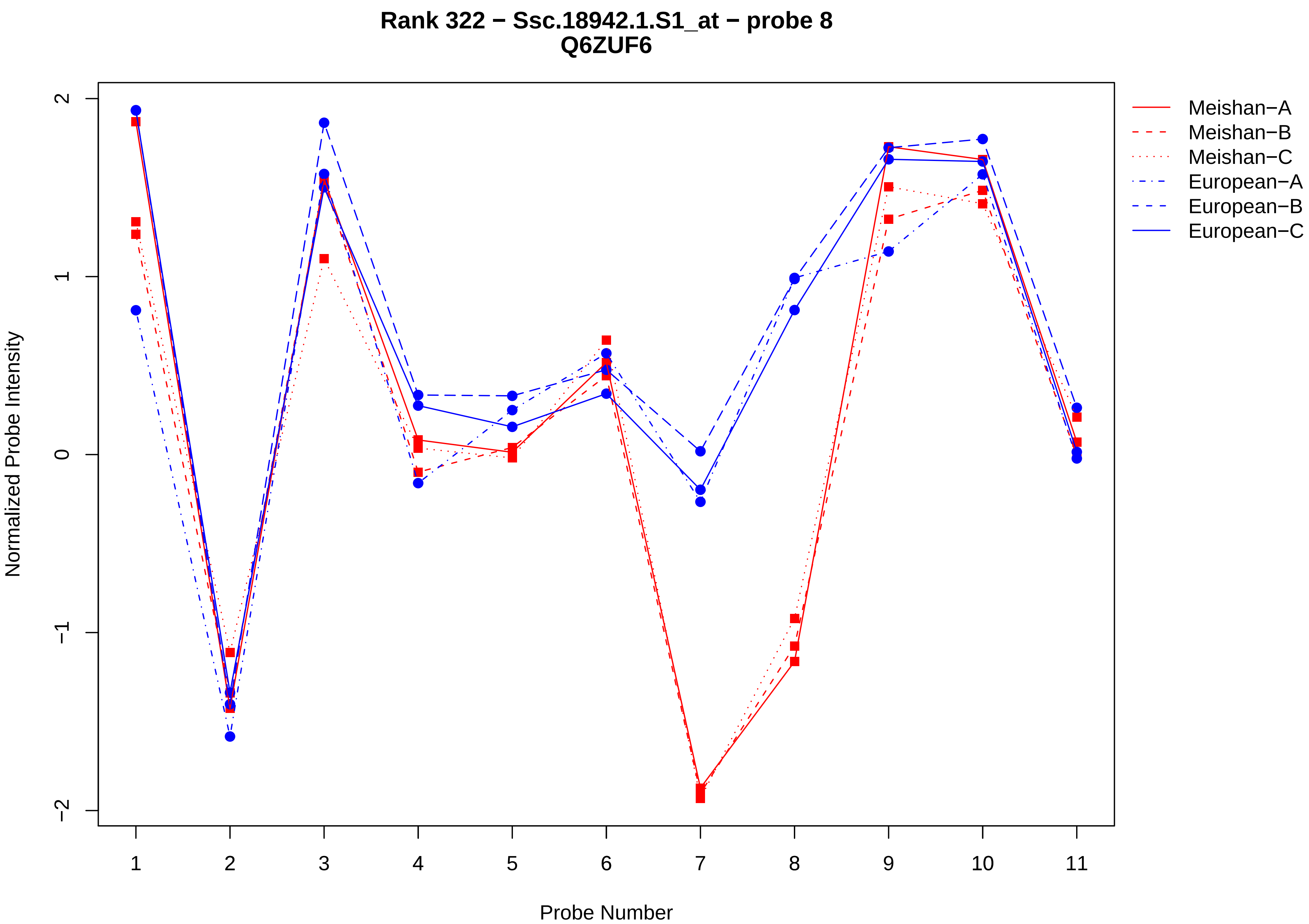

Rank 322 − Ssc.18942.1.S1_at − probe 8
Q6ZUF6
Normalized Probe Intensity
Probe Number
Meishan−A
Meishan−B
Meishan−C
European−A
European−B
European−C
−2
−1
0
1
2
1
2
3
4
5
6
7
8
9
10
11

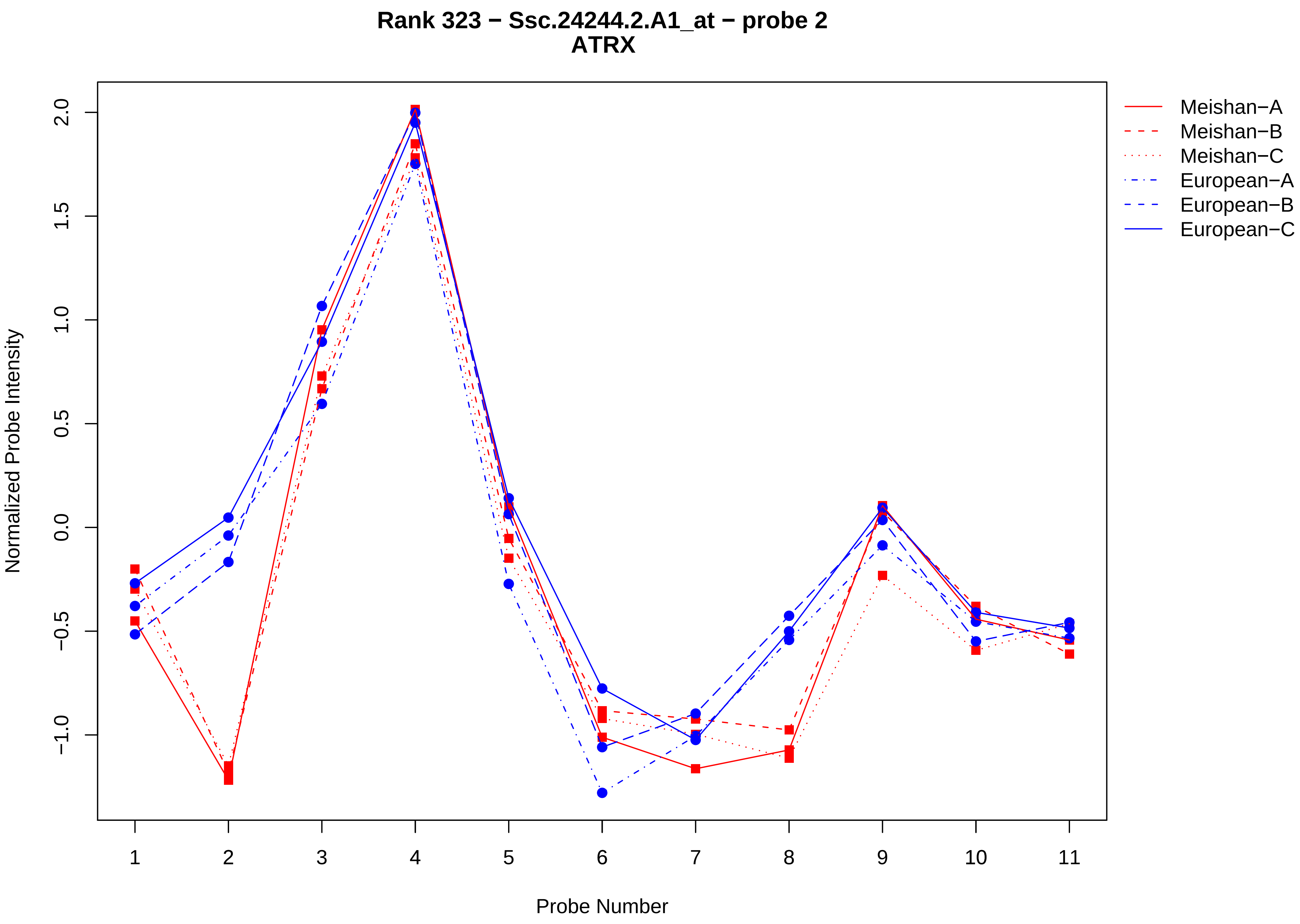
Rank 323 − Ssc.24244.2.A1_at − probe 2
ATRX
Normalized Probe Intensity
Probe Number
−1.0
−0.5
0.0
0.5
1.0
1.5
2.0
1
2
3
4
5
6
7
8
9
10
11
Meishan−A
Meishan−B
Meishan−C
European−A
European−B
European−C

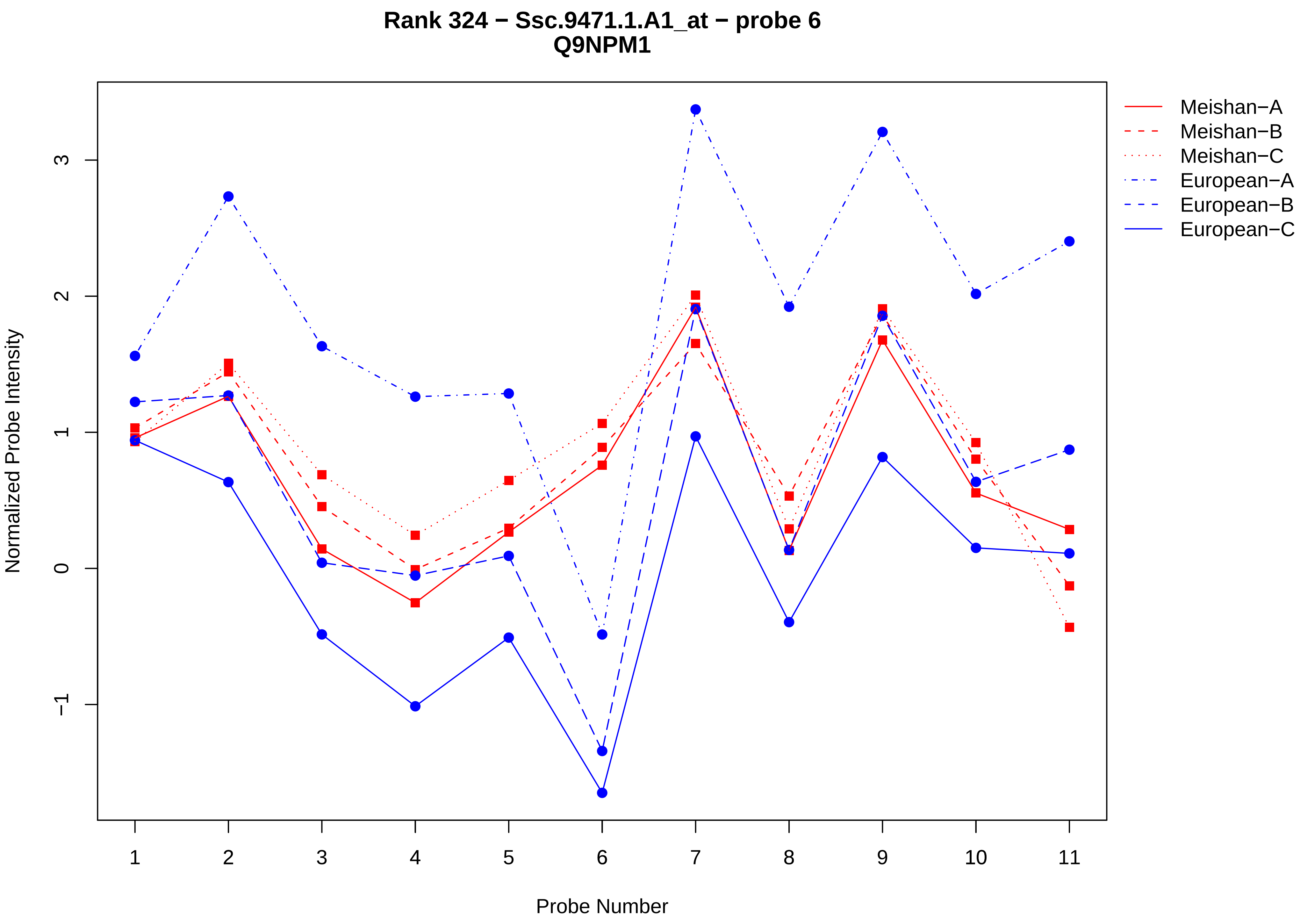
Rank 324 − Ssc.9471.1.A1_at − probe 6
Q9NPM1
Normalized Probe Intensity
Probe Number
Meishan−A
Meishan−B
Meishan−C
European−A
European−B
European−C
−1
0
1
2
3
1
2
3
4
5
6
7
8
9
10
11

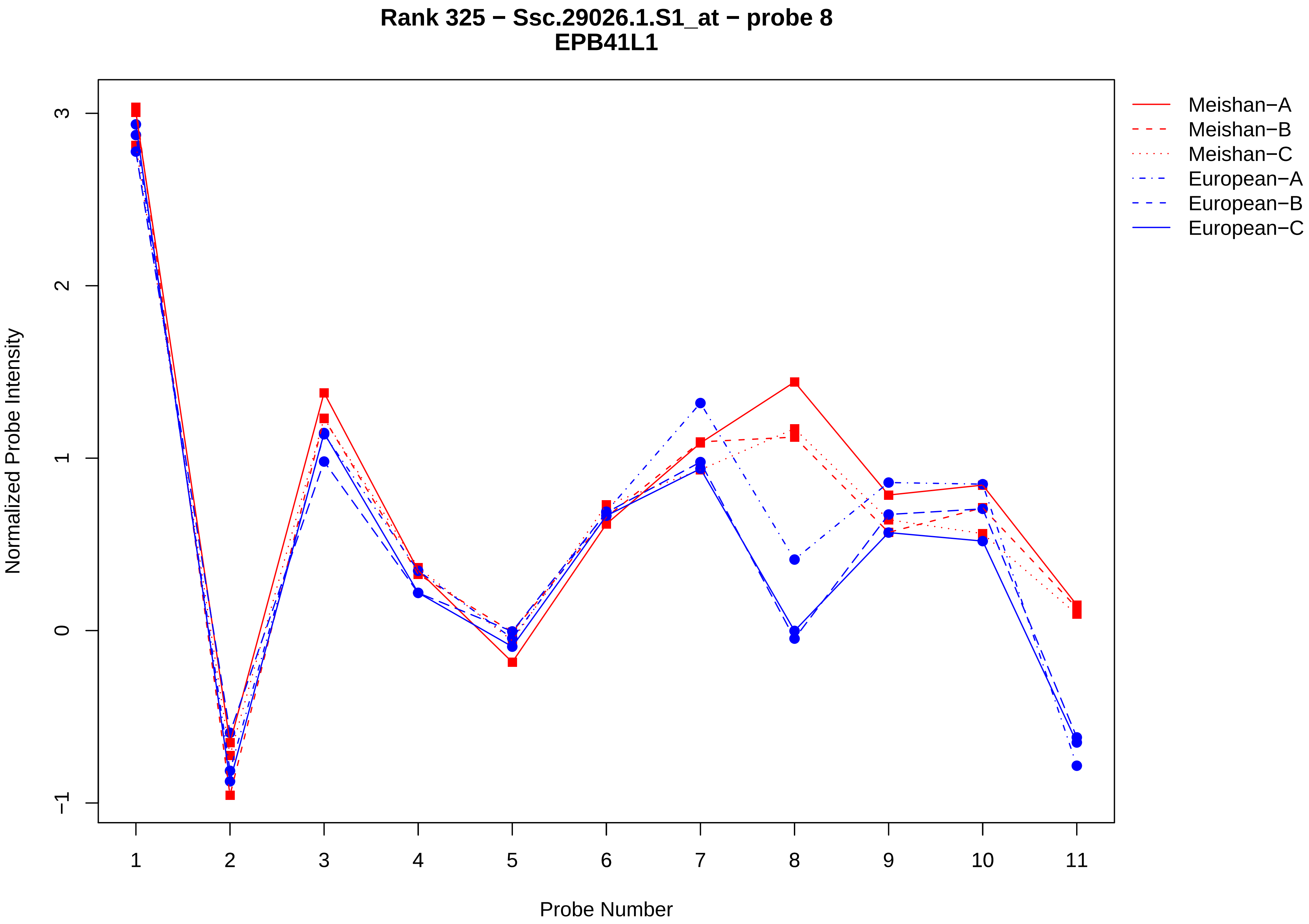

Rank 325 − Ssc.29026.1.S1_at − probe 8
EPB41L1
Normalized Probe Intensity
Probe Number
−1
0
1
2
3
1
2
3
4
5
6
7
8
9
10
11
Meishan−A
Meishan−B
Meishan−C
European−A
European−B
European−C

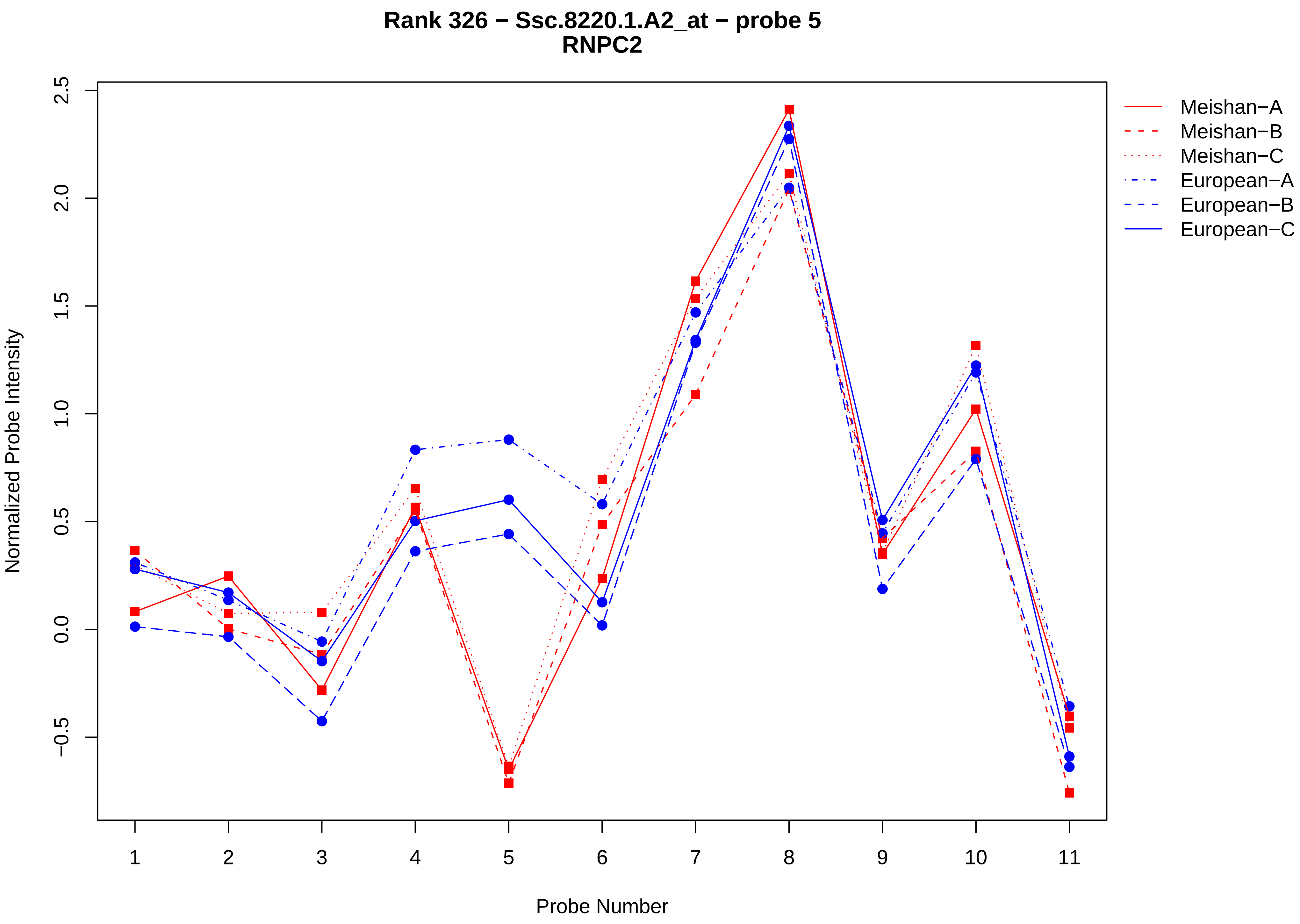

Rank 326 − Ssc.8220.1.A2_at − probe 5
RNPC2
Normalized Probe Intensity
Probe Number
−0.5
0.0
0.5
1.0
1.5
2.0
2.5
1
2
3
4
5
6
7
8
9
10
11
Meishan−A
Meishan−B
Meishan−C
European−A
European−B
European−C

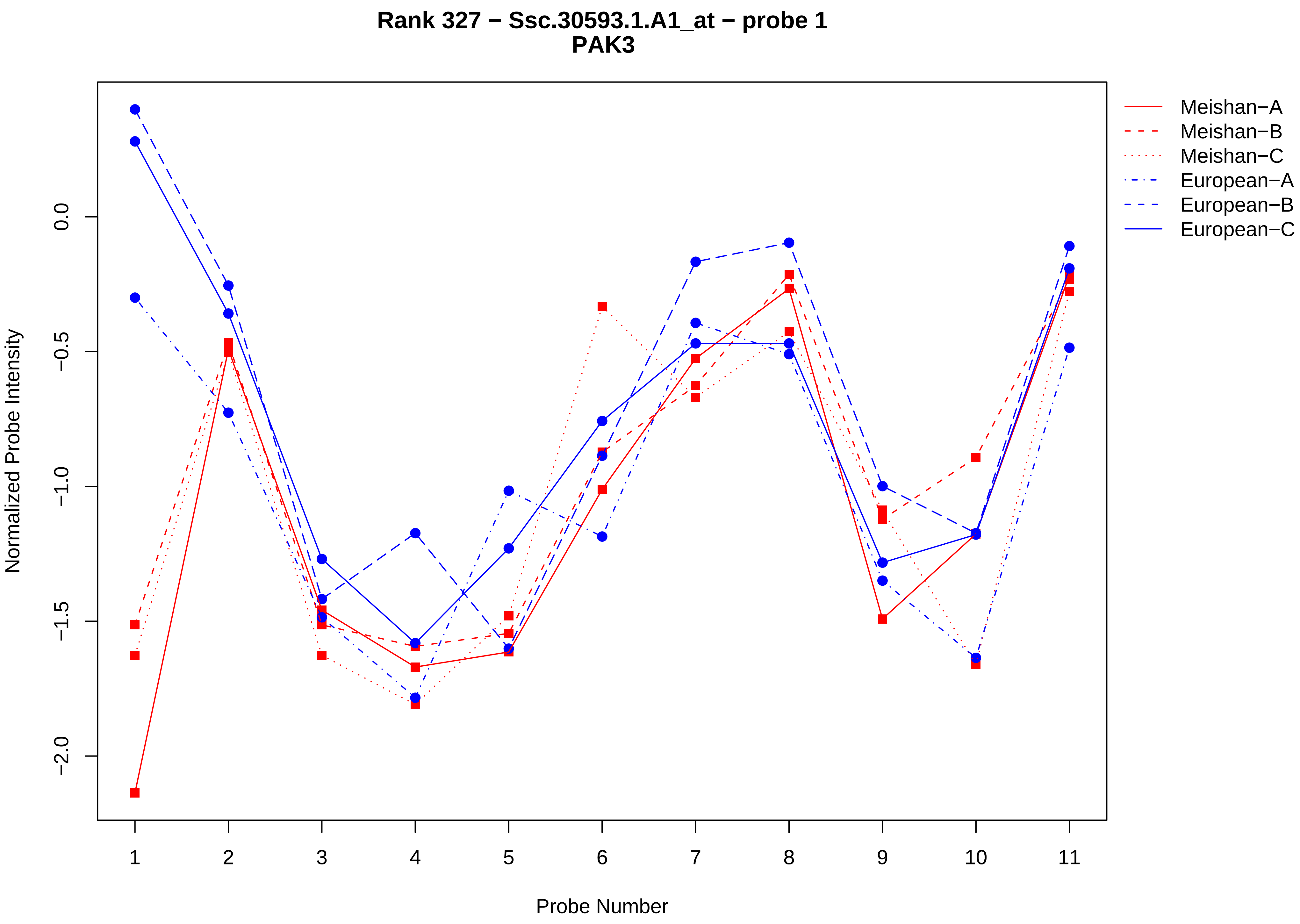
Rank 327 − Ssc.30593.1.A1_at − probe 1
PAK3
Normalized Probe Intensity
0.0
−0.5
−1.0
−1.5
−2.0
Probe Number
1
2
3
4
5
6
7
8
9
10
11
Meishan−A
Meishan−B
Meishan−C
European−A
European−B
European−C

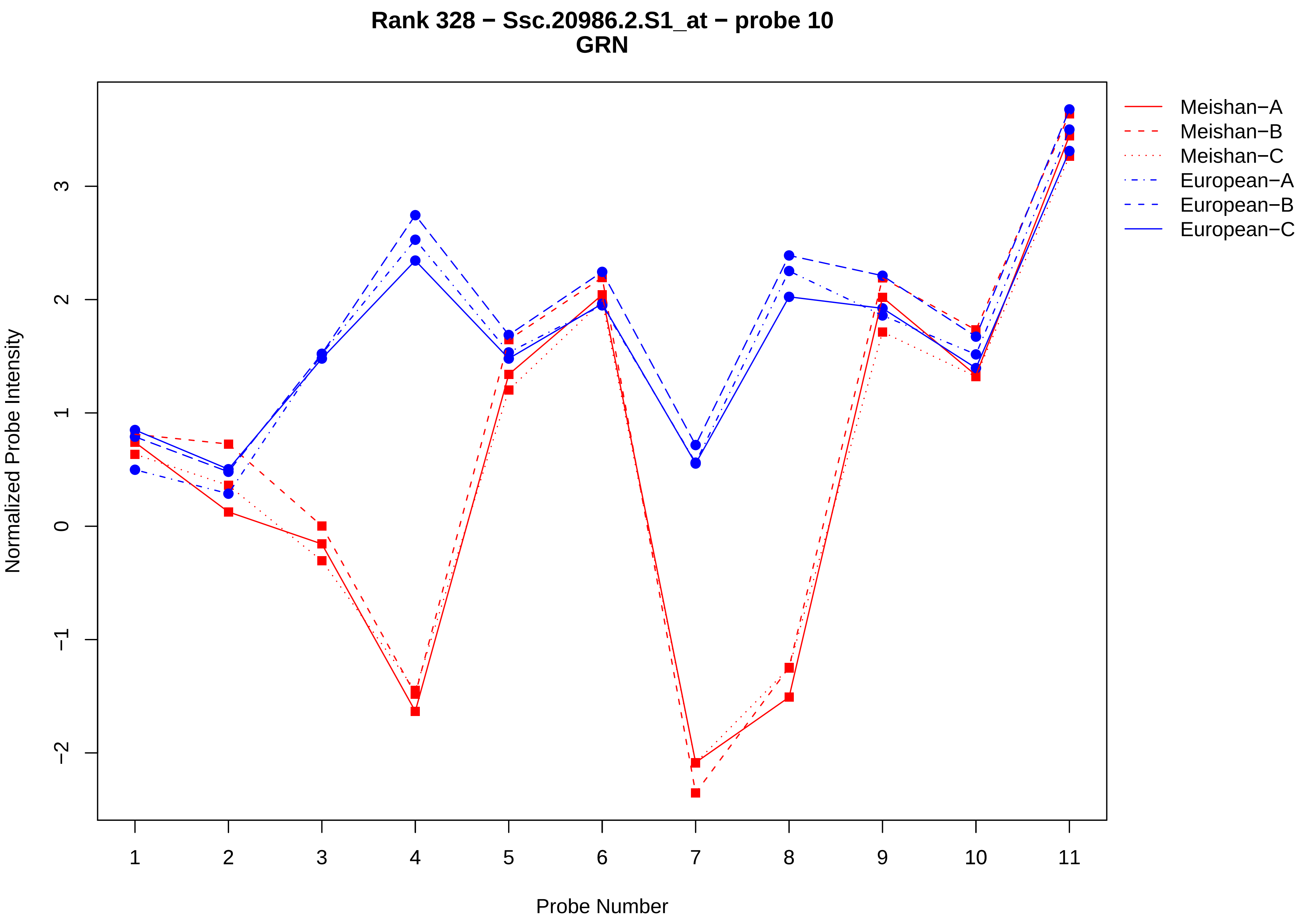

Rank 328 − Ssc.20986.2.S1_at − probe 10
GRN
Normalized Probe Intensity
Probe Number
Meishan−A
Meishan−B
Meishan−C
European−A
European−B
European−C

Rank 329 − Ssc.5895.1.A2_at − probe 2
COL6A1

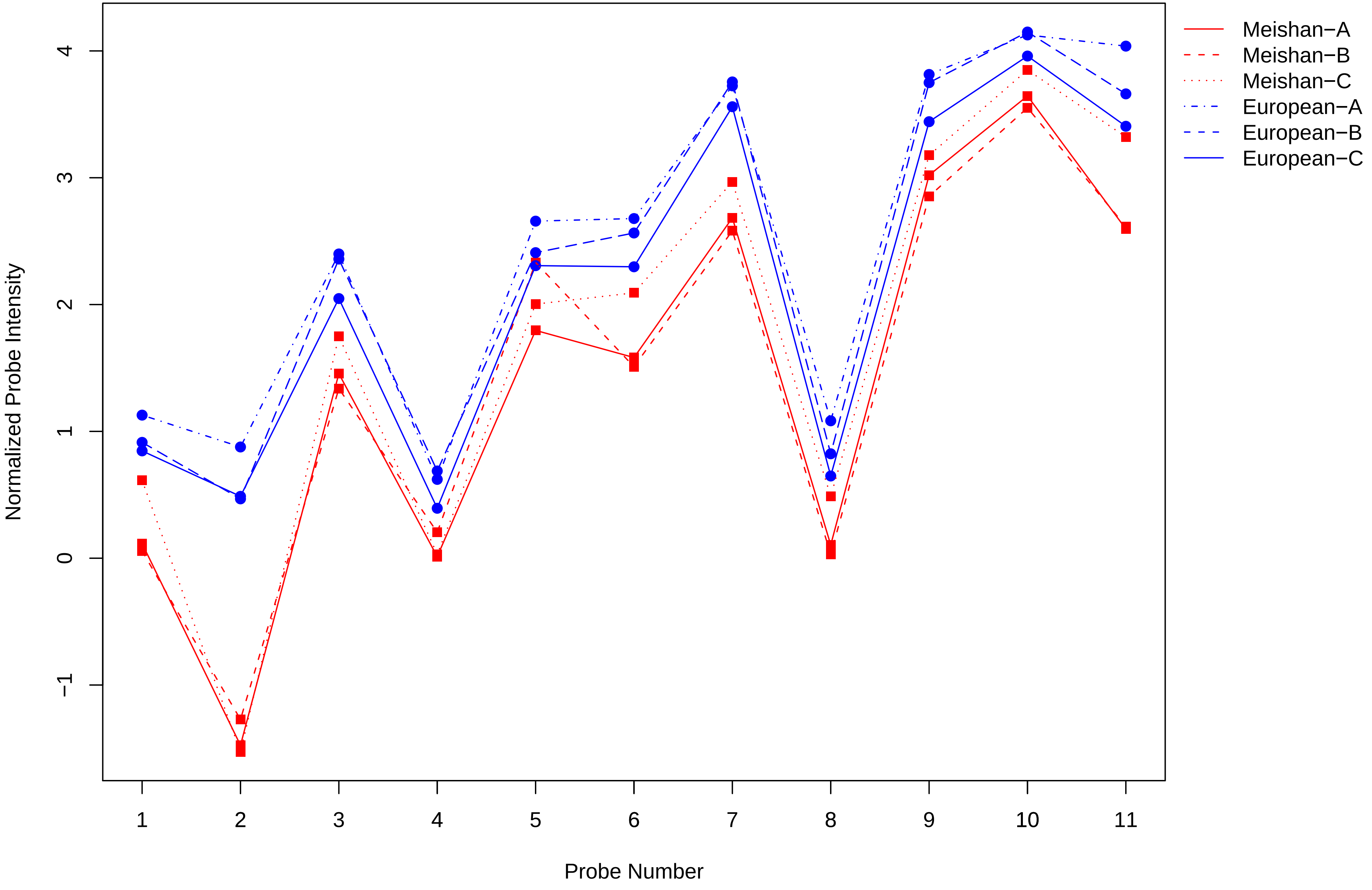

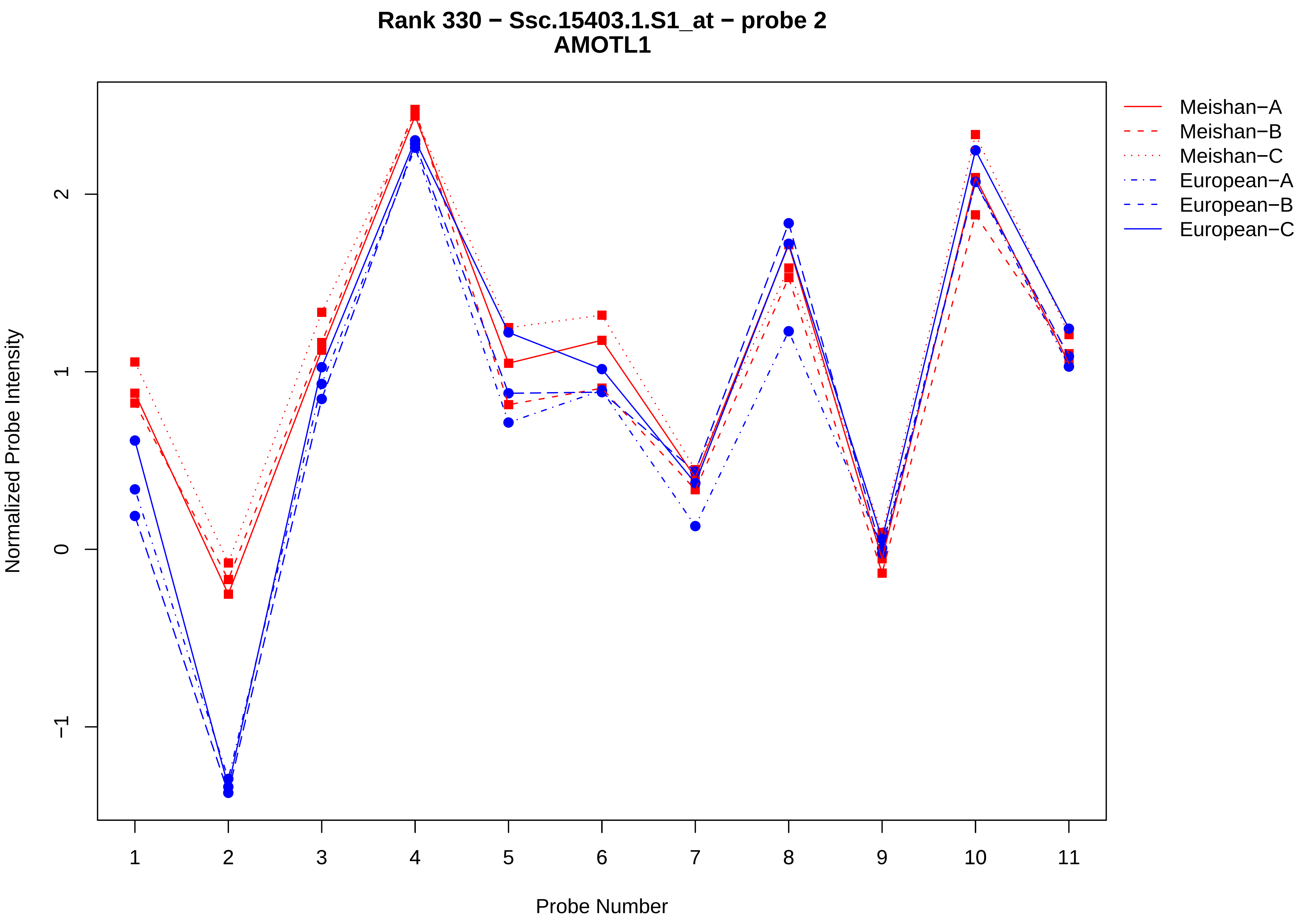

Rank 330 − Ssc.15403.1.S1_at − probe 2
AMOTL1
Normalized Probe Intensity
Probe Number
−1
0
1
2
1
2
3
4
5
6
7
8
9
10
11
Meishan−A
Meishan−B
Meishan−C
European−A
European−B
European−C

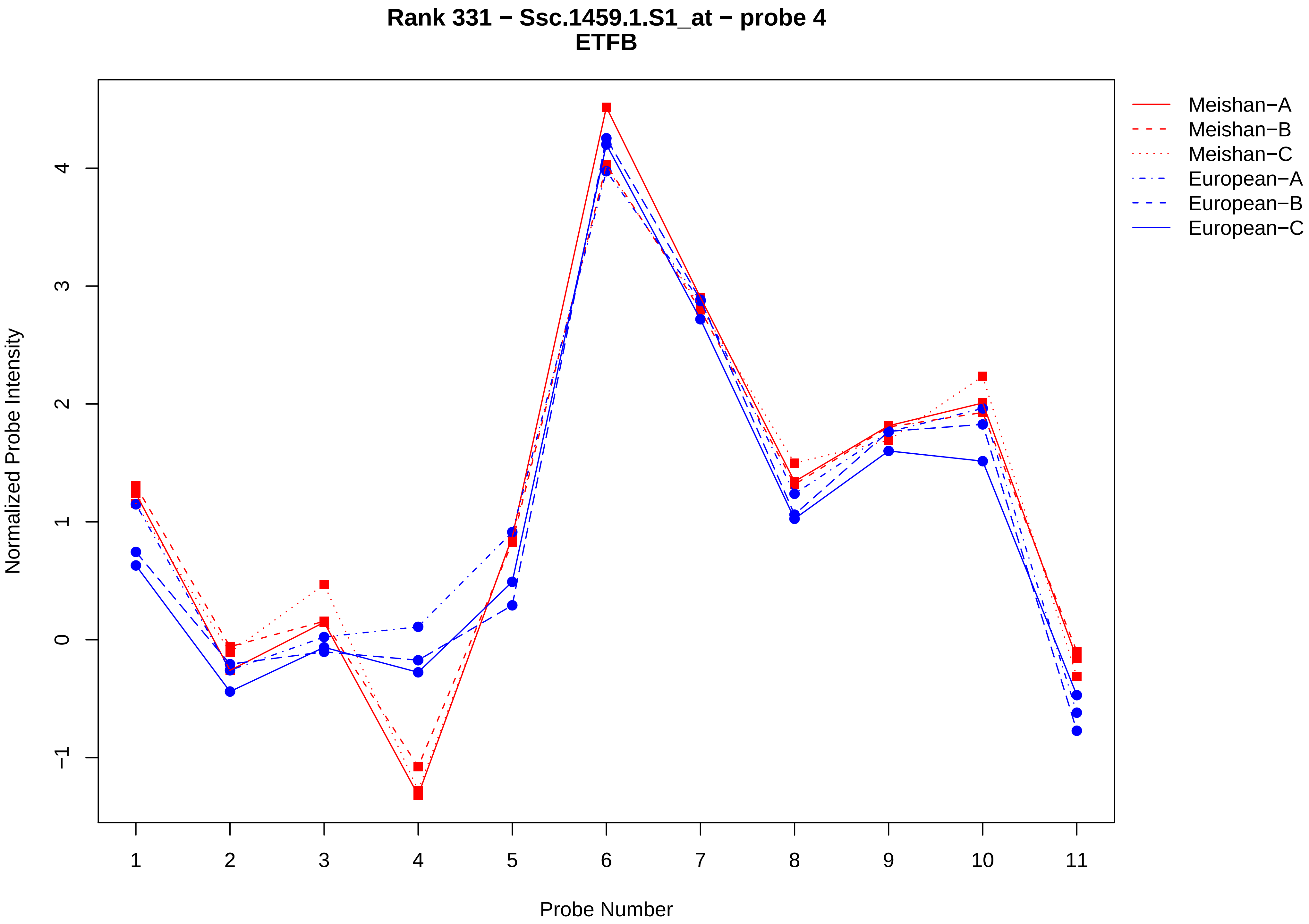

Rank 331 − Ssc.1459.1.S1_at − probe 4
ETFB
Normalized Probe Intensity
Probe Number
−1
0
1
2
3
4
1
2
3
4
5
6
7
8
9
10
11
Meishan−A
Meishan−B
Meishan−C
European−A
European−B
European−C

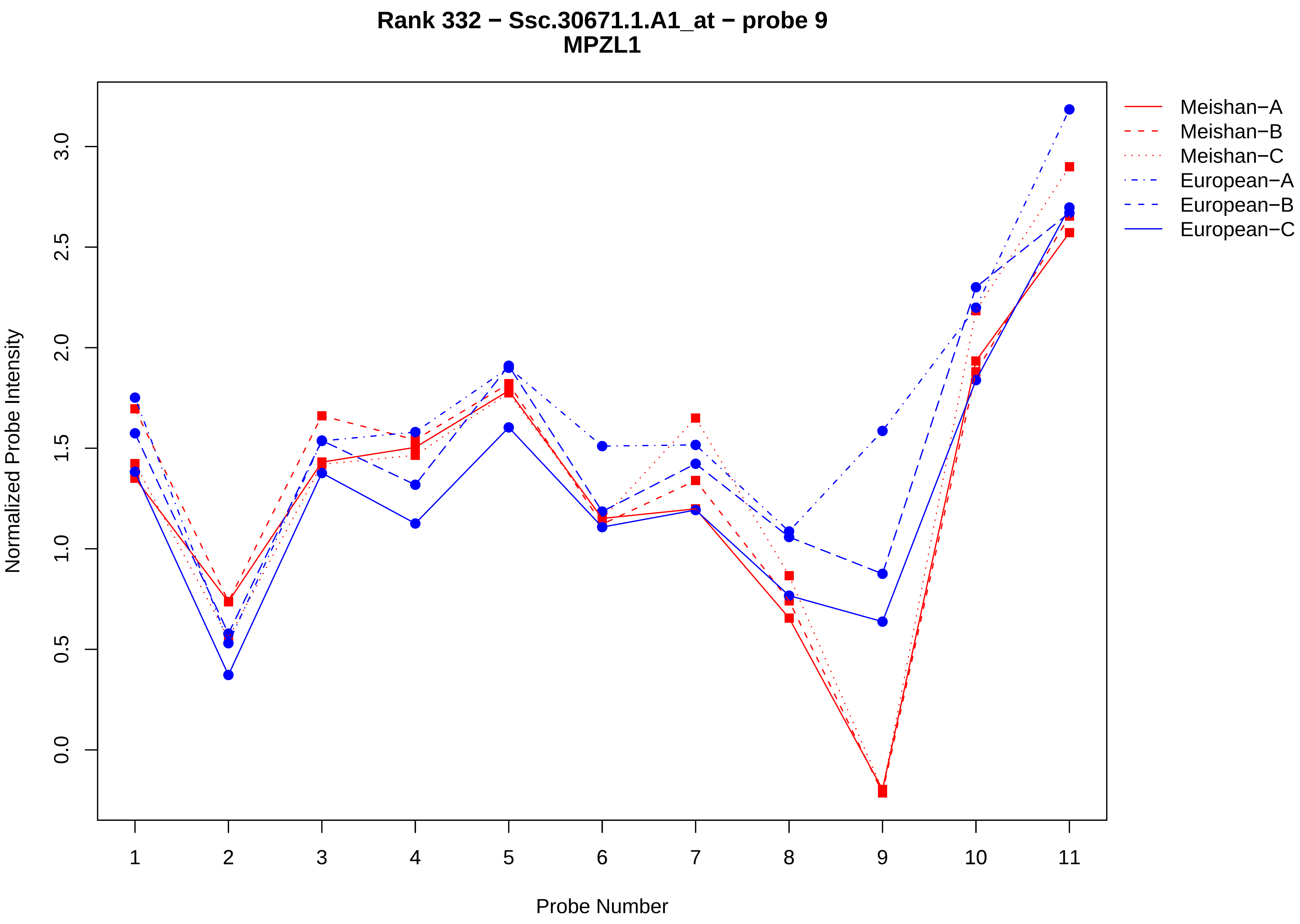
Rank 332 − Ssc.30671.1.A1_at − probe 9
MPZL1
Normalized Probe Intensity
Probe Number
0.0
0.5
1.0
1.5
2.0
2.5
3.0
1
2
3
4
5
6
7
8
9
10
11
Meishan−A
Meishan−B
Meishan−C
European−A
European−B
European−C

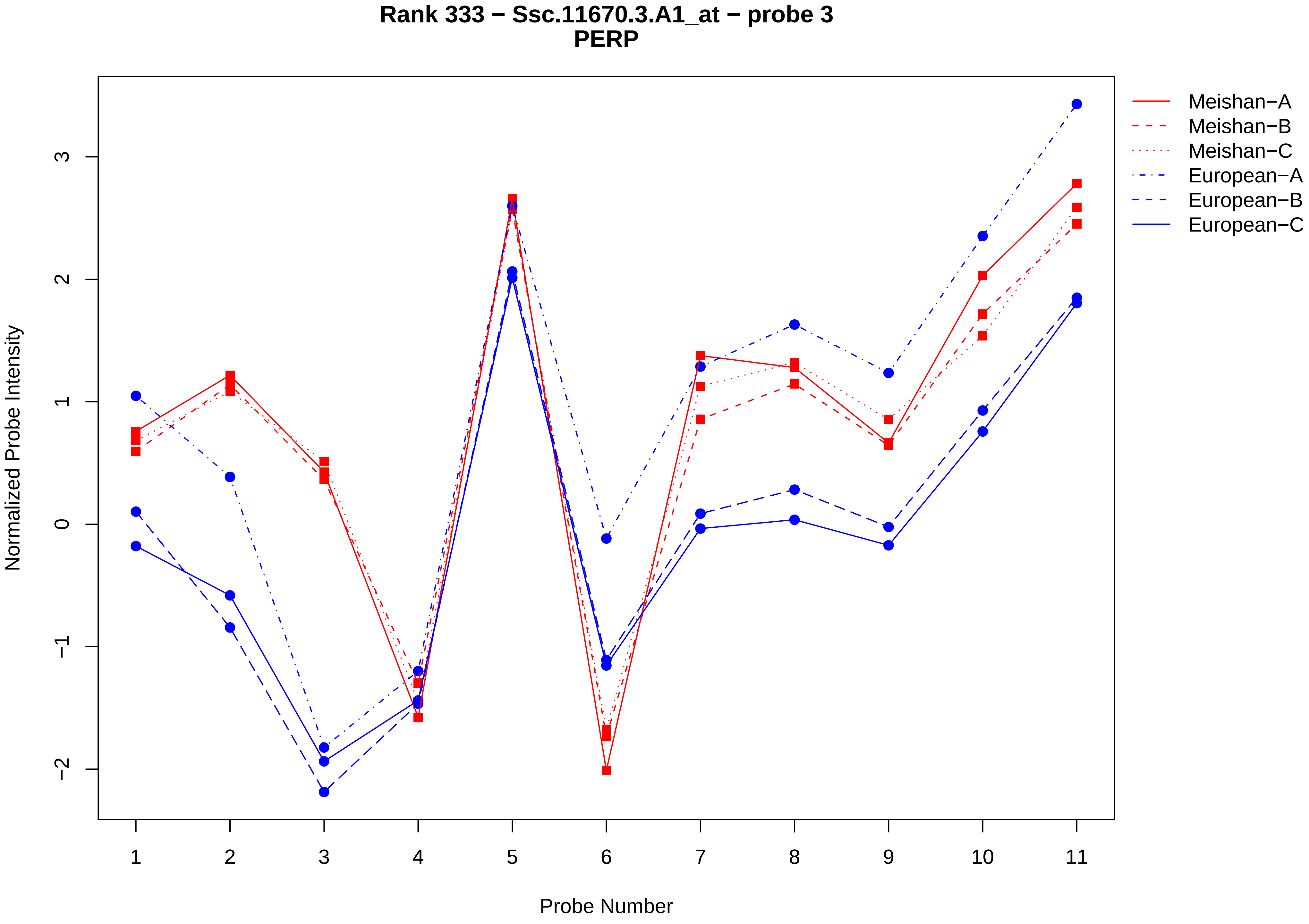
Rank 333 − Ssc.11670.3.A1_at − probe 3
PERP
Normalized Probe Intensity
Probe Number
−2
−1
0
1
2
3
1
2
3
4
5
6
7
8
9
10
11
Meishan−A
Meishan−B
Meishan−C
European−A
European−B
European−C

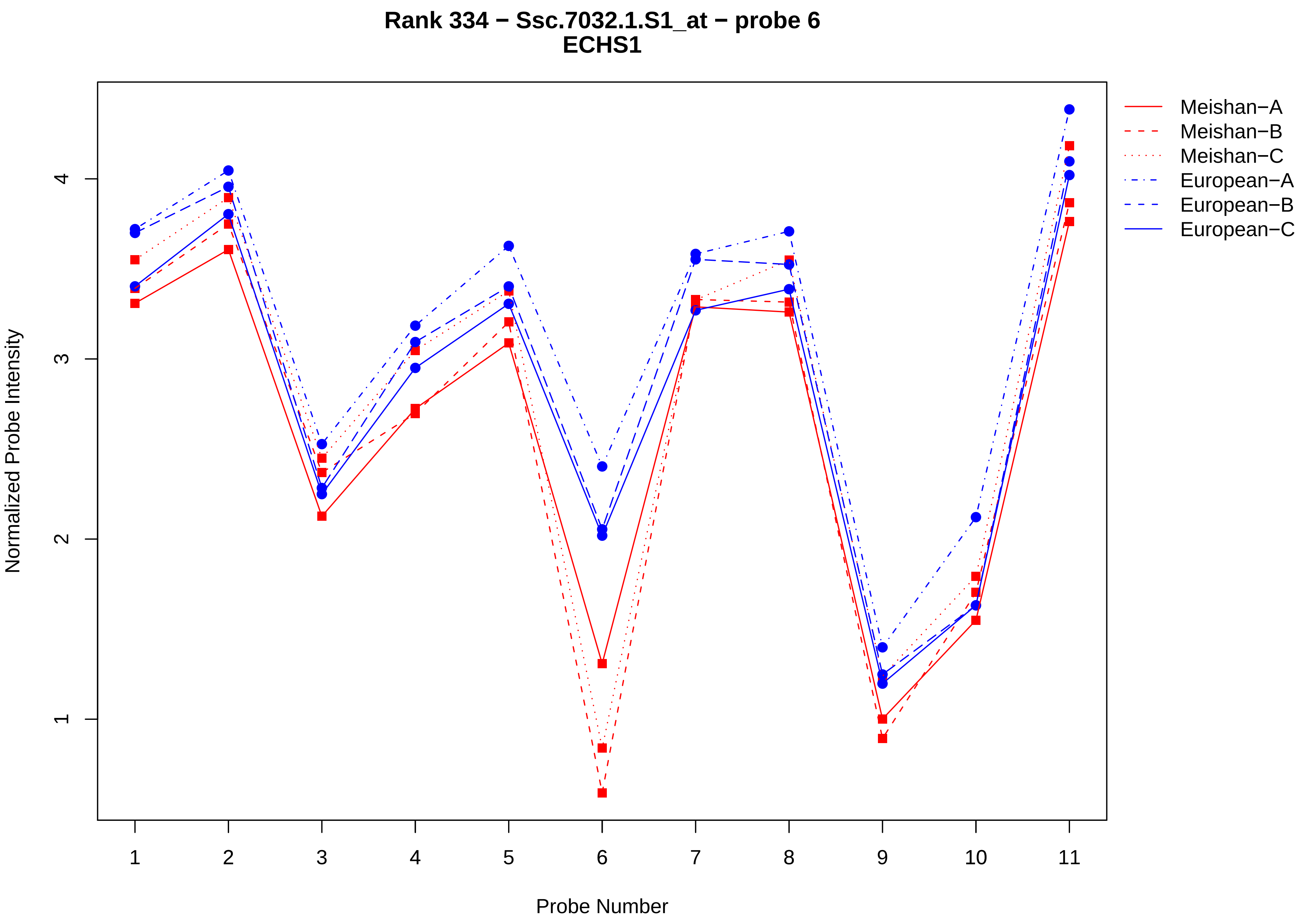

Rank 334 − Ssc.7032.1.S1_at − probe 6
ECHS1
Normalized Probe Intensity
Probe Number
1
2
3
4
1
2
3
4
5
6
7
8
9
10
11
Meishan−A
Meishan−B
Meishan−C
European−A
European−B
European−C

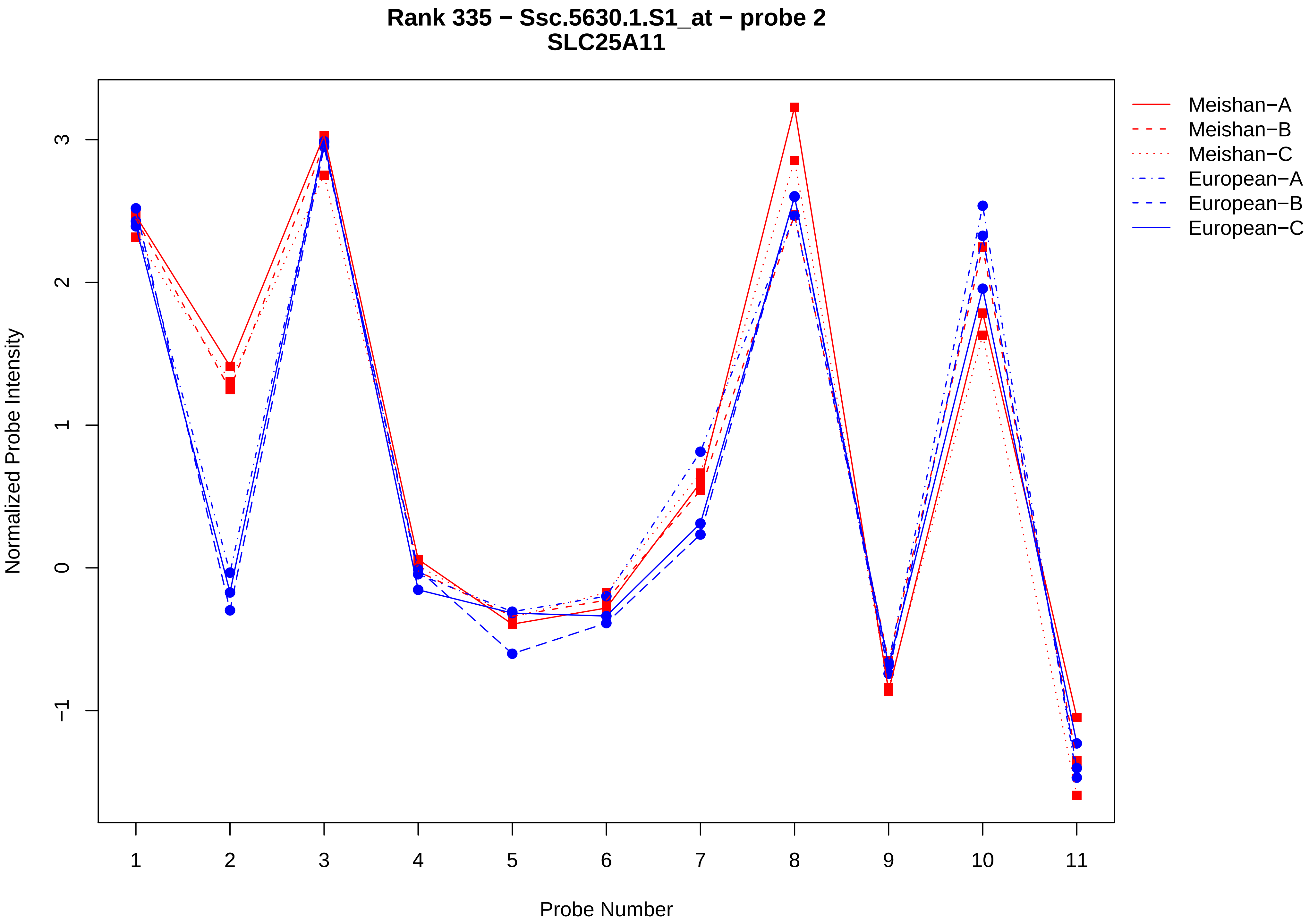
Rank 335 − Ssc.5630.1.S1_at − probe 2
SLC25A11
Normalized Probe Intensity
Probe Number
Meishan−A
Meishan−B
Meishan−C
European−A
European−B
European−C
−1
0
1
2
3
1
2
3
4
5
6
7
8
9
10
11

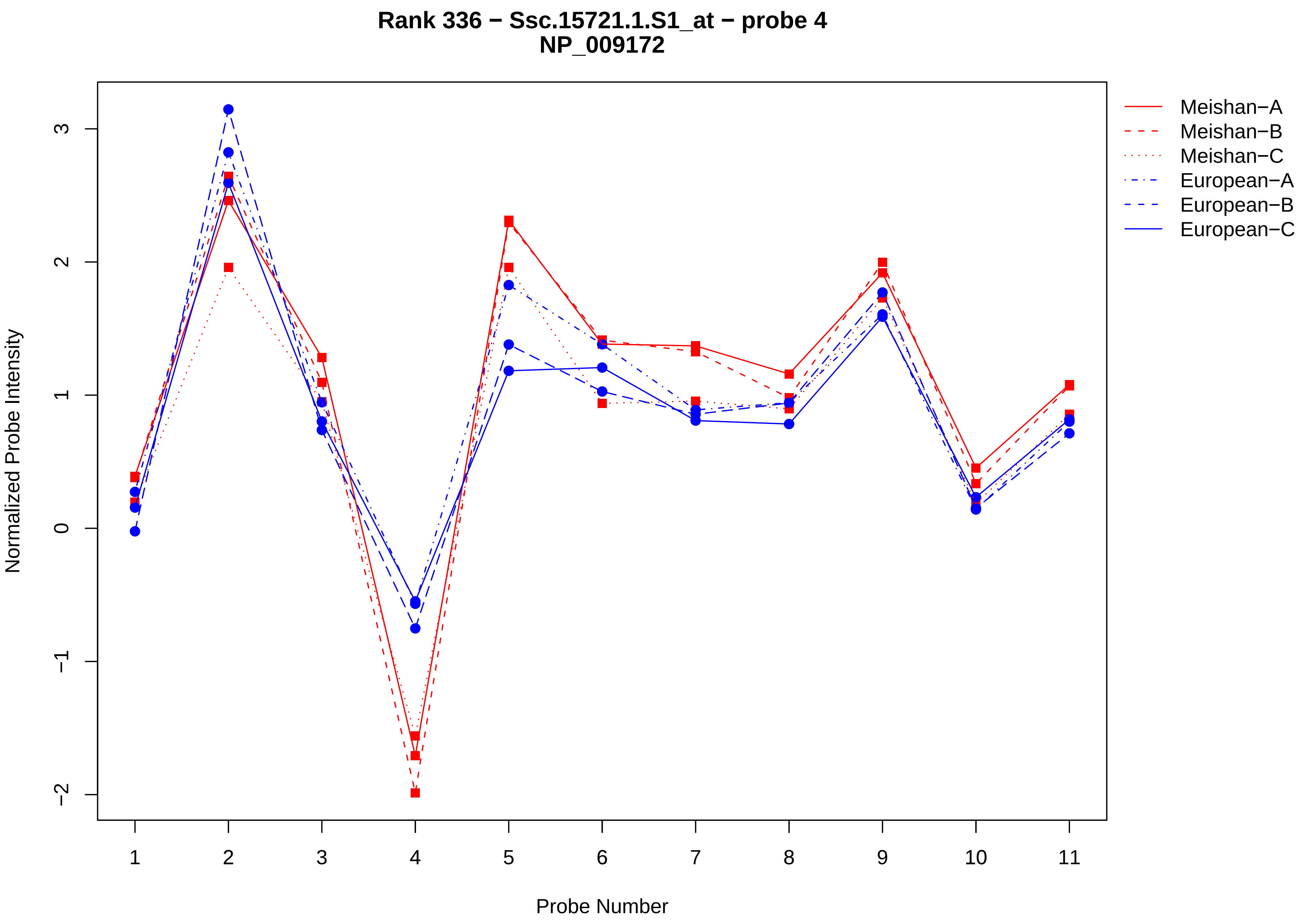

Rank 336 − Ssc.15721.1.S1_at − probe 4
NP_009172
Normalized Probe Intensity
Probe Number
Meishan−A
Meishan−B
Meishan−C
European−A
European−B
European−C
−2
−1
0
1
2
3
1
2
3
4
5
6
7
8
9
10
11

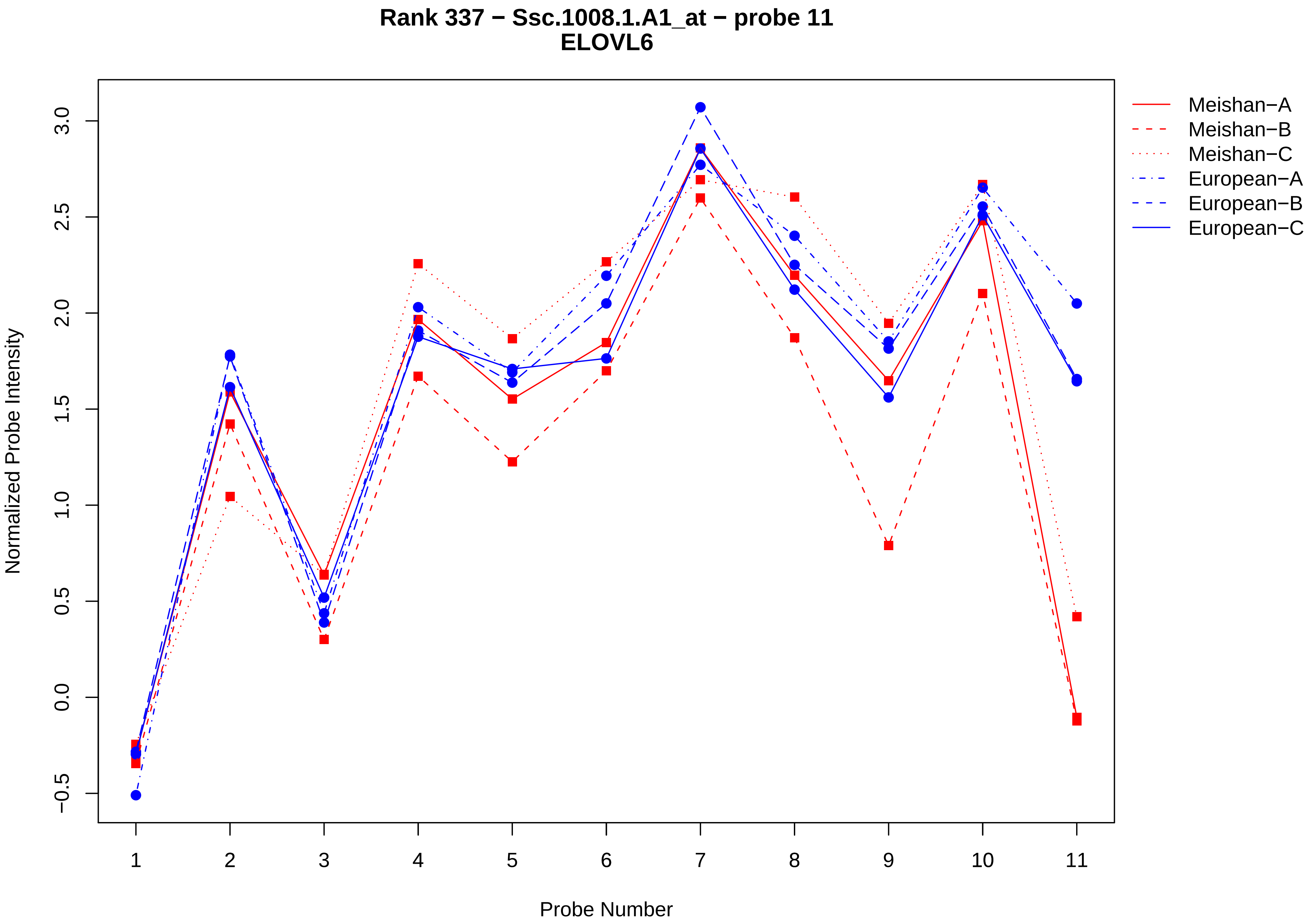

Rank 337 − Ssc.1008.1.A1_at − probe 11
ELOVL6
Normalized Probe Intensity
Probe Number
−0.5
0.0
0.5
1.0
1.5
2.0
2.5
3.0
1
2
3
4
5
6
7
8
9
10
11
Meishan−A
Meishan−B
Meishan−C
European−A
European−B
European−C

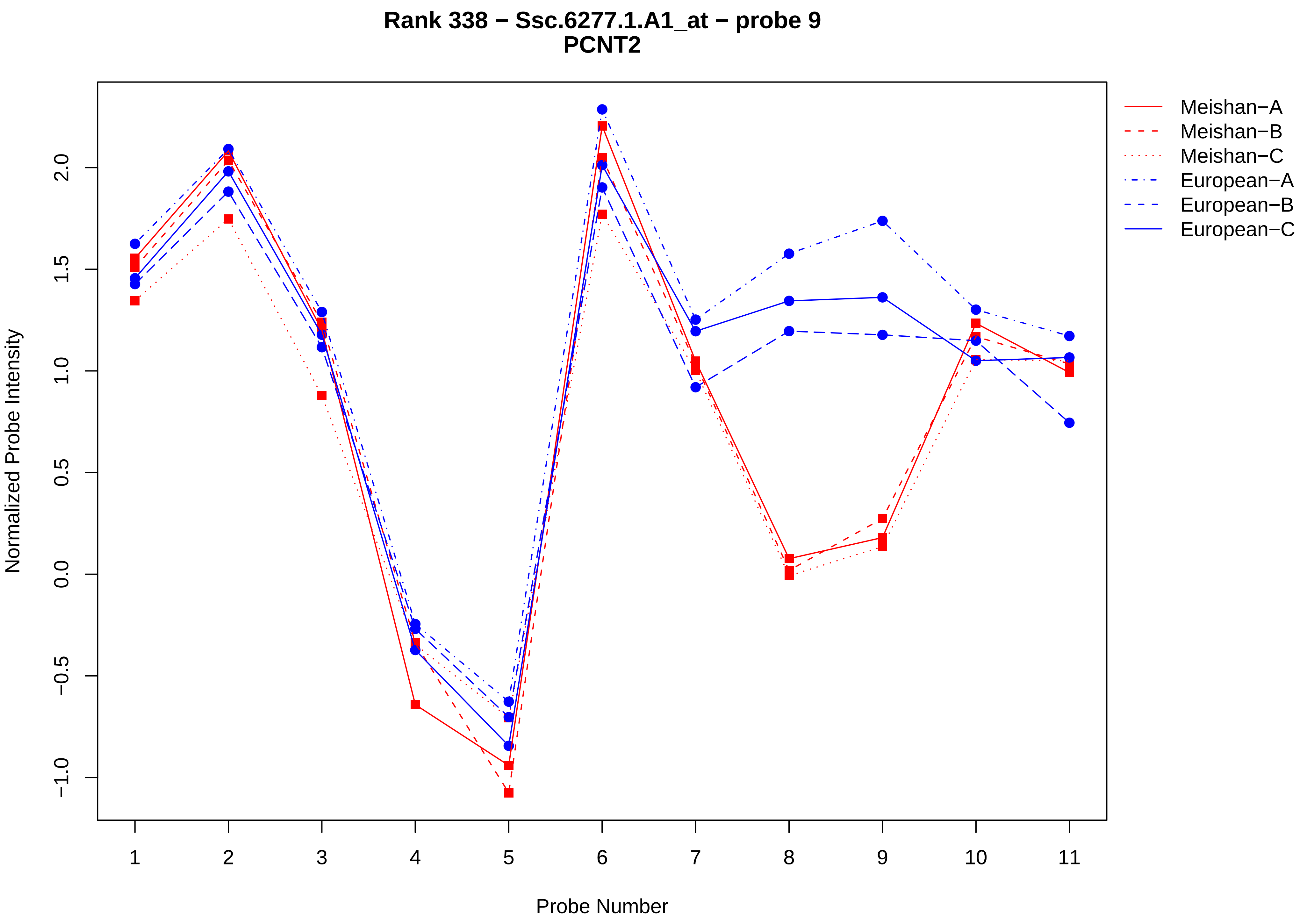
Rank 338 − Ssc.6277.1.A1_at − probe 9
PCNT2
Normalized Probe Intensity
Probe Number
Meishan−A
Meishan−B
Meishan−C
European−A
European−B
European−C
−1.0
−0.5
0.0
0.5
1.0
1.5
2.0
1
2
3
4
5
6
7
8
9
10
11

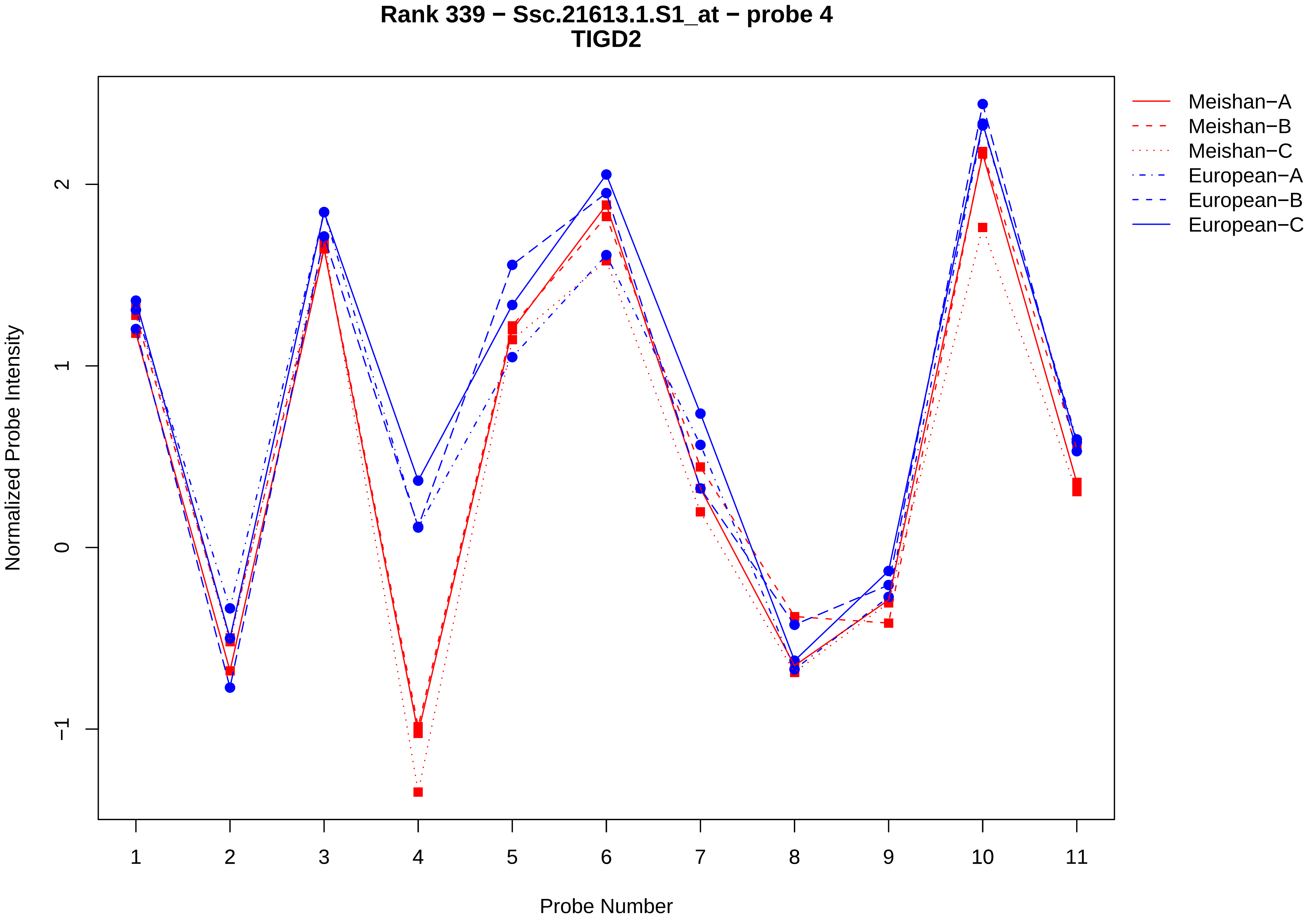

Rank 339 − Ssc.21613.1.S1_at − probe 4
TIGD2
Normalized Probe Intensity
Probe Number
−1
0
1
2
1
2
3
4
5
6
7
8
9
10
11
Meishan−A
Meishan−B
Meishan−C
European−A
European−B
European−C

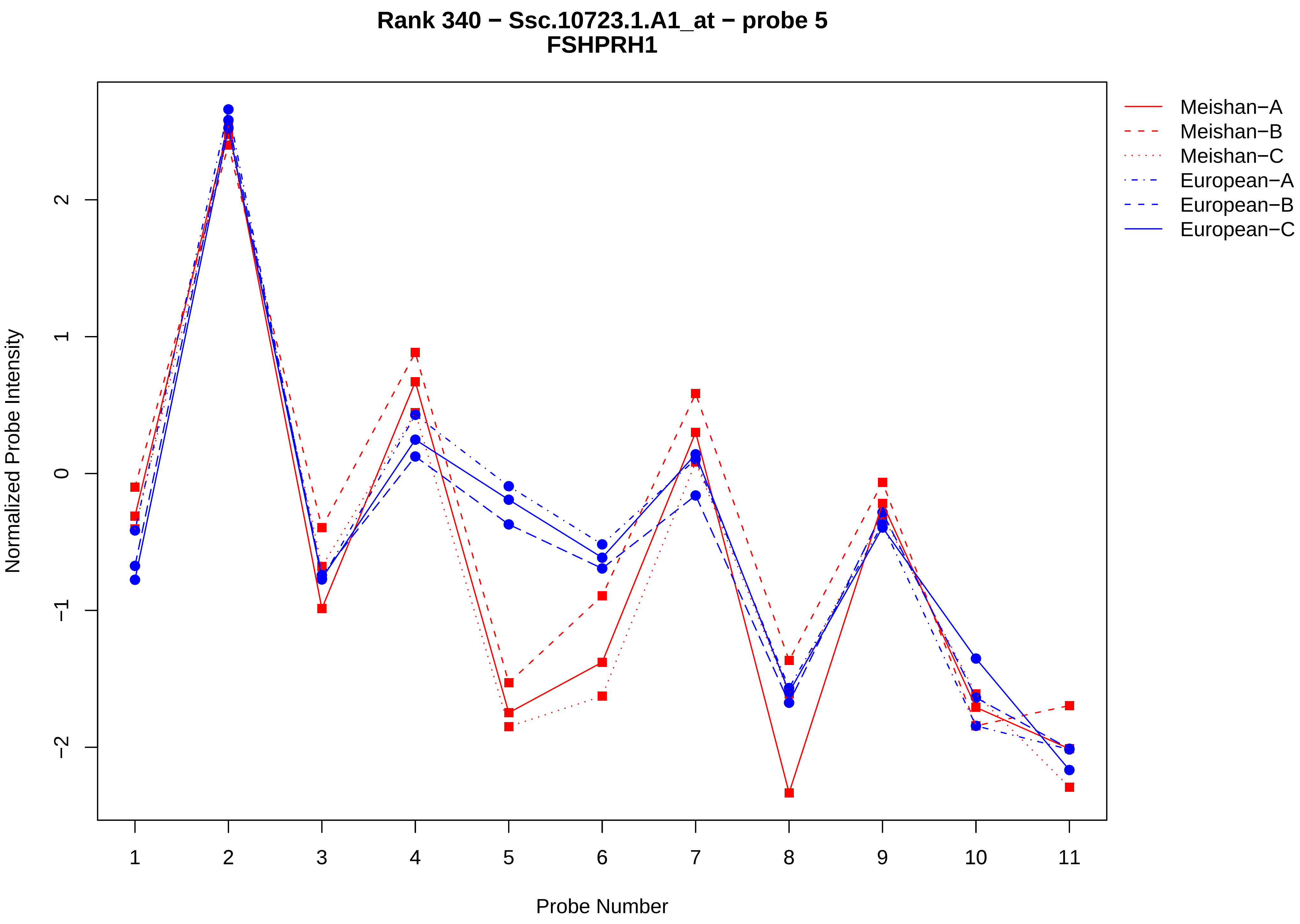
Rank 340 − Ssc.10723.1.A1_at − probe 5
FSHPRH1
Normalized Probe Intensity
Probe Number
Meishan−A
Meishan−B
Meishan−C
European−A
European−B
European−C

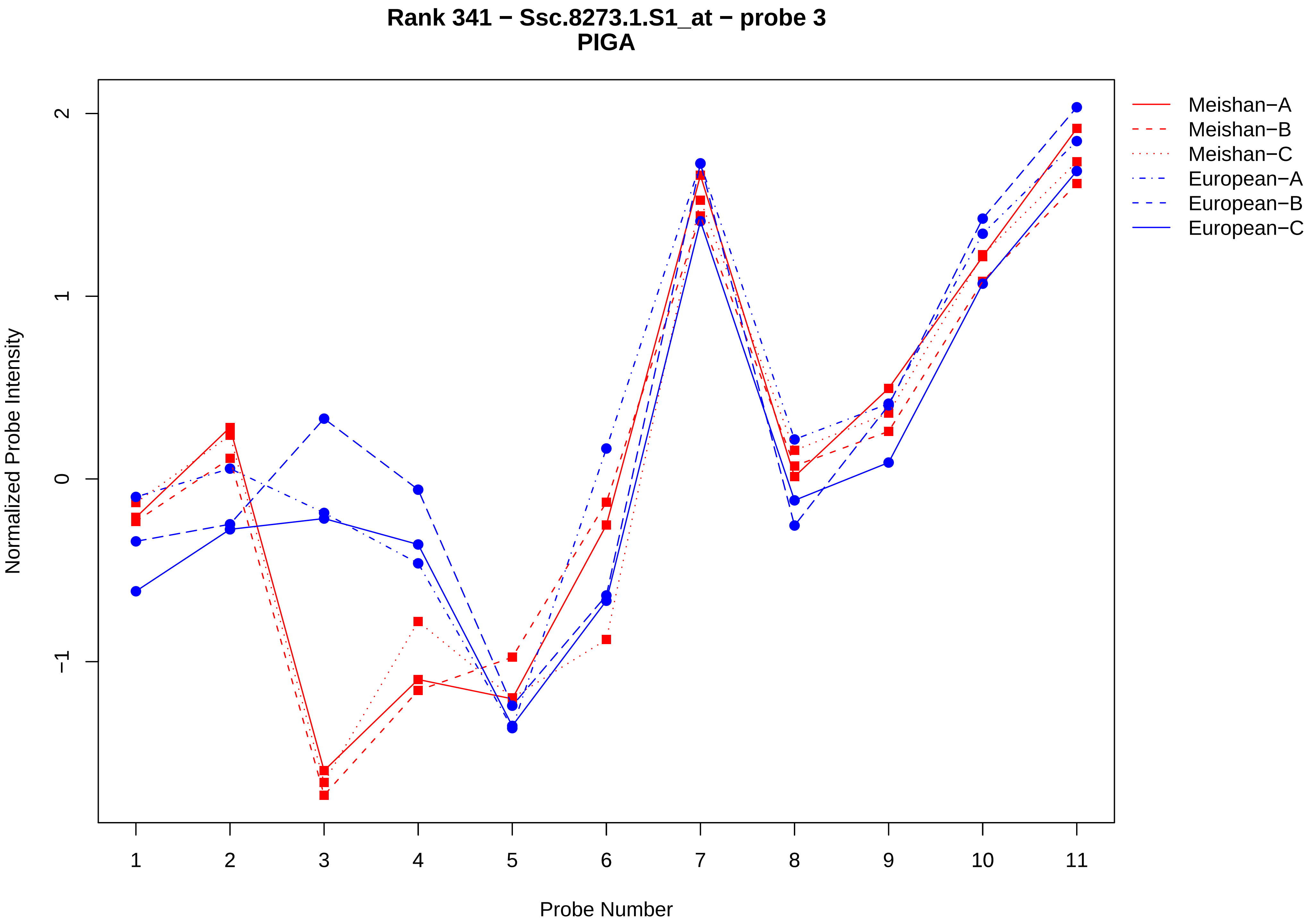

Rank 341 − Ssc.8273.1.S1_at − probe 3
PIGA
Normalized Probe Intensity
Probe Number
Meishan−A
Meishan−B
Meishan−C
European−A
European−B
European−C
−1
0
1
2
1
2
3
4
5
6
7
8
9
10
11

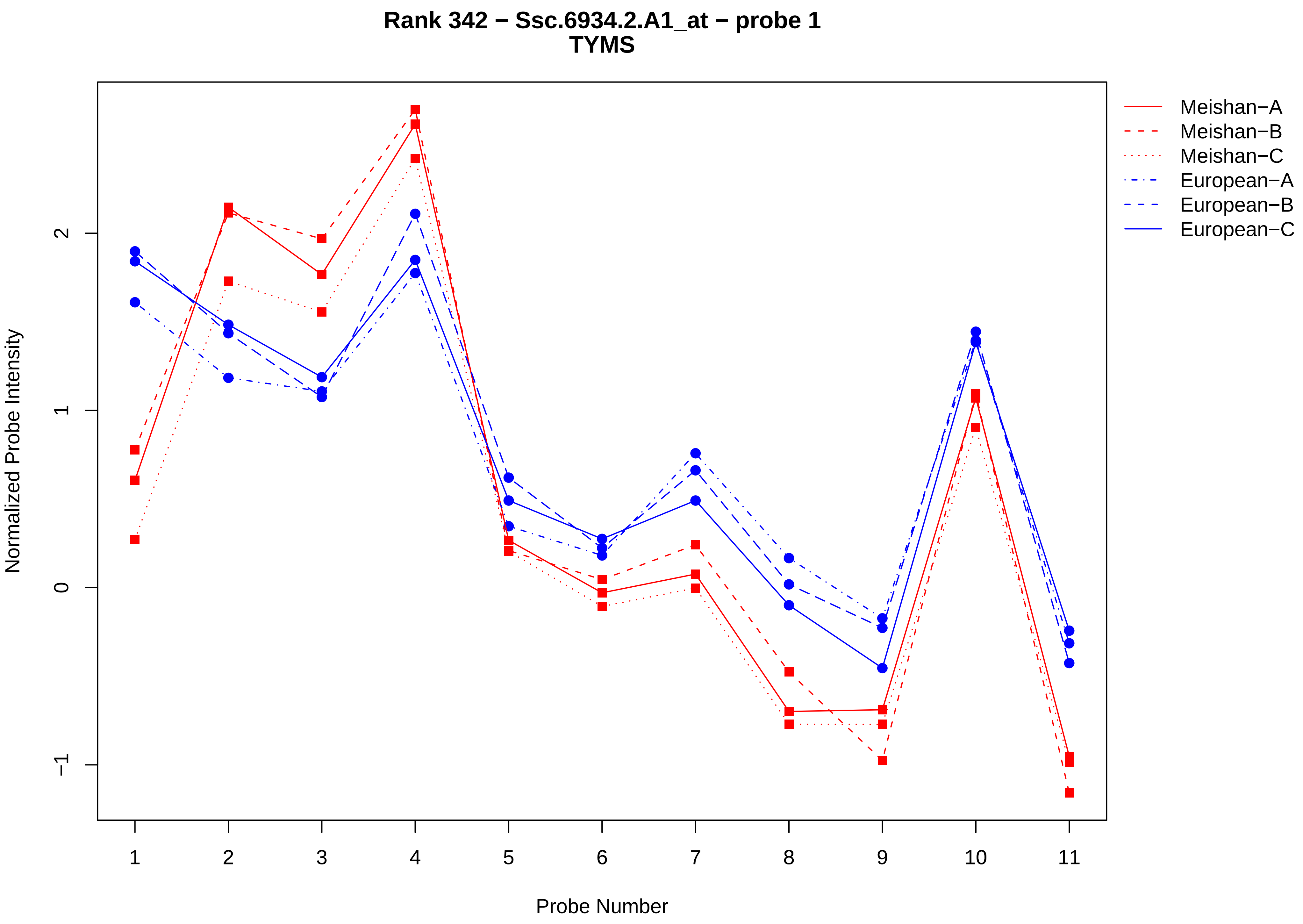
Rank 342 − Ssc.6934.2.A1_at − probe 1
TYMS
Normalized Probe Intensity
Probe Number
−1
0
1
2
1
2
3
4
5
6
7
8
9
10
11
Meishan−A
Meishan−B
Meishan−C
European−A
European−B
European−C

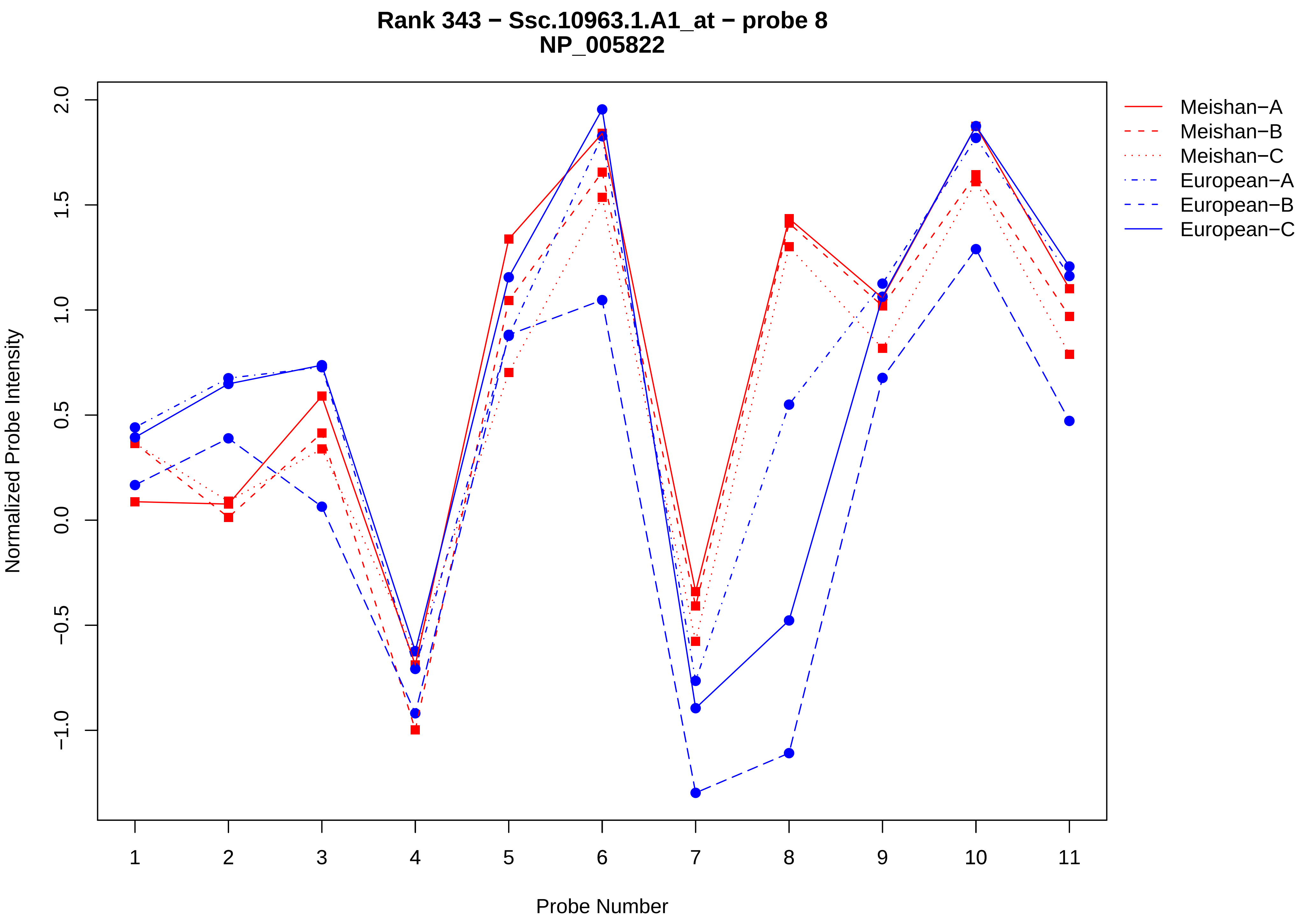

Rank 343 − Ssc.10963.1.A1_at − probe 8
NP_005822
Normalized Probe Intensity
Probe Number
−1.0
−0.5
0.0
0.5
1.0
1.5
2.0
1
2
3
4
5
6
7
8
9
10
11
Meishan−A
Meishan−B
Meishan−C
European−A
European−B
European−C

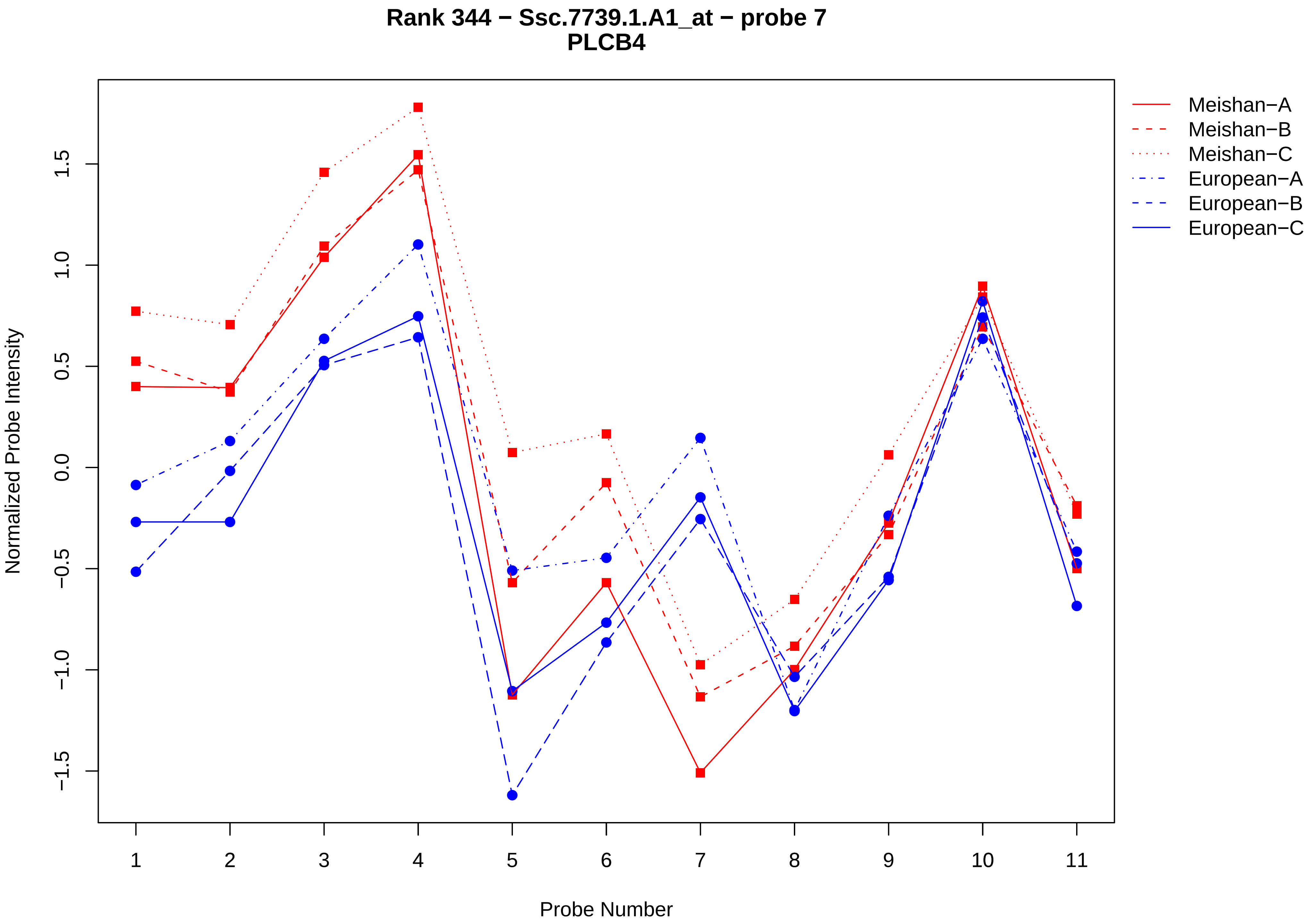

Rank 344 − Ssc.7739.1.A1_at − probe 7
PLCB4
Normalized Probe Intensity
Probe Number
Meishan−A
Meishan−B
Meishan−C
European−A
European−B
European−C
−1.5
−1.0
−0.5
0.0
0.5
1.0
1.5
1
2
3
4
5
6
7
8
9
10
11

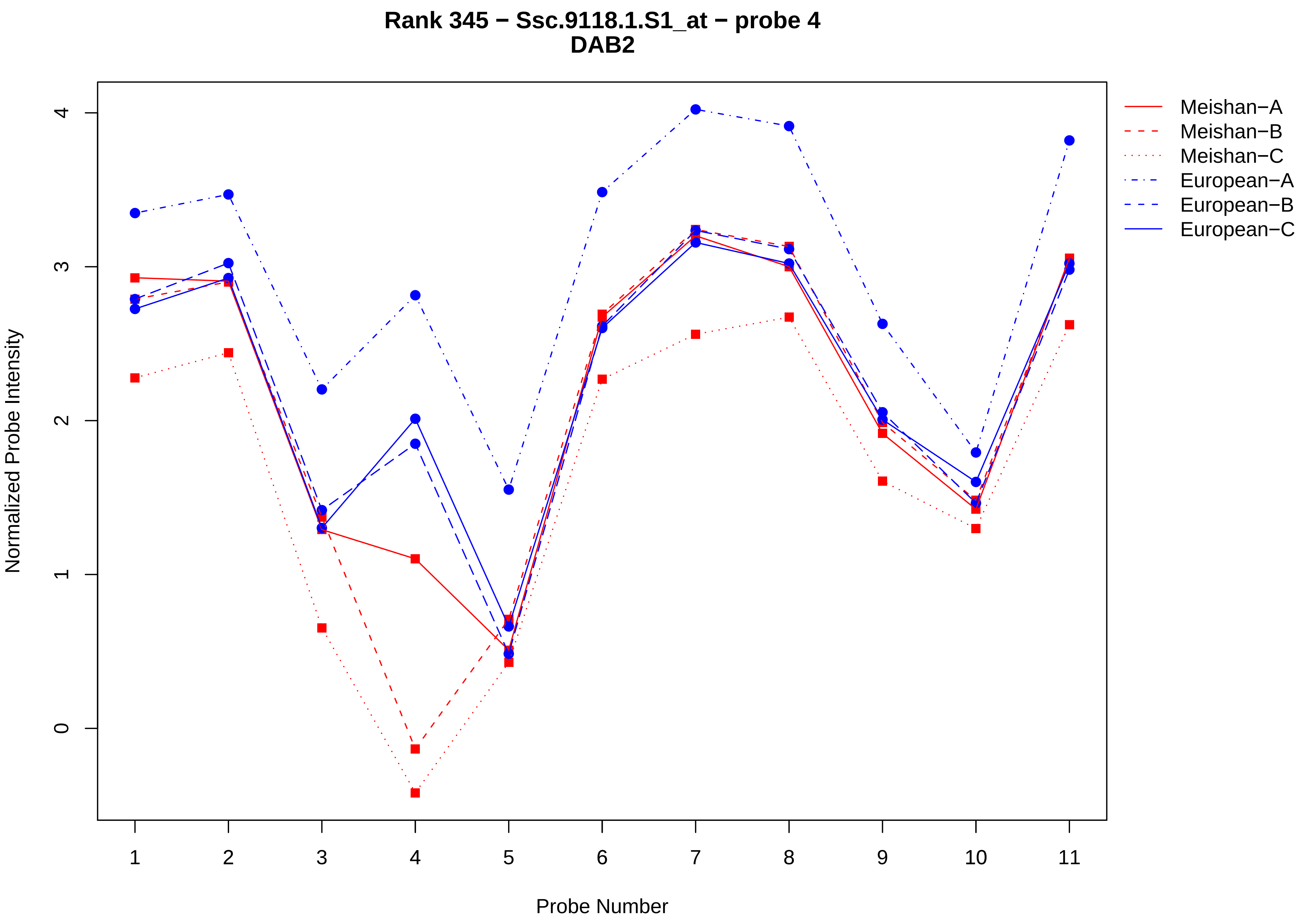

Rank 345 − Ssc.9118.1.S1_at − probe 4
DAB2
Normalized Probe Intensity
0
1
2
3
4
1
2
3
4
5
6
7
8
9
10
11
Probe Number
Meishan−A
Meishan−B
Meishan−C
European−A
European−B
European−C

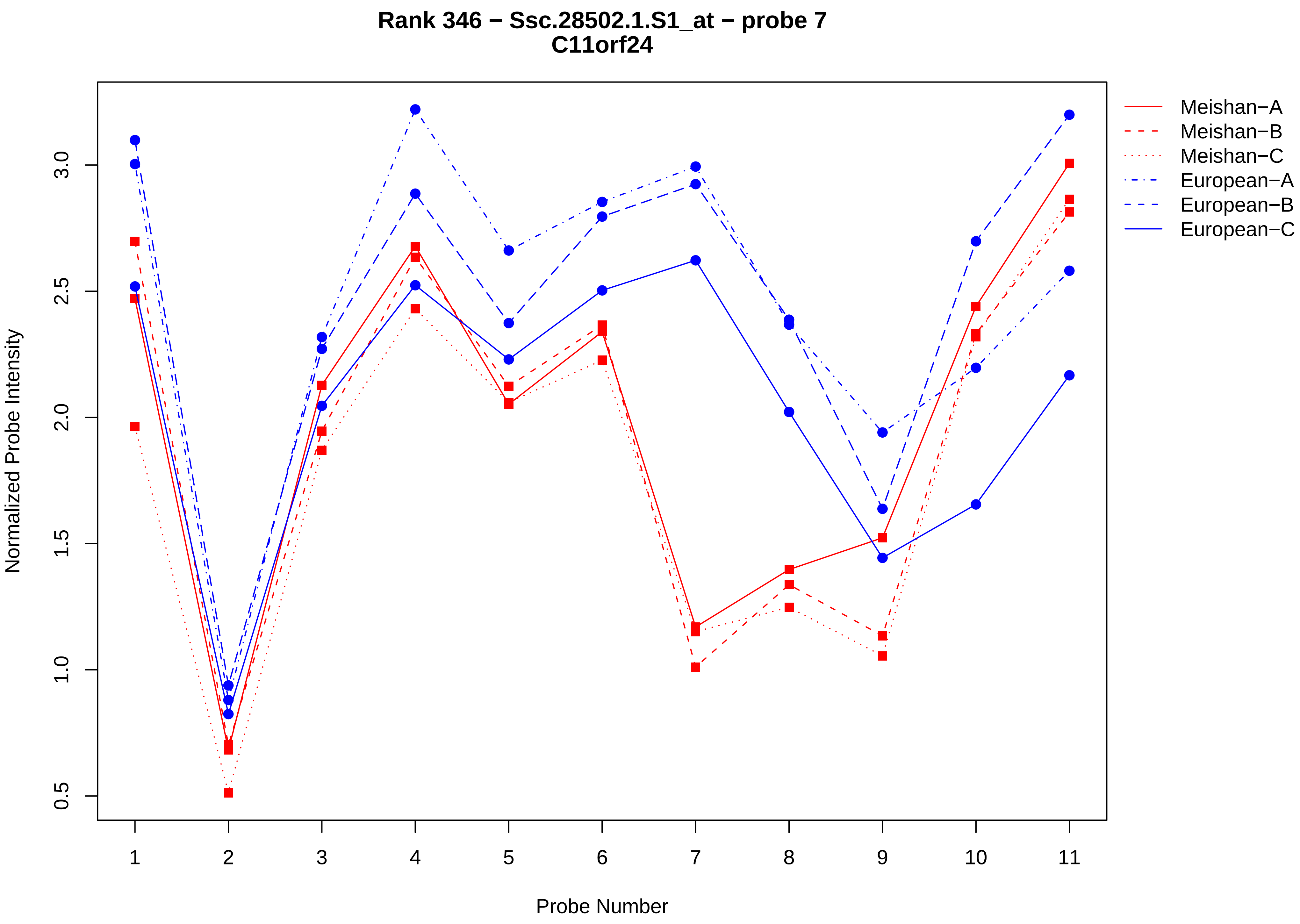

Rank 346 − Ssc.28502.1.S1_at − probe 7
C11orf24
Normalized Probe Intensity
Probe Number
Meishan−A
Meishan−B
Meishan−C
European−A
European−B
European−C
0.5
1.0
1.5
2.0
2.5
3.0
1
2
3
4
5
6
7
8
9
10
11

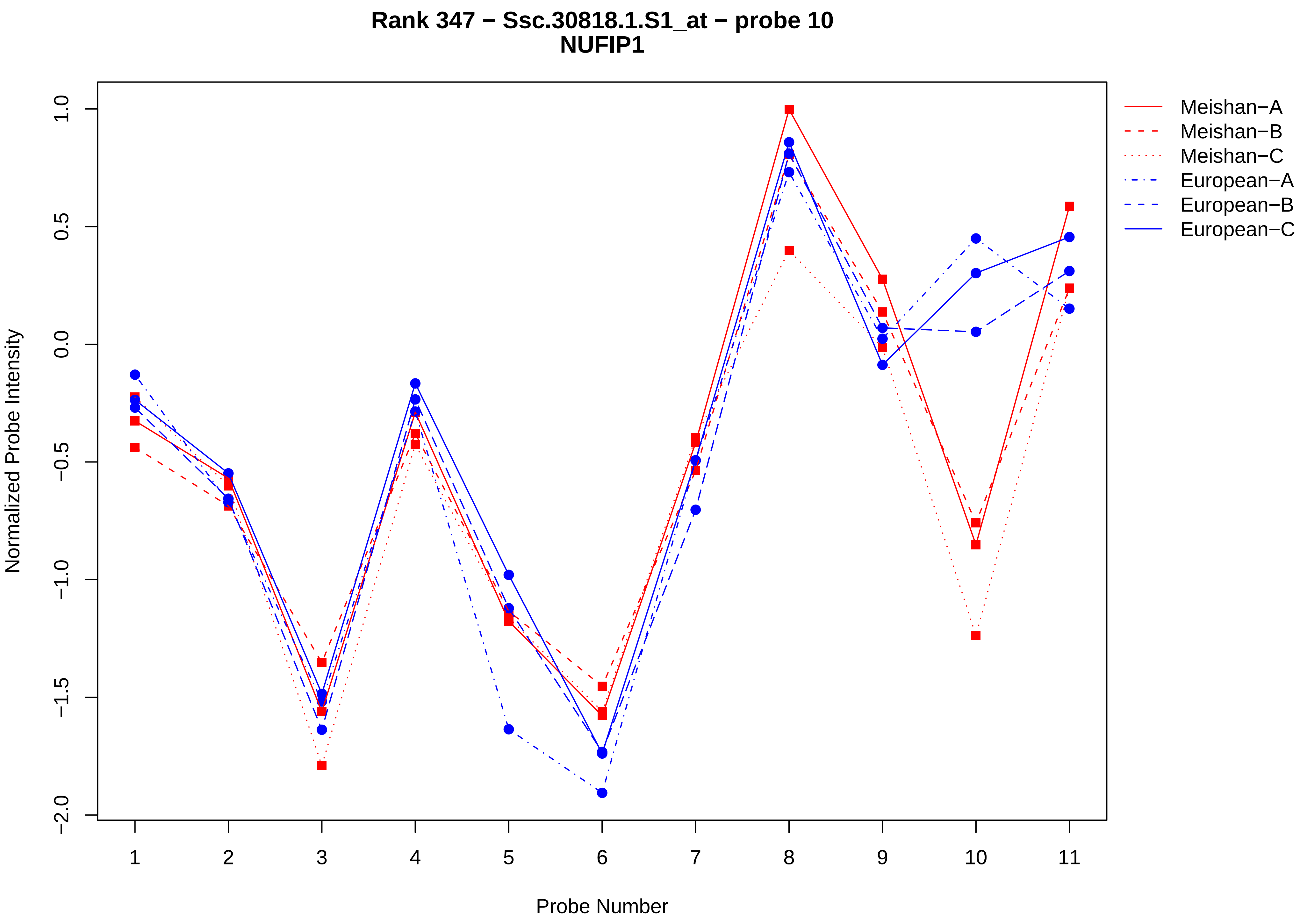

Rank 347 − Ssc.30818.1.S1_at − probe 10
NUFIP1
Normalized Probe Intensity
Probe Number
1.0
0.5
0.0
−0.5
−1.0
−1.5
−2.0
1
2
3
4
5
6
7
8
9
10
11
Meishan−A
Meishan−B
Meishan−C
European−A
European−B
European−C

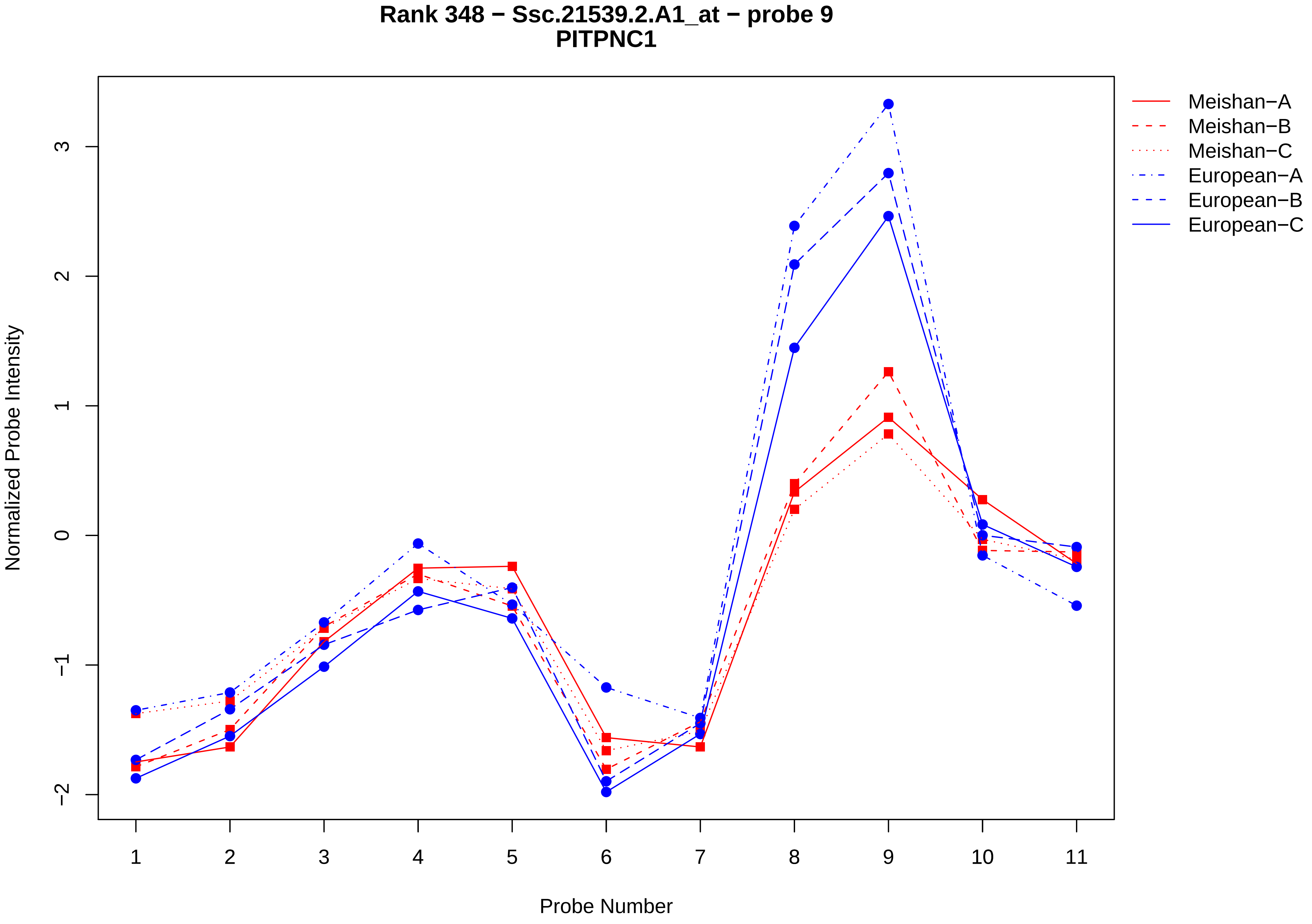
Rank 348 − Ssc.21539.2.A1_at − probe 9
PITPNC1
Normalized Probe Intensity
Probe Number
−2
−1
0
1
2
3
1
2
3
4
5
6
7
8
9
10
11
Meishan−A
Meishan−B
Meishan−C
European−A
European−B
European−C

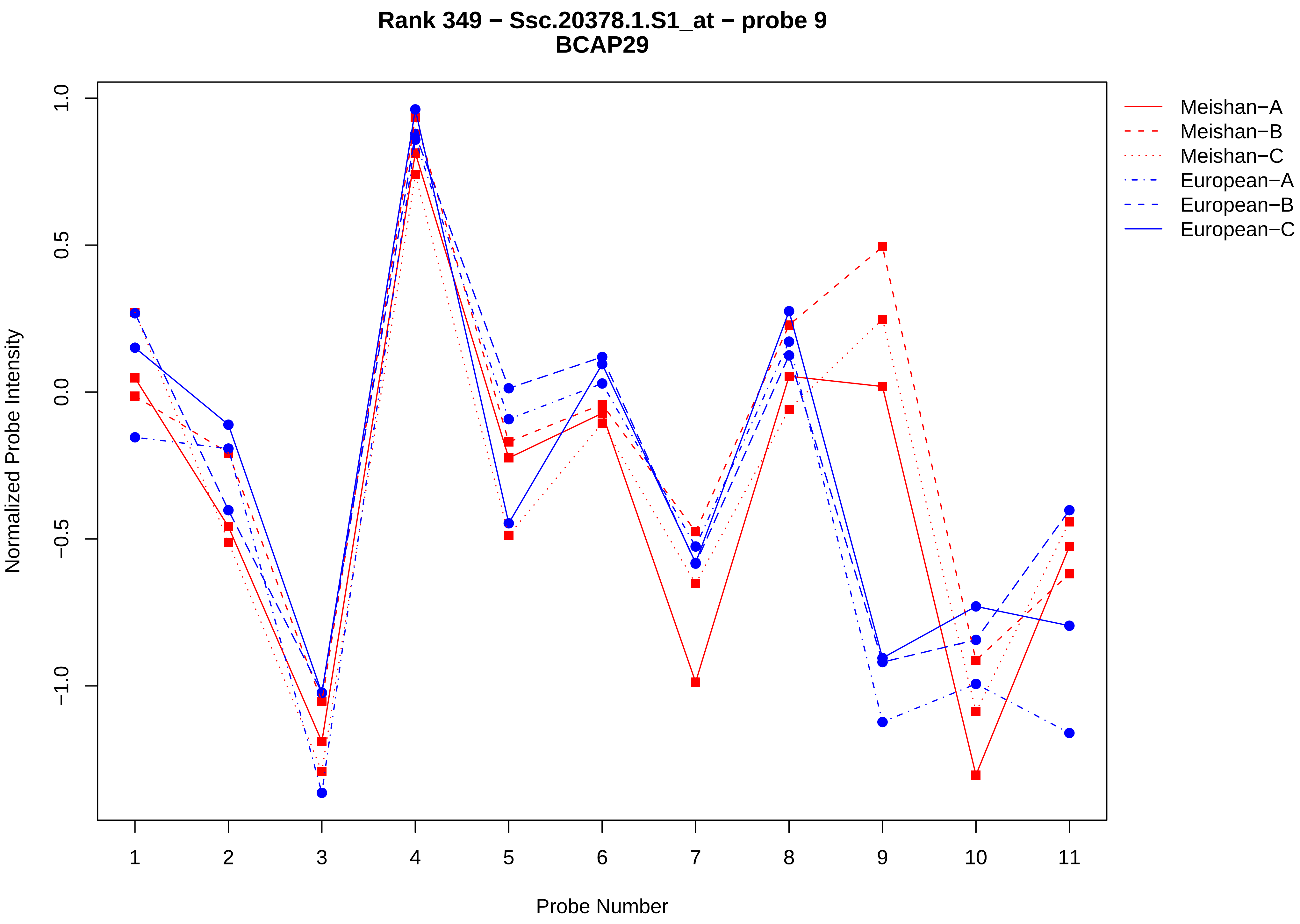

Rank 349 − Ssc.20378.1.S1_at − probe 9
BCAP29
Normalized Probe Intensity
Probe Number
1.0
0.5
0.0
−0.5
−1.0
1
2
3
4
5
6
7
8
9
10
11
Meishan−A
Meishan−B
Meishan−C
European−A
European−B
European−C

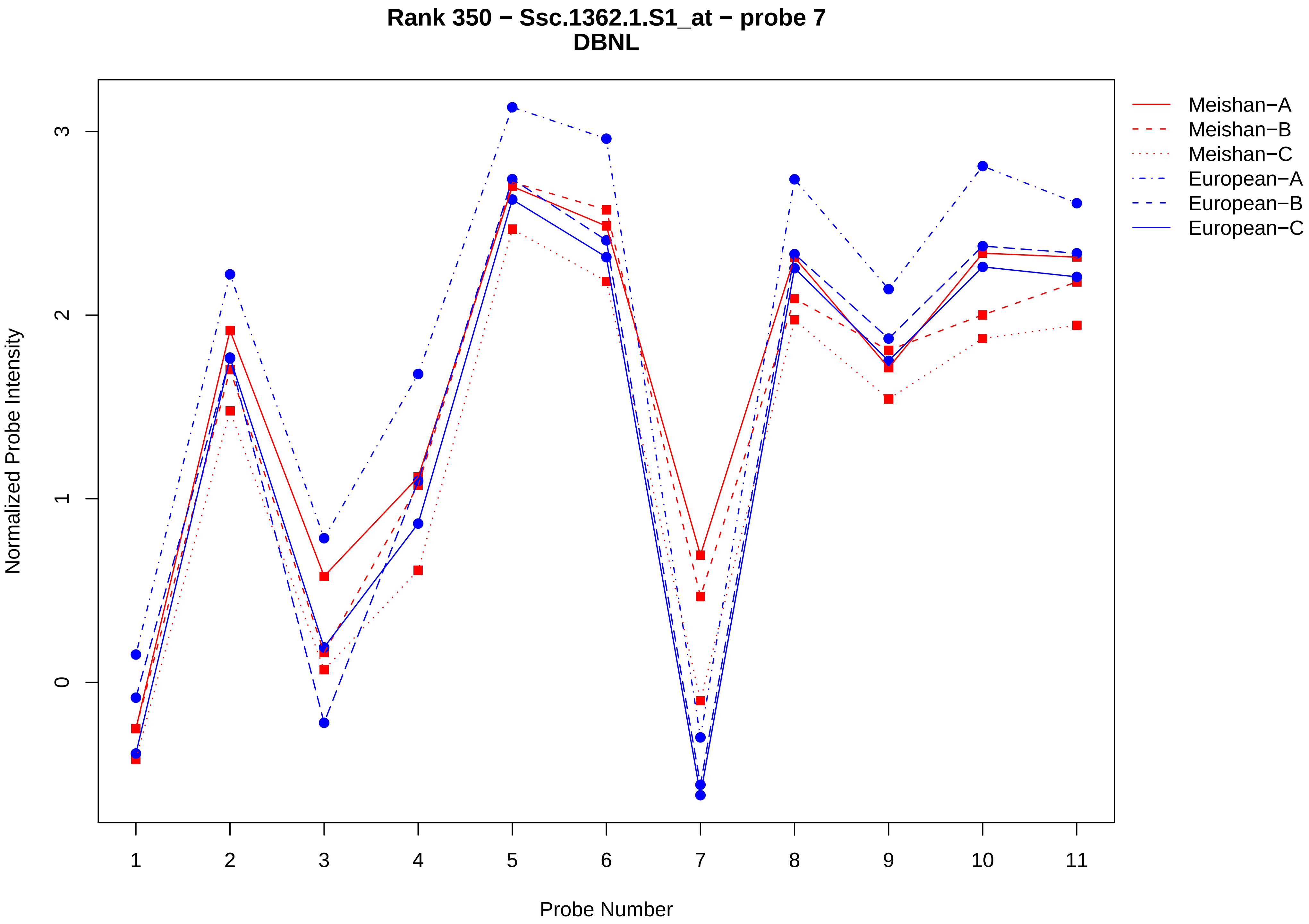

Rank 350 − Ssc.1362.1.S1_at − probe 7
DBNL
Normalized Probe Intensity
Probe Number
0
1
2
3
1 2 3 4 5 6 7 8 9 10 11
Meishan−A
Meishan−B
Meishan−C
European−A
European−B
European−C

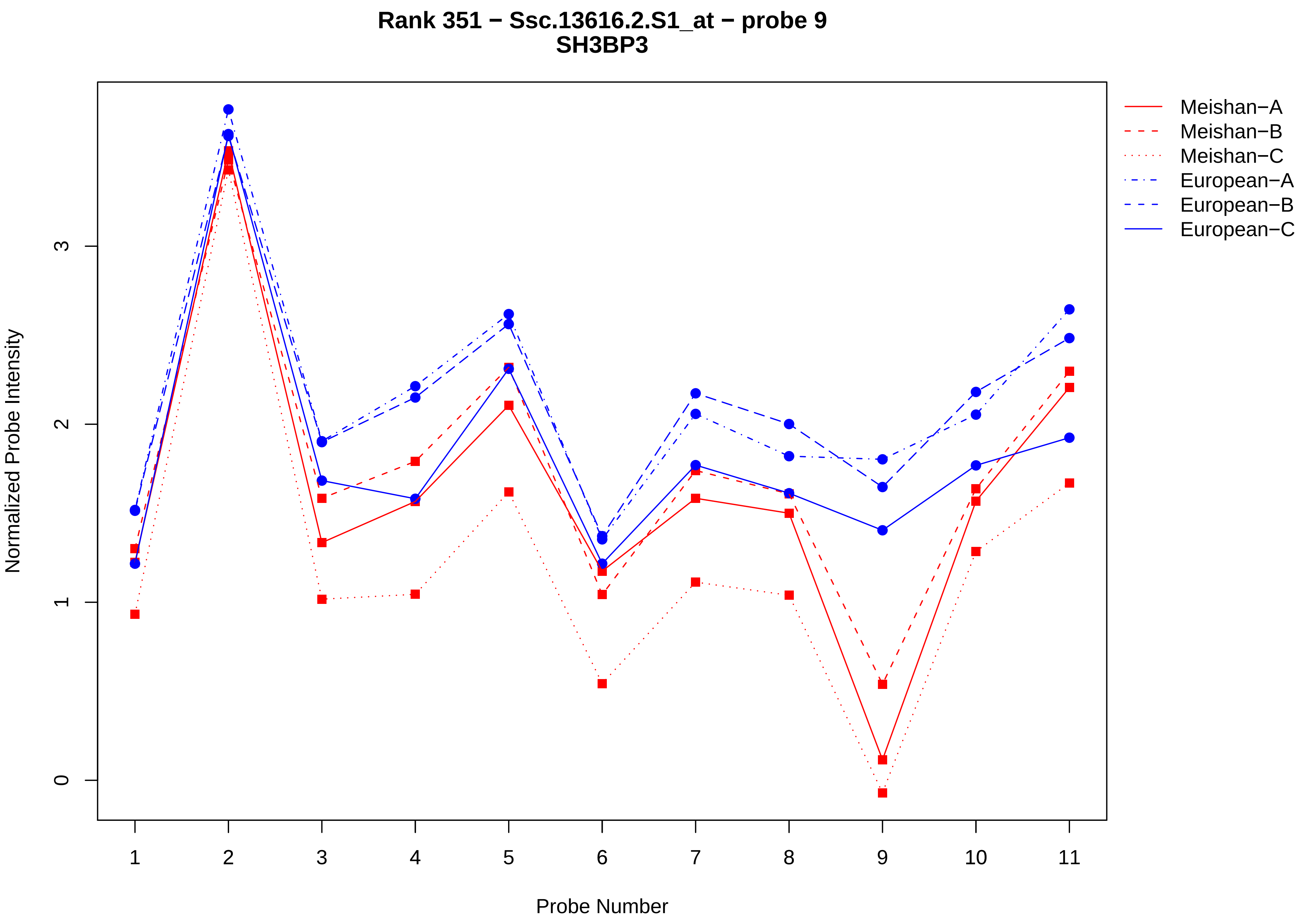
Rank 351 − Ssc.13616.2.S1_at − probe 9
SH3BP3
Normalized Probe Intensity
Probe Number
0
1
2
3
1
2
3
4
5
6
7
8
9
10
11
Meishan−A
Meishan−B
Meishan−C
European−A
European−B
European−C

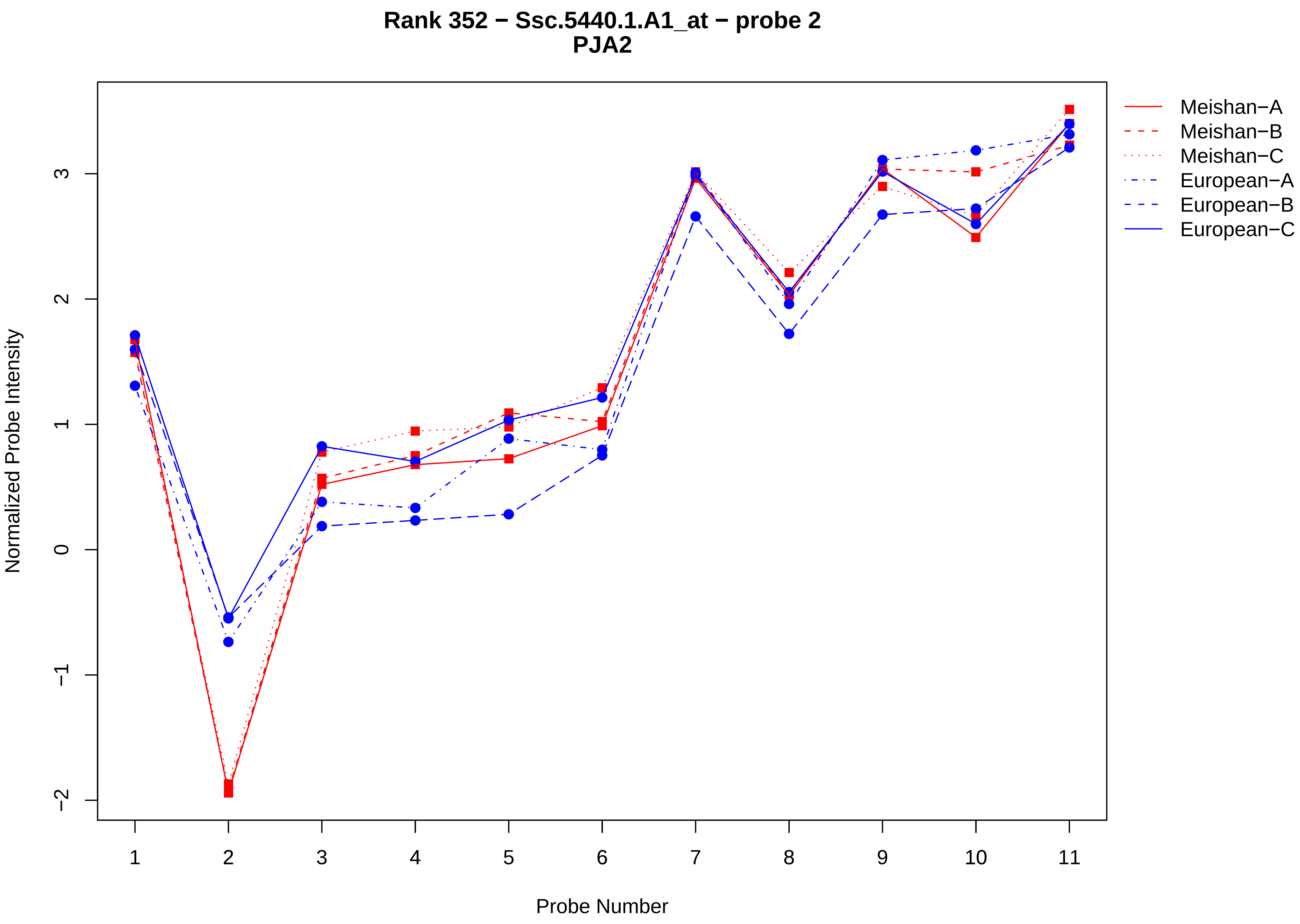

Rank 352 − Ssc.5440.1.A1_at − probe 2
PJA2
Normalized Probe Intensity
Probe Number
Meishan−A
Meishan−B
Meishan−C
European−A
European−B
European−C
−2
−1
0
1
2
3
1
2
3
4
5
6
7
8
9
10
11

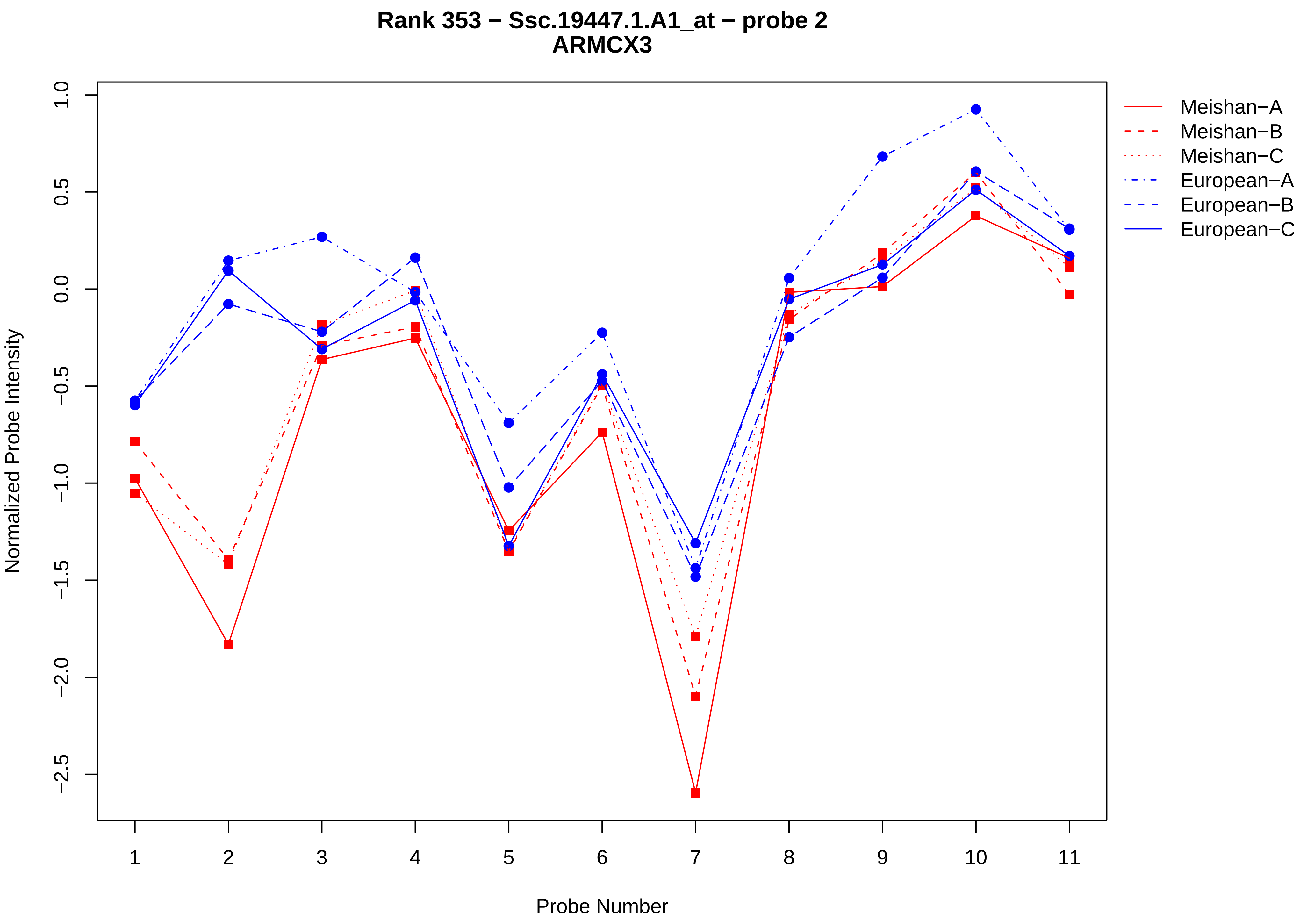

Rank 353 − Ssc.19447.1.A1_at − probe 2
ARMCX3
Normalized Probe Intensity
Probe Number
1.0
0.5
0.0
−0.5
−1.0
−1.5
−2.0
−2.5
1
2
3
4
5
6
7
8
9
10
11
Meishan−A
Meishan−B
Meishan−C
European−A
European−B
European−C

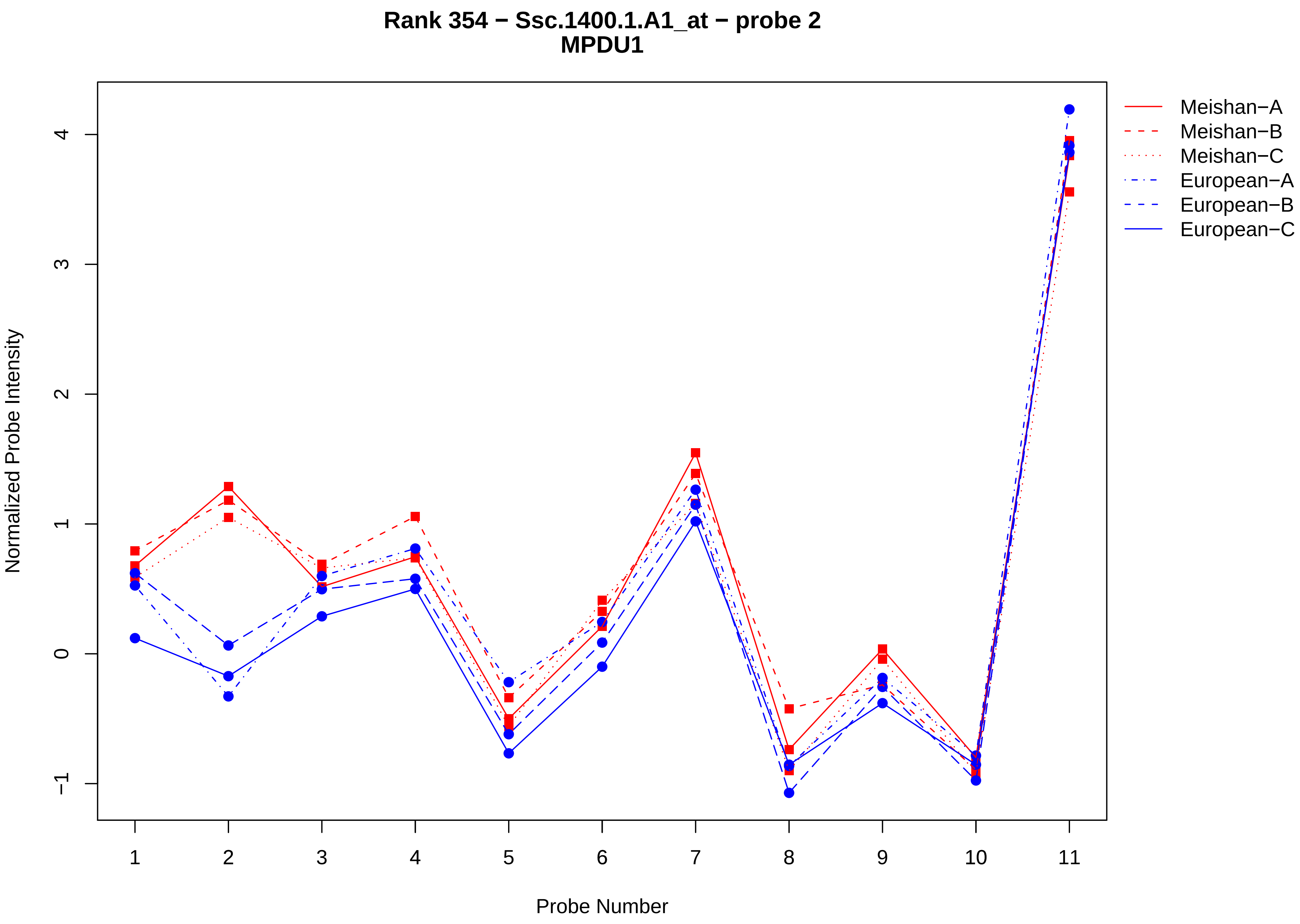

Rank 354 − Ssc.1400.1.A1_at − probe 2
MPDU1
Normalized Probe Intensity
Probe Number
−1
0
1
2
3
4
1
2
3
4
5
6
7
8
9
10
11
Meishan−A
Meishan−B
Meishan−C
European−A
European−B
European−C

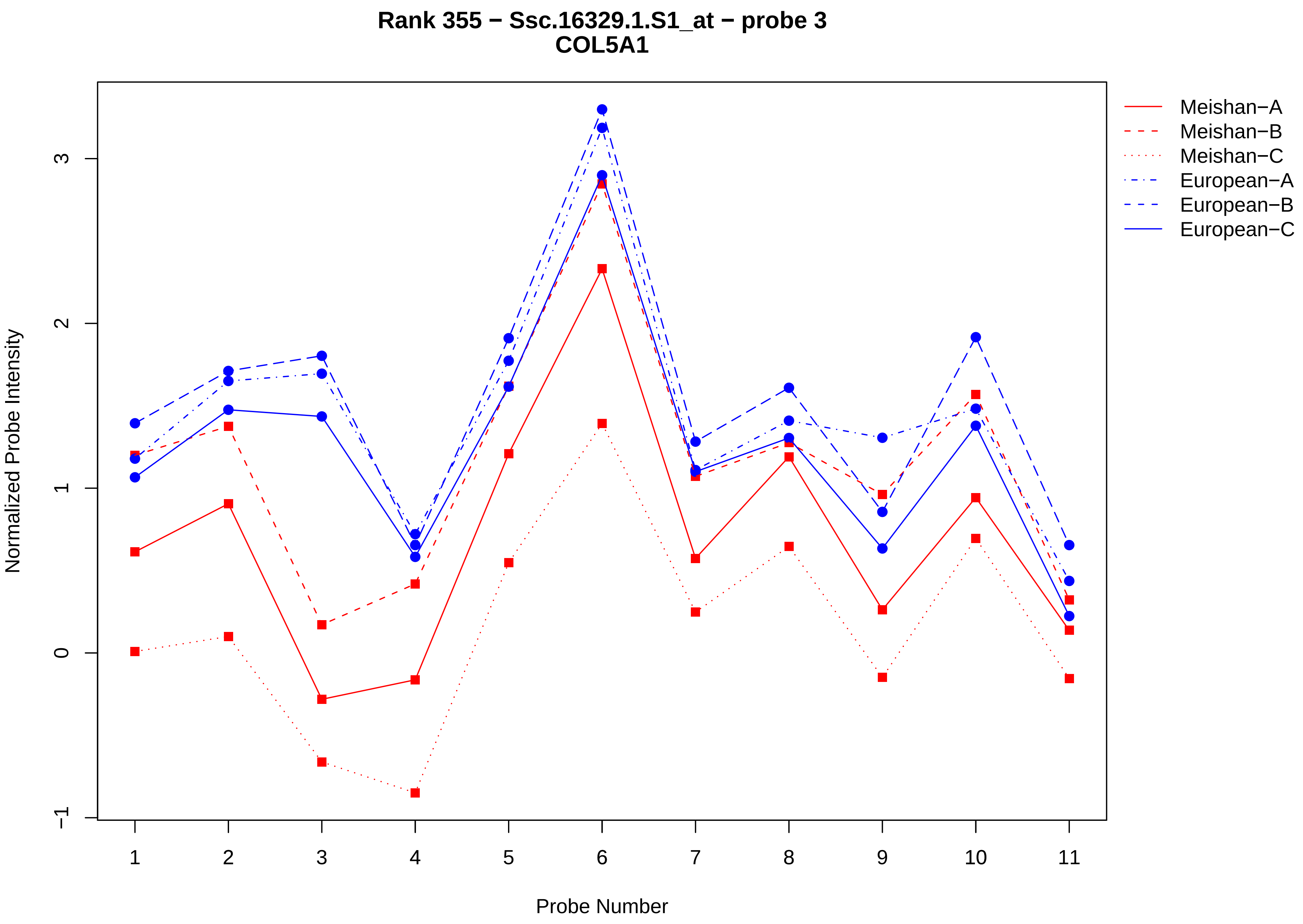
Rank 355 − Ssc.16329.1.S1_at − probe 3
COL5A1
Normalized Probe Intensity
Probe Number
−1
0
1
2
3
1
2
3
4
5
6
7
8
9
10
11
Meishan−A
Meishan−B
Meishan−C
European−A
European−B
European−C

Rank 356 − Ssc.15245.2.S1_a_at − probe 8
NP_057729

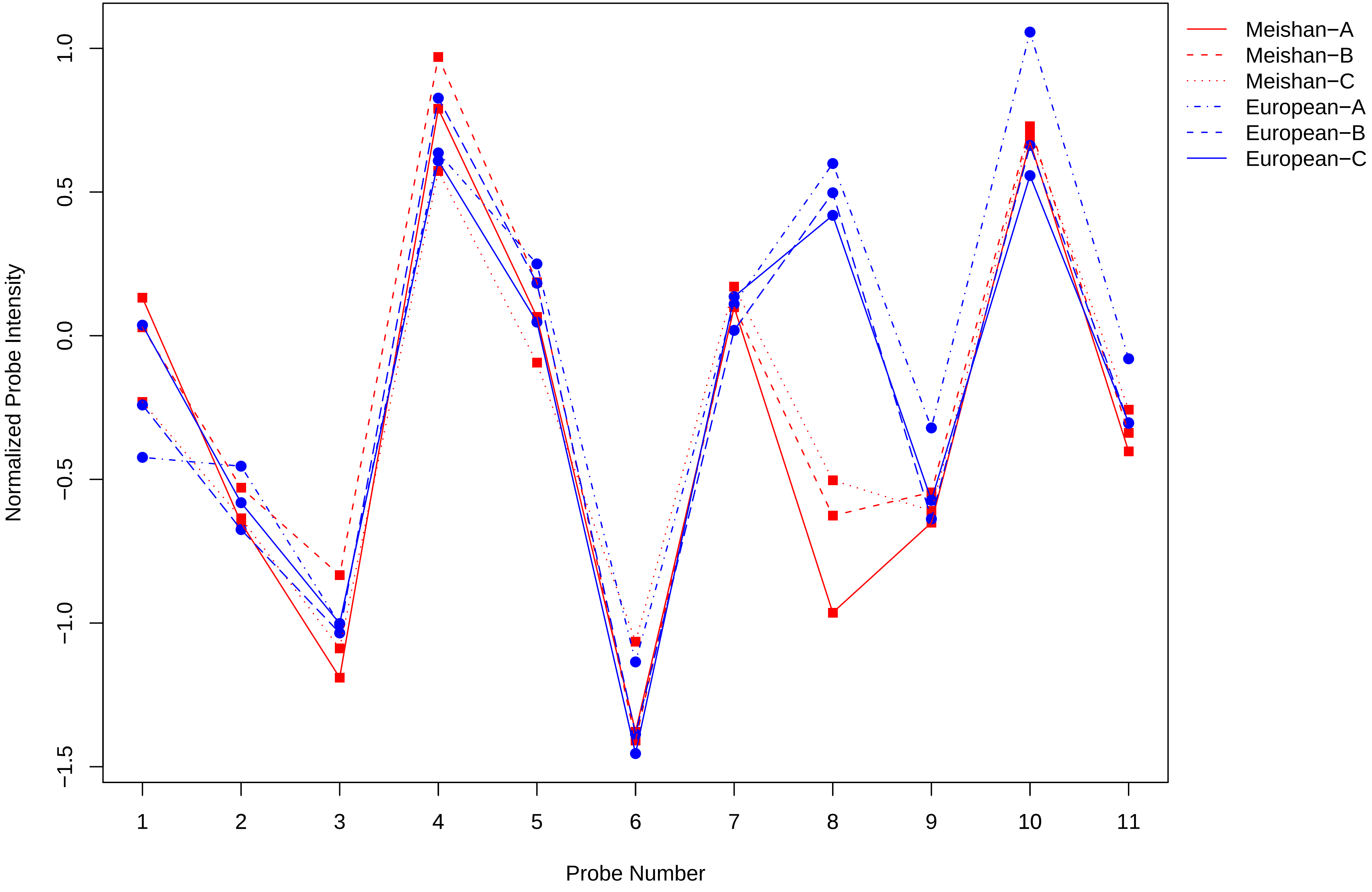

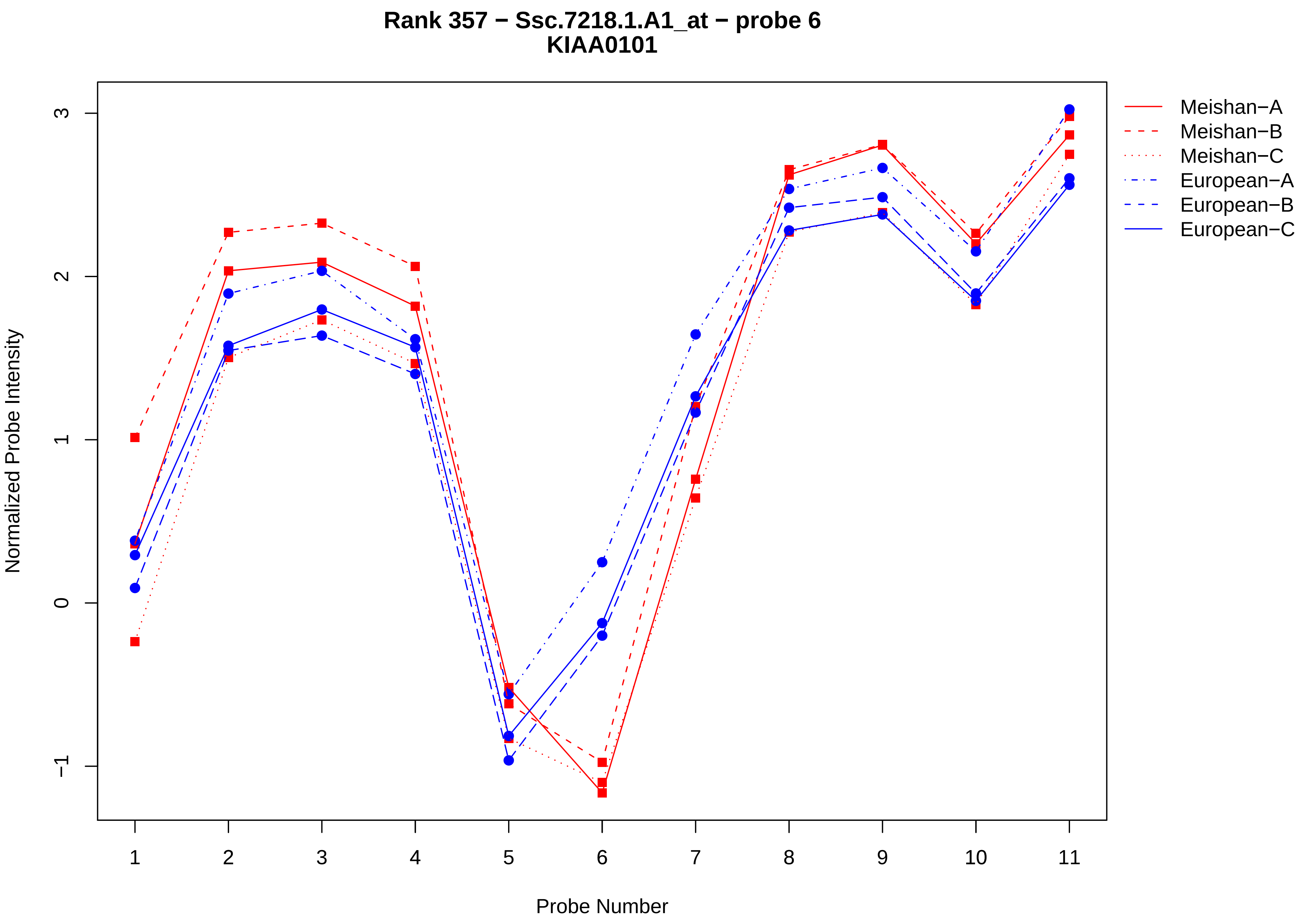
Rank 357 − Ssc.7218.1.A1_at − probe 6
KIAA0101
Normalized Probe Intensity
Probe Number
−1
0
1
2
3
1
2
3
4
5
6
7
8
9
10
11
Meishan−A
Meishan−B
Meishan−C
European−A
European−B
European−C

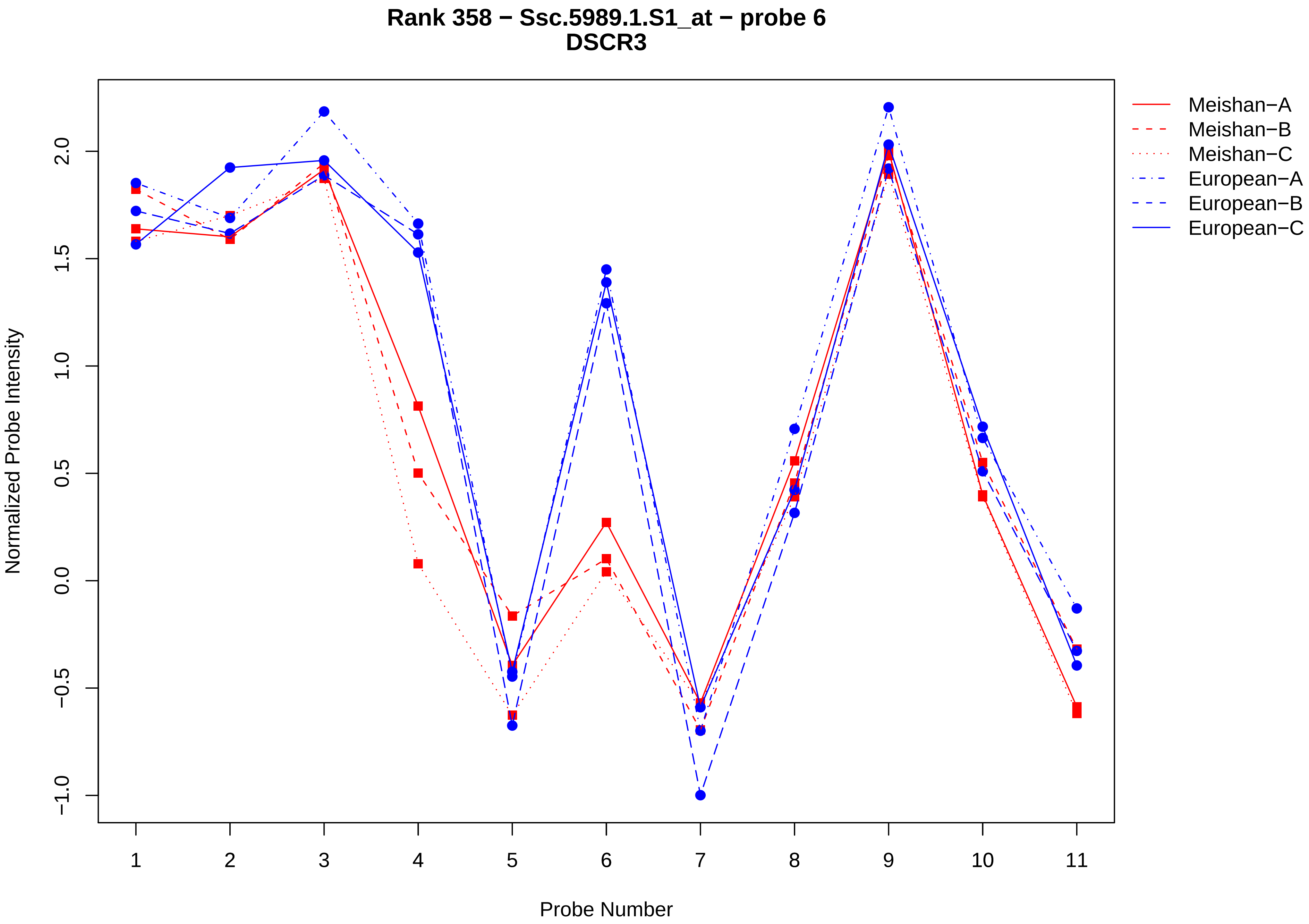

Rank 358 − Ssc.5989.1.S1_at − probe 6
DSCR3
Normalized Probe Intensity
Probe Number
−1.0
−0.5
0.0
0.5
1.0
1.5
2.0
1
2
3
4
5
6
7
8
9
10
11
Meishan−A
Meishan−B
Meishan−C
European−A
European−B
European−C

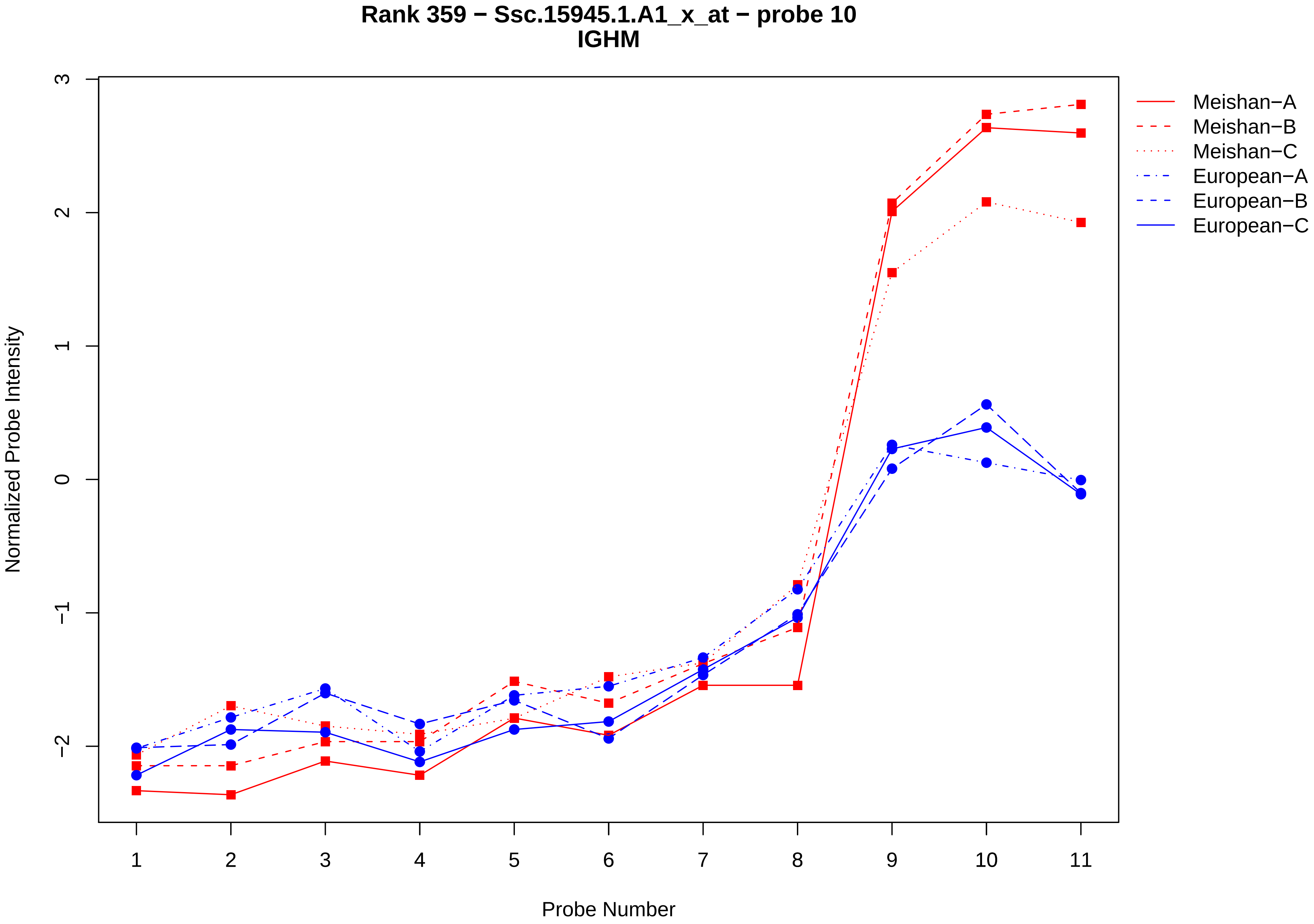
Rank 359 − Ssc.15945.1.A1_x_at − probe 10
IGHM
Normalized Probe Intensity
Probe Number
Meishan−A
Meishan−B
Meishan−C
European−A
European−B
European−C
−2
−1
0
1
2
3
1
2
3
4
5
6
7
8
9
10
11

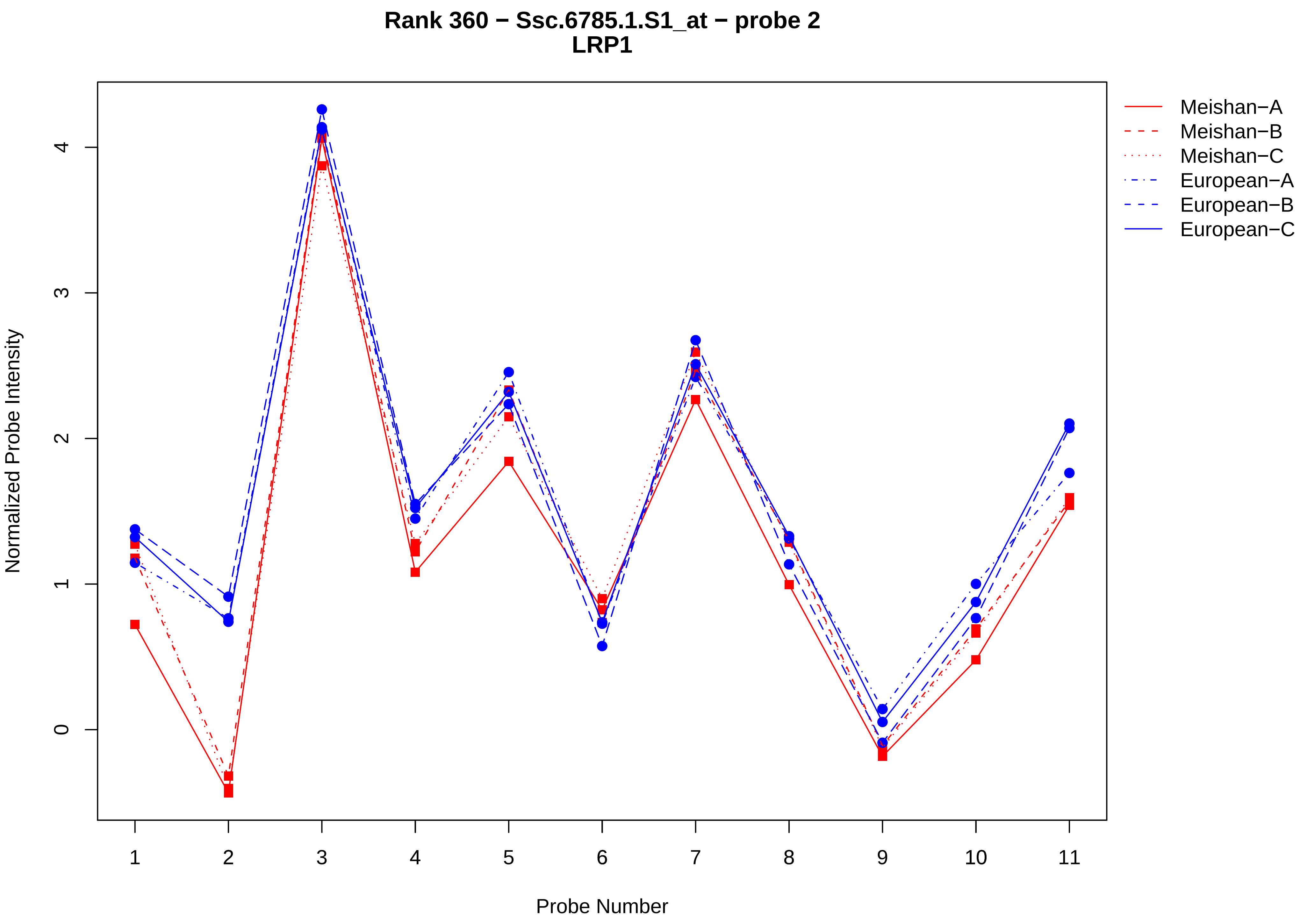

Rank 360 − Ssc.6785.1.S1_at − probe 2
LRP1
Normalized Probe Intensity
Probe Number
0
1
2
3
4
1
2
3
4
5
6
7
8
9
10
11
Meishan−A
Meishan−B
Meishan−C
European−A
European−B
European−C

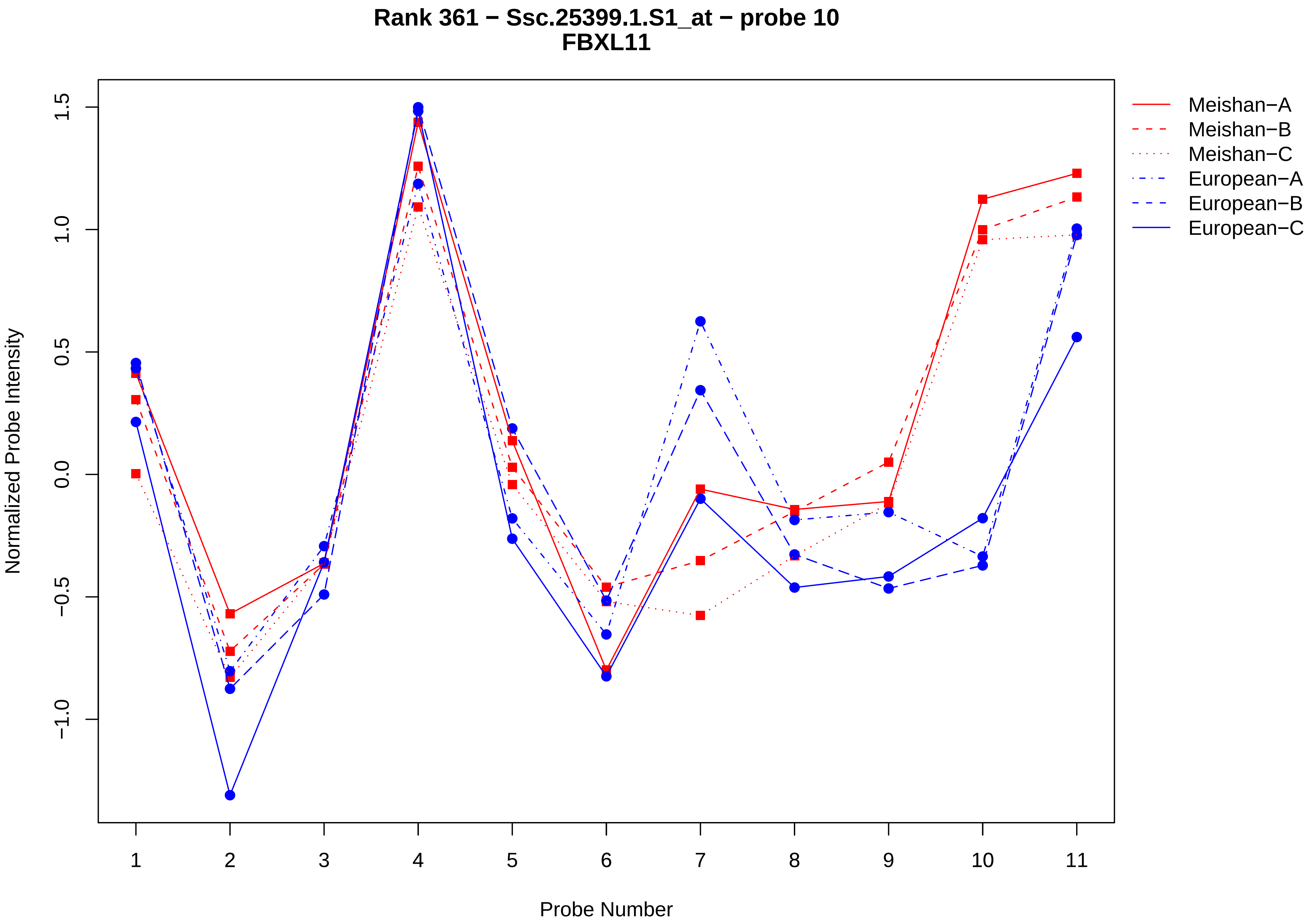

Rank 361 − Ssc.25399.1.S1_at − probe 10
FBXL11
Normalized Probe Intensity
Probe Number
−1.0
−0.5
0.0
0.5
1.0
1.5
1
2
3
4
5
6
7
8
9
10
11
Meishan−A
Meishan−B
Meishan−C
European−A
European−B
European−C

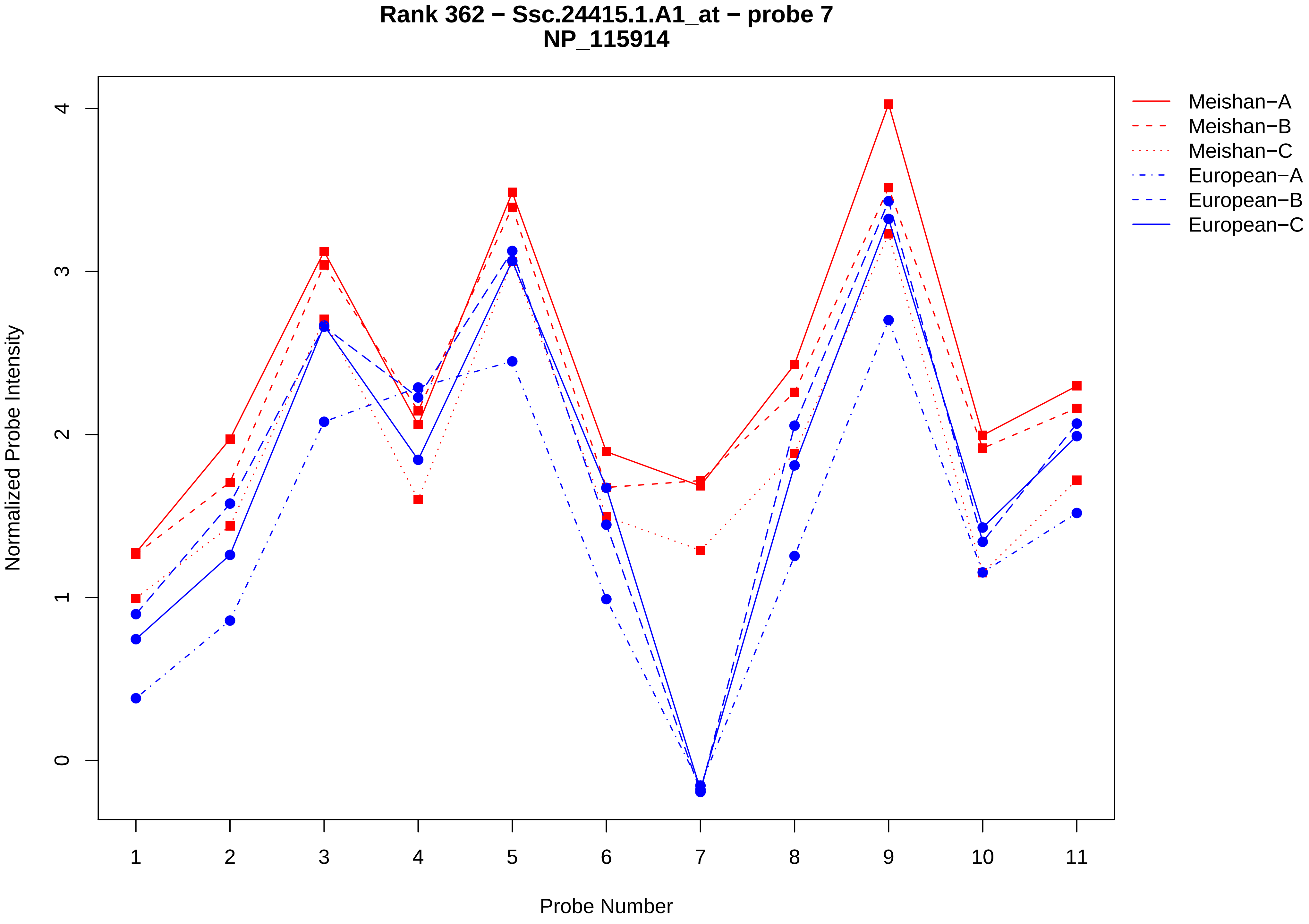

Rank 362 − Ssc.24415.1.A1_at − probe 7
NP_115914
Normalized Probe Intensity
Probe Number
0
1
2
3
4
1
2
3
4
5
6
7
8
9
10
11
Meishan−A
Meishan−B
Meishan−C
European−A
European−B
European−C

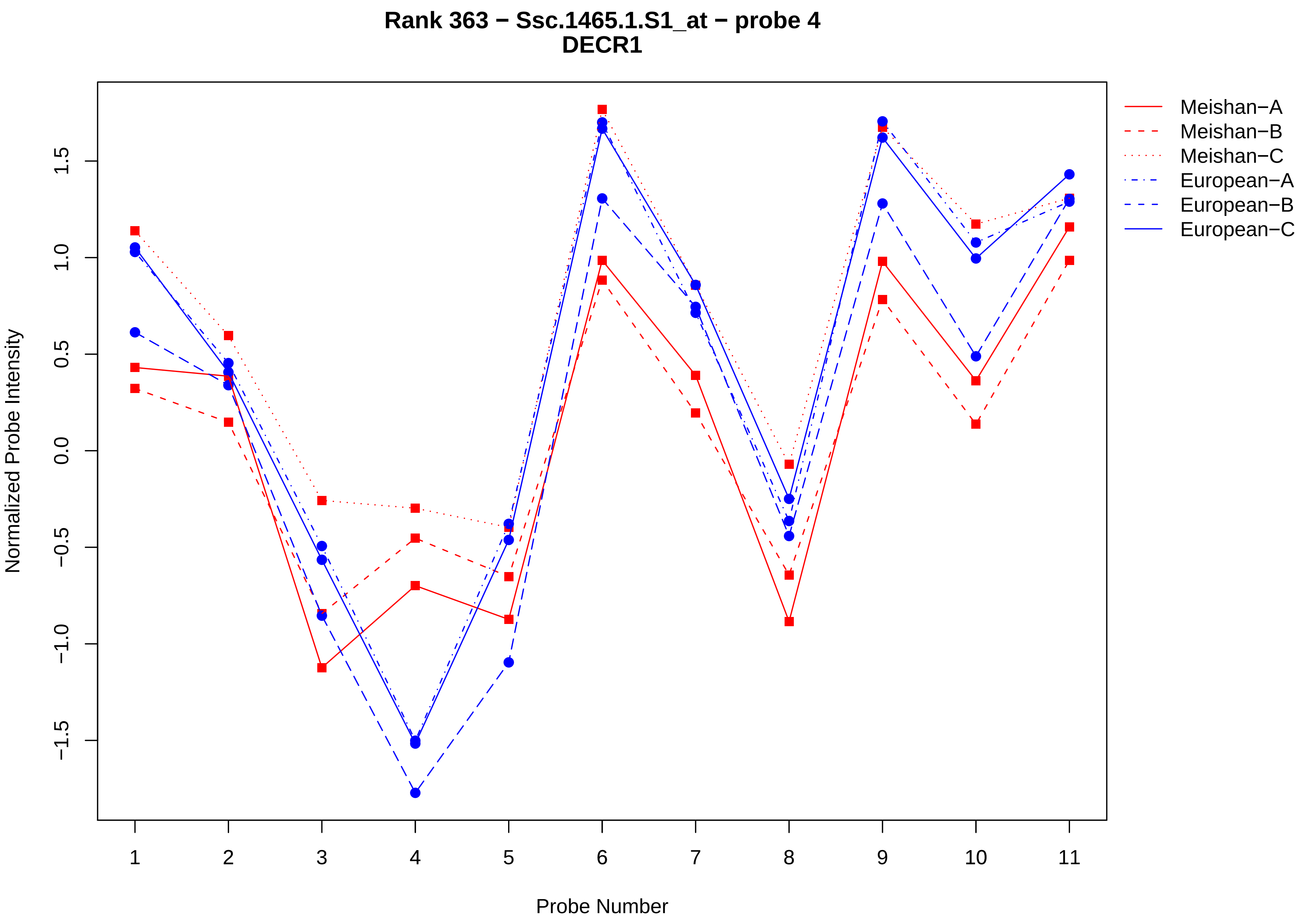

Rank 363 − Ssc.1465.1.S1_at − probe 4
DECR1
Normalized Probe Intensity
Probe Number
−1.5
−1.0
−0.5
0.0
0.5
1.0
1.5
1
2
3
4
5
6
7
8
9
10
11
Meishan−A
Meishan−B
Meishan−C
European−A
European−B
European−C

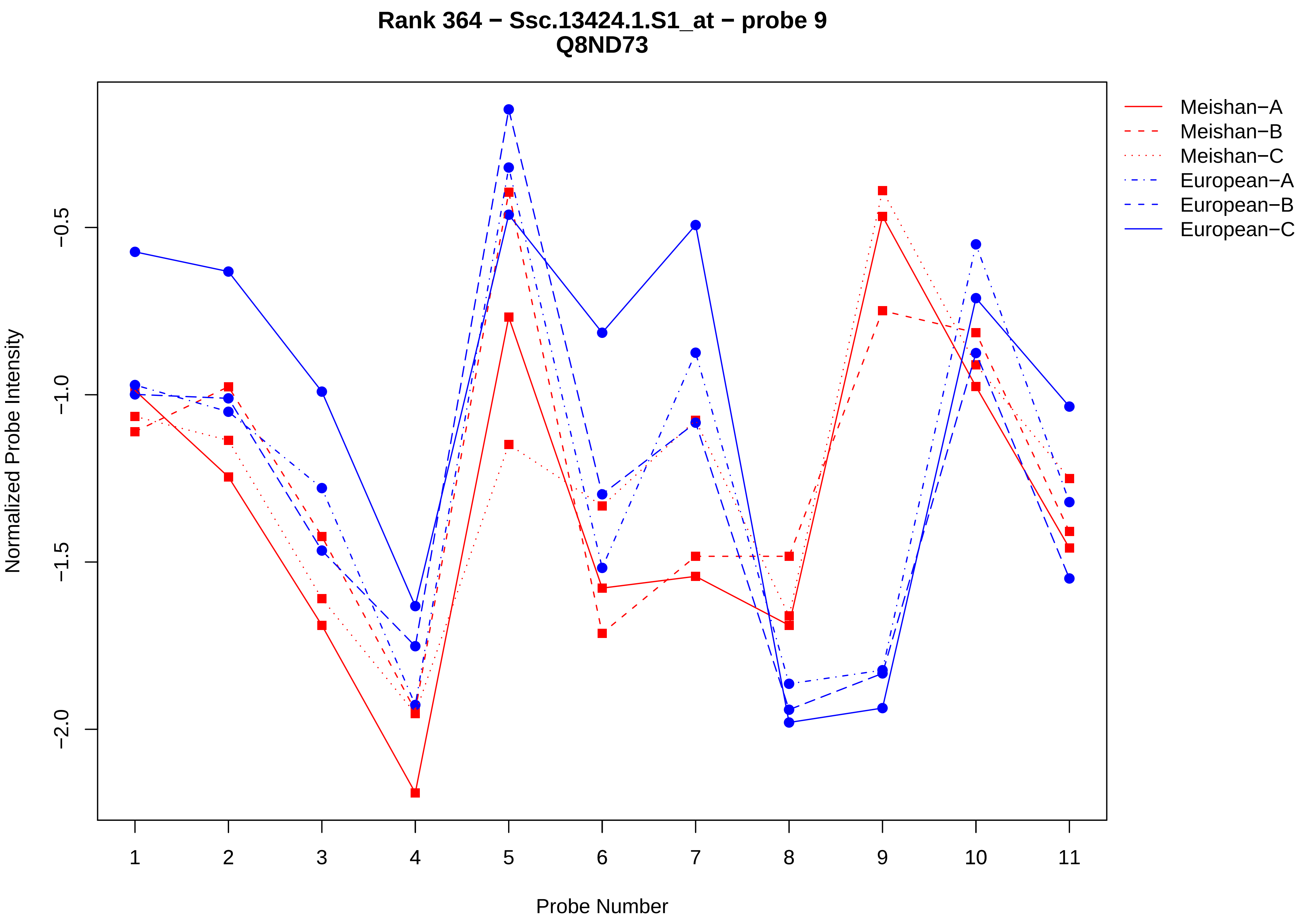

Rank 364 − Ssc.13424.1.S1_at − probe 9
Q8ND73
Normalized Probe Intensity
Probe Number
−0.5
−1.0
−1.5
−2.0
1
2
3
4
5
6
7
8
9
10
11
Meishan−A
Meishan−B
Meishan−C
European−A
European−B
European−C

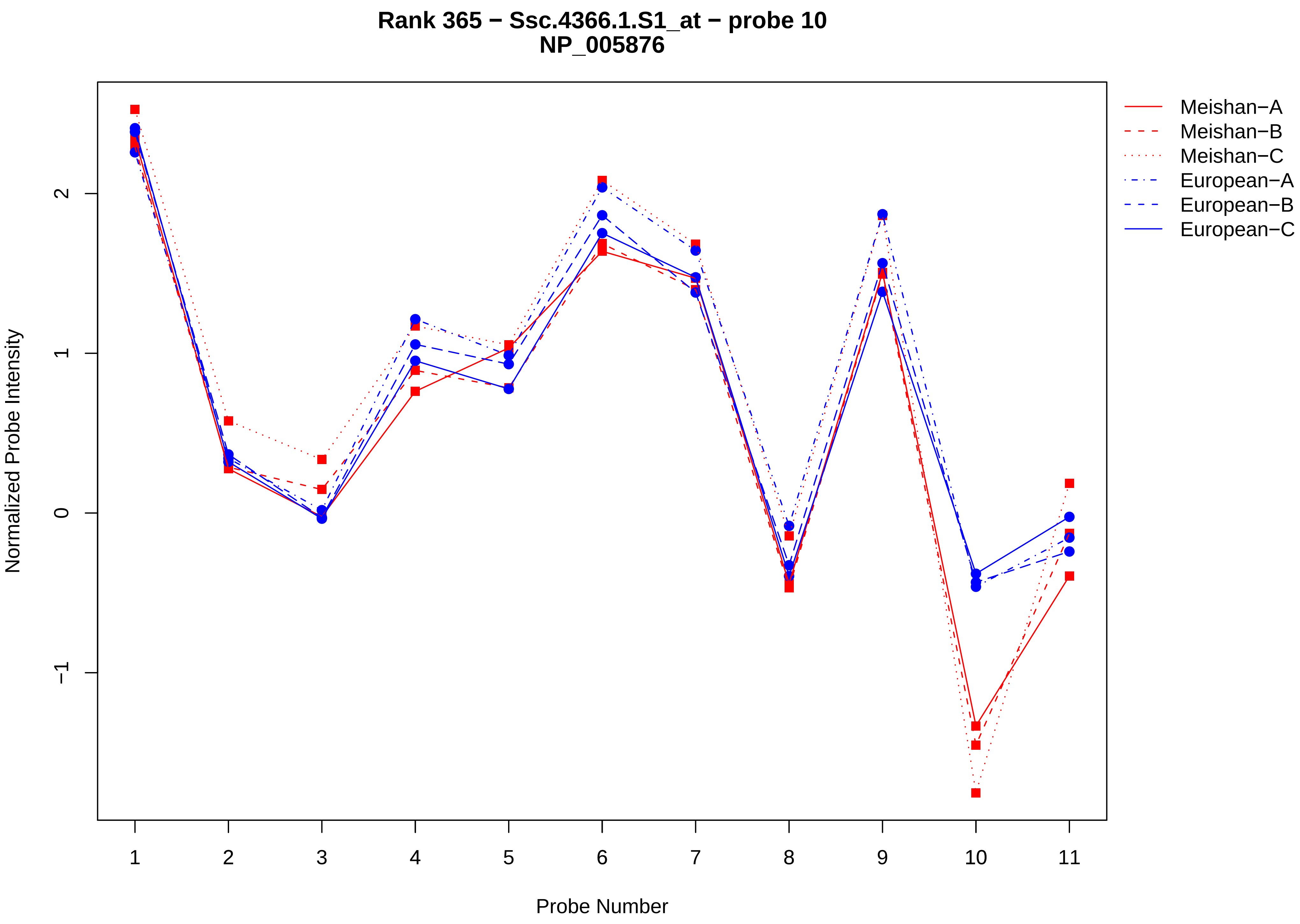

Rank 365 − Ssc.4366.1.S1_at − probe 10
NP_005876
Normalized Probe Intensity
Probe Number
−1
0
1
2
1
2
3
4
5
6
7
8
9
10
11
Meishan−A
Meishan−B
Meishan−C
European−A
European−B
European−C

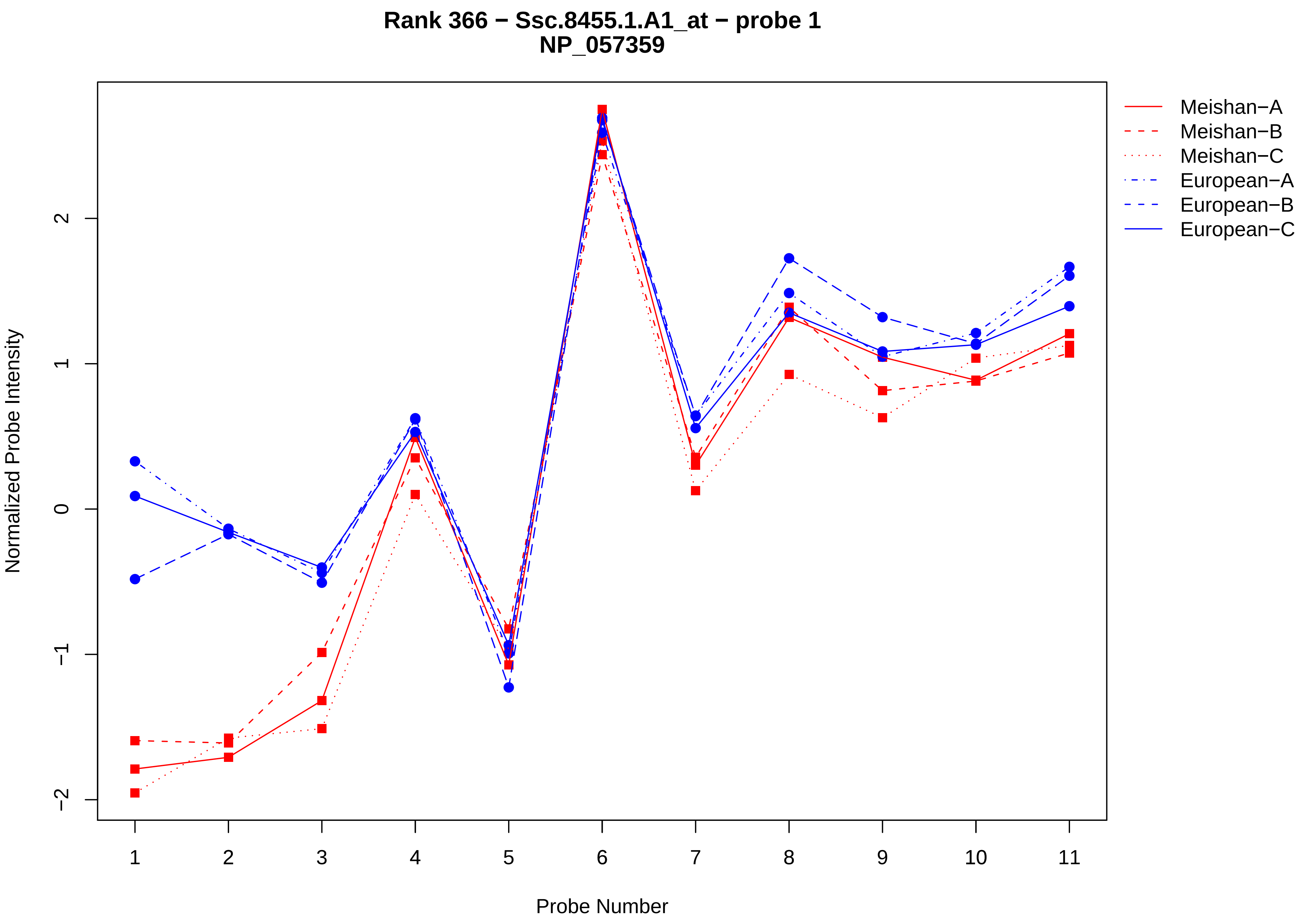

Rank 366 − Ssc.8455.1.A1_at − probe 1
NP_057359
Normalized Probe Intensity
Probe Number
−2
−1
0
1
2
1
2
3
4
5
6
7
8
9
10
11
Meishan−A
Meishan−B
Meishan−C
European−A
European−B
European−C

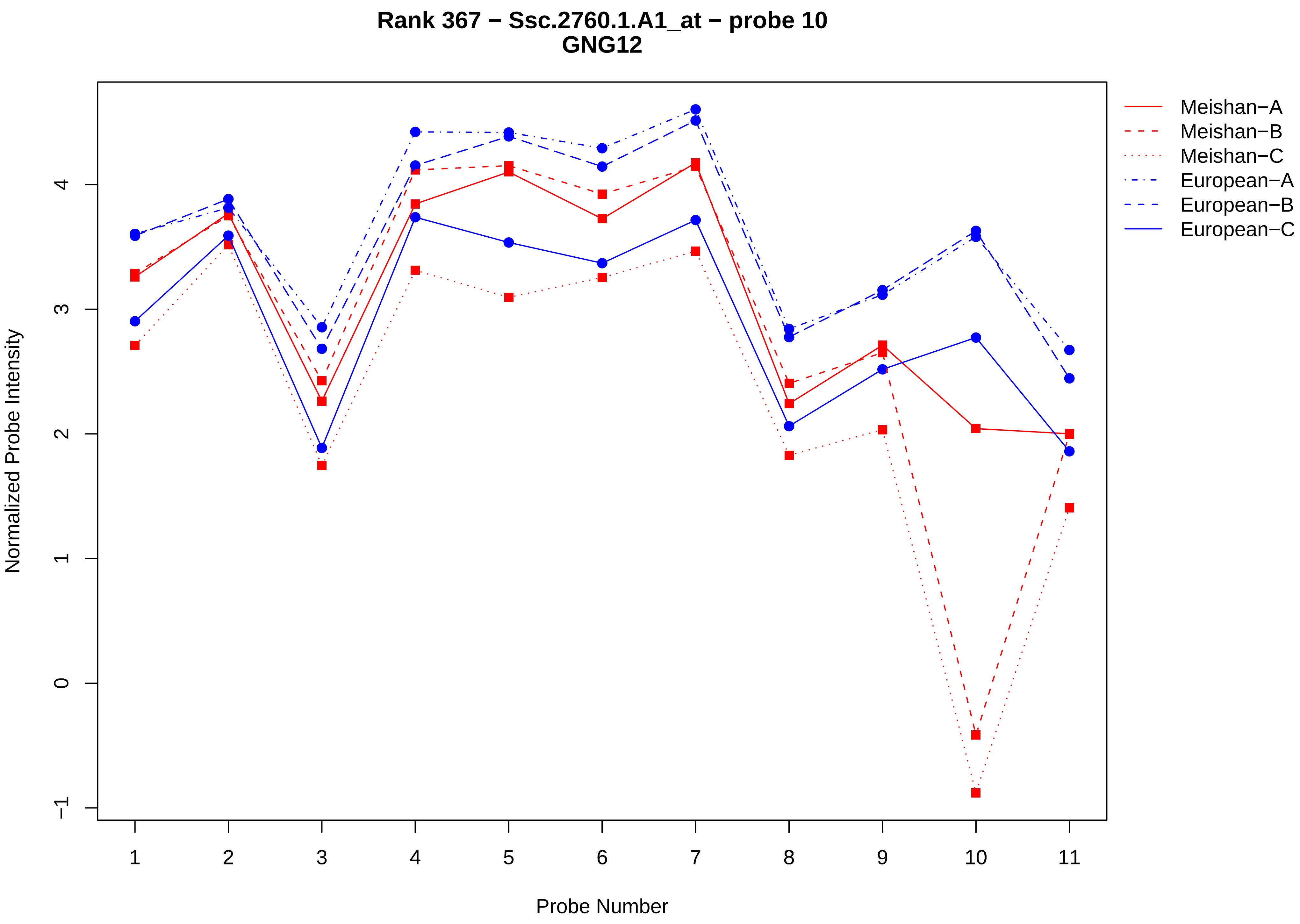
Rank 367 − Ssc.2760.1.A1_at − probe 10
GNG12
Normalized Probe Intensity
Probe Number
−1
0
1
2
3
4
1
2
3
4
5
6
7
8
9
10
11
Meishan−A
Meishan−B
Meishan−C
European−A
European−B
European−C

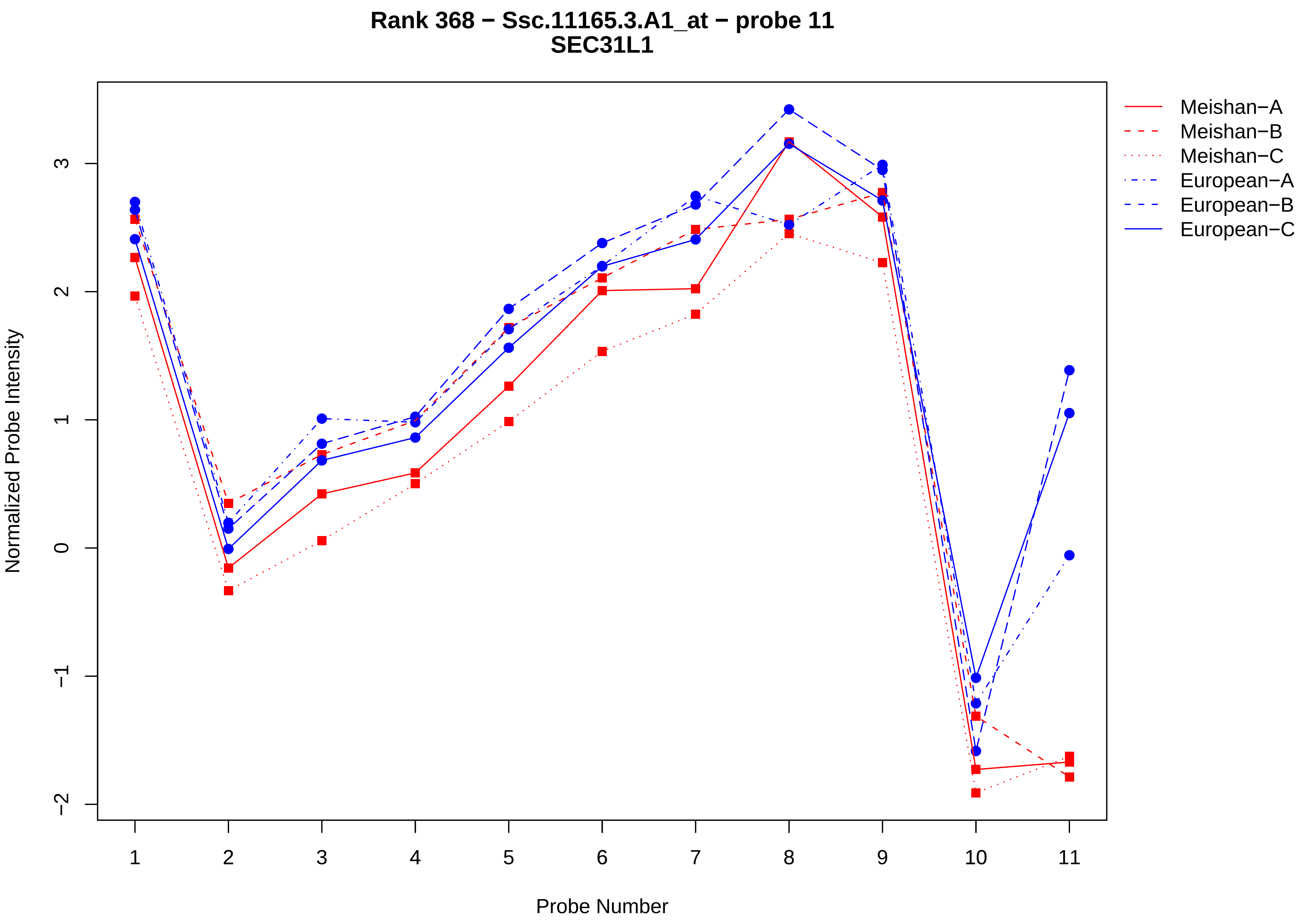

Rank 368 − Ssc.11165.3.A1_at − probe 11
SEC31L1
Normalized Probe Intensity
Probe Number
−2
−1
0
1
2
3
1
2
3
4
5
6
7
8
9
10
11
Meishan−A
Meishan−B
Meishan−C
European−A
European−B
European−C

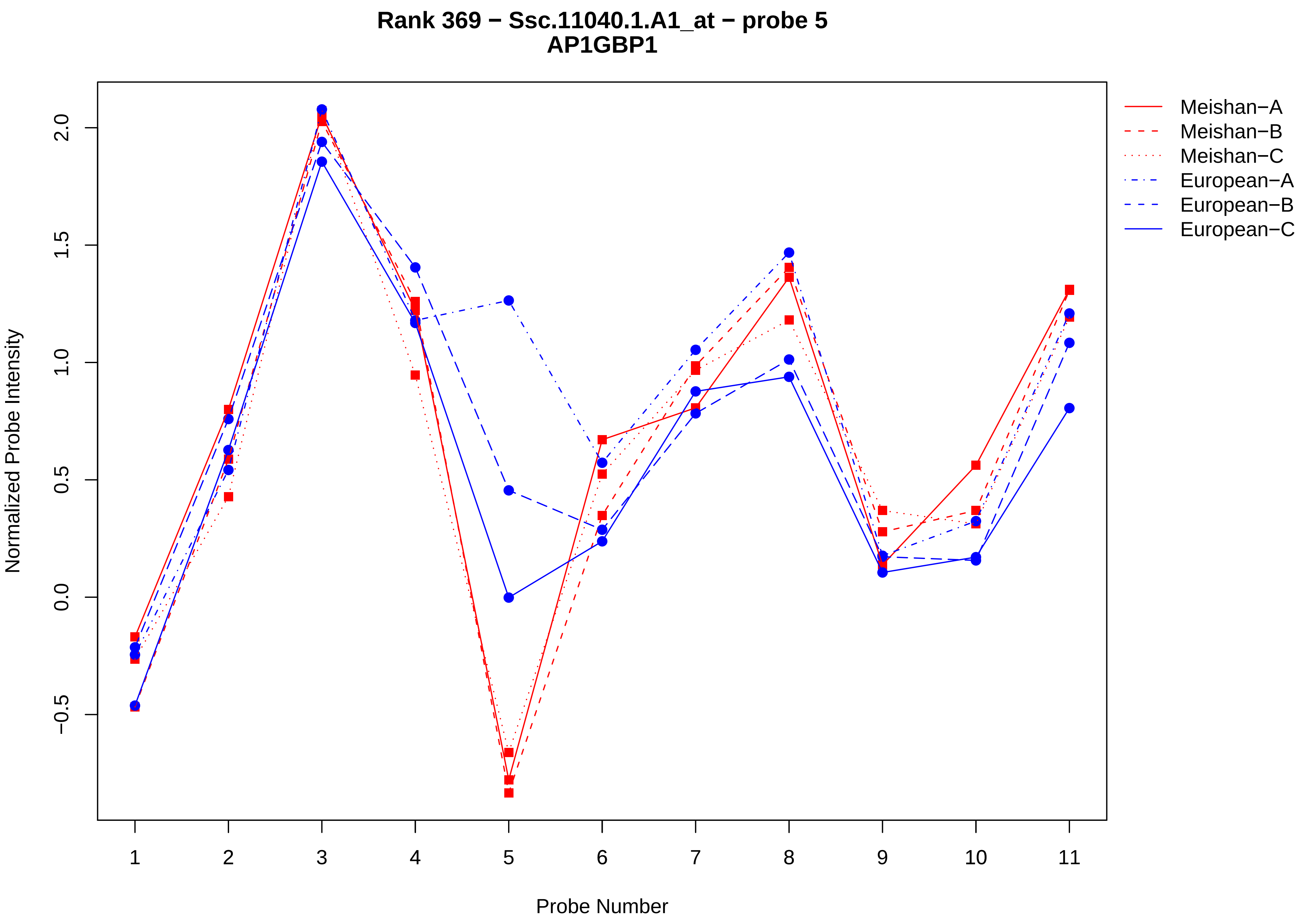

Rank 369 − Ssc.11040.1.A1_at − probe 5
AP1GBP1
Normalized Probe Intensity
Probe Number
−0.5
0.0
0.5
1.0
1.5
2.0
1
2
3
4
5
6
7
8
9
10
11
Meishan−A
Meishan−B
Meishan−C
European−A
European−B
European−C

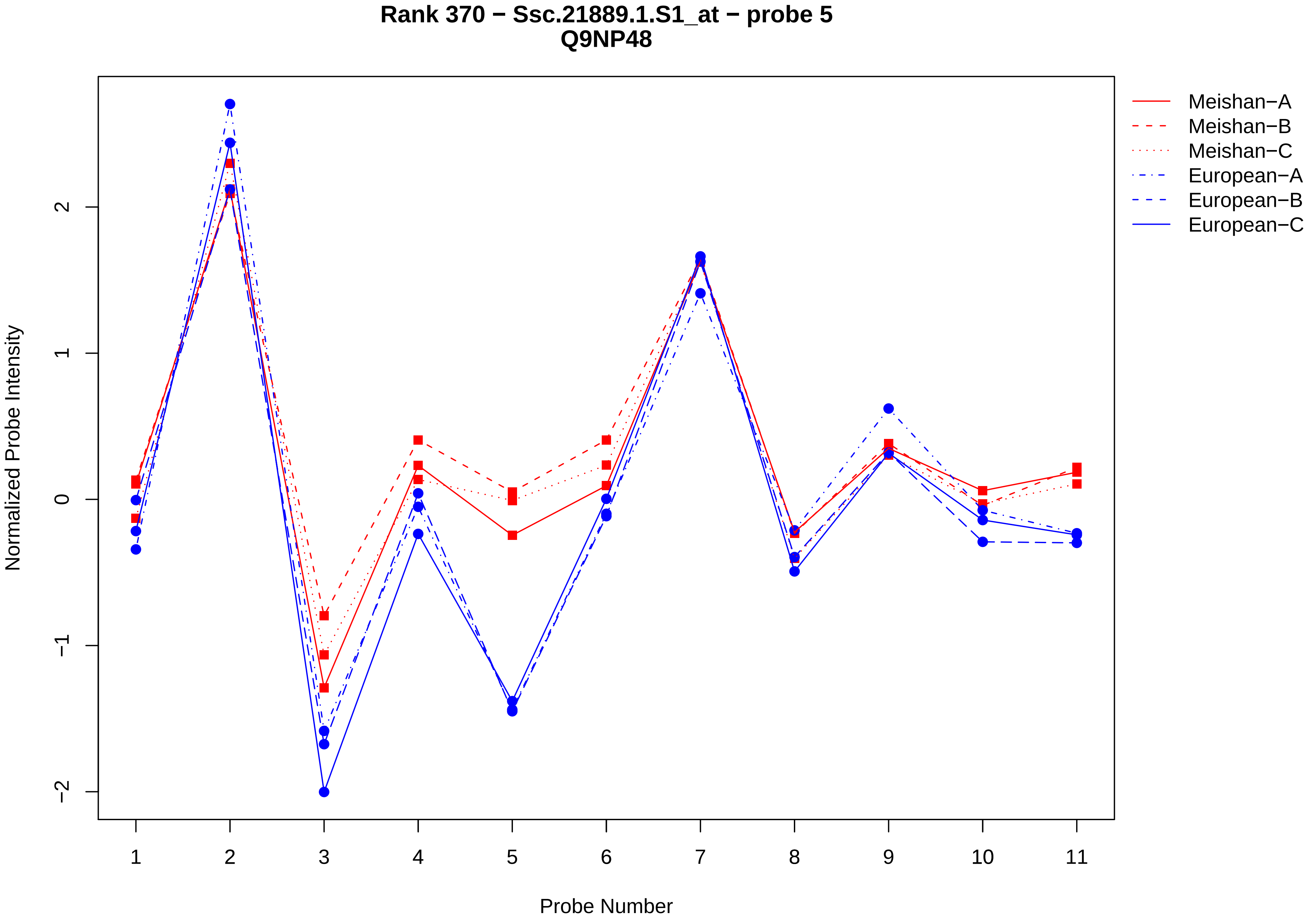

Rank 370 − Ssc.21889.1.S1_at − probe 5
Q9NP48
Normalized Probe Intensity
Probe Number
−2
−1
0
1
2
1
2
3
4
5
6
7
8
9
10
11
Meishan−A
Meishan−B
Meishan−C
European−A
European−B
European−C

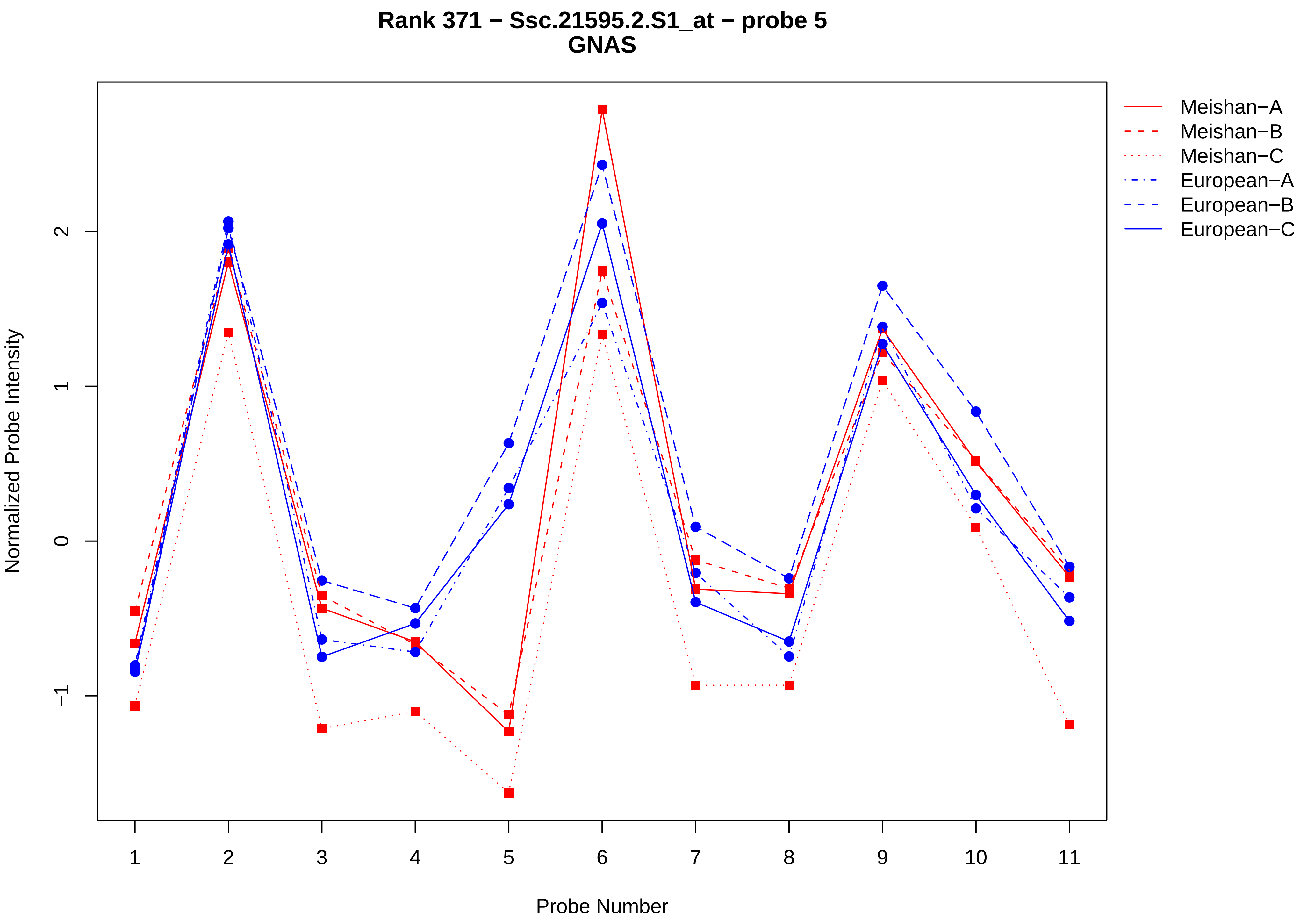

Rank 371 − Ssc.21595.2.S1_at − probe 5
GNAS
Normalized Probe Intensity
Probe Number
−1
0
1
2
1
2
3
4
5
6
7
8
9
10
11
Meishan−A
Meishan−B
Meishan−C
European−A
European−B
European−C

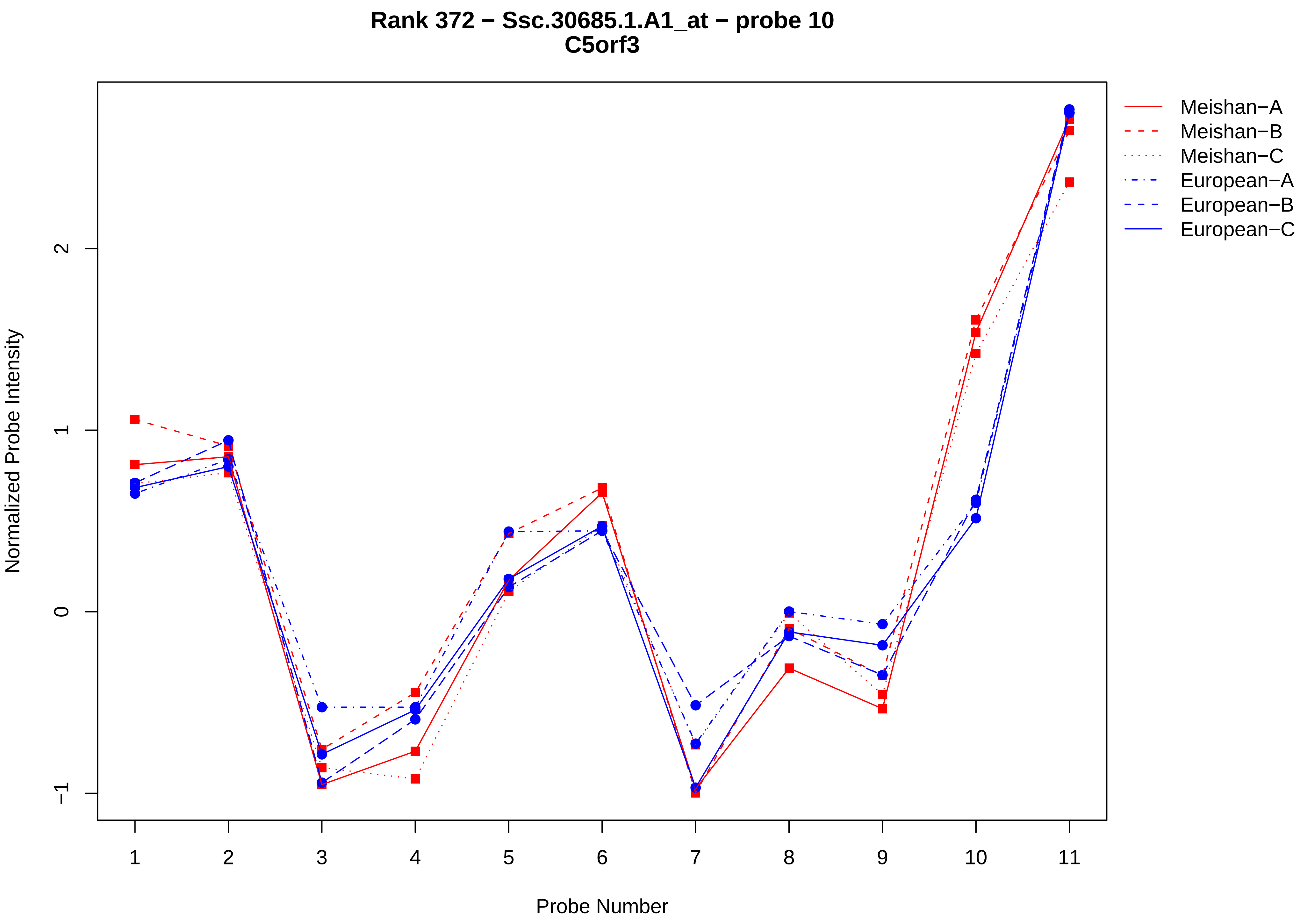

Rank 372 − Ssc.30685.1.A1_at − probe 10
C5orf3
Normalized Probe Intensity
Probe Number
−1
0
1
2
1
2
3
4
5
6
7
8
9
10
11
Meishan−A
Meishan−B
Meishan−C
European−A
European−B
European−C

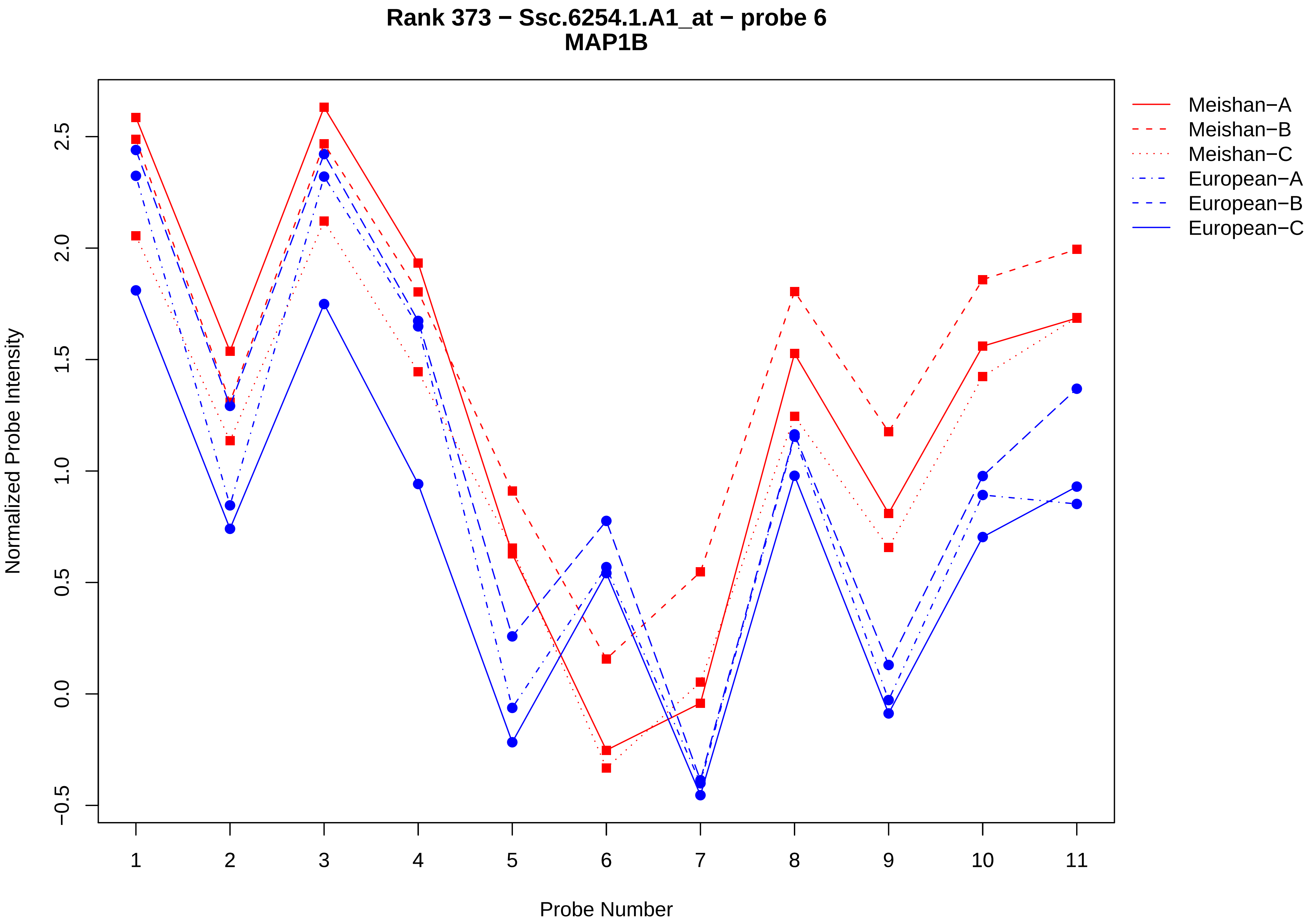
Rank 373 − Ssc.6254.1.A1_at − probe 6
MAP1B
Normalized Probe Intensity
Probe Number
−0.5
0.0
0.5
1.0
1.5
2.0
2.5
1
2
3
4
5
6
7
8
9
10
11
Meishan−A
Meishan−B
Meishan−C
European−A
European−B
European−C

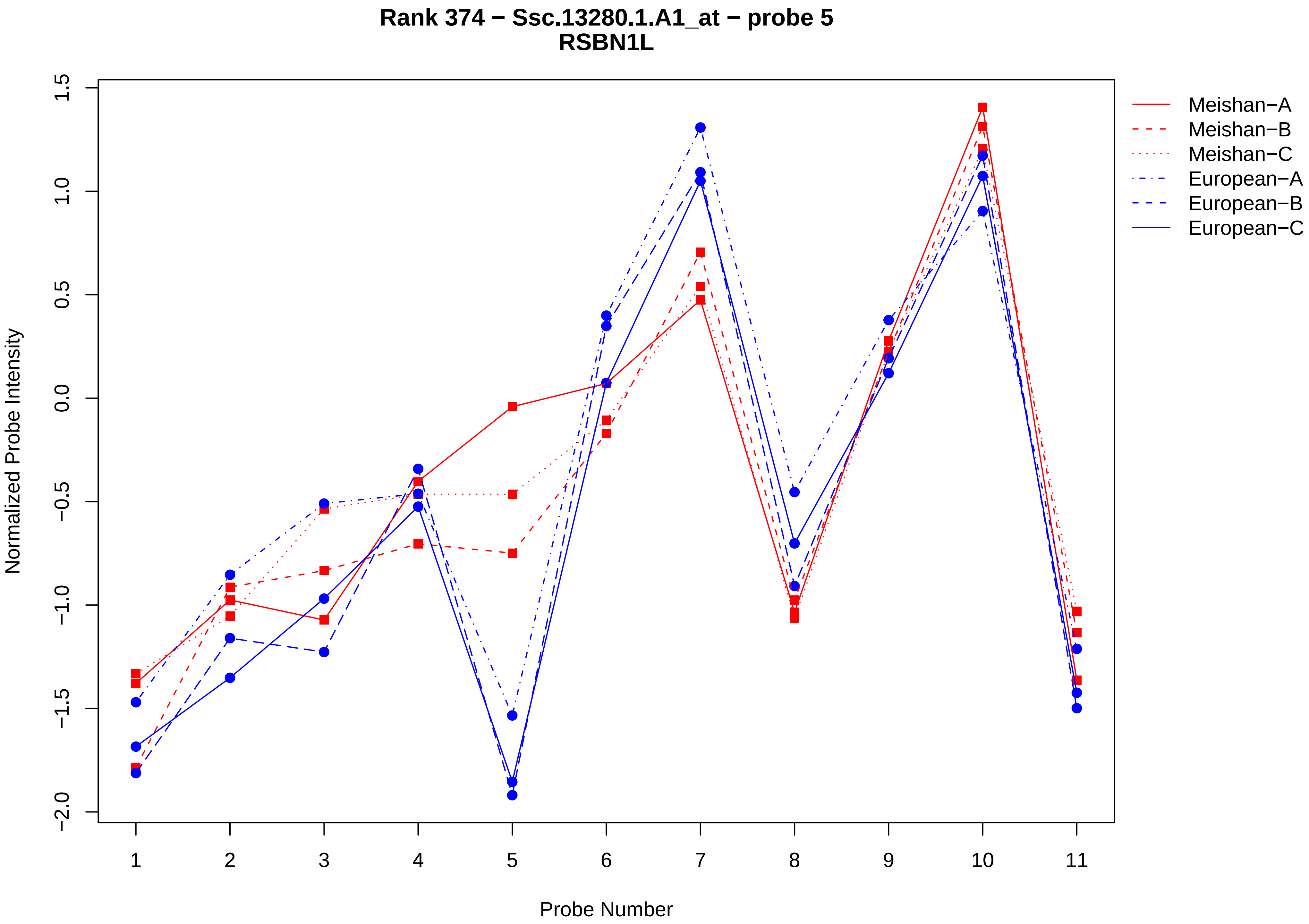

Rank 374 − Ssc.13280.1.A1_at − probe 5
RSBN1L
Normalized Probe Intensity
Probe Number
1.5
1.0
0.5
0.0
−0.5
−1.0
−1.5
−2.0
1
2
3
4
5
6
7
8
9
10
11
Meishan−A
Meishan−B
Meishan−C
European−A
European−B
European−C

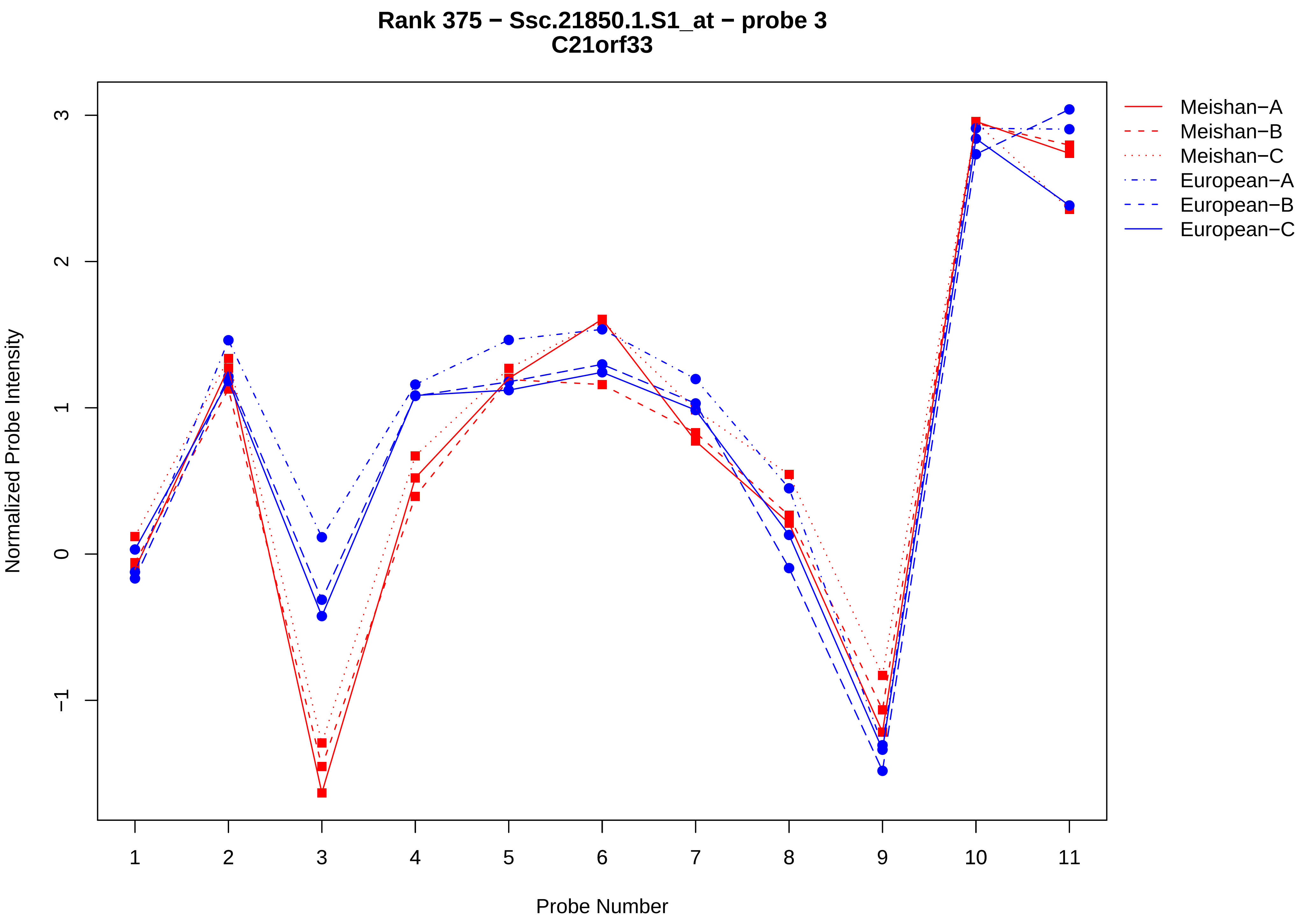
Rank 375 − Ssc.21850.1.S1_at − probe 3
C21orf33
Normalized Probe Intensity
Probe Number
Meishan−A
Meishan−B
Meishan−C
European−A
European−B
European−C
−1
0
1
2
3
1
2
3
4
5
6
7
8
9
10
11

Rank 376 − Ssc.19636.1.S1_at − probe 2
TBL1X

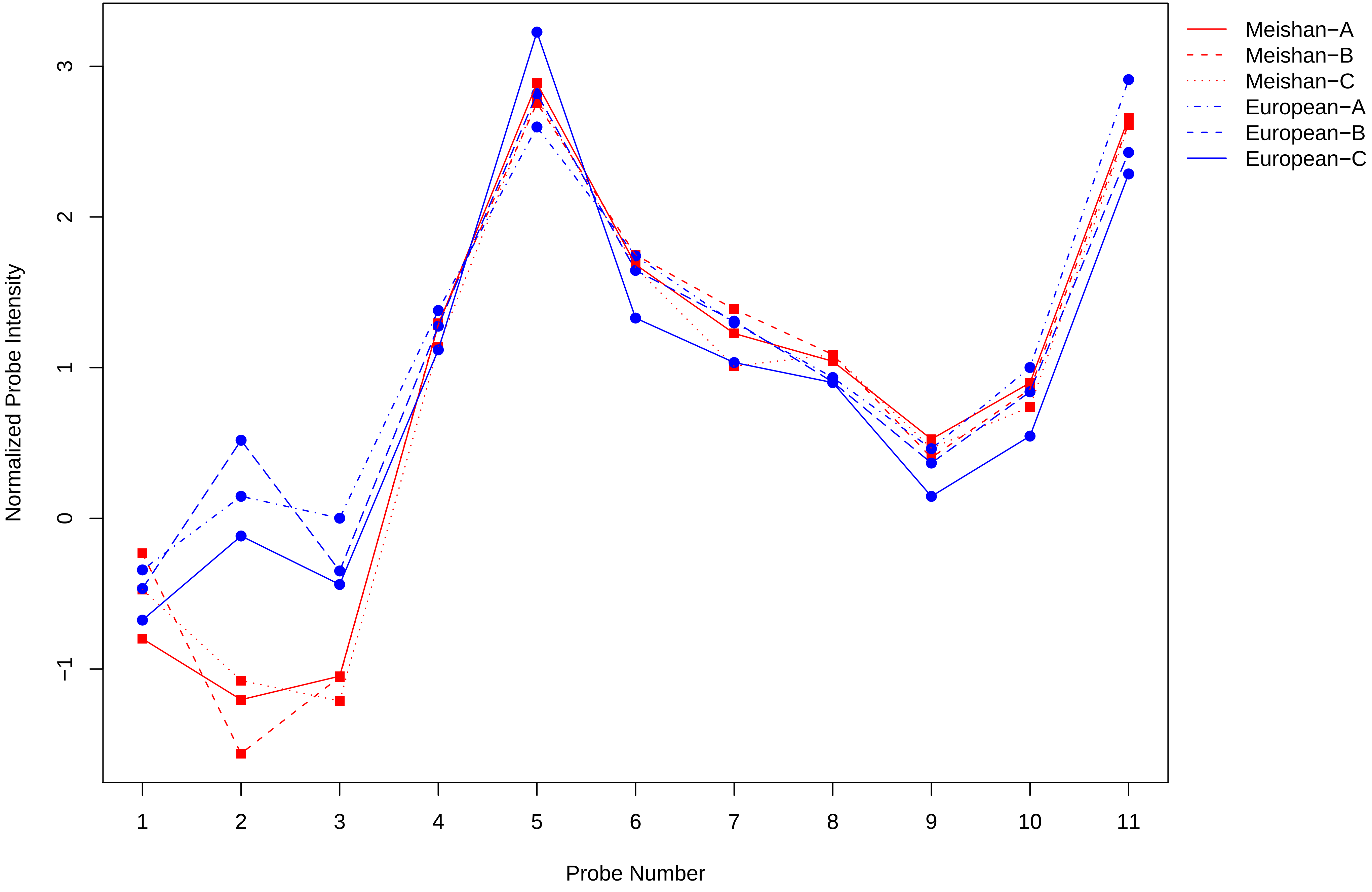

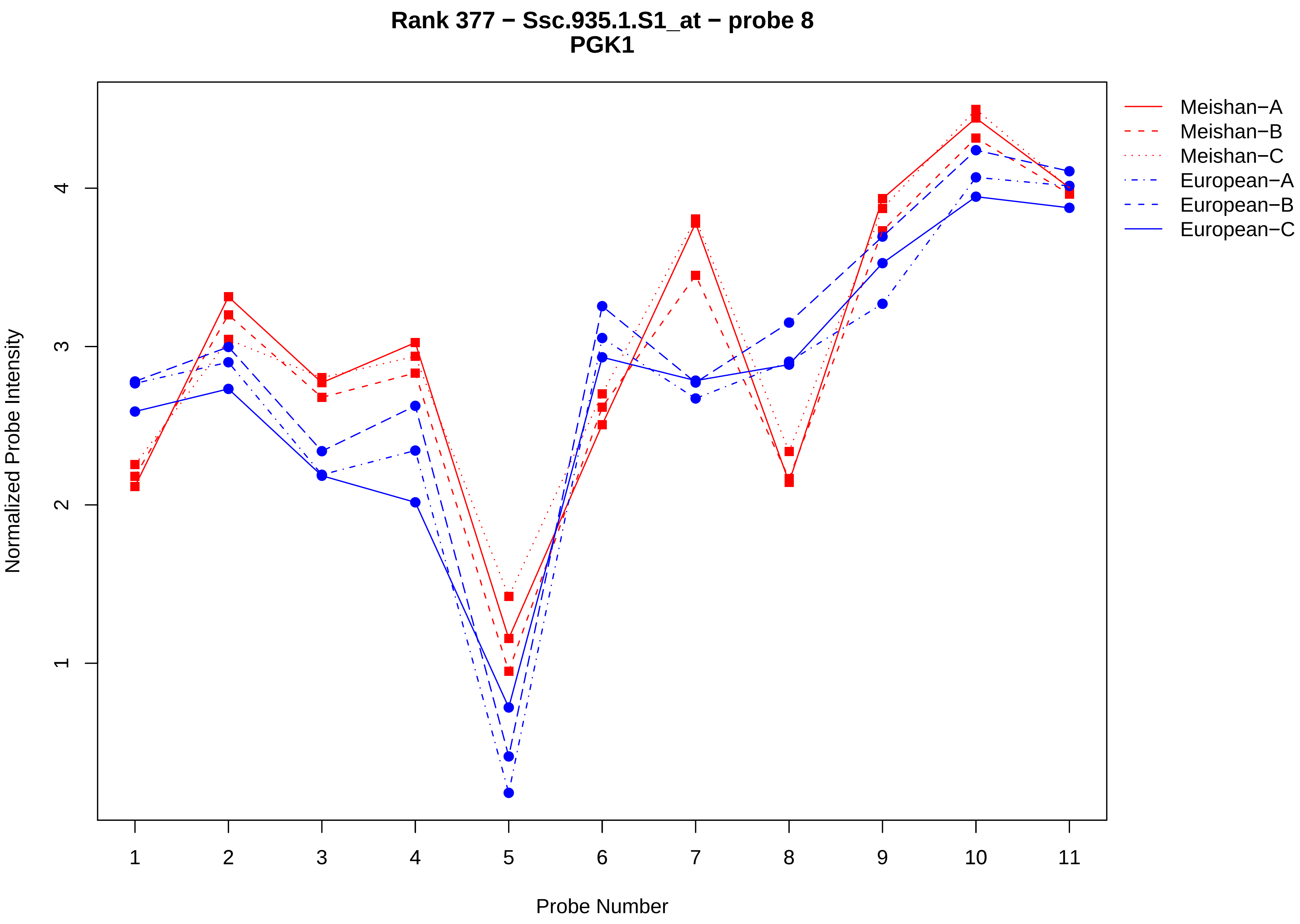

Rank 377 − Ssc.935.1.S1_at − probe 8
PGK1
Normalized Probe Intensity
Probe Number
Meishan−A
Meishan−B
Meishan−C
European−A
European−B
European−C
1
2
3
4
1
2
3
4
5
6
7
8
9
10
11

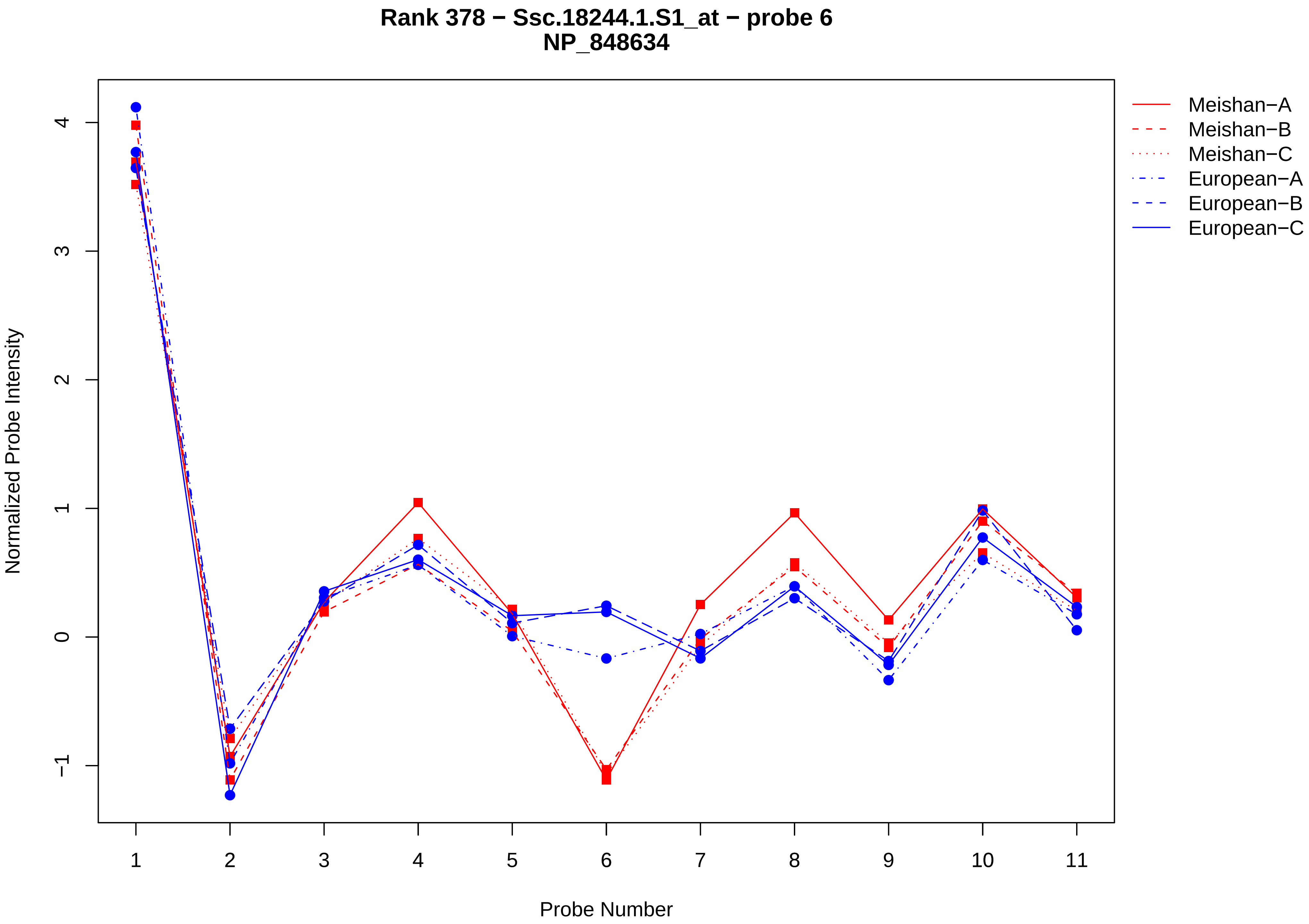

Rank 378 − Ssc.18244.1.S1_at − probe 6
NP_848634
Normalized Probe Intensity
Probe Number
−1
0
1
2
3
4
1
2
3
4
5
6
7
8
9
10
11
Meishan−A
Meishan−B
Meishan−C
European−A
European−B
European−C

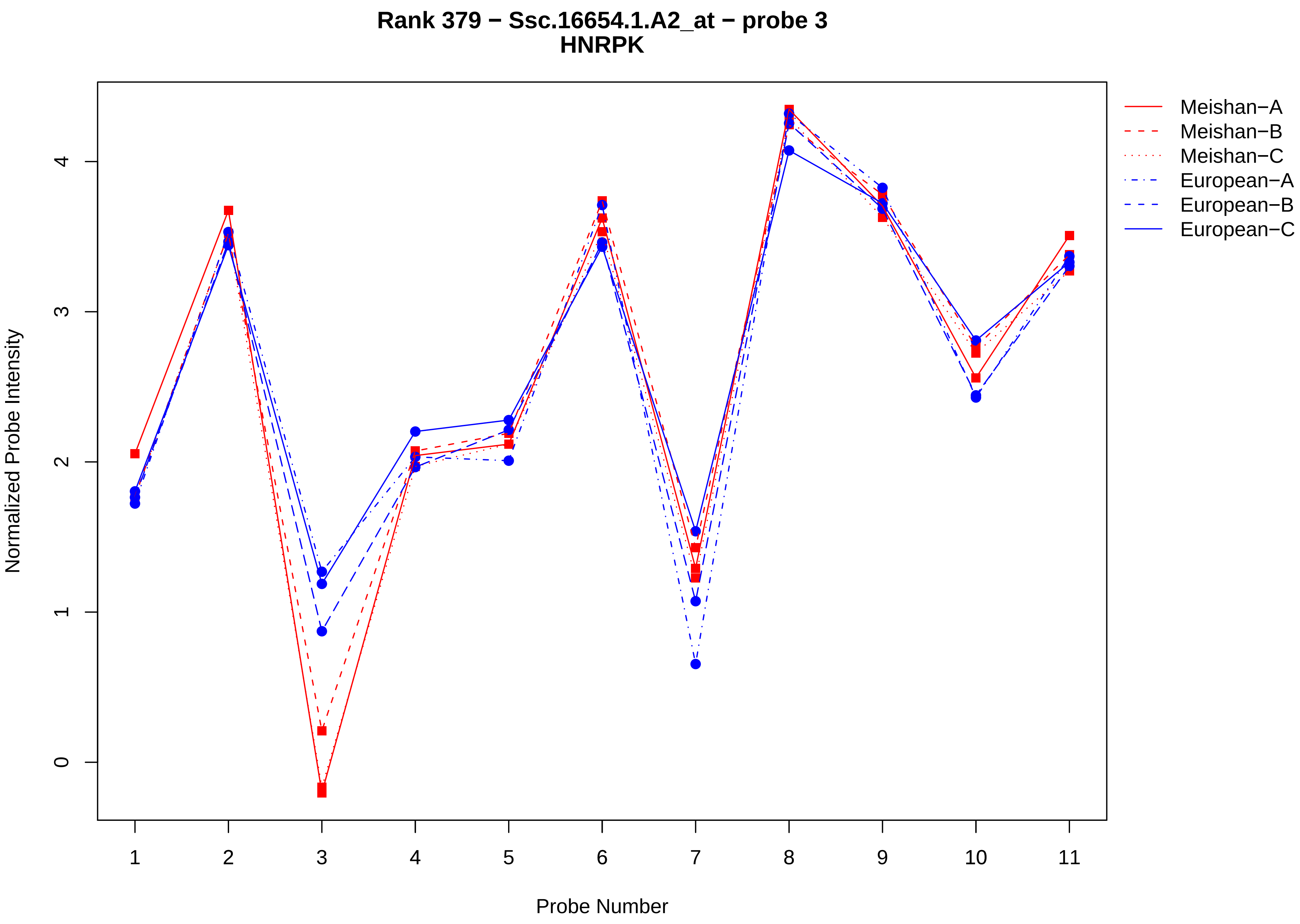
Rank 379 − Ssc.16654.1.A2_at − probe 3
HNRPK
Normalized Probe Intensity
Probe Number
0
1
2
3
4
1
2
3
4
5
6
7
8
9
10
11
Meishan−A
Meishan−B
Meishan−C
European−A
European−B
European−C

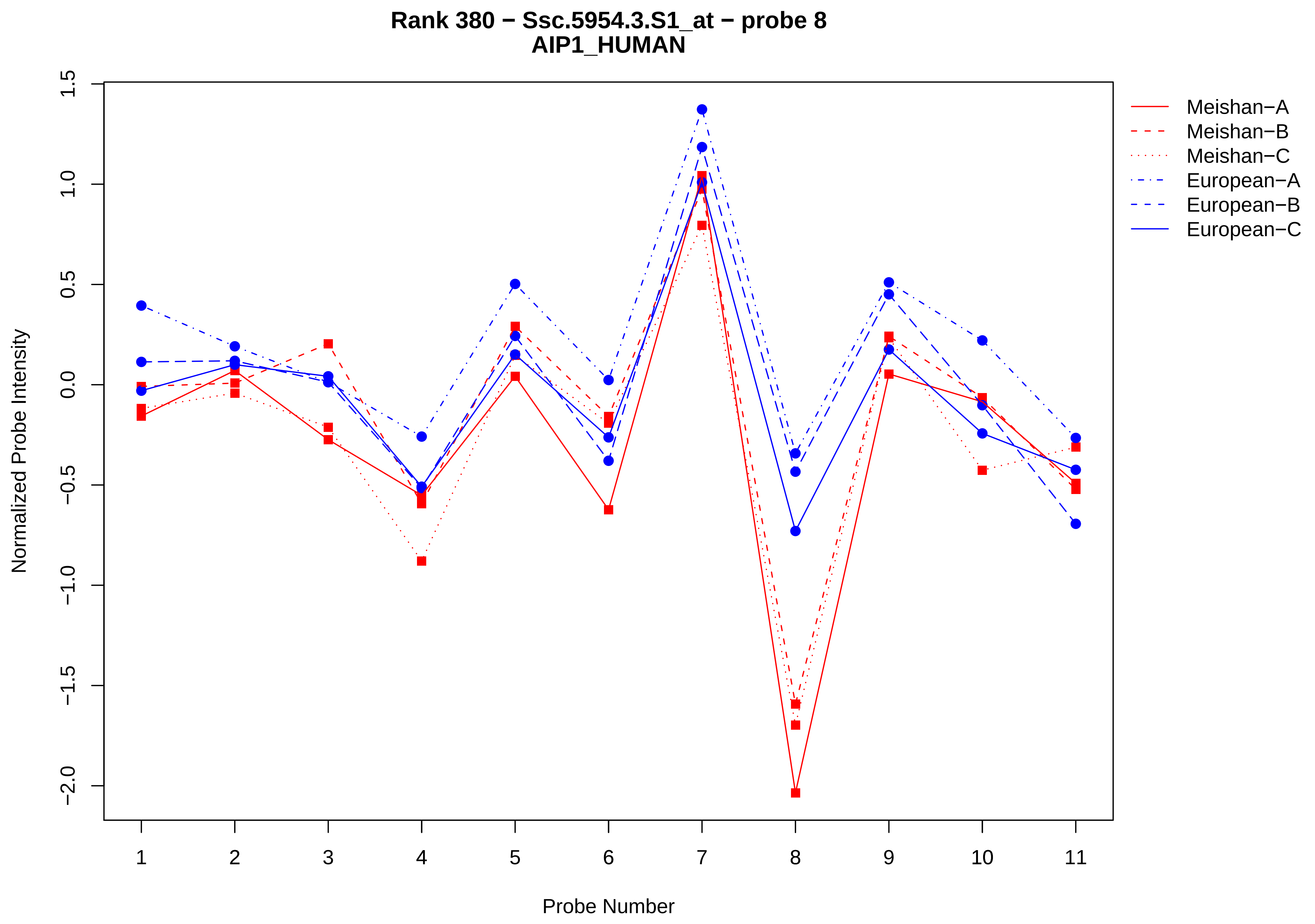

Rank 380 − Ssc.5954.3.S1_at − probe 8
AIP1_HUMAN
Normalized Probe Intensity
Probe Number
1.5
1.0
0.5
0.0
−0.5
−1.0
−1.5
−2.0
1
2
3
4
5
6
7
8
9
10
11
Meishan−A
Meishan−B
Meishan−C
European−A
European−B
European−C

Rank 381 − Ssc.29097.1.A1_at − probe 7
Q9H3X3

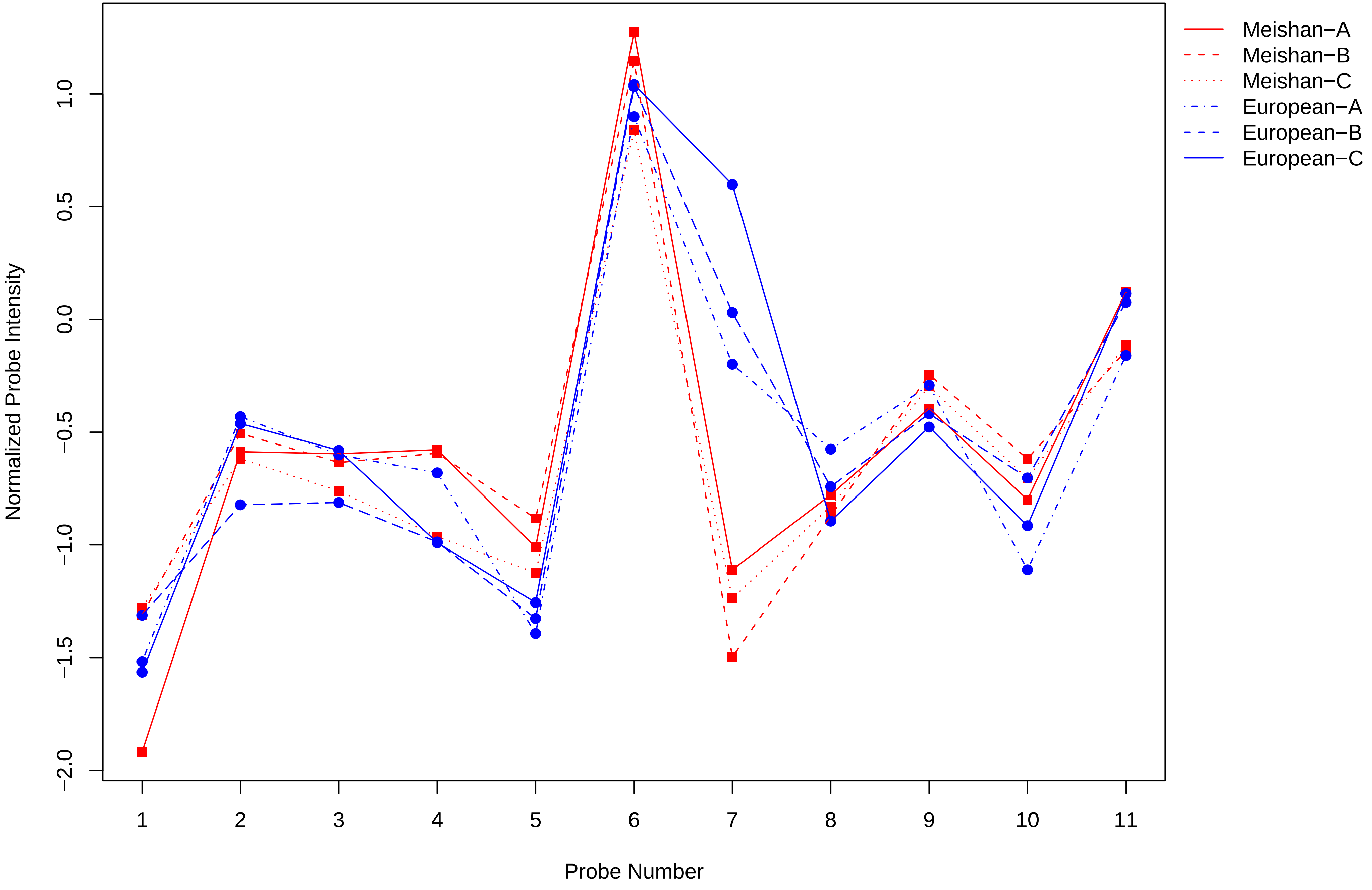

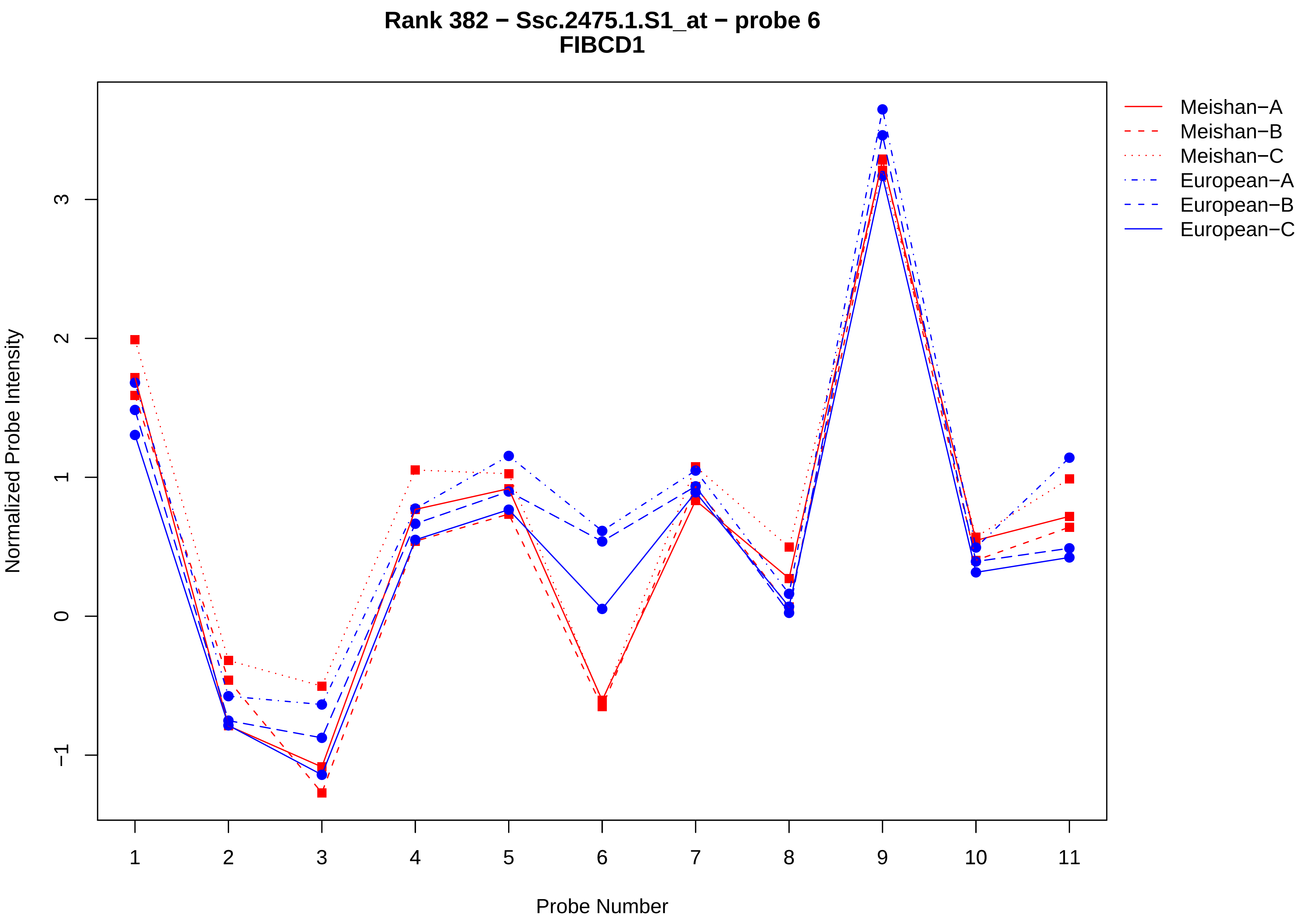

Rank 382 − Ssc.2475.1.S1_at − probe 6
FIBCD1
Normalized Probe Intensity
Probe Number
−1
0
1
2
3
1
2
3
4
5
6
7
8
9
10
11
Meishan−A
Meishan−B
Meishan−C
European−A
European−B
European−C

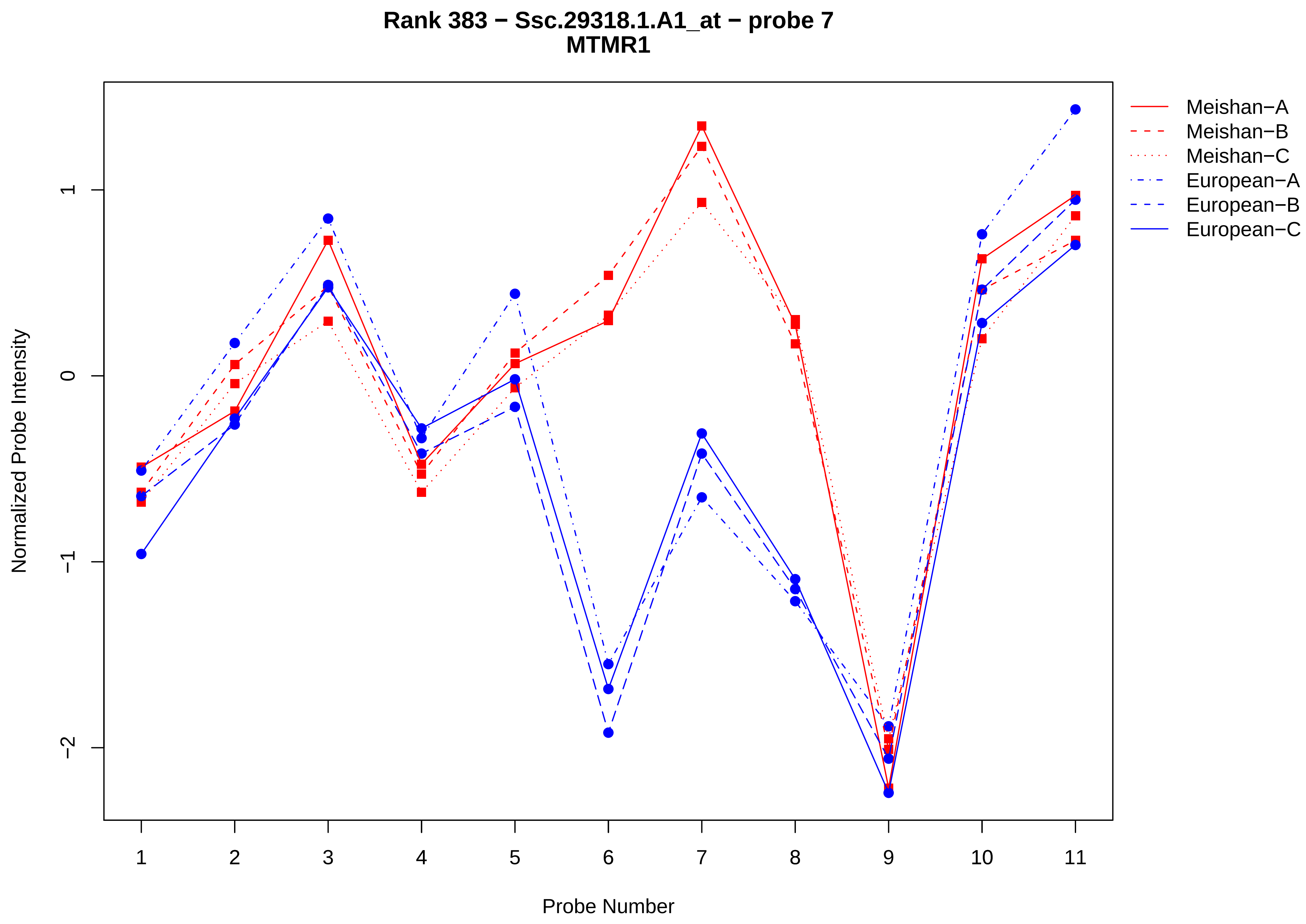
Rank 383 − Ssc.29318.1.A1_at − probe 7
MTMR1
Normalized Probe Intensity
Probe Number
−2
−1
0
1
1
2
3
4
5
6
7
8
9
10
11
Meishan−A
Meishan−B
Meishan−C
European−A
European−B
European−C

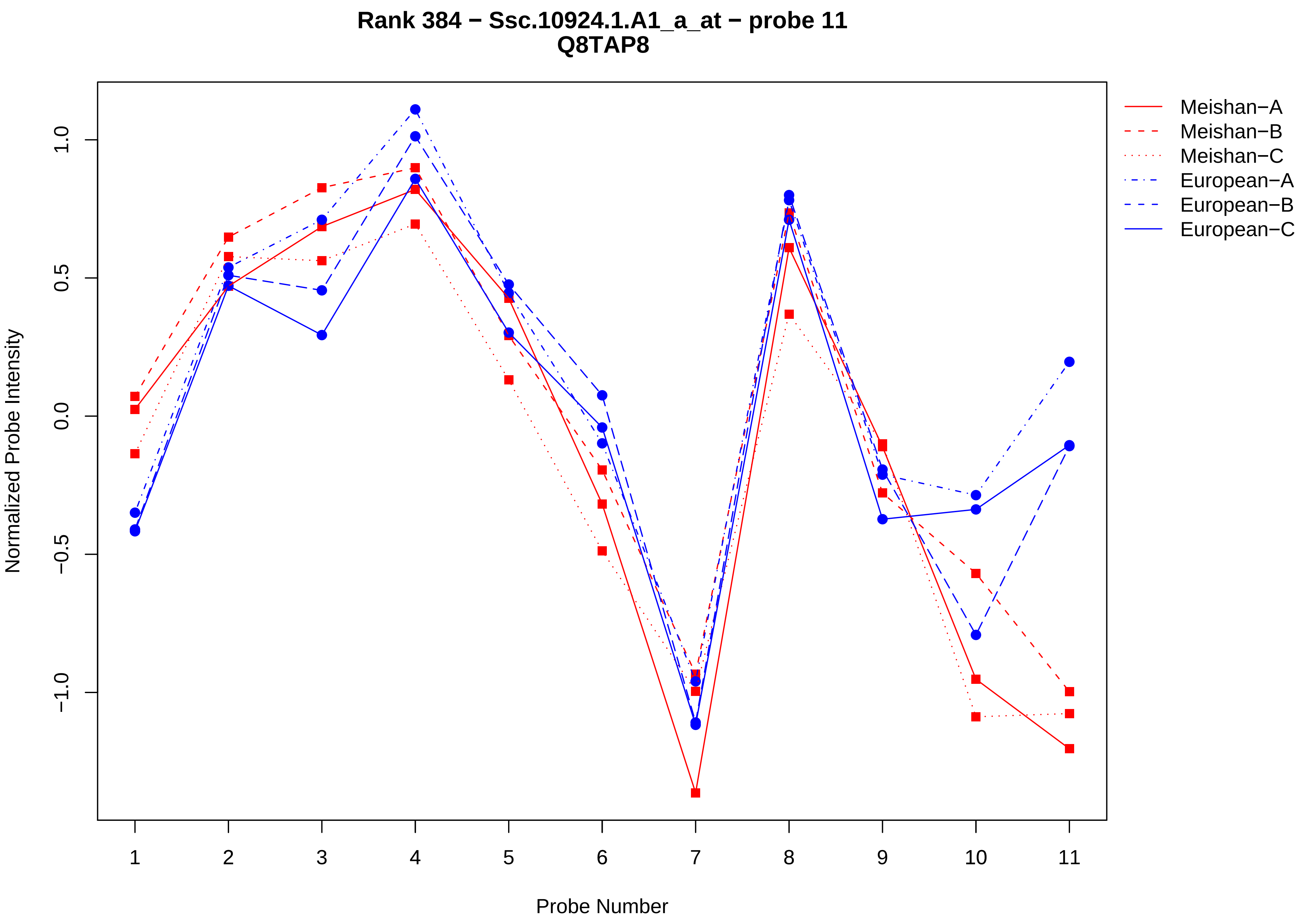

Rank 384 − Ssc.10924.1.A1_a_at − probe 11
Q8TAP8
Normalized Probe Intensity
Probe Number
−1.0
−0.5
0.0
0.5
1.0
1
2
3
4
5
6
7
8
9
10
11
Meishan−A
Meishan−B
Meishan−C
European−A
European−B
European−C

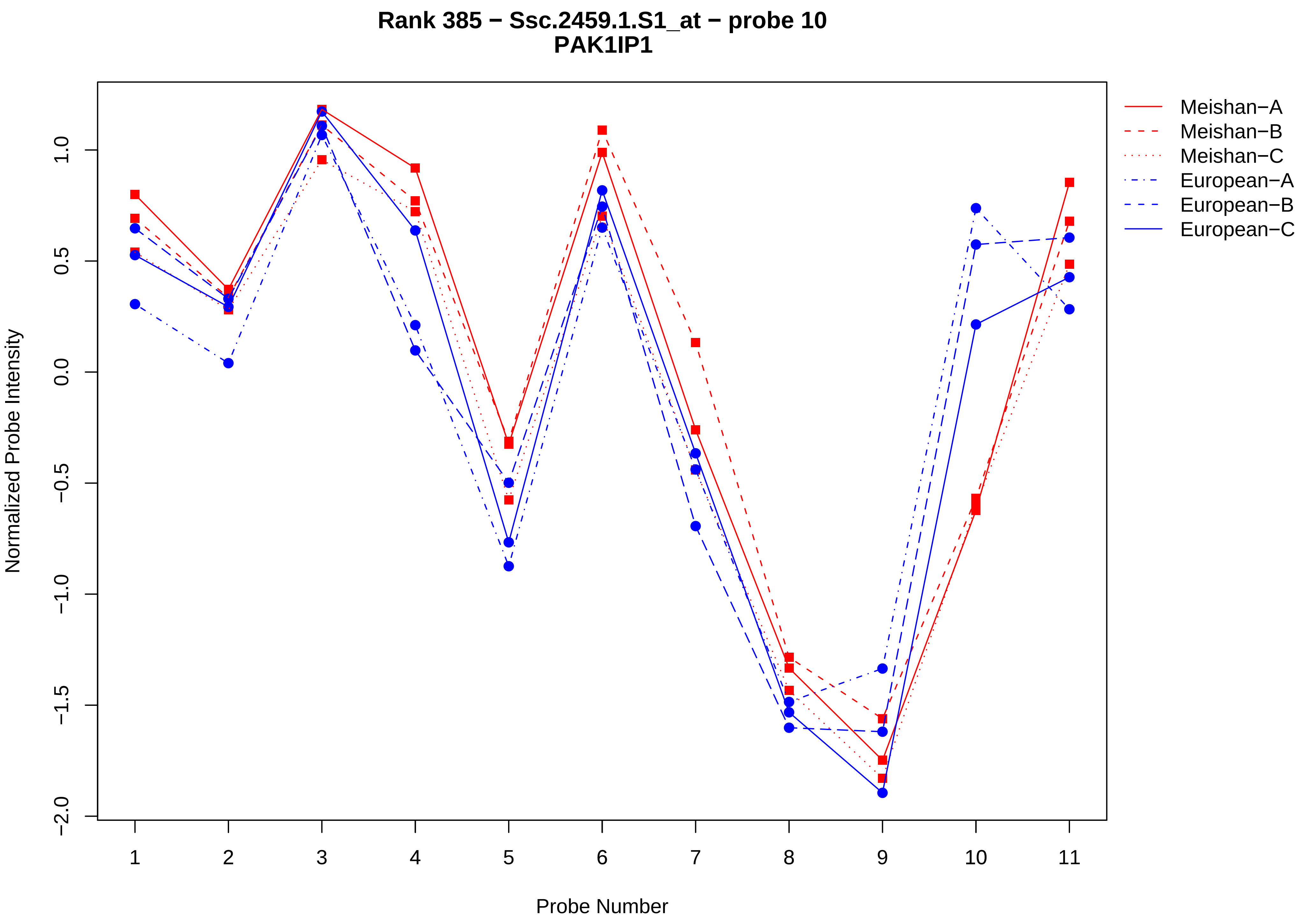

Rank 385 − Ssc.2459.1.S1_at − probe 10
PAK1IP1
Normalized Probe Intensity
Probe Number
1.0
0.5
0.0
−0.5
−1.0
−1.5
−2.0
1
2
3
4
5
6
7
8
9
10
11
Meishan−A
Meishan−B
Meishan−C
European−A
European−B
European−C

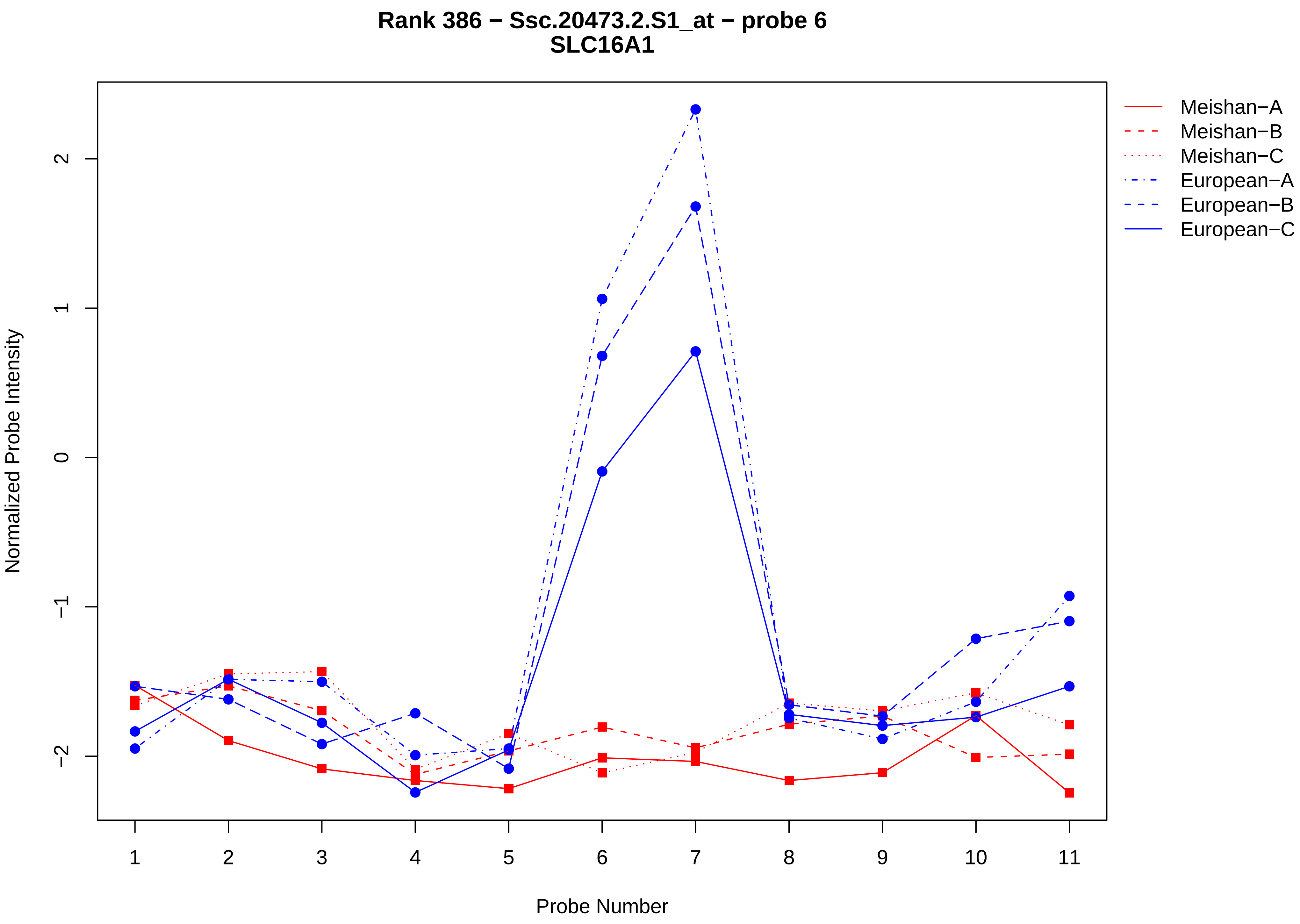
Rank 386 − Ssc.20473.2.S1_at − probe 6
SLC16A1
Normalized Probe Intensity
Probe Number
2
1
0
−1
−2
1
2
3
4
5
6
7
8
9
10
11
Meishan−A
Meishan−B
Meishan−C
European−A
European−B
European−C

Rank 387 − Ssc.8331.1.S1_at − probe 3
HINT1

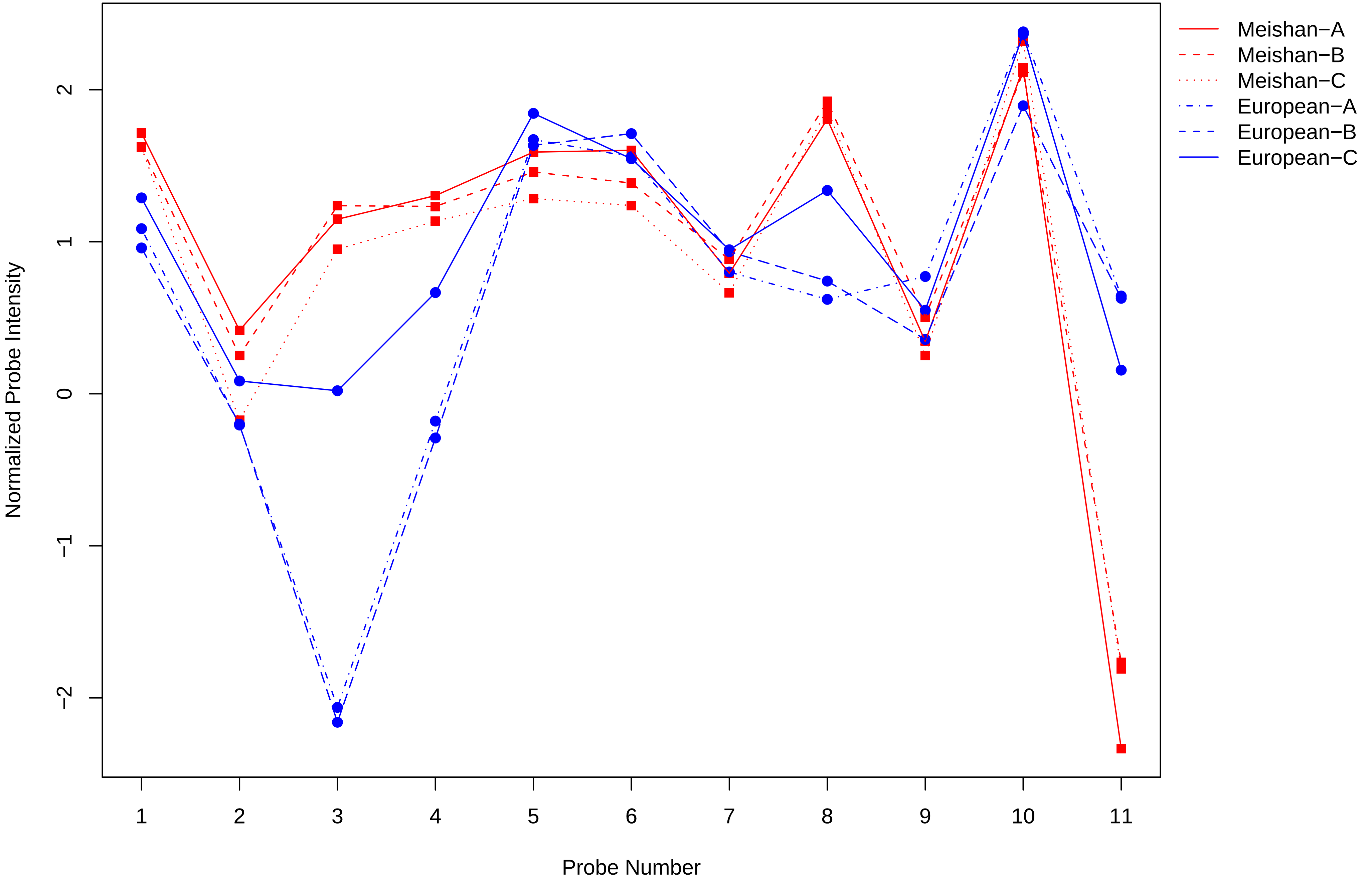

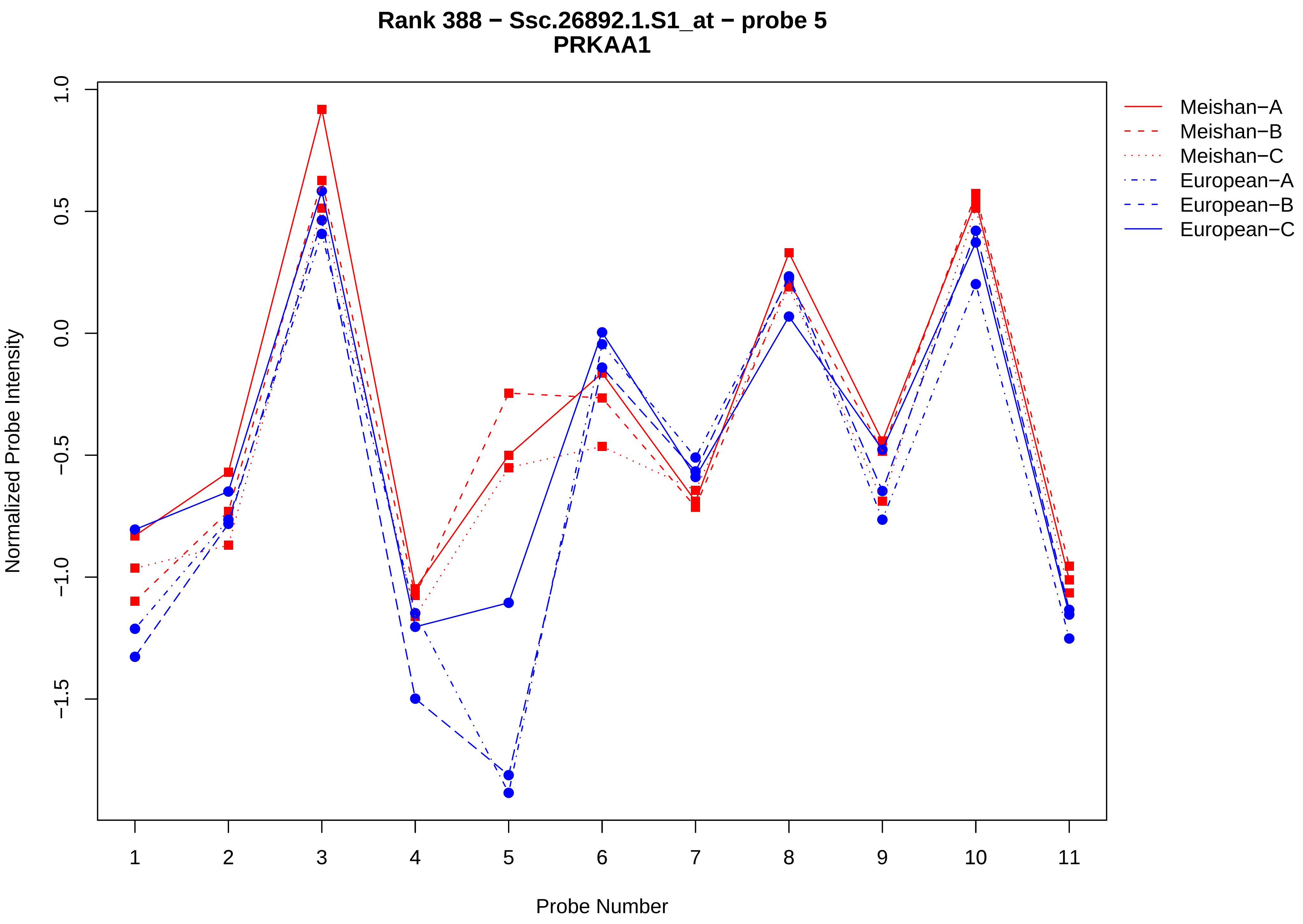
Rank 388 − Ssc.26892.1.S1_at − probe 5
PRKAA1
Normalized Probe Intensity
Probe Number
1.0
0.5
0.0
−0.5
−1.0
−1.5
1
2
3
4
5
6
7
8
9
10
11
Meishan−A
Meishan−B
Meishan−C
European−A
European−B
European−C

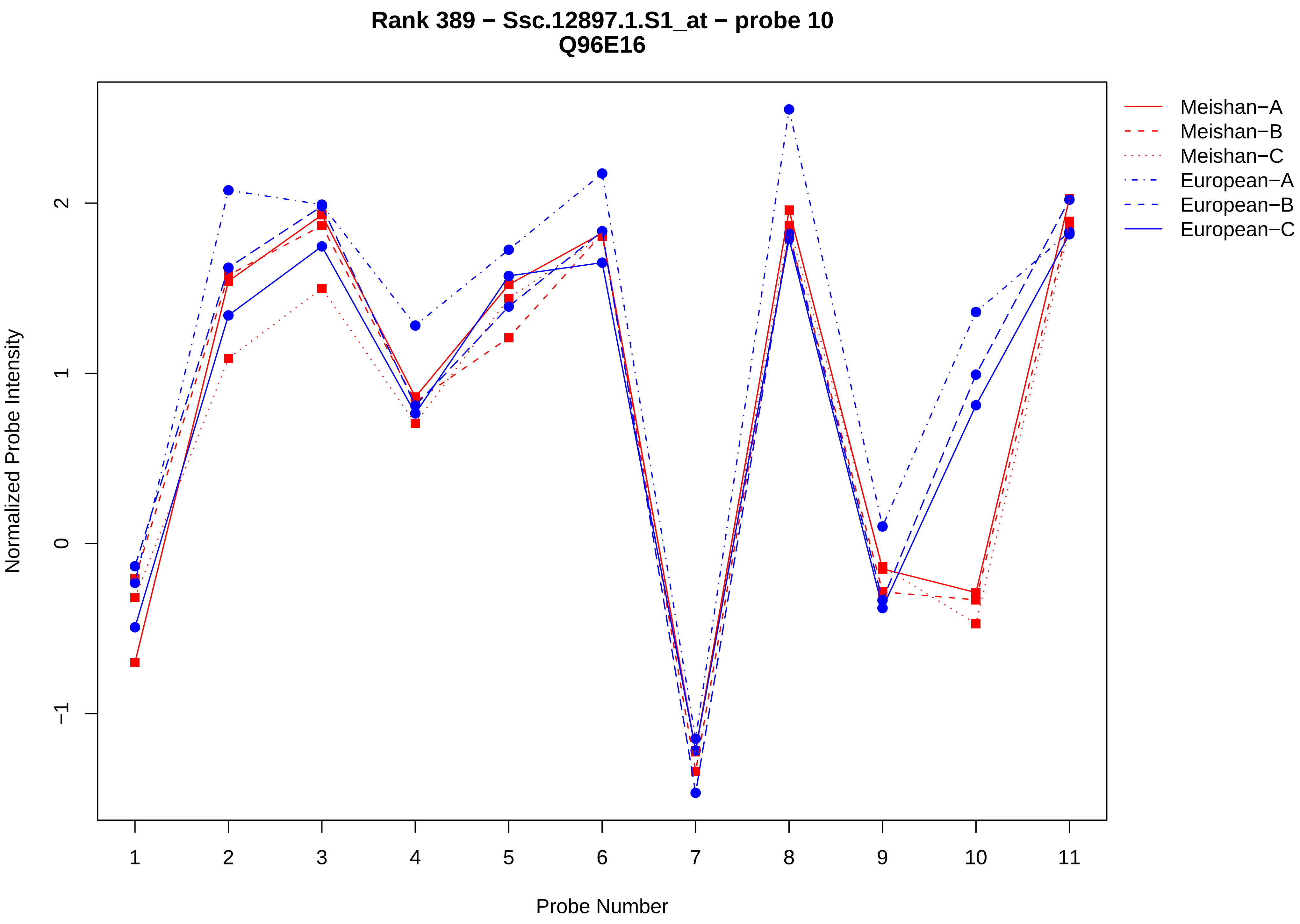
Rank 389 − Ssc.12897.1.S1_at − probe 10
Q96E16
Normalized Probe Intensity
Probe Number
−1
0
1
2
1
2
3
4
5
6
7
8
9
10
11
Meishan−A
Meishan−B
Meishan−C
European−A
European−B
European−C

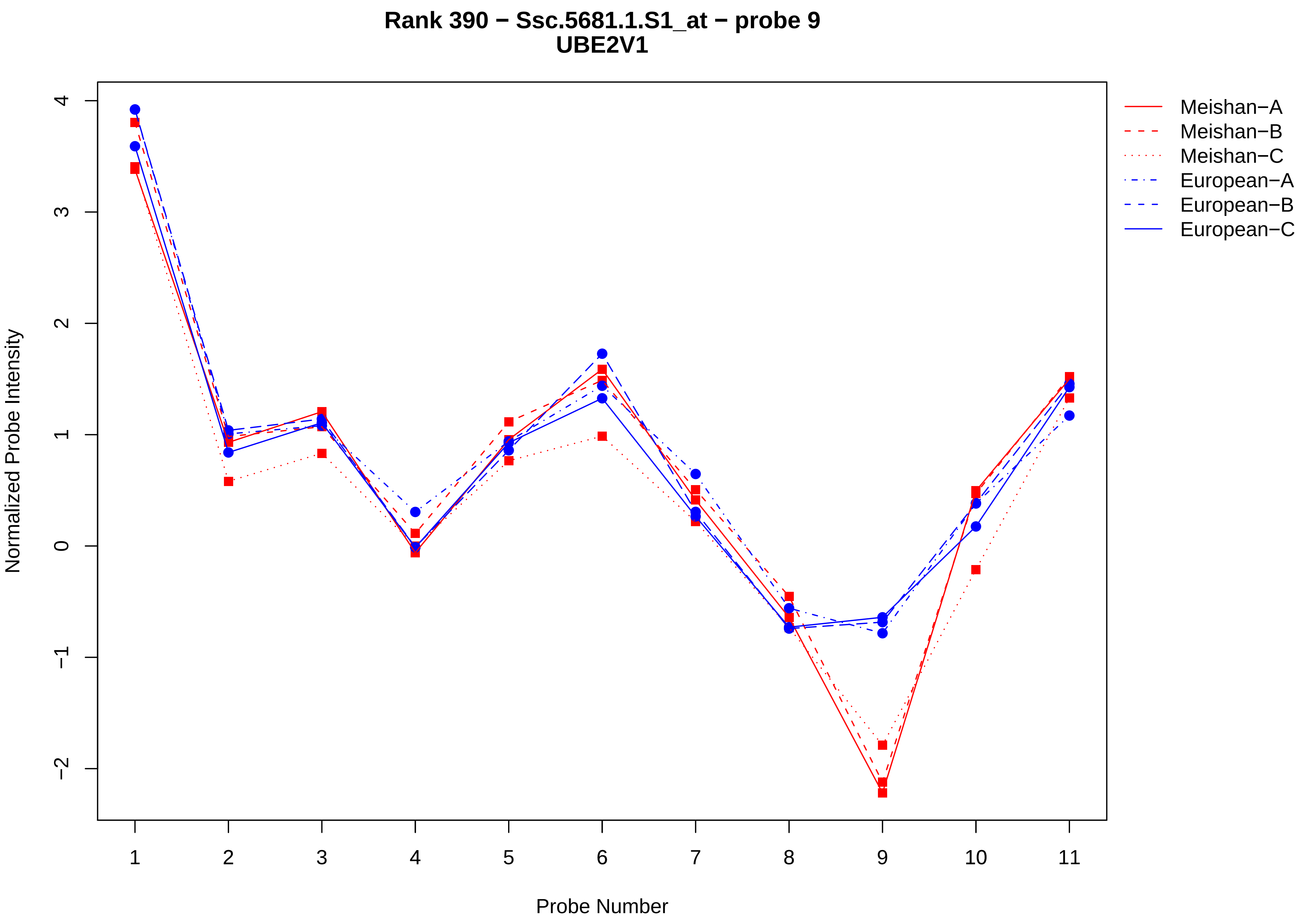

Rank 390 − Ssc.5681.1.S1_at − probe 9
UBE2V1
Normalized Probe Intensity
Probe Number
Meishan−A
Meishan−B
Meishan−C
European−A
European−B
European−C

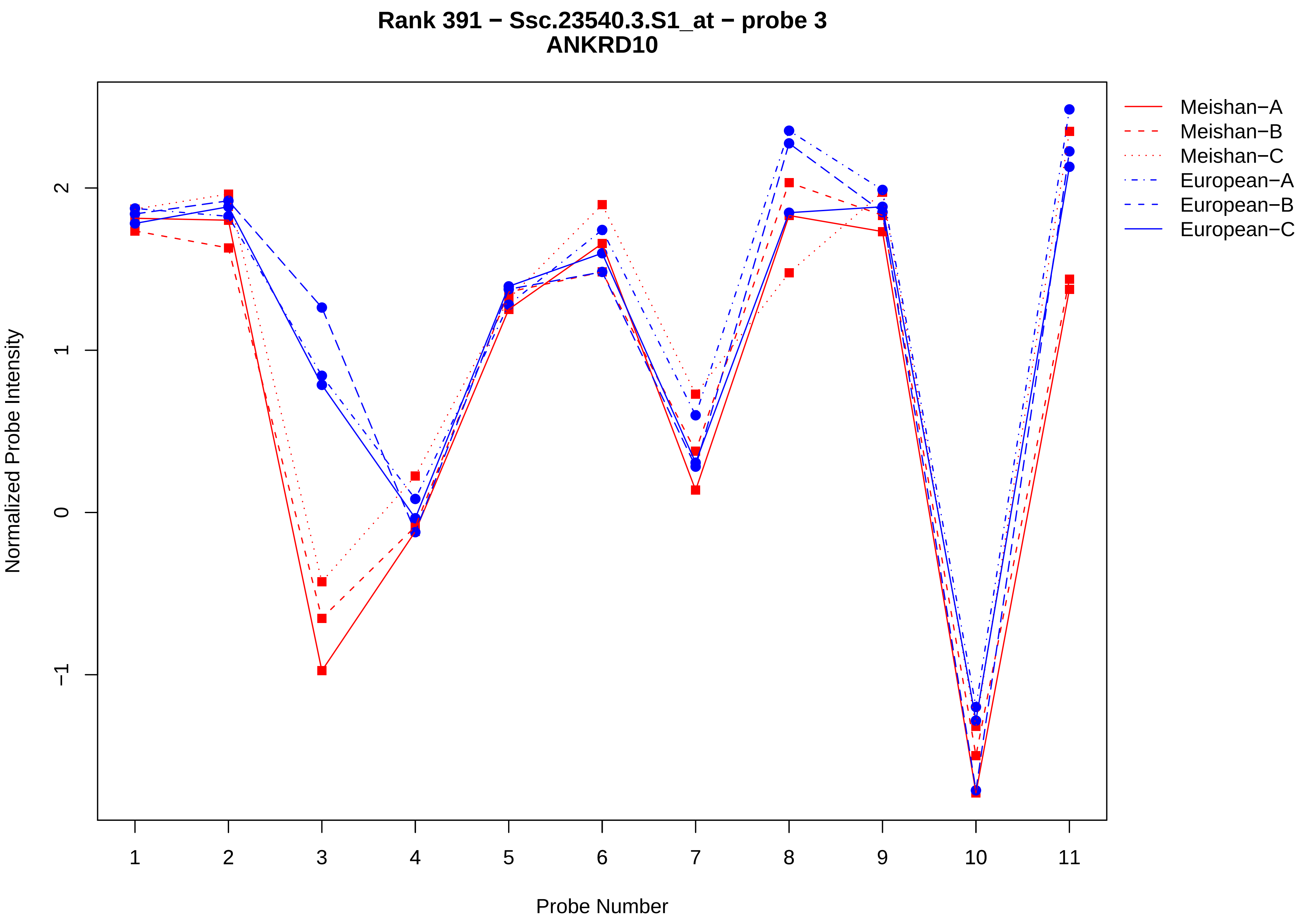
Rank 391 − Ssc.23540.3.S1_at − probe 3
ANKRD10
Normalized Probe Intensity
Probe Number
−1
0
1
2
1
2
3
4
5
6
7
8
9
10
11
Meishan−A
Meishan−B
Meishan−C
European−A
European−B
European−C

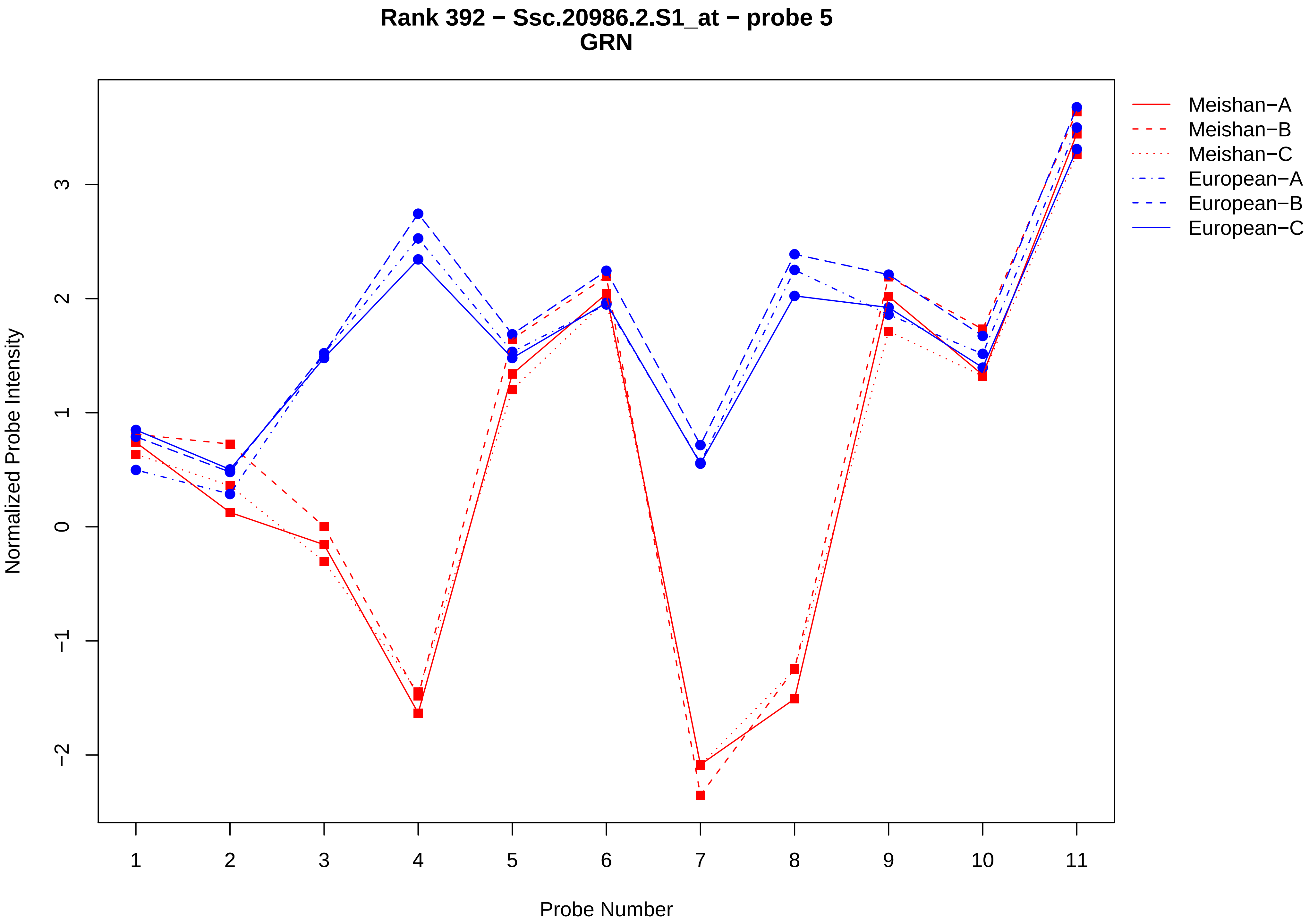

Rank 392 − Ssc.20986.2.S1_at − probe 5
GRN
Normalized Probe Intensity
Probe Number
−2
−1
0
1
2
3
1
2
3
4
5
6
7
8
9
10
11
Meishan−A
Meishan−B
Meishan−C
European−A
European−B
European−C

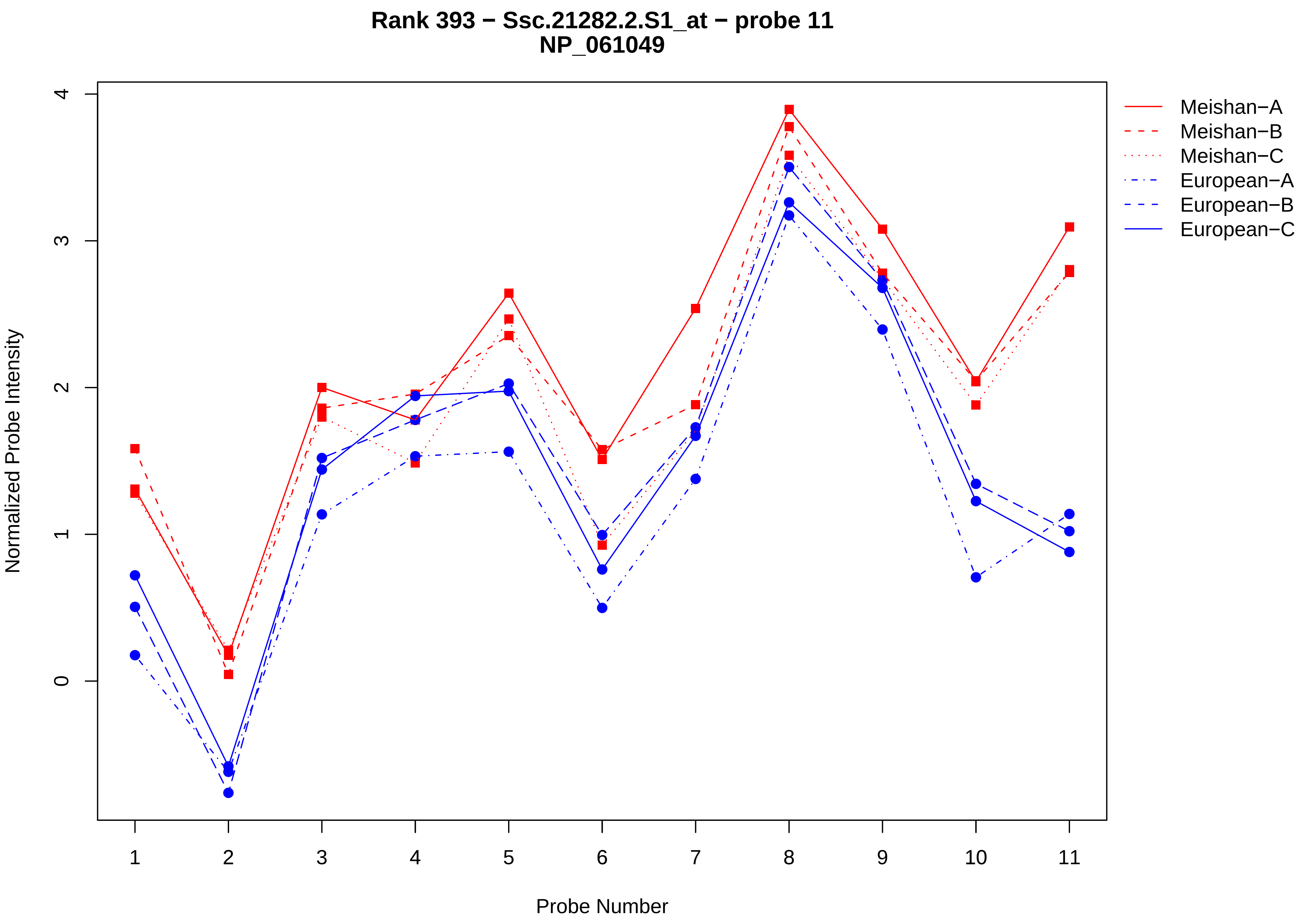

Rank 393 − Ssc.21282.2.S1_at − probe 11
NP_061049
Normalized Probe Intensity
0
1
2
3
4
1
2
3
4
5
6
7
8
9
10
11
Probe Number
Meishan−A
Meishan−B
Meishan−C
European−A
European−B
European−C

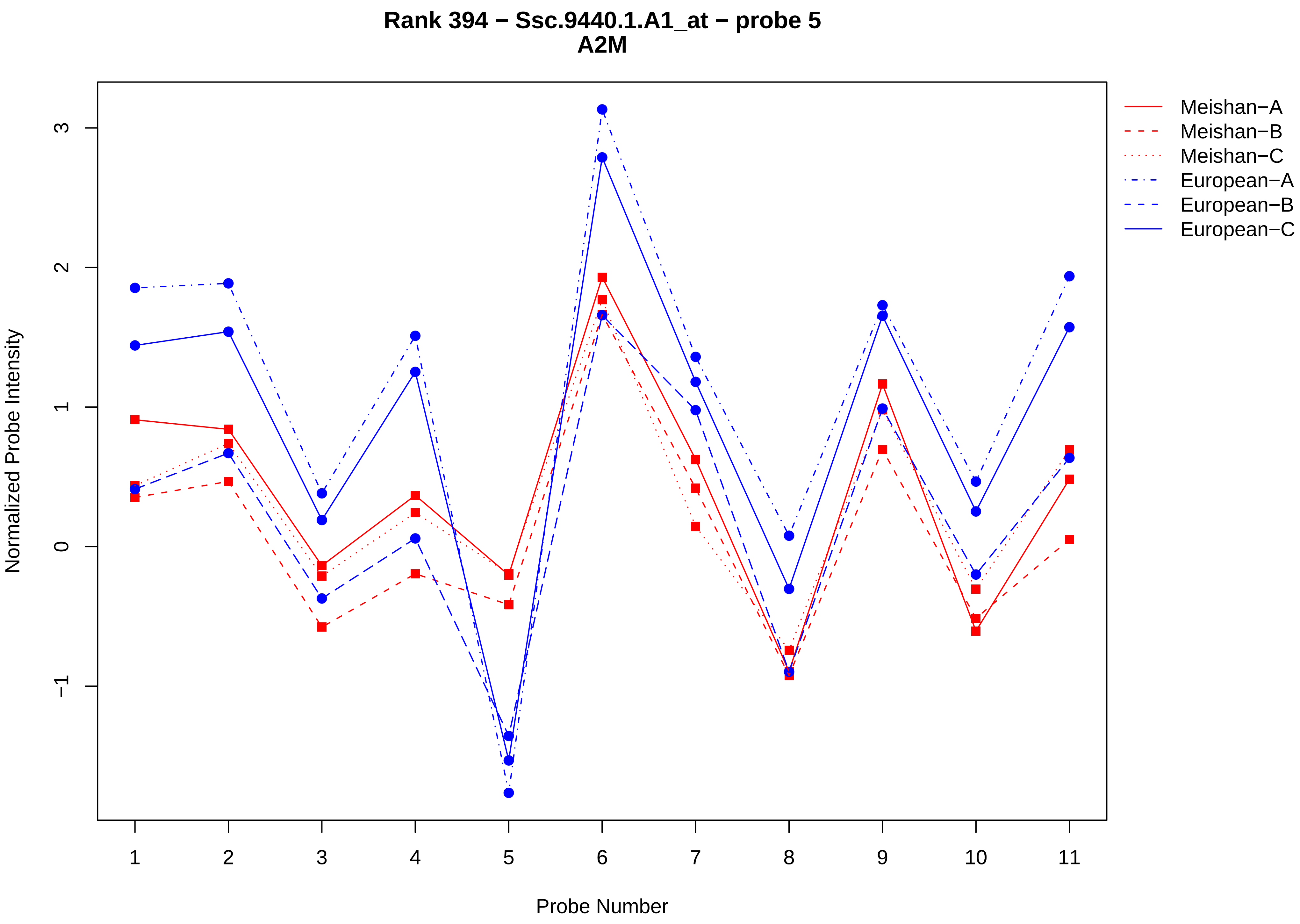

Rank 394 − Ssc.9440.1.A1_at − probe 5
A2M
Normalized Probe Intensity
Probe Number
Meishan−A
Meishan−B
Meishan−C
European−A
European−B
European−C

Rank 395 − Ssc.23501.1.S1_s_at − probe 10

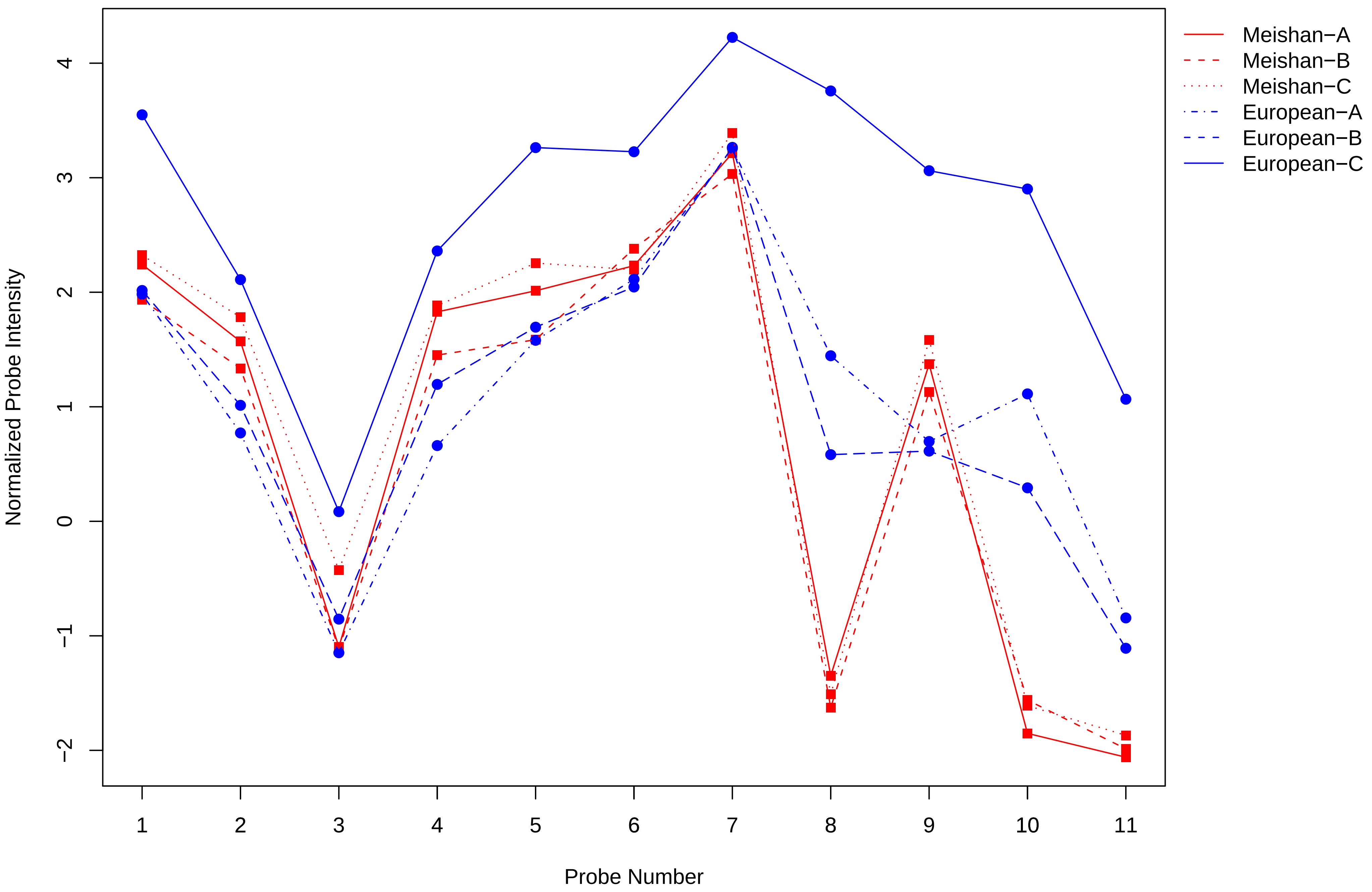

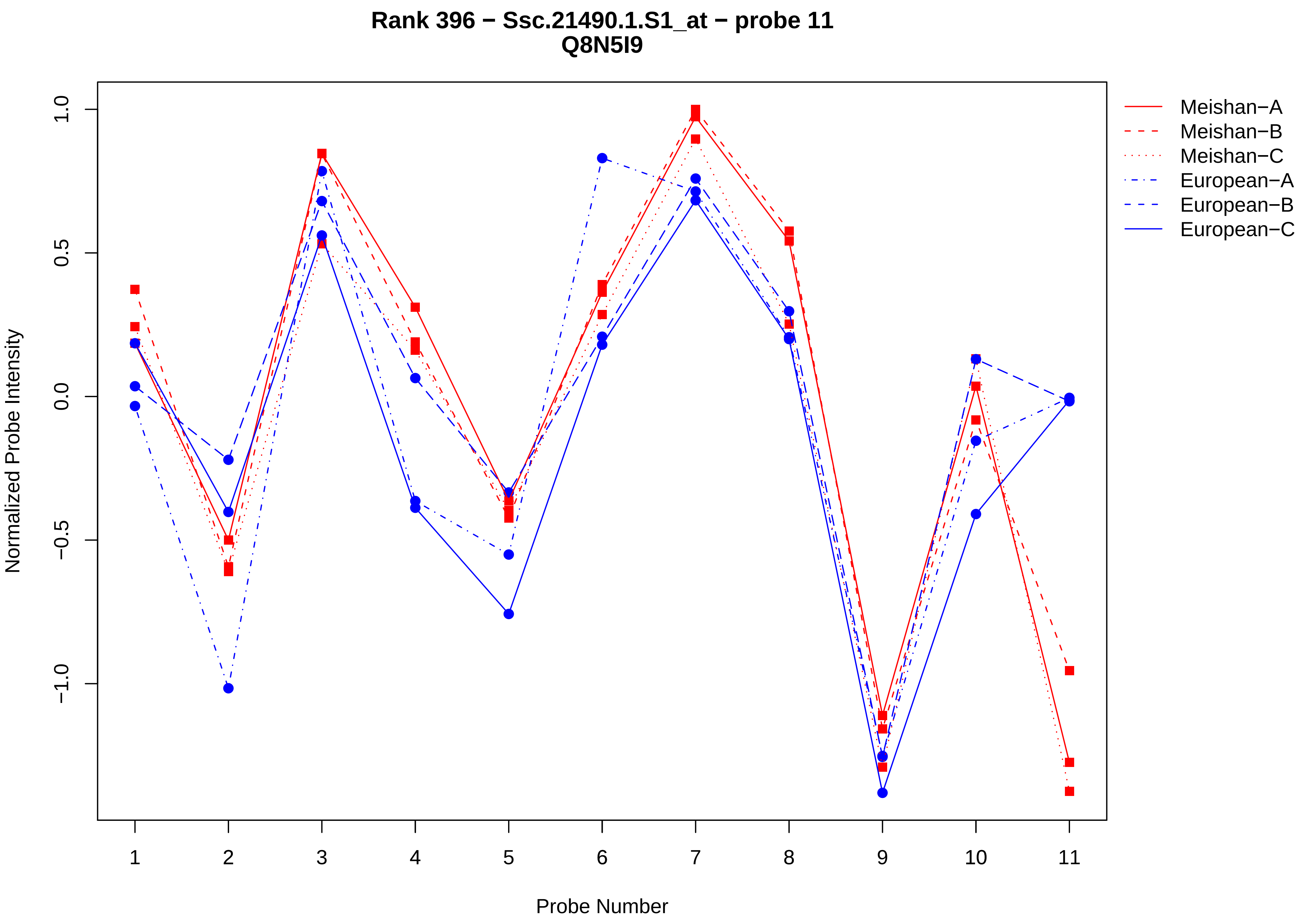
Rank 396 − Ssc.21490.1.S1_at − probe 11
Q8N5I9
Normalized Probe Intensity
Probe Number
1.0
0.5
0.0
−0.5
−1.0
1
2
3
4
5
6
7
8
9
10
11
Meishan−A
Meishan−B
Meishan−C
European−A
European−B
European−C

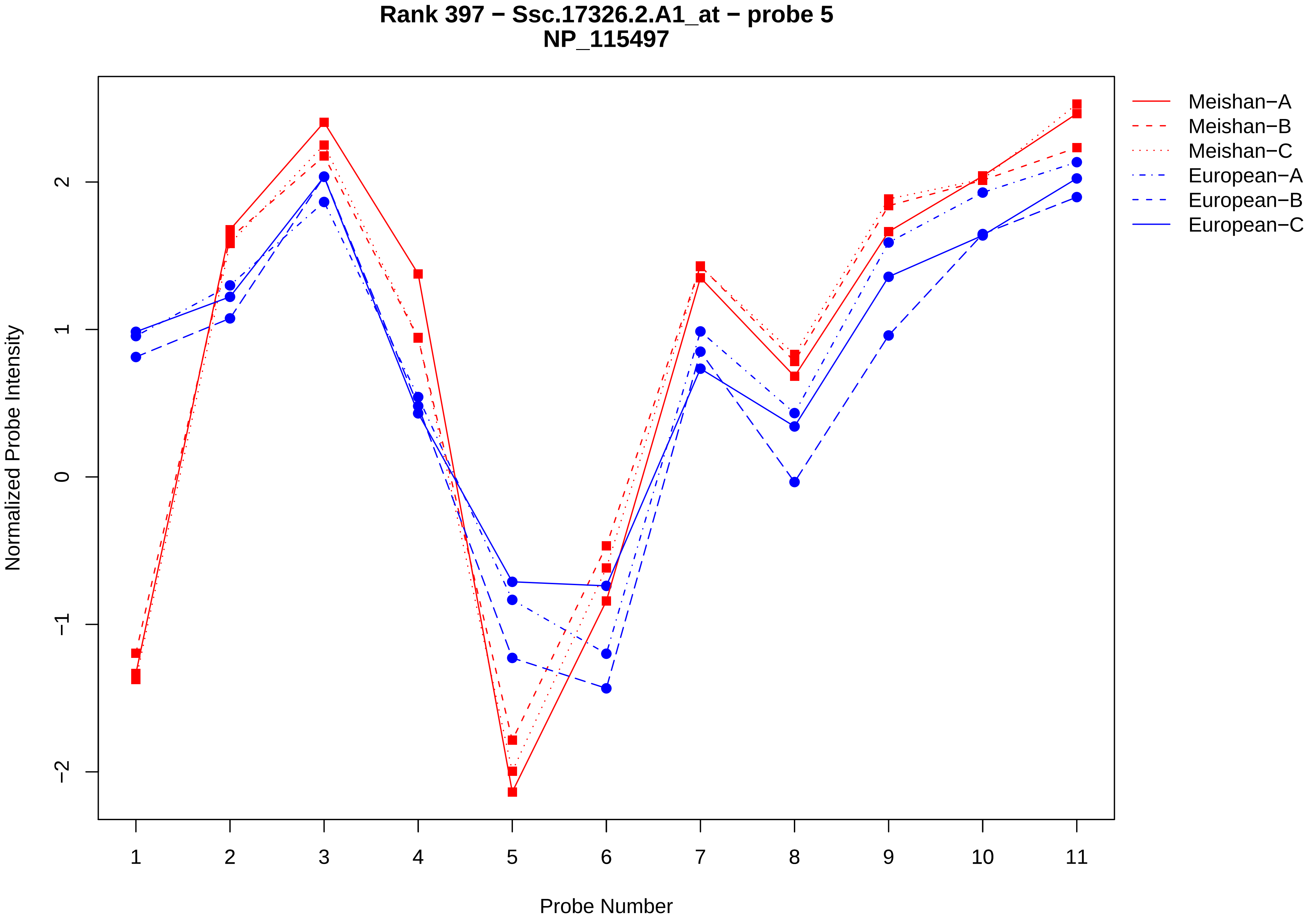

Rank 397 − Ssc.17326.2.A1_at − probe 5
NP_115497
Normalized Probe Intensity
Probe Number
Meishan−A
Meishan−B
Meishan−C
European−A
European−B
European−C
−2
−1
0
1
2
1
2
3
4
5
6
7
8
9
10
11

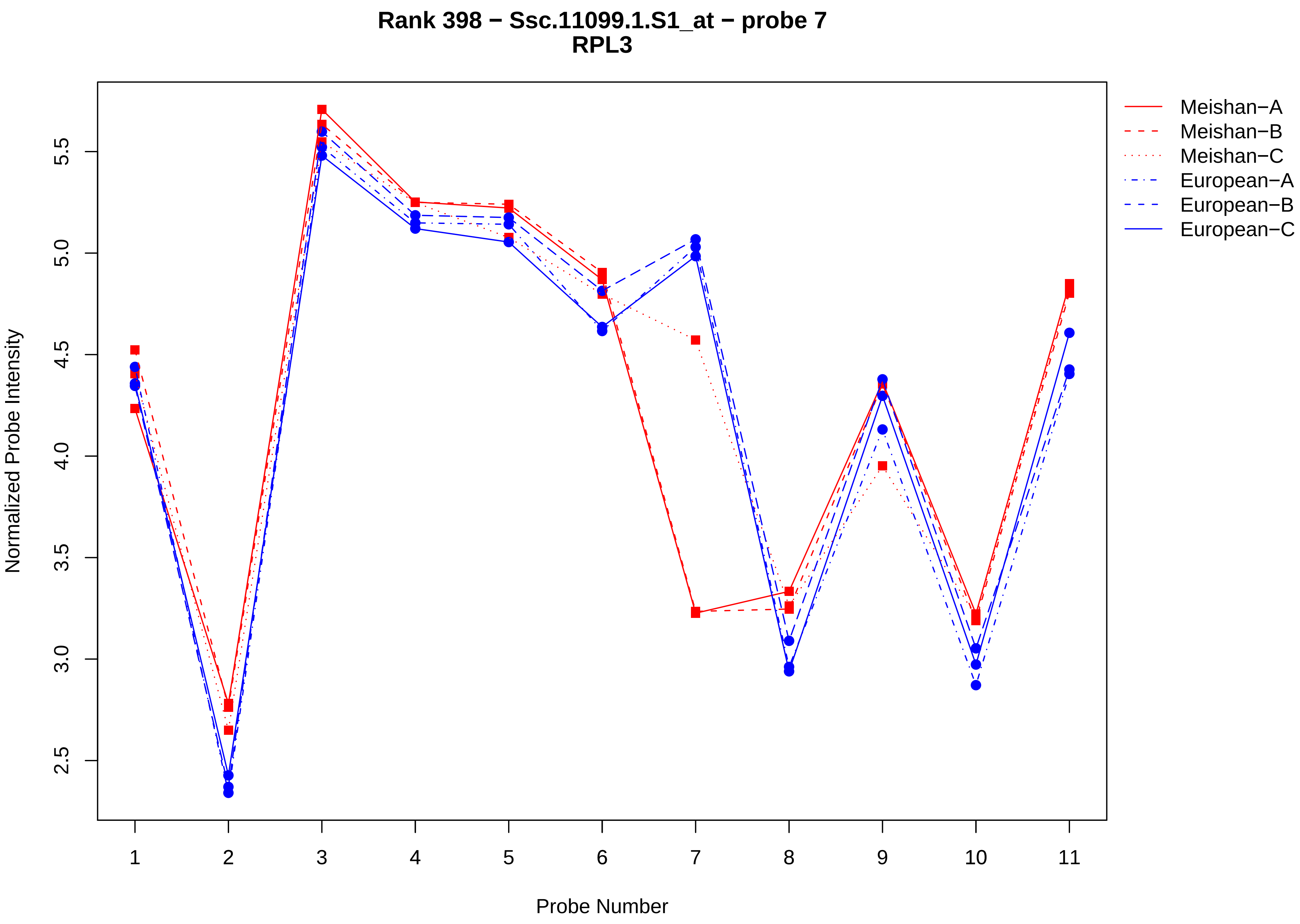

Rank 398 − Ssc.11099.1.S1_at − probe 7
RPL3
Normalized Probe Intensity
Probe Number
2.5
3.0
3.5
4.0
4.5
5.0
5.5
1
2
3
4
5
6
7
8
9
10
11
Meishan−A
Meishan−B
Meishan−C
European−A
European−B
European−C

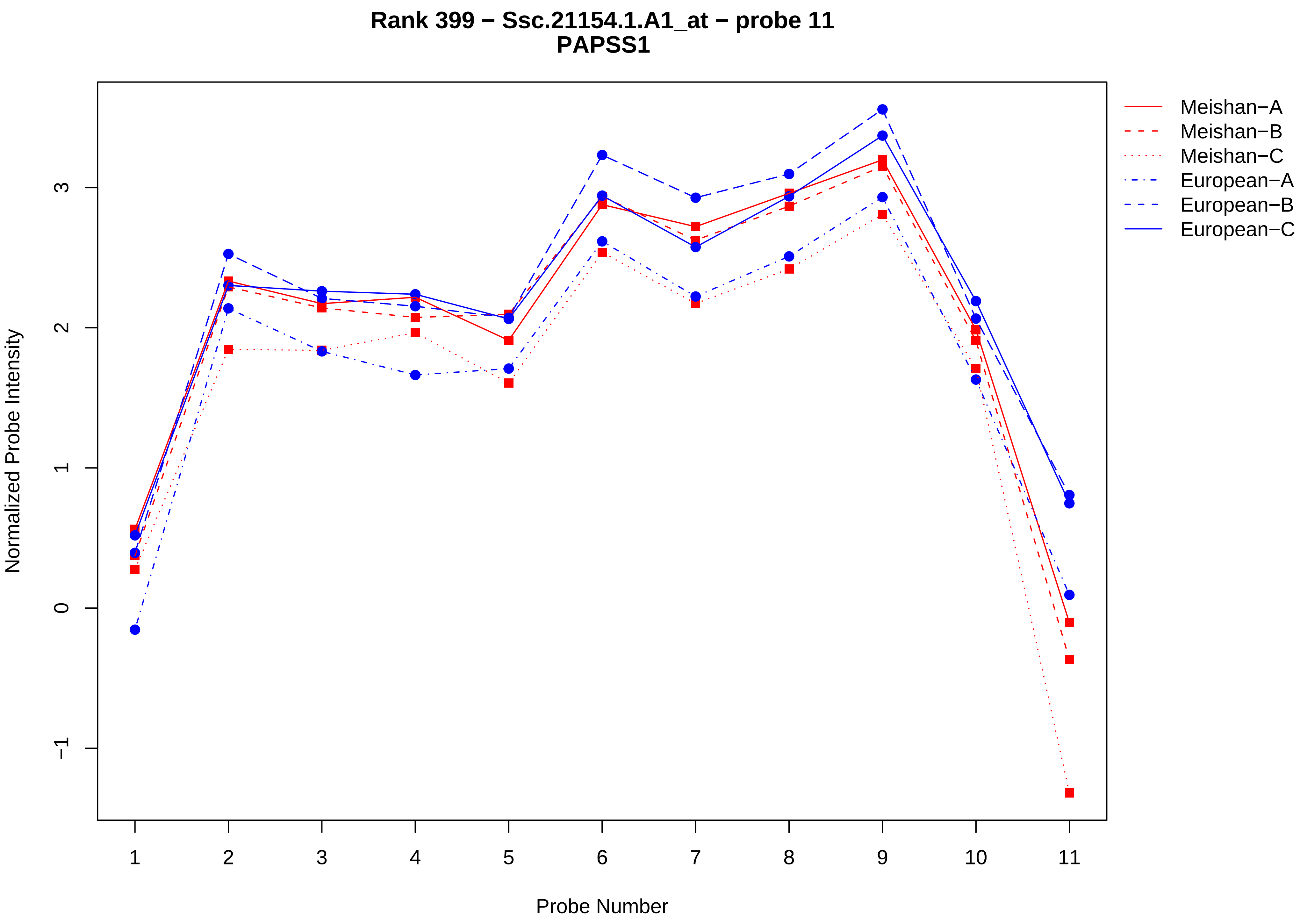
Rank 399 − Ssc.21154.1.A1_at − probe 11
PAPSS1
Normalized Probe Intensity
Probe Number
−1
0
1
2
3
1
2
3
4
5
6
7
8
9
10
11
Meishan−A
Meishan−B
Meishan−C
European−A
European−B
European−C

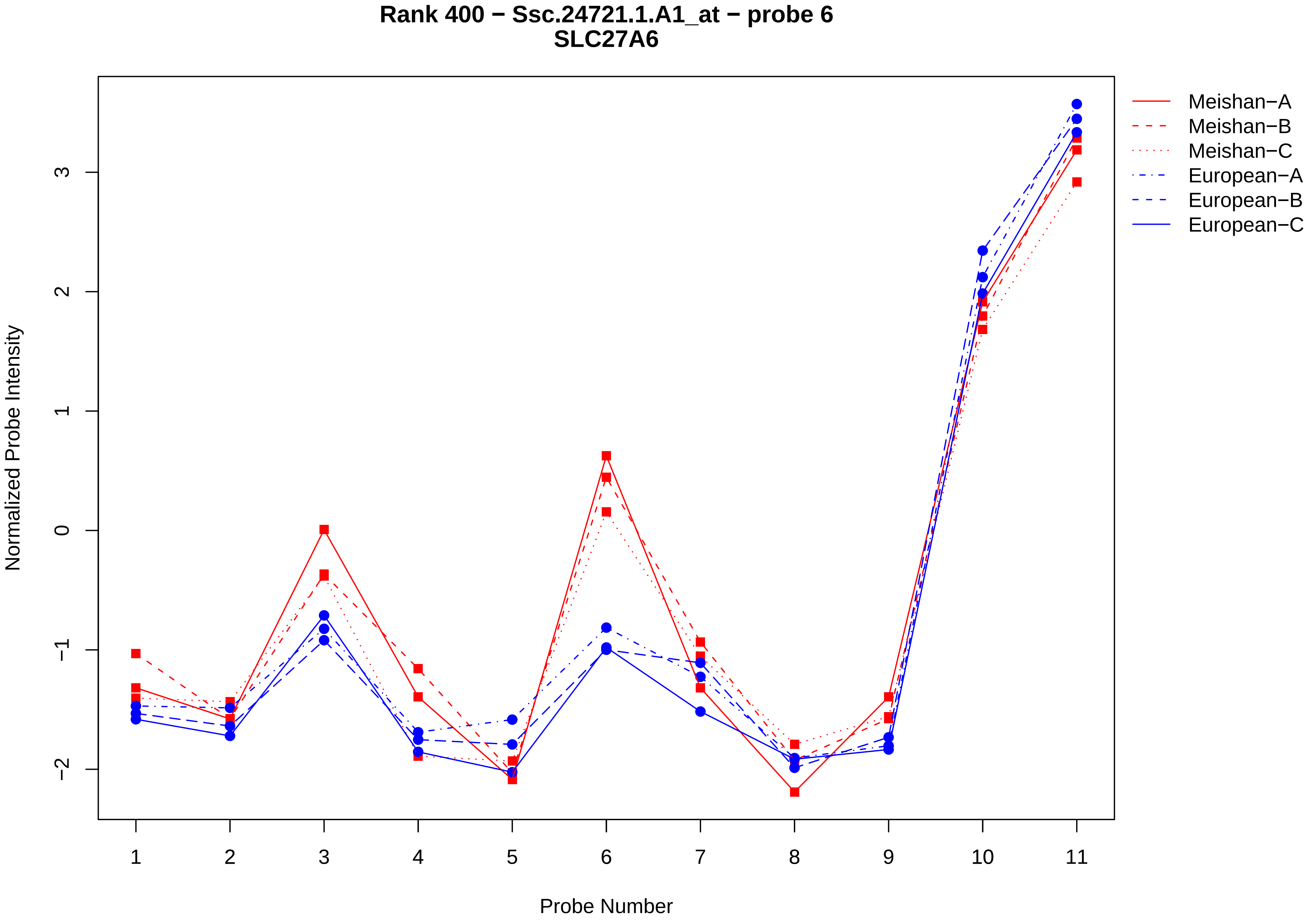

Rank 400 − Ssc.24721.1.A1_at − probe 6
SLC27A6
Normalized Probe Intensity
Probe Number
Meishan−A
Meishan−B
Meishan−C
European−A
European−B
European−C

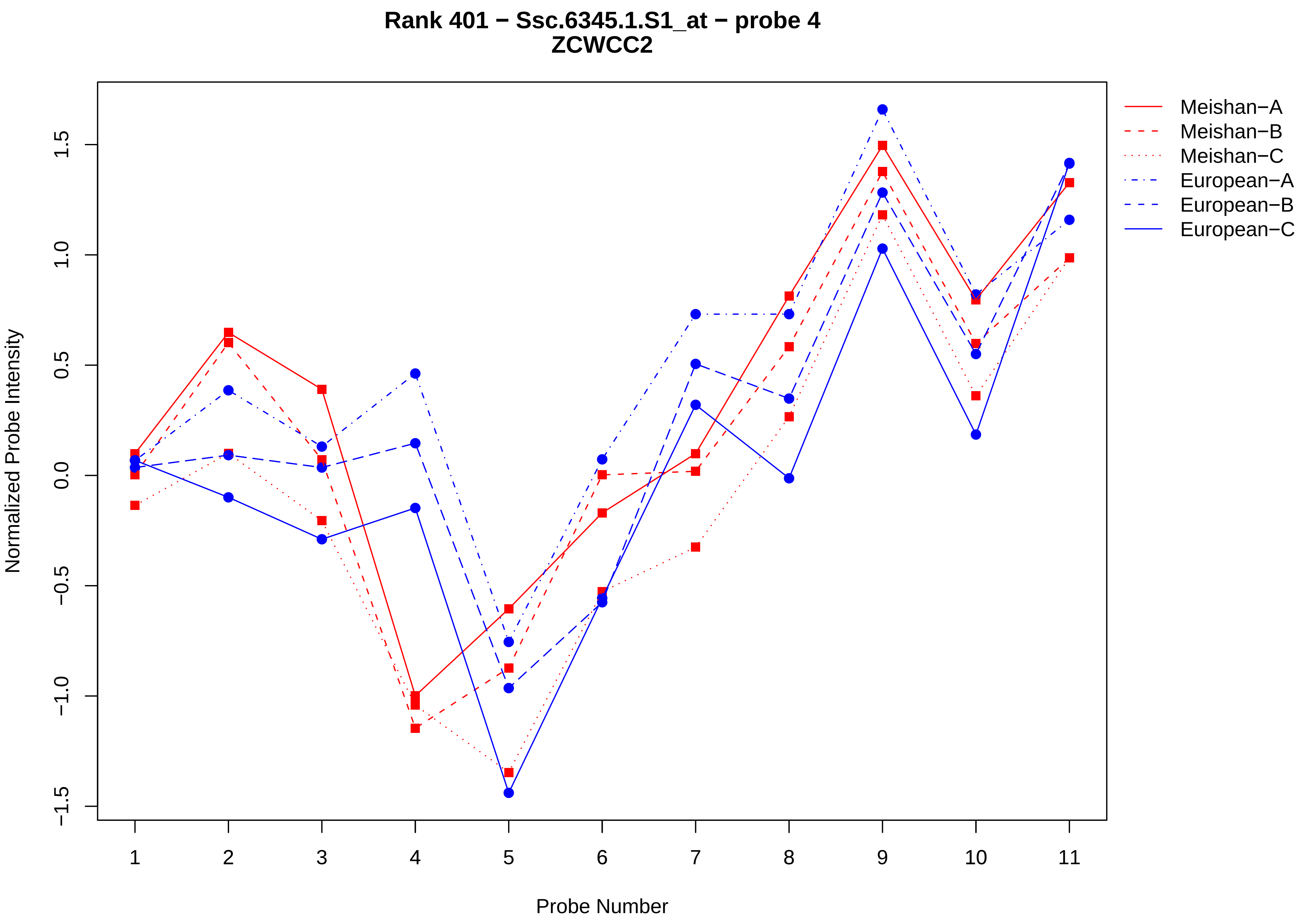

Rank 401 − Ssc.6345.1.S1_at − probe 4
ZCWCC2
Normalized Probe Intensity
Probe Number
−1.5
−1.0
−0.5
0.0
0.5
1.0
1.5
1
2
3
4
5
6
7
8
9
10
11
Meishan−A
Meishan−B
Meishan−C
European−A
European−B
European−C

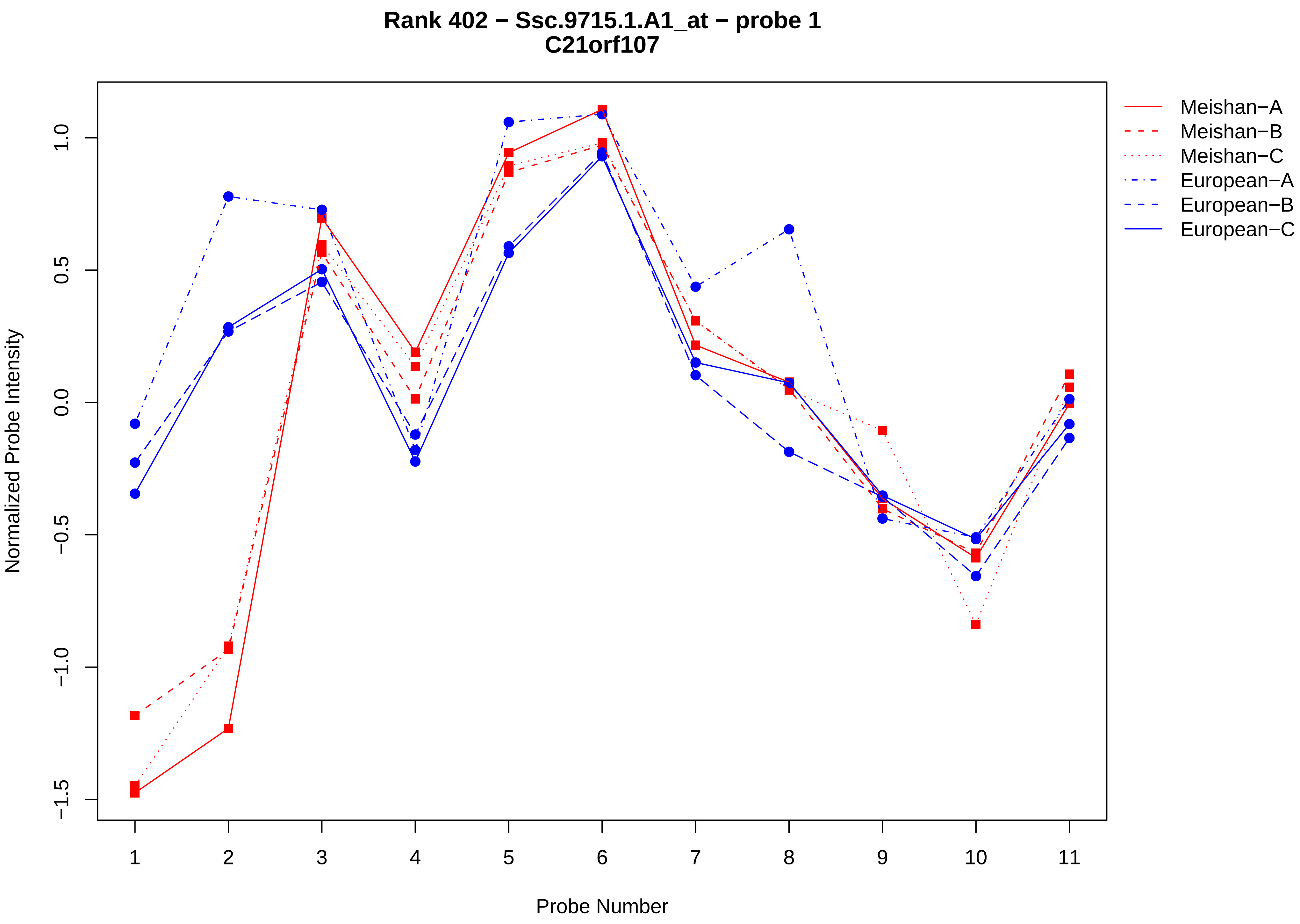

Rank 402 − Ssc.9715.1.A1_at − probe 1
C21orf107
Normalized Probe Intensity
Probe Number
1.0
0.5
0.0
−0.5
−1.0
−1.5
1
2
3
4
5
6
7
8
9
10
11
Meishan−A
Meishan−B
Meishan−C
European−A
European−B
European−C

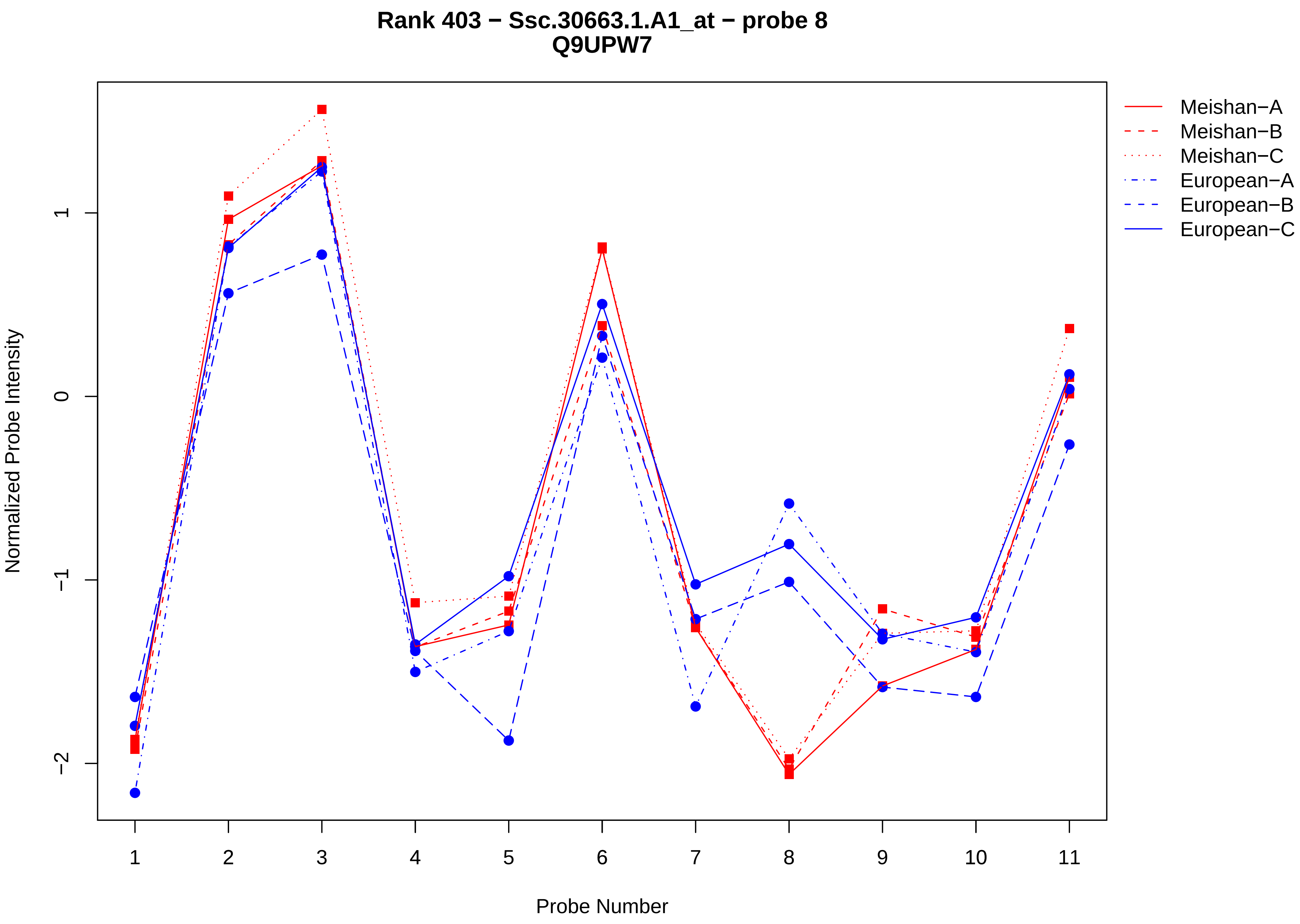

Rank 403 − Ssc.30663.1.A1_at − probe 8
Q9UPW7
Normalized Probe Intensity
Probe Number
−2
−1
0
1
1
2
3
4
5
6
7
8
9
10
11
Meishan−A
Meishan−B
Meishan−C
European−A
European−B
European−C

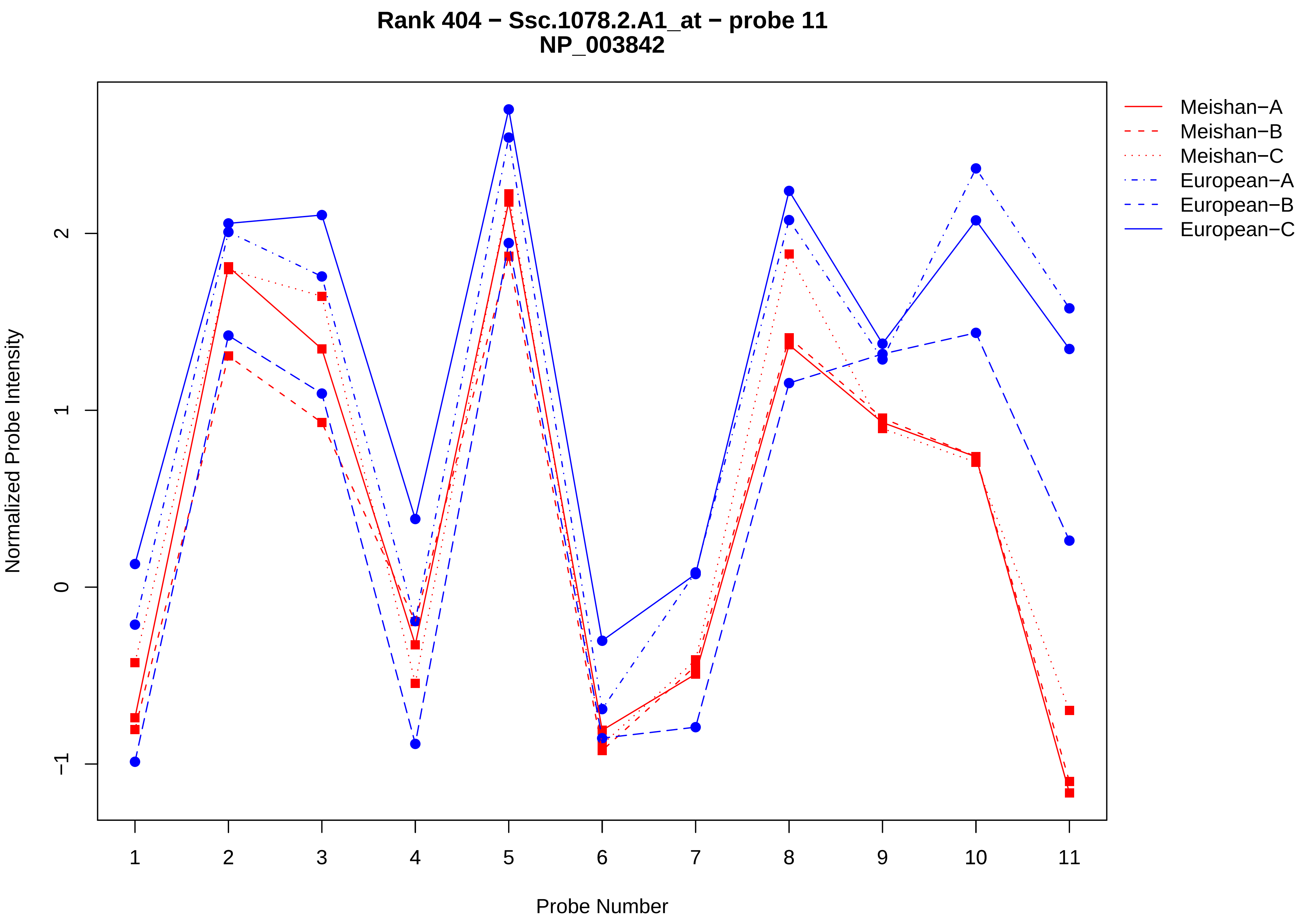

Rank 404 − Ssc.1078.2.A1_at − probe 11
NP_003842
Normalized Probe Intensity
Probe Number
−1
0
1
2
1
2
3
4
5
6
7
8
9
10
11
Meishan−A
Meishan−B
Meishan−C
European−A
European−B
European−C

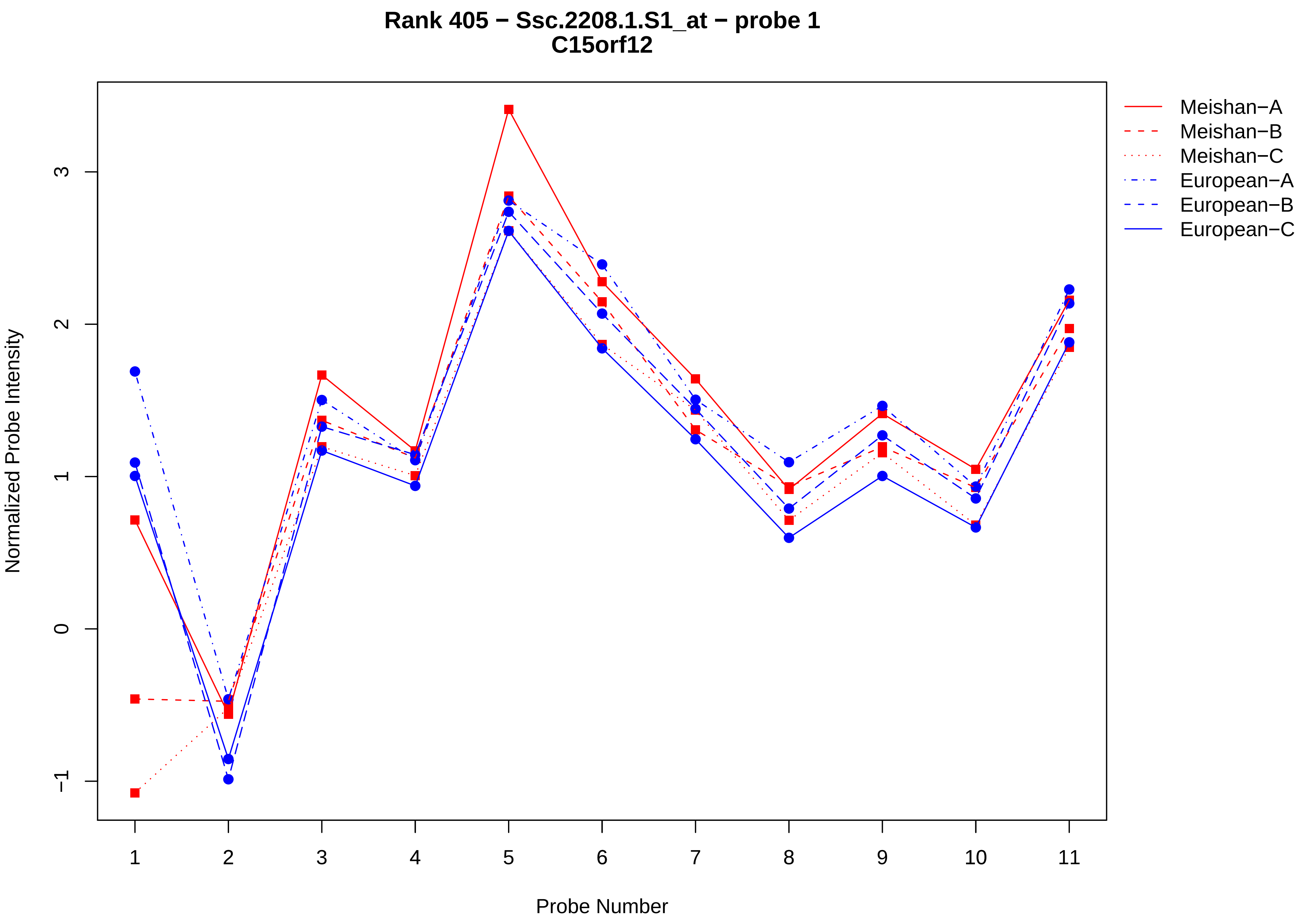

Rank 405 − Ssc.2208.1.S1_at − probe 1
C15orf12
Normalized Probe Intensity
Probe Number
−1
0
1
2
3
1
2
3
4
5
6
7
8
9
10
11
Meishan−A
Meishan−B
Meishan−C
European−A
European−B
European−C

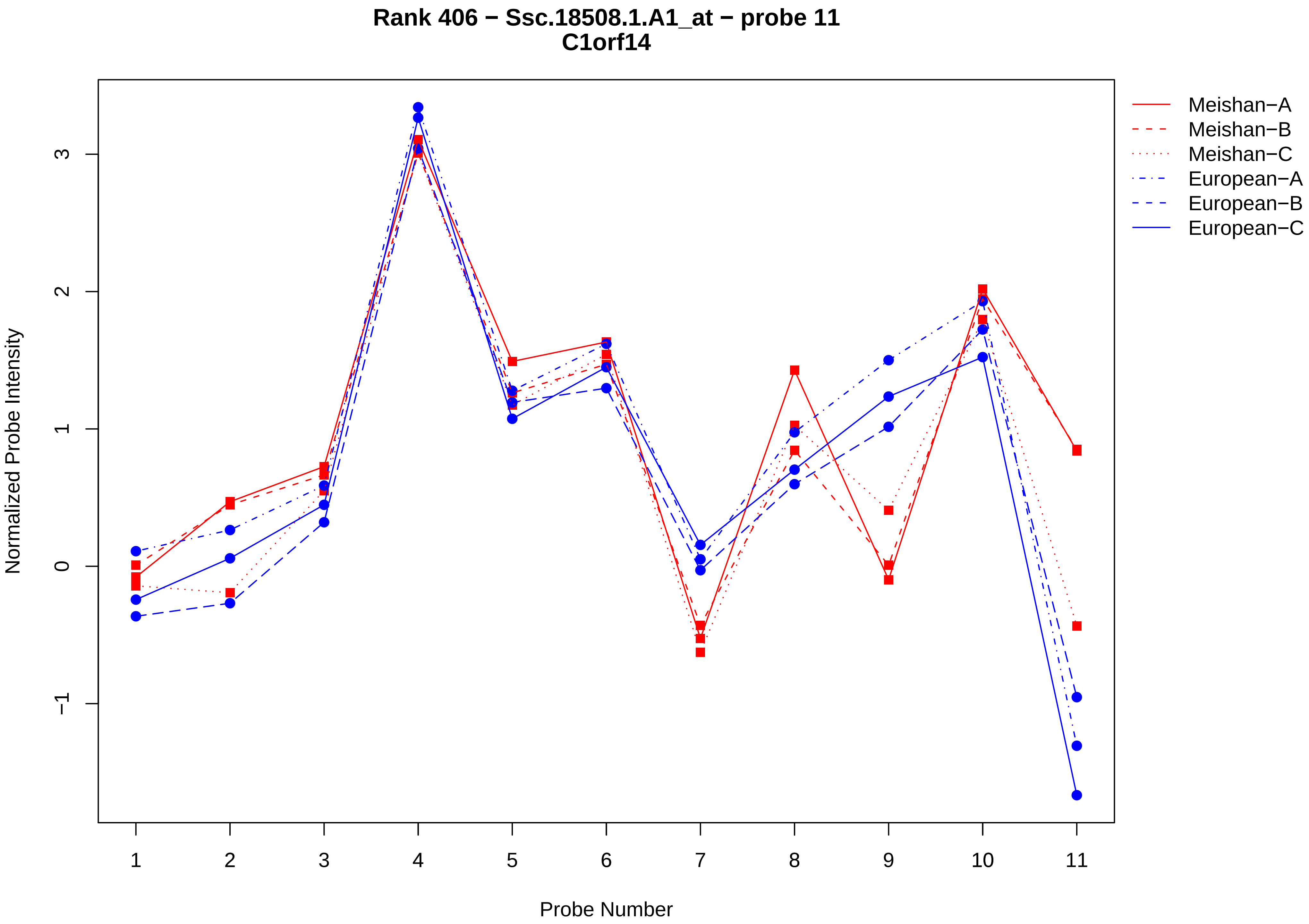
Rank 406 − Ssc.18508.1.A1_at − probe 11
C1orf14
Normalized Probe Intensity
Probe Number
−1
0
1
2
3
1
2
3
4
5
6
7
8
9
10
11
Meishan−A
Meishan−B
Meishan−C
European−A
European−B
European−C

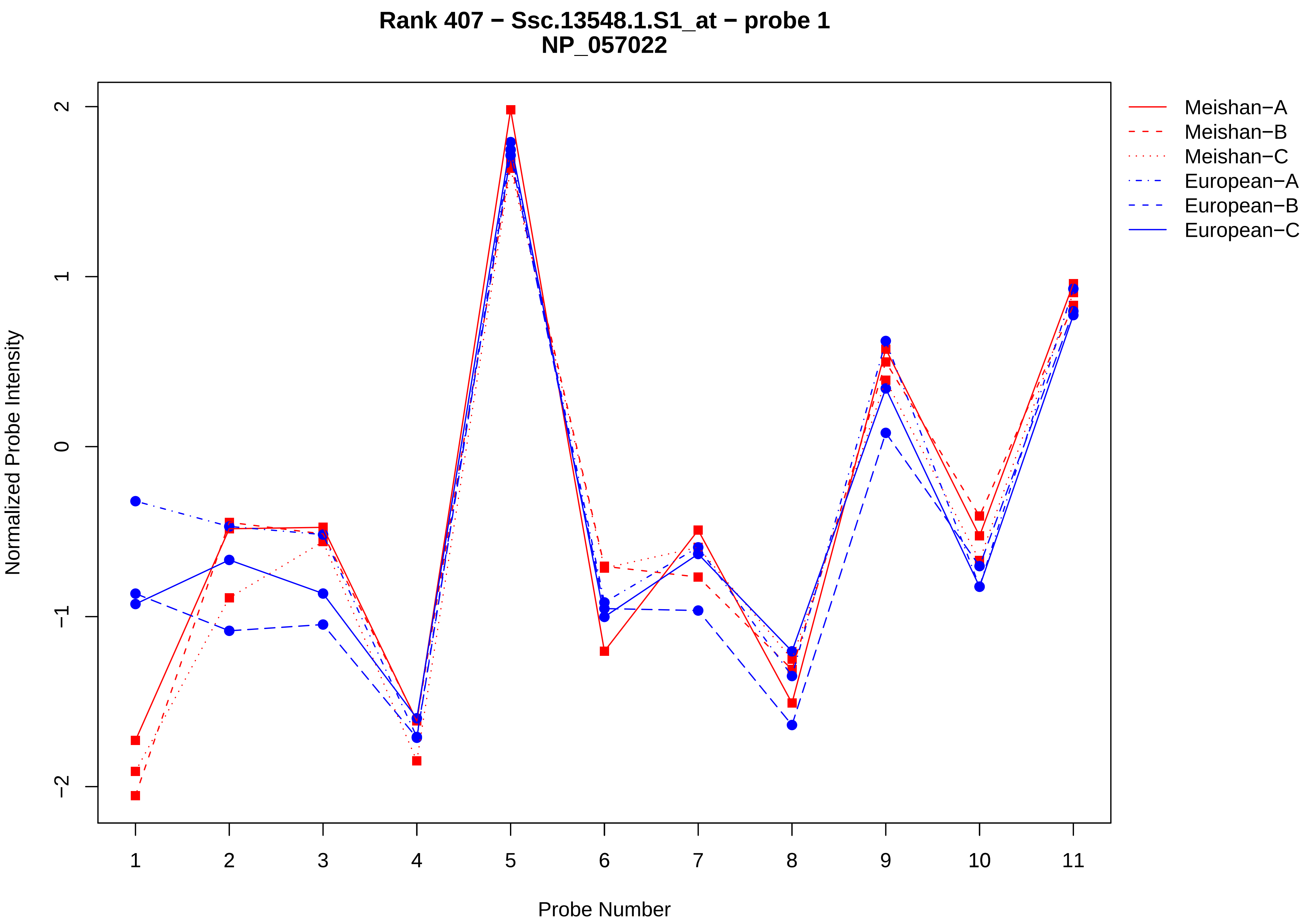

Rank 407 − Ssc.13548.1.S1_at − probe 1
NP_057022
Normalized Probe Intensity
Probe Number
2
1
0
−1
−2
1
2
3
4
5
6
7
8
9
10
11
Meishan−A
Meishan−B
Meishan−C
European−A
European−B
European−C

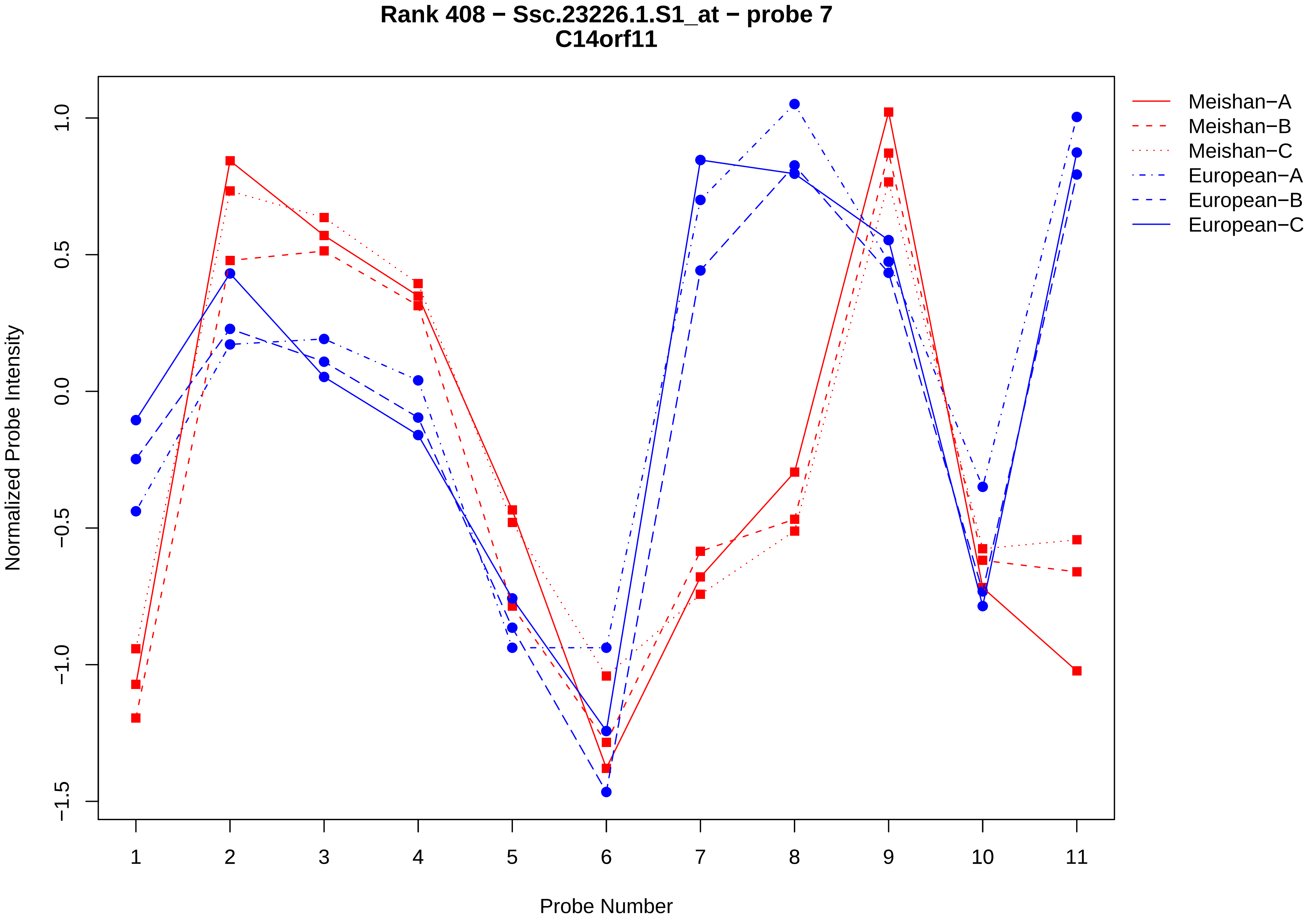

Rank 408 − Ssc.23226.1.S1_at − probe 7
C14orf11
Normalized Probe Intensity
Probe Number
1.0
0.5
0.0
−0.5
−1.0
−1.5
1
2
3
4
5
6
7
8
9
10
11
Meishan−A
Meishan−B
Meishan−C
European−A
European−B
European−C

Rank 409 − Ssc.30972.1.S1_at − probe 2
C12orf14

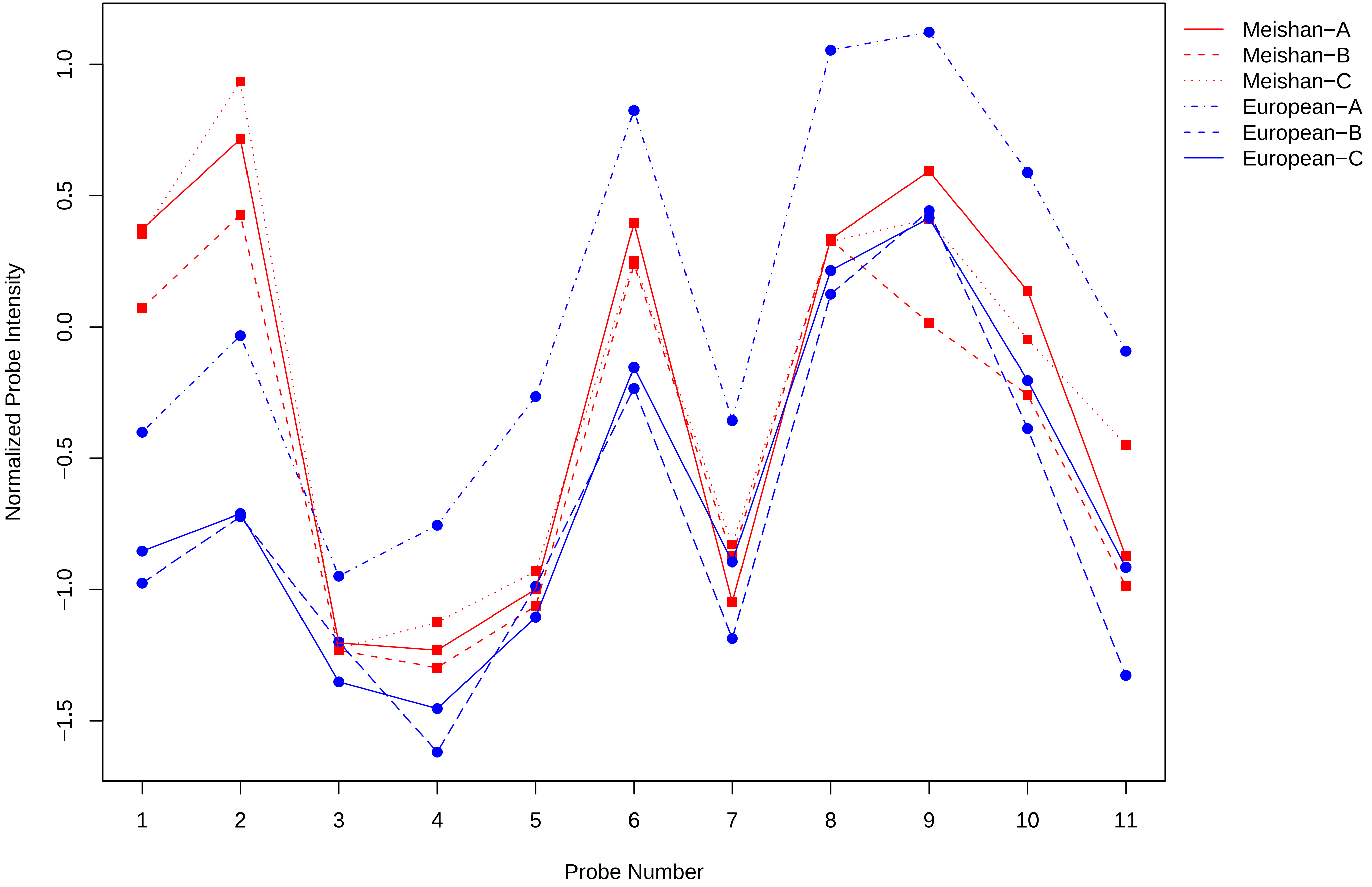

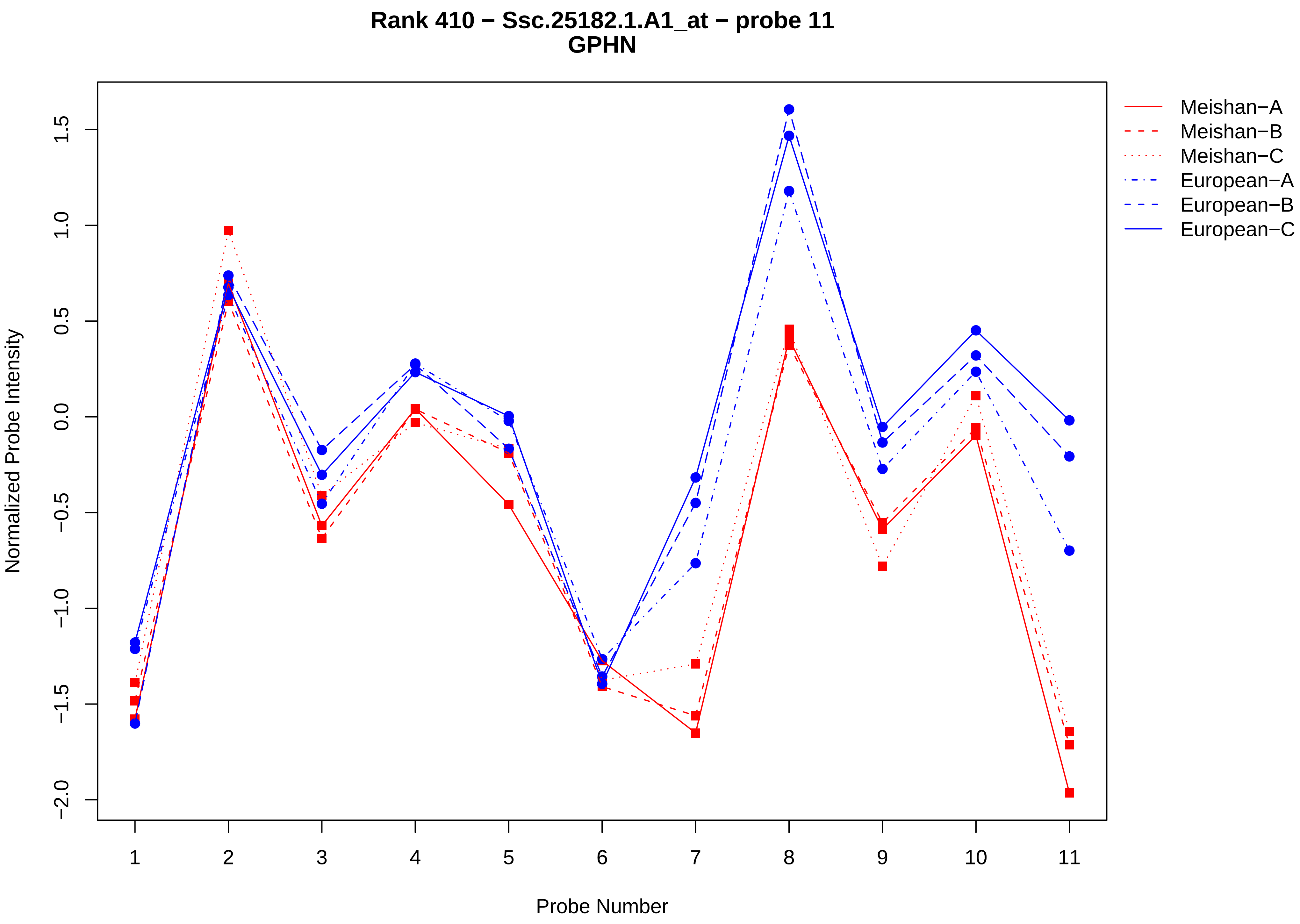

Rank 410 − Ssc.25182.1.A1_at − probe 11
GPHN
Normalized Probe Intensity
Probe Number
−2.0
−1.5
−1.0
−0.5
0.0
0.5
1.0
1.5
1
2
3
4
5
6
7
8
9
10
11
Meishan−A
Meishan−B
Meishan−C
European−A
European−B
European−C

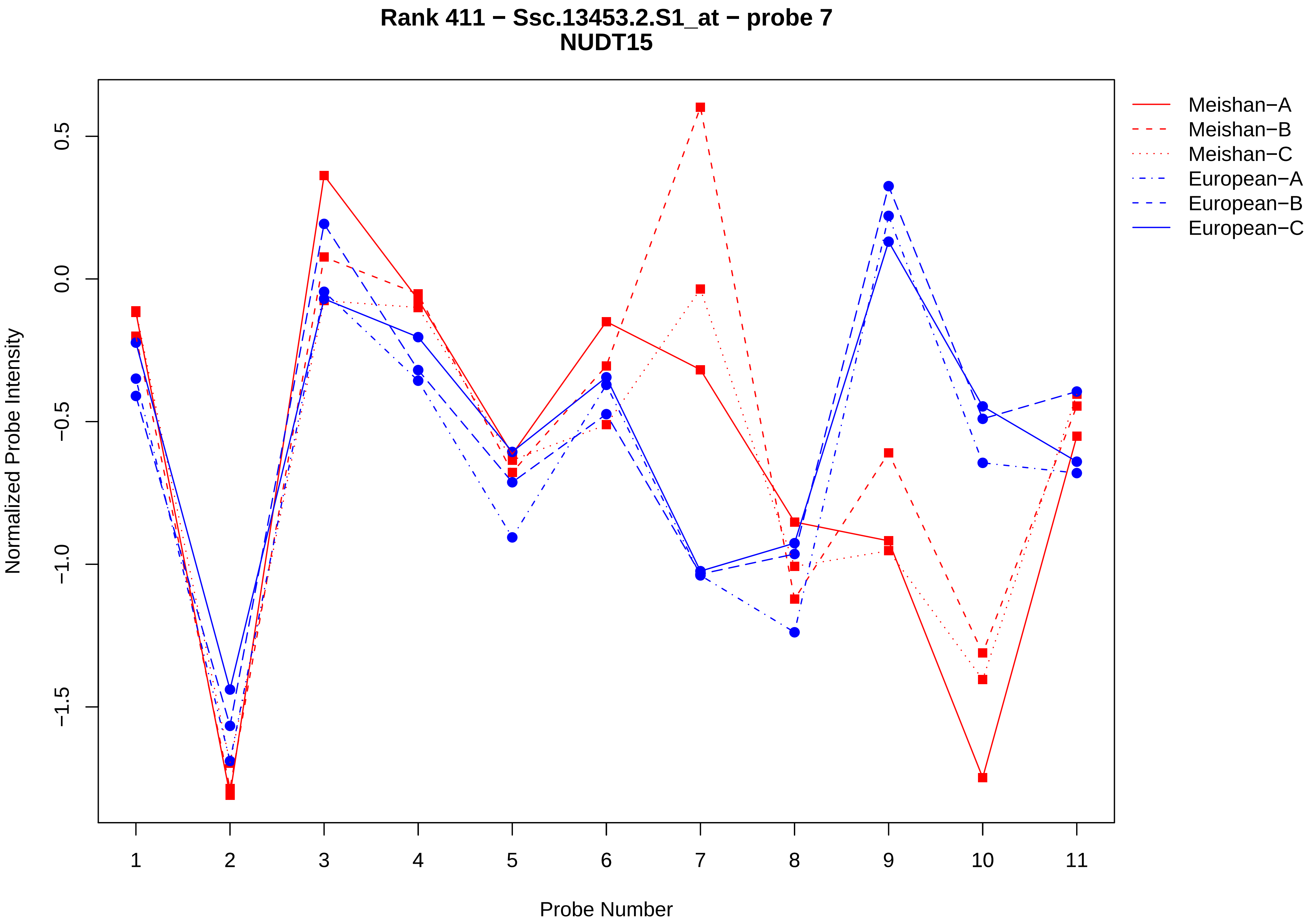

Rank 411 − Ssc.13453.2.S1_at − probe 7
NUDT15
Normalized Probe Intensity
Probe Number
0.5
0.0
−0.5
−1.0
−1.5
1
2
3
4
5
6
7
8
9
10
11
Meishan−A
Meishan−B
Meishan−C
European−A
European−B
European−C

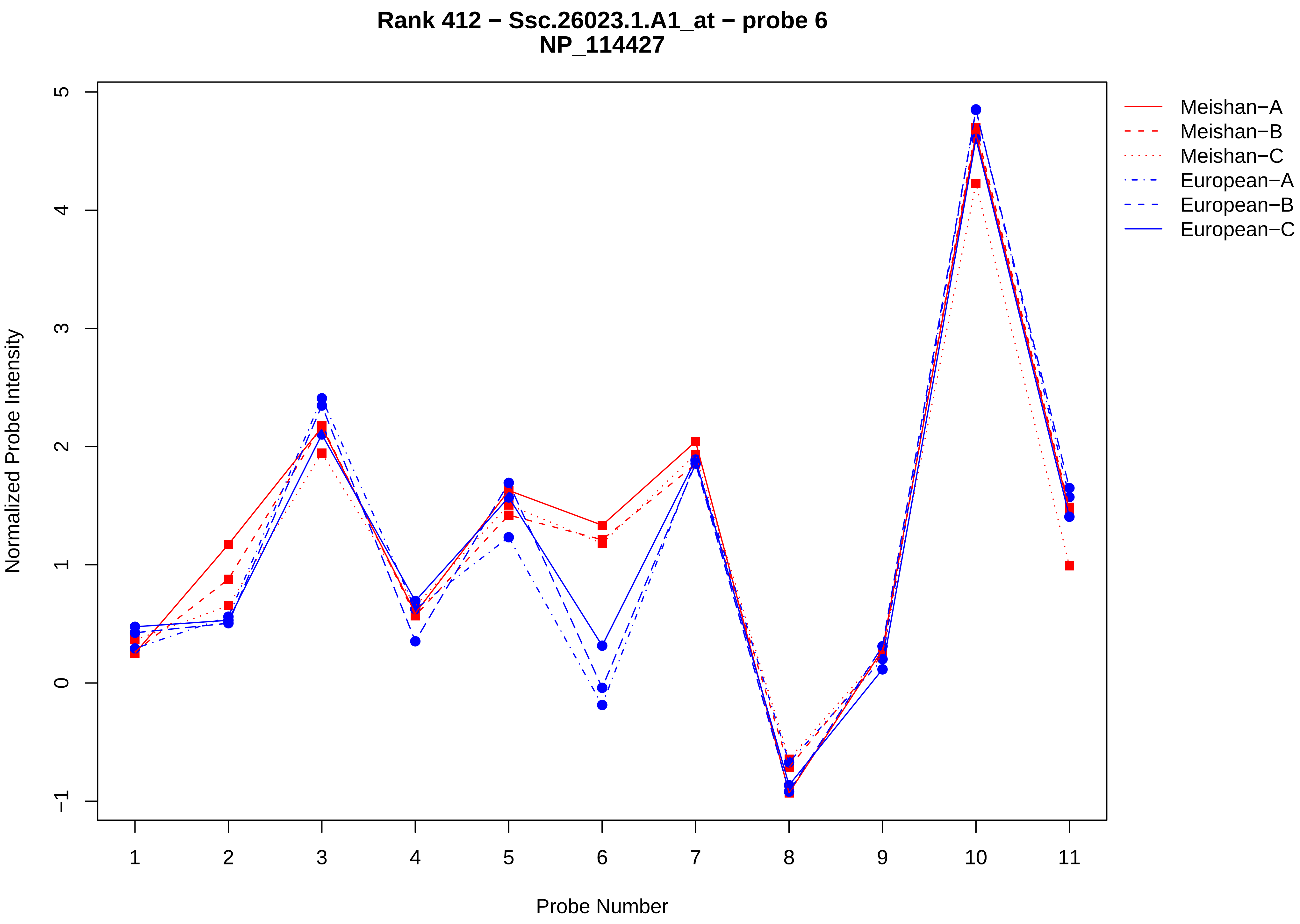

Rank 412 − Ssc.26023.1.A1_at − probe 6
NP_114427
Normalized Probe Intensity
Probe Number
−1
0
1
2
3
4
5
1
2
3
4
5
6
7
8
9
10
11
Meishan−A
Meishan−B
Meishan−C
European−A
European−B
European−C

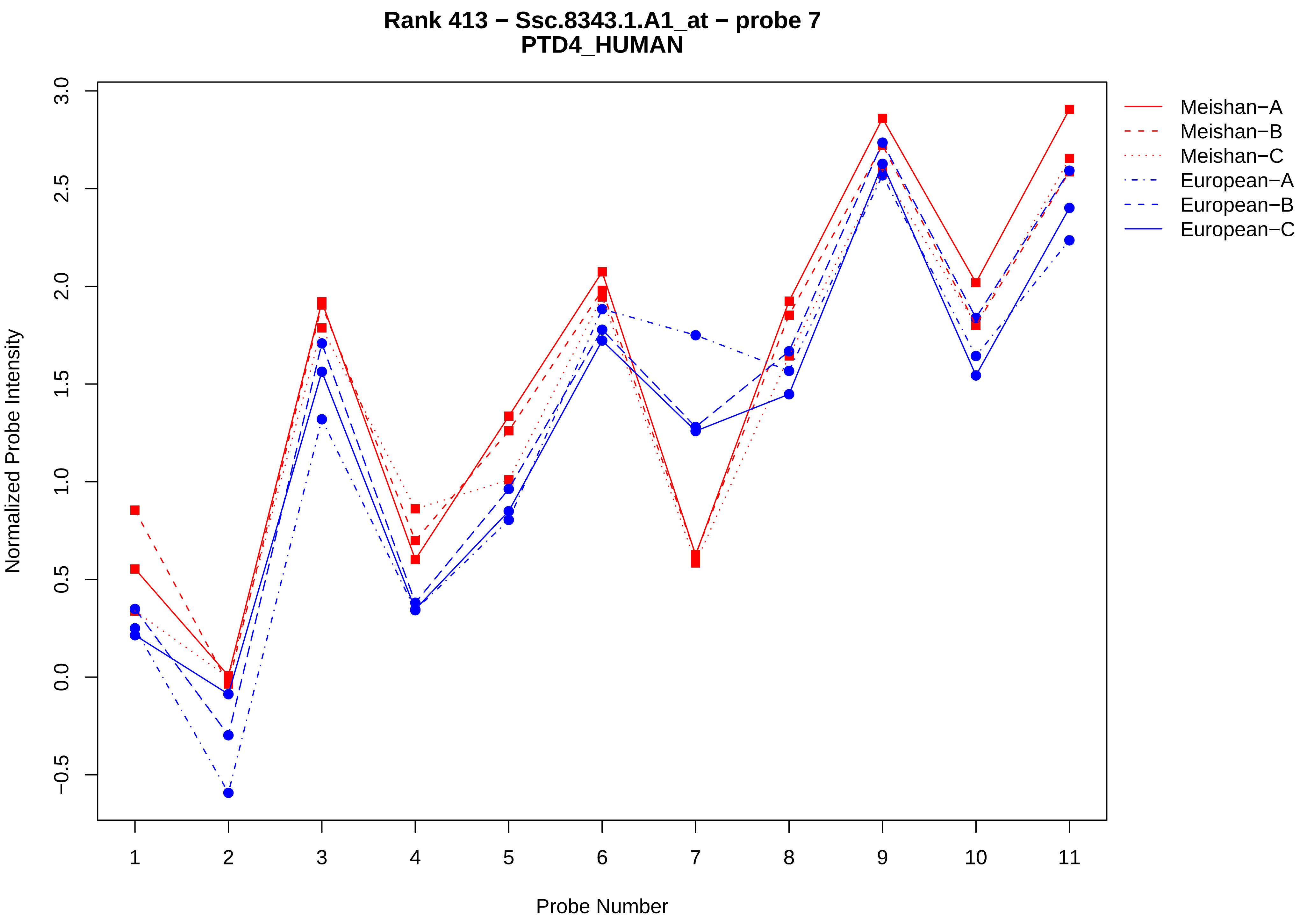

Rank 413 − Ssc.8343.1.A1_at − probe 7
PTD4_HUMAN
Normalized Probe Intensity
Probe Number
−0.5
0.0
0.5
1.0
1.5
2.0
2.5
3.0
1
2
3
4
5
6
7
8
9
10
11
Meishan−A
Meishan−B
Meishan−C
European−A
European−B
European−C

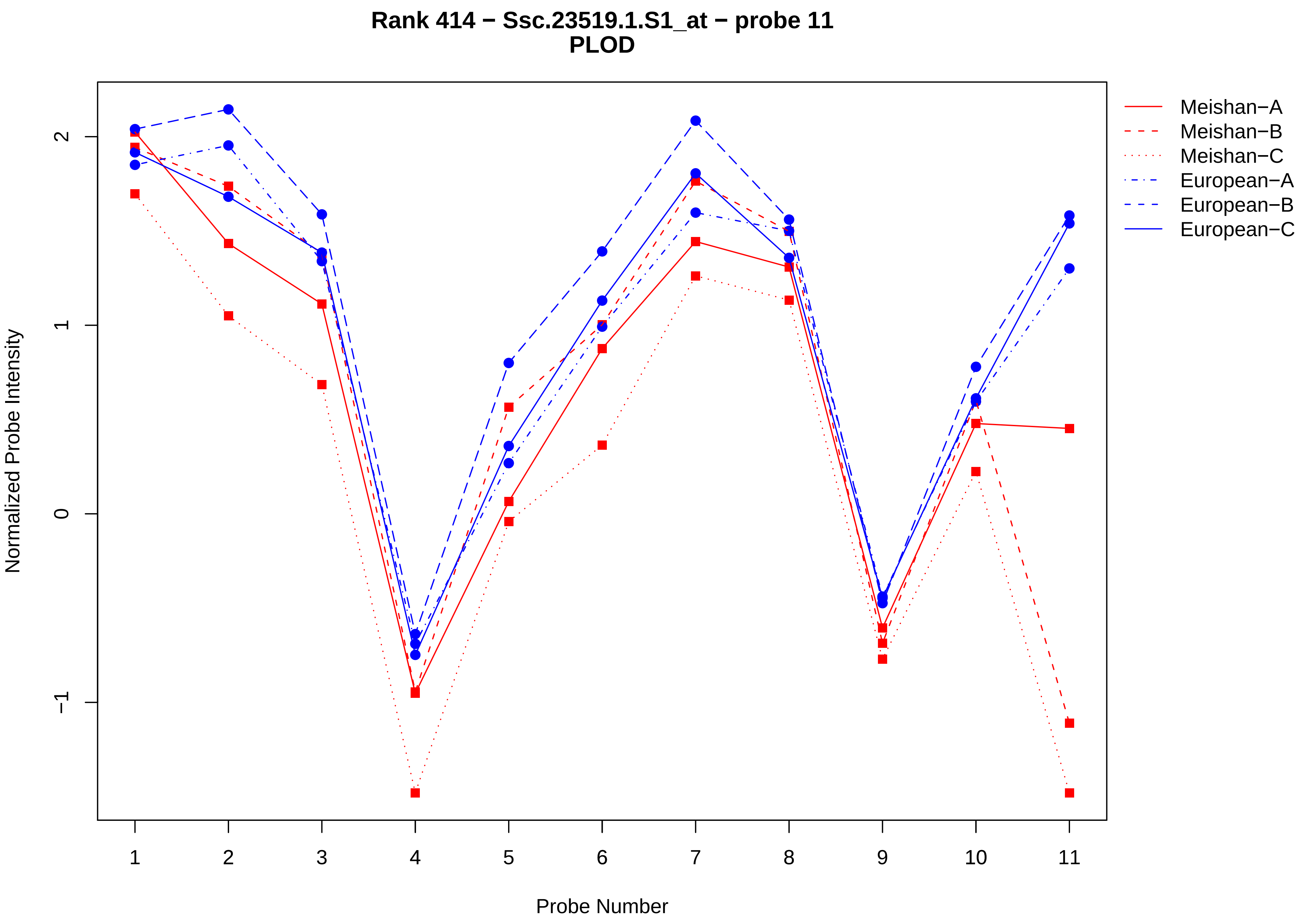
Rank 414 − Ssc.23519.1.S1_at − probe 11
PLOD
Normalized Probe Intensity
Probe Number
2
1
0
−1
1
2
3
4
5
6
7
8
9
10
11
Meishan−A
Meishan−B
Meishan−C
European−A
European−B
European−C

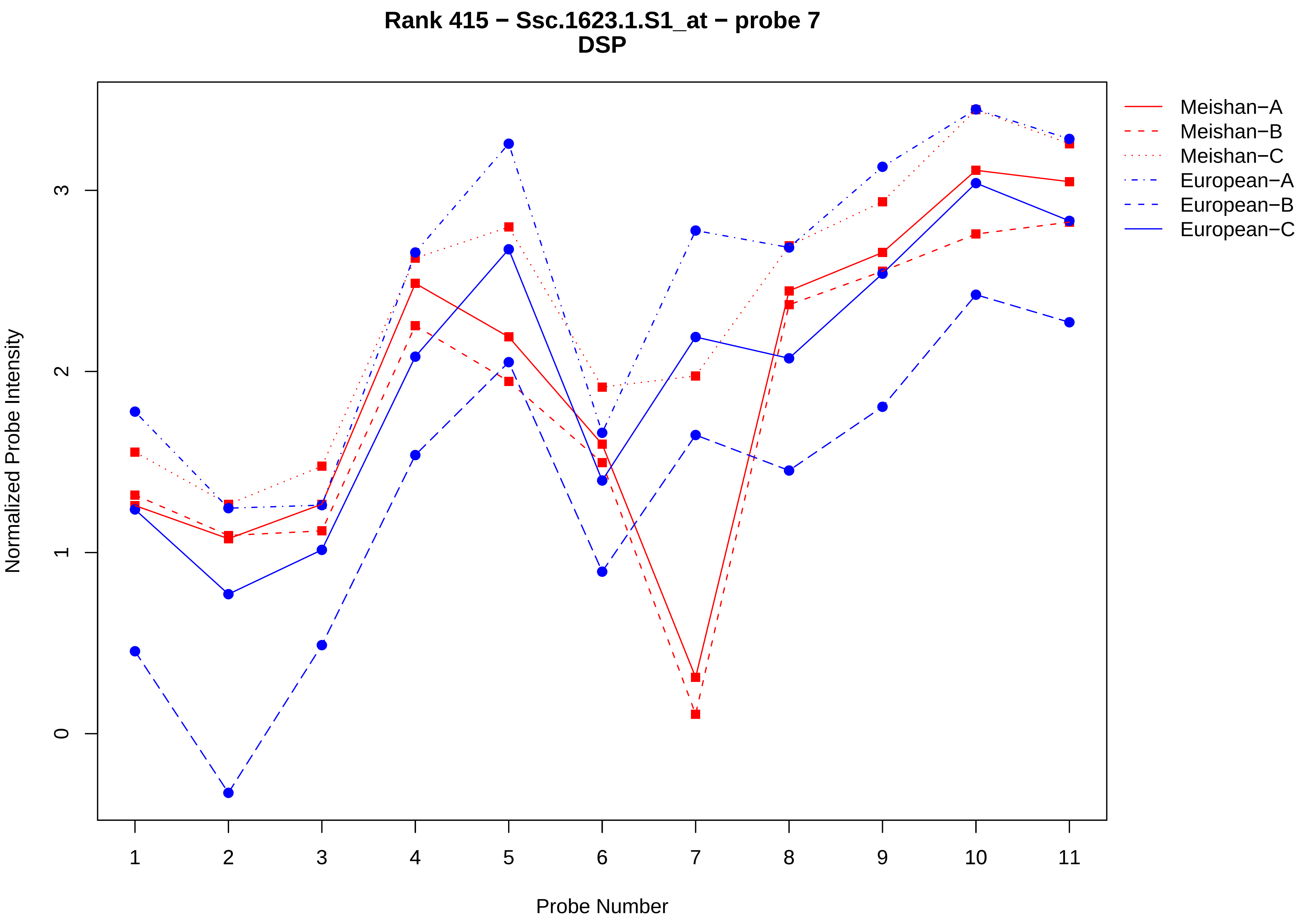

Rank 415 − Ssc.1623.1.S1_at − probe 7
DSP
Normalized Probe Intensity
Probe Number
0
1
2
3
1
2
3
4
5
6
7
8
9
10
11
Meishan−A
Meishan−B
Meishan−C
European−A
European−B
European−C

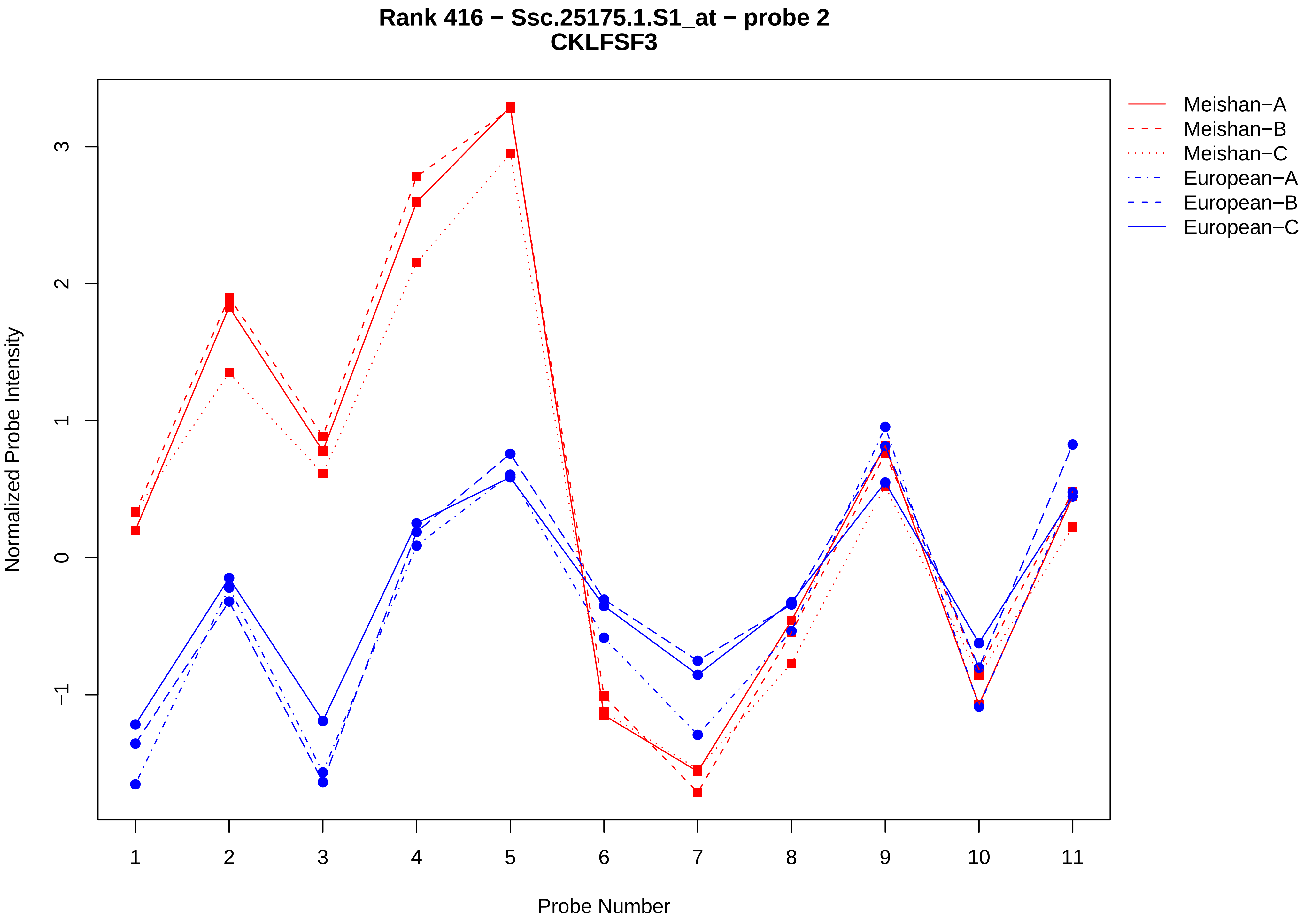

Rank 416 − Ssc.25175.1.S1_at − probe 2
CKLFSF3
Normalized Probe Intensity
Probe Number
−1
0
1
2
3
1
2
3
4
5
6
7
8
9
10
11
Meishan−A
Meishan−B
Meishan−C
European−A
European−B
European−C

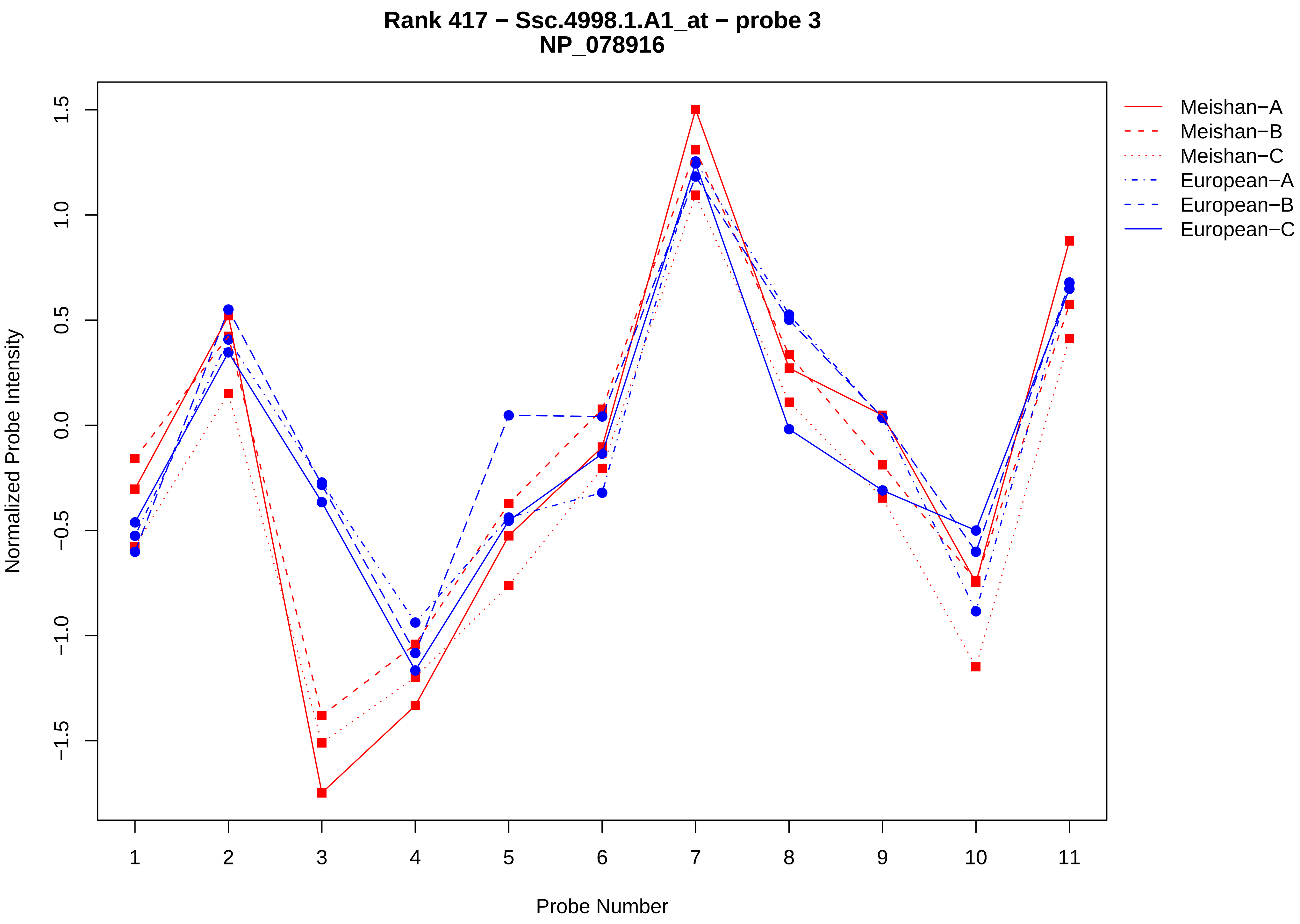

Rank 417 − Ssc.4998.1.A1_at − probe 3
NP_078916
Normalized Probe Intensity
Probe Number
−1.5
−1.0
−0.5
0.0
0.5
1.0
1.5
1
2
3
4
5
6
7
8
9
10
11
Meishan−A
Meishan−B
Meishan−C
European−A
European−B
European−C

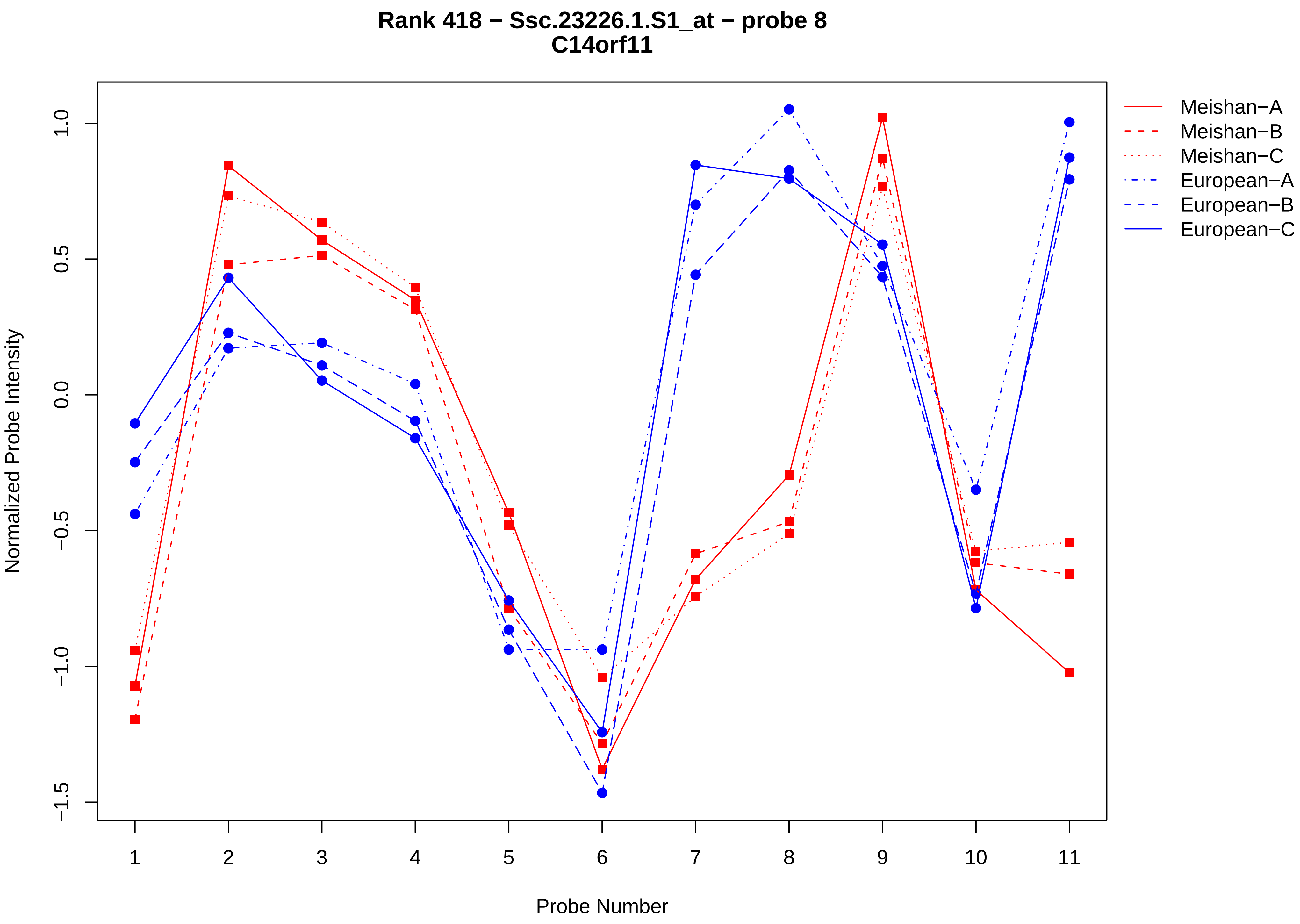

Rank 418 − Ssc.23226.1.S1_at − probe 8
C14orf11
Normalized Probe Intensity
Probe Number
Meishan−A
Meishan−B
Meishan−C
European−A
European−B
European−C
1.0
0.5
0.0
−0.5
−1.0
−1.5
1
2
3
4
5
6
7
8
9
10
11

Rank 419 − Ssc.6356.1.S1_at − probe 5
ODC1

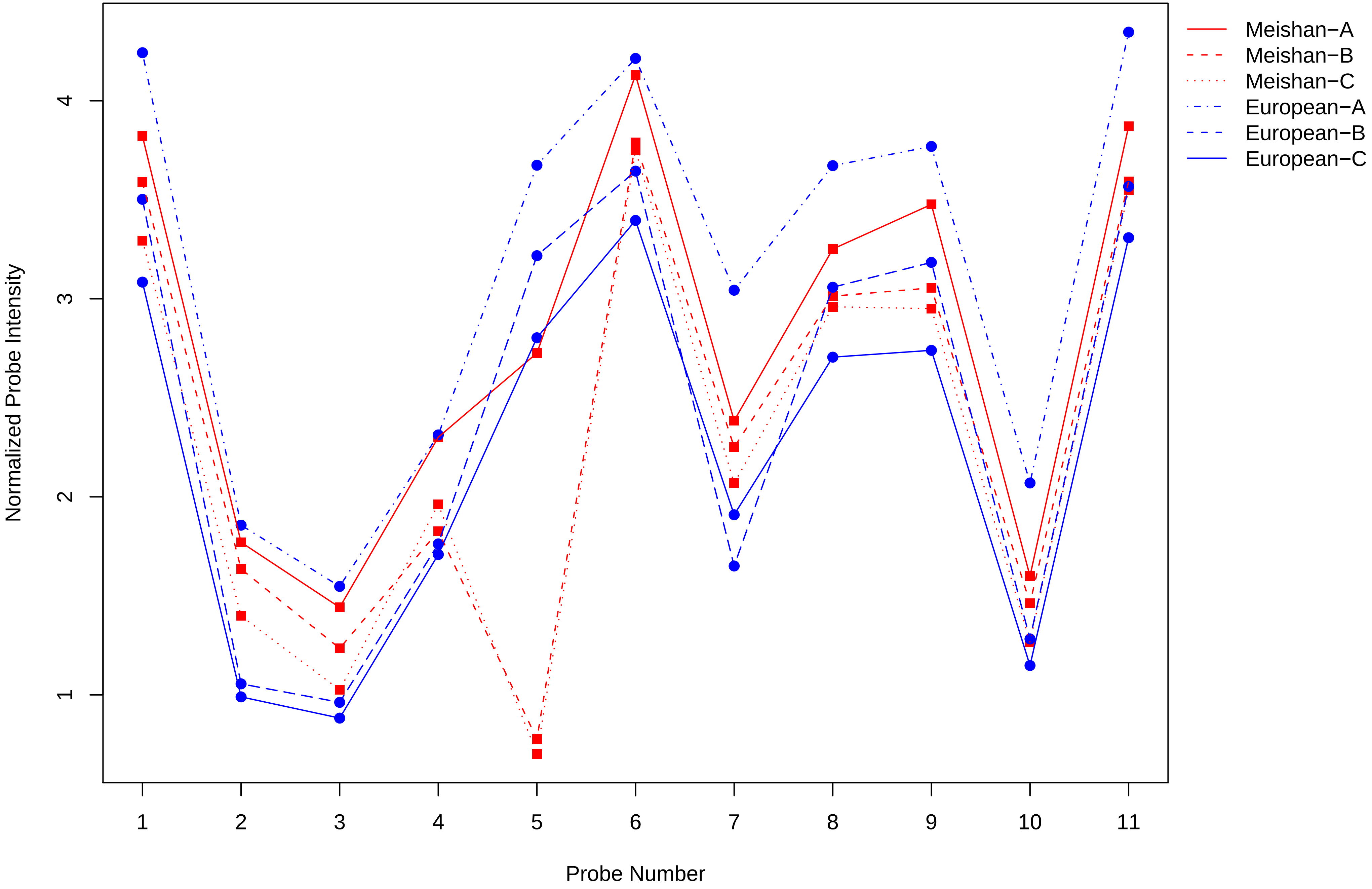

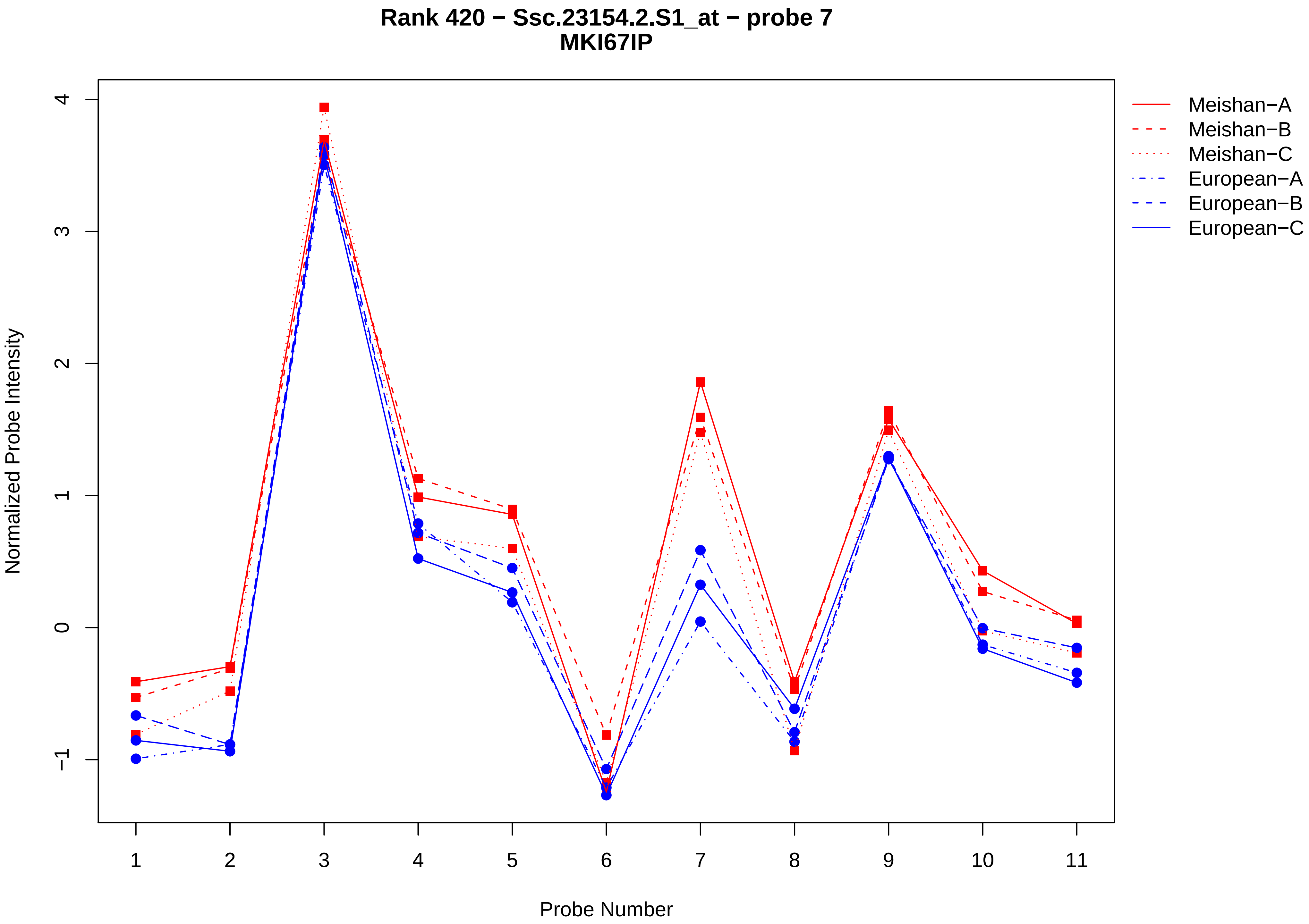

Rank 420 − Ssc.23154.2.S1_at − probe 7
MKI67IP
Normalized Probe Intensity
Probe Number
−1
0
1
2
3
4
1
2
3
4
5
6
7
8
9
10
11
Meishan−A
Meishan−B
Meishan−C
European−A
European−B
European−C

Rank 421 − Ssc.15924.1.S1_at − probe 9
ACVR2

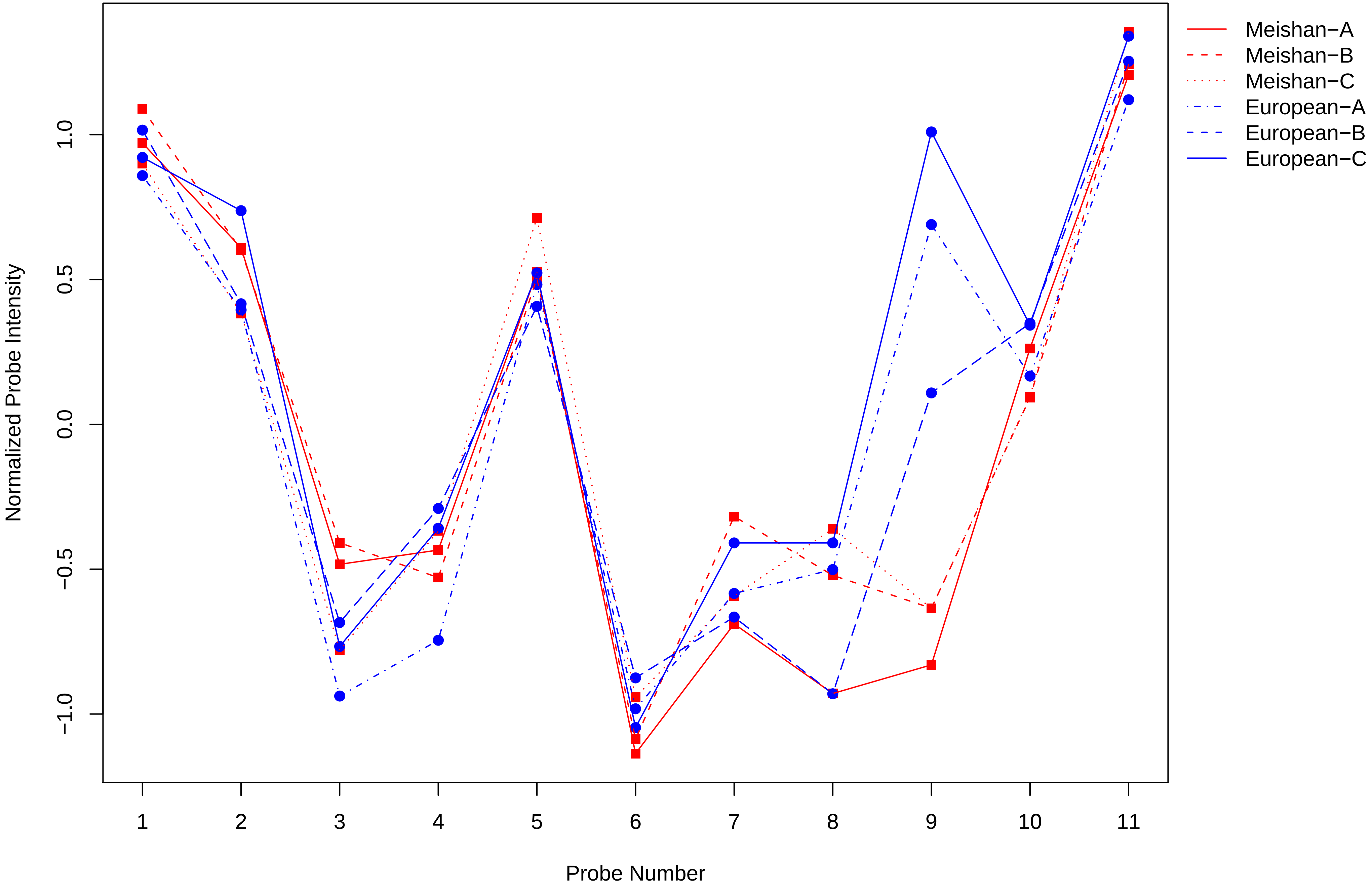

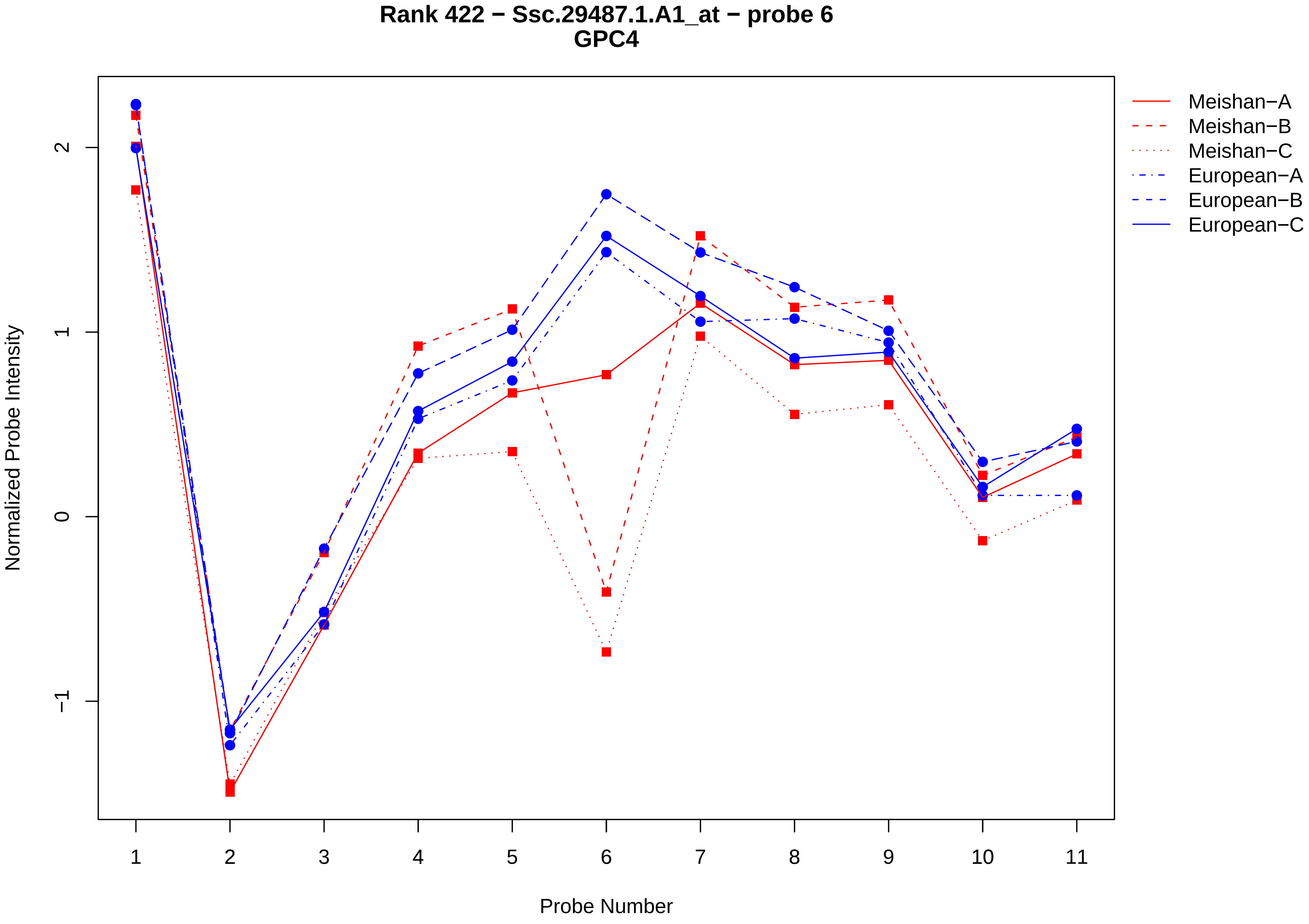

Rank 422 − Ssc.29487.1.A1_at − probe 6
GPC4
Normalized Probe Intensity
Probe Number
Meishan−A
Meishan−B
Meishan−C
European−A
European−B
European−C

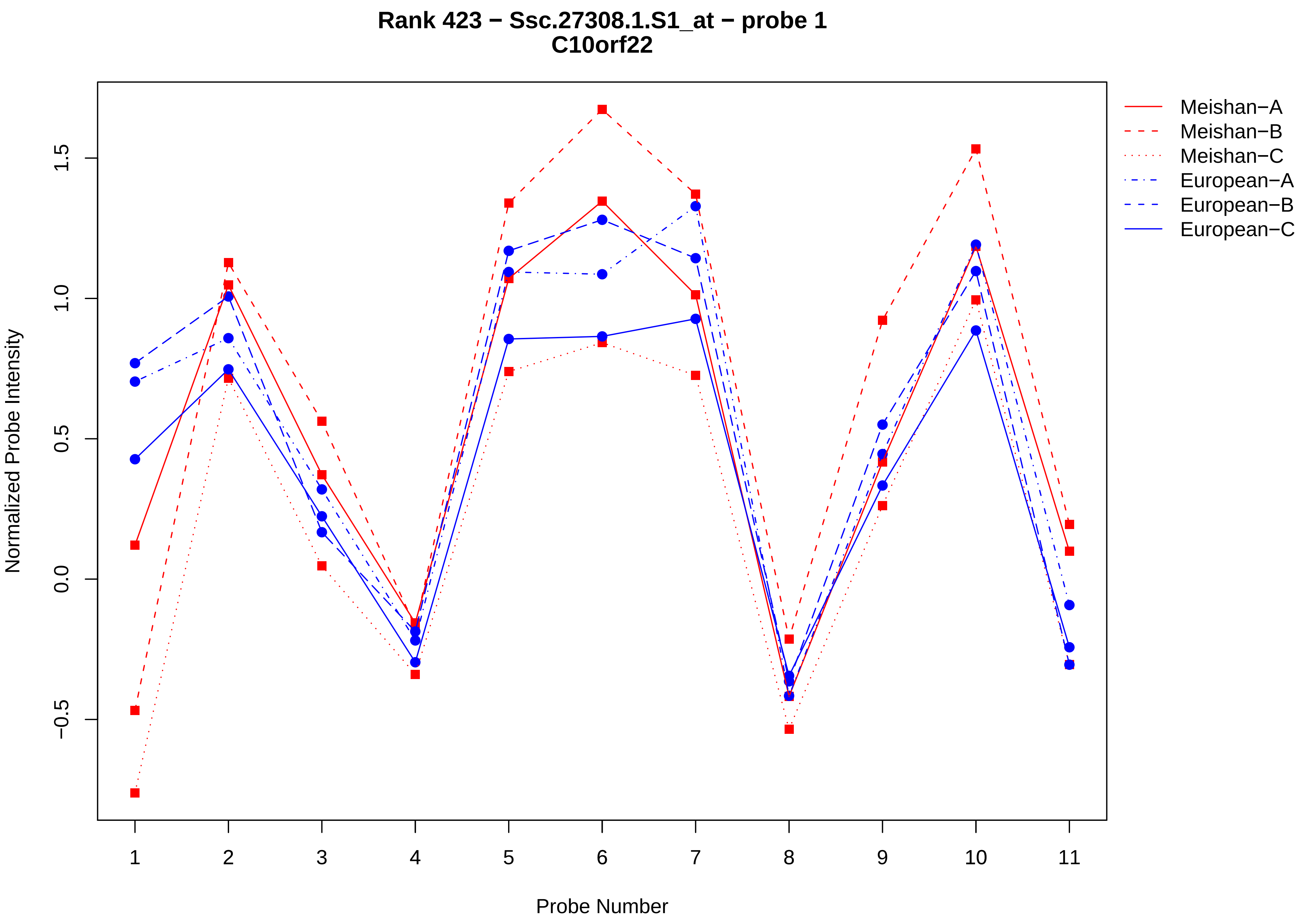

Rank 423 − Ssc.27308.1.S1_at − probe 1
C10orf22
Normalized Probe Intensity
Probe Number
−0.5
0.0
0.5
1.0
1.5
1
2
3
4
5
6
7
8
9
10
11
Meishan−A
Meishan−B
Meishan−C
European−A
European−B
European−C

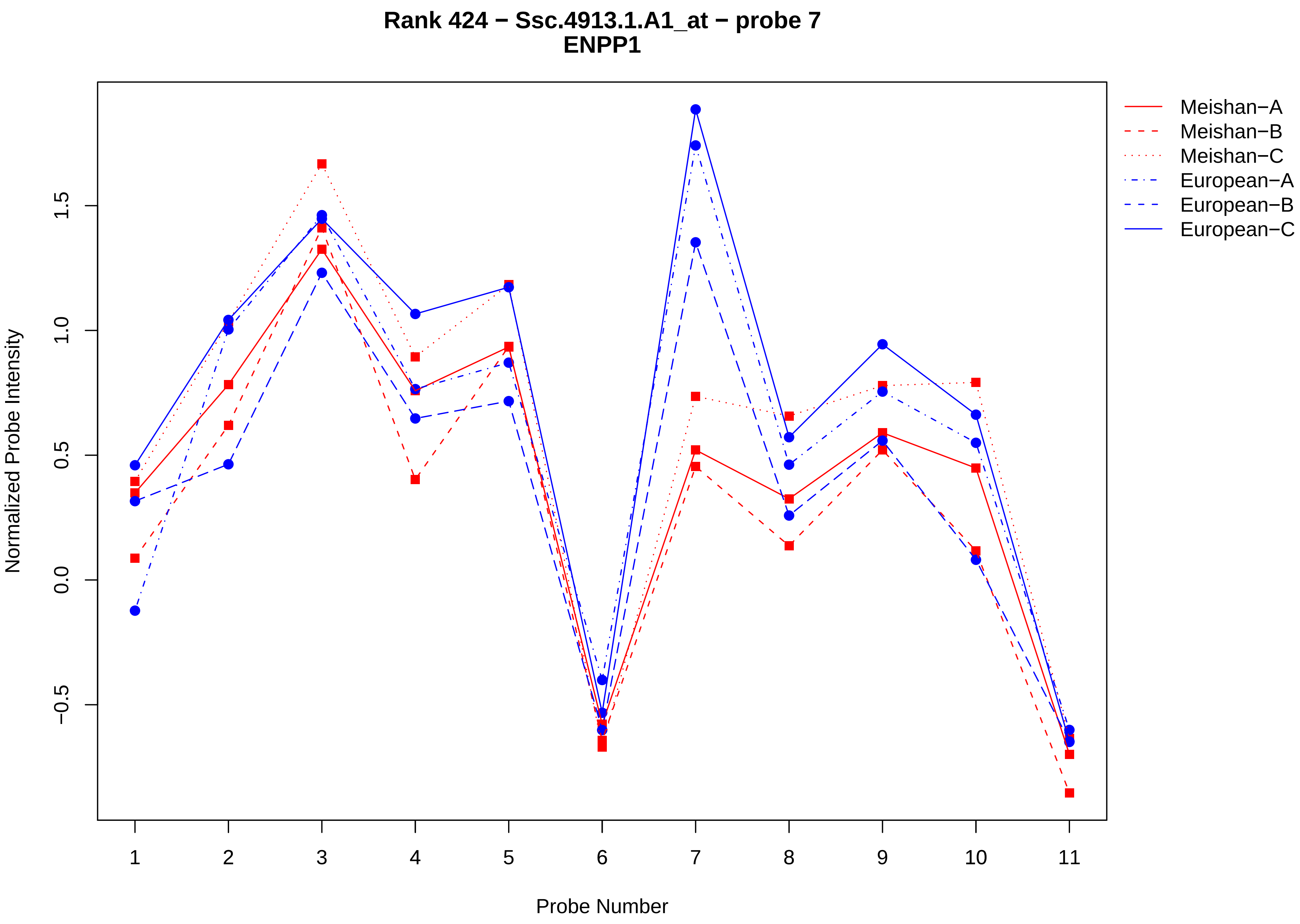
Rank 424 − Ssc.4913.1.A1_at − probe 7
ENPP1
Normalized Probe Intensity
Probe Number
−0.5
0.0
0.5
1.0
1.5
1
2
3
4
5
6
7
8
9
10
11
Meishan−A
Meishan−B
Meishan−C
European−A
European−B
European−C

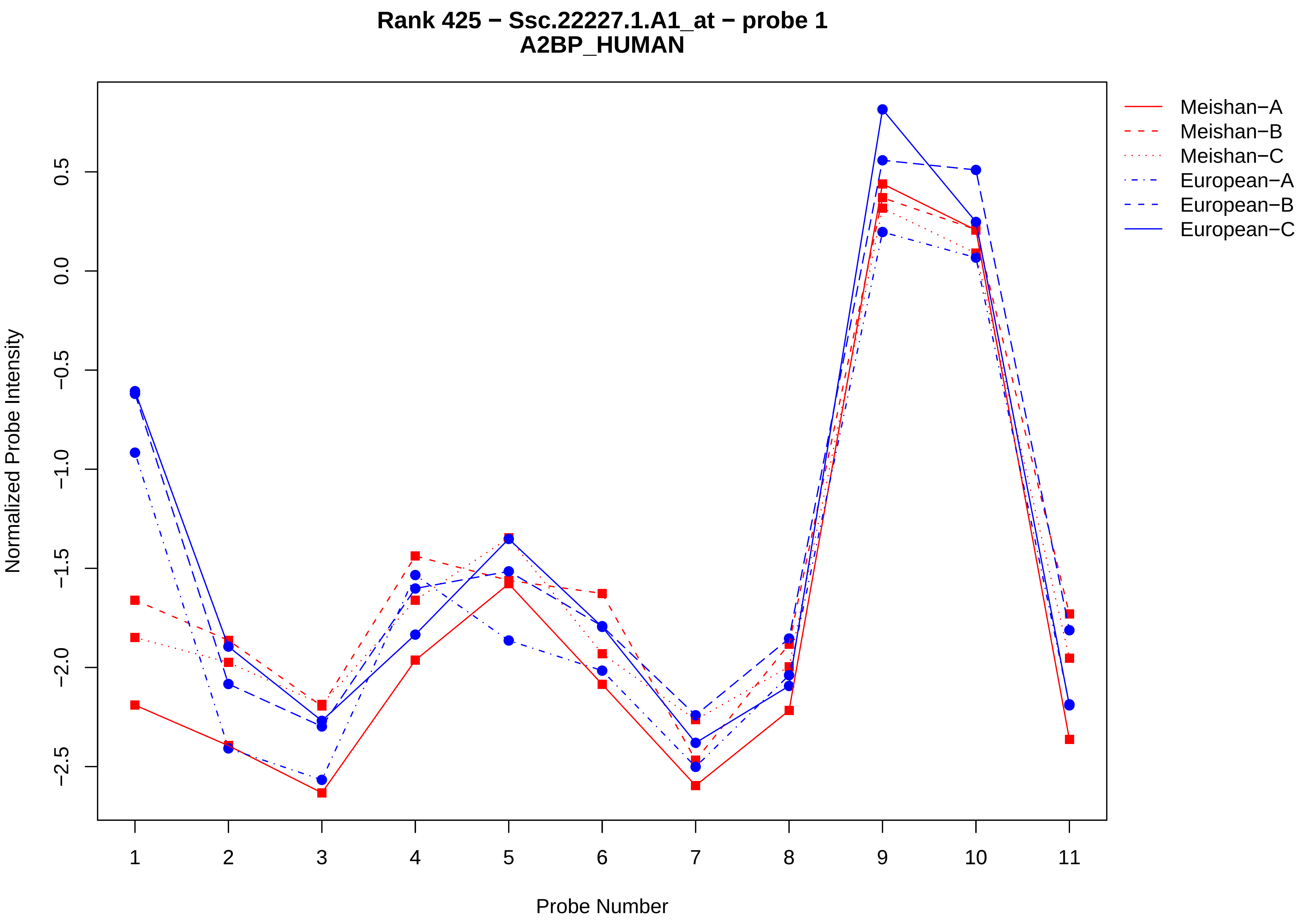

Rank 425 − Ssc.22227.1.A1_at − probe 1
A2BP_HUMAN
Normalized Probe Intensity
Probe Number
0.5
0.0
−0.5
−1.0
−1.5
−2.0
−2.5
1
2
3
4
5
6
7
8
9
10
11
Meishan−A
Meishan−B
Meishan−C
European−A
European−B
European−C

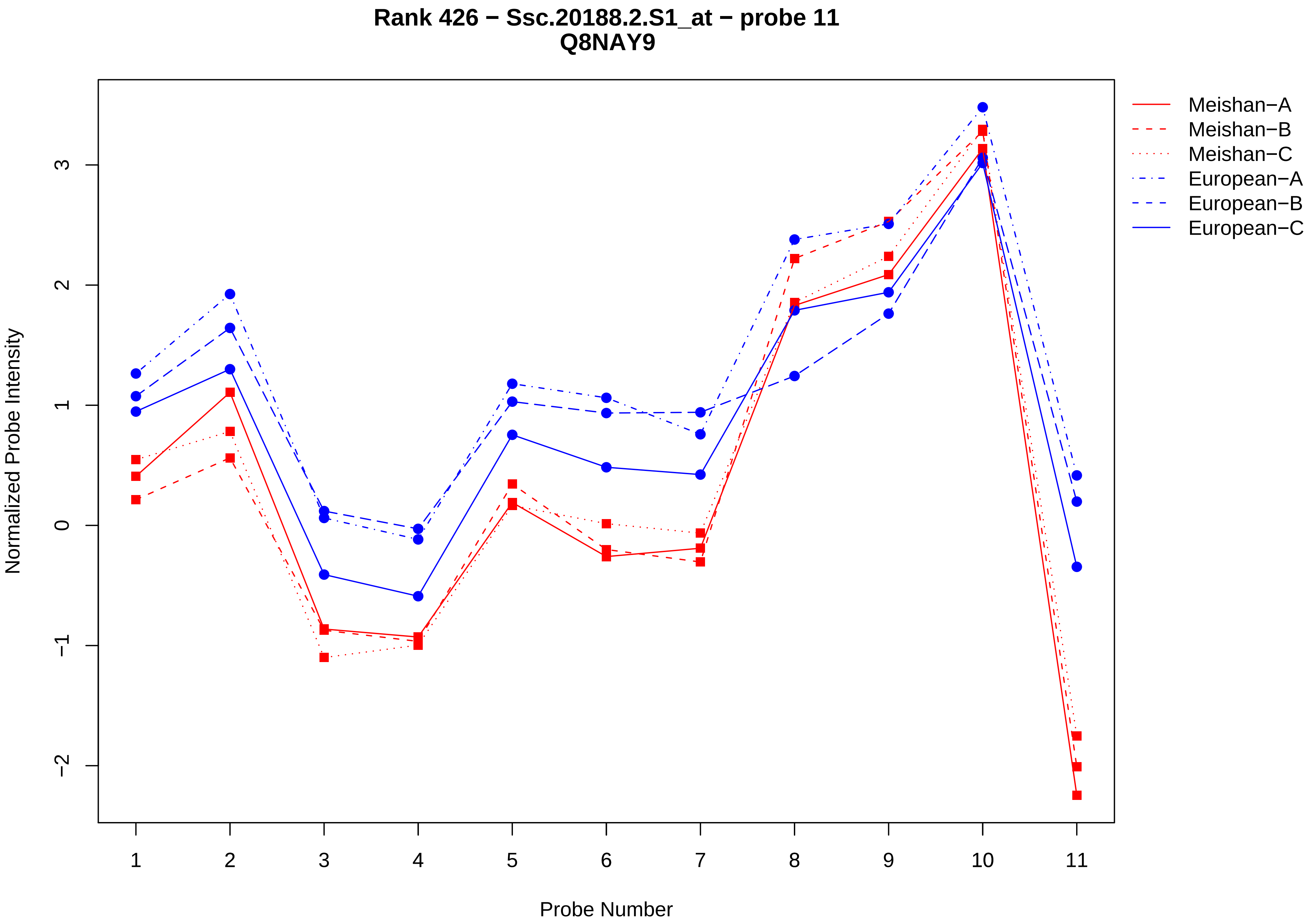

Rank 426 − Ssc.20188.2.S1_at − probe 11
Q8NAY9
Normalized Probe Intensity
−2
−1
0
1
2
3
1
2
3
4
5
6
7
8
9
10
11
Probe Number
Meishan−A
Meishan−B
Meishan−C
European−A
European−B
European−C

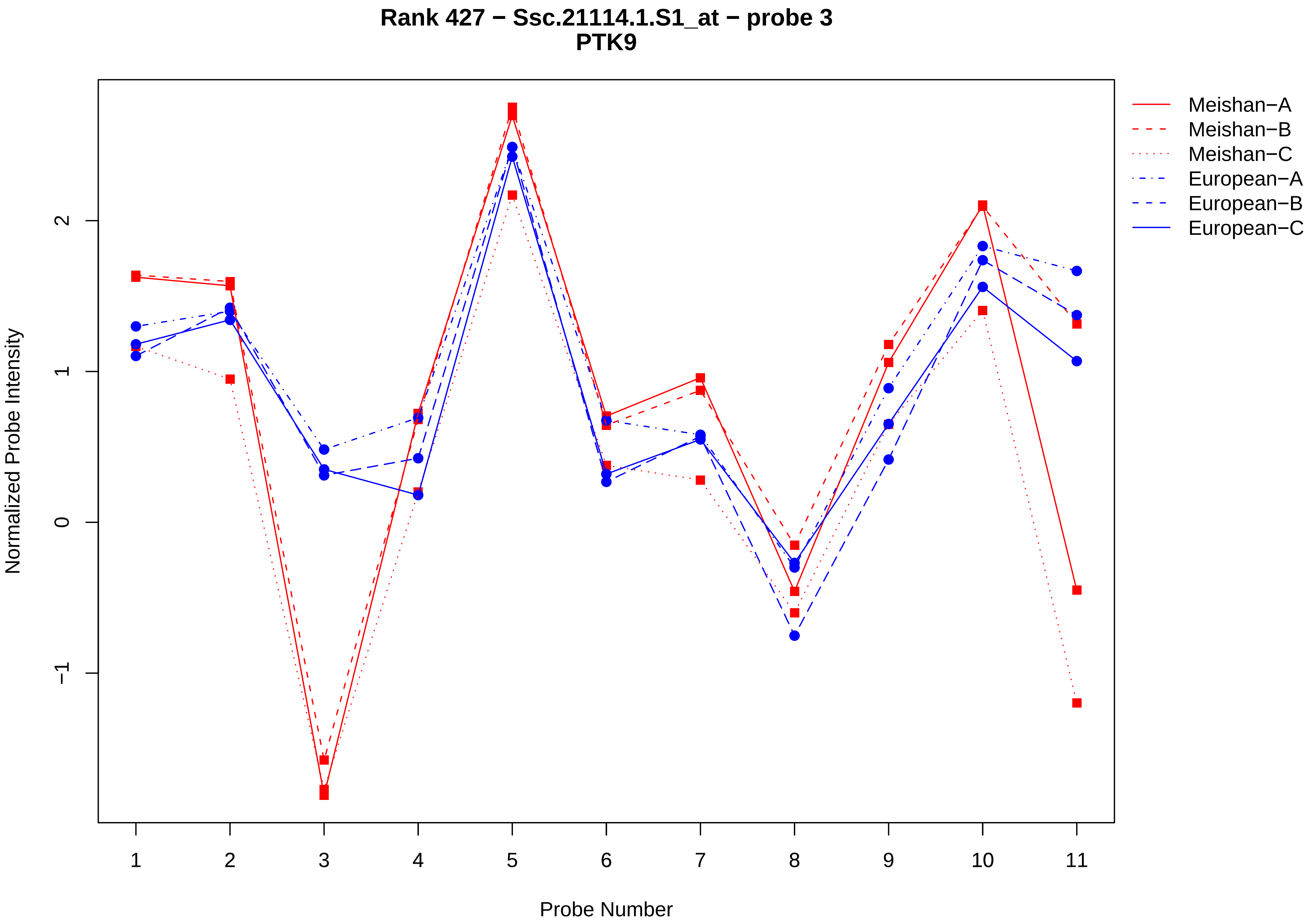

Rank 427 − Ssc.21114.1.S1_at − probe 3
PTK9
Normalized Probe Intensity
Probe Number
−1
0
1
2
1
2
3
4
5
6
7
8
9
10
11
Meishan−A
Meishan−B
Meishan−C
European−A
European−B
European−C

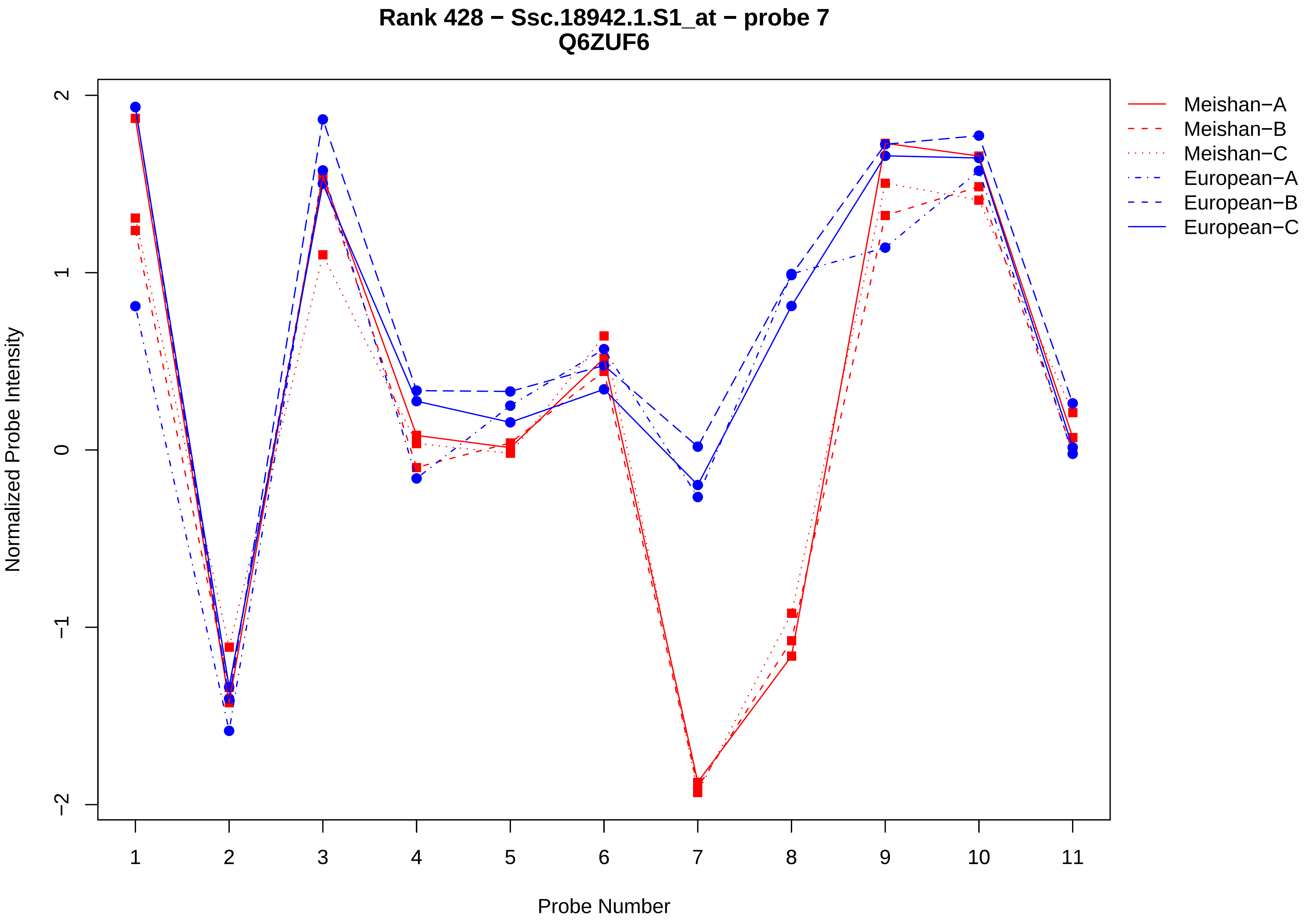

Rank 428 − Ssc.18942.1.S1_at − probe 7
Q6ZUF6
Normalized Probe Intensity
Probe Number
Meishan−A
Meishan−B
Meishan−C
European−A
European−B
European−C

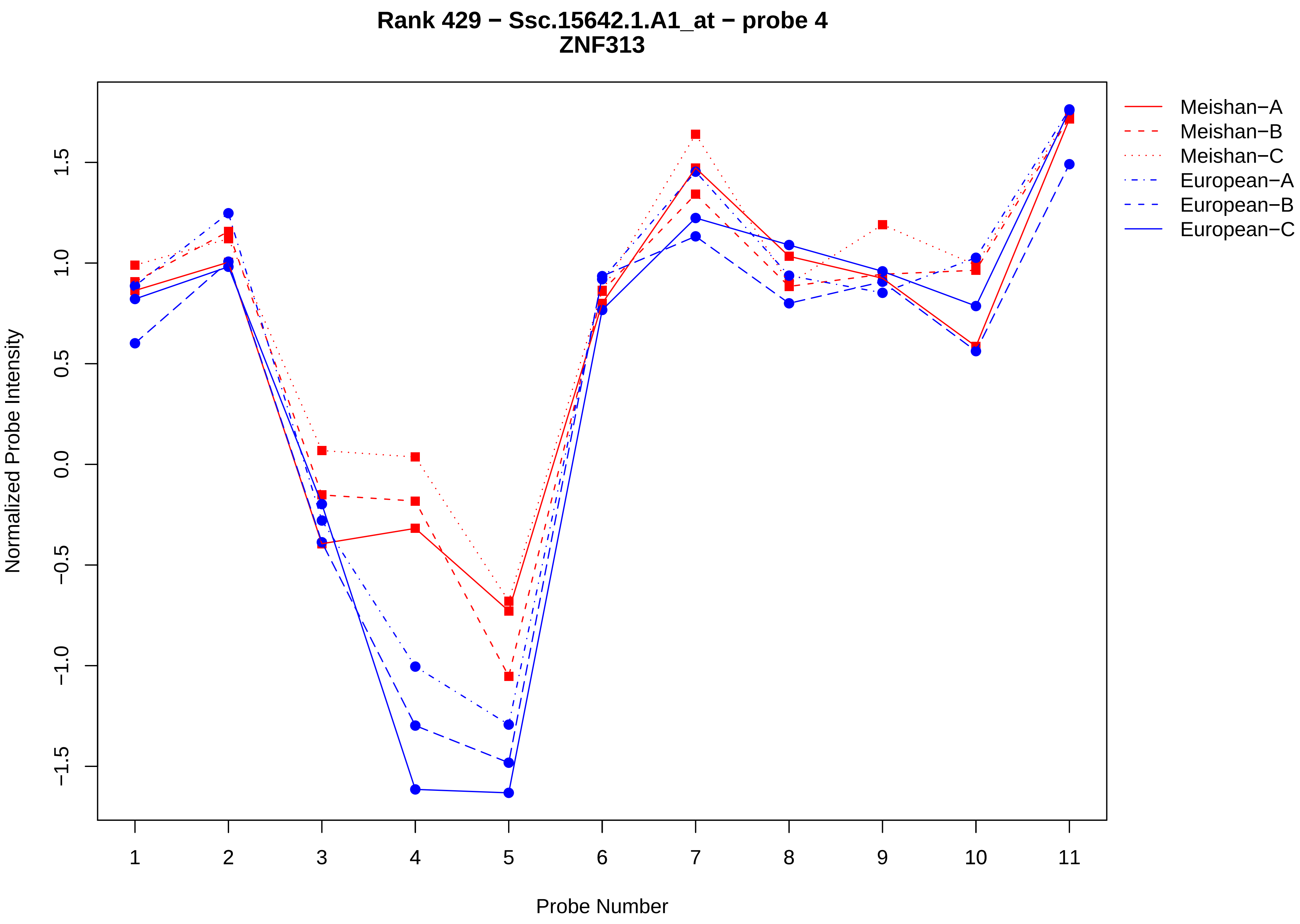

Rank 429 − Ssc.15642.1.A1_at − probe 4
ZNF313
Normalized Probe Intensity
Probe Number
−1.5
−1.0
−0.5
0.0
0.5
1.0
1.5
1
2
3
4
5
6
7
8
9
10
11
Meishan−A
Meishan−B
Meishan−C
European−A
European−B
European−C

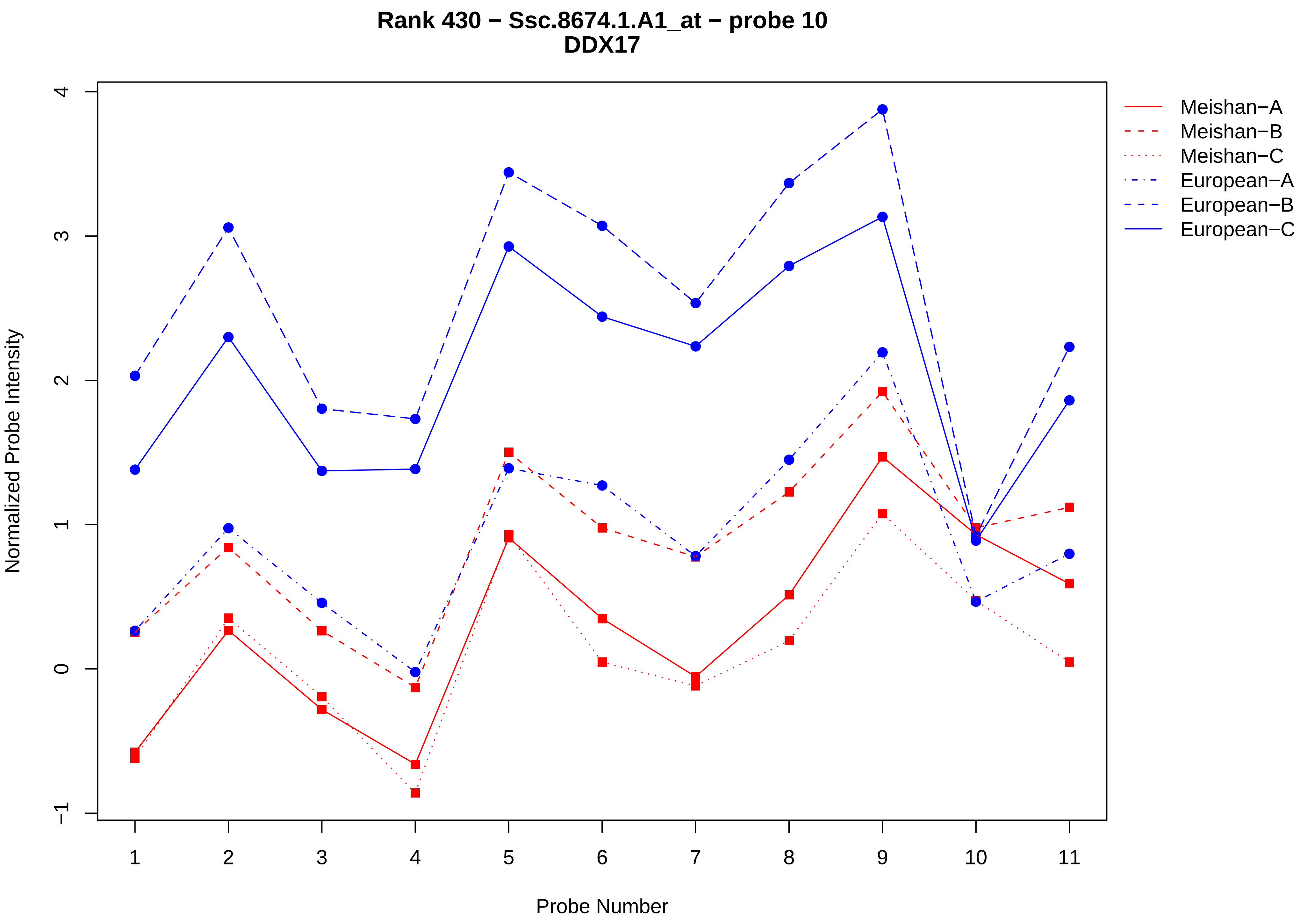

Rank 430 − Ssc.8674.1.A1_at − probe 10
DDX17
Normalized Probe Intensity
Probe Number
−1
0
1
2
3
4
1
2
3
4
5
6
7
8
9
10
11
Meishan−A
Meishan−B
Meishan−C
European−A
European−B
European−C

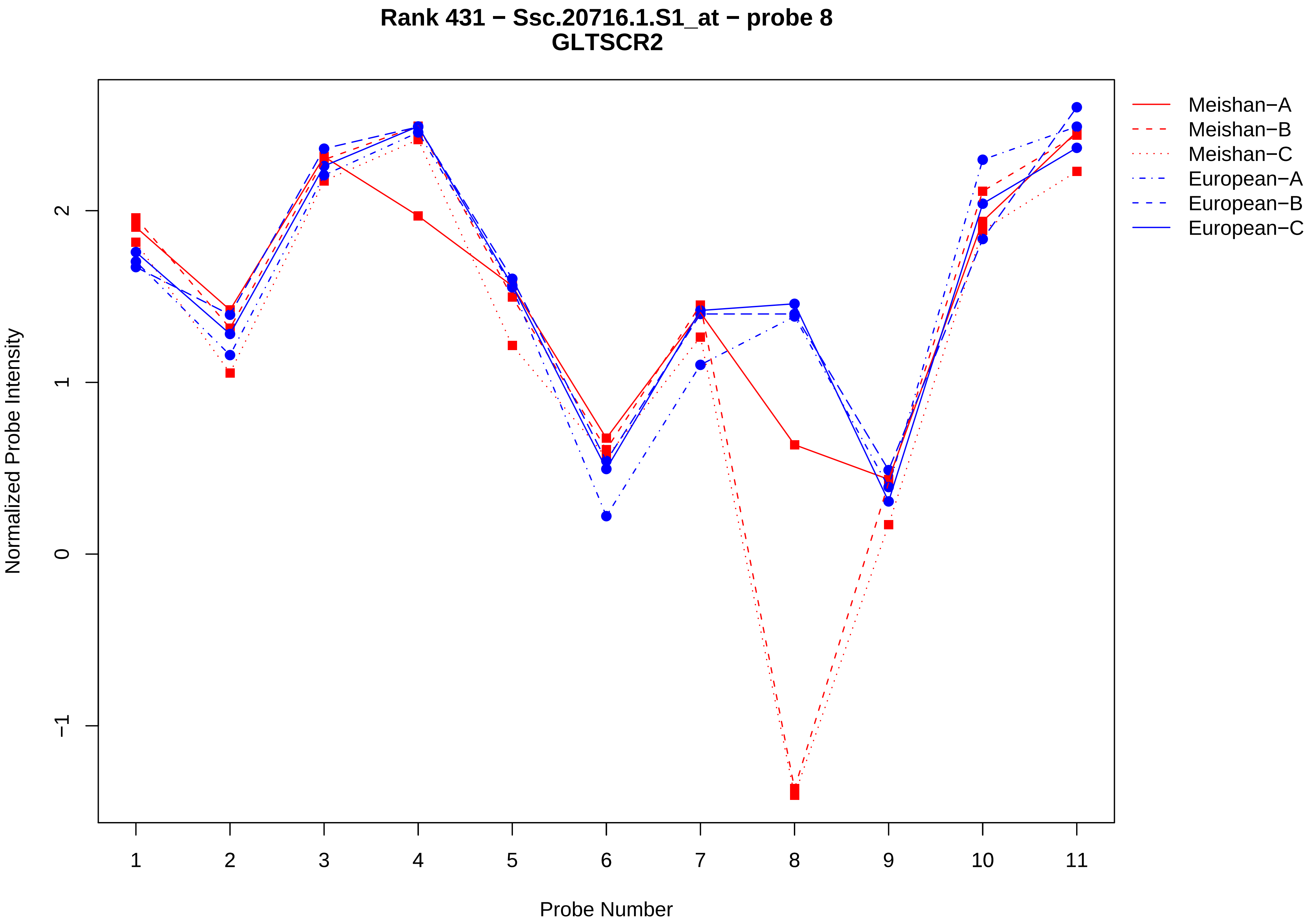

Rank 431 − Ssc.20716.1.S1_at − probe 8
GLTSCR2
Normalized Probe Intensity
Probe Number
−1
0
1
2
1
2
3
4
5
6
7
8
9
10
11
Meishan−A
Meishan−B
Meishan−C
European−A
European−B
European−C

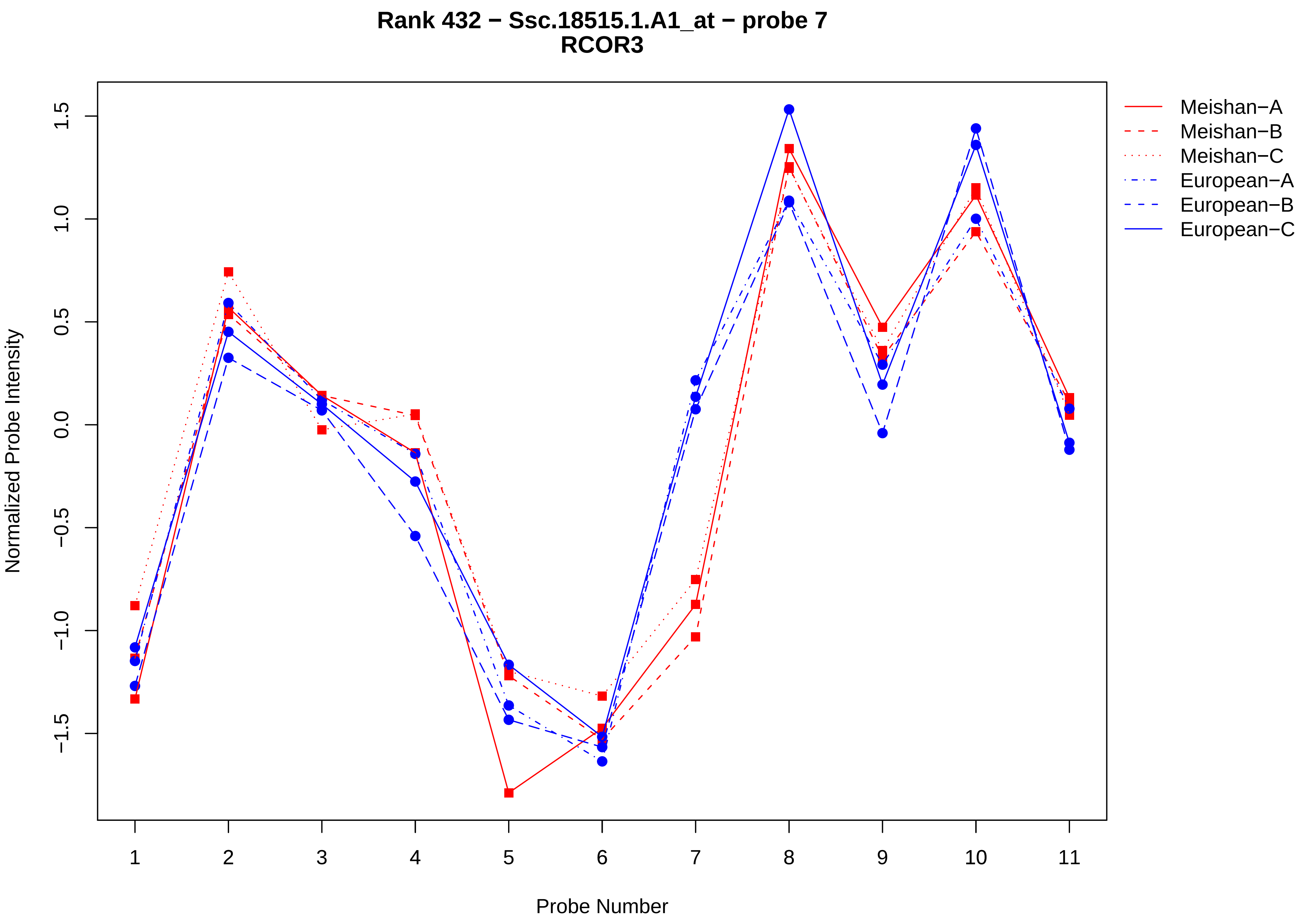

Rank 432 − Ssc.18515.1.A1_at − probe 7
RCOR3
Normalized Probe Intensity
Probe Number
Meishan−A
Meishan−B
Meishan−C
European−A
European−B
European−C

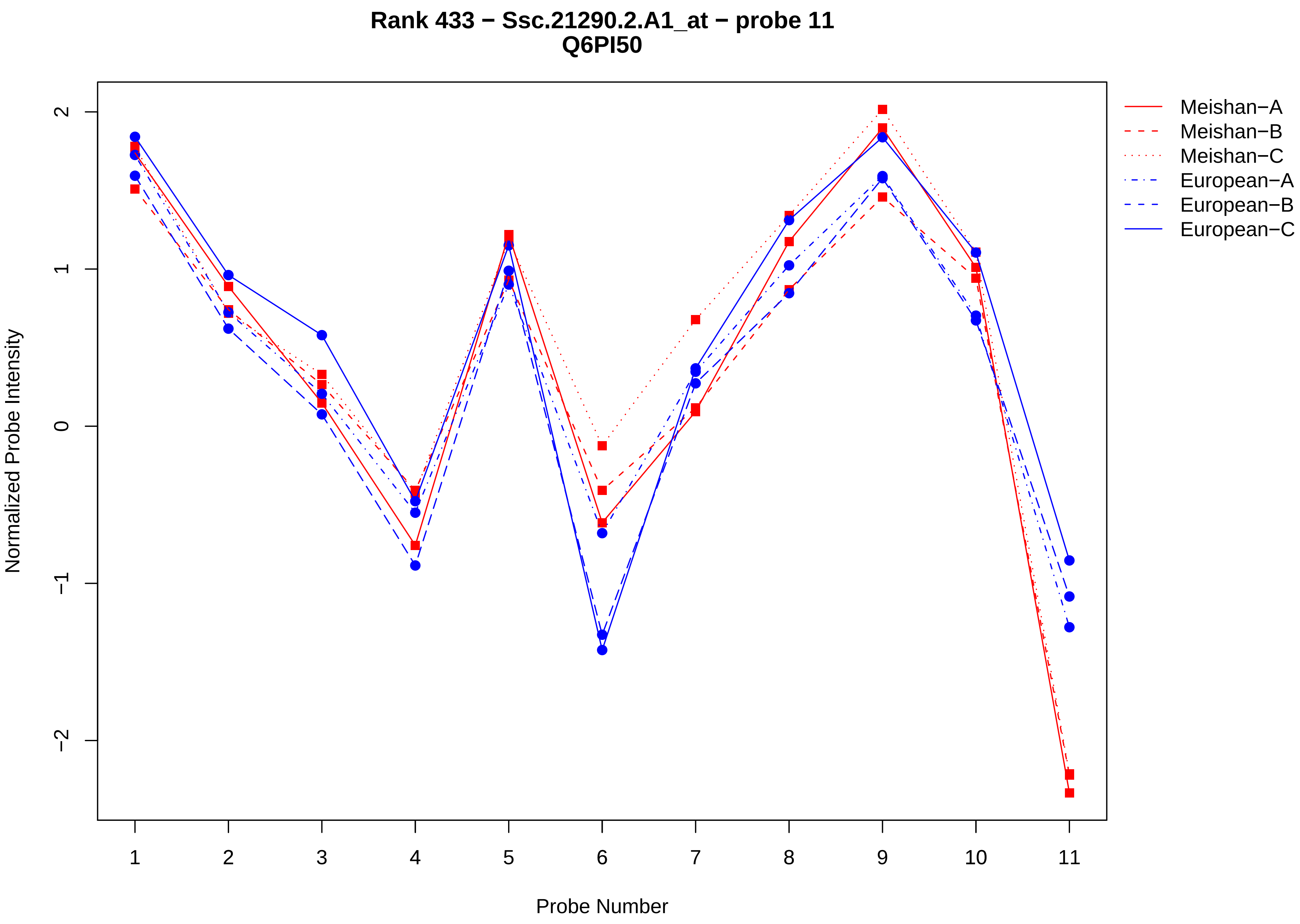
Rank 433 − Ssc.21290.2.A1_at − probe 11
Q6PI50
Normalized Probe Intensity
Probe Number
Meishan−A
Meishan−B
Meishan−C
European−A
European−B
European−C
2
1
0
−1
−2
1
2
3
4
5
6
7
8
9
10
11

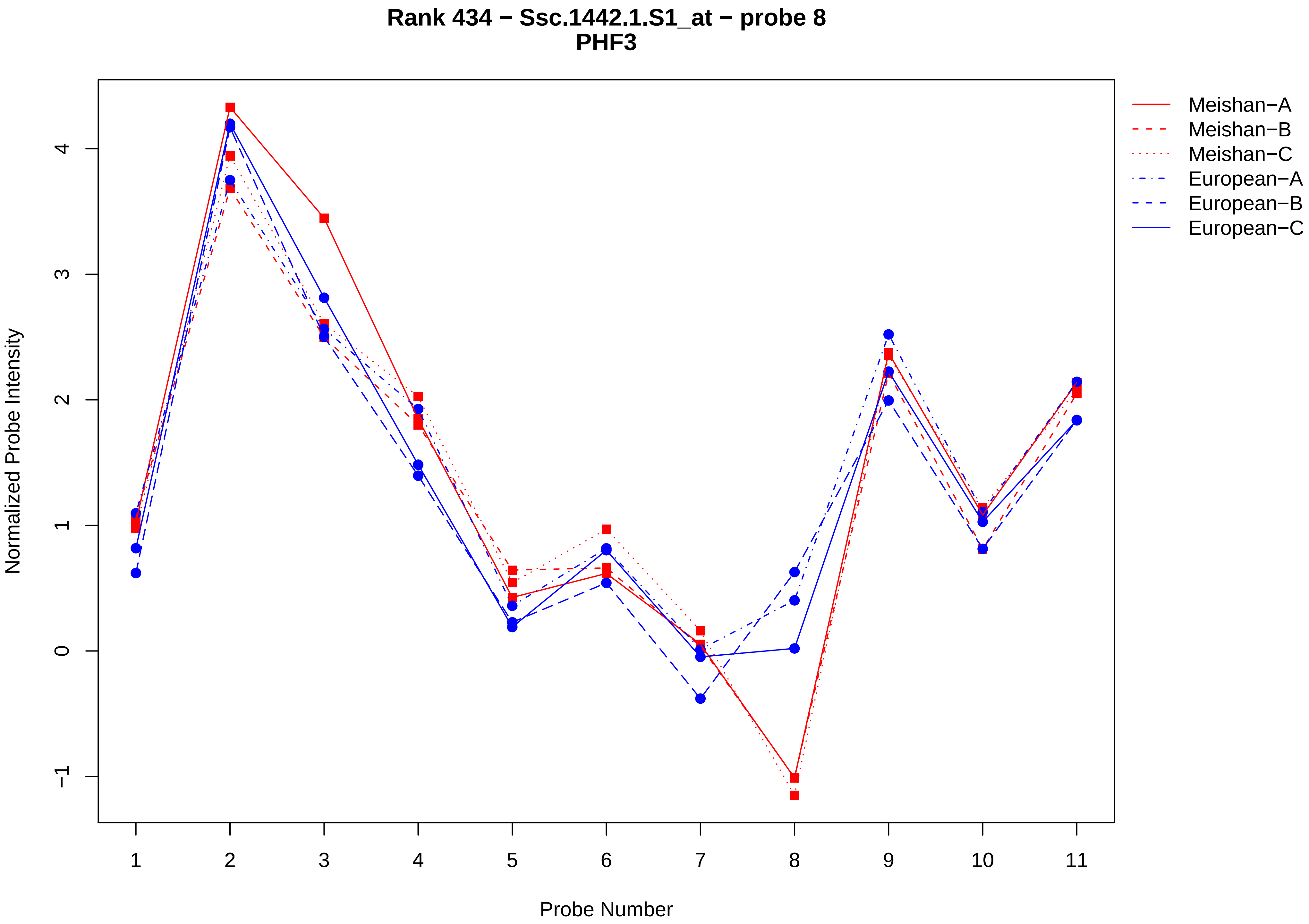

Rank 434 − Ssc.1442.1.S1_at − probe 8
PHF3
Normalized Probe Intensity
Probe Number
−1
0
1
2
3
4
1
2
3
4
5
6
7
8
9
10
11
Meishan−A
Meishan−B
Meishan−C
European−A
European−B
European−C

Rank 435 − Ssc.12005.1.A1_at − probe 11
ATP6V1G2

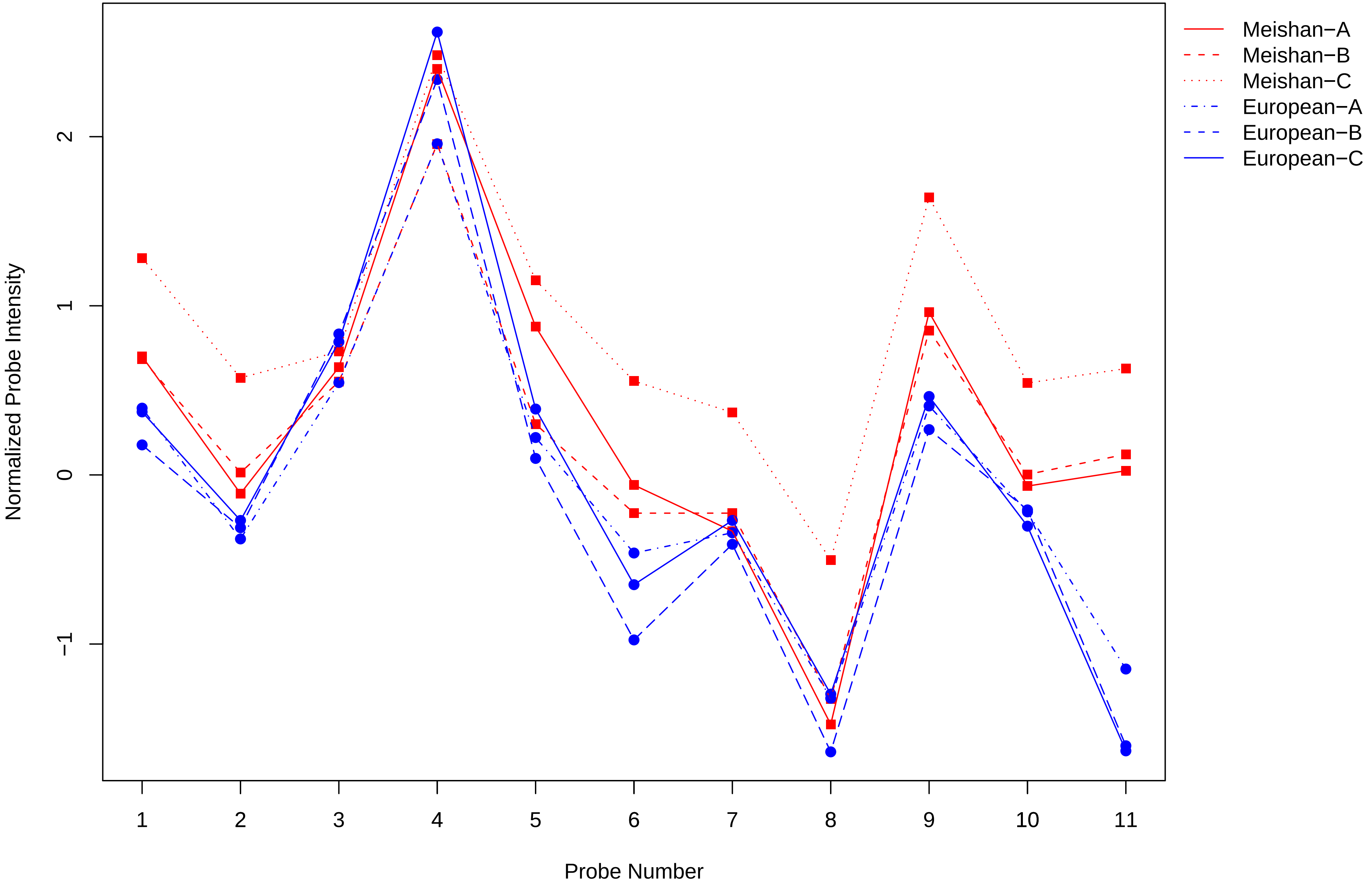

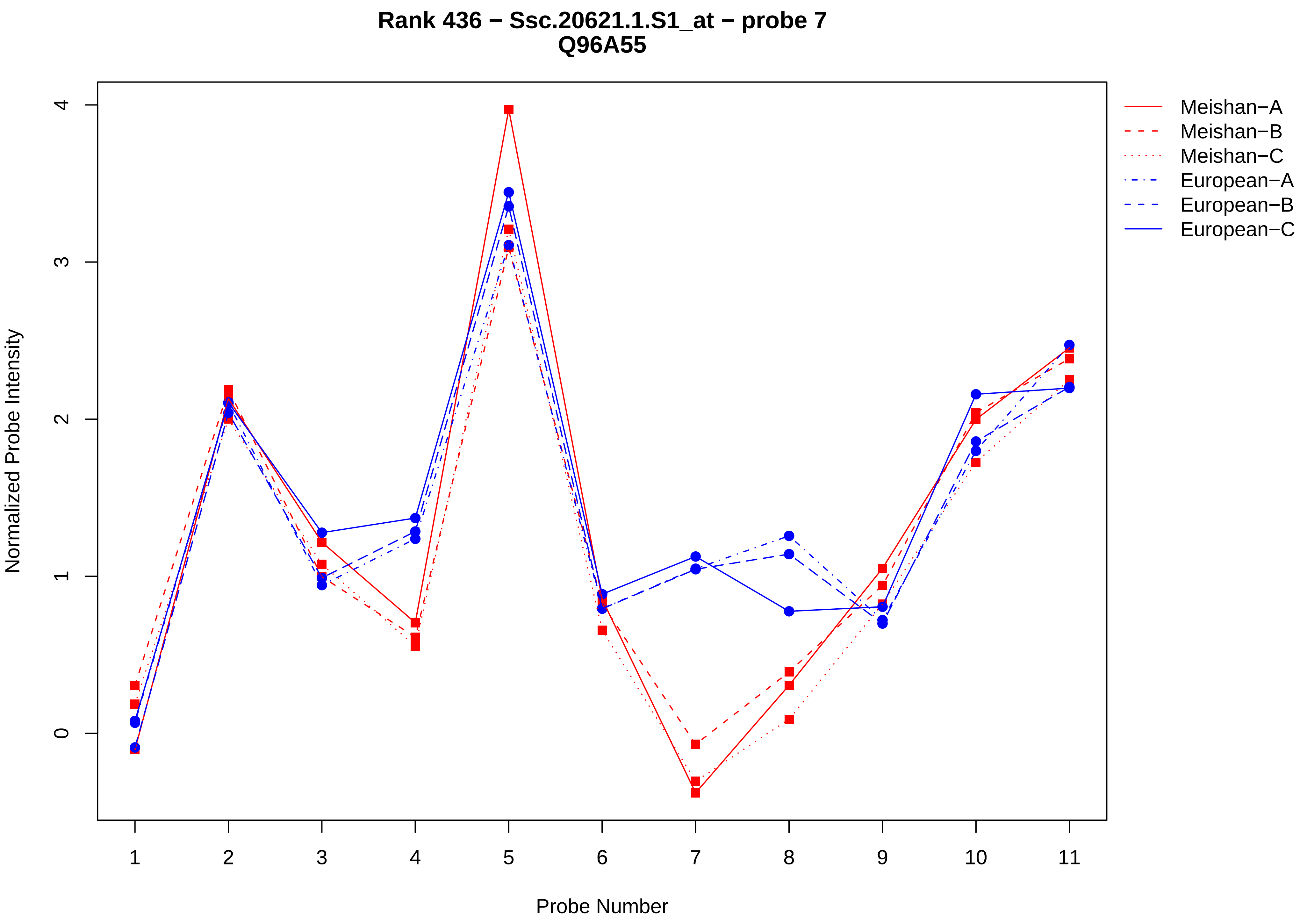

Rank 436 − Ssc.20621.1.S1_at − probe 7
Q96A55
Normalized Probe Intensity
Probe Number
Meishan−A
Meishan−B
Meishan−C
European−A
European−B
European−C

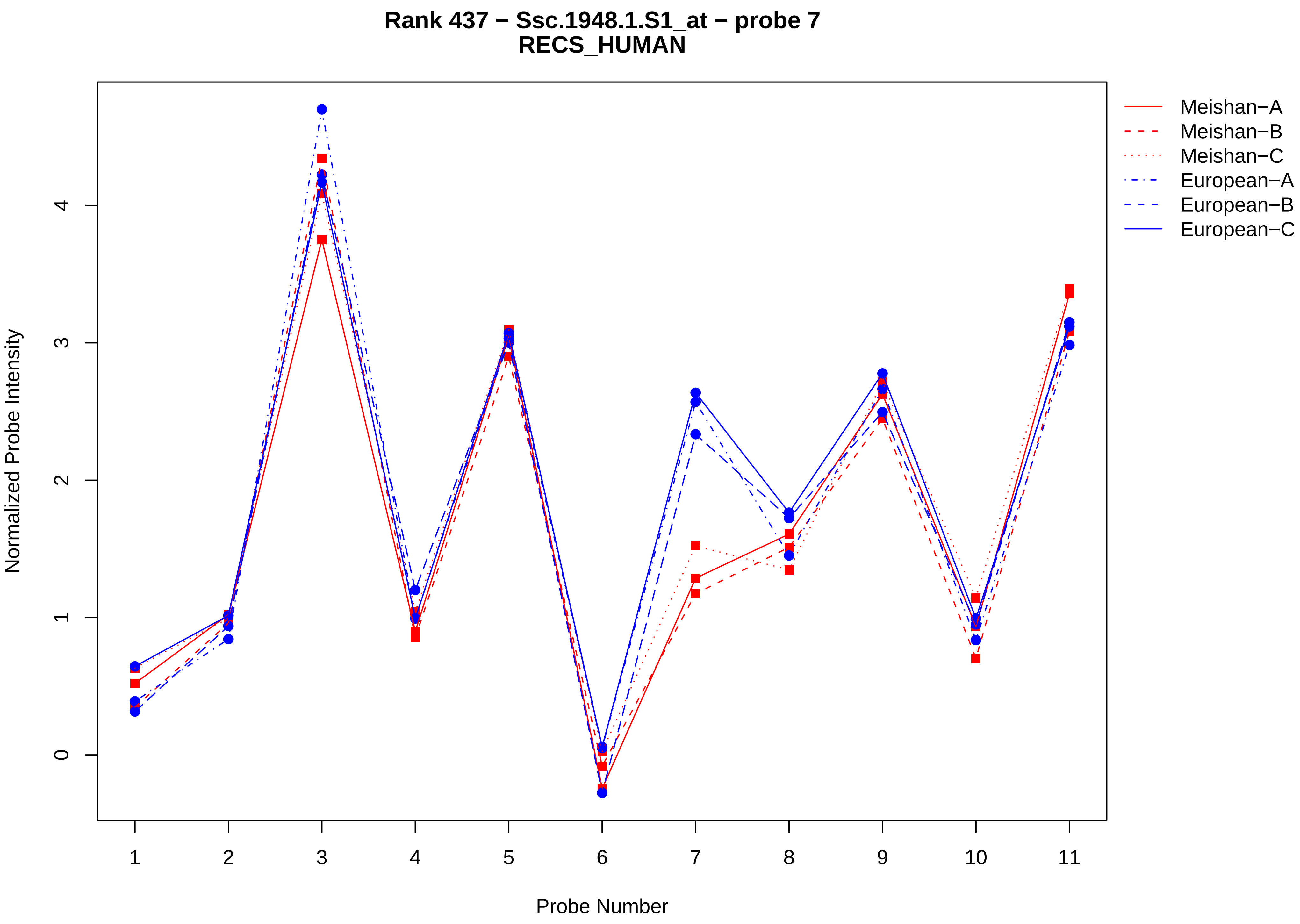

Rank 437 − Ssc.1948.1.S1_at − probe 7
RECS_HUMAN
Normalized Probe Intensity
Probe Number
0
1
2
3
4
1
2
3
4
5
6
7
8
9
10
11
Meishan−A
Meishan−B
Meishan−C
European−A
European−B
European−C

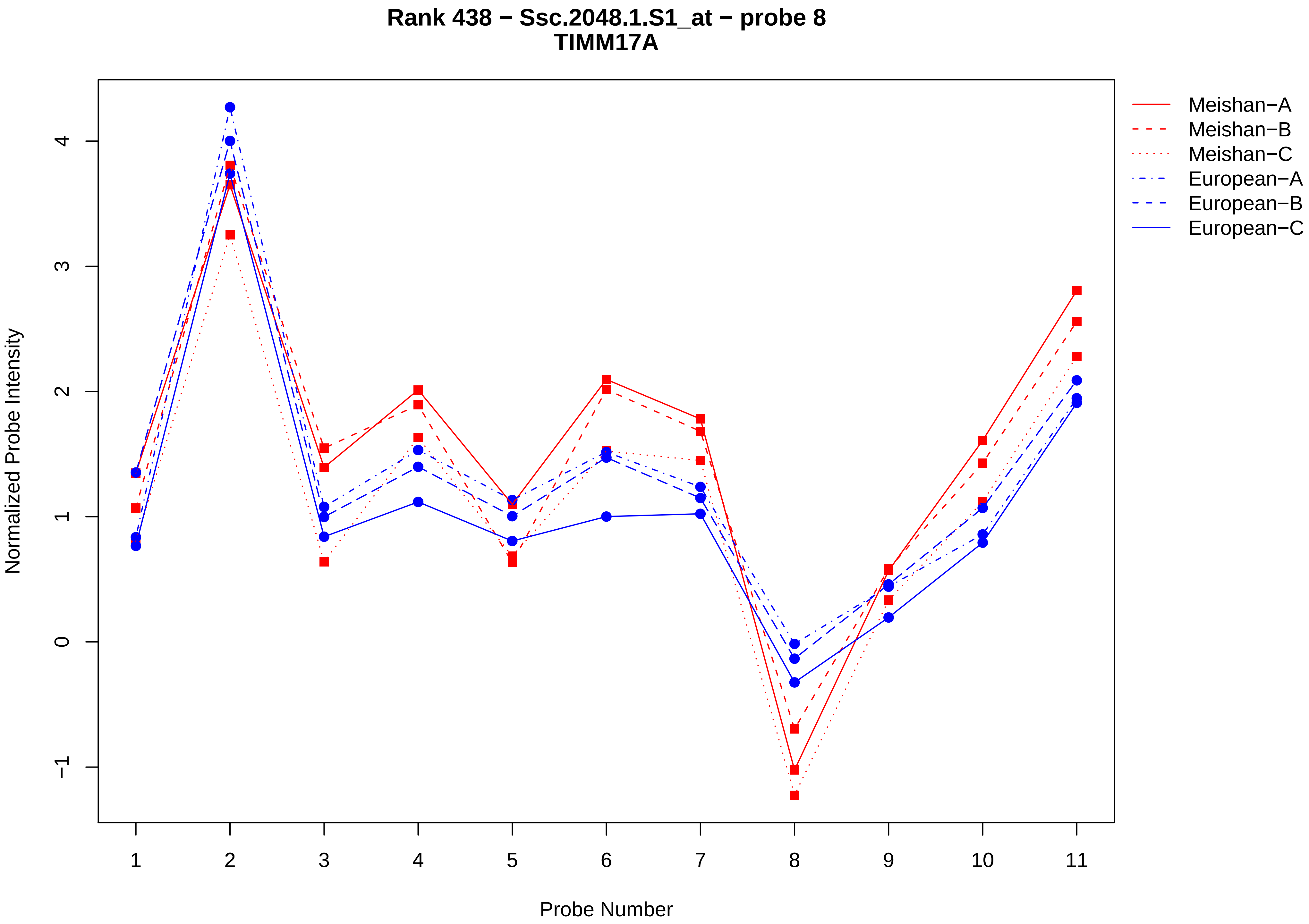

Rank 438 − Ssc.2048.1.S1_at − probe 8
TIMM17A
Normalized Probe Intensity
Probe Number
−1
0
1
2
3
4
1
2
3
4
5
6
7
8
9
10
11
Meishan−A
Meishan−B
Meishan−C
European−A
European−B
European−C

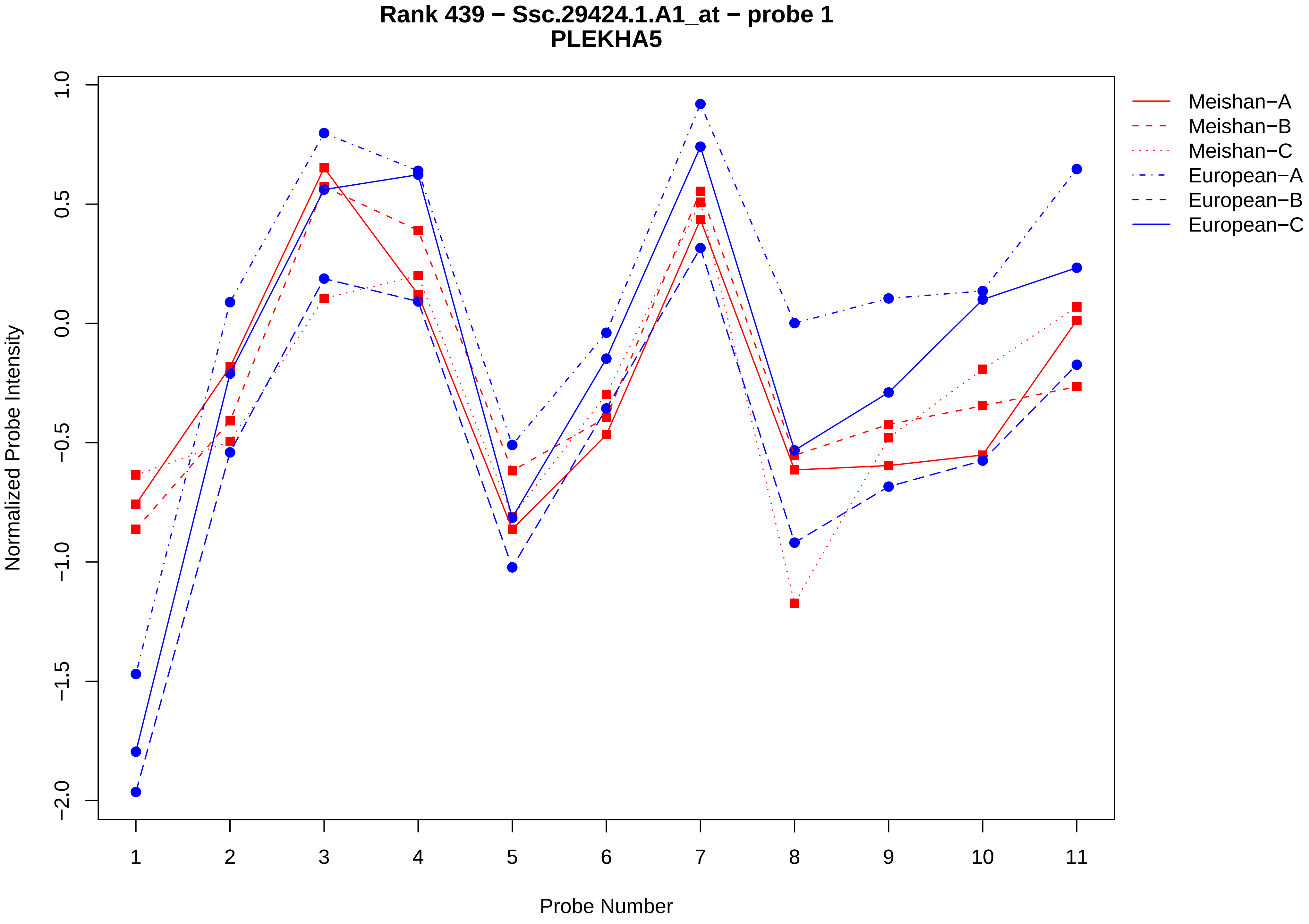

Rank 439 − Ssc.29424.1.A1_at − probe 1
PLEKHA5
Normalized Probe Intensity
Probe Number
1.0
0.5
0.0
−0.5
−1.0
−1.5
−2.0
1
2
3
4
5
6
7
8
9
10
11
Meishan−A
Meishan−B
Meishan−C
European−A
European−B
European−C

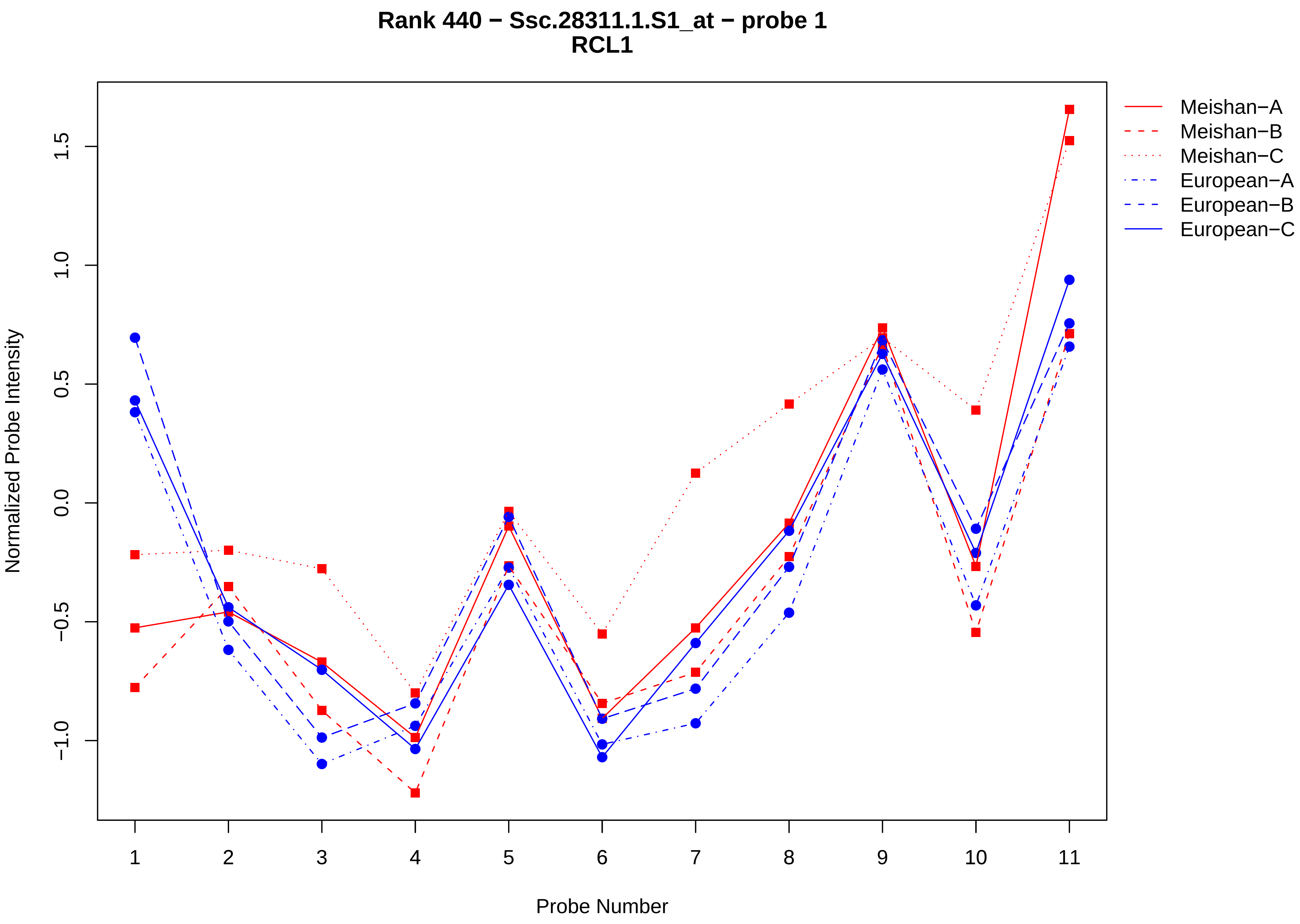

Rank 440 − Ssc.28311.1.S1_at − probe 1
RCL1
Normalized Probe Intensity
Probe Number
−1.0
−0.5
0.0
0.5
1.0
1.5
1
2
3
4
5
6
7
8
9
10
11
Meishan−A
Meishan−B
Meishan−C
European−A
European−B
European−C

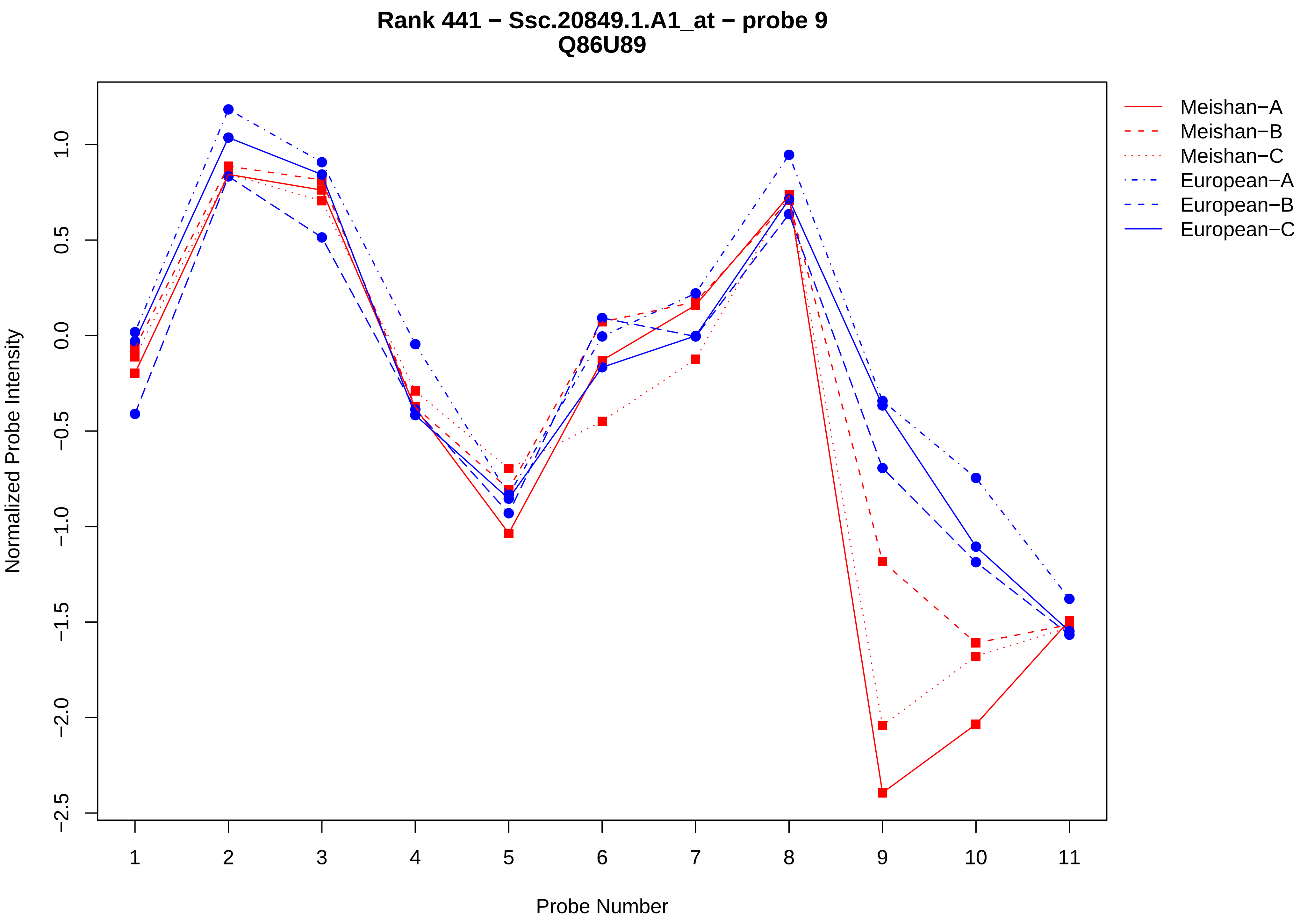

Rank 441 − Ssc.20849.1.A1_at − probe 9
Q86U89
Normalized Probe Intensity
Probe Number
1.0
0.5
0.0
−0.5
−1.0
−1.5
−2.0
−2.5
1
2
3
4
5
6
7
8
9
10
11
Meishan−A
Meishan−B
Meishan−C
European−A
European−B
European−C

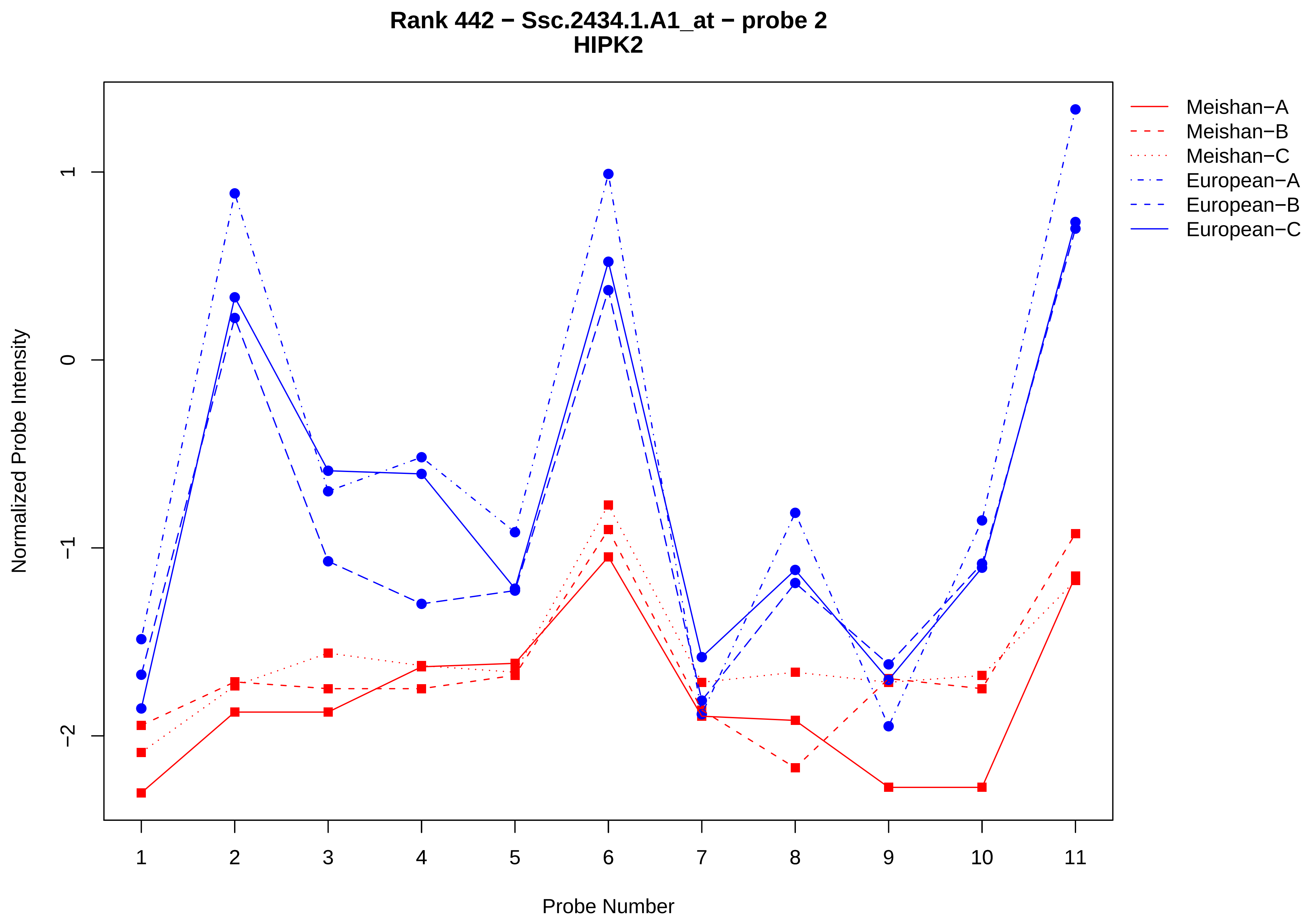

Rank 442 − Ssc.2434.1.A1_at − probe 2
HIPK2
Normalized Probe Intensity
Probe Number
1
0
−1
−2
1
2
3
4
5
6
7
8
9
10
11
Meishan−A
Meishan−B
Meishan−C
European−A
European−B
European−C

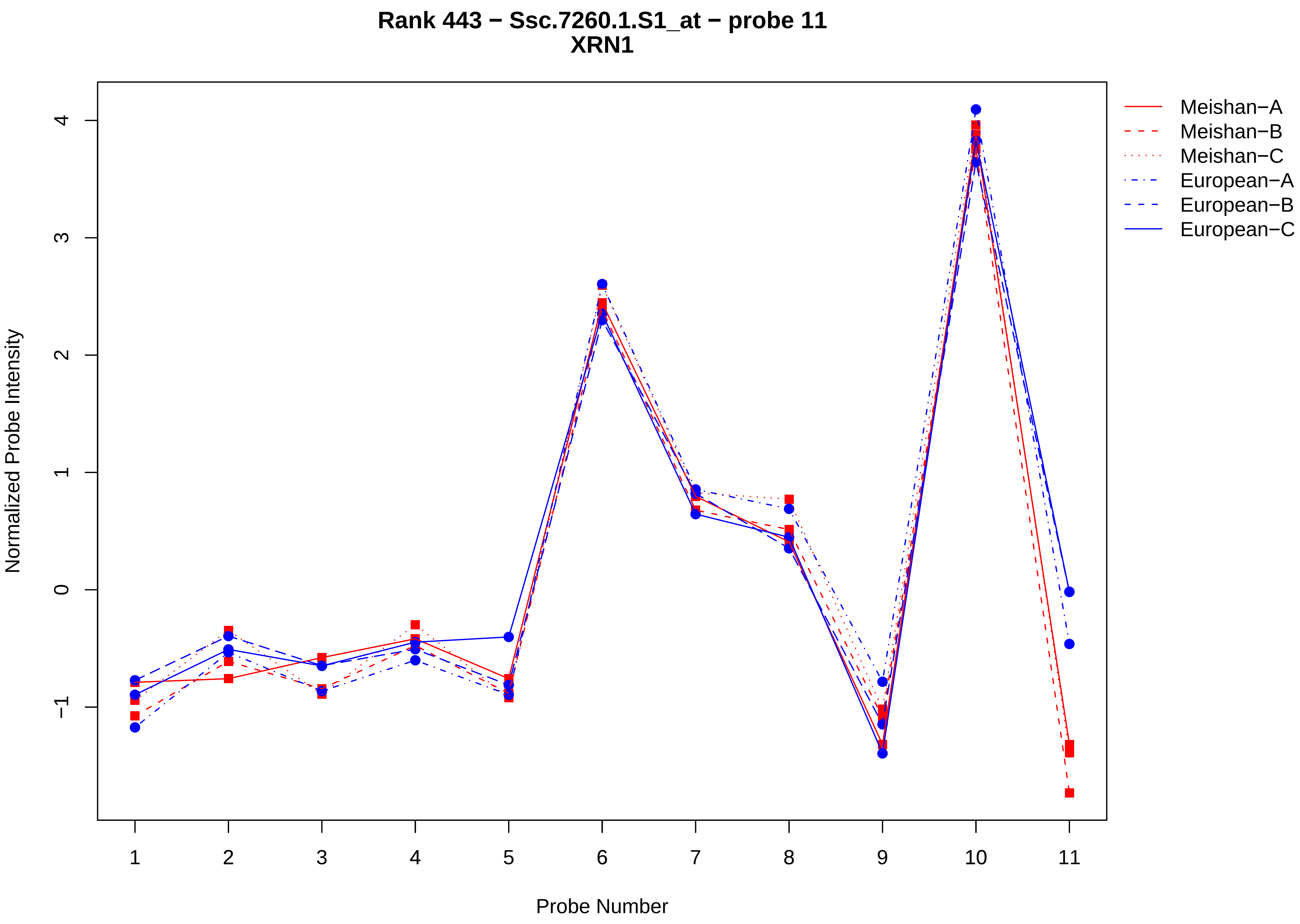

Rank 443 − Ssc.7260.1.S1_at − probe 11
XRN1
Normalized Probe Intensity
−1
0
1
2
3
4
Probe Number
1
2
3
4
5
6
7
8
9
10
11
Meishan−A
Meishan−B
Meishan−C
European−A
European−B
European−C

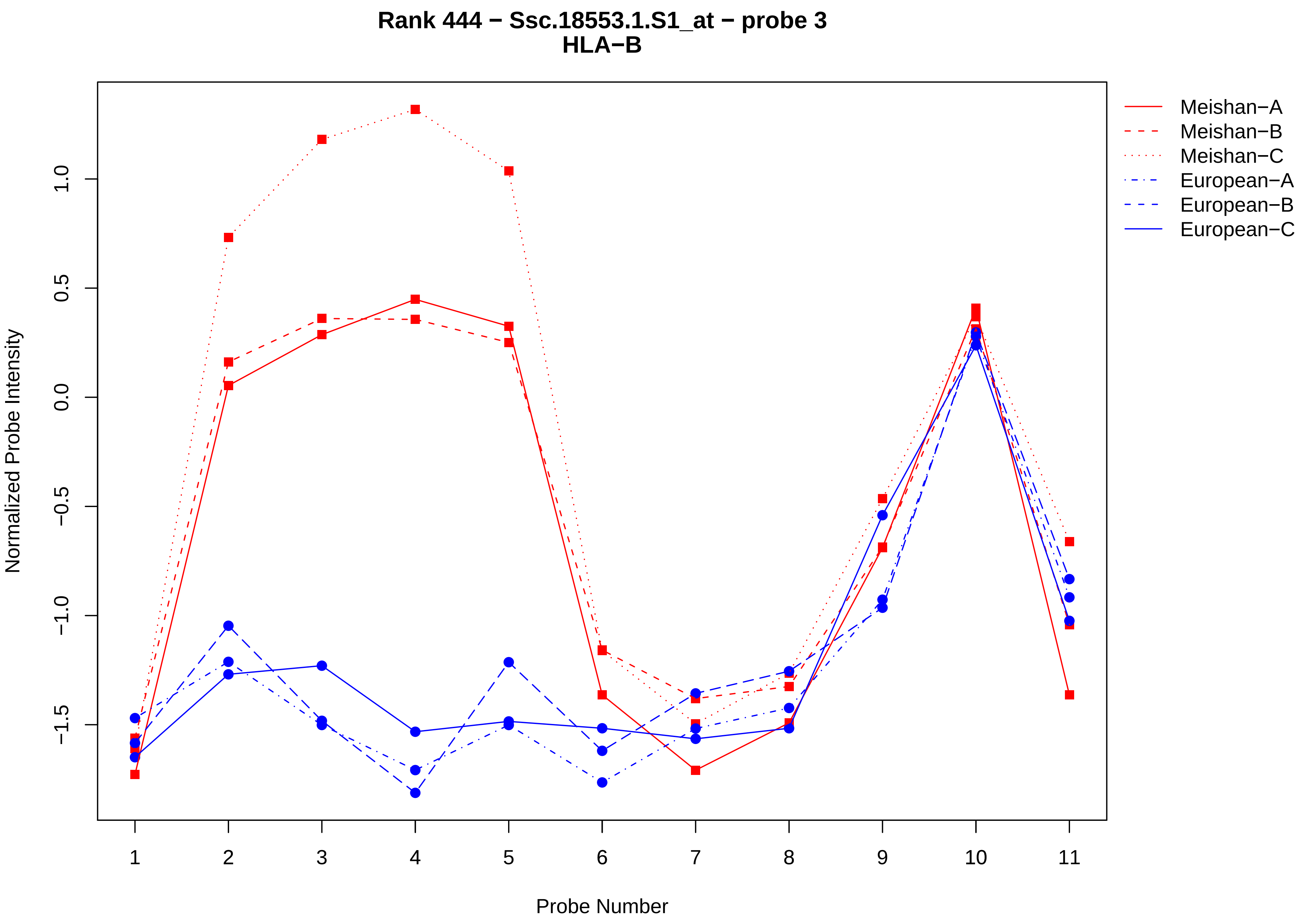

Rank 444 − Ssc.18553.1.S1_at − probe 3
HLA−B
Normalized Probe Intensity
Probe Number
1.0
0.5
0.0
−0.5
−1.0
−1.5
1
2
3
4
5
6
7
8
9
10
11
Meishan−A
Meishan−B
Meishan−C
European−A
European−B
European−C

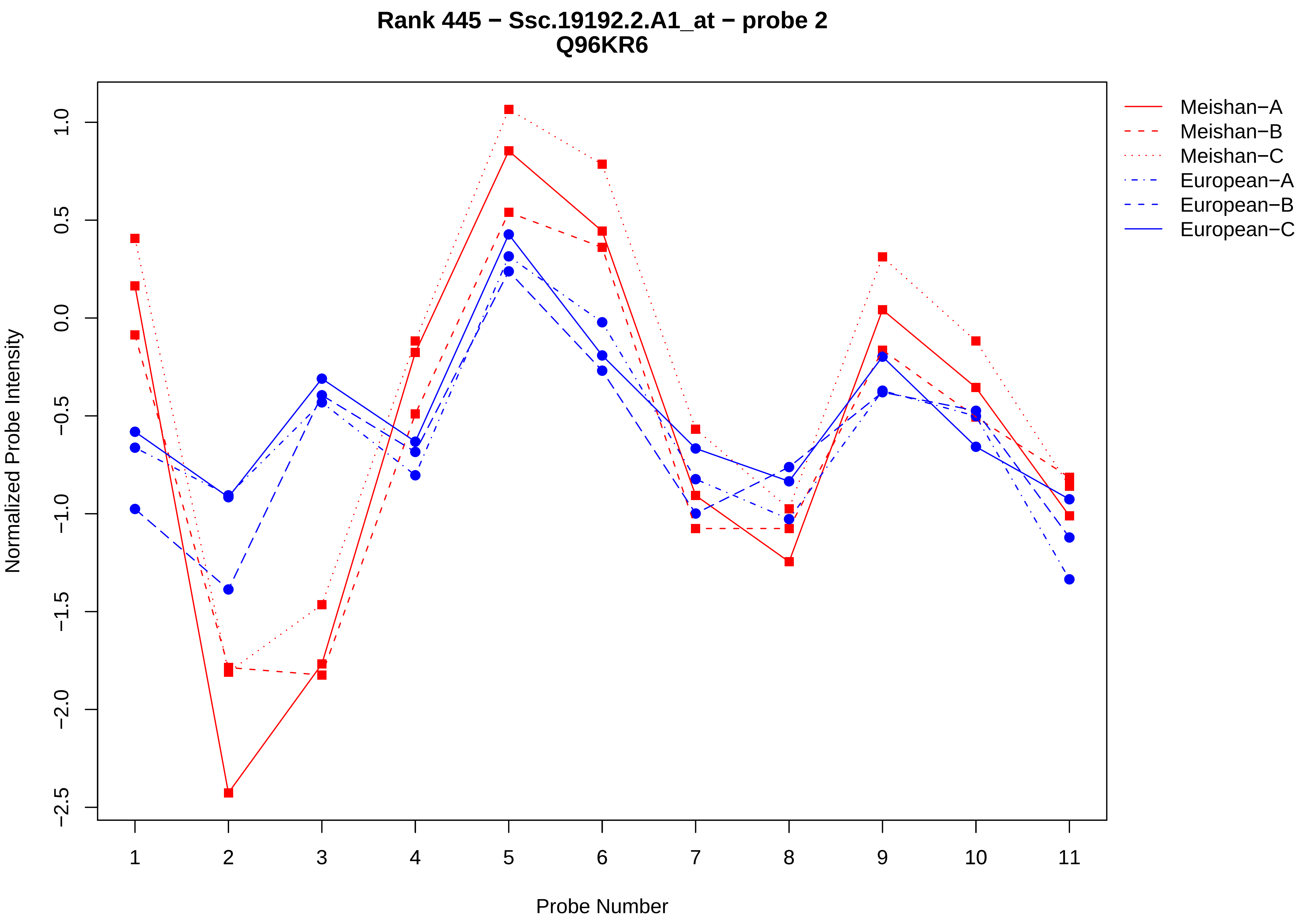

Rank 445 − Ssc.19192.2.A1_at − probe 2
Q96KR6
Normalized Probe Intensity
Probe Number
1.0
0.5
0.0
−0.5
−1.0
−1.5
−2.0
−2.5
1
2
3
4
5
6
7
8
9
10
11
Meishan−A
Meishan−B
Meishan−C
European−A
European−B
European−C

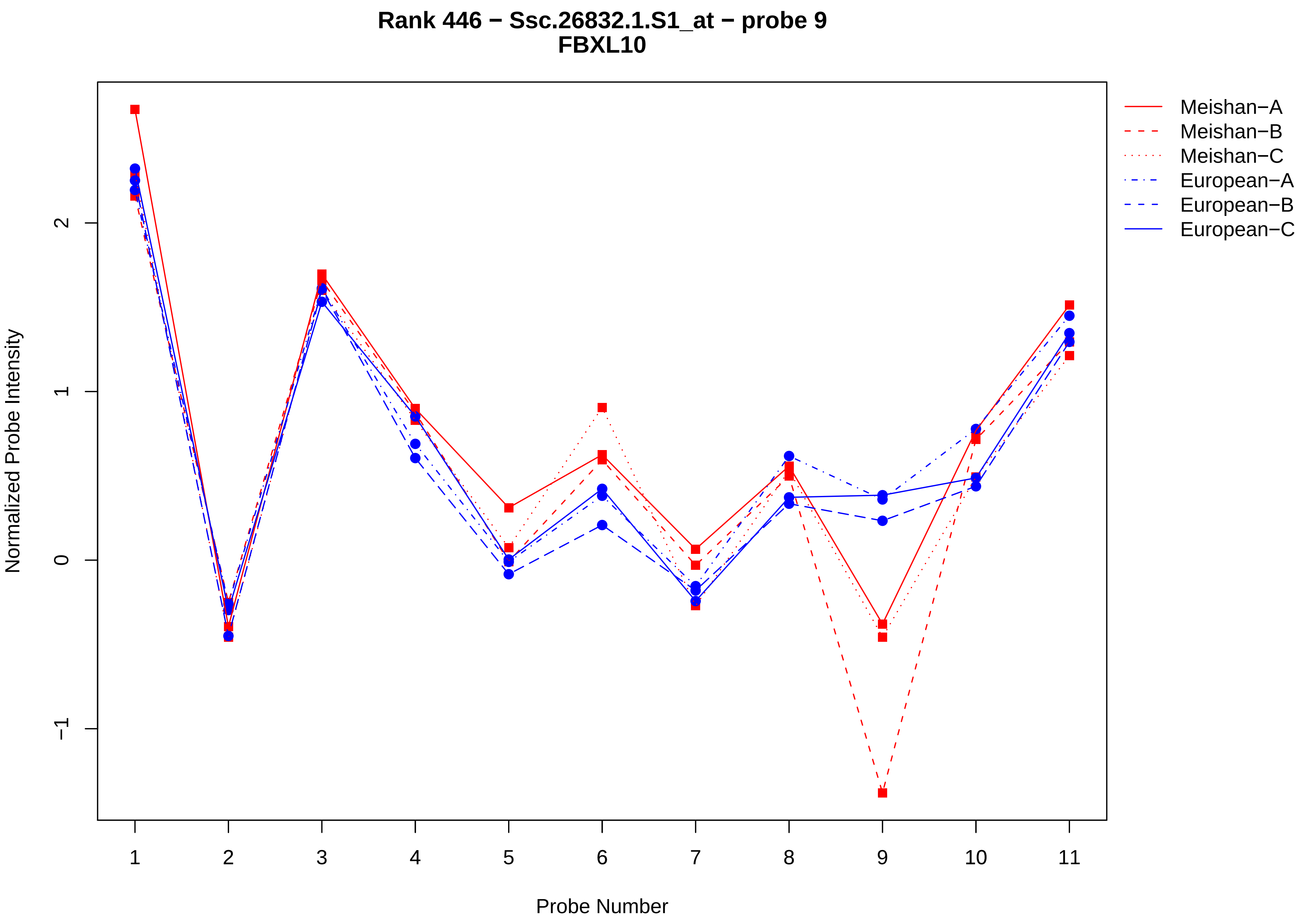

Rank 446 − Ssc.26832.1.S1_at − probe 9
FBXL10
Normalized Probe Intensity
Probe Number
−1
0
1
2
1
2
3
4
5
6
7
8
9
10
11
Meishan−A
Meishan−B
Meishan−C
European−A
European−B
European−C

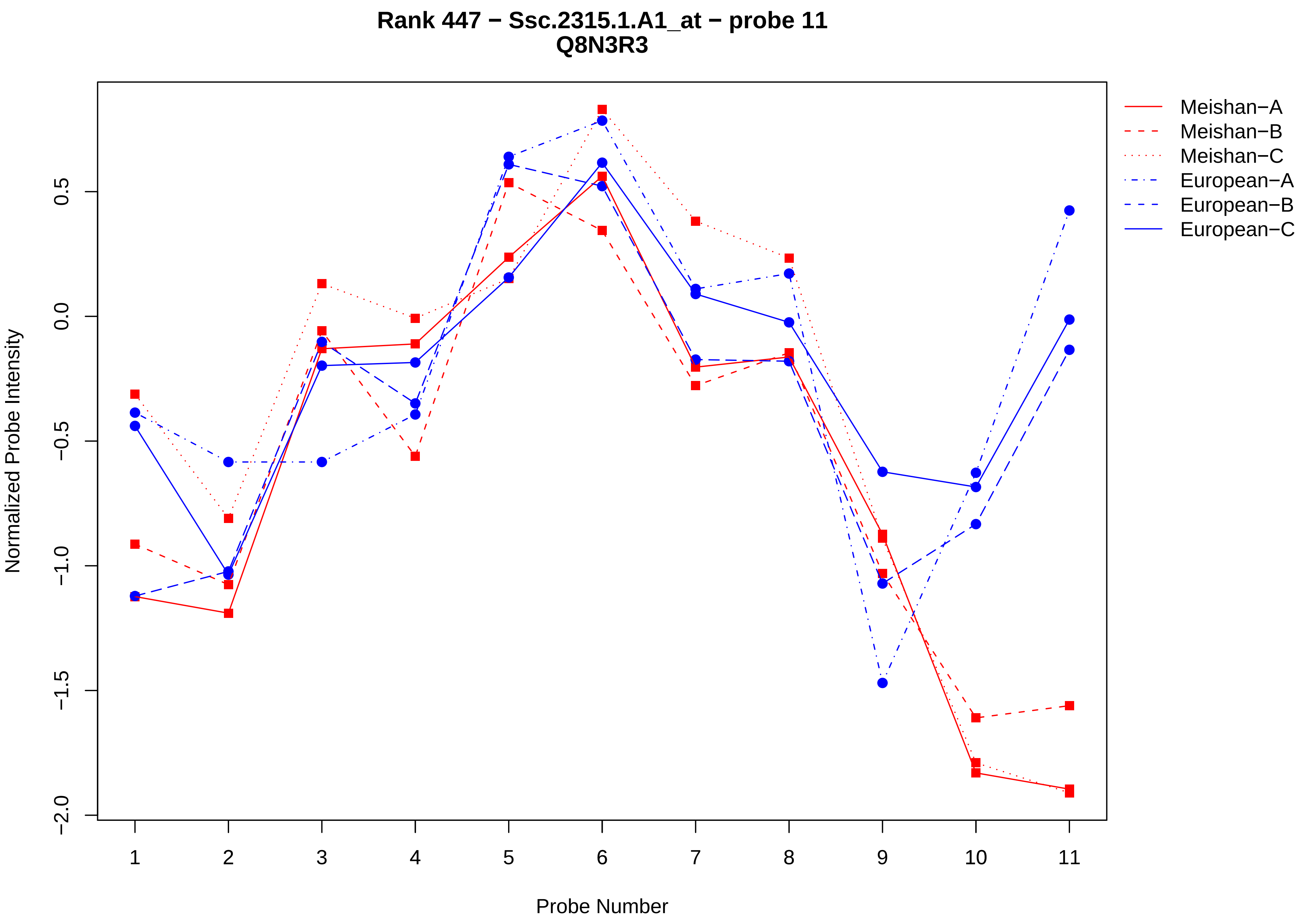

Rank 447 − Ssc.2315.1.A1_at − probe 11
Q8N3R3
Normalized Probe Intensity
Probe Number
Meishan−A
Meishan−B
Meishan−C
European−A
European−B
European−C
−2.0
−1.5
−1.0
−0.5
0.0
0.5
1
2
3
4
5
6
7
8
9
10
11

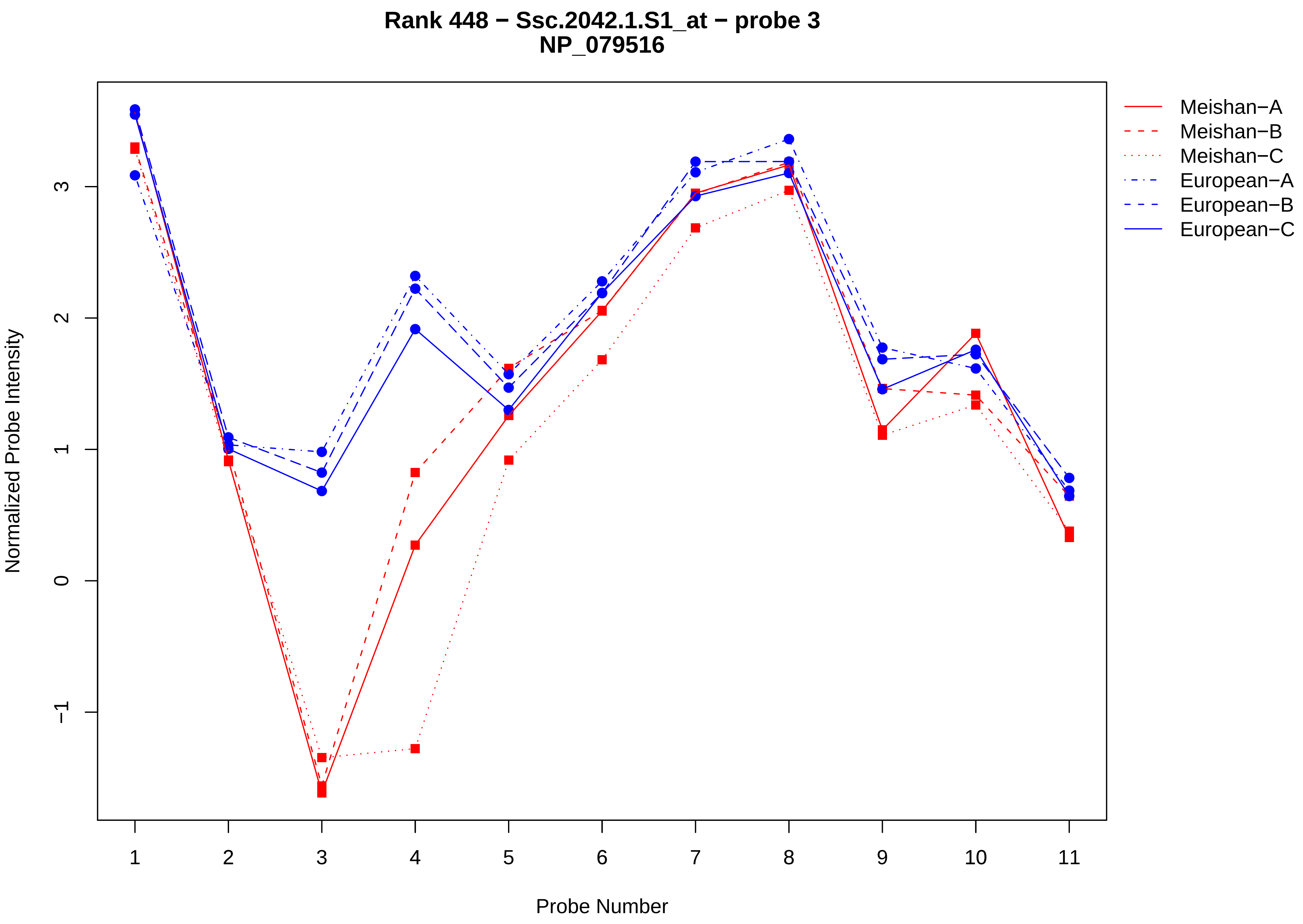
Rank 448 − Ssc.2042.1.S1_at − probe 3
NP_079516
Normalized Probe Intensity
Probe Number
−1
0
1
2
3
1
2
3
4
5
6
7
8
9
10
11
Meishan−A
Meishan−B
Meishan−C
European−A
European−B
European−C

Rank 449 − Ssc.27200.1.A1_at − probe 10
RP9

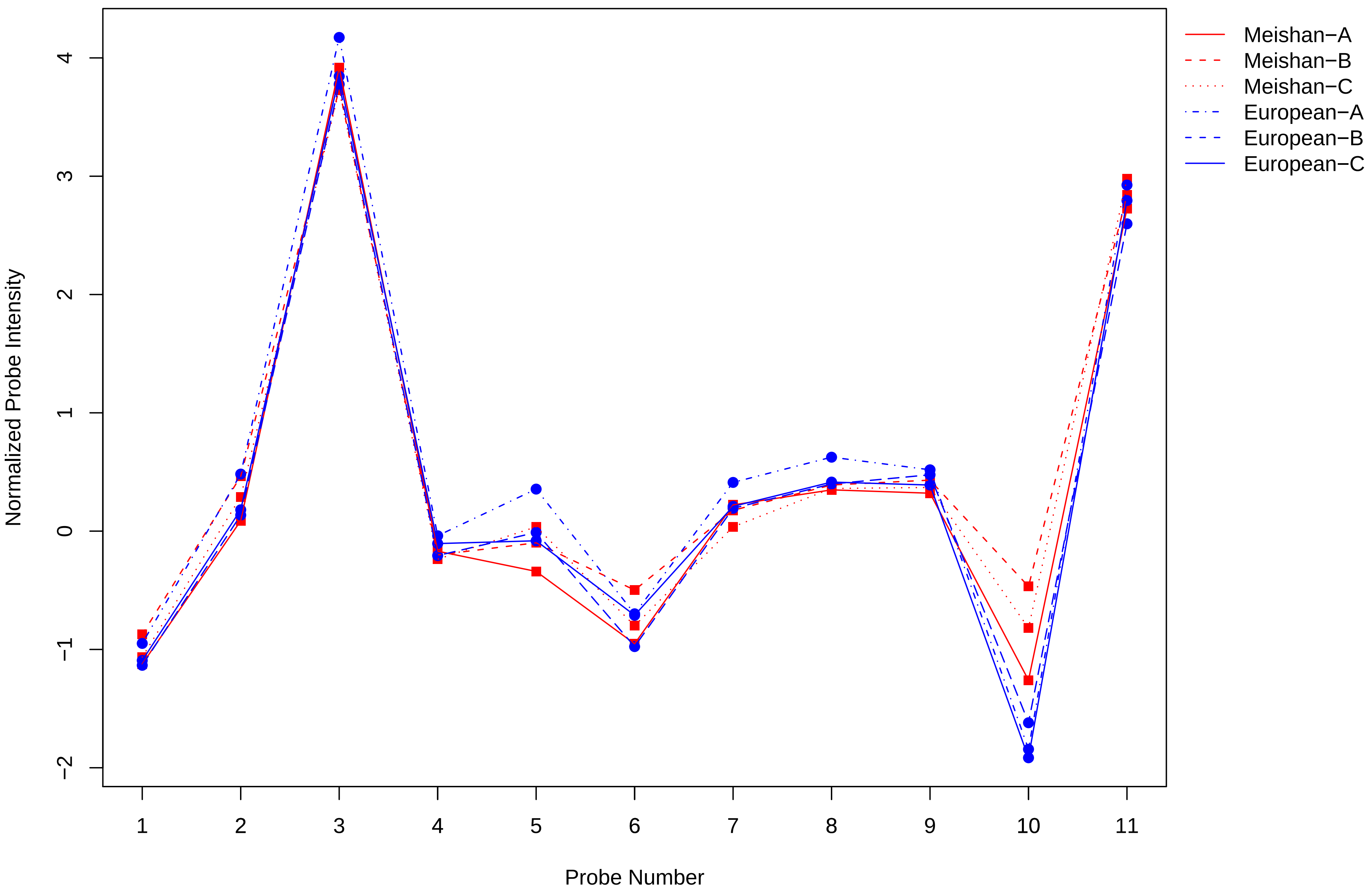

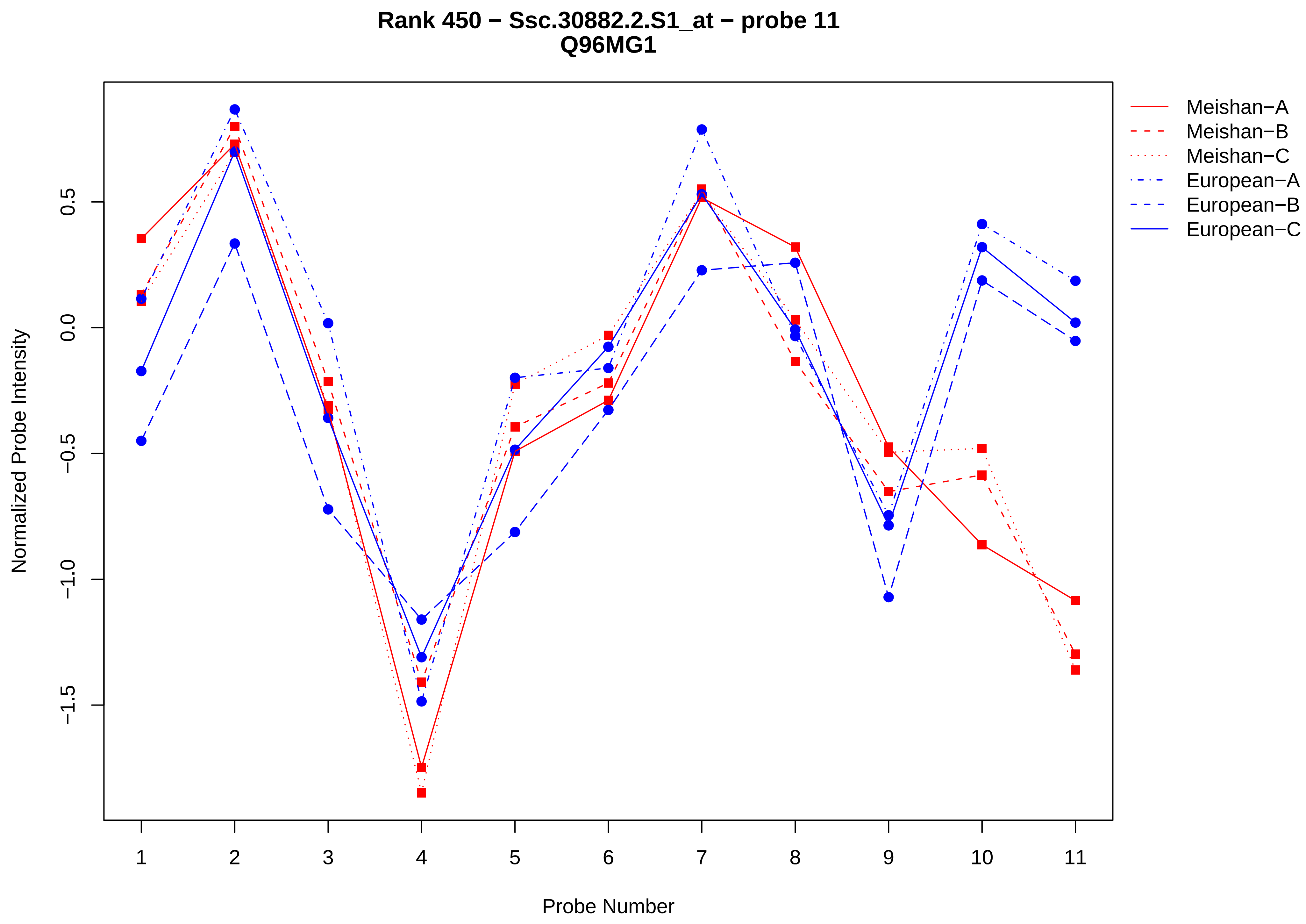

Rank 450 − Ssc.30882.2.S1_at − probe 11
Q96MG1
Normalized Probe Intensity
Probe Number
Meishan−A
Meishan−B
Meishan−C
European−A
European−B
European−C
0.5
0.0
−0.5
−1.0
−1.5
1
2
3
4
5
6
7
8
9
10
11

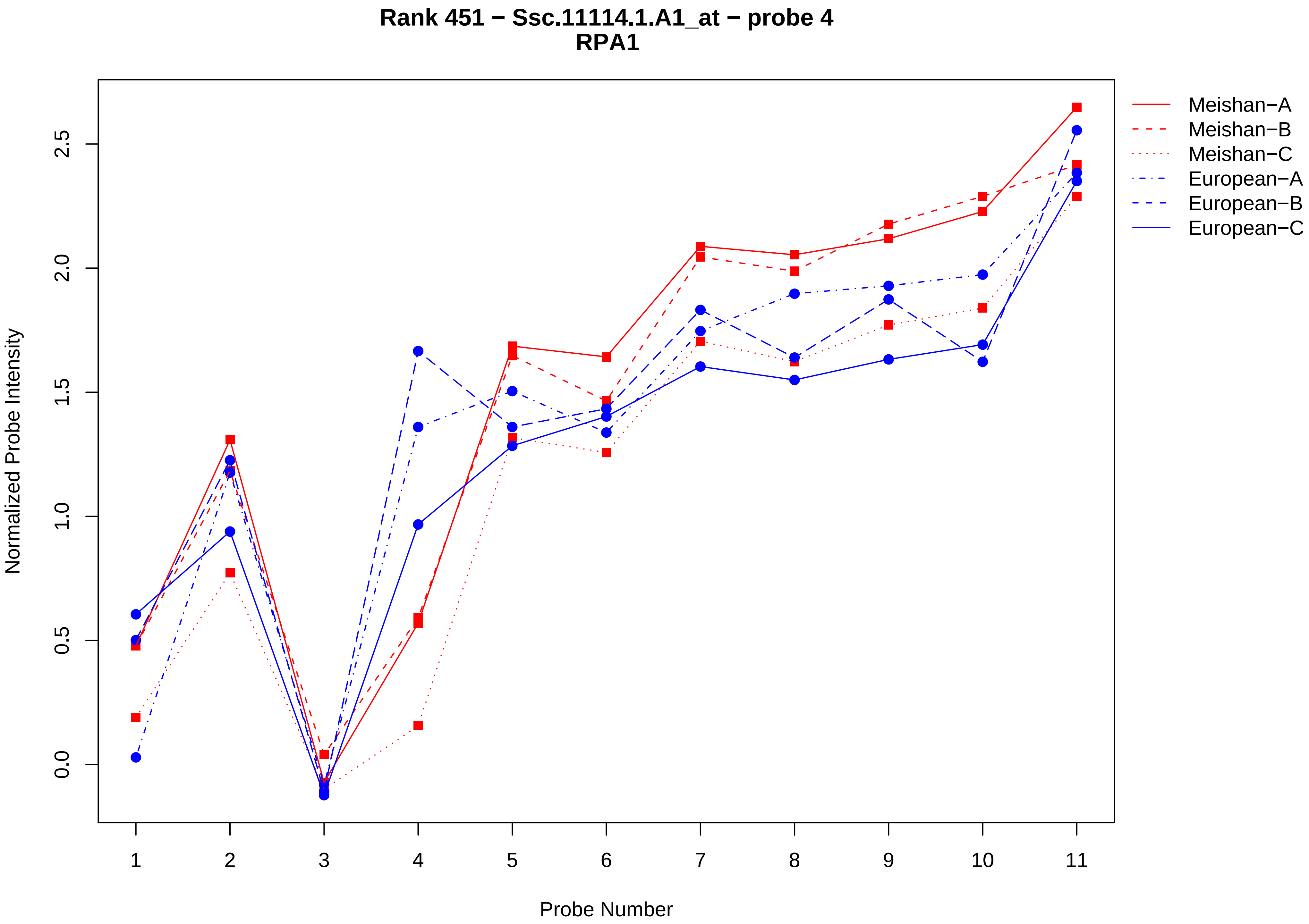

Rank 451 − Ssc.11114.1.A1_at − probe 4
RPA1
Normalized Probe Intensity
0.0
0.5
1.0
1.5
2.0
2.5
1
2
3
4
5
6
7
8
9
10
11
Probe Number
Meishan−A
Meishan−B
Meishan−C
European−A
European−B
European−C

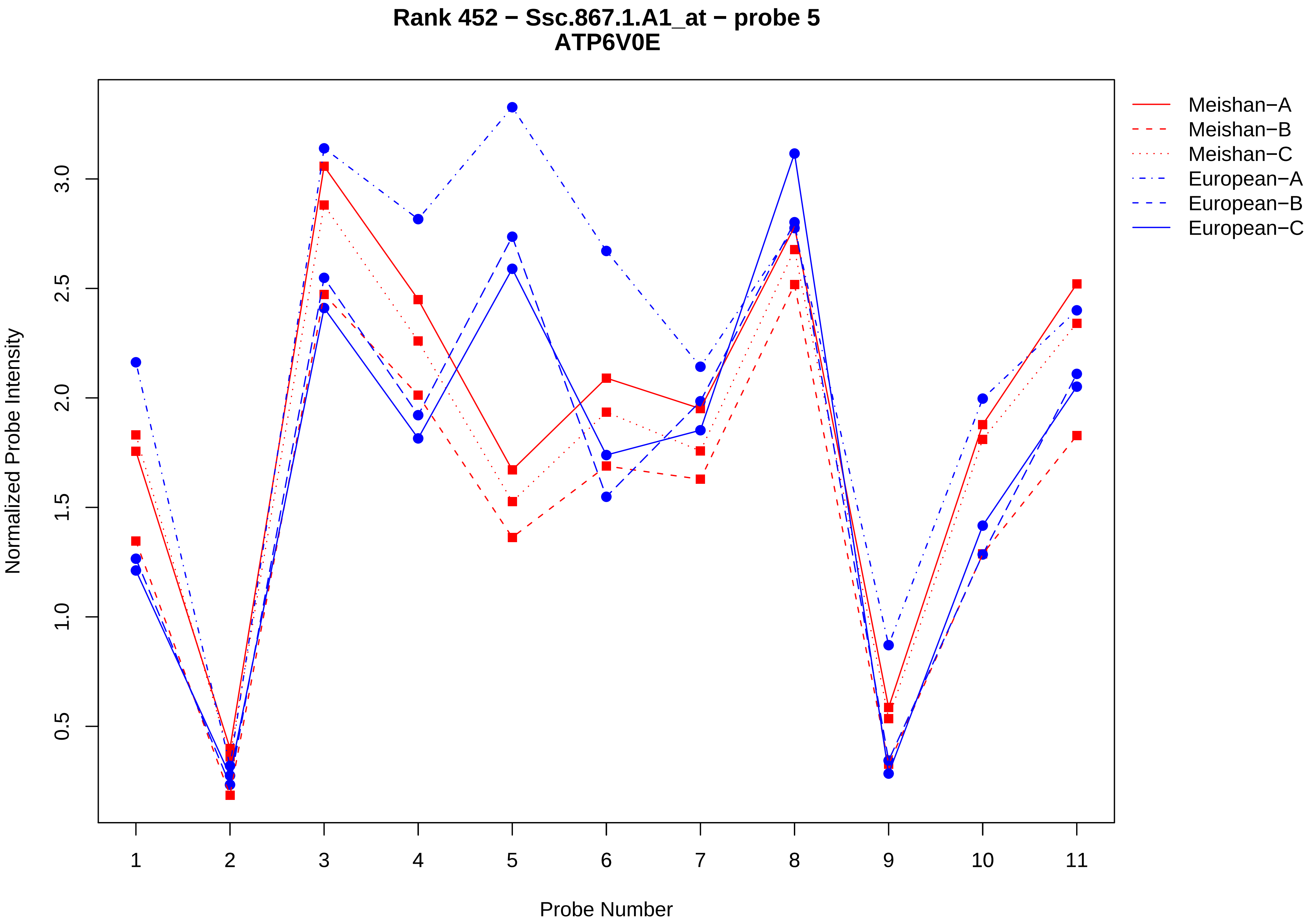

Rank 452 − Ssc.867.1.A1_at − probe 5
ATP6V0E
Normalized Probe Intensity
Probe Number
Meishan−A
Meishan−B
Meishan−C
European−A
European−B
European−C

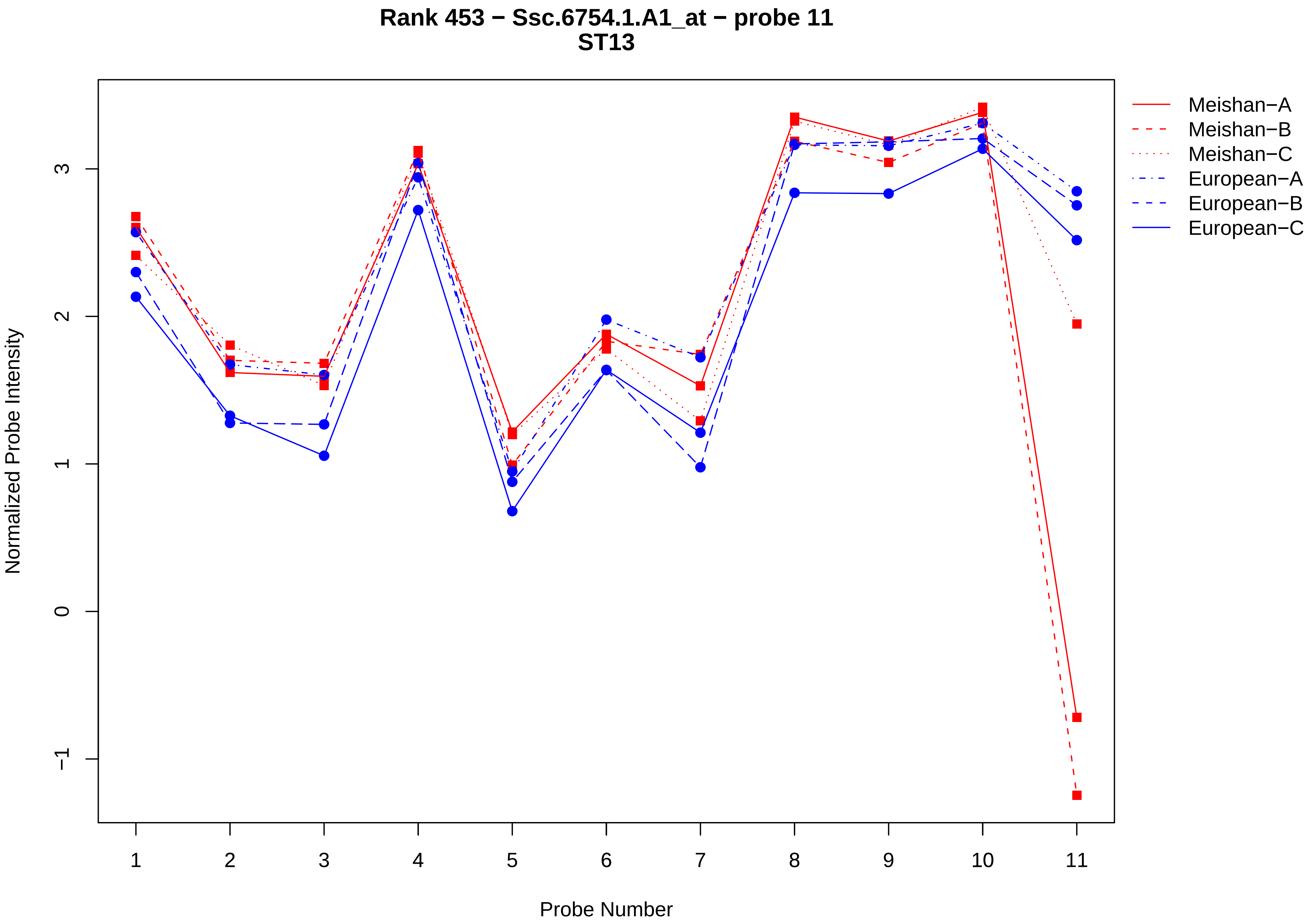
Rank 453 − Ssc.6754.1.A1_at − probe 11
ST13
Normalized Probe Intensity
Probe Number
−1
0
1
2
3
1
2
3
4
5
6
7
8
9
10
11
Meishan−A
Meishan−B
Meishan−C
European−A
European−B
European−C

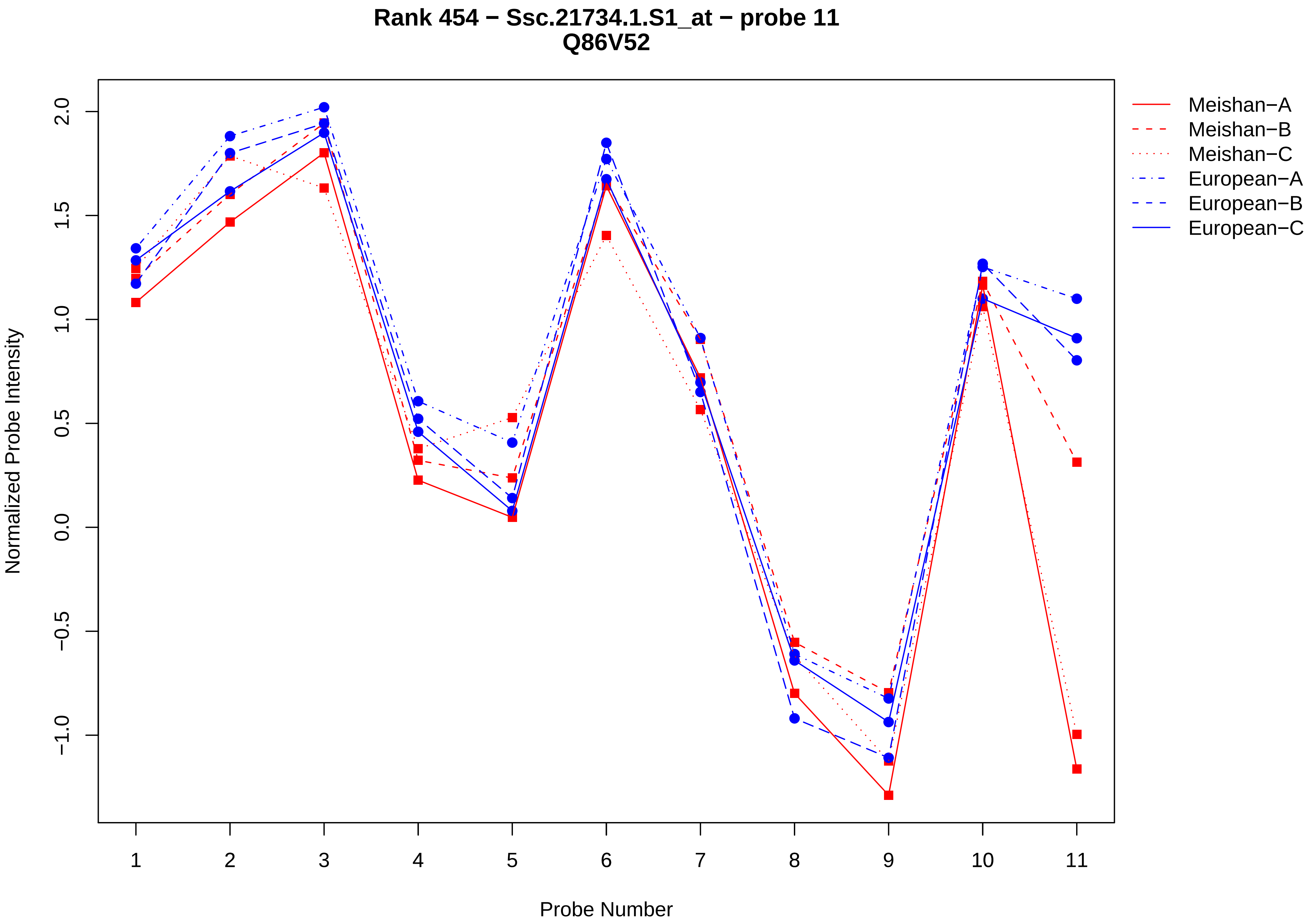

Rank 454 − Ssc.21734.1.S1_at − probe 11
Q86V52
Normalized Probe Intensity
Probe Number
Meishan−A
Meishan−B
Meishan−C
European−A
European−B
European−C
2.0
1.5
1.0
0.5
0.0
−0.5
−1.0
1
2
3
4
5
6
7
8
9
10
11

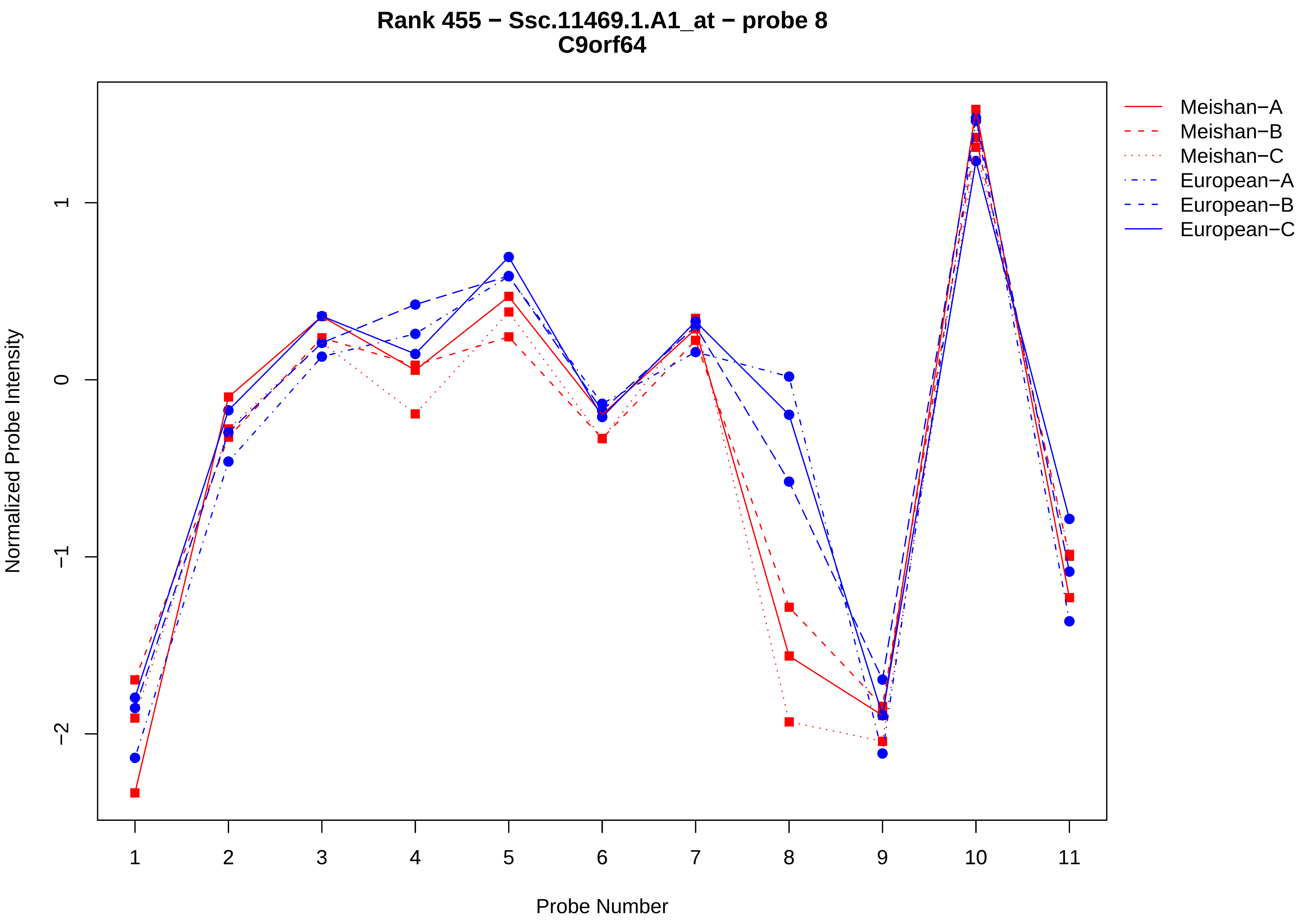

Rank 455 − Ssc.11469.1.A1_at − probe 8
C9orf64
Normalized Probe Intensity
Probe Number
1
0
−1
−2
1
2
3
4
5
6
7
8
9
10
11
Meishan−A
Meishan−B
Meishan−C
European−A
European−B
European−C

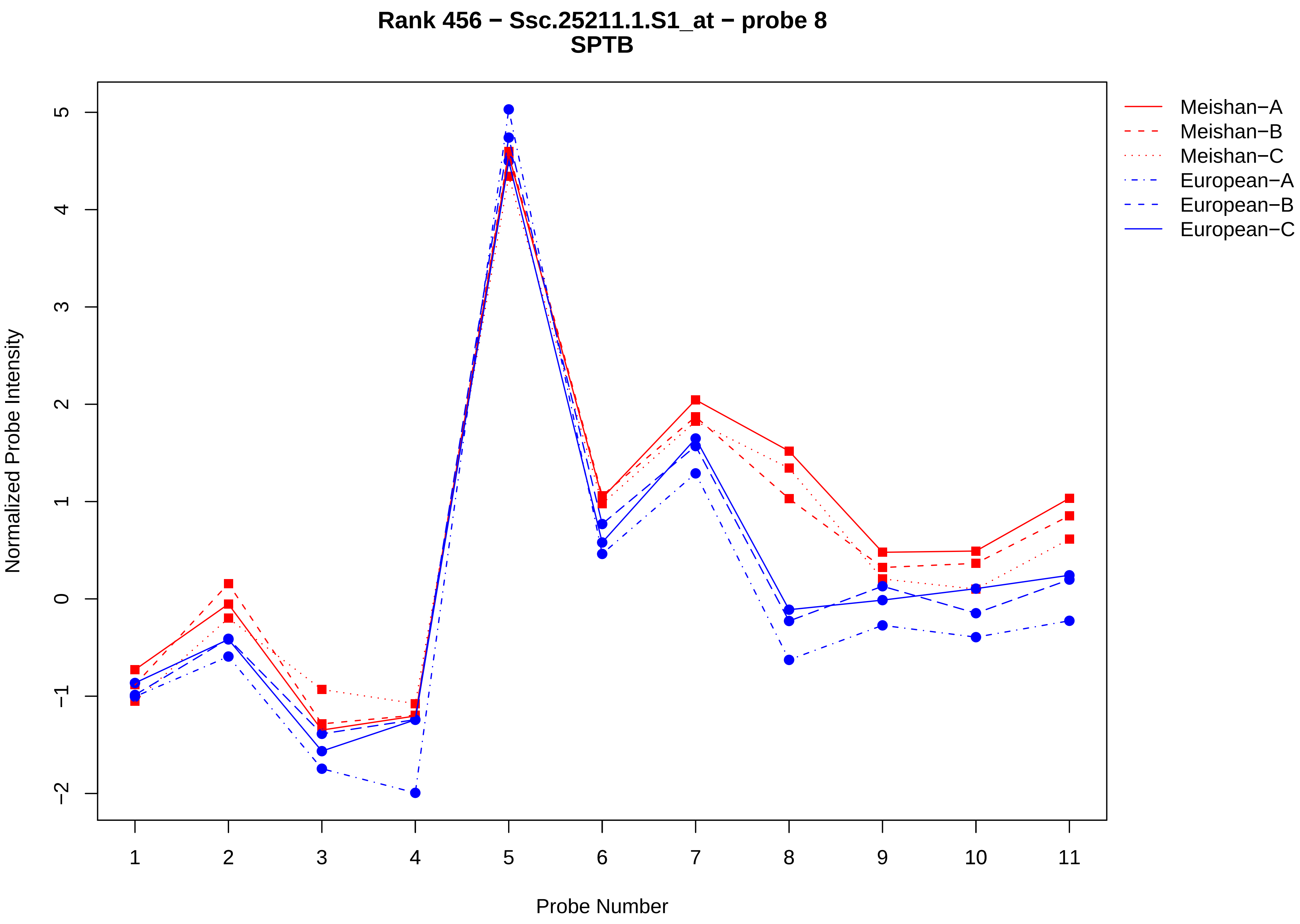

Rank 456 − Ssc.25211.1.S1_at − probe 8
SPTB
Normalized Probe Intensity
Probe Number
Meishan−A
Meishan−B
Meishan−C
European−A
European−B
European−C

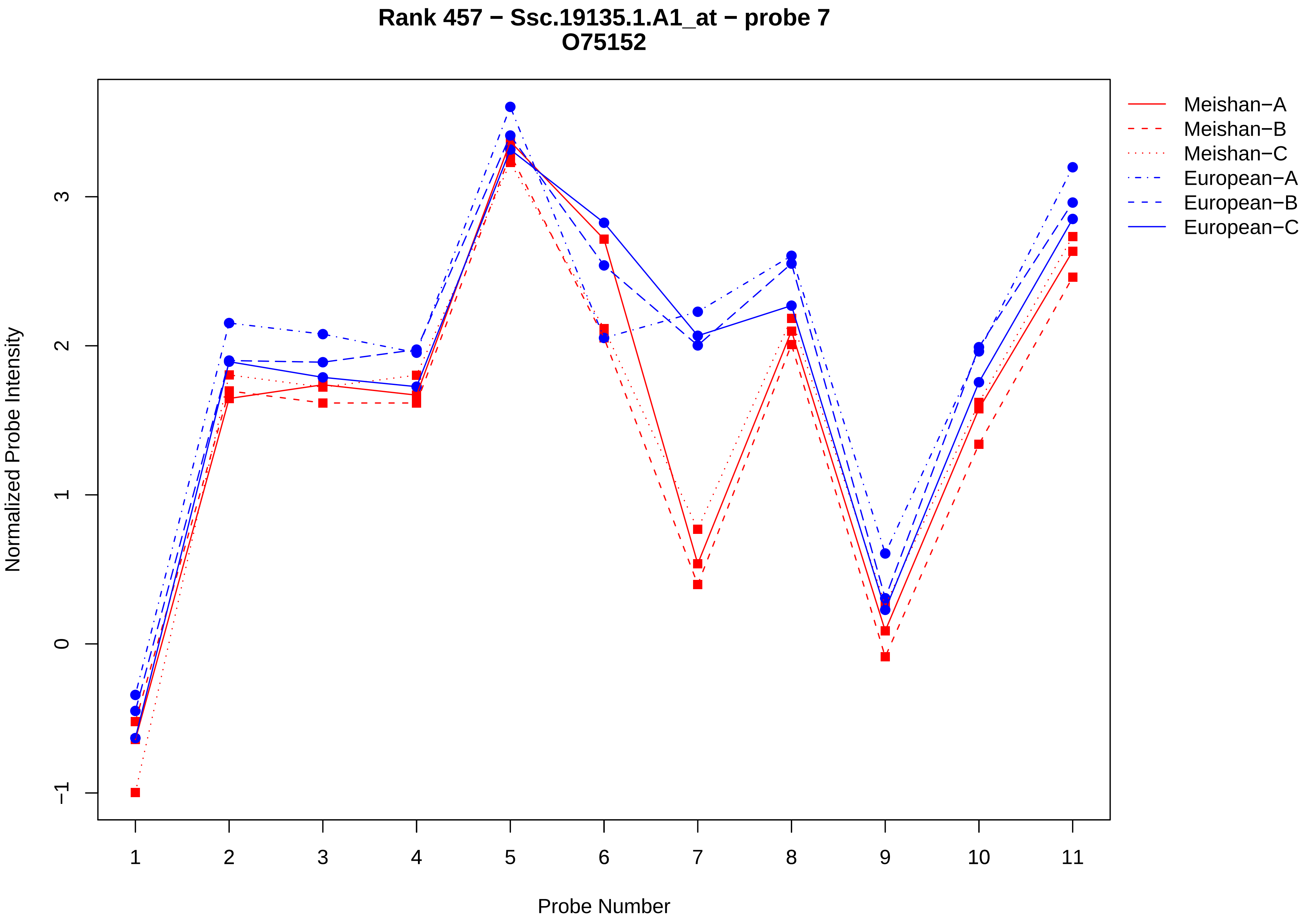
Rank 457 − Ssc.19135.1.A1_at − probe 7
O75152
Normalized Probe Intensity
Probe Number
−1
0
1
2
3
1
2
3
4
5
6
7
8
9
10
11
Meishan−A
Meishan−B
Meishan−C
European−A
European−B
European−C

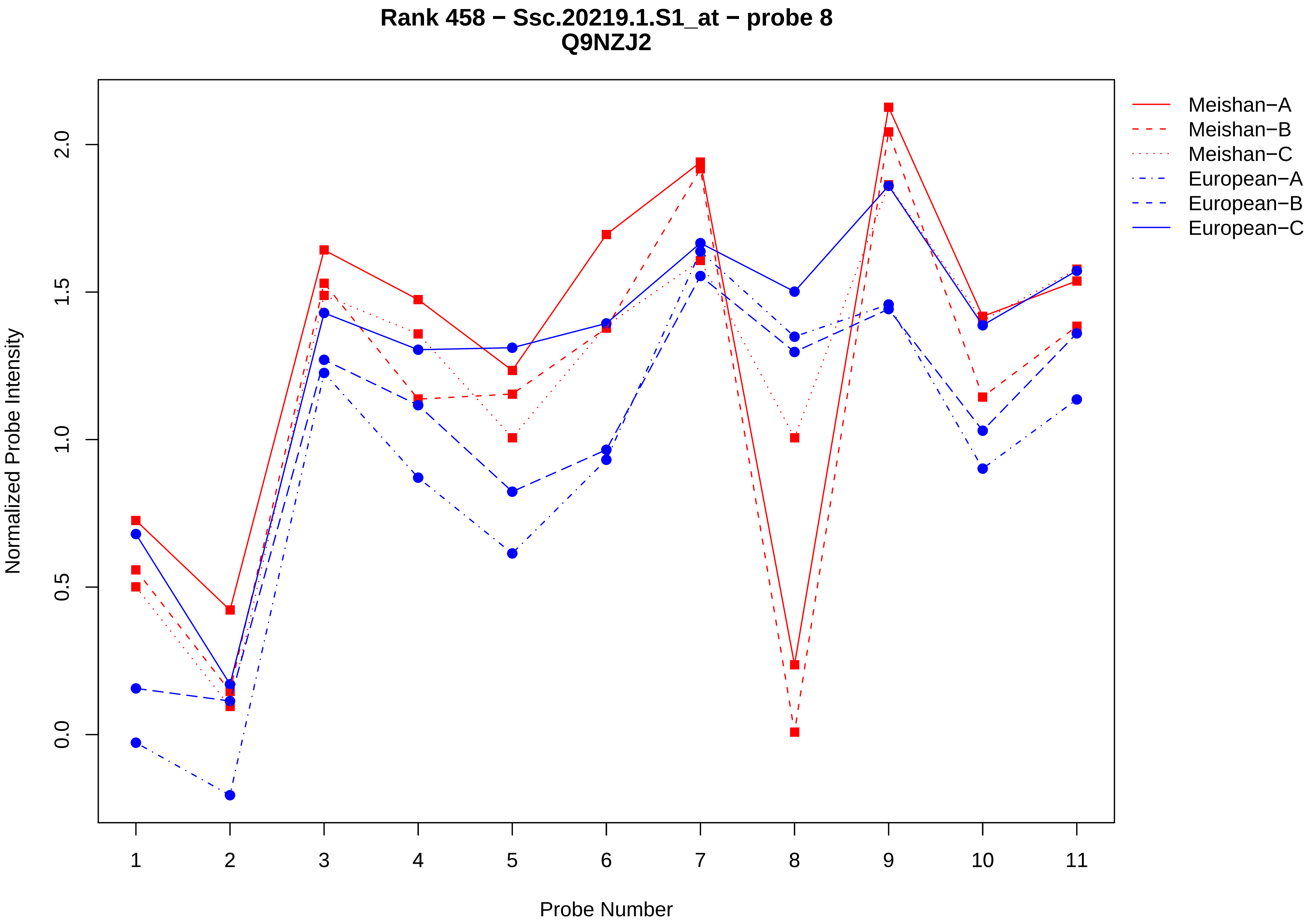

Rank 458 − Ssc.20219.1.S1_at − probe 8
Q9NZJ2
Normalized Probe Intensity
0.0
0.5
1.0
1.5
2.0
1
2
3
4
5
6
7
8
9
10
11
Probe Number
Meishan−A
Meishan−B
Meishan−C
European−A
European−B
European−C

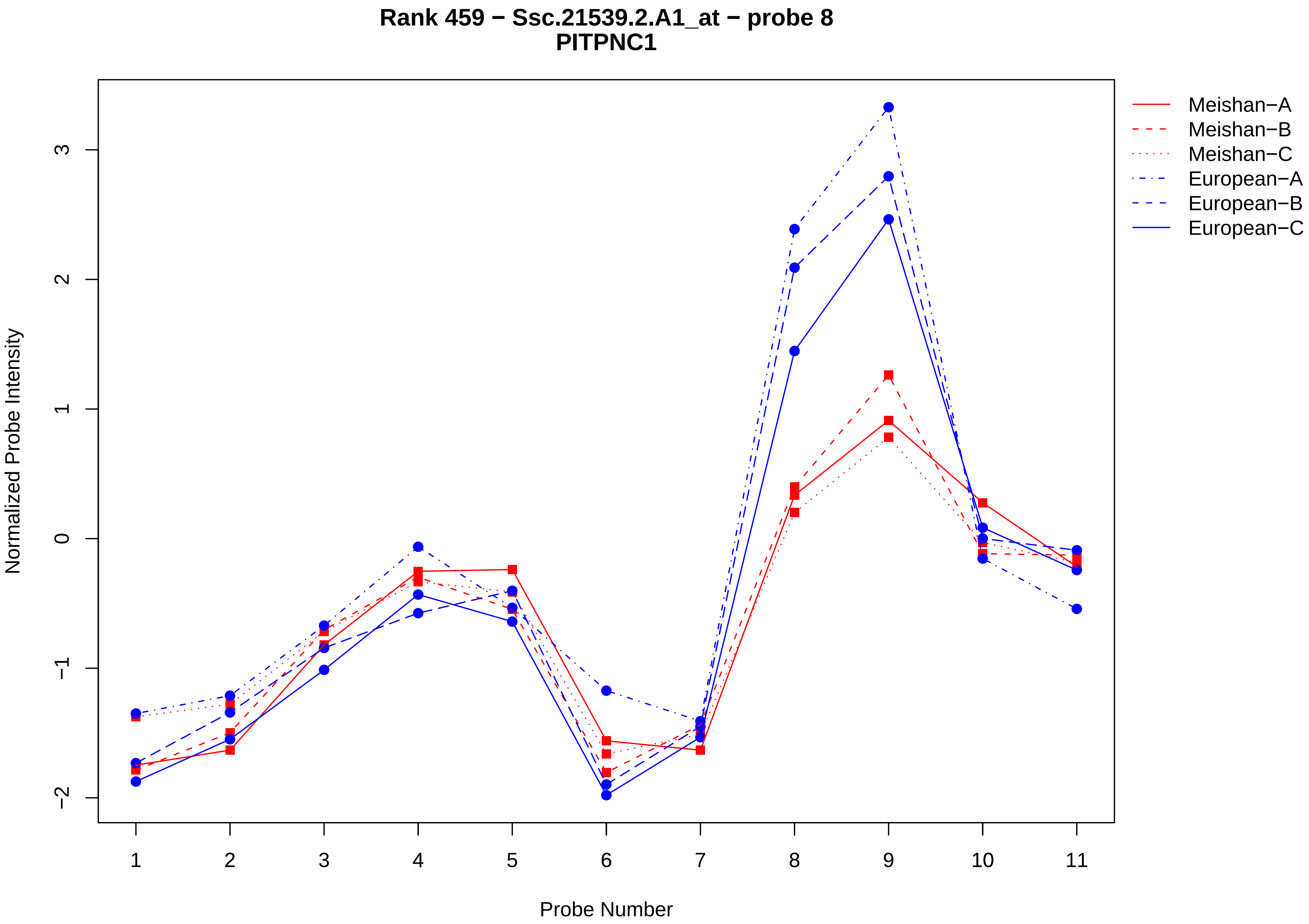

Rank 459 − Ssc.21539.2.A1_at − probe 8
PITPNC1
Normalized Probe Intensity
Probe Number
−2
−1
0
1
2
3
1
2
3
4
5
6
7
8
9
10
11
Meishan−A
Meishan−B
Meishan−C
European−A
European−B
European−C

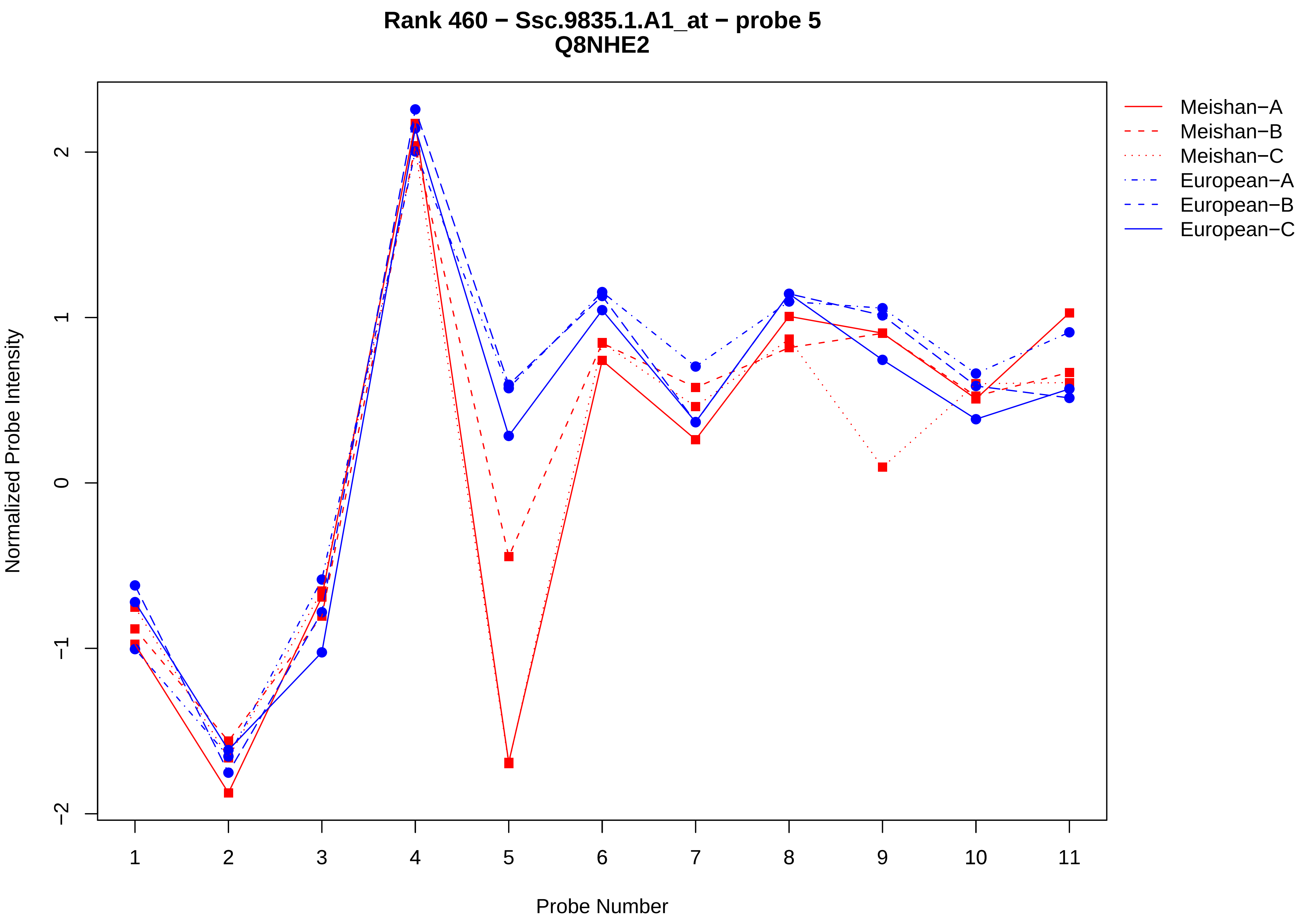

Rank 460 − Ssc.9835.1.A1_at − probe 5
Q8NHE2
Normalized Probe Intensity
Probe Number
−2
−1
0
1
2
1
2
3
4
5
6
7
8
9
10
11
Meishan−A
Meishan−B
Meishan−C
European−A
European−B
European−C

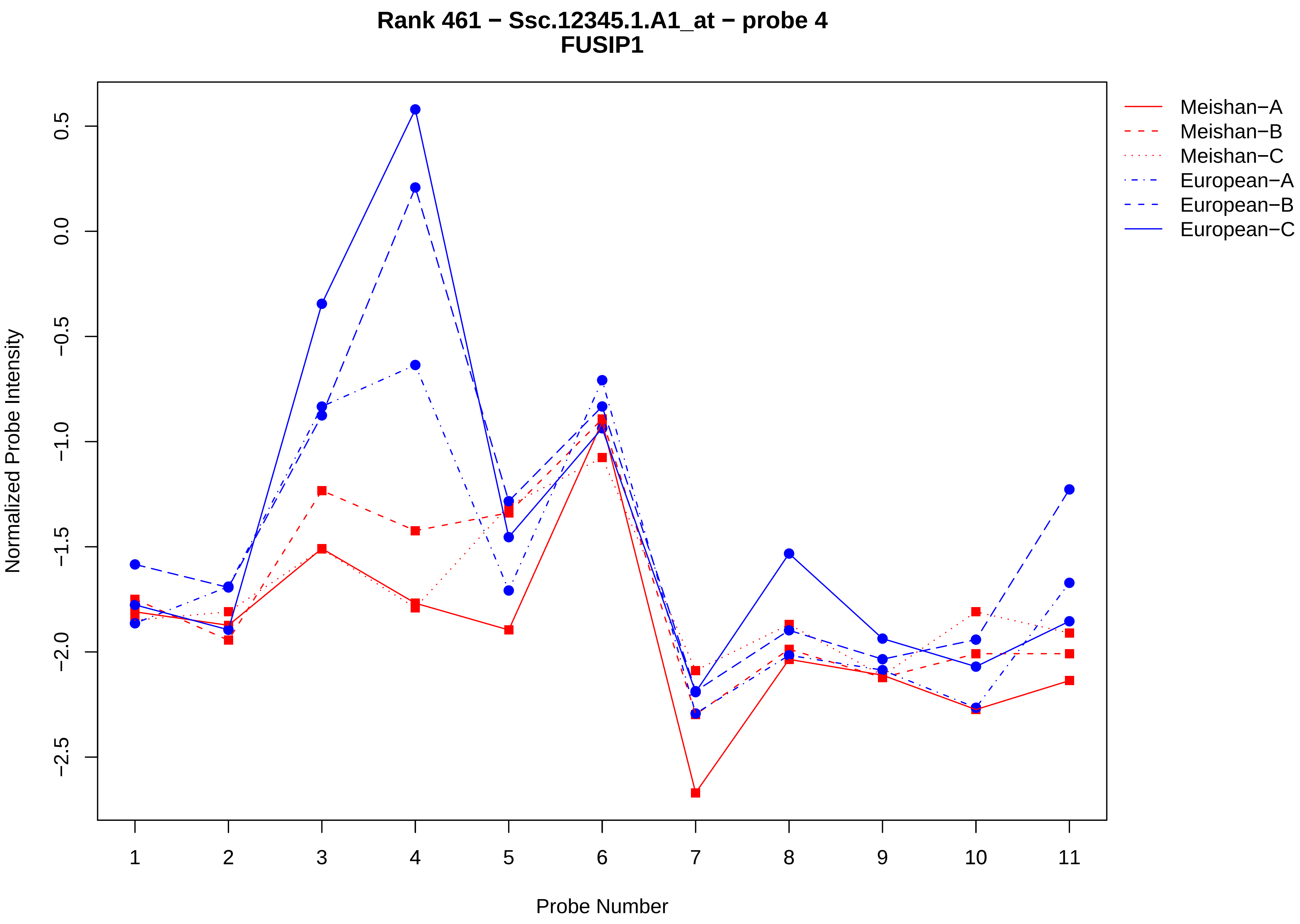
Rank 461 − Ssc.12345.1.A1_at − probe 4
FUSIP1
Normalized Probe Intensity
Probe Number
0.5
0.0
−0.5
−1.0
−1.5
−2.0
−2.5
1
2
3
4
5
6
7
8
9
10
11
Meishan−A
Meishan−B
Meishan−C
European−A
European−B
European−C

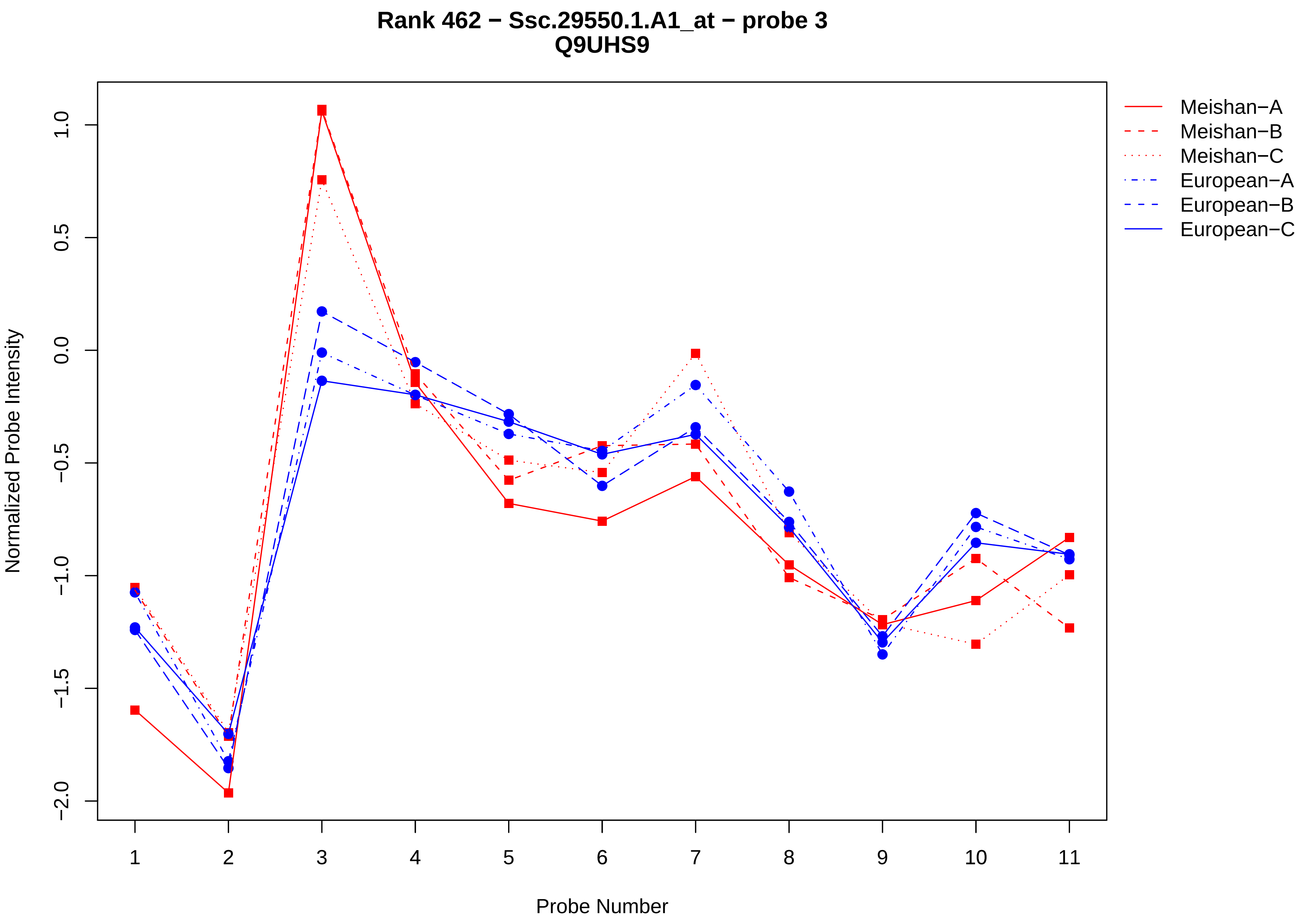
Rank 462 − Ssc.29550.1.A1_at − probe 3
Q9UHS9
Normalized Probe Intensity
1.0
0.5
0.0
−0.5
−1.0
−1.5
−2.0
1
2
3
4
5
6
7
8
9
10
11
Probe Number
Meishan−A
Meishan−B
Meishan−C
European−A
European−B
European−C

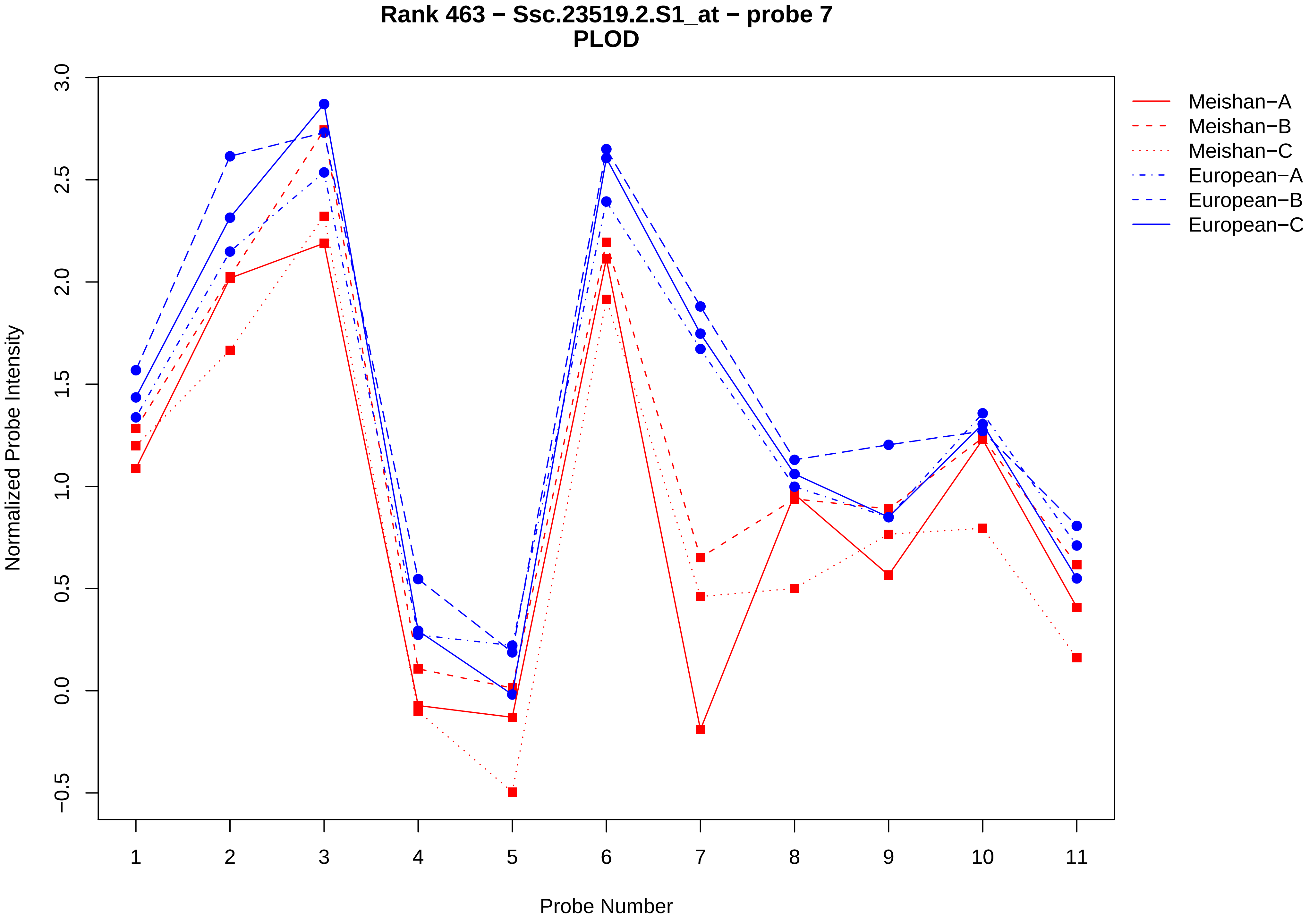

Rank 463 − Ssc.23519.2.S1_at − probe 7
PLOD
Normalized Probe Intensity
Probe Number
−0.5
0.0
0.5
1.0
1.5
2.0
2.5
3.0
1
2
3
4
5
6
7
8
9
10
11
Meishan−A
Meishan−B
Meishan−C
European−A
European−B
European−C

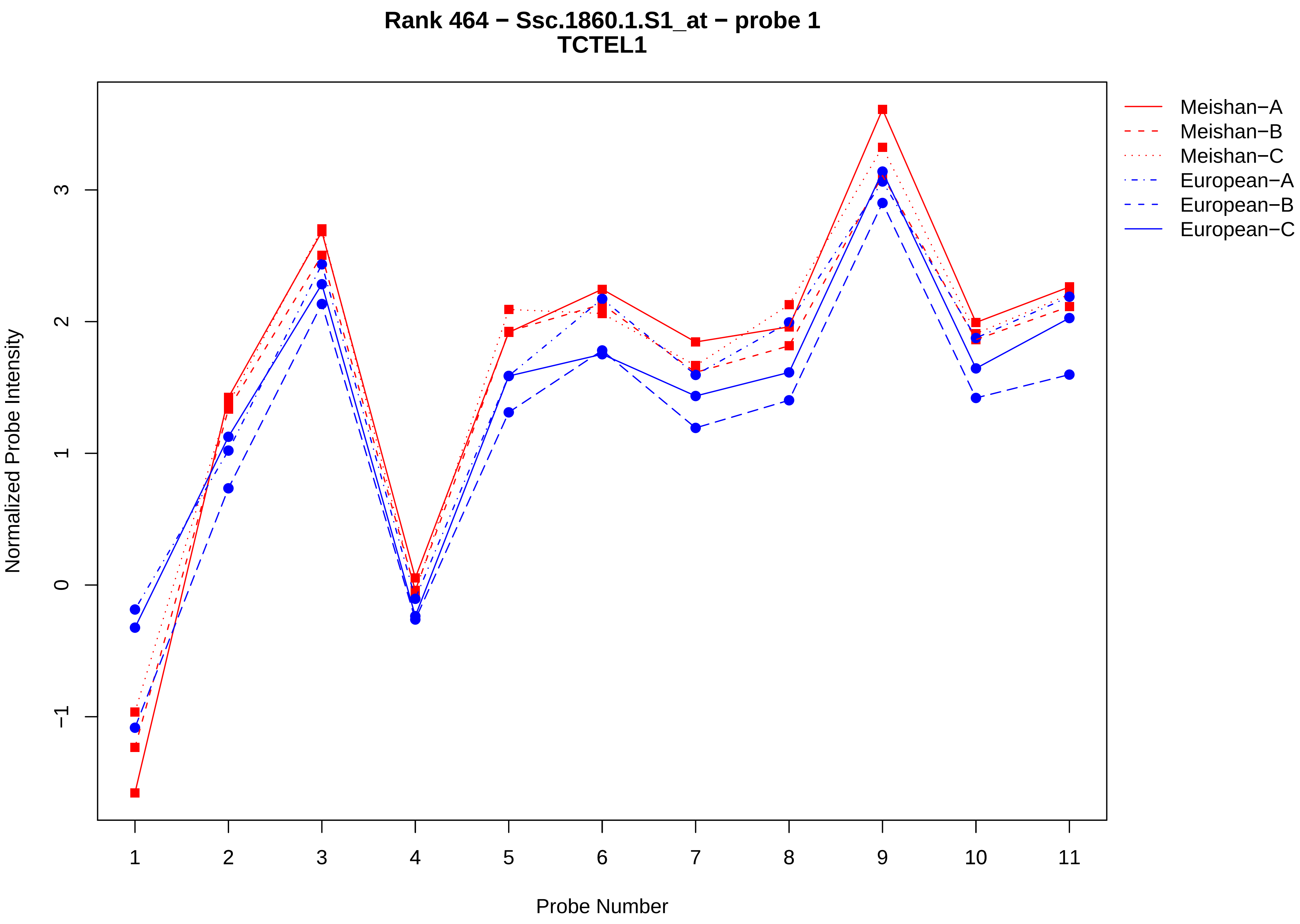

Rank 464 − Ssc.1860.1.S1_at − probe 1
TCTEL1
Normalized Probe Intensity
Probe Number
−1
0
1
2
3
1
2
3
4
5
6
7
8
9
10
11
Meishan−A
Meishan−B
Meishan−C
European−A
European−B
European−C

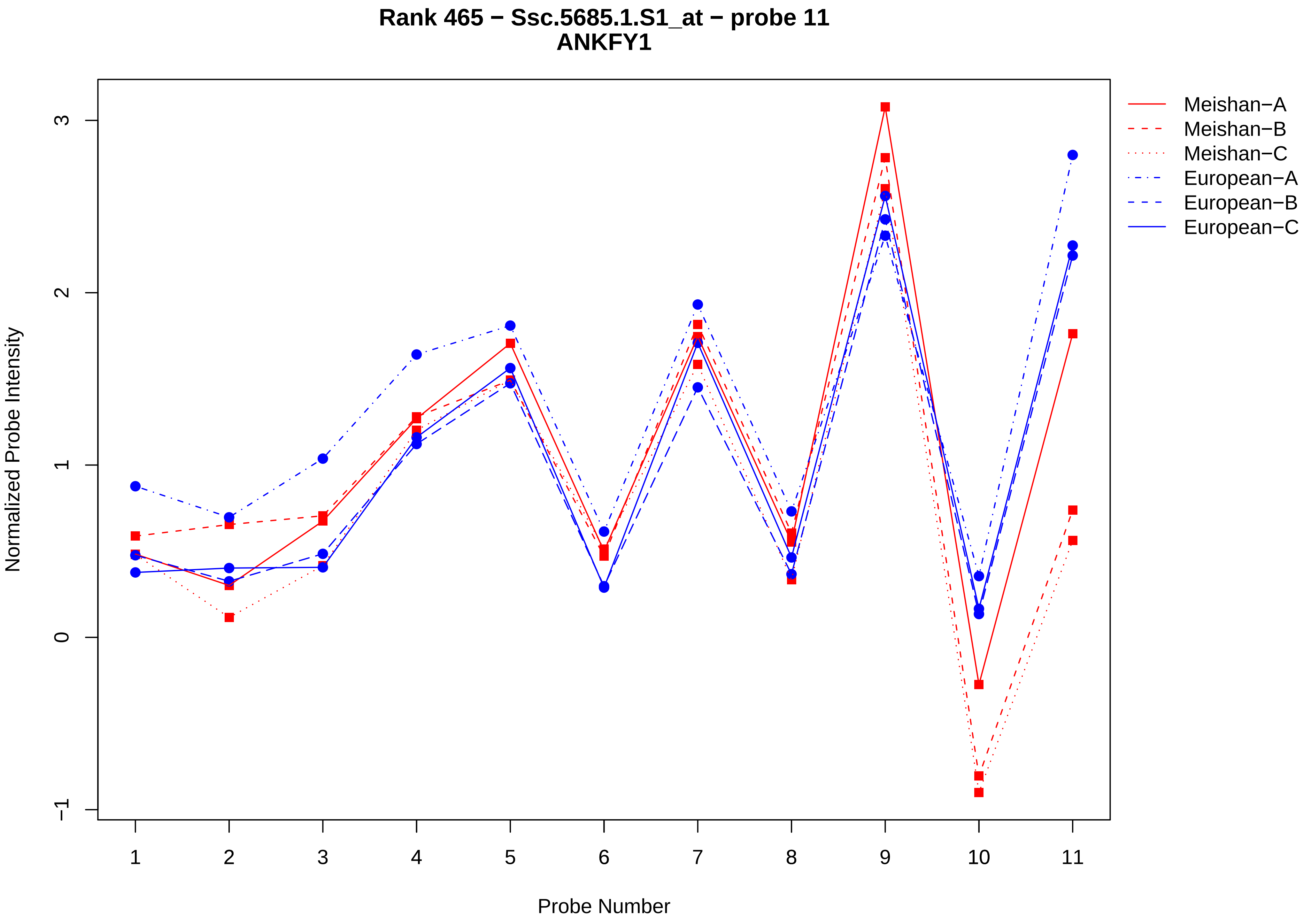

Rank 465 − Ssc.5685.1.S1_at − probe 11
ANKFY1
Normalized Probe Intensity
Probe Number
−1
0
1
2
3
1
2
3
4
5
6
7
8
9
10
11
Meishan−A
Meishan−B
Meishan−C
European−A
European−B
European−C

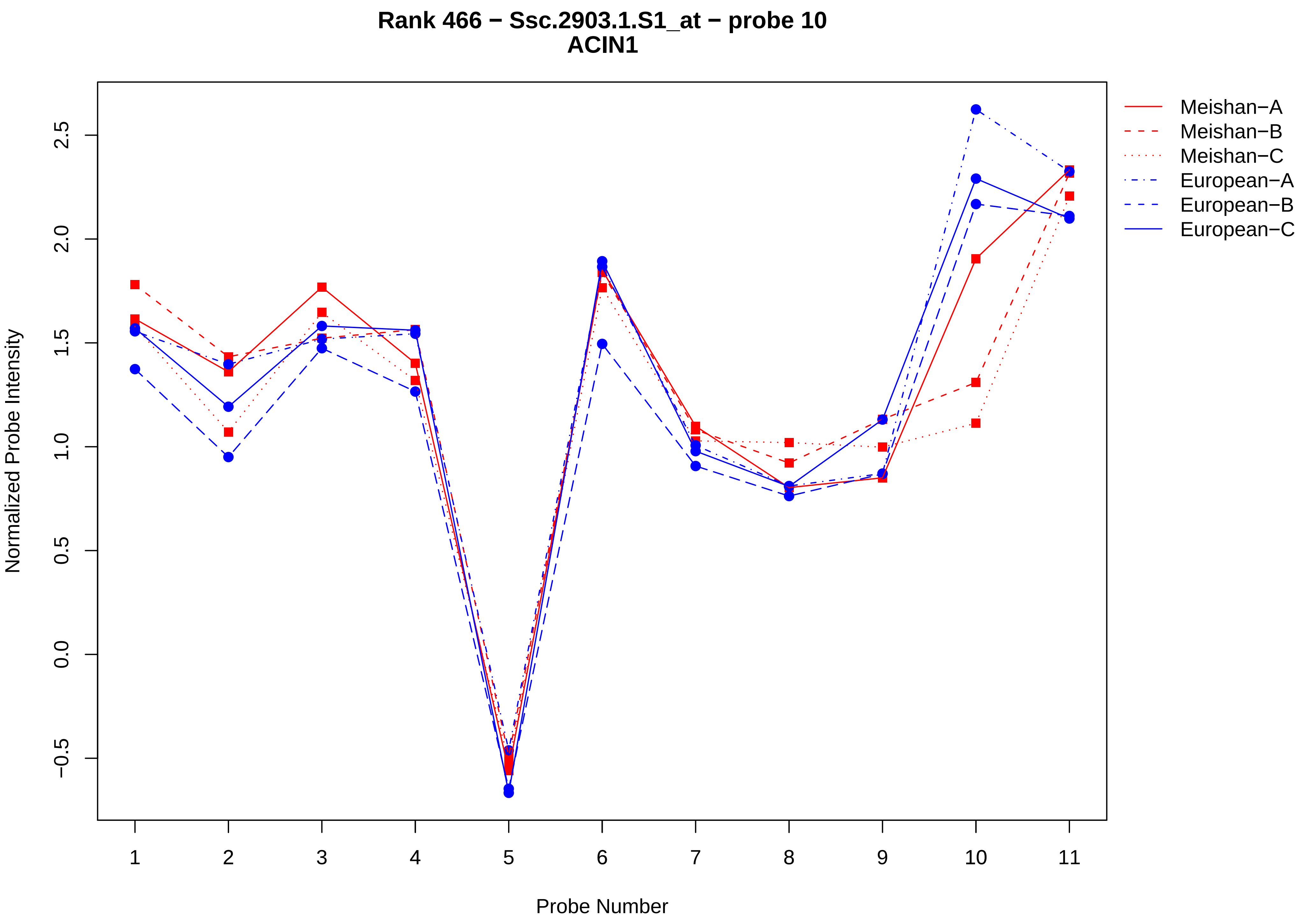

Rank 466 − Ssc.2903.1.S1_at − probe 10
ACIN1
Normalized Probe Intensity
Probe Number
−0.5
0.0
0.5
1.0
1.5
2.0
2.5
1
2
3
4
5
6
7
8
9
10
11
Meishan−A
Meishan−B
Meishan−C
European−A
European−B
European−C

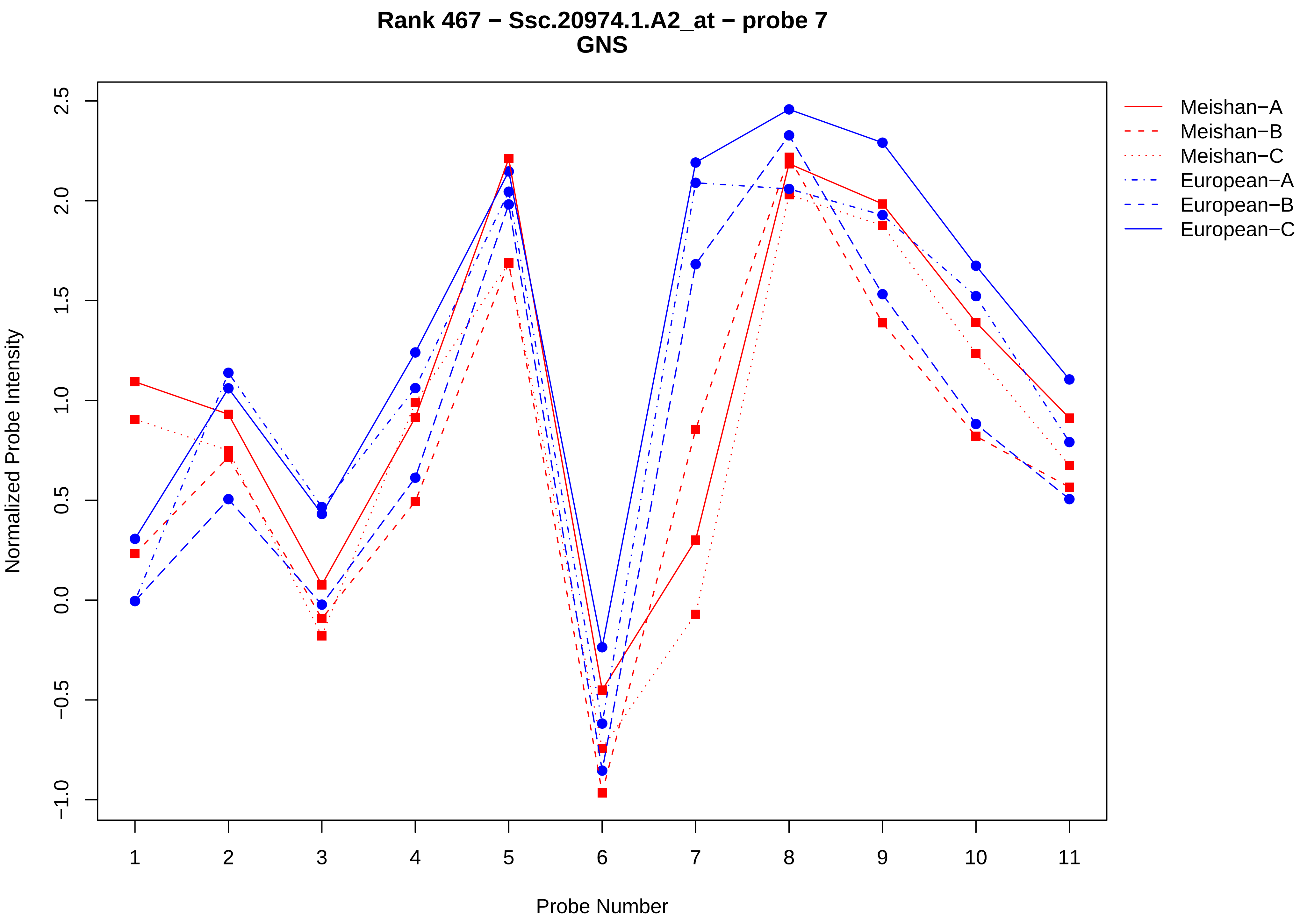

Rank 467 − Ssc.20974.1.A2_at − probe 7
GNS
Normalized Probe Intensity
Probe Number
−1.0
−0.5
0.0
0.5
1.0
1.5
2.0
2.5
1
2
3
4
5
6
7
8
9
10
11
Meishan−A
Meishan−B
Meishan−C
European−A
European−B
European−C

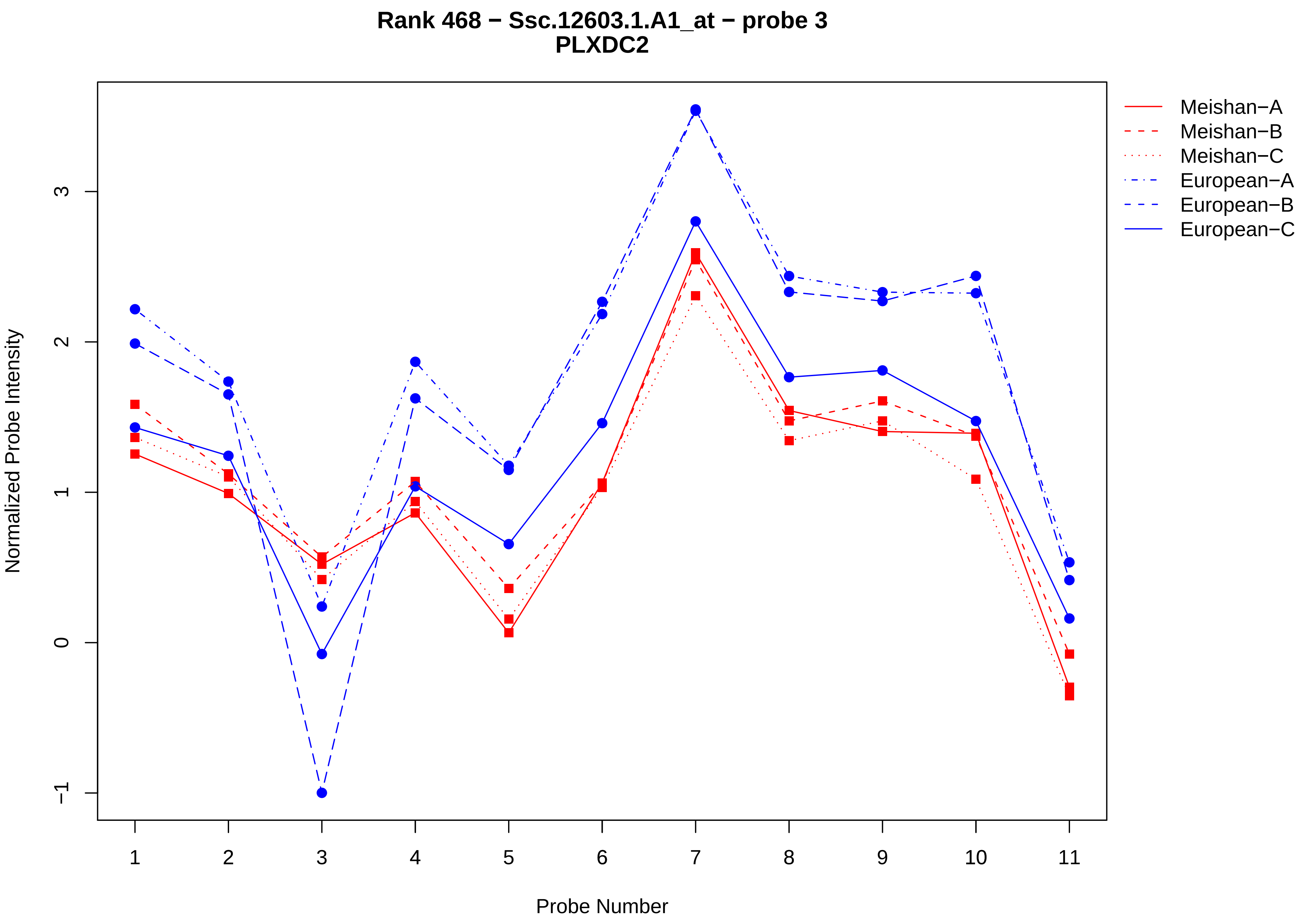

Rank 468 − Ssc.12603.1.A1_at − probe 3
PLXDC2
Normalized Probe Intensity
Probe Number
−1
0
1
2
3
1
2
3
4
5
6
7
8
9
10
11
Meishan−A
Meishan−B
Meishan−C
European−A
European−B
European−C

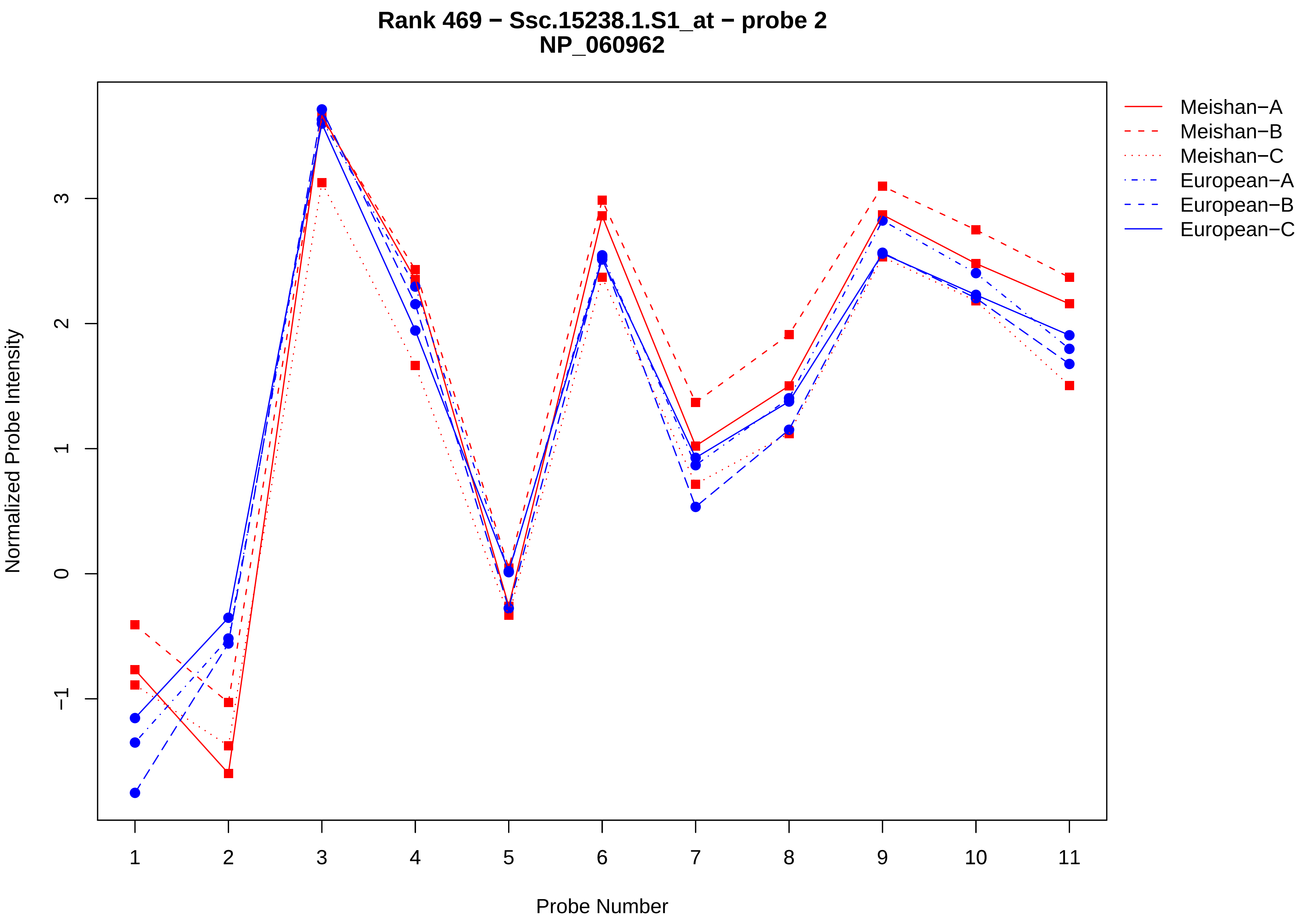

Rank 469 − Ssc.15238.1.S1_at − probe 2
NP_060962
Normalized Probe Intensity
Probe Number
Meishan−A
Meishan−B
Meishan−C
European−A
European−B
European−C
−1
0
1
2
3
1
2
3
4
5
6
7
8
9
10
11

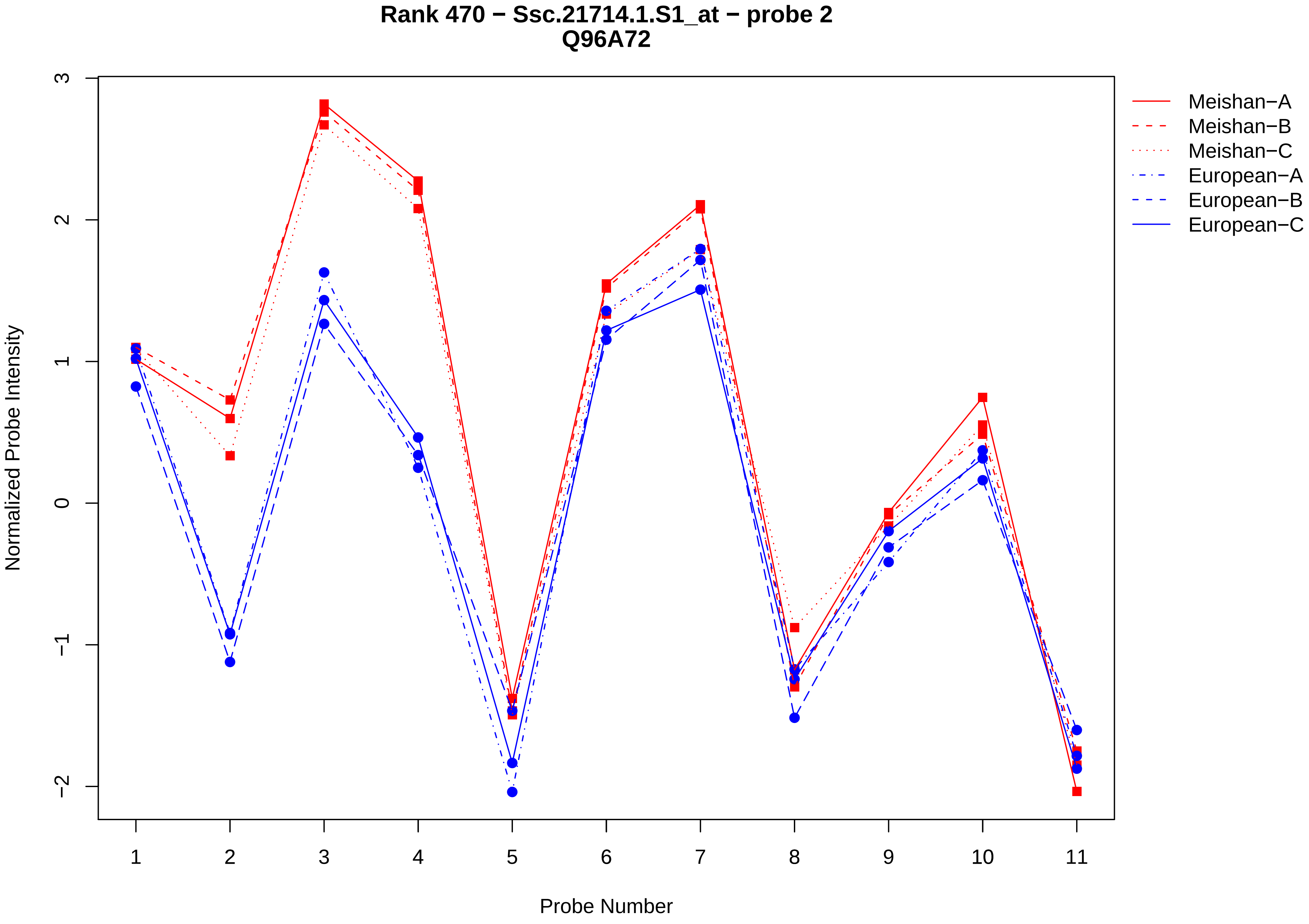
Rank 470 − Ssc.21714.1.S1_at − probe 2
Q96A72
Normalized Probe Intensity
Probe Number
Meishan−A
Meishan−B
Meishan−C
European−A
European−B
European−C
−2
−1
0
1
2
3
1
2
3
4
5
6
7
8
9
10
11

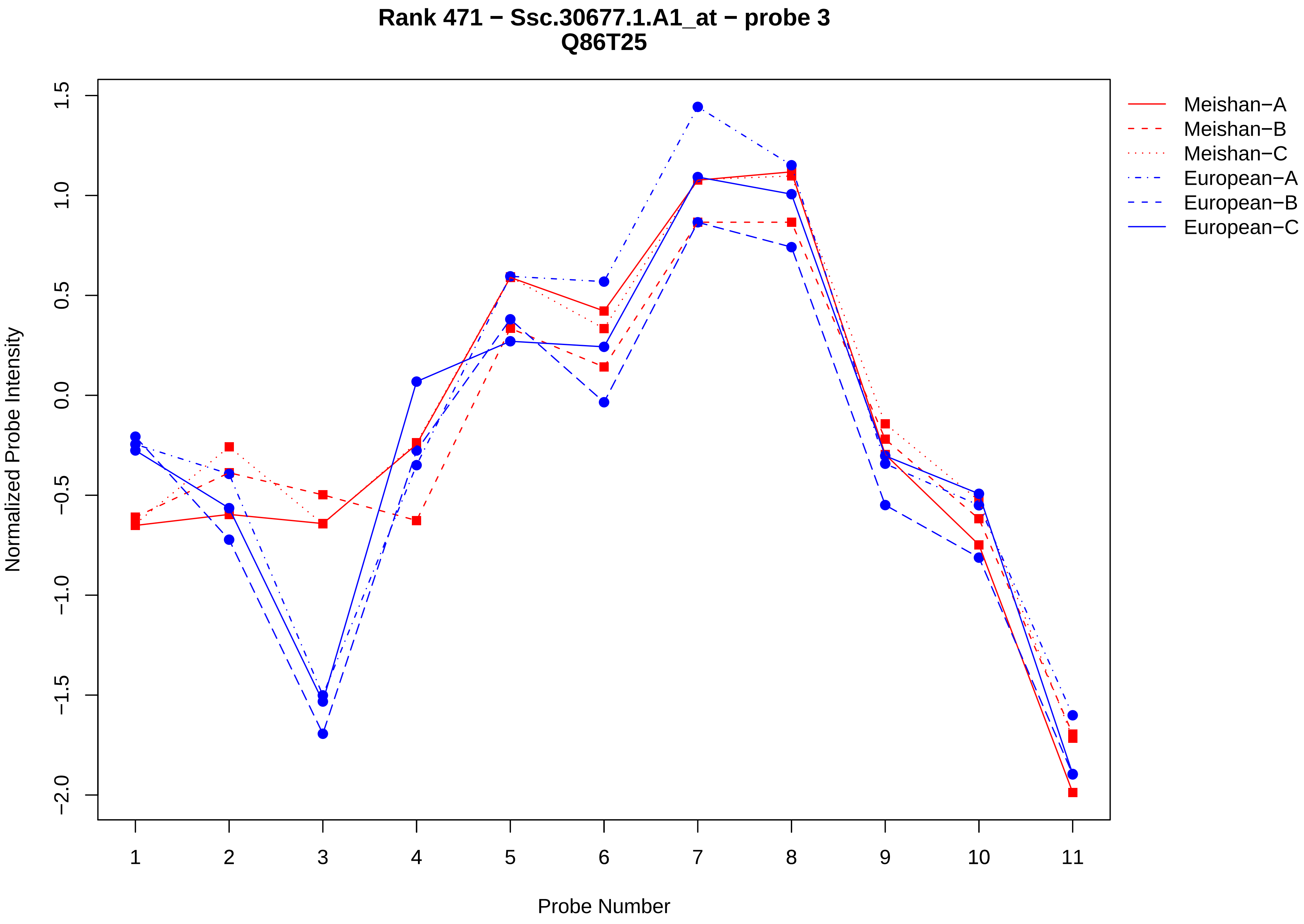

Rank 471 − Ssc.30677.1.A1_at − probe 3
Q86T25
Normalized Probe Intensity
Probe Number
1.5
1.0
0.5
0.0
−0.5
−1.0
−1.5
−2.0
1
2
3
4
5
6
7
8
9
10
11
Meishan−A
Meishan−B
Meishan−C
European−A
European−B
European−C

Rank 472 − Ssc.13529.1.A1_at − probe 4
ZNF6

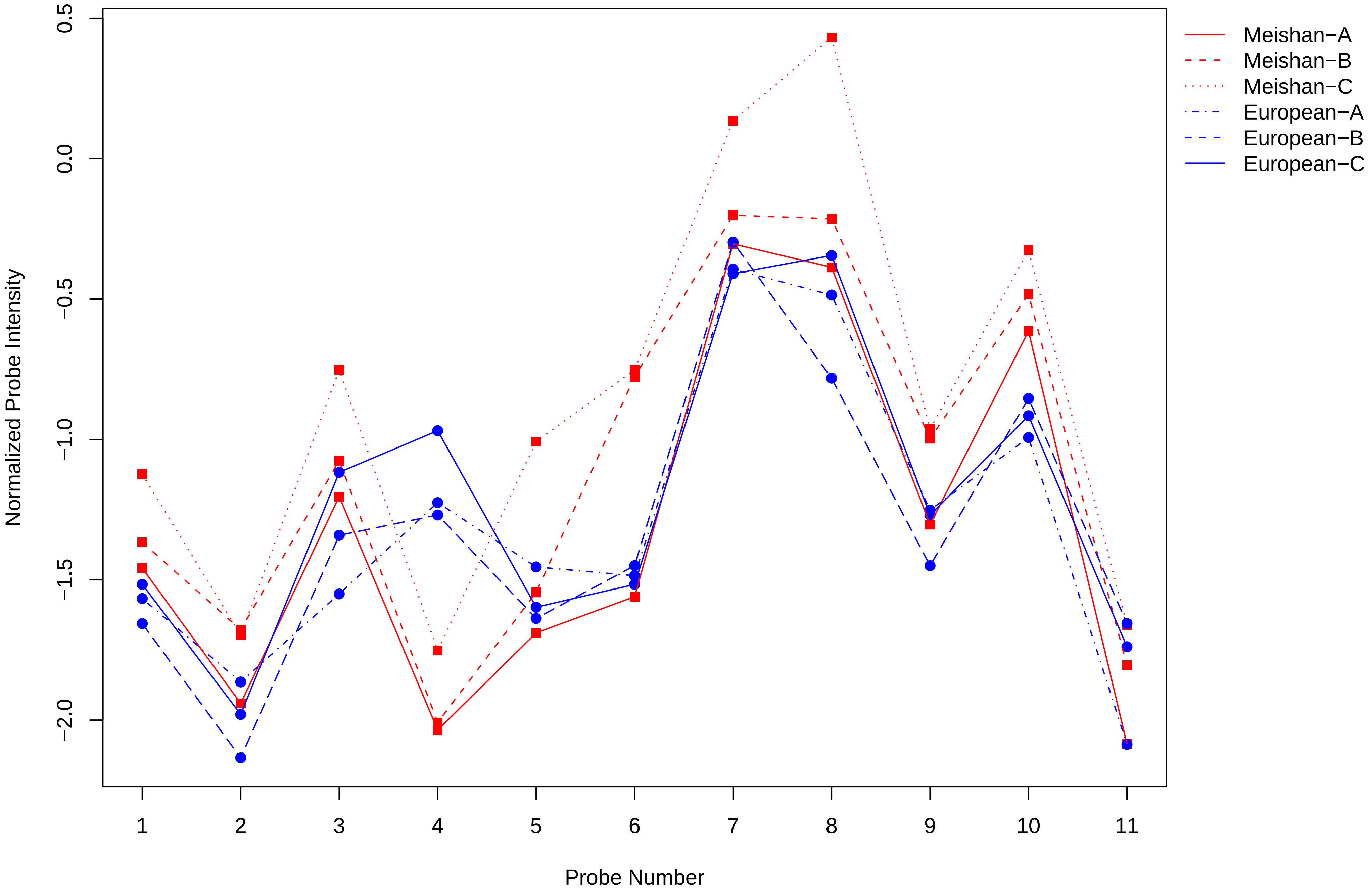

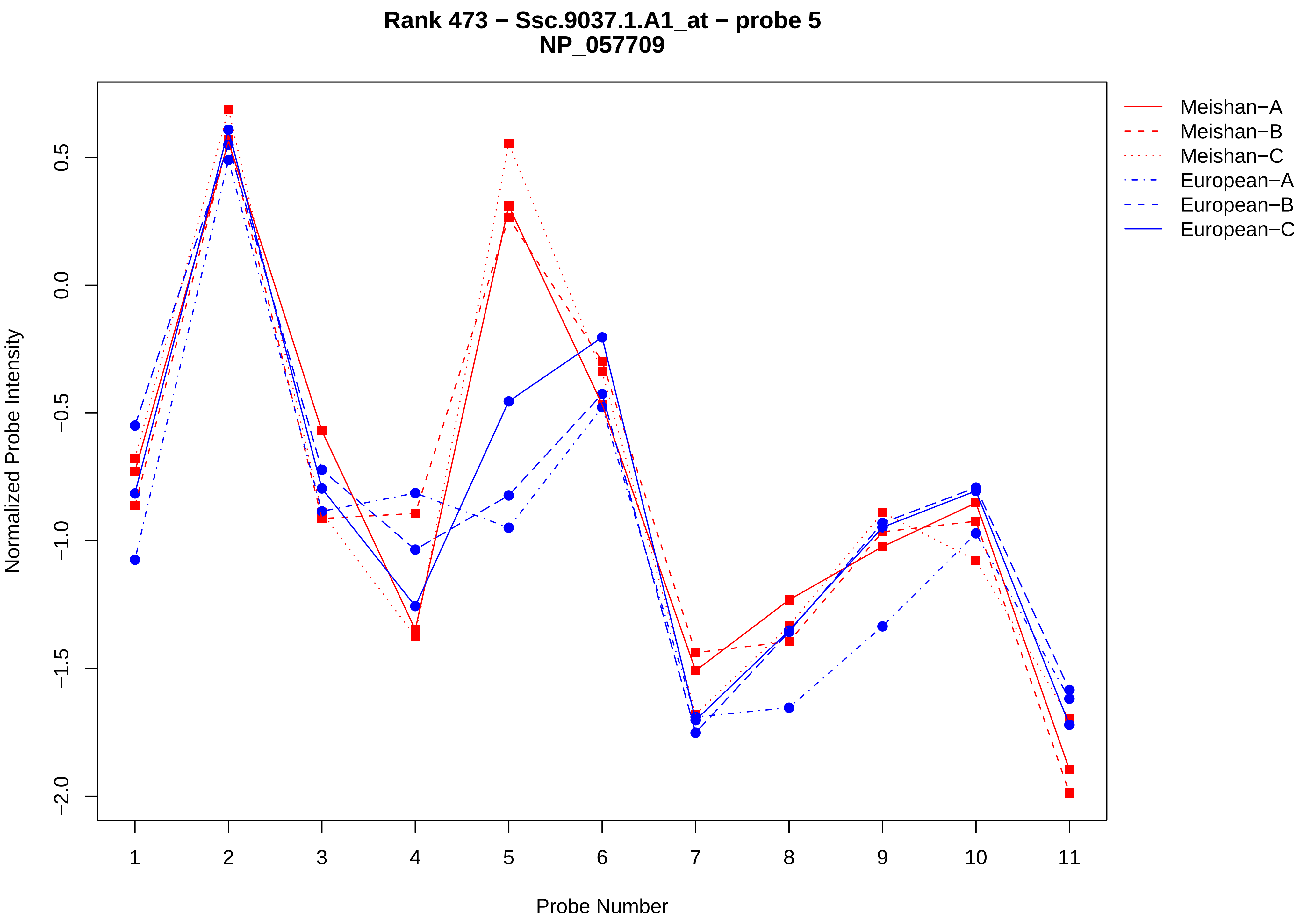

Rank 473 − Ssc.9037.1.A1_at − probe 5
NP_057709
Normalized Probe Intensity
Probe Number
0.5
0.0
−0.5
−1.0
−1.5
−2.0
1
2
3
4
5
6
7
8
9
10
11
Meishan−A
Meishan−B
Meishan−C
European−A
European−B
European−C

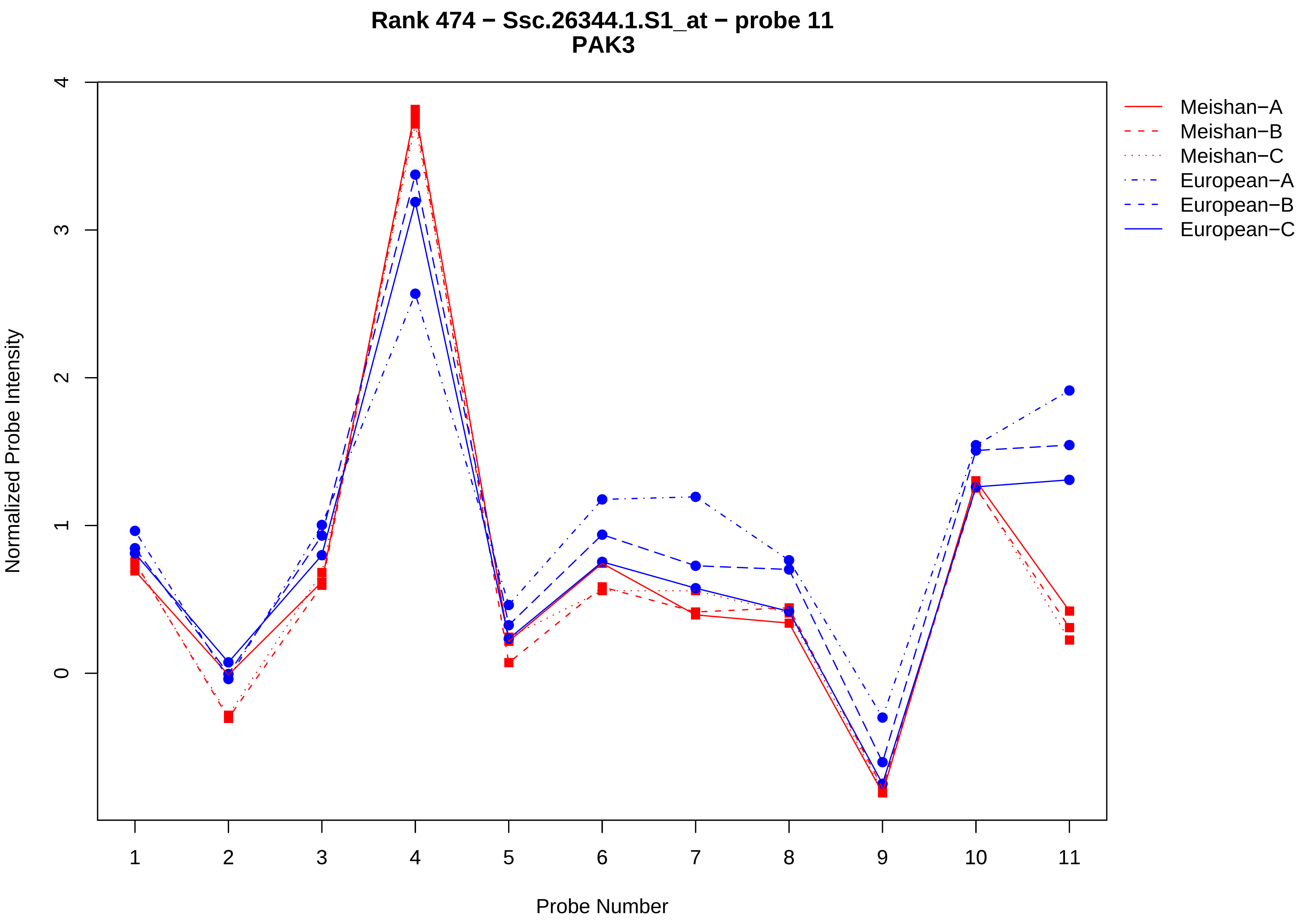

Rank 474 − Ssc.26344.1.S1_at − probe 11
PAK3
Normalized Probe Intensity
Probe Number
0
1
2
3
4
1
2
3
4
5
6
7
8
9
10
11
Meishan−A
Meishan−B
Meishan−C
European−A
European−B
European−C

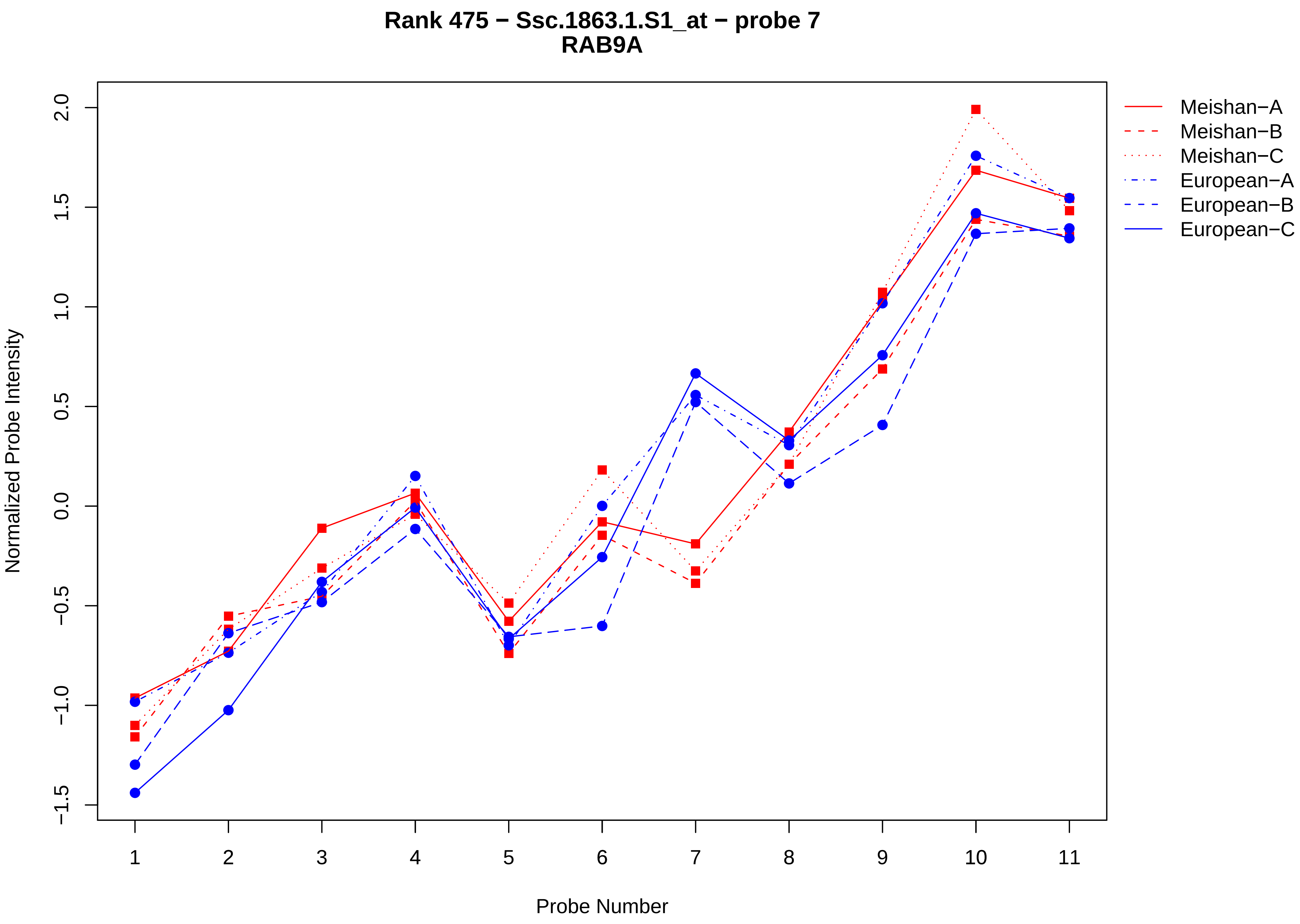

Rank 475 − Ssc.1863.1.S1_at − probe 7
RAB9A
Normalized Probe Intensity
Probe Number
−1.5
−1.0
−0.5
0.0
0.5
1.0
1.5
2.0
1
2
3
4
5
6
7
8
9
10
11
Meishan−A
Meishan−B
Meishan−C
European−A
European−B
European−C

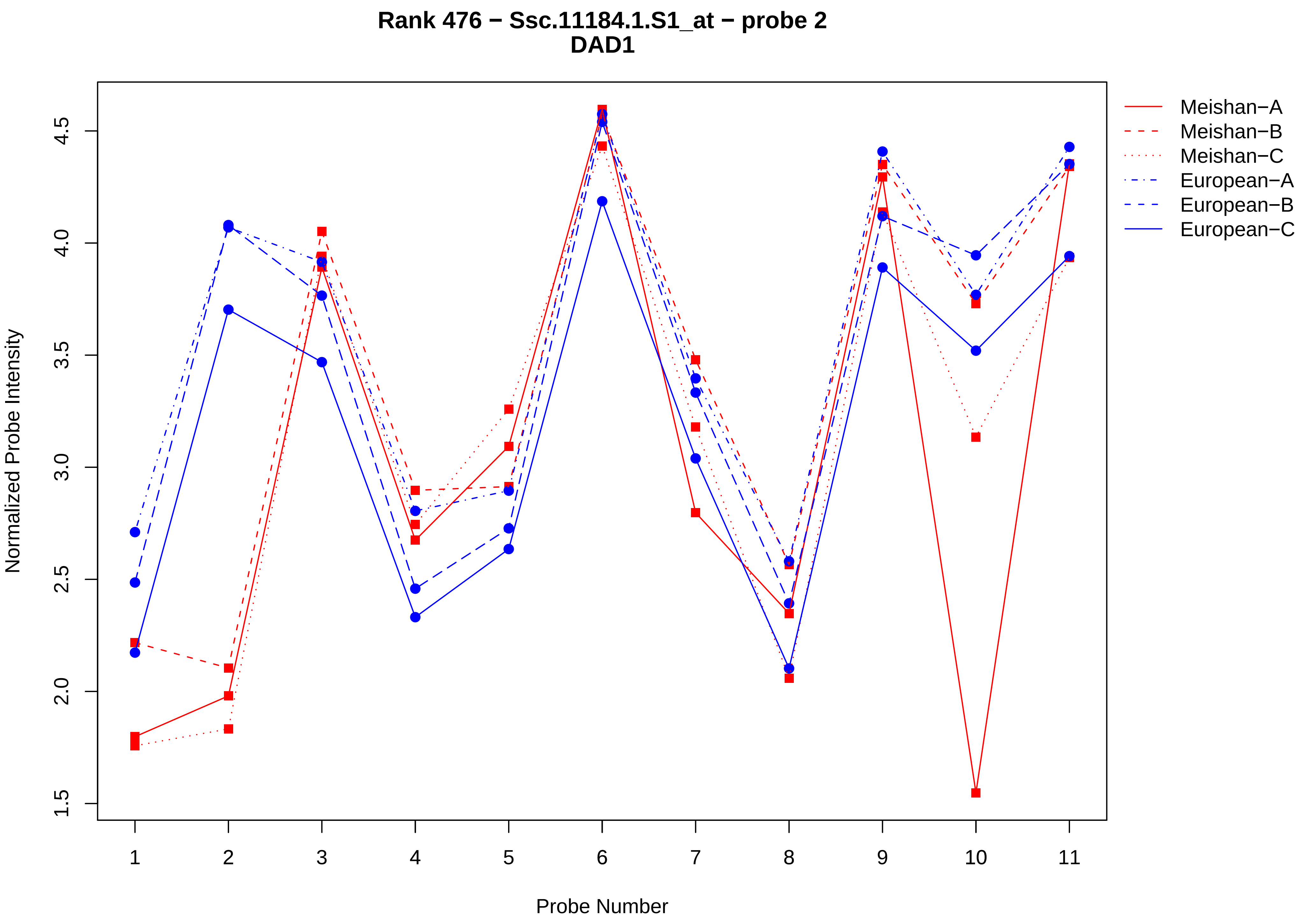
Rank 476 − Ssc.11184.1.S1_at − probe 2
DAD1
Normalized Probe Intensity
Probe Number
1.5
2.0
2.5
3.0
3.5
4.0
4.5
1
2
3
4
5
6
7
8
9
10
11
Meishan−A
Meishan−B
Meishan−C
European−A
European−B
European−C

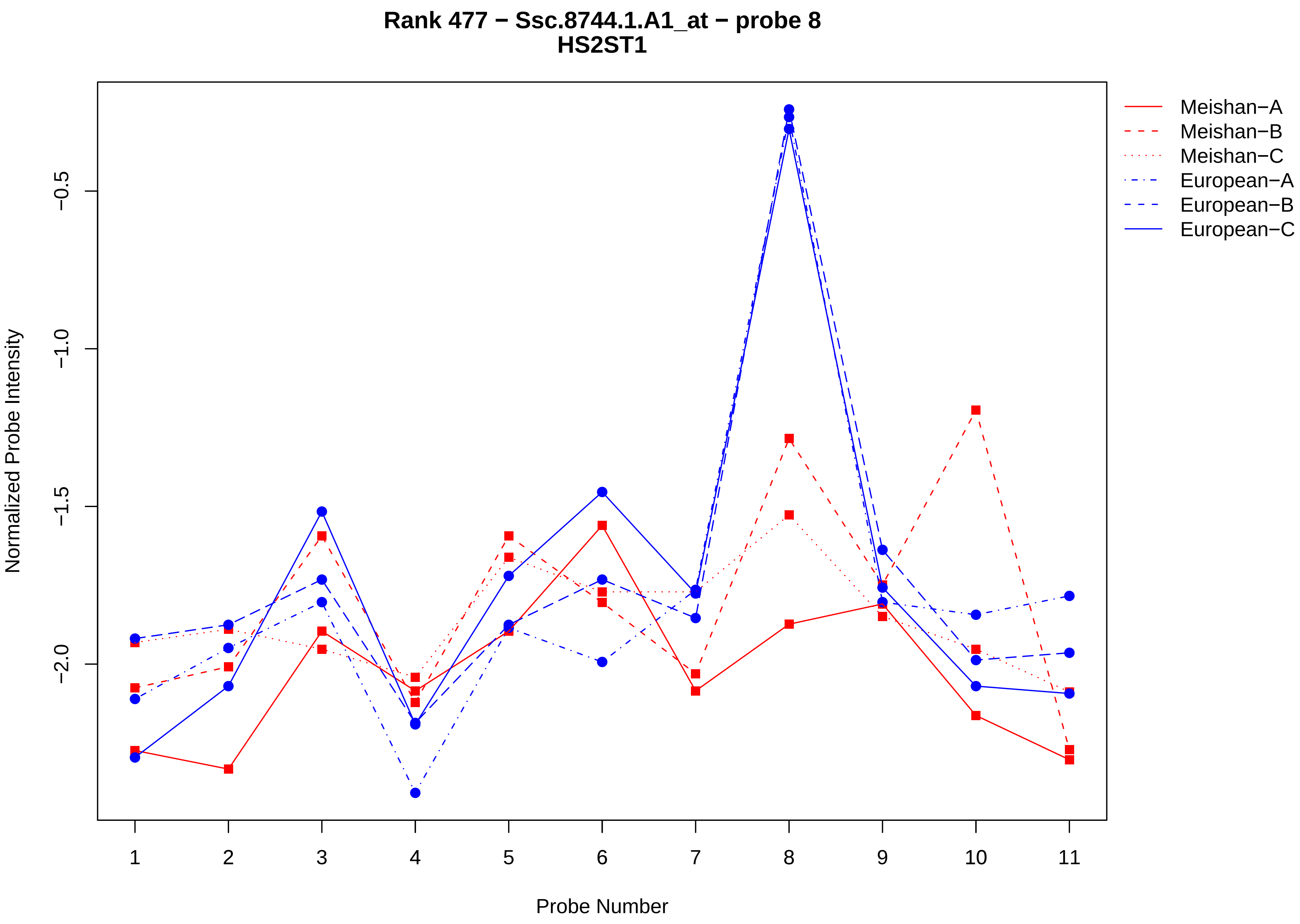
Rank 477 − Ssc.8744.1.A1_at − probe 8
HS2ST1
Normalized Probe Intensity
Probe Number
−0.5
−1.0
−1.5
−2.0
1
2
3
4
5
6
7
8
9
10
11
Meishan−A
Meishan−B
Meishan−C
European−A
European−B
European−C

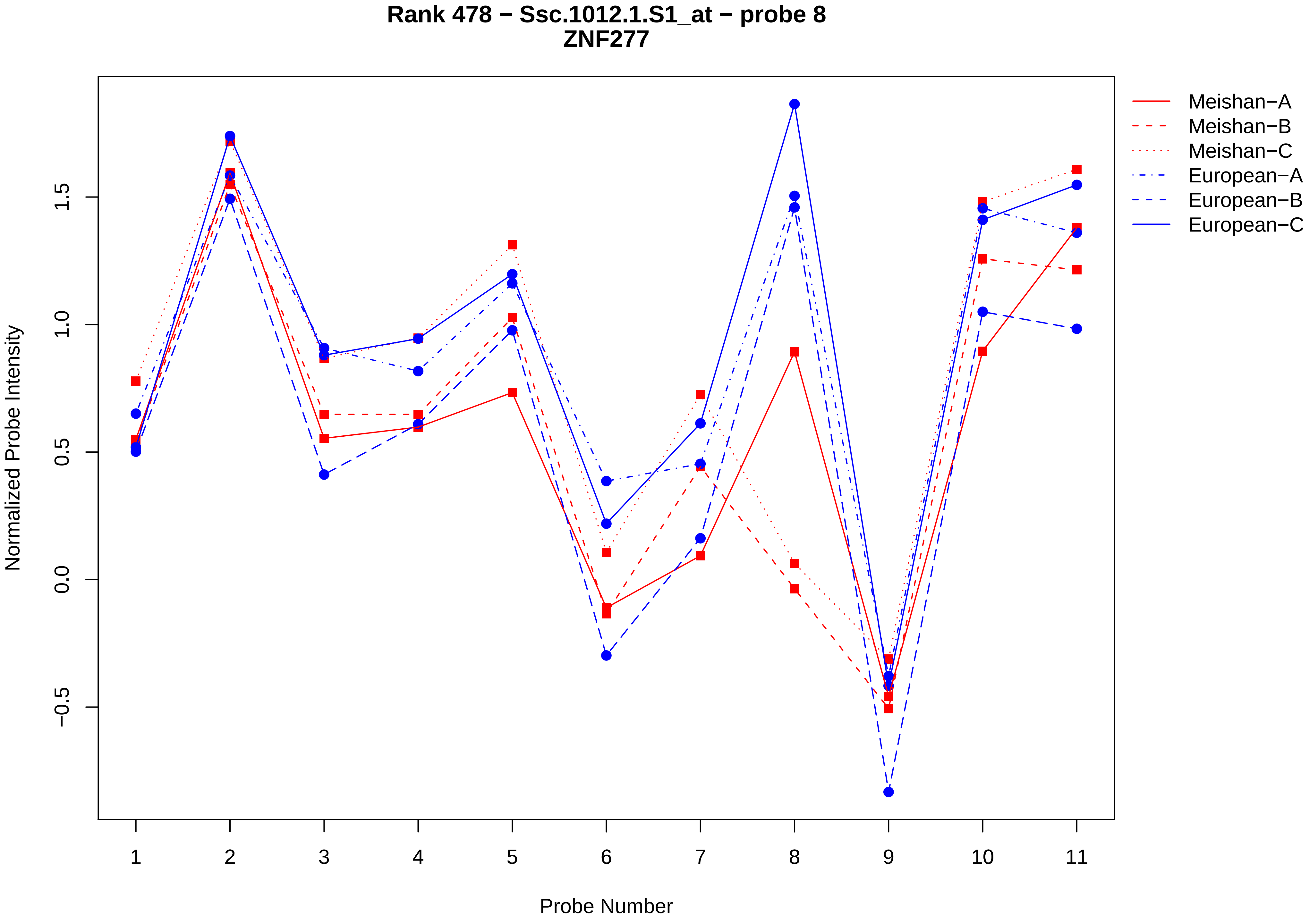

Rank 478 − Ssc.1012.1.S1_at − probe 8
ZNF277
Normalized Probe Intensity
Probe Number
−0.5
0.0
0.5
1.0
1.5
1
2
3
4
5
6
7
8
9
10
11
Meishan−A
Meishan−B
Meishan−C
European−A
European−B
European−C

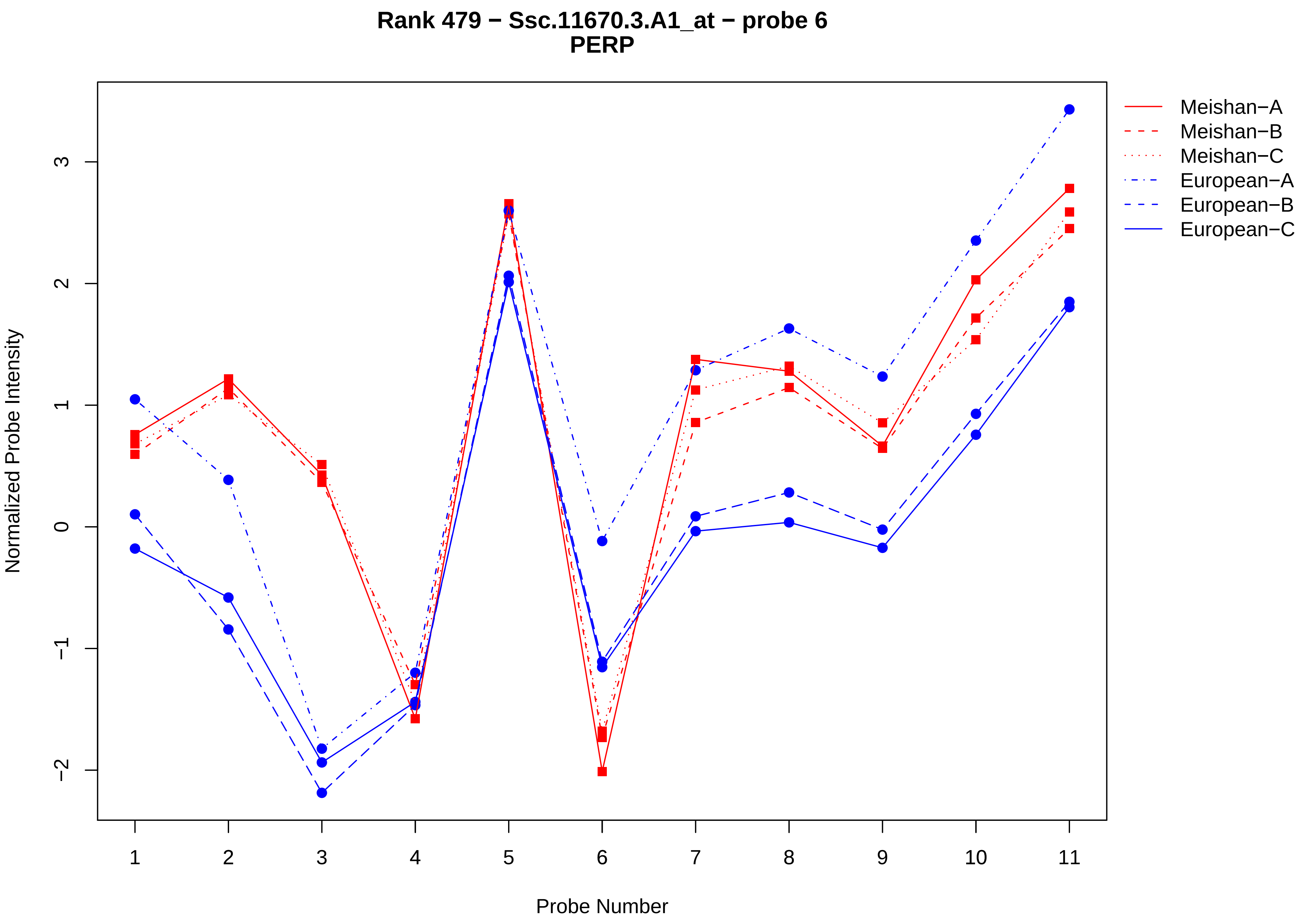

Rank 479 − Ssc.11670.3.A1_at − probe 6
PERP
Normalized Probe Intensity
Probe Number
−2
−1
0
1
2
3
1
2
3
4
5
6
7
8
9
10
11
Meishan−A
Meishan−B
Meishan−C
European−A
European−B
European−C

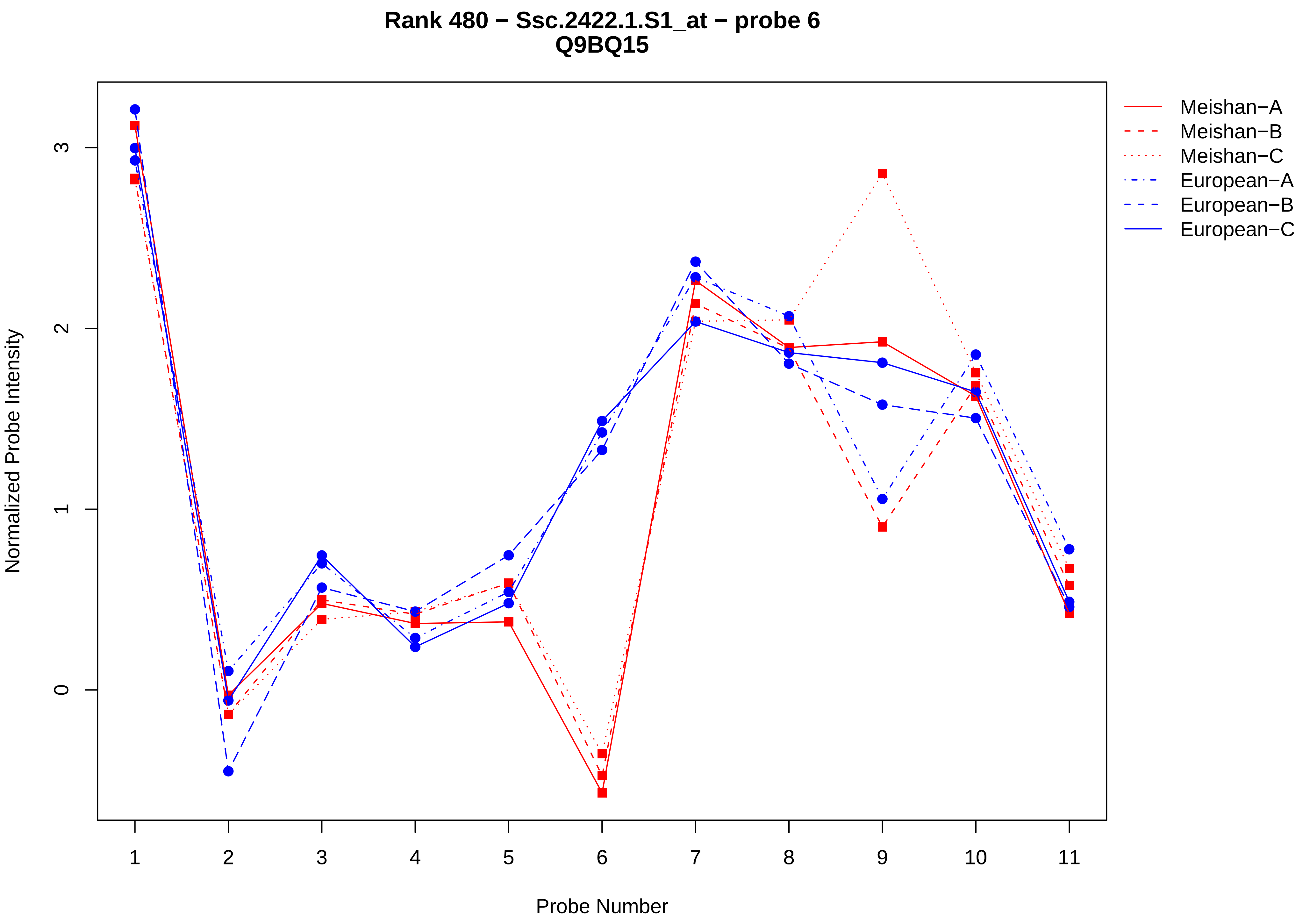
Rank 480 − Ssc.2422.1.S1_at − probe 6
Q9BQ15
Normalized Probe Intensity
Probe Number
0
1
2
3
1
2
3
4
5
6
7
8
9
10
11
Meishan−A
Meishan−B
Meishan−C
European−A
European−B
European−C

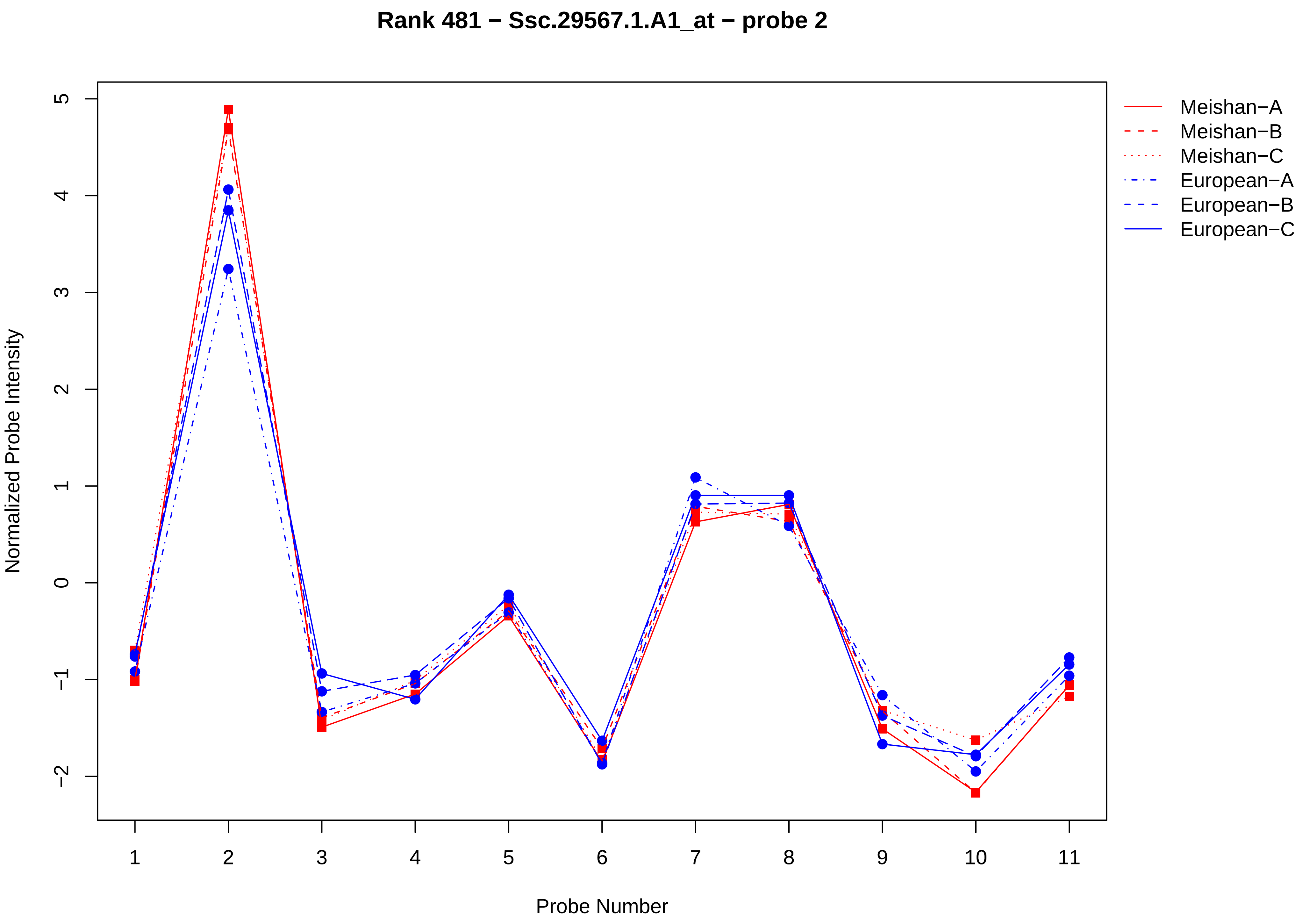
Rank 481 − Ssc.29567.1.A1_at − probe 2
Normalized Probe Intensity
Probe Number
Meishan−A
Meishan−B
Meishan−C
European−A
European−B
European−C

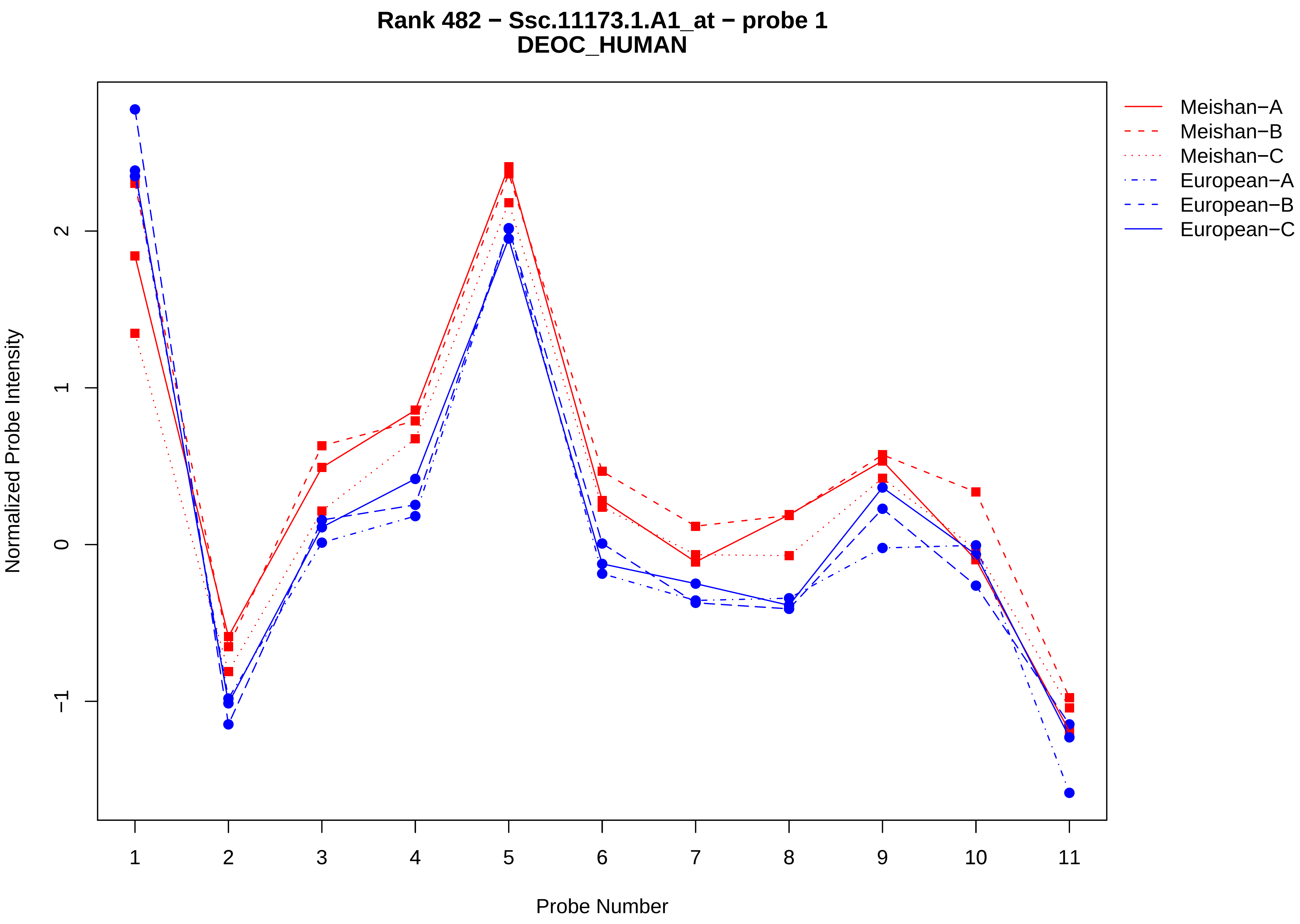

Rank 482 − Ssc.11173.1.A1_at − probe 1
DEOC_HUMAN
Normalized Probe Intensity
Probe Number
−1
0
1
2
1
2
3
4
5
6
7
8
9
10
11
Meishan−A
Meishan−B
Meishan−C
European−A
European−B
European−C

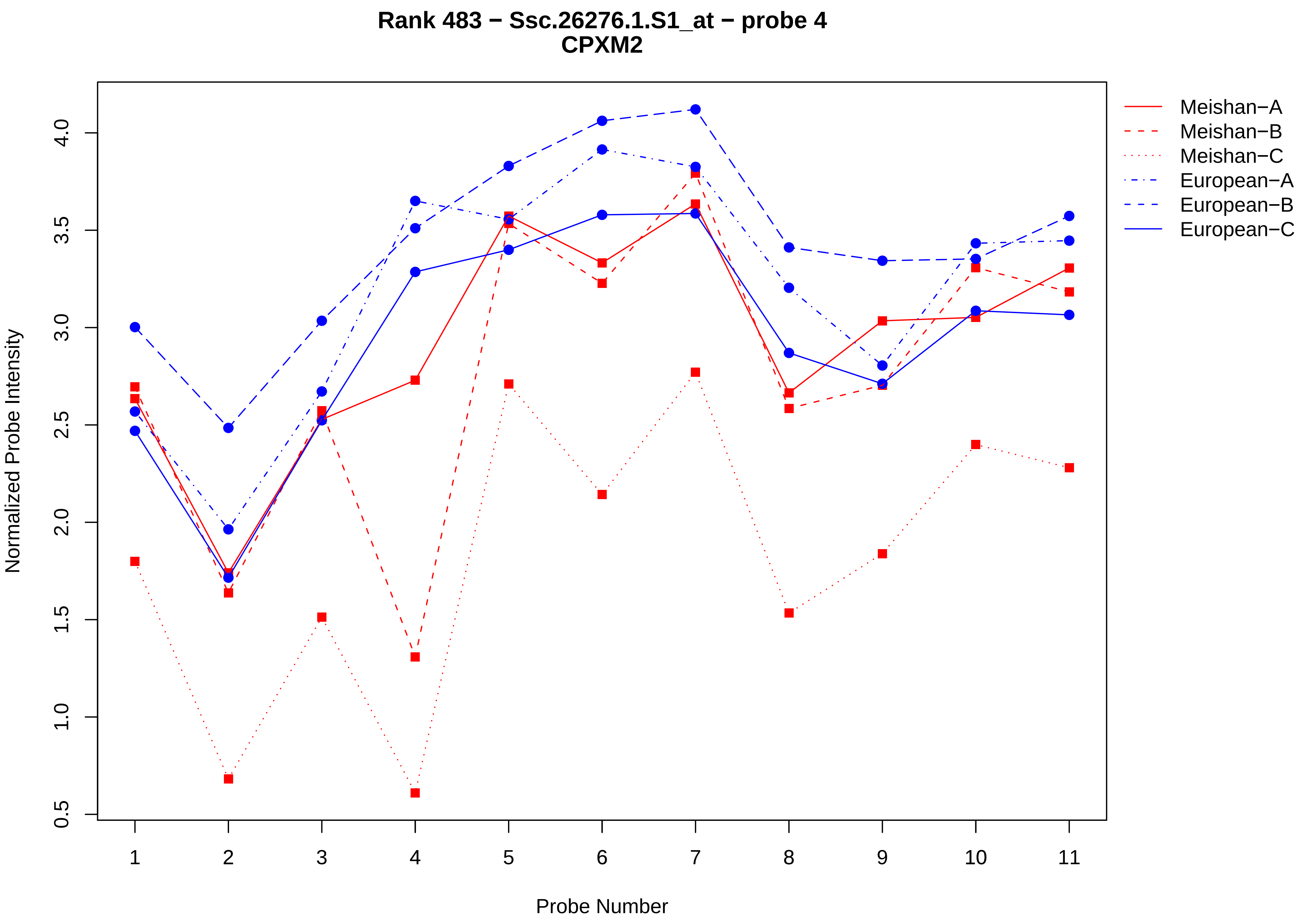
Rank 483 − Ssc.26276.1.S1_at − probe 4
CPXM2
Normalized Probe Intensity
Probe Number
Meishan−A
Meishan−B
Meishan−C
European−A
European−B
European−C
0.5
1.0
1.5
2.0
2.5
3.0
3.5
4.0
1
2
3
4
5
6
7
8
9
10
11

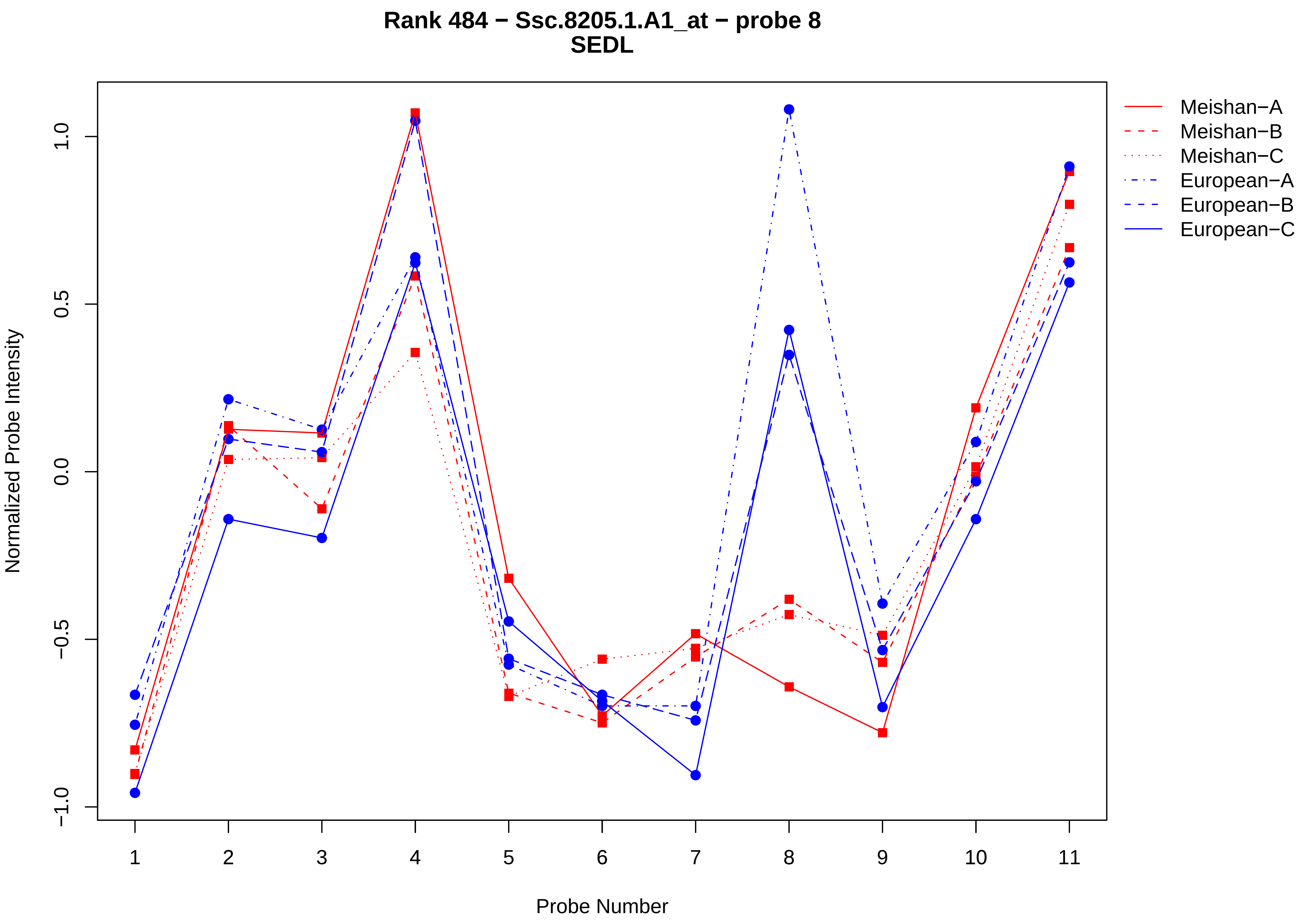

Rank 484 − Ssc.8205.1.A1_at − probe 8
SEDL
Normalized Probe Intensity
Probe Number
Meishan−A
Meishan−B
Meishan−C
European−A
European−B
European−C
−1.0
−0.5
0.0
0.5
1.0
1
2
3
4
5
6
7
8
9
10
11

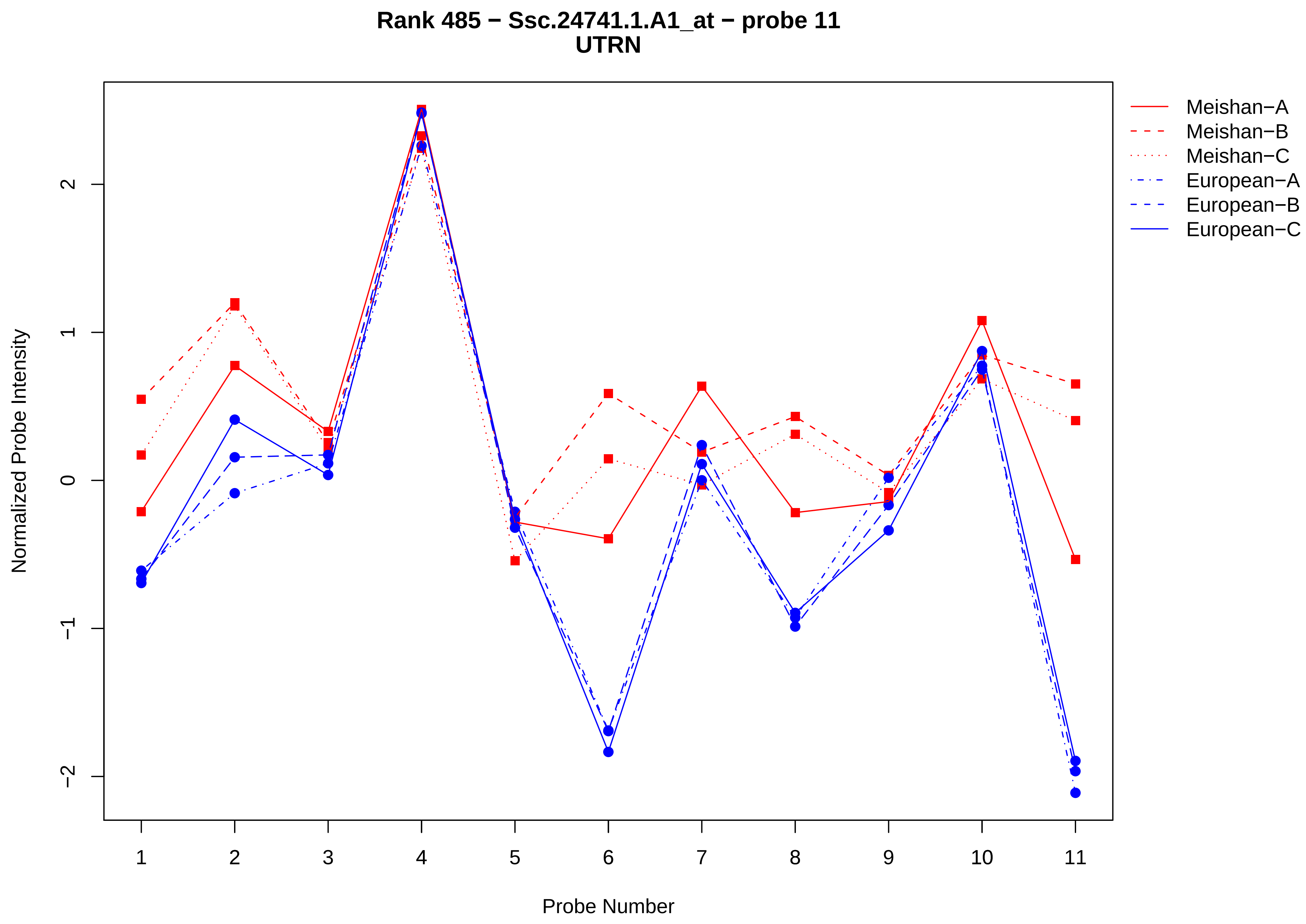

Rank 485 − Ssc.24741.1.A1_at − probe 11
UTRN
Normalized Probe Intensity
Probe Number
−2
−1
0
1
2
1
2
3
4
5
6
7
8
9
10
11
Meishan−A
Meishan−B
Meishan−C
European−A
European−B
European−C

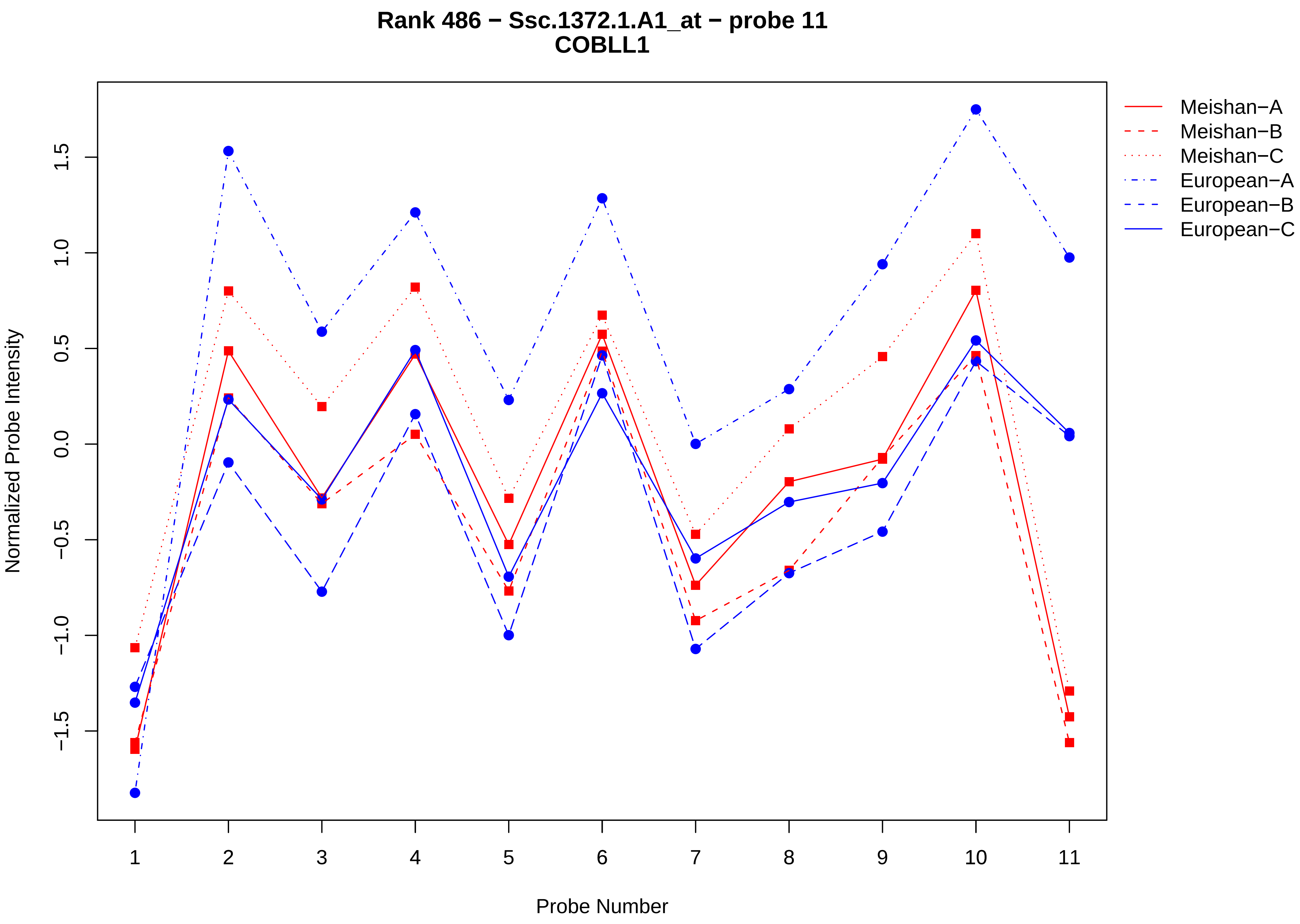

Rank 486 − Ssc.1372.1.A1_at − probe 11
COBLL1
Normalized Probe Intensity
Probe Number
Meishan−A
Meishan−B
Meishan−C
European−A
European−B
European−C
−1.5
−1.0
−0.5
0.0
0.5
1.0
1.5
1
2
3
4
5
6
7
8
9
10
11

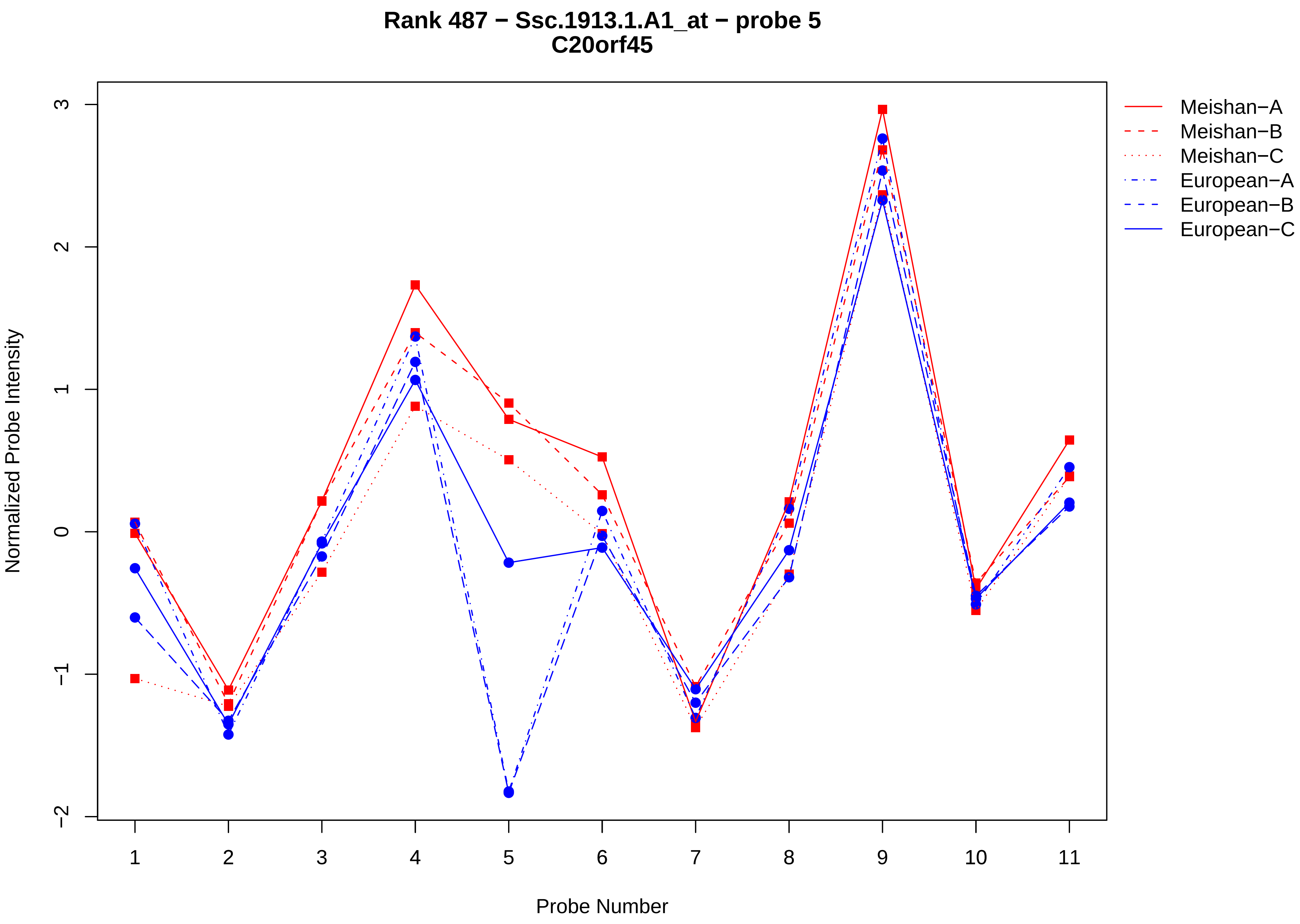

Rank 487 − Ssc.1913.1.A1_at − probe 5
C20orf45
Normalized Probe Intensity
Probe Number
−2
−1
0
1
2
3
1
2
3
4
5
6
7
8
9
10
11
Meishan−A
Meishan−B
Meishan−C
European−A
European−B
European−C

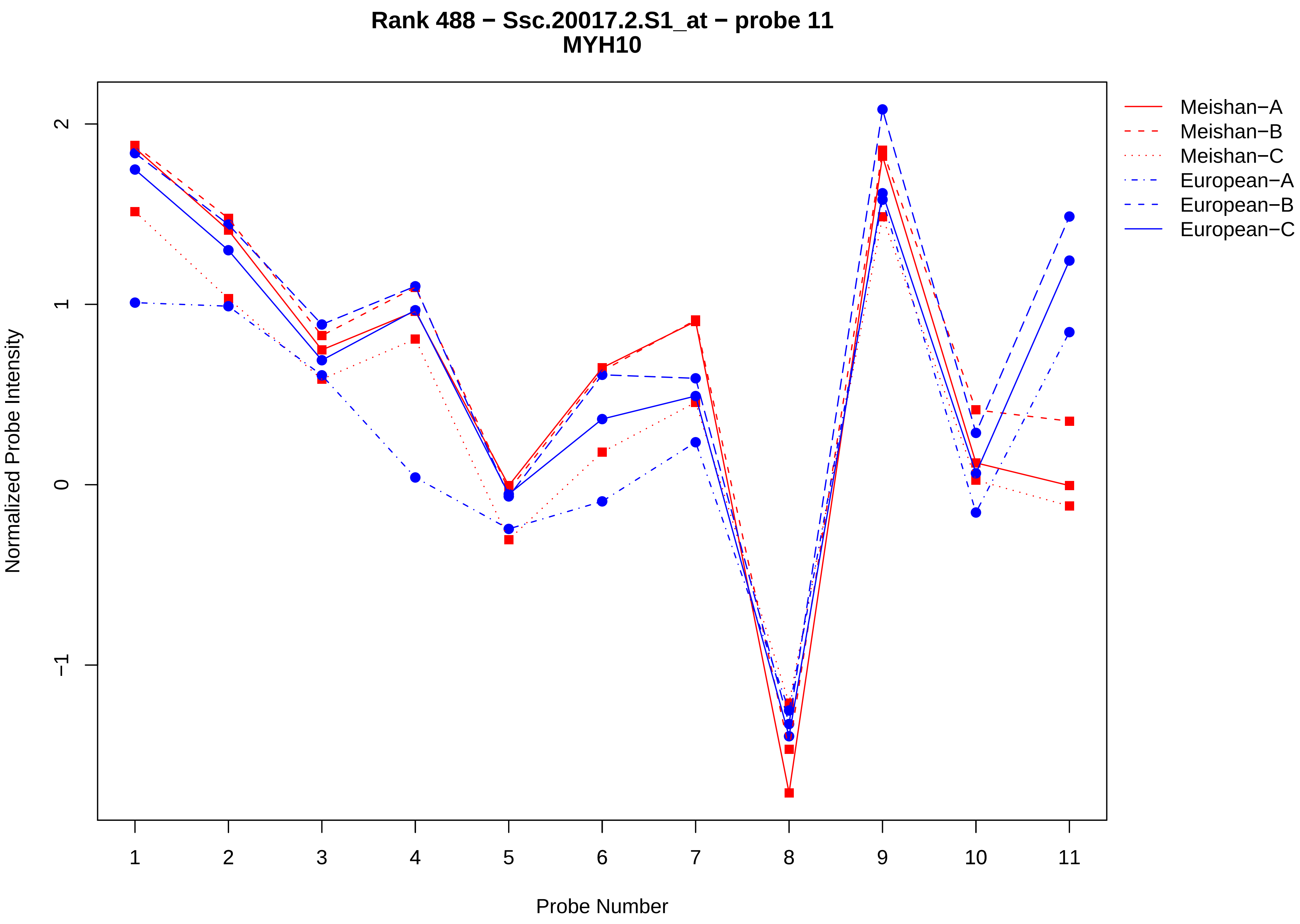

Rank 488 − Ssc.20017.2.S1_at − probe 11
MYH10
Normalized Probe Intensity
Probe Number
−1
0
1
2
1
2
3
4
5
6
7
8
9
10
11
Meishan−A
Meishan−B
Meishan−C
European−A
European−B
European−C

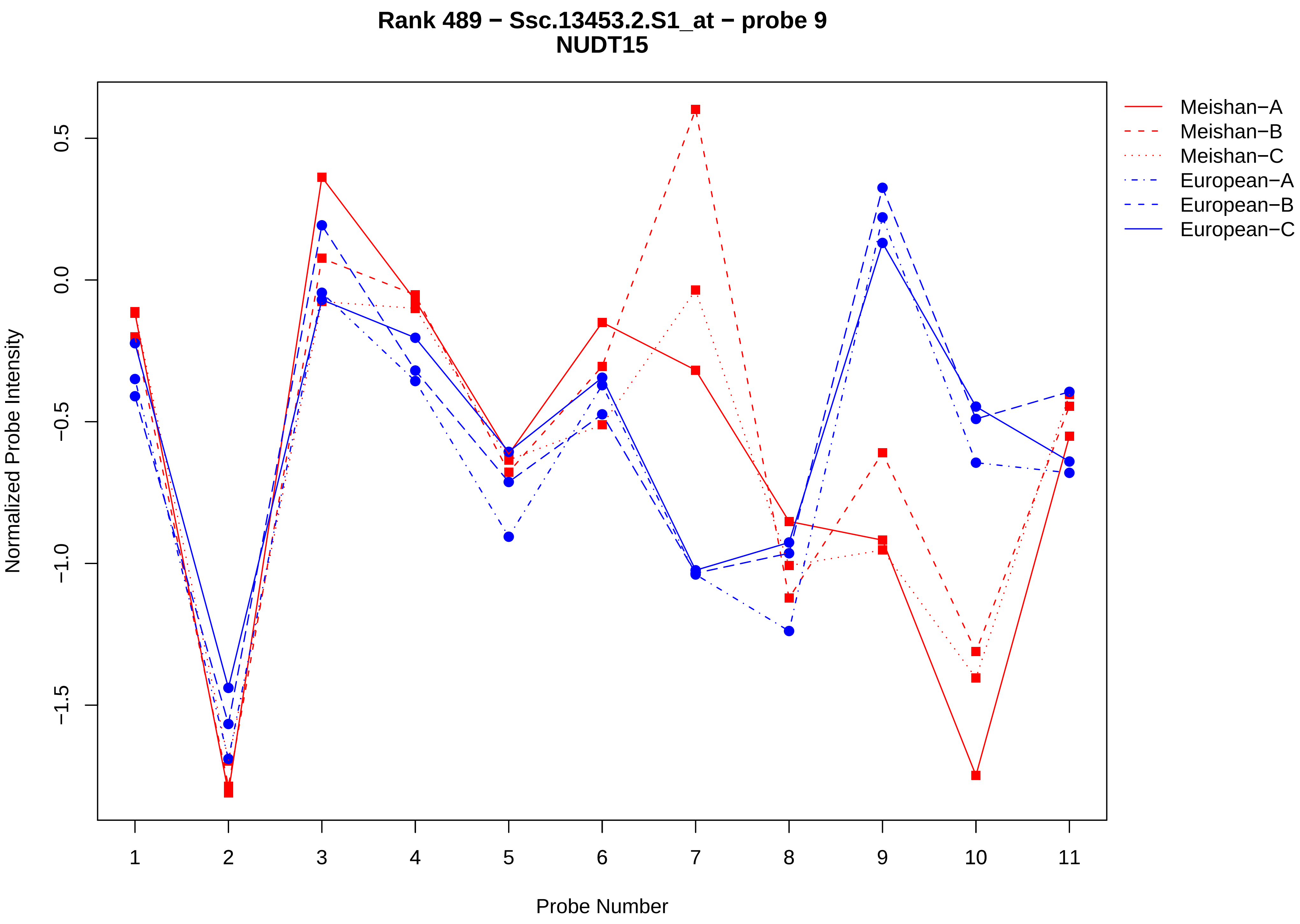

Rank 489 − Ssc.13453.2.S1_at − probe 9
NUDT15
Normalized Probe Intensity
Probe Number
0.5
0.0
−0.5
−1.0
−1.5
1
2
3
4
5
6
7
8
9
10
11
Meishan−A
Meishan−B
Meishan−C
European−A
European−B
European−C

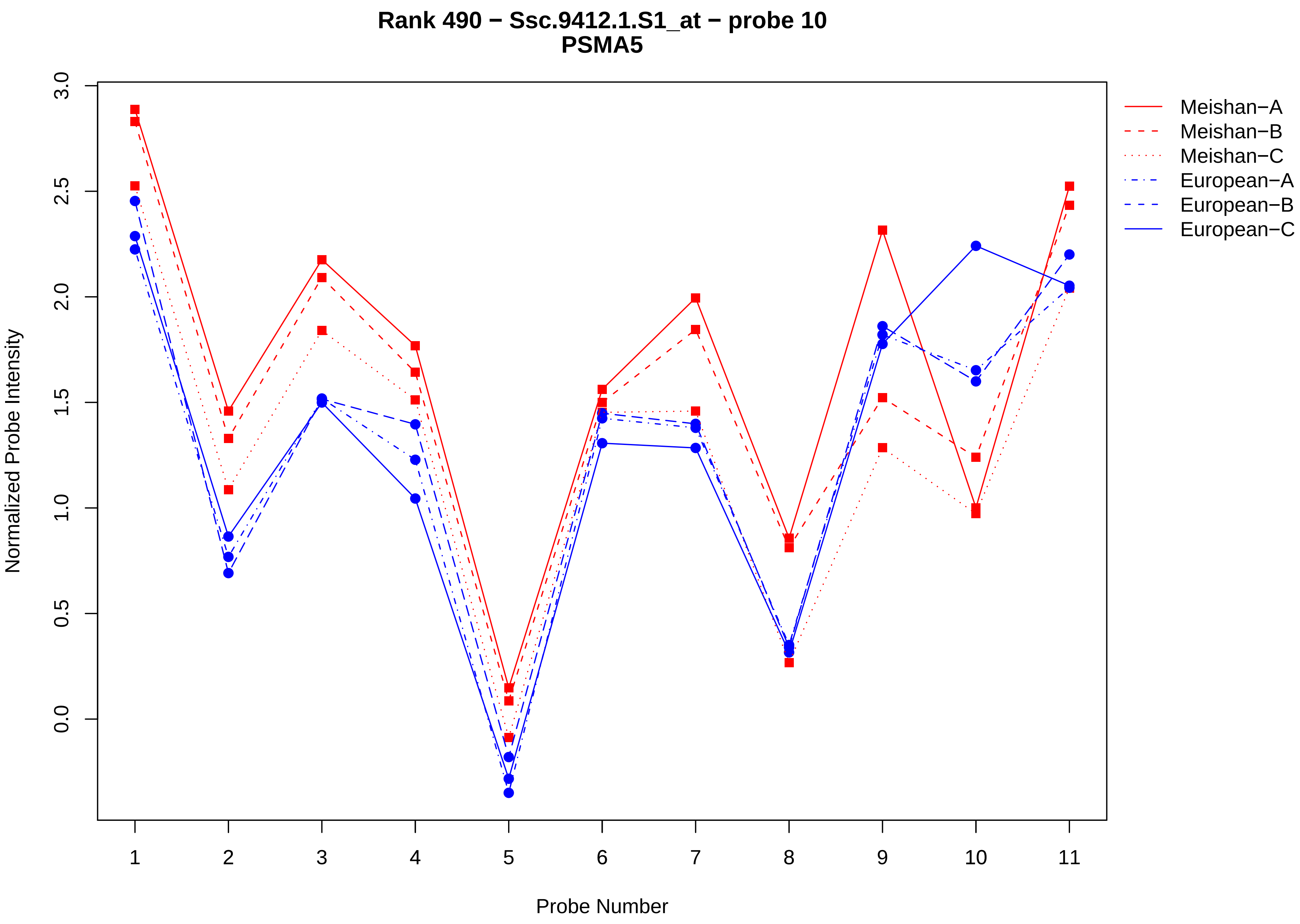

Rank 490 − Ssc.9412.1.S1_at − probe 10
PSMA5
Normalized Probe Intensity
0.0
0.5
1.0
1.5
2.0
2.5
3.0
1
2
3
4
5
6
7
8
9
10
11
Probe Number
Meishan−A
Meishan−B
Meishan−C
European−A
European−B
European−C

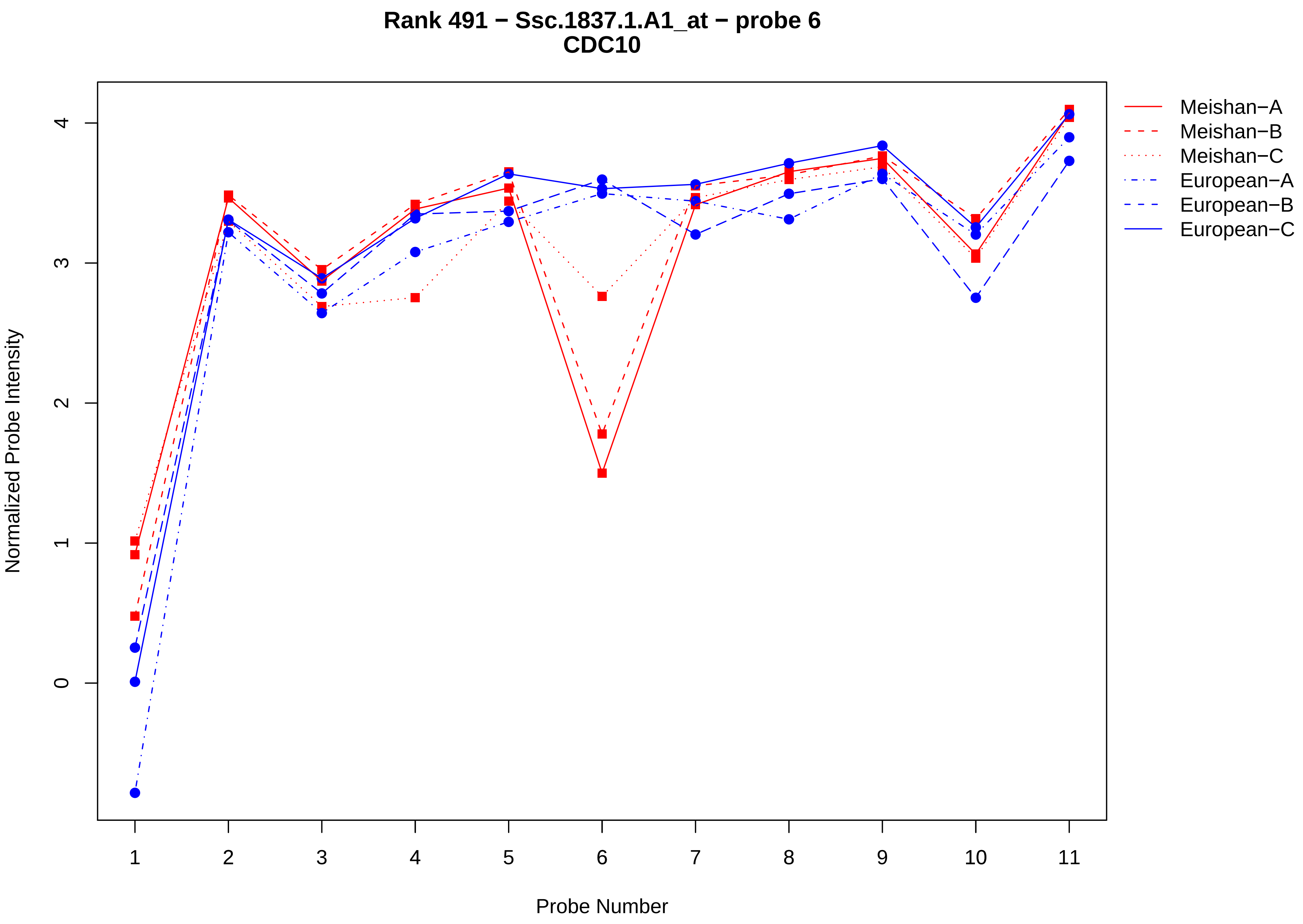

Rank 491 − Ssc.1837.1.A1_at − probe 6
CDC10
Normalized Probe Intensity
Probe Number
0
1
2
3
4
1
2
3
4
5
6
7
8
9
10
11
Meishan−A
Meishan−B
Meishan−C
European−A
European−B
European−C

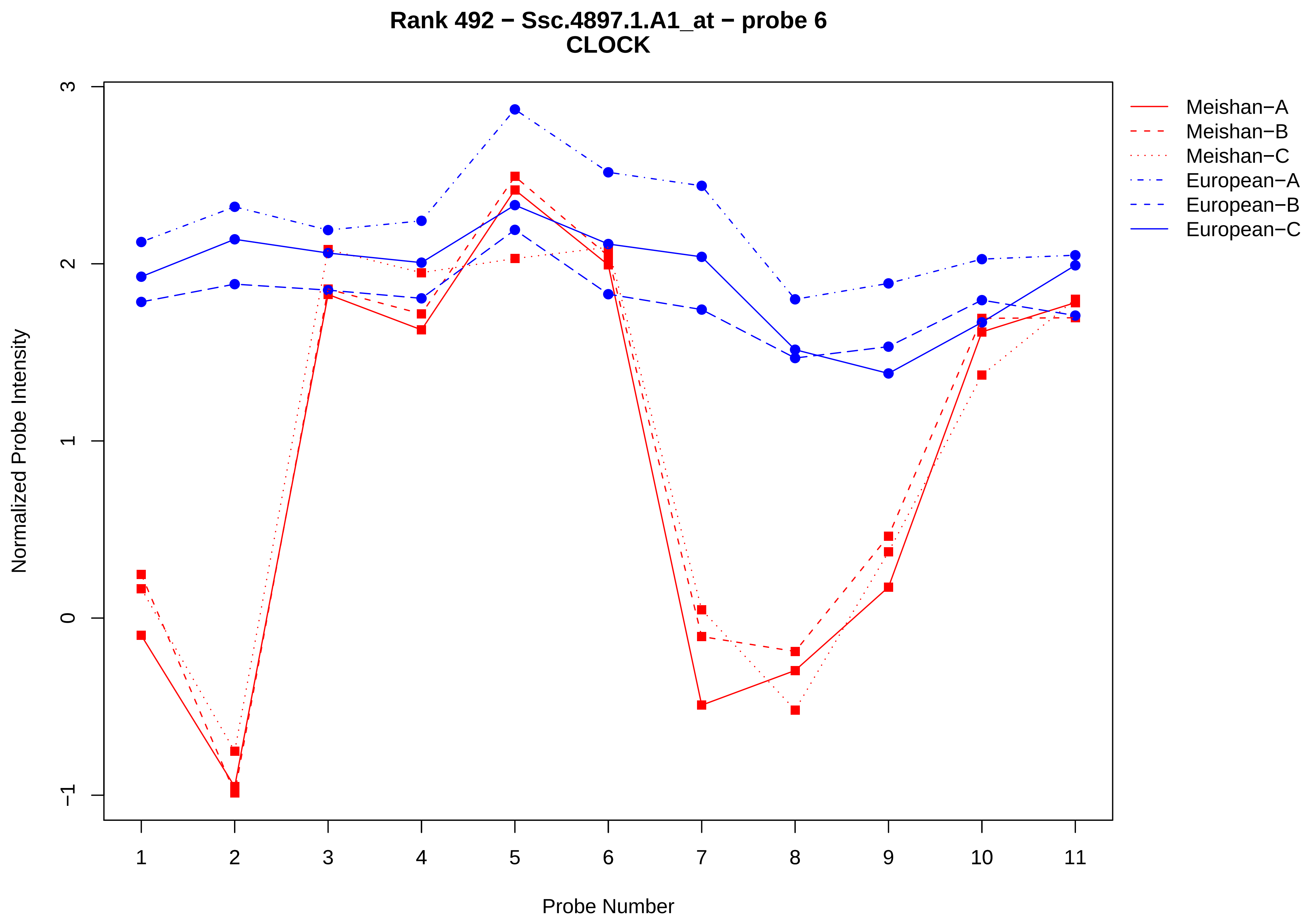

Rank 492 − Ssc.4897.1.A1_at − probe 6
CLOCK
Normalized Probe Intensity
Probe Number
−1
0
1
2
3
1
2
3
4
5
6
7
8
9
10
11
Meishan−A
Meishan−B
Meishan−C
European−A
European−B
European−C

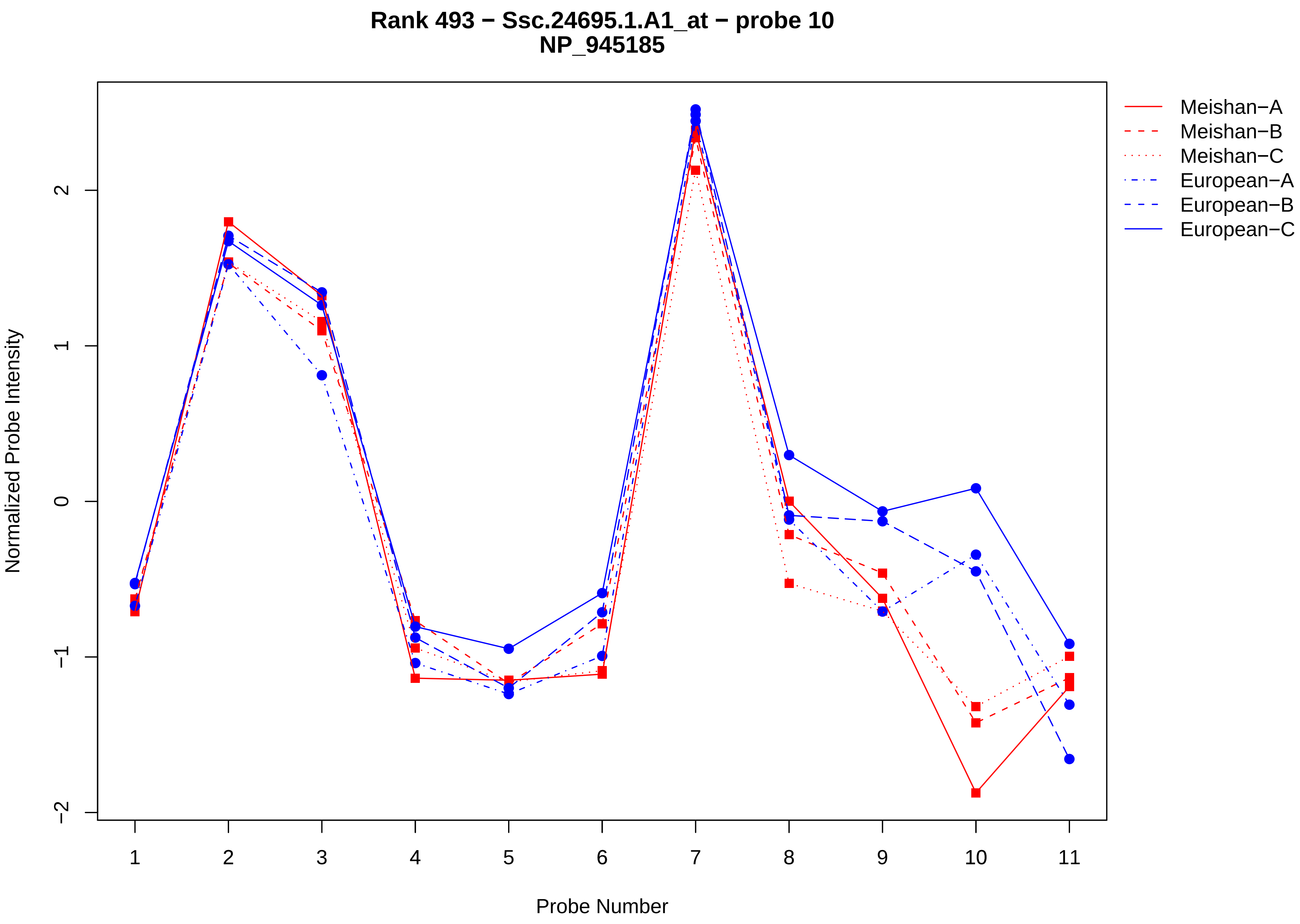

Rank 493 − Ssc.24695.1.A1_at − probe 10
NP_945185
Normalized Probe Intensity
Probe Number
−2
−1
0
1
2
1
2
3
4
5
6
7
8
9
10
11
Meishan−A
Meishan−B
Meishan−C
European−A
European−B
European−C

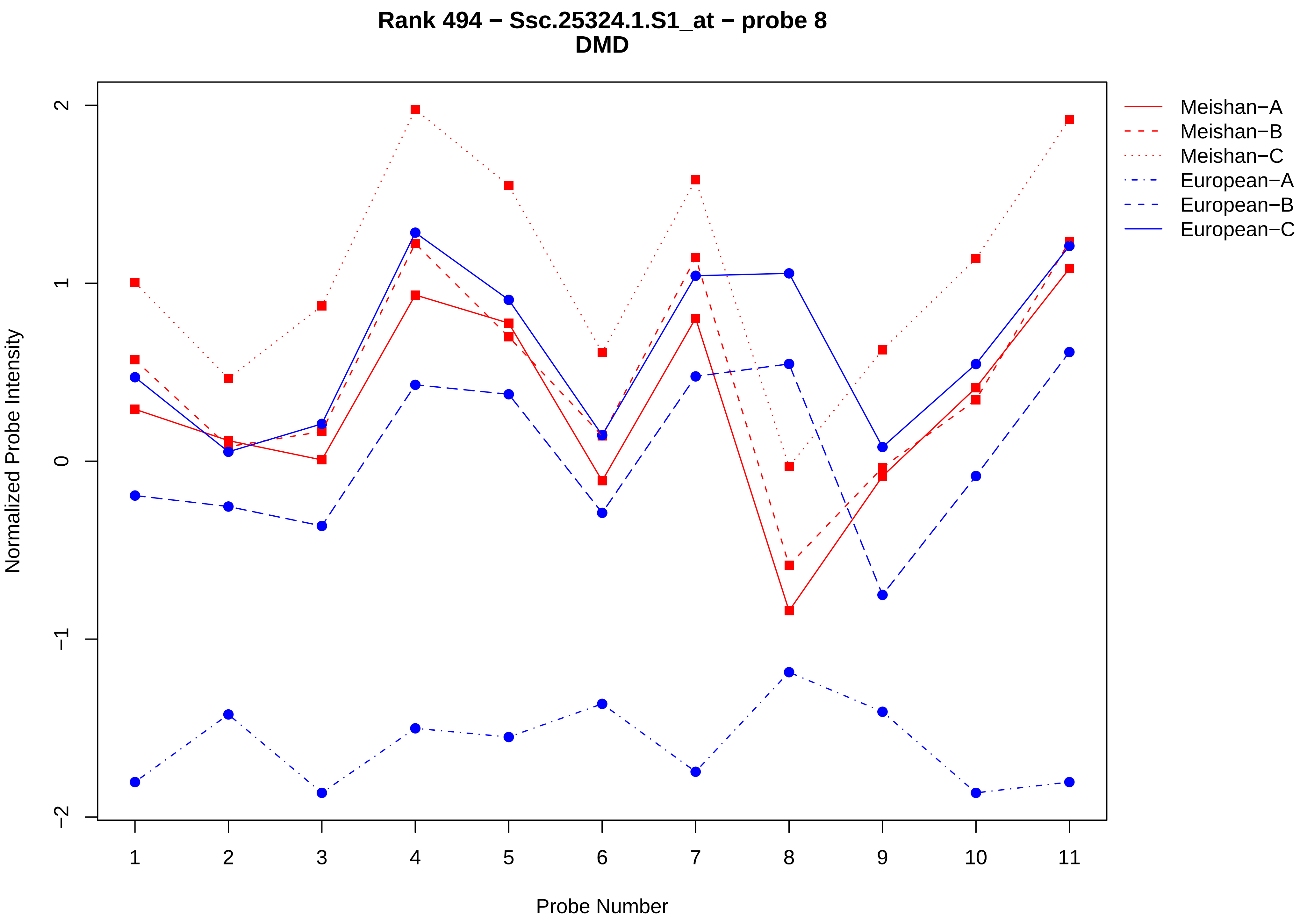

Rank 494 − Ssc.25324.1.S1_at − probe 8
DMD
Normalized Probe Intensity
Probe Number
−2
−1
0
1
2
1
2
3
4
5
6
7
8
9
10
11
Meishan−A
Meishan−B
Meishan−C
European−A
European−B
European−C

Rank 495 − Ssc.11061.1.S1_at − probe 11
Q9P007

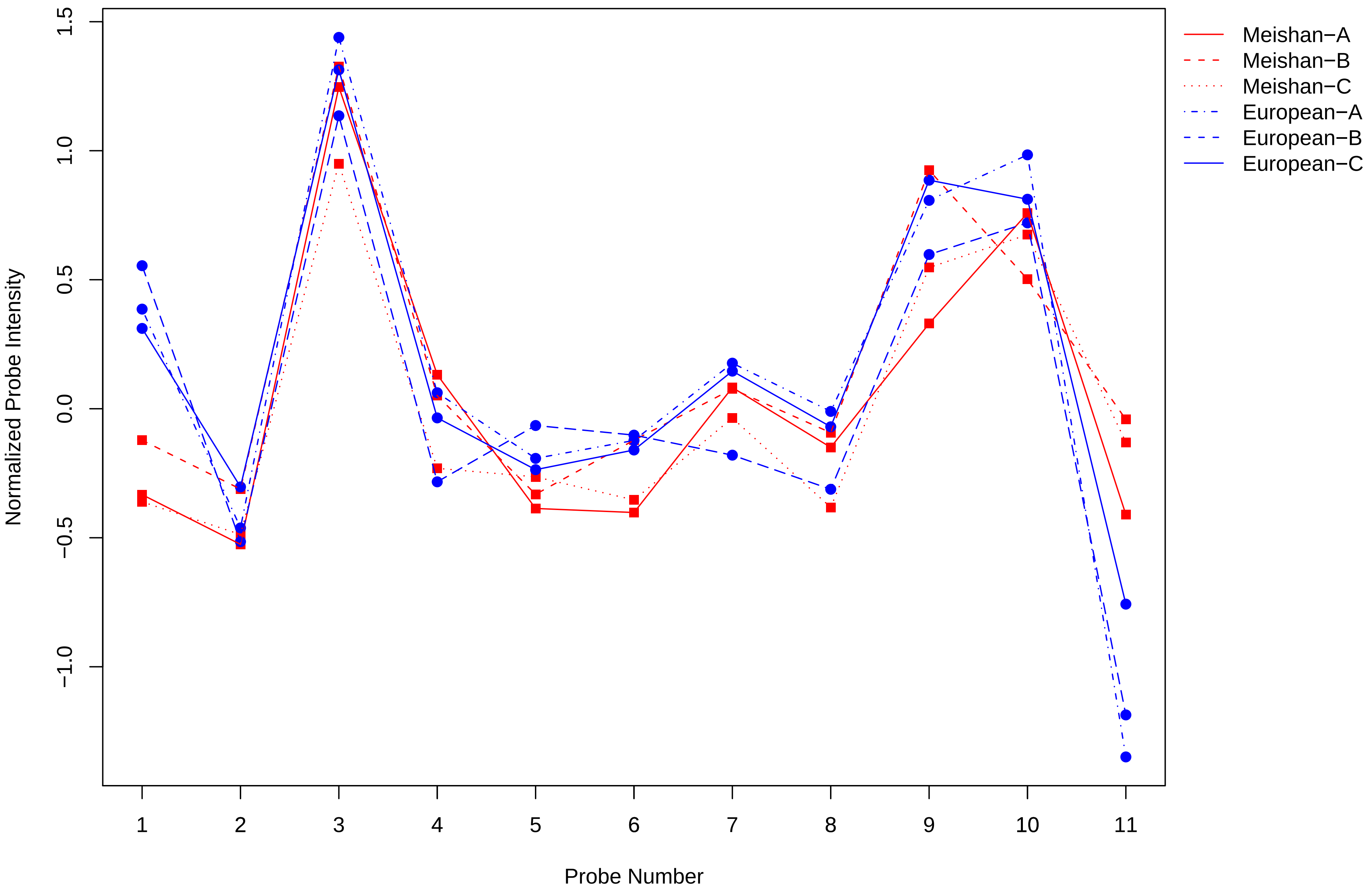

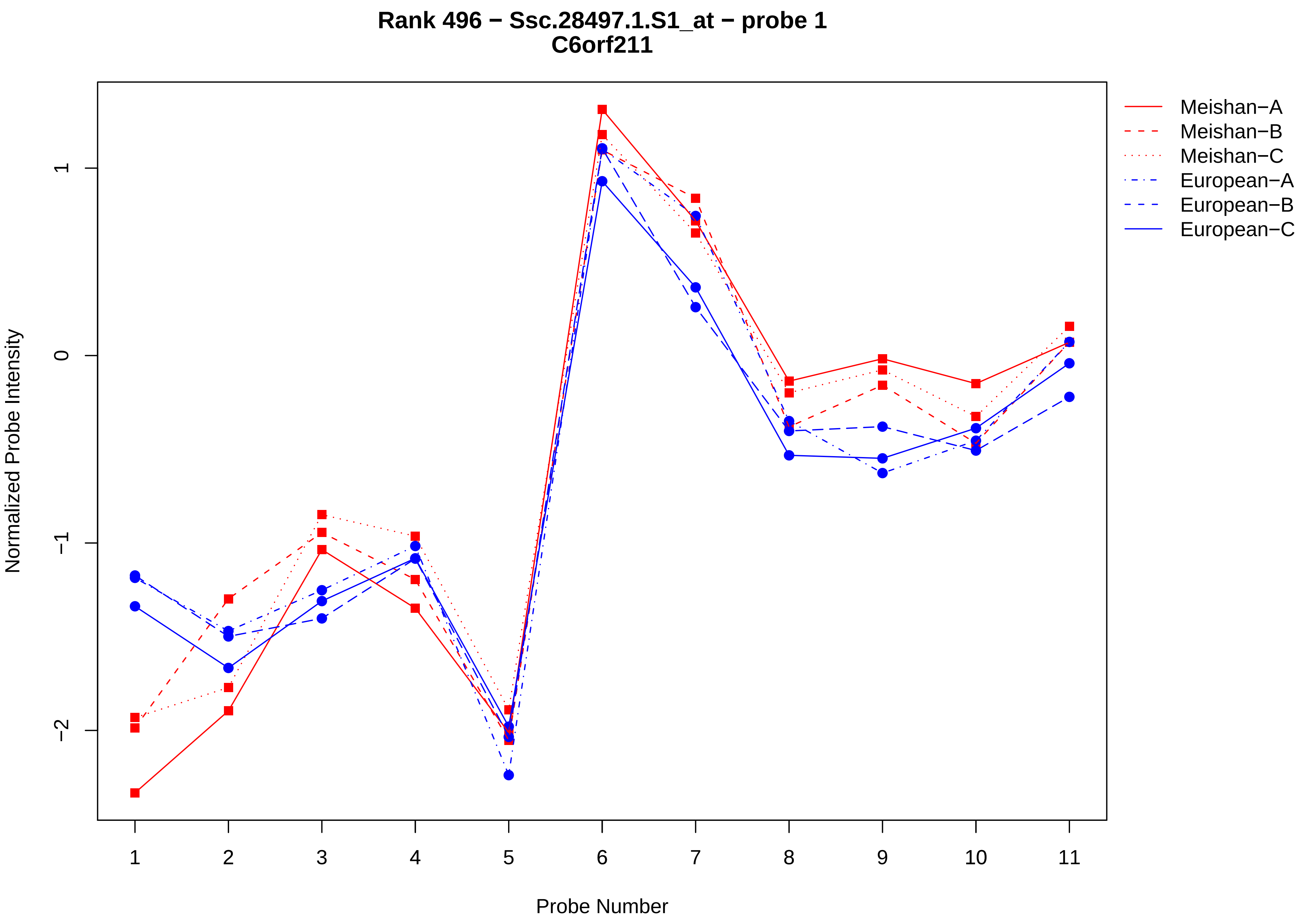

Rank 496 − Ssc.28497.1.S1_at − probe 1
C6orf211
Normalized Probe Intensity
Probe Number
−2
−1
0
1
1
2
3
4
5
6
7
8
9
10
11
Meishan−A
Meishan−B
Meishan−C
European−A
European−B
European−C

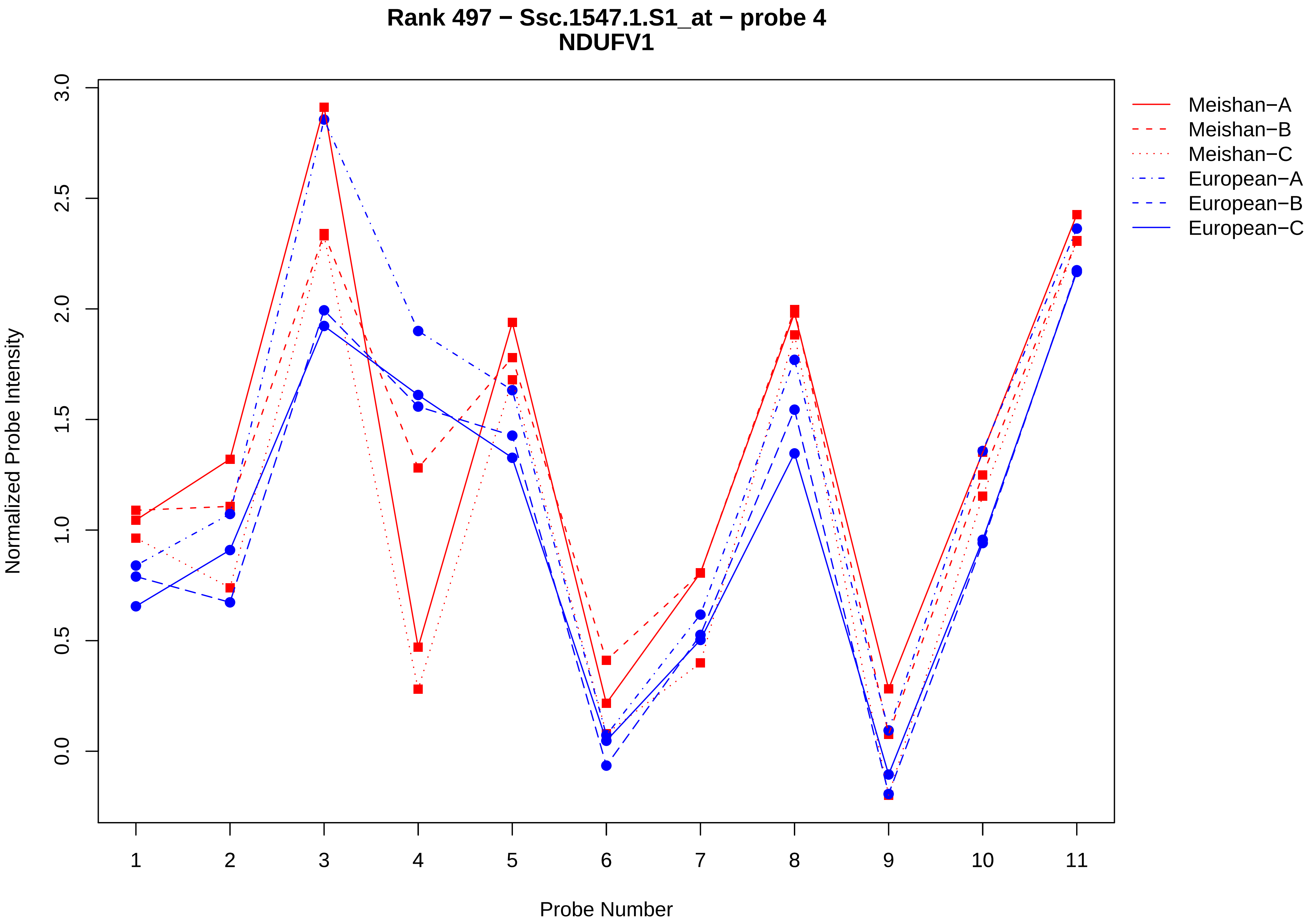

Rank 497 − Ssc.1547.1.S1_at − probe 4
NDUFV1
Normalized Probe Intensity
Probe Number
0.0
0.5
1.0
1.5
2.0
2.5
3.0
1
2
3
4
5
6
7
8
9
10
11
Meishan−A
Meishan−B
Meishan−C
European−A
European−B
European−C

Rank 498 − Ssc.4897.1.A1_at − probe 3
CLOCK

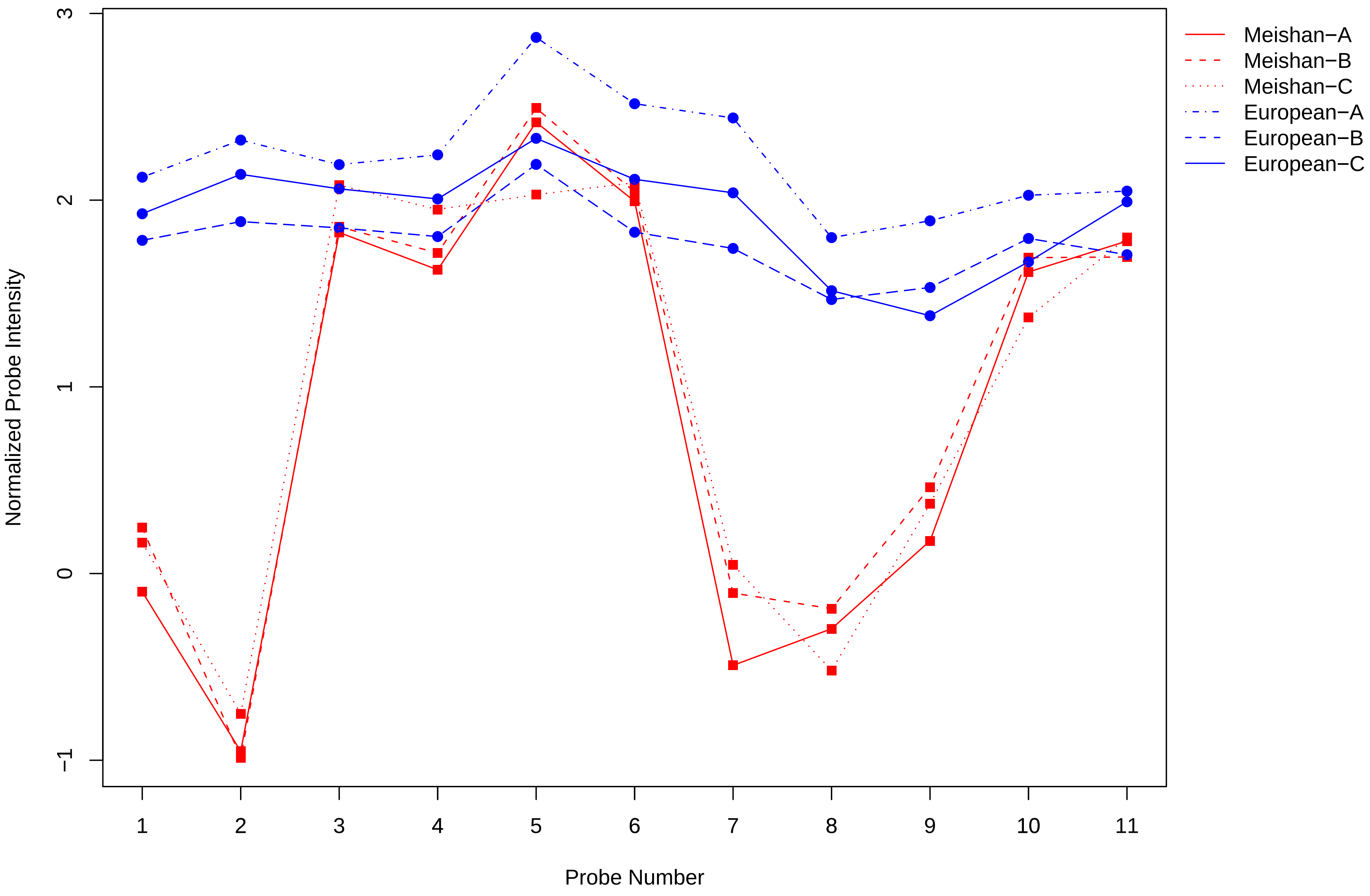

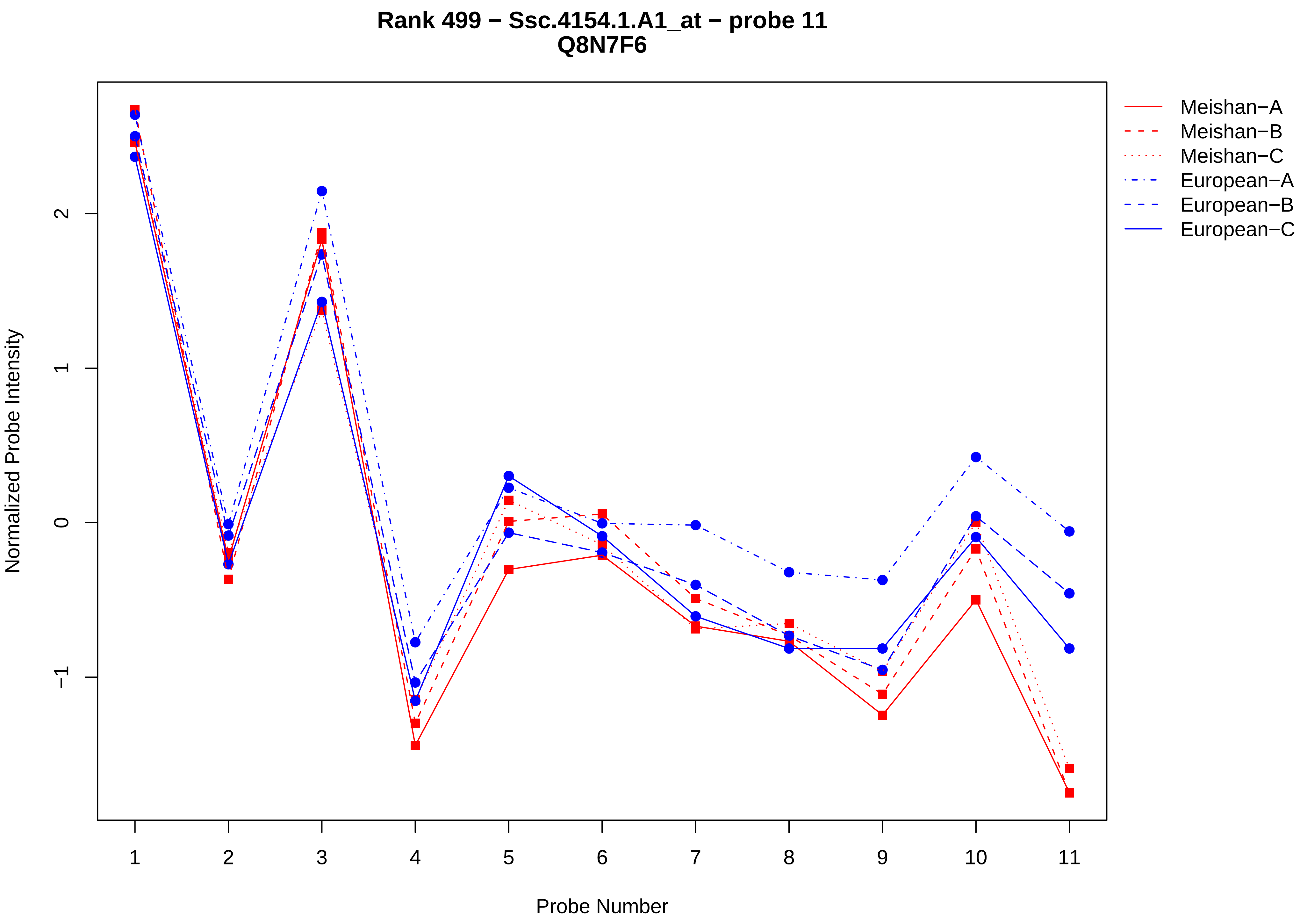

Rank 499 − Ssc.4154.1.A1_at − probe 11
Q8N7F6
Normalized Probe Intensity
Probe Number
−1
0
1
2
1
2
3
4
5
6
7
8
9
10
11
Meishan−A
Meishan−B
Meishan−C
European−A
European−B
European−C

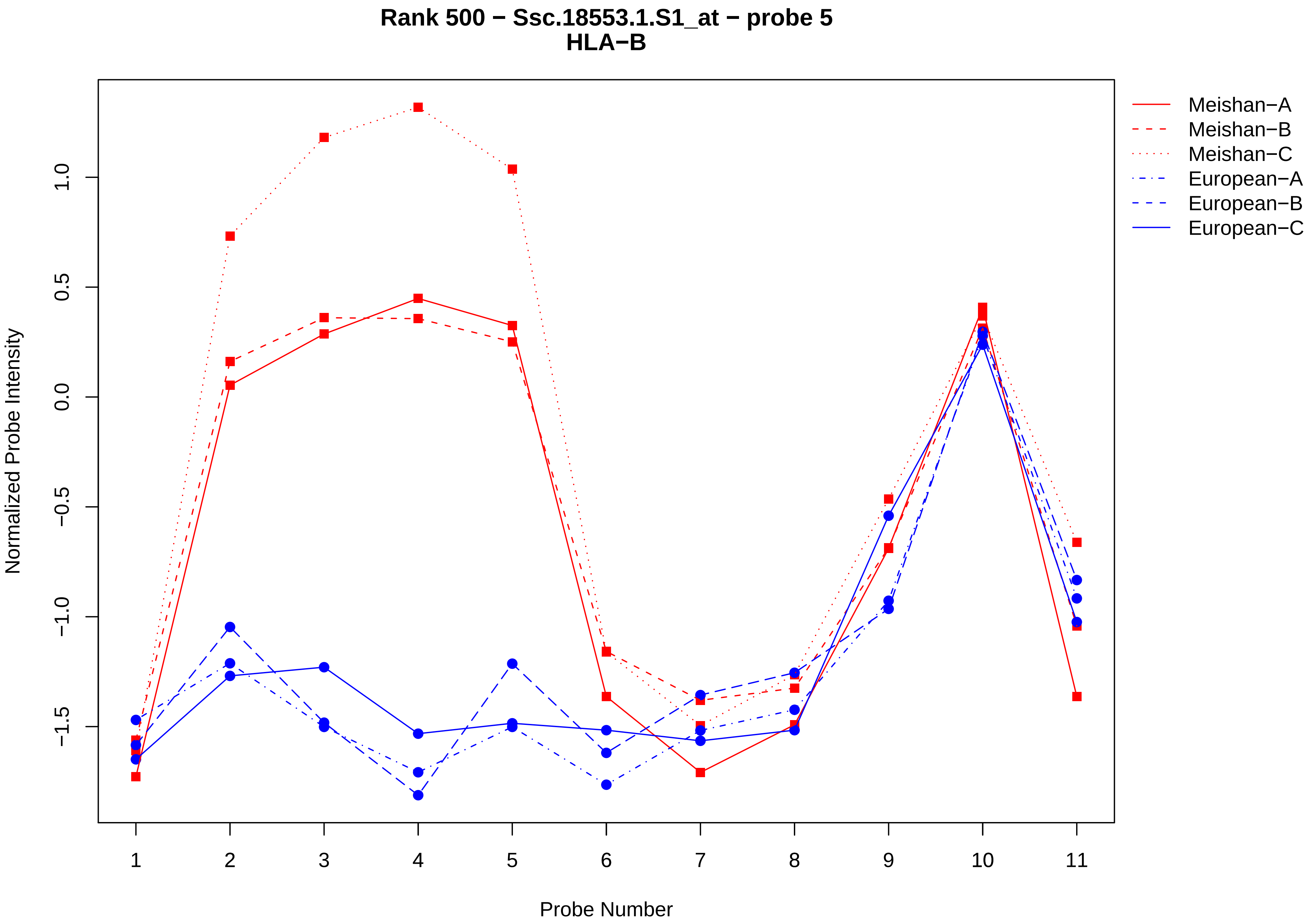

Rank 500 − Ssc.18553.1.S1_at − probe 5
HLA−B
Normalized Probe Intensity
Probe Number
1.0
0.5
0.0
−0.5
−1.0
−1.5
1
2
3
4
5
6
7
8
9
10
11
Meishan−A
Meishan−B
Meishan−C
European−A
European−B
European−C

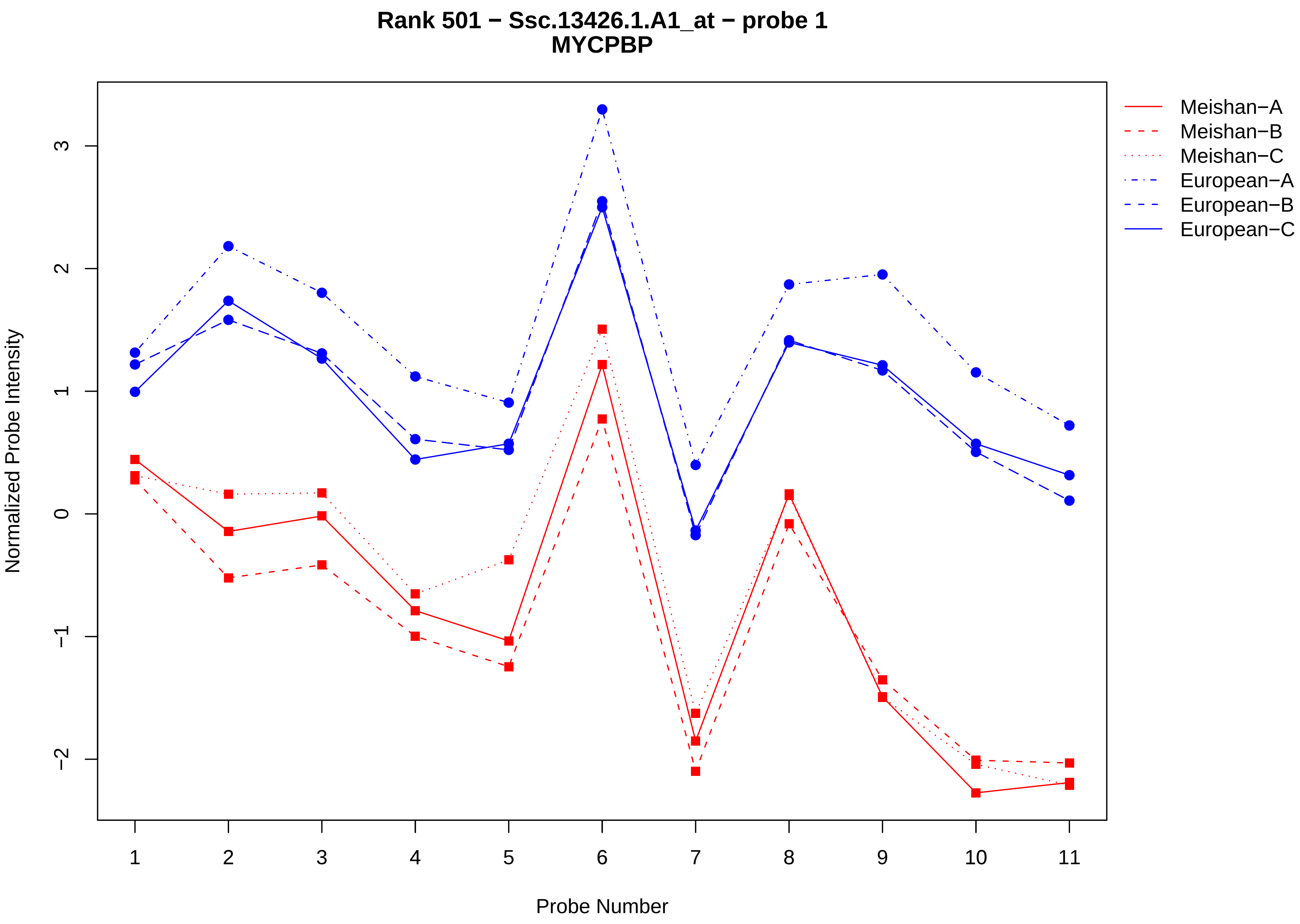
Rank 501 − Ssc.13426.1.A1_at − probe 1
MYCPBP
Normalized Probe Intensity
Probe Number
Meishan−A
Meishan−B
Meishan−C
European−A
European−B
European−C
−2
−1
0
1
2
3
1
2
3
4
5
6
7
8
9
10
11

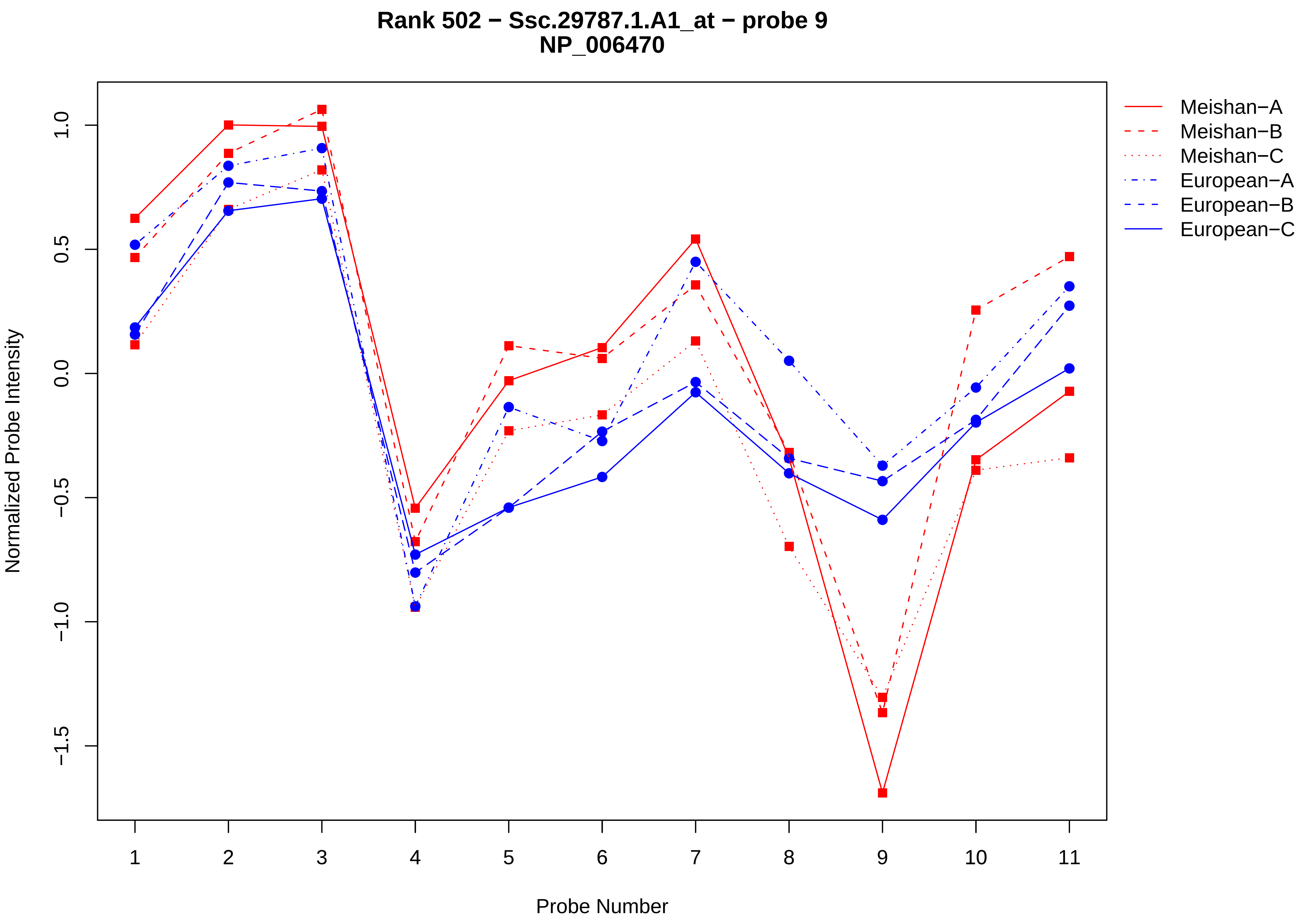

Rank 502 − Ssc.29787.1.A1_at − probe 9
NP_006470
Normalized Probe Intensity
Probe Number
1.0
0.5
0.0
−0.5
−1.0
−1.5
1
2
3
4
5
6
7
8
9
10
11
Meishan−A
Meishan−B
Meishan−C
European−A
European−B
European−C

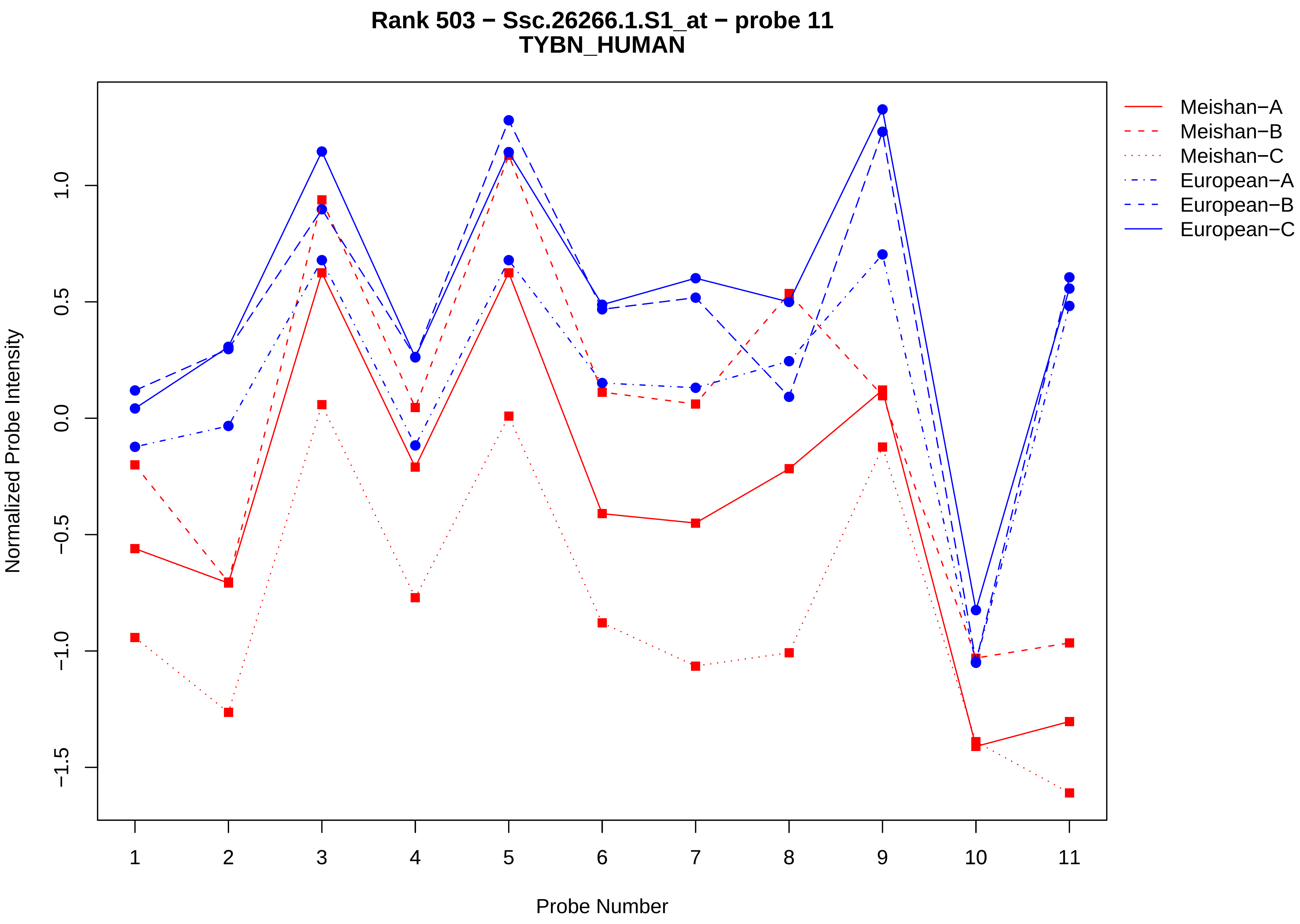

Rank 503 − Ssc.26266.1.S1_at − probe 11
TYBN_HUMAN
Normalized Probe Intensity
Probe Number
1.0
0.5
0.0
−0.5
−1.0
−1.5
1
2
3
4
5
6
7
8
9
10
11
Meishan−A
Meishan−B
Meishan−C
European−A
European−B
European−C

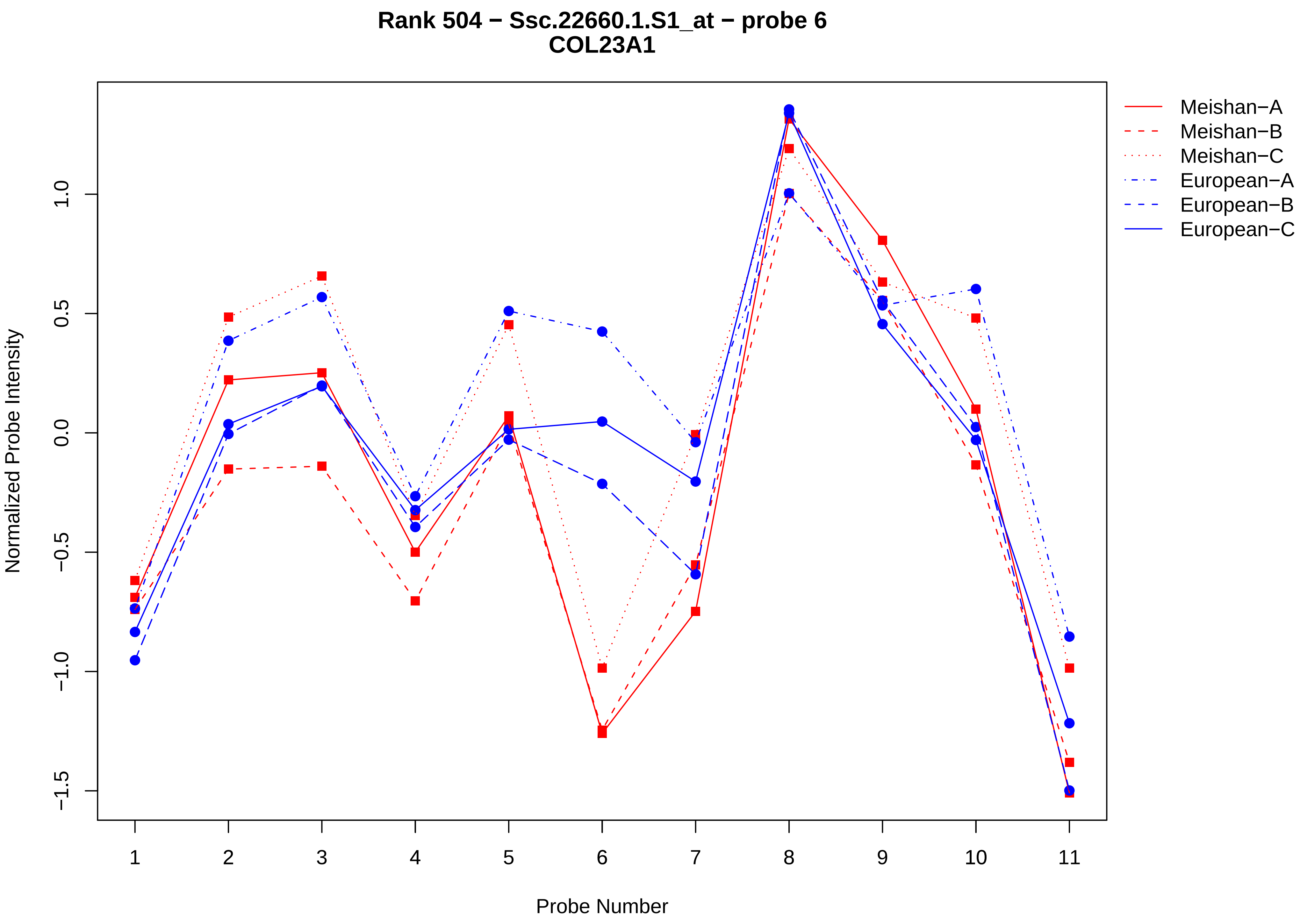
Rank 504 − Ssc.22660.1.S1_at − probe 6
COL23A1
Normalized Probe Intensity
−1.5
−1.0
−0.5
0.0
0.5
1.0
Probe Number
1
2
3
4
5
6
7
8
9
10
11
Meishan−A
Meishan−B
Meishan−C
European−A
European−B
European−C

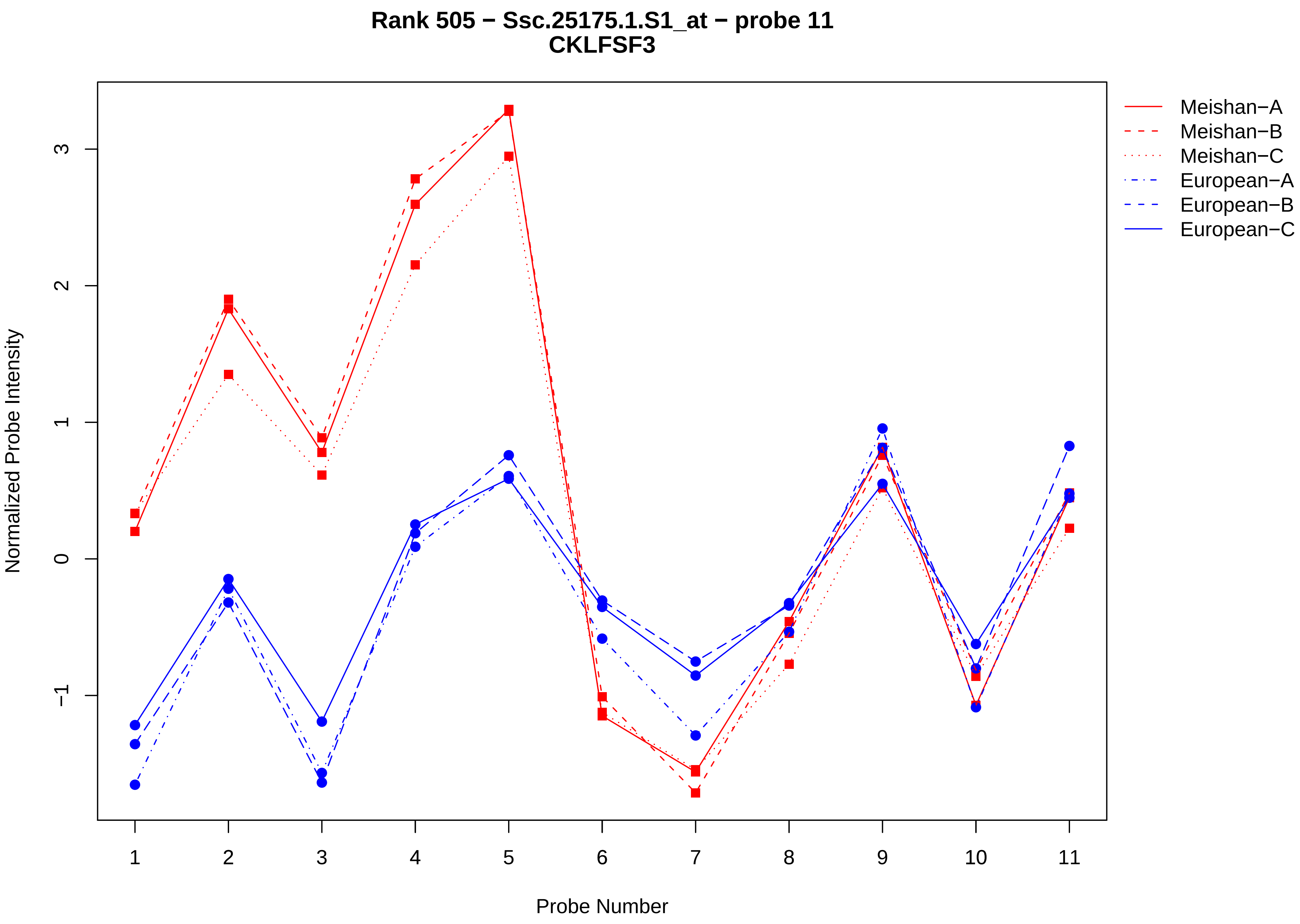

Rank 505 − Ssc.25175.1.S1_at − probe 11
CKLFSF3
Normalized Probe Intensity
Probe Number
Meishan−A
Meishan−B
Meishan−C
European−A
European−B
European−C
−1
0
1
2
3
1
2
3
4
5
6
7
8
9
10
11

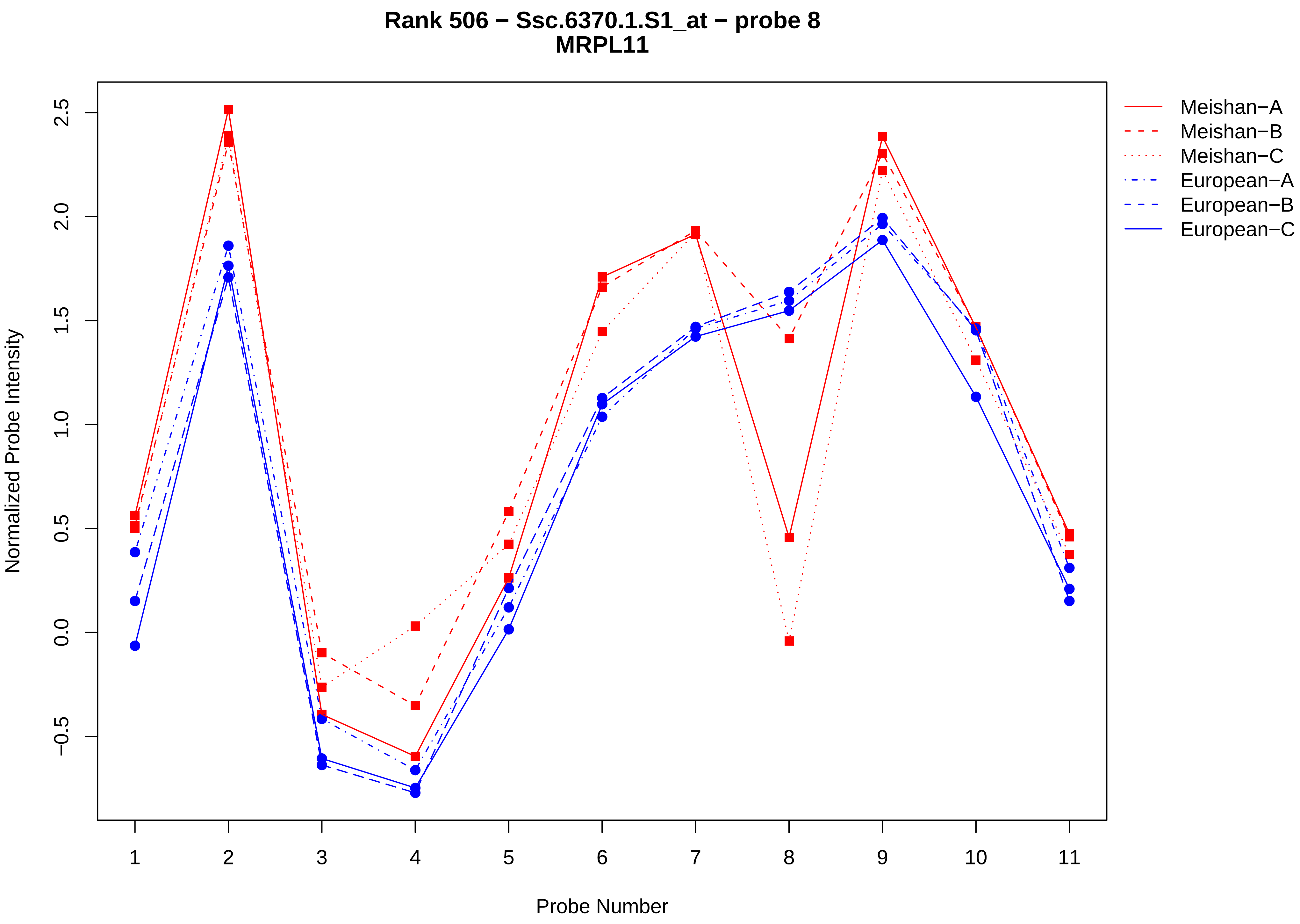
Rank 506 − Ssc.6370.1.S1_at − probe 8
MRPL11
Normalized Probe Intensity
Probe Number
Meishan−A
Meishan−B
Meishan−C
European−A
European−B
European−C
−0.5
0.0
0.5
1.0
1.5
2.0
2.5
1
2
3
4
5
6
7
8
9
10
11

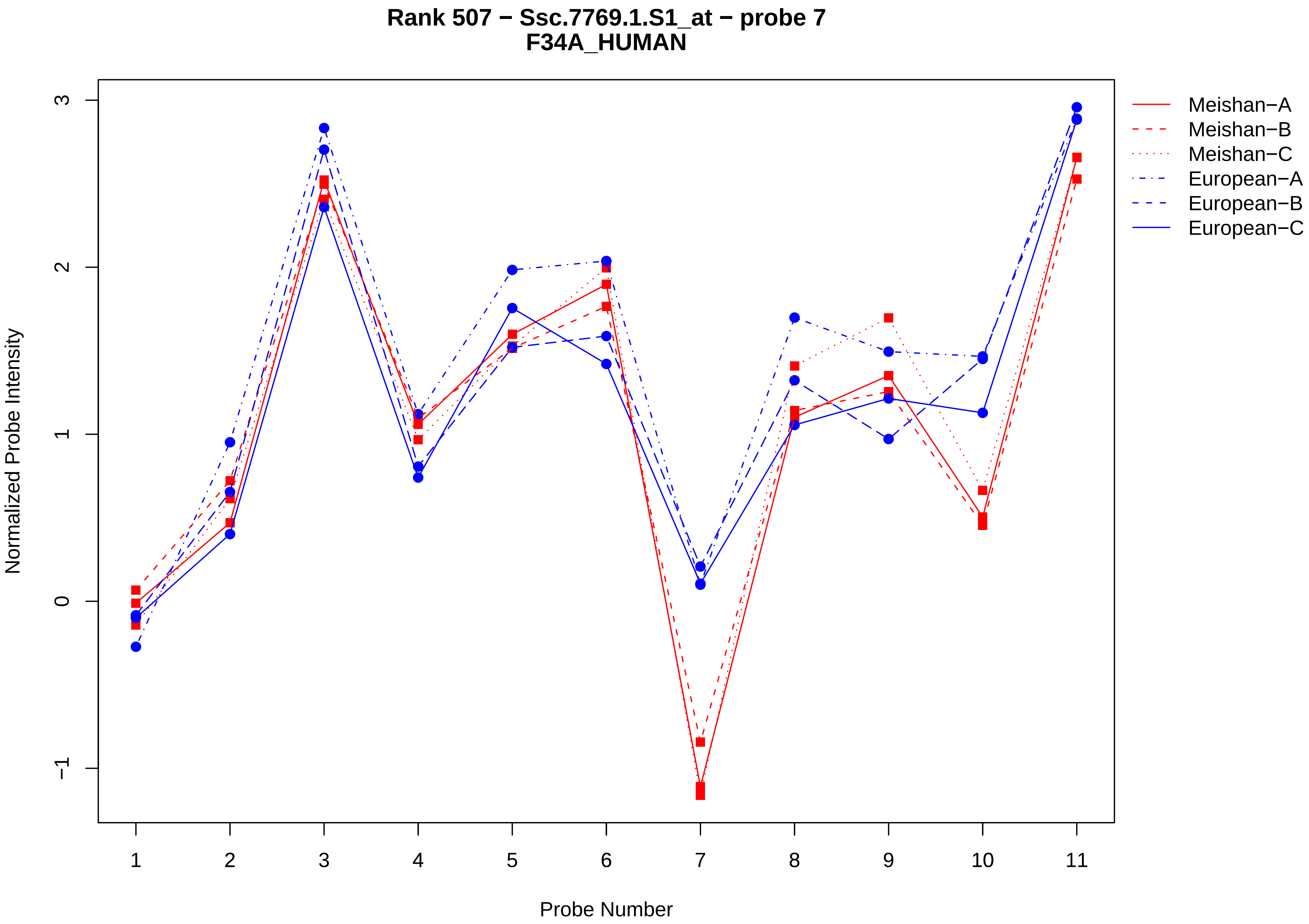

Rank 507 − Ssc.7769.1.S1_at − probe 7
F34A_HUMAN
Normalized Probe Intensity
Probe Number
−1
0
1
2
3
1
2
3
4
5
6
7
8
9
10
11
Meishan−A
Meishan−B
Meishan−C
European−A
European−B
European−C

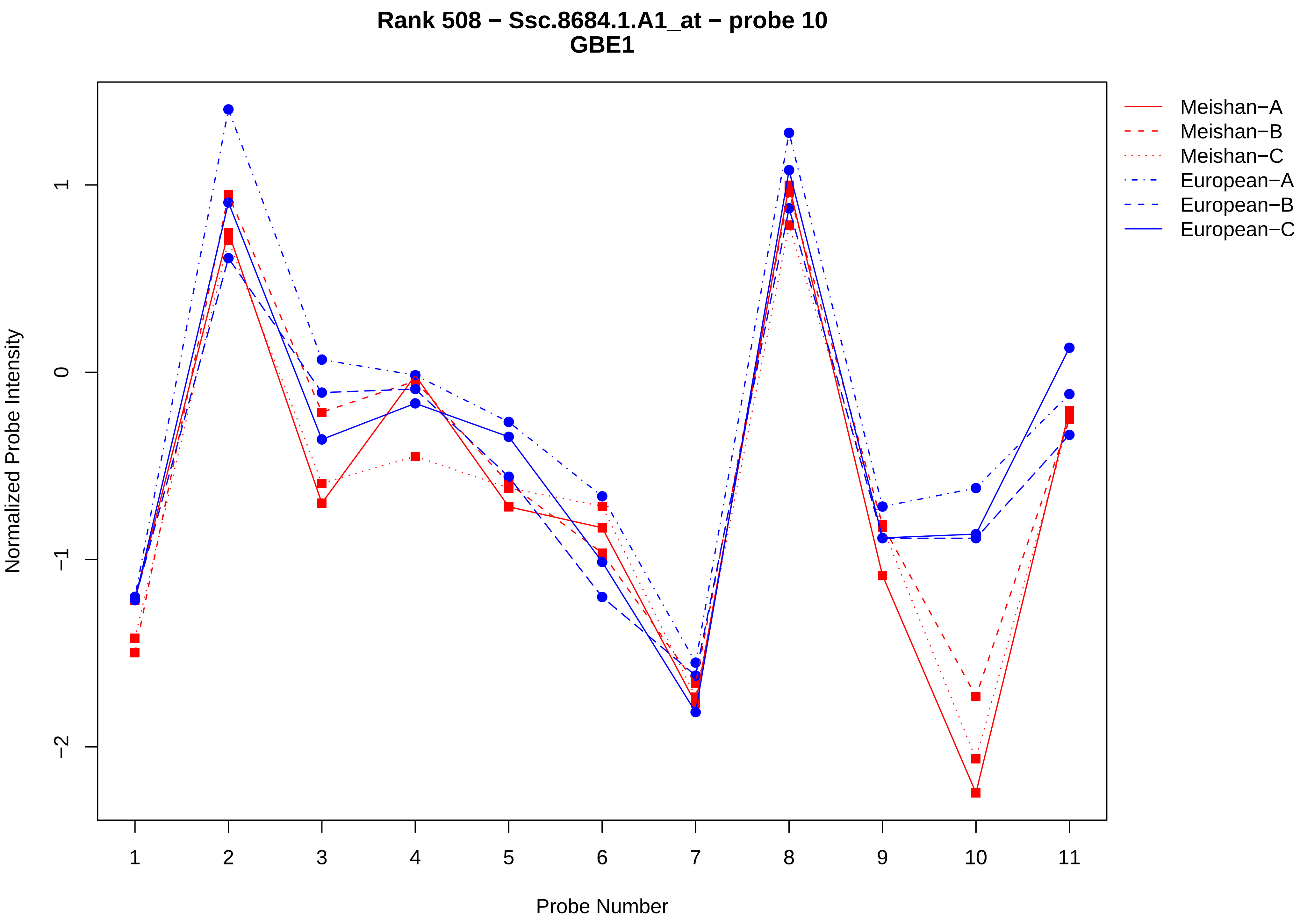
Rank 508 − Ssc.8684.1.A1_at − probe 10
GBE1
Normalized Probe Intensity
Probe Number
Meishan−A
Meishan−B
Meishan−C
European−A
European−B
European−C

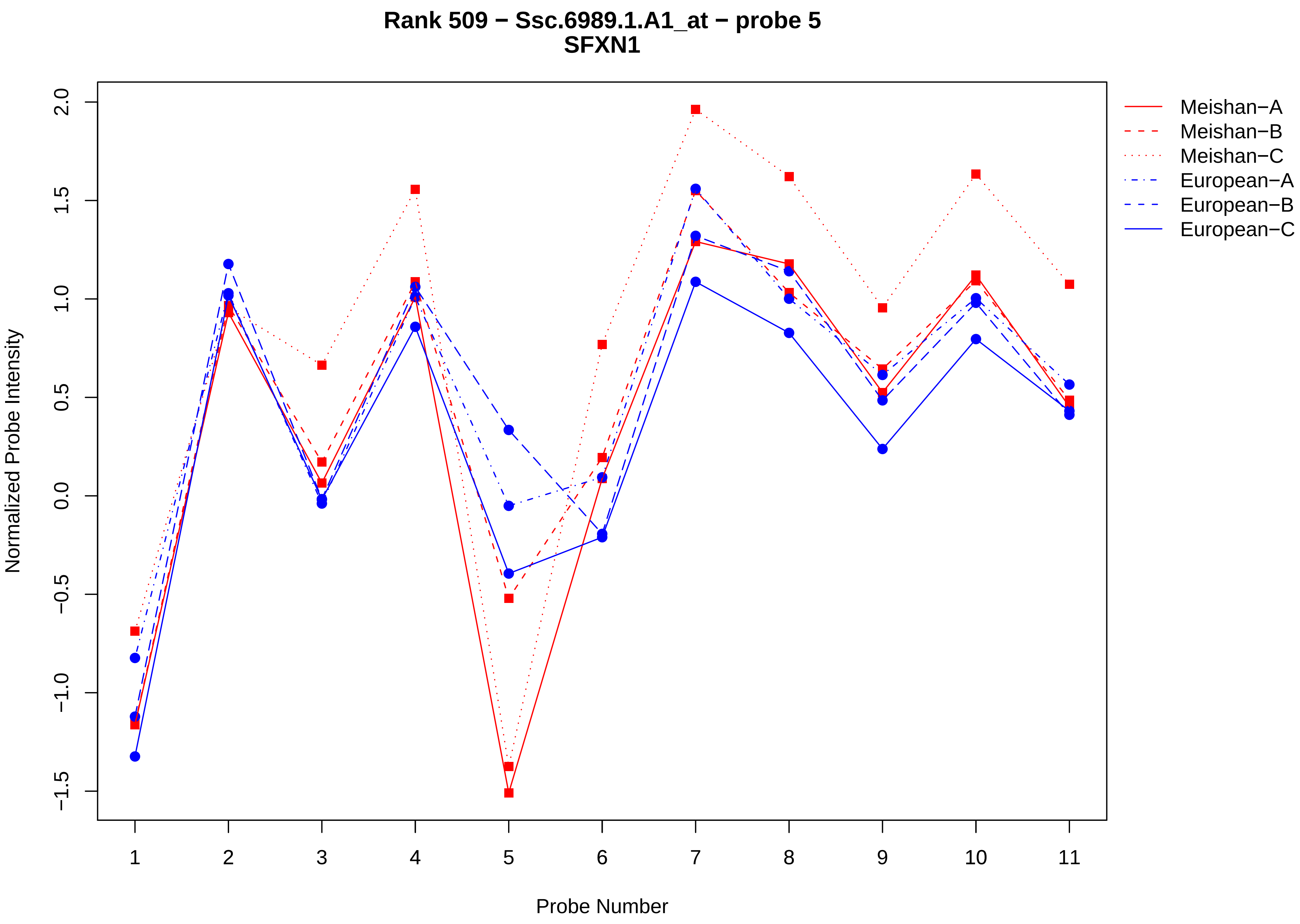
Rank 509 − Ssc.6989.1.A1_at − probe 5
SFXN1
Normalized Probe Intensity
Probe Number
Meishan−A
Meishan−B
Meishan−C
European−A
European−B
European−C
2.0
1.5
1.0
0.5
0.0
−0.5
−1.0
−1.5
1
2
3
4
5
6
7
8
9
10
11

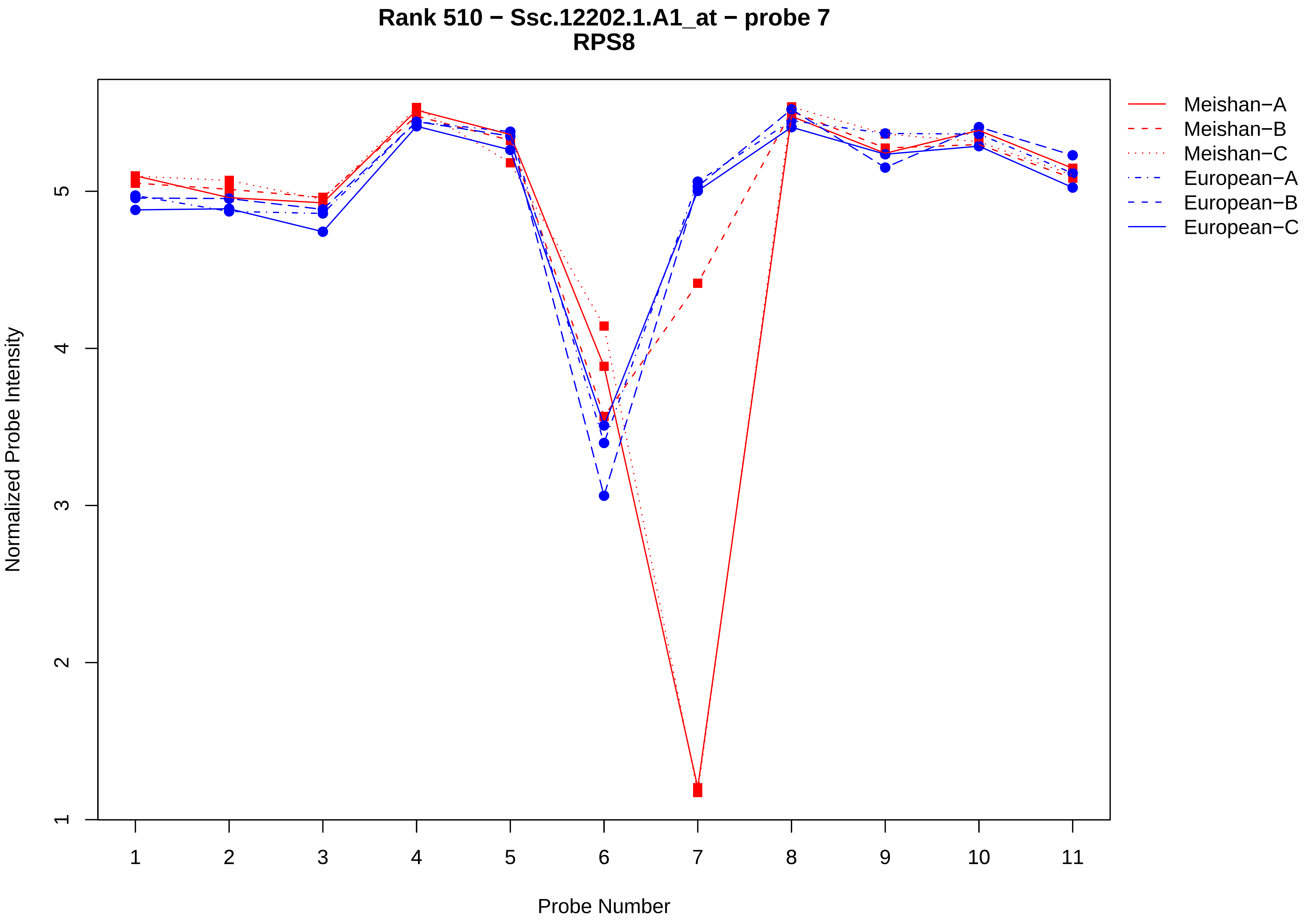

Rank 510 − Ssc.12202.1.A1_at − probe 7
RPS8
Normalized Probe Intensity
Probe Number
1
2
3
4
5
1
2
3
4
5
6
7
8
9
10
11
Meishan−A
Meishan−B
Meishan−C
European−A
European−B
European−C

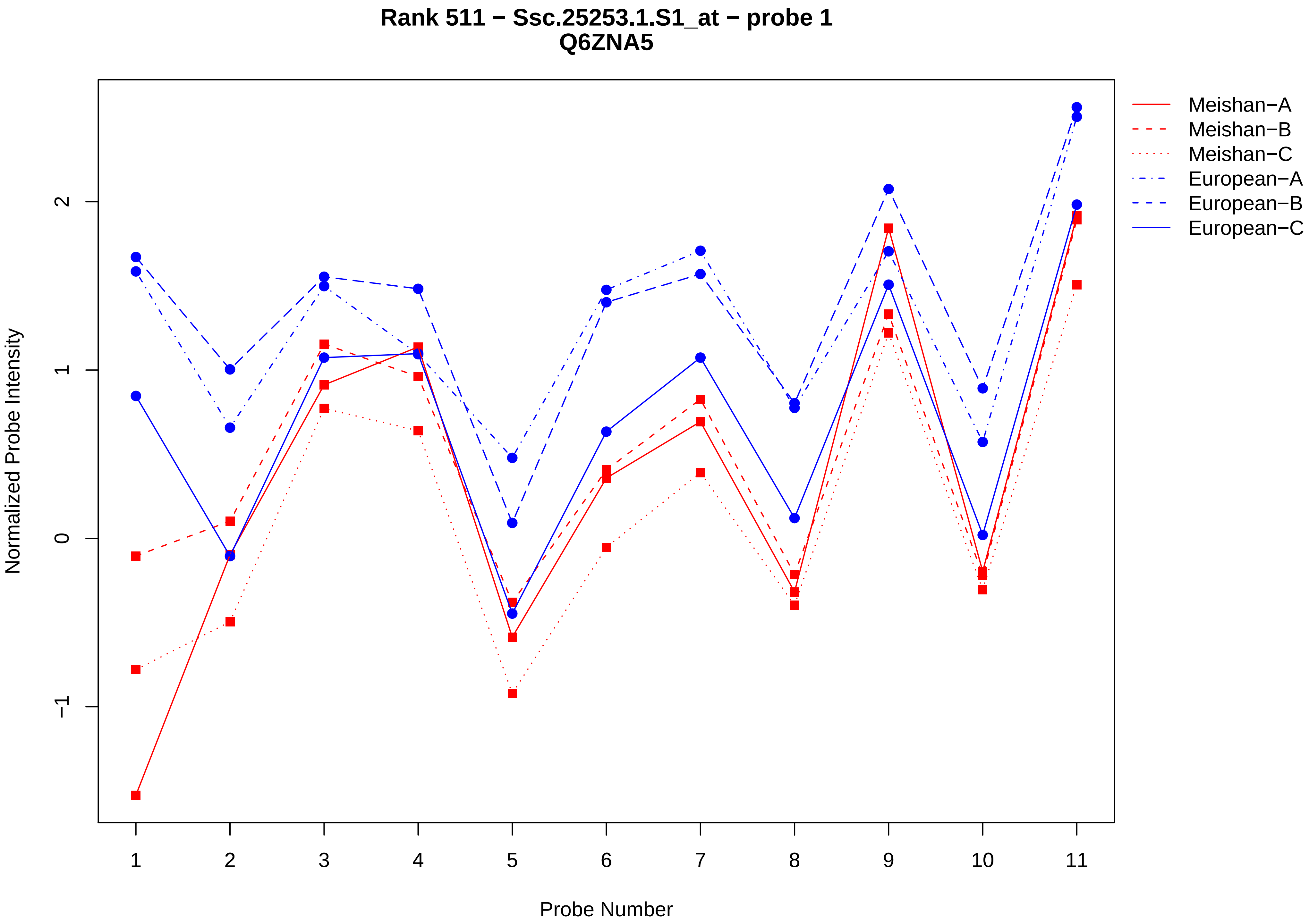

Rank 511 − Ssc.25253.1.S1_at − probe 1
Q6ZNA5
Normalized Probe Intensity
Probe Number
Meishan−A
Meishan−B
Meishan−C
European−A
European−B
European−C
−1
0
1
2
1
2
3
4
5
6
7
8
9
10
11

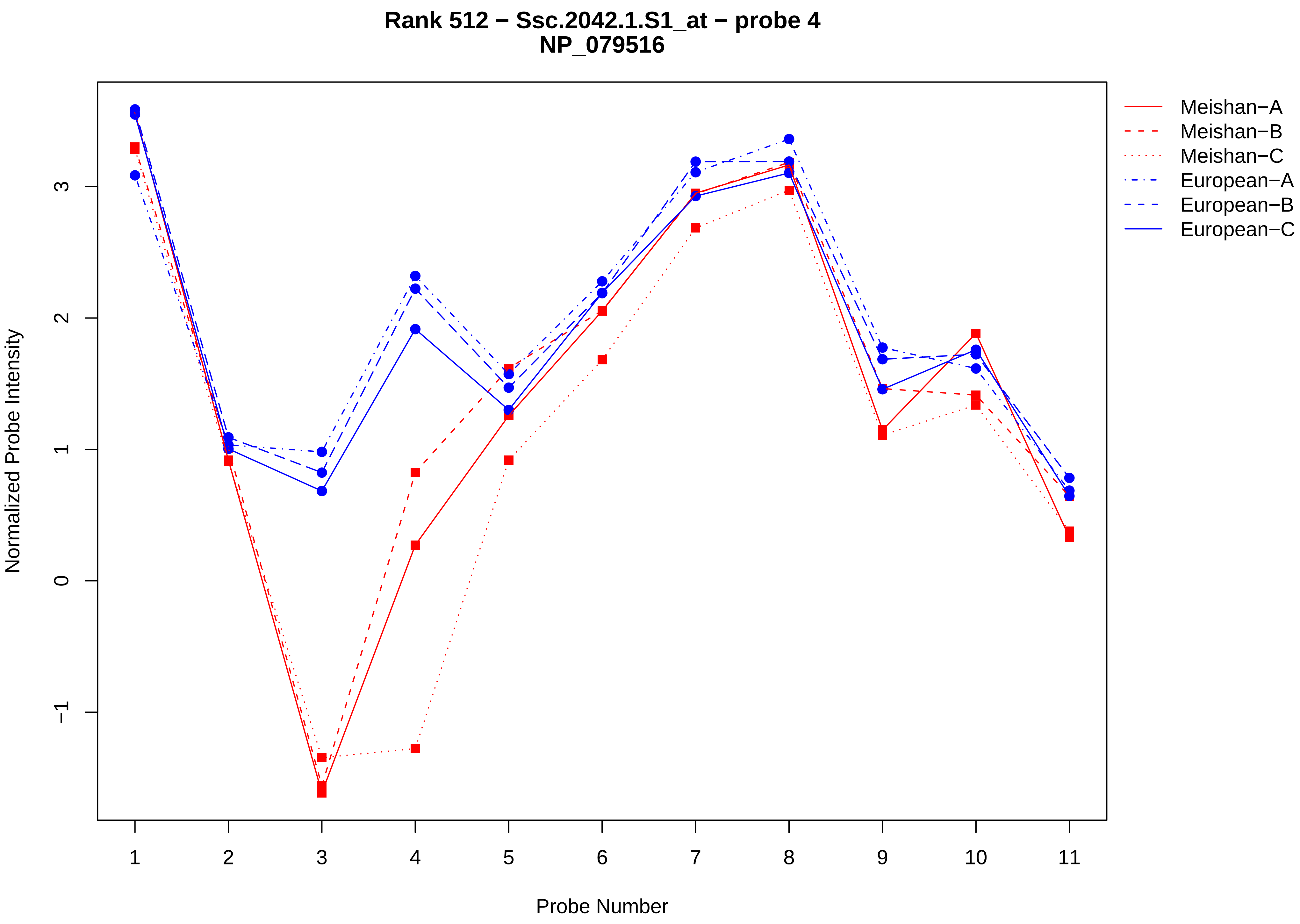

Rank 512 − Ssc.2042.1.S1_at − probe 4
NP_079516
Normalized Probe Intensity
Probe Number
Meishan−A
Meishan−B
Meishan−C
European−A
European−B
European−C
−1
0
1
2
3
1
2
3
4
5
6
7
8
9
10
11

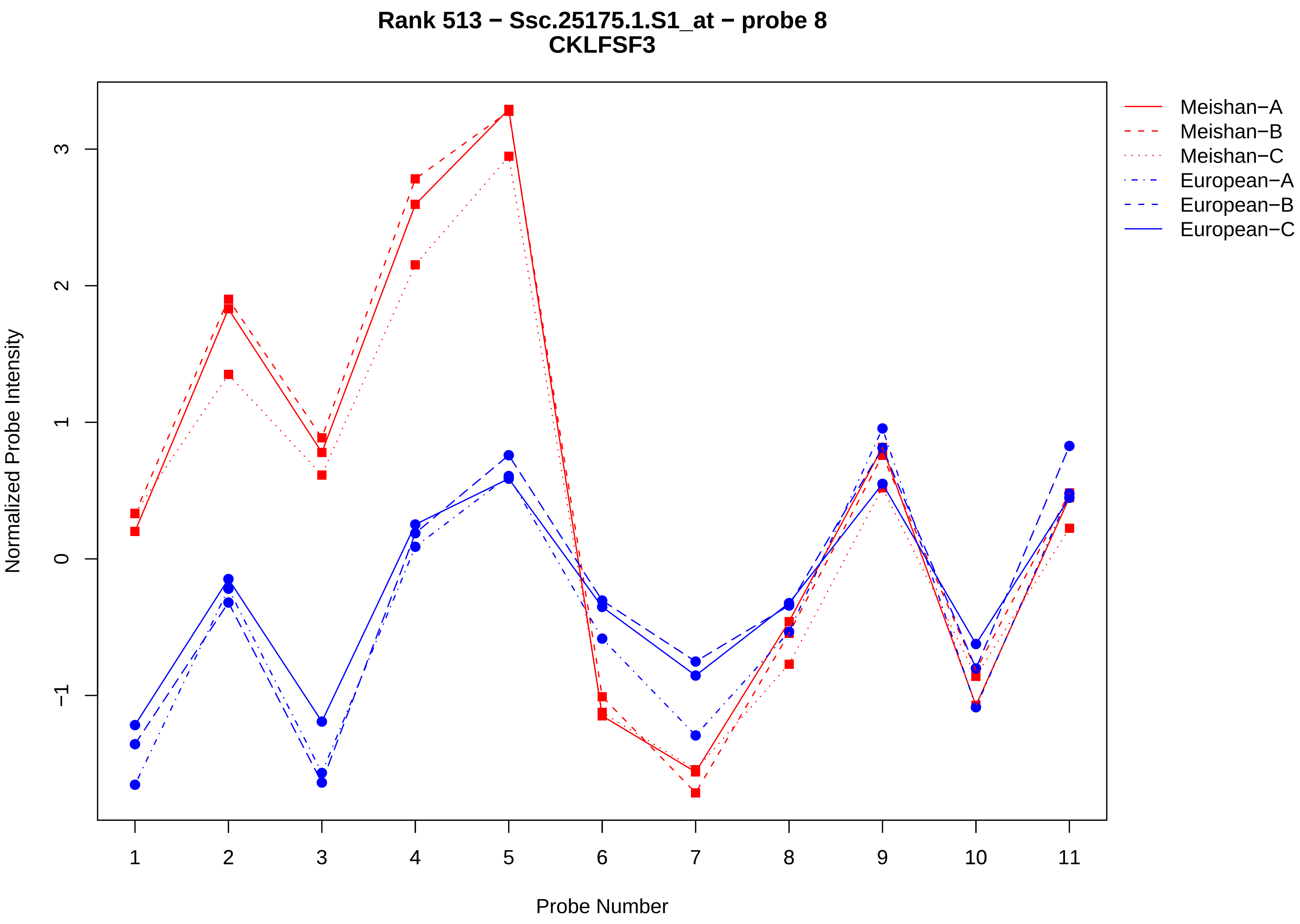

Rank 513 − Ssc.25175.1.S1_at − probe 8
CKLFSF3
Normalized Probe Intensity
Probe Number
−1
0
1
2
3
1
2
3
4
5
6
7
8
9
10
11
Meishan−A
Meishan−B
Meishan−C
European−A
European−B
European−C

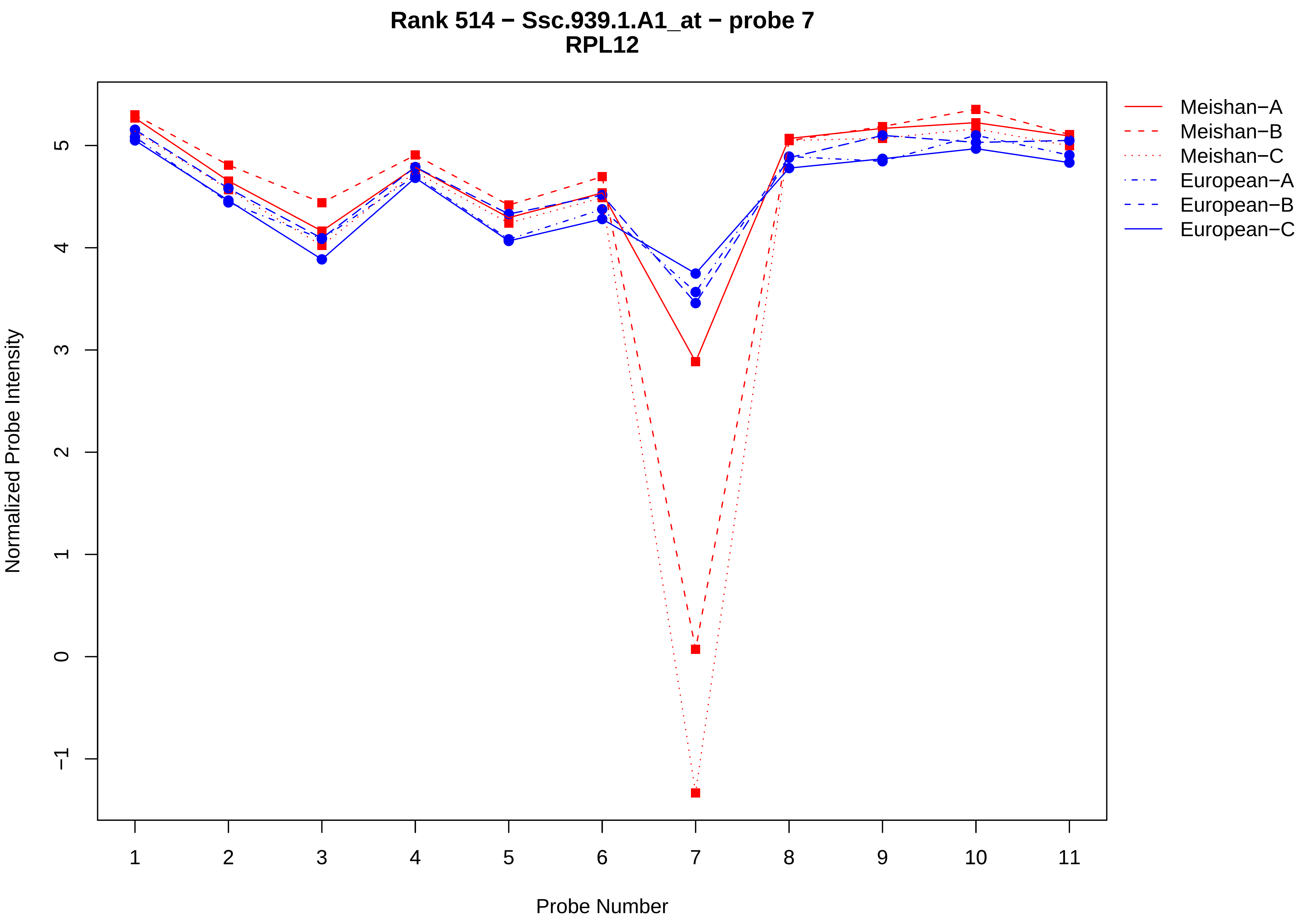
Rank 514 − Ssc.939.1.A1_at − probe 7
RPL12
Normalized Probe Intensity
Probe Number
−1
0
1
2
3
4
5
1
2
3
4
5
6
7
8
9
10
11
Meishan−A
Meishan−B
Meishan−C
European−A
European−B
European−C

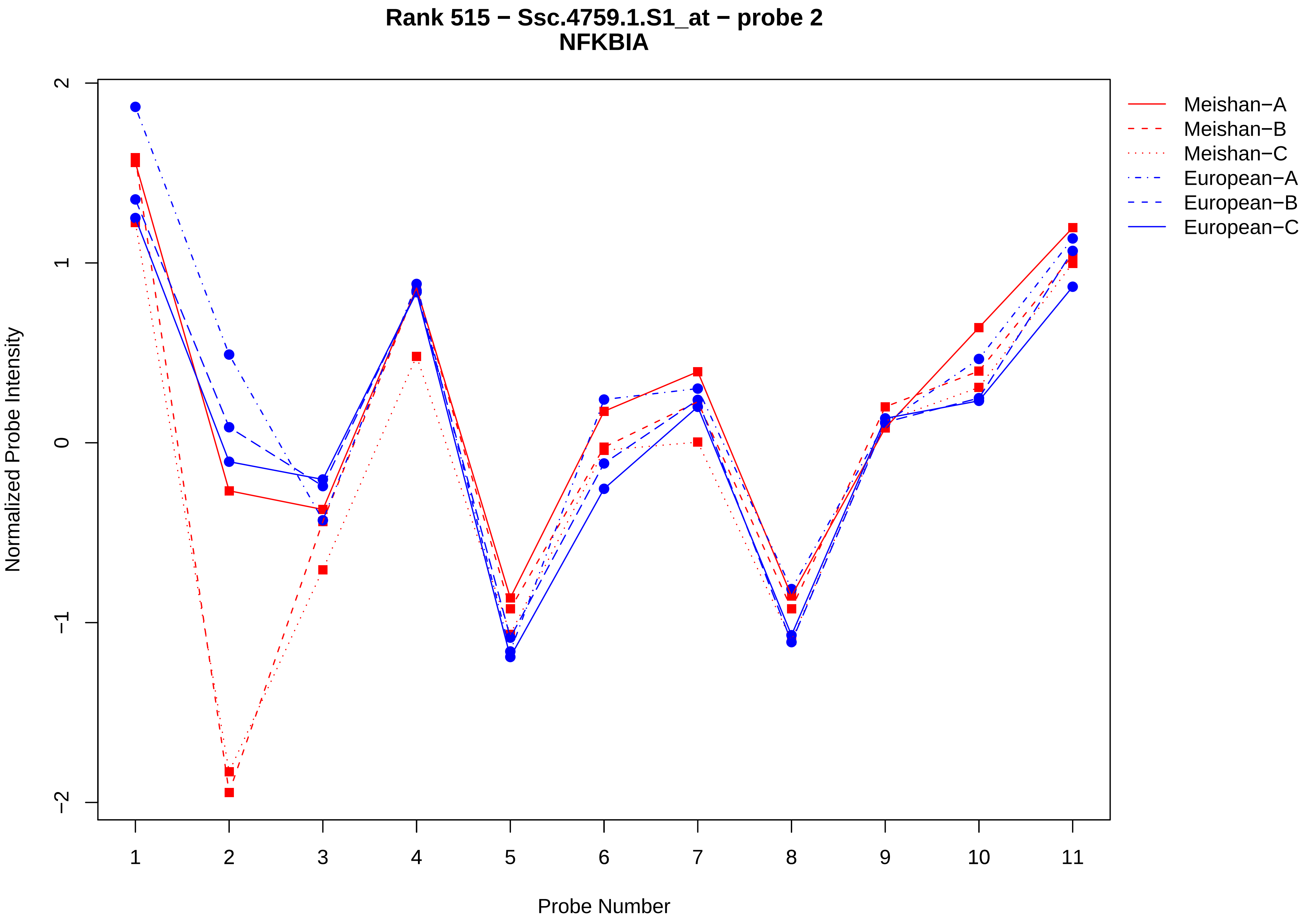
Rank 515 − Ssc.4759.1.S1_at − probe 2
NFKBIA
Normalized Probe Intensity
Probe Number
Meishan−A
Meishan−B
Meishan−C
European−A
European−B
European−C
2
1
0
−1
−2
1
2
3
4
5
6
7
8
9
10
11

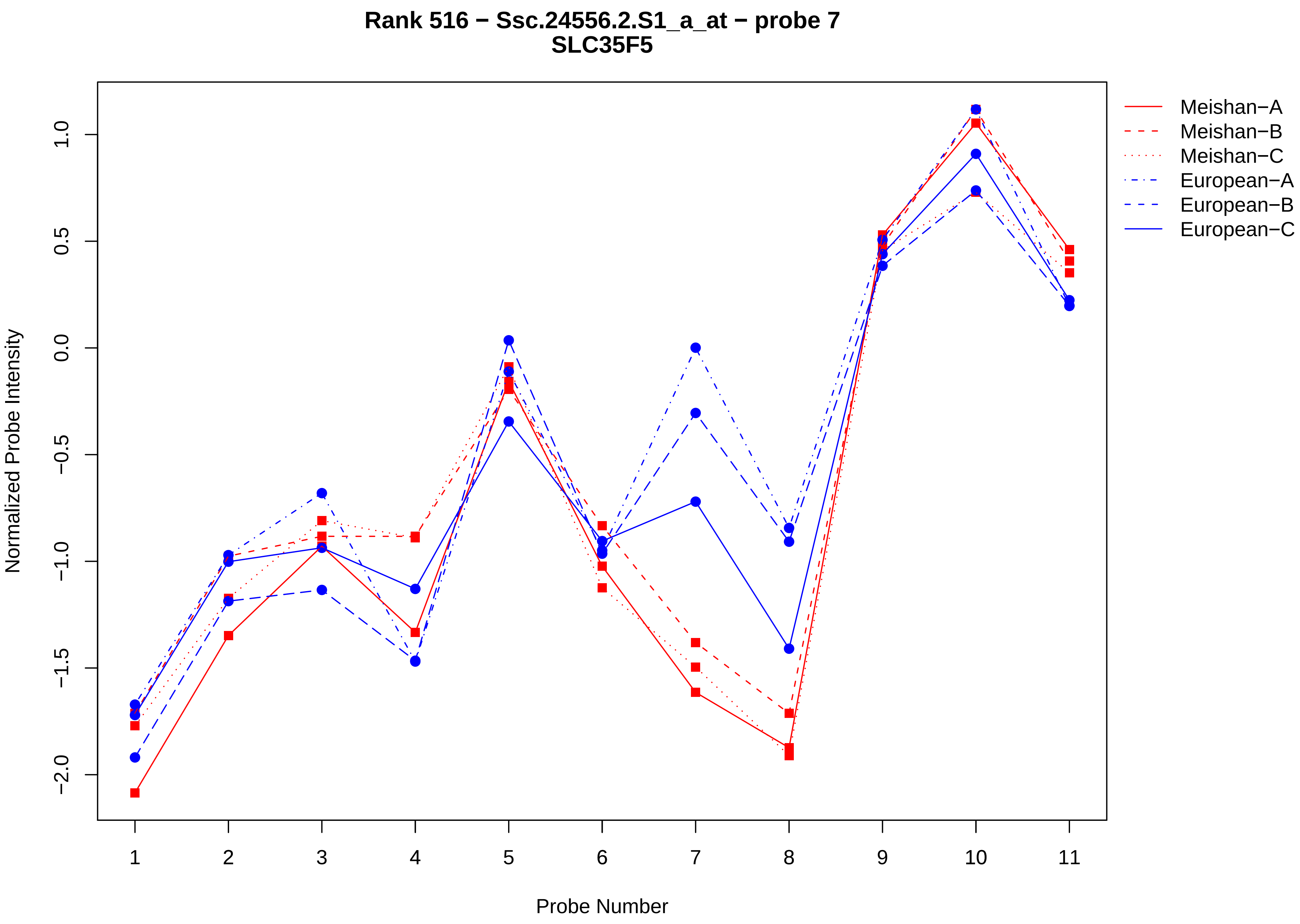

Rank 516 − Ssc.24556.2.S1_a_at − probe 7
SLC35F5
Normalized Probe Intensity
Probe Number
1.0
0.5
0.0
−0.5
−1.0
−1.5
−2.0
1
2
3
4
5
6
7
8
9
10
11
Meishan−A
Meishan−B
Meishan−C
European−A
European−B
European−C

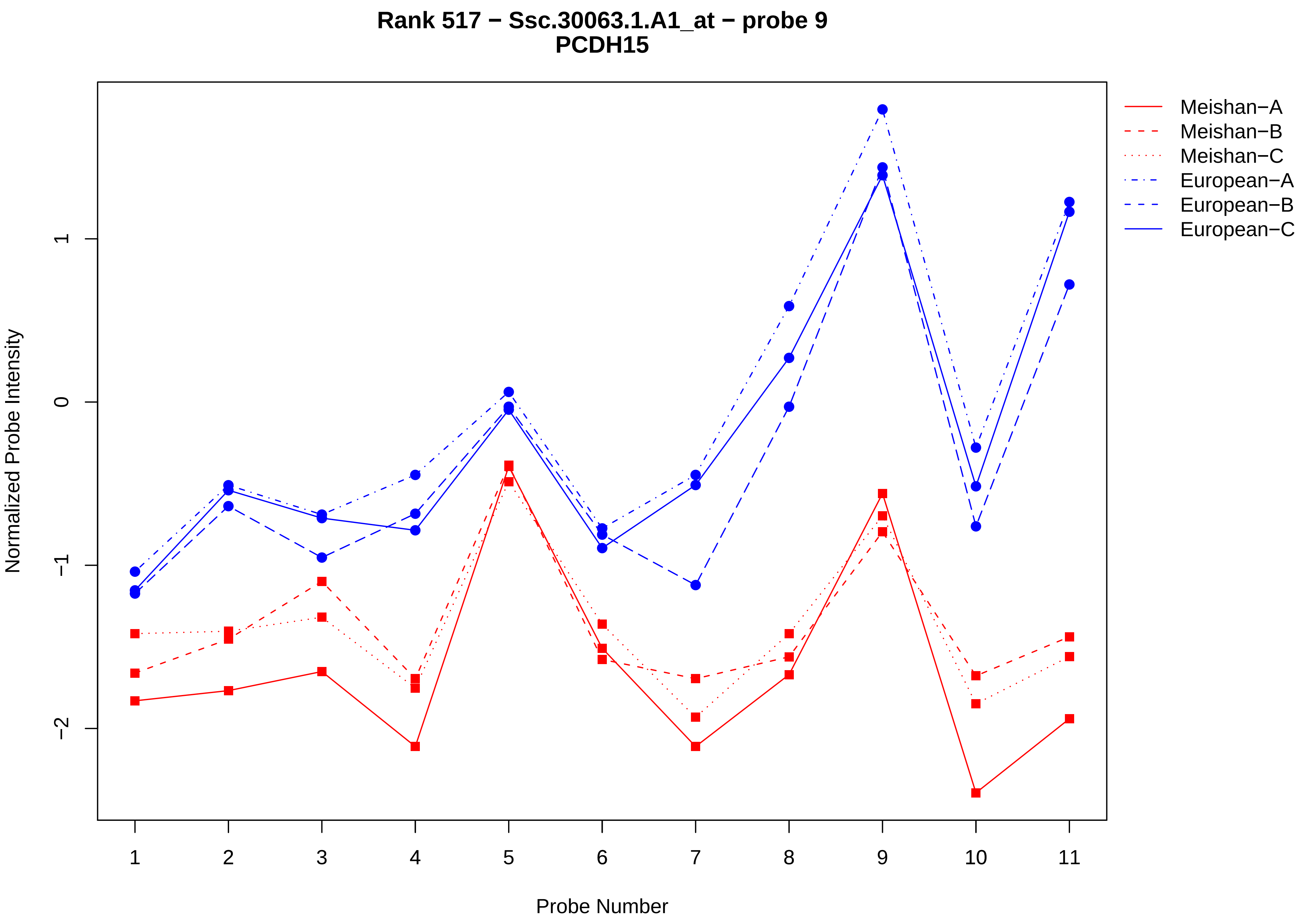
Rank 517 − Ssc.30063.1.A1_at − probe 9
PCDH15
Normalized Probe Intensity
Probe Number
−2
−1
0
1
1
2
3
4
5
6
7
8
9
10
11
Meishan−A
Meishan−B
Meishan−C
European−A
European−B
European−C

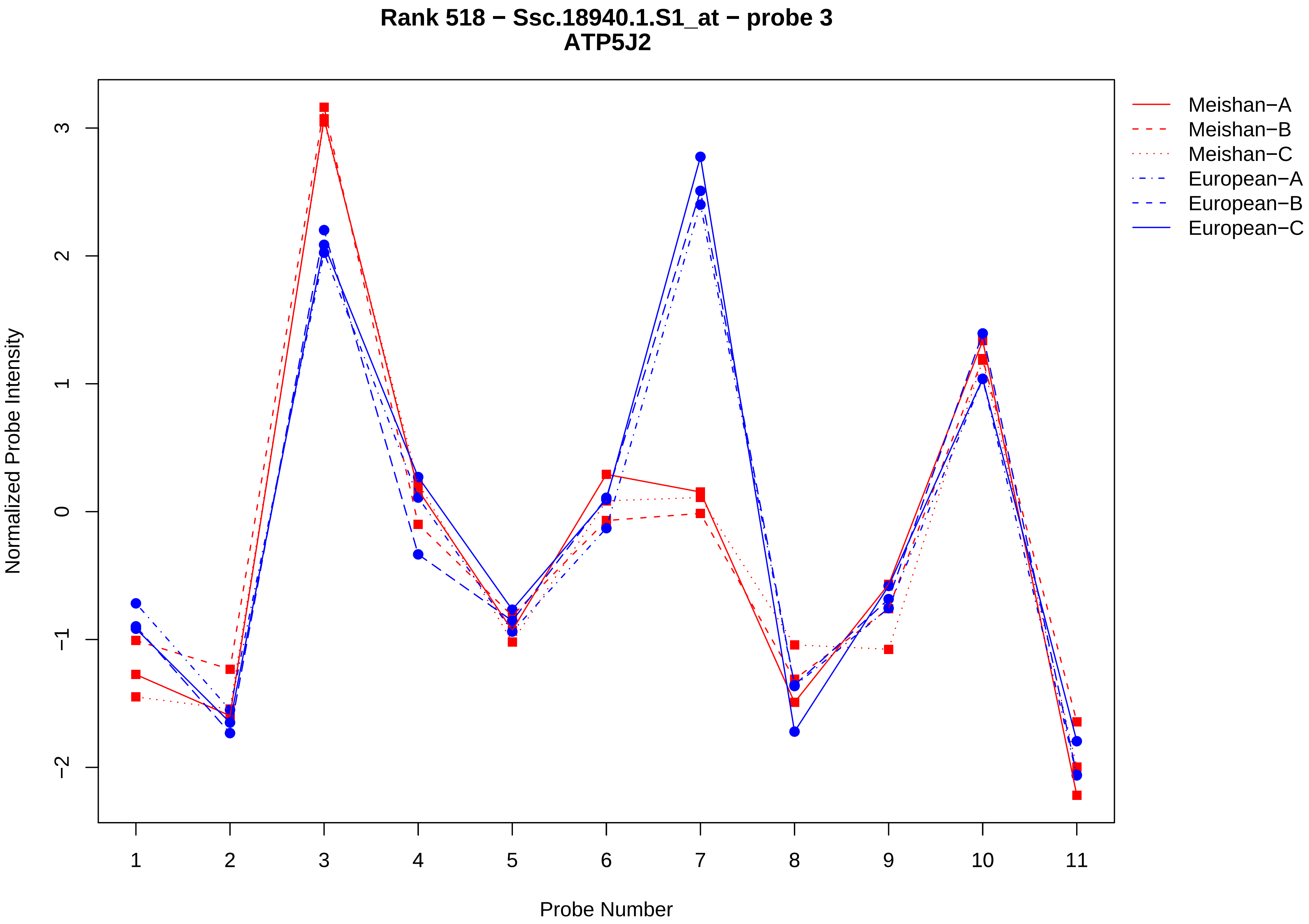

Rank 518 − Ssc.18940.1.S1_at − probe 3
ATP5J2
Normalized Probe Intensity
Probe Number
Meishan−A
Meishan−B
Meishan−C
European−A
European−B
European−C
−2
−1
0
1
2
3
1
2
3
4
5
6
7
8
9
10
11

Rank 519 − Ssc.24981.1.S1_at − probe 3
Q8TE89

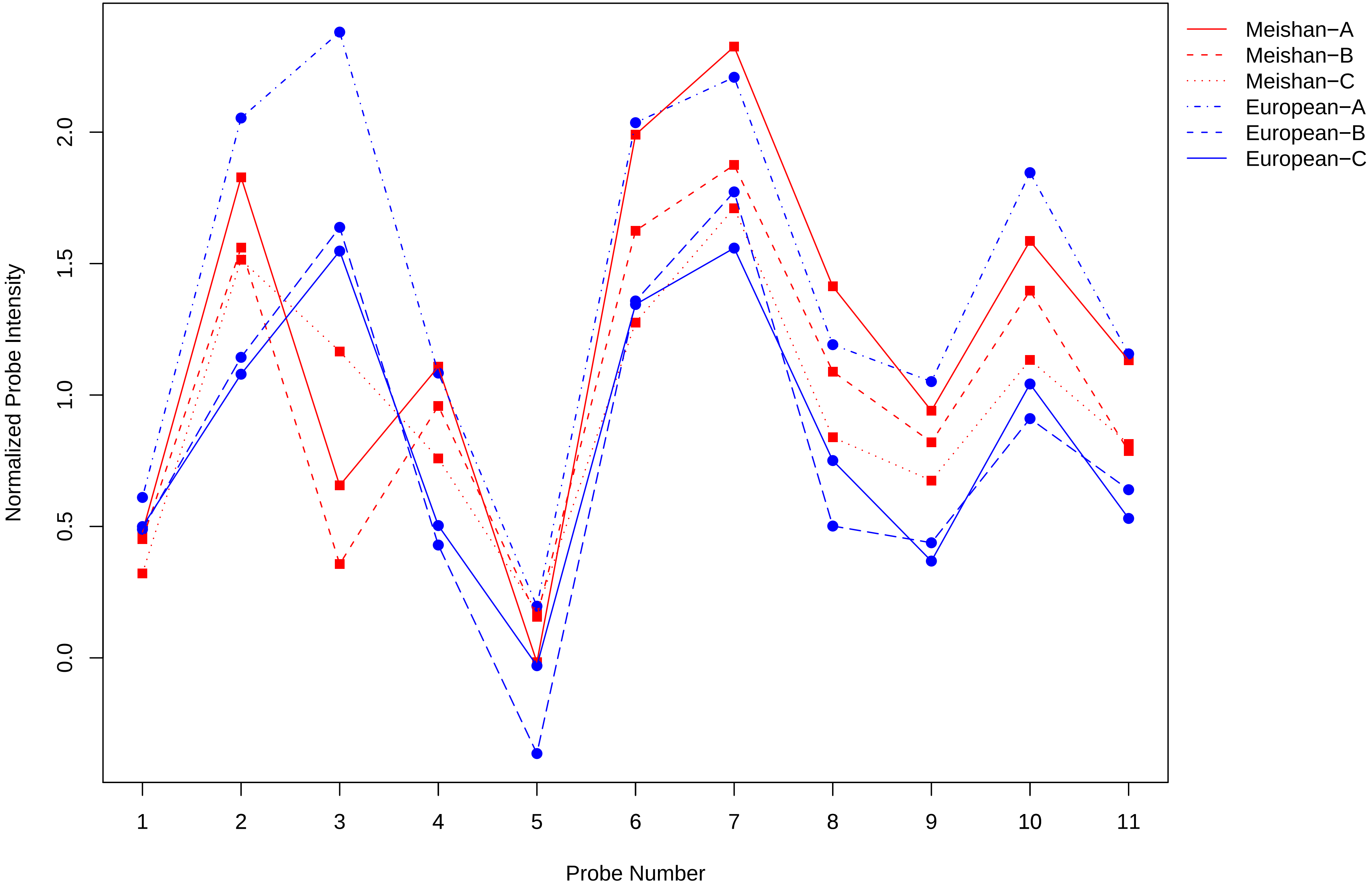

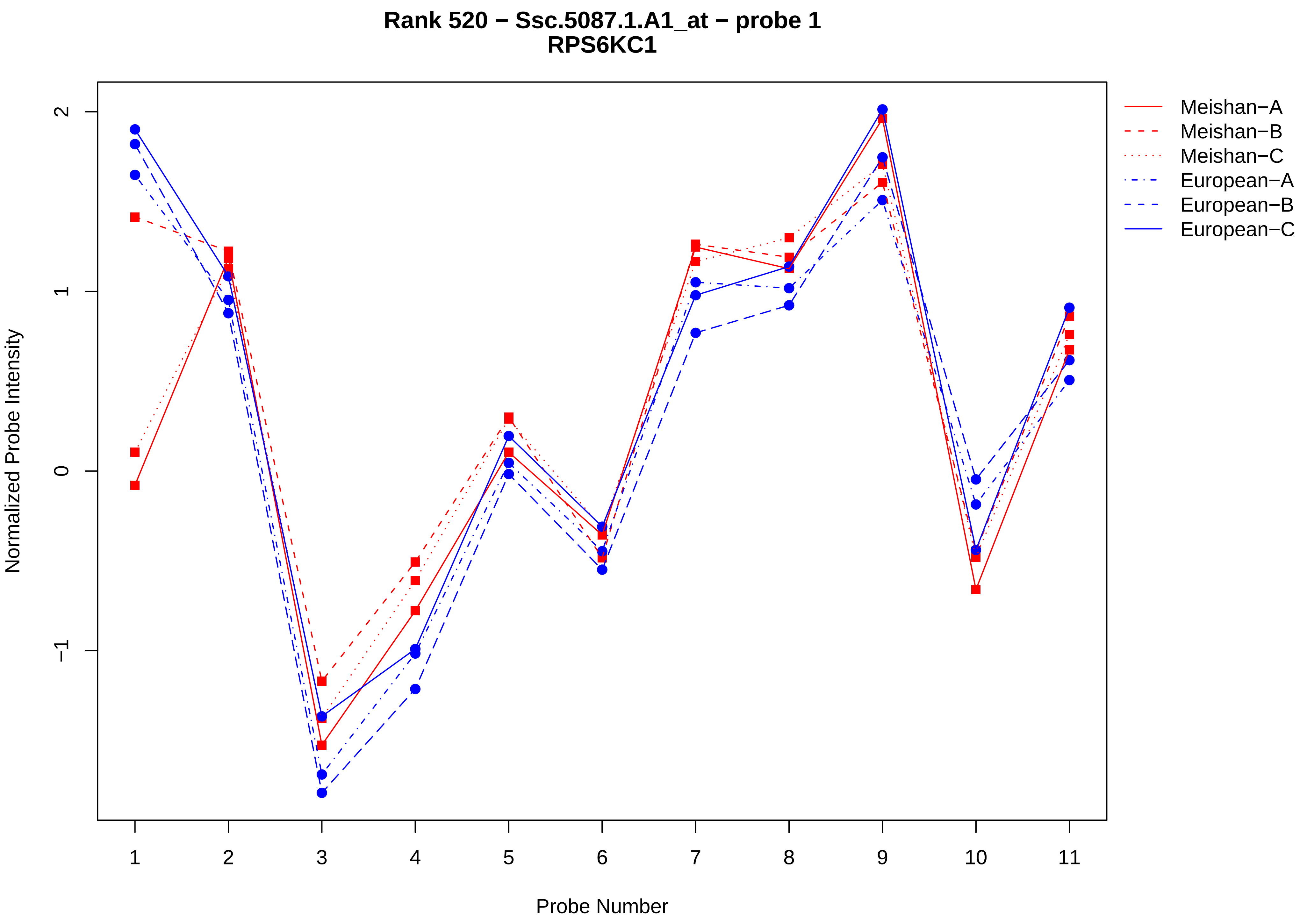

Rank 520 − Ssc.5087.1.A1_at − probe 1
RPS6KC1
Normalized Probe Intensity
Probe Number
−1
0
1
2
1
2
3
4
5
6
7
8
9
10
11
Meishan−A
Meishan−B
Meishan−C
European−A
European−B
European−C

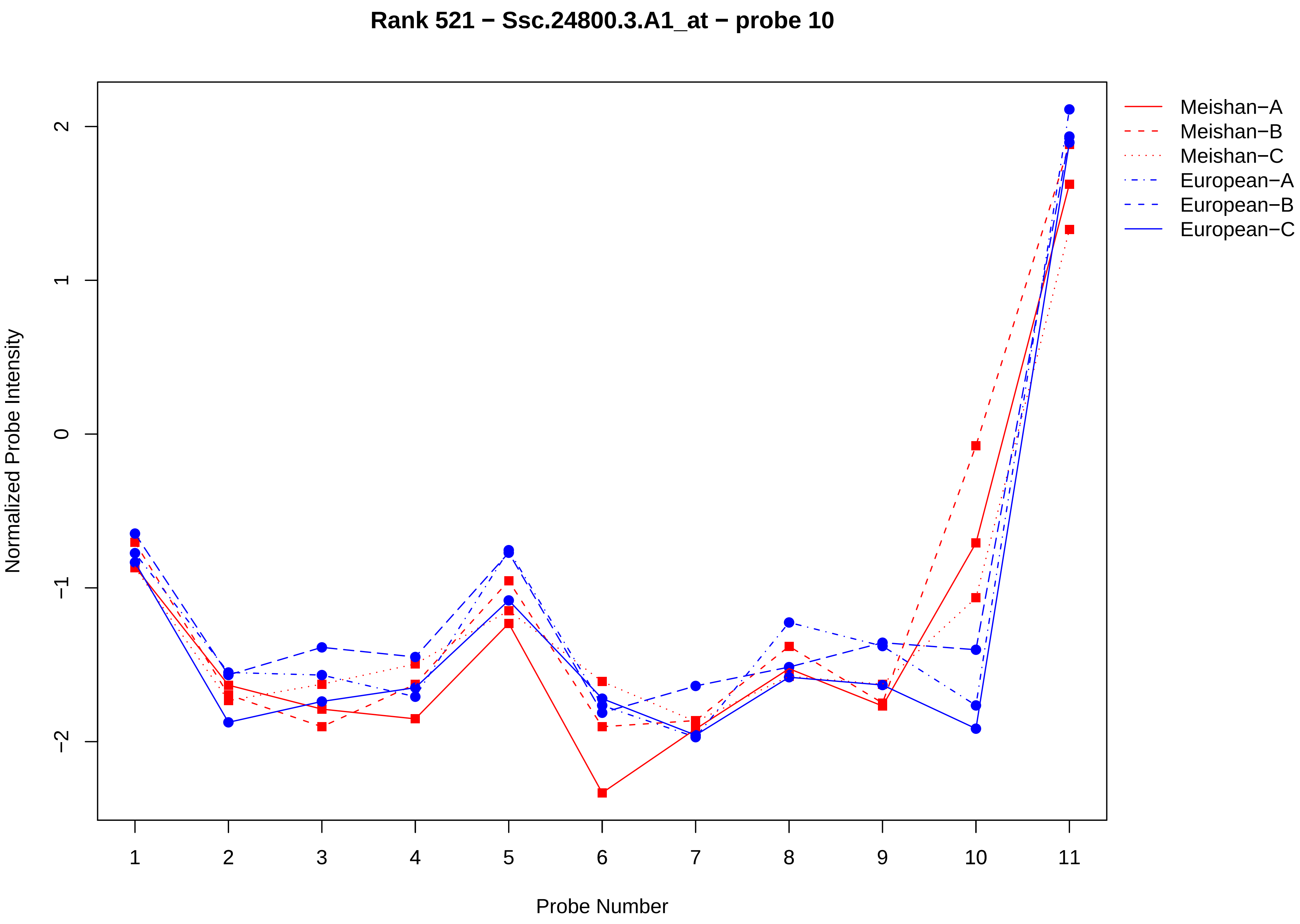

Rank 521 − Ssc.24800.3.A1_at − probe 10
Normalized Probe Intensity
Probe Number
Meishan−A
Meishan−B
Meishan−C
European−A
European−B
European−C

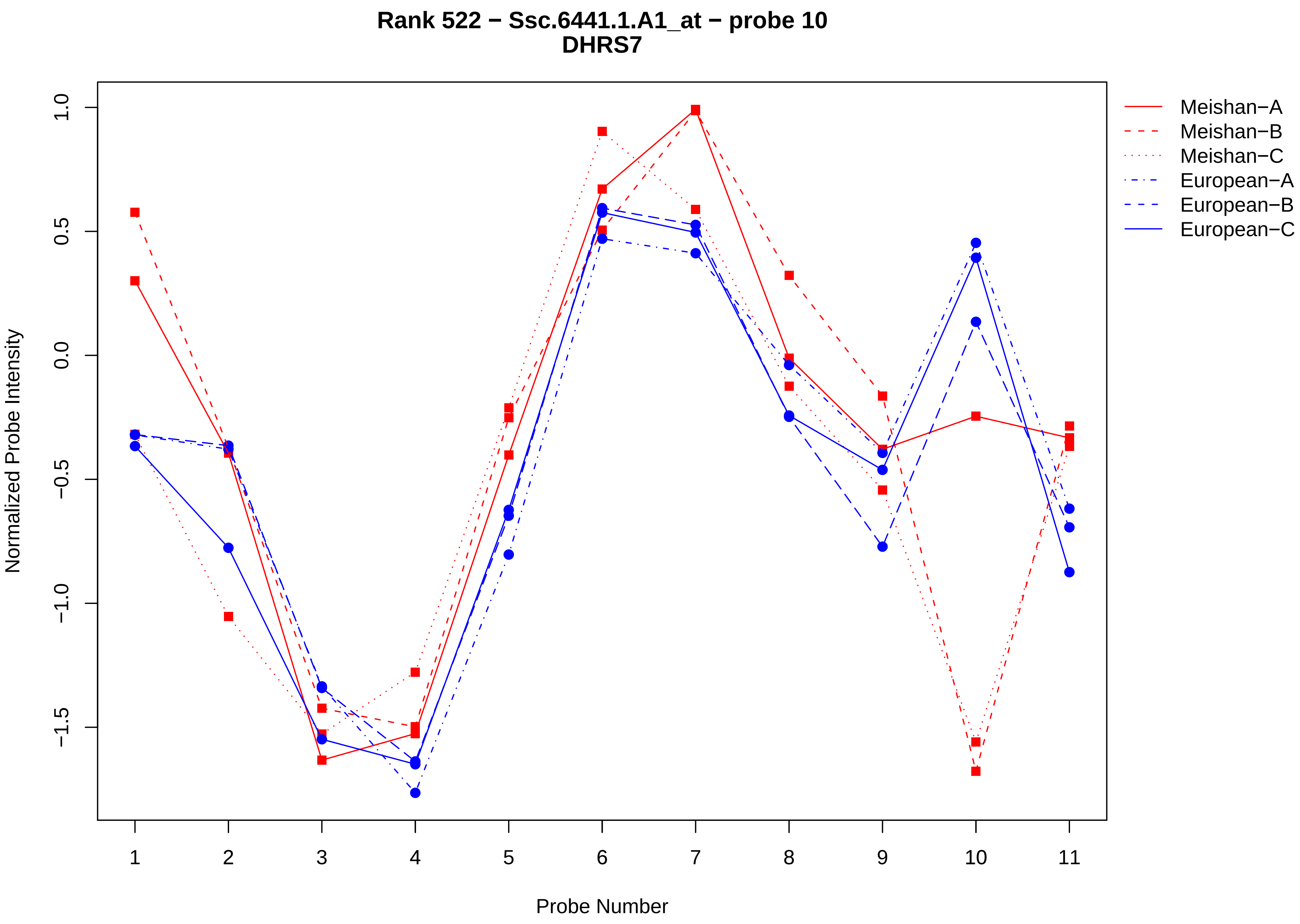
Rank 522 − Ssc.6441.1.A1_at − probe 10
DHRS7
Normalized Probe Intensity
Probe Number
1.0
0.5
0.0
−0.5
−1.0
−1.5
1
2
3
4
5
6
7
8
9
10
11
Meishan−A
Meishan−B
Meishan−C
European−A
European−B
European−C

Rank 523 − Ssc.26344.1.S1_at − probe 4
PAK3

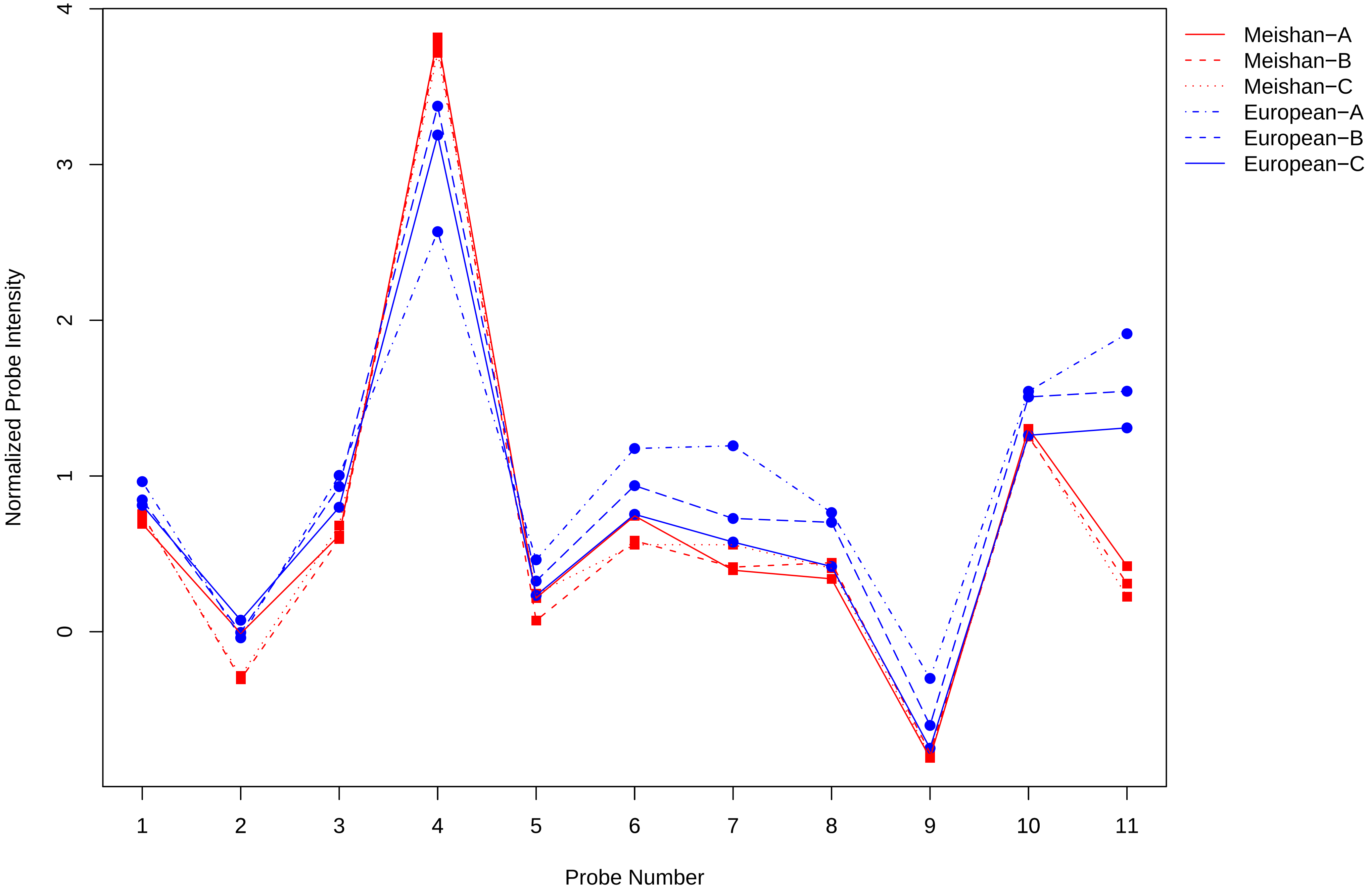

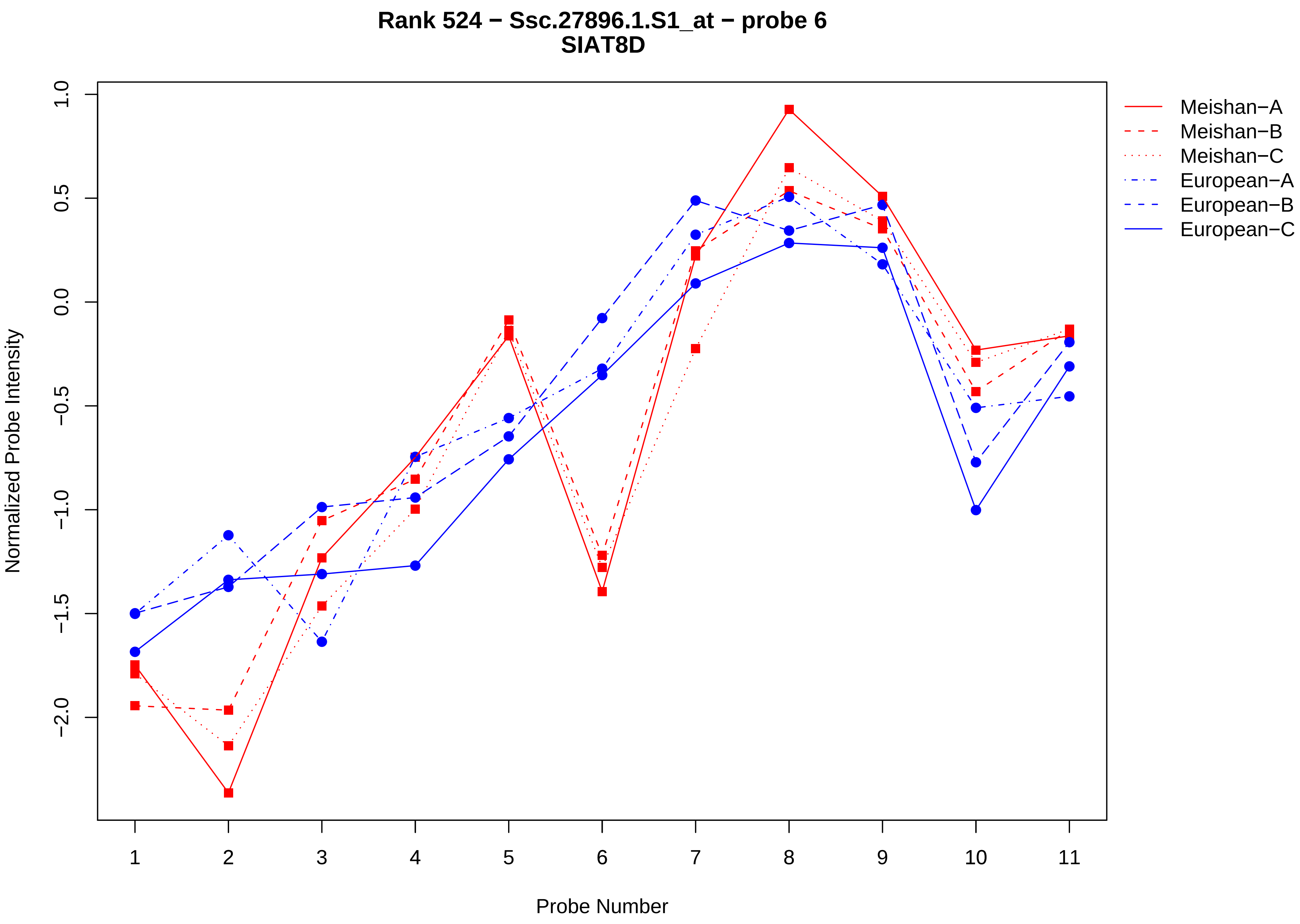
Rank 524 − Ssc.27896.1.S1_at − probe 6
SIAT8D
Normalized Probe Intensity
Probe Number
1.0
0.5
0.0
−0.5
−1.0
−1.5
−2.0
1
2
3
4
5
6
7
8
9
10
11
Meishan−A
Meishan−B
Meishan−C
European−A
European−B
European−C

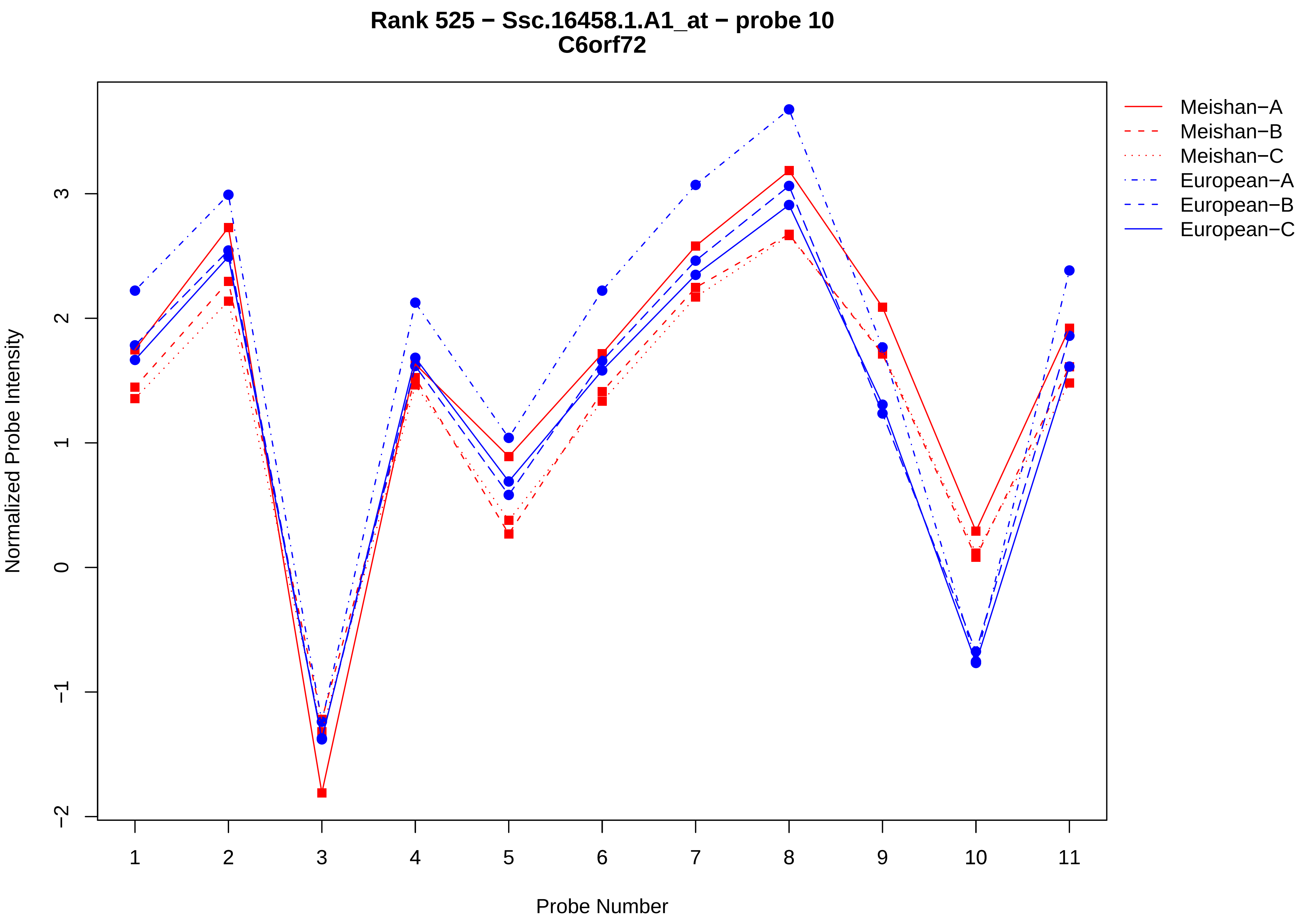

Rank 525 − Ssc.16458.1.A1_at − probe 10
C6orf72
Normalized Probe Intensity
Probe Number
−2
−1
0
1
2
3
1
2
3
4
5
6
7
8
9
10
11
Meishan−A
Meishan−B
Meishan−C
European−A
European−B
European−C

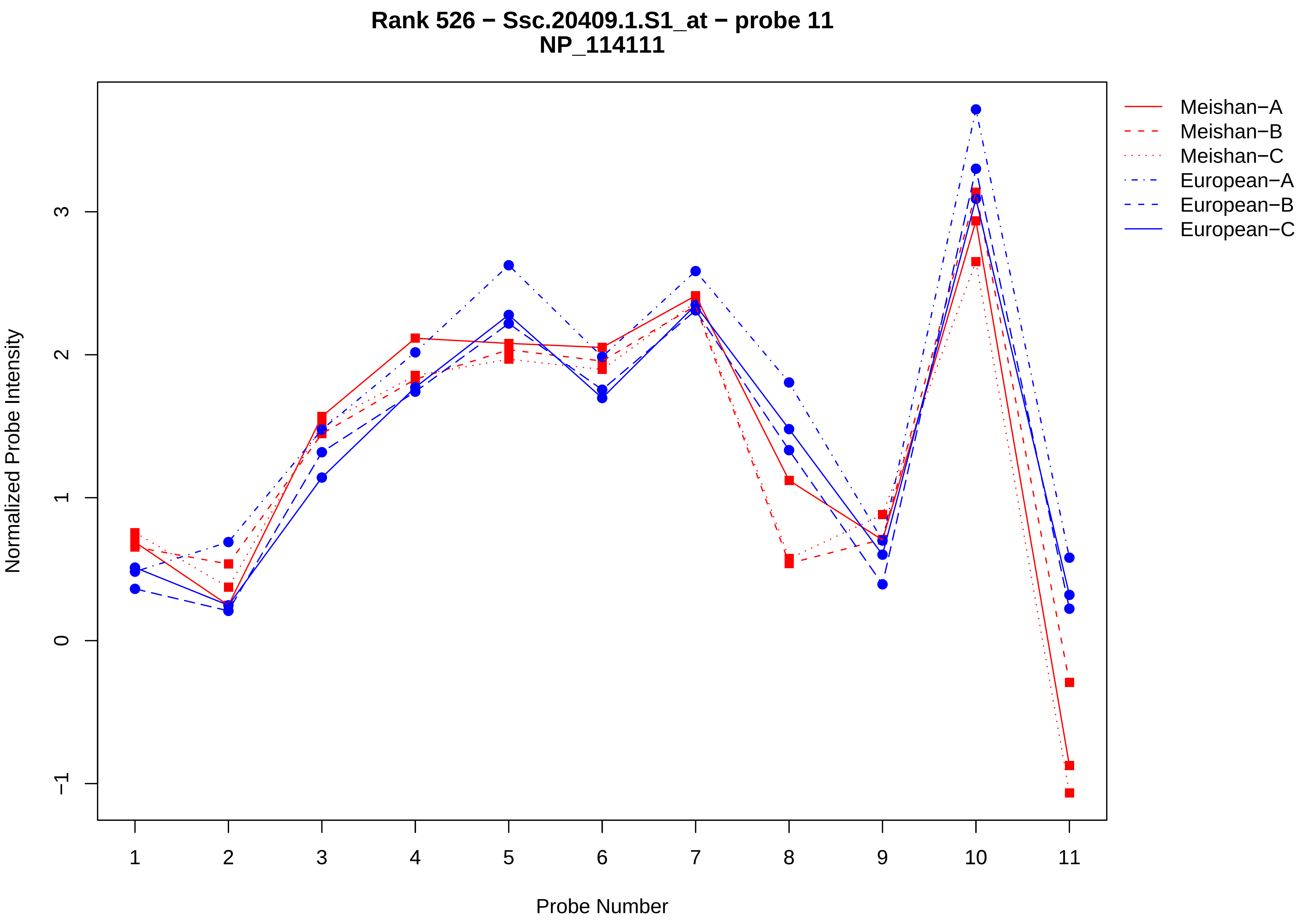
Rank 526 − Ssc.20409.1.S1_at − probe 11
NP_114111
Normalized Probe Intensity
Probe Number
Meishan−A
Meishan−B
Meishan−C
European−A
European−B
European−C
−1
0
1
2
3
1
2
3
4
5
6
7
8
9
10
11

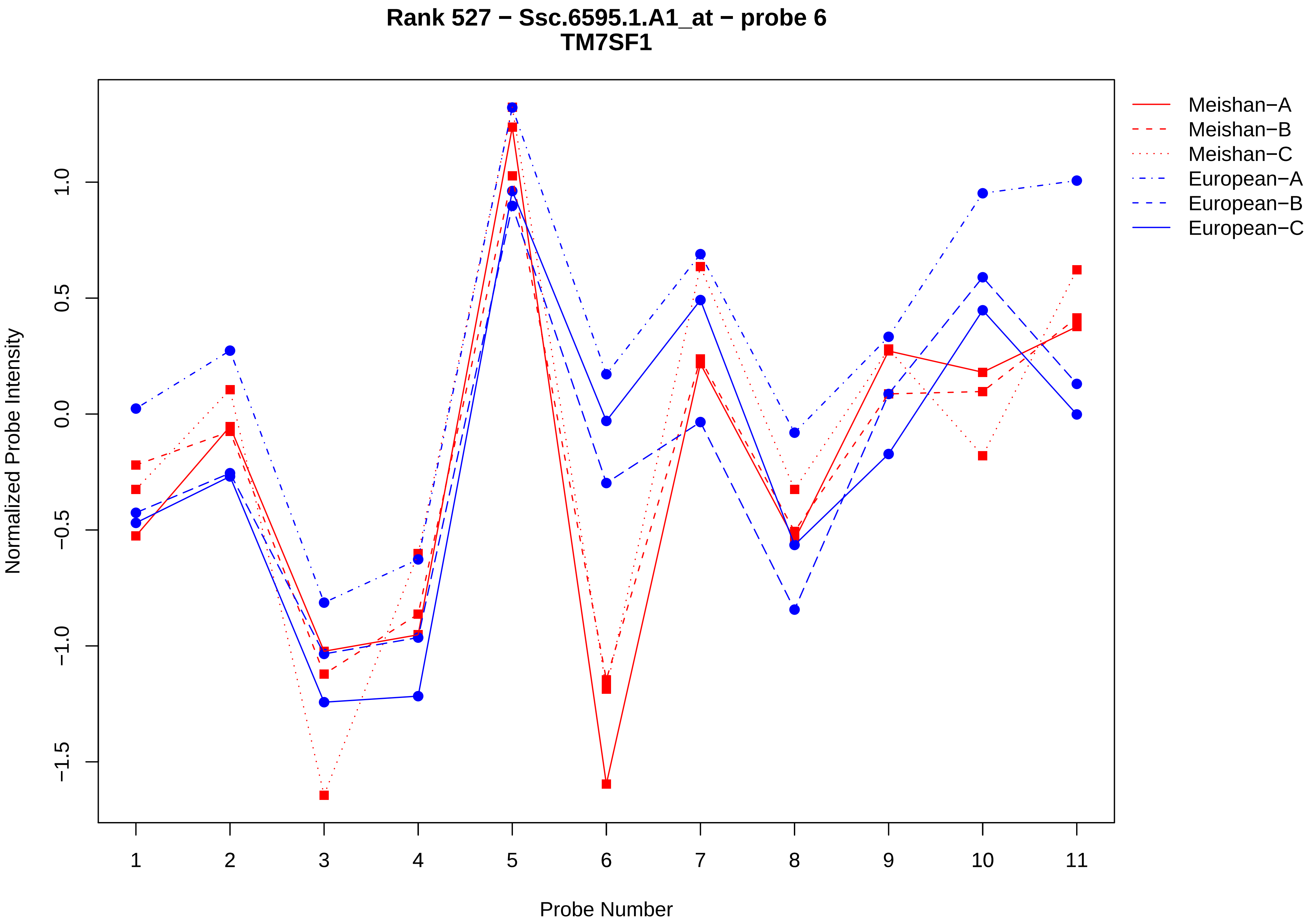

Rank 527 − Ssc.6595.1.A1_at − probe 6
TM7SF1
Normalized Probe Intensity
Probe Number
1.0
0.5
0.0
−0.5
−1.0
−1.5
1
2
3
4
5
6
7
8
9
10
11
Meishan−A
Meishan−B
Meishan−C
European−A
European−B
European−C

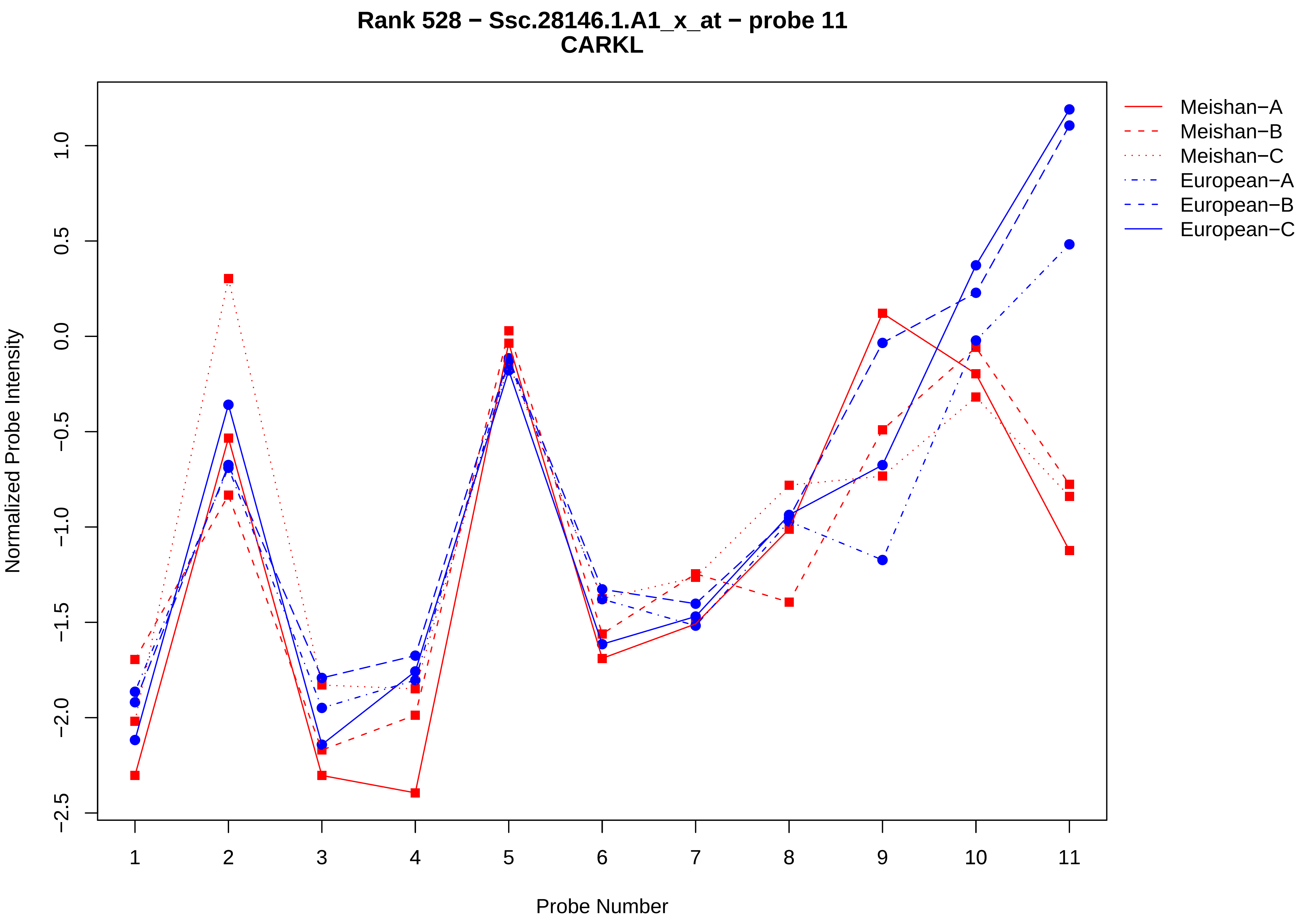
Rank 528 − Ssc.28146.1.A1_x_at − probe 11
CARKL
Normalized Probe Intensity
Probe Number
−2.5
−2.0
−1.5
−1.0
−0.5
0.0
0.5
1.0
1
2
3
4
5
6
7
8
9
10
11
Meishan−A
Meishan−B
Meishan−C
European−A
European−B
European−C

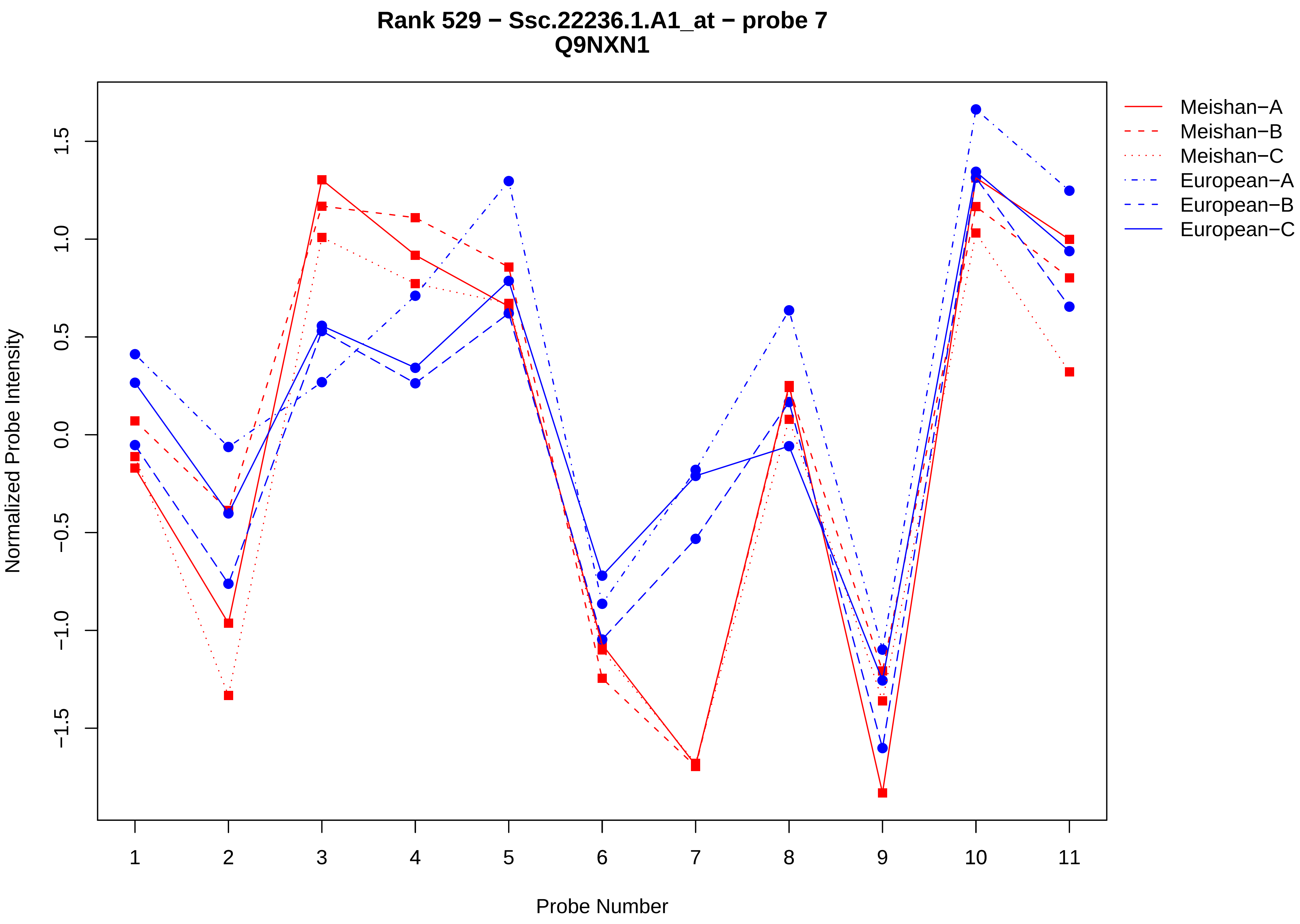

Rank 529 − Ssc.22236.1.A1_at − probe 7
Q9NXN1
Normalized Probe Intensity
Probe Number
Meishan−A
Meishan−B
Meishan−C
European−A
European−B
European−C
1.5
1.0
0.5
0.0
−0.5
−1.0
−1.5
1
2
3
4
5
6
7
8
9
10
11

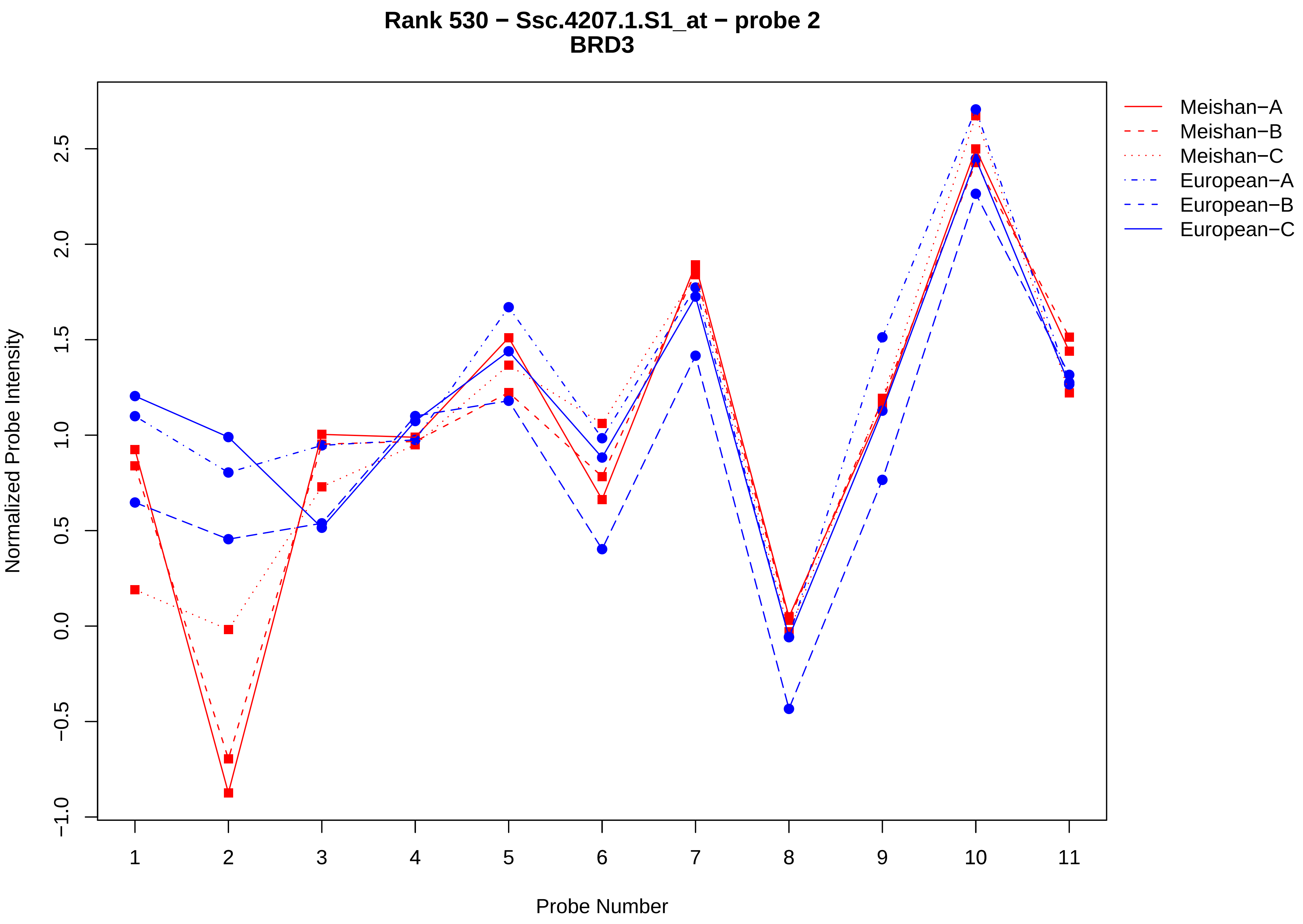

Rank 530 − Ssc.4207.1.S1_at − probe 2
BRD3
Normalized Probe Intensity
Probe Number
−1.0
−0.5
0.0
0.5
1.0
1.5
2.0
2.5
1
2
3
4
5
6
7
8
9
10
11
Meishan−A
Meishan−B
Meishan−C
European−A
European−B
European−C

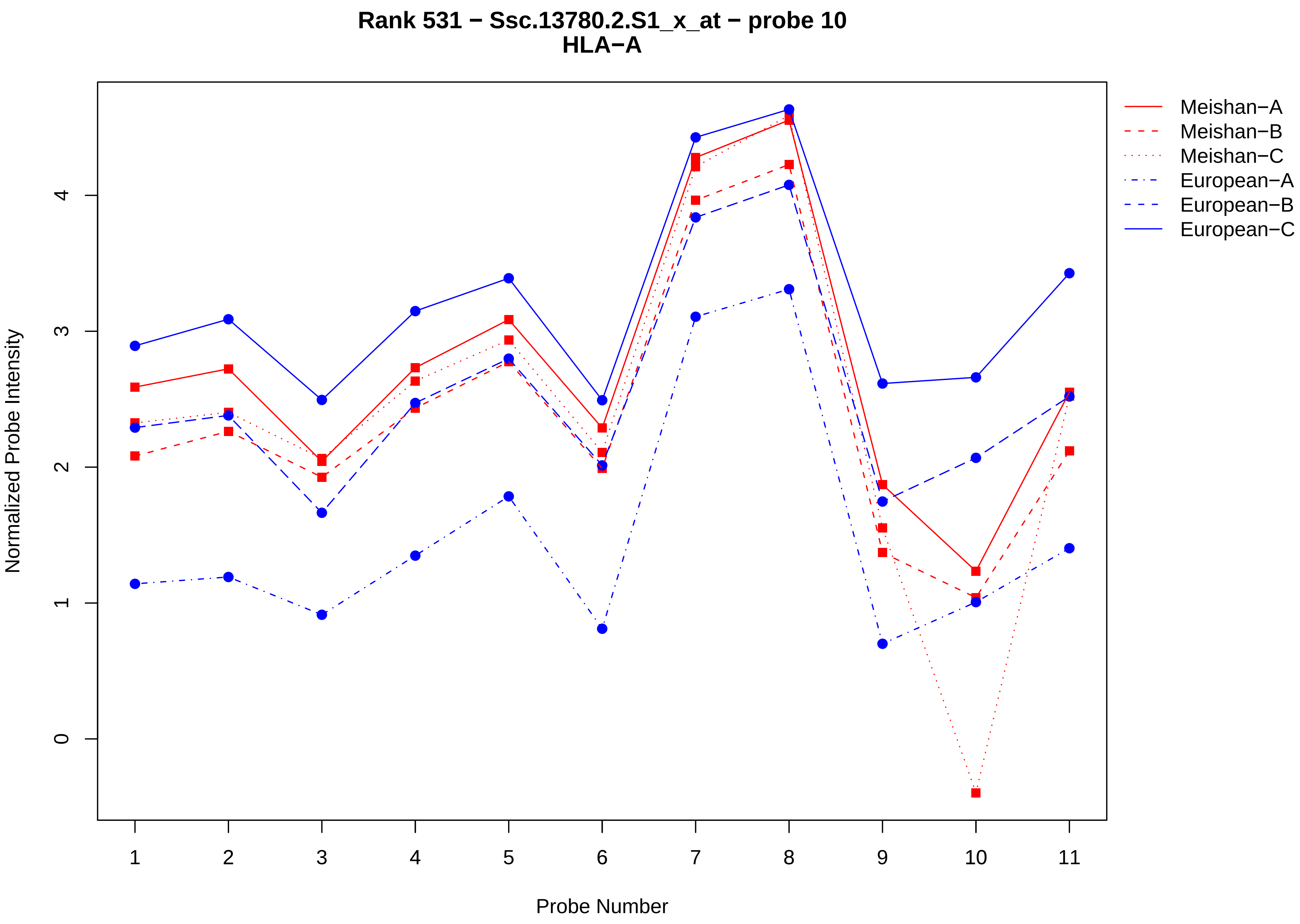

Rank 531 − Ssc.13780.2.S1_x_at − probe 10
HLA−A
Normalized Probe Intensity
Probe Number
Meishan−A
Meishan−B
Meishan−C
European−A
European−B
European−C

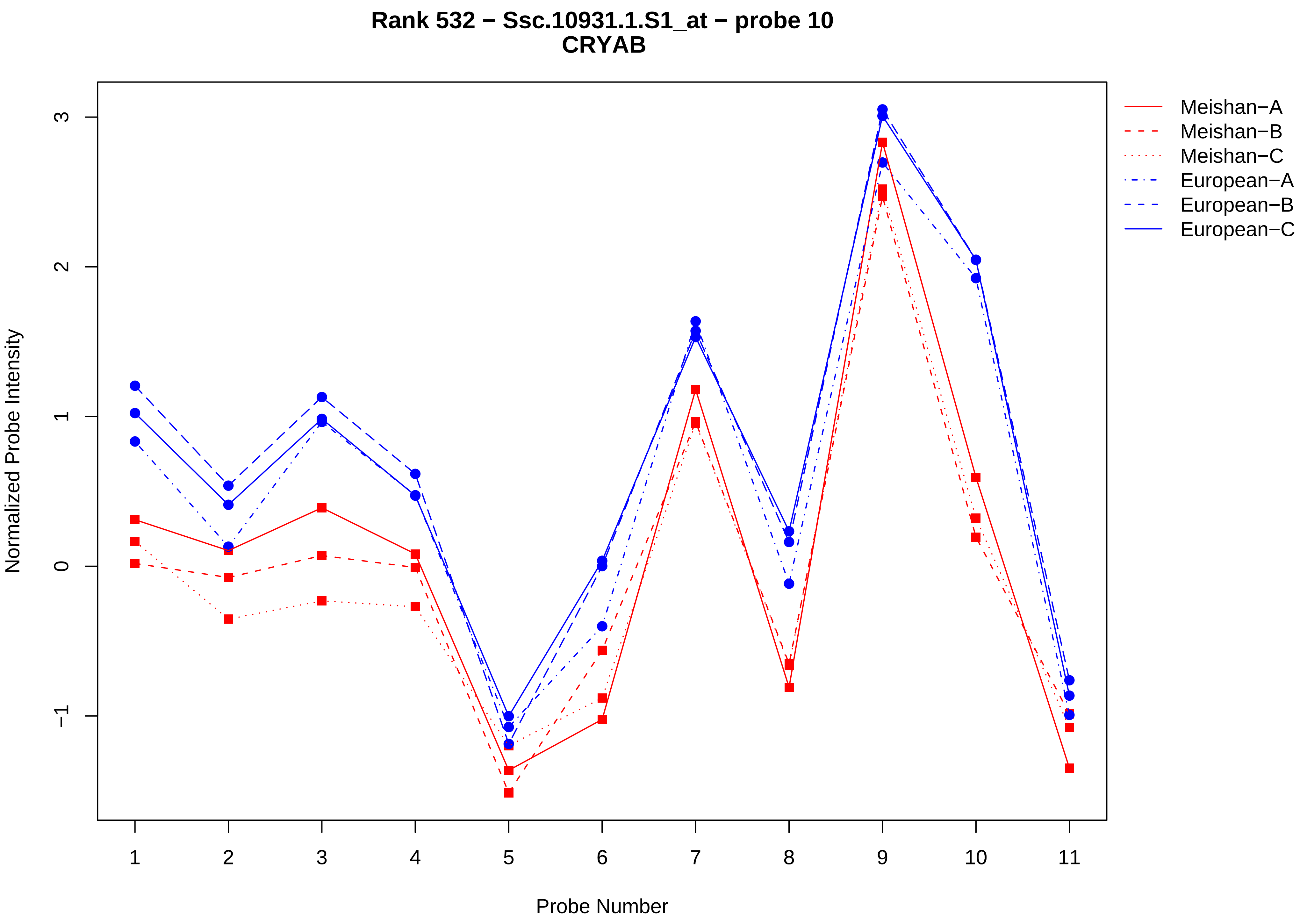

Rank 532 − Ssc.10931.1.S1_at − probe 10
CRYAB
Normalized Probe Intensity
Probe Number
−1
0
1
2
3
1
2
3
4
5
6
7
8
9
10
11
Meishan−A
Meishan−B
Meishan−C
European−A
European−B
European−C

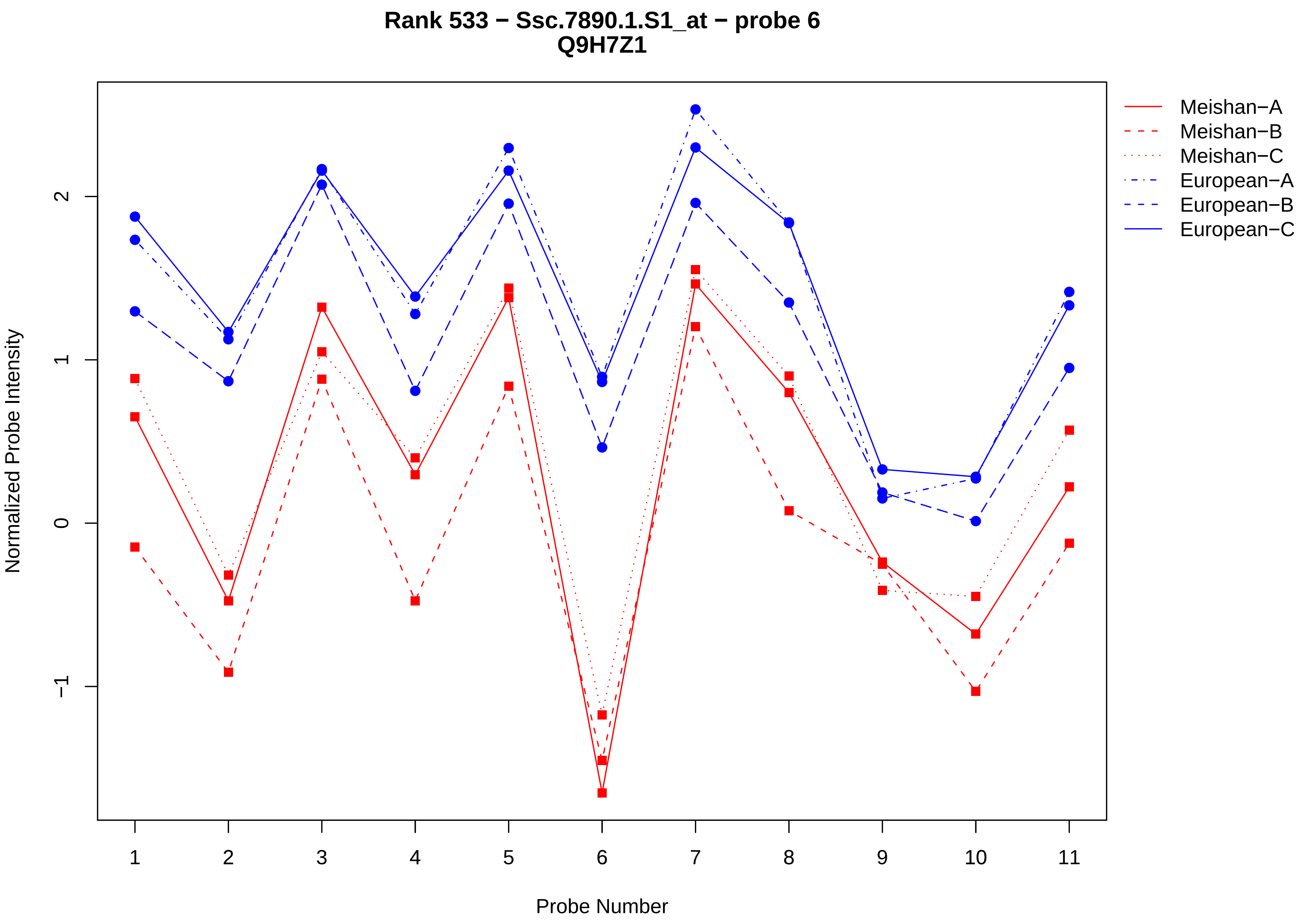

Rank 533 − Ssc.7890.1.S1_at − probe 6
Q9H7Z1
Normalized Probe Intensity
Probe Number
Meishan−A
Meishan−B
Meishan−C
European−A
European−B
European−C
−1
0
1
2
1
2
3
4
5
6
7
8
9
10
11

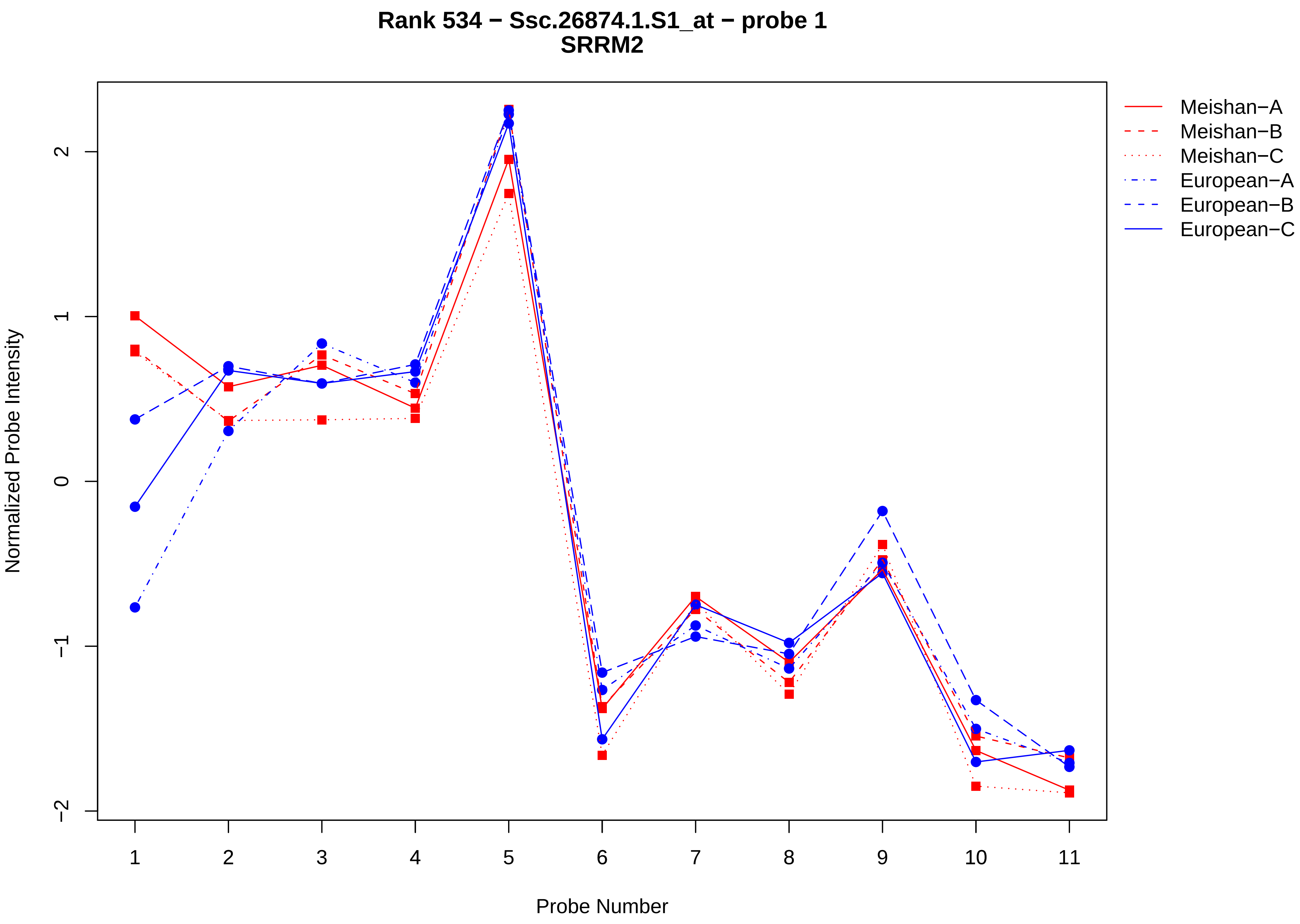

Rank 534 − Ssc.26874.1.S1_at − probe 1
SRRM2
Normalized Probe Intensity
Probe Number
Meishan−A
Meishan−B
Meishan−C
European−A
European−B
European−C

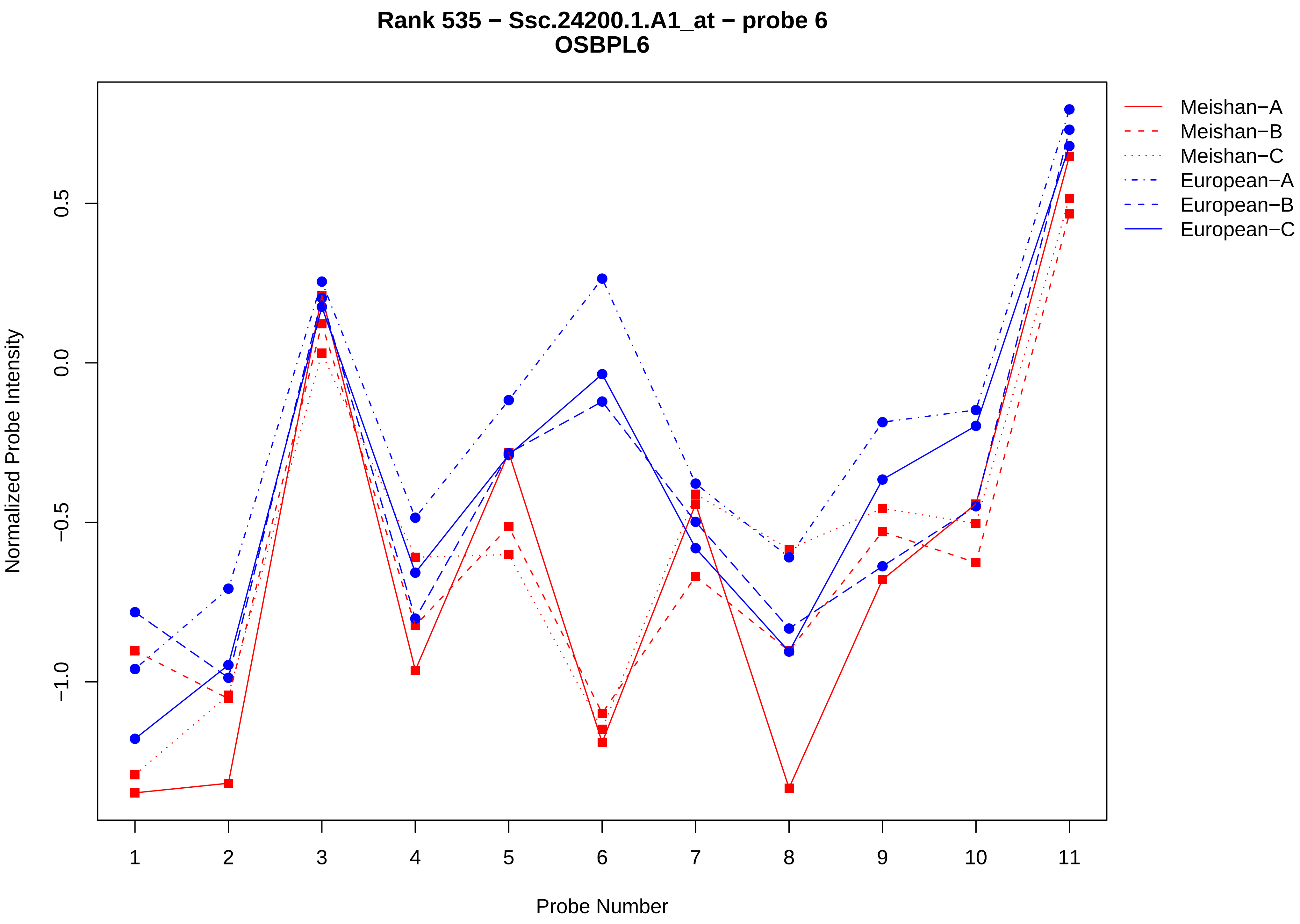

Rank 535 − Ssc.24200.1.A1_at − probe 6
OSBPL6
Normalized Probe Intensity
Probe Number
0.5
0.0
−0.5
−1.0
1
2
3
4
5
6
7
8
9
10
11
Meishan−A
Meishan−B
Meishan−C
European−A
European−B
European−C

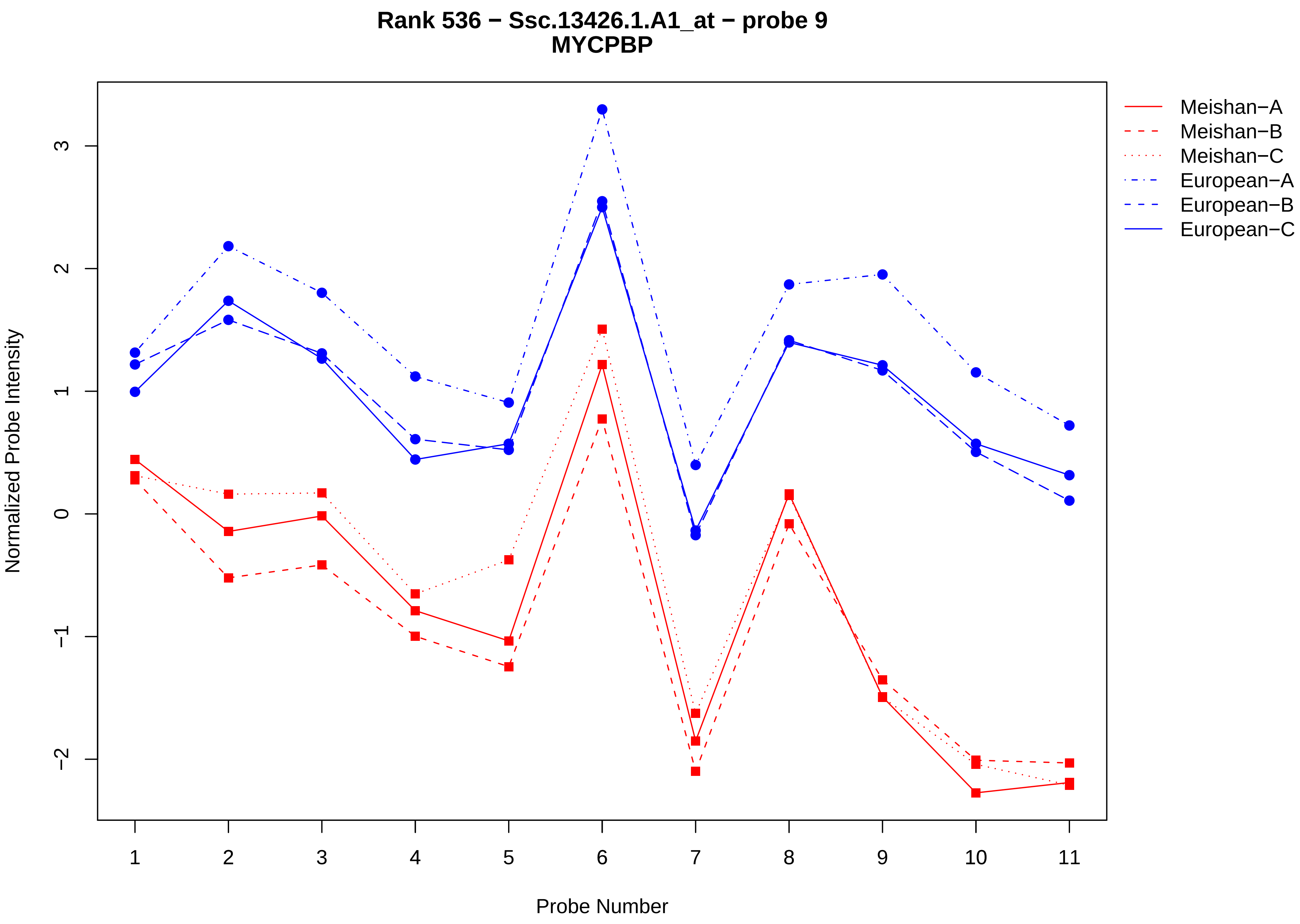
Rank 536 − Ssc.13426.1.A1_at − probe 9
MYCPBP
Normalized Probe Intensity
Probe Number
Meishan−A
Meishan−B
Meishan−C
European−A
European−B
European−C
3
2
1
0
−1
−2
1
2
3
4
5
6
7
8
9
10
11

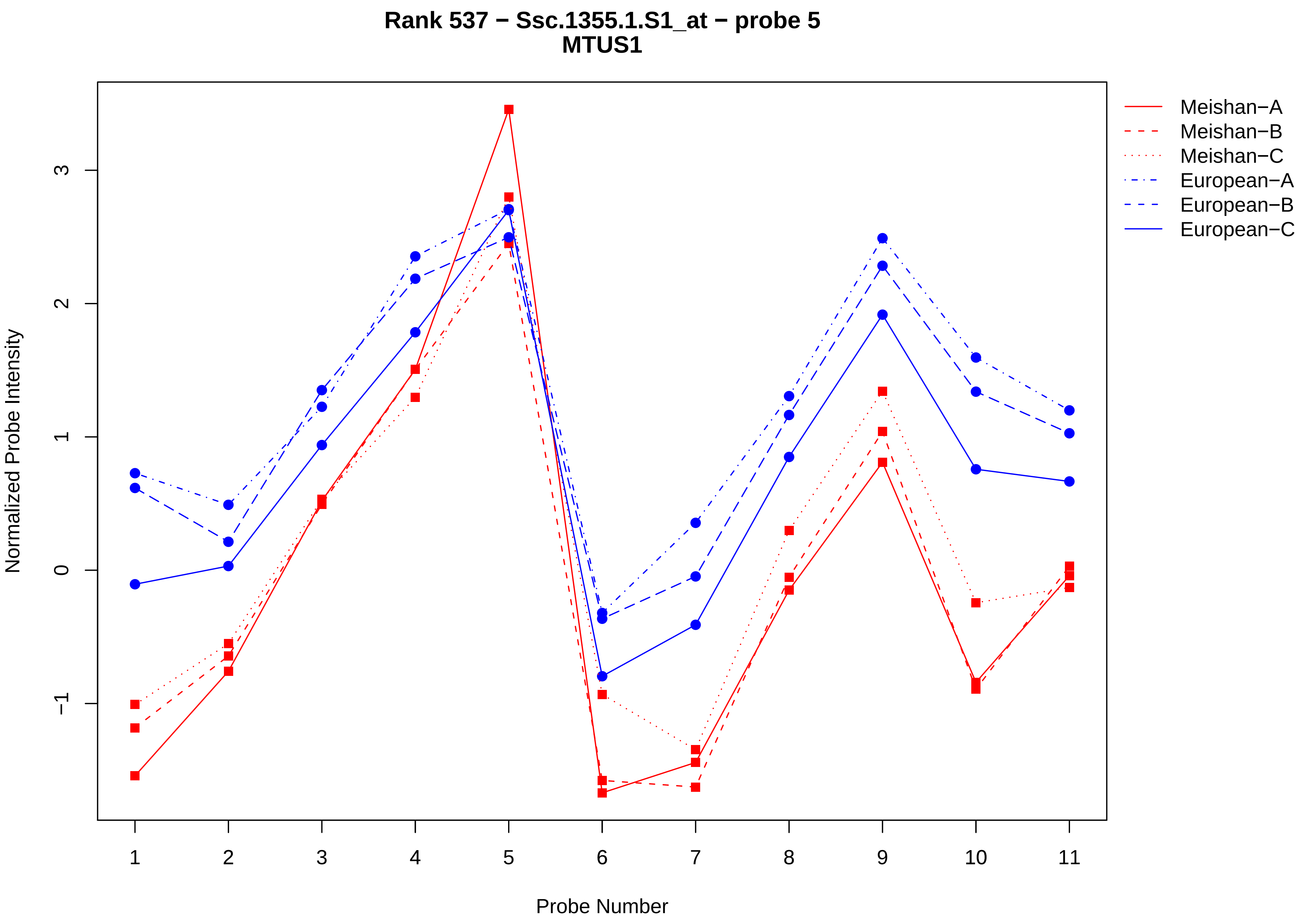
Rank 537 − Ssc.1355.1.S1_at − probe 5
MTUS1
Normalized Probe Intensity
Probe Number
Meishan−A
Meishan−B
Meishan−C
European−A
European−B
European−C

Rank 538 − Ssc.489.1.A1_at − probe 4
Q9HB73

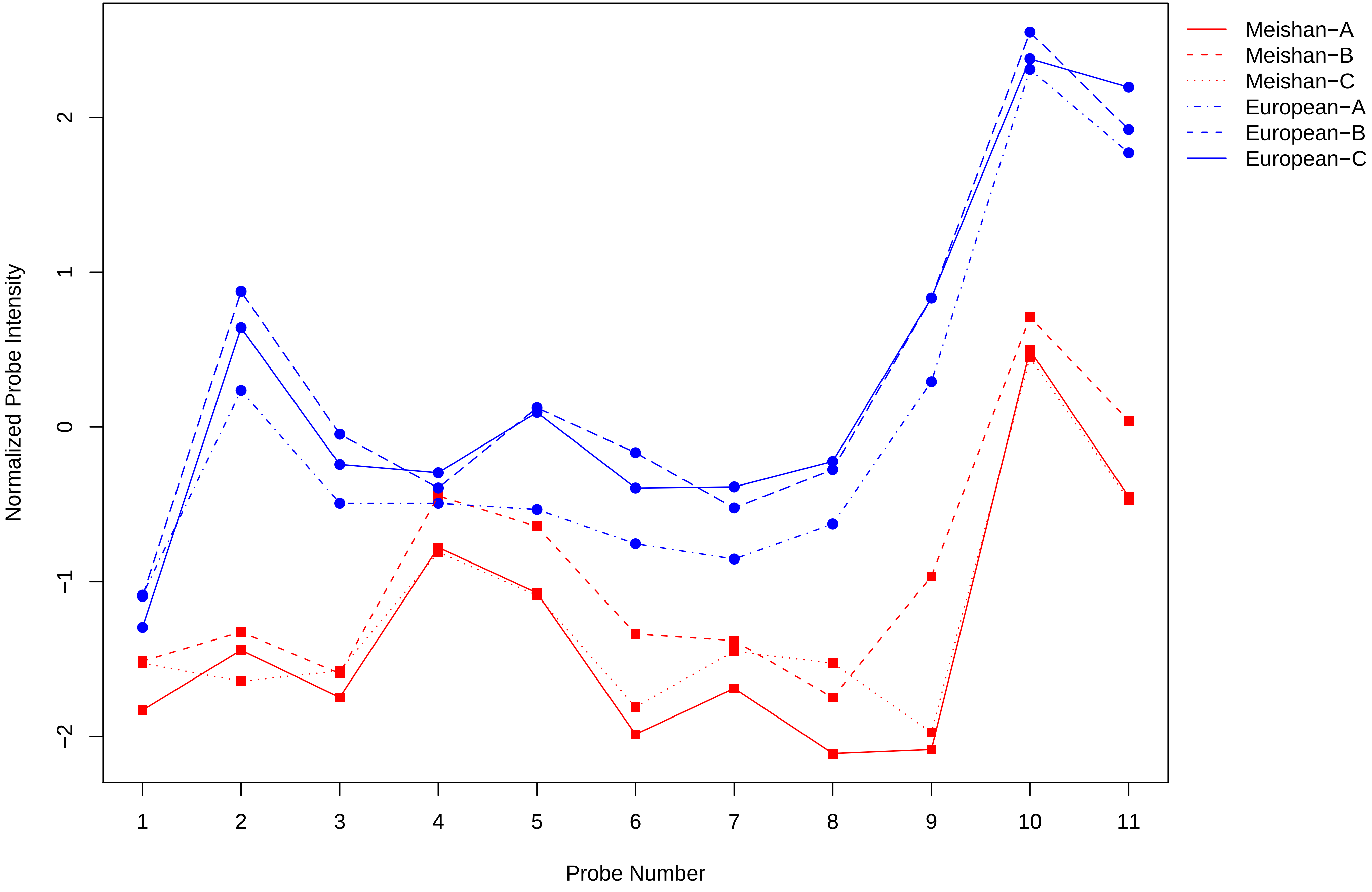

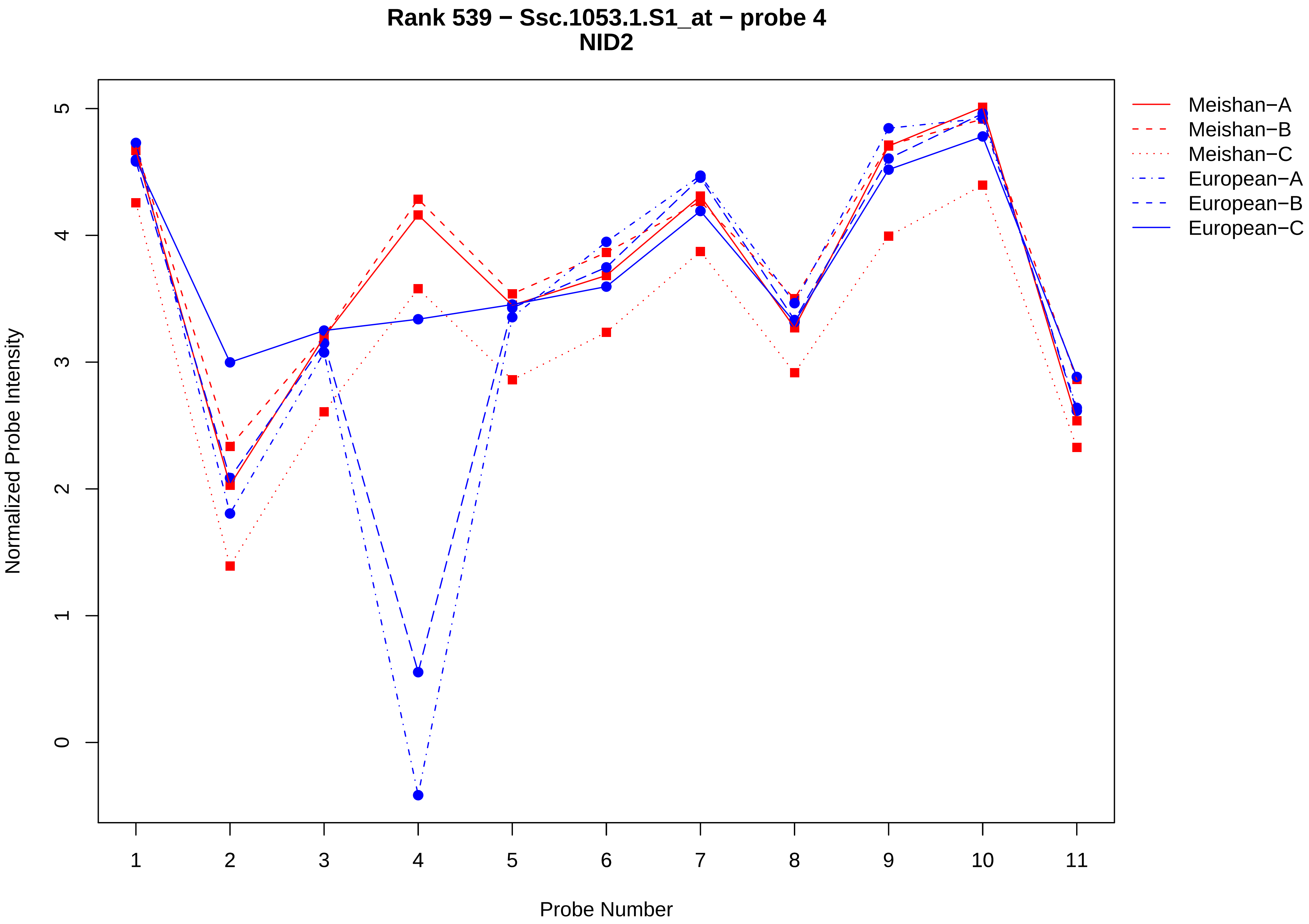
Rank 539 − Ssc.1053.1.S1_at − probe 4
NID2
Normalized Probe Intensity
Probe Number
0
1
2
3
4
5
1
2
3
4
5
6
7
8
9
10
11
Meishan−A
Meishan−B
Meishan−C
European−A
European−B
European−C

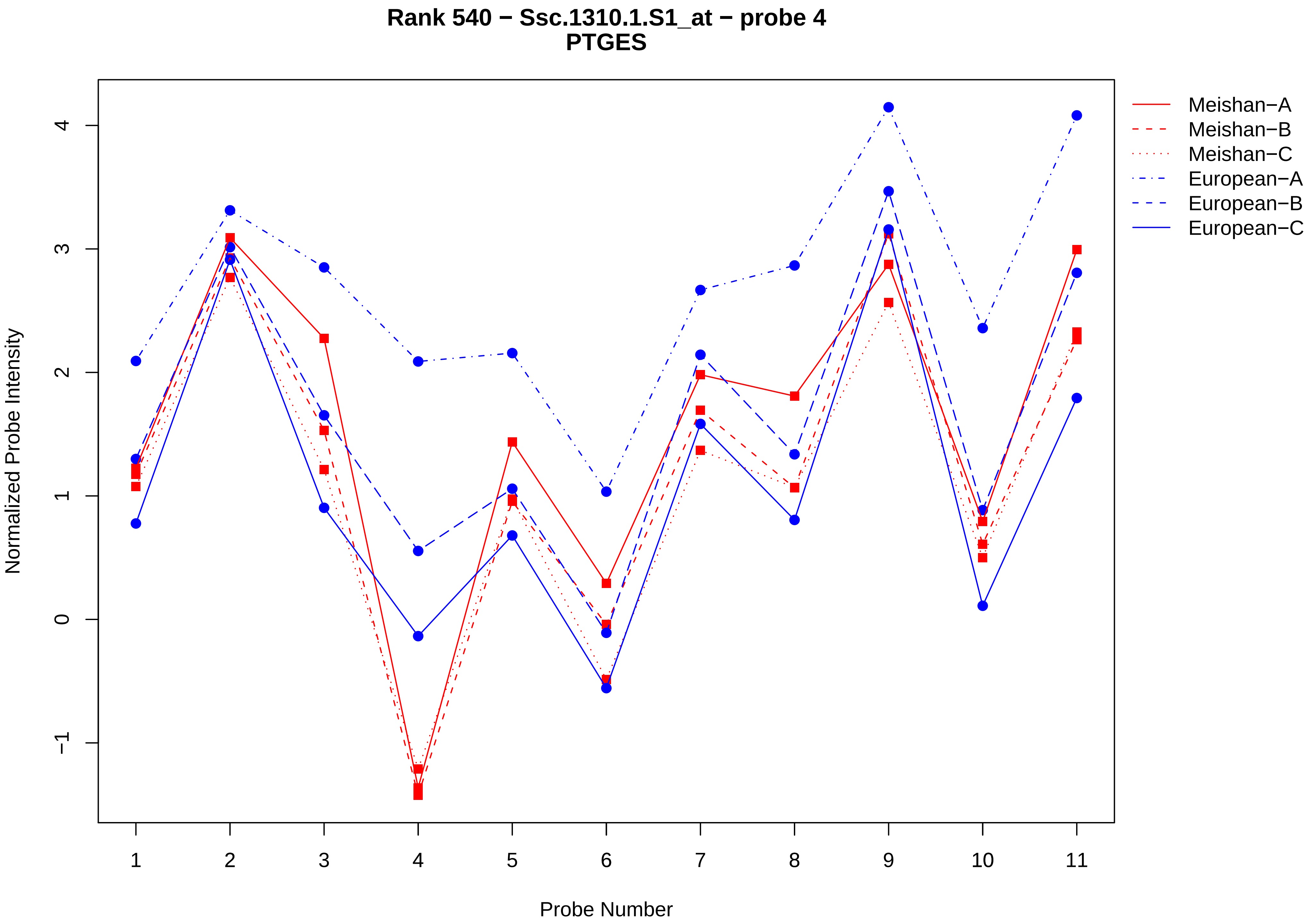

Rank 540 − Ssc.1310.1.S1_at − probe 4
PTGES
Normalized Probe Intensity
Probe Number
Meishan−A
Meishan−B
Meishan−C
European−A
European−B
European−C
−1
0
1
2
3
4
1
2
3
4
5
6
7
8
9
10
11

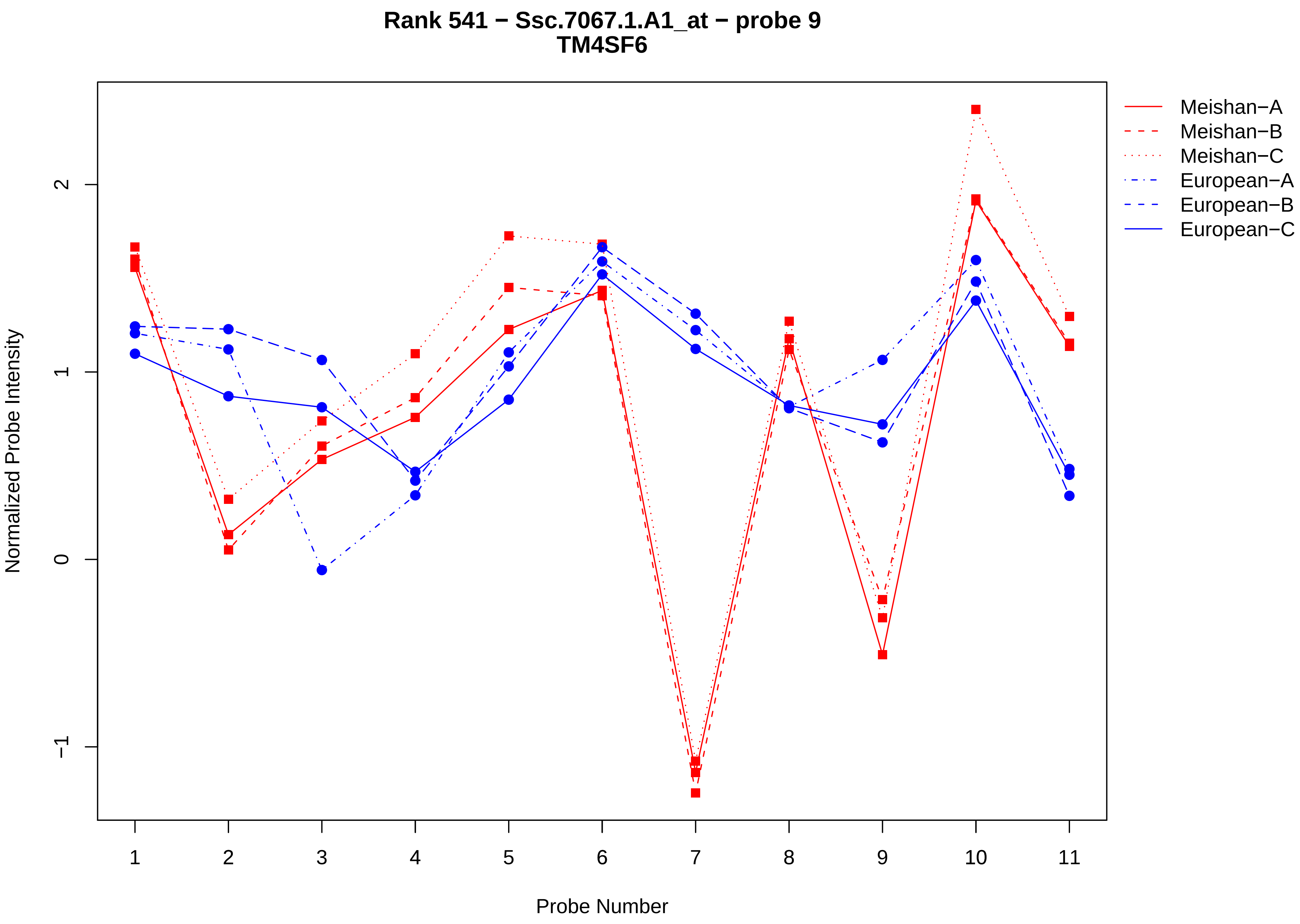

Rank 541 − Ssc.7067.1.A1_at − probe 9
TM4SF6
Normalized Probe Intensity
Probe Number
−1
0
1
2
1
2
3
4
5
6
7
8
9
10
11
Meishan−A
Meishan−B
Meishan−C
European−A
European−B
European−C

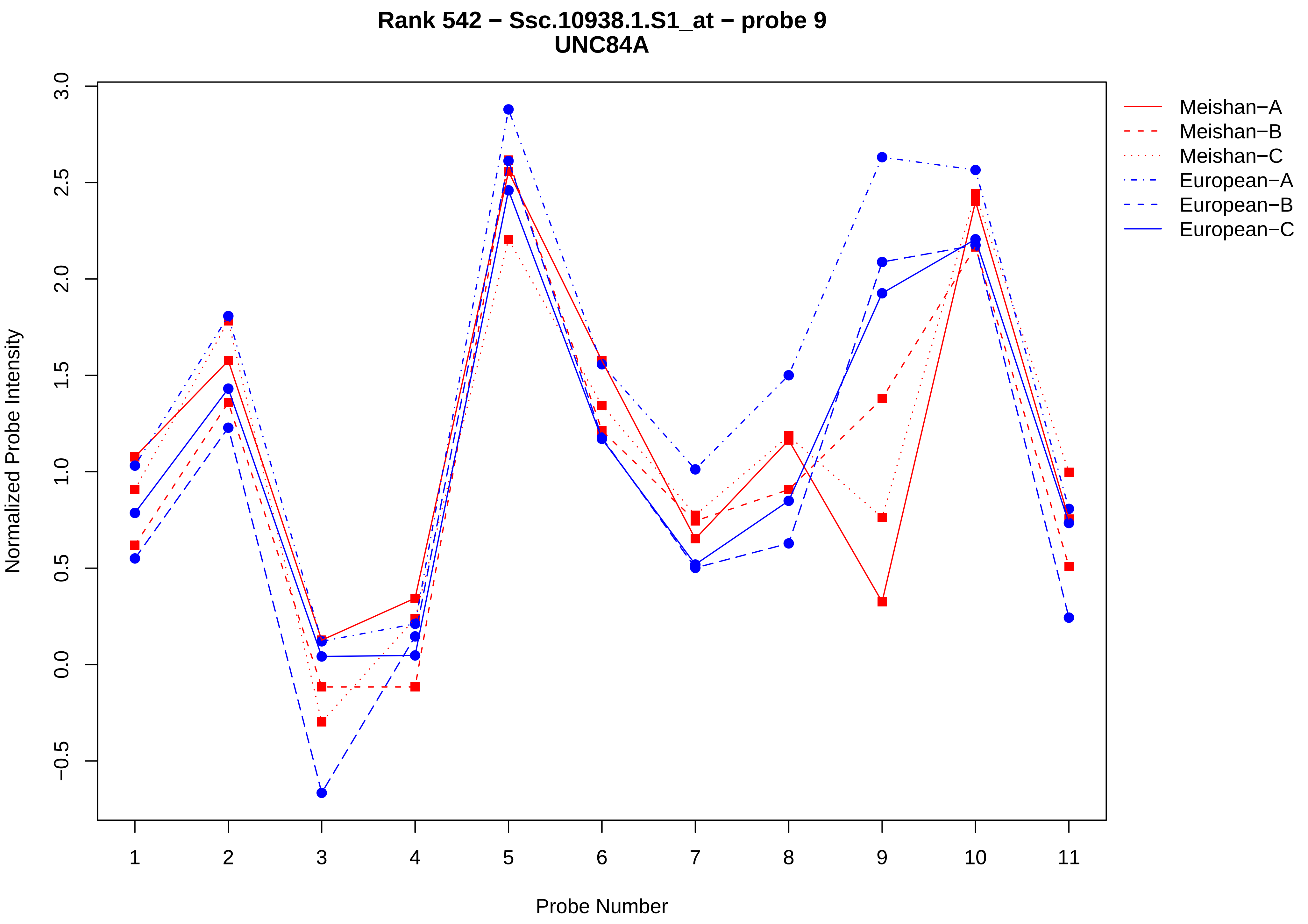

Rank 542 − Ssc.10938.1.S1_at − probe 9
UNC84A
Normalized Probe Intensity
Probe Number
−0.5
0.0
0.5
1.0
1.5
2.0
2.5
3.0
1
2
3
4
5
6
7
8
9
10
11
Meishan−A
Meishan−B
Meishan−C
European−A
European−B
European−C

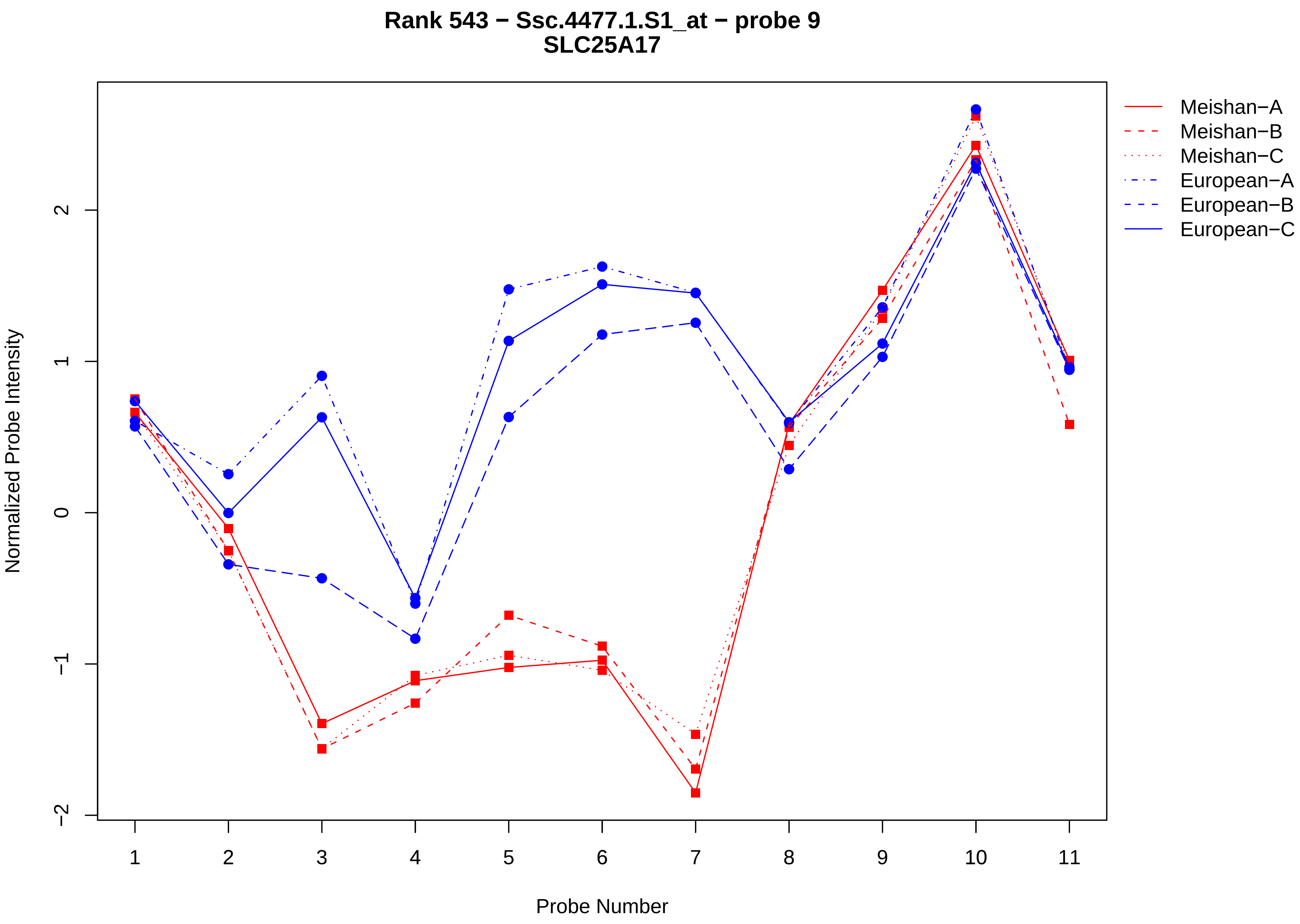

Rank 543 − Ssc.4477.1.S1_at − probe 9
SLC25A17
Normalized Probe Intensity
Probe Number
−2
−1
0
1
2
1
2
3
4
5
6
7
8
9
10
11
Meishan−A
Meishan−B
Meishan−C
European−A
European−B
European−C

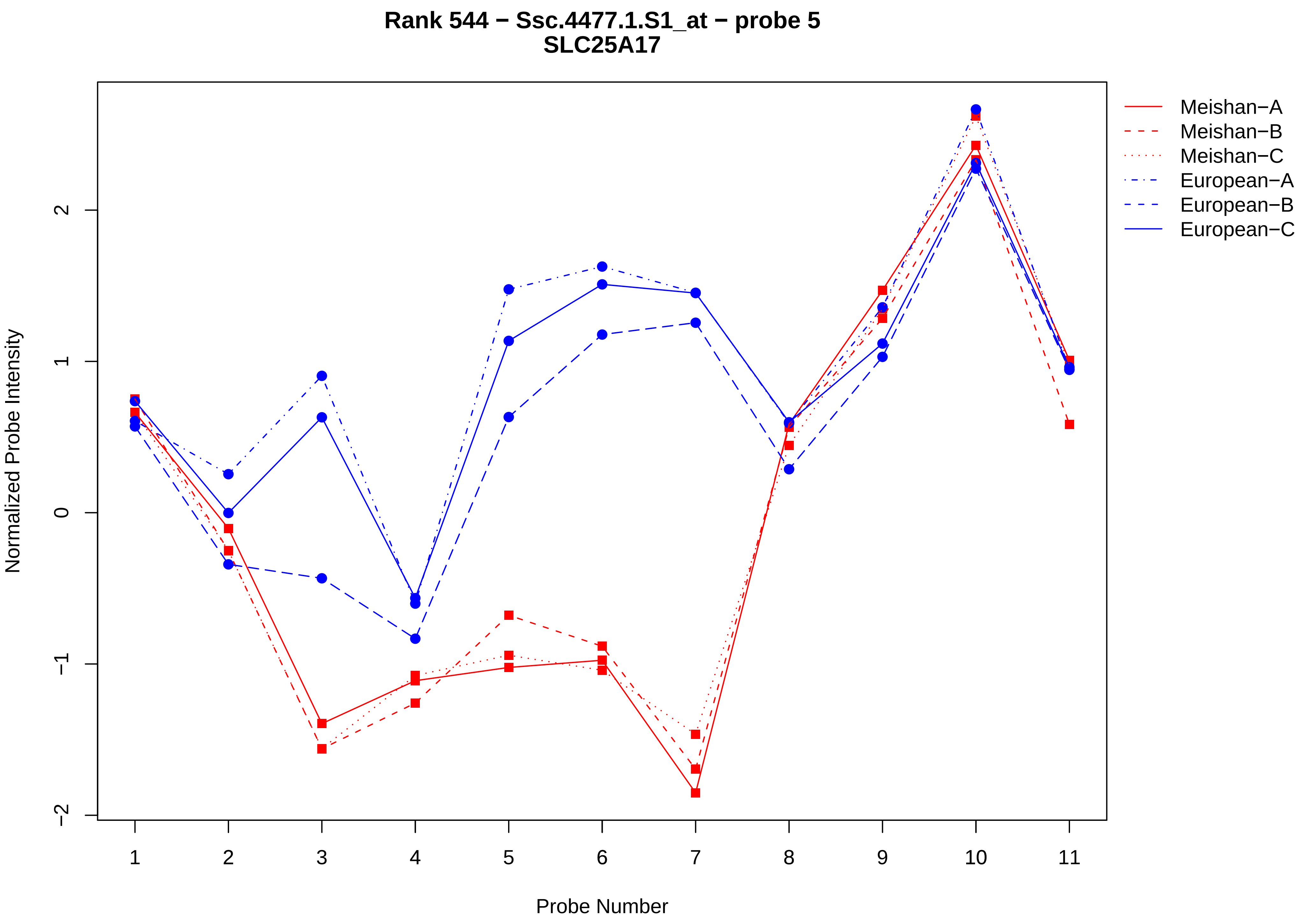
Rank 544 − Ssc.4477.1.S1_at − probe 5
SLC25A17
Normalized Probe Intensity
Probe Number
−2
−1
0
1
2
1
2
3
4
5
6
7
8
9
10
11
Meishan−A
Meishan−B
Meishan−C
European−A
European−B
European−C

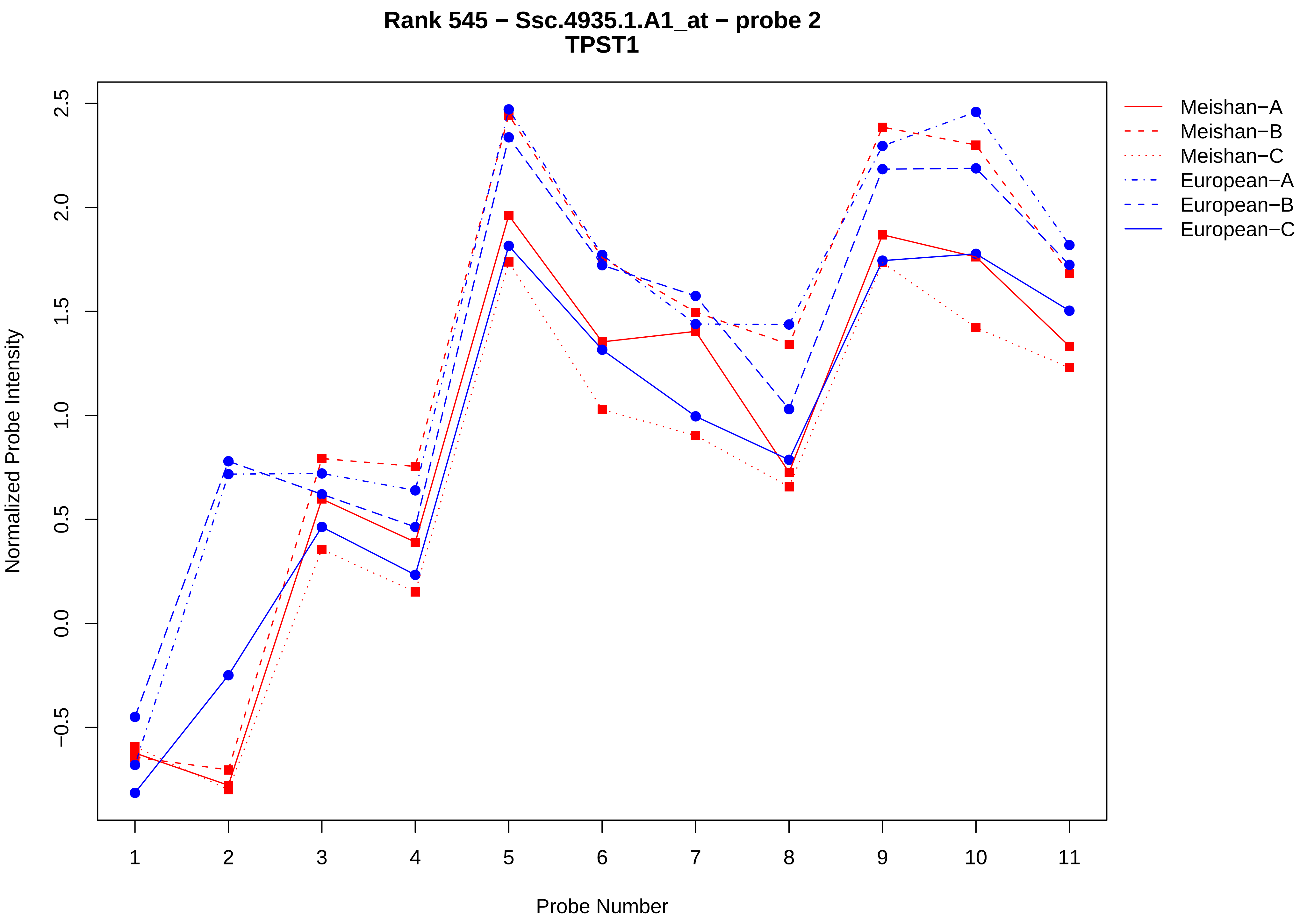

Rank 545 − Ssc.4935.1.A1_at − probe 2
TPST1
Normalized Probe Intensity
−0.5
0.0
0.5
1.0
1.5
2.0
2.5
1
2
3
4
5
6
7
8
9
10
11
Probe Number
Meishan−A
Meishan−B
Meishan−C
European−A
European−B
European−C

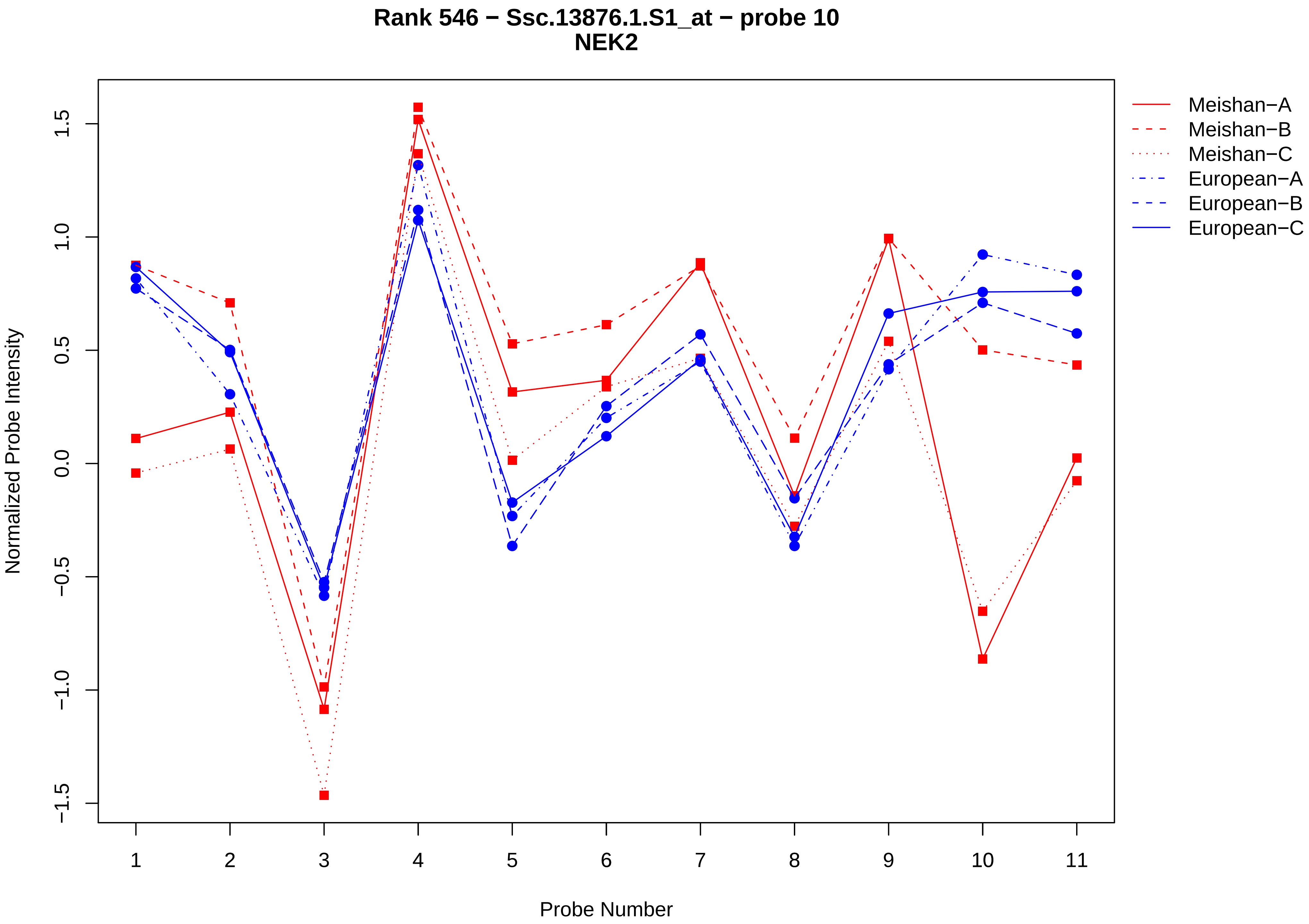

Rank 546 − Ssc.13876.1.S1_at − probe 10
NEK2
Normalized Probe Intensity
Probe Number
Meishan−A
Meishan−B
Meishan−C
European−A
European−B
European−C
1.5
1.0
0.5
0.0
−0.5
−1.0
−1.5
1
2
3
4
5
6
7
8
9
10
11

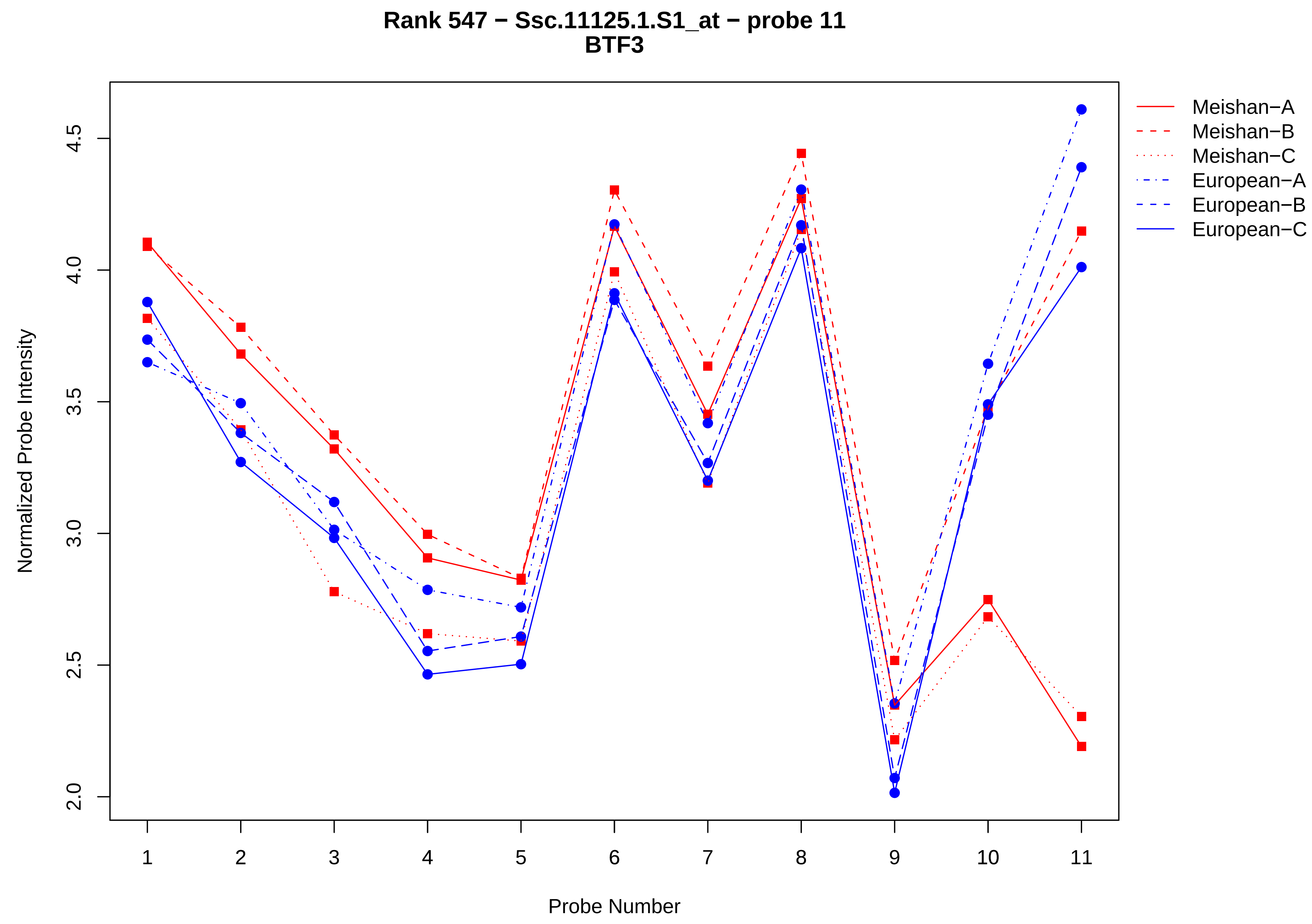

Rank 547 − Ssc.11125.1.S1_at − probe 11
BTF3
Normalized Probe Intensity
Probe Number
Meishan−A
Meishan−B
Meishan−C
European−A
European−B
European−C
2.0
2.5
3.0
3.5
4.0
4.5
1
2
3
4
5
6
7
8
9
10
11

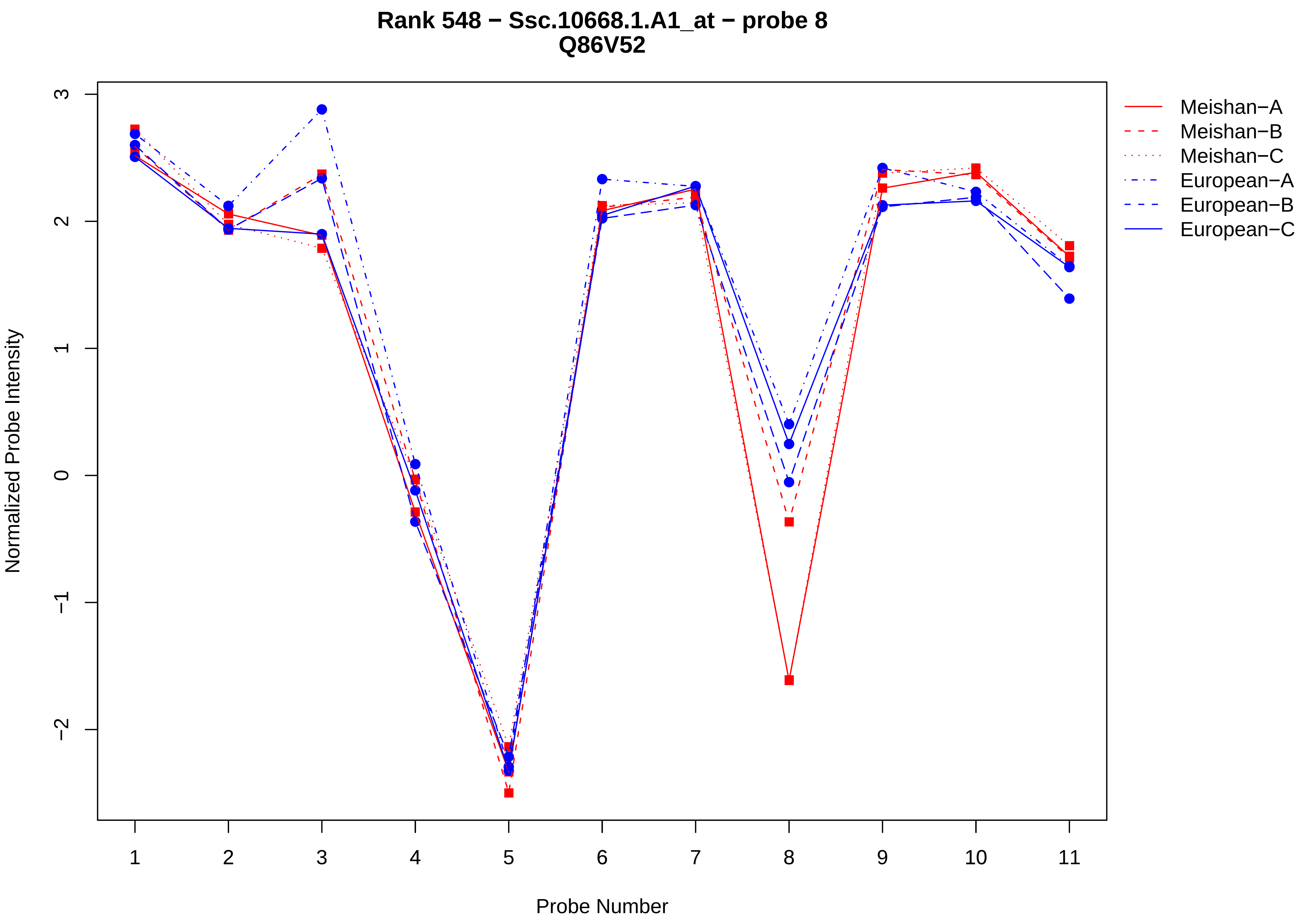

Rank 548 − Ssc.10668.1.A1_at − probe 8
Q86V52
Normalized Probe Intensity
Probe Number
−2
−1
0
1
2
3
1
2
3
4
5
6
7
8
9
10
11
Meishan−A
Meishan−B
Meishan−C
European−A
European−B
European−C

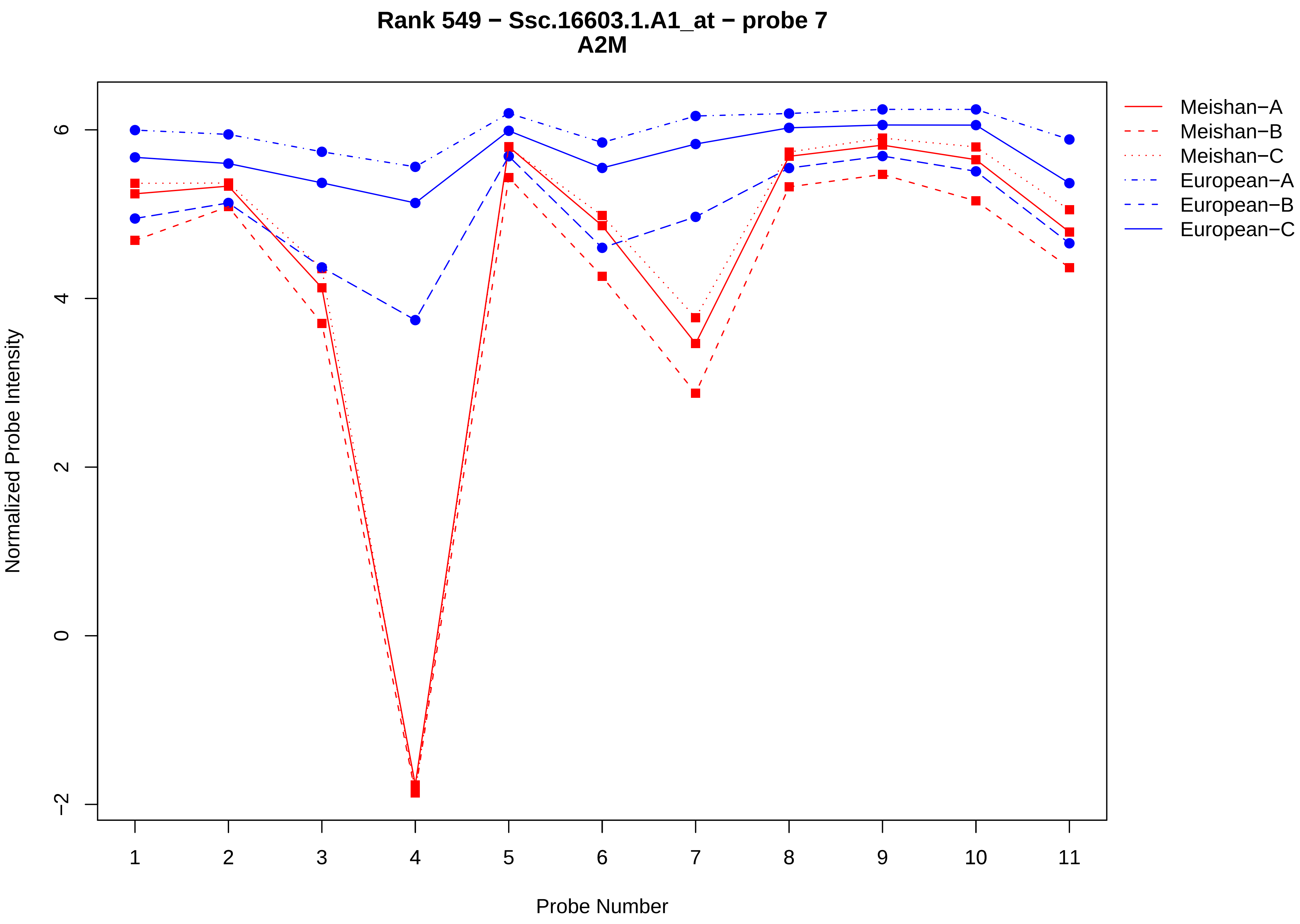

Rank 549 − Ssc.16603.1.A1_at − probe 7
A2M
Normalized Probe Intensity
Probe Number
−2
0
2
4
6
1
2
3
4
5
6
7
8
9
10
11
Meishan−A
Meishan−B
Meishan−C
European−A
European−B
European−C

Rank 550 − Ssc.10054.1.S1_at − probe 3
O94936

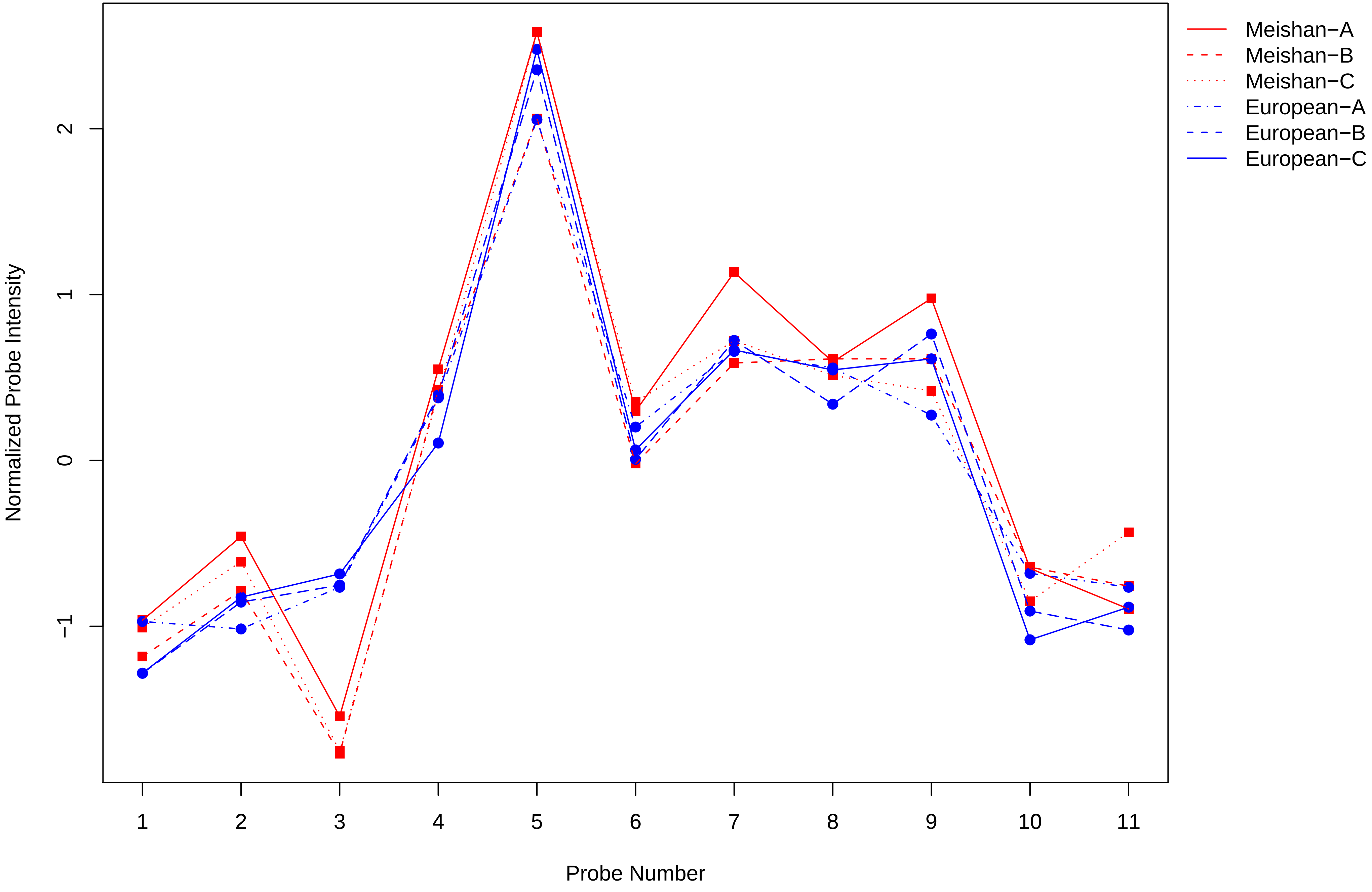

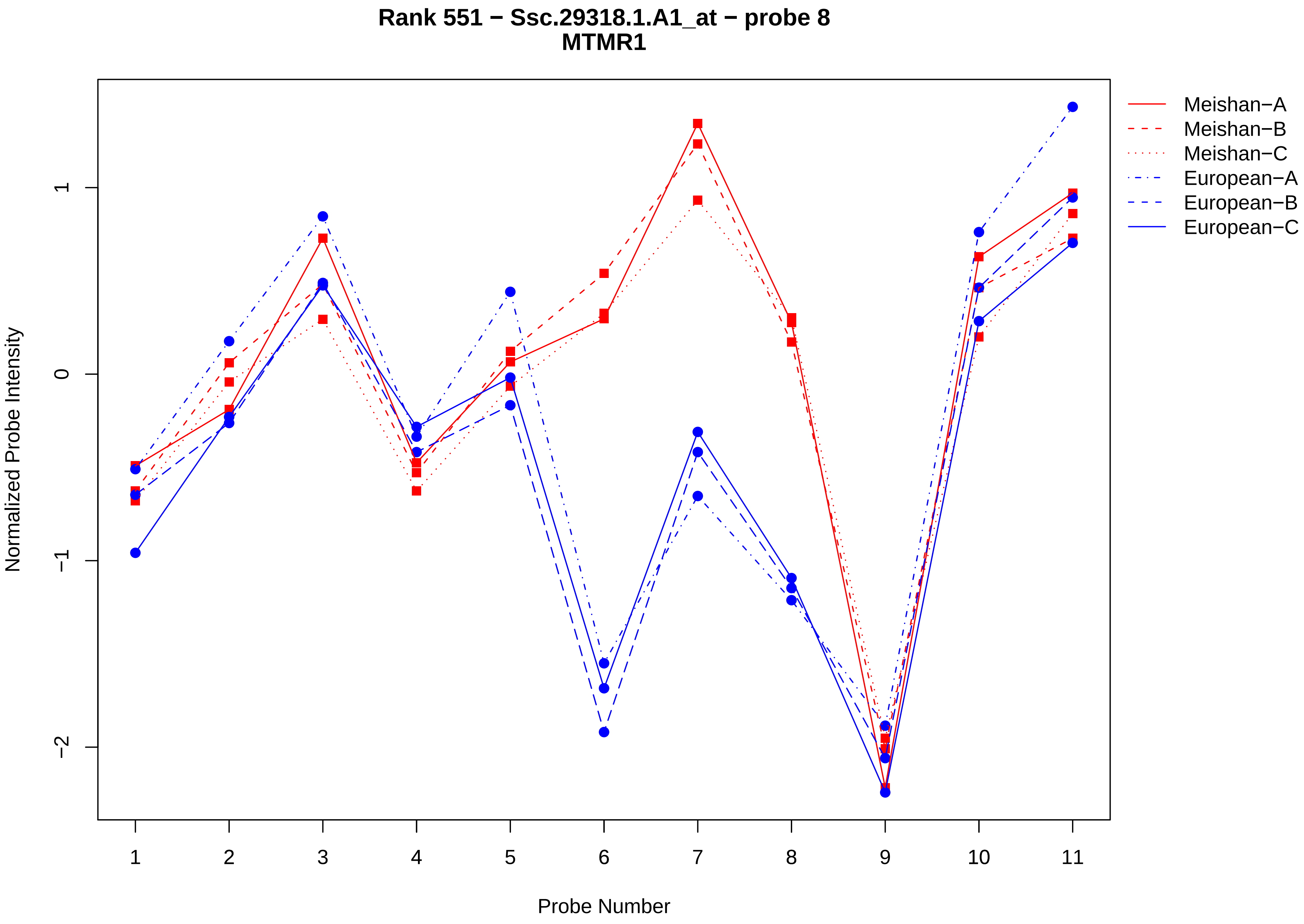

Rank 551 − Ssc.29318.1.A1_at − probe 8
MTMR1
Normalized Probe Intensity
Probe Number
−2
−1
0
1
1
2
3
4
5
6
7
8
9
10
11
Meishan−A
Meishan−B
Meishan−C
European−A
European−B
European−C

Rank 552 − Ssc.1742.1.S1_at − probe 11
Q15453

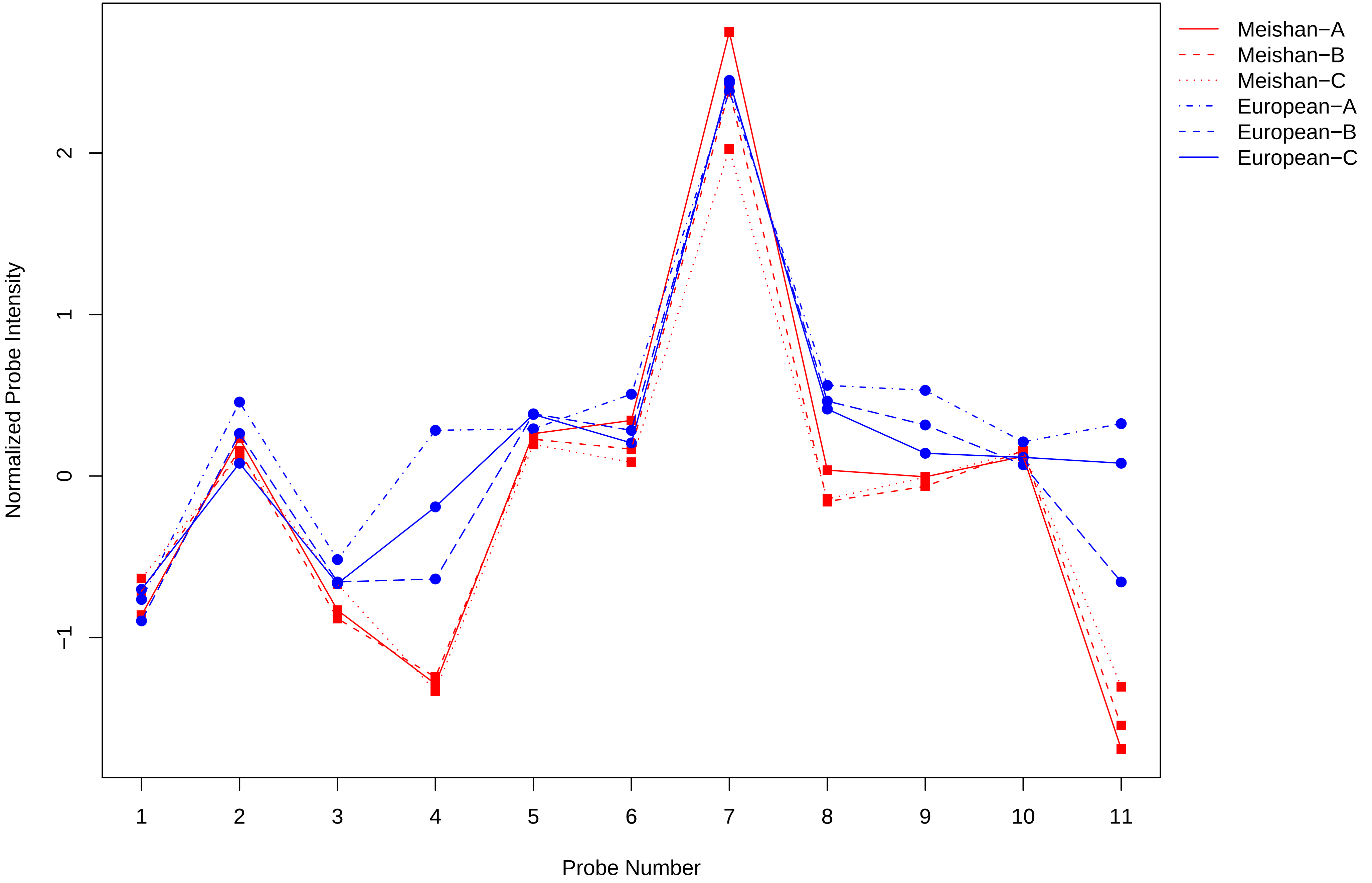

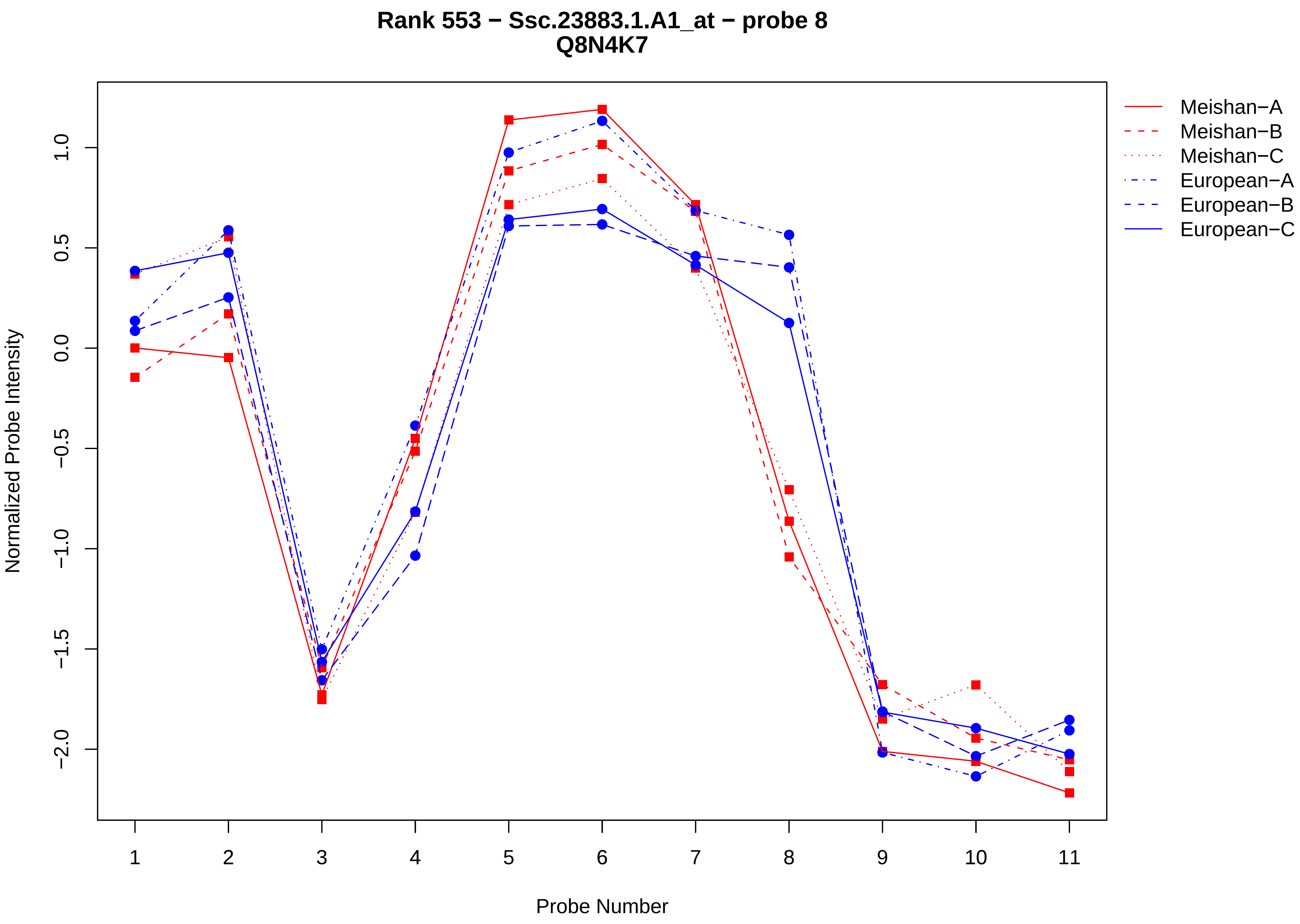
Rank 553 − Ssc.23883.1.A1_at − probe 8
Q8N4K7
Normalized Probe Intensity
Probe Number
Meishan−A
Meishan−B
Meishan−C
European−A
European−B
European−C

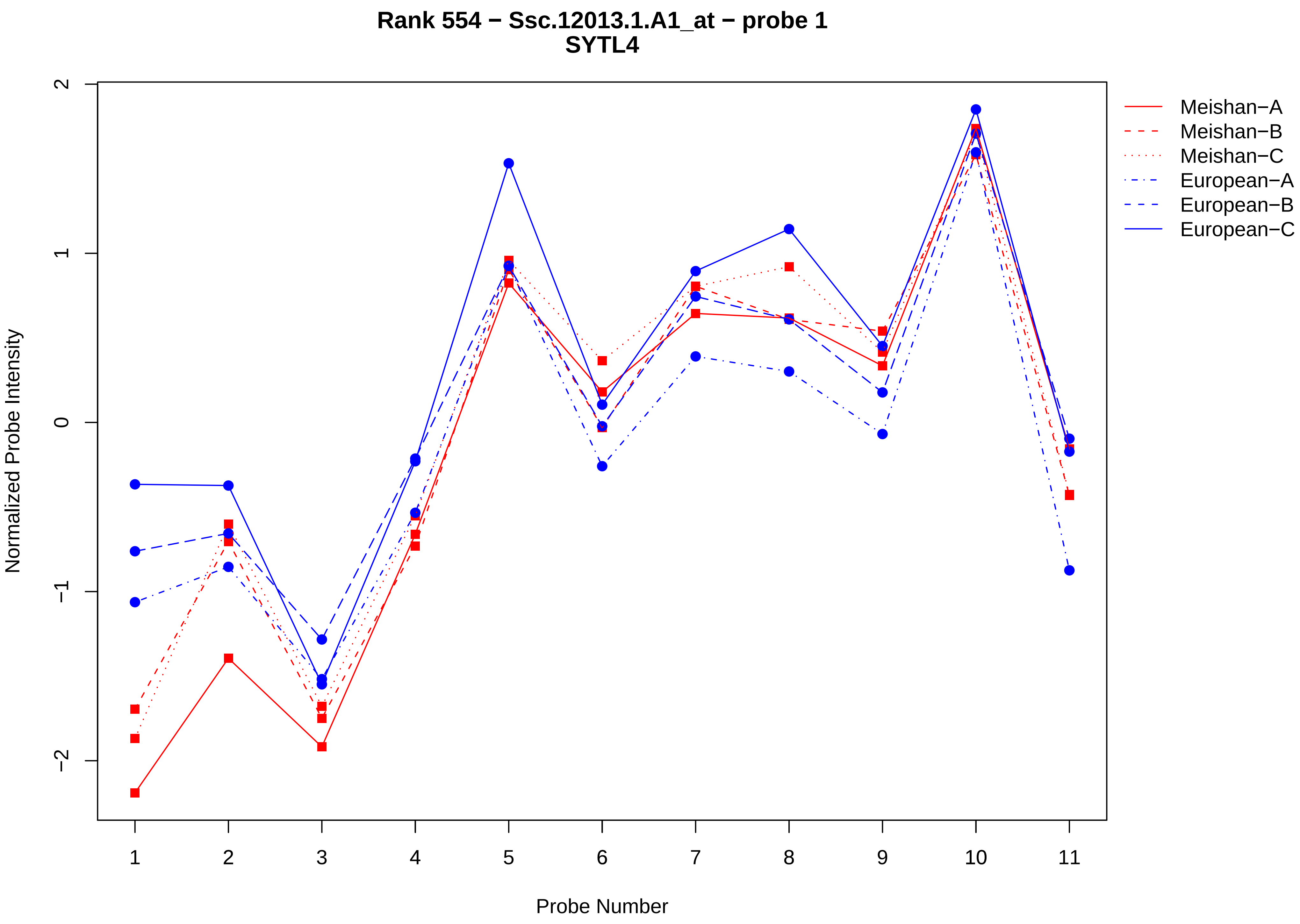

Rank 554 − Ssc.12013.1.A1_at − probe 1
SYTL4
Normalized Probe Intensity
Probe Number
Meishan−A
Meishan−B
Meishan−C
European−A
European−B
European−C

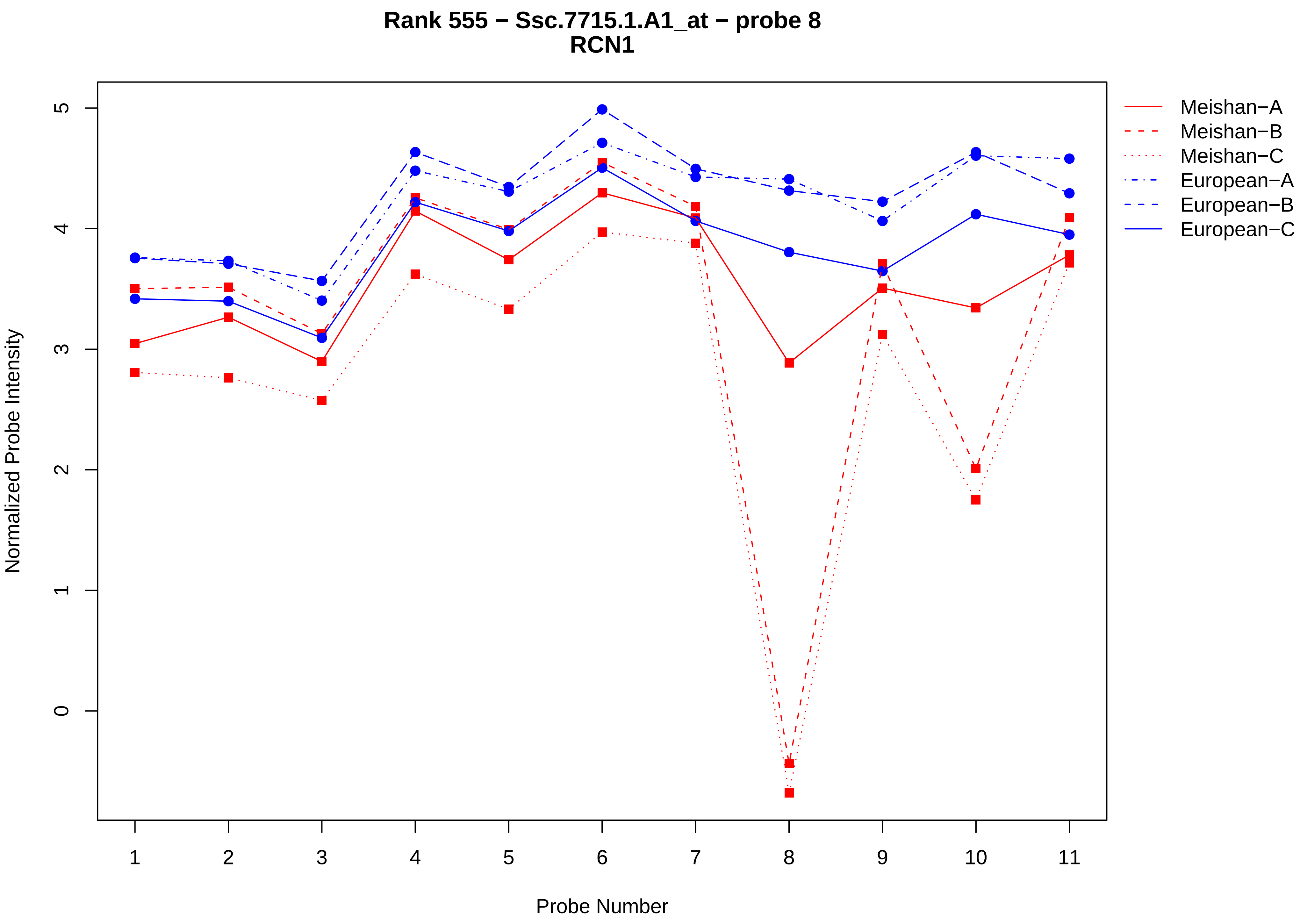

Rank 555 − Ssc.7715.1.A1_at − probe 8
RCN1
Normalized Probe Intensity
Probe Number
0
1
2
3
4
5
1
2
3
4
5
6
7
8
9
10
11
Meishan−A
Meishan−B
Meishan−C
European−A
European−B
European−C

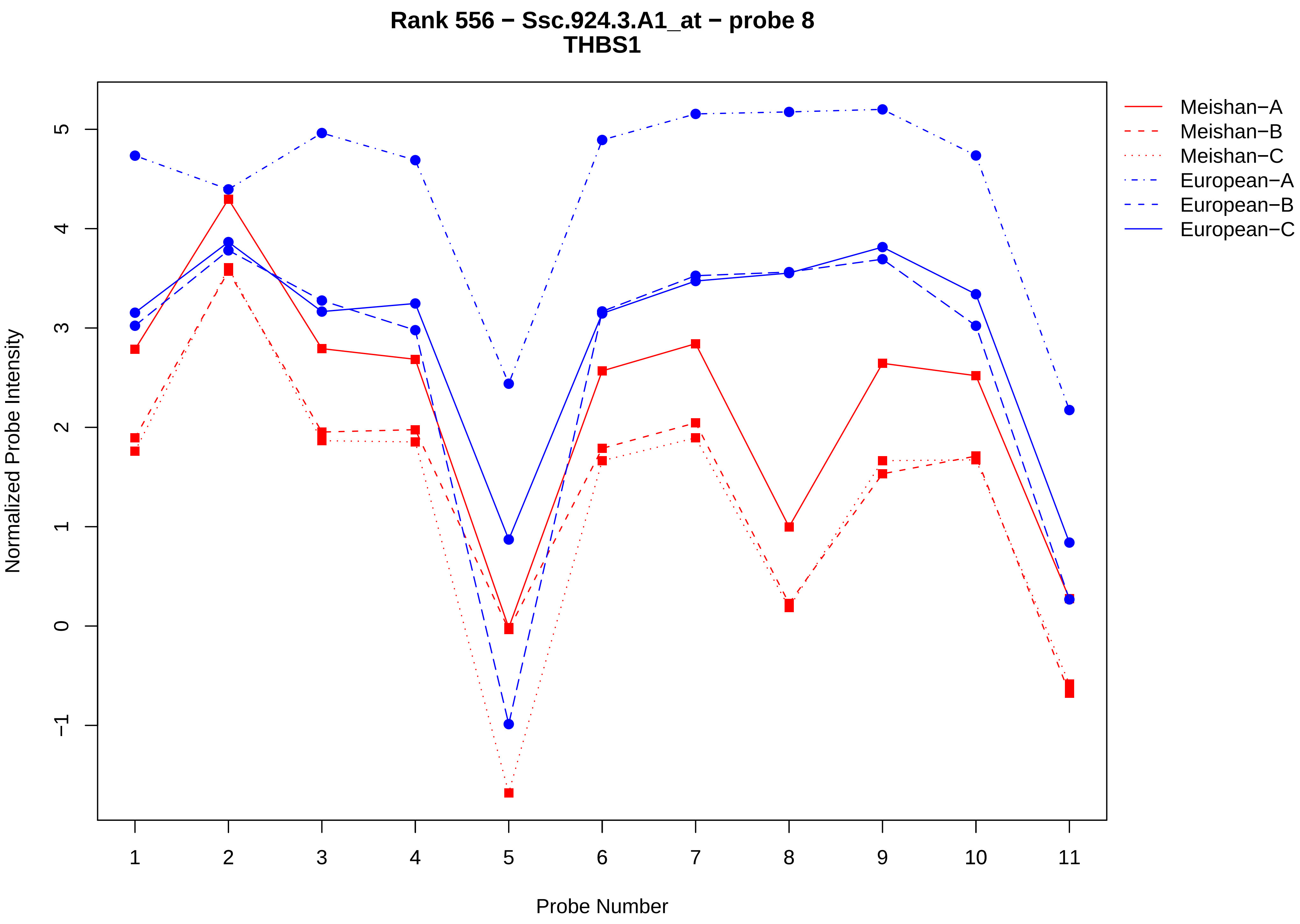
Rank 556 − Ssc.924.3.A1_at − probe 8
THBS1
Normalized Probe Intensity
Probe Number
Meishan−A
Meishan−B
Meishan−C
European−A
European−B
European−C
−1
0
1
2
3
4
5
1
2
3
4
5
6
7
8
9
10
11

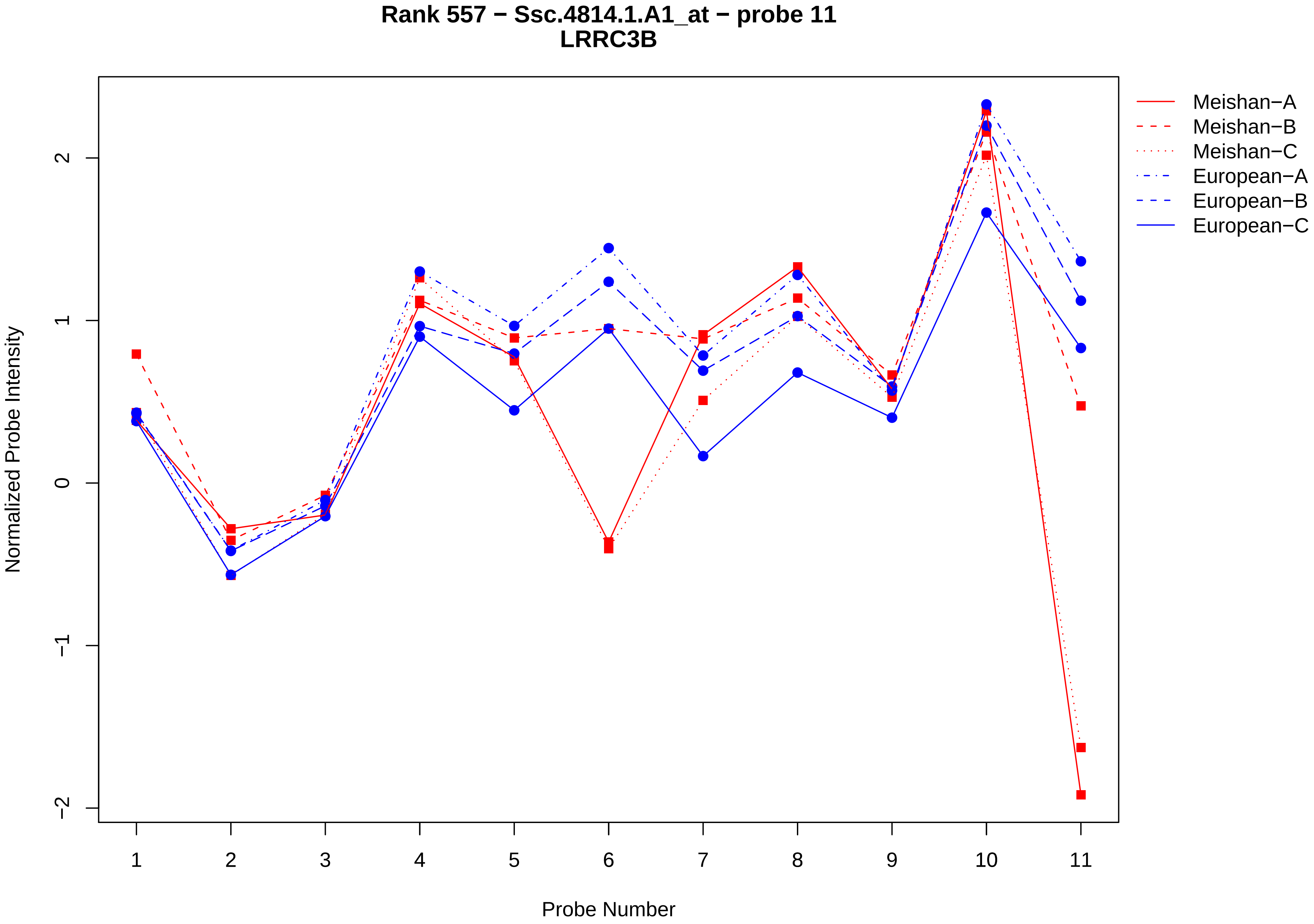

Rank 557 − Ssc.4814.1.A1_at − probe 11
LRRC3B
Normalized Probe Intensity
Probe Number
Meishan−A
Meishan−B
Meishan−C
European−A
European−B
European−C
−2
−1
0
1
2
1
2
3
4
5
6
7
8
9
10
11

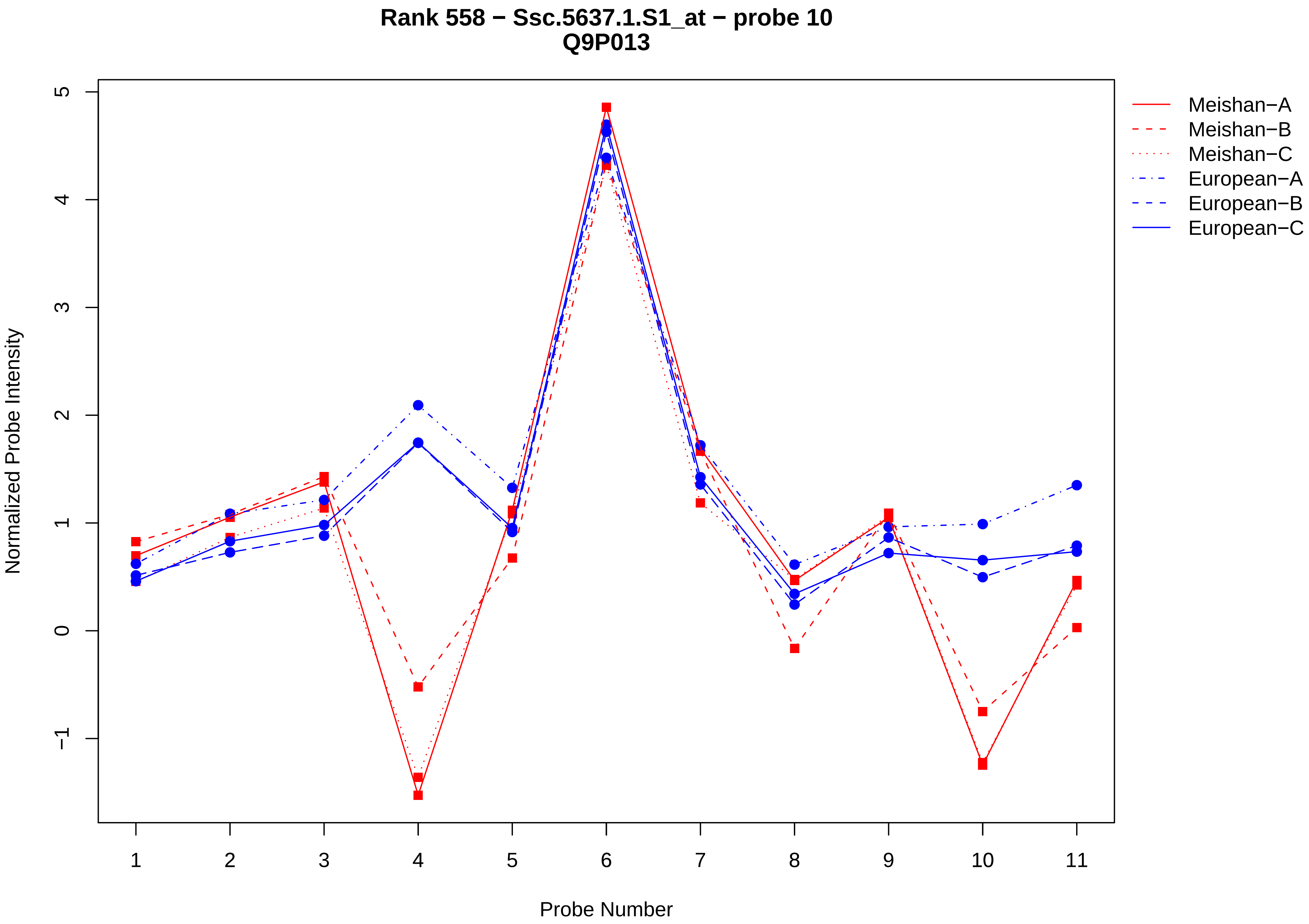
Rank 558 − Ssc.5637.1.S1_at − probe 10
Q9P013
Normalized Probe Intensity
Probe Number
Meishan−A
Meishan−B
Meishan−C
European−A
European−B
European−C
−1
0
1
2
3
4
5
1
2
3
4
5
6
7
8
9
10
11

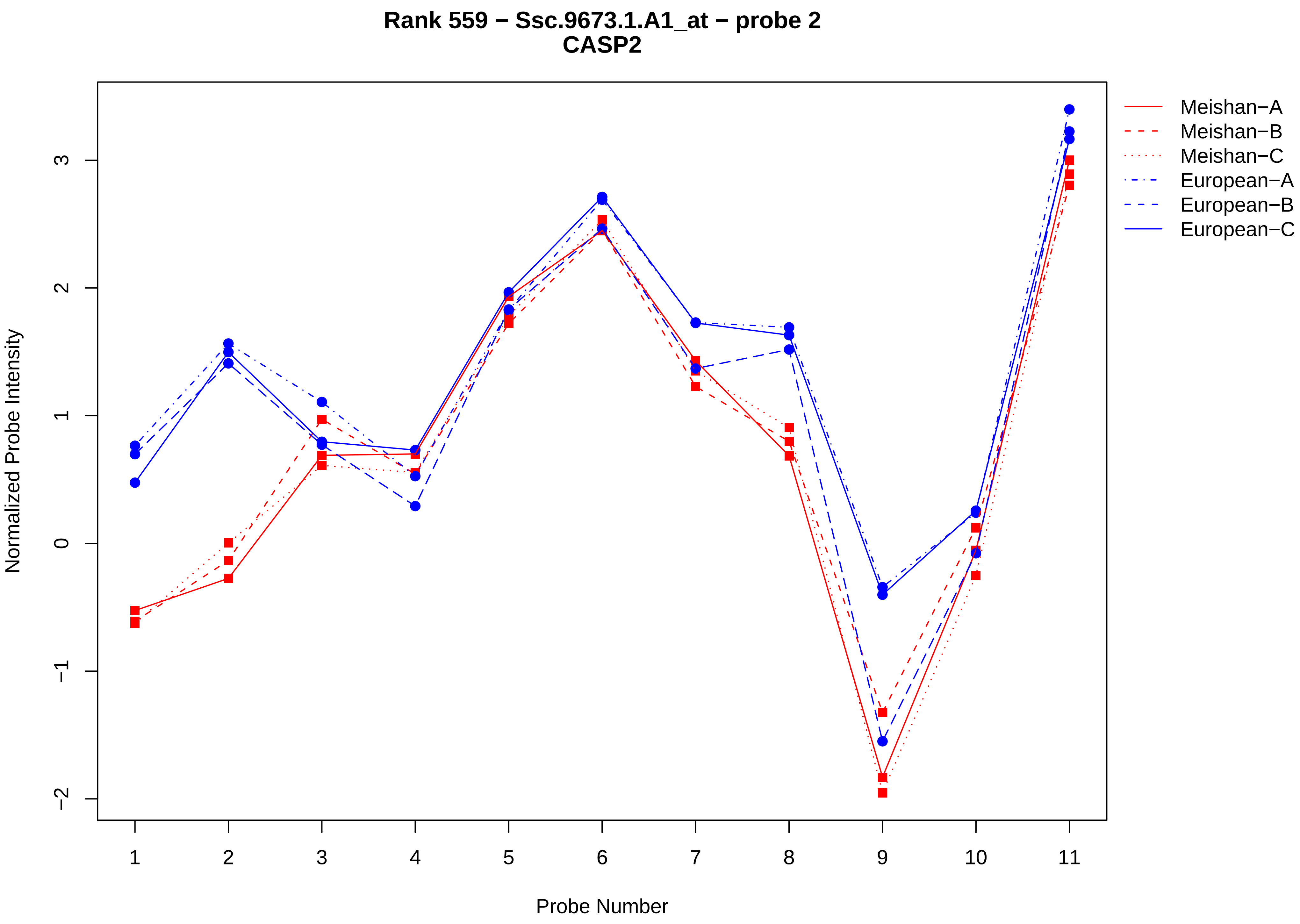
Rank 559 − Ssc.9673.1.A1_at − probe 2
CASP2
Normalized Probe Intensity
Probe Number
−2
−1
0
1
2
3
1
2
3
4
5
6
7
8
9
10
11
Meishan−A
Meishan−B
Meishan−C
European−A
European−B
European−C

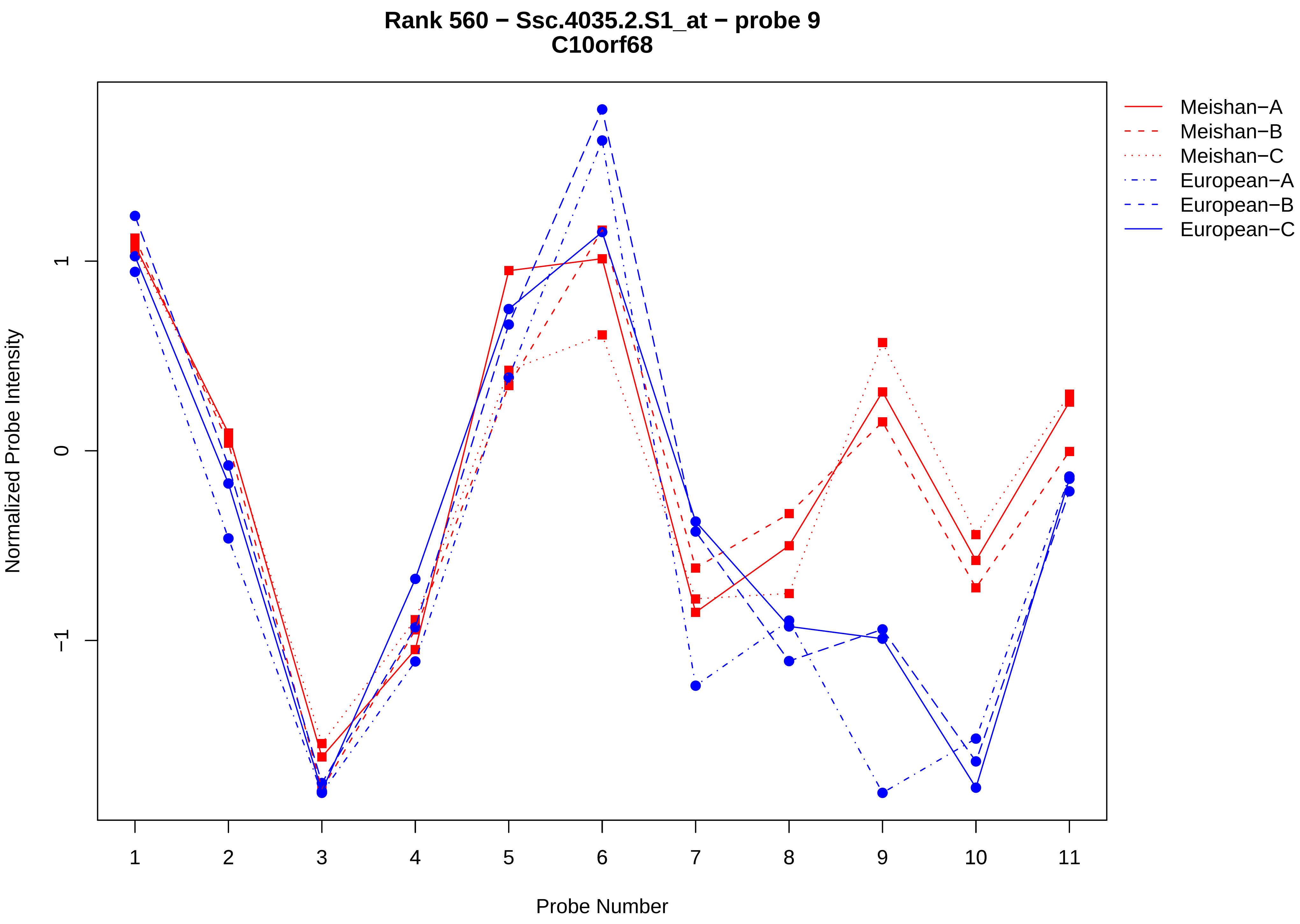

Rank 560 − Ssc.4035.2.S1_at − probe 9
C10orf68
Normalized Probe Intensity
Probe Number
1
0
−1
1
2
3
4
5
6
7
8
9
10
11
Meishan−A
Meishan−B
Meishan−C
European−A
European−B
European−C

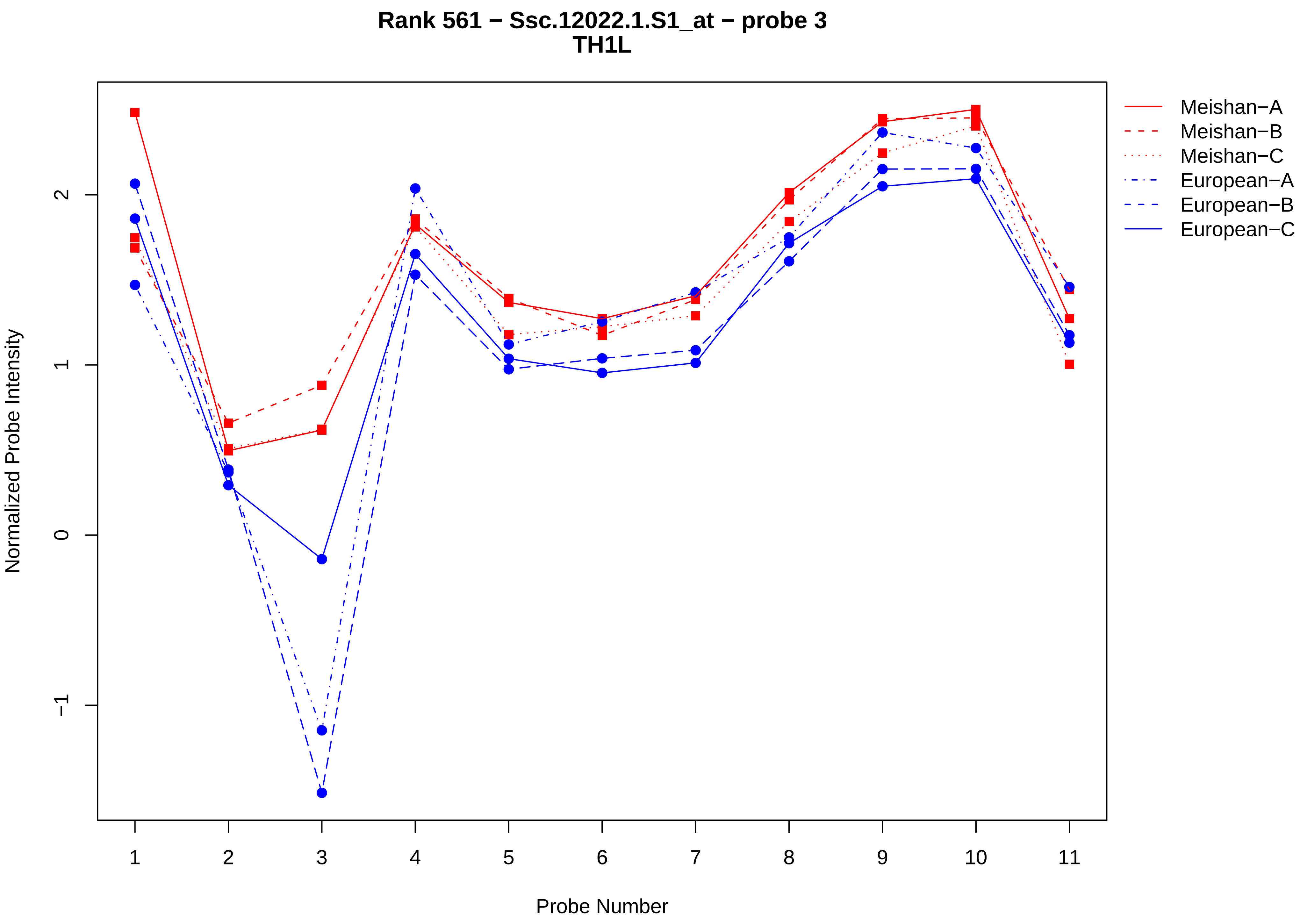

Rank 561 − Ssc.12022.1.S1_at − probe 3
TH1L
Normalized Probe Intensity
Probe Number
−1
0
1
2
1
2
3
4
5
6
7
8
9
10
11
Meishan−A
Meishan−B
Meishan−C
European−A
European−B
European−C

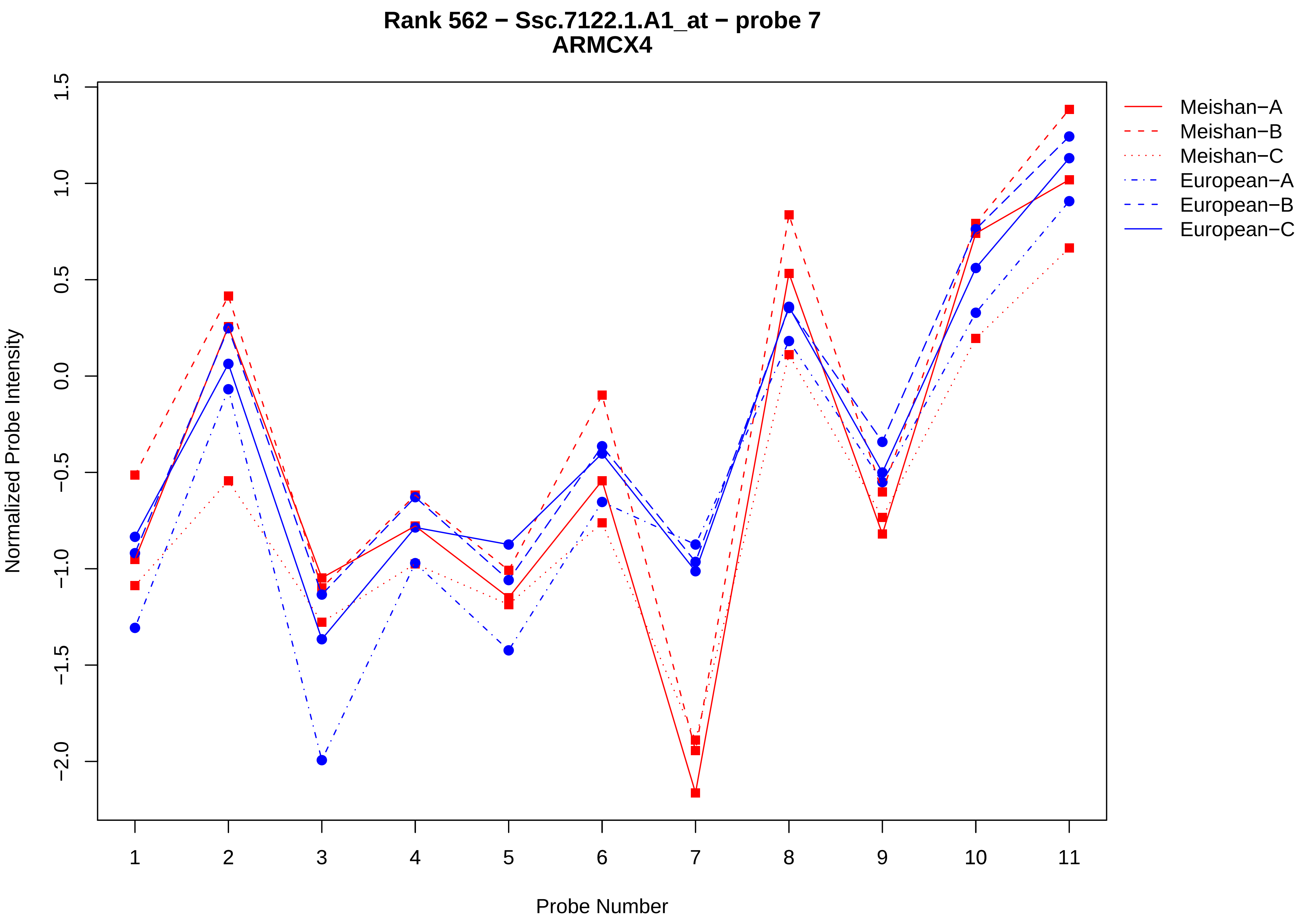

Rank 562 − Ssc.7122.1.A1_at − probe 7
ARMCX4
Normalized Probe Intensity
Probe Number
1.5
1.0
0.5
0.0
−0.5
−1.0
−1.5
−2.0
1
2
3
4
5
6
7
8
9
10
11
Meishan−A
Meishan−B
Meishan−C
European−A
European−B
European−C

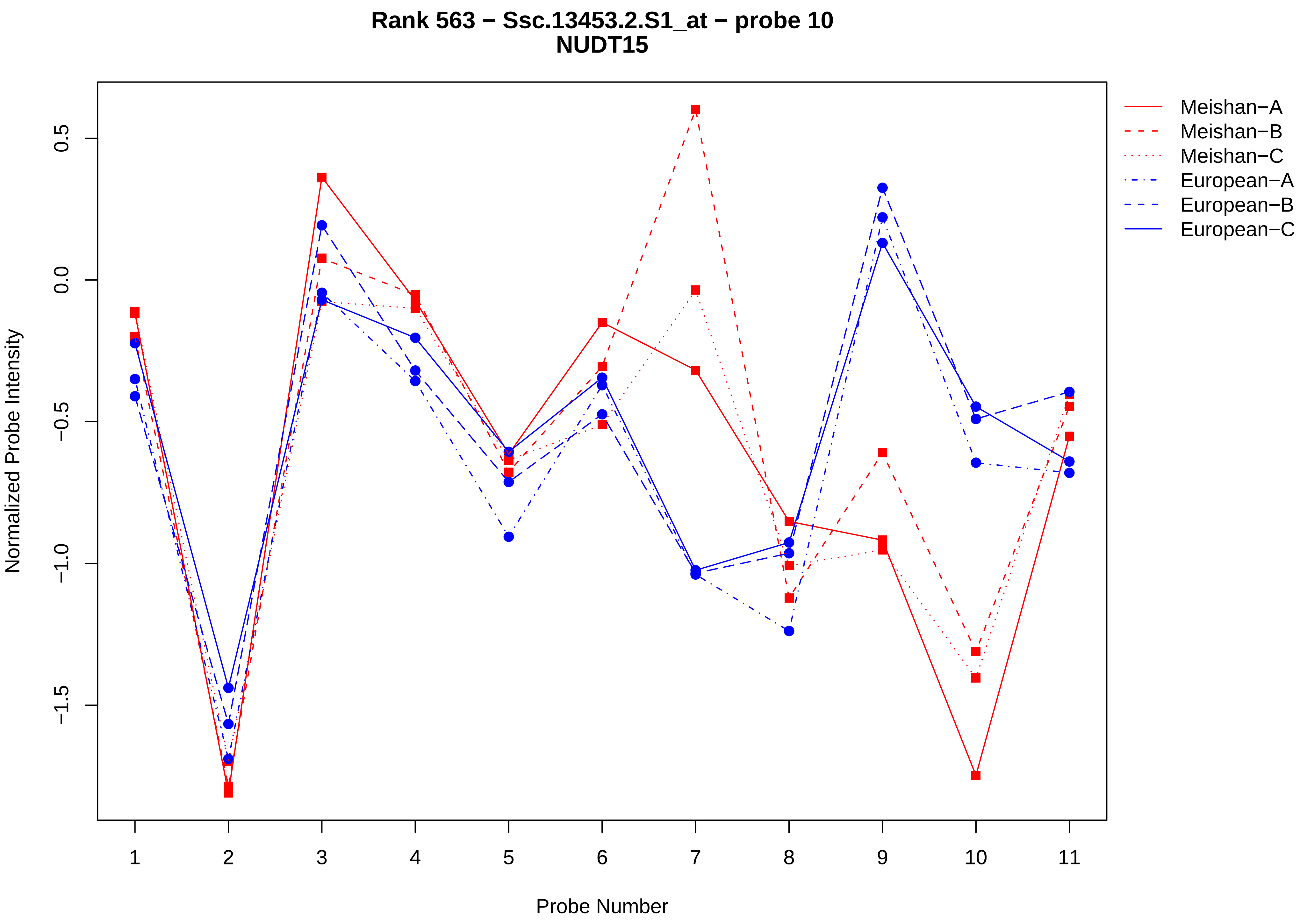

Rank 563 − Ssc.13453.2.S1_at − probe 10
NUDT15
Normalized Probe Intensity
Probe Number
0.5
0.0
−0.5
−1.0
−1.5
1
2
3
4
5
6
7
8
9
10
11
Meishan−A
Meishan−B
Meishan−C
European−A
European−B
European−C

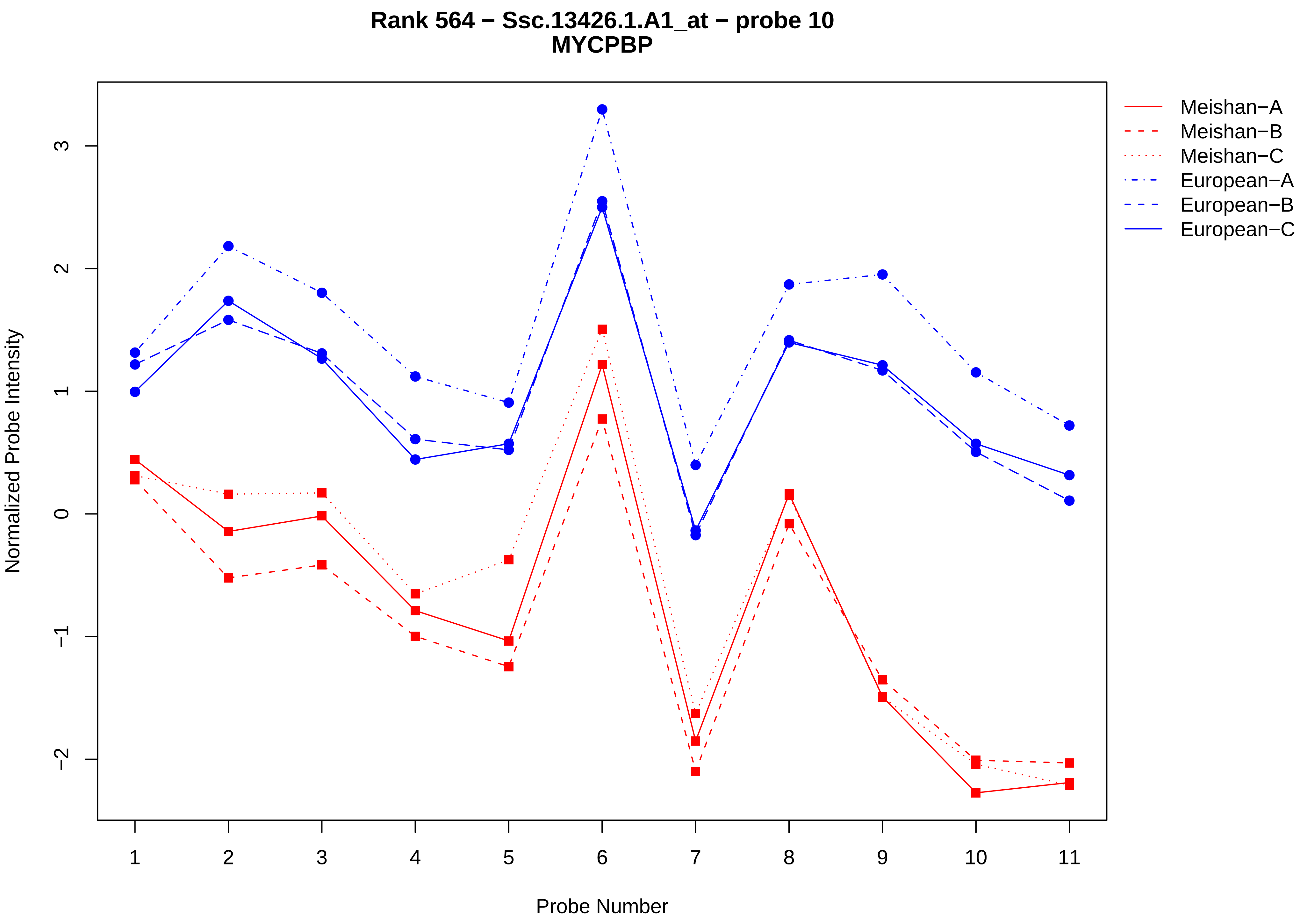

Rank 564 − Ssc.13426.1.A1_at − probe 10
MYCPBP
Normalized Probe Intensity
Probe Number
Meishan−A
Meishan−B
Meishan−C
European−A
European−B
European−C

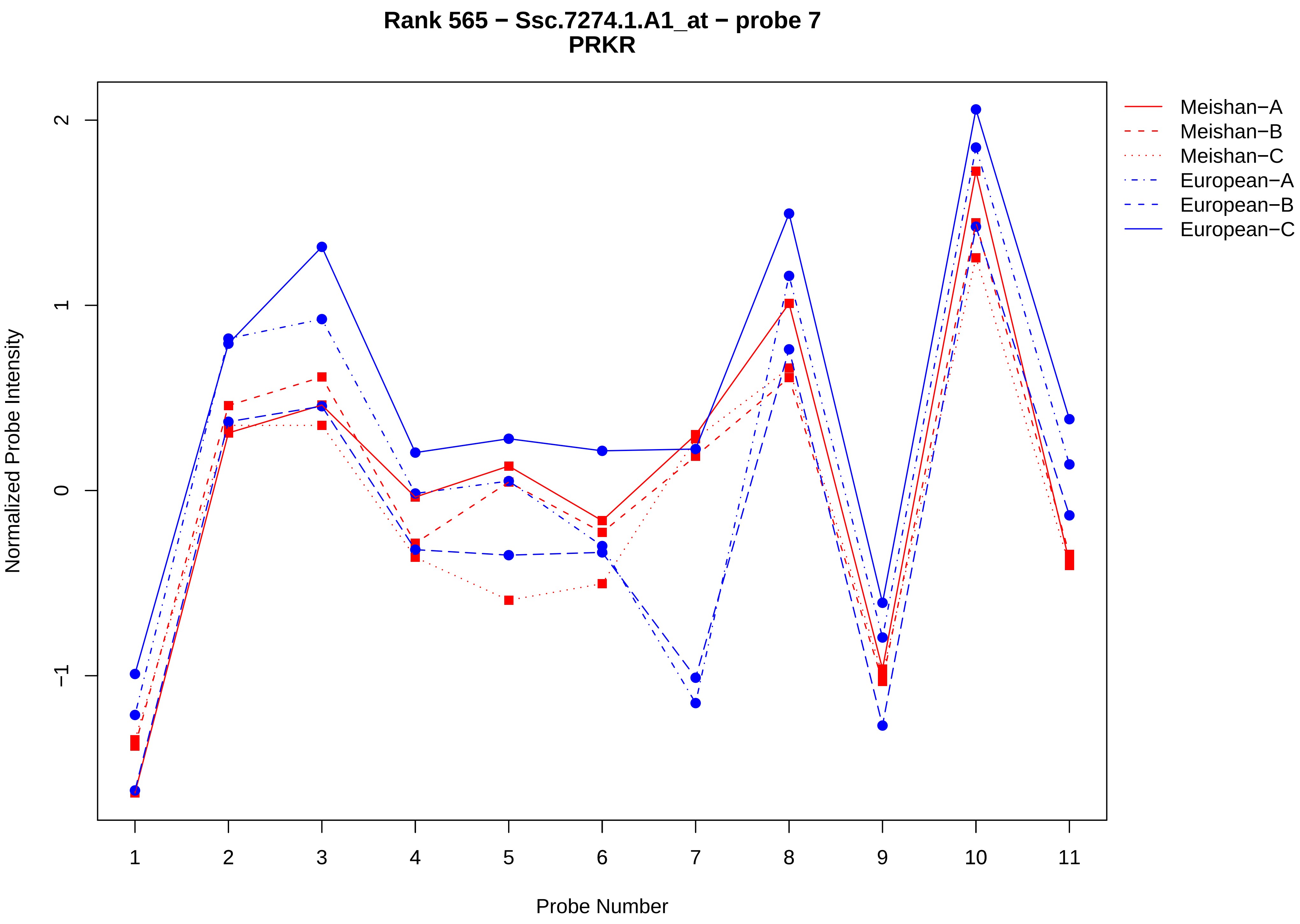

Rank 565 − Ssc.7274.1.A1_at − probe 7
PRKR
Normalized Probe Intensity
Probe Number
−1
0
1
2
1
2
3
4
5
6
7
8
9
10
11
Meishan−A
Meishan−B
Meishan−C
European−A
European−B
European−C

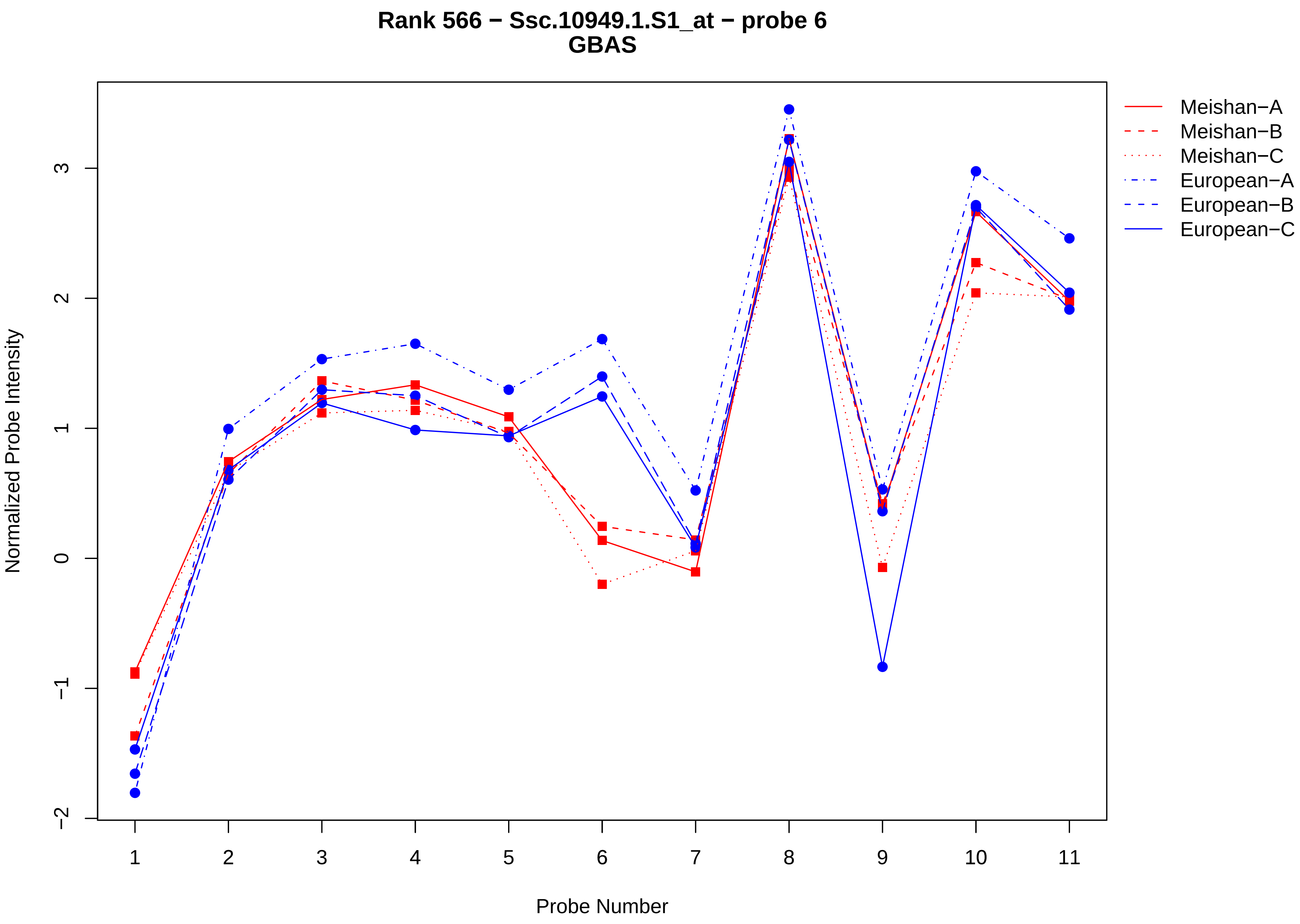
Rank 566 − Ssc.10949.1.S1_at − probe 6
GBAS
Normalized Probe Intensity
Probe Number
−2
−1
0
1
2
3
1
2
3
4
5
6
7
8
9
10
11
Meishan−A
Meishan−B
Meishan−C
European−A
European−B
European−C

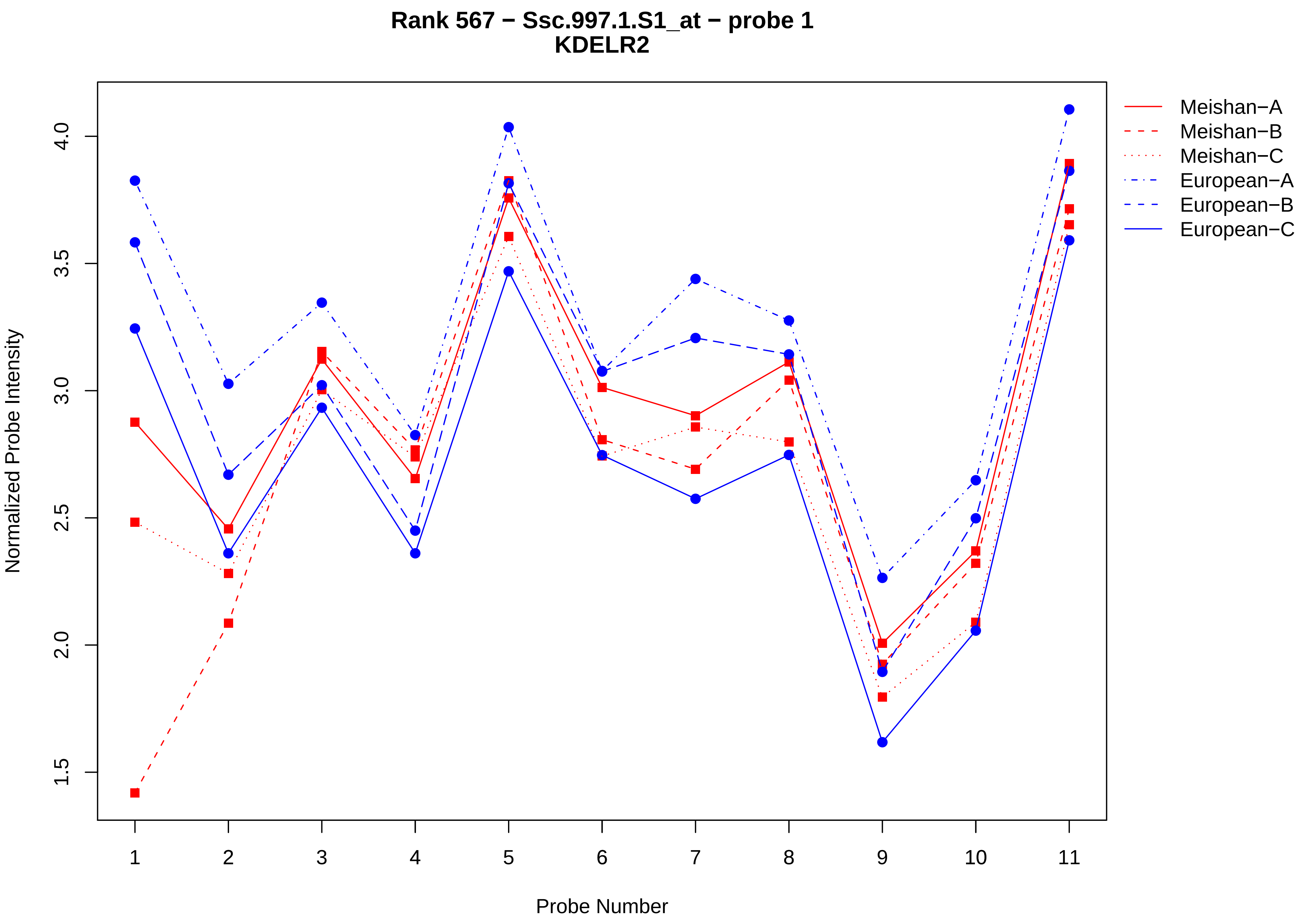

Rank 567 − Ssc.997.1.S1_at − probe 1
KDELR2
Normalized Probe Intensity
Probe Number
1.5
2.0
2.5
3.0
3.5
4.0
1
2
3
4
5
6
7
8
9
10
11
Meishan−A
Meishan−B
Meishan−C
European−A
European−B
European−C

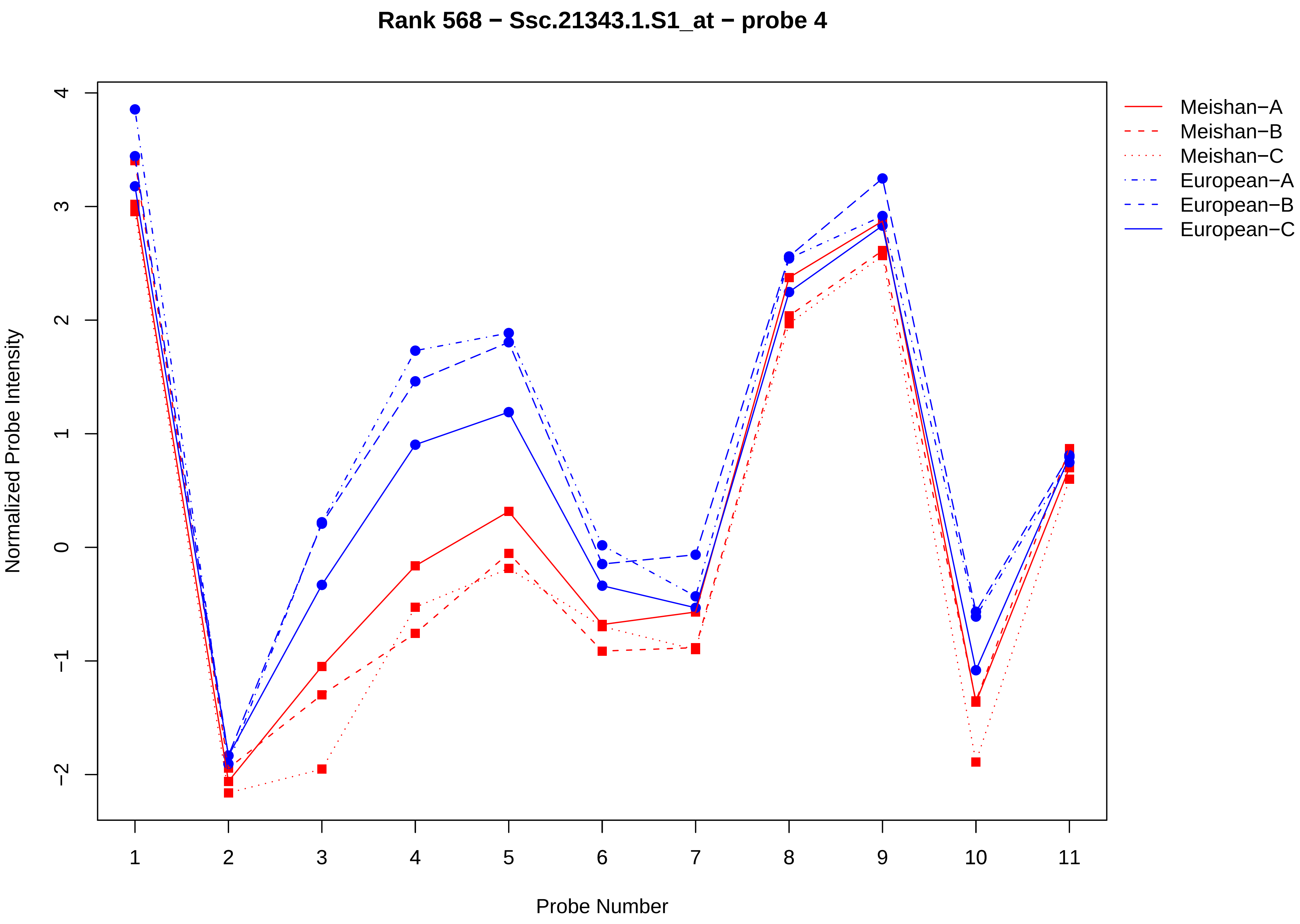
Rank 568 − Ssc.21343.1.S1_at − probe 4
Normalized Probe Intensity
Probe Number
Meishan−A
Meishan−B
Meishan−C
European−A
European−B
European−C
−2
−1
0
1
2
3
4
1
2
3
4
5
6
7
8
9
10
11

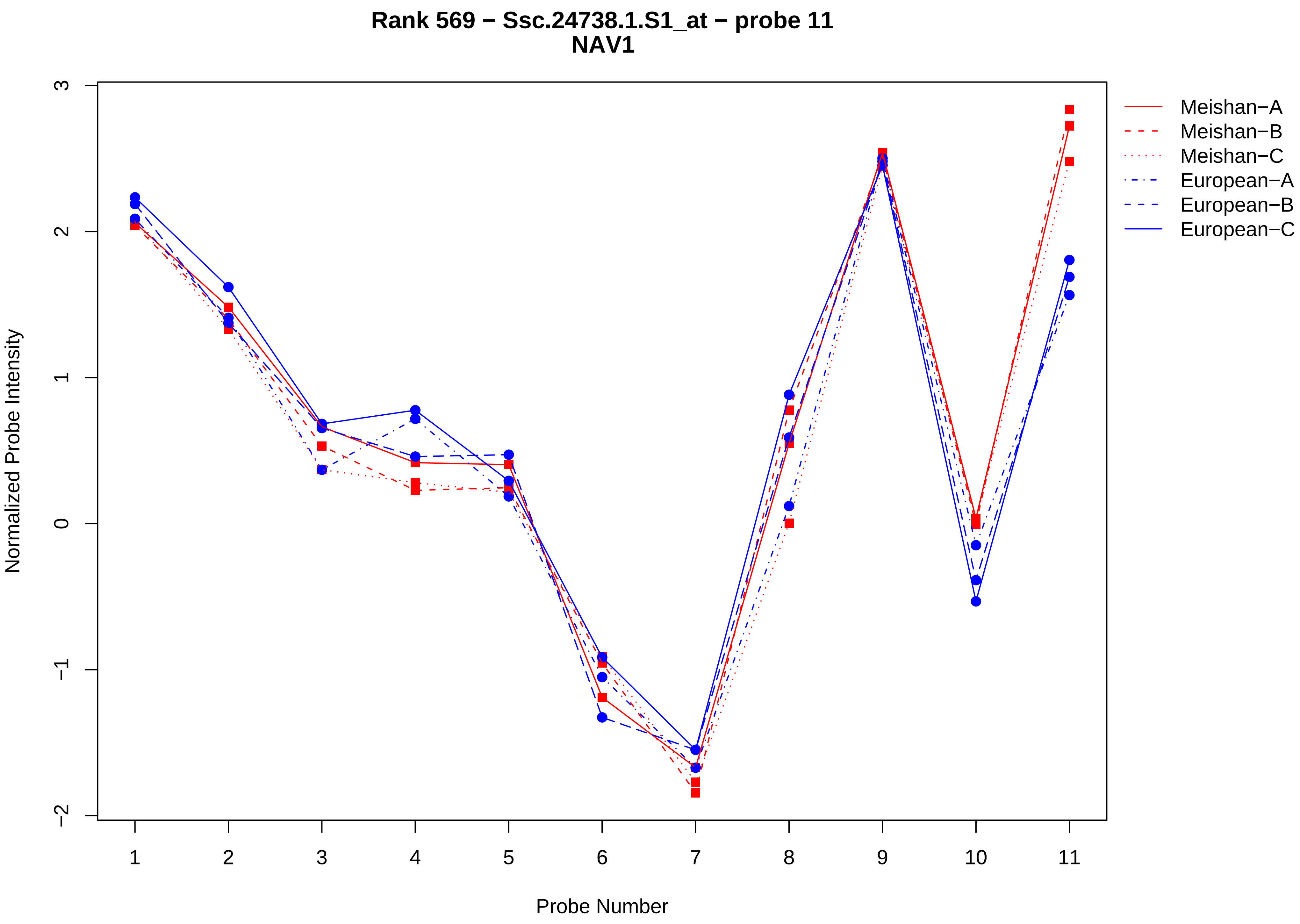

Rank 569 − Ssc.24738.1.S1_at − probe 11
NAV1
Normalized Probe Intensity
Probe Number
−2
−1
0
1
2
3
1
2
3
4
5
6
7
8
9
10
11
Meishan−A
Meishan−B
Meishan−C
European−A
European−B
European−C

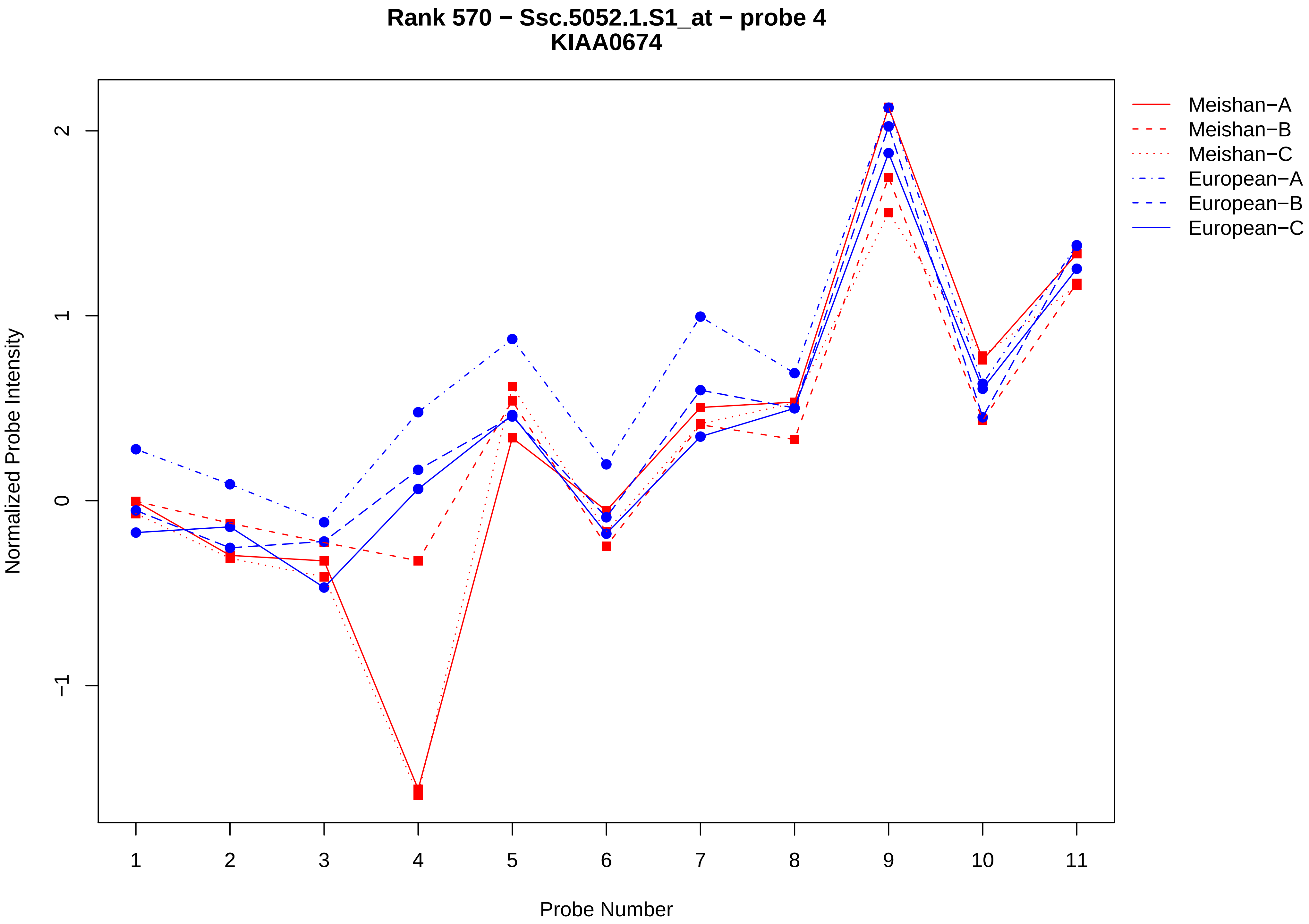

Rank 570 − Ssc.5052.1.S1_at − probe 4
KIAA0674
Normalized Probe Intensity
Probe Number
−1
0
1
2
1
2
3
4
5
6
7
8
9
10
11
Meishan−A
Meishan−B
Meishan−C
European−A
European−B
European−C

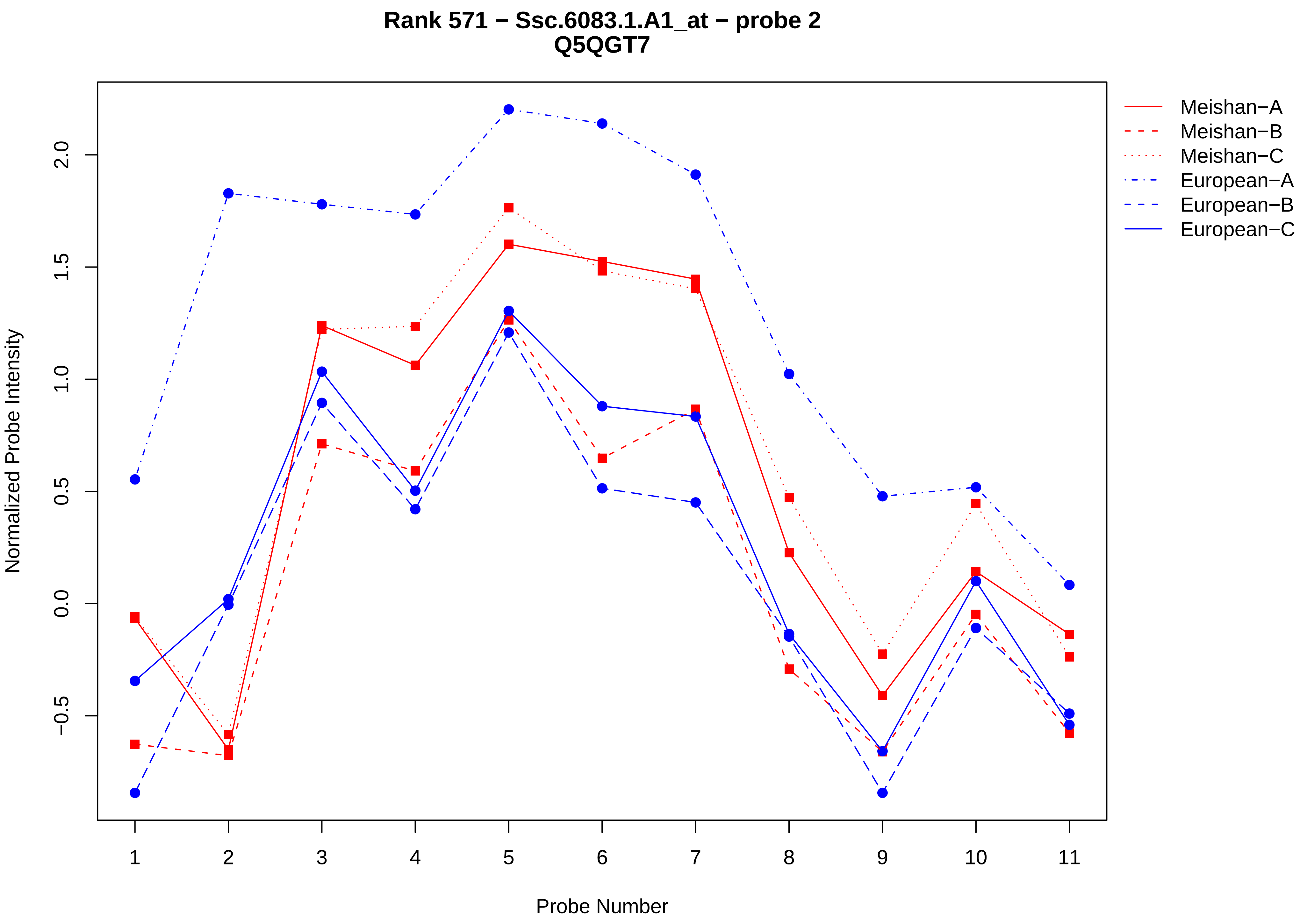

Rank 571 − Ssc.6083.1.A1_at − probe 2
Q5QGT7
Normalized Probe Intensity
−0.5
0.0
0.5
1.0
1.5
2.0
Probe Number
1
2
3
4
5
6
7
8
9
10
11
Meishan−A
Meishan−B
Meishan−C
European−A
European−B
European−C

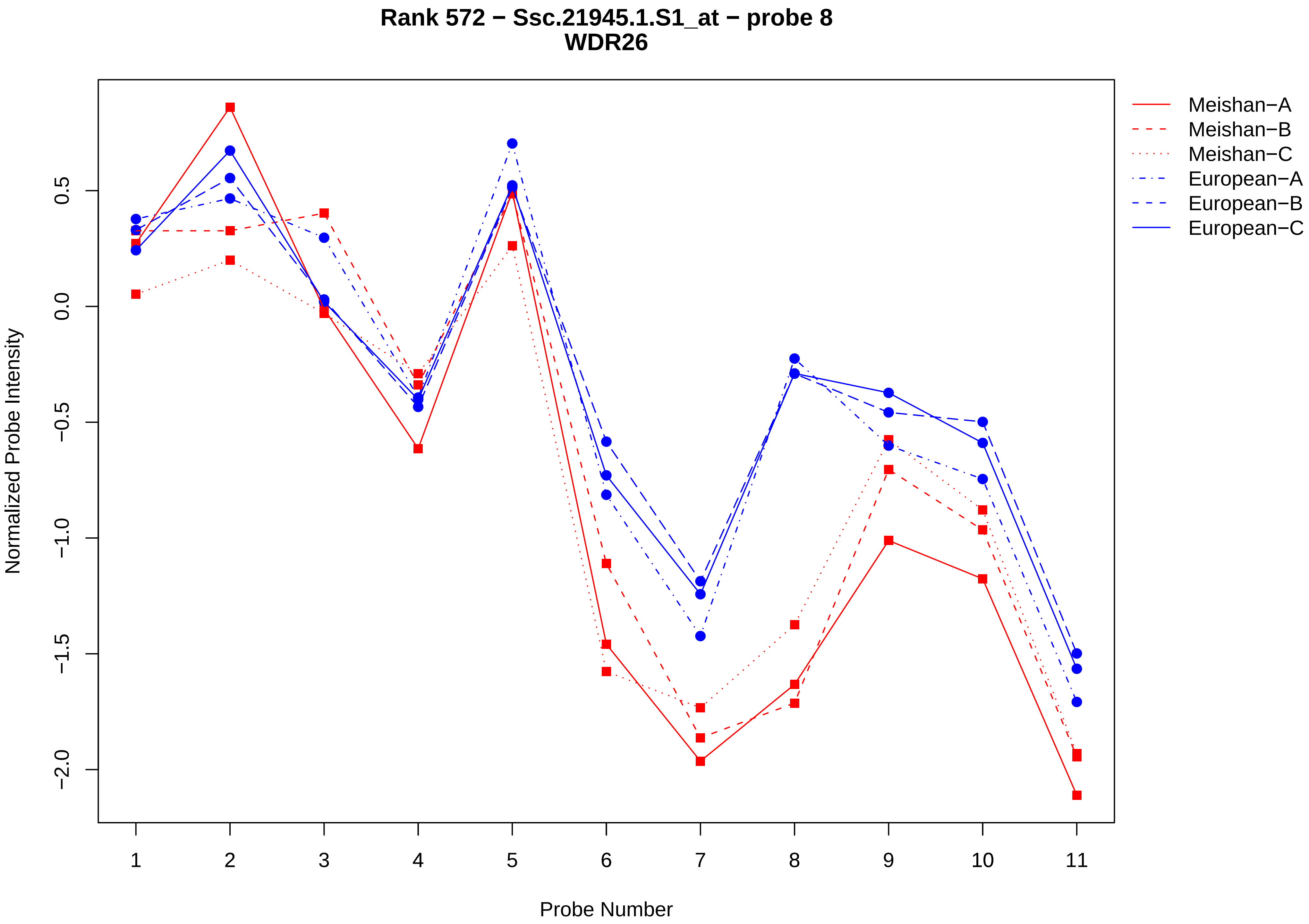
Rank 572 − Ssc.21945.1.S1_at − probe 8
WDR26
Normalized Probe Intensity
Probe Number
0.5
0.0
−0.5
−1.0
−1.5
−2.0
1
2
3
4
5
6
7
8
9
10
11
Meishan−A
Meishan−B
Meishan−C
European−A
European−B
European−C

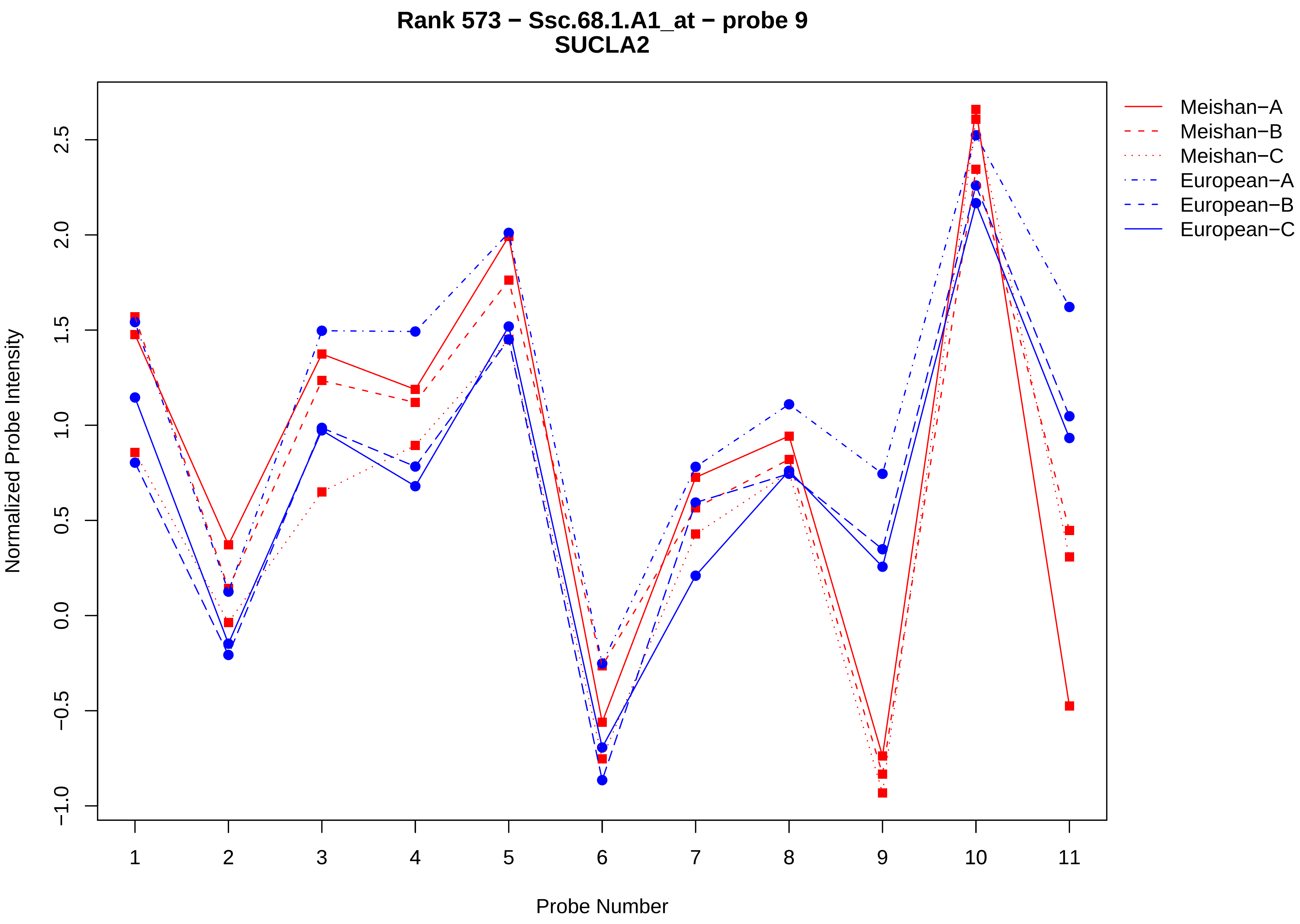

Rank 573 − Ssc.68.1.A1_at − probe 9
SUCLA2
Normalized Probe Intensity
Probe Number
−1.0
−0.5
0.0
0.5
1.0
1.5
2.0
2.5
1
2
3
4
5
6
7
8
9
10
11
Meishan−A
Meishan−B
Meishan−C
European−A
European−B
European−C

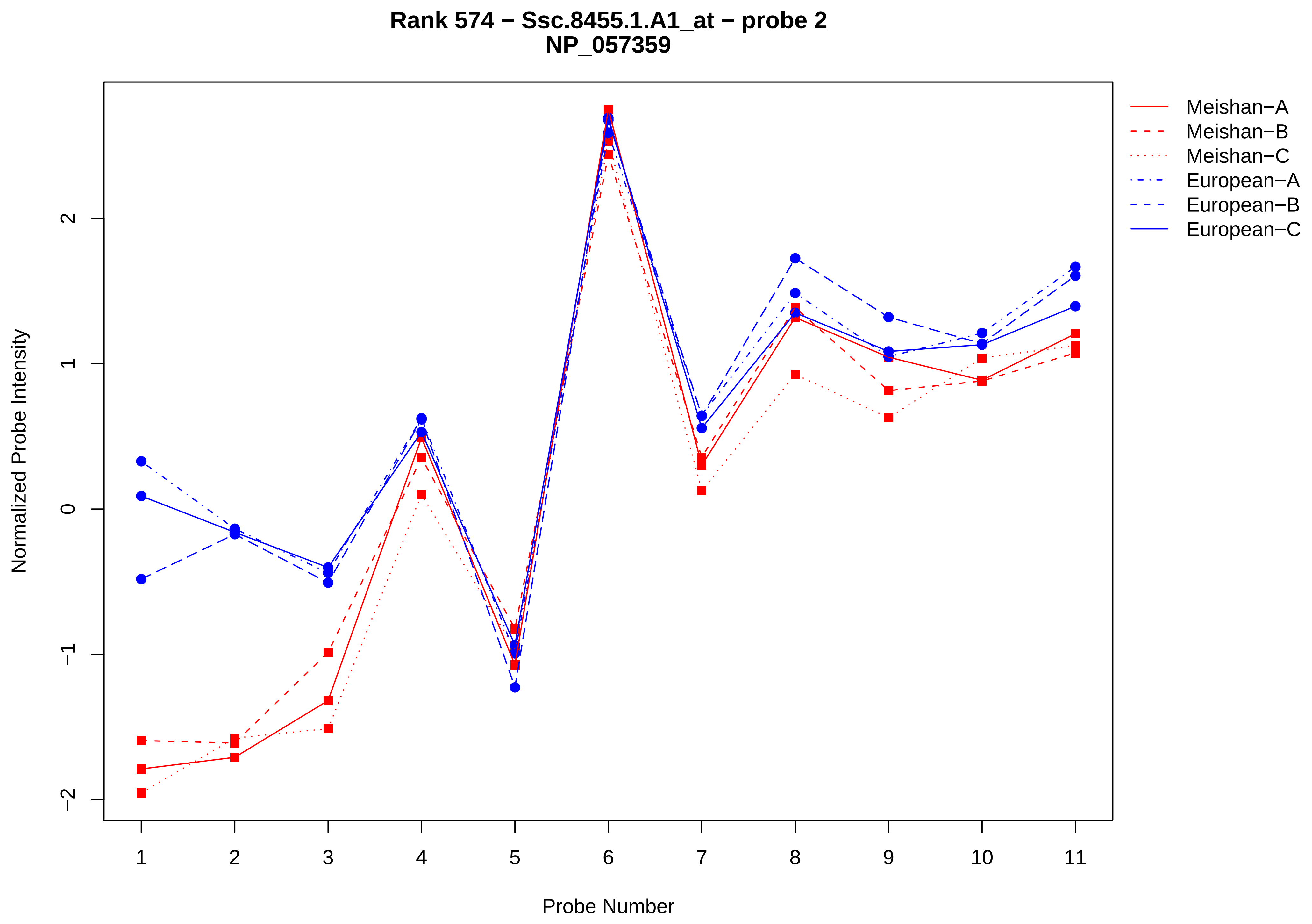
Rank 574 − Ssc.8455.1.A1_at − probe 2
NP_057359
Normalized Probe Intensity
Probe Number
−2
−1
0
1
2
1
2
3
4
5
6
7
8
9
10
11
Meishan−A
Meishan−B
Meishan−C
European−A
European−B
European−C

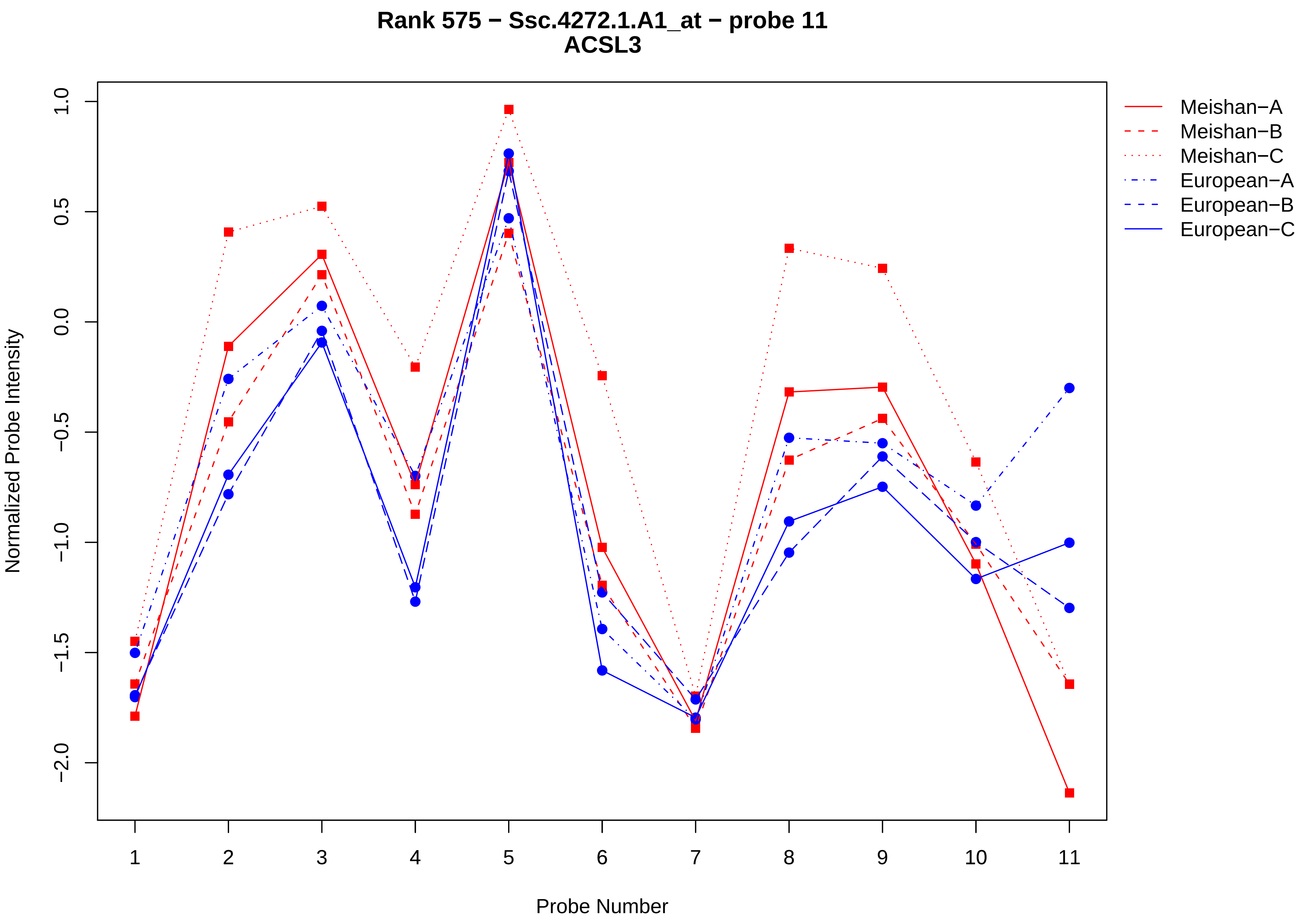

Rank 575 − Ssc.4272.1.A1_at − probe 11
ACSL3
Normalized Probe Intensity
Probe Number
1.0
0.5
0.0
−0.5
−1.0
−1.5
−2.0
1
2
3
4
5
6
7
8
9
10
11
Meishan−A
Meishan−B
Meishan−C
European−A
European−B
European−C

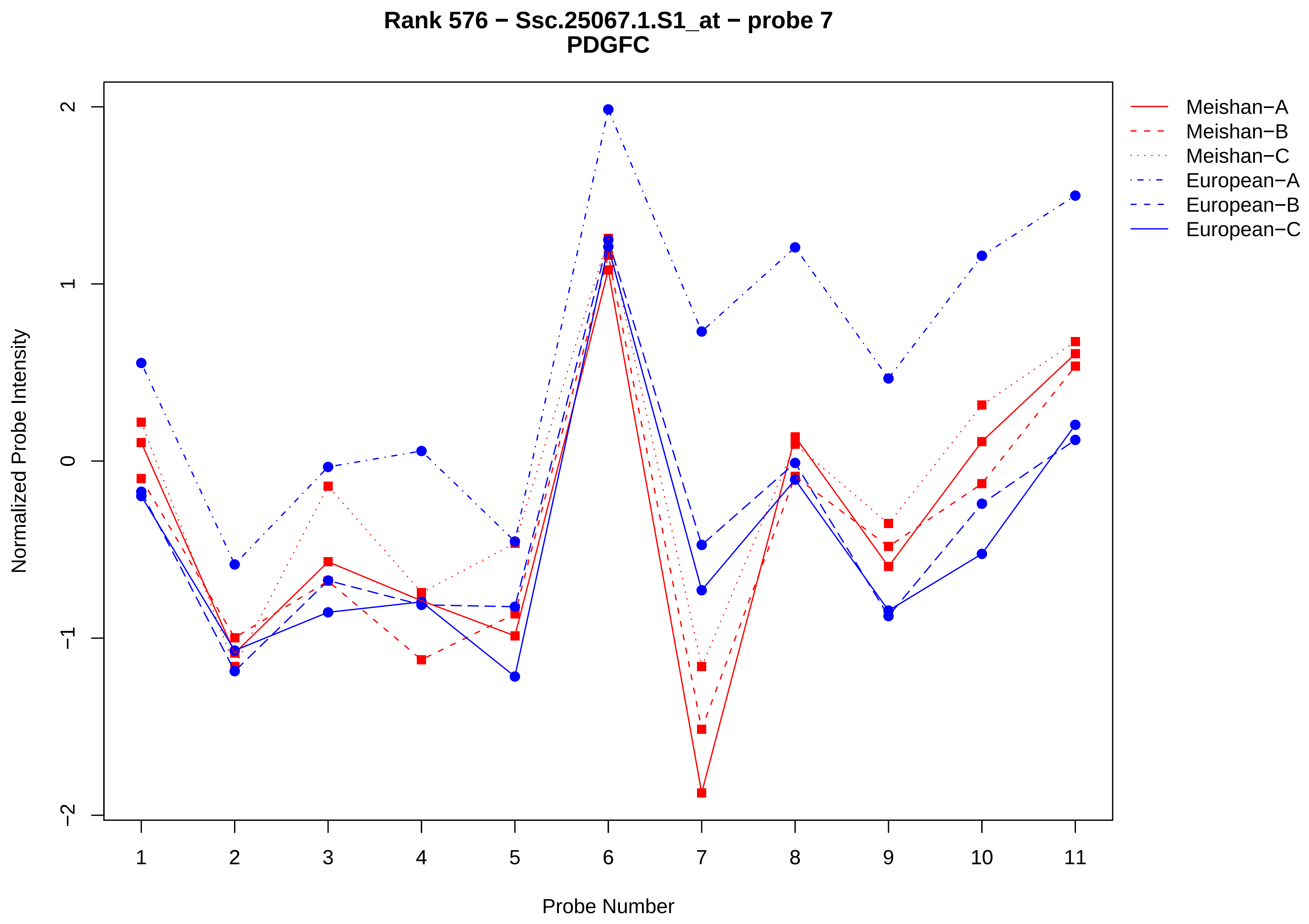

Rank 576 − Ssc.25067.1.S1_at − probe 7
PDGFC
Normalized Probe Intensity
Probe Number
−2
−1
0
1
2
1
2
3
4
5
6
7
8
9
10
11
Meishan−A
Meishan−B
Meishan−C
European−A
European−B
European−C

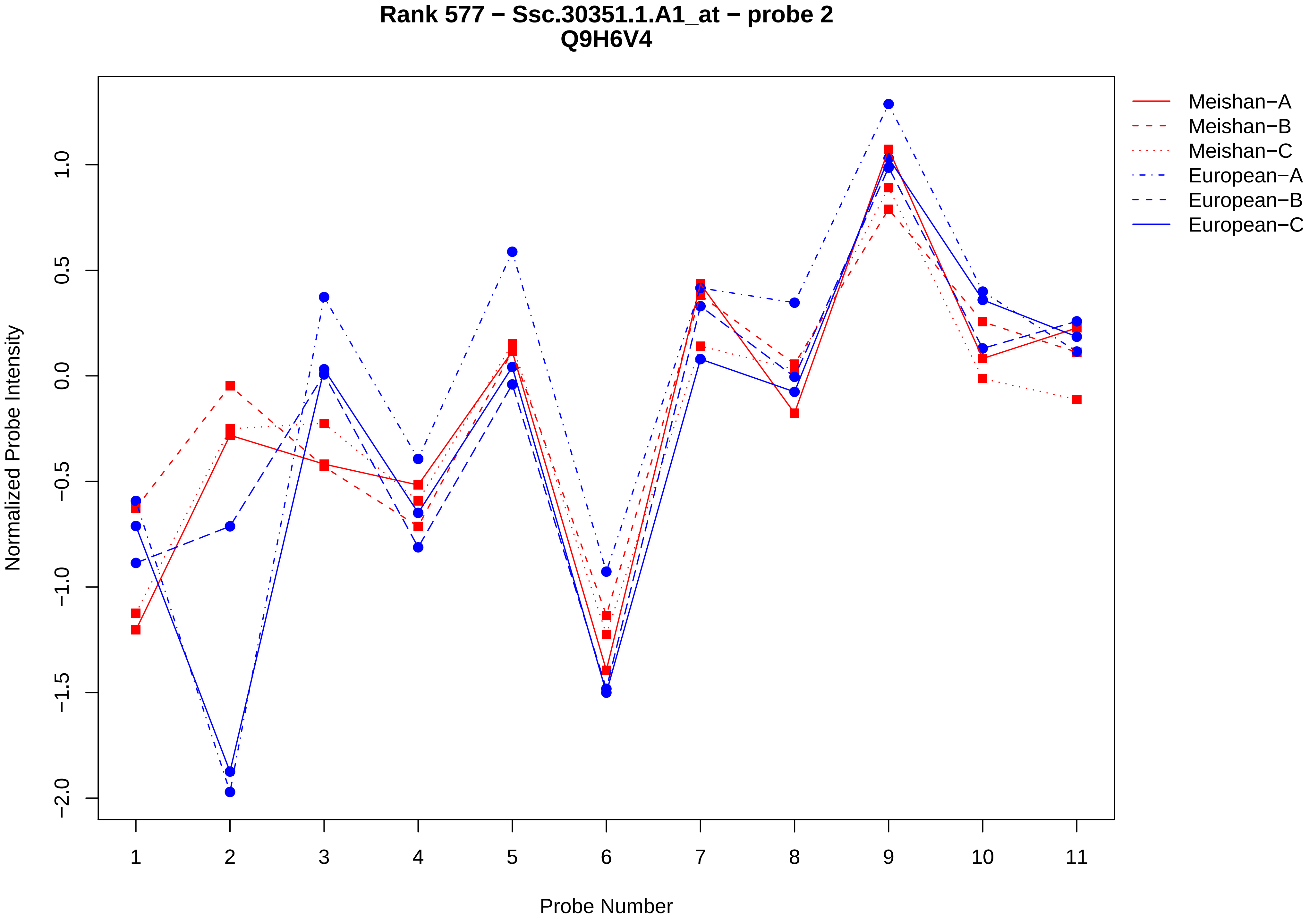

Rank 577 − Ssc.30351.1.A1_at − probe 2
Q9H6V4
Normalized Probe Intensity
Probe Number
1.0
0.5
0.0
−0.5
−1.0
−1.5
−2.0
1
2
3
4
5
6
7
8
9
10
11
Meishan−A
Meishan−B
Meishan−C
European−A
European−B
European−C

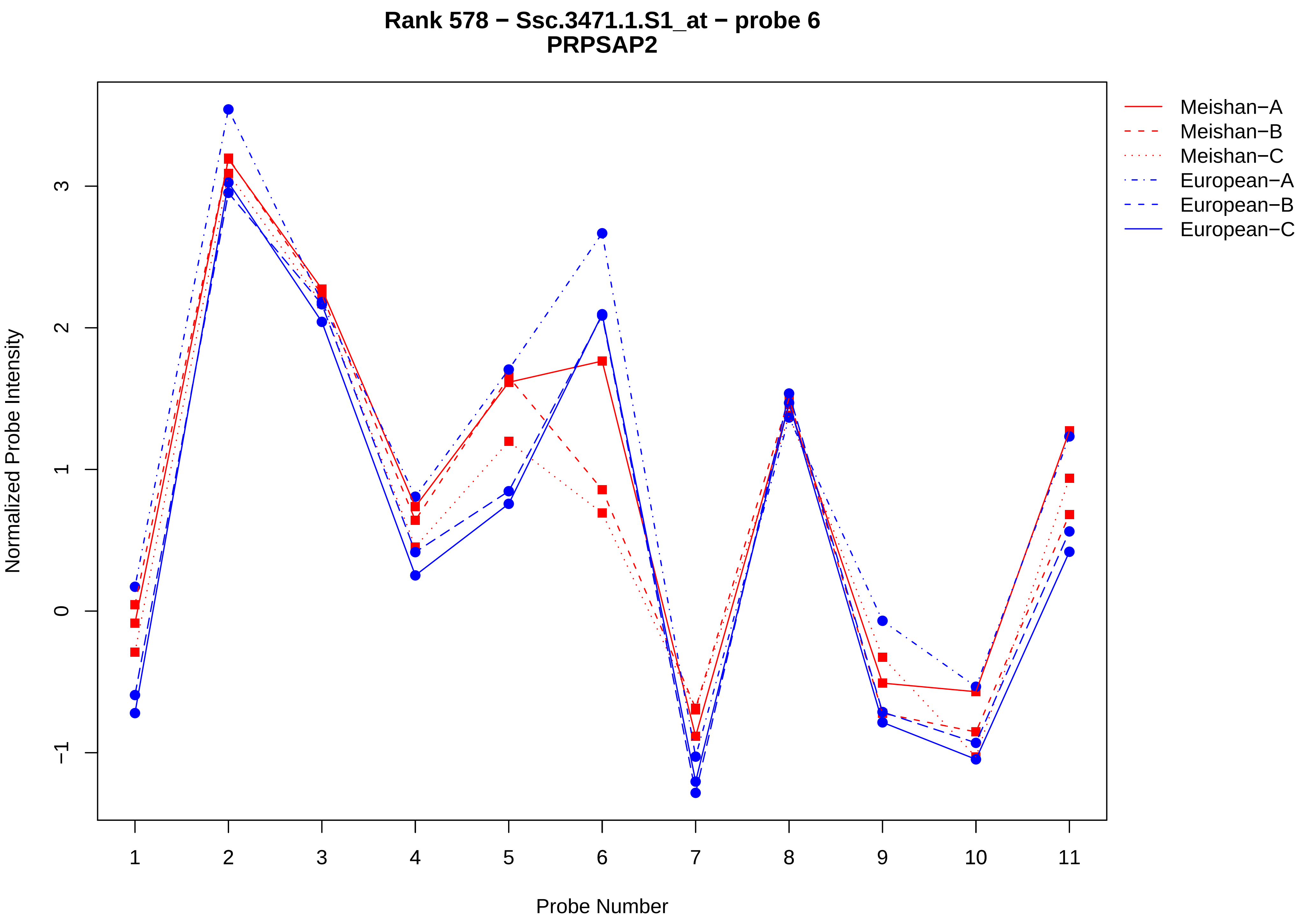

Rank 578 − Ssc.3471.1.S1_at − probe 6
PRPSAP2
Normalized Probe Intensity
Probe Number
−1
0
1
2
3
1
2
3
4
5
6
7
8
9
10
11
Meishan−A
Meishan−B
Meishan−C
European−A
European−B
European−C

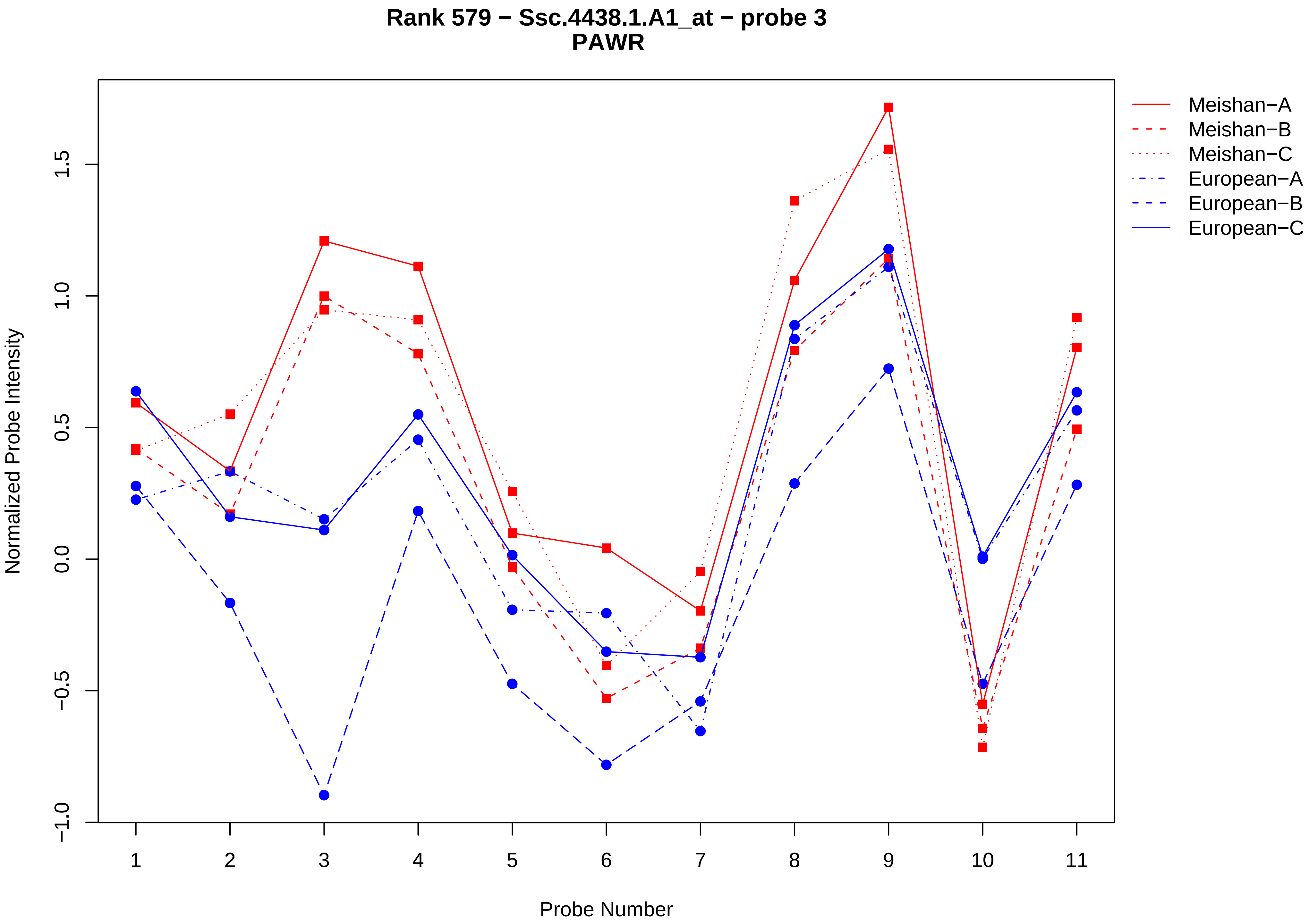

Rank 579 − Ssc.4438.1.A1_at − probe 3
PAWR
Normalized Probe Intensity
Probe Number
−1.0
−0.5
0.0
0.5
1.0
1.5
1
2
3
4
5
6
7
8
9
10
11
Meishan−A
Meishan−B
Meishan−C
European−A
European−B
European−C

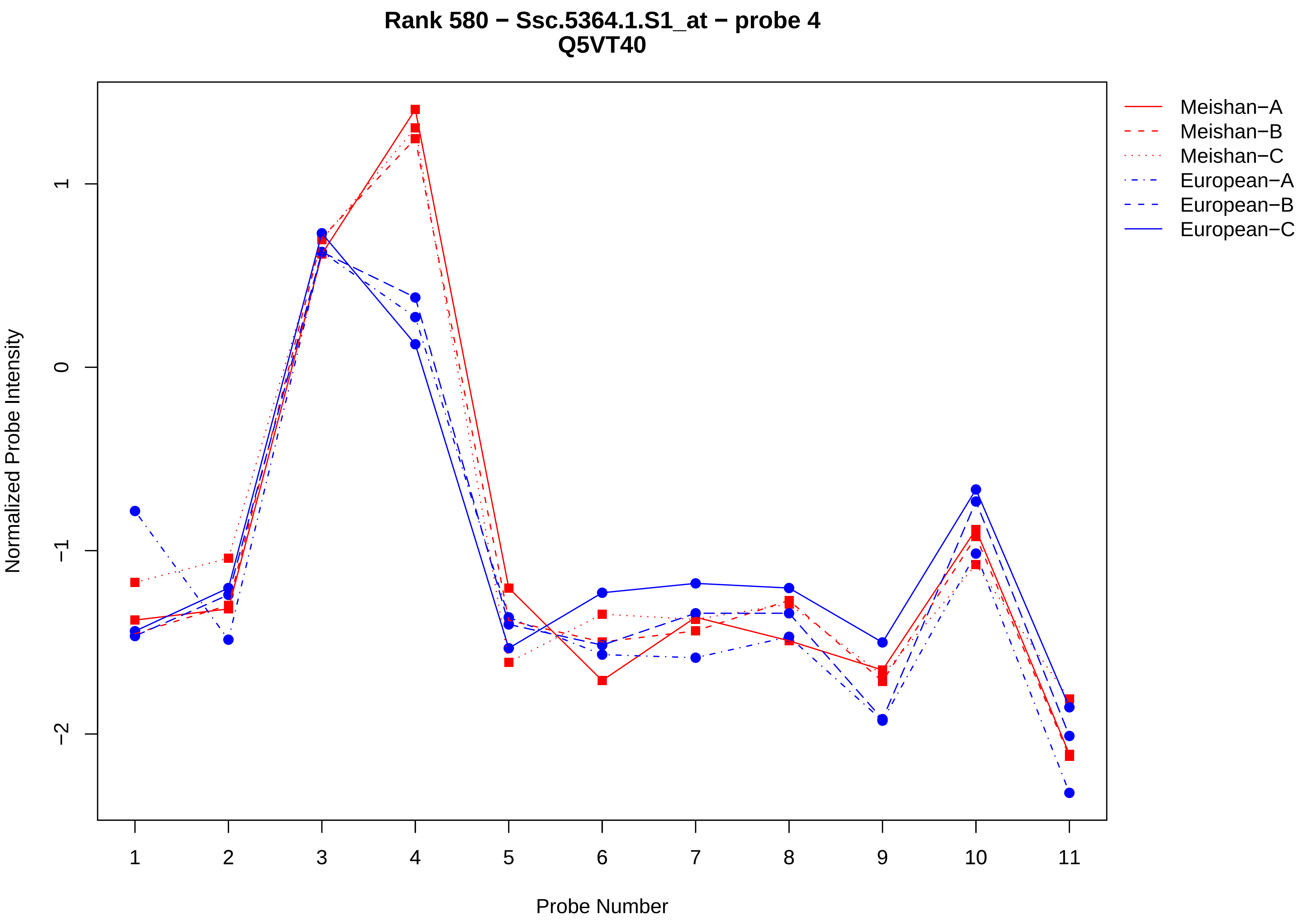

Rank 580 − Ssc.5364.1.S1_at − probe 4
Q5VT40
Normalized Probe Intensity
−2
−1
0
1
1
2
3
4
5
6
7
8
9
10
11
Probe Number
Meishan−A
Meishan−B
Meishan−C
European−A
European−B
European−C

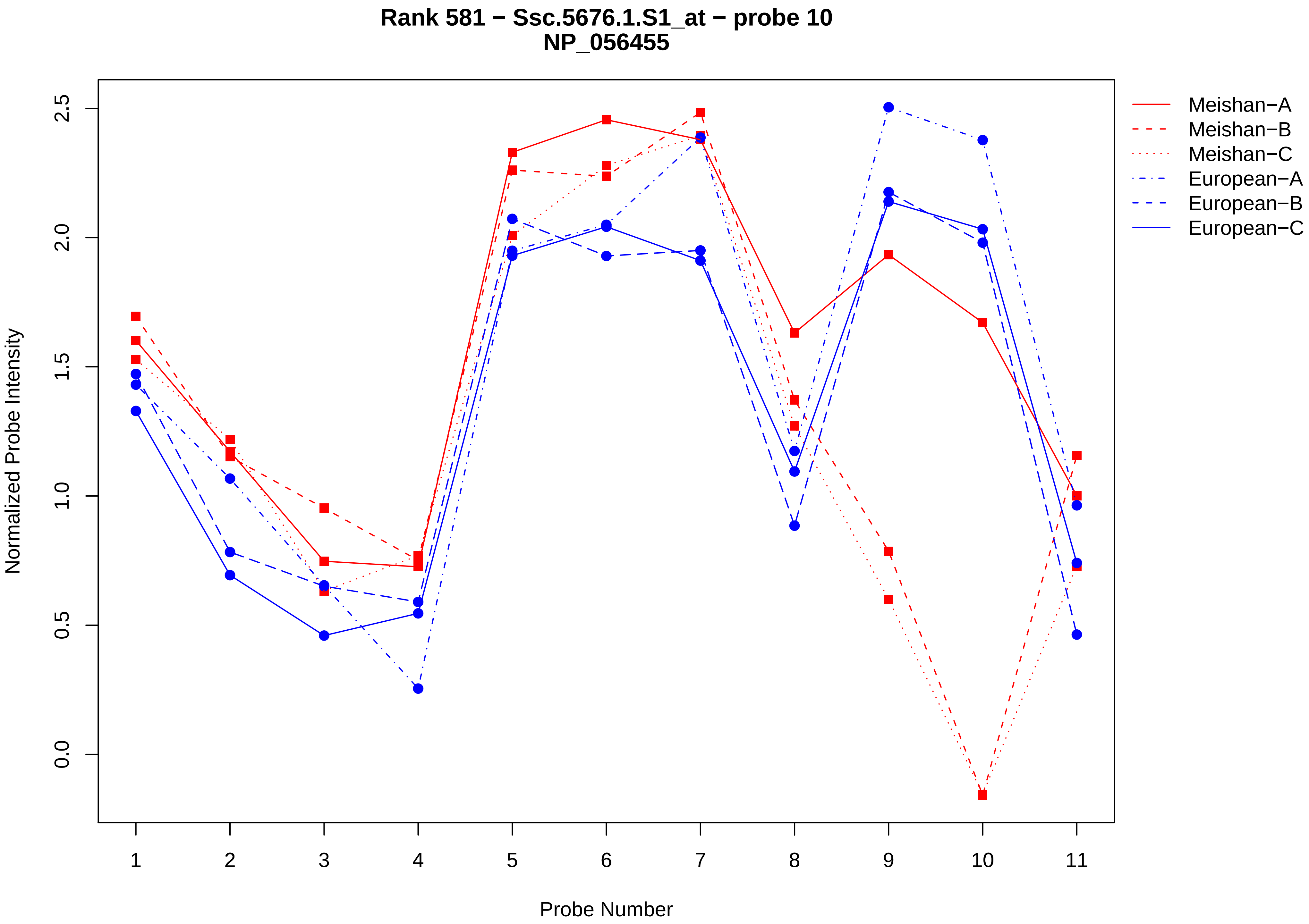

Rank 581 − Ssc.5676.1.S1_at − probe 10
NP_056455
Normalized Probe Intensity
Probe Number
0.0
0.5
1.0
1.5
2.0
2.5
1
2
3
4
5
6
7
8
9
10
11
Meishan−A
Meishan−B
Meishan−C
European−A
European−B
European−C

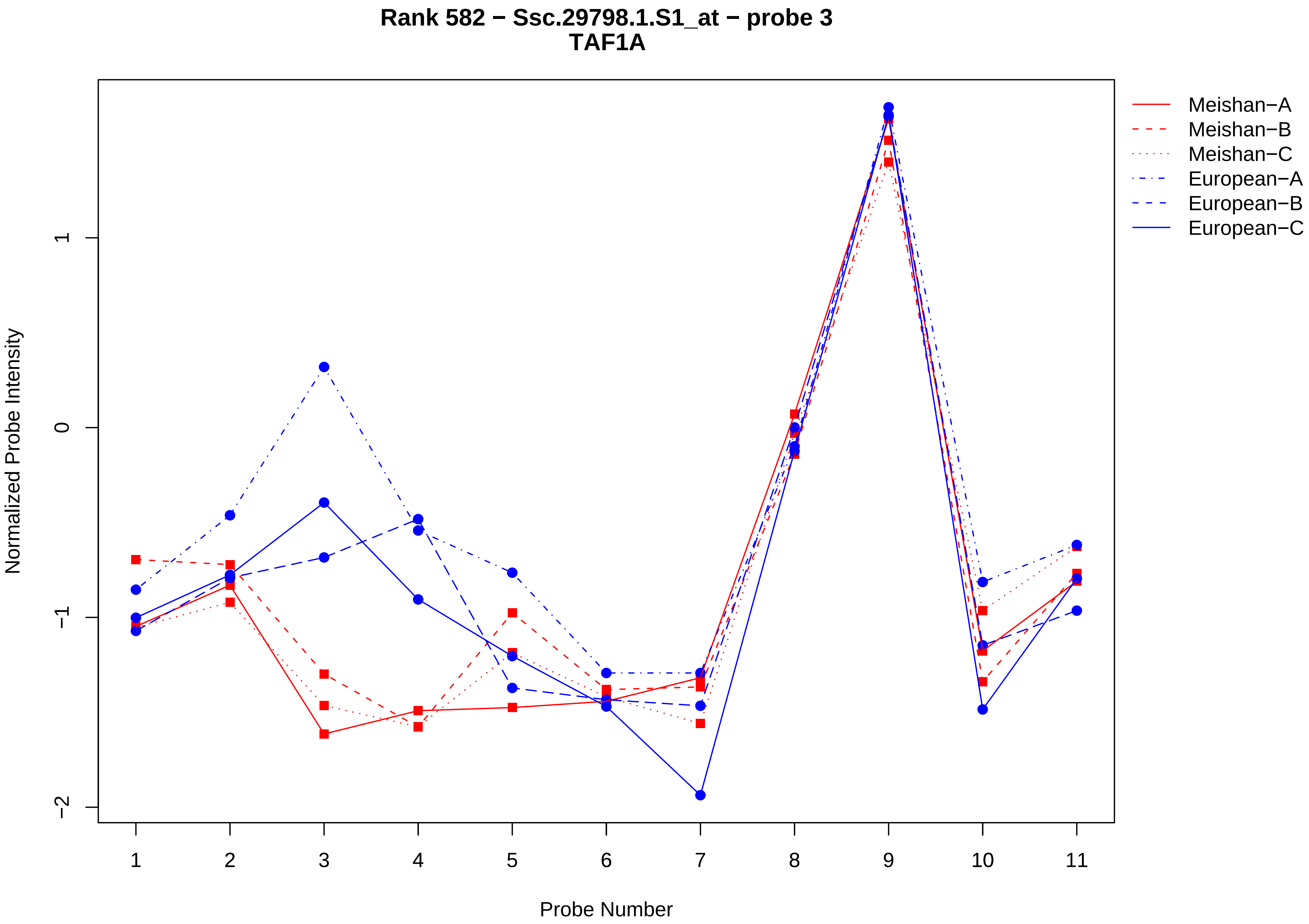
Rank 582 − Ssc.29798.1.S1_at − probe 3
TAF1A
Normalized Probe Intensity
Probe Number
−2
−1
0
1
1
2
3
4
5
6
7
8
9
10
11
Meishan−A
Meishan−B
Meishan−C
European−A
European−B
European−C

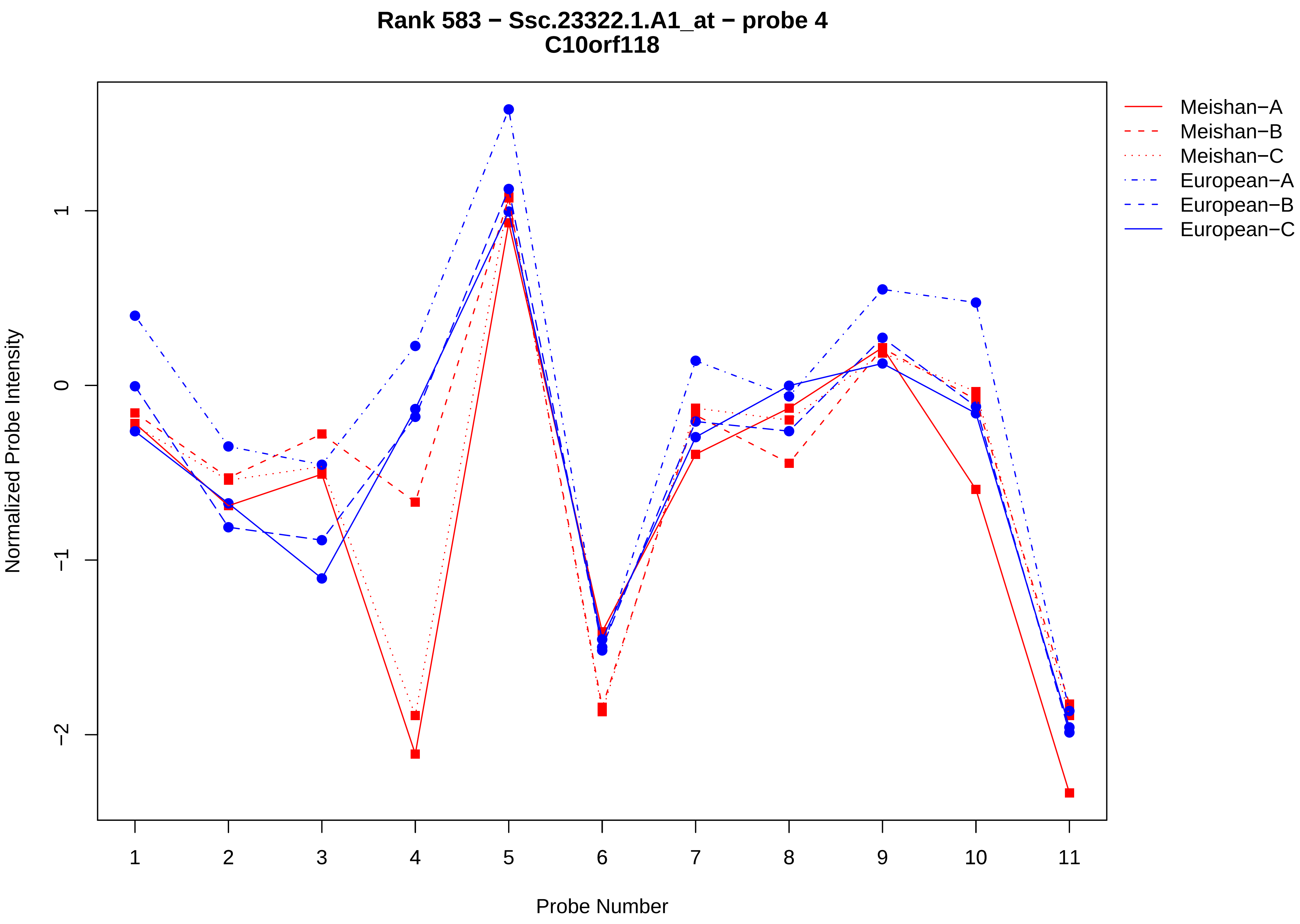

Rank 583 − Ssc.23322.1.A1_at − probe 4
C10orf118
Normalized Probe Intensity
Probe Number
1
0
−1
−2
1
2
3
4
5
6
7
8
9
10
11
Meishan−A
Meishan−B
Meishan−C
European−A
European−B
European−C

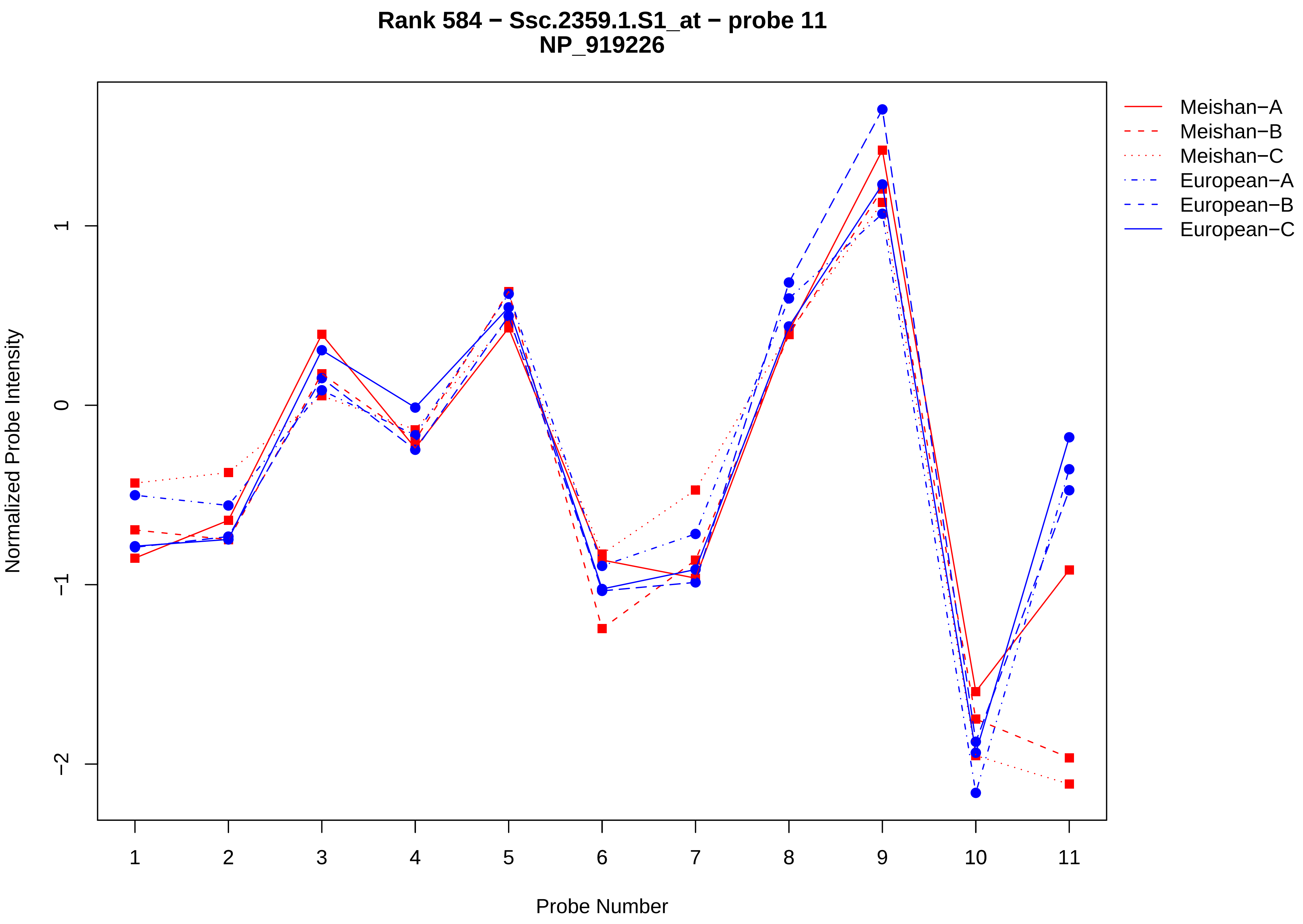

Rank 584 − Ssc.2359.1.S1_at − probe 11
NP_919226
Normalized Probe Intensity
Probe Number
Meishan−A
Meishan−B
Meishan−C
European−A
European−B
European−C
1
0
−1
−2
1
2
3
4
5
6
7
8
9
10
11

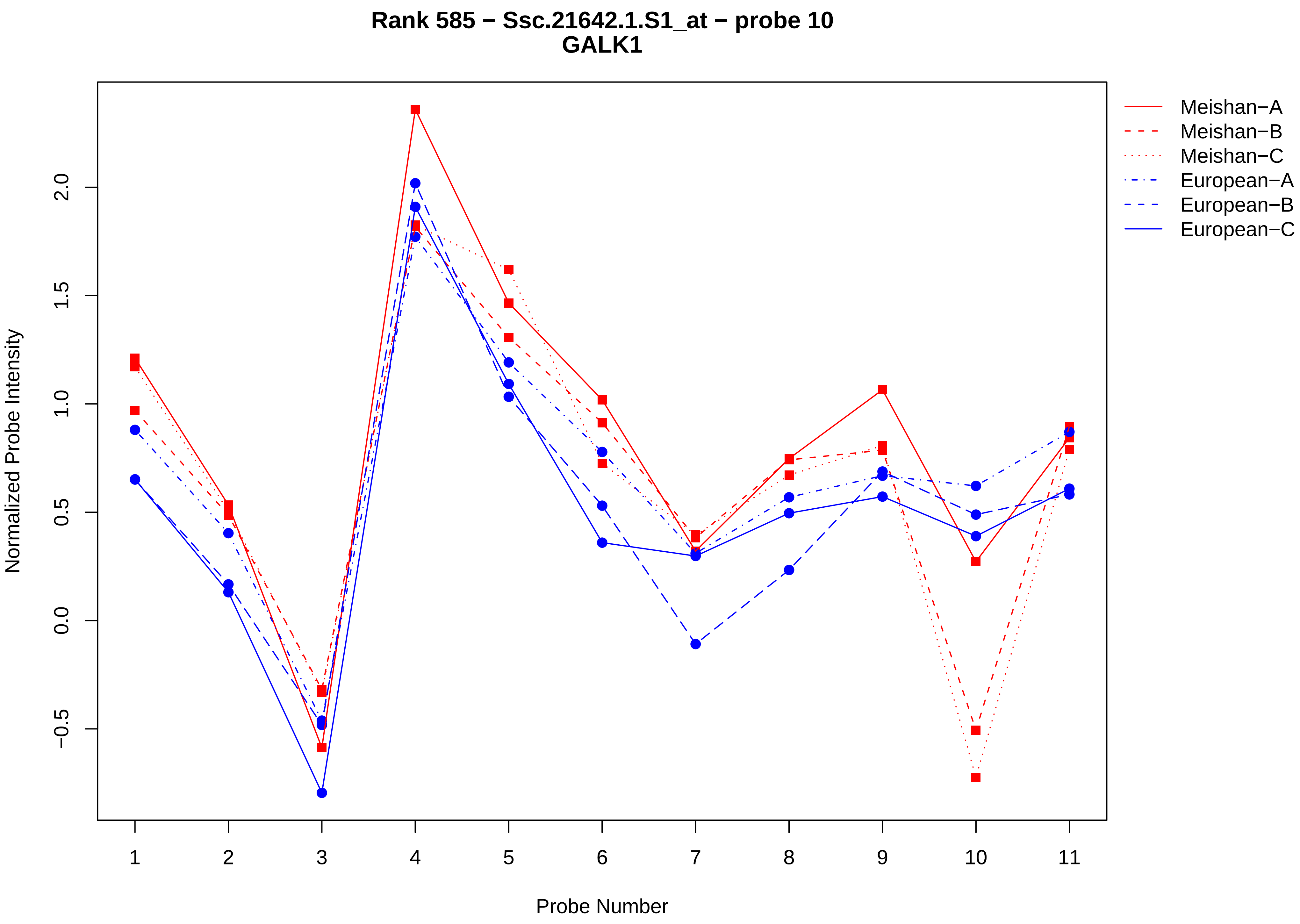

Rank 585 − Ssc.21642.1.S1_at − probe 10
GALK1
Normalized Probe Intensity
Probe Number
−0.5
0.0
0.5
1.0
1.5
2.0
1
2
3
4
5
6
7
8
9
10
11
Meishan−A
Meishan−B
Meishan−C
European−A
European−B
European−C

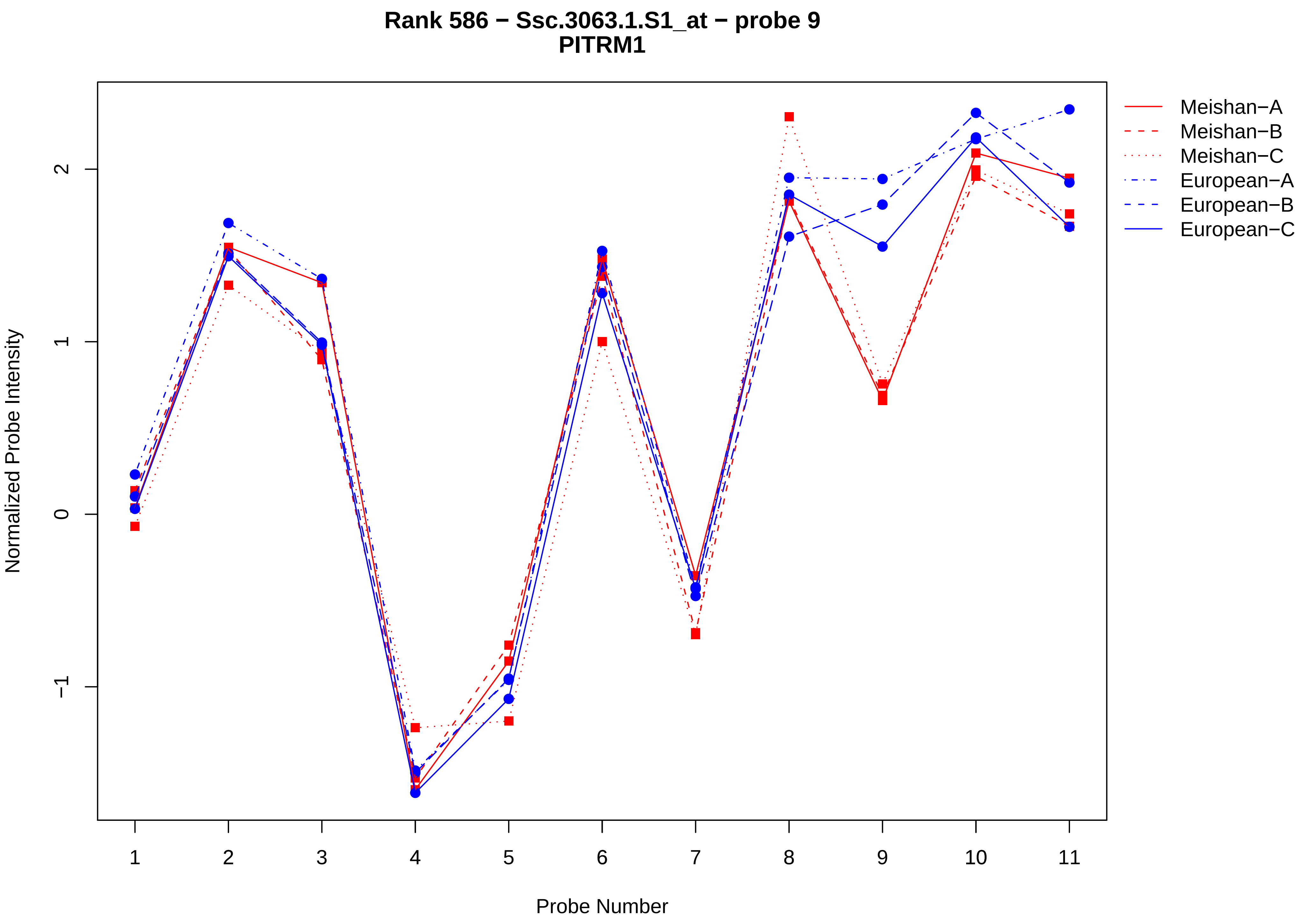
Rank 586 − Ssc.3063.1.S1_at − probe 9
PITRM1
Normalized Probe Intensity
Probe Number
−1
0
1
2
1
2
3
4
5
6
7
8
9
10
11
Meishan−A
Meishan−B
Meishan−C
European−A
European−B
European−C

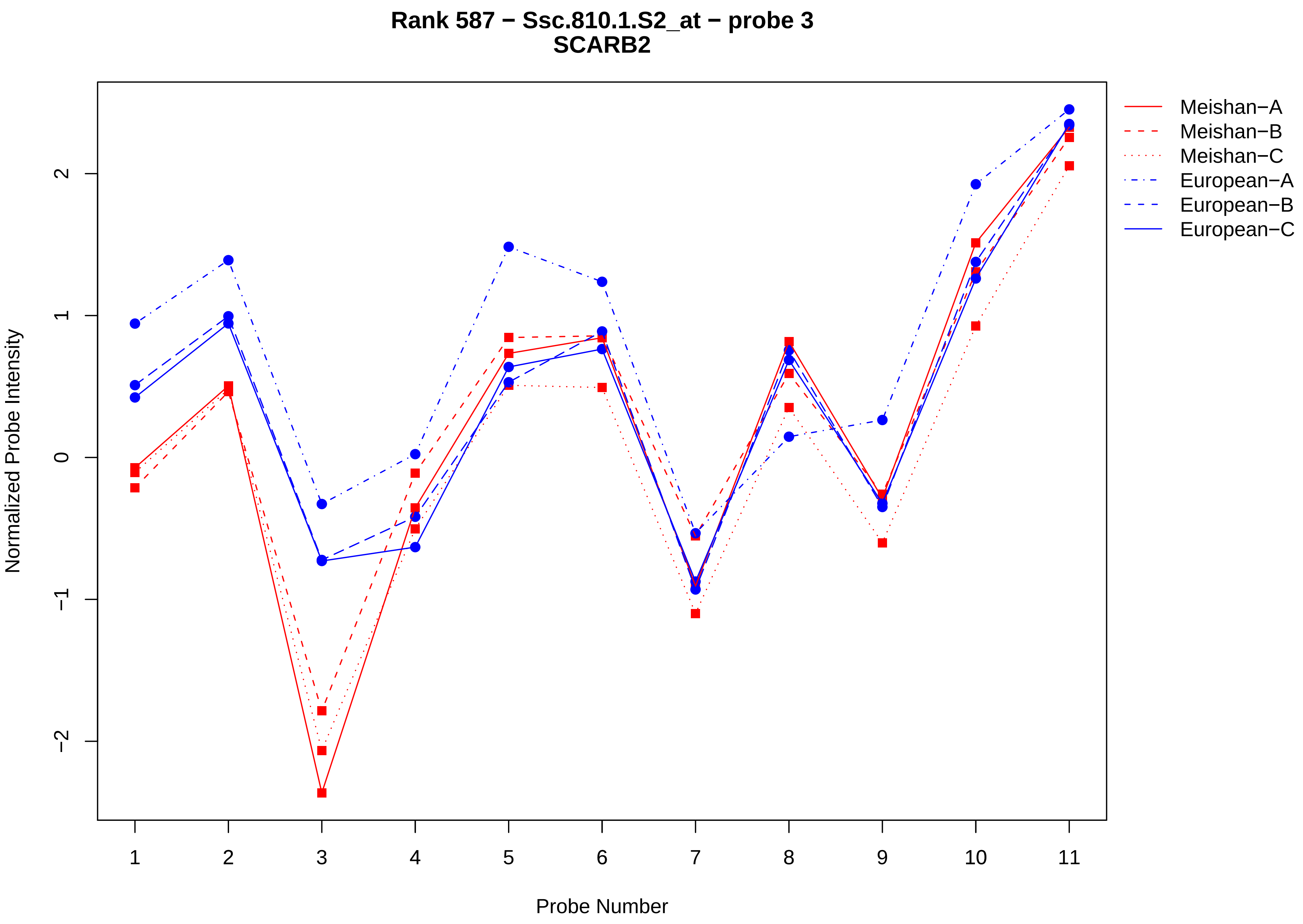

Rank 587 − Ssc.810.1.S2_at − probe 3
SCARB2
Normalized Probe Intensity
Probe Number
−2
−1
0
1
2
1
2
3
4
5
6
7
8
9
10
11
Meishan−A
Meishan−B
Meishan−C
European−A
European−B
European−C

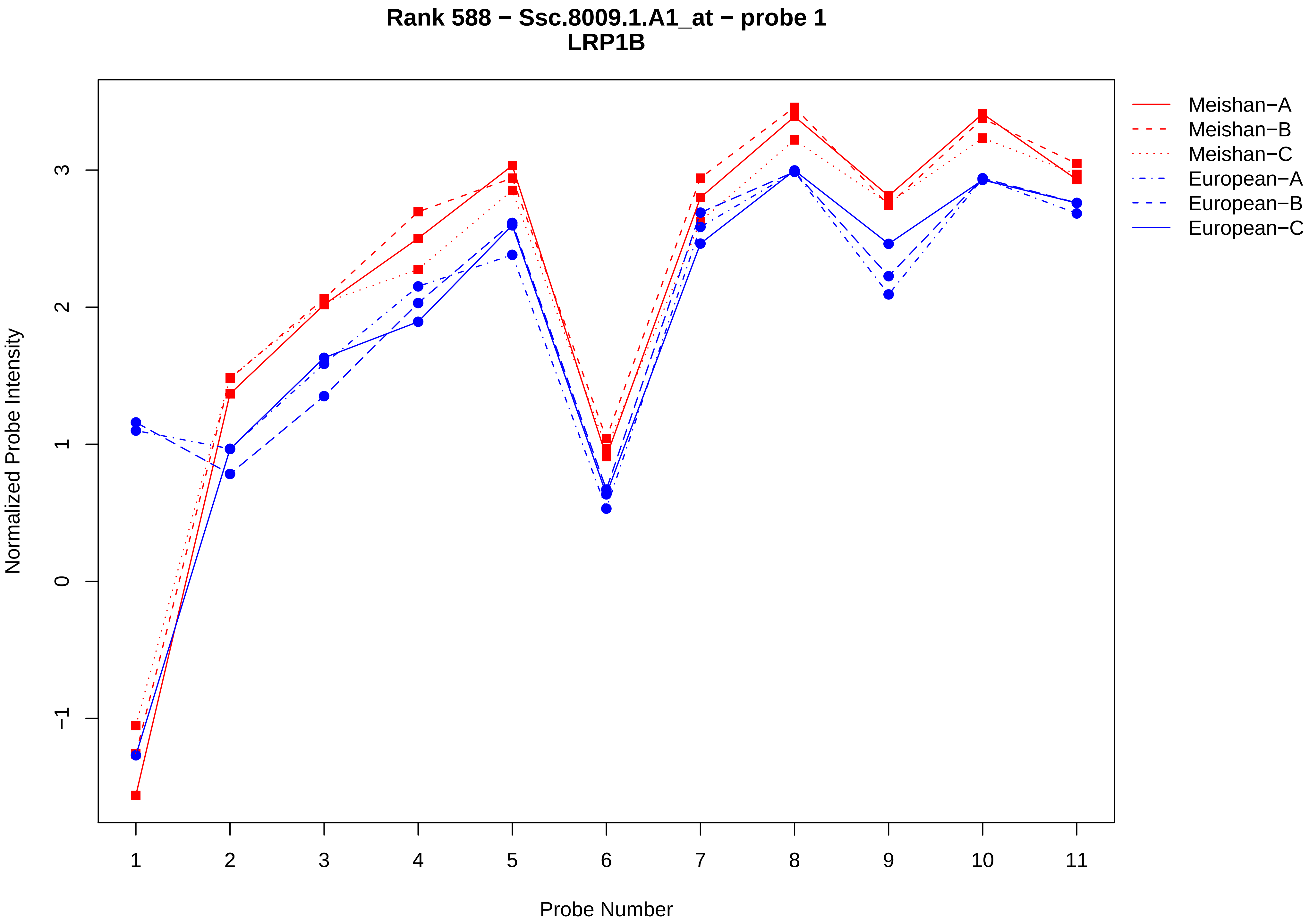

Rank 588 − Ssc.8009.1.A1_at − probe 1
LRP1B
Normalized Probe Intensity
Probe Number
Meishan−A
Meishan−B
Meishan−C
European−A
European−B
European−C
−1
0
1
2
3
1
2
3
4
5
6
7
8
9
10
11

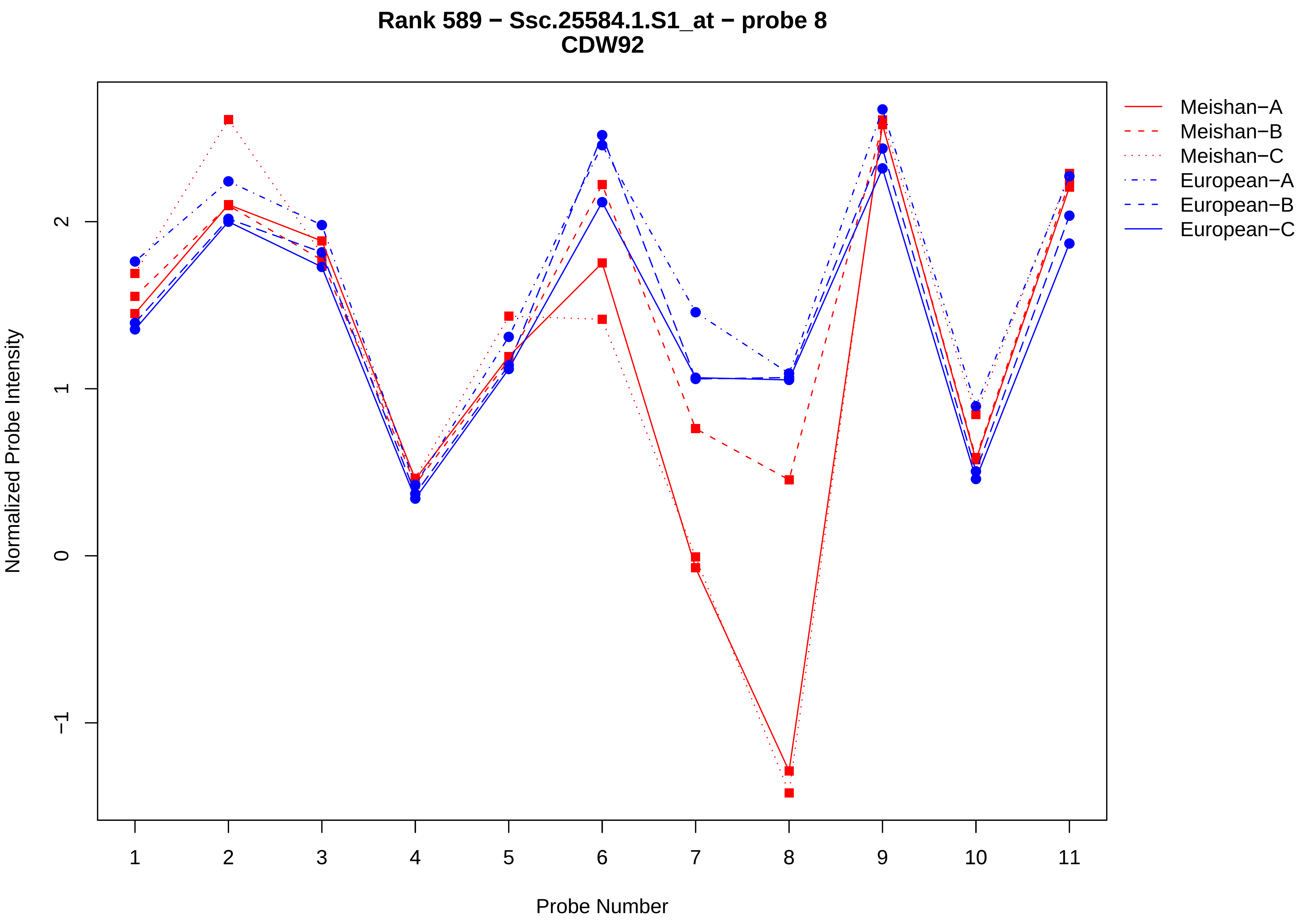

Rank 589 − Ssc.25584.1.S1_at − probe 8
CDW92
Normalized Probe Intensity
Probe Number
−1
0
1
2
1
2
3
4
5
6
7
8
9
10
11
Meishan−A
Meishan−B
Meishan−C
European−A
European−B
European−C

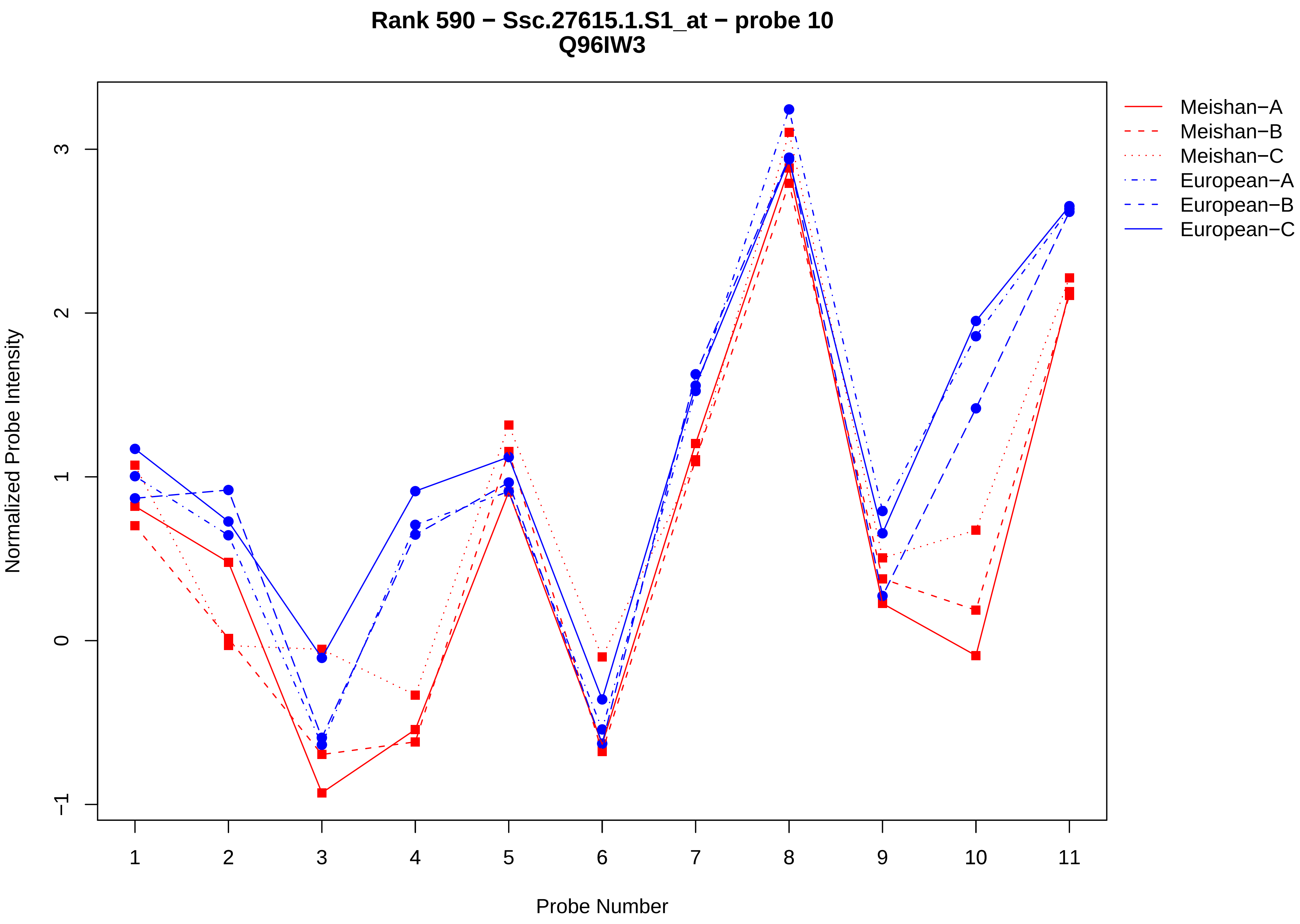

Rank 590 − Ssc.27615.1.S1_at − probe 10
Q96IW3
Normalized Probe Intensity
Probe Number
−1
0
1
2
3
1
2
3
4
5
6
7
8
9
10
11
Meishan−A
Meishan−B
Meishan−C
European−A
European−B
European−C

Rank 591 − Ssc.23897.1.S1_a_at − probe 6
CENTG2

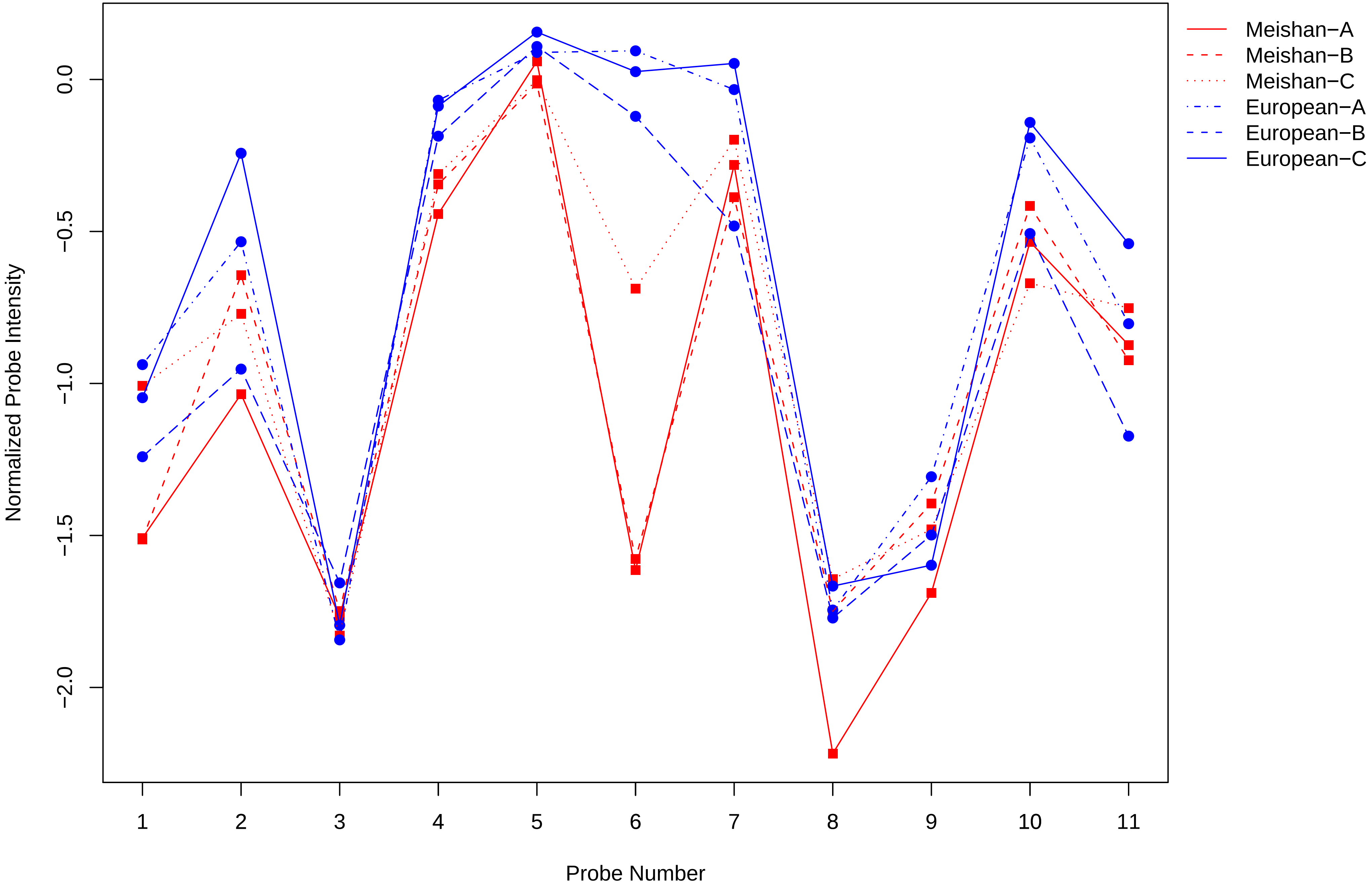

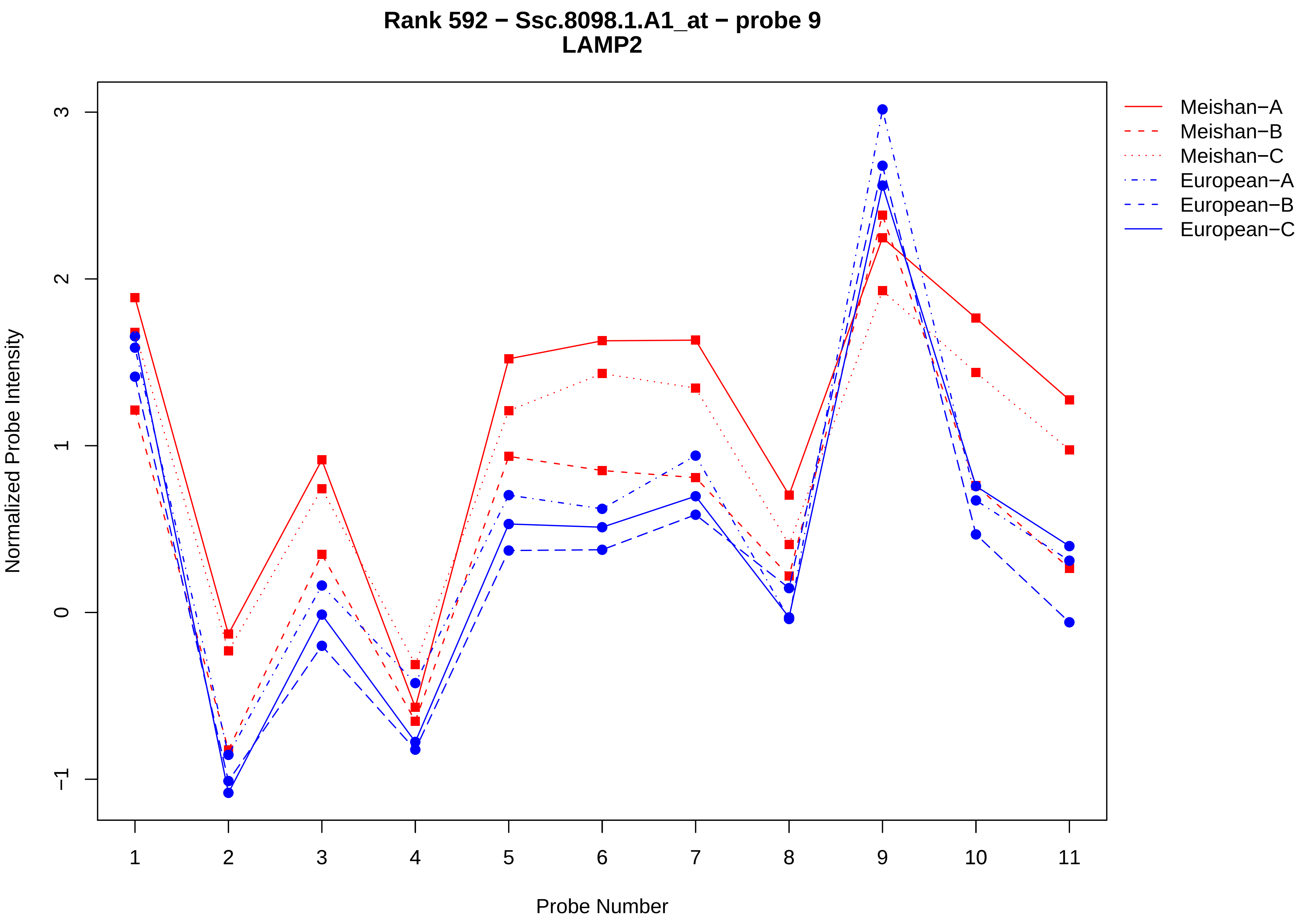

Rank 592 − Ssc.8098.1.A1_at − probe 9
LAMP2
Normalized Probe Intensity
Probe Number
Meishan−A
Meishan−B
Meishan−C
European−A
European−B
European−C

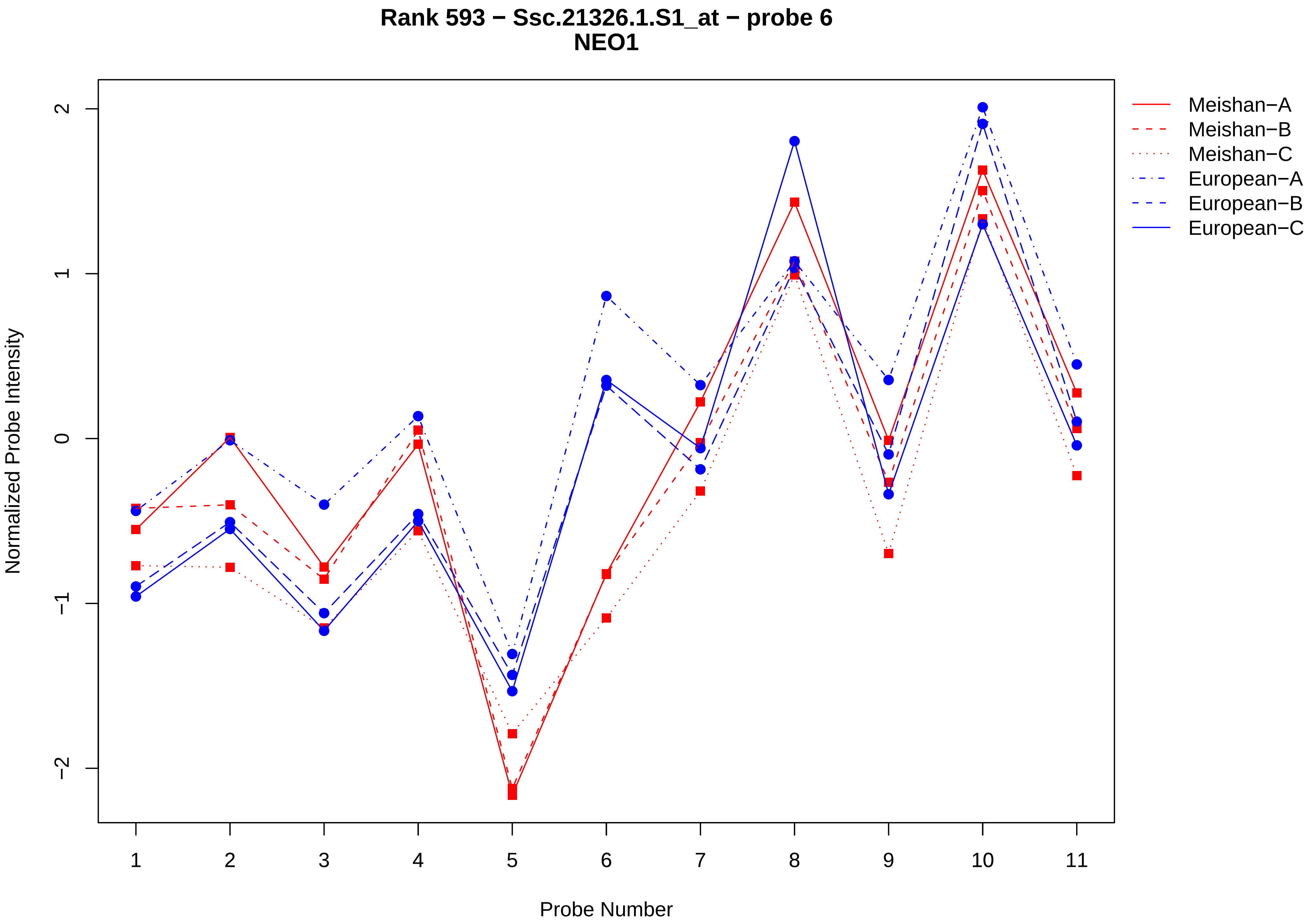
Rank 593 − Ssc.21326.1.S1_at − probe 6
NEO1
Normalized Probe Intensity
Probe Number
Meishan−A
Meishan−B
Meishan−C
European−A
European−B
European−C
−2
−1
0
1
2
1
2
3
4
5
6
7
8
9
10
11

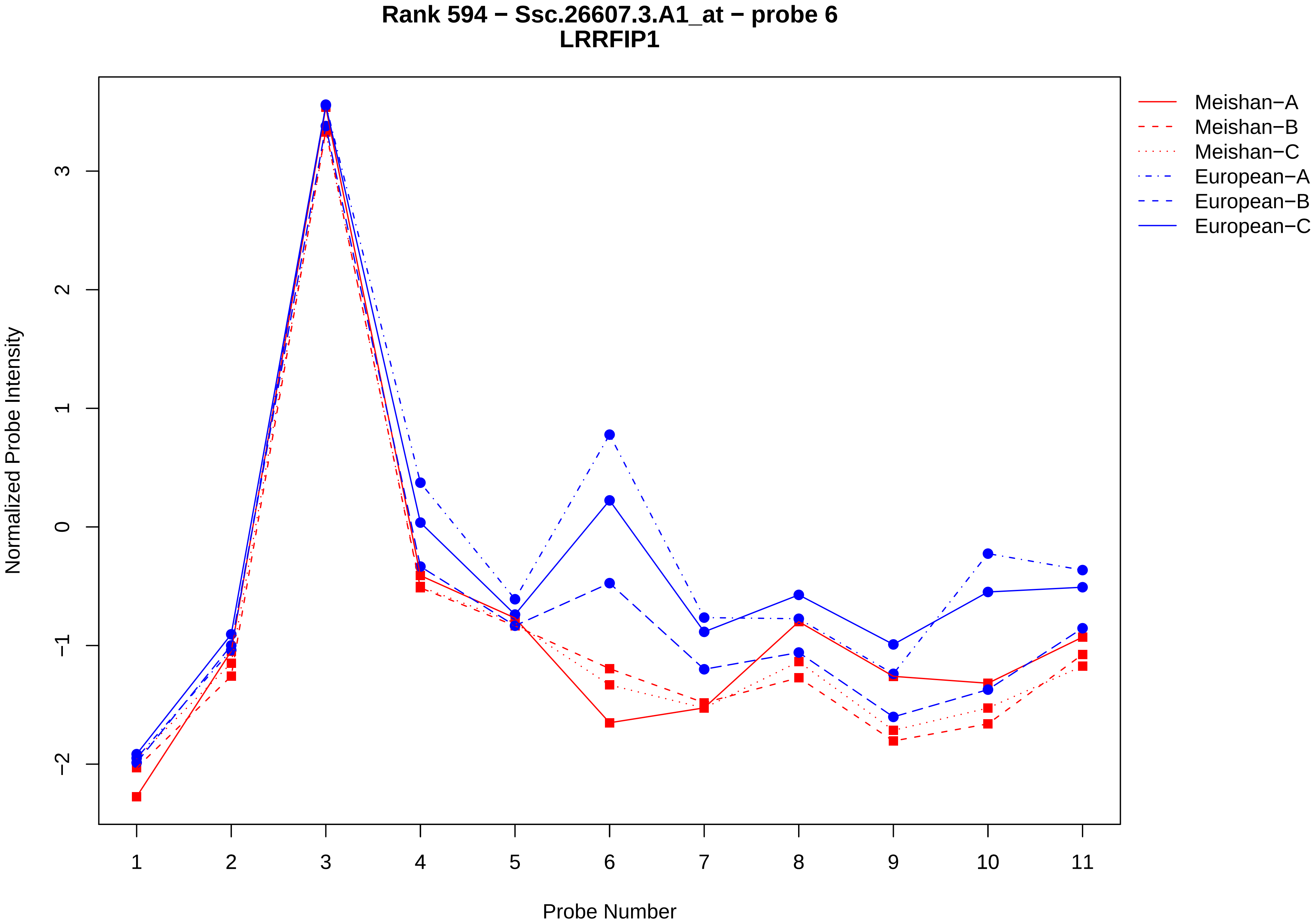

Rank 594 − Ssc.26607.3.A1_at − probe 6
LRRFIP1
Normalized Probe Intensity
Probe Number
−2
−1
0
1
2
3
1
2
3
4
5
6
7
8
9
10
11
Meishan−A
Meishan−B
Meishan−C
European−A
European−B
European−C

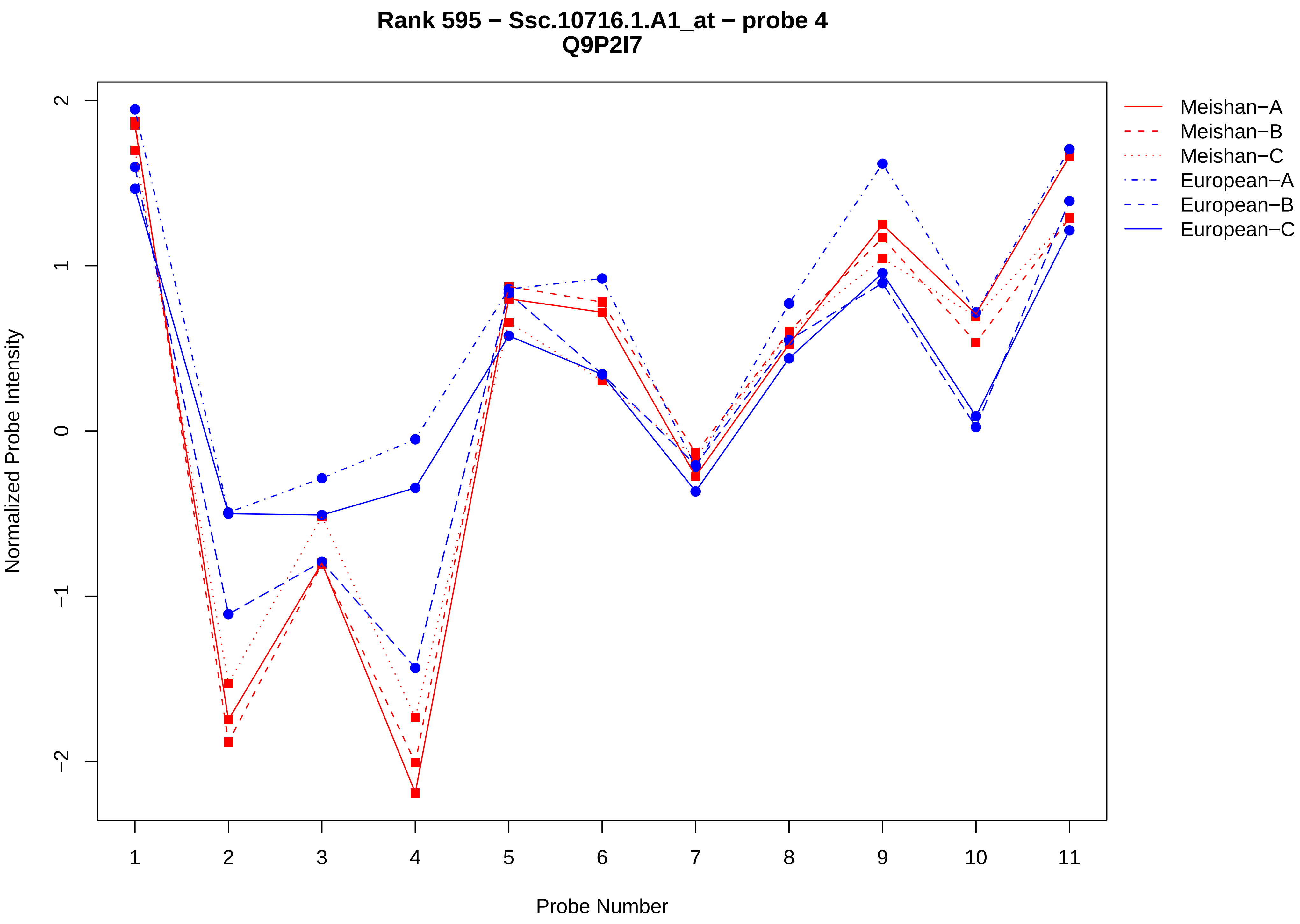

Rank 595 − Ssc.10716.1.A1_at − probe 4
Q9P2I7
Normalized Probe Intensity
Probe Number
Meishan−A
Meishan−B
Meishan−C
European−A
European−B
European−C

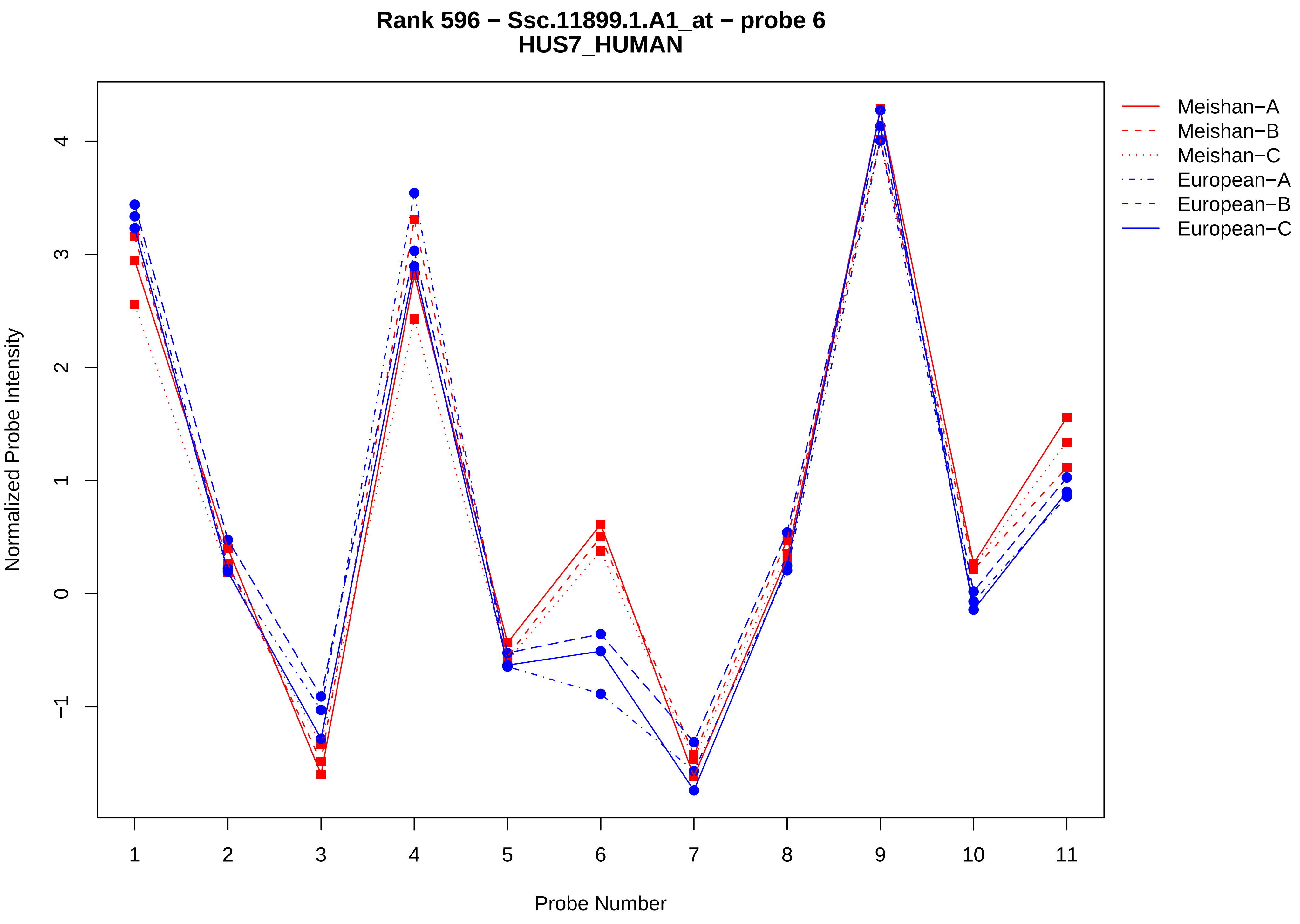

Rank 596 − Ssc.11899.1.A1_at − probe 6
HUS7_HUMAN
Normalized Probe Intensity
Probe Number
−1
0
1
2
3
4
1
2
3
4
5
6
7
8
9
10
11
Meishan−A
Meishan−B
Meishan−C
European−A
European−B
European−C

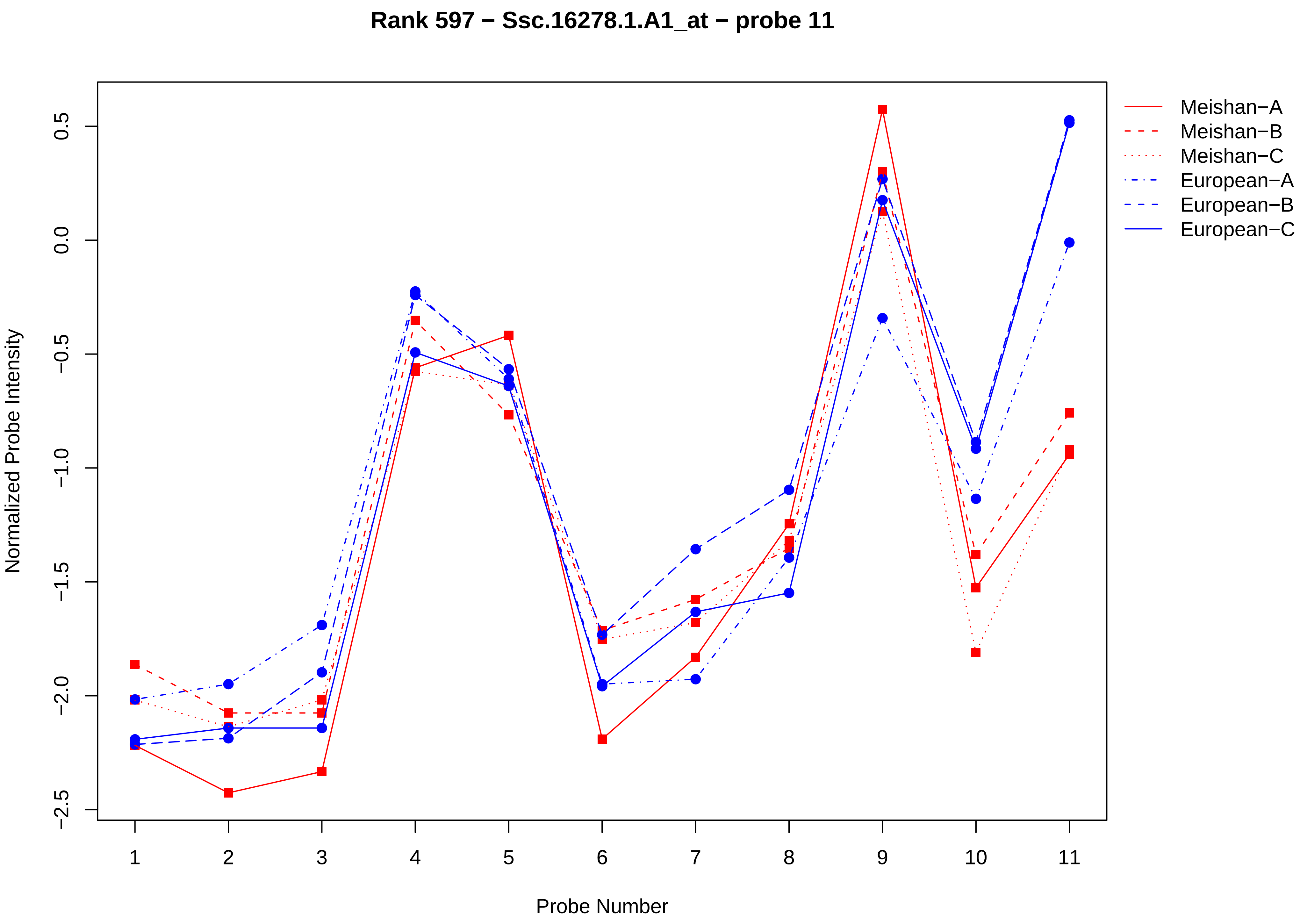
Rank 597 − Ssc.16278.1.A1_at − probe 11
Normalized Probe Intensity
Probe Number
0.5
0.0
−0.5
−1.0
−1.5
−2.0
−2.5
1
2
3
4
5
6
7
8
9
10
11
Meishan−A
Meishan−B
Meishan−C
European−A
European−B
European−C

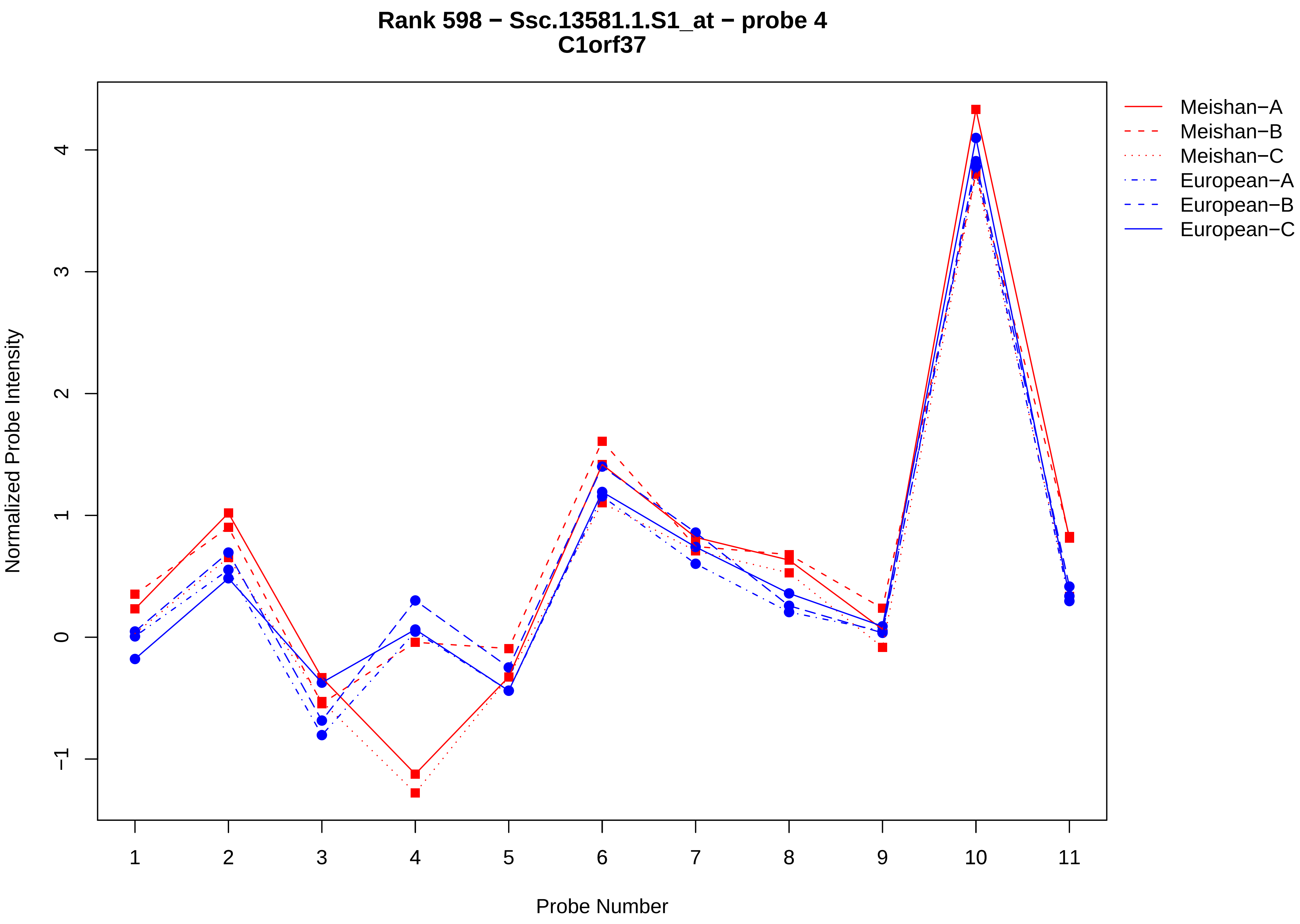

Rank 598 − Ssc.13581.1.S1_at − probe 4
C1orf37
Normalized Probe Intensity
Probe Number
−1
0
1
2
3
4
1
2
3
4
5
6
7
8
9
10
11
Meishan−A
Meishan−B
Meishan−C
European−A
European−B
European−C

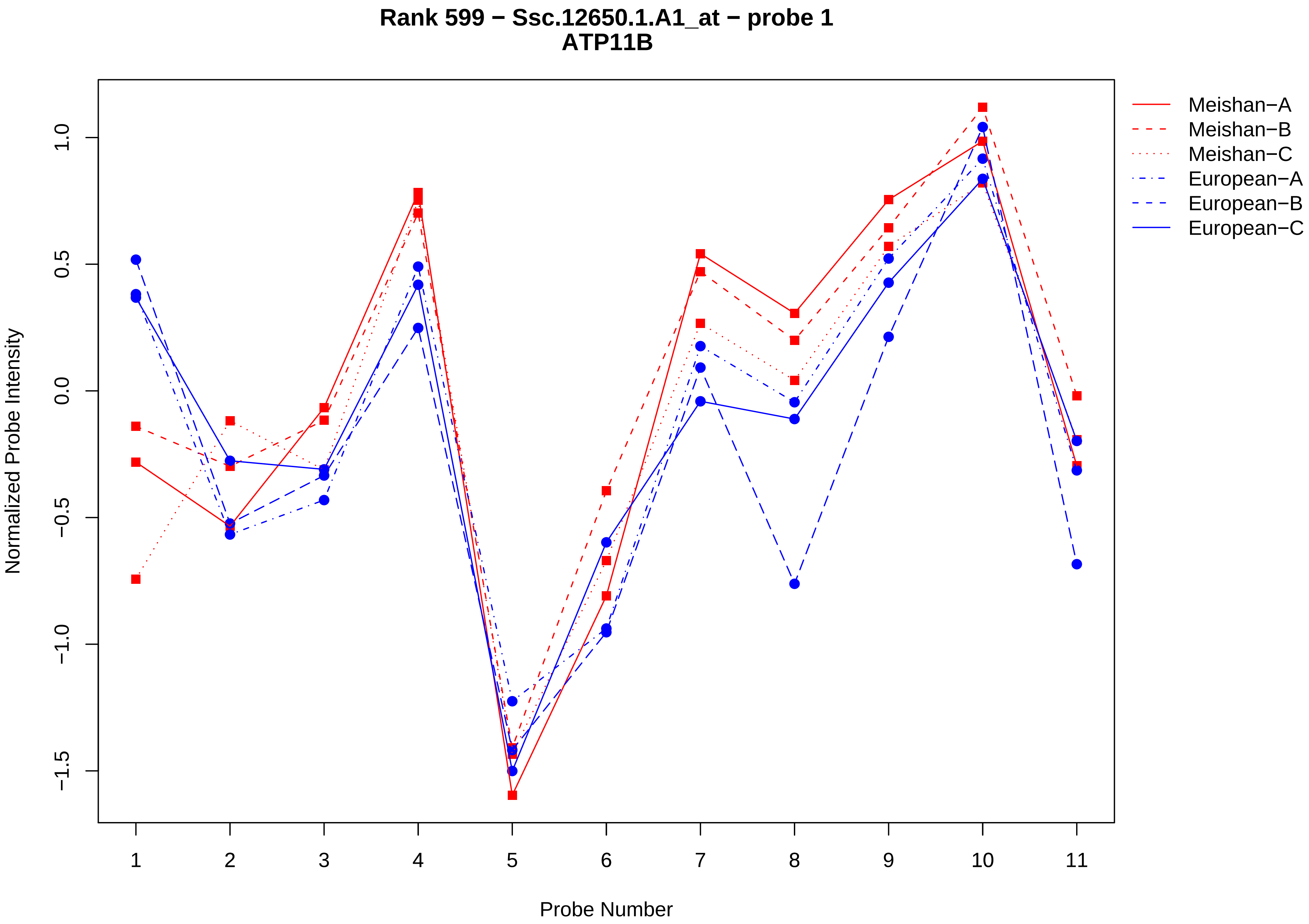
Rank 599 − Ssc.12650.1.A1_at − probe 1
ATP11B
Normalized Probe Intensity
Probe Number
1.0
0.5
0.0
−0.5
−1.0
−1.5
1
2
3
4
5
6
7
8
9
10
11
Meishan−A
Meishan−B
Meishan−C
European−A
European−B
European−C

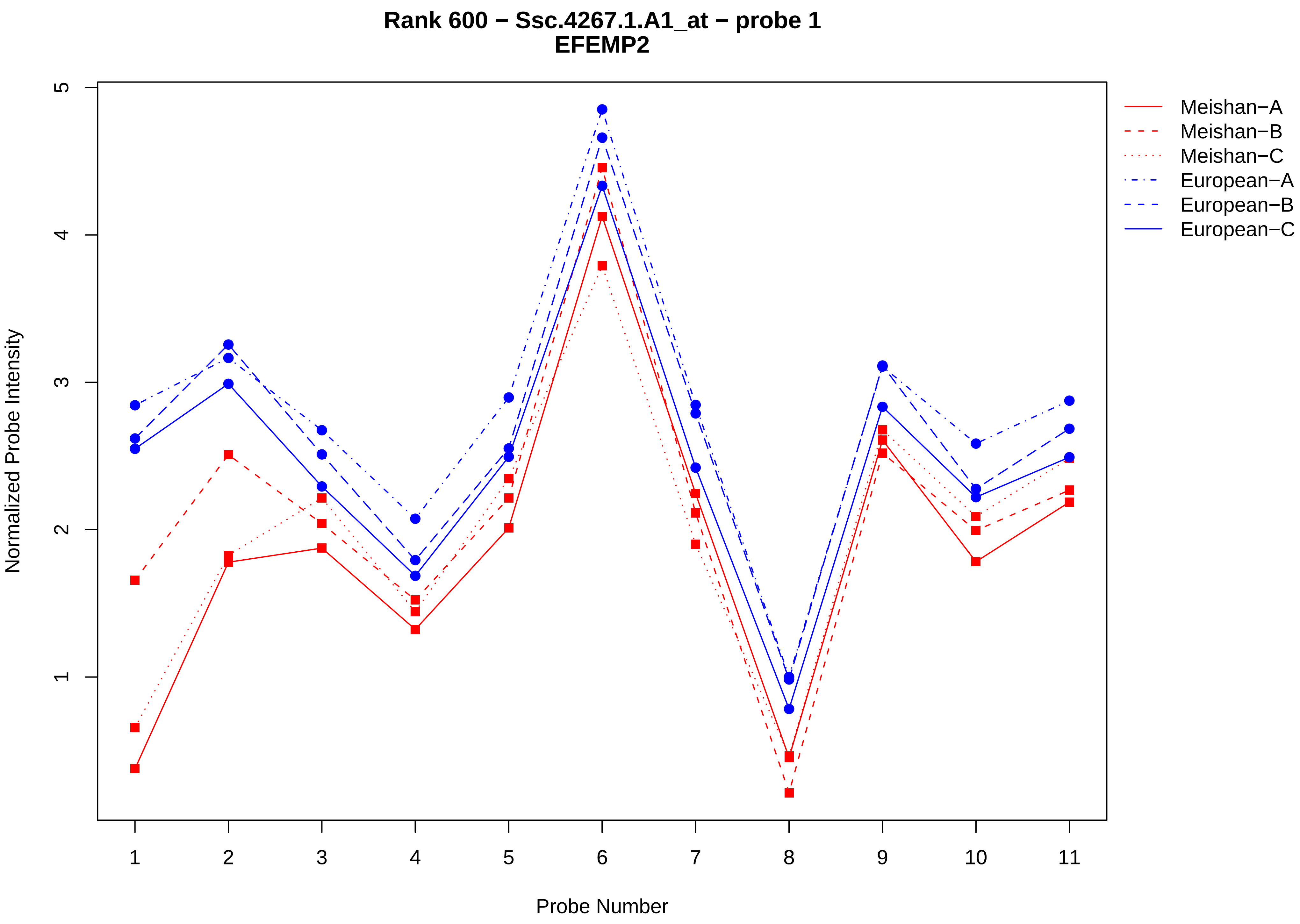

Rank 600 − Ssc.4267.1.A1_at − probe 1
EFEMP2
Normalized Probe Intensity
Probe Number
1
2
3
4
5
1
2
3
4
5
6
7
8
9
10
11
Meishan−A
Meishan−B
Meishan−C
European−A
European−B
European−C

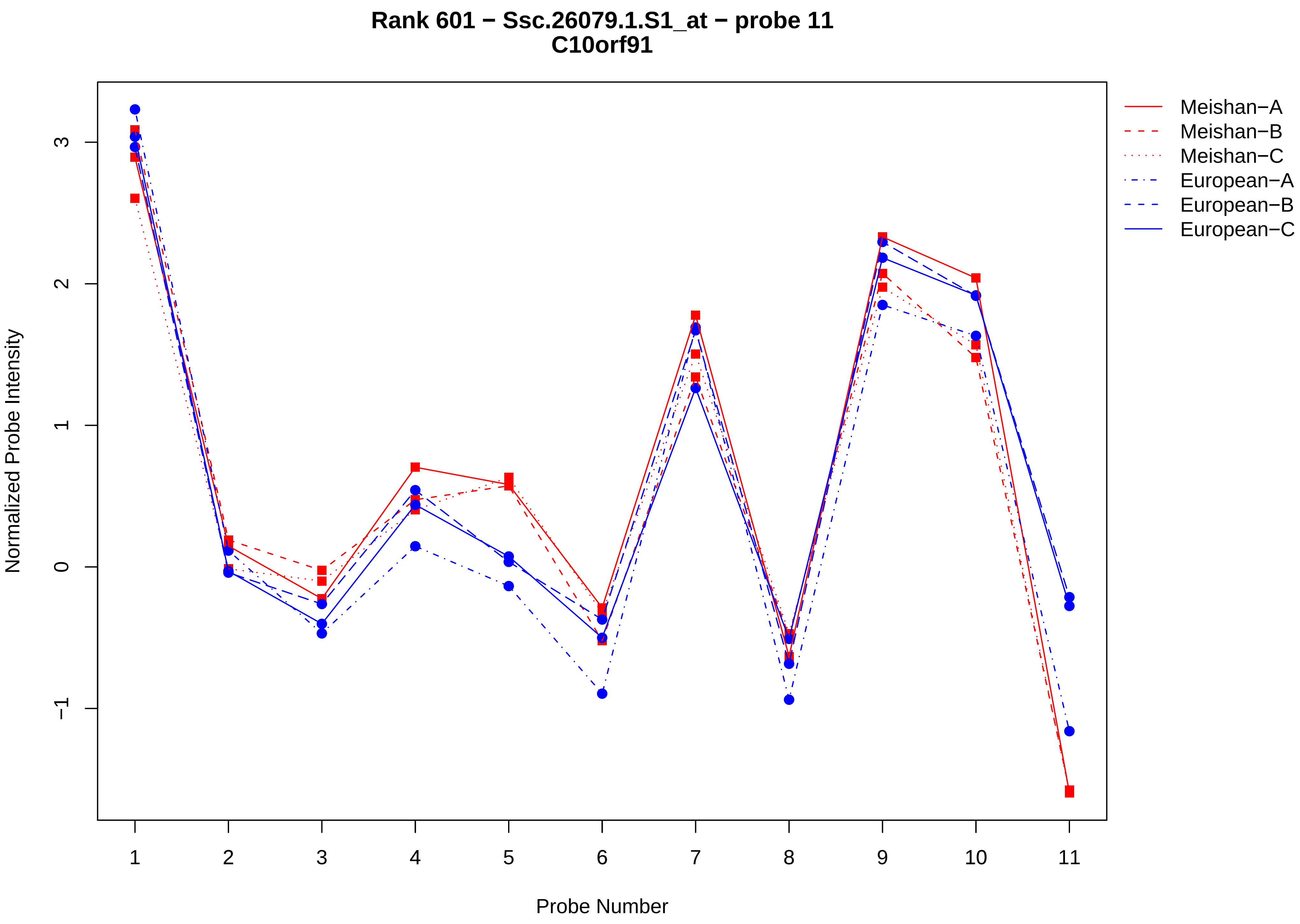

Rank 601 − Ssc.26079.1.S1_at − probe 11
C10orf91
Normalized Probe Intensity
Probe Number
Meishan−A
Meishan−B
Meishan−C
European−A
European−B
European−C

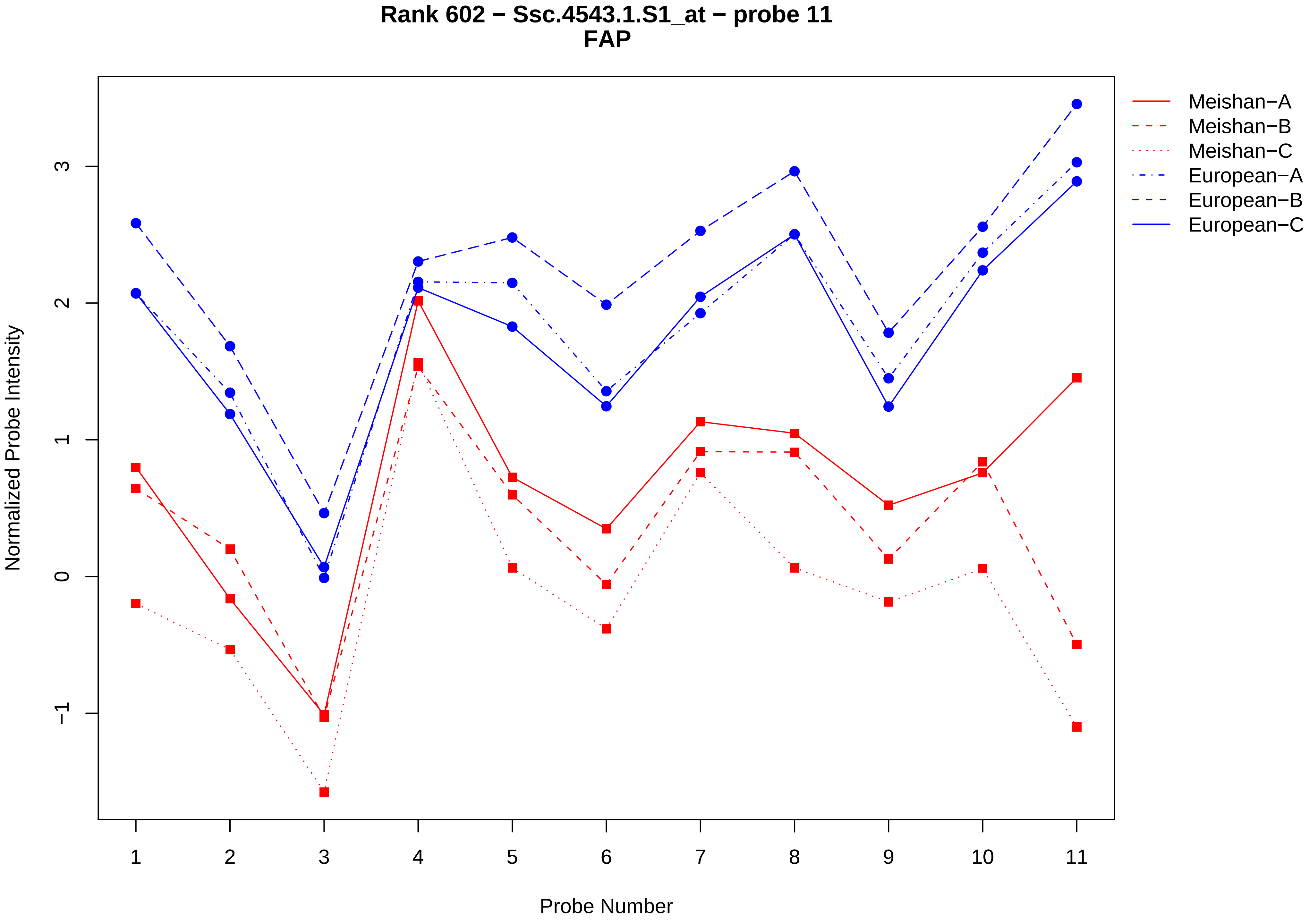
Rank 602 − Ssc.4543.1.S1_at − probe 11
FAP
Normalized Probe Intensity
Probe Number
−1
0
1
2
3
1
2
3
4
5
6
7
8
9
10
11
Meishan−A
Meishan−B
Meishan−C
European−A
European−B
European−C

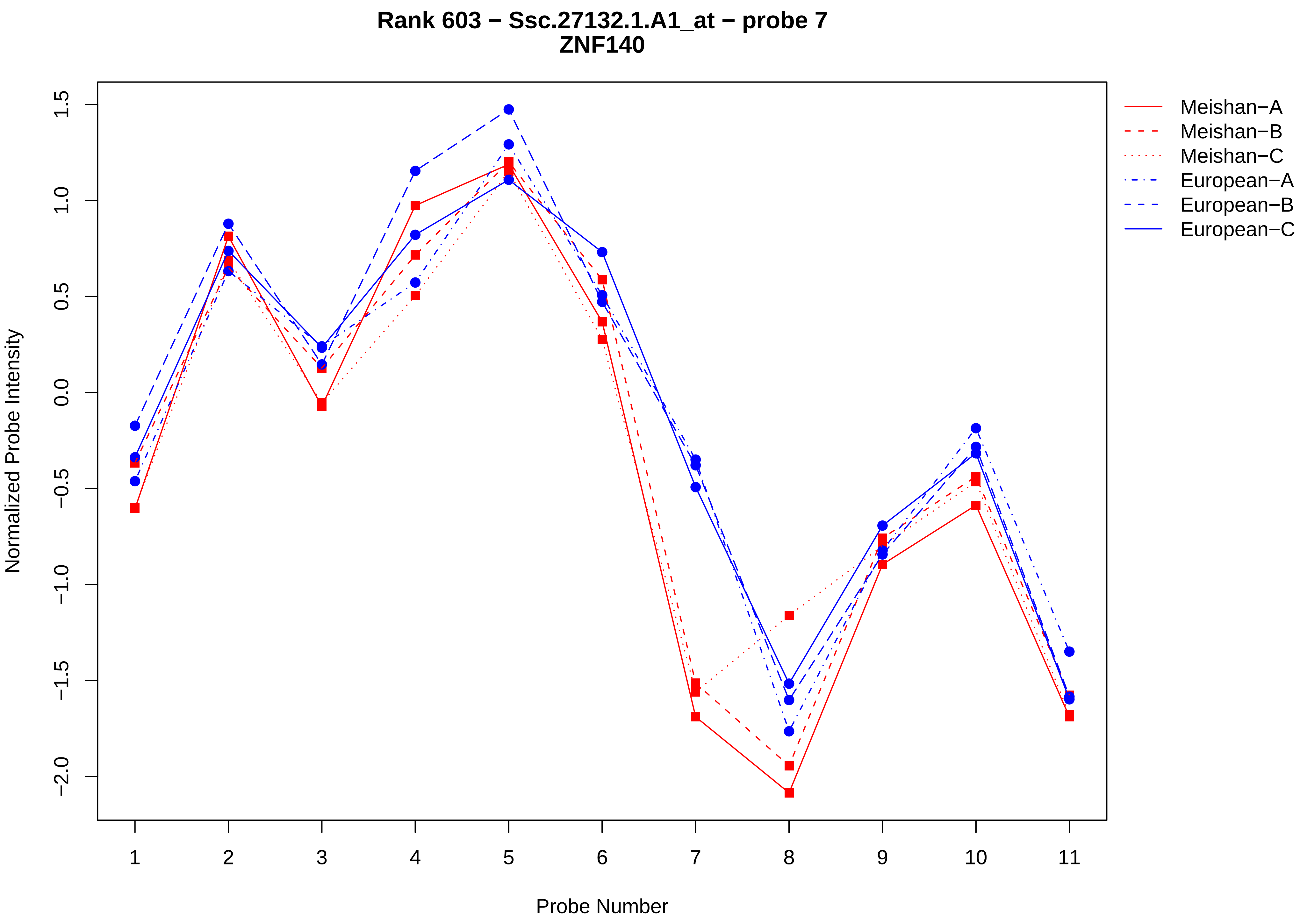
Rank 603 − Ssc.27132.1.A1_at − probe 7
ZNF140
Normalized Probe Intensity
Probe Number
1.5
1.0
0.5
0.0
−0.5
−1.0
−1.5
−2.0
1
2
3
4
5
6
7
8
9
10
11
Meishan−A
Meishan−B
Meishan−C
European−A
European−B
European−C

Rank 604 − Ssc.10727.1.A1_at − probe 3
RBL2

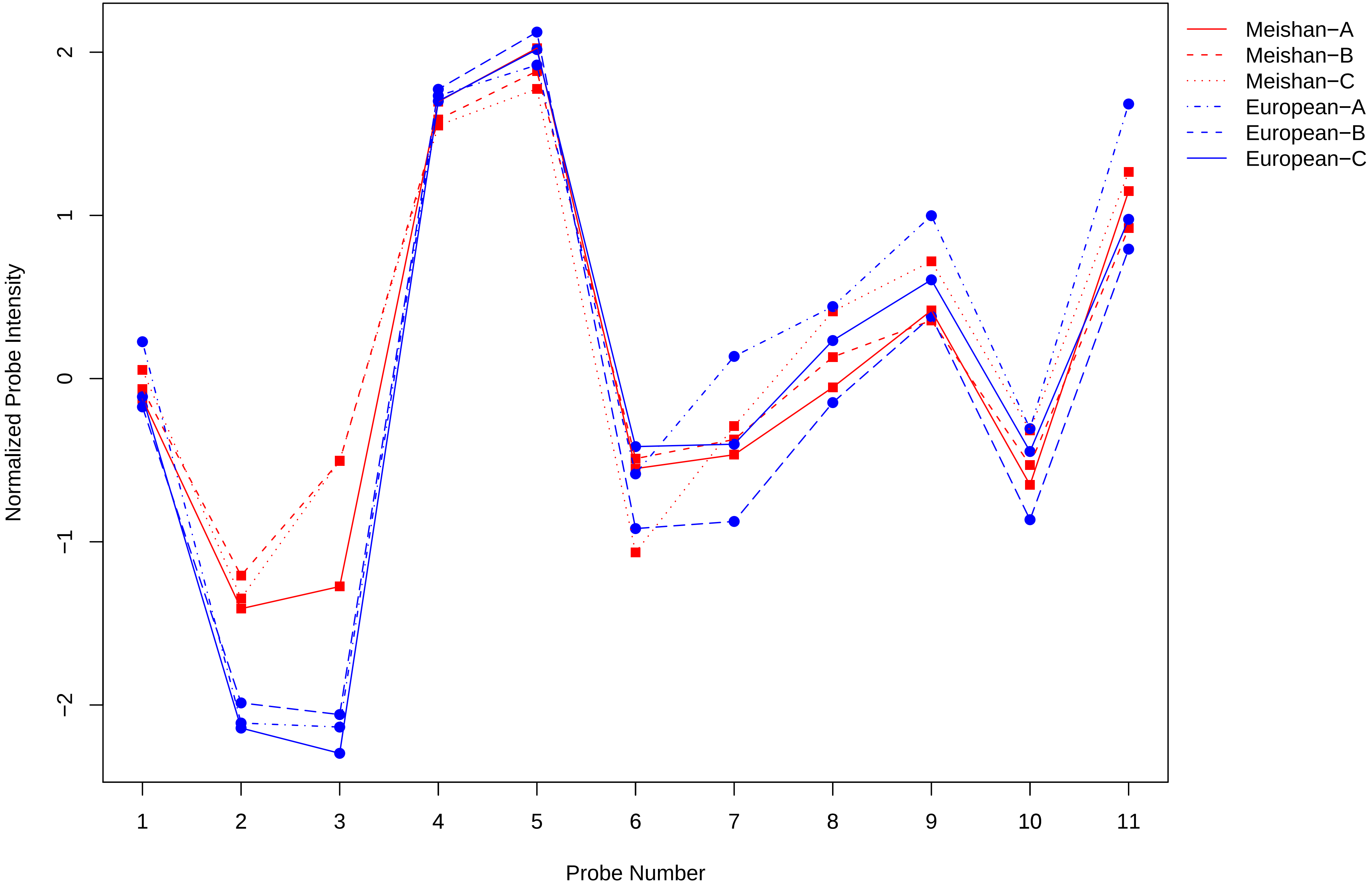

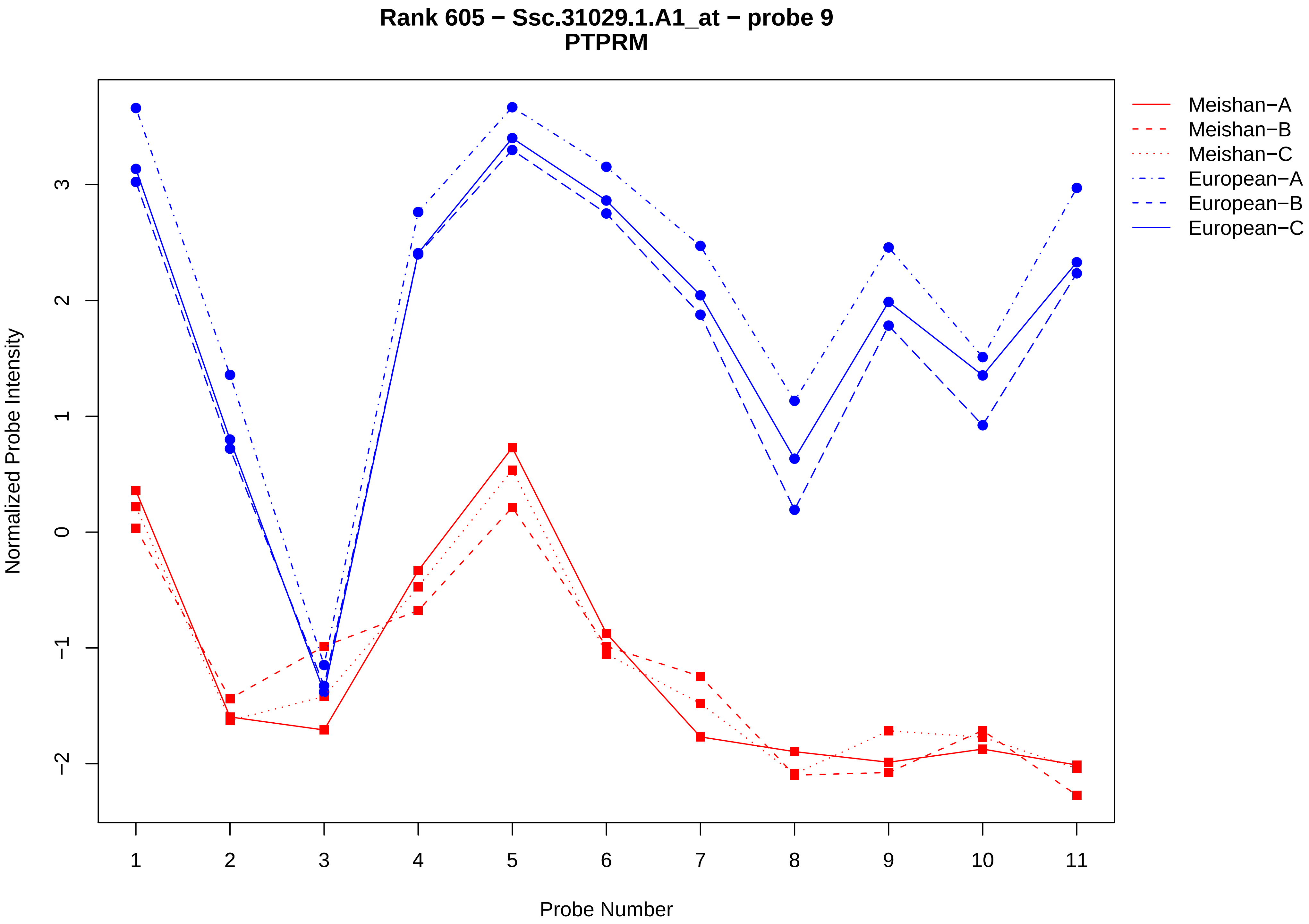

Rank 605 − Ssc.31029.1.A1_at − probe 9
PTPRM
Normalized Probe Intensity
Probe Number
Meishan−A
Meishan−B
Meishan−C
European−A
European−B
European−C
−2
−1
0
1
2
3
1
2
3
4
5
6
7
8
9
10
11

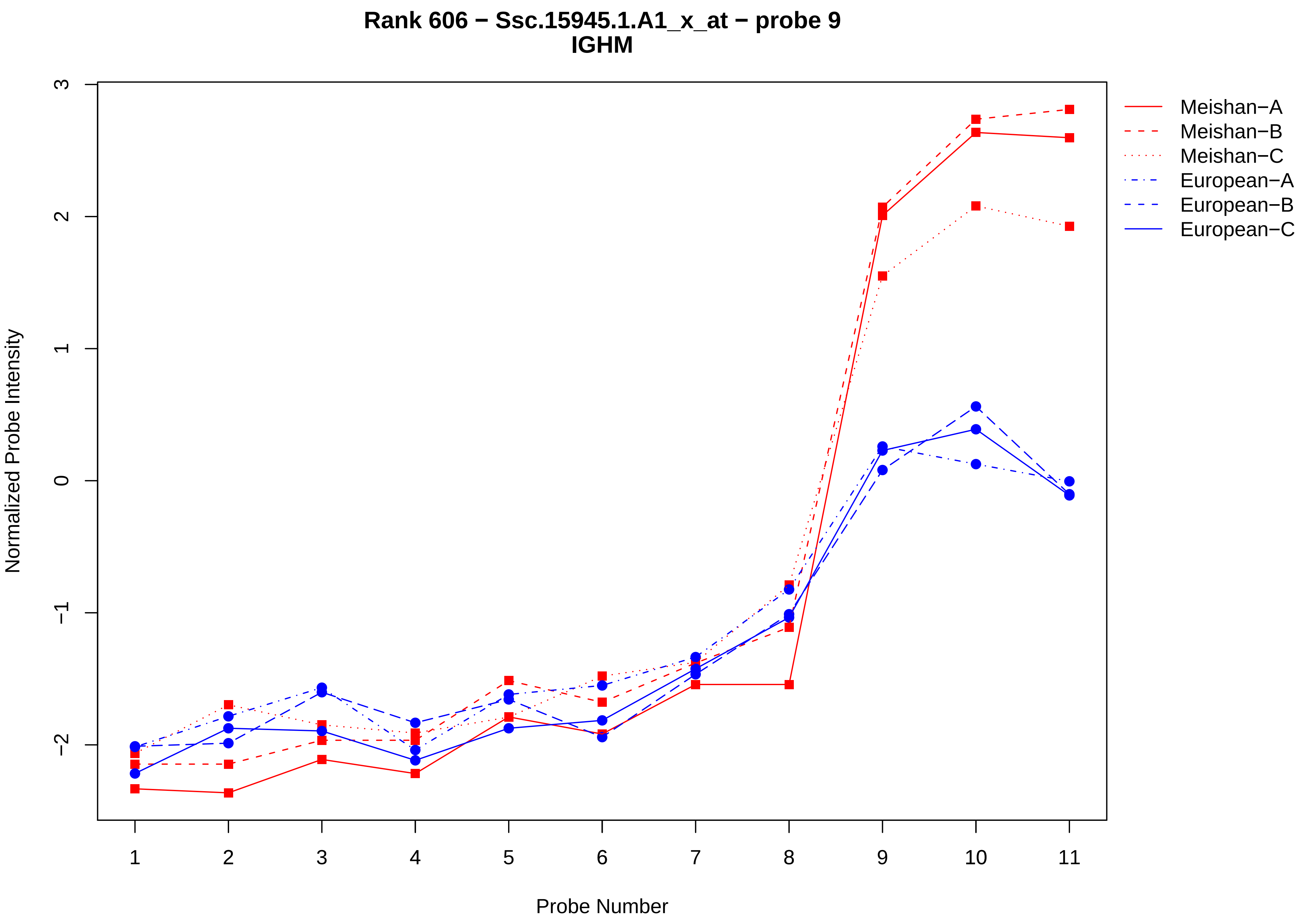

Rank 606 − Ssc.15945.1.A1_x_at − probe 9
IGHM
Normalized Probe Intensity
Probe Number
Meishan−A
Meishan−B
Meishan−C
European−A
European−B
European−C
3
2
1
0
−1
−2
1
2
3
4
5
6
7
8
9
10
11

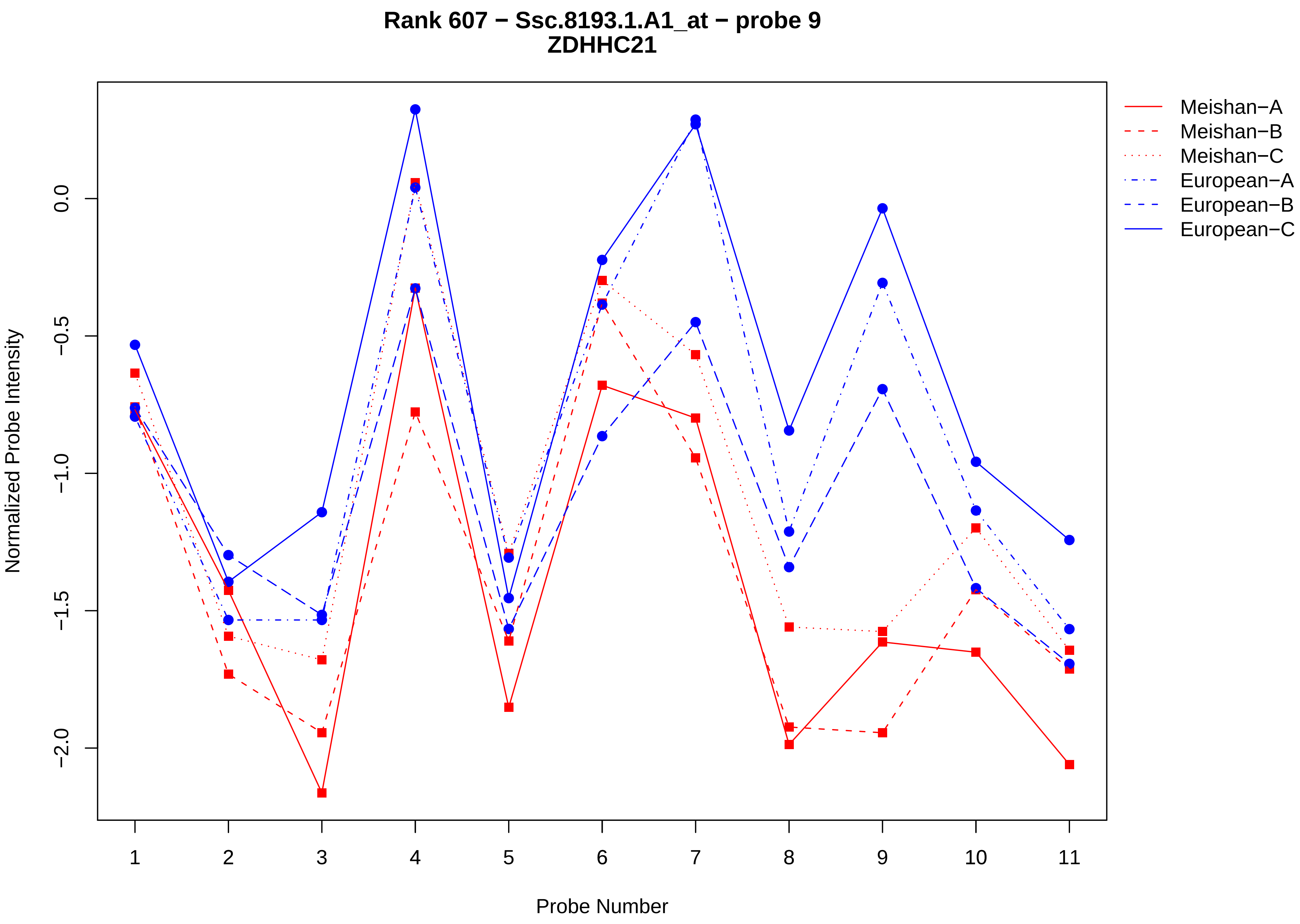

Rank 607 − Ssc.8193.1.A1_at − probe 9
ZDHHC21
Normalized Probe Intensity
Probe Number
0.0
−0.5
−1.0
−1.5
−2.0
1
2
3
4
5
6
7
8
9
10
11
Meishan−A
Meishan−B
Meishan−C
European−A
European−B
European−C

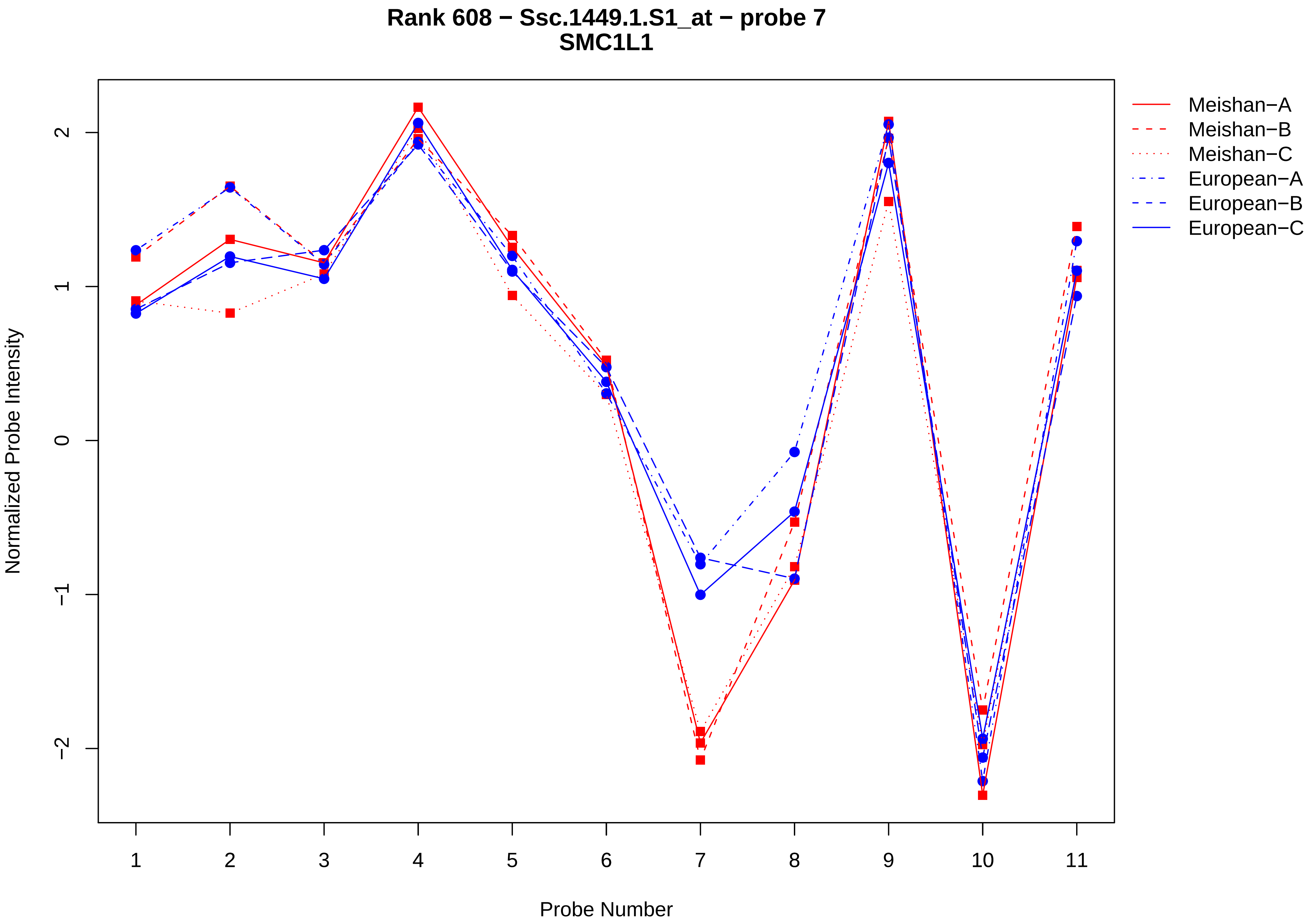
Rank 608 − Ssc.1449.1.S1_at − probe 7
SMC1L1
Normalized Probe Intensity
Probe Number
−2
−1
0
1
2
1
2
3
4
5
6
7
8
9
10
11
Meishan−A
Meishan−B
Meishan−C
European−A
European−B
European−C

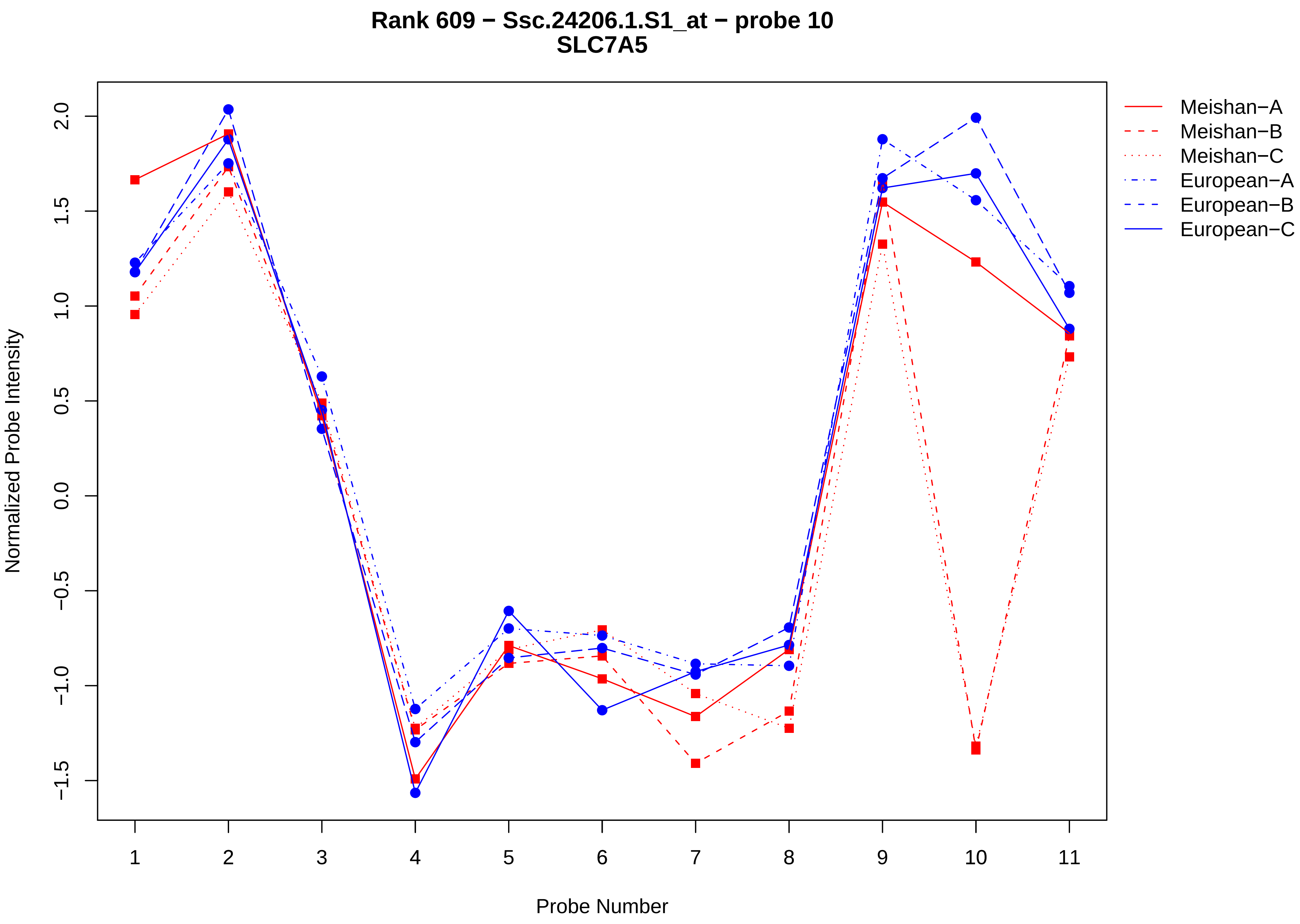
Rank 609 − Ssc.24206.1.S1_at − probe 10
SLC7A5
Normalized Probe Intensity
Probe Number
Meishan−A
Meishan−B
Meishan−C
European−A
European−B
European−C
2.0
1.5
1.0
0.5
0.0
−0.5
−1.0
−1.5
1
2
3
4
5
6
7
8
9
10
11

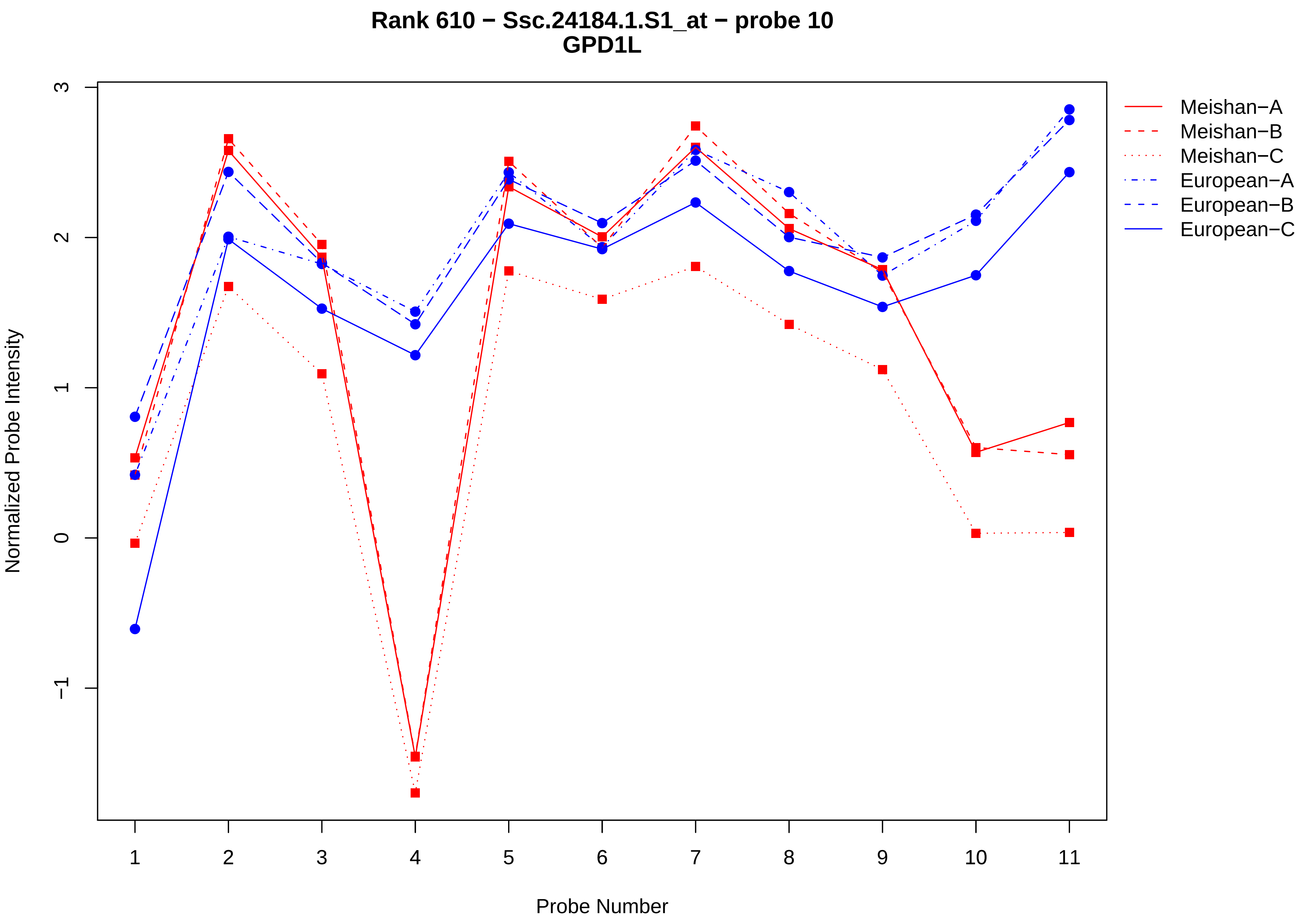

Rank 610 − Ssc.24184.1.S1_at − probe 10
GPD1L
Normalized Probe Intensity
Probe Number
−1
0
1
2
3
1
2
3
4
5
6
7
8
9
10
11
Meishan−A
Meishan−B
Meishan−C
European−A
European−B
European−C

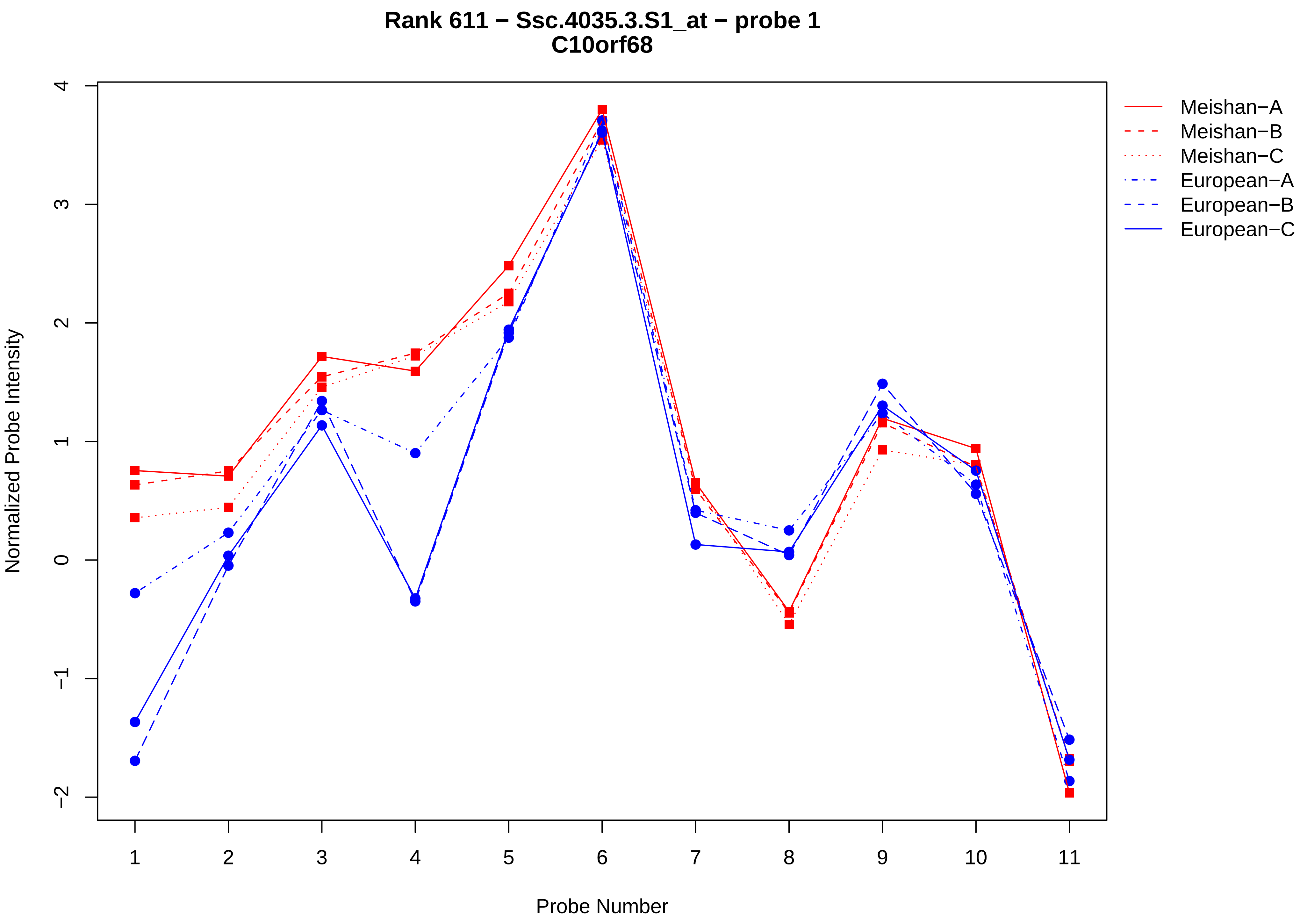
Rank 611 − Ssc.4035.3.S1_at − probe 1
C10orf68
Normalized Probe Intensity
Probe Number
Meishan−A
Meishan−B
Meishan−C
European−A
European−B
European−C

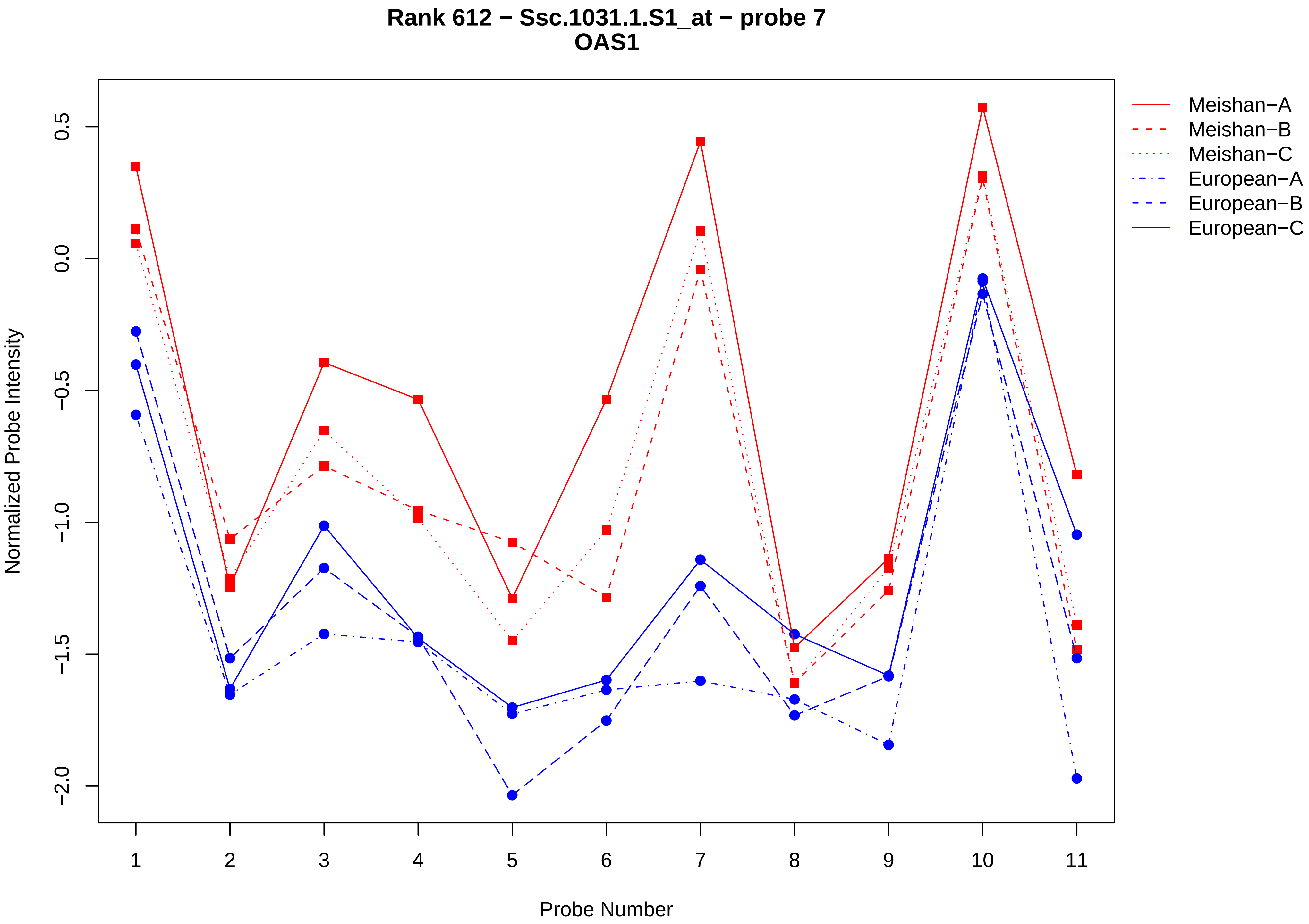

Rank 612 − Ssc.1031.1.S1_at − probe 7
OAS1
Normalized Probe Intensity
Probe Number
Meishan−A
Meishan−B
Meishan−C
European−A
European−B
European−C
0.5
0.0
−0.5
−1.0
−1.5
−2.0
1
2
3
4
5
6
7
8
9
10
11

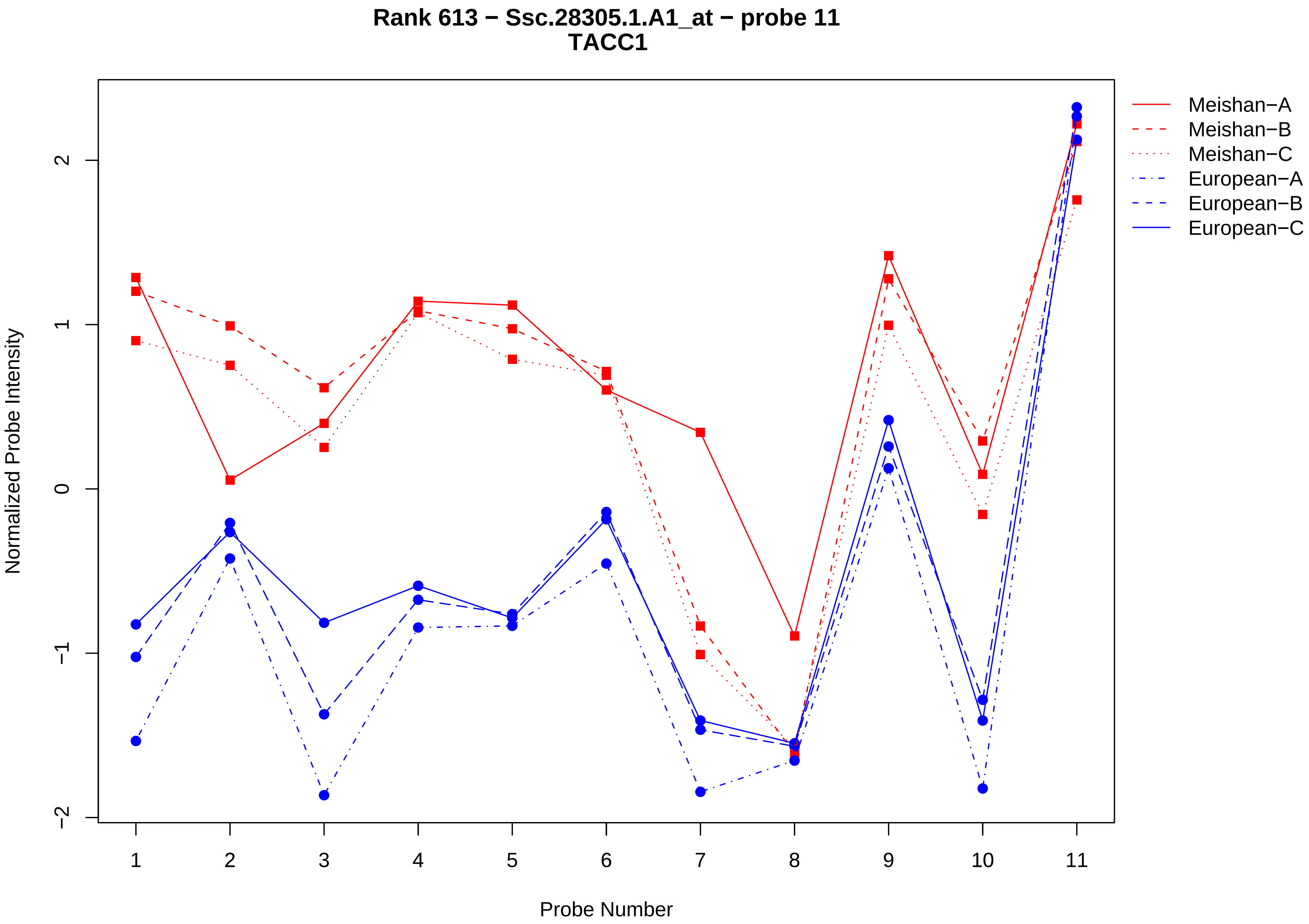
Rank 613 − Ssc.28305.1.A1_at − probe 11
TACC1
Normalized Probe Intensity
Probe Number
Meishan−A
Meishan−B
Meishan−C
European−A
European−B
European−C
−2
−1
0
1
2
1
2
3
4
5
6
7
8
9
10
11

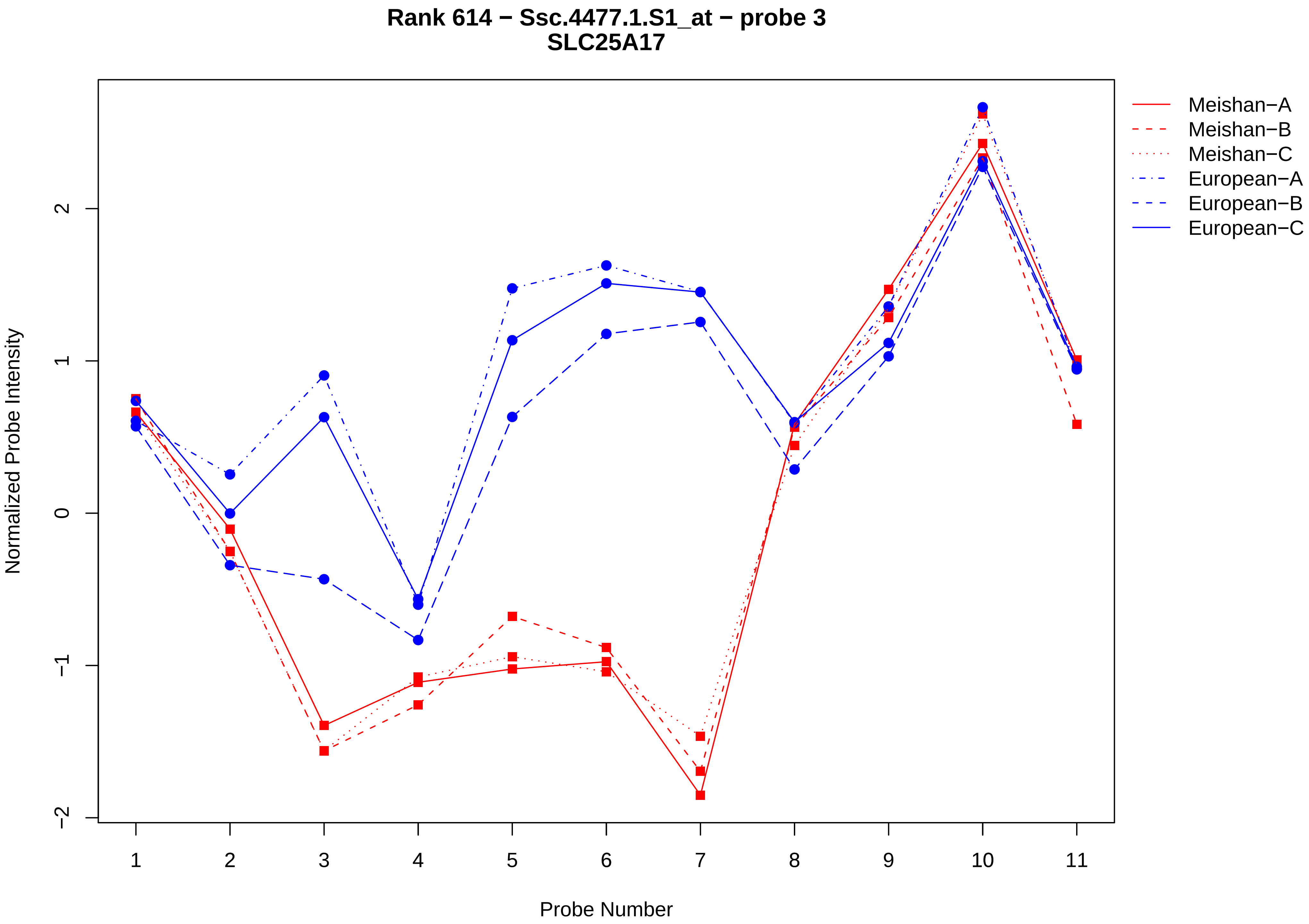

Rank 614 − Ssc.4477.1.S1_at − probe 3
SLC25A17
Normalized Probe Intensity
Probe Number
Meishan−A
Meishan−B
Meishan−C
European−A
European−B
European−C
−2
−1
0
1
2
1
2
3
4
5
6
7
8
9
10
11

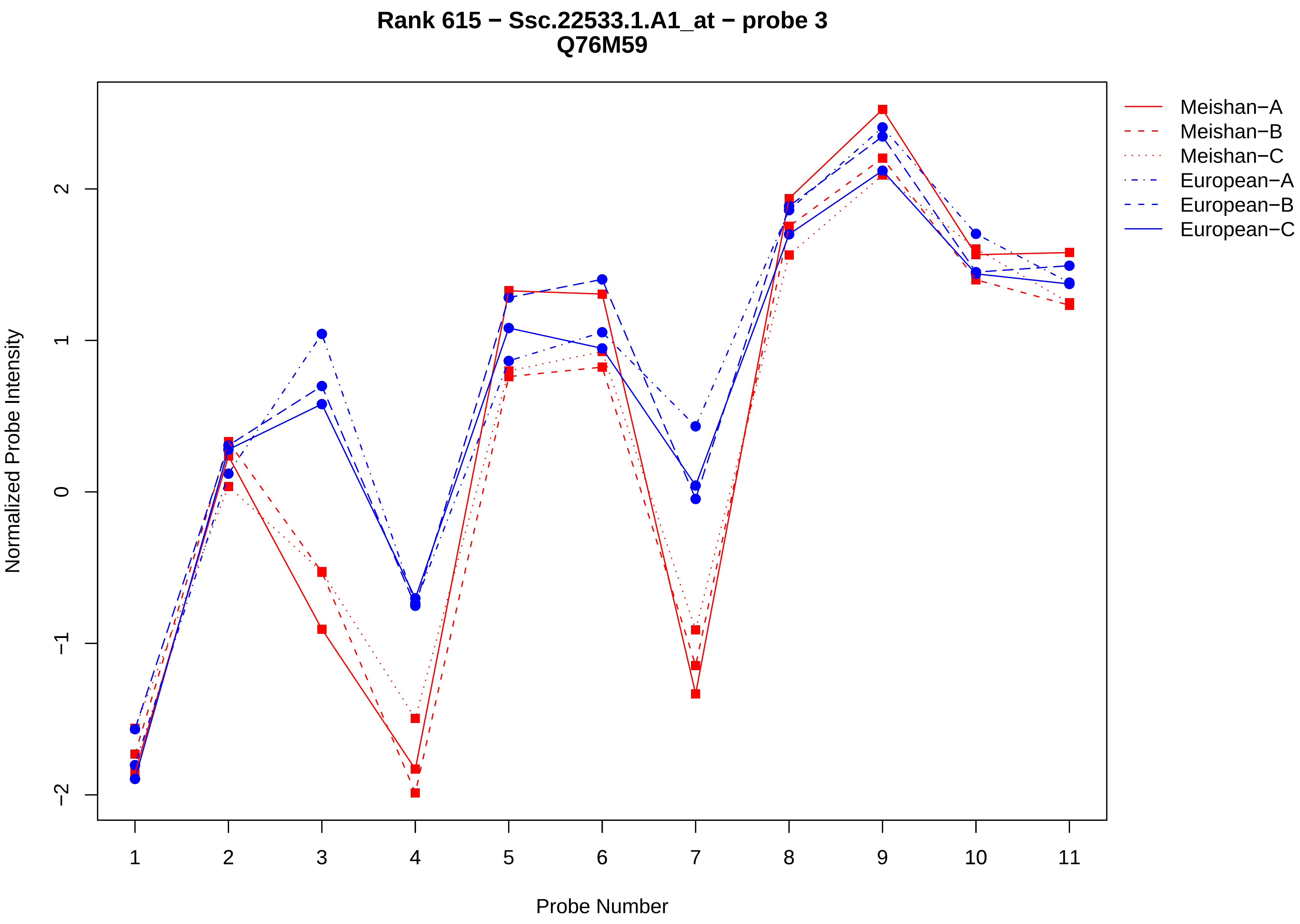

Rank 615 − Ssc.22533.1.A1_at − probe 3
Q76M59
Normalized Probe Intensity
Probe Number
Meishan−A
Meishan−B
Meishan−C
European−A
European−B
European−C
−2
−1
0
1
2
1
2
3
4
5
6
7
8
9
10
11

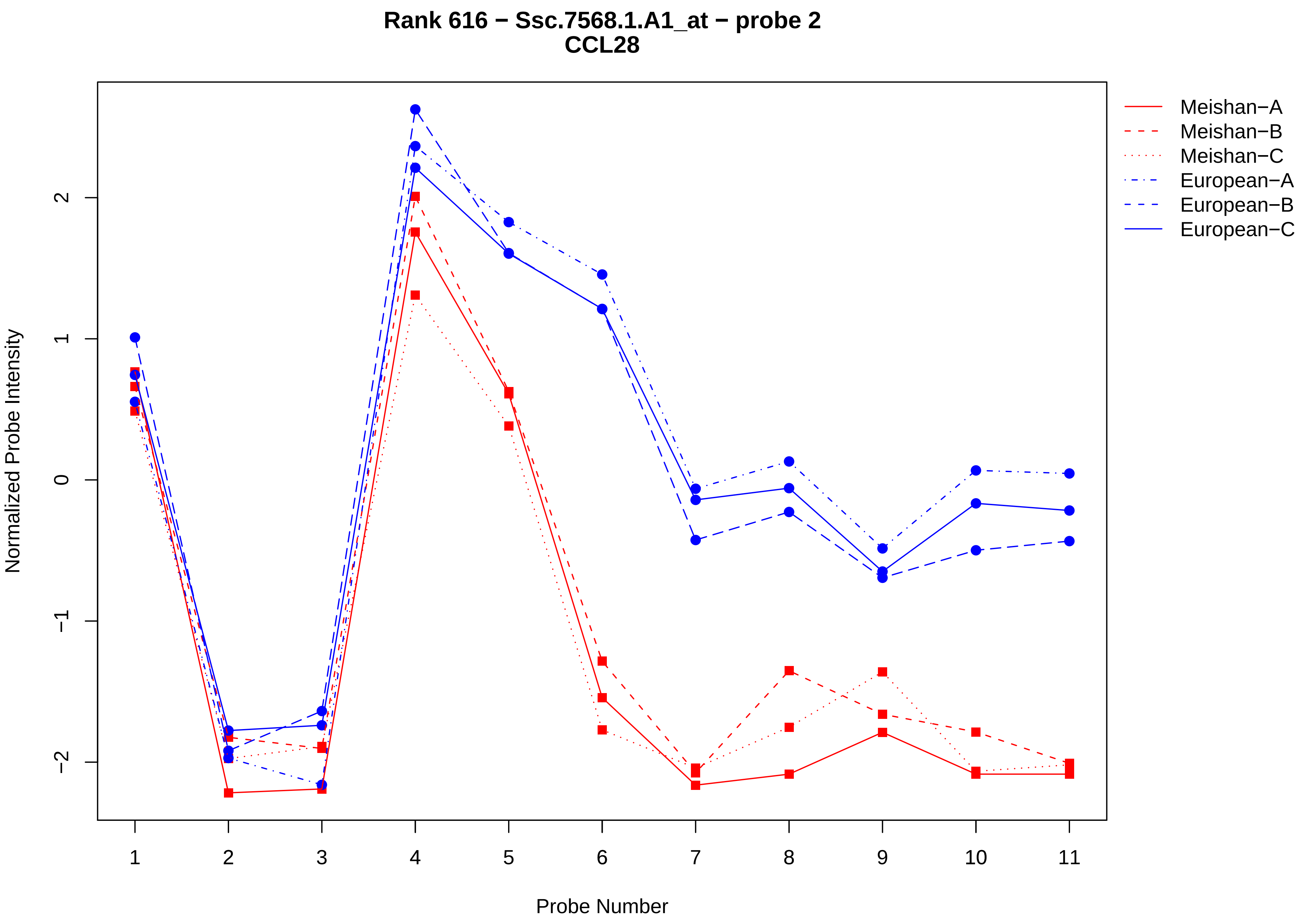

Rank 616 − Ssc.7568.1.A1_at − probe 2
CCL28
Normalized Probe Intensity
Probe Number
Meishan−A
Meishan−B
Meishan−C
European−A
European−B
European−C
−2
−1
0
1
2
1
2
3
4
5
6
7
8
9
10
11

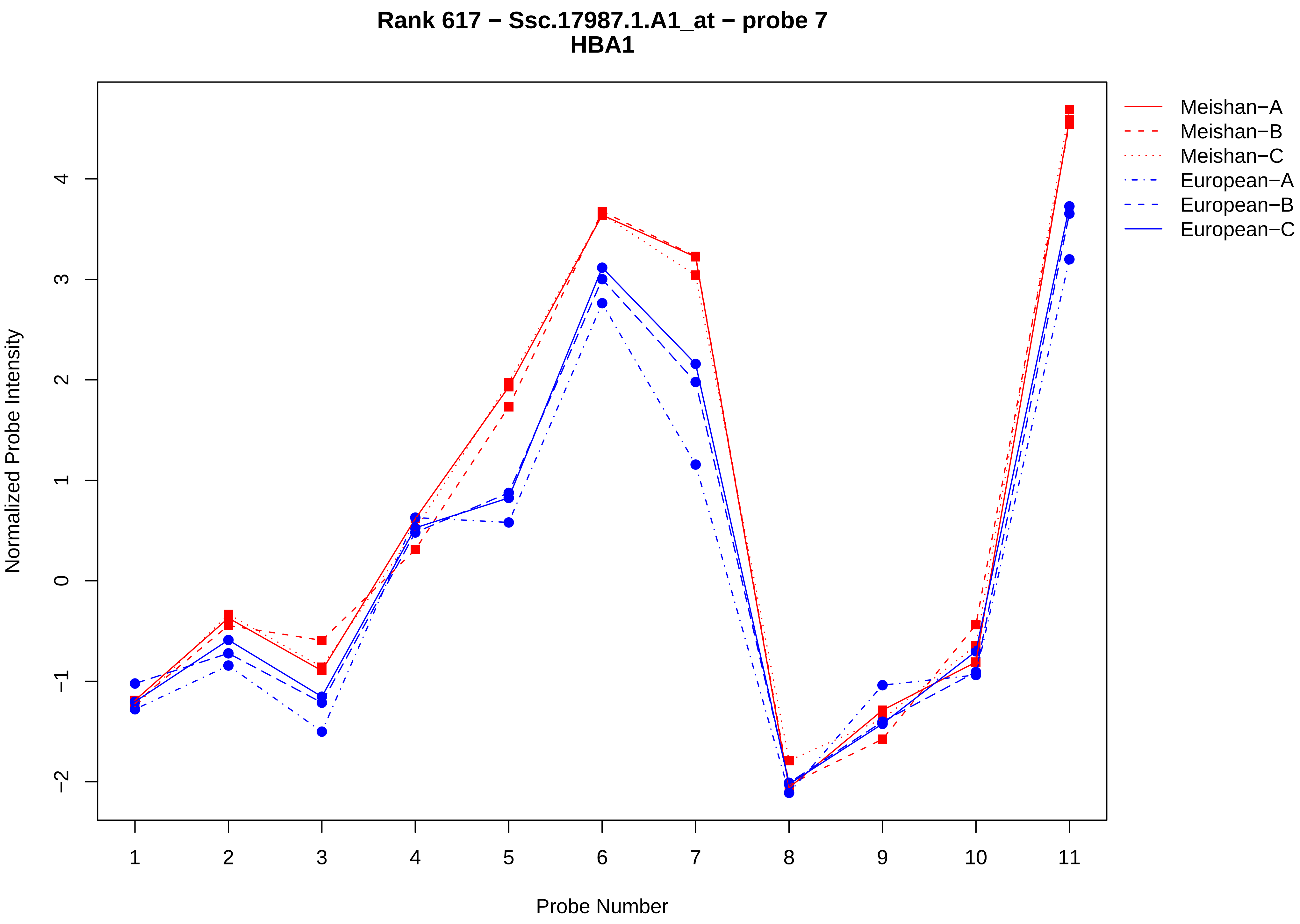
Rank 617 − Ssc.17987.1.A1_at − probe 7
HBA1
Normalized Probe Intensity
Probe Number
−2
−1
0
1
2
3
4
1
2
3
4
5
6
7
8
9
10
11
Meishan−A
Meishan−B
Meishan−C
European−A
European−B
European−C

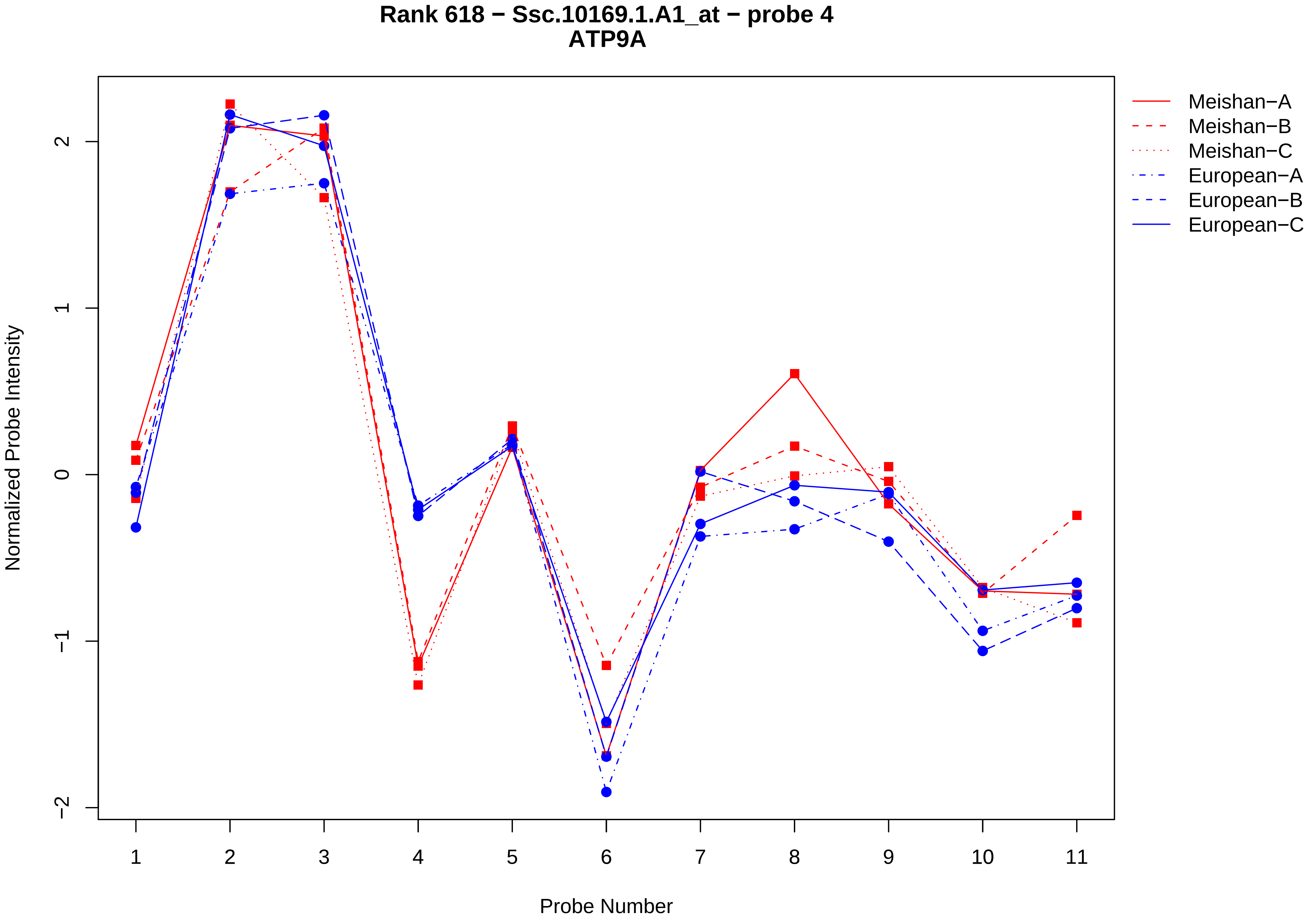

Rank 618 − Ssc.10169.1.A1_at − probe 4
ATP9A
Normalized Probe Intensity
Probe Number
−2
−1
0
1
2
1
2
3
4
5
6
7
8
9
10
11
Meishan−A
Meishan−B
Meishan−C
European−A
European−B
European−C

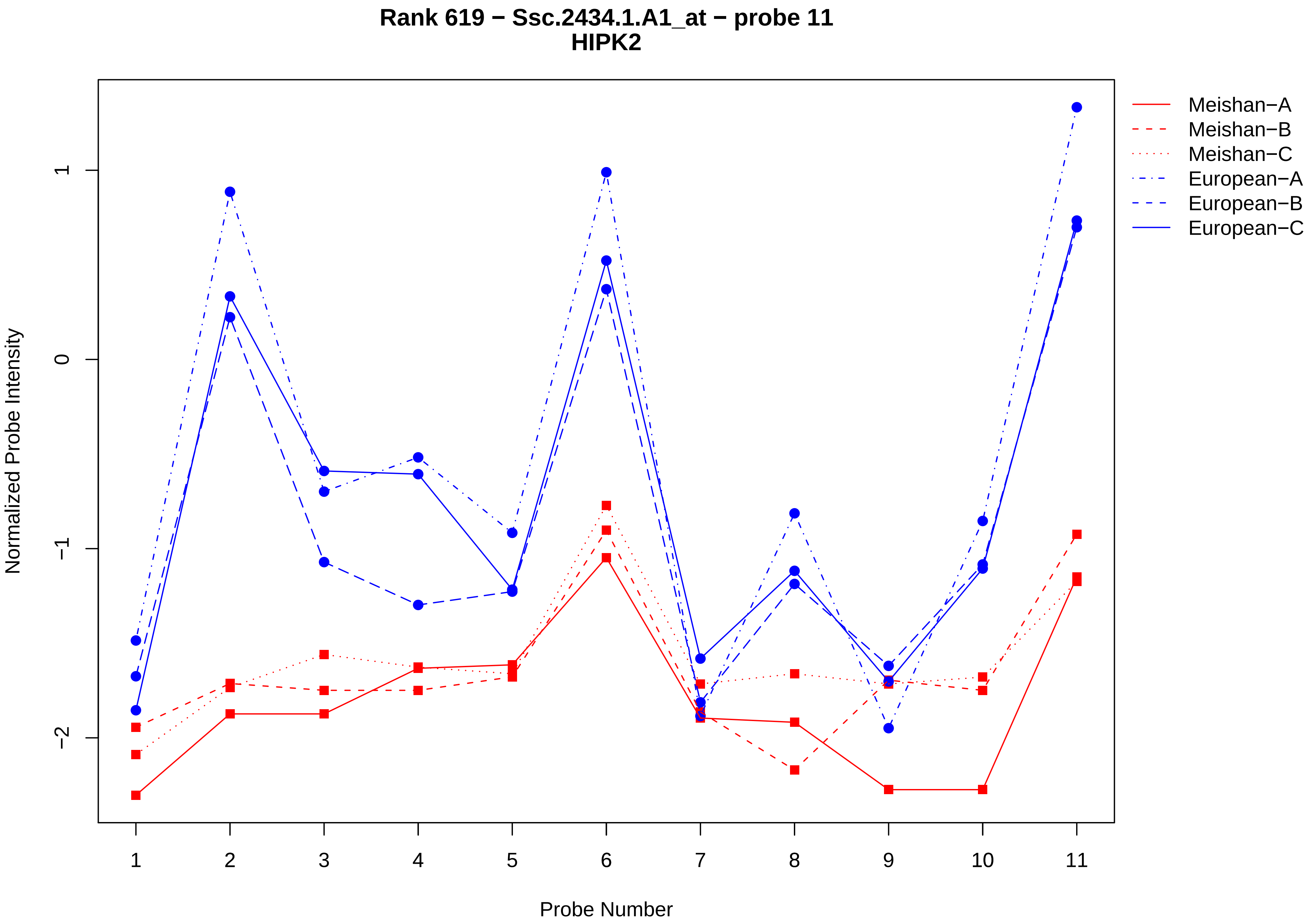

Rank 619 − Ssc.2434.1.A1_at − probe 11
HIPK2
Normalized Probe Intensity
Probe Number
1
0
−1
−2
1
2
3
4
5
6
7
8
9
10
11
Meishan−A
Meishan−B
Meishan−C
European−A
European−B
European−C

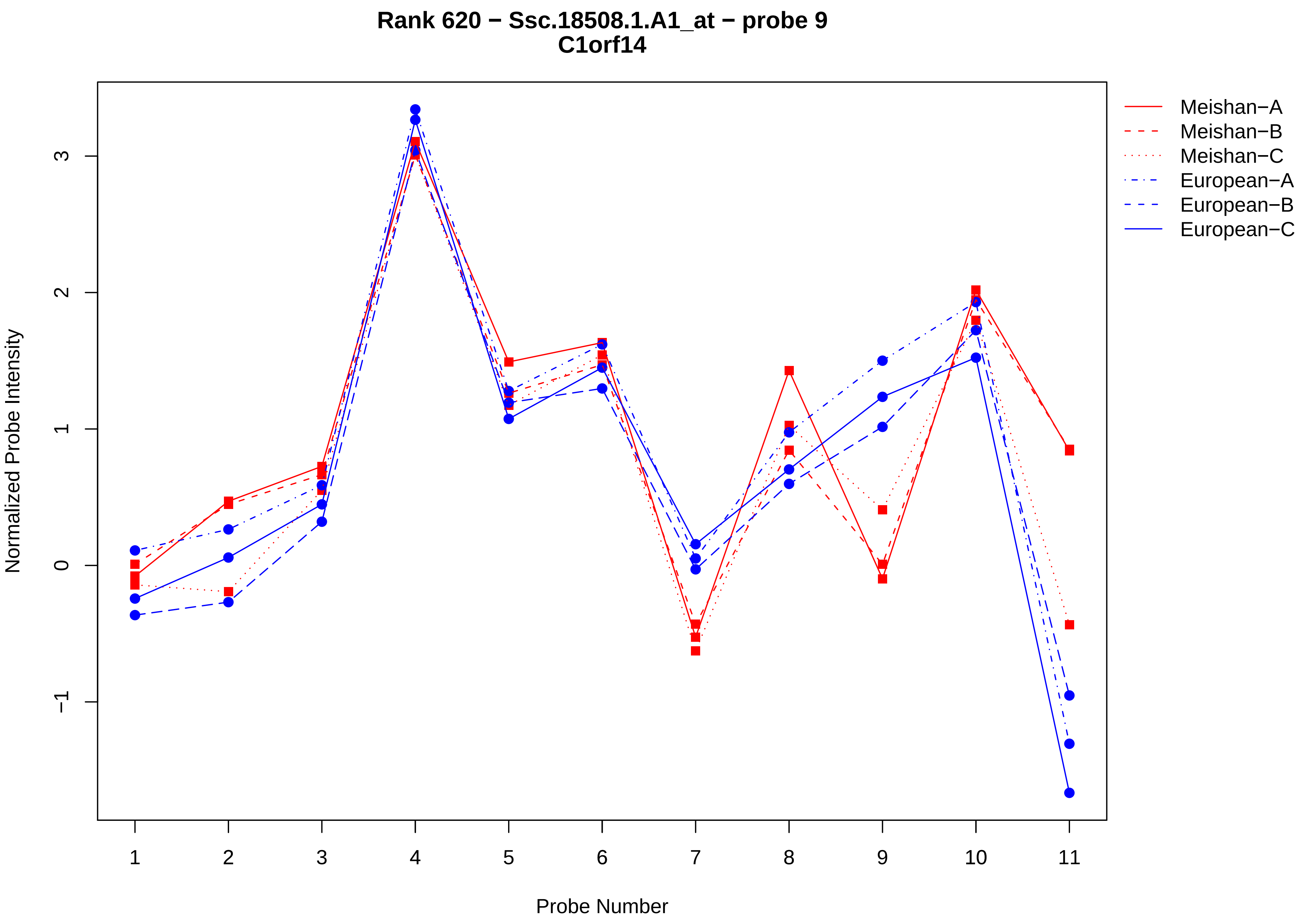
Rank 620 − Ssc.18508.1.A1_at − probe 9
C1orf14
Normalized Probe Intensity
Probe Number
Meishan−A
Meishan−B
Meishan−C
European−A
European−B
European−C

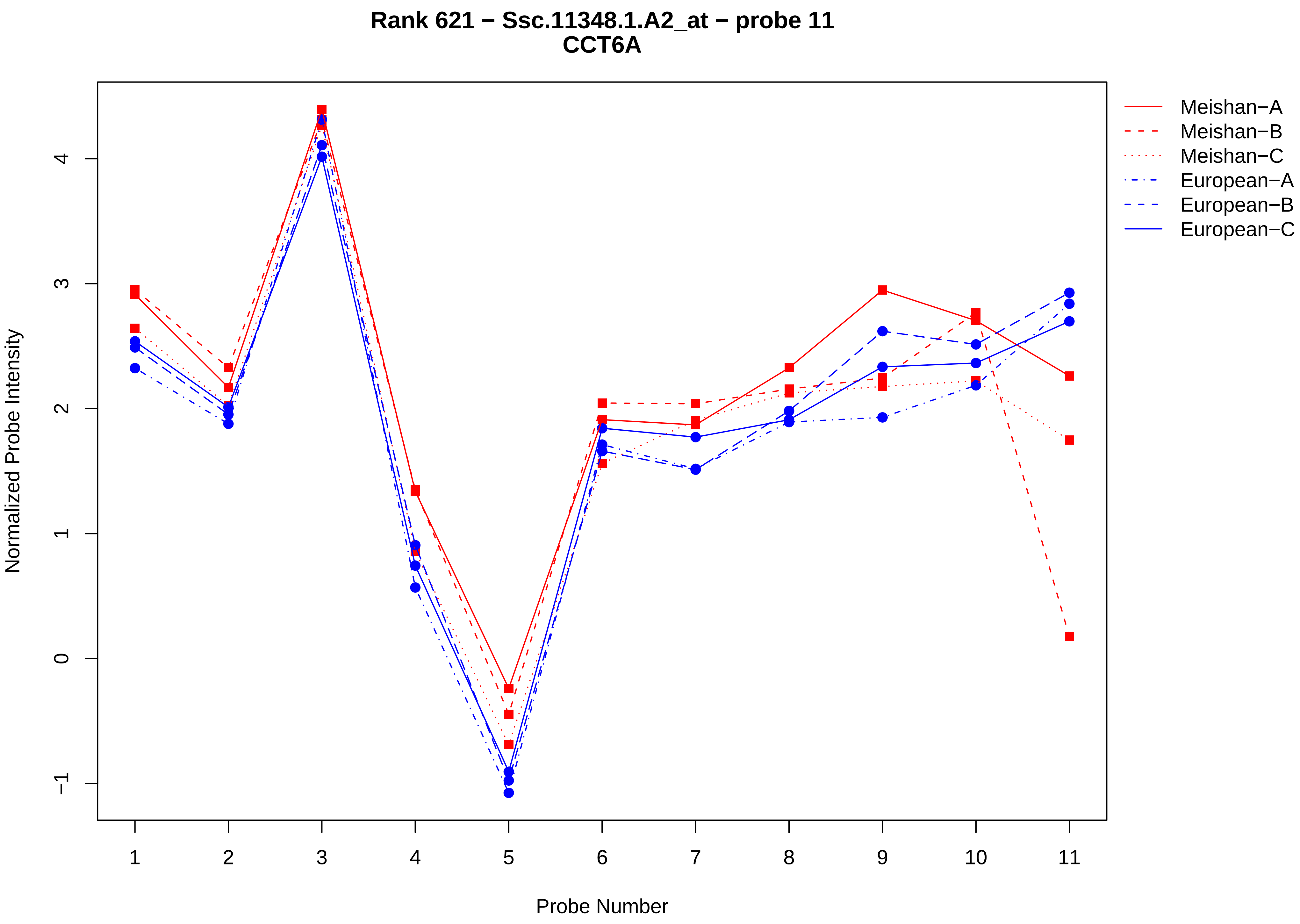

Rank 621 − Ssc.11348.1.A2_at − probe 11
CCT6A
Normalized Probe Intensity
Probe Number
−1
0
1
2
3
4
1
2
3
4
5
6
7
8
9
10
11
Meishan−A
Meishan−B
Meishan−C
European−A
European−B
European−C

Rank 622 − Ssc.11248.1.A1_at − probe 10
MTMR6

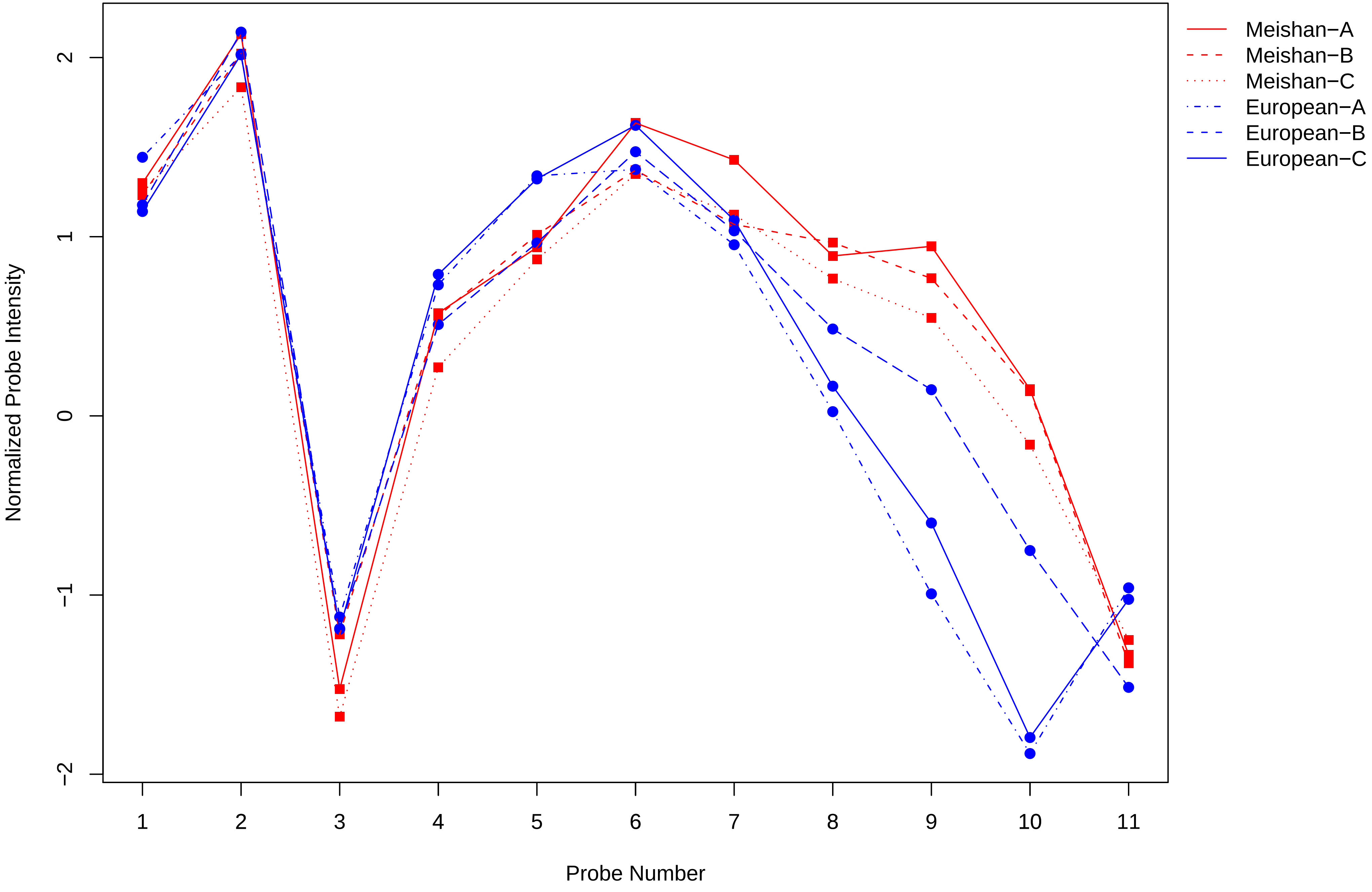

Rank 623 − Ssc.7568.1.A1_at − probe 1
CCL28

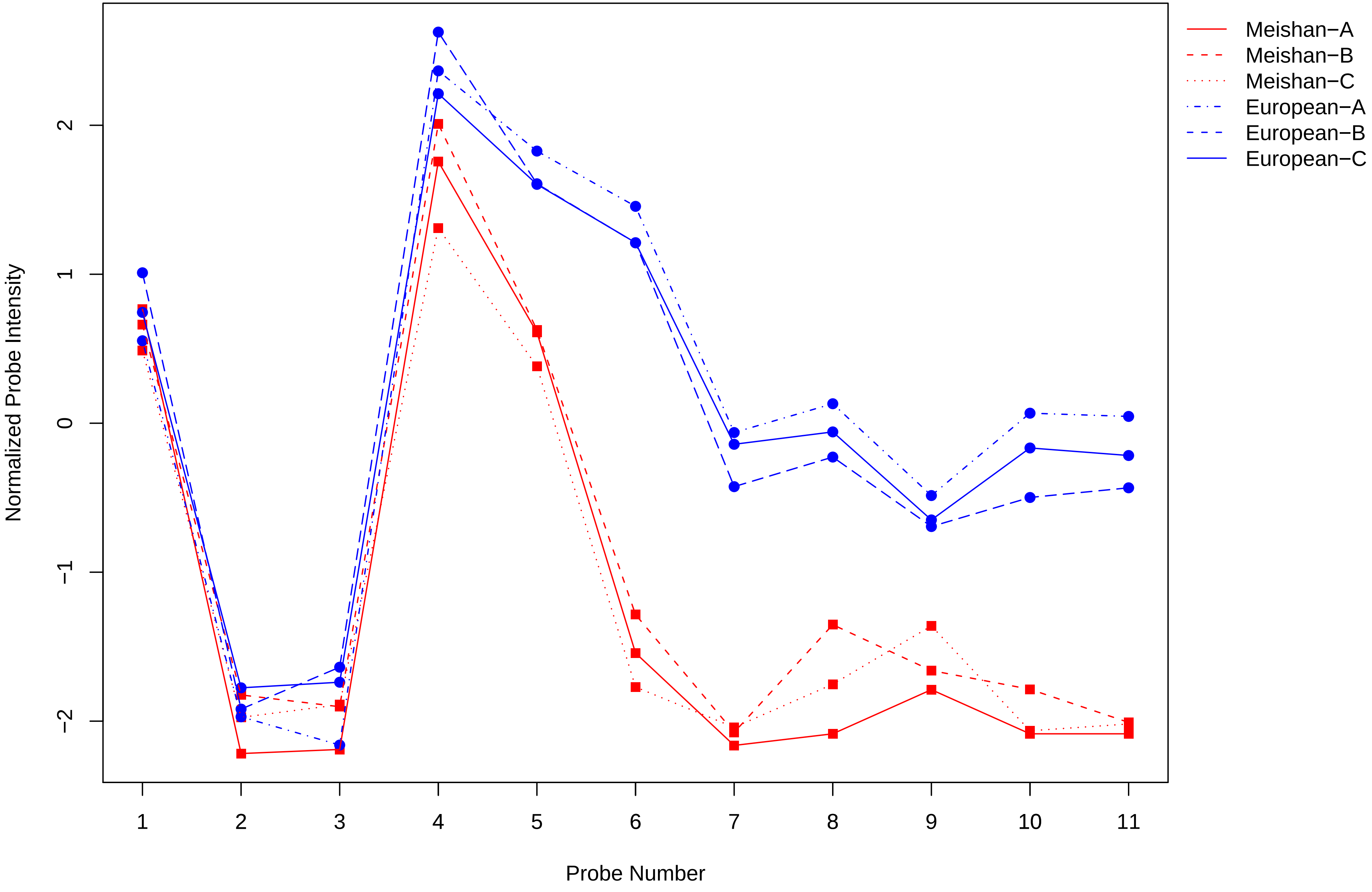

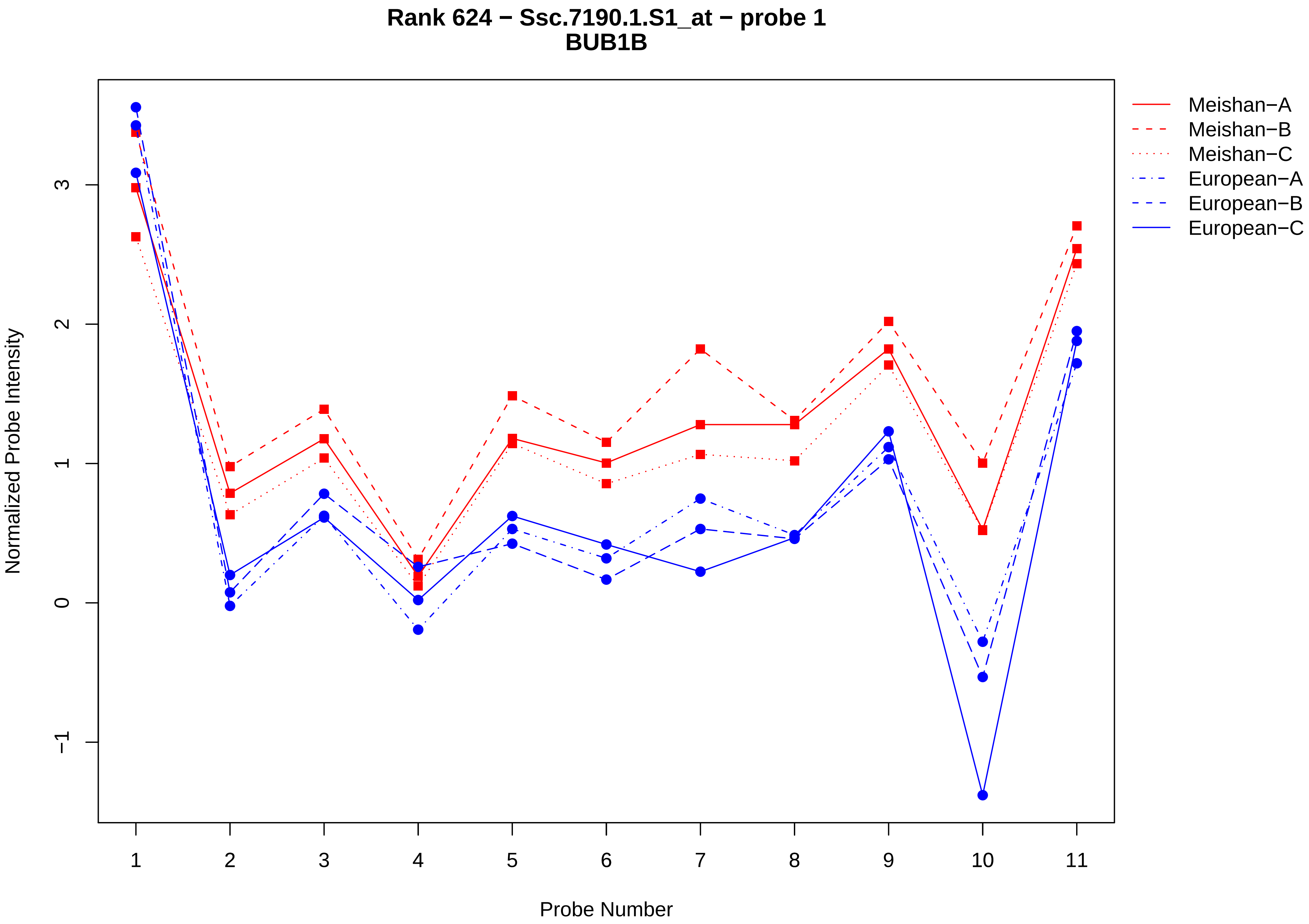

Rank 624 − Ssc.7190.1.S1_at − probe 1
BUB1B
Normalized Probe Intensity
Probe Number
−1
0
1
2
3
1
2
3
4
5
6
7
8
9
10
11
Meishan−A
Meishan−B
Meishan−C
European−A
European−B
European−C

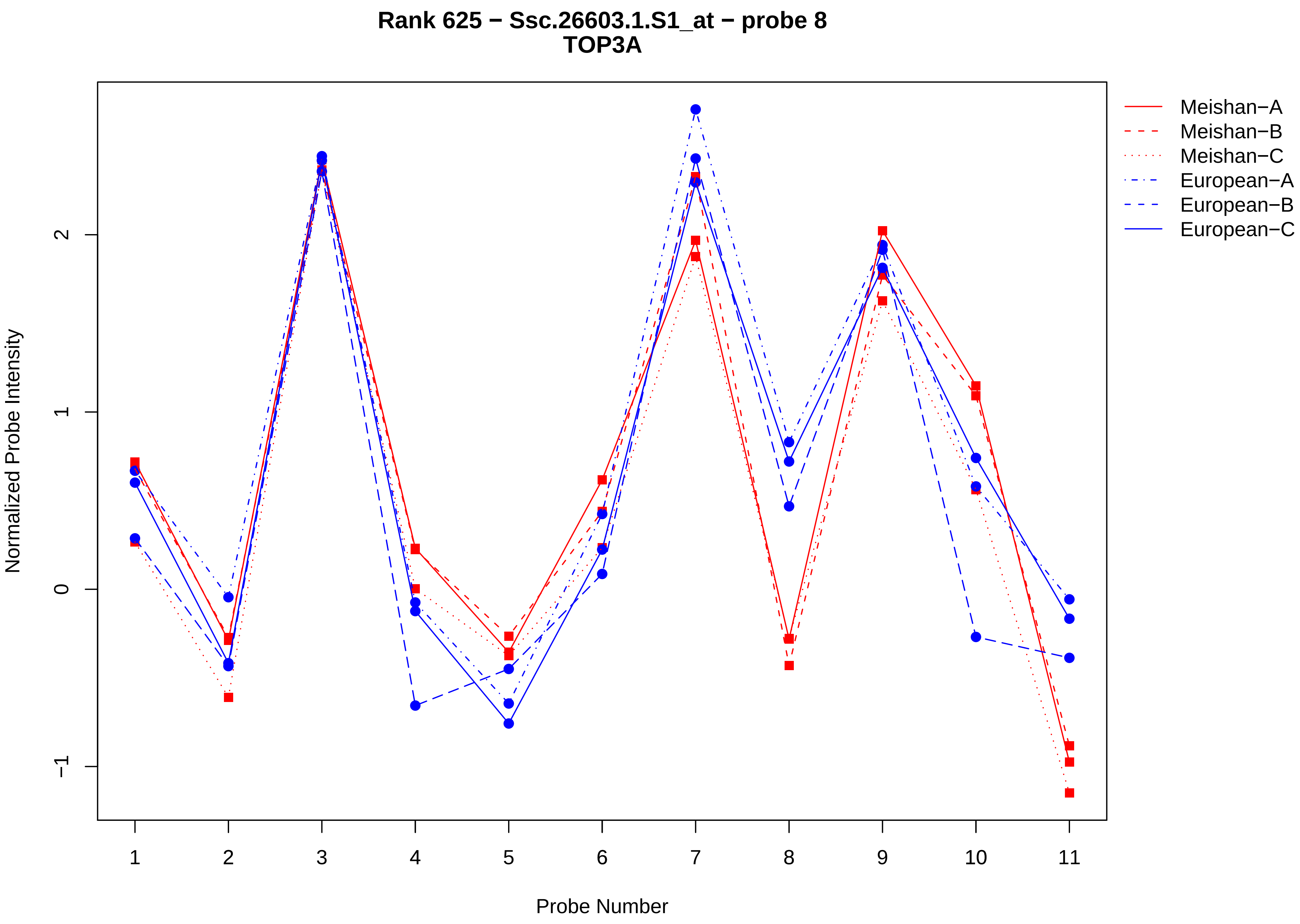

Rank 625 − Ssc.26603.1.S1_at − probe 8
TOP3A
Normalized Probe Intensity
Probe Number
−1
0
1
2
1
2
3
4
5
6
7
8
9
10
11
Meishan−A
Meishan−B
Meishan−C
European−A
European−B
European−C

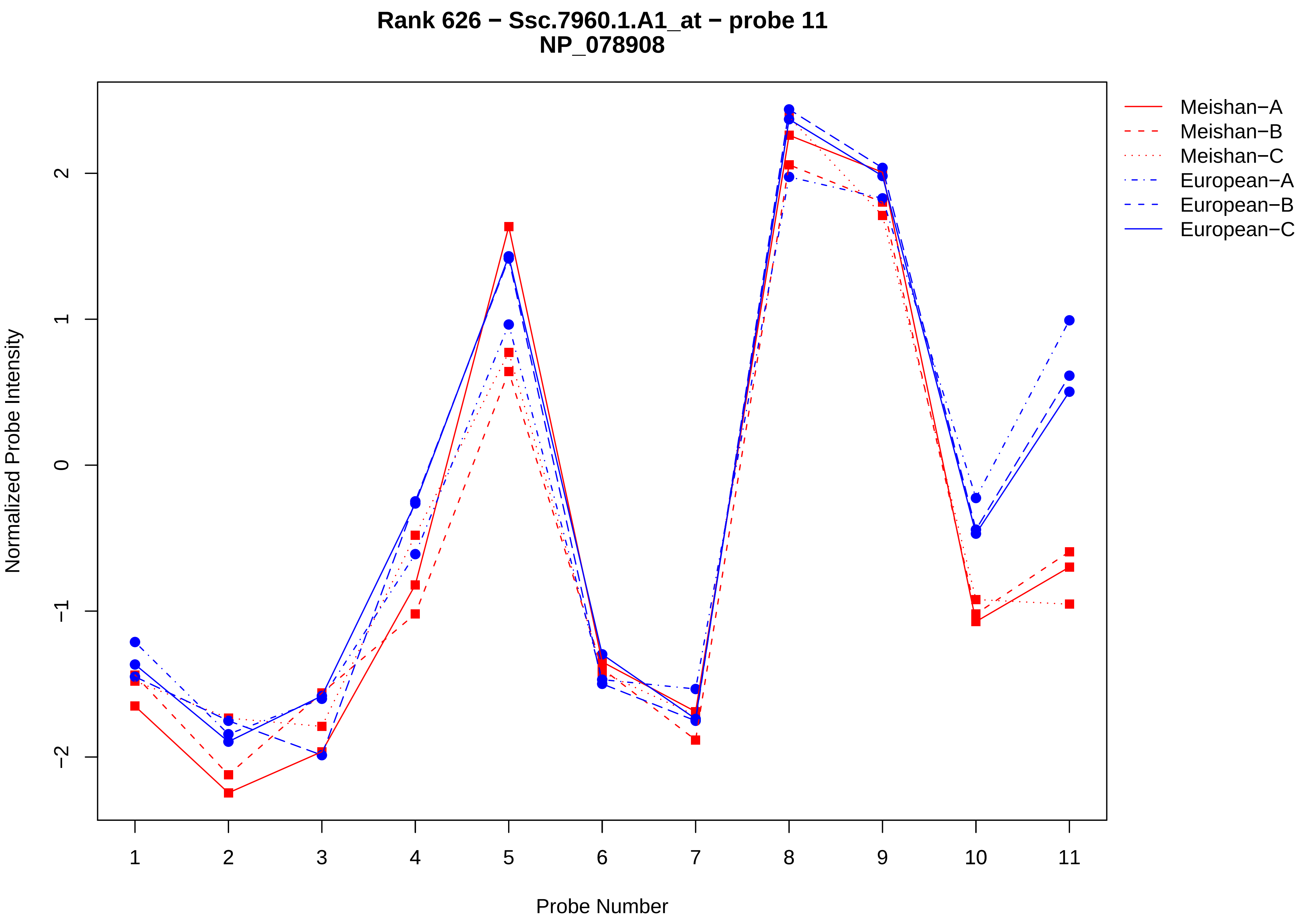

Rank 626 − Ssc.7960.1.A1_at − probe 11
NP_078908
Normalized Probe Intensity
Probe Number
−2
−1
0
1
2
1
2
3
4
5
6
7
8
9
10
11
Meishan−A
Meishan−B
Meishan−C
European−A
European−B
European−C

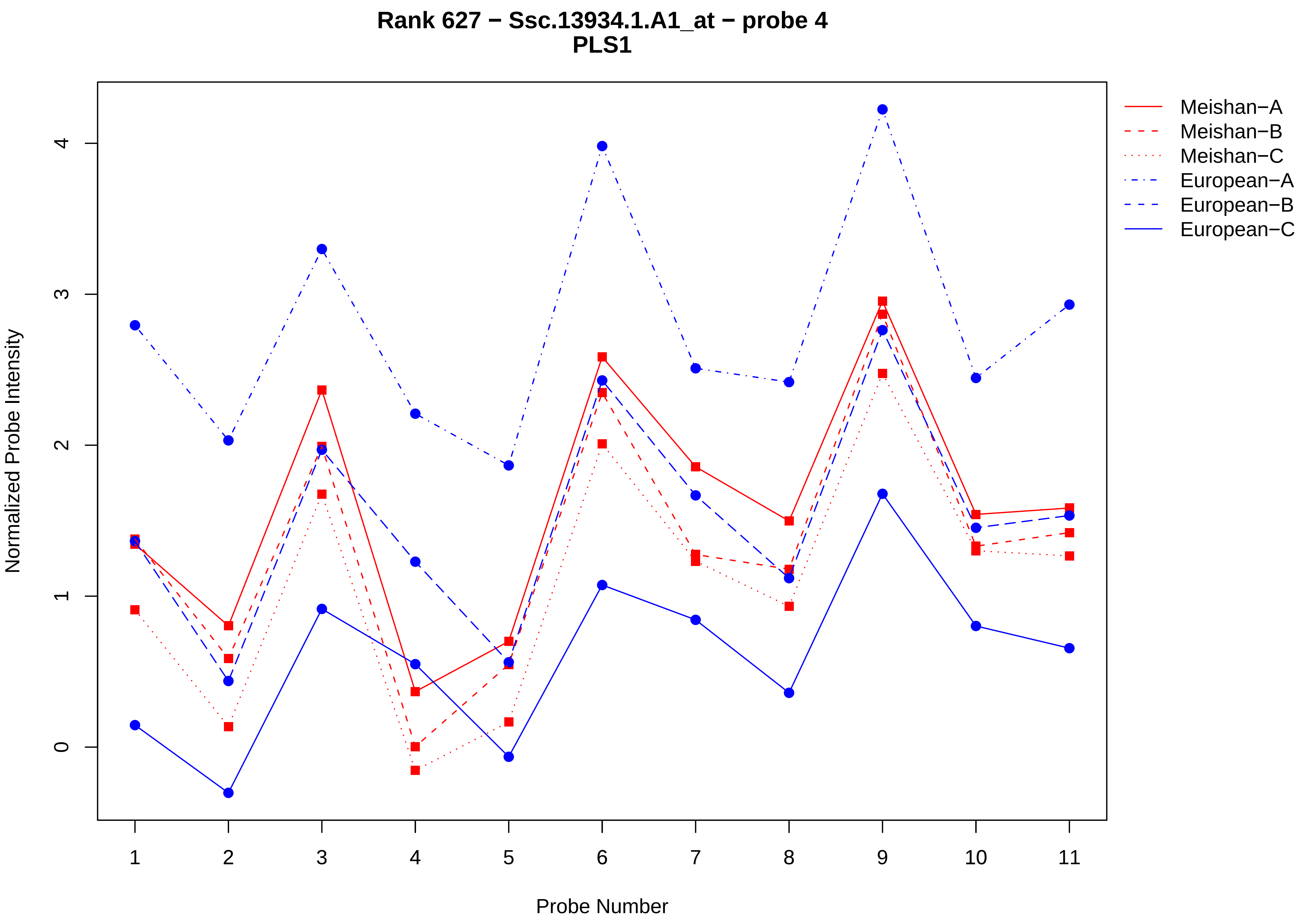

Rank 627 − Ssc.13934.1.A1_at − probe 4
PLS1
Normalized Probe Intensity
Probe Number
0
1
2
3
4
1
2
3
4
5
6
7
8
9
10
11
Meishan−A
Meishan−B
Meishan−C
European−A
European−B
European−C

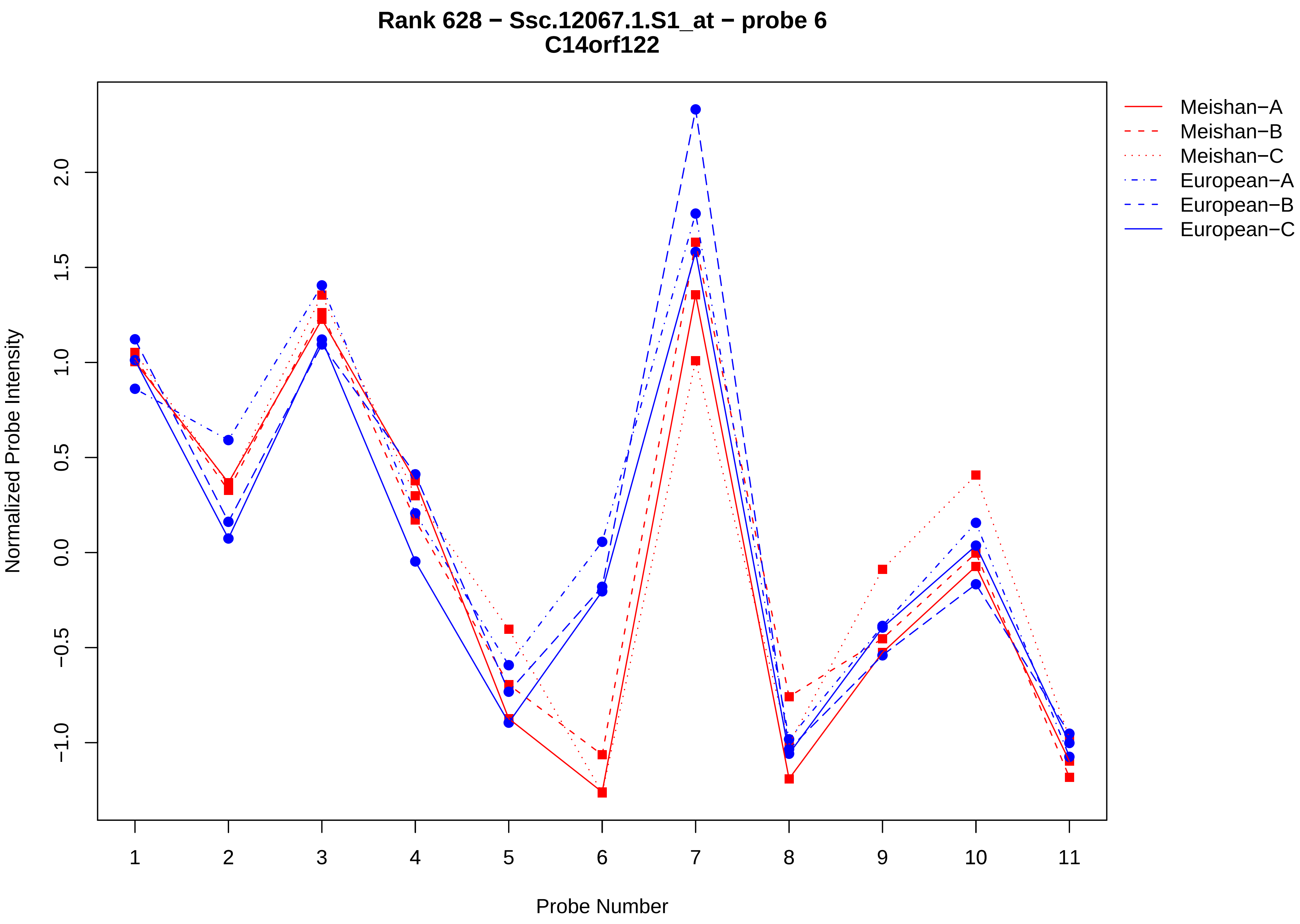
Rank 628 − Ssc.12067.1.S1_at − probe 6
C14orf122
Normalized Probe Intensity
−1.0
−0.5
0.0
0.5
1.0
1.5
2.0
1
2
3
4
5
6
7
8
9
10
11
Probe Number
Meishan−A
Meishan−B
Meishan−C
European−A
European−B
European−C

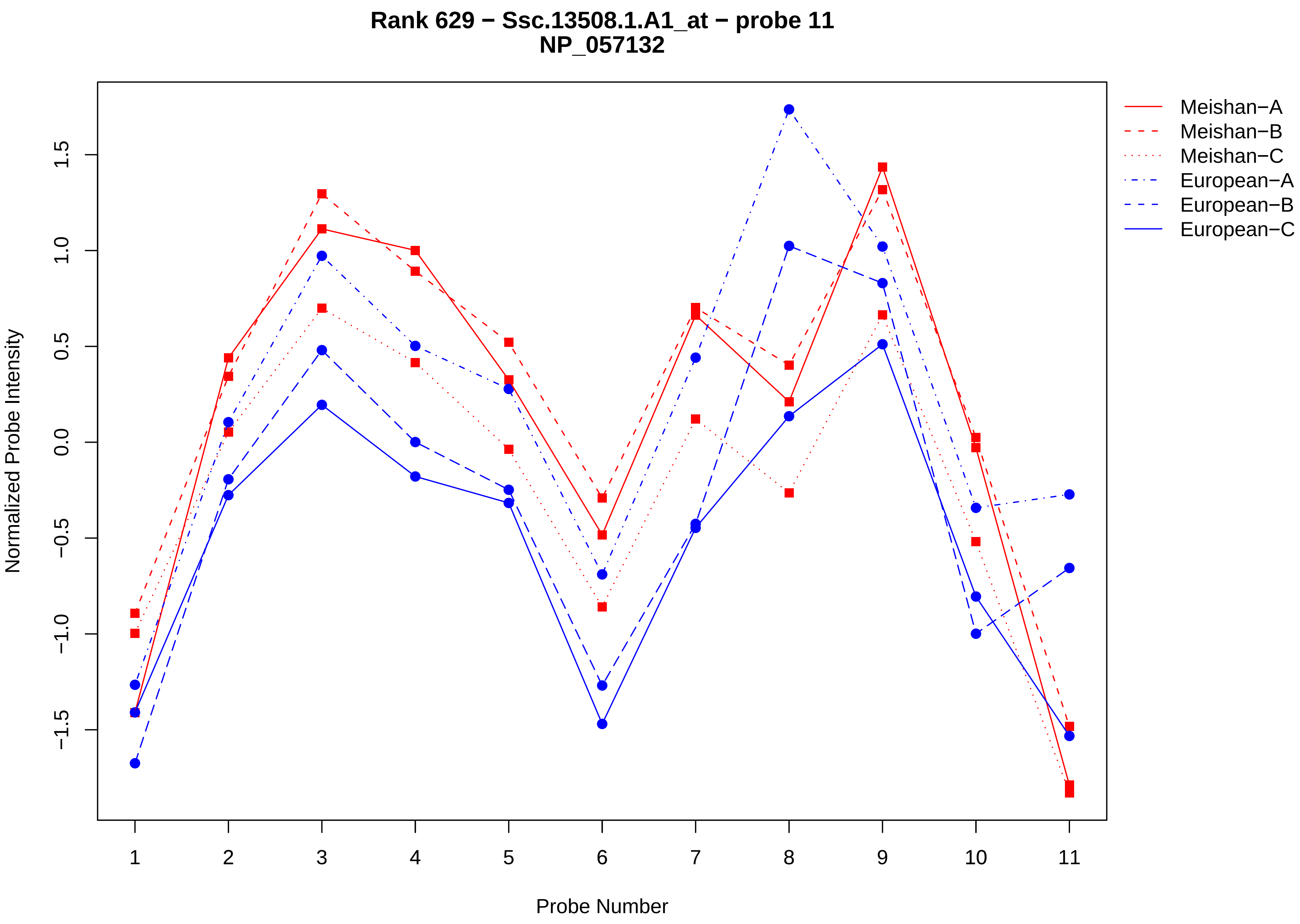
Rank 629 − Ssc.13508.1.A1_at − probe 11
NP_057132
Normalized Probe Intensity
−1.5
−1.0
−0.5
0.0
0.5
1.0
1.5
1
2
3
4
5
6
7
8
9
10
11
Probe Number
Meishan−A
Meishan−B
Meishan−C
European−A
European−B
European−C

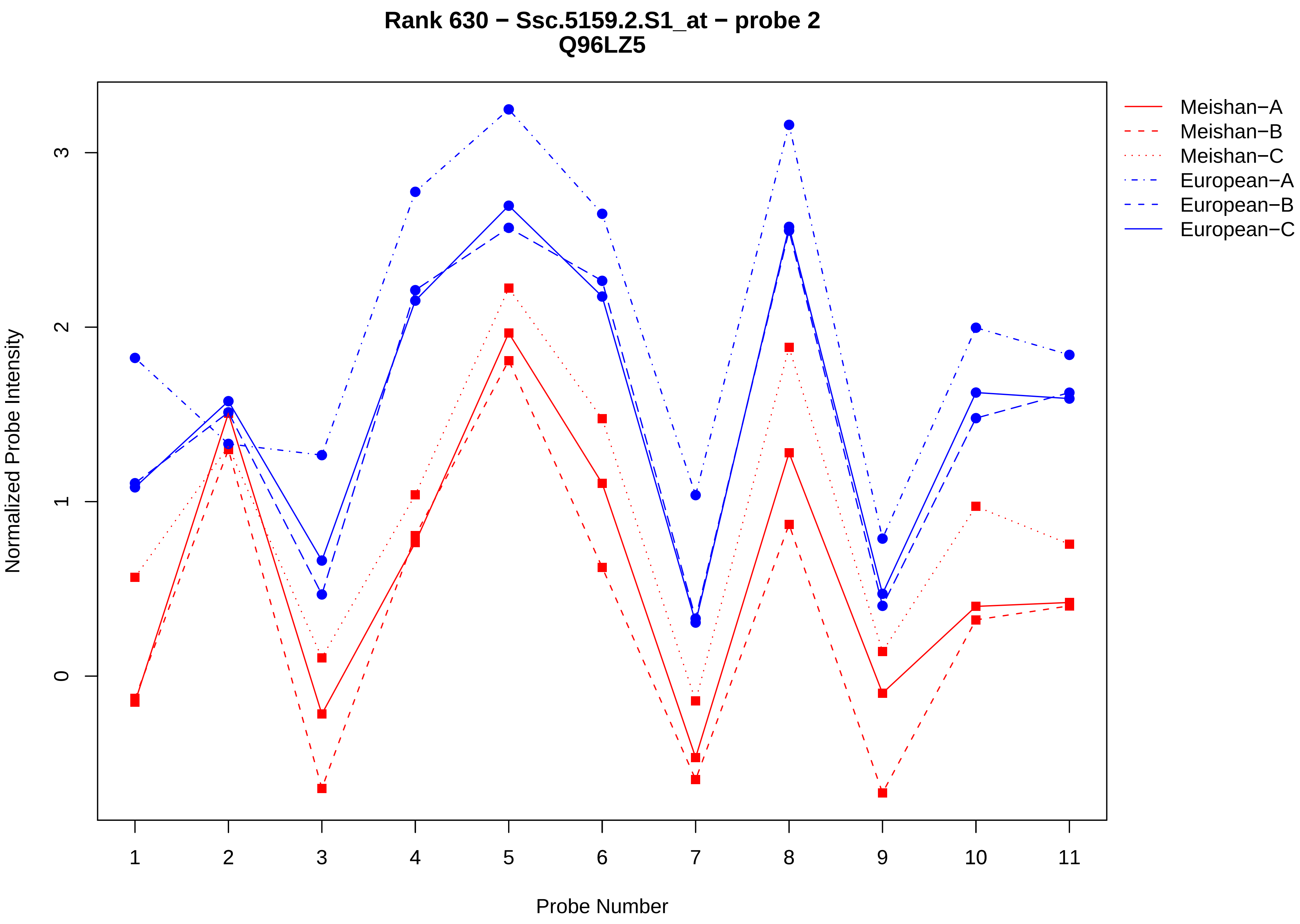
Rank 630 − Ssc.5159.2.S1_at − probe 2
Q96LZ5
Normalized Probe Intensity
Probe Number
Meishan−A
Meishan−B
Meishan−C
European−A
European−B
European−C
0
1
2
3
1
2
3
4
5
6
7
8
9
10
11

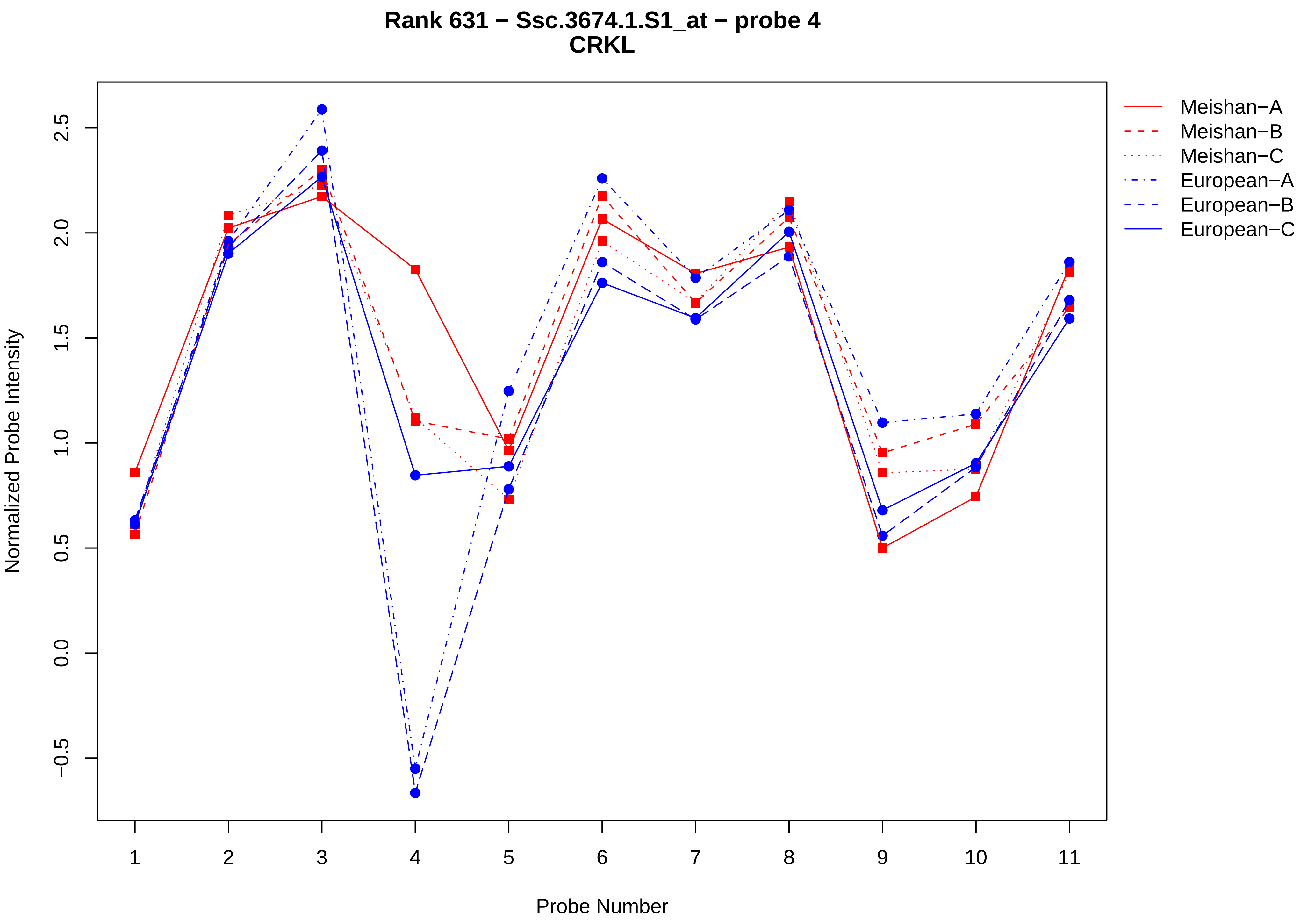

Rank 631 − Ssc.3674.1.S1_at − probe 4
CRKL
Normalized Probe Intensity
Probe Number
−0.5
0.0
0.5
1.0
1.5
2.0
2.5
1
2
3
4
5
6
7
8
9
10
11
Meishan−A
Meishan−B
Meishan−C
European−A
European−B
European−C

Rank 632 − Ssc.489.1.A1_at − probe 9
Q9HB73

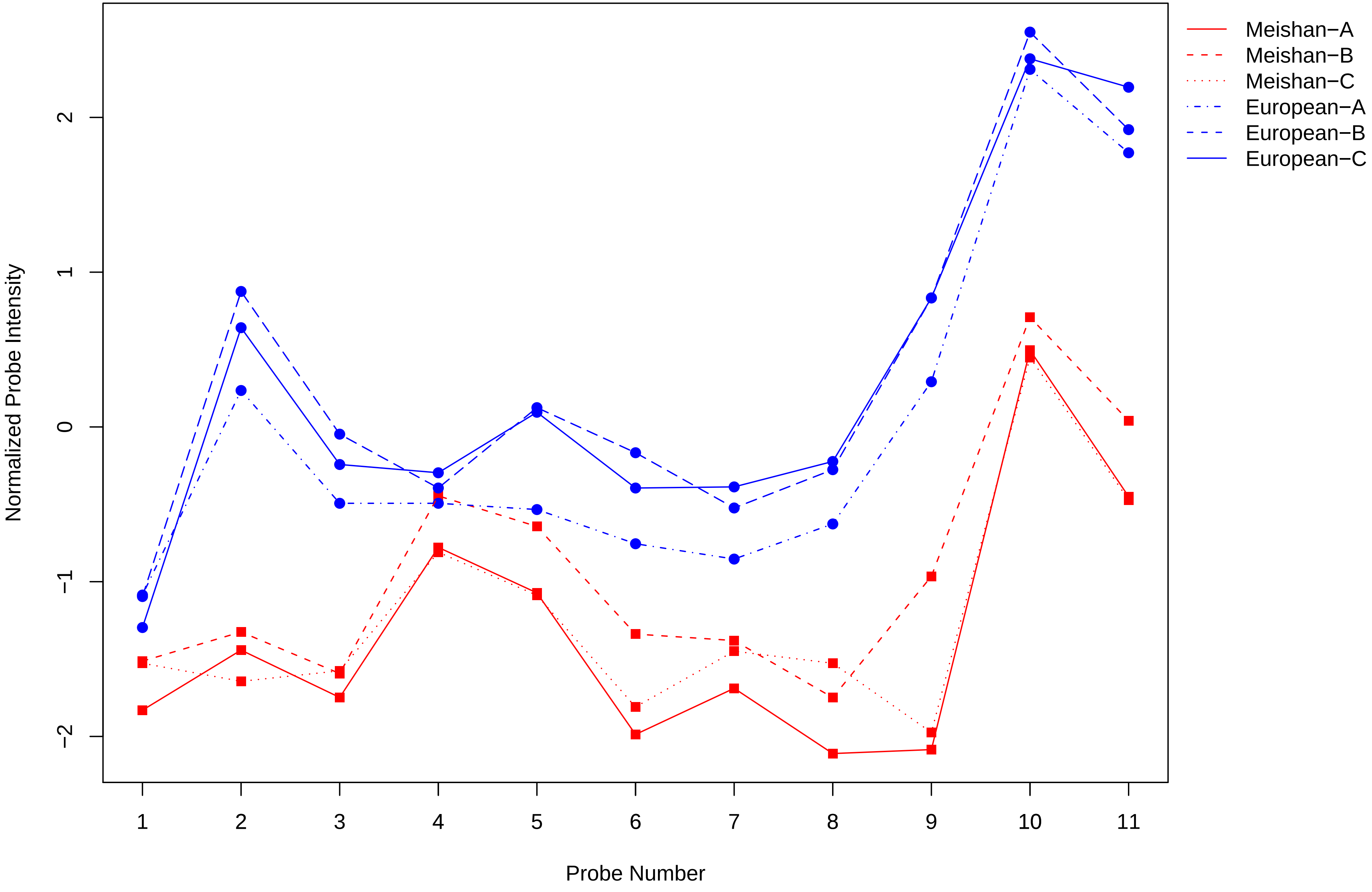

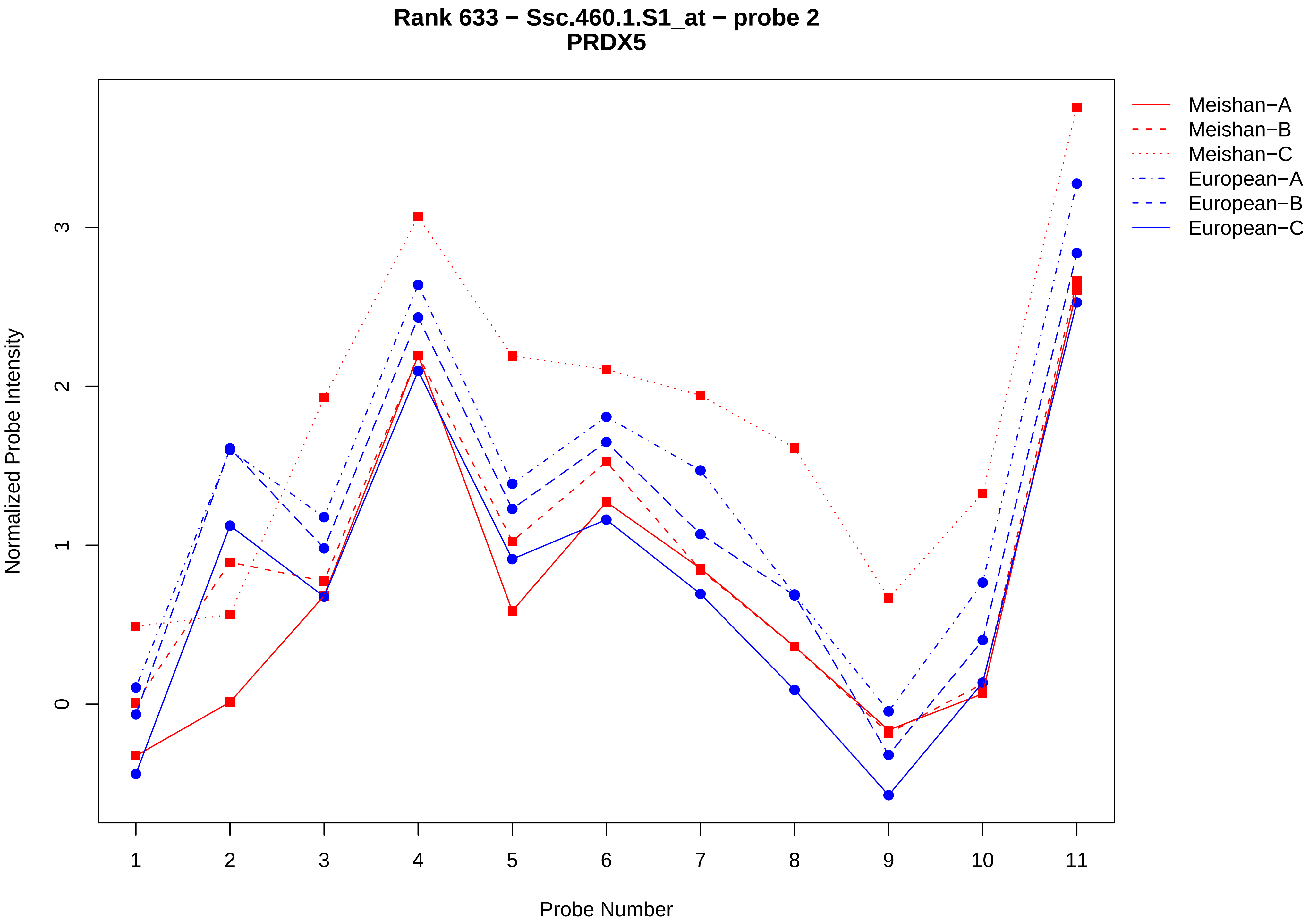

Rank 633 − Ssc.460.1.S1_at − probe 2
PRDX5
Normalized Probe Intensity
Probe Number
0
1
2
3
1
2
3
4
5
6
7
8
9
10
11
Meishan−A
Meishan−B
Meishan−C
European−A
European−B
European−C

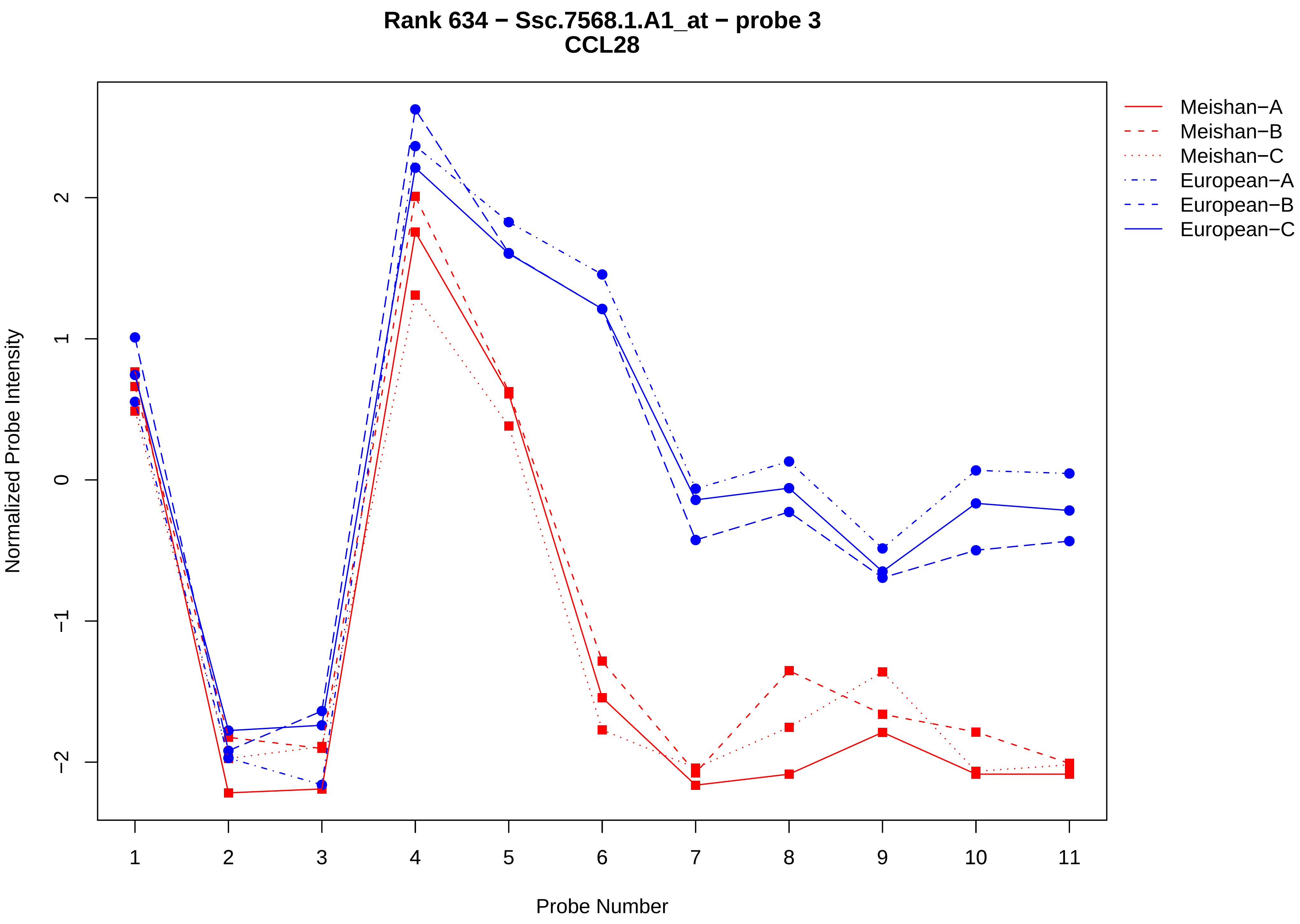

Rank 634 − Ssc.7568.1.A1_at − probe 3
CCL28
Normalized Probe Intensity
Probe Number
−2
−1
0
1
2
1
2
3
4
5
6
7
8
9
10
11
Meishan−A
Meishan−B
Meishan−C
European−A
European−B
European−C

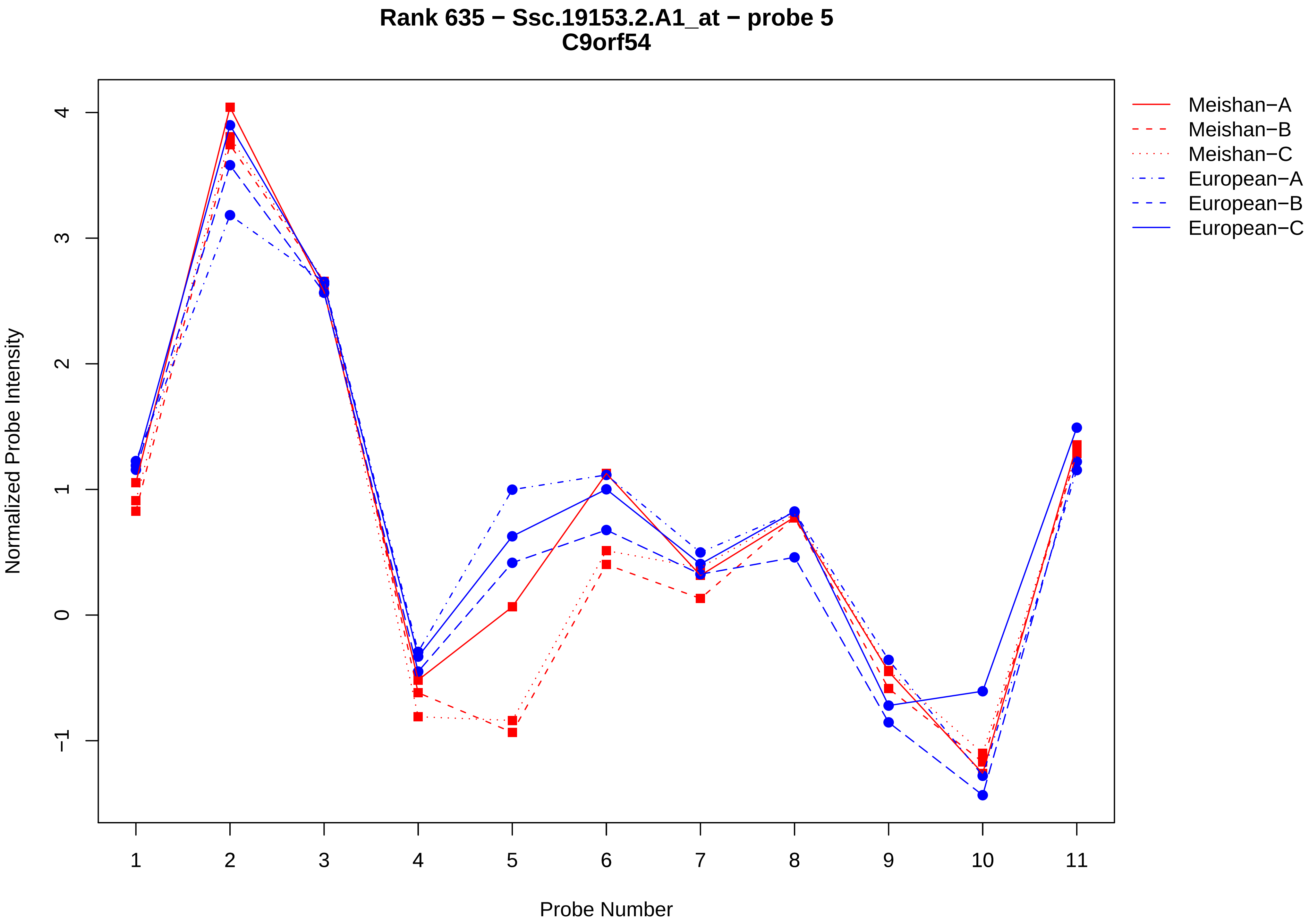

Rank 635 − Ssc.19153.2.A1_at − probe 5
C9orf54
Normalized Probe Intensity
Probe Number
−1
0
1
2
3
4
1
2
3
4
5
6
7
8
9
10
11
Meishan−A
Meishan−B
Meishan−C
European−A
European−B
European−C

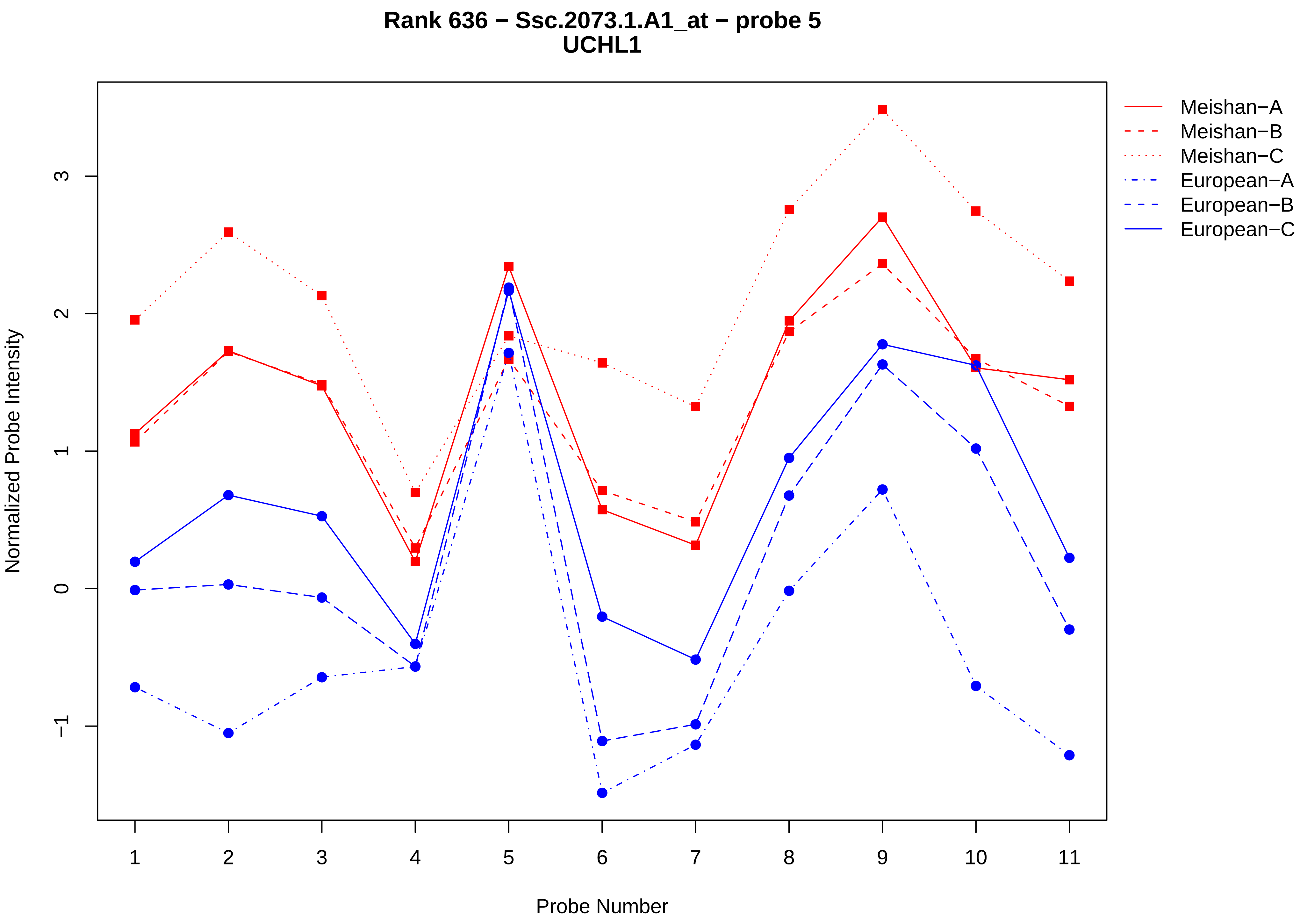

Rank 636 − Ssc.2073.1.A1_at − probe 5
UCHL1
Normalized Probe Intensity
Probe Number
−1
0
1
2
3
1
2
3
4
5
6
7
8
9
10
11
Meishan−A
Meishan−B
Meishan−C
European−A
European−B
European−C

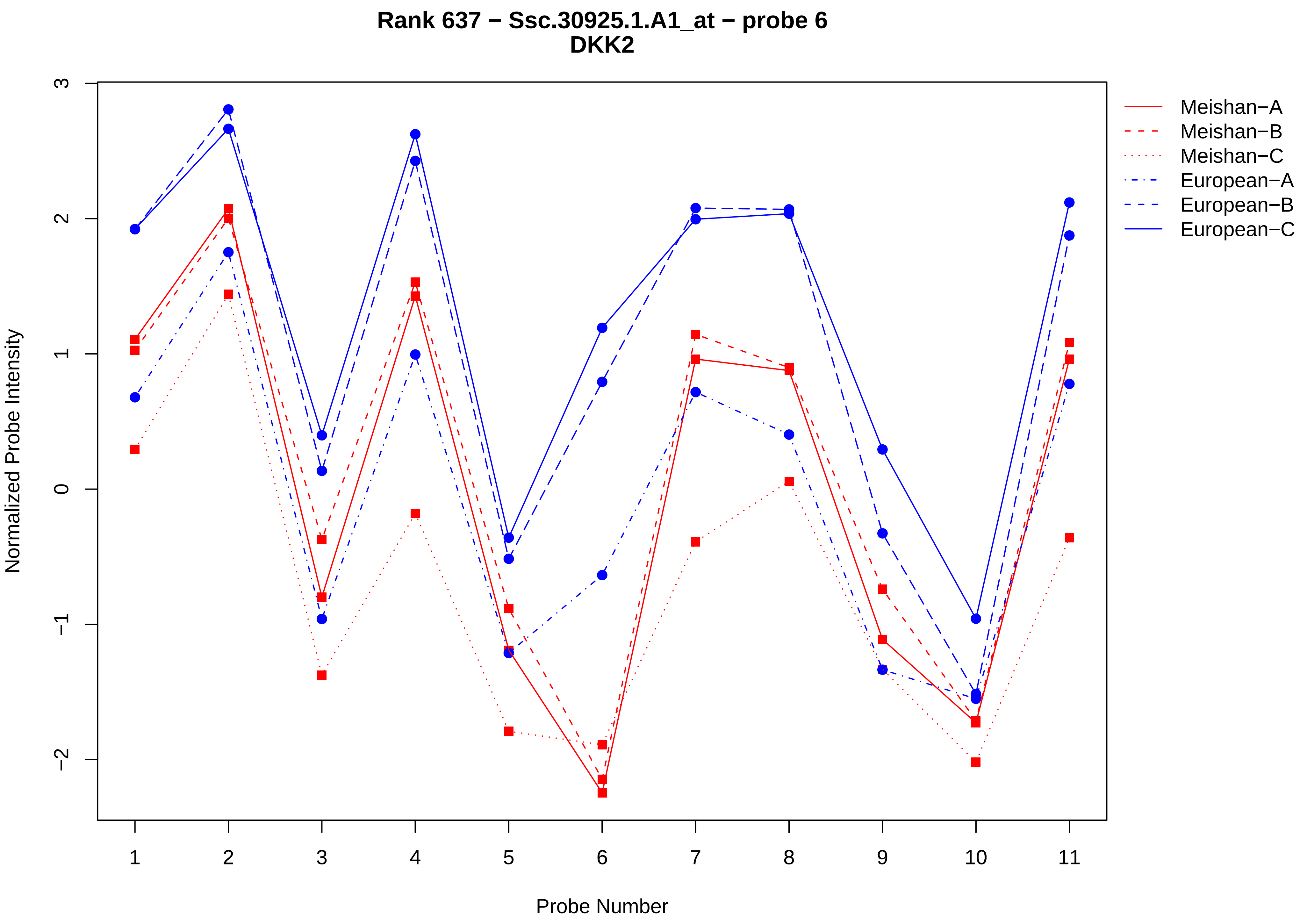

Rank 637 − Ssc.30925.1.A1_at − probe 6
DKK2
Normalized Probe Intensity
Probe Number
−2
−1
0
1
2
3
1
2
3
4
5
6
7
8
9
10
11
Meishan−A
Meishan−B
Meishan−C
European−A
European−B
European−C

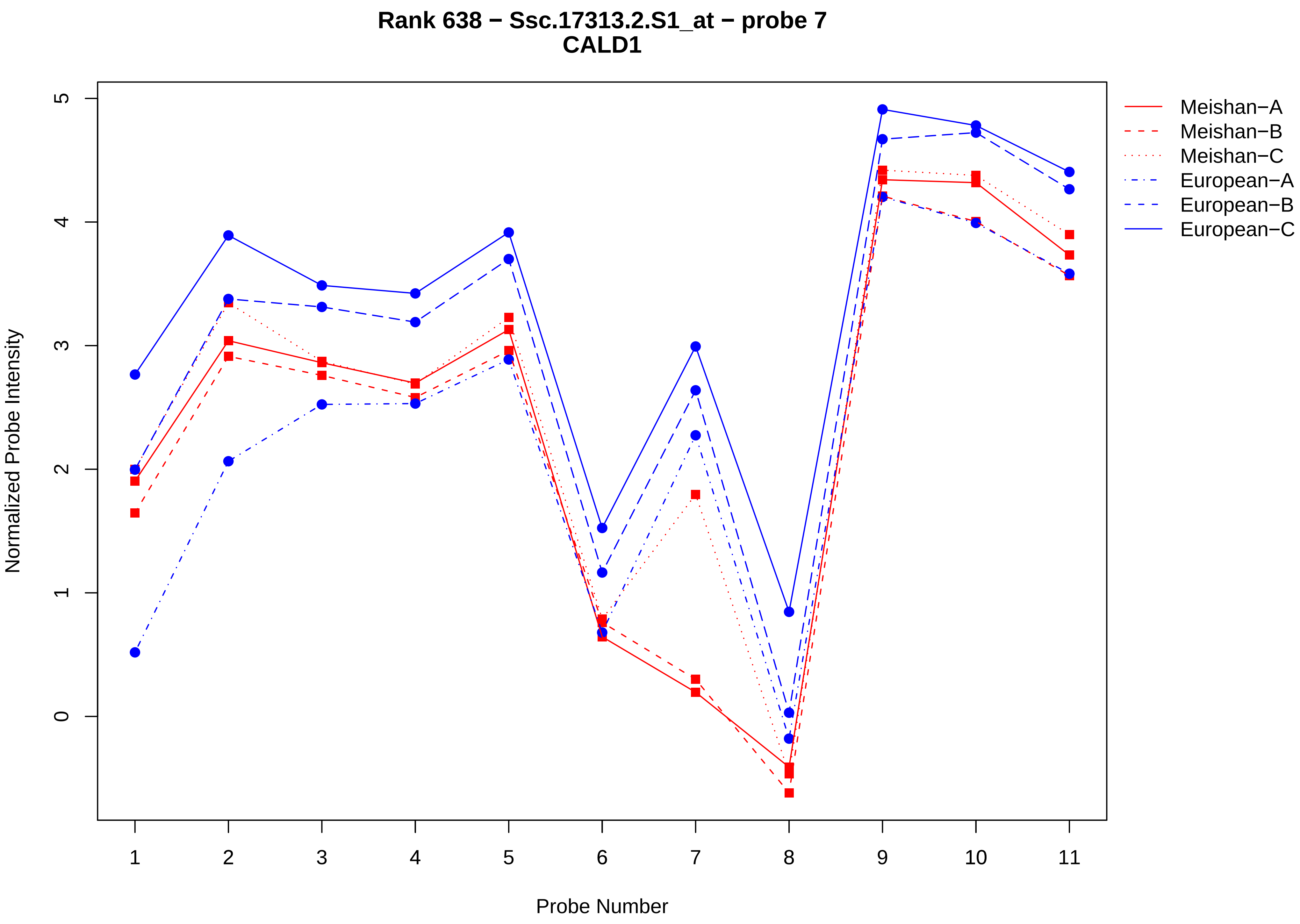

Rank 638 − Ssc.17313.2.S1_at − probe 7
CALD1
Normalized Probe Intensity
0
1
2
3
4
5
1
2
3
4
5
6
7
8
9
10
11
Probe Number
Meishan−A
Meishan−B
Meishan−C
European−A
European−B
European−C

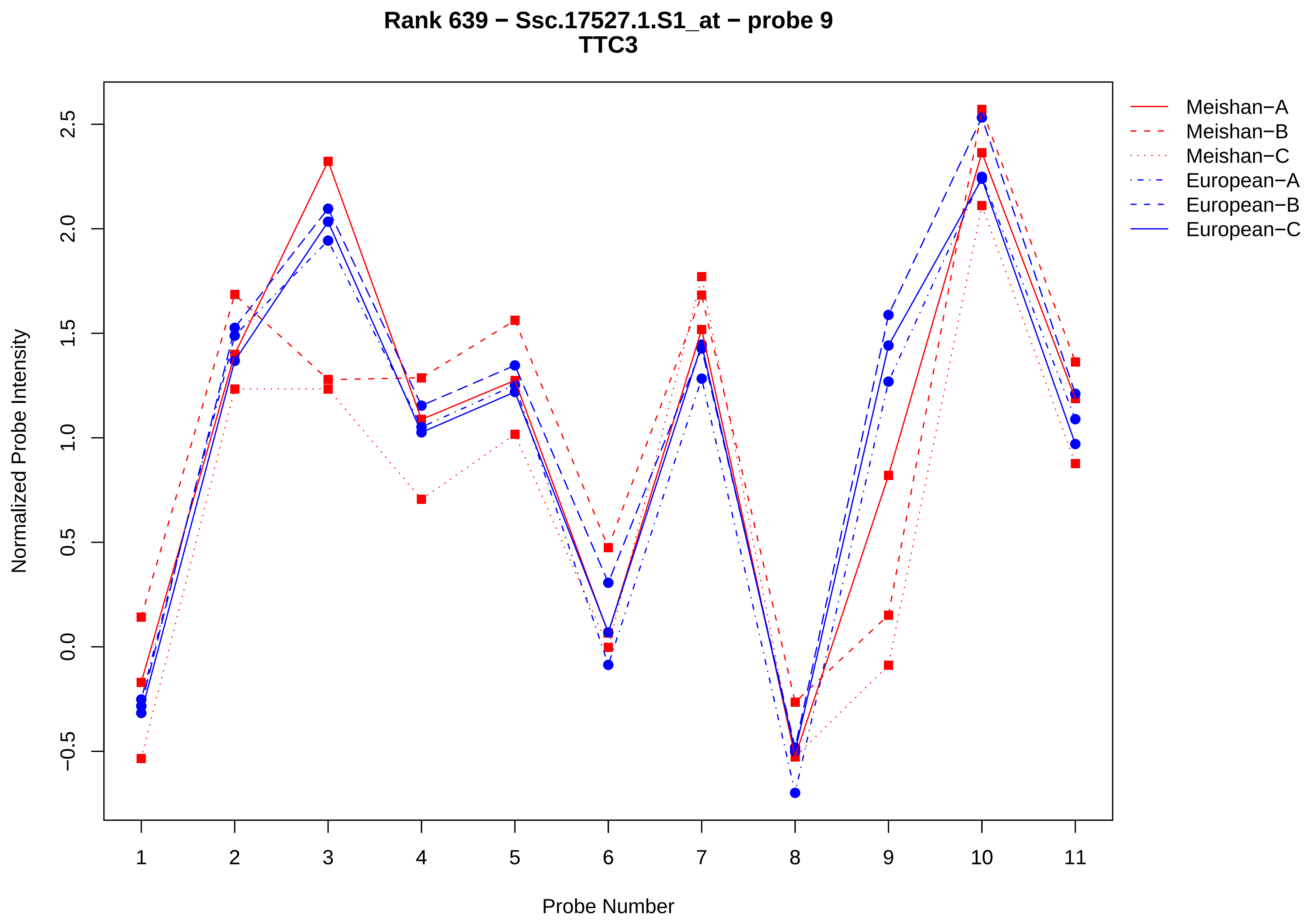

Rank 639 − Ssc.17527.1.S1_at − probe 9
TTC3
Normalized Probe Intensity
Probe Number
−0.5
0.0
0.5
1.0
1.5
2.0
2.5
1
2
3
4
5
6
7
8
9
10
11
Meishan−A
Meishan−B
Meishan−C
European−A
European−B
European−C

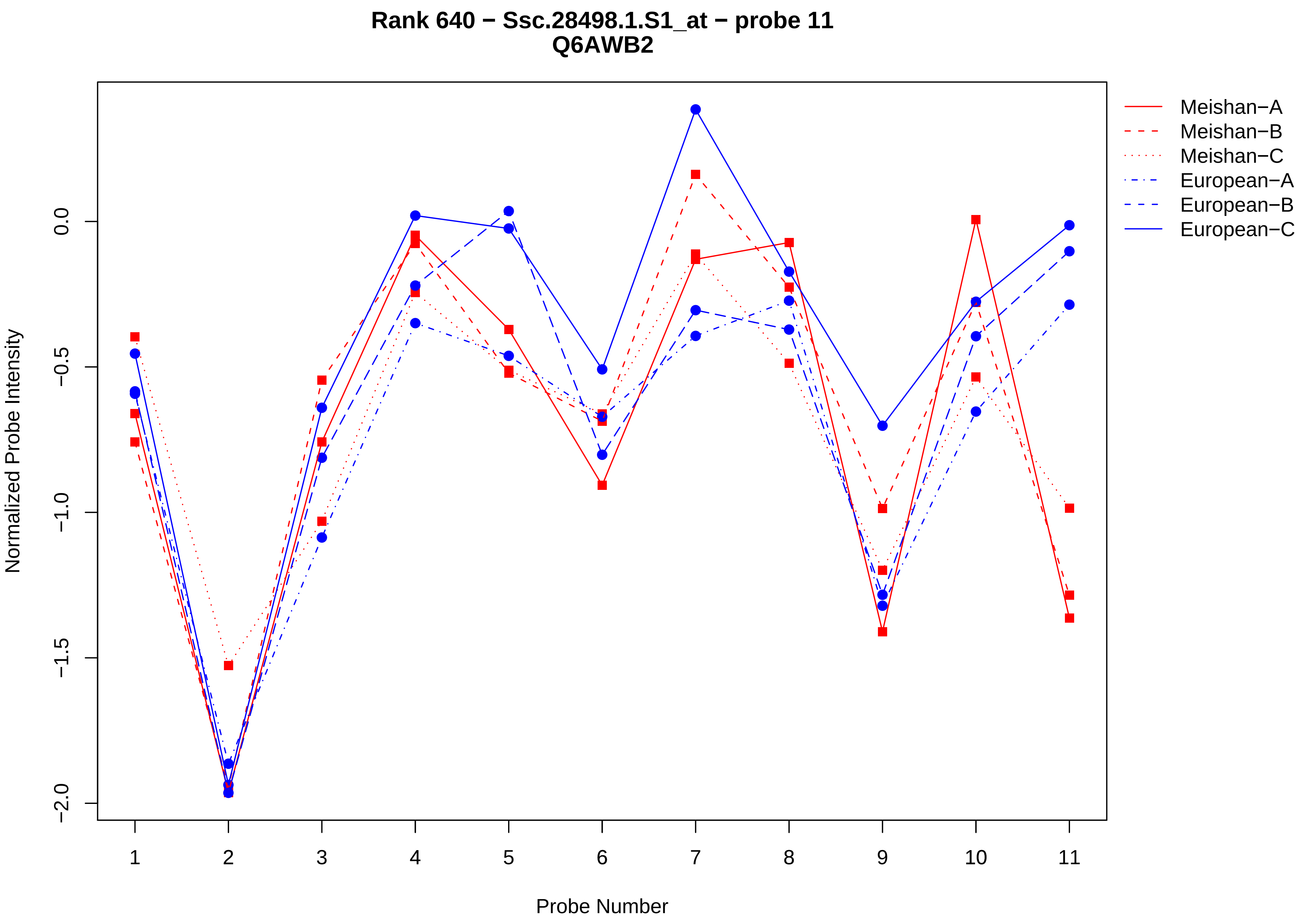

Rank 640 − Ssc.28498.1.S1_at − probe 11
Q6AWB2
Normalized Probe Intensity
Probe Number
0.0
−0.5
−1.0
−1.5
−2.0
1
2
3
4
5
6
7
8
9
10
11
Meishan−A
Meishan−B
Meishan−C
European−A
European−B
European−C

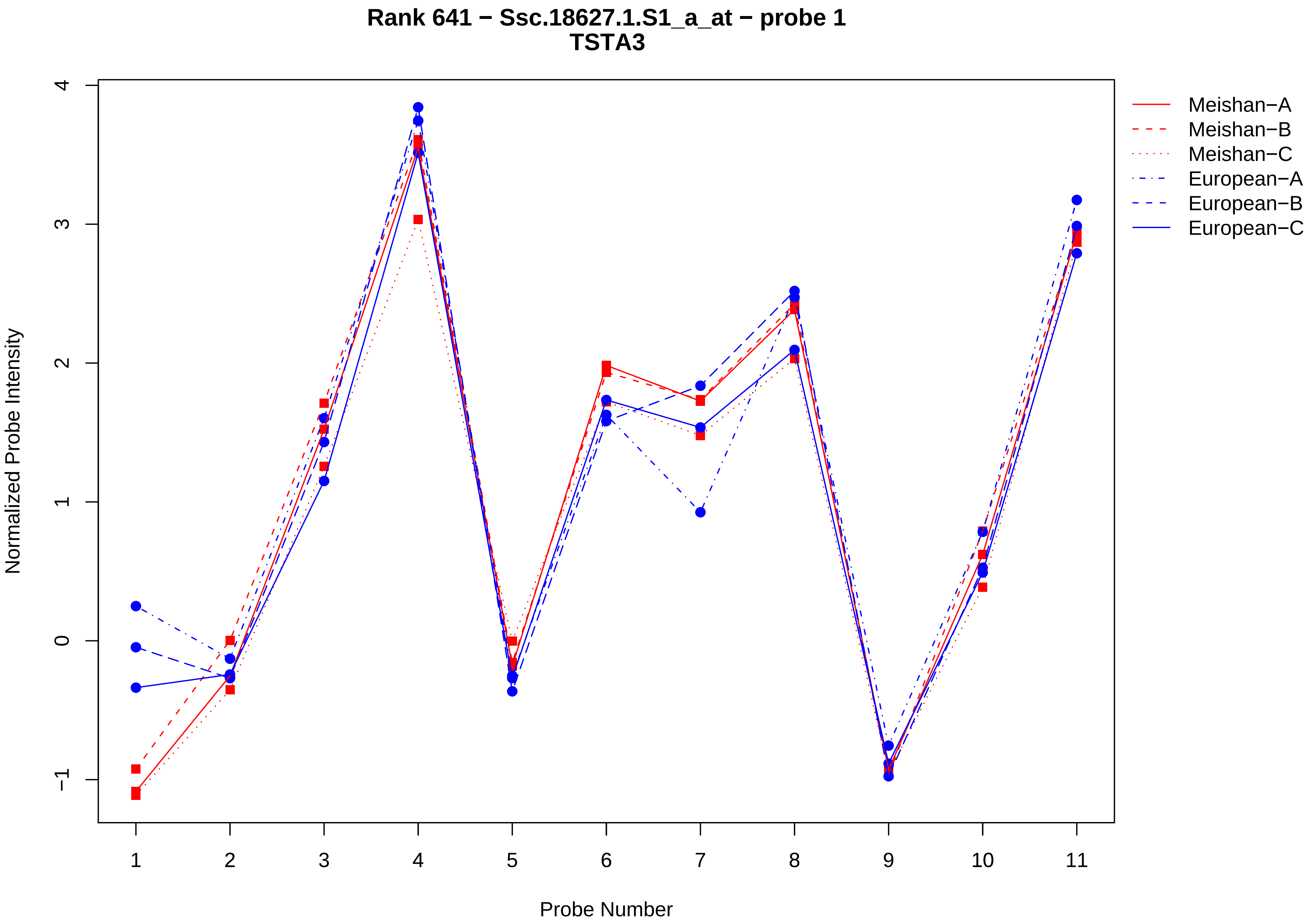

Rank 641 − Ssc.18627.1.S1_a_at − probe 1
TSTA3
Normalized Probe Intensity
Probe Number
−1
0
1
2
3
4
1
2
3
4
5
6
7
8
9
10
11
Meishan−A
Meishan−B
Meishan−C
European−A
European−B
European−C

Rank 642 − Ssc.2471.1.A1_at − probe 4
C2orf25

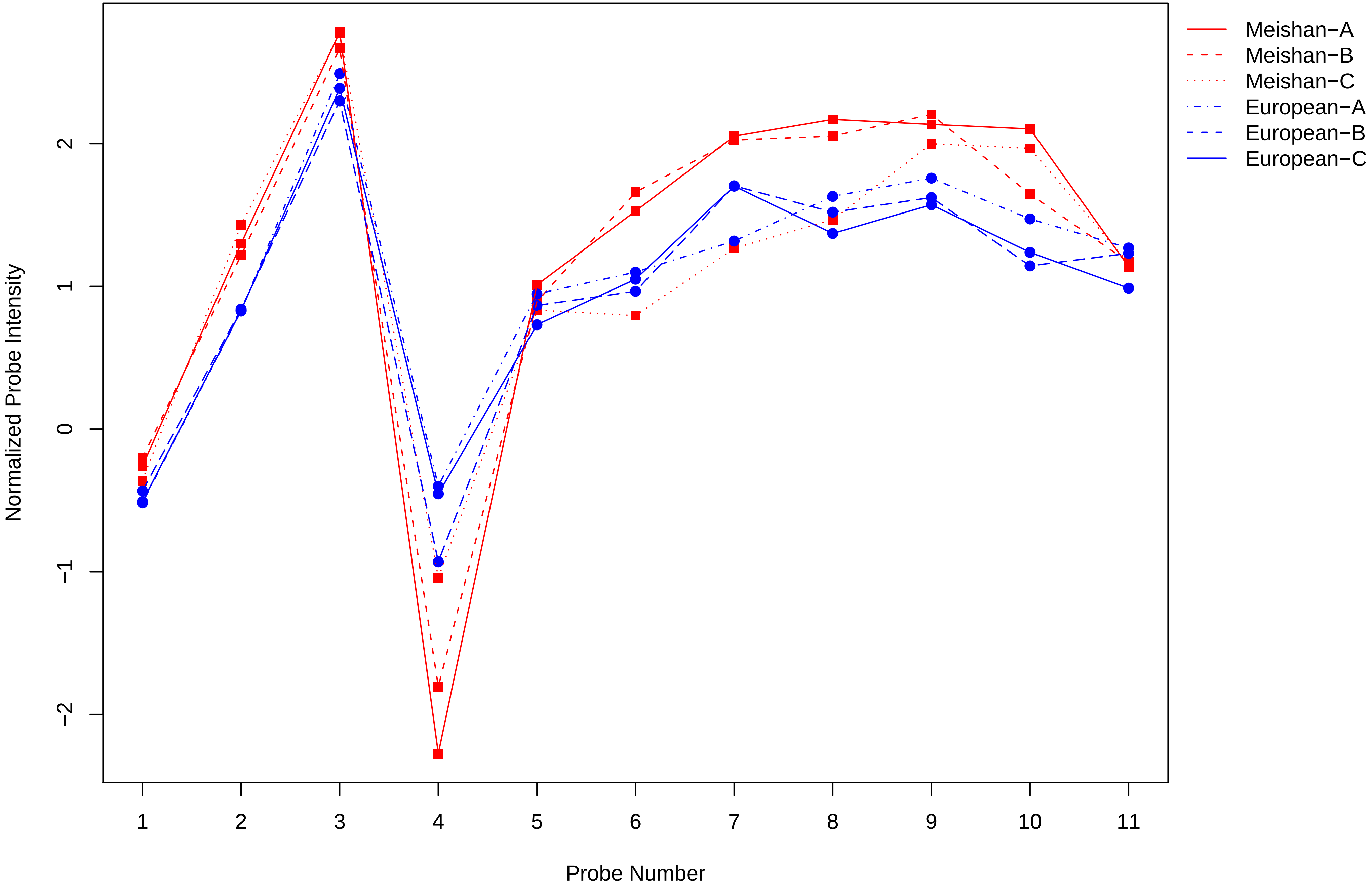

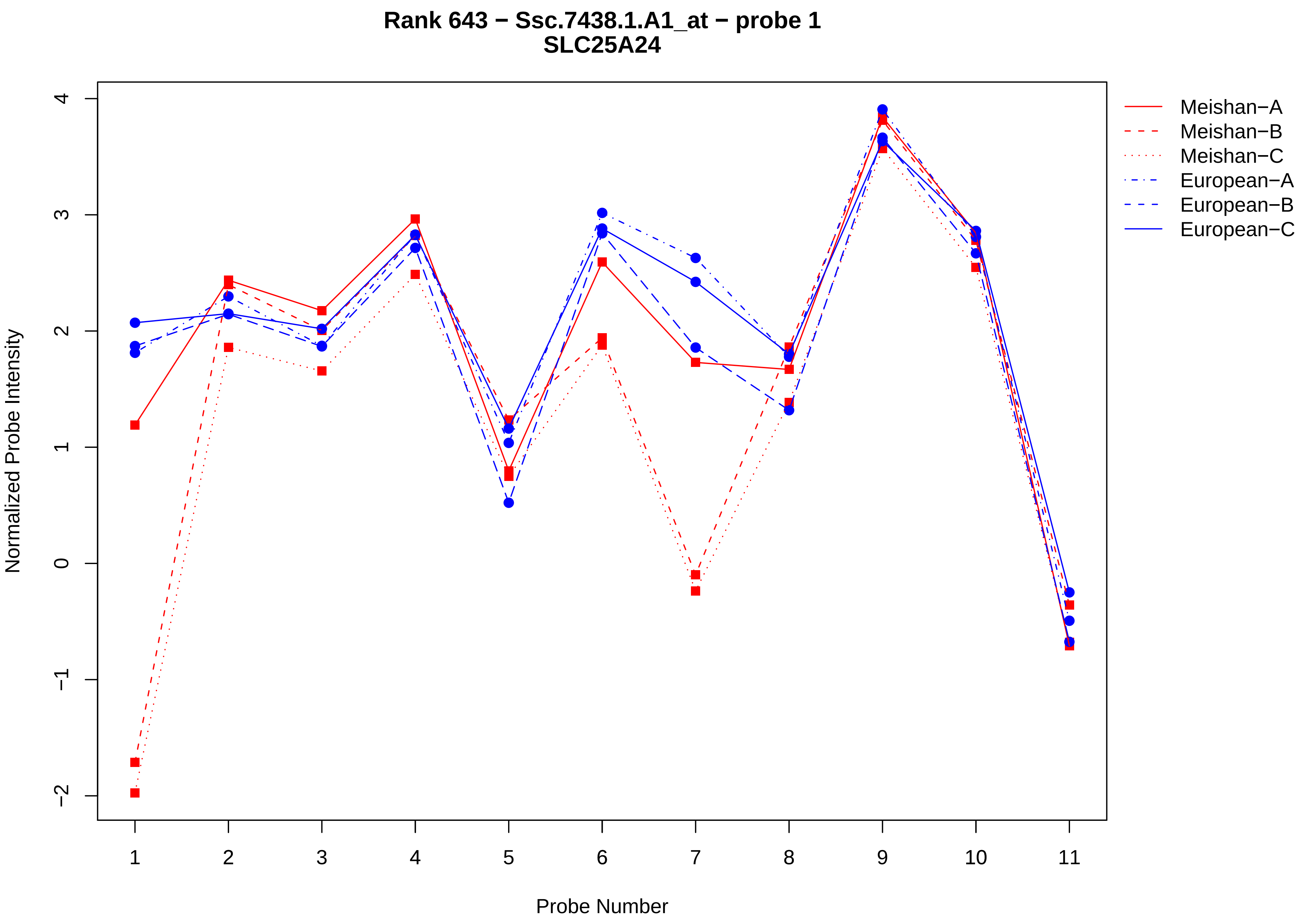

Rank 643 − Ssc.7438.1.A1_at − probe 1
SLC25A24
Normalized Probe Intensity
Probe Number
Meishan−A
Meishan−B
Meishan−C
European−A
European−B
European−C

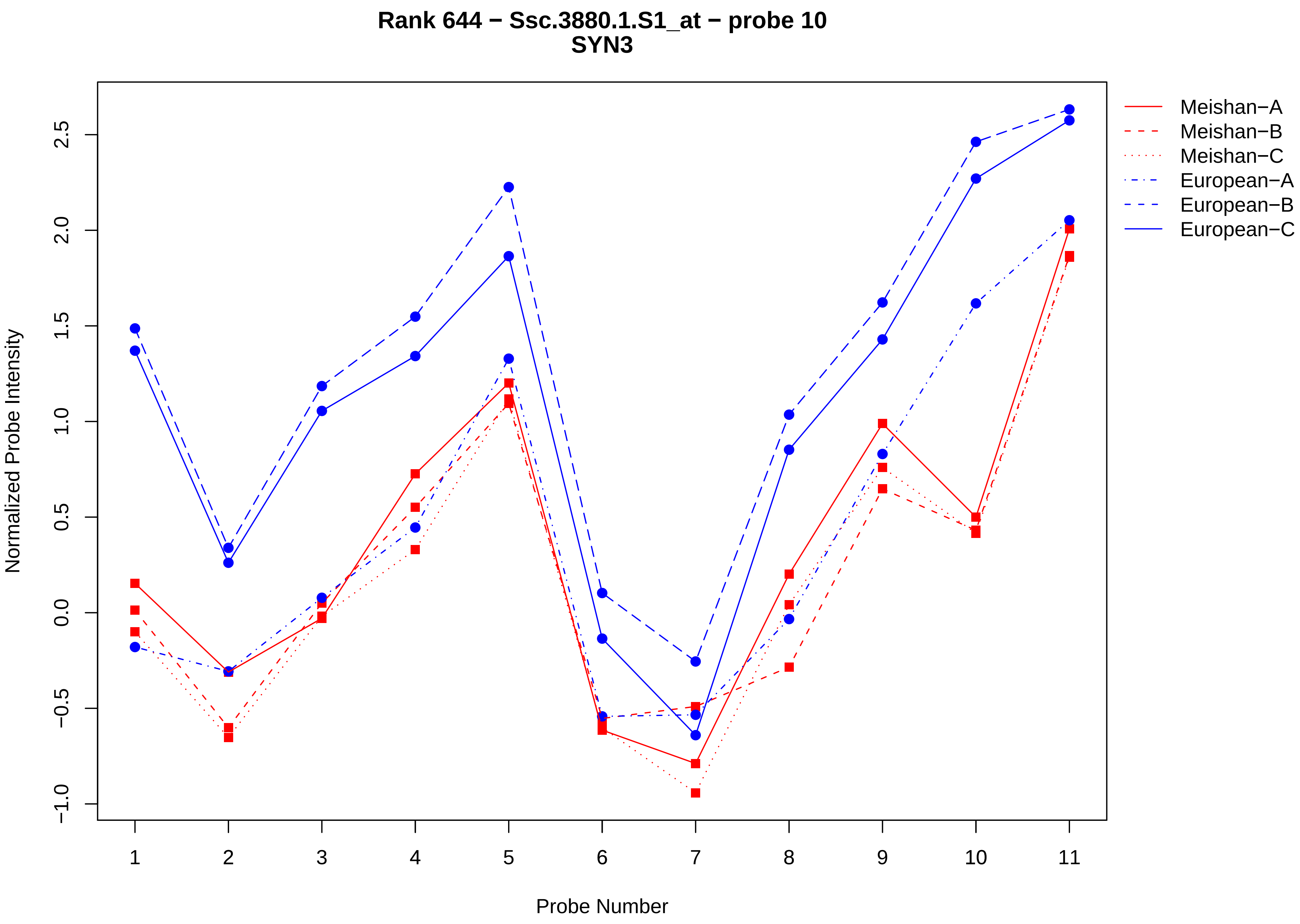
Rank 644 − Ssc.3880.1.S1_at − probe 10
SYN3
Normalized Probe Intensity
Probe Number
−1.0
−0.5
0.0
0.5
1.0
1.5
2.0
2.5
1
2
3
4
5
6
7
8
9
10
11
Meishan−A
Meishan−B
Meishan−C
European−A
European−B
European−C

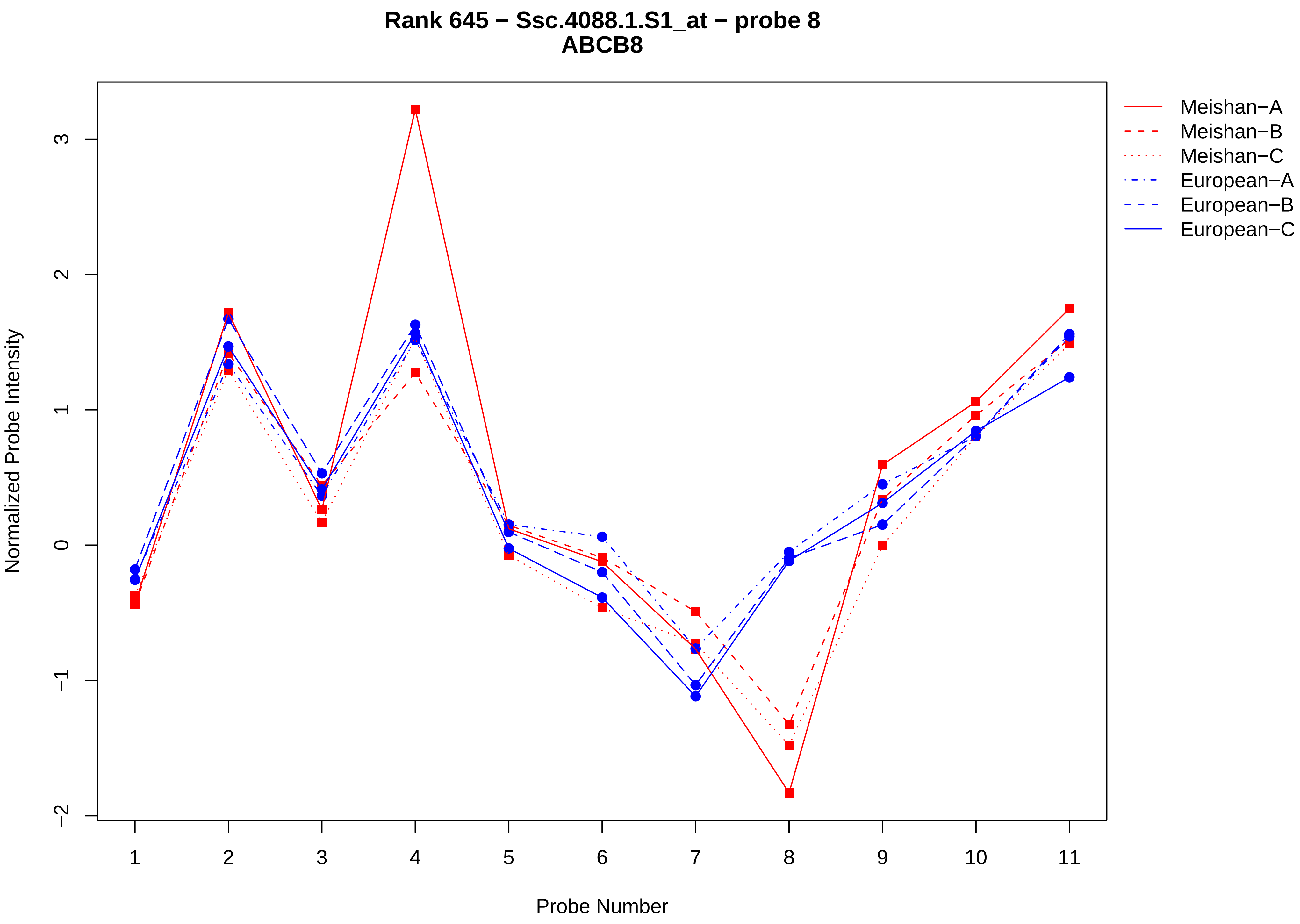

Rank 645 − Ssc.4088.1.S1_at − probe 8
ABCB8
Normalized Probe Intensity
Probe Number
−2
−1
0
1
2
3
1
2
3
4
5
6
7
8
9
10
11
Meishan−A
Meishan−B
Meishan−C
European−A
European−B
European−C

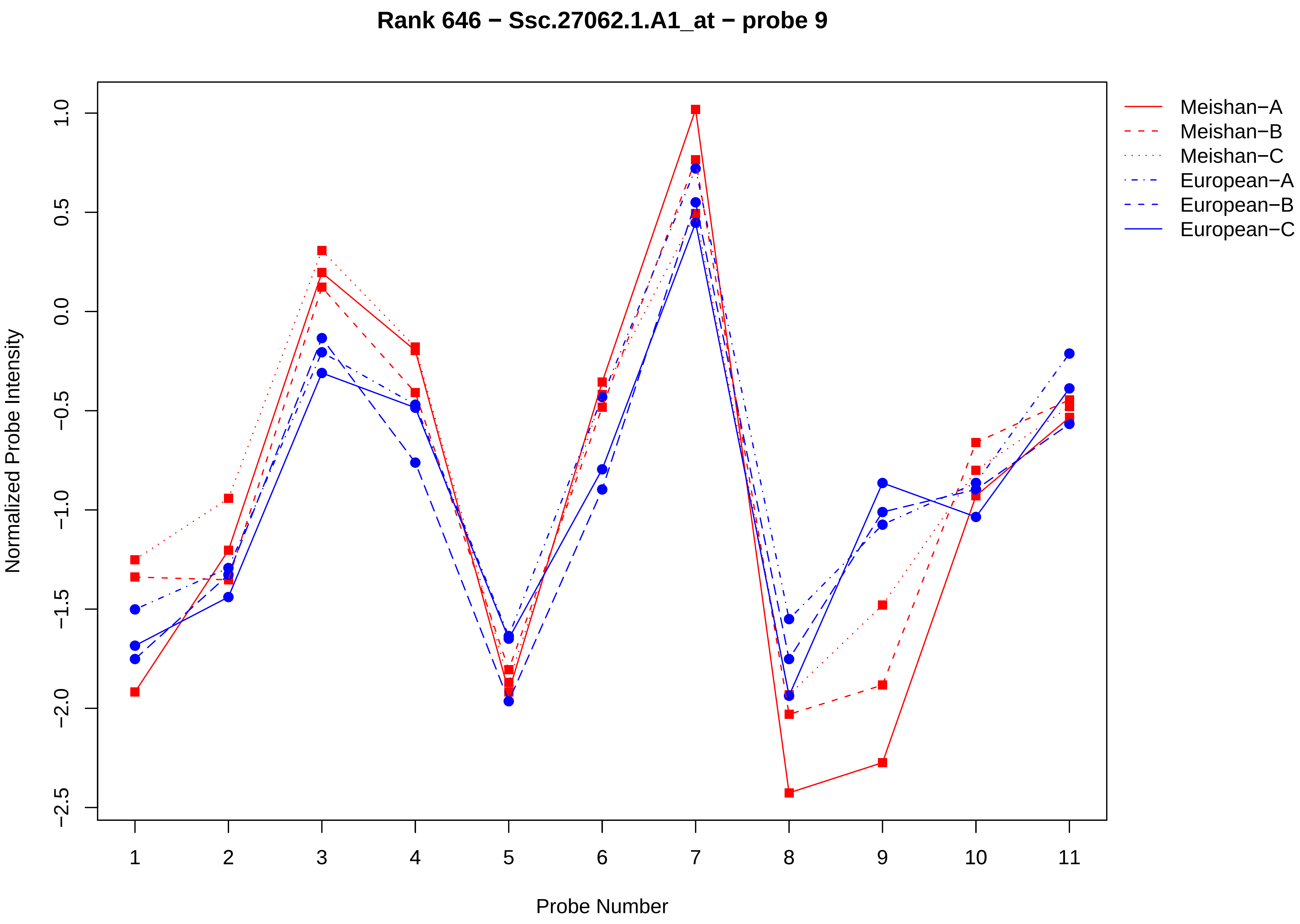
Rank 646 − Ssc.27062.1.A1_at − probe 9
Normalized Probe Intensity
1.0
0.5
0.0
−0.5
−1.0
−1.5
−2.0
−2.5
1
2
3
4
5
6
7
8
9
10
11
Probe Number
Meishan−A
Meishan−B
Meishan−C
European−A
European−B
European−C

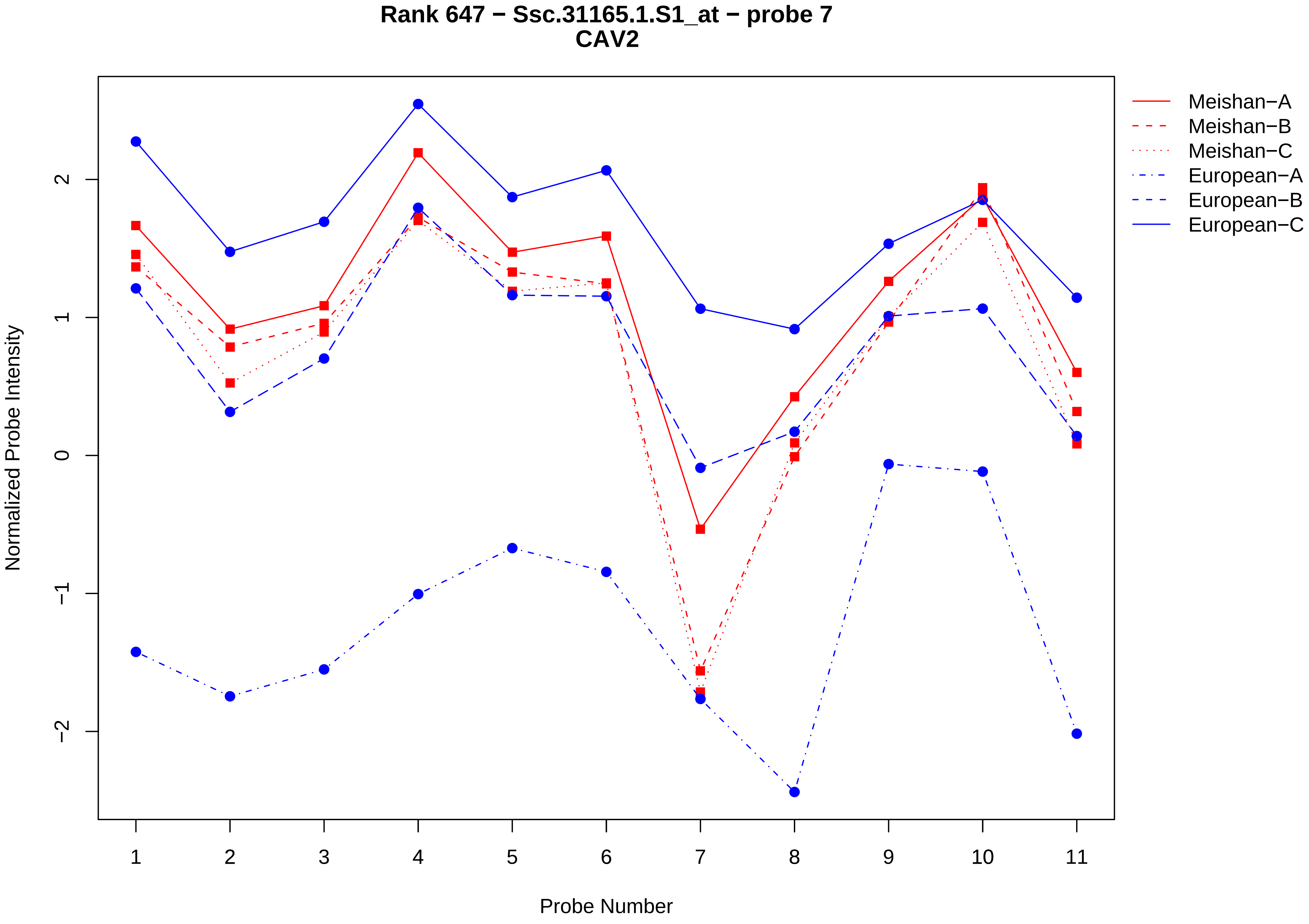

Rank 647 − Ssc.31165.1.S1_at − probe 7
CAV2
Normalized Probe Intensity
Probe Number
−2
−1
0
1
2
1
2
3
4
5
6
7
8
9
10
11
Meishan−A
Meishan−B
Meishan−C
European−A
European−B
European−C

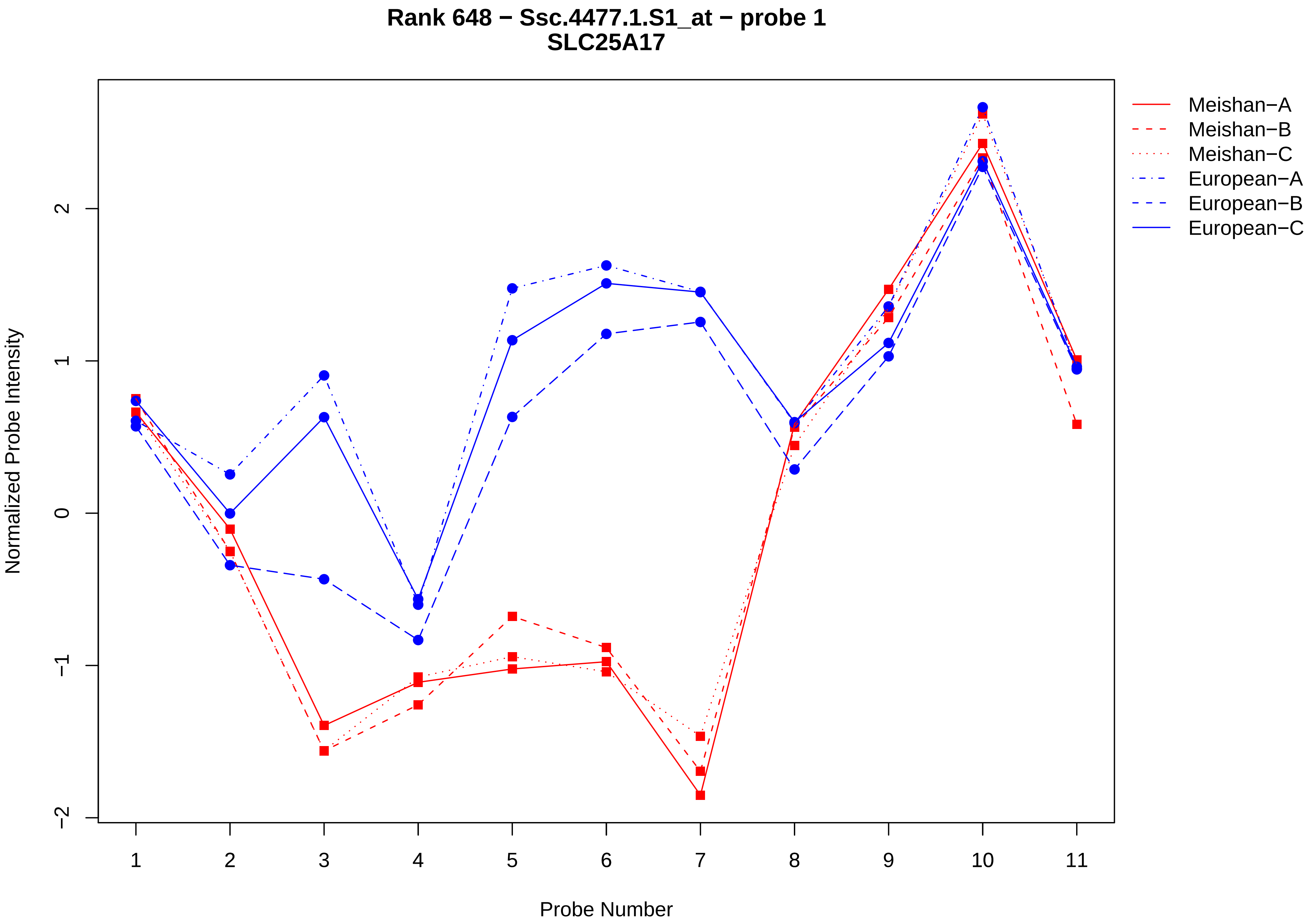

Rank 648 − Ssc.4477.1.S1_at − probe 1
SLC25A17
Normalized Probe Intensity
Probe Number
−2
−1
0
1
2
1
2
3
4
5
6
7
8
9
10
11
Meishan−A
Meishan−B
Meishan−C
European−A
European−B
European−C

Rank 649 − Ssc.13508.1.A1_at − probe 8
NP_057132

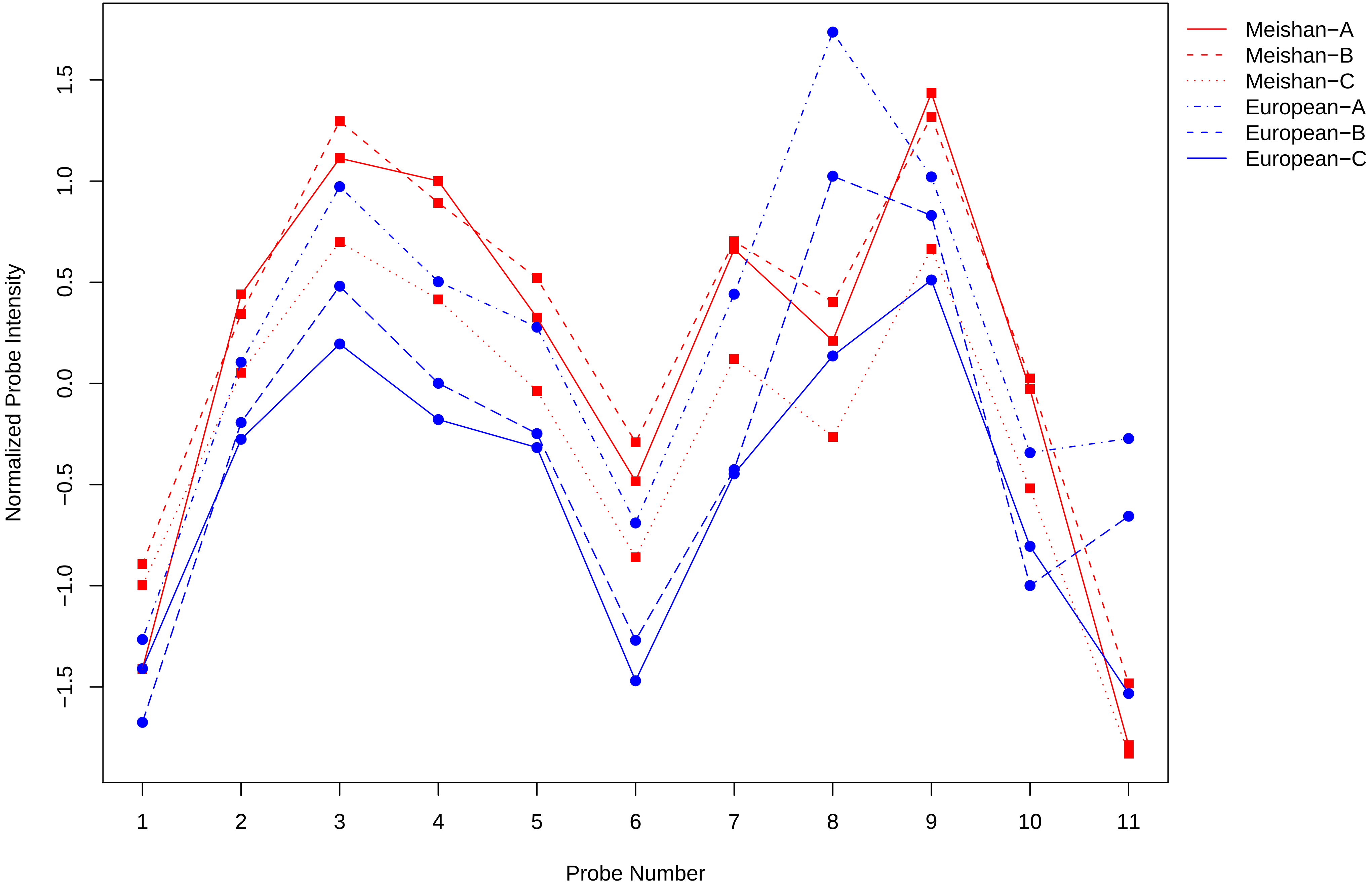

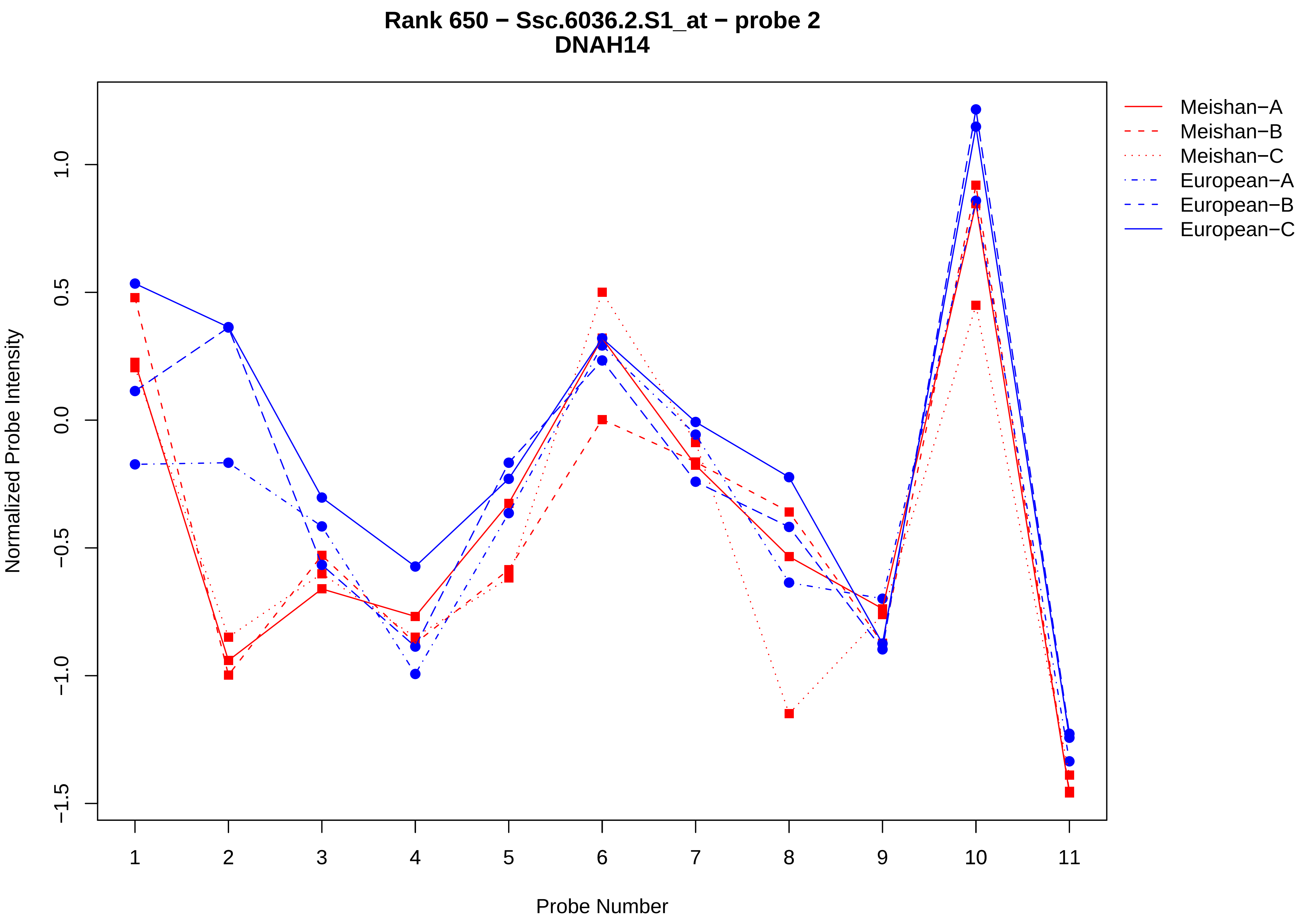

Rank 650 − Ssc.6036.2.S1_at − probe 2
DNAH14
Normalized Probe Intensity
Probe Number
−1.5
−1.0
−0.5
0.0
0.5
1.0
1
2
3
4
5
6
7
8
9
10
11
Meishan−A
Meishan−B
Meishan−C
European−A
European−B
European−C

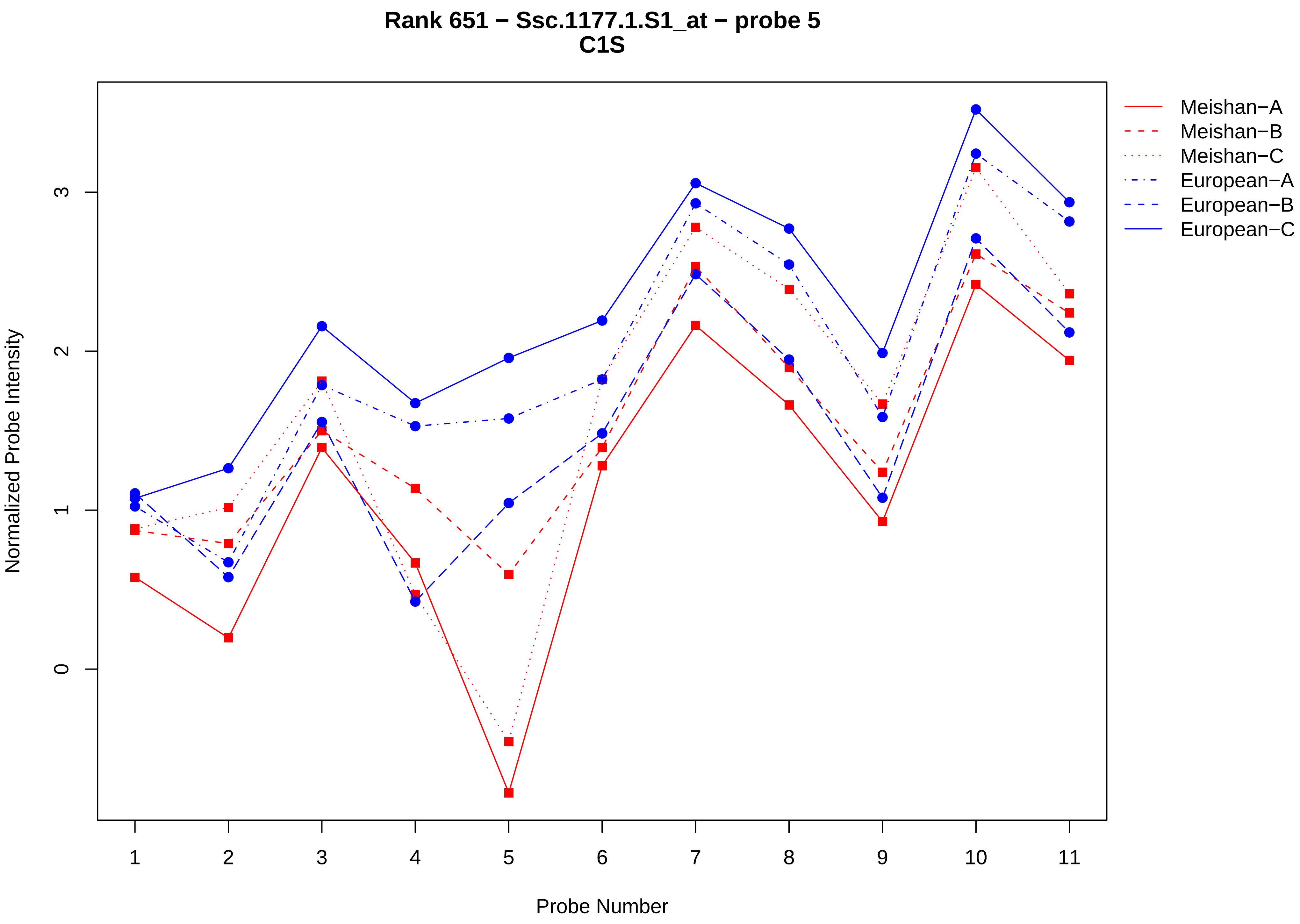
Rank 651 − Ssc.1177.1.S1_at − probe 5
C1S
Normalized Probe Intensity
0
1
2
3
Probe Number
1
2
3
4
5
6
7
8
9
10
11
Meishan−A
Meishan−B
Meishan−C
European−A
European−B
European−C

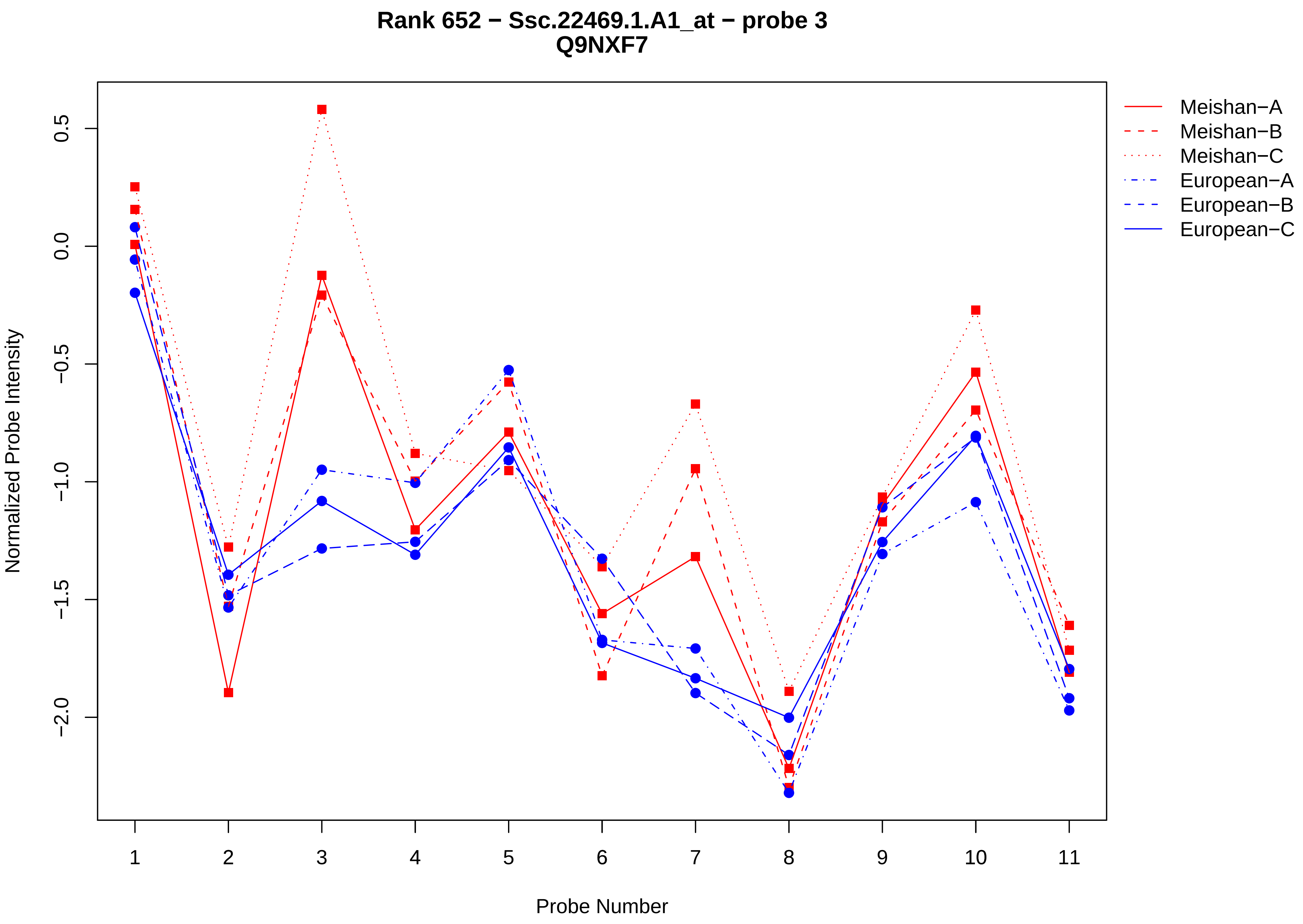

Rank 652 − Ssc.22469.1.A1_at − probe 3
Q9NXF7
Normalized Probe Intensity
Probe Number
0.5
0.0
−0.5
−1.0
−1.5
−2.0
1
2
3
4
5
6
7
8
9
10
11
Meishan−A
Meishan−B
Meishan−C
European−A
European−B
European−C

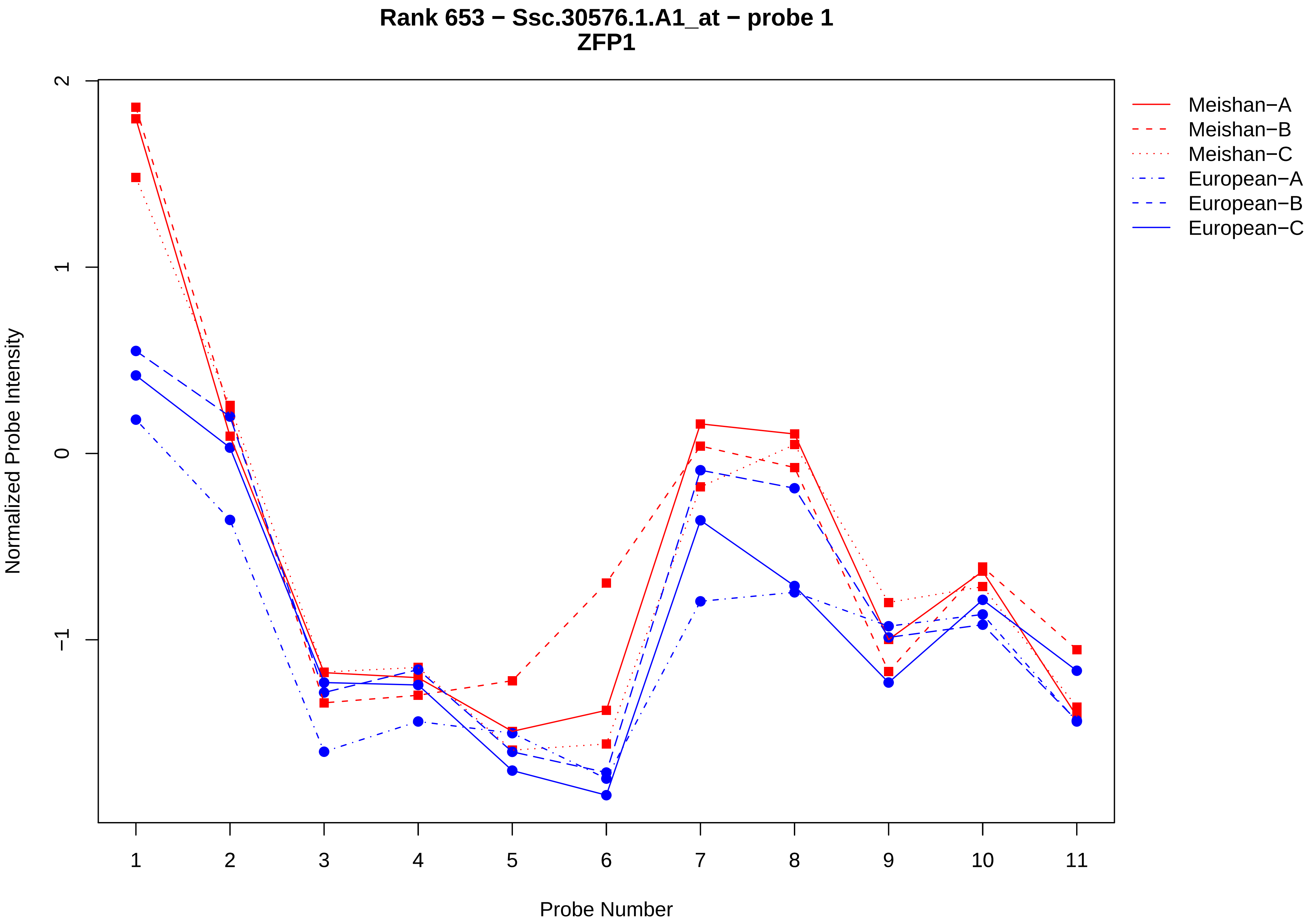

Rank 653 − Ssc.30576.1.A1_at − probe 1
ZFP1
Normalized Probe Intensity
Probe Number
Meishan−A
Meishan−B
Meishan−C
European−A
European−B
European−C
2
1
0
−1
1
2
3
4
5
6
7
8
9
10
11

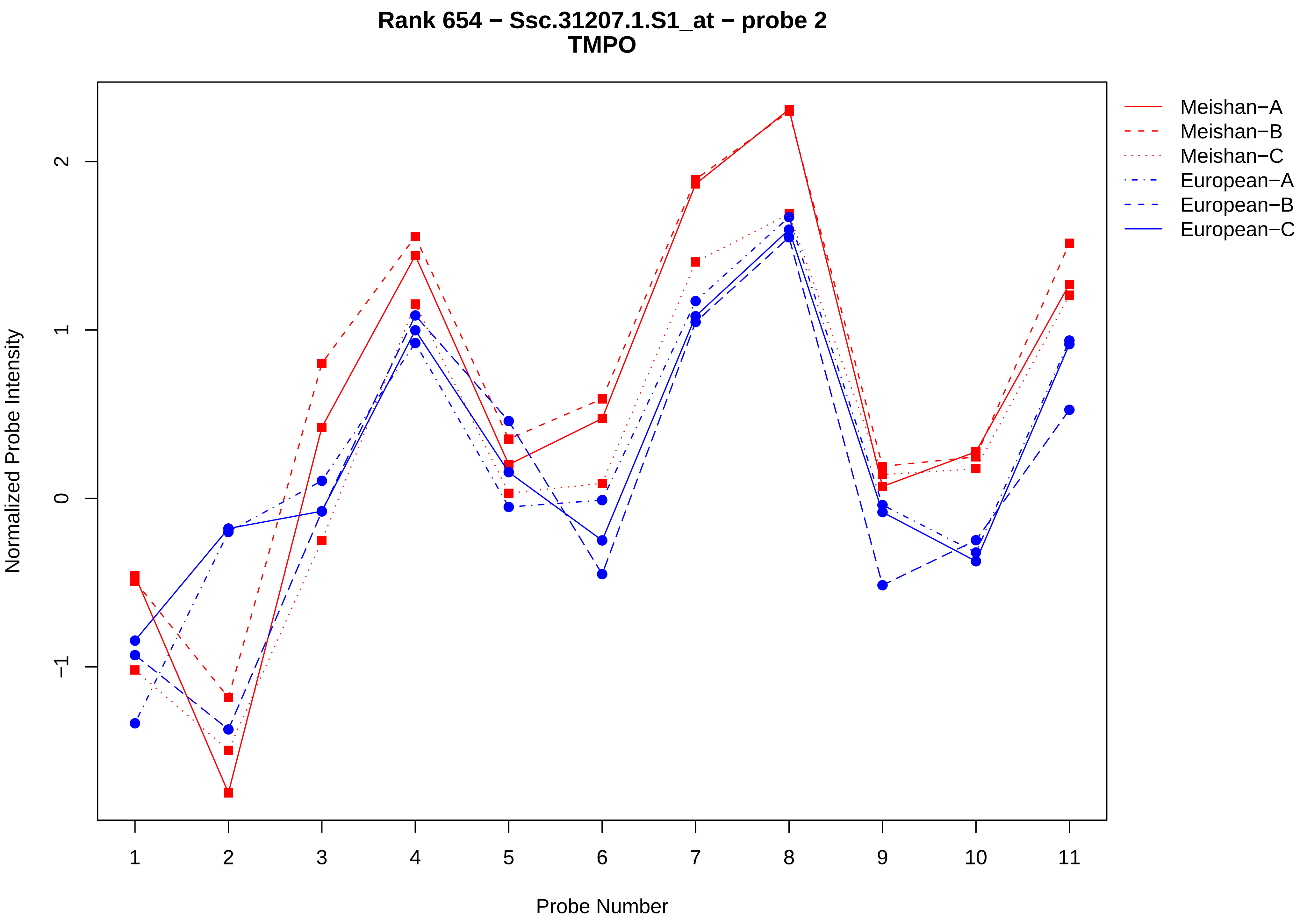

Rank 654 − Ssc.31207.1.S1_at − probe 2
TMPO
Normalized Probe Intensity
Probe Number
−1
0
1
2
1
2
3
4
5
6
7
8
9
10
11
Meishan−A
Meishan−B
Meishan−C
European−A
European−B
European−C

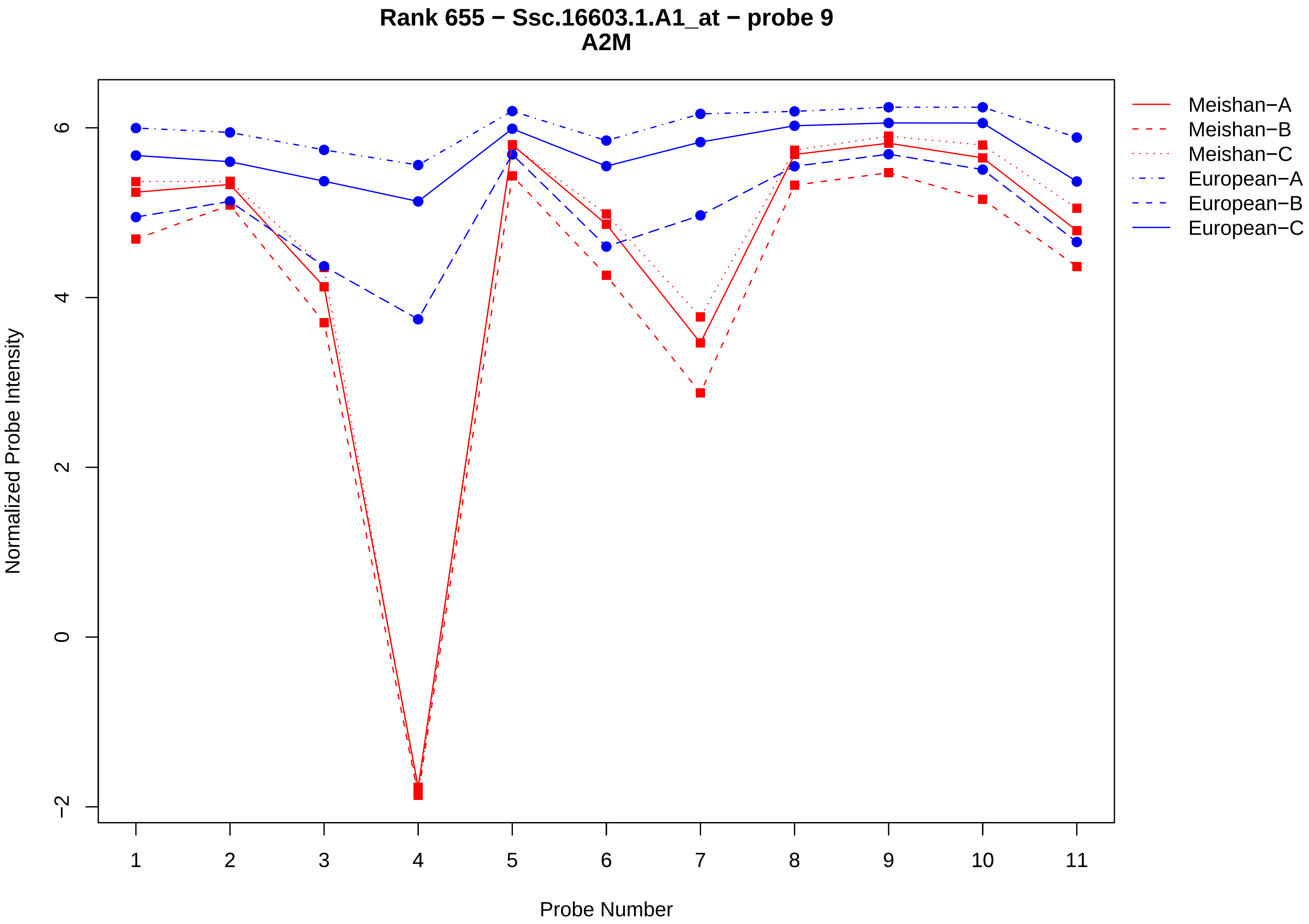
Rank 655 − Ssc.16603.1.A1_at − probe 9
A2M
Normalized Probe Intensity
Probe Number
−2
0
2
4
6
1
2
3
4
5
6
7
8
9
10
11
Meishan−A
Meishan−B
Meishan−C
European−A
European−B
European−C

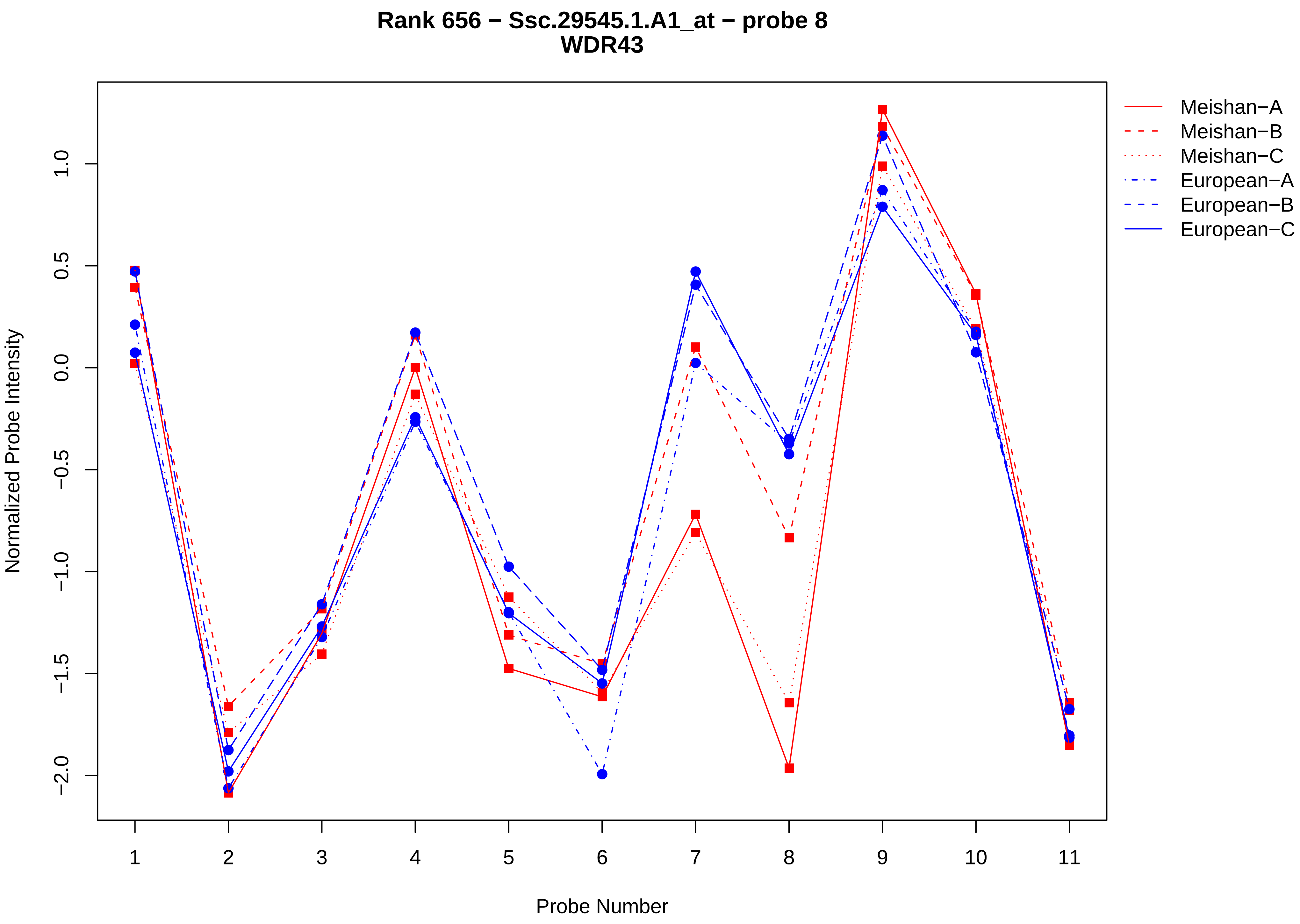

Rank 656 − Ssc.29545.1.A1_at − probe 8
WDR43
Normalized Probe Intensity
Probe Number
Meishan−A
Meishan−B
Meishan−C
European−A
European−B
European−C

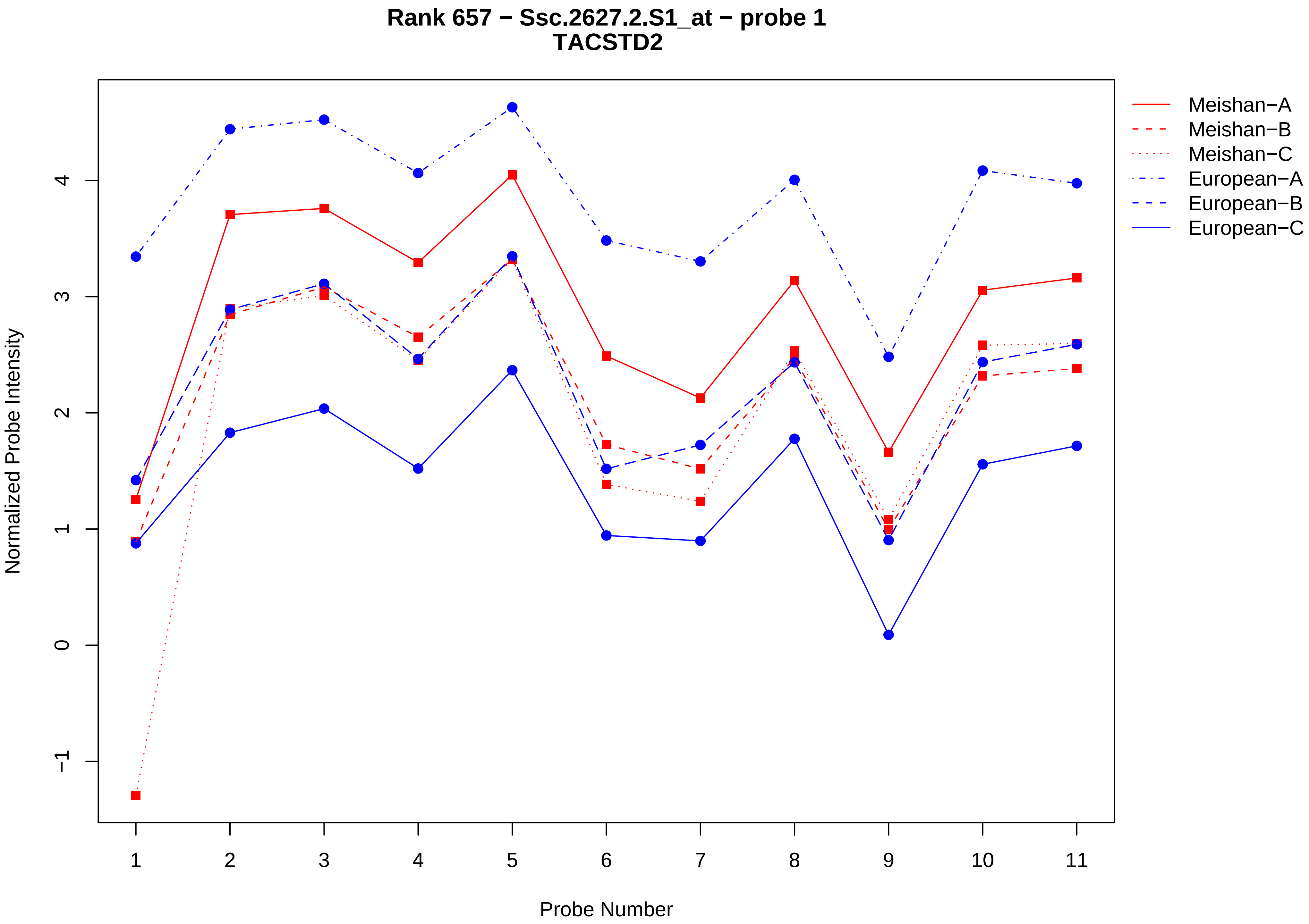
Rank 657 − Ssc.2627.2.S1_at − probe 1
TACSTD2
Normalized Probe Intensity
Probe Number
−1
0
1
2
3
4
1
2
3
4
5
6
7
8
9
10
11
Meishan−A
Meishan−B
Meishan−C
European−A
European−B
European−C

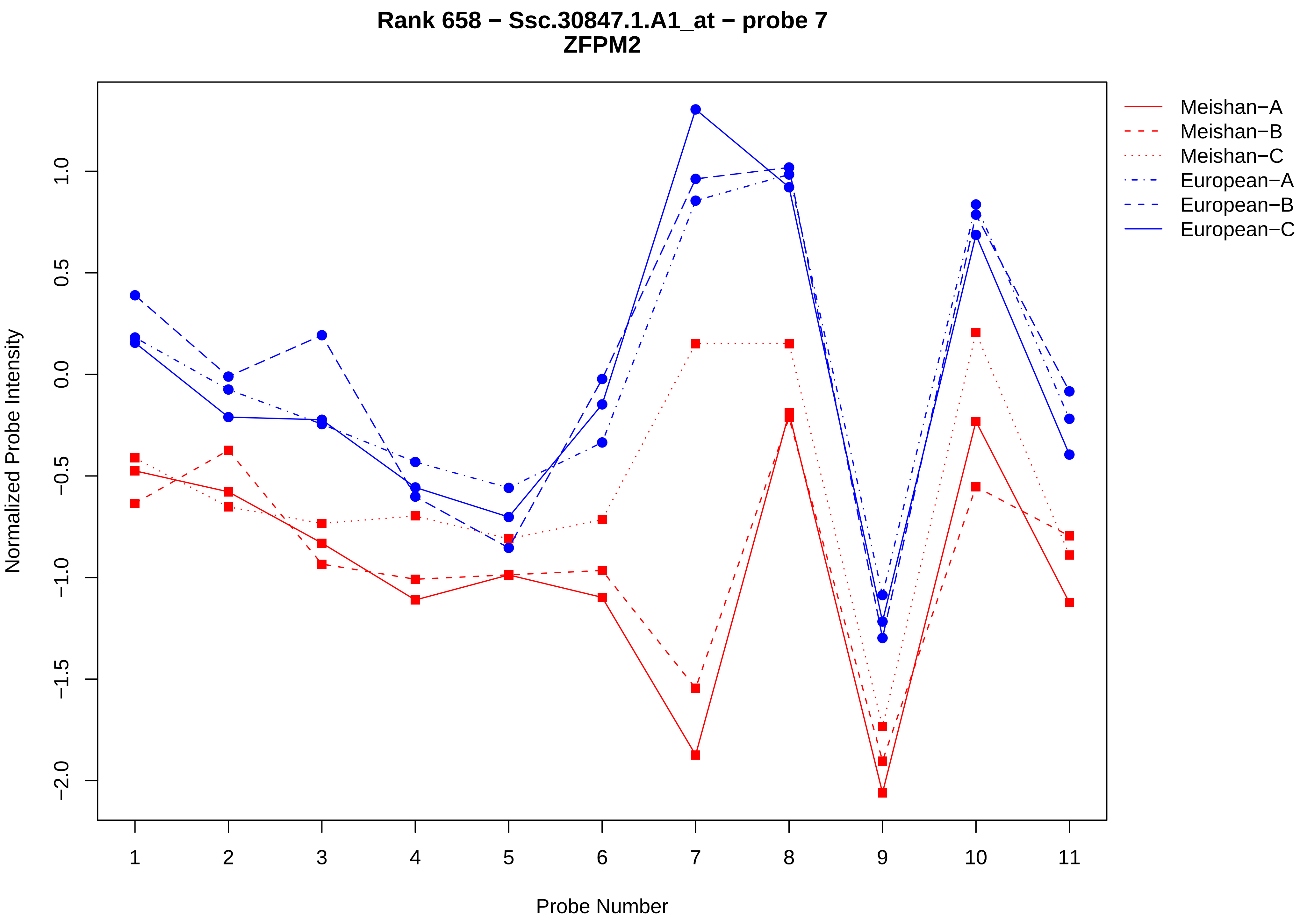

Rank 658 − Ssc.30847.1.A1_at − probe 7
ZFPM2
Normalized Probe Intensity
Probe Number
1.0
0.5
0.0
−0.5
−1.0
−1.5
−2.0
1
2
3
4
5
6
7
8
9
10
11
Meishan−A
Meishan−B
Meishan−C
European−A
European−B
European−C

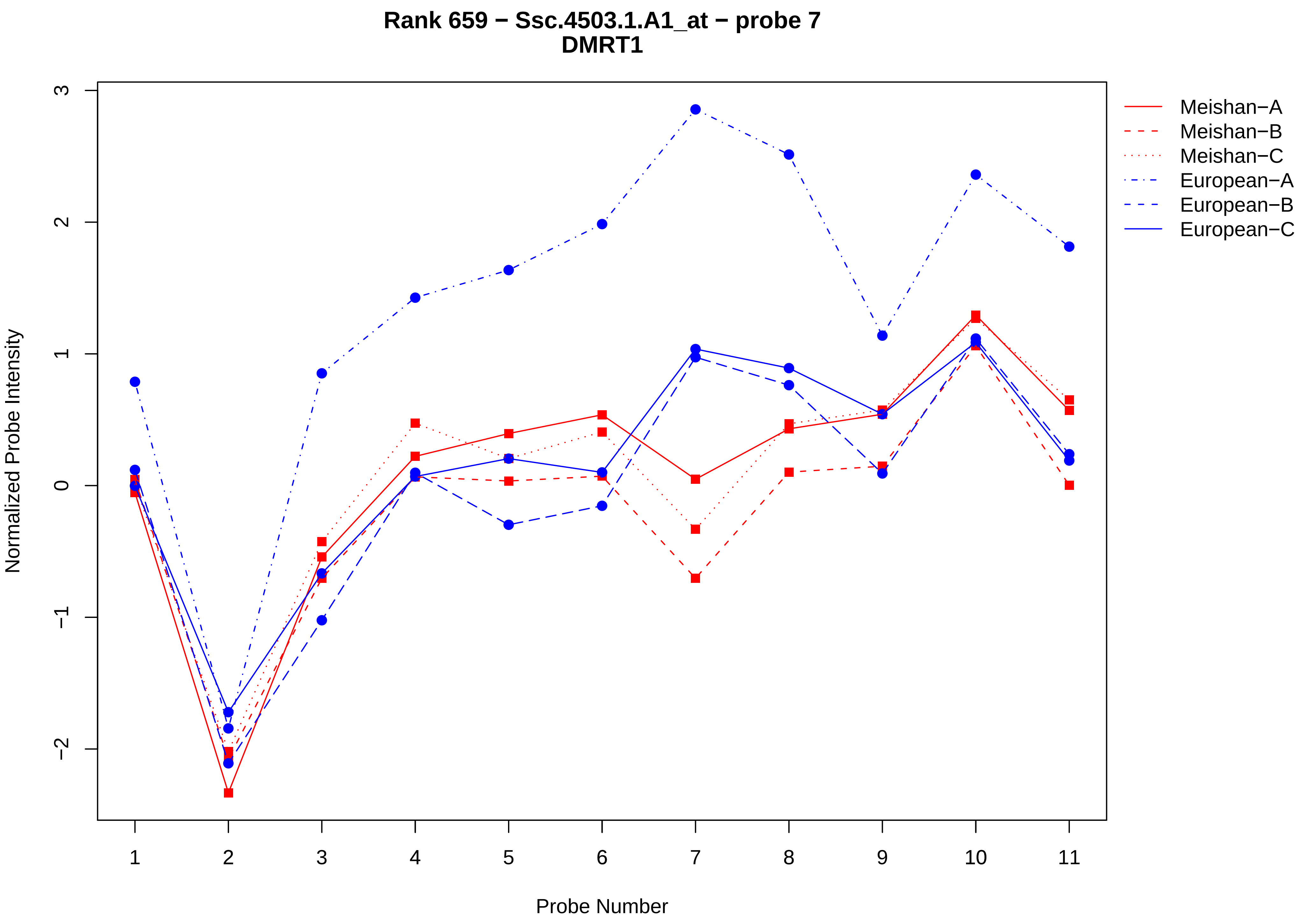
Rank 659 − Ssc.4503.1.A1_at − probe 7
DMRT1
Normalized Probe Intensity
Probe Number
−2
−1
0
1
2
3
1
2
3
4
5
6
7
8
9
10
11
Meishan−A
Meishan−B
Meishan−C
European−A
European−B
European−C

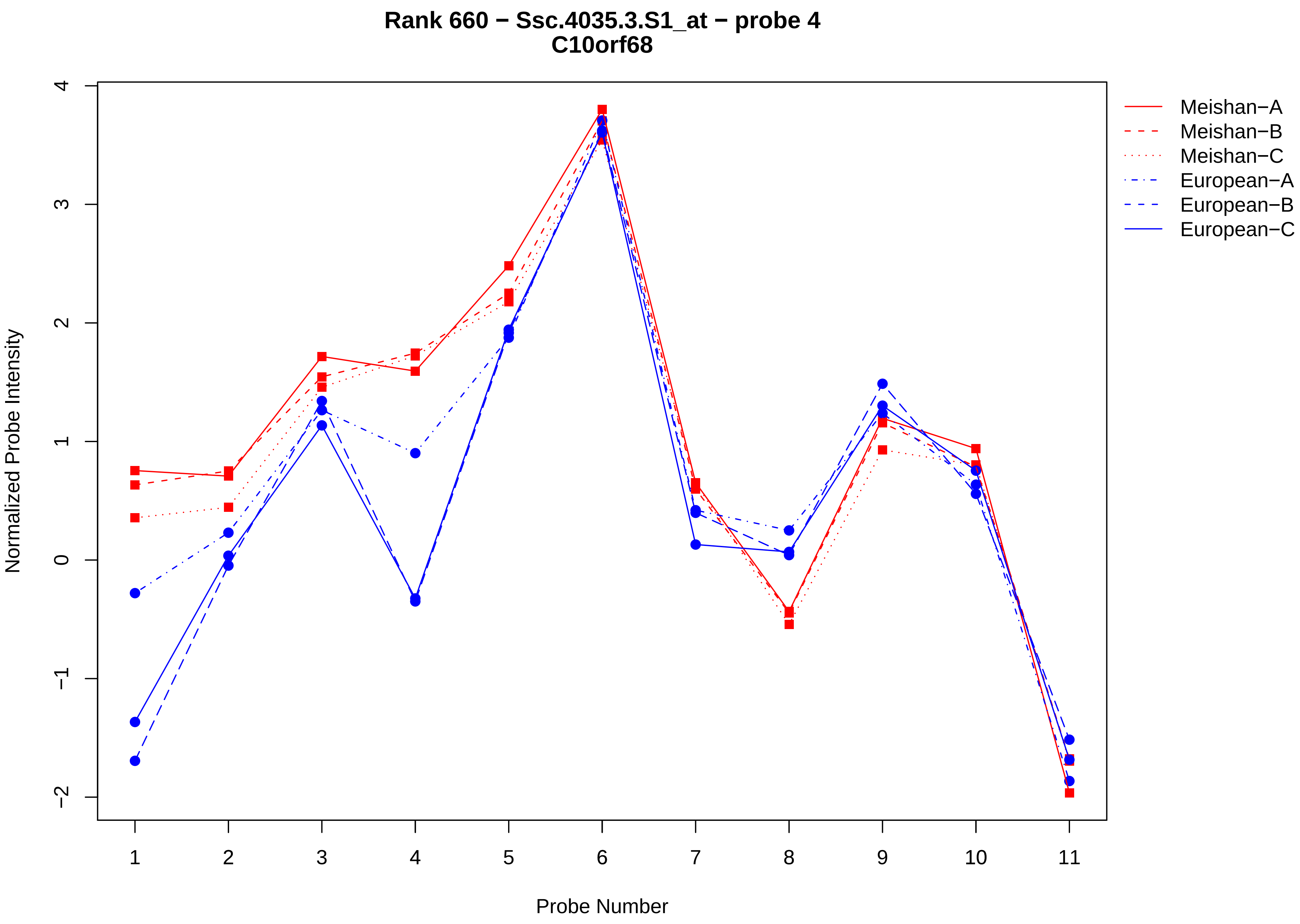

Rank 660 − Ssc.4035.3.S1_at − probe 4
C10orf68
Normalized Probe Intensity
Probe Number
Meishan−A
Meishan−B
Meishan−C
European−A
European−B
European−C

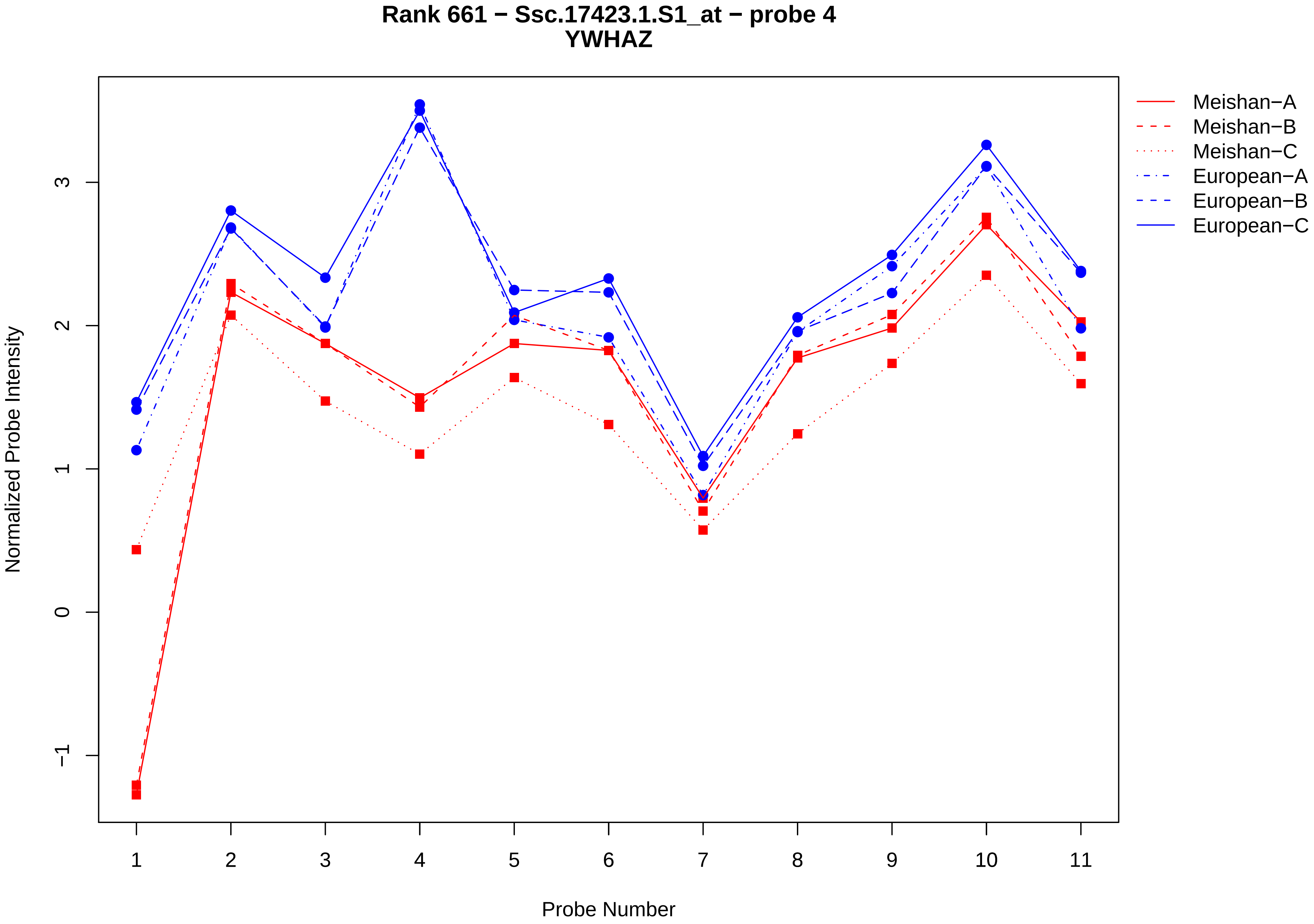

Rank 661 − Ssc.17423.1.S1_at − probe 4
YWHAZ
Normalized Probe Intensity
Probe Number
−1
0
1
2
3
1
2
3
4
5
6
7
8
9
10
11
Meishan−A
Meishan−B
Meishan−C
European−A
European−B
European−C

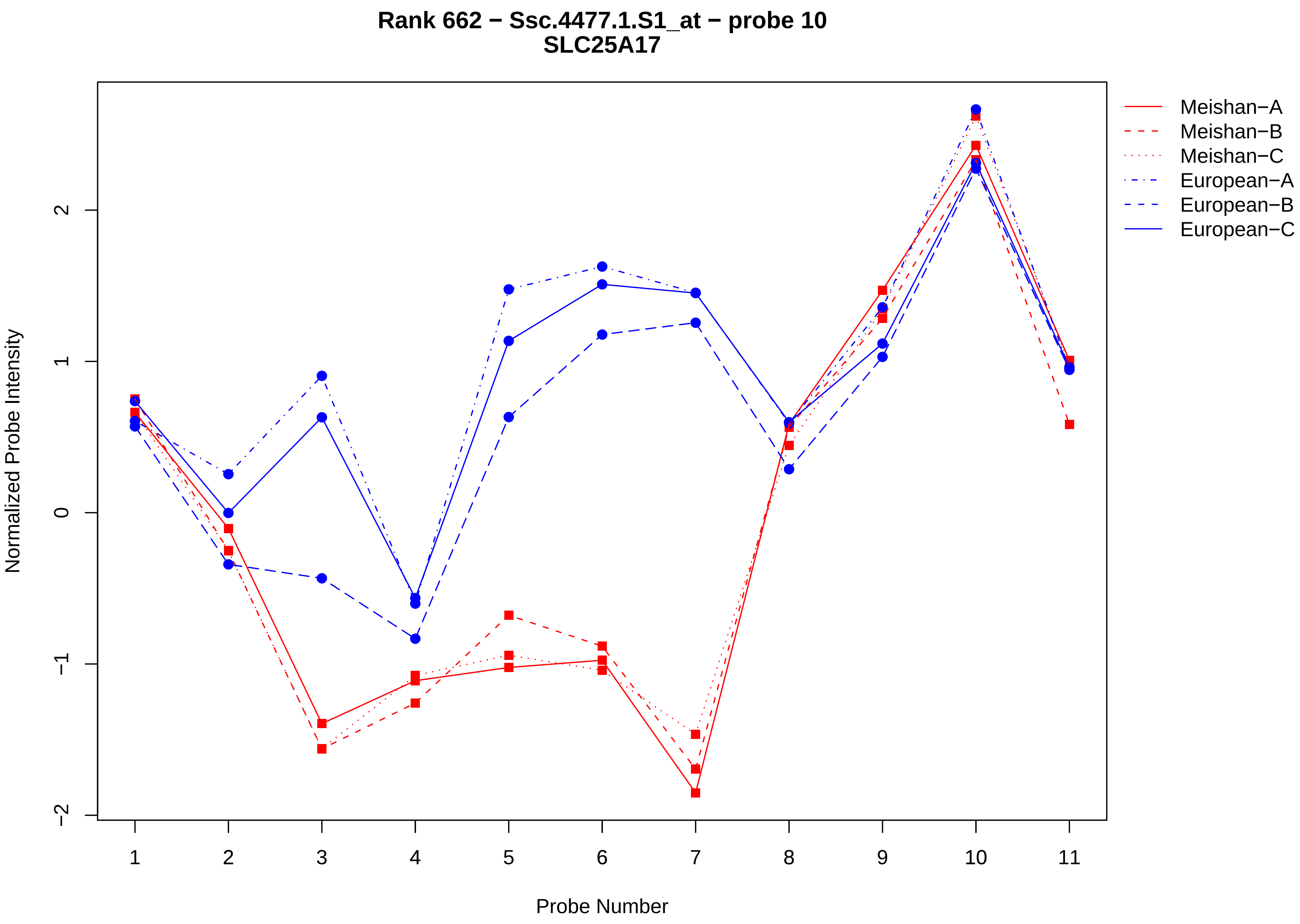

Rank 662 − Ssc.4477.1.S1_at − probe 10
SLC25A17
Normalized Probe Intensity
Probe Number
−2
−1
0
1
2
1
2
3
4
5
6
7
8
9
10
11
Meishan−A
Meishan−B
Meishan−C
European−A
European−B
European−C

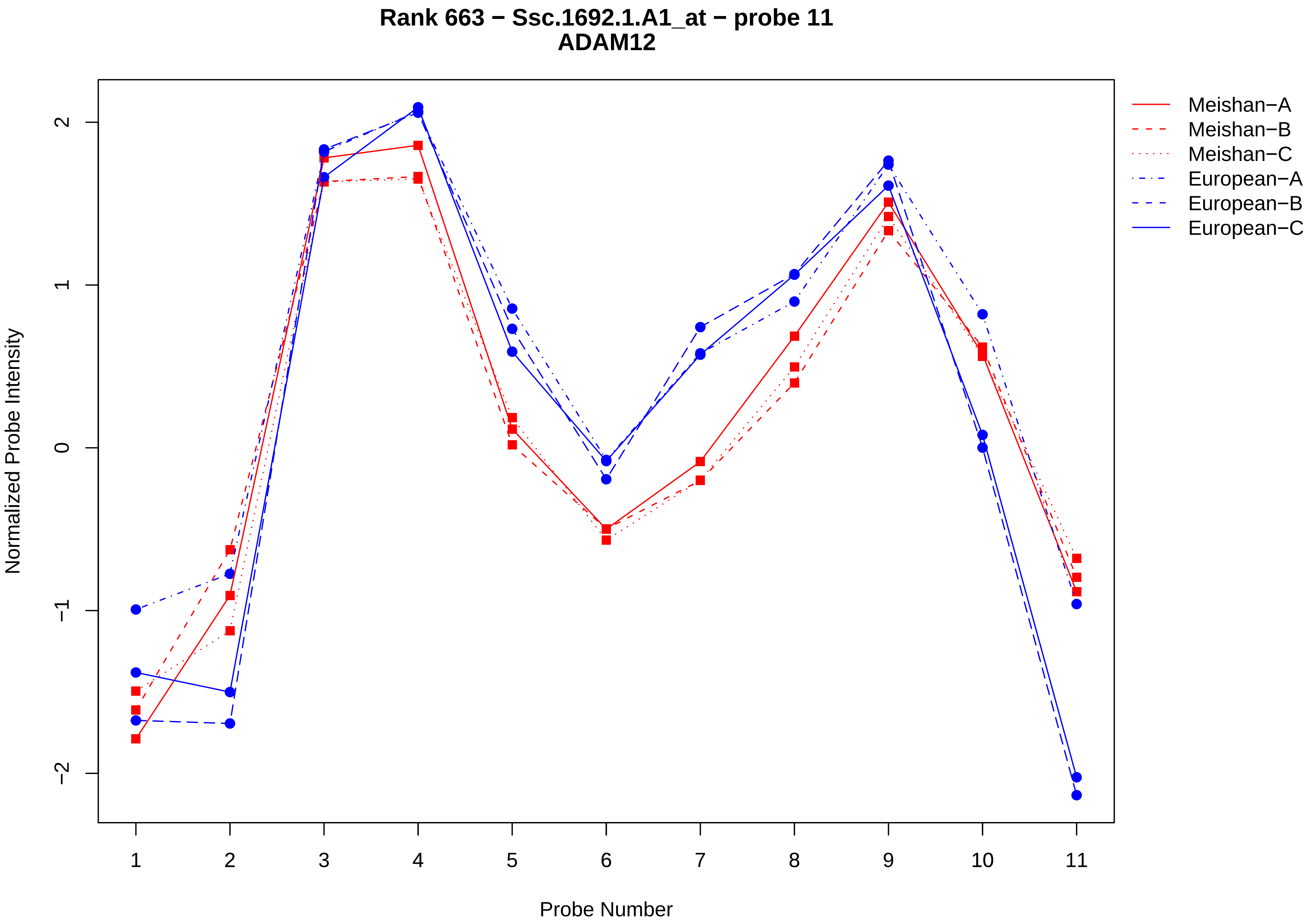

Rank 663 − Ssc.1692.1.A1_at − probe 11
ADAM12
Normalized Probe Intensity
Probe Number
−2
−1
0
1
2
1
2
3
4
5
6
7
8
9
10
11
Meishan−A
Meishan−B
Meishan−C
European−A
European−B
European−C

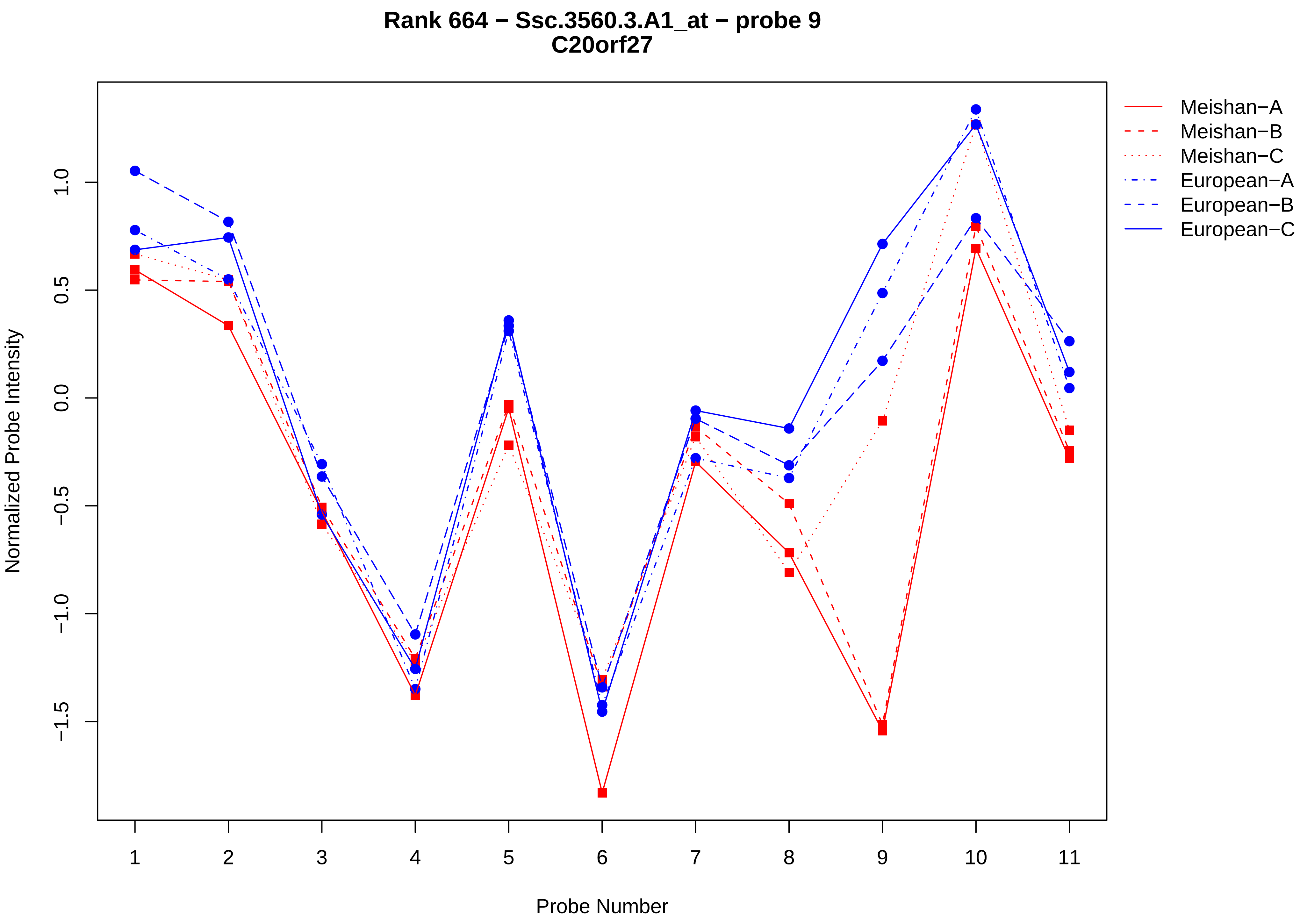
Rank 664 − Ssc.3560.3.A1_at − probe 9
C20orf27
Normalized Probe Intensity
Probe Number
Meishan−A
Meishan−B
Meishan−C
European−A
European−B
European−C
1.0
0.5
0.0
−0.5
−1.0
−1.5
1
2
3
4
5
6
7
8
9
10
11

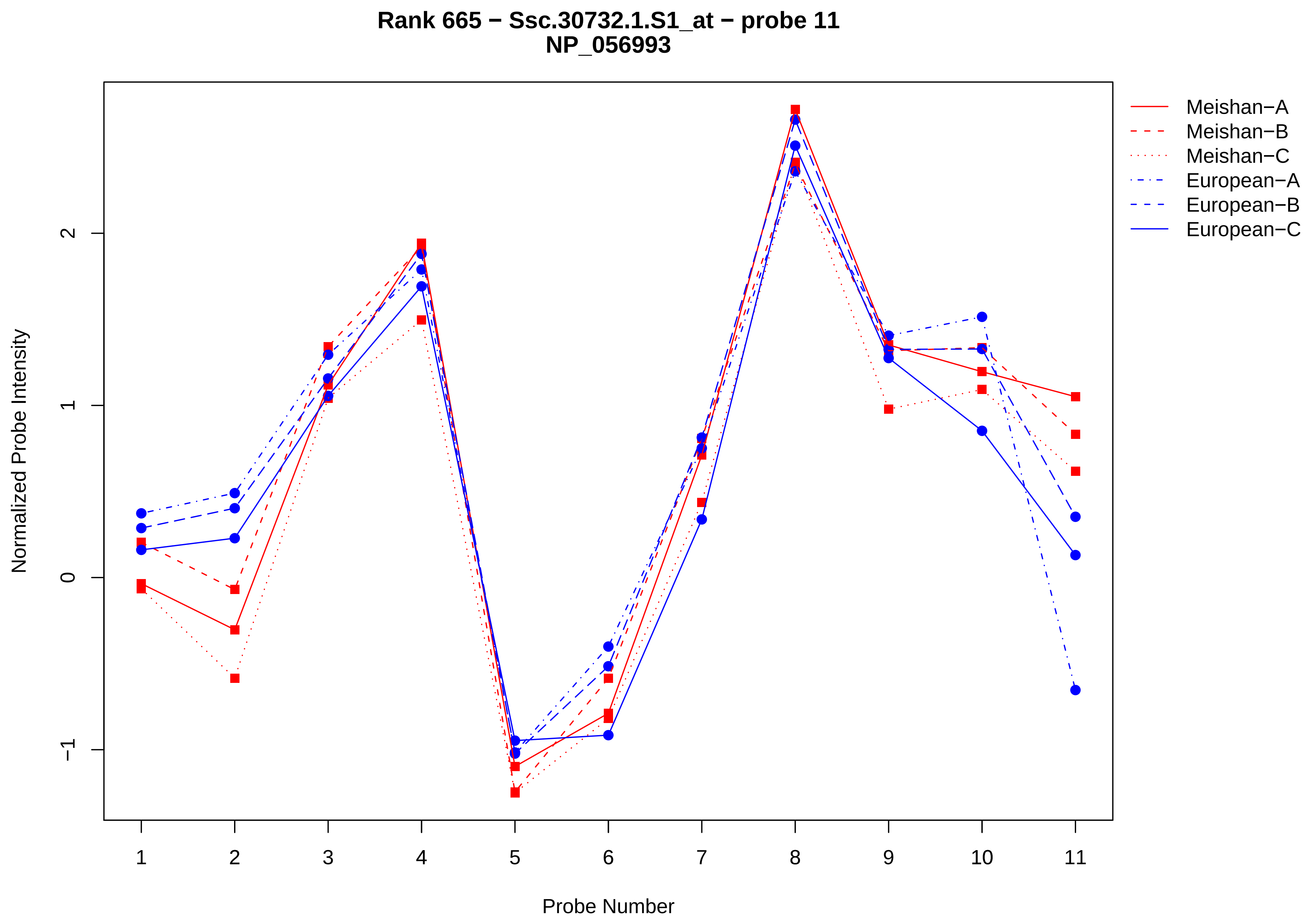
Rank 665 − Ssc.30732.1.S1_at − probe 11
NP_056993
Normalized Probe Intensity
Probe Number
−1
0
1
2
1
2
3
4
5
6
7
8
9
10
11
Meishan−A
Meishan−B
Meishan−C
European−A
European−B
European−C

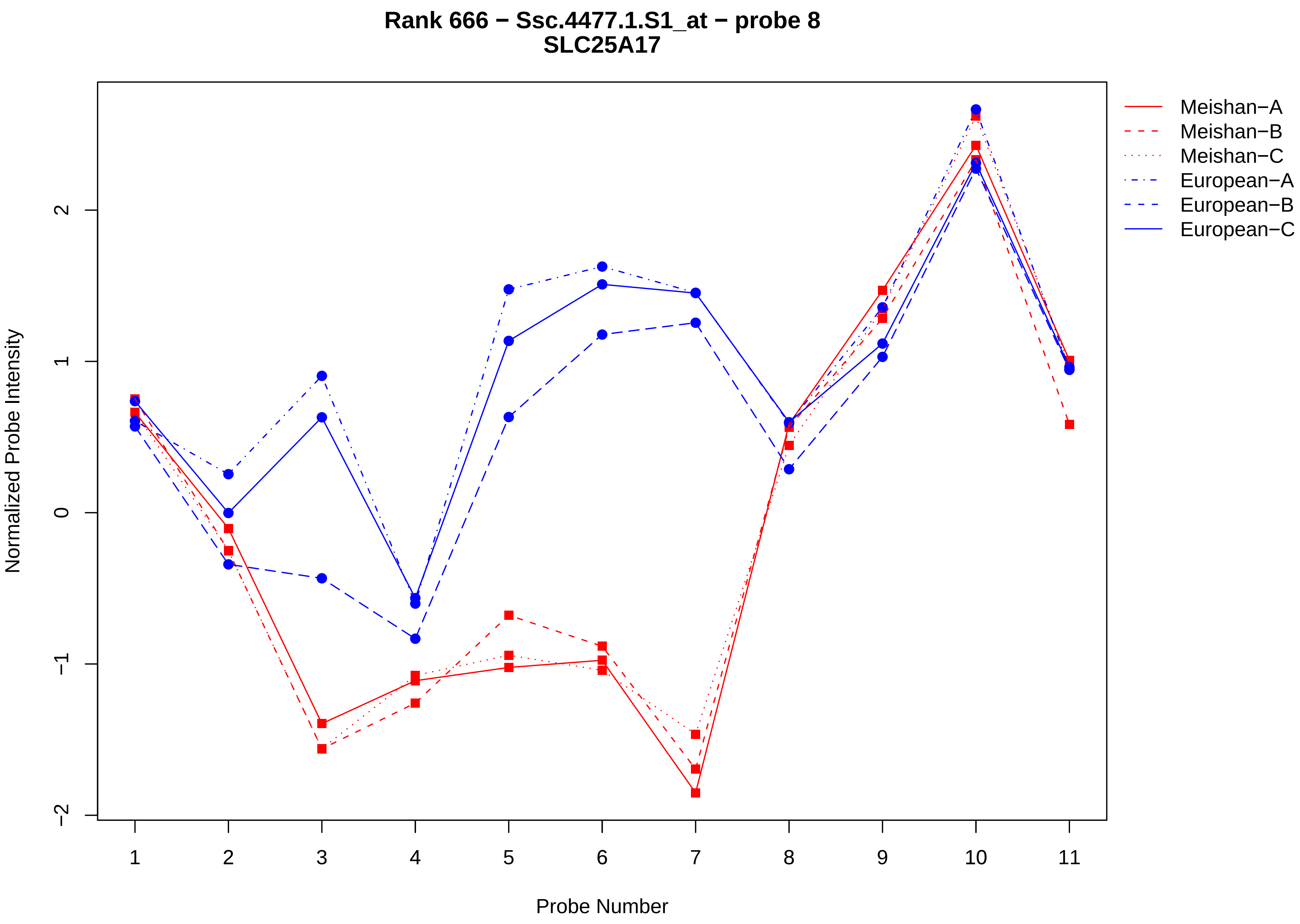

Rank 666 − Ssc.4477.1.S1_at − probe 8
SLC25A17
Normalized Probe Intensity
Probe Number
−2
−1
0
1
2
1
2
3
4
5
6
7
8
9
10
11
Meishan−A
Meishan−B
Meishan−C
European−A
European−B
European−C

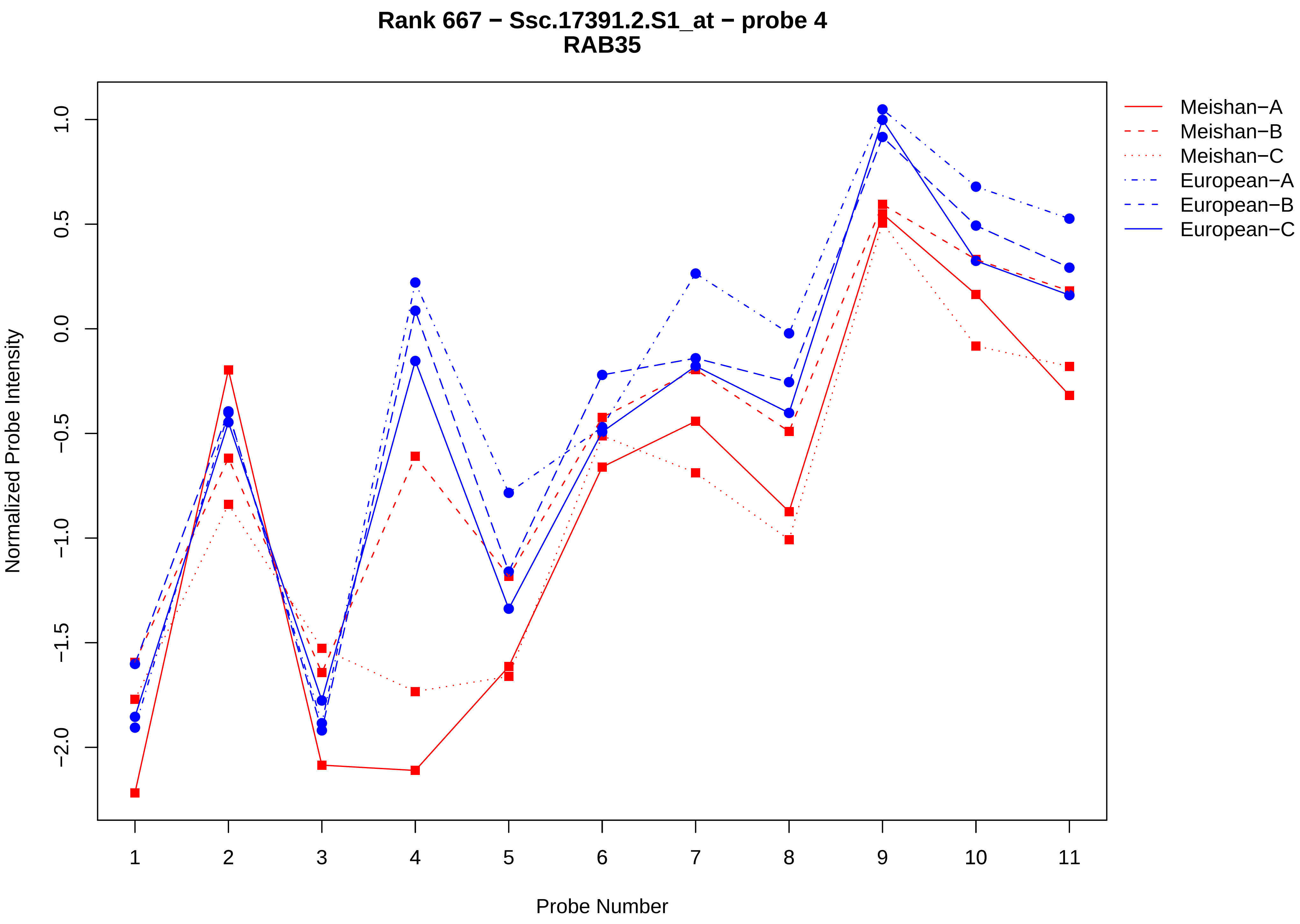

Rank 667 − Ssc.17391.2.S1_at − probe 4
RAB35
Normalized Probe Intensity
Probe Number
Meishan−A
Meishan−B
Meishan−C
European−A
European−B
European−C
1.0
0.5
0.0
−0.5
−1.0
−1.5
−2.0
1
2
3
4
5
6
7
8
9
10
11

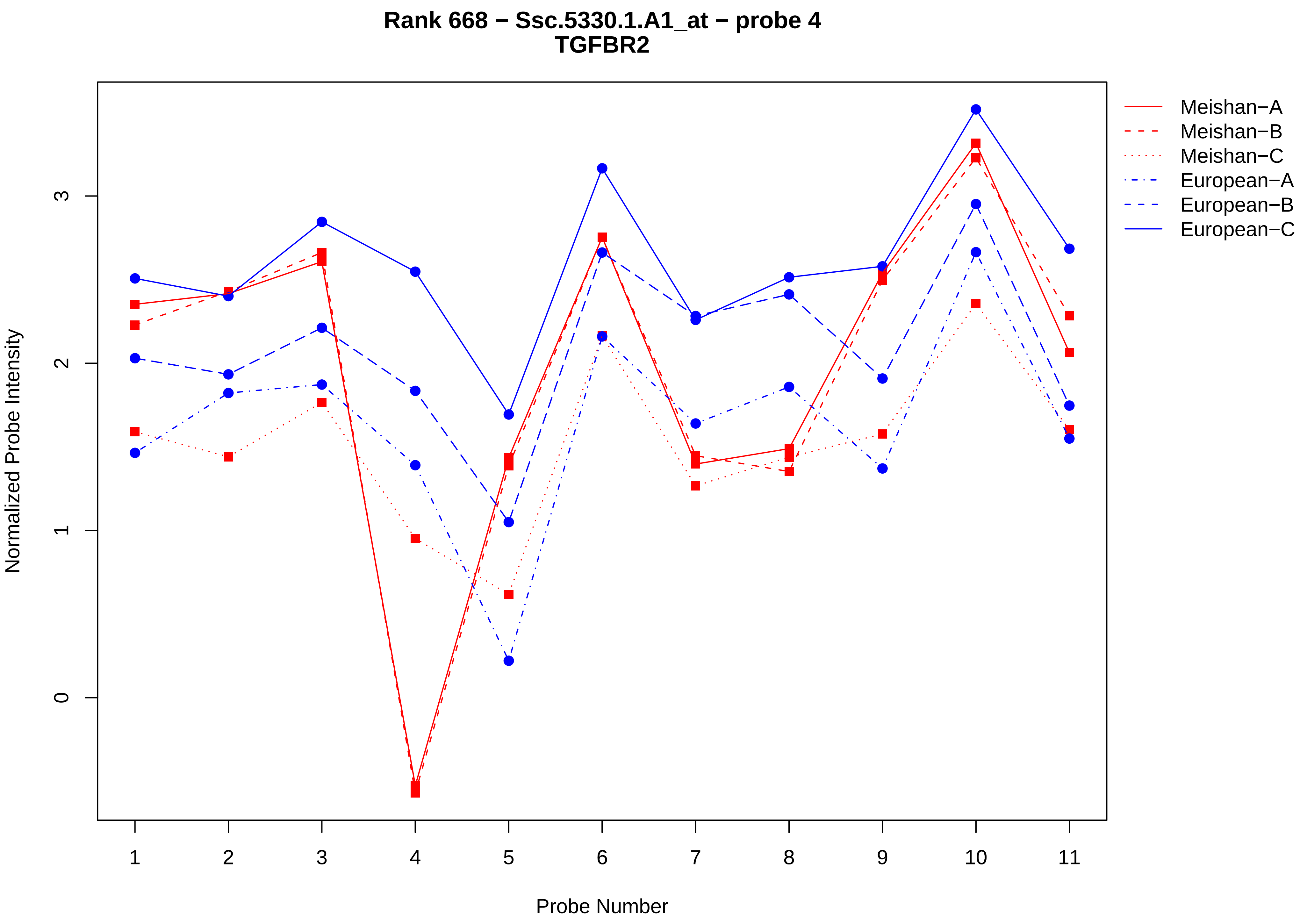
Rank 668 − Ssc.5330.1.A1_at − probe 4
TGFBR2
Normalized Probe Intensity
Probe Number
0
1
2
3
1
2
3
4
5
6
7
8
9
10
11
Meishan−A
Meishan−B
Meishan−C
European−A
European−B
European−C

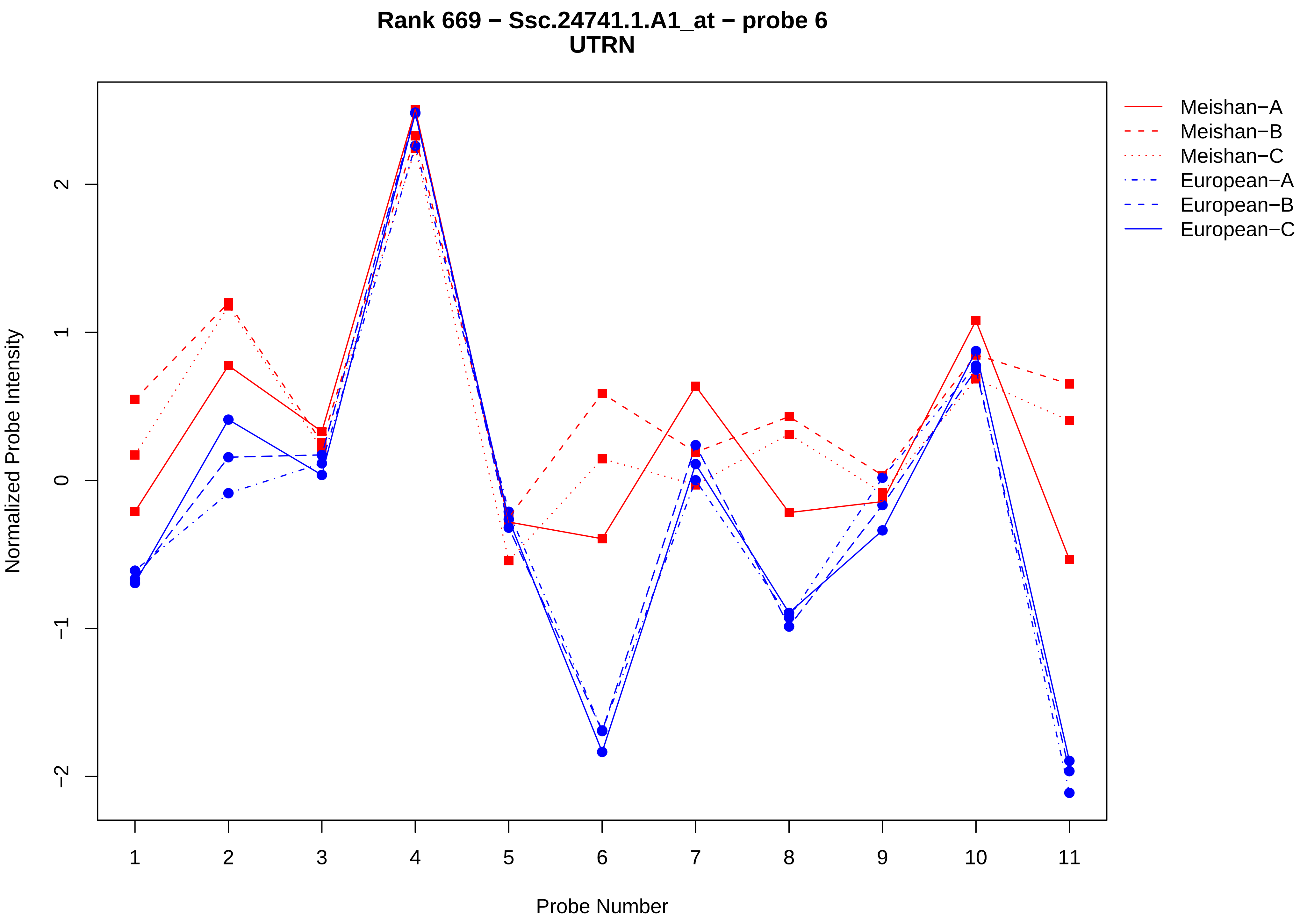

Rank 669 − Ssc.24741.1.A1_at − probe 6
UTRN
Normalized Probe Intensity
Probe Number
Meishan−A
Meishan−B
Meishan−C
European−A
European−B
European−C
−2
−1
0
1
2
1
2
3
4
5
6
7
8
9
10
11

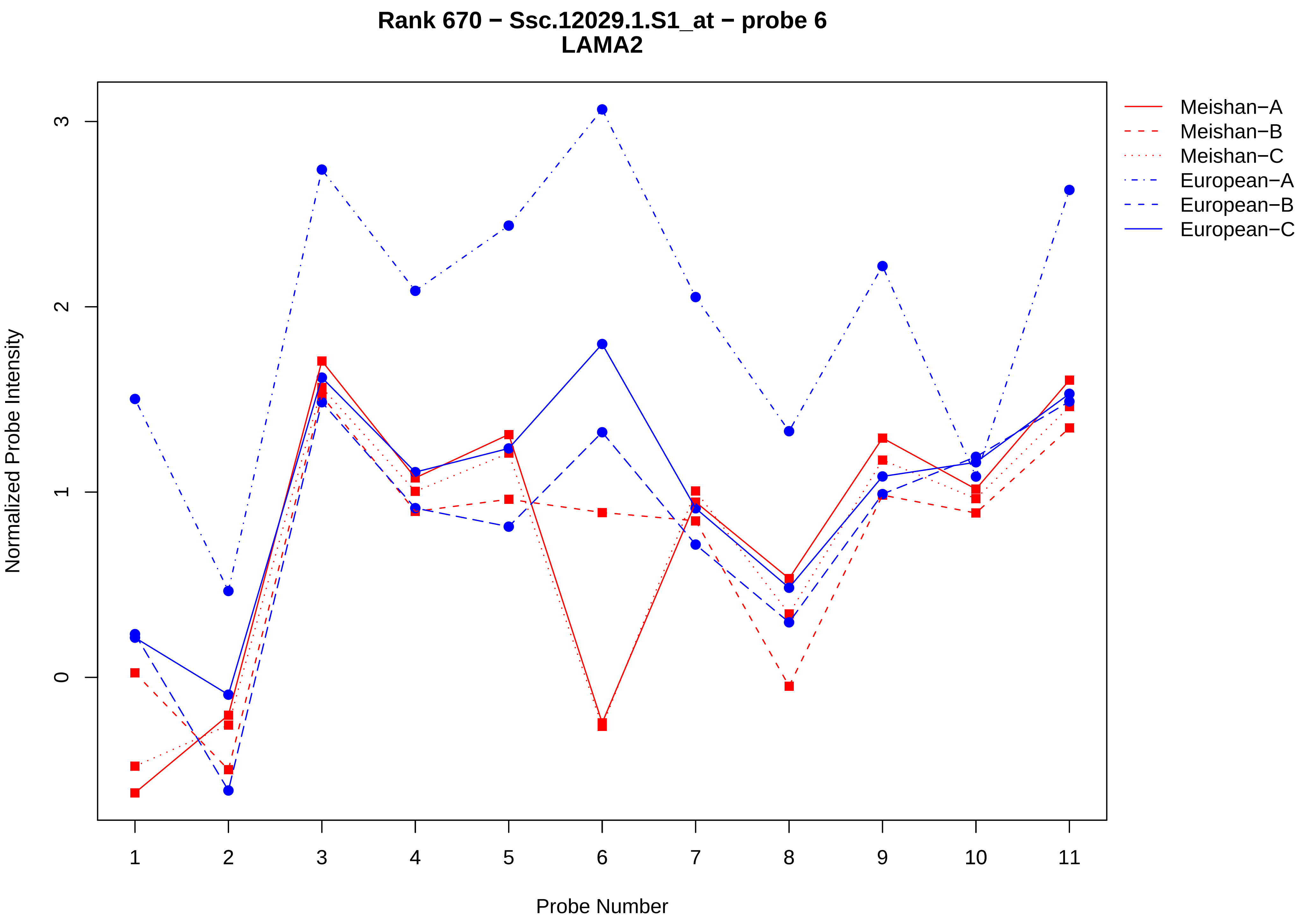

Rank 670 − Ssc.12029.1.S1_at − probe 6
LAMA2
Normalized Probe Intensity
Probe Number
0
1
2
3
1
2
3
4
5
6
7
8
9
10
11
Meishan−A
Meishan−B
Meishan−C
European−A
European−B
European−C

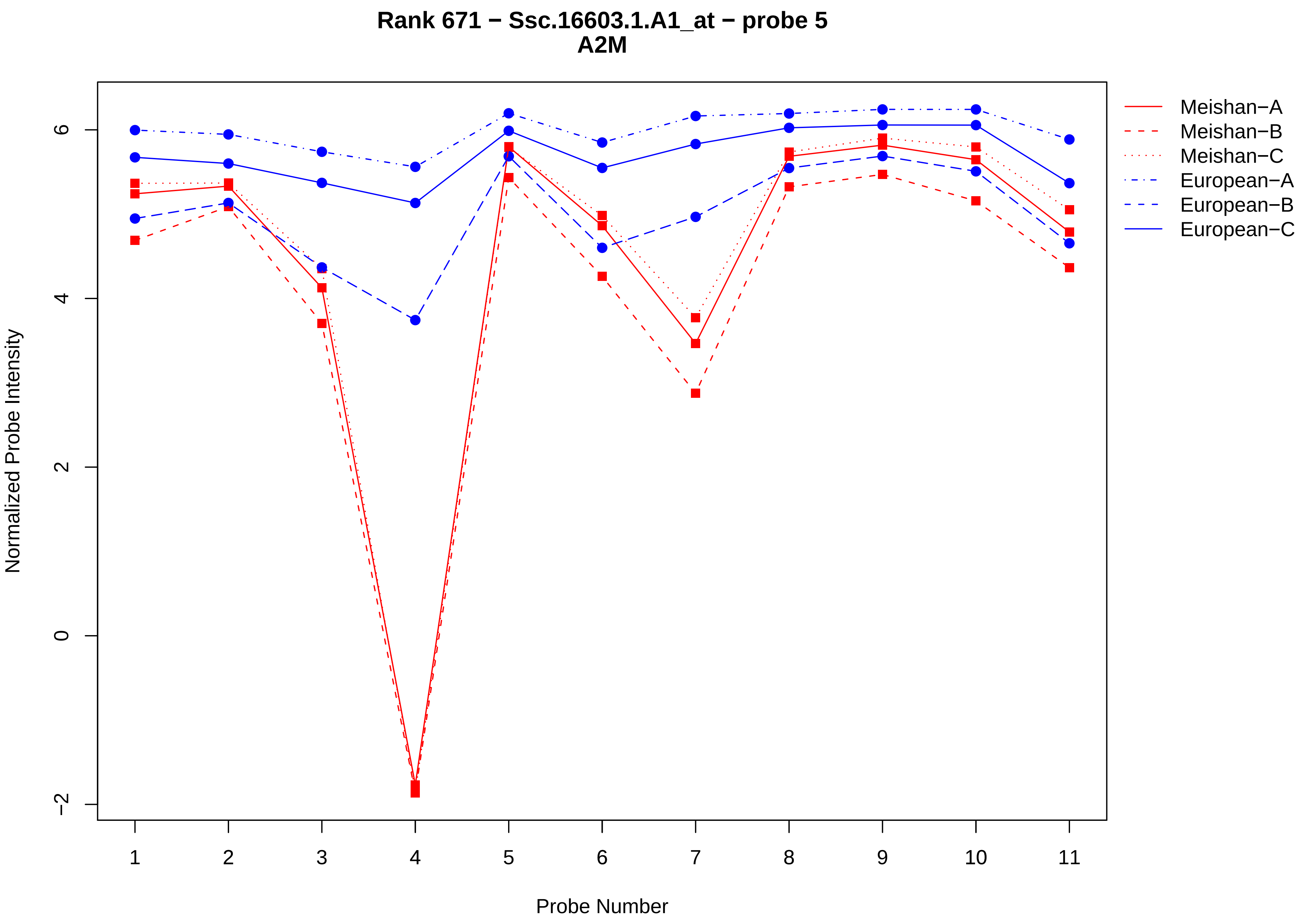
Rank 671 − Ssc.16603.1.A1_at − probe 5
A2M
Normalized Probe Intensity
Probe Number
−2
0
2
4
6
1
2
3
4
5
6
7
8
9
10
11
Meishan−A
Meishan−B
Meishan−C
European−A
European−B
European−C

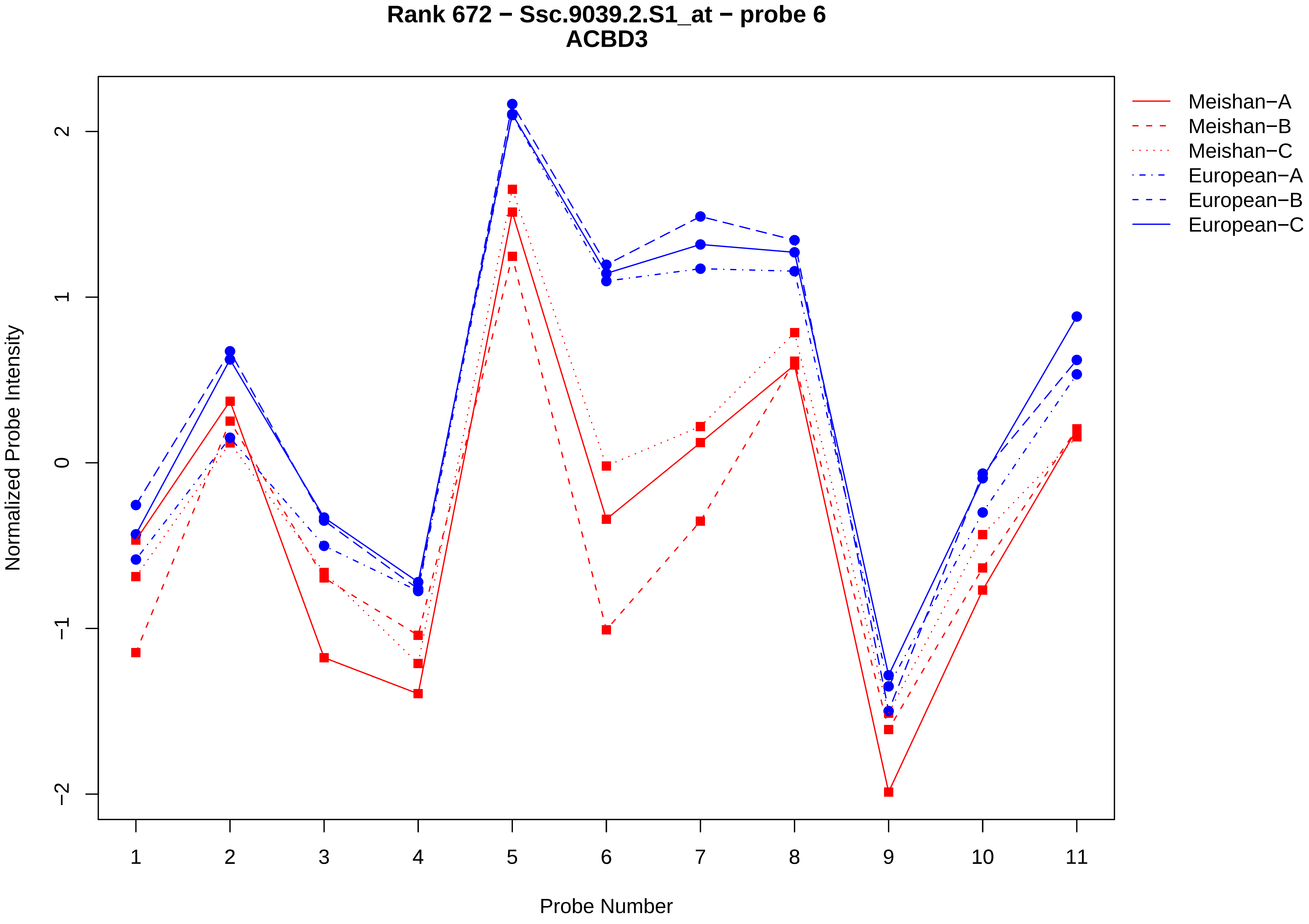
Rank 672 − Ssc.9039.2.S1_at − probe 6
ACBD3
Normalized Probe Intensity
Probe Number
−2
−1
0
1
2
1
2
3
4
5
6
7
8
9
10
11
Meishan−A
Meishan−B
Meishan−C
European−A
European−B
European−C

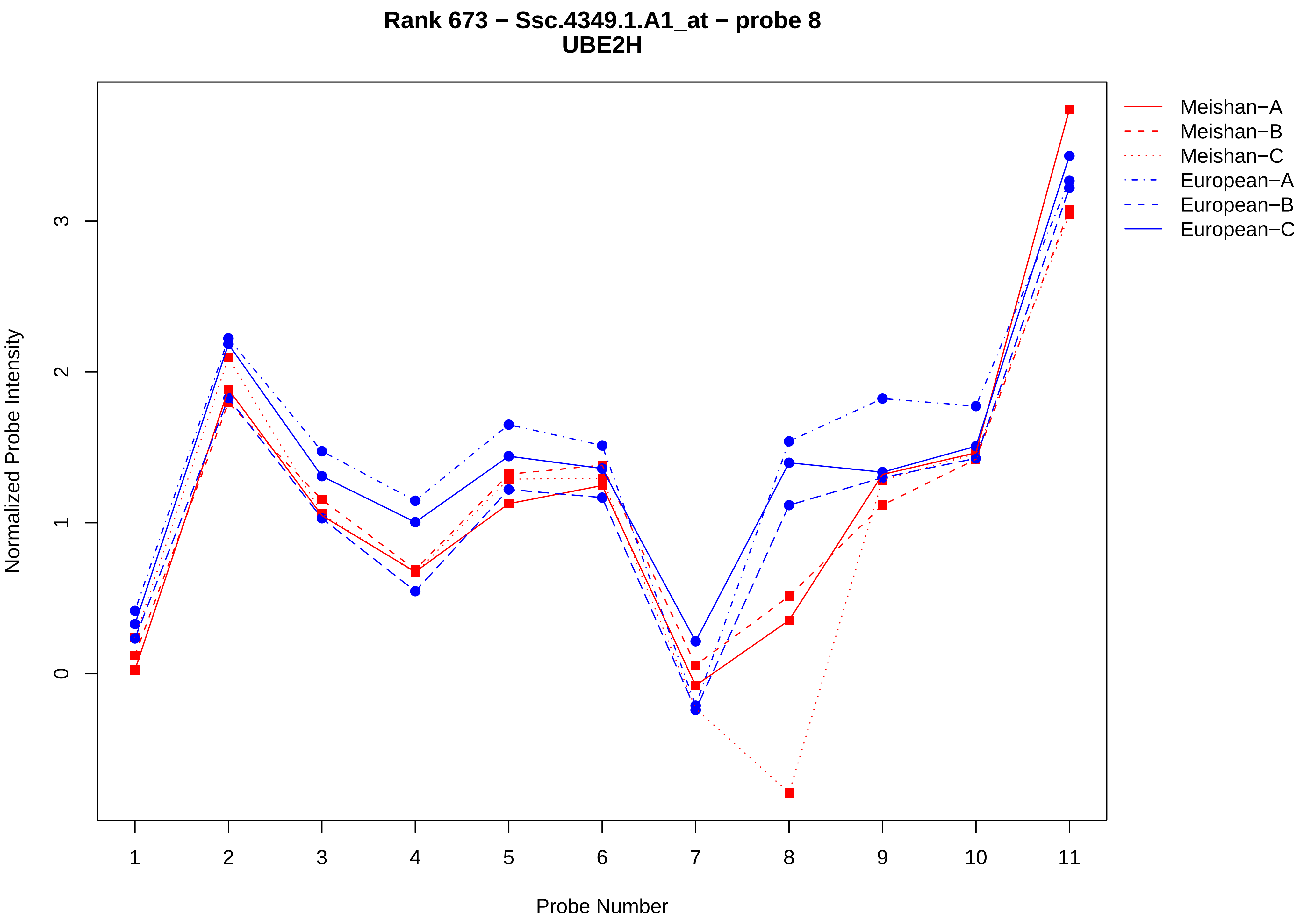

Rank 673 − Ssc.4349.1.A1_at − probe 8
UBE2H
Normalized Probe Intensity
0
1
2
3
1
2
3
4
5
6
7
8
9
10
11
Probe Number
Meishan−A
Meishan−B
Meishan−C
European−A
European−B
European−C

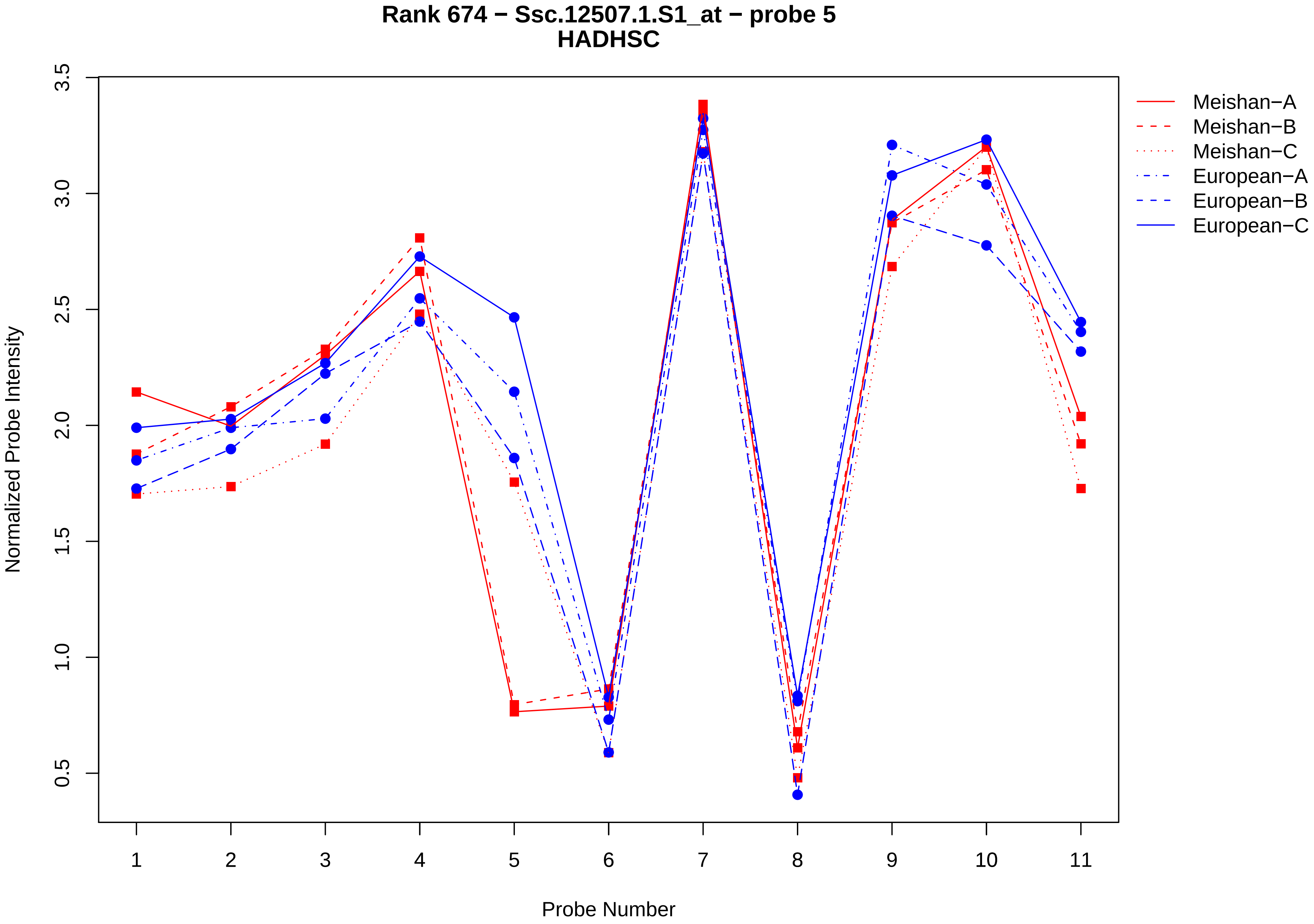

Rank 674 − Ssc.12507.1.S1_at − probe 5
HADHSC
Normalized Probe Intensity
0.5
1.0
1.5
2.0
2.5
3.0
3.5
Probe Number
1
2
3
4
5
6
7
8
9
10
11
Meishan−A
Meishan−B
Meishan−C
European−A
European−B
European−C

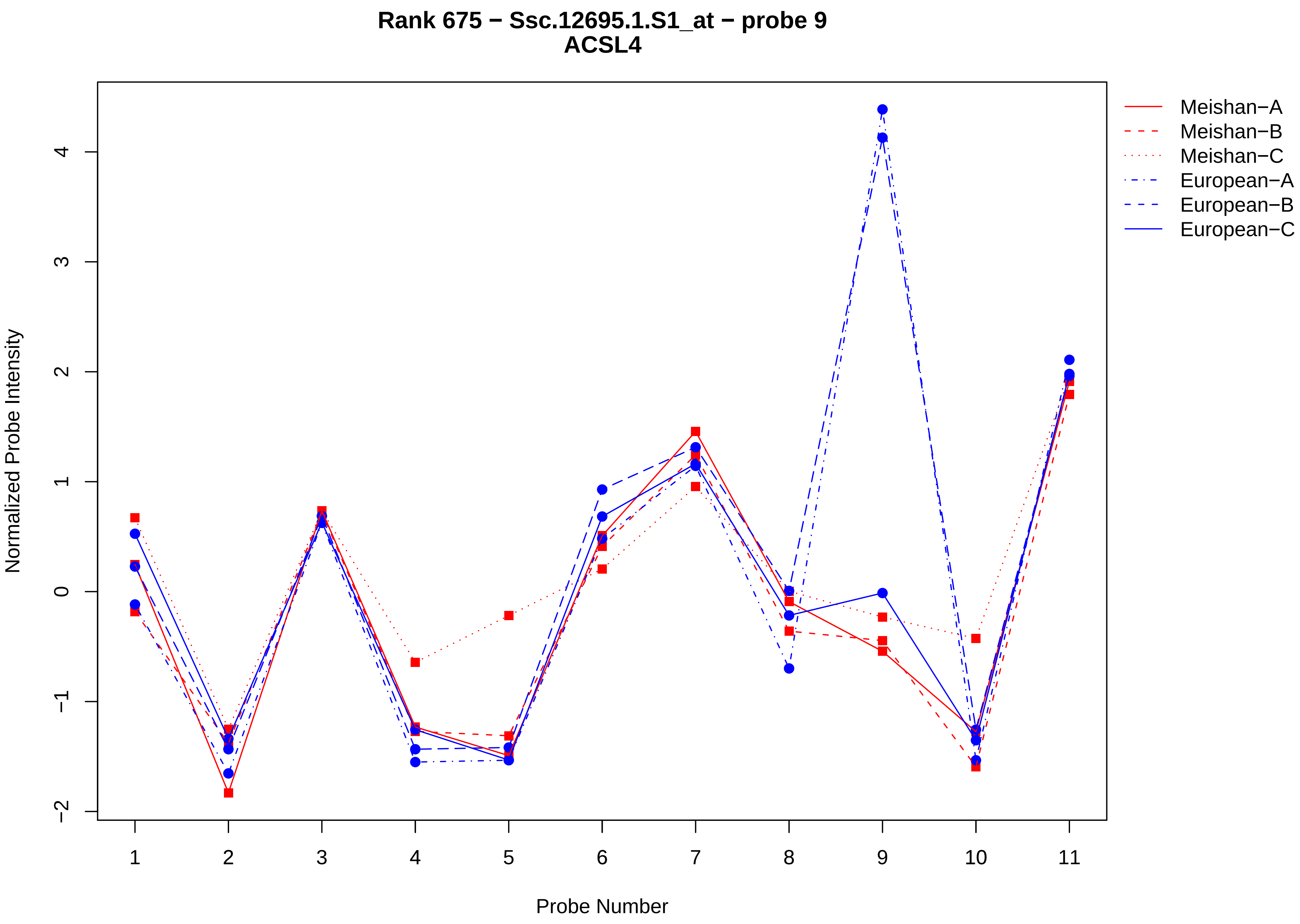

Rank 675 − Ssc.12695.1.S1_at − probe 9
ACSL4
Normalized Probe Intensity
Probe Number
Meishan−A
Meishan−B
Meishan−C
European−A
European−B
European−C
−2
−1
0
1
2
3
4
1
2
3
4
5
6
7
8
9
10
11

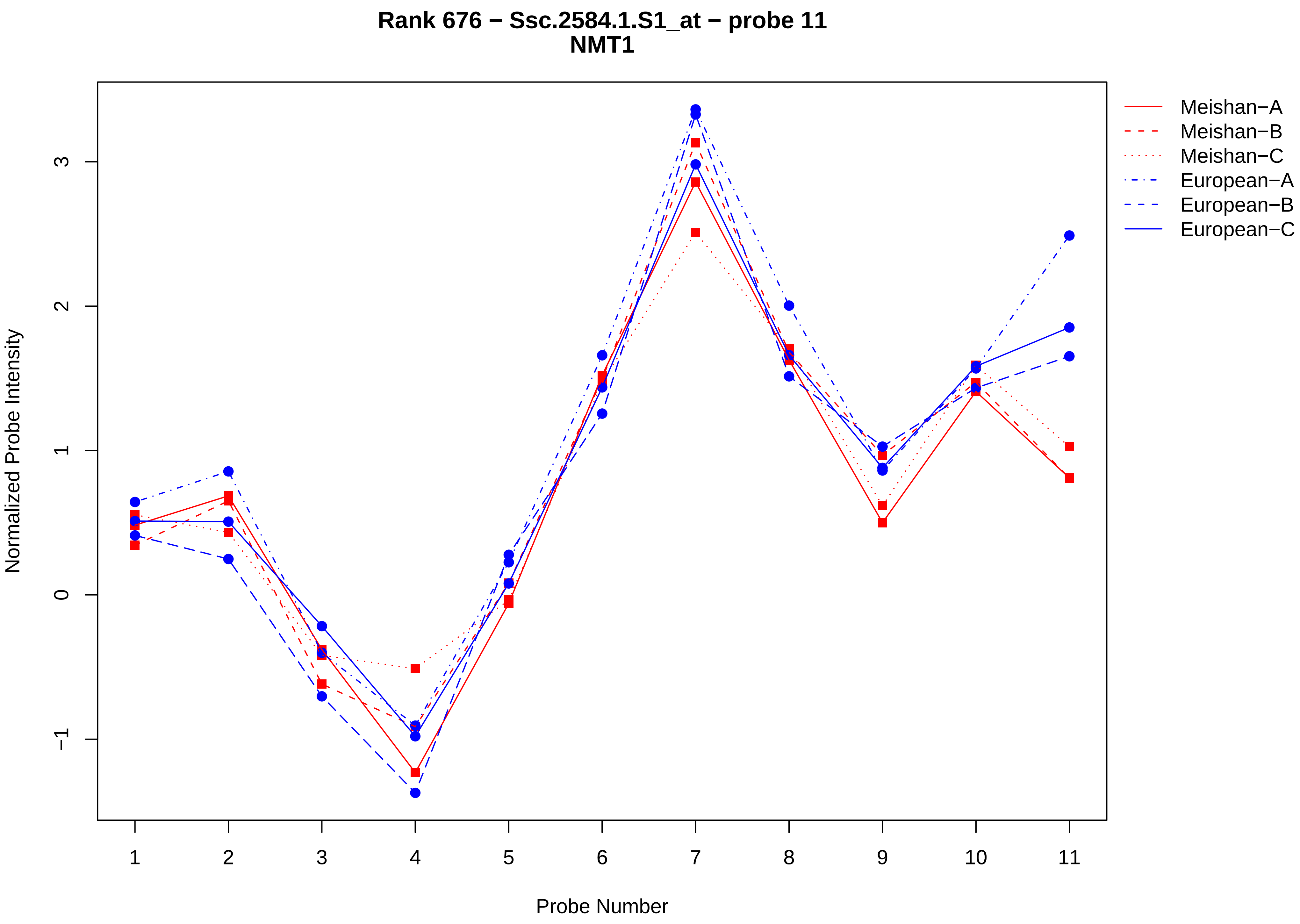

Rank 676 − Ssc.2584.1.S1_at − probe 11
NMT1
Normalized Probe Intensity
Probe Number
−1
0
1
2
3
1
2
3
4
5
6
7
8
9
10
11
Meishan−A
Meishan−B
Meishan−C
European−A
European−B
European−C

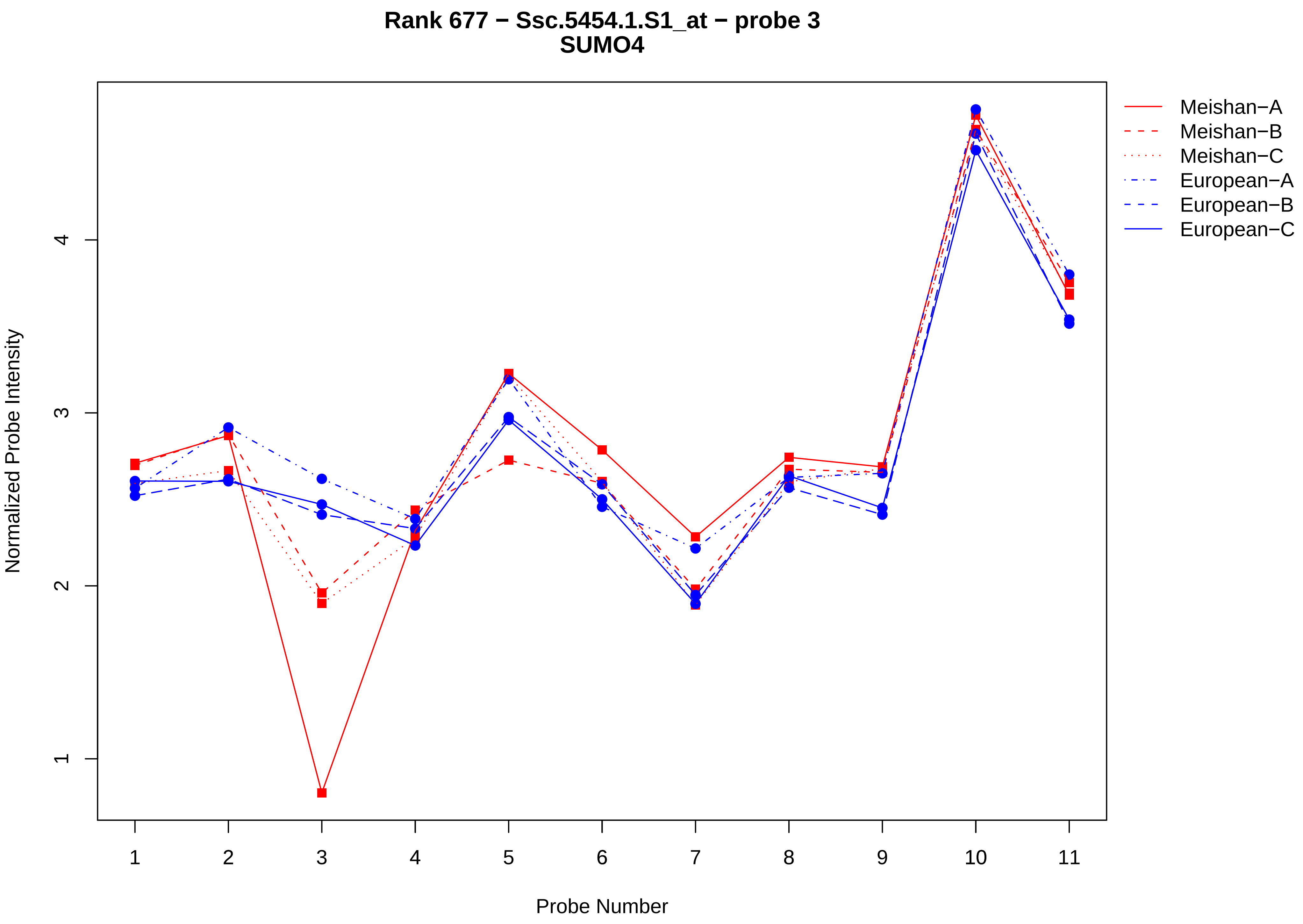

Rank 677 − Ssc.5454.1.S1_at − probe 3
SUMO4
Normalized Probe Intensity
Probe Number
1
2
3
4
1
2
3
4
5
6
7
8
9
10
11
Meishan−A
Meishan−B
Meishan−C
European−A
European−B
European−C

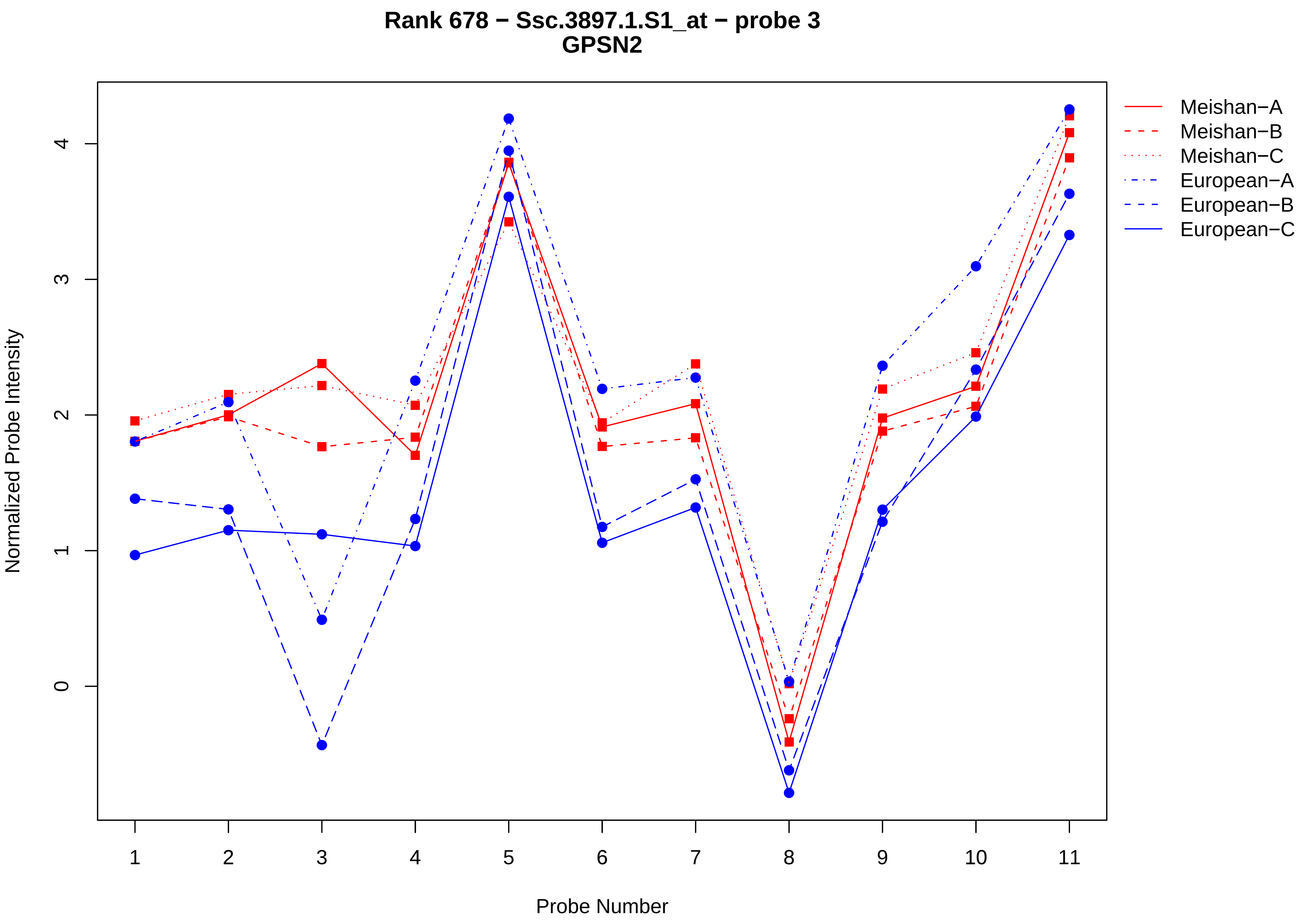

Rank 678 − Ssc.3897.1.S1_at − probe 3
GPSN2
Normalized Probe Intensity
Probe Number
0
1
2
3
4
1
2
3
4
5
6
7
8
9
10
11
Meishan−A
Meishan−B
Meishan−C
European−A
European−B
European−C

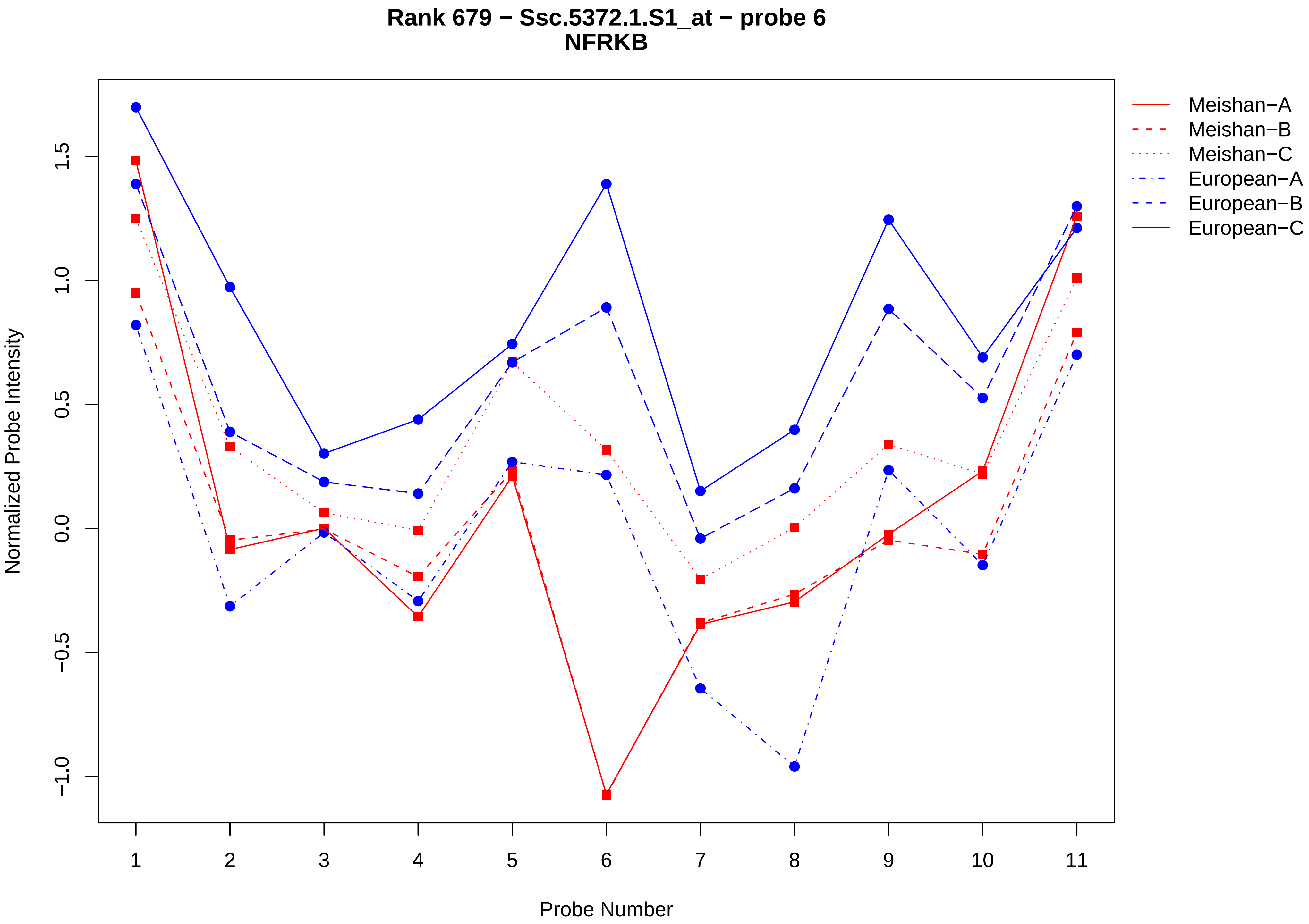

Rank 679 − Ssc.5372.1.S1_at − probe 6
NFRKB
Normalized Probe Intensity
Probe Number
Meishan−A
Meishan−B
Meishan−C
European−A
European−B
European−C
−1.0
−0.5
0.0
0.5
1.0
1.5
1
2
3
4
5
6
7
8
9
10
11

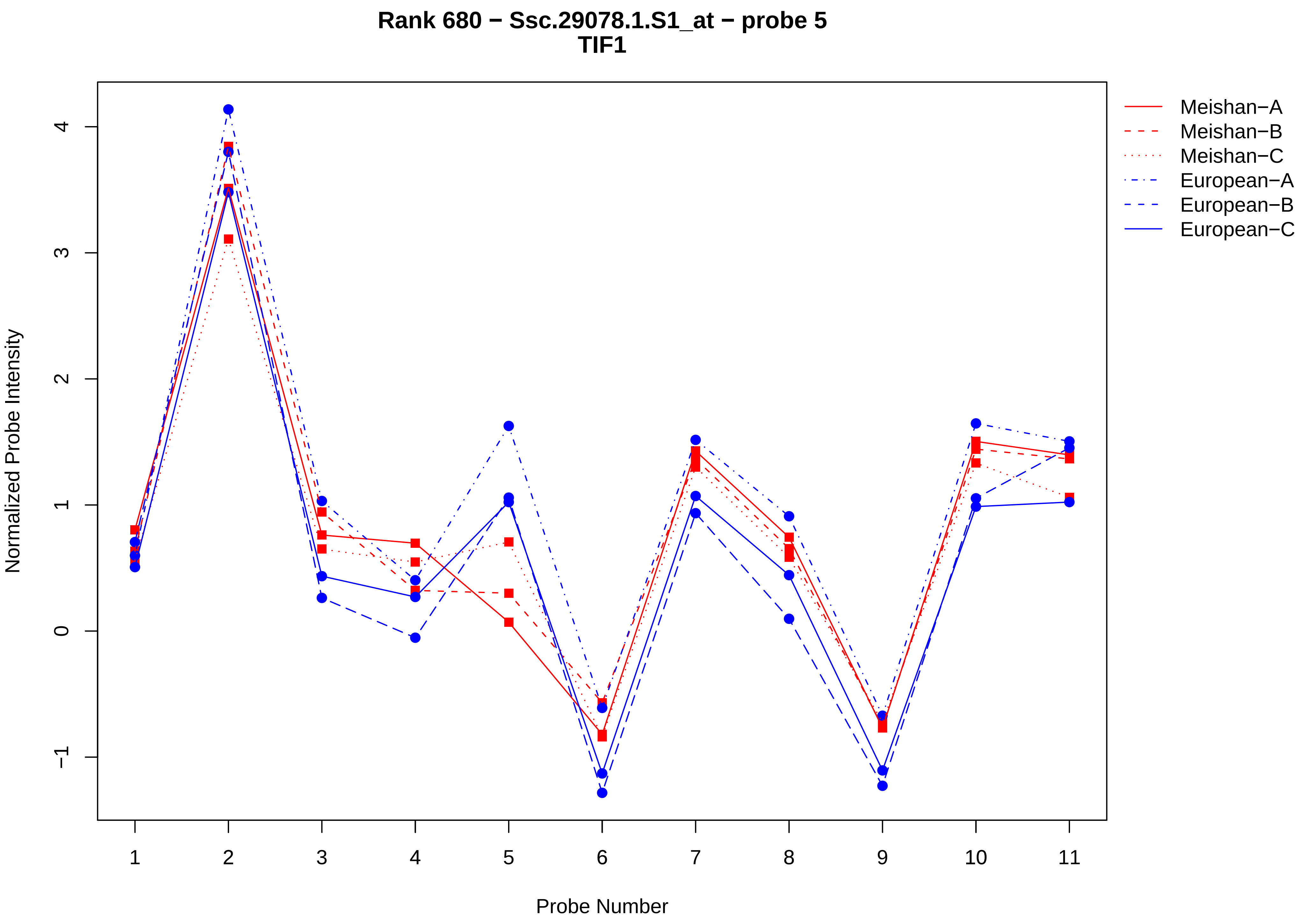

Rank 680 − Ssc.29078.1.S1_at − probe 5
TIF1
Normalized Probe Intensity
Probe Number
Meishan−A
Meishan−B
Meishan−C
European−A
European−B
European−C

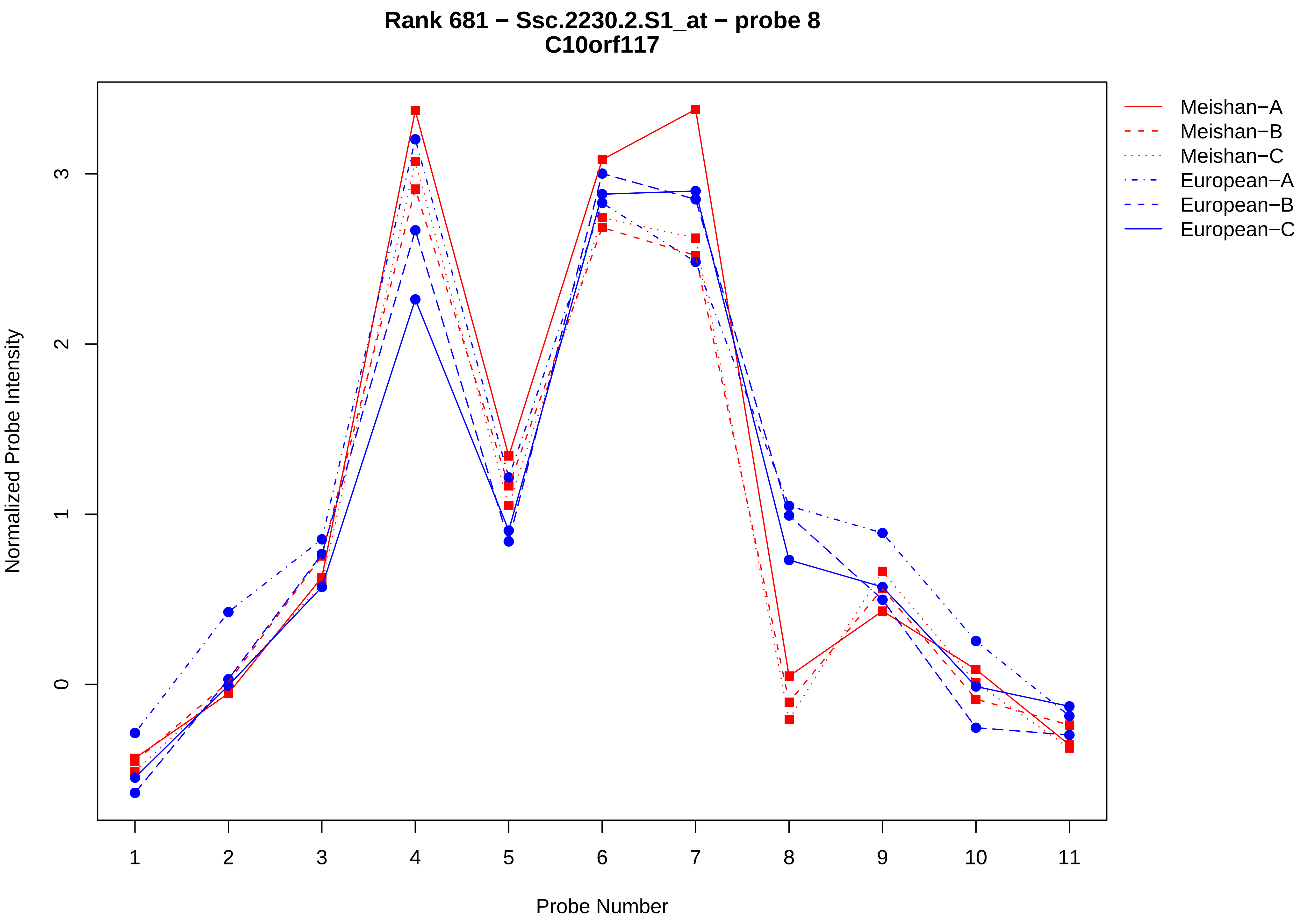
Rank 681 − Ssc.2230.2.S1_at − probe 8
C10orf117
Normalized Probe Intensity
0
1
2
3
1
2
3
4
5
6
7
8
9
10
11
Probe Number
Meishan−A
Meishan−B
Meishan−C
European−A
European−B
European−C

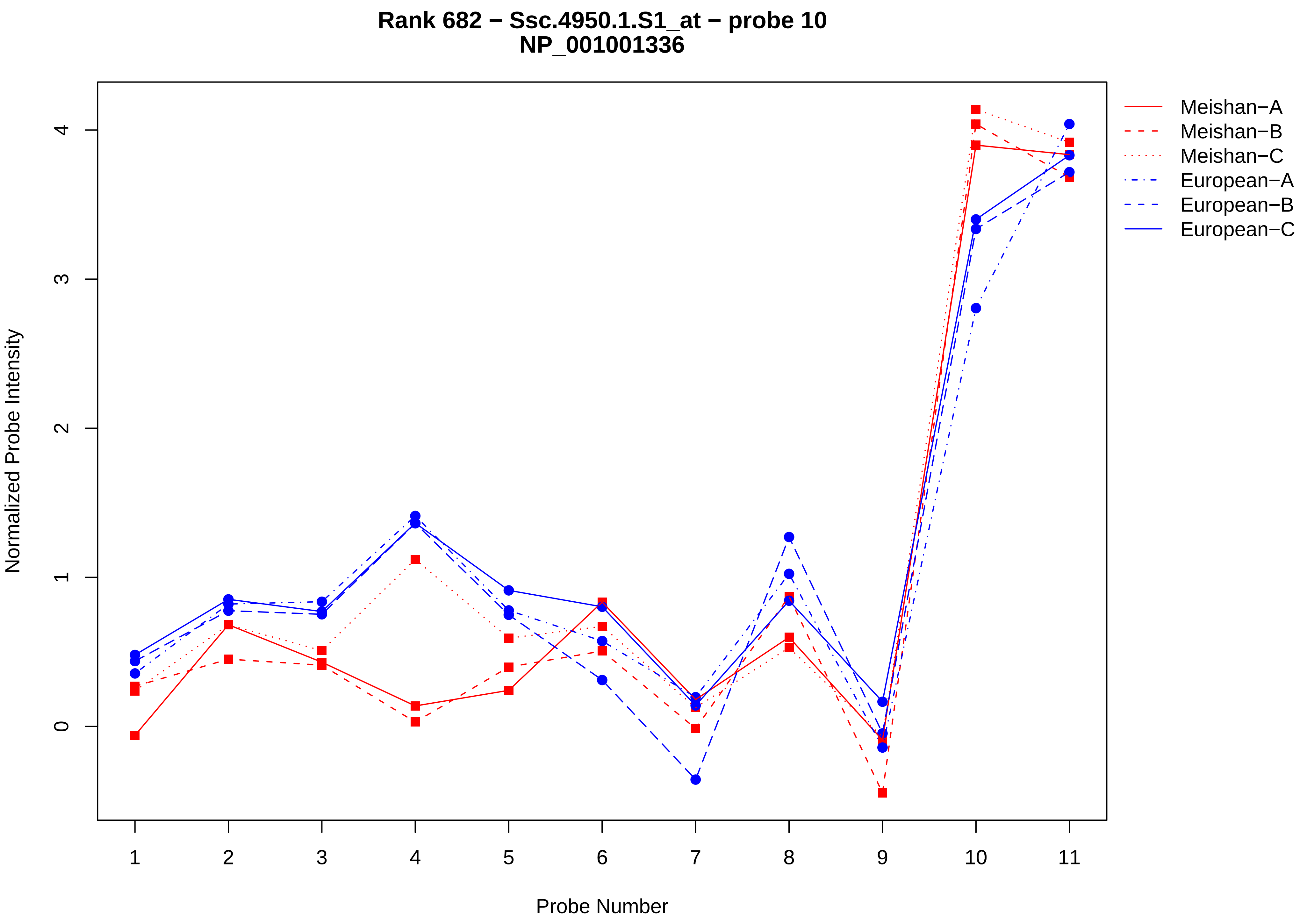

Rank 682 − Ssc.4950.1.S1_at − probe 10
NP_001001336
Normalized Probe Intensity
0
1
2
3
4
1
2
3
4
5
6
7
8
9
10
11
Probe Number
Meishan−A
Meishan−B
Meishan−C
European−A
European−B
European−C

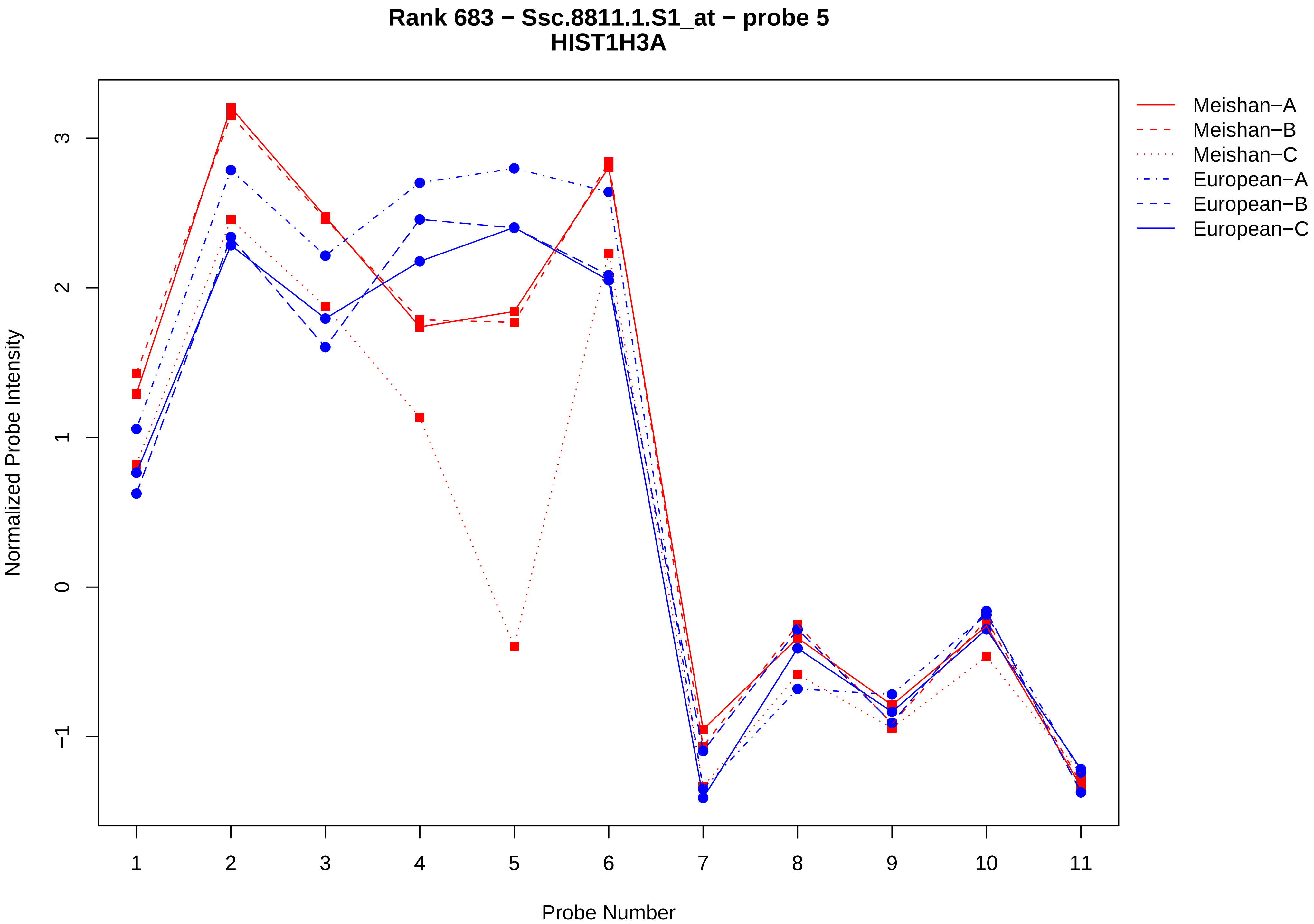

Rank 683 − Ssc.8811.1.S1_at − probe 5
HIST1H3A
Normalized Probe Intensity
Probe Number
Meishan−A
Meishan−B
Meishan−C
European−A
European−B
European−C
−1
0
1
2
3
1
2
3
4
5
6
7
8
9
10
11

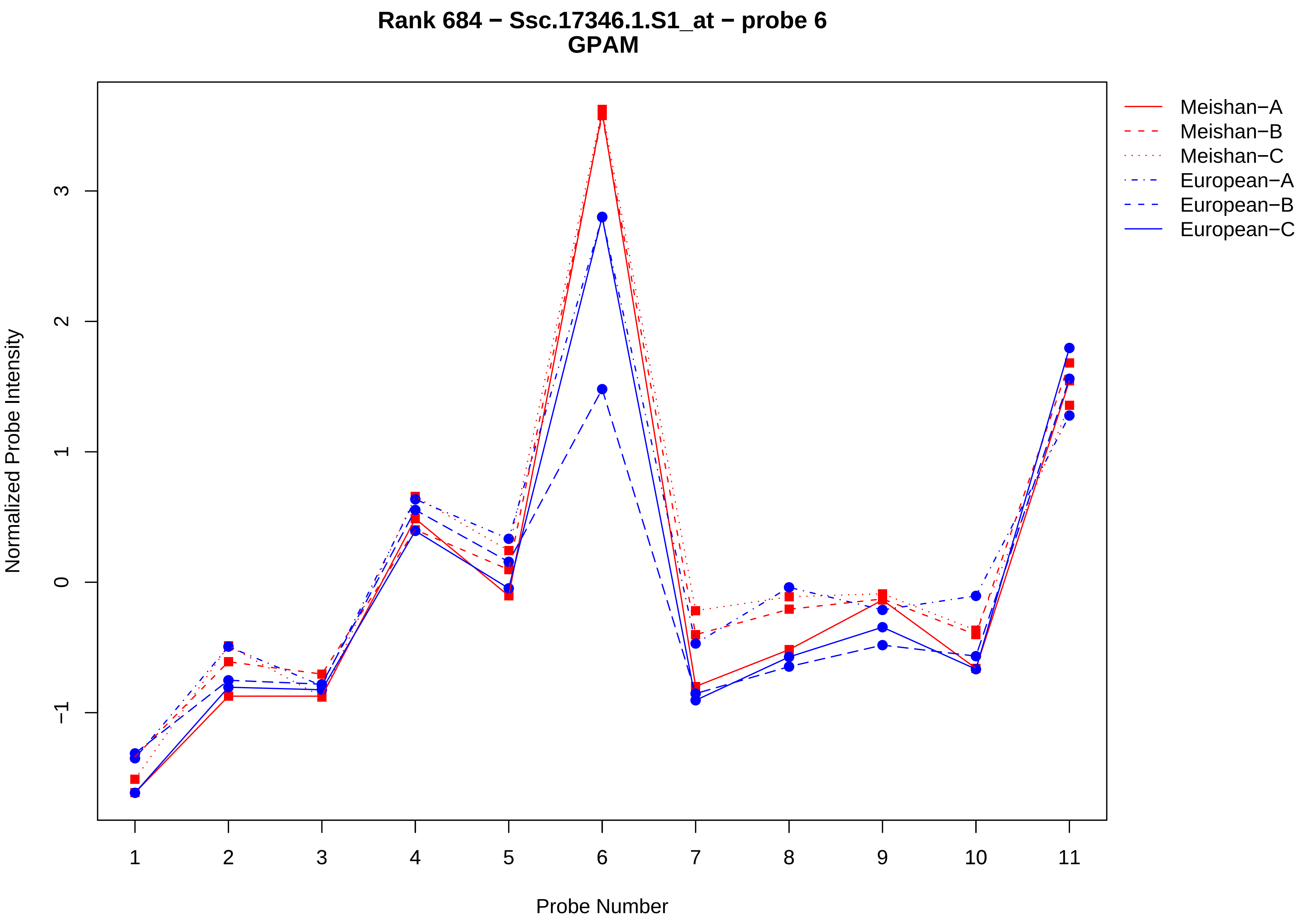

Rank 684 − Ssc.17346.1.S1_at − probe 6
GPAM
Normalized Probe Intensity
Probe Number
−1
0
1
2
3
1
2
3
4
5
6
7
8
9
10
11
Meishan−A
Meishan−B
Meishan−C
European−A
European−B
European−C

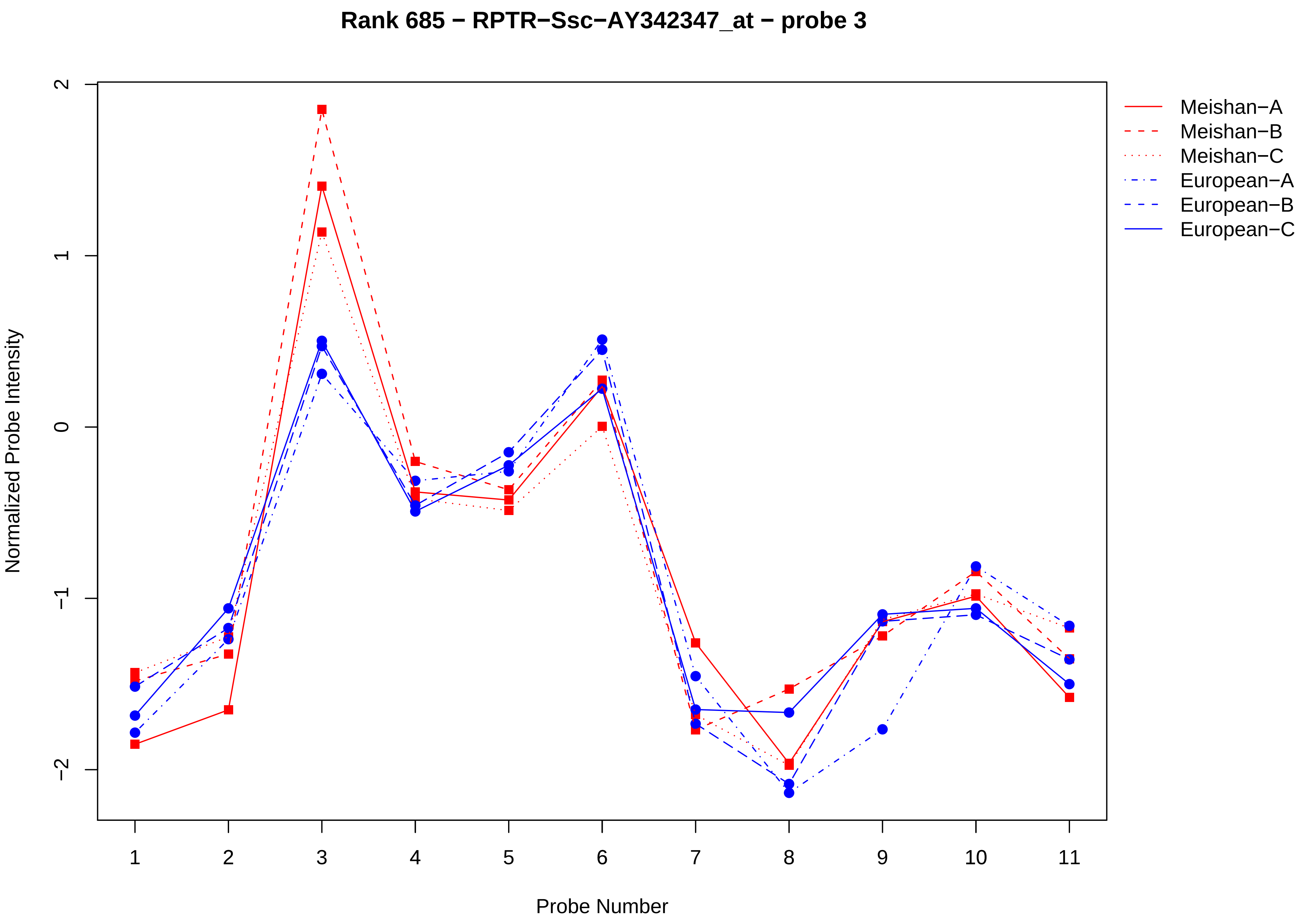

Rank 685 − RPTR−Ssc−AY342347_at − probe 3
Normalized Probe Intensity
Probe Number
2
1
0
−1
−2
1
2
3
4
5
6
7
8
9
10
11
Meishan−A
Meishan−B
Meishan−C
European−A
European−B
European−C

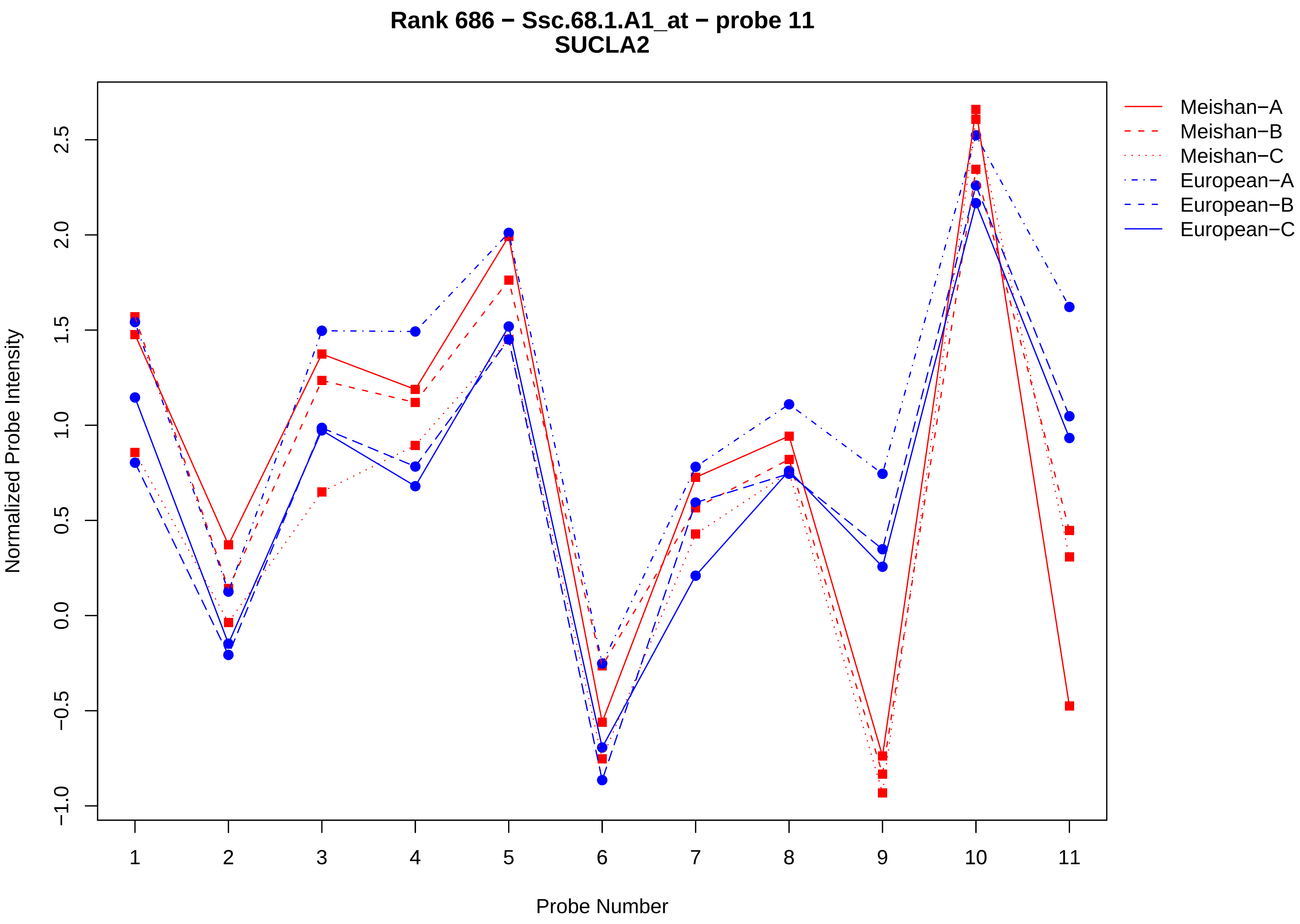

Rank 686 − Ssc.68.1.A1_at − probe 11
SUCLA2
Normalized Probe Intensity
Probe Number
−1.0
−0.5
0.0
0.5
1.0
1.5
2.0
2.5
1
2
3
4
5
6
7
8
9
10
11
Meishan−A
Meishan−B
Meishan−C
European−A
European−B
European−C

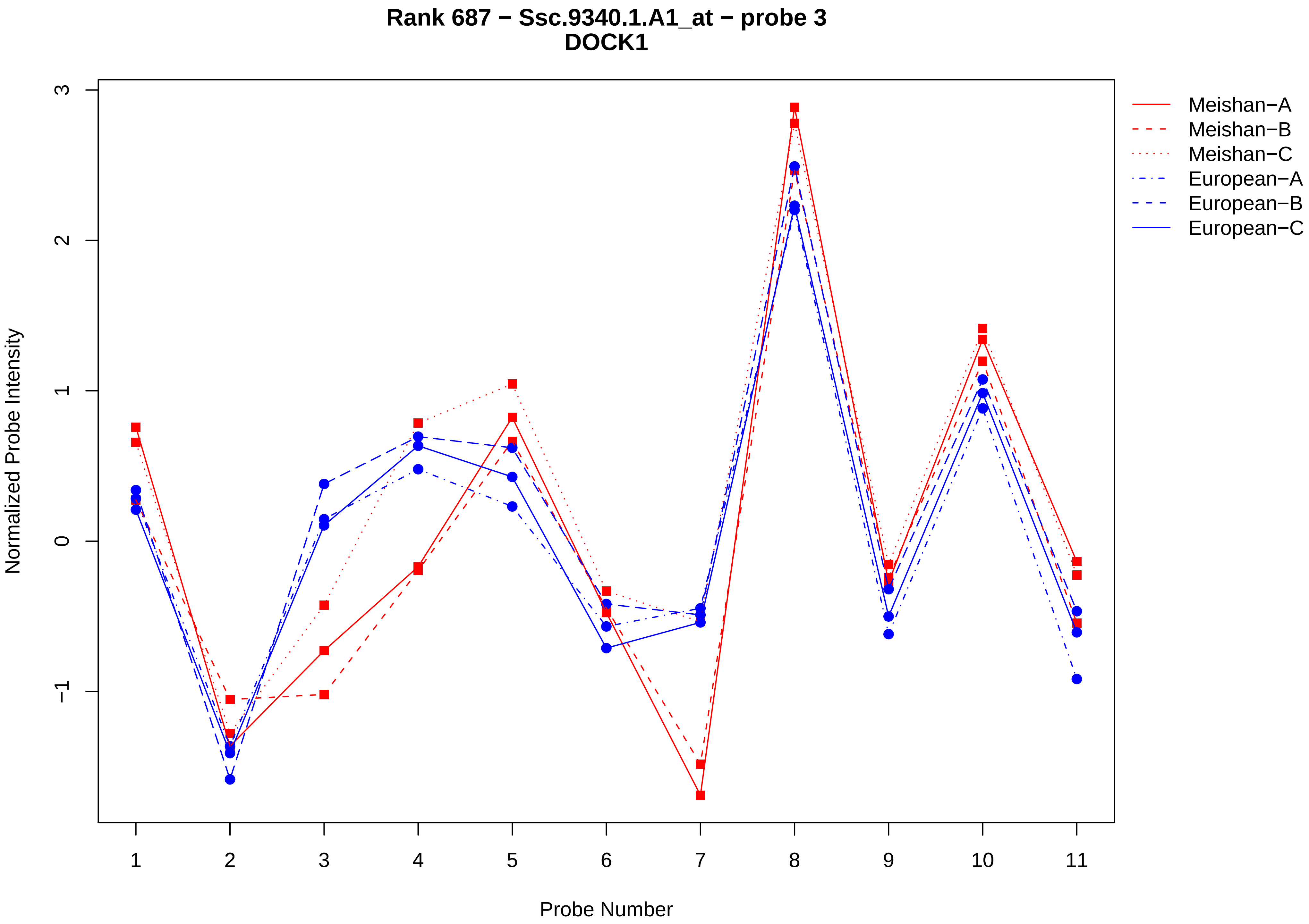

Rank 687 − Ssc.9340.1.A1_at − probe 3
DOCK1
Normalized Probe Intensity
Probe Number
Meishan−A
Meishan−B
Meishan−C
European−A
European−B
European−C
-1
0
1
2
3
1
2
3
4
5
6
7
8
9
10
11

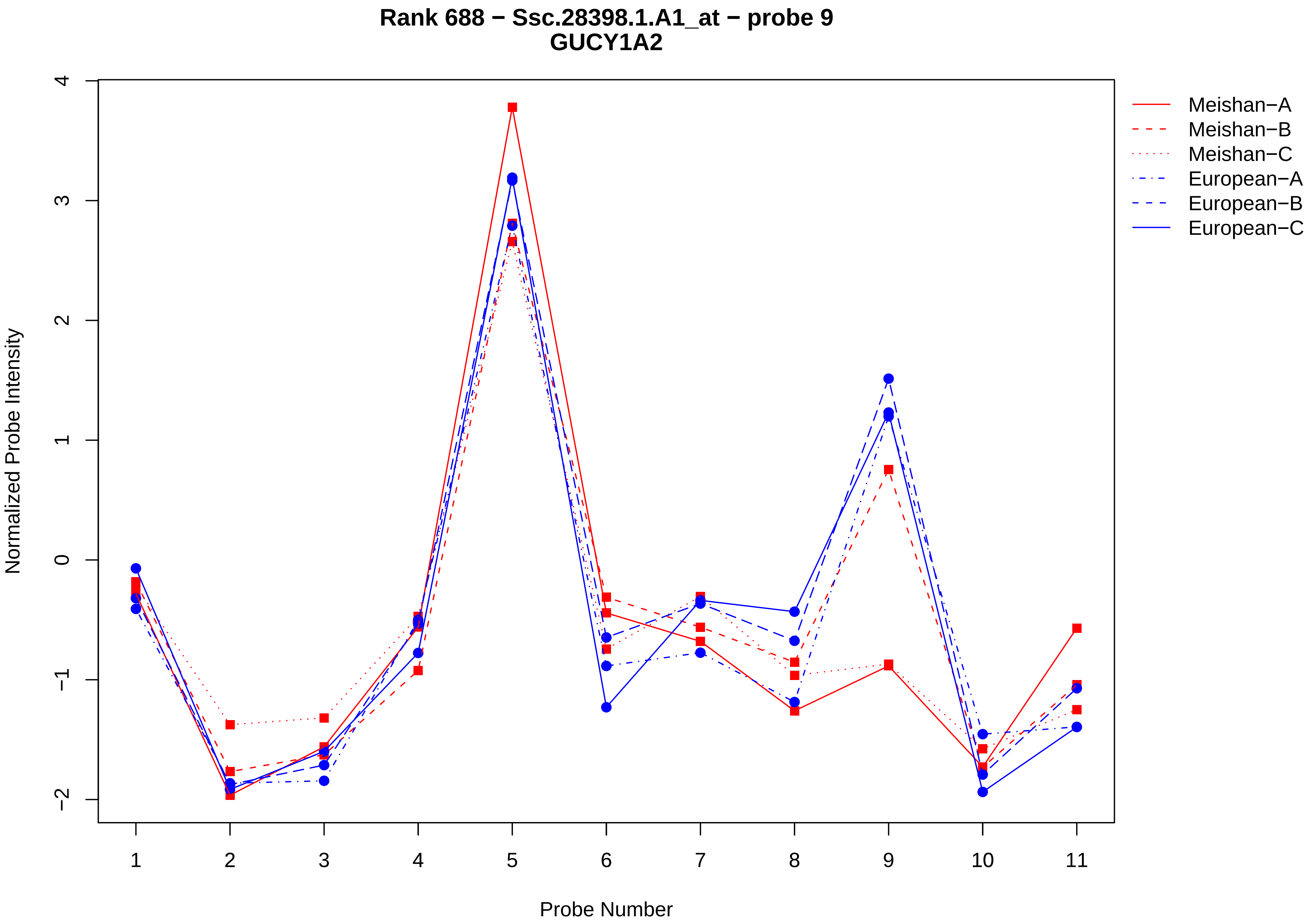

Rank 688 − Ssc.28398.1.A1_at − probe 9
GUCY1A2
Normalized Probe Intensity
Probe Number
−2
−1
0
1
2
3
4
1
2
3
4
5
6
7
8
9
10
11
Meishan−A
Meishan−B
Meishan−C
European−A
European−B
European−C

Rank 689 − Ssc.5322.1.S1_at − probe 10
Q5SR20

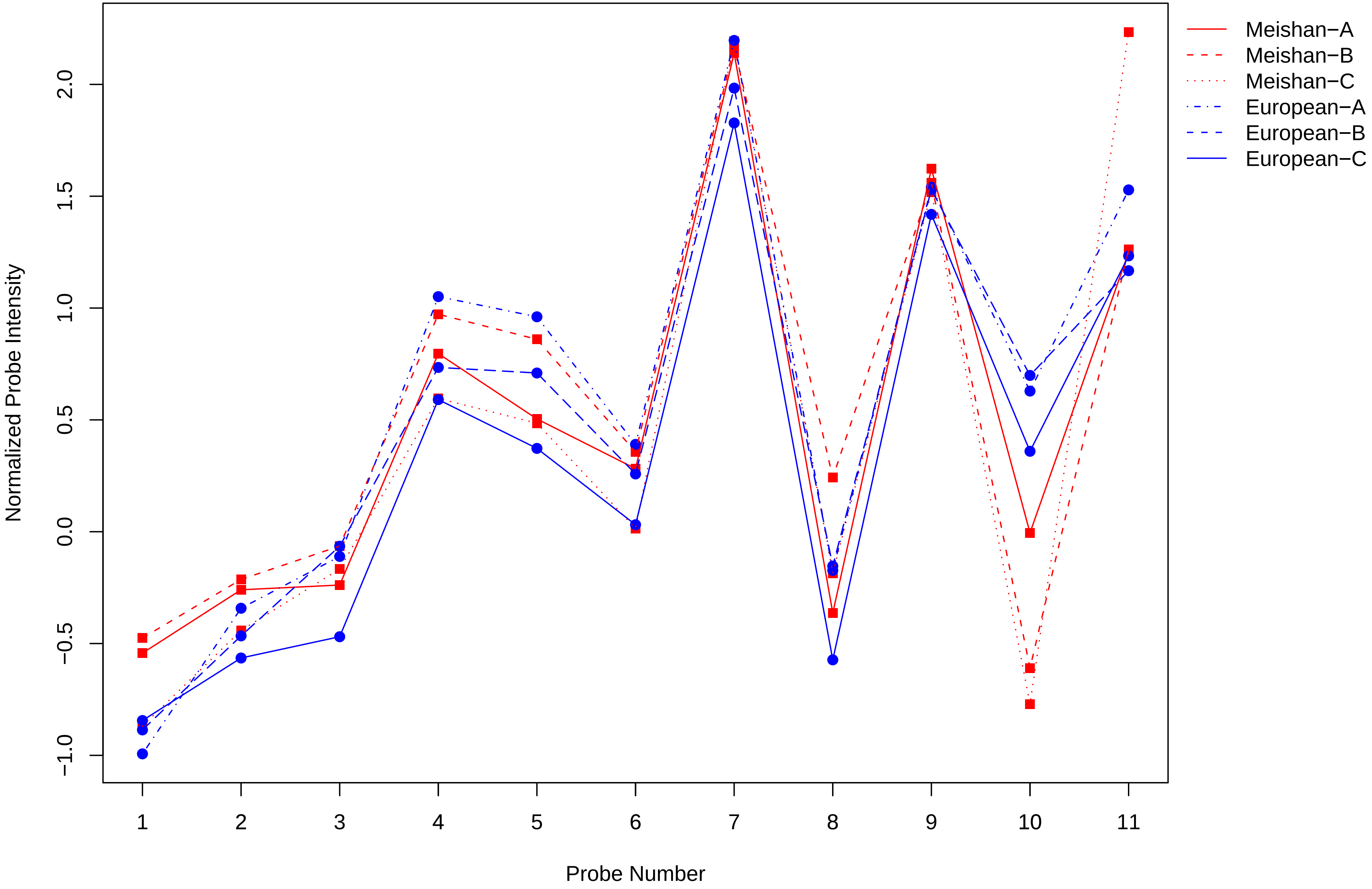

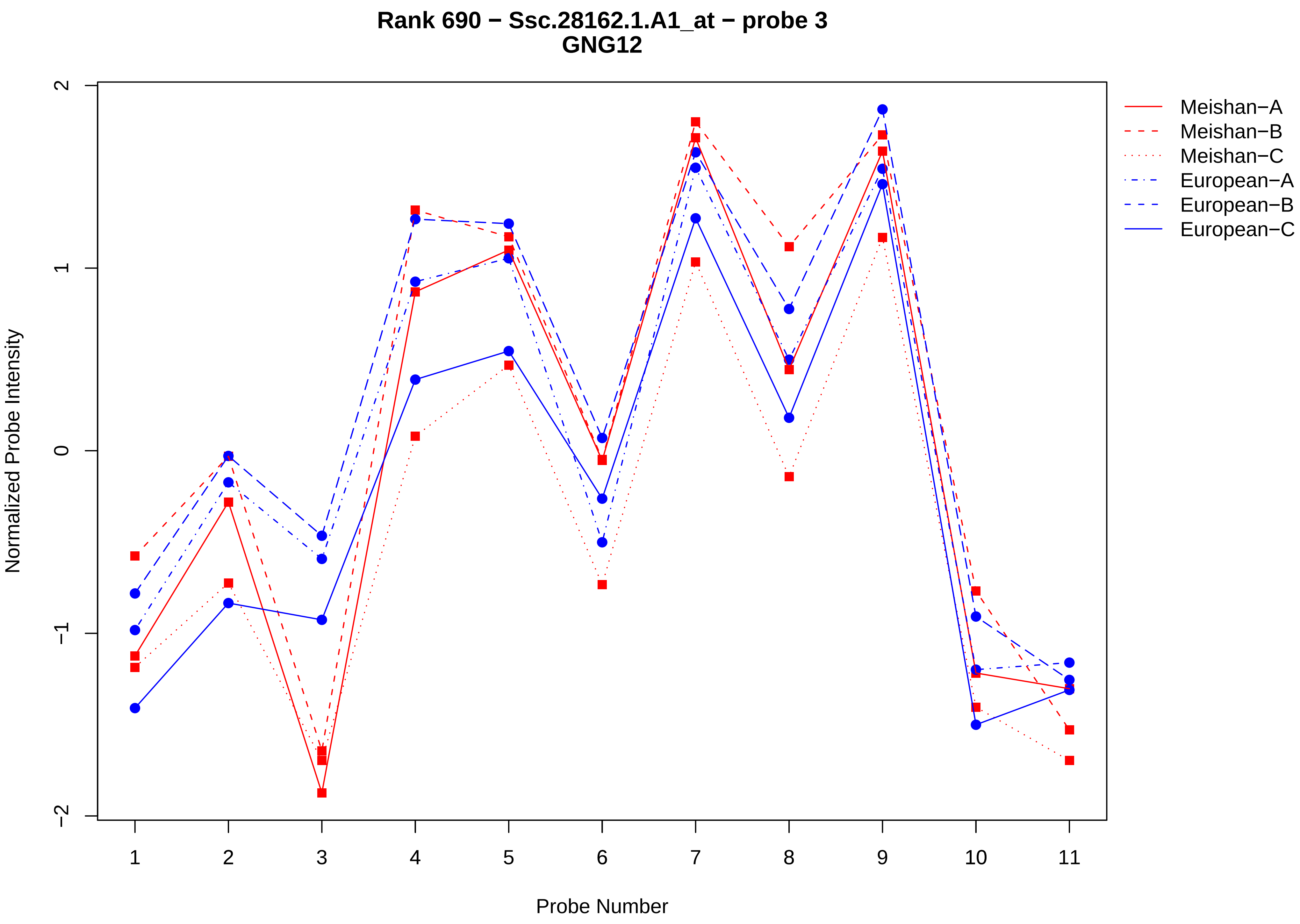

Rank 690 − Ssc.28162.1.A1_at − probe 3
GNG12
Normalized Probe Intensity
Probe Number
2
1
0
−1
−2
1
2
3
4
5
6
7
8
9
10
11
Meishan−A
Meishan−B
Meishan−C
European−A
European−B
European−C

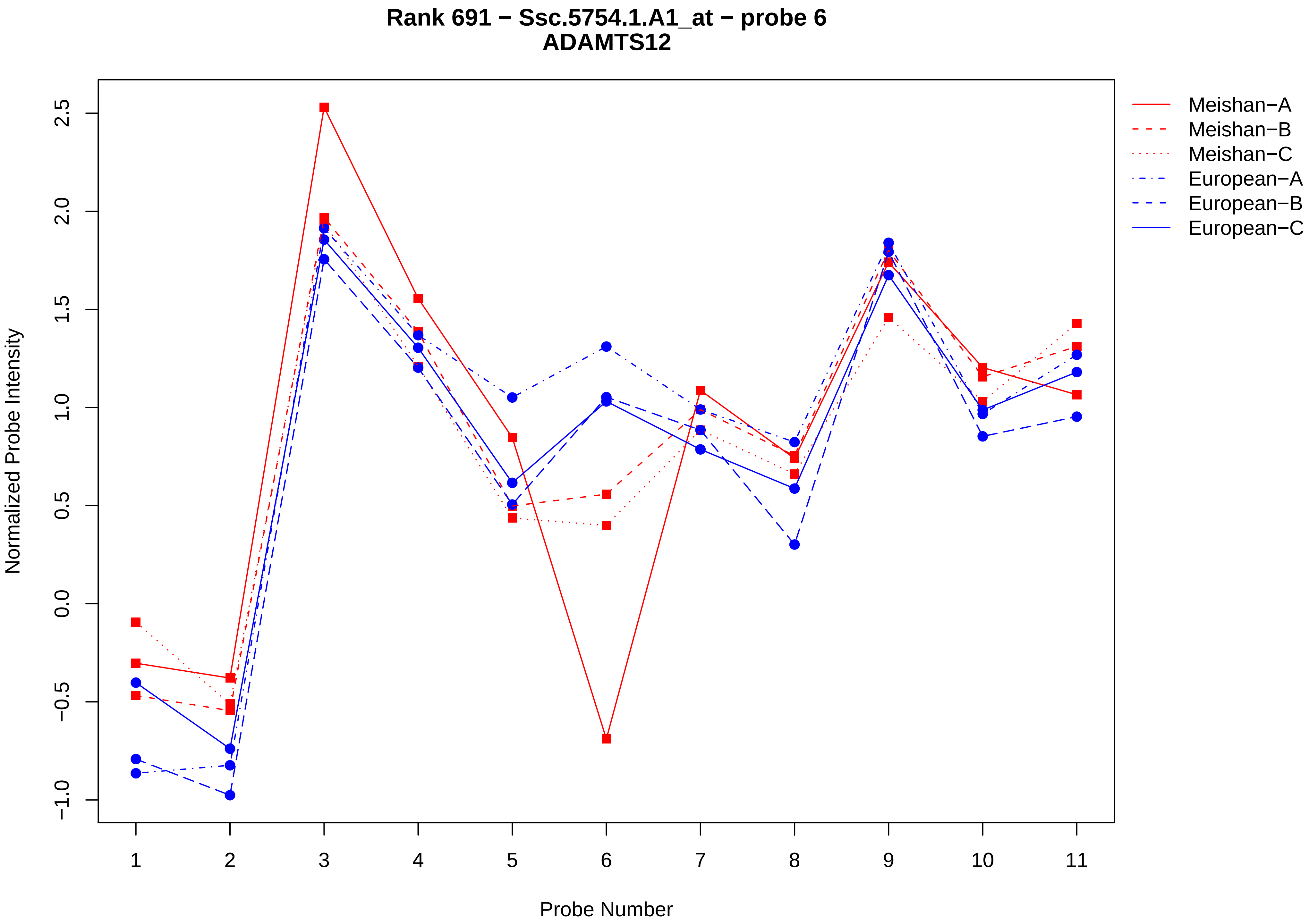
Rank 691 − Ssc.5754.1.A1_at − probe 6
ADAMTS12
Normalized Probe Intensity
Probe Number
Meishan−A
Meishan−B
Meishan−C
European−A
European−B
European−C
−1.0
−0.5
0.0
0.5
1.0
1.5
2.0
2.5
1
2
3
4
5
6
7
8
9
10
11

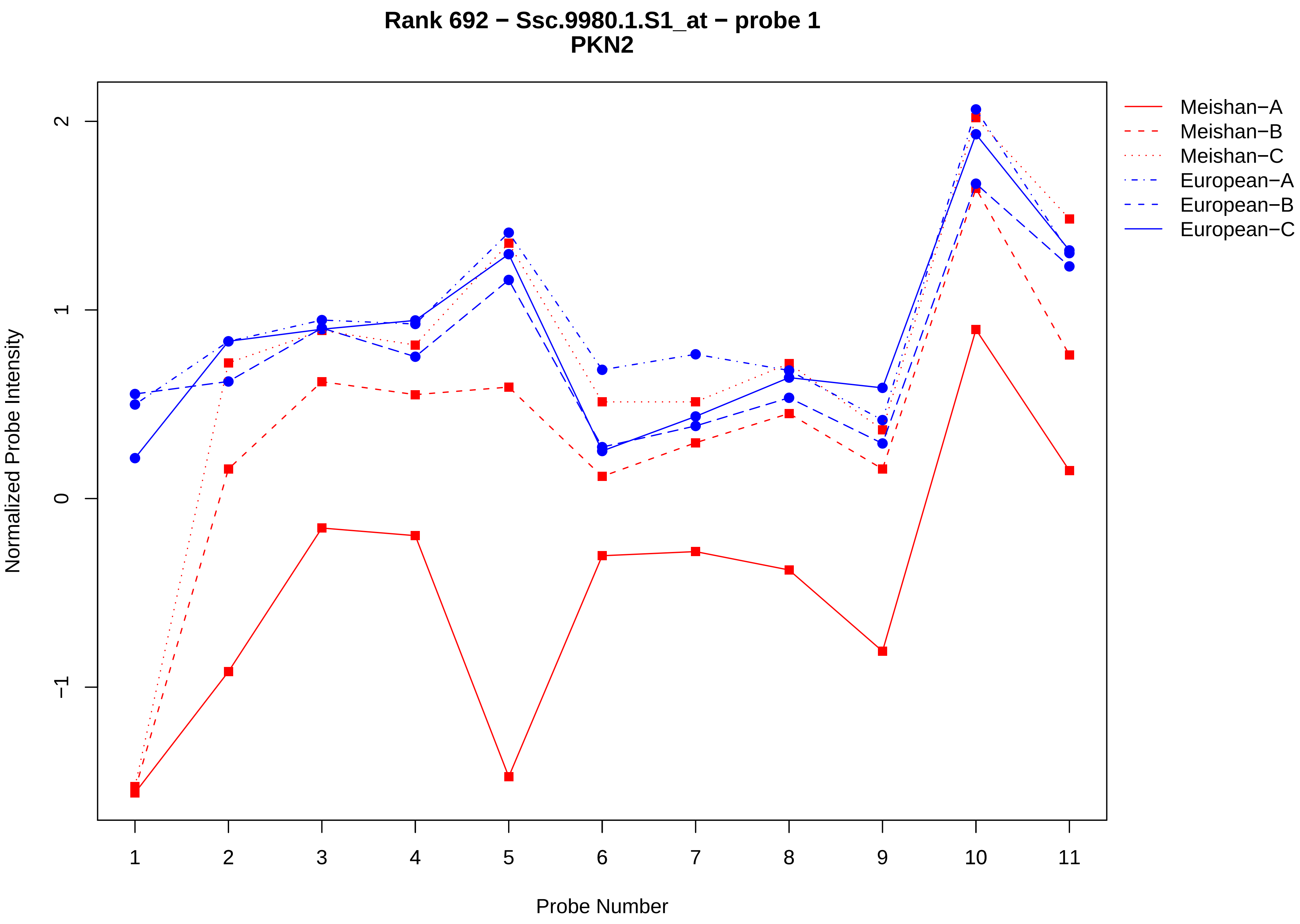
Rank 692 − Ssc.9980.1.S1_at − probe 1
PKN2
Normalized Probe Intensity
Probe Number
−1
0
1
2
1
2
3
4
5
6
7
8
9
10
11
Meishan−A
Meishan−B
Meishan−C
European−A
European−B
European−C

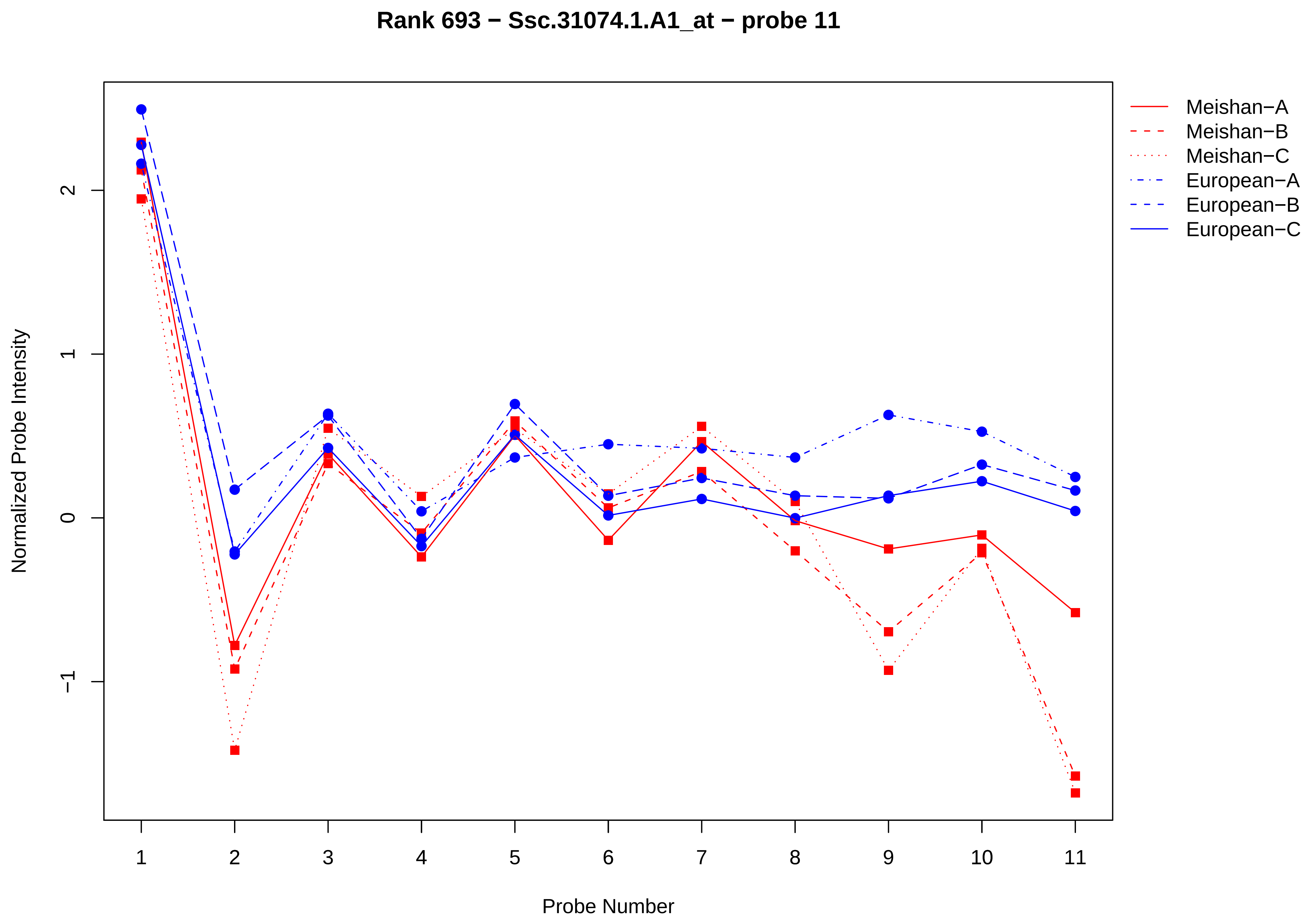

Rank 693 − Ssc.31074.1.A1_at − probe 11
Normalized Probe Intensity
Probe Number
−1
0
1
2
1
2
3
4
5
6
7
8
9
10
11
Meishan−A
Meishan−B
Meishan−C
European−A
European−B
European−C

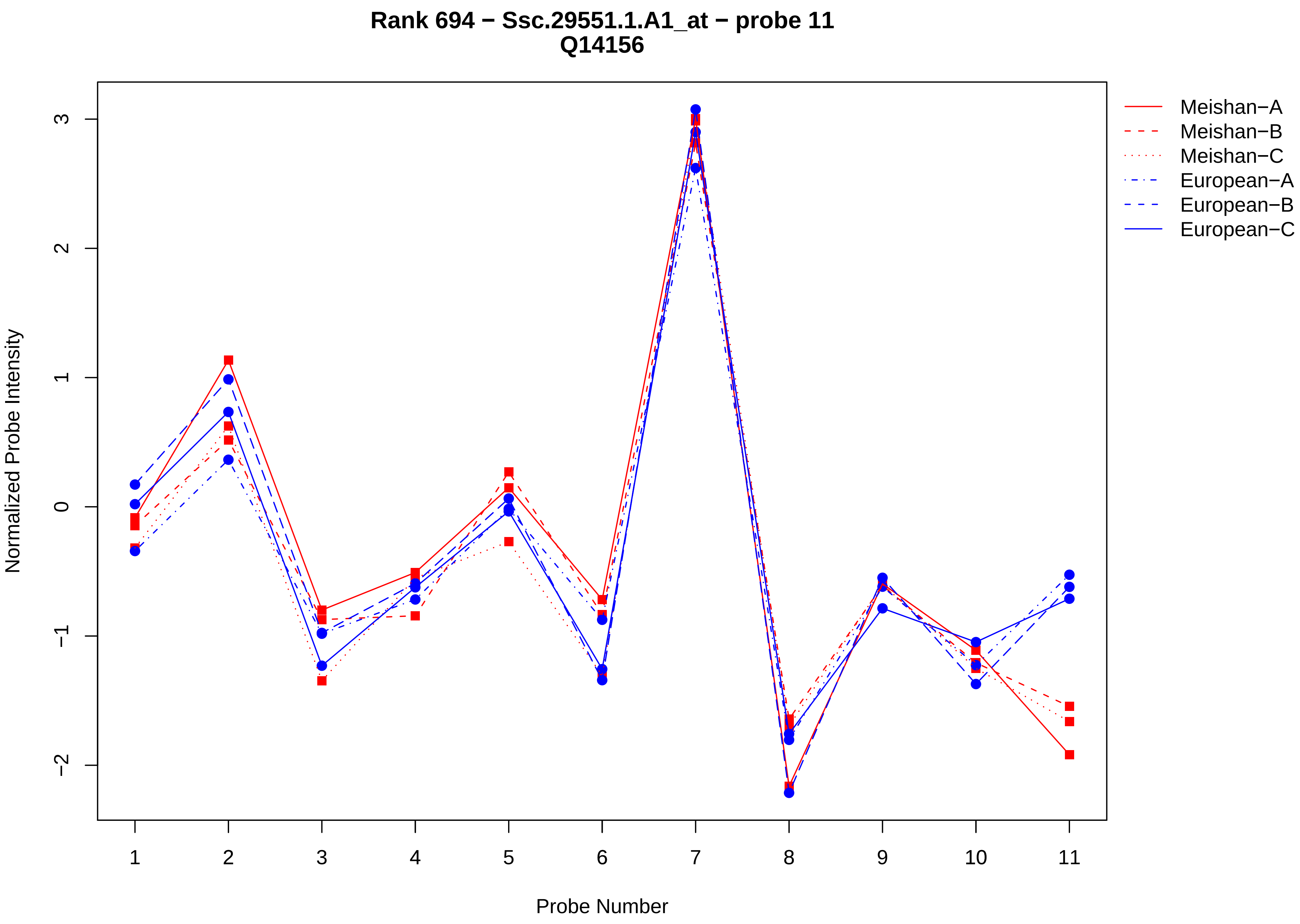

Rank 694 − Ssc.29551.1.A1_at − probe 11
Q14156
Normalized Probe Intensity
Probe Number
Meishan−A
Meishan−B
Meishan−C
European−A
European−B
European−C

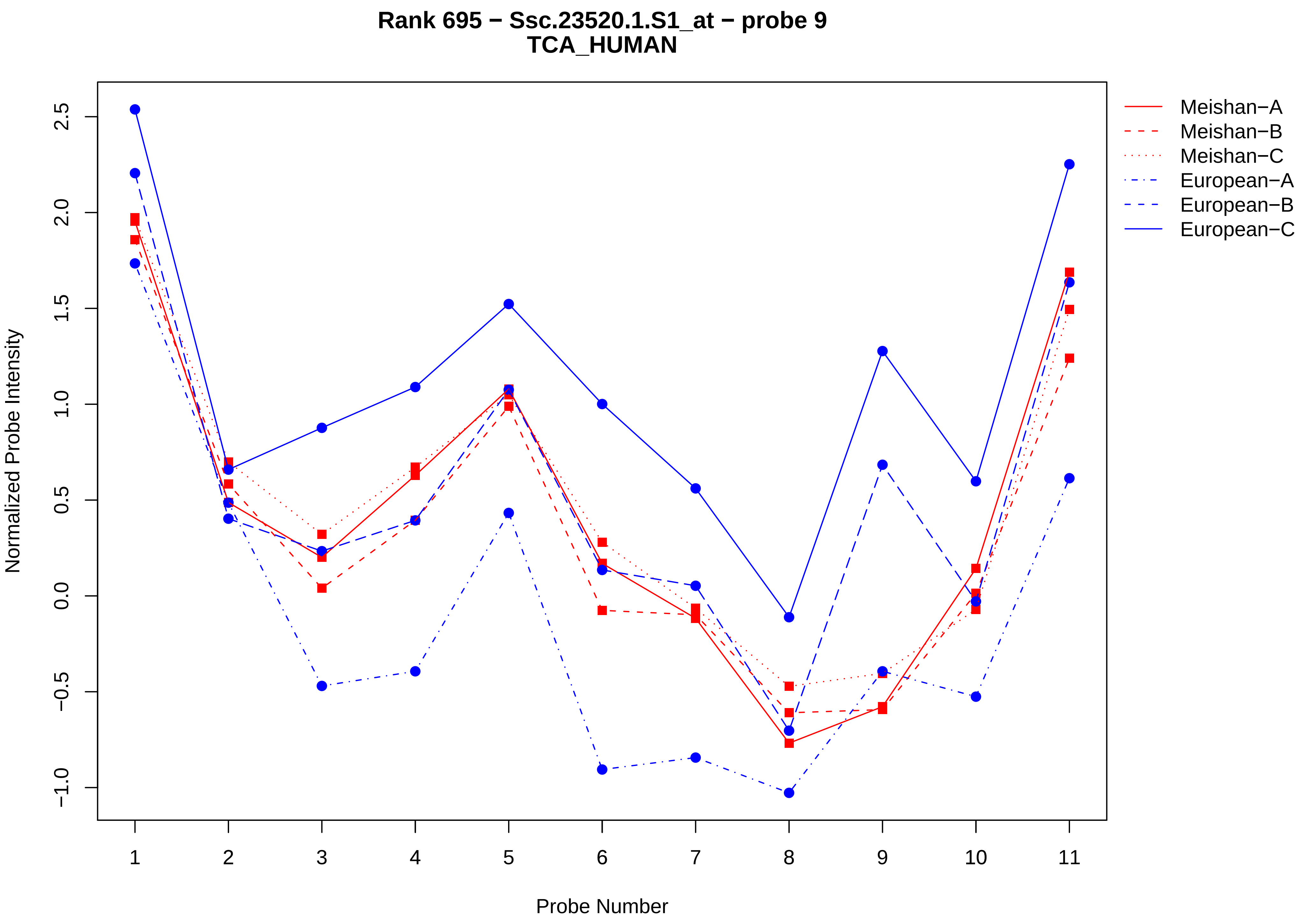

Rank 695 − Ssc.23520.1.S1_at − probe 9
TCA_HUMAN
Normalized Probe Intensity
Probe Number
Meishan−A
Meishan−B
Meishan−C
European−A
European−B
European−C

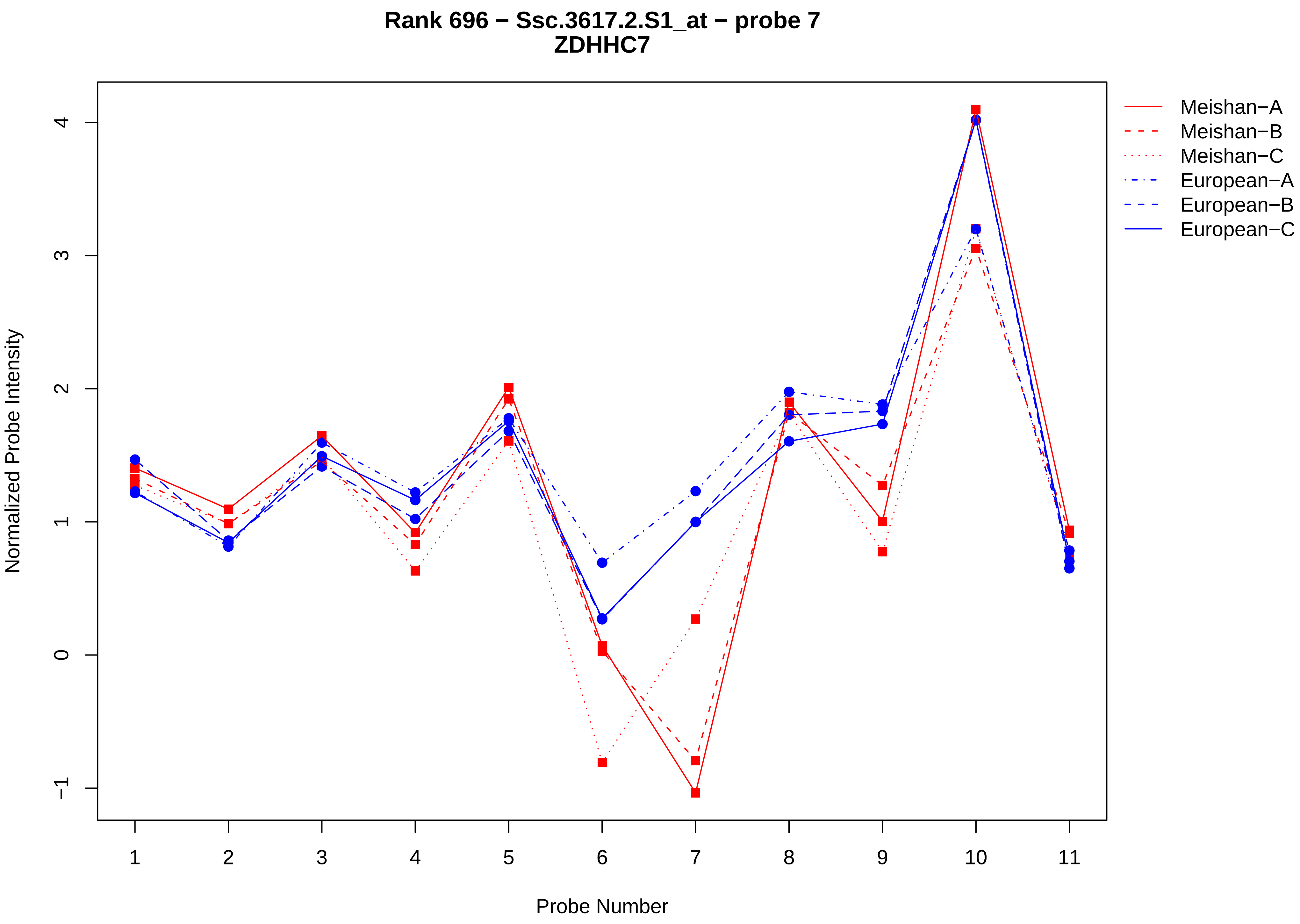
Rank 696 − Ssc.3617.2.S1_at − probe 7
ZDHHC7
Normalized Probe Intensity
Probe Number
−1
0
1
2
3
4
1
2
3
4
5
6
7
8
9
10
11
Meishan−A
Meishan−B
Meishan−C
European−A
European−B
European−C

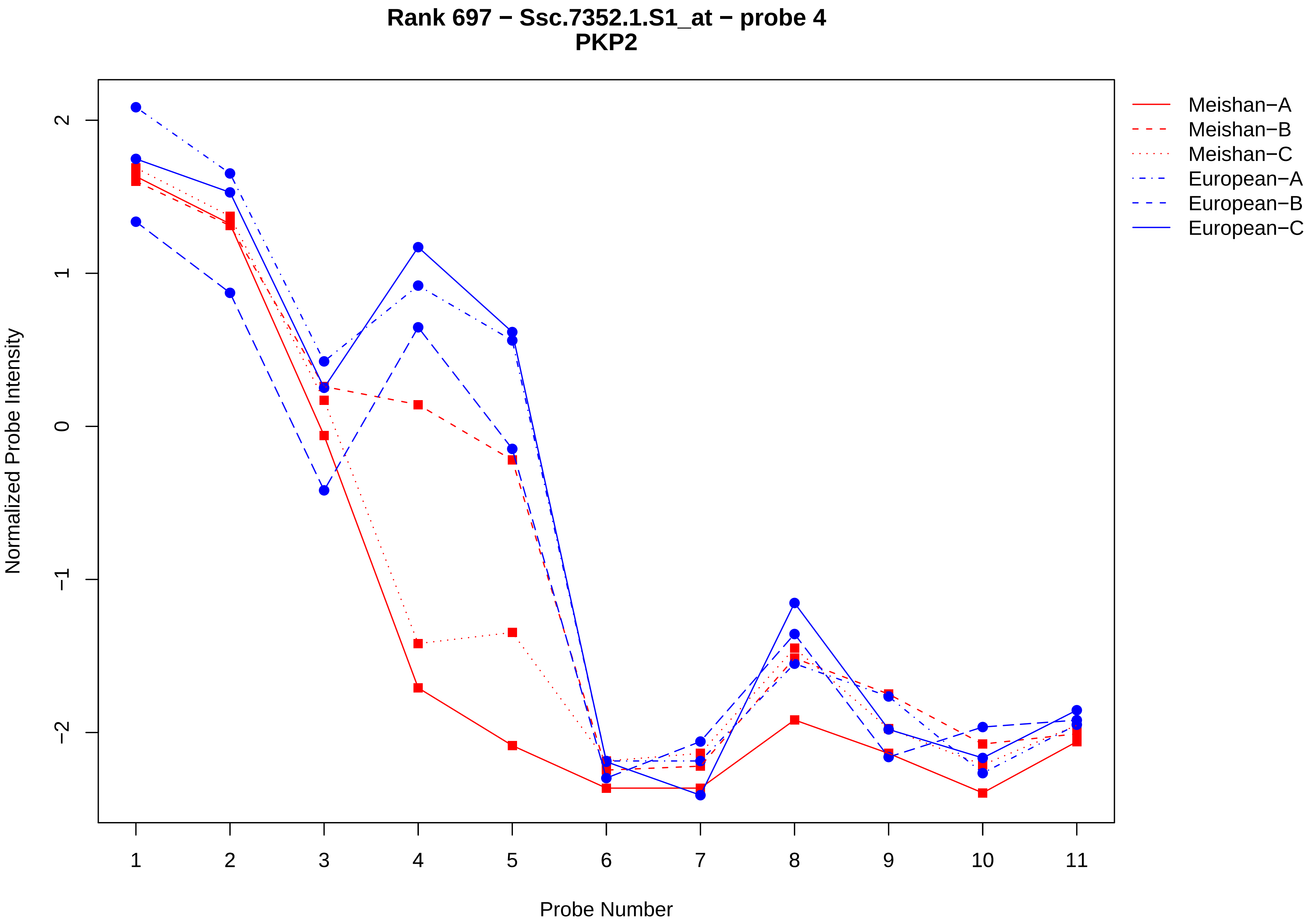

Rank 697 − Ssc.7352.1.S1_at − probe 4
PKP2
Normalized Probe Intensity
Probe Number
−2
−1
0
1
2
1
2
3
4
5
6
7
8
9
10
11
Meishan−A
Meishan−B
Meishan−C
European−A
European−B
European−C

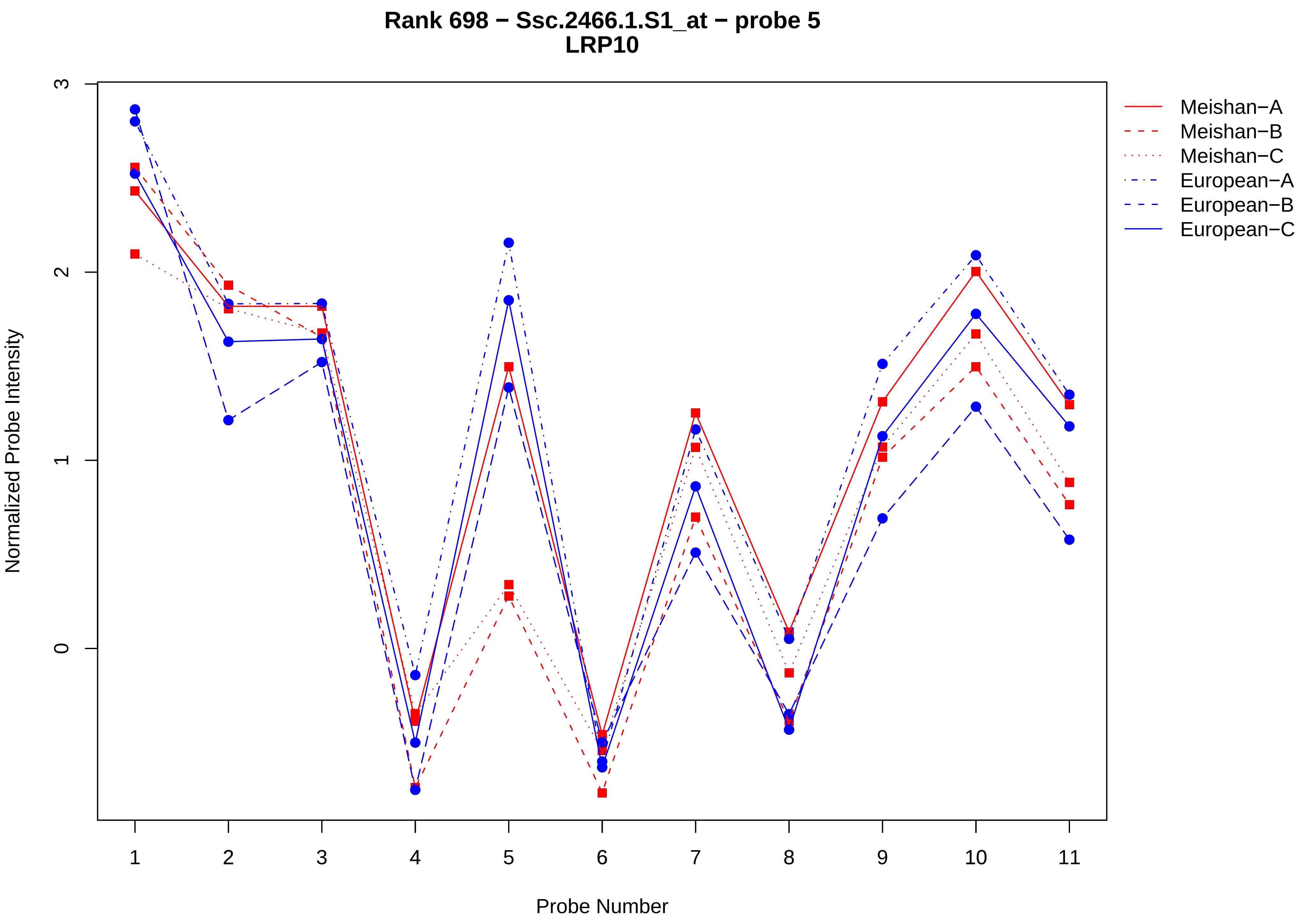

Rank 698 − Ssc.2466.1.S1_at − probe 5
LRP10
Normalized Probe Intensity
Probe Number
0
1
2
3
1
2
3
4
5
6
7
8
9
10
11
Meishan−A
Meishan−B
Meishan−C
European−A
European−B
European−C

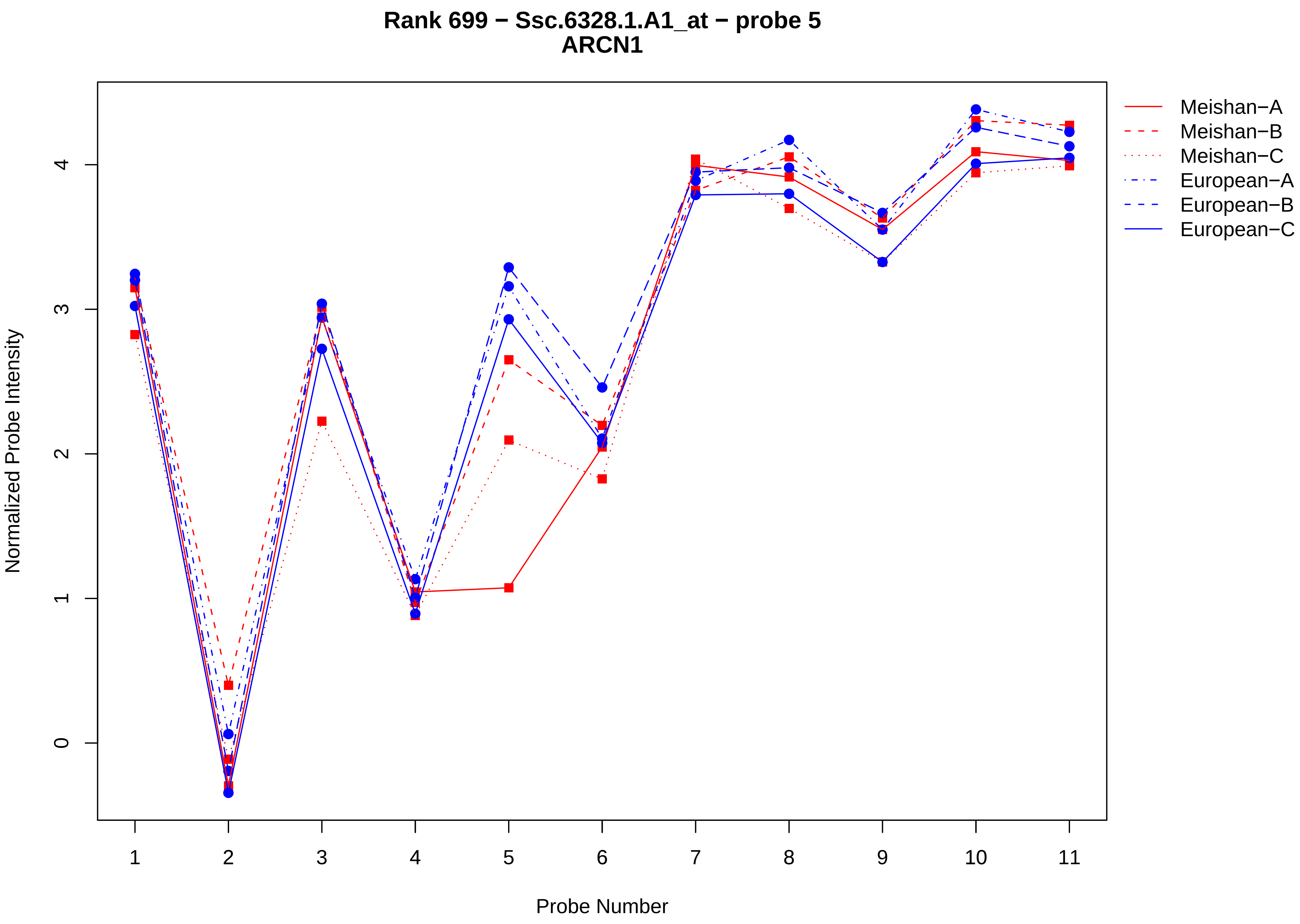
Rank 699 − Ssc.6328.1.A1_at − probe 5
ARCN1
Normalized Probe Intensity
Probe Number
Meishan−A
Meishan−B
Meishan−C
European−A
European−B
European−C
0
1
2
3
4
1
2
3
4
5
6
7
8
9
10
11

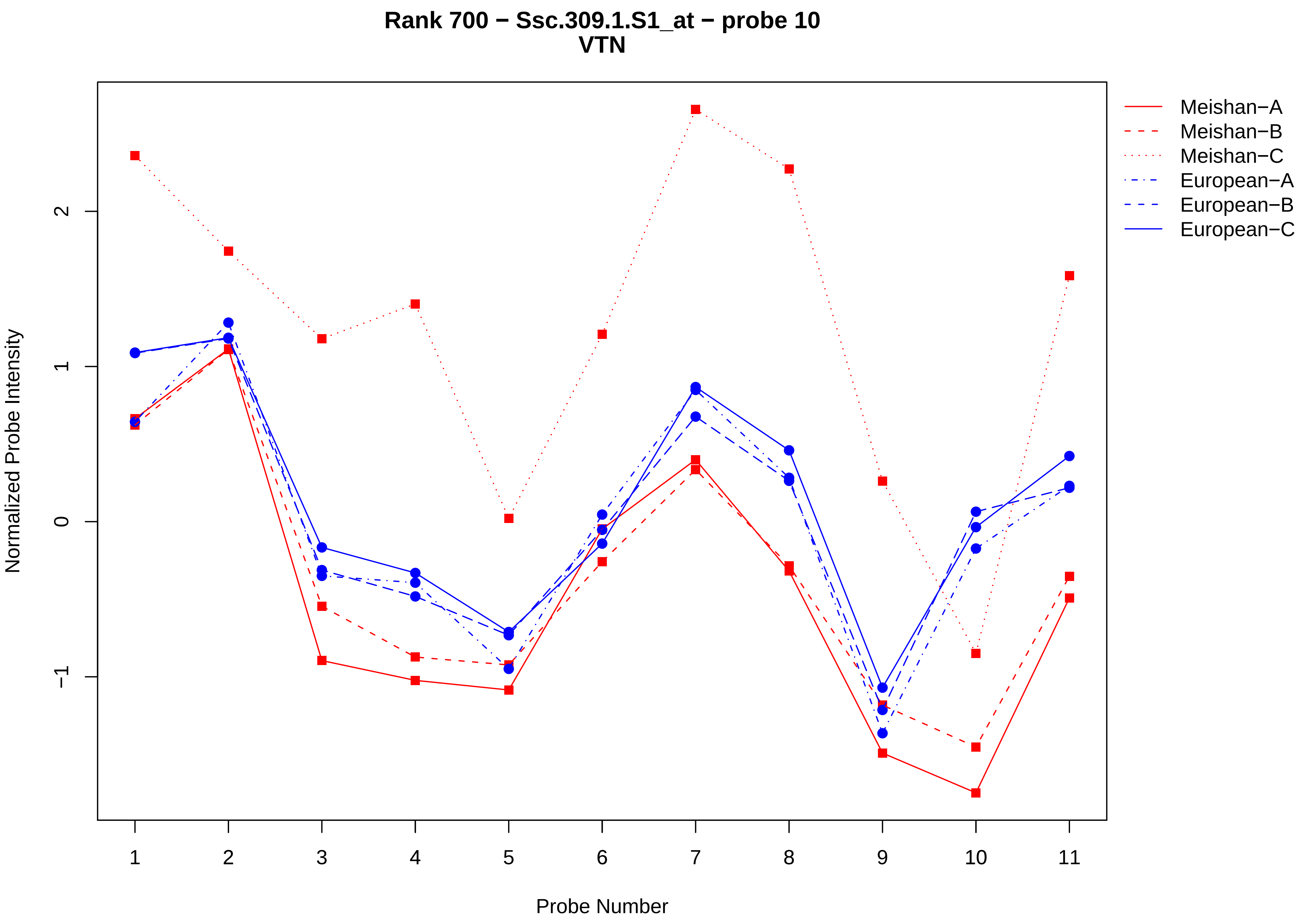

Rank 700 − Ssc.309.1.S1_at − probe 10
VTN
Normalized Probe Intensity
Probe Number
−1
0
1
2
1
2
3
4
5
6
7
8
9
10
11
Meishan−A
Meishan−B
Meishan−C
European−A
European−B
European−C

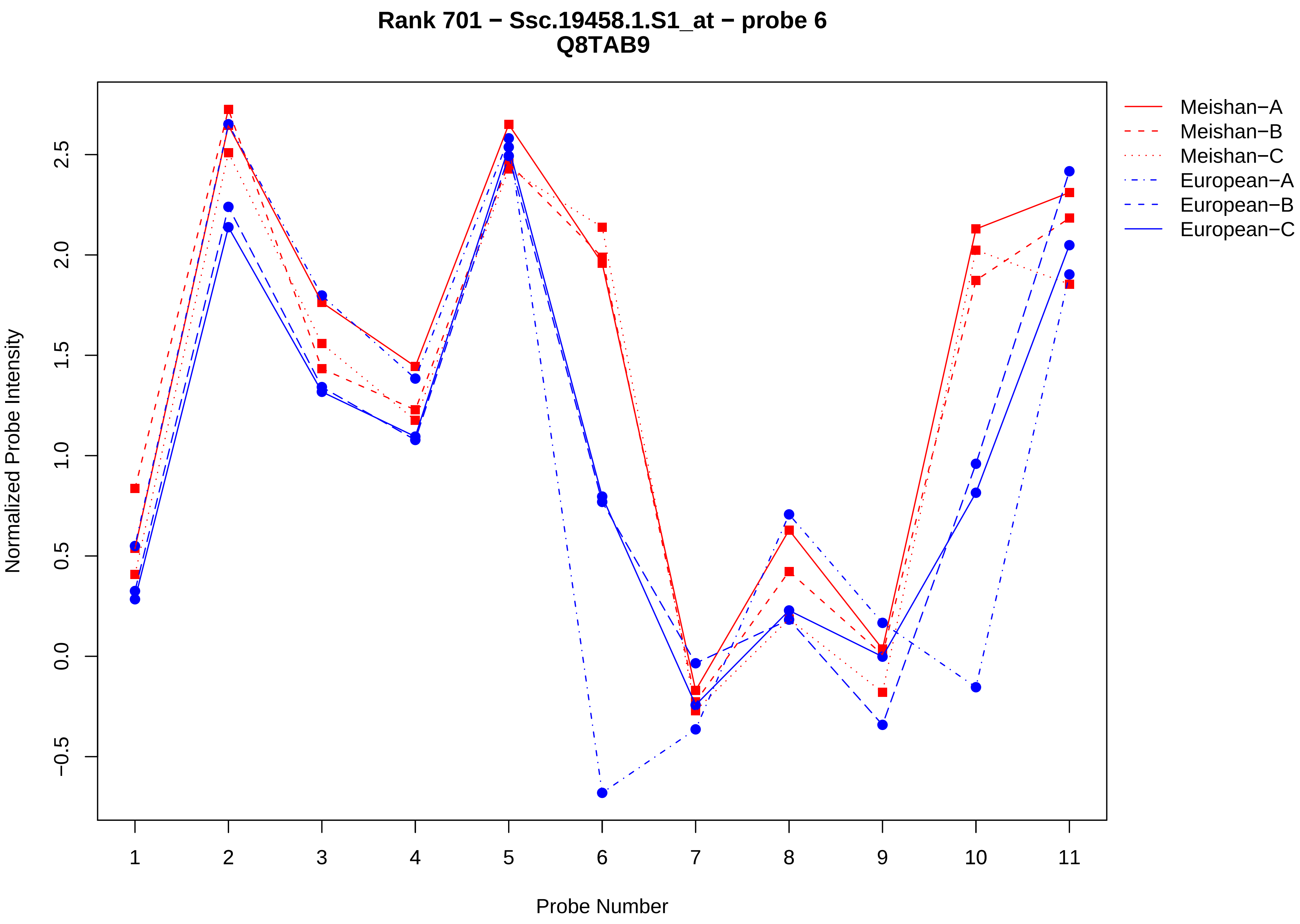

Rank 701 − Ssc.19458.1.S1_at − probe 6
Q8TAB9
Normalized Probe Intensity
Probe Number
−0.5
0.0
0.5
1.0
1.5
2.0
2.5
1
2
3
4
5
6
7
8
9
10
11
Meishan−A
Meishan−B
Meishan−C
European−A
European−B
European−C

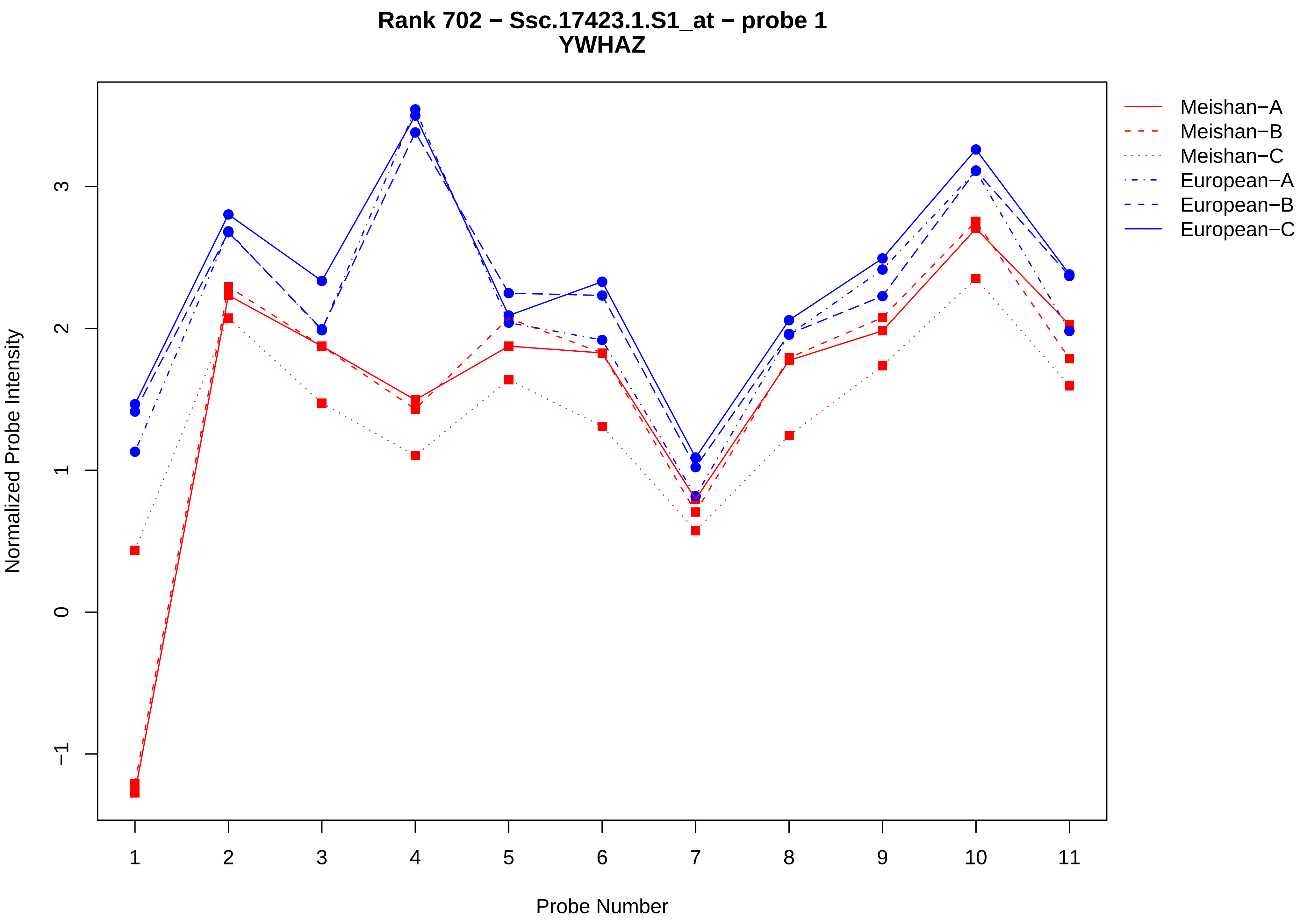

Rank 702 − Ssc.17423.1.S1_at − probe 1
YWHAZ
Normalized Probe Intensity
Probe Number
−1
0
1
2
3
1
2
3
4
5
6
7
8
9
10
11
Meishan−A
Meishan−B
Meishan−C
European−A
European−B
European−C

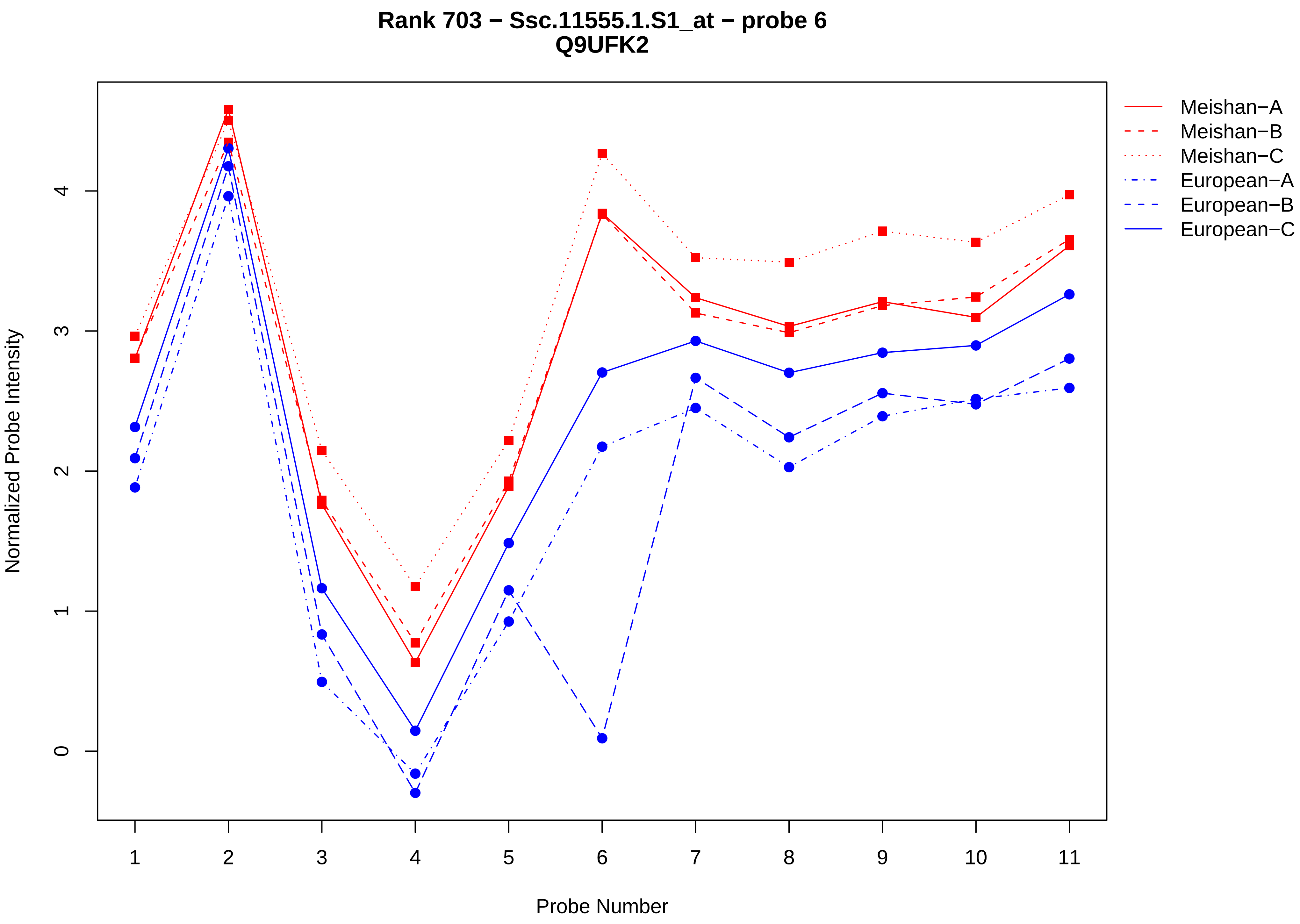

Rank 703 − Ssc.11555.1.S1_at − probe 6
Q9UFK2
Normalized Probe Intensity
Probe Number
0
1
2
3
4
1
2
3
4
5
6
7
8
9
10
11
Meishan−A
Meishan−B
Meishan−C
European−A
European−B
European−C

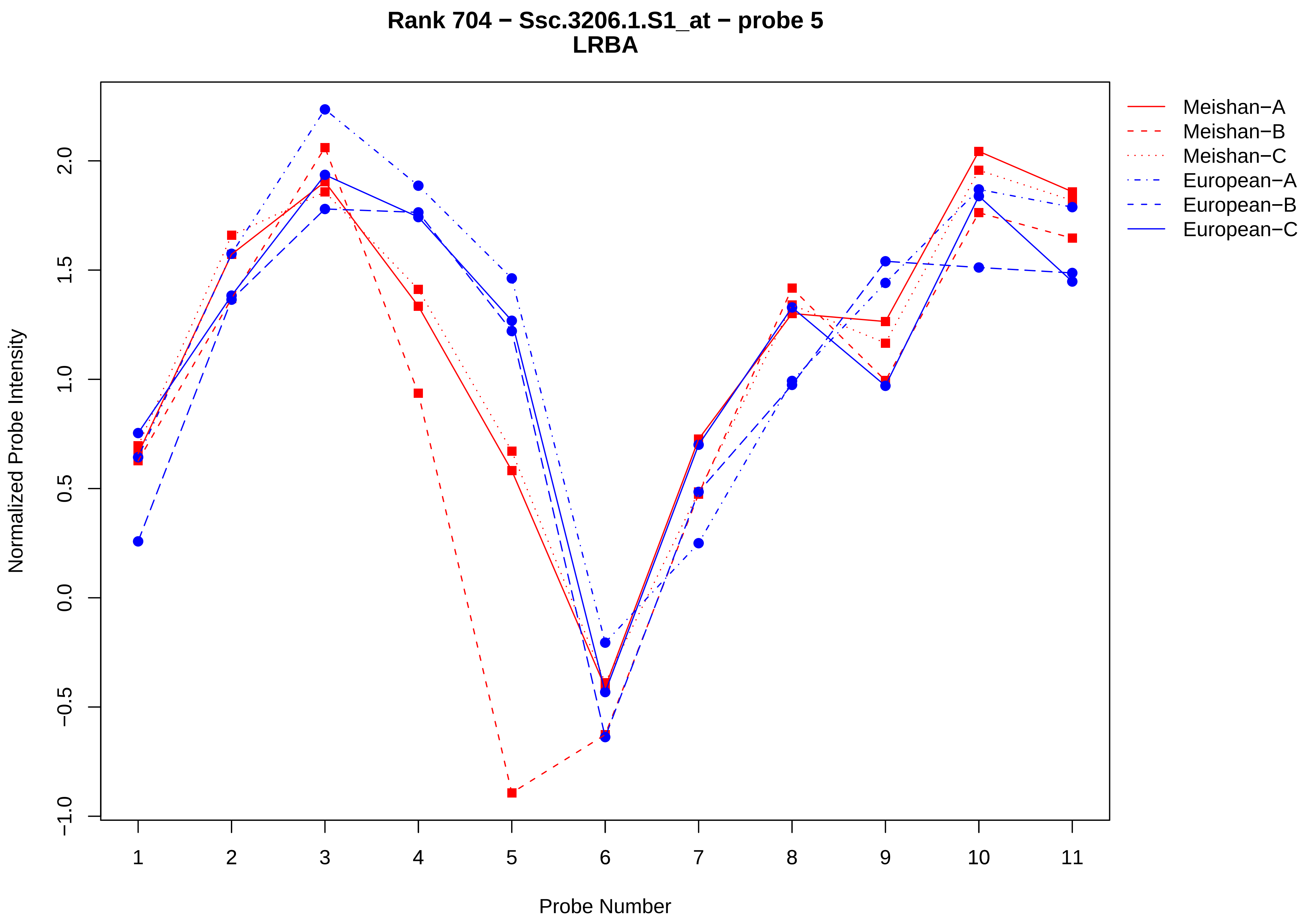

Rank 704 − Ssc.3206.1.S1_at − probe 5
LRBA
Normalized Probe Intensity
Probe Number
−1.0
−0.5
0.0
0.5
1.0
1.5
2.0
1
2
3
4
5
6
7
8
9
10
11
Meishan−A
Meishan−B
Meishan−C
European−A
European−B
European−C

Rank 705 − Ssc.21114.1.S1_at − probe 11
PTK9

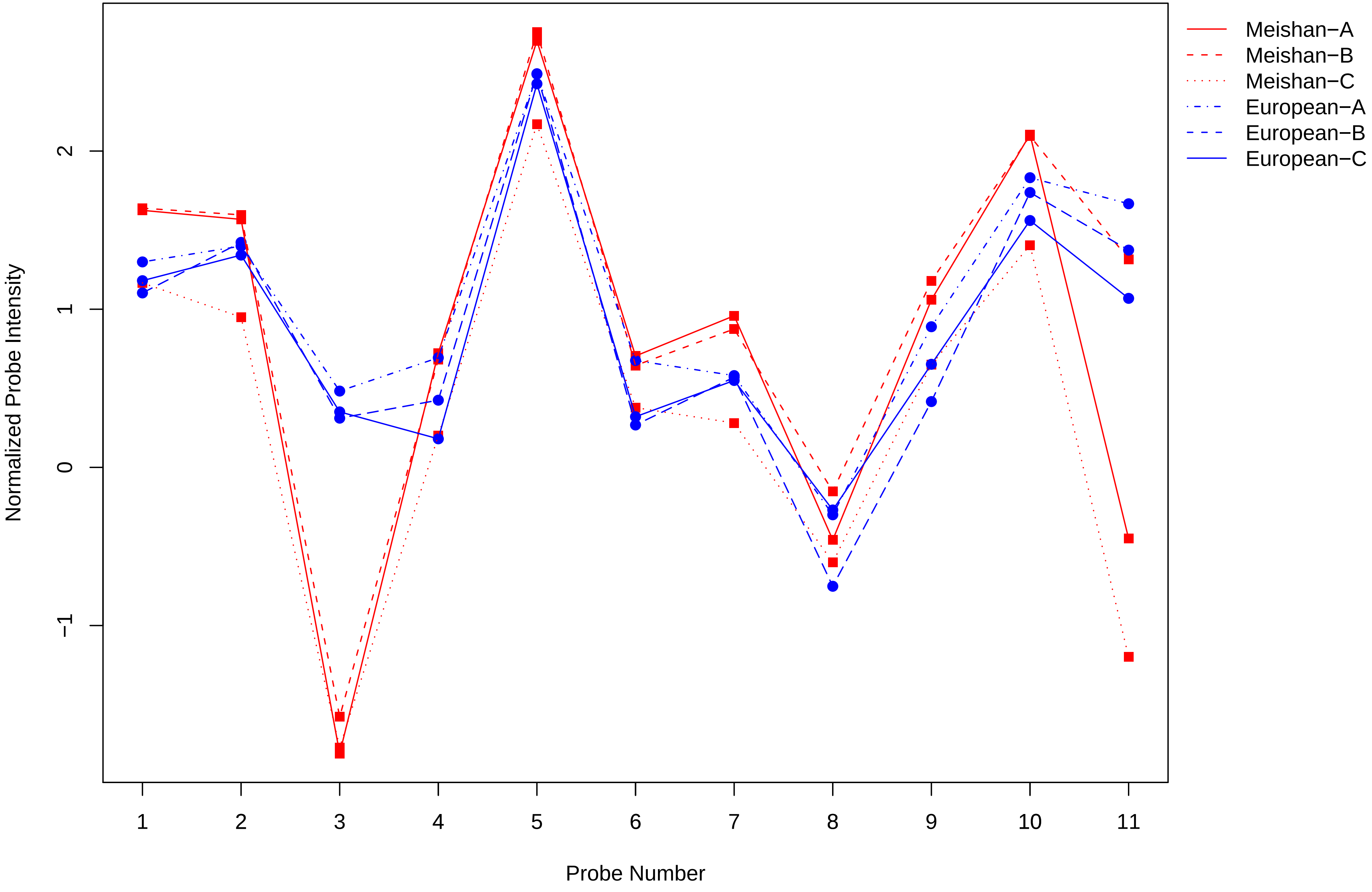

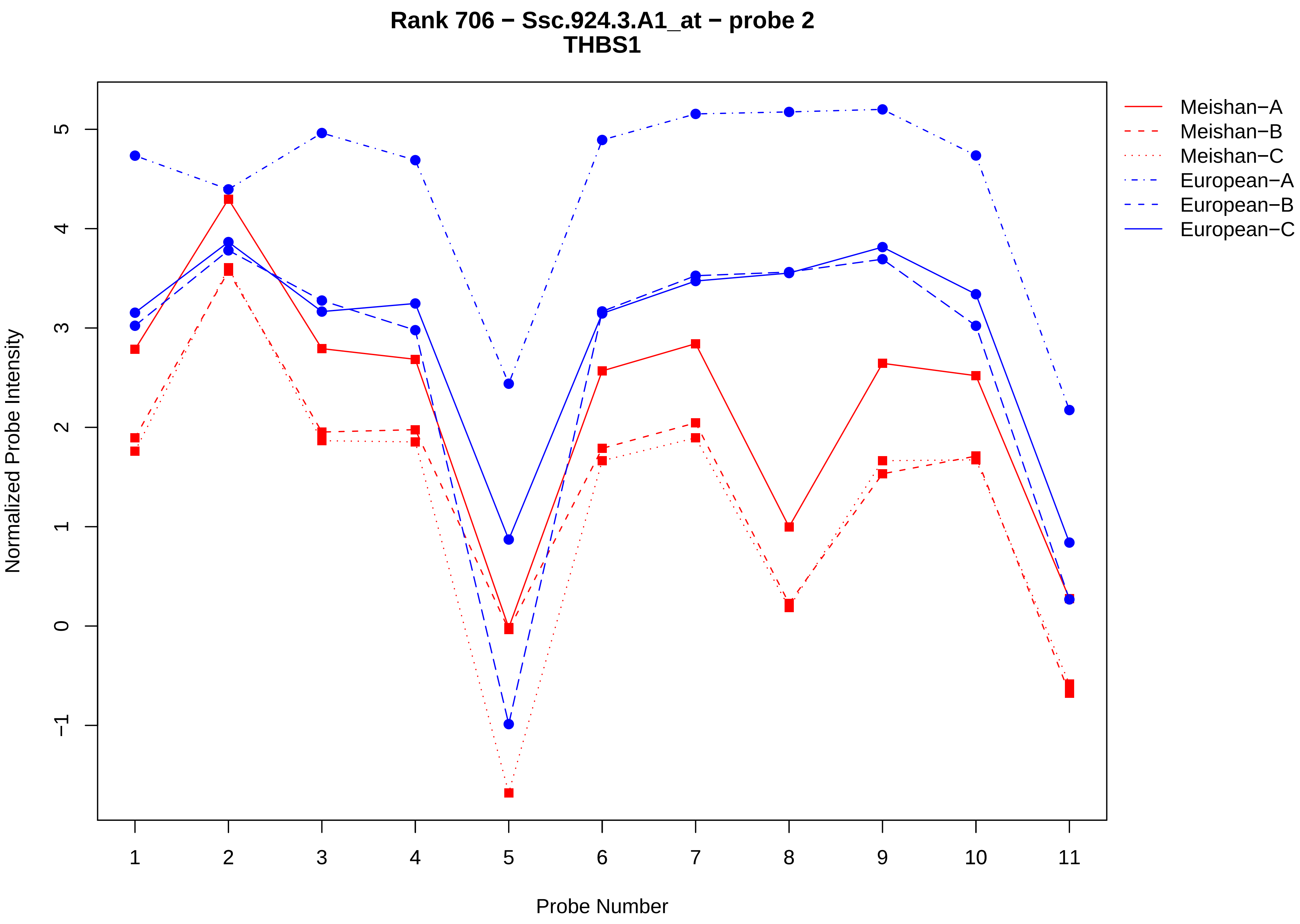
Rank 706 − Ssc.924.3.A1_at − probe 2
THBS1
Normalized Probe Intensity
Probe Number
−1
0
1
2
3
4
5
1
2
3
4
5
6
7
8
9
10
11
Meishan−A
Meishan−B
Meishan−C
European−A
European−B
European−C

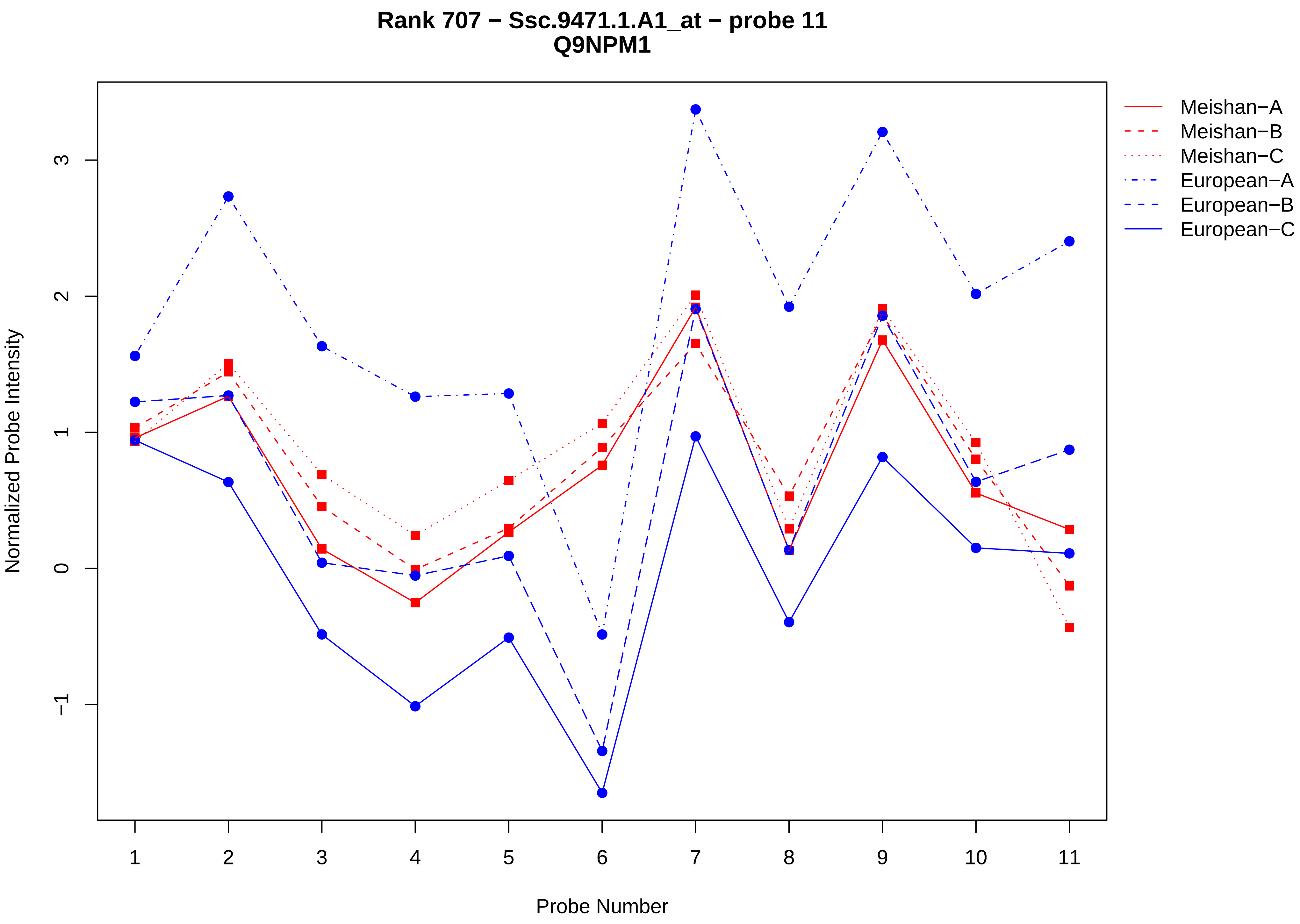

Rank 707 − Ssc.9471.1.A1_at − probe 11
Q9NPM1
Normalized Probe Intensity
Probe Number
−1
0
1
2
3
1
2
3
4
5
6
7
8
9
10
11
Meishan−A
Meishan−B
Meishan−C
European−A
European−B
European−C

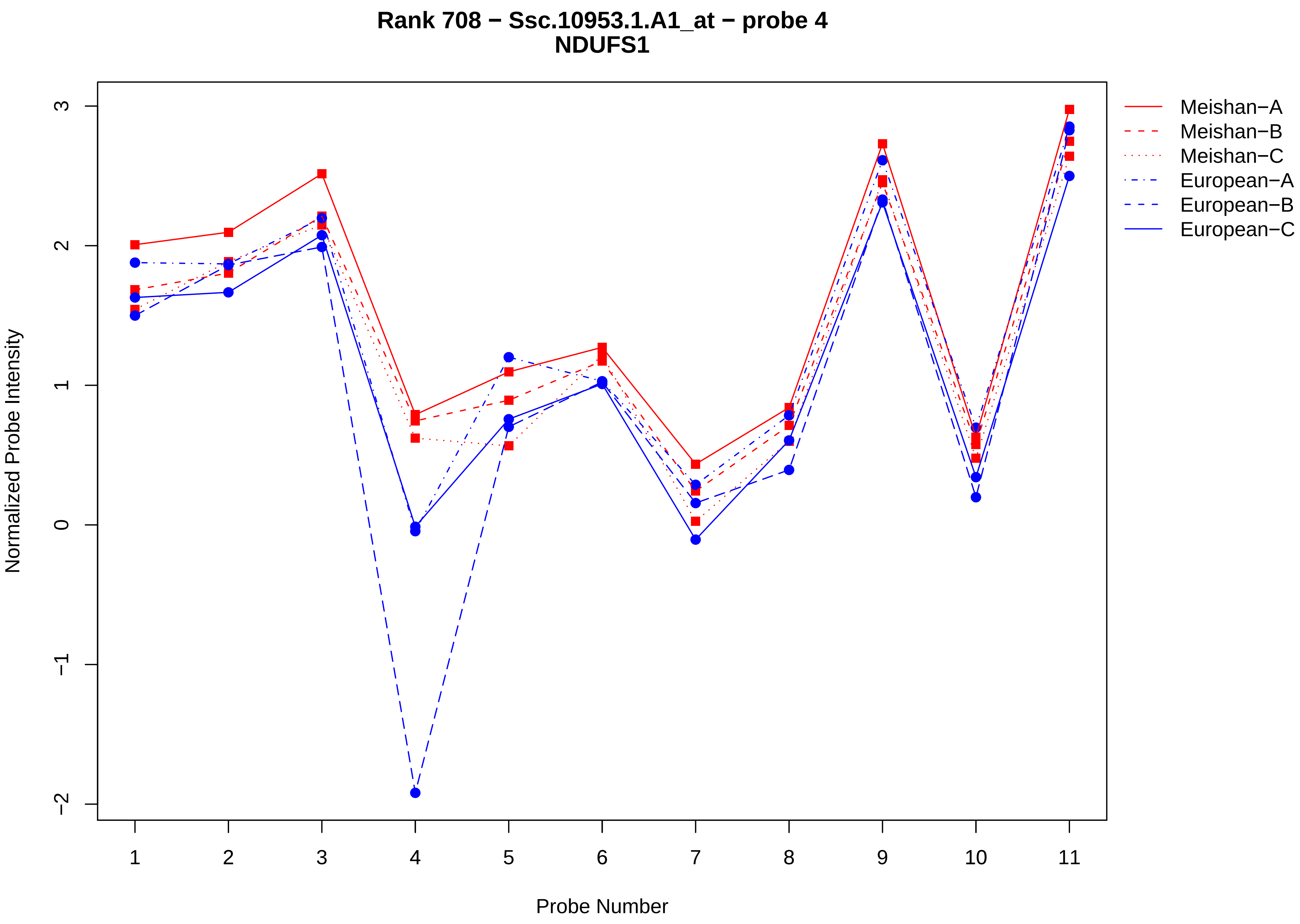

Rank 708 − Ssc.10953.1.A1_at − probe 4
NDUFS1
Normalized Probe Intensity
Probe Number
Meishan−A
Meishan−B
Meishan−C
European−A
European−B
European−C
−2
−1
0
1
2
3
1
2
3
4
5
6
7
8
9
10
11

Rank 709 − Ssc.3216.2.A1_at − probe 8
HNRPH2

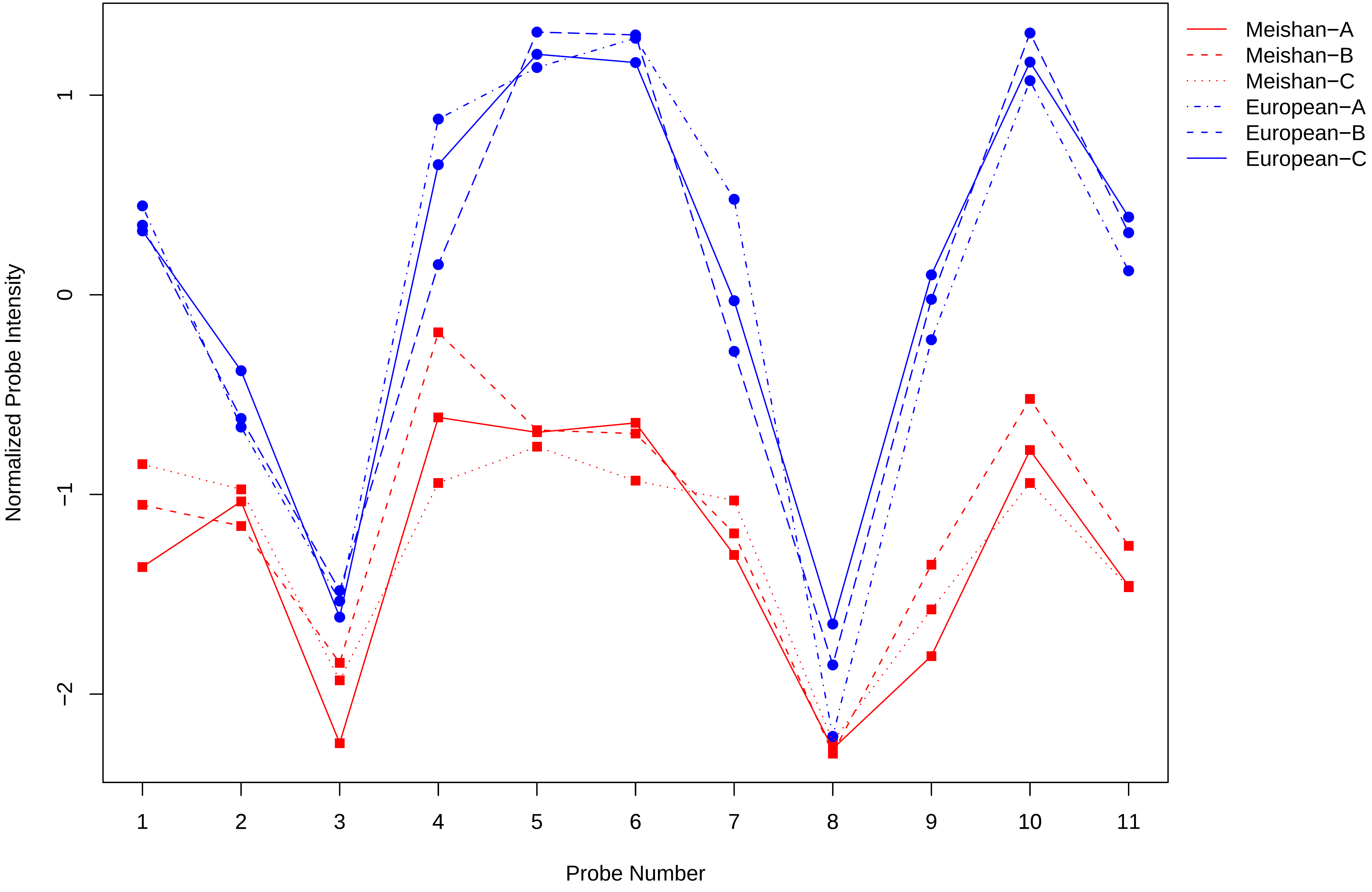

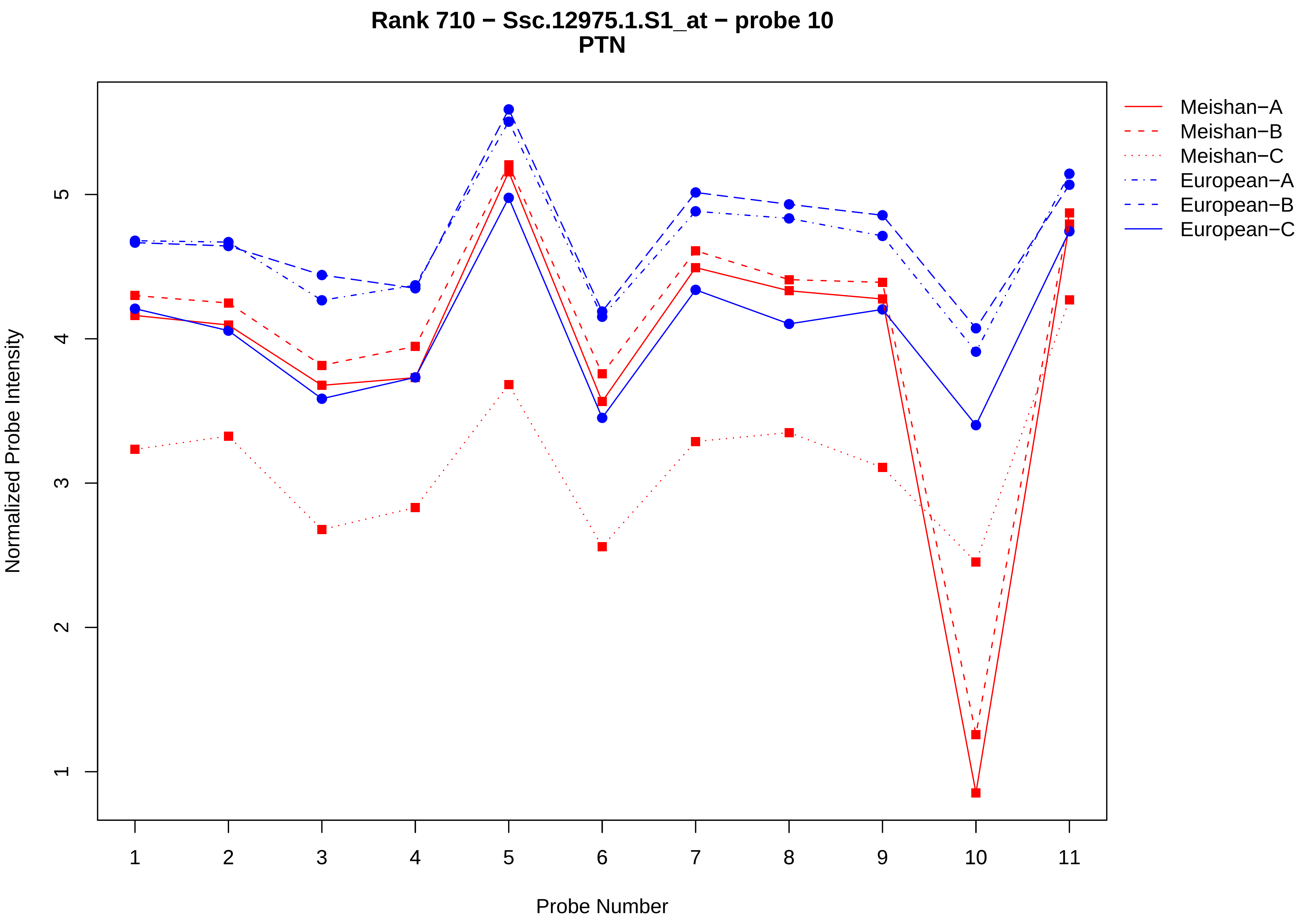

Rank 710 − Ssc.12975.1.S1_at − probe 10
PTN
Normalized Probe Intensity
Probe Number
1
2
3
4
5
6
7
8
9
10
11
Meishan−A
Meishan−B
Meishan−C
European−A
European−B
European−C

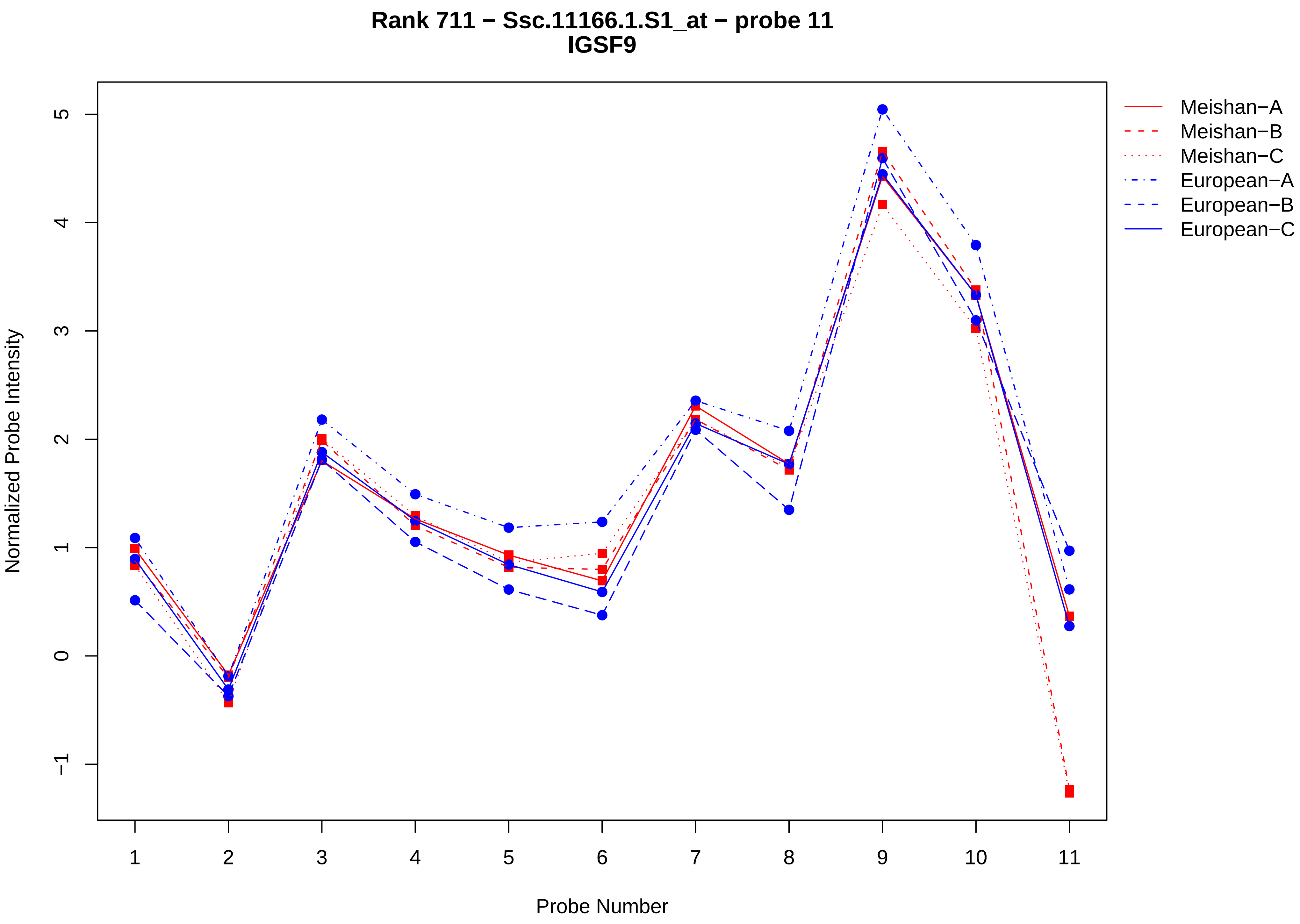

Rank 711 − Ssc.11166.1.S1_at − probe 11
IGSF9
Normalized Probe Intensity
Probe Number
Meishan−A
Meishan−B
Meishan−C
European−A
European−B
European−C

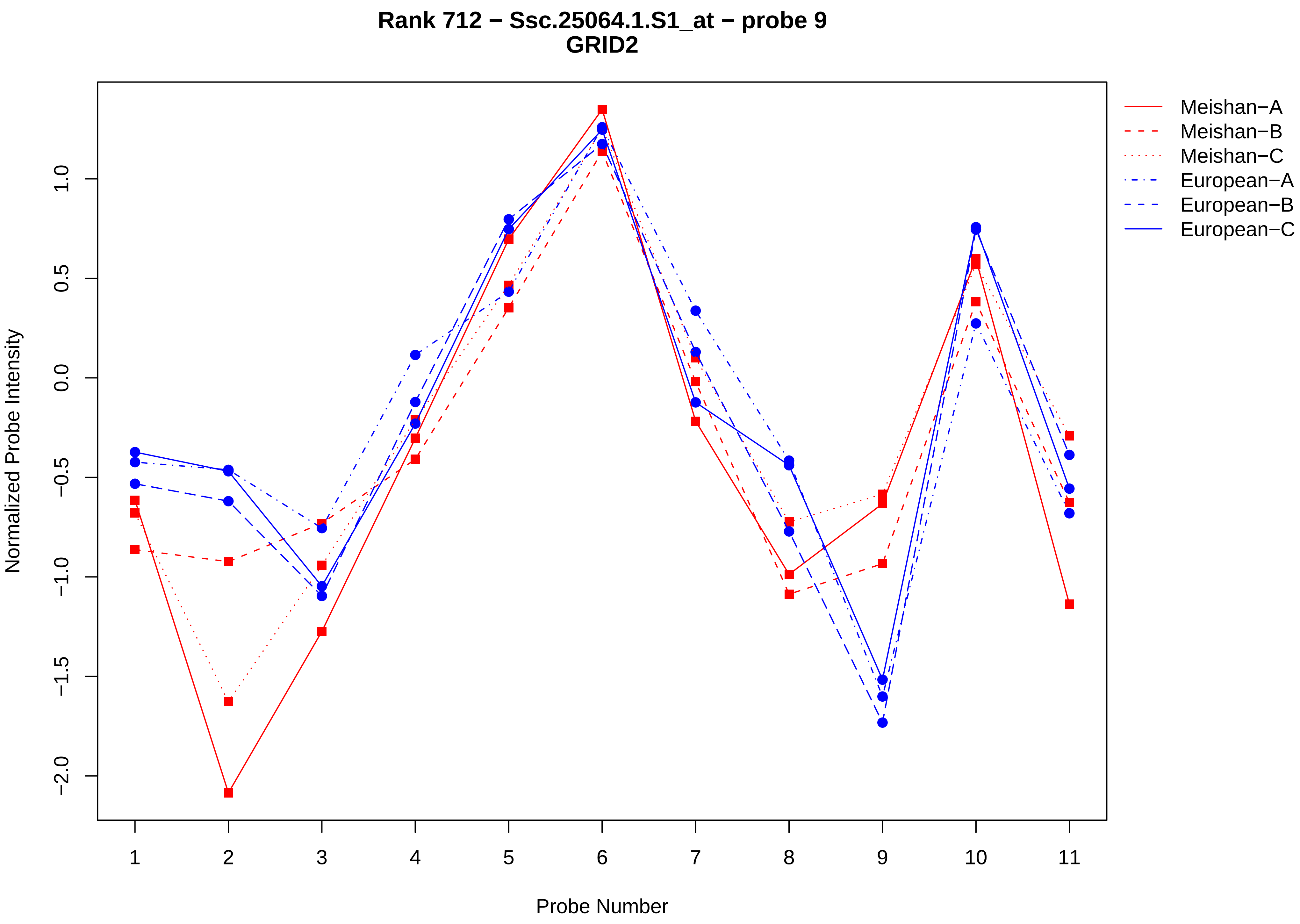
Rank 712 − Ssc.25064.1.S1_at − probe 9
GRID2
Normalized Probe Intensity
Probe Number
Meishan−A
Meishan−B
Meishan−C
European−A
European−B
European−C
−2.0
−1.5
−1.0
−0.5
0.0
0.5
1.0
1
2
3
4
5
6
7
8
9
10
11

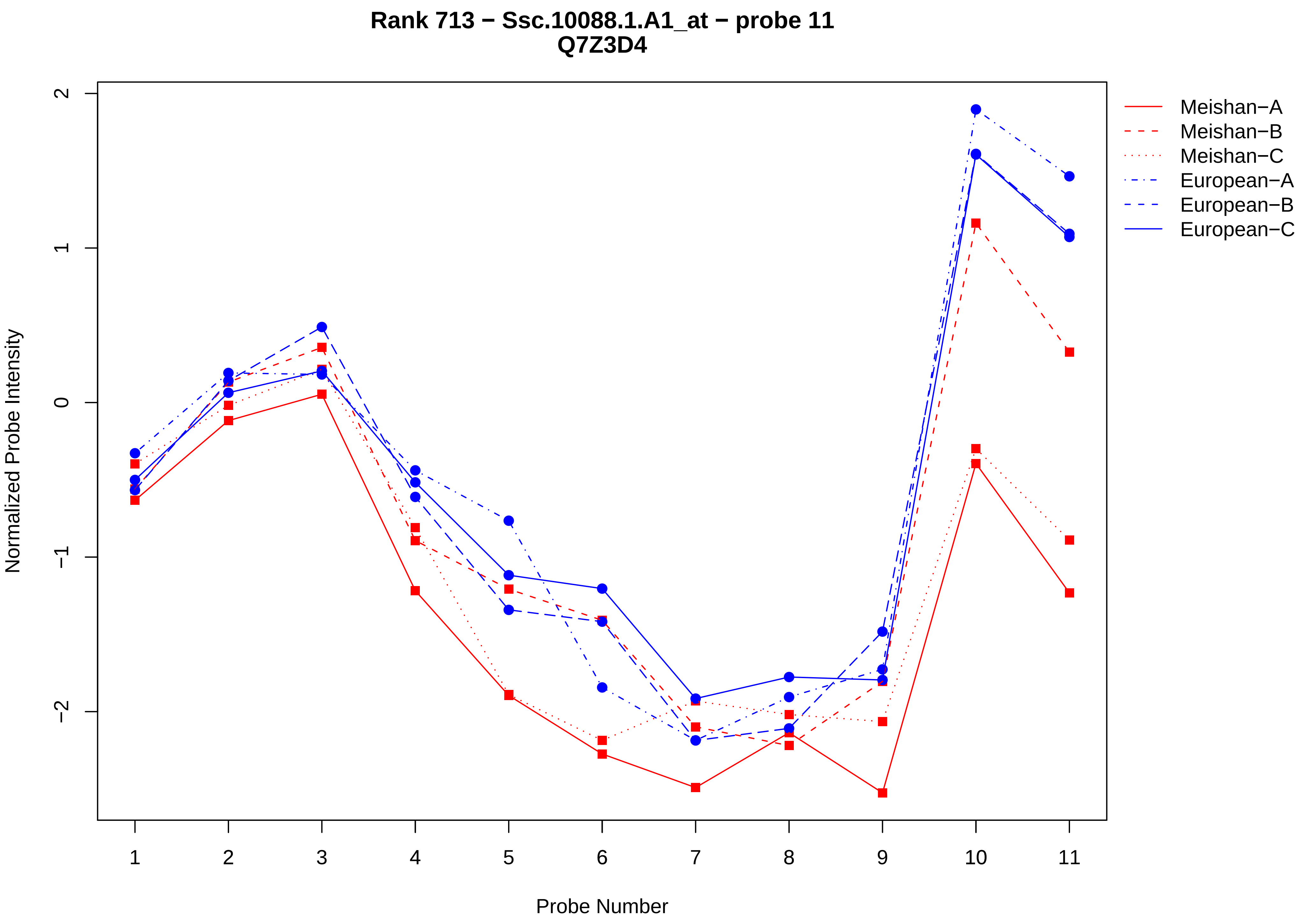

Rank 713 − Ssc.10088.1.A1_at − probe 11
Q7Z3D4
Normalized Probe Intensity
Probe Number
−2
−1
0
1
2
1
2
3
4
5
6
7
8
9
10
11
Meishan−A
Meishan−B
Meishan−C
European−A
European−B
European−C

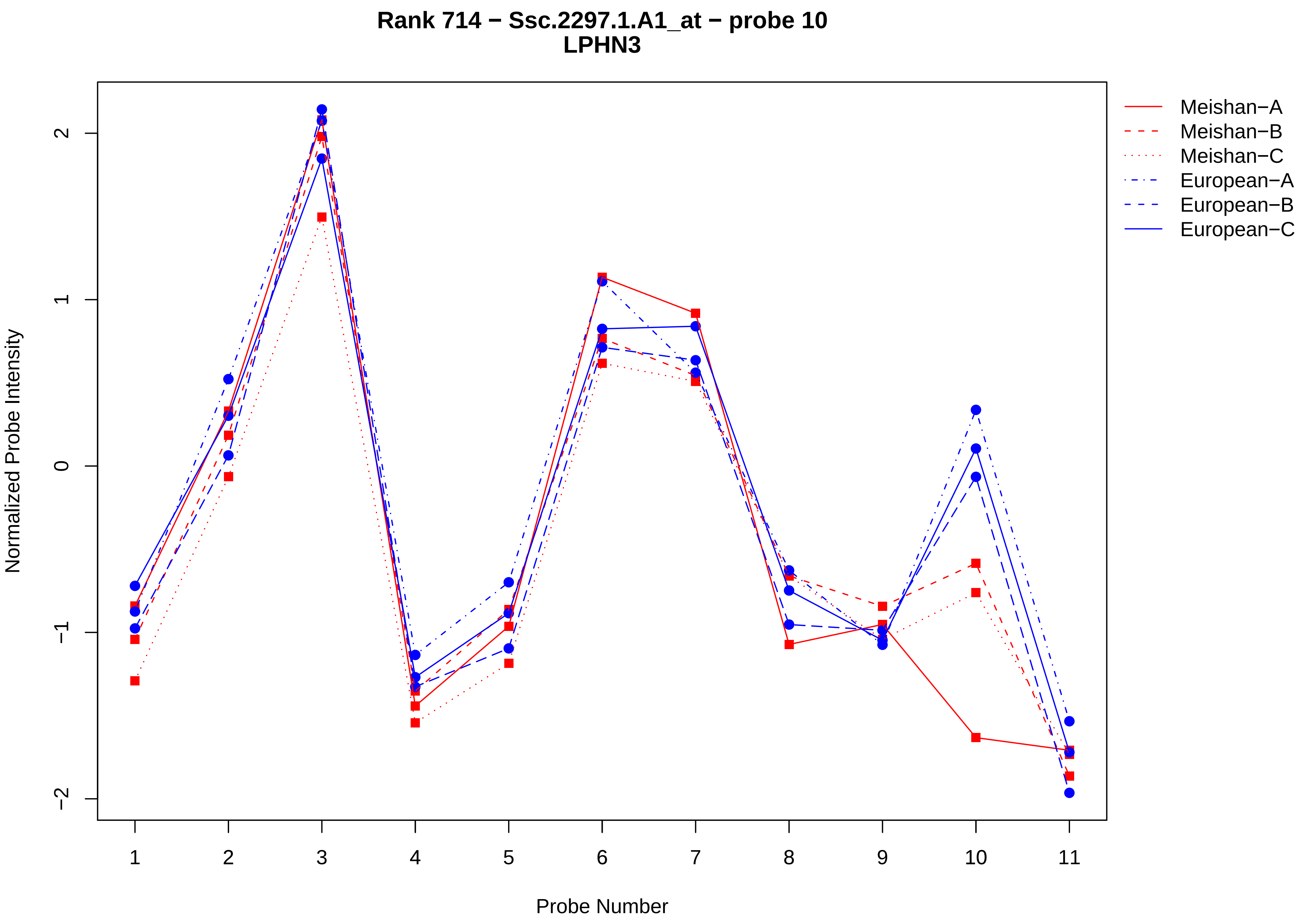

Rank 714 − Ssc.2297.1.A1_at − probe 10
LPHN3
Normalized Probe Intensity
Probe Number
−2
−1
0
1
2
1
2
3
4
5
6
7
8
9
10
11
Meishan−A
Meishan−B
Meishan−C
European−A
European−B
European−C

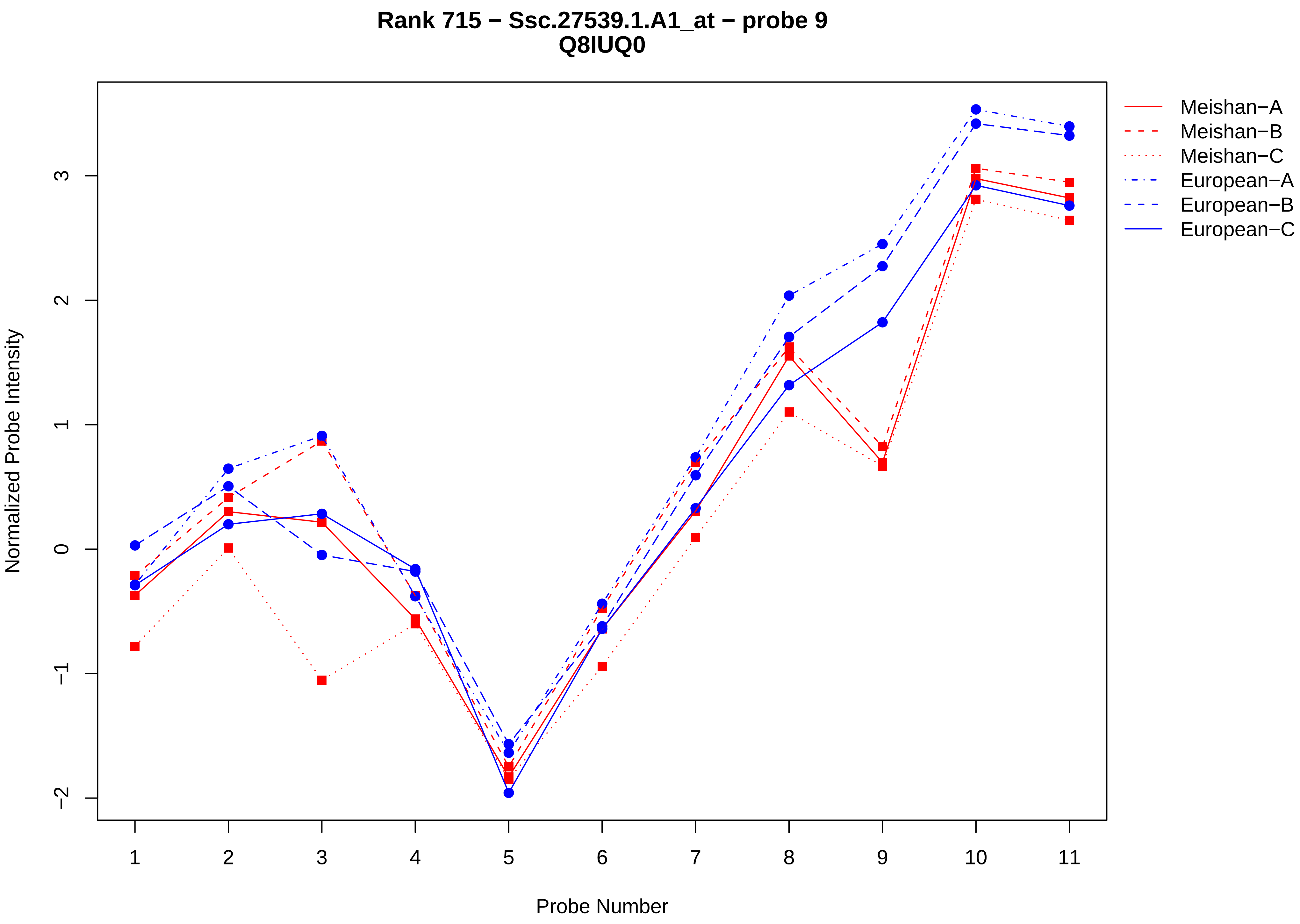

Rank 715 − Ssc.27539.1.A1_at − probe 9
Q8IUQ0
Normalized Probe Intensity
Probe Number
−2
−1
0
1
2
3
1
2
3
4
5
6
7
8
9
10
11
Meishan−A
Meishan−B
Meishan−C
European−A
European−B
European−C

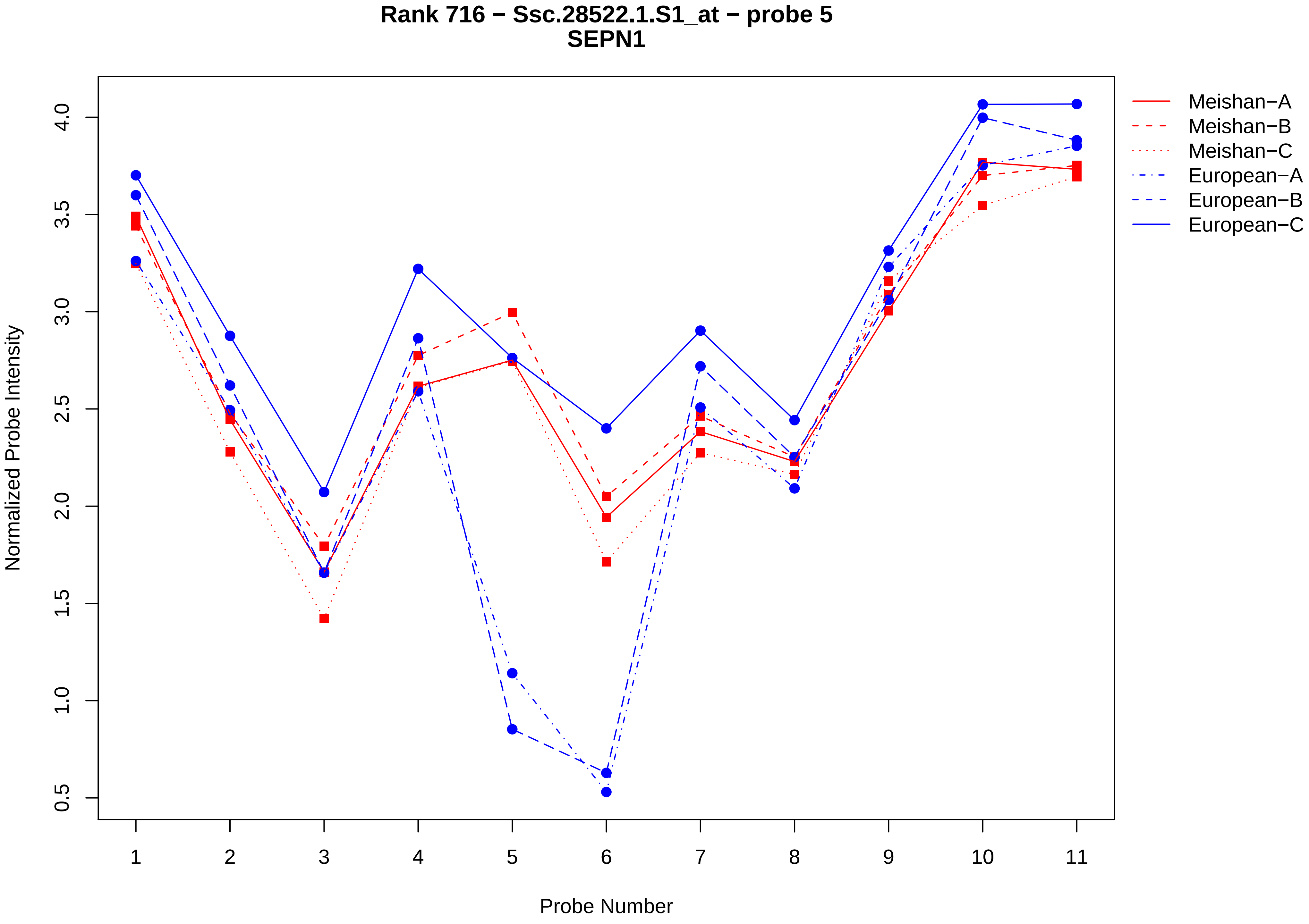

Rank 716 − Ssc.28522.1.S1_at − probe 5
SEPN1
Normalized Probe Intensity
Probe Number
0.5
1.0
1.5
2.0
2.5
3.0
3.5
4.0
1
2
3
4
5
6
7
8
9
10
11
Meishan−A
Meishan−B
Meishan−C
European−A
European−B
European−C

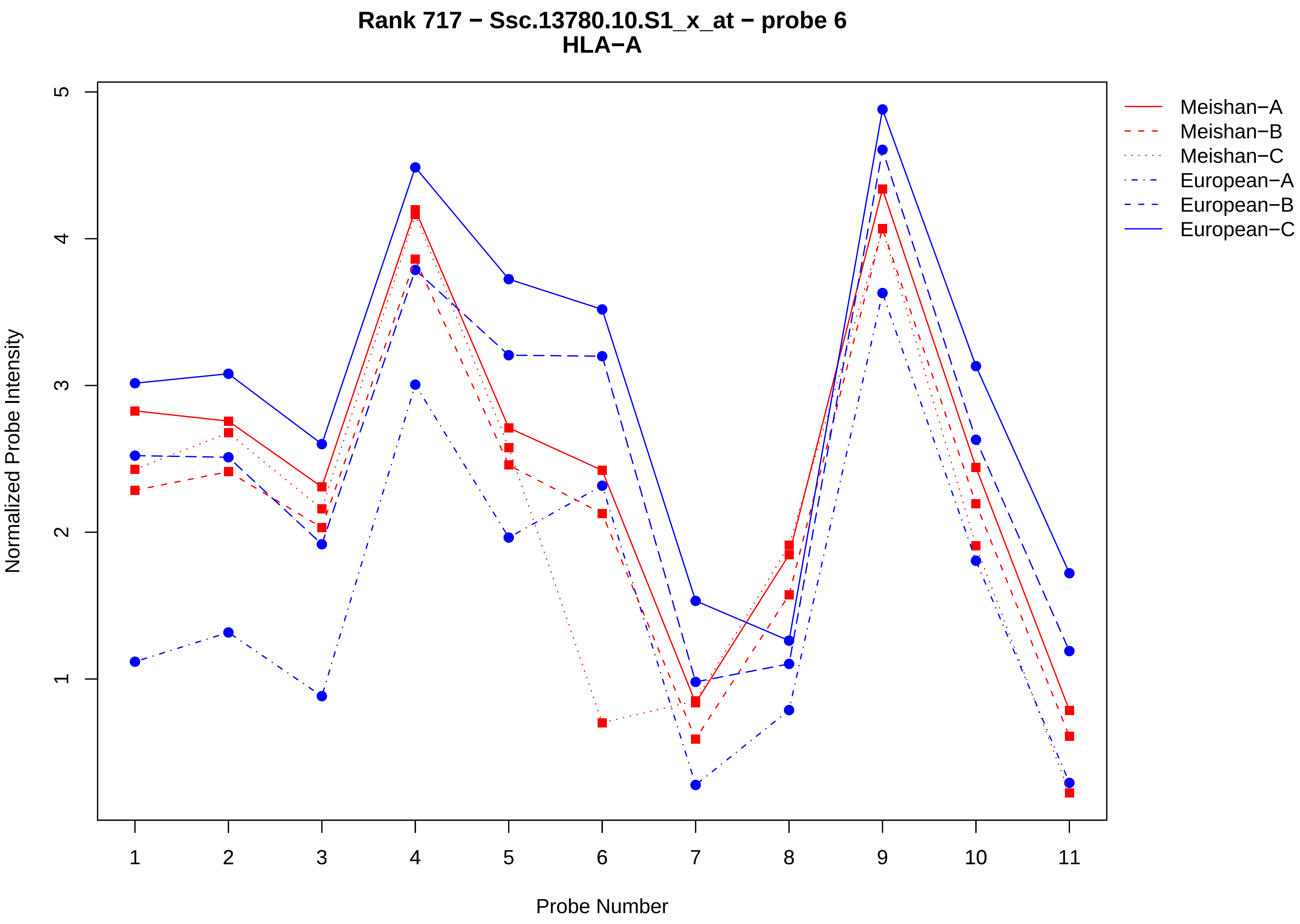

Rank 717 − Ssc.13780.10.S1_x_at − probe 6
HLA−A
Normalized Probe Intensity
Probe Number
1
2
3
4
5
6
7
8
9
10
11
5
4
3
2
1
Meishan−A
Meishan−B
Meishan−C
European−A
European−B
European−C

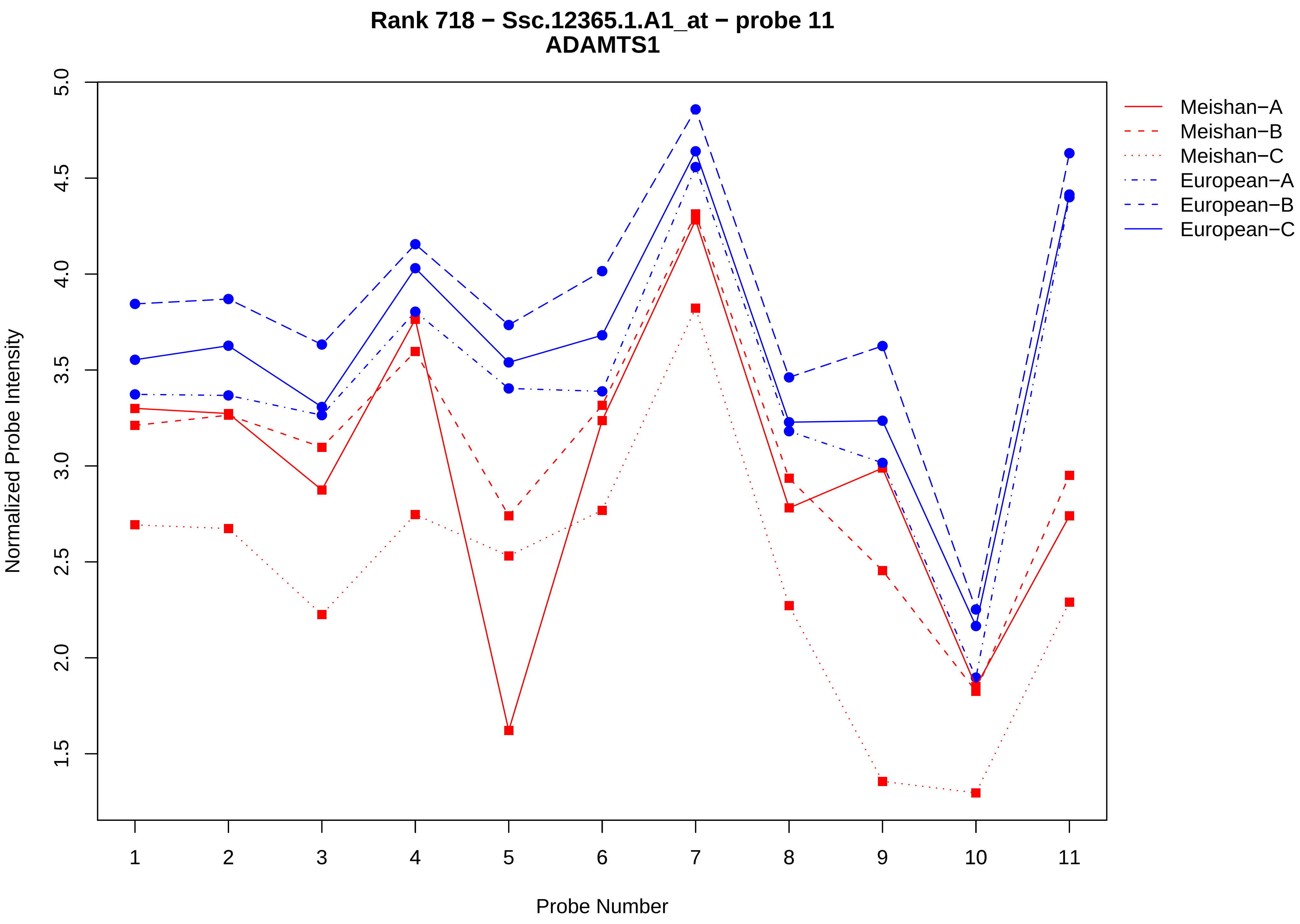

Rank 718 − Ssc.12365.1.A1_at − probe 11
ADAMTS1
Normalized Probe Intensity
Probe Number
Meishan−A
Meishan−B
Meishan−C
European−A
European−B
European−C
1.5
2.0
2.5
3.0
3.5
4.0
4.5
5.0
1
2
3
4
5
6
7
8
9
10
11

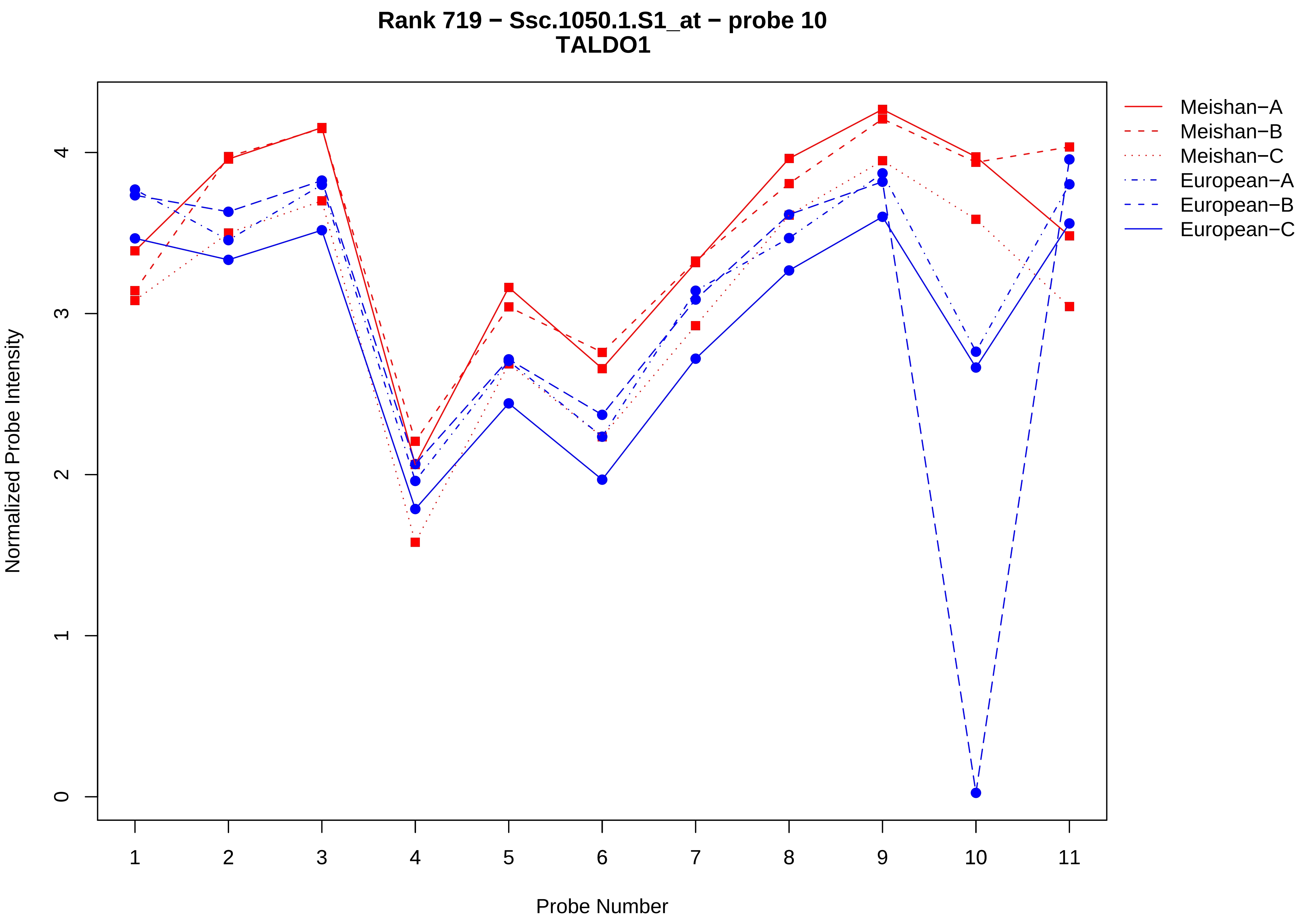
Rank 719 − Ssc.1050.1.S1_at − probe 10
TALDO1
Normalized Probe Intensity
Probe Number
0
1
2
3
4
1
2
3
4
5
6
7
8
9
10
11
Meishan−A
Meishan−B
Meishan−C
European−A
European−B
European−C

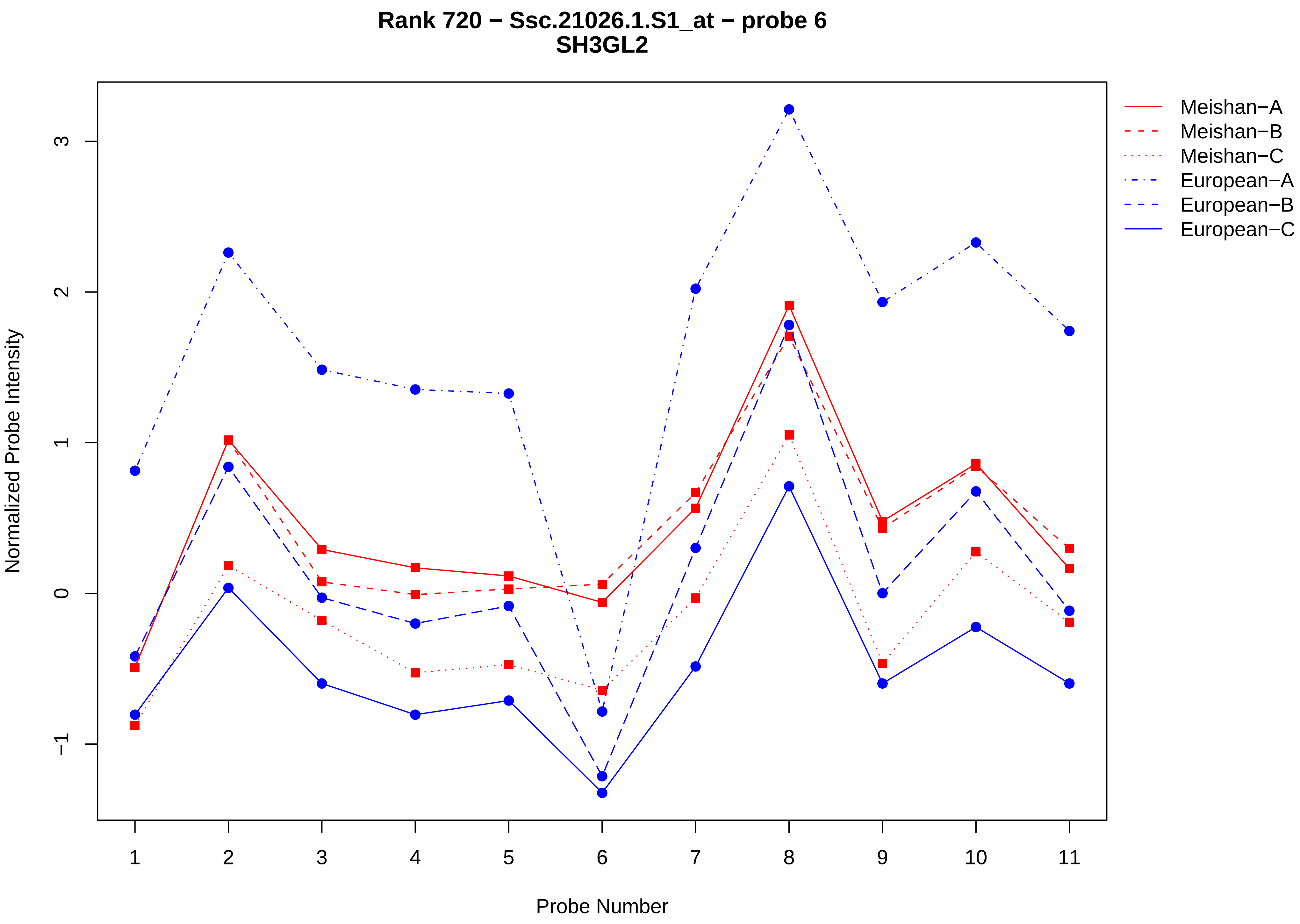

Rank 720 − Ssc.21026.1.S1_at − probe 6
SH3GL2
Normalized Probe Intensity
Probe Number
−1
0
1
2
3
1
2
3
4
5
6
7
8
9
10
11
Meishan−A
Meishan−B
Meishan−C
European−A
European−B
European−C

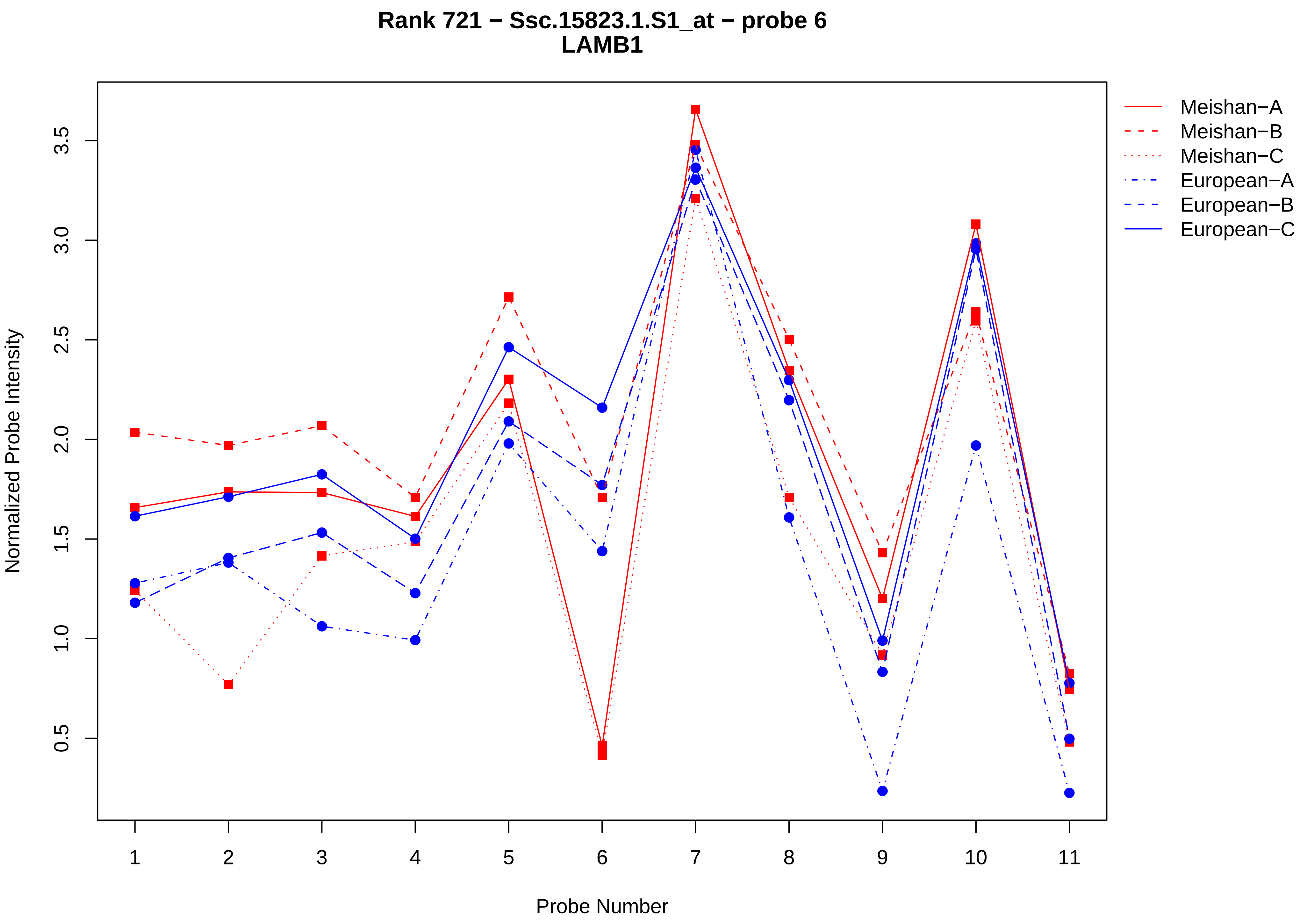

Rank 721 − Ssc.15823.1.S1_at − probe 6
LAMB1
Normalized Probe Intensity
0.5
1.0
1.5
2.0
2.5
3.0
3.5
Probe Number
1
2
3
4
5
6
7
8
9
10
11
Meishan−A
Meishan−B
Meishan−C
European−A
European−B
European−C

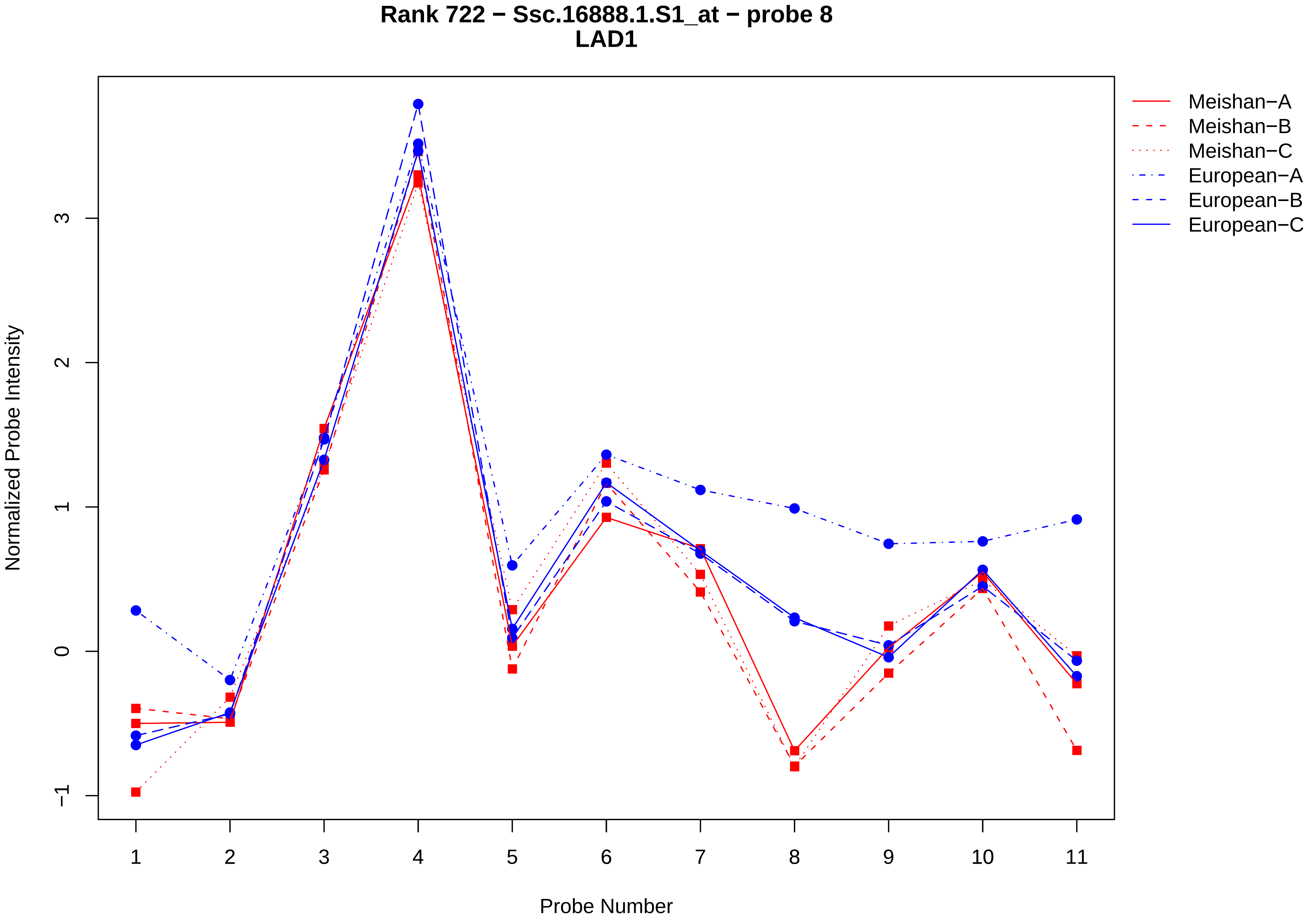

Rank 722 − Ssc.16888.1.S1_at − probe 8
LAD1
Normalized Probe Intensity
Probe Number
−1
0
1
2
3
1
2
3
4
5
6
7
8
9
10
11
Meishan−A
Meishan−B
Meishan−C
European−A
European−B
European−C

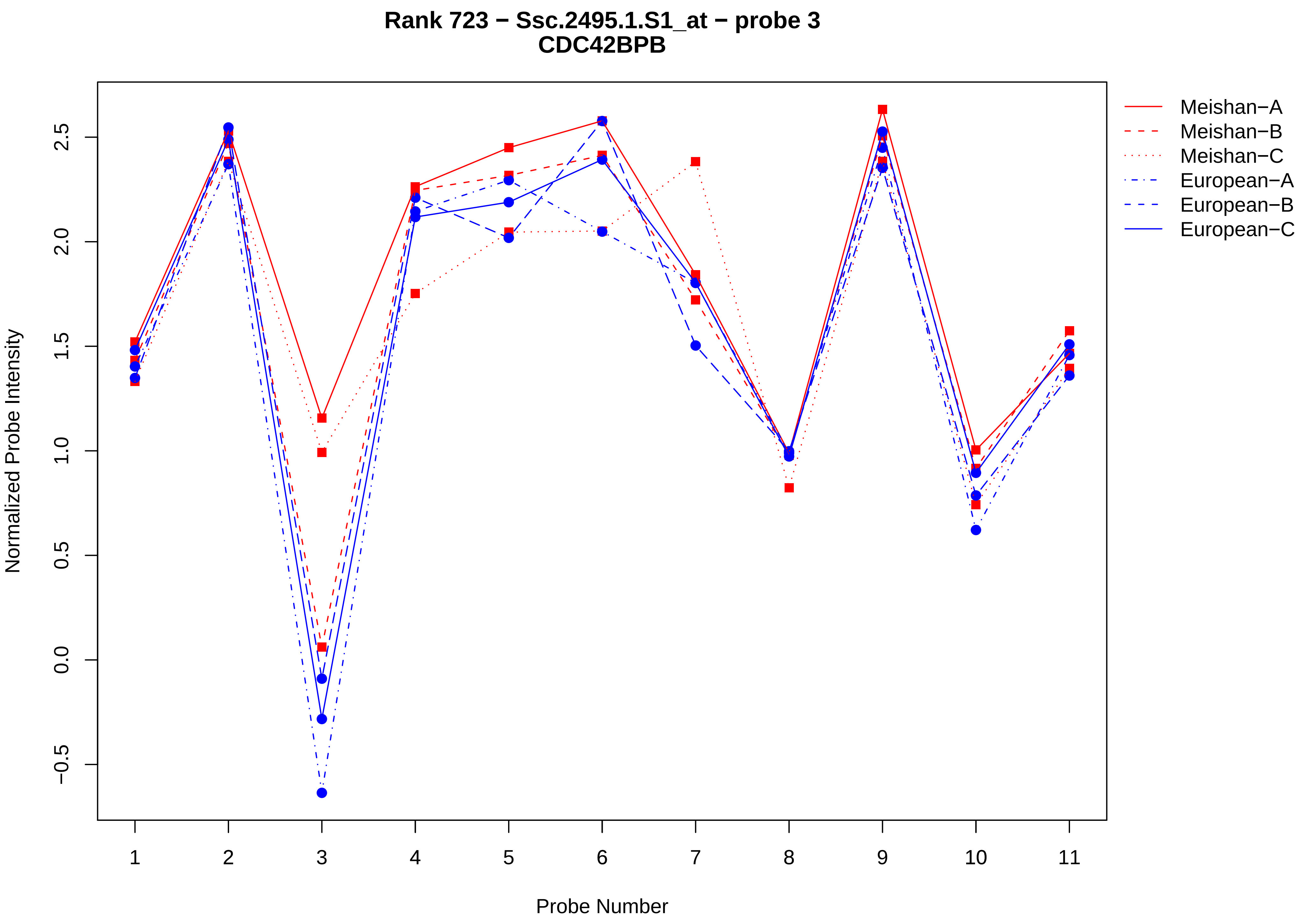

Rank 723 − Ssc.2495.1.S1_at − probe 3
CDC42BPB
Normalized Probe Intensity
Probe Number
Meishan−A
Meishan−B
Meishan−C
European−A
European−B
European−C
−0.5
0.0
0.5
1.0
1.5
2.0
2.5
1
2
3
4
5
6
7
8
9
10
11

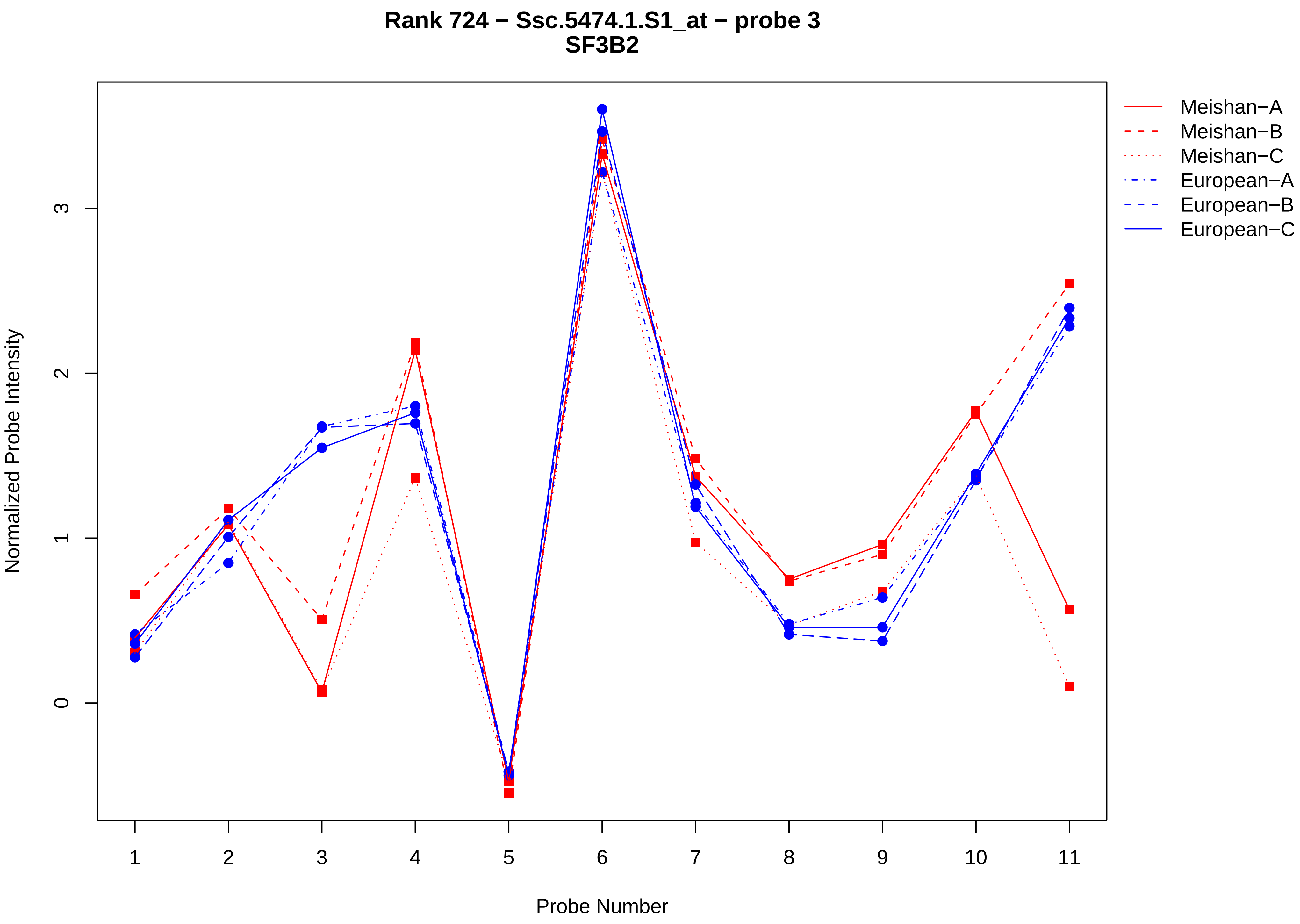
Rank 724 − Ssc.5474.1.S1_at − probe 3
SF3B2
Normalized Probe Intensity
Probe Number
0
1
2
3
1
2
3
4
5
6
7
8
9
10
11
Meishan−A
Meishan−B
Meishan−C
European−A
European−B
European−C

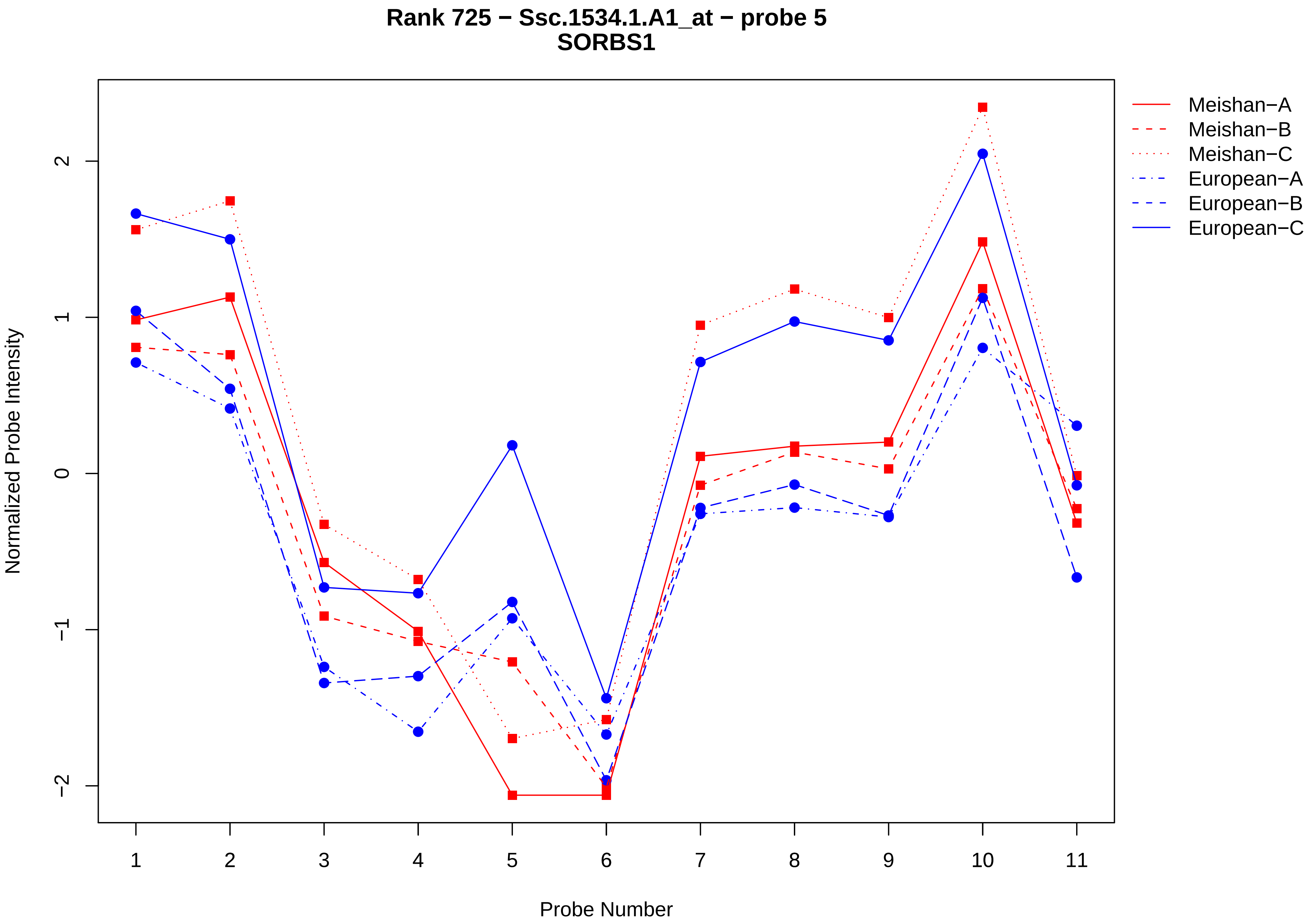

Rank 725 − Ssc.1534.1.A1_at − probe 5
SORBS1
Normalized Probe Intensity
Probe Number
Meishan−A
Meishan−B
Meishan−C
European−A
European−B
European−C
−2
−1
0
1
2
1
2
3
4
5
6
7
8
9
10
11

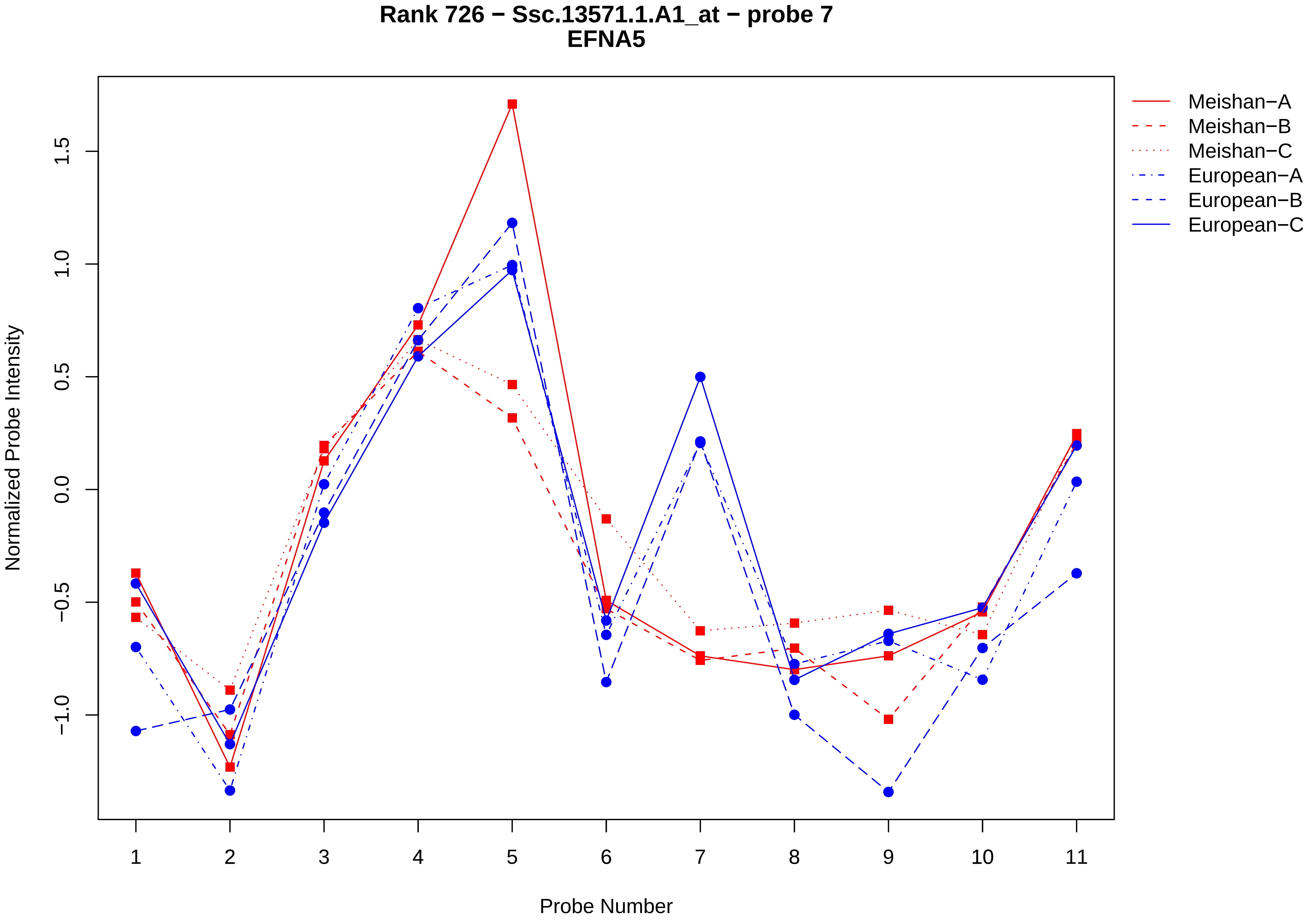

Rank 726 − Ssc.13571.1.A1_at − probe 7
EFNA5
Meishan−A
Meishan−B
Meishan−C
European−A
European−B
European−C
Normalized Probe Intensity
Probe Number
−1.0
−0.5
0.0
0.5
1.0
1.5
1
2
3
4
5
6
7
8
9
10
11

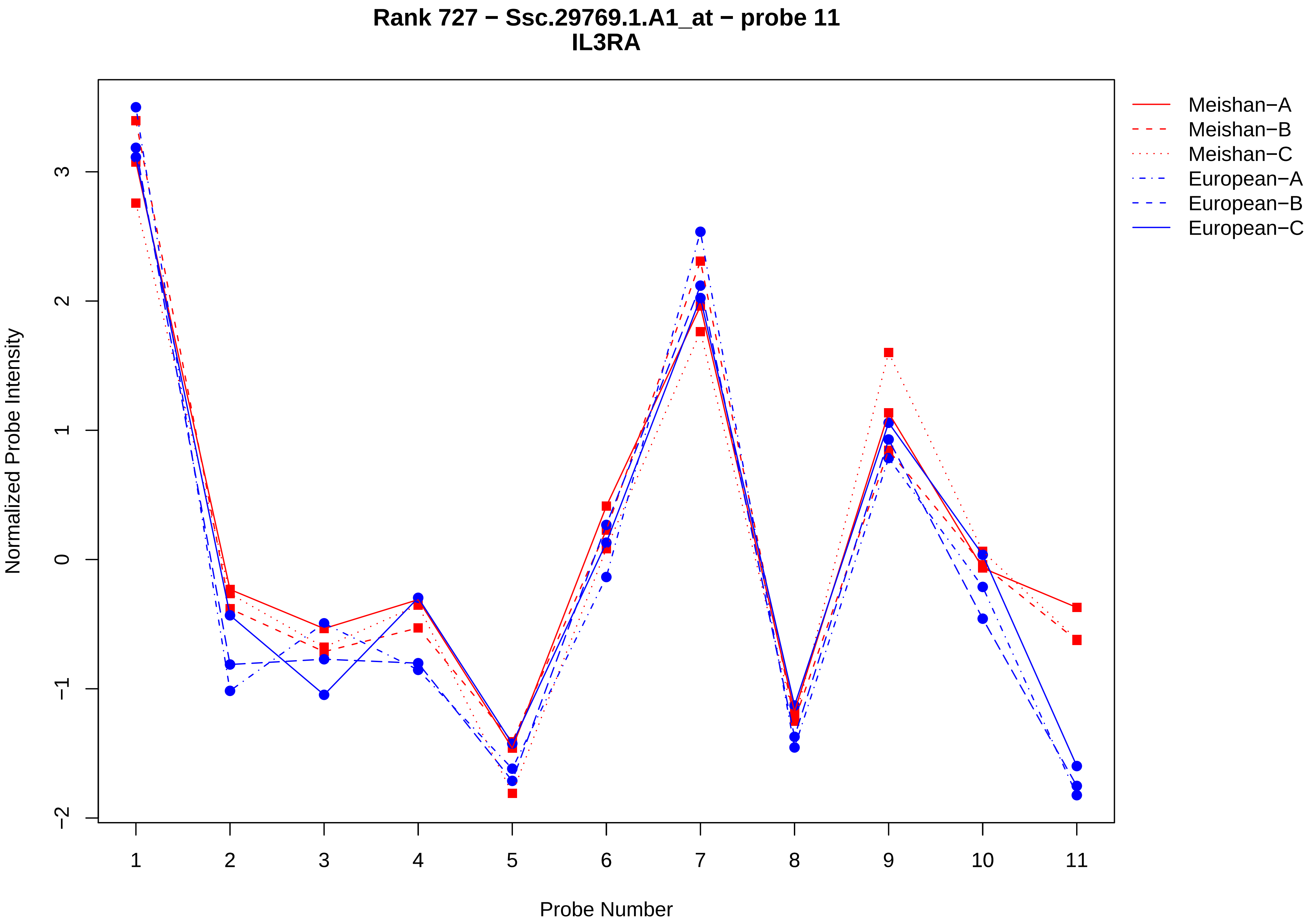

Rank 727 − Ssc.29769.1.A1_at − probe 11
IL3RA
Normalized Probe Intensity
Probe Number
−2
−1
0
1
2
3
1
2
3
4
5
6
7
8
9
10
11
Meishan−A
Meishan−B
Meishan−C
European−A
European−B
European−C

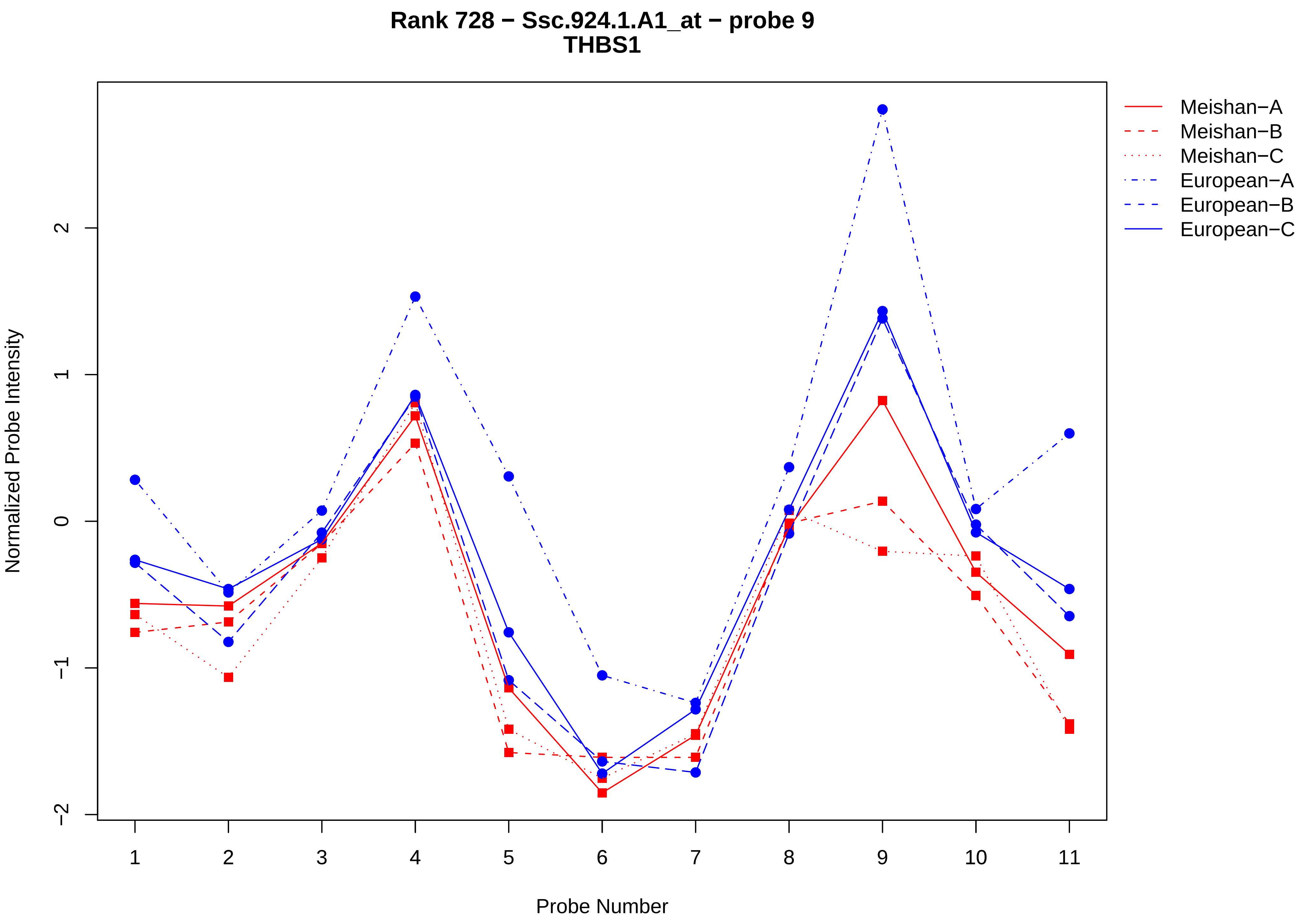

Rank 728 − Ssc.924.1.A1_at − probe 9
THBS1
Normalized Probe Intensity
Probe Number
−2
−1
0
1
2
1
2
3
4
5
6
7
8
9
10
11
Meishan−A
Meishan−B
Meishan−C
European−A
European−B
European−C

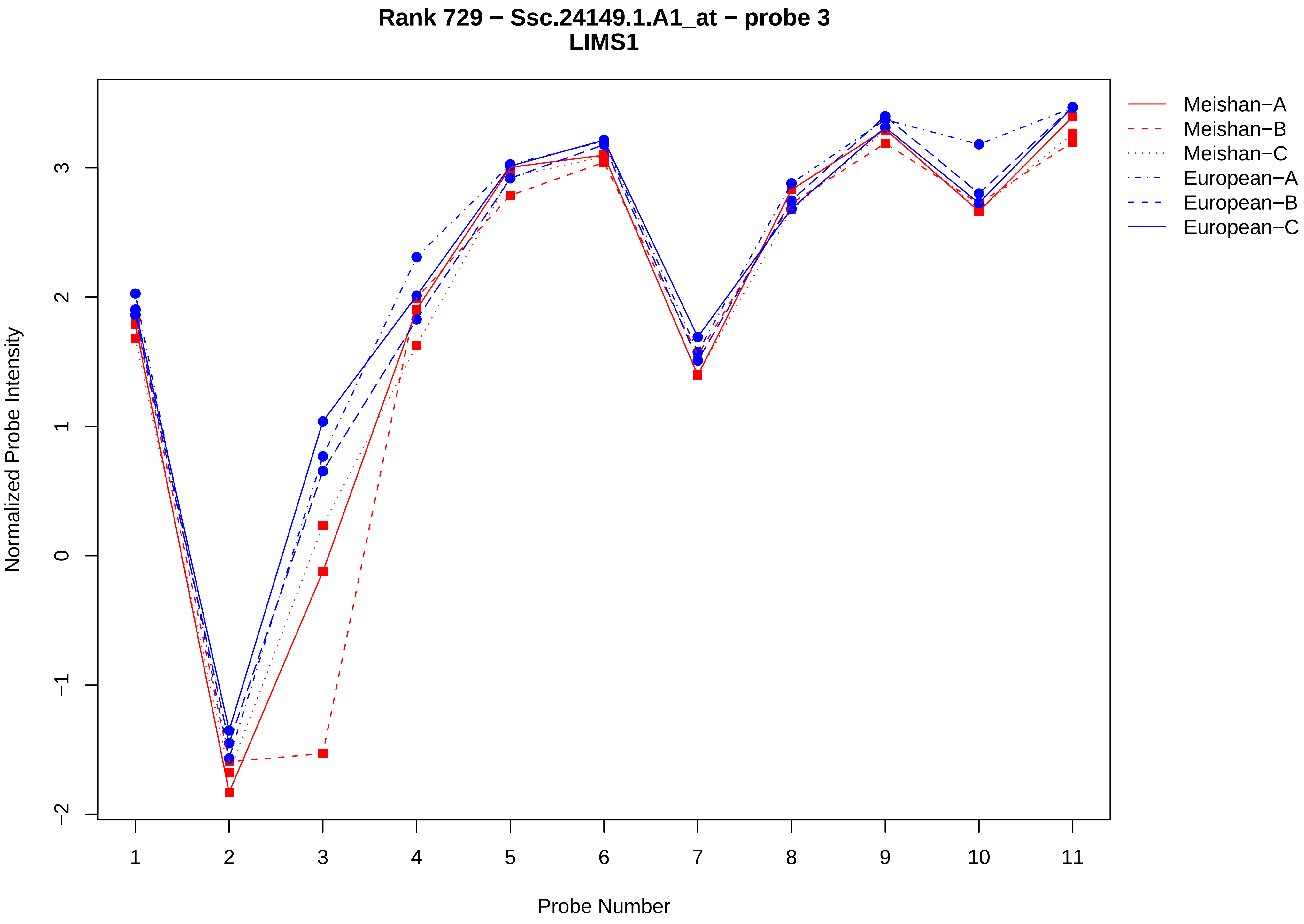

Rank 729 − Ssc.24149.1.A1_at − probe 3
LIMS1
Normalized Probe Intensity
Probe Number
−2
−1
0
1
2
3
1
2
3
4
5
6
7
8
9
10
11
Meishan−A
Meishan−B
Meishan−C
European−A
European−B
European−C

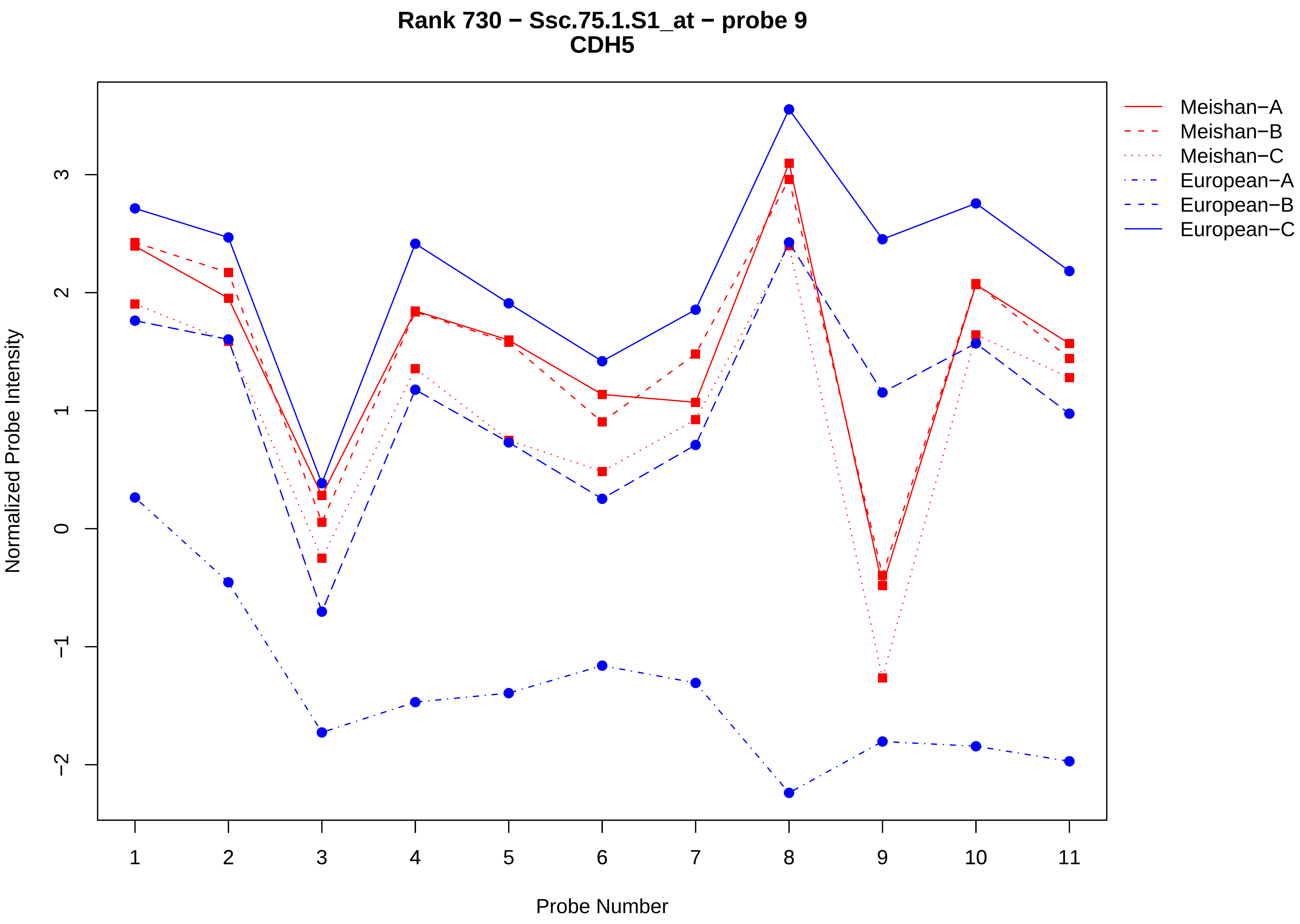

Rank 730 − Ssc.75.1.S1_at − probe 9
CDH5
Normalized Probe Intensity
Probe Number
Meishan−A
Meishan−B
Meishan−C
European−A
European−B
European−C

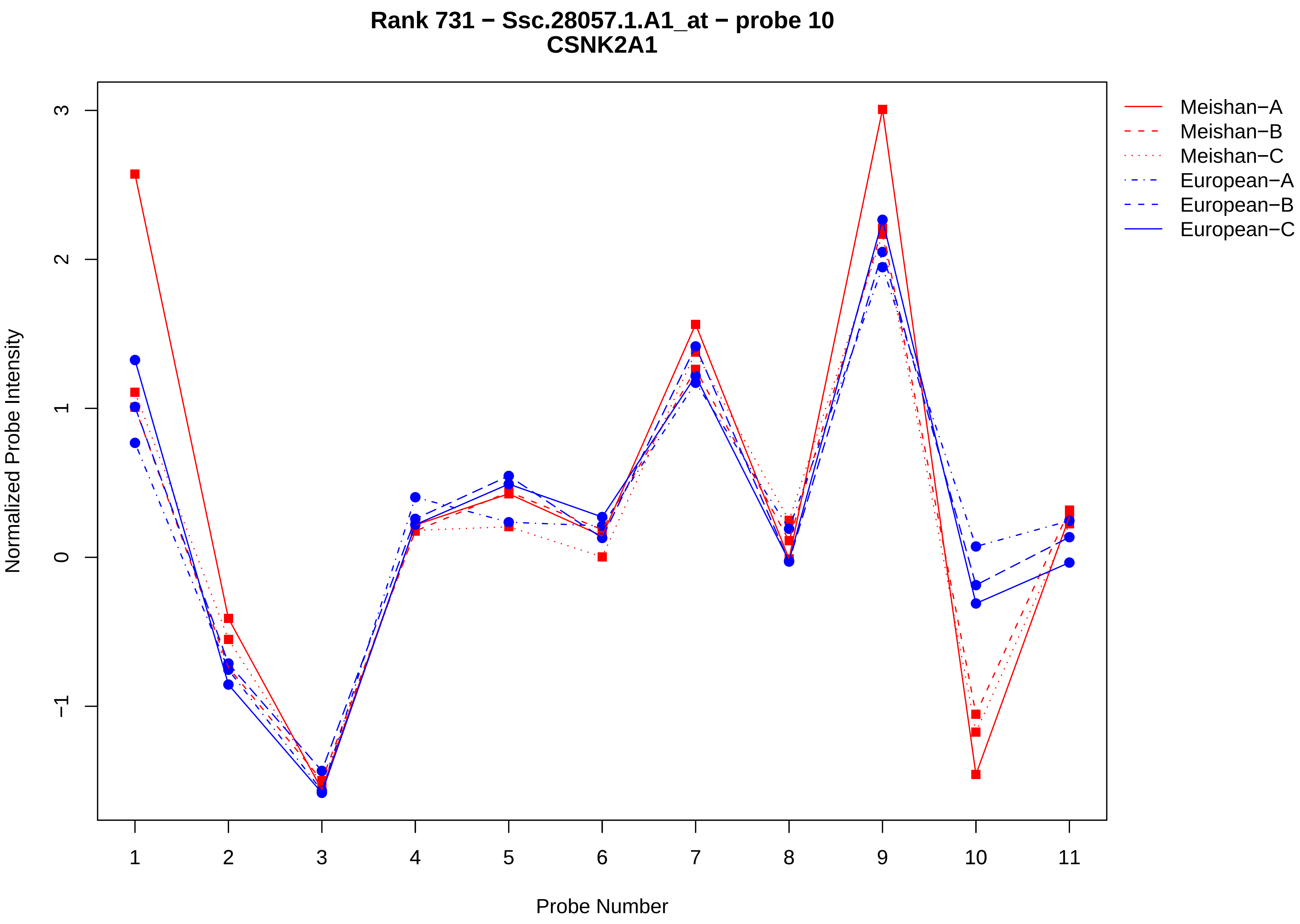

Rank 731 − Ssc.28057.1.A1_at − probe 10
CSNK2A1
Normalized Probe Intensity
Probe Number
−1
0
1
2
3
1
2
3
4
5
6
7
8
9
10
11
Meishan−A
Meishan−B
Meishan−C
European−A
European−B
European−C

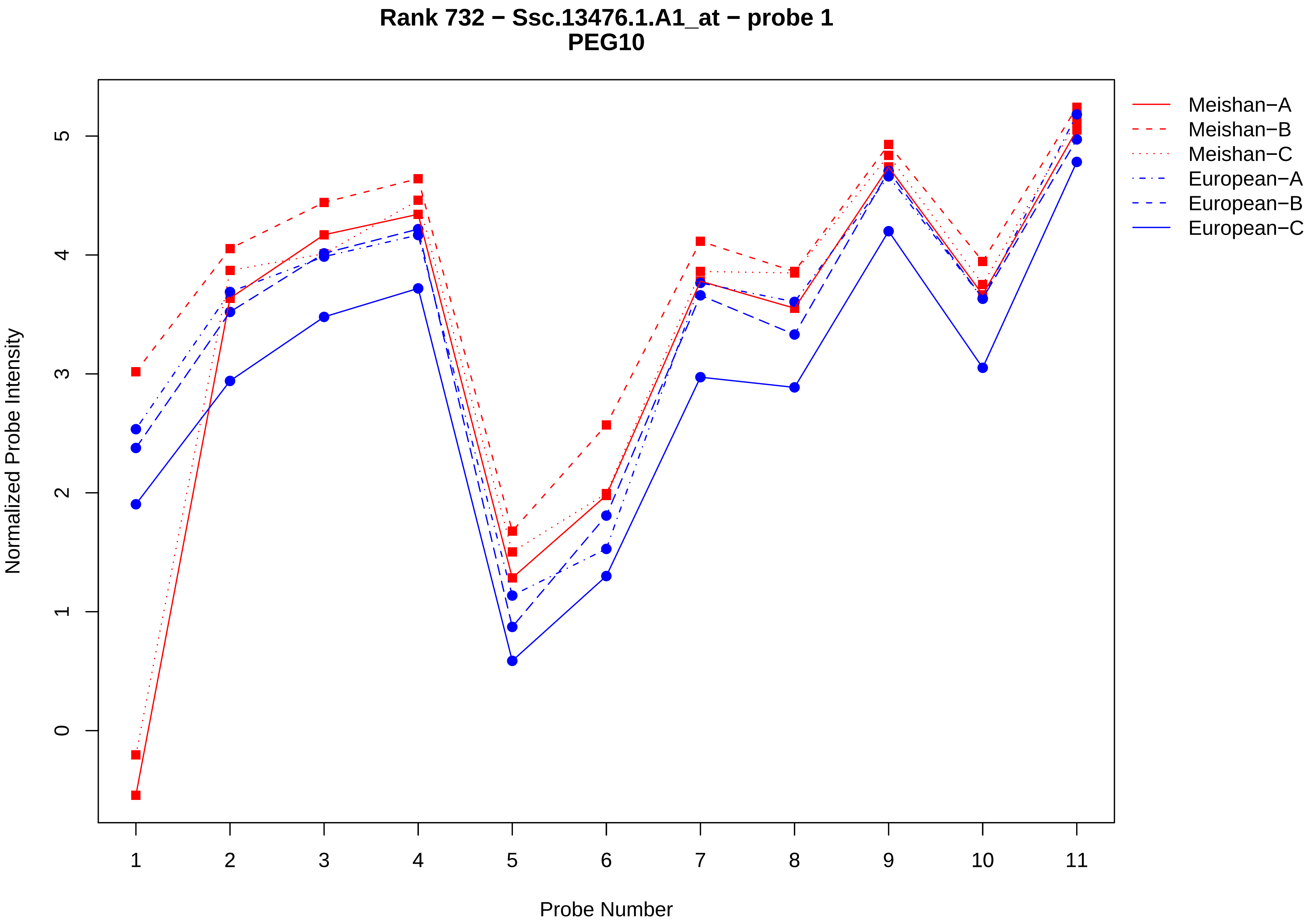

Rank 732 − Ssc.13476.1.A1_at − probe 1
PEG10
Normalized Probe Intensity
Probe Number
0
1
2
3
4
5
1
2
3
4
5
6
7
8
9
10
11
Meishan−A
Meishan−B
Meishan−C
European−A
European−B
European−C

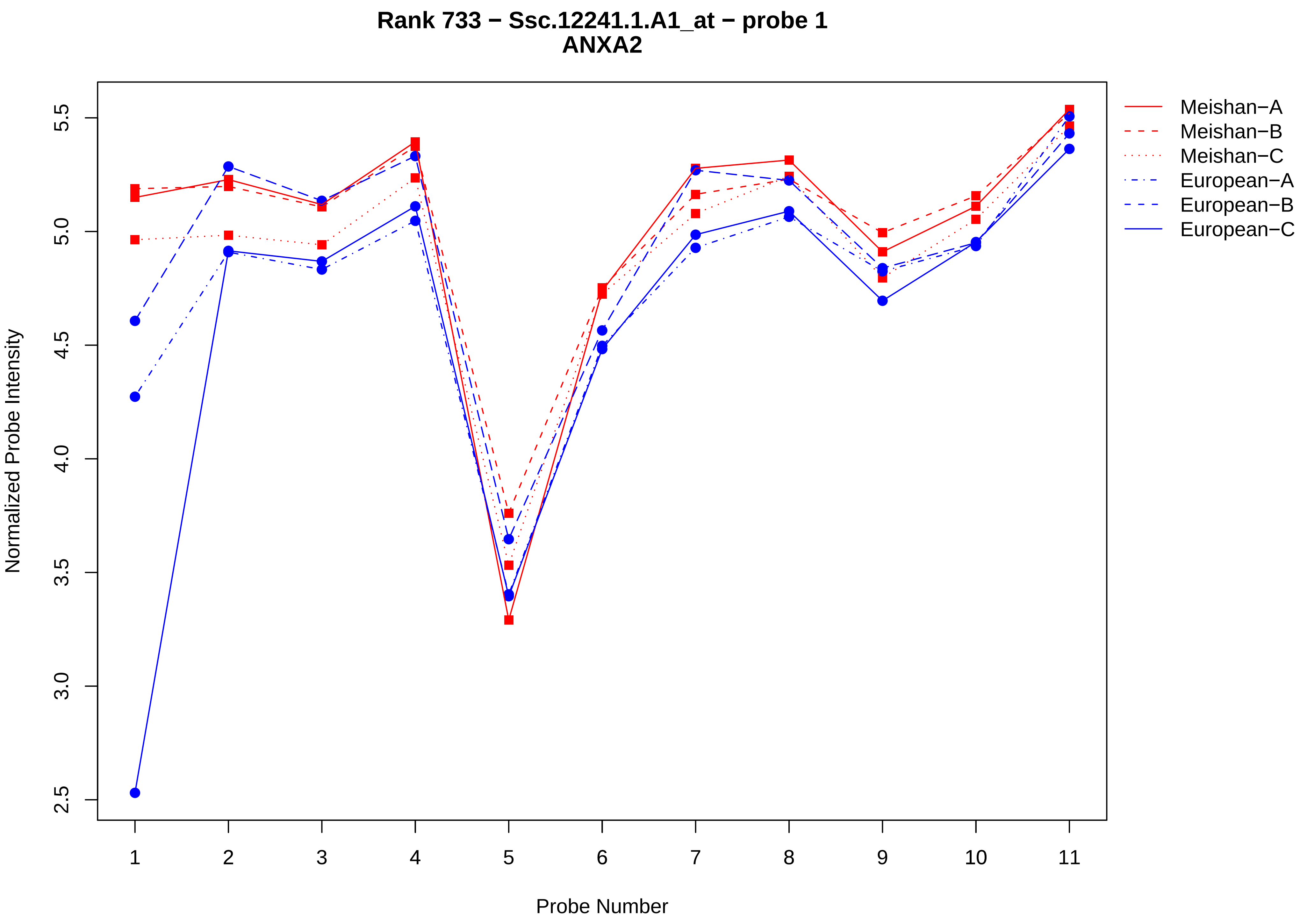

Rank 733 − Ssc.12241.1.A1_at − probe 1
ANXA2
Normalized Probe Intensity
2.5
3.0
3.5
4.0
4.5
5.0
5.5
Probe Number
1
2
3
4
5
6
7
8
9
10
11
Meishan−A
Meishan−B
Meishan−C
European−A
European−B
European−C

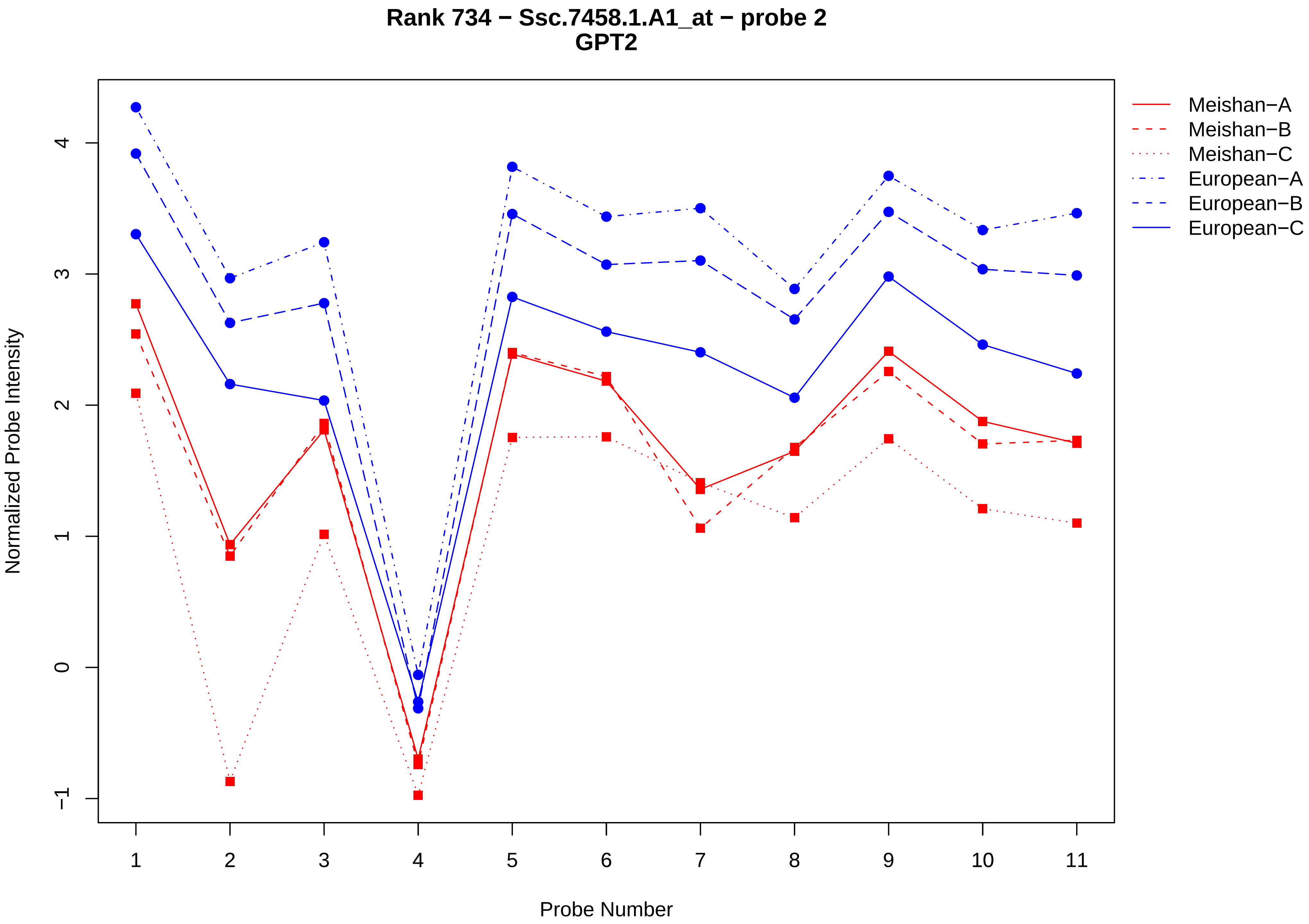
Rank 734 − Ssc.7458.1.A1_at − probe 2
GPT2
Normalized Probe Intensity
Probe Number
−1
0
1
2
3
4
1
2
3
4
5
6
7
8
9
10
11
Meishan−A
Meishan−B
Meishan−C
European−A
European−B
European−C

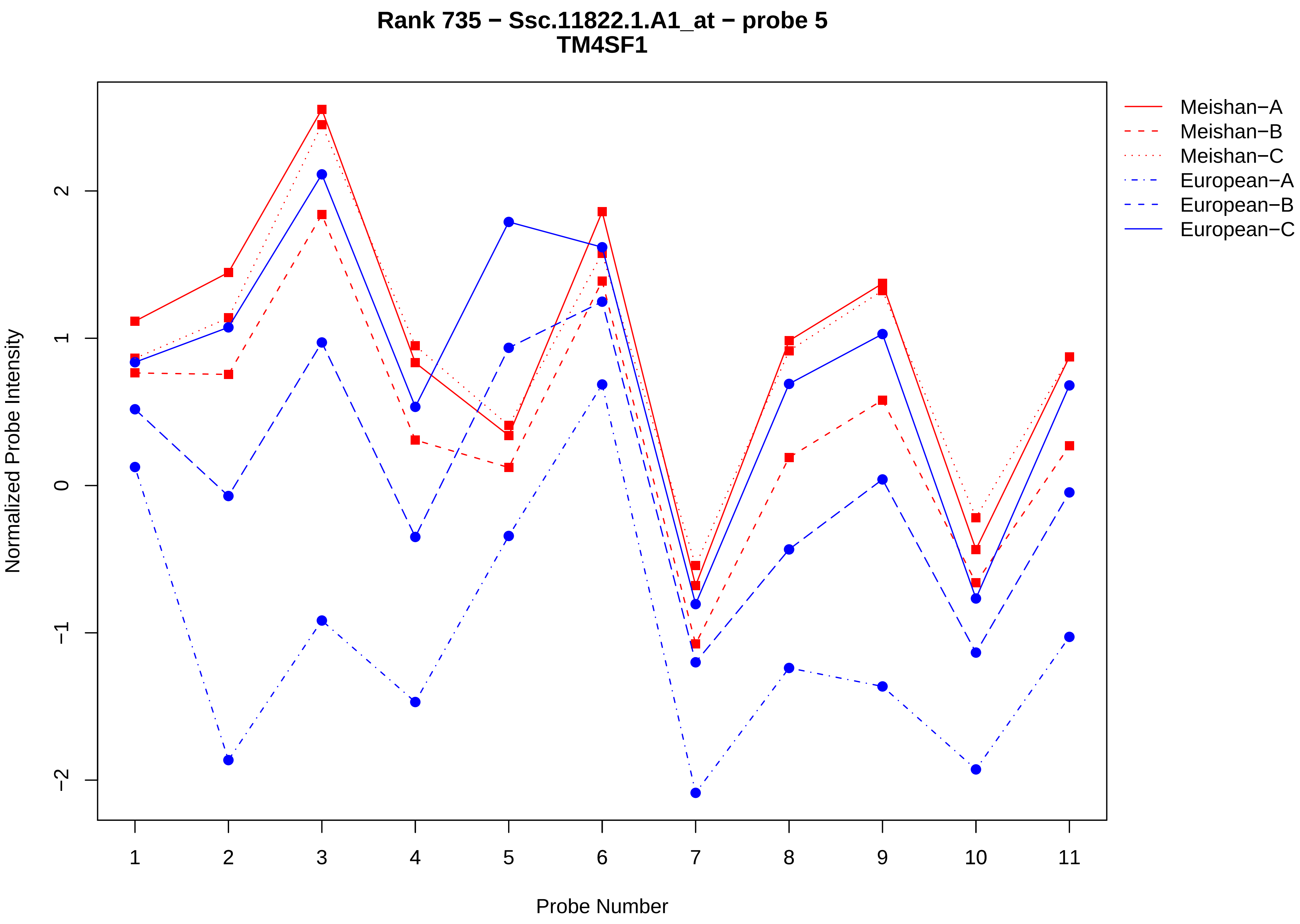

Rank 735 − Ssc.11822.1.A1_at − probe 5
TM4SF1
Normalized Probe Intensity
Probe Number
−2
−1
0
1
2
1
2
3
4
5
6
7
8
9
10
11
Meishan−A
Meishan−B
Meishan−C
European−A
European−B
European−C

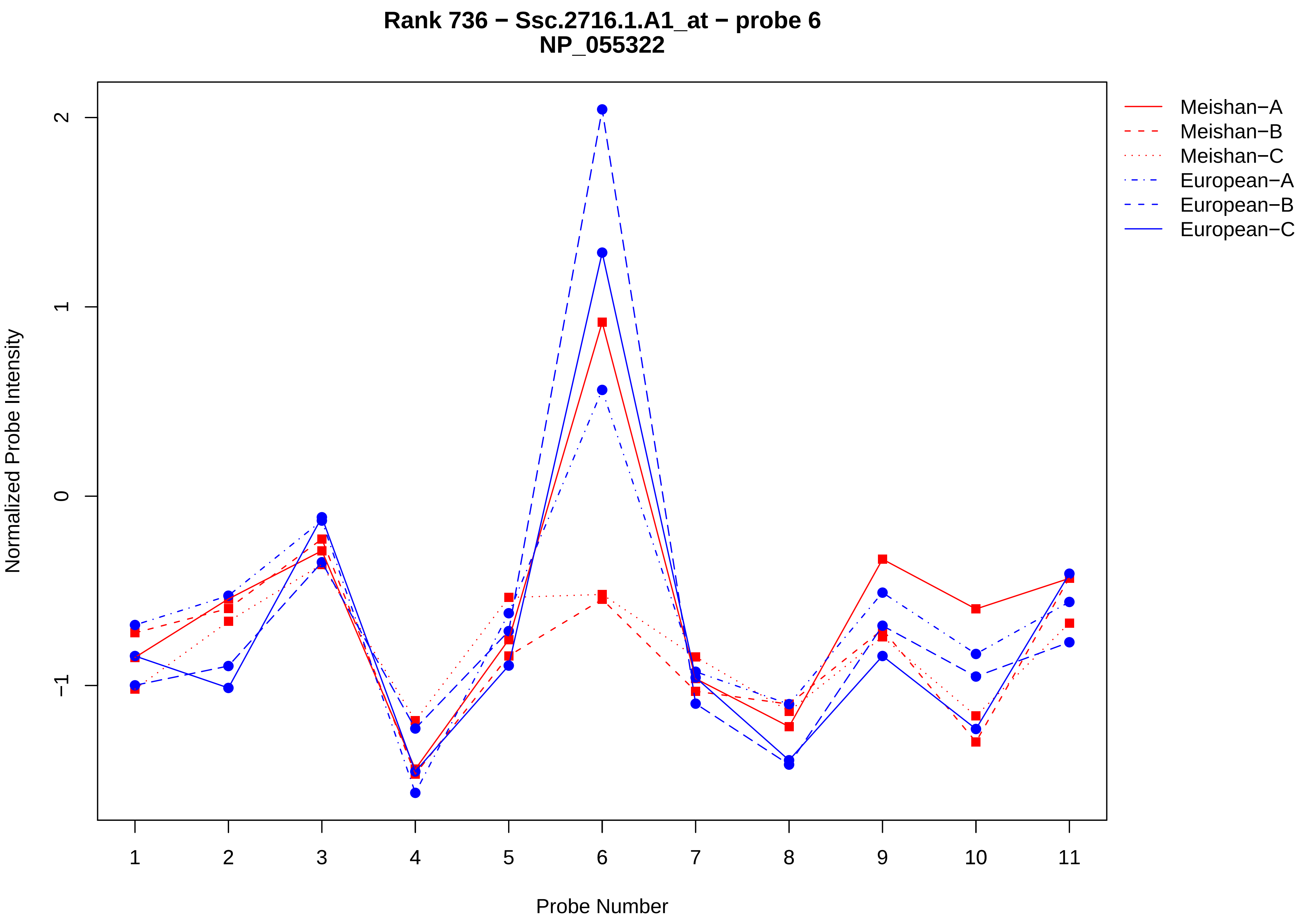

Rank 736 − Ssc.2716.1.A1_at − probe 6
NP_055322
Normalized Probe Intensity
Probe Number
Meishan−A
Meishan−B
Meishan−C
European−A
European−B
European−C
−1
0
1
2
1
2
3
4
5
6
7
8
9
10
11

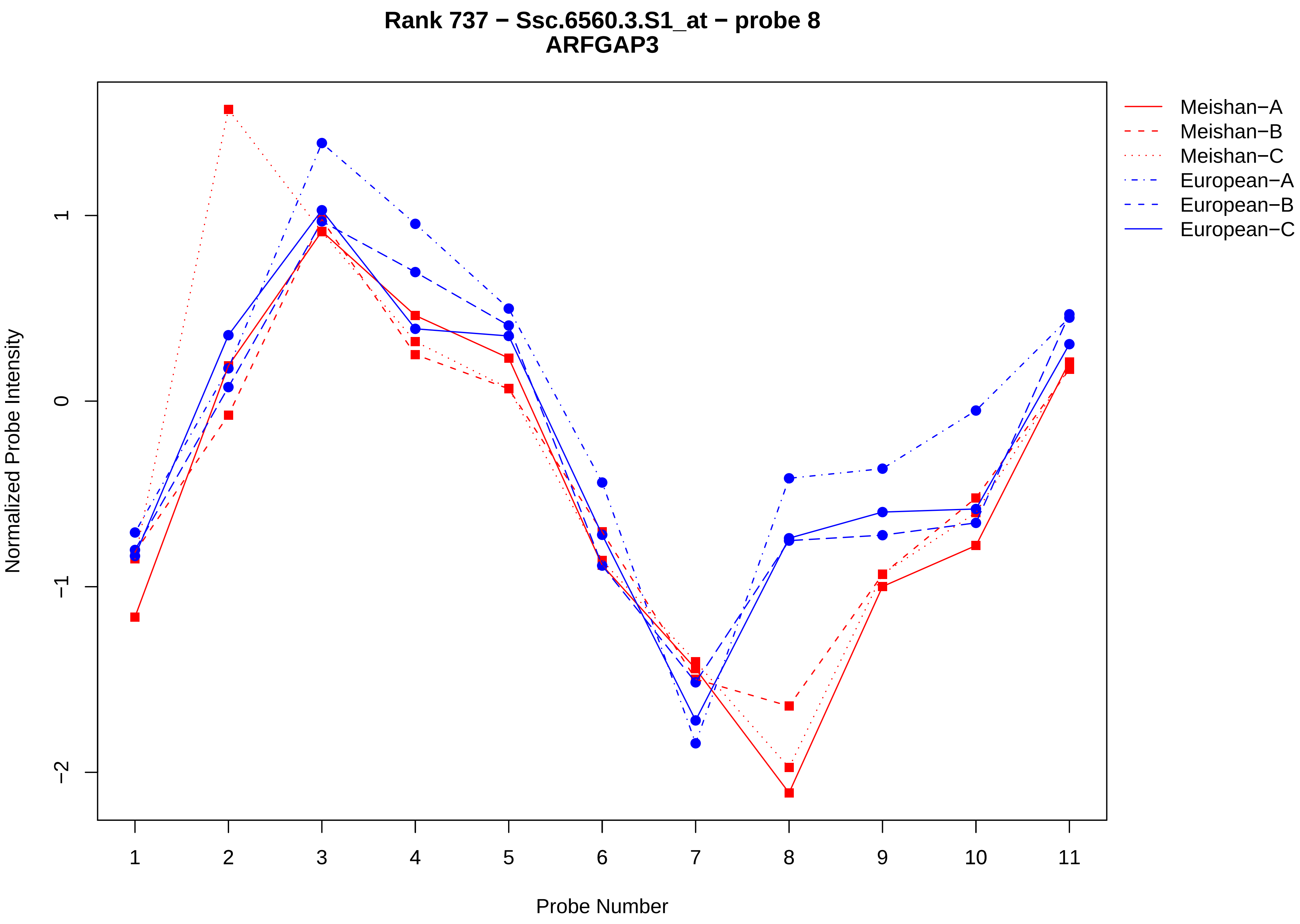

Rank 737 − Ssc.6560.3.S1_at − probe 8
ARFGAP3
Normalized Probe Intensity
Probe Number
−2
−1
0
1
1
2
3
4
5
6
7
8
9
10
11
Meishan−A
Meishan−B
Meishan−C
European−A
European−B
European−C

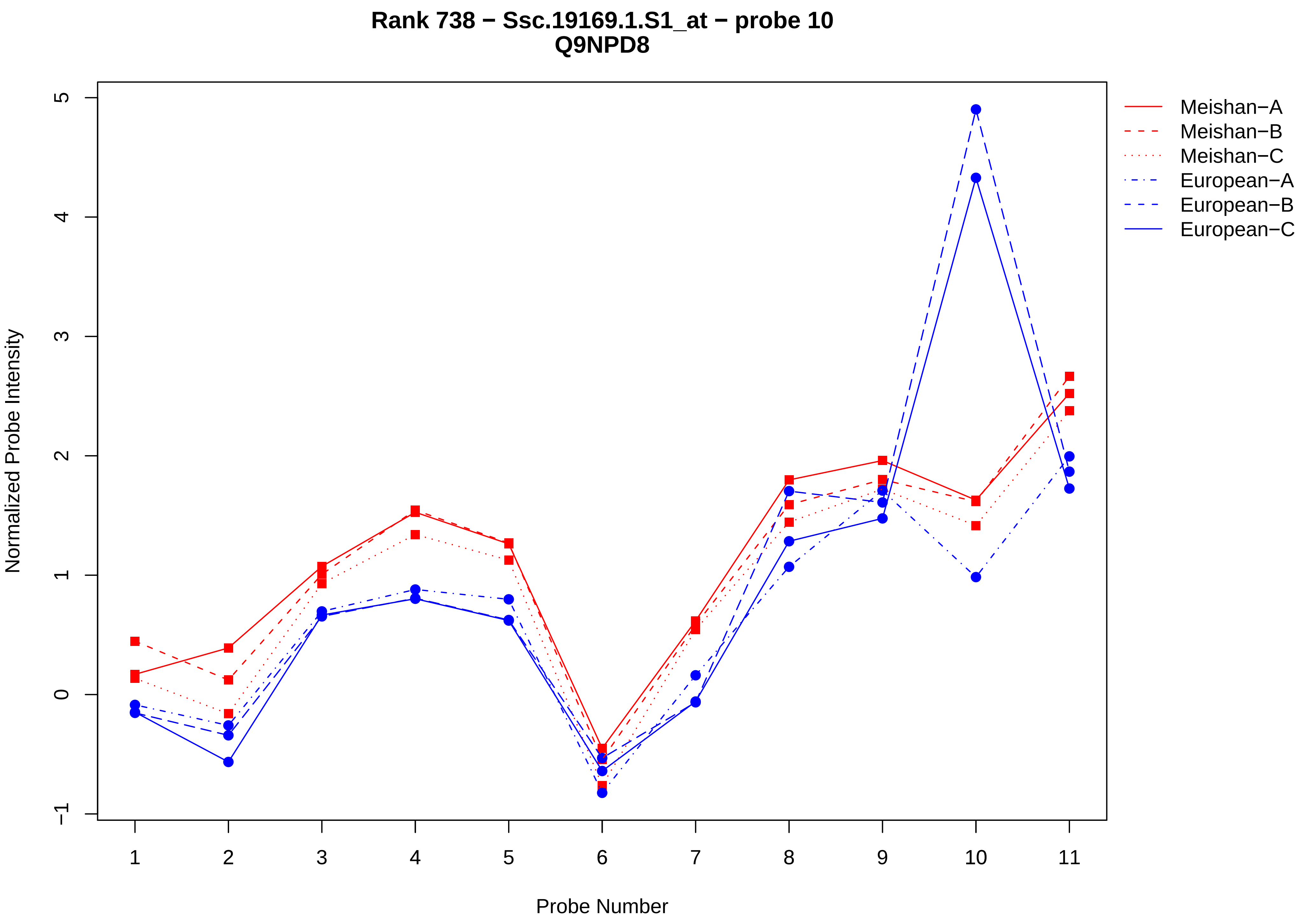
Rank 738 − Ssc.19169.1.S1_at − probe 10
Q9NPD8
Normalized Probe Intensity
Probe Number
−1
0
1
2
3
4
5
1
2
3
4
5
6
7
8
9
10
11
Meishan−A
Meishan−B
Meishan−C
European−A
European−B
European−C

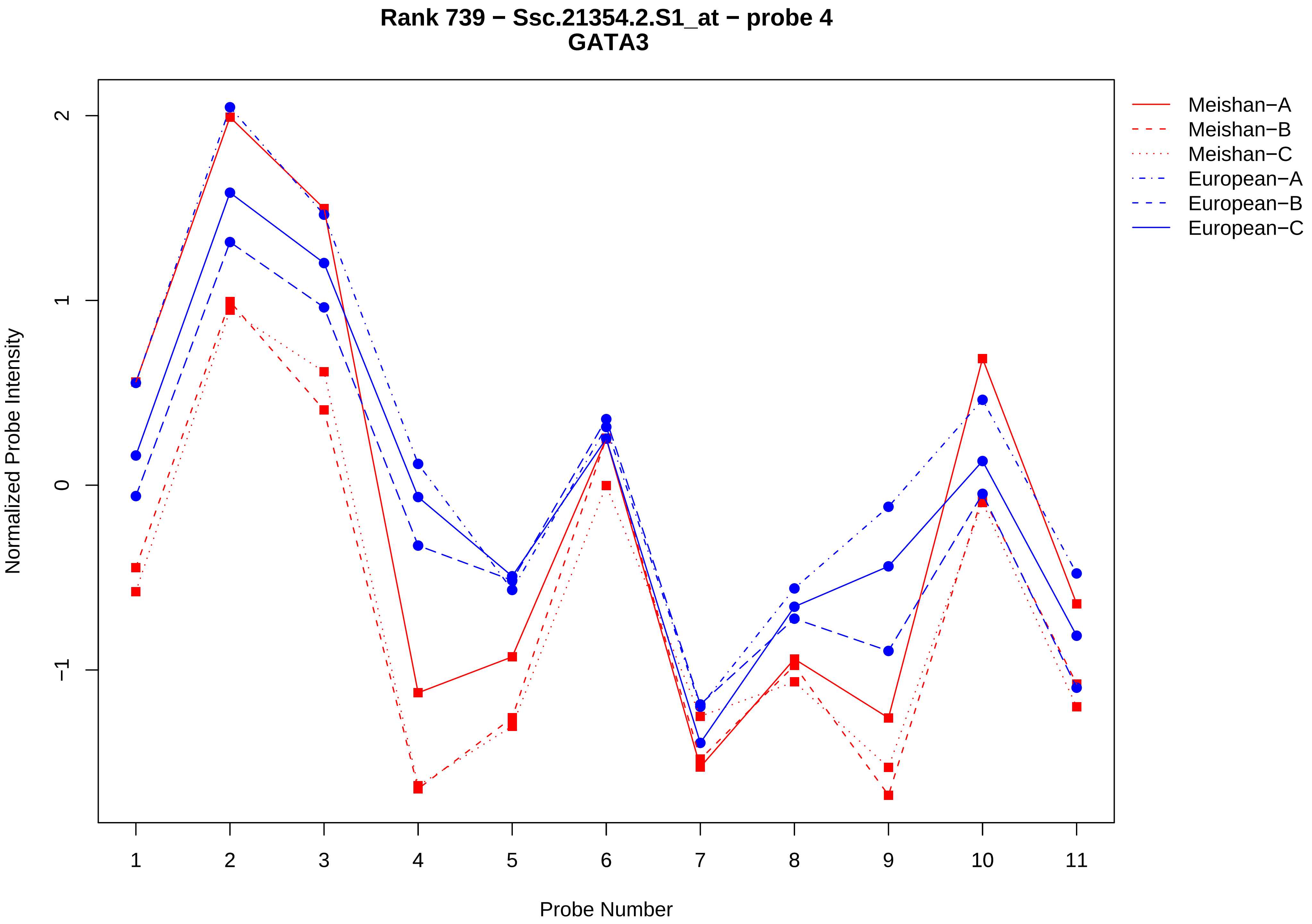

Rank 739 − Ssc.21354.2.S1_at − probe 4
GATA3
Normalized Probe Intensity
Probe Number
2
1
0
−1
1
2
3
4
5
6
7
8
9
10
11
Meishan−A
Meishan−B
Meishan−C
European−A
European−B
European−C

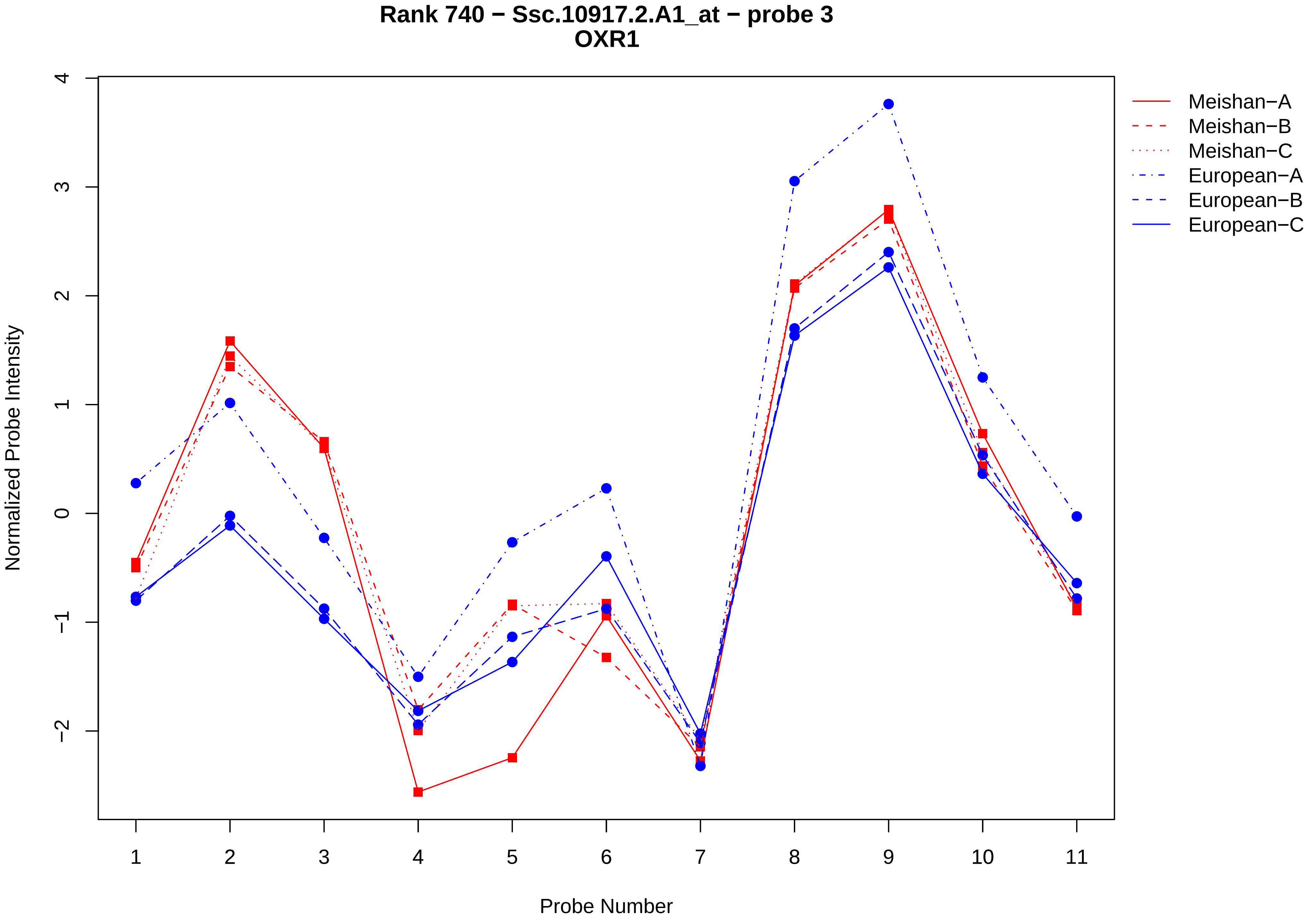

Rank 740 − Ssc.10917.2.A1_at − probe 3
OXR1
Normalized Probe Intensity
Probe Number
Meishan−A
Meishan−B
Meishan−C
European−A
European−B
European−C

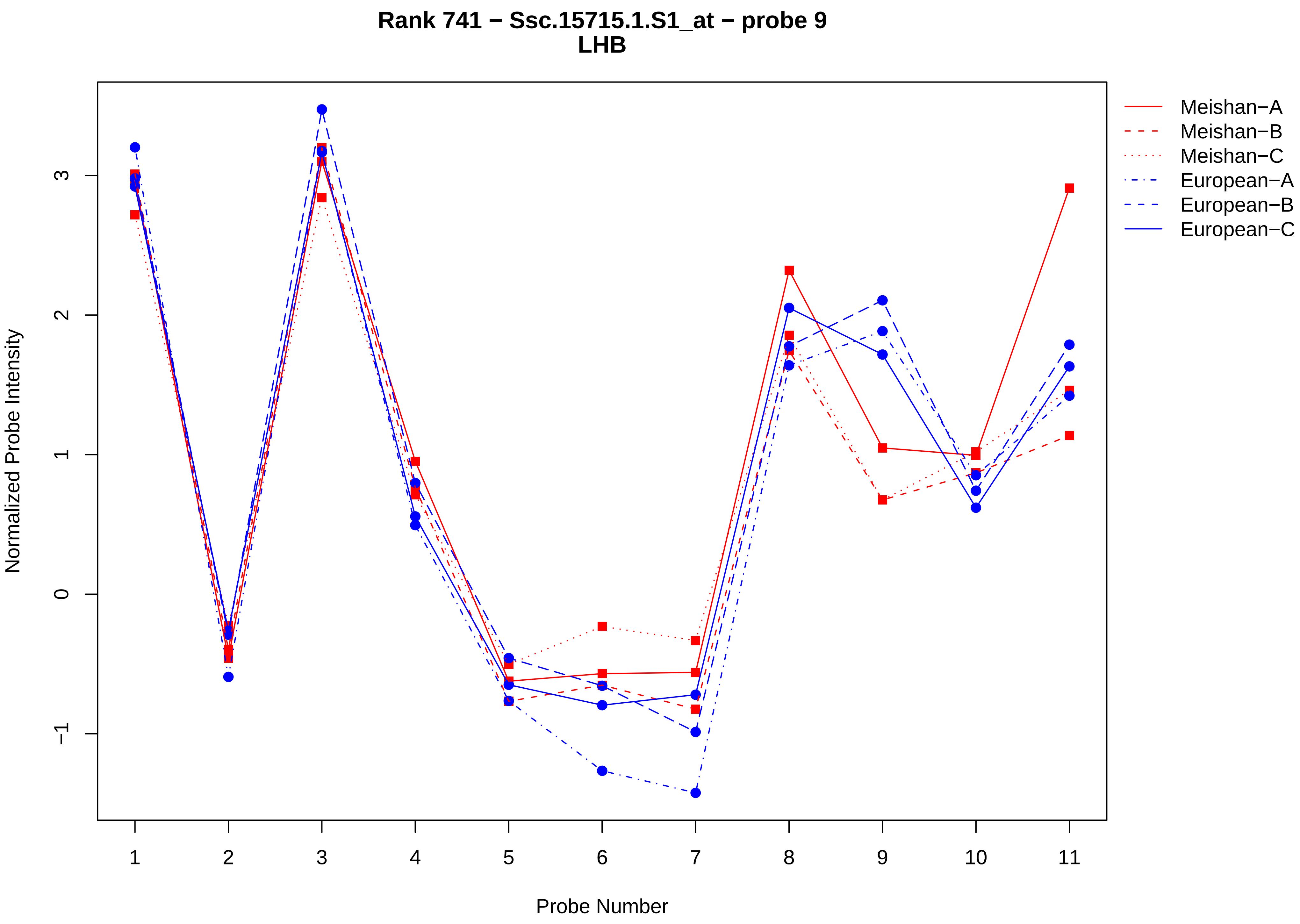
Rank 741 − Ssc.15715.1.S1_at − probe 9
LHB
Normalized Probe Intensity
Probe Number
−1
0
1
2
3
1
2
3
4
5
6
7
8
9
10
11
Meishan−A
Meishan−B
Meishan−C
European−A
European−B
European−C

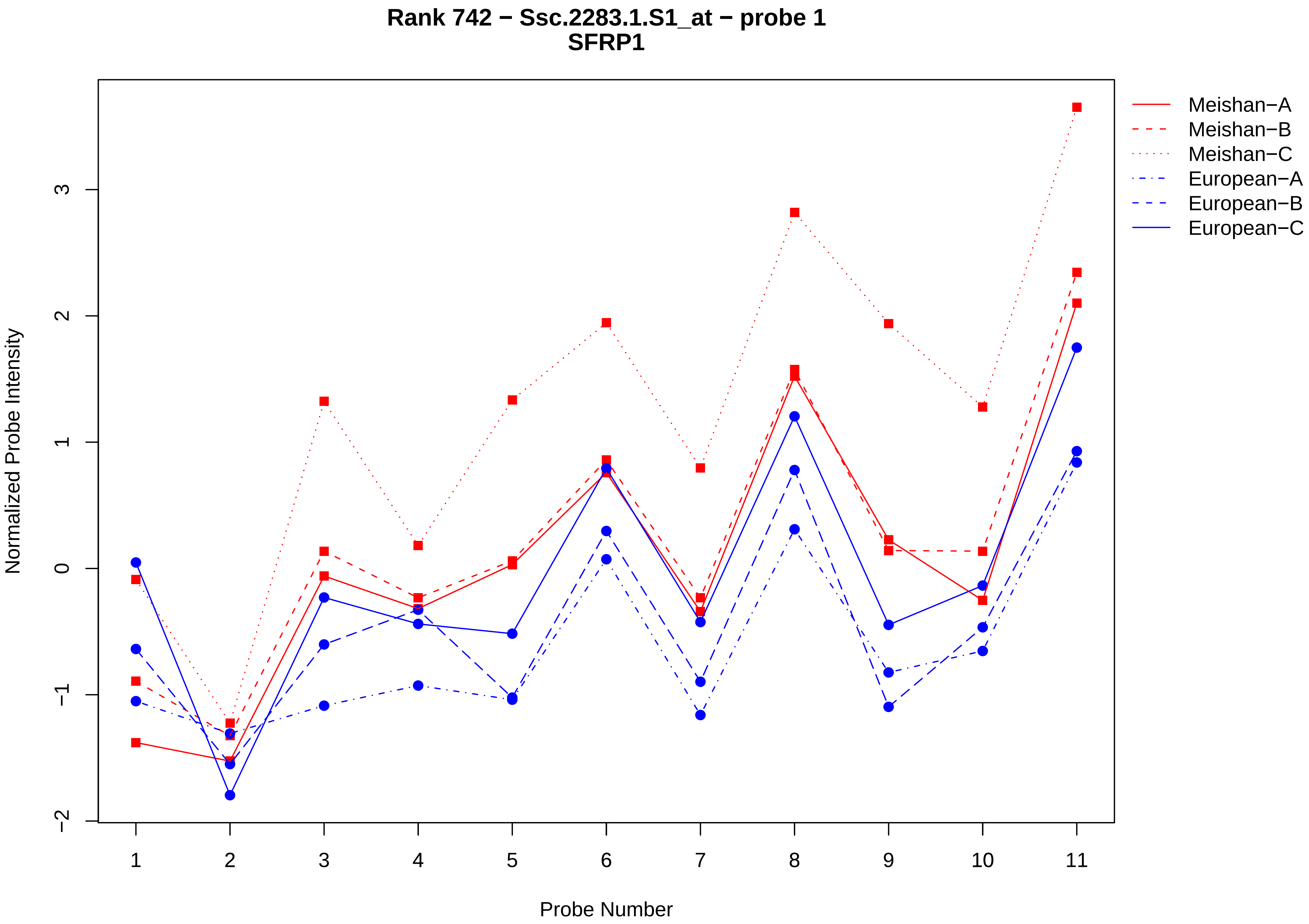

Rank 742 − Ssc.2283.1.S1_at − probe 1
SFRP1
Normalized Probe Intensity
Probe Number
−2
−1
0
1
2
3
1
2
3
4
5
6
7
8
9
10
11
Meishan−A
Meishan−B
Meishan−C
European−A
European−B
European−C

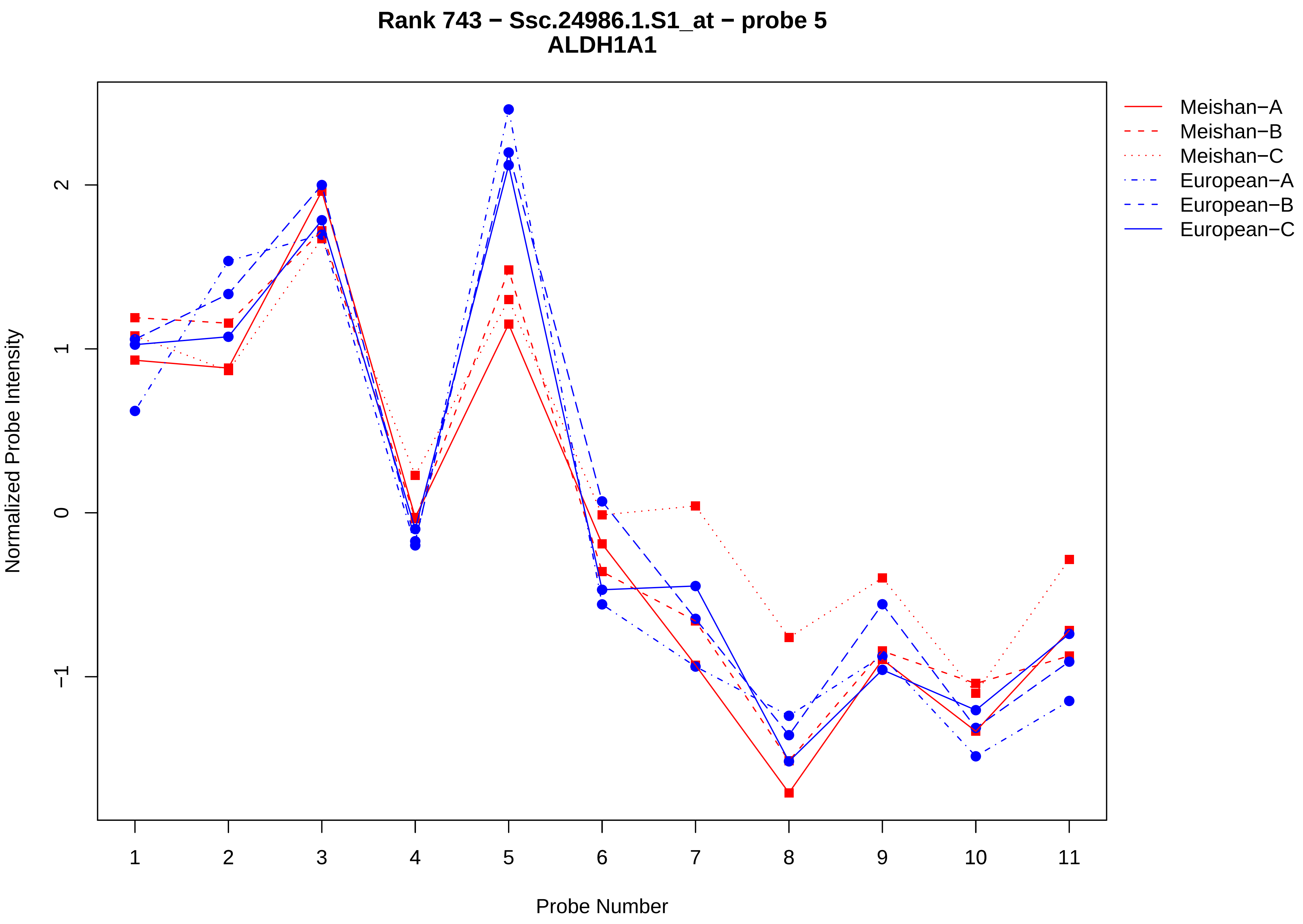
Rank 743 − Ssc.24986.1.S1_at − probe 5
ALDH1A1
Normalized Probe Intensity
Probe Number
−1
0
1
2
1
2
3
4
5
6
7
8
9
10
11
Meishan−A
Meishan−B
Meishan−C
European−A
European−B
European−C

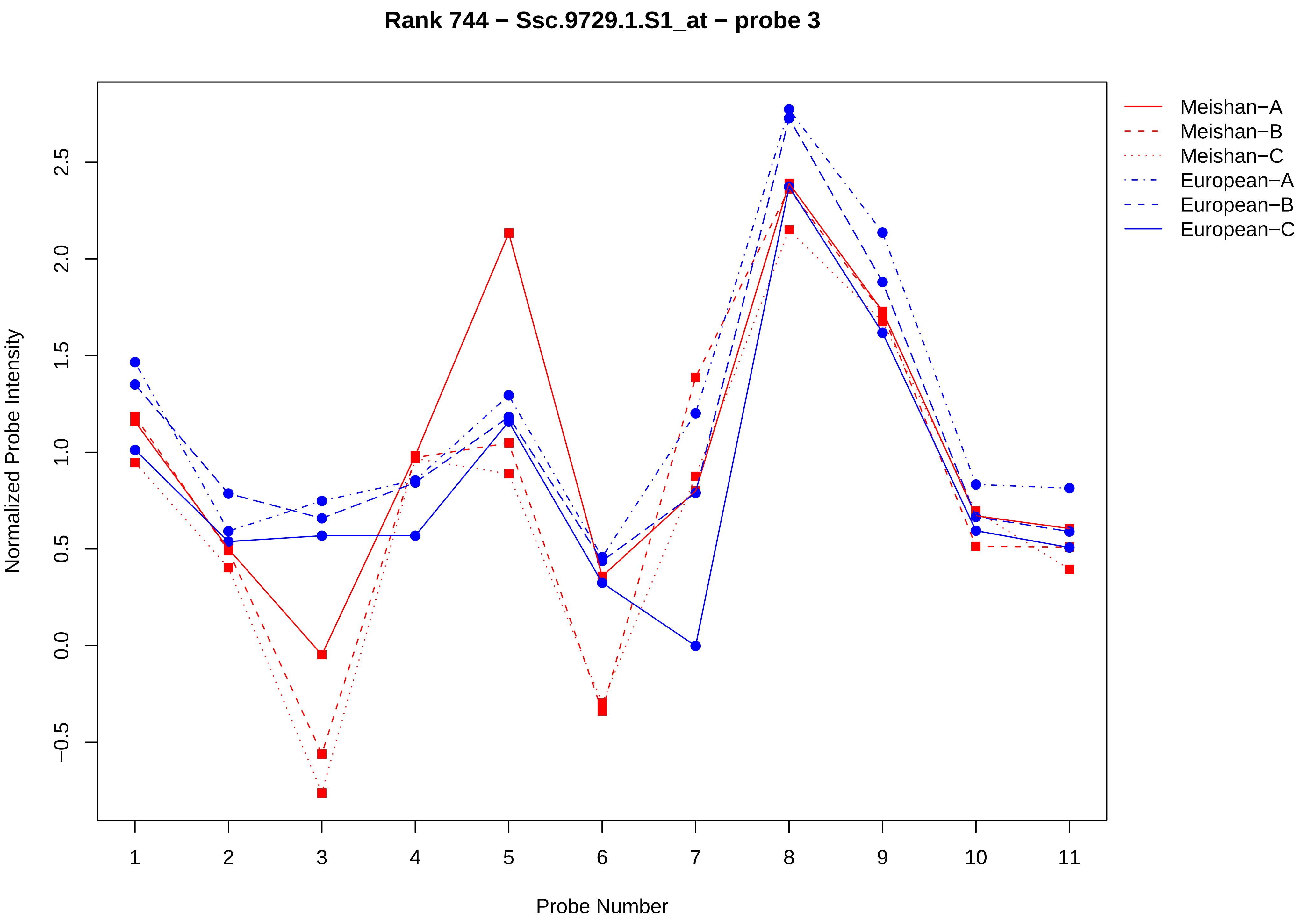

Rank 744 − Ssc.9729.1.S1_at − probe 3
Meishan−A
Meishan−B
Meishan−C
European−A
European−B
European−C
Normalized Probe Intensity
−0.5
0.0
0.5
1.0
1.5
2.0
2.5
1
2
3
4
5
6
7
8
9
10
11
Probe Number

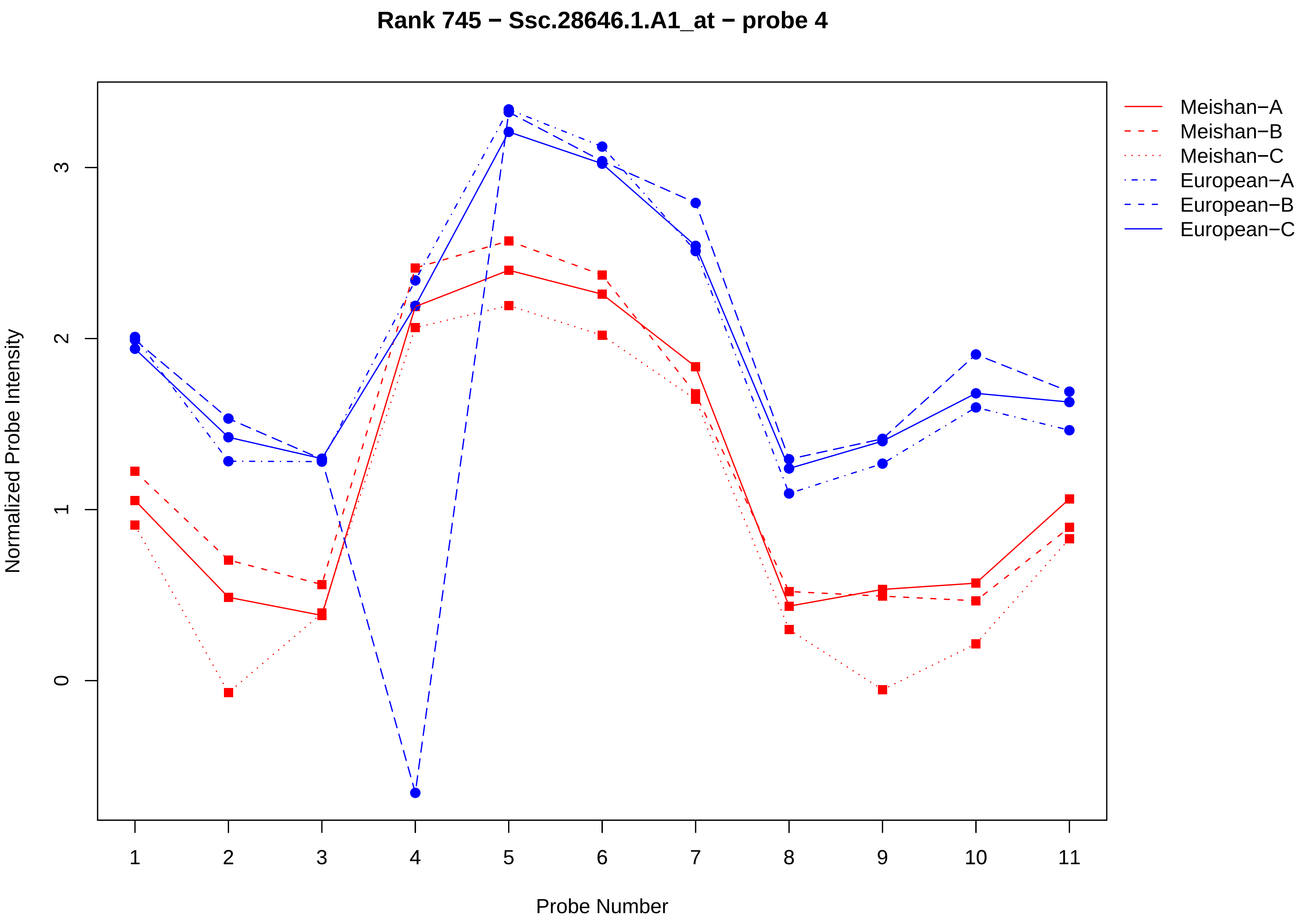
Rank 745 − Ssc.28646.1.A1_at − probe 4
Normalized Probe Intensity
0
1
2
3
1
2
3
4
5
6
7
8
9
10
11
Probe Number
Meishan−A
Meishan−B
Meishan−C
European−A
European−B
European−C

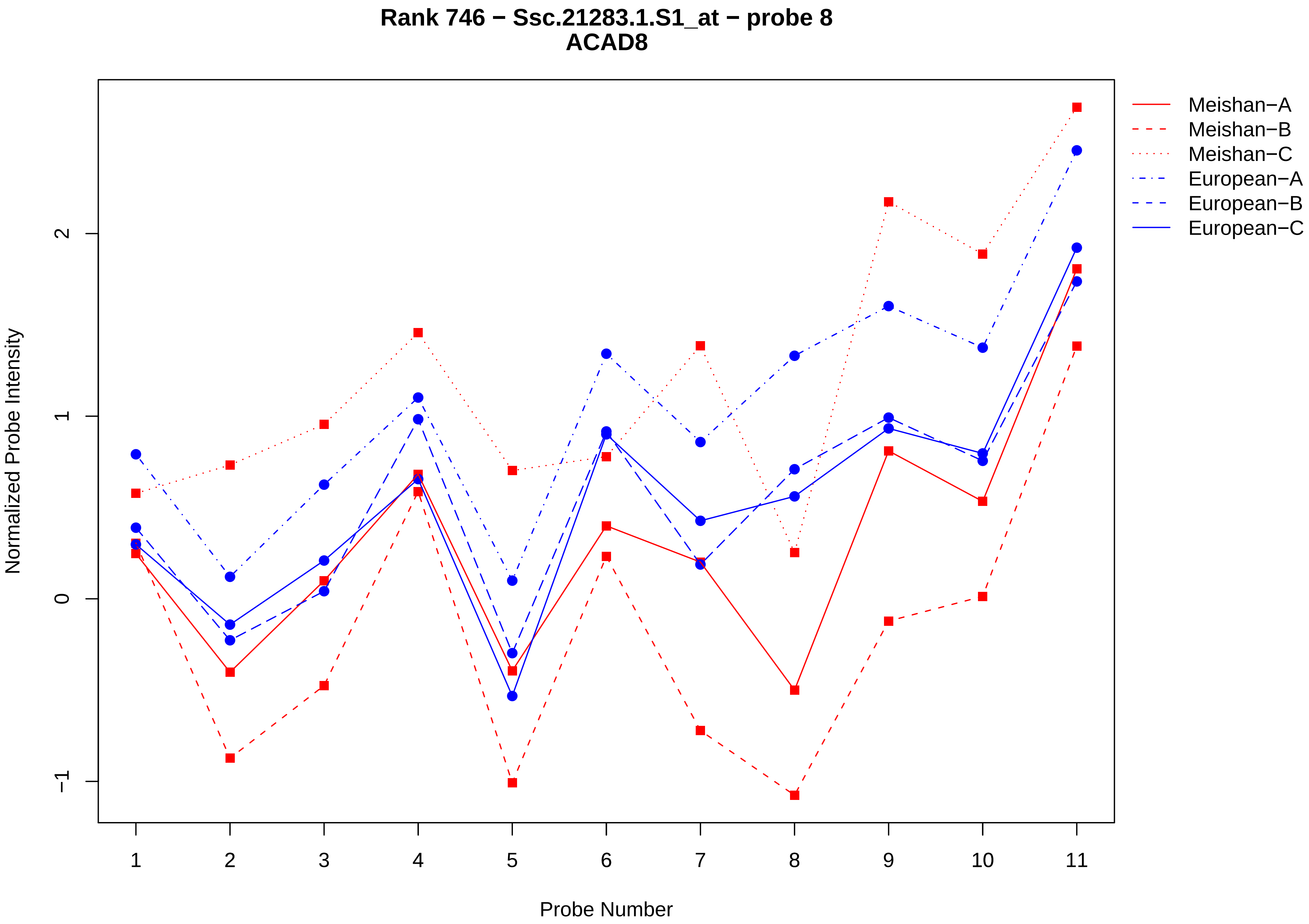
Rank 746 − Ssc.21283.1.S1_at − probe 8
ACAD8
Normalized Probe Intensity
Probe Number
−1
0
1
2
1
2
3
4
5
6
7
8
9
10
11
Meishan−A
Meishan−B
Meishan−C
European−A
European−B
European−C

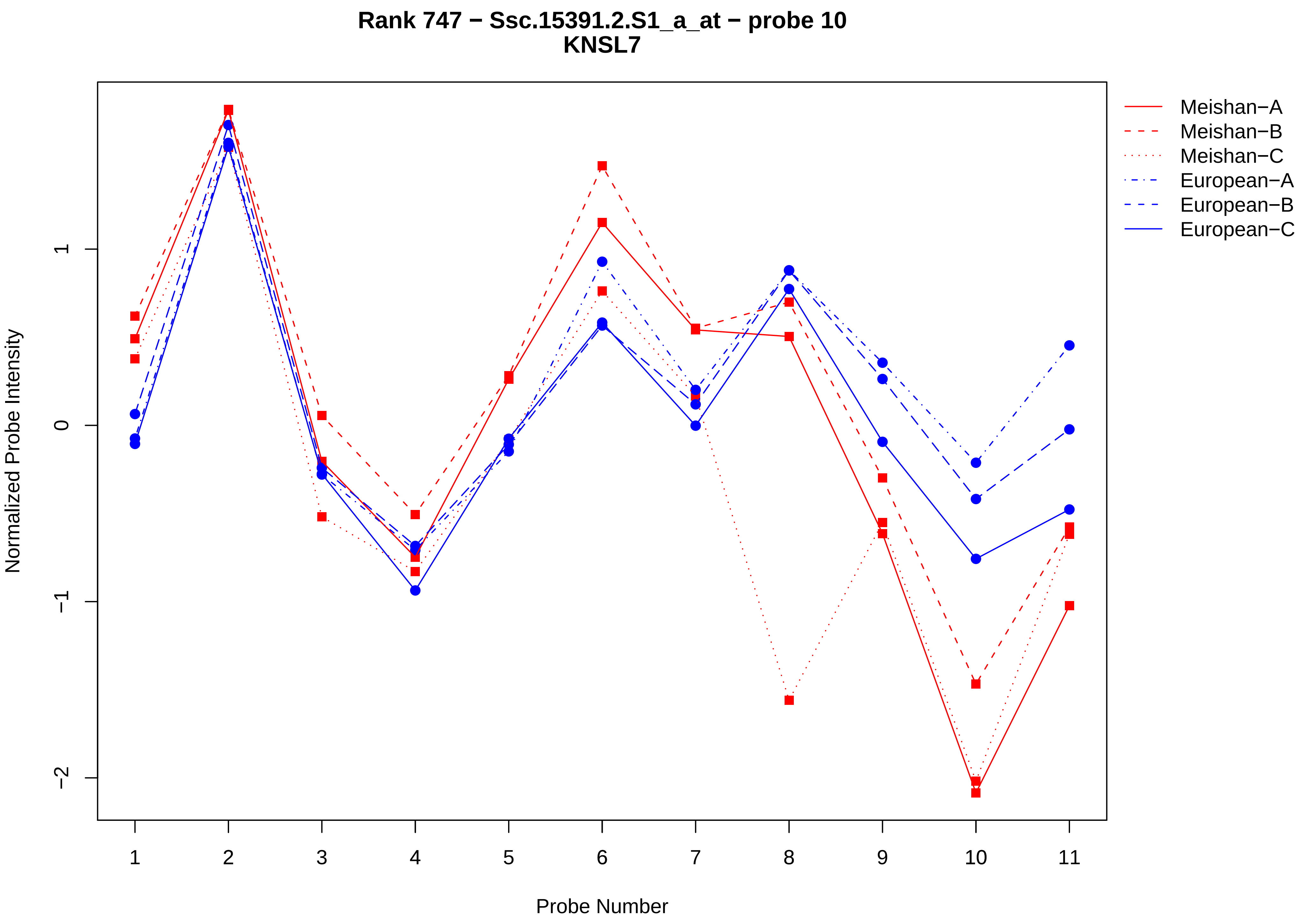

Rank 747 − Ssc.15391.2.S1_a_at − probe 10
KNSL7
Normalized Probe Intensity
Probe Number
1
0
−1
−2
1
2
3
4
5
6
7
8
9
10
11
Meishan−A
Meishan−B
Meishan−C
European−A
European−B
European−C

Rank 748 − Ssc.11248.1.A1_at − probe 9
MTMR6

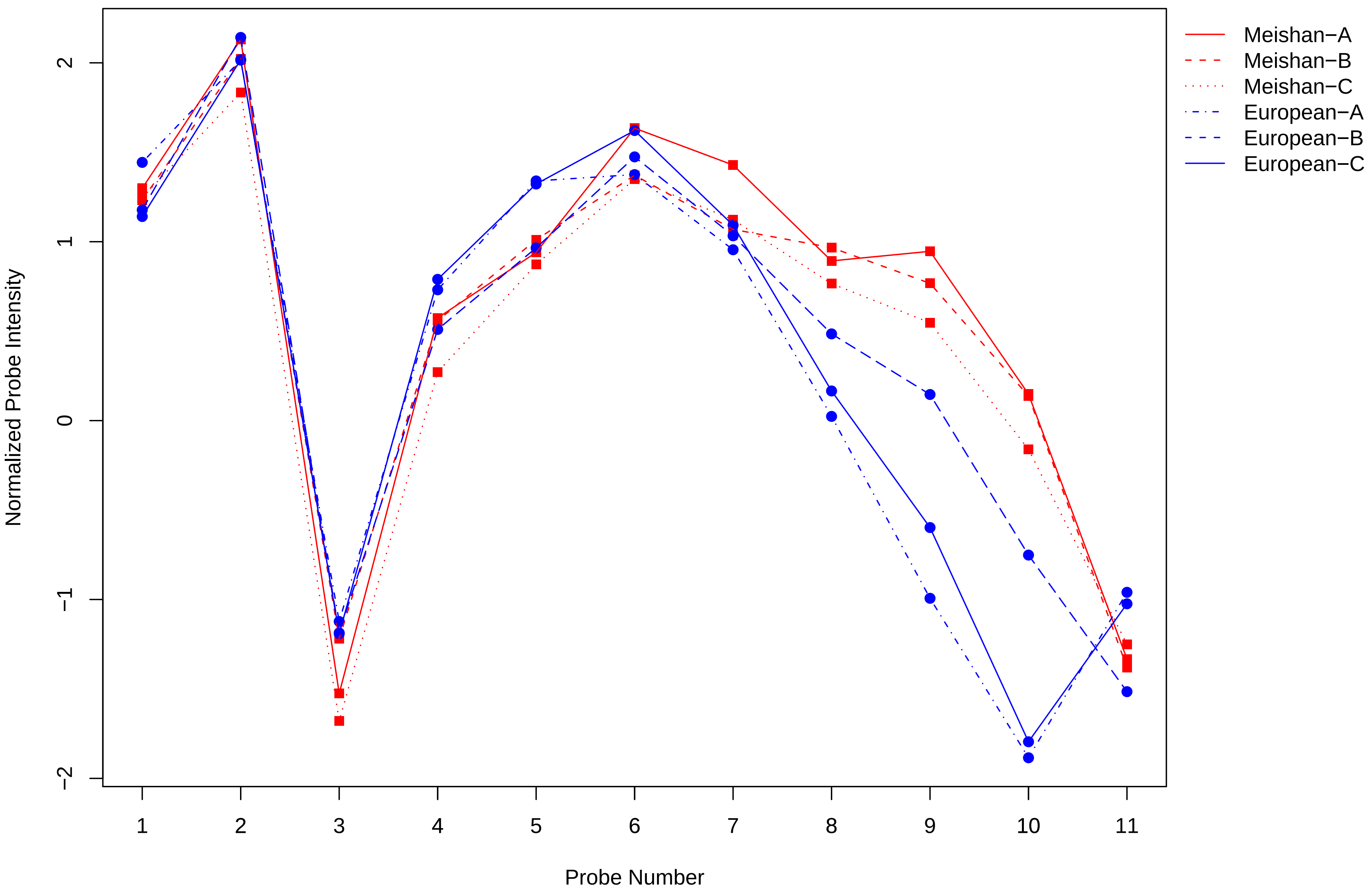

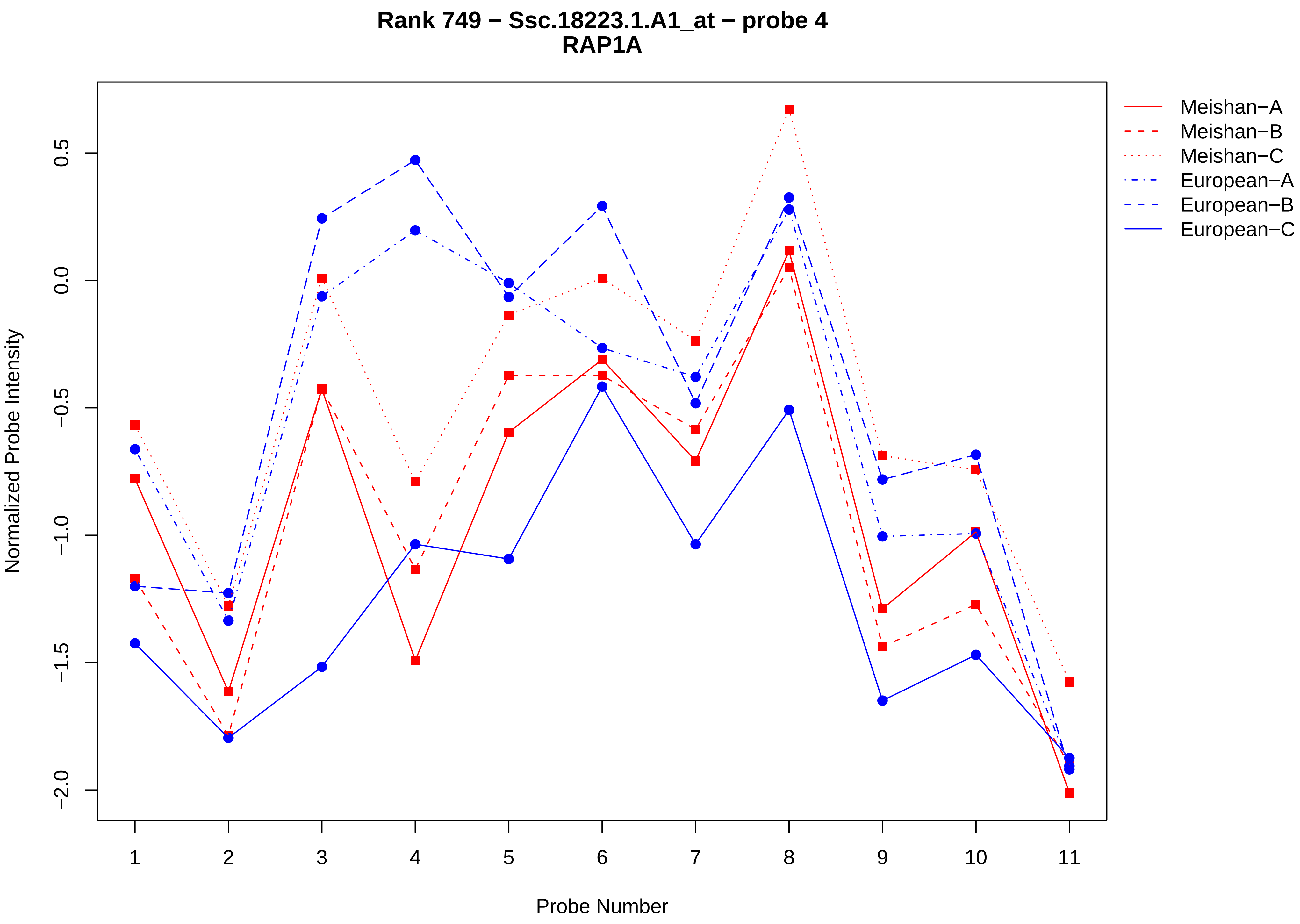
Rank 749 − Ssc.18223.1.A1_at − probe 4
RAP1A
Normalized Probe Intensity
Probe Number
0.5
0.0
−0.5
−1.0
−1.5
−2.0
1
2
3
4
5
6
7
8
9
10
11
Meishan−A
Meishan−B
Meishan−C
European−A
European−B
European−C

Rank 750 − Ssc.28305.1.A1_at − probe 1
TACC1

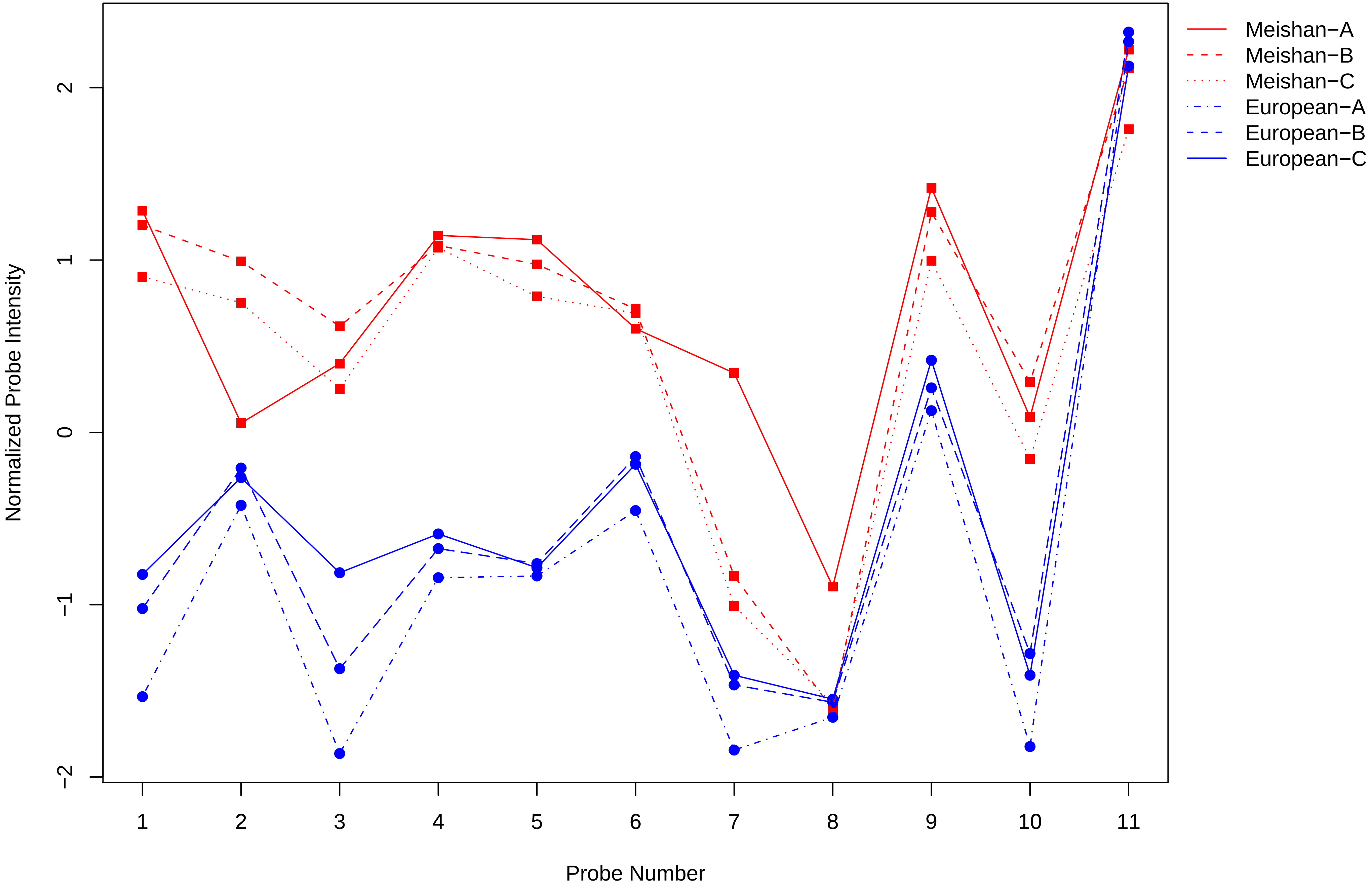

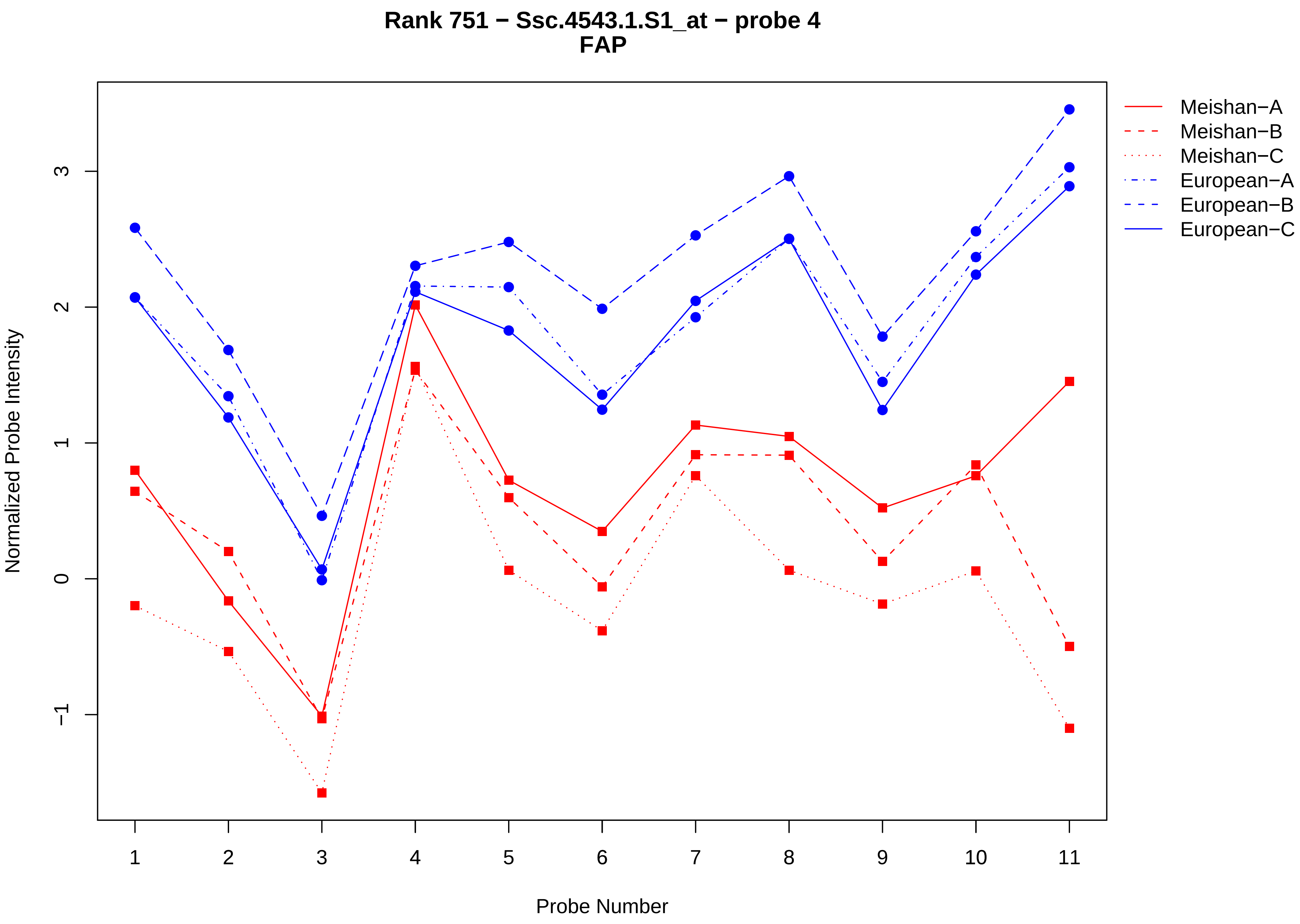

Rank 751 − Ssc.4543.1.S1_at − probe 4
FAP
Normalized Probe Intensity
Probe Number
Meishan−A
Meishan−B
Meishan−C
European−A
European−B
European−C
−1
0
1
2
3
1
2
3
4
5
6
7
8
9
10
11

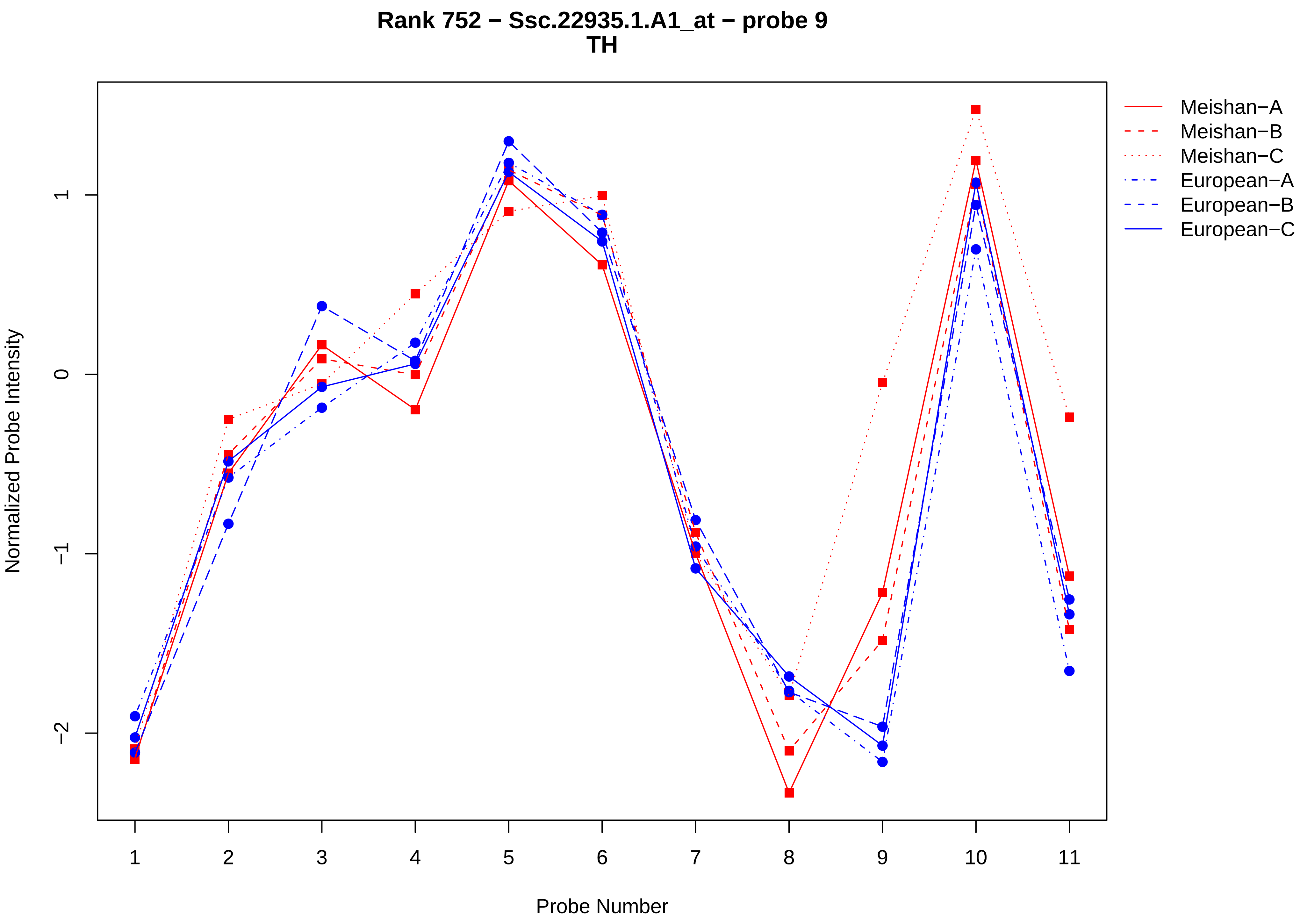

Rank 752 − Ssc.22935.1.A1_at − probe 9
TH
Normalized Probe Intensity
Probe Number
Meishan−A
Meishan−B
Meishan−C
European−A
European−B
European−C

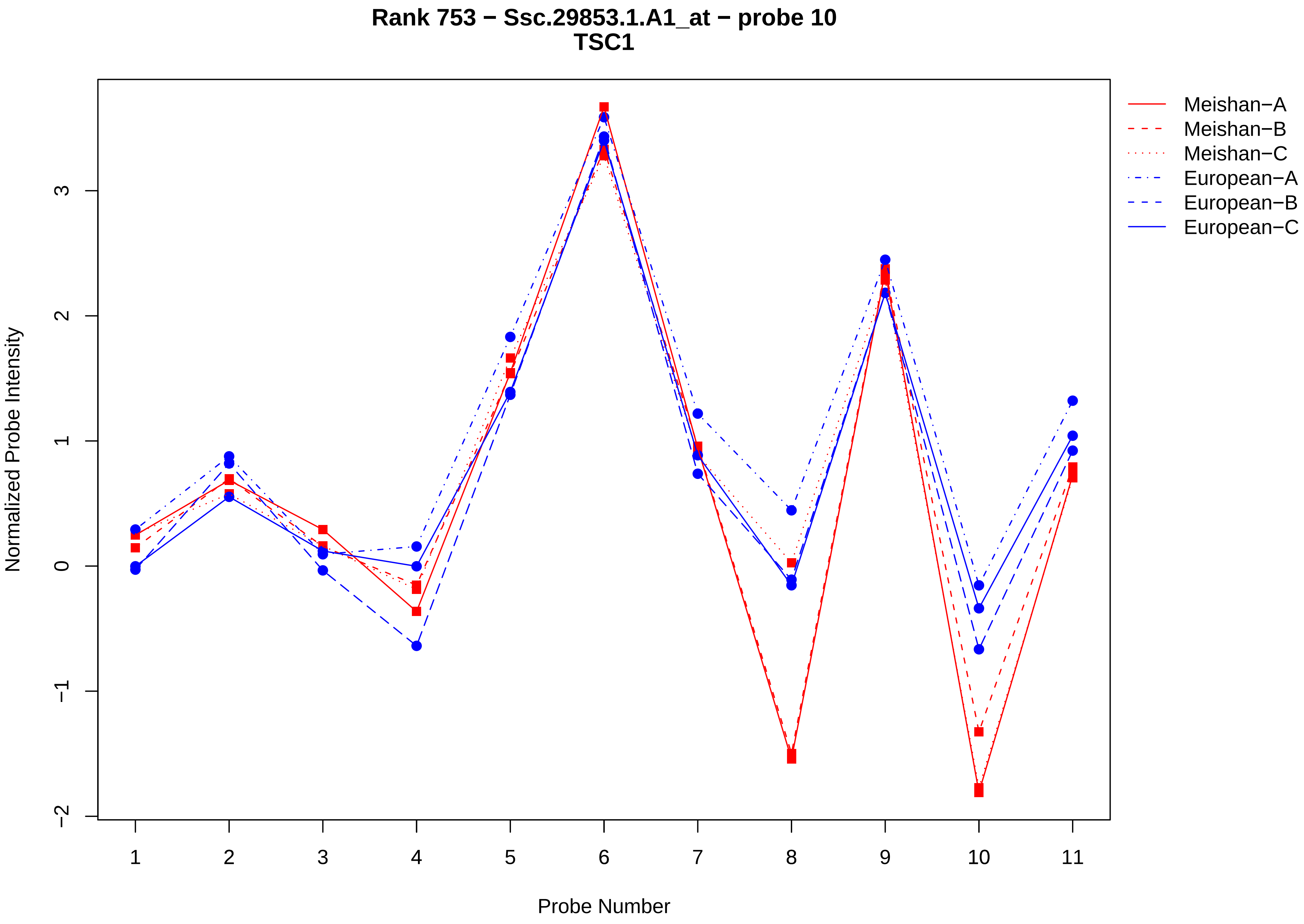

Rank 753 − Ssc.29853.1.A1_at − probe 10
TSC1
Normalized Probe Intensity
Probe Number
−2
−1
0
1
2
3
1
2
3
4
5
6
7
8
9
10
11
Meishan−A
Meishan−B
Meishan−C
European−A
European−B
European−C

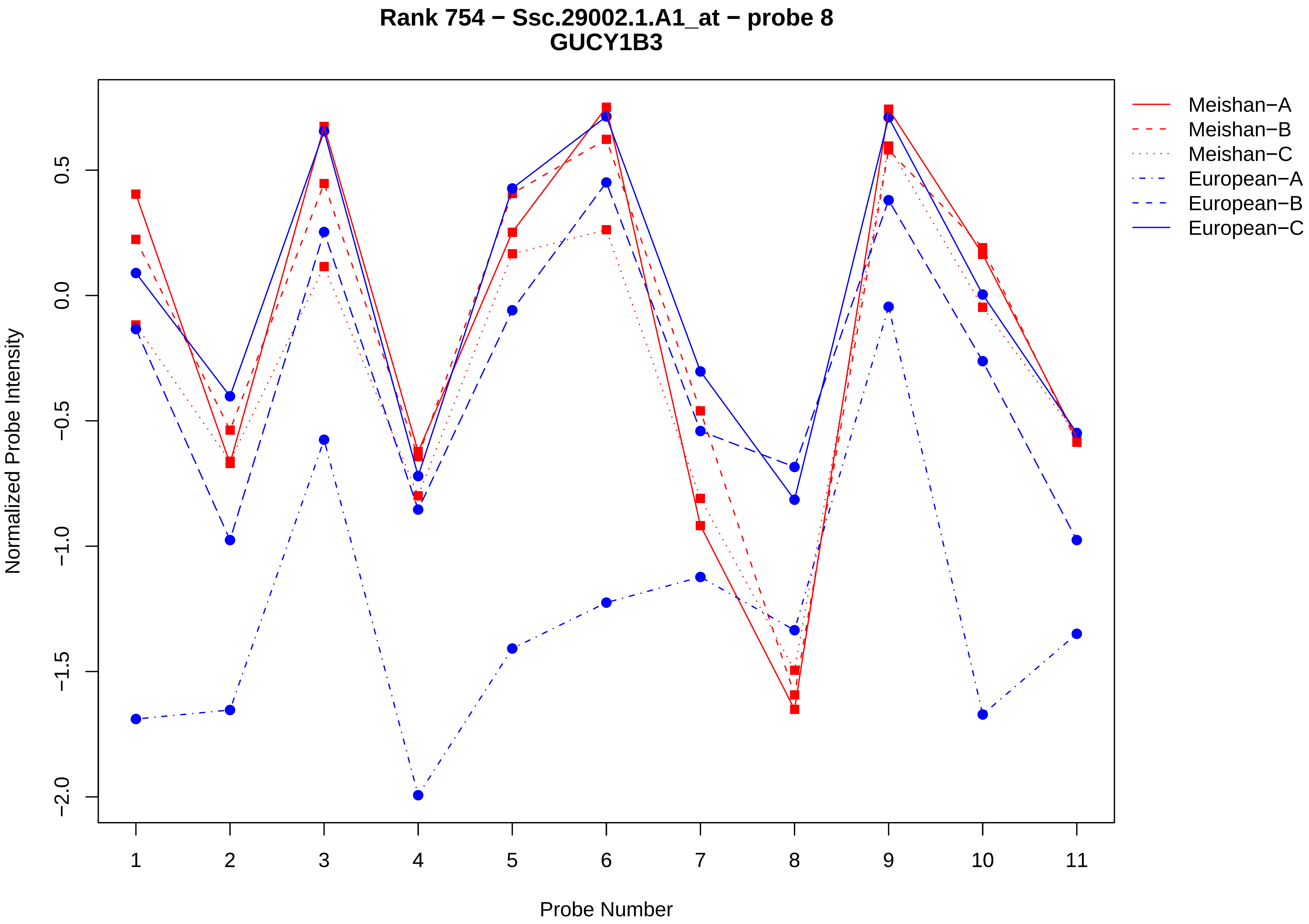

Rank 754 − Ssc.29002.1.A1_at − probe 8
GUCY1B3
Normalized Probe Intensity
Probe Number
0.5
0.0
−0.5
−1.0
−1.5
−2.0
1
2
3
4
5
6
7
8
9
10
11
Meishan−A
Meishan−B
Meishan−C
European−A
European−B
European−C

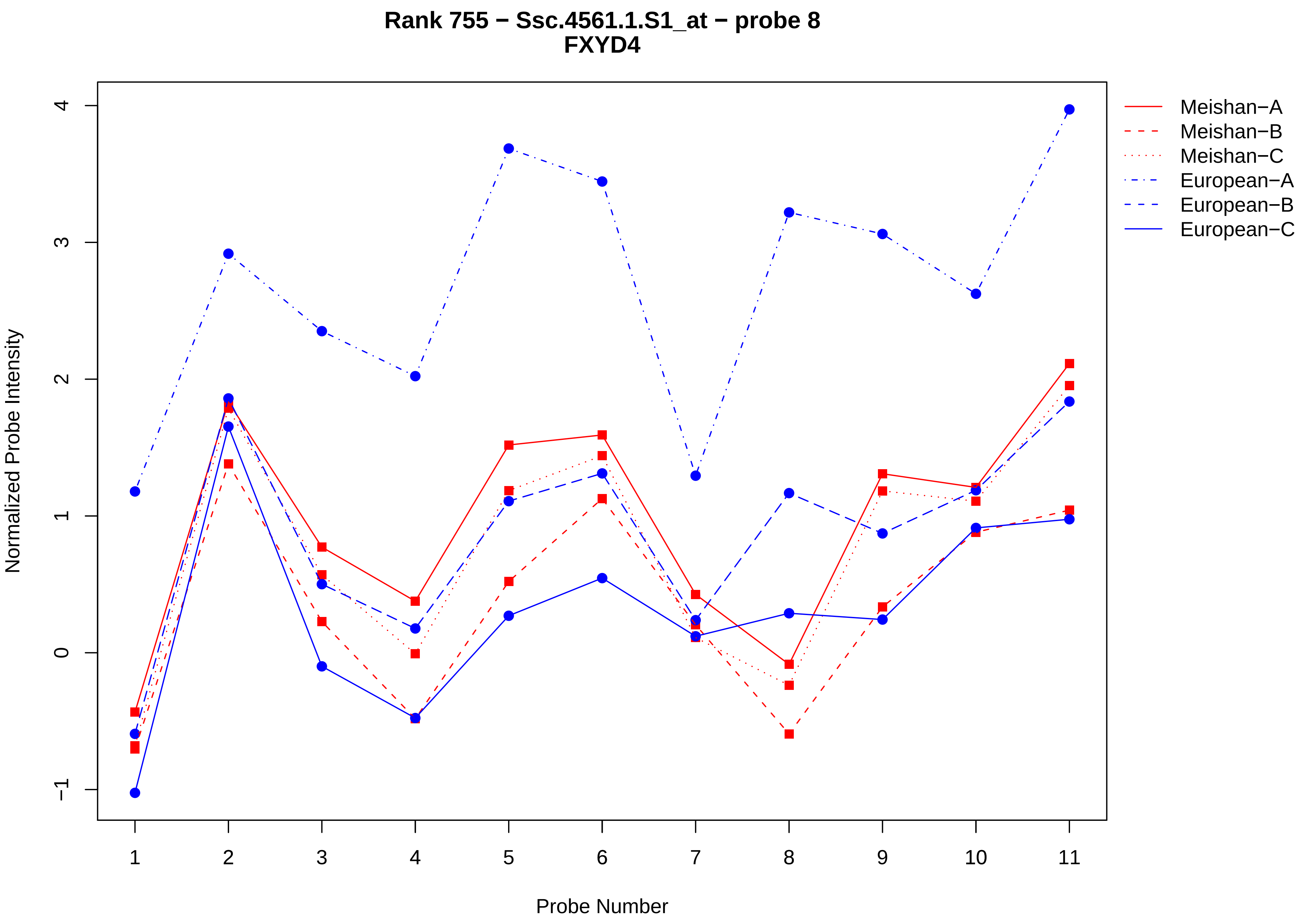

Rank 755 − Ssc.4561.1.S1_at − probe 8
FXYD4
Normalized Probe Intensity
Probe Number
Meishan−A
Meishan−B
Meishan−C
European−A
European−B
European−C

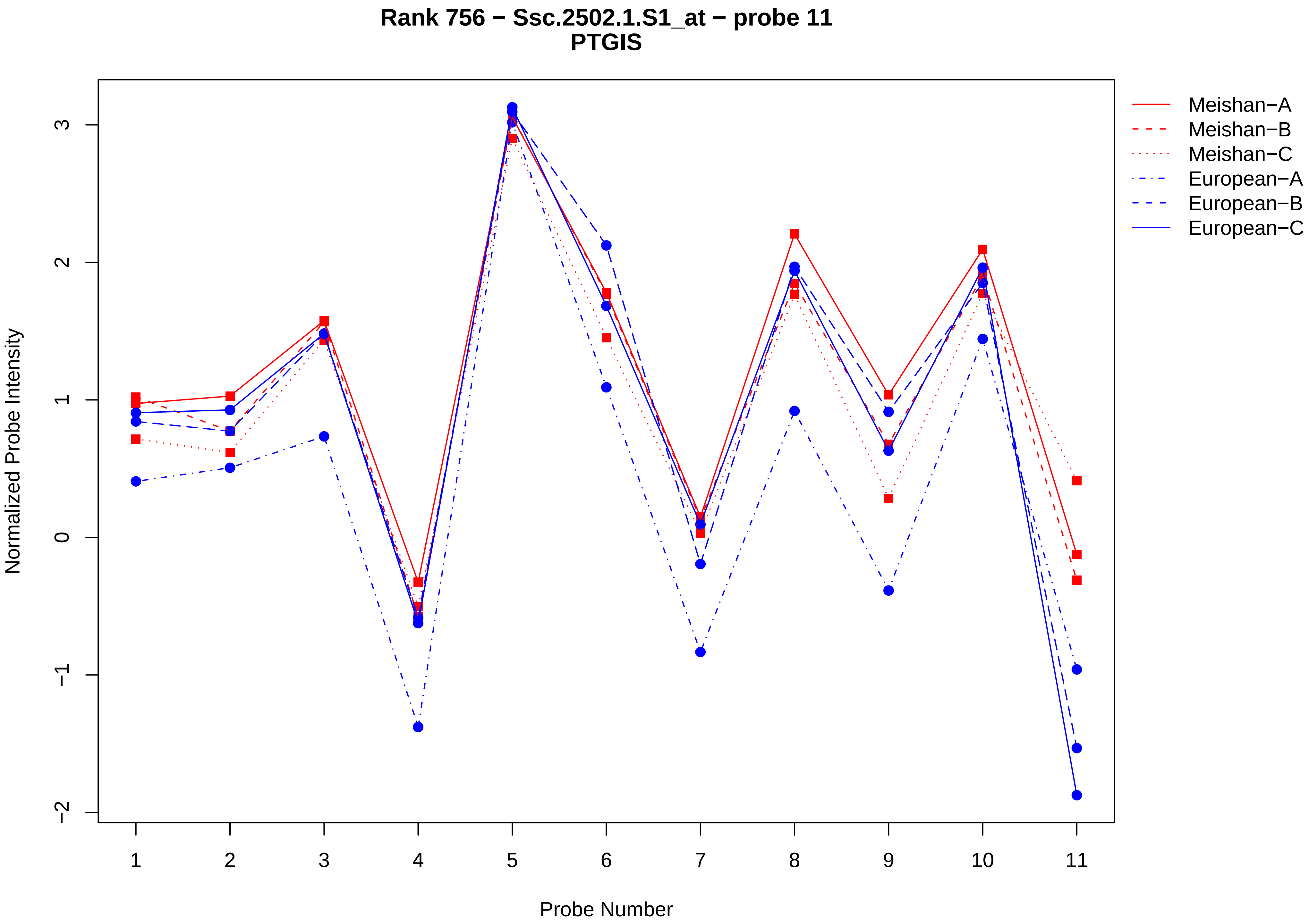

Rank 756 − Ssc.2502.1.S1_at − probe 11
PTGIS
Normalized Probe Intensity
Probe Number
3
2
1
0
−1
−2
1
2
3
4
5
6
7
8
9
10
11
Meishan−A
Meishan−B
Meishan−C
European−A
European−B
European−C

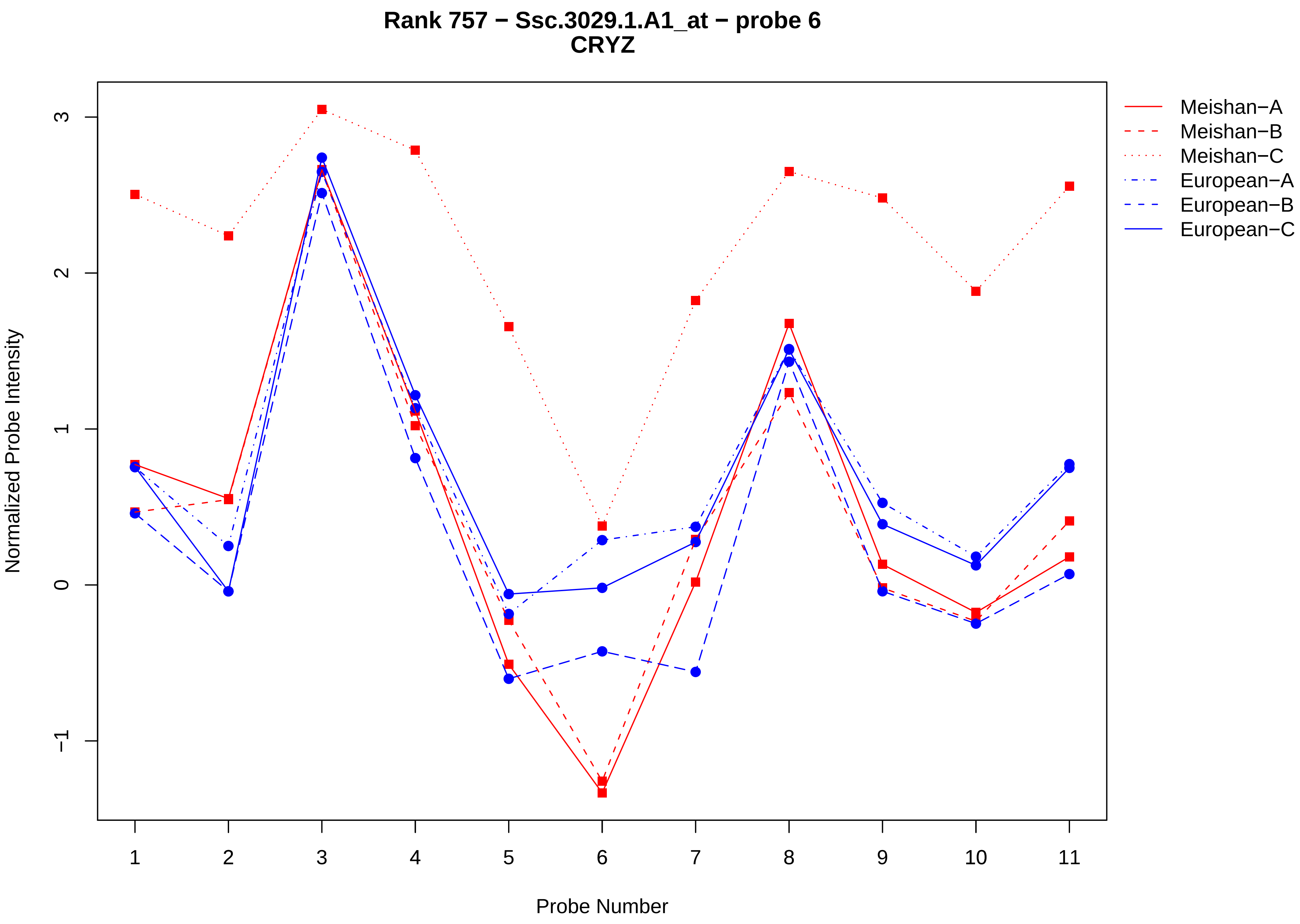

Rank 757 − Ssc.3029.1.A1_at − probe 6
CRYZ
Normalized Probe Intensity
Probe Number
Meishan−A
Meishan−B
Meishan−C
European−A
European−B
European−C

Rank 758 − Ssc.18850.1.S1_at − probe 2
GOLPH2

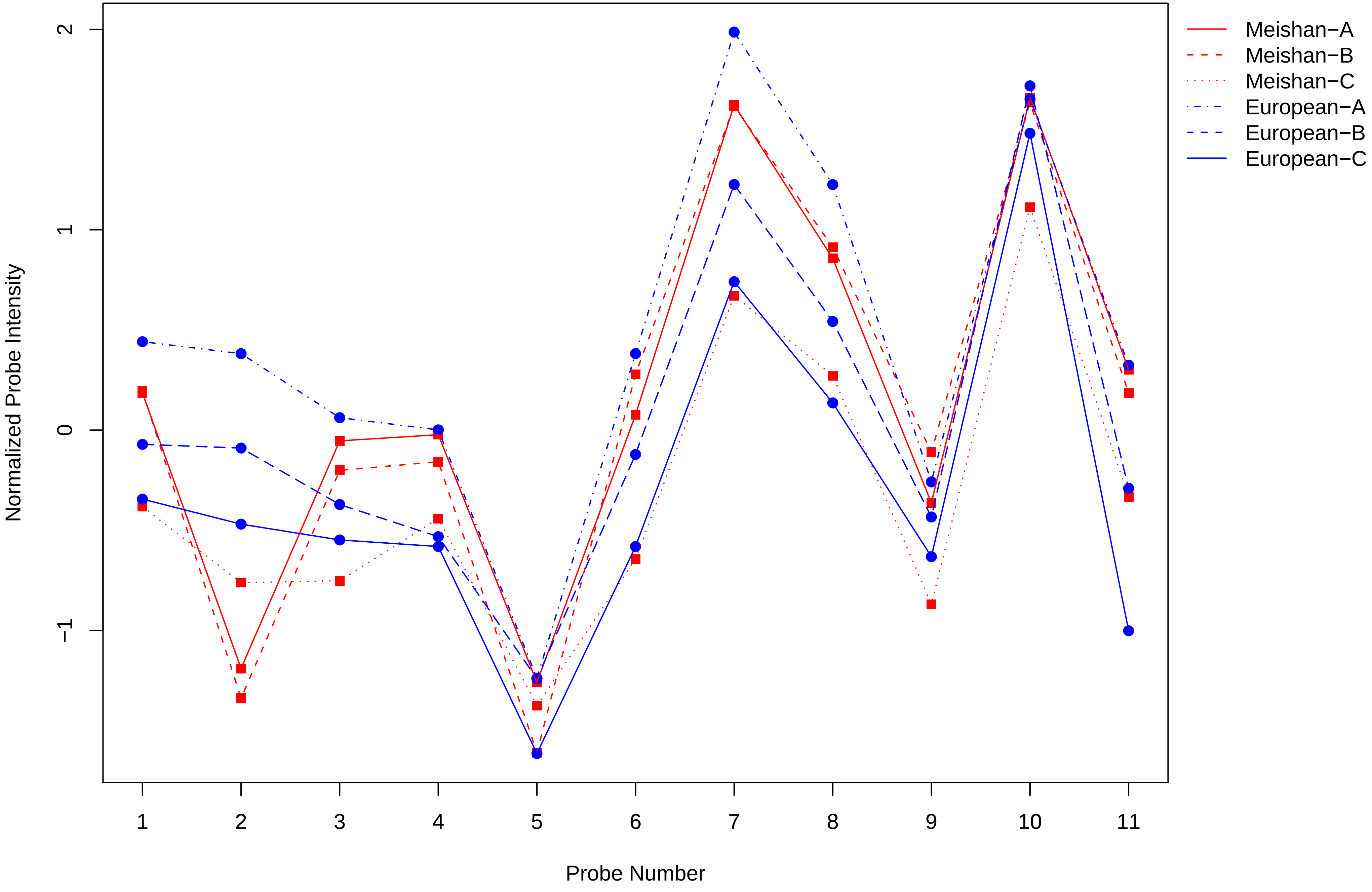

Rank 759 − Ssc.7884.1.A1_at − probe 5
IBTK

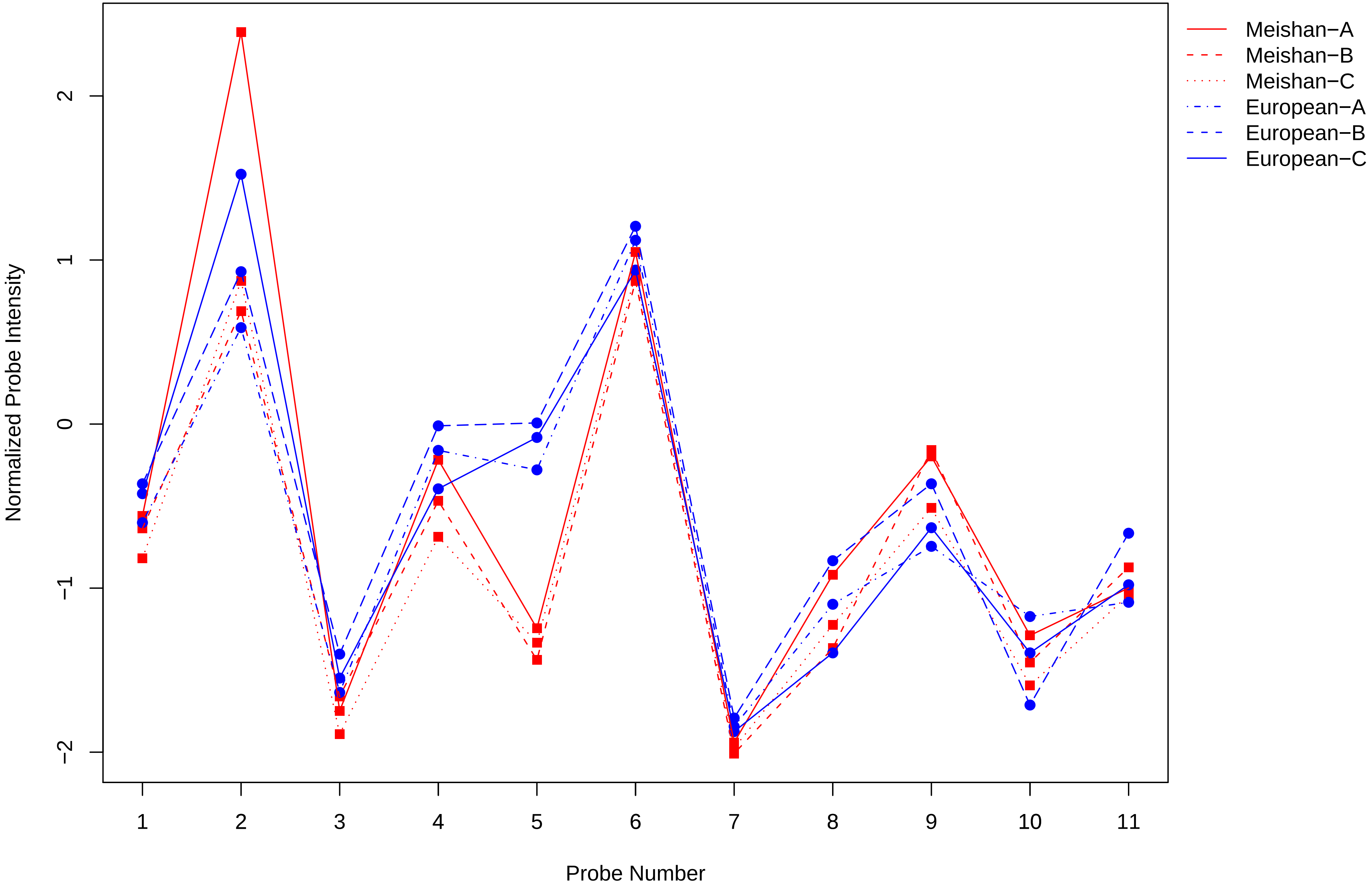

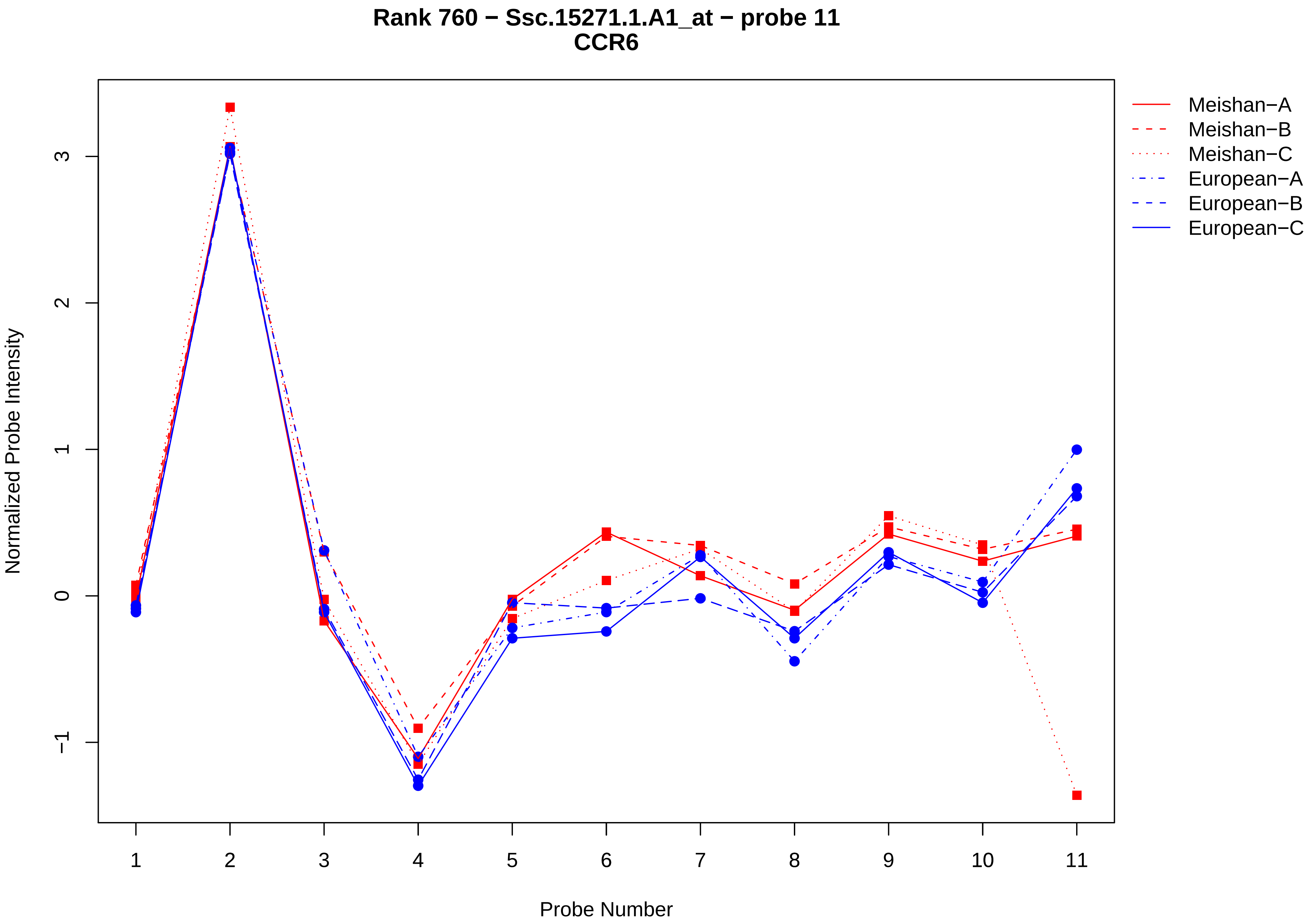

Rank 760 − Ssc.15271.1.A1_at − probe 11
CCR6
Normalized Probe Intensity
Probe Number
−1
0
1
2
3
1
2
3
4
5
6
7
8
9
10
11
Meishan−A
Meishan−B
Meishan−C
European−A
European−B
European−C

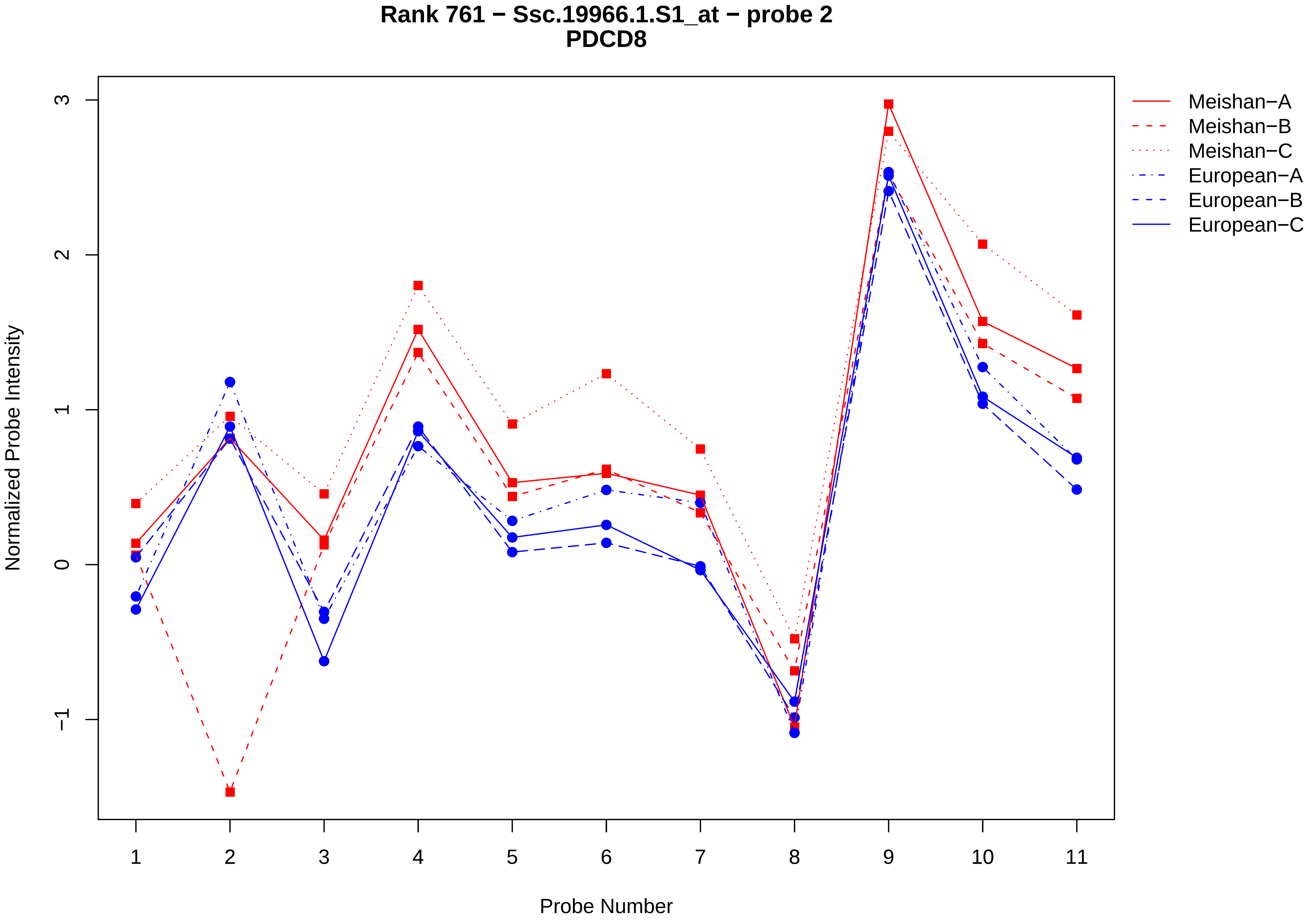

Rank 761 − Ssc.19966.1.S1_at − probe 2
PDCD8
Normalized Probe Intensity
Probe Number
Meishan−A
Meishan−B
Meishan−C
European−A
European−B
European−C

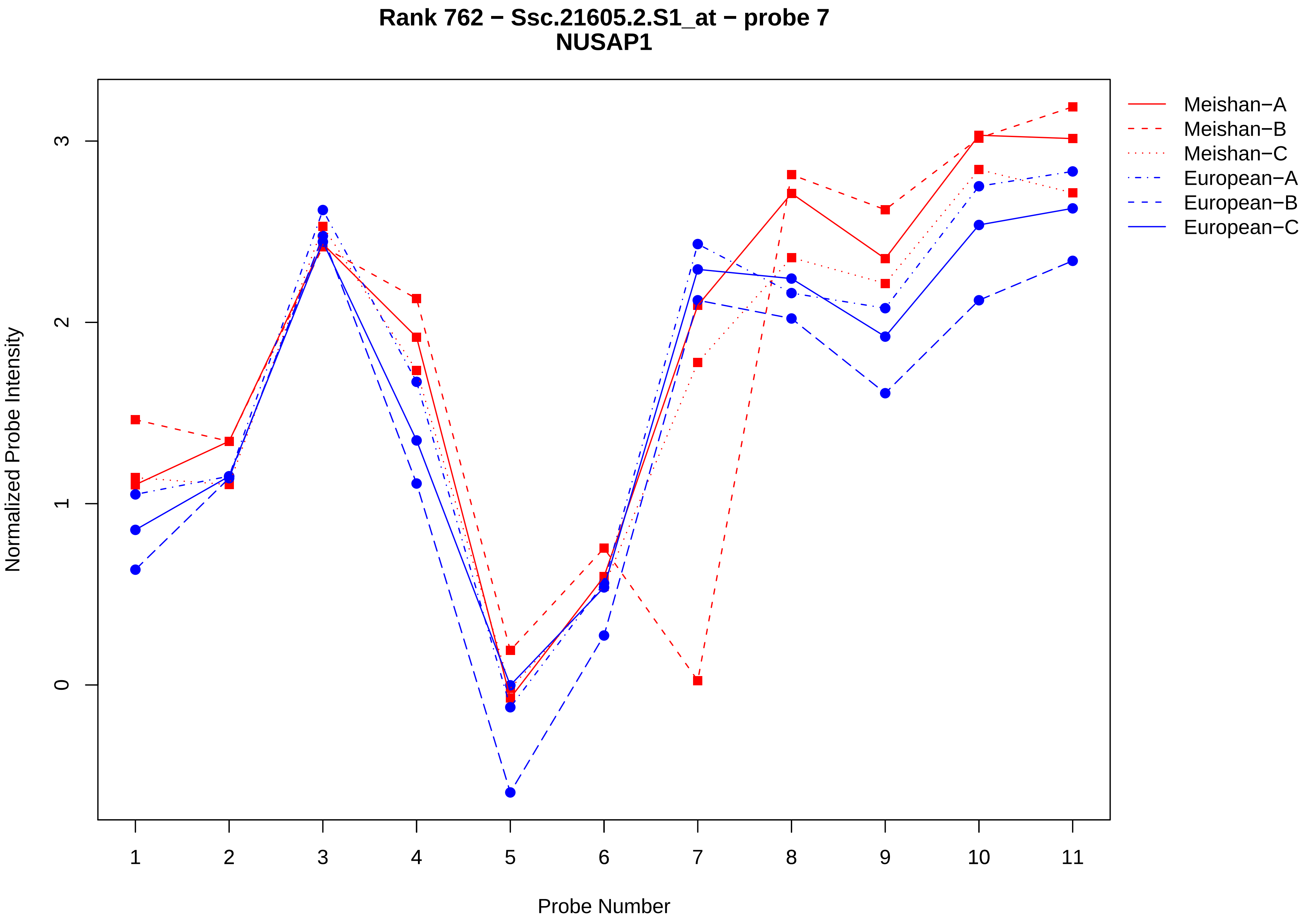
Rank 762 − Ssc.21605.2.S1_at − probe 7
NUSAP1
Normalized Probe Intensity
0
1
2
3
1
2
3
4
5
6
7
8
9
10
11
Probe Number
Meishan−A
Meishan−B
Meishan−C
European−A
European−B
European−C

Rank 763 − Ssc.13780.12.S1_x_at − probe 5
HLA−B

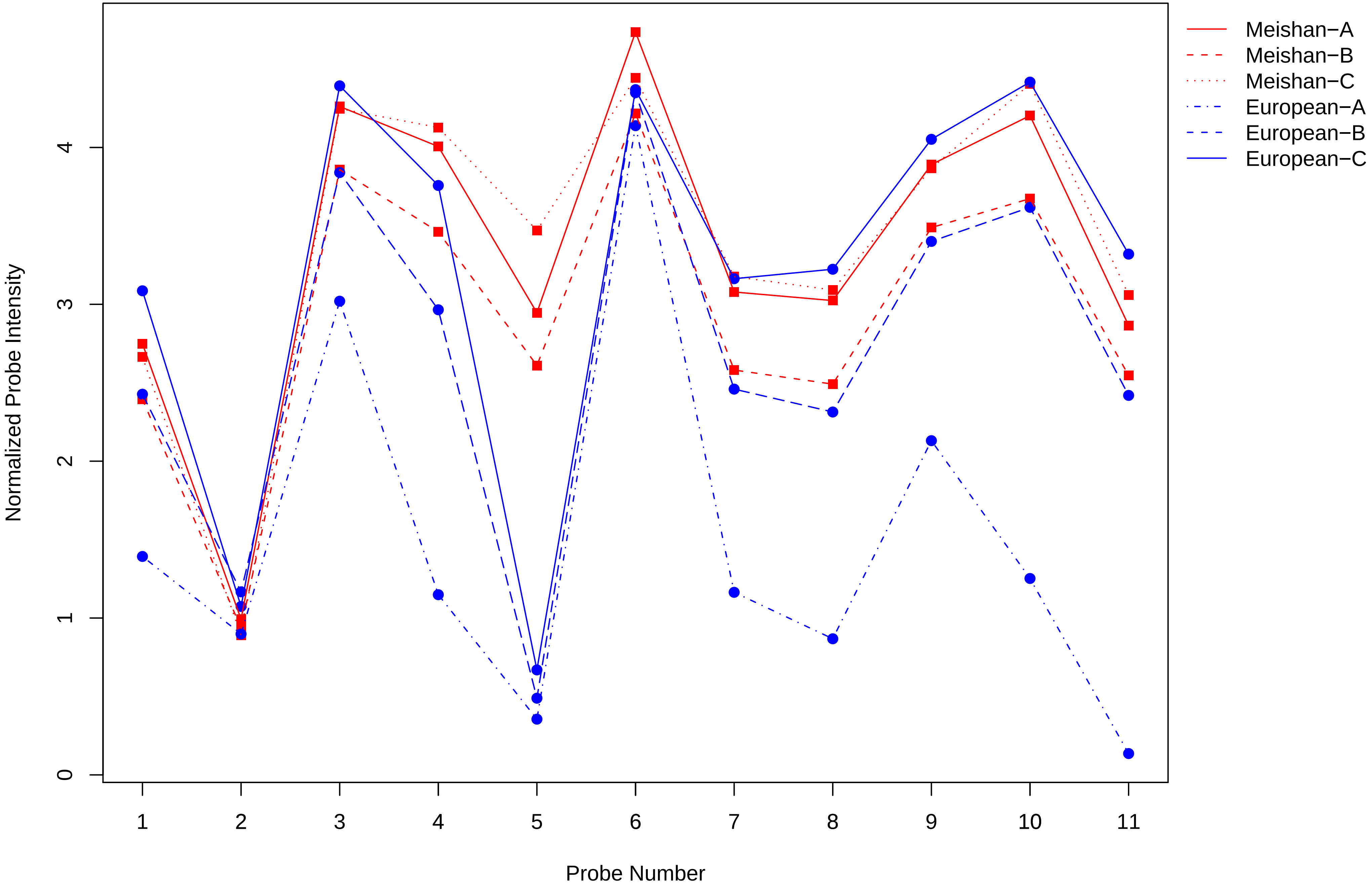

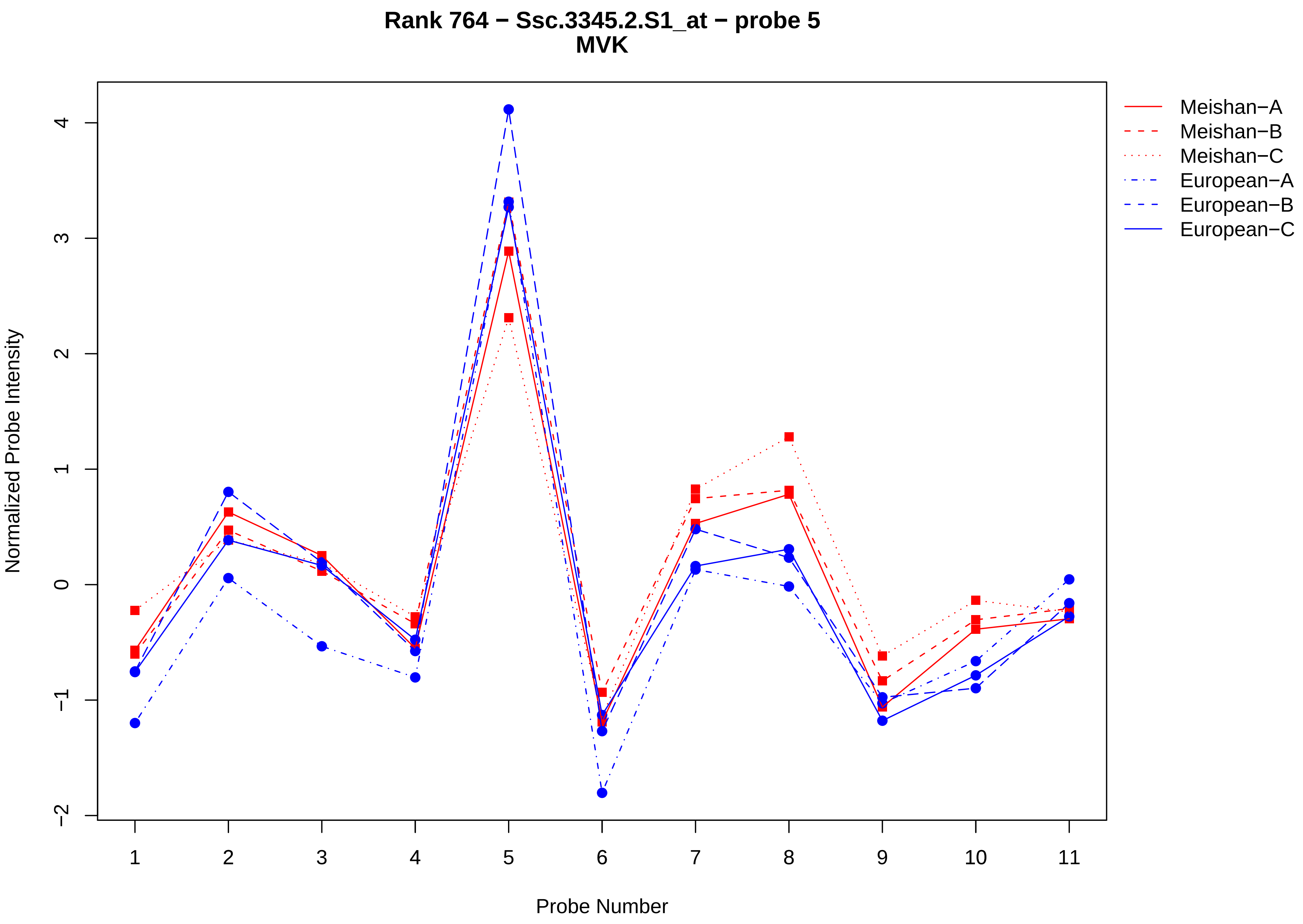

Rank 764 − Ssc.3345.2.S1_at − probe 5
MVK
Normalized Probe Intensity
Probe Number
−2
−1
0
1
2
3
4
1
2
3
4
5
6
7
8
9
10
11
Meishan−A
Meishan−B
Meishan−C
European−A
European−B
European−C

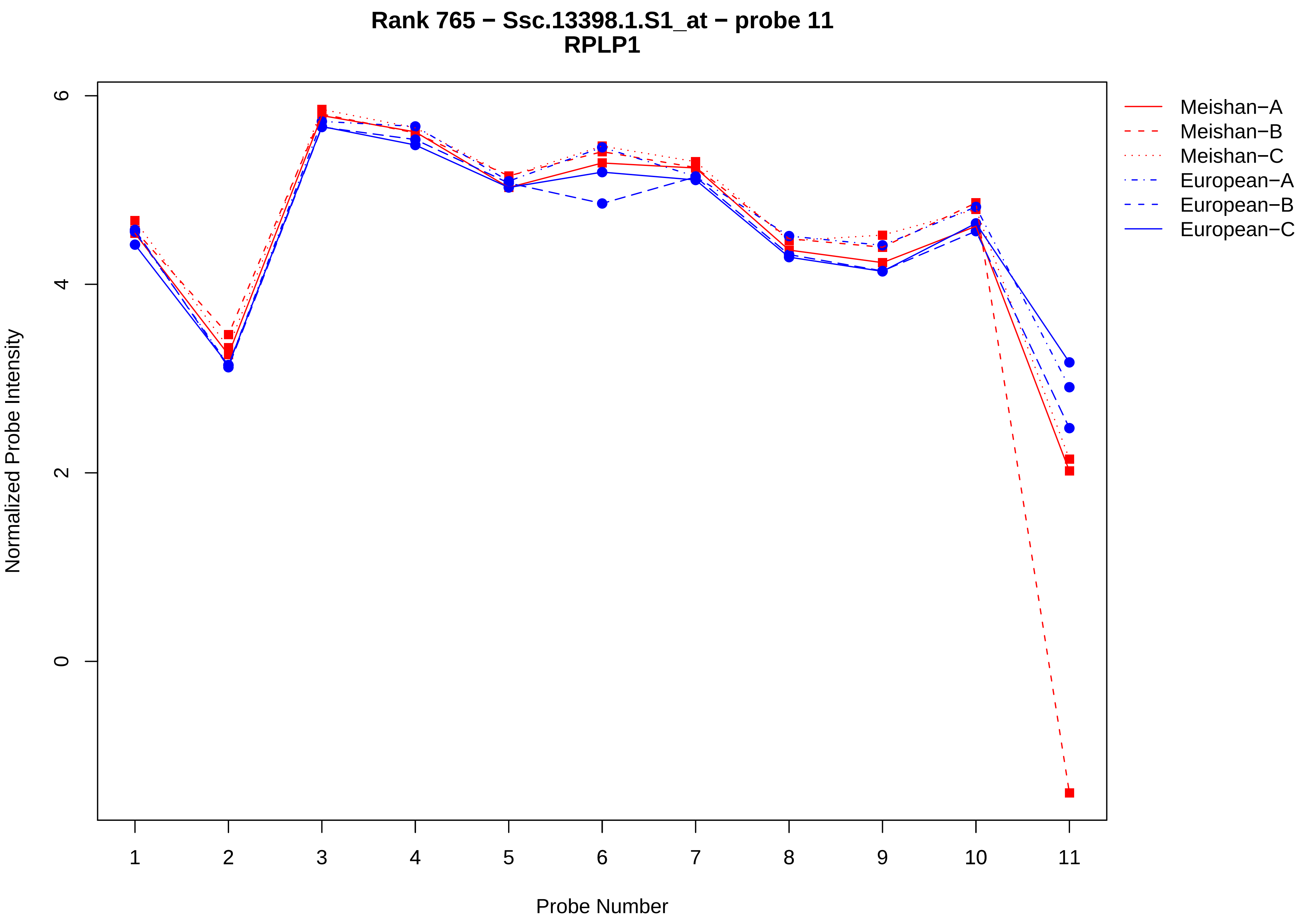

Rank 765 − Ssc.13398.1.S1_at − probe 11
RPLP1
Normalized Probe Intensity
Probe Number
0
2
4
6
1
2
3
4
5
6
7
8
9
10
11
Meishan−A
Meishan−B
Meishan−C
European−A
European−B
European−C

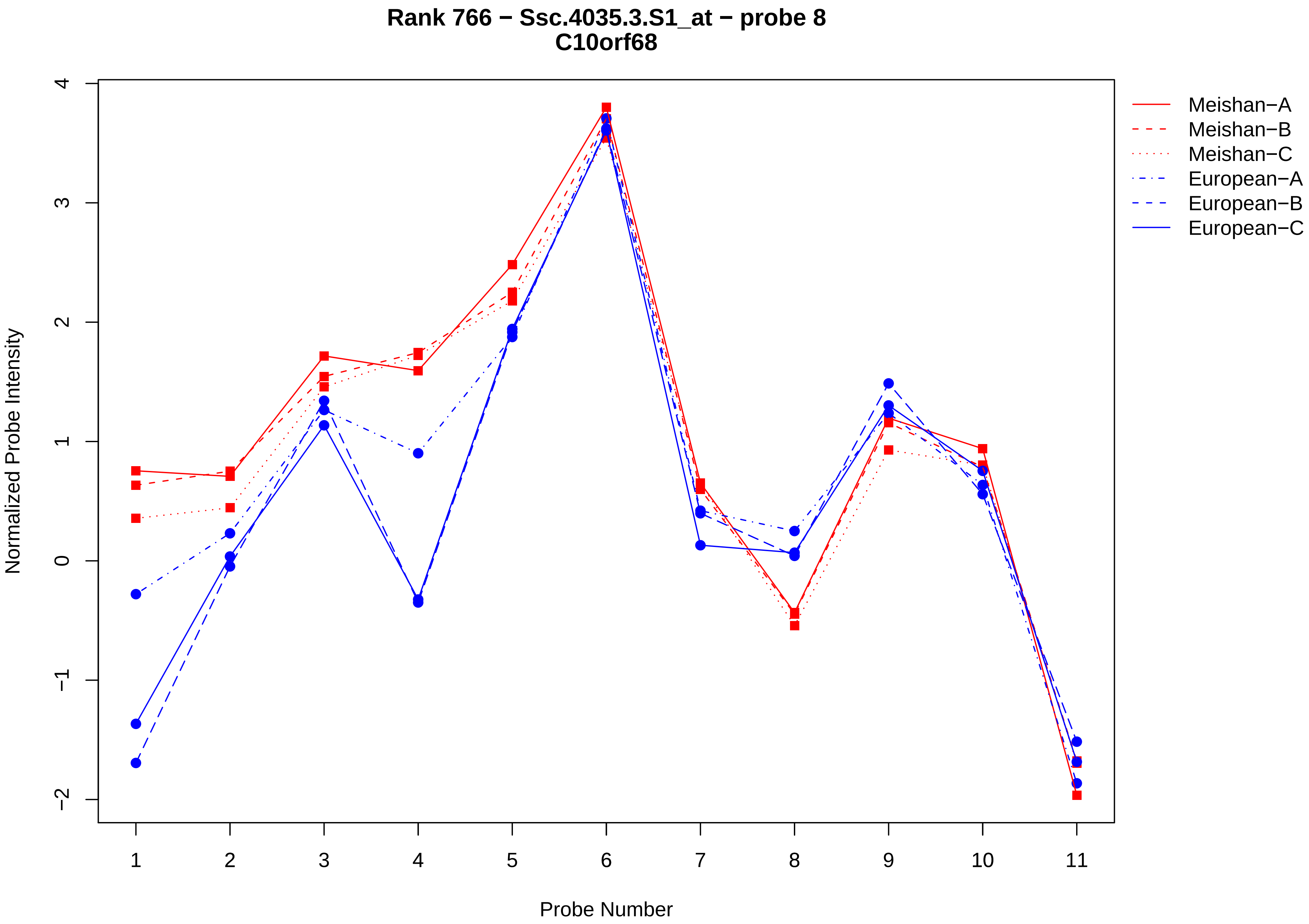

Rank 766 − Ssc.4035.3.S1_at − probe 8
C10orf68
Normalized Probe Intensity
Probe Number
−2
−1
0
1
2
3
4
1
2
3
4
5
6
7
8
9
10
11
Meishan−A
Meishan−B
Meishan−C
European−A
European−B
European−C

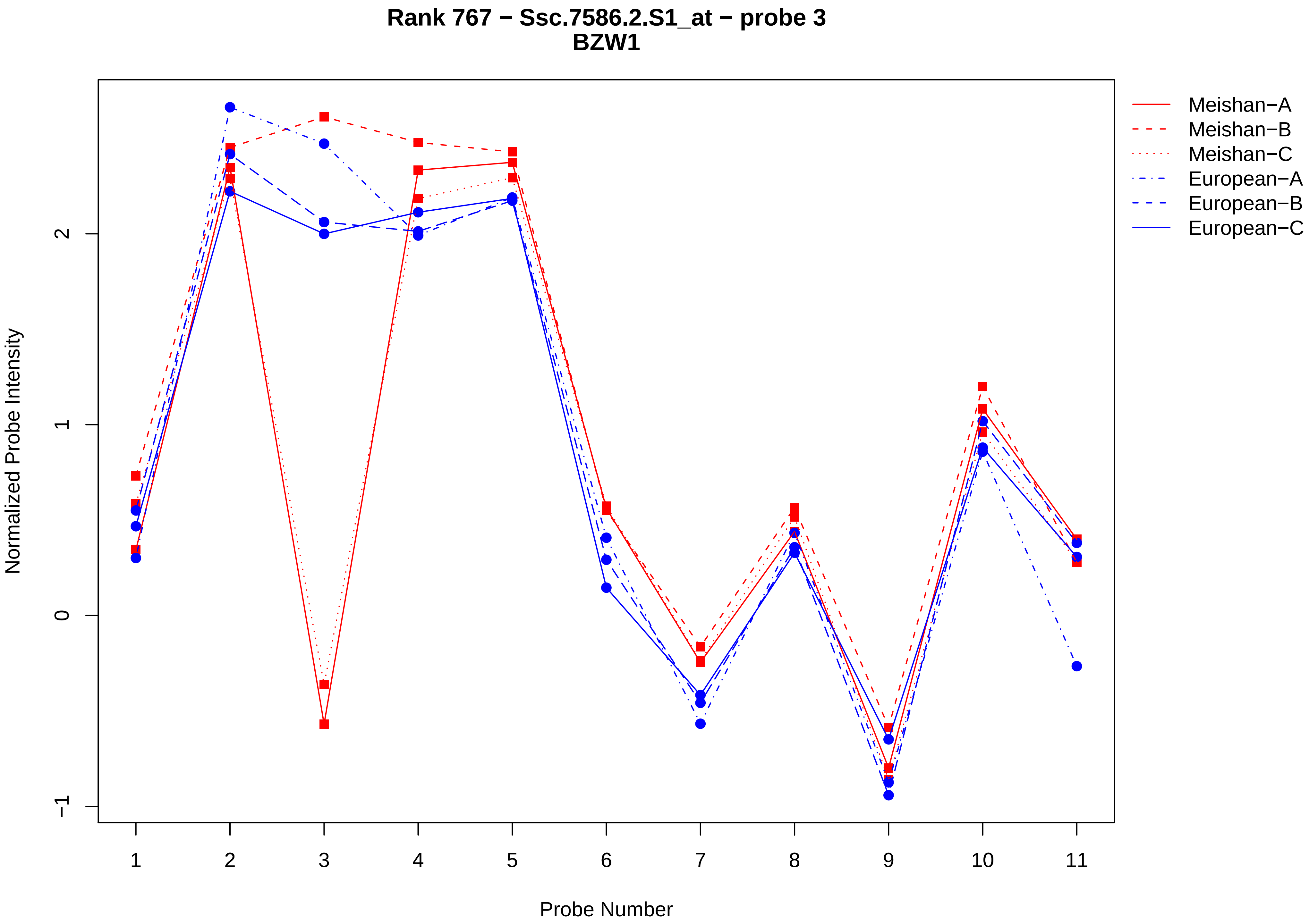

Rank 767 − Ssc.7586.2.S1_at − probe 3
BZW1
Normalized Probe Intensity
Probe Number
−1
0
1
2
1
2
3
4
5
6
7
8
9
10
11
Meishan−A
Meishan−B
Meishan−C
European−A
European−B
European−C

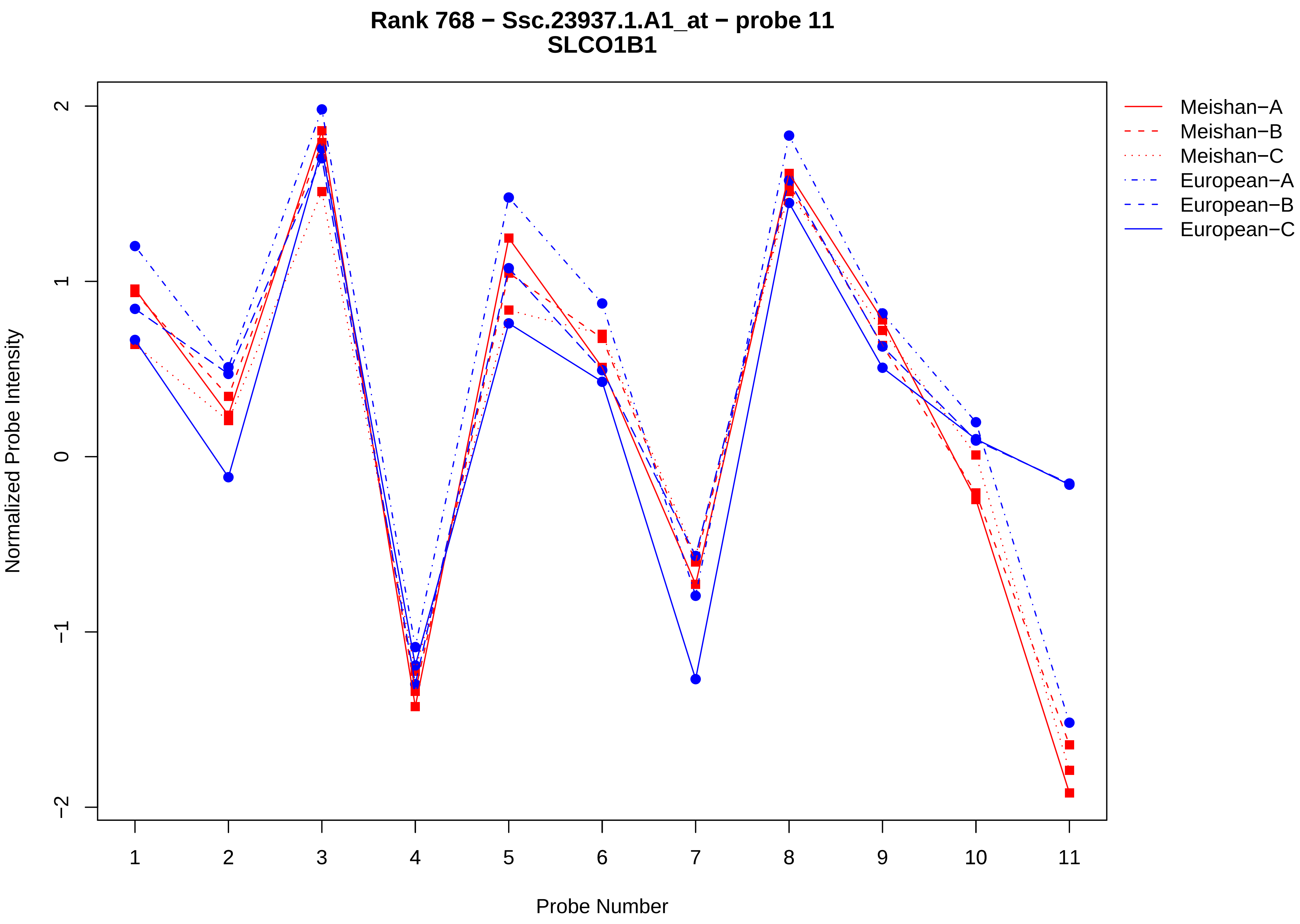

Rank 768 − Ssc.23937.1.A1_at − probe 11
SLCO1B1
Normalized Probe Intensity
Probe Number
−2
−1
0
1
2
1
2
3
4
5
6
7
8
9
10
11
Meishan−A
Meishan−B
Meishan−C
European−A
European−B
European−C

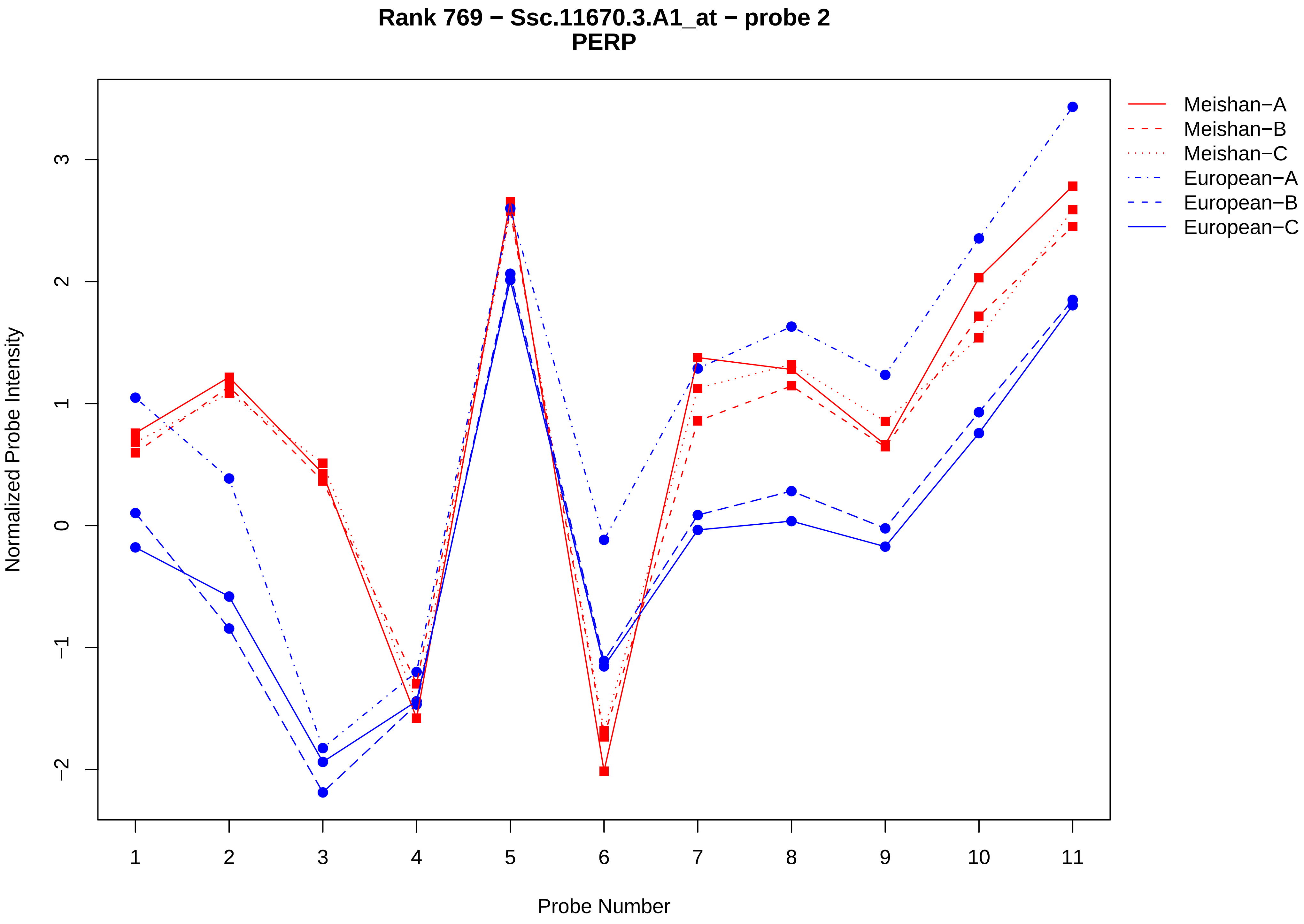

Rank 769 − Ssc.11670.3.A1_at − probe 2
PERP
Normalized Probe Intensity
Probe Number
Meishan−A
Meishan−B
Meishan−C
European−A
European−B
European−C
−2
−1
0
1
2
3
1
2
3
4
5
6
7
8
9
10
11

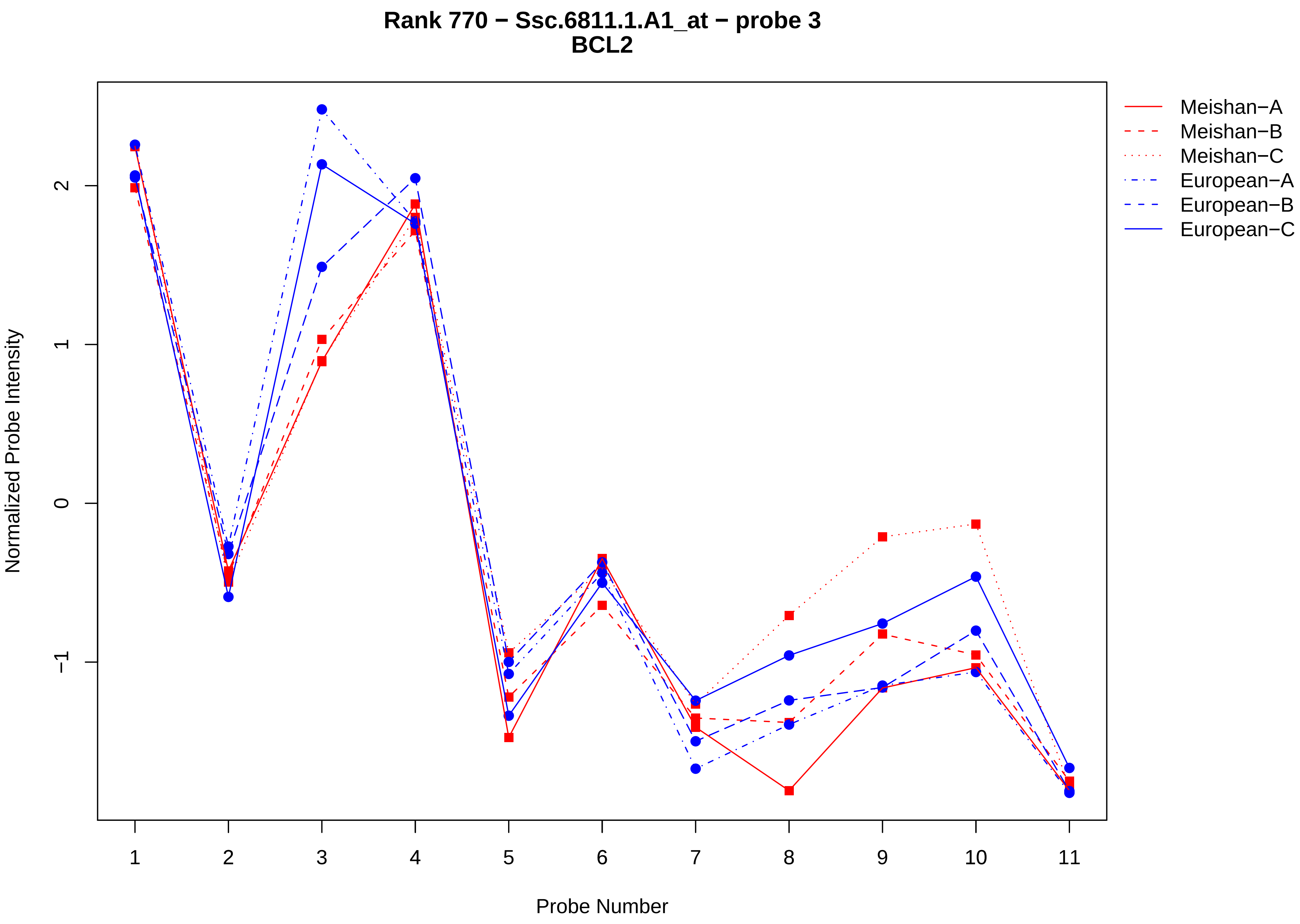

Rank 770 − Ssc.6811.1.A1_at − probe 3
BCL2
Normalized Probe Intensity
Probe Number
−1
0
1
2
1
2
3
4
5
6
7
8
9
10
11
Meishan−A
Meishan−B
Meishan−C
European−A
European−B
European−C

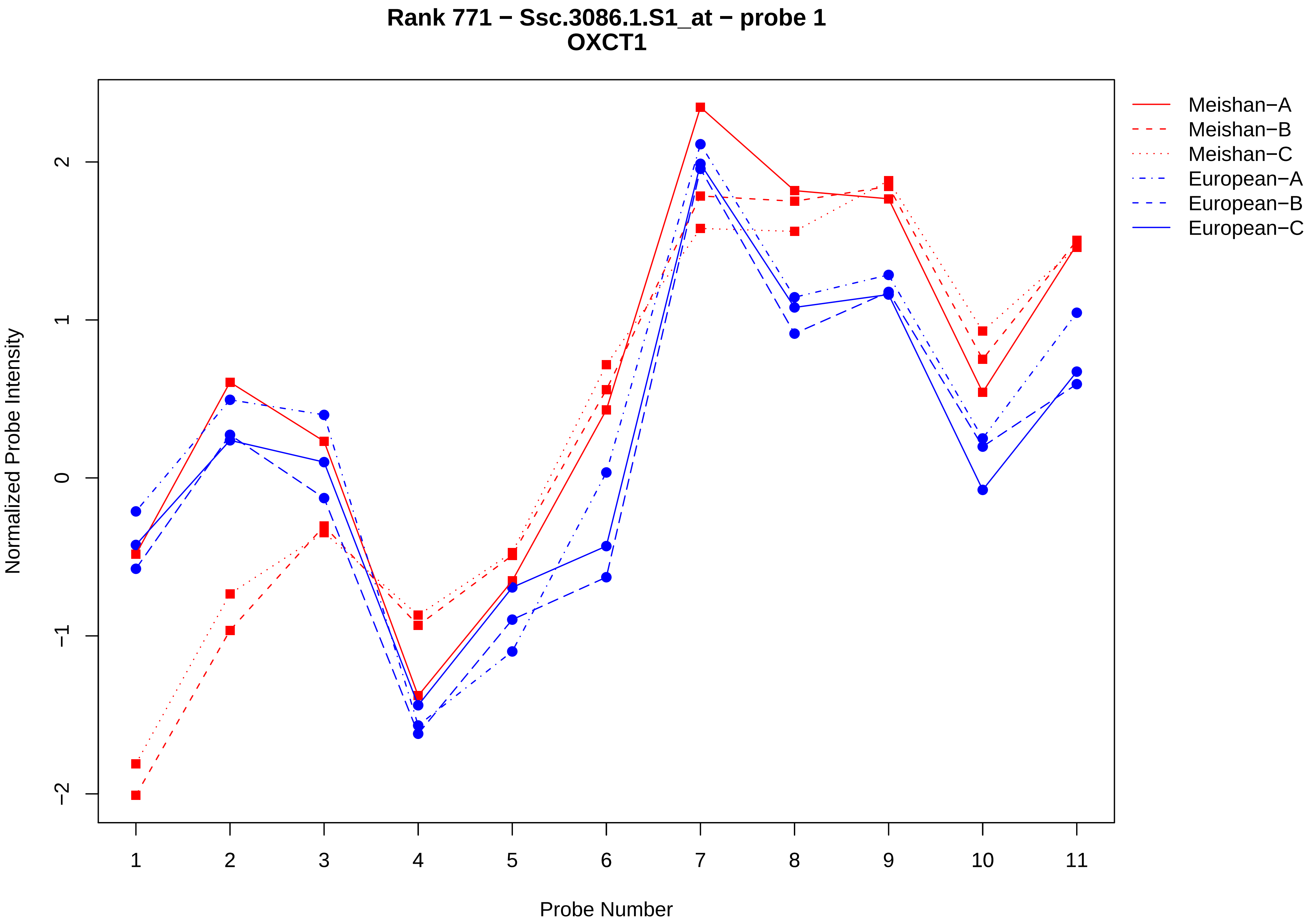

Rank 771 − Ssc.3086.1.S1_at − probe 1
OXCT1
Normalized Probe Intensity
Probe Number
−2
−1
0
1
2
1
2
3
4
5
6
7
8
9
10
11
Meishan−A
Meishan−B
Meishan−C
European−A
European−B
European−C

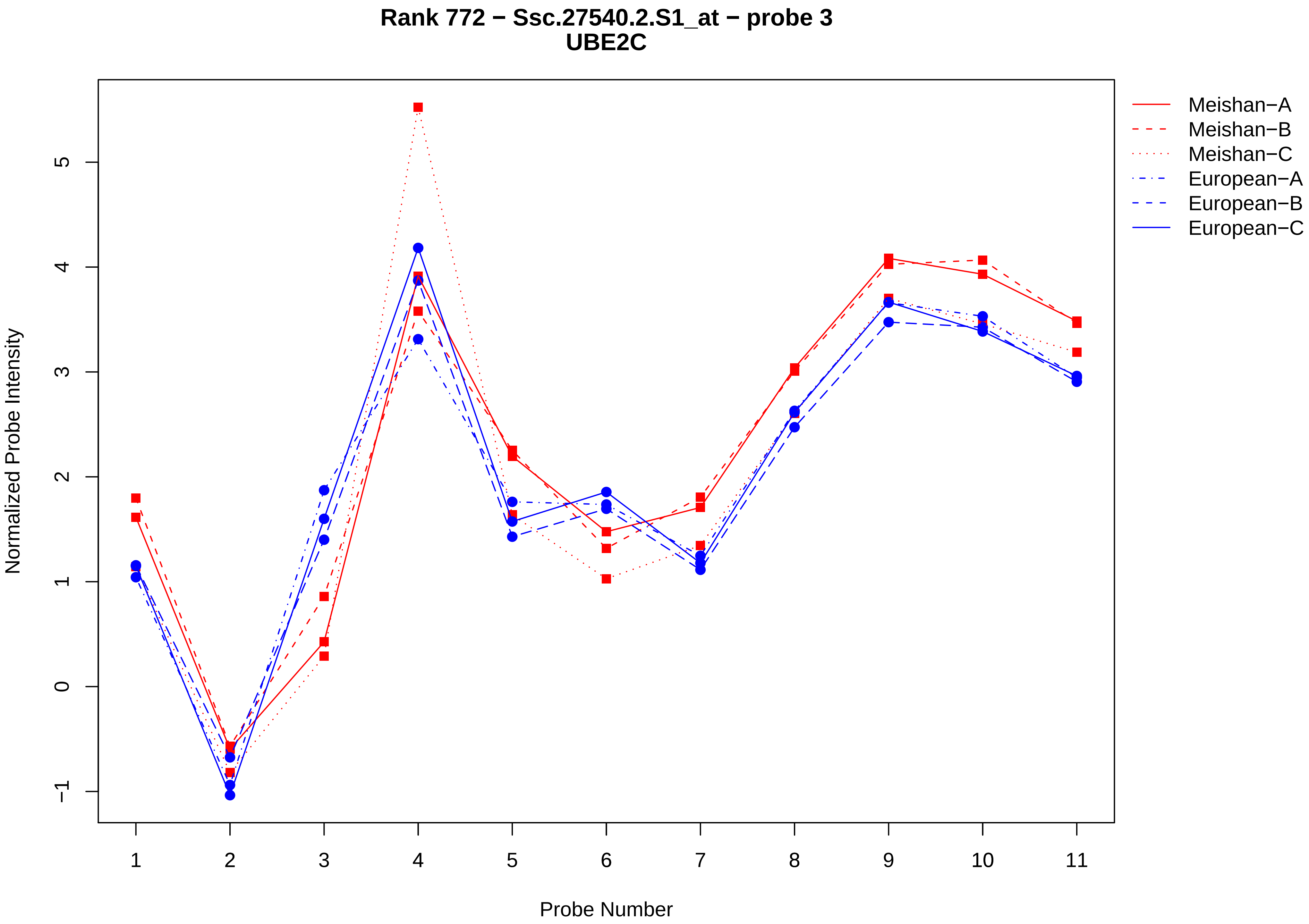

Rank 772 − Ssc.27540.2.S1_at − probe 3
UBE2C
Normalized Probe Intensity
Probe Number
−1
0
1
2
3
4
5
1
2
3
4
5
6
7
8
9
10
11
Meishan−A
Meishan−B
Meishan−C
European−A
European−B
European−C

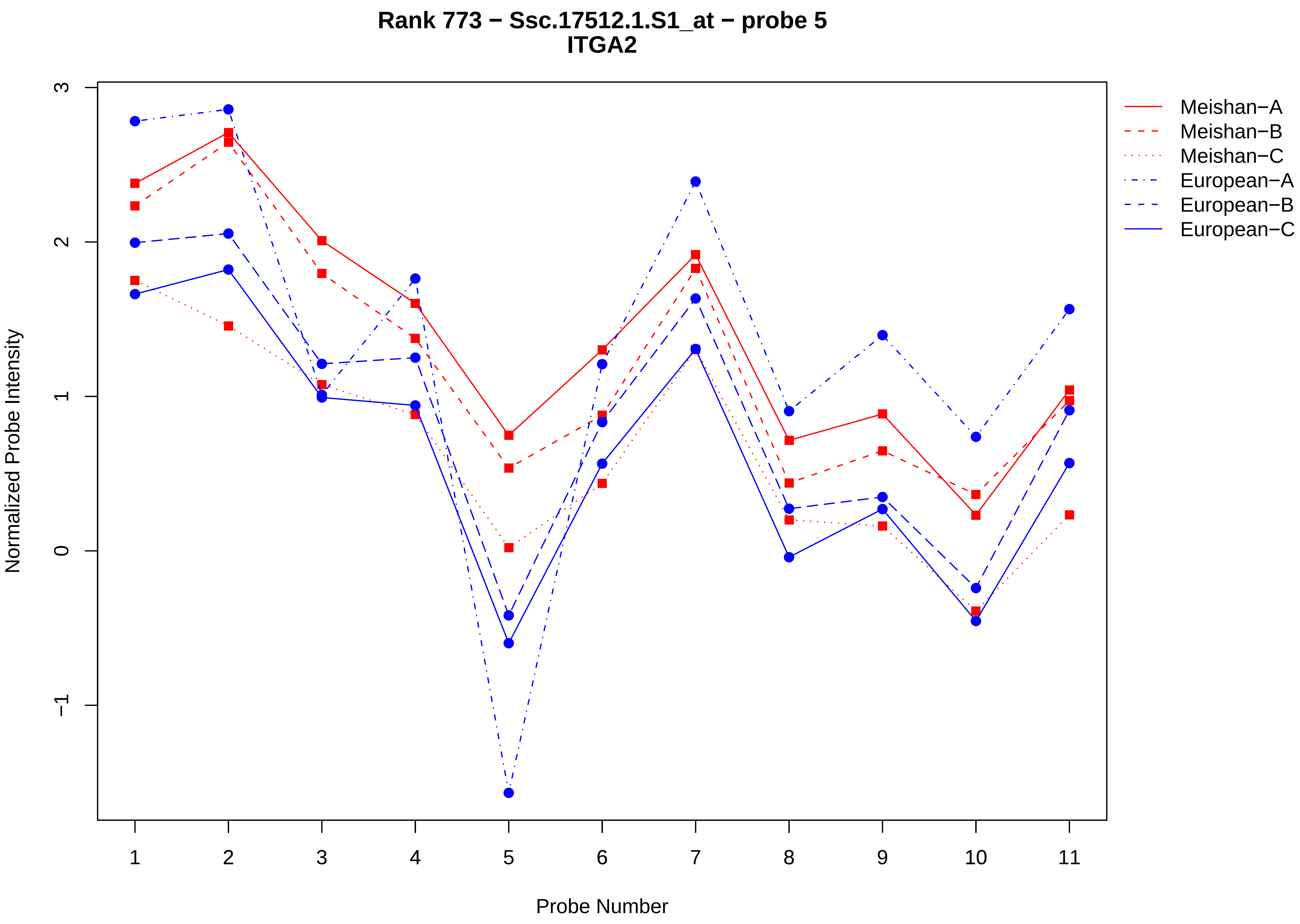

Rank 773 − Ssc.17512.1.S1_at − probe 5
ITGA2
Normalized Probe Intensity
Probe Number
Meishan−A
Meishan−B
Meishan−C
European−A
European−B
European−C
−1
0
1
2
3
1
2
3
4
5
6
7
8
9
10
11

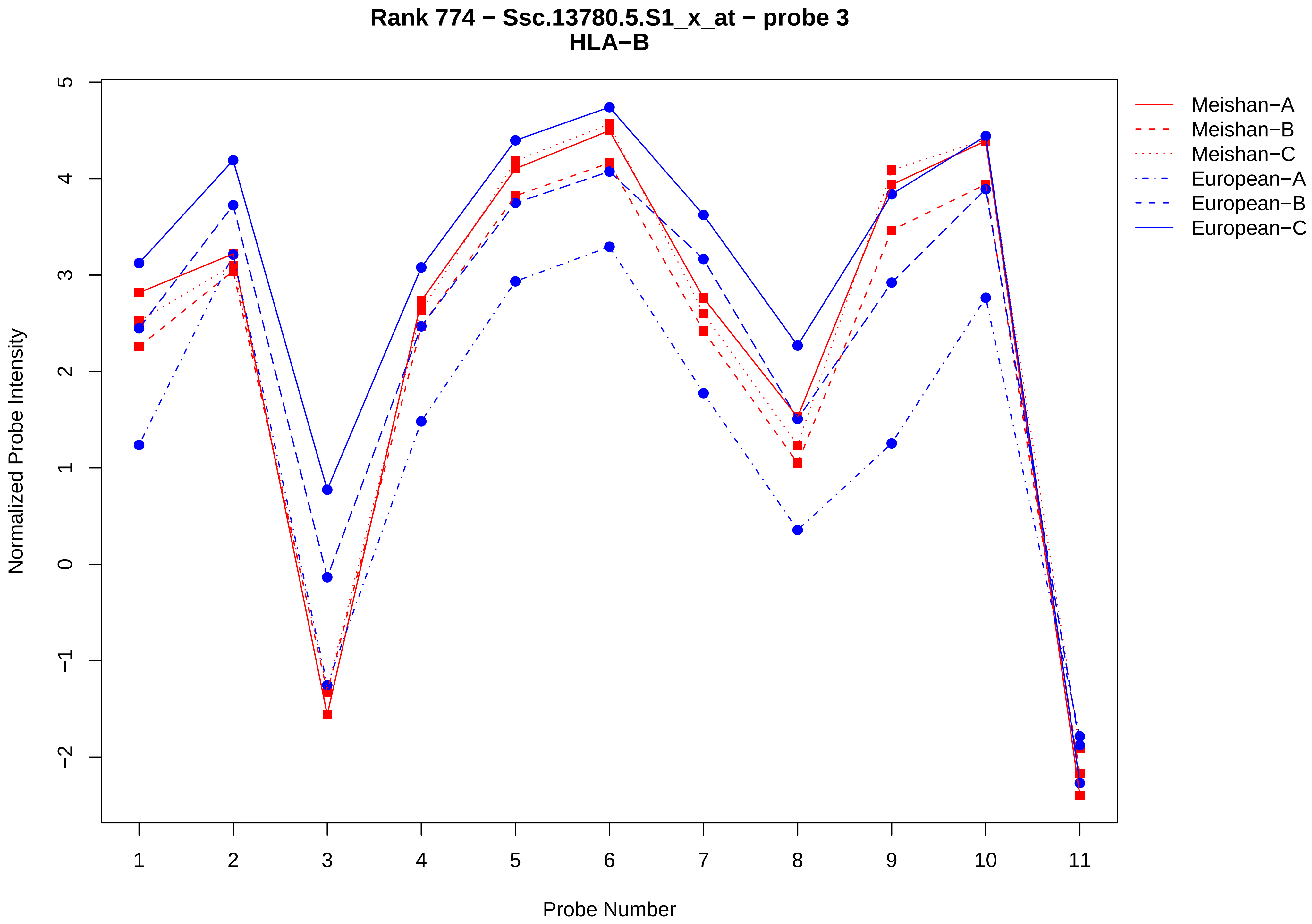

Rank 774 − Ssc.13780.5.S1_x_at − probe 3
HLA−B
Normalized Probe Intensity
Probe Number
5
4
3
2
1
0
−1
−2
1
2
3
4
5
6
7
8
9
10
11
Meishan−A
Meishan−B
Meishan−C
European−A
European−B
European−C

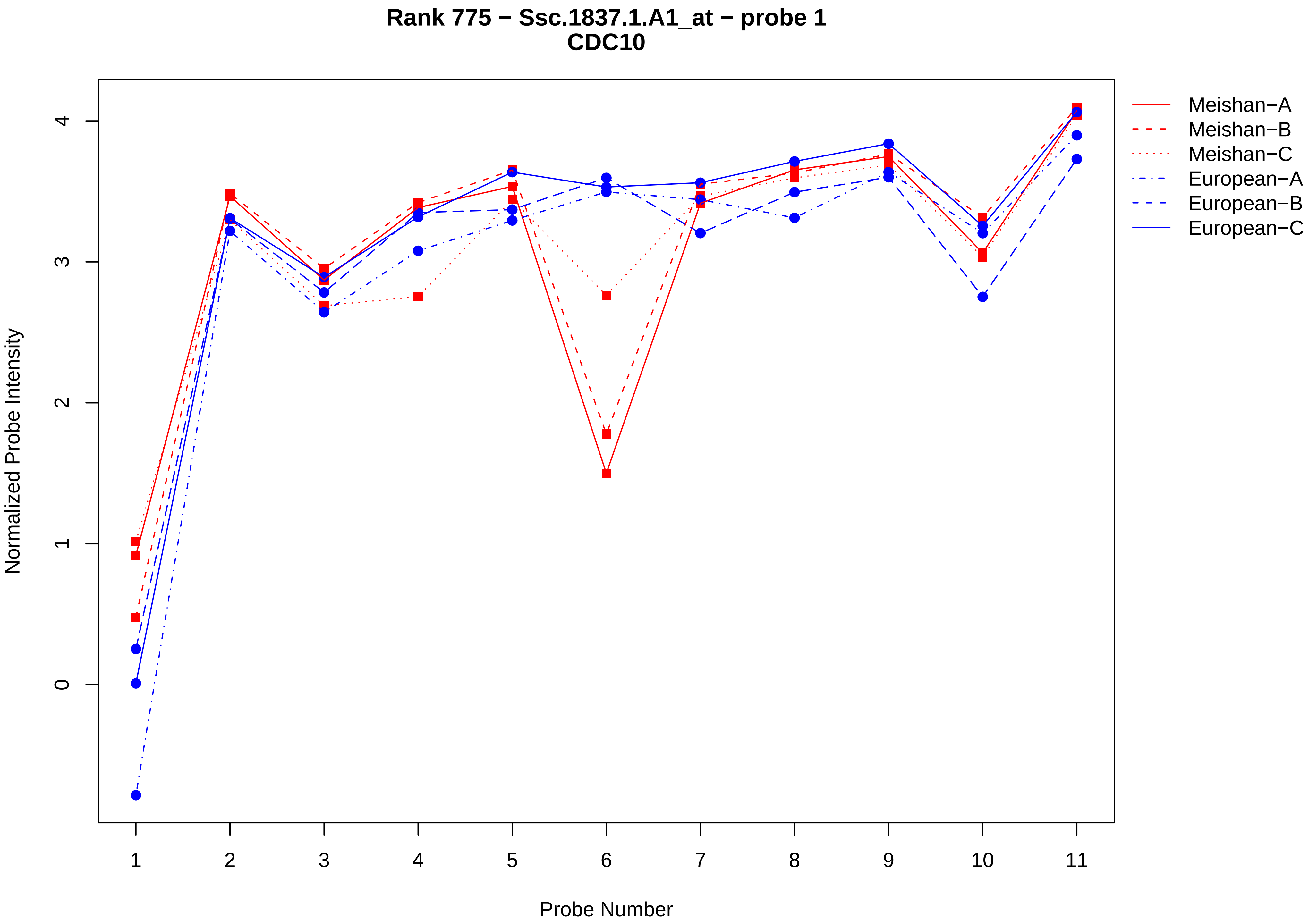

Rank 775 − Ssc.1837.1.A1_at − probe 1
CDC10
Normalized Probe Intensity
Probe Number
0
1
2
3
4
1
2
3
4
5
6
7
8
9
10
11
Meishan−A
Meishan−B
Meishan−C
European−A
European−B
European−C

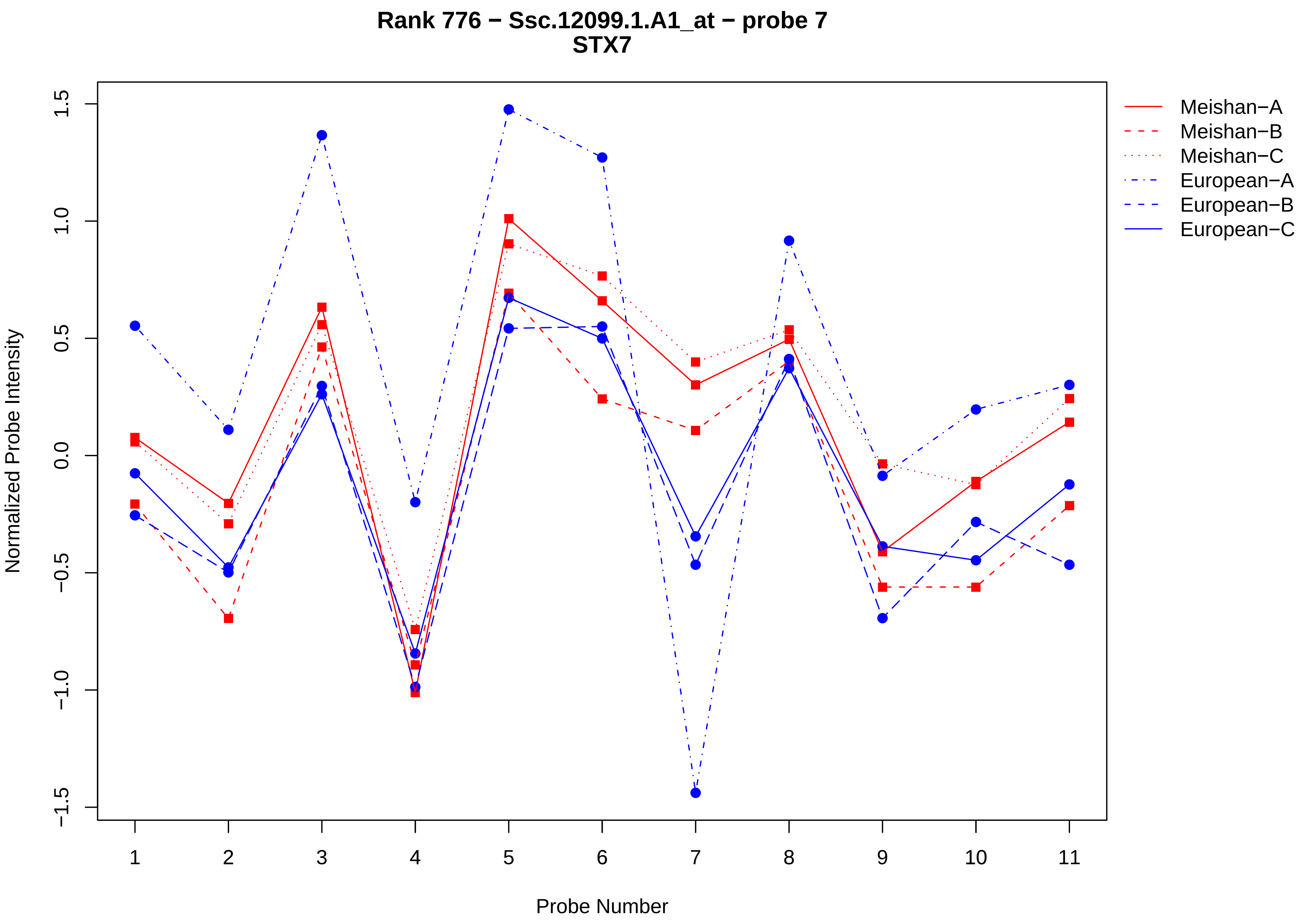

Rank 776 − Ssc.12099.1.A1_at − probe 7
STX7
Normalized Probe Intensity
Probe Number
Meishan−A
Meishan−B
Meishan−C
European−A
European−B
European−C

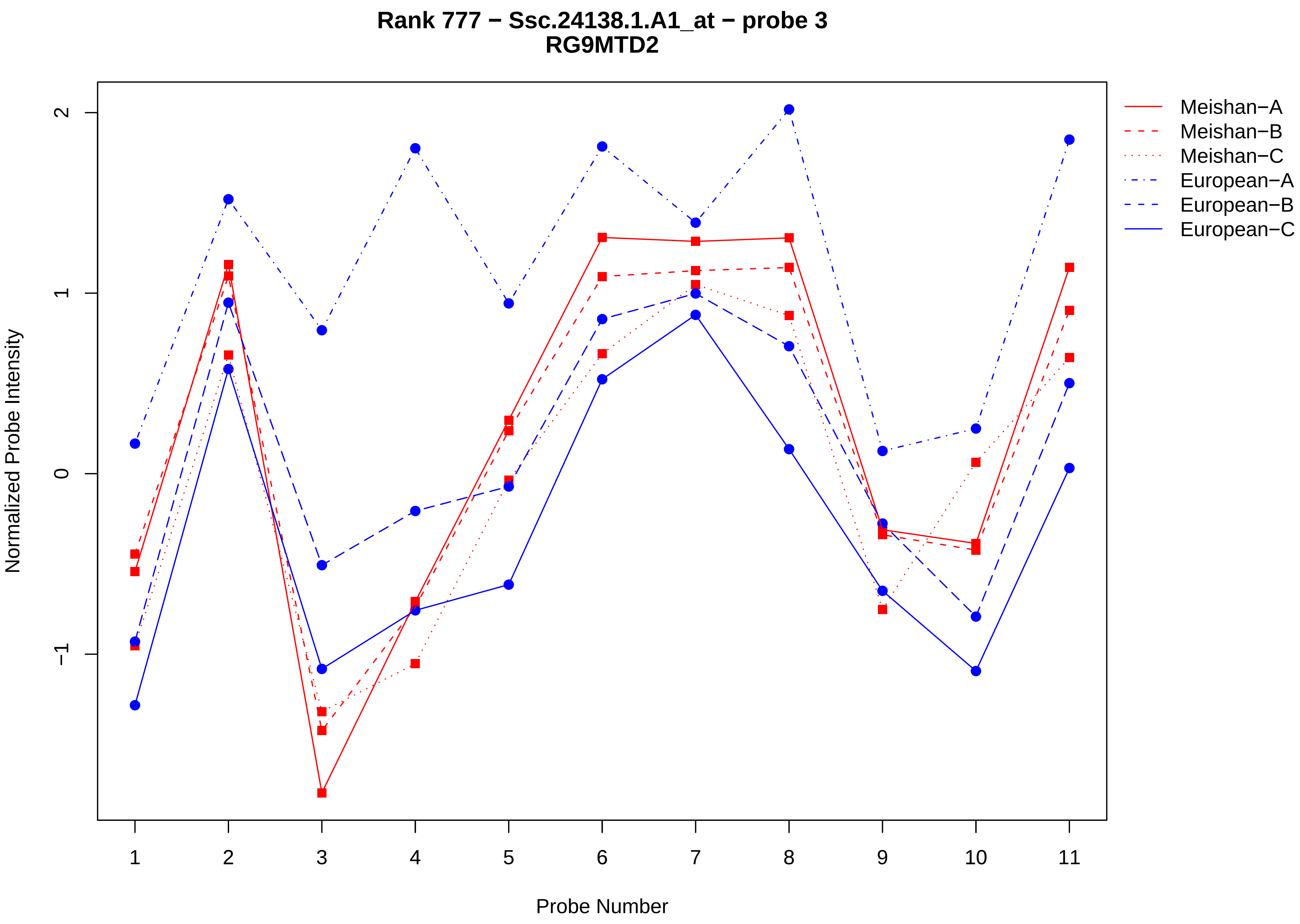
Rank 777 − Ssc.24138.1.A1_at − probe 3
RG9MTD2
Normalized Probe Intensity
Probe Number
−1
0
1
2
1
2
3
4
5
6
7
8
9
10
11
Meishan−A
Meishan−B
Meishan−C
European−A
European−B
European−C

Rank 778 − Ssc.1407.3.S1_at − probe 6
AKAP2

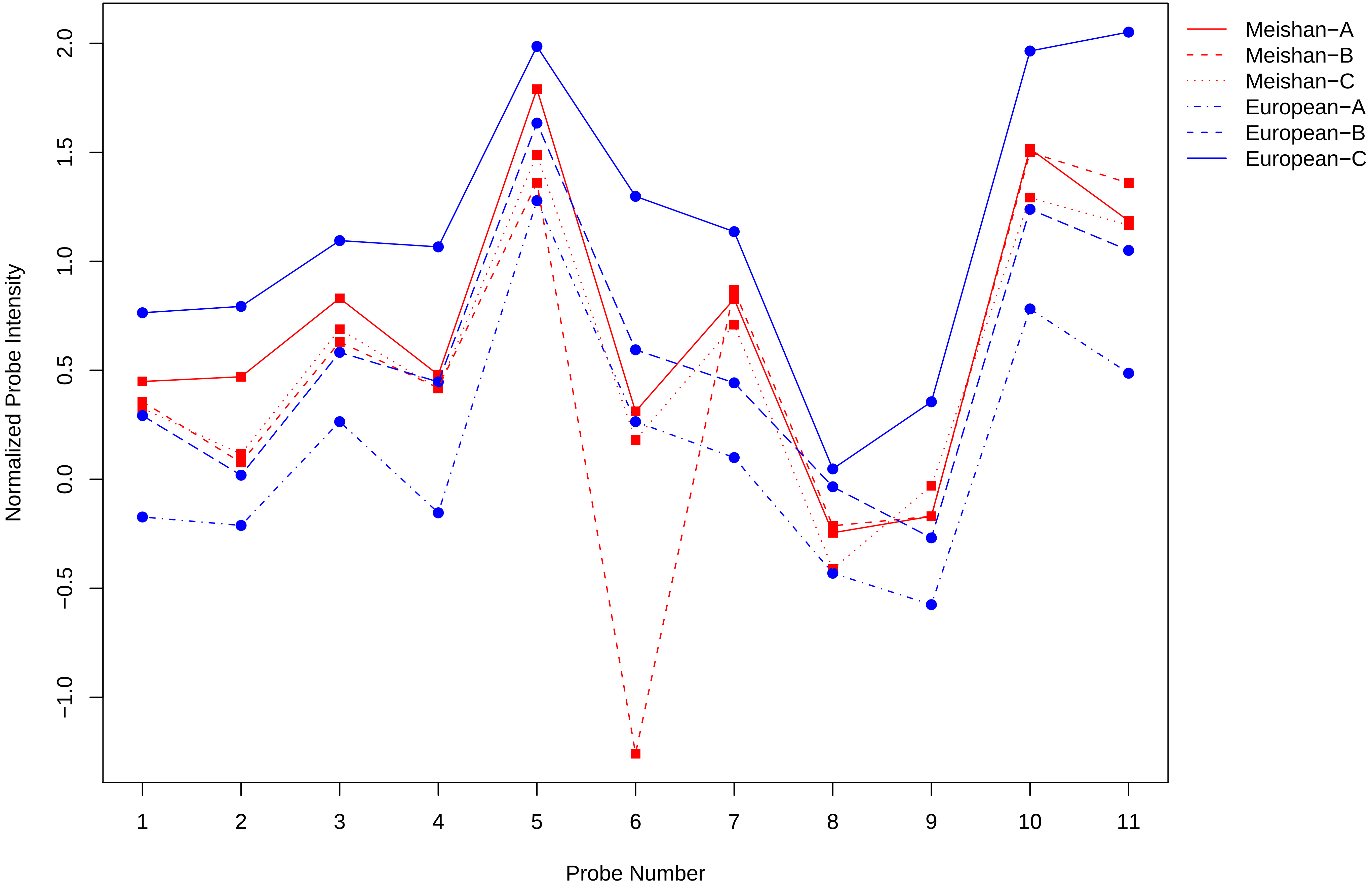

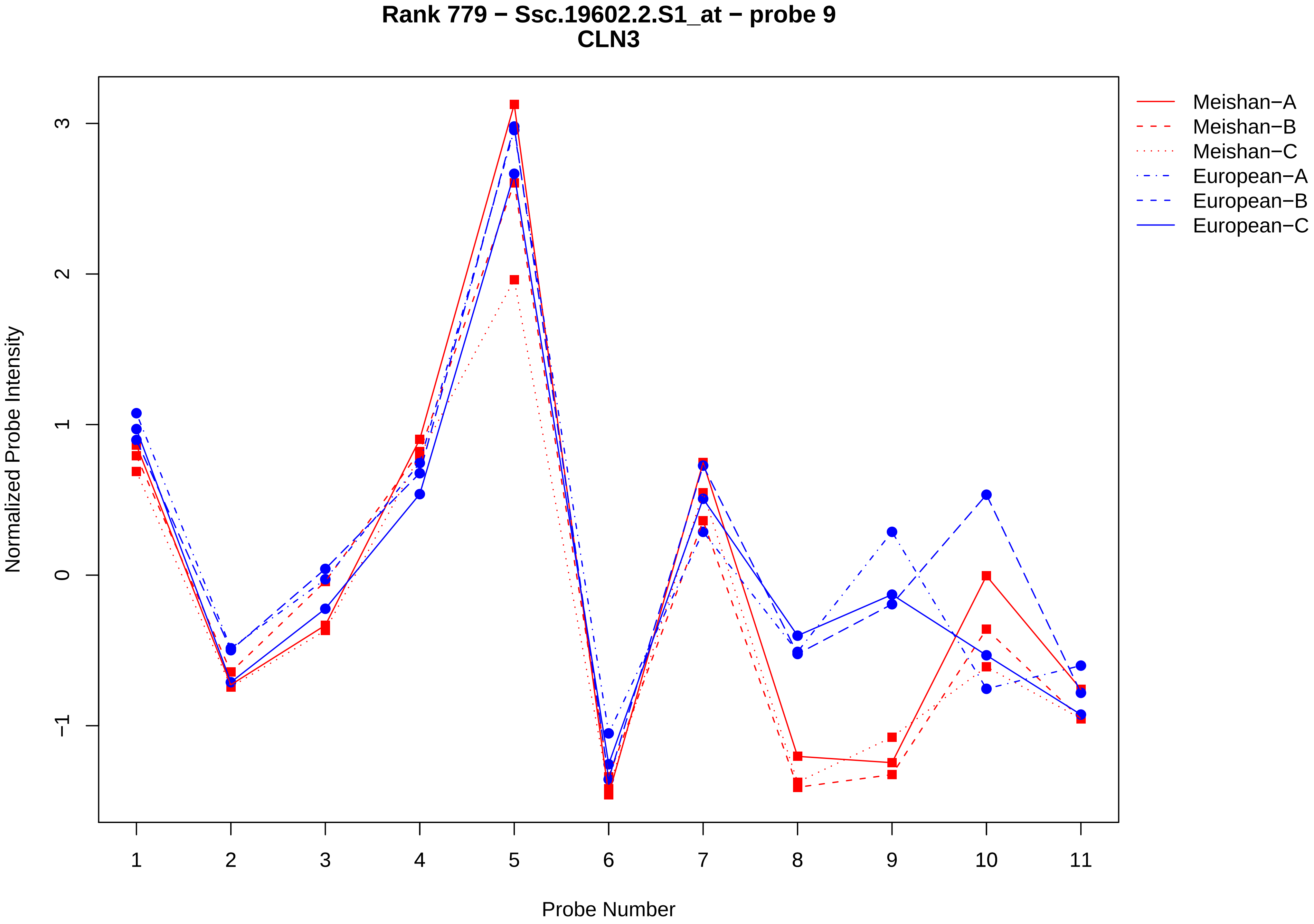

Rank 779 − Ssc.19602.2.S1_at − probe 9
CLN3
Normalized Probe Intensity
Probe Number
Meishan−A
Meishan−B
Meishan−C
European−A
European−B
European−C
−1
0
1
2
3
1
2
3
4
5
6
7
8
9
10
11

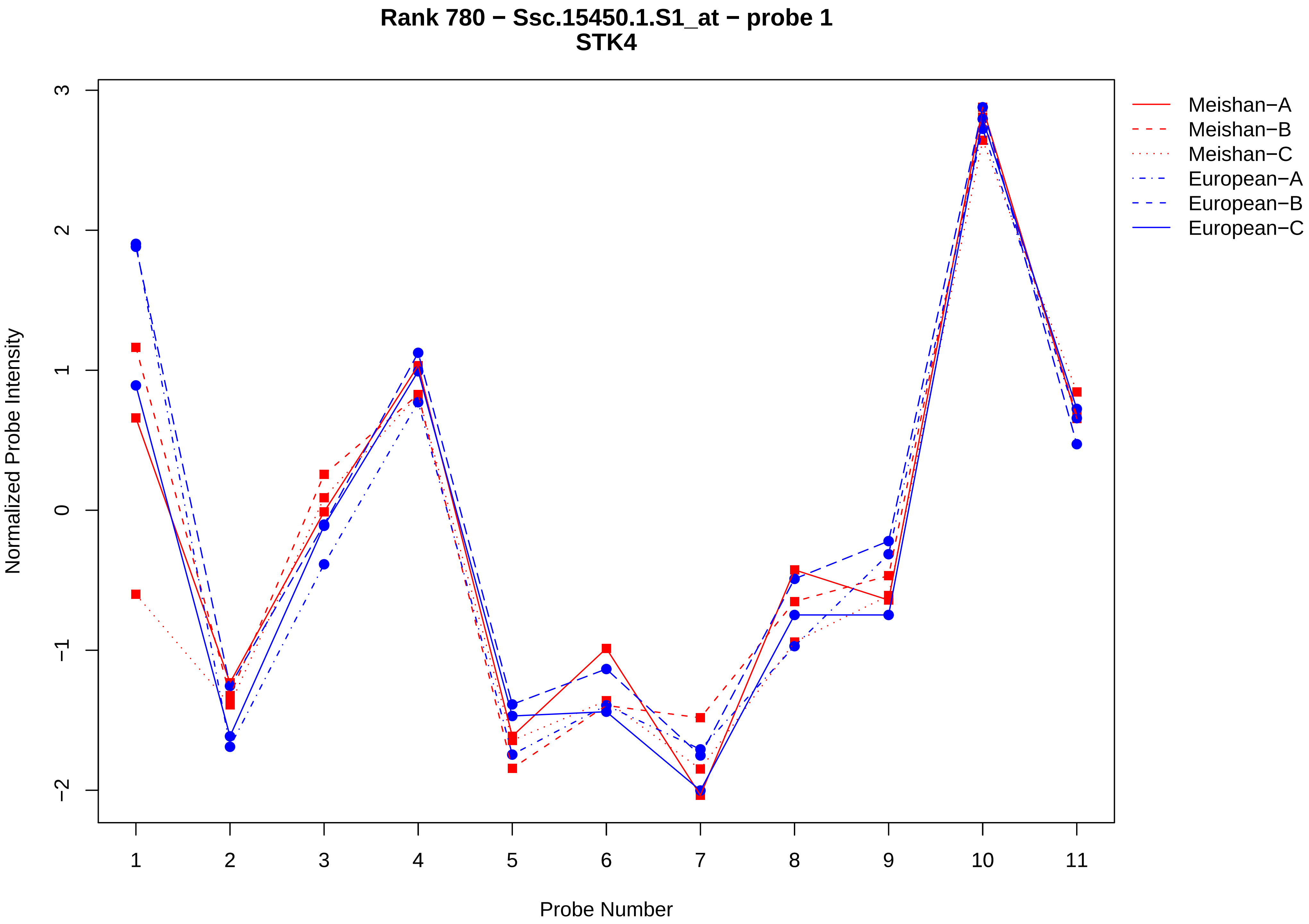

Rank 780 − Ssc.15450.1.S1_at − probe 1
STK4
Normalized Probe Intensity
Probe Number
−2
−1
0
1
2
3
1
2
3
4
5
6
7
8
9
10
11
Meishan−A
Meishan−B
Meishan−C
European−A
European−B
European−C

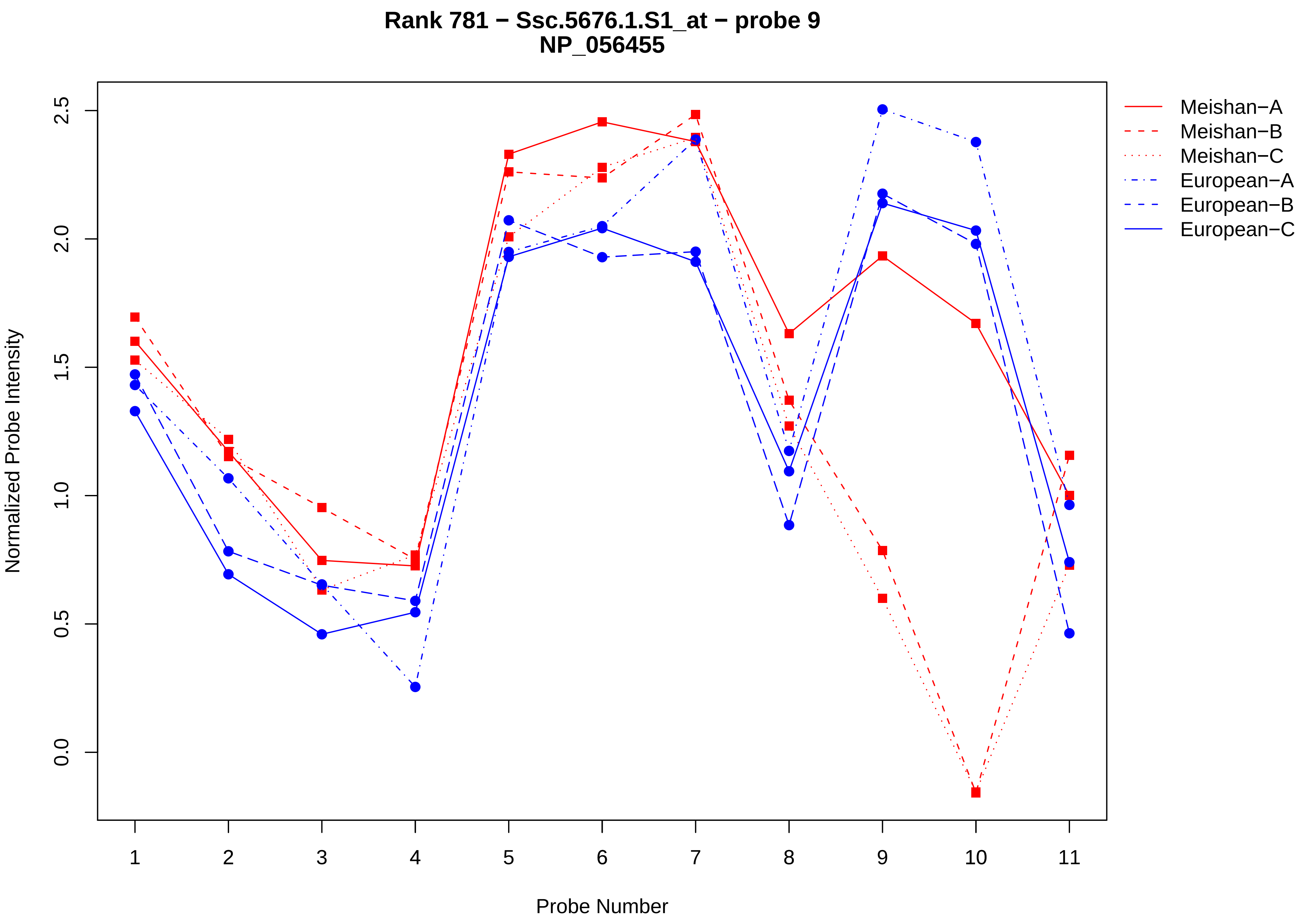

Rank 781 − Ssc.5676.1.S1_at − probe 9
NP_056455
Normalized Probe Intensity
Probe Number
0.0
0.5
1.0
1.5
2.0
2.5
1
2
3
4
5
6
7
8
9
10
11
Meishan−A
Meishan−B
Meishan−C
European−A
European−B
European−C

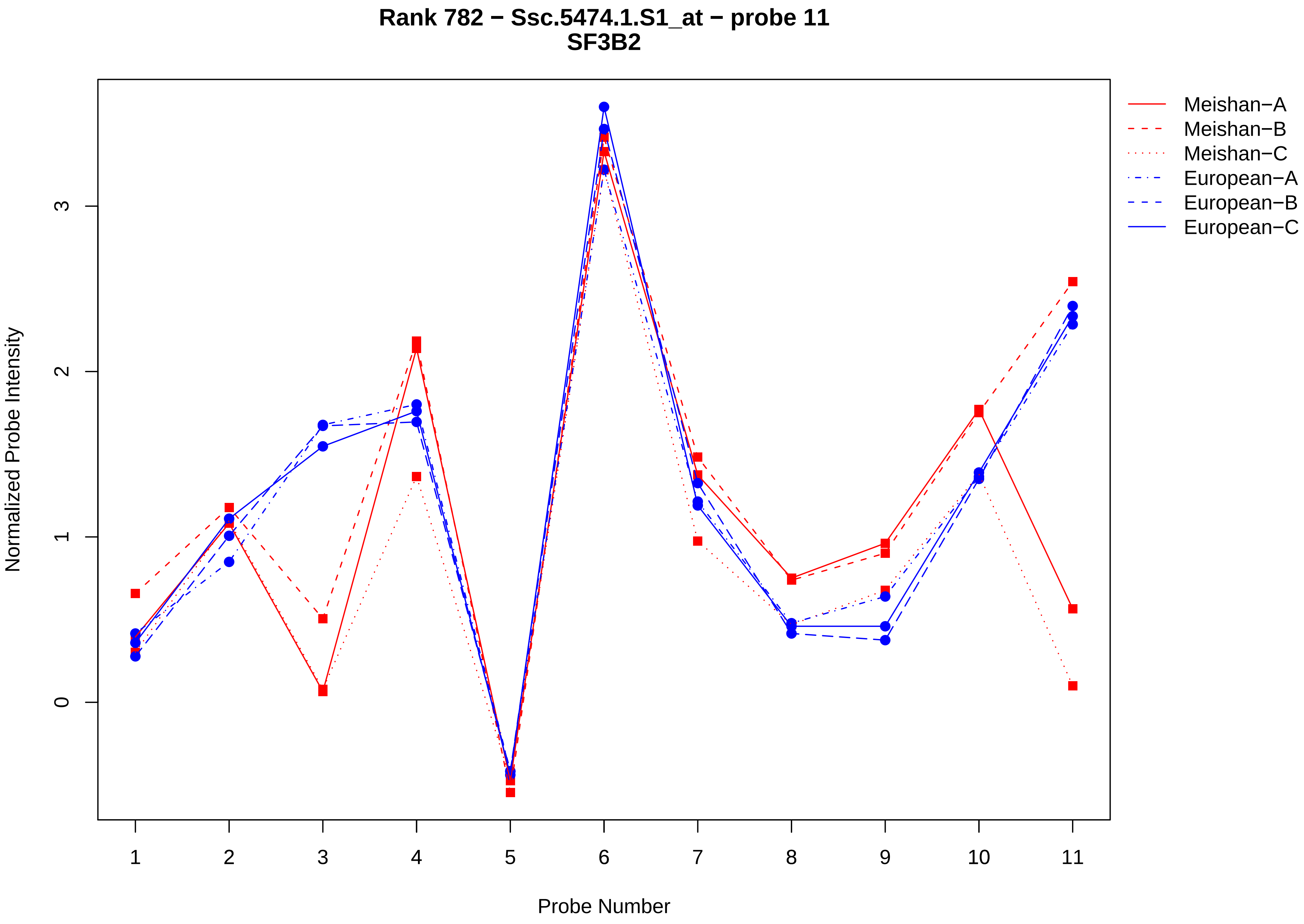
Rank 782 − Ssc.5474.1.S1_at − probe 11
SF3B2
Normalized Probe Intensity
Probe Number
0
1
2
3
1
2
3
4
5
6
7
8
9
10
11
Meishan−A
Meishan−B
Meishan−C
European−A
European−B
European−C

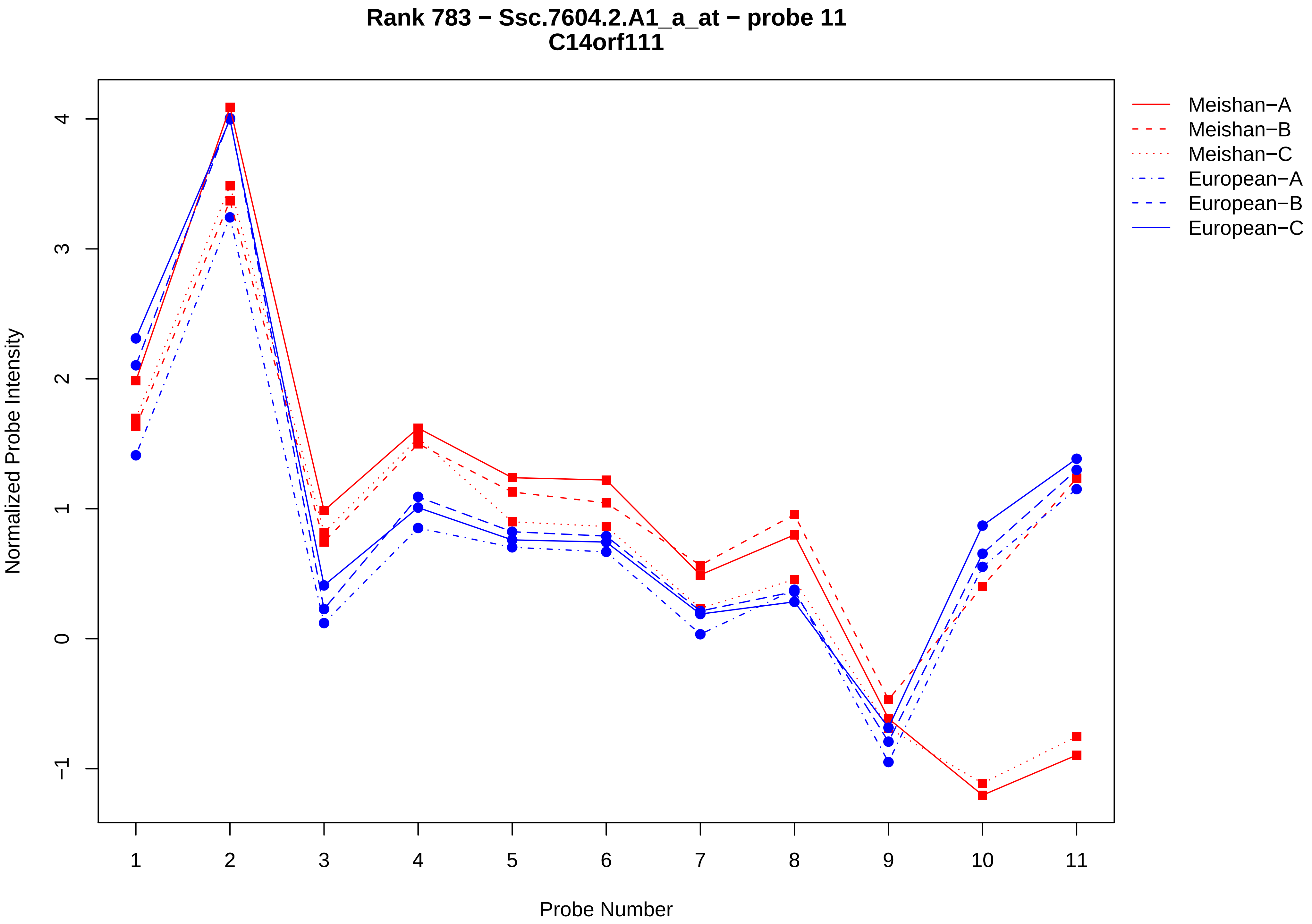

Rank 783 − Ssc.7604.2.A1_a_at − probe 11
C14orf111
Normalized Probe Intensity
Probe Number
−1
0
1
2
3
4
1
2
3
4
5
6
7
8
9
10
11
Meishan−A
Meishan−B
Meishan−C
European−A
European−B
European−C

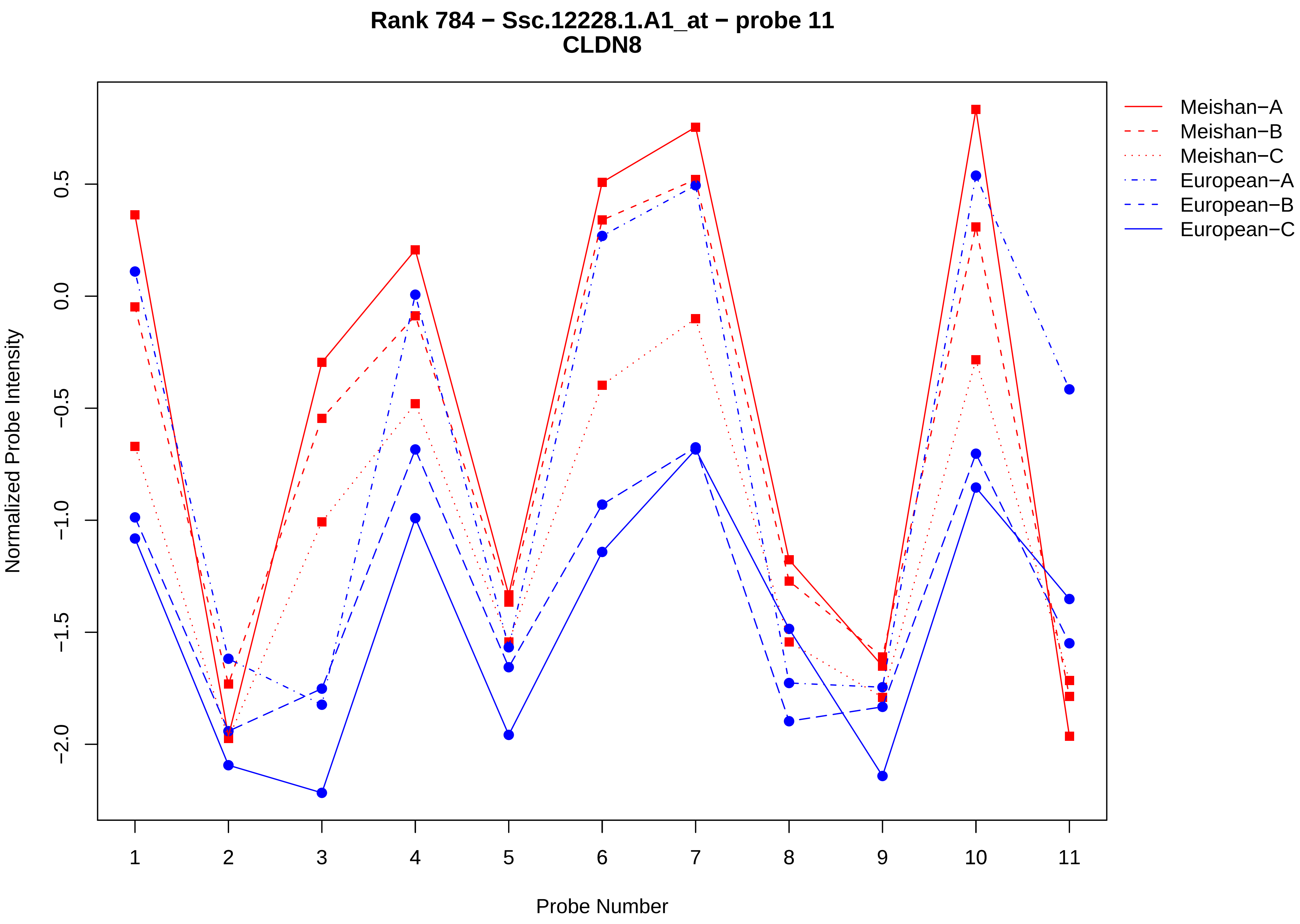

Rank 784 − Ssc.12228.1.A1_at − probe 11
CLDN8
Normalized Probe Intensity
Probe Number
Meishan−A
Meishan−B
Meishan−C
European−A
European−B
European−C
0.5
0.0
−0.5
−1.0
−1.5
−2.0
1
2
3
4
5
6
7
8
9
10
11

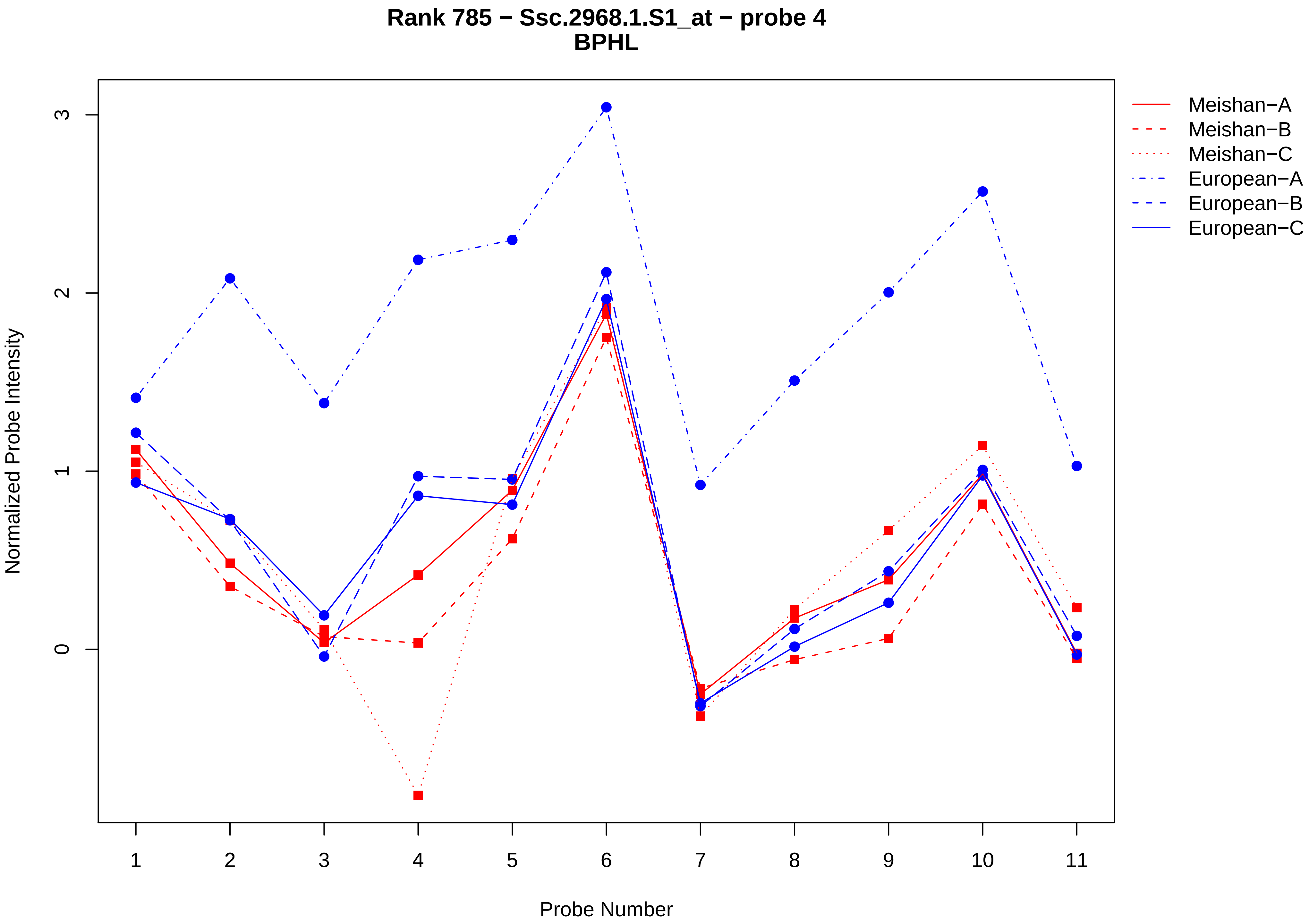

Rank 785 − Ssc.2968.1.S1_at − probe 4
BPHL
Normalized Probe Intensity
Probe Number
0
1
2
3
1
2
3
4
5
6
7
8
9
10
11
Meishan−A
Meishan−B
Meishan−C
European−A
European−B
European−C

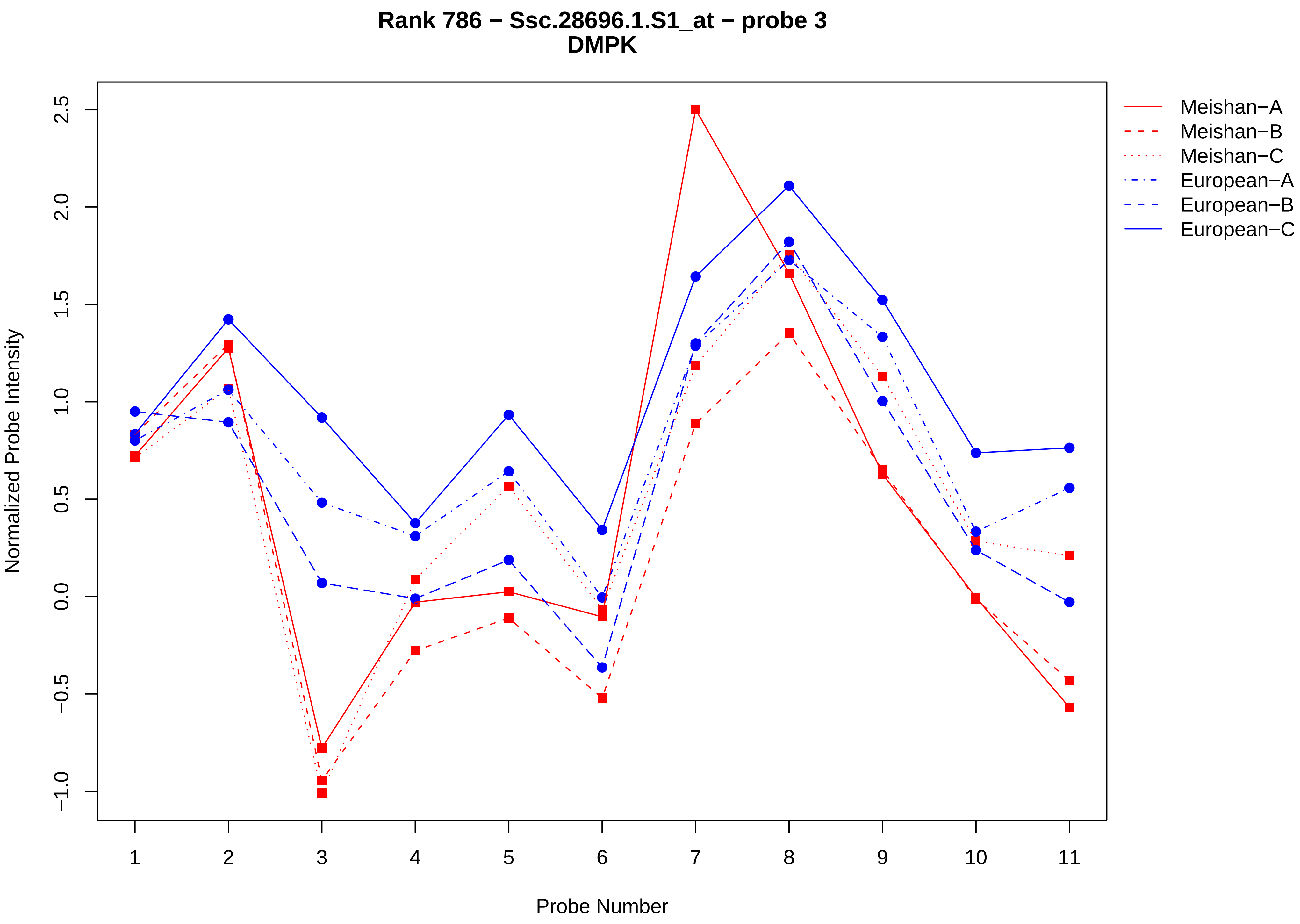

Rank 786 − Ssc.28696.1.S1_at − probe 3
DMPK
Normalized Probe Intensity
Probe Number
Meishan−A
Meishan−B
Meishan−C
European−A
European−B
European−C
−1.0
−0.5
0.0
0.5
1.0
1.5
2.0
2.5
1
2
3
4
5
6
7
8
9
10
11

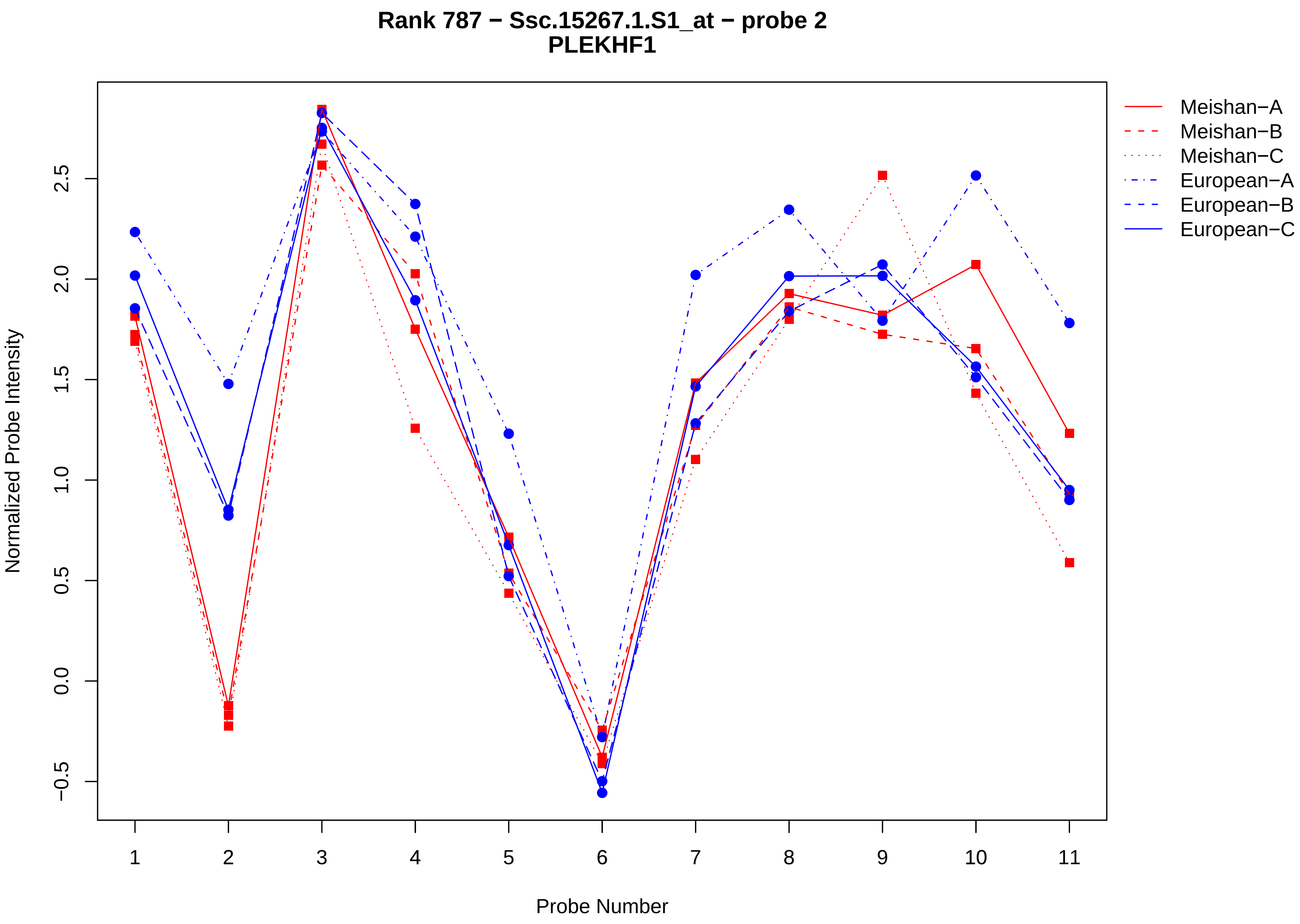

Rank 787 − Ssc.15267.1.S1_at − probe 2
PLEKHF1
Normalized Probe Intensity
−0.5
0.0
0.5
1.0
1.5
2.0
2.5
1
2
3
4
5
6
7
8
9
10
11
Probe Number
Meishan−A
Meishan−B
Meishan−C
European−A
European−B
European−C

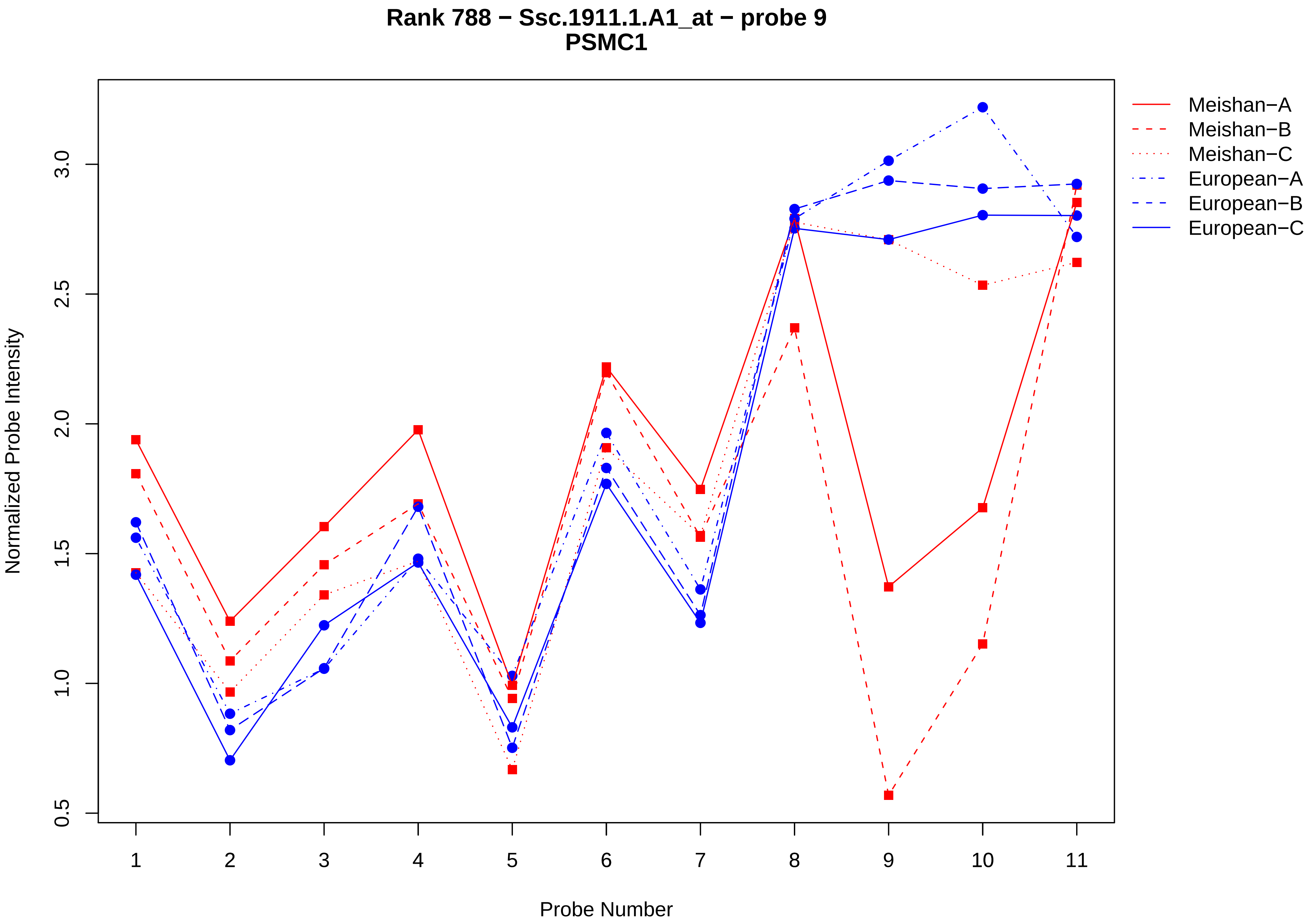

Rank 788 − Ssc.1911.1.A1_at − probe 9
PSMC1
Normalized Probe Intensity
Probe Number
0.5
1.0
1.5
2.0
2.5
3.0
1
2
3
4
5
6
7
8
9
10
11
Meishan−A
Meishan−B
Meishan−C
European−A
European−B
European−C

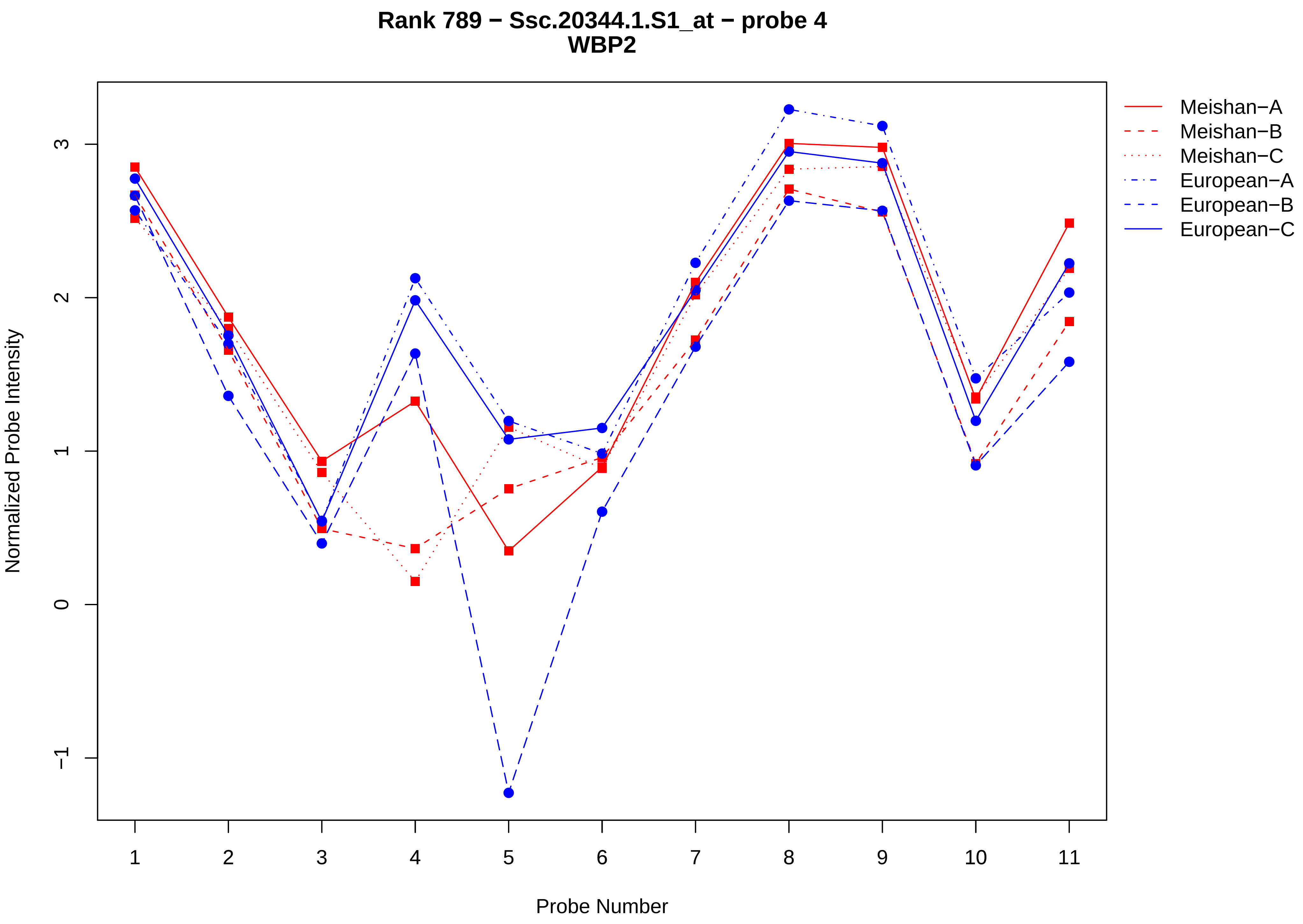
Rank 789 − Ssc.20344.1.S1_at − probe 4
WBP2
Normalized Probe Intensity
Probe Number
Meishan−A
Meishan−B
Meishan−C
European−A
European−B
European−C

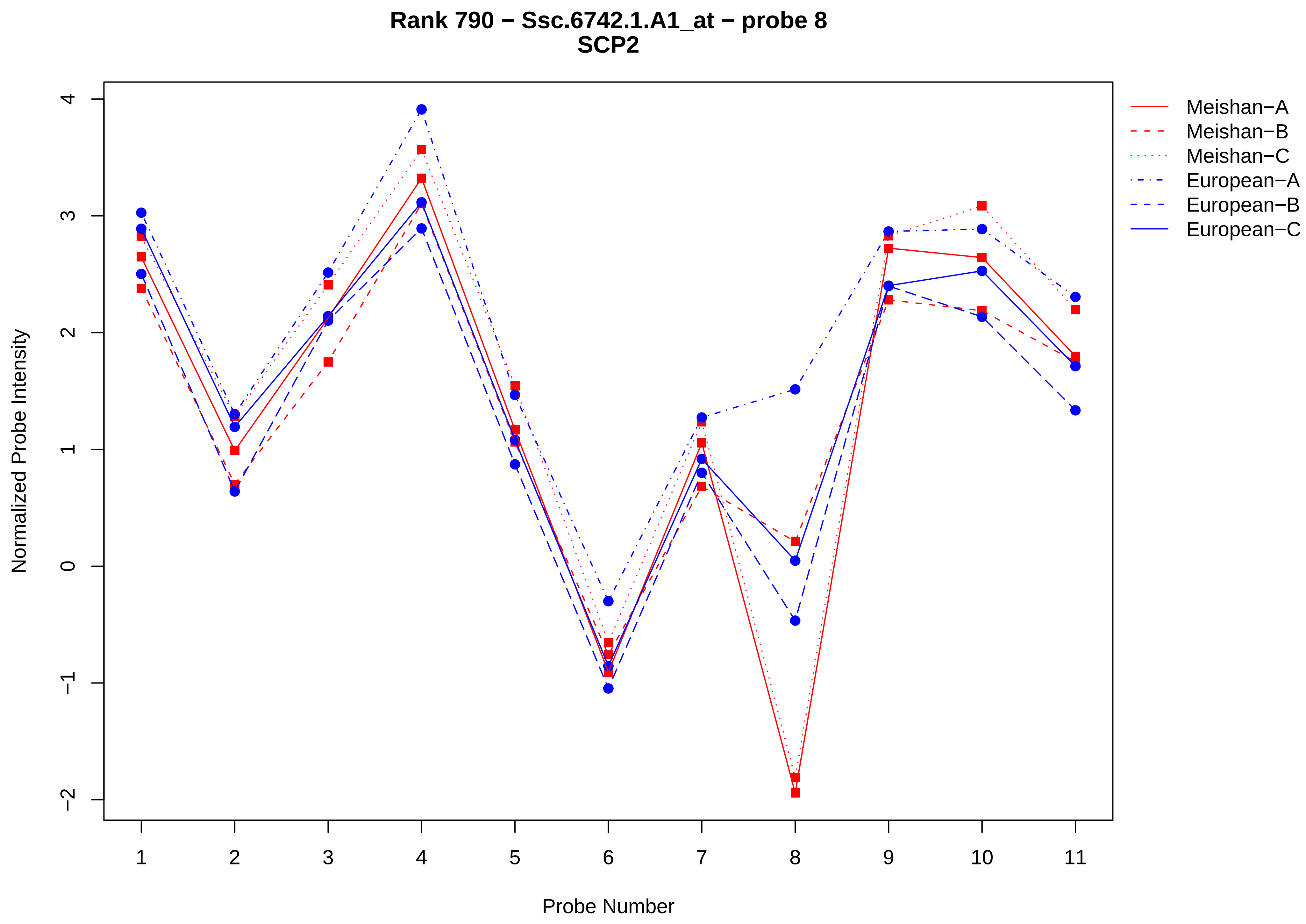

Rank 790 − Ssc.6742.1.A1_at − probe 8
SCP2
Normalized Probe Intensity
Probe Number
Meishan−A
Meishan−B
Meishan−C
European−A
European−B
European−C

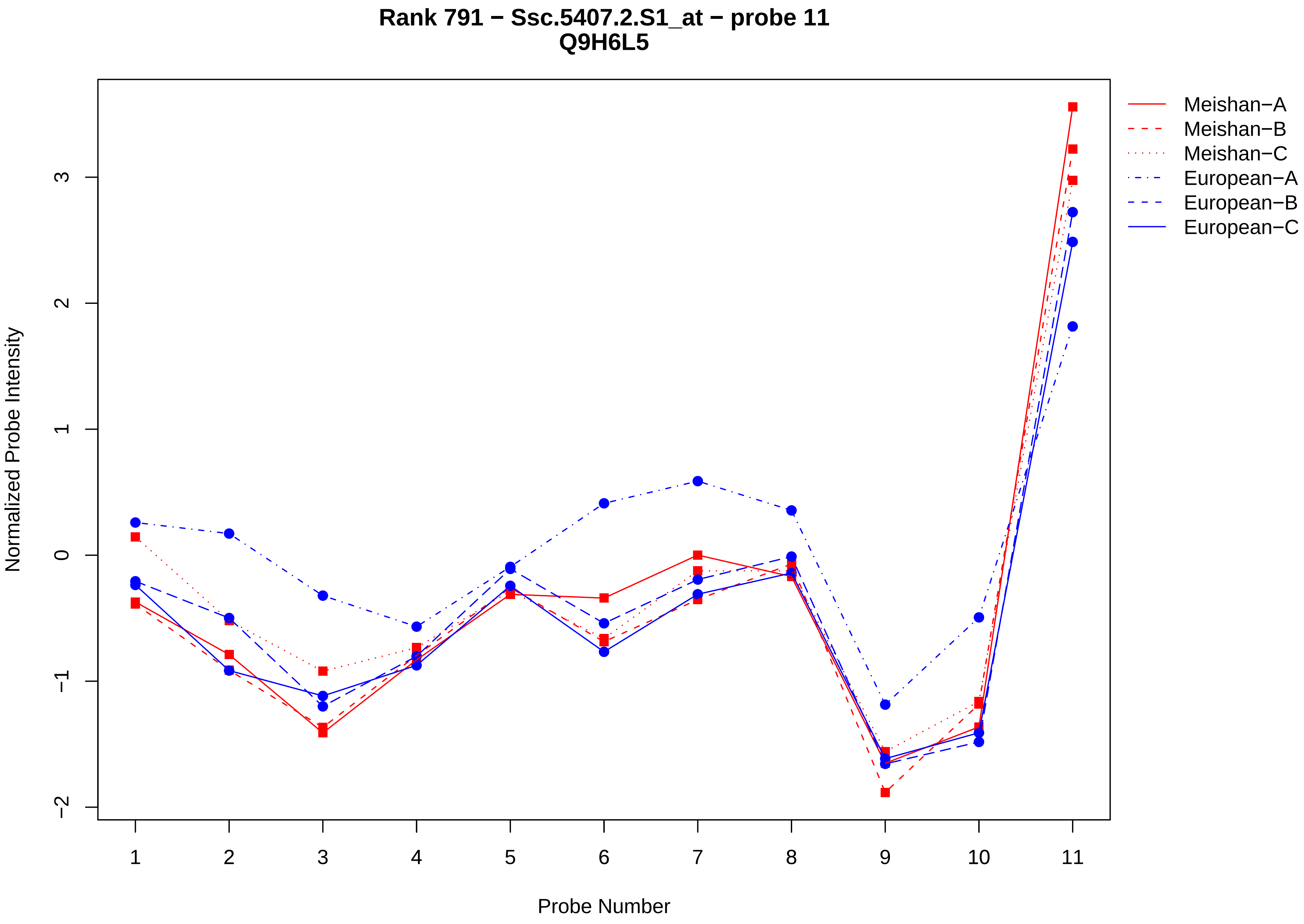

Rank 791 − Ssc.5407.2.S1_at − probe 11
Q9H6L5
Normalized Probe Intensity
Probe Number
−2
−1
0
1
2
3
1
2
3
4
5
6
7
8
9
10
11
Meishan−A
Meishan−B
Meishan−C
European−A
European−B
European−C

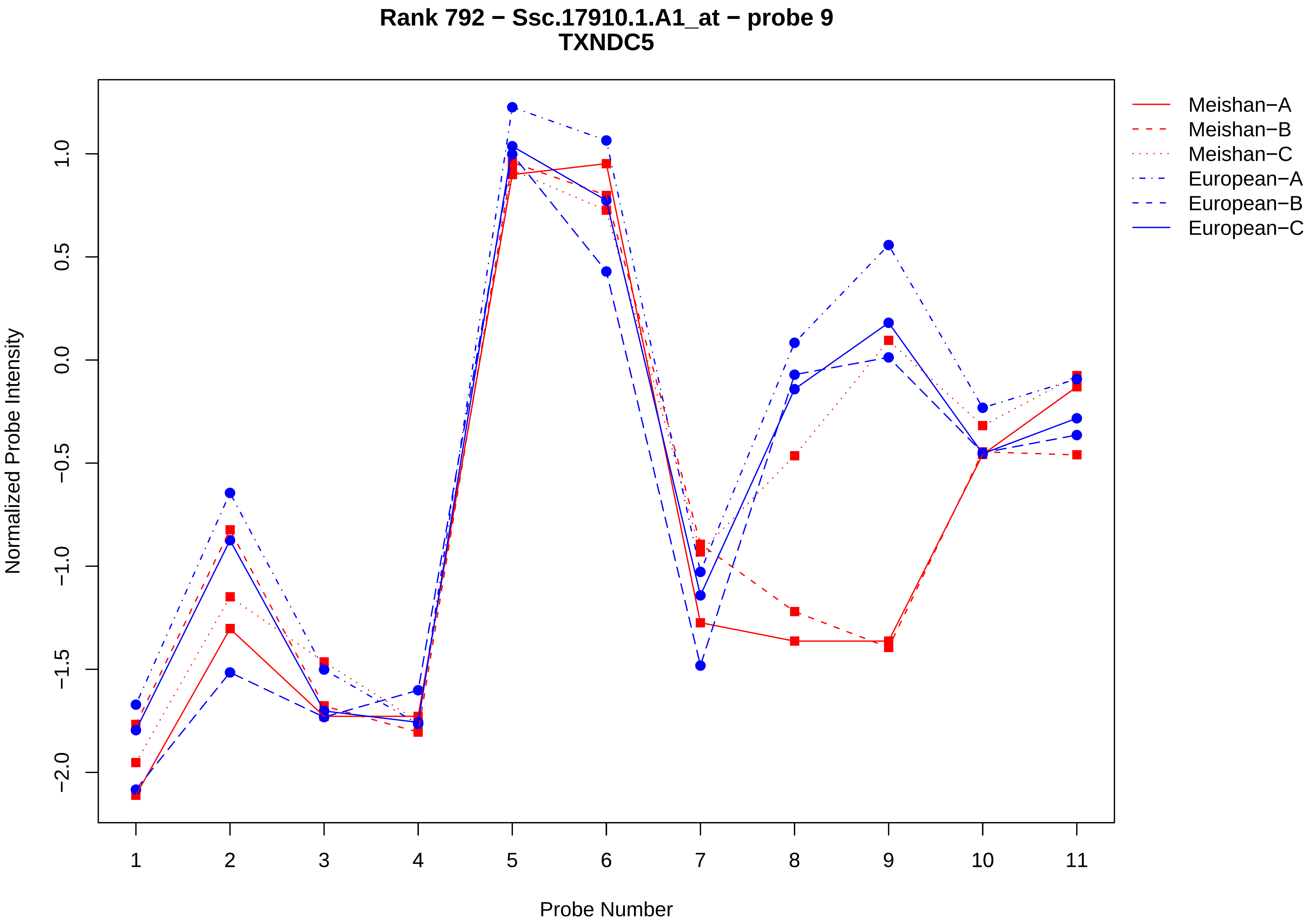

Rank 792 − Ssc.17910.1.A1_at − probe 9
TXNDC5
Normalized Probe Intensity
Probe Number
−2.0
−1.5
−1.0
−0.5
0.0
0.5
1.0
1
2
3
4
5
6
7
8
9
10
11
Meishan−A
Meishan−B
Meishan−C
European−A
European−B
European−C

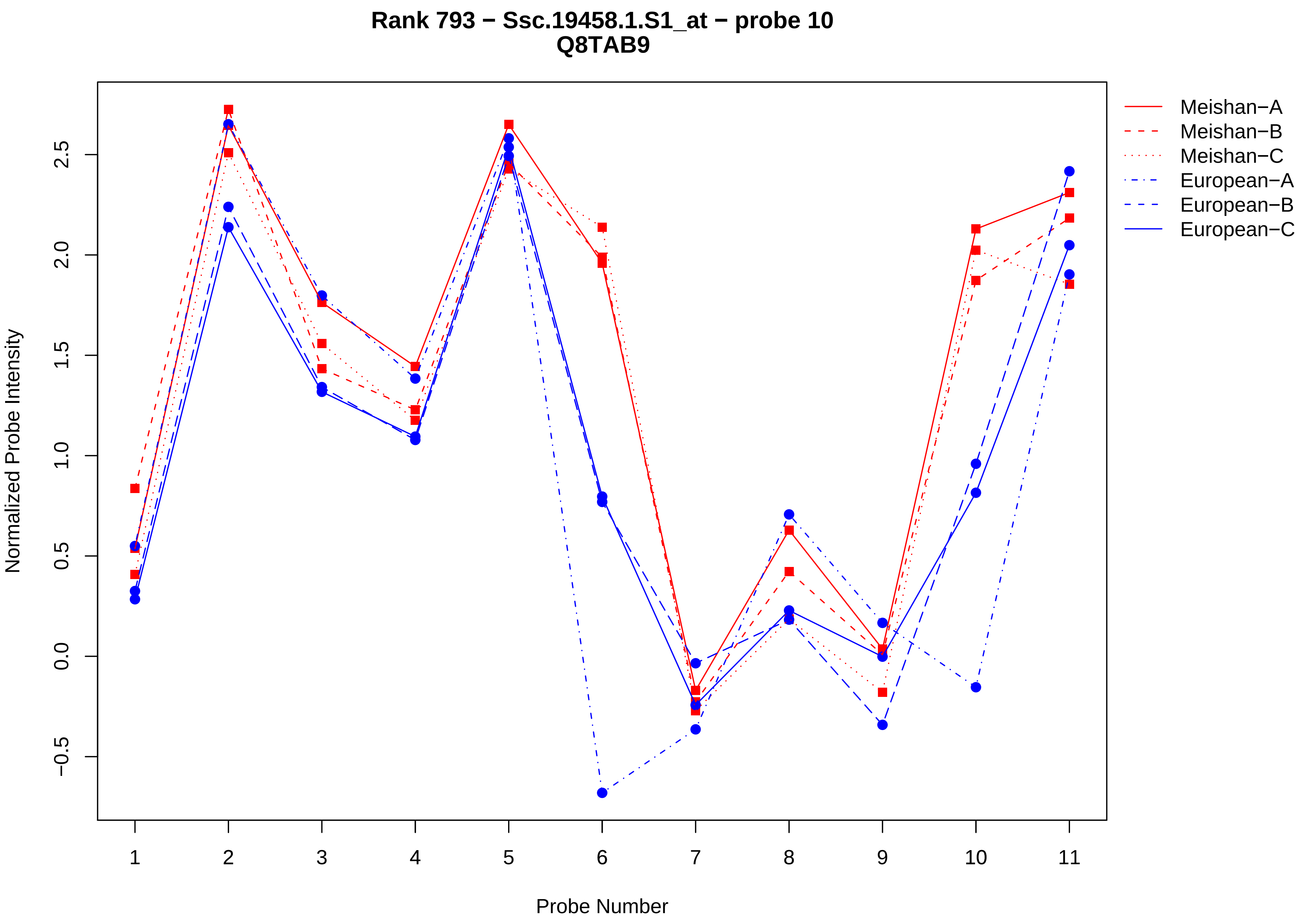

Rank 793 − Ssc.19458.1.S1_at − probe 10
Q8TAB9
Normalized Probe Intensity
Probe Number
−0.5
0.0
0.5
1.0
1.5
2.0
2.5
1
2
3
4
5
6
7
8
9
10
11
Meishan−A
Meishan−B
Meishan−C
European−A
European−B
European−C

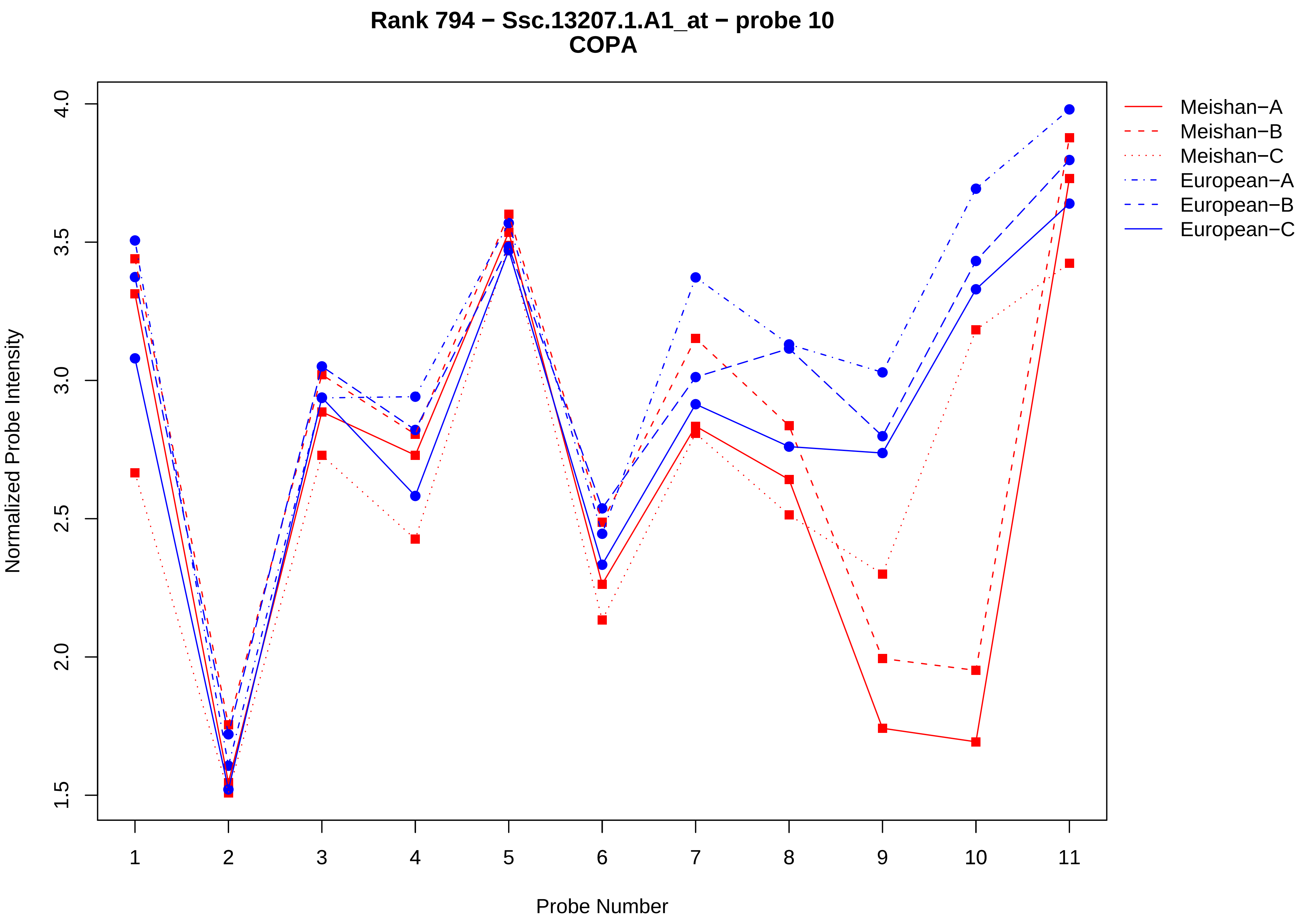

Rank 794 − Ssc.13207.1.A1_at − probe 10
COPA
Normalized Probe Intensity
Probe Number
1.5
2.0
2.5
3.0
3.5
4.0
1
2
3
4
5
6
7
8
9
10
11
Meishan−A
Meishan−B
Meishan−C
European−A
European−B
European−C

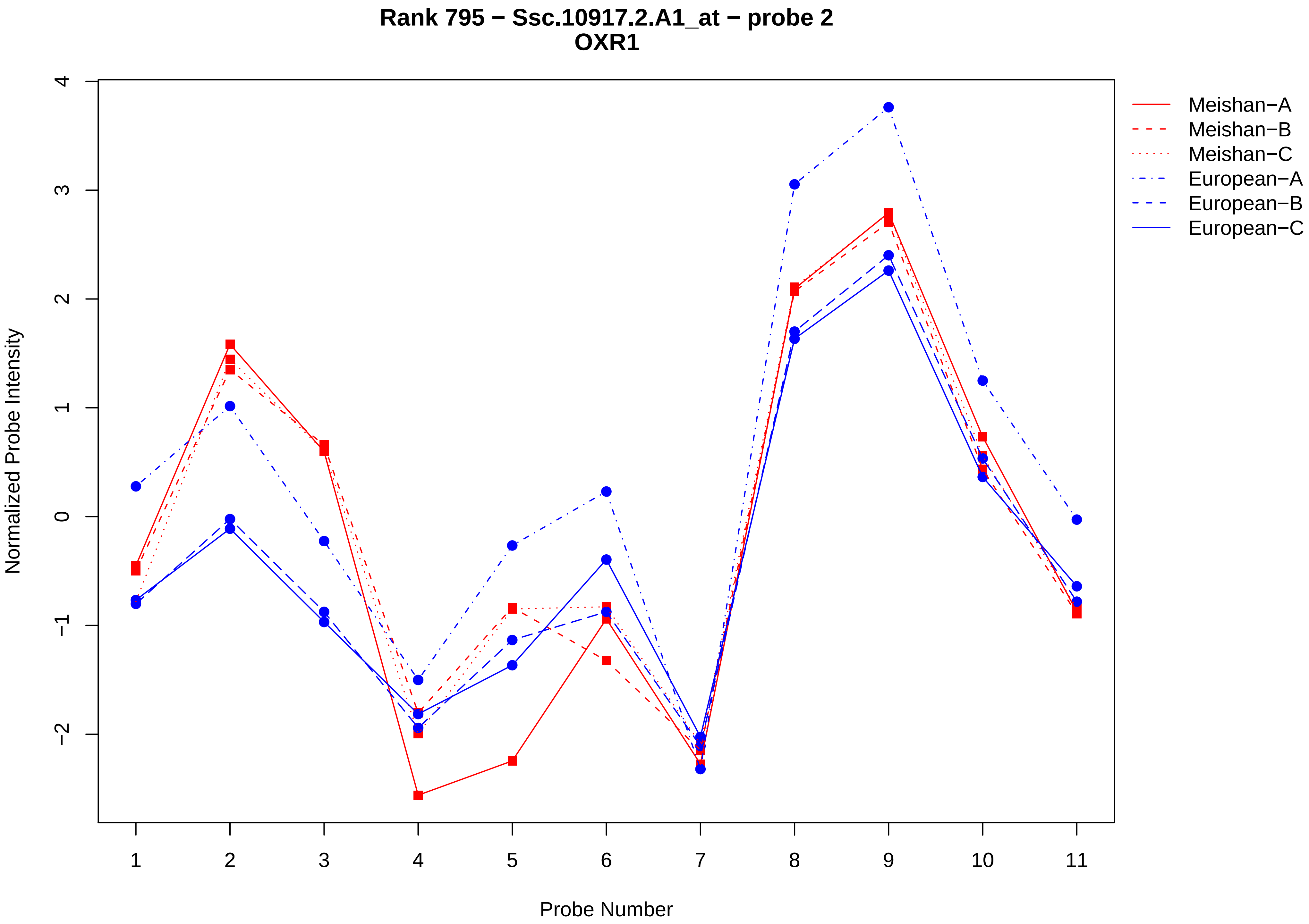

Rank 795 − Ssc.10917.2.A1_at − probe 2
OXR1
Normalized Probe Intensity
Probe Number
Meishan−A
Meishan−B
Meishan−C
European−A
European−B
European−C
4
3
2
1
0
−1
−2
1
2
3
4
5
6
7
8
9
10
11

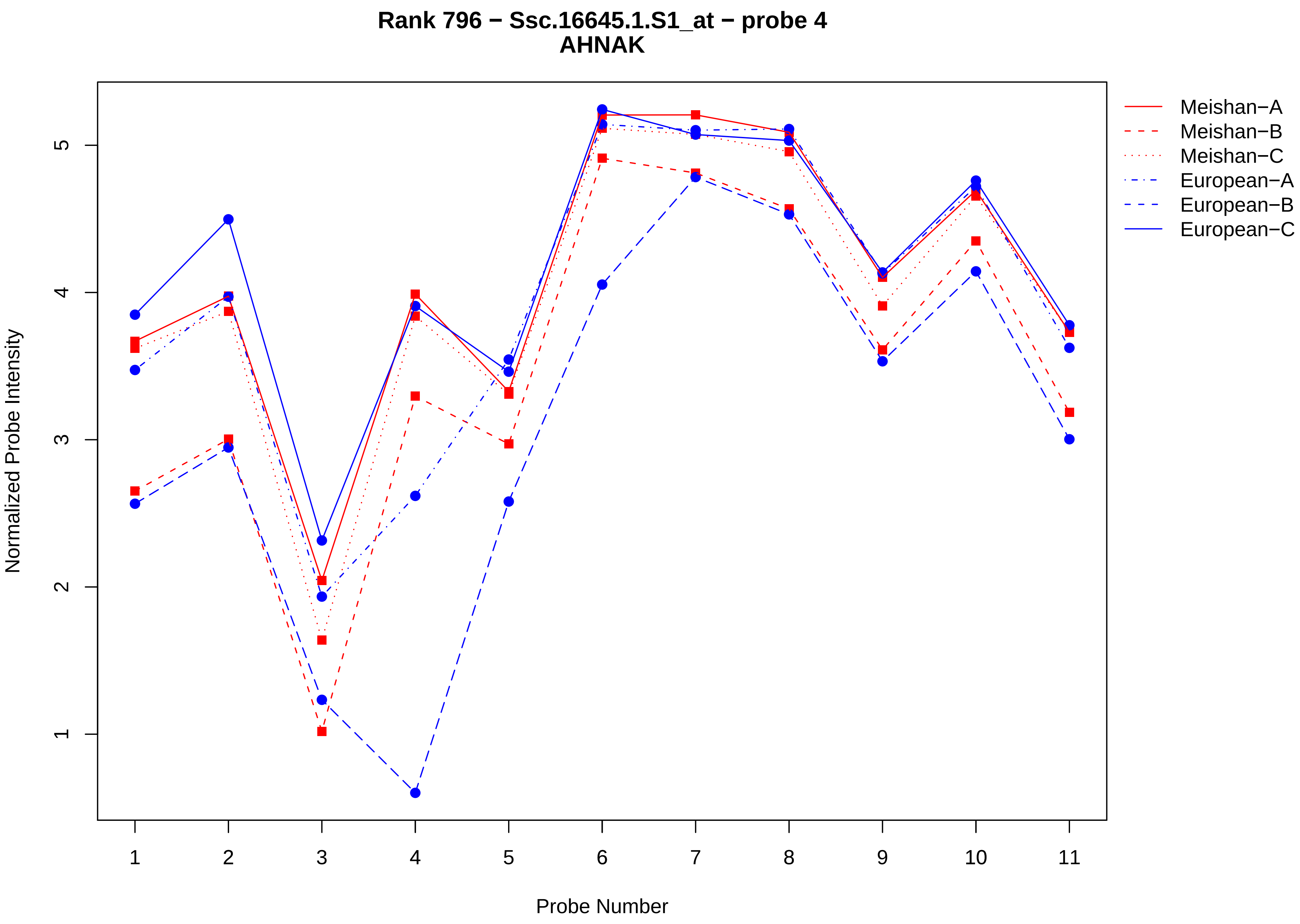
Rank 796 − Ssc.16645.1.S1_at − probe 4
AHNAK
Normalized Probe Intensity
Probe Number
1
2
3
4
5
6
7
8
9
10
11
Meishan−A
Meishan−B
Meishan−C
European−A
European−B
European−C

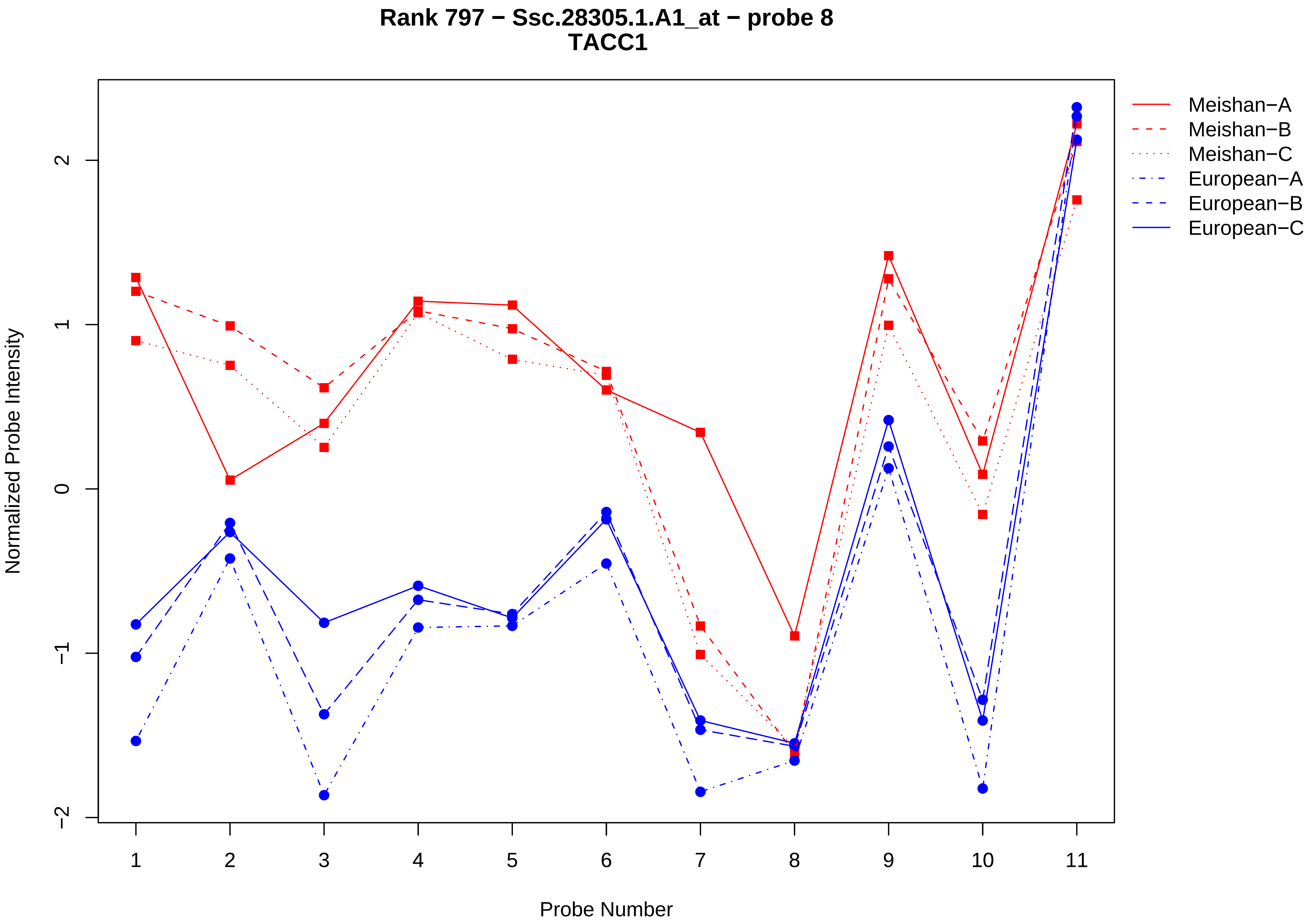
Rank 797 − Ssc.28305.1.A1_at − probe 8
TACC1
Normalized Probe Intensity
Probe Number
−2
−1
0
1
2
1
2
3
4
5
6
7
8
9
10
11
Meishan−A
Meishan−B
Meishan−C
European−A
European−B
European−C

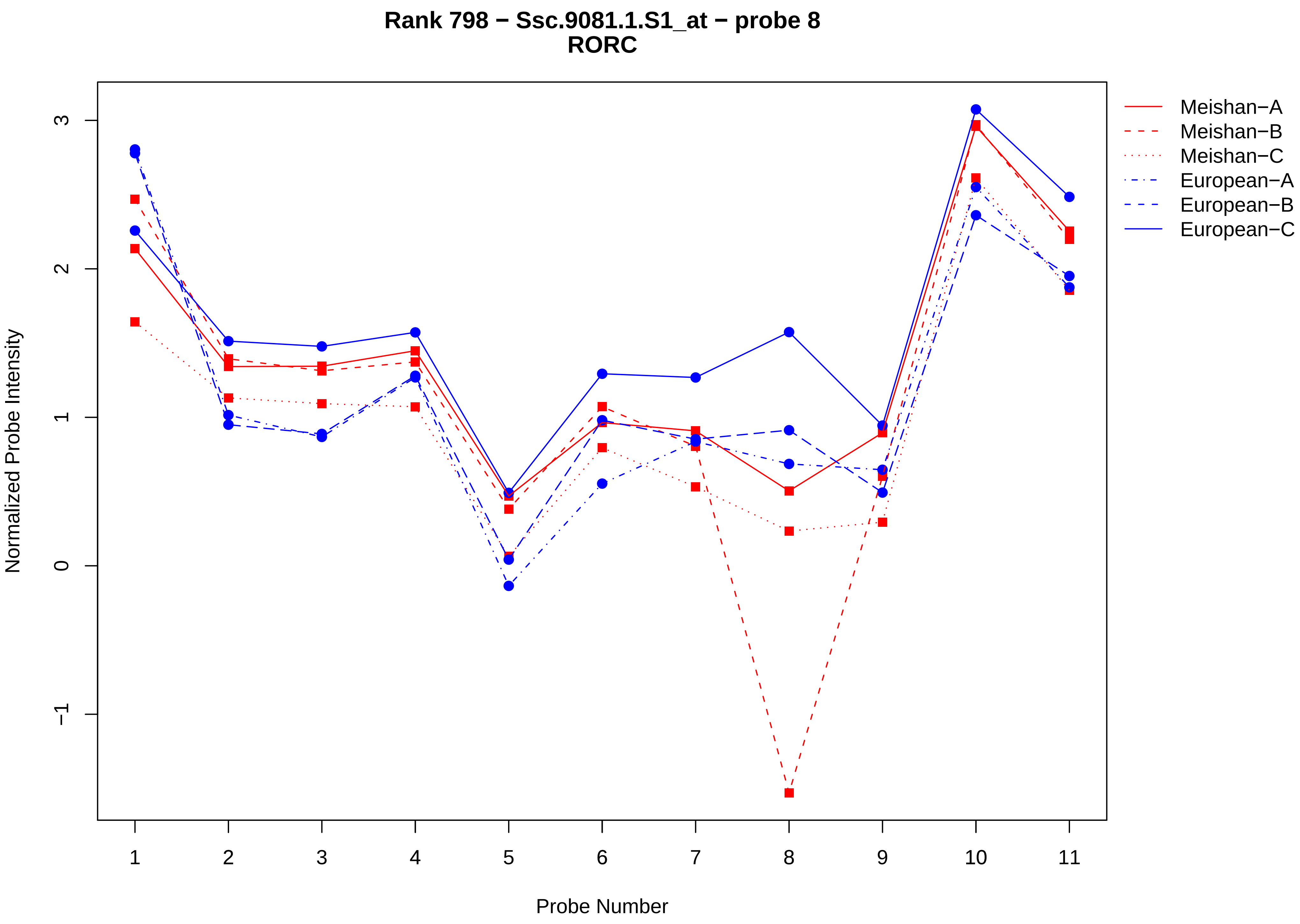

Rank 798 − Ssc.9081.1.S1_at − probe 8
RORC
Normalized Probe Intensity
Probe Number
−1
0
1
2
3
1
2
3
4
5
6
7
8
9
10
11
Meishan−A
Meishan−B
Meishan−C
European−A
European−B
European−C

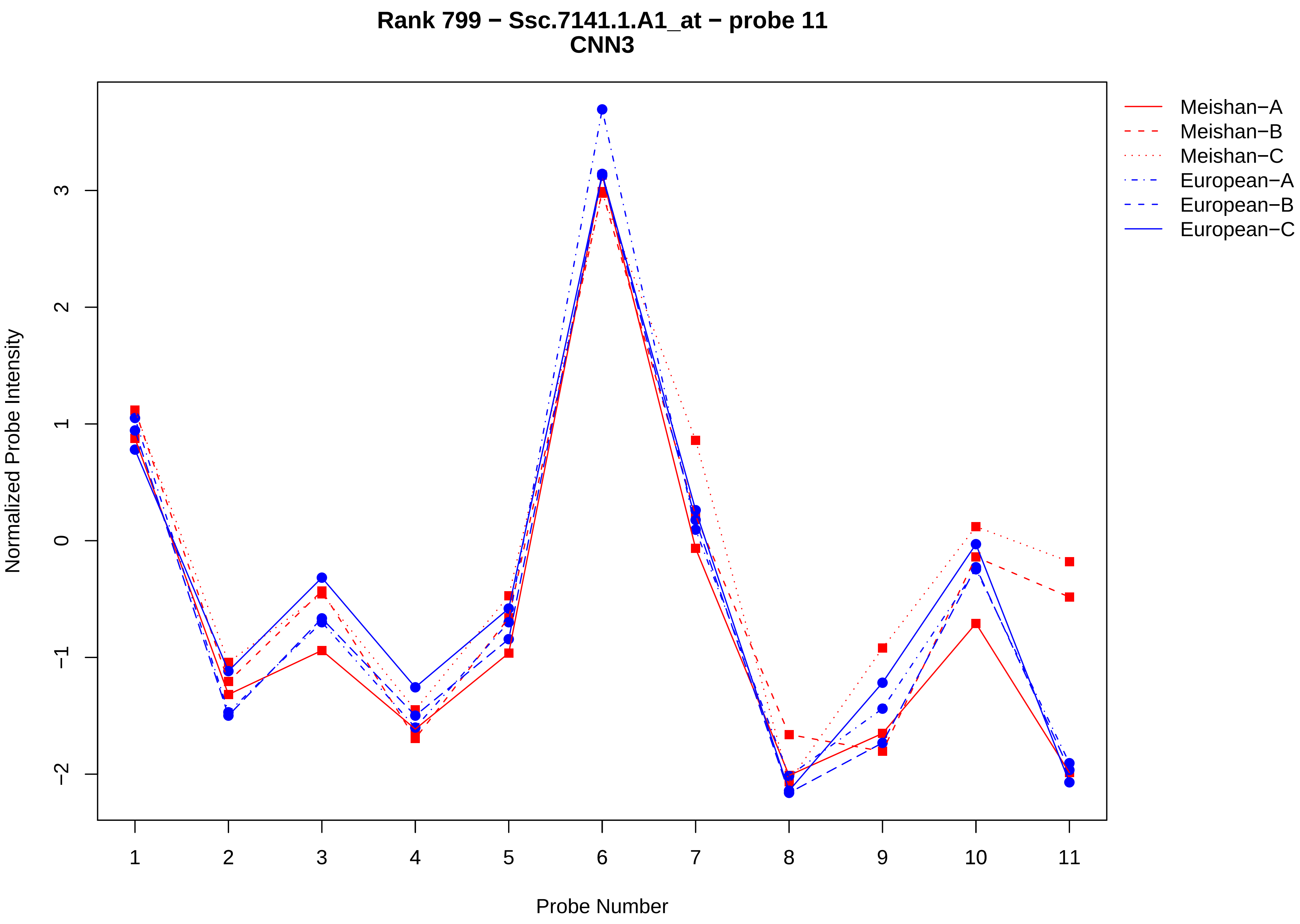
Rank 799 − Ssc.7141.1.A1_at − probe 11
CNN3
Normalized Probe Intensity
Probe Number
Meishan−A
Meishan−B
Meishan−C
European−A
European−B
European−C
−2
−1
0
1
2
3
1
2
3
4
5
6
7
8
9
10
11

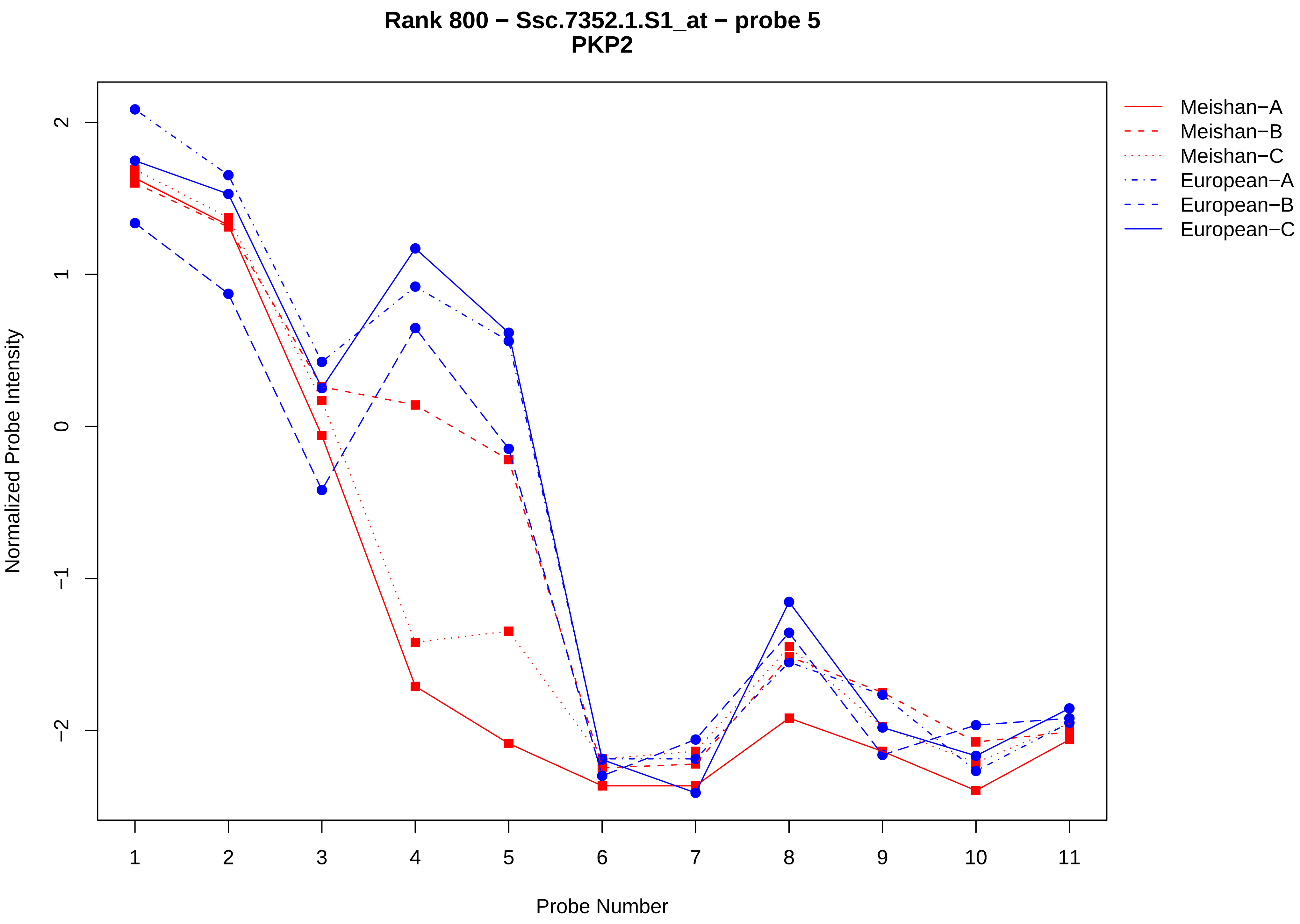

Rank 800 − Ssc.7352.1.S1_at − probe 5
PKP2
Normalized Probe Intensity
2
1
0
−1
−2
1
2
3
4
5
6
7
8
9
10
11
Probe Number
Meishan−A
Meishan−B
Meishan−C
European−A
European−B
European−C

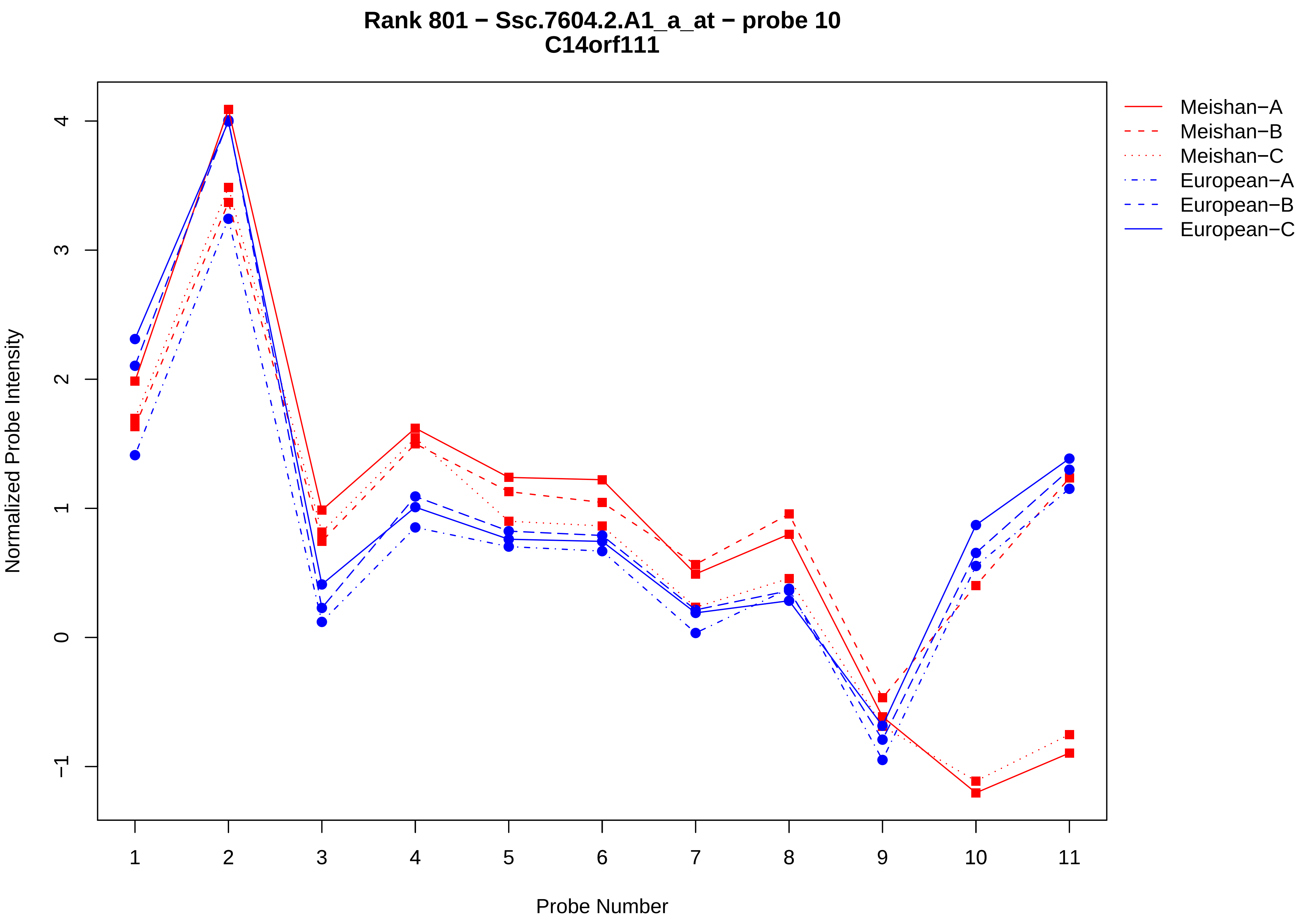

Rank 801 − Ssc.7604.2.A1_a_at − probe 10
C14orf111
Normalized Probe Intensity
Probe Number
Meishan−A
Meishan−B
Meishan−C
European−A
European−B
European−C
−1
0
1
2
3
4
1
2
3
4
5
6
7
8
9
10
11

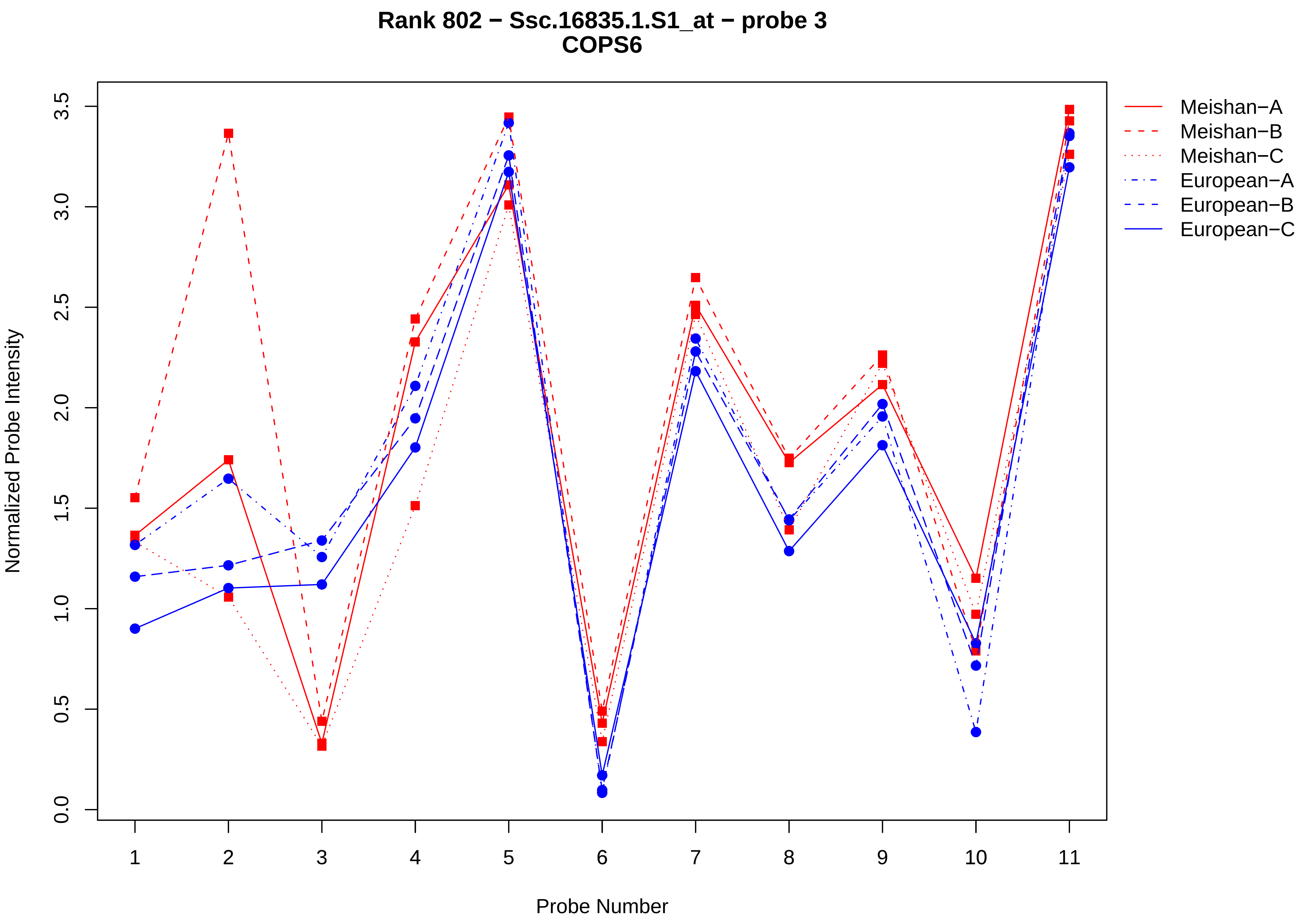

Rank 802 − Ssc.16835.1.S1_at − probe 3
COPS6
Normalized Probe Intensity
0.0
0.5
1.0
1.5
2.0
2.5
3.0
3.5
1
2
3
4
5
6
7
8
9
10
11
Probe Number
Meishan−A
Meishan−B
Meishan−C
European−A
European−B
European−C

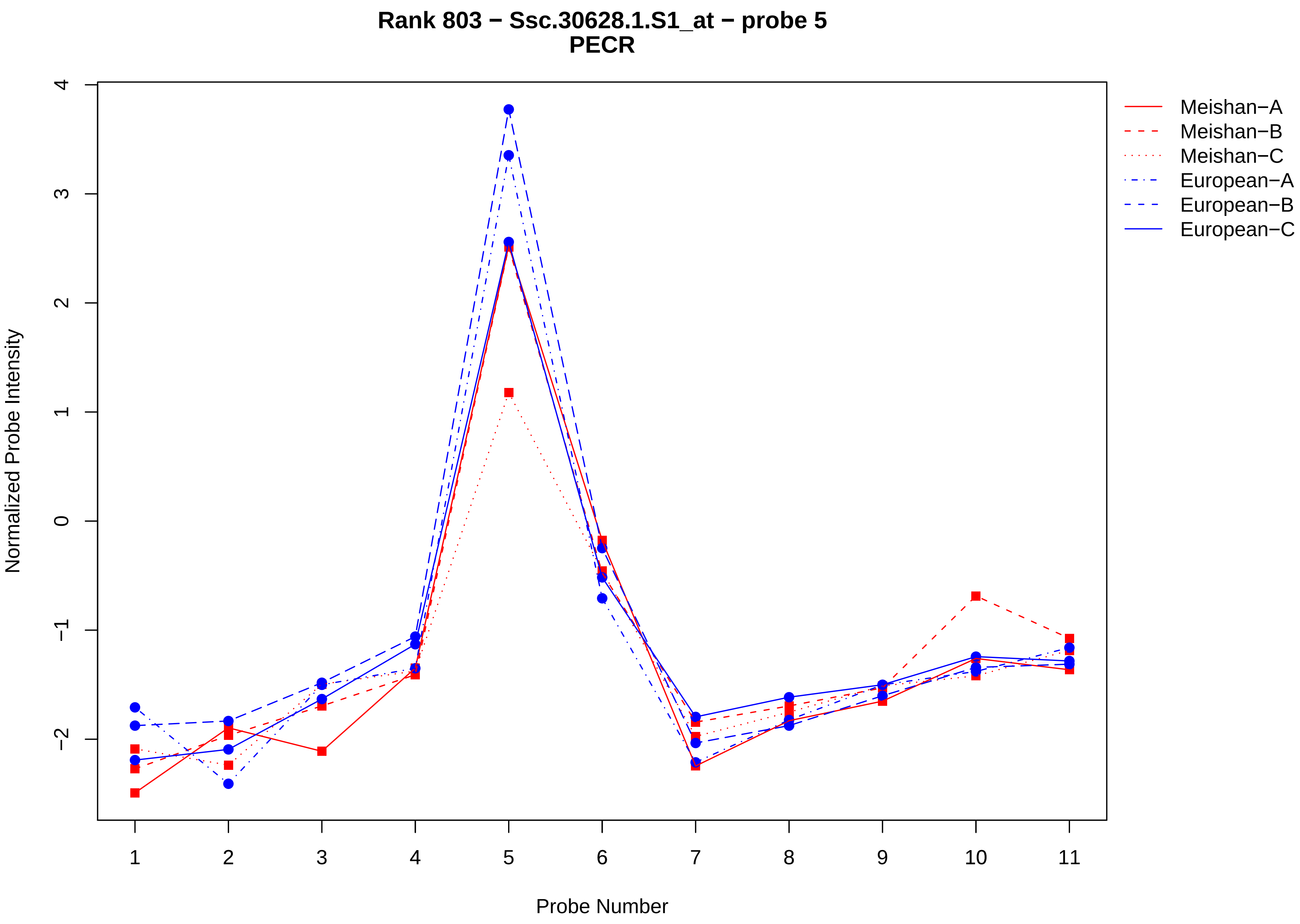
Rank 803 − Ssc.30628.1.S1_at − probe 5
PECR
Normalized Probe Intensity
Probe Number
4
3
2
1
0
−1
−2
1
2
3
4
5
6
7
8
9
10
11
Meishan−A
Meishan−B
Meishan−C
European−A
European−B
European−C

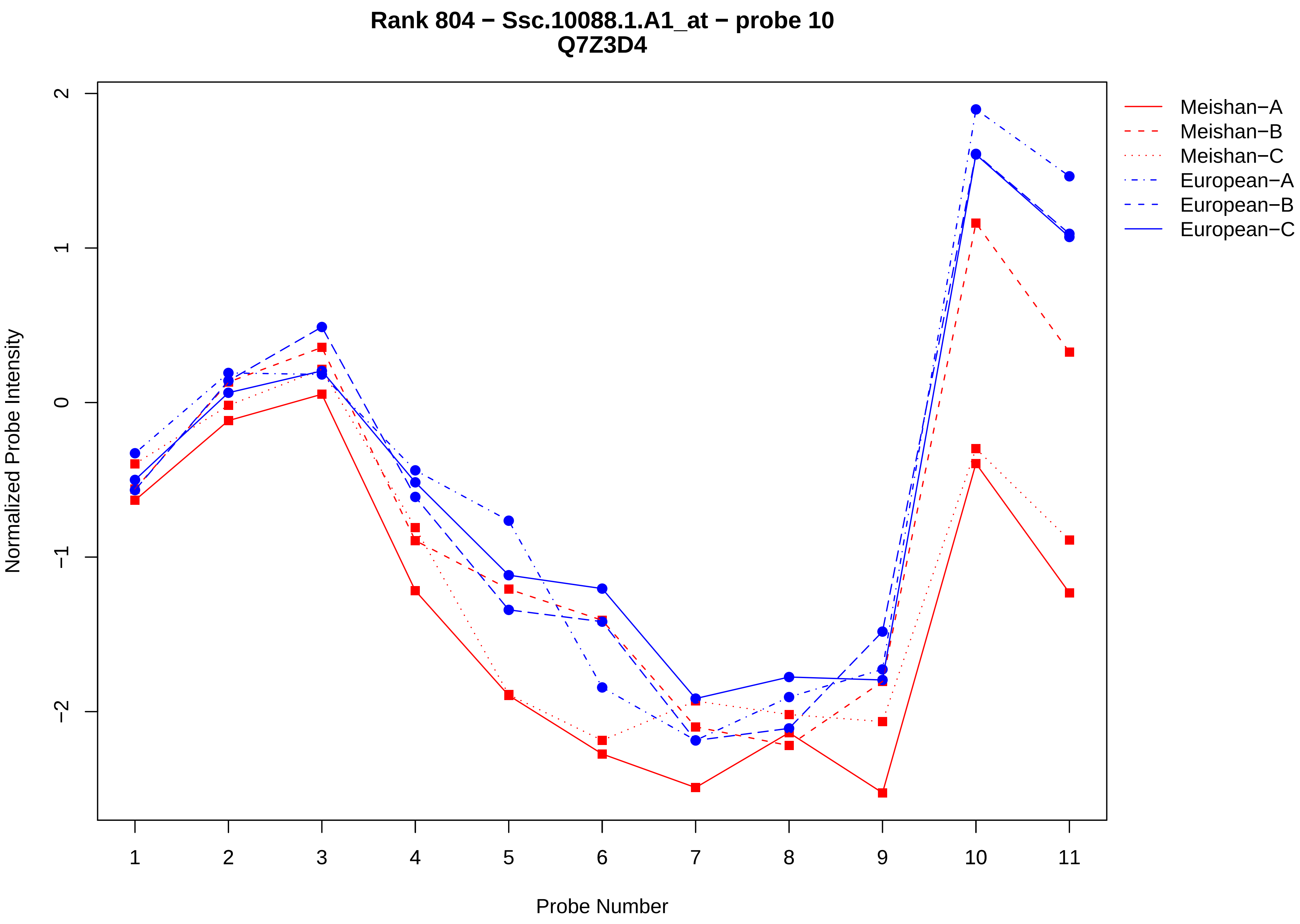
Rank 804 − Ssc.10088.1.A1_at − probe 10
Q7Z3D4
Normalized Probe Intensity
Probe Number
2
1
0
−1
−2
1
2
3
4
5
6
7
8
9
10
11
Meishan−A
Meishan−B
Meishan−C
European−A
European−B
European−C

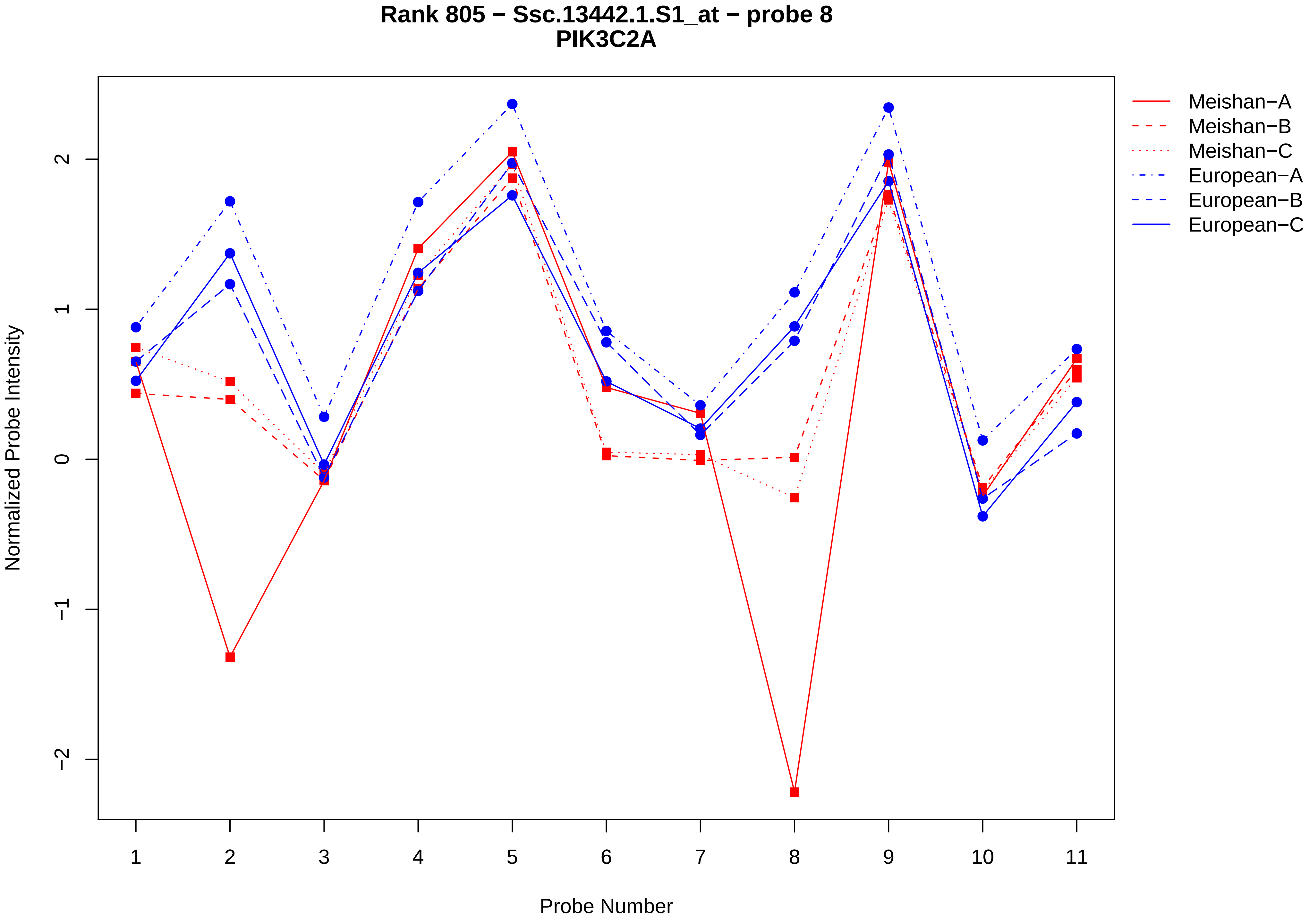
Rank 805 − Ssc.13442.1.S1_at − probe 8
PIK3C2A
Normalized Probe Intensity
Probe Number
−2
−1
0
1
2
1
2
3
4
5
6
7
8
9
10
11
Meishan−A
Meishan−B
Meishan−C
European−A
European−B
European−C

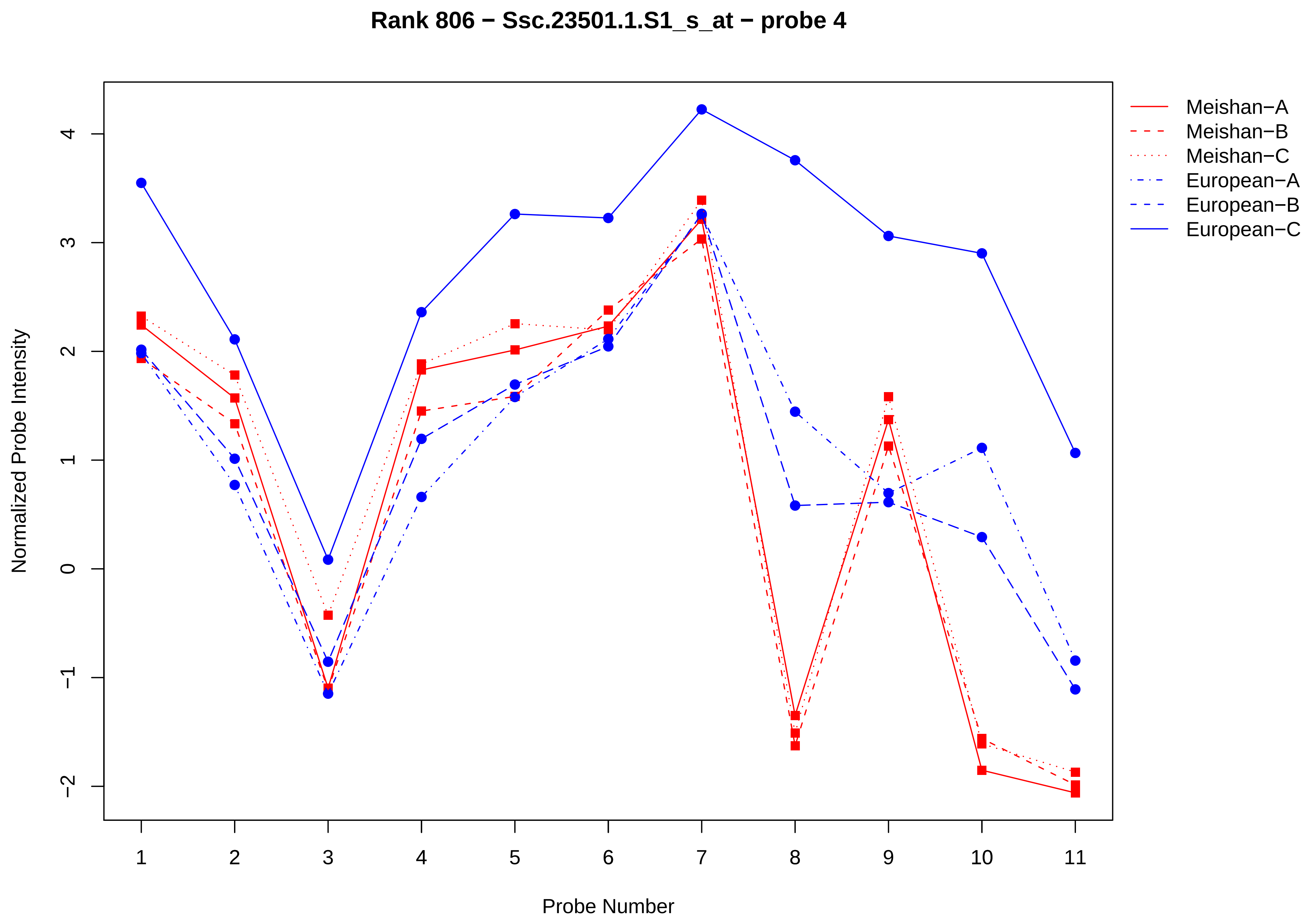

Rank 806 − Ssc.23501.1.S1_s_at − probe 4
Normalized Probe Intensity
Probe Number
−2
−1
0
1
2
3
4
1
2
3
4
5
6
7
8
9
10
11
Meishan−A
Meishan−B
Meishan−C
European−A
European−B
European−C

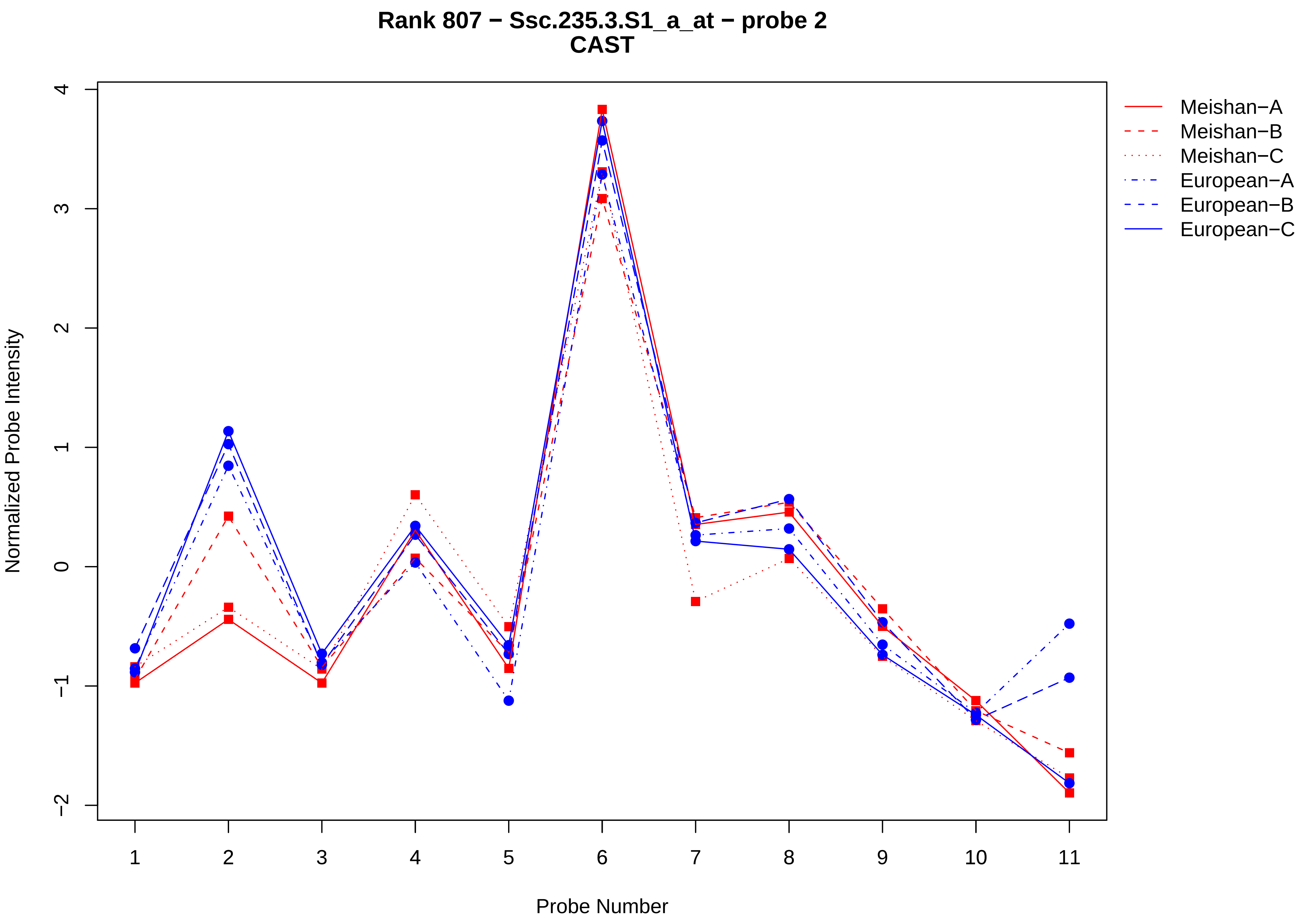
Rank 807 − Ssc.235.3.S1_a_at − probe 2
CAST
Normalized Probe Intensity
Probe Number
Meishan−A
Meishan−B
Meishan−C
European−A
European−B
European−C

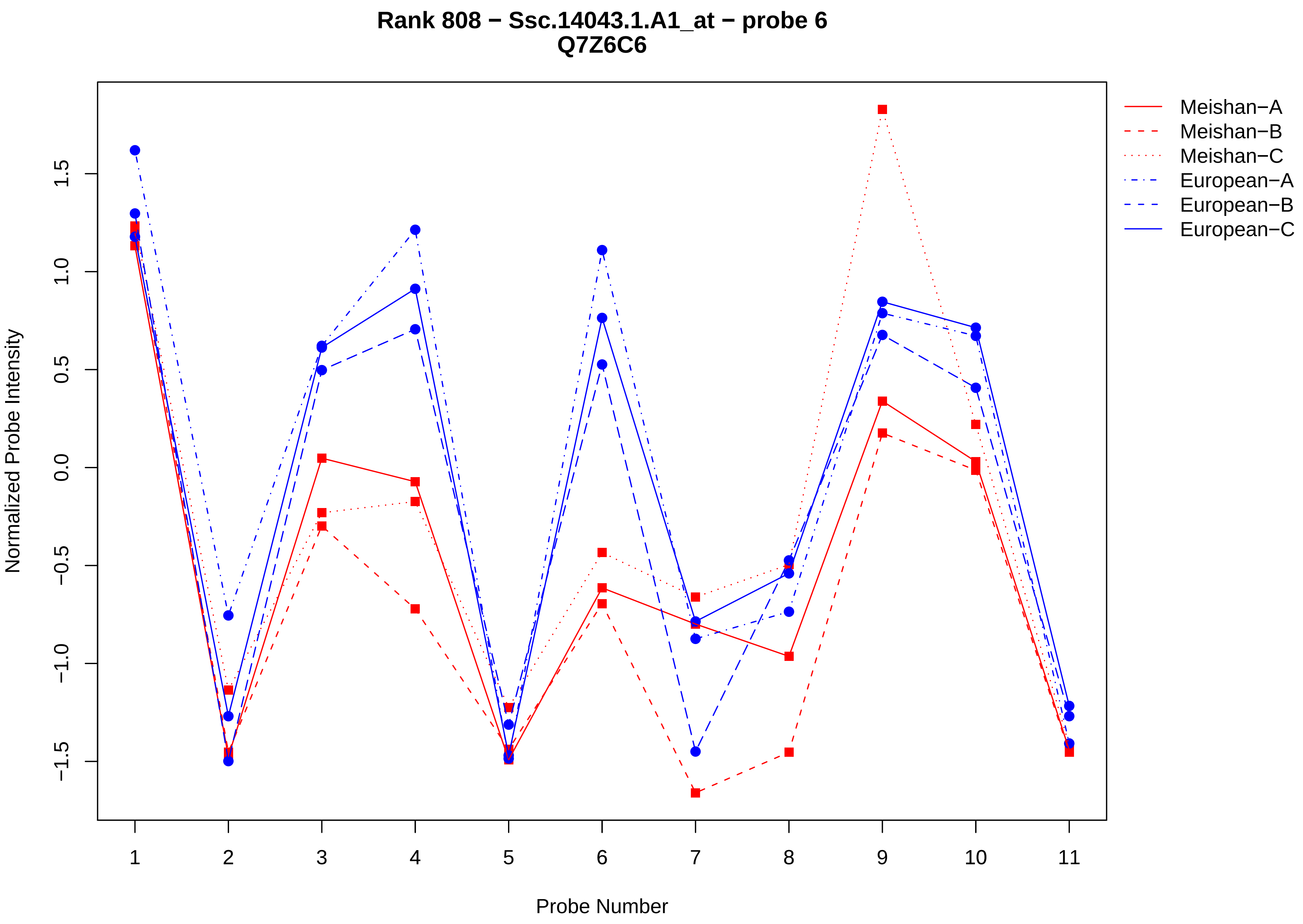

Rank 808 − Ssc.14043.1.A1_at − probe 6
Q7Z6C6
Normalized Probe Intensity
Probe Number
1.5
1.0
0.5
0.0
−0.5
−1.0
−1.5
1
2
3
4
5
6
7
8
9
10
11
Meishan−A
Meishan−B
Meishan−C
European−A
European−B
European−C

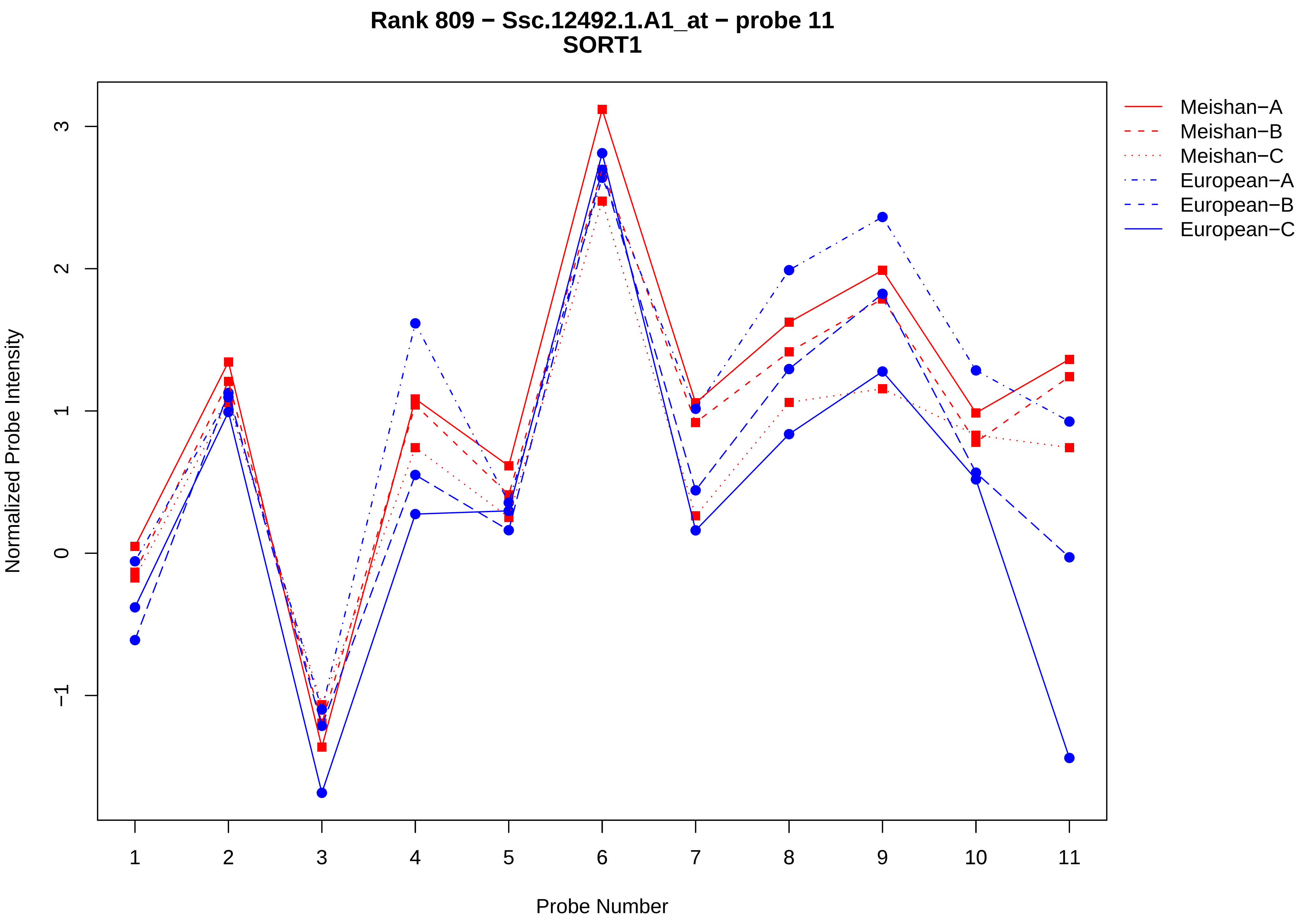
Rank 809 − Ssc.12492.1.A1_at − probe 11
SORT1
Normalized Probe Intensity
Probe Number
Meishan−A
Meishan−B
Meishan−C
European−A
European−B
European−C

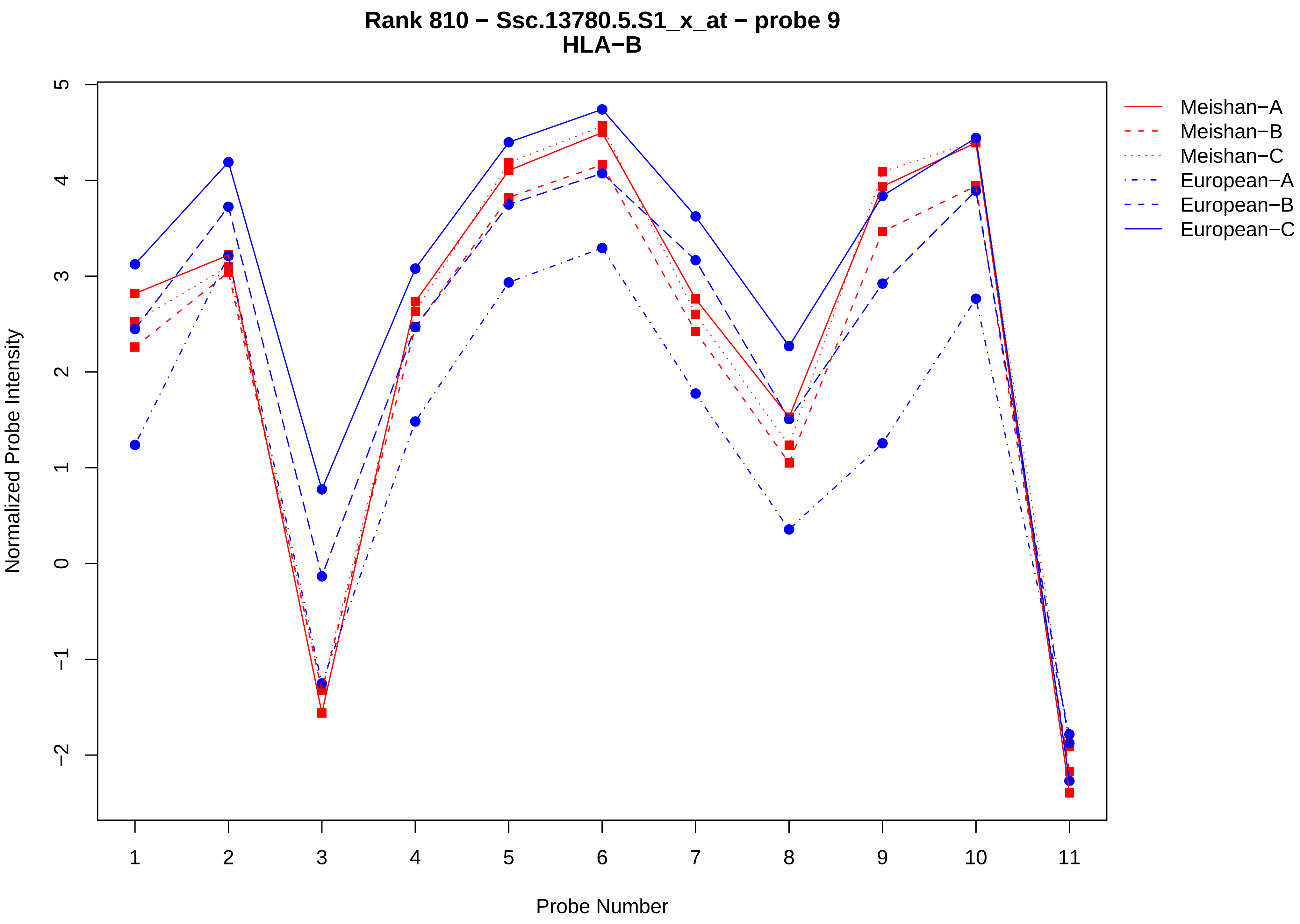

Rank 810 − Ssc.13780.5.S1_x_at − probe 9
HLA−B
Normalized Probe Intensity
Probe Number
Meishan−A
Meishan−B
Meishan−C
European−A
European−B
European−C

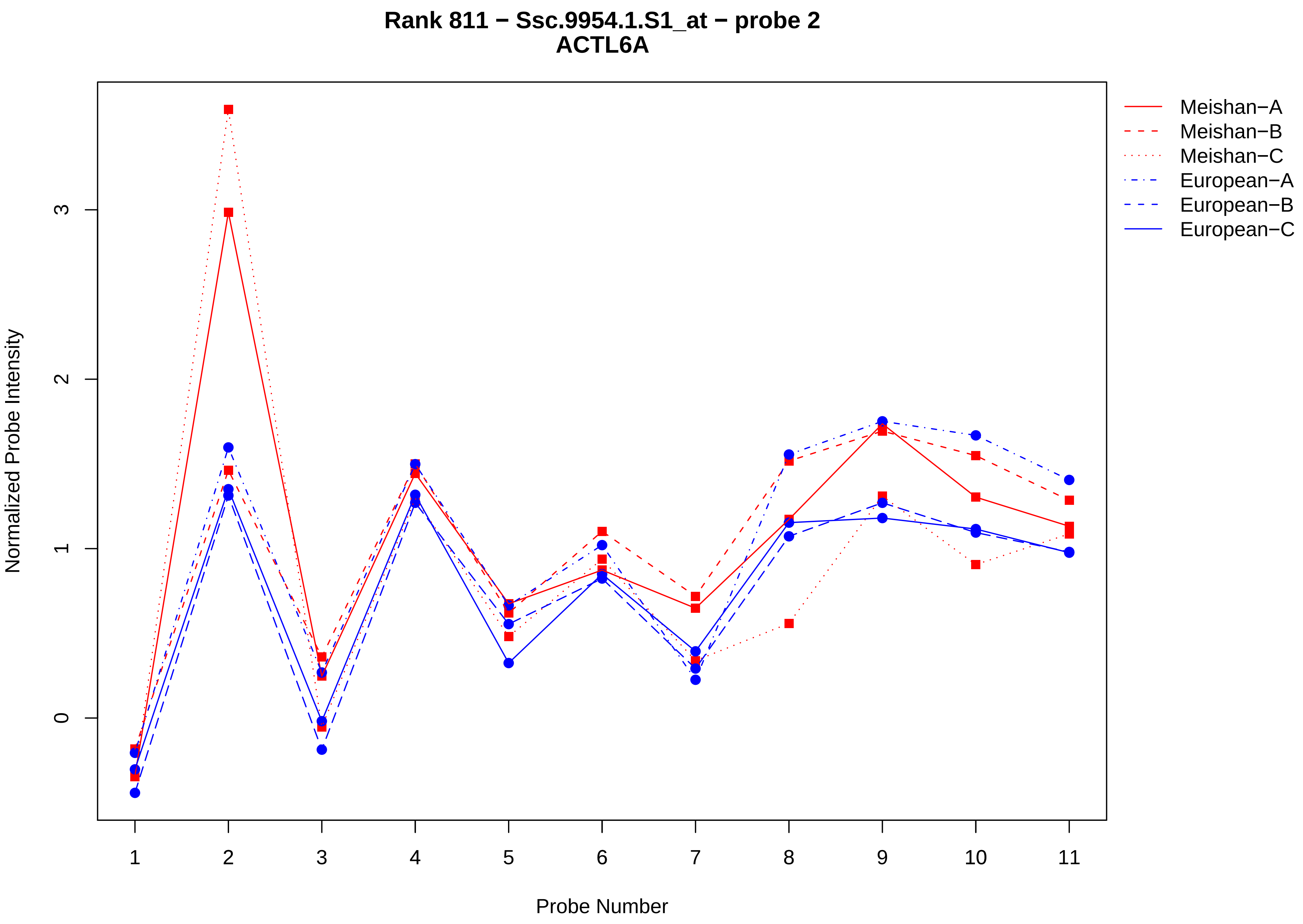

Rank 811 − Ssc.9954.1.S1_at − probe 2
ACTL6A
Normalized Probe Intensity
Probe Number
0
1
2
3
1
2
3
4
5
6
7
8
9
10
11
Meishan−A
Meishan−B
Meishan−C
European−A
European−B
European−C

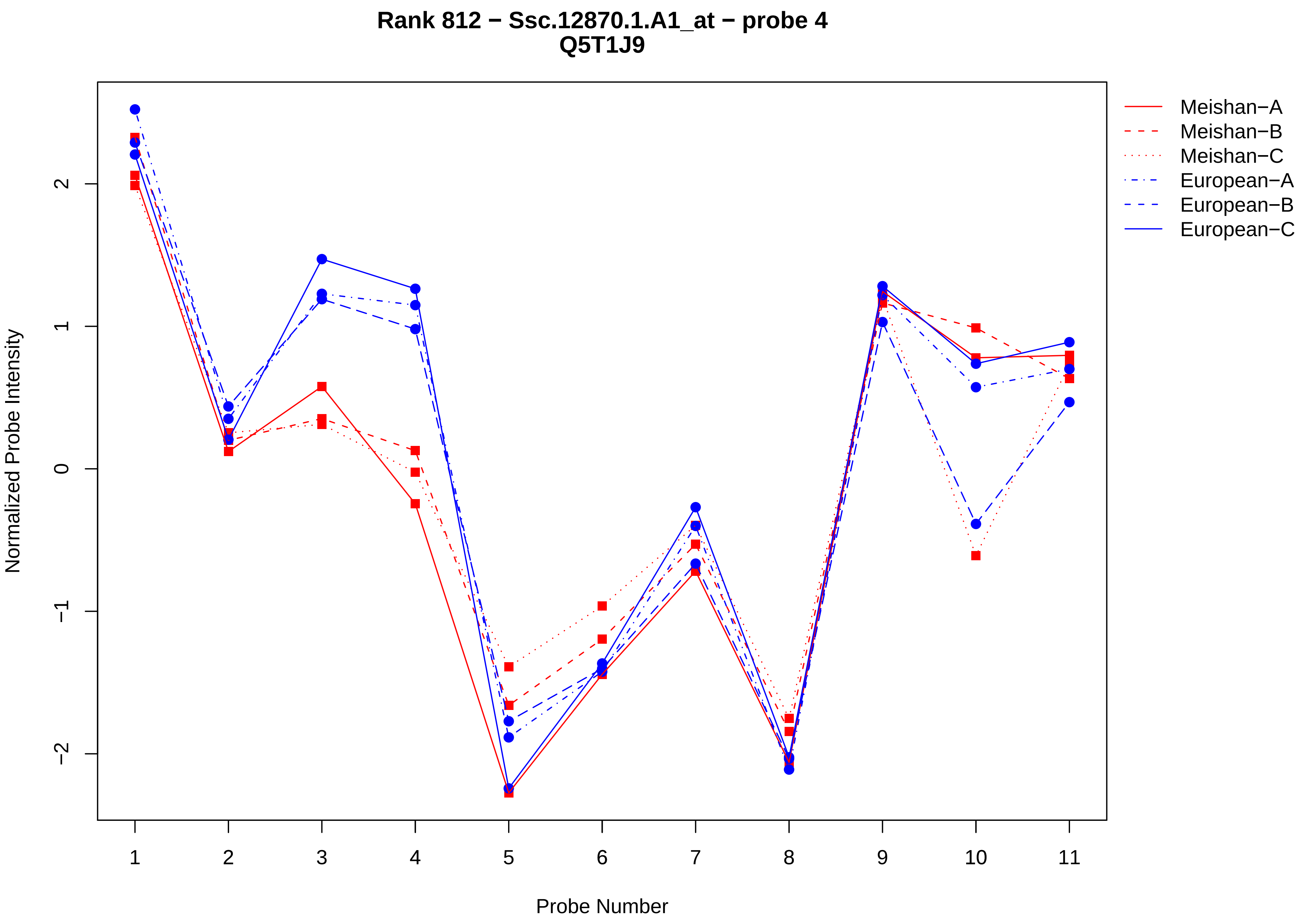
Rank 812 − Ssc.12870.1.A1_at − probe 4
Q5T1J9
Normalized Probe Intensity
Probe Number
−2
−1
0
1
2
1
2
3
4
5
6
7
8
9
10
11
Meishan−A
Meishan−B
Meishan−C
European−A
European−B
European−C

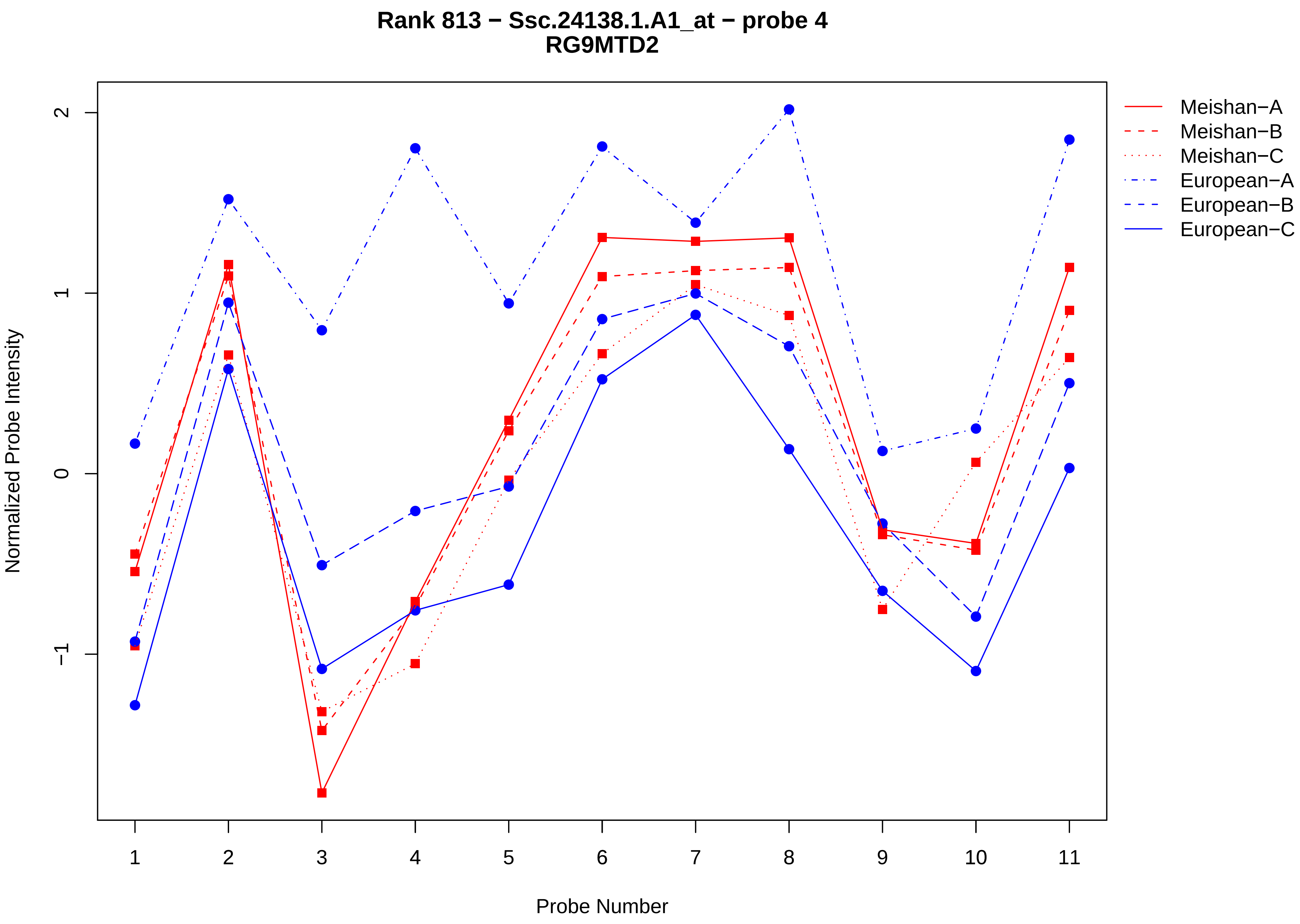
Rank 813 − Ssc.24138.1.A1_at − probe 4
RG9MTD2
Normalized Probe Intensity
Probe Number
Meishan−A
Meishan−B
Meishan−C
European−A
European−B
European−C
−1
0
1
2
1
2
3
4
5
6
7
8
9
10
11

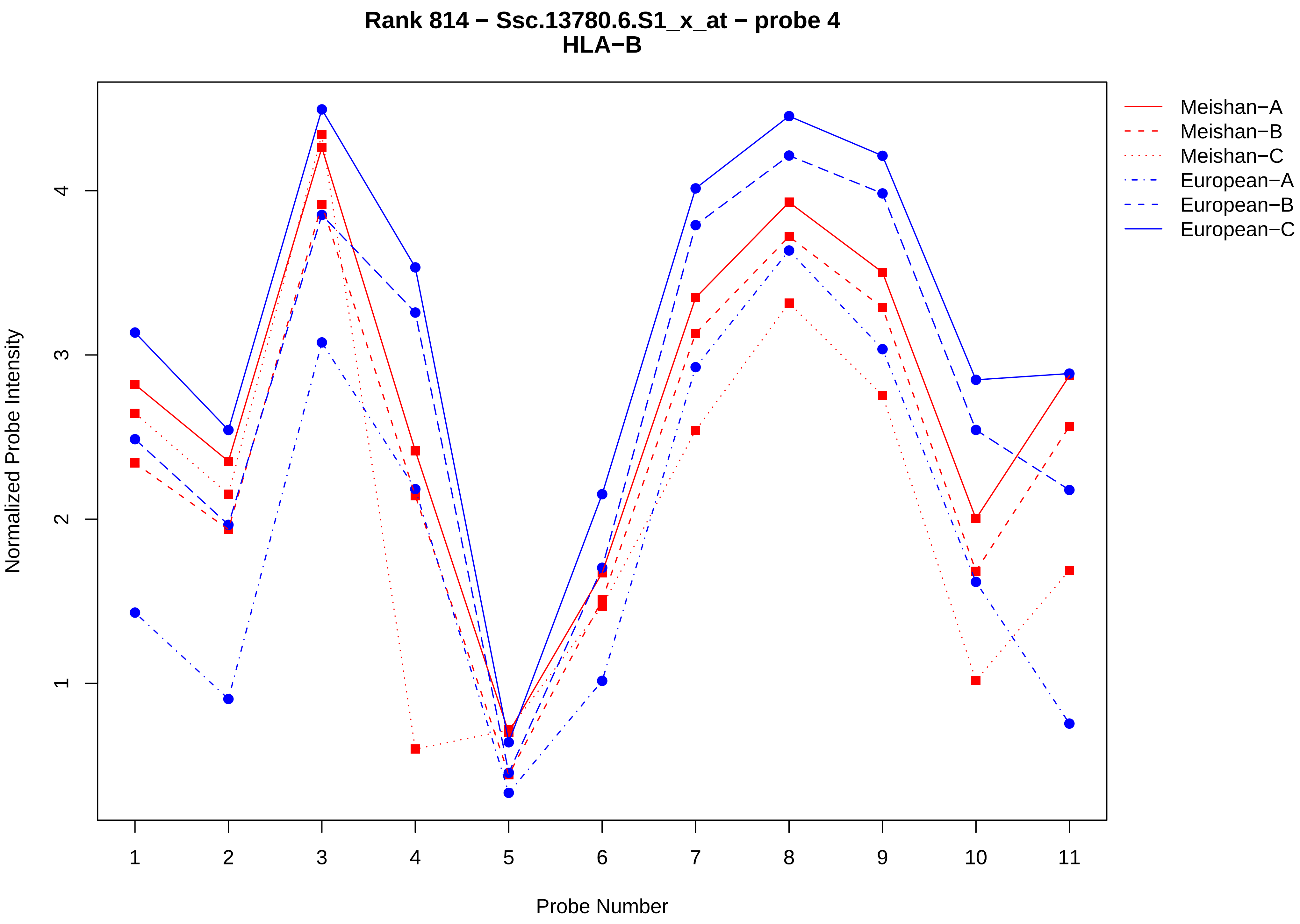

Rank 814 − Ssc.13780.6.S1_x_at − probe 4
HLA−B
Normalized Probe Intensity
Probe Number
1
2
3
4
1
2
3
4
5
6
7
8
9
10
11
Meishan−A
Meishan−B
Meishan−C
European−A
European−B
European−C

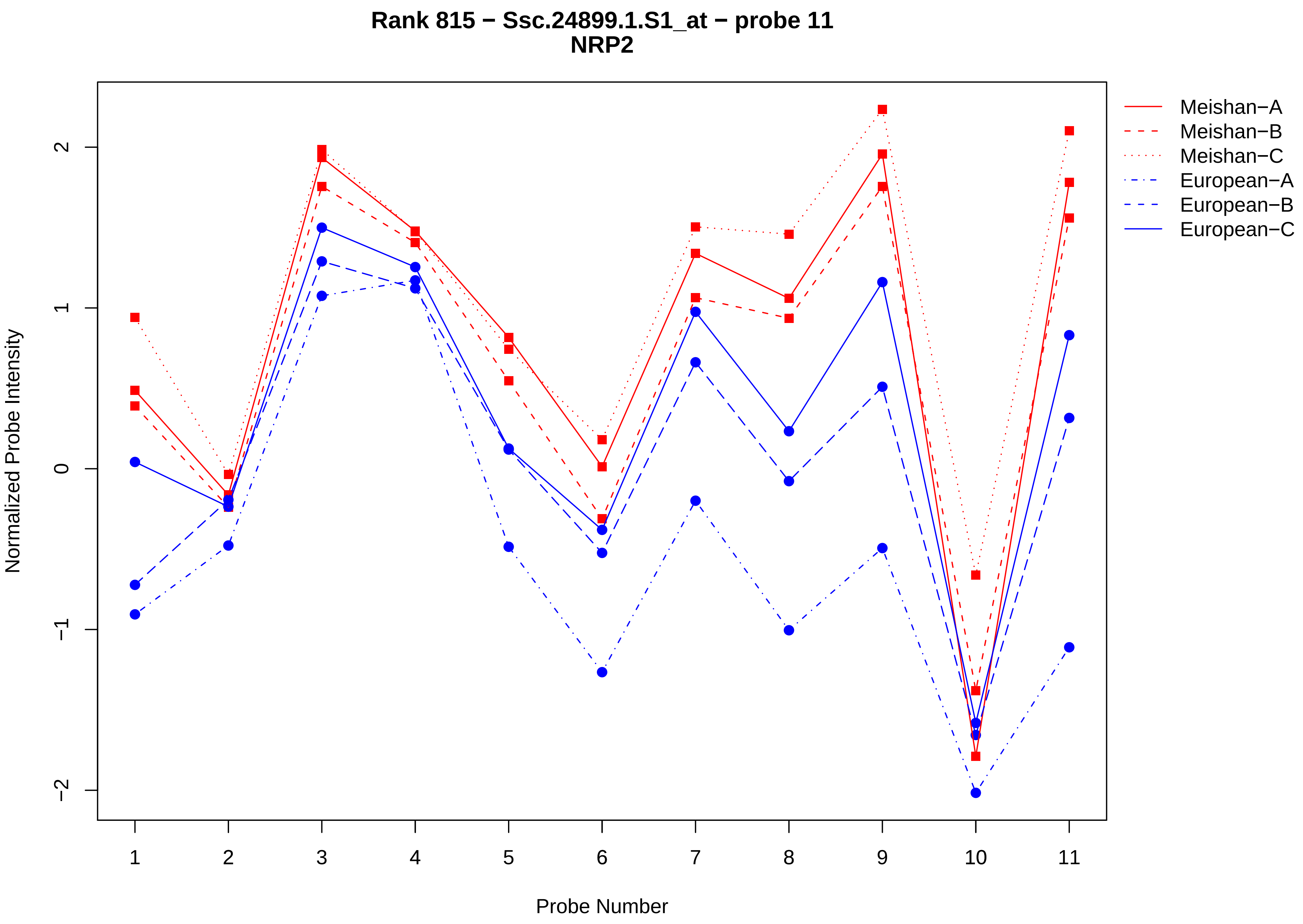
Rank 815 − Ssc.24899.1.S1_at − probe 11
NRP2
Normalized Probe Intensity
Probe Number
−2
−1
0
1
2
1
2
3
4
5
6
7
8
9
10
11
Meishan−A
Meishan−B
Meishan−C
European−A
European−B
European−C

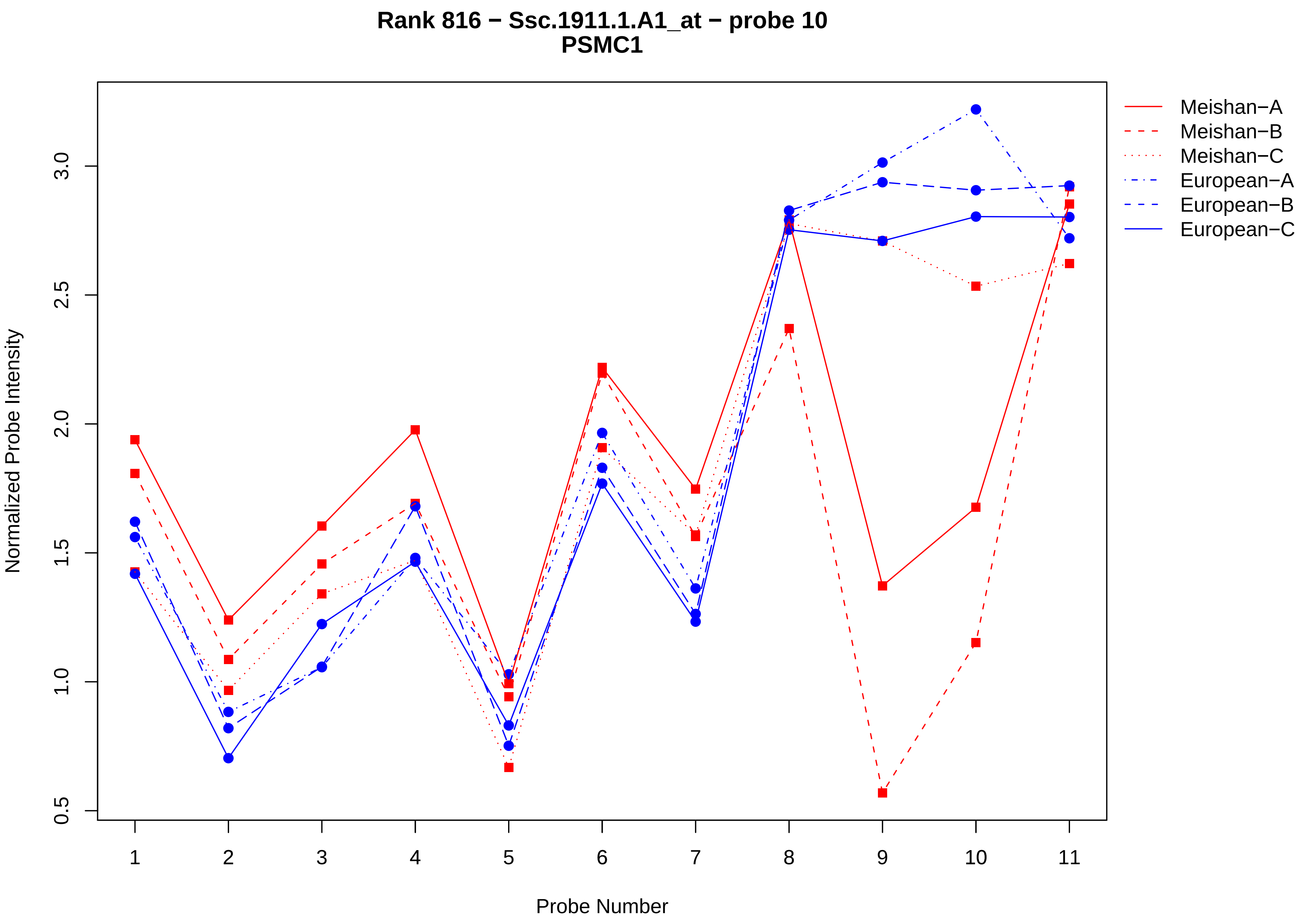

Rank 816 − Ssc.1911.1.A1_at − probe 10
PSMC1
Normalized Probe Intensity
0.5
1.0
1.5
2.0
2.5
3.0
Probe Number
1
2
3
4
5
6
7
8
9
10
11
Meishan−A
Meishan−B
Meishan−C
European−A
European−B
European−C

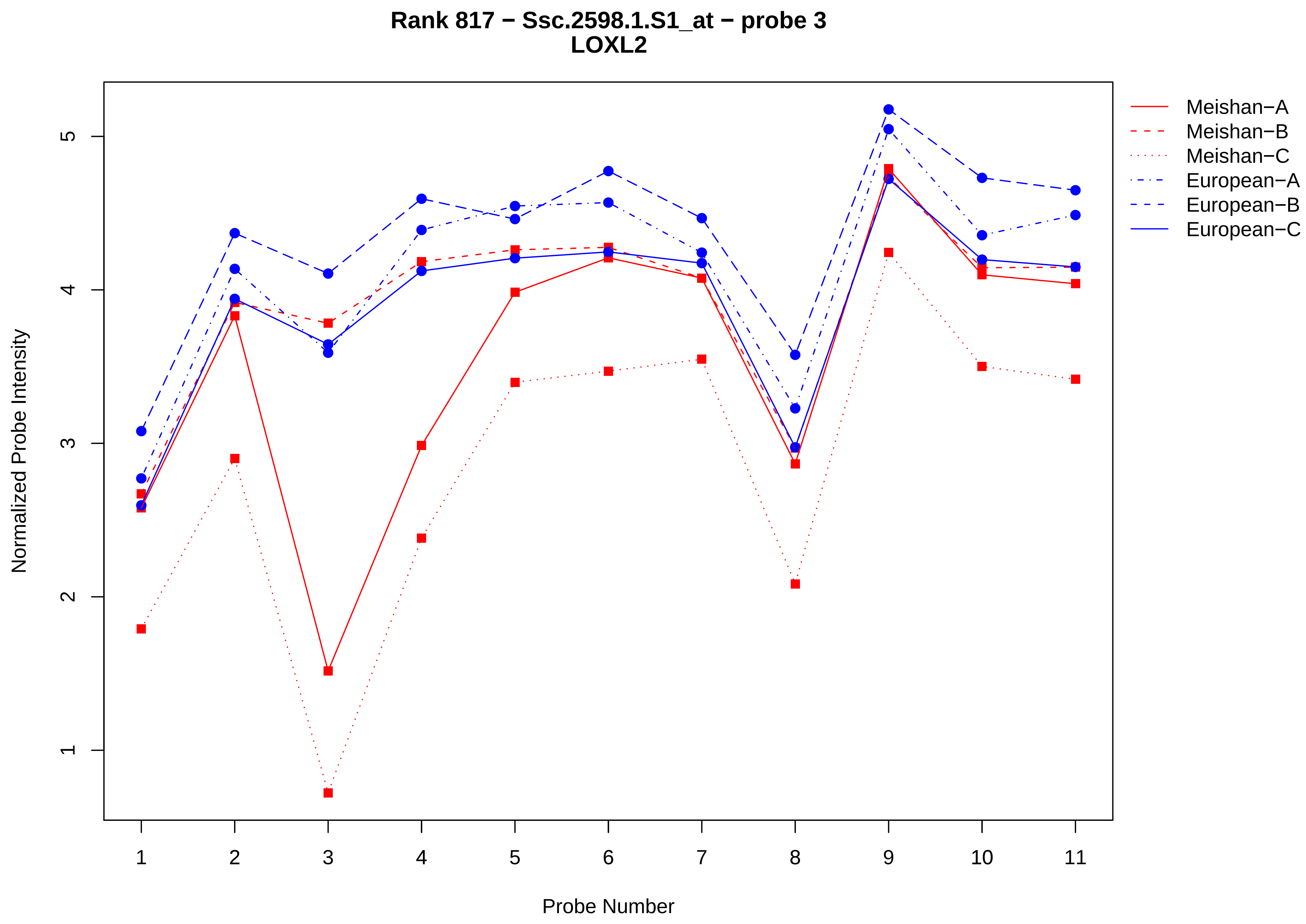

Rank 817 − Ssc.2598.1.S1_at − probe 3
LOXL2
Normalized Probe Intensity
Probe Number
1
2
3
4
5
1
2
3
4
5
6
7
8
9
10
11
Meishan−A
Meishan−B
Meishan−C
European−A
European−B
European−C

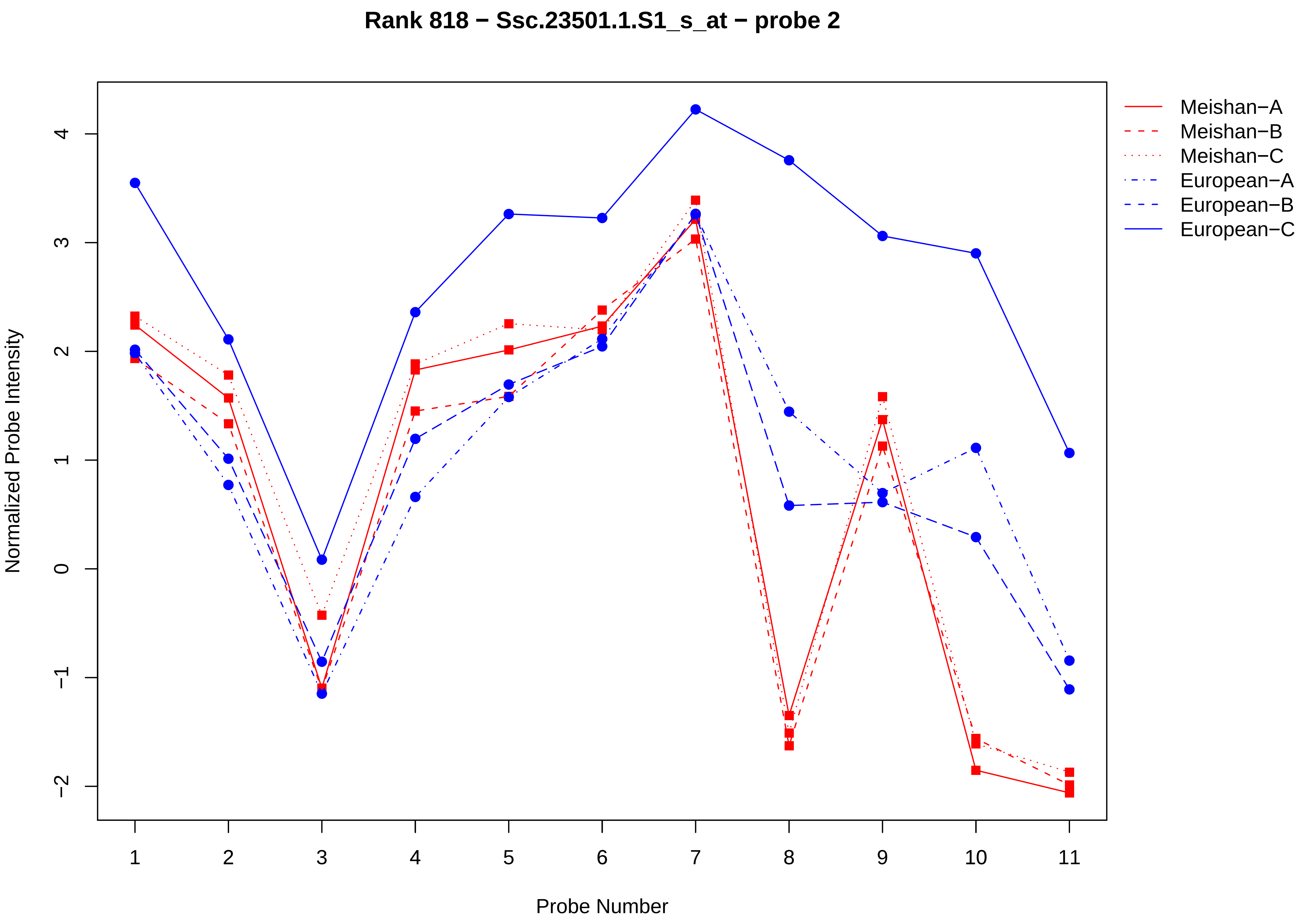

Rank 818 − Ssc.23501.1.S1_s_at − probe 2
Normalized Probe Intensity
Probe Number
−2
−1
0
1
2
3
4
1
2
3
4
5
6
7
8
9
10
11
Meishan−A
Meishan−B
Meishan−C
European−A
European−B
European−C

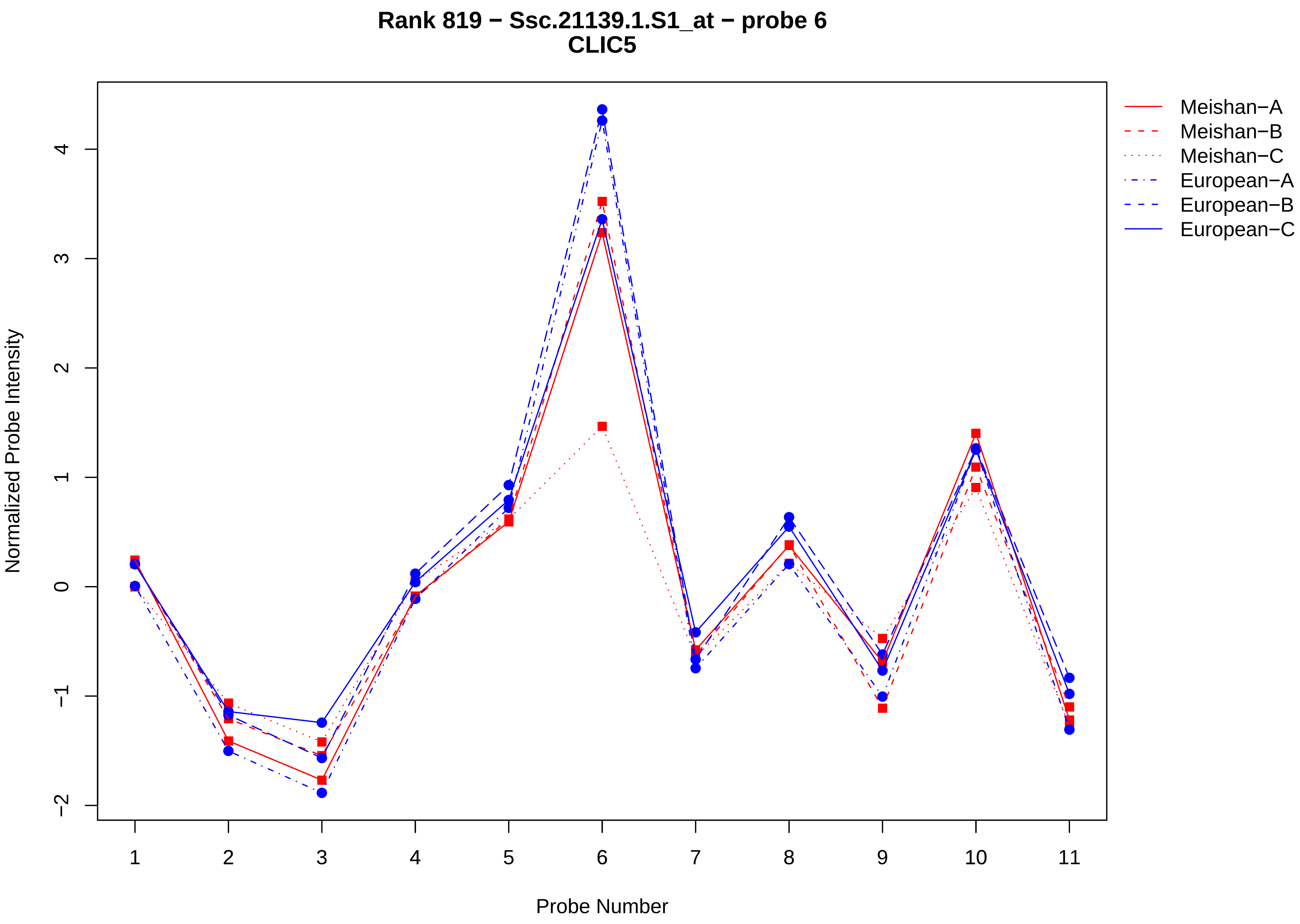
Rank 819 − Ssc.21139.1.S1_at − probe 6
CLIC5
Normalized Probe Intensity
Probe Number
−2
−1
0
1
2
3
4
1
2
3
4
5
6
7
8
9
10
11
Meishan−A
Meishan−B
Meishan−C
European−A
European−B
European−C

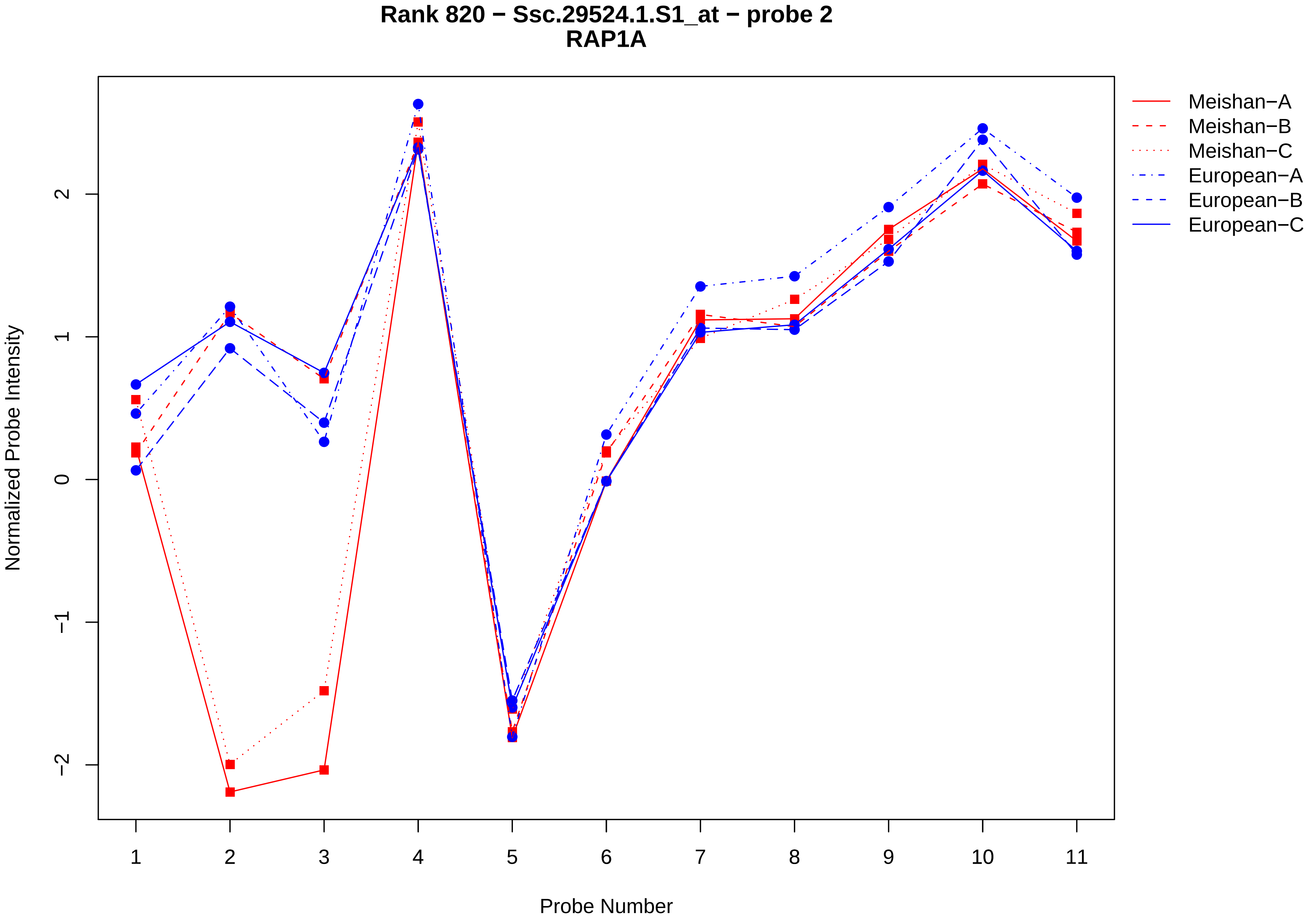

Rank 820 − Ssc.29524.1.S1_at − probe 2
RAP1A
Normalized Probe Intensity
Probe Number
−2
−1
0
1
2
1
2
3
4
5
6
7
8
9
10
11
Meishan−A
Meishan−B
Meishan−C
European−A
European−B
European−C

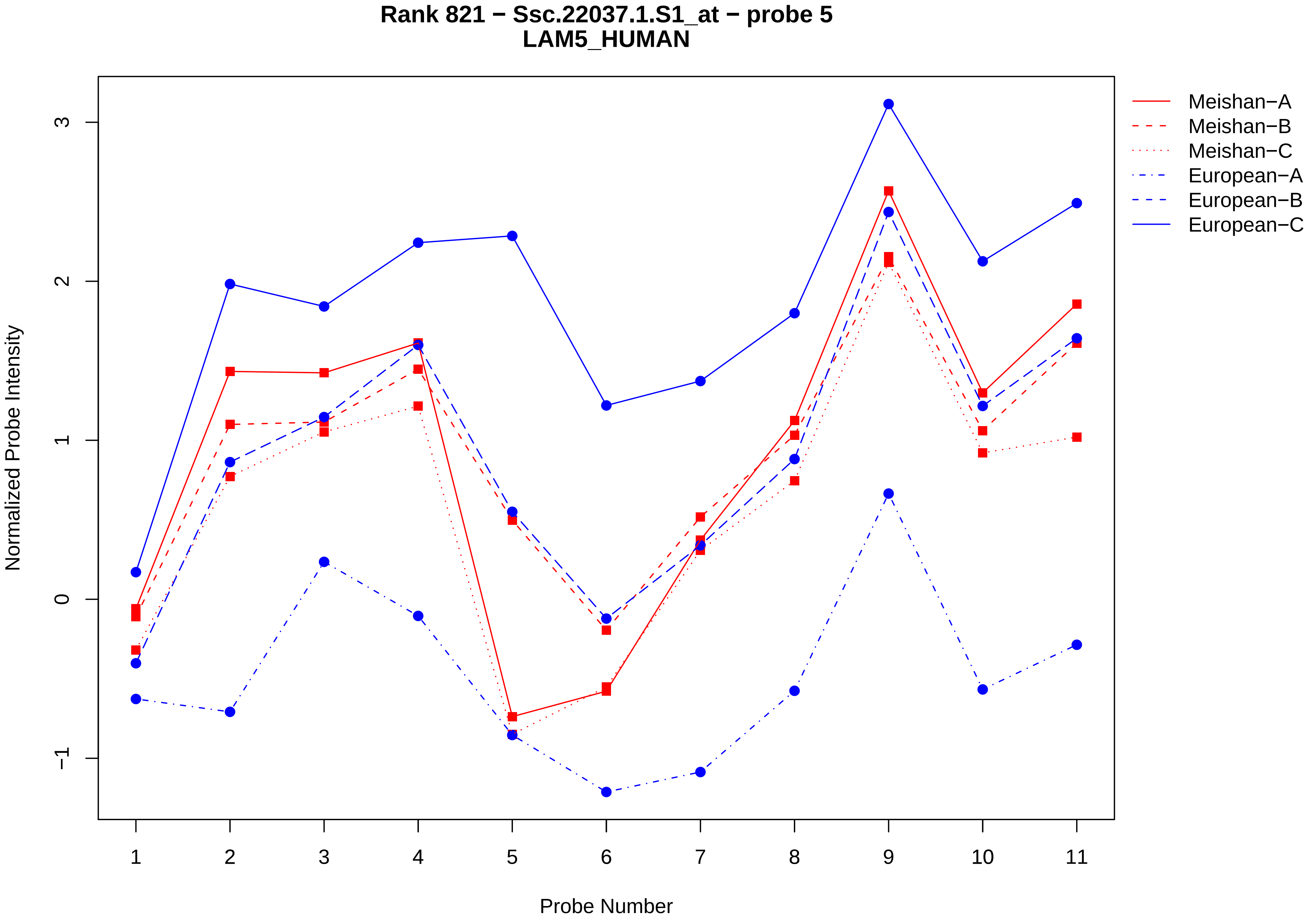

Rank 821 − Ssc.22037.1.S1_at − probe 5
LAM5_HUMAN
Normalized Probe Intensity
Probe Number
−1
0
1
2
3
1
2
3
4
5
6
7
8
9
10
11
Meishan−A
Meishan−B
Meishan−C
European−A
European−B
European−C

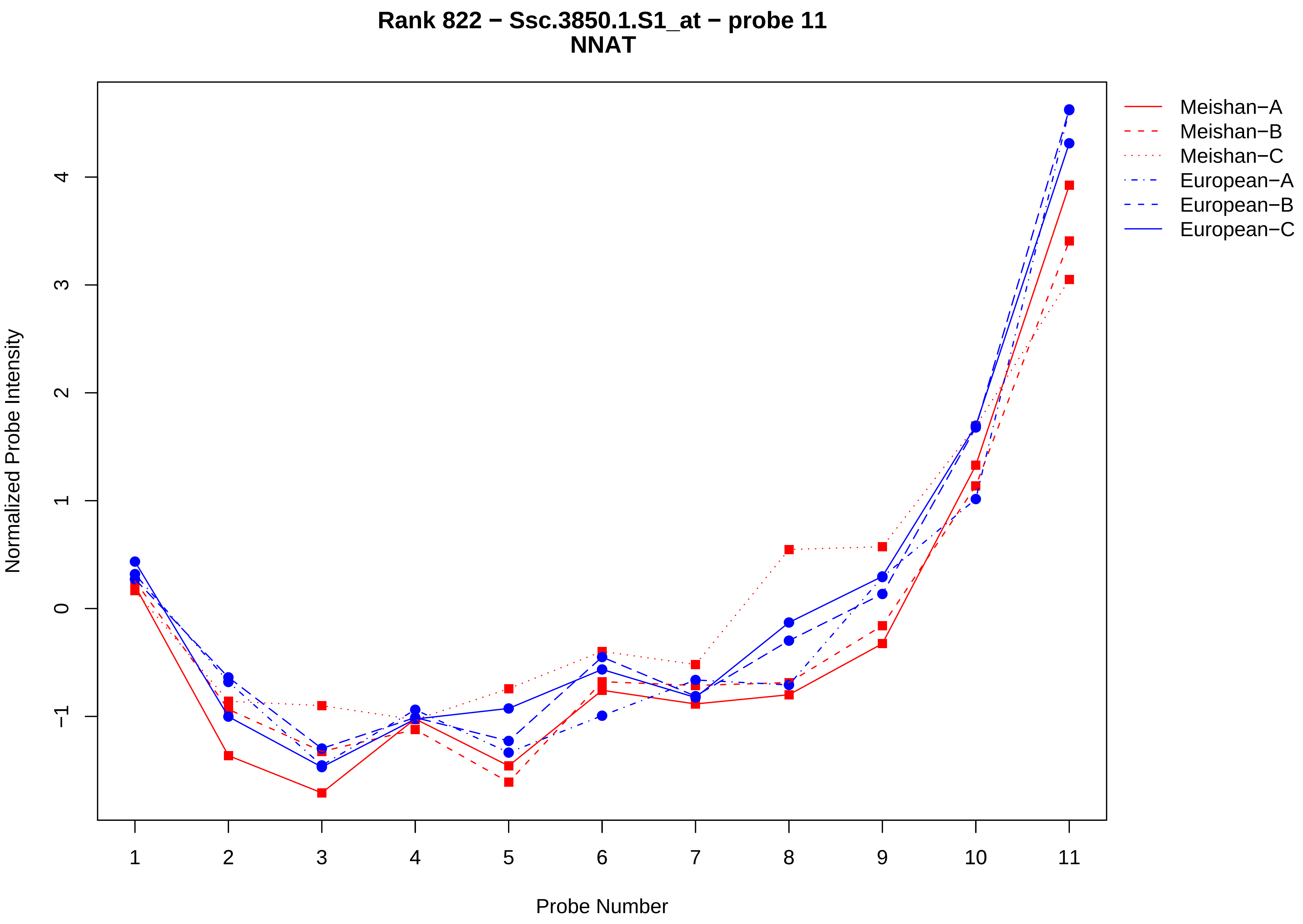
Rank 822 − Ssc.3850.1.S1_at − probe 11
NNAT
Normalized Probe Intensity
Probe Number
−1
0
1
2
3
4
1
2
3
4
5
6
7
8
9
10
11
Meishan−A
Meishan−B
Meishan−C
European−A
European−B
European−C

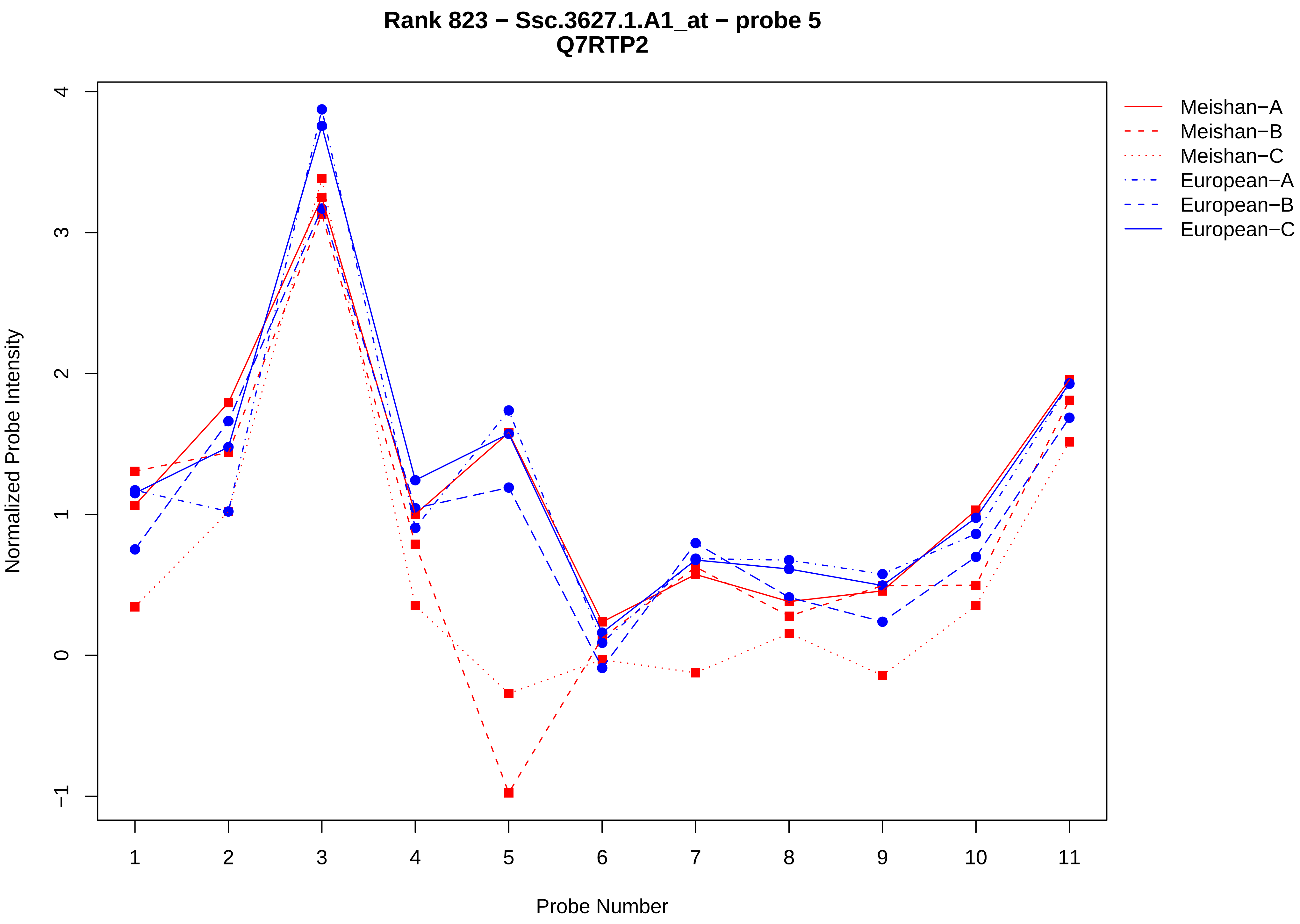

Rank 823 − Ssc.3627.1.A1_at − probe 5
Q7RTP2
Normalized Probe Intensity
Probe Number
−1
0
1
2
3
4
1
2
3
4
5
6
7
8
9
10
11
Meishan−A
Meishan−B
Meishan−C
European−A
European−B
European−C

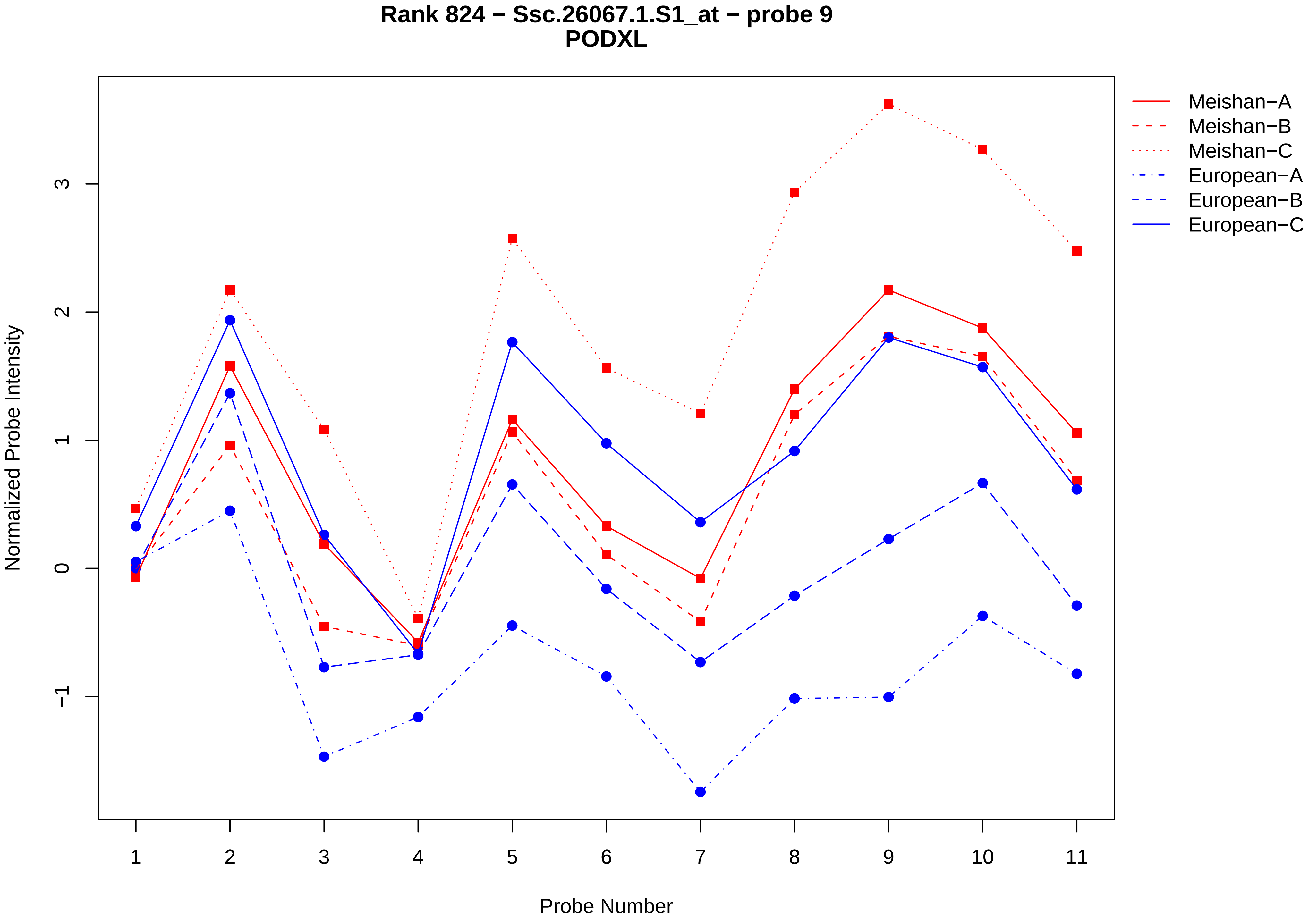

Rank 824 − Ssc.26067.1.S1_at − probe 9
PODXL
Normalized Probe Intensity
Probe Number
−1
0
1
2
3
1
2
3
4
5
6
7
8
9
10
11
Meishan−A
Meishan−B
Meishan−C
European−A
European−B
European−C

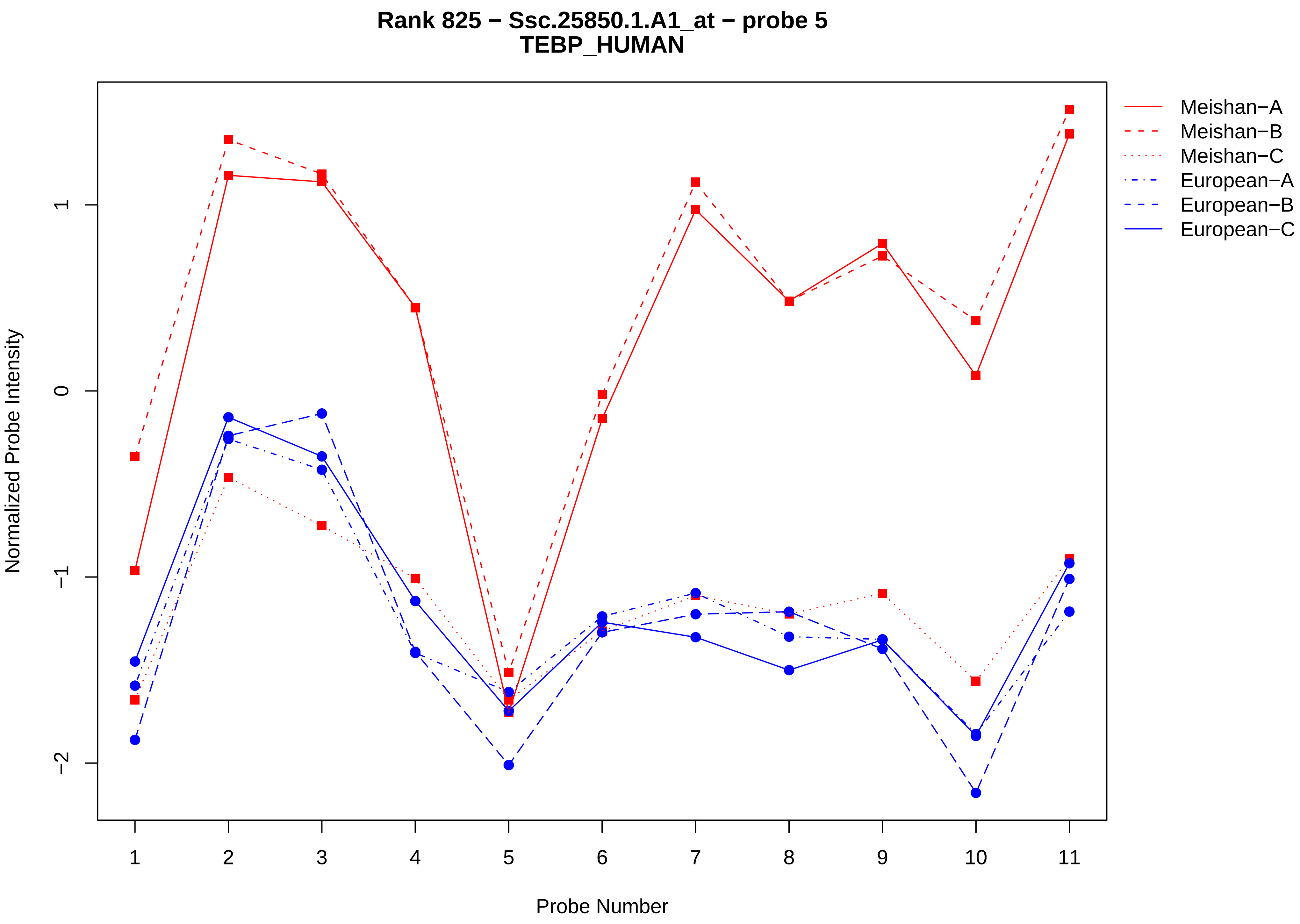
Rank 825 − Ssc.25850.1.A1_at − probe 5
TEBP_HUMAN
Normalized Probe Intensity
Probe Number
−2
−1
0
1
1
2
3
4
5
6
7
8
9
10
11
Meishan−A
Meishan−B
Meishan−C
European−A
European−B
European−C

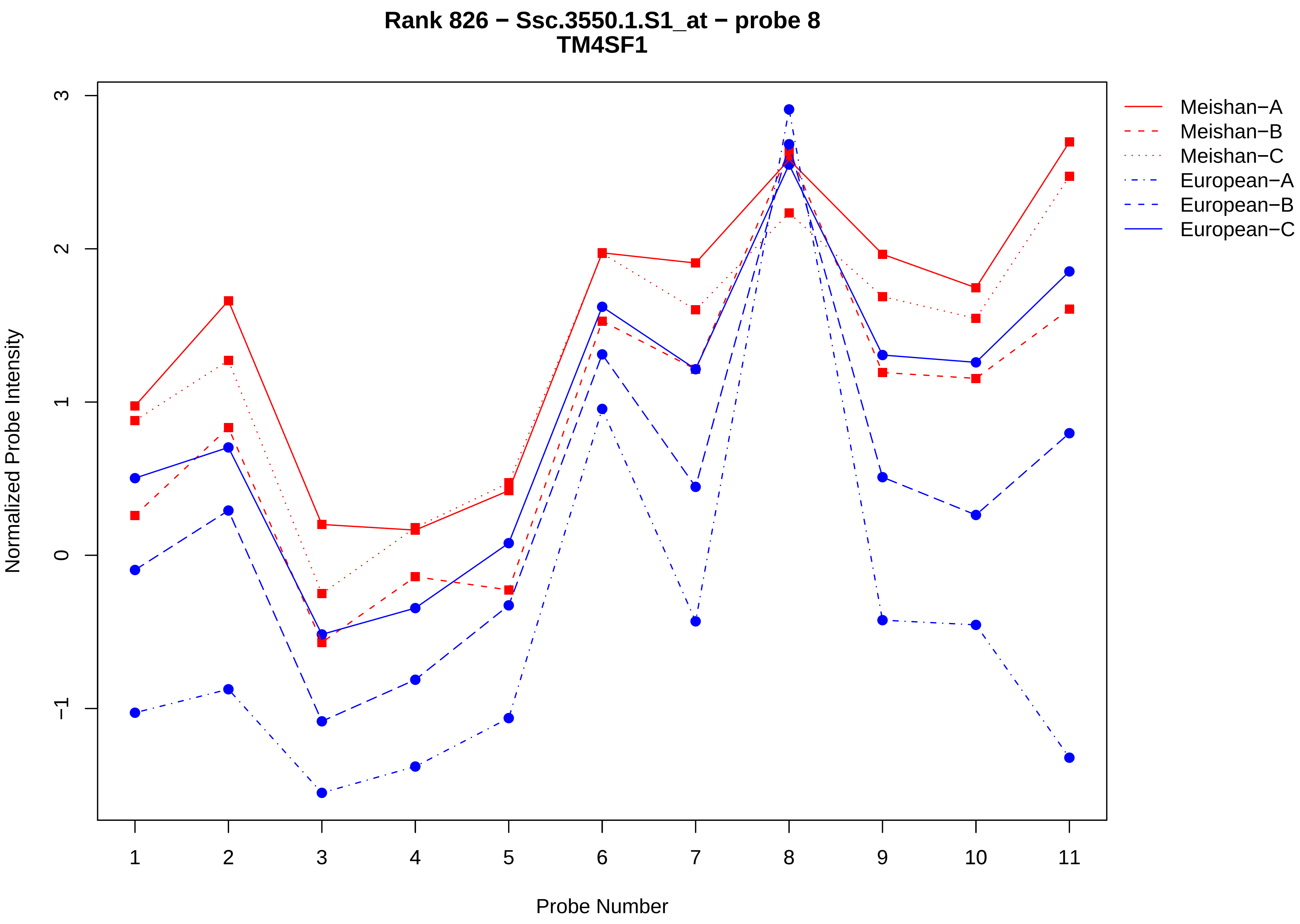

Rank 826 − Ssc.3550.1.S1_at − probe 8
TM4SF1
Normalized Probe Intensity
Probe Number
−1
0
1
2
3
1
2
3
4
5
6
7
8
9
10
11
Meishan−A
Meishan−B
Meishan−C
European−A
European−B
European−C

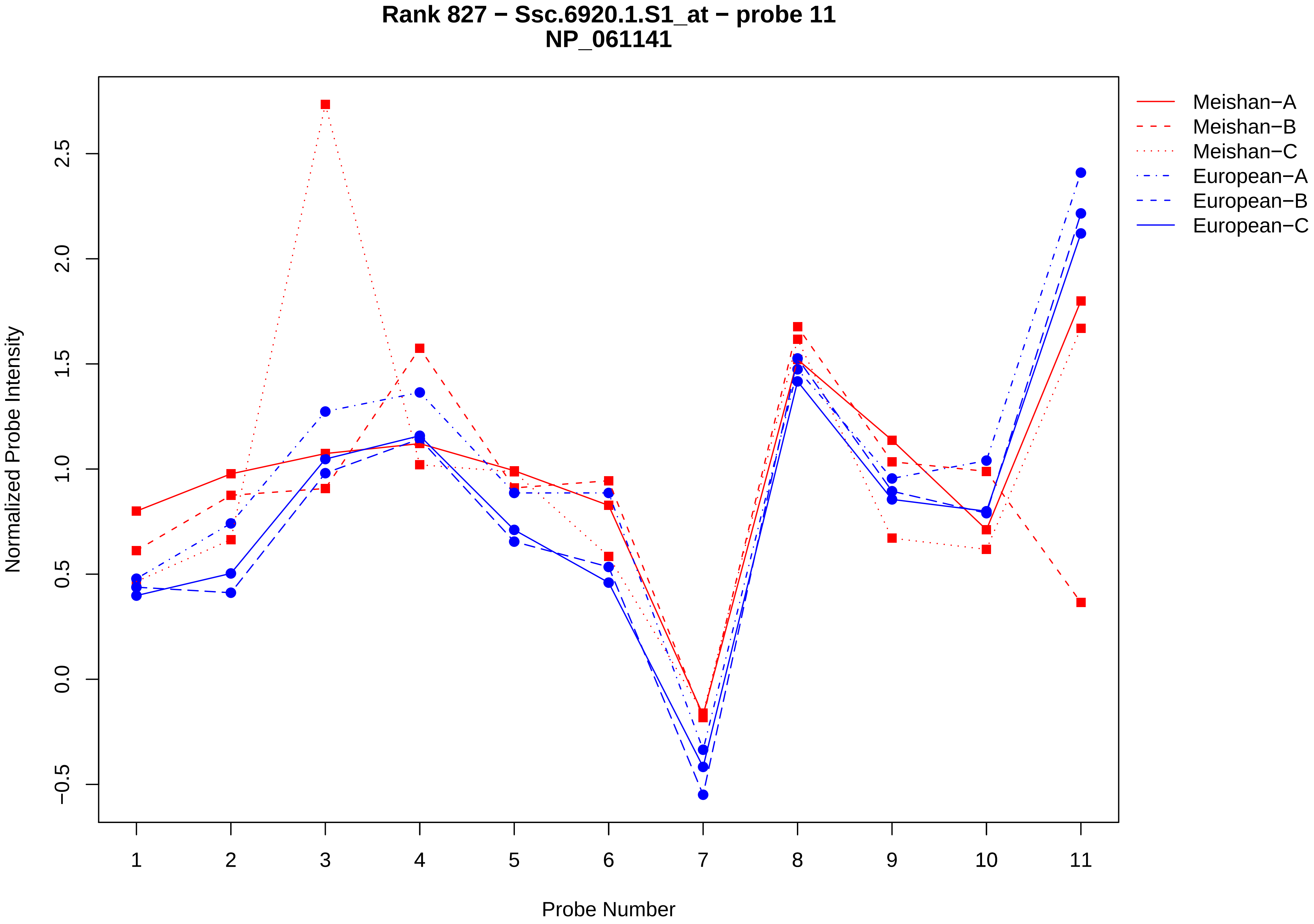

Rank 827 − Ssc.6920.1.S1_at − probe 11
NP_061141
Normalized Probe Intensity
Probe Number
Meishan−A
Meishan−B
Meishan−C
European−A
European−B
European−C
−0.5
0.0
0.5
1.0
1.5
2.0
2.5
1
2
3
4
5
6
7
8
9
10
11

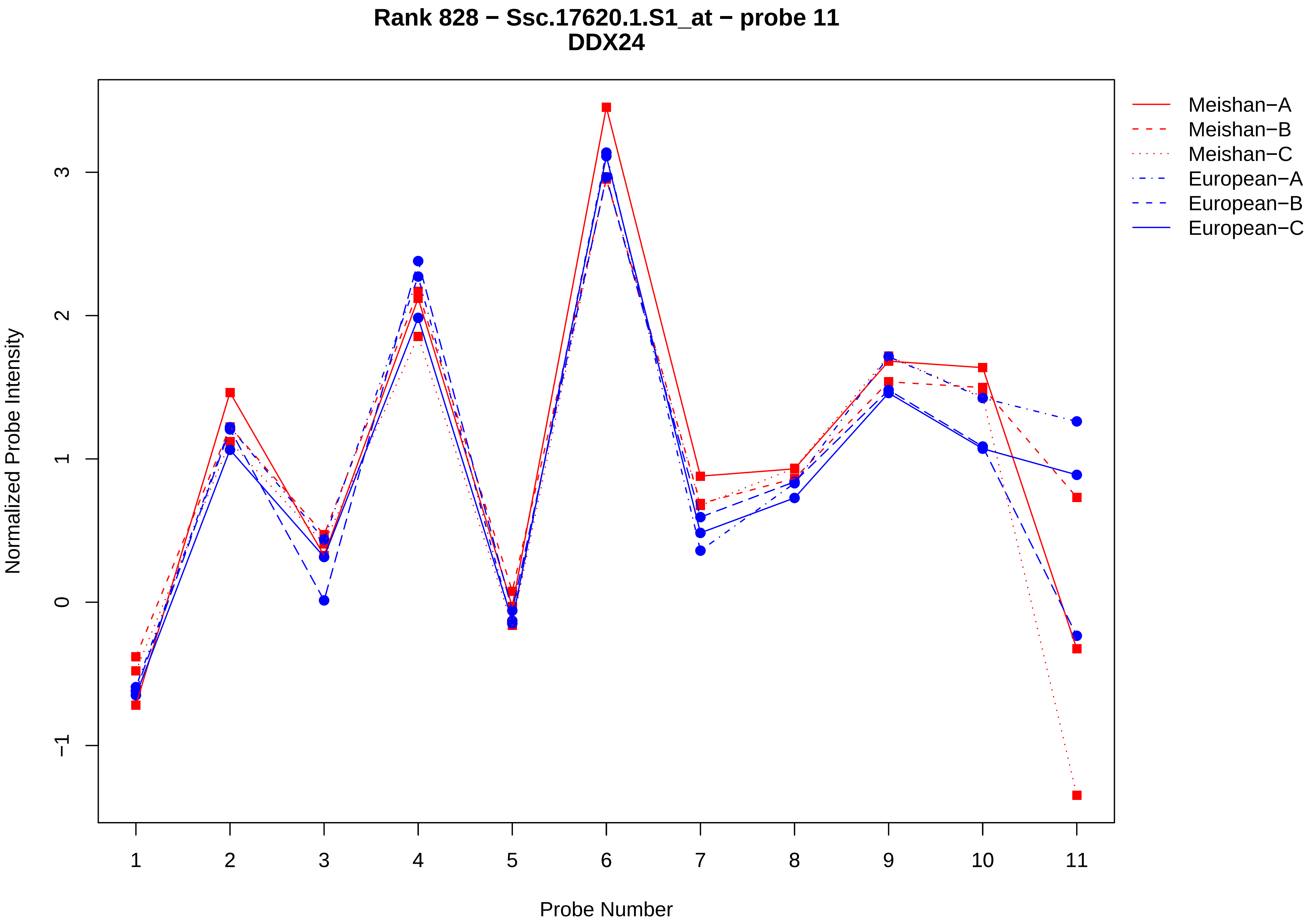

Rank 828 − Ssc.17620.1.S1_at − probe 11
DDX24
Normalized Probe Intensity
Probe Number
−1
0
1
2
3
1
2
3
4
5
6
7
8
9
10
11
Meishan−A
Meishan−B
Meishan−C
European−A
European−B
European−C

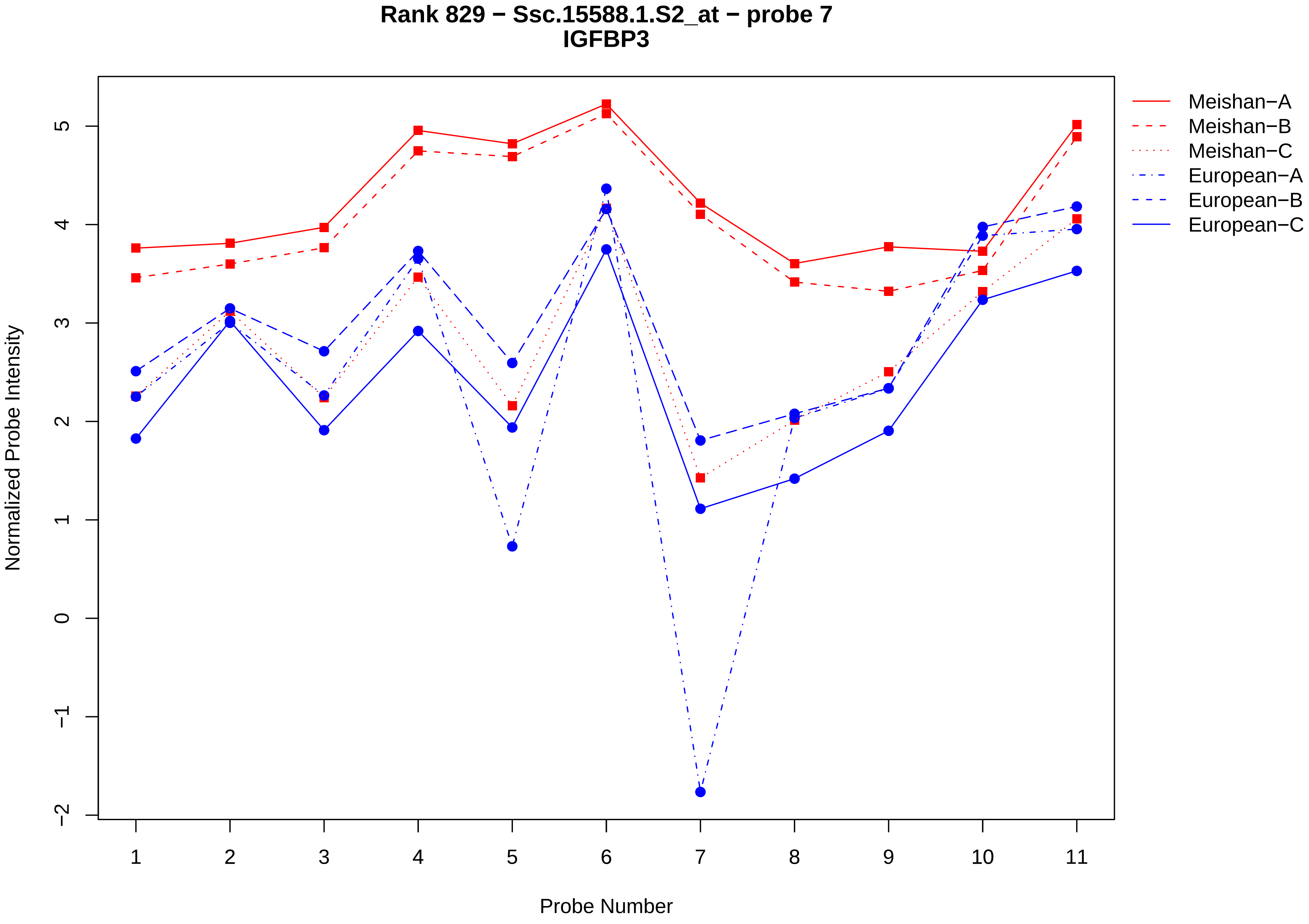
Rank 829 − Ssc.15588.1.S2_at − probe 7
IGFBP3
Normalized Probe Intensity
Probe Number
−2
−1
0
1
2
3
4
5
1
2
3
4
5
6
7
8
9
10
11
Meishan−A
Meishan−B
Meishan−C
European−A
European−B
European−C

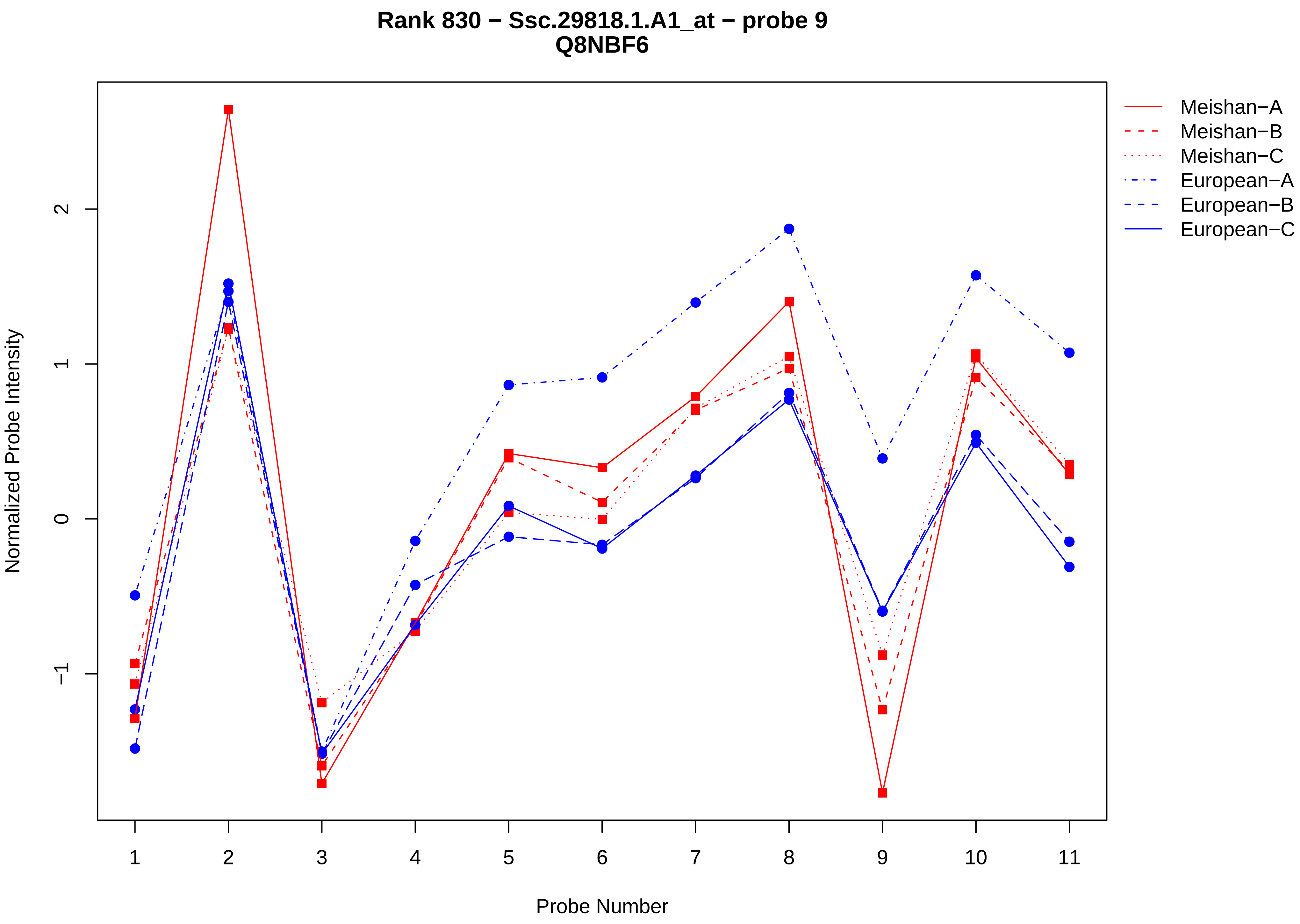

Rank 830 − Ssc.29818.1.A1_at − probe 9
Q8NBF6
Normalized Probe Intensity
Probe Number
Meishan−A
Meishan−B
Meishan−C
European−A
European−B
European−C
−1
0
1
2
1
2
3
4
5
6
7
8
9
10
11

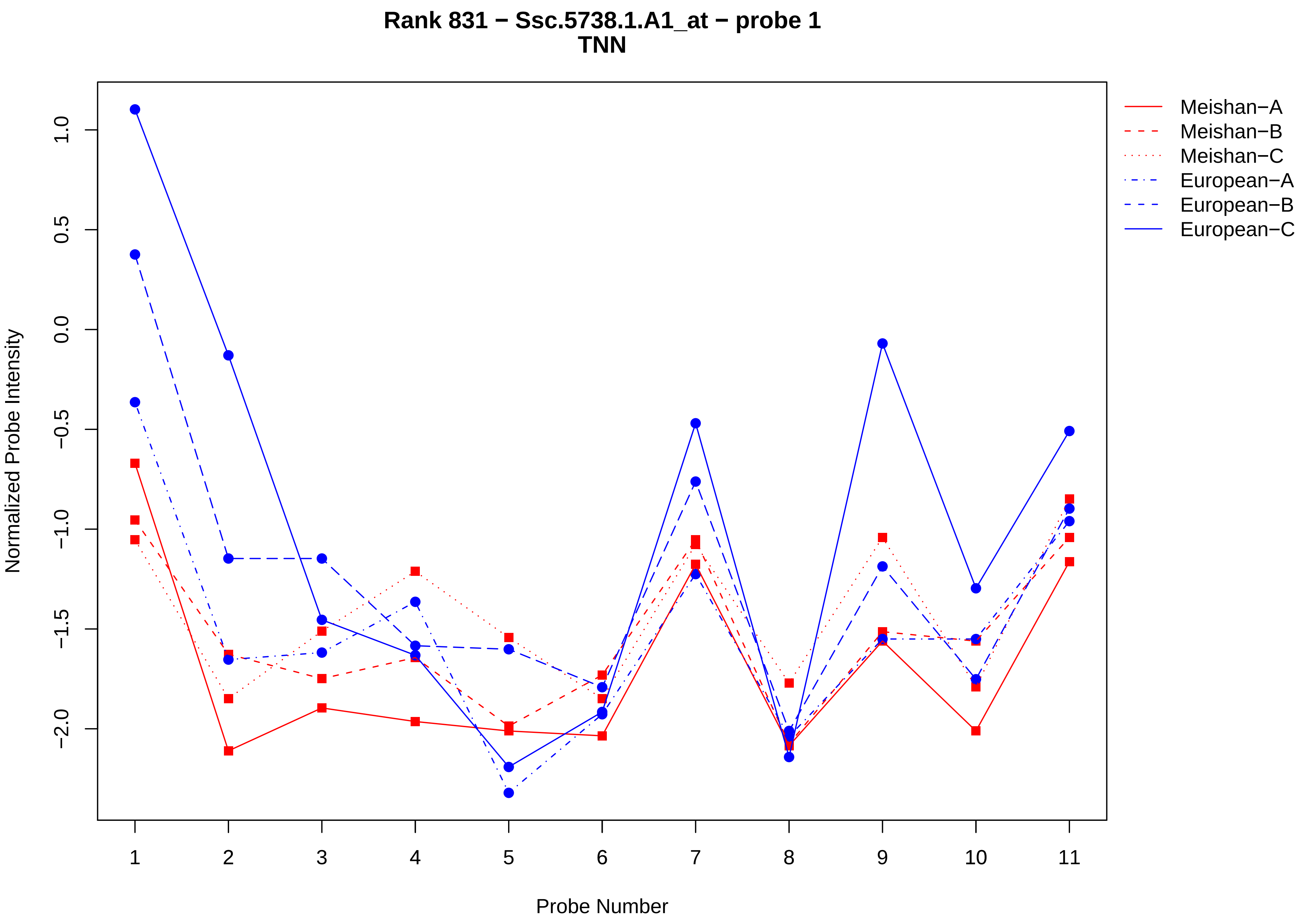

Rank 831 − Ssc.5738.1.A1_at − probe 1
TNN
Normalized Probe Intensity
Probe Number
Meishan−A
Meishan−B
Meishan−C
European−A
European−B
European−C
1.0
0.5
0.0
−0.5
−1.0
−1.5
−2.0
1
2
3
4
5
6
7
8
9
10
11

Rank 832 − Ssc.7438.1.A1_at − probe 7
SLC25A24

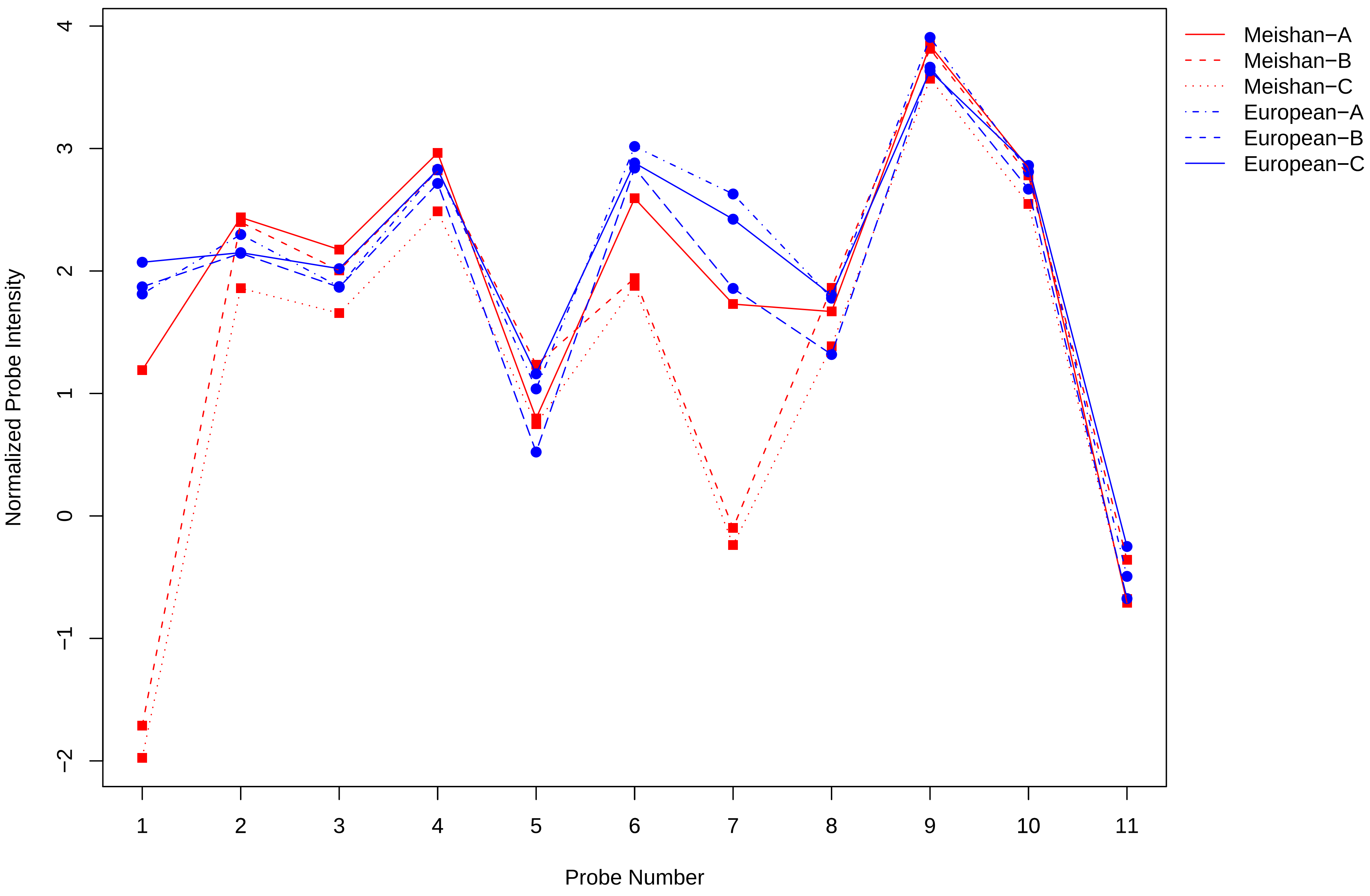

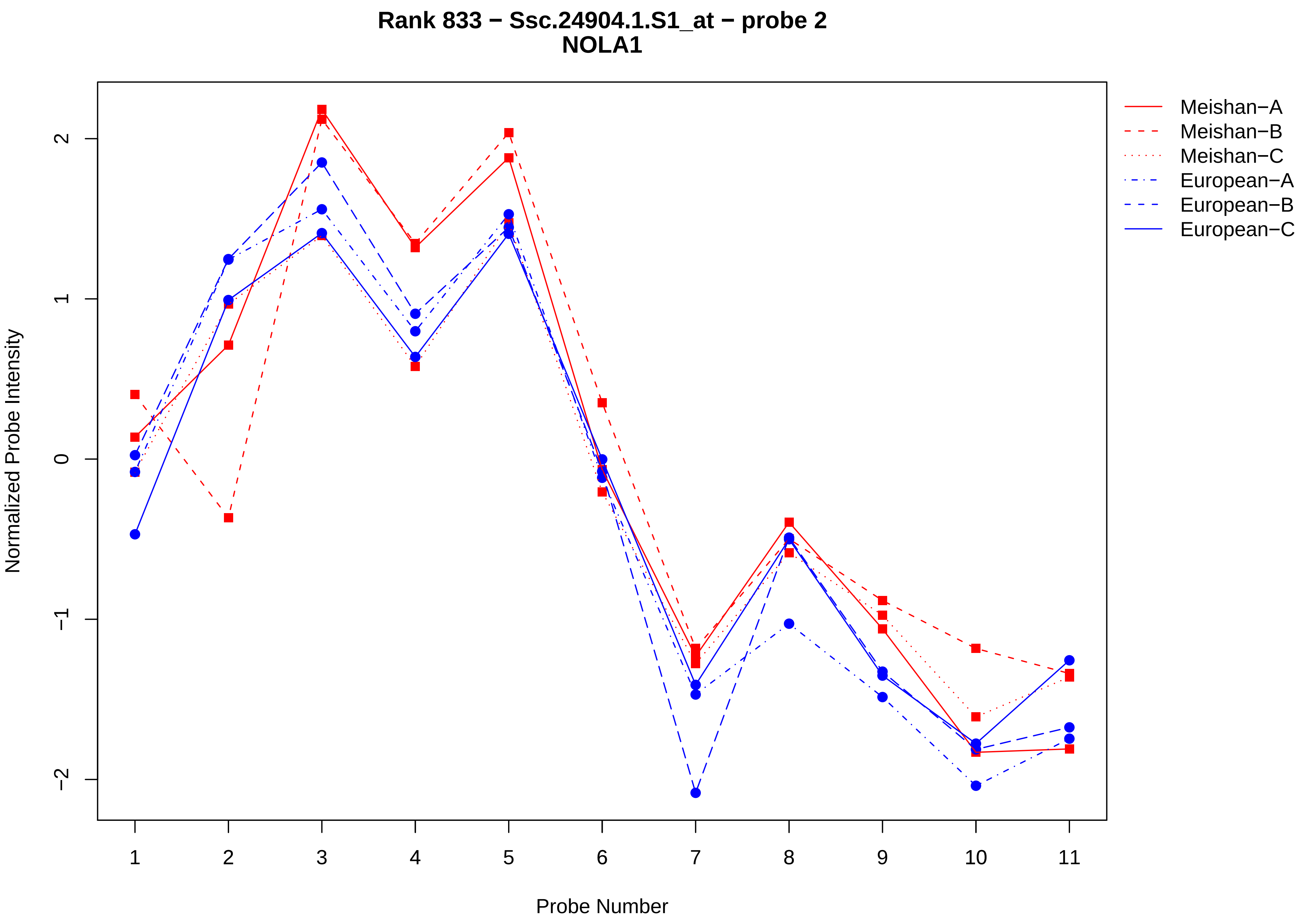

Rank 833 − Ssc.24904.1.S1_at − probe 2
NOLA1
Normalized Probe Intensity
Probe Number
Meishan−A
Meishan−B
Meishan−C
European−A
European−B
European−C

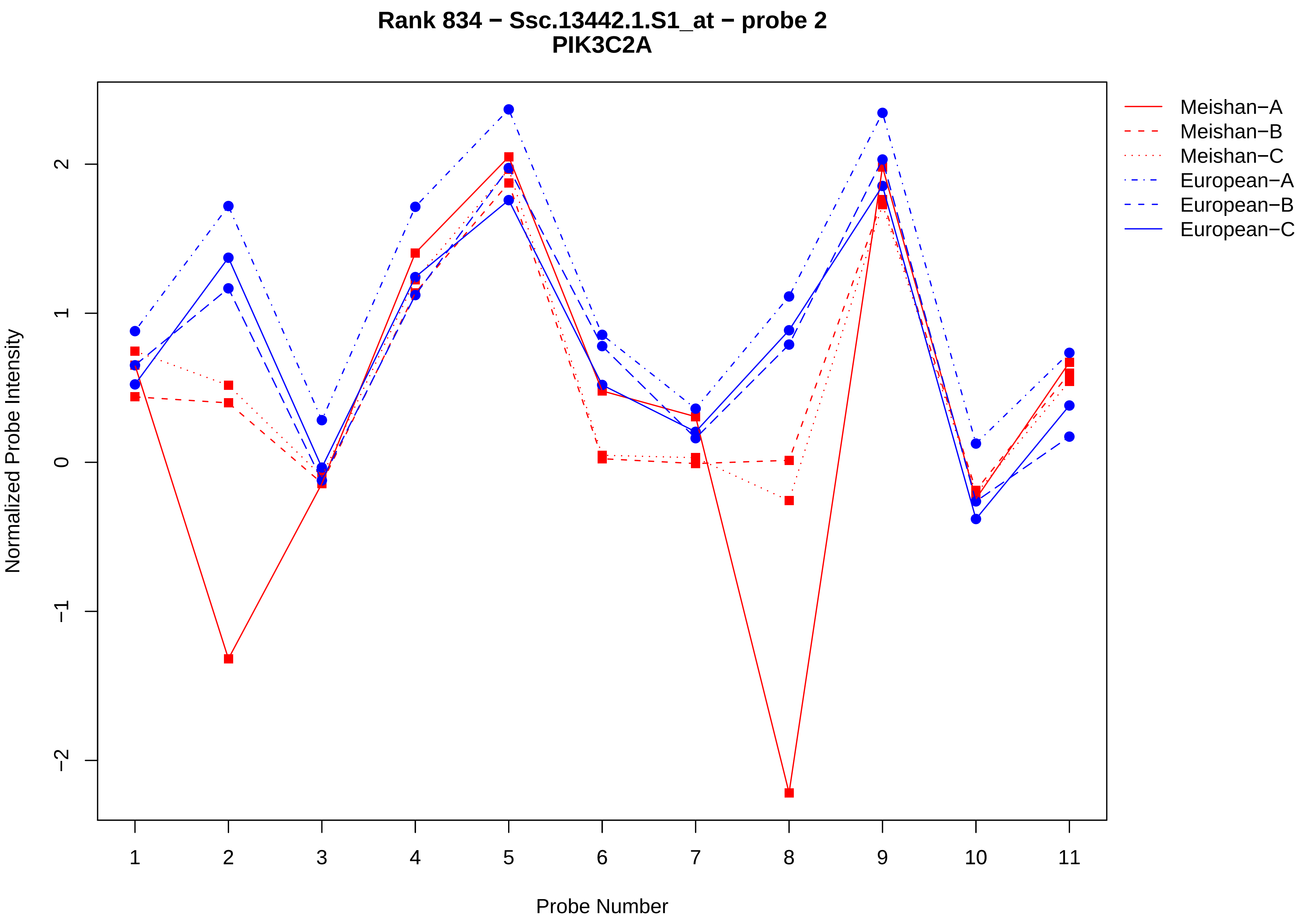

Rank 834 − Ssc.13442.1.S1_at − probe 2
PIK3C2A
Normalized Probe Intensity
Probe Number
−2
−1
0
1
2
1
2
3
4
5
6
7
8
9
10
11
Meishan−A
Meishan−B
Meishan−C
European−A
European−B
European−C

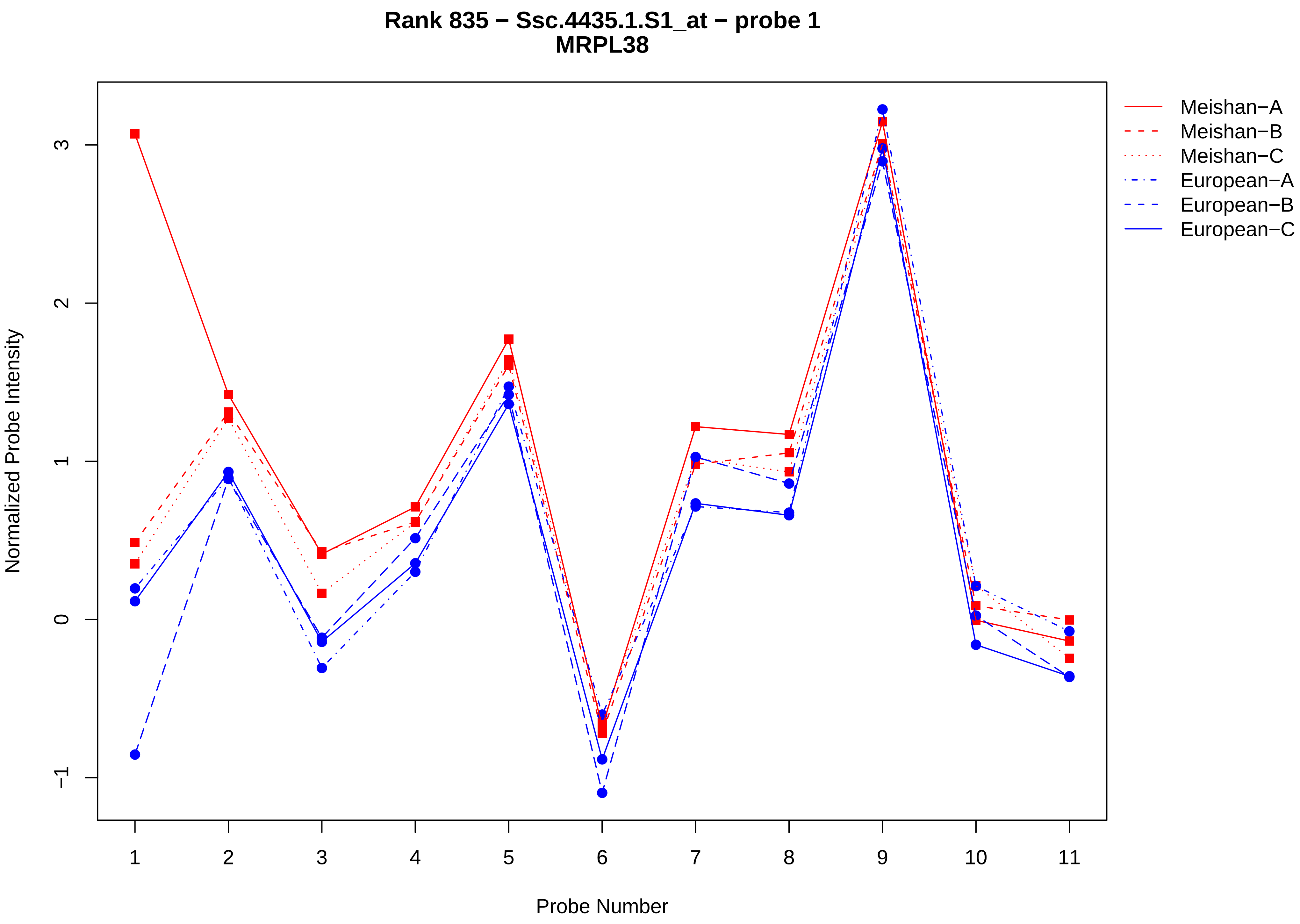

Rank 835 − Ssc.4435.1.S1_at − probe 1
MRPL38
Normalized Probe Intensity
Probe Number
Meishan−A
Meishan−B
Meishan−C
European−A
European−B
European−C
−1
0
1
2
3
1
2
3
4
5
6
7
8
9
10
11

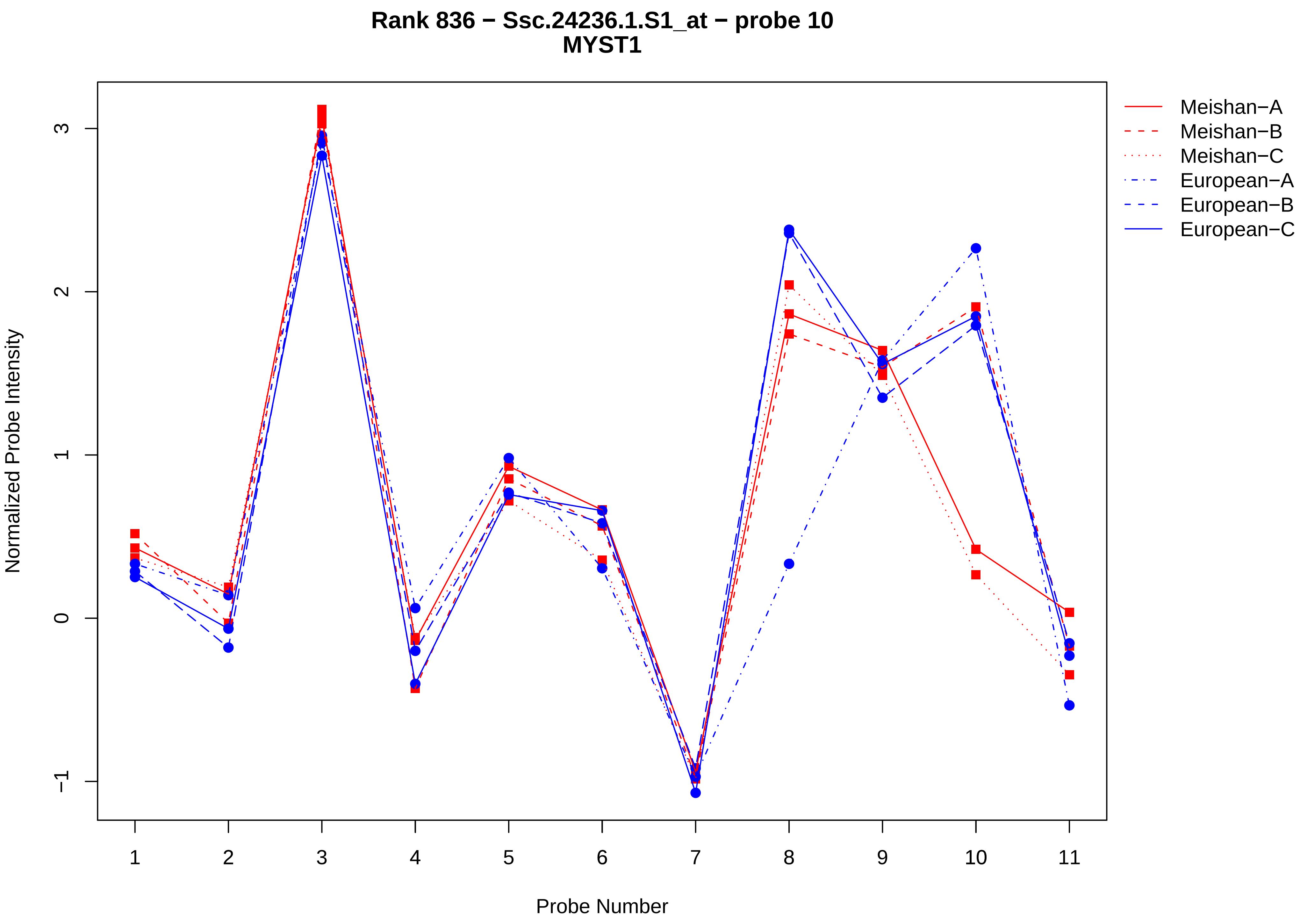
Rank 836 − Ssc.24236.1.S1_at − probe 10
MYST1
Normalized Probe Intensity
Probe Number
−1
0
1
2
3
1
2
3
4
5
6
7
8
9
10
11
Meishan−A
Meishan−B
Meishan−C
European−A
European−B
European−C

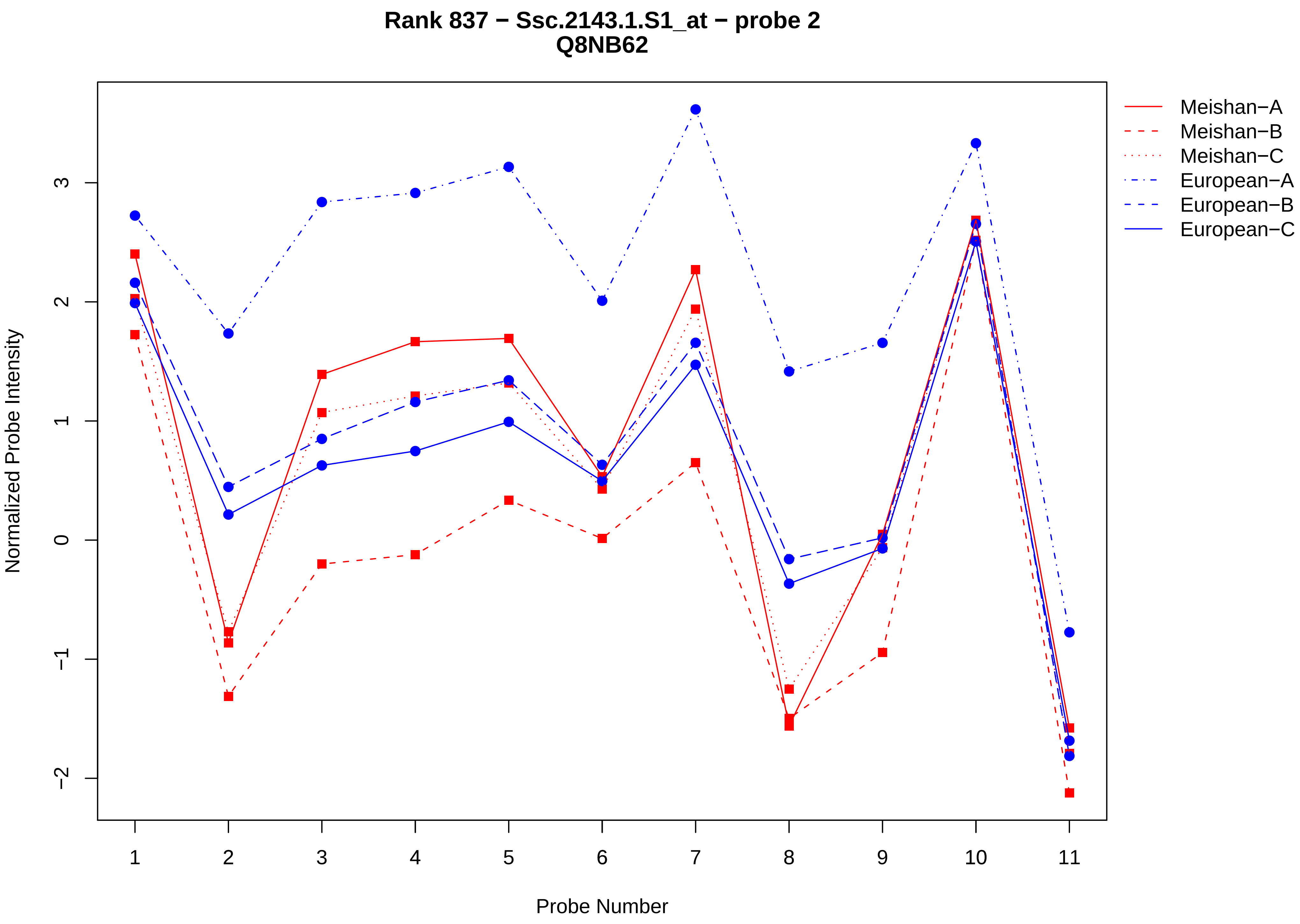
Rank 837 − Ssc.2143.1.S1_at − probe 2
Q8NB62
Normalized Probe Intensity
Probe Number
−2
−1
0
1
2
3
1
2
3
4
5
6
7
8
9
10
11
Meishan−A
Meishan−B
Meishan−C
European−A
European−B
European−C

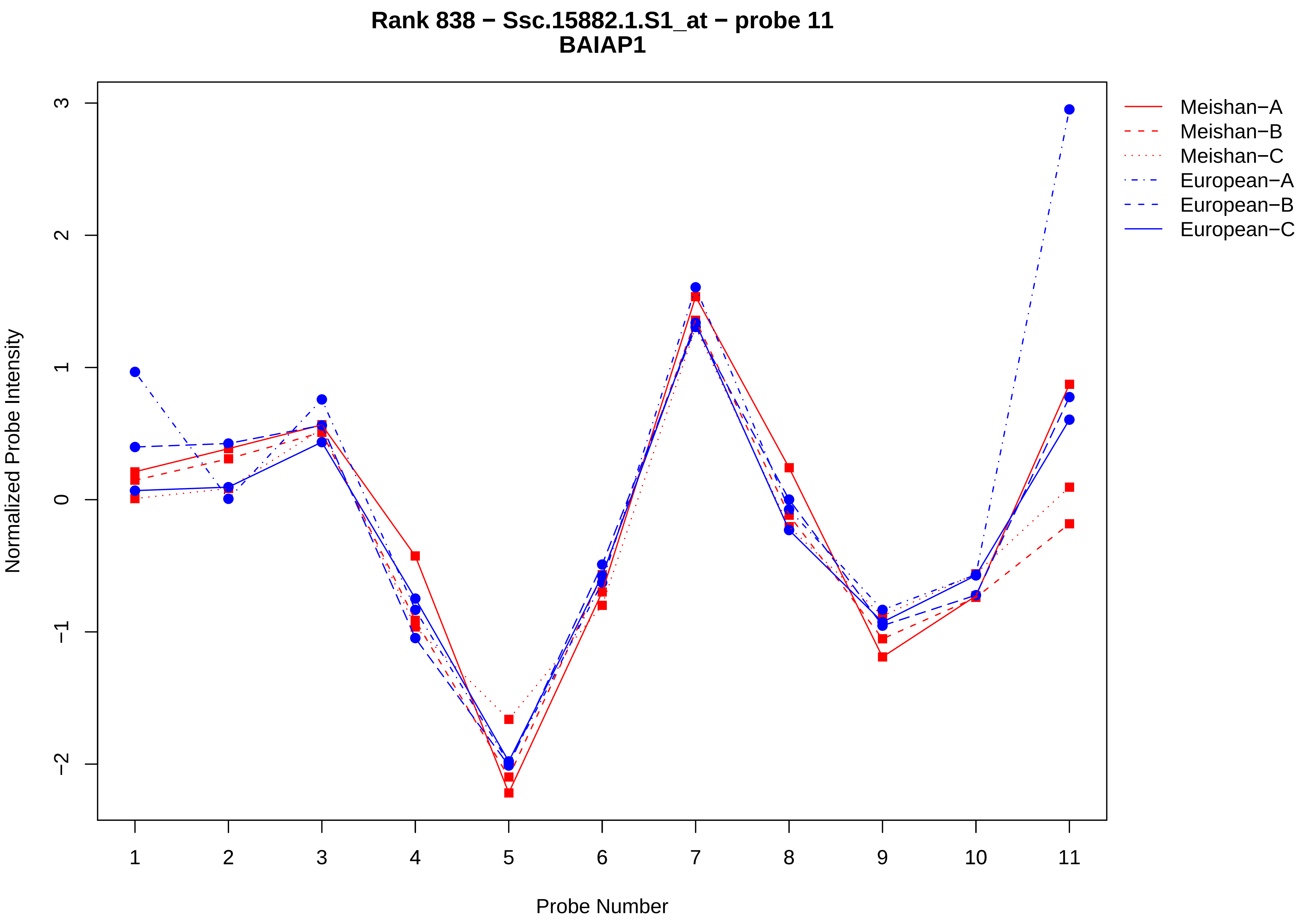

Rank 838 − Ssc.15882.1.S1_at − probe 11
BAIAP1
Normalized Probe Intensity
Probe Number
Meishan−A
Meishan−B
Meishan−C
European−A
European−B
European−C

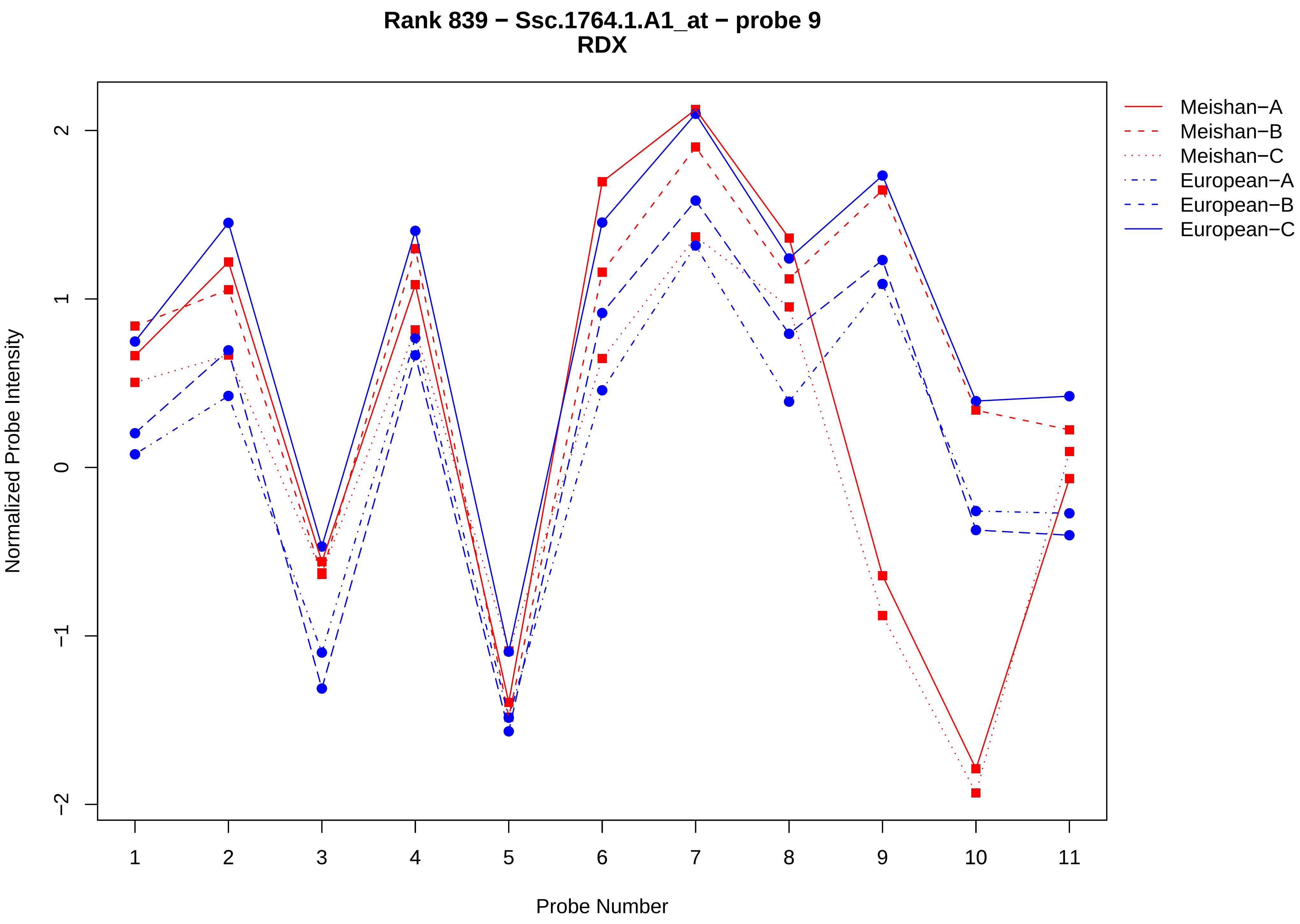
Rank 839 − Ssc.1764.1.A1_at − probe 9
RDX
Normalized Probe Intensity
Probe Number
Meishan−A
Meishan−B
Meishan−C
European−A
European−B
European−C
−2
−1
0
1
2
1
2
3
4
5
6
7
8
9
10
11

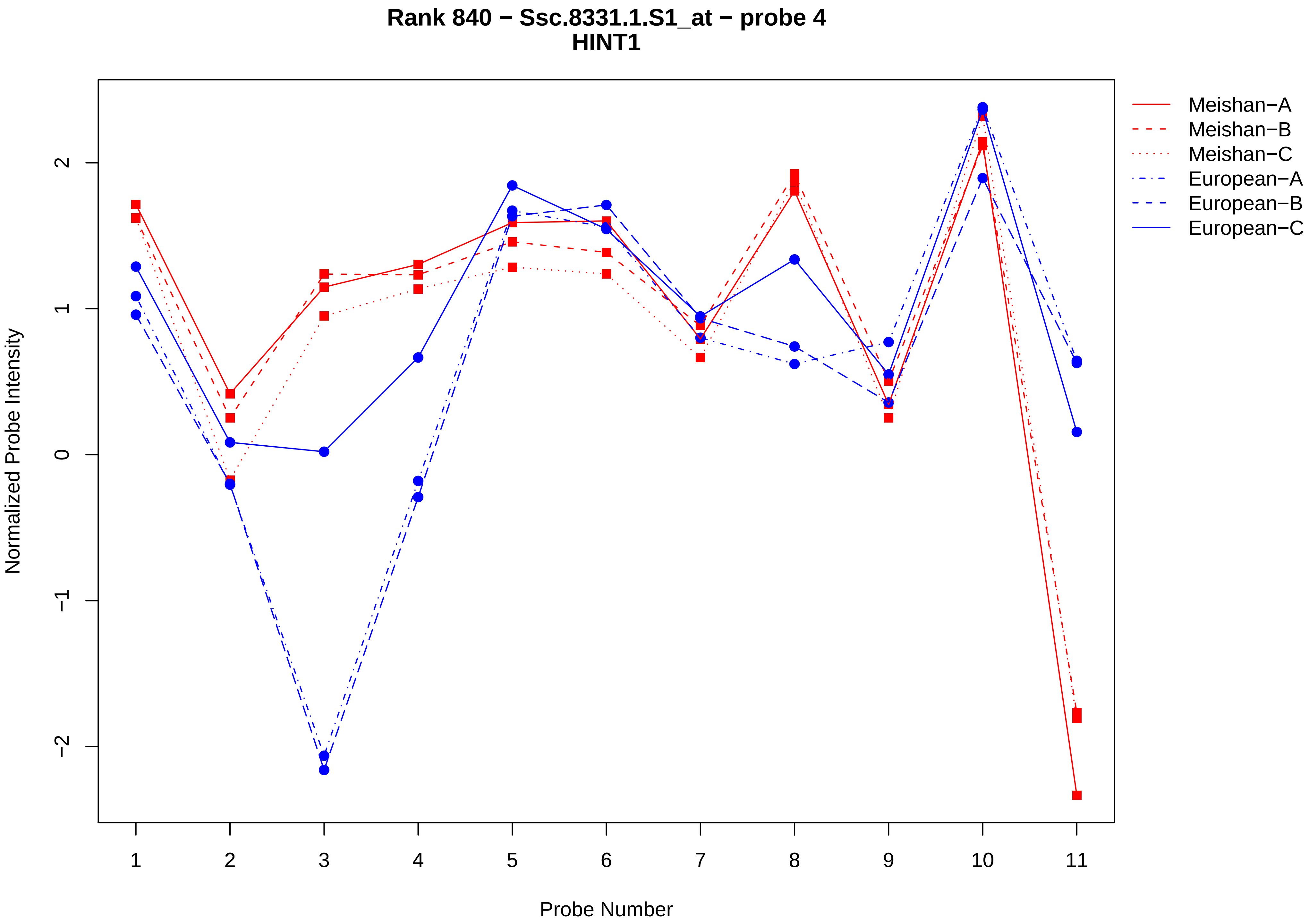
Rank 840 − Ssc.8331.1.S1_at − probe 4
HINT1
Normalized Probe Intensity
Probe Number
Meishan−A
Meishan−B
Meishan−C
European−A
European−B
European−C
−2
−1
0
1
2
1
2
3
4
5
6
7
8
9
10
11

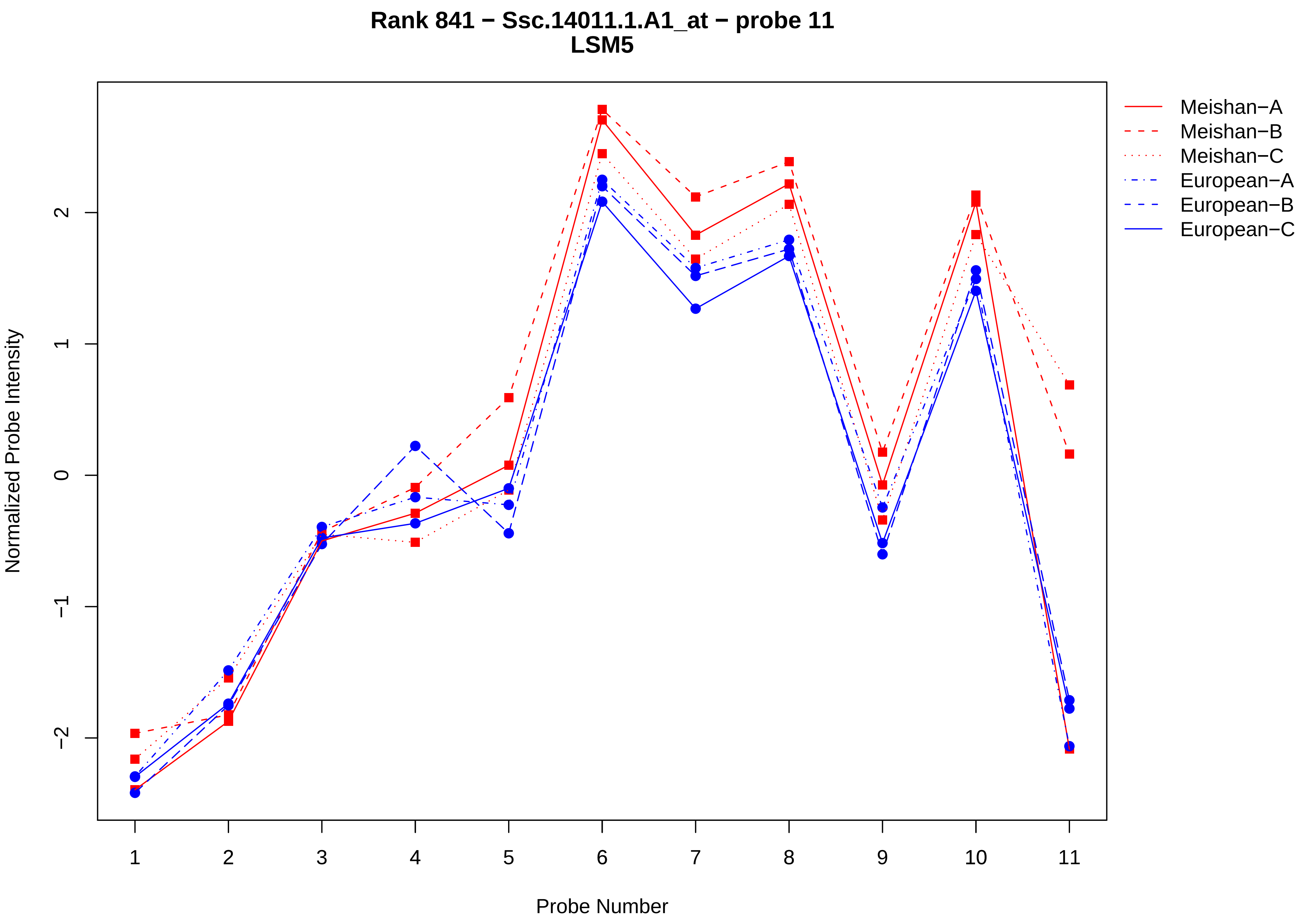

Rank 841 − Ssc.14011.1.A1_at − probe 11
LSM5
Normalized Probe Intensity
Probe Number
−2
−1
0
1
2
1
2
3
4
5
6
7
8
9
10
11
Meishan−A
Meishan−B
Meishan−C
European−A
European−B
European−C

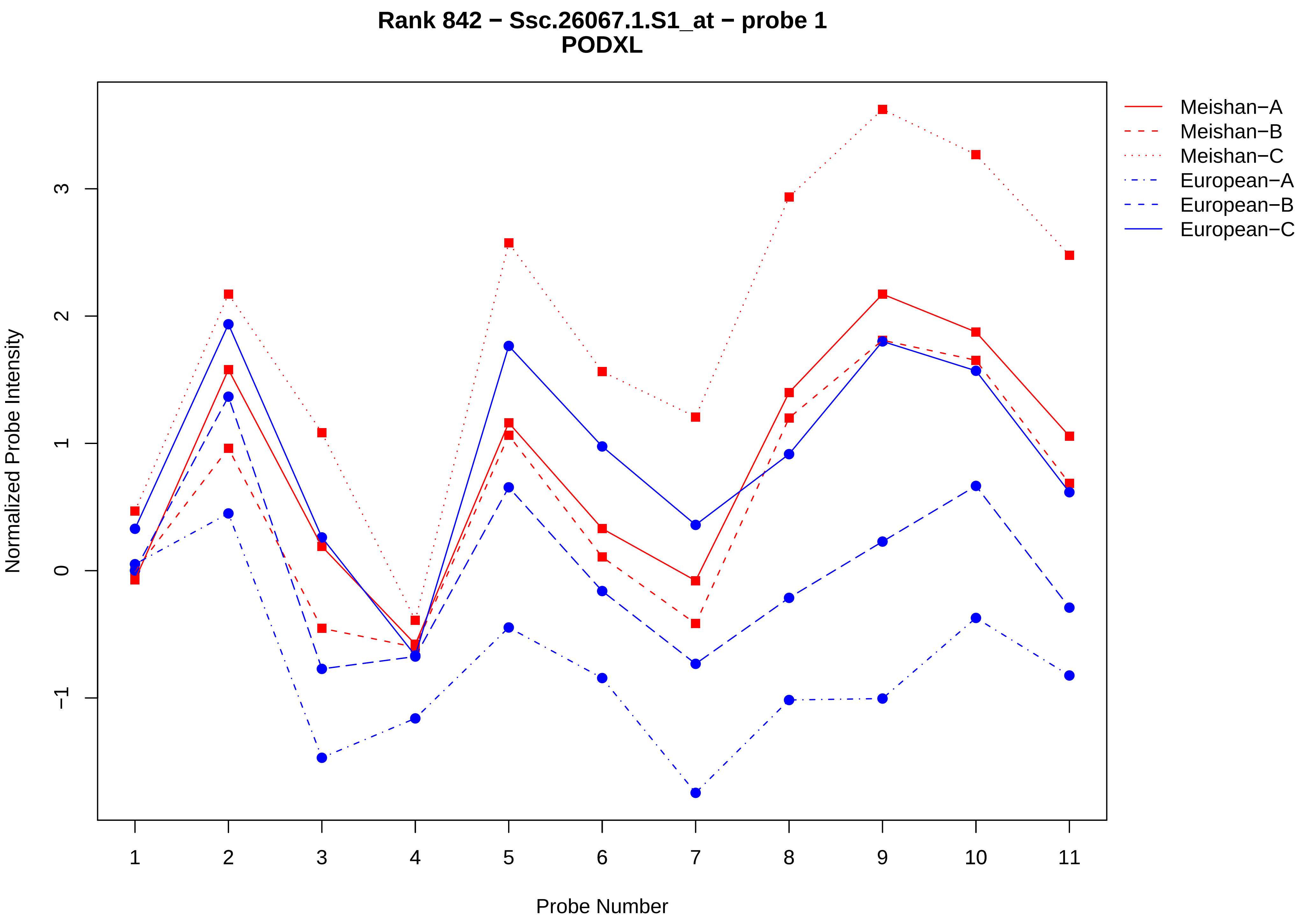
Rank 842 − Ssc.26067.1.S1_at − probe 1
PODXL
Normalized Probe Intensity
Probe Number
Meishan−A
Meishan−B
Meishan−C
European−A
European−B
European−C
−1
0
1
2
3
1
2
3
4
5
6
7
8
9
10
11

Rank 843 − Ssc.2506.1.S1_at − probe 6
SLCO1A2

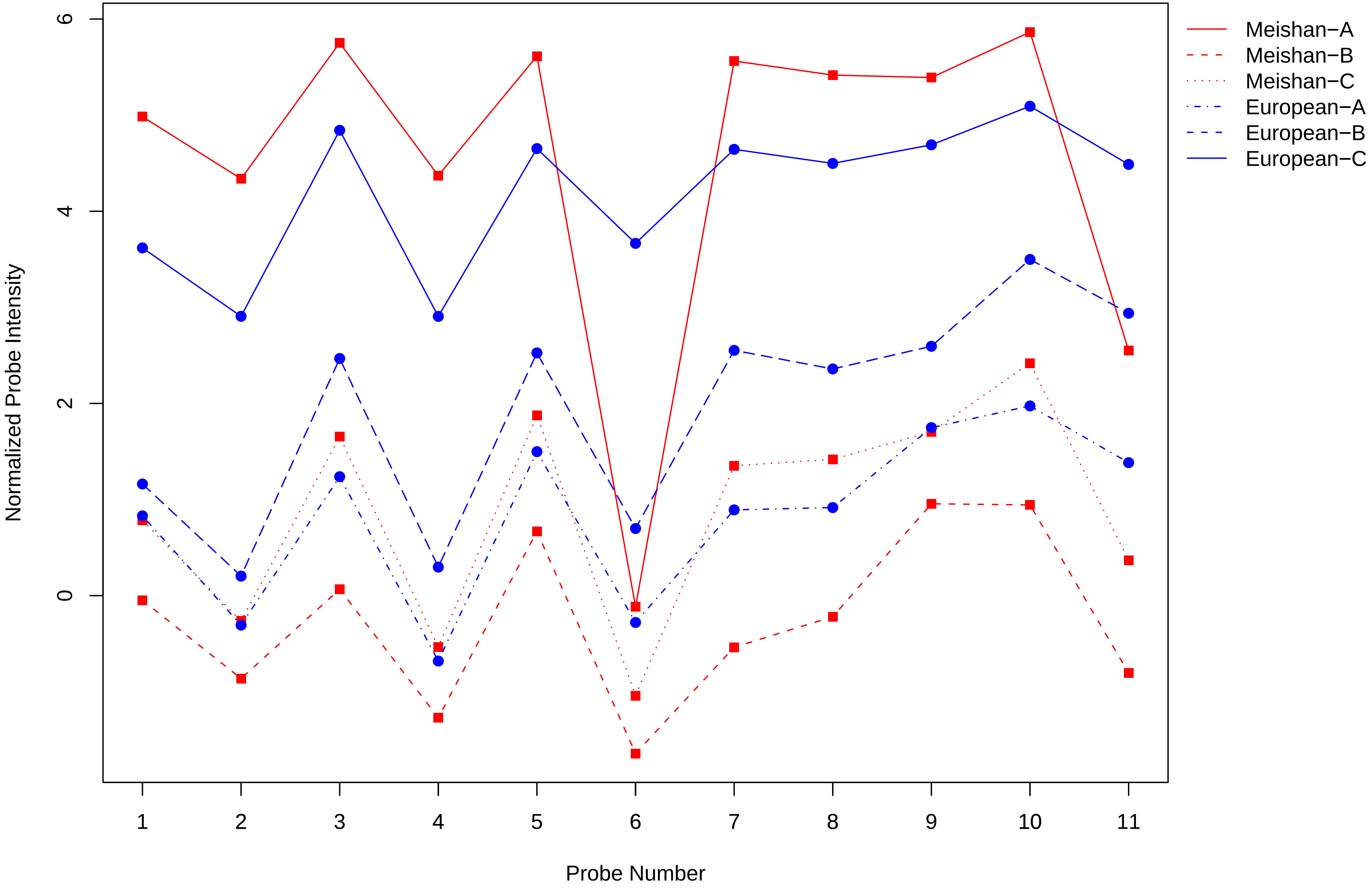

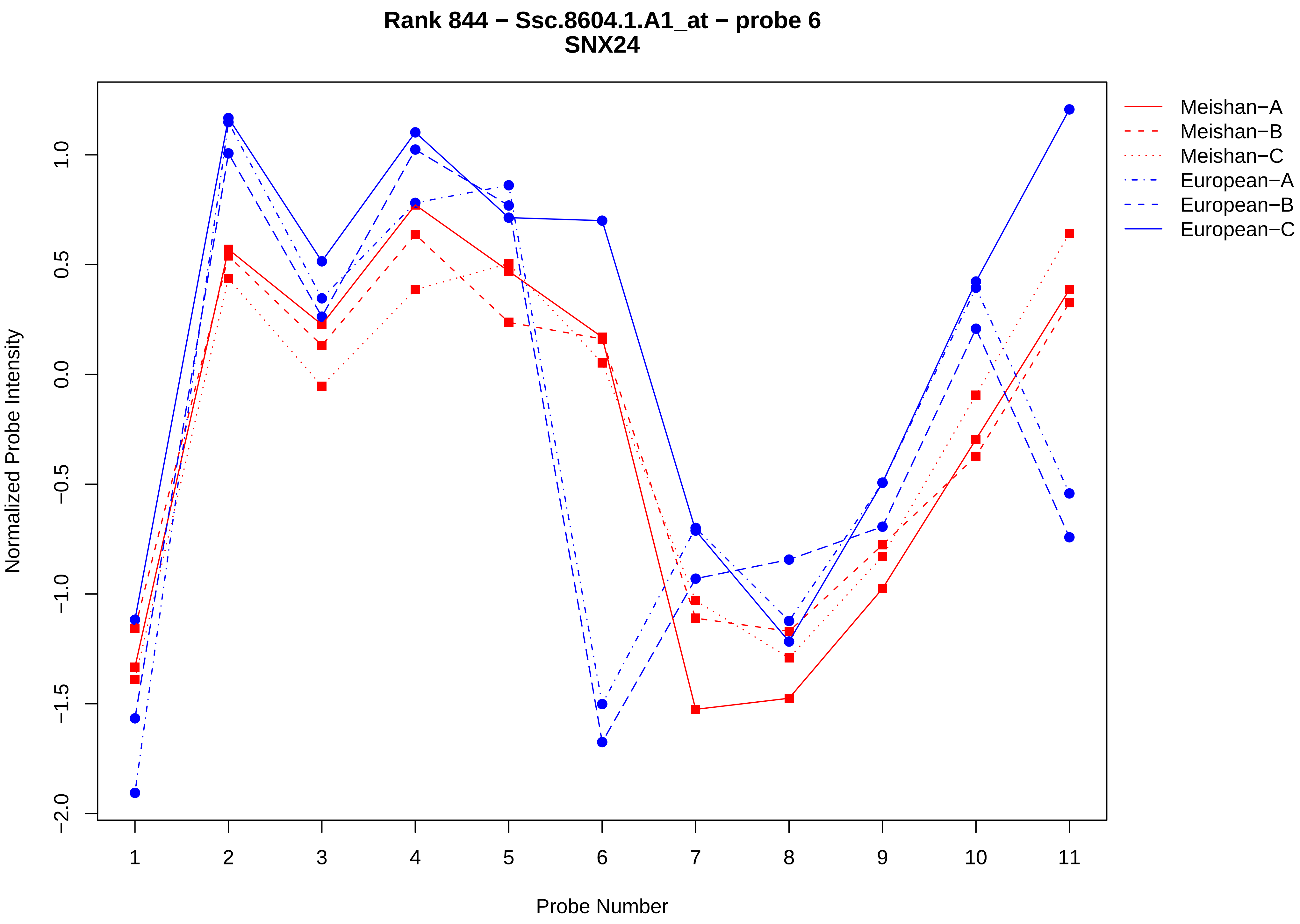
Rank 844 − Ssc.8604.1.A1_at − probe 6
SNX24
Normalized Probe Intensity
Probe Number
Meishan−A
Meishan−B
Meishan−C
European−A
European−B
European−C
1.0
0.5
0.0
−0.5
−1.0
−1.5
−2.0
1
2
3
4
5
6
7
8
9
10
11

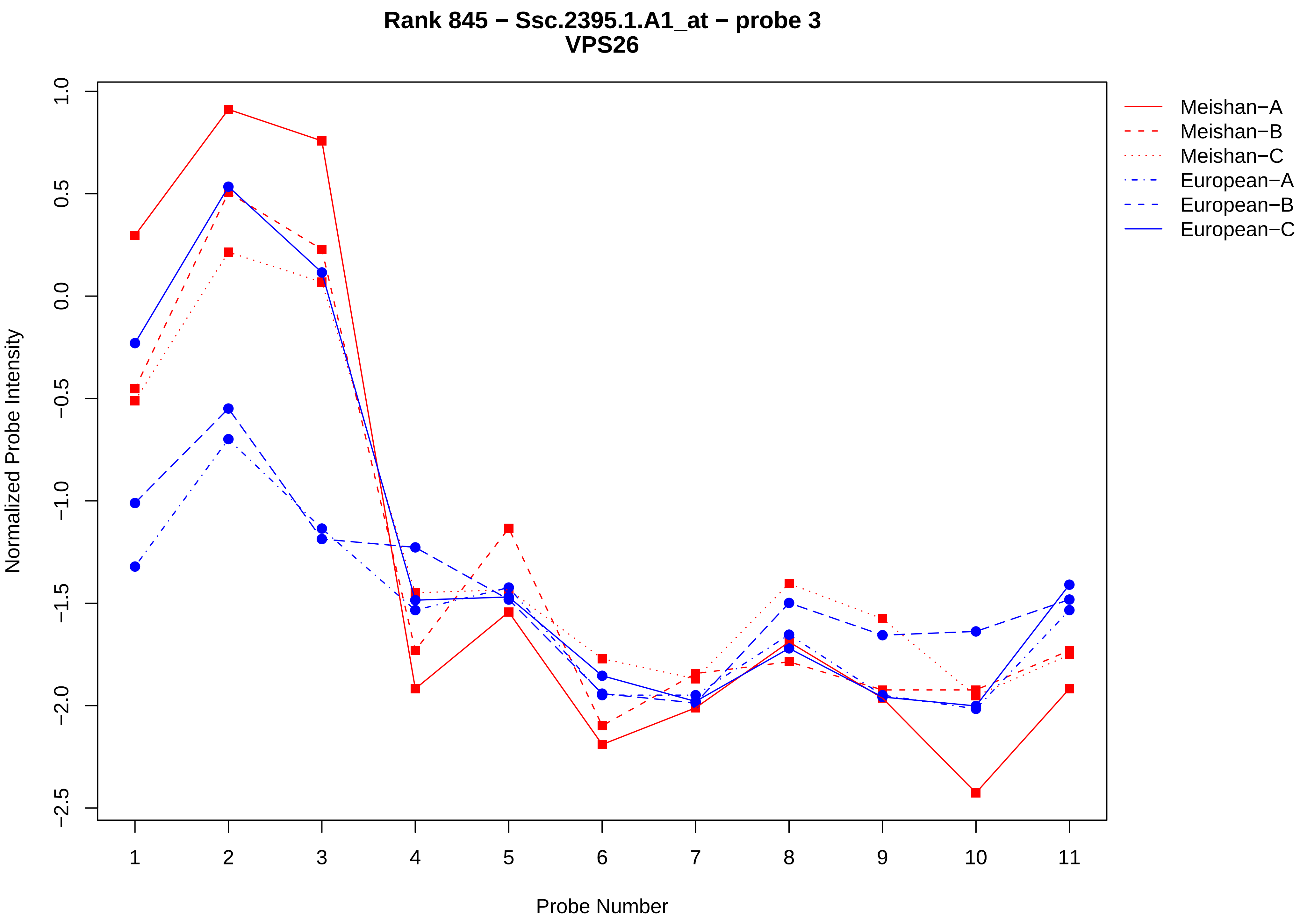

Rank 845 − Ssc.2395.1.A1_at − probe 3
VPS26
Normalized Probe Intensity
Probe Number
Meishan−A
Meishan−B
Meishan−C
European−A
European−B
European−C
1.0
0.5
0.0
−0.5
−1.0
−1.5
−2.0
−2.5
1
2
3
4
5
6
7
8
9
10
11

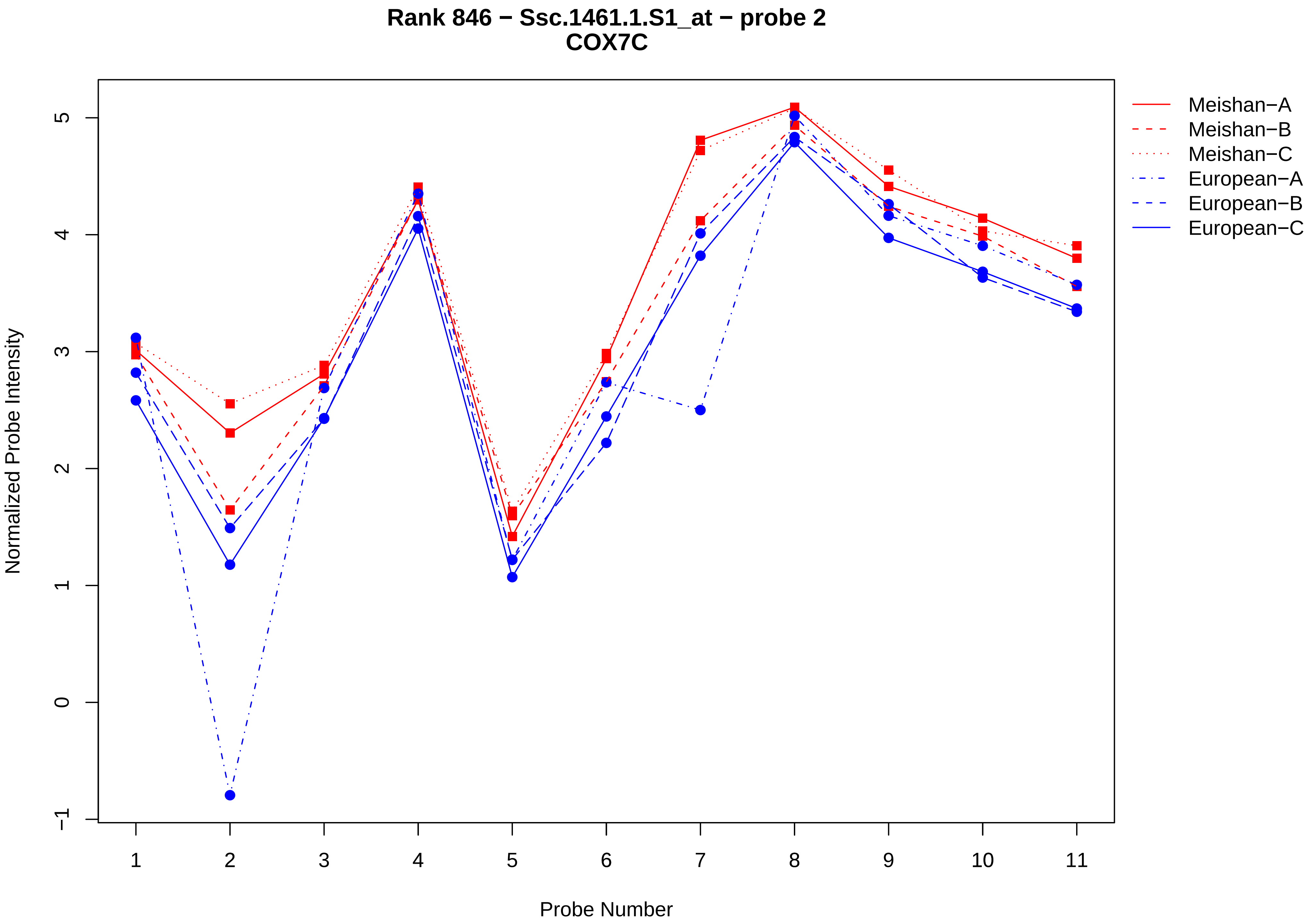

Rank 846 − Ssc.1461.1.S1_at − probe 2
COX7C
Normalized Probe Intensity
Probe Number
Meishan−A
Meishan−B
Meishan−C
European−A
European−B
European−C
−1
0
1
2
3
4
5
1
2
3
4
5
6
7
8
9
10
11

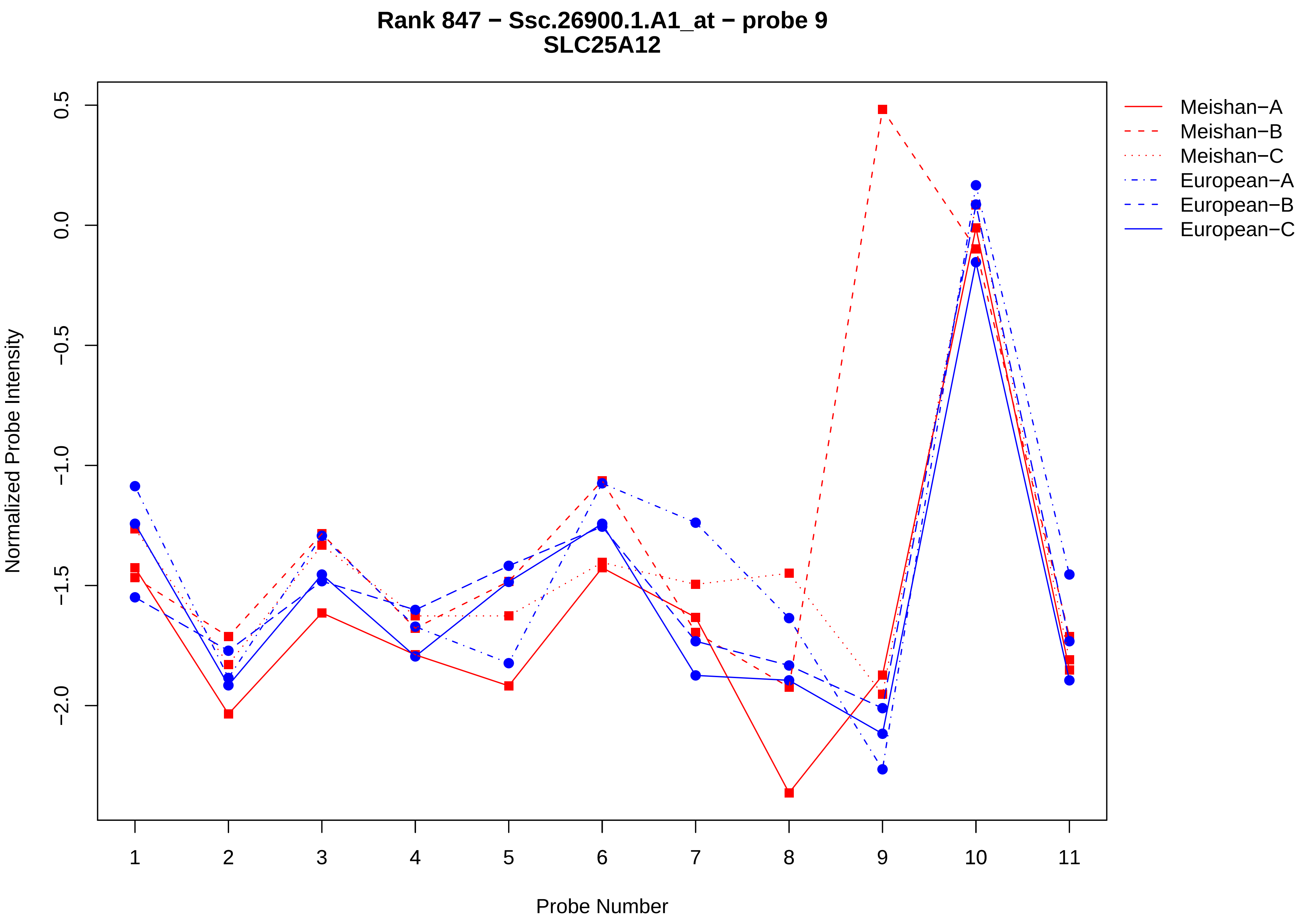

Rank 847 − Ssc.26900.1.A1_at − probe 9
SLC25A12
Normalized Probe Intensity
0.5
0.0
−0.5
−1.0
−1.5
−2.0
1
2
3
4
5
6
7
8
9
10
11
Probe Number
Meishan−A
Meishan−B
Meishan−C
European−A
European−B
European−C

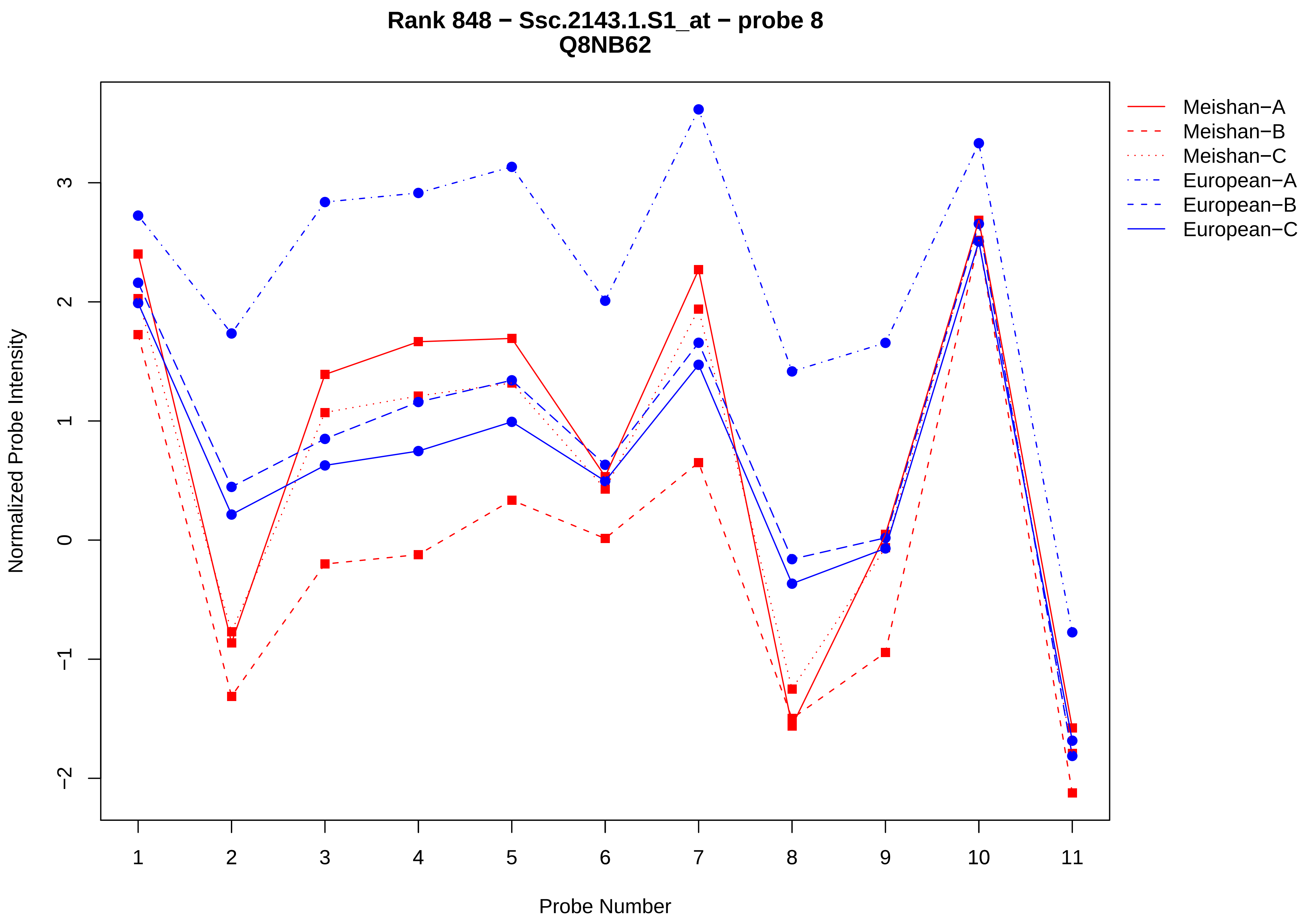

Rank 848 − Ssc.2143.1.S1_at − probe 8
Q8NB62
Normalized Probe Intensity
Probe Number
−2
−1
0
1
2
3
1
2
3
4
5
6
7
8
9
10
11
Meishan−A
Meishan−B
Meishan−C
European−A
European−B
European−C

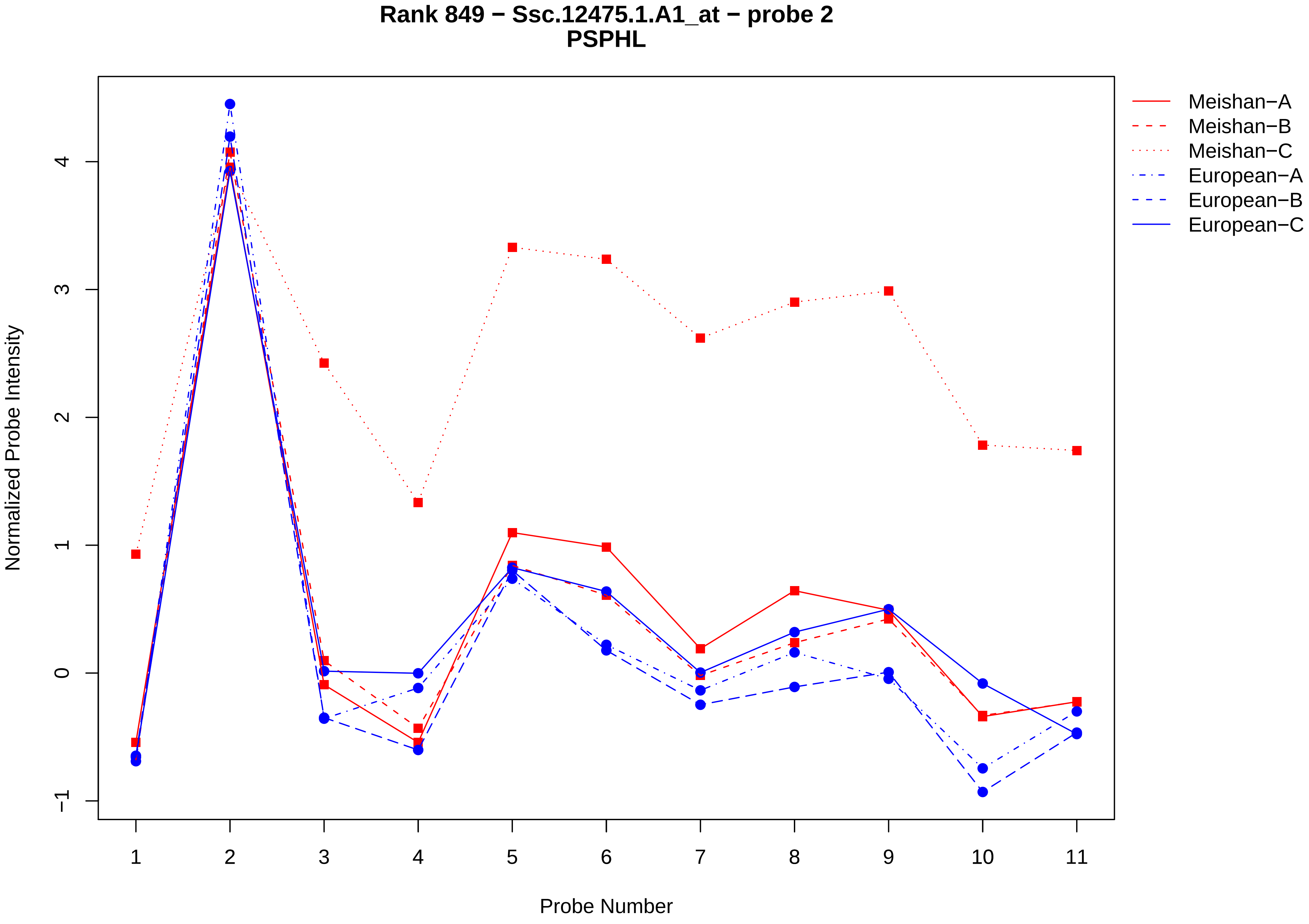
Rank 849 − Ssc.12475.1.A1_at − probe 2
PSPHL
Normalized Probe Intensity
Probe Number
−1
0
1
2
3
4
1
2
3
4
5
6
7
8
9
10
11
Meishan−A
Meishan−B
Meishan−C
European−A
European−B
European−C

Rank 850 − Ssc.4814.1.A1_at − probe 6
LRRC3B

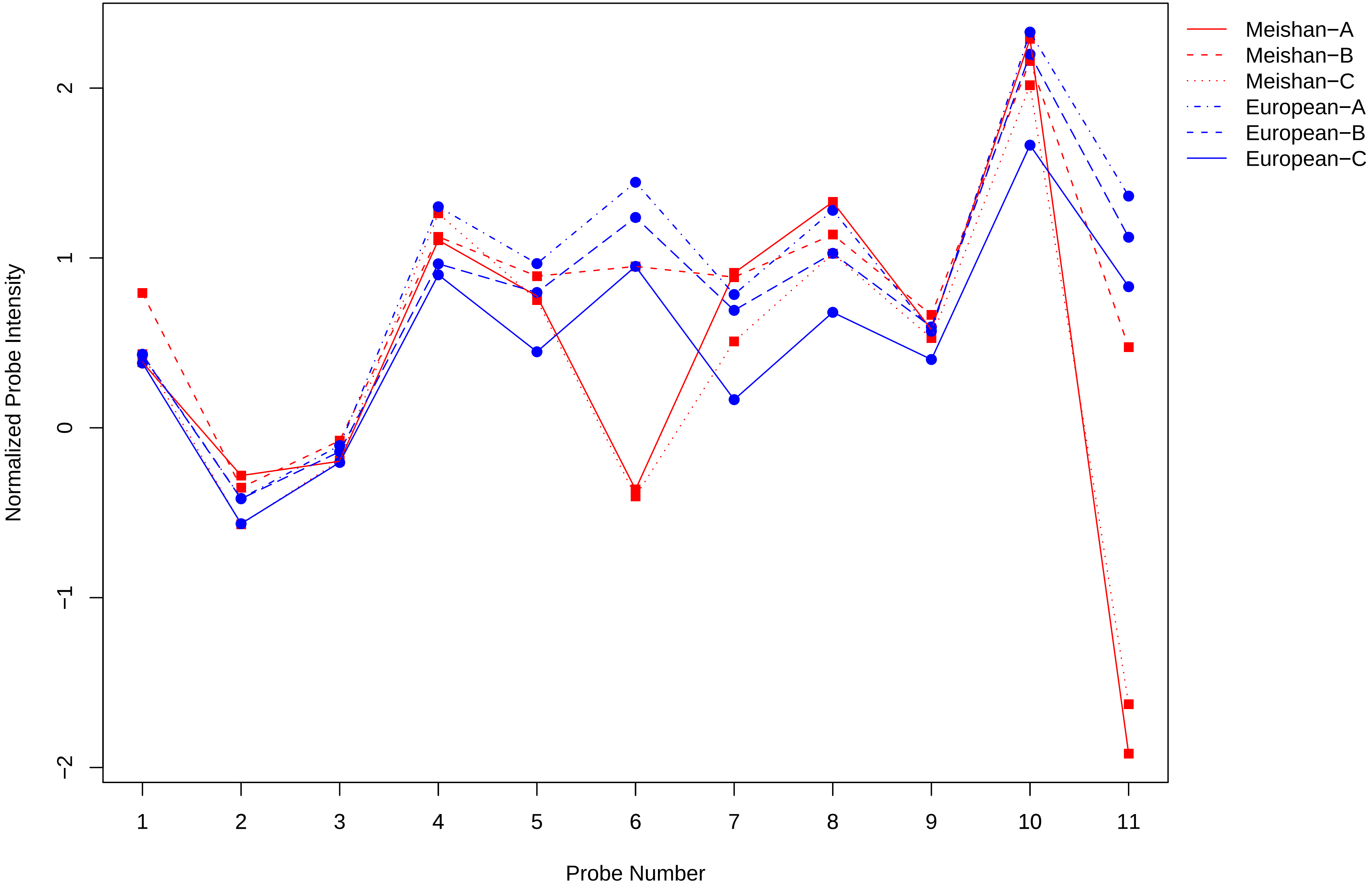

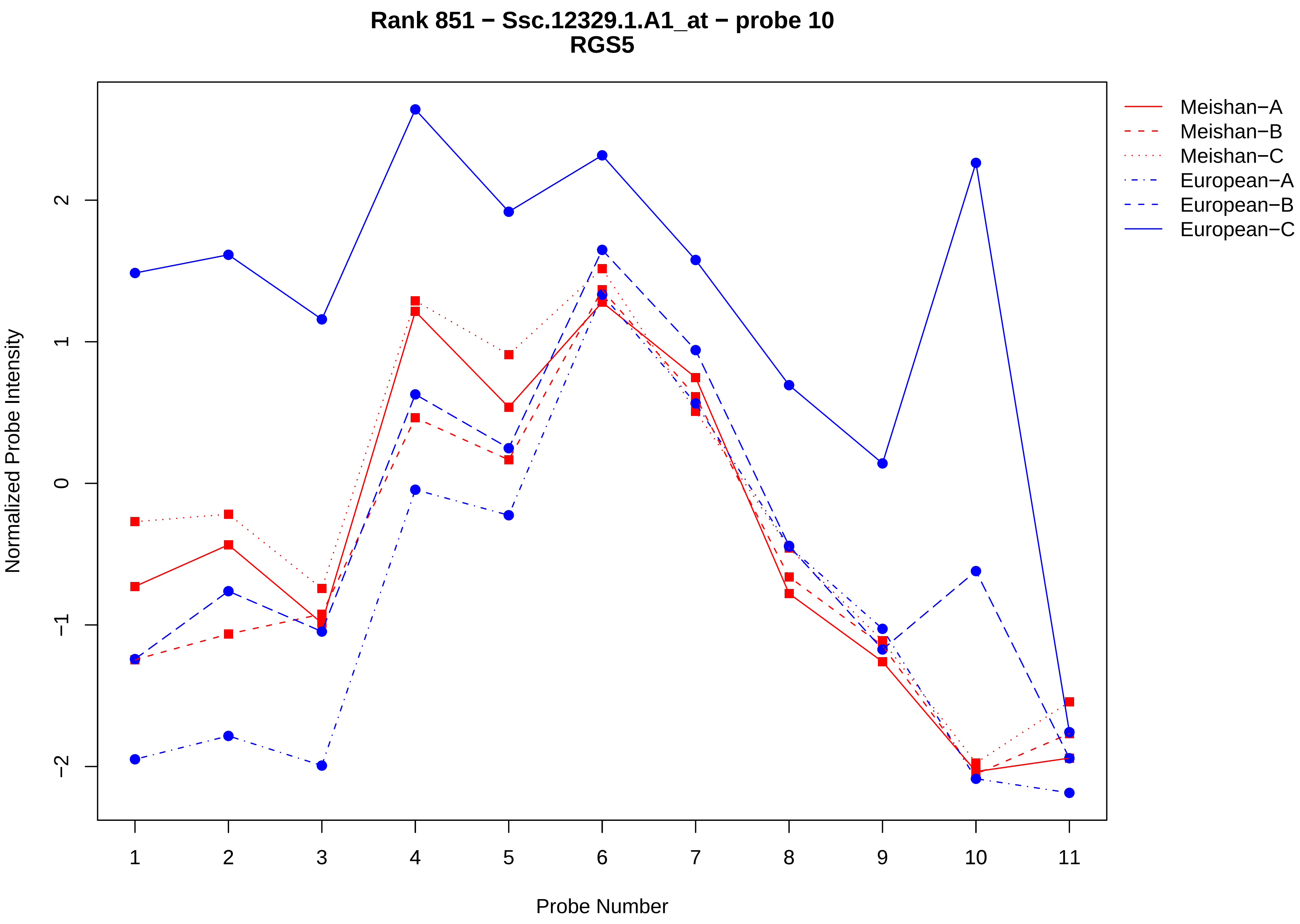

Rank 851 − Ssc.12329.1.A1_at − probe 10
RGS5
Normalized Probe Intensity
Probe Number
−2
−1
0
1
2
1
2
3
4
5
6
7
8
9
10
11
Meishan−A
Meishan−B
Meishan−C
European−A
European−B
European−C

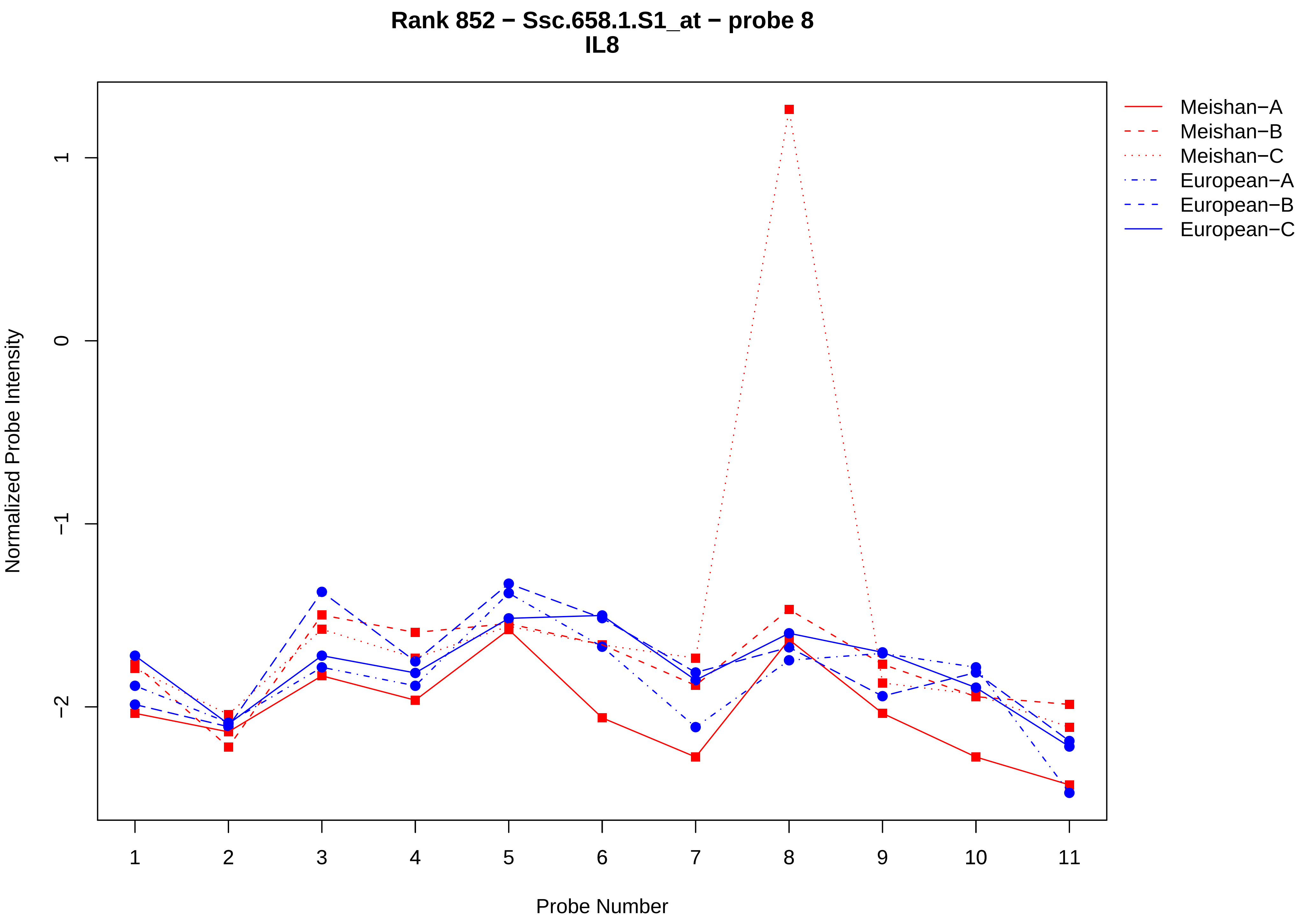

Rank 852 − Ssc.658.1.S1_at − probe 8
IL8
Normalized Probe Intensity
Probe Number
1
0
−1
−2
1
2
3
4
5
6
7
8
9
10
11
Meishan−A
Meishan−B
Meishan−C
European−A
European−B
European−C

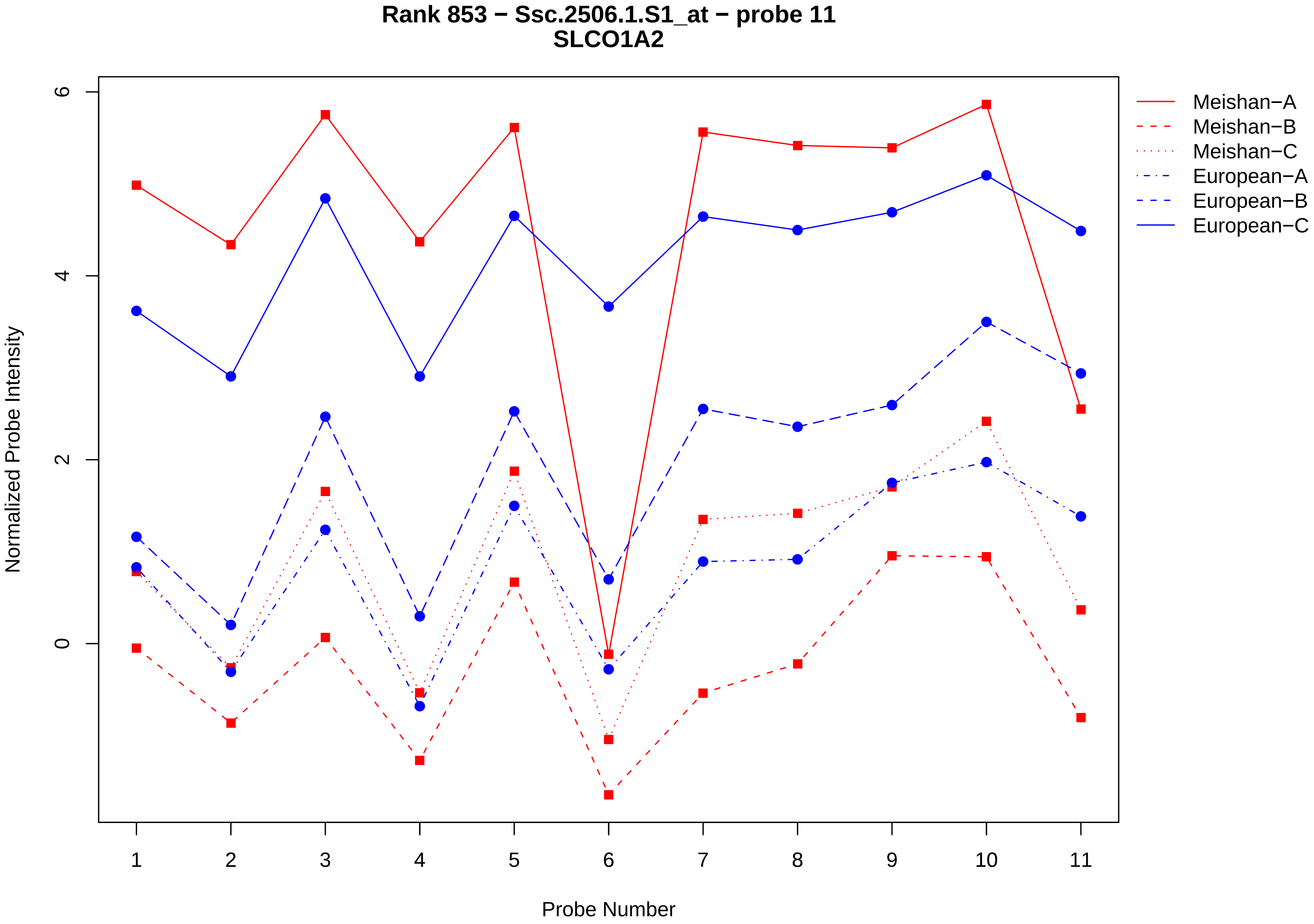

Rank 853 − Ssc.2506.1.S1_at − probe 11
SLCO1A2
Normalized Probe Intensity
Probe Number
0
2
4
6
1
2
3
4
5
6
7
8
9
10
11
Meishan−A
Meishan−B
Meishan−C
European−A
European−B
European−C

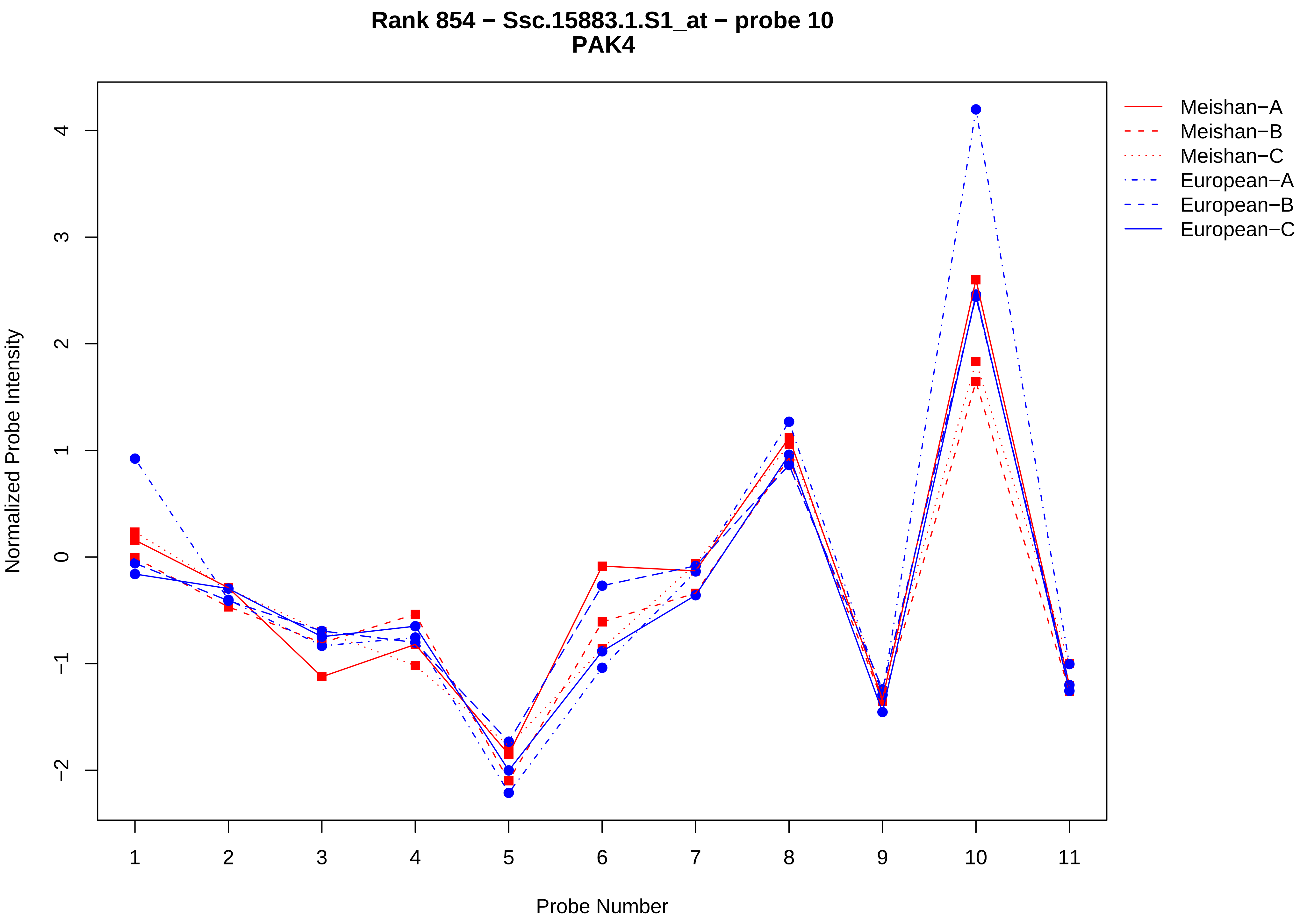

Rank 854 − Ssc.15883.1.S1_at − probe 10
PAK4
Normalized Probe Intensity
Probe Number
Meishan−A
Meishan−B
Meishan−C
European−A
European−B
European−C

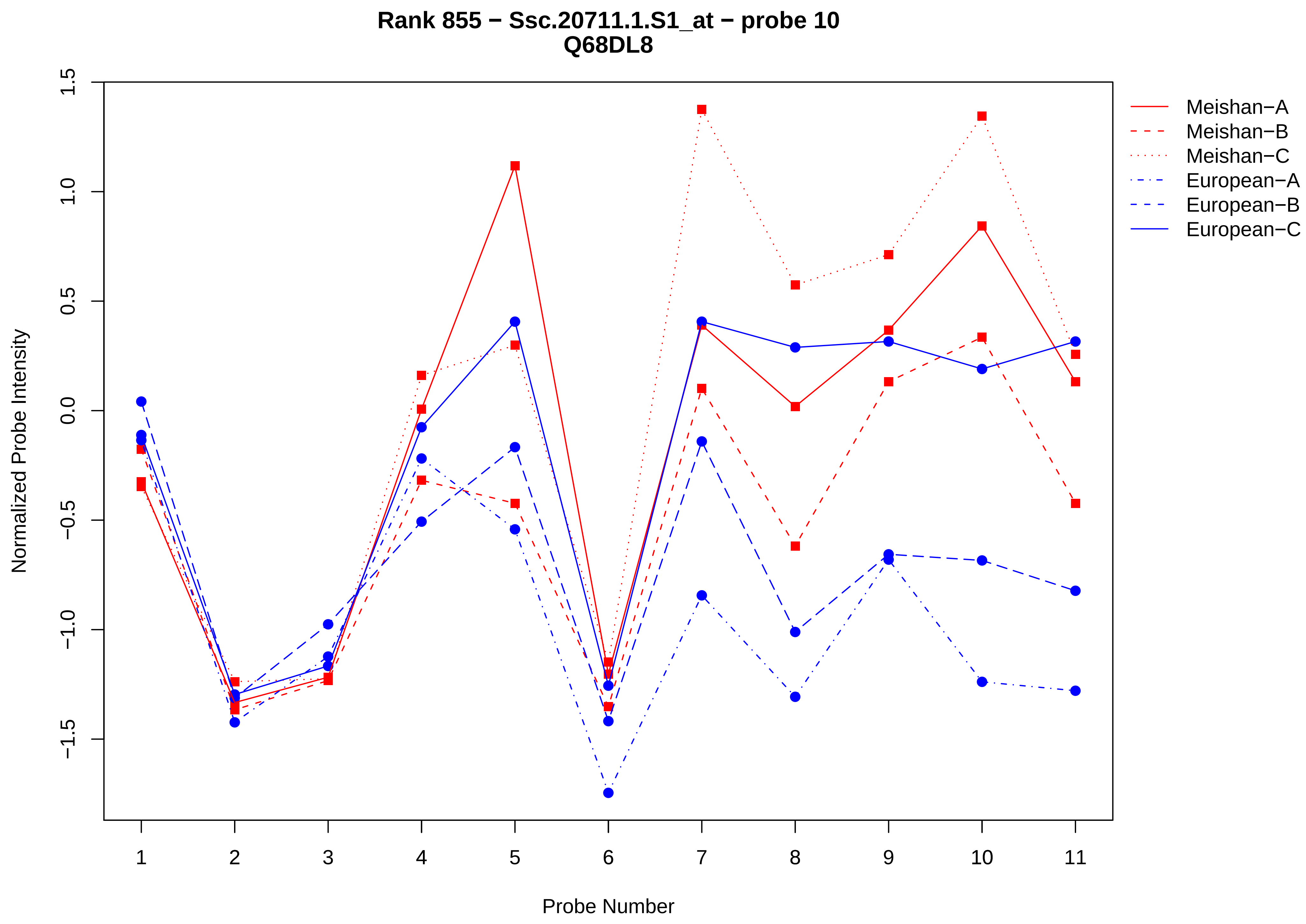
Rank 855 − Ssc.20711.1.S1_at − probe 10
Q68DL8
Normalized Probe Intensity
Probe Number
1.5
1.0
0.5
0.0
−0.5
−1.0
−1.5
1
2
3
4
5
6
7
8
9
10
11
Meishan−A
Meishan−B
Meishan−C
European−A
European−B
European−C

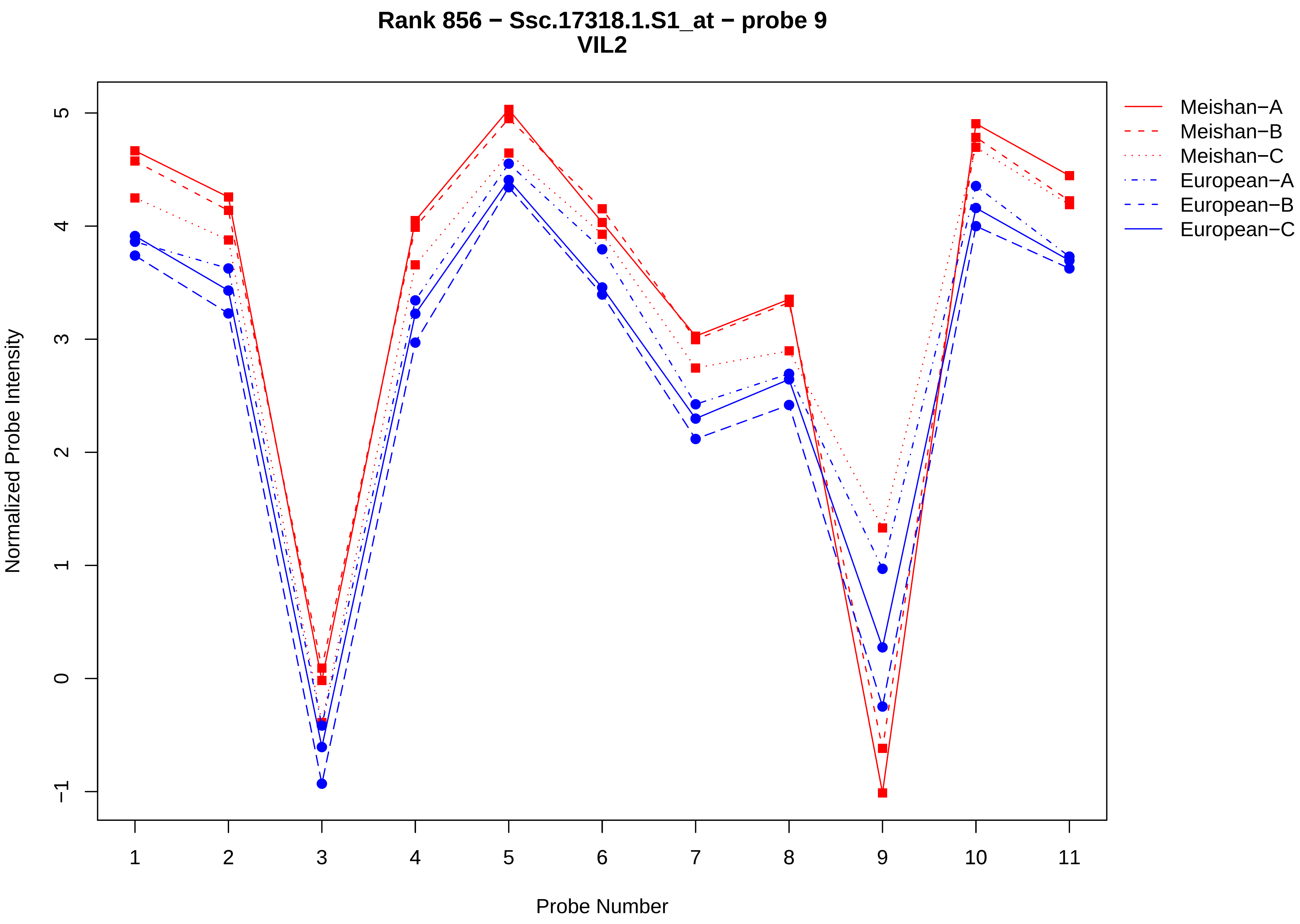

Rank 856 − Ssc.17318.1.S1_at − probe 9
VIL2
Normalized Probe Intensity
Probe Number
−1
0
1
2
3
4
5
1
2
3
4
5
6
7
8
9
10
11
Meishan−A
Meishan−B
Meishan−C
European−A
European−B
European−C

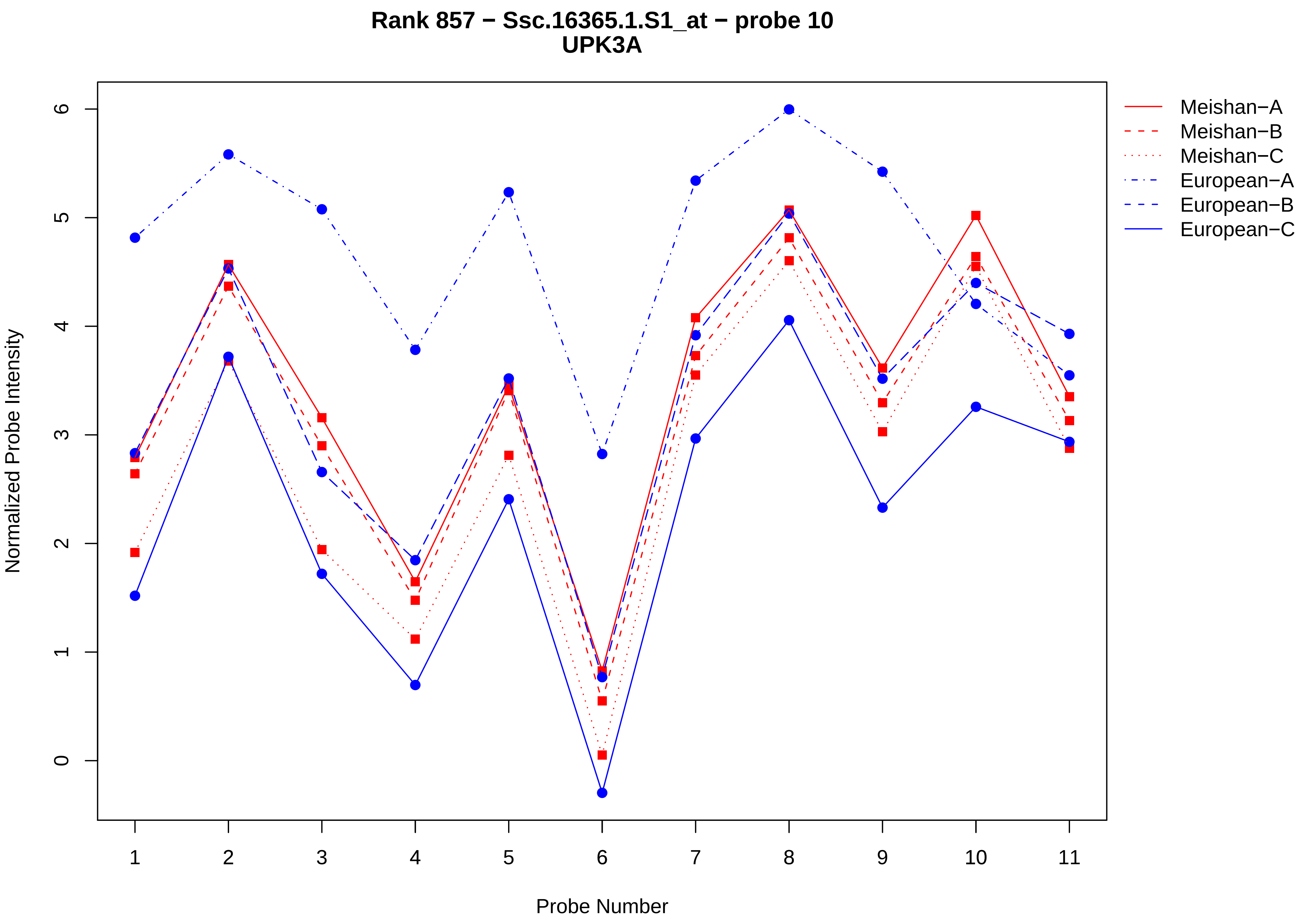

Rank 857 − Ssc.16365.1.S1_at − probe 10
UPK3A
Normalized Probe Intensity
0
1
2
3
4
5
6
Probe Number
1
2
3
4
5
6
7
8
9
10
11
Meishan−A
Meishan−B
Meishan−C
European−A
European−B
European−C
